# Proximity Labeling Proteomics Reveals Kv1.3 Potassium Channel Immune Interactors in Microglia

**DOI:** 10.1016/j.mcpro.2024.100809

**Published:** 2024-06-25

**Authors:** Christine A. Bowen, Hai M. Nguyen, Young Lin, Pritha Bagchi, Aditya Natu, Claudia Espinosa-Garcia, Erica Werner, Rashmi Kumari, Amanda Dabdab Brandelli, Prateek Kumar, Brendan R. Tobin, Levi Wood, Victor Faundez, Heike Wulff, Nicholas T. Seyfried, Srikant Rangaraju

**Affiliations:** 1 Center for Neurodegenerative Diseases, Emory University, Atlanta, Georgia, USA; 2 Department of Biochemistry, Emory University, Atlanta, Georgia, USA; 3 Department of Pharmacology, University of California – Davis, Davis, California, USA; 4 Emory Integrated Proteomics Core, Emory University, Atlanta, Georgia, USA; 5 Department of Human Genetics, Emory University, Atlanta, Georgia, USA; 6 School of Medicine, Yale University, New Haven, Connecticut, USA; 7 Department of Cell Biology, Emory University, Atlanta, Georgia, USA; 8 School of Chemical and Biomolecular Engineering, Georgia Institute of Technology, Atlanta, Georgia, USA; 9 George W. Woodruff School of Mechanical Engineering, Wallace H. Coulter Department of Biomedical Enigneering, and Parker H. Petit Institute for Bioengineering and Bioscience, Georgia Institute of Technology, Atlanta, Georgia, USA

**Keywords:** neurodegeneration, Kv1.3 potassium channel, microglia, proximity labeling, neuroinflammation

## Abstract

Microglia are resident immune cells of the brain and regulate its inflammatory state. In neurodegenerative diseases, microglia transition from a homeostatic state to a state referred to as disease-associated microglia (DAM). DAM express higher levels of proinflammatory signaling molecules, like STAT1 and TLR2, and show transitions in mitochondrial activity toward a more glycolytic response. Inhibition of Kv1.3 decreases the proinflammatory signature of DAM, though how Kv1.3 influences the response is unknown. Our goal was to identify the potential proteins interacting with Kv1.3 during transition to DAM. We utilized TurboID, a biotin ligase, fused to Kv1.3 to evaluate potential interacting proteins with Kv1.3 *via* mass spectrometry in BV-2 microglia following TLR4-mediated activation. Electrophysiology, Western blotting, and flow cytometry were used to evaluate Kv1.3 channel presence and TurboID biotinylation activity. We hypothesized that Kv1.3 contains domain-specific interactors that vary during a TLR4-induced inflammatory response, some of which are dependent on the PDZ-binding domain on the C terminus. We determined that the N terminus of Kv1.3 is responsible for trafficking Kv1.3 to the cell surface and mitochondria (*e.g.*, NUDC, TIMM50). Whereas, the C terminus interacts with immune signaling proteins in a lipopolysaccharide-induced inflammatory response (*e.g.*, STAT1, TLR2, and C3). There are 70 proteins that rely on the C-terminal PDZ-binding domain to interact with Kv1.3 (*e.g.*, ND3, Snx3, and Sun1). Furthermore, we used Kv1.3 blockade to verify functional coupling between Kv1.3 and interferon-mediated STAT1 activation. Overall, we highlight that the Kv1.3 potassium channel functions beyond conducting the outward flux of potassium ions in an inflammatory context and that Kv1.3 modulates the activity of key immune signaling proteins, such as STAT1 and C3.

## Highlights

• Kv1.3 channel interactions with proteins may regulate microglial functions.
• Several novel proteins interact with the N and C terminus of Kv1.3 channels.
• During homeostasis, the N terminus of Kv1.3 interacts with protein trafficking machinery.
• The PDZ-binding region was an important determinant of the C-terminal interactome.
• During an inflammatory response, the Kv1.3 C terminus interacts with immune proteins.

## Graphical Abstract

**Figure:**
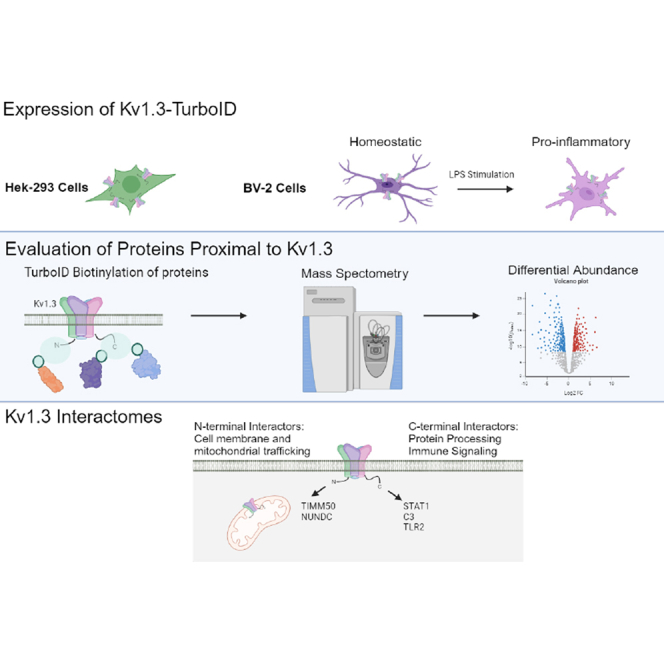

## In Brief

Kv1.3 channels are abundant in proinflammatory microglia in neurological diseases. Kv1.3 may have functions beyond movement of potassium across the membrane. Using proximity-based proteomics, we identified several novel domain-specific proteins that interact with the N and C terminus of Kv1.3 channels. Under homeostatic conditions, the N terminus of Kv1.3 interacts with proteins involved in protein trafficking to the cell surface and mitochondria. During an LPS-induced inflammatory response, the C terminus of Kv1.3 interacts with immune and signaling proteins of disease relevance, including STAT1.

Microglia are the resident myeloid immune cells of the central nervous system and are recognized to play critical roles in the pathogenesis of several neurological disorders. Microglia are involved in synaptic pruning, phagocytosis and clearance of cellular debris and protein aggregates, release of trophic and toxic factors and extracellular vesicles (1, 2). Recent advances in transcriptomic profiling have revealed heterogeneity within microglia, where some homeostatic microglia adopt a disease-associated microglia (DAM) signature (3, 4). DAM signatures appear to be conserved across several chronic neuroinflammatory and neurodegenerative disorders, including Alzheimer’s disease (AD), Parkinson’s disease (PD), multiple sclerosis, and ischemic stroke (5, 6, 7). Within the DAM population, functional heterogeneity is also present, such that a subset of DAM phenotypes may have detrimental effects *via* increased synaptic phagocytosis and release of neurotoxic factors that have damaging effects on neurons and overall neurological function, by promoting premature cellular death and impaired pathological protein clearance (8). Regulators of these proinflammatory DAM functions in neurological diseases, therefore, represent potential therapeutic targets for neuroimmunomodulation. The Kv1.3 potassium channel has emerged as a regulator of proinflammatory functions of DAM. Pharmacological blockade of Kv1.3 channels has also been shown to reduce neuropathology in mouse models of AD, PD, and stroke models (5, 9, 10, 11). However, the molecular mechanisms that allow Kv1.3 channels to regulate immune functions of microglia are not fully understood.

The *Kcna3* gene encodes the Kv1.3 potassium channel protein, which homotetramerizes to form functional voltage-activated outward-rectifying K^+^ channels (12, 13). K^+^ efflux *via* Kv1.3 fine tunes membrane potential following membrane depolarization, which in turn regulates calcium flux in immune cells such as effector memory T cells and microglia (14). Beyond the cell surface, there is also biophysical evidence for the presence of Kv1.3 channels in the inner mitochondrial membrane, which may allow intracellular Kv1.3 channels to regulate metabolism, apoptosis, and immune functions (15, 16). While Kv1.3 channels can regulate membrane potential and calcium flux, Kv1.3 channels may also regulate immune pathways *via* direct protein–protein interactions (PPIs) at the plasma membrane (9, 17). Based on the observed colocalization of Kv1.3 channels with integrins, receptors, and immune signaling proteins in T cells, there is a strong possibility that Kv1.3 also regulates immune signaling in microglia through directly interacting with proteins associated with immune function (18, 19).

Each Kv1.3 monomeric subunit contains six transmembrane domains, with both the N and C termini facing the cytosol (12, 20, 21). The N terminus of Kv1.3 contains domains responsible for channel tetramerization, subunit assembly, and localization to the membrane. The C terminus contains a PDZ-binding domain, which can interact with PSD-95 and signaling proteins, which form scaffolds for extracellular signal–regulated kinase (ERK) signaling (17, 22). Removal of the PDZ-binding domain results in changes of Kv1.3 function and localization (23). The Kv1.3 channel also interacts with a homotetramer of Kvβ proteins (Kvβ1–3), which further extends the breadth of potential interactors of Kv1.3 channels (22). Many mechanistic aspects of Kv1.3 channel interactors and function have been investigated in T cells. However, the protein interactions with the N and C termini of Kv1.3 channels in microglia are less well characterized. Given the molecular and functional heterogeneity in microglial responses within DAM, it is also possible that the protein–protein interactome of Kv1.3 channels may vary, based on the activation context of microglia.

To test this hypothesis, we investigated the N- and C-terminal interactomes of Kv1.3 channels in microglia and their relationship to the activation state of microglia. We hypothesized that Kv1.3 contains domain-specific interactors, some of which are dependent on the PDZ-binding domain on the C terminus, and shift toward immune function–annotated proteins during a lipopolysaccharide (LPS) response in microglia. To accomplish this, we utilized proximity-dependent biotinylation of Kv1.3 by fusing the biotin ligase TurboID to the N or C terminus of Kv1.3 in human embryonic kidney-293 (HEK-293) and BV-2 microglial cell lines (24). Utilizing the BV-2 microglia cell line is more efficient than primary microglia for stable cell line transduction and generating sufficient material for proteomics studies (25). Biotinylated proteins were then enriched and quantified using mass spectrometry (MS)–based proteomics. After verifying that these TurboID-Kv1.3 fusions did not impact channel localization and function, we identified N- and C-terminal protein–protein interactomes, highlighting several novel domain-specific associations between Kv1.3 channels and immune function. We also imposed an inflammatory challenge with LPS to induce a proinflammatory microglial state, which mimics the Toll-like receptor (TLR) activation that occurs in AD and PD (26, 27). We assessed the relationship between microglial proinflammatory activation and the Kv1.3 channel interactomes. Our studies identified several novel domain-specific immune protein interactions of Kv1.3 channels in microglia, including STAT1 and C3 at the C terminus and TIMM50 at the N terminus, which may explain how microglial Kv1.3 channels participate in regulating diverse immune mechanisms of neurological diseases.

## Experimental Procedures

### Plasmid and Lentivirus Design

Five plasmid constructs were designed through the Emory University custom cloning core using cloning strategies summarized in Table 1.

**Table 1:** Constructs designed for experiments conducted

Construct	Restriction sites	Primer sequences
MetRS	NheI/BstBI	5′-ctagagctagcgccaccatgggcaagcccatccccaaccccctgctgggcctggacagcaccAGACTGTTCGTGAGCGAGGGTTCCCC-3′ 5′-gatcgttcgaaTCACTTTTTCTTCTTGCCTTTAGGAGTT-3′
TurboID	BstBI/BamHI	5′-gcgcctactctagagctagcgaattcgaagccaccatgggcaagcccatccccaa-3′ 5′-agaaggcacagtcggcggccgcggatccttagtccagggtcaggcgctccagggg-3′
Kv1.3 N-term Fusion	XbaI/AgeI	5′-CAGGTCGACTCTAGagccaccatgggcaagcccatccccaaccccctgct-3′ 5′-gtggcaccggtGCTGCCACCGCCACCGCTTCCACCCCCGCCTGAGCCCCCTCCGCCcttttcggcagaccgcagactgattt—3′
Kv1.3 C-term Fusion	BamHI/BsrGI	5′-ACTTAAGCTTGGTACCGAGCTCGGATCCgccaccatgaccgtggtgcccggggaccacct-3′ 5′-tggctgtacaAGGCCCGGGGTTGCTCTCAACATCTCCAGCCAATTTCAAGAGAGCATAGTTAGTACACTGcttttcggcagaccgcagactgatt-3′
Kv1.3 C-term Fusion ΔPDZ	NheI/BamHI	5′-ACTTAAGCTTGGTACCGAGCTCGGATCCgccaccatgaccgtggtgcccggggaccacct-3′ 5′-ctgttcgccactatggaactcgcc-3′

The TurboID construct was previously described by Sunna *et al.* (28). The V5-TurboID-NES plasmid (Addgene, #107169) was transformed using a competent *Escherichia coli* strain DH5α according to the manufacturer protocols. QIAfilter Plasmid kits (Midi prep kit; Qiagen; catalog no.: 12243) were utilized to purify plasmid DNAs following the manufacturer's protocol. Restriction sites were introduced *via* the PCR primers, and the V5-TurboID-NES sequence was subcloned into pCDH-EF1-MCS-BGH-PGK-GFP-T2A-Puro (CD550A-1) and sequenced (Table 1). This construct was utilized as a positive control for biotinylation of proteins.

The construct utilized for a transfection control comprised of an overexpression of methionine-tRNA synthetase (AddGene, pMarsL274G) was created and sequenced using the protocol described previously.

Each of the *Kcna3*-based constructs was on a FUGW backbone with *Kcna3* isolated from complementary DNA (cDNA) with a GS-rich 15-amino acid linker (5′-GGCGGAGGGGGCTCA-3′x3), a V5 tag, and TurboID either on the 3′ or 5′ end of the *Kcna3* gene. These constructs were formed *via* protocols described by Sunna *et al.* (29).

The Kv1.3 C-terminal FusionΔPDZ construct had a truncated *Kcna3*. The deletion of TDV, which are the last three amino acids, is described as essential to Kv1.3 function and localization (23).This truncated *Kcna3* was fused with the same 15-amino acid linker, a V5 tag, and TurboID. Puromycin resistance was used as a selection marker at a separate location of the plasmid. DNA sequencing was used to confirm plasmid orientation and correct insertion.

Plasmids were packaged into lentivirus (LV) by the Emory University Viral Vector Core and purified as described later. HEK-293FT (Invitrogen) cells were maintained in complete medium (4.5 g/l glucose and l-glutamine containing Dulbecco's modified Eagle's medium [DMEM] supplemented with 10% fetal bovine serum [FBS] and 1% penicillin/streptomycin [P/S]) and incubated at 37 °C, 5% CO_2_. One day before transfection, HEK-293FT cells were seeded onto five 150 mm plates at a density of 1 × 10^7^ cells per plate in 20 ml of complete medium. The cells were approximately 70 to 80% confluent at the time of transfection. The day of transfection, the DNA mixture was prepared as per the following: 53 μg of lentiviral plasmid, 35 μg of pCMVΔ 8.9, and 17.5 μg of pVSVG in 4.5 ml of ddH_2_O, 0.5 ml of 1.5 M NaCl. The polyethyleneimine (PEI) mixture was prepared as the following: 0.84 ml of 7.5 mM PEI and 0.5 ml of 1.5 M NaCl in 3.66 ml of ddH_2_O. The DNA mixture and PEI mixture were then combined. This 10 ml solution was vortexed 20 s and incubated for 20 min at room temperature. Then 2 ml of the mixture was added dropwise to each dish and then incubated for 48 h before harvesting.

For plasmid purification, the supernatants (media) containing LV were collected 48 h and 72 h post-transfection, combined, and then centrifuged at 500*g* for 5 min at 4 °C, followed by passage through a 0.45 μm low protein-binding filter. The total 200 ml of supernatant was centrifuged at 28,000 rpm for 2 h at 40 °C in a 45Ti rotor (Beckman), which can sustain faster speeds. The virus pellets were resuspended in 500 μl of PBS, incubated on ice for 30 min, resuspended virus particles were combined and loaded into a 12 ml of SW 41 tube, 3 ml of 20% sucrose cushion, and centrifuged at 28,000 rpm for 2 h at 4 °C in a SW 41 rotor (Beckman). The virus pellet was resuspended in 60 μl of PBS and then stored at −80 °C.

For transduction of BV-2 cells, purified virus was added to cells at a multiplicity of infection of 10 along with 8 μg/ml polybrene for 24 h. Remaining LV media were then removed, and cells were allowed to grow in DMEM-F12 (10% FBS, 1% P/S) for 5 days. Puromycin was then added at 2 μg/ml for 7 days. The presence of *Kcna3* gene was confirmed with quantitative PCR.

### Cell Culture and Maintenance

Mycoplasma-free HEK-293 cells (obtained from Seyfried Lab) were grown in DMEM-F12 (10% FBS and 1% P/S) and plated at 1 million cells in a 10 cm dish for protein lysis and 100,000 cells/well on poly-l-lysine-coated coverslips (coating protocol followed *via* manufacturer’s procedure) in 24-well plates for immunofluorescence. Mycoplasma-free BV-2 cells (Obtained from Tansey Lab) were grown in DMEM-F12 (10% FBS and 1% P/S) at 1 million cells in a 10 cm dish. Cells were allowed to adhere to the plate for 24 h prior to experiments.

### Dosing and Immune Stimulation

HEK-293 cells were transfected with plasmids using the JETPRIME transfection reagent for 24 h according to the manufacturer’s protocol. For biotinylation, HEK-293 cells underwent a full media change with the addition of 200 μM biotin in DMEM-F12 (10% FBS and 1% P/S) for 24 h.

BV-2 cells were treated with either 100 ng/ml LPS or PBS for 24 h. At 23 h of LPS or PBS incubation, 200 μM biotin was added in without a media change for 1 h. In contrast to 24 h of biotinylation used in HEK-293 studies, we limited the biotinylation duration to 1 h, to increase the stringency of the interactomes of Kv1.3.

### Electrophysiology

Electrophysiological experiments were conducted on transfected HEK-293 and transduced BV-2 cells that were plated on polylysine-coated glass coverslips. The cells were allowed to attach for 10 min at 37 °C before starting the electrophysiological measurements using the whole-cell configuration of the patch-clamp technique at room temperature with an EPC-10 HEKA amplifier. Transfected HEK-293 cells were visualized by epifluorescence microscopy of green fluorescent protein by eEGFP-C1 plasmid cotransfection (Addgene, 2487, discontinued). The Ringer solution used contained 160 mM NaCl, 4.5 mM KCl, 2 mM CaCl_2_, 1 mM MgCl_2_, 10 mM Hepes, pH 7.4, and had an osmolarity of 300 mOsm. Patch pipettes were made from soda lime glass (microhematocrit tubes, Kimble Chase) and had resistances of 2 to 3 MΩ when submerged in the bath solution. The pipettes were filled with an internal solution containing 145 mM kF, 1 mM Hepes, 10 mM EGTA, 2 mM MgCl_2_, pH 7.2, and 300 mOsm. Series resistance and whole-cell capacitance compensation were used as quality criteria for electrophysiology. Current amplitudes were recorded in voltage-clamp mode and elicited using a 200-ms voltage step from −80 to +40 mV at a frequency of 0.1 Hz. Use-dependency was determined using the same protocol but with a pulse frequency of 1 Hz for 10 pulses. The fractional current of the last pulse was normalized to the first pulse to determine the extent of cumulative (use-dependent) inactivation. Whole-cell patch-clamp data are presented as mean ± SD, and statistical significance was determined using a paired Student's *t* test for direct comparison between WT and TurboID-fusion constructs.

The voltage dependence of activation was examined using a step protocol where cells were depolarized for 200 ms from a holding potential of −80 mV to a range of potentials from −80 to +40 mV in 10-mV increments, with an interpulse duration of 30 s. The peak currents were normalized to the maximum peak current and plotted against the voltage. The reversal potential was calculated by individually fitting the resulting I–V curve of each dataset with the equation: I = z ∗ (V – Vr), where I is the normalized current amplitude, z is the apparent gating charge, V is the potential of the given pulse, and Vr is the reversal potential. Conductance was then directly calculated using the equation: G = I/(V – Vr), where G is conductance and I, V, and Vr are as described previously. The conductance values were fit with the two-state Boltzmann equation: G = [1 + exp(−0.03937 × z × (V – V_1/2_))] − 1, where z is the apparent gating charge and V is the potential of the given pulse, and V_1/2_ is the potential for half-maximal activation.

The activation and inactivation kinetics were examined using currents elicited by the same step protocol used for determining the current amplitude at +40 mV above. The activation and inactivation time constants were calculated using the Chebyshev method to fit the activating phase and inactivating phase, respectively, of each trace with a single exponential equation: I = A × exp[–(t – K)/|τ] + C, where I is the current, A represents the relative proportions of current activating with the time constants τ, K is the time shift, and C is the steady-state asymptote. Only the constants τ are reported.

### Quantitative RT–PCR

#### RNA Extraction

BV-2 cells were washed 2× with cold 1× PBS, then incubated in Trizol for 5 min. A chloroform–phenol extraction was performed according to the manufacturer’s protocol (Invitrogen). RNA was suspended in 50 μl of diethylpyrocarbonate (DPEC)-treated water. Purity and quantity of RNA was evaluated using a Nanodrop 2000 (Thermo Scientific). A260/280 above 1.3 and A260/230 ≥ 2.0 were considered acceptable purity levels for RNA.

#### cDNA Synthesis

About 2 μg of RNA was mixed with 5 μl 10x RT buffer, 2.5 μl Multiscribe reverse transcriptase, 2 μl dNTP mix, 5 μl 10x RT random primers, and DPEC H_2_O for a 50 μl reaction and incubated in an Applied Biosystems 2720 Thermocycler according to Applied Biosystems Protocol. cDNA was diluted 1:10 prior to qRT–PCR.

#### Real-Time qRT–PCR

A 20 μl reaction was utilized with 10 μLTaqman Universal Master Mix II, 1 μl designated primer (listed in Table 2), 4 μl DPEC H_2_O, and 5 μl cDNA (diluted 1:10) according to the Applied Biosystems protocol in the MicroAmp Fast-96-well Reaction Plate sealed with MicroAmp Optical Adhesive Film. The Applied Biosystems 7500 Fast Real-Time PCR System was utilized. cDNA was probed with either *Kcna3* or the housekeeping gene *Gapdh*. Fold change (FC) was calculated on CT values and normalized to housekeeping genes and negative control. Statistical significance was calculated based on unpaired *t* test and graphed on PRISM (version 10.1.0).

**Table 2:** Reagent list

Reagent	Manufacturer	Catalog number
Dulbecco’s modified Eagle's medium (DMEM-F12)	Gibco	11965-092
Penicillin–streptomycin	Gibco	15140-122
Fetal bovine serum (FBS)	Gibco	26140-079
JETPRIME	Polyplus transfection	101000046
Lipopolysaccharide (LPS)	Sigma–Aldrich	L4391, 1 mg
Polybrene	Sigma–Aldrich	S2667
Puromycin	Gibco	A11138-02
Recombinant mouse IFN-γ	R&D Systems	485-MI-100
0.05% Trypsin–EDTA	Gibco	253000054
Biotin	Sigma–Aldrich	B4639-100mg
Trizol	Invitrogen	15596018
10x RT buffer	Applied Biosystems	4319981
Multiscribe reverse transcriptase	Applied Biosystems	4308228
dNTP mix	Applied Biosystems	4367381
10x RT random Primers	Applied Biosystems	4319979
Taqman Universal Master Mix II, no UNG	Applied Biosystems	4440040
MicroAmp Fast-96-well Reaction Plate	Applied Biosystems	4346907
MicroAmp Optical Adhesive Film	Applied Biosystems	4311971
*Kcna3* qPCR primer	Applied Biosystems	Mm00434599_s1
*Gapdh* qPCR primer	Applied Biosystems	Mm99999915_g1
Rabbit Anti-V5 tag	Abcam	ab206566
Donkey anti-Rabbit, FITC	Invitrogen	A16030
Streptavidin-594 antibody	Invitrogen	21842
DAPI	Sigma–Aldrich	10236276001
Prolong Diamond	Invitrogen	p36970
1x fixation buffer	eBioscience	00-8222-49
1x permeabilization buffer	eBioscience	00-8333-56
Streptavidin, Alexa-Fluor 488 conjugate	Invitrogen	21832
Unstained OneComp ebeads compensation beads	eBioscience	01-111-42
HALT protease & phosphatase inhibitor cocktail	ThermoFisher	1861284
BCA colorimetric assay	ThermoFisher	23227
Bovine serum albumin standards	ThermoFisher	23208
4x Laemmli buffer	Bio-Rad	1610747
BOLT 4%–12% Bis–Tris Gels	Invitrogen	NW04125BOX
250 kDa Protein Ladder	BioLabs	P7719S
iBLOT mini stack system	Invitrogen	IB23002
StartingBlock T20	ThermoFisher	37543
Streptavidin, Alexa-Fluor 680 conjugate	Invitrogen	S32358
Silver stain	ThermoFisher	24612
Lysyl endopeptidase	Wako	125-05061
Trypsin	Promega	V5111
HLB columns	Oasis	186003908
Reagent A	ThermoFisher	23222
Reagent B	ThermoFisher	23224
Complement proteins Luminex immunoassay	Millipore	HCMP2MAG-19K-06
MAPK phosphoproteins Luminex immunoassay	Millipore	48-660MaG
HSP60, 60 KDa heat-shock protein	Cell Signaling	12165
β-actin	Santa Cruz Biotechnology	sc-47778
IRDye800	LI-COR	925-32211
HRP anti-mouse	Jackson ImmunoResearch	115-035-033
PAP-1	Synthesized in Wulff lab as described (37)	N/A
Anti-pSTAT1	Cell Signaling	9167S
Anti-STAT1	Cell Signaling	14994

### Immunofluorescence Microscopy

Cells were washed in plate twice with 1x PBS prior to fixing with 4% paraformaldehyde for 30 min at room temperature and then washed three times with 1x PBS for 5 min (n = 2). Cells were permeabilized in 0.1% Triton X-100 + 2% horse serum in 1x PBS for 30 min at room temperature. Cells were incubated with rabbit anti-V5 (1:500 dilution) in 2% horse serum in 1× PBS for 1 h at room temperature and then washed three times with 1× PBS for 5 min at room temperature. Cells were further incubated with a secondary antibody mix consisting of 1:500 Donkey anti-Rabbit FITC, and 1:500 Streptavidin-594 for 1 h at room temperature followed by three 1x PBS 5 min washes at room temperature. Coverslips were mounted on slides using mounting media (Prolong Diamond) and dried for 48 h. Slides were sealed with clear nail polish for 24 h and imaged using a Nikon A1R HD25 inverted confocal microscope using a 60× objective lens using NIS-Elements Imaging software. Image analysis and processing was completed *via* ImageJ (NIH, https://imagej.net/ij/).

### Flow Cytometry

Cells (BV-2 or HEK-293) were scraped and collected using ice-cold PBS in a centrifuge tube and washed by adding extra PBS (n = 3). To determine Kv1.3 presence, the cells were transferred into a flow tube and incubated with ShK-F6CA at a concentration of 5.5 μM in 100 μl PBS on ice for 30 min in the dark, followed by 3 PBS washes (30). For each PBS washing step, 1 ml of PBS was added to the flow tube and centrifuged at 2200 rpm for 3 min, followed by the removal of the supernatant. The cells were kept in the dark on ice until flow cytometry was performed. To determine biotin presence in BV-2 cells, cells were first fixed in 1× fixation buffer for 30 min on ice, then washed thrice with cold 1× PBS. Cells were then permeabilized for 30 min using 1x permeabilization buffer on ice. To determine the presence of biotin, fixed and permeabilized cells were incubated with Streptavidin-488 (1:500 in permeabilization buffer) and incubated for 1 h on ice in the dark. After incubation, the cells were washed, as mentioned previously. After the last wash, 200 μl of PBS was added, vortexed, and kept on ice in the dark until flow cytometry was performed. Unstained OneComp beads and beads stained with Alexa Fluoro-488, ShK-F6CA, and unstained cells were used as a control. All flow cytometry data were collected on the BD Aria II instrument and analyzed using the FlowJo software (BD Biosciences).

### Cell Lysis and Protein Processing

BV-2 and HEK-293 cells were rinsed twice with 1x PBS prior to scraping in PBS and then spun at 800*g* for 5 min at room temperature (n = 3). Cells were lysed in 8 M urea in Tris–NaH_2_PO_4_ with Halt protease inhibitor (1:100 dilution). Lysates were probe sonicated at 30% amplitude three times for 5 s on 10 s off. Lysates were centrifuged at 15,000*g* for 15 min. Supernatants, containing solubilized proteins, were processed for affinity purification (AP) and MS.

#### Western Blot

Protein amount in each cell lysate was quantified using a BCA colorimetric assay (n = 3). To confirm protein biotinylation, 10 μg of protein were added to 4× Laemmli buffer (1:50 beta-mercaptoethanol) and boiled for 10 min at 95 ^°^C and resolved on a BOLT 4%–12% Bis–Tris gel at a current of 80 V for 15 min followed by 120 V for 40 min along with a 250 kDa ladder. Proteins were transferred to a nitrocellulose membrane using the iBLOT mini stack system. Ponceau staining for 2 min was used to confirm equal loading. The membrane was washed using 1x Tris-buffered saline (TBS) for 15 min and blocked for 30 min using StartingBlock T20 (TBS) blocking buffer at room temperature. Blots were then incubated with Streptavidin-680 (1:10,000 dilution in blocking buffer) for 1 h at room temperature protected from light. Blots were washed twice in TBS with Tween-20 for 10 min at room temperature and twice in TBS for 5 min at room temperature. Membranes were then imaged using the Odyssey Li-COR system.

### Affinity Purification of Biotinylated Proteins

About 83 μl of Pierce streptavidin magnetic beads (Thermo Scientific; catalog no.: 83817) were washed with 1 ml radioimmunoprecipitation assay (RIPA) buffer for 2 min on rotation at room temperature. Protein lysates, 1 mg for HEK-293 cells and 0.5 mg for BV-2, were brought up in 500 μl RIPA buffer and incubated with magnetic beads on rotation at 4 ^°^C for 1 h. Beads were washed twice with 1 ml of RIPA buffer for 8 min followed by one wash with 1 ml of 1 M KCl for 8 min on rotation at room temperature. Beads were then washed with 1 ml of 0.1 M Na_2_CO_3_ for 10 s and 1 ml of 2 M urea in Tris–HCl buffer (10 mM, pH:8) for 10 s at room temperature. The beads were then washed twice with RIPA buffer for 8 min followed by two PBS washes for 2 min each. For each wash, samples were spun down and incubated on magnetic rack for 2 min to allow full attachment of beads to the magnet. After the final PBS wash, PBS was removed completely and the beads were dissolved in 80 μl of PSB. About 8 μl of beads (10% of total) were resuspended in 30 μl of 2x Laemmli buffer supplemented with 2 mM biotin and 20 mM DTT and boiled at 95 ^°^C for 15 min. Western blot and silver stain were used to check for streptavidin labeling and protein abundance post-AP. Protein-bound beads were stored at 20 °C until on-bead digestion.

### Protein Digestion and Peptide Clean Up

For MS, proteins were digested from cell lysates (input; represents protein present in whole cell) and on protein attached to the beads (represents biotin-enriched proteins). To prepare biotin-enriched samples for MS, protein-bound streptavidin beads were washed three times with 1× PBS and then resuspended with 150 μl 50 mM ammonium bicarbonate (ABC, NH_4_HCO_3_). About 1 mM DTT was added to reduce samples for 30 min on a rotor (800 rpm) at room temperature. About 5 mM iodoacetamide was added to alkylate cysteines for 30 min on a rotor at room temperature, protected from light. Proteins were digested overnight with 0.5 μg of lysyl endopeptidase on rotation (800 rpm) at room temperature. Proteins were further digested by 1 μg trypsin overnight on rotation (800 rpm) at room temperature. Samples were acidified to 1% formic acid and 0.1% TFA, desalted using an HLB column, and dried using cold vacuum centrifugation (SpeedVac Vacuum Concentrator).

To prepare cell lysates for MS, different concentrations were optimized for digestion. About 100 μg of pooled cell lysates were reduced with 1 mM DTT for 30 min and alkylated with 5 mM iodoacetamide for 30 min protected from light. Samples were diluted twofold in ABC and digested with 2 μg of lysyl endopeptidase overnight on rotation at room temperature. Samples were further diluted to a final urea concentration of 1 M and digested by 4 μg trypsin overnight on rotation at room temperature. Samples were acidified to 1% formic acid and 0.1% TFA, desalted using an HLB column, and dried down using cold vacuum centrifugation (SpeedVac Vacuum Concentrator). Digestion protocol is also outlined in the study by Sunna *et al*. and Rayaprolu *et al*. (28, 31).

### Mass Spectrometry

Derived peptides were resuspended in the loading buffer (0.1% TFA) and were separated on a Water's Charged Surface Hybrid column (150 μm internal diameter × 15 cm; particle size: 1.7 μm). HEK-293 and BV-2 samples were ran on the same mass spectrometer with similar chromatography settings, only differing by number of samples run. BV-2 cell samples were run on an EVOSEP liquid chromatography system using the 15 samples per day preset gradient (88 min) and were monitored on a Q-Exactive Plus Hybrid Quadrupole-Orbitrap Mass Spectrometer (ThermoFisher Scientific). Whereas, HEK-293 cell samples were run on an EVOSEP liquid chromatography system using the 30 samples per day preset gradient (44 min) and were monitored on a Q-Exactive Plus Hybrid Quadrupole-Orbitrap Mass Spectrometer (ThermoFisher Scientific). The mass spectrometer cycle was programmed to collect one full MS scan followed by 20 data-dependent MS–MS scans. The MS scans (400–1600 *m/z* range, 3 × 10^6^ automatic gain control target, 100 ms maximum ion time) were collected at a resolution of 70,000 at *m/z* 200 in profile mode. The higher-energy collisional dissociation MS–MS spectra (1.6 *m/z* isolation width, 28% collision energy, 1 x 10^5^ automatic gain control target, 100 ms maximum ion time) were acquired at a resolution of 17,500 at *m/z* 200. Dynamic exclusion was set to exclude previously sequenced precursor ions for 30 s. Precursor ions with +1, and +7, +8, or higher charge states were excluded from sequencing. MS protocols are previously outlined (28, 31).

### Protein Identification and Quantification

MS raw files were uploaded into MaxQuant software (version 2.4.9.0), where HEK-293 data were searched against the human UniProt 2017 database (90,412 proteins), and BV-2 data were searched against the 2020 mouse UniProt proteome database (91,441 proteins), both of which were modified to contain target sequences for TurboID. Methionine oxidation, protein N-terminal acetylation, and deamination were variable modifications; carbamidomethyl was a fixed modification; the maximum number of modifications to a protein was 5. Label-free quantification minimum ratio count was set to 1. Requantification, a process in MaxQuant to double check peptides, was used. The minimum peptide length was set to 6 amino acids, with a maximum peptide mass of 6000 Da. Identifications were matched between runs. For protein quantification, the label minimum ratio count was set to 1, peptides were quantified using unique and razor peptides. Fourier transformed MS match tolerance was set to 0.05 Da, and ion trap MS was set to 0.6 Da. The false discovery rate was set to 1%. The maximum number of missed cleavages is set to 1.

For both the enriched samples and cell lysates, MaxQuant intensities were uploaded into Perseus (version 1.6.15). Categorical variables, such as potential contaminants, reverse peptides, and samples only identified by one site were all removed. Data were log_2_ transformed and filtered for missingness such that at least two samples had nonmissing values within an experimental group. After this threshold, missing data were imputed based on normal distribution and matched to UniPort gene names (Supplemental Datasheets S1 and S2). In enriched samples, protein groups were normalized to TurboID abundance (Supplemental Datasheets S3 and S4). The same process was applied to all cell types and conditions described later.

### Data Analysis

#### Analysis of TurboID Biotinylated Proteomes of HEK-293 Cells

The analysis was divided into four groups, which are “Control,” “Cterm,” “Nterm,” and “TurboID,” each with an n = 3 and a final dataset of 2122 proteins. The C-term fusion ΔPDZ was excluded from analysis because of inconsistent labeling. Principal component analysis (PCA) of affinity purified data was performed. Furthermore, to identify genes of interest that were differentially abundant, we performed a one-way ANOVA on all samples (n = 12), comparing the groups are “Control,” “Cterm,” “Nterm,” and “TurboID” (n = 3 each). The code for the one-way ANOVA was adapted from the "parANOVA" repository on GitHub (https://github.com/edammer/parANOVA). Volcano plot comparisons were generated for the following data. The proteins that have a raw *p* value of ≤0.05 and a Log_2_FC of ±1 are considered statistically significant.

#### Analysis of TurboID Biotinylated Proteomes of BV-2 Cells

The finalized dataset of 1412 proteins were identified. A smaller number of proteins were identified in BV-2s likely just because there were less proteins biotinylated. We performed PCA for analyzing high-dimensional datasets, which helped to distinguish patterns, relationships in the dataset, and identify the main sources of variation.

Differential enrichment analysis (DEA) was performed. Significantly differentially abundant proteins were identified with an unadjusted *p* value ≤0.05 (nominal *p* values are widely accepted for volcano analysis and hence were chosen for this analysis over BH corrected to avoid overfiltering of genes and data) and a Log_2_FC of ±1 for up and decreased proteins considered statistically significant, respectively. Following the roadmap for statistical analysis, the "parANOVA" repository on GitHub (https://github.com/edammer/parANOVA) was referenced for the one-way ANOVA code and was implemented. Volcano plots were created to represent results from differential abundance analyses. Morpheus (https://software.broadinstitute.org/morpheus) was used to create visual heat maps of protein abundance. Individual proteins were colored based on z-score, where the darker shades of red indicate +1 and the darker shades of blue indicates −1. Hierarchical clustering arranged proteins based on groups.

#### Analysis of Whole BV-2 Cell Lysates

The input data were loaded and shared in the supplemental file. Postprocessing, the finalized dataset identified 4152 observations of proteins. To distinguish patterns, relationships in the dataset and identify the main sources of variation, we performed PCA for analyzing high-dimensional datasets.

DEA was performed. Significantly differentially enriched proteins were identified *via* unadjusted *p* value ≤0.05 and Log2FC of ±1. The "parANOVA" repository on GitHub (https://github.com/edammer/parANOVA) was referenced for the one-way ANOVA code and was implemented for analysis. The PBS group was compared to the LPS group with an n = 5 of each, and Volcano plot was generated for the comparison. “KCNA3” and “TurboID” were highlighted as significant proteins to clarify biological insight from the pertinent dataset.

### Gene Set Enrichment Analysis

#### HEK-293 Cells

Gene set enrichment analysis (GSEA) was performed utilizing the software AltAnalyze (version 2.1.4) (32, 33) currently run by the Cincinnati Children's Hosptial Medical Center and the University of Cincinnati. Z-score of higher than 1.96 was used to identify significantly enriched genes. In the HEK-293 cells, the DEA comparison between N-terminal and C-terminal regions were used to create an input list that was unique to the N terminus of Kv1.3 and a list of proteins enriched with the C terminus of Kv1.3. These lists were compared to all the proteins captured normalized to TurboID abundance. The most abundant genes were listed along with the STRING analysis.

#### BV-2 Microglial Cells

GSEA was performed utilizing the software (version 2.1.4) (32, 33). Z-score of higher than 1.96 was used to identify significantly enriched genes The DEA comparison of both N and C termini compared to global TurboID presence was used to calculate an input list of all microglial Kv1.3 interactors. The DEA comparison between N and C termini was used to create an input list specifically for Kv1.3 N-terminal interactors. For the LPS conditions, the DEA comparison for the C-terminal fusion with PBS or LPS was used to create lists of proteins more abundant in the PBS conditioned media or LPS conditioned media. Lists were created for proteins that are enriched and depleted with the deletion of the PDZ-binding domain on the C terminus. Lists were referenced to all proteins abundant in MS data that were normalized to TurboID abundance. The top 10 genes lists were selected based on highest z-score. The GSEA results were plotted with a bar graph of z-score and colored based on type of process using PRISM (version 10.1.0).

### Protein–Protein Interaction Network Analysis (STRING)

#### HEK-293 Cells

Interacting networks were made using the STRING analysis software (34). Network nodes represent proteins. Node color represents gene term member, where dark colors indicate significant z-score in GSEA list and light color indicates not significant. Disconnected nodes were excluded from STRING network. Edges represent protein–protein association, where edge thickness indicates confidence, thickest line indicates highest confidence (0.900), whereas thinnest line indicates low confidence (0.150).

#### BV-2 Cells

Interacting networks were made using the STRING analysis software (34). Network nodes represent proteins of Kv1.3 interactors or Kv1.3 interactors overlapping with MITOCARTA. Node color represents GSEA list. Disconnected nodes were excluded from STRING network. Edges represent protein–protein association, where edge thickness indicates confidence, thickest line indicates highest confidence (0.900), whereas thinnest line indicates low confidence (0.150).

### Experimental Design and Statistical Rationale

Sample conditions were prepared by cell type (either BV-2 or HEK-293), inflammatory challenge (LPS, PBS), type of TurboID (control—absent of turboID, Kv1.3 with an N-terminal fusion of TurboID, Kv1.3 with a C-terminal fusion of TurboID, truncated Kv1.3 with the PDZ-binding domain removed and a C-terminal fusion of TurboID), and biotin enrichment (streptavidin affinity purified or lysate). For MS, AP samples were run in three biological replicates and lysates were pooled for each experimental condition. Overall, a total of 46 samples were analyzed and described in the results. Maximum number of samples was based on budget allowance, and *a priori* power analysis was not performed. Sample acquisition order was randomized by nonbiotinylated samples followed by biotin-containing samples, as to prevent contamination of biotin-labeled proteins. Statistical rationals and analysis is further described in the “Data analysis,” “GSEA,” and “protein–protein network interactions analysis (STRING).”

#### Luminex Assays

BV-2 cells were grown at 50,000 cells/well in 12-well plates and allowed to adhere to bottom of plate for 24 h. Cells were then treated with LPS (100 ng/ml) for 24 h and then exposed to interferon gamma (IFN-γ) (100 ng/ml) for 60 min. Lysates were prepared in 8 M urea as previously described (28).

Complement proteins and mitogen-activated protein kinase (MAPK) phosphoproteins were quantified using multiplexed Luminex immunoassays; these have been previously used without crossreactivity (31). The analytes measured with the human complement panel are complement C1q, complement C3, complement C3b/iC3B, complement C4, complement factor B, and complement factor H. To determine cross-species reactivity between the human complement panel analytes and the murine cell line lysates, linear ranging was performed. Only analytes obeying a linear response were evaluated. Analytes detected with the MAPK panel are pATF2 (Thr71), pErk (Thr185/Tyr187), pHSP27 (Ser78), pJNK (Thr183/Tyr185), p-c-Jun (Ser73), pMEK1 (Ser222), pMSK1 (Ser212), p38 (Thr180/Tyr182), p53 (Ser15), and pSTAT1 (phosphorylated STAT1; Tyr701). Standard protocols from the manufacturer were followed, with quarter volume loading of reagents to reduce excess antigen. Sample loadings were normalized to total protein *via* Pierce BCA. To identify samples biotinylated *in vitro*, an adapted Luminex protocol was followed as previously reported (31). The overarching mechanism in the standard protocol is an analyte is immobilized using a magnetic bead specific to the analyte. The analytes are then tagged with specific biotinylated antibodies and biotinylation is detected with a streptavidin fluorophore; streptavidin–phycoerythrin. The adapted assay protocol utilizes the biotin-ligated proteome and thus omits the biotinylated antibody. Appropriate protein loadings for both standard and adapted assays were determined following a linear ranging to ensure signal above noise/background levels and to mitigate potential impact of the hook effect (false negatives) (31). Average net mean fluorescence intensity values were utilized; negative values were imputed to zero. Statistical significance was calculated based on unpaired *t* test and graphed in PRISM (version 10.1.0).

### Preparation of Mitochondrial-Enriched Fractions from BV-2 Microglia Cells

Method for mitochondrial enrichment was adapted from Wieckowski *et al*. (35). The BV-2 cell lines Described Previously (BV-2, TurboID, N-terminal fusion, C-terminal fusion, and the C-terminal fusion ΔPDZ) were cultured at 5% CO_2_ in DMEM-F12 (10% FBS, 1% P/S). Untransduced cells were removed by adding 2 μg/ml puromycin to the complete medium. After 5 to 7 days of selection, all cell lines were seeded in 150 mm Petri dishes and allowed to grow until 90% of confluency in complete medium. Cells were exposed to 200 μM biotin for 24 h before harvesting. Approximately 40 million cells were used to generate mitochondrial-enriched fractions by differential centrifugation as described previously (35). Experiments were performed in triplicates at the same time. Briefly, cells were detached by trypsinization, then washed with cold 1× PBS, and pelleted at 800*g* for 10 min at 4 °C. After aspirating PBS, pellets were resuspended in 1.6 ml IB-1 buffer (225 mM mannitol, 75 mM sucrose, 0.1 EGTA, and 30 mM Tris–HCl [pH 7.4]) with HALT protease and phosphatase inhibitor cocktail and homogenized using a Teflon potter homogenizer with 25 strokes on ice. Total homogenates (fraction A0) were transferred to a clean tube and then centrifuged twice at 600*g* for 5 min at 4 °C, pellets containing unbroken cells, nuclei, and heavy membranes were fraction A1. Supernatants were pelleted at 7000*g* for 10 min at 4 °C, and the resulting pellets (fraction A2) correspond to heavy membrane organelles, likely containing the largest presence of Kv1.3. Pellets were washed in 200 μl 1B-2 buffer (225 mM mannitol, 75 mM sucrose, 30 mM Tris–HCl [pH 7.4]) and centrifuged at 7000*g* for 10 min at 4 °C, resulting pellets are crude mitochondria (fraction A3). For each fraction, pellets and homogenates were diluted in 200 μl of 8 M urea buffer and sonicated (5 s on–off pulses, amplitude 30%) for 45 s and then centrifuged at 21,130*g* for 10 min at 4 °C. Supernatants were collected, and protein concentration was determined by BCA assay. About 10 μg of protein were used to verify mitochondrial enrichment (HSP60, 1:1000 dilution), TurboID fusion (anti-V5 tag, 1:250 dilution, IRDye800, 1:10,000 dilution) by Western blot.

### Evaluating Changes in STAT1 Phosphorylation with Kv1.3 Blockade

N-terminal KV1.3-TurboID BV-2 cells were seeded in a 6-well plate at a density of 1 million cells in DMEM-F12 (10% FBS and 1% P/S) for 24 h. Media were changed, and PAP-1 (1 μM) was added for 30 min followed by induction with IFN-γ (10 ng/ml) in the same media for 30 min. Cells were washed in chilled PBS and harvested in urea lysis buffer with protease and phosphatase inhibitor cocktail. After cell lysis, protein quantification was performed using BCA colorimetric assay. Samples were then subjected to Western blot with 30 μg loading per well as described earlier. Blots were probed for pSTAT1, Tyr701 (1:1000 dilution), total STAT1 (1:1000 dilution), and β-actin (1:10,000 dilution) as loading control. Band density was analyzed using ImageJ software.

## Results

### Validation of N and C Terminus TurboID Fusion Constructs for Mapping the Kv1.3 Interactome in HEK-293 Cells

**Fig. 1:**
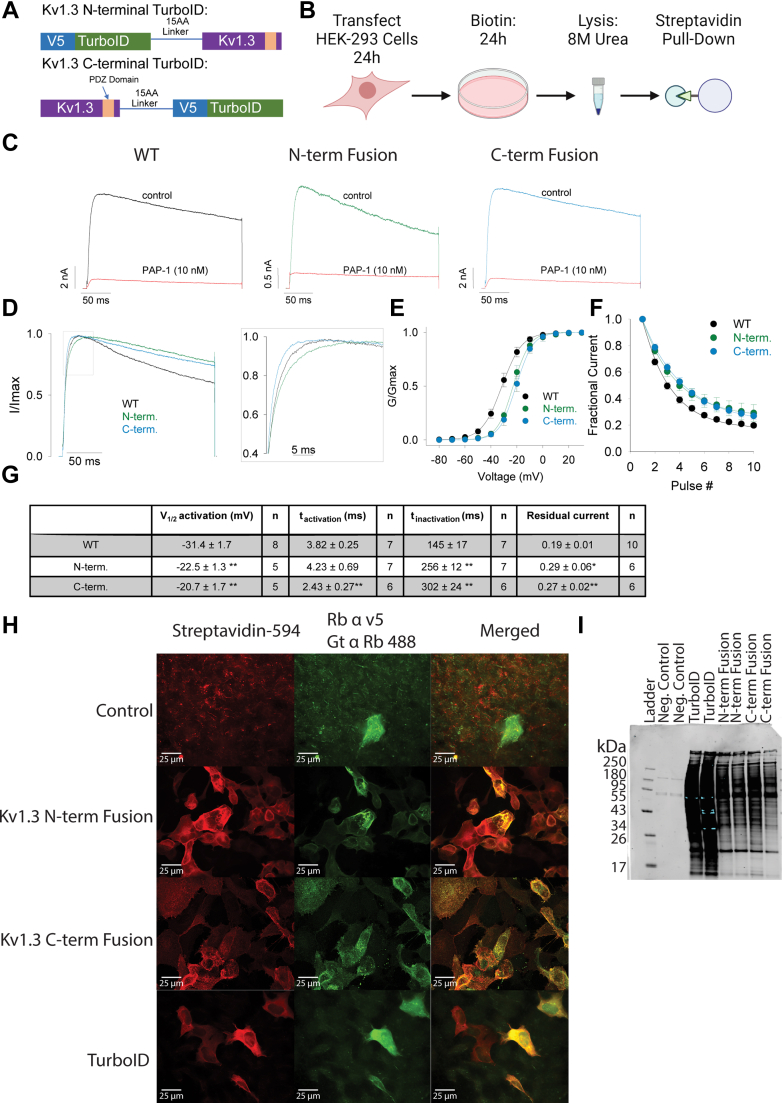
**Transfected cells show Kv1.3 channel activity and biotinylation of proximal proteins.***A*, schematic of experimental design. HEK-293 cells were transfected with Kv1.3-turboID fusion constructs for 24 h and then exposed to biotin for 24 h. Cells were then lysed in 8 M urea and pulled-down using magnetic beads fused to streptavidin prior to mass spectrometry. *B*, constructs of Kv1.3 fusion with TurboID. Each fusion construct contains TurboID, a 15-amino acid linker, a V5 tag, and Kv1.3. The N-terminal fusion has TurboID located on the N terminus of Kv1.3, and the C-terminal fusion has TurboID located on the C-term of Kv1.3. *C*, electrophysiology of HEK-293 cells transfected with Kv1.3-TurboID constructs shows similar biophysical properties and pharmacological responses to the Kv1.3 inhibitor PAP-1. *D*, averaged current traces showing N-terminal and C-terminal fusions induce a slight slowing of current inactivation, whereas the C-terminal fusion enhances the activation compared with WT control. *E*, the voltage dependence of activation of Kv1.3 channels fused to TurboID shows a small shift in the depolarized direction compared with the WT control. *F*, TurboID fusing reduces use-dependent inactivation. *G*, table highlighting changes in biophysical properties with TurboID fusion to Kv1.3. Statistical significance denotes *p* < 0.05 (∗) and *p* < 0.01 (∗∗). *H*, immunofluorescence (IF) of HEK-293 cells transfected with Kv1.3-TurboID constructs. IF highlights colocalization of biotinylated proteins (tagged with streptavidin) with V5-tagged TurboID. *I*, streptavidin (680) Western blot shows high biotin labeling with the presence of TurboID (n = 3). HEK-293, human embryonic kidney 293 cell line.

To identify Kv1.3 interacting proteins, we employed proximity labeling *via* TurboID, wherein TurboID was fused to the N terminus or C terminus of Kv1.3 and then expressed in mammalian cells *in vitro* for proteomic labeling and MS-based quantification of biotinylated proteins. TurboID is a biotin ligase that rapidly and promiscuously biotinylates proteins within a 10 to 30 nm radius (24). TurboID constructs were generated such that TurboID was fused to the N terminus or C terminus of Kv1.3 with an intervening 15-amino acid linker and a V5 tag (Fig. 1, *A* and *B*). A construct that globally expresses TurboID in the cytoplasm was used as a positive control. Transfection with plasmid containing an unrelated V5-tagged protein (MetRS) with an otherwise identical plasmid was a transfection control. These constructs were inserted into plasmids and transfected into HEK-293 cells. After 24 h of transfection, cells were then exposed to biotin for 24 h (Fig. 1*B*). We specifically chose HEK-293 cells based on their negligible basal levels of Kv1.3 channel expression, providing an optimal system for initial validation of the TurboID-Kv1.3 fusion constructs (36).

We conducted electrophysiological studies on transfected HEK-293 cells using the whole-cell patch-clamp technique to confirm the functional expression of recombinant Kv1.3 channels at the cell surface. The current amplitude produced by the transiently transfected fusion constructs was similar to that of the transfected WT Kv1.3, indicating that the fusion with TurboID on either the N terminus or C terminus had no effect on trafficking and insertion of the channel into the plasma membrane. (Fig. 1*C* and Supplemental Fig. S1*A*). At 10 nM, PAP-1, a selective Kv1.3 blocker, potently inhibited more than 90% of Kv1.3 currents in HEK-293 cells overexpressing WT Kv1.3 or Kv1.3-TurboID fusions, confirming that all currents detected were indeed associated with transfected Kv1.3 (Fig. 1*C* and Supplemental Fig. S1*A*). Interestingly, both constructs fused with TurboID exhibited a small shift in the voltage dependence of activation toward more positive potentials (Fig. 1, *D* and *G*), a delayed decay of use-dependent current upon repeated stimulation (Fig. 1, *E* and *G*), and slower inactivation decay (Fig. 1, *F* and *G*). The C-terminal fused Kv1.3 showed delayed activation kinetics compared with positive controls of HEK-293 cells transfected with unmodified Kv1.3 (Fig. 1, *D* and *G*). Overall, these studies highlighted that our Kv1.3-TurboID fusion constructs are properly inserted and retained functional and pharmacological properties at the cell surface but exhibit minor changes in activation/inactivation kinetics (35).

Immunofluorescence microscopy was performed to confirm Kv1.3 channel localization to the cell surface and inside the cell, along with colocalization with biotinylated proteins (Fig. 1*H*). Transfection control, with a non-TurboID plasmid, showed a presence of the V5-tagged methionine-tRNA synthetase and no biotinylation (Fig. 1*H*, control row). The N-terminal construct and the C-terminal construct showed similar localization of V5 and biotinylation to the cell surface, which confirmed localization of Kv1.3 and biotinylation of membrane-associated proteins that was comparable across Kv1.3 TurboID-fusion constructs. In contrast with Kv1.3-TurboID fusion constructs, we also overexpressed cytosolic TurboID (TurboID-NES not fused to any protein) (24) in HEK-293 cells, which showed expected V5 localization and biotinylation to the cytosol. These indicated that the expression of the Kv1.3 channel is likely on the plasma membrane and that the biotinylation of proteins corresponded with membrane localization of Kv1.3 channels.

Cells were then lysed in 8 M urea lysis buffer, and biotinylated proteins were affinity purified using streptavidin magnetic beads. Western blot of inputs (before streptavidin AP) and after AP showed a higher level of biotinylated proteins in N-terminal and C-terminal fusions compared with negative control (Fig. 1*I* and Supplemental Fig. S1, *B* and *C*). In contrast, the biotinylation pattern of cytosolic TurboID-transfected cells was distinct from that of the Kv1.3-TurboID fusion–transfected cells (Fig. 1*I* and Supplemental Fig. S1, *B* and *C*). These experiments established that Kv1.3 is present and functional on the surface, and that TurboID is able to biotinylate proteins in HEK-293 cells in a pattern distinct from global cytosolic TurboID expression. We proceeded with MS of biotinylated proteins to identify Kv1.3 N-terminal and C-terminal interacting proteins in HEK-293 cells.

### Kv1.3 Amino and Carboxyl Terminal Fusions with TurboID Identifies Distinct Domain-Interacting Proteins in HEK-293 Cells

**Fig. 2:**
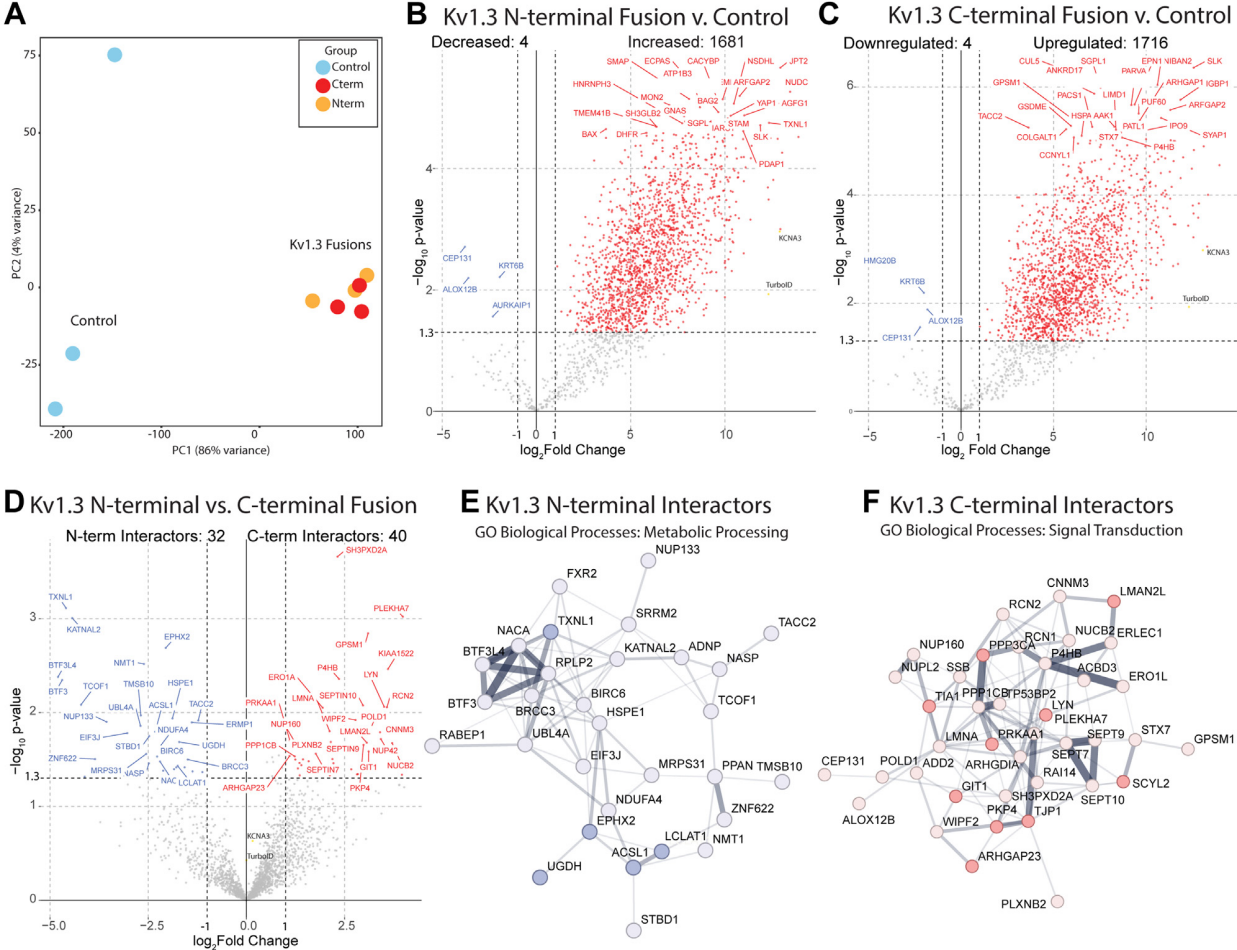
**Kv1.3 interactors in HEK-293 cells show that the N terminus is associated with protein processing and the C terminus is involved in signaling.***A*, principal component analysis (PCA) of mass spectrometry of biotinylated proteins shows distinct separation between control and Kv1.3-TurboID transfected cells. *B*, differential abundance analysis of N-terminal interactors shows about 1600 protein interacting with Kv1.3. *C*, differential abundance analysis of C-terminal interactors shows about 1700 proteins interacting with Kv1.3. *D*, differential abundance comparison between N- and C-terminal interactors shows 32 proteins interacting more with the N terminus of Kv1.3 and 40 proteins interacting with the C terminus of Kv1.3. *E*, STRING analysis shows close associations with N-terminal interactors. Gene Ontology highlights that many of these proteins are associated with metabolic processing. *F*, STRING analysis and GSEA analysis show that many of the C-terminal interactors are associated with signal transduction. Darker colors represent statistical significance in GSEA results, lighter color indicates an interactor that is not present in GSEA lists but interacting with Kv1.3, and edge thickness represents confidence of interactions based on the literature. Differential abundant proteins were calculated using paired *t* test, where log *p* value >1.3 and Log_2_ fold change (FC) of ±1 were considered significant. n = 3. ∗∗*p* < 0.01, ∗∗∗ *p*< 0.001. GSEA, gene set enrichment analysis; HEK-293, human embryonic kidney 293 cell line.

After confirmation of Kv1.3 channel activity and TurboID biotinylation of proteins, we wanted to evaluate which proteins were interacting with Kv1.3. AP proteins were assessed by silver stain and Western blot to check for quality of proteins available and efficiency of AP, a higher efficiency meaning less overall protein in controls in the silver stain (Supplemental Fig. S1*C*). To evaluate the Kv1.3 interactome, AP proteomes from all Kv1.3-TurboID fusion transfections were first normalized to TurboID protein abundance to account for any uneven transfection or efficiency of SA enrichment. These normalized data were visualized using PCA. Principal component 1 (PC1) accounted for 79% variance and clearly separated samples containing biotinylated proteins *via* TurboID, from non-TurboID controls (Fig. 2*A*). PC1 therefore represents proteins that were labeled by TurboID across all experimental conditions.

We then performed DEA to identify proteins that interact with Kv1.3 that appear both in the N- and C-terminal proteomes (*i.e.*, proteins within labeling radius of TurboID) as well as proteins that show selective interactions with the N or C terminus of Kv1.3. Differential enrichment showed 1681 N-terminal interacting proteins (Fig. 2*B*) and 1716 C-terminal interacting proteins (Fig. 2*C*). A large proportion of these N-terminal and C-terminal interactomes overlapped (Supplemental Fig. S1), probably indicative of general Kv1.3 channel protein interactors that were within the 10-20 nm labeling radius of TurboID, regardless of N terminus or C terminus. While the N and C terminus of one Kv1.3 monomer may be separated in space, N and C termini of the adjacent Kv1.3 monomers in the tetrameric complex are likely closer to each other potentially explaining this overlap. The surprisingly large numbers of Kv1.3 interactors is also likely because of TurboID biotinylating proteins at all steps of Kv1.3 being processed, through the endoplasmic reticulum (ER) before being localized to the cell membrane. Accordingly, many of the interactors listed were associated with ER processing of proteins (*e.g.*, SEC24A, SEC16A, HSPA5).

Although the majority of the N-terminal and C-terminal interactomes of Kv1.3 overlapped, some proteins were uniquely enriched in a domain-specific manner. DEA comparing N-terminal and C-terminal interactors directly, showed 32 proteins associated with the N terminus of Kv1.3 and 40 proteins associated with the C terminus of Kv1.3 (Fig. 2*D*). GSEA highlighted biological processes associated with the N terminus, involved in metabolic processing (*e.g.*, TNXL1, ACSL1, and EPHX2) (Fig. 2*D*). The proteins that interacted with the N terminus were likely associated with processing proteins to the ER such as some of the key metabolic interactors. In contrast, the C-terminal–specific interactome of Kv1.3 was enriched in proteins associated with signal transduction (*e.g.*, SMAD3, RAC1, and CNOT1) (Fig. 2*D*). We also used Search Tool for the Retrieval of Interacting Genes/Proteins (STRING) to visualize known PPIs within these domain-specific Kv1.3 interactors (Fig. 2, *E* and *F*). STRING analysis showed that Kv1.3 N-terminal interactors were involved in processing of proteins from the ER to the cell membrane, for example, SRRM2, NACA, and TXNL1 (Fig. 2*E*). Kv1.3 C-terminal interactors were largely associated with cell signaling (Fig. 2*F*). These results in HEK-293 cells indicate that distinct protein interactors of Kv1.3 channels can be defined using N- and C-terminal TurboID fusion, without significant disruption of channel physiology. Our analyses of both the protein lists and GSEA suggest that the N terminus of Kv1.3 is primarily associated with protein processing, whereas the C terminus is associated with signaling proteins. Based on these studies in HEK-293 cells, we next applied TurboID-based proximity labeling to examine Kv1.3 channel interactomes in microglia.

### Generation and Validation of Stably Transduced BV-2 Microglial Lines Expressing N and C Term Kv1.3-TurboID Fusions

**Fig. 3:**
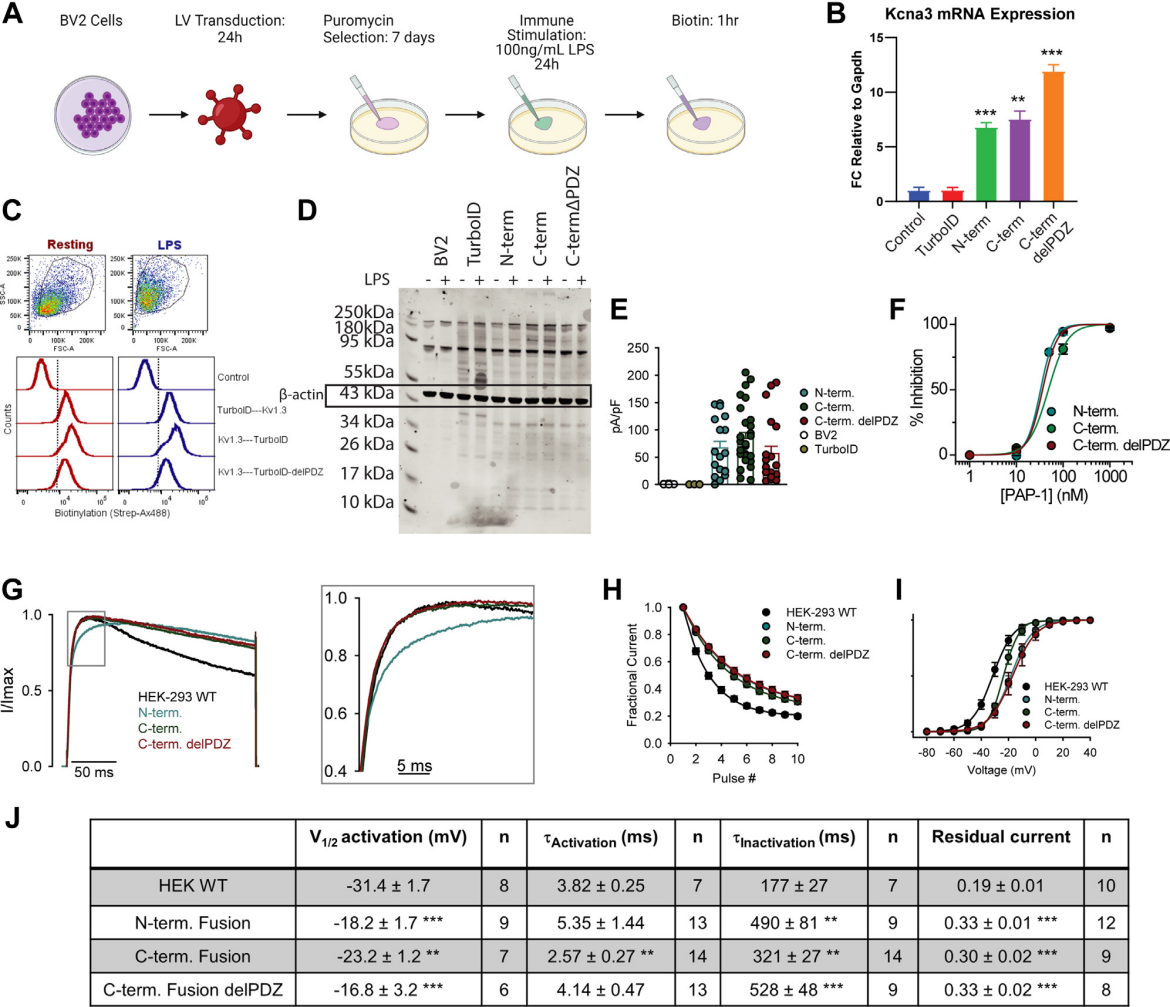
**BV-2 cells transduced with Kv1.3-TurboID constructs show the presence of biotinylation and channel activity.***A*, schematic of experimental design. BV-2 cells were transduced with Kv1-3 constructs described in Fig 1B and selected for plasmid uptake using puromycin. Cells were then exposed to LPS to induce an inflammatory response and biotin to allow for biotinylation of proximal proteins. *B*, quantitative RT–PCR of *Kcna3* transcript shows increased *Kcna3* mRNA expression in Kv1.3-TurboID transduced cell lines compared with control and *Gapdh*. *C*, flow cytometry of biotinylated proteins using Streptavidin-488 shows high biotinylation in Kv1.3-TurboID transduced cell lines independent of LPS exposure. *D*, Western blot depicting Streptavidin-680 labeling, which highlights biotinylation increase in the presence of TurboID. Ponceau staining shows no change in protein concentration across samples. *E*, scattered plot shows increased channel density in BV-2 cells transduced with Kv1.3-TurboID constructs. *F*, inhibition of Kv1.3 by PAP-1, a Kv1.3-selective small molecule inhibitor, following transduction o f Kv1.3-TurboID constructs. *G*, exemplifying current traces showin g changes in inactivation and activation kinetics in transduced Kv1.3-TurboID cells. *H*, fractional currents show reduced use-dependent current reduction of Kv1.3 currents in Kv1.3-TurboID transduced cells. *I*, voltage-dependent activation of Kv1.3 is shown to be shifted in the depolarized direction in cells transduced by Kv1.3-TurboID constructs. *J*, table summarizing electrophysiology results. Statistical significance denotes *p* < 0.05 (∗), *p* < 0.01 (∗∗), and *p* < 0.001 (∗∗∗). LPS, lipolysaccharide.

We chose the mouse-derived immortalized BV-2 microglial cell line as a model system to first identify proteins that interact with Kv1.3 channels in a N and C term-specific manner and then test whether these domain-specific interactions were altered when microglia adopt proinflammatory phenotypes. BV-2 cells were transduced for 24 h with LVs or no LV for control, encoding one of three constructs confirmed in HEK-293 cells: a Kv1.3 Fusion protein with TurboID on the N terminus, Kv1.3 fusion with TurboID on the C terminus, and an Kv1.3-TurboID C-terminal fusion with the PDZ-binding domain removed (Fig. 3*A*). TurboID globally expressed in the cytoplasm was used as a positive control, and untransduced BV-2 cells were utilized as a negative control. Following puromycin selection for 7 days, transduced cell lines were either exposed to LPS (100 ng/ml) or PBS for 24 h and then exposed to biotin for 1 h to increase the stringency of the interactomes of Kv1.3 (Fig. 3*A*). LPS induction was used to induce a proinflammatory-like state in BV-2 microglia. We first performed qRT–PCR and confirmed that *Kcna3* mRNA was highly and comparably transcribed across all three lines as compared with sham-transduced/control BV-2 cells (Fig. 3*B*). Western blot and flow cytometry showed biotinylation in Kv1.3-TurboID-transduced microglia, which was absent in control BV-2 cells (Fig. 3, *C* and *D*).

To determine whether stably transduced BV-2 lines exhibited functional Kv1.3 channel currents on the cell surface, we performed electrophysiological studies confirming that comparable and high Kv1.3 current densities were present across all cell lines (Fig. 3*E*). Similar to results observed in HEK-293 cells, BV-2 cells transduced with either of the three constructs fused with TurboID exhibited a positive shift in the voltage dependence of activation, less use-dependent current decay, and delayed inactivation kinetics (Fig. 3, *G*–*J*). The C-terminal fused Kv1.3 also showed enhanced activation kinetics compared with positive controls of HEK-293 cells transfected with unmodified Kv1.3 (Fig. 3, *G* and *J*). PAP-1 also blocked Kv1.3 currents (37) (Fig. 3*I* and Supplemental Fig. S2*A*) confirming the identity of the overexpressed Kv1.3 channels in BV-2 cells. These experiments showed that Kv1.3 is functionally active at comparable levels across all BV-2 Kv1.3-TurboID cell lines, with small, yet significant, increase in current with BV-2 cells transduced with Kv1.3 compared to a HEK-293 cell. These studies therefore lay the foundation for proteomic assessments of Kv1.3 channel interactomes in microglia using the proximity-labeling approach.

### Identification of Kv1.3 Channel Domain-Specific Protein Interactors and Molecular Pathways in BV-2 Microglia

**Fig. 4:**
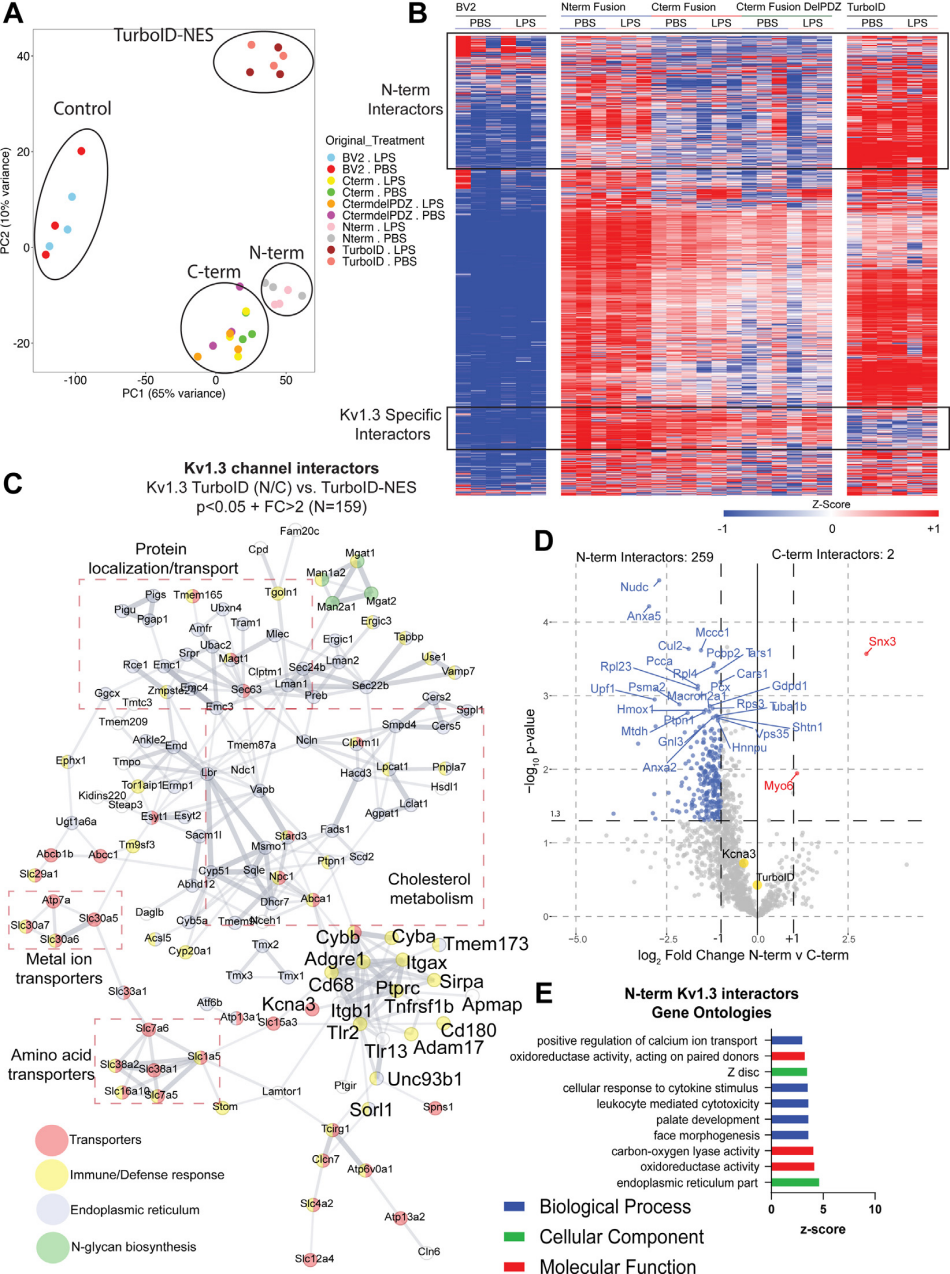
**Microglial Kv1.3 have distinct N-terminal and C-terminal interactors.***A*, principal component analysis (PCA) of mass spectrometry of biotinylated proteins shows distinct clustering of controls, TurboID, Kv1.3 N-term fusion, and Kv1.3 C-term fusion. *B*, heat map of biotinylated proteins, designed by Morpheus, shows distinct clusters of Kv1.3-specific interactors, Kv1.3 N-terminal specific interactors, and Kv1.3 C-terminal interactors. Individual proteins were colored based on z-score, where the darker shades of red indicate +1 and the darker shades of blue indicates −1. Hierarchical clustering arranged proteins based on groups. *C*, STRING analysis highlights proteins differentially interacting with Kv1.3 over global TurboID expression. Color indicates protein type. Associated protein groups are *boxed*. *D*, differential enrichment analysis (DEA) of proteins enriched with Kv1.3 N-terminal fusion and Kv1.3 C-terminal fusion indicates about 250 N-terminal interactors and 2 C-terminal interacIs. *E*, gene set enrichment analysis (GSEA) analysis shows most of the interacting proteins with the N terminus of Kv1.3 appear to be a part of calcium transport and oxidoreductase activity. Differential abundant proteins were calculated using paired *t* test, where log *p* value >1.3 and Log_2_ fold change (FC) of ±1 were considered significant (n = 3).

In order to evaluate proteins that interact with Kv1.3 in microglia, biotinylated proteins were enriched from whole cell lysates of BV-2 lines by AP with streptavidin magnetic beads. Affinity-purified proteins were verified by Western blot and silver stain (Supplemental Fig. S2*B*). *Via* PCA of the cell lysates, we confirmed that LPS induction accounted for 23% of the variance of proteins in the total samples, whereas the addition of Kv1.3 did not alter variance within the samples (Supplemental Fig. S3*A*). This indicated that overexpression of Kv1.3 did not significantly alter proteins present in each sample. DEA of proteins present in the cell lysates highlighted that LPS induced an increase of proteins associated with a neuroinflammatory response, including TLR2, IL-1α, and STAT1 (Supplemental Fig. S3*B*). TurboID-normalized AP samples (n = 3) were clustered using PCA. PC1 accounted for 65% variance and separated the samples with TurboID present from the untransduced BV-2 controls as well as N-terminal fusions from C-terminal fusions (Fig. 4*A*). Separation of controls and all TurboID samples indicated that both biotinylation and enrichment was successful. PC2 accounted for 10% of the variance and separated the different cell types: overexpression of Kv1.3, untransduced control, and global TurboID, which acts as a positive control for biotinylation but does not have Kv1.3 attached (Fig. 4*A*). This finding highlighted that the proteins biotinylated in BV-2 cells transduced with Kv1.3 are unique compared with untransduced controls or the biotinylated proteome of BV-2 cells with global (cytosolic) TurboID localization.

We hypothesized that Kv1.3 would have distinct interactors between the N and C termini of the channel in microglia. There were many proteins present in the samples transduced with Kv1.3-TurboID that were absent in the untransduced controls (Fig. 4*B*). In total, 991 proteins interacted either with the N or C terminus of Kv1.3 and were distinctly present in Kv1.3-TurboID transduced (Fig. 4*B* and Supplemental Fig. S3, C–*E*). GSEA and STRING analyses of these Kv1.3-specific interactors identified protein localization/transport, metal ion transporters, cholesterol metabolism, amino acid transporters, *N*-glycan biosynthesis, and immune/defense proteins (Fig. 4*C*). The most represented pathway in the Kv1.3 interactome in KEGG 2021 was protein processing in ER (corrected p 4.84E-22). These include ER translocon and translocation machinery, such as SEC61A1, SEC61B, SEC61G, SEC63, and HSPA5. The largest interaction groups with Kv1.3 in general were associated with cholesterol metabolism (*e.g.*, NPC1 and ABCA1) and protein localization/transport (*e.g.*, Tmem165 and MAGT1) (Fig. 4*C*). We also identified an immune/defense response cluster of Kv1.3 channel interactors including integrins (ITGB1, ITGAX), TLRs (TLR2, TLR13), receptor tyrosine phosphatases (*e.g.*, PTPRC/CD45), lysosomal protein CD68, and AD-related proteins (*e.g.*, SORL1, ITGAX) (Fig. 4*C*). Next, we compared how Kv1.3 terminal interactors differ between the termini.

Comparing the Kv1.3 N terminus to C terminus in BV-2-transduced cells, identified 284 proteins that preferentially interacted with the Kv1.3 N terminus. These included proteins like TIMM50, a mitochondrial translocase protein, and NUDC and TXNL1, associated with protein processing (Fig. 4*D*). In comparison, far fewer exclusive C-terminal interactors were identified, including SNX3 and MYO6, both associated with intracellular processing (Fig. 4*D*). GSEA of the Kv1.3 N-terminal interactors show processes associated with calcium ion transport and oxidoreductase activity (Fig. 4*E*). In contrast to N- and C-terminal interactors in HEK-293 cells (albeit human), the N-terminal interactome of Kv1.3 in BV-2 cells was much larger, whereas the C-terminal interactome was smaller. Our analyses in BV-2 cells identified the distinct Kv1.3 N-terminal and C-terminal interactors, among which the C-terminal interactome was most impacted by LPS treatment of BV-2 cells, indicative of context-dependent changes of the Kv1.3 channel interactome.

### Proinflammatory Activation Preferentially Modifies the C-Terminal Interactome of Kv1.3 Channels in Microglia

**Fig. 5:**
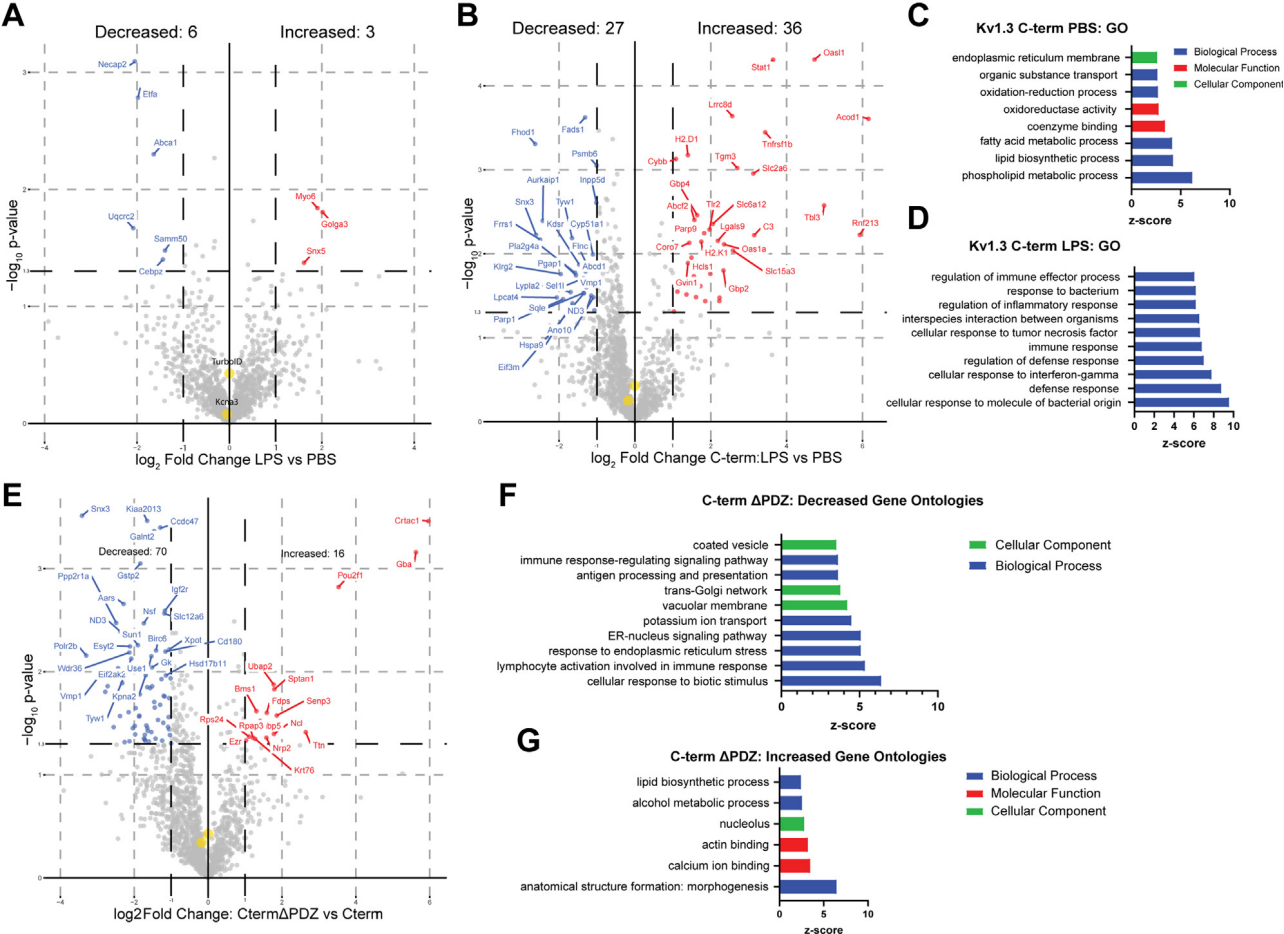
**Inflammatory exposure results in a PDZ-binding domain–dependent interactions with the C terminus of Kv1.3.***A*, differential enrichment analysis (DEA) between N-terminal interactors and N-terminal interactors with LPS exposure highlights minimal change in N-terminal interactors with LPS exposure. *B*, DEA between C-terminal interactors and C-terminal interactors shows 27 proteins interacting with the C-term of Kv1.3 during homeostasis and 36 proteins interacting with the C terminus during an LPS-induced inflammatory response. *C*, gene set enrichment analysis (GSEA) shows protein transport and processing terms downregulated during an LPS response. *D*, GSEA shows an upregulation of immune signaling terms associated with the C terminus of Kv1.3 during LPS immune stimulation. *E*, DEA comparison of Kv1.3 C-terminal interactors and C-terminal interactors with the PDZ-binding domain removed shows 70 proteins downregulated and 16 upregulated with deletion of the PDZ-binding domain. *F*, GSEA of proteins downregulated with the deletion of the PDZ-binding domain are mostly associated with immune-signaling response and protein packaging. *G*, GSEA of proteins upregulated with deletion of the Kv1.3 C-terminal PDZ-binding domain shows terms associated with lipid biosynthesis and alcohol metabolism. Differential abundant proteins were calculated using paired *t* test, where log *p* value >1.3 and Log_2_ fold change (FC) of ±1 were considered significant. LPS, lipopolysaccharide.

Microglia are well known to adopt distinct inflammatory profiles when exposed to immune stimuli, and their cellular functions are context dependent (38, 39). We therefore next assessed whether LPS proinflammatory activation impacted the Kv1.3 channel interactome in a domain-specific manner. We found that there were many LPS-induced C-terminal interactors that were also highly abundant in cells with an LPS response, although the N-terminal interactome was not impacted by LPS treatment (Fig. 4*B*). In the presence of LPS, we hypothesized the interactors of Kv1.3 would shift toward inflammatory signaling. DEA showed negligible effects of LPS on the Kv1.3 N-terminal interactome, even though LPS effects were noted on the whole cell proteome (Fig. 5*A*). DEA showed that in the presence of LPS there were 36 proteins increased and 27 proteins decreased in the C-terminal interactome (Fig. 5*B*). Proteins with increased interaction with the C terminus upon LPS activation included STAT1, C3, and TLR2, often associated with a proinflammatory response in microglia, whereas the decreased proteins include SNX3 and HSPA9. GSEA showed the C-terminal proteins that interacted with Kv1.3 in the absence of LPS, associated with the ER and transport (Fig. 5*C*). In the presence of LPS, Kv1.3 C-terminal interactors transitioned toward inflammatory proteins and immune effector processes (Fig. 5*D*). This increase in C-terminal interactors by LPS cannot be explained by increased total protein abundance itself, because this increase was largely absent in the N terminus as well as in the global TurboID conditions. In addition, what makes this interactome even more interesting is that there is a paucity of secreted proteins in the unstimulated Kv1.3 interactome, which suggested that these results may be selective. In fact, the overlap between the Kv1.3 interactome and the human secretome is just 63 proteins. Therefore, we conclude that the proinflammatory context of microglial activation specifically modifies the C-terminal interactome of Kv1.3 channels, potentially linking channel function with immune signaling machinery.

### Kv1.3 C-Terminal Inflammatory Interactors Dependent on the PDZ-Binding Domain

The Kv1.3 PDZ-binding domain has been shown to be essential to Kv1.3 function (23). Since the C terminus of Kv1.3 interacted with signaling proteins across two mammalian cell lines, we hypothesized that deletion of the PDZ-binding domain of the C terminus of Kv1.3 would result in a reduction of interacting proteins with the C terminus. DEA identified 70 Kv1.3 C-terminal interacting proteins with reduced interactions with the C terminus upon deletion of the PDZ-binding domain, including SNX3, ND3, and NSF. Conversely, we found 16 proteins with increased interactions with the C terminus when the PDZ-binding domain was removed, including GBA, NRP2, and CRTAC1 (Fig. 5*E*). GSEA of the proteins that interacted with the C terminus in a PDZ-dependent manner (proteins with reduced interactions with Kv1.3 upon PDZ-binding domain removal) showed an association with the cellular component of coated vesicles and the biological processes of antigen processing and presentation and immune response–regulating signaling pathway (Fig. 5*F*). Proteins that showed increased C-terminal interaction upon PDZ-binding domain removal were enriched in ontologies related to lipid biosynthesis and alcohol metabolic processing (Fig. 5*G*). These results highlighted several proteins and pathways related to the C-terminal Kv1.3 channel interactome that require the PDZ-binding domain to interact with Kv1.3.

### Kv1.3 Channel is Present in Mitochondria-Enriched Fractions in Microglia

**Fig. 6:**
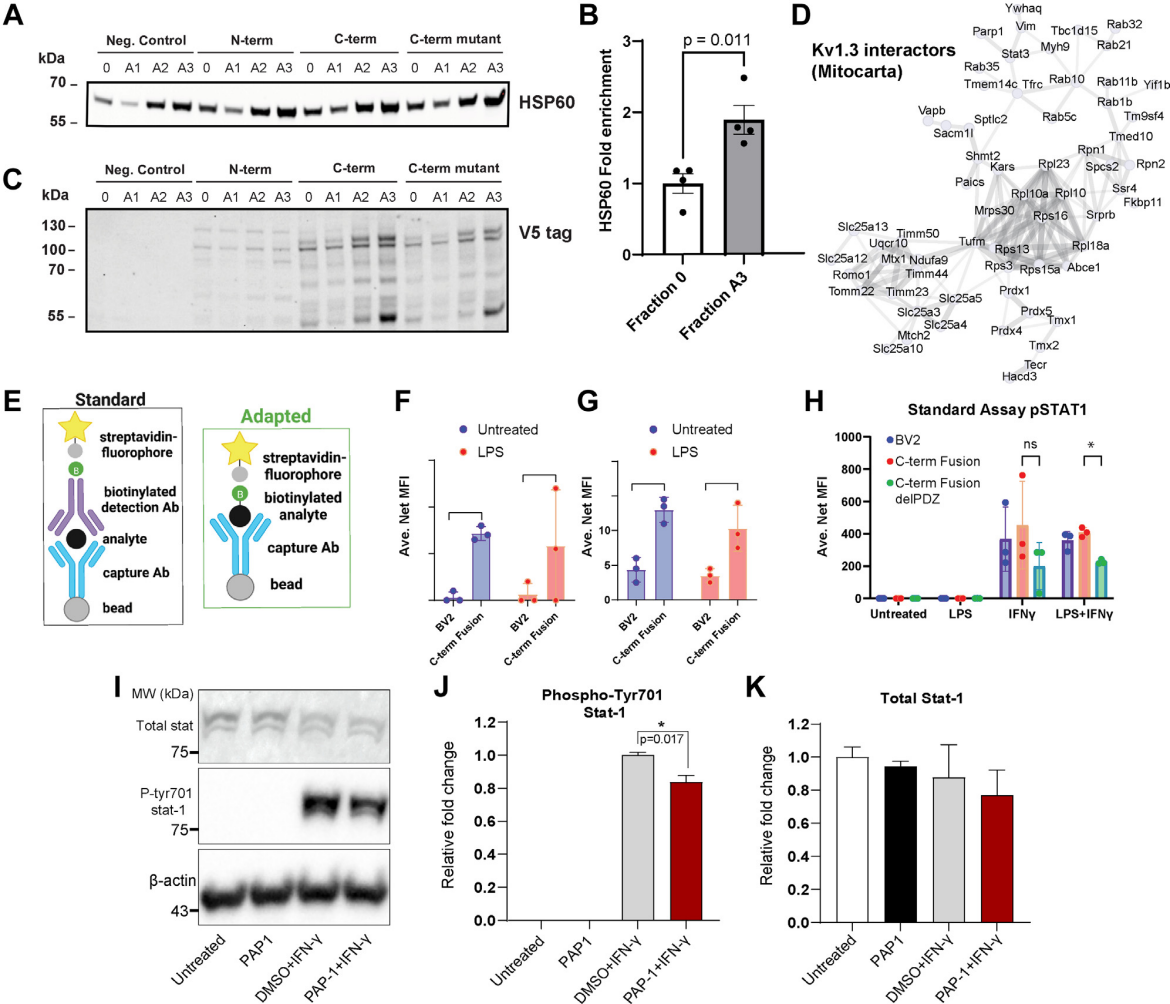
**Confirmation of Kv1.3 interactions with mitochondria and immune signaling.***A*, validation of mitochondria enrichment in the subcellular fractions obtained during the isolation process from untransduced BV-2 cells (negative control) and BV-2 cells transduced with Kv1.3-TurboID constructs (N-term, C-term, and C-termΔPDZ). Total homogenates (0) were fractionated to heavy membranes (A1), crude mitochondria (A2), and mitochondria-enriched fractions (A3) by sequential centrifugation and then analyzed by Western blot using HSP60 as a mitochondrial marker. *B*, quantification of HSP60 comparing total homogenates (0) to mitochondria-enriched fractions (A3). *p* Value for two-sided unpaired *t* test is indicated. *C*, representative blot of V5 tag (TurboID fusion) in fractions 0–A3 from BV-2 cells transduced with Kv1.3-TurboID constructs. *D*, STRING analysis of Kv1.3 interactors crossreferenced to MITOCARTA 3.0 database highlights many proteins interacting with Kv1.3 associated with the mitochondria and the functional and physical interactions. *E*, schematic of standard and adapted Luminex assay. Standard assay includes a bead attached to a capture antibody that binds to the protein of interest, then a biotinylated antibody binds to the protein of interest to form a sandwich. Streptavidin with a fluorophore binds to the biotin. This estimates how much of a protein is in a sample. The adapted assay includes a capture antibody with bead and then adds streptavidin with the fluorophore directly to the protein of interest. The adapted assay captures abundance of proteins interacting with Kv1.3 directly. *F*, adapted Luminex assay of intracellular C3 shows an increased interaction with Kv1.3 at a resting state. *G*, standard Luminex assay of proteins isolated from BV-2 cells transduced with Kv1.3-TurboID highlights an increase in intracellular C3 with the overexpression of Kv1.3, independent of LPS exposure. *H*, standard LuMINEX assay of pSTAT1 shows activation of pSTAT1 in the presence of IFN-γ, a known activator of the STAT1 signaling pathway. In the presence of LPS and IFN-γ, the removal of the PDZ-binding domain in Kv1.3 leads to a decrease in the presence of pSTAT1. *I*, Western blot analysis of whole cell lysate from BV-2 cells expressing N-terminal Kv1.3-TurboID shows reduced STAT1 phosphorylation upon induction with IFN-γ post Kv1.3 blockade with PAP-1. No changes were observed in the total STAT level. *J*, Quantification of pSTAT1 shows decrease of IFN-γ response with PAP-1 exposure using densitometry. *K*, densitometry analysis shows total STAT1 is unchanged by IFN-γ exposure or PAP-1 exposure. Significance was calculated utilizing unpaired *t* test. ∗ *p* < 0.05, ∗∗*p* < 0.01, ∗∗∗*p* < 0.001, ∗∗∗∗*p* < 0.0001. n = 3. IFN-γ, interferon gamma, LPS, lipopolysaccharide; pSTAT1, phosphorylated STAT1.

The Kv1.3 channel can be detected at the plasma as well as the inner mitochondrial membranes in lymphocytes and cancer cells (15, 40, 41). To assess the presence of Kv1.3 in mitochondria from BV-2 cells transduced with Kv1.3-TurboID constructs, we prepared subcellular fractions from total cell homogenates (fraction 0) by sequential centrifugation. Fraction A1 contained light membranes, fraction A2 contained crude mitochondria, and fraction A3 was enriched in mitochondria (Fig. 6*A*). Western blot analysis of the mitochondrial marker HSP60 showed that HSP60 levels were increased in fraction A3 compared with fraction 0, confirming that mitochondrial enrichment of fraction A3 was successful (Fig. 6*B*). Next, Western blot analysis of these fractions, probed for V5 (fused to Kv1.3-TurboID or to TurboID), detected V5 bands that corresponded to the predicted molecular weights of fusion proteins in all subcellular fractions from BV-2 cells transduced with Kv1.3-TurboID constructs (N-term, C-term, and C-term ΔPDZ), including the mitochondria-enriched fractions A3 (Fig. 6*C*). Small variabilities between V5-tagged N terminus and C terminus intensities may be due to small variability in TurboID expression. No bands were detected in untransduced BV-2 cells (negative control) (Fig. 6*C*). STRING analysis of Kv1.3 interactors that are present in the MITOCARTA 3.0 database shows 73 proteins associated with the mitochondria (Fig. 6*D*). These include proteins associated with transport of proteins to the mitochondria and mitochondrial ribosomal machinery (*e.g.*, TIMM50, MRPS30, and TMX1). Our results provide evidence for mitochondrial Kv1.3 protein presence in mitochondrial fractions from BV-2 cells transduced with Kv1.3-TurboID constructs.

### Verification of Interactions of Kv1.3 with C3 and pSTAT1 *via* the C-Terminal Domain

Among the immune-related protein interactors of Kv1.3 that were measured by our MS studies, we nominated C3 and STAT1 as proteins of interest to be validated by Luminex based on their known role in several neuroinflammatory and neurodegenerative diseases (42, 43, 44, 45, 46, 47). The standard Luminex approach immobilizes the protein of interest on to a bead *via* capture antibody and then uses a biotinylated detection antibody followed by streptavidin–fluorophore conjugate to quantify abundance of the target protein (31). We adapted this approach by omission of the detection antibody, so that all C3/pSTAT1 would be captured but only their biotinylated forms would be detected, which proved a direct quantification of the biotinylated forms of these proteins from a total cell lysate without enrichment (Fig. 6*E*).

The adapted assay was able to detect biotinylated C3 in cell lysates from the C-term TurboID Kv1.3 BV-2 cells, regardless of LPS stimulation, confirming the MS result of an interaction between the C terminus of Kv1.3 and C3 (Fig. 6*F*). In comparison to the adapted assay, which only detects biotinylated C3 in cell lysates, the standard assay for C3 also showed an overall increased level of C3 in C terminus Kv1.3 independent of global expression of TurboID (Fig. 6*G*). This finding can be explained by either increased C3 protein abundance in microglia when Kv1.3 is overexpressed, or, an increased C3 signal because of detection of the protein *via* the capture antibody as well as detection of the biotinylated C3 *via* streptavidin–fluorophore. Importantly, the adapted C3 levels detected in cell lysates from Kv1.3-tranduced BV-2 cells were greater than 50% of the C3 signal from the standard assay. This suggested that majority of C3 in BV-2 TurboID cells is biotinylated by TurboID, consistent with C3 being identified as an important Kv1.3 interactor. Since C3 is not traditionally a membrane-attached protein, this result most likely represents an interaction between Kv1.3 and C3 in the processing stage of C3 before its secretion, potentially at the stage of processing in the ER translocon. This was consistent with several other ER translocon proteins identified as Kv1.3 interactors (*e.g.*, SEC61A1, SEC61B, SEC61G, SEC63, HSPA5), and enrichment of protein processing in the ER, as a major over-represented pathway in the Kv1.3 interactome.

We also measured levels of pSTAT1, Tyr701 to reflect activated Stat1 in BV-2 cells. Stat1 phosphorylation is triggered by type 1 and 2 interferons, leading to activation of interferon-related gene expression, which are important in antiviral immune responses, as well as in neurological diseases (48). Previously, a functional relationship between Kv1.3 channel activity and pSTAT1 phosphorylation was also suggested (9). Our MS studies suggested that the C terminus of Kv1.3 interacts with STAT1. We utilized the Luminex assay to detect pSTAT1 levels in cell lysates from BV-2 cells exposed to LPS and IFN-γ, a proinflammatory conditioning stimulus and an activator of type II interferon signaling, respectively. We also examined whether the PDZ-binding domain is required for this interaction. Standard Luminex measurements of pSTAT1 showed that IFN-γ treated BV-2 cells, regardless of preincubation with LPS, responded *via* increased pSTAT1 levels to IFN-γ, and this effect of IFN-γ was decreased by half when the PDZ-binding domain of the C terminus Kv1.3 was deleted (Fig. 6*H*).

Since our Luminex data indicated the role of Kv1.3 in the regulation of STAT1 phosphorylation, we also investigated the effect of inhibition of Kv1.3 channel on STAT1 phosphorylation levels postinterferon induction. PAP-1, a small molecule inhibitor of KV1.3, was used for channel blockade in N-terminal Kv1.3-TurboID BV-2 cells followed by IFN-γ induction. IFN-γ induction resulted in a significant increase in pSTAT1, whereas the total STAT1 was unchanged (Fig. 6, *I*–*K* and Supplemental Fig. S5). Blockade of Kv1.3 significantly reduced STAT1 Tyr701 phosphorylation (Fig. 6, *I* and *J*). However, the total STAT1 level did not show significant changes upon treatment (Fig. 6, *I* and *K*). This result supported an important role of the C-terminal domain, particularly the PDZ-binding domain, in the regulation of the interaction between Kv1.3 and STAT1 signaling proteins in microglia.

## Discussion

### Kv1.3 Interacts with Immune Signaling Proteins in Microglia, Exhibiting Domain-Specific as Well as Context-Dependent Interaction Patterns

Among several immune targets for neuroimmune modulation in neurological diseases, the Kv1.3 channel has emerged as a promising target that is highly expressed in proinflammatory subsets of DAM. To address gaps in our current understanding of how Kv1.3 channels regulate immune functions of microglia, we applied a proximity-labeling approach to label the protein–protein interactome of Kv1.3 channels in mammalian cells, including mouse microglia *in vitro*. The TurboID proximity-labeling technique has emerged as a tool to determine what proteins are interacting with in a close proximity to other proteins. By fusing the biotin ligase TurboID to the C or N terminus of Kv1.3 channels in microglia, we labeled the C- and N-terminal interactomes of Kv1.3 and used MS to quantify these biotinylated proteins after streptavidin-based enrichment. We identified over 900 proteins that interact with Kv1.3 channels in both HEK-293 cells and BV-2 microglia.

**Fig. 7:**
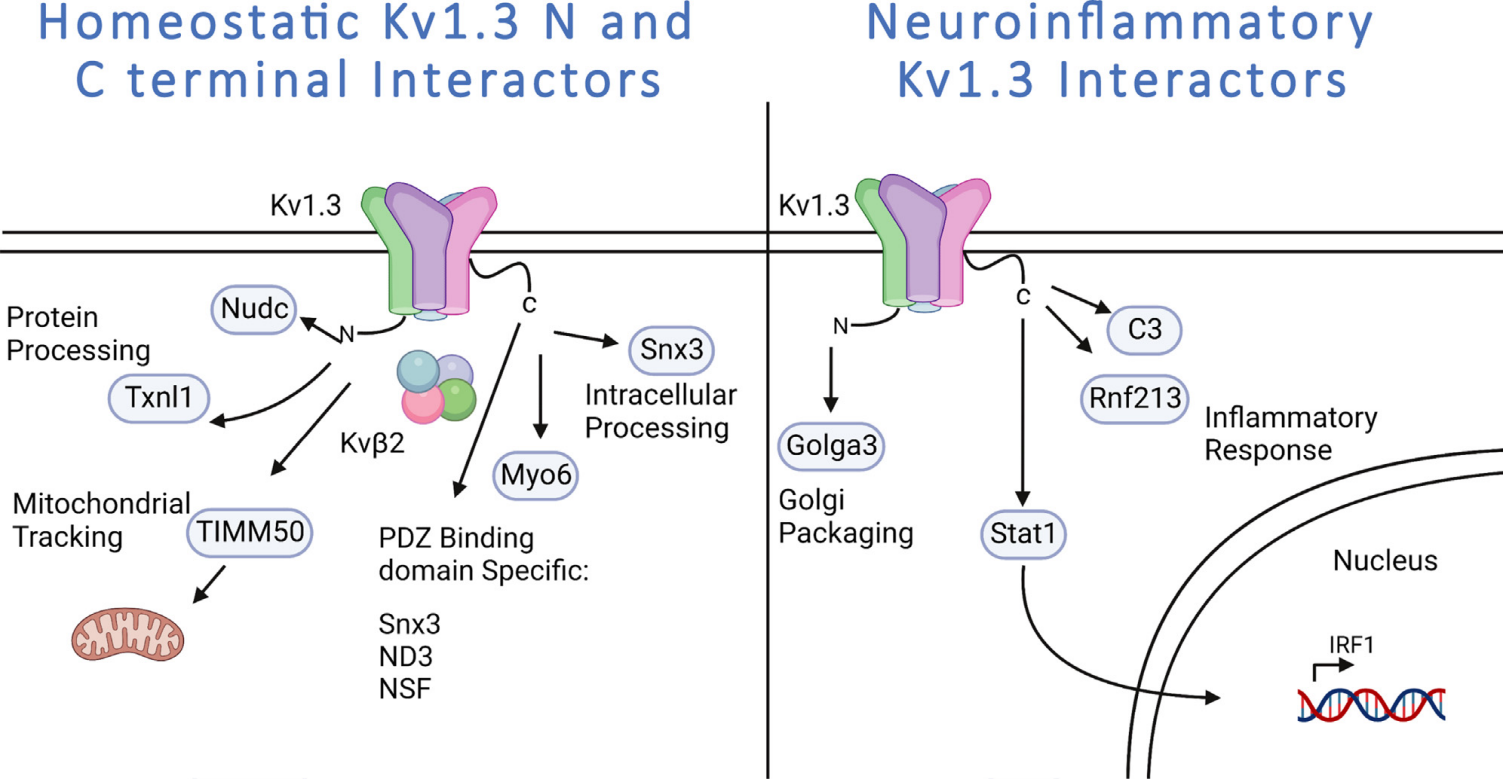
**Schematic of Kv1.3 interactors in microglia during homeostatic and neuroinflammatory states.** During homeostasis, many of the interactors of Kv1.3 are shared. The N terminus appears to be responsible for protein processing and tracking to the mitochondria, whereas the C terminus has some PDZ-binding domain–specific interactors that are largely associated with intracellular processing. During LPS stimulation of an immune response, the N terminus of Kv1.3 has very little change in the interacting partners, whereas the C terminus has PDZ-binding domain–dependent interactors associated with immune signaling. LPS, lipopolysaccharide.

Using a combination of electrophysiology, biochemical, and immunofluorescence microscopy approaches, we confirmed that fusion of TurboID to the N- or C-terminal domains of Kv1.3 *via* a flexible linker has minimal impact on channel localization and biophysical properties. During a homeostatic state in HEK-293 and BV-2 cells, it appears that the majority of interactors with Kv1.3 are indistinguishable between the N terminus and the C terminus but distinct from a global exposure to TurboID. LPS acts through TLRs, which are involved in pathogenesis of both AD and PD (26, 27). Using LPS allows a more precise pathway-specific Kv1.3 interactome analysis. The Kv1.3 interactors are strongly associated with many disease-associated signaling pathways, including proteins like TLR2 and ITGAX, many of which are associated with immune responses and the DAM phenotype of microglia neurodegeneration (26, 27, 49). Within MS-quantified Kv1.3 interacting proteins, we identified distinct groups of proteins that preferentially interact with N- and C-terminal domains of Kv1.3. It appears that the N terminus is responsible primarily for interacting with proteins associated with protein processing, whereas the C terminus interacts with signaling proteins (Fig. 7).

Given the context dependency of microglial states and functions in disease, we assessed whether the Kv1.3 channel interactome is altered when microglia adopt proinflammatory states, using LPS as a well-known proinflammatory stimulus (38, 39). We found that while the N-terminal interactome was not impacted by LPS, the C-terminal interactome underwent significant reorganization, such that immune and signaling proteins showed higher levels of interaction, indicative of a domain-specific and context-specific effect of LPS. The proteins with higher interaction with the C terminus during LPS activation included immune signaling proteins and immune proteins that are part of the TLR and MHC complex as well as STAT1 and C3 (Fig. 7). It is very likely that the presence and activation of the Kv1.3 increases pSTAT1 activity and aids in the induction of an inflammatory response in microglia. Similarly, in HEK-293 cells, the C terminus of Kv1.3 also interacts with key signaling proteins such as MEK, a kinase that activates ERK signaling. We also found that a large fraction of the C-terminal interactome is dependent on the PDZ-binding domain (50). Removal of the PDZ-binding domain resulted in a reduction of immune signaling protein interactions, suggesting that this binding domain is necessary for physical interactions between Kv1.3 and these proteins (Fig. 7). Proteins such as STAT1 and C3 are independent of the PDZ-binding domain, whereas others like CD190 and SUN1 are PDZ-binding domain dependent. While both status and the PDZ-binding domain interacting proteins are involved in signaling, the PDZ-binding regulation does not influence the interaction of proteins during an activate state. This means that other domains of Kv1.3 on the C terminus (or another intermediary interactor) may be determining how immune status impacts C-terminal interactions.

These traits of Kv1.3 provide insight into how Kv1.3 may regulate microglial immune functions, and how regulation of the Kv1.3 channel might be useful as a therapeutic target. Perhaps, the observed beneficial effects of blocking Kv1.3 channels in a plethora of neurological disease models may be explained in part by the interaction between Kv1.3 channels and immune signaling proteins that coassemble in activated microglia.

### Kv1.3 Presence in the Mitochondria in Microglia

Our MS-based studies also suggested an interaction between Kv1.3 and mitochondrial proteins. Specifically, we found that the N-terminal interactome of Kv1.3 includes proteins such as TIMM50, suggesting that some Kv1.3 channels in microglia are transported to the mitochondria, where they are likely present in the inner membrane (15, 51). To validate this biochemically, we performed mitochondrial fractionation from whole cell lysates and found that Kv1.3 protein (detected *via* the V5 tag fused to Kv1.3) was indeed enriched in the mitochondrial fractions along with a known canonical mitochondrial marker (HSP60). Our finding of Kv1.3 protein presence in mitochondria of microglia aligns with prior observations that Kv1.3 channels can localize to the mitochondria in T cells, where they may regulate apoptosis, mitochondrial potential, and proton flux. Future proteomics studies of the Kv1.3 channel interactome specifically in microglial mitochondria, and mechanistic studies investigating the functional implications of these interactors, are therefore warranted. These functional implications include how Kv1.3 in the mitochondria is changed during LPS exposure and how Kv1.3 could regulate mitochondrial function during inflammation.

### Kv1.3 Potassium Channels May Regulate Immune Function of Microglia *via* Protein–Protein Interactions

Microglia in neurological diseases have a higher expression of Kv1.3 (5, 7, 52). Kv1.3 potassium channel activity is increased when membrane potential is reduced by the influx of calcium into microglia during an inflammatory response (53). Activity of Kv1.3 clearly plays an important role in regulating immune function in microglia; however, it appears that Kv1.3 is also directly interacting with immune proteins independent of its channel activity. This is evident by the fact that Kv1.3 has multiple binding locations including the SH3-binding region located on the N terminus and the PDZ-binding domain located on the C terminus (30). Previous studies have shown some immune signaling proteins directly interacting with Kv1.3, including MEK, a necessary part of the ERK activation pathway (50). The ERK activation pathway is strongly linked to neurological diseases and is a necessary step in the activation of microglia.

Our findings strengthen previous connections between Kv1.3 and immune interactors specifically highlighting the potential for physical interactors of Kv1.3 with the TLR proteins in an immune stimulation complex. Prior work in mouse microglia cells, a murine cell line endogenously expressing Kv1.3, and primary microglia shows that blockade of Kv1.3 using Shk-223 results in p38 and MAPK regulation (9, 54). The current article describes that pSTAT1 is regulated by the C terminus, cooperating with our previous data showing that Stat1 phosphorylation is regulated by Kv1.3 function in BV-2, and Kv1.3 may interact with TLR (11). This work highlights that the C-terminal interactome has several immune proteins including TLR2, MHC proteins, and STAT1, further supporting previous predictions (9). These data and previous reports highlight that Kv1.3 channels may regulate or be functionally coupled with type 1/2 IFN signaling in microglia. IFN signaling is important in neuroinflammatory diseases like AD and aging, and in microglia, where IFN-based signatures have been identified in human brain (55, 56). The data presented in this article provides rational for further evaluation of Kv1.3 influence in immune signaling *in vivo* and *in vitro*. Based on interactions with anchoring proteins (*e.g.*, cortactin, integrins, etc.), Kv1.3 likely becomes a part of a much larger macromolecular complex of proteins that come together as an immune complex.

### Limitations of This Study

There are many desirable benefits to utilizing an *in vitro* system to gain insights into Kv1.3’s interactomes, including complete control over both the concentration and duration of stimulus in a highly pathway-specific manner; however, this model system does present some limitations. While BV-2 cells retain many properties of primary mouse microglia, they are not a physiological replicate of *in vivo* microglia. In general, BV-2 cells are known to have adopted a more reactive phenotype compared with primary microglia but do have similar inflammatory responses (46). Endogenously, BV-2 cells and HEK-293 cells express minimal Kv1.3; however, we utilized transduced or transiently transfected overexpression models of Kv1.3. There is an inherent risk that the overexpression model itself impacts levels of proteins that could be interacting with Kv1.3 channels, compared with cells that inherently express Kv1.3 channels.

TurboID is a proximity-labeling technique, meaning that biotinylation of proteins can occur as long as it is within the 10 to 30 nm radius of labeling (24). As a result, TurboID can biotinylate proteins that are just within proximity and not physically interacting with Kv1.3. Fortunately, the radius of labeling for TurboID fused with Kv1.3 should be within the range of direct and immediate interactors, including those are transient or fixed interactors. In addition, TurboID can biotinylate proteins that are near Kv1.3 as it is being packaged to the cell surface, such as labeling while Kv1.3 is present in the ER, which could explain the interactions observed with secreted proteins such as C3 (24). In support of this, we found ER translocon/chaperone proteins such as HSP90 and HSPA5 as interactors of Kv1.3. It will be important to determine whether these interactions are dependent or independent of K^+^ conductance *via* the pore of Kv1.3 channels. It is necessary to ascertain whether the Kv1.3 initiates the formation of the immune signaling complex or activates in responses to the formation of the immune signaling complex. With the establishment of the potential interactors of Kv1.3 in microglia, future studies are needed to determine how regulation of Kv1.3 alters in immune and disease response of microglia *in vivo* and how the directionality of immune interactions can be determined.

## Conclusions

We used proximity-based proteomics to identify the interactome of Kv1.3 channels, which is a critical regulator of microglial function. Our analysis revealed several novel protein interactors of Kv1.3 channels in BV-2 microglia, including domain-specific interactors of the N terminus (*e.g*., TIMM50) and C terminus (*e.g.*, STAT1 and C3) of the channel. While the N-terminal interactome is larger and includes anchoring, localization, and metabolic processing–related proteins, the C-terminal interactome is enriched in immune signaling proteins, and many of these are directly governed by the immune status of microglia, and some are dependent on the PDZ-binding domain. Collectively, our data emphasize the pleiotropic nature of Kv1.3 and highlight its relevance as a therapeutic target for modulating microglial function.

## Data Availability

The MS proteomics data have been deposited to the ProteomeXchange Consortium *via* the PRIDE partner repository with the dataset identifier PXD049433.

## Supplemental data

This article contains supplemental data.

## Conflict of interest

H. W. is an inventor on a University of California patent claiming PAP-1 for immunosuppression. This patent has been abandoned because of its short remaining patent life. The authors declare no competing interests.

## Supplemental Data

Supplemental Figure Legends

Hek_AP

Hek_cell Lysate

BV2_AP

BV2_cell lysates

OHW_BV2_AP

OHW_HEK_Lysate

OHW_BV2_lysate

OHW_HEK_AP
