# Supplementary material for: Proximity Labeling Proteomics Reveals Kv1.3 Potassium Channel Immune Interactors in Microglia
Source: Mol Cell Proteomics. 2024 Jun 25;23(8):100809. doi: 10.1016/j.mcpro.2024.100809 (PMC11780389; doi:10.1016/j.mcpro.2024.100809)
Supplement: Supplemental Figure Legends [file mmc2.docx]

**Proximity labeling proteomics reveals Kv1.3 potassium channel immune interactors in microglia.**

Christine A Bowen^1,2^, Hai M Nguyen^3^, Young Lin^1,2^, Pritha Bagchi^2,4^, Aditya Natu^5^, Claudia Espinosa-Garcia^6 *^, Erica Werner^7^, Rashmi Kumari^6^, Amanda Dabdab Brandelli^6^, Prateek Kumar^6 *^, Brendan R Tobin^8^, Levi Wood^9^, Victor Faundez^7^, Heike Wulff^3^, Nicholas T Seyfried^1,2^, Srikant Rangaraju^1,6 *^

*1. Center for Neurodegenerative Diseases, Emory University, Atlanta, GA 30322, USA*

*2. Department of Biochemistry, Emory University, Atlanta, GA 30322, USA*

*3. Department of Pharmacology, University of California – Davis, Davis, CA, 95616, USA*

*4. Emory Integrated Proteomics Core, Emory University, Atlanta, GA 30322, USA*

*5. Department of Human Genetics, Emory University, Atlanta, GA, 30322, USA*

*6. School of Medicine, Yale University, New Haven, CT, 62481, USA*

*7. Department of Cell Biology, Emory University, Atlanta, GA 30322, USA*

*8. School of Chemical and Biomolecular Engineering, Georgia Institute of Technology, Atlanta, GA, 30322, USA*

*9. George W. Woodruff School of Mechanical Engineering, Wallace H. Coulter Department of Biomedical Enigneering, and Parker H. Petit Institute for Bioengineering and Bioscience, Georgia Institute of Technology, Atlanta, GA 30322, USA*

*Author’s work was completed at Emory University. Author has since transitioned to Yale University.

**Corresponding author:** Srikant Rangaraju MD MS, [Srikant.rangaraju@yale.edu](mailto:Srikant.rangaraju@yale.edu)

Supplemental Figures: 4

Supplemental Datasheets: 4

**SUPPLEMENTAL FIGURE LEGENDS**

**
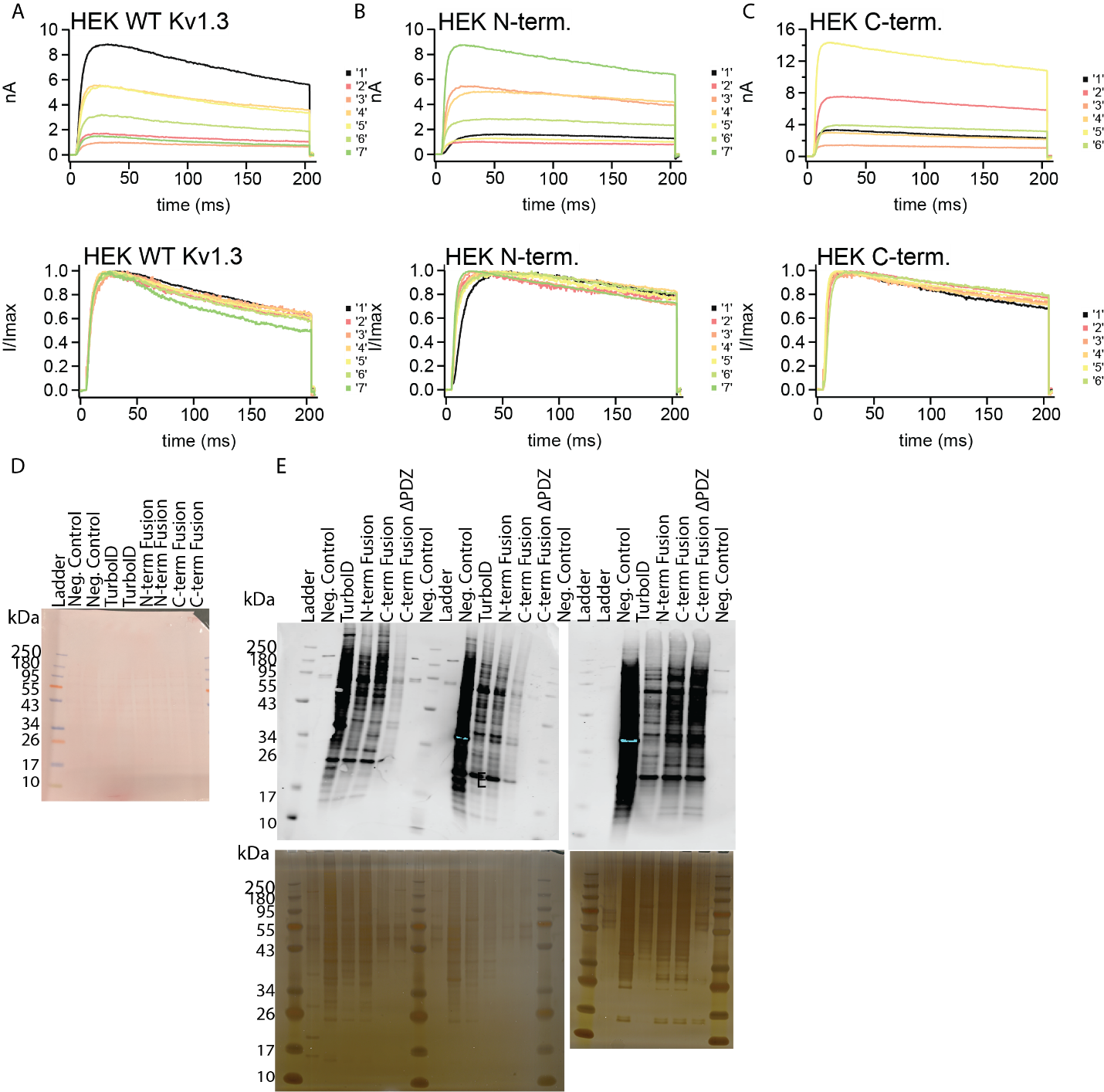
**

**Supplemental Figure 1: Confirmation of Hek-293 cell Kv1.3 function and TurboID activity. (A)** Traces of Kv1.3 electrophysiology of HEK-293 transfected with WT Kv1.3. **(B)** Traces of Kv1.3 activity of HEK-293 cells transfected with TurboID fused to the N-terminus of Kv1.3. **(C)** Traces of Kv1.3 activity of HEK-293 cells transfected with TurboID fused to the C-terminus of Kv1.3. **(D)** Ponceau staining of HEK-293 showing even protein loading across western blots prior to affinity purification. **(E)** Post-affinity purification western blot and silverstain show streptavidin labeling of proteins transfected with TurboID and proper affinity purification. **(F)** DEA comparison of HEK-293 Kv1.3 interactors on the N-terminus and C-terminus. Differential Abundant proteins were calculated using paired t-test, where log P-value > 1.3 and Log_2_ Fold Change (FC) of +/-1 were considered significant. n=3


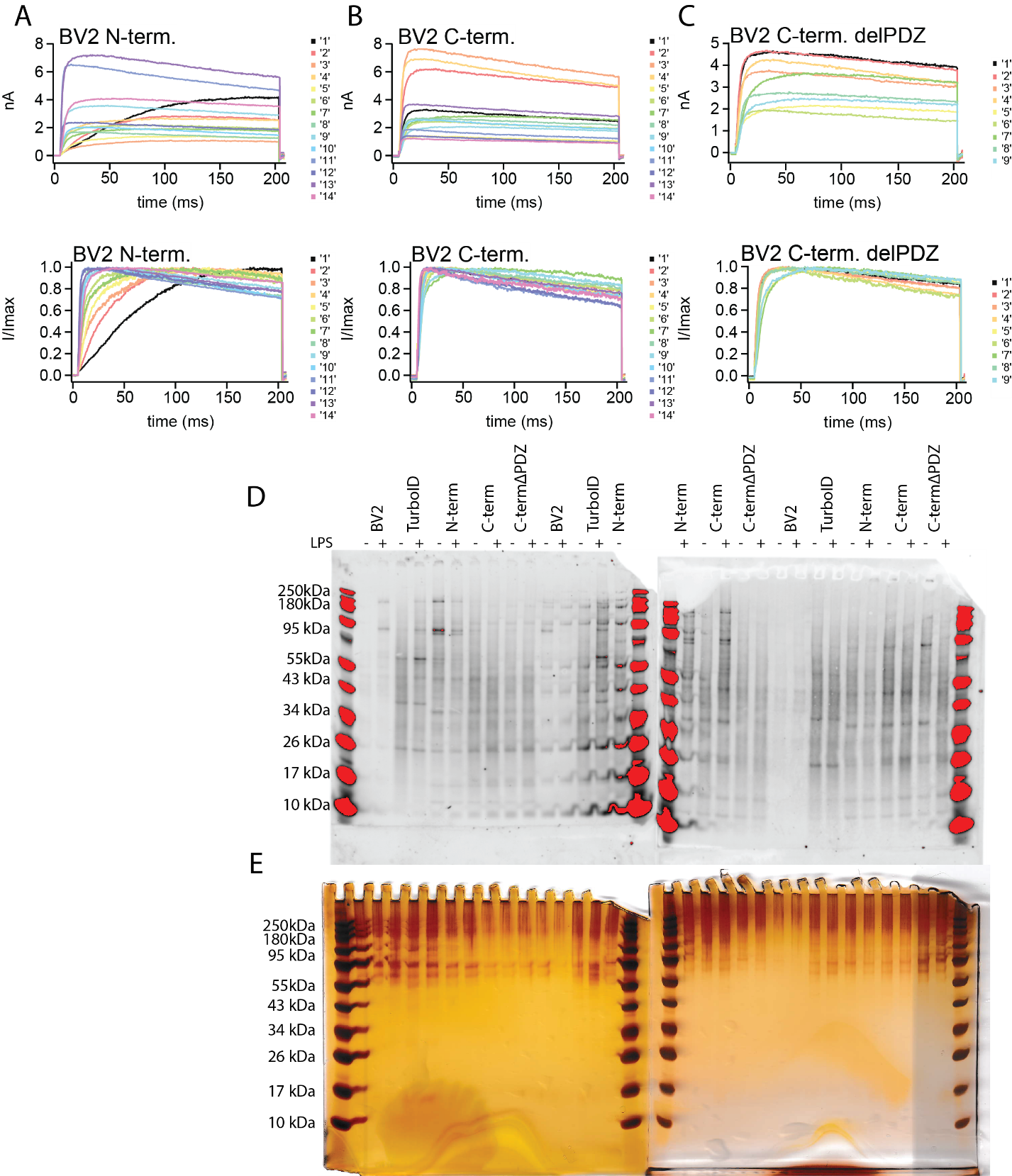


**Supplemental Figure 2: Confirmation of BV-2 cell Kv1.3 function and TurboID activity. (A)** Traces of Kv1.3 electrophysiology of BV-2 cells transduced with TurboID fused to the N-terminus of Kv1.3. **(B)** Traces of Kv1.3 activity of BV-2 cells transduced with TurboID fused to the C-terminus of Kv1.3. **(C)** Traces of Kv1.3 activity of BV-2 cells transduced with TurboID fused to the C-terminus of Kv1.3 with the PDZ-binding domain removed. **(D)** Post-affinity purification western blot and silverstain show streptavidin labeling of proteins transfected with TurboID and proper affinity purification. n=3


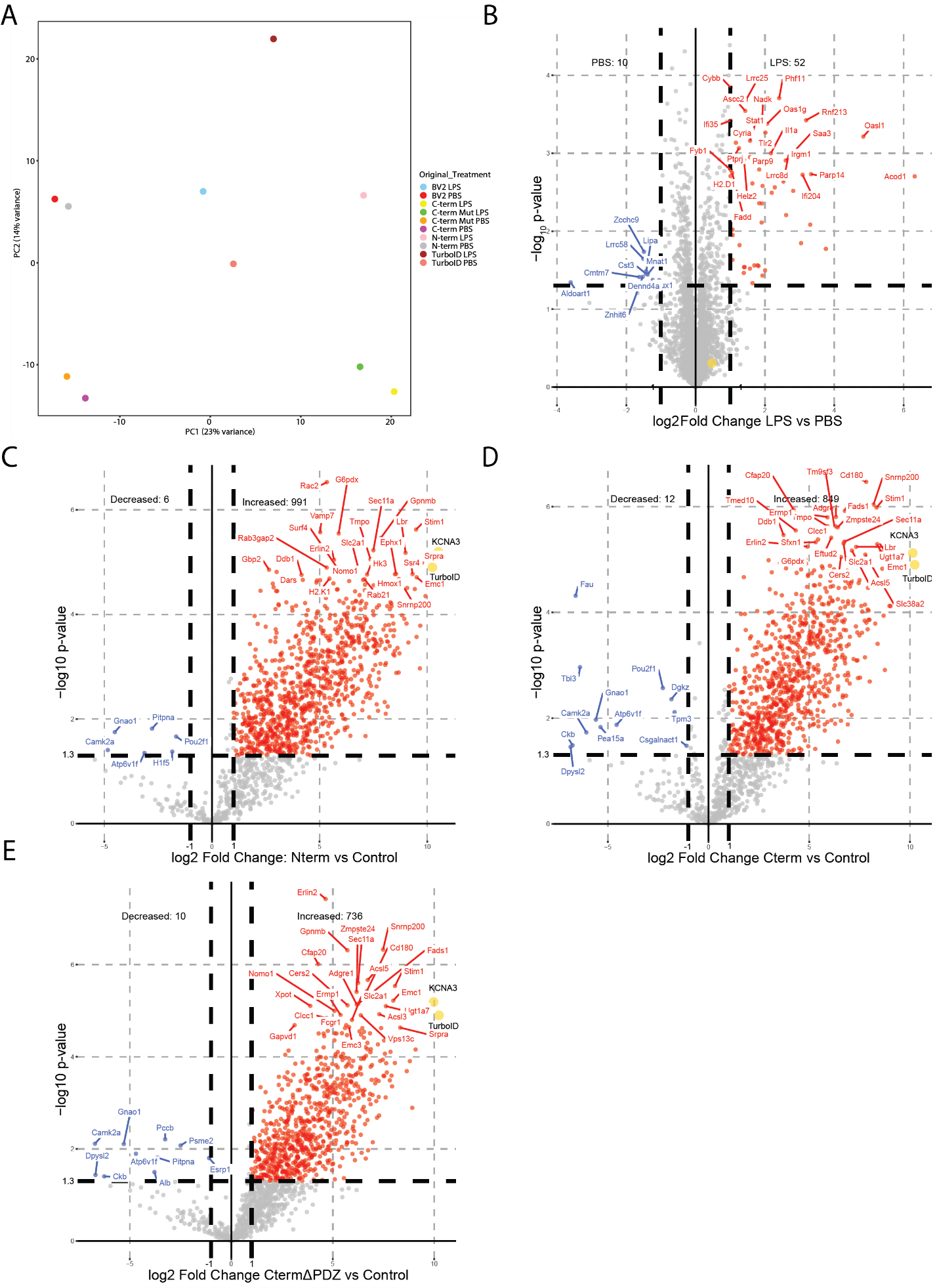


**Supplemental Figure 3: BV-2 cells successfully produce inflammatory responses and cells transduced with Kv1.3 have distinct interactors. (A)** Principal Component Analysis (PCA) of mass spectrometry of BV-2 cell lysates shows distinct separation between cells exposed to LPS compared to PBS. There is little variance explained in the cell lysate proteomes that show difference between overexpression of Kv1.3. **(B)** DEA of whole cell lysates of BV-2 cells induced with LPS have increased presence of key inflammatory proteins. **(C)** DEA of affinity purified BV-2 cells with an N-terminal fusion of TurboID to Kv1.3 compared to controls shows presence of proteins interacting with the Kv1.3 channel. **(D)** DEA of affinity purified BV-2 cells with an C-terminal fusion of TurboID to Kv1.3 compared to controls shows presence of proteins interacting with the Kv1.3 channel. **(E)** DEA of affinity purified BV-2 cells with an C-terminal fusion of TurboID to Kv1.3 with a deletion of the PDZ-binding domain compared to controls shows presence of proteins interacting with the Kv1.3 channel. Differential Abundant proteins were calculated using paired t-test, where log P-value > 1.3 and Log_2_ Fold Change (FC) of +/-1 were considered significant. n=3

**
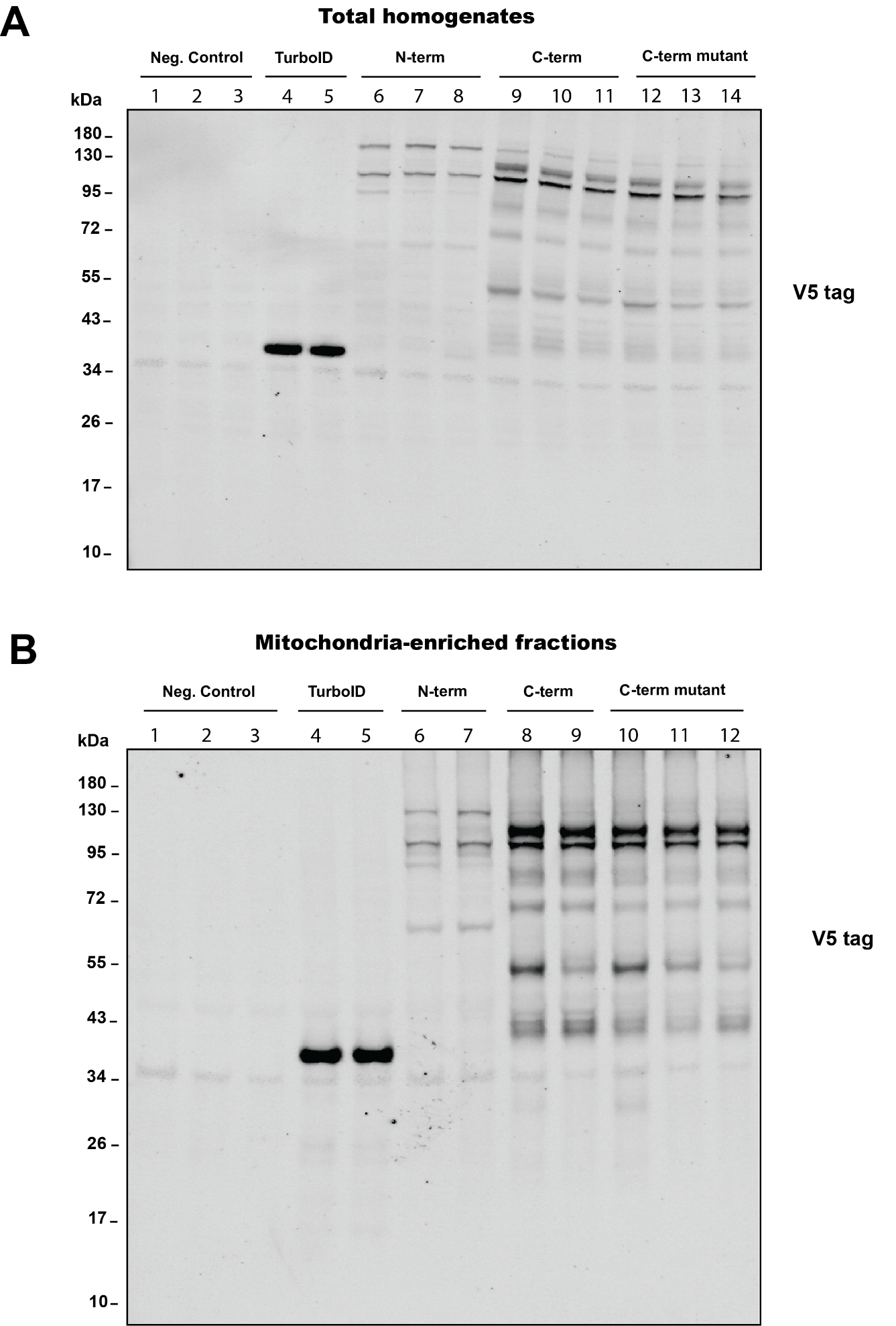
**

**Supplemental Figure 4: Full-length Western blot images of V5 tag in total homogenates (A) and mitochondria-enriched fractions (B).** Fractionation experiments were run in triplicates and TurboID was included as positive control for anti-V5 tag antibody.


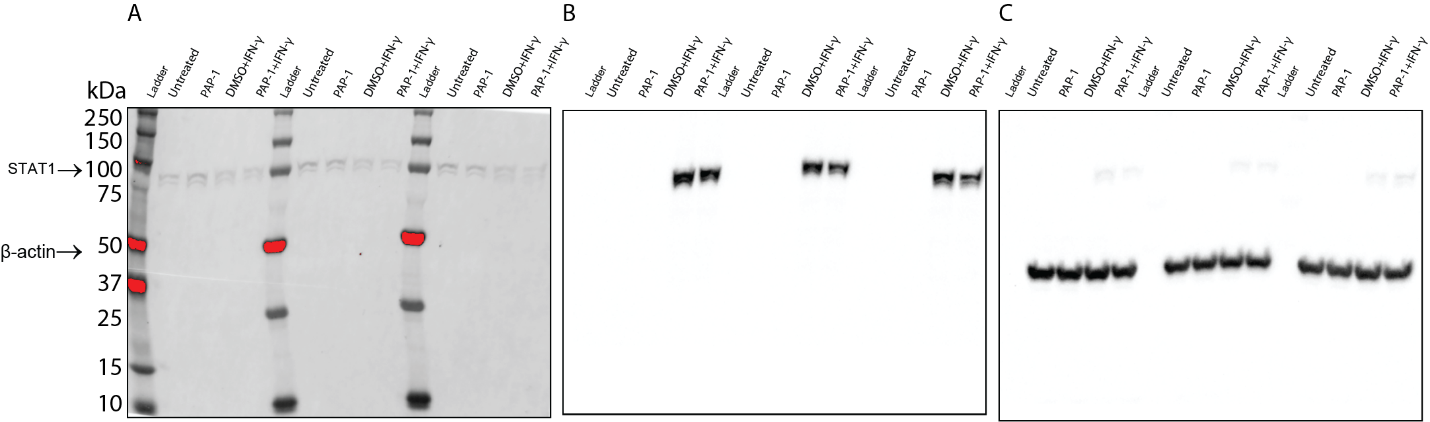


**Supplemental Figure 5:  Full-length WB images of STAT1(A), pSTAT1(B), β-actin (C).** The molecular weights of STAT1, pSTAT1, and β-actin are 84kDa, 84kDa, and 45kDa, respectively. Triplicate experiment showing the western blot analysis of whole cell lysate from BV-2 cells expressing N-terminal KV1.3 -TurboID. There is a consistent reduction of STAT1 phosphorylation upon induction with IFN-γ post KV1.3 blockade with PAP1 in all three experiments.
