# Supplementary material for: Proximity Labeling Proteomics Reveals Kv1.3 Potassium Channel Immune Interactors in Microglia
Source: Mol Cell Proteomics. 2024 Jun 25;23(8):100809. doi: 10.1016/j.mcpro.2024.100809 (PMC11780389; doi:10.1016/j.mcpro.2024.100809)
Supplement: OHW_BV2_AP [file mmc7.pdf]

QDLSPEEAYDLGR

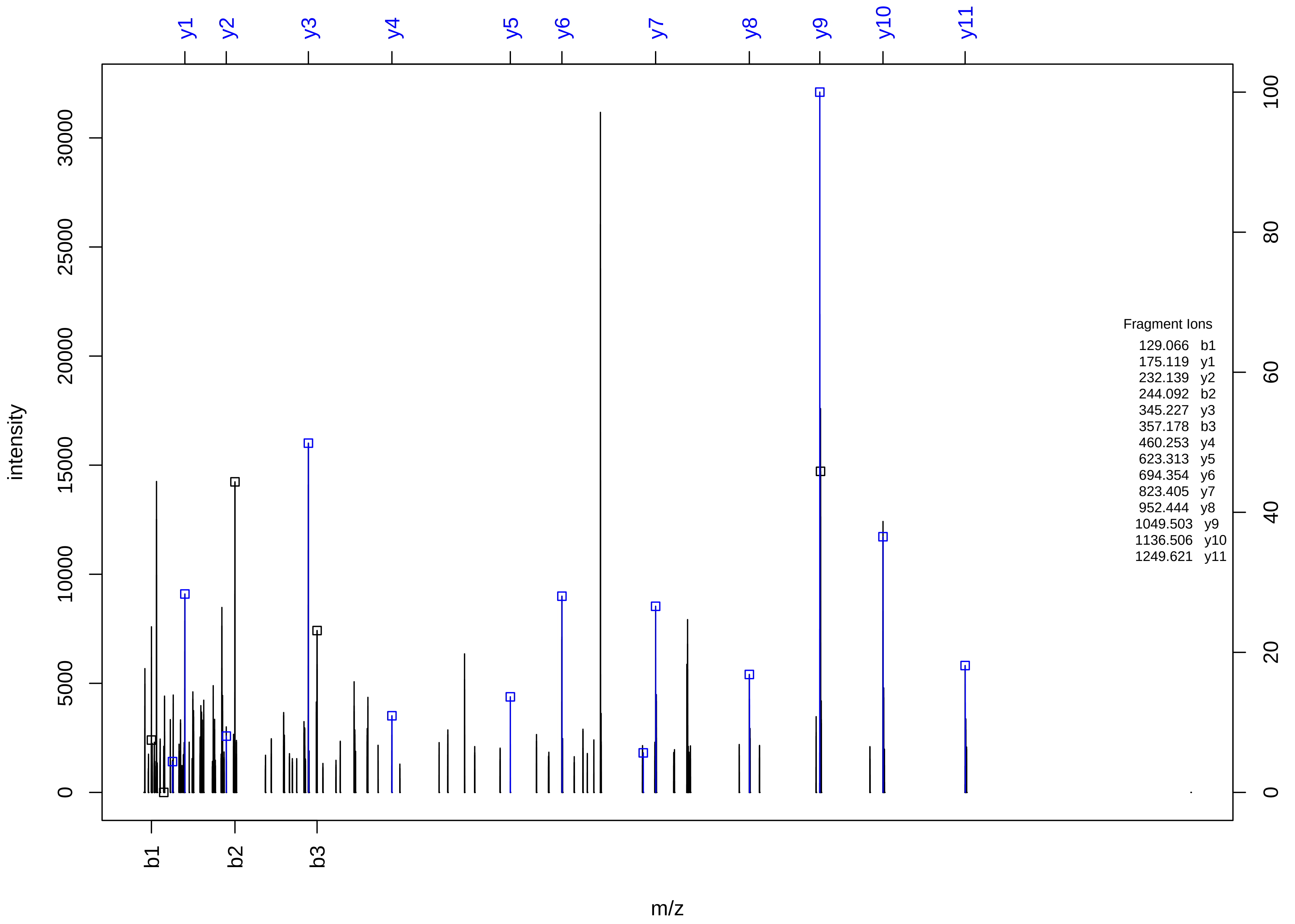

QDLSPEEAYDLGR / File: christine_30turboips_ip23.raw; SpectrumID: ; scans: 9699

VPFLPGDSDLDQLTR

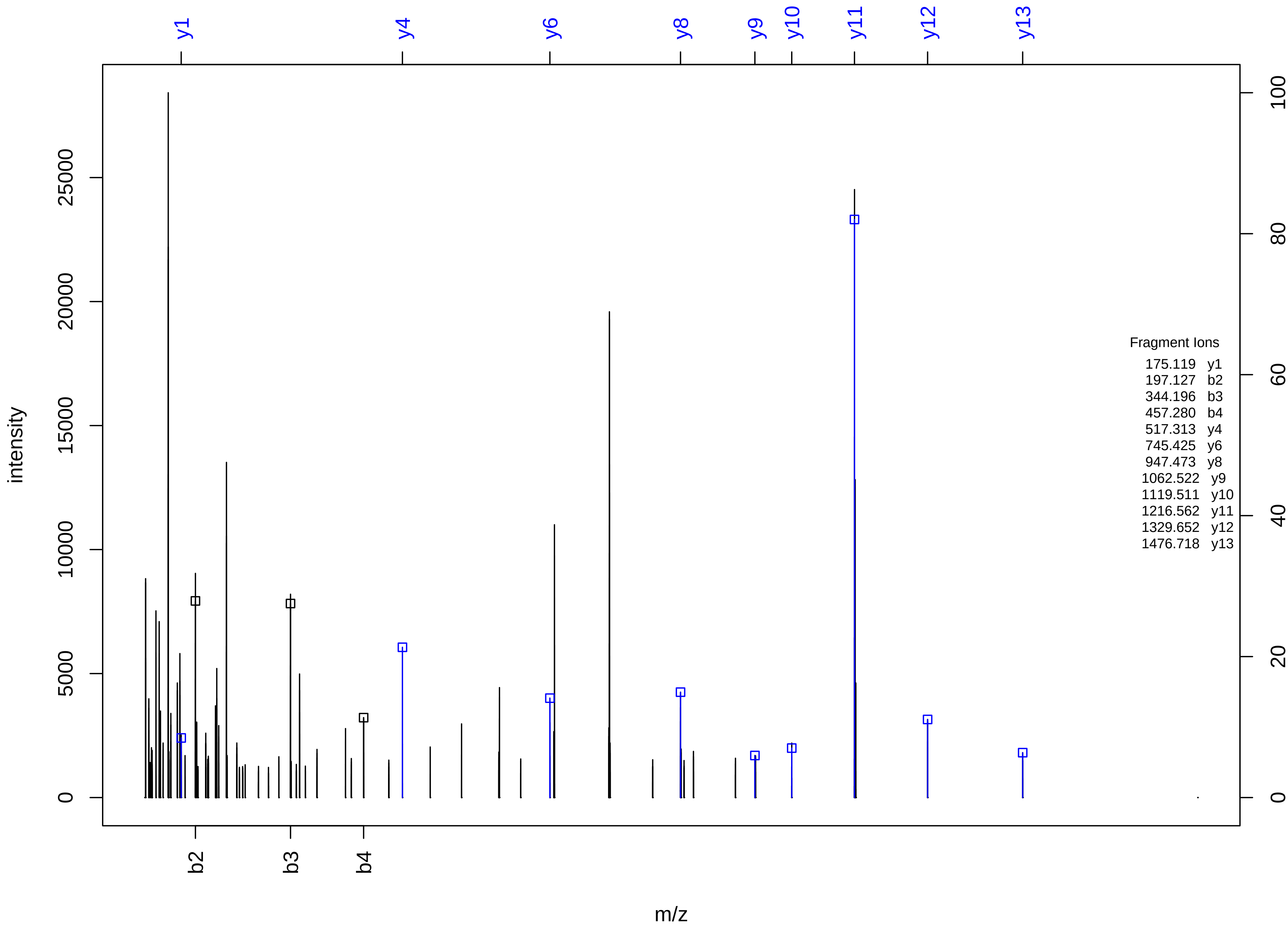

VPFLPGDSDLDQLTR / File: christine_30turboips_ip23.raw; SpectrumID: ; scans: 20837

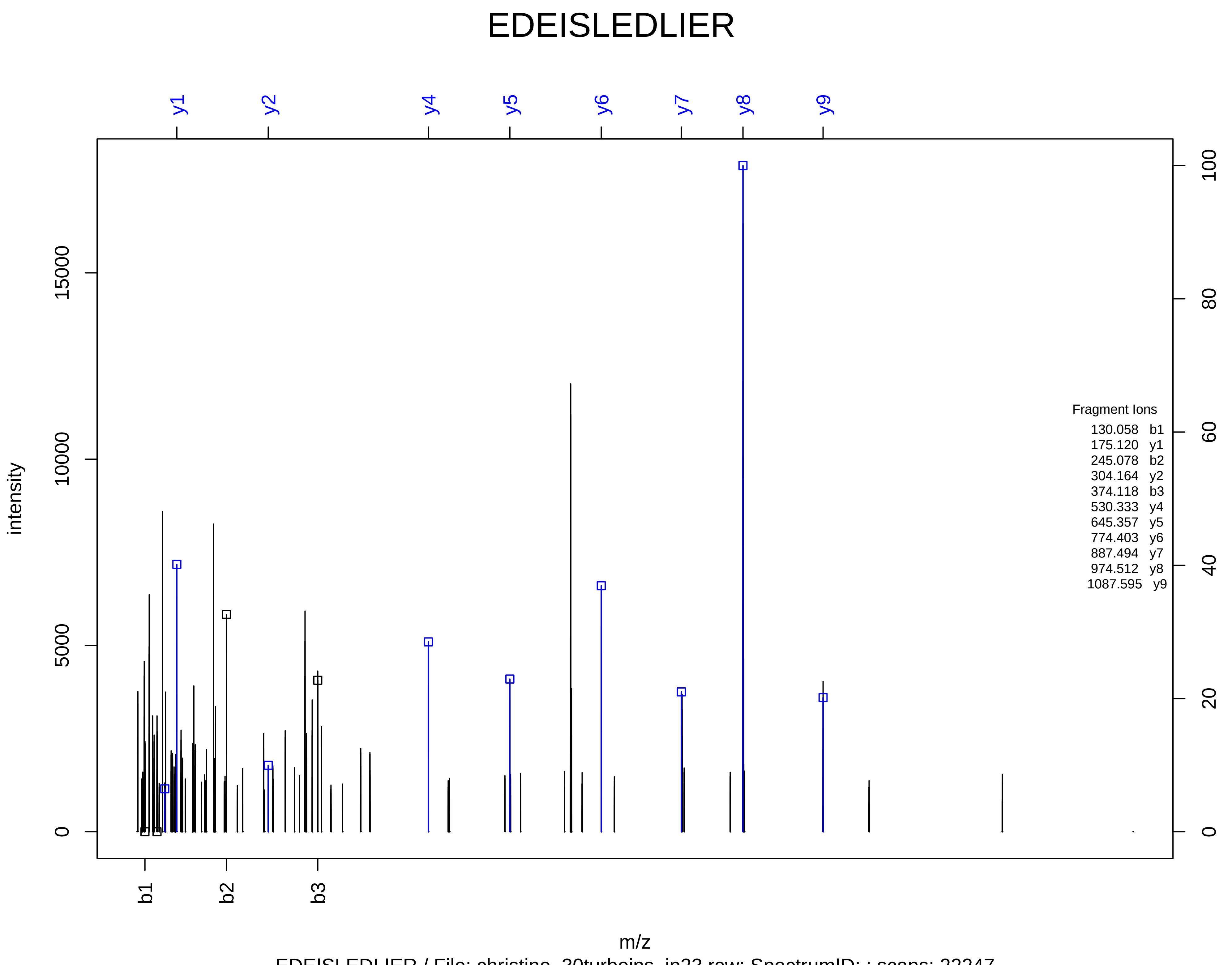
EDEISLEDLIER
y1
y2
y4
y5
y6
y7
y8
y9
intensity
0
5000
10000
15000
0
20
40
60
80
100
Fragment Ions
130.058 b1
175.120 y1
245.078 b2
304.164 y2
374.118 b3
530.333 y4
645.357 y5
774.403 y6
887.494 y7
974.512 y8
1087.595 y9
b1
b2
b3
m/z
EDEISLEDLIER / File: christine_30turboips_ip23.raw; SpectrumID: ; scans: 22247

LPVPQNEVITDASVTK

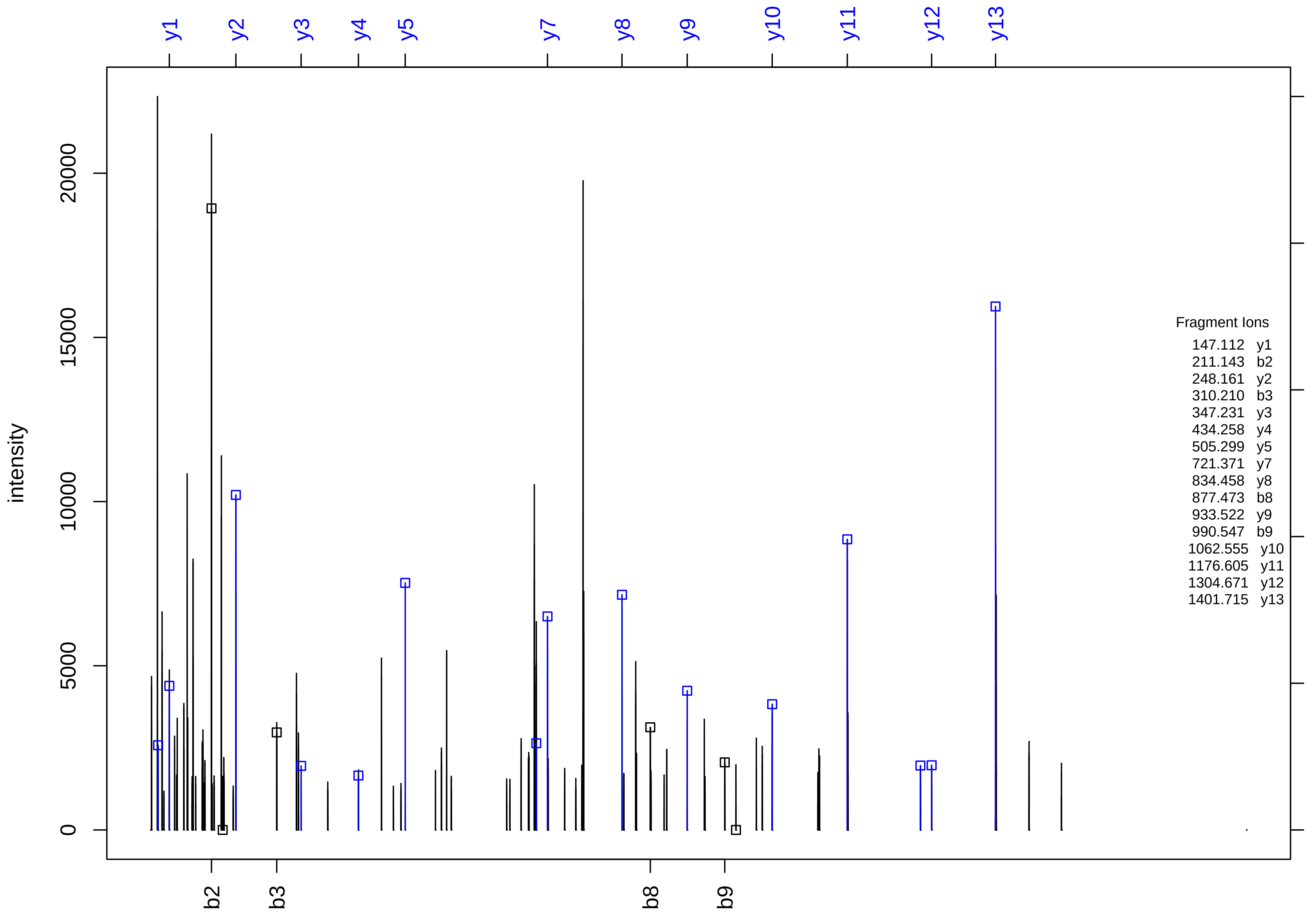

LPVPQNEVITDASVTK / File: christine_30turboips_ip23.raw; SpectrumID: ; scans: 11214

VVSWLVSSDTPQPEVAPPAHESR

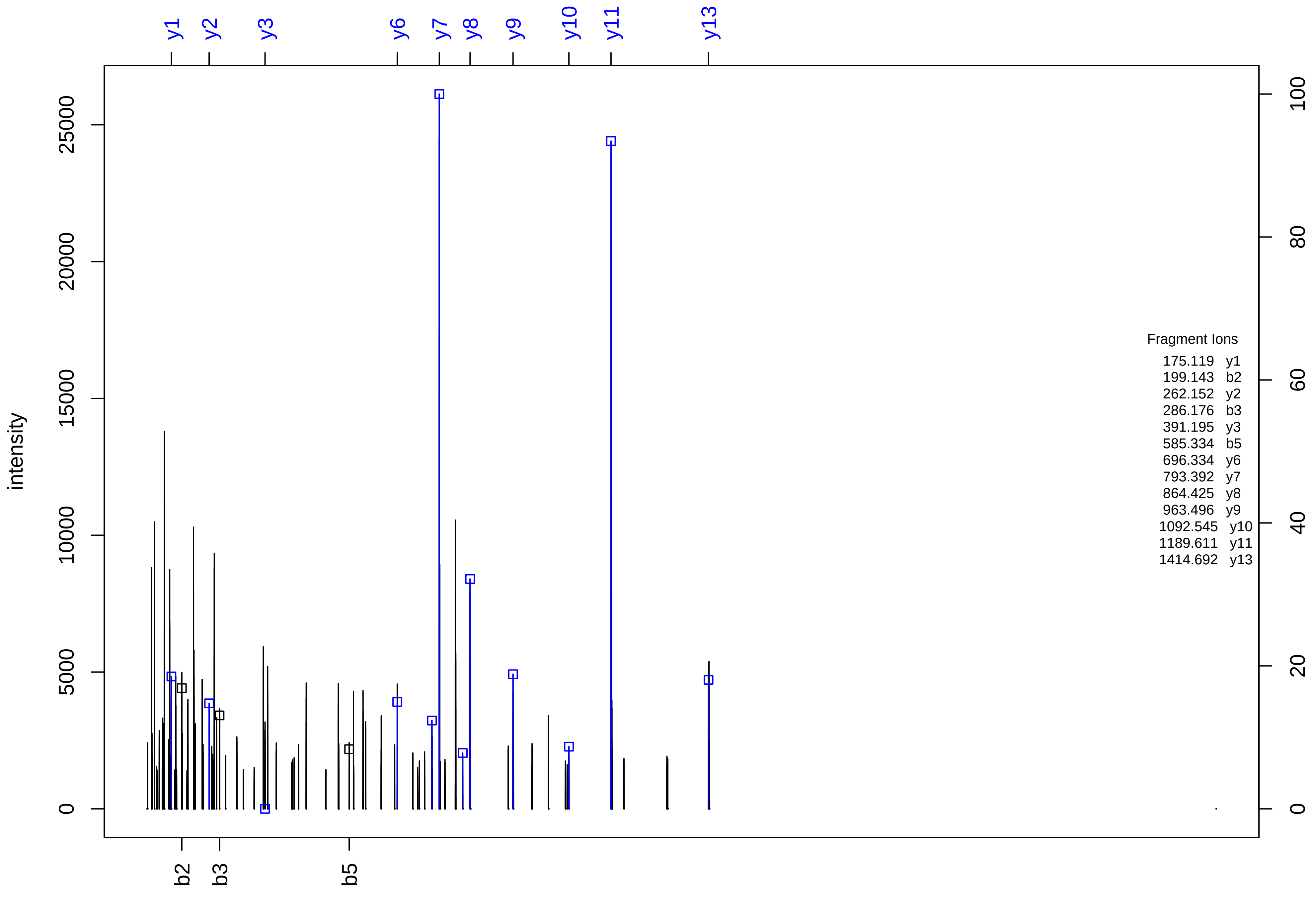

VVSWLVSSDTPQPEVAPPAHESR / File: christine_30turboips_ip23.raw; SpectrumID: ; scans: 13052

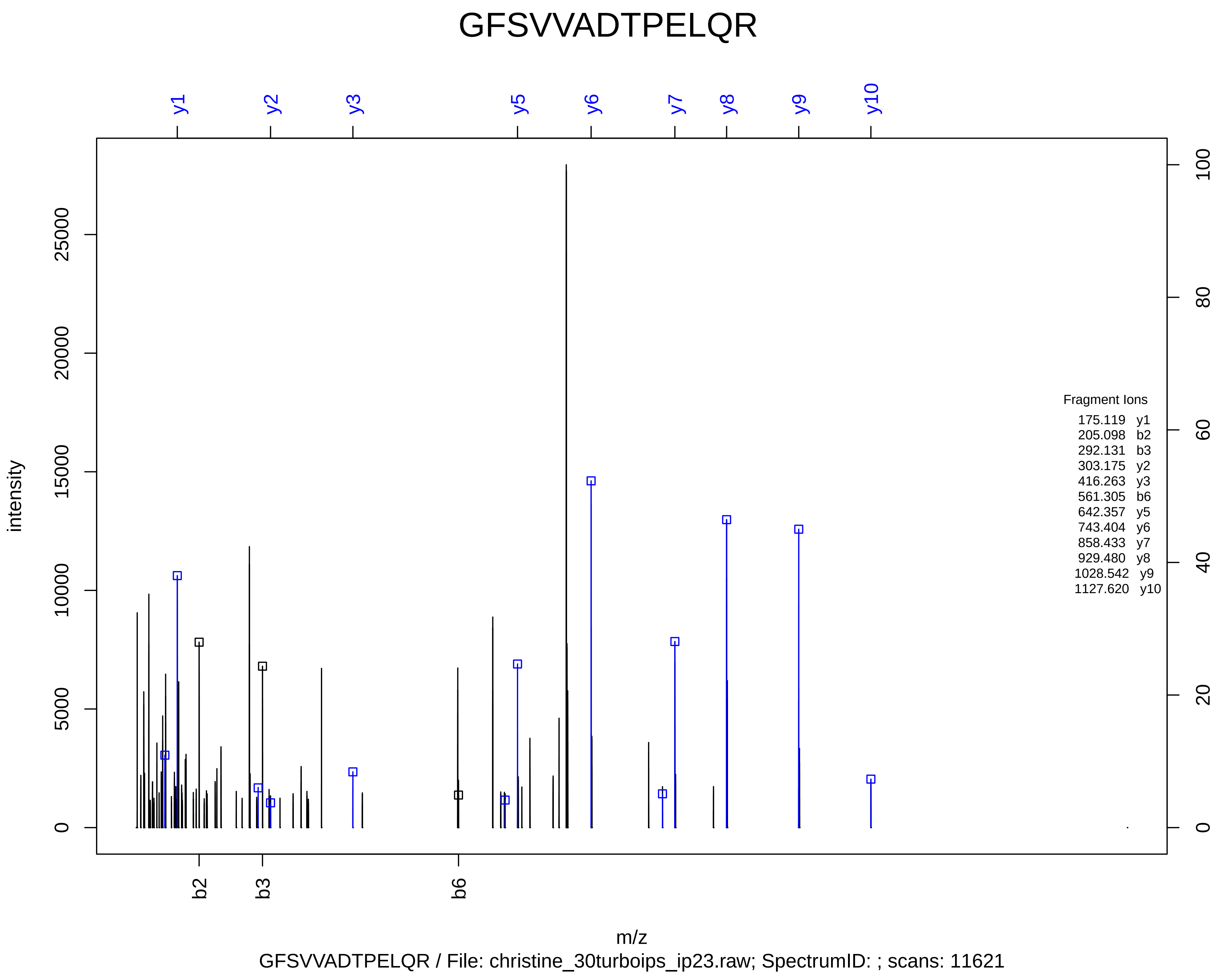
GFSVVADTPELQR
y1
y2
y3
y5
y6
y7
y8
y9
y10
intensity
0
5000
10000
15000
20000
25000
0
20
40
60
80
100
Fragment Ions
175.119 y1
205.098 b2
292.131 b3
303.175 y2
416.263 y3
561.305 b6
642.357 y5
743.404 y6
858.433 y7
929.480 y8
1028.542 y9
1127.620 y10
b2
b3
b6
m/z
GFSVVADTPELQR / File: christine_30turboips_ip23.raw; SpectrumID: ; scans: 11621

VPEPCQPK

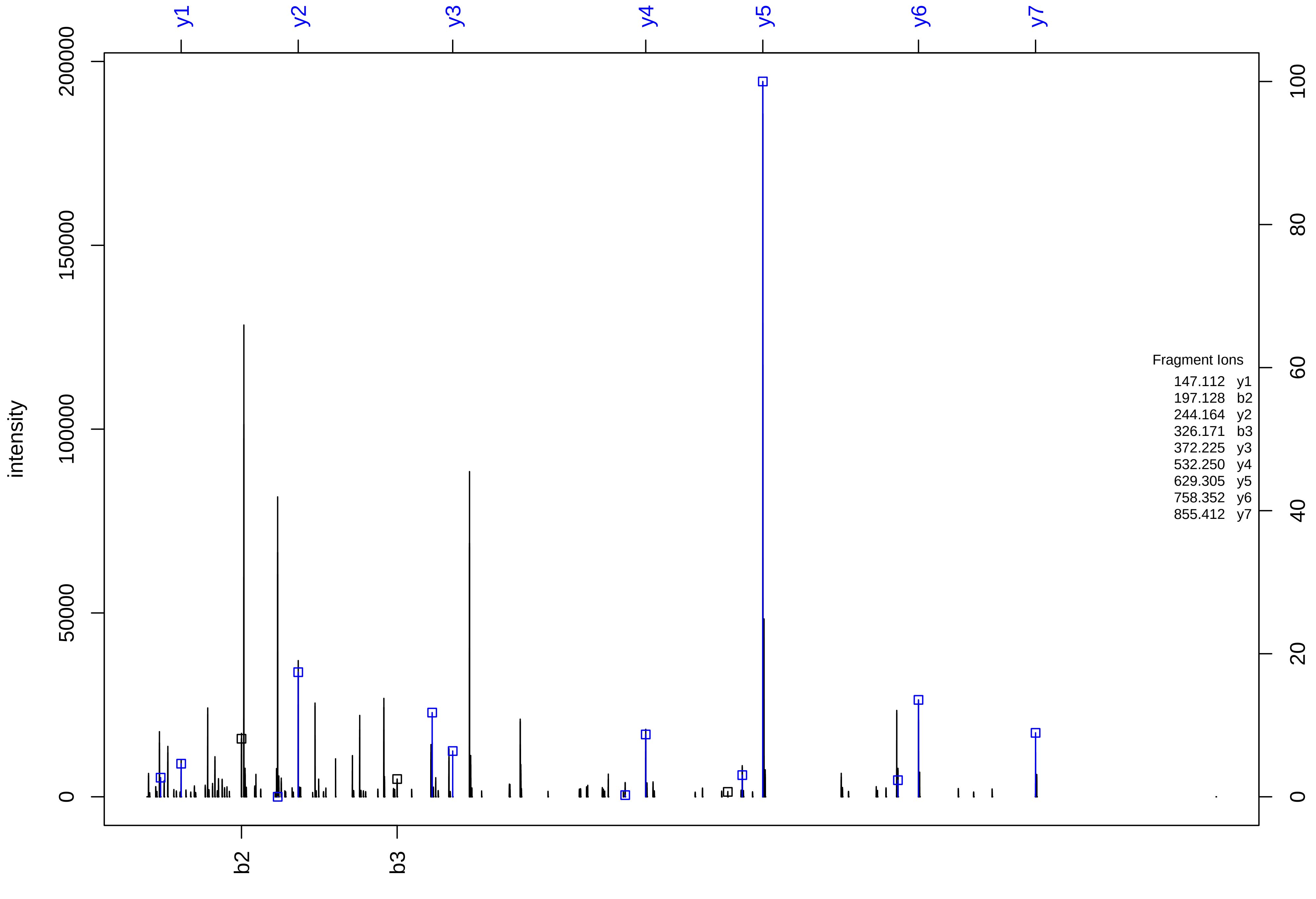

VPEPCQPK / File: christine_30turboips_ip23.raw; SpectrumID: ; scans: 947

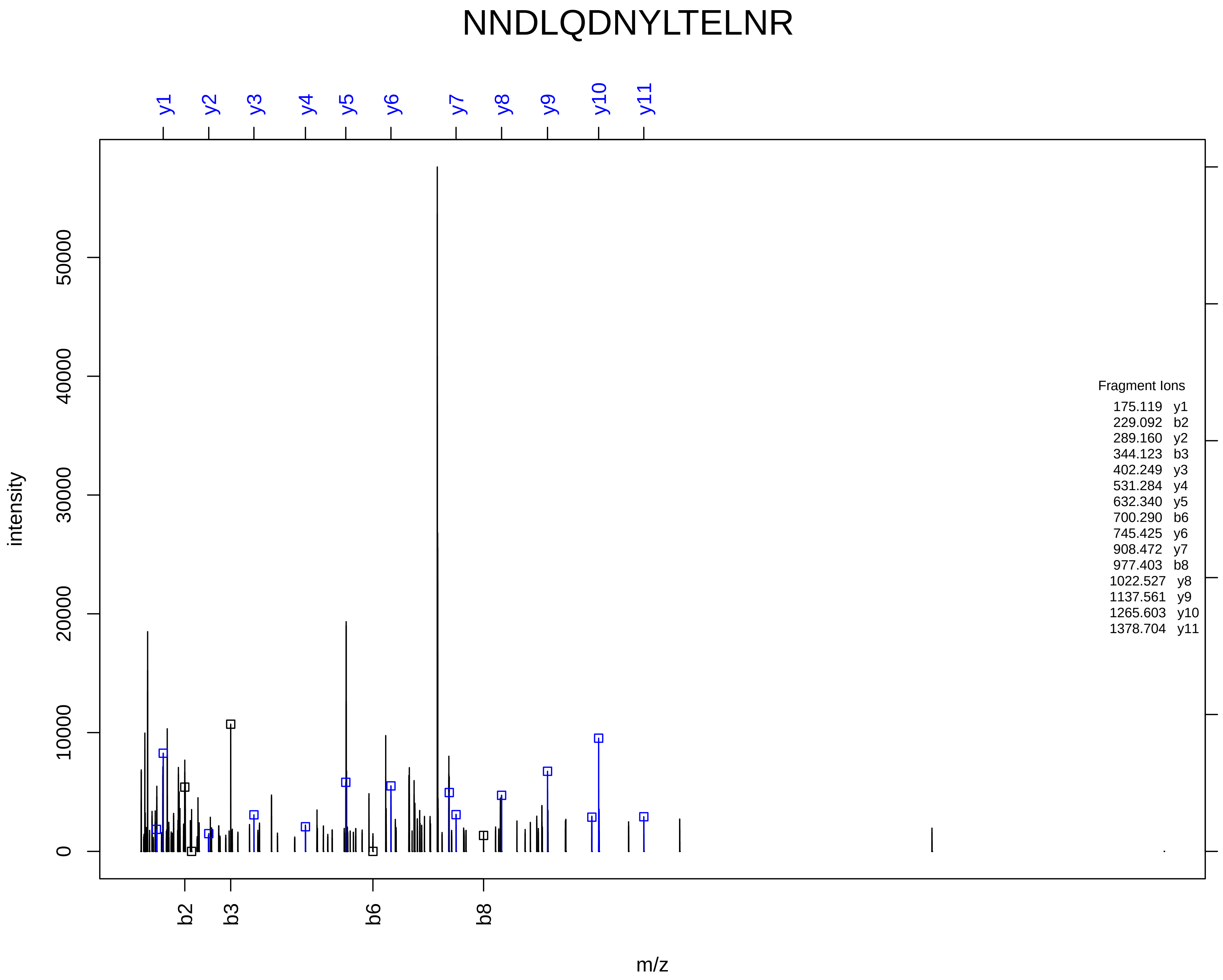

NNDLQDNYLTELNR / File: christine_30turboips_ip23.raw; SpectrumID: ; scans: 13002

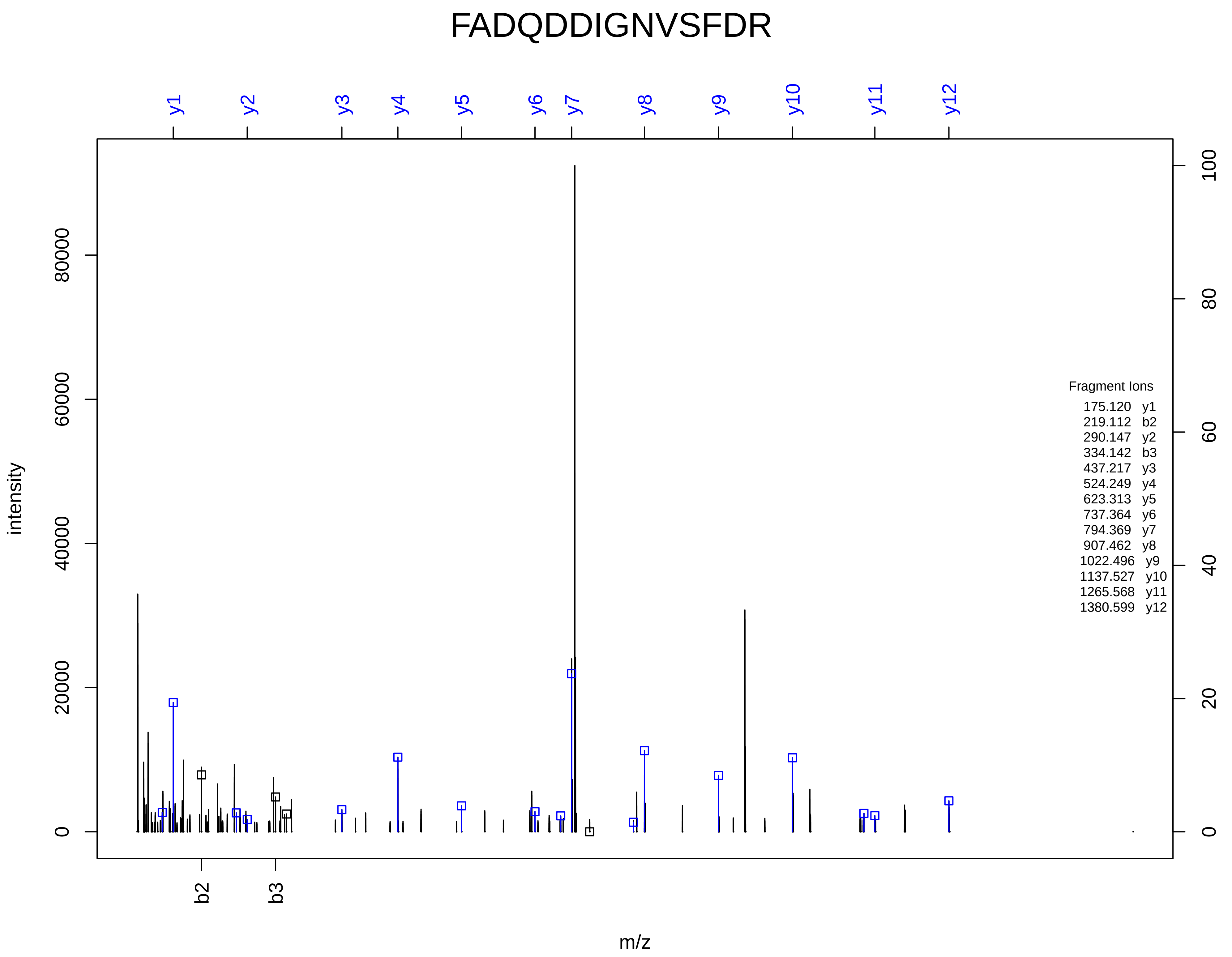
FADQDDIGNVSFDR
y1
y2
y3
y4
y5
y6
y7
y8
y9
y10
y11
y12
intensity
0
20000
40000
60000
80000
0
20
40
60
80
100
b2
b3
Fragment Ions
175.120 y1
219.112 b2
290.147 y2
334.142 b3
437.217 y3
524.249 y4
623.313 y5
737.364 y6
794.369 y7
907.462 y8
1022.496 y9
1137.527 y10
1265.568 y11
1380.599 y12
m/z
FADQDDIGNVSFDR / File: christine_30turboips_ip23.raw; SpectrumID: ; scans: 11255

YLEAGAAGLR

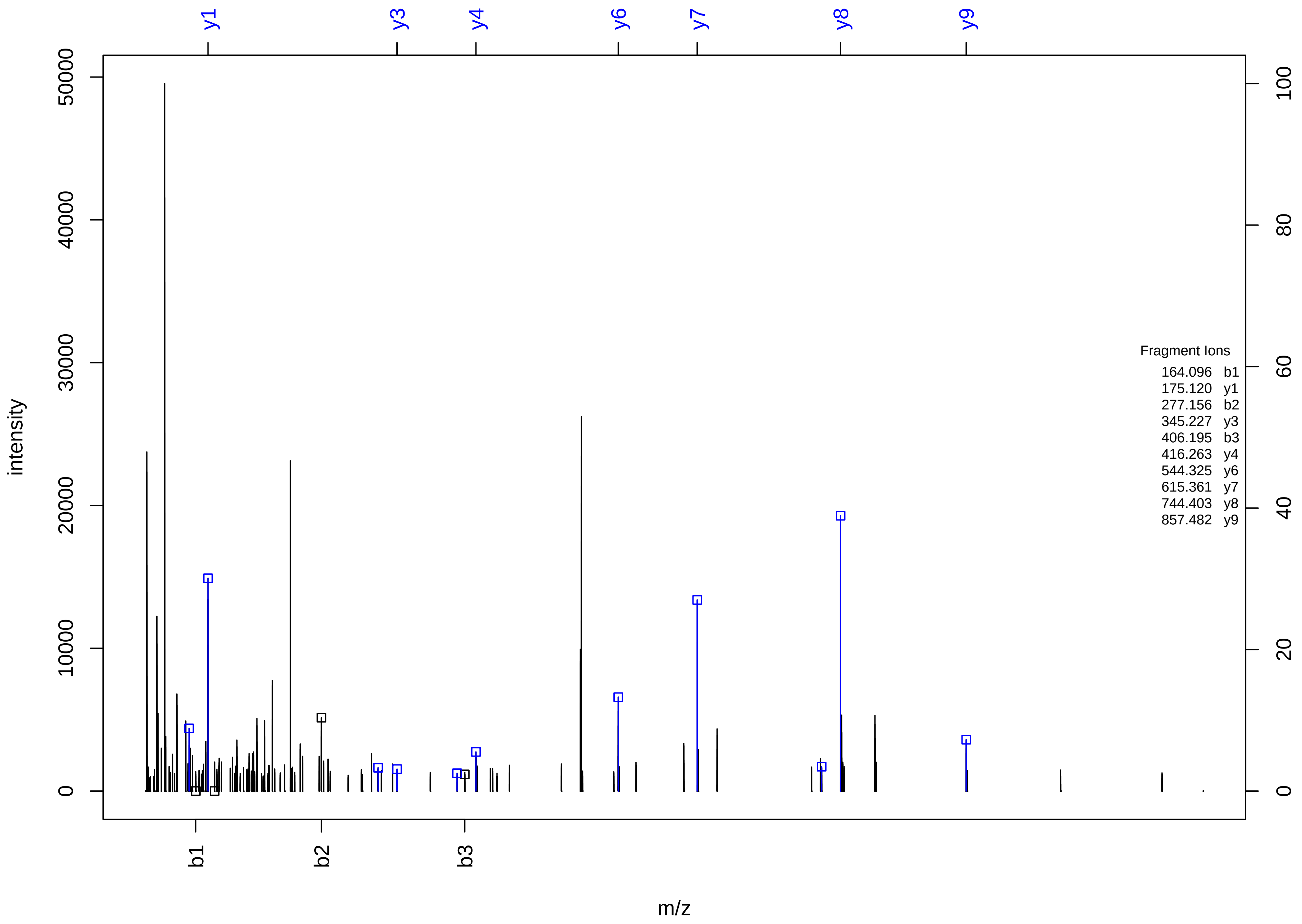

YLEAGAAGLR / File: christine_30turboips_ip23.raw; SpectrumID: ; scans: 3905

EALENANTNTEVLK

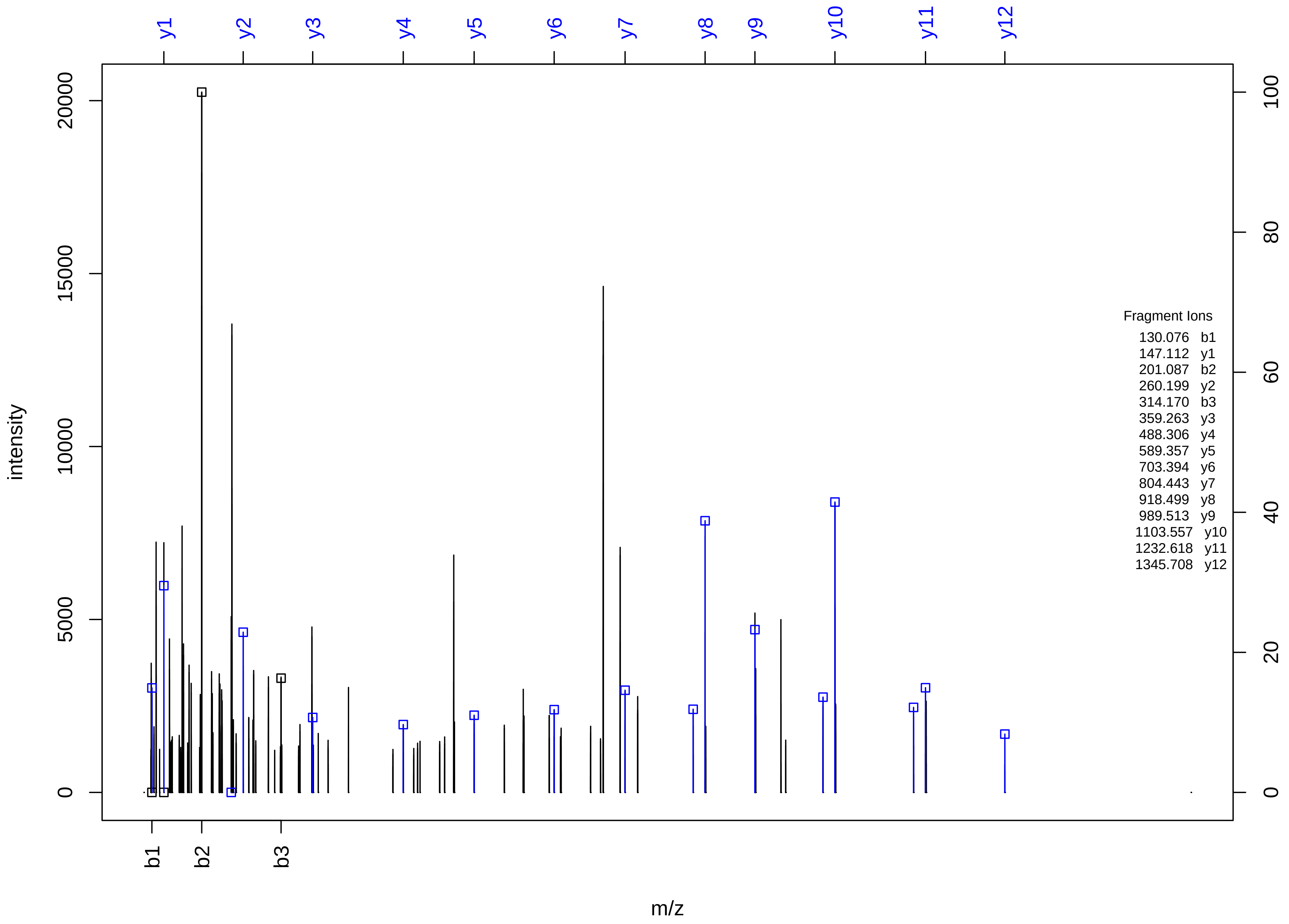

EALENANTNTEVLK / File: christine_30turboips_ip23.raw; SpectrumID: ; scans: 4110

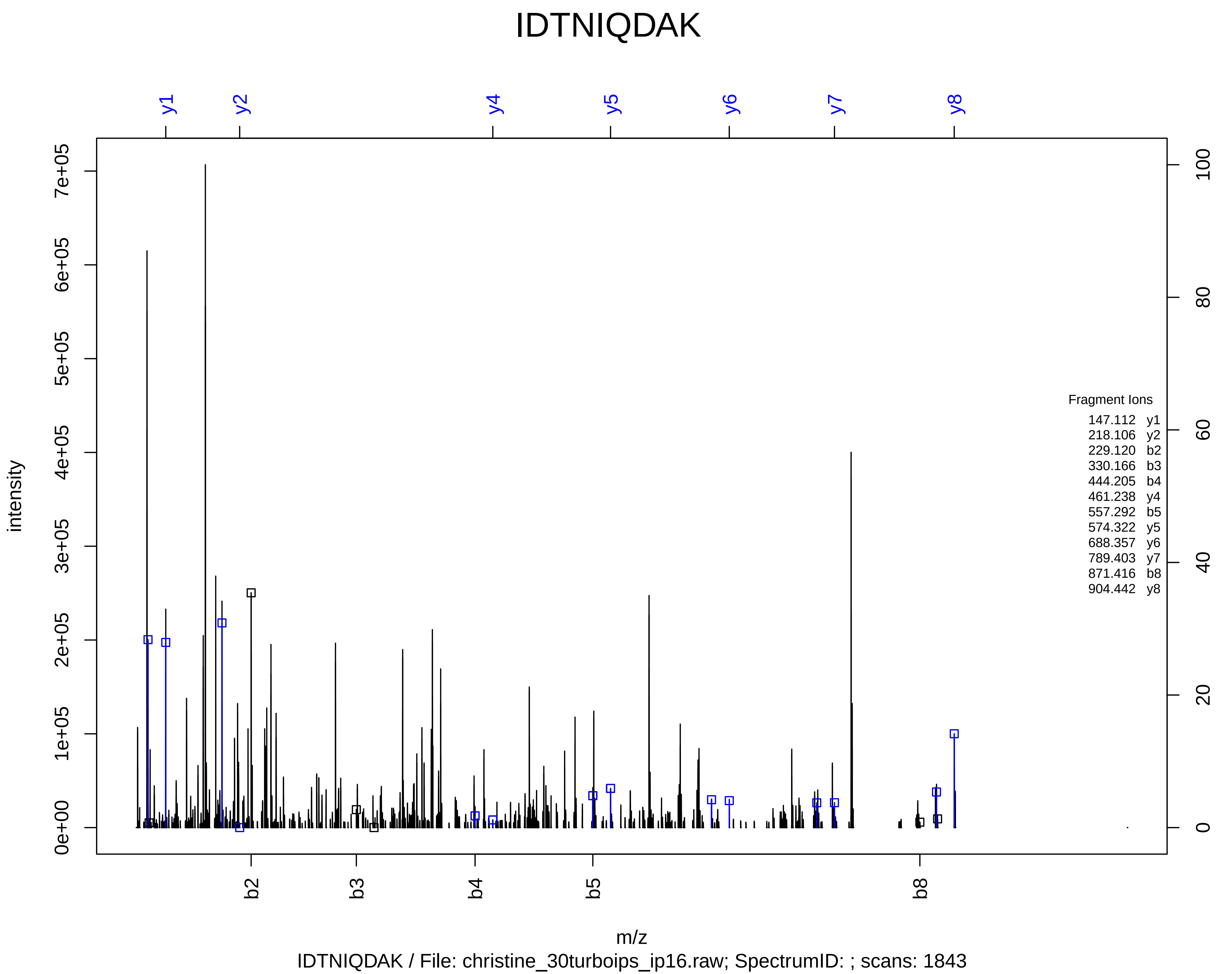

IDTNIQDAK
y1
y2
y4
y5
y6
y7
y8
intensity
0e+00
1e+05
2e+05
3e+05
4e+05
5e+05
6e+05
7e+05
0
20
40
60
80
100
Fragment Ions
147.112 y1
218.106 y2
229.120 b2
330.166 b3
444.205 b4
461.238 y4
557.292 b5
574.322 y5
688.357 y6
789.403 y7
871.416 b8
904.442 y8
b2
b3
b4
b5
b8
m/z
IDTNIQDAK / File: christine_30turboips_ip16.raw; SpectrumID: ; scans: 1843

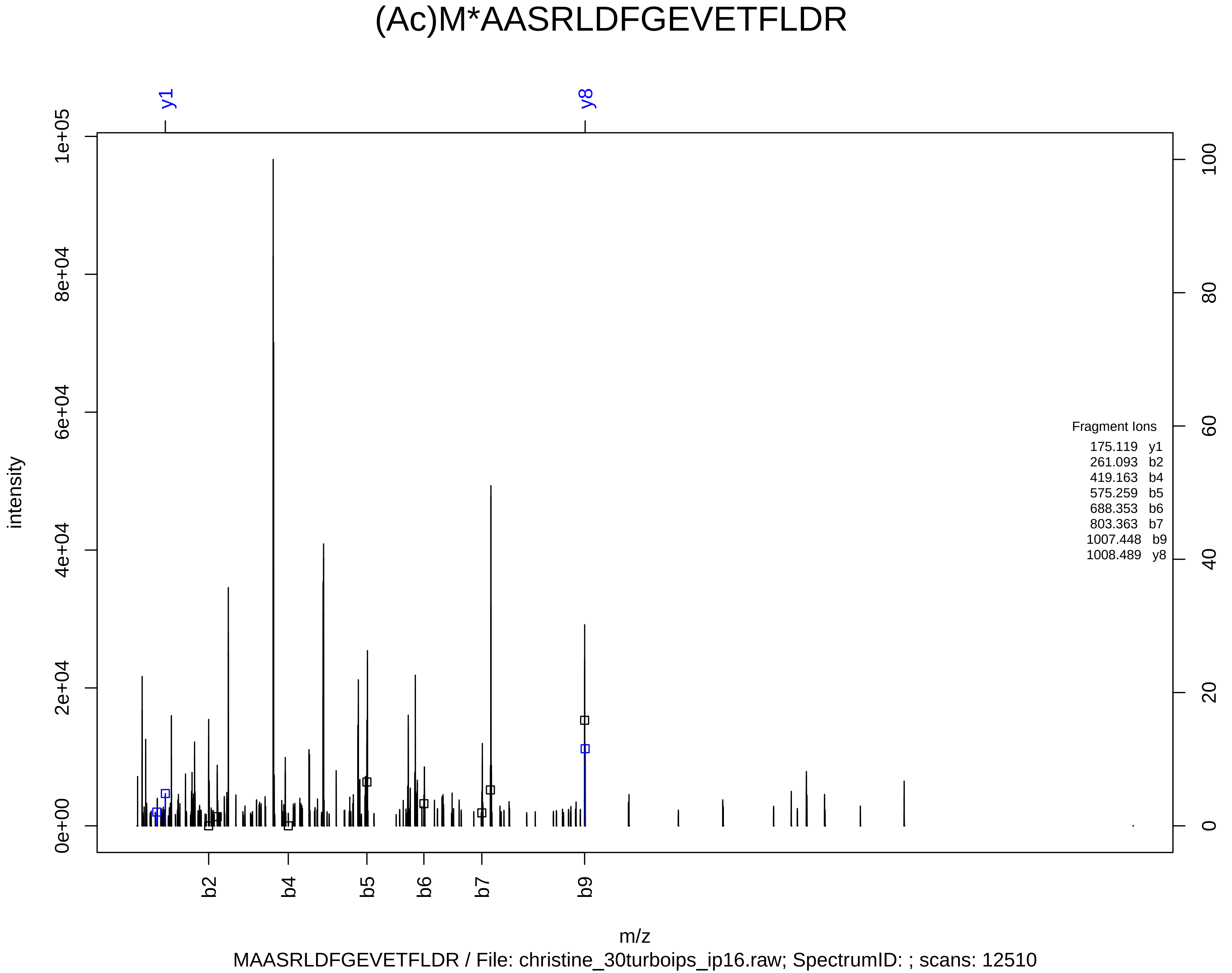
(Ac)M*AASRLDFGEVETFLDR
y1
y8
intensity
0e+00
2e+04
4e+04
6e+04
8e+04
1e+05
0
20
40
60
80
100
Fragment Ions
175.119 y1
261.093 b2
419.163 b4
575.259 b5
688.353 b6
803.363 b7
1007.448 b9
1008.489 y8
b2
b4
b5
b6
b7
b9
m/z
MAASRLDFGEVETFLDR / File: christine_30turboips_ip16.raw; SpectrumID: ; scans: 12510

(Ac)M*DIAIHHPWIRRPFFPFHSPSR

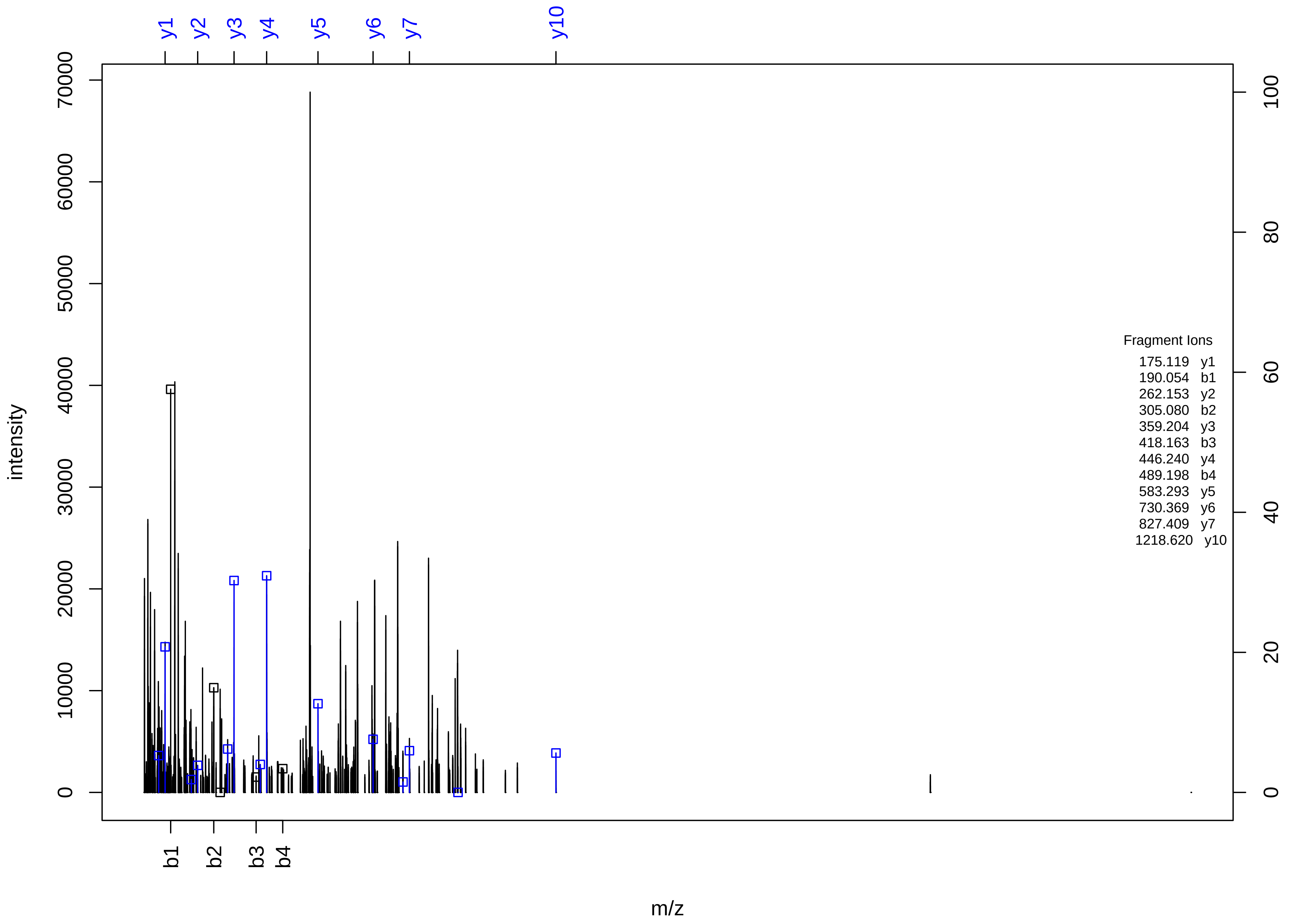

MDIAIHHPWIRRPFFPFHSPSR / File: christine_30turboips_ip16.raw; SpectrumID: ; scans: 15996

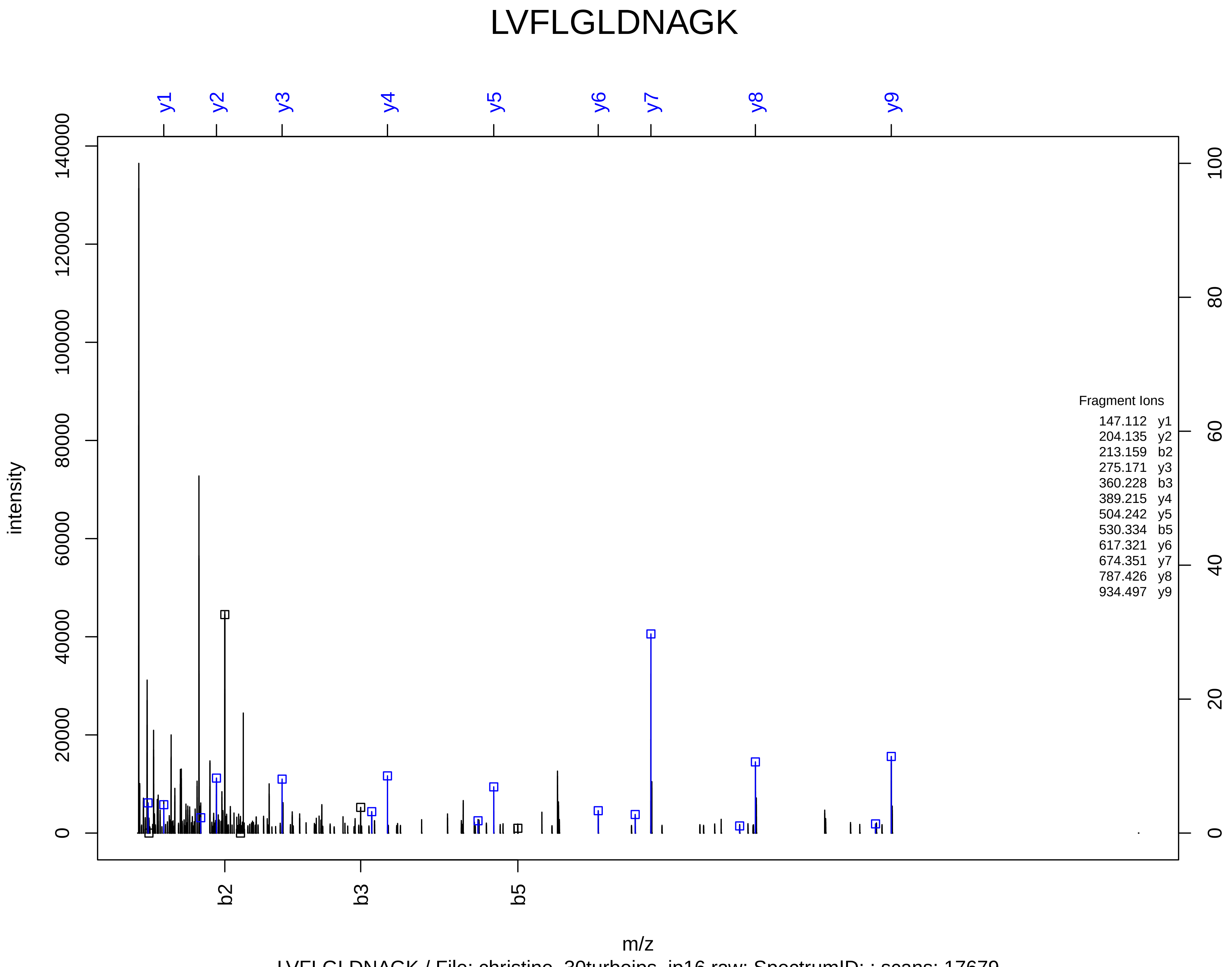
LVFLGLDNAGK
y1
y2
y3
y4
y5
y6
y7
y8
y9
intensity
0
20000
40000
60000
80000
100000
120000
140000
0
20
40
60
80
100
b2
b3
b5
m/z
Fragment Ions
147.112 y1
204.135 y2
213.159 b2
275.171 y3
360.228 b3
389.215 y4
504.242 y5
530.334 b5
617.321 y6
674.351 y7
787.426 y8
934.497 y9
LVFLGLDNAGK / File: christine_30turboips_ip16.raw; SpectrumID: ; scans: 17679

IAILDIEPQTLK

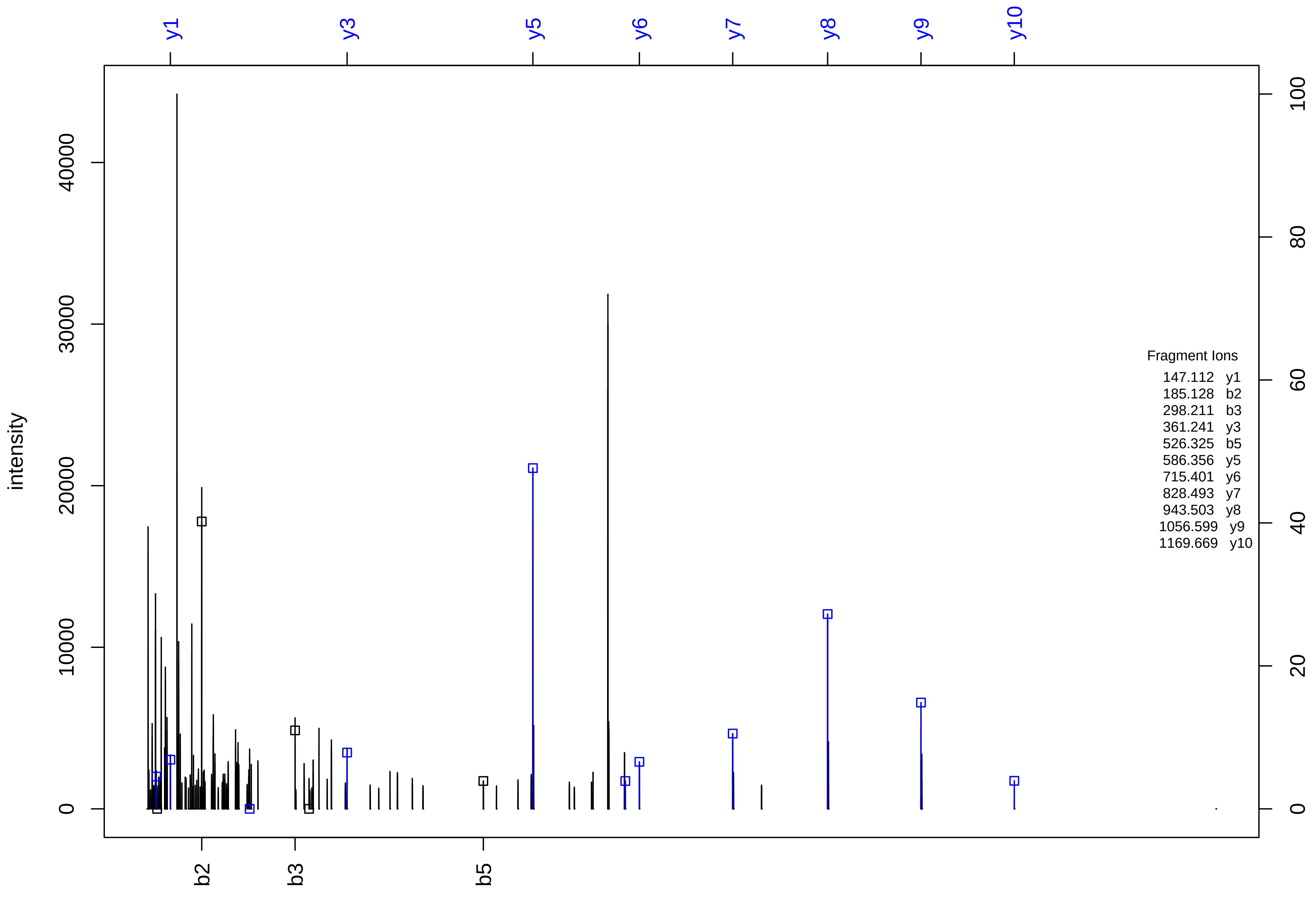

IAILDIEPQTLK / File: christine_30turboips_ip16.raw; SpectrumID: ; scans: 20544

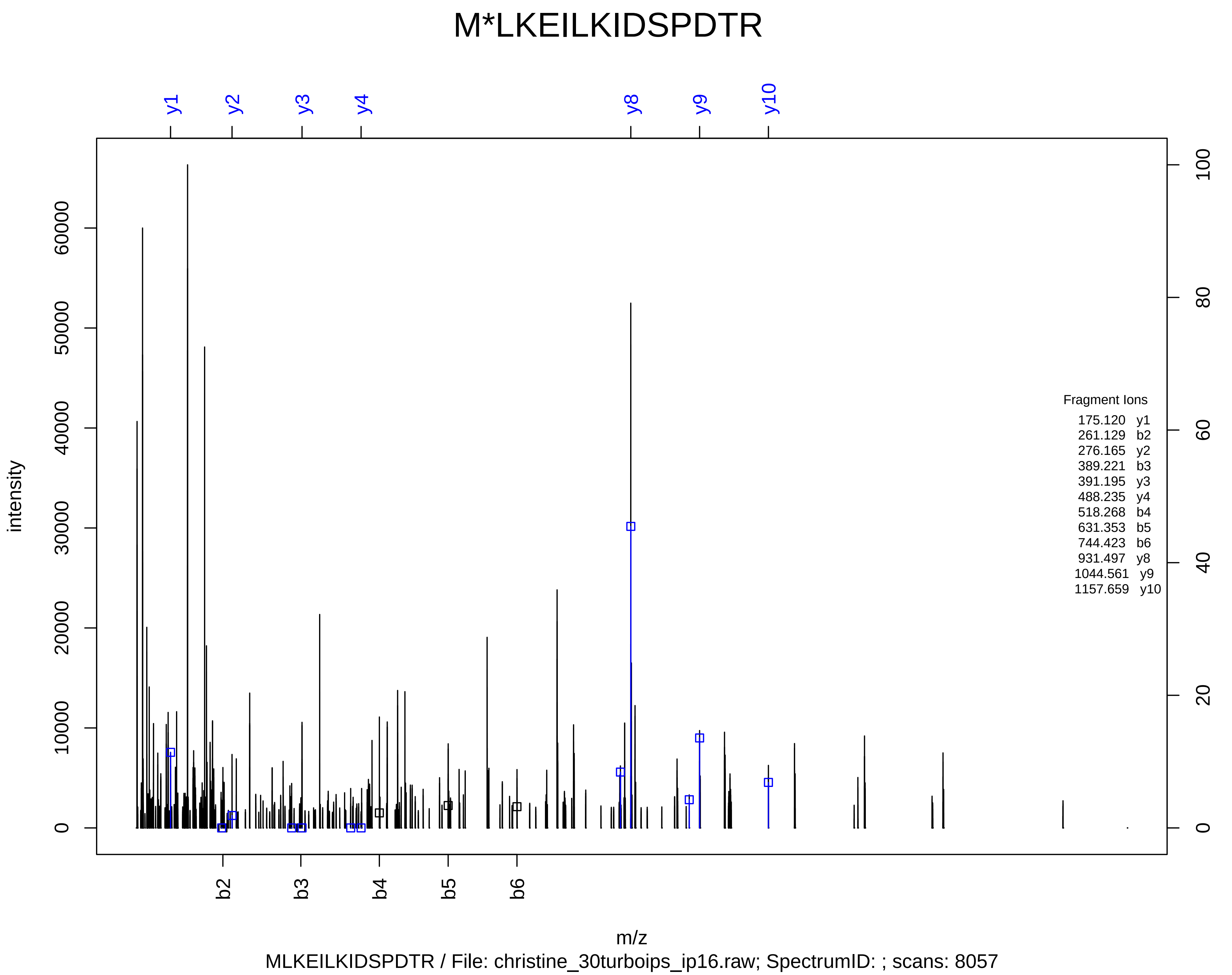
M*LKEILKIDSPDTR
y1
y2
y3
y4
y8
y9
y10
intensity
0
10000
20000
30000
40000
50000
60000
0
20
40
60
80
100
Fragment Ions
175.120 y1
261.129 b2
276.165 y2
389.221 b3
391.195 y3
488.235 y4
518.268 b4
631.353 b5
744.423 b6
931.497 y8
1044.561 y9
1157.659 y10
b2
b3
b4
b5
b6
m/z
MLKEILKIDSPDTR / File: christine_30turboips_ip16.raw; SpectrumID: ; scans: 8057

ELGVGLALR

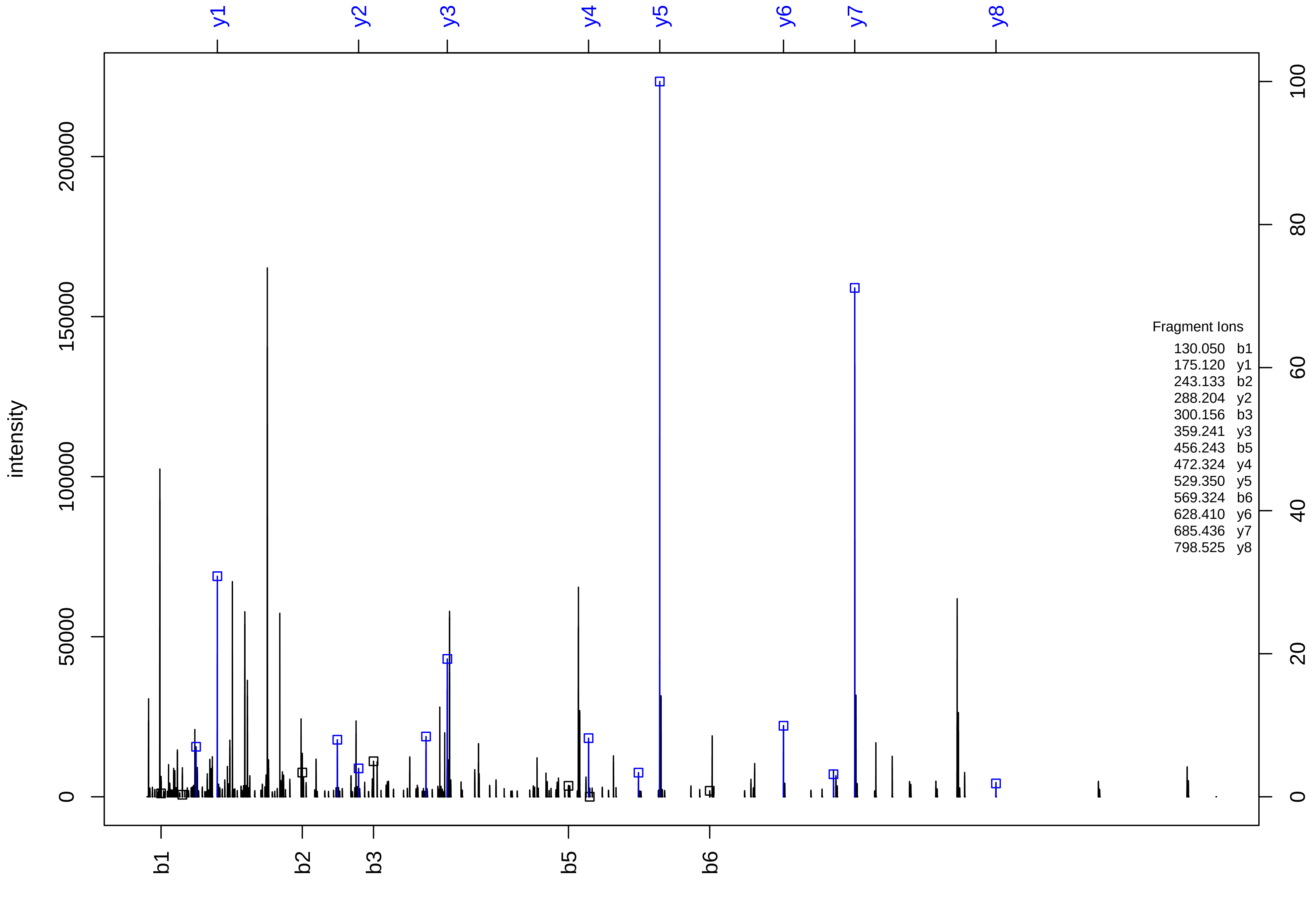

ELGVGLALR / File: christine_30turboips_ip16.raw; SpectrumID: ; scans: 12654

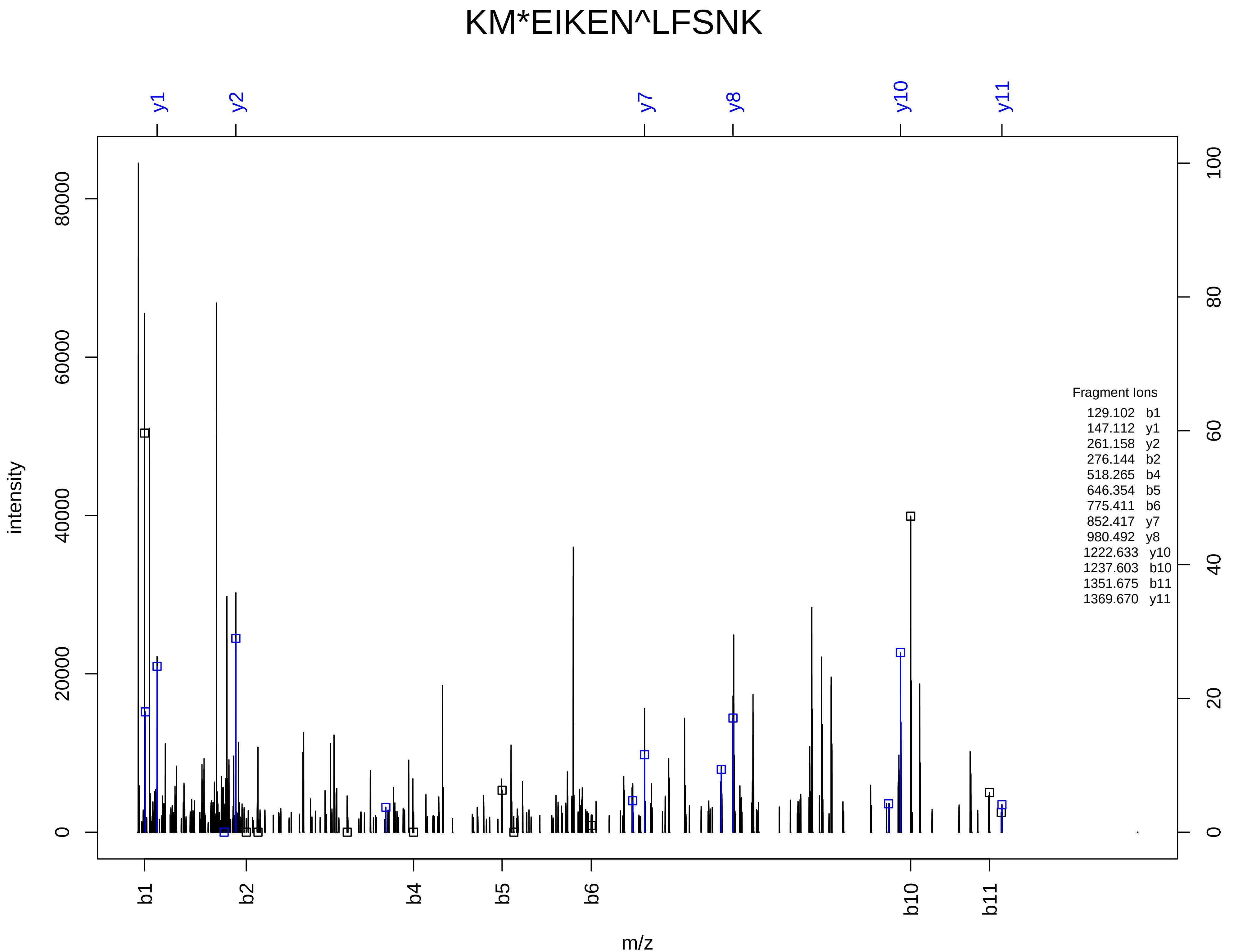

KMEIKENLFSNK / File: christine_30turboips_ip16.raw; SpectrumID: ; scans: 6291

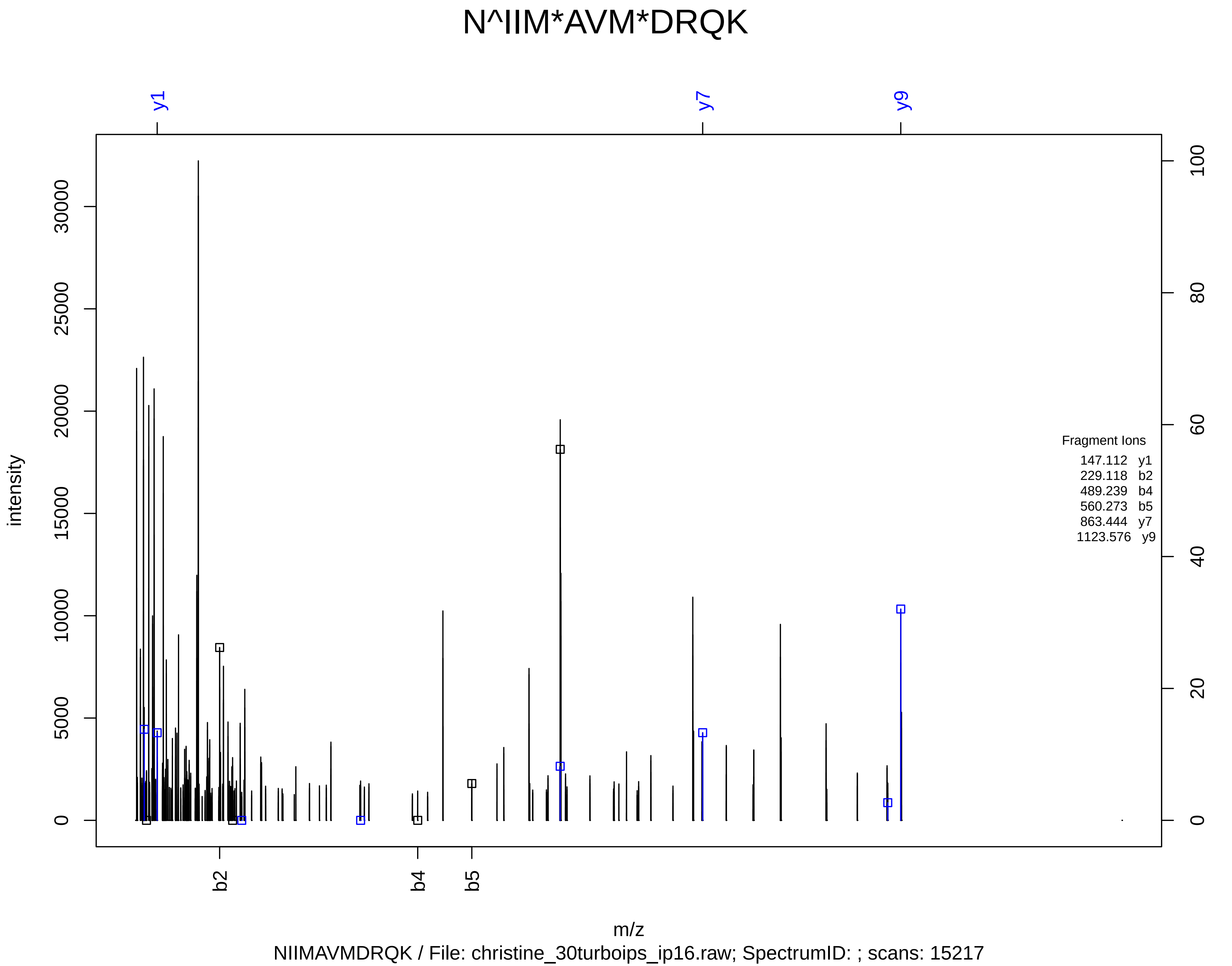

N^IIM*AVM*DRQK
y1
y7
y9
intensity
30000
25000
20000
15000
10000
5000
0
100
80
60
40
20
0
Fragment Ions
147.112 y1
229.118 b2
489.239 b4
560.273 b5
863.444 y7
1123.576 y9
b2
b4
b5
m/z
NIIMAVMDRQK / File: christine_30turboips_ip16.raw; SpectrumID: ; scans: 15217

EDFVANKLGDEVQK

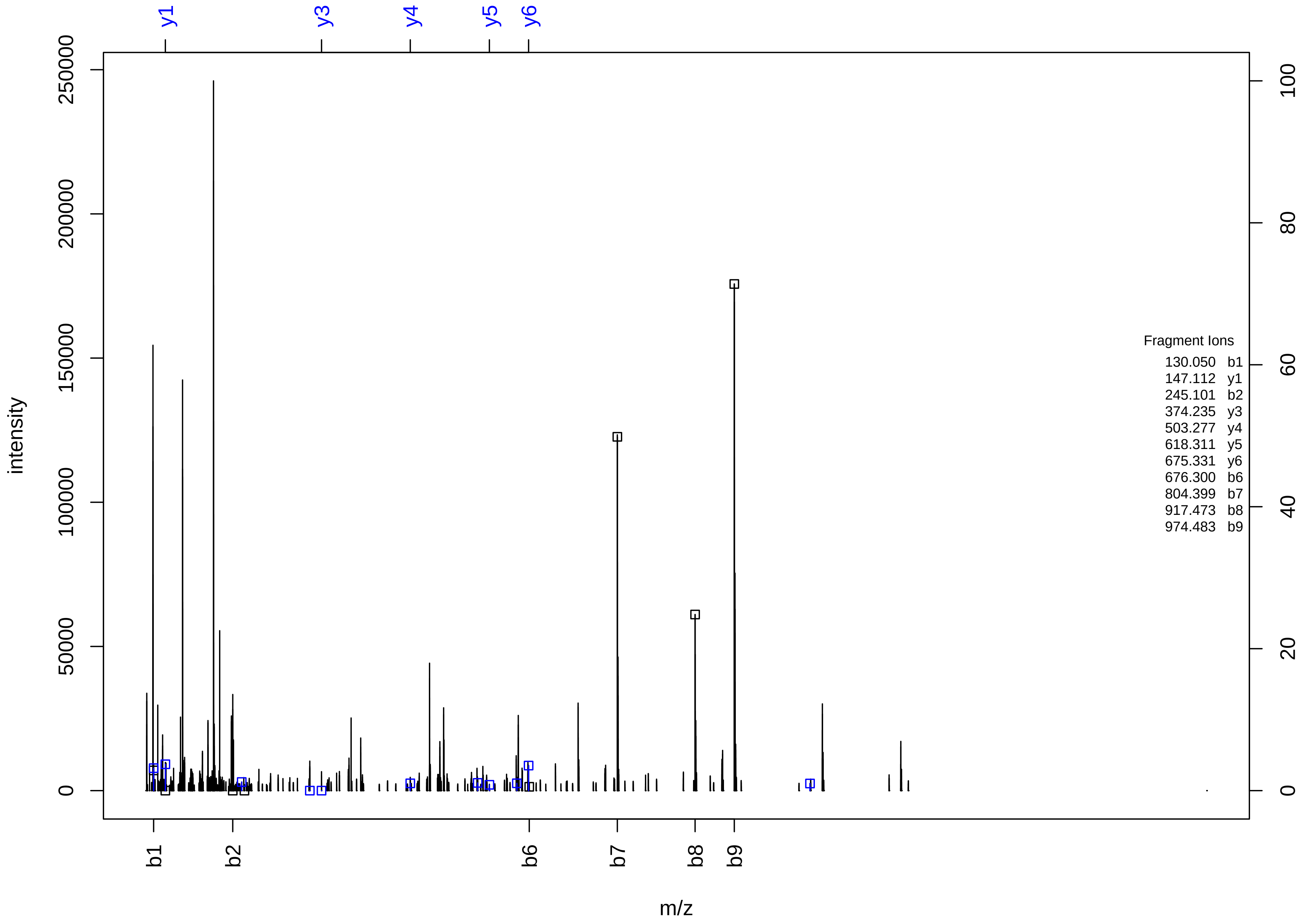

EDFVANKLGDEVQK / File: christine_30turboips_ip16.raw; SpectrumID: ; scans: 4357

VLELEPDNFEATNELR

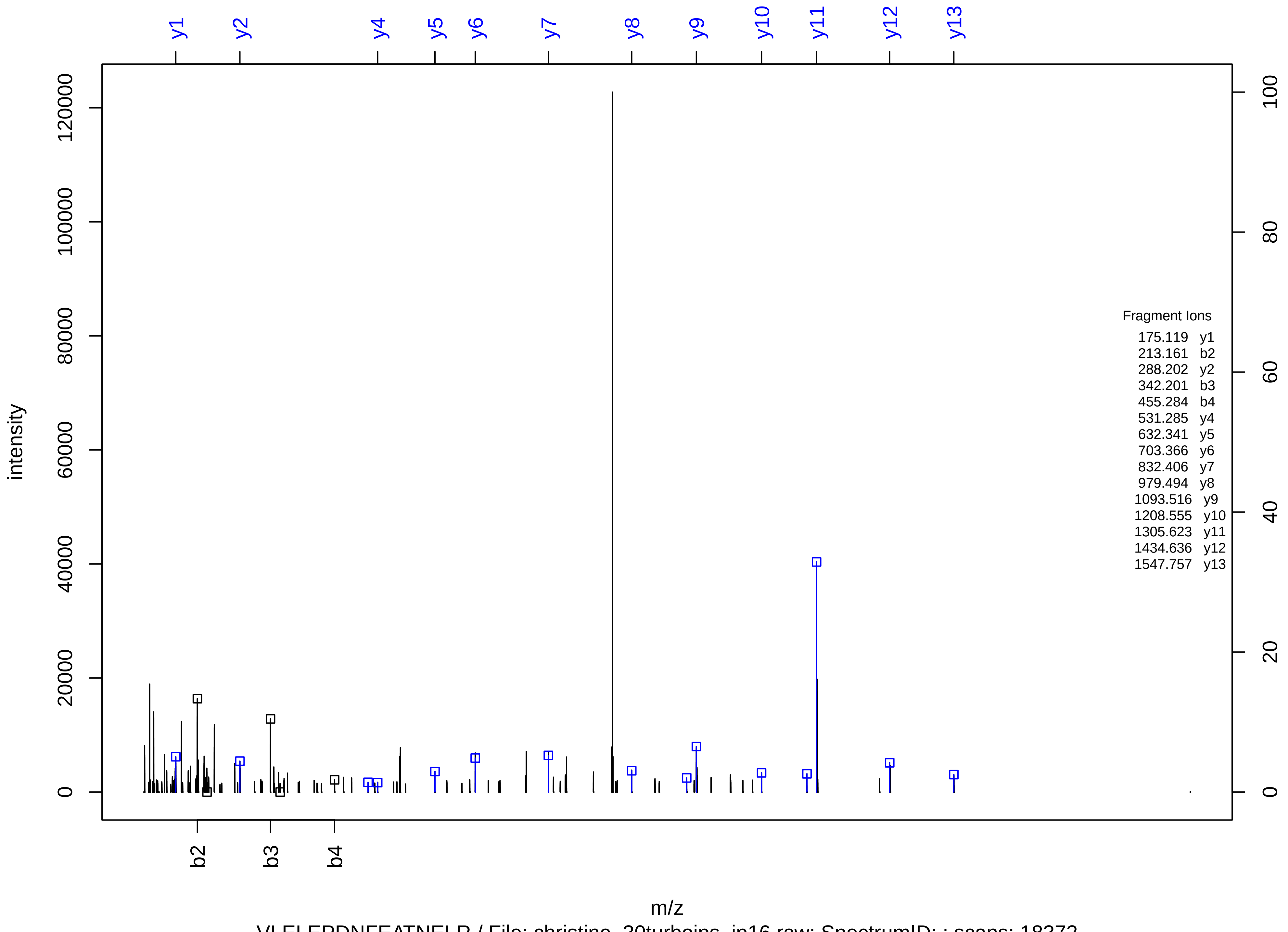

VLELEPDNFEATNELR / File: christine_30turboips_ip16.raw; SpectrumID: ; scans: 18372

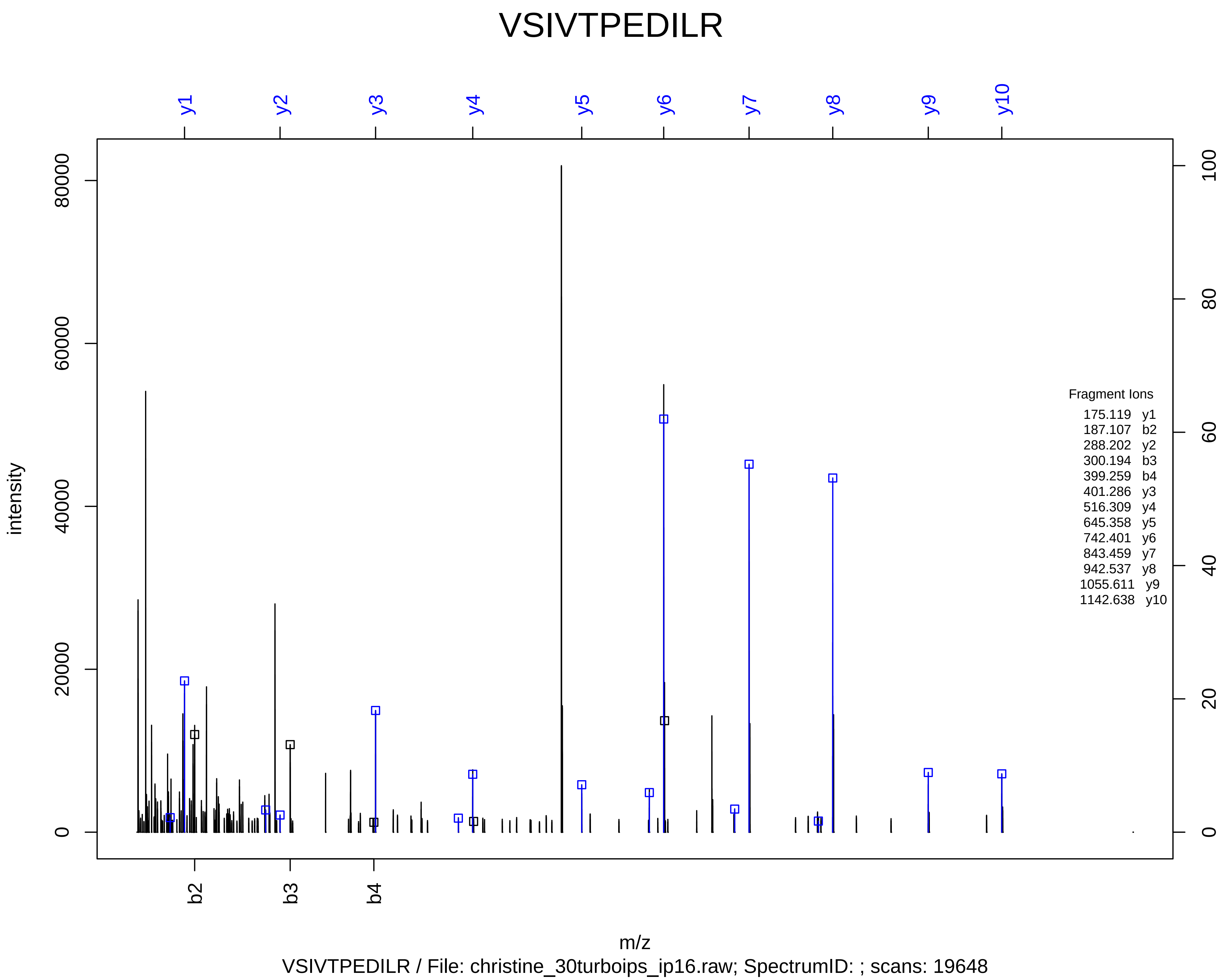
VSIVTPEDILR
y1
y2
y3
y4
y5
y6
y7
y8
y9
y10
b2
b3
b4
intensity
0
20000
40000
60000
80000
0
20
40
60
80
100
m/z
Fragment Ions
175.119 y1
187.107 b2
288.202 y2
300.194 b3
399.259 b4
401.286 y3
516.309 y4
645.358 y5
742.401 y6
843.459 y7
942.537 y8
1055.611 y9
1142.638 y10
VSIVTPEDILR / File: christine_30turboips_ip16.raw; SpectrumID: ; scans: 19648

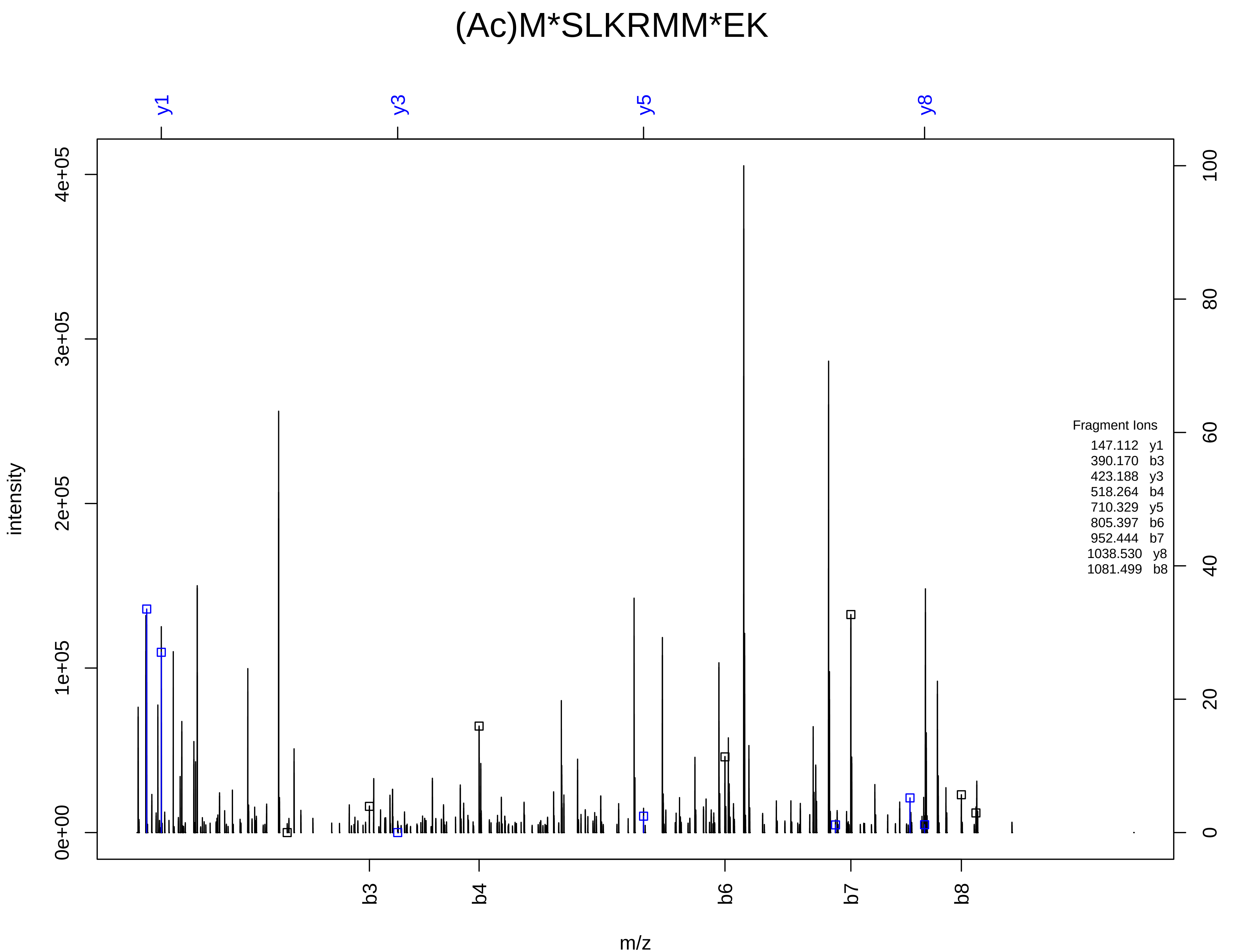

(Ac)M*SLKRMM*EK
y1
y3
y5
y8
intensity
4e+05
3e+05
2e+05
1e+05
0e+00
100
80
60
40
20
0
Fragment Ions
147.112 y1
390.170 b3
423.188 y3
518.264 b4
710.329 y5
805.397 b6
952.444 b7
1038.530 y8
1081.499 b8
b3
b4
b6
b7
b8
m/z
MSLKRMMEK / File: christine_30turboips_ip16.raw; SpectrumID: ; scans: 1832

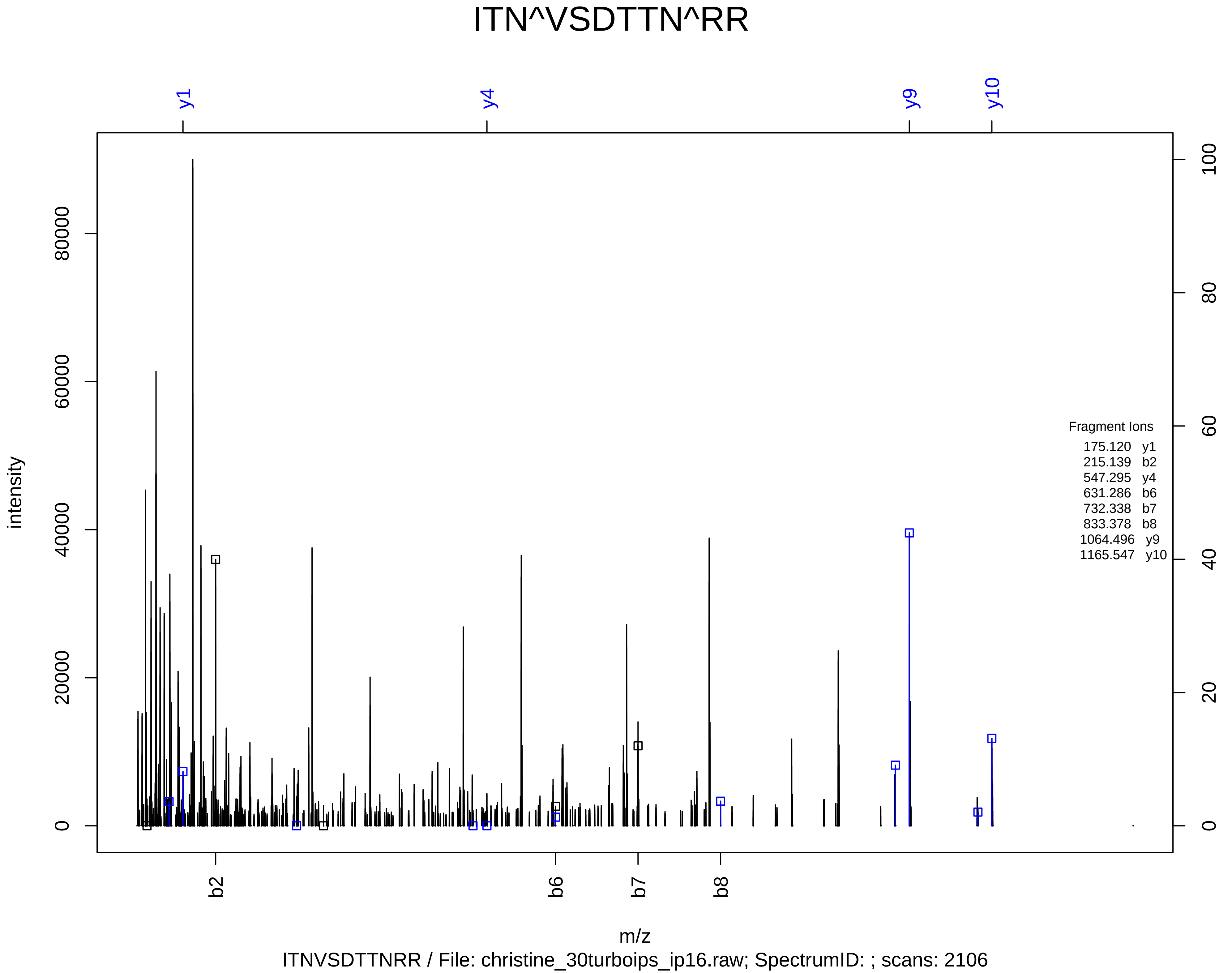

ITN^VSDTTN^RR
y1
y4
y9
y10
intensity
80000
60000
40000
20000
0
100
80
60
40
20
0
Fragment Ions
175.120 y1
215.139 b2
547.295 y4
631.286 b6
732.338 b7
833.378 b8
1064.496 y9
1165.547 y10
b2
b6
b7
b8
m/z
ITNVSDTTNRR / File: christine_30turboips_ip16.raw; SpectrumID: ; scans: 2106

EVAPETEVATAAR

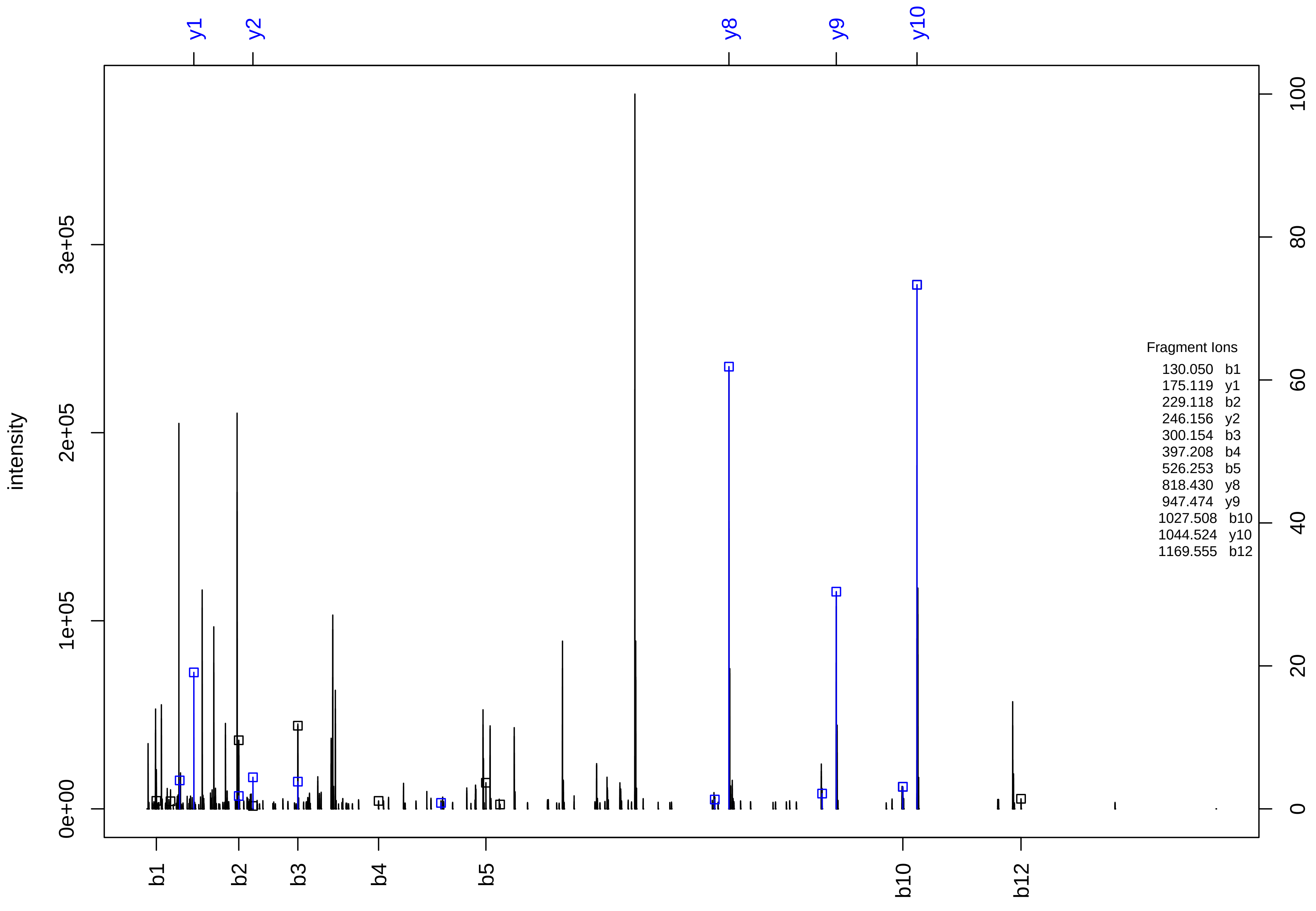

EVAPETEVATAAR / File: christine_30turboips_ip16.raw; SpectrumID: ; scans: 7982

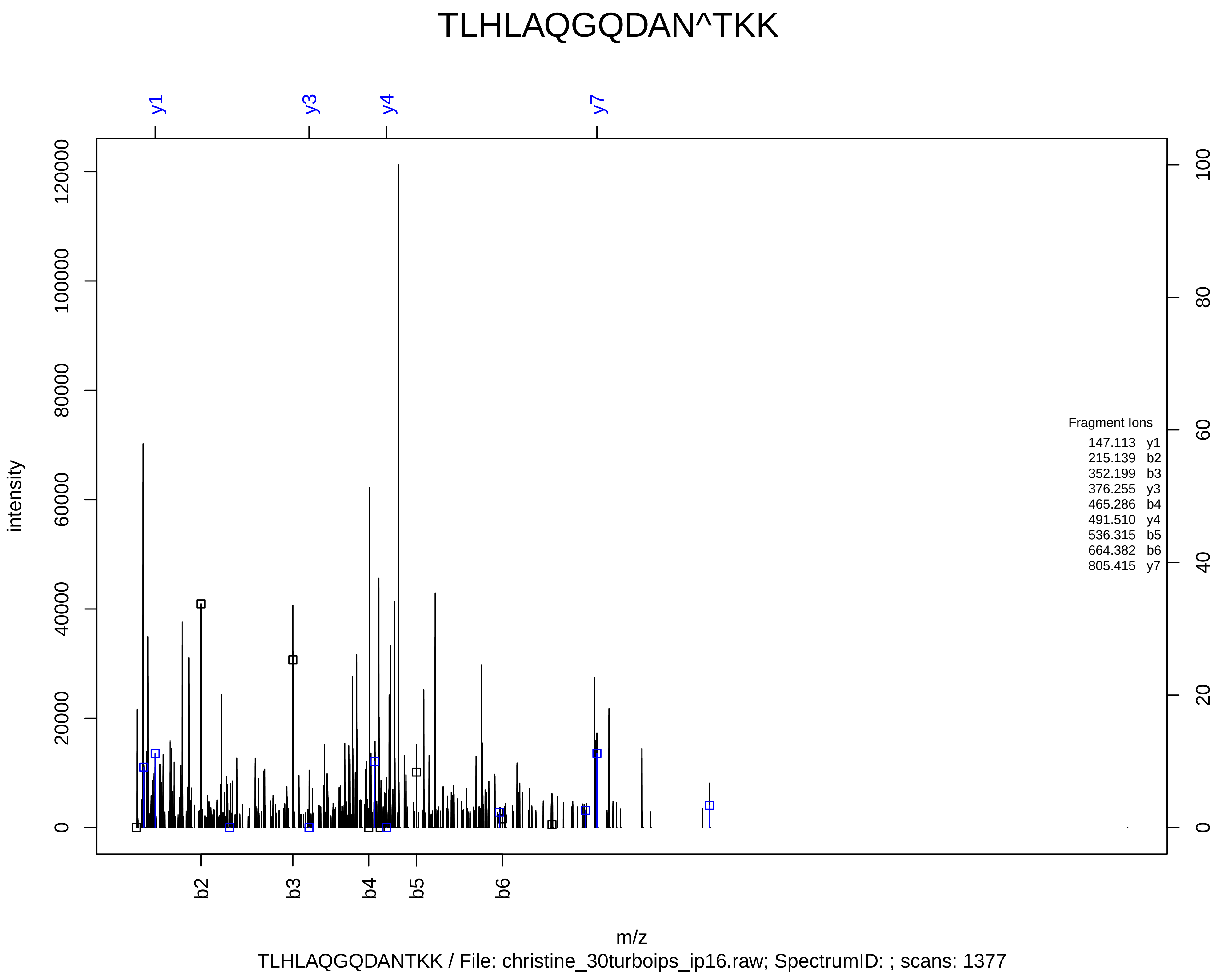

TLHLAQGQDAN^TKK
y1
y3
y4
y7
intensity
120000
100000
80000
60000
40000
20000
0
100
80
60
40
20
0
Fragment Ions
147.113 y1
215.139 b2
352.199 b3
376.255 y3
465.286 b4
491.510 y4
536.315 b5
664.382 b6
805.415 y7
b2
b3
b4
b5
b6
m/z
TLHLAQGQDANTKK / File: christine_30turboips_ip16.raw; SpectrumID: ; scans: 1377

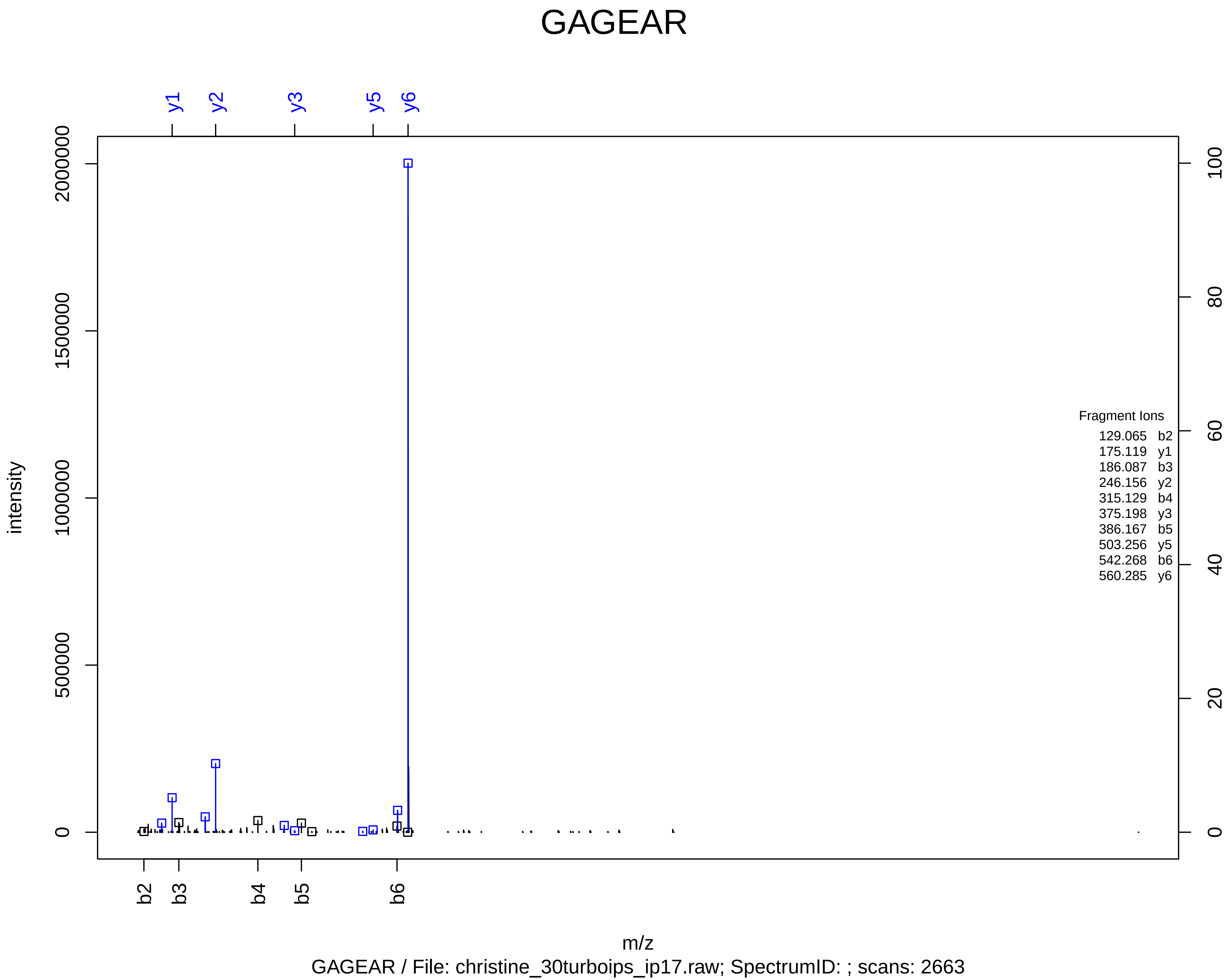
GAGEAR
y1
y2
y3
y5
y6
intensity
2000000
1500000
1000000
500000
0
100
80
60
40
20
0
Fragment Ions
129.065 b2
175.119 y1
186.087 b3
246.156 y2
315.129 b4
375.198 y3
386.167 b5
503.256 y5
542.268 b6
560.285 y6
b2
b3
b4
b5
b6
m/z
GAGEAR / File: christine_30turboips_ip17.raw; SpectrumID: ; scans: 2663

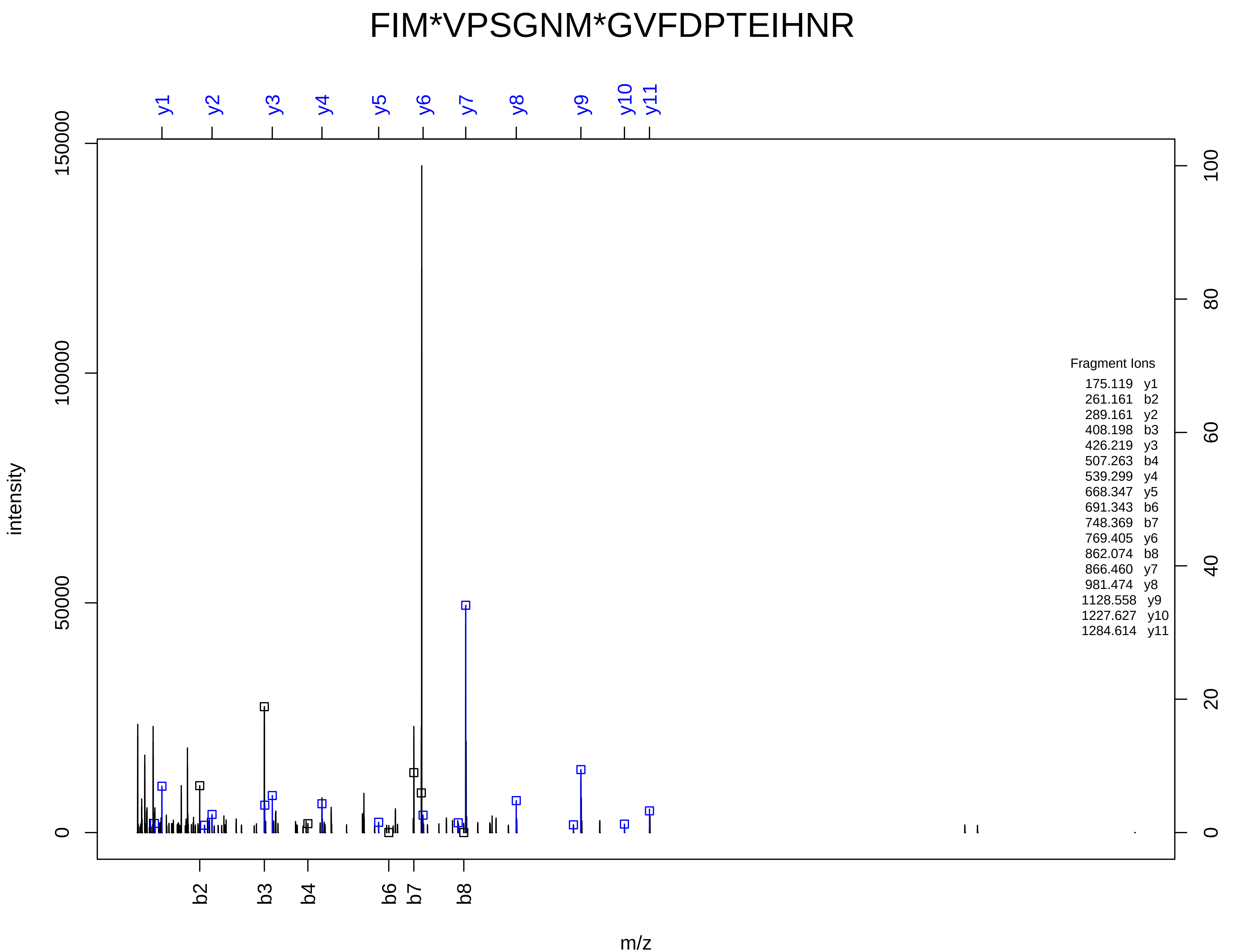
FIM*VPSGNM*GVFDPTEIHNR
intensity
m/z
y1 y2 y3 y4 y5 y6 y7 y8 y9 y10 y11
b2 b3 b4 b6 b7 b8
0 50000 100000 150000
0 20 40 60 80 100
Fragment Ions
175.119 y1
261.161 b2
289.161 y2
408.198 b3
426.219 y3
507.263 b4
539.299 y4
668.347 y5
691.343 b6
748.369 b7
769.405 y6
862.074 b8
866.460 y7
981.474 y8
1128.558 y9
1227.627 y10
1284.614 y11
FIMVPSGNMGVFDPTEIHNR / File: christine_30turboips_ip17.raw; SpectrumID: ; scans: 14026

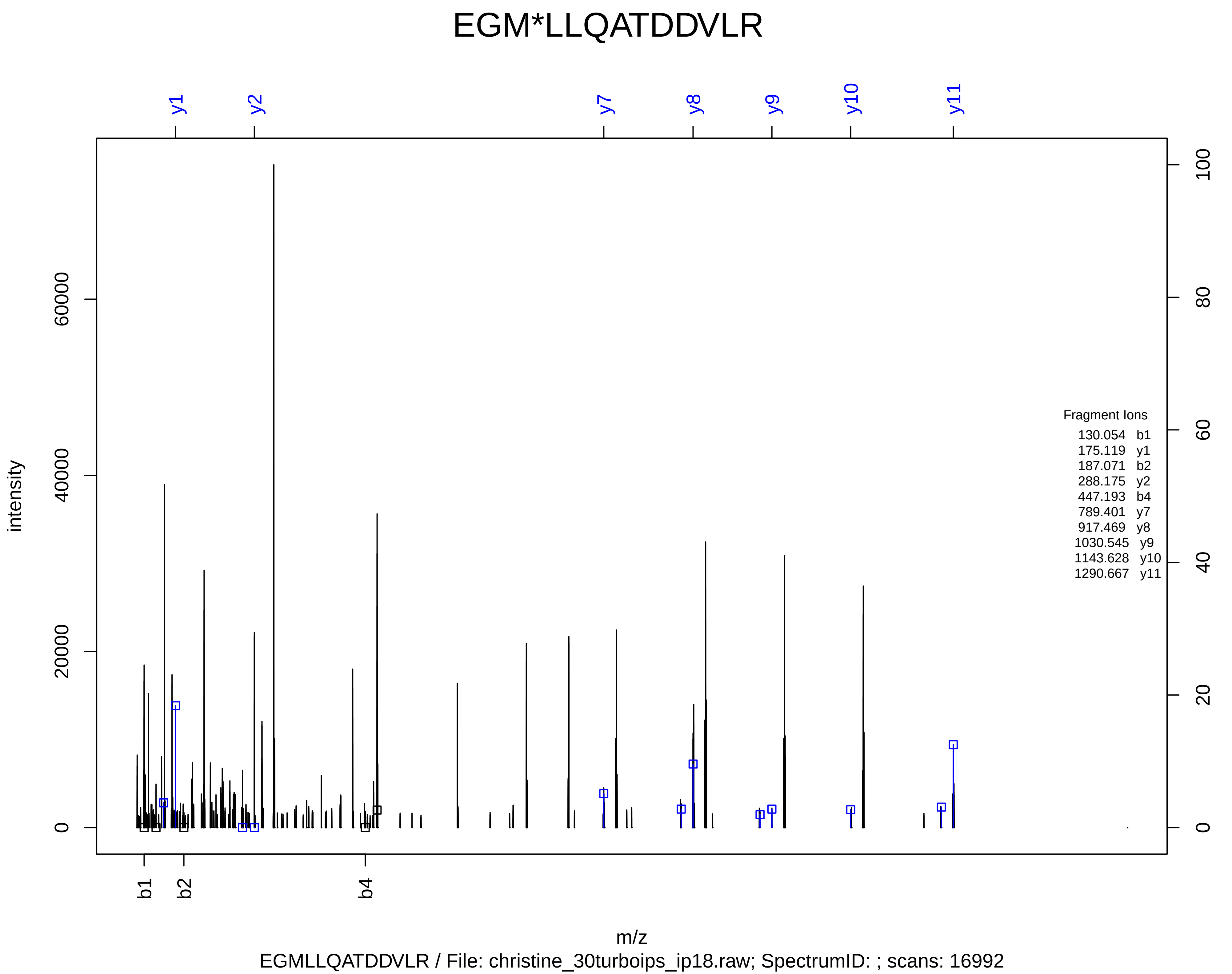
EGM*LLQATDDVLR
y1
y2
y7
y8
y9
y10
y11
intensity
0
20000
40000
60000
0
20
40
60
80
100
Fragment Ions
130.054 b1
175.119 y1
187.071 b2
288.175 y2
447.193 b4
789.401 y7
917.469 y8
1030.545 y9
1143.628 y10
1290.667 y11
b1
b2
b4
m/z
EGMLLQATDDVLR / File: christine_30turboips_ip18.raw; SpectrumID: ; scans: 16992

AYDYVECPVTGAR

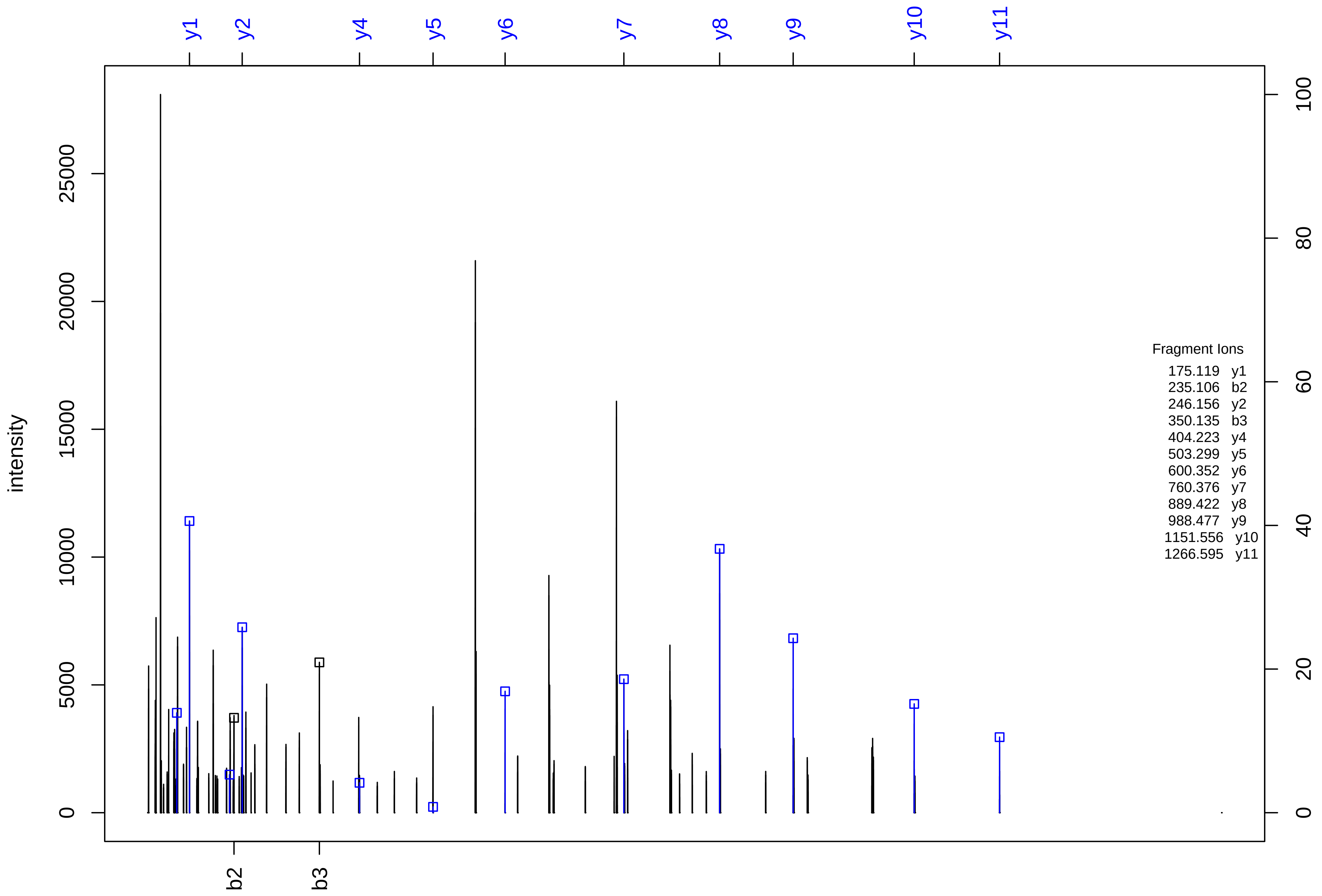

AYDYVECPVTGAR / File: christine_30turboips_ip18.raw; SpectrumID: ; scans: 6034

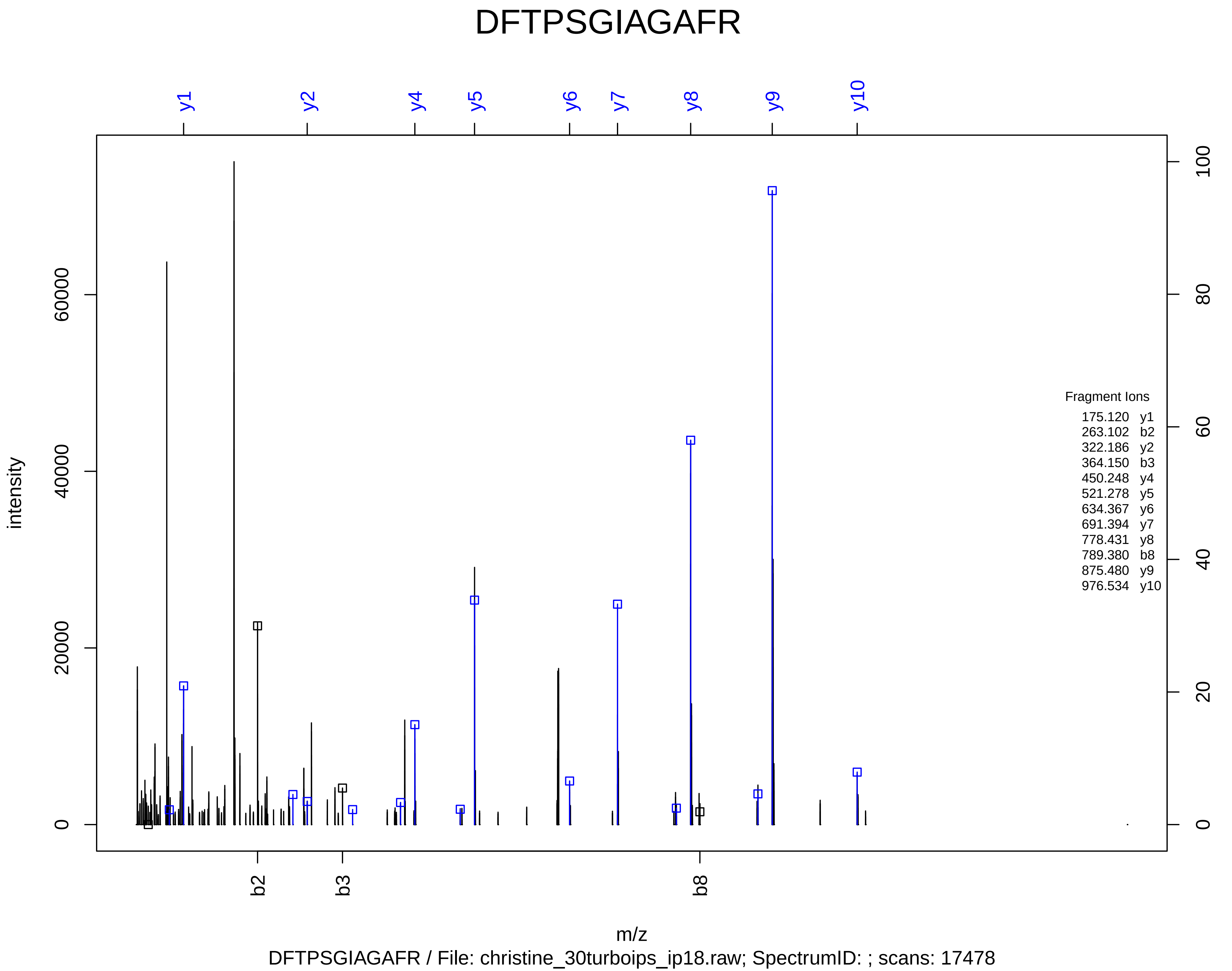

DFTPSGIAGAFR
y1
y2
y4
y5
y6
y7
y8
y9
y10
intensity
0
20000
40000
60000
0
20
40
60
80
100
Fragment Ions
175.120 y1
263.102 b2
322.186 y2
364.150 b3
450.248 y4
521.278 y5
634.367 y6
691.394 y7
778.431 y8
789.380 b8
875.480 y9
976.534 y10
b2
b3
b8
m/z
DFTPSGIAGAFR / File: christine_30turboips_ip18.raw; SpectrumID: ; scans: 17478

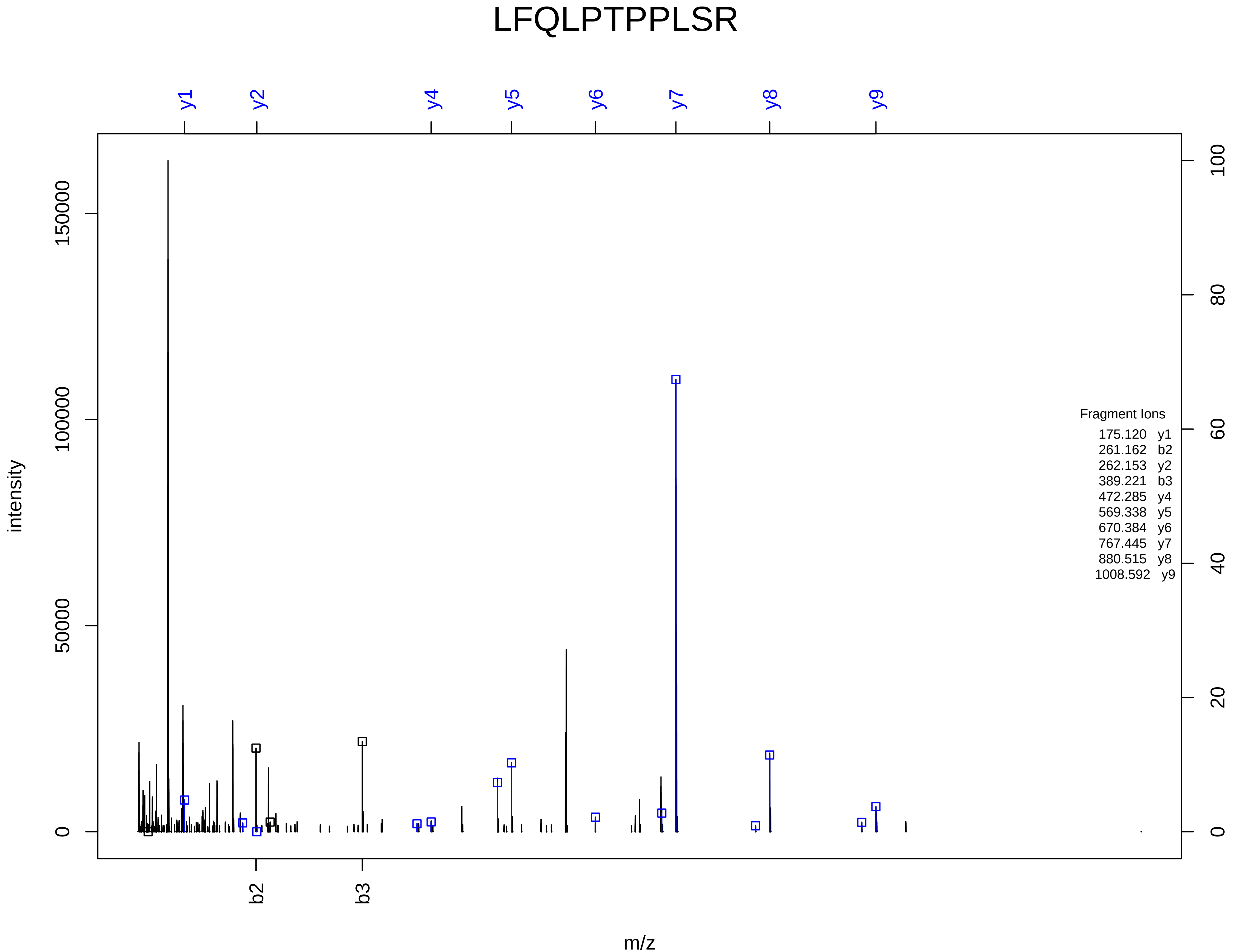

LFQLPTPPLSR / File: christine_30turboips_ip18.raw; SpectrumID: ; scans: 19490

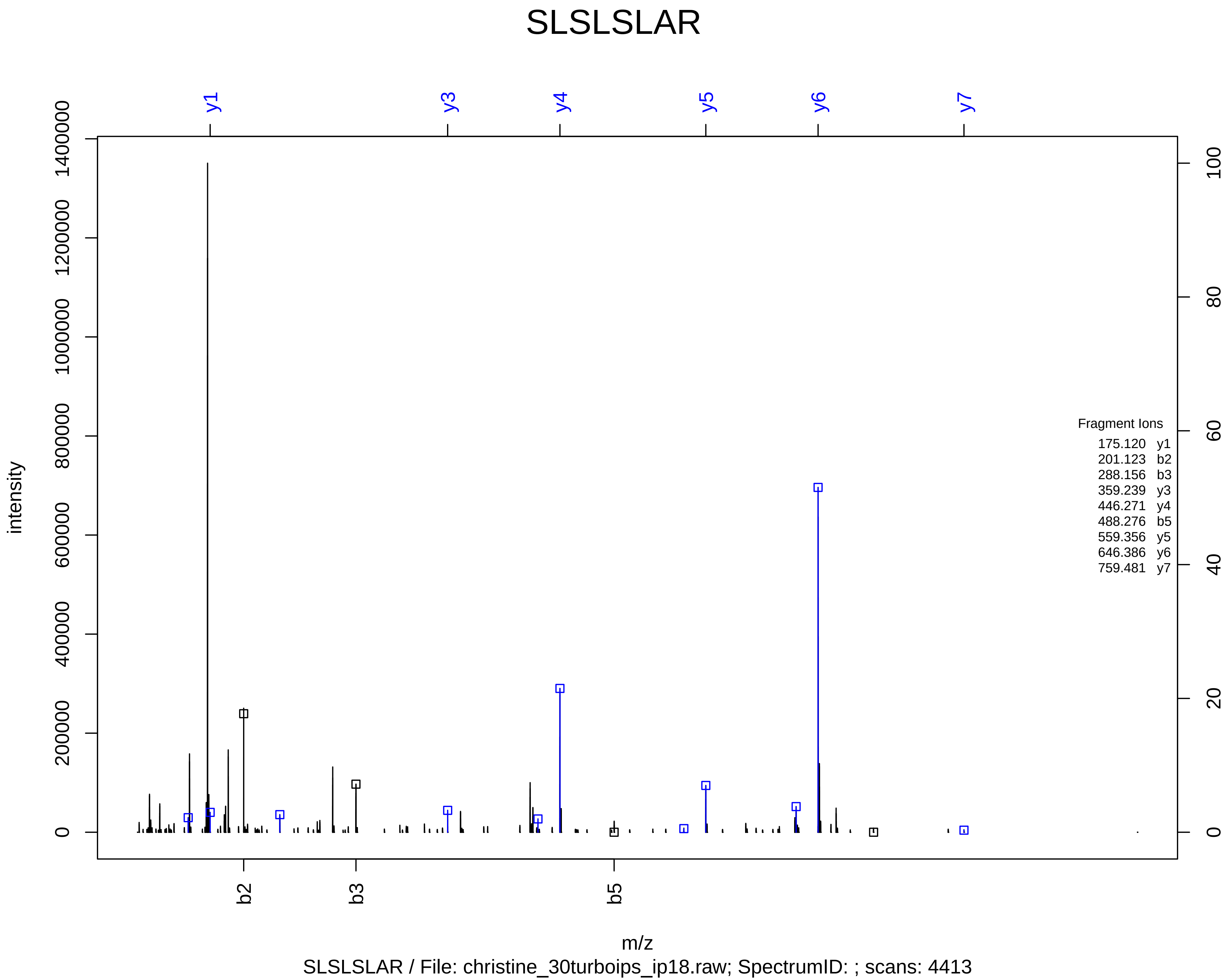
SLSLSLAR
y1
y3
y4
y5
y6
y7
intensity
0
200000
400000
600000
800000
1000000
1200000
1400000
0
20
40
60
80
100
Fragment Ions
175.120 y1
201.123 b2
288.156 b3
359.239 y3
446.271 y4
488.276 b5
559.356 y5
646.386 y6
759.481 y7
b2
b3
b5
m/z
SLSLSLAR / File: christine_30turboips_ip18.raw; SpectrumID: ; scans: 4413

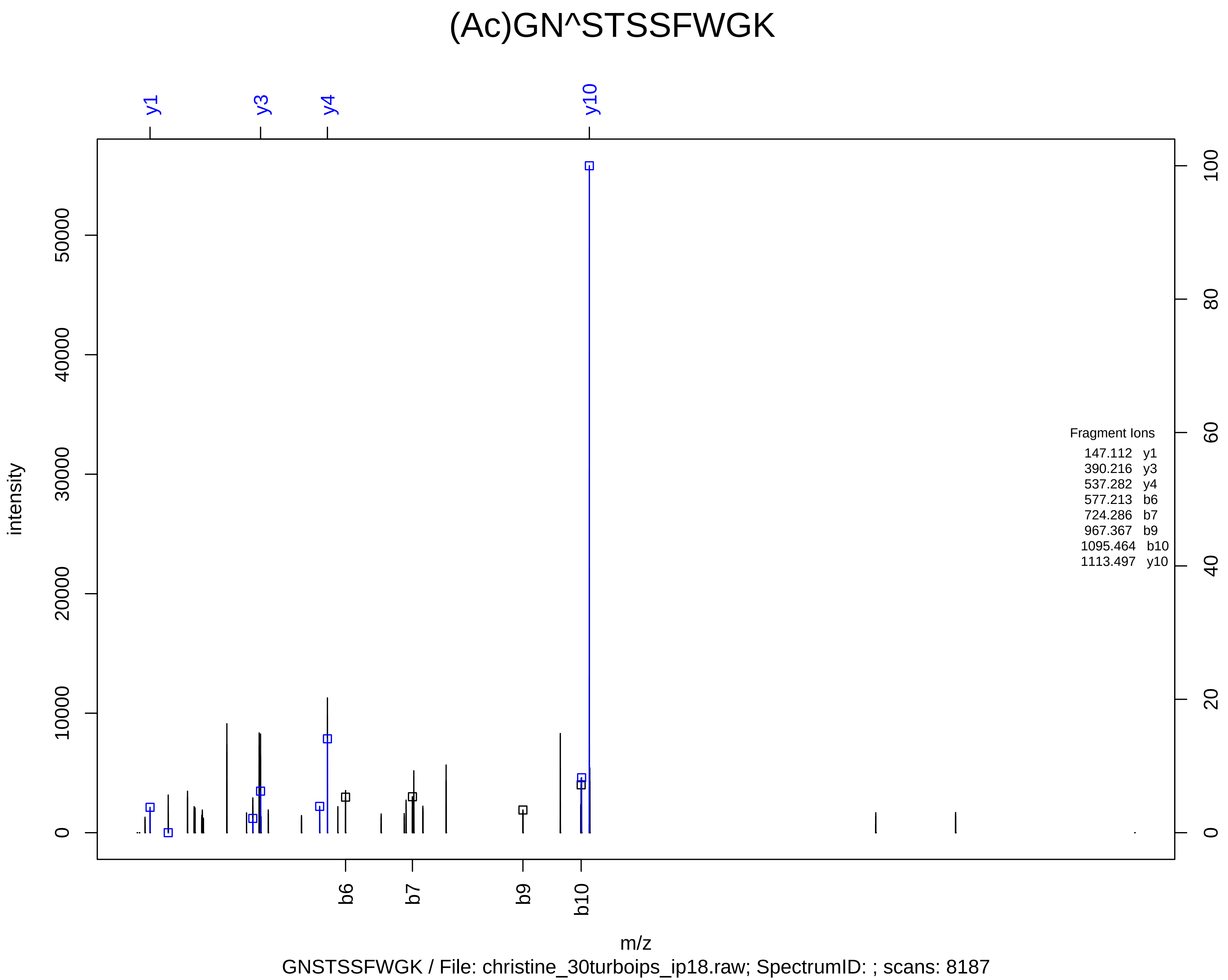

(Ac)GN^STSSFWGK
y1
y3
y4
y10
intensity
0
10000
20000
30000
40000
50000
0
20
40
60
80
100
Fragment Ions
147.112 y1
390.216 y3
537.282 y4
577.213 b6
724.286 b7
967.367 b9
1095.464 b10
1113.497 y10
b6
b7
b9
b10
m/z
GNSTSSFWGK / File: christine_30turboips_ip18.raw; SpectrumID: ; scans: 8187

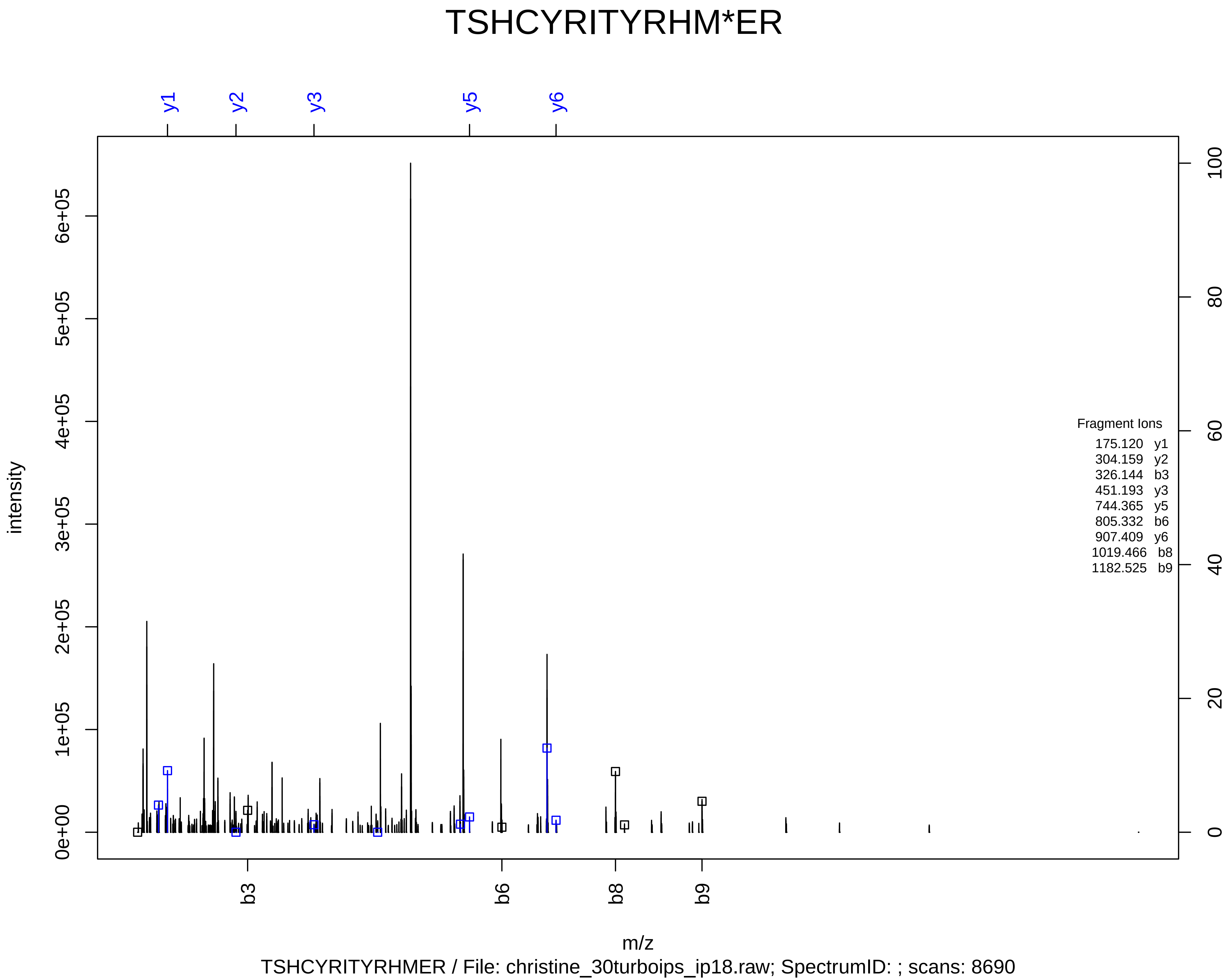
TSHCYRITYRHM*ER
y1
y2
y3
y5
y6
intensity
0e+00
1e+05
2e+05
3e+05
4e+05
5e+05
6e+05
0
20
40
60
80
100
Fragment Ions
175.120 y1
304.159 y2
326.144 b3
451.193 y3
744.365 y5
805.332 b6
907.409 y6
1019.466 b8
1182.525 b9
b3
b6
b8
b9
m/z
TSHCYRITYRHMER / File: christine_30turboips_ip18.raw; SpectrumID: ; scans: 8690

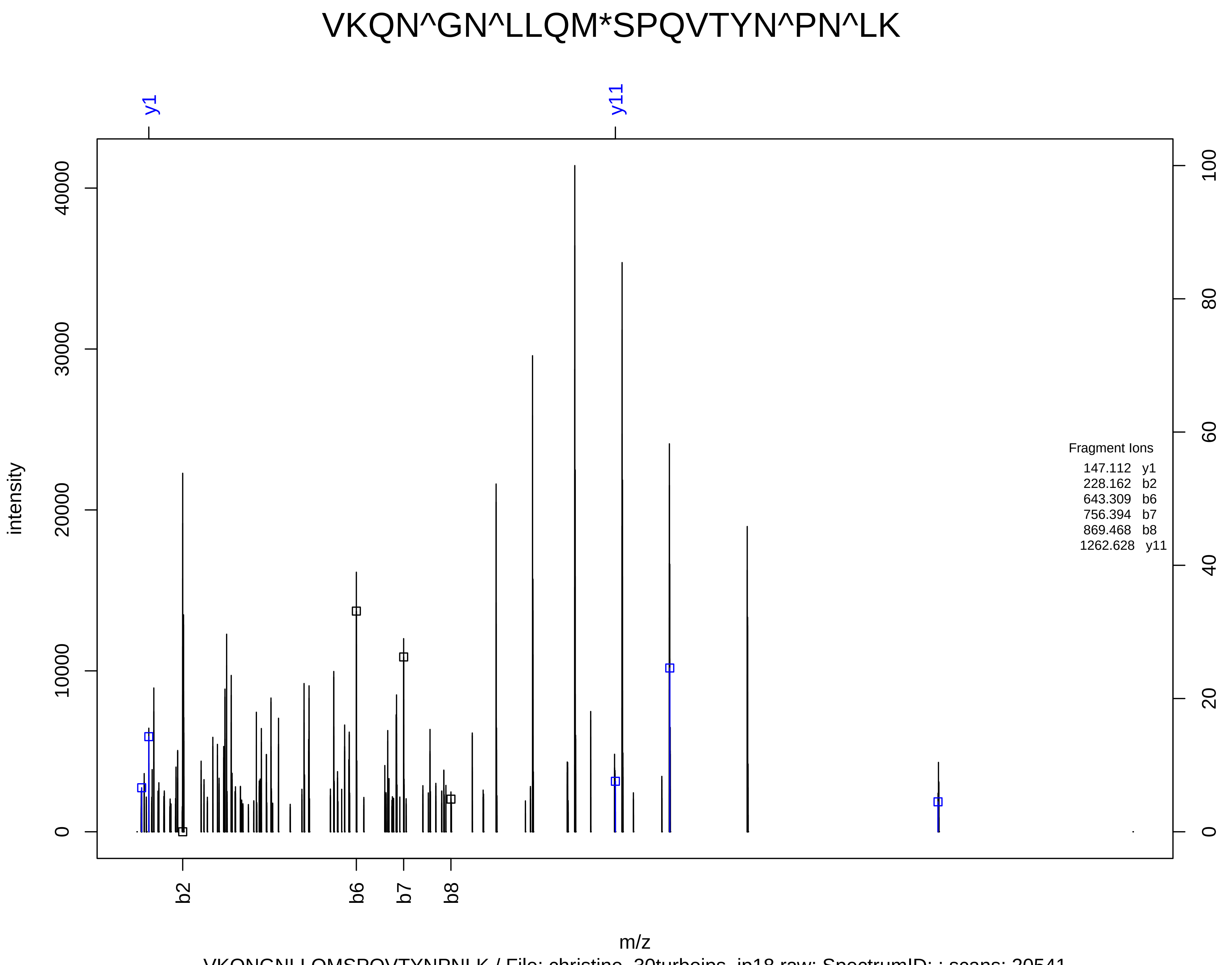
VKQN^GN^LLQM*SPQVTYN^PN^LK
y1
y11
intensity
40000
30000
20000
10000
0
100
80
60
40
20
0
Fragment Ions
147.112 y1
228.162 b2
643.309 b6
756.394 b7
869.468 b8
1262.628 y11
b2
b6
b7
b8
m/z
VKQNGNLLQMSPQVTYNPNLK / File: christine_30turboips_ip18.raw; SpectrumID: ; scans: 20541

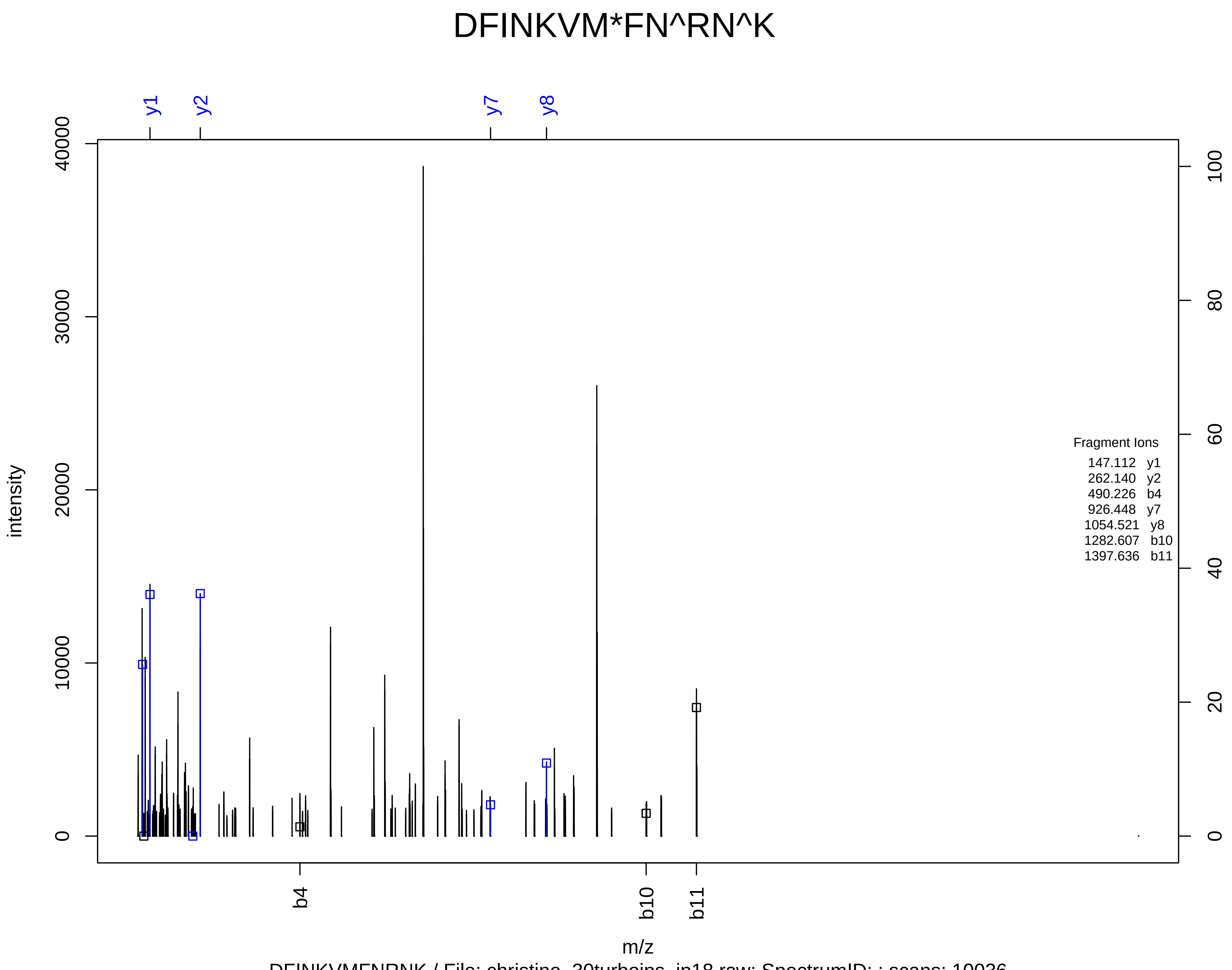
DFINKVM*FN^RN^K
y1
y2
y7
y8
intensity
40000
30000
20000
10000
0
100
80
60
40
20
0
Fragment Ions
147.112 y1
262.140 y2
490.226 b4
926.448 y7
1054.521 y8
1282.607 b10
1397.636 b11
b4
b10
b11
m/z
DFINKVMFNRNK / File: christine_30turboips_ip18.raw; SpectrumID: ; scans: 10036

CVVGSCAGMDVEGLQR

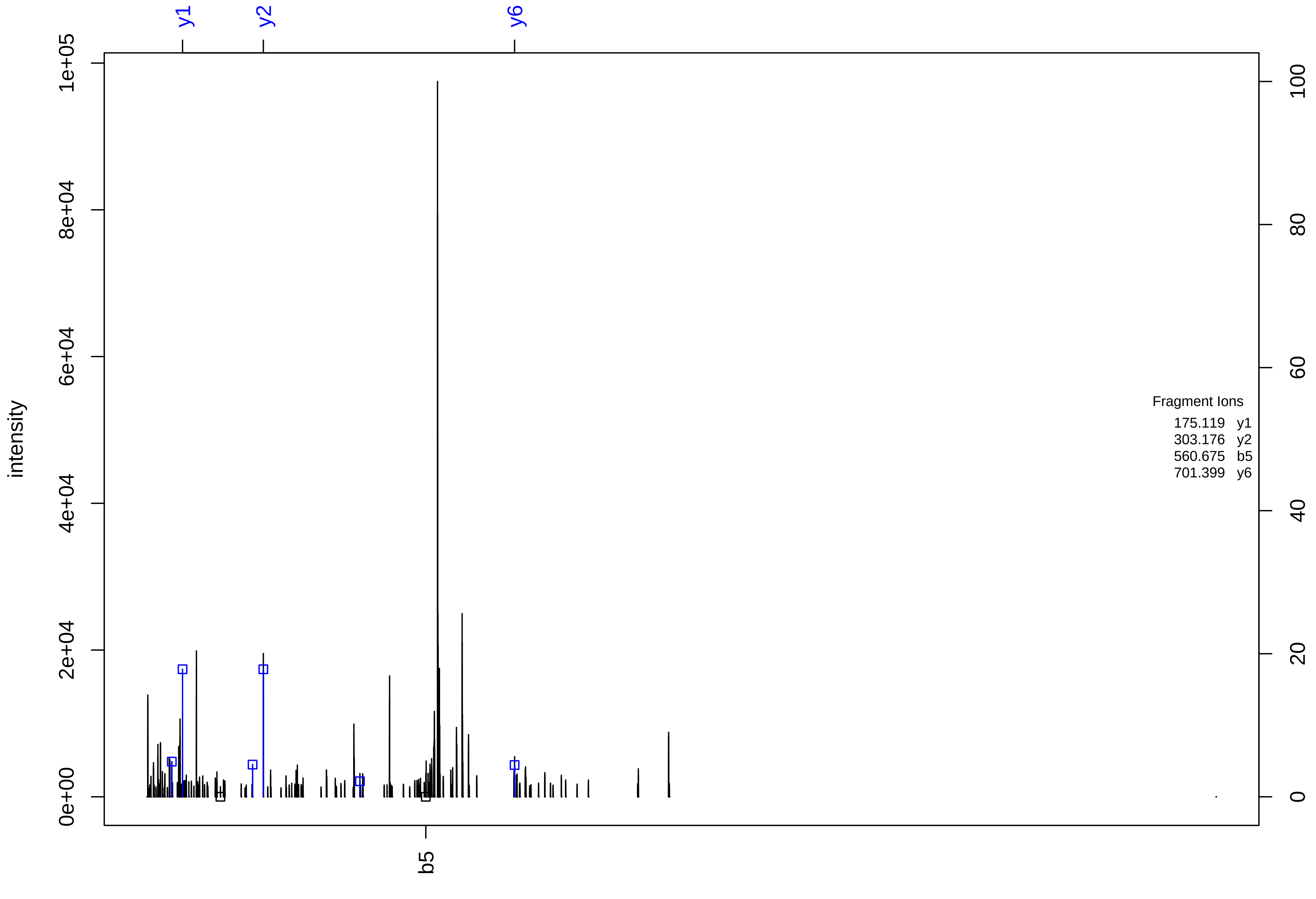

CVVGSCAGMDVEGLQR / File: christine_30turboips_ip19.raw; SpectrumID: ; scans: 17421

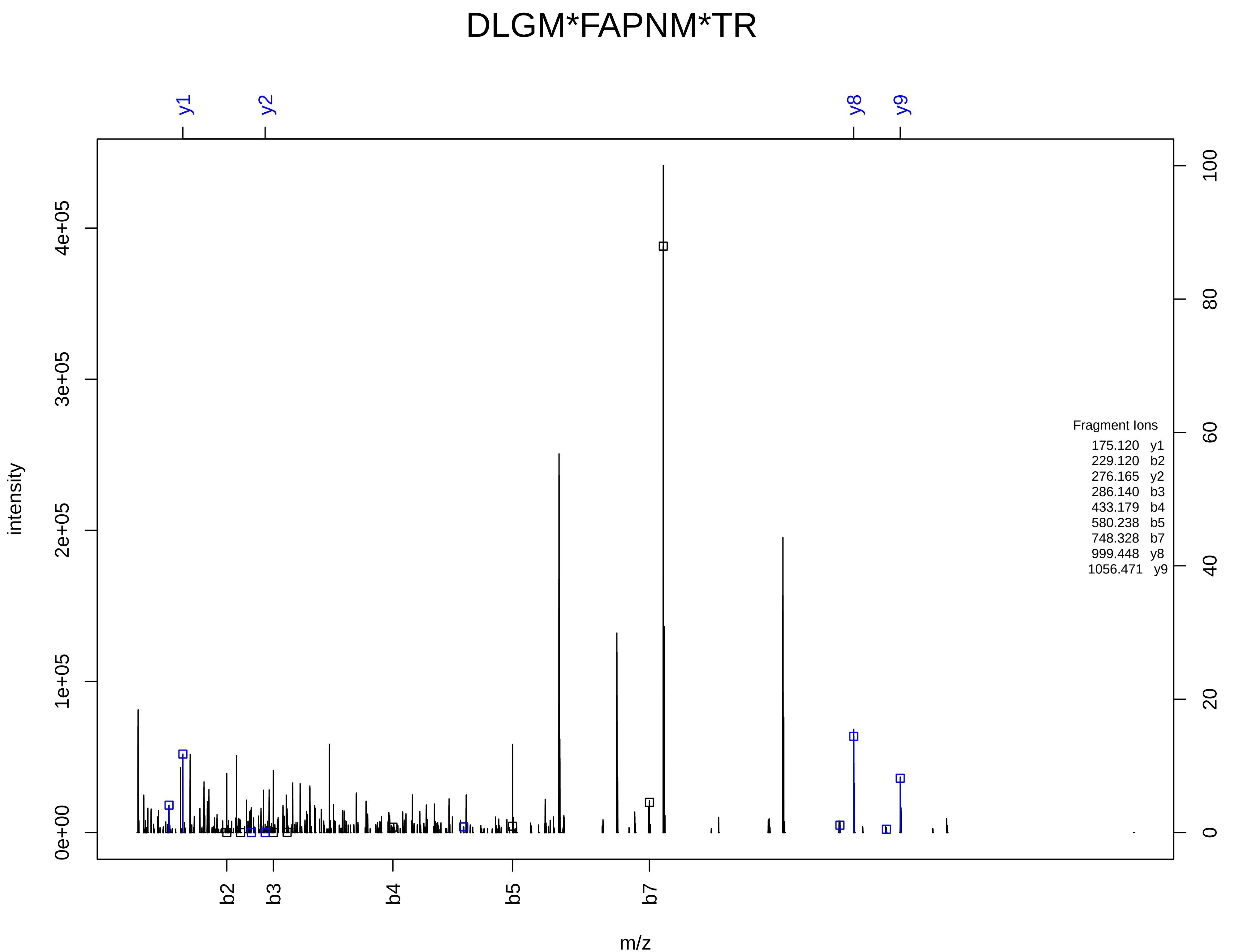

DLGM*FAPNM*TR
y1
y2
y8
y9
intensity
0e+00
1e+05
2e+05
3e+05
4e+05
0
20
40
60
80
100
b2
b3
b4
b5
b7
m/z
Fragment Ions
175.120 y1
229.120 b2
276.165 y2
286.140 b3
433.179 b4
580.238 b5
748.328 b7
999.448 y8
1056.471 y9
DLGMFAPNMTR / File: christine_30turboips_ip19.raw; SpectrumID: ; scans: 2254

EMETLVEAVN^K

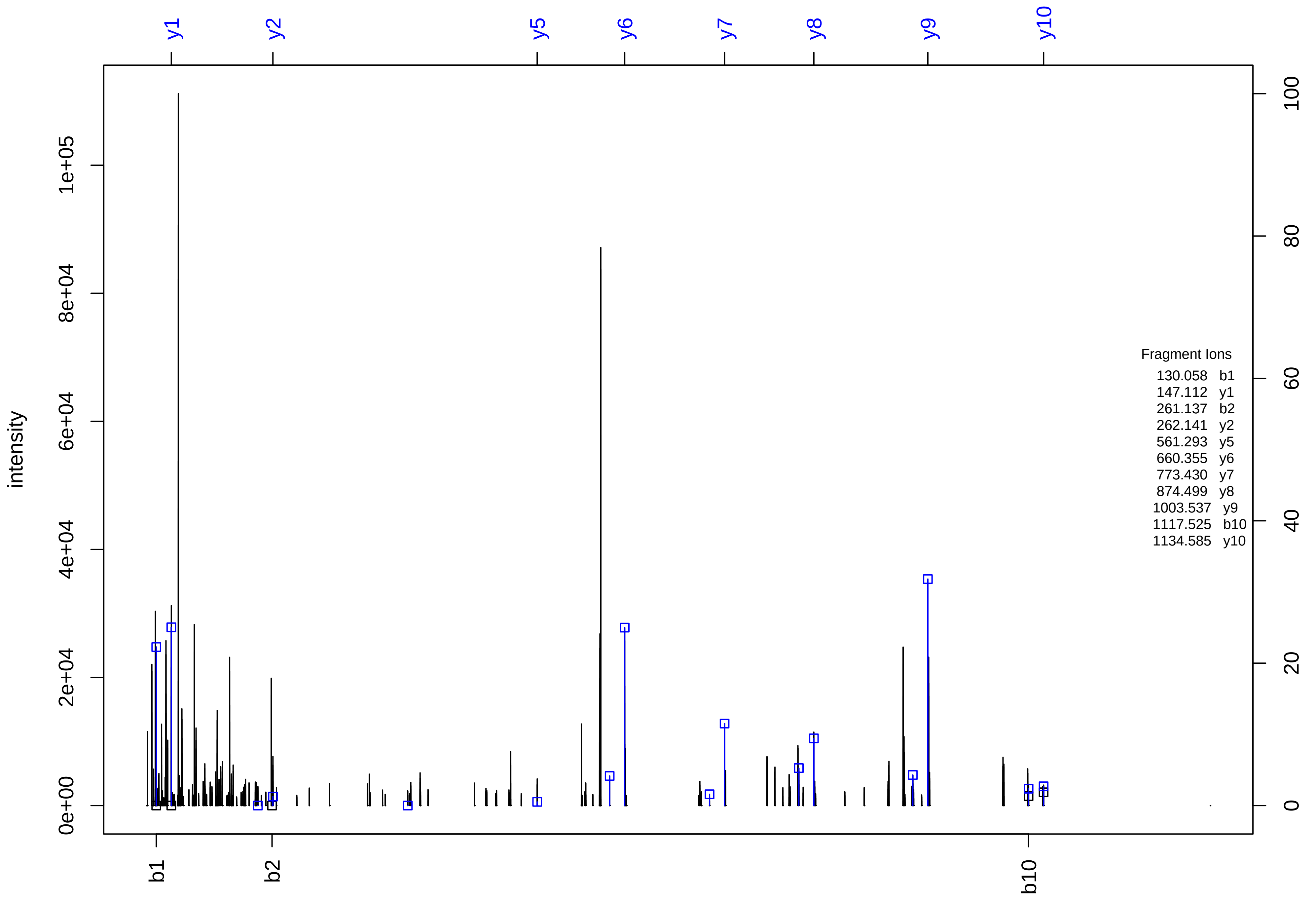

EMETLVEAVNK / File: christine_30turboips_ip19.raw; SpectrumID: ; scans: 13523

QN^IHSLFPVLIDTKN^VTK

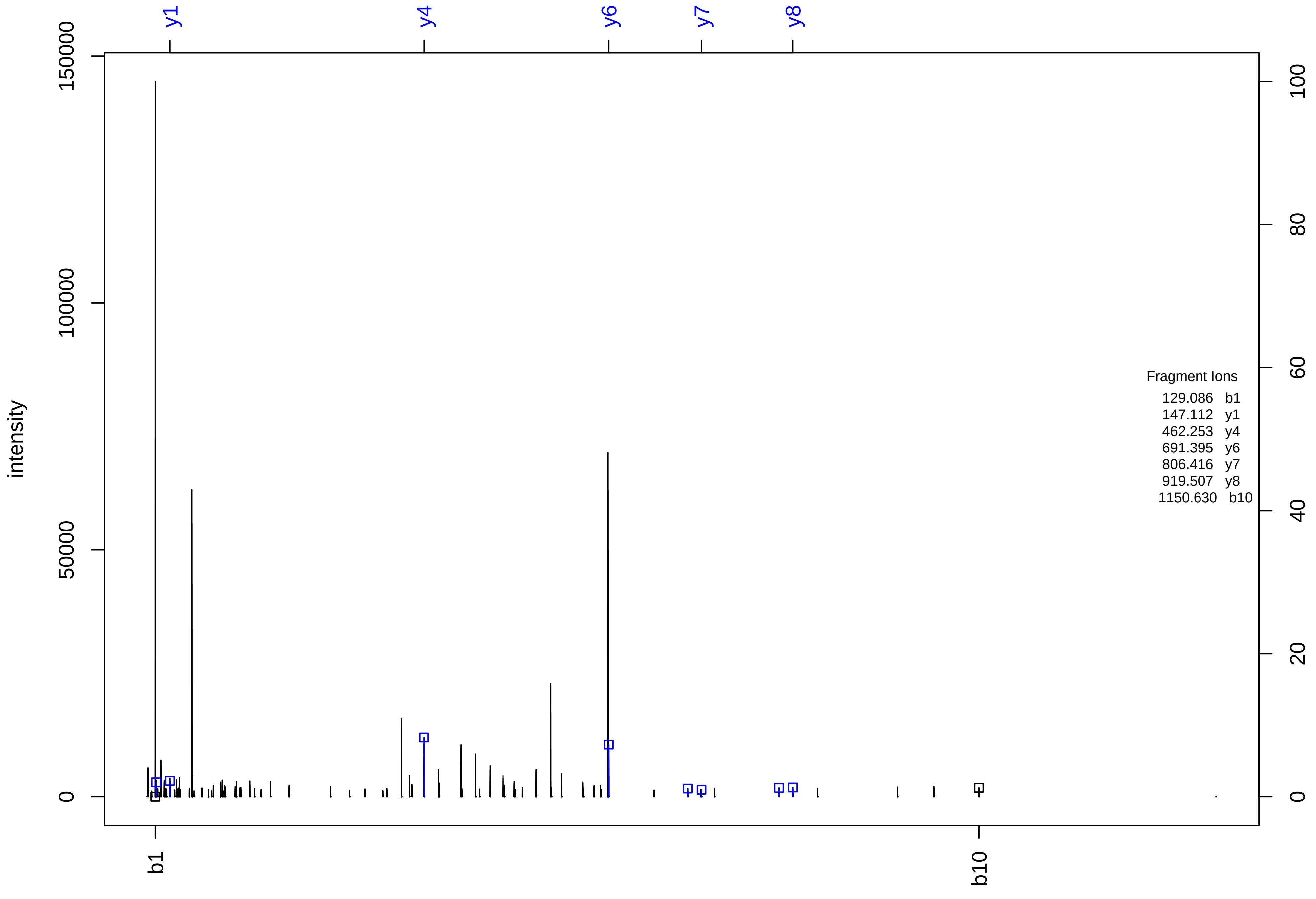

QNIHSLFPVLIDTKNVTK / File: christine_30turboips_ip19.raw; SpectrumID: ; scans: 12144

ANPYECGFDPTSSAR

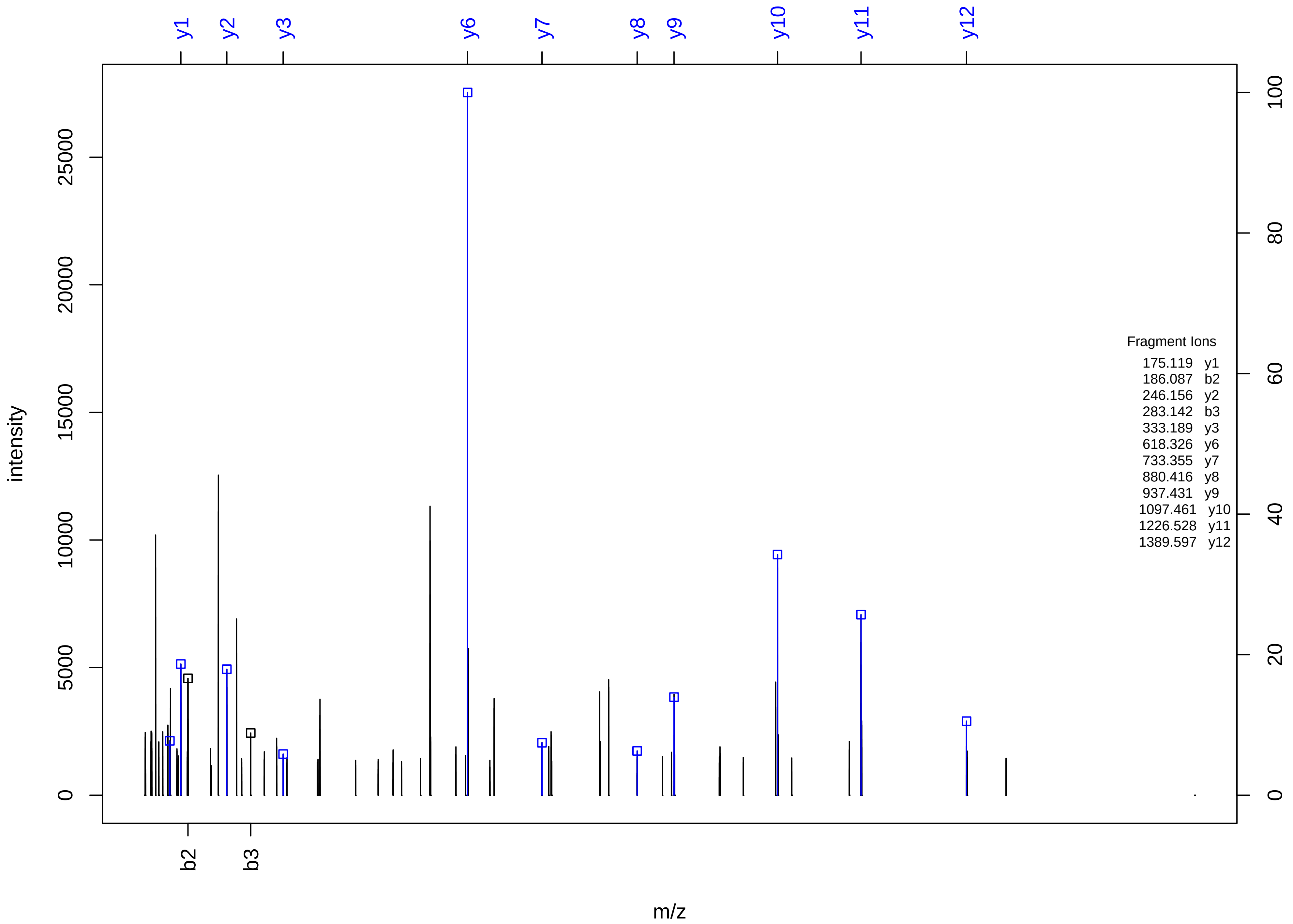

ANPYECGFDPTSSAR / File: christine_30turboips_ip19.raw; SpectrumID: ; scans: 6493

VDATEESDLAQQYGVR

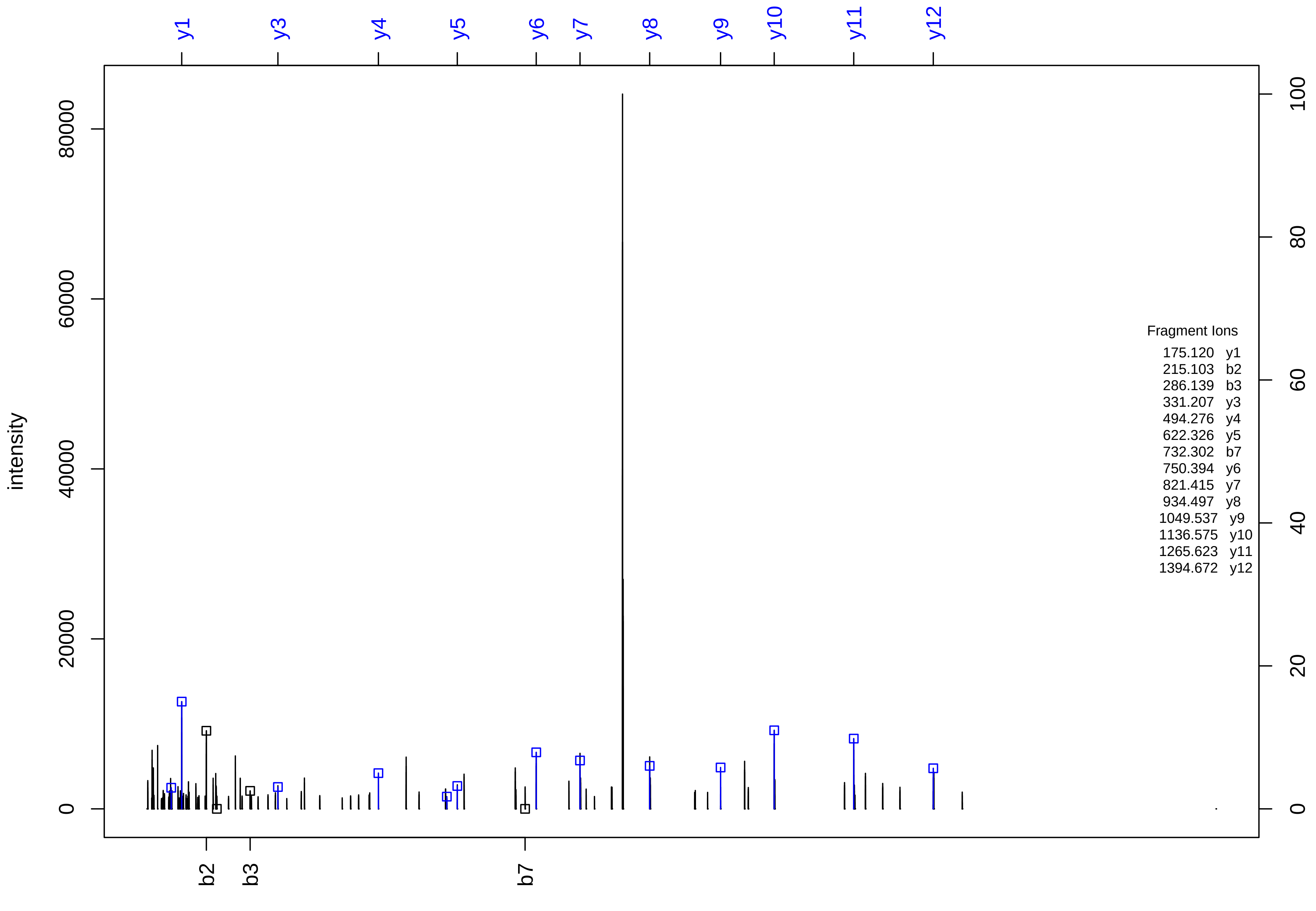

VDATEESDLAQQYGVR / File: christine_30turboips_ip19.raw; SpectrumID: ; scans: 9543

LIHIPINNIIVGG

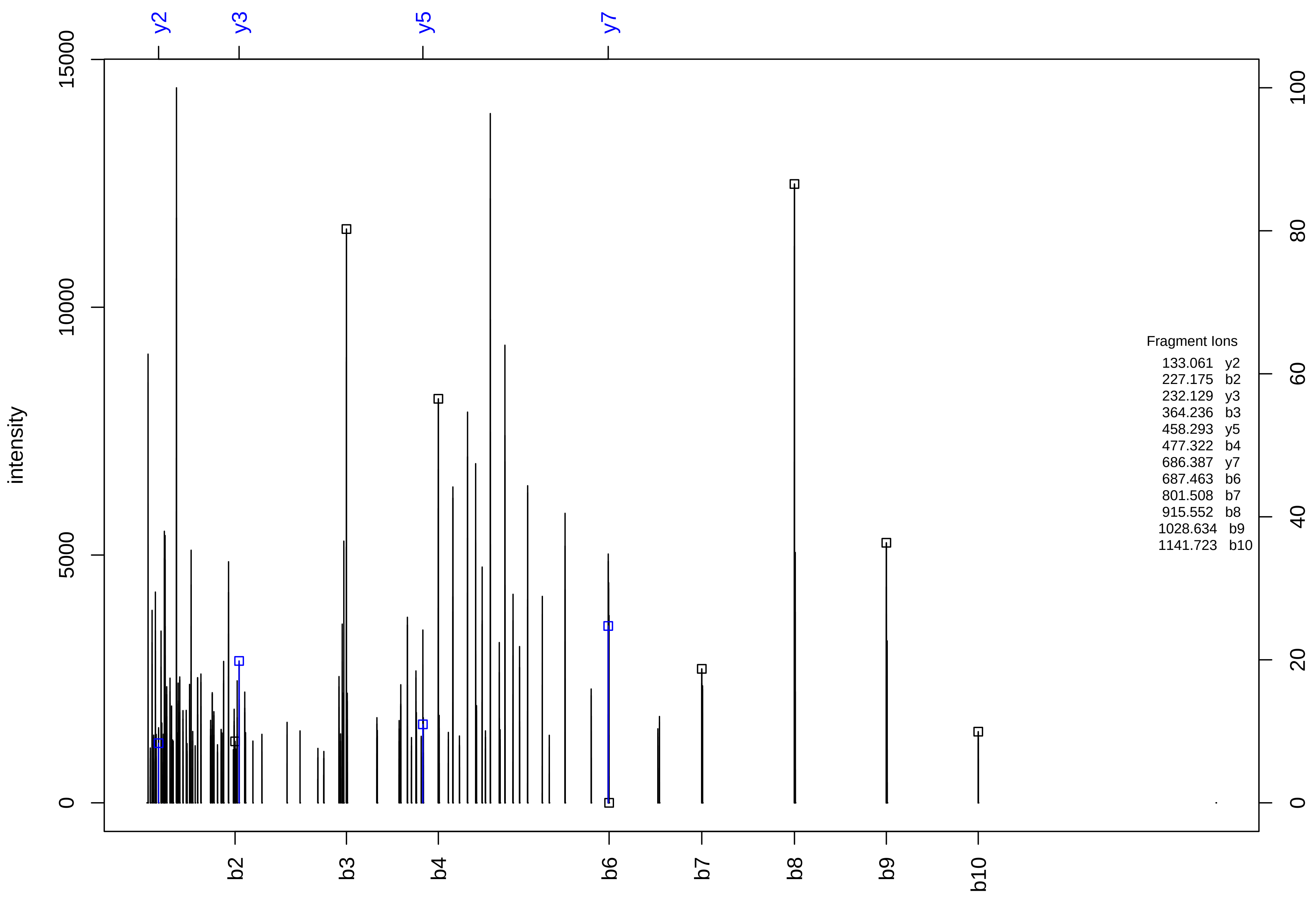

LIHIPINNIIVGG / File: christine_30turboips_ip19.raw; SpectrumID: ; scans: 23487

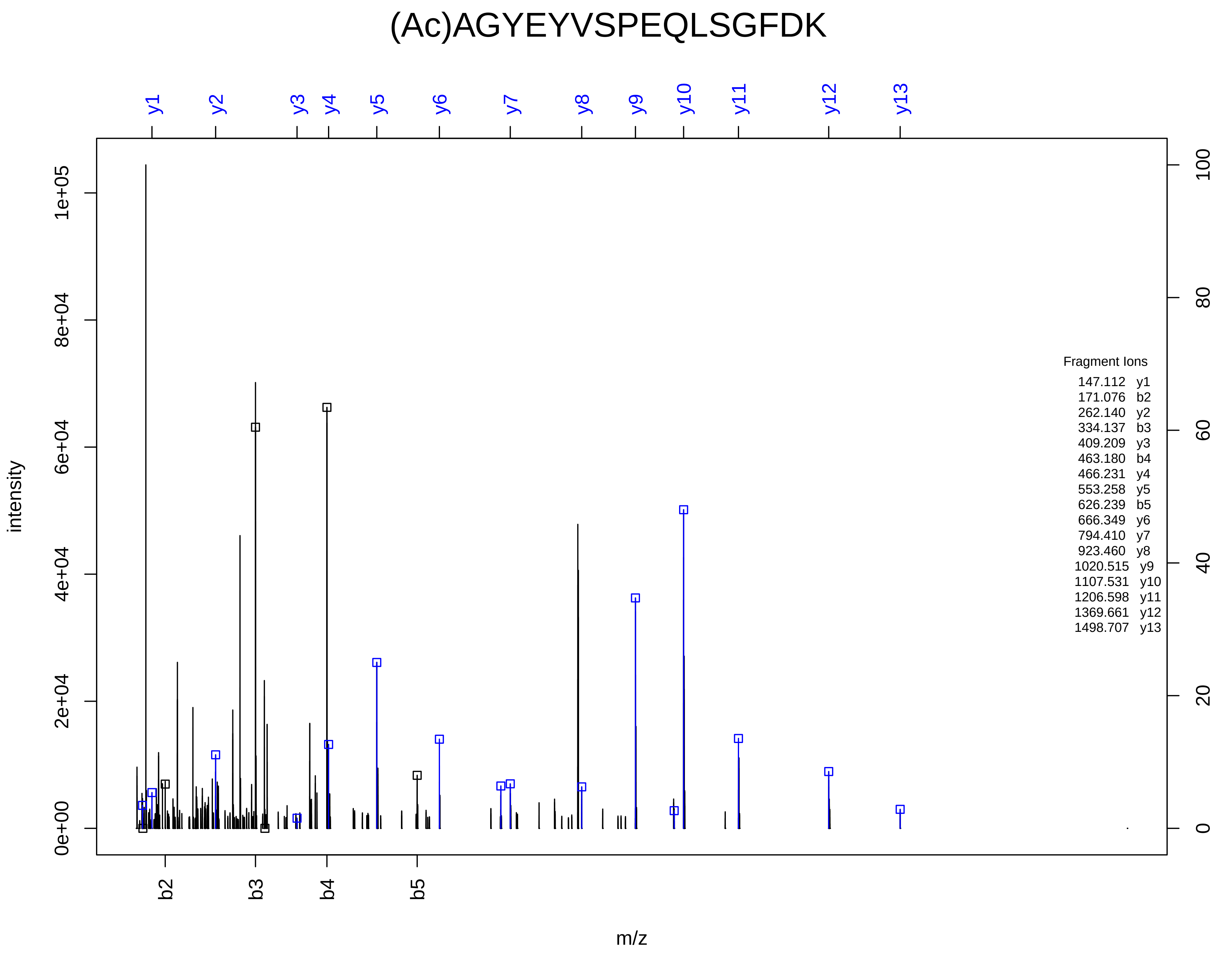

(Ac)AGYEYVSPEQLSGFDK
y1
y2
y3
y4
y5
y6
y7
y8
y9
y10
y11
y12
y13
intensity
0e+00
2e+04
4e+04
6e+04
8e+04
1e+05
0
20
40
60
80
100
b2
b3
b4
b5
m/z
Fragment Ions
147.112 y1
171.076 b2
262.140 y2
334.137 b3
409.209 y3
463.180 b4
466.231 y4
553.258 y5
626.239 b5
666.349 y6
794.410 y7
923.460 y8
1020.515 y9
1107.531 y10
1206.598 y11
1369.661 y12
1498.707 y13
AGYEYVSPEQLSGFDK / File: christine_30turboips_ip19.raw; SpectrumID: ; scans: 22179

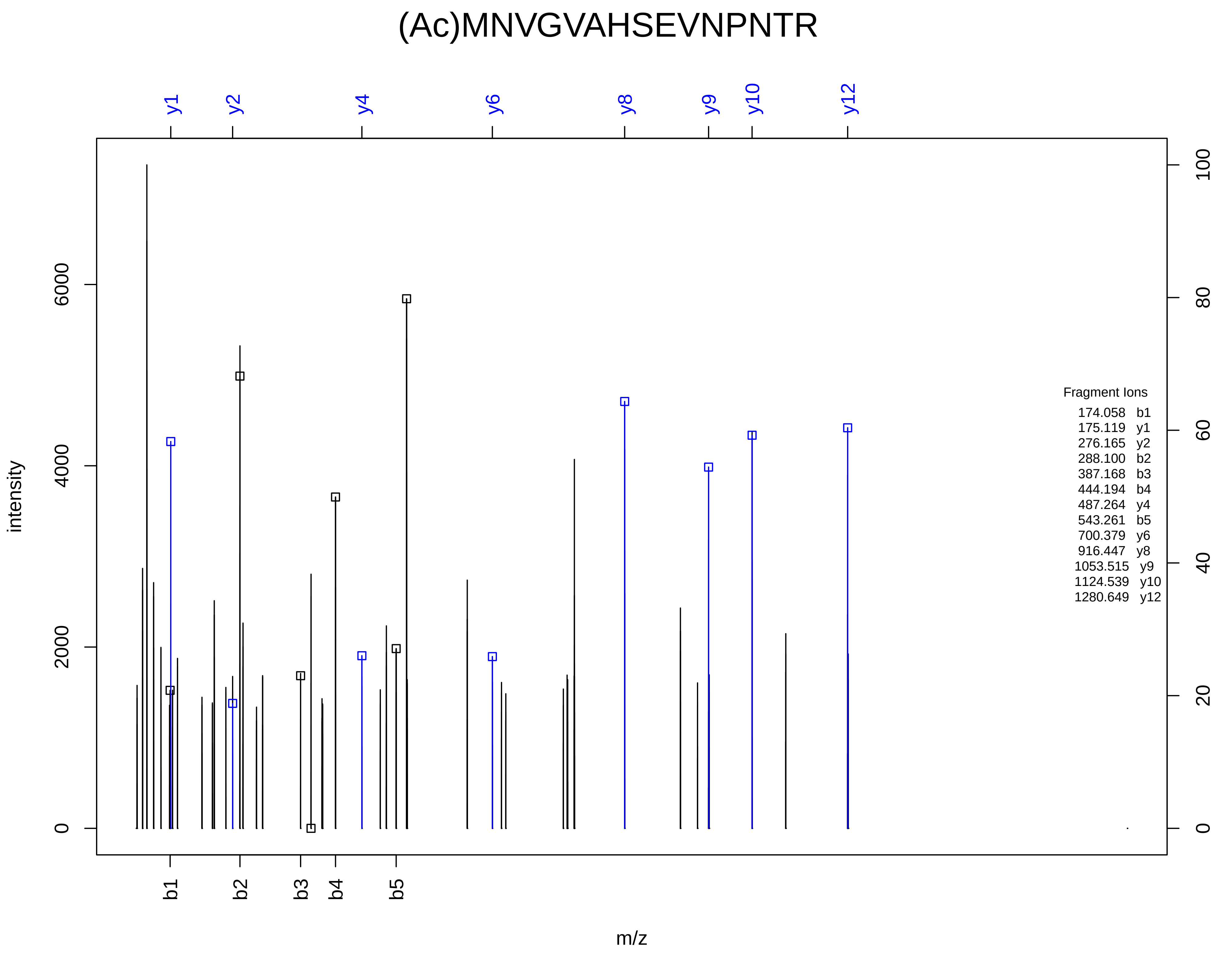

MNVGVAHSEVNPNTR / File: christine_30turboips_ip19.raw; SpectrumID: ; scans: 5868

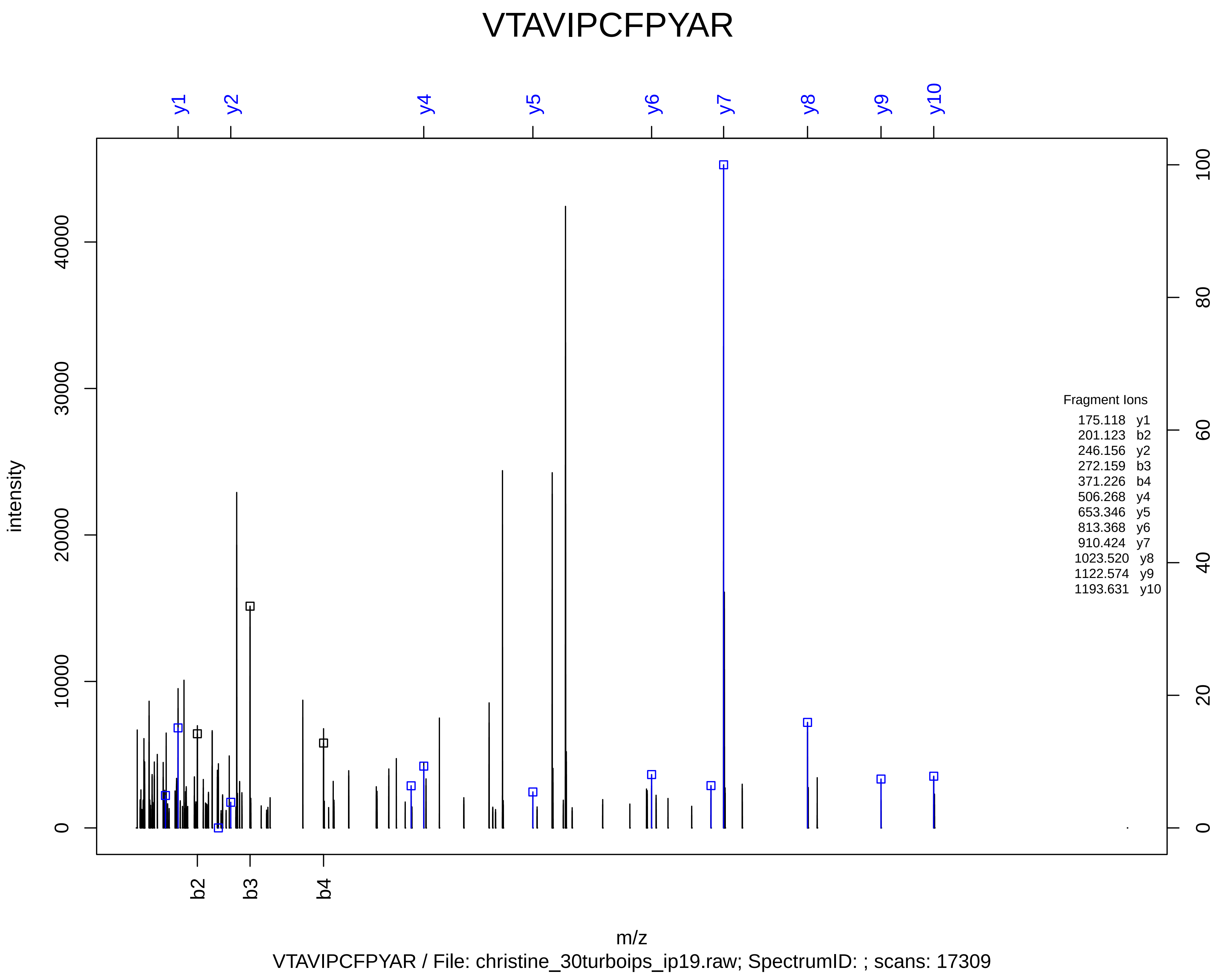
VTAVIPCFPYAR
y1
y2
y4
y5
y6
y7
y8
y9
y10
b2
b3
b4
intensity
0
10000
20000
30000
40000
0
20
40
60
80
100
Fragment Ions
175.118 y1
201.123 b2
246.156 y2
272.159 b3
371.226 b4
506.268 y4
653.346 y5
813.368 y6
910.424 y7
1023.520 y8
1122.574 y9
1193.631 y10
m/z
VTAVIPCFPYAR / File: christine_30turboips_ip19.raw; SpectrumID: ; scans: 17309

SANAEDAQEFSDVER

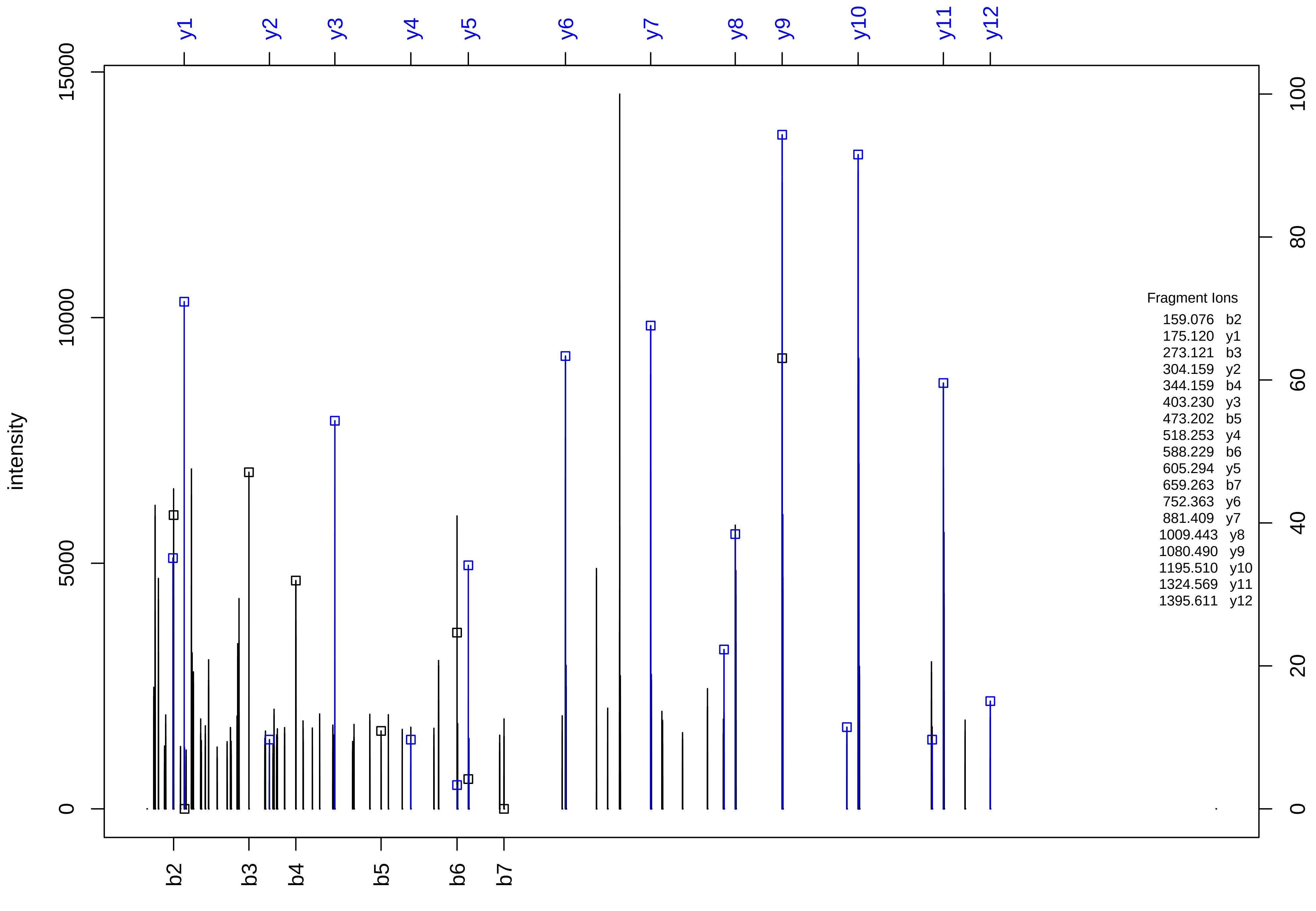

SANAEDAQEFSDVER / File: christine_30turboips_ip19.raw; SpectrumID: ; scans: 3978

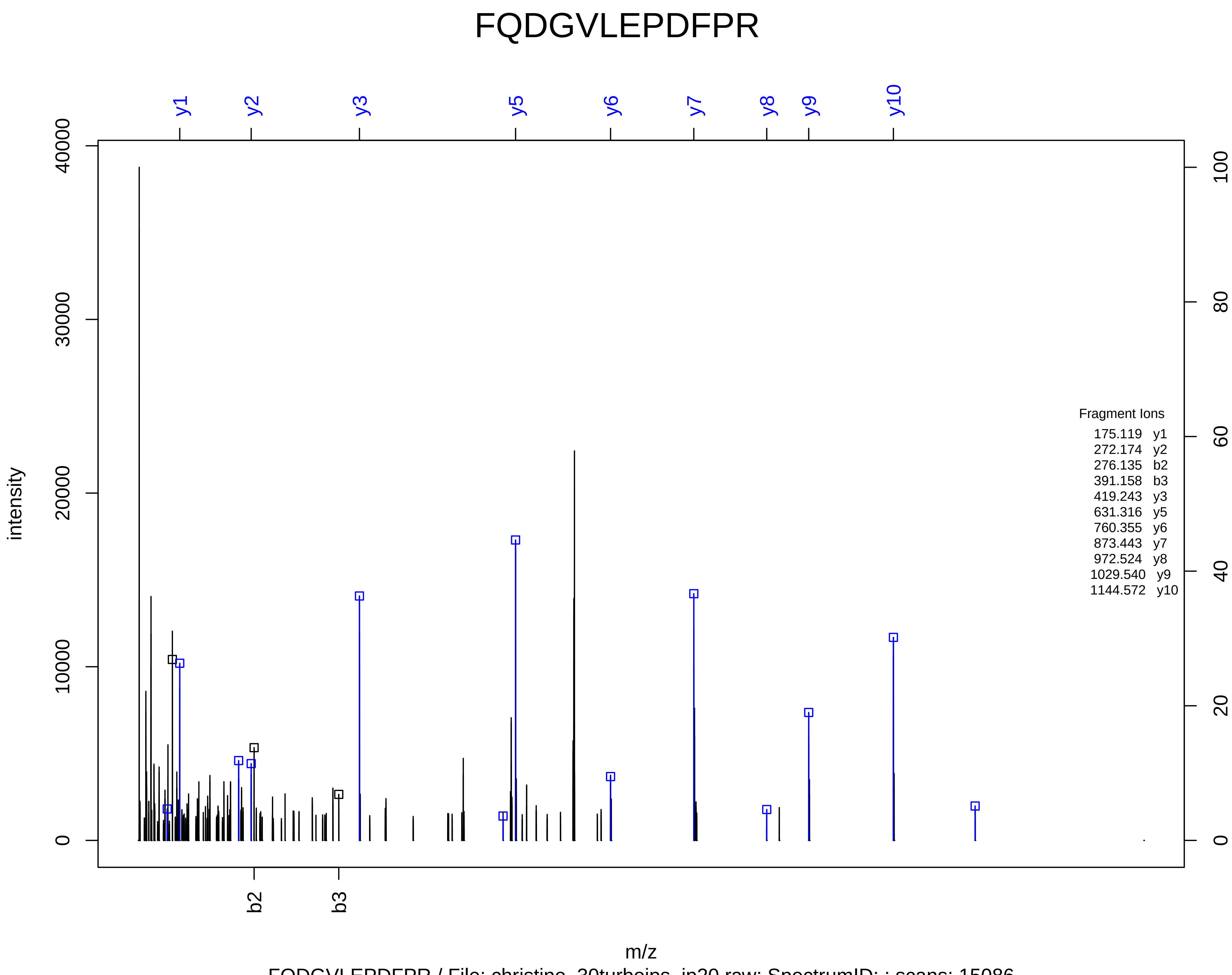
FQDGVLEPDFPR
y1
y2
y3
y5
y6
y7
y8
y9
y10
b2
b3
intensity
0
10000
20000
30000
40000
0
20
40
60
80
100
m/z
Fragment Ions
175.119 y1
272.174 y2
276.135 b2
391.158 b3
419.243 y3
631.316 y5
760.355 y6
873.443 y7
972.524 y8
1029.540 y9
1144.572 y10
FQDGVLEPDFPR / File: christine_30turboips_ip20.raw; SpectrumID: ; scans: 15086

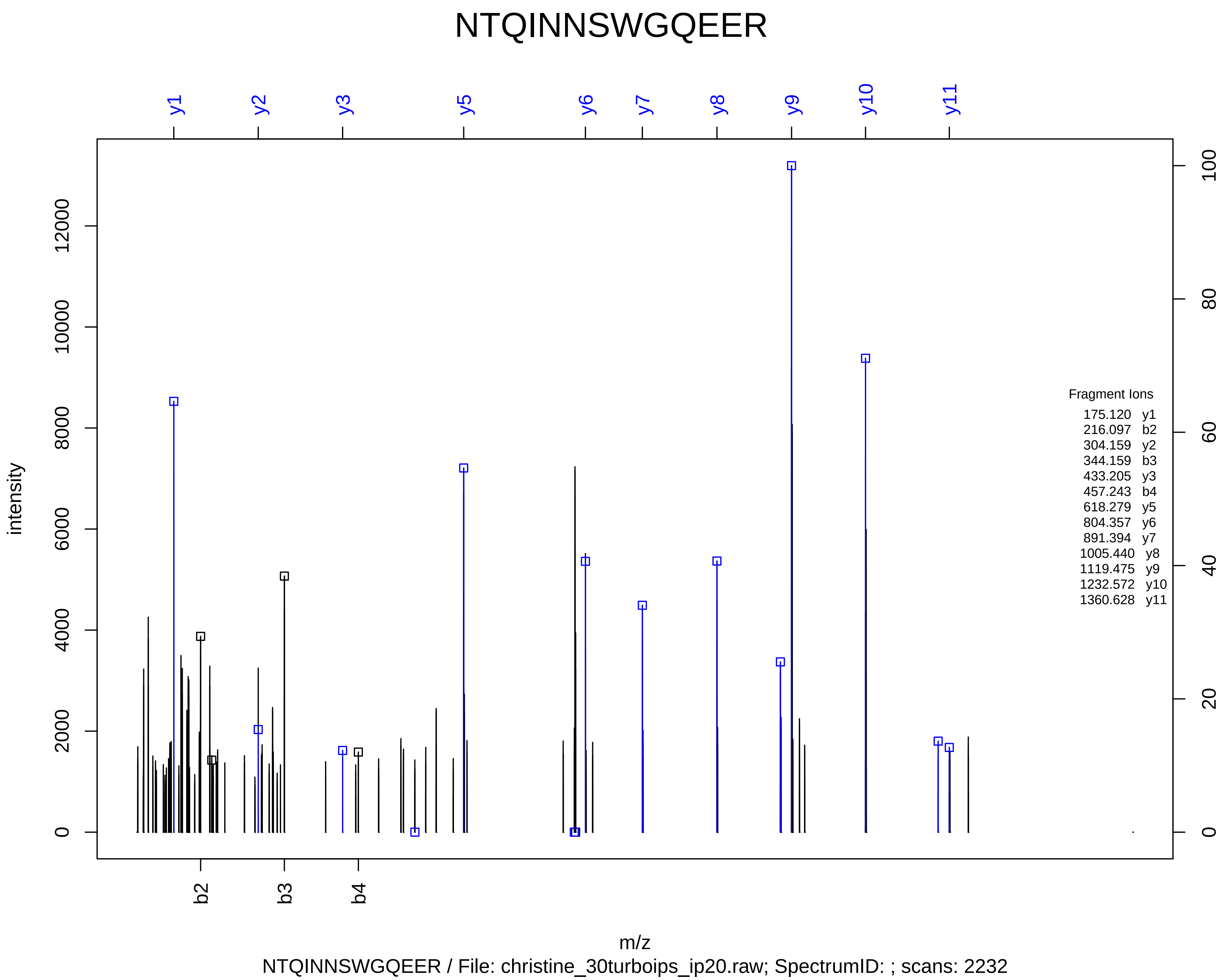
NTQINNSWGQEER
y1
y2
y3
y5
y6
y7
y8
y9
y10
y11
intensity
0
2000
4000
6000
8000
10000
12000
0
20
40
60
80
100
b2
b3
b4
m/z
Fragment Ions
175.120 y1
216.097 b2
304.159 y2
344.159 b3
433.205 y3
457.243 b4
618.279 y5
804.357 y6
891.394 y7
1005.440 y8
1119.475 y9
1232.572 y10
1360.628 y11
NTQINNSWGQEER / File: christine_30turboips_ip20.raw; SpectrumID: ; scans: 2232

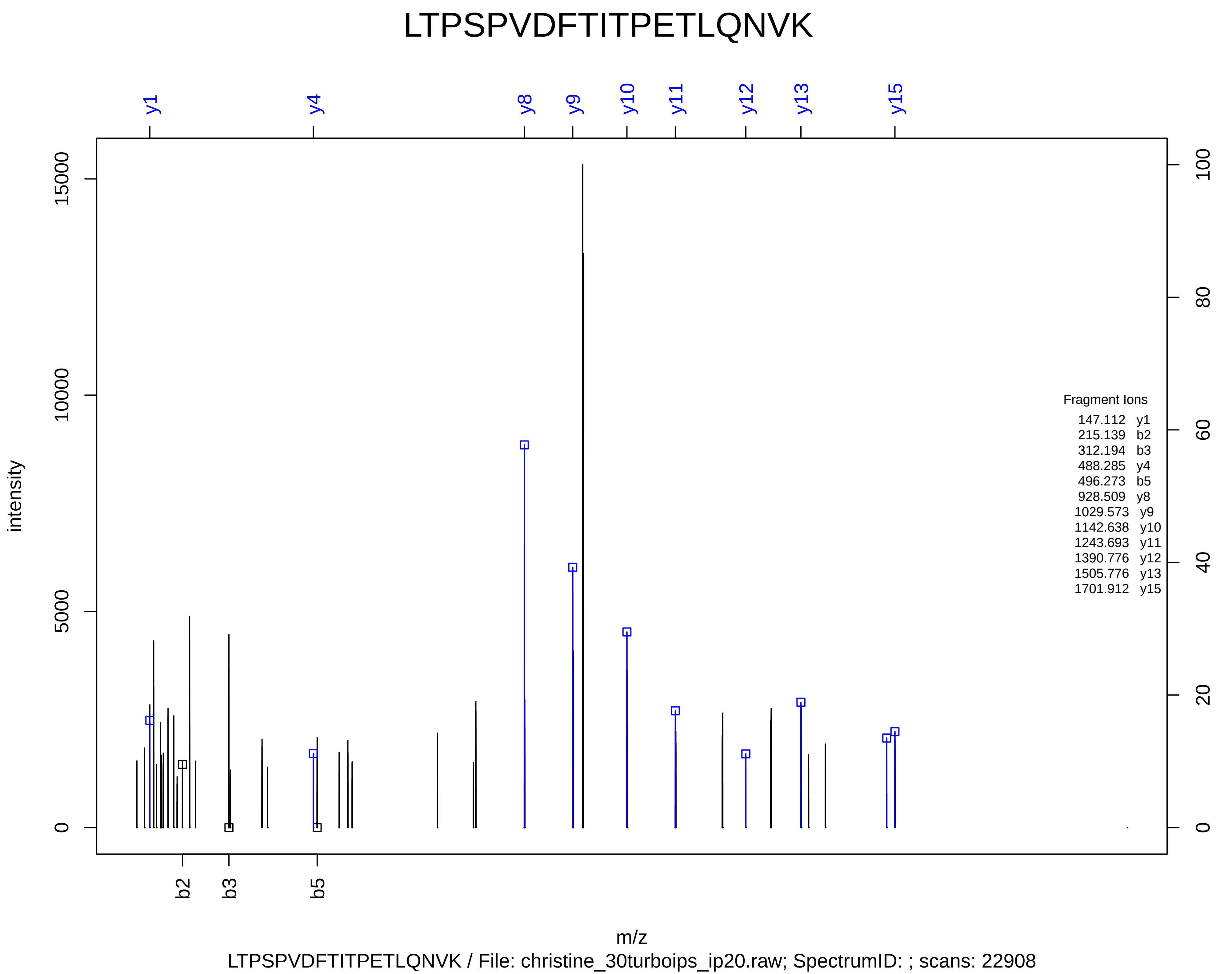

LTPSPVDFTITPETLQNVK
y1
y4
y8
y9
y10
y11
y12
y13
y15
intensity
0
5000
10000
15000
0
20
40
60
80
100
b2
b3
b5
Fragment Ions
147.112 y1
215.139 b2
312.194 b3
488.285 y4
496.273 b5
928.509 y8
1029.573 y9
1142.638 y10
1243.693 y11
1390.776 y12
1505.776 y13
1701.912 y15
m/z
LTPSPVDFTITPETLQNVK / File: christine_30turboips_ip20.raw; SpectrumID: ; scans: 22908

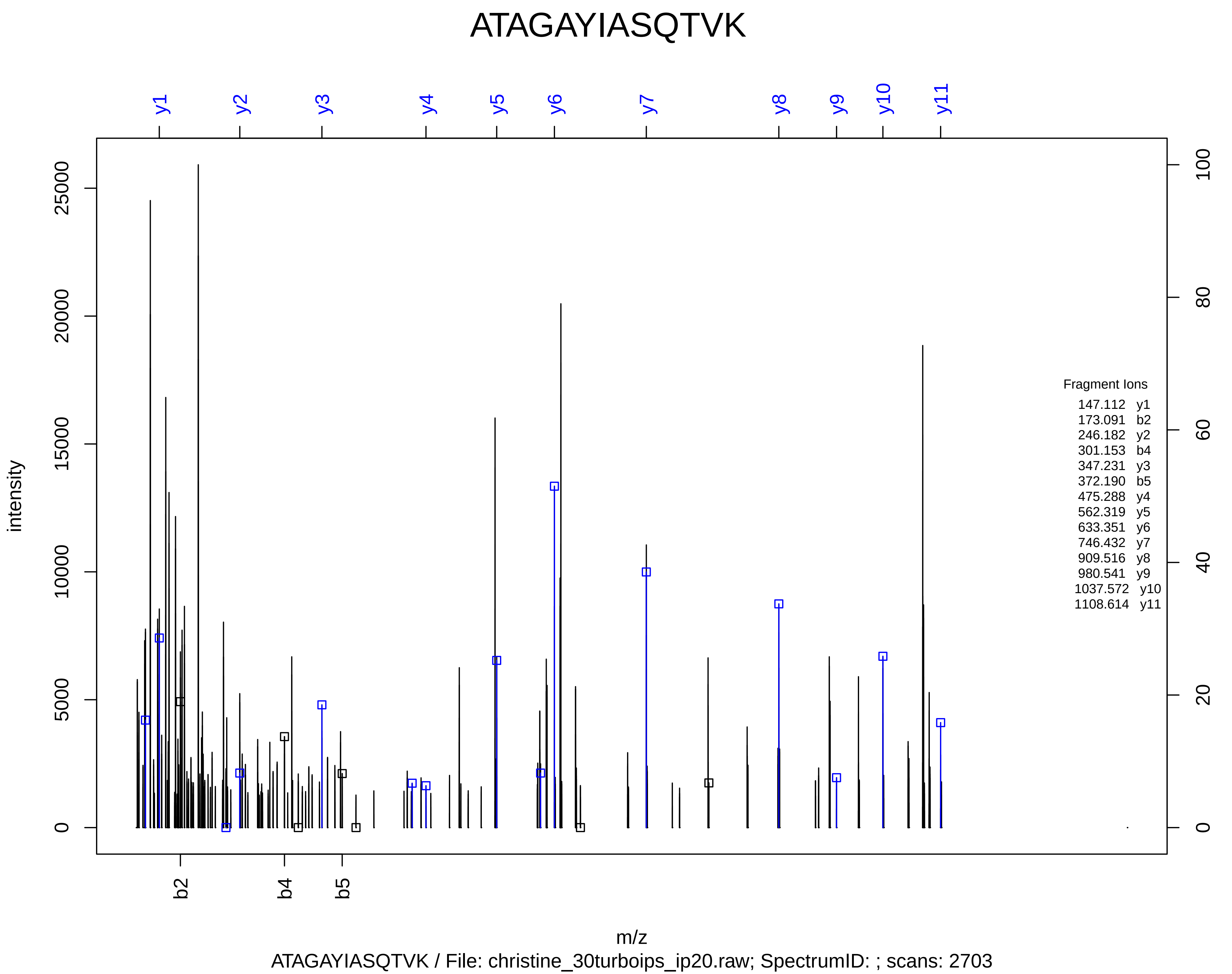
ATAGAYIASQTVK
y1
y2
y3
y4
y5
y6
y7
y8
y9
y10
y11
b2
b4
b5
intensity
m/z
0
5000
10000
15000
20000
25000
0
20
40
60
80
100
Fragment Ions
147.112 y1
173.091 b2
246.182 y2
301.153 b4
347.231 y3
372.190 b5
475.288 y4
562.319 y5
633.351 y6
746.432 y7
909.516 y8
980.541 y9
1037.572 y10
1108.614 y11
ATAGAYIASQTVK / File: christine_30turboips_ip20.raw; SpectrumID: ; scans: 2703

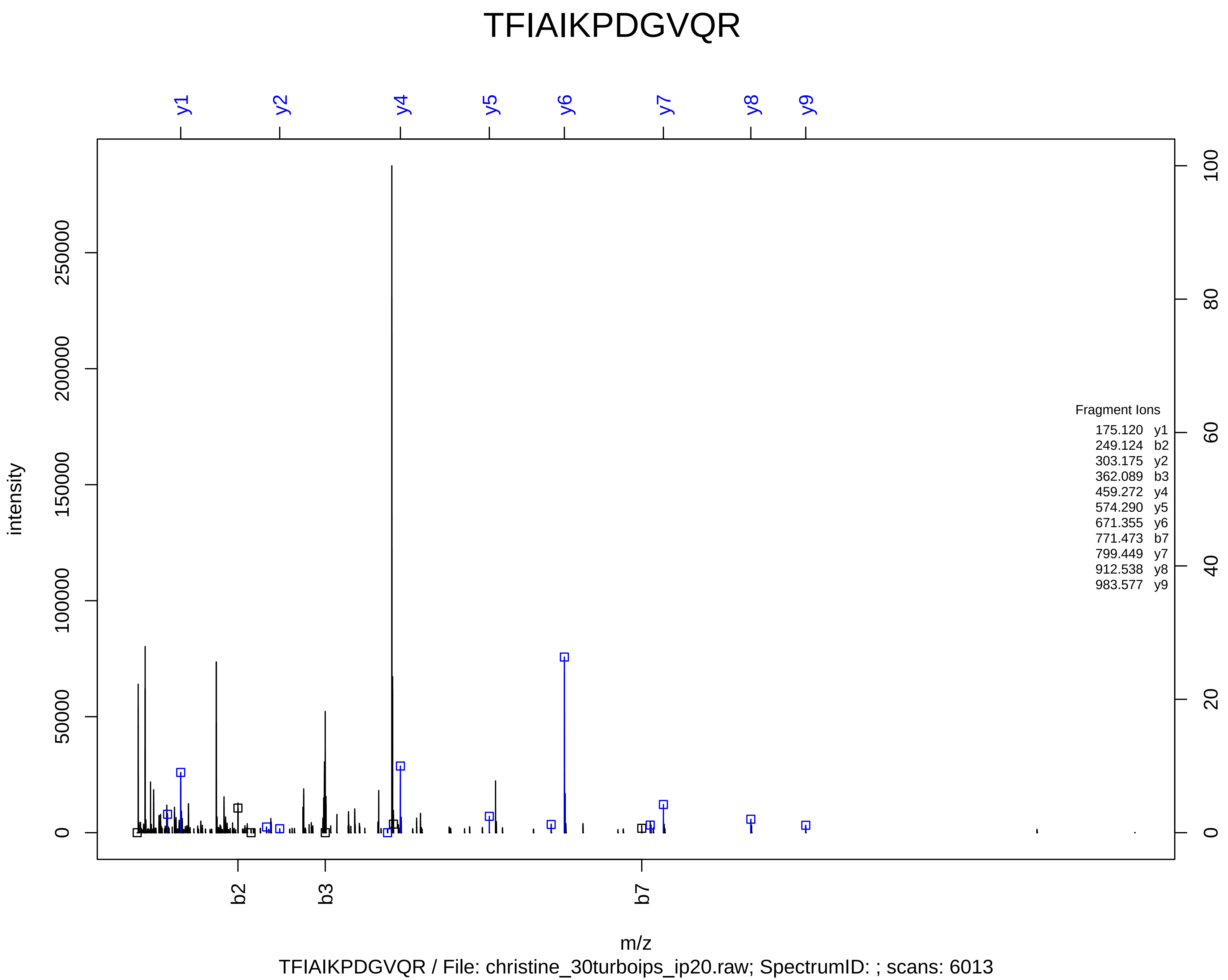
TFIAIKPDGVQR
y1
y2
y4
y5
y6
y7
y8
y9
b2
b3
b7
intensity
m/z
Fragment Ions
175.120 y1
249.124 b2
303.175 y2
362.089 b3
459.272 y4
574.290 y5
671.355 y6
771.473 b7
799.449 y7
912.538 y8
983.577 y9
TFIAIKPDGVQR / File: christine_30turboips_ip20.raw; SpectrumID: ; scans: 6013

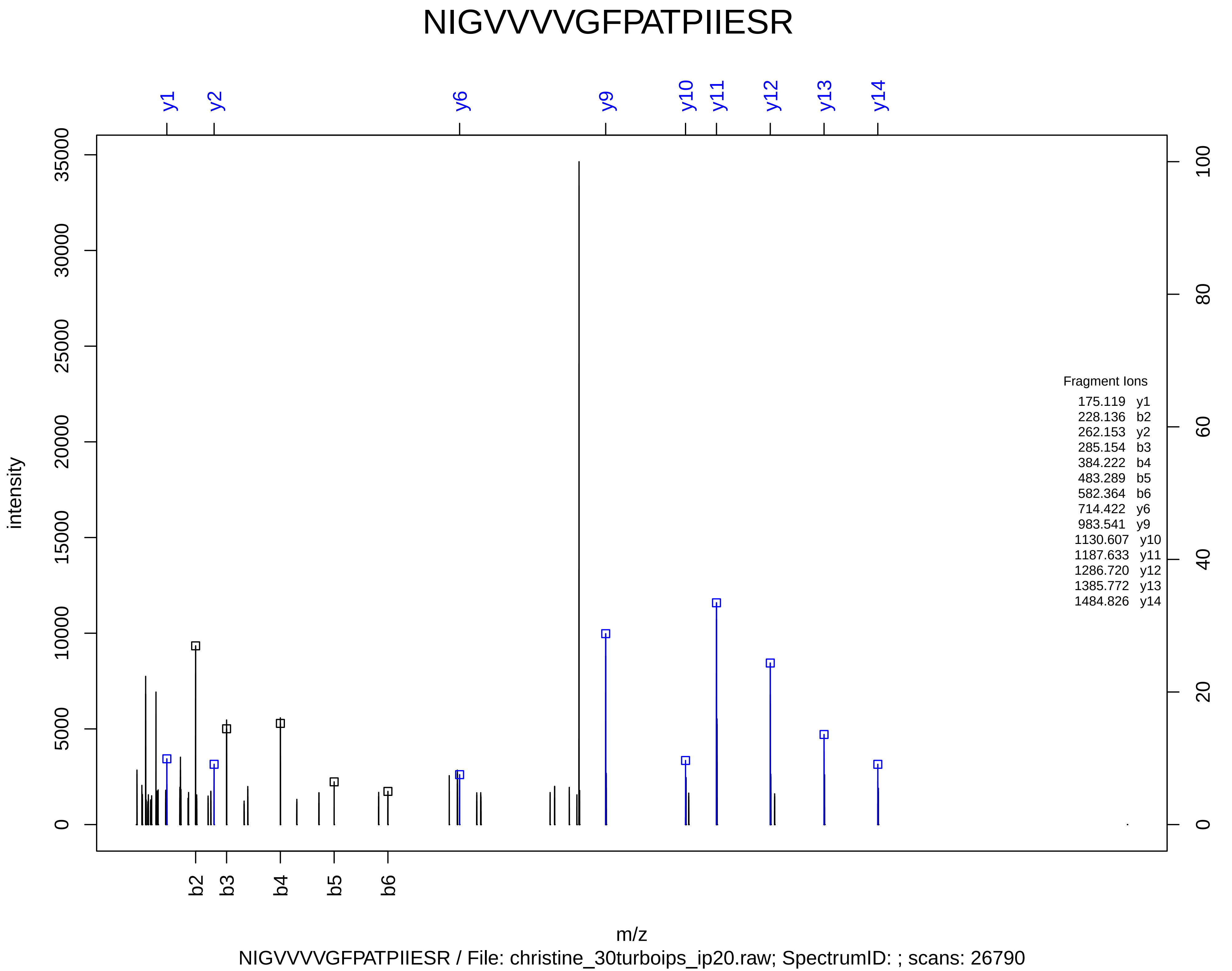
NIGVVVVGFPATPIIESR
intensity
m/z
y1
y2
y6
y9
y10
y11
y12
y13
y14
b2
b3
b4
b5
b6
Fragment Ions
175.119 y1
228.136 b2
262.153 y2
285.154 b3
384.222 b4
483.289 b5
582.364 b6
714.422 y6
983.541 y9
1130.607 y10
1187.633 y11
1286.720 y12
1385.772 y13
1484.826 y14
NIGVVVVGFPATPIIESR / File: christine_30turboips_ip20.raw; SpectrumID: ; scans: 26790

AGQTLVLLDTEGLEDVEK

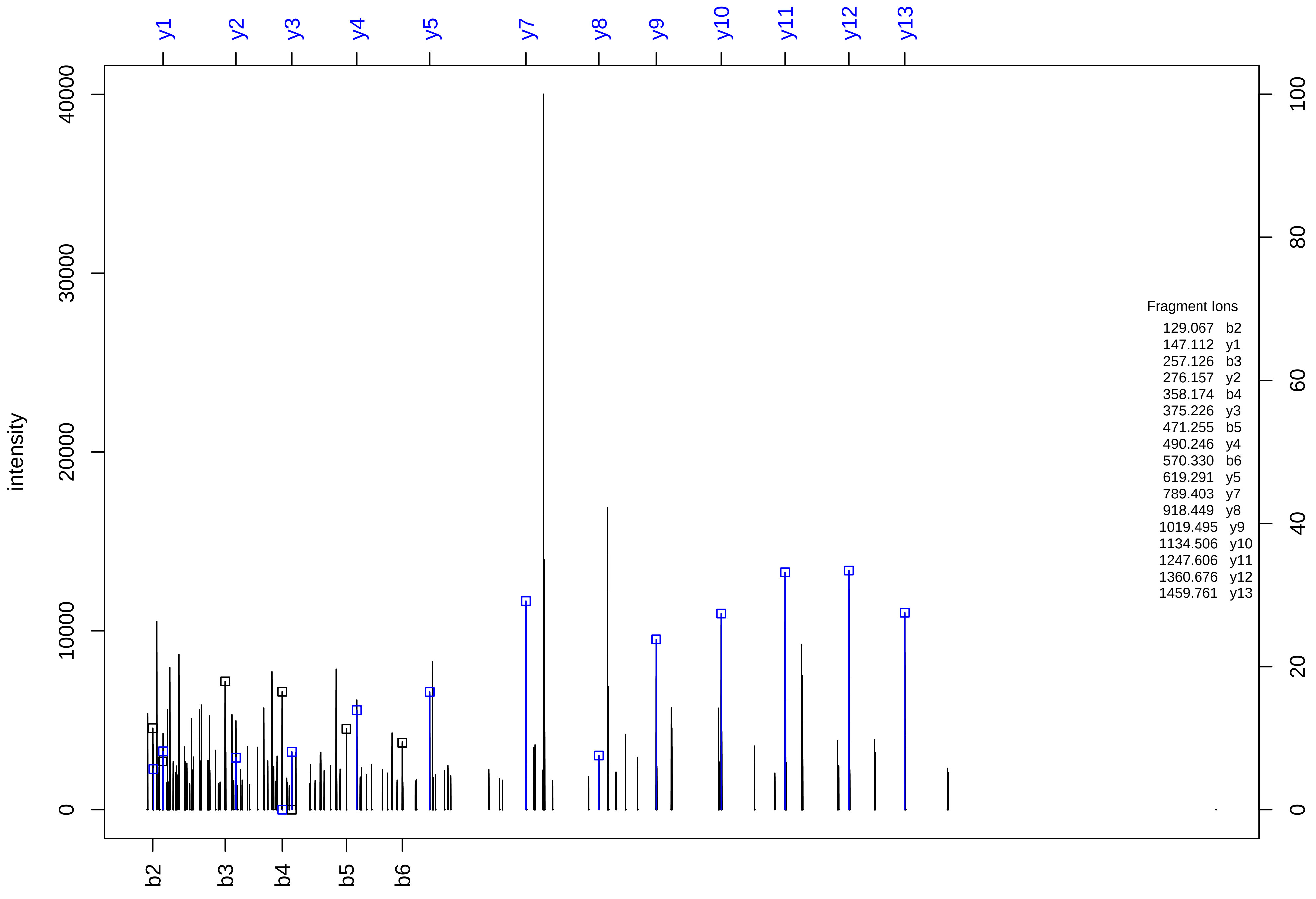

AGQTLVLLDTEGLEDVEK / File: christine_30turboips_ip20.raw; SpectrumID: ; scans: 22088

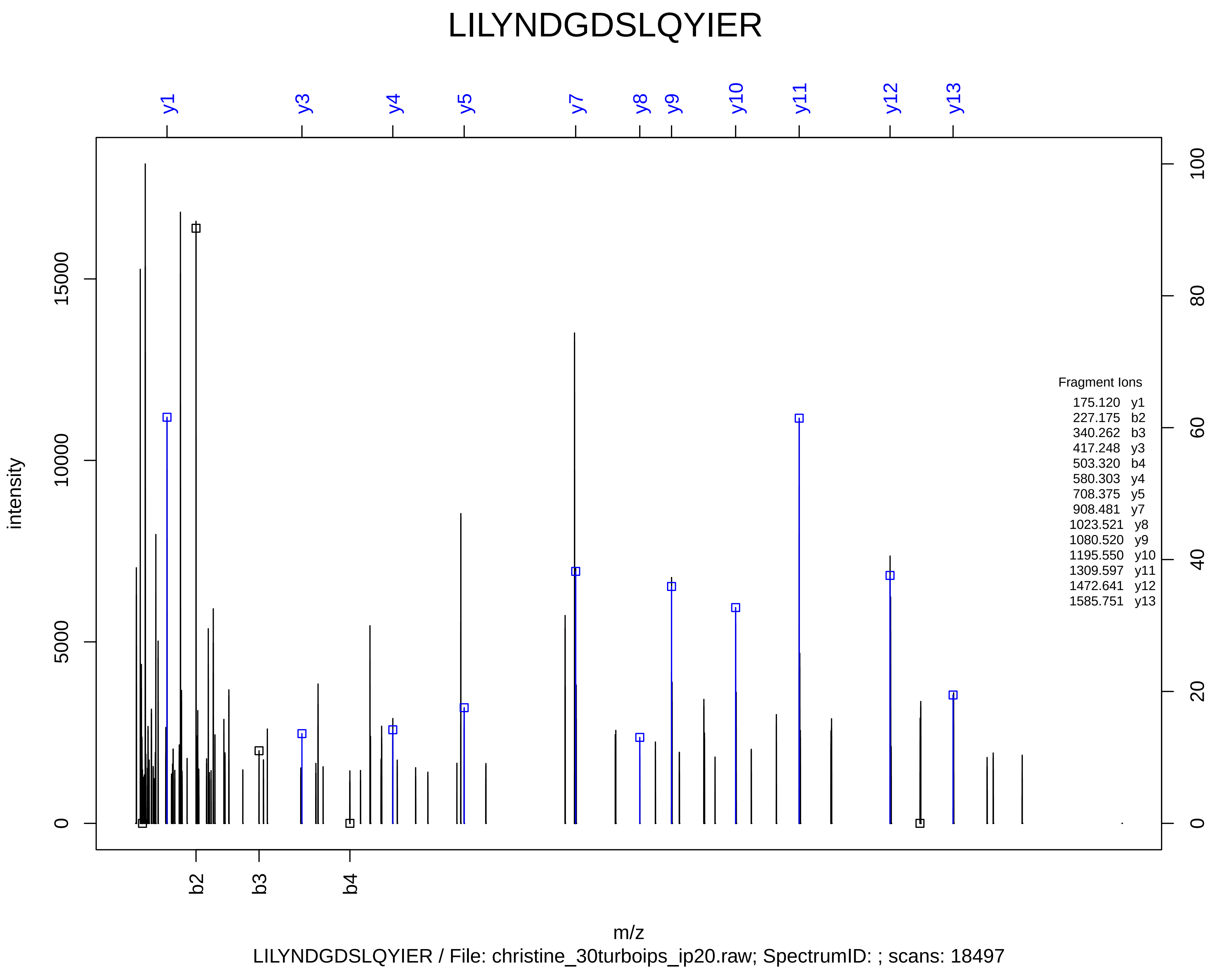
LILYNDGDSLQYIER
y1
y3
y4
y5
y7
y8
y9
y10
y11
y12
y13
intensity
0
5000
10000
15000
0
20
40
60
80
100
Fragment Ions
175.120 y1
227.175 b2
340.262 b3
417.248 y3
503.320 b4
580.303 y4
708.375 y5
908.481 y7
1023.521 y8
1080.520 y9
1195.550 y10
1309.597 y11
1472.641 y12
1585.751 y13
b2
b3
b4
m/z
LILYNDGDSLQYIER / File: christine_30turboips_ip20.raw; SpectrumID: ; scans: 18497

ETNEAQPPGPAPSDDAPLPGPGPSDVSDVAAEK

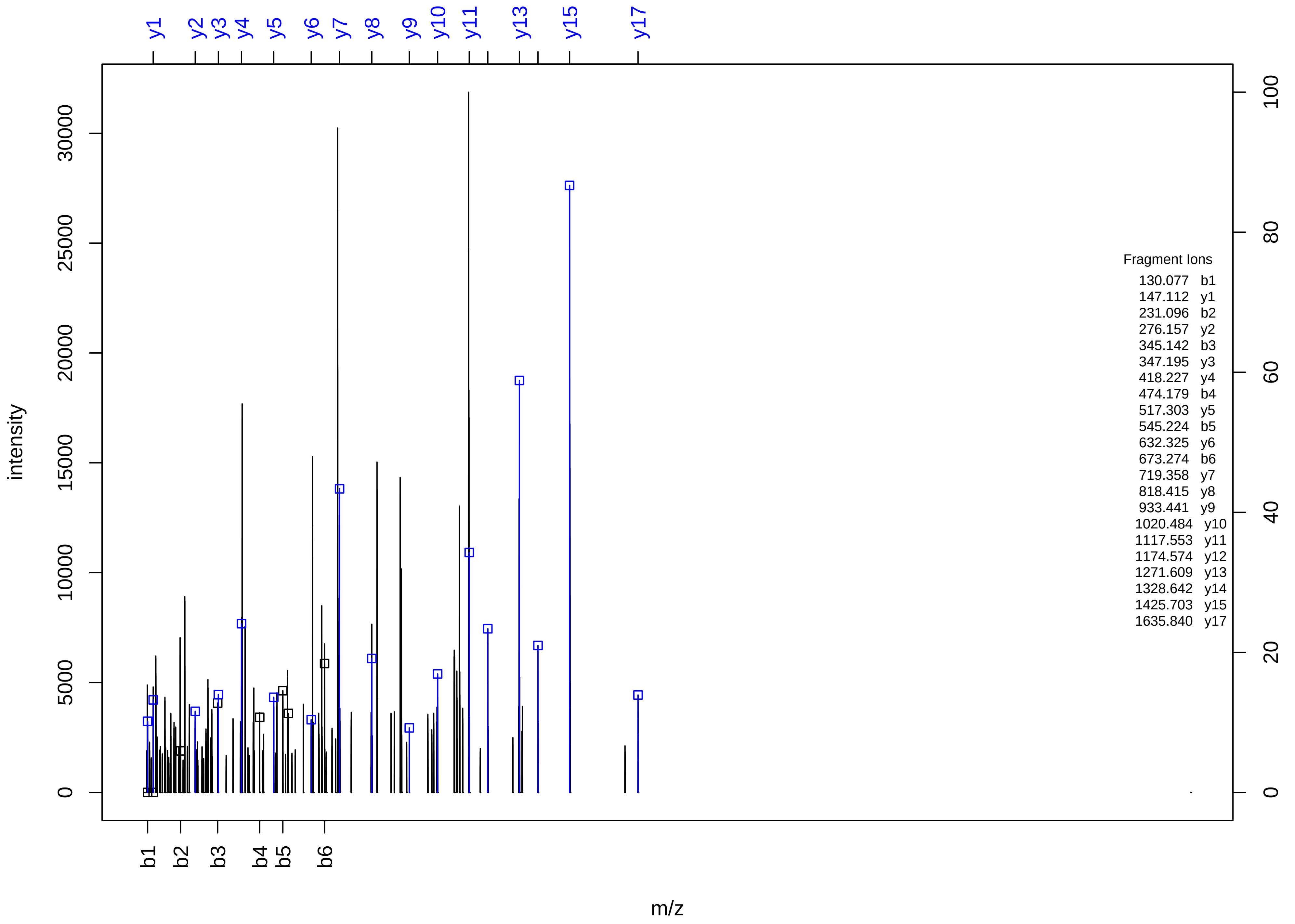

ETNEAQPPGPAPSDDAPLPGPGPSDVSDVAAEK / File: christine_30turboips_ip20.raw; SpectrumID: ; scans: 12856

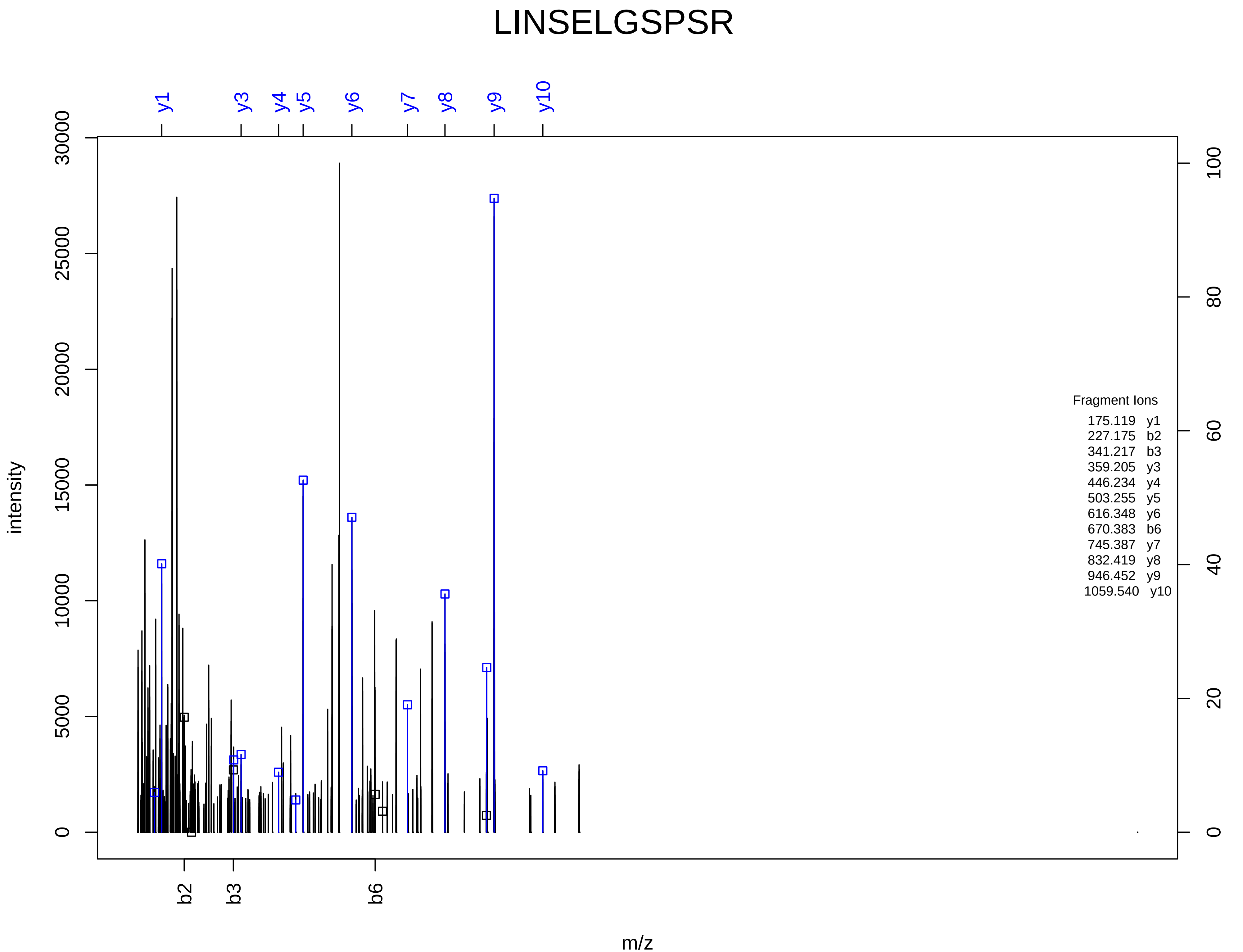

LINSELGSPSR
y1
y3
y4
y5
y6
y7
y8
y9
y10
intensity
0
5000
10000
15000
20000
25000
30000
0
20
40
60
80
100
b2
b3
b6
m/z
Fragment Ions
175.119 y1
227.175 b2
341.217 b3
359.205 y3
446.234 y4
503.255 y5
616.348 y6
670.383 b6
745.387 y7
832.419 y8
946.452 y9
1059.540 y10
LINSELGSPSR / File: christine_30turboips_ip20.raw; SpectrumID: ; scans: 2735

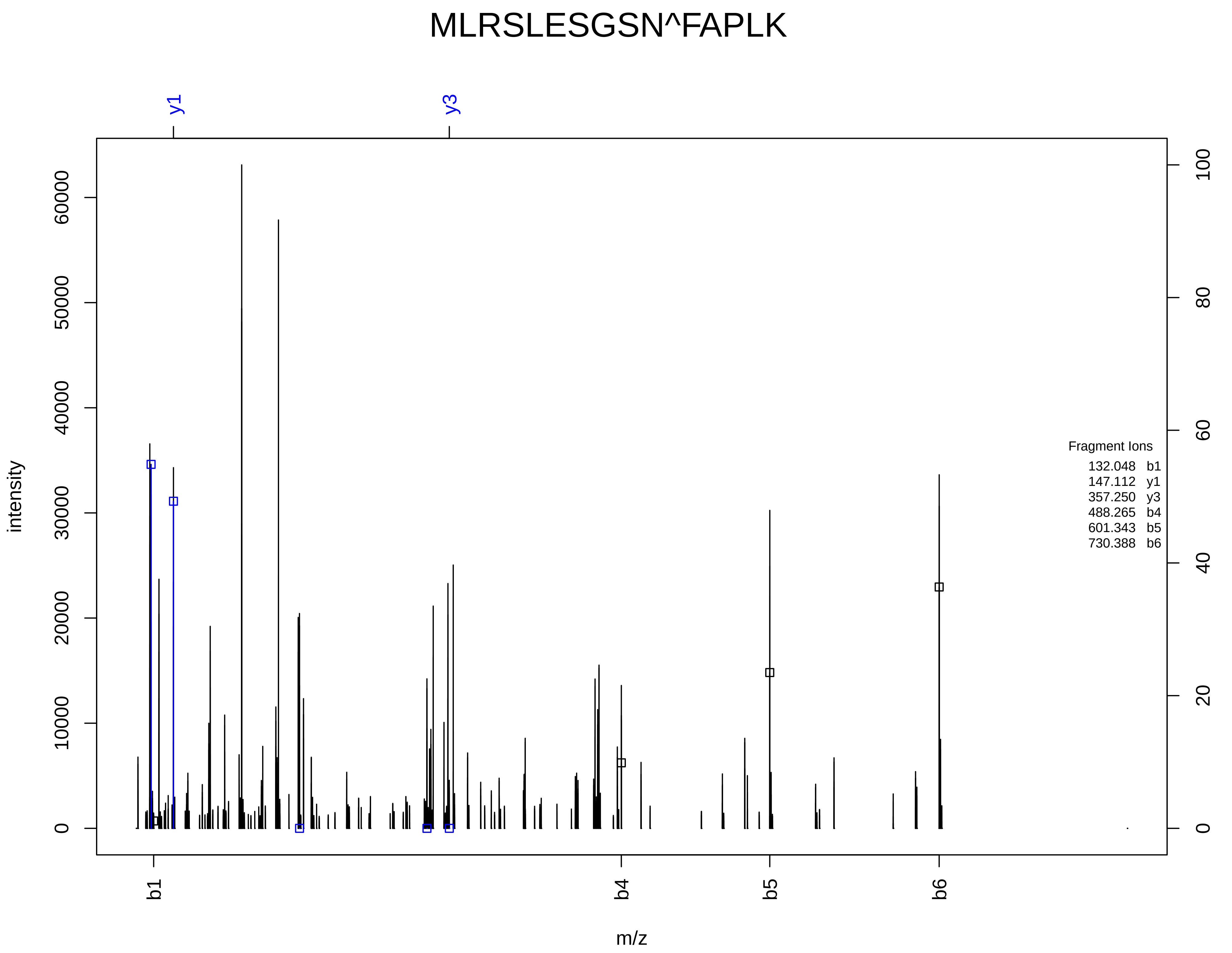
MLRSLESGSN^FAPLK
y1
y3
intensity
Fragment Ions
132.048 b1
147.112 y1
357.250 y3
488.265 b4
601.343 b5
730.388 b6
b1
b4
b5
b6
m/z
MLRSLESGSNFAPLK / File: christine_30turboips_ip20.raw; SpectrumID: ; scans: 943

LLLQIENVPENQLER

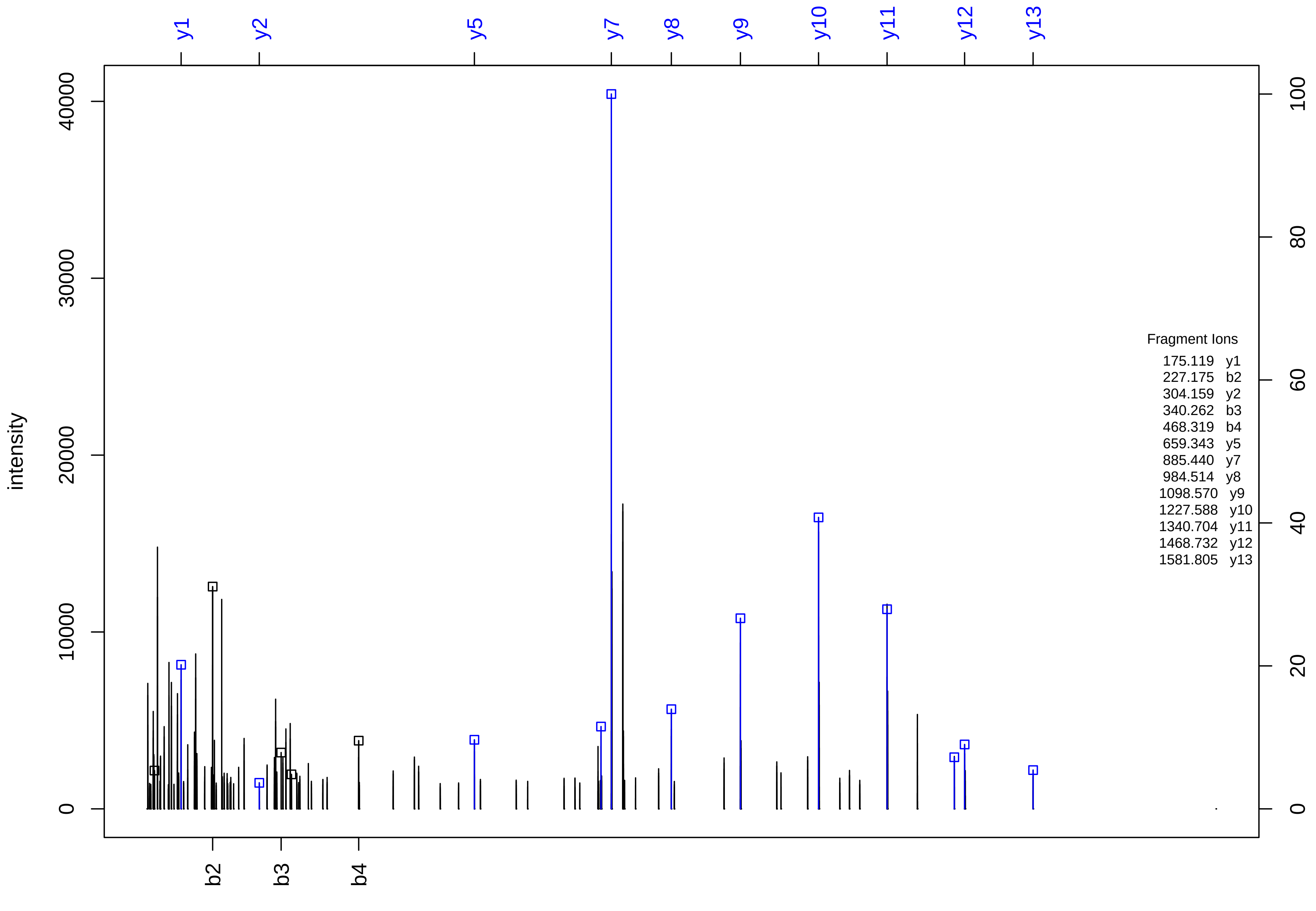

LLLQIENVPENQLER / File: christine_30turboips_ip20.raw; SpectrumID: ; scans: 18544

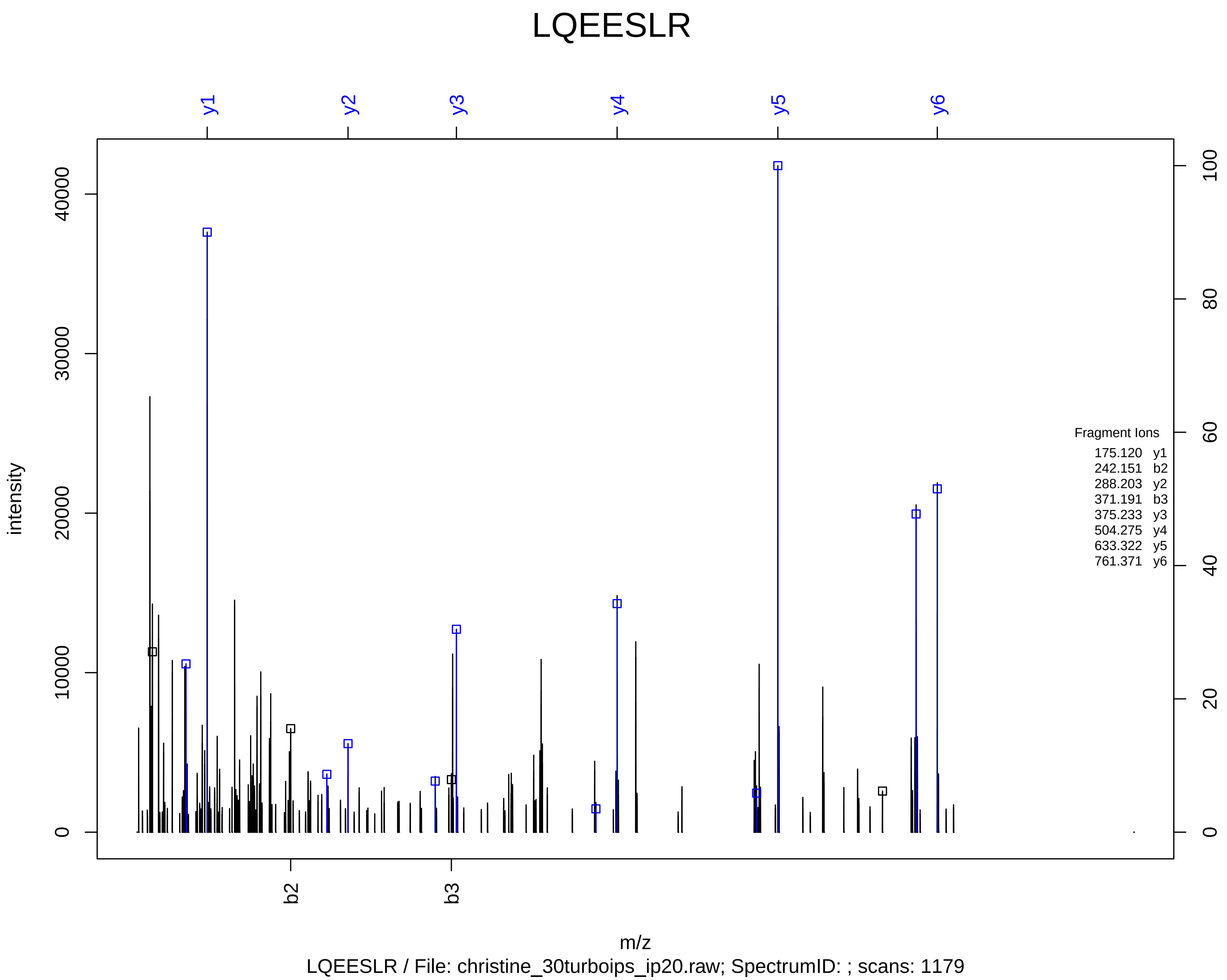
LQEESLR
y1
y2
y3
y4
y5
y6
intensity
0
10000
20000
30000
40000
0
20
40
60
80
100
b2
b3
m/z
Fragment Ions
175.120 y1
242.151 b2
288.203 y2
371.191 b3
375.233 y3
504.275 y4
633.322 y5
761.371 y6
LQEESLR / File: christine_30turboips_ip20.raw; SpectrumID: ; scans: 1179

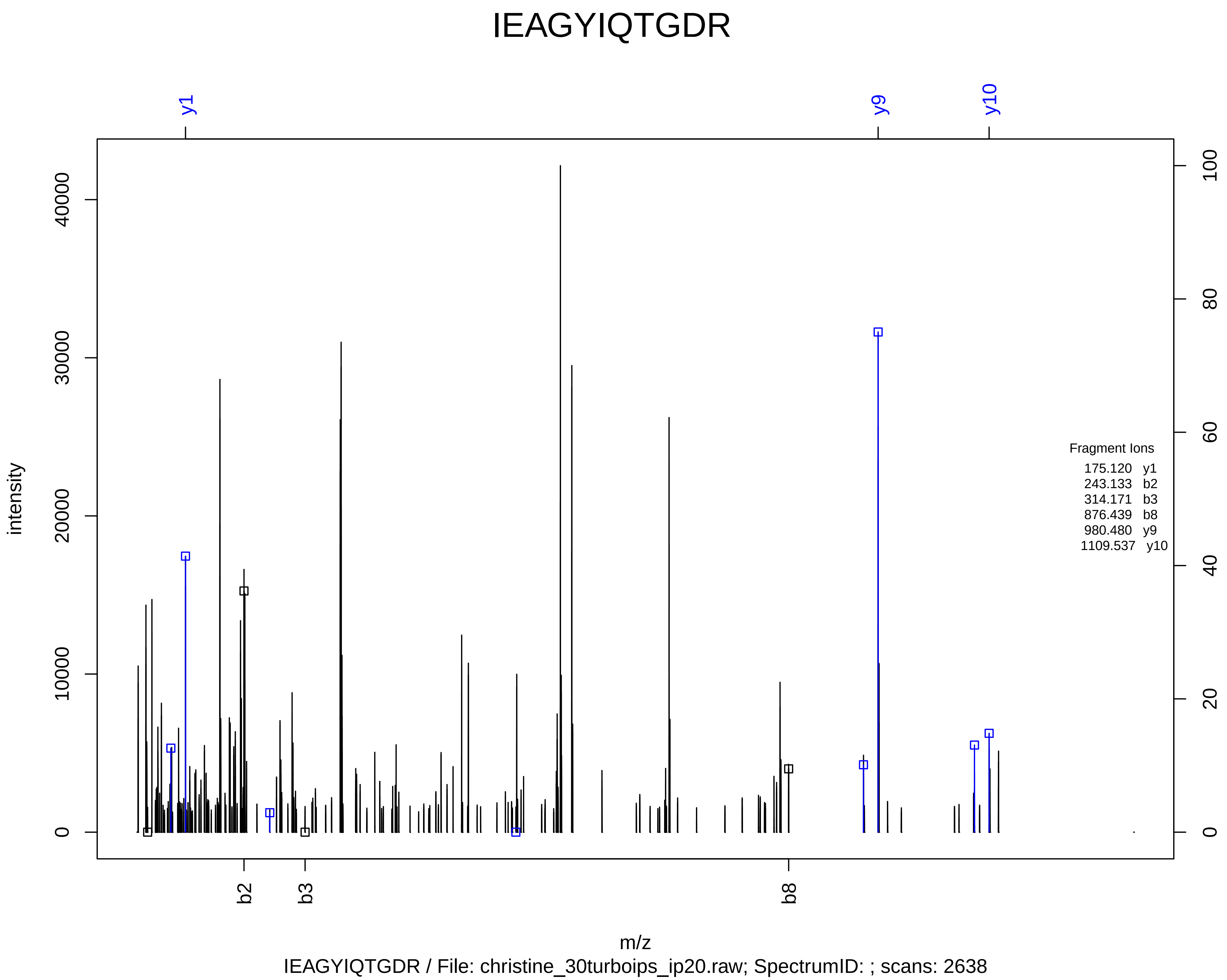
IEAGYIQTGDR
y1
y9
y10
intensity
0
10000
20000
30000
40000
0
20
40
60
80
100
Fragment Ions
175.120 y1
243.133 b2
314.171 b3
876.439 b8
980.480 y9
1109.537 y10
b2
b3
b8
m/z
IEAGYIQTGDR / File: christine_30turboips_ip20.raw; SpectrumID: ; scans: 2638

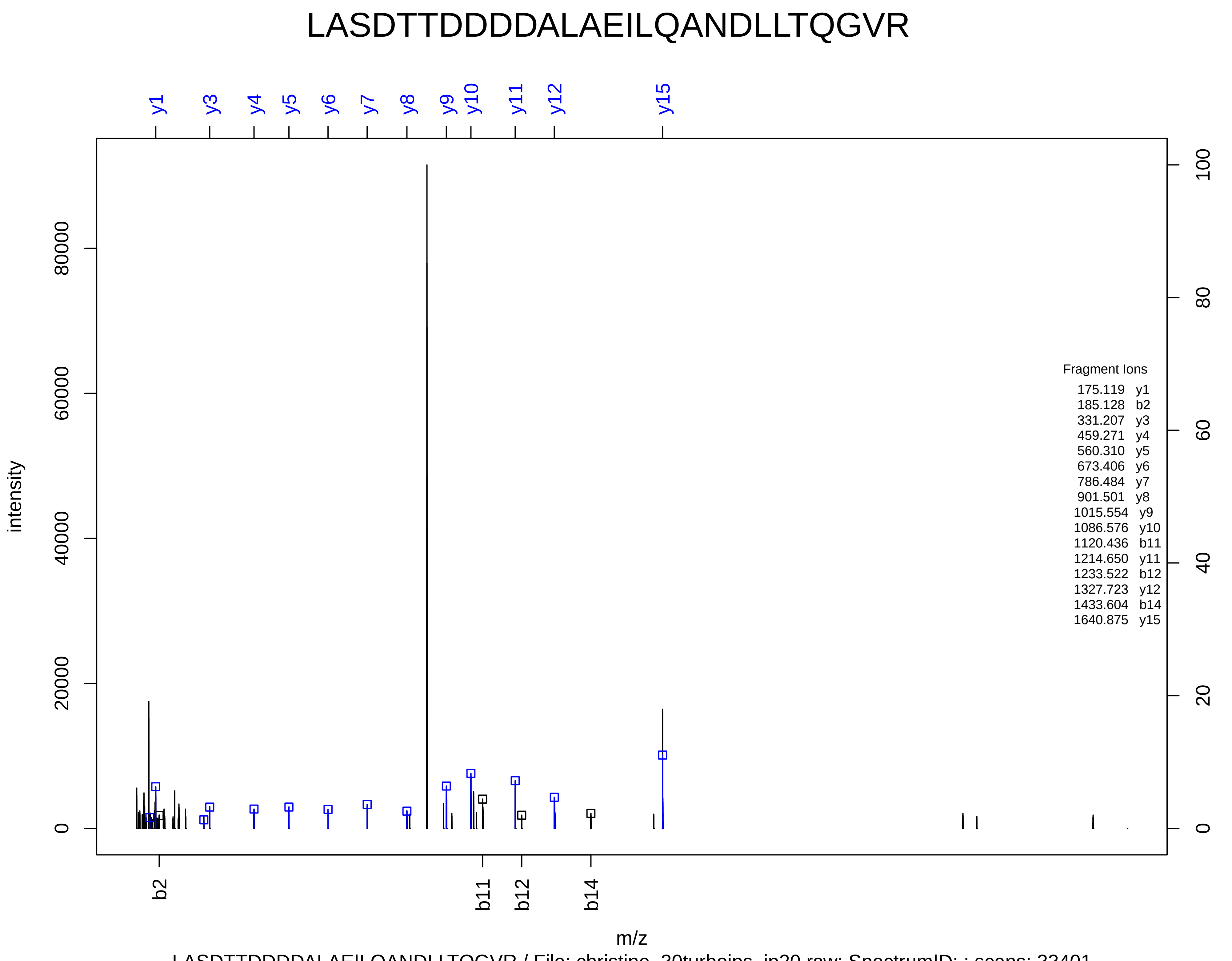

LASDTTDDDDALAEILQANDLLTQGVR
y1
y3
y4
y5
y6
y7
y8
y9
y10
y11
y12
y15
intensity
0
20000
40000
60000
80000
0
20
40
60
80
100
Fragment Ions
175.119 y1
185.128 b2
331.207 y3
459.271 y4
560.310 y5
673.406 y6
786.484 y7
901.501 y8
1015.554 y9
1086.576 y10
1120.436 b11
1214.650 y11
1233.522 b12
1327.723 y12
1433.604 b14
1640.875 y15
b2
b11
b12
b14
m/z
LASDTTDDDDALAEILQANDLLTQGVR / File: christine_30turboips_ip20.raw; SpectrumID: ; scans: 33401

AVFDETYPDPVR

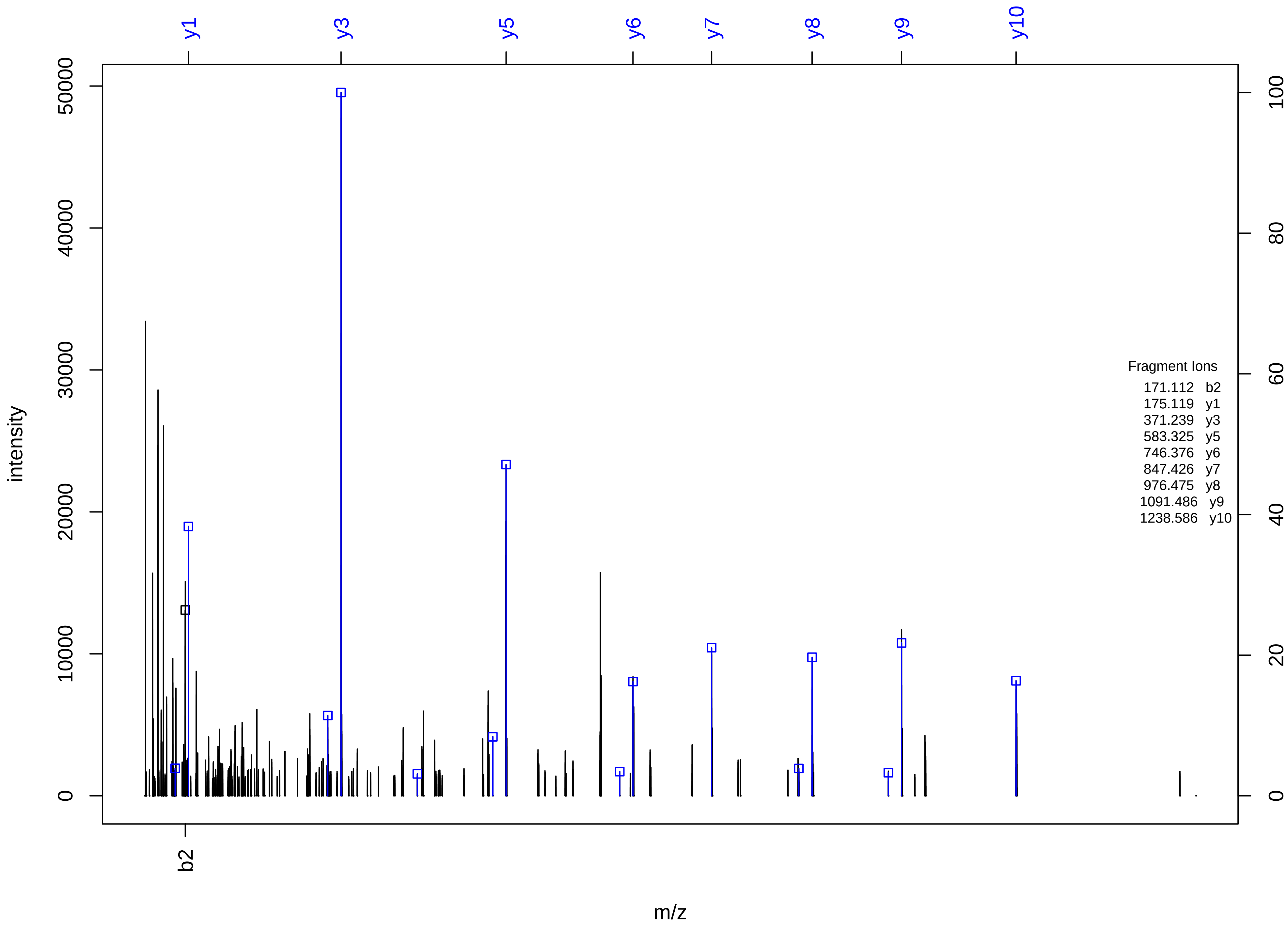

AVFDETYPDPVR / File: christine_30turboips_ip20.raw; SpectrumID: ; scans: 11480

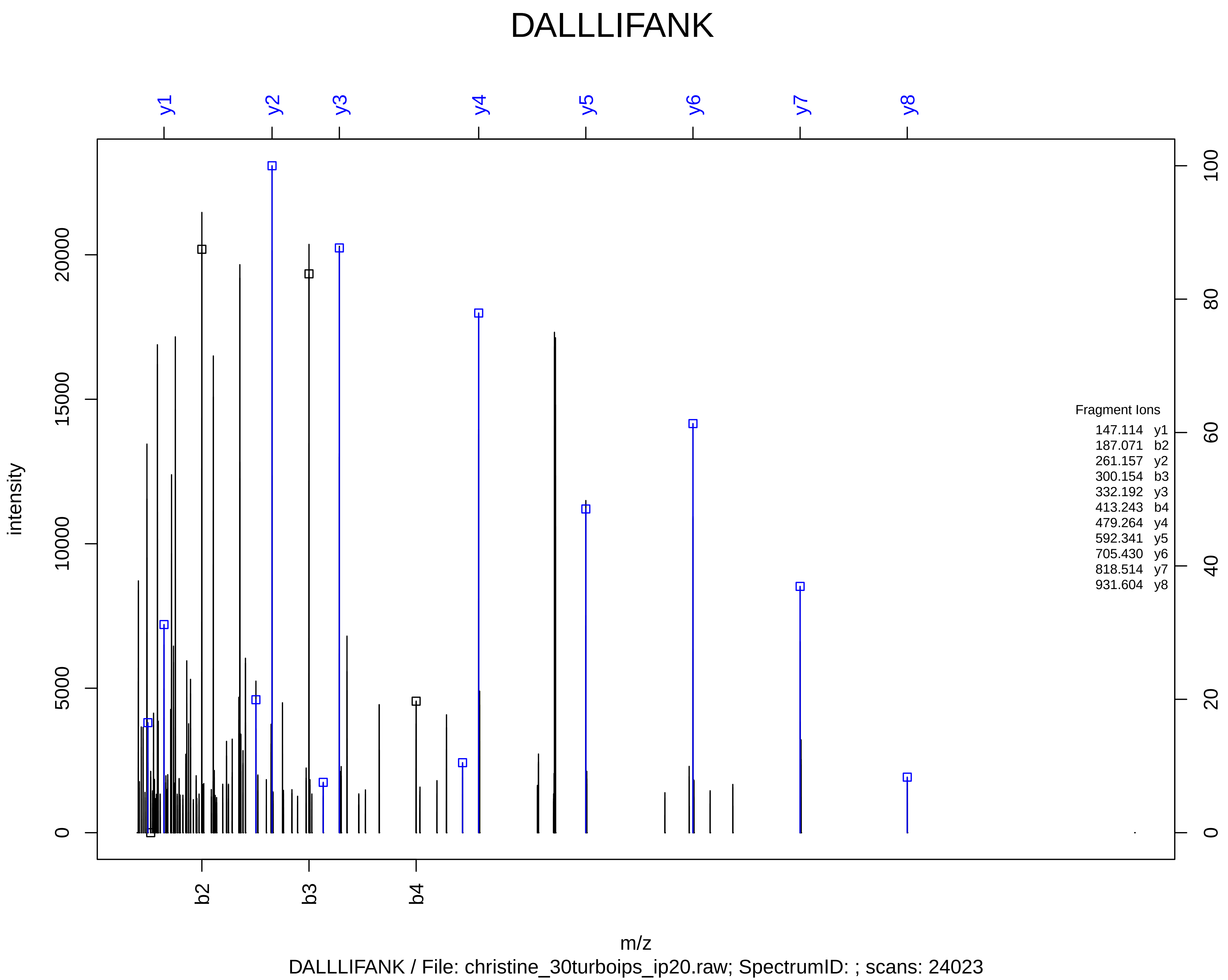
DALLLIFANK
y1
y2
y3
y4
y5
y6
y7
y8
b2
b3
b4
intensity
m/z
0
5000
10000
15000
20000
0
20
40
60
80
100
Fragment Ions
147.114 y1
187.071 b2
261.157 y2
300.154 b3
332.192 y3
413.243 b4
479.264 y4
592.341 y5
705.430 y6
818.514 y7
931.604 y8
DALLLIFANK / File: christine_30turboips_ip20.raw; SpectrumID: ; scans: 24023

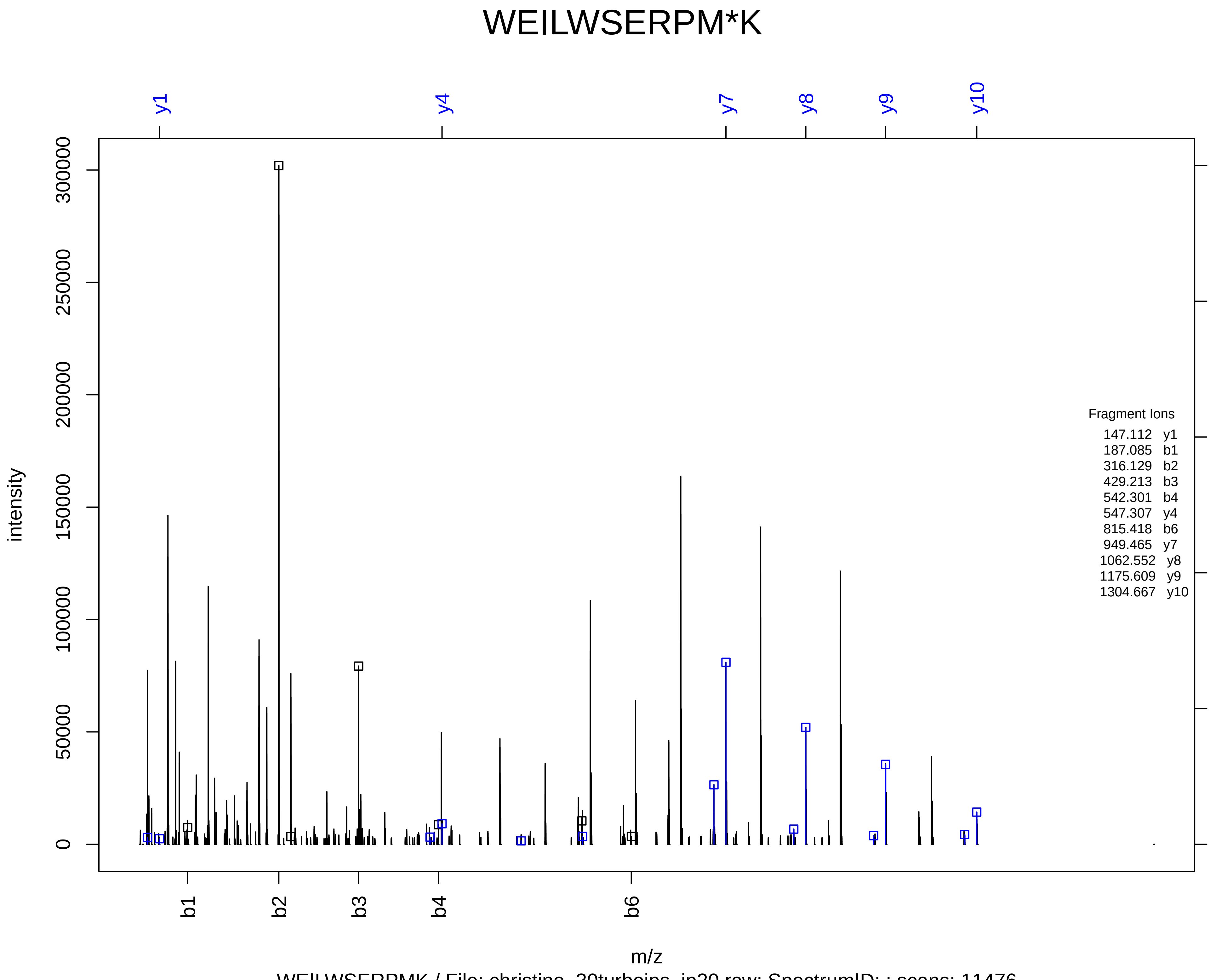
WEILWSERPM*K
y1
y4
y7
y8
y9
y10
intensity
0
50000
100000
150000
200000
250000
300000
0
20
40
60
80
100
Fragment Ions
147.112 y1
187.085 b1
316.129 b2
429.213 b3
542.301 b4
547.307 y4
815.418 b6
949.465 y7
1062.552 y8
1175.609 y9
1304.667 y10
b1
b2
b3
b4
b6
m/z
WEILWSERPMK / File: christine_30turboips_ip20.raw; SpectrumID: ; scans: 11476

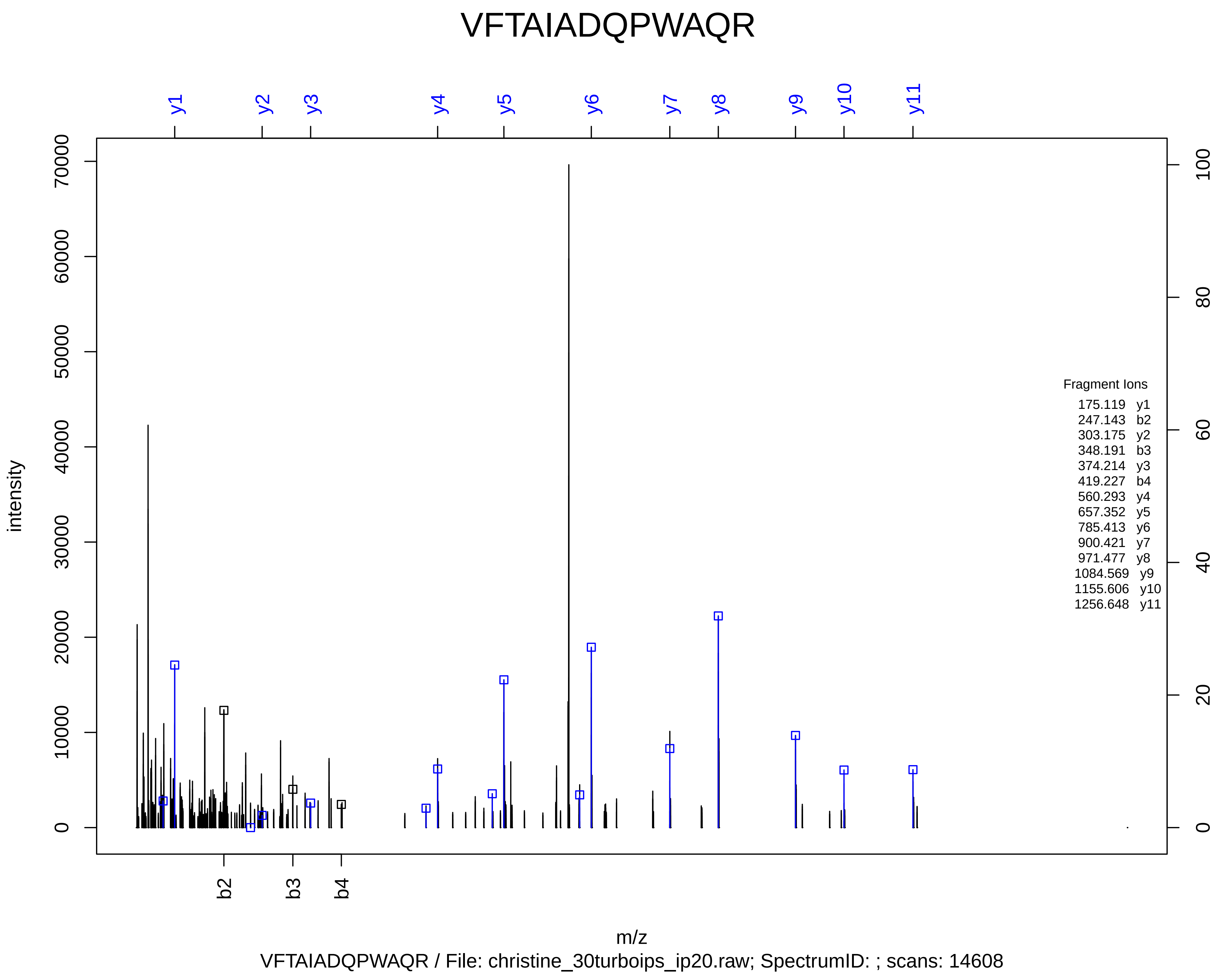
VFTAIADQPWAQR
y1
y2
y3
y4
y5
y6
y7
y8
y9
y10
y11
b2
b3
b4
intensity
m/z
0
10000
20000
30000
40000
50000
60000
70000
0
20
40
60
80
100
Fragment Ions
175.119 y1
247.143 b2
303.175 y2
348.191 b3
374.214 y3
419.227 b4
560.293 y4
657.352 y5
785.413 y6
900.421 y7
971.477 y8
1084.569 y9
1155.606 y10
1256.648 y11
VFTAIADQPWAQR / File: christine_30turboips_ip20.raw; SpectrumID: ; scans: 14608

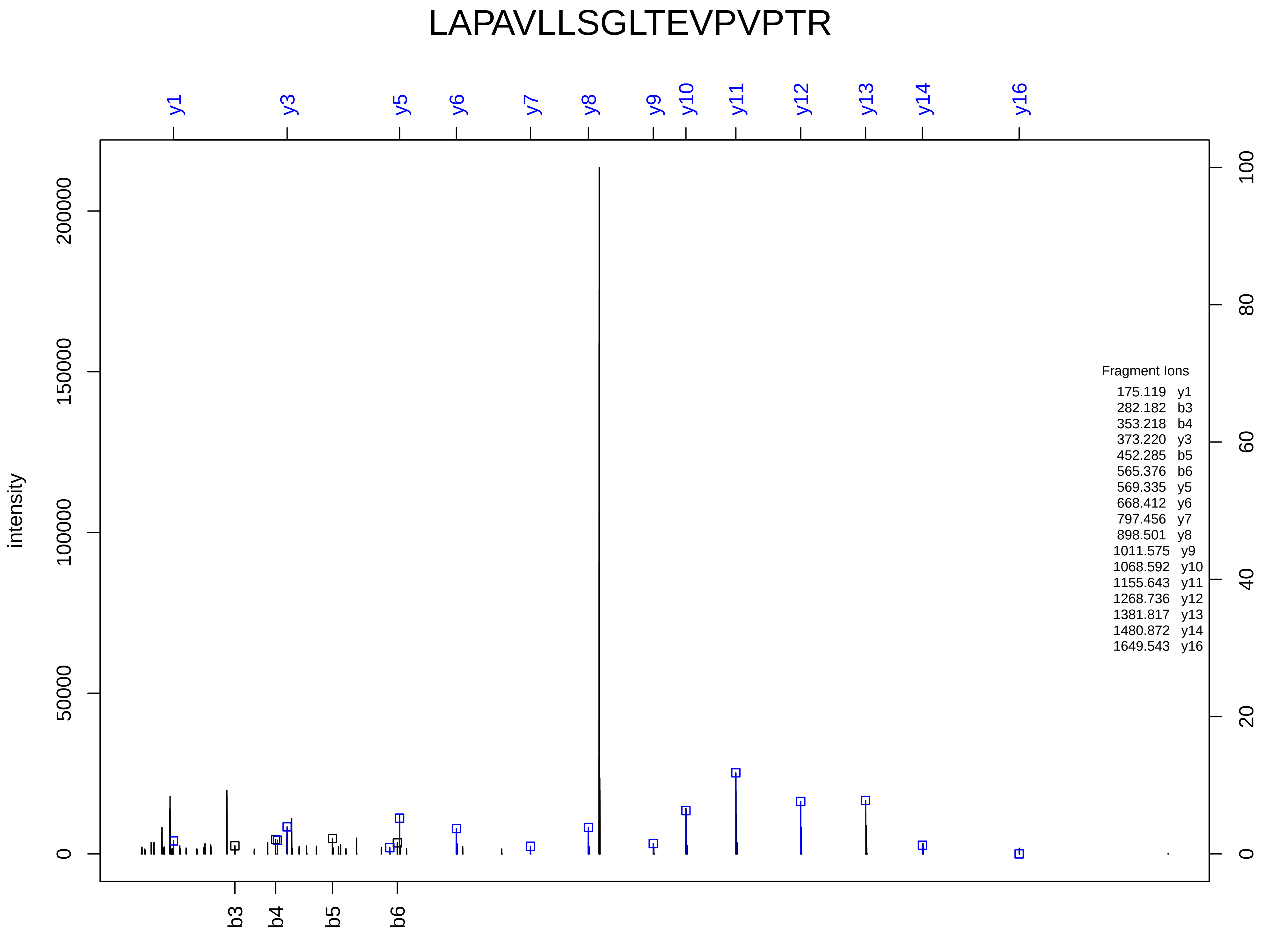

LAPAVLLSGLTEVPVPTR / File: christine_30turboips_ip20.raw; SpectrumID: ; scans: 29077

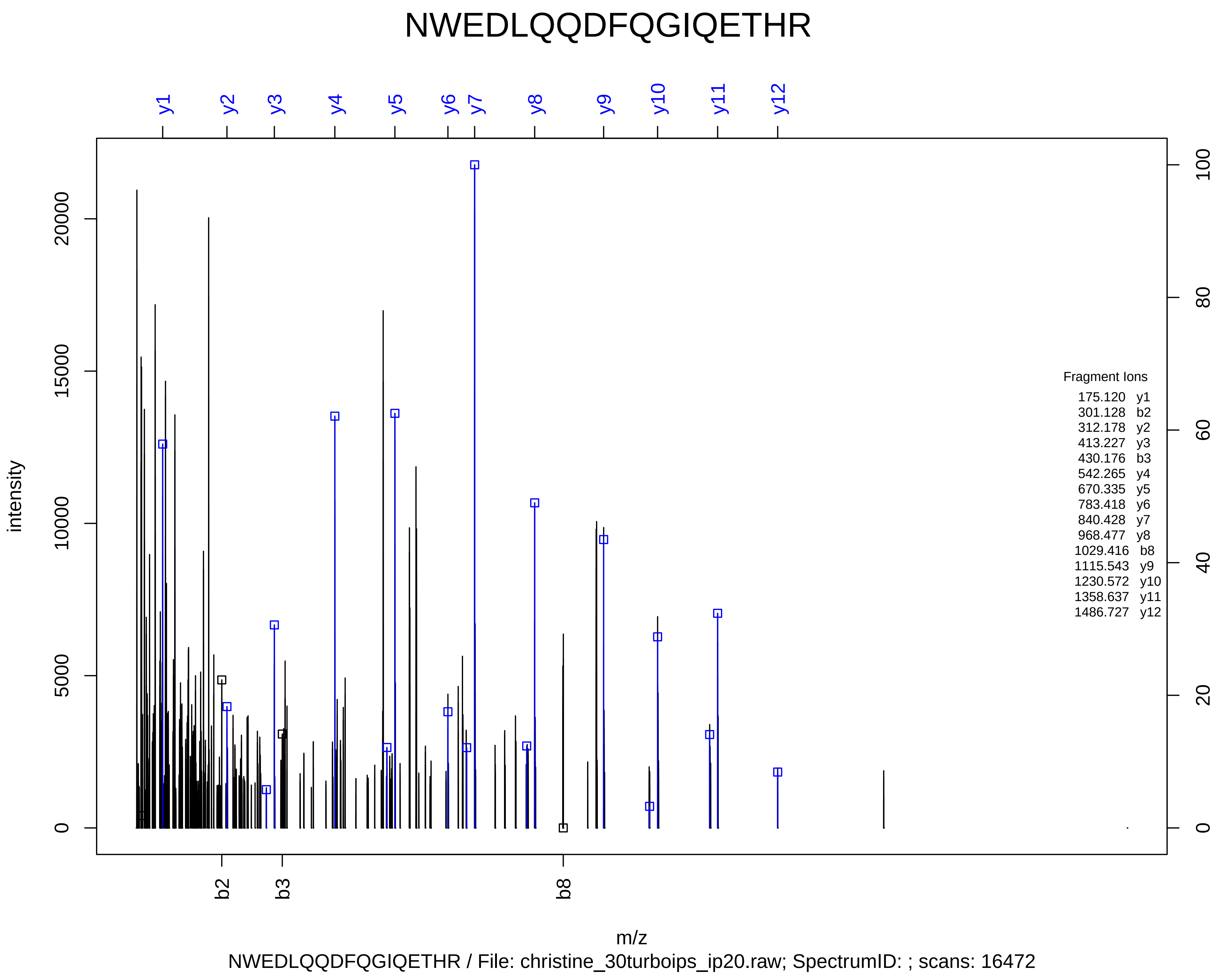
NWEDLQQDFQGIQETHR
y1
y2
y3
y4
y5
y6
y7
y8
y9
y10
y11
y12
intensity
0
5000
10000
15000
20000
0
20
40
60
80
100
b2
b3
b8
m/z
Fragment Ions
175.120 y1
301.128 b2
312.178 y2
413.227 y3
430.176 b3
542.265 y4
670.335 y5
783.418 y6
840.428 y7
968.477 y8
1029.416 b8
1115.543 y9
1230.572 y10
1358.637 y11
1486.727 y12
NWEDLQQDFQGIQETHR / File: christine_30turboips_ip20.raw; SpectrumID: ; scans: 16472

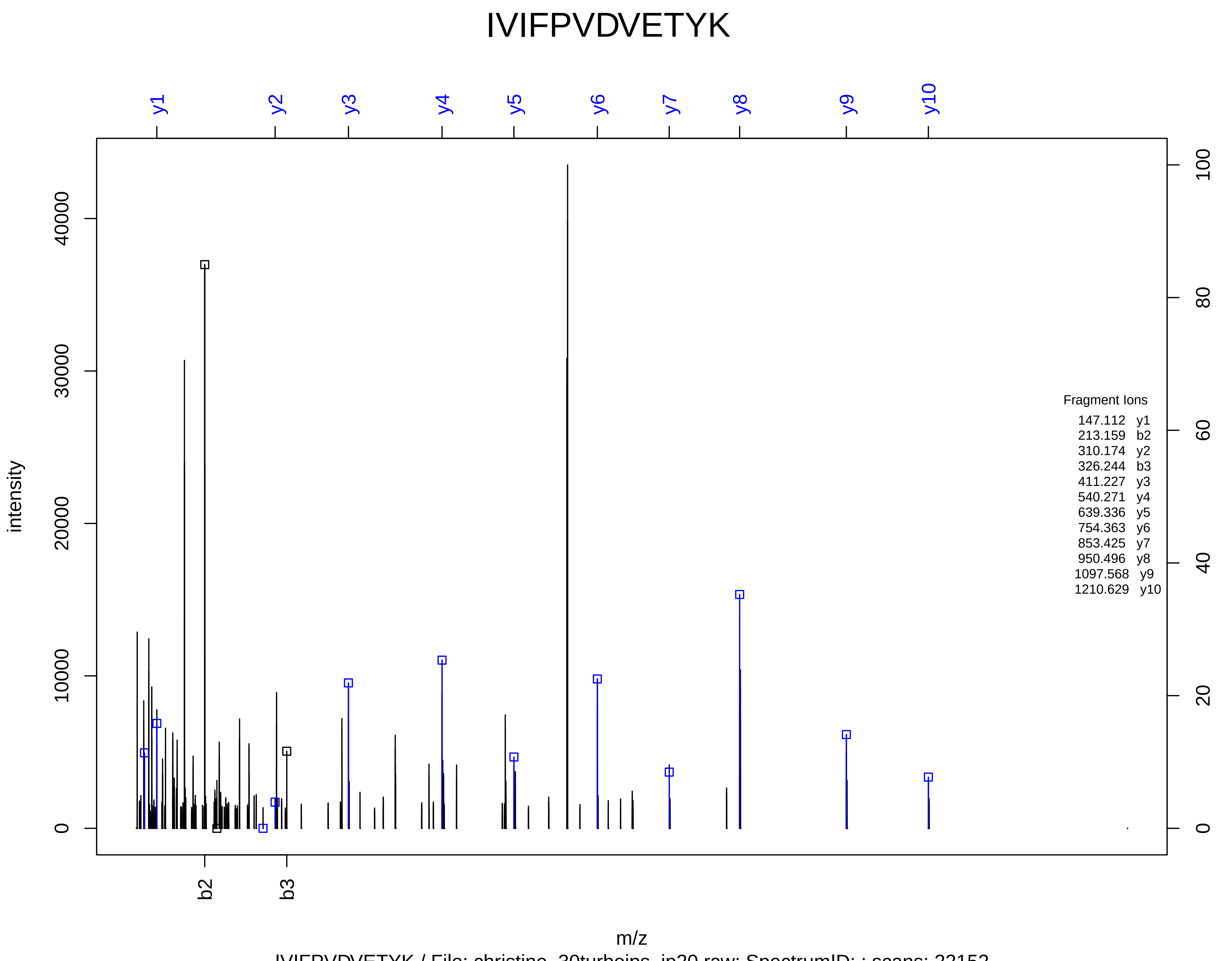
IVIFPVDVETYK
y1
y2
y3
y4
y5
y6
y7
y8
y9
y10
intensity
0
10000
20000
30000
40000
0
20
40
60
80
100
b2
b3
m/z
Fragment Ions
147.112 y1
213.159 b2
310.174 y2
326.244 b3
411.227 y3
540.271 y4
639.336 y5
754.363 y6
853.425 y7
950.496 y8
1097.568 y9
1210.629 y10
IVIFPVDVETYK / File: christine_30turboips_ip20.raw; SpectrumID: ; scans: 22152

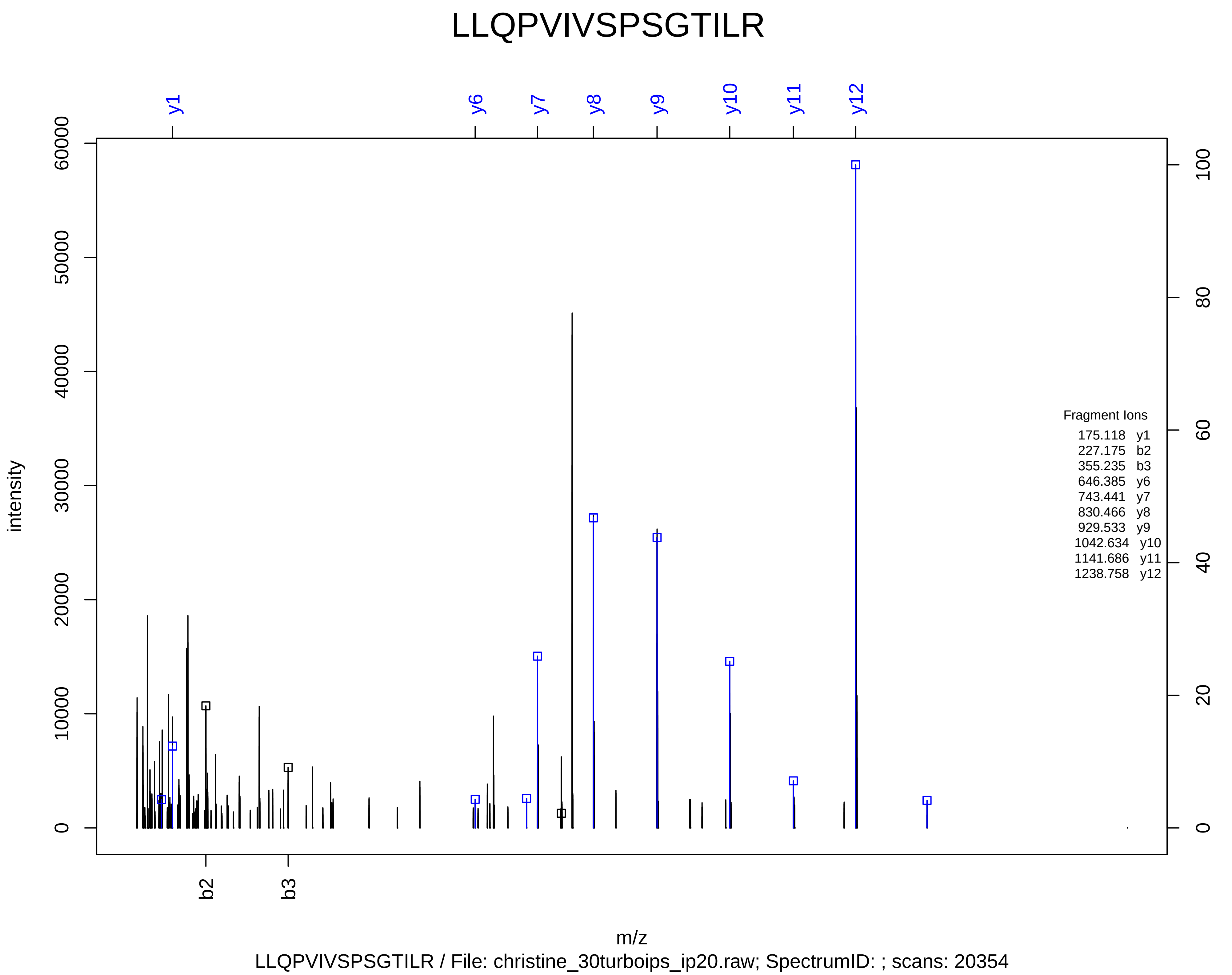
LLQPVIVSPSGTILR
y1
y6
y7
y8
y9
y10
y11
y12
b2
b3
intensity
60000
50000
40000
30000
20000
10000
0
100
80
60
40
20
0
Fragment Ions
175.118 y1
227.175 b2
355.235 b3
646.385 y6
743.441 y7
830.466 y8
929.533 y9
1042.634 y10
1141.686 y11
1238.758 y12
m/z
LLQPVIVSPSGTILR / File: christine_30turboips_ip20.raw; SpectrumID: ; scans: 20354

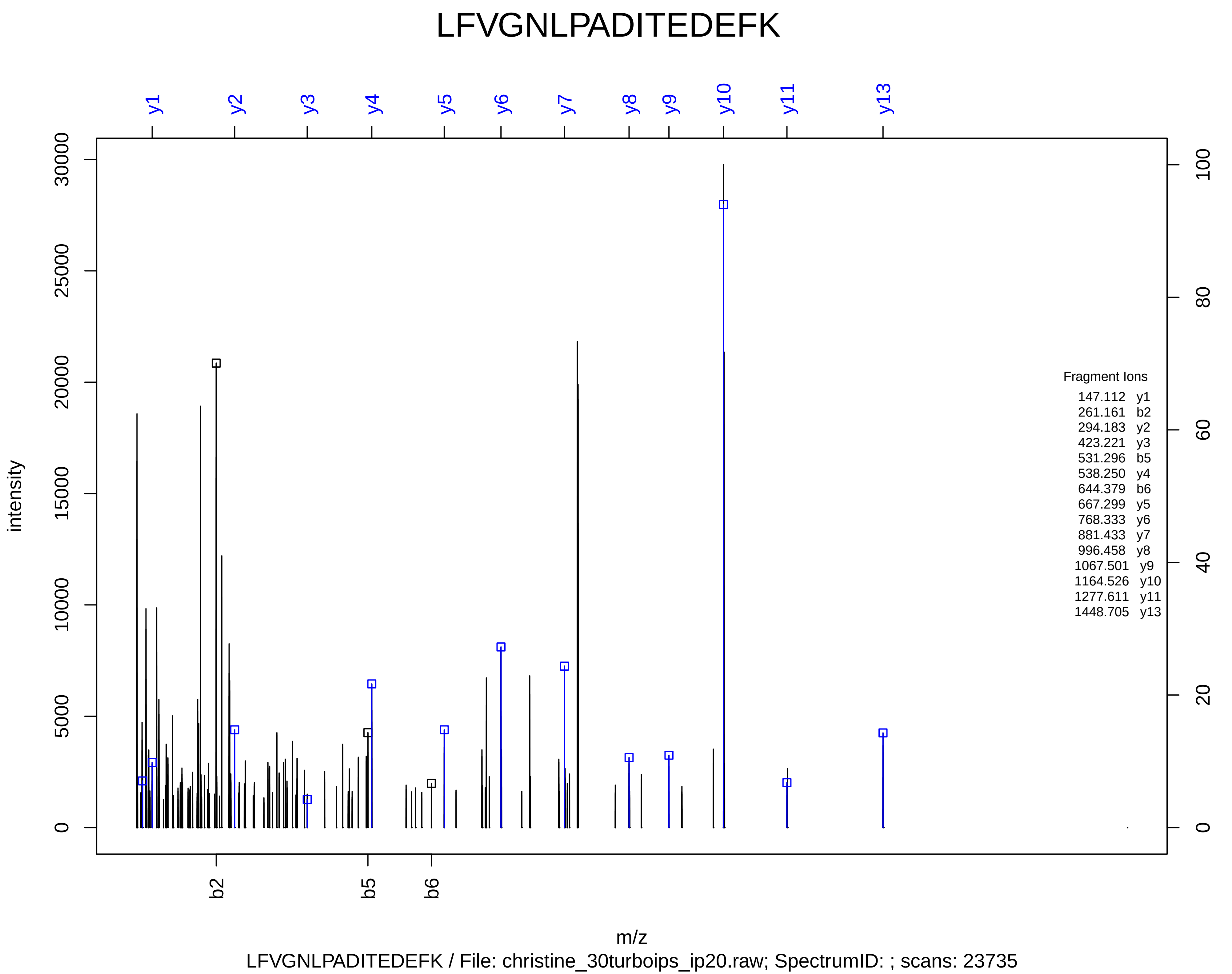
LFVGNLPADITEDEFK
y1
y2
y3
y4
y5
y6
y7
y8
y9
y10
y11
y13
intensity
30000
25000
20000
15000
10000
5000
0
100
80
60
40
20
0
b2
b5
b6
m/z
Fragment Ions
147.112 y1
261.161 b2
294.183 y2
423.221 y3
531.296 b5
538.250 y4
644.379 b6
667.299 y5
768.333 y6
881.433 y7
996.458 y8
1067.501 y9
1164.526 y10
1277.611 y11
1448.705 y13
LFVGNLPADITEDEFK / File: christine_30turboips_ip20.raw; SpectrumID: ; scans: 23735

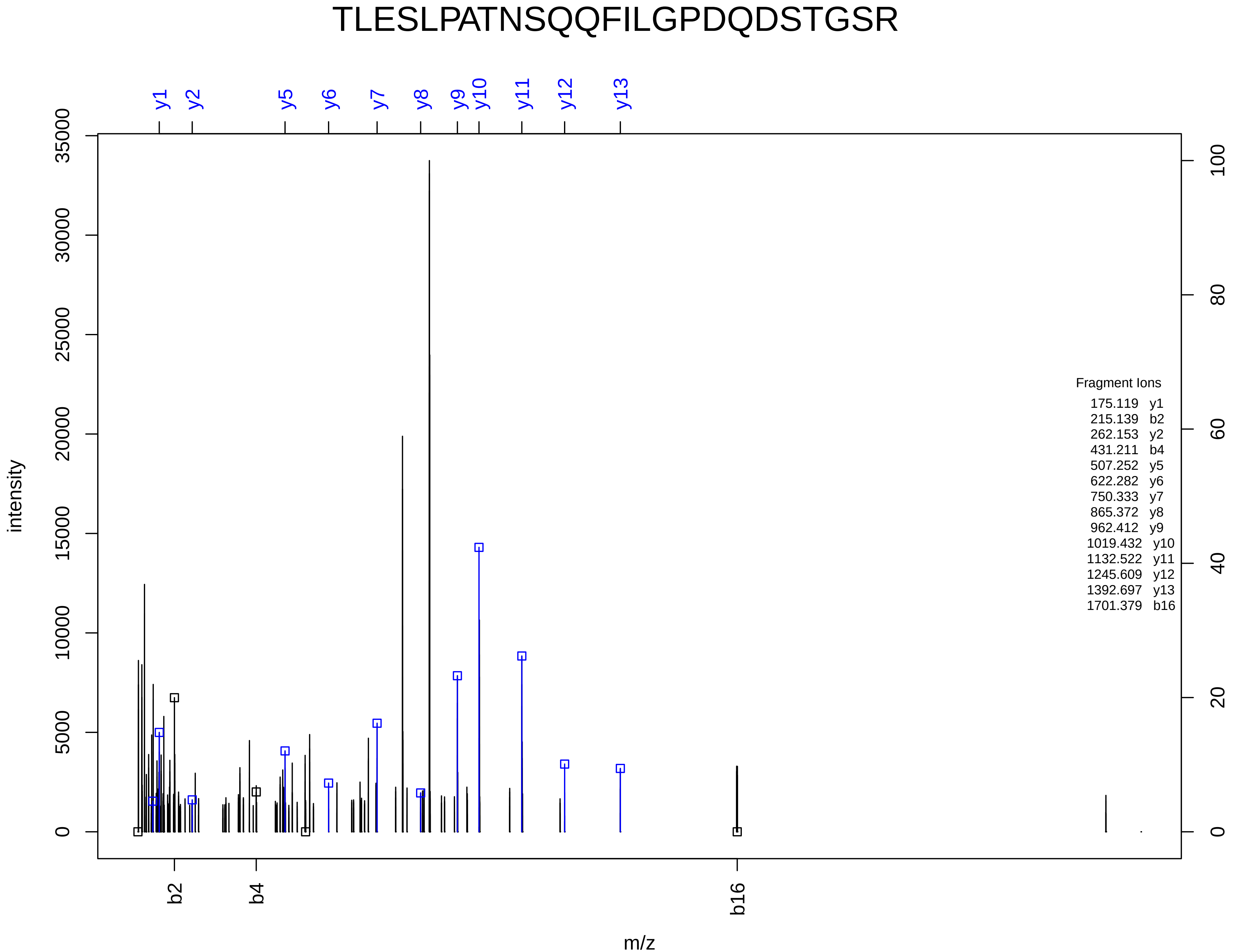

TLESLPATNSQQFILGPDQDSTGSR / File: christine_30turboips_ip20.raw; SpectrumID: ; scans: 17782

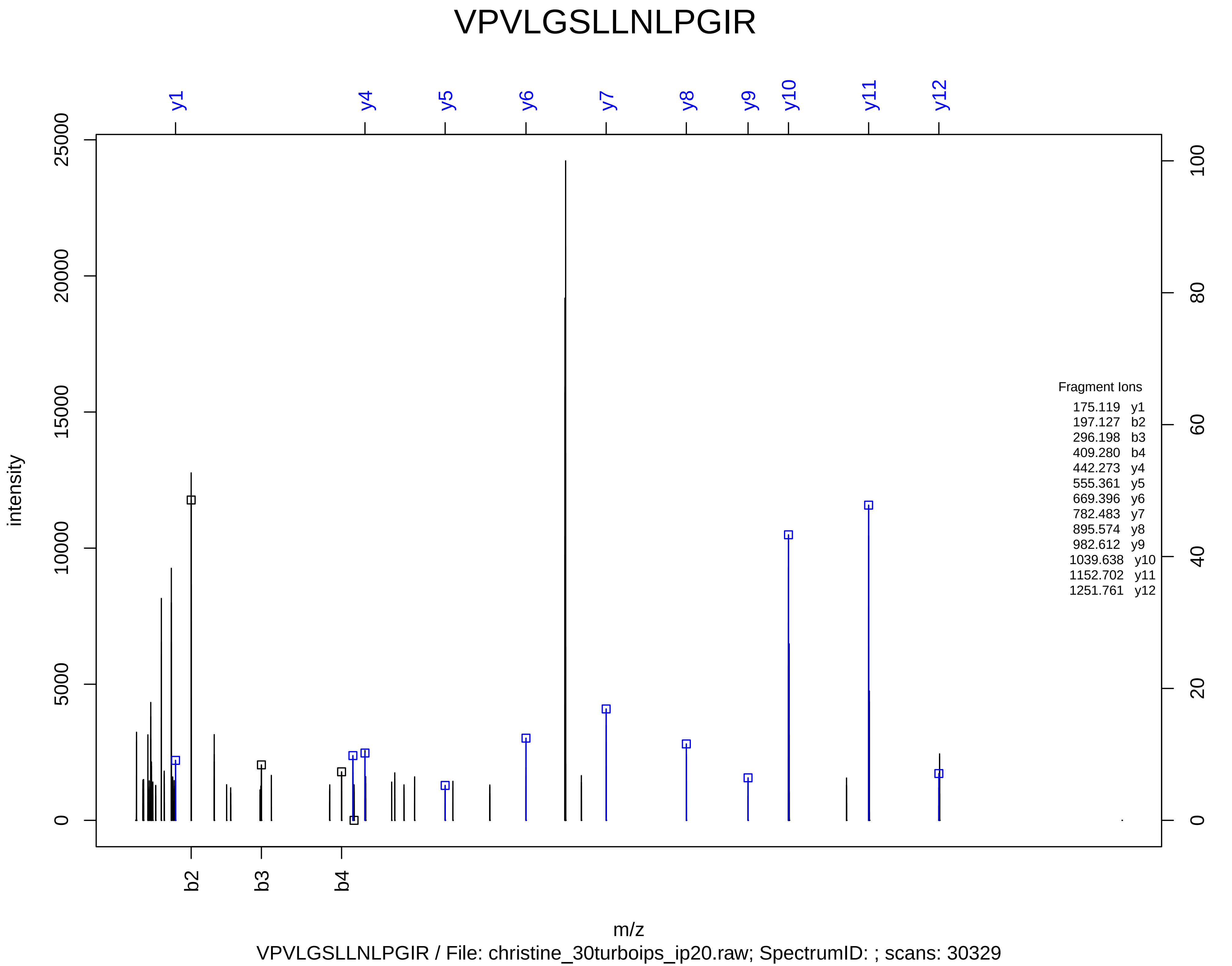
VPVLGSLLNLPGIR
y1
y4
y5
y6
y7
y8
y9
y10
y11
y12
intensity
0
5000
10000
15000
20000
25000
0
20
40
60
80
100
b2
b3
b4
Fragment Ions
175.119 y1
197.127 b2
296.198 b3
409.280 b4
442.273 y4
555.361 y5
669.396 y6
782.483 y7
895.574 y8
982.612 y9
1039.638 y10
1152.702 y11
1251.761 y12
m/z
VPVLGSLLNLPGIR / File: christine_30turboips_ip20.raw; SpectrumID: ; scans: 30329

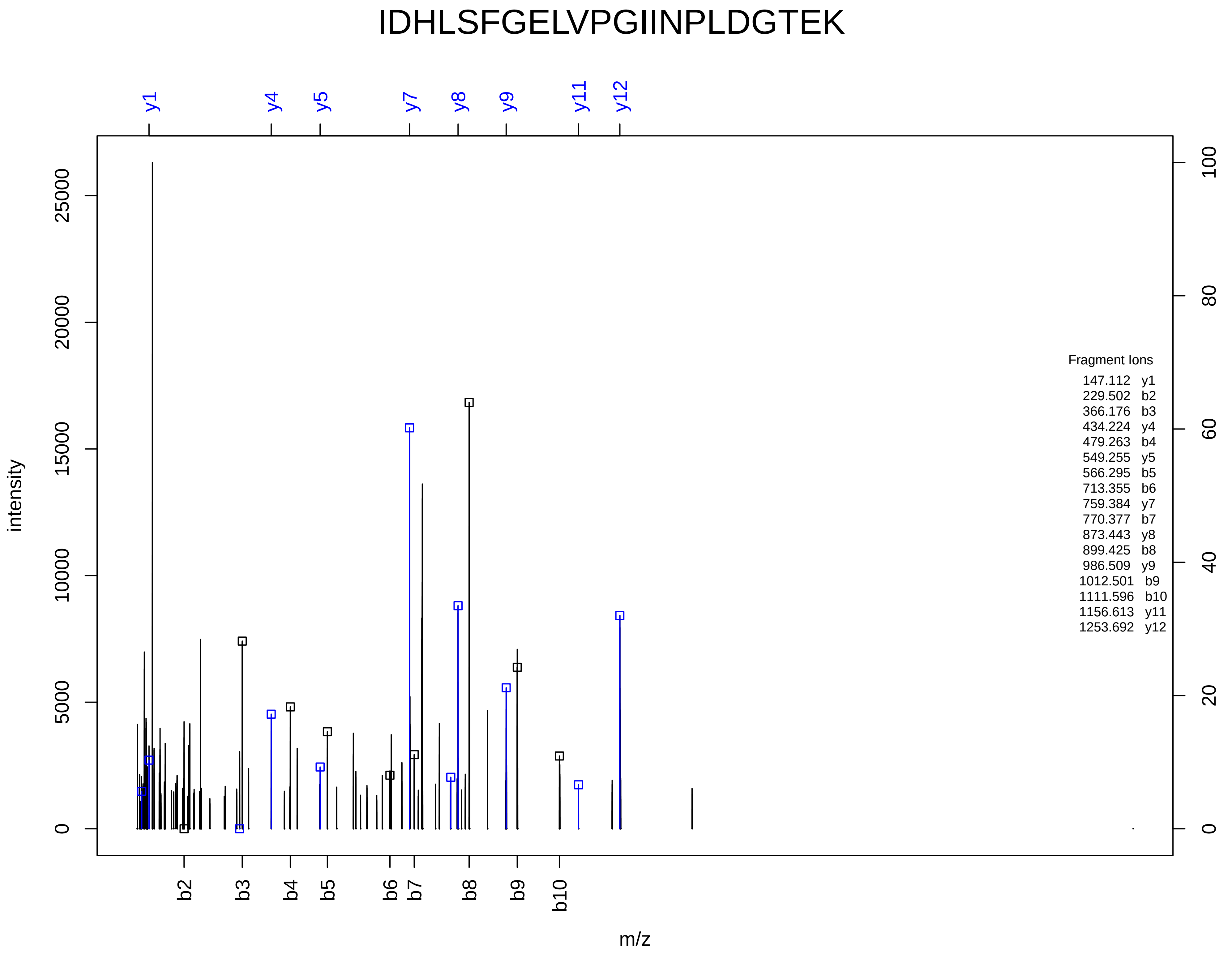
IDHLSFGELVPGIINPLDGTEK
y1
y4
y5
y7
y8
y9
y11
y12
intensity
0
5000
10000
15000
20000
25000
0
20
40
60
80
100
Fragment Ions
147.112 y1
229.502 b2
366.176 b3
434.224 y4
479.263 b4
549.255 y5
566.295 b5
713.355 b6
759.384 y7
770.377 b7
873.443 y8
899.425 b8
986.509 y9
1012.501 b9
1111.596 b10
1156.613 y11
1253.692 y12
b2
b3
b4
b5
b6
b7
b8
b9
b10
m/z
IDHLSFGELVPGIINPLDGTEK / File: christine_30turboips_ip20.raw; SpectrumID: ; scans: 28714

IQQILTSTGFSDK

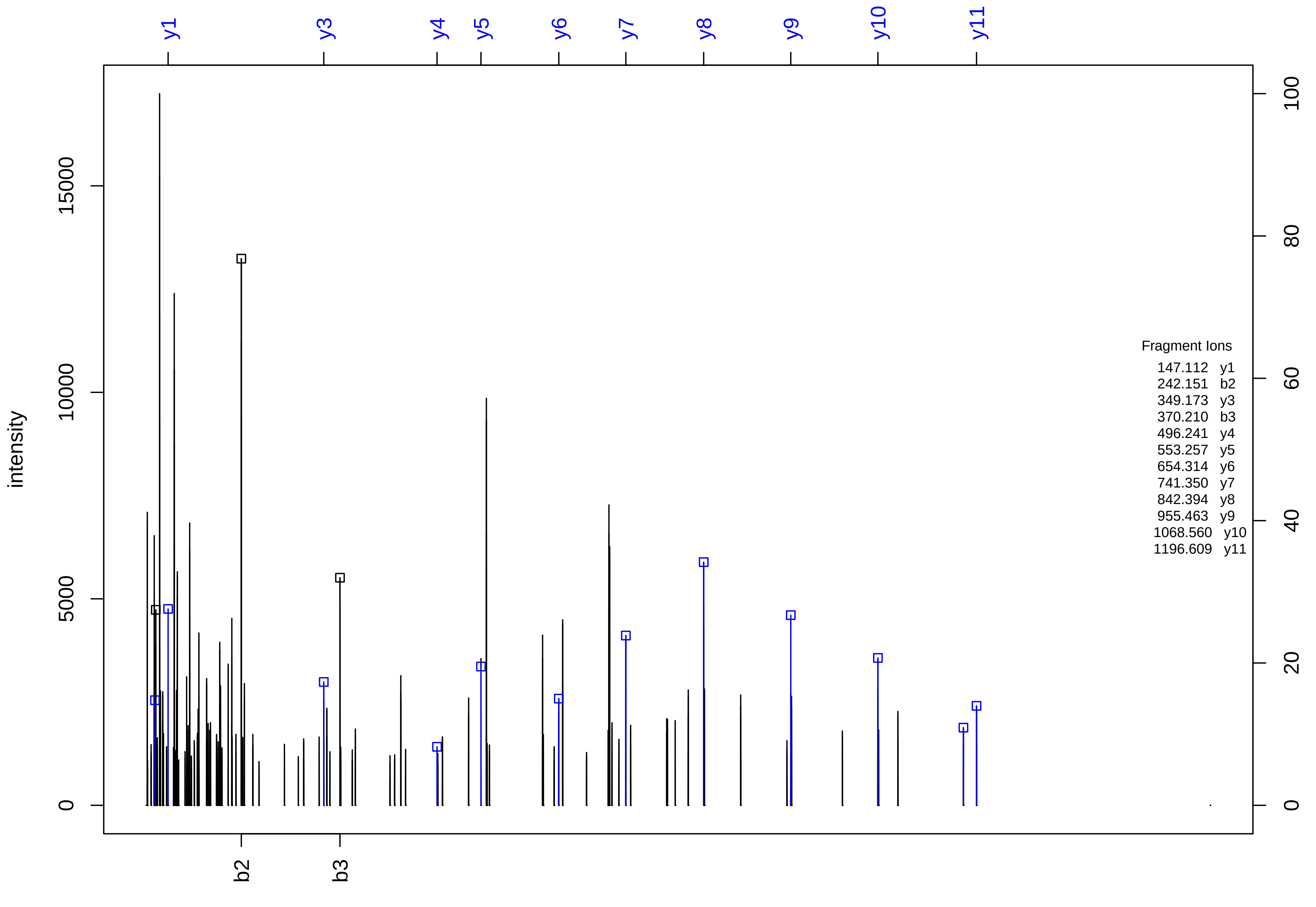

IQQILTSTGFSDK / File: christine_30turboips_ip20.raw; SpectrumID: ; scans: 10176

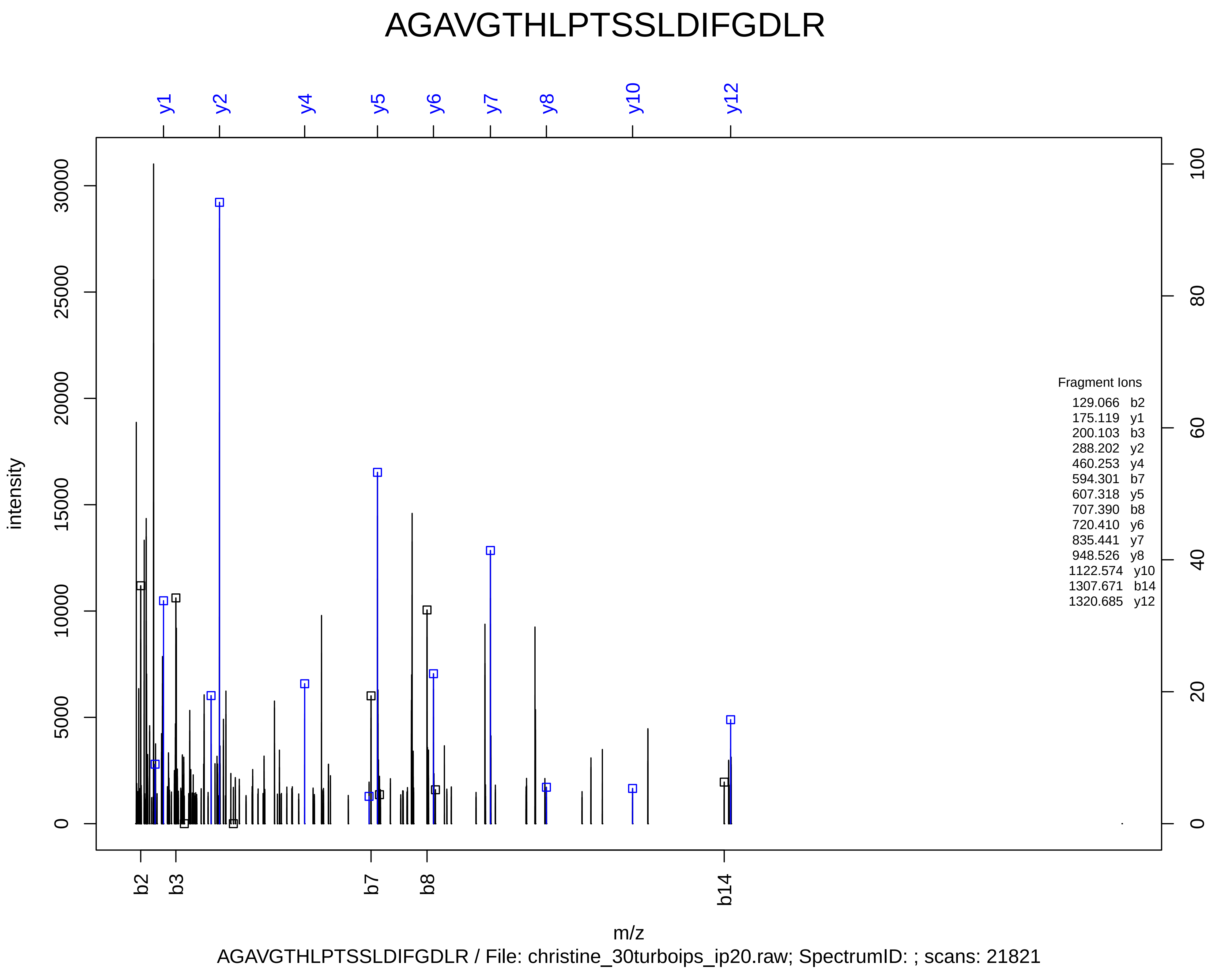

AGAVGTHLPTSSLDIFGDLR
y1
y2
y4
y5
y6
y7
y8
y10
y12
intensity
0
5000
10000
15000
20000
25000
30000
0
20
40
60
80
100
b2
b3
b7
b8
b14
m/z
Fragment Ions
129.066 b2
175.119 y1
200.103 b3
288.202 y2
460.253 y4
594.301 b7
607.318 y5
707.390 b8
720.410 y6
835.441 y7
948.526 y8
1122.574 y10
1307.671 b14
1320.685 y12
AGAVGTHLPTSSLDIFGDLR / File: christine_30turboips_ip20.raw; SpectrumID: ; scans: 21821

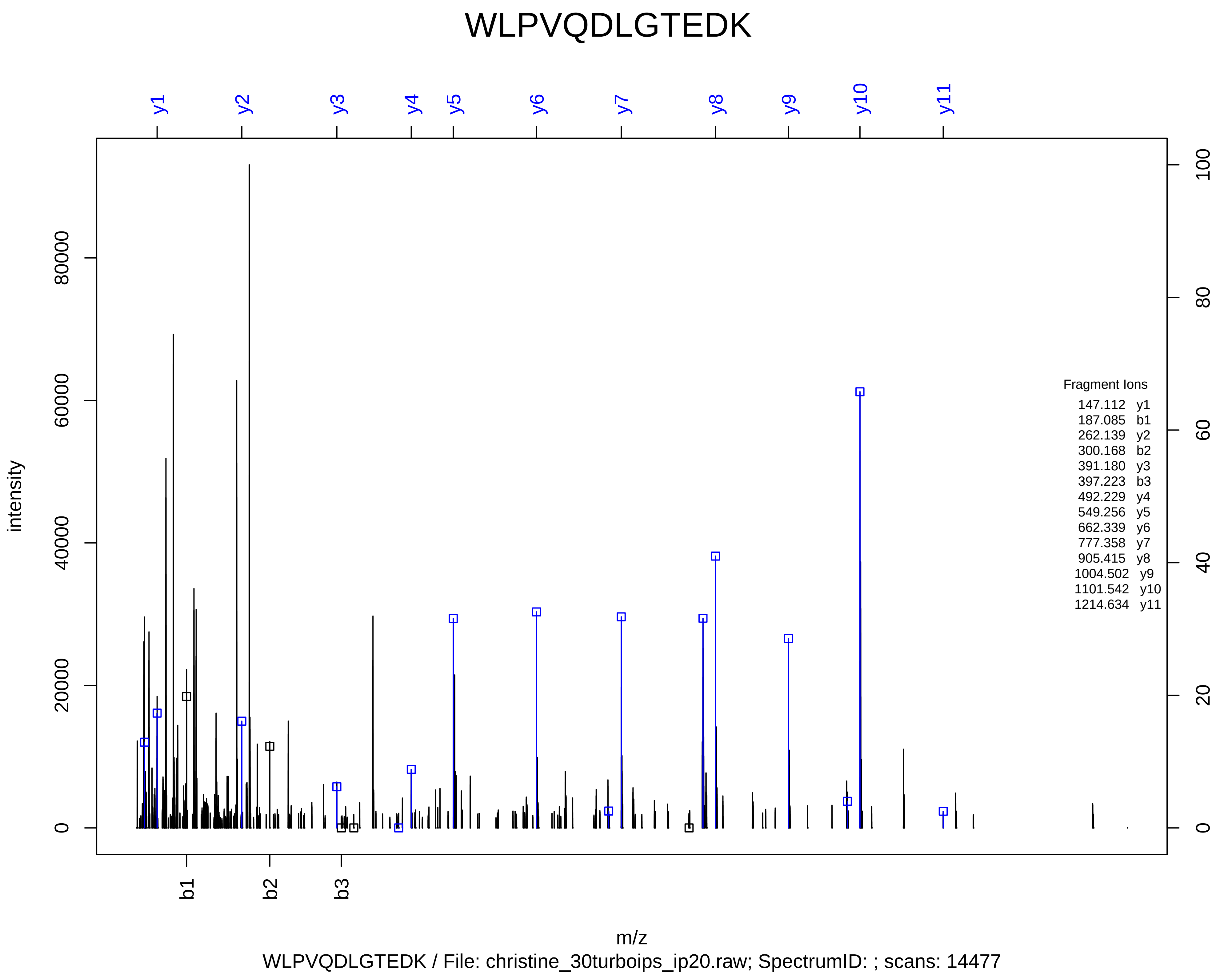
WLPVQDLGTEDK
y1
y2
y3
y4
y5
y6
y7
y8
y9
y10
y11
b1
b2
b3
intensity
m/z
Fragment Ions
147.112 y1
187.085 b1
262.139 y2
300.168 b2
391.180 y3
397.223 b3
492.229 y4
549.256 y5
662.339 y6
777.358 y7
905.415 y8
1004.502 y9
1101.542 y10
1214.634 y11
WLPVQDLGTEDK / File: christine_30turboips_ip20.raw; SpectrumID: ; scans: 14477

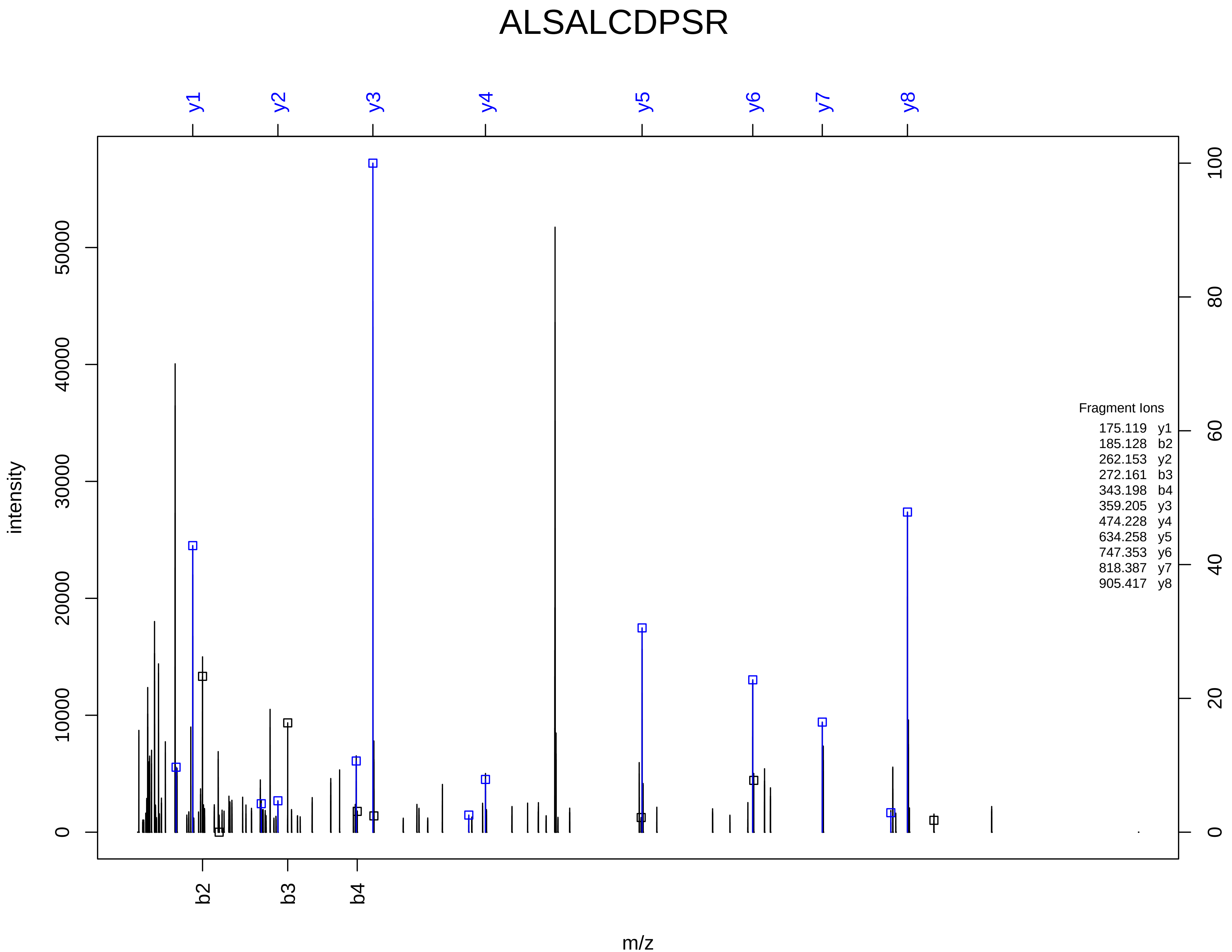

ALSALCDPSR
y1
y2
y3
y4
y5
y6
y7
y8
b2
b3
b4
intensity
0
10000
20000
30000
40000
50000
0
20
40
60
80
100
Fragment Ions
175.119 y1
185.128 b2
262.153 y2
272.161 b3
343.198 b4
359.205 y3
474.228 y4
634.258 y5
747.353 y6
818.387 y7
905.417 y8
m/z
ALSALCDPSR / File: christine_30turboips_ip20.raw; SpectrumID: ; scans: 2896

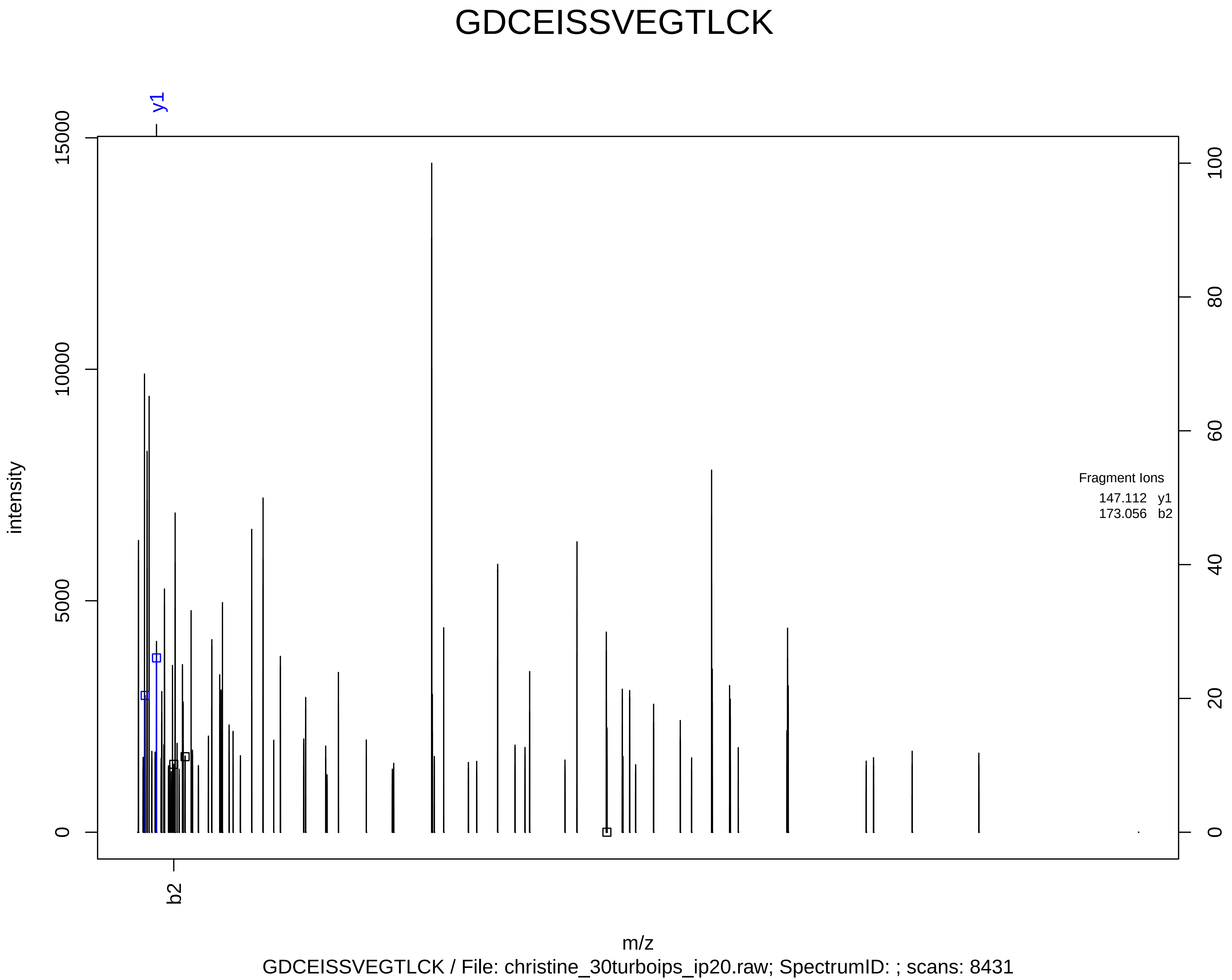
GDCEISSVEGTLCK
y1
b2
intensity
m/z
15000
10000
5000
0
100
80
60
40
20
0
Fragment Ions
147.112 y1
173.056 b2
GDCEISSVEGTLCK / File: christine_30turboips_ip20.raw; SpectrumID: ; scans: 8431

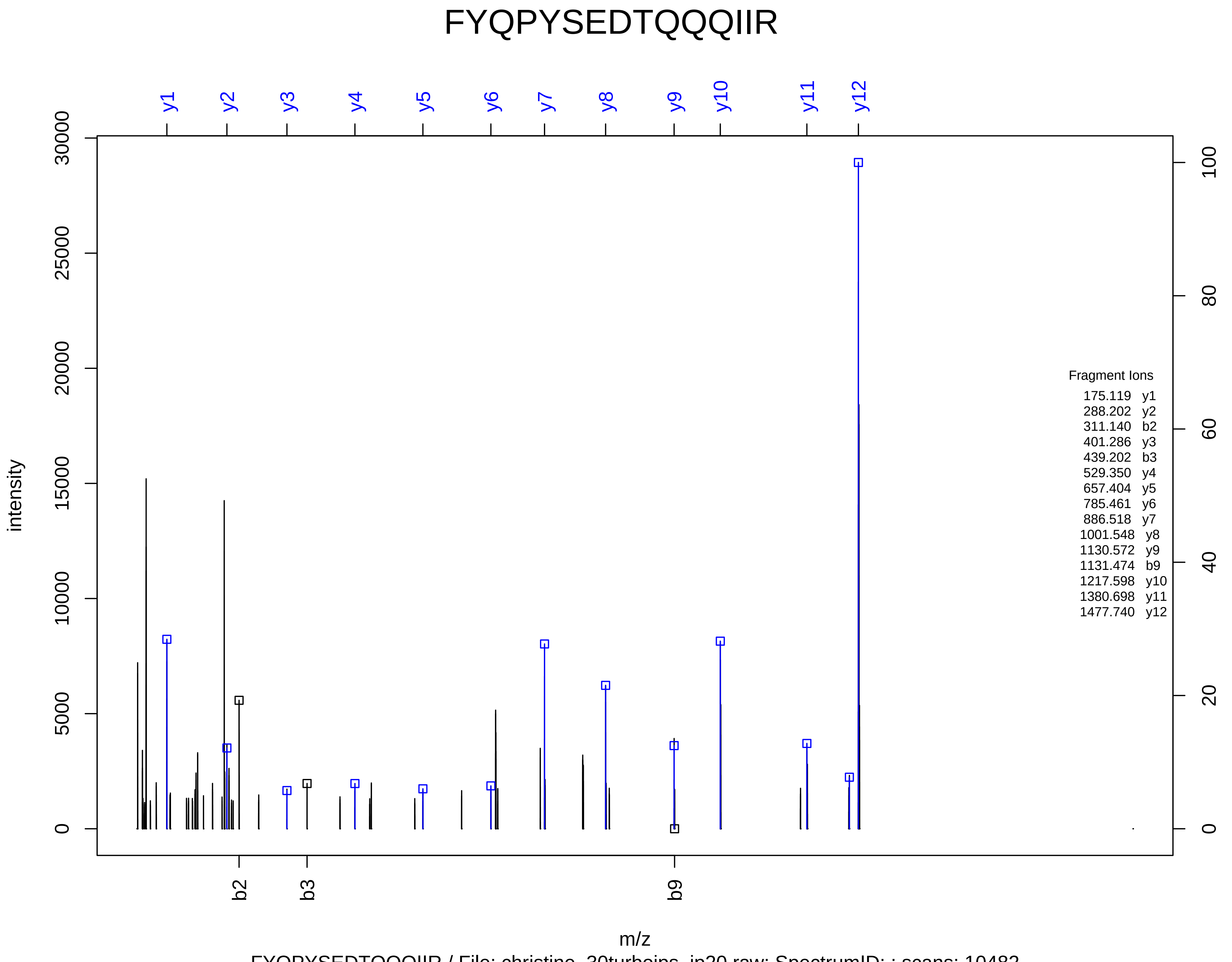
FYQPYSEDTQQQIIR
y1
y2
y3
y4
y5
y6
y7
y8
y9
y10
y11
y12
intensity
0
5000
10000
15000
20000
25000
30000
0
20
40
60
80
100
b2
b3
b9
m/z
Fragment Ions
175.119 y1
288.202 y2
311.140 b2
401.286 y3
439.202 b3
529.350 y4
657.404 y5
785.461 y6
886.518 y7
1001.548 y8
1130.572 y9
1131.474 b9
1217.598 y10
1380.698 y11
1477.740 y12
FYQPYSEDTQQQIIR / File: christine_30turboips_ip20.raw; SpectrumID: ; scans: 10482

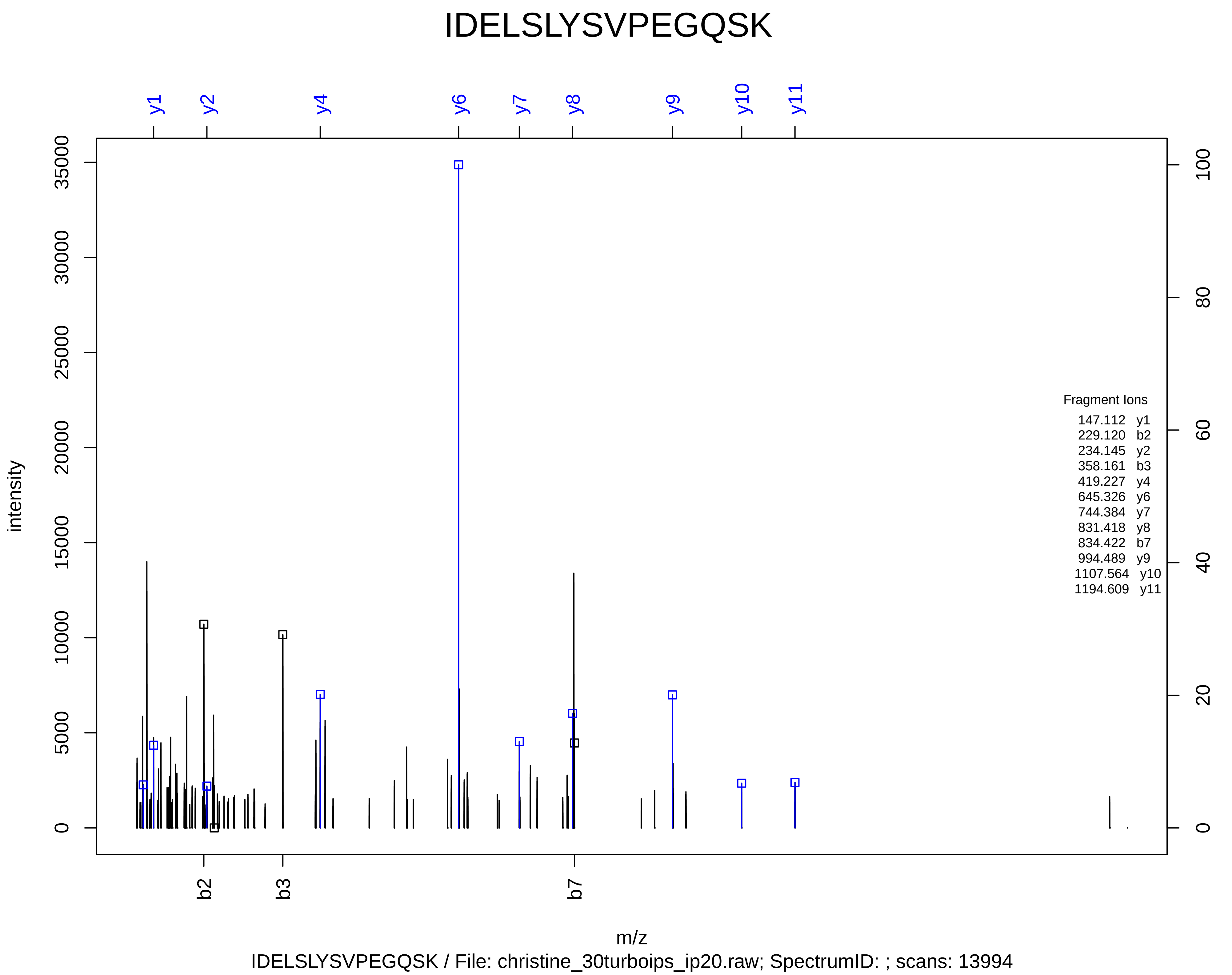
IDELSLYSVPEGQSK
y1
y2
y4
y6
y7
y8
y9
y10
y11
intensity
0
5000
10000
15000
20000
25000
30000
35000
0
20
40
60
80
100
b2
b3
b7
m/z
Fragment Ions
147.112 y1
229.120 b2
234.145 y2
358.161 b3
419.227 y4
645.326 y6
744.384 y7
831.418 y8
834.422 b7
994.489 y9
1107.564 y10
1194.609 y11
IDELSLYSVPEGQSK / File: christine_30turboips_ip20.raw; SpectrumID: ; scans: 13994

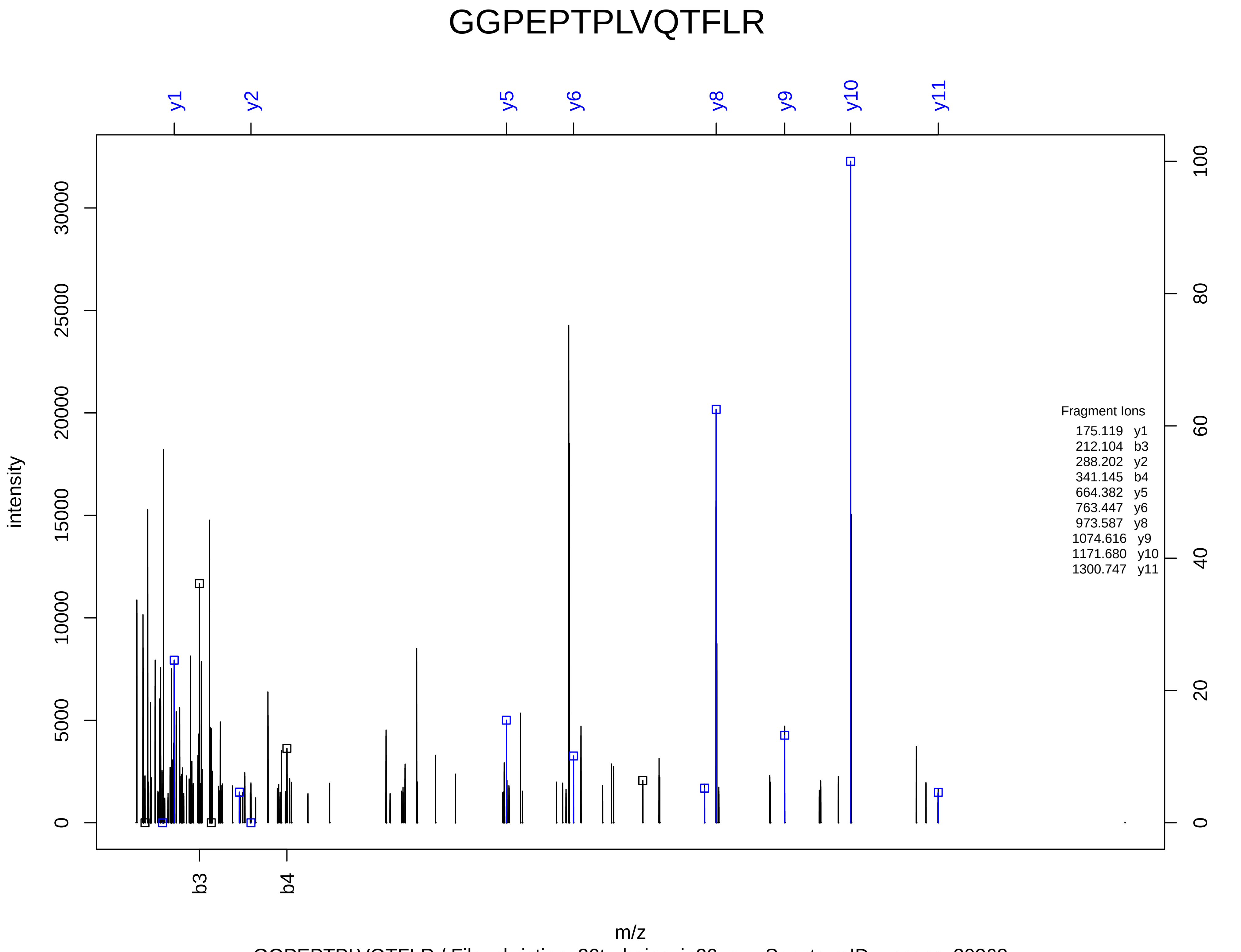

GGPEPTPLVQTFLR / File: christine_30turboips_ip20.raw; SpectrumID: ; scans: 20368

LTFSCLGGSDNFK

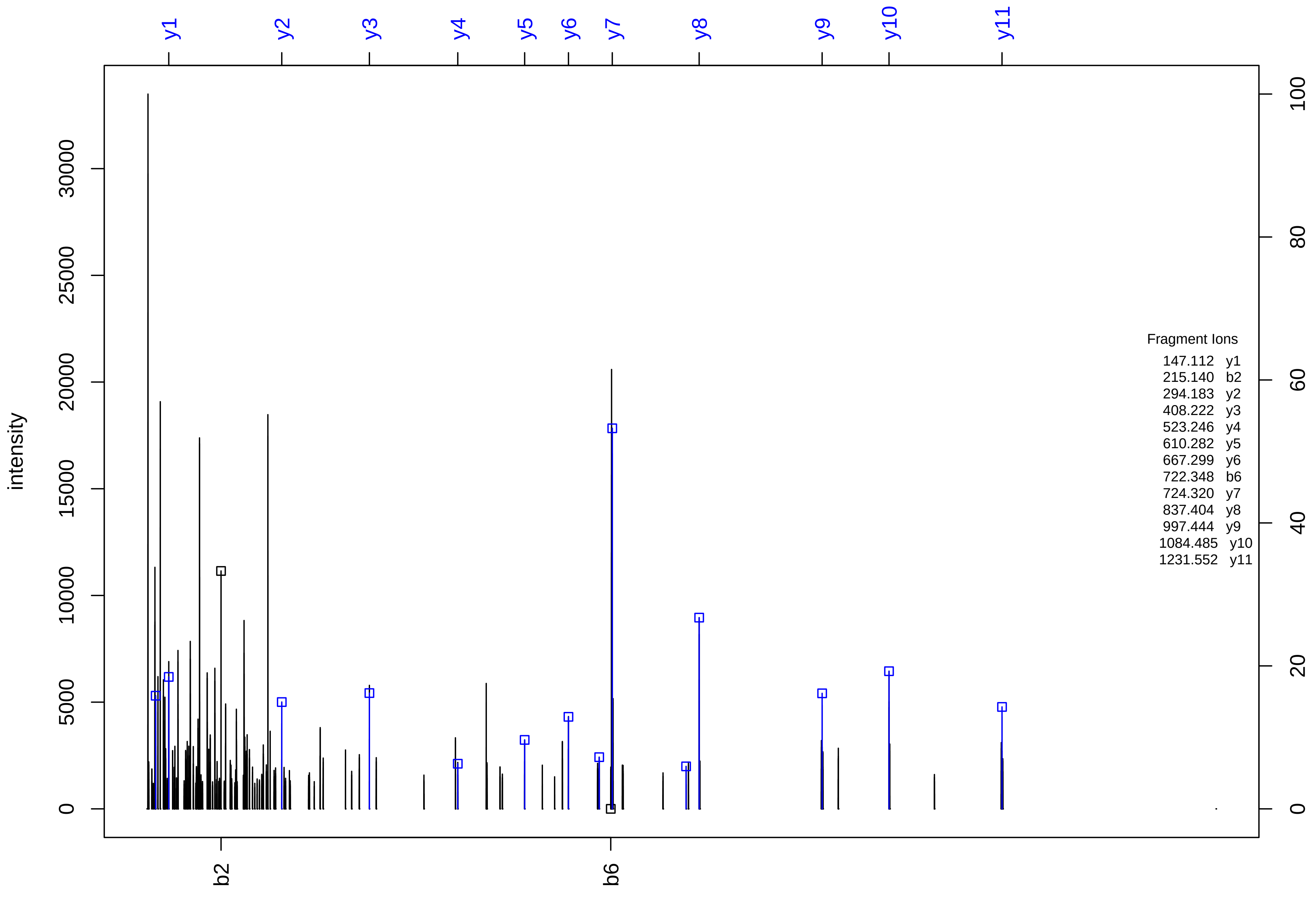

LTFSCLGGSDNFK / File: christine_30turboips_ip20.raw; SpectrumID: ; scans: 13222

SSPLYEYIQEQR

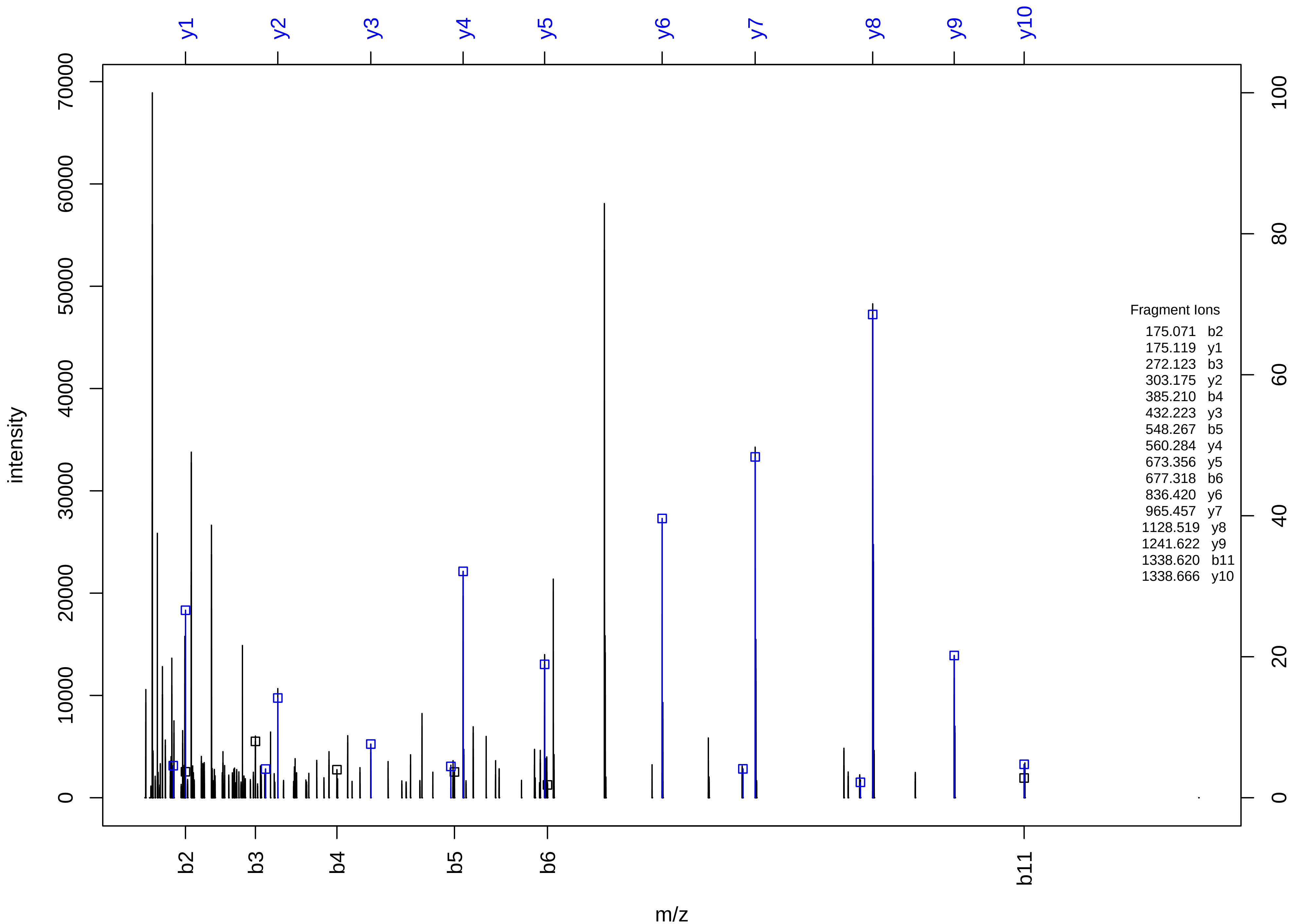

SSPLYEYIQEQR / File: christine_30turboips_ip20.raw; SpectrumID: ; scans: 12853

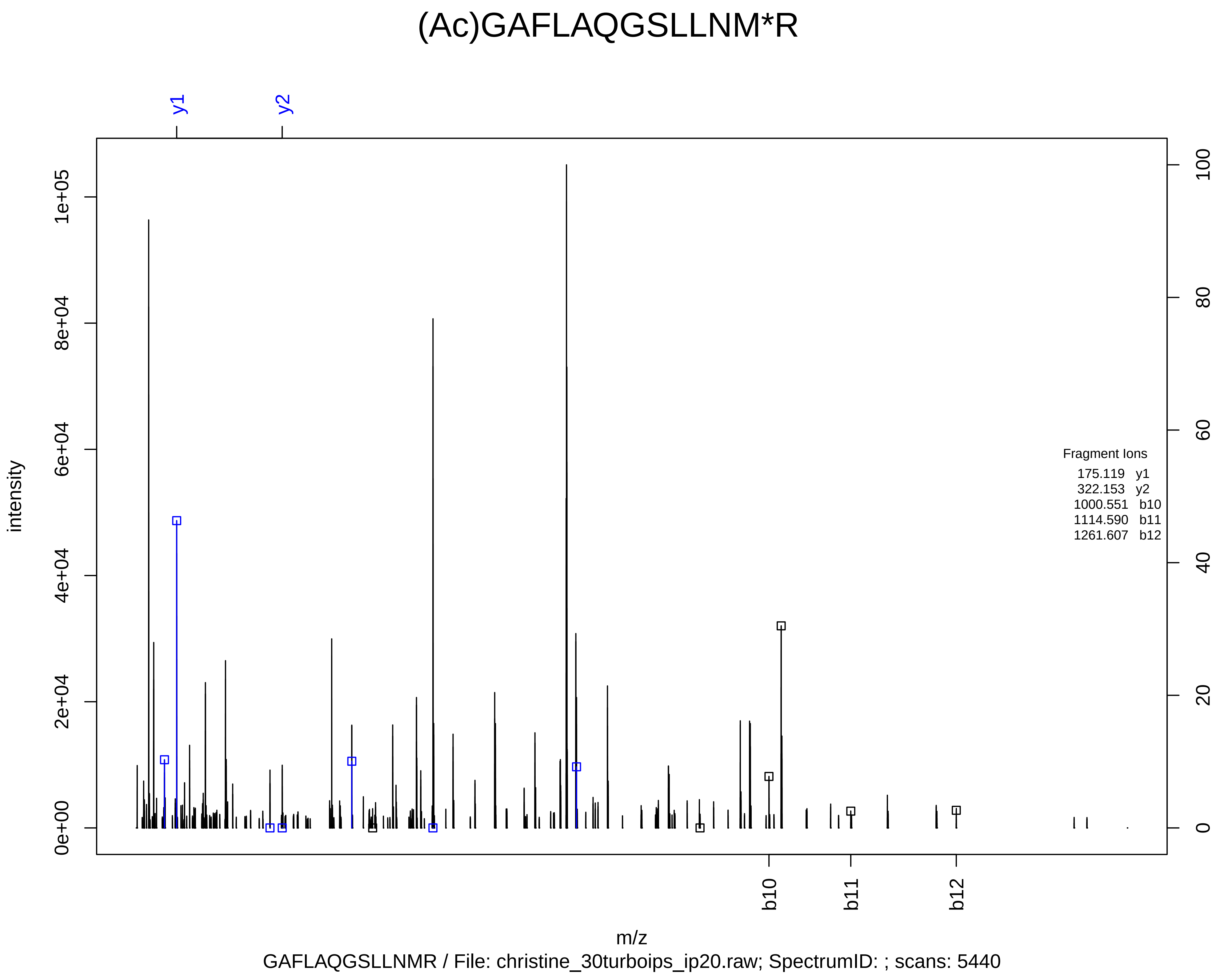
(Ac)GAFLAQGSLLNM*R
y1
y2
intensity
0e+00
2e+04
4e+04
6e+04
8e+04
1e+05
0
20
40
60
80
100
Fragment Ions
175.119 y1
322.153 y2
1000.551 b10
1114.590 b11
1261.607 b12
b10
b11
b12
m/z
GAFLAQGSLLNMR / File: christine_30turboips_ip20.raw; SpectrumID: ; scans: 5440

AIEFLNNPPEEAPR

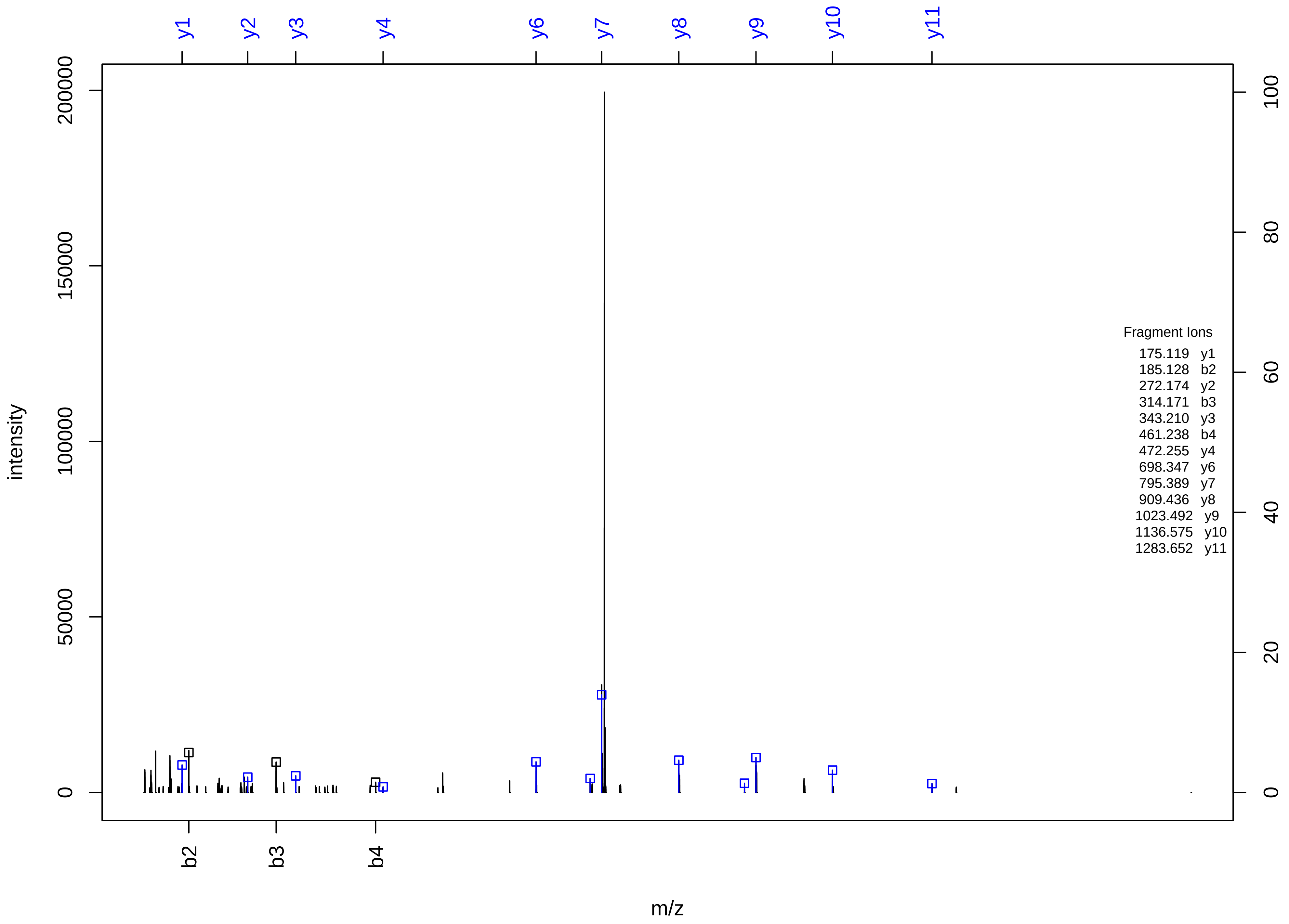

AIEFLNNPPEEAPR / File: christine_30turboips_ip21.raw; SpectrumID: ; scans: 11540

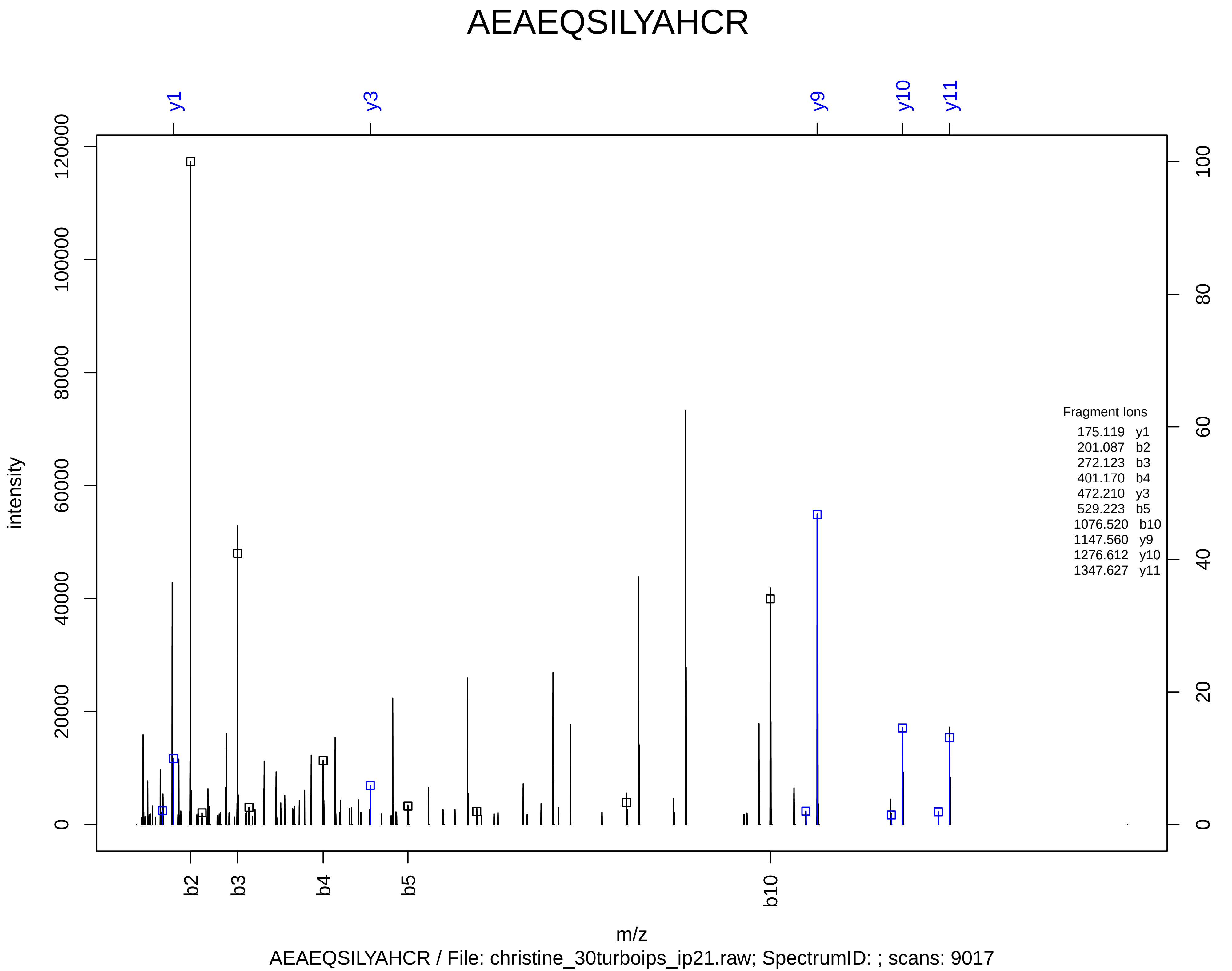

AEAEQSILYAHCR
intensity
m/z
y1
y3
y9
y10
y11
b2
b3
b4
b5
b10
0
20000
40000
60000
80000
100000
120000
20
40
60
80
100
Fragment Ions
175.119 y1
201.087 b2
272.123 b3
401.170 b4
472.210 y3
529.223 b5
1076.520 b10
1147.560 y9
1276.612 y10
1347.627 y11
AEAEQSILYAHCR / File: christine_30turboips_ip21.raw; SpectrumID: ; scans: 9017

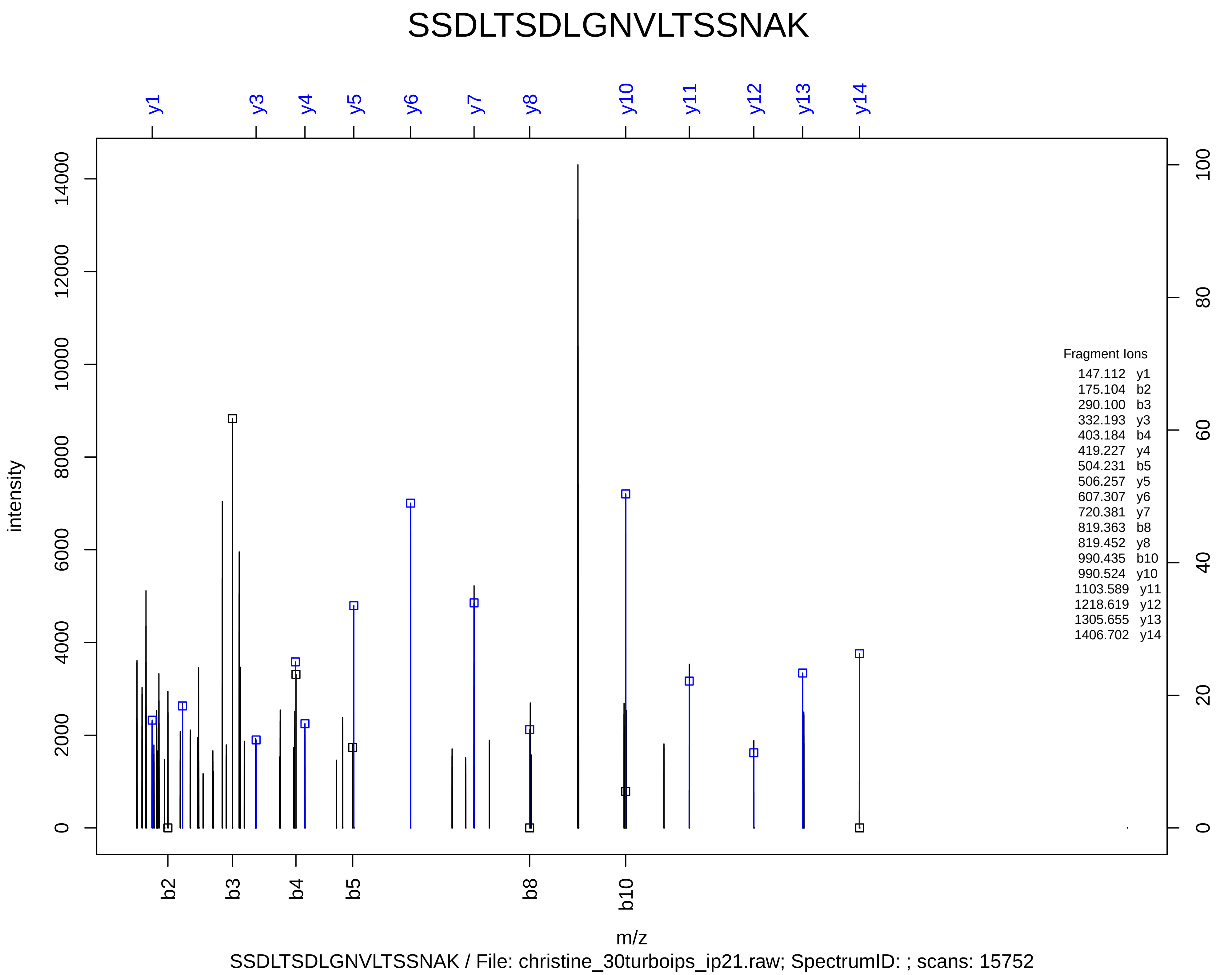
SSDLTSDLGNVLTSSNAK
intensity
m/z
Fragment Ions
147.112 y1
175.104 b2
290.100 b3
332.193 y3
403.184 b4
419.227 y4
504.231 b5
506.257 y5
607.307 y6
720.381 y7
819.363 b8
819.452 y8
990.435 b10
990.524 y10
1103.589 y11
1218.619 y12
1305.655 y13
1406.702 y14
SSDLTSDLGNVLTSSNAK / File: christine_30turboips_ip21.raw; SpectrumID: ; scans: 15752

NPVSEVVCATGPLR

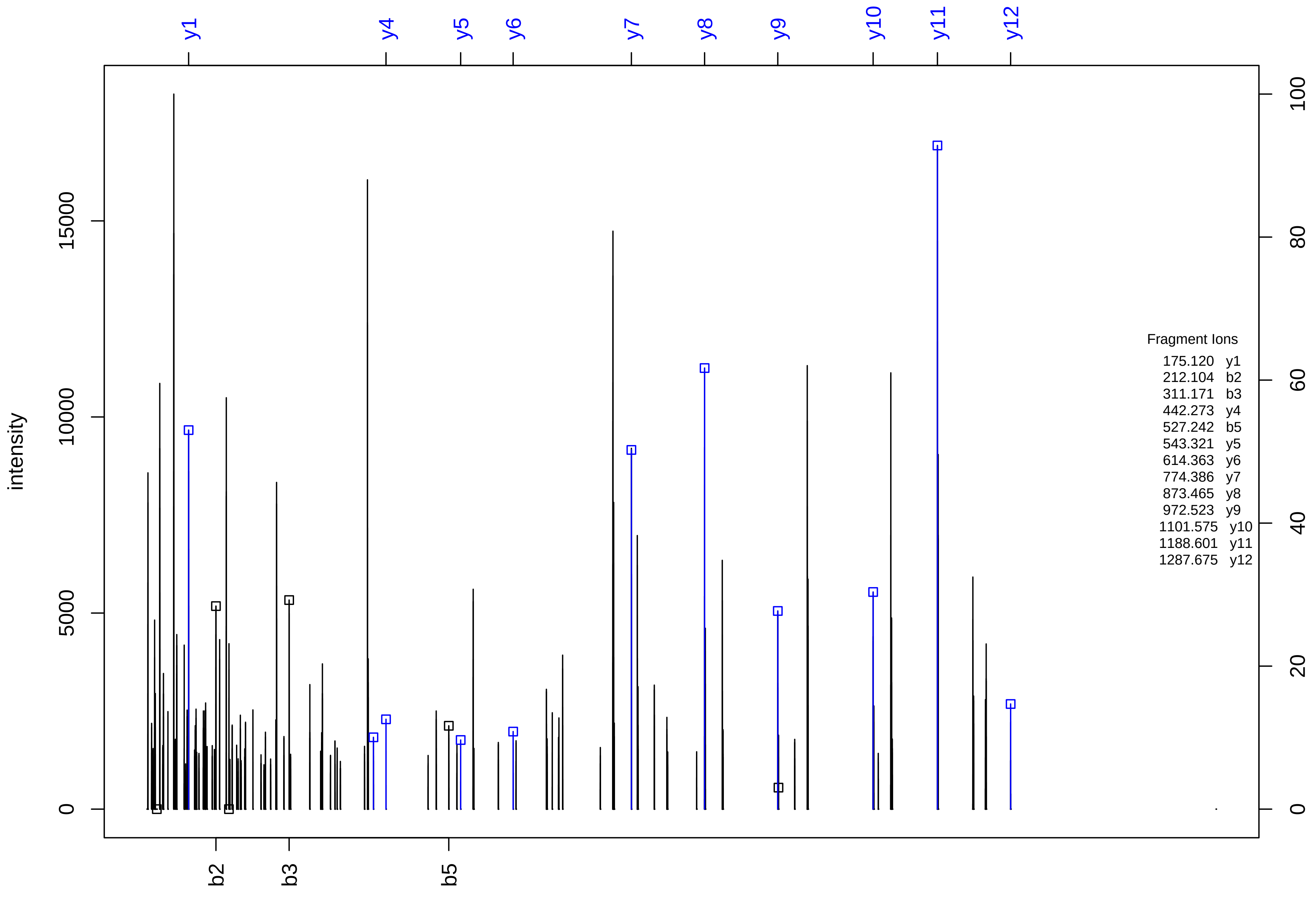

NPVSEVVCATGPLR / File: christine_30turboips_ip21.raw; SpectrumID: ; scans: 12315

(Ac)M*SLM*ENNTK

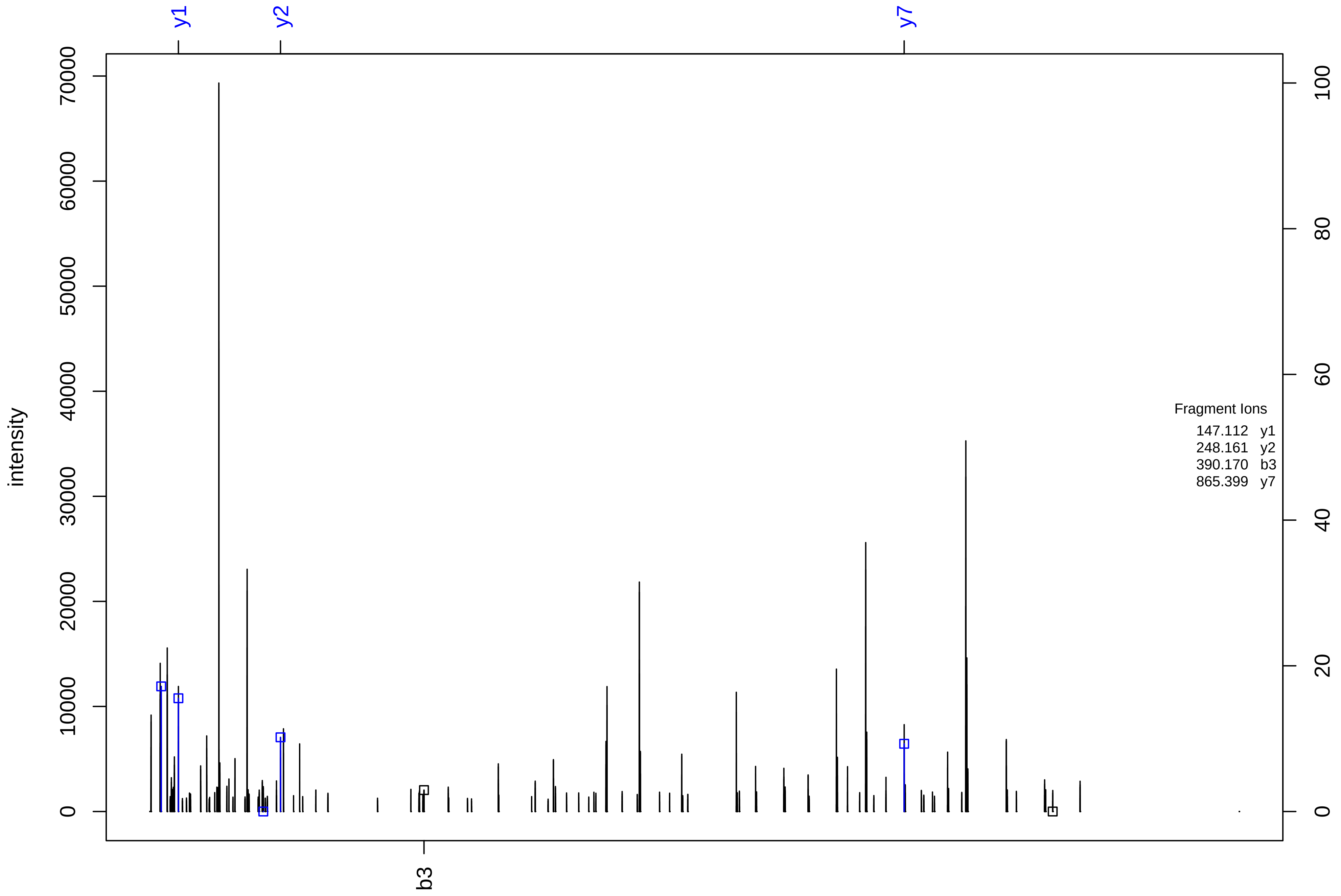

MSLMENNTK / File: christine_30turboips_ip21.raw; SpectrumID: ; scans: 1516

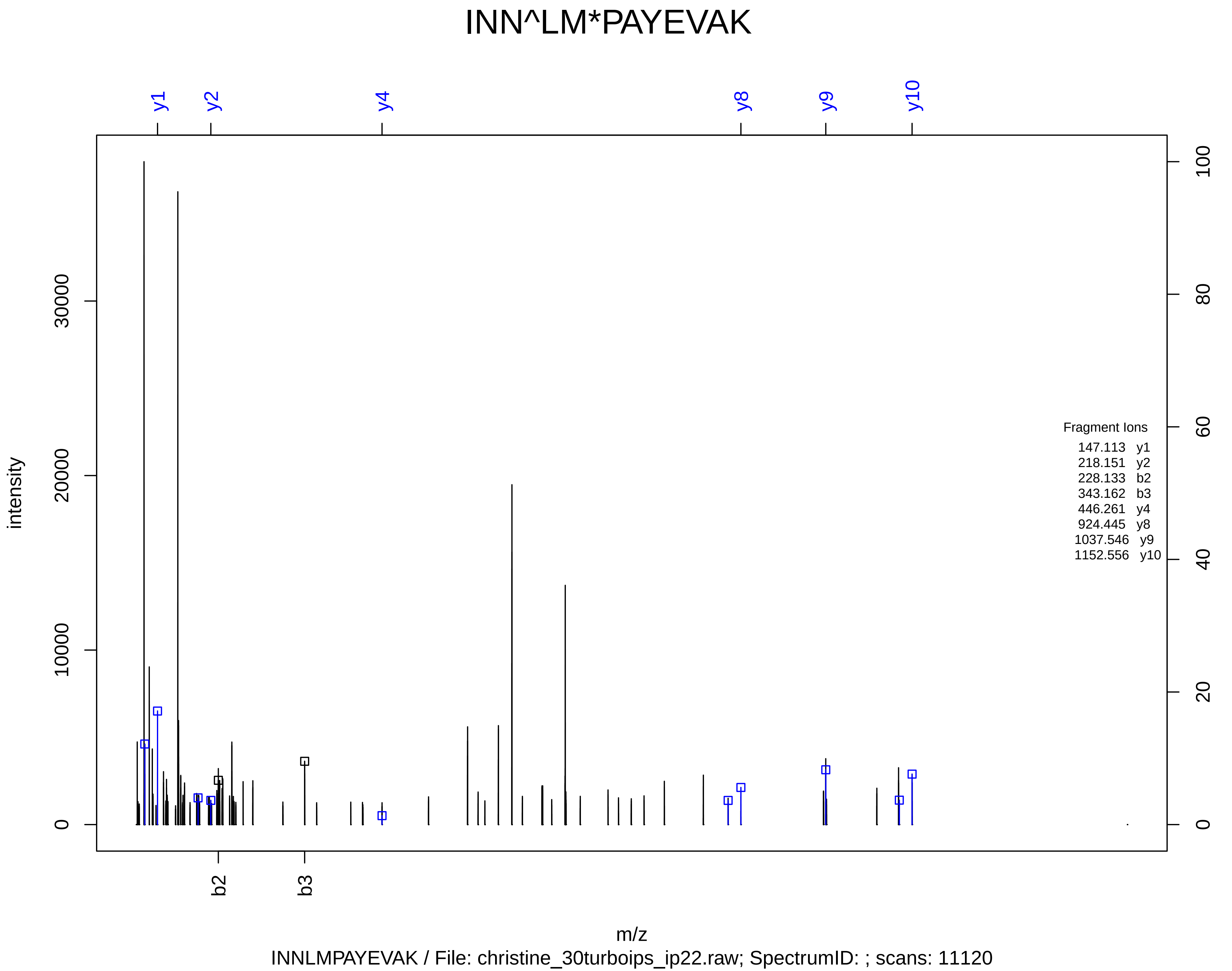

INN^LM*PAYEVAK
y1
y2
y4
y8
y9
y10
intensity
0
10000
20000
30000
0
20
40
60
80
100
Fragment Ions
147.113 y1
218.151 y2
228.133 b2
343.162 b3
446.261 y4
924.445 y8
1037.546 y9
1152.556 y10
b2
b3
m/z
INNLMPAYEVAK / File: christine_30turboips_ip22.raw; SpectrumID: ; scans: 11120

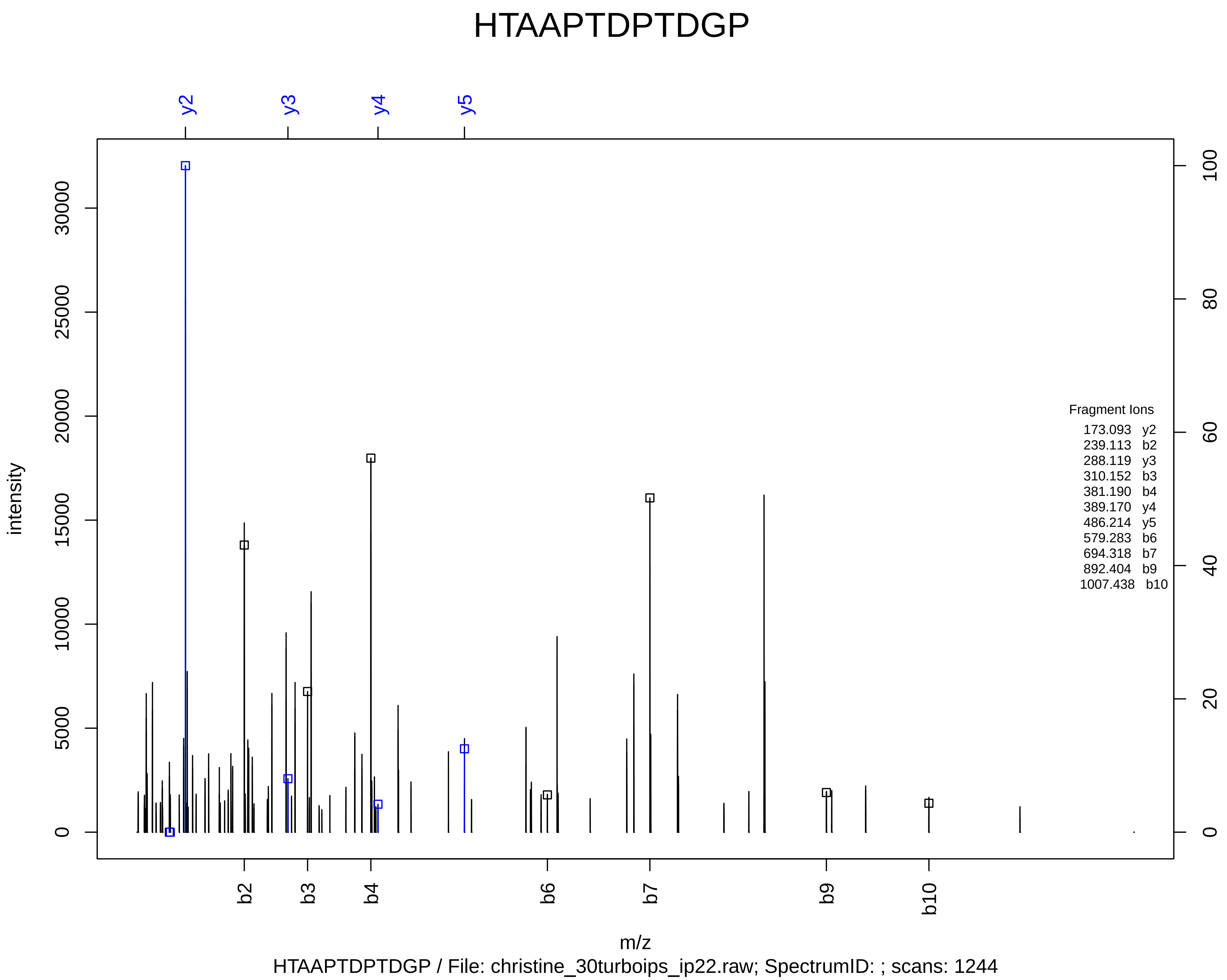

HTAAPTDPTDGP
y2
y3
y4
y5
intensity
0
5000
10000
15000
20000
25000
30000
0
20
40
60
80
100
b2
b3
b4
b6
b7
b9
b10
m/z
Fragment Ions
173.093 y2
239.113 b2
288.119 y3
310.152 b3
381.190 b4
389.170 y4
486.214 y5
579.283 b6
694.318 b7
892.404 b9
1007.438 b10
HTAAPTDPTDGP / File: christine_30turboips_ip22.raw; SpectrumID: ; scans: 1244

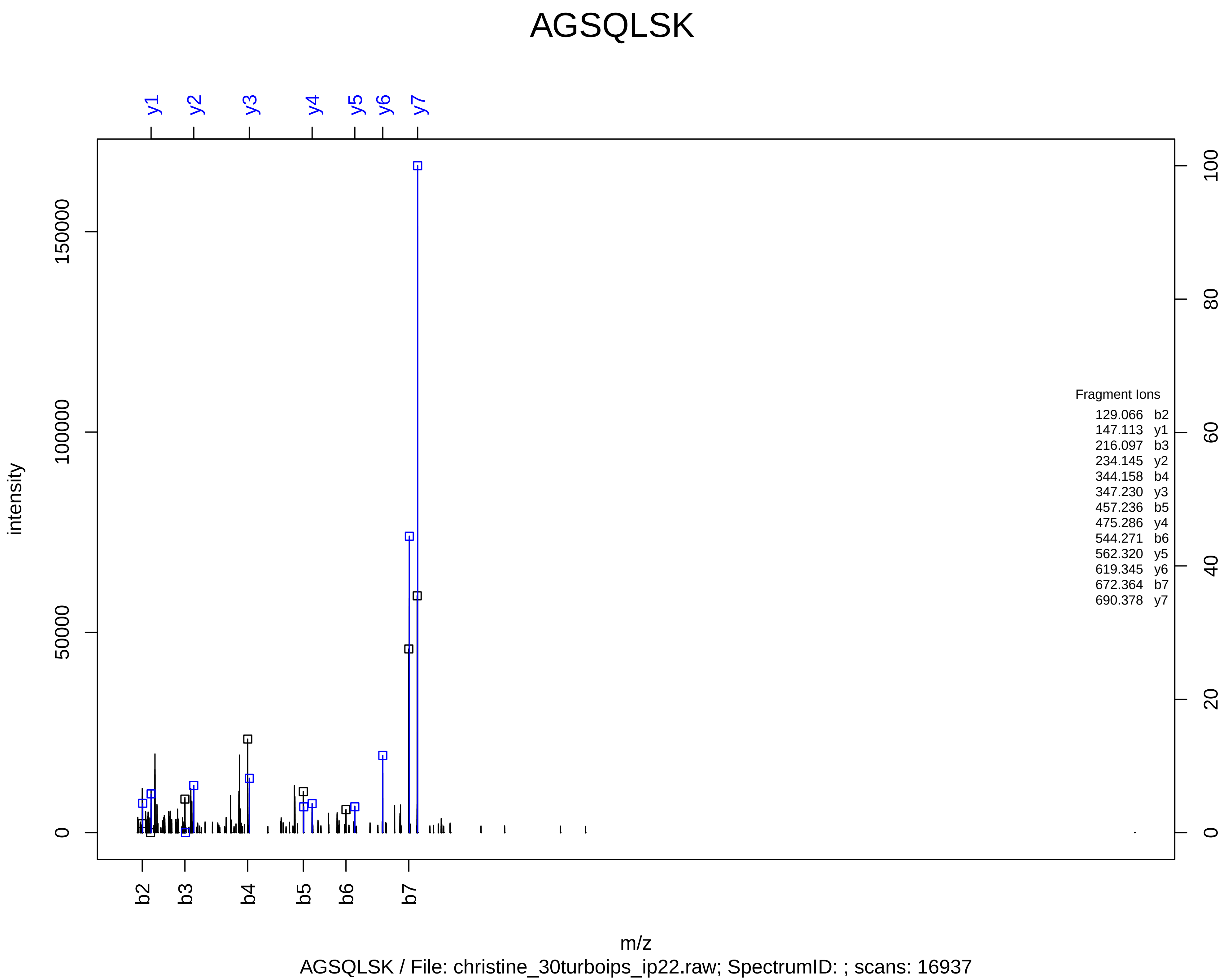
AGSQLSK
y1
y2
y3
y4
y5
y6
y7
intensity
0
50000
100000
150000
0
20
40
60
80
100
Fragment Ions
129.066 b2
147.113 y1
216.097 b3
234.145 y2
344.158 b4
347.230 y3
457.236 b5
475.286 y4
544.271 b6
562.320 y5
619.345 y6
672.364 b7
690.378 y7
b2
b3
b4
b5
b6
b7
m/z
AGSQLSK / File: christine_30turboips_ip22.raw; SpectrumID: ; scans: 16937

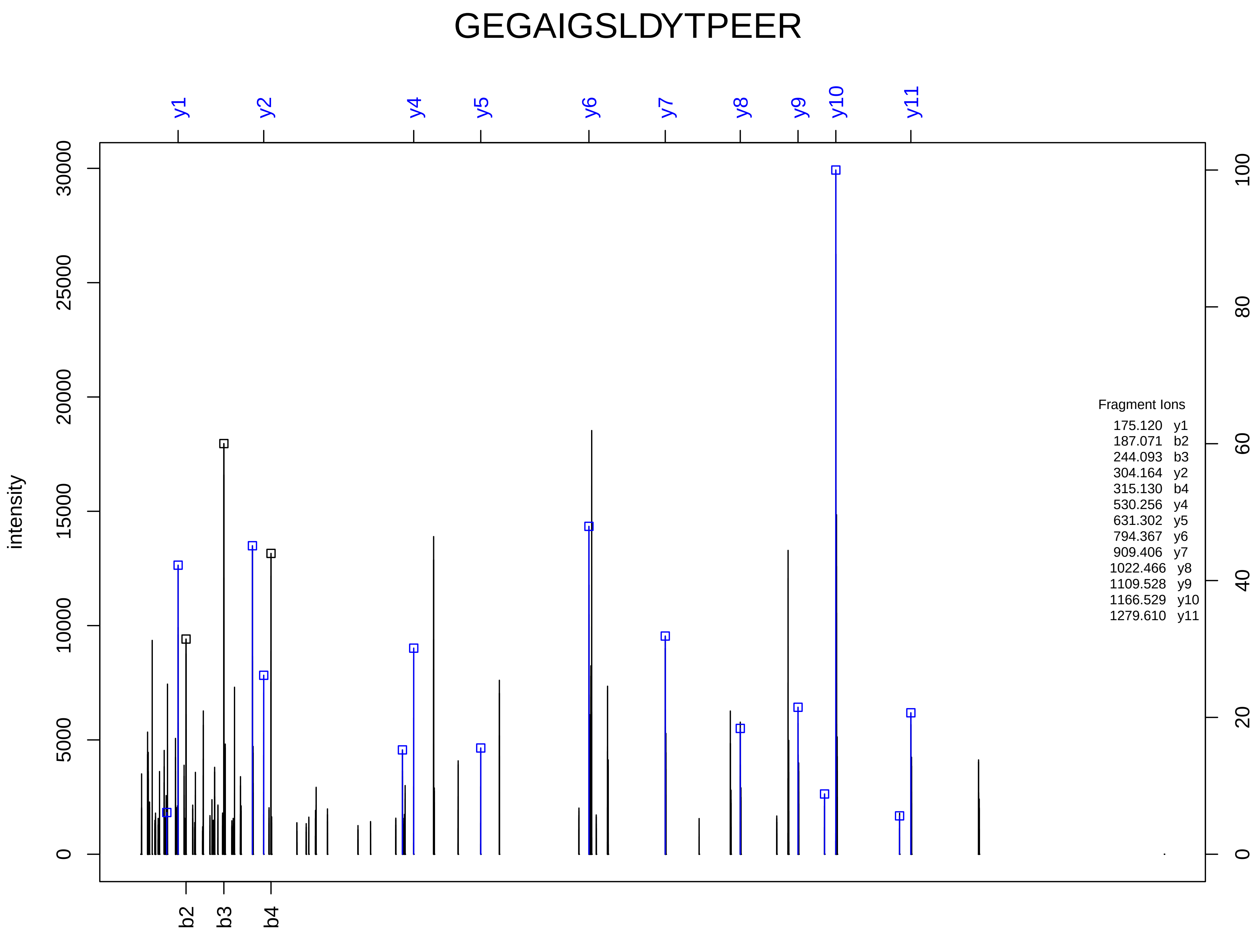
GEGAIGSLDYTPEER
y1
y2
y4
y5
y6
y7
y8
y9
y10
y11
intensity
0
5000
10000
15000
20000
25000
30000
0
20
40
60
80
100
b2
b3
b4
m/z
Fragment Ions
175.120 y1
187.071 b2
244.093 b3
304.164 y2
315.130 b4
530.256 y4
631.302 y5
794.367 y6
909.406 y7
1022.466 y8
1109.528 y9
1166.529 y10
1279.610 y11
GEGAIGSLDYTPEER / File: christine_30turboips_ip13.raw; SpectrumID: ; scans: 8446

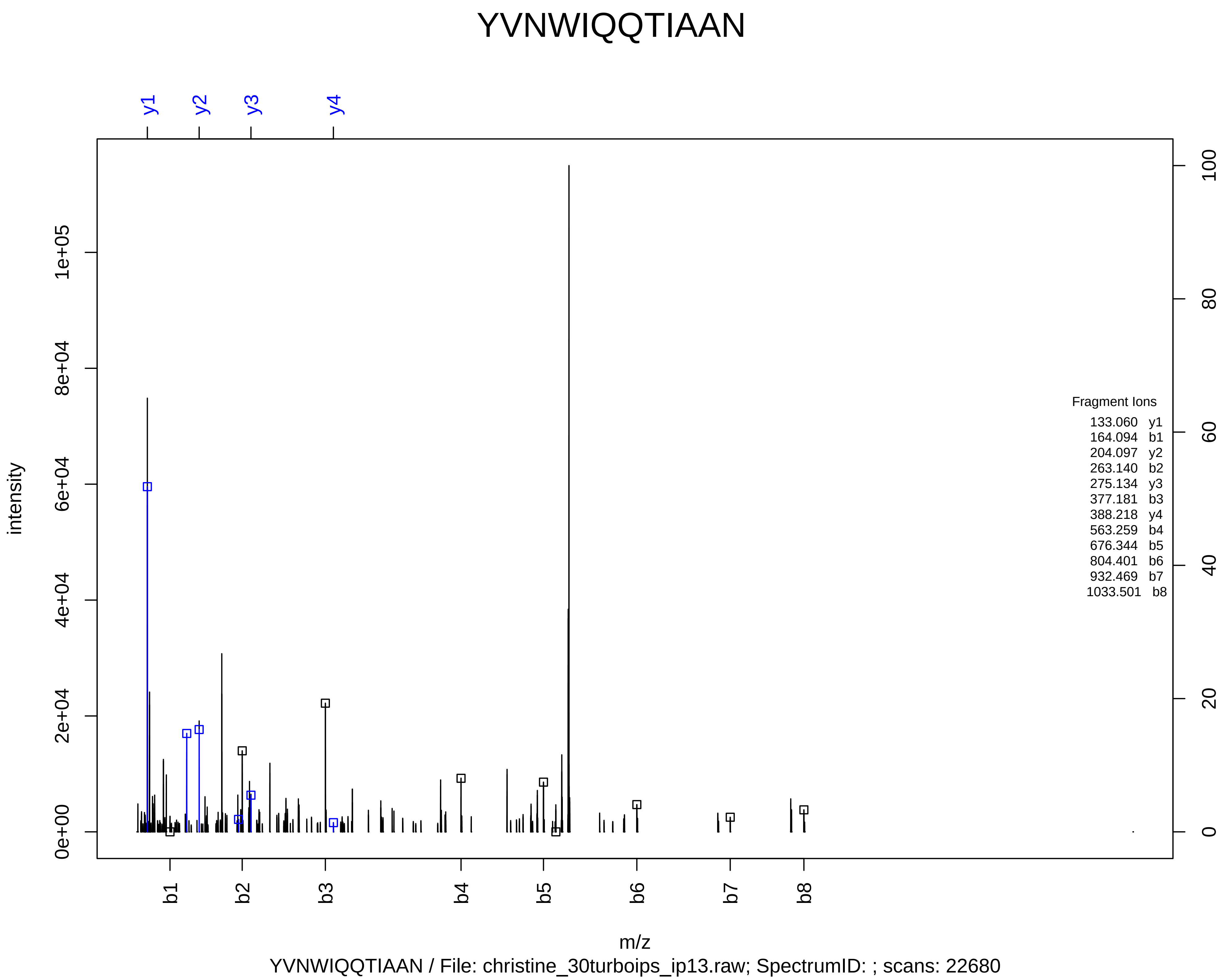
YVNWIQQTIAAN
y1
y2
y3
y4
b1
b2
b3
b4
b5
b6
b7
b8
intensity
0e+00
2e+04
4e+04
6e+04
8e+04
1e+05
0
20
40
60
80
100
m/z
Fragment Ions
133.060 y1
164.094 b1
204.097 y2
263.140 b2
275.134 y3
377.181 b3
388.218 y4
563.259 b4
676.344 b5
804.401 b6
932.469 b7
1033.501 b8
YVNWIQQTIAAN / File: christine_30turboips_ip13.raw; SpectrumID: ; scans: 22680

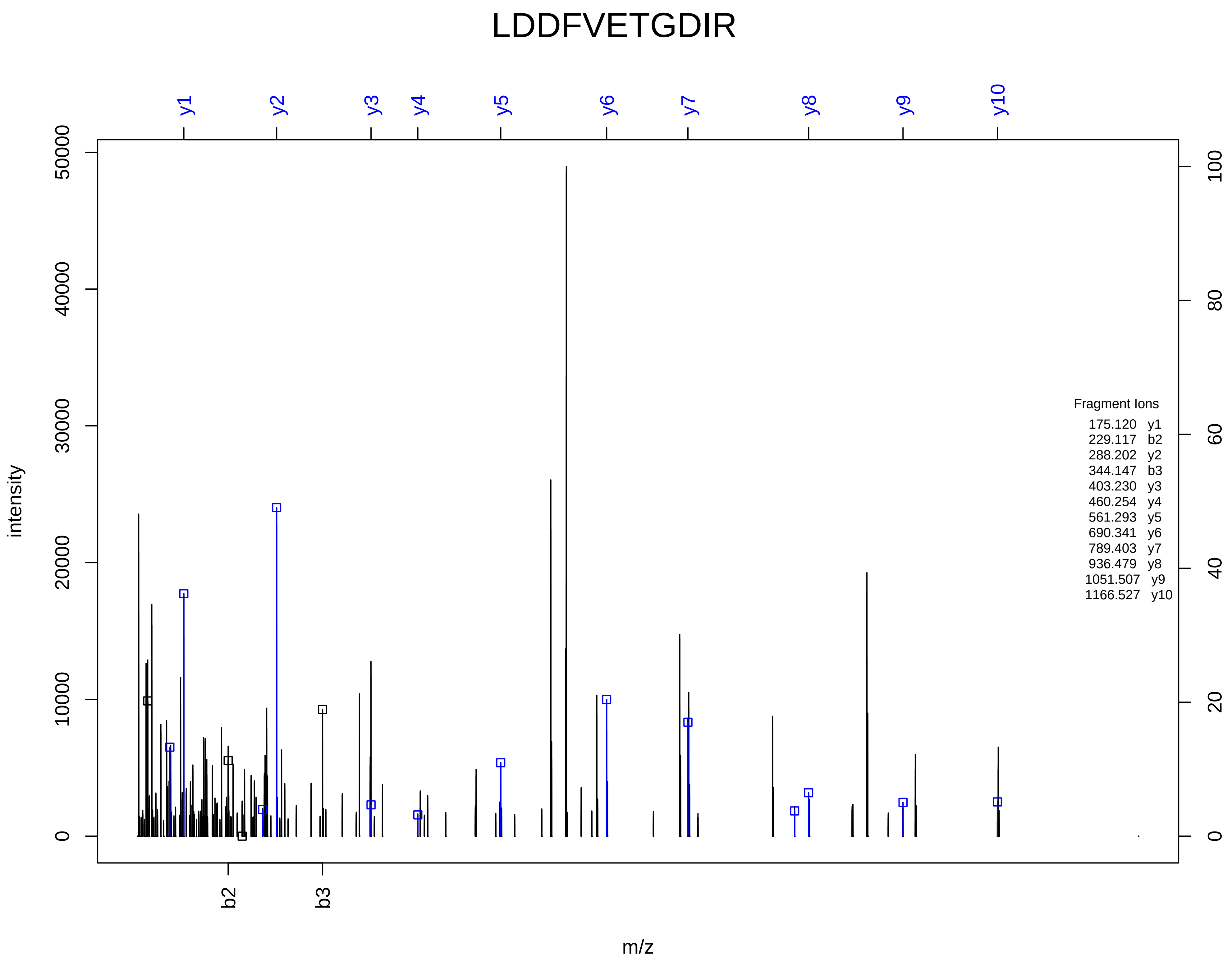

LDDFVETGDIR / File: christine_30turboips_ip12.raw; SpectrumID: ; scans: 10866

VPVCEPEVELAEPEPEPELEHVR

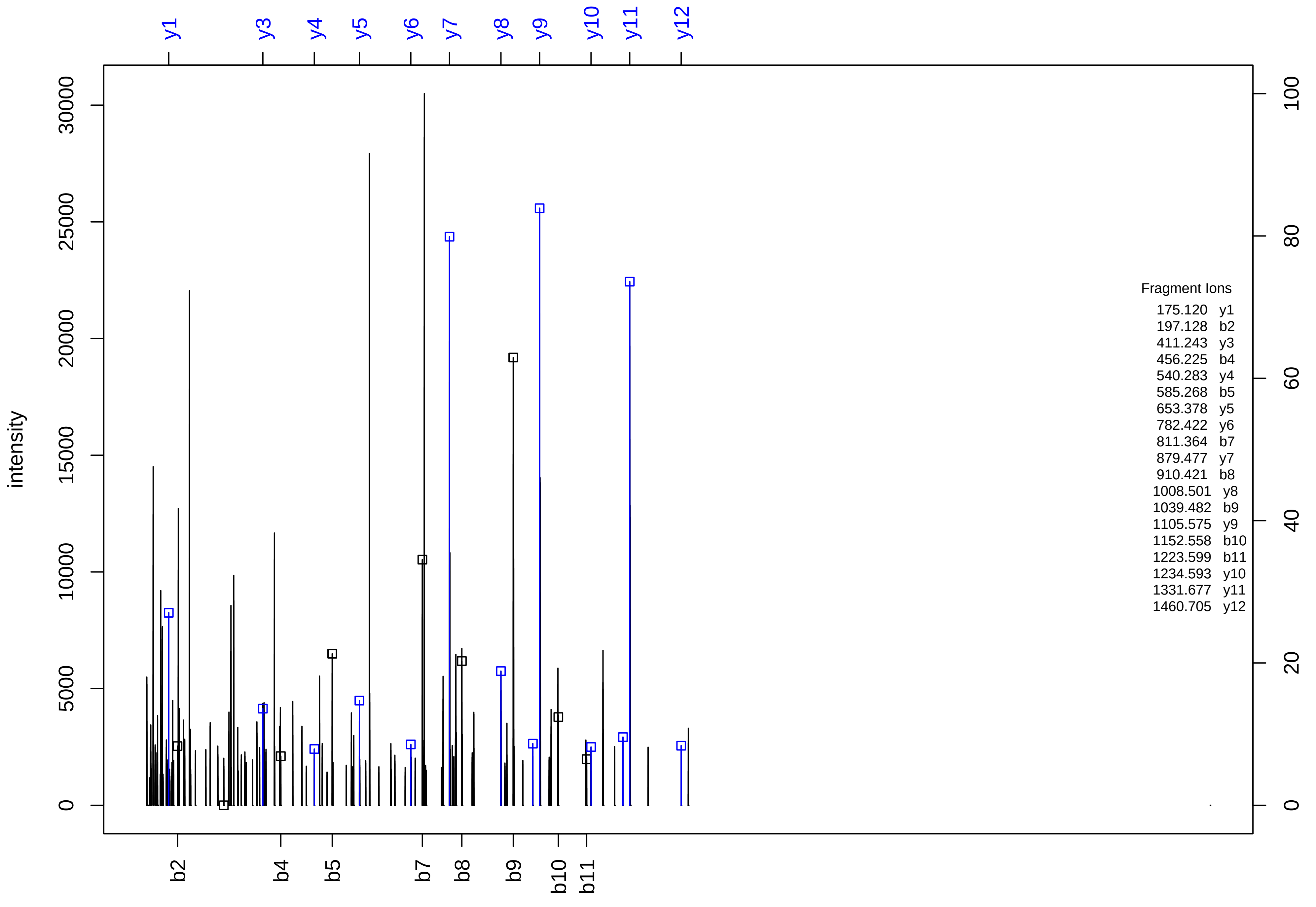

VPVCEPEVELAEPEPEPELEHVR / File: christine_30turboips_ip12.raw; SpectrumID: ; scans: 14606

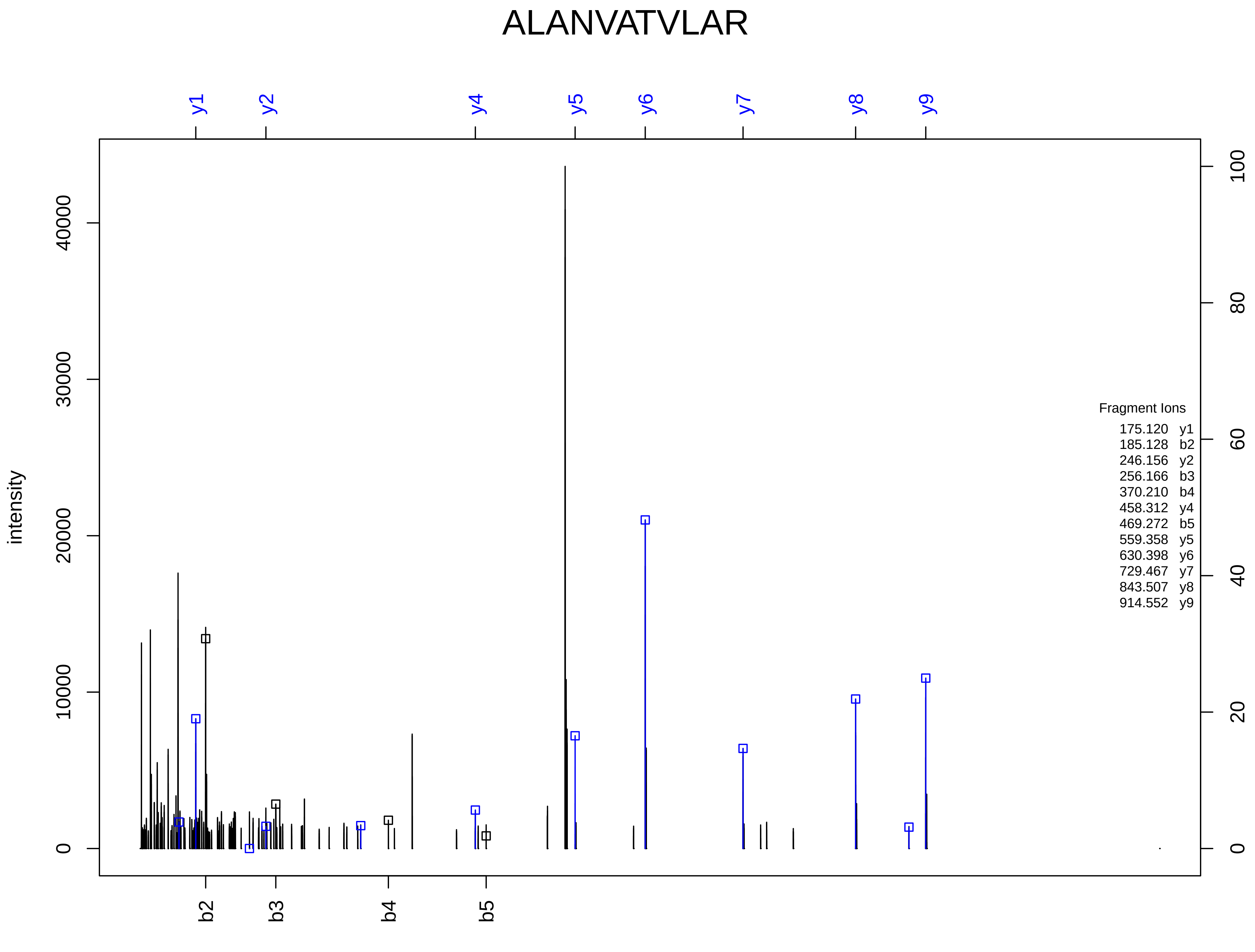

ALANVATVLAR / File: christine_30turboips_ip12.raw; SpectrumID: ; scans: 12391

DGPVKPQSPEEEK

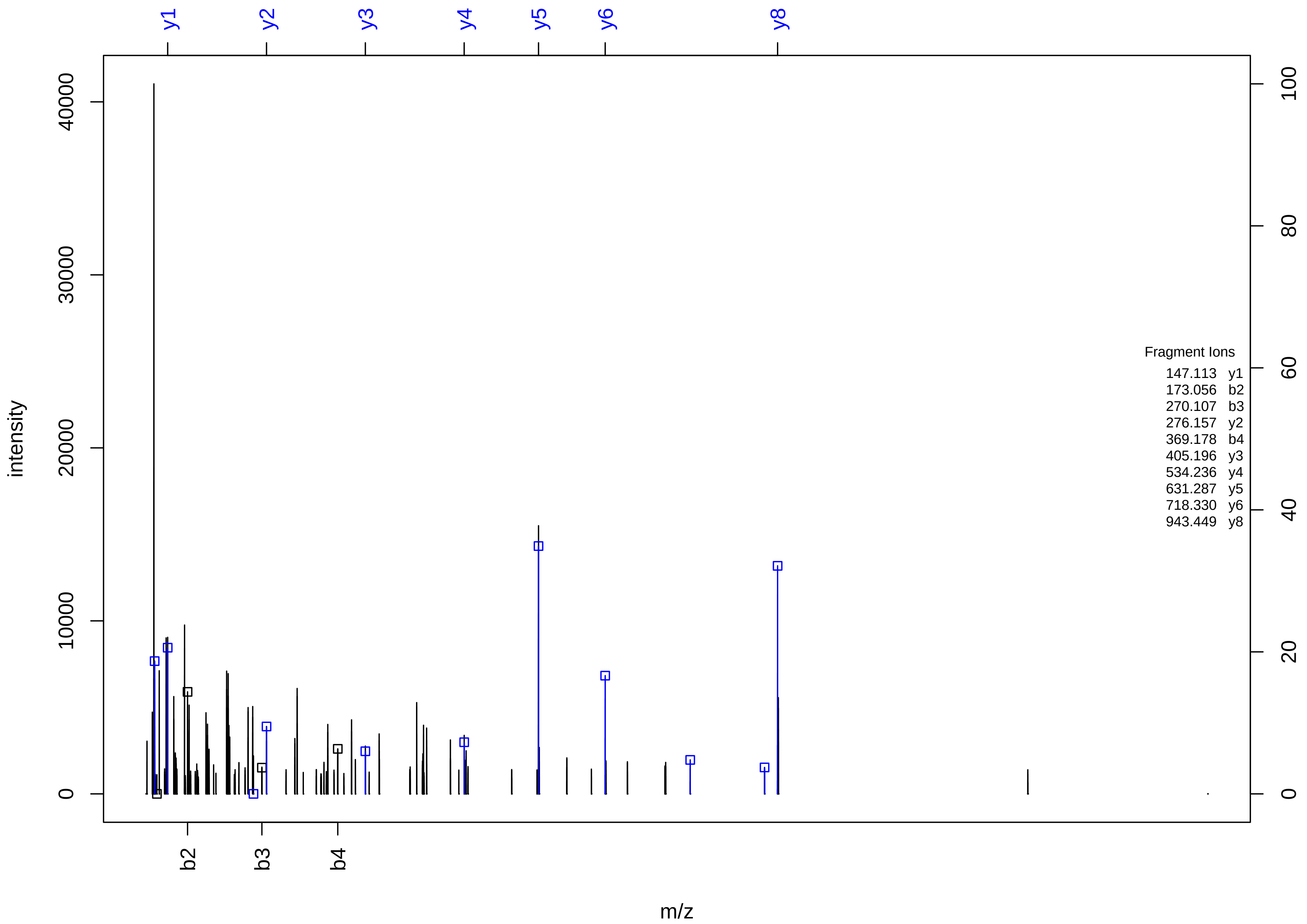

DGPVKPQSPEEEK / File: christine_30turboips_ip12.raw; SpectrumID: ; scans: 937

GLLDSSPAPTPTEDLTPGSVEEAEEAEPDEEFK

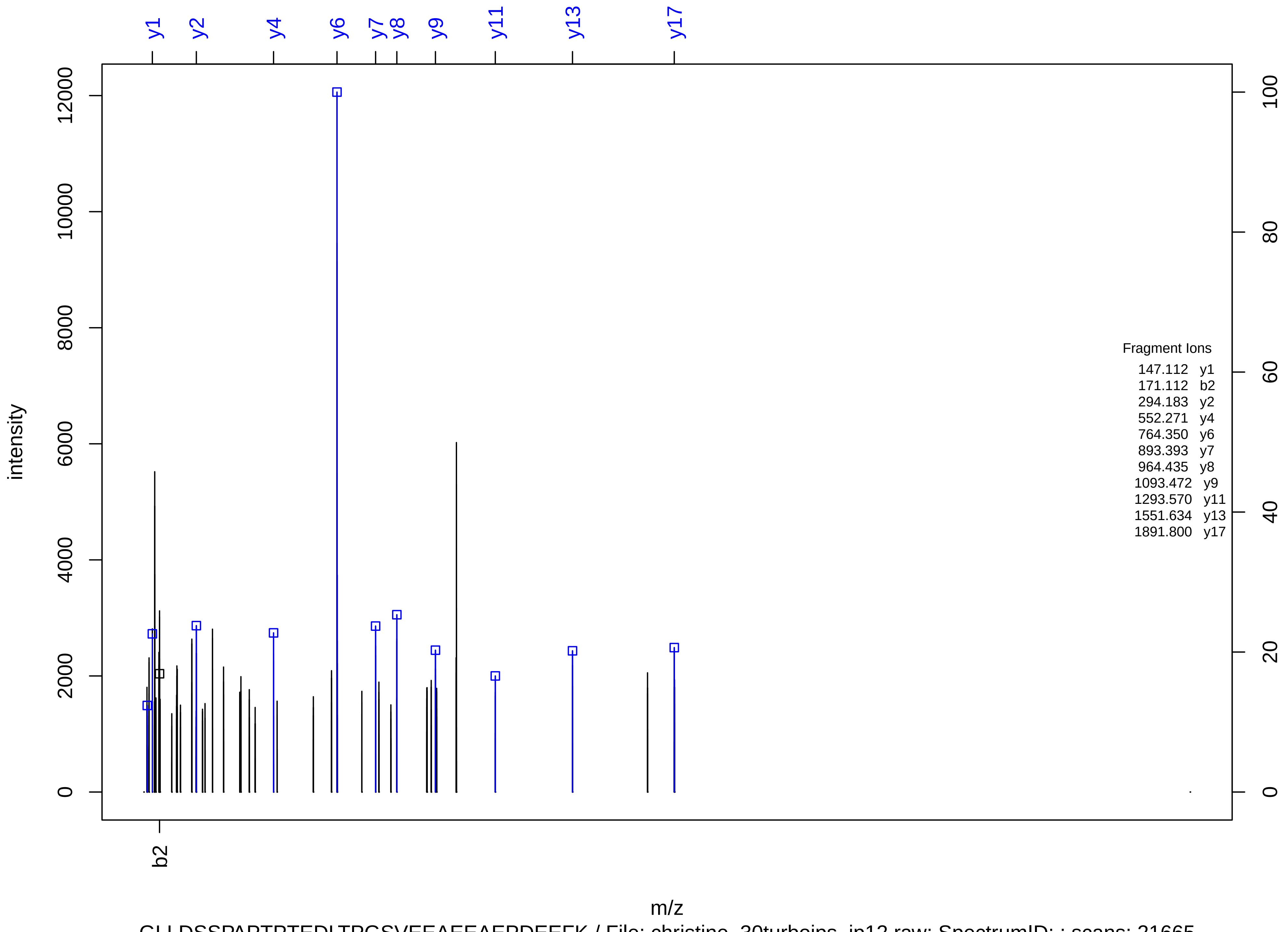

GLLDSSPAPTPTEDLTPGSVEEAEEAEPDEEFK / File: christine_30turboips_ip12.raw; SpectrumID: ; scans: 21665

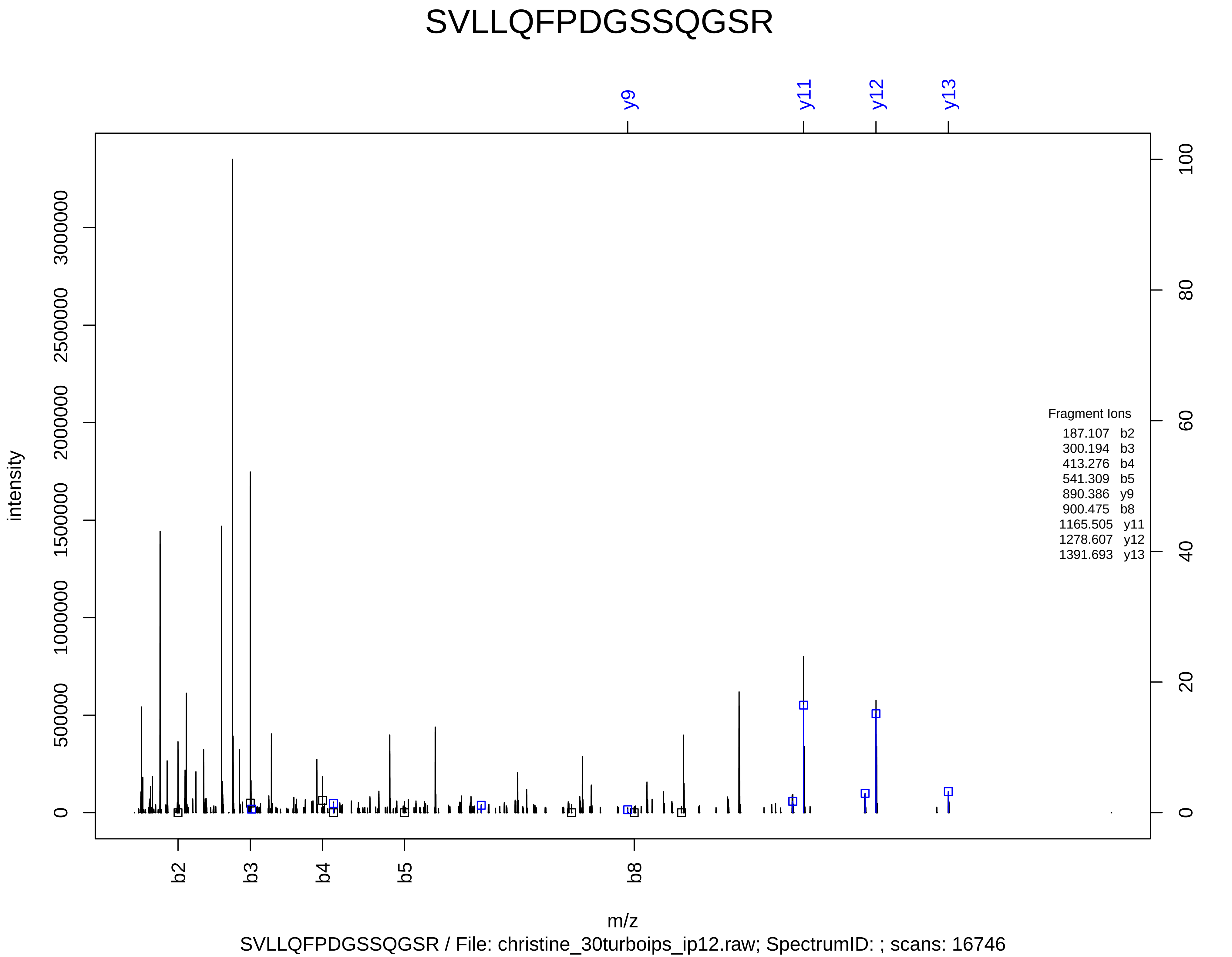
SVLLQFPDGSSQGSR
y9
y11
y12
y13
intensity
0
500000
1000000
1500000
2000000
2500000
3000000
0
20
40
60
80
100
Fragment Ions
187.107 b2
300.194 b3
413.276 b4
541.309 b5
890.386 y9
900.475 b8
1165.505 y11
1278.607 y12
1391.693 y13
b2
b3
b4
b5
b8
m/z
SVLLQFPDGSSQGSR / File: christine_30turboips_ip12.raw; SpectrumID: ; scans: 16746

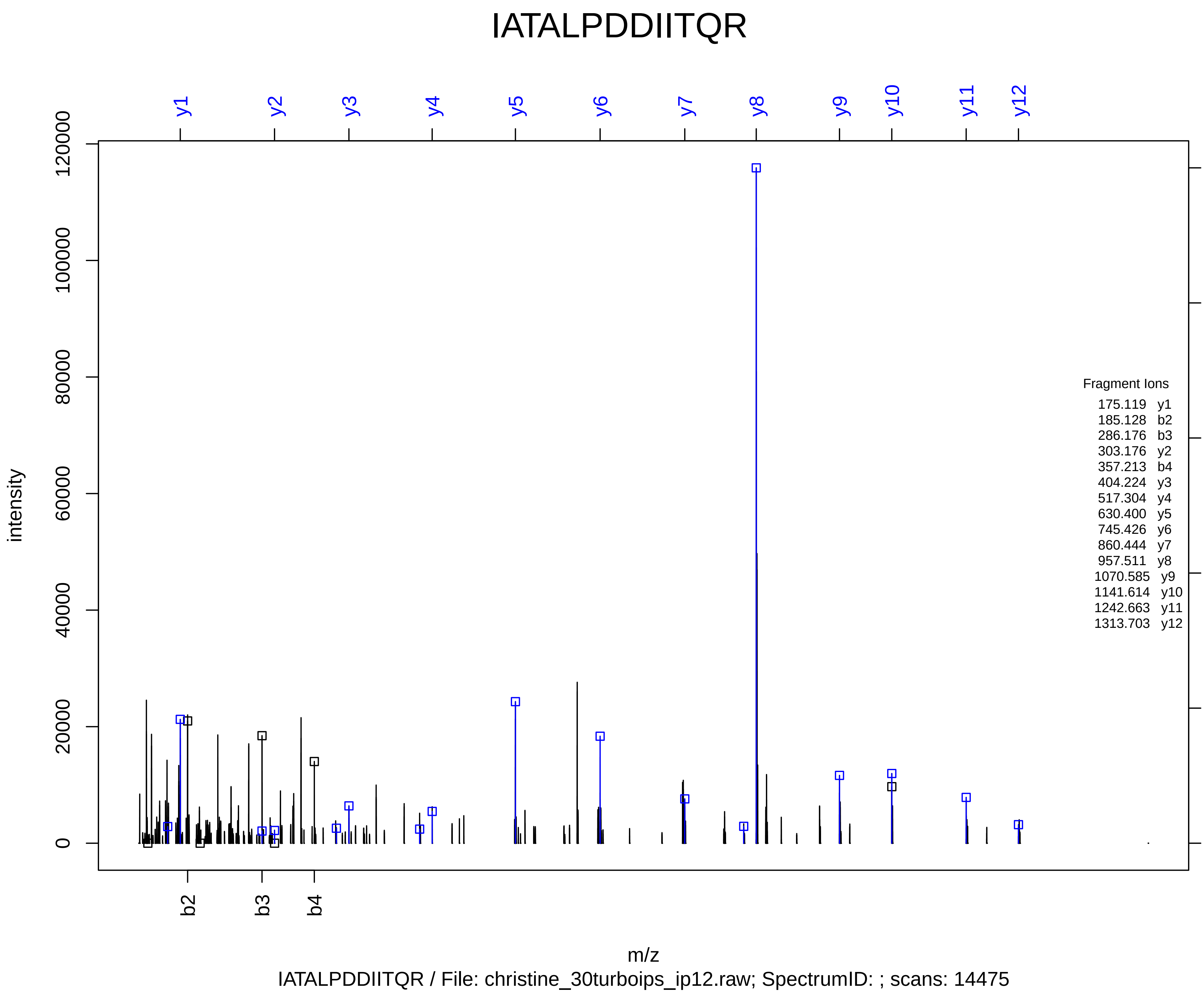
IATALPDDIITQR
y1
y2
y3
y4
y5
y6
y7
y8
y9
y10
y11
y12
intensity
0
20000
40000
60000
80000
100000
120000
0
20
40
60
80
100
b2
b3
b4
m/z
Fragment Ions
175.119 y1
185.128 b2
286.176 b3
303.176 y2
357.213 b4
404.224 y3
517.304 y4
630.400 y5
745.426 y6
860.444 y7
957.511 y8
1070.585 y9
1141.614 y10
1242.663 y11
1313.703 y12
IATALPDDIITQR / File: christine_30turboips_ip12.raw; SpectrumID: ; scans: 14475

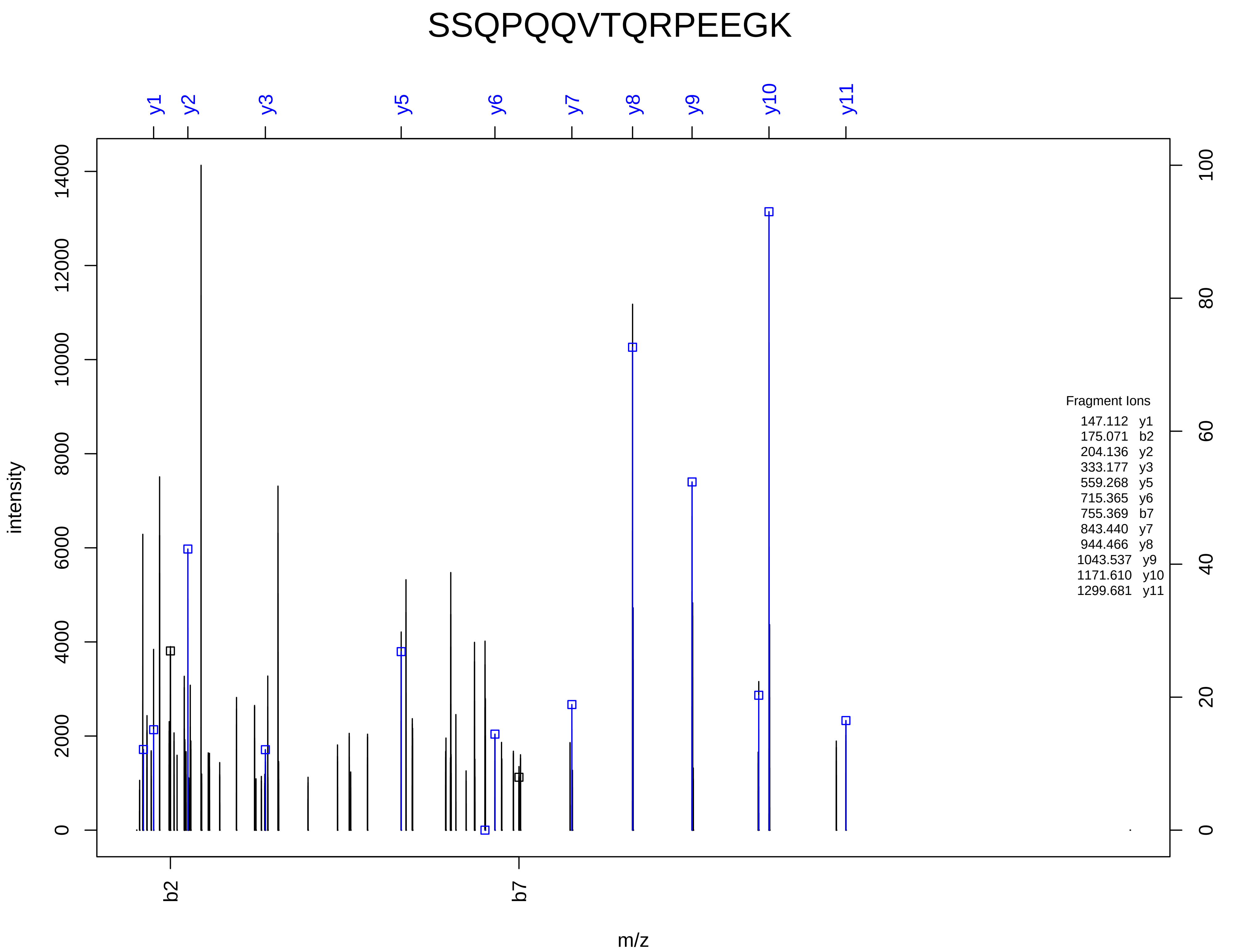

SSQPQQVTQRPEEGK / File: christine_30turboips_ip12.raw; SpectrumID: ; scans: 713

(Ac)M*LLSSPTTPSR

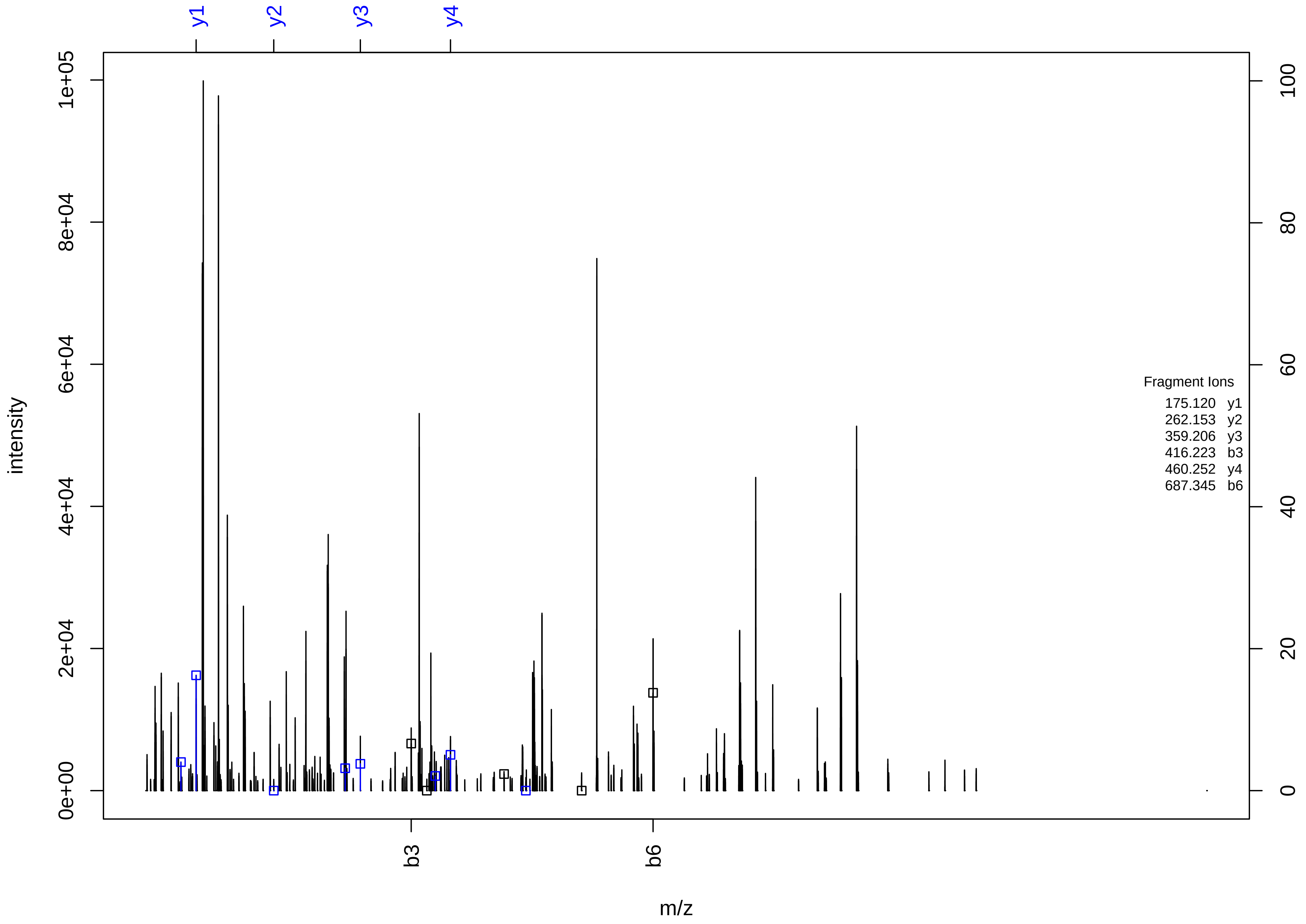

MLLSSPTTPSR / File: christine_30turboips_ip12.raw; SpectrumID: ; scans: 2274

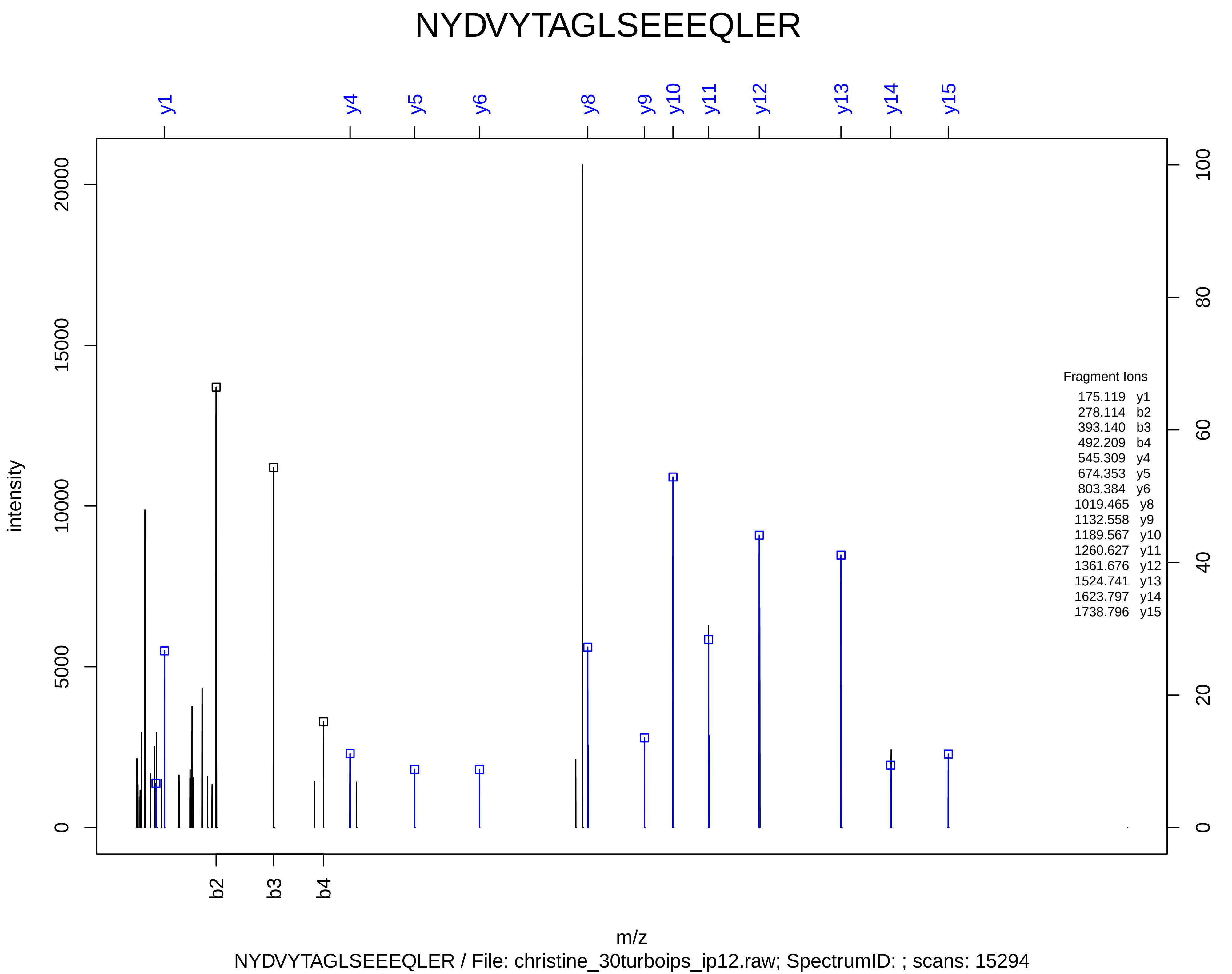
NYDVYTAGLSEEEQLER
y1
y4
y5
y6
y8
y9
y10
y11
y12
y13
y14
y15
intensity
20000
15000
10000
5000
0
100
80
60
40
20
0
b2
b3
b4
m/z
Fragment Ions
175.119 y1
278.114 b2
393.140 b3
492.209 b4
545.309 y4
674.353 y5
803.384 y6
1019.465 y8
1132.558 y9
1189.567 y10
1260.627 y11
1361.676 y12
1524.741 y13
1623.797 y14
1738.796 y15
NYDVYTAGLSEEEQLER / File: christine_30turboips_ip12.raw; SpectrumID: ; scans: 15294

SDFINIGGFDLDIK

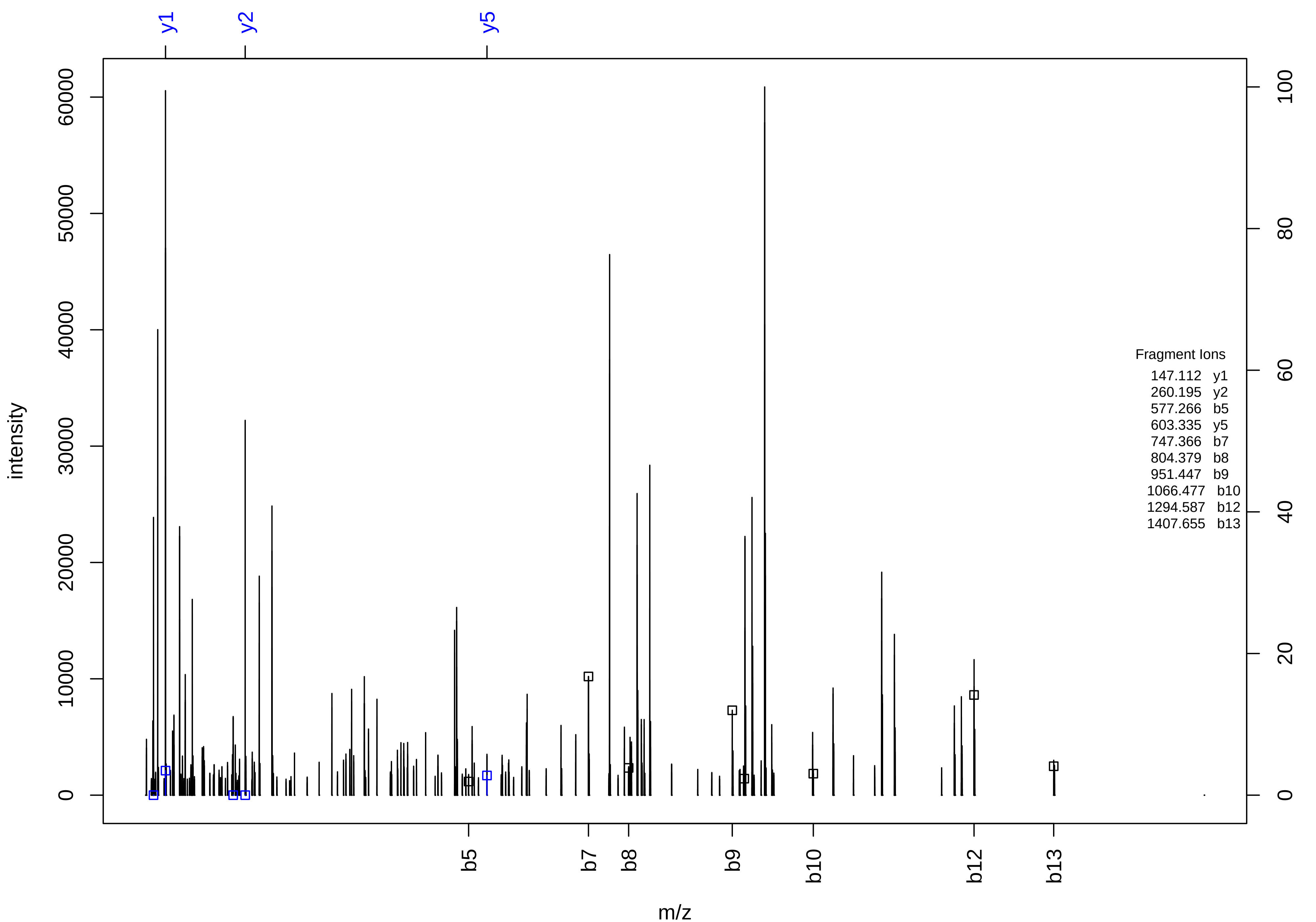

SDFINIGGFDLDIK / File: christine_30turboips_ip12.raw; SpectrumID: ; scans: 7114

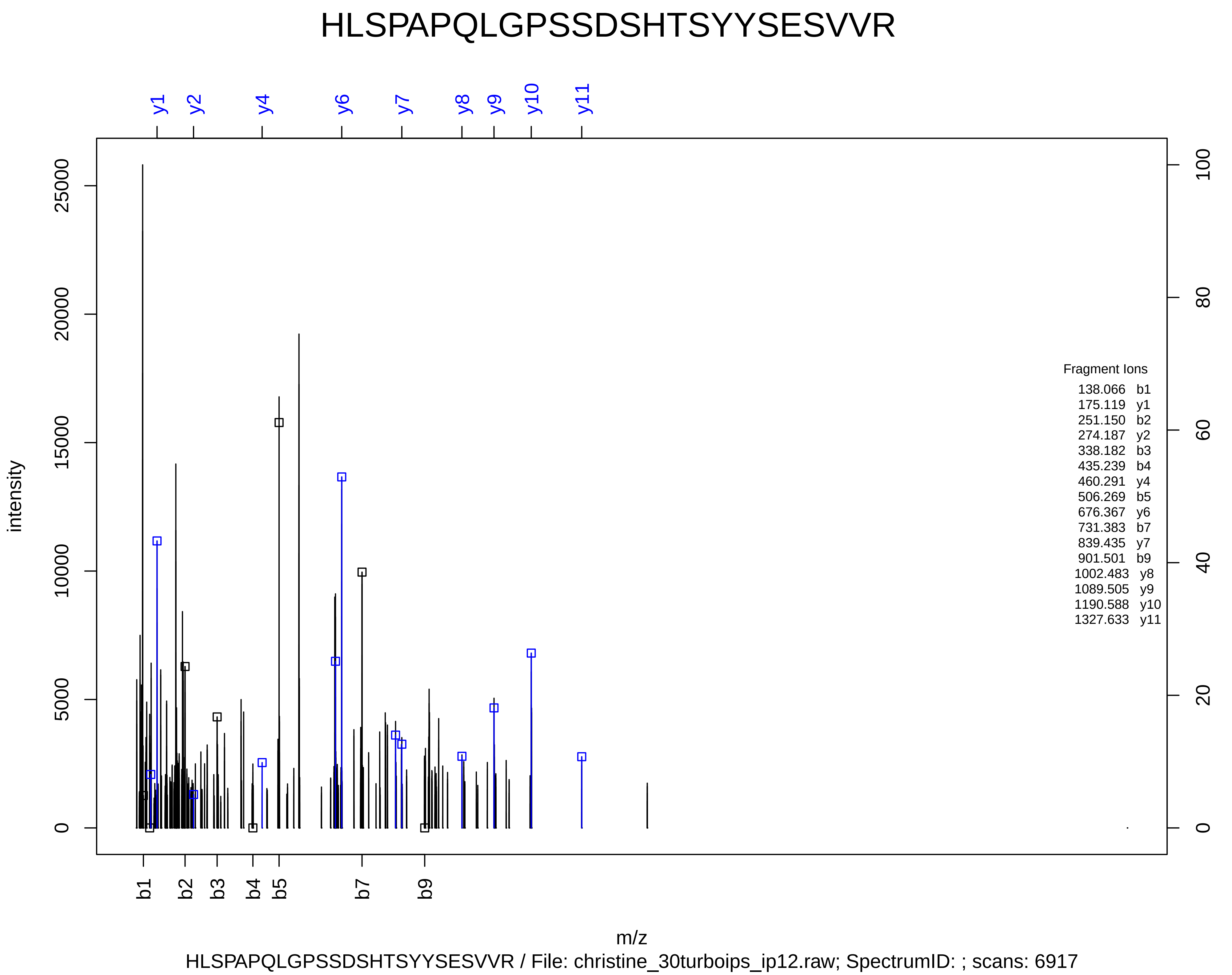
HLSPAPQLGPSSDSHTSYYSESVVR
y1
y2
y4
y6
y7
y8
y9
y10
y11
intensity
0
5000
10000
15000
20000
25000
0
20
40
60
80
100
b1
b2
b3
b4
b5
b7
b9
m/z
Fragment Ions
138.066 b1
175.119 y1
251.150 b2
274.187 y2
338.182 b3
435.239 b4
460.291 y4
506.269 b5
676.367 y6
731.383 b7
839.435 y7
901.501 b9
1002.483 y8
1089.505 y9
1190.588 y10
1327.633 y11
HLSPAPQLGPSSDSHTSYYSESVVR / File: christine_30turboips_ip12.raw; SpectrumID: ; scans: 6917

DPTSLLFPESELSIR

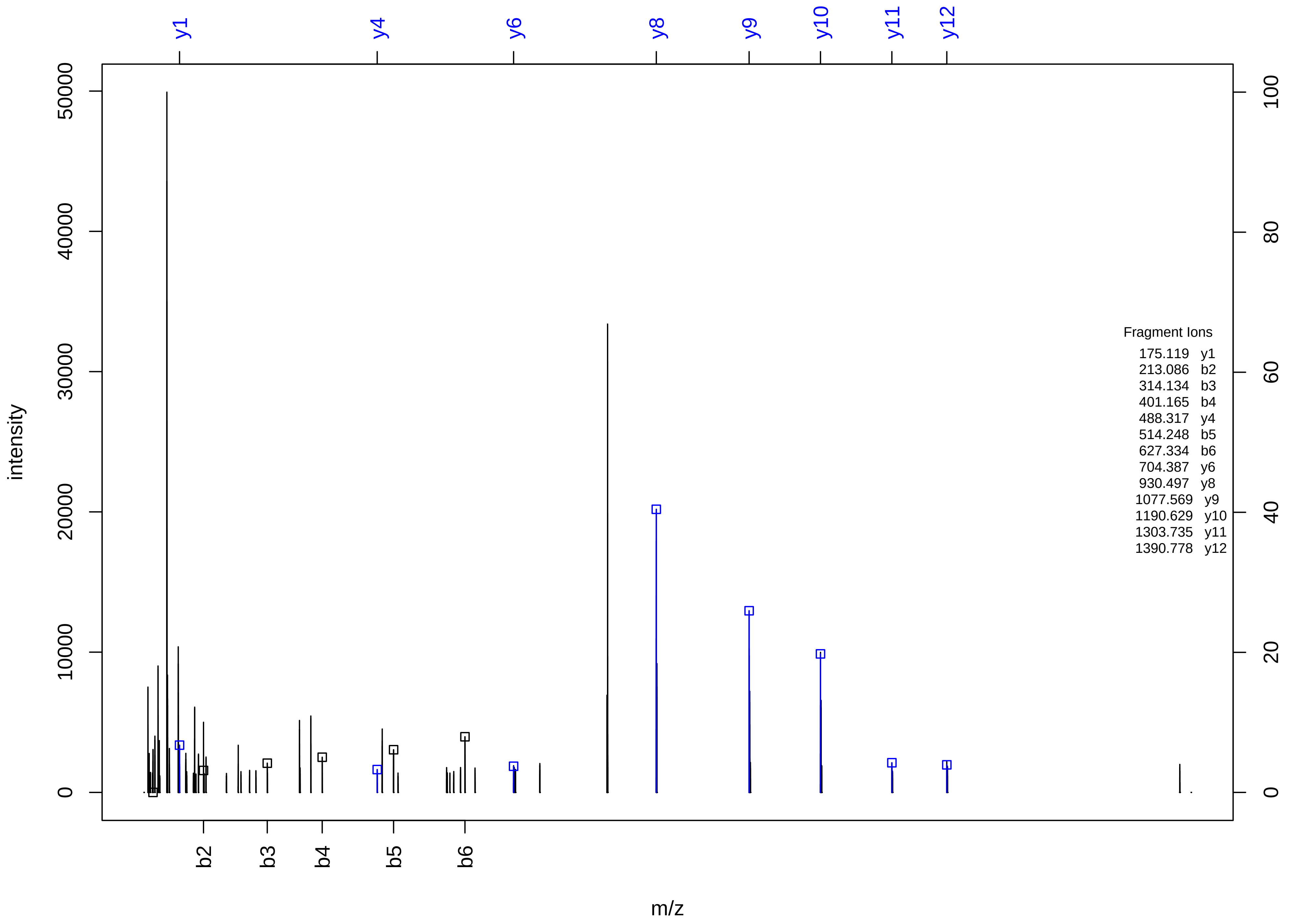

DPTSLLFPESELSIR / File: christine_30turboips_ip12.raw; SpectrumID: ; scans: 26462

TALNNTLDLANVK

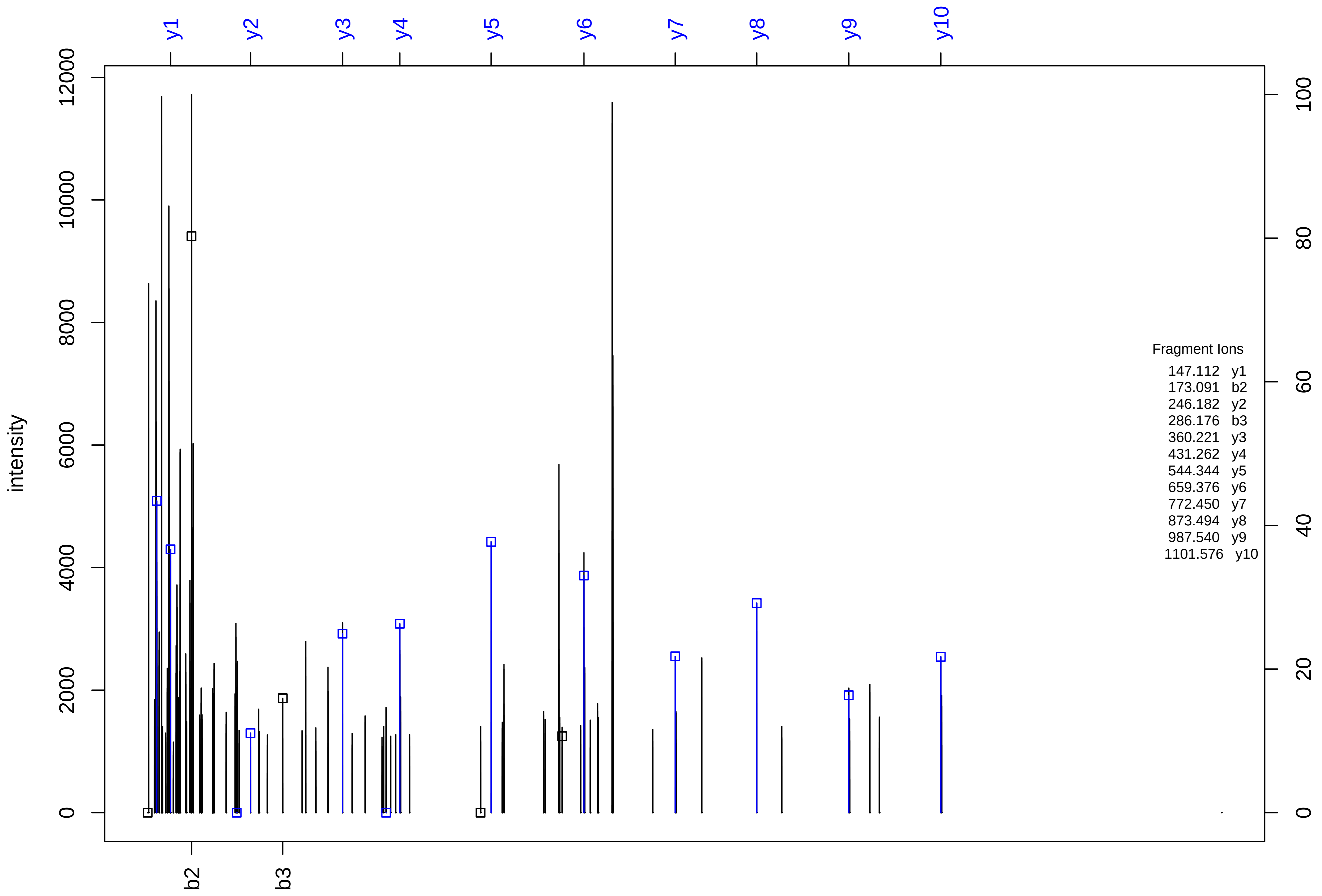

TALNNTLDLANVK / File: christine_30turboips_ip12.raw; SpectrumID: ; scans: 12096

DPTSLEEEIK

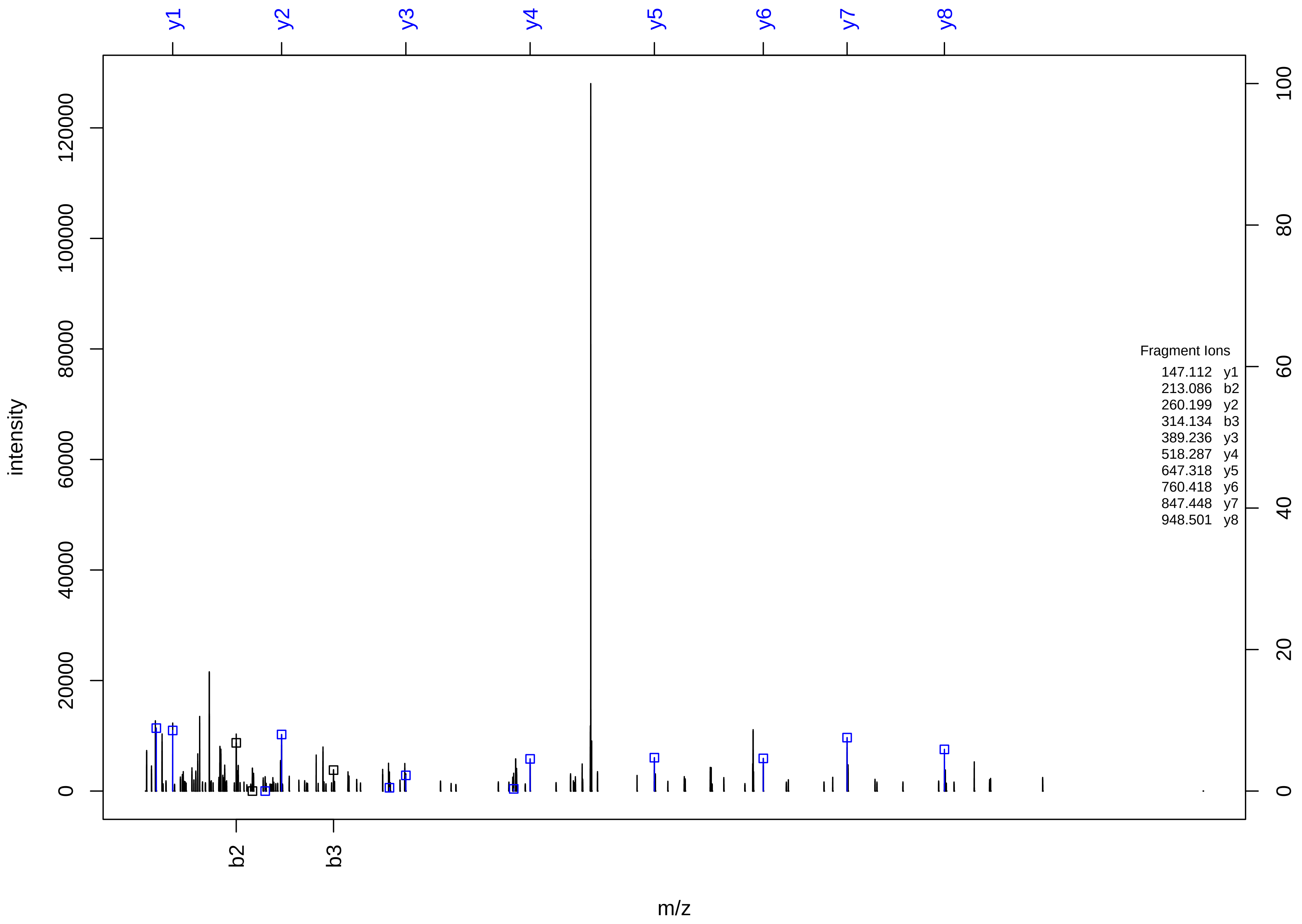

DPTSLEEEIK / File: christine_30turboips_ip12.raw; SpectrumID: ; scans: 8131

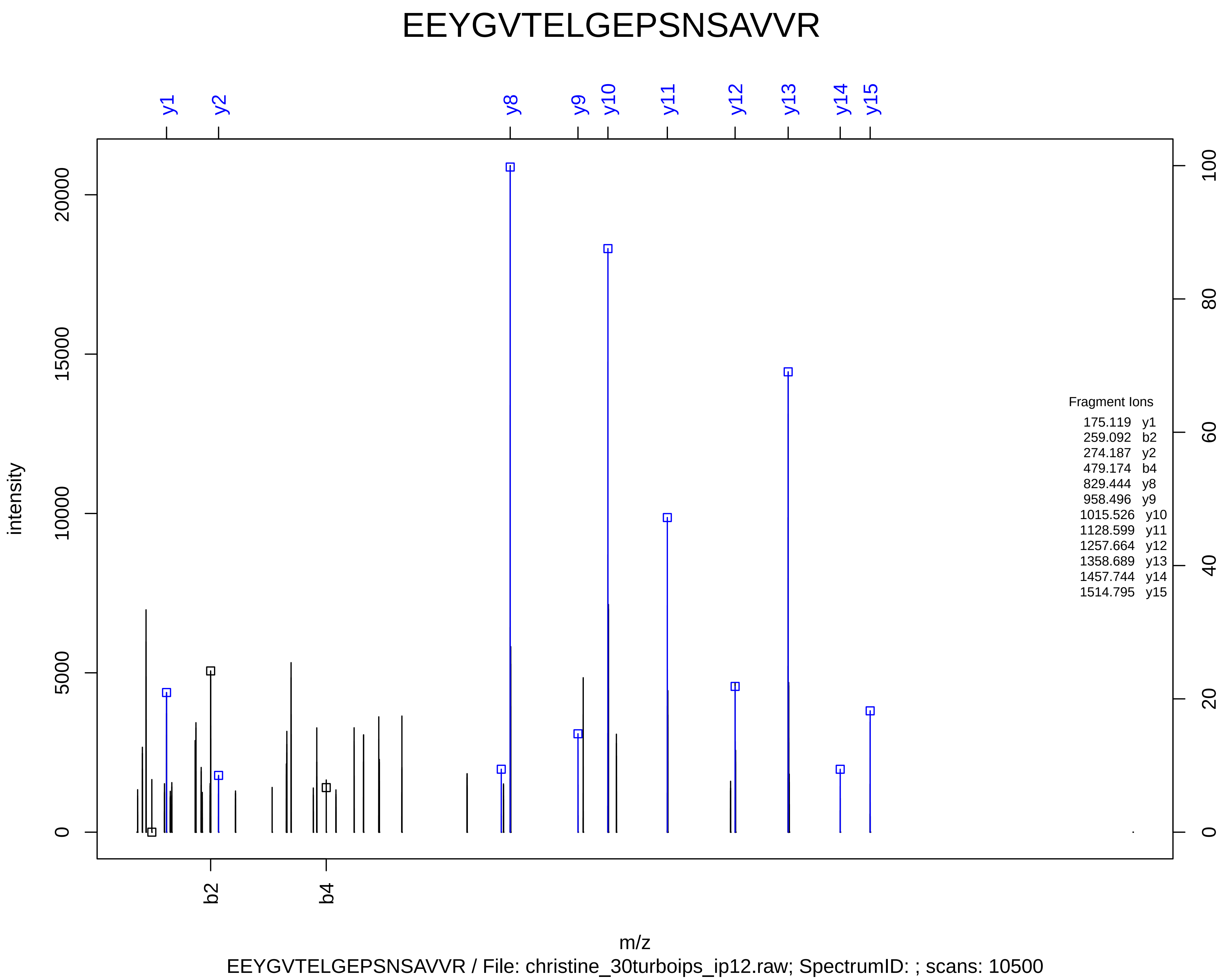
EEYGVTELGEPSNSAVVR
y1
y2
y8
y9
y10
y11
y12
y13
y14
y15
intensity
0
5000
10000
15000
20000
0
20
40
60
80
100
b2
b4
m/z
Fragment Ions
175.119 y1
259.092 b2
274.187 y2
479.174 b4
829.444 y8
958.496 y9
1015.526 y10
1128.599 y11
1257.664 y12
1358.689 y13
1457.744 y14
1514.795 y15
EEYGVTELGEPSNSAVVR / File: christine_30turboips_ip12.raw; SpectrumID: ; scans: 10500

(Ac)STNTDLSLSSYDEGQGSK

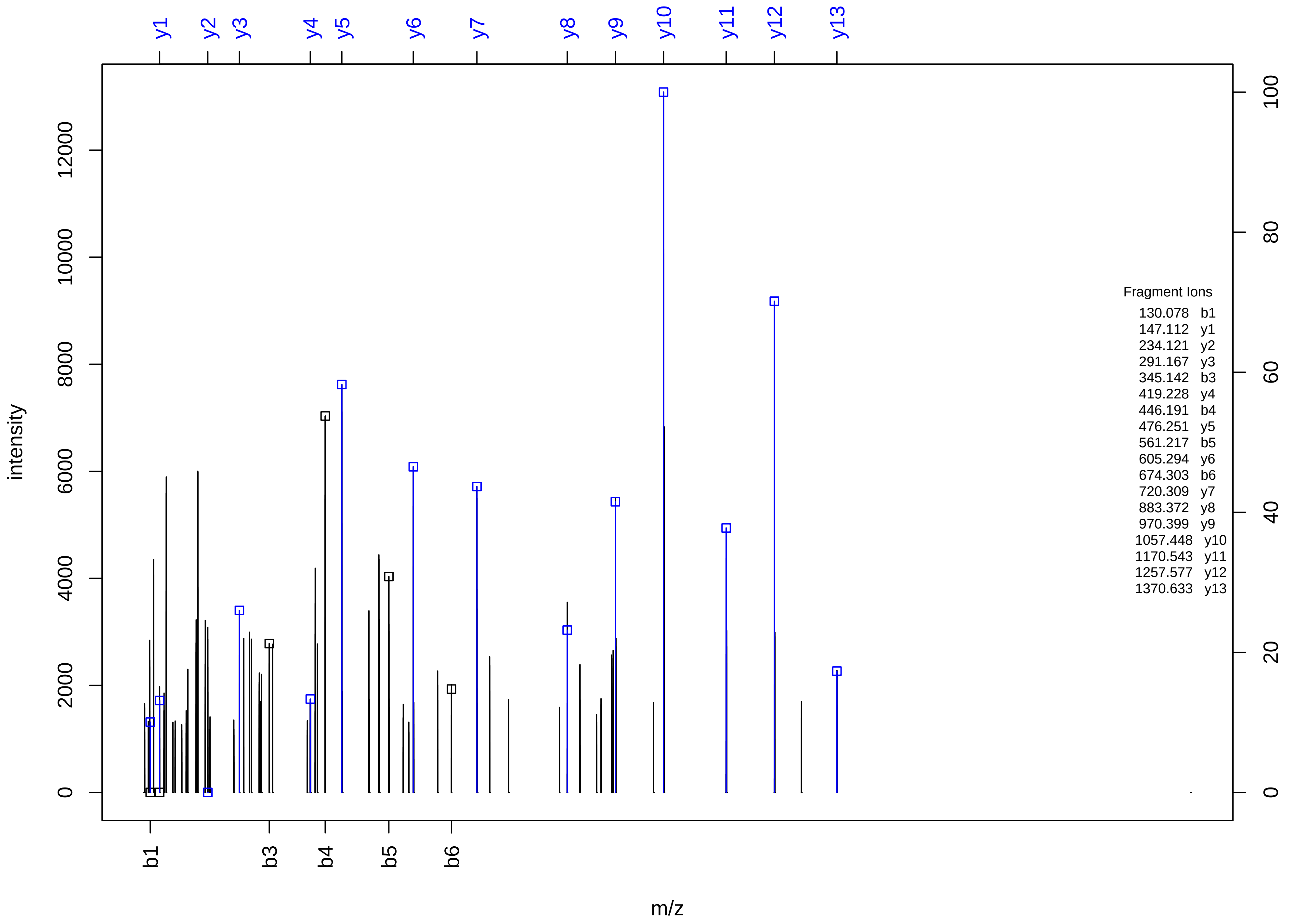

STNTDLSLSSYDEGQGSK / File: christine_30turboips_ip12.raw; SpectrumID: ; scans: 11961

AIASASASGPCIHQQPPGQPPPPQLSSR

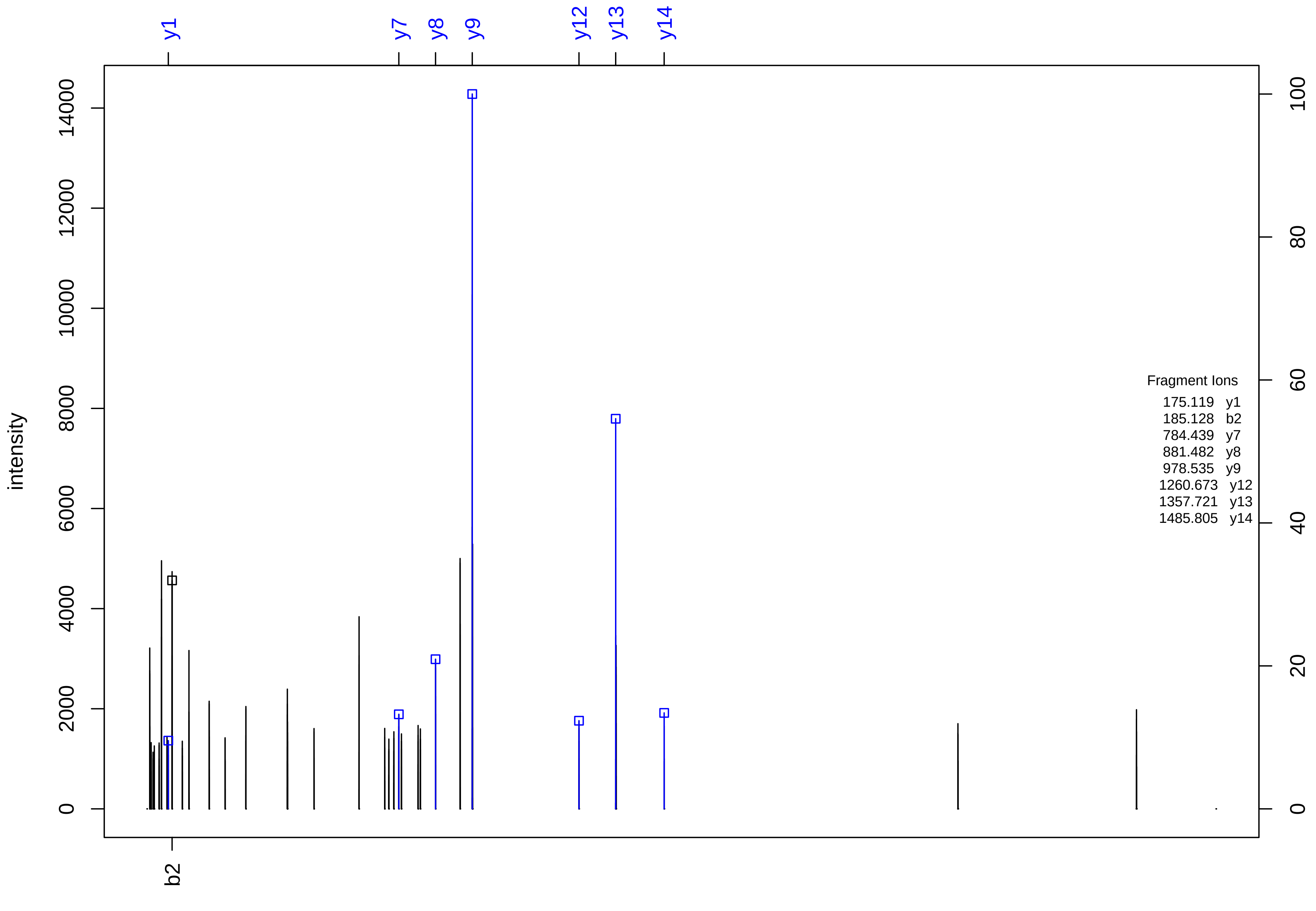

AIASASASGPCIHQQPPGQPPPPQLSSR / File: christine_30turboips_ip12.raw; SpectrumID: ; scans: 5094

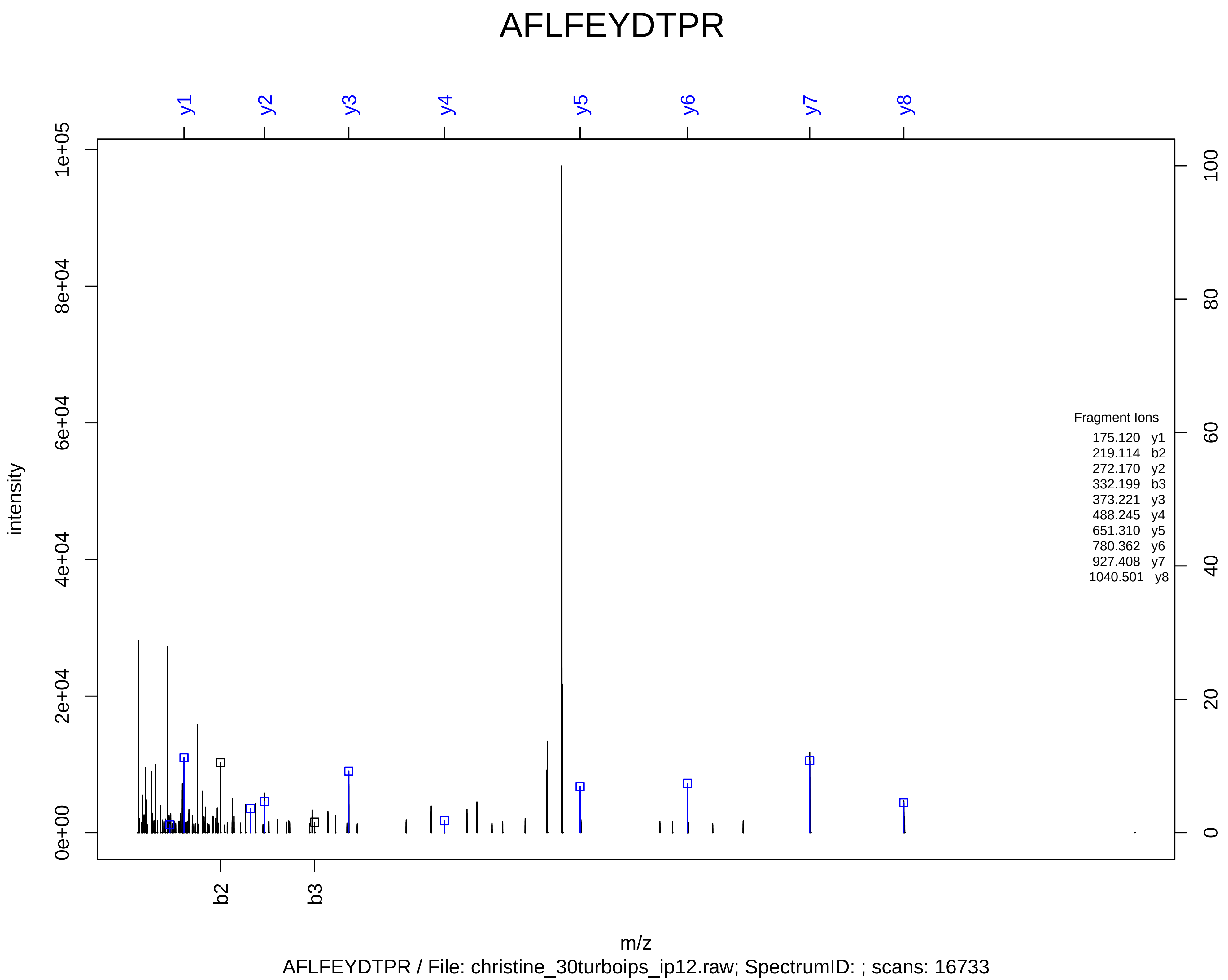
AFLFEYDTPR
y1
y2
y3
y4
y5
y6
y7
y8
b2
b3
intensity
0e+00
2e+04
4e+04
6e+04
8e+04
1e+05
0
20
40
60
80
100
Fragment Ions
175.120 y1
219.114 b2
272.170 y2
332.199 b3
373.221 y3
488.245 y4
651.310 y5
780.362 y6
927.408 y7
1040.501 y8
m/z
AFLFEYDTPR / File: christine_30turboips_ip12.raw; SpectrumID: ; scans: 16733

DLEAAVSSEDDTANLER

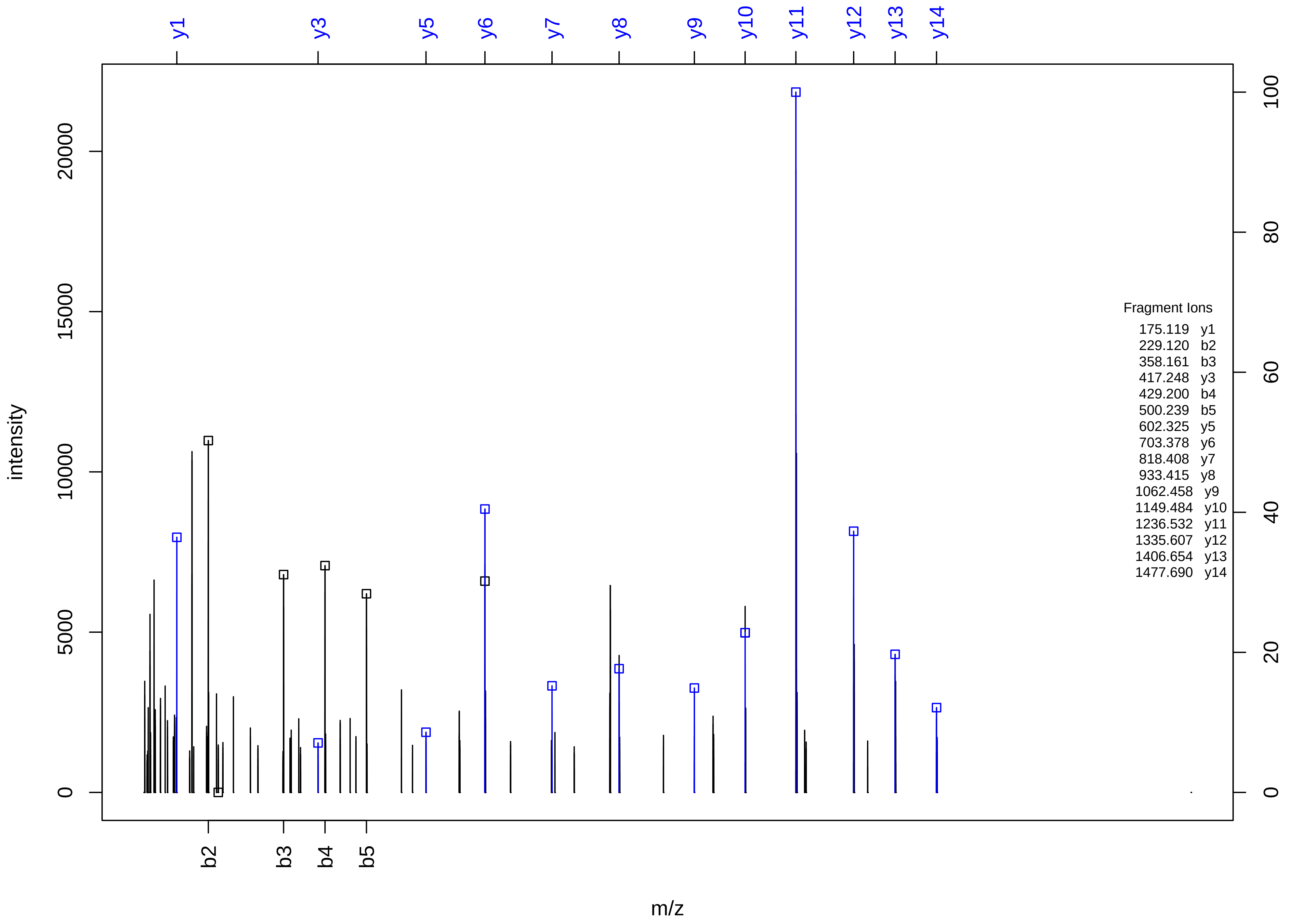

DLEAAVSSEDDTANLER / File: christine_30turboips_ip12.raw; SpectrumID: ; scans: 10941

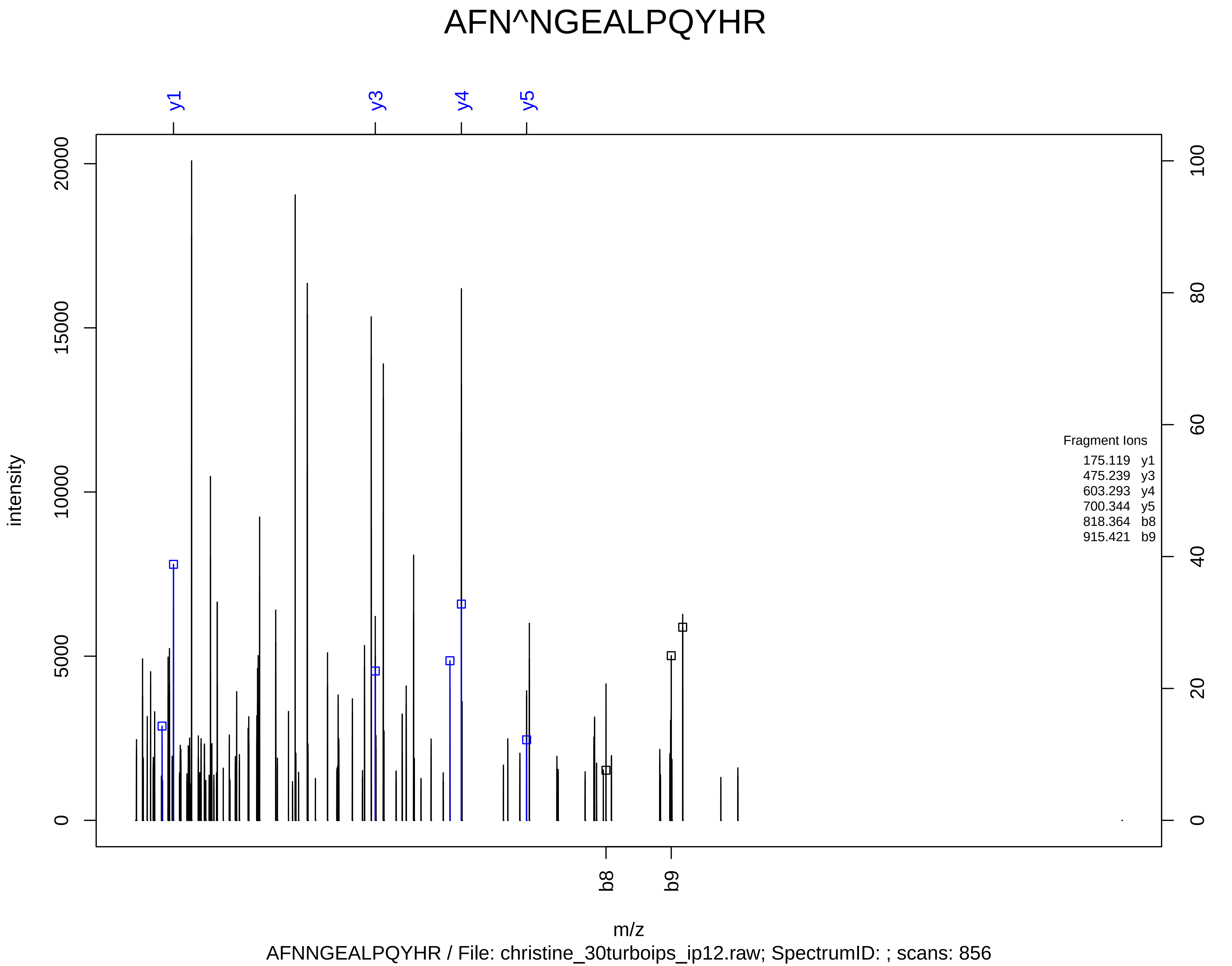

AFN^NGEALPQYHR
y1
y3
y4
y5
intensity
0
5000
10000
15000
20000
0
20
40
60
80
100
Fragment Ions
175.119 y1
475.239 y3
603.293 y4
700.344 y5
818.364 b8
915.421 b9
b8
b9
m/z
AFNNGEALPQYHR / File: christine_30turboips_ip12.raw; SpectrumID: ; scans: 856

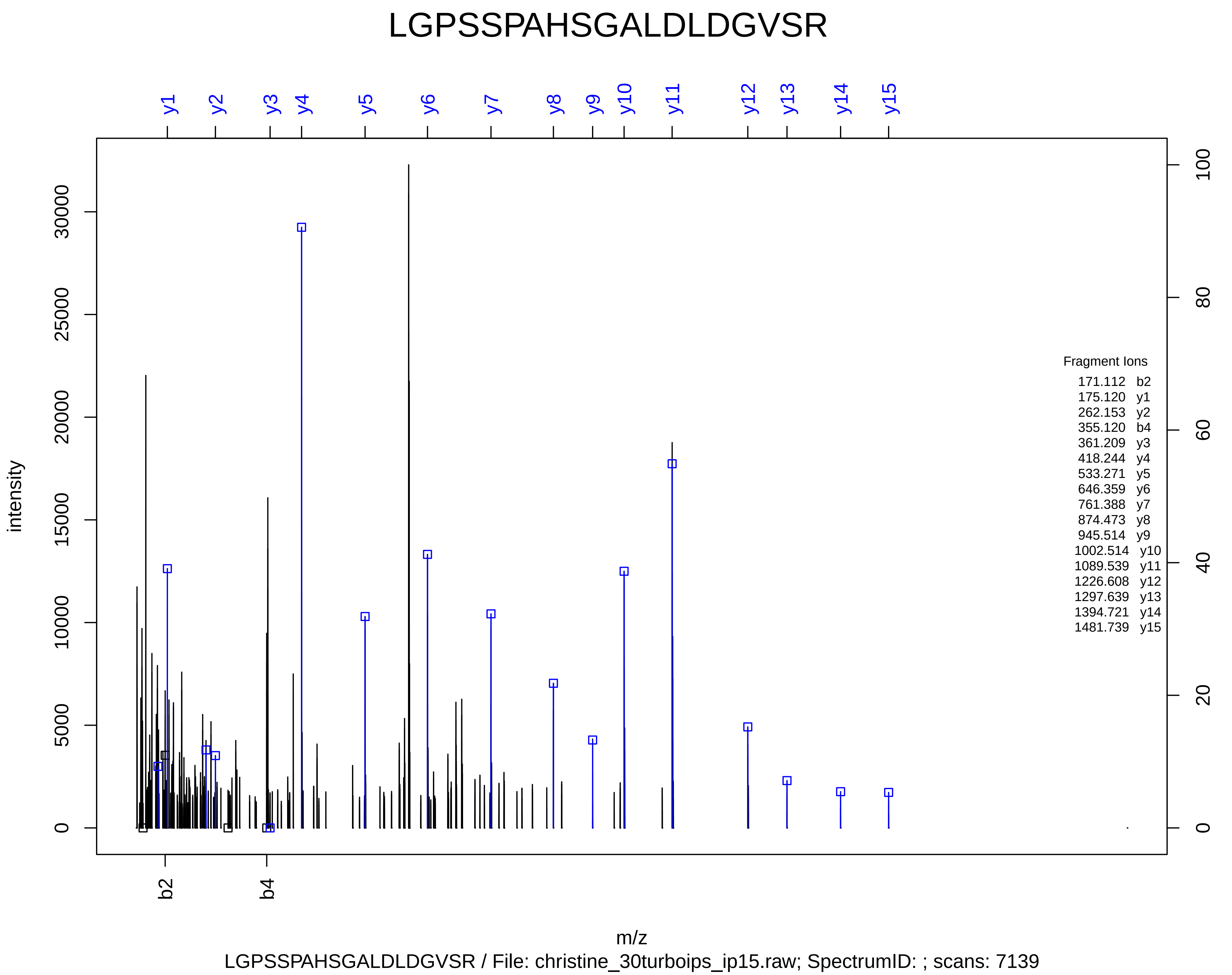
LGPSSPAHSGALDLDGVSR
y1 y2 y3 y4 y5 y6 y7 y8 y9 y10 y11 y12 y13 y14 y15
b2 b4
intensity
m/z
Fragment Ions
171.112 b2
175.120 y1
262.153 y2
355.120 b4
361.209 y3
418.244 y4
533.271 y5
646.359 y6
761.388 y7
874.473 y8
945.514 y9
1002.514 y10
1089.539 y11
1226.608 y12
1297.639 y13
1394.721 y14
1481.739 y15
LGPSSPAHSGALDLDGVSR / File: christine_30turboips_ip15.raw; SpectrumID: ; scans: 7139

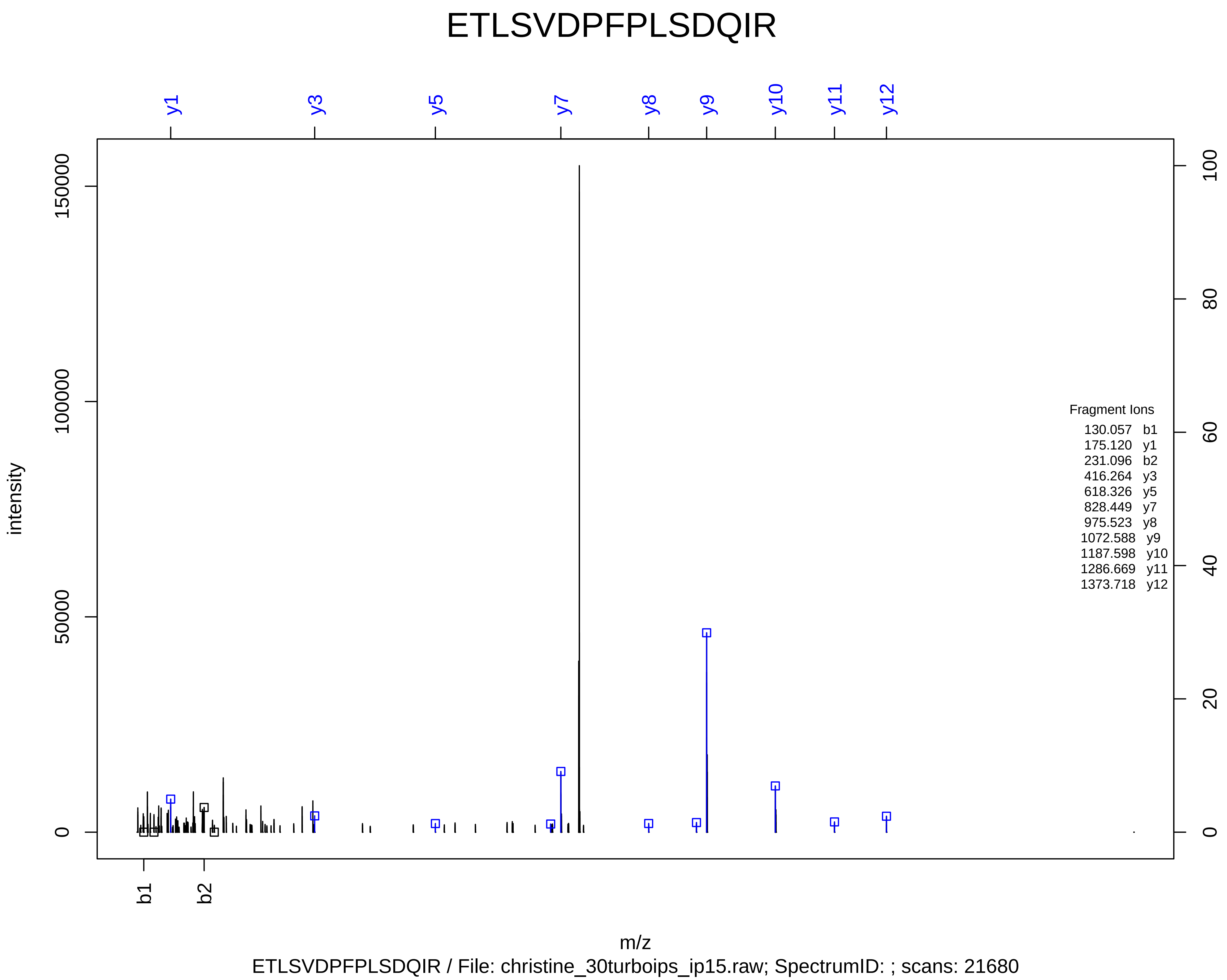
ETLSVDPFPLSDQIR
y1
y3
y5
y7
y8
y9
y10
y11
y12
intensity
150000
100000
50000
0
100
80
60
40
20
0
Fragment Ions
130.057 b1
175.120 y1
231.096 b2
416.264 y3
618.326 y5
828.449 y7
975.523 y8
1072.588 y9
1187.598 y10
1286.669 y11
1373.718 y12
b1
b2
m/z
ETLSVDPFPLSDQIR / File: christine_30turboips_ip15.raw; SpectrumID: ; scans: 21680

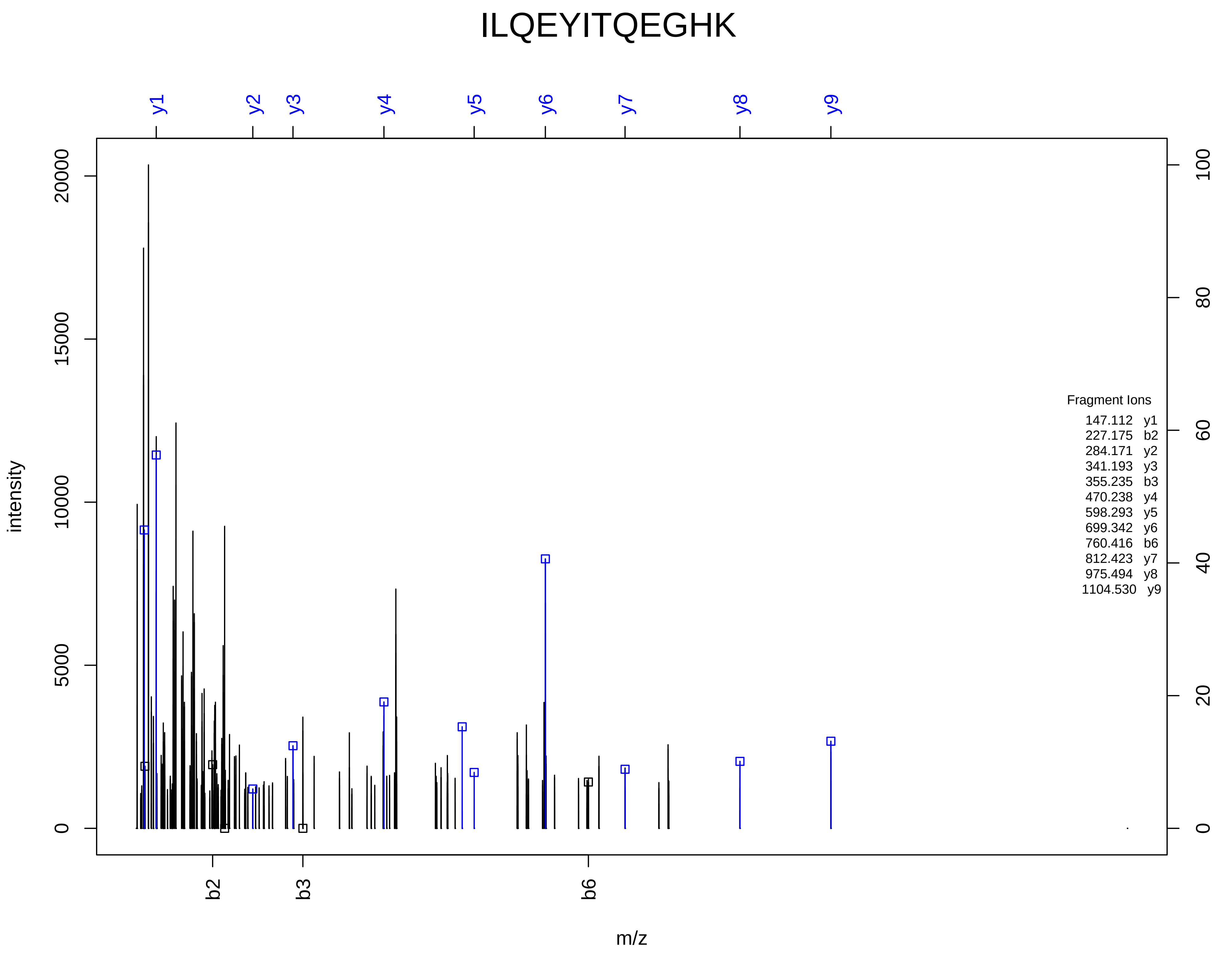

ILQEYITQEGHK / File: christine_30turboips_ip15.raw; SpectrumID: ; scans: 3447

GYDFESETDTETIAK

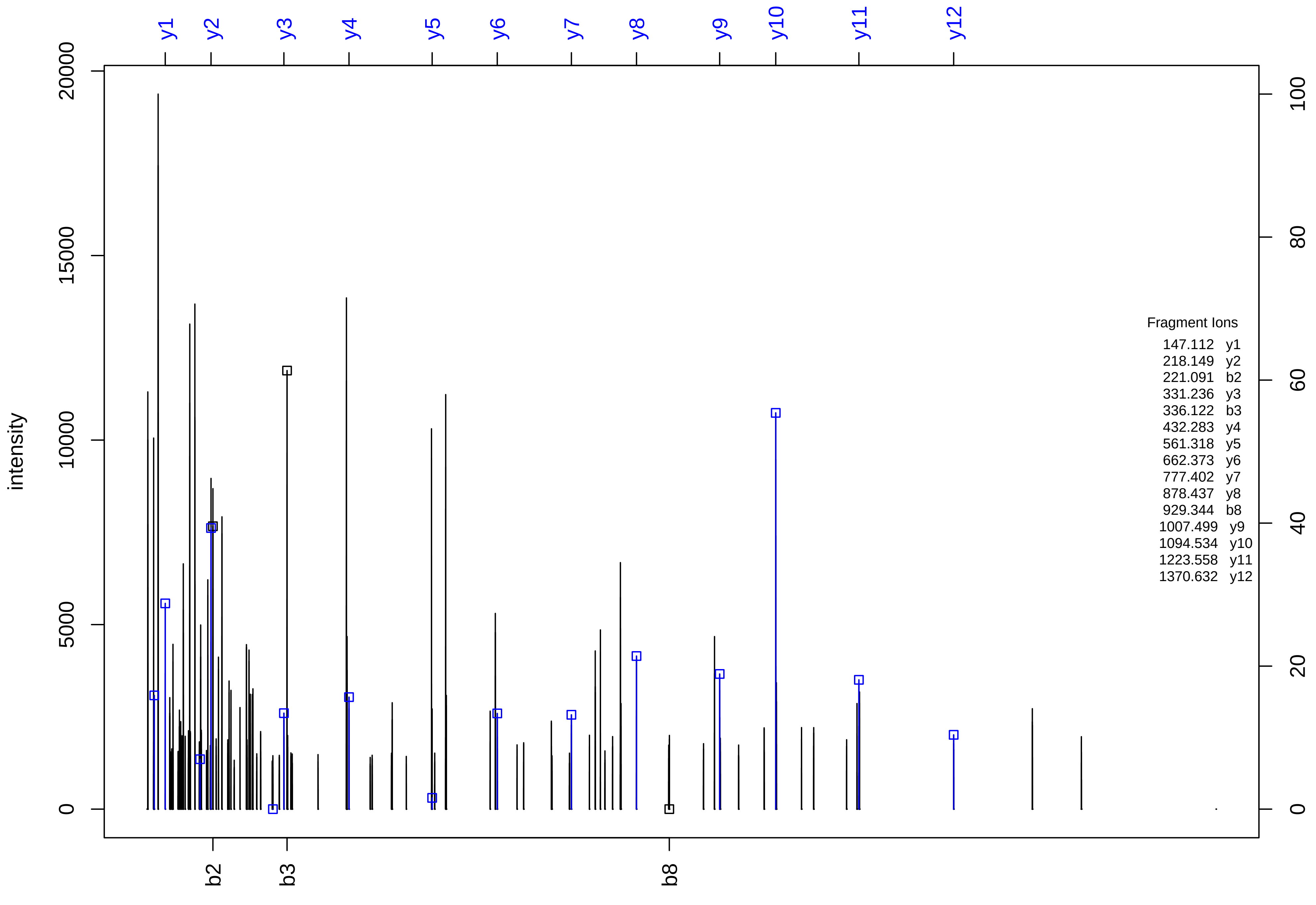

GYDFESETDTETIAK / File: christine_30turboips_ip15.raw; SpectrumID: ; scans: 8815

VLEDPAIVSDENFGPVK

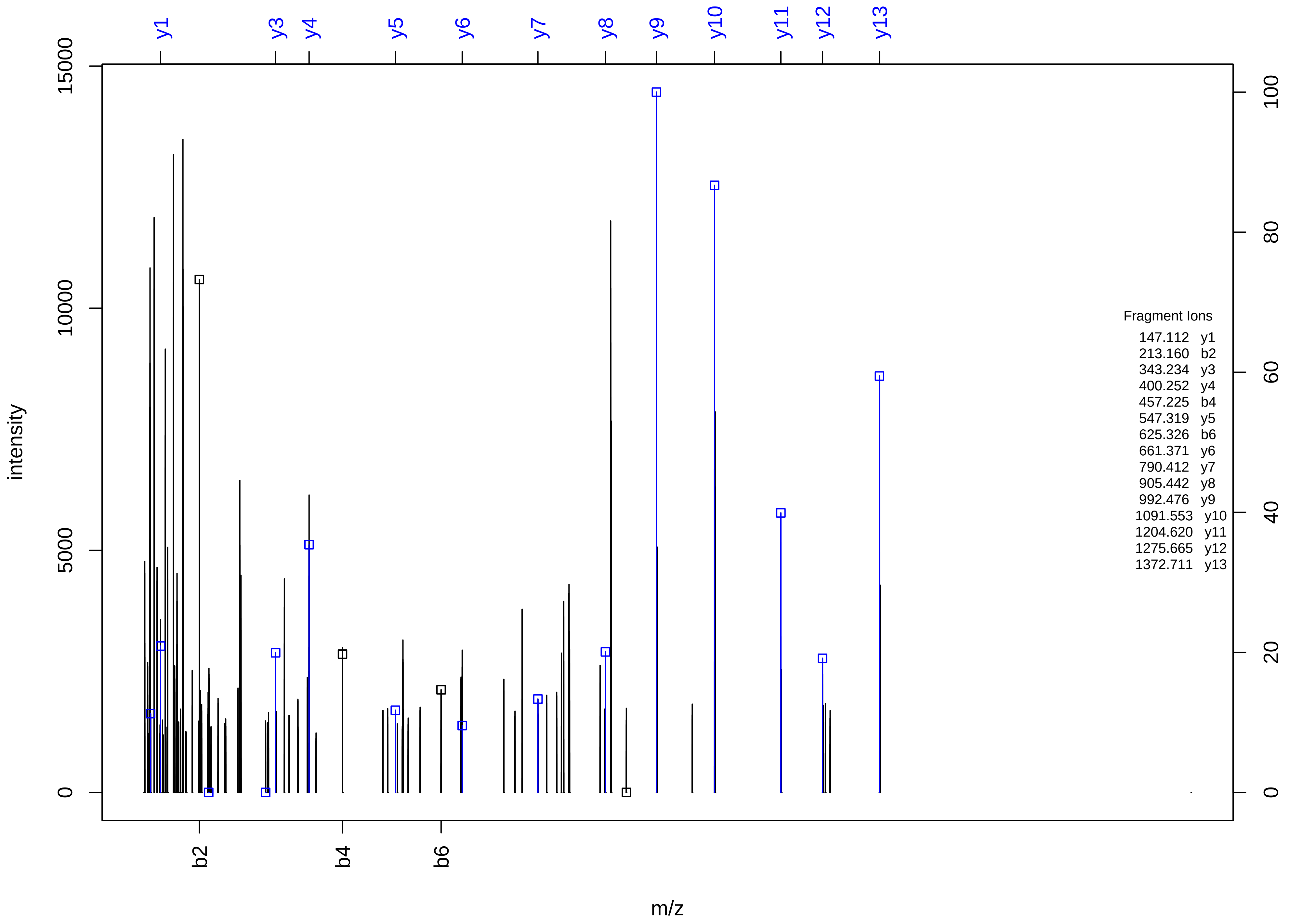

VLEDPAIVSDENFGPVK / File: christine_30turboips_ip15.raw; SpectrumID: ; scans: 16440

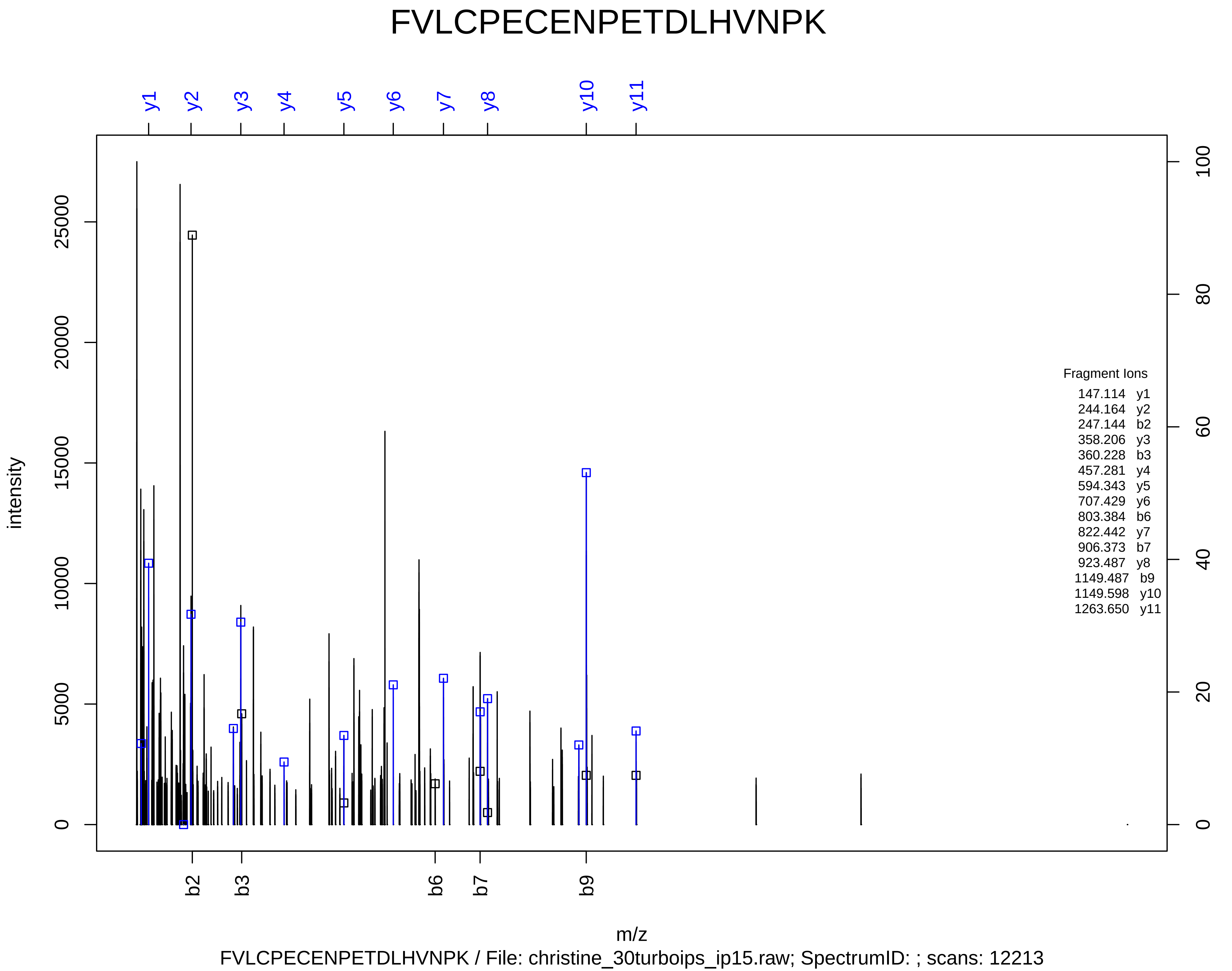
FVLCPECENPETDLHVNPK
y1
y2
y3
y4
y5
y6
y7
y8
y10
y11
intensity
0
5000
10000
15000
20000
25000
0
20
40
60
80
100
b2
b3
b6
b7
b9
m/z
Fragment Ions
147.114 y1
244.164 y2
247.144 b2
358.206 y3
360.228 b3
457.281 y4
594.343 y5
707.429 y6
803.384 b6
822.442 y7
906.373 b7
923.487 y8
1149.487 b9
1149.598 y10
1263.650 y11
FVLCPECENPETDLHVNPK / File: christine_30turboips_ip15.raw; SpectrumID: ; scans: 12213

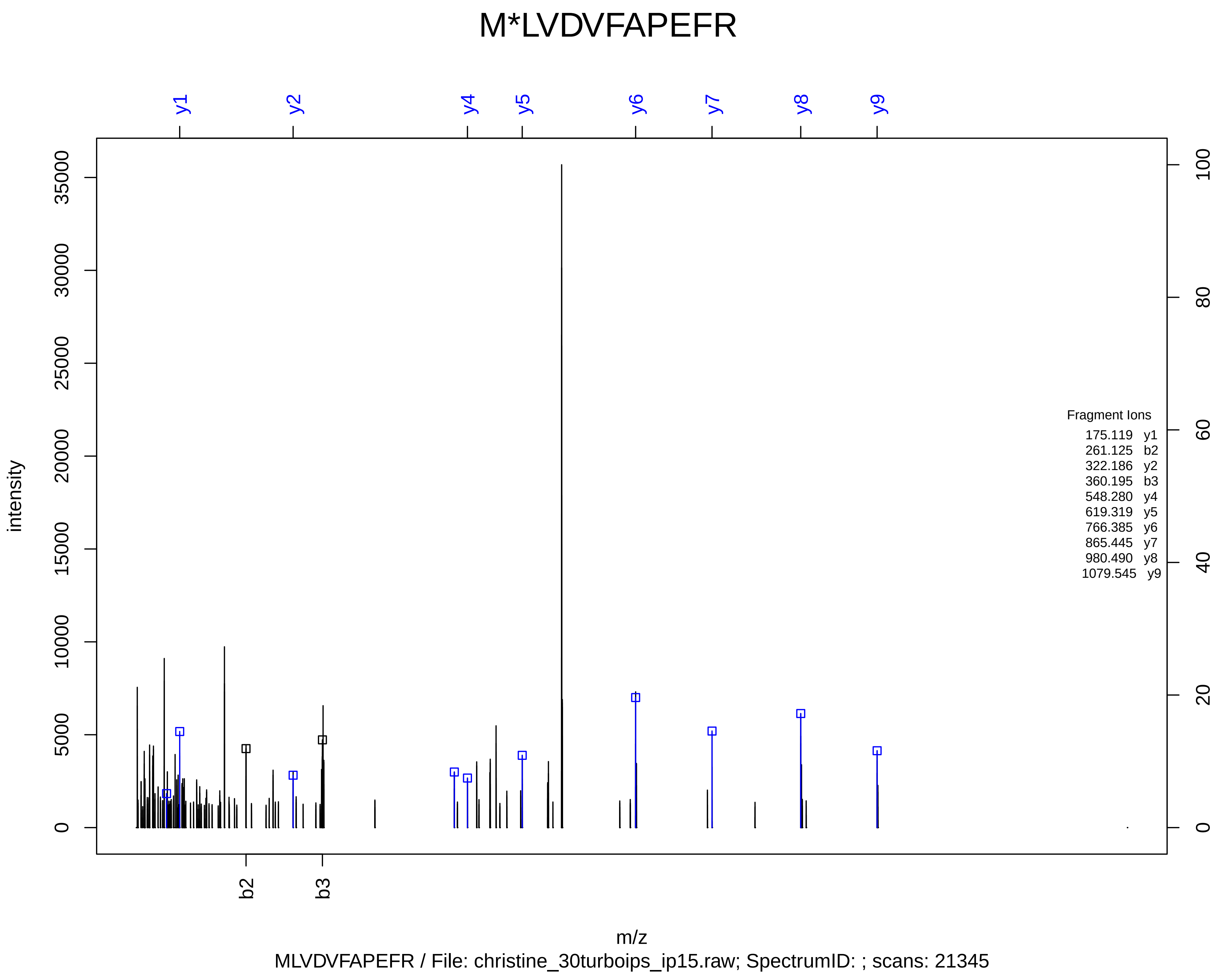
M*LVDVFAPEFR
y1
y2
y4
y5
y6
y7
y8
y9
b2
b3
intensity
m/z
0
5000
10000
15000
20000
25000
30000
35000
0
20
40
60
80
100
Fragment Ions
175.119 y1
261.125 b2
322.186 y2
360.195 b3
548.280 y4
619.319 y5
766.385 y6
865.445 y7
980.490 y8
1079.545 y9
MLVDVFAPEFR / File: christine_30turboips_ip15.raw; SpectrumID: ; scans: 21345

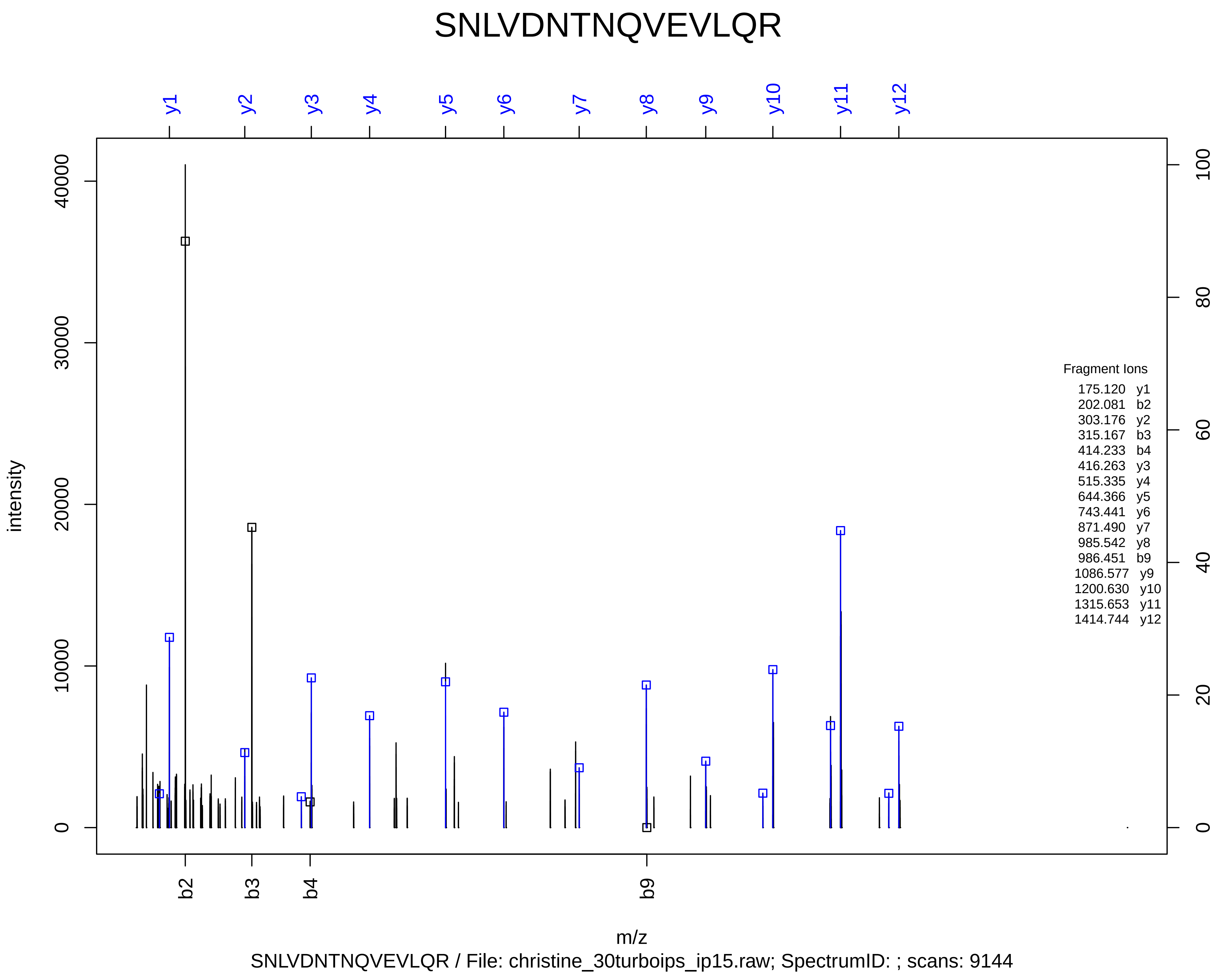
SNLVDNTNQVEVLQR
y1
y2
y3
y4
y5
y6
y7
y8
y9
y10
y11
y12
b2
b3
b4
b9
intensity
m/z
Fragment Ions
175.120 y1
202.081 b2
303.176 y2
315.167 b3
414.233 b4
416.263 y3
515.335 y4
644.366 y5
743.441 y6
871.490 y7
985.542 y8
986.451 b9
1086.577 y9
1200.630 y10
1315.653 y11
1414.744 y12
SNLVDNTNQVEVLQR / File: christine_30turboips_ip15.raw; SpectrumID: ; scans: 9144

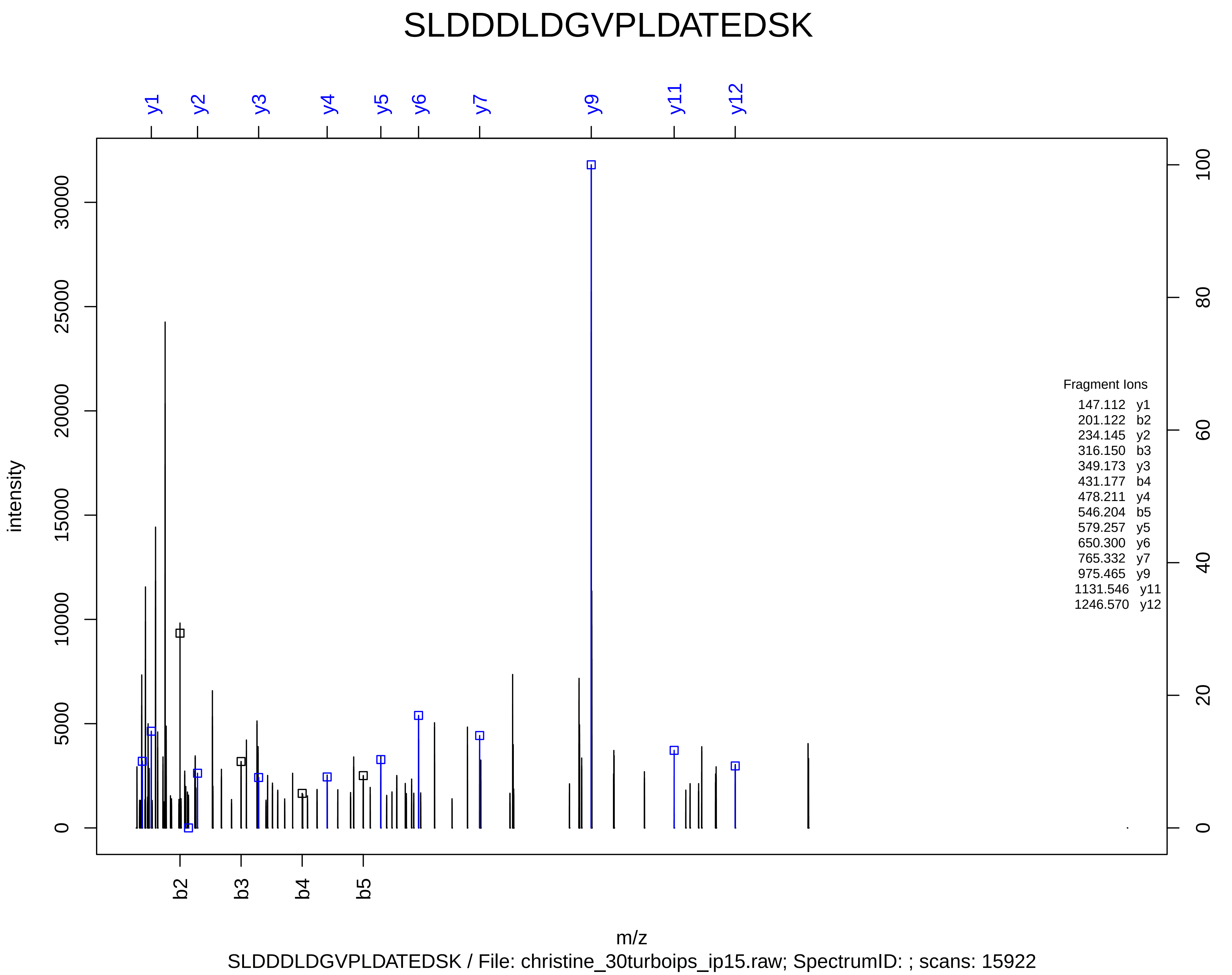
SLDDDLDGVPLDATEDSK
y1
y2
y3
y4
y5
y6
y7
y9
y11
y12
b2
b3
b4
b5
intensity
m/z
Fragment Ions
147.112 y1
201.122 b2
234.145 y2
316.150 b3
349.173 y3
431.177 b4
478.211 y4
546.204 b5
579.257 y5
650.300 y6
765.332 y7
975.465 y9
1131.546 y11
1246.570 y12
SLDDDLDGVPLDATEDSK / File: christine_30turboips_ip15.raw; SpectrumID: ; scans: 15922

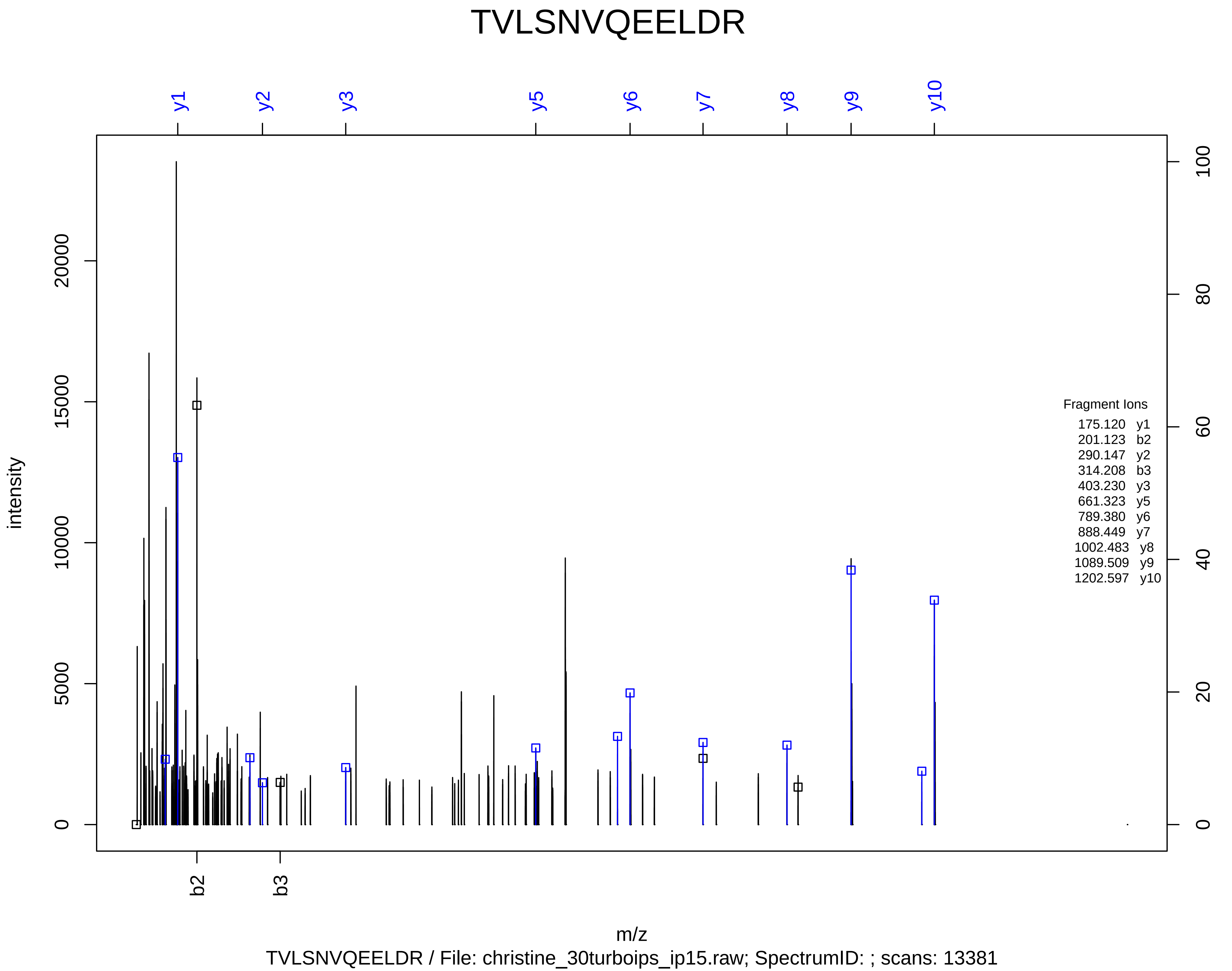
TVLSNVQEELDR
y1
y2
y3
y5
y6
y7
y8
y9
y10
b2
b3
intensity
m/z
0
5000
10000
15000
20000
0
20
40
60
80
100
Fragment Ions
175.120 y1
201.123 b2
290.147 y2
314.208 b3
403.230 y3
661.323 y5
789.380 y6
888.449 y7
1002.483 y8
1089.509 y9
1202.597 y10
TVLSNVQEELDR / File: christine_30turboips_ip15.raw; SpectrumID: ; scans: 13381

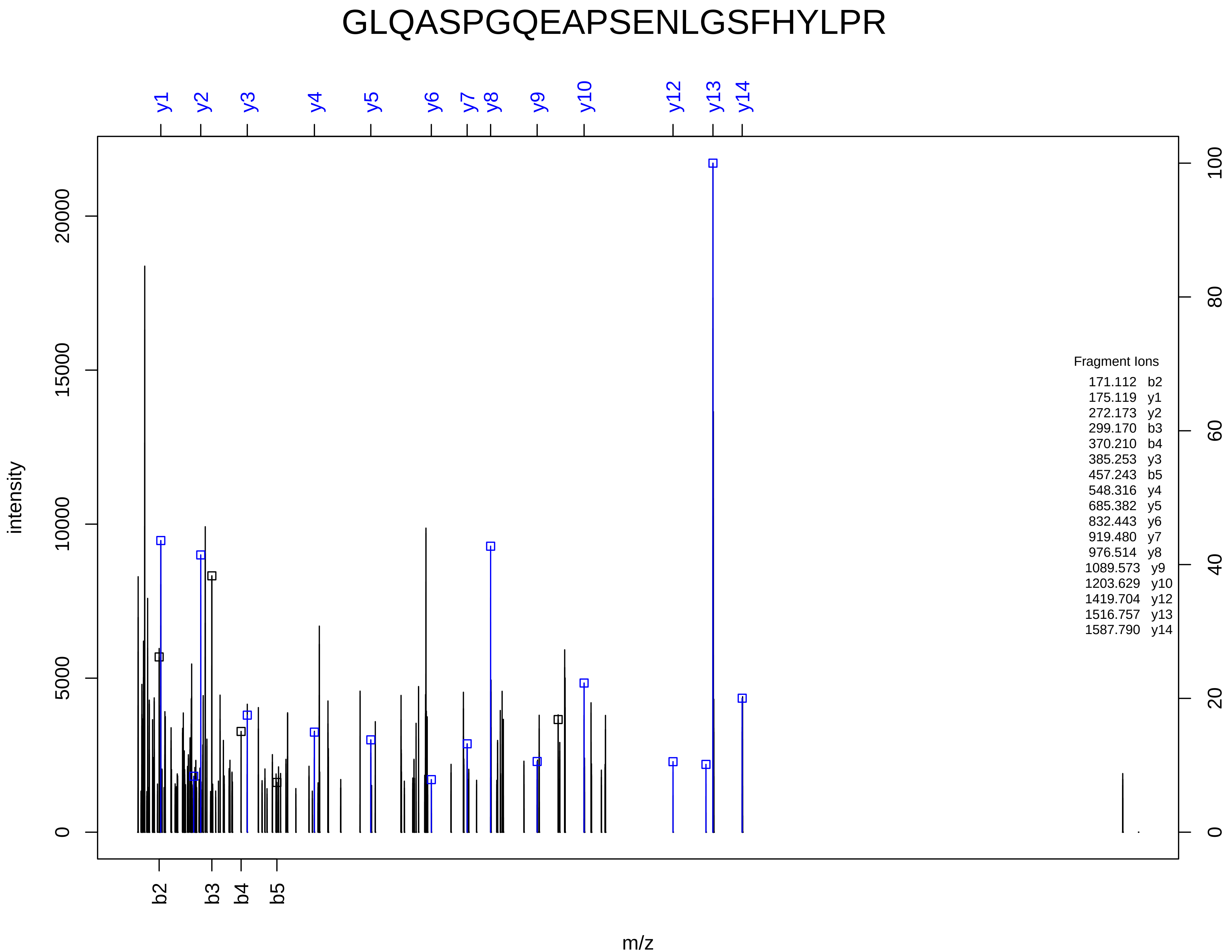

GLQASPGQEAPSENLGSFHYLPR / File: christine_30turboips_ip15.raw; SpectrumID: ; scans: 12615

AVLESNPESPITITEPYR

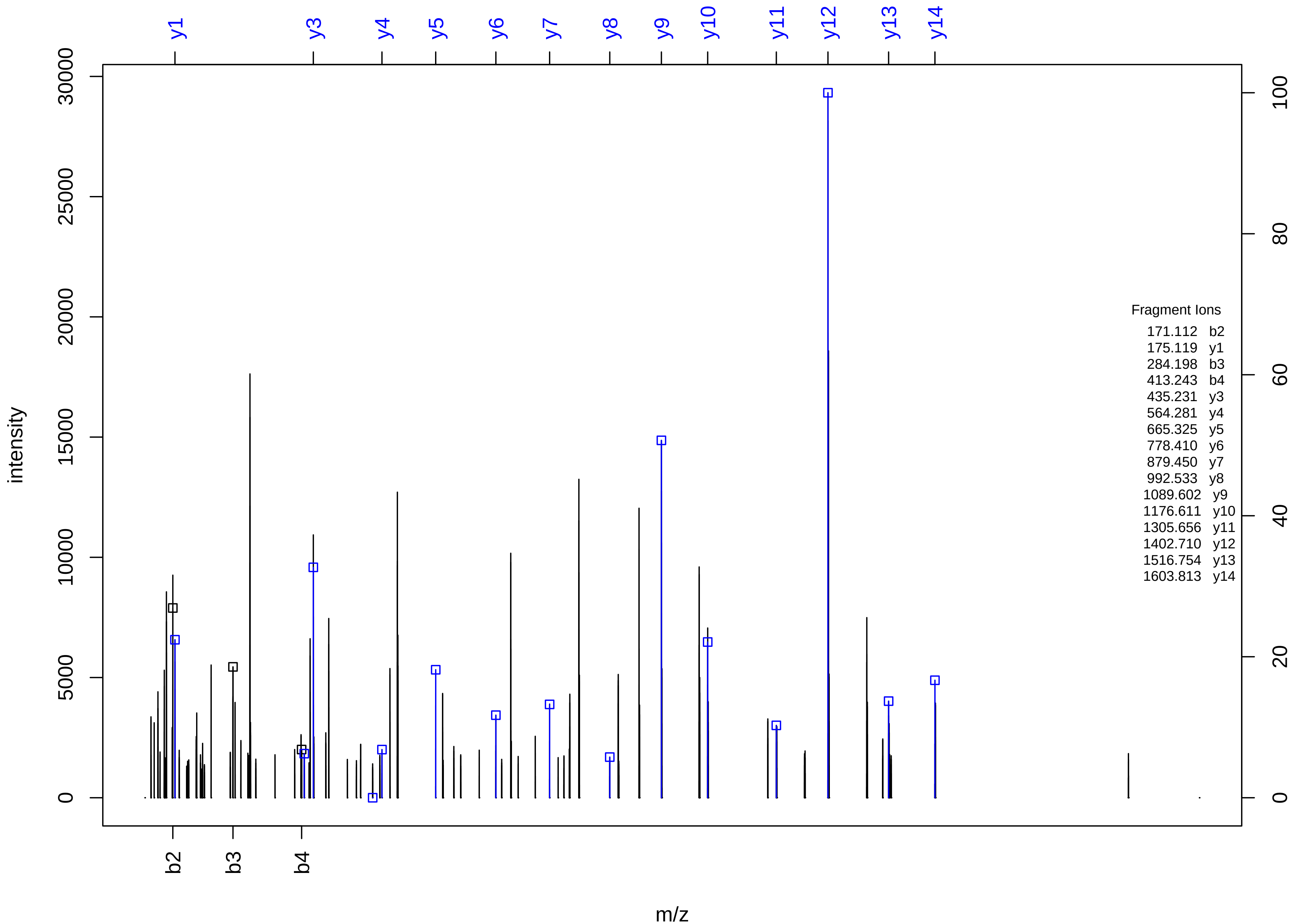

AVLESNPESPITITEPYR / File: christine_30turboips_ip15.raw; SpectrumID: ; scans: 15529

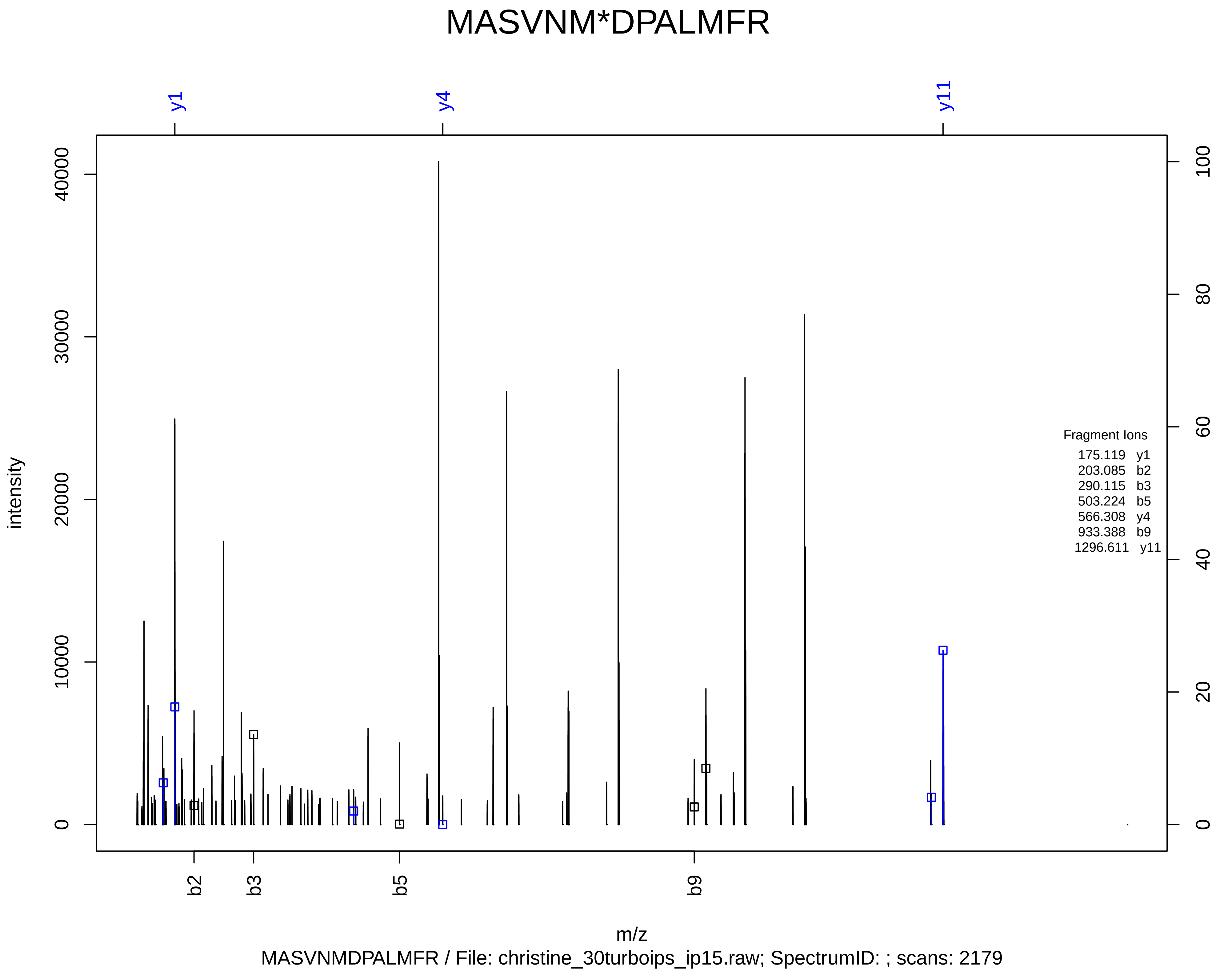
MASVNM*DPALMFR
y1
y4
y11
intensity
0
10000
20000
30000
40000
0
20
40
60
80
100
b2
b3
b5
b9
m/z
Fragment Ions
175.119 y1
203.085 b2
290.115 b3
503.224 b5
566.308 y4
933.388 b9
1296.611 y11
MASVNMDPALMFR / File: christine_30turboips_ip15.raw; SpectrumID: ; scans: 2179

VIAINVDDPDAANYK

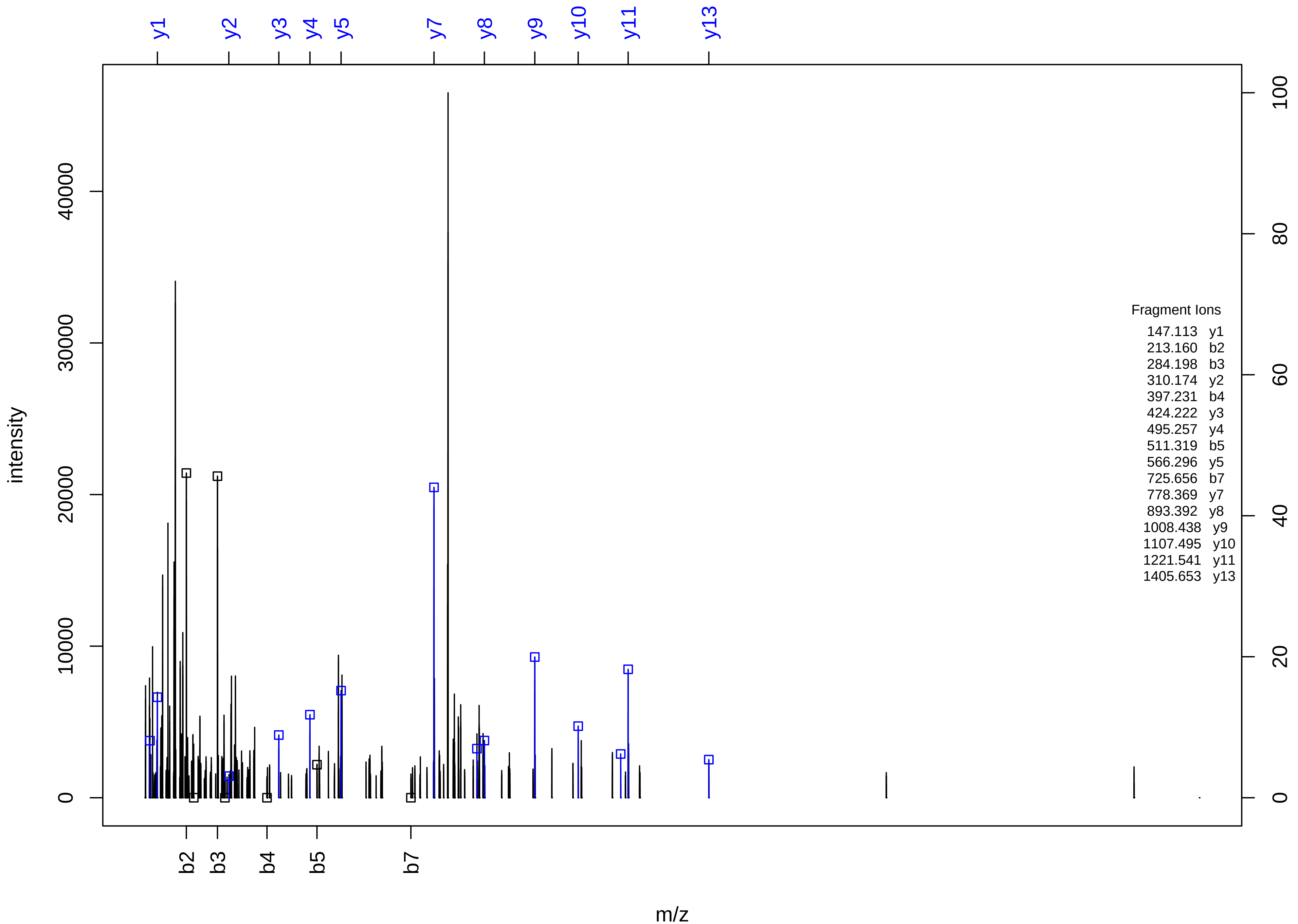

VIAINVDDPDAANYK / File: christine_30turboips_ip15.raw; SpectrumID: ; scans: 11019

DILEPSAPEPEPECFGSFK

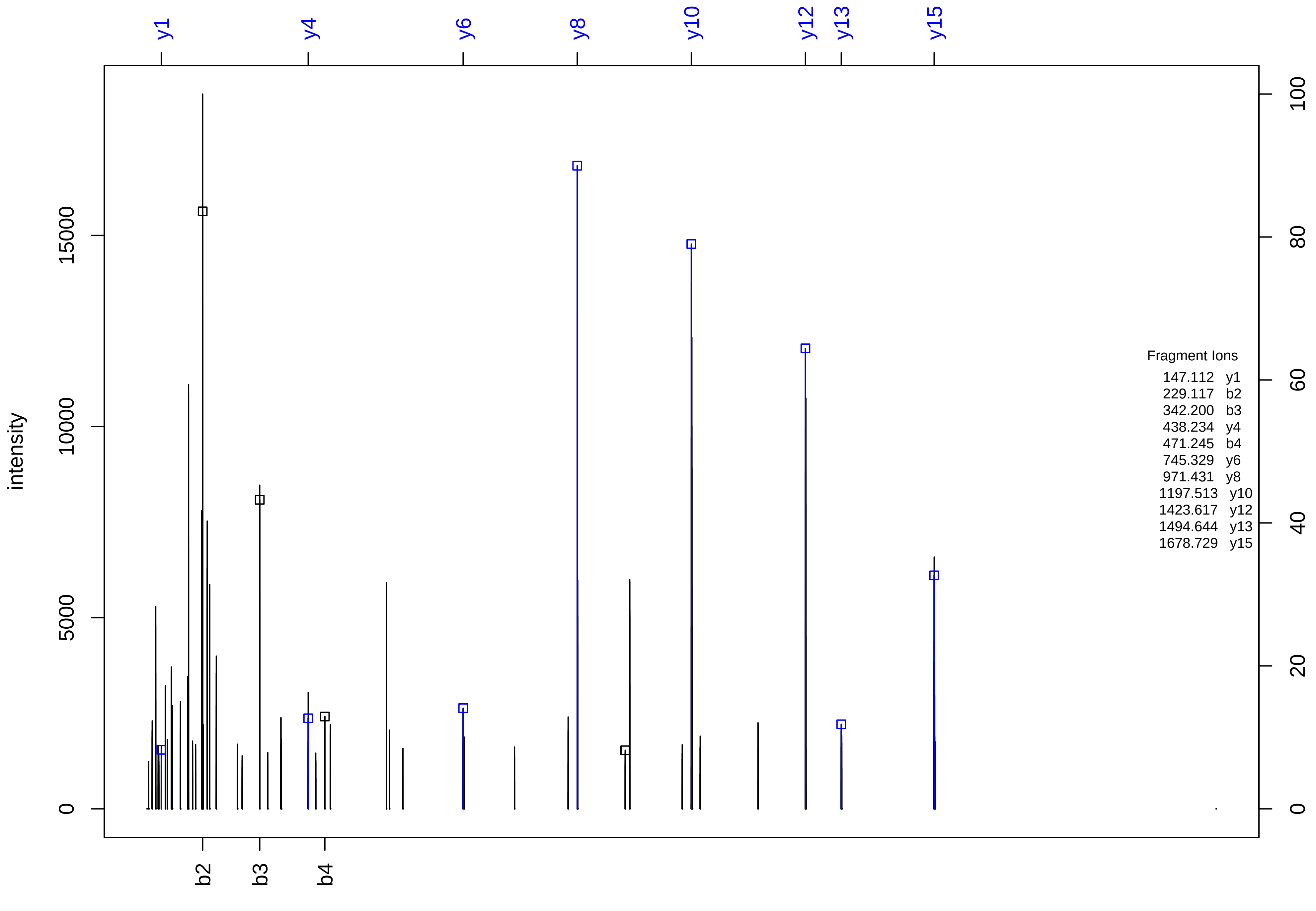

DILEPSAPEPEPECFGSFK / File: christine_30turboips_ip15.raw; SpectrumID: ; scans: 19220

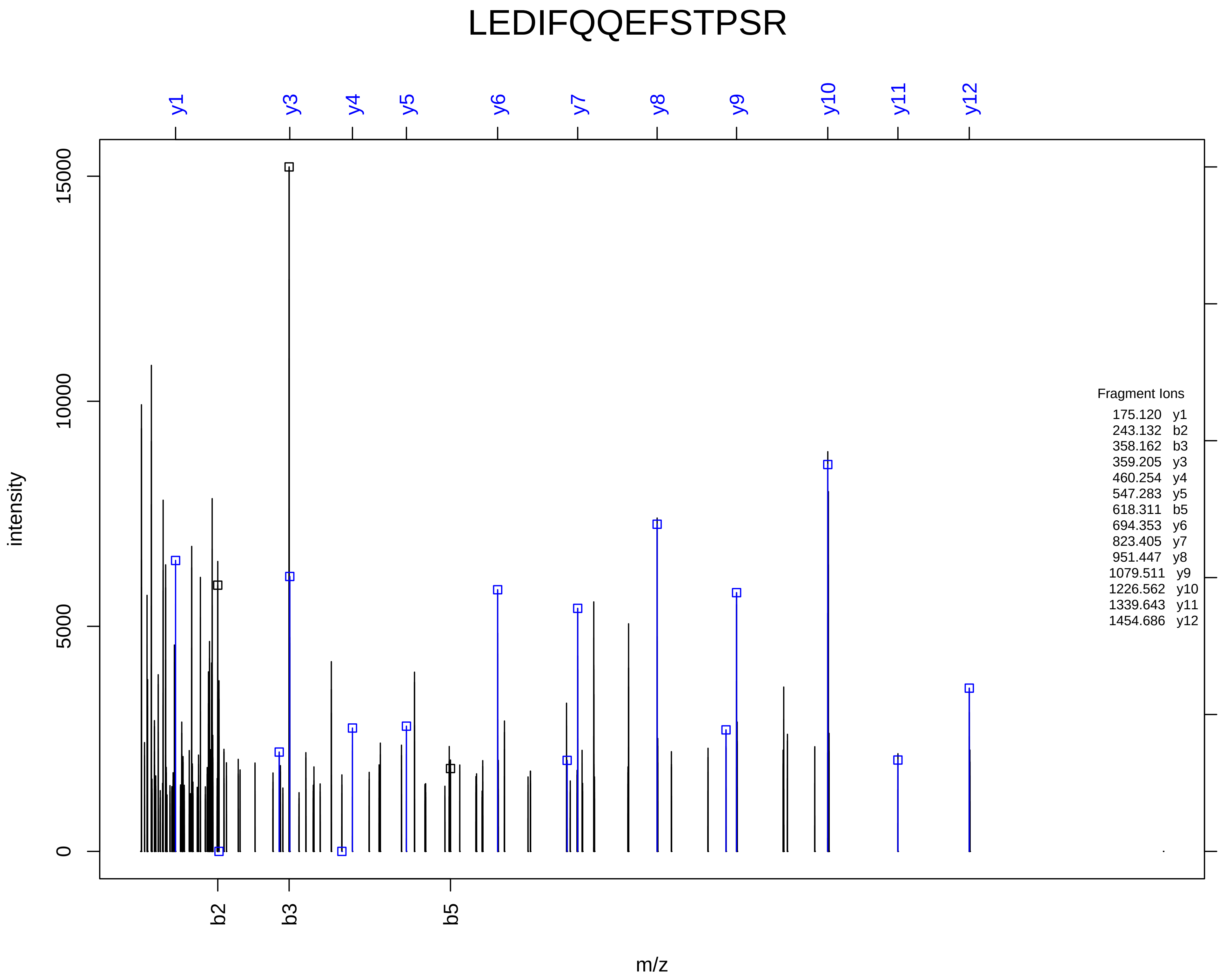

LEDIFQQEFSTPSR / File: christine_30turboips_ip15.raw; SpectrumID: ; scans: 17868

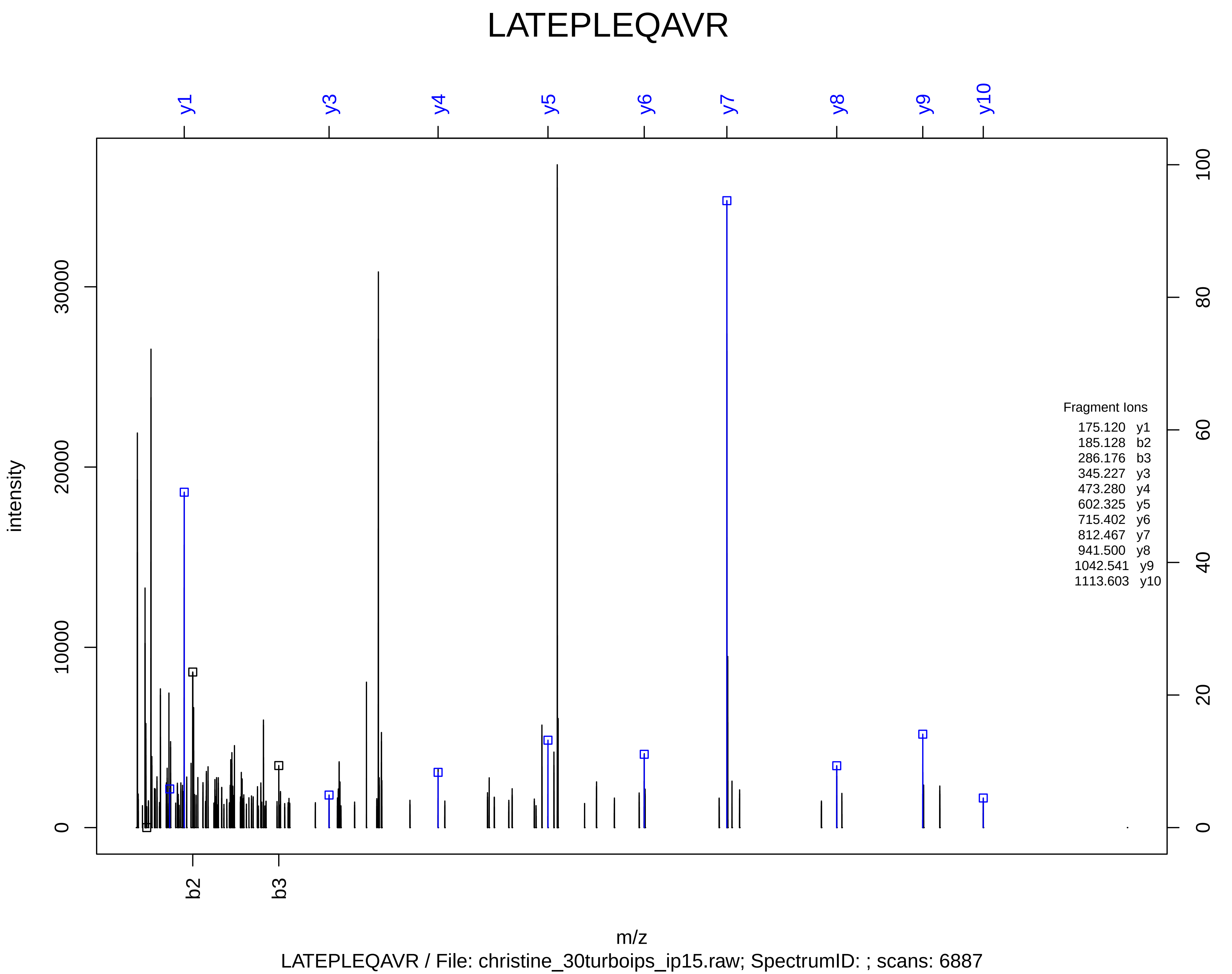

LATEPLEQAVR
y1
y3
y4
y5
y6
y7
y8
y9
y10
b2
b3
intensity
m/z
0
10000
20000
30000
0
20
40
60
80
100
Fragment Ions
175.120 y1
185.128 b2
286.176 b3
345.227 y3
473.280 y4
602.325 y5
715.402 y6
812.467 y7
941.500 y8
1042.541 y9
1113.603 y10
LATEPLEQAVR / File: christine_30turboips_ip15.raw; SpectrumID: ; scans: 6887

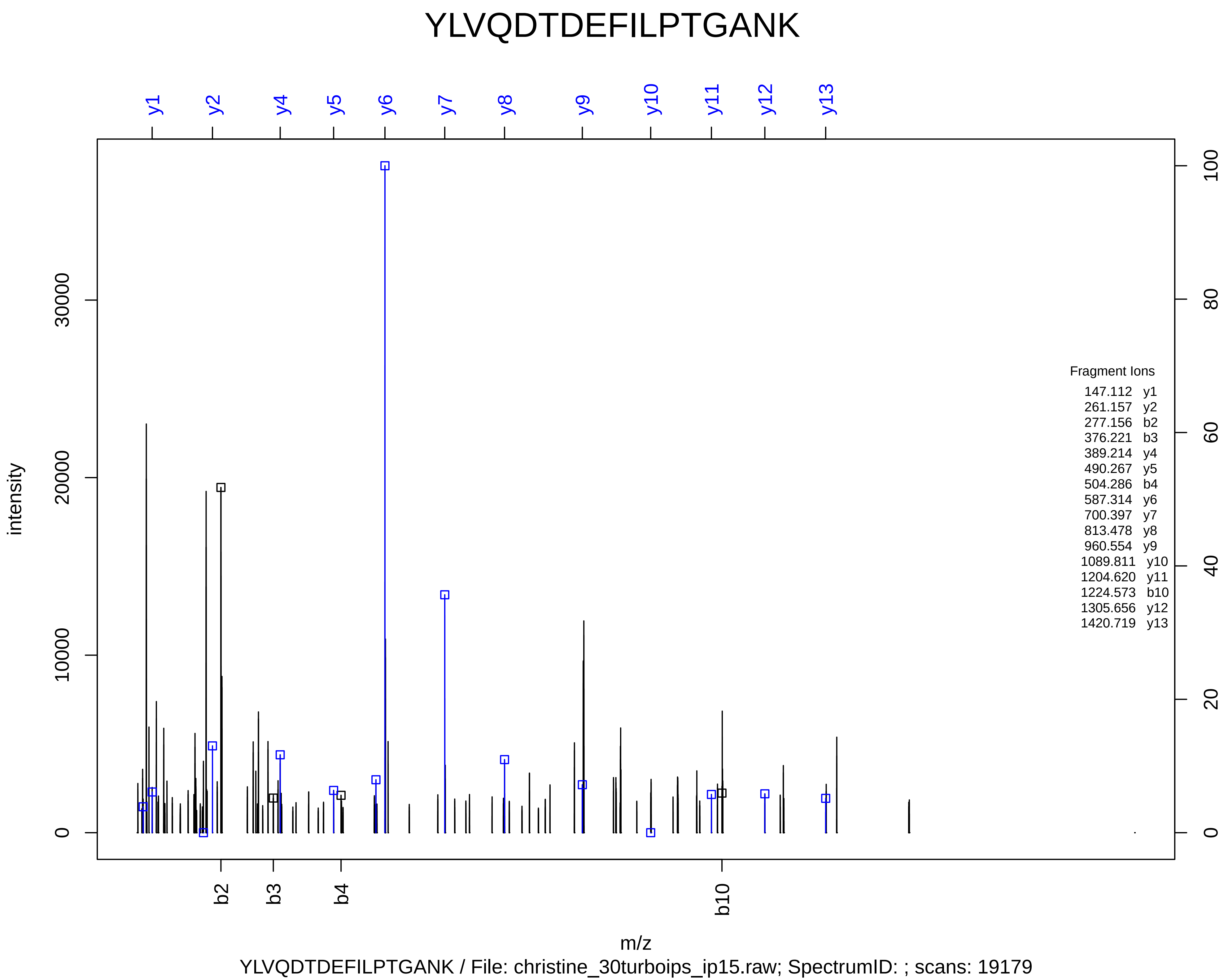
YLVQDTDEFILPTGANK
y1
y2
y4
y5
y6
y7
y8
y9
y10
y11
y12
y13
intensity
0
10000
20000
30000
0
20
40
60
80
100
b2
b3
b4
b10
m/z
Fragment Ions
147.112 y1
261.157 y2
277.156 b2
376.221 b3
389.214 y4
490.267 y5
504.286 b4
587.314 y6
700.397 y7
813.478 y8
960.554 y9
1089.811 y10
1204.620 y11
1224.573 b10
1305.656 y12
1420.719 y13
YLVQDTDEFILPTGANK / File: christine_30turboips_ip15.raw; SpectrumID: ; scans: 19179

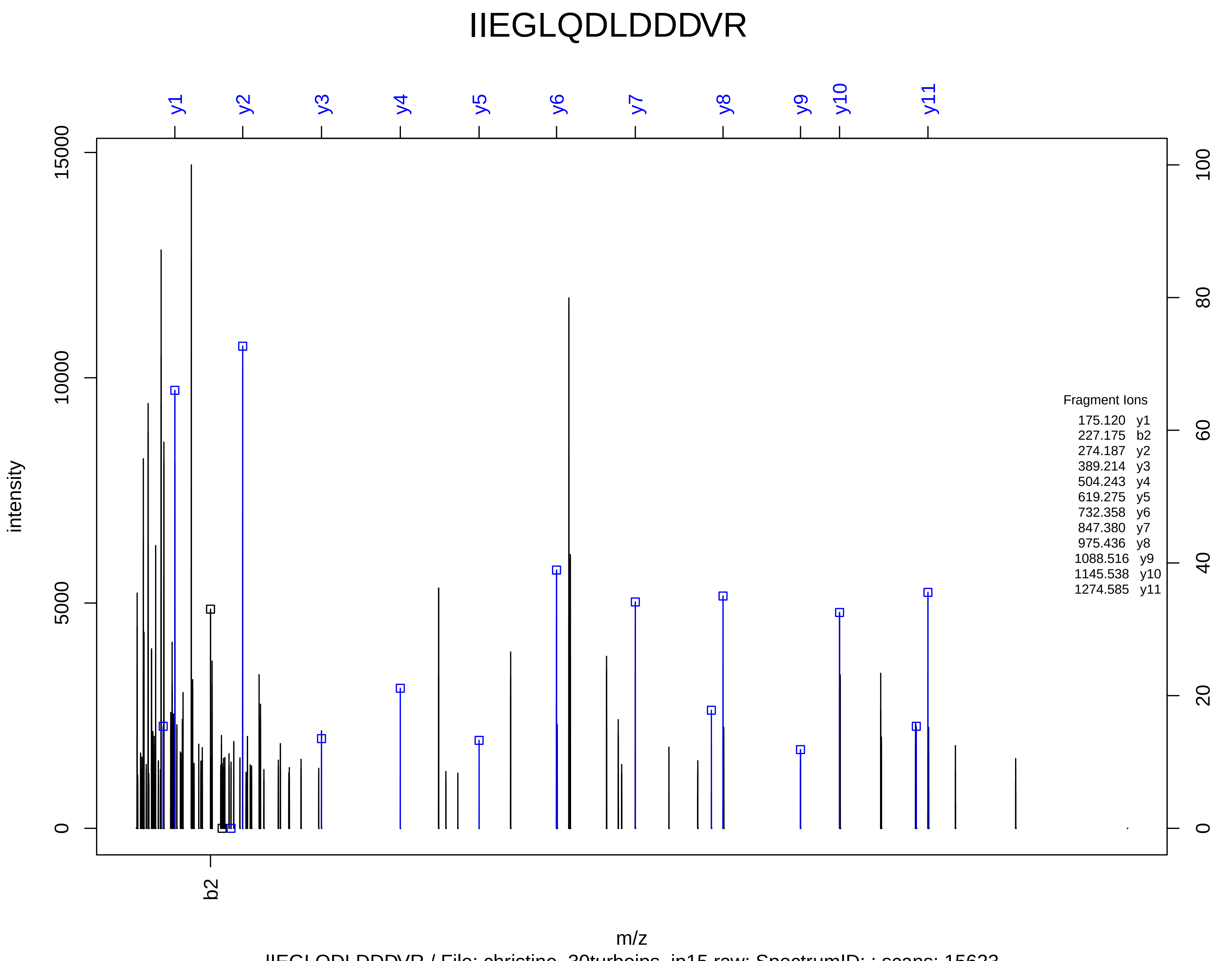
IIEGLQDLDDDVR
y1
y2
y3
y4
y5
y6
y7
y8
y9
y10
y11
b2
intensity
m/z
Fragment Ions
175.120 y1
227.175 b2
274.187 y2
389.214 y3
504.243 y4
619.275 y5
732.358 y6
847.380 y7
975.436 y8
1088.516 y9
1145.538 y10
1274.585 y11
IIEGLQDLDDDVR / File: christine_30turboips_ip15.raw; SpectrumID: ; scans: 15623

LLEEAQLQDLEK

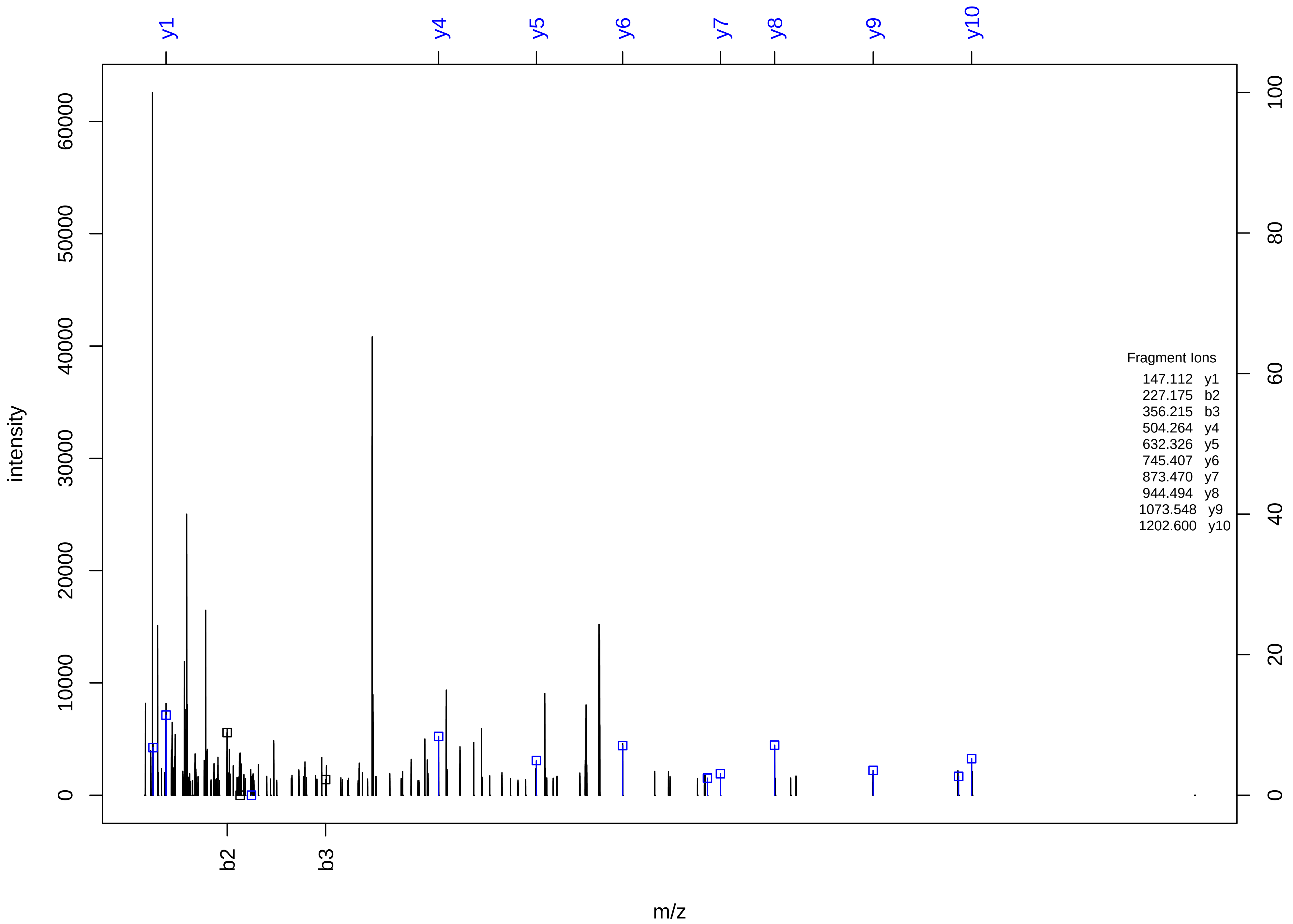

LLEEAQLQDLEK / File: christine_30turboips_ip15.raw; SpectrumID: ; scans: 12872

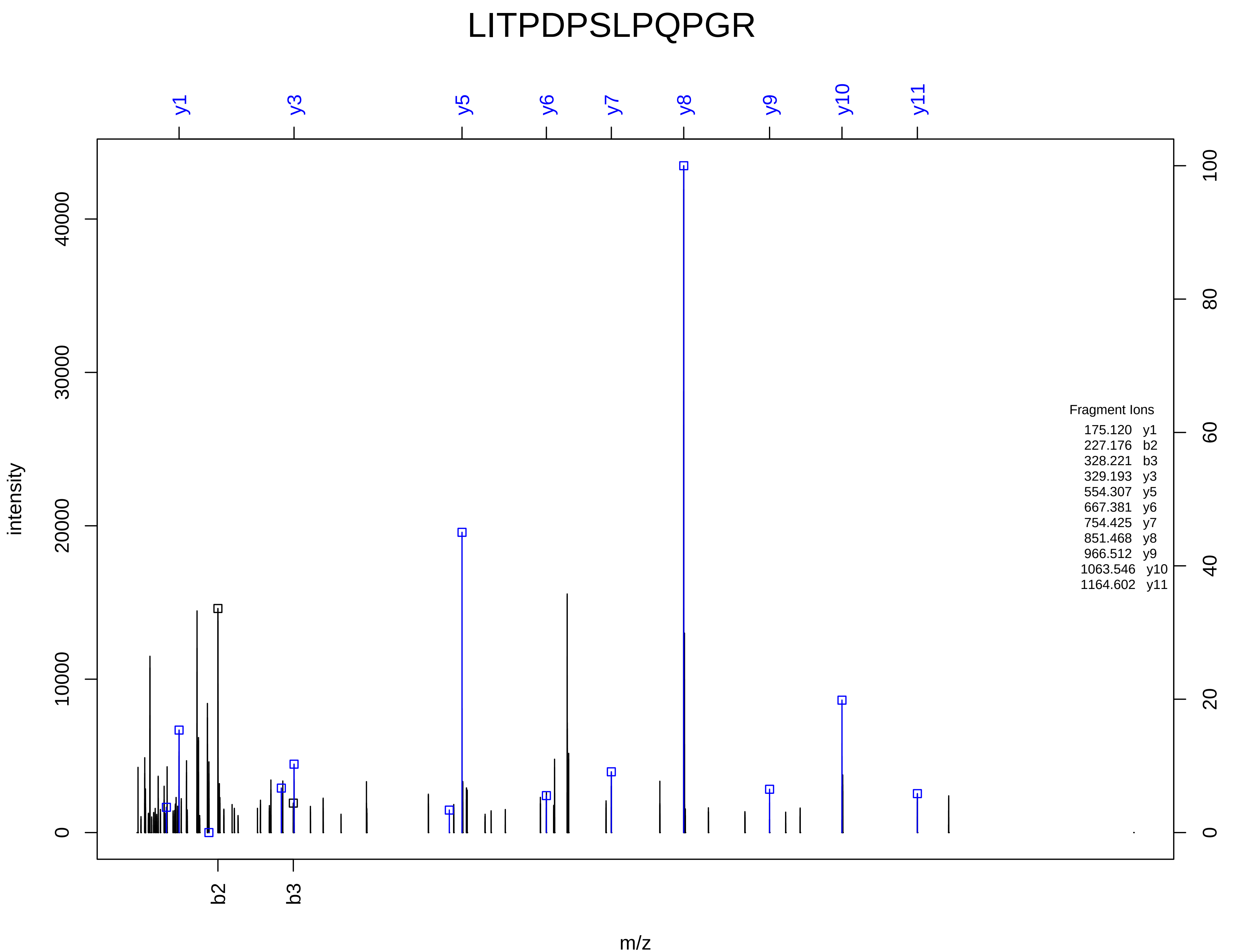

LITPDPSLPQPGR
y1
y3
y5
y6
y7
y8
y9
y10
y11
intensity
0
10000
20000
30000
40000
0
20
40
60
80
100
Fragment Ions
175.120 y1
227.176 b2
328.221 b3
329.193 y3
554.307 y5
667.381 y6
754.425 y7
851.468 y8
966.512 y9
1063.546 y10
1164.602 y11
b2
b3
m/z
LITPDPSLPQPGR / File: christine_30turboips_ip14.raw; SpectrumID: ; scans: 10221

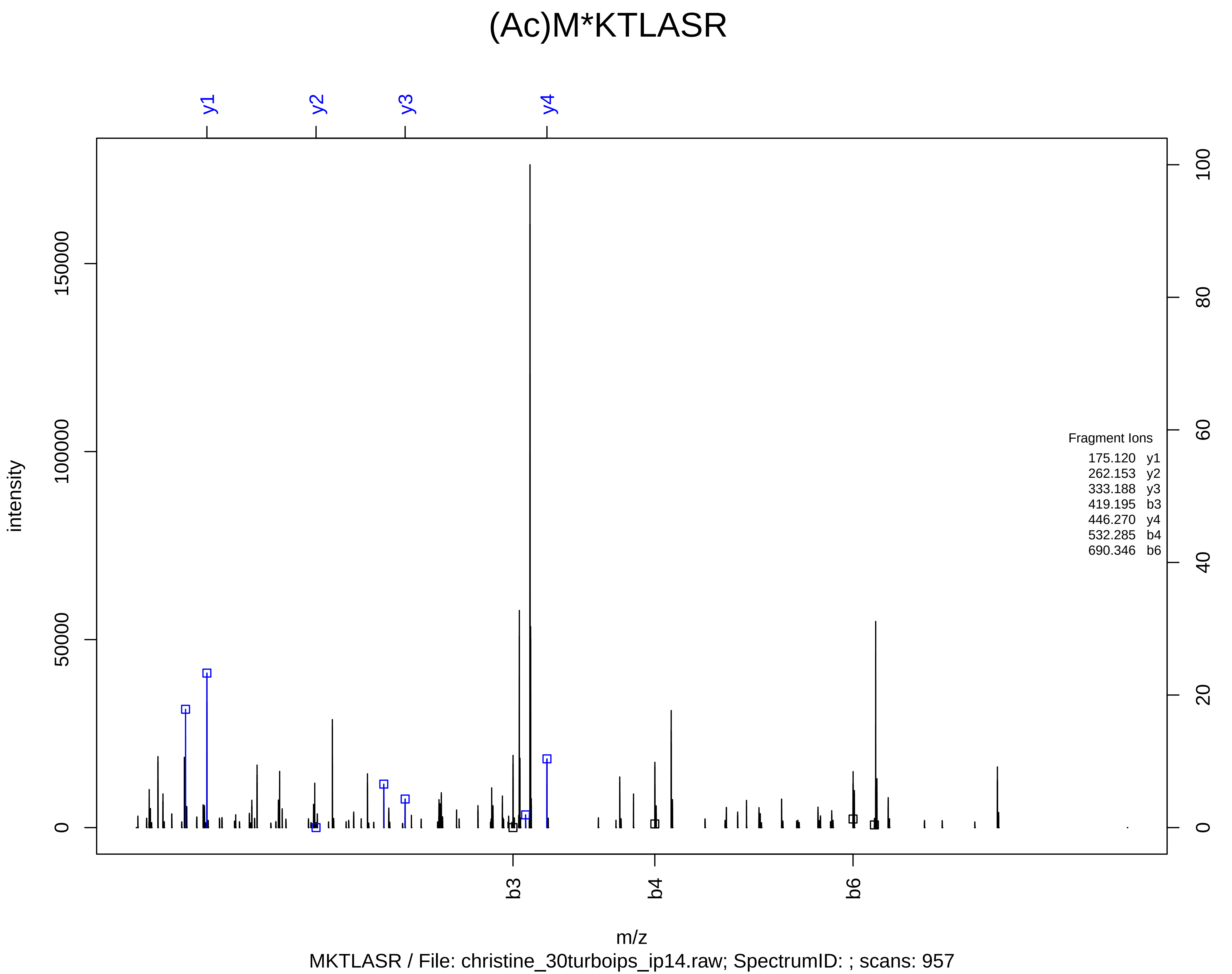
(Ac)M*KTLASR
y1
y2
y3
y4
intensity
0
50000
100000
150000
0
20
40
60
80
100
b3
b4
b6
m/z
Fragment Ions
175.120 y1
262.153 y2
333.188 y3
419.195 b3
446.270 y4
532.285 b4
690.346 b6
MKTLASR / File: christine_30turboips_ip14.raw; SpectrumID: ; scans: 957

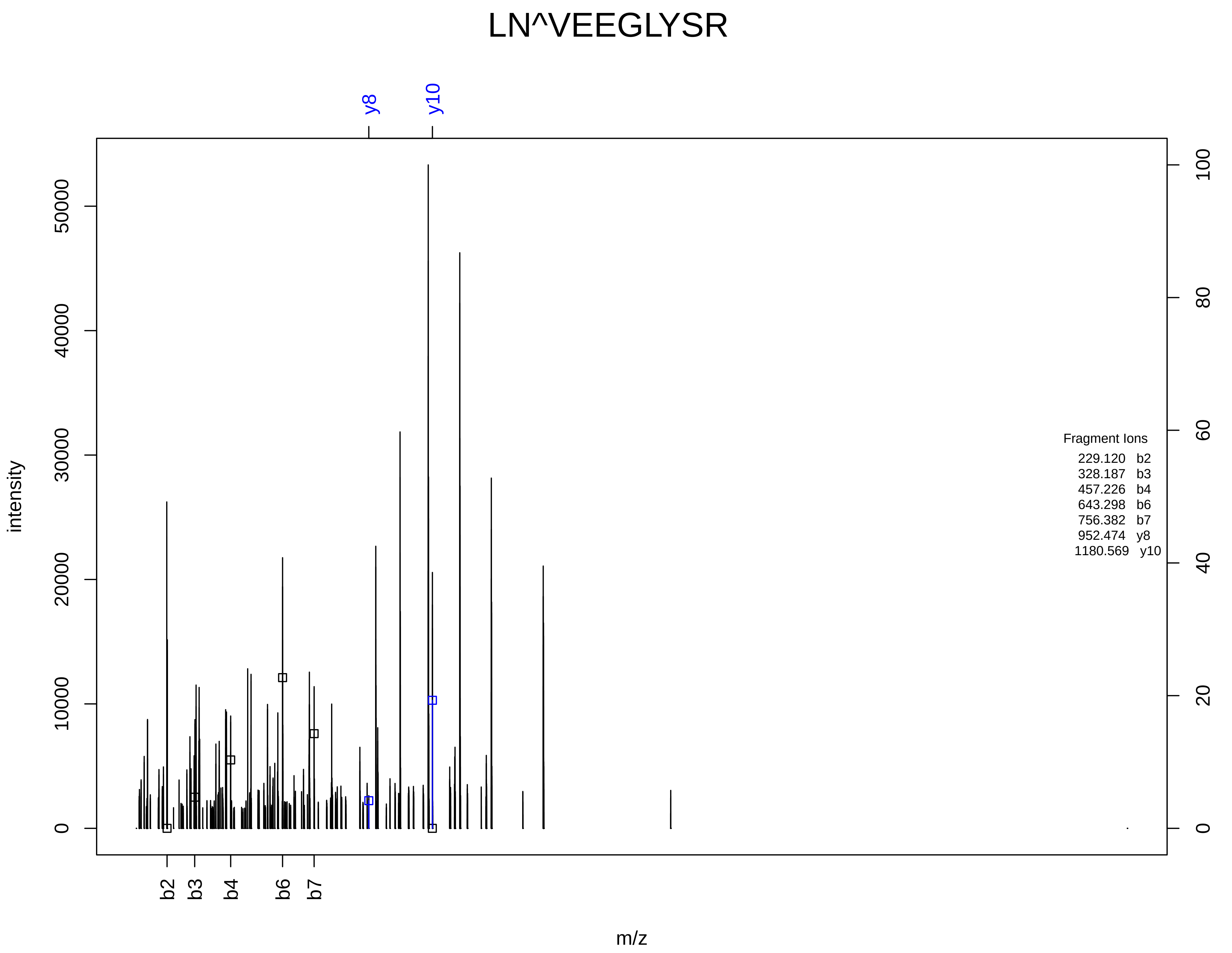

LN^VEEGLYSR
y8
y10
intensity
0
10000
20000
30000
40000
50000
0
20
40
60
80
100
b2
b3
b4
b6
b7
m/z
Fragment Ions
229.120 b2
328.187 b3
457.226 b4
643.298 b6
756.382 b7
952.474 y8
1180.569 y10
LNVEEGLYSR / File: christine_30turboips_ip14.raw; SpectrumID: ; scans: 19943

FYDGVVQTVK

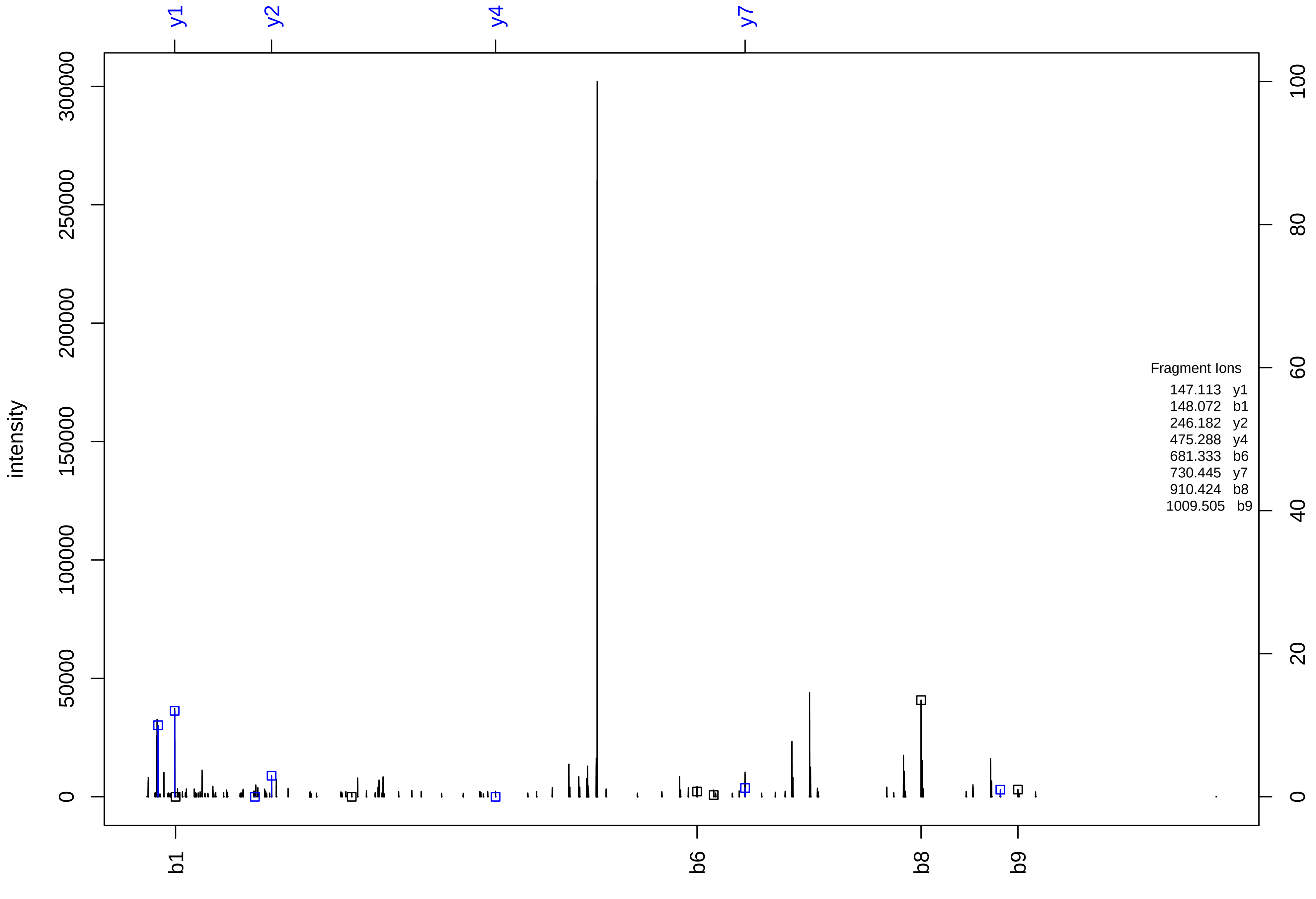

FYDGVVQTVK / File: christine_30turboips_ip14.raw; SpectrumID: ; scans: 7460

LN^M*ELEVN^KK

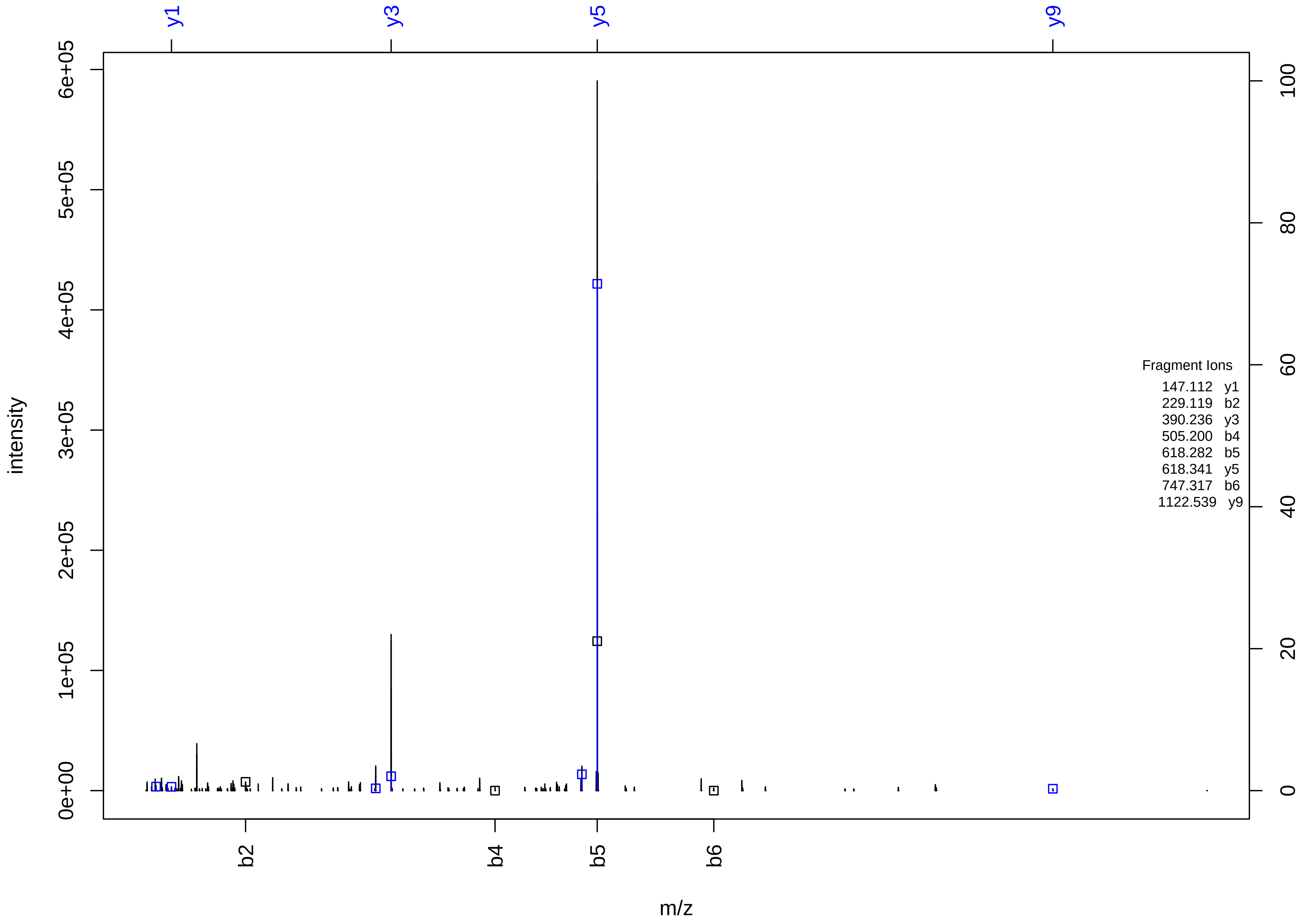

LNMELEVNKK / File: christine_30turboips_ip14.raw; SpectrumID: ; scans: 8586

VLAQQGEYSEAIPILR

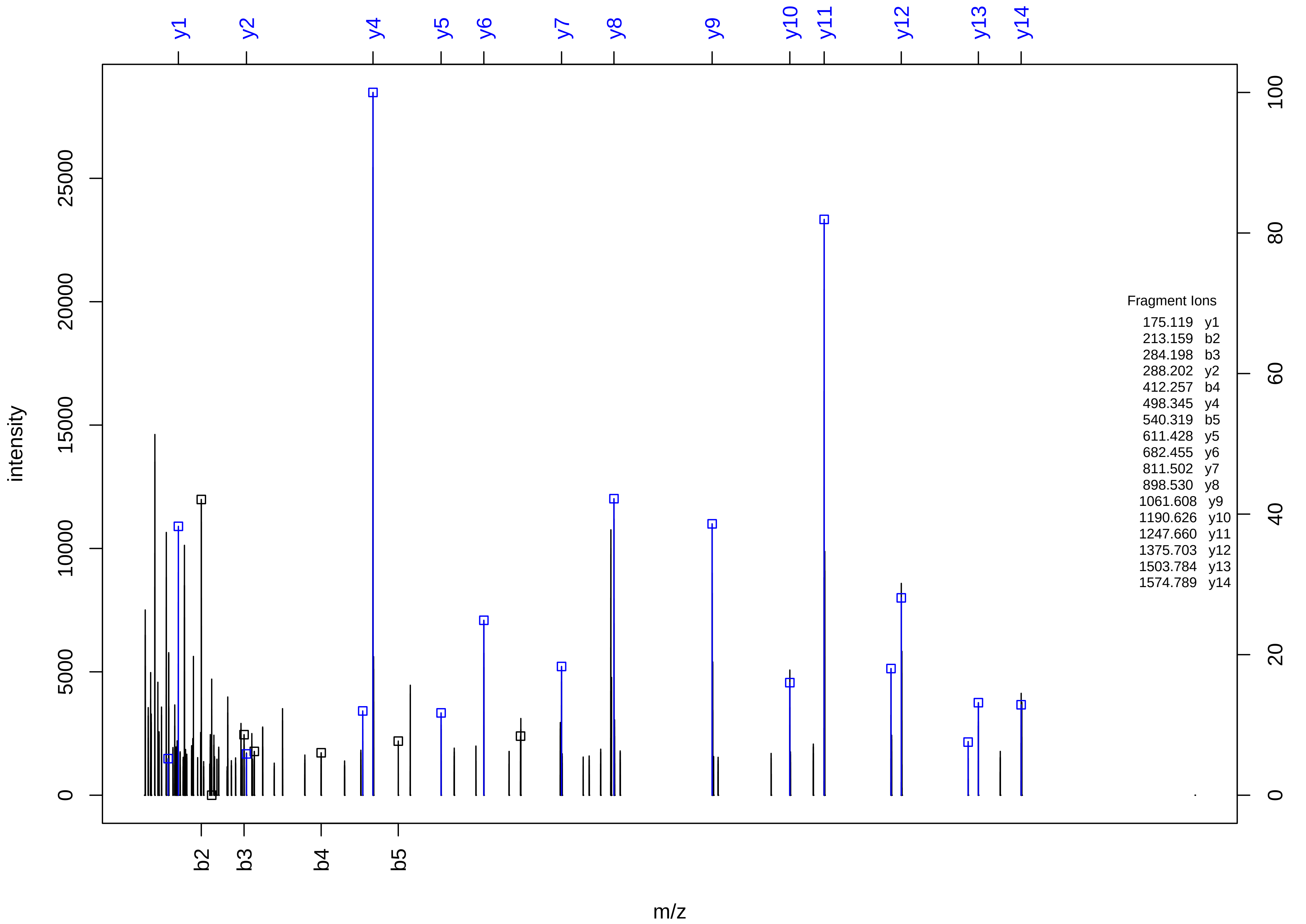

VLAQQGEYSEAIPILR / File: christine_30turboips_ip11.raw; SpectrumID: ; scans: 17730

(Ac)ASALEQFVNSVR

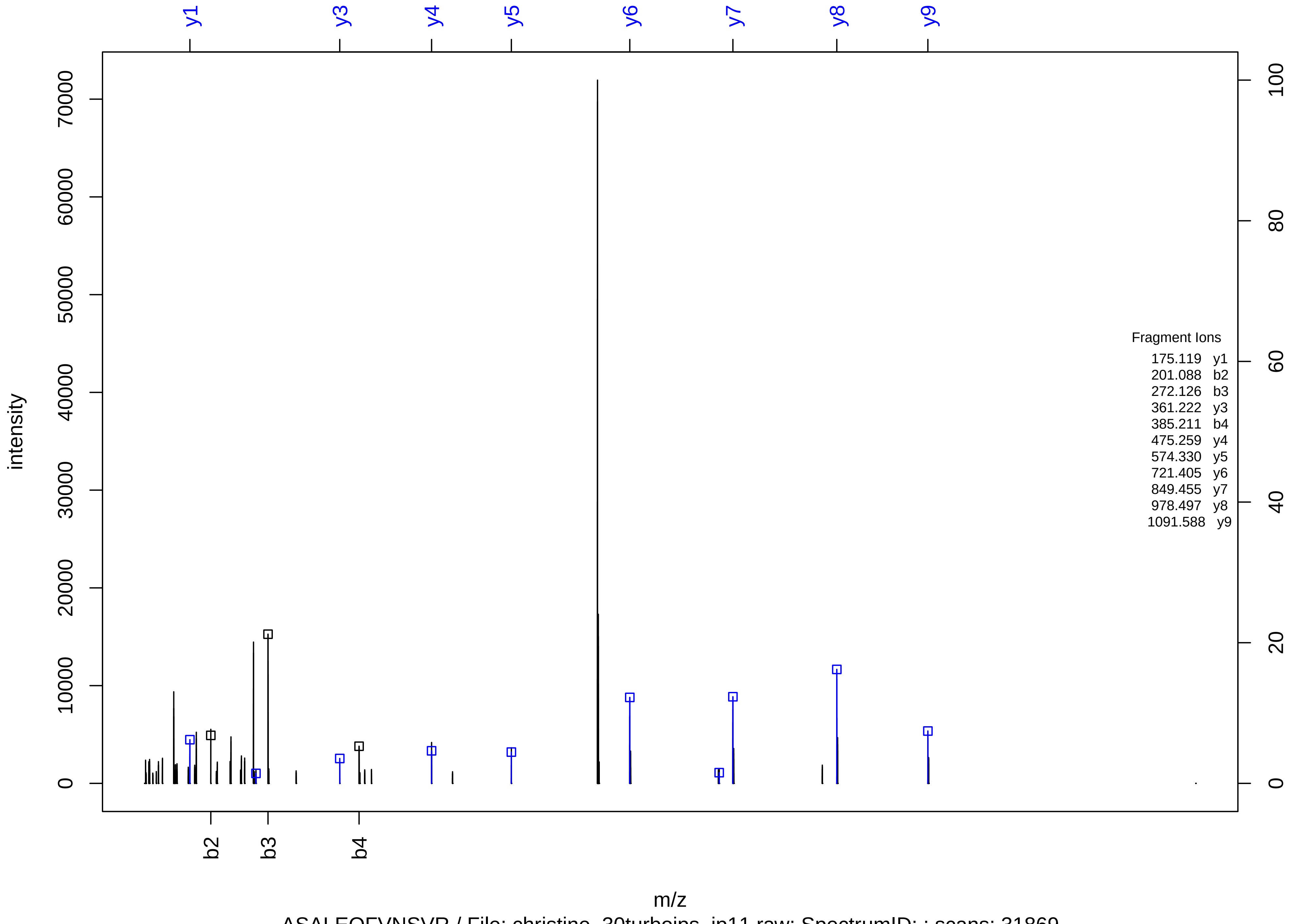

ASALEQFVNSVR / File: christine_30turboips_ip11.raw; SpectrumID: ; scans: 31869

VIIQSQLPIGTLK

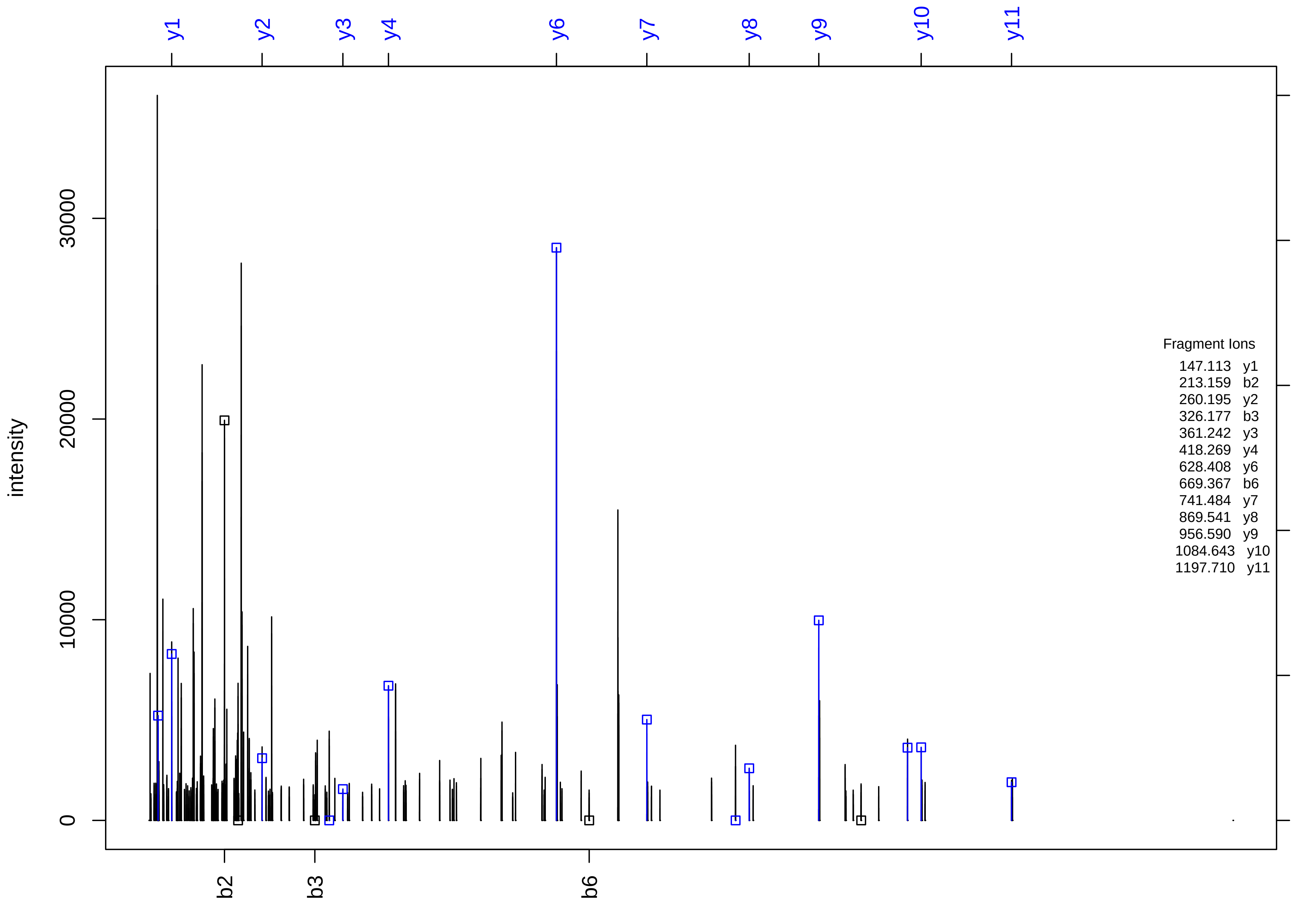

VIIQSQLPIGTLK / File: christine_30turboips_ip11.raw; SpectrumID: ; scans: 17031

ILYPIQGGR

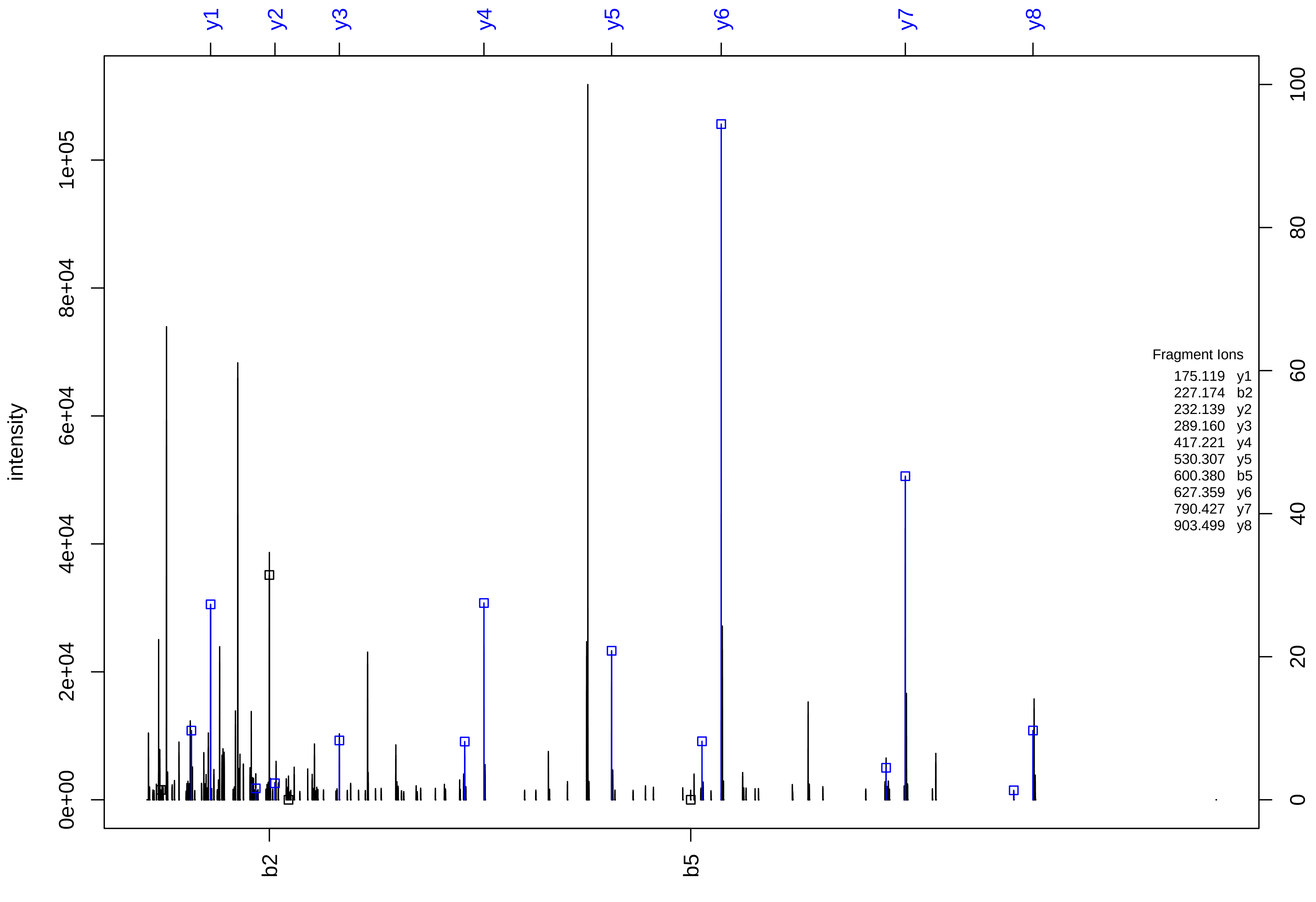

ILYPIQGGR / File: christine_30turboips_ip11.raw; SpectrumID: ; scans: 6467

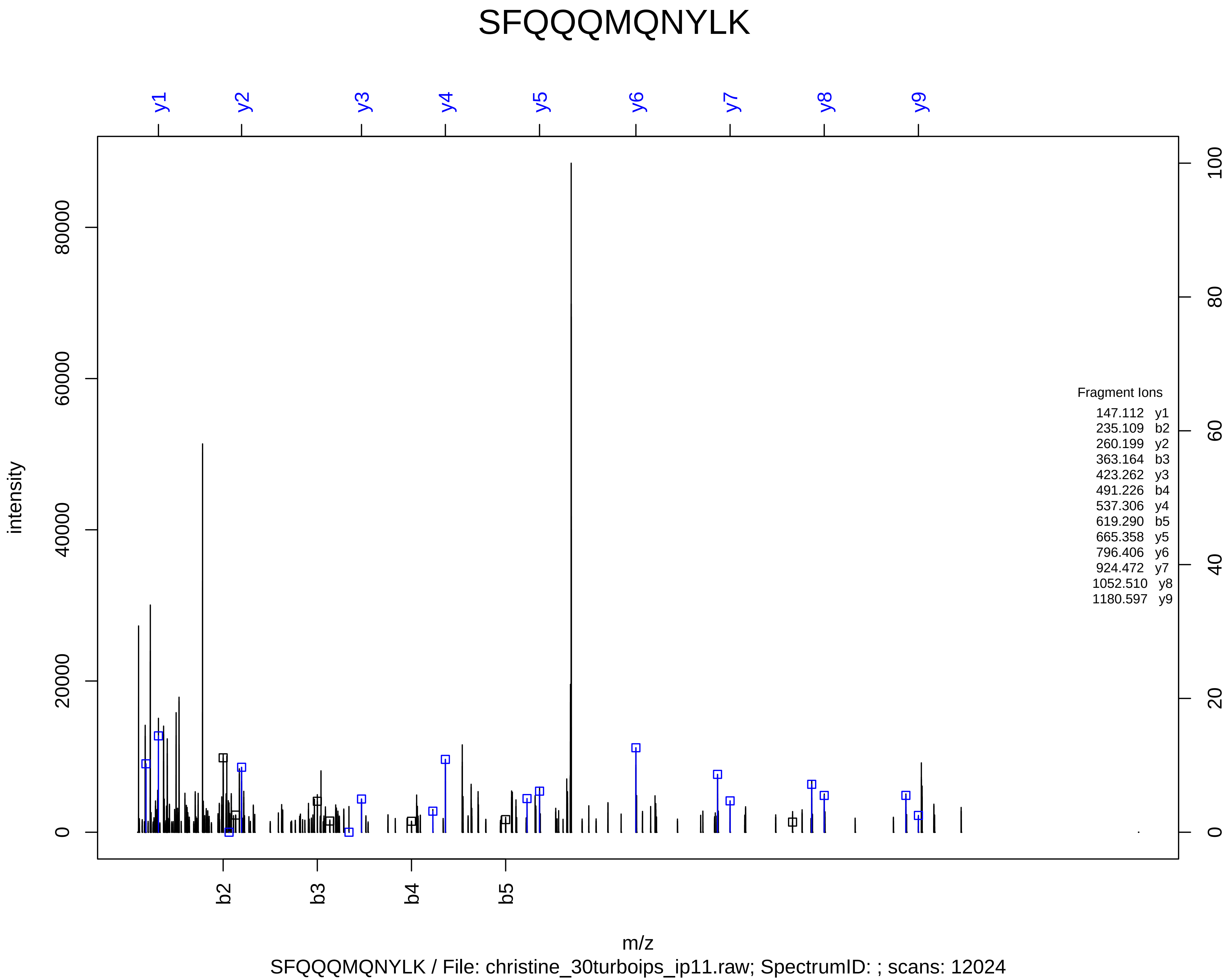
SFQQQMQNYLK
y1
y2
y3
y4
y5
y6
y7
y8
y9
b2
b3
b4
b5
intensity
m/z
Fragment Ions
147.112 y1
235.109 b2
260.199 y2
363.164 b3
423.262 y3
491.226 b4
537.306 y4
619.290 b5
665.358 y5
796.406 y6
924.472 y7
1052.510 y8
1180.597 y9
SFQQQMQNYLK / File: christine_30turboips_ip11.raw; SpectrumID: ; scans: 12024

EGDVLTLLESER

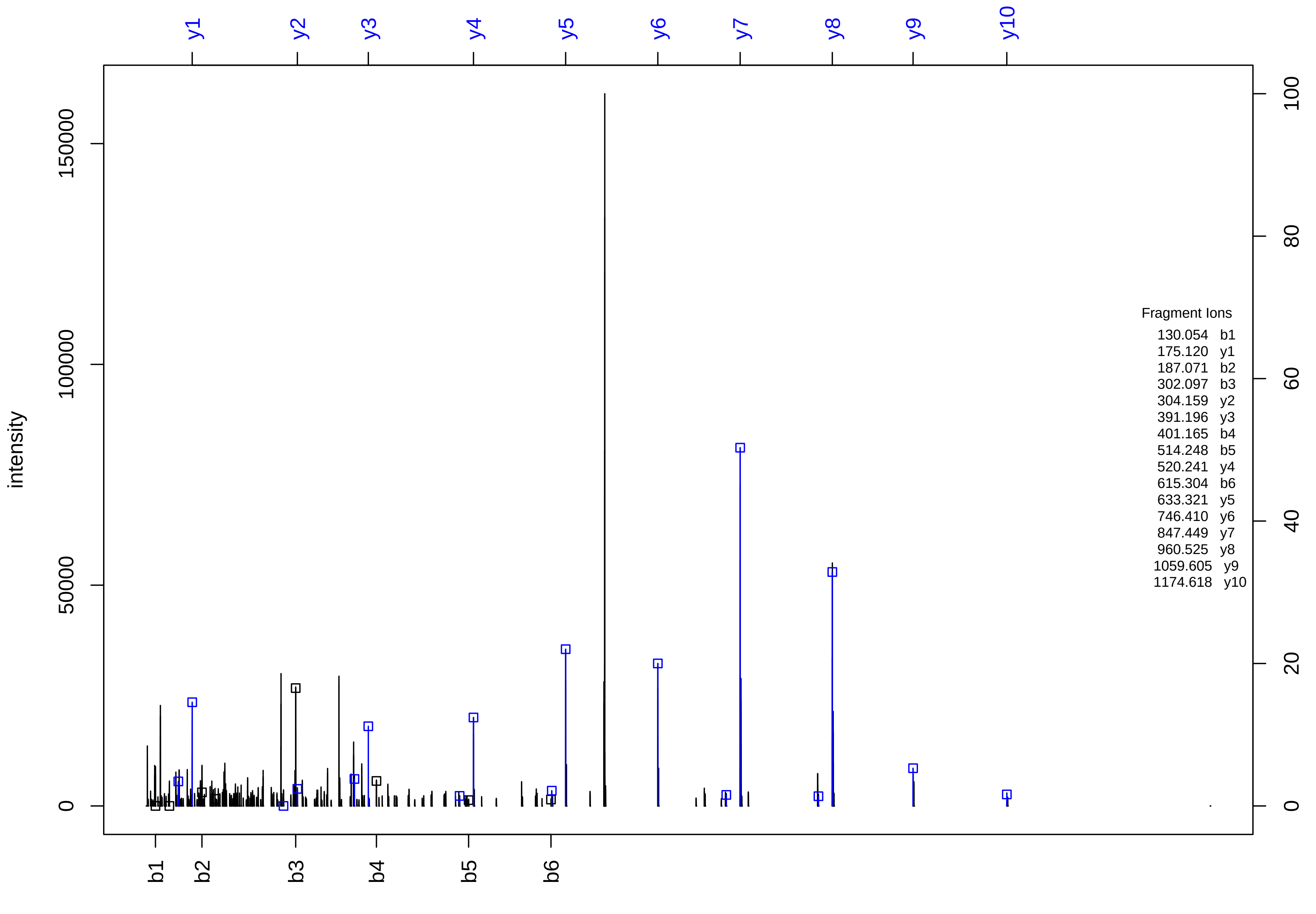

EGDVLTLLESER / File: christine_30turboips_ip11.raw; SpectrumID: ; scans: 20803

IIYQAASPDEGALVR

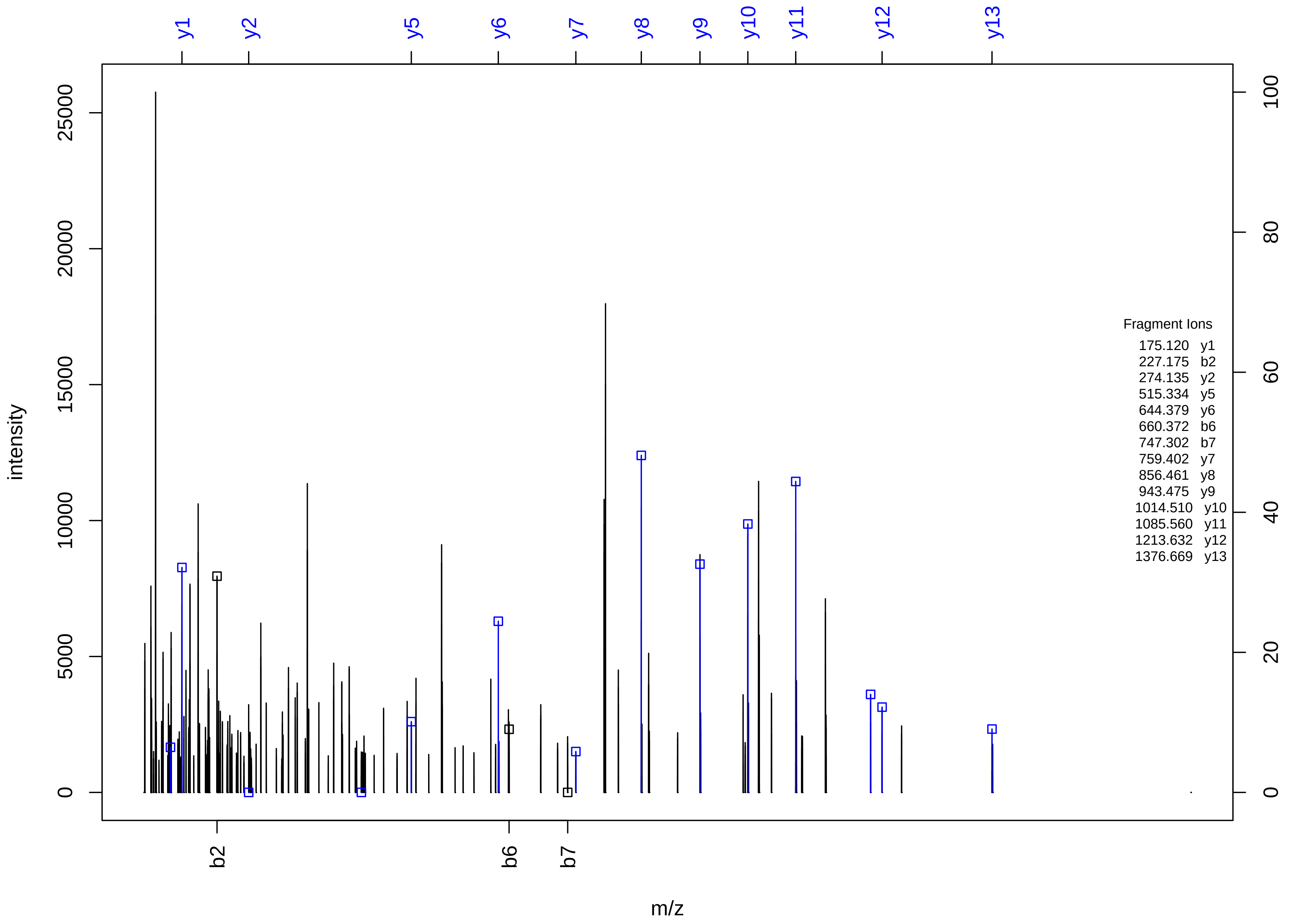

IIYQAASPDEGALVR / File: christine_30turboips_ip11.raw; SpectrumID: ; scans: 9770

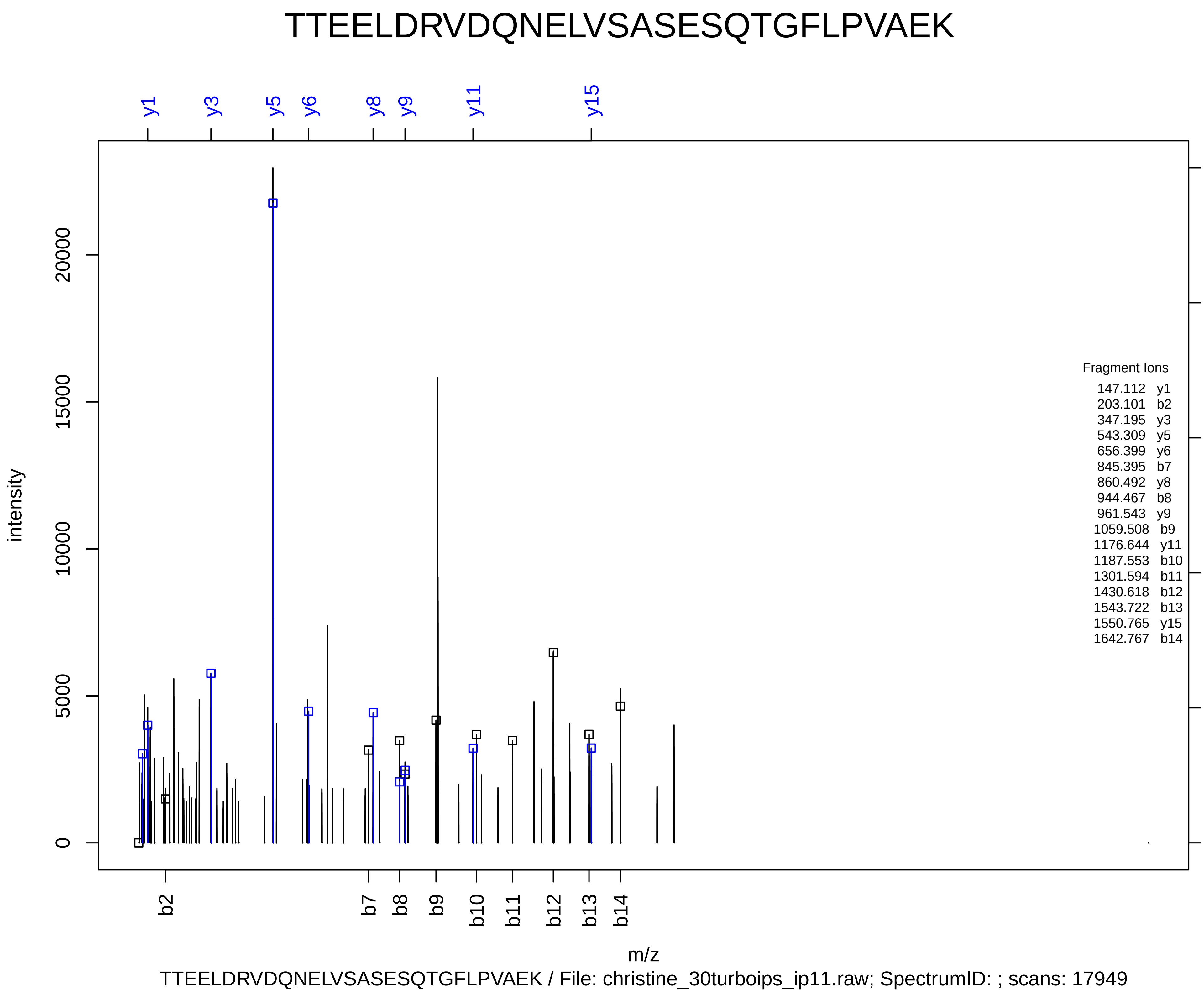
TTEELDRVDQNELVSASESQTGFLPVAEK
y1
y3
y5
y6
y8
y9
y11
y15
intensity
0
5000
10000
15000
20000
0
20
40
60
80
100
b2
b7
b8
b9
b10
b11
b12
b13
b14
m/z
Fragment Ions
147.112 y1
203.101 b2
347.195 y3
543.309 y5
656.399 y6
845.395 b7
860.492 y8
944.467 b8
961.543 y9
1059.508 b9
1176.644 y11
1187.553 b10
1301.594 b11
1430.618 b12
1543.722 b13
1550.765 y15
1642.767 b14
TTEELDRVDQNELVSASESQTGFLPVAEK / File: christine_30turboips_ip11.raw; SpectrumID: ; scans: 17949

GVVGPGPAAIAAPGGGGAGPPAVGGGGGR

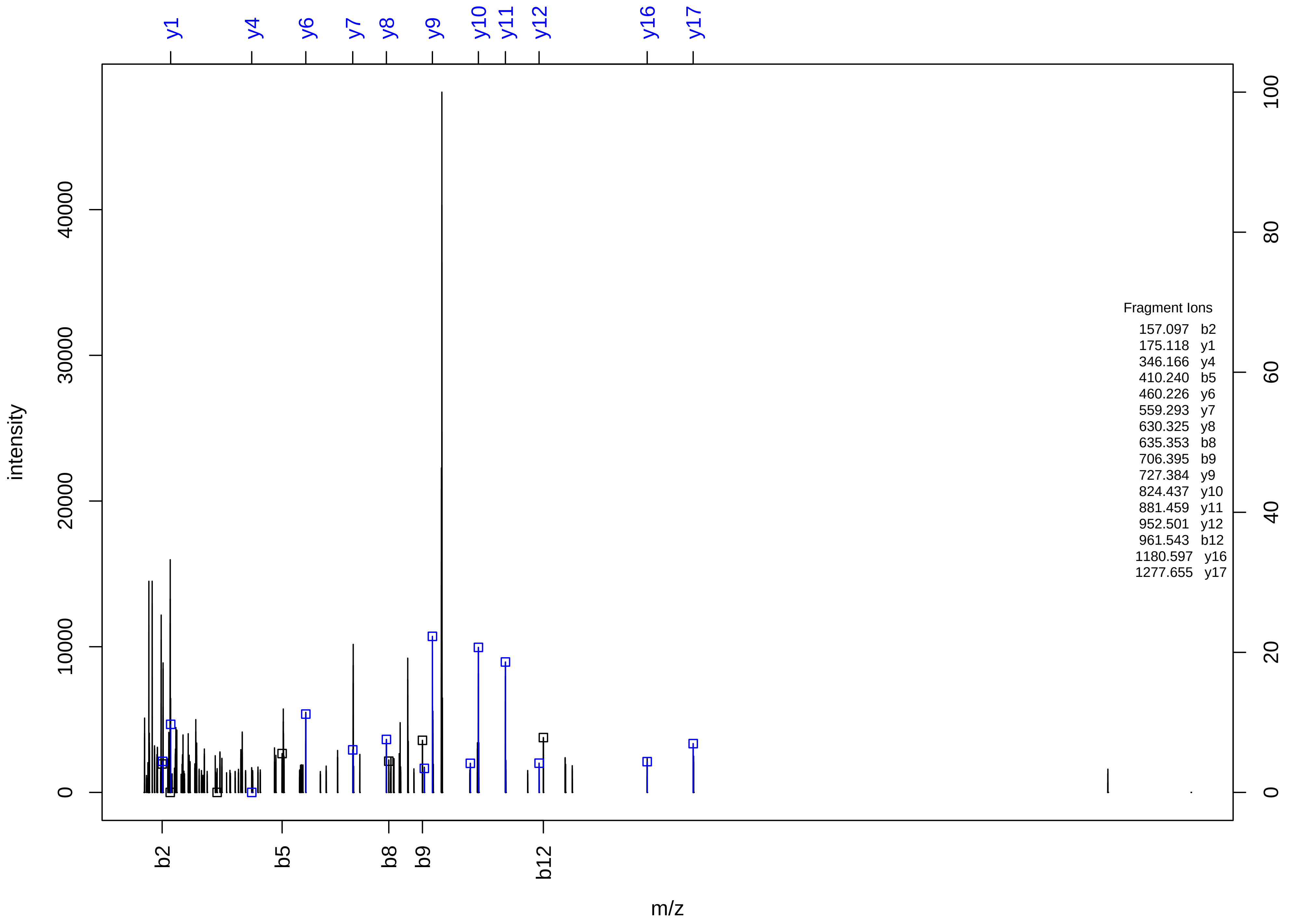

GVVGPGPAAIAAPGGGGAGPPAVGGGGGR / File: christine_30turboips_ip11.raw; SpectrumID: ; scans: 10977

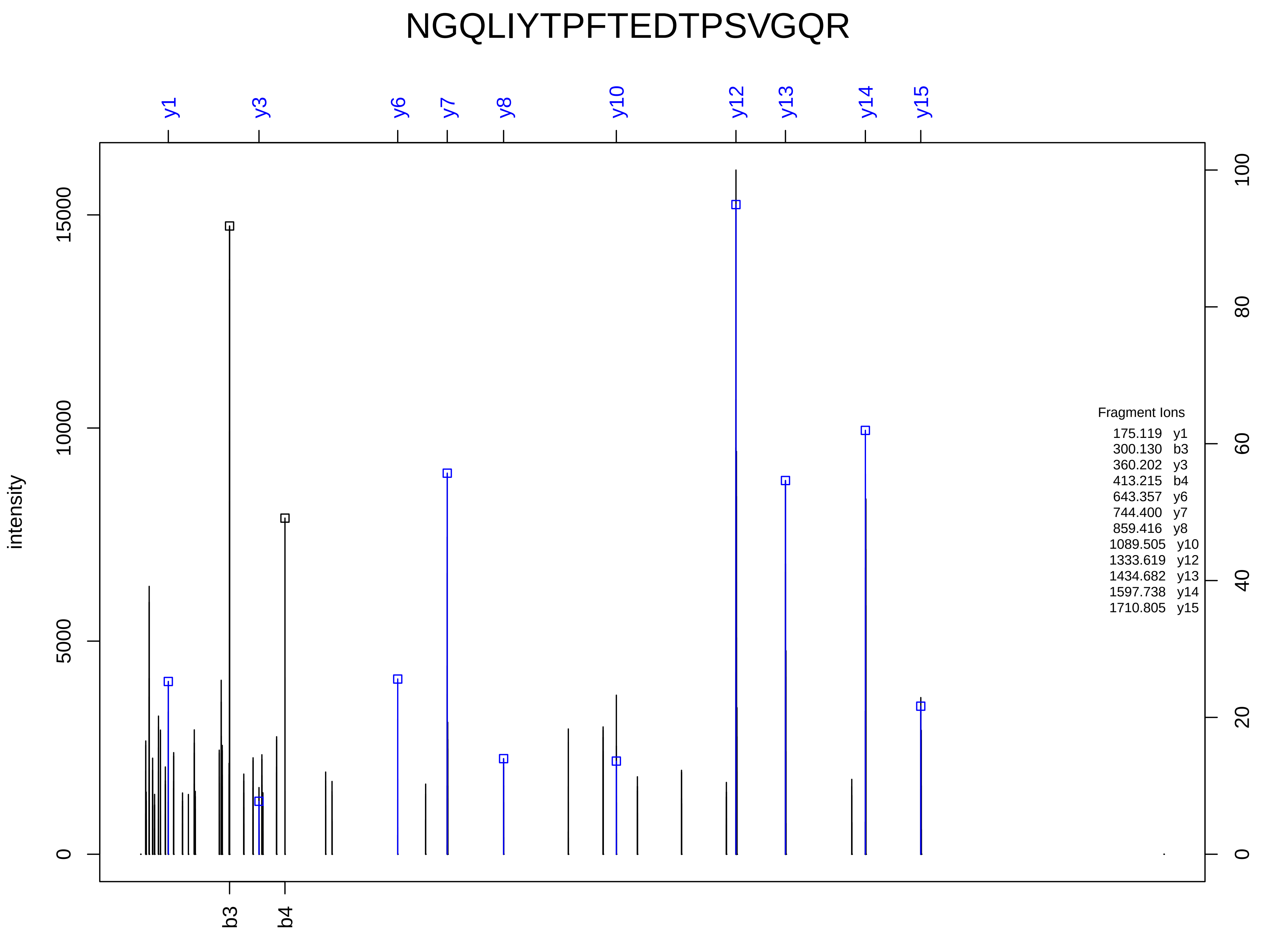

NGQLIYTPFTEDTPSVGQR / File: christine_30turboips_ip11.raw; SpectrumID: ; scans: 17878

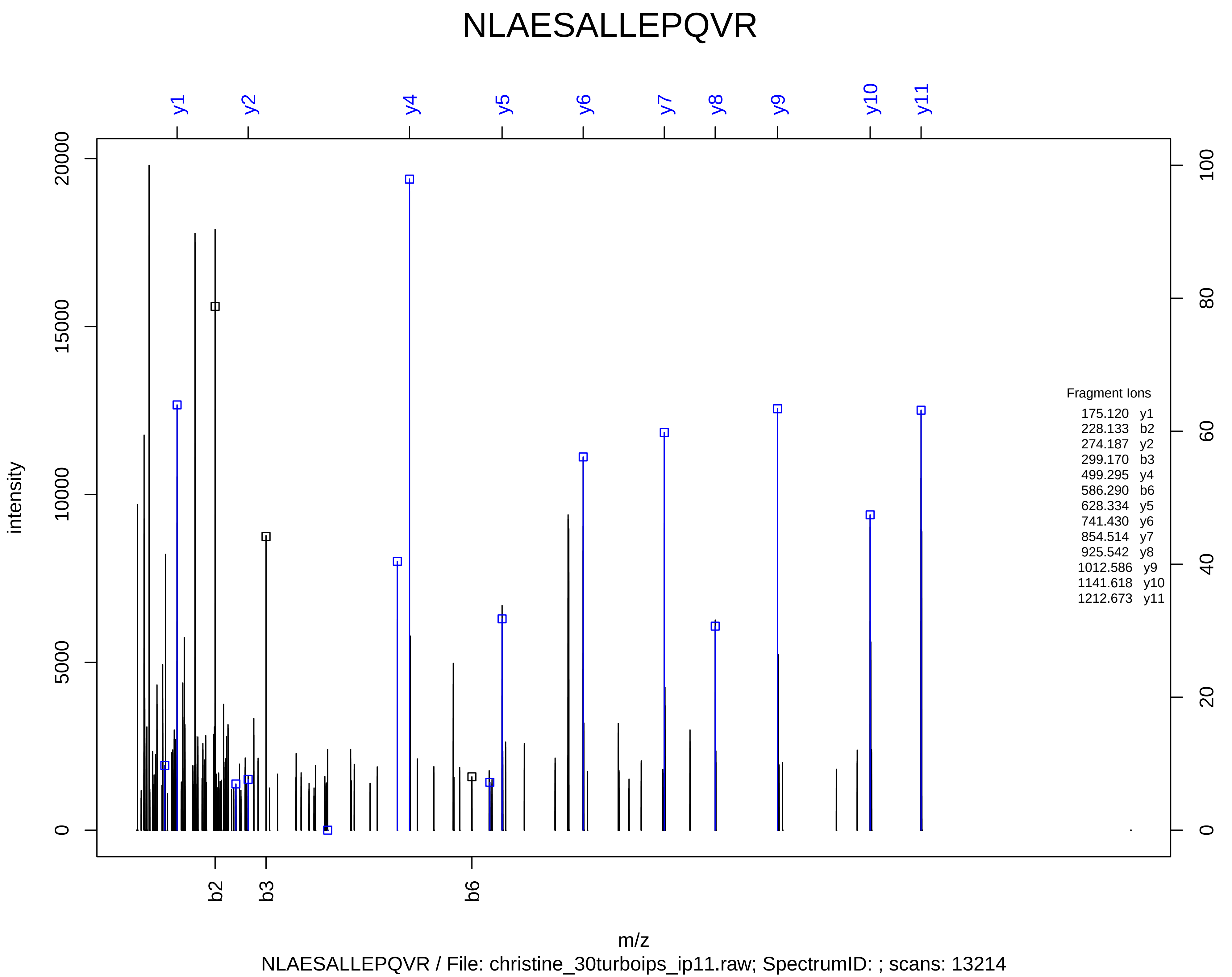
NLAESALLEPQVR
y1
y2
y4
y5
y6
y7
y8
y9
y10
y11
b2
b3
b6
intensity
m/z
Fragment Ions
175.120 y1
228.133 b2
274.187 y2
299.170 b3
499.295 y4
586.290 b6
628.334 y5
741.430 y6
854.514 y7
925.542 y8
1012.586 y9
1141.618 y10
1212.673 y11
NLAESALLEPQVR / File: christine_30turboips_ip11.raw; SpectrumID: ; scans: 13214

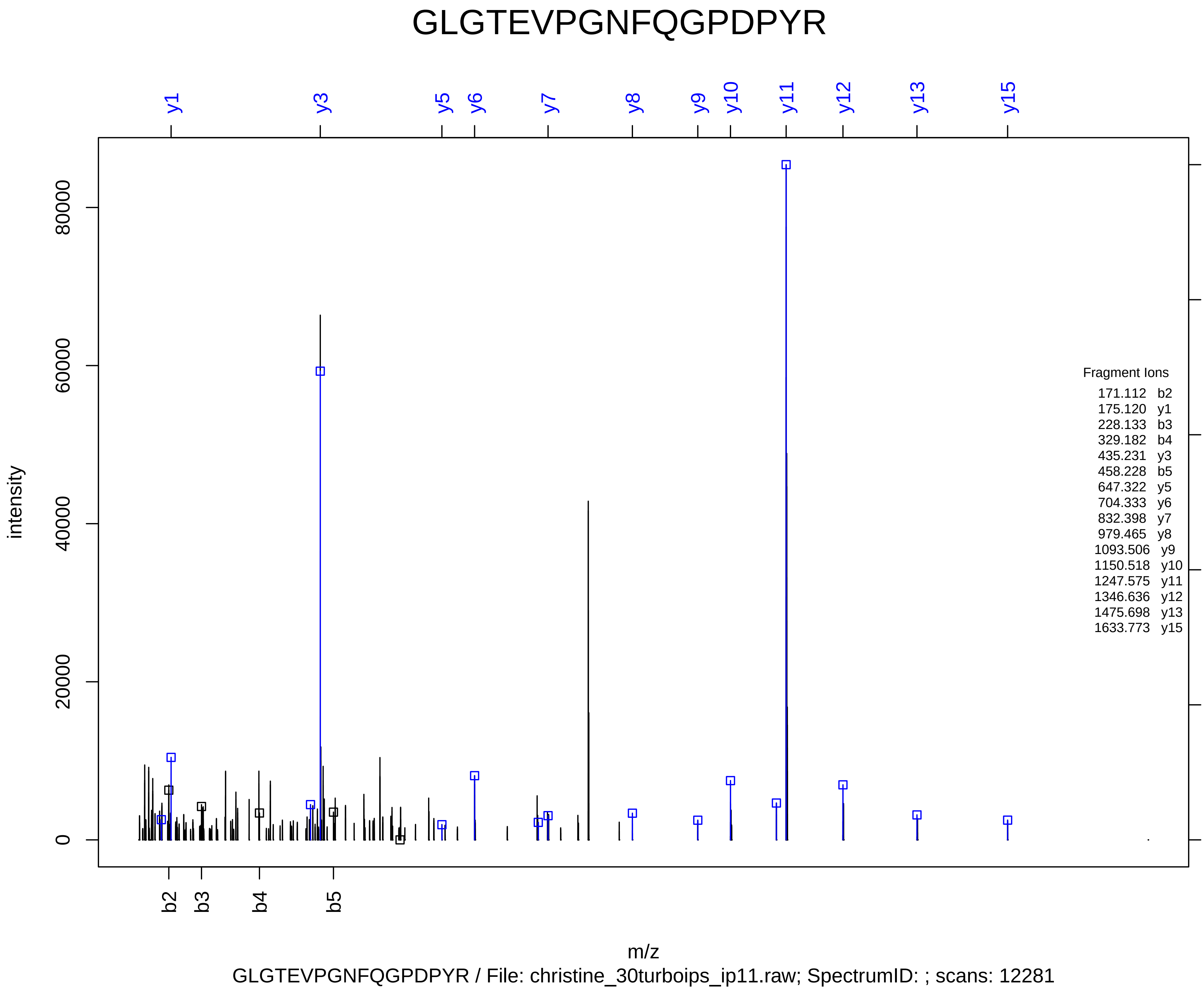
GLGTEVPGNFQGPDPYR
y1
y3
y5
y6
y7
y8
y9
y10
y11
y12
y13
y15
b2
b3
b4
b5
intensity
m/z
Fragment Ions
171.112 b2
175.120 y1
228.133 b3
329.182 b4
435.231 y3
458.228 b5
647.322 y5
704.333 y6
832.398 y7
979.465 y8
1093.506 y9
1150.518 y10
1247.575 y11
1346.636 y12
1475.698 y13
1633.773 y15
GLGTEVPGNFQGPDPYR / File: christine_30turboips_ip11.raw; SpectrumID: ; scans: 12281

LLLDPSSTPTK

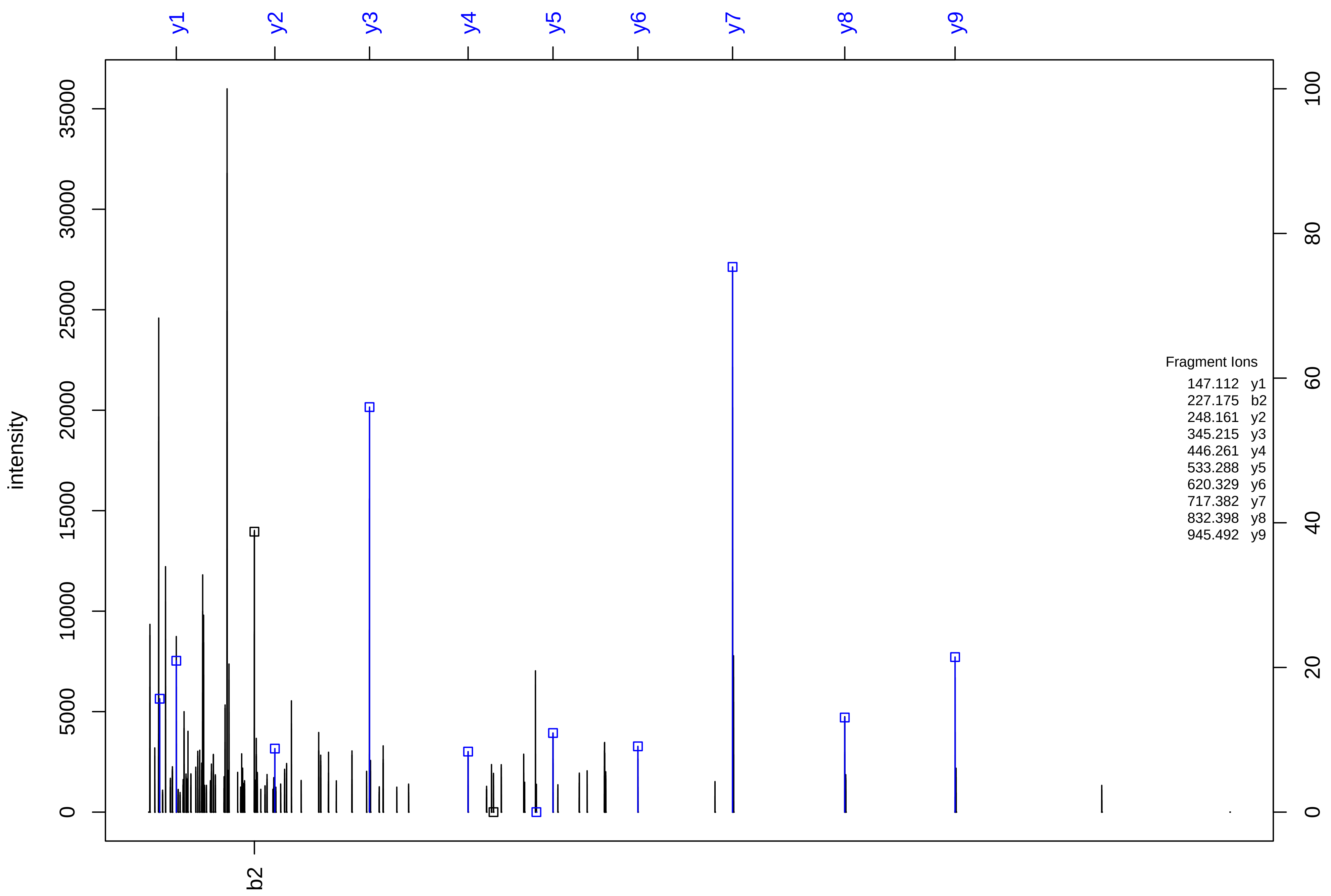

LLLDPSSTPTK / File: christine_30turboips_ip11.raw; SpectrumID: ; scans: 7868

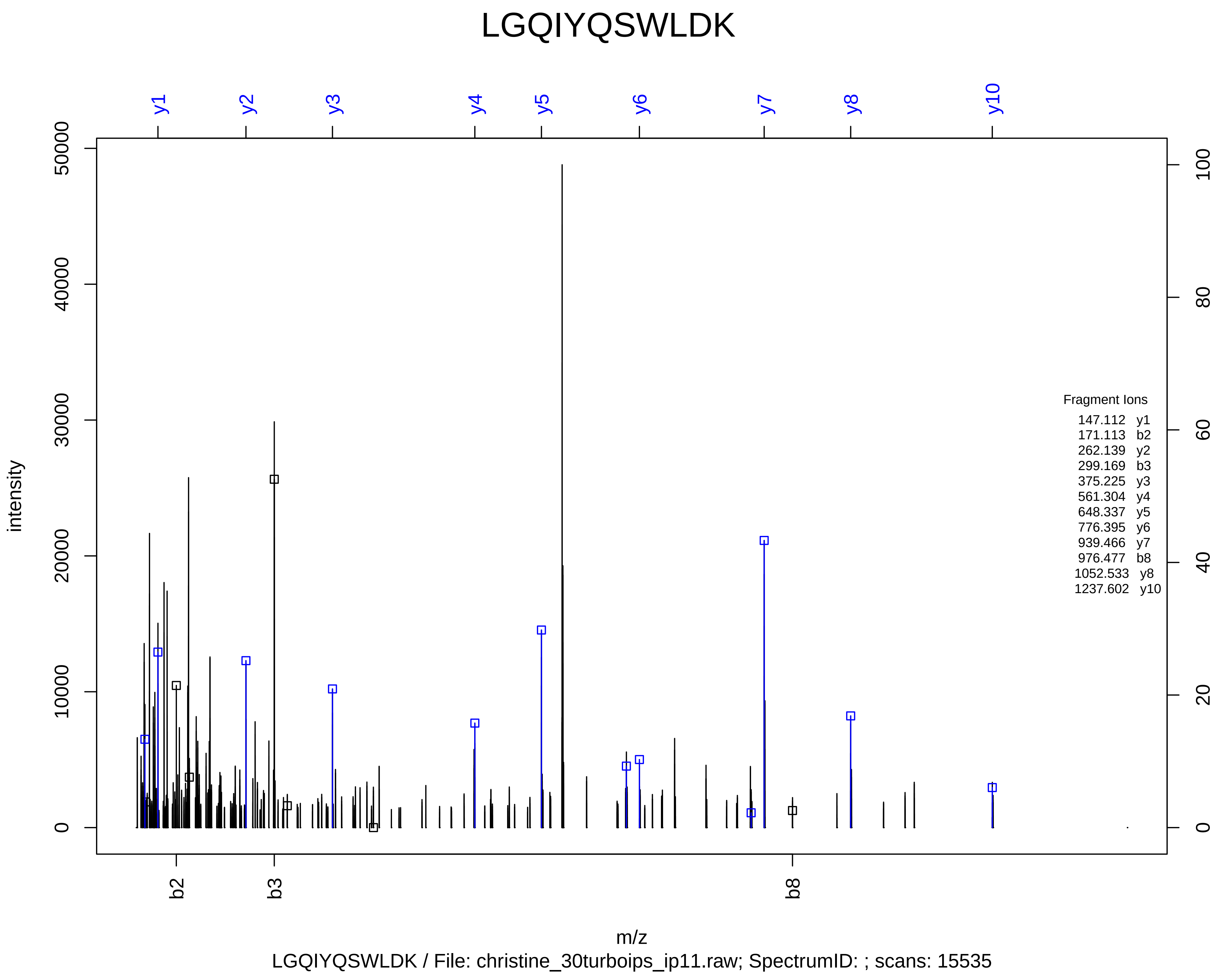
LGQIYQSWLDK
y1
y2
y3
y4
y5
y6
y7
y8
y10
intensity
0
10000
20000
30000
40000
50000
0
20
40
60
80
100
b2
b3
b8
m/z
Fragment Ions
147.112 y1
171.113 b2
262.139 y2
299.169 b3
375.225 y3
561.304 y4
648.337 y5
776.395 y6
939.466 y7
976.477 b8
1052.533 y8
1237.602 y10
LGQIYQSWLDK / File: christine_30turboips_ip11.raw; SpectrumID: ; scans: 15535

QQSEEDLLLQDFSR

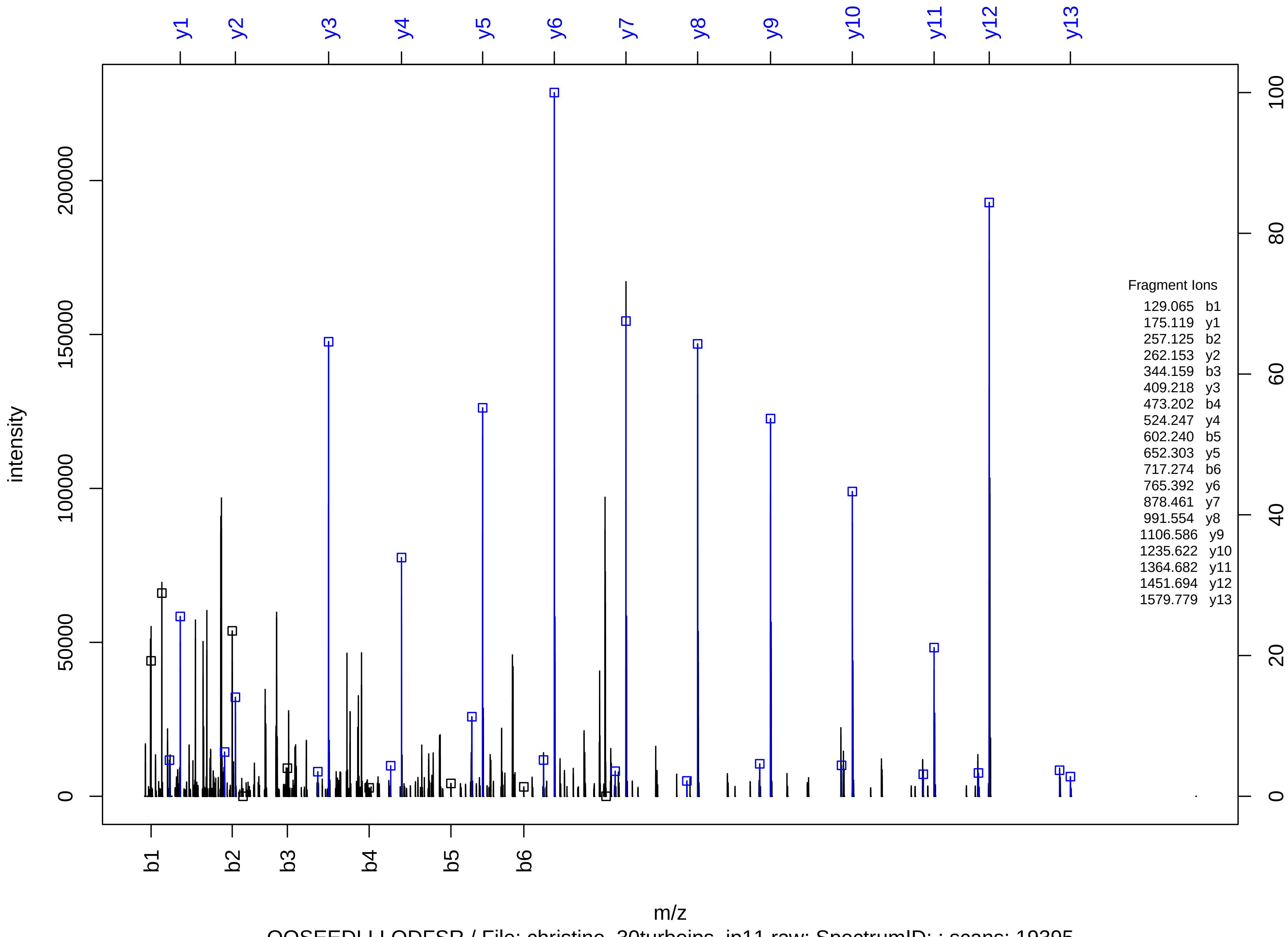

QQSEEDLLLQDFSR / File: christine_30turboips_ip11.raw; SpectrumID: ; scans: 19395

LETVDPTYIFHPR

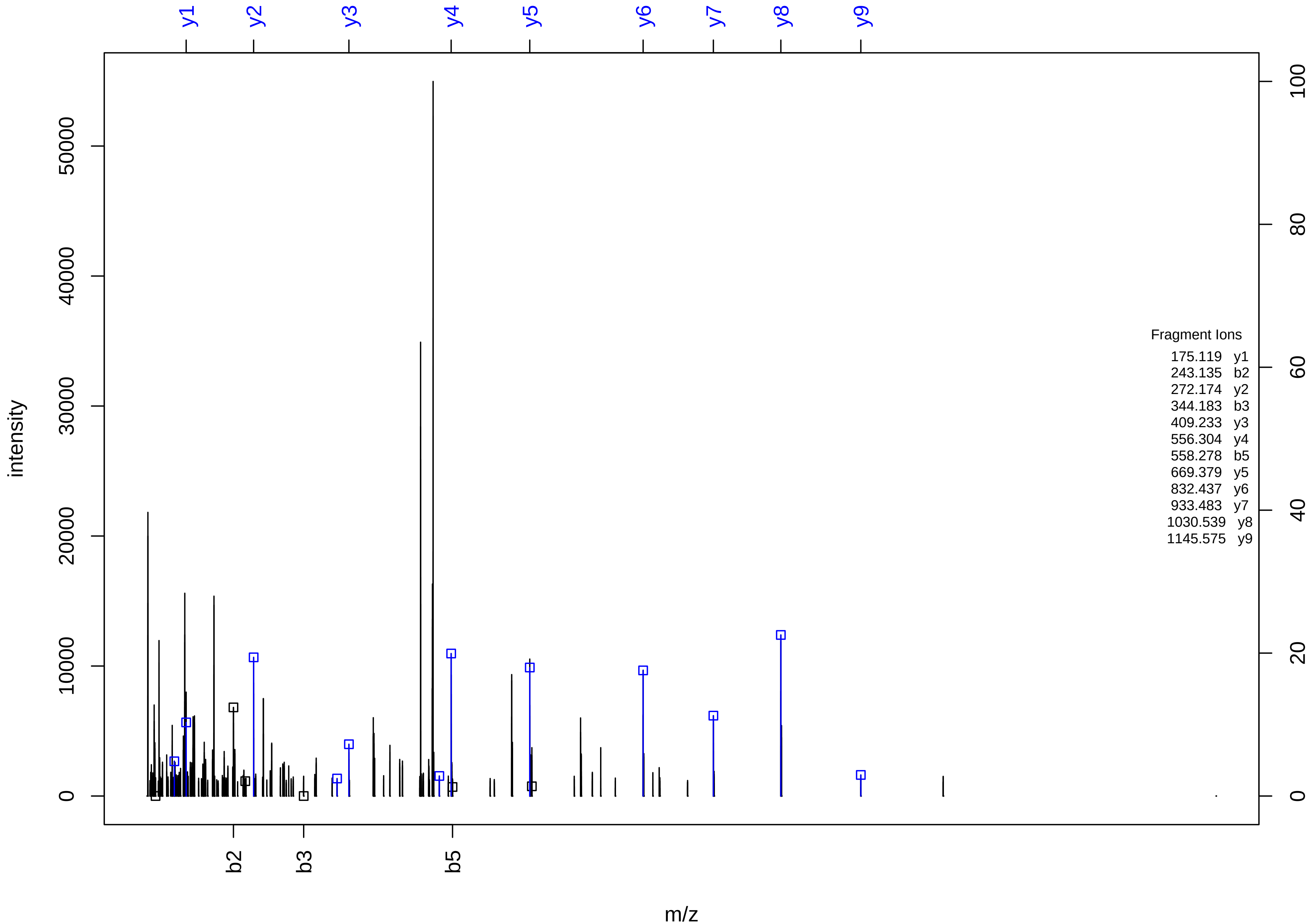

LETVDPTYIFHPR / File: christine_30turboips_ip11.raw; SpectrumID: ; scans: 15752

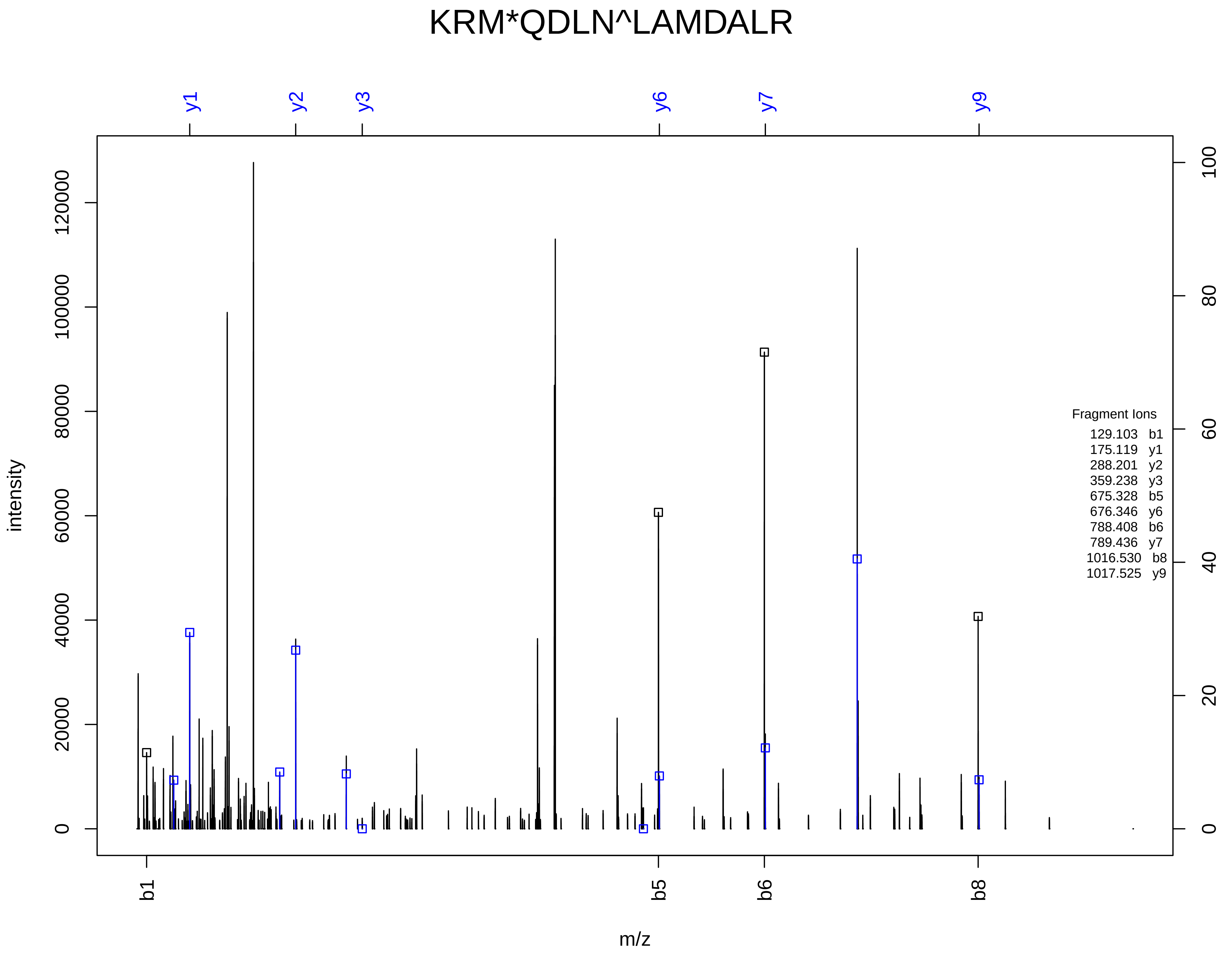

KRMQDLNLAMDALR / File: christine_30turboips_ip11.raw; SpectrumID: ; scans: 11741

(Ac)TTSGALFPSLVPGSR

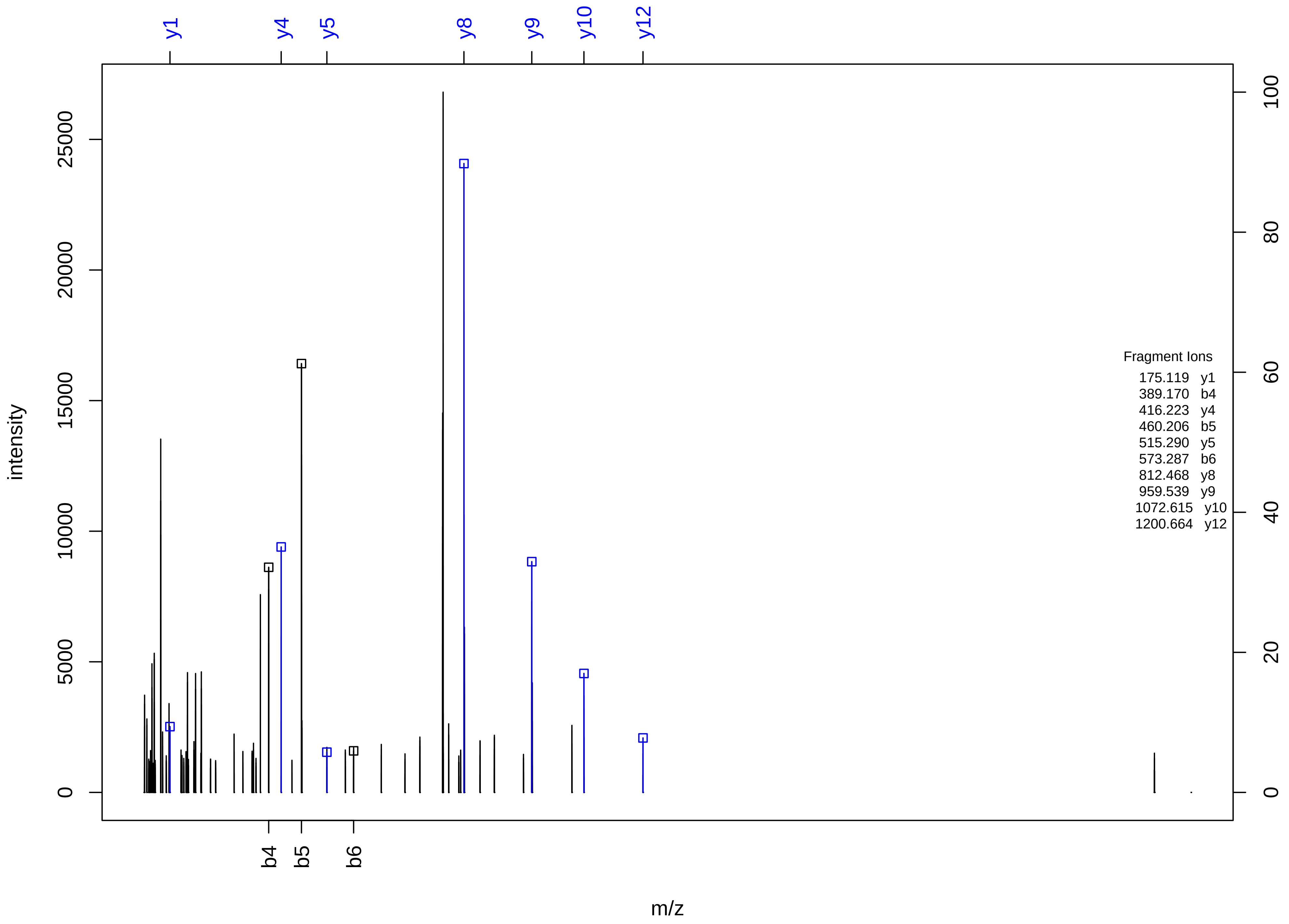

TTSGALFPSLVPGSR / File: christine_30turboips_ip11.raw; SpectrumID: ; scans: 26438

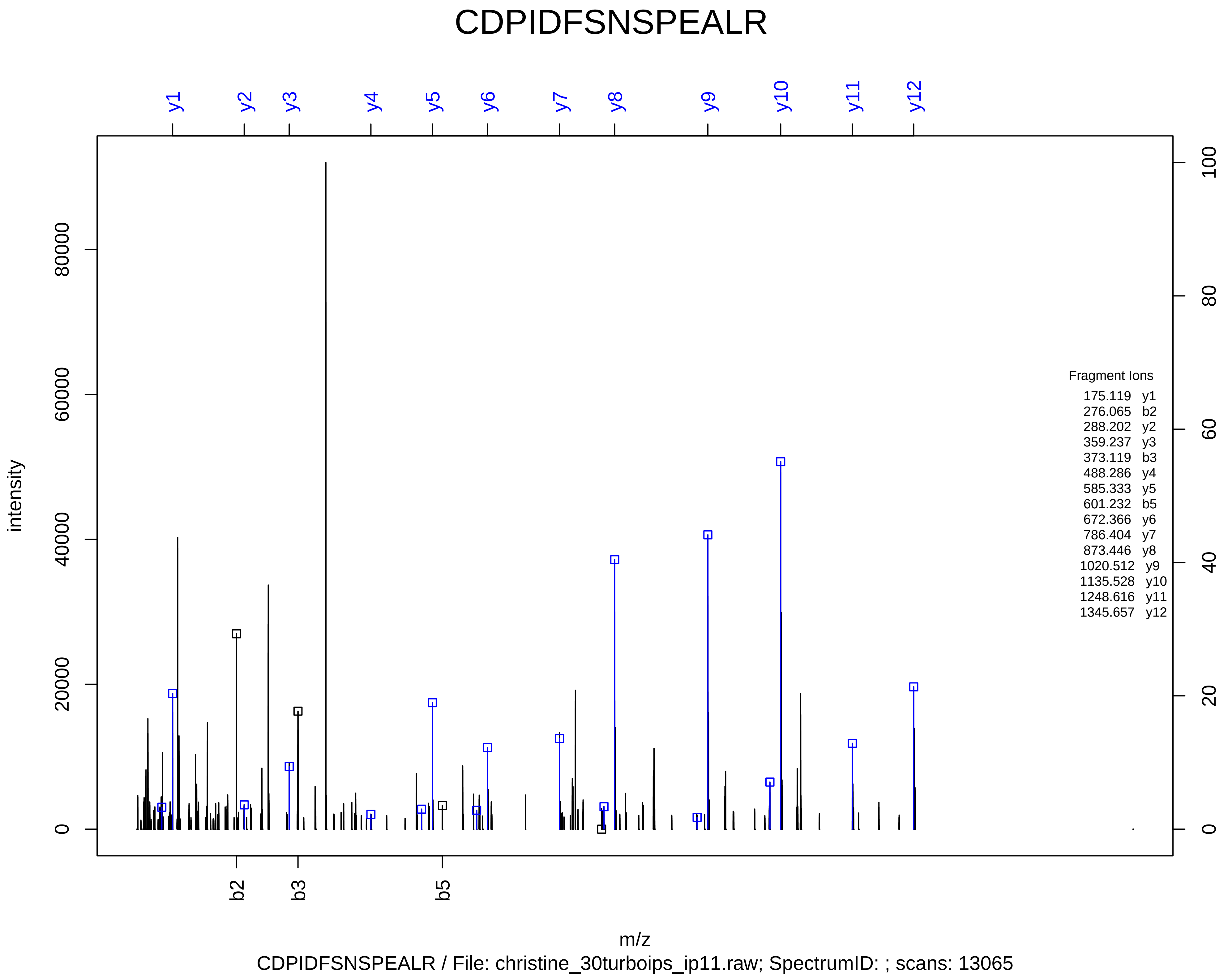

CDPIDFSNSPEALR
y1
y2
y3
y4
y5
y6
y7
y8
y9
y10
y11
y12
b2
b3
b5
intensity
0
20000
40000
60000
80000
100
80
60
40
20
0
m/z
Fragment Ions
175.119 y1
276.065 b2
288.202 y2
359.237 y3
373.119 b3
488.286 y4
585.333 y5
601.232 b5
672.366 y6
786.404 y7
873.446 y8
1020.512 y9
1135.528 y10
1248.616 y11
1345.657 y12
CDPIDFSNSPEALR / File: christine_30turboips_ip11.raw; SpectrumID: ; scans: 13065

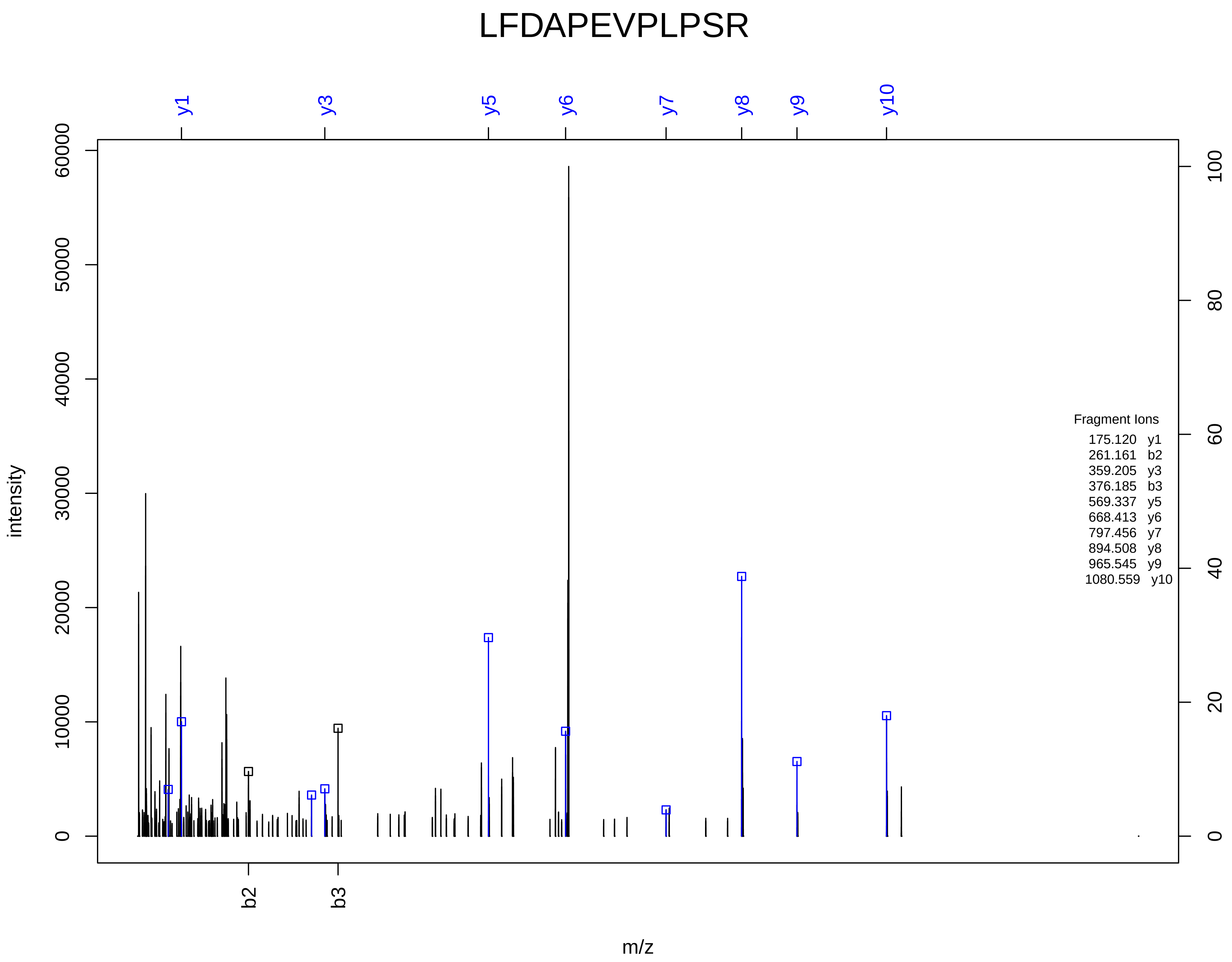

LFDAPEVPLPSR / File: christine_30turboips_ip11.raw; SpectrumID: ; scans: 17208

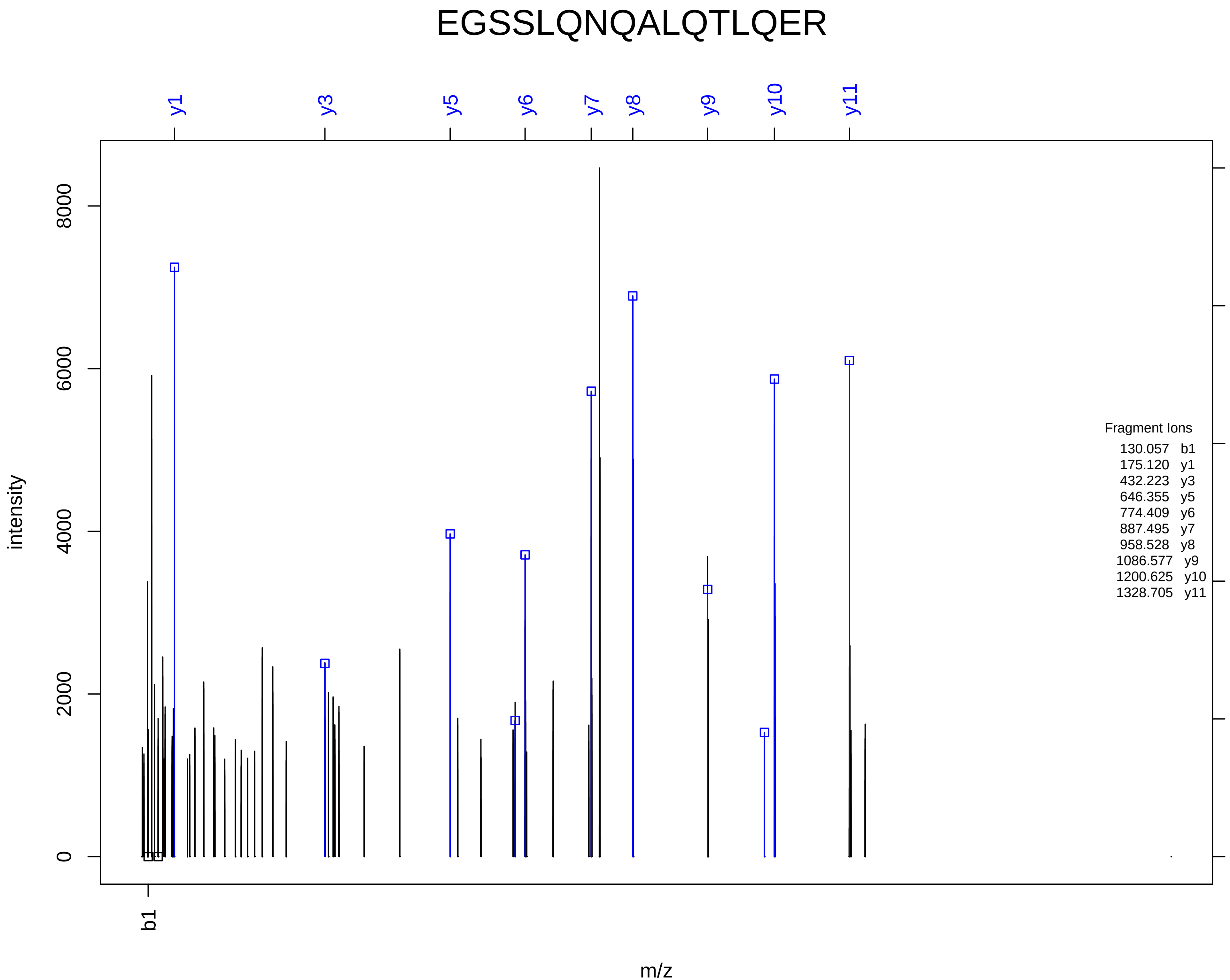

EGSSLQNQALQTLQER / File: christine_30turboips_ip11.raw; SpectrumID: ; scans: 11185

VAVEYLDPSPEVQK

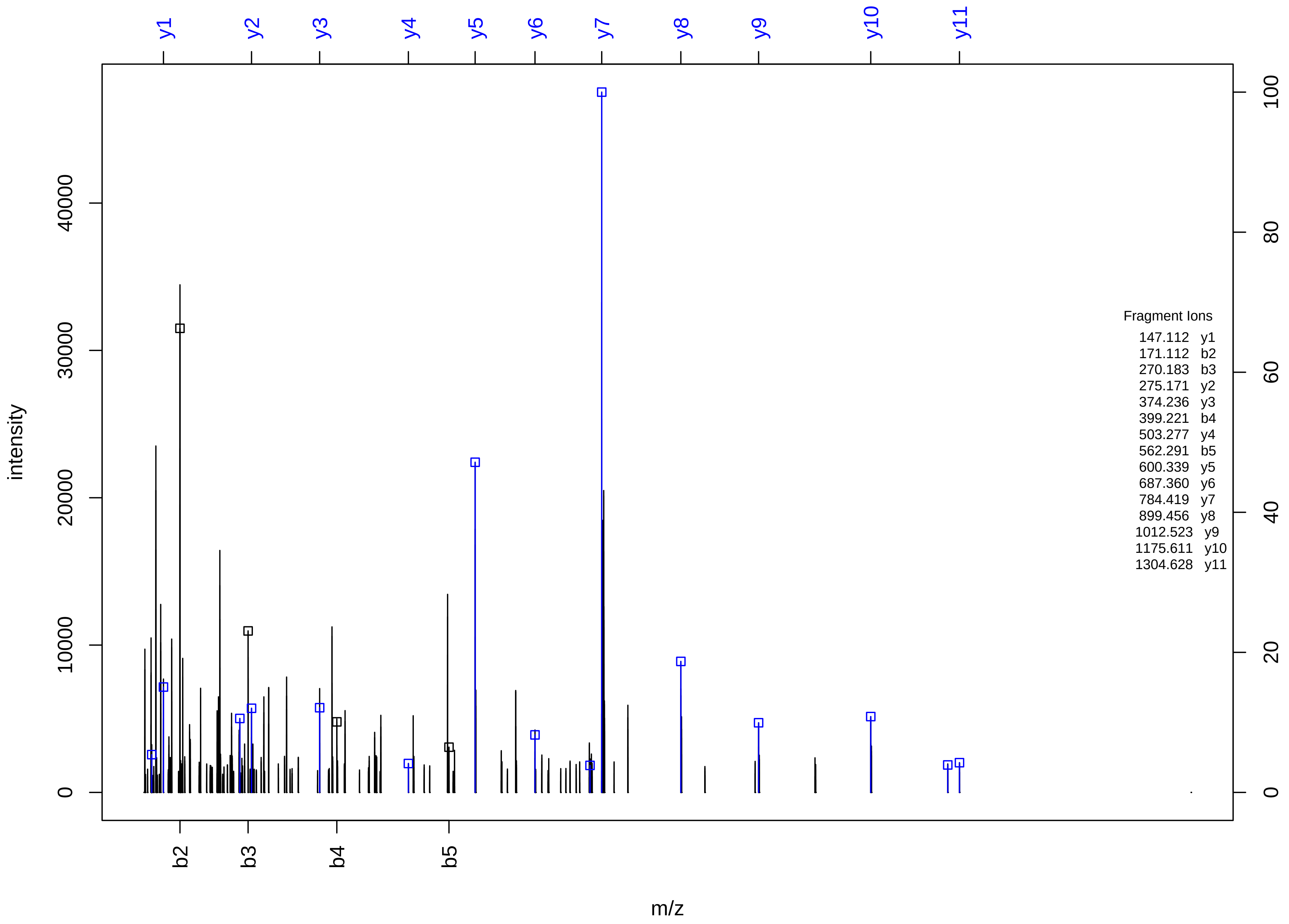

VAVEYLDPSPEVQK / File: christine_30turboips_ip11.raw; SpectrumID: ; scans: 10122

VVAGQIFVDSEEAEVESLLQDEEDSSK

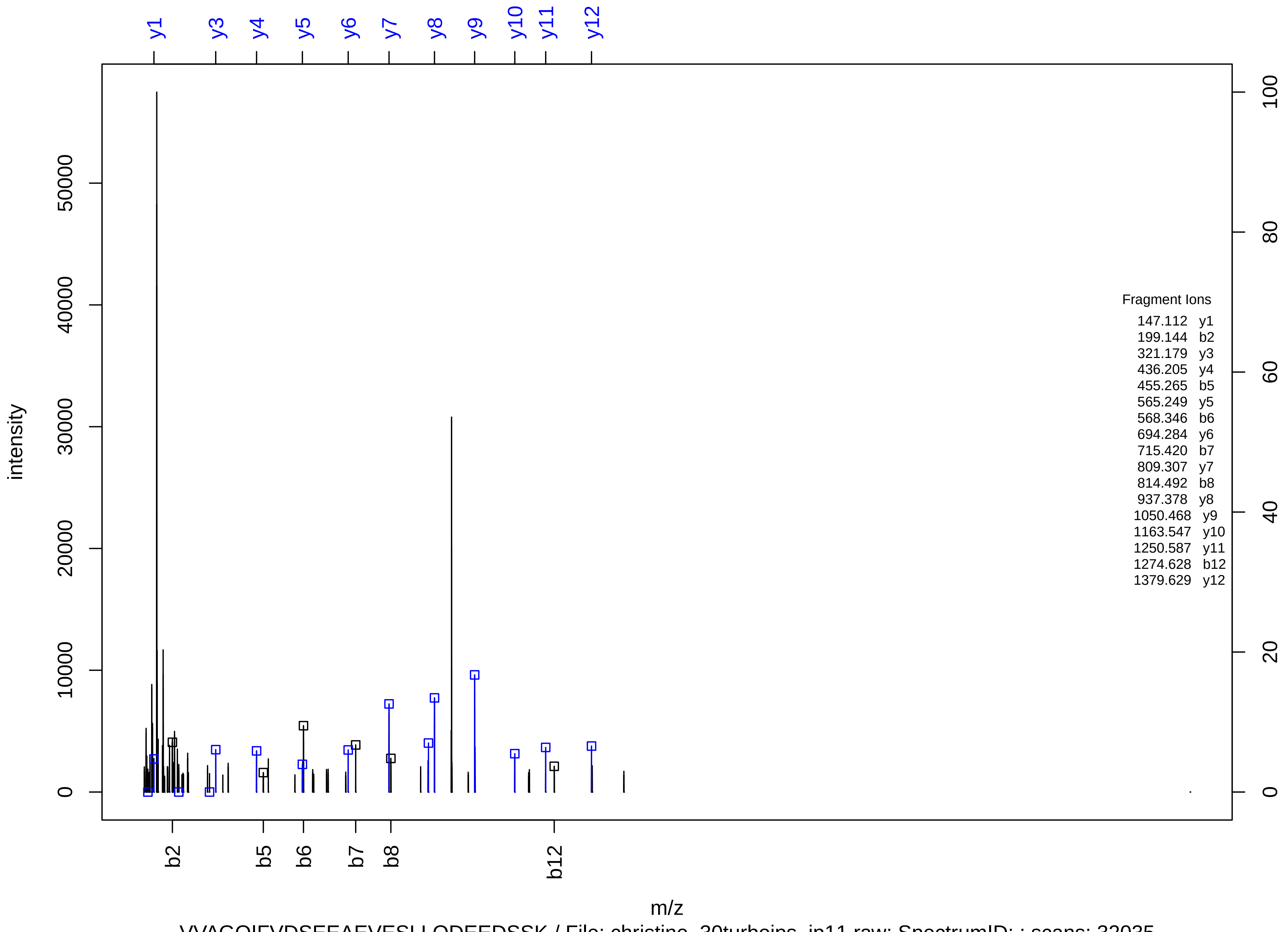

VVAGQIFVDSEEAEVESLLQDEEDSSK / File: christine_30turboips_ip11.raw; SpectrumID: ; scans: 32035

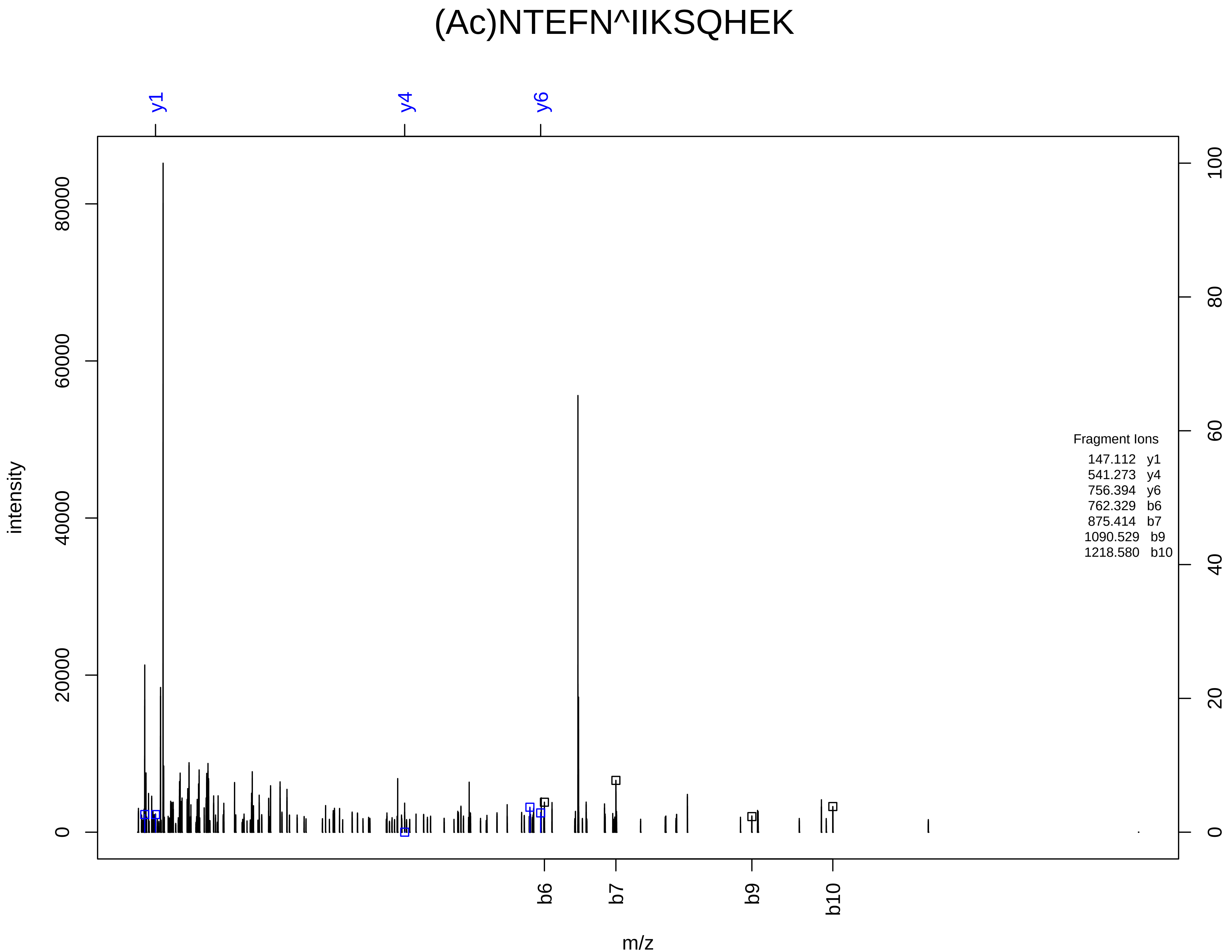
(Ac)NTEFN^IIKSQHEK
y1
y4
y6
intensity
80000
60000
40000
20000
0
100
80
60
40
20
0
Fragment Ions
147.112 y1
541.273 y4
756.394 y6
762.329 b6
875.414 b7
1090.529 b9
1218.580 b10
b6
b7
b9
b10
m/z
NTEFNIIKSQHEK / File: christine_30turboips_ip11.raw; SpectrumID: ; scans: 33013

GKPSLLAAARPM*R

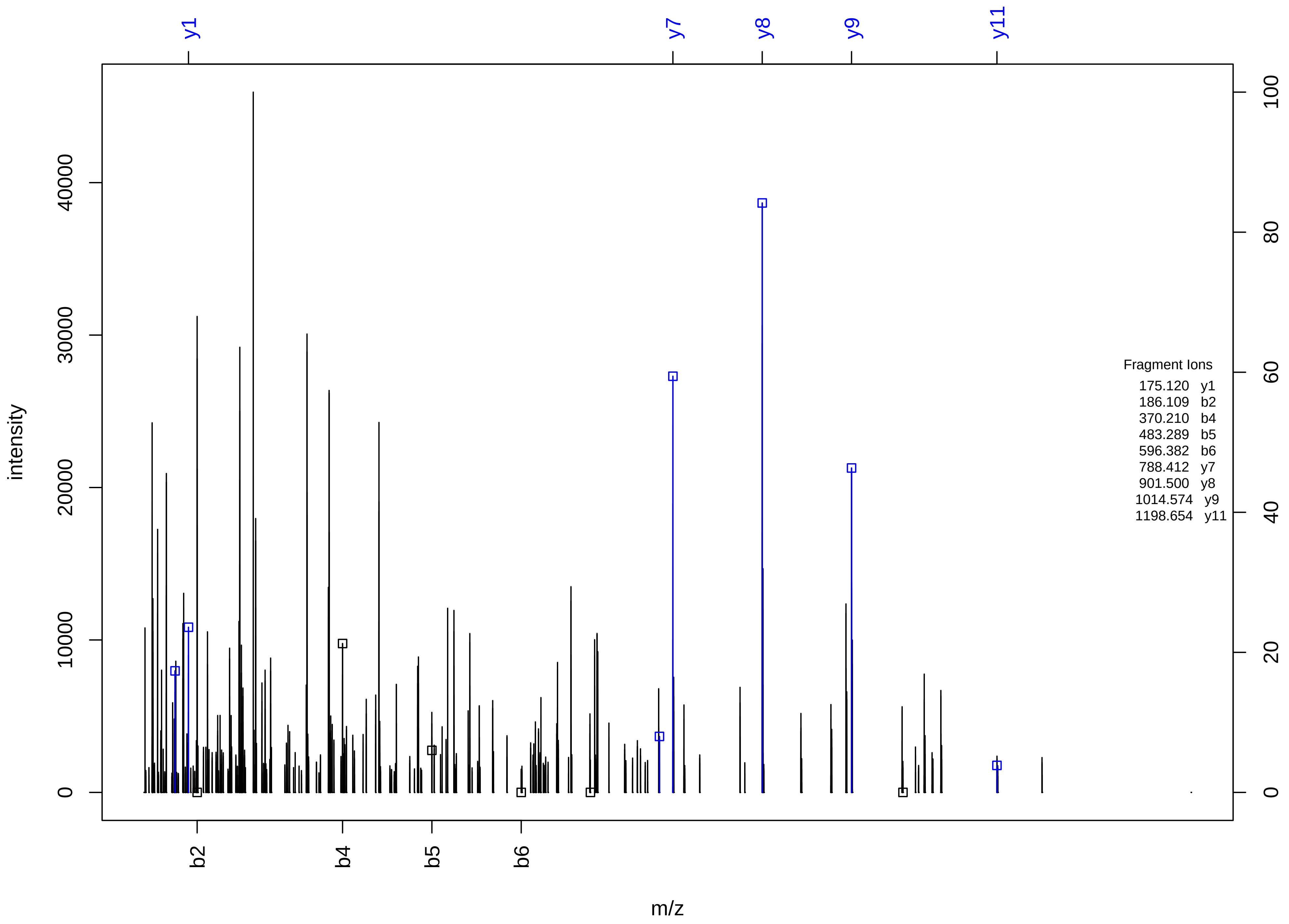

GKPSLLAAARPMR / File: christine_30turboips_ip11.raw; SpectrumID: ; scans: 14224

(Ac)N^AHSKEM*VPLM*GKR

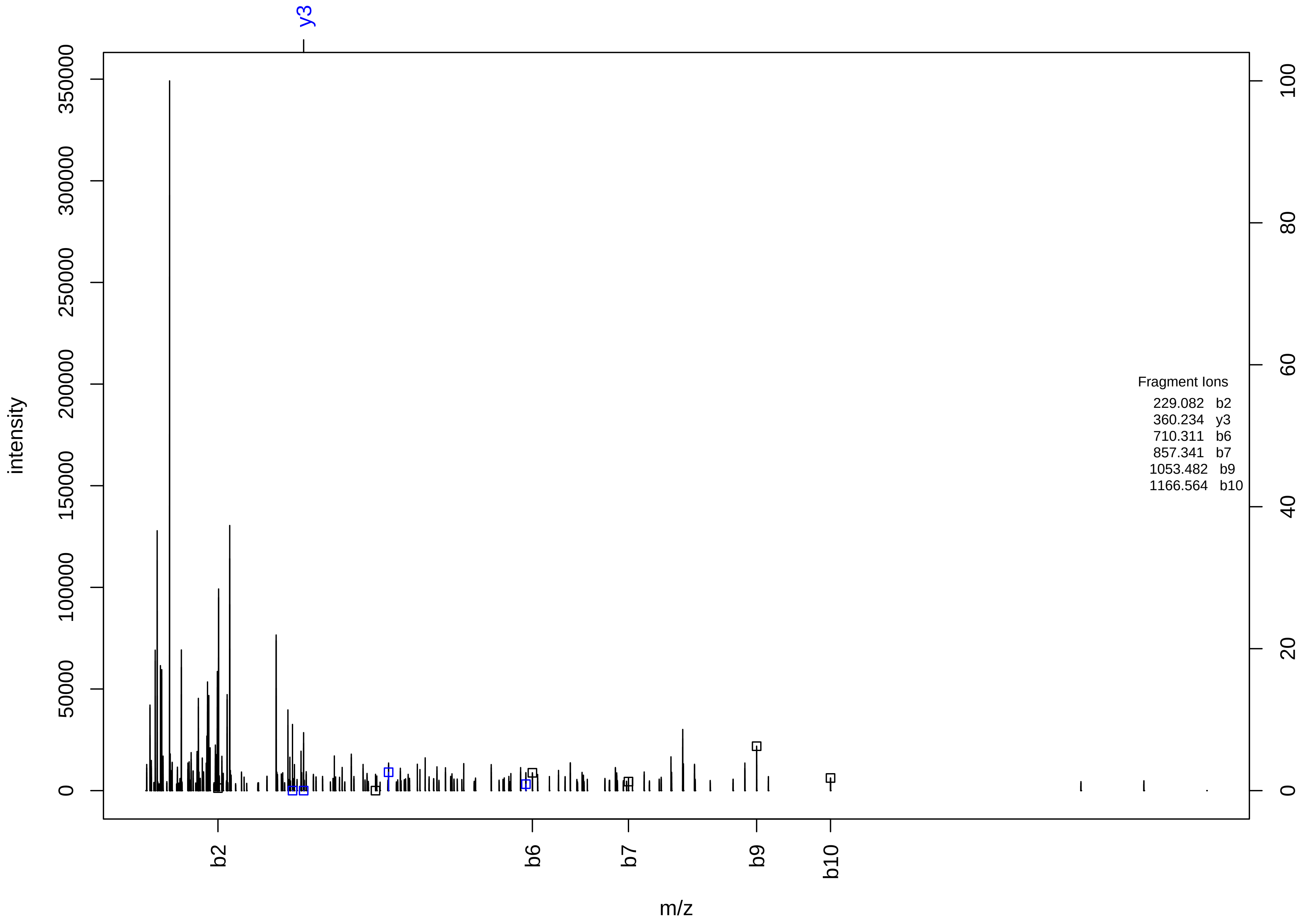

NAHSKEMVPLMGKR / File: christine_30turboips_ip11.raw; SpectrumID: ; scans: 26367

LGEHNIN^VLEGNEQFIDAANIIKHPK

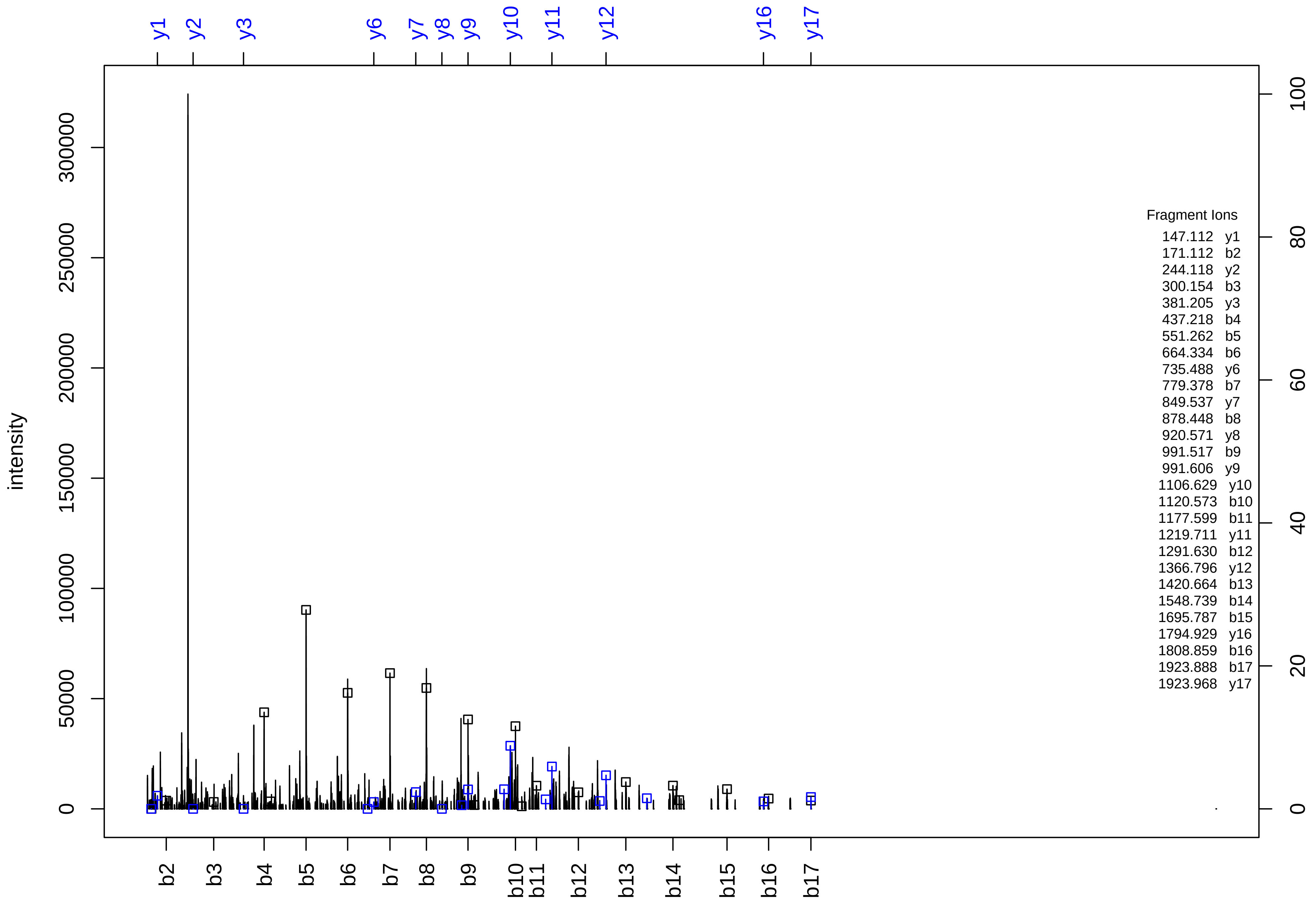

LGEHNINVLEGNEQFIDAANIIKHPK / File: christine_30turboips_ip11.raw; SpectrumID: ; scans: 21006

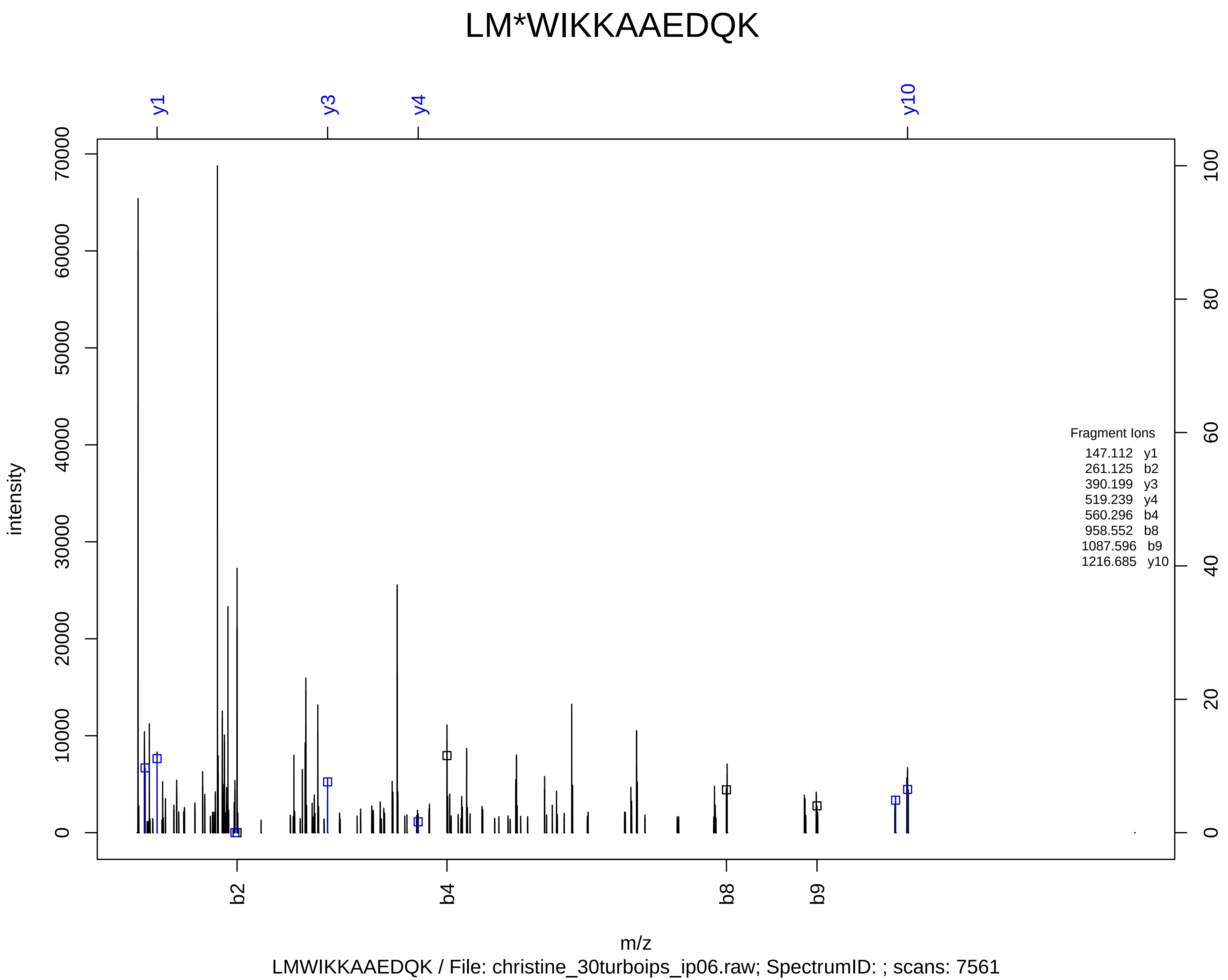
LM*WIKKAAEDQK
y1
y3
y4
y10
intensity
0
10000
20000
30000
40000
50000
60000
70000
0
20
40
60
80
100
Fragment Ions
147.112 y1
261.125 b2
390.199 y3
519.239 y4
560.296 b4
958.552 b8
1087.596 b9
1216.685 y10
b2
b4
b8
b9
m/z
LMWIKKAAEDQK / File: christine_30turboips_ip06.raw; SpectrumID: ; scans: 7561

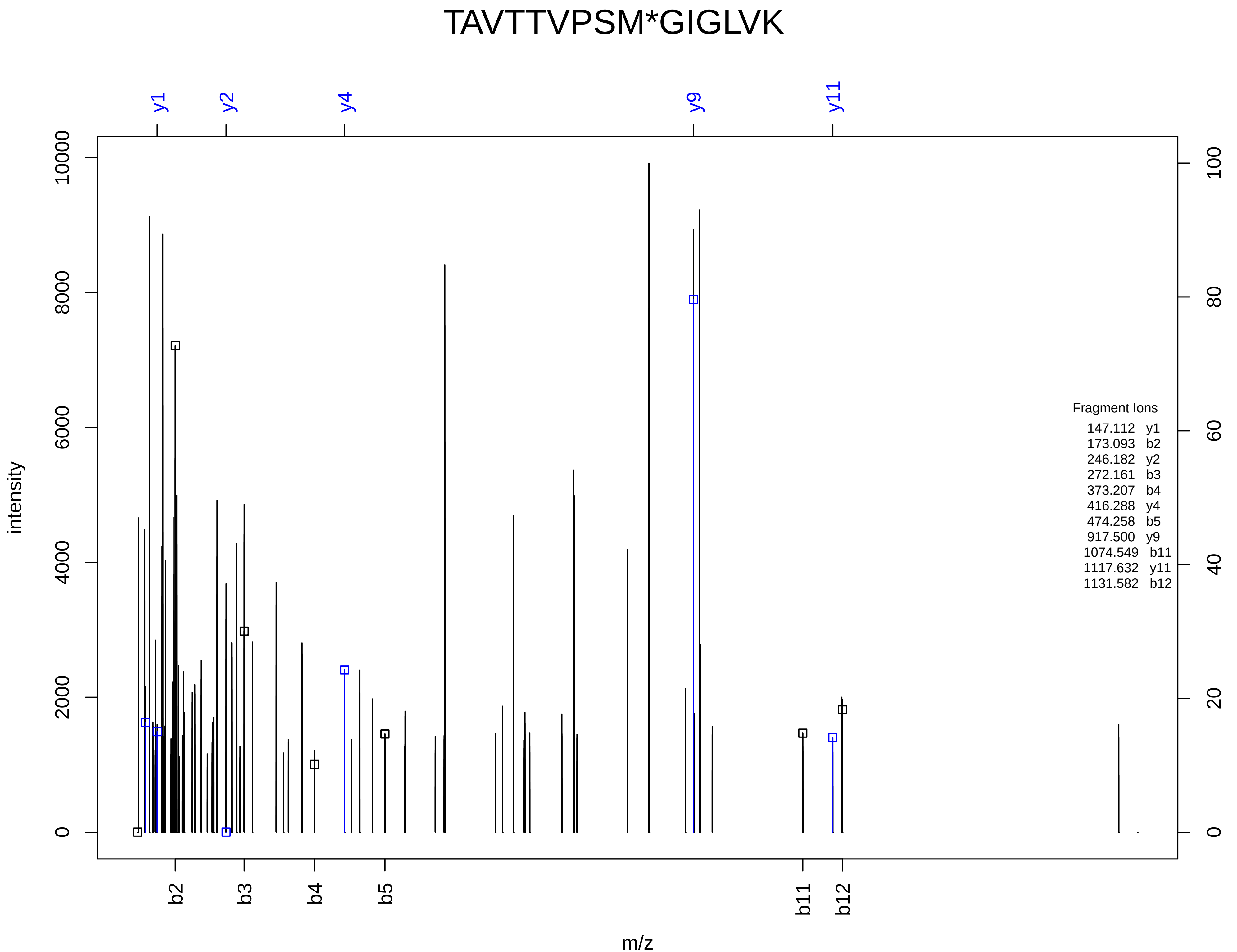

TAVTTVPSMGIGLVK / File: christine_30turboips_ip06.raw; SpectrumID: ; scans: 10329

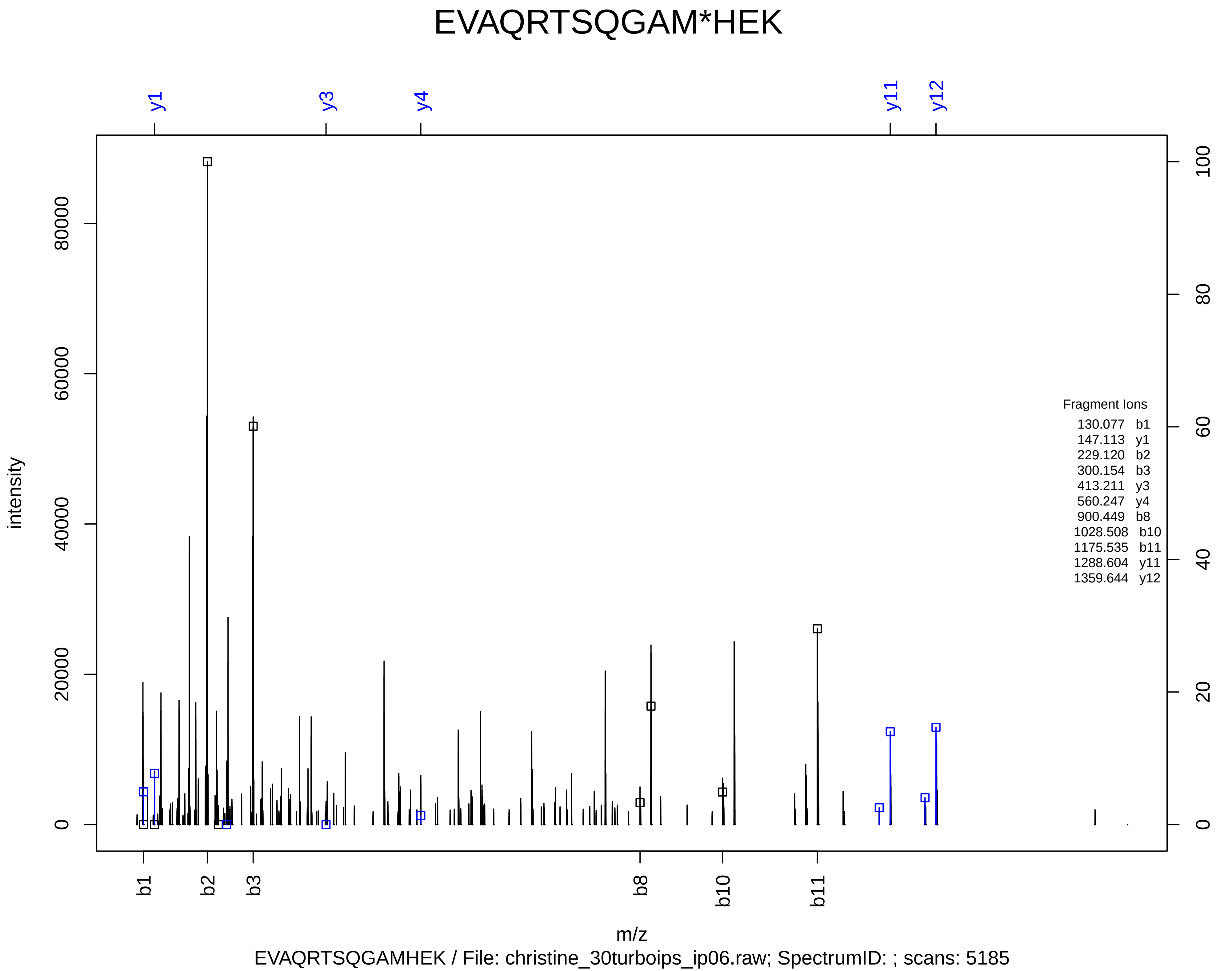

EVAQRTSQGAM*HEK
y1
y3
y4
y11
y12
intensity
80000
60000
40000
20000
0
100
80
60
40
20
0
Fragment Ions
130.077 b1
147.113 y1
229.120 b2
300.154 b3
413.211 y3
560.247 y4
900.449 b8
1028.508 b10
1175.535 b11
1288.604 y11
1359.644 y12
b1
b2
b3
b8
b10
b11
m/z
EVAQRTSQGAMHEK / File: christine_30turboips_ip06.raw; SpectrumID: ; scans: 5185

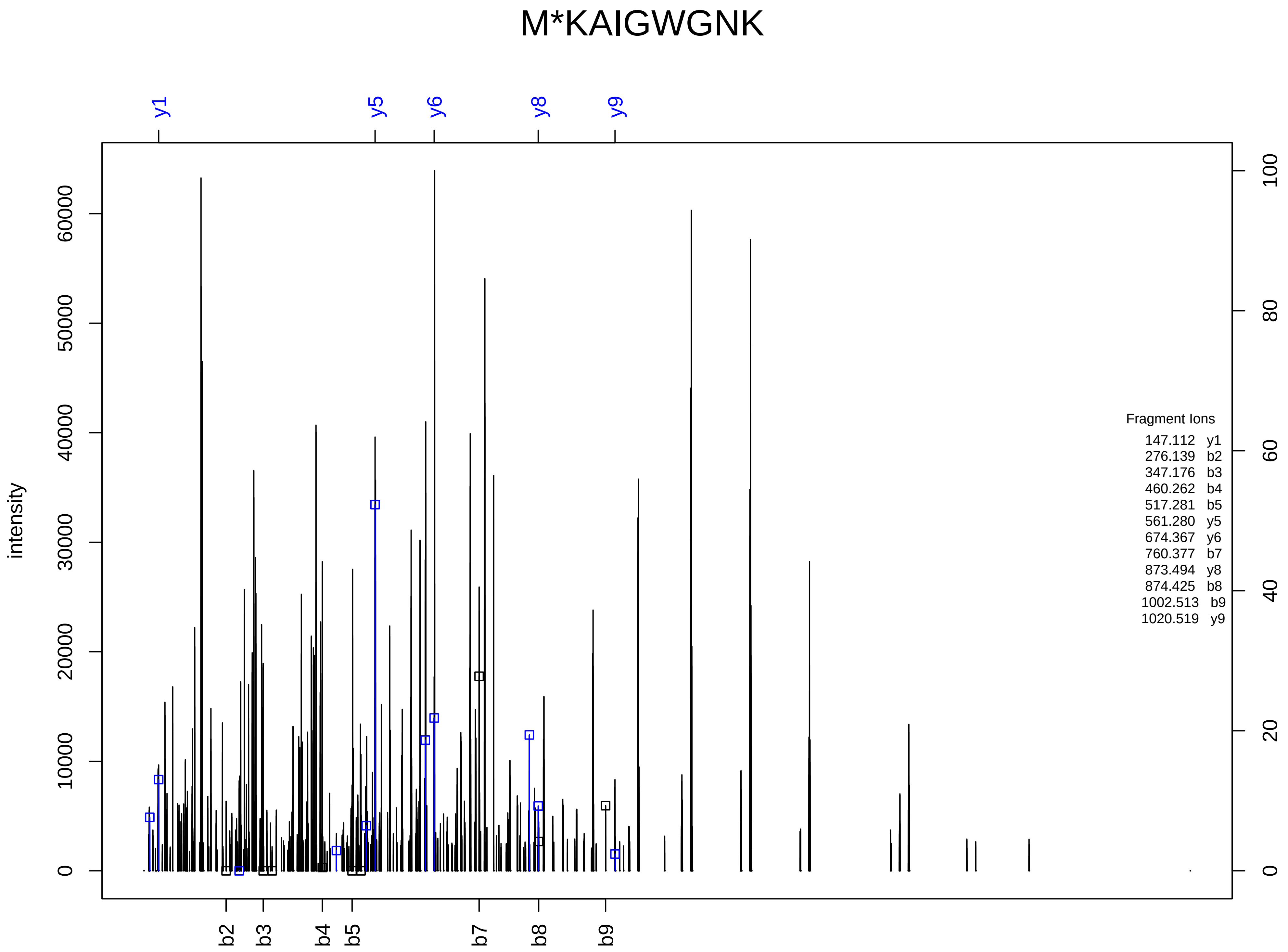

MKAIGWGNK / File: christine_30turboips_ip05.raw; SpectrumID: ; scans: 17902

ENGRAN^ISR

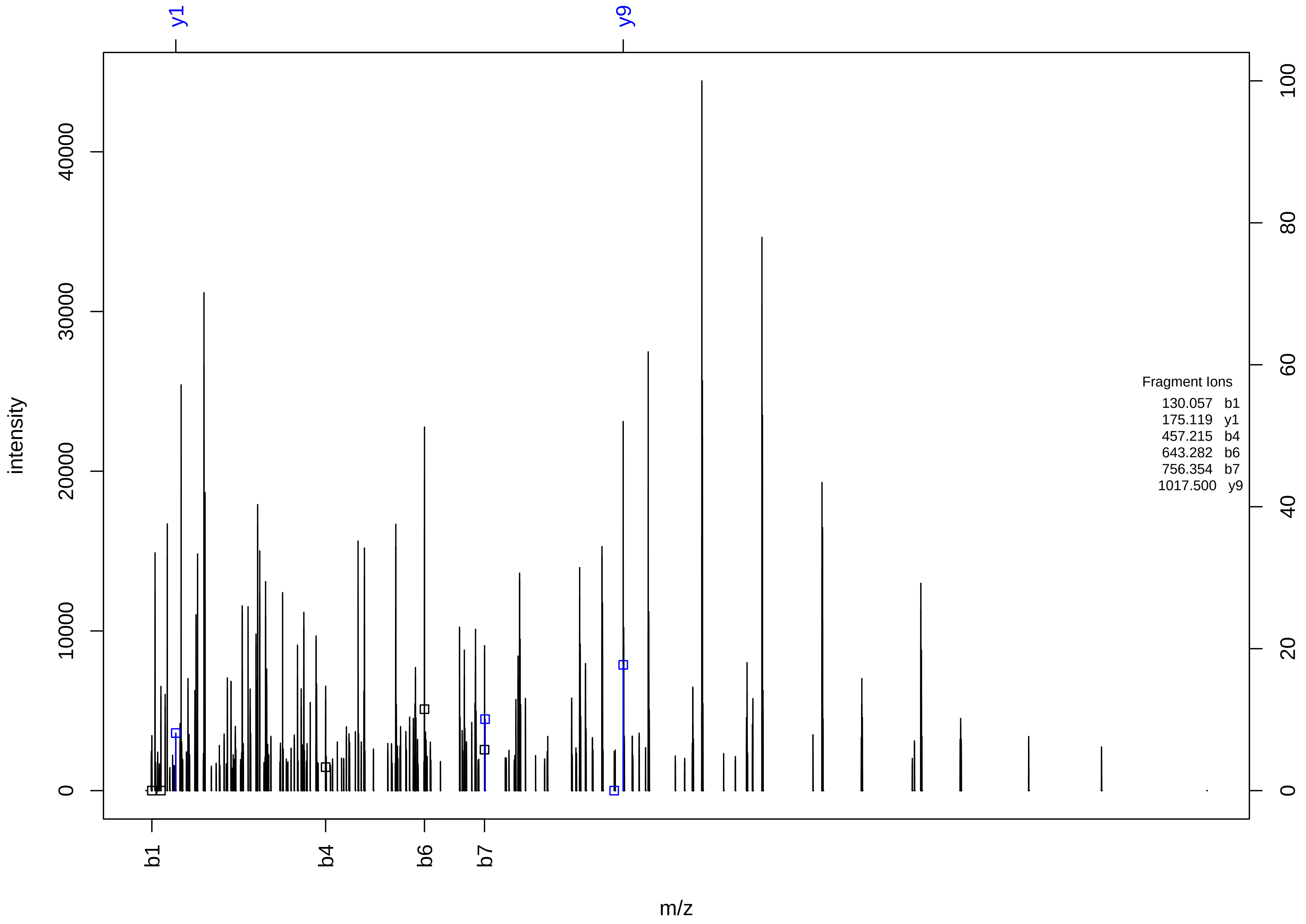

ENGRANISR / File: christine_30turboips_ip05.raw; SpectrumID: ; scans: 20457

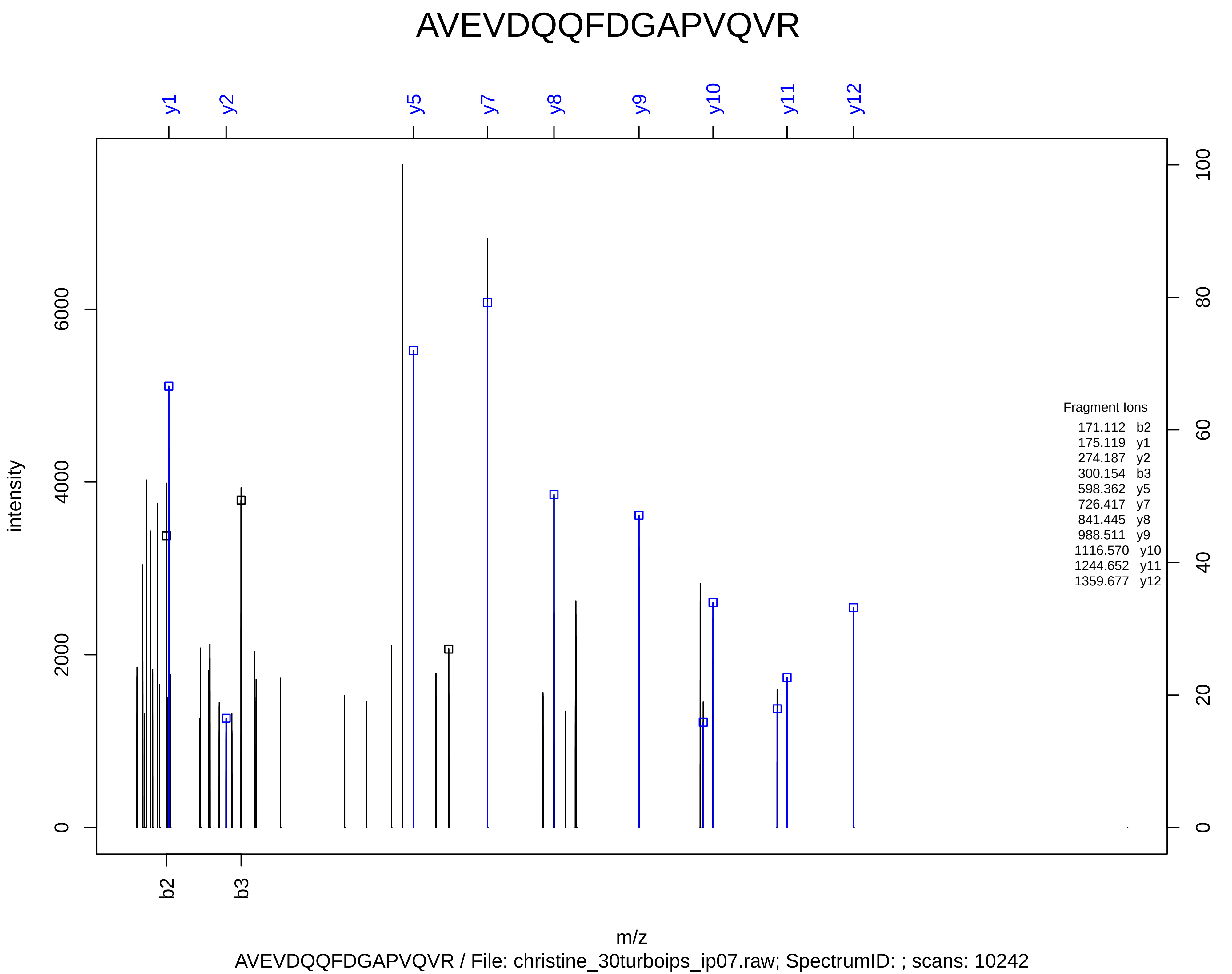
AVEVDQQFDGAPVQVR
y1
y2
y5
y7
y8
y9
y10
y11
y12
intensity
0
2000
4000
6000
0
20
40
60
80
100
b2
b3
Fragment Ions
171.112 b2
175.119 y1
274.187 y2
300.154 b3
598.362 y5
726.417 y7
841.445 y8
988.511 y9
1116.570 y10
1244.652 y11
1359.677 y12
m/z
AVEVDQQFDGAPVQVR / File: christine_30turboips_ip07.raw; SpectrumID: ; scans: 10242

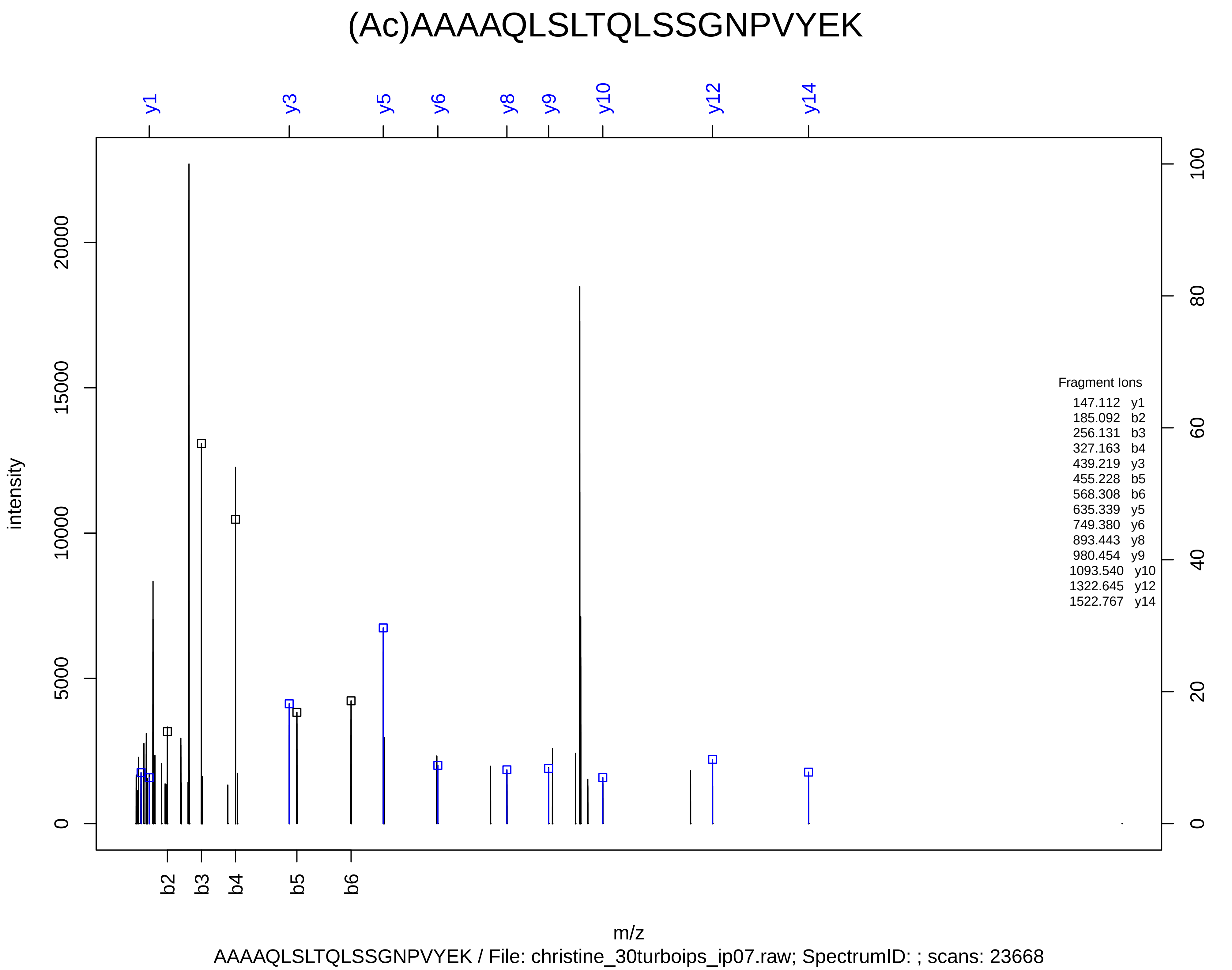
(Ac)AAAAQLSLTQLSSGNPVYEK
y1
y3
y5
y6
y8
y9
y10
y12
y14
intensity
0
5000
10000
15000
20000
0
20
40
60
80
100
b2
b3
b4
b5
b6
m/z
Fragment Ions
147.112 y1
185.092 b2
256.131 b3
327.163 b4
439.219 y3
455.228 b5
568.308 b6
635.339 y5
749.380 y6
893.443 y8
980.454 y9
1093.540 y10
1322.645 y12
1522.767 y14
AAAAQLSLTQLSSGNPVYEK / File: christine_30turboips_ip07.raw; SpectrumID: ; scans: 23668

PVAVGPYGQSQPSCFDR

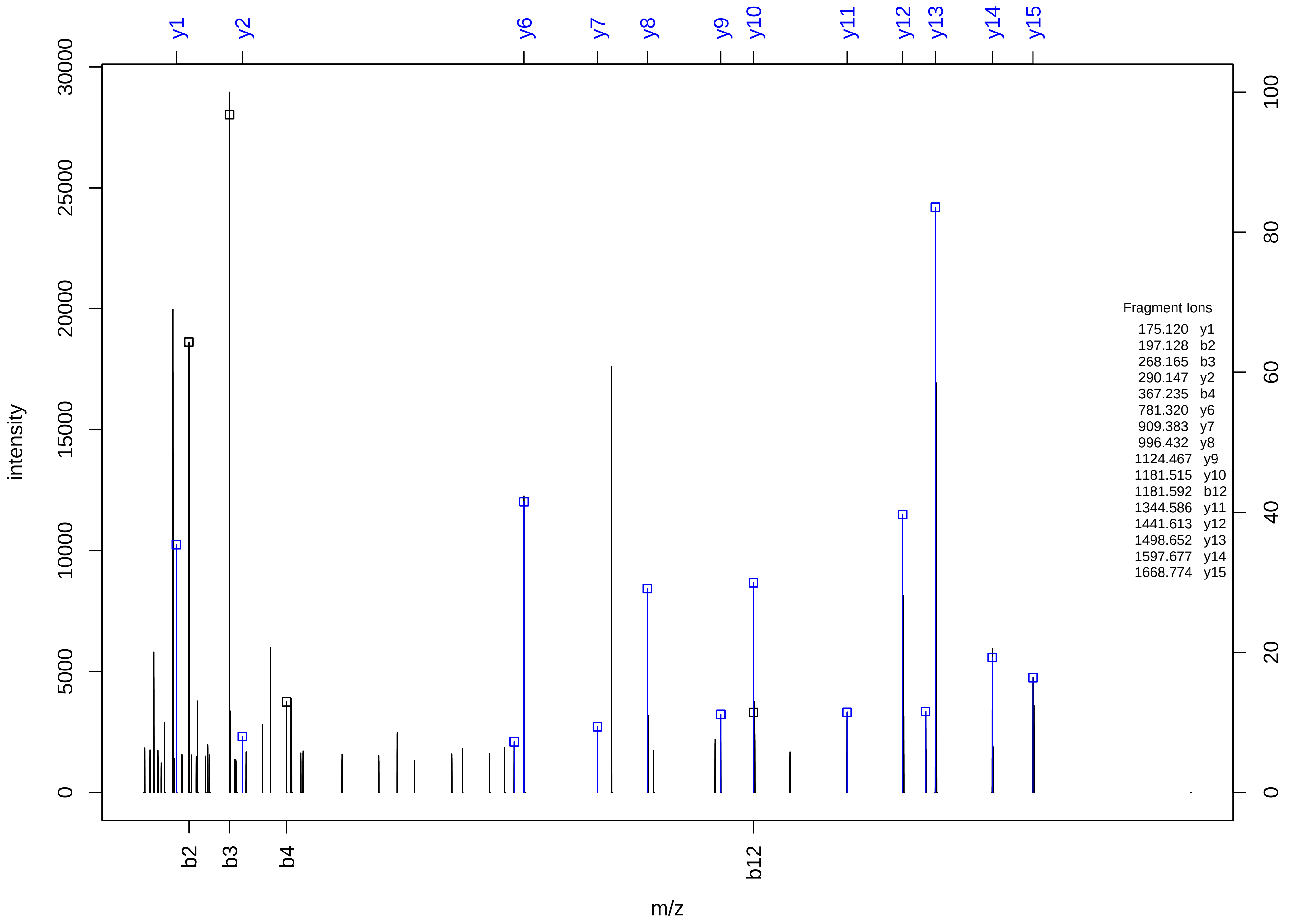

PVAVGPYGQSQPSCFDR / File: christine_30turboips_ip07.raw; SpectrumID: ; scans: 7398

GDSVIVVLR

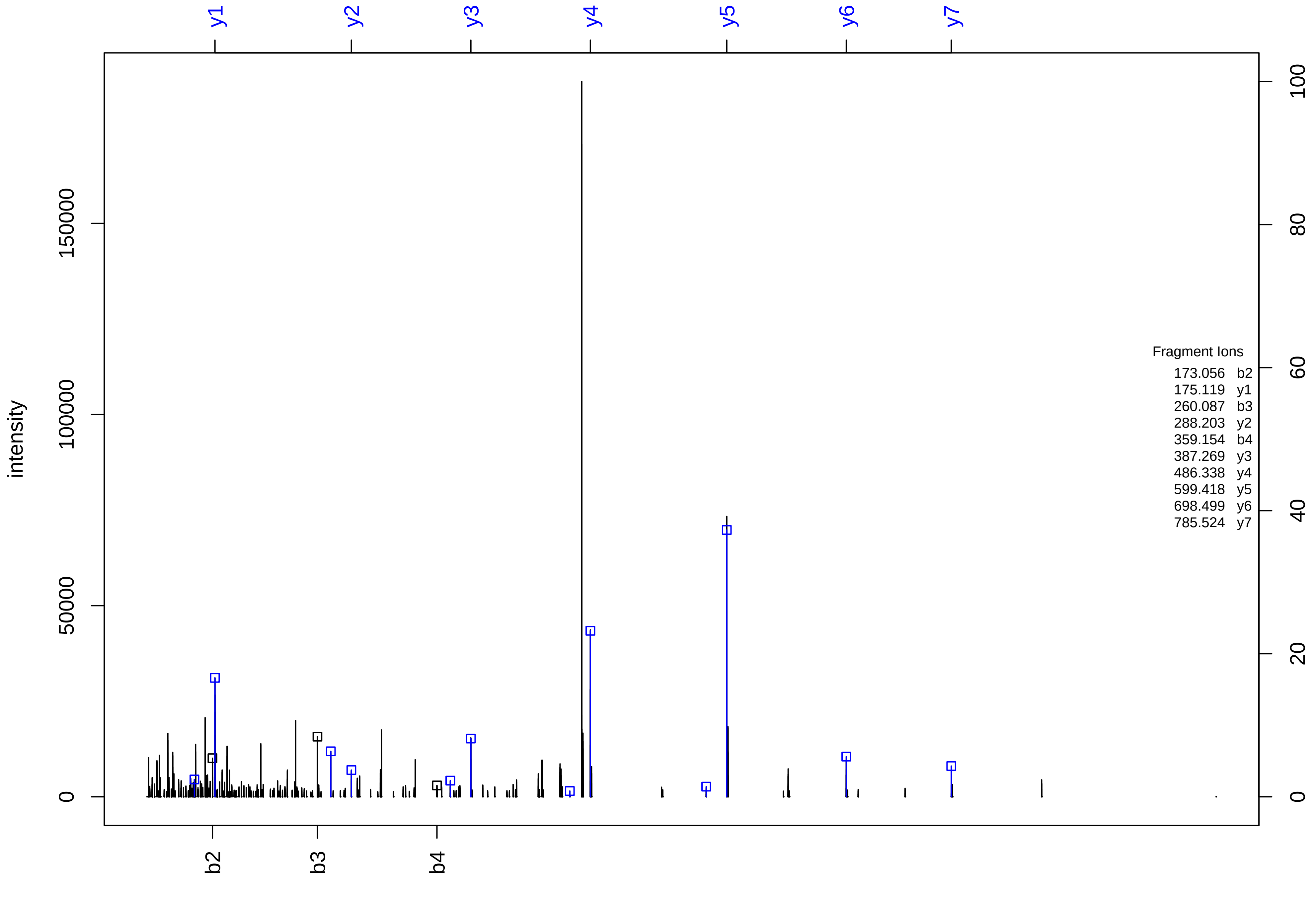

GDSVIVVLR / File: christine_30turboips_ip07.raw; SpectrumID: ; scans: 10835

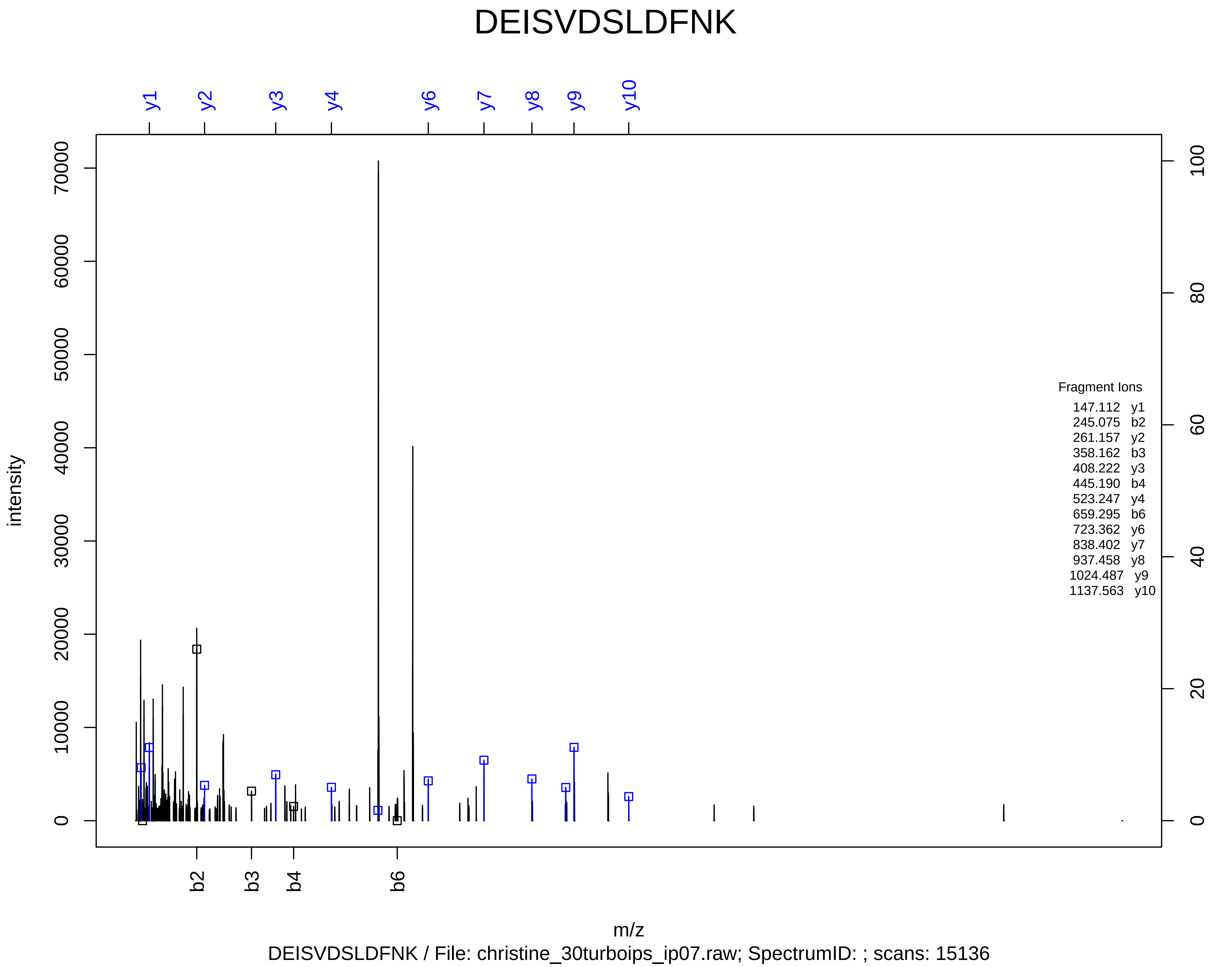

DEISVDSLDFNK
y1
y2
y3
y4
y6
y7
y8
y9
y10
b2
b3
b4
b6
intensity
m/z
0
10000
20000
30000
40000
50000
60000
70000
0
20
40
60
80
100
Fragment Ions
147.112 y1
245.075 b2
261.157 y2
358.162 b3
408.222 y3
445.190 b4
523.247 y4
659.295 b6
723.362 y6
838.402 y7
937.458 y8
1024.487 y9
1137.563 y10
DEISVDSLDFNK / File: christine_30turboips_ip07.raw; SpectrumID: ; scans: 15136

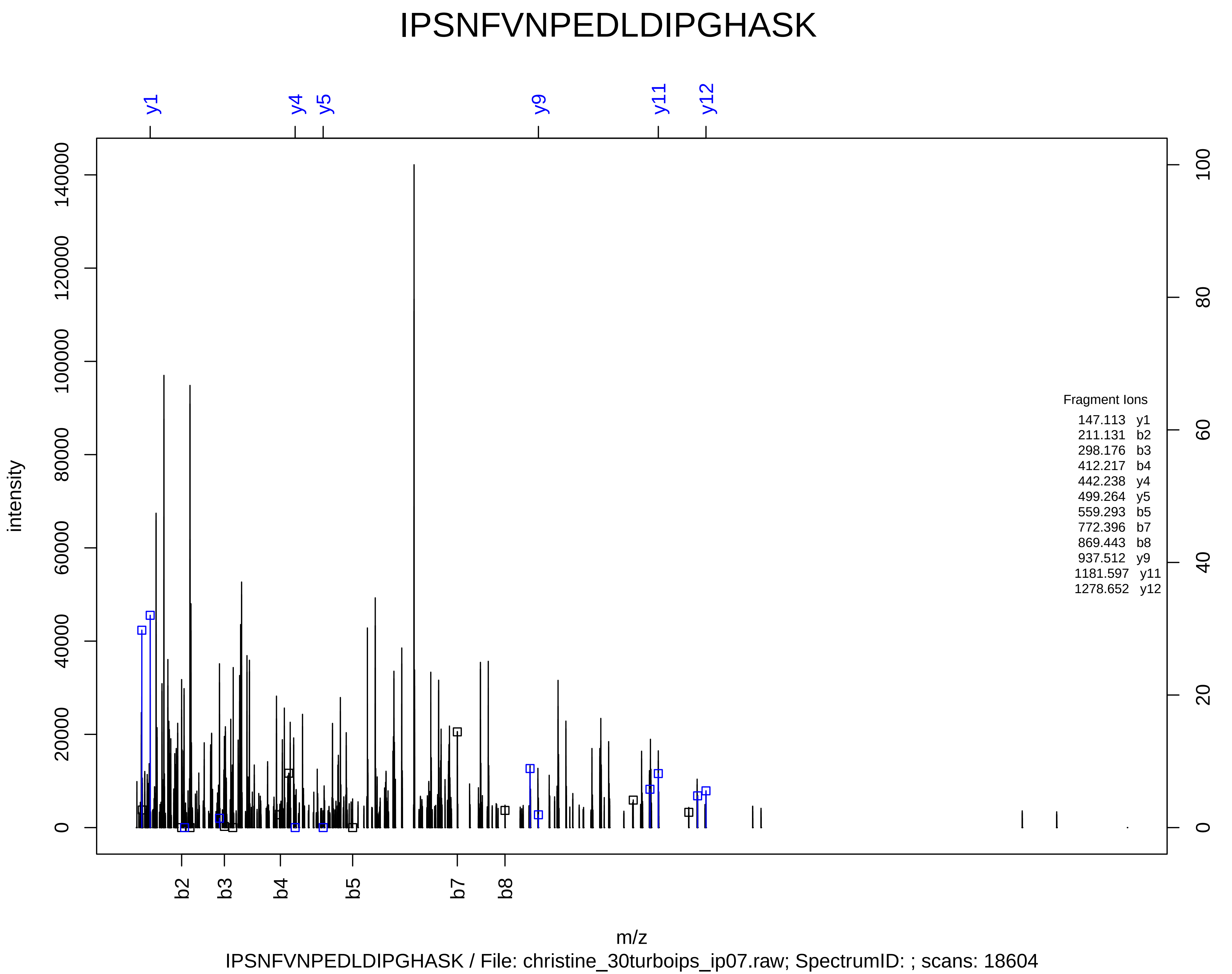
IPSNFVNPEDLDIPGHASK
intensity
m/z
y1
y4
y5
y9
y11
y12
b2
b3
b4
b5
b7
b8
0
20000
40000
60000
80000
100000
120000
140000
0
20
40
60
80
100
Fragment Ions
147.113 y1
211.131 b2
298.176 b3
412.217 b4
442.238 y4
499.264 y5
559.293 b5
772.396 b7
869.443 b8
937.512 y9
1181.597 y11
1278.652 y12
IPSNFVNPEDLDIPGHASK / File: christine_30turboips_ip07.raw; SpectrumID: ; scans: 18604

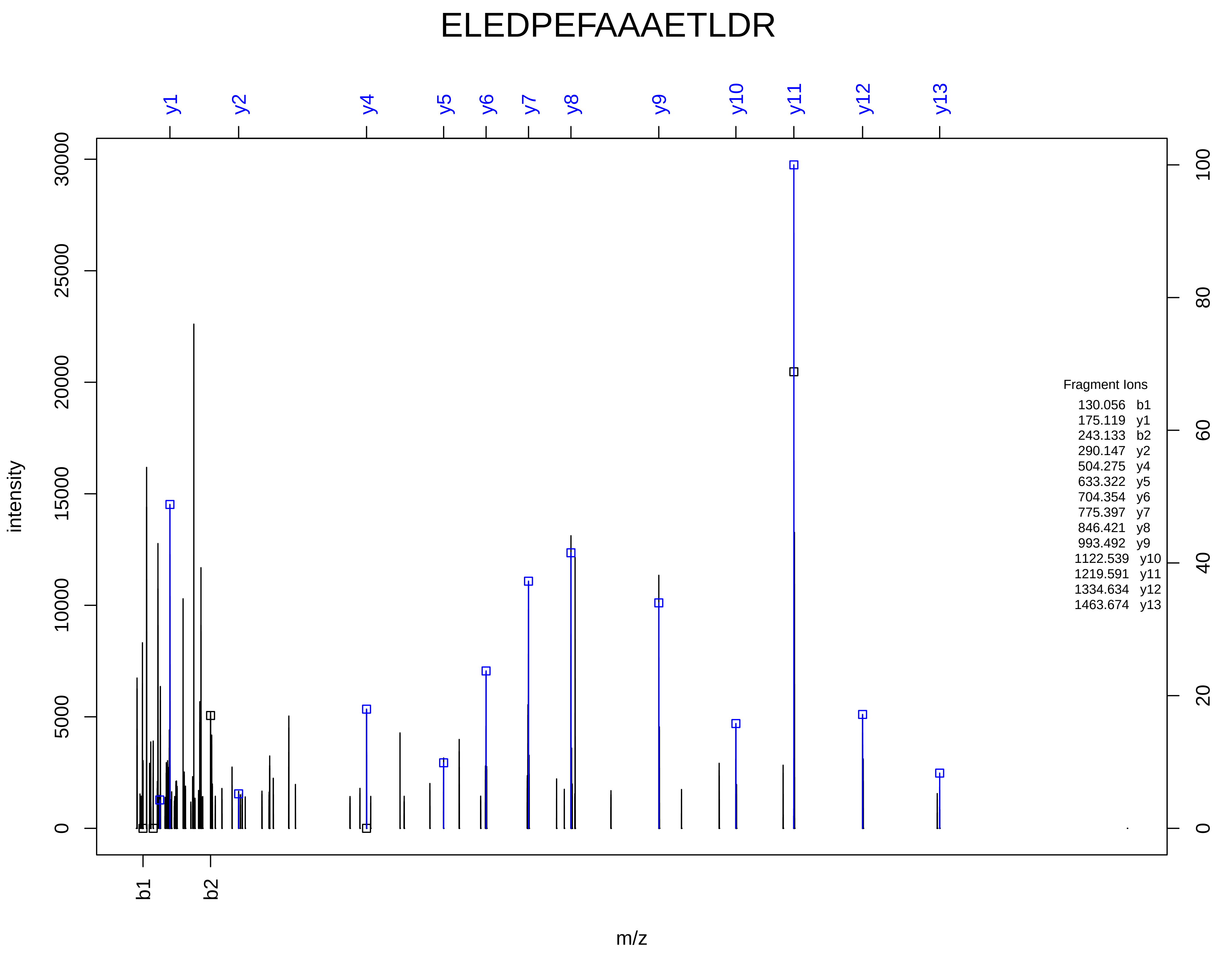
ELEDPEFAAAETLDR
y1 y2 y4 y5 y6 y7 y8 y9 y10 y11 y12 y13
b1 b2
intensity
m/z
Fragment Ions
130.056 b1
175.119 y1
243.133 b2
290.147 y2
504.275 y4
633.322 y5
704.354 y6
775.397 y7
846.421 y8
993.492 y9
1122.539 y10
1219.591 y11
1334.634 y12
1463.674 y13
ELEDPEFAAAETLDR / File: christine_30turboips_ip07.raw; SpectrumID: ; scans: 16750

FIYVDVLPEEAVGPVRPSR

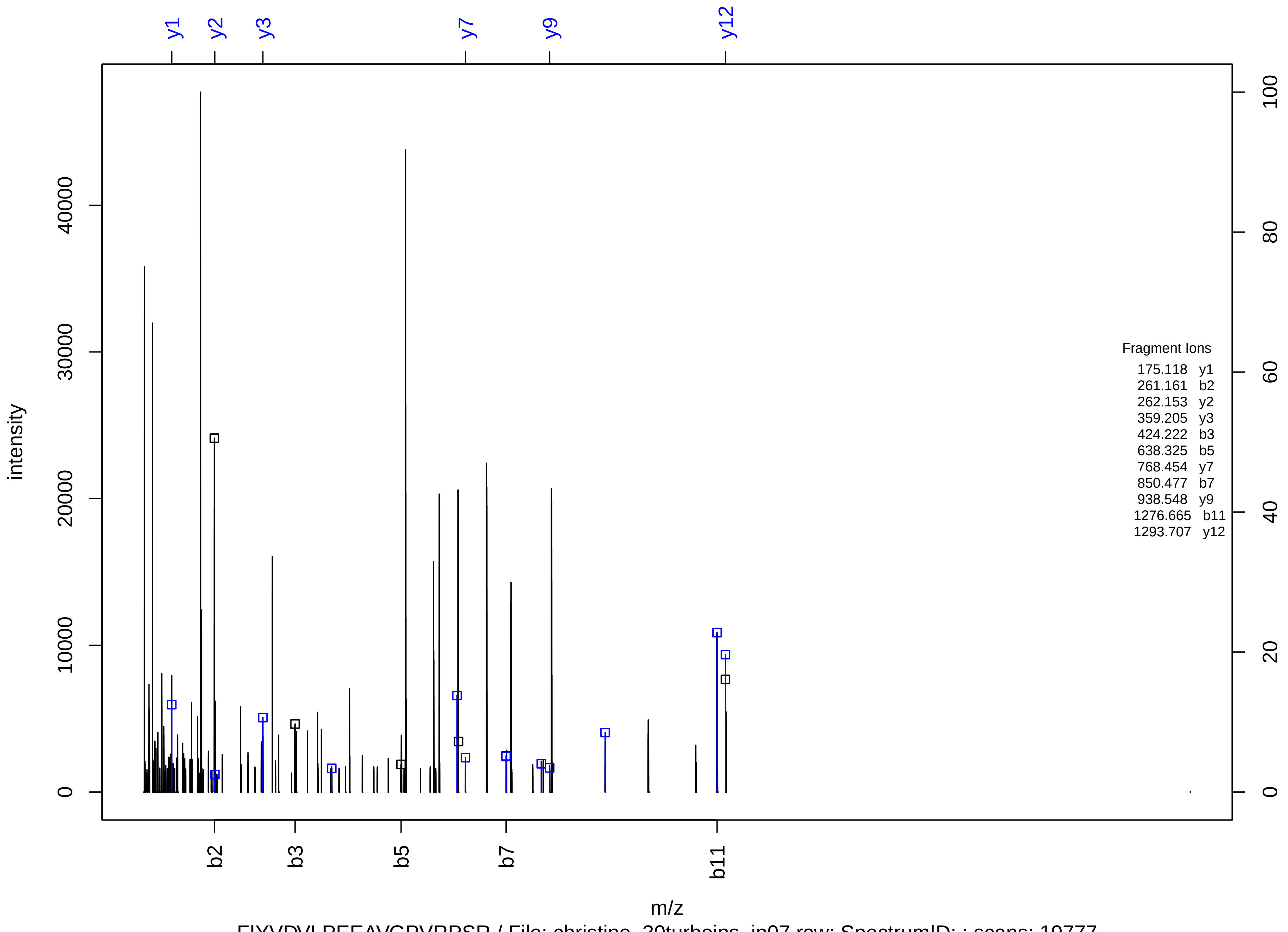

FIYVDVLPEEAVGPVRPSR / File: christine_30turboips_ip07.raw; SpectrumID: ; scans: 19777

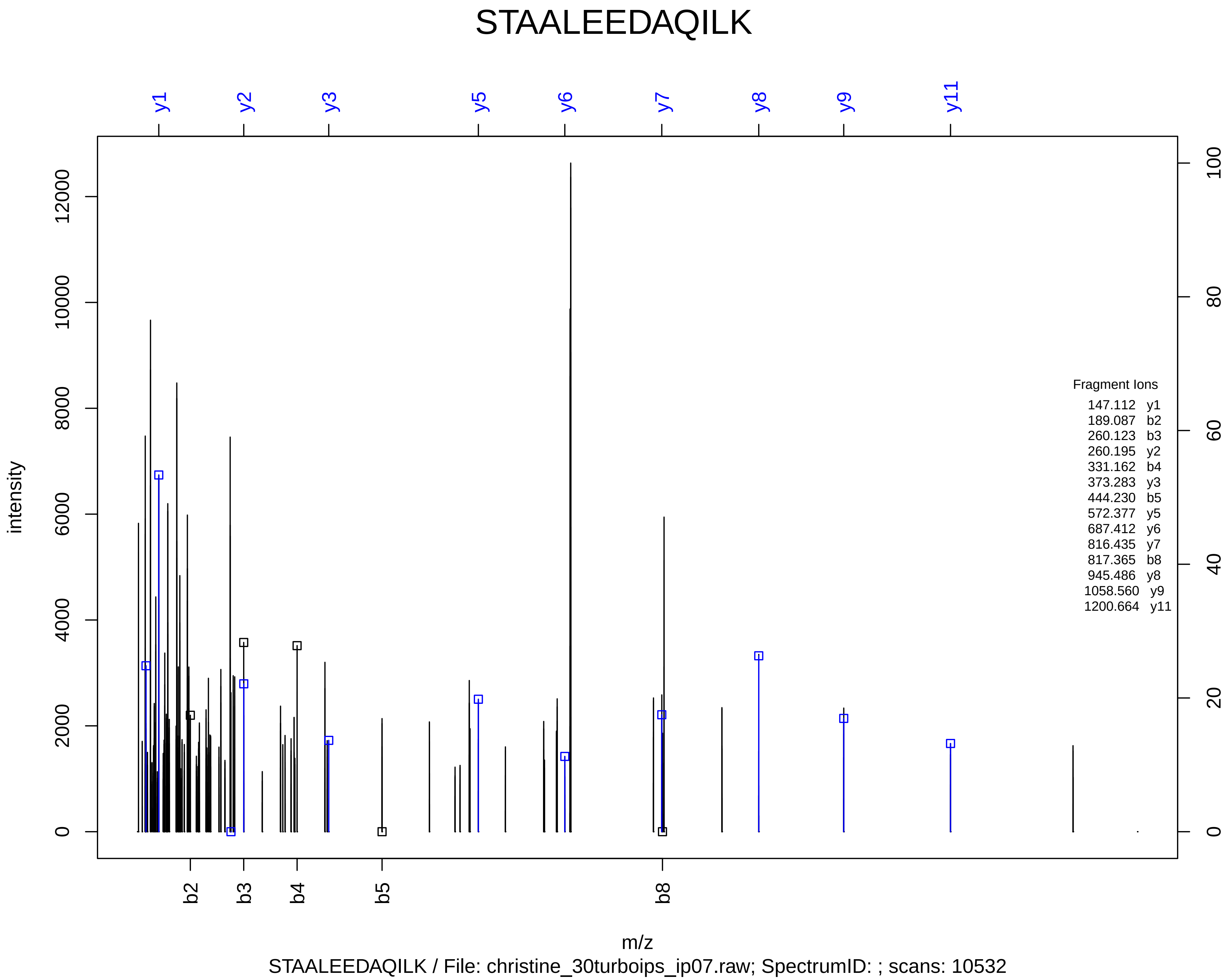

STAALEEDAQILK
y1
y2
y3
y5
y6
y7
y8
y9
y11
b2
b3
b4
b5
b8
intensity
m/z
0
2000
4000
6000
8000
10000
12000
0
20
40
60
80
100
Fragment Ions
147.112 y1
189.087 b2
260.123 b3
260.195 y2
331.162 b4
373.283 y3
444.230 b5
572.377 y5
687.412 y6
816.435 y7
817.365 b8
945.486 y8
1058.560 y9
1200.664 y11
STAALEEDAQILK / File: christine_30turboips_ip07.raw; SpectrumID: ; scans: 10532

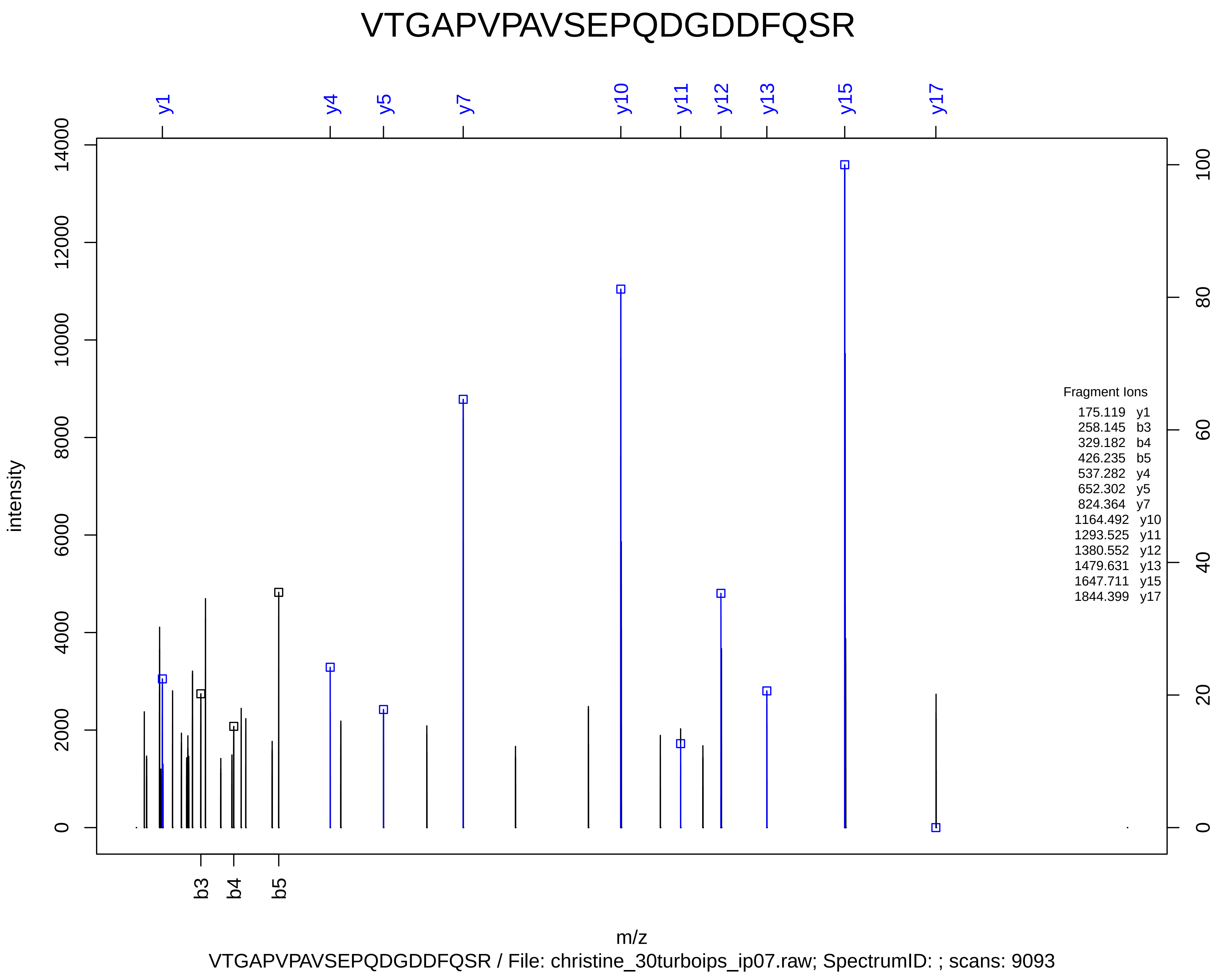

VTGAPVPAVSEPQDGDDFQSR
y1
y4
y5
y7
y10
y11
y12
y13
y15
y17
intensity
0
2000
4000
6000
8000
10000
12000
14000
0
20
40
60
80
100
b3
b4
b5
m/z
Fragment Ions
175.119 y1
258.145 b3
329.182 b4
426.235 b5
537.282 y4
652.302 y5
824.364 y7
1164.492 y10
1293.525 y11
1380.552 y12
1479.631 y13
1647.711 y15
1844.399 y17
VTGAPVPAVSEPQDGDDFQSR / File: christine_30turboips_ip07.raw; SpectrumID: ; scans: 9093

GFGHIGIAVPDVYSACK

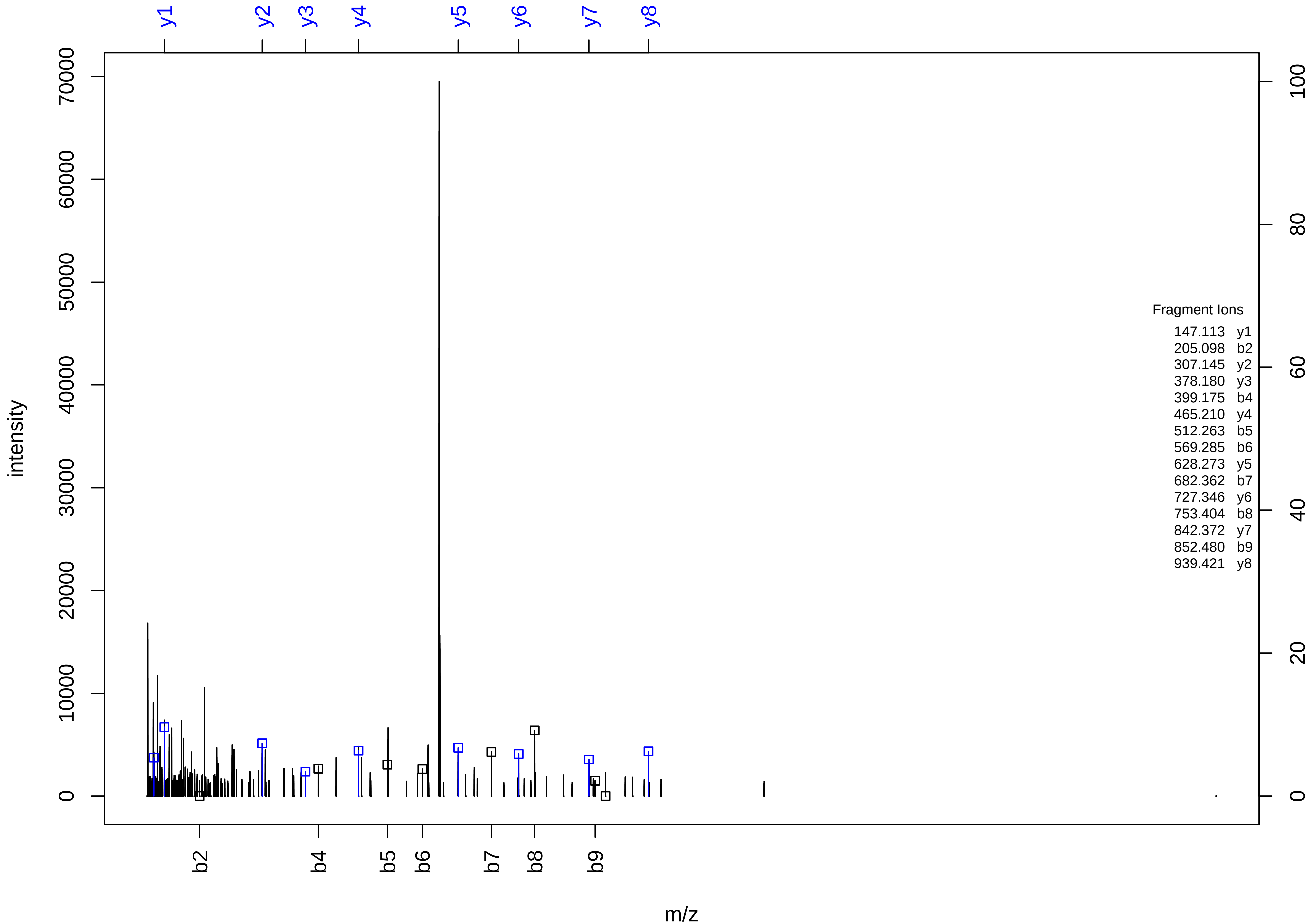

GFGHIGIAVPDVYSACK / File: christine_30turboips_ip07.raw; SpectrumID: ; scans: 15303

APLVPPGSPVVNALFR

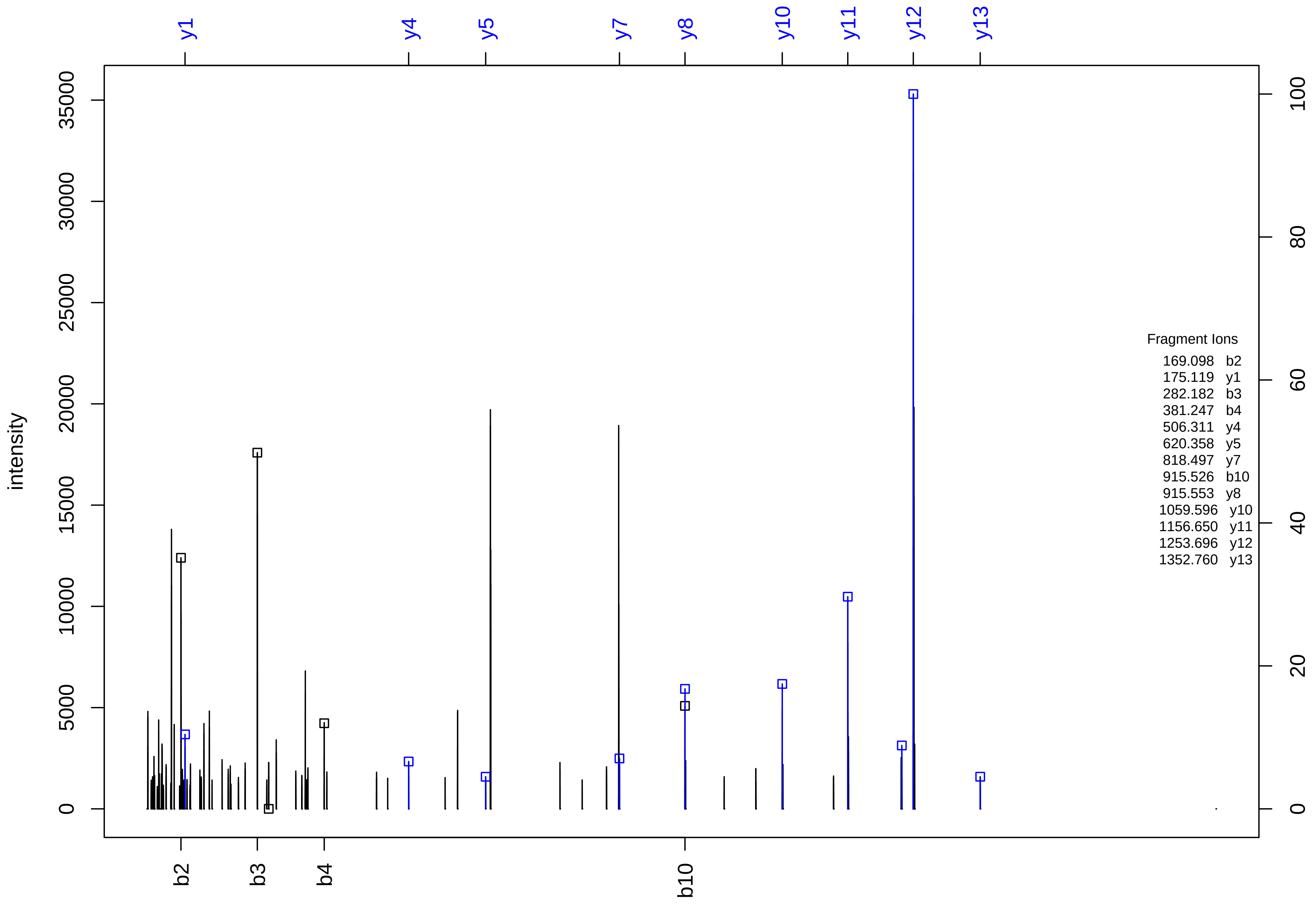

APLVPPGSPVVNALFR / File: christine_30turboips_ip07.raw; SpectrumID: ; scans: 24231

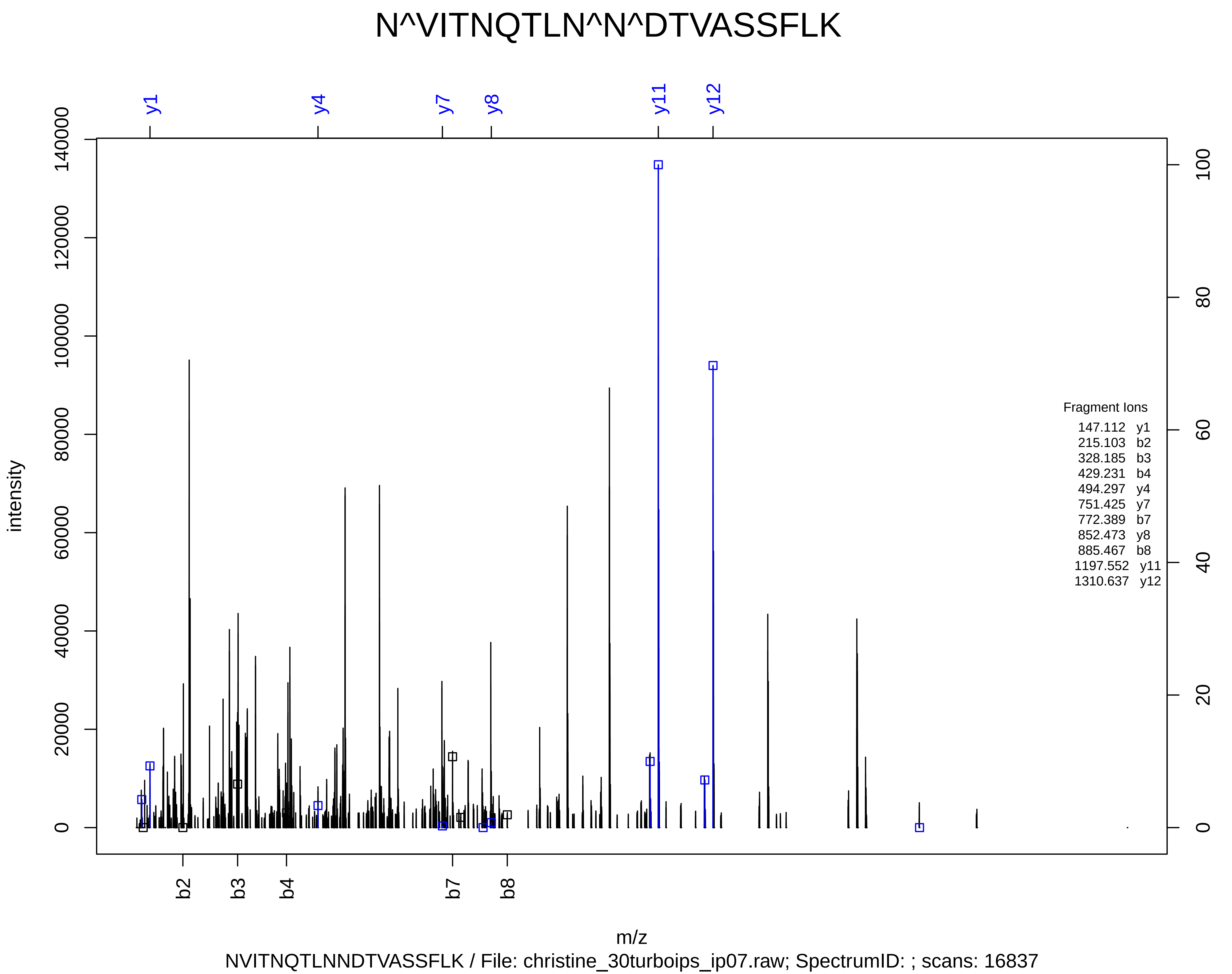
N^VITNQTLN^N^DTVASSFLK
intensity
m/z
y1
y4
y7
y8
y11
y12
b2
b3
b4
b7
b8
Fragment Ions
147.112 y1
215.103 b2
328.185 b3
429.231 b4
494.297 y4
751.425 y7
772.389 b7
852.473 y8
885.467 b8
1197.552 y11
1310.637 y12
NVITNQTLNNDTVASSFLK / File: christine_30turboips_ip07.raw; SpectrumID: ; scans: 16837

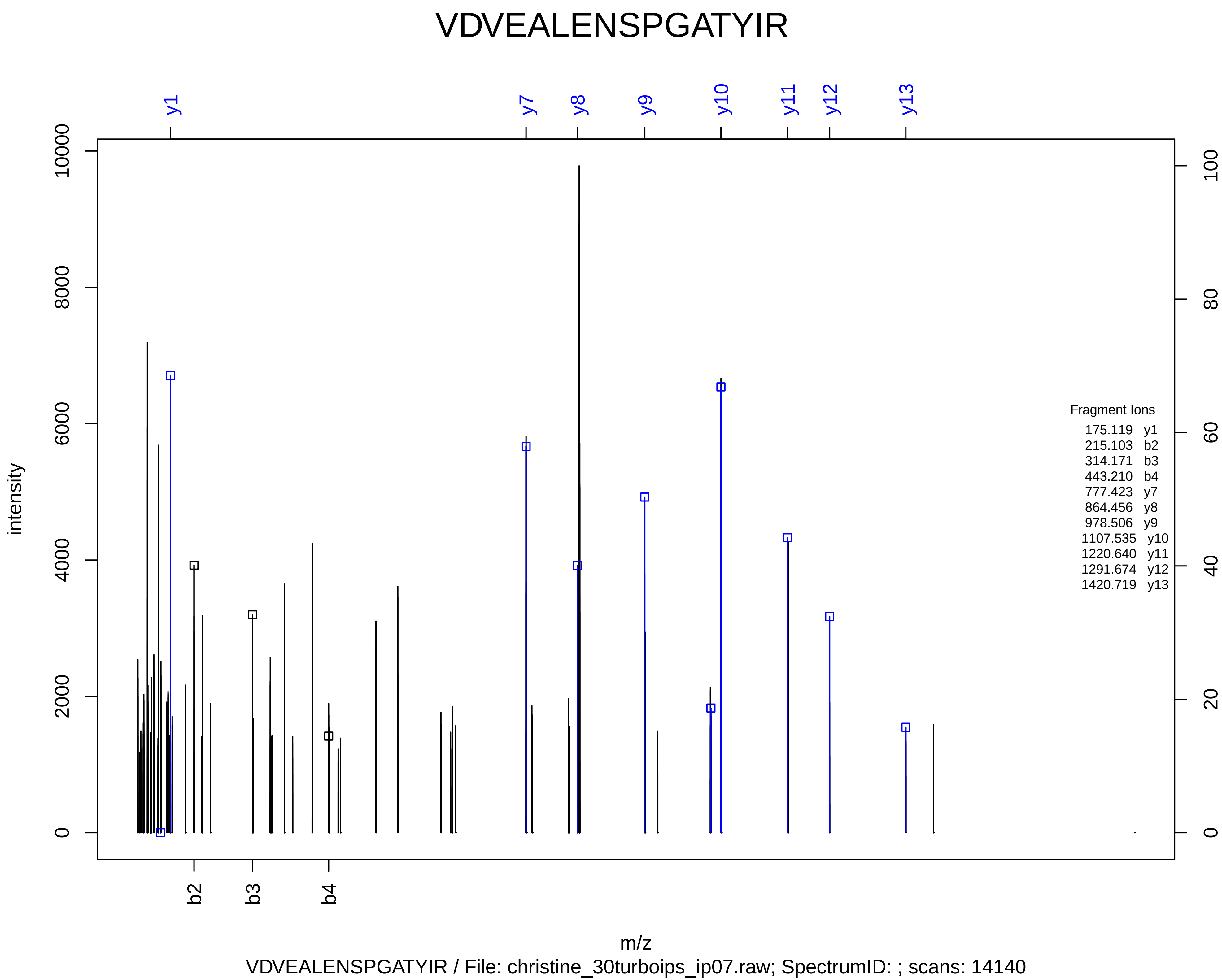
VDVEALENSPGATYIR
intensity
m/z
y1
y7
y8
y9
y10
y11
y12
y13
b2
b3
b4
0
2000
4000
6000
8000
10000
20
40
60
80
100
Fragment Ions
175.119 y1
215.103 b2
314.171 b3
443.210 b4
777.423 y7
864.456 y8
978.506 y9
1107.535 y10
1220.640 y11
1291.674 y12
1420.719 y13
VDVEALENSPGATYIR / File: christine_30turboips_ip07.raw; SpectrumID: ; scans: 14140

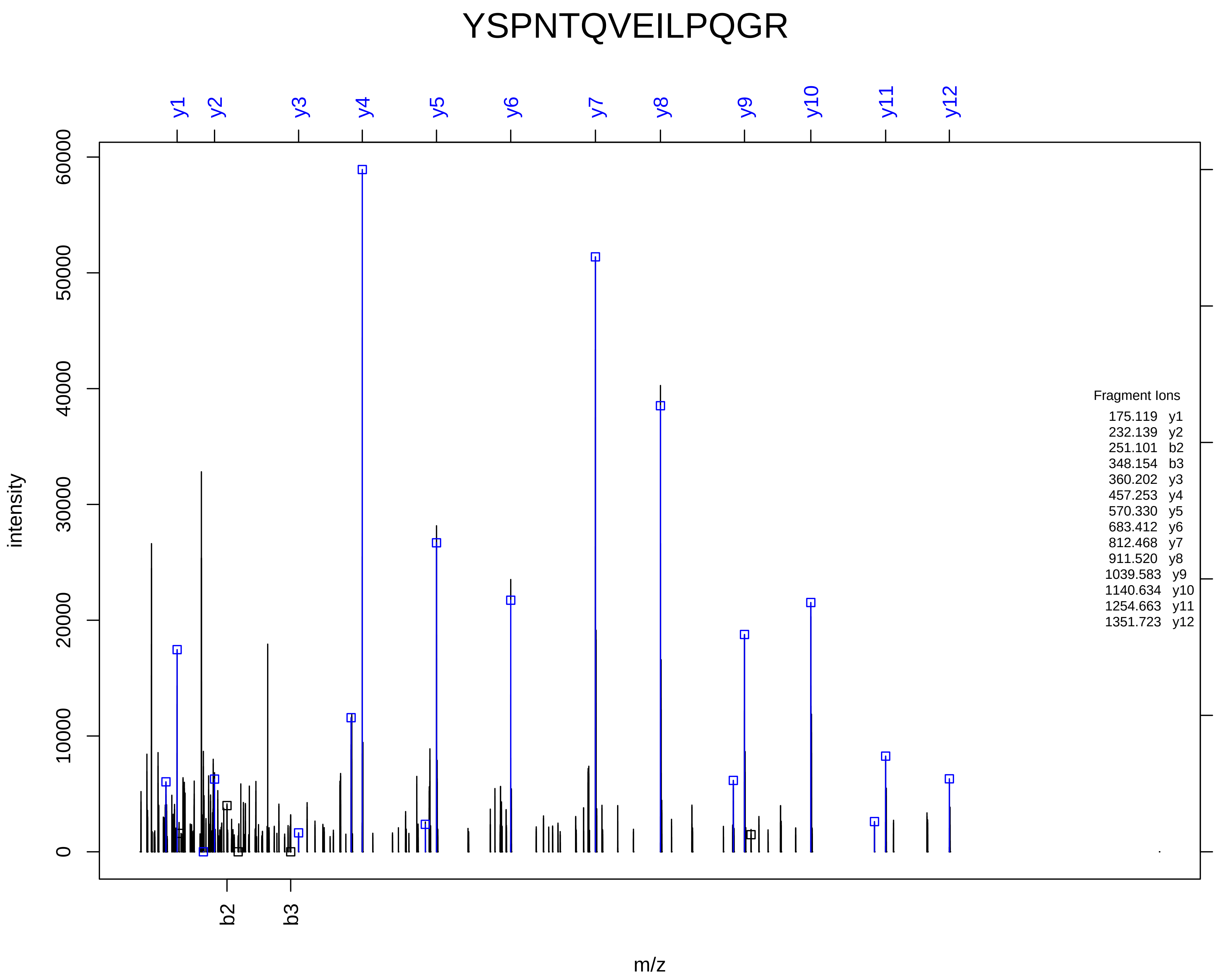
YSPNTQVEILPQGR
y1
y2
y3
y4
y5
y6
y7
y8
y9
y10
y11
y12
b2
b3
intensity
m/z
Fragment Ions
175.119 y1
232.139 y2
251.101 b2
348.154 b3
360.202 y3
457.253 y4
570.330 y5
683.412 y6
812.468 y7
911.520 y8
1039.583 y9
1140.634 y10
1254.663 y11
1351.723 y12
YSPNTQVEILPQGR / File: christine_30turboips_ip08.raw; SpectrumID: ; scans: 9173

VITNFNSAHDTDR

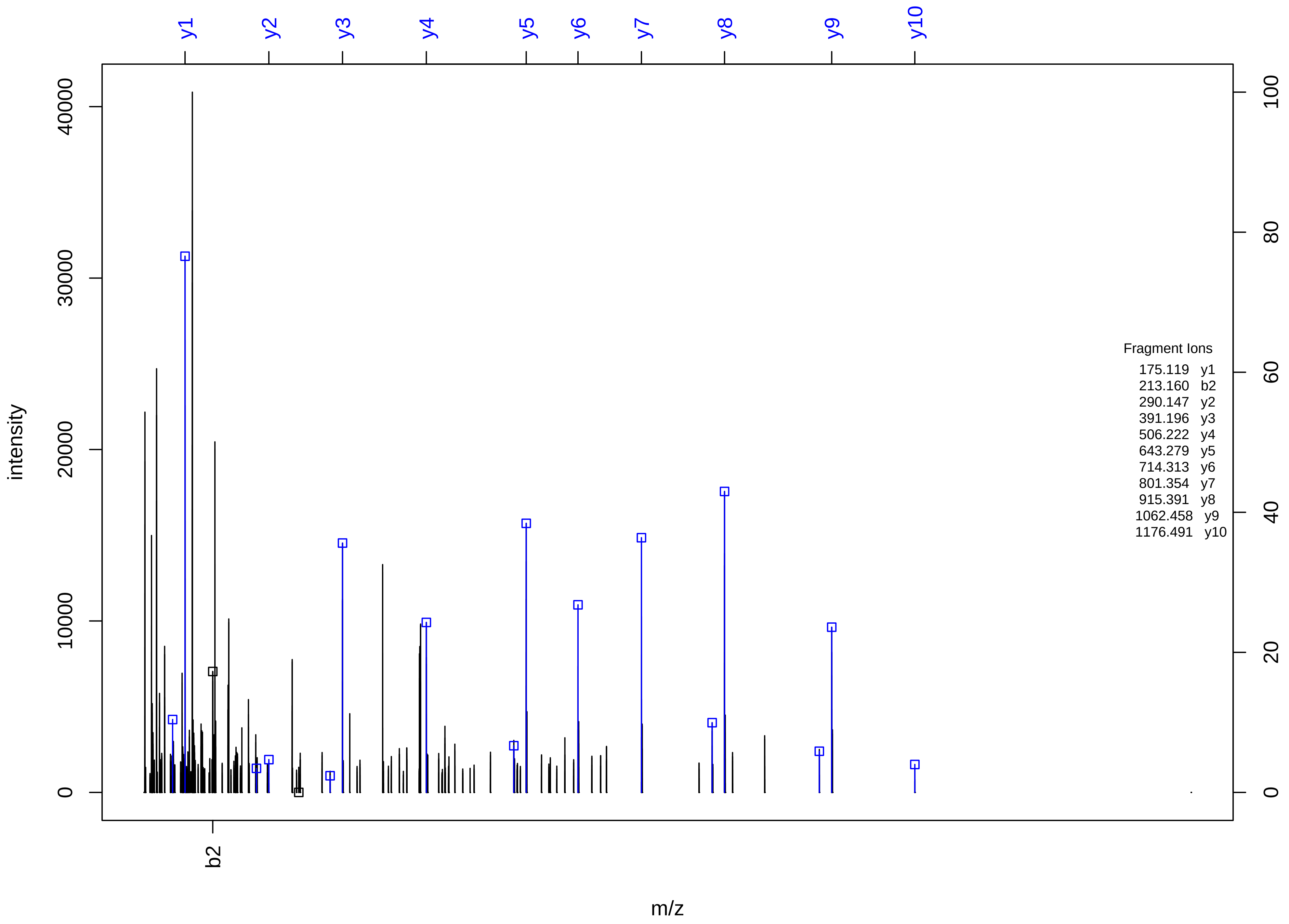

VITNFNSAHDTDR / File: christine_30turboips_ip08.raw; SpectrumID: ; scans: 1398

FVIGGPQGDAGLTGR

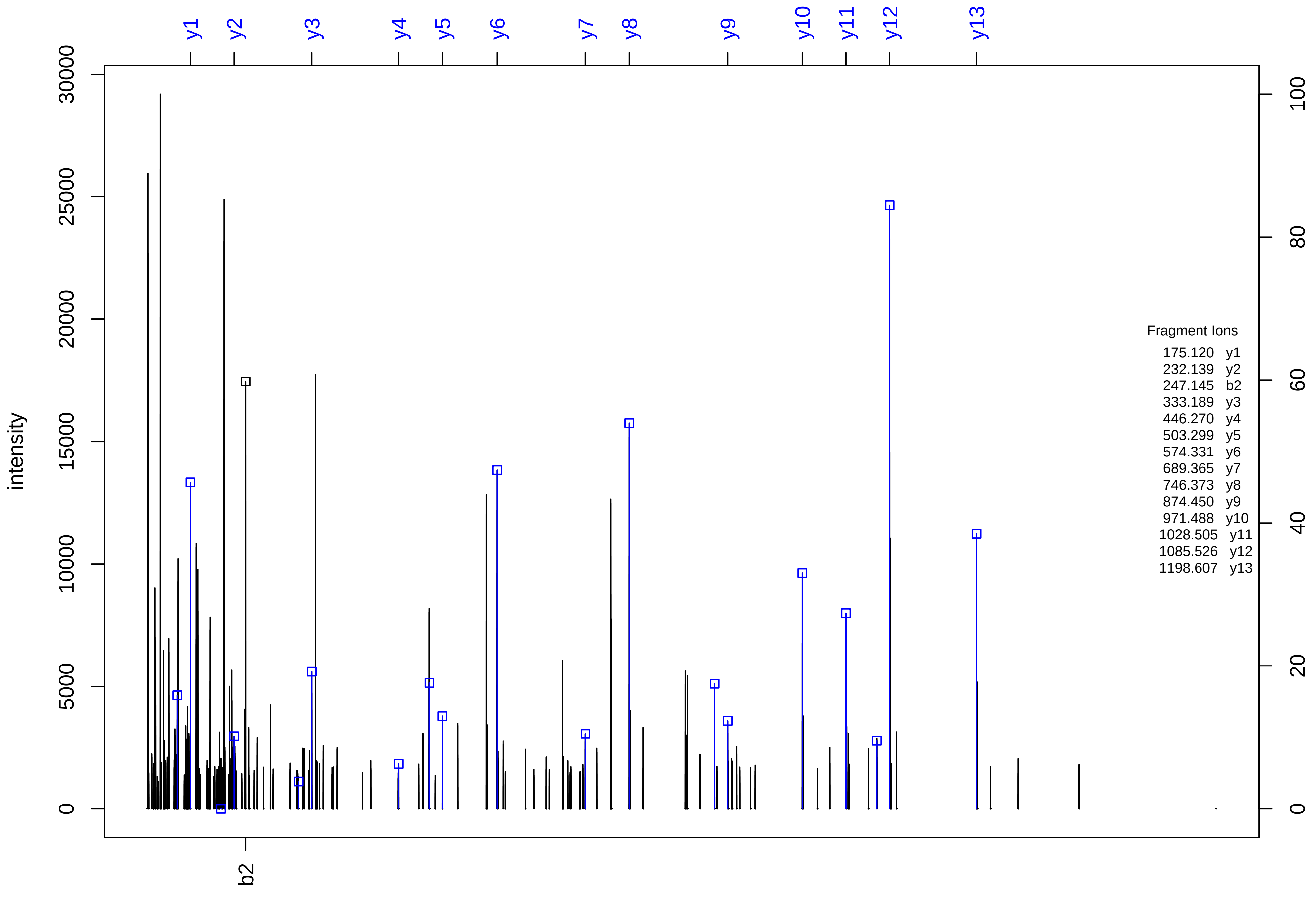

FVIGGPQGDAGLTGR / File: christine_30turboips_ip08.raw; SpectrumID: ; scans: 10833

YPSAEM*MLIPM*KGEFSR

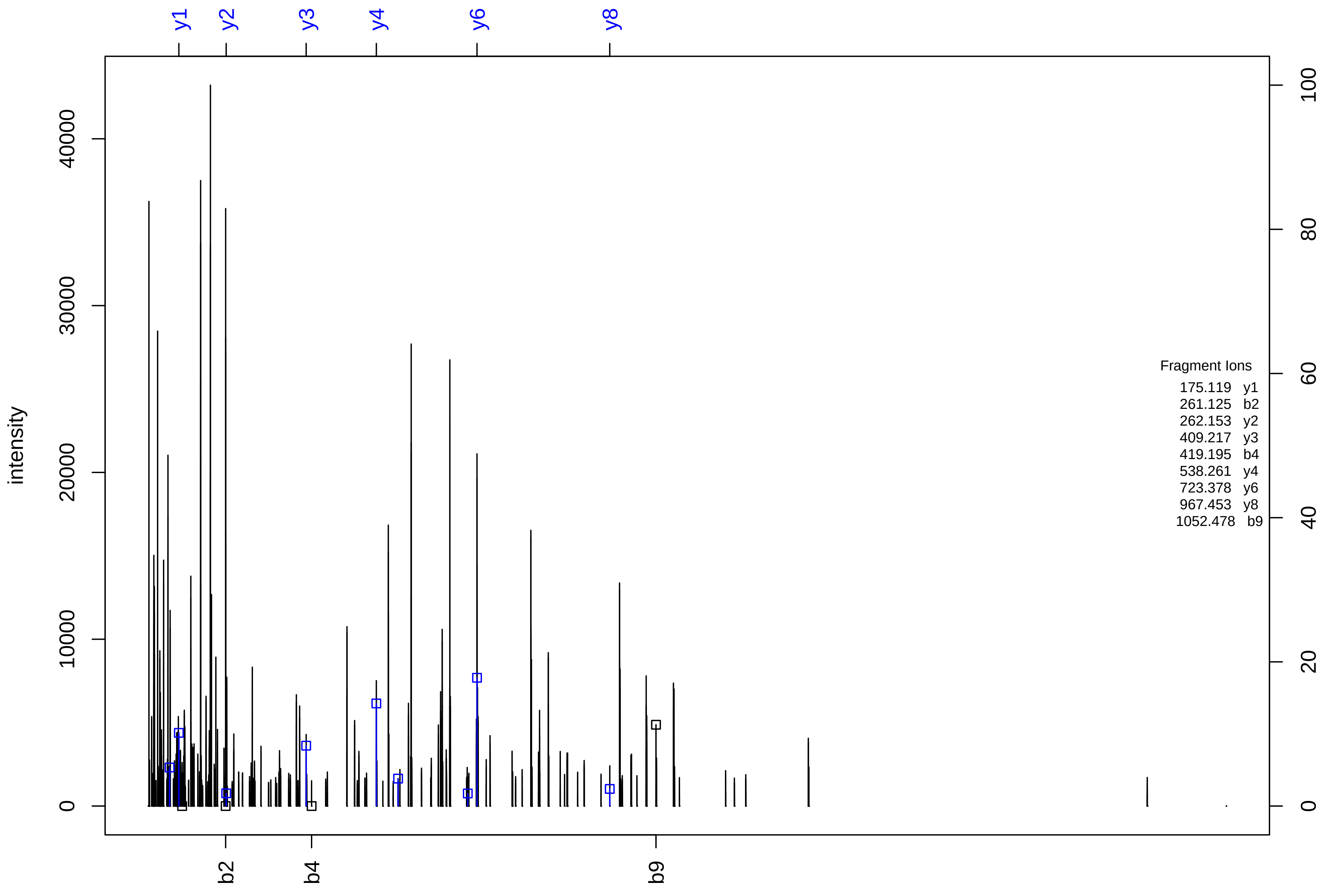

YPSAEMMLIPMKGEFSR / File: christine_30turboips_ip08.raw; SpectrumID: ; scans: 18251

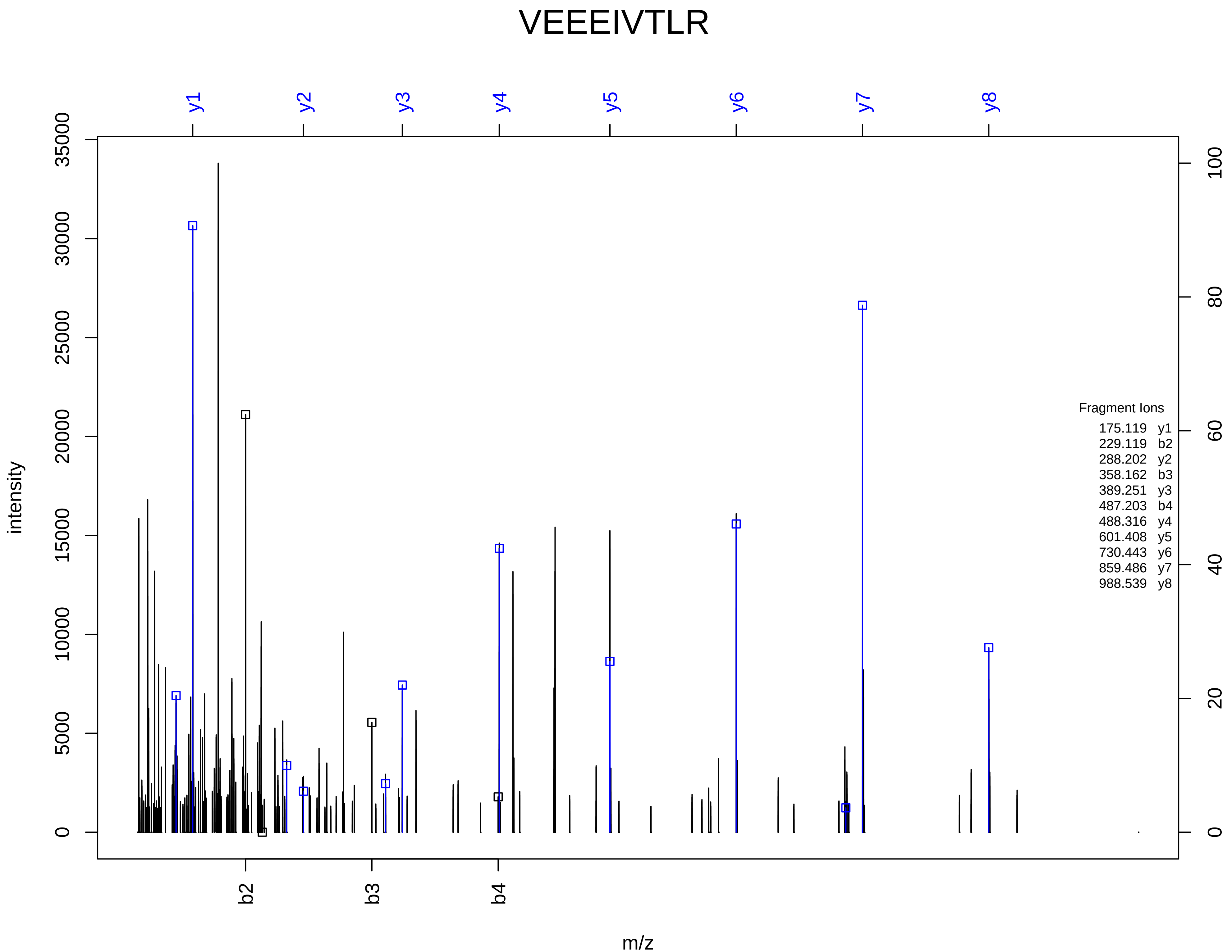

VEEEIVTLR / File: christine_30turboips_ip08.raw; SpectrumID: ; scans: 7806

(Ac)M*DLGASRNTSR

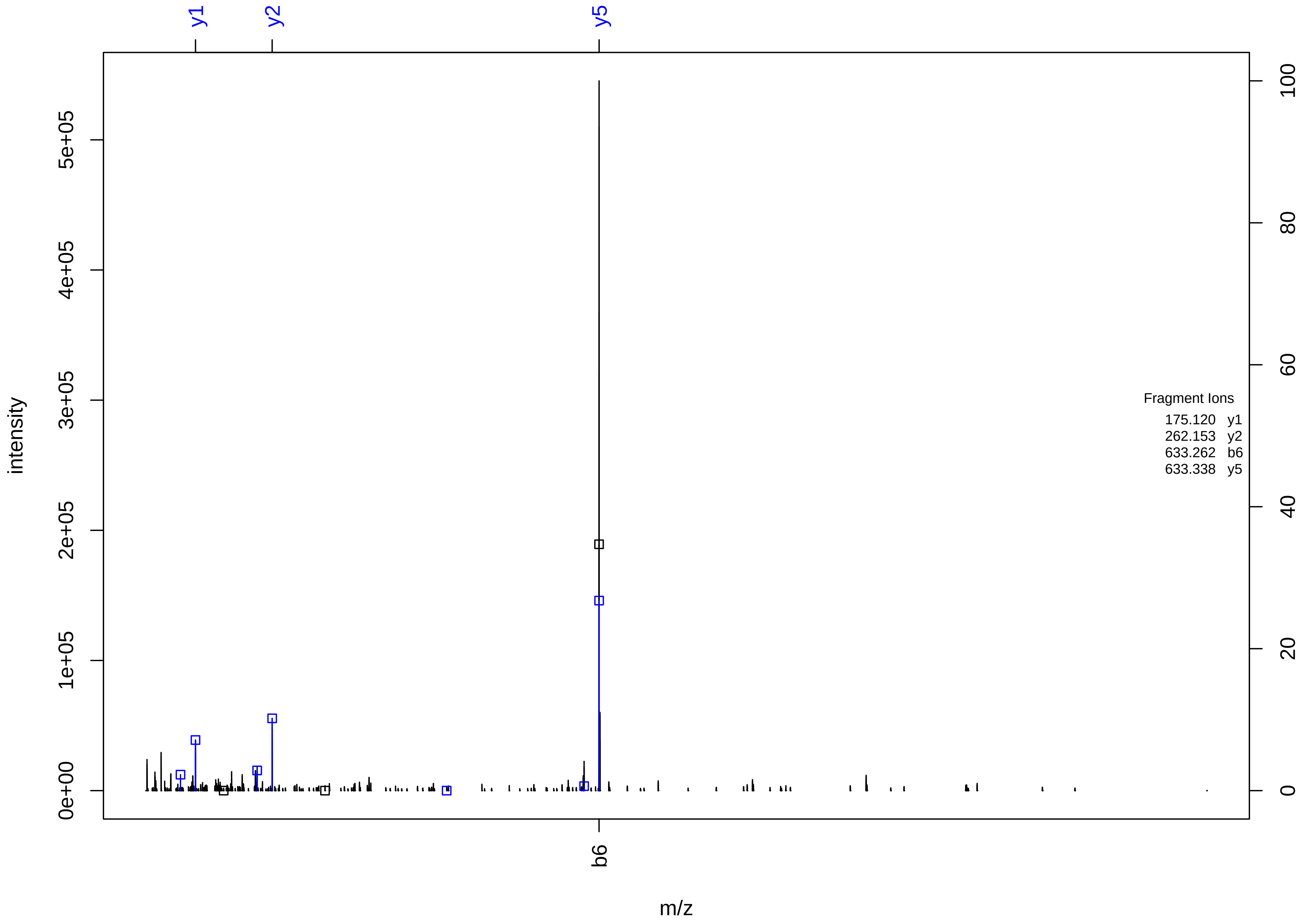

MDLGASRNTSR / File: christine_30turboips_ip08.raw; SpectrumID: ; scans: 9290

IQFN^AHIWTK

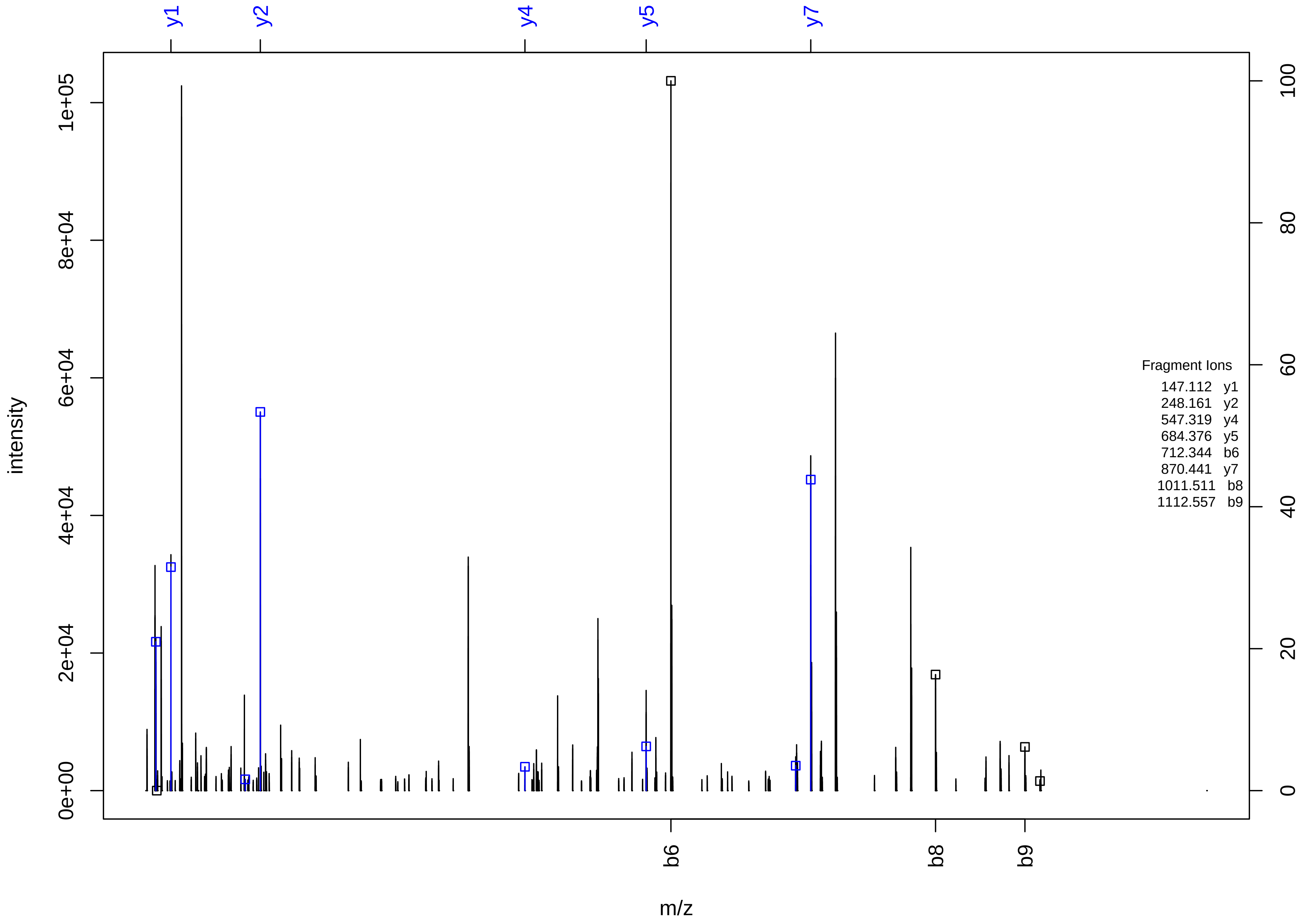

IQFNAHIWTK / File: christine_30turboips_ip09.raw; SpectrumID: ; scans: 1759

QLSSGVSEIR

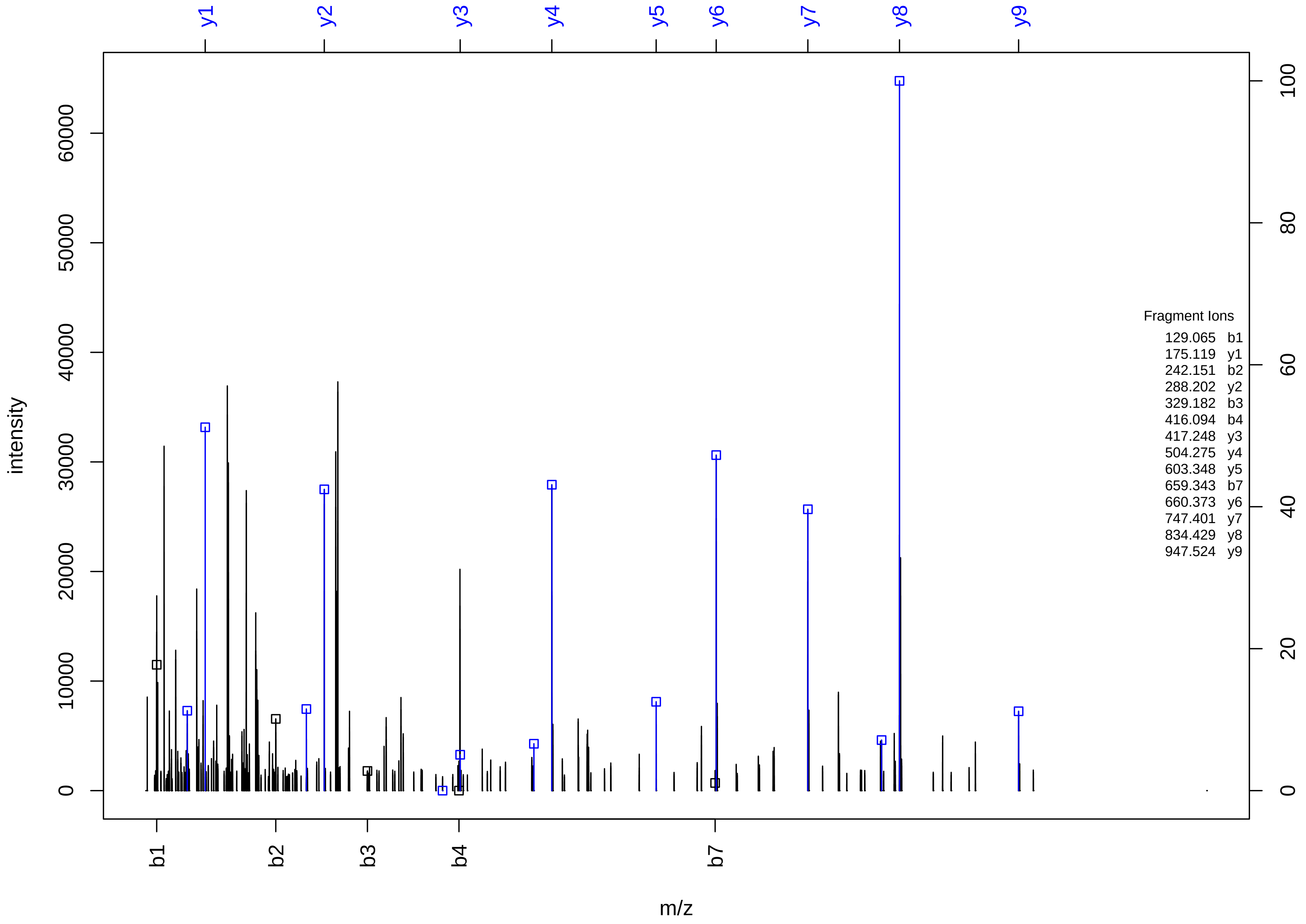

QLSSGVSEIR / File: christine_30turboips_ip09.raw; SpectrumID: ; scans: 2021

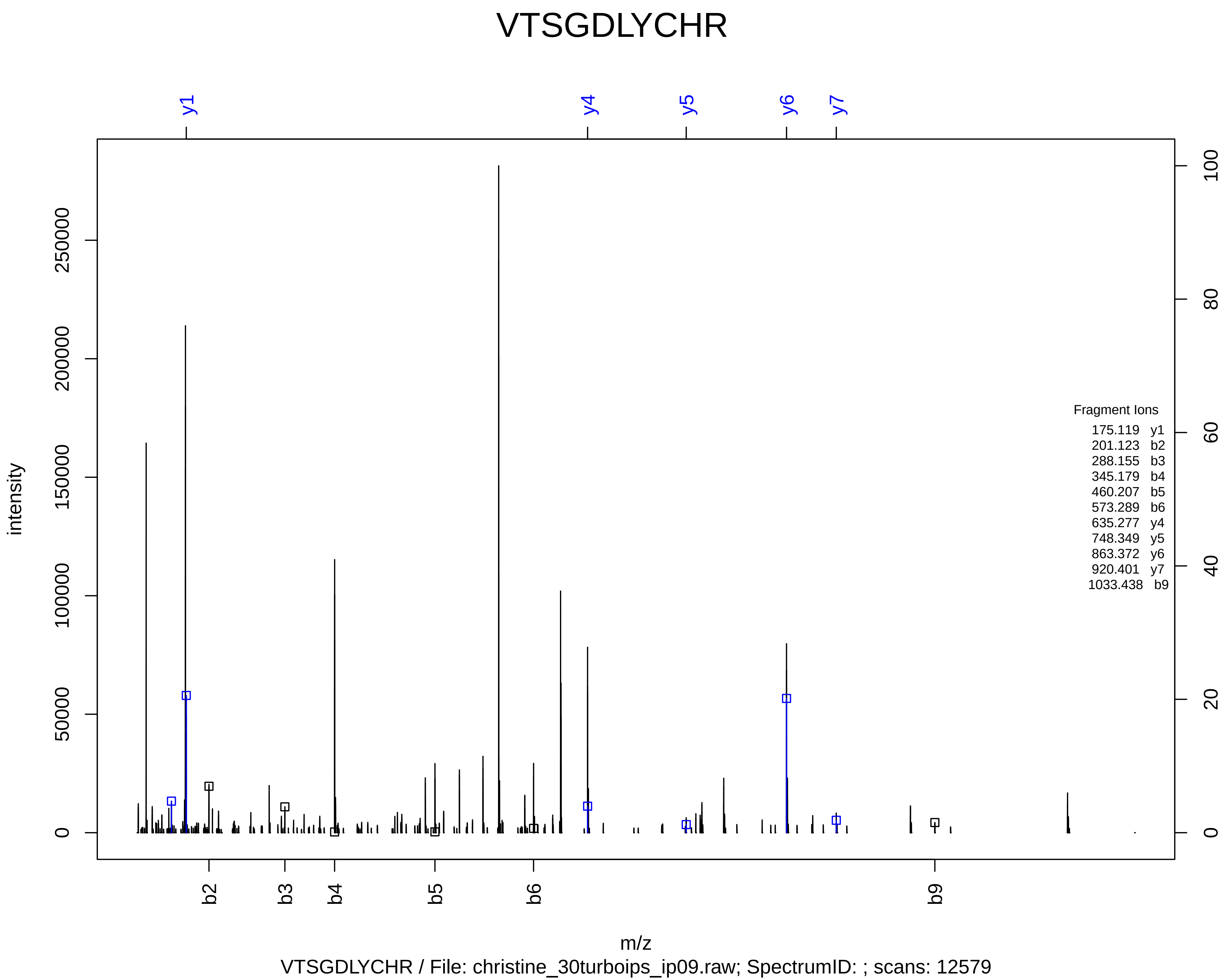

VTSGDLYCHR
y1
y4
y5
y6
y7
intensity
0
50000
100000
150000
200000
250000
0
20
40
60
80
100
b2
b3
b4
b5
b6
b9
m/z
Fragment Ions
175.119 y1
201.123 b2
288.155 b3
345.179 b4
460.207 b5
573.289 b6
635.277 y4
748.349 y5
863.372 y6
920.401 y7
1033.438 b9
VTSGDLYCHR / File: christine_30turboips_ip09.raw; SpectrumID: ; scans: 12579

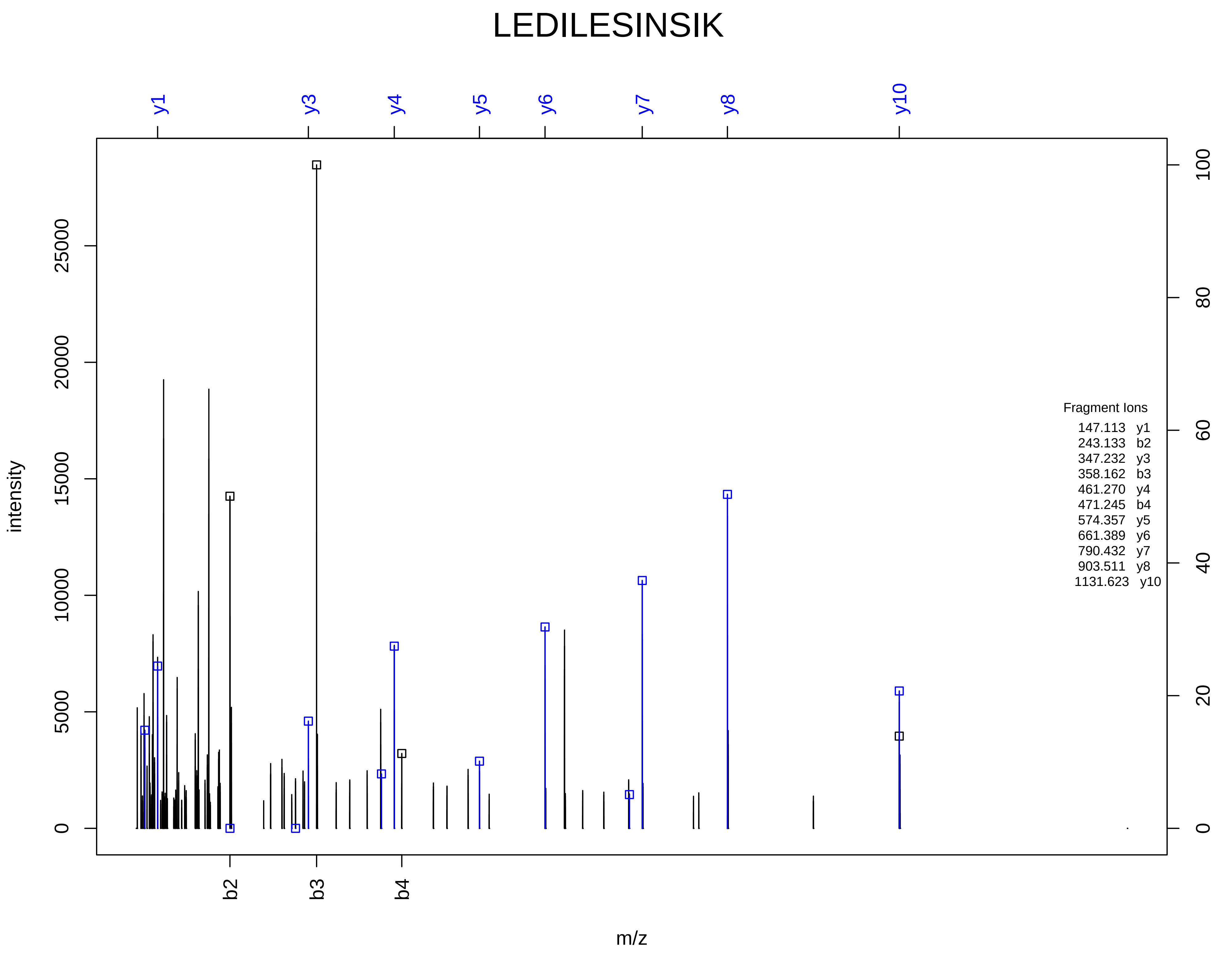
LEDILESINSIK
y1
y3
y4
y5
y6
y7
y8
y10
intensity
0
5000
10000
15000
20000
25000
0
20
40
60
80
100
b2
b3
b4
m/z
Fragment Ions
147.113 y1
243.133 b2
347.232 y3
358.162 b3
461.270 y4
471.245 b4
574.357 y5
661.389 y6
790.432 y7
903.511 y8
1131.623 y10
LEDILESINSIK / File: christine_30turboips_ip09.raw; SpectrumID: ; scans: 21105

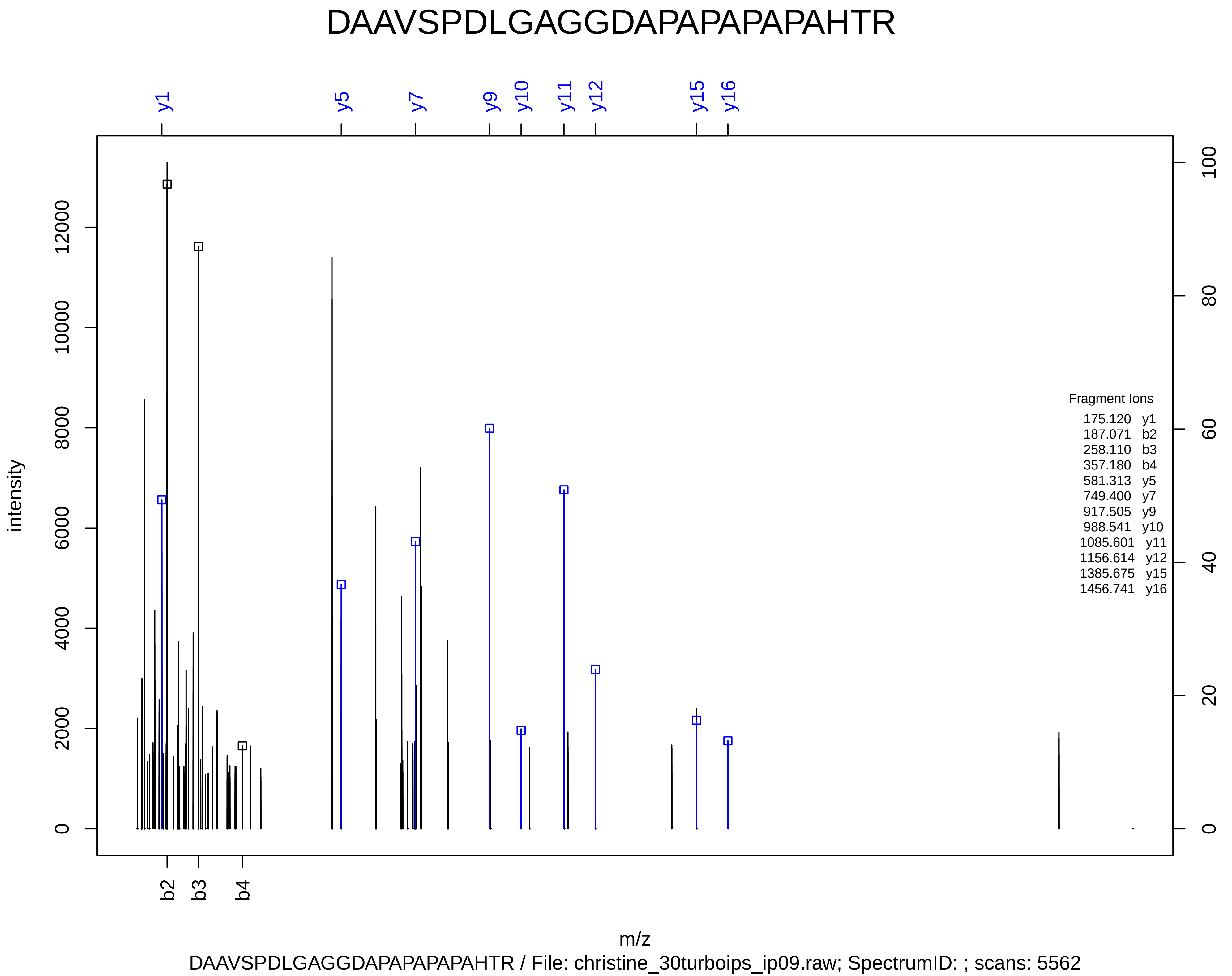
DAAVSPDLGAGGDAPAPAPAPAHTR
y1
y5
y7
y9
y10
y11
y12
y15
y16
b2
b3
b4
intensity
0
2000
4000
6000
8000
10000
12000
0
20
40
60
80
100
Fragment Ions
175.120 y1
187.071 b2
258.110 b3
357.180 b4
581.313 y5
749.400 y7
917.505 y9
988.541 y10
1085.601 y11
1156.614 y12
1385.675 y15
1456.741 y16
m/z
DAAVSPDLGAGGDAPAPAPAPAHTR / File: christine_30turboips_ip09.raw; SpectrumID: ; scans: 5562

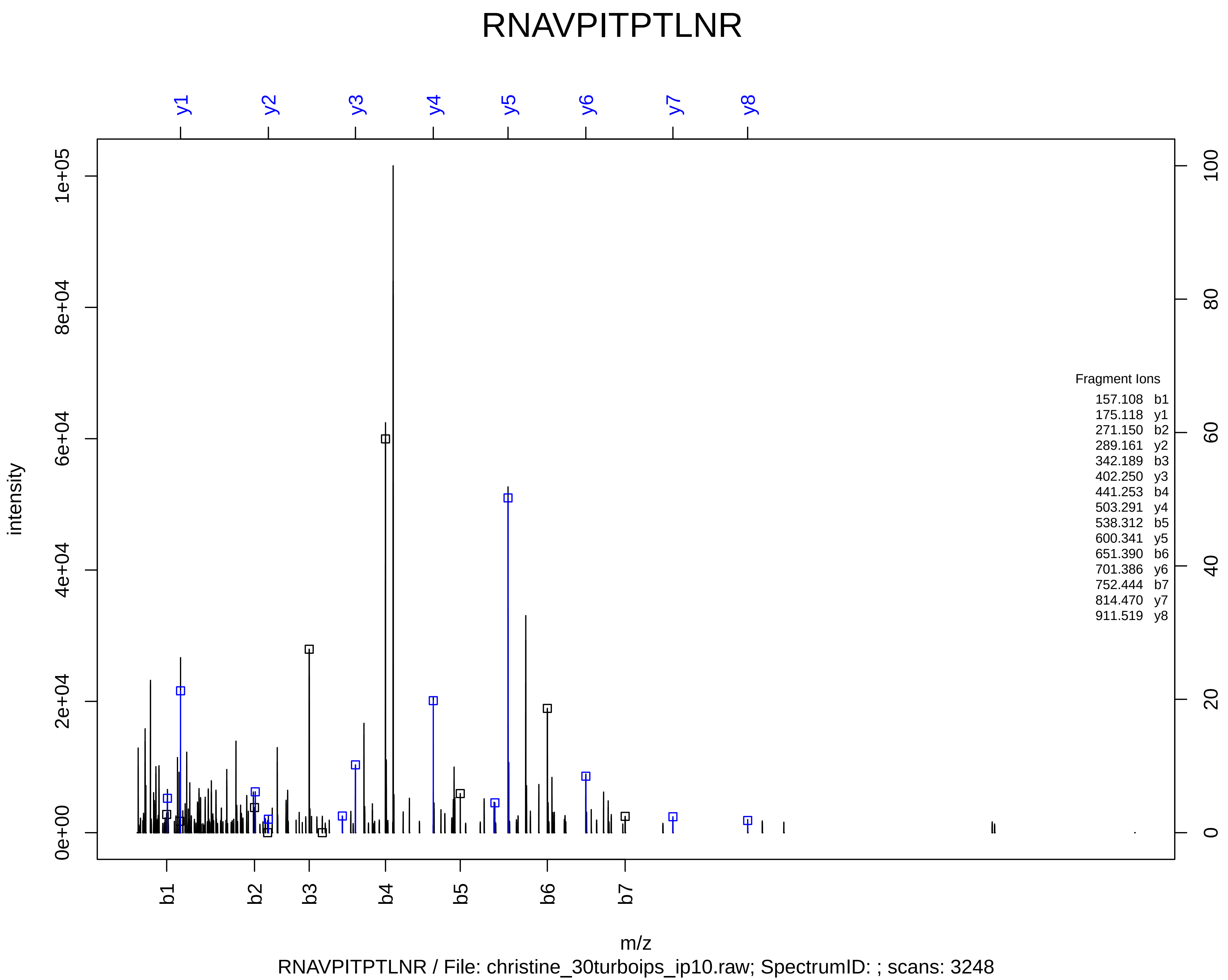

RNAVPITPTLNR
y1
y2
y3
y4
y5
y6
y7
y8
intensity
0e+00
2e+04
4e+04
6e+04
8e+04
1e+05
0
20
40
60
80
100
b1
b2
b3
b4
b5
b6
b7
m/z
Fragment Ions
157.108 b1
175.118 y1
271.150 b2
289.161 y2
342.189 b3
402.250 y3
441.253 b4
503.291 y4
538.312 b5
600.341 y5
651.390 b6
701.386 y6
752.444 b7
814.470 y7
911.519 y8
RNAVPITPTLNR / File: christine_30turboips_ip10.raw; SpectrumID: ; scans: 3248

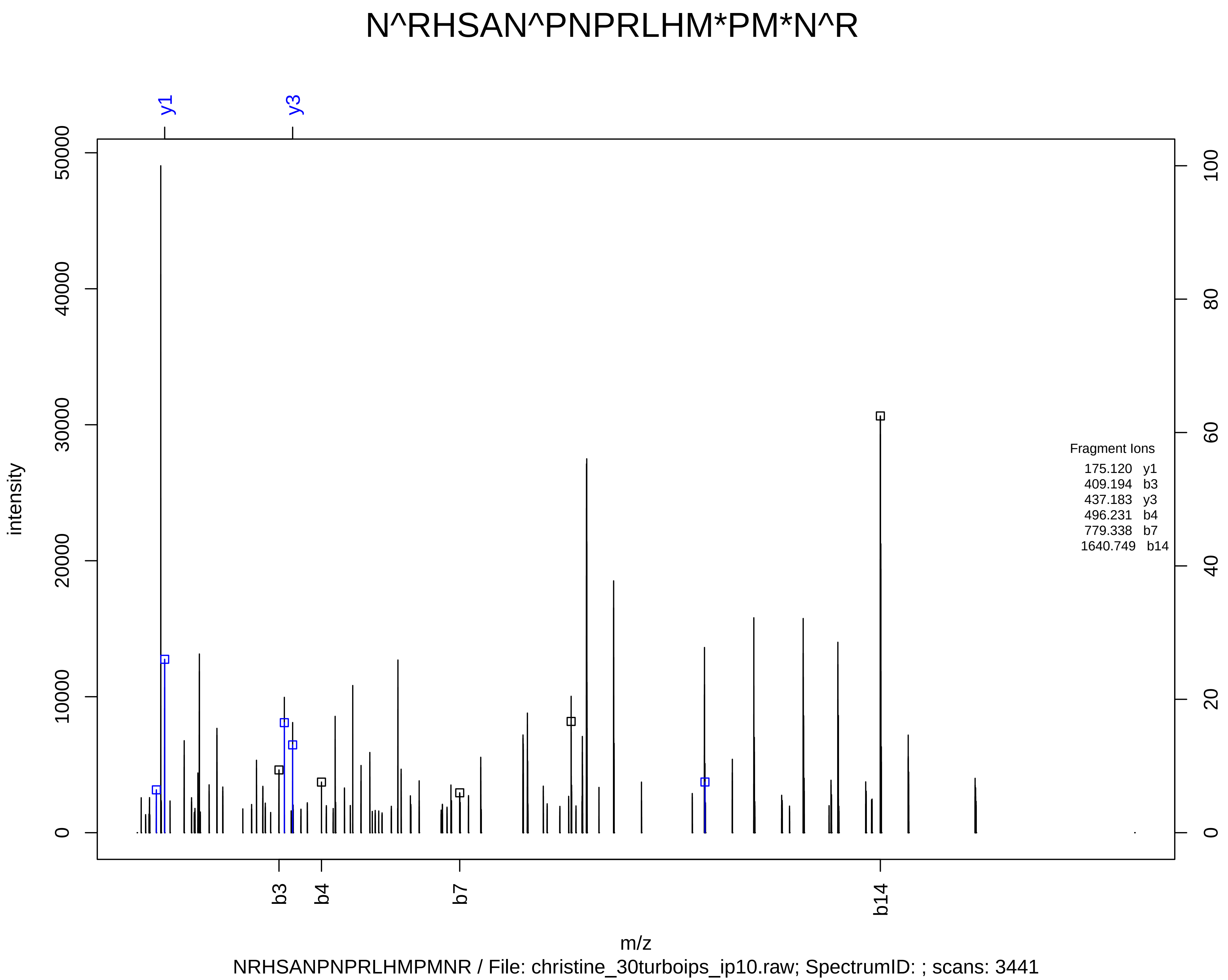

N^RHSAN^PNPRLHM*PM*N^R
y1
y3
intensity
50000
40000
30000
20000
10000
0
100
80
60
40
20
0
Fragment Ions
175.120 y1
409.194 b3
437.183 y3
496.231 b4
779.338 b7
1640.749 b14
b3
b4
b7
b14
m/z
NRHSANPNPRLHMPMNR / File: christine_30turboips_ip10.raw; SpectrumID: ; scans: 3441

NVLSLLHQR

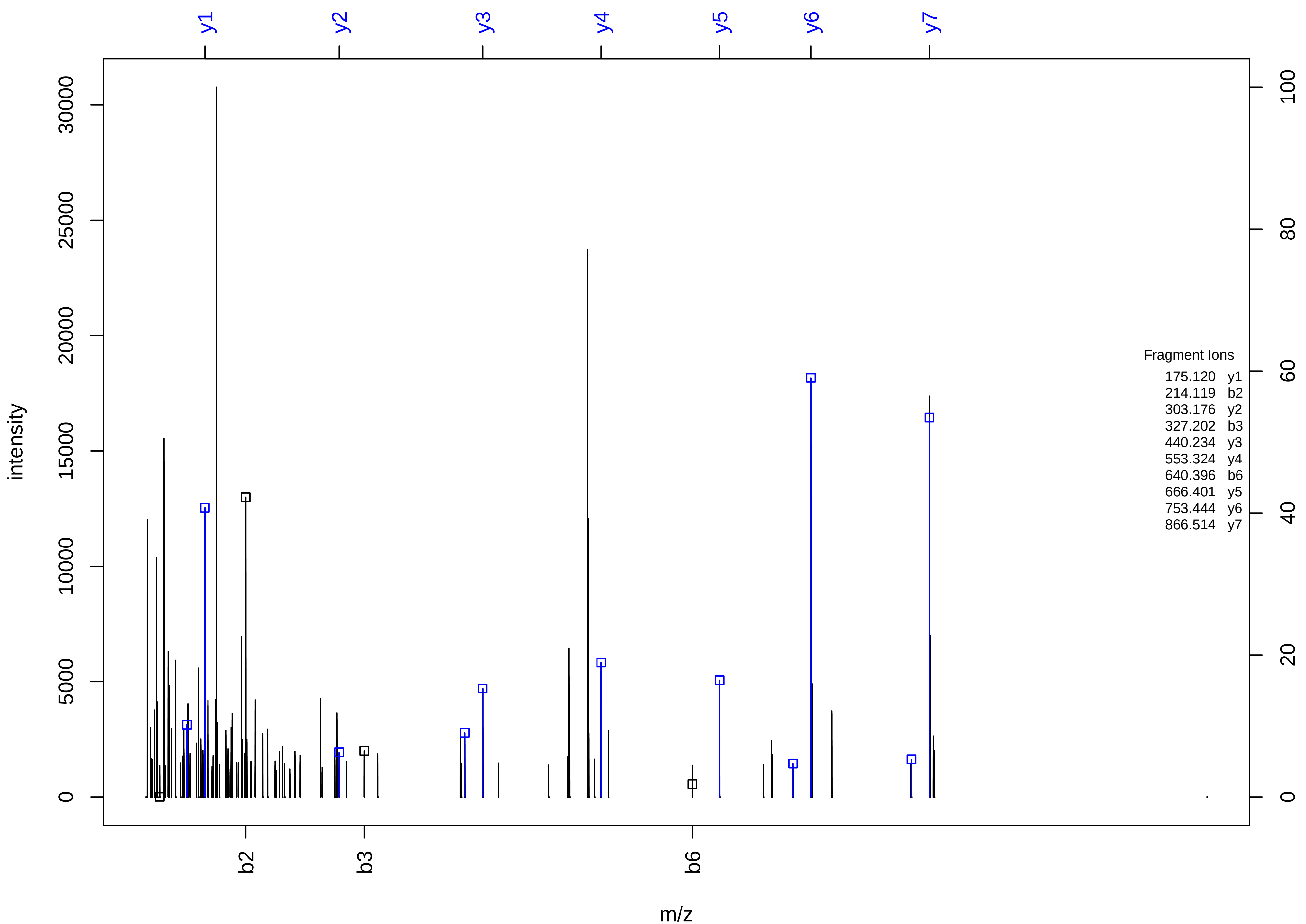

NVLSLLHQR / File: christine_30turboips_ip10.raw; SpectrumID: ; scans: 8862

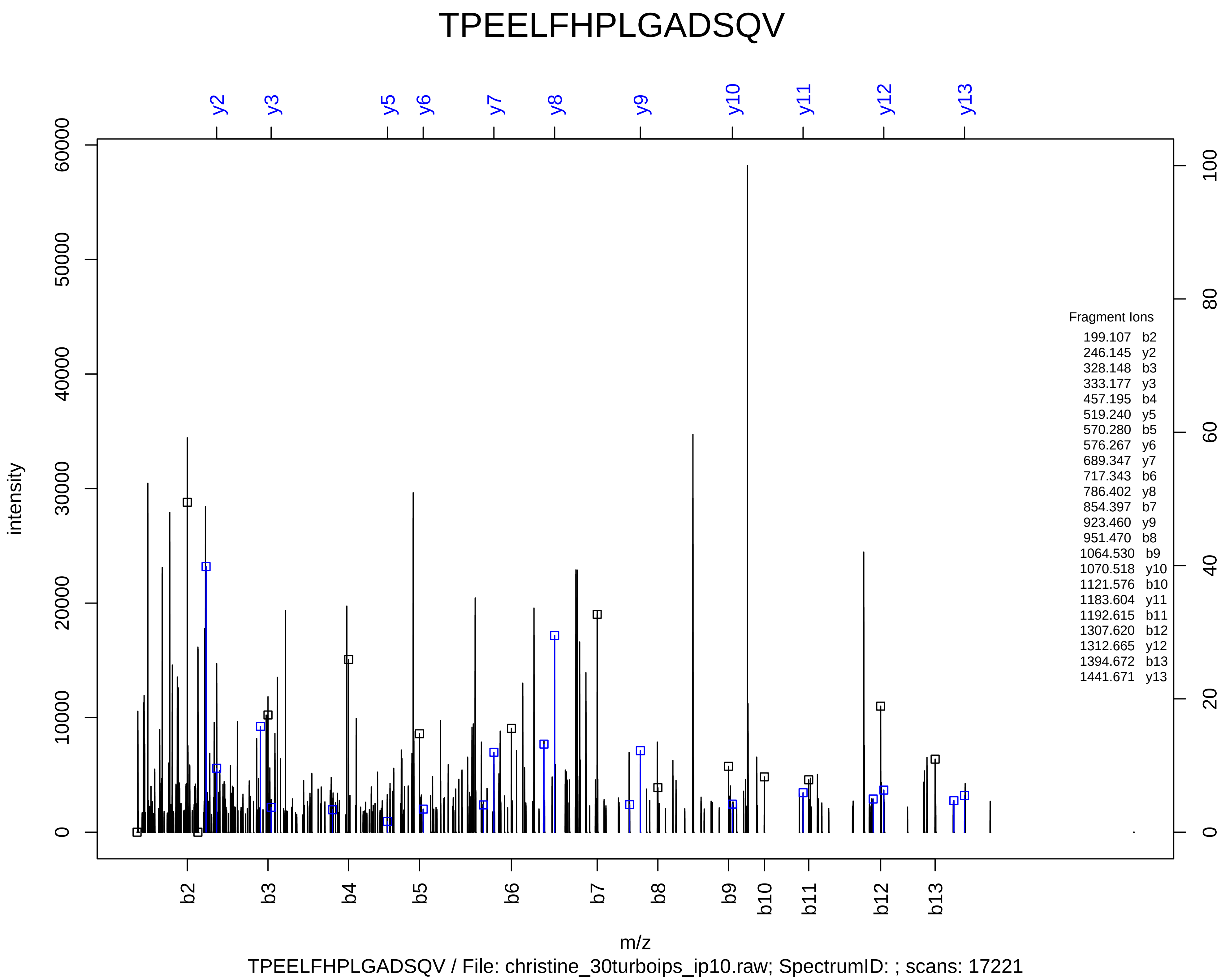
TPEELFHPLGADSQV
y2
y3
y5
y6
y7
y8
y9
y10
y11
y12
y13
intensity
0
10000
20000
30000
40000
50000
60000
0
20
40
60
80
100
Fragment Ions
199.107 b2
246.145 y2
328.148 b3
333.177 y3
457.195 b4
519.240 y5
570.280 b5
576.267 y6
689.347 y7
717.343 b6
786.402 y8
854.397 b7
923.460 y9
951.470 b8
1064.530 b9
1070.518 y10
1121.576 b10
1183.604 y11
1192.615 b11
1307.620 b12
1312.665 y12
1394.672 b13
1441.671 y13
b2
b3
b4
b5
b6
b7
b8
b9
b10
b11
b12
b13
m/z
TPEELFHPLGADSQV / File: christine_30turboips_ip10.raw; SpectrumID: ; scans: 17221

VNPSGTVLFR

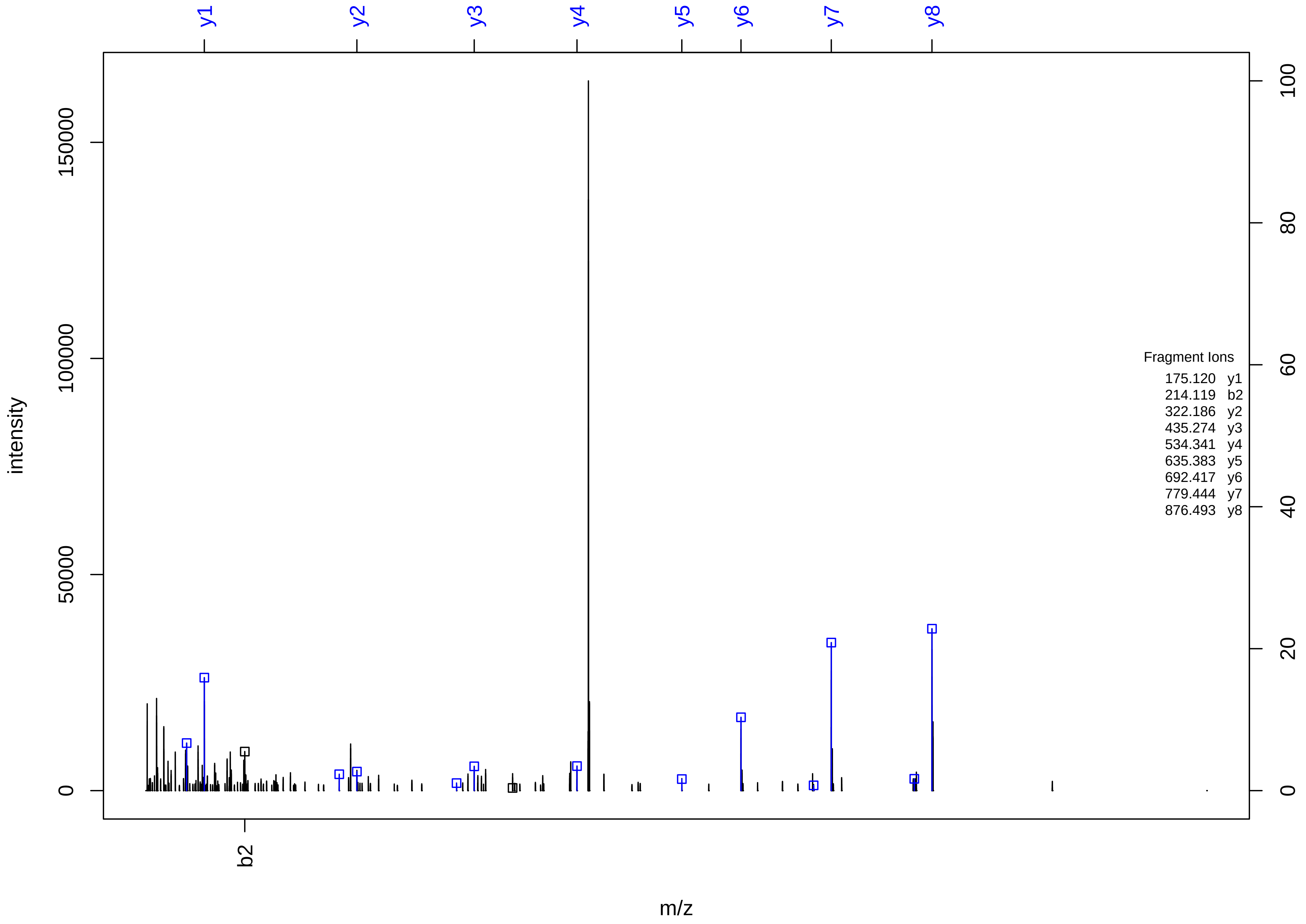

VNPSGTVLFR / File: christine_30turboips_ip10.raw; SpectrumID: ; scans: 7974

NQVIAPLTEELVFR

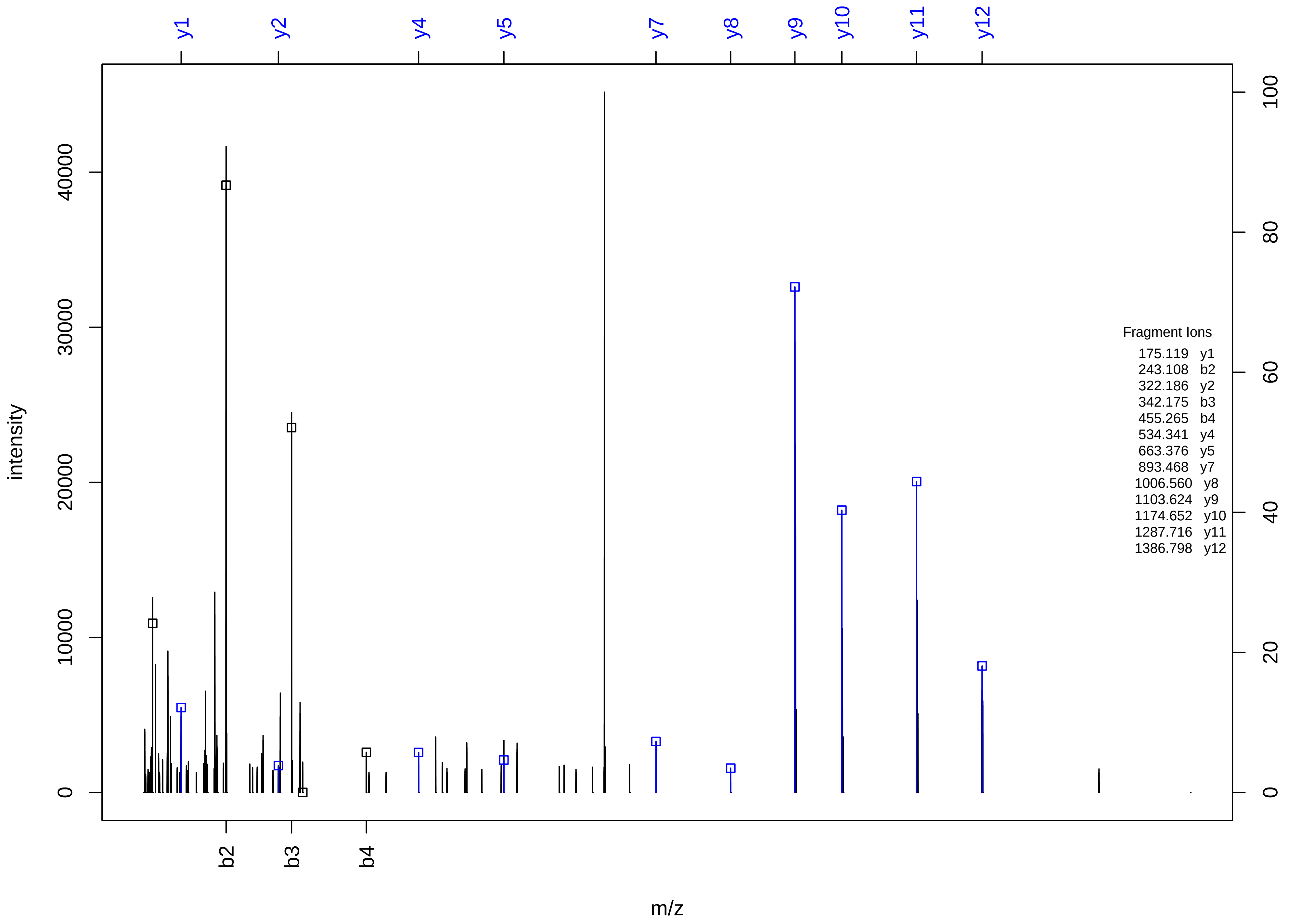

NQVIAPLTEELVFR / File: christine_30turboips_ip10.raw; SpectrumID: ; scans: 23941

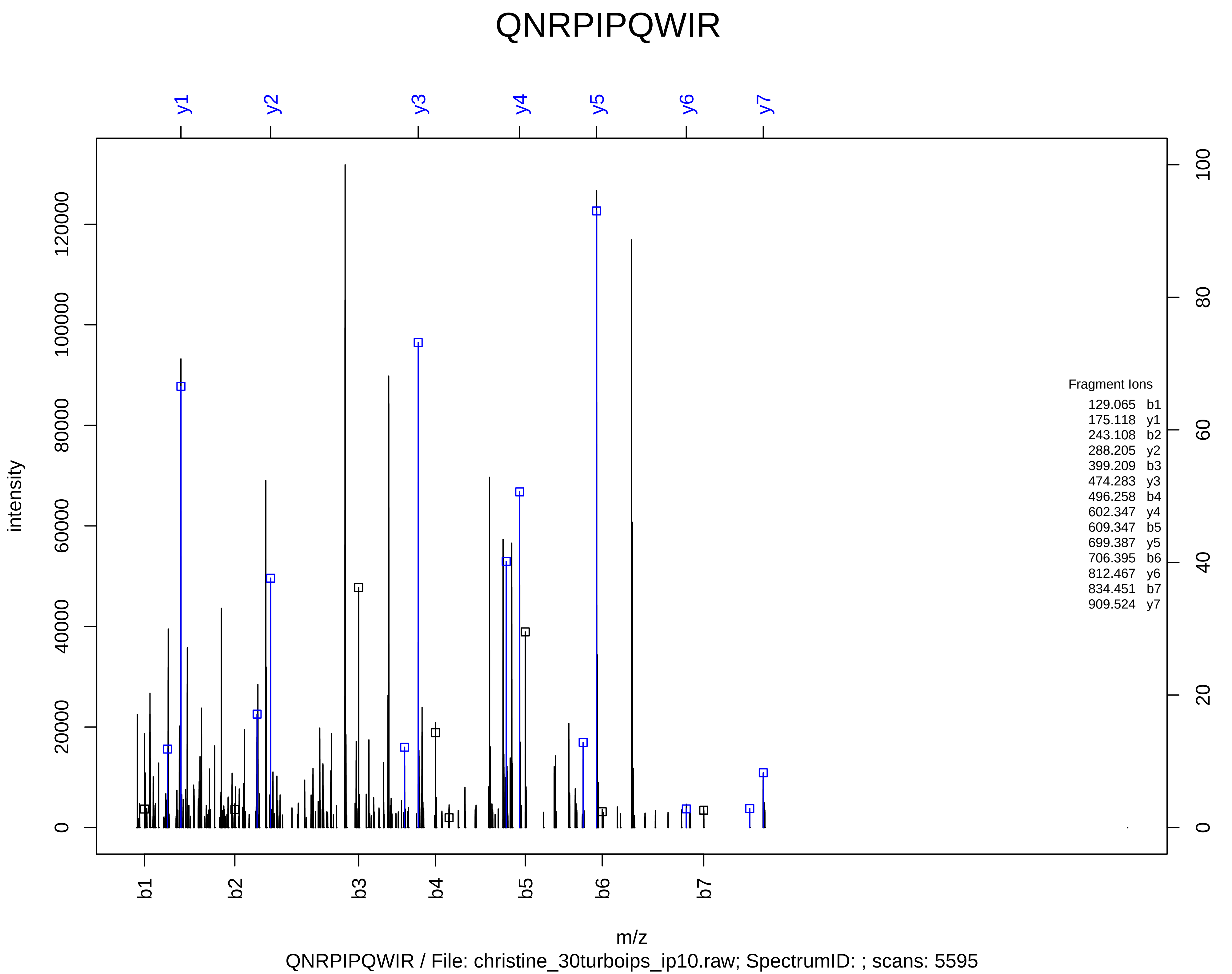
QNRPIPQWIR
y1
y2
y3
y4
y5
y6
y7
intensity
0
20000
40000
60000
80000
100000
120000
0
20
40
60
80
100
Fragment Ions
129.065 b1
175.118 y1
243.108 b2
288.205 y2
399.209 b3
474.283 y3
496.258 b4
602.347 y4
609.347 b5
699.387 y5
706.395 b6
812.467 y6
834.451 b7
909.524 y7
b1
b2
b3
b4
b5
b6
b7
m/z
QNRPIPQWIR / File: christine_30turboips_ip10.raw; SpectrumID: ; scans: 5595

ISSTLYQATAPVLTPAK

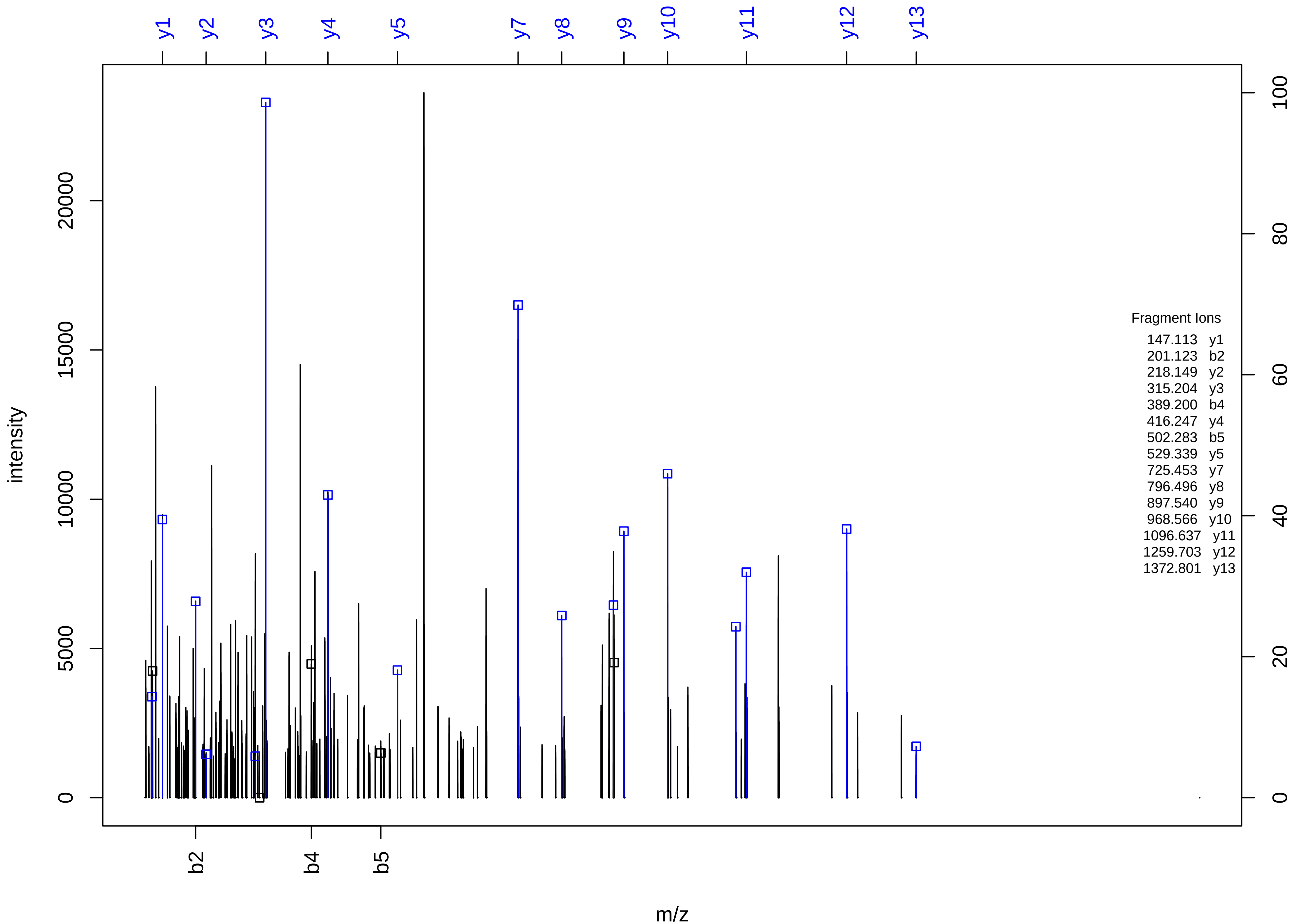

ISSTLYQATAPVLTPAK / File: christine_30turboips_ip10.raw; SpectrumID: ; scans: 14244

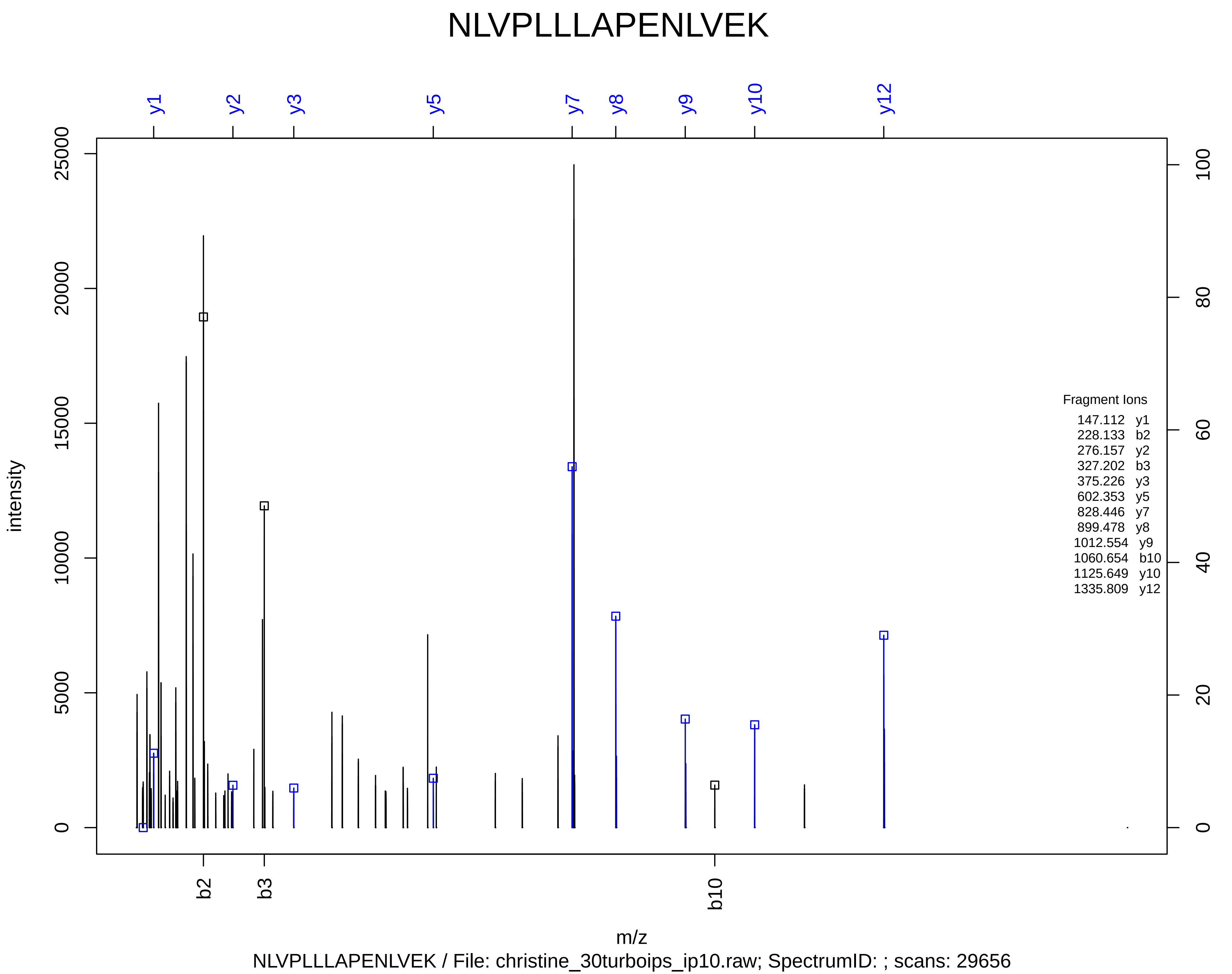

NLVPLLLAPENLVEK
y1
y2
y3
y5
y7
y8
y9
y10
y12
b2
b3
b10
intensity
m/z
Fragment Ions
147.112 y1
228.133 b2
276.157 y2
327.202 b3
375.226 y3
602.353 y5
828.446 y7
899.478 y8
1012.554 y9
1060.654 b10
1125.649 y10
1335.809 y12
NLVPLLLAPENLVEK / File: christine_30turboips_ip10.raw; SpectrumID: ; scans: 29656

LITALGSSEVQPQFTR

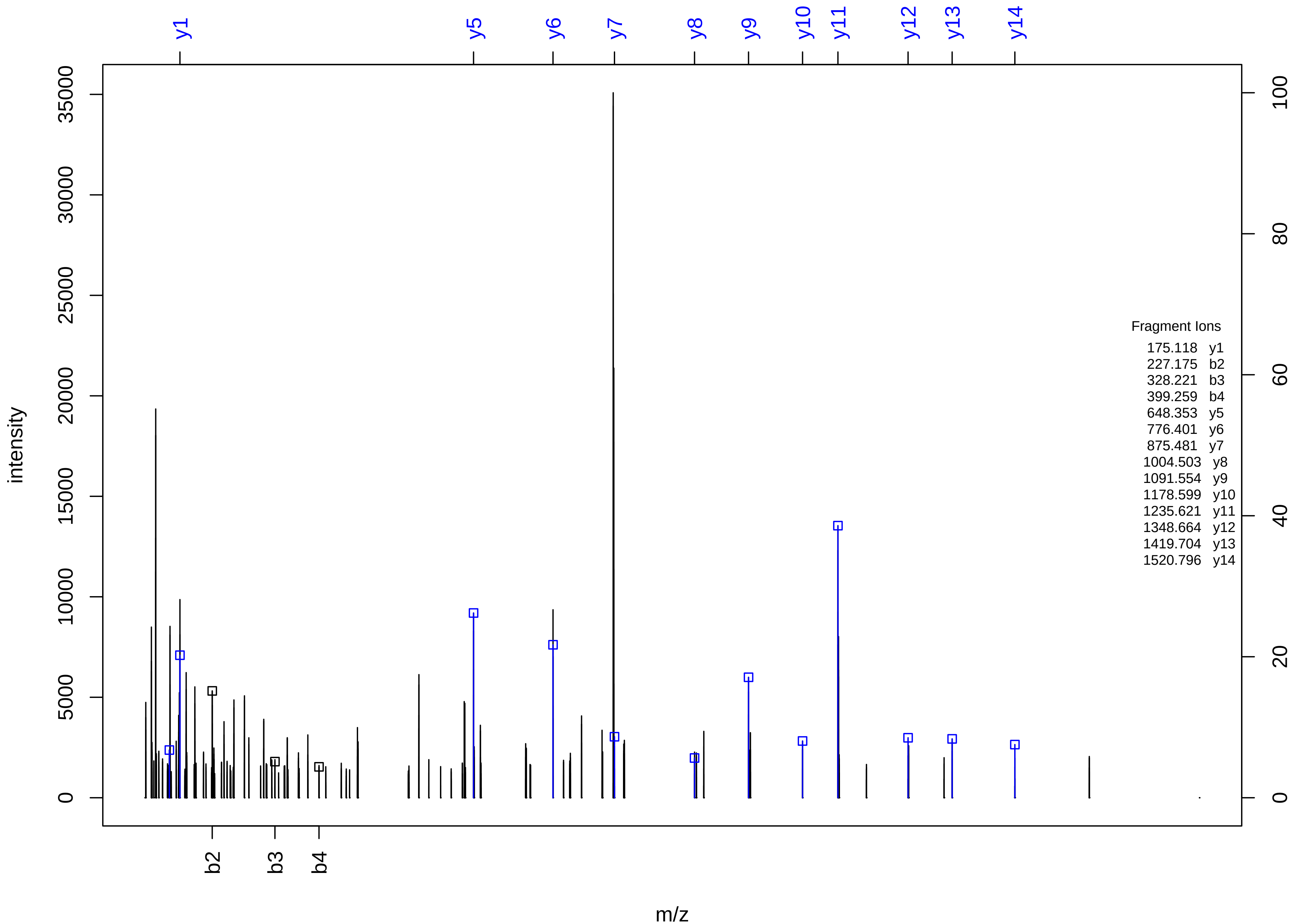

LITALGSSEVQPQFTR / File: christine_30turboips_ip10.raw; SpectrumID: ; scans: 14160

LNLGVSGDFIK

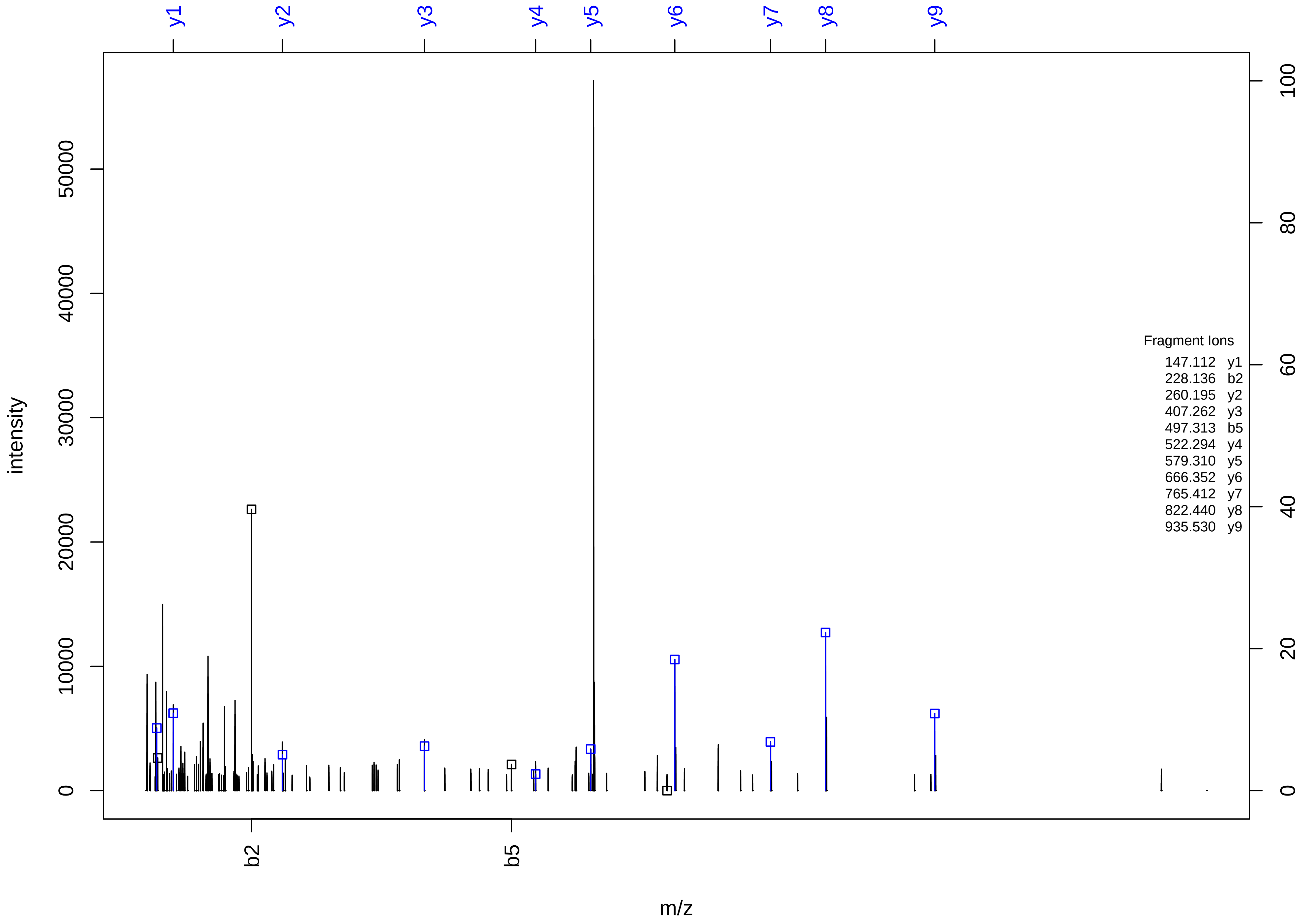

LNLGVSGDFIK / File: christine_30turboips_ip10.raw; SpectrumID: ; scans: 16157

LENTEDIEEVEQHIQTIR

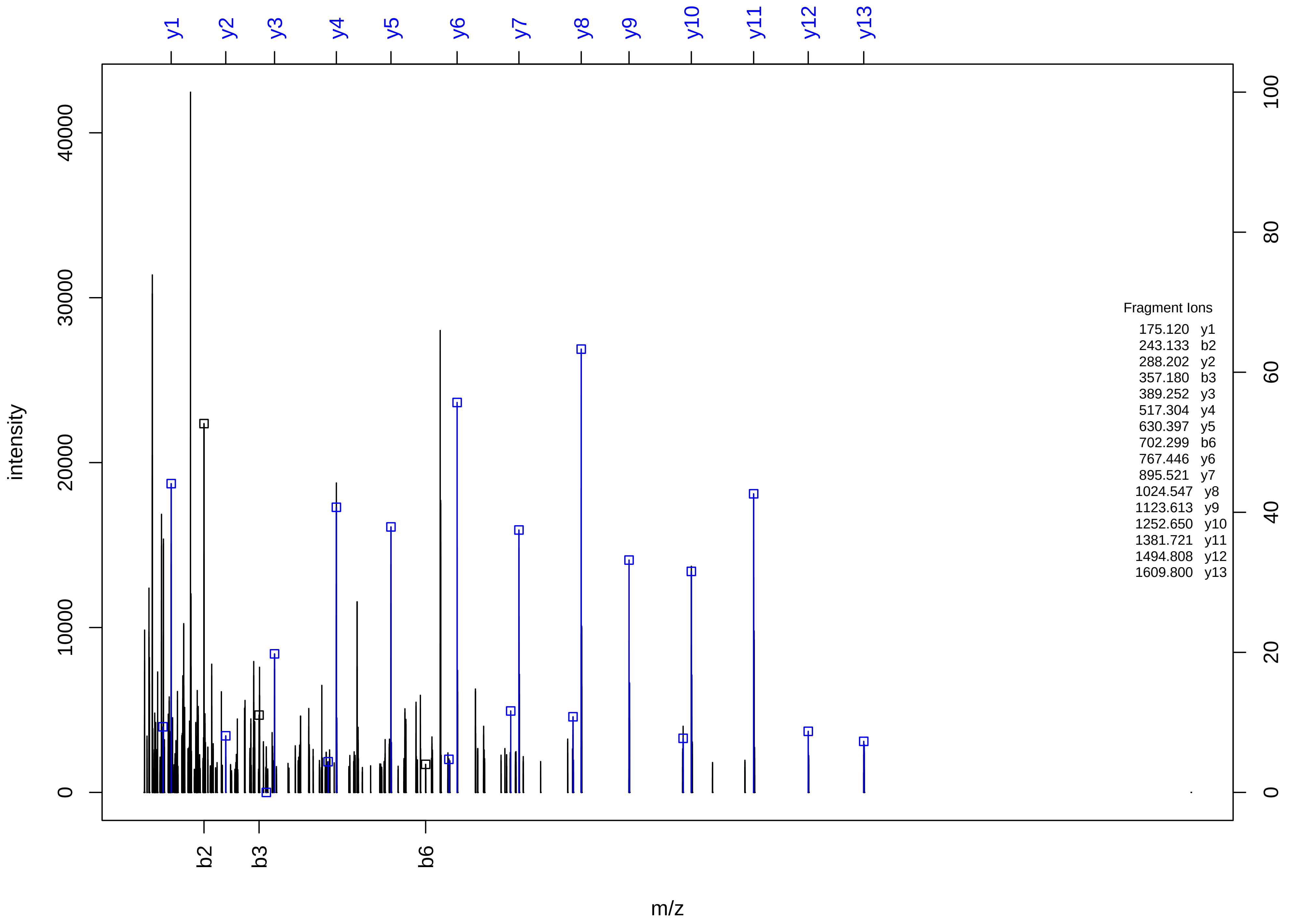

LENTEDIEEVEQHIQTIR / File: christine_30turboips_ip10.raw; SpectrumID: ; scans: 15468

AFDQGADAIYEHINEGK

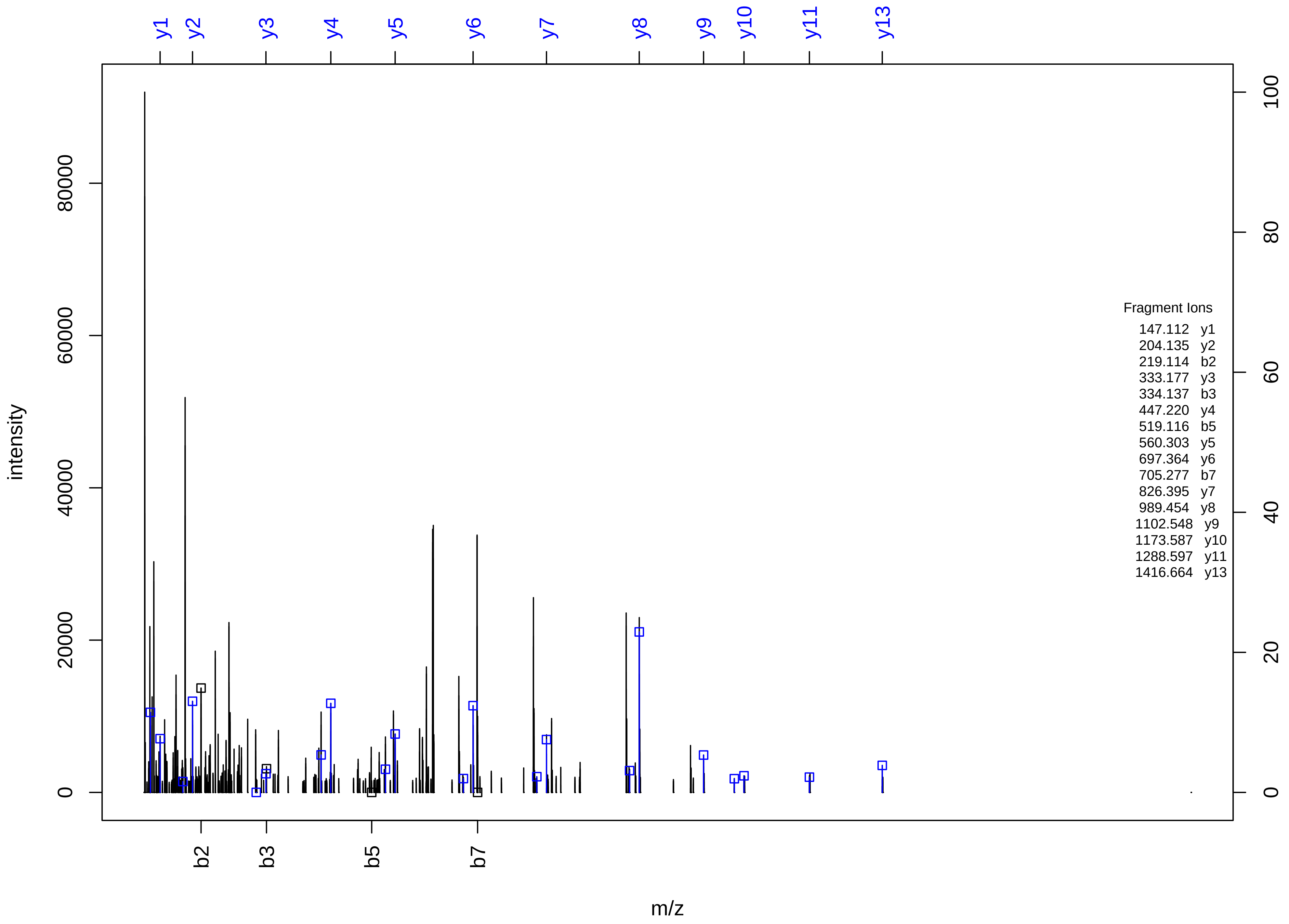

AFDQGADAIYEHINEGK / File: christine_30turboips_ip10.raw; SpectrumID: ; scans: 10739

TYLDM*N^R

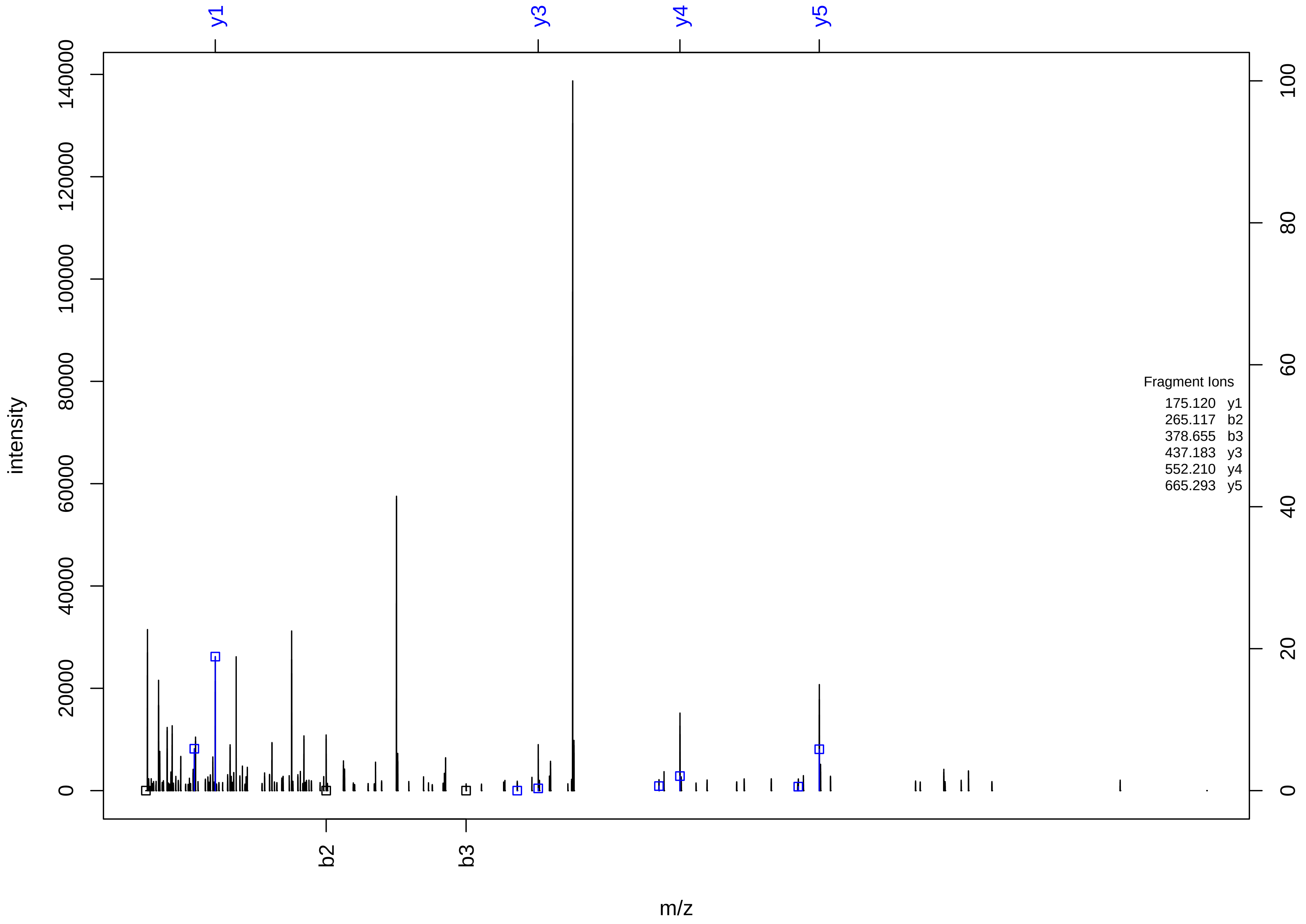

TYLDMNR / File: christine_30turboips_ip10.raw; SpectrumID: ; scans: 4858

AWDDFFPGSDR

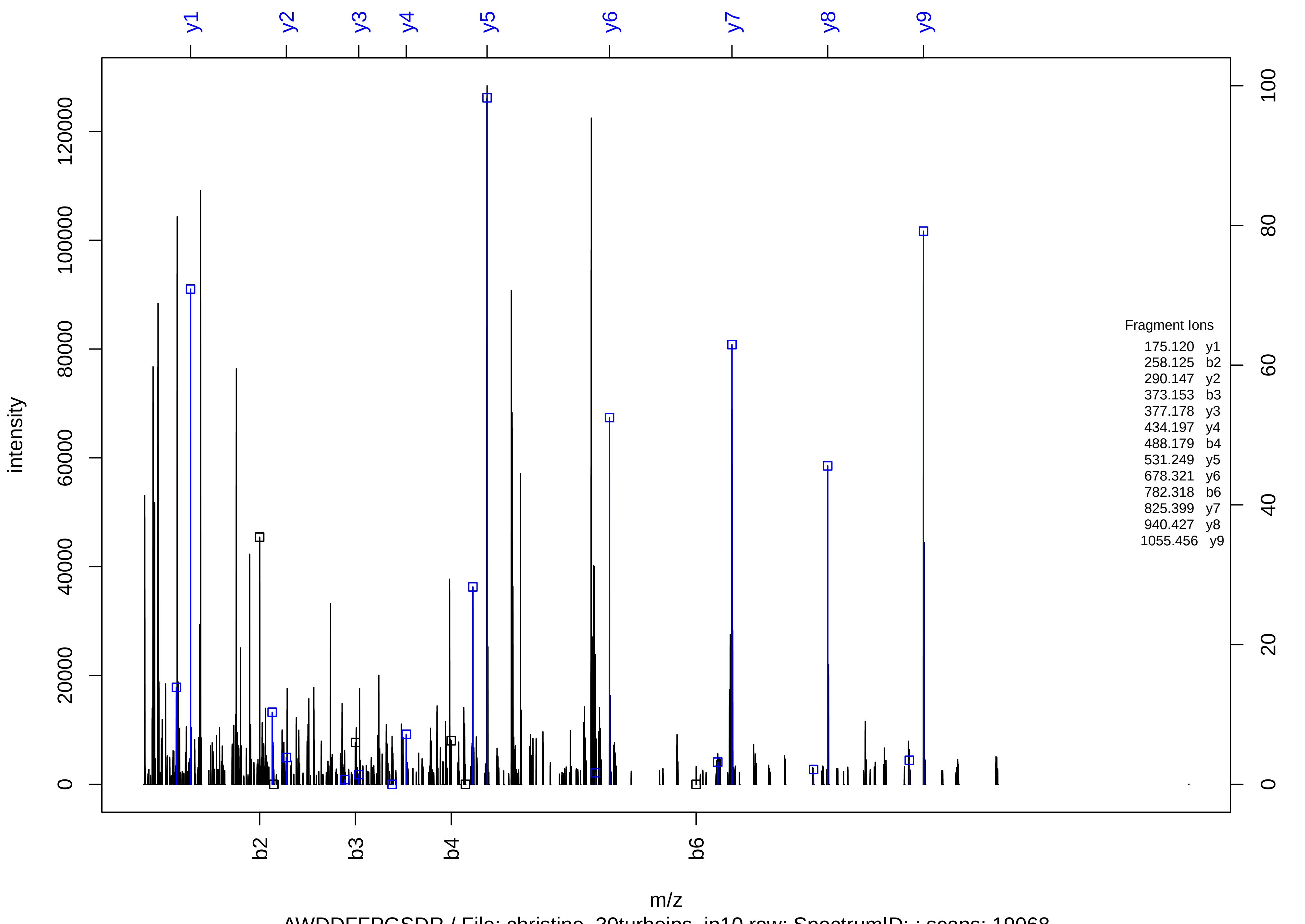

AWDDFFPGSDR / File: christine_30turboips_ip10.raw; SpectrumID: ; scans: 19068

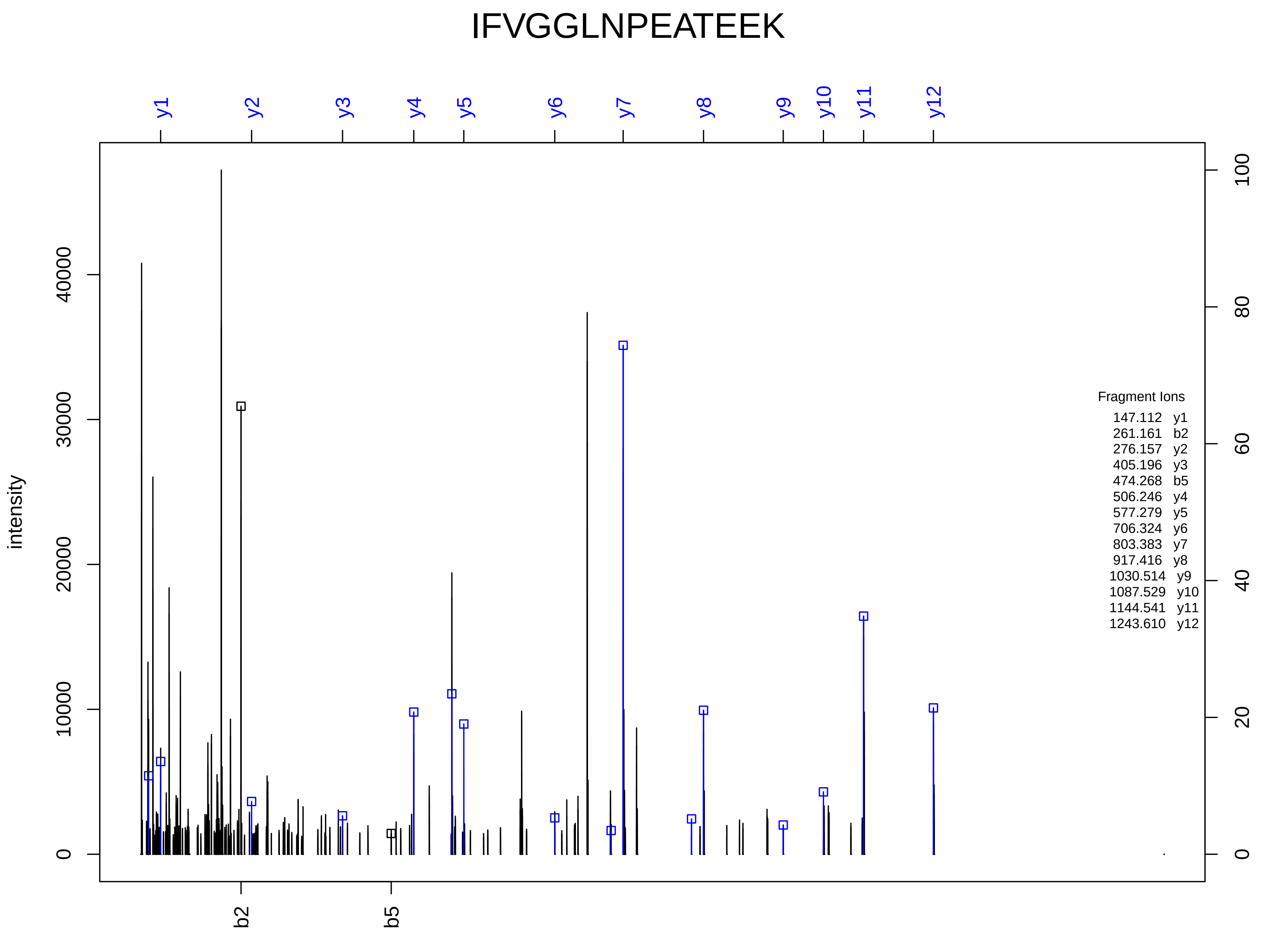

IFVGGLNPEATEEK
y1
y2
y3
y4
y5
y6
y7
y8
y9
y10
y11
y12
intensity
0
10000
20000
30000
40000
0
20
40
60
80
100
b2
b5
m/z
Fragment Ions
147.112 y1
261.161 b2
276.157 y2
405.196 y3
474.268 b5
506.246 y4
577.279 y5
706.324 y6
803.383 y7
917.416 y8
1030.514 y9
1087.529 y10
1144.541 y11
1243.610 y12
IFVGGLNPEATEEK / File: christine_30turboips_ip10.raw; SpectrumID: ; scans: 11074

(Ac)AAGAAAALAFLNQESR

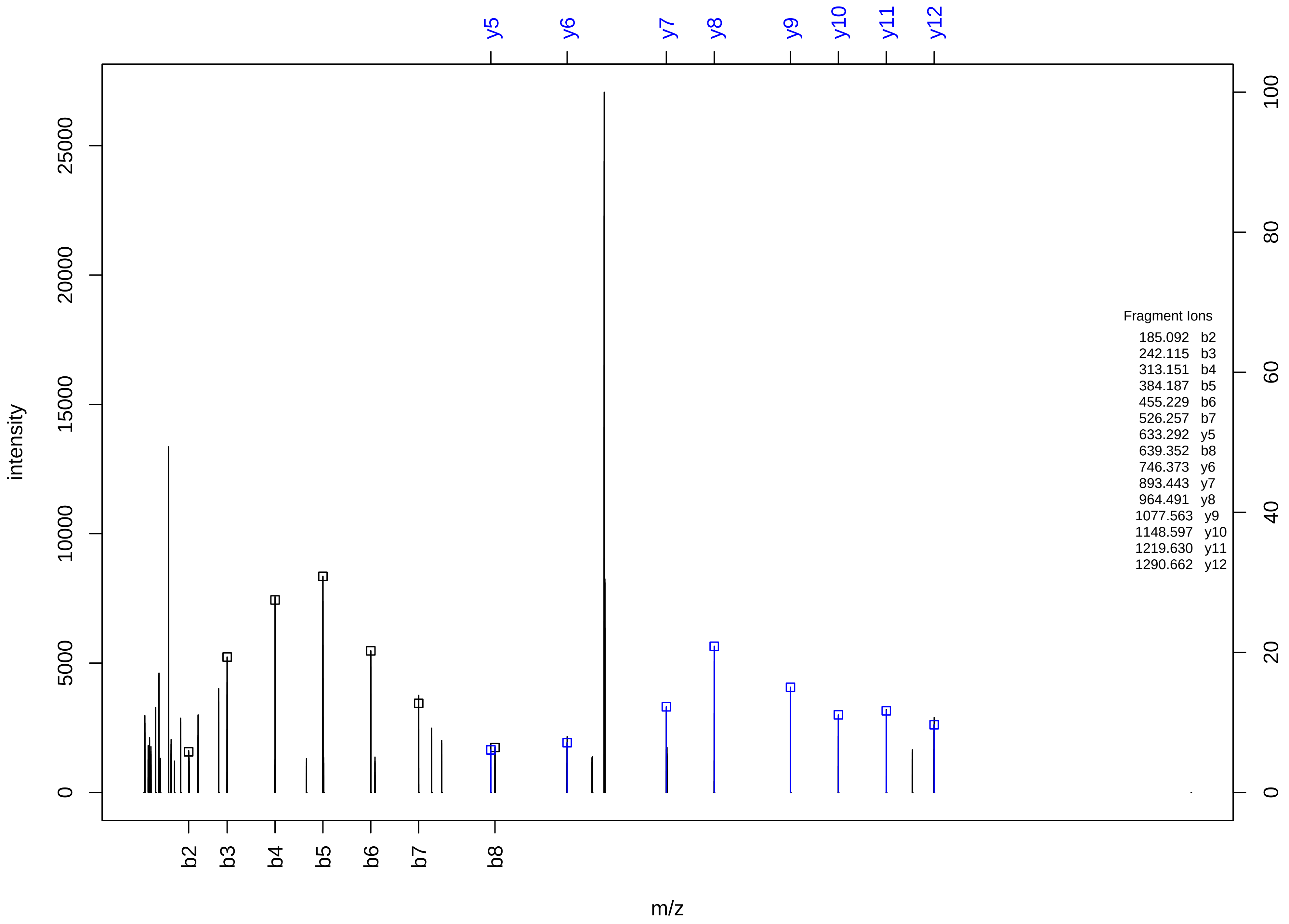

AAGAAAALAFLNQESR / File: christine_30turboips_ip10.raw; SpectrumID: ; scans: 31935

VELVPPTPAEIPTAIQSVK

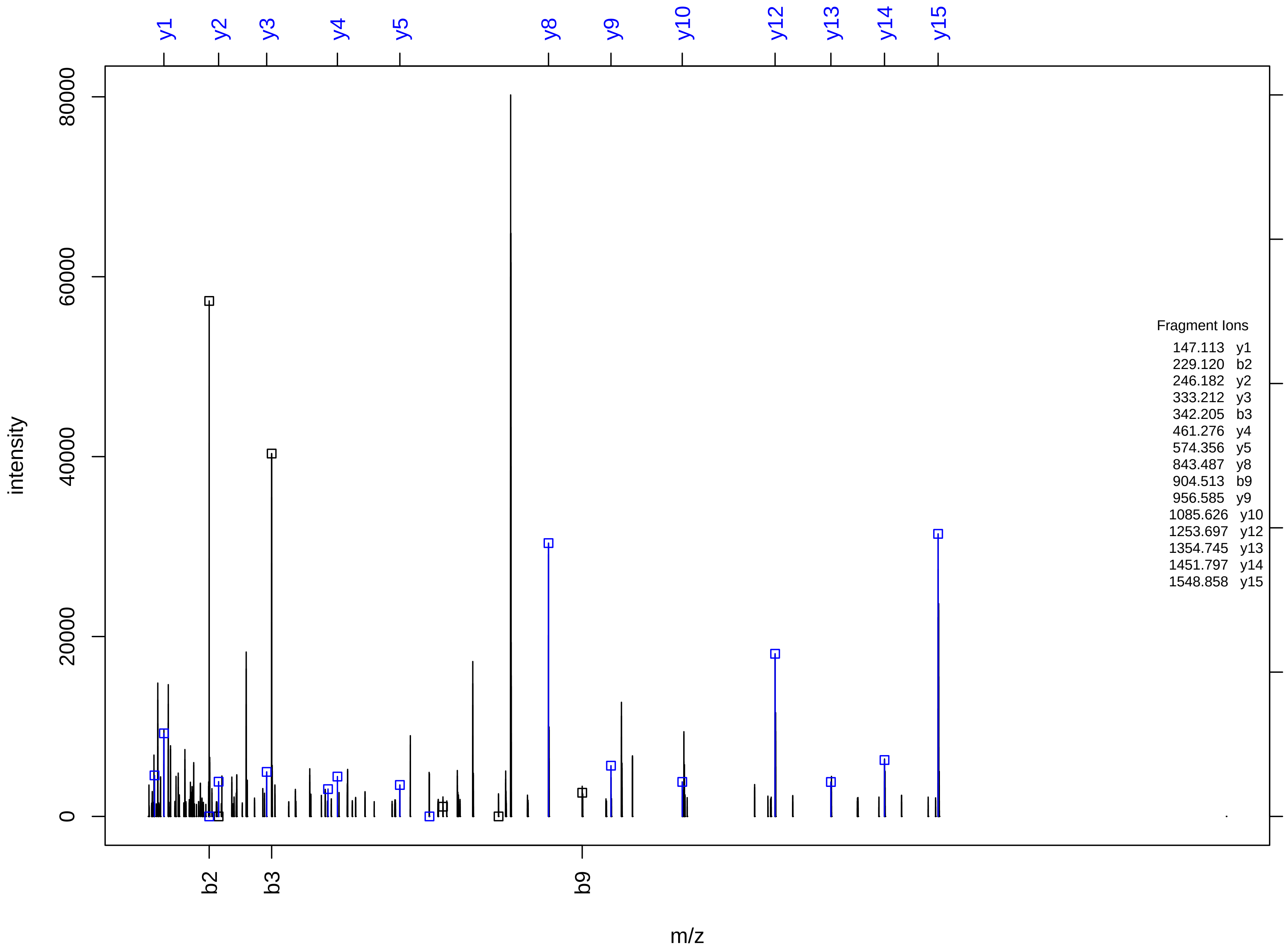

VELVPPTPAEIPTAIQSVK / File: christine_30turboips_ip10.raw; SpectrumID: ; scans: 22762

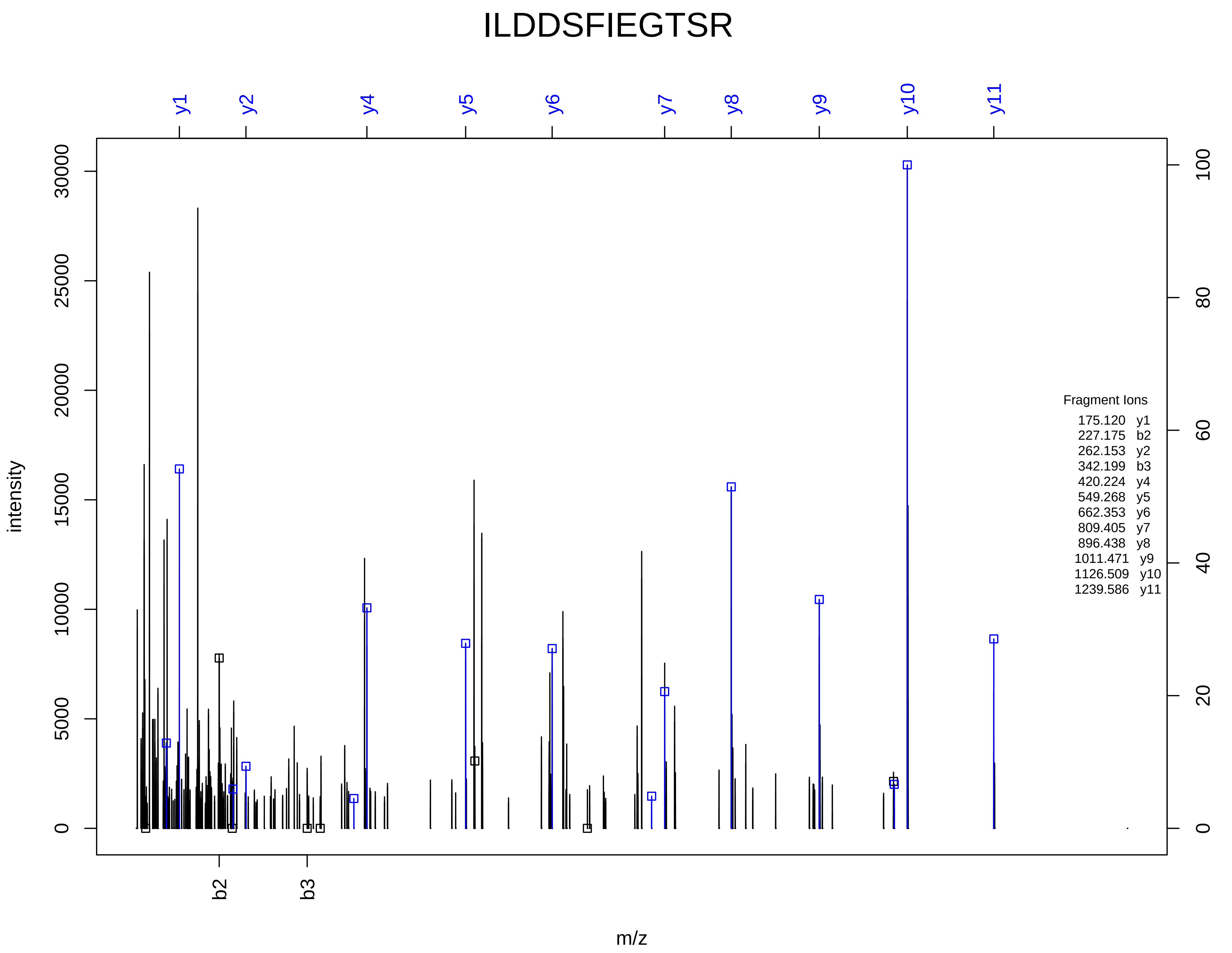

ILDDSFIEGTSR / File: christine_30turboips_ip10.raw; SpectrumID: ; scans: 12854

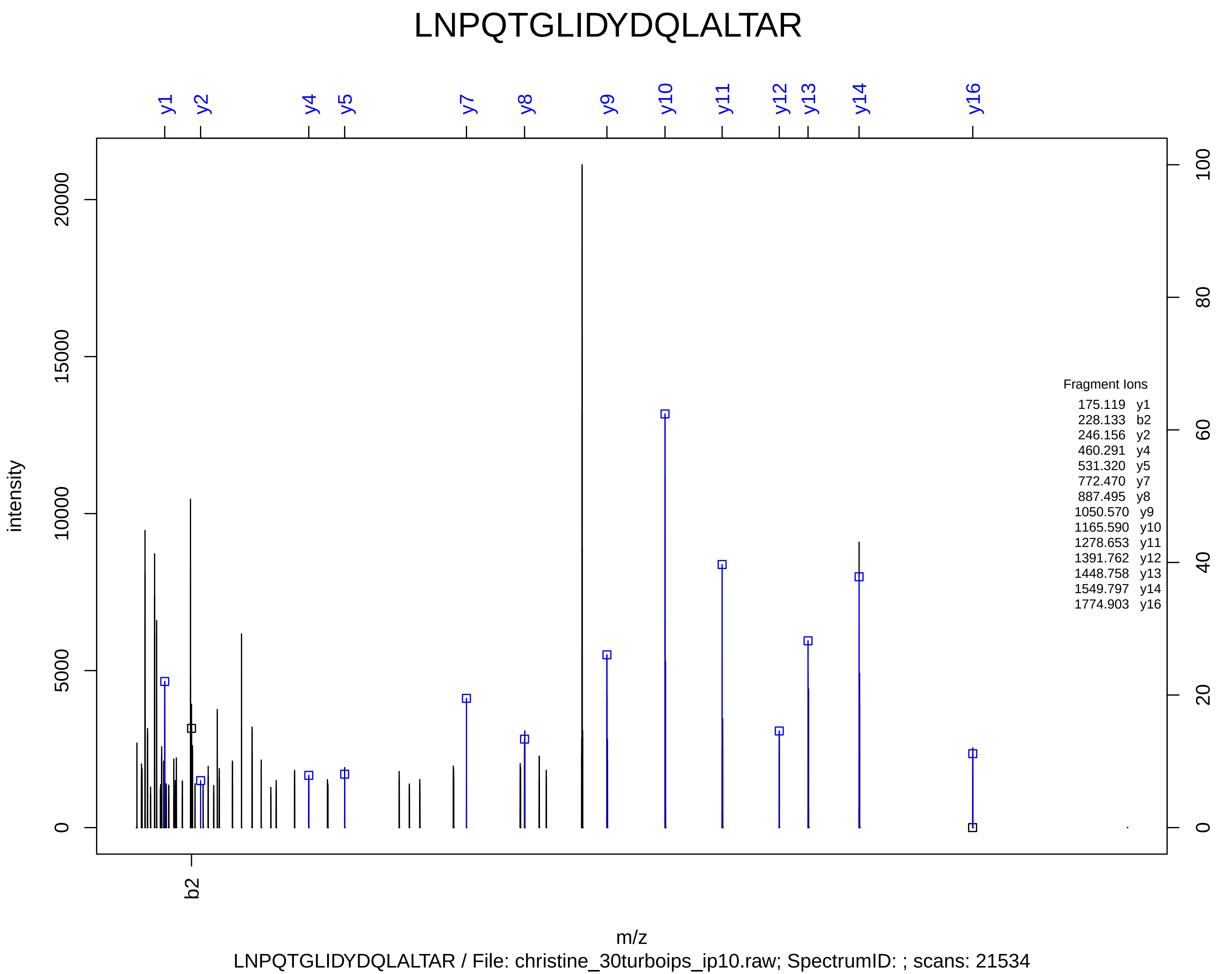
LNPQTGLIDYDQLALTAR
y1
y2
y4
y5
y7
y8
y9
y10
y11
y12
y13
y14
y16
intensity
0
5000
10000
15000
20000
0
20
40
60
80
100
b2
m/z
Fragment Ions
175.119 y1
228.133 b2
246.156 y2
460.291 y4
531.320 y5
772.470 y7
887.495 y8
1050.570 y9
1165.590 y10
1278.653 y11
1391.762 y12
1448.758 y13
1549.797 y14
1774.903 y16
LNPQTGLIDYDQLALTAR / File: christine_30turboips_ip10.raw; SpectrumID: ; scans: 21534

IQIQLTQSFEK

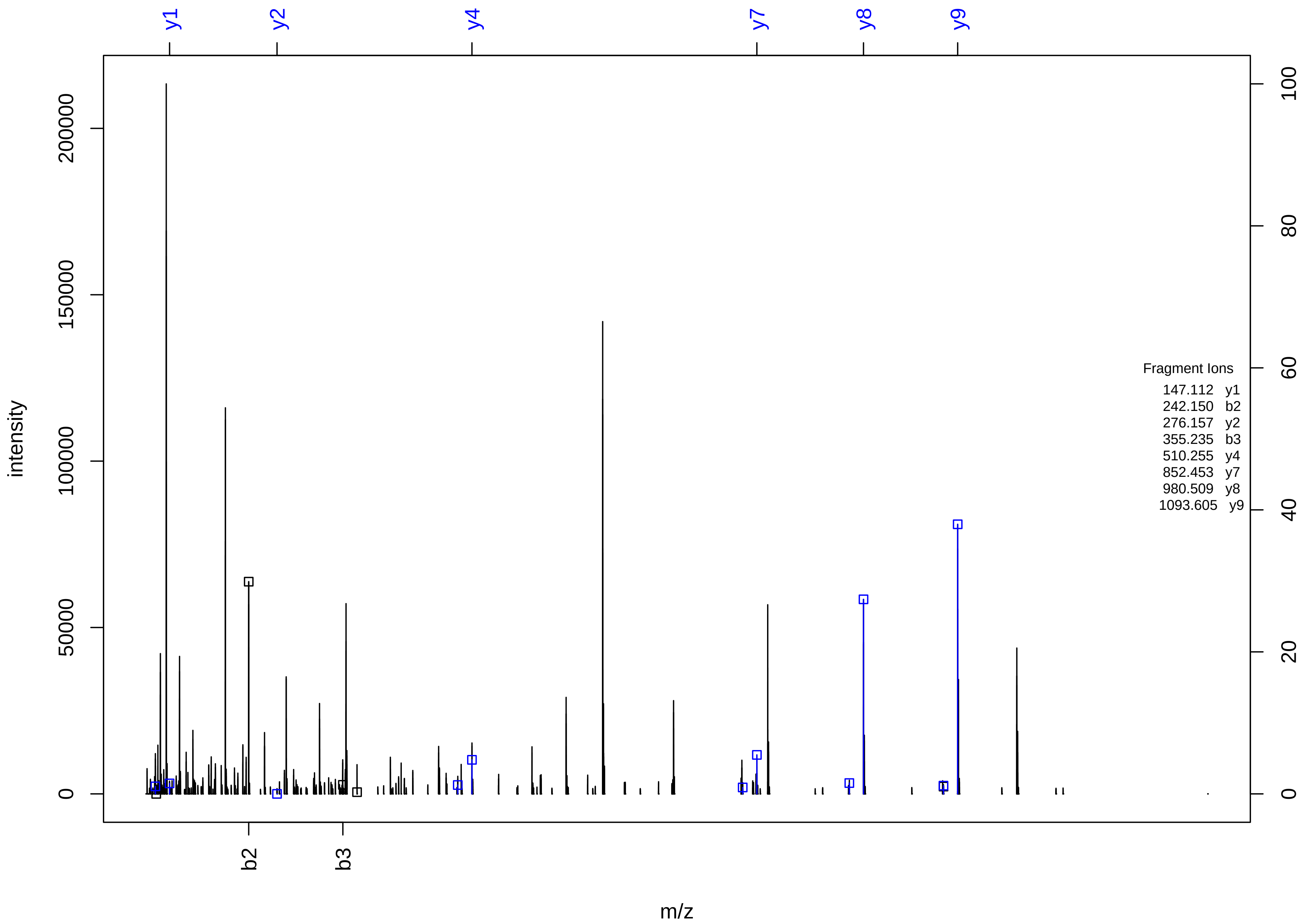

IQIQLTQSFEK / File: christine_30turboips_ip10.raw; SpectrumID: ; scans: 21242

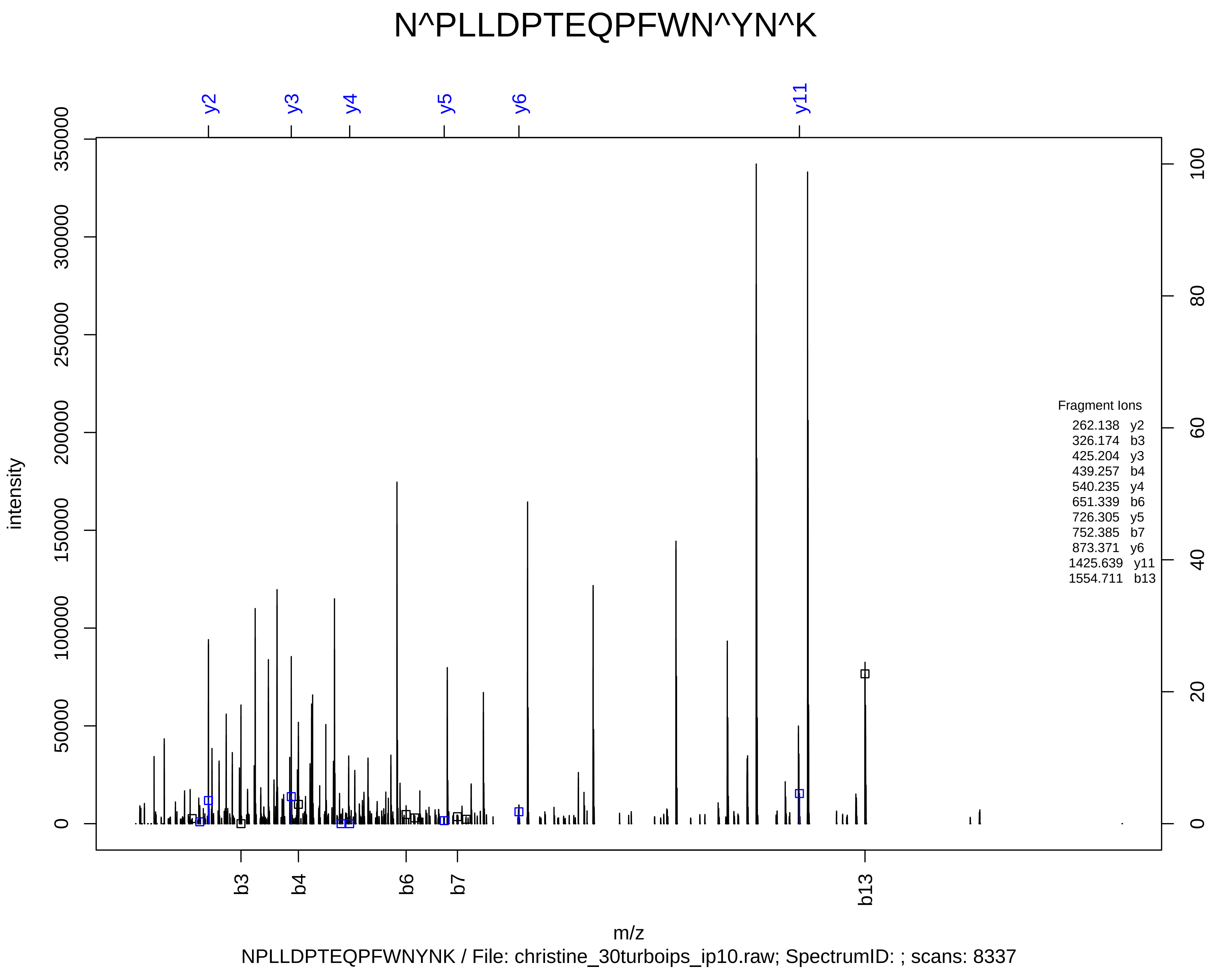

N^PLLDPTEQPFWN^YN^K
intensity
m/z
y2
y3
y4
y5
y6
y11
b3
b4
b6
b7
b13
0
50000
100000
150000
200000
250000
300000
350000
0
20
40
60
80
100
Fragment Ions
262.138 y2
326.174 b3
425.204 y3
439.257 b4
540.235 y4
651.339 b6
726.305 y5
752.385 b7
873.371 y6
1425.639 y11
1554.711 b13
NPLLDPTEQPFWNYNK / File: christine_30turboips_ip10.raw; SpectrumID: ; scans: 8337

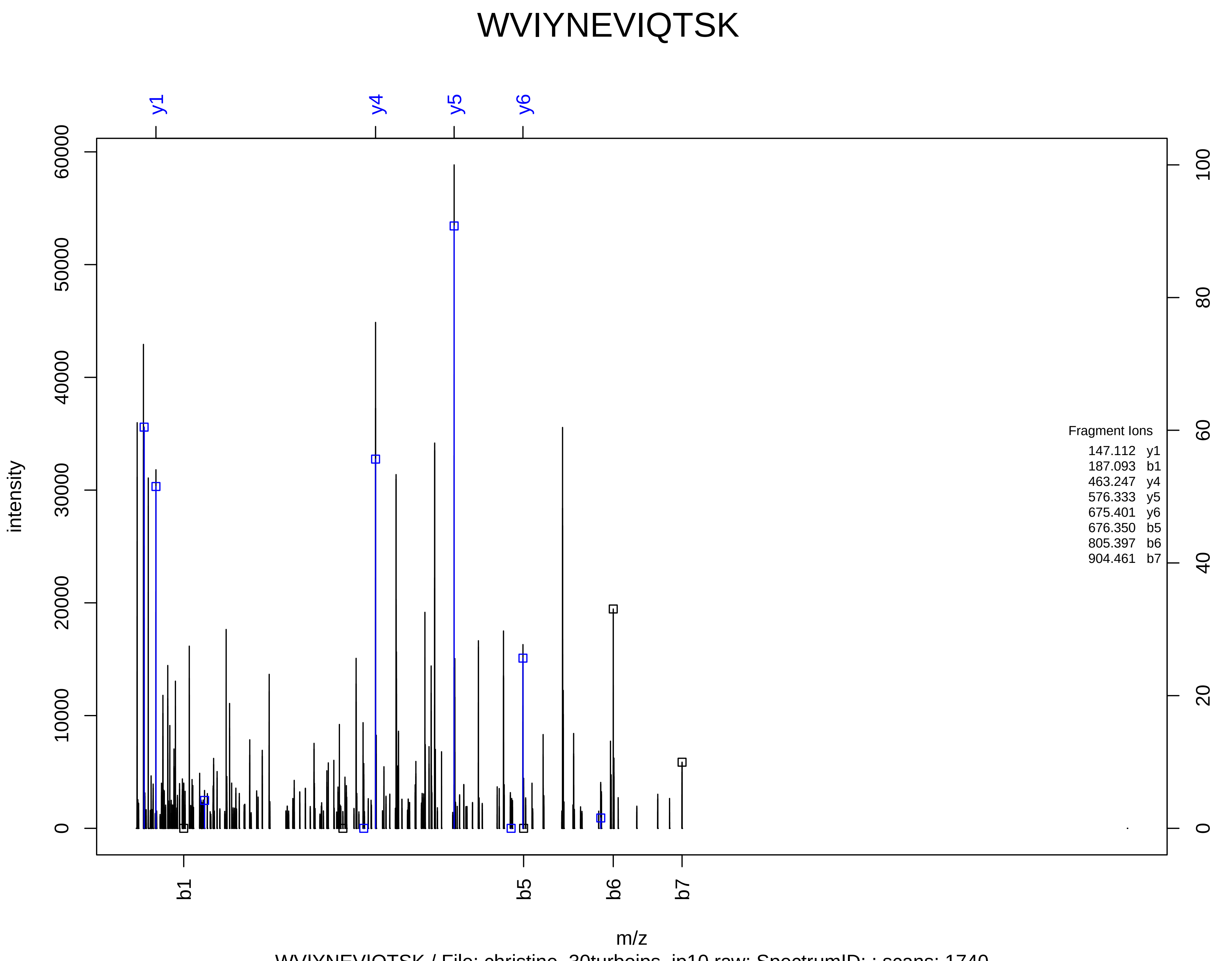

WVIYNEVIQTSK
y1
y4
y5
y6
intensity
0
10000
20000
30000
40000
50000
60000
0
20
40
60
80
100
b1
b5
b6
b7
m/z
Fragment Ions
147.112 y1
187.093 b1
463.247 y4
576.333 y5
675.401 y6
676.350 b5
805.397 b6
904.461 b7
WVIYNEVIQTSK / File: christine_30turboips_ip10.raw; SpectrumID: ; scans: 1740

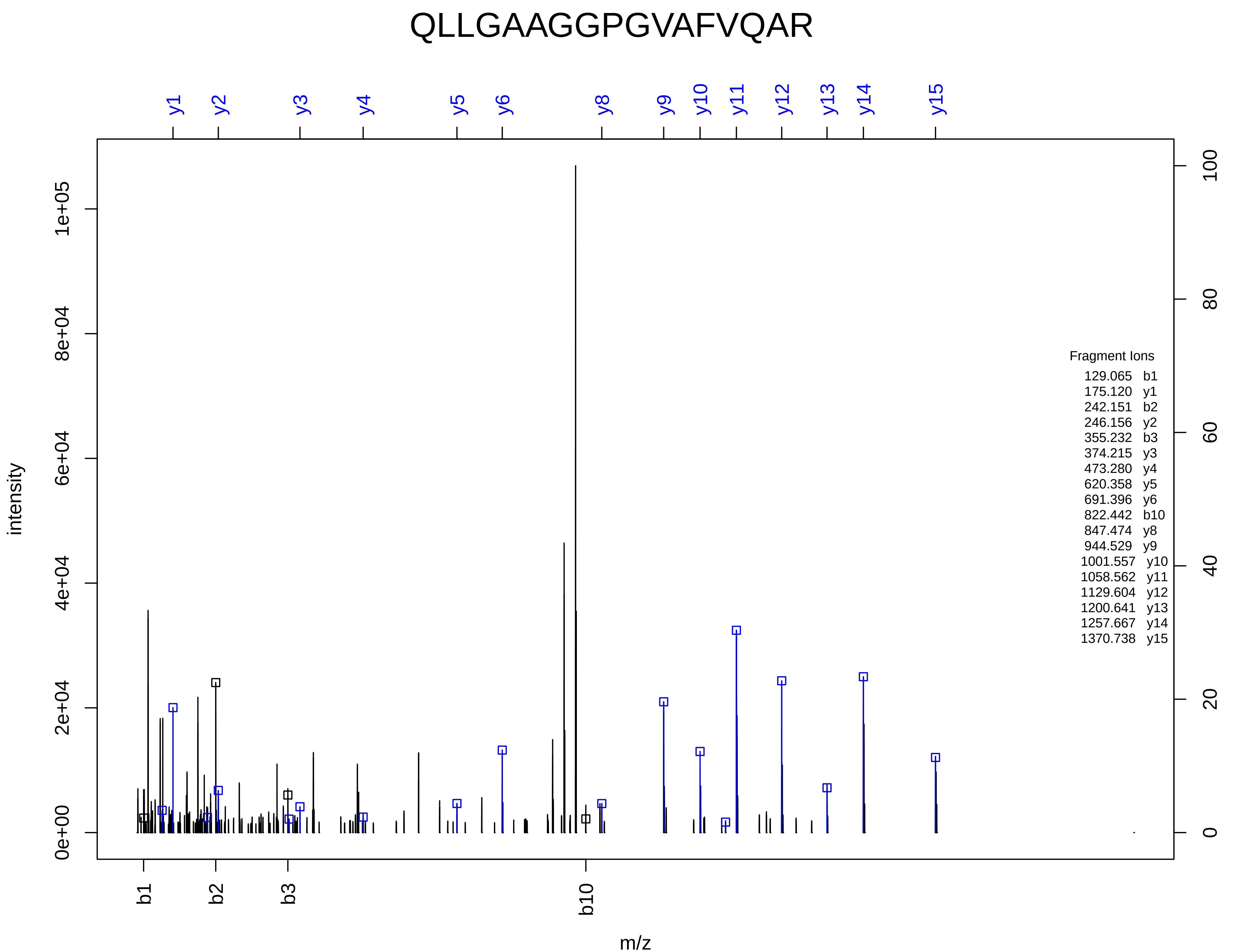
QLLGAAGGPGVAFVQAR
y1 y2 y3 y4 y5 y6 y8 y9 y10 y11 y12 y13 y14 y15
intensity
0e+00 2e+04 4e+04 6e+04 8e+04 1e+05
0 20 40 60 80 100
Fragment Ions
129.065 b1
175.120 y1
242.151 b2
246.156 y2
355.232 b3
374.215 y3
473.280 y4
620.358 y5
691.396 y6
822.442 b10
847.474 y8
944.529 y9
1001.557 y10
1058.562 y11
1129.604 y12
1200.641 y13
1257.667 y14
1370.738 y15
b1 b2 b3 b10
m/z
QLLGAAGGPGVAFVQAR / File: christine_30turboips_ip10.raw; SpectrumID: ; scans: 15886

(Ac)AEEGIAAGGVMDVN^TALQEVLK

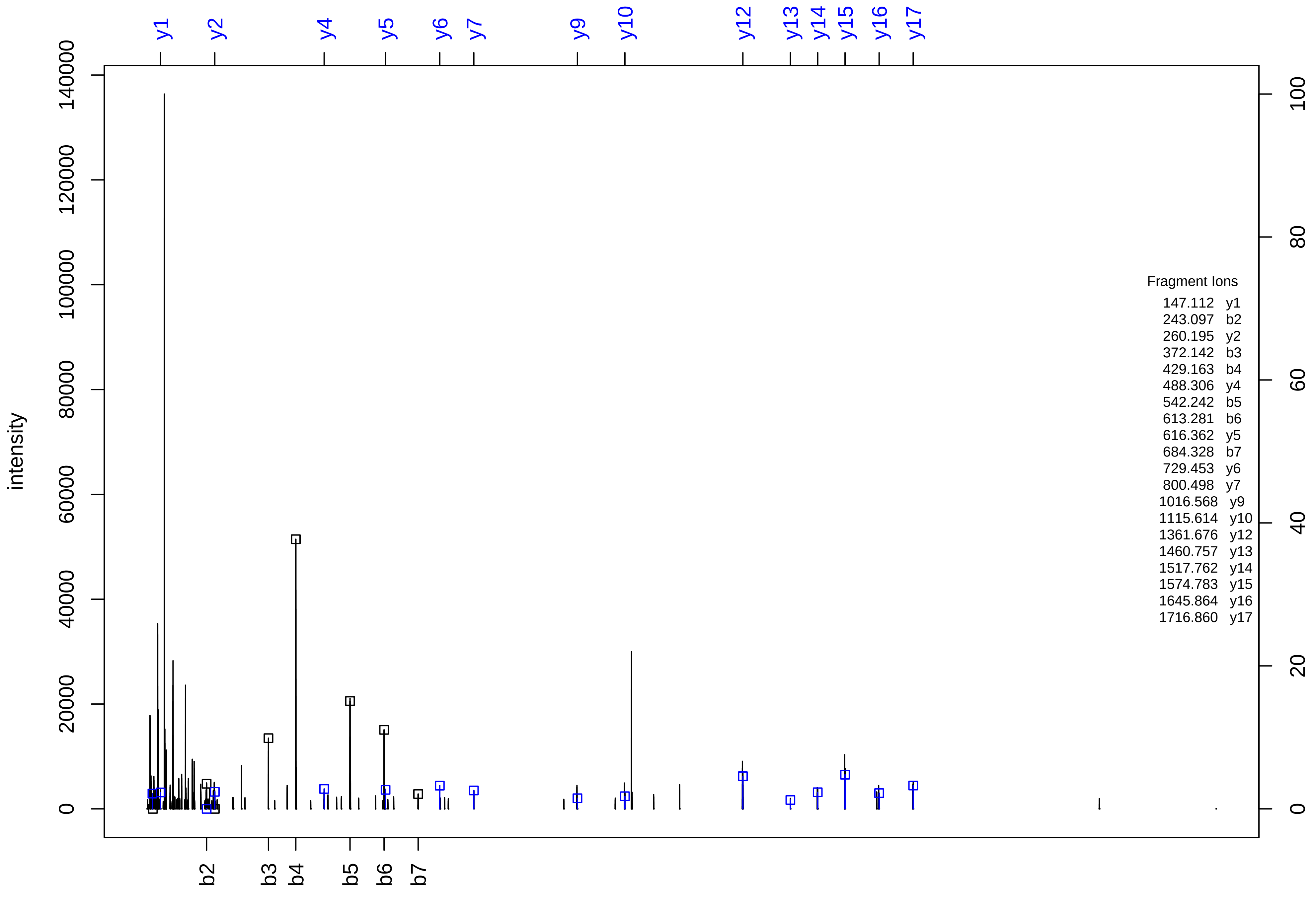

AEEGIAAGGVMDVNTALQEVLK / File: christine_30turboips_ip03.raw; SpectrumID: ; scans: 30326

INTQWR

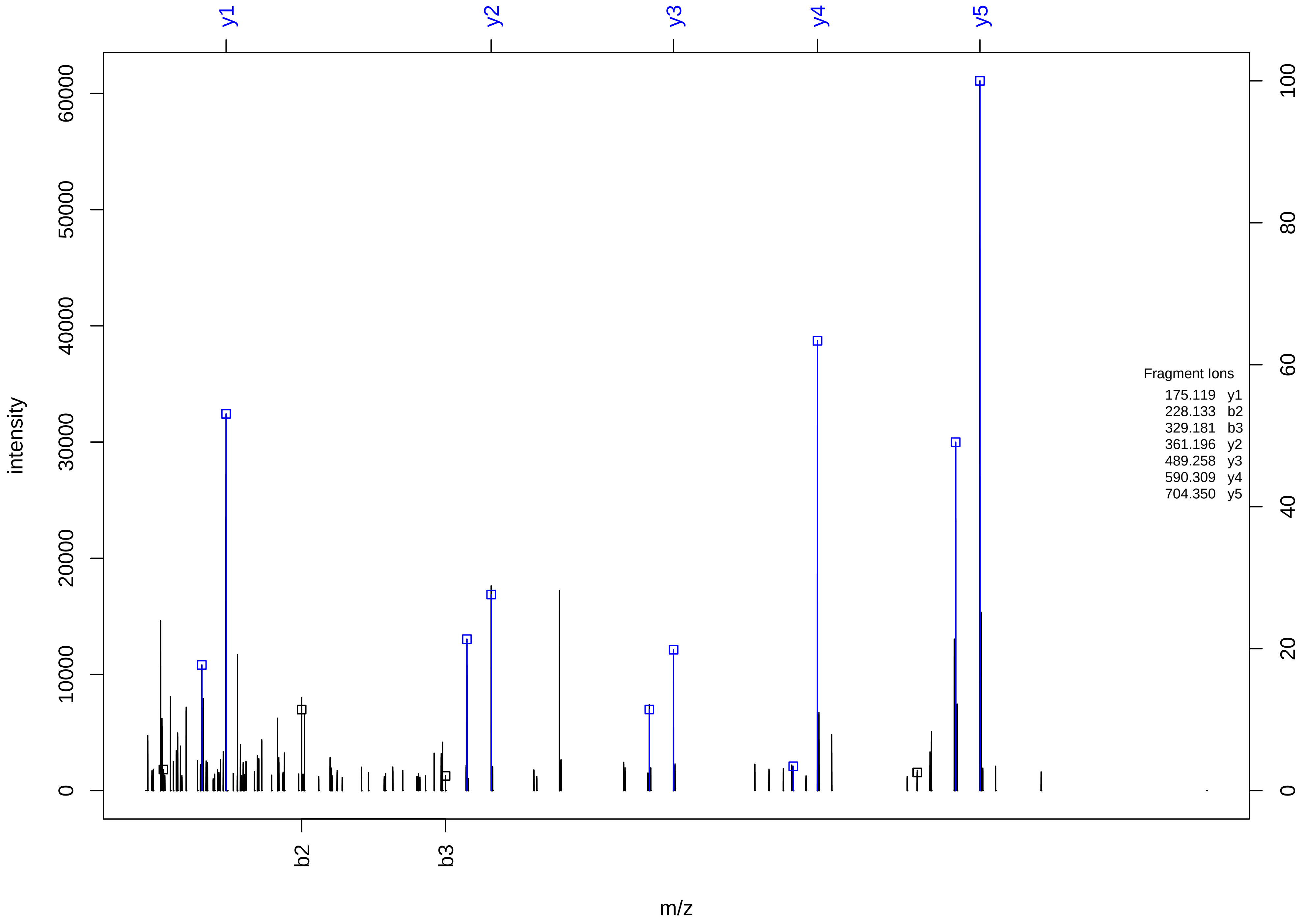

INTQWR / File: christine_30turboips_ip03.raw; SpectrumID: ; scans: 1454

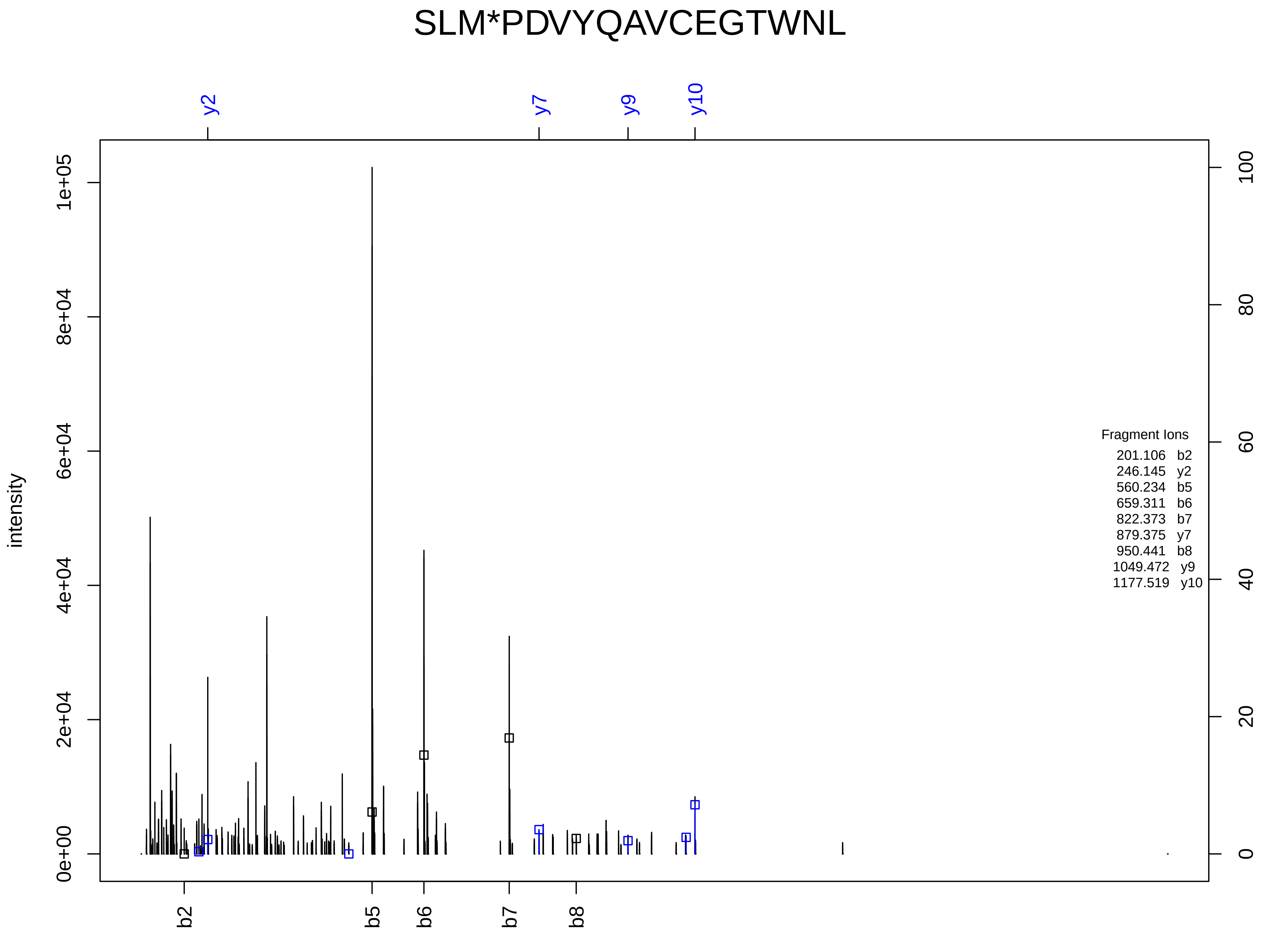

SLMPDVYQAVCEGTWNL / File: christine_30turboips_ip04.raw; SpectrumID: ; scans: 1105

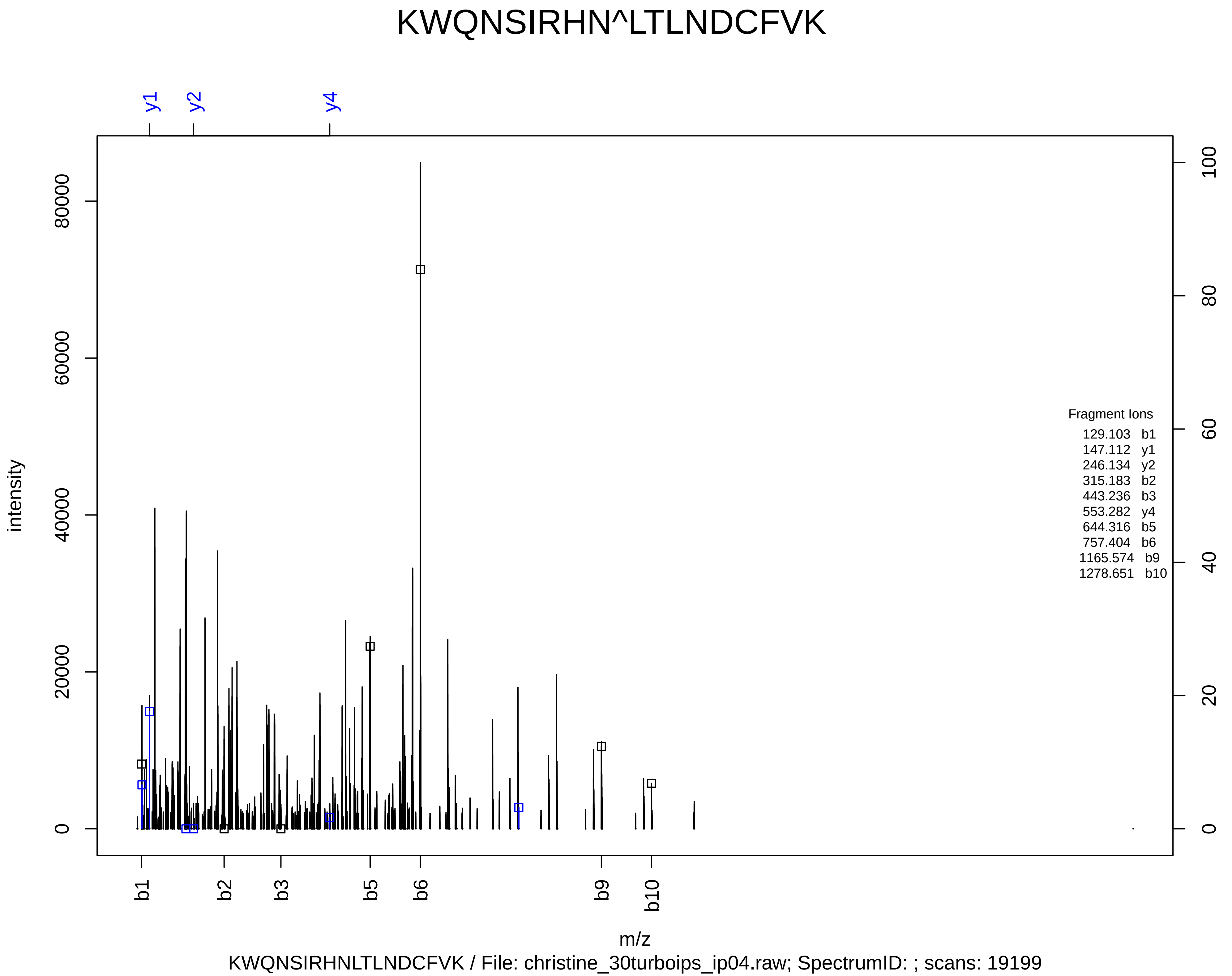

KWQNSIRHN^LTLNDCFVK
y1
y2
y4
intensity
m/z
b1
b2
b3
b5
b6
b9
b10
Fragment Ions
129.103 b1
147.112 y1
246.134 y2
315.183 b2
443.236 b3
553.282 y4
644.316 b5
757.404 b6
1165.574 b9
1278.651 b10
KWQNSIRHNLTLNDCFVK / File: christine_30turboips_ip04.raw; SpectrumID: ; scans: 19199

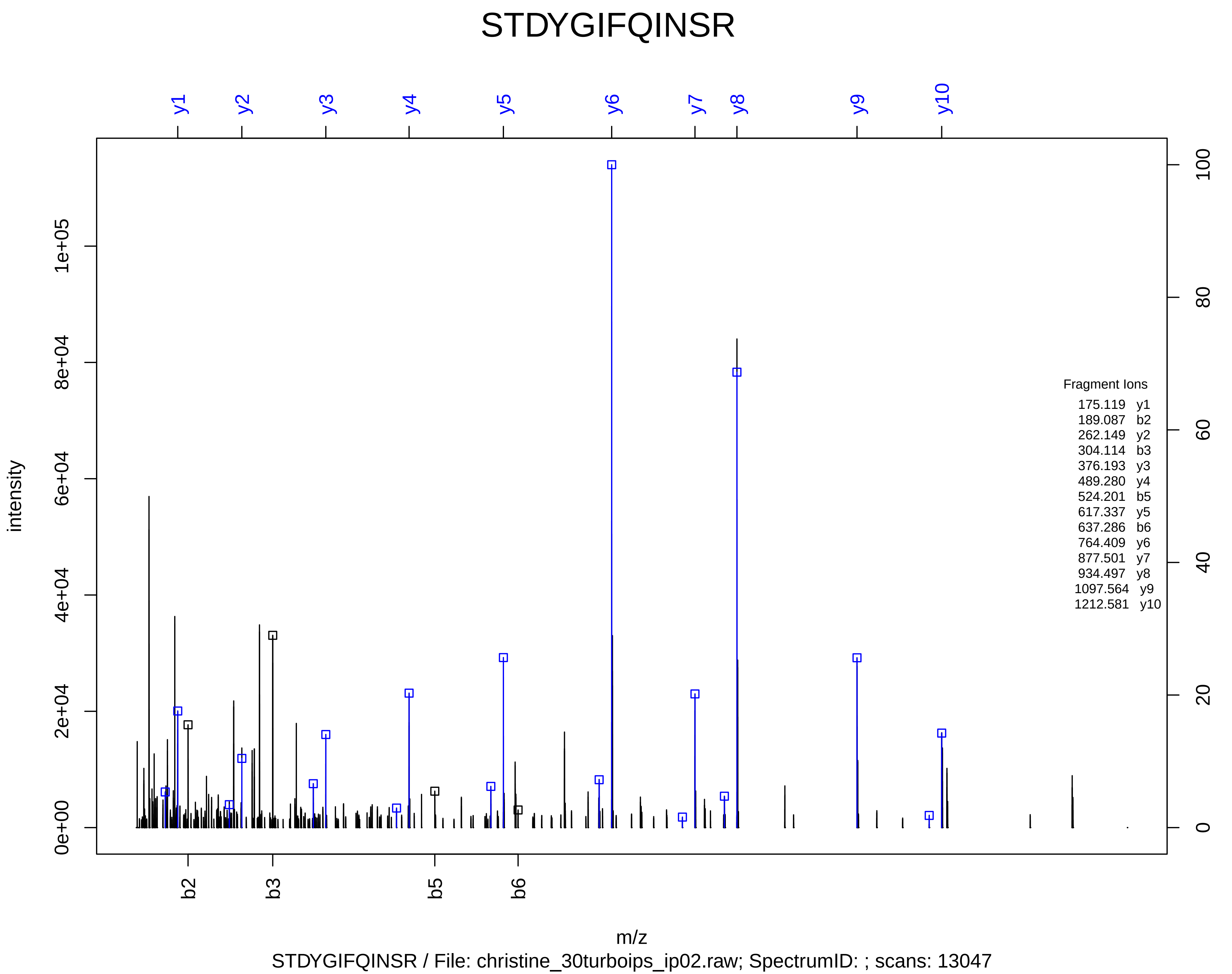
STDYGIFQINSR
y1 y2 y3 y4 y5 y6 y7 y8 y9 y10
intensity
0e+00 2e+04 4e+04 6e+04 8e+04 1e+05
0 20 40 60 80 100
b2 b3 b5 b6
m/z
Fragment Ions
175.119 y1
189.087 b2
262.149 y2
304.114 b3
376.193 y3
489.280 y4
524.201 b5
617.337 y5
637.286 b6
764.409 y6
877.501 y7
934.497 y8
1097.564 y9
1212.581 y10
STDYGIFQINSR / File: christine_30turboips_ip02.raw; SpectrumID: ; scans: 13047

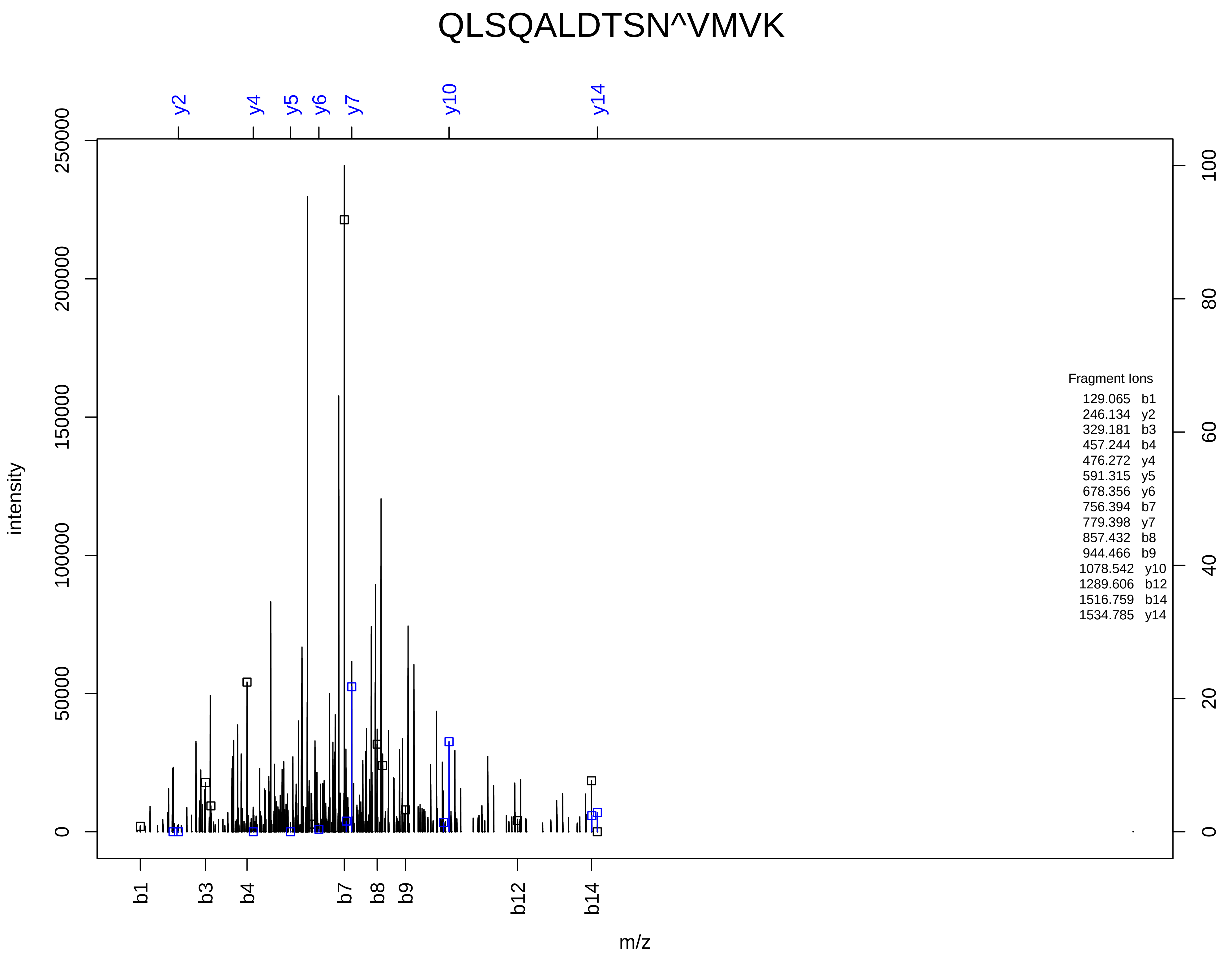

QLSQALDTSNVMVK / File: christine_30turboips_ip02.raw; SpectrumID: ; scans: 20509

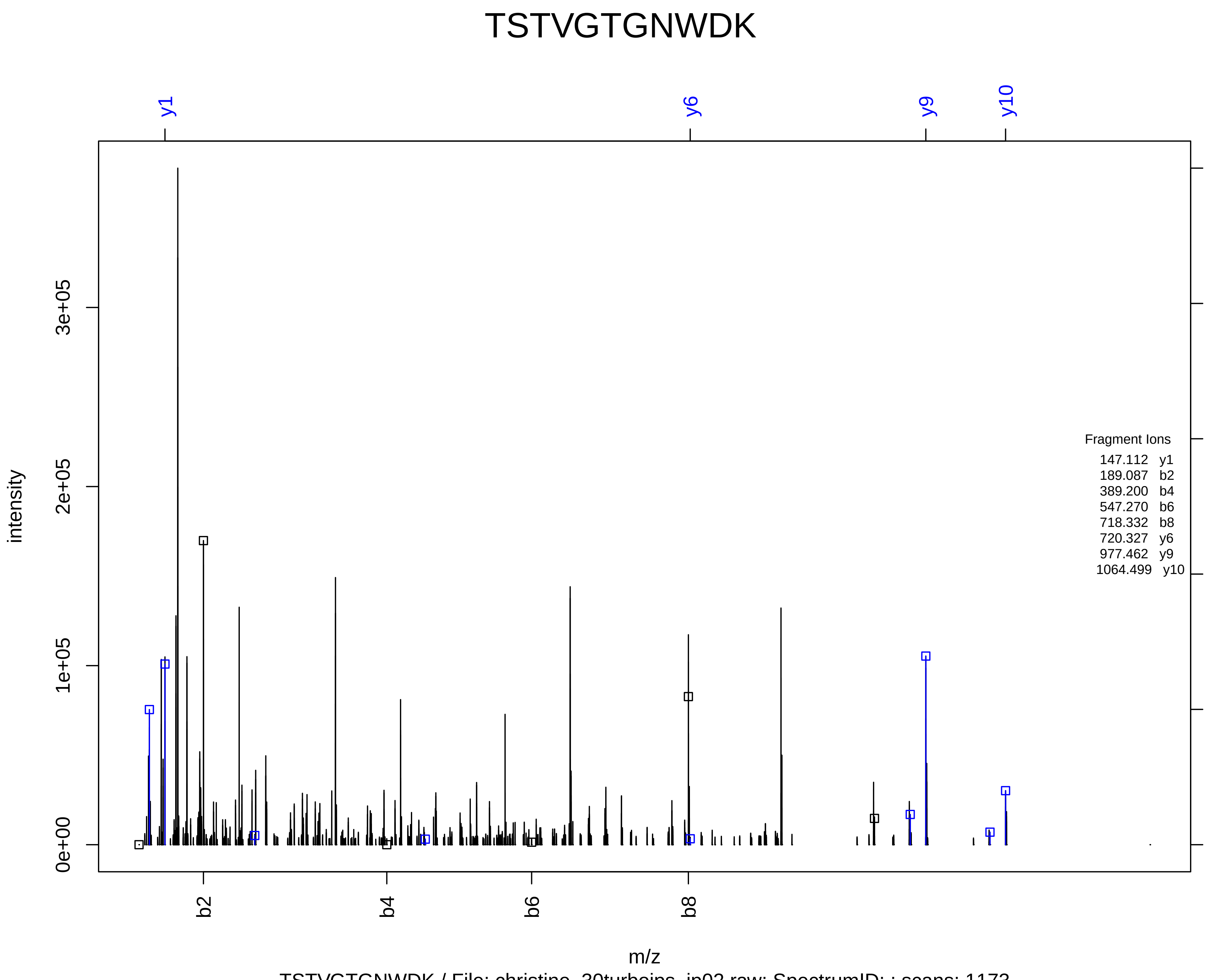
TSTVGTGNWDK
y1
y6
y9
y10
intensity
0e+00
1e+05
2e+05
3e+05
0
20
40
60
80
100
b2
b4
b6
b8
m/z
Fragment Ions
147.112 y1
189.087 b2
389.200 b4
547.270 b6
718.332 b8
720.327 y6
977.462 y9
1064.499 y10
TSTVGTGNWDK / File: christine_30turboips_ip02.raw; SpectrumID: ; scans: 1173

IAAAQYSVTGSAVAR

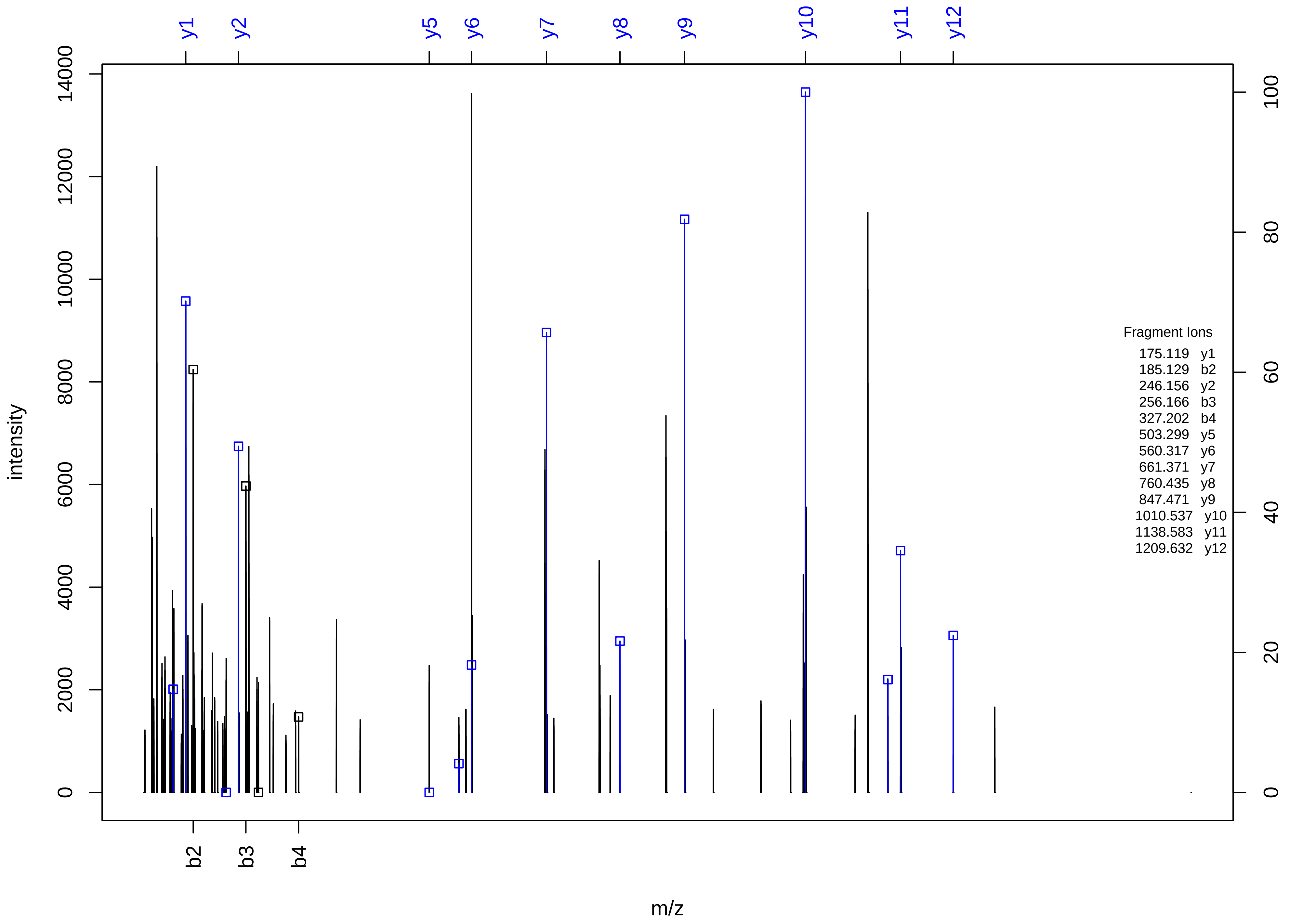

IAAAQYSVTGSAVAR / File: christine_30turboips_ip02.raw; SpectrumID: ; scans: 4284

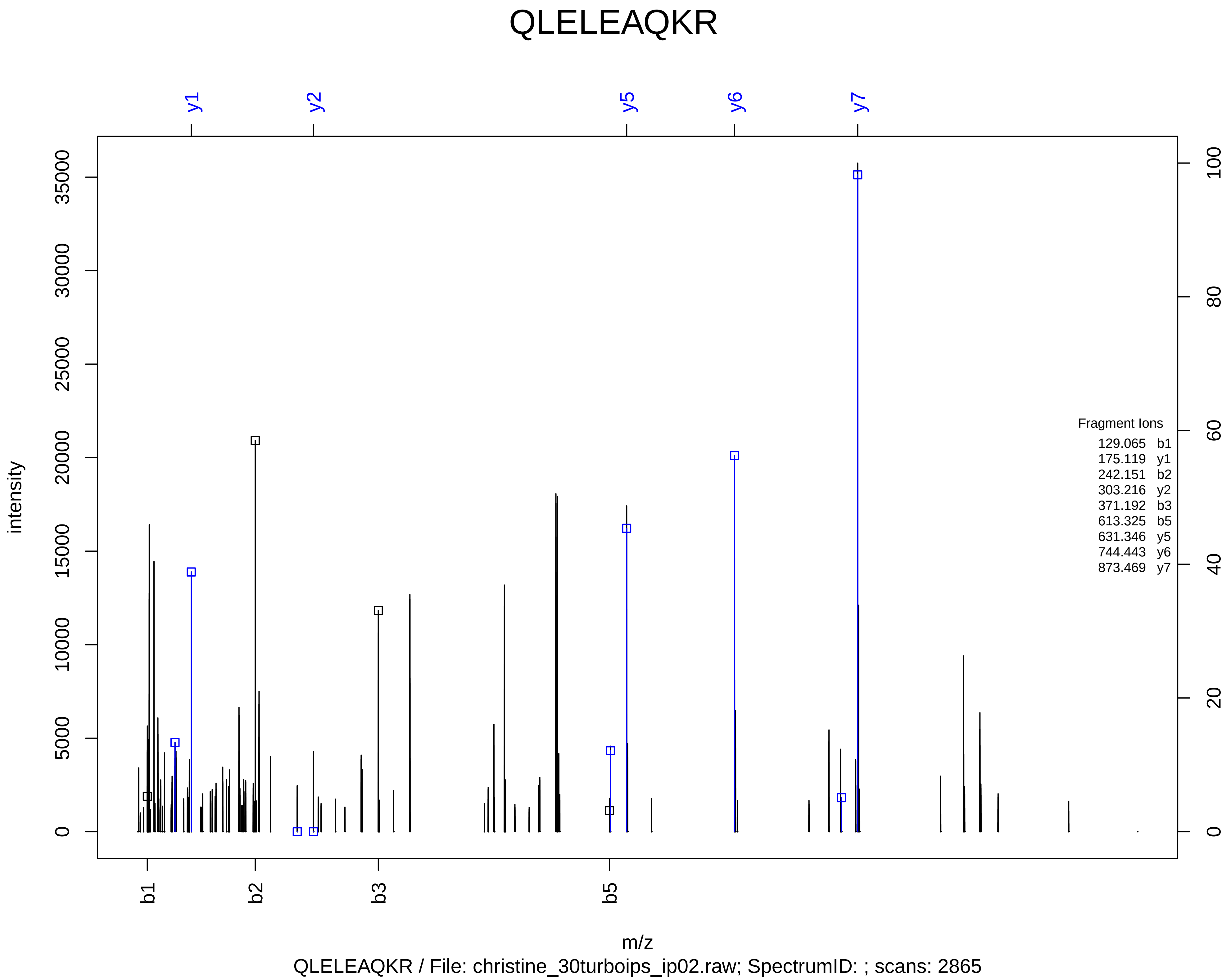

QLELEAQKR
y1
y2
y5
y6
y7
intensity
0
5000
10000
15000
20000
25000
30000
35000
0
20
40
60
80
100
b1
b2
b3
b5
m/z
Fragment Ions
129.065 b1
175.119 y1
242.151 b2
303.216 y2
371.192 b3
613.325 b5
631.346 y5
744.443 y6
873.469 y7
QLELEAQKR / File: christine_30turboips_ip02.raw; SpectrumID: ; scans: 2865

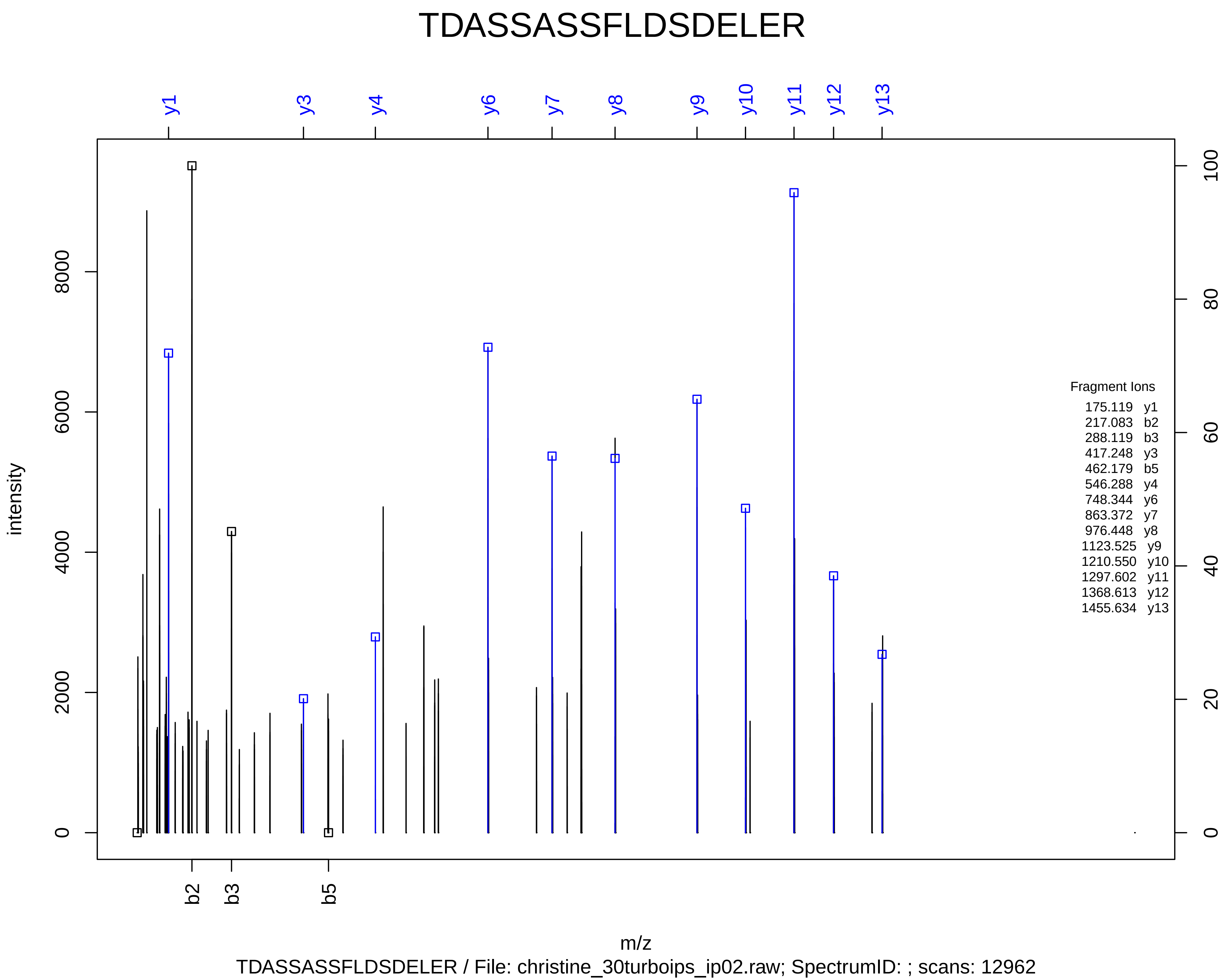

TDASSASSFLDSDELER
y1
y3
y4
y6
y7
y8
y9
y10
y11
y12
y13
b2
b3
b5
intensity
0
2000
4000
6000
8000
0
20
40
60
80
100
Fragment Ions
175.119 y1
217.083 b2
288.119 b3
417.248 y3
462.179 b5
546.288 y4
748.344 y6
863.372 y7
976.448 y8
1123.525 y9
1210.550 y10
1297.602 y11
1368.613 y12
1455.634 y13
m/z
TDASSASSFLDSDELER / File: christine_30turboips_ip02.raw; SpectrumID: ; scans: 12962

TVAAPSVFIFPPSDEQLK

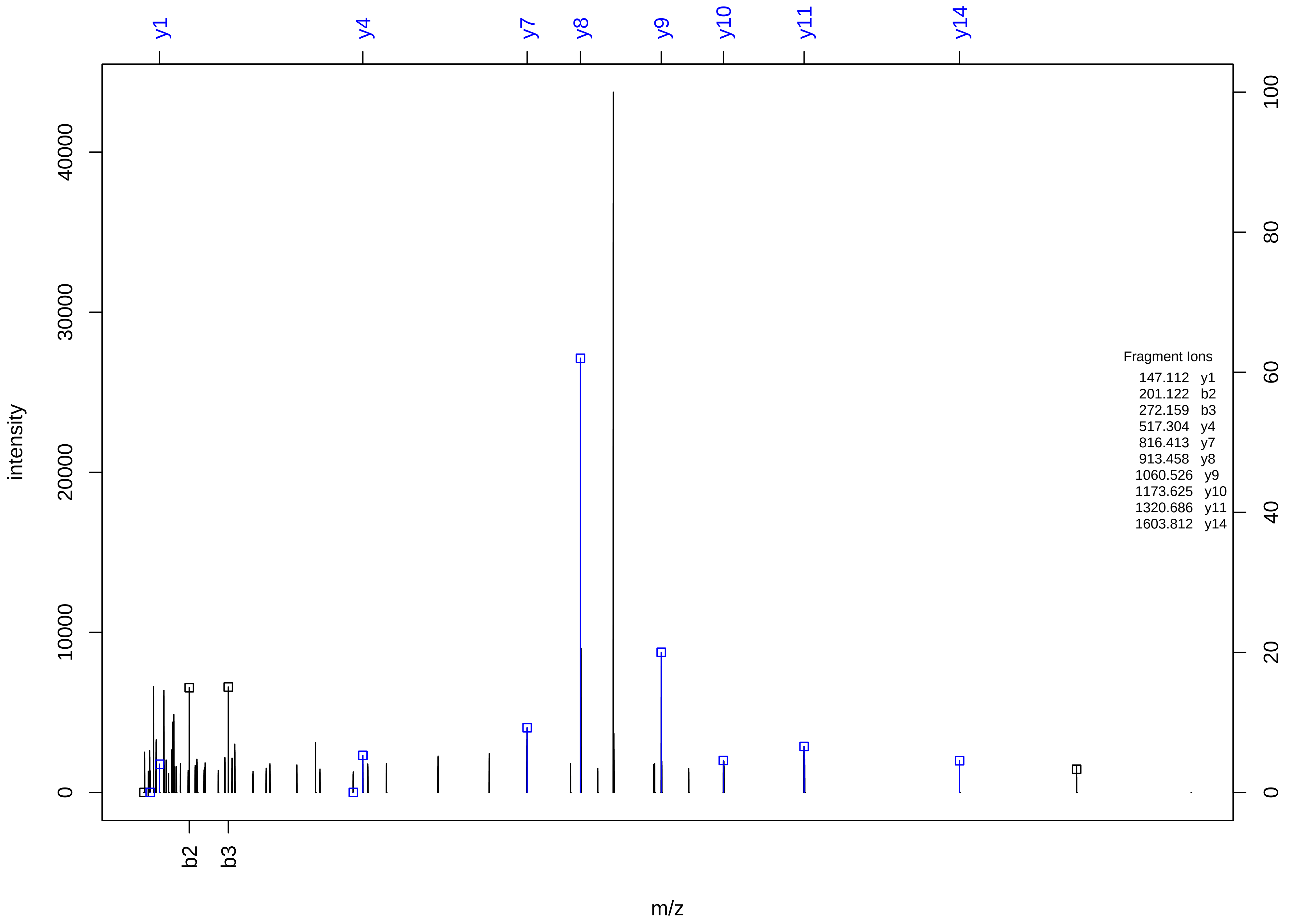

TVAAPSVFIFPPSDEQLK / File: christine_30turboips_ip02.raw; SpectrumID: ; scans: 21880

VGFYVNTFQSIAGLEENFHK

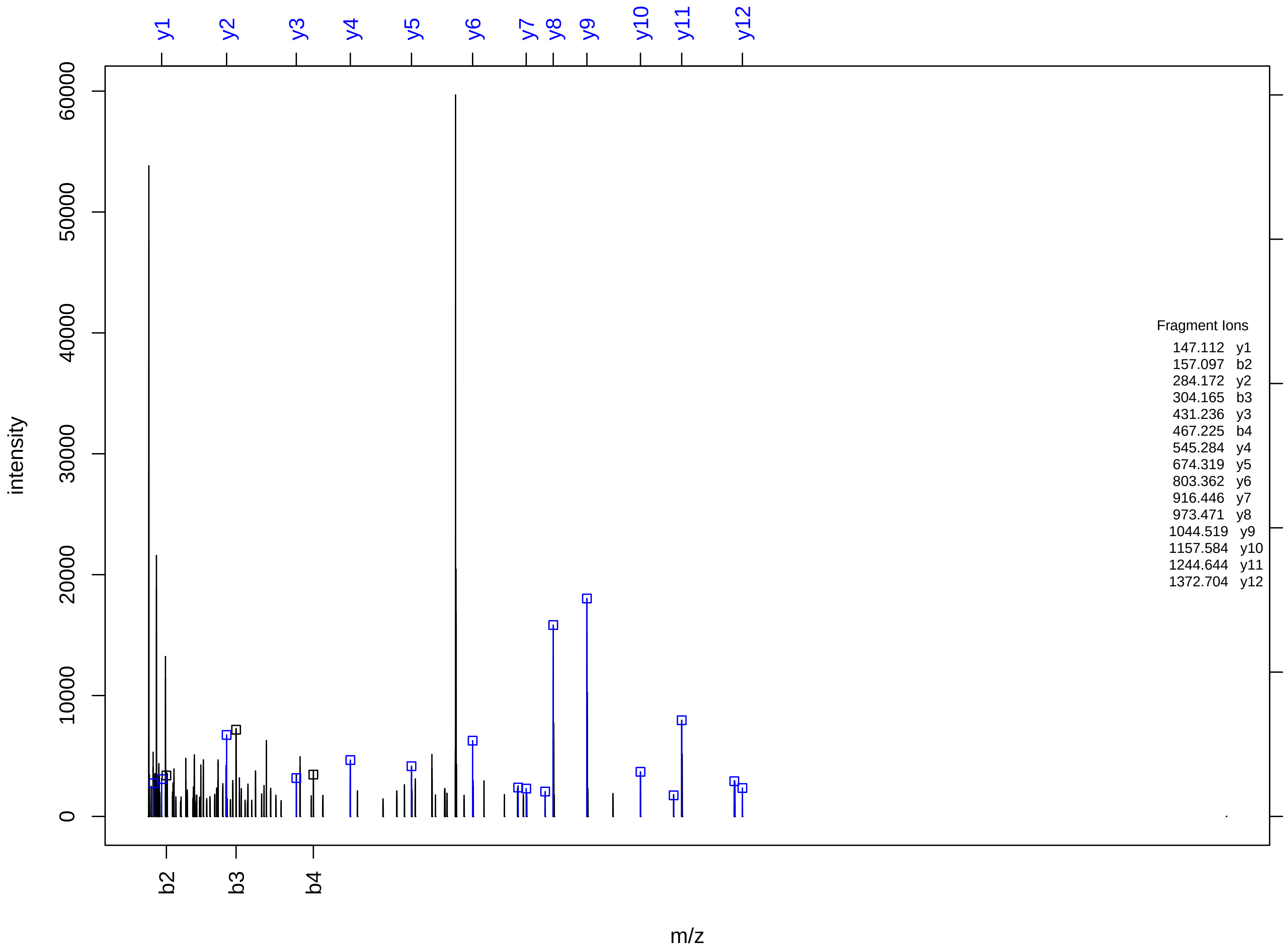

VGFYVNTFQSIAGLEENFHK / File: christine_30turboips_ip01.raw; SpectrumID: ; scans: 23482

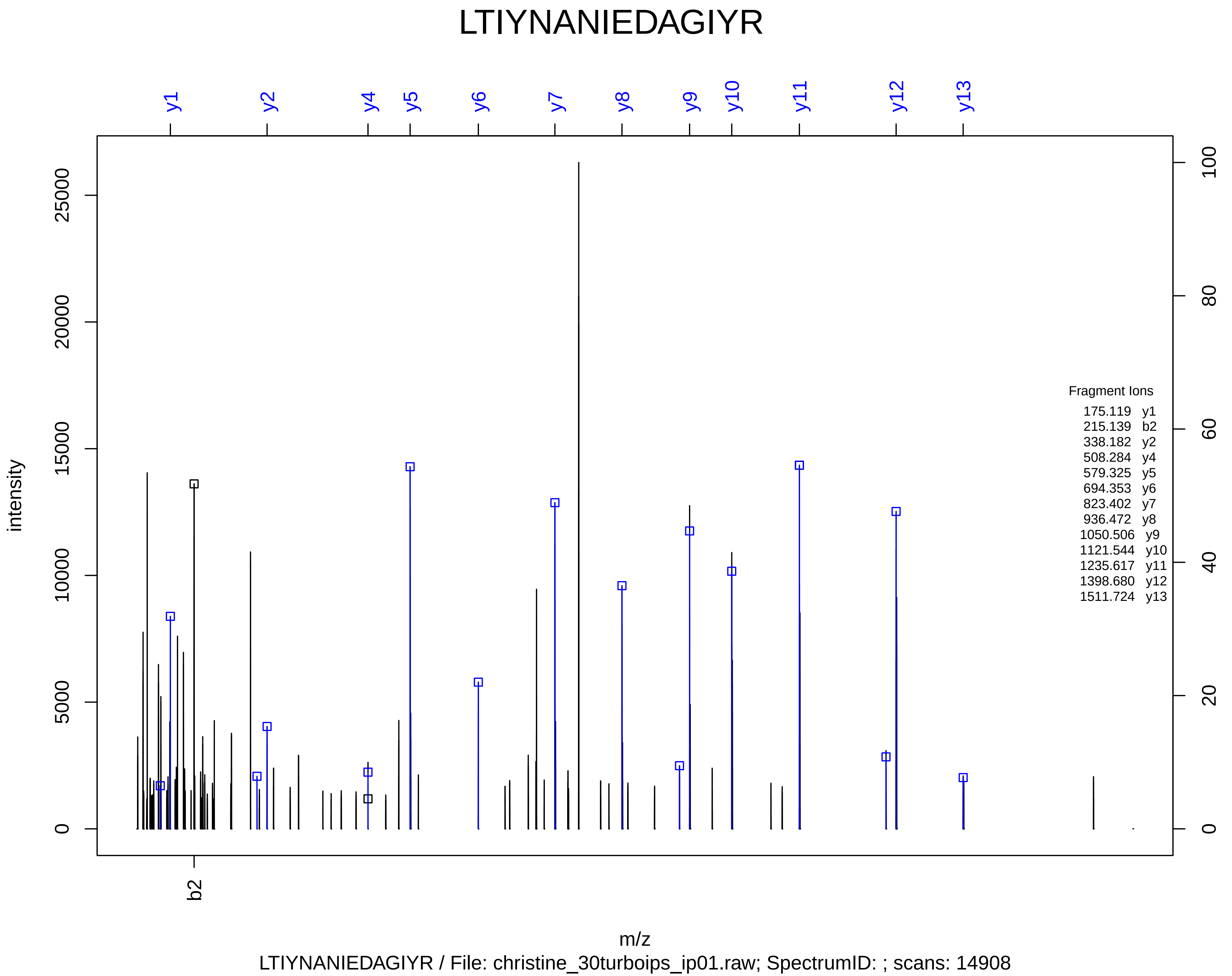

LTIYNANIEDAGIYR
y1
y2
y4
y5
y6
y7
y8
y9
y10
y11
y12
y13
intensity
0
5000
10000
15000
20000
25000
0
20
40
60
80
100
b2
m/z
Fragment Ions
175.119 y1
215.139 b2
338.182 y2
508.284 y4
579.325 y5
694.353 y6
823.402 y7
936.472 y8
1050.506 y9
1121.544 y10
1235.617 y11
1398.680 y12
1511.724 y13
LTIYNANIEDAGIYR / File: christine_30turboips_ip01.raw; SpectrumID: ; scans: 14908

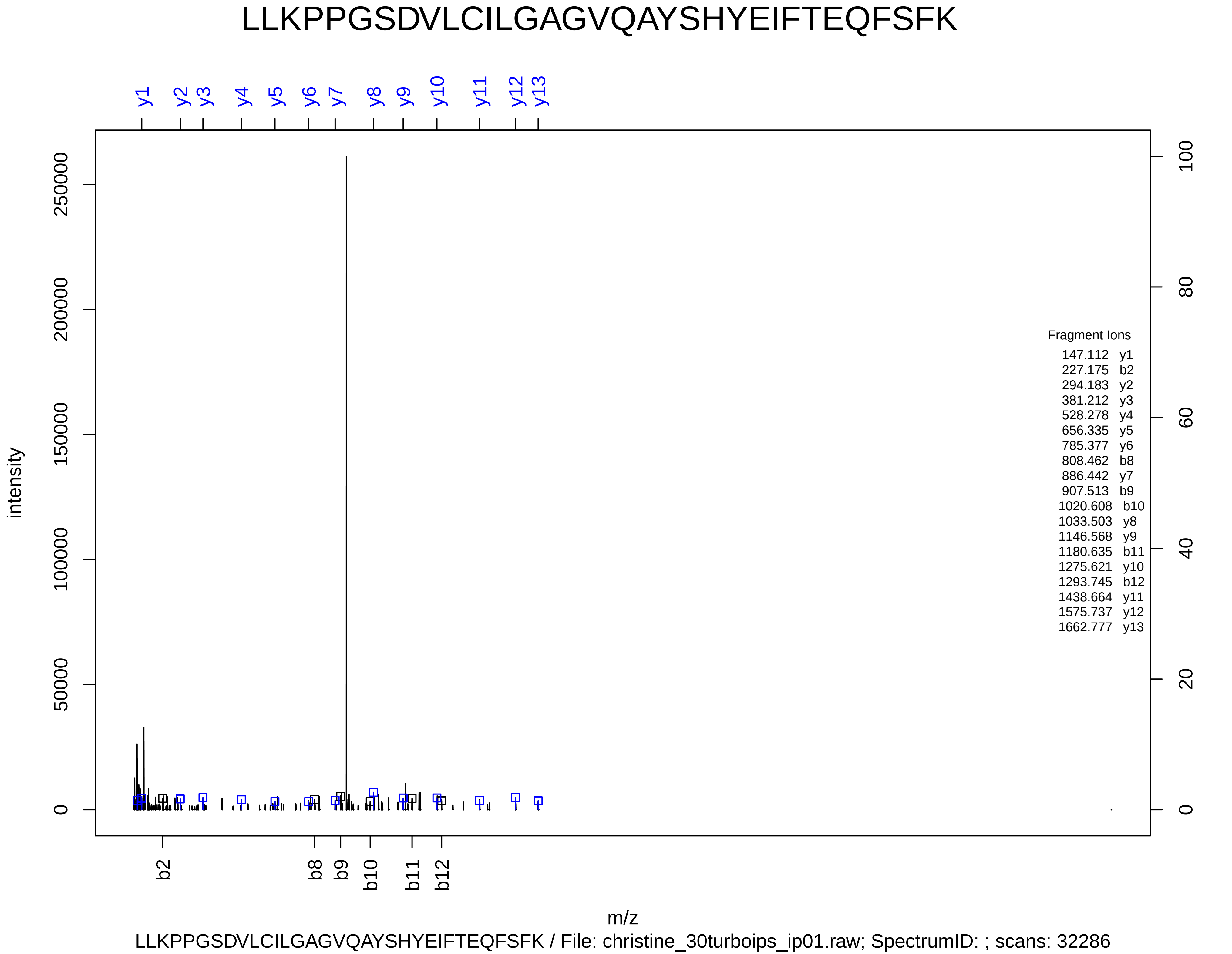
LLKPPGSDVLCILGAGVQAYSHYEIFTEQFSFK
y1 y2 y3 y4 y5 y6 y7 y8 y9 y10 y11 y12 y13
b2 b8 b9 b10 b11 b12
intensity
0
50000
100000
150000
200000
250000
0
20
40
60
80
100
m/z
Fragment Ions
147.112 y1
227.175 b2
294.183 y2
381.212 y3
528.278 y4
656.335 y5
785.377 y6
808.462 b8
886.442 y7
907.513 b9
1020.608 b10
1033.503 y8
1146.568 y9
1180.635 b11
1275.621 y10
1293.745 b12
1438.664 y11
1575.737 y12
1662.777 y13
LLKPPGSDVLCILGAGVQAYSHYEIFTEQFSFK / File: christine_30turboips_ip01.raw; SpectrumID: ; scans: 32286

EQVTNVGGAVVTGVTAVAQK

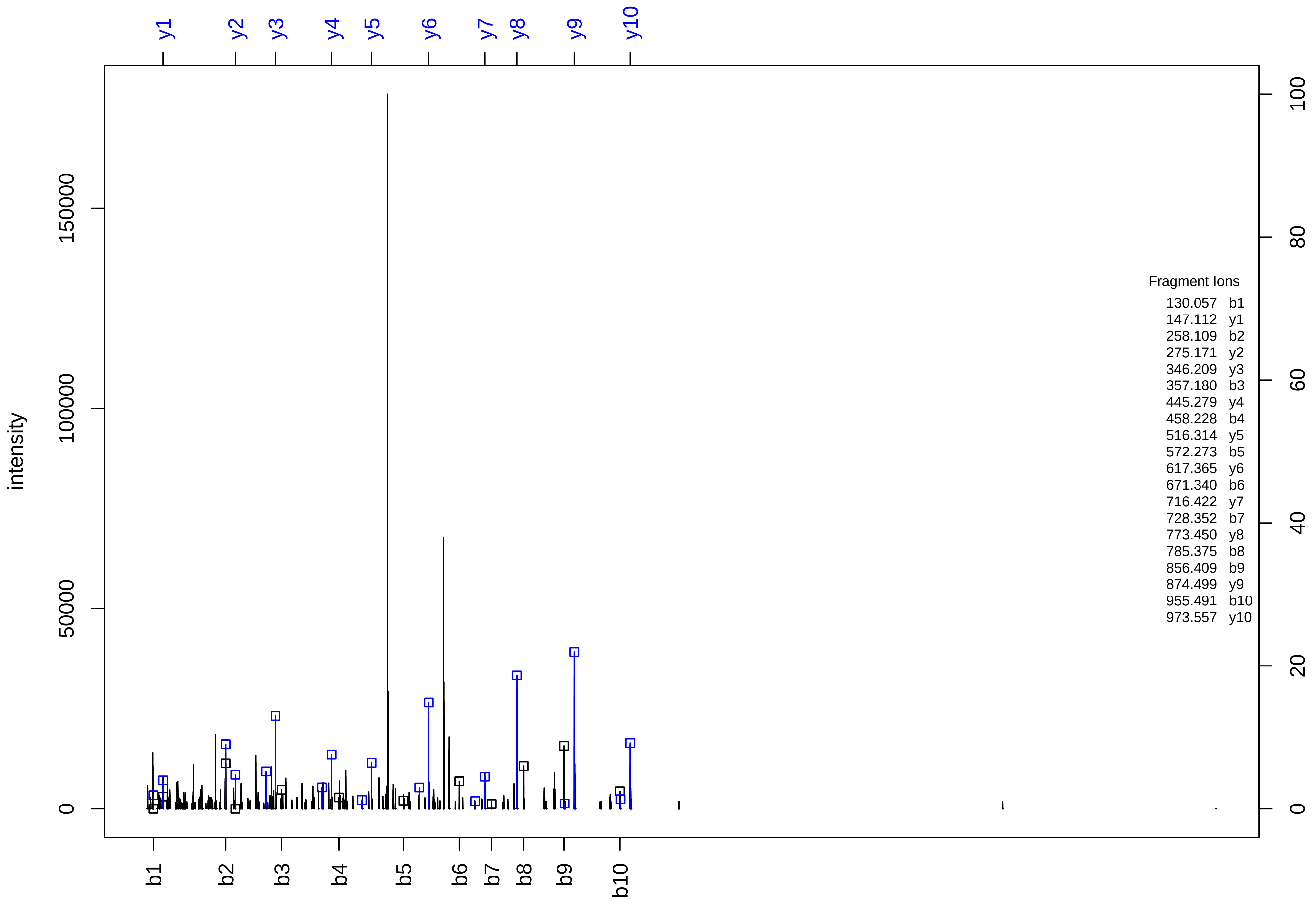

EQVTNVGGAVVTGVTAVAQK / File: christine_30turboips_ip01.raw; SpectrumID: ; scans: 13504

EIYSHFTCATDTSNIQFVFDAVTDVIIQNNLK

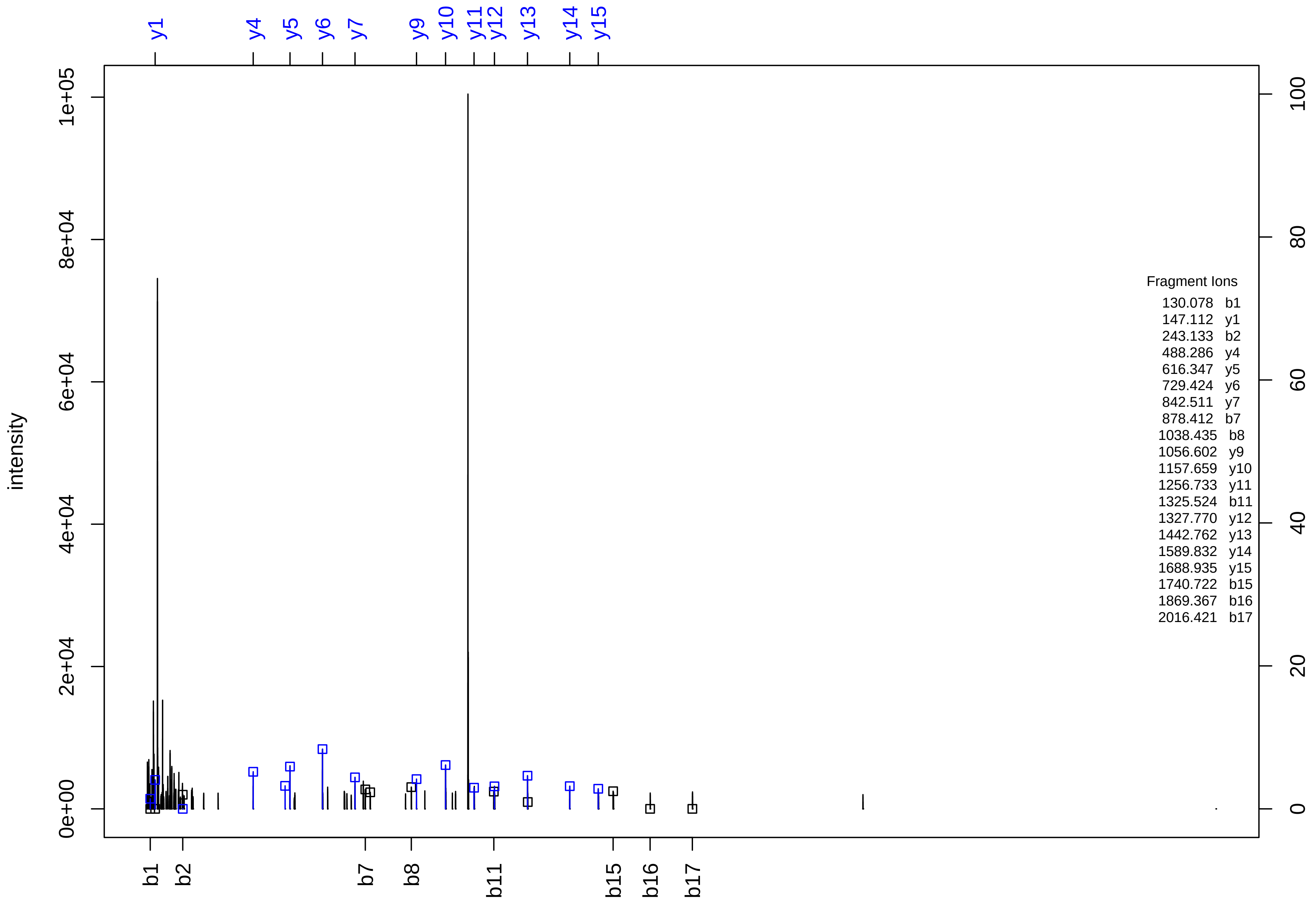

EIYSHFTCATDTSNIQFVFDAVTDVIIQNNLK / File: christine_30turboips_ip01.raw; SpectrumID: ; scans: 32642

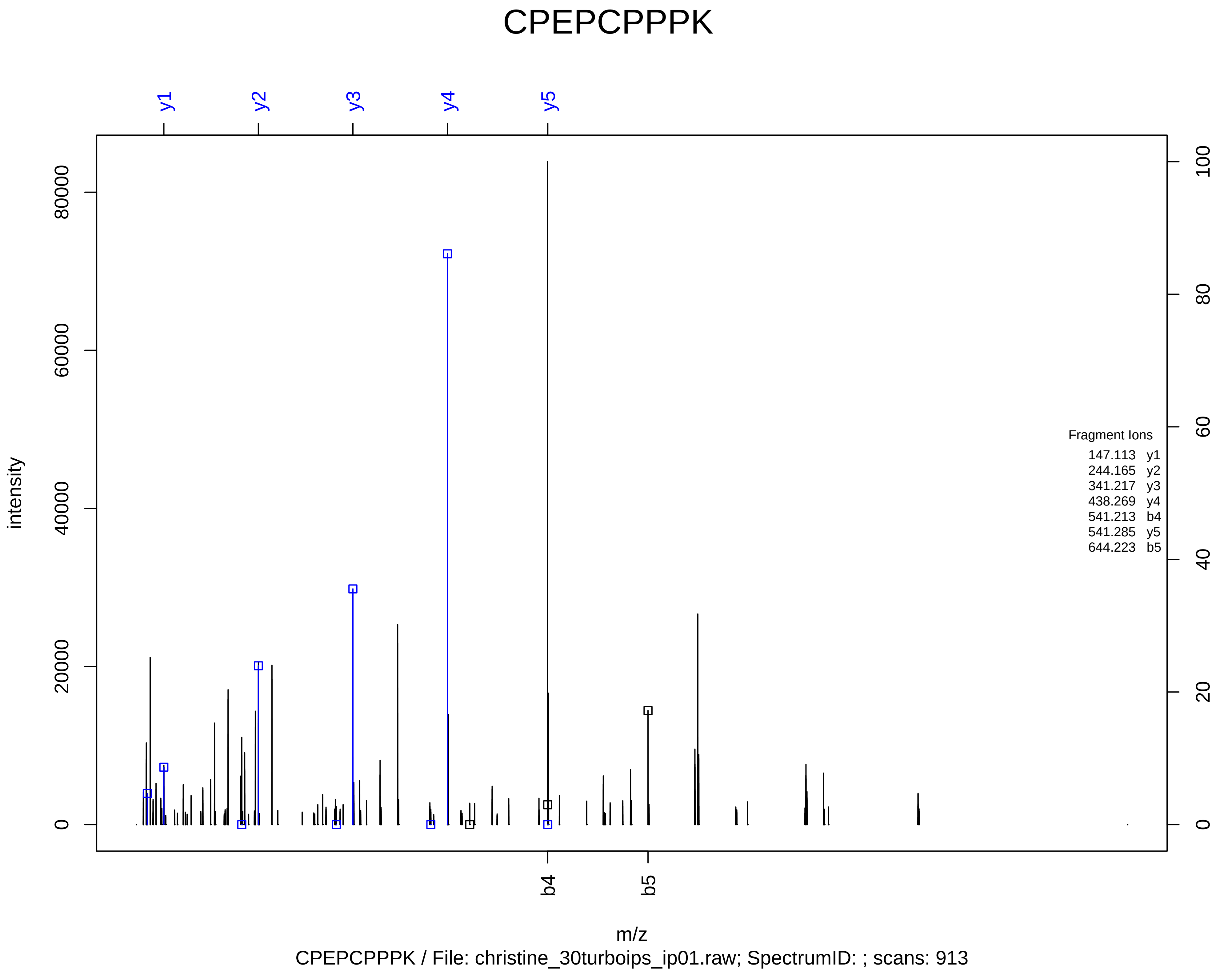
CPEPCPPPK
y1
y2
y3
y4
y5
intensity
0
20000
40000
60000
80000
0
20
40
60
80
100
Fragment Ions
147.113 y1
244.165 y2
341.217 y3
438.269 y4
541.213 b4
541.285 y5
644.223 b5
b4
b5
m/z
CPEPCPPPK / File: christine_30turboips_ip01.raw; SpectrumID: ; scans: 913

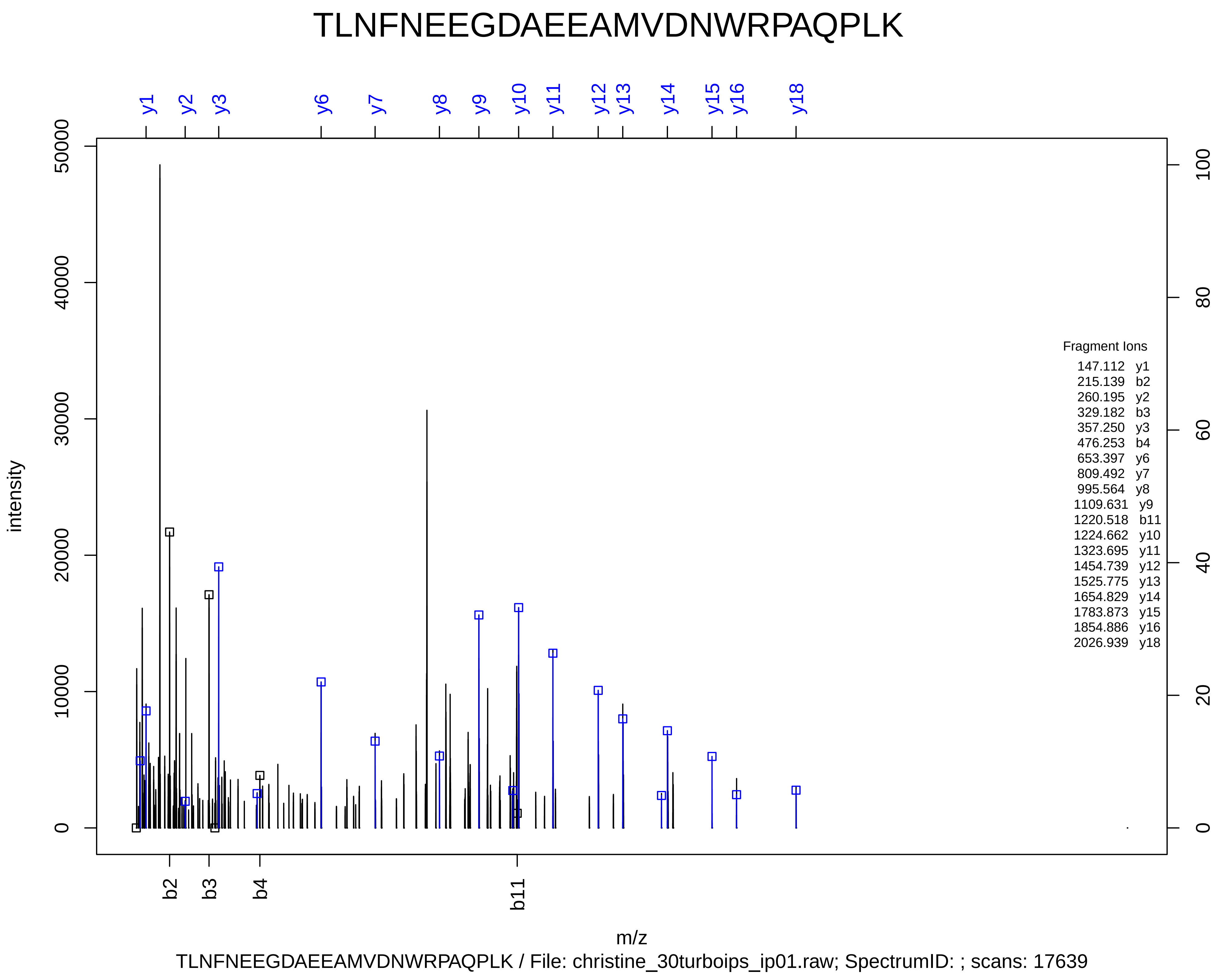

TLNFNEEGDAEEAMVDNWRPAQPLK
y1
y2
y3
y6
y7
y8
y9
y10
y11
y12
y13
y14
y15
y16
y18
intensity
0
10000
20000
30000
40000
50000
0
20
40
60
80
100
b2
b3
b4
b11
m/z
Fragment Ions
147.112 y1
215.139 b2
260.195 y2
329.182 b3
357.250 y3
476.253 b4
653.397 y6
809.492 y7
995.564 y8
1109.631 y9
1220.518 b11
1224.662 y10
1323.695 y11
1454.739 y12
1525.775 y13
1654.829 y14
1783.873 y15
1854.886 y16
2026.939 y18
TLNFNEEGDAEEAMVDNWRPAQPLK / File: christine_30turboips_ip01.raw; SpectrumID: ; scans: 17639

LVFFAEDVGSNK

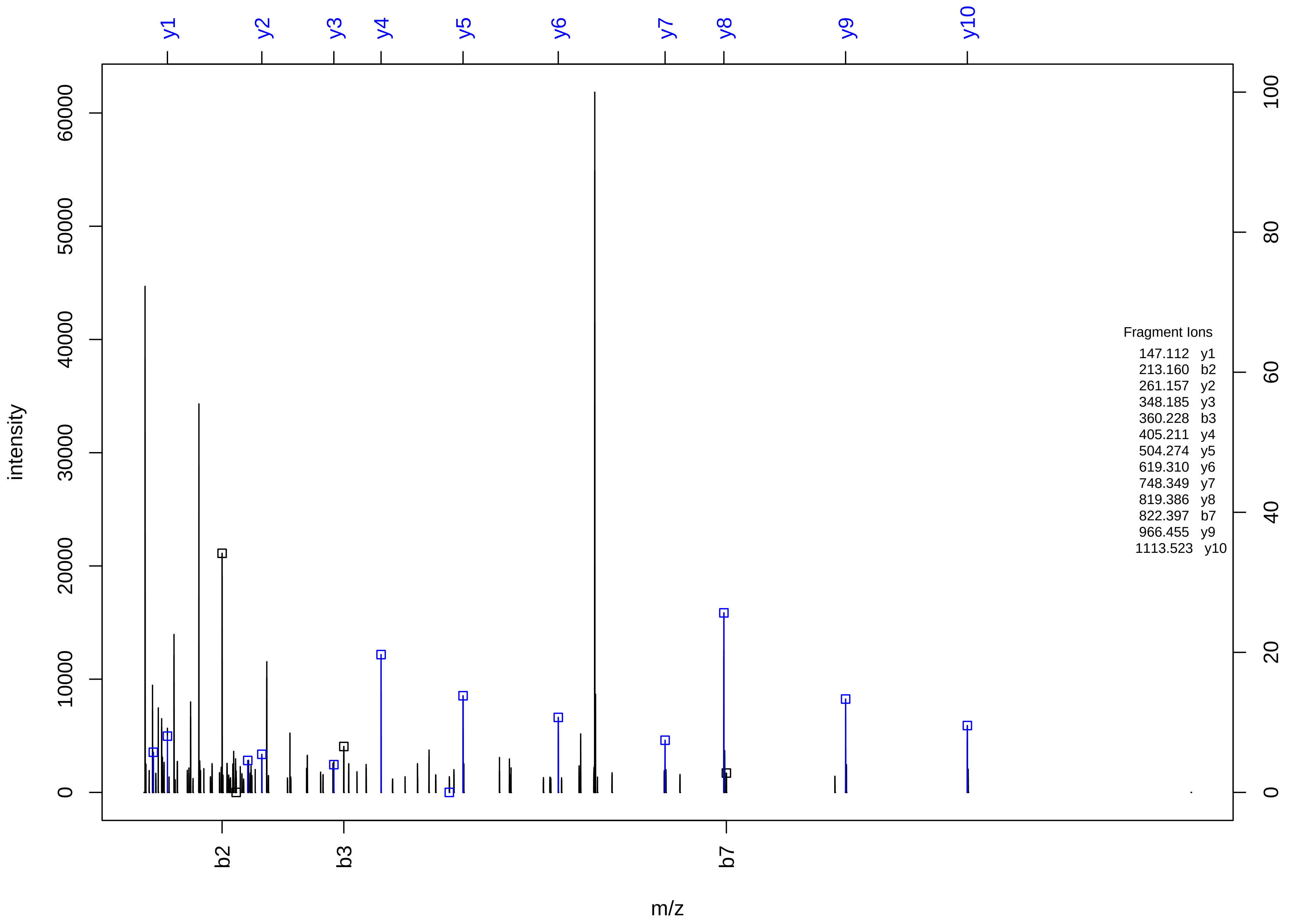

LVFFAEDVGSNK / File: christine_30turboips_ip01.raw; SpectrumID: ; scans: 14290

WGTDEAQFIYILGNR

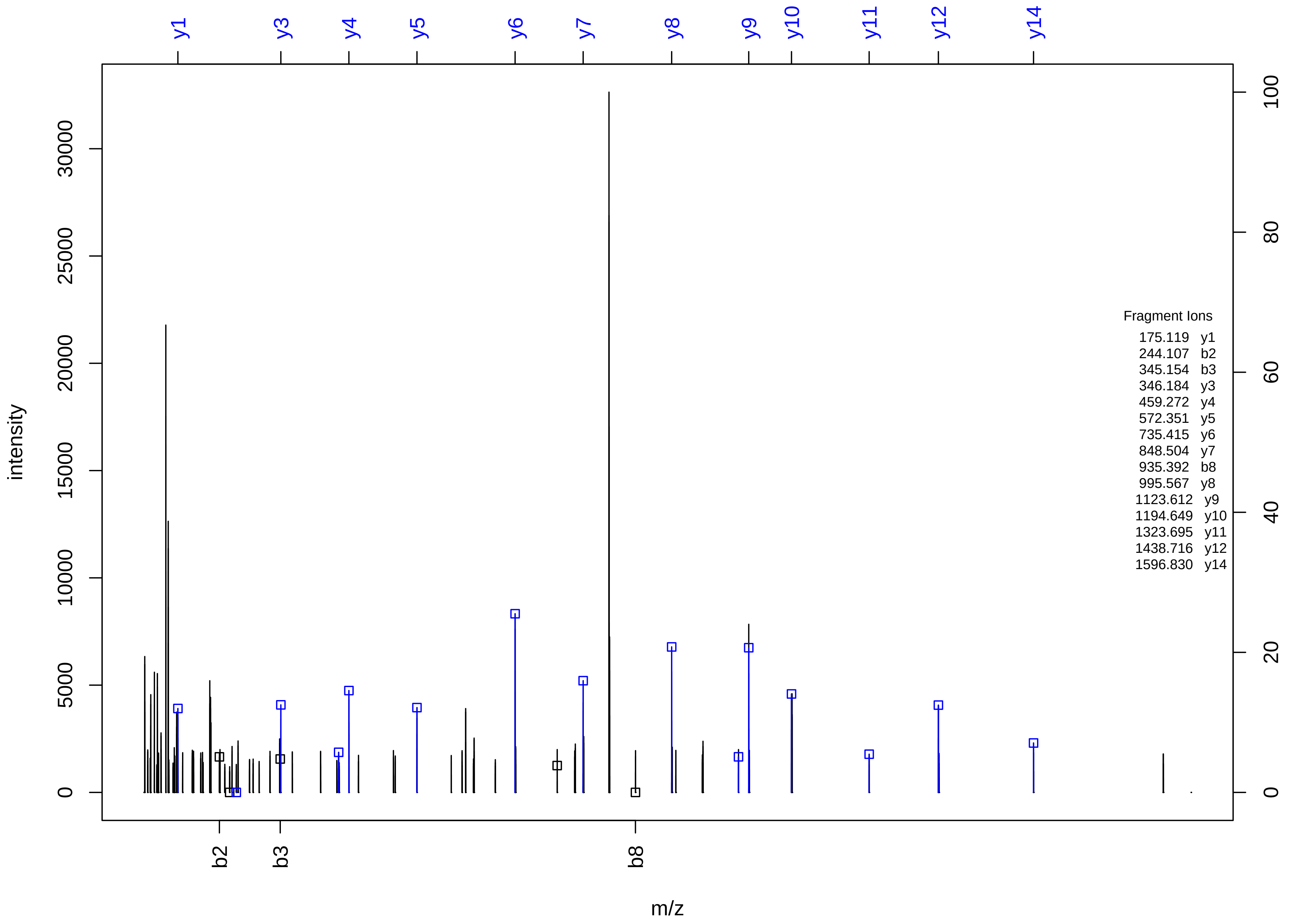

WGTDEAQFIYILGNR / File: christine_30turboips_ip01.raw; SpectrumID: ; scans: 23892

AHVTLGCAADVQPVQTGLDLLDILQQVK

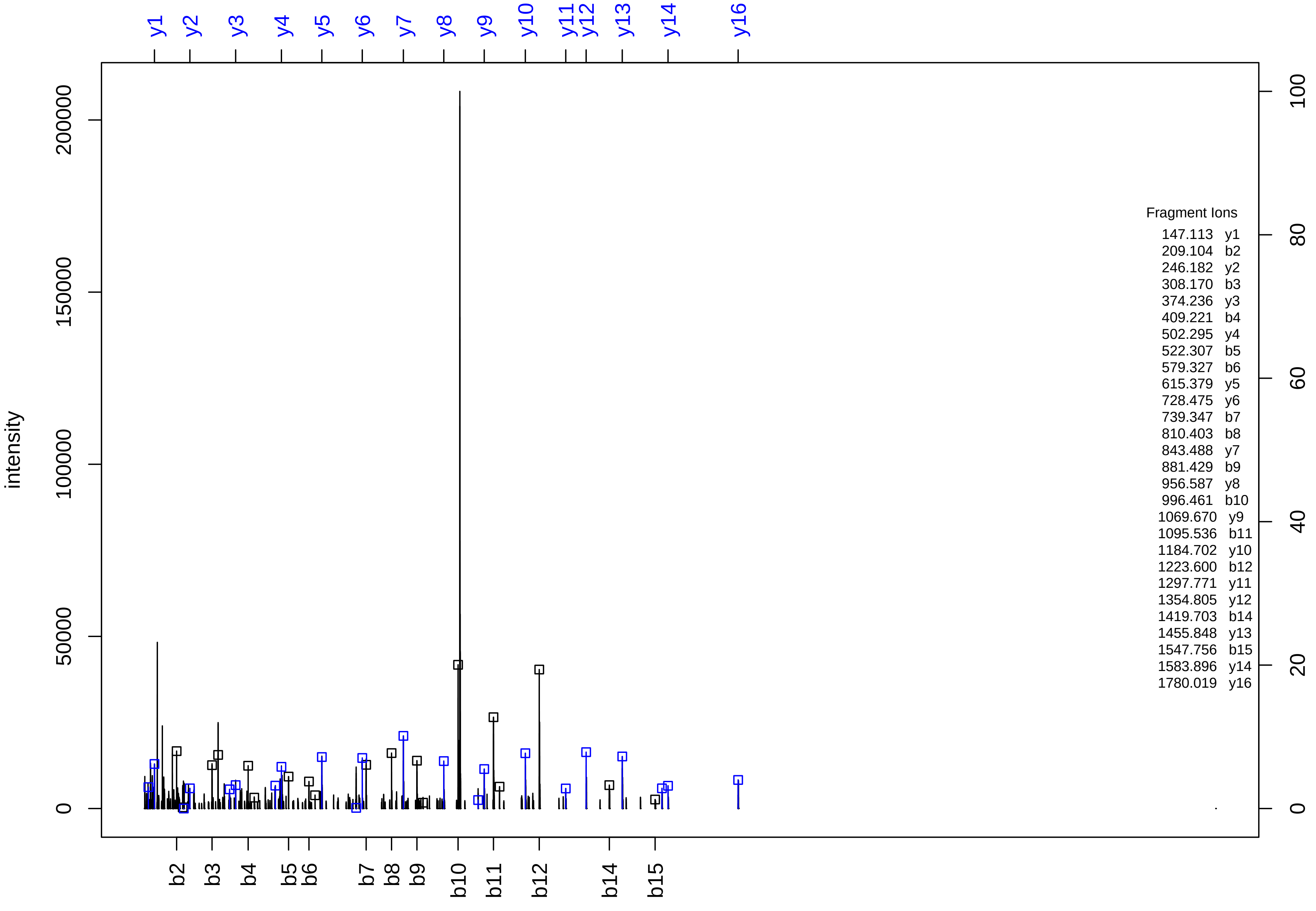

AHVTLGCAADVQPVQTGLDLLDILQQVK / File: christine_30turboips_ip01.raw; SpectrumID: ; scans: 32257

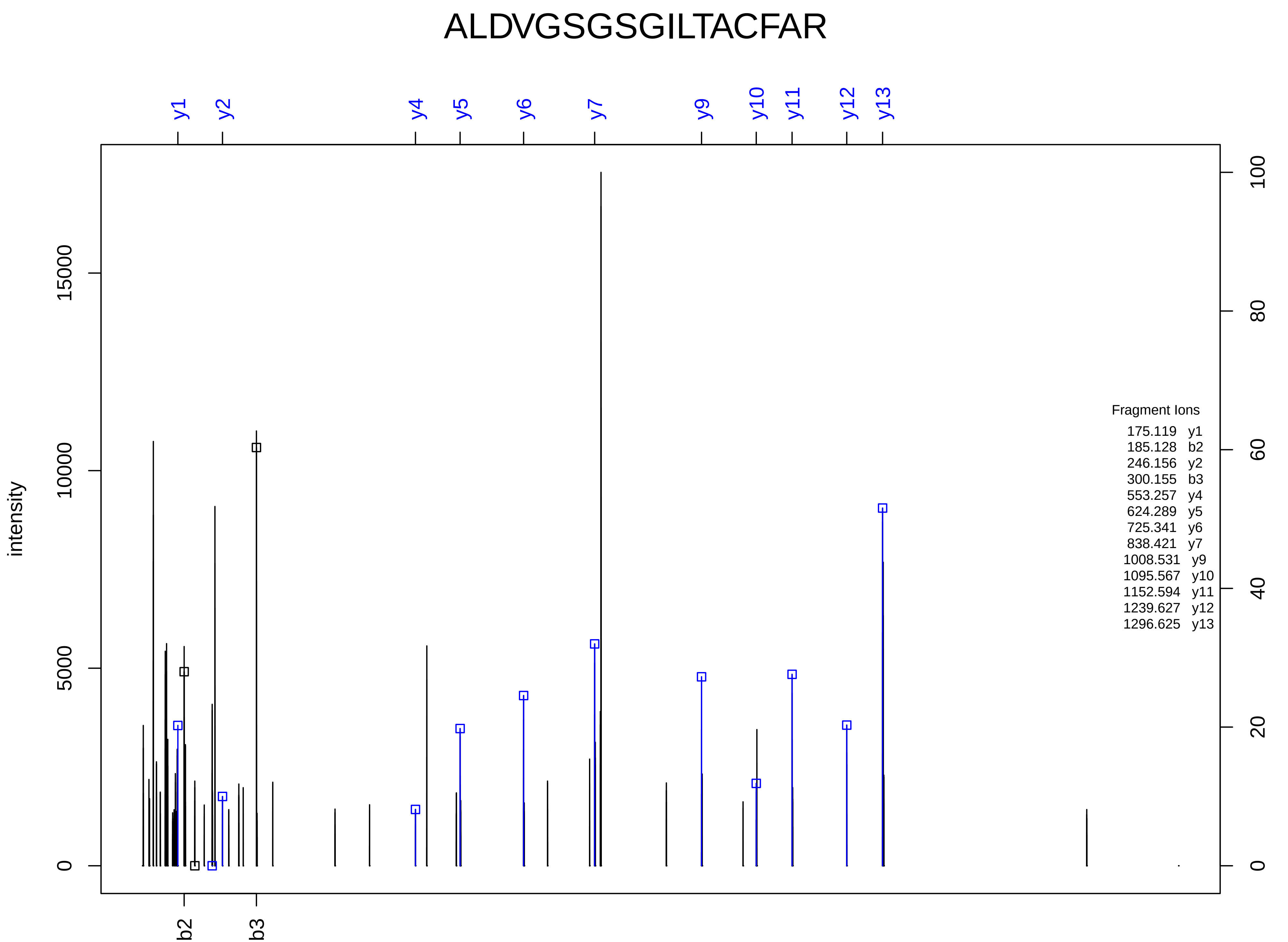

ALDVGSGSGILTACFAR / File: christine_30turboips_ip01.raw; SpectrumID: ; scans: 17278

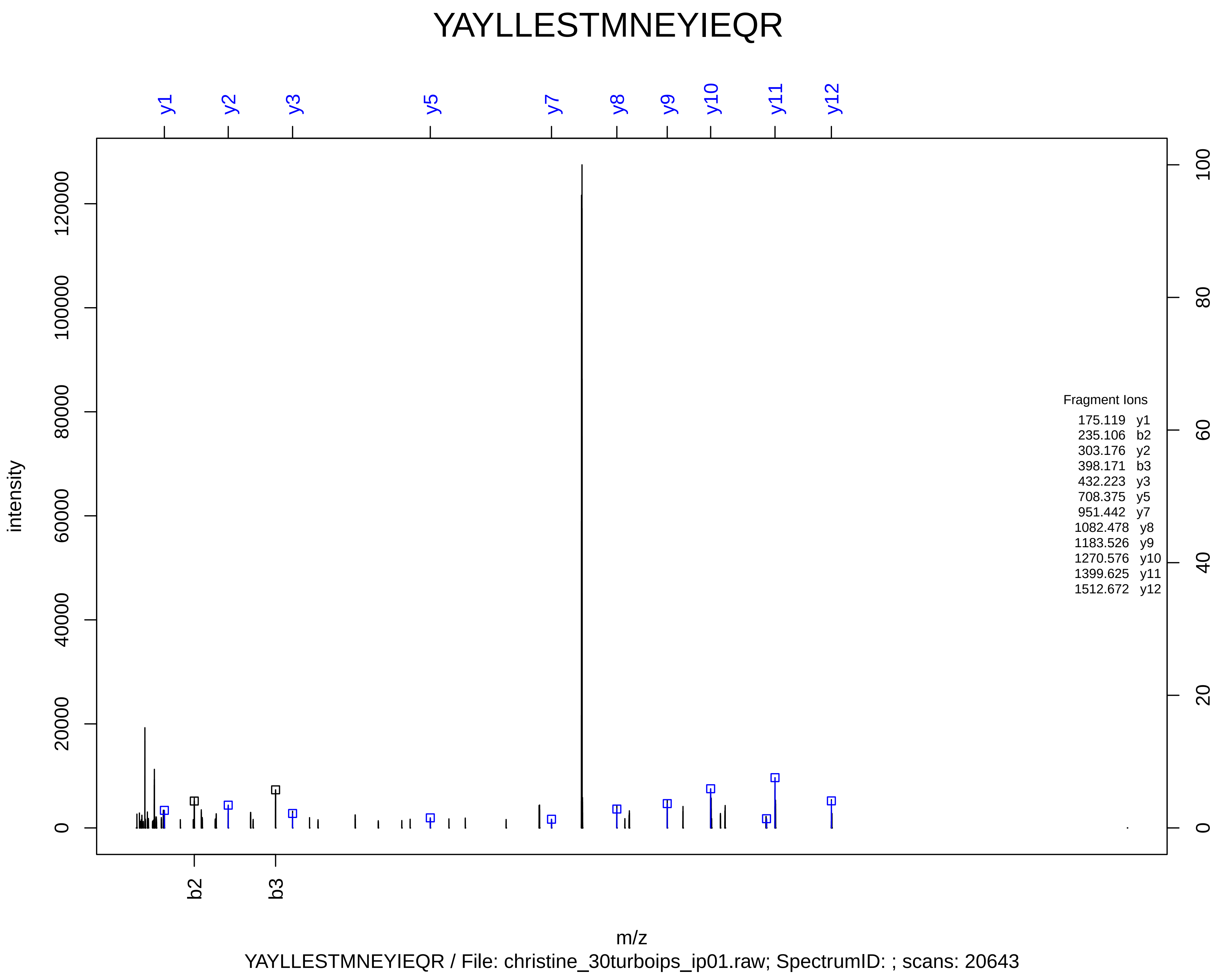
YAYLLESTMNEYIEQR
y1
y2
y3
y5
y7
y8
y9
y10
y11
y12
intensity
0
20000
40000
60000
80000
100000
120000
0
20
40
60
80
100
b2
b3
m/z
Fragment Ions
175.119 y1
235.106 b2
303.176 y2
398.171 b3
432.223 y3
708.375 y5
951.442 y7
1082.478 y8
1183.526 y9
1270.576 y10
1399.625 y11
1512.672 y12
YAYLLESTMNEYIEQR / File: christine_30turboips_ip01.raw; SpectrumID: ; scans: 20643

(Ac)SDAAVDTSSEITTK

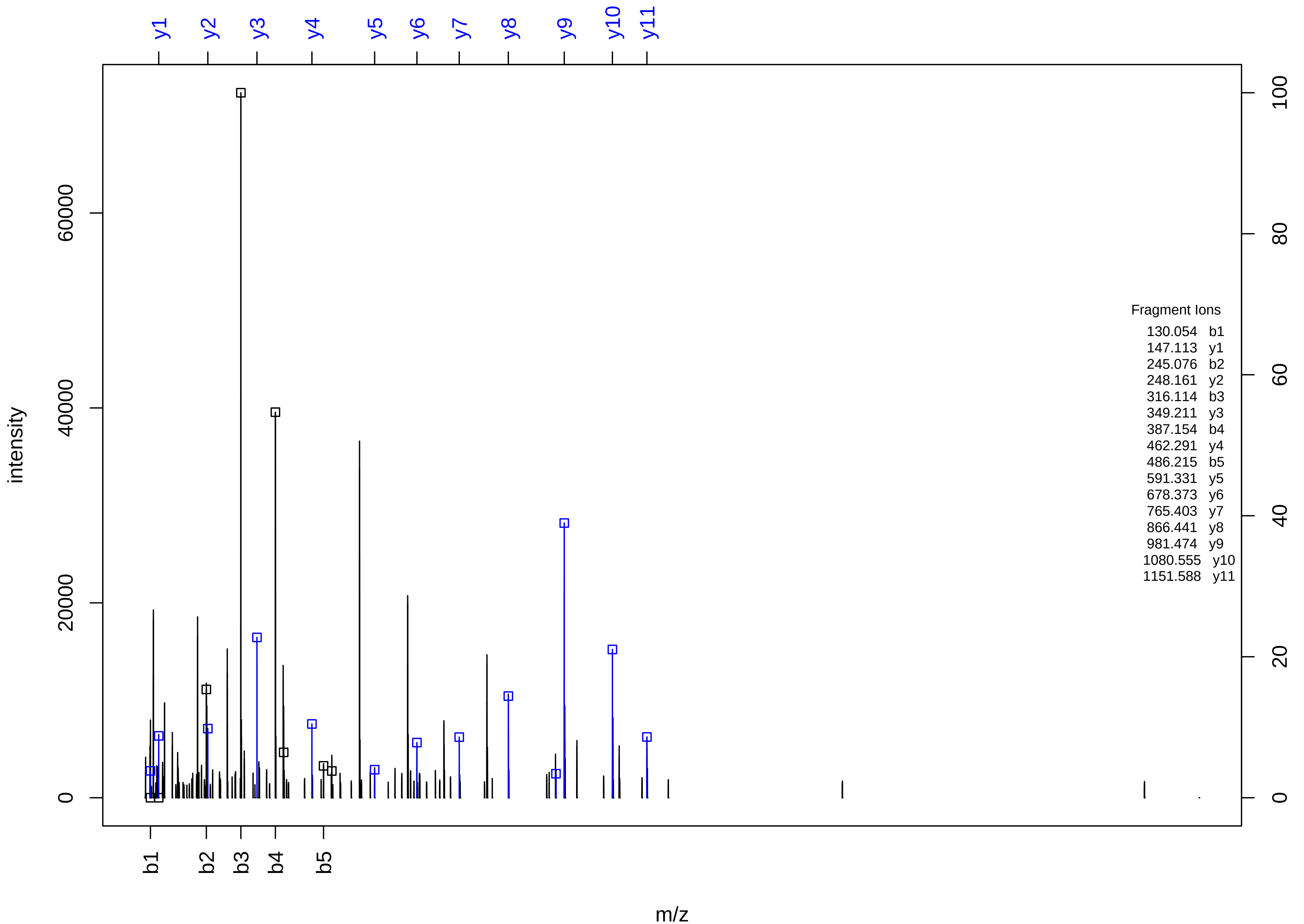

SDAAVDTSSEITTK / File: christine_30turboips_ip01.raw; SpectrumID: ; scans: 6176

GWEEGVAQMSVGQR

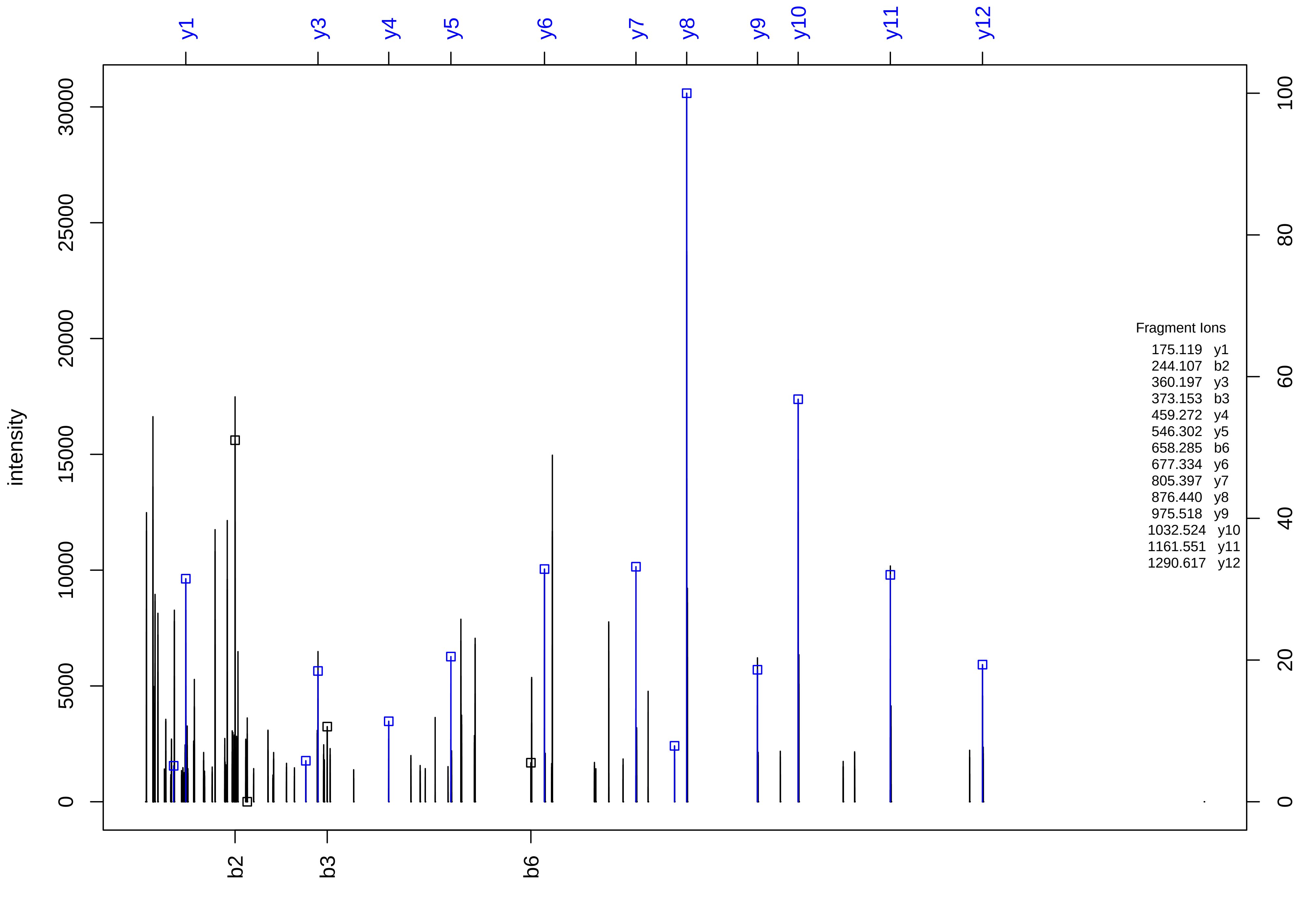

GWEEGVAQMSVGQR / File: christine_30turboips_ip01.raw; SpectrumID: ; scans: 9427

TTPTGWTLDQCIQTGVDNPGHPFIK

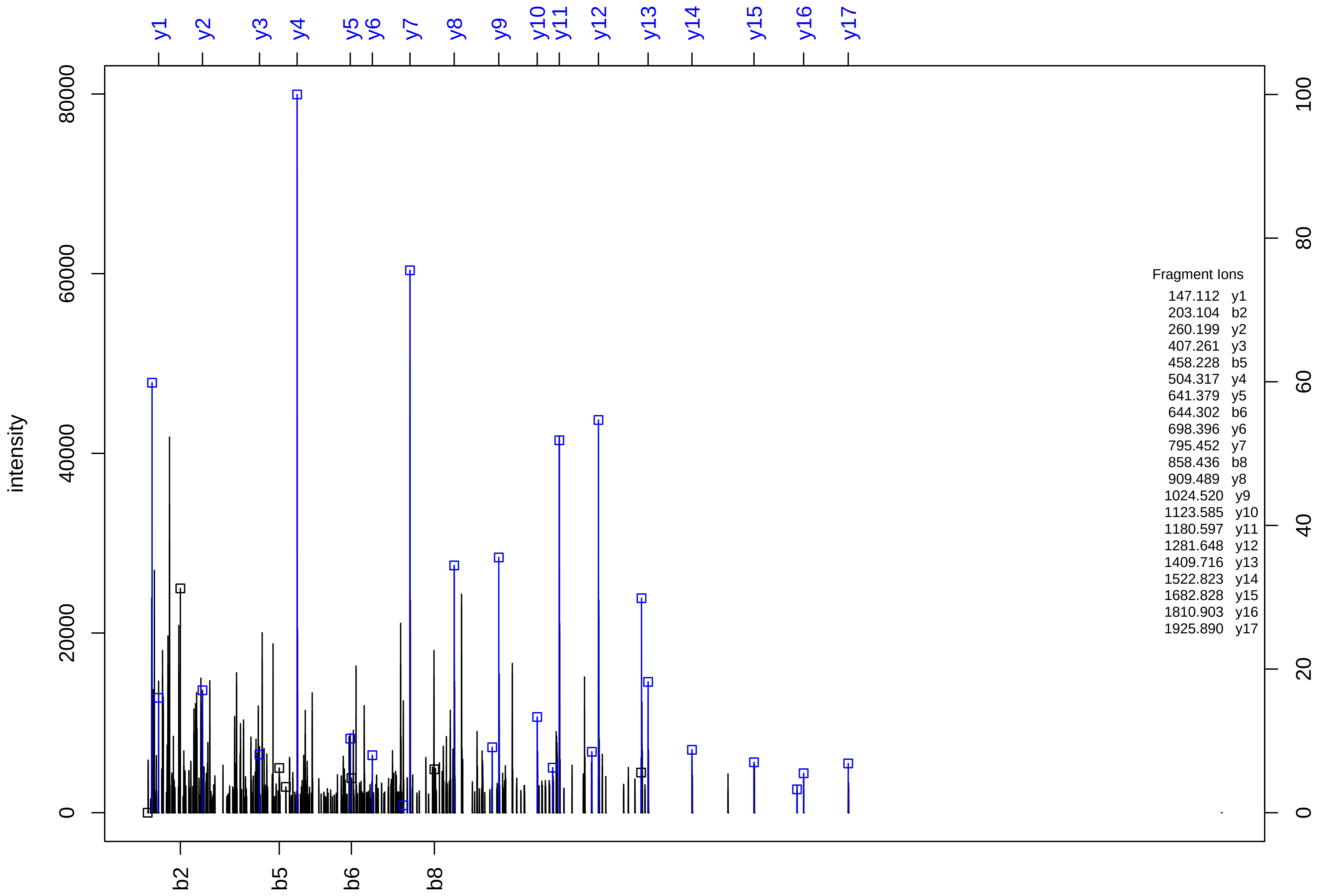

TTPTGWTLDQCIQTGVDNPGHPFIK / File: christine_30turboips_ip01.raw; SpectrumID: ; scans: 16384

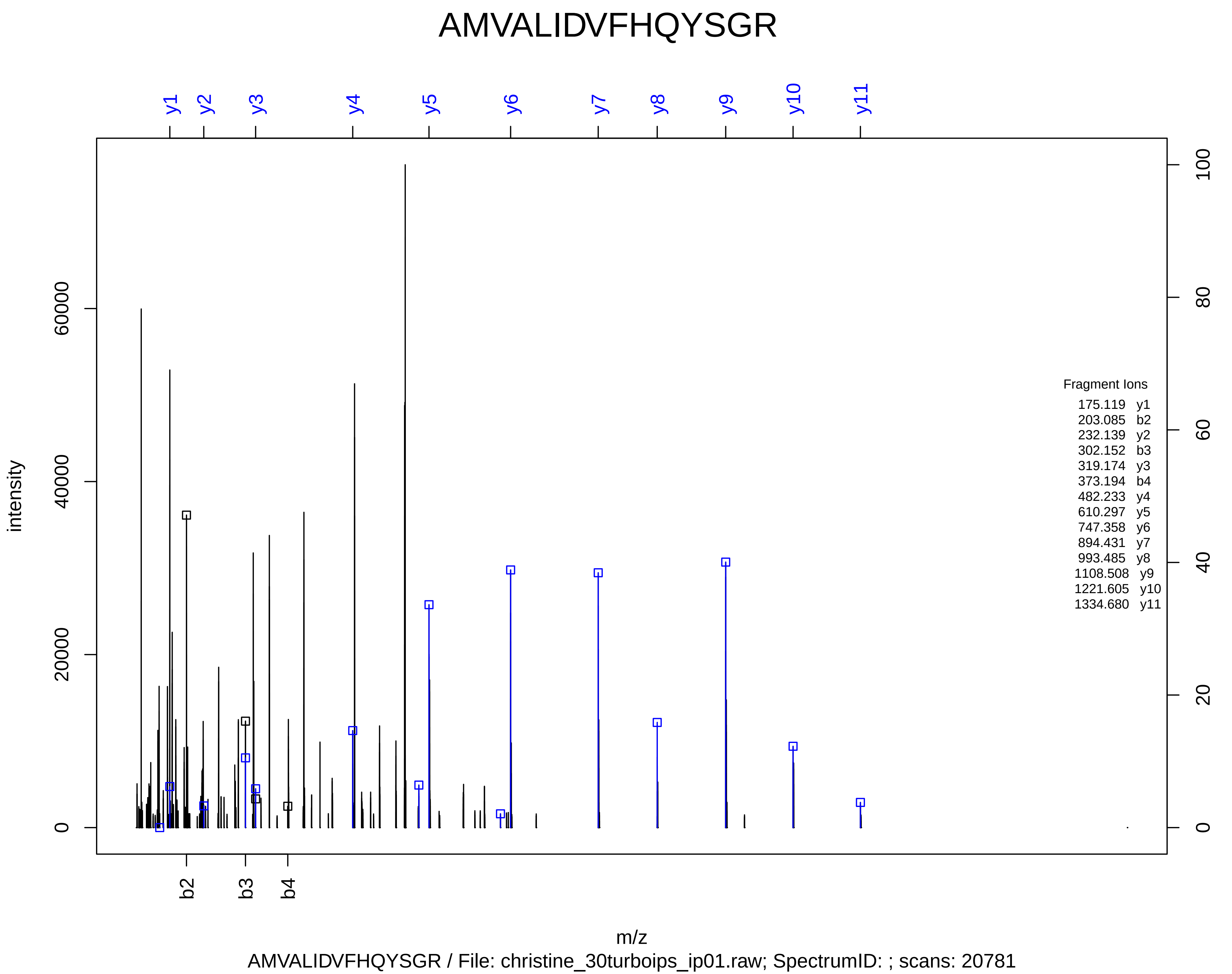
AMVALIDVFHQYSGR
y1 y2 y3 y4 y5 y6 y7 y8 y9 y10 y11
b2 b3 b4
intensity
0
20000
40000
60000
0
20
40
60
80
100
m/z
Fragment Ions
175.119 y1
203.085 b2
232.139 y2
302.152 b3
319.174 y3
373.194 b4
482.233 y4
610.297 y5
747.358 y6
894.431 y7
993.485 y8
1108.508 y9
1221.605 y10
1334.680 y11
AMVALIDVFHQYSGR / File: christine_30turboips_ip01.raw; SpectrumID: ; scans: 20781

DNVAGVTLPVFEHYHEGTDSYELTGLAR

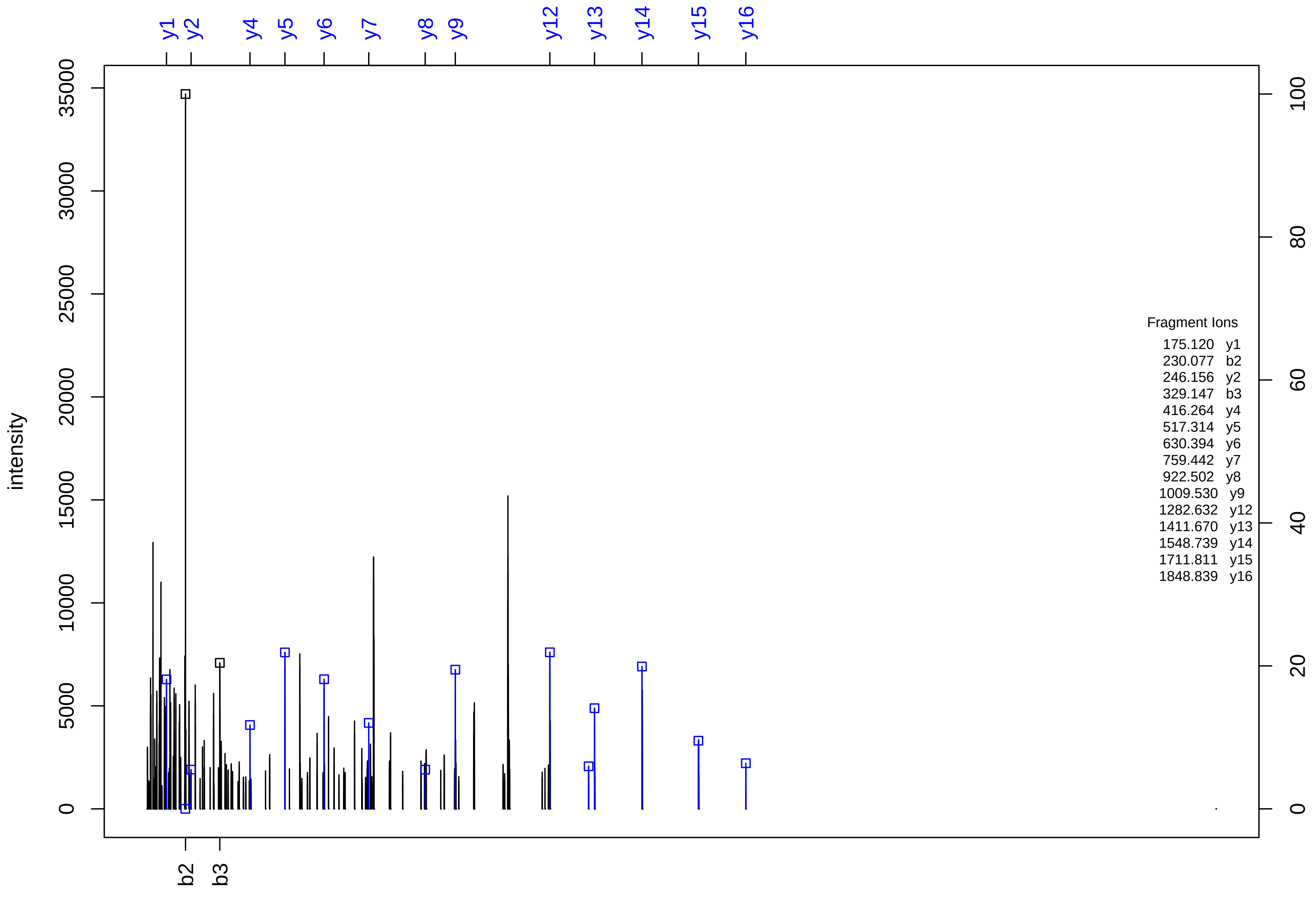

DNVAGVTLPVFEHYHEGTDSYELTGLAR / File: christine_30turboips_ip01.raw; SpectrumID: ; scans: 19679

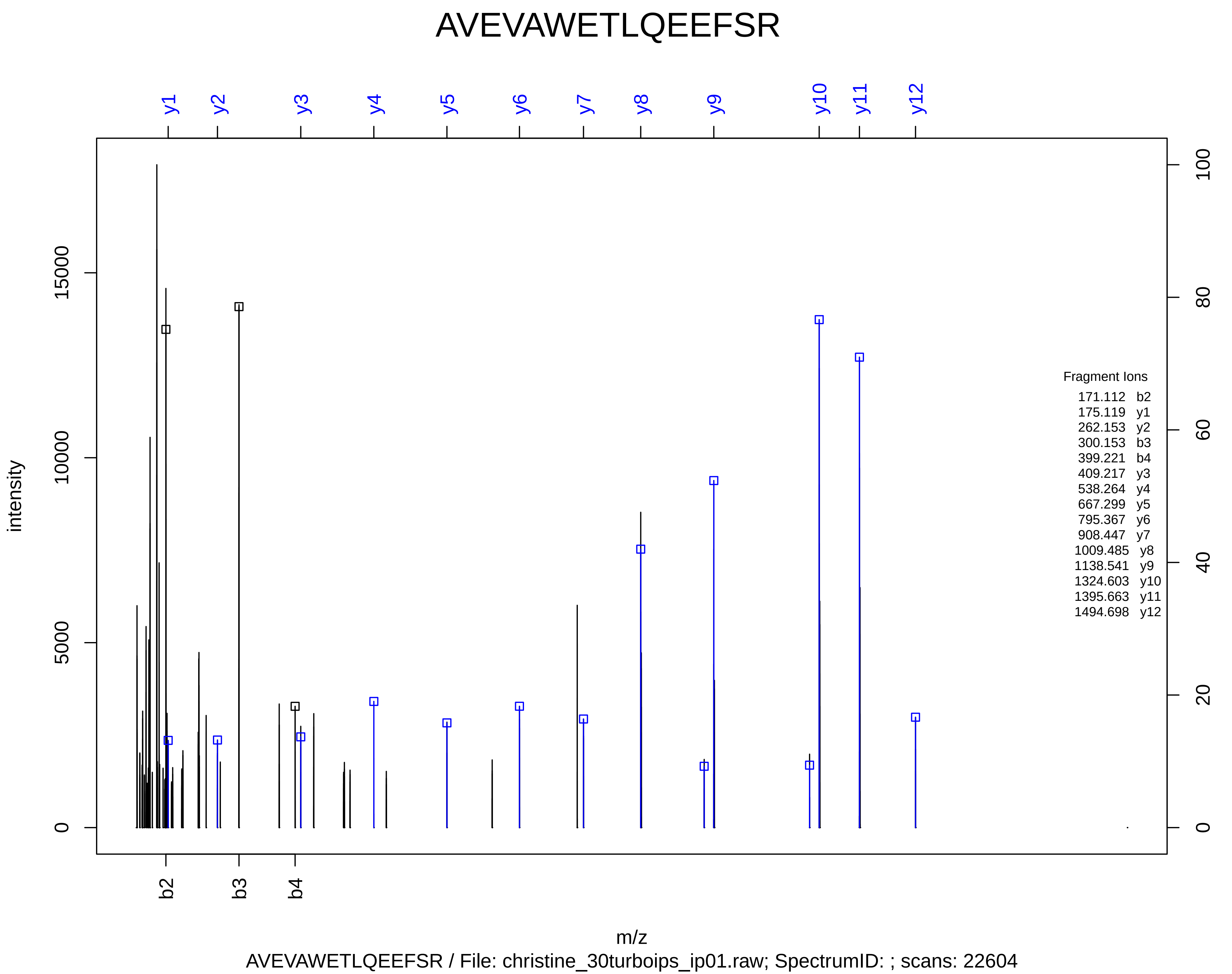
AVEVAWETLQEEFSR
y1
y2
y3
y4
y5
y6
y7
y8
y9
y10
y11
y12
intensity
0
5000
10000
15000
0
20
40
60
80
100
Fragment Ions
171.112 b2
175.119 y1
262.153 y2
300.153 b3
399.221 b4
409.217 y3
538.264 y4
667.299 y5
795.367 y6
908.447 y7
1009.485 y8
1138.541 y9
1324.603 y10
1395.663 y11
1494.698 y12
b2
b3
b4
m/z
AVEVAWETLQEEFSR / File: christine_30turboips_ip01.raw; SpectrumID: ; scans: 22604

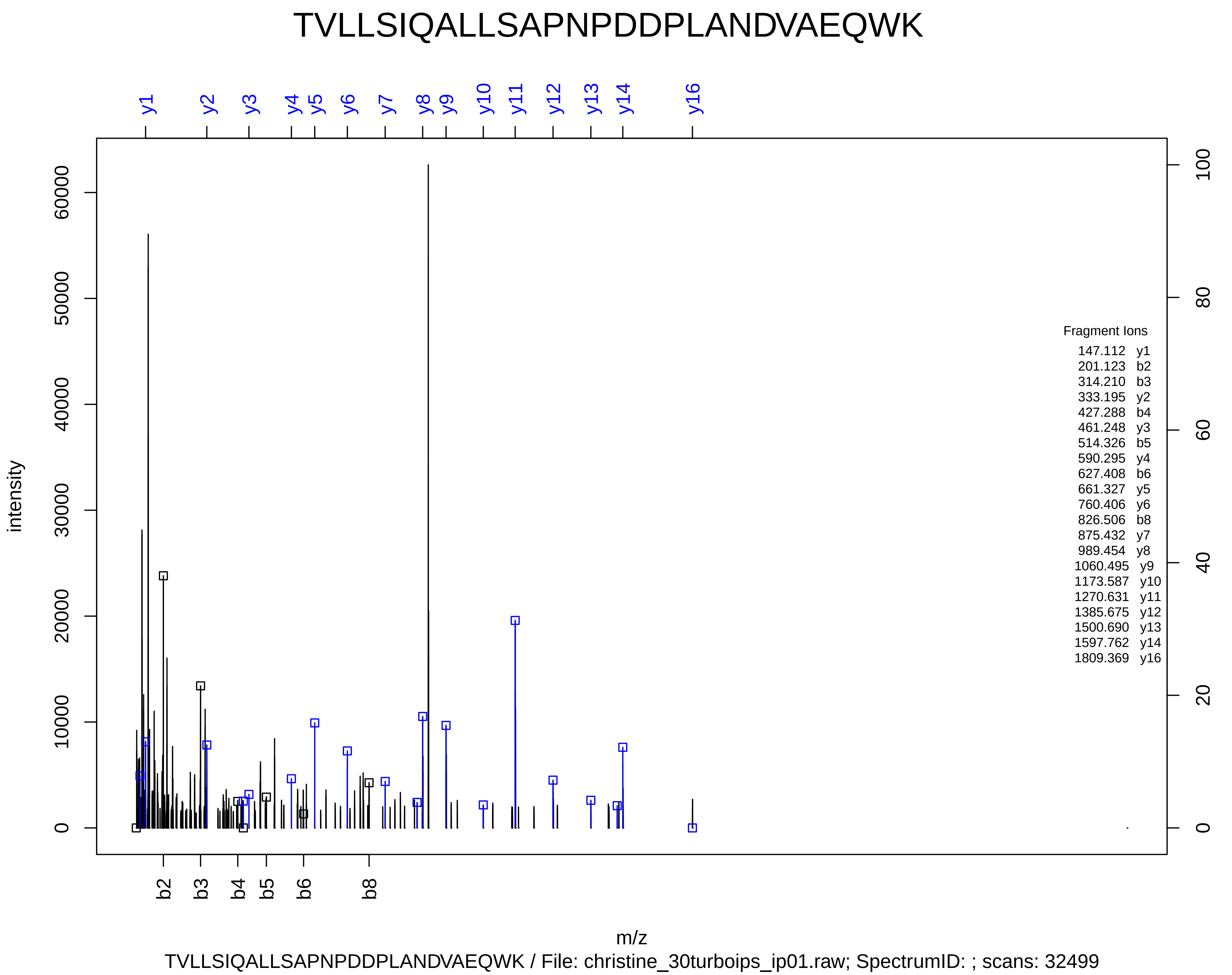

TVLLSIQALLSAPNPDDPLANDVAEQWK
y1
y2
y3
y4
y5
y6
y7
y8
y9
y10
y11
y12
y13
y14
y16
intensity
0
10000
20000
30000
40000
50000
60000
0
20
40
60
80
100
b2
b3
b4
b5
b6
b8
Fragment Ions
147.112 y1
201.123 b2
314.210 b3
333.195 y2
427.288 b4
461.248 y3
514.326 b5
590.295 y4
627.408 b6
661.327 y5
760.406 y6
826.506 b8
875.432 y7
989.454 y8
1060.495 y9
1173.587 y10
1270.631 y11
1385.675 y12
1500.690 y13
1597.762 y14
1809.369 y16
m/z
TVLLSIQALLSAPNPDDPLANDVAEQWK / File: christine_30turboips_ip01.raw; SpectrumID: ; scans: 32499

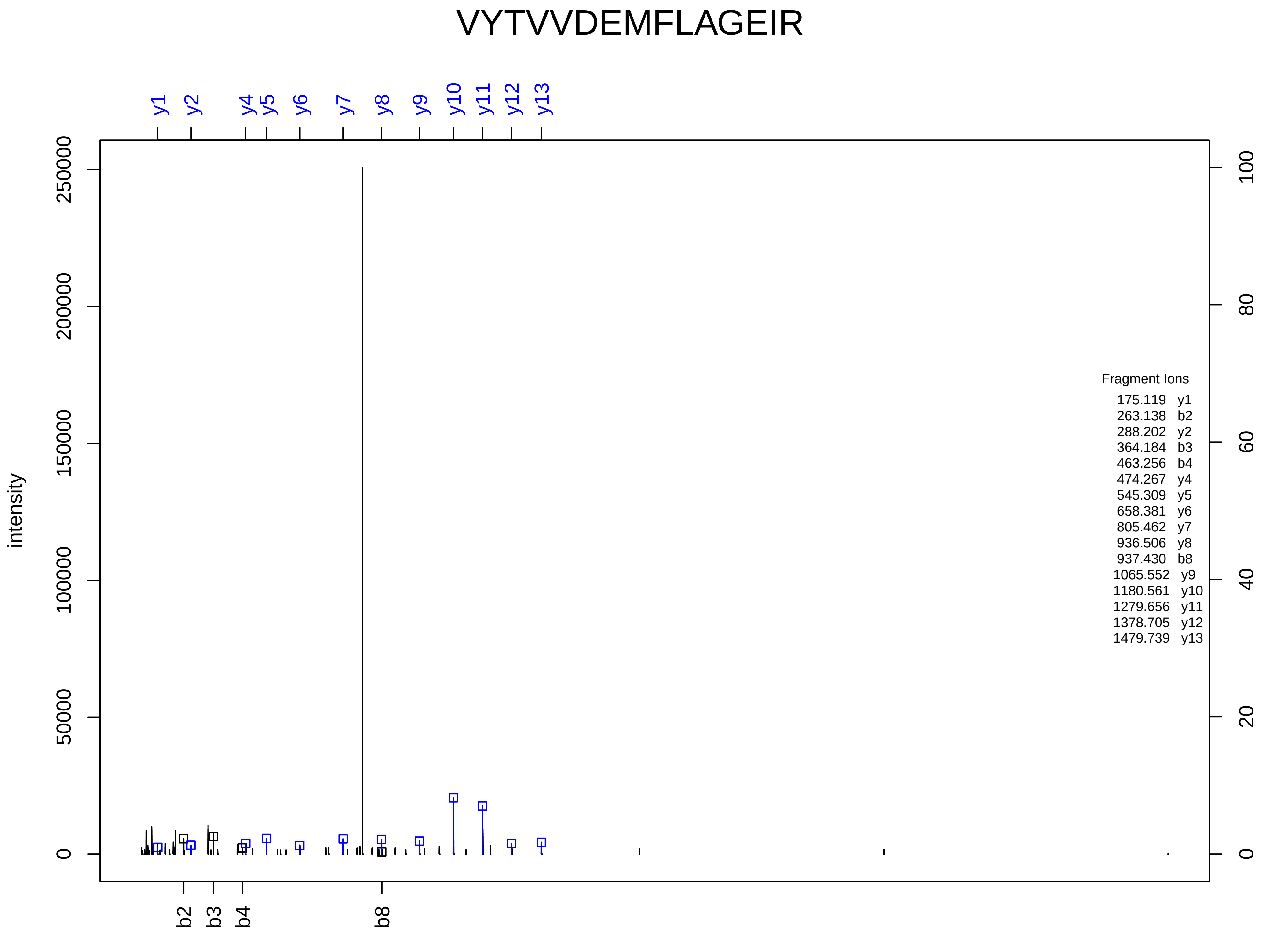

VYTVVDEMFLAGEIR / File: christine_30turboips_ip01.raw; SpectrumID: ; scans: 25641

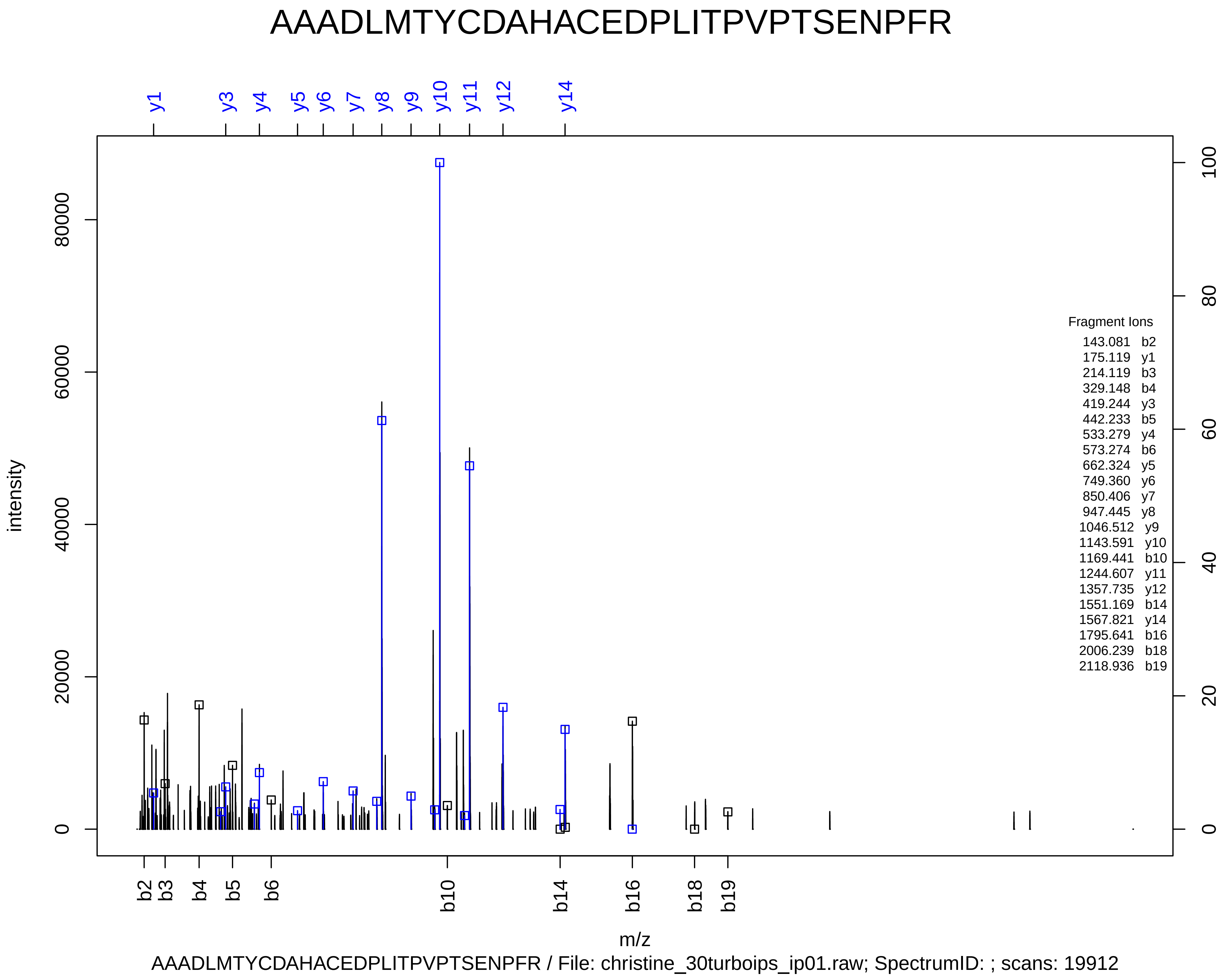

AAADLMTYCDAHACEDPLITPVPTSENPFR
y1
y3
y4
y5
y6
y7
y8
y9
y10
y11
y12
y14
intensity
0
20000
40000
60000
80000
0
20
40
60
80
100
Fragment Ions
143.081 b2
175.119 y1
214.119 b3
329.148 b4
419.244 y3
442.233 b5
533.279 y4
573.274 b6
662.324 y5
749.360 y6
850.406 y7
947.445 y8
1046.512 y9
1143.591 y10
1169.441 b10
1244.607 y11
1357.735 y12
1551.169 b14
1567.821 y14
1795.641 b16
2006.239 b18
2118.936 b19
b2
b3
b4
b5
b6
b10
b14
b16
b18
b19
m/z
AAADLMTYCDAHACEDPLITPVPTSENPFR / File: christine_30turboips_ip01.raw; SpectrumID: ; scans: 19912

LVLASIDQADFQGFTYVNPDFVHPDAR

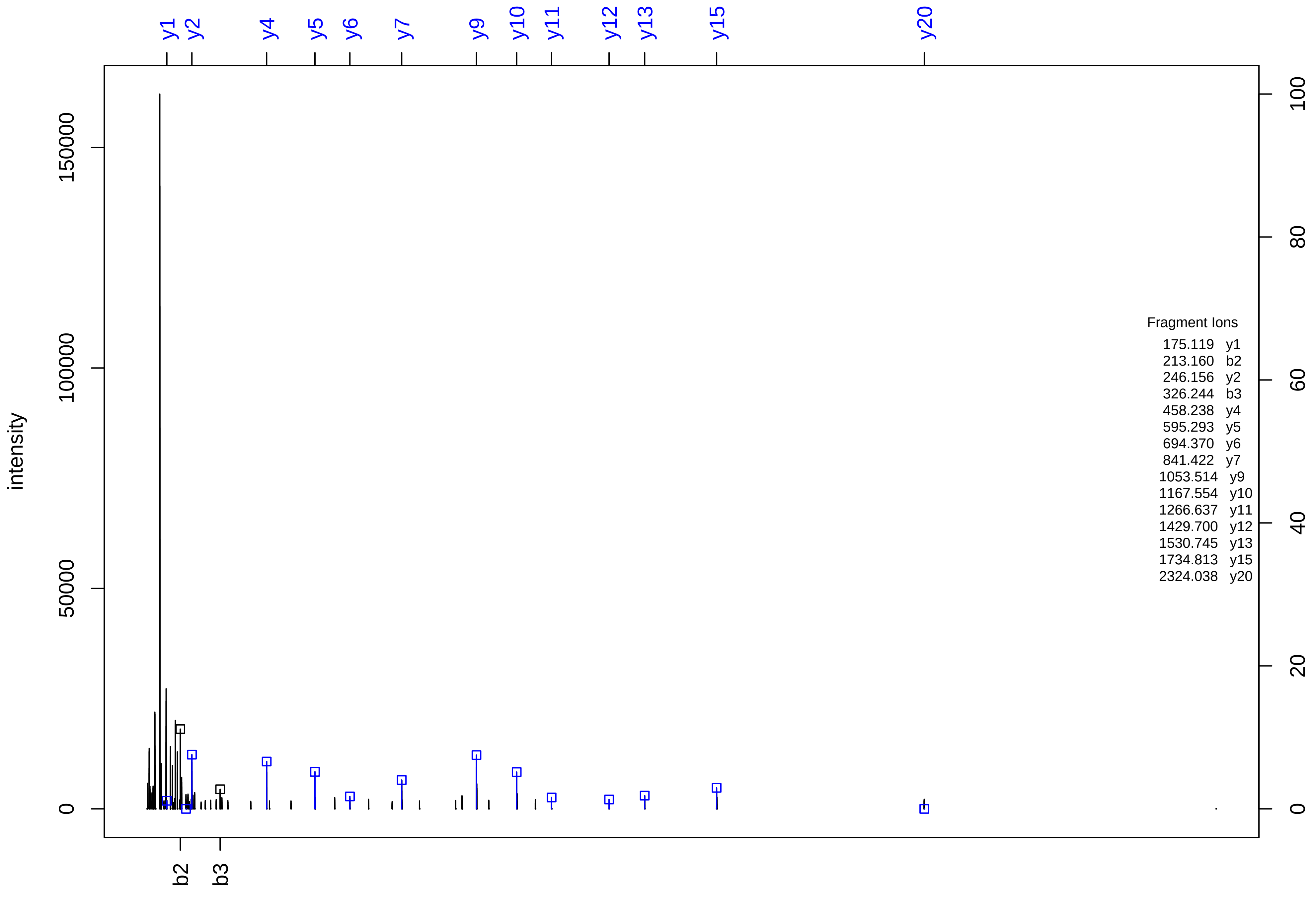

LVLASIDQADFQGFTYVNPDFVHPDAR / File: christine_30turboips_ip01.raw; SpectrumID: ; scans: 25258

NIQVDEANLLTWQGLIVPDNPPYDK

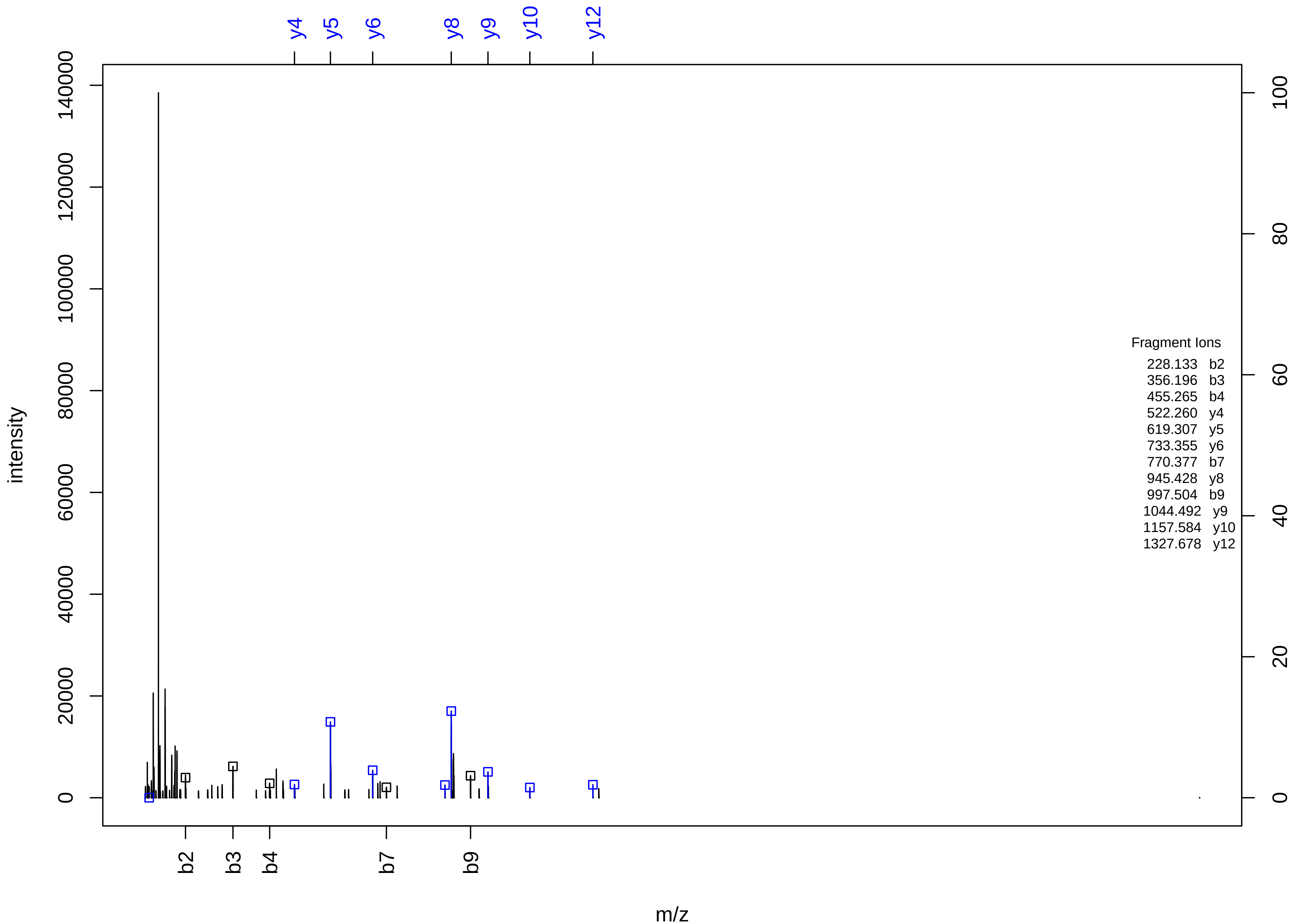

NIQVDEANLLTWQGLIVPDNPPYDK / File: christine_30turboips_ip01.raw; SpectrumID: ; scans: 29582

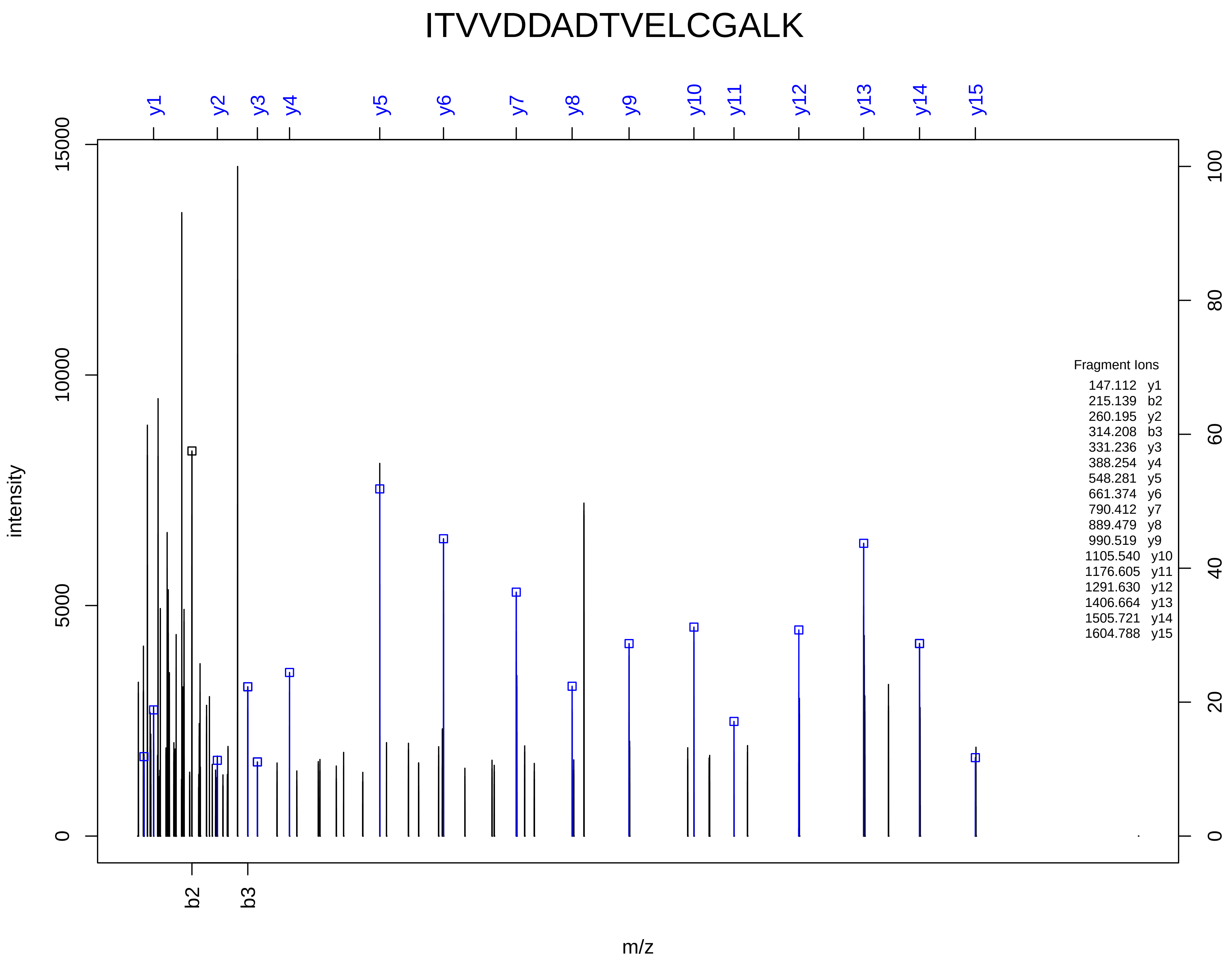
ITVVDDADTVELCGALK
y1
y2
y3
y4
y5
y6
y7
y8
y9
y10
y11
y12
y13
y14
y15
b2
b3
intensity
m/z
Fragment Ions
147.112 y1
215.139 b2
260.195 y2
314.208 b3
331.236 y3
388.254 y4
548.281 y5
661.374 y6
790.412 y7
889.479 y8
990.519 y9
1105.540 y10
1176.605 y11
1291.630 y12
1406.664 y13
1505.721 y14
1604.788 y15
ITVVDDADTVELCGALK / File: christine_30turboips_ip01.raw; SpectrumID: ; scans: 16034

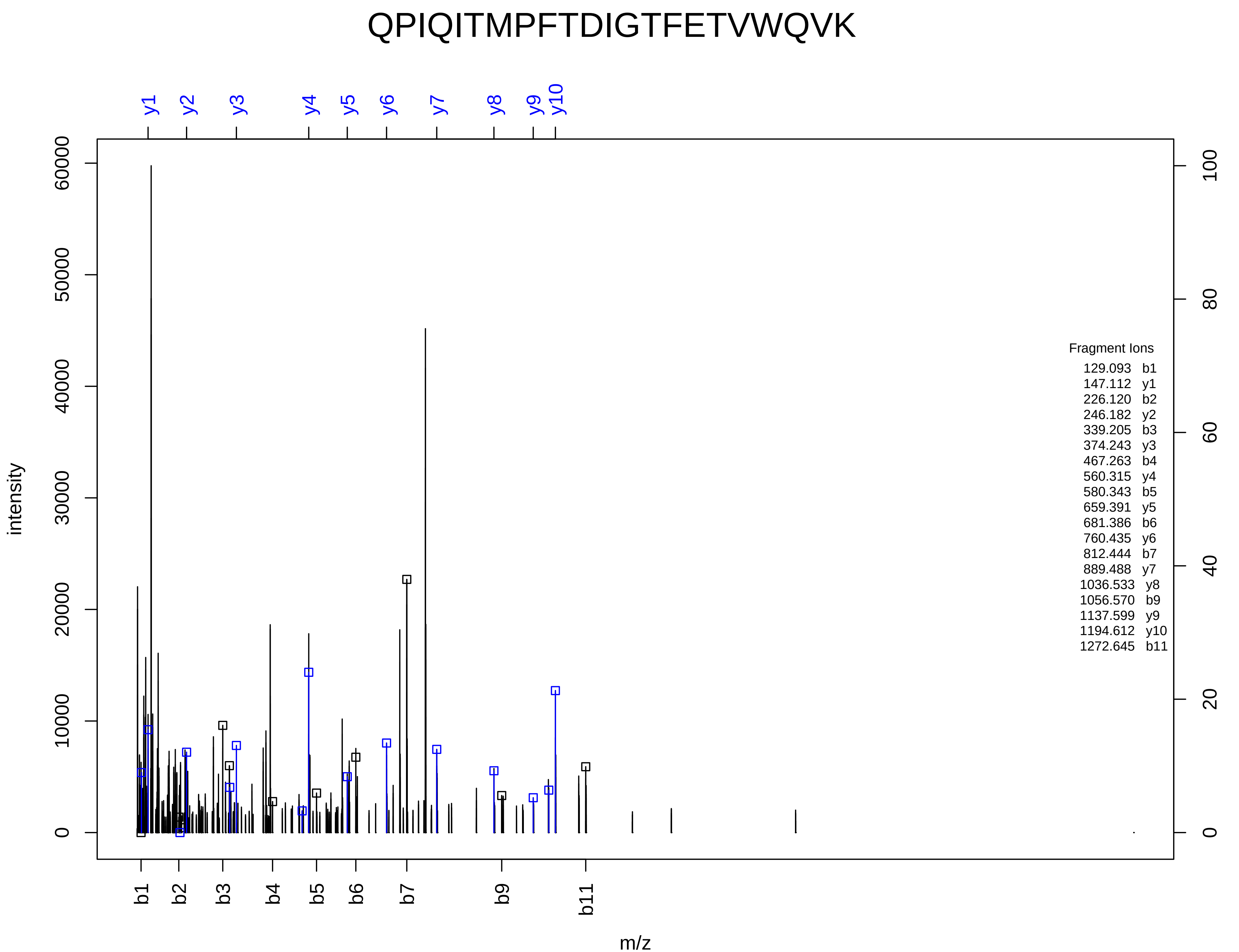

QPIQITMPFTDIGTFETVWQVK / File: christine_30turboips_ip01.raw; SpectrumID: ; scans: 32409

TVDWALAEYMAFGSLLK

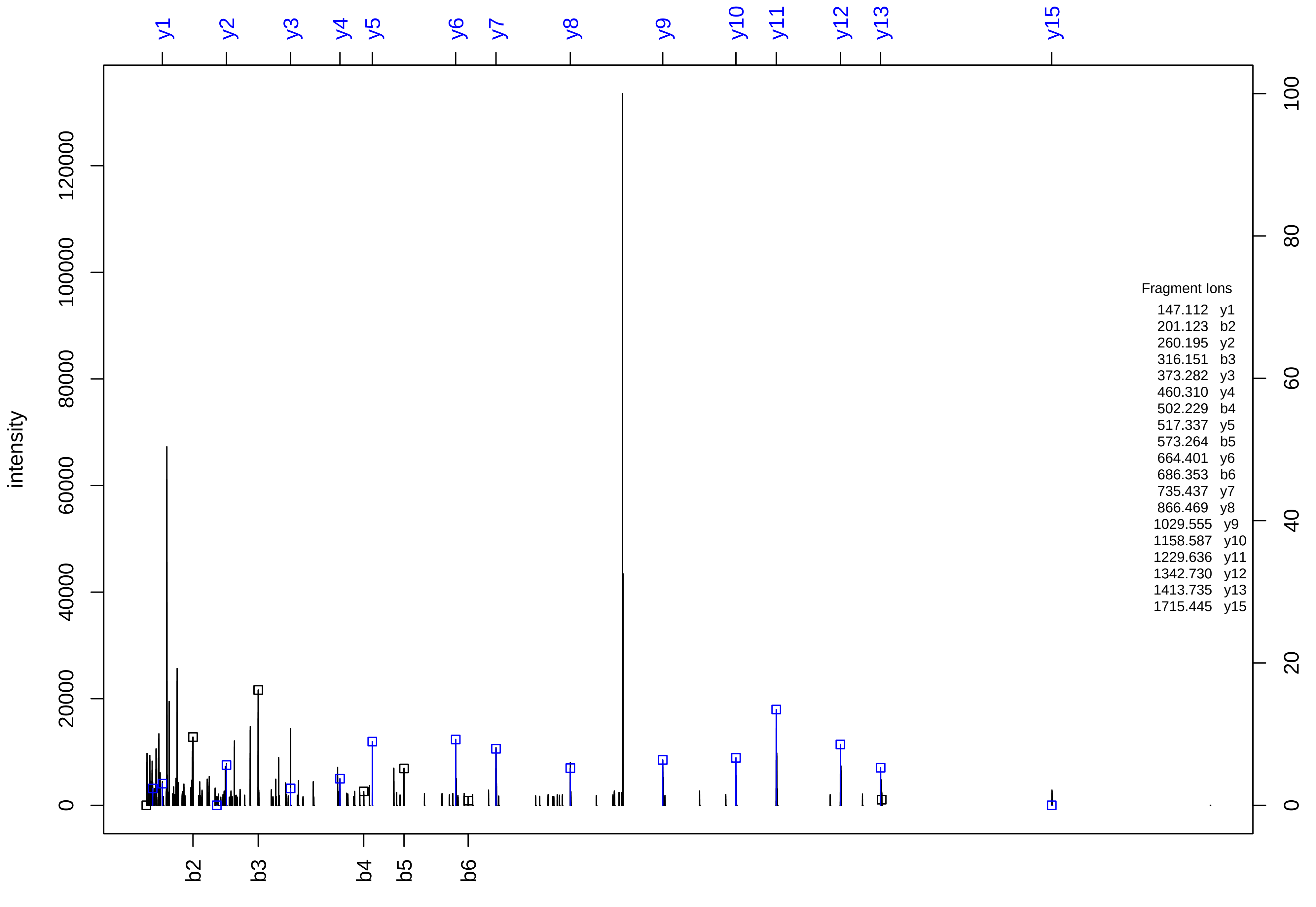

TVDWALAEYMAFGSLLK / File: christine_30turboips_ip01.raw; SpectrumID: ; scans: 32639

VIVVWVGTNNHENTAEEVAGGIEAIVQLINTR

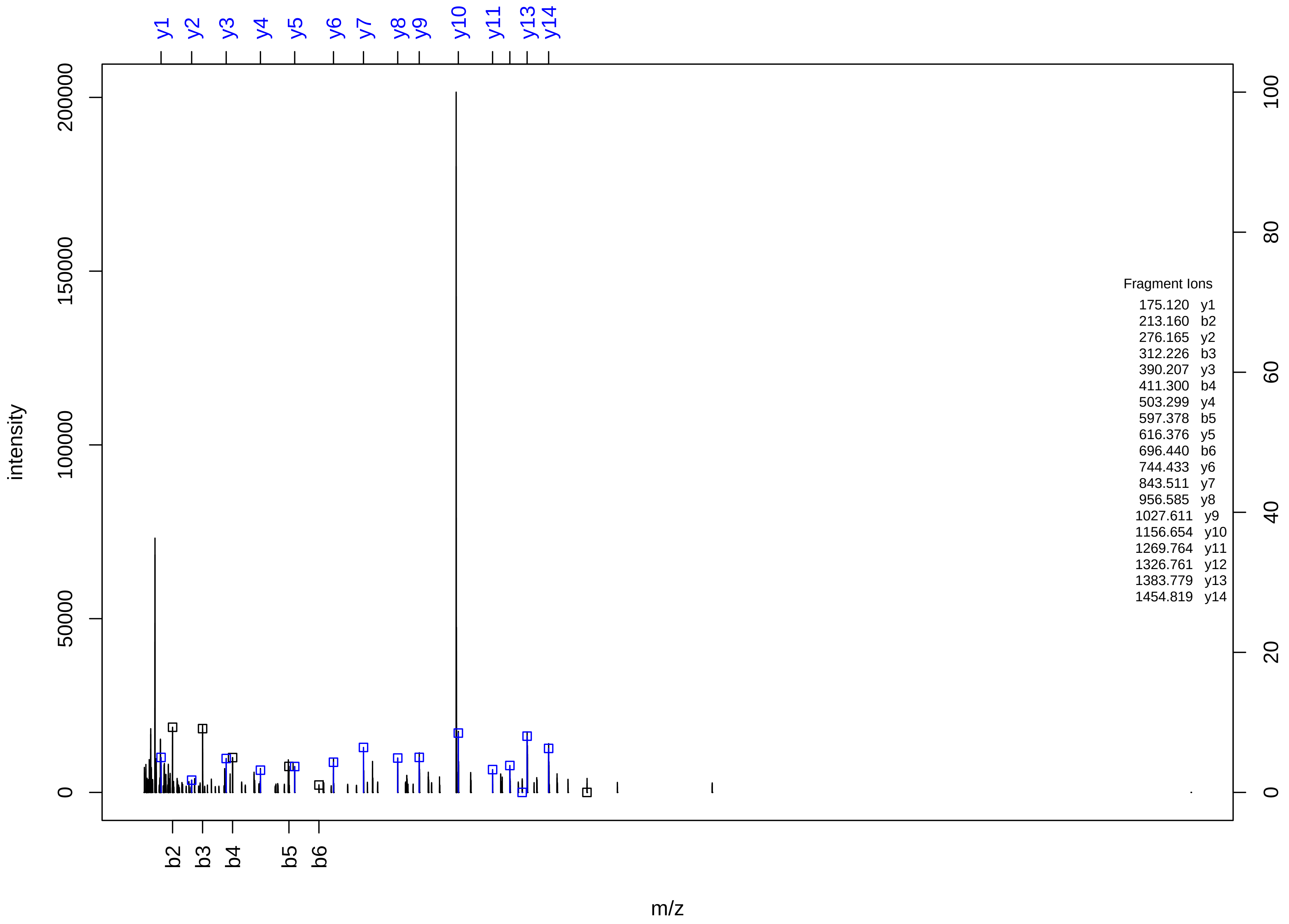

VIVVWVGTNNHENTAEEVAGGIEAIVQLINTR / File: christine_30turboips_ip01.raw; SpectrumID: ; scans: 32668

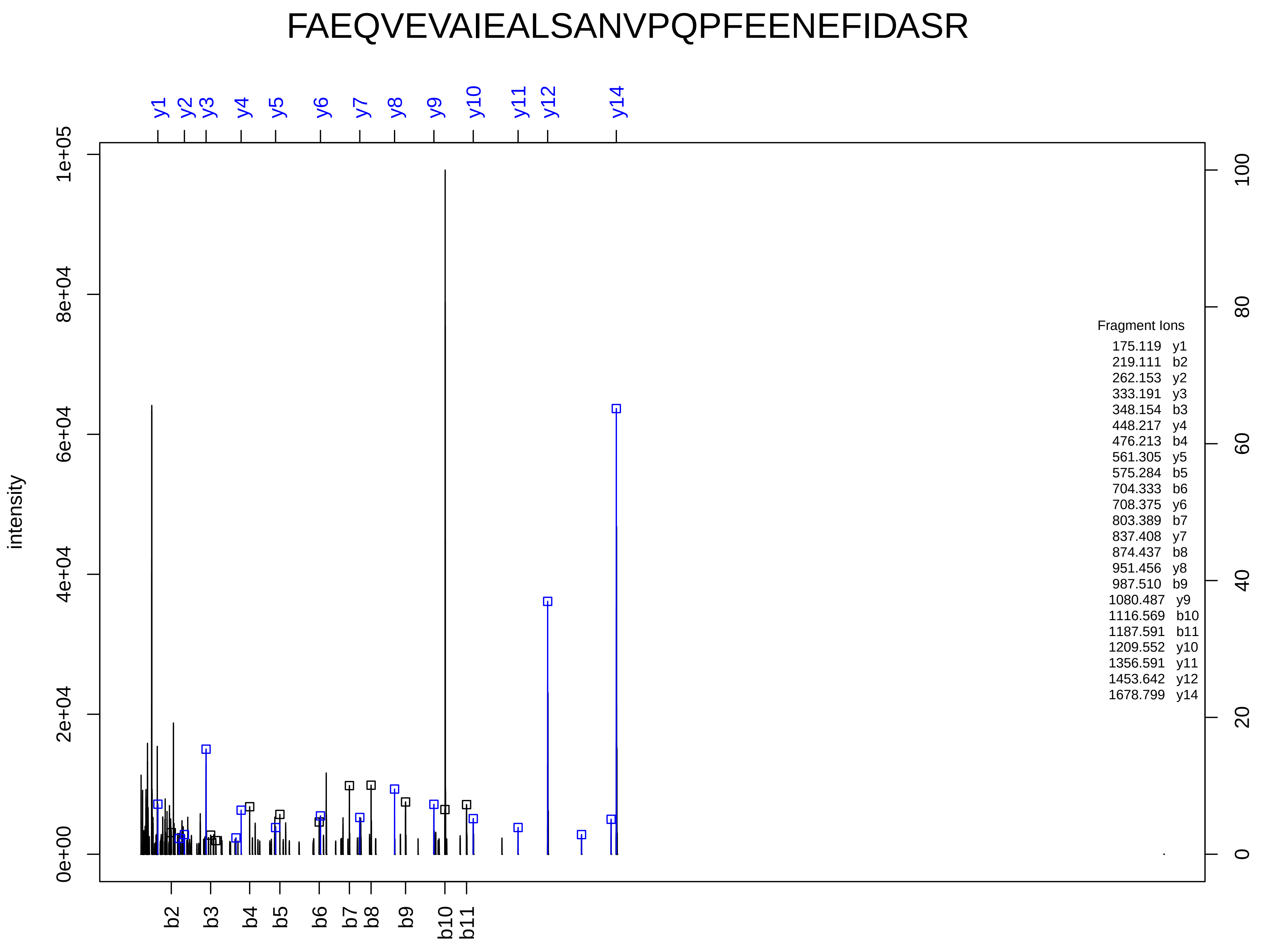

FAEQVEVAIEALSANVPQPFEENEFIDASR / File: christine_30turboips_ip01.raw; SpectrumID: ; scans: 32478

ALYDYDGQEQDELSFK

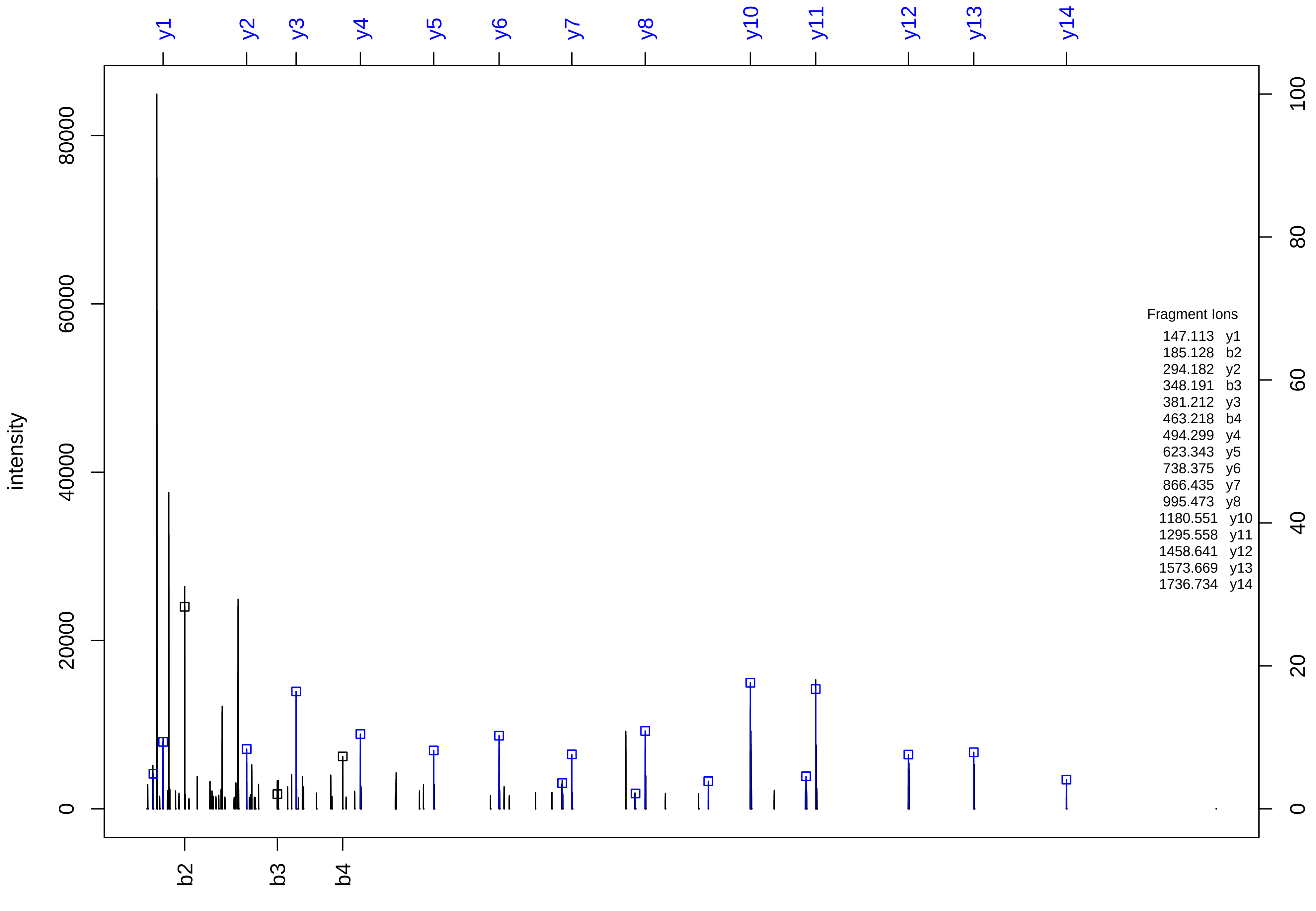

ALYDYDGQEQDELSFK / File: christine_30turboips_ip01.raw; SpectrumID: ; scans: 13182

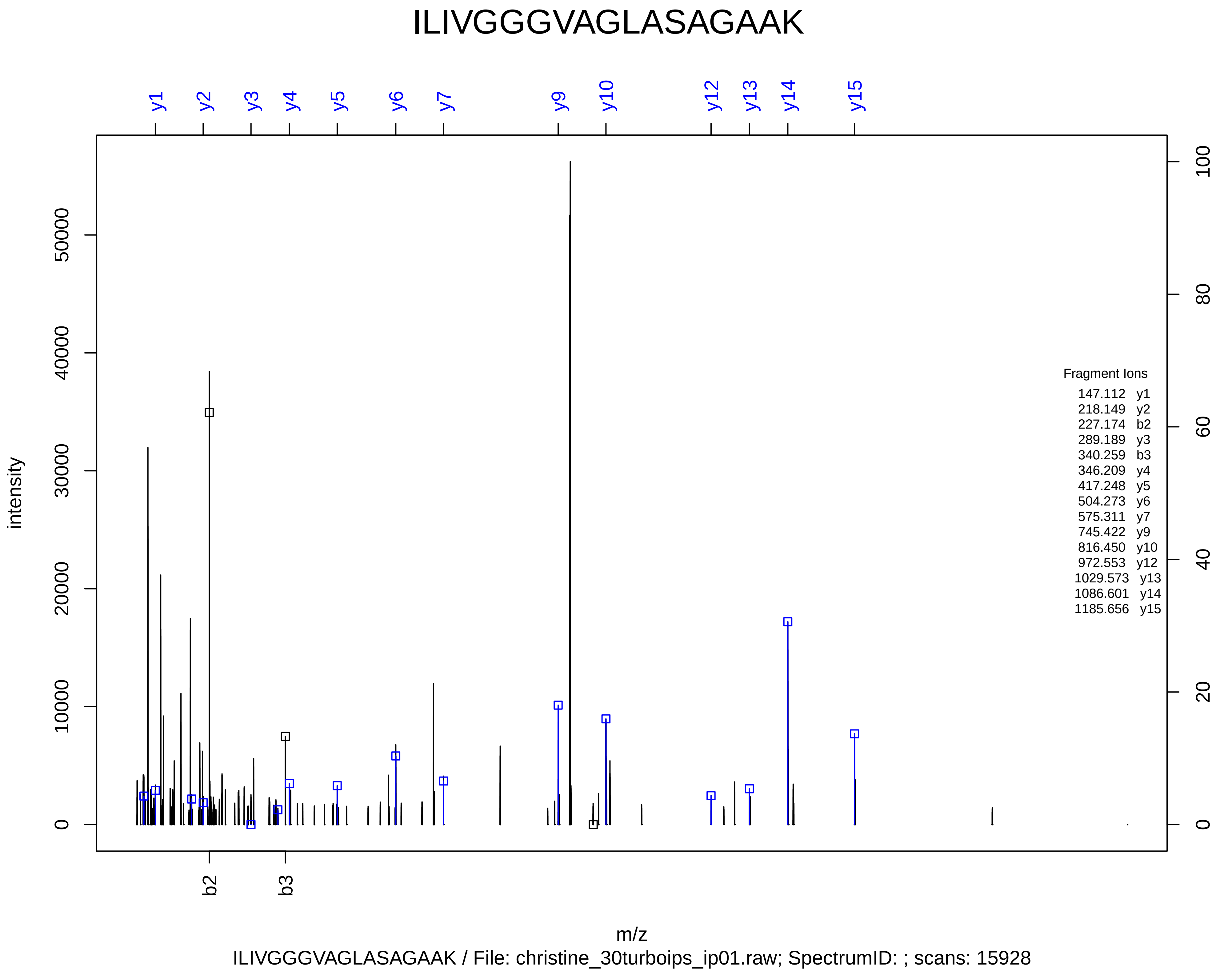

ILIVGGGVAGLASAGAAK
y1
y2
y3
y4
y5
y6
y7
y9
y10
y12
y13
y14
y15
b2
b3
intensity
m/z
Fragment Ions
147.112 y1
218.149 y2
227.174 b2
289.189 y3
340.259 b3
346.209 y4
417.248 y5
504.273 y6
575.311 y7
745.422 y9
816.450 y10
972.553 y12
1029.573 y13
1086.601 y14
1185.656 y15
ILIVGGGVAGLASAGAAK / File: christine_30turboips_ip01.raw; SpectrumID: ; scans: 15928

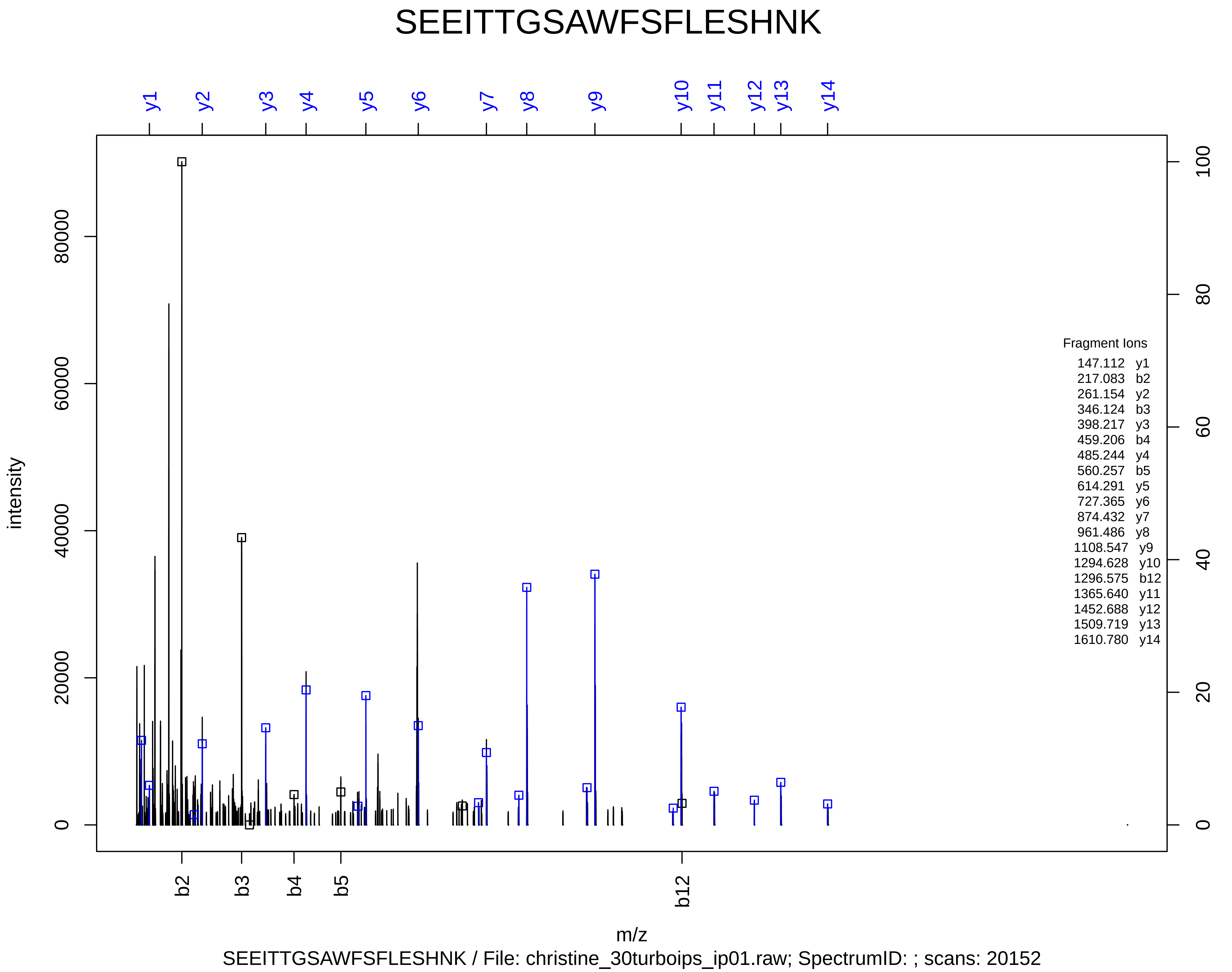
SEEITTGSAWFSFLESHNK
y1 y2 y3 y4 y5 y6 y7 y8 y9 y10 y11 y12 y13 y14
intensity
0 20000 40000 60000 80000
0 20 40 60 80 100
b2 b3 b4 b5 b12
m/z
Fragment Ions
147.112 y1
217.083 b2
261.154 y2
346.124 b3
398.217 y3
459.206 b4
485.244 y4
560.257 b5
614.291 y5
727.365 y6
874.432 y7
961.486 y8
1108.547 y9
1294.628 y10
1296.575 b12
1365.640 y11
1452.688 y12
1509.719 y13
1610.780 y14
SEEITTGSAWFSFLESHNK / File: christine_30turboips_ip01.raw; SpectrumID: ; scans: 20152

LATVGELQAAWR

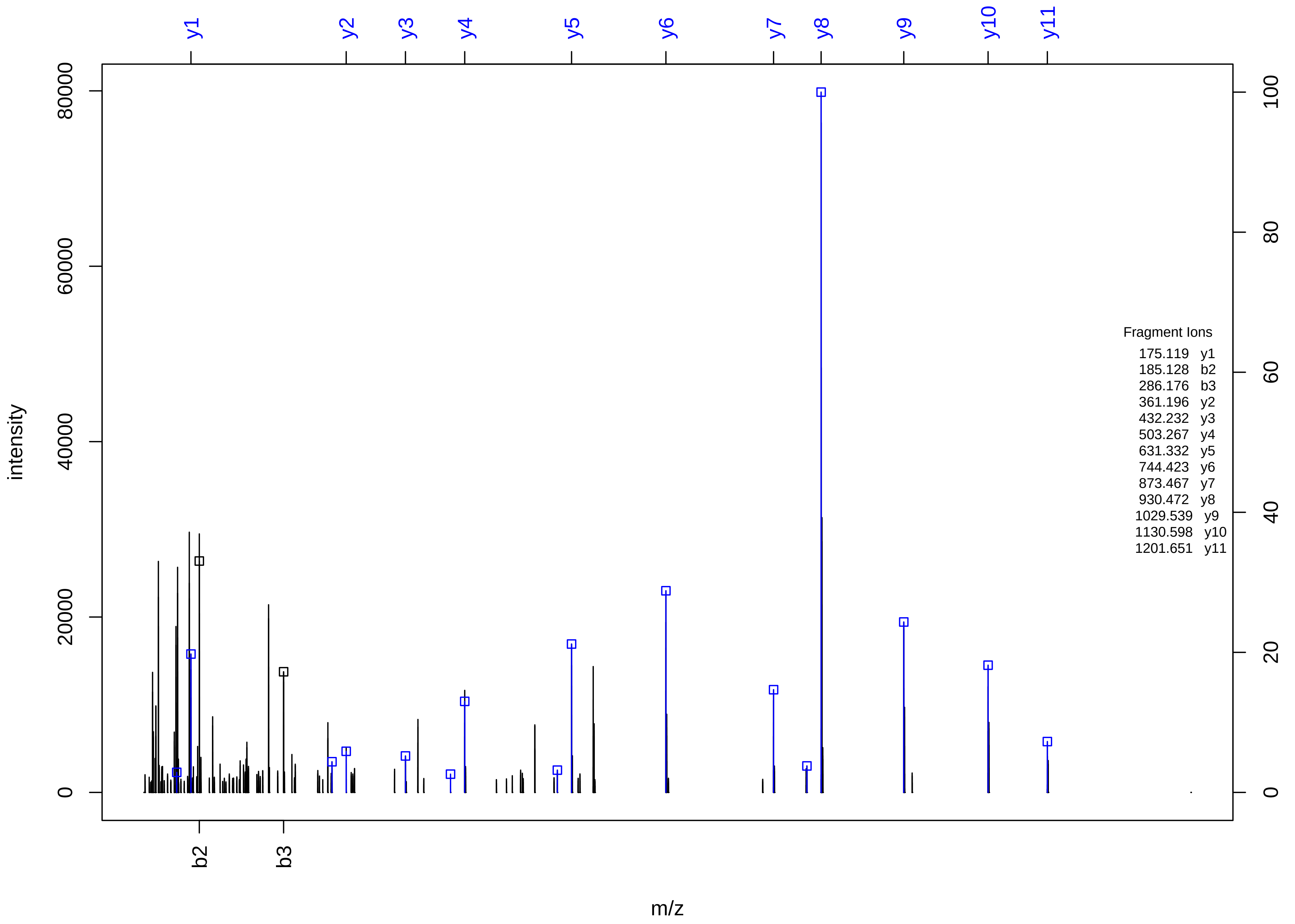

LATVGELQAAWR / File: christine_30turboips_ip01.raw; SpectrumID: ; scans: 13665

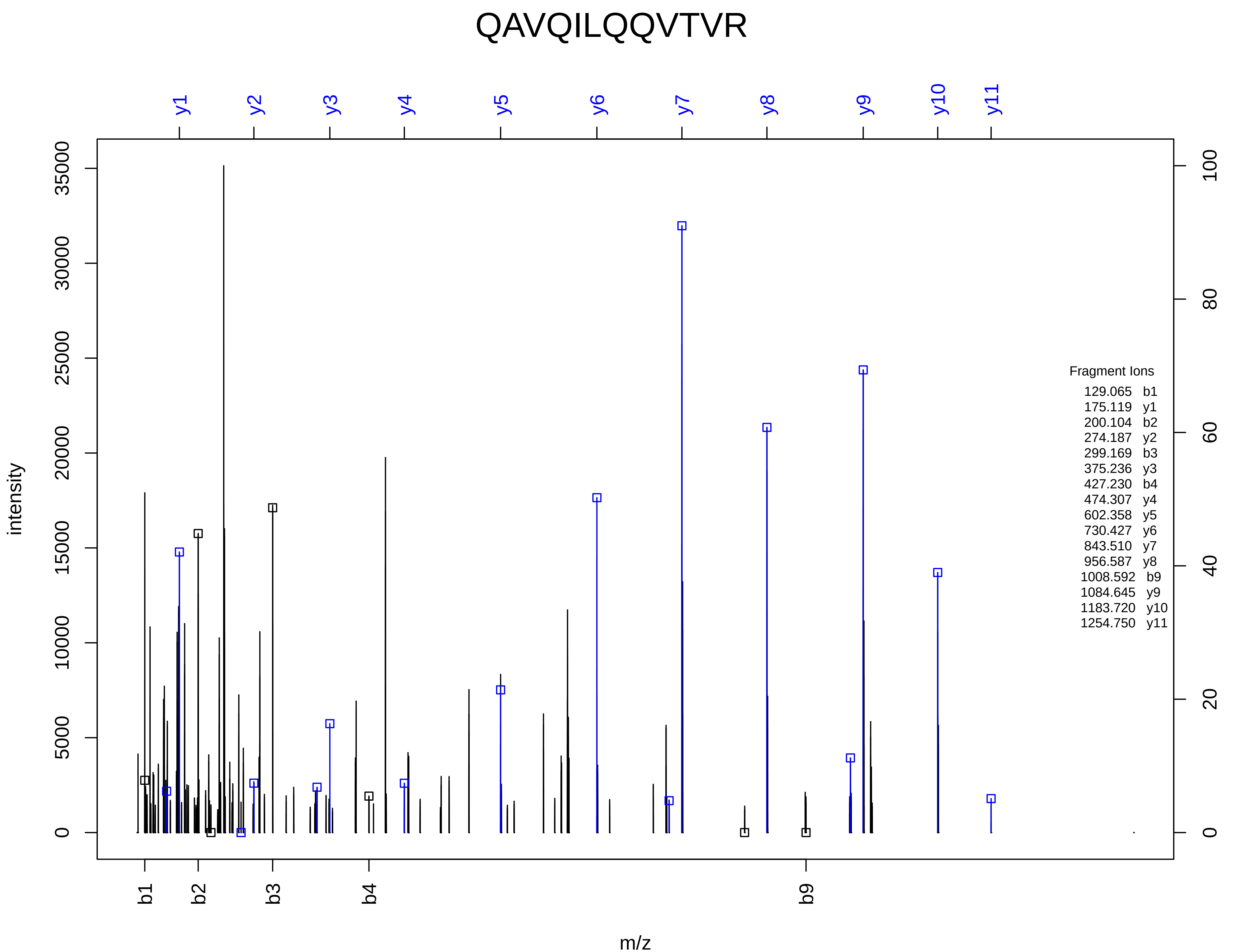

QAVQILQQVTVR / File: christine_30turboips_ip01.raw; SpectrumID: ; scans: 11493

FICTSVPVDADMCAASVAAGGAEELR

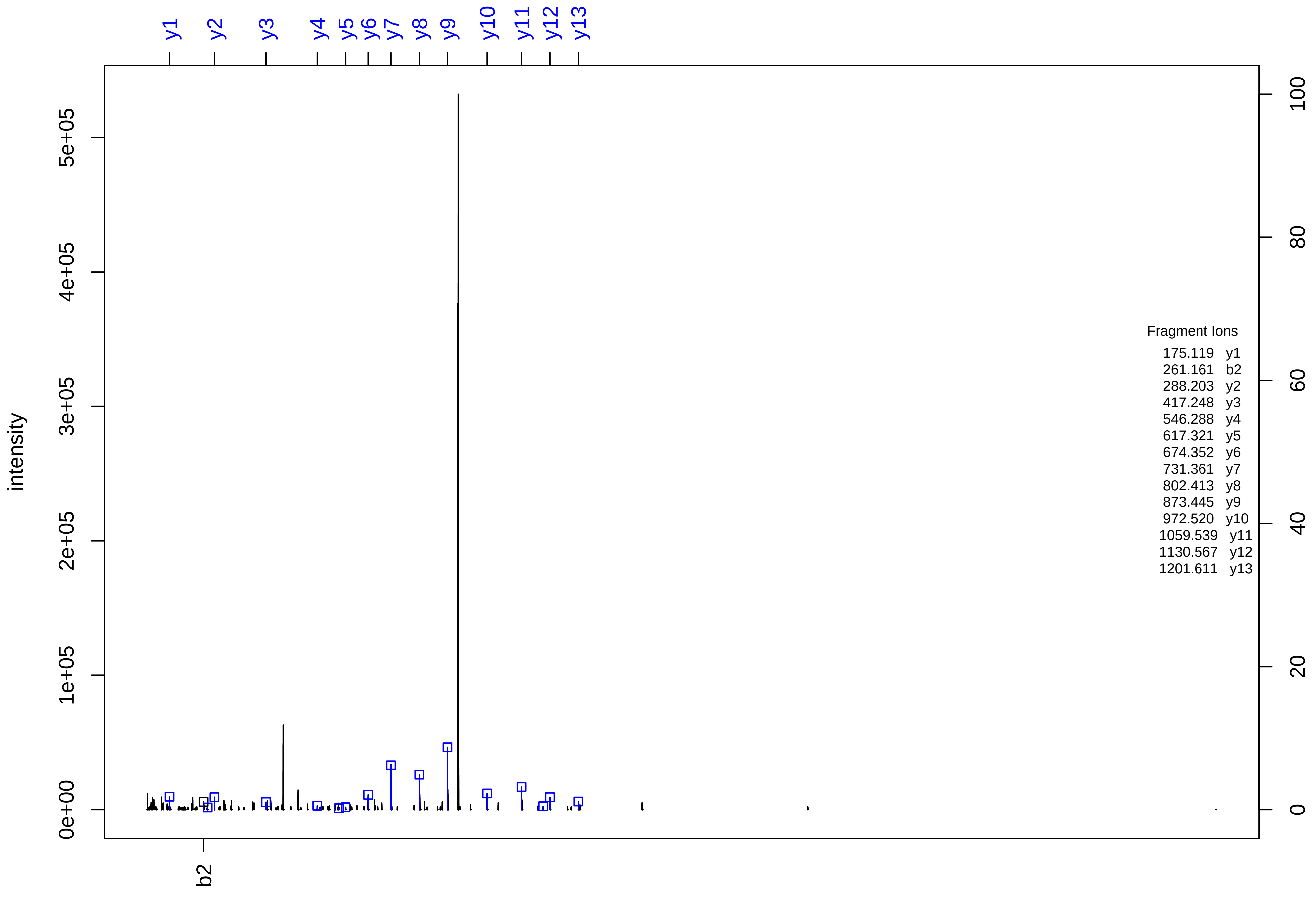

FICTSVPVDADMCAASVAAGGAEELR / File: christine_30turboips_ip01.raw; SpectrumID: ; scans: 19797

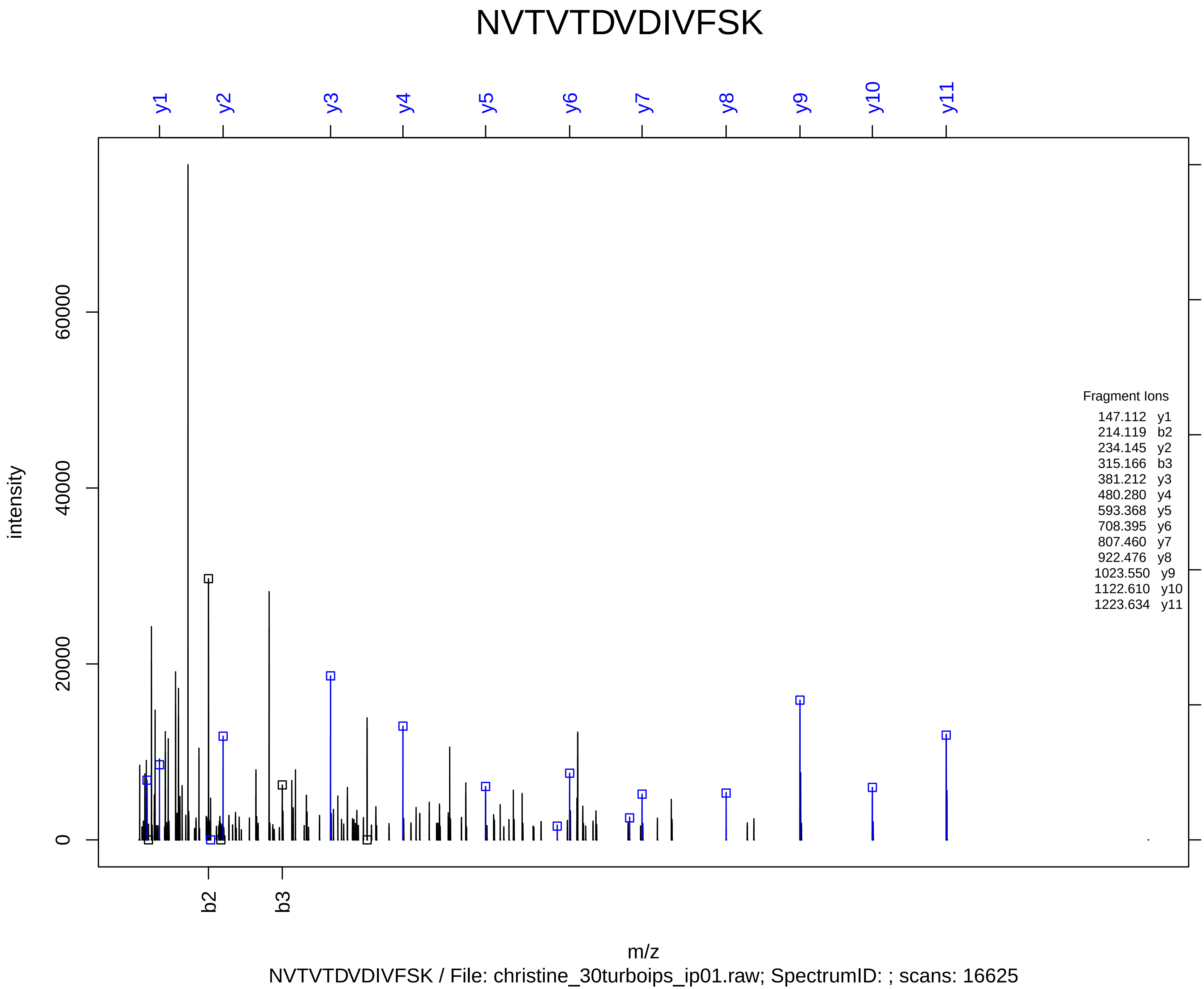
NVTVTDVDIVFSK
y1
y2
y3
y4
y5
y6
y7
y8
y9
y10
y11
intensity
0
20000
40000
60000
0
20
40
60
80
100
b2
b3
m/z
Fragment Ions
147.112 y1
214.119 b2
234.145 y2
315.166 b3
381.212 y3
480.280 y4
593.368 y5
708.395 y6
807.460 y7
922.476 y8
1023.550 y9
1122.610 y10
1223.634 y11
NVTVTDVDIVFSK / File: christine_30turboips_ip01.raw; SpectrumID: ; scans: 16625

GDVVLVVPSDSEADQDAGWLVGVK

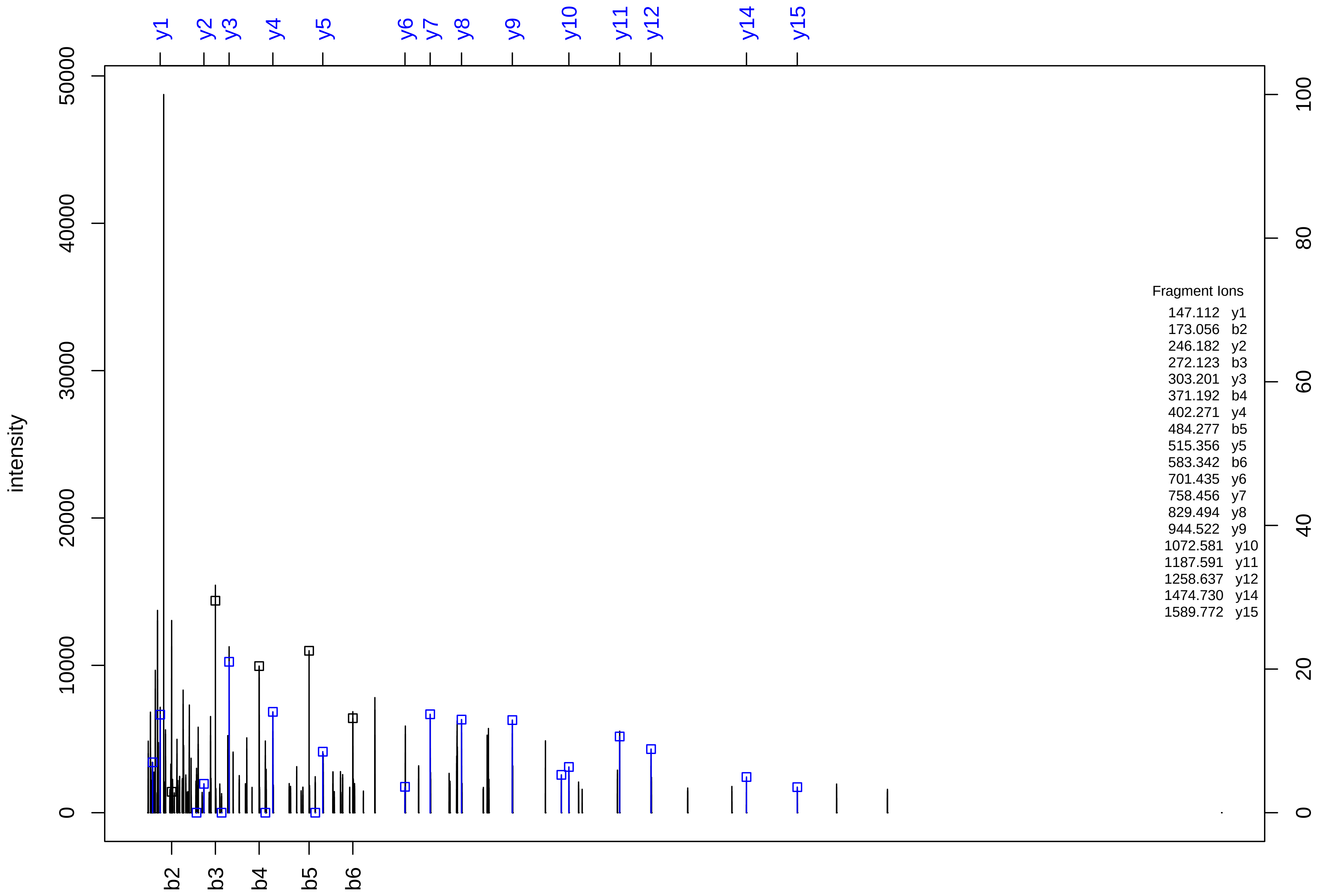

GDVVLVVPSDSEADQDAGWLVGVK / File: christine_30turboips_ip01.raw; SpectrumID: ; scans: 24069

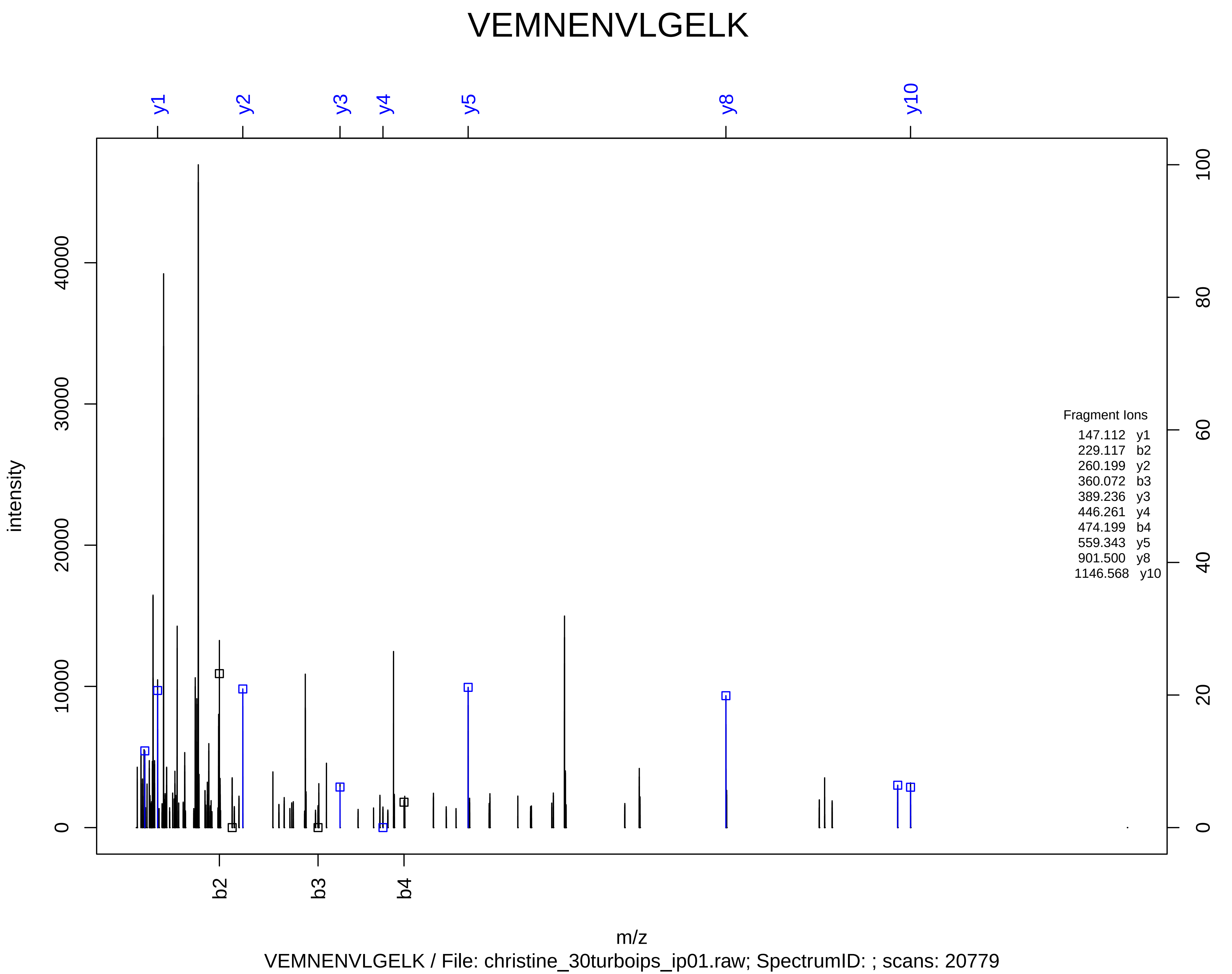

VEMNENVLGELK
y1
y2
y3
y4
y5
y8
y10
intensity
0
10000
20000
30000
40000
0
20
40
60
80
100
b2
b3
b4
m/z
Fragment Ions
147.112 y1
229.117 b2
260.199 y2
360.072 b3
389.236 y3
446.261 y4
474.199 b4
559.343 y5
901.500 y8
1146.568 y10
VEMNENVLGELK / File: christine_30turboips_ip01.raw; SpectrumID: ; scans: 20779

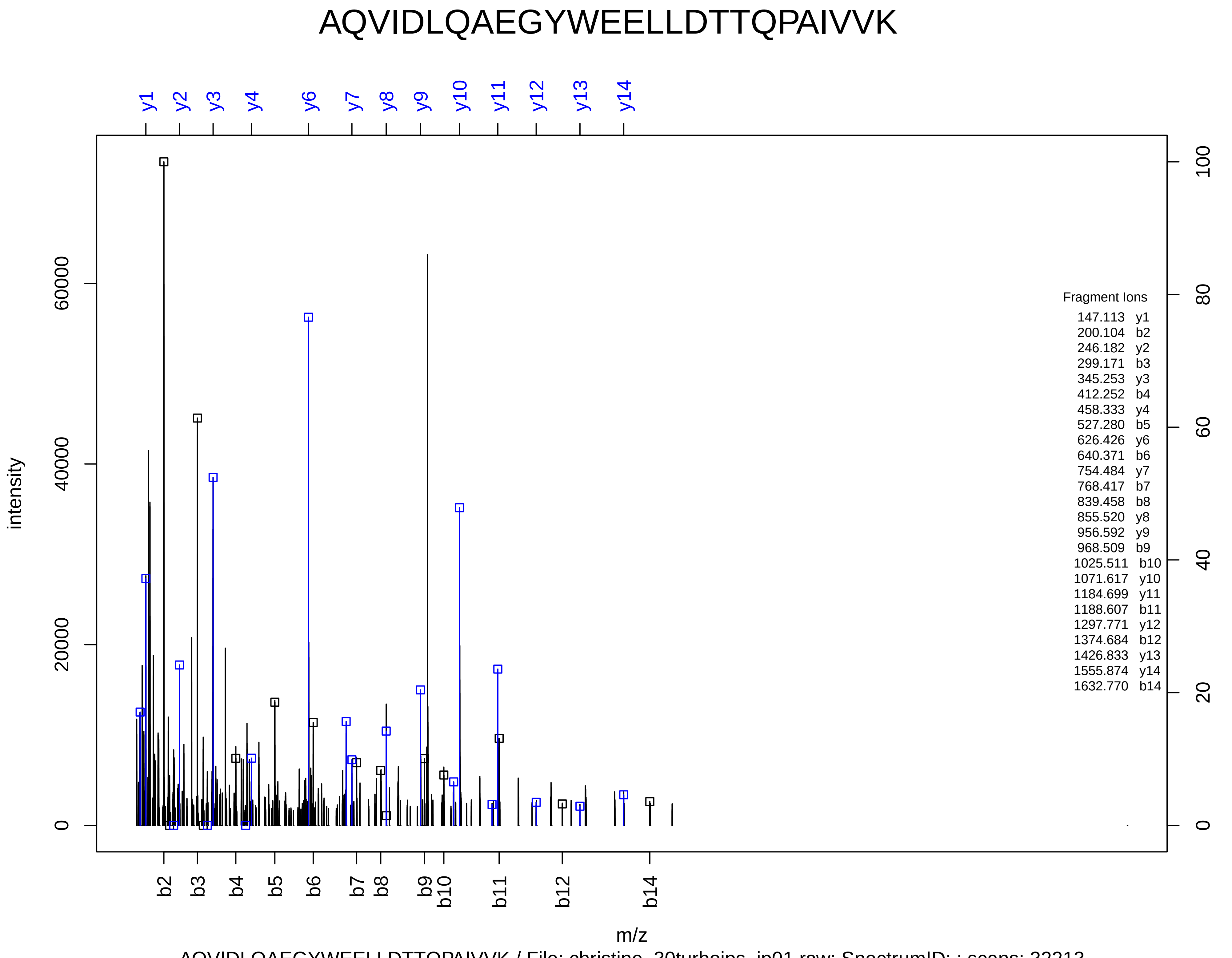
AQVIDLQAEGYWEELLDTTQPAIVVK
y1 y2 y3 y4 y6 y7 y8 y9 y10 y11 y12 y13 y14
intensity
0
20000
40000
60000
0
20
40
60
80
100
Fragment Ions
147.113 y1
200.104 b2
246.182 y2
299.171 b3
345.253 y3
412.252 b4
458.333 y4
527.280 b5
626.426 y6
640.371 b6
754.484 y7
768.417 b7
839.458 b8
855.520 y8
956.592 y9
968.509 b9
1025.511 b10
1071.617 y10
1184.699 y11
1188.607 b11
1297.771 y12
1374.684 b12
1426.833 y13
1555.874 y14
1632.770 b14
b2 b3 b4 b5 b6 b7 b8 b9 b10 b11 b12 b14
m/z
AQVIDLQAEGYWEELLDTTQPAIVVK / File: christine_30turboips_ip01.raw; SpectrumID: ; scans: 32213

RDYSLQIQNVDVTDDGPYTCSVQTQHTPR

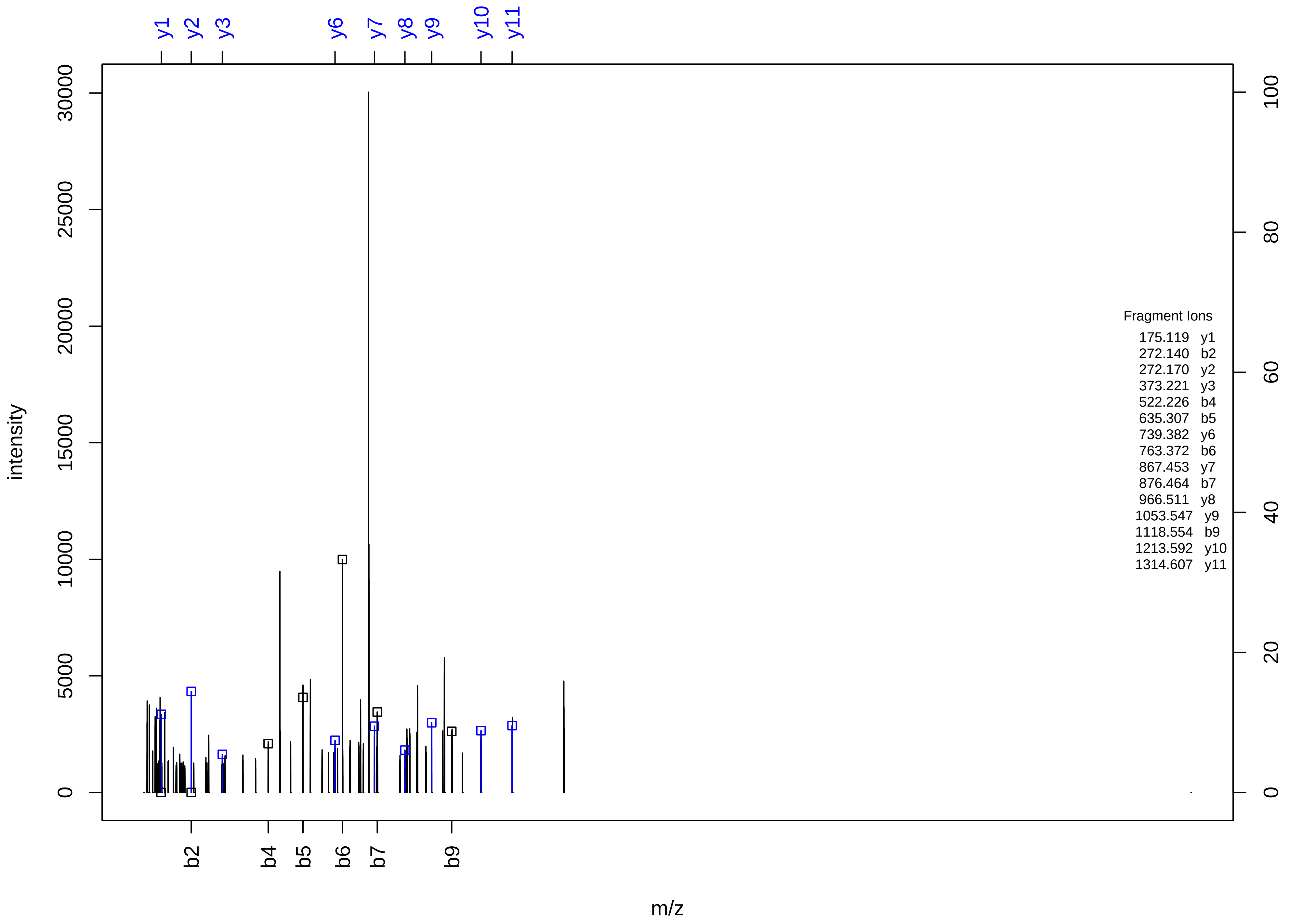

RDYSLQIQNVDVTDDGPYTCSVQTQHTPR / File: christine_30turboips_ip01.raw; SpectrumID: ; scans: 9842

RVSQGHN^GDLYFSNVMLQDMQTDYSCNAR

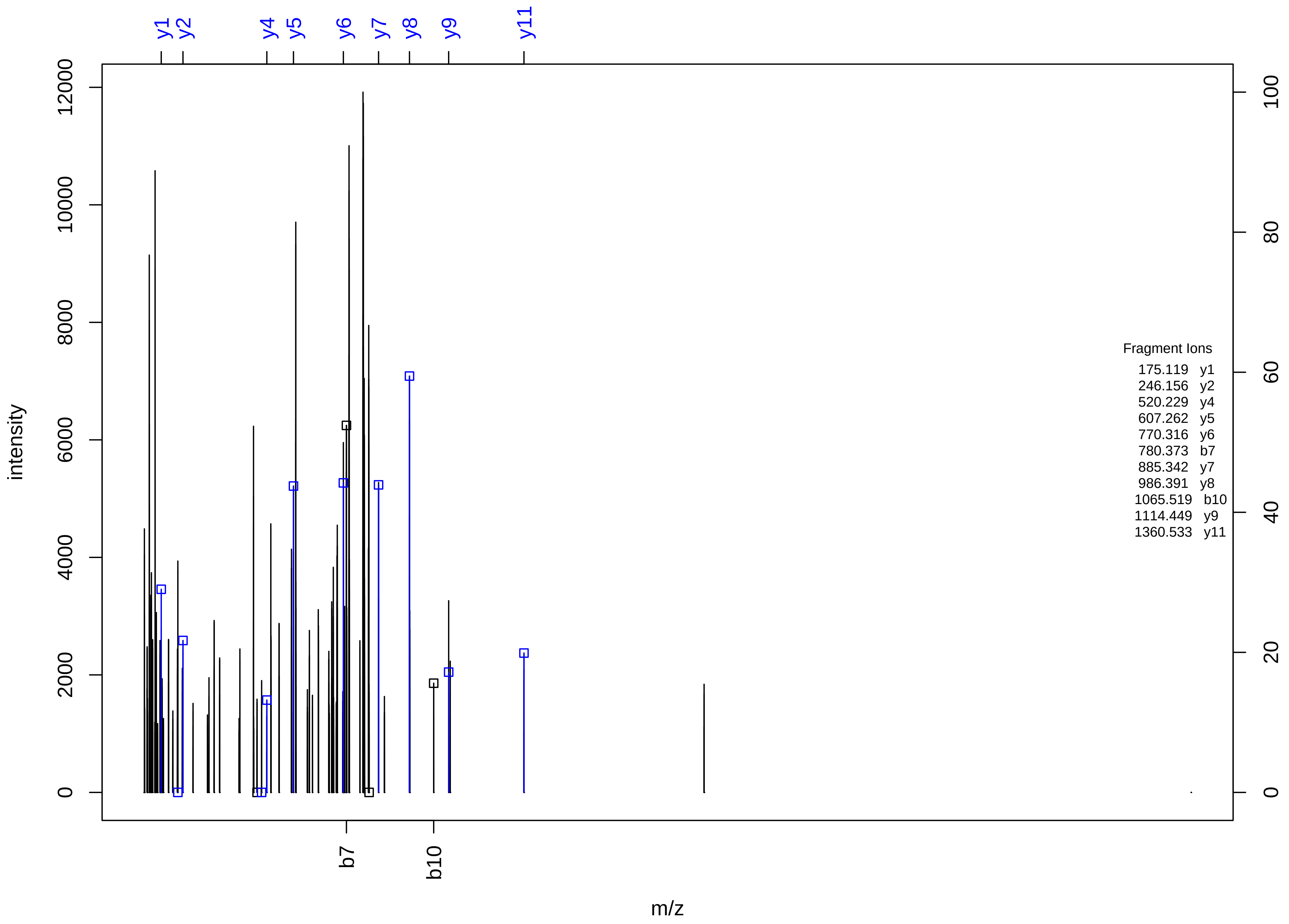

RVSQGHNGDLYFSNVMLQDMQTDYSCNAR / File: christine_30turboips_ip01.raw; SpectrumID: ; scans: 16968

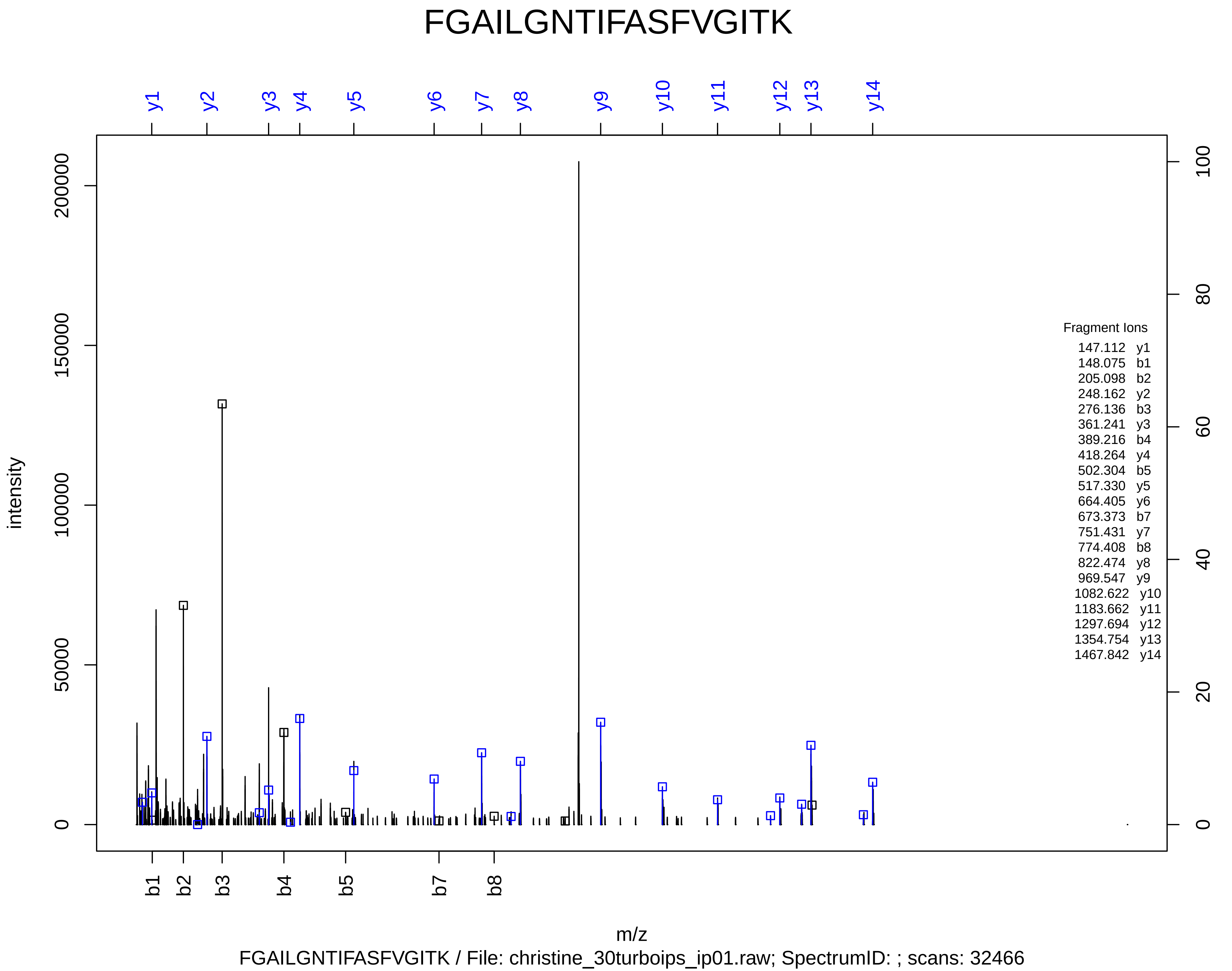
FGAILGNTIFASFVGITK
y1 y2 y3 y4 y5 y6 y7 y8 y9 y10 y11 y12 y13 y14
b1 b2 b3 b4 b5 b7 b8
intensity
m/z
Fragment Ions
147.112 y1
148.075 b1
205.098 b2
248.162 y2
276.136 b3
361.241 y3
389.216 b4
418.264 y4
502.304 b5
517.330 y5
664.405 y6
673.373 b7
751.431 y7
774.408 b8
822.474 y8
969.547 y9
1082.622 y10
1183.662 y11
1297.694 y12
1354.754 y13
1467.842 y14
FGAILGNTIFASFVGITK / File: christine_30turboips_ip01.raw; SpectrumID: ; scans: 32466

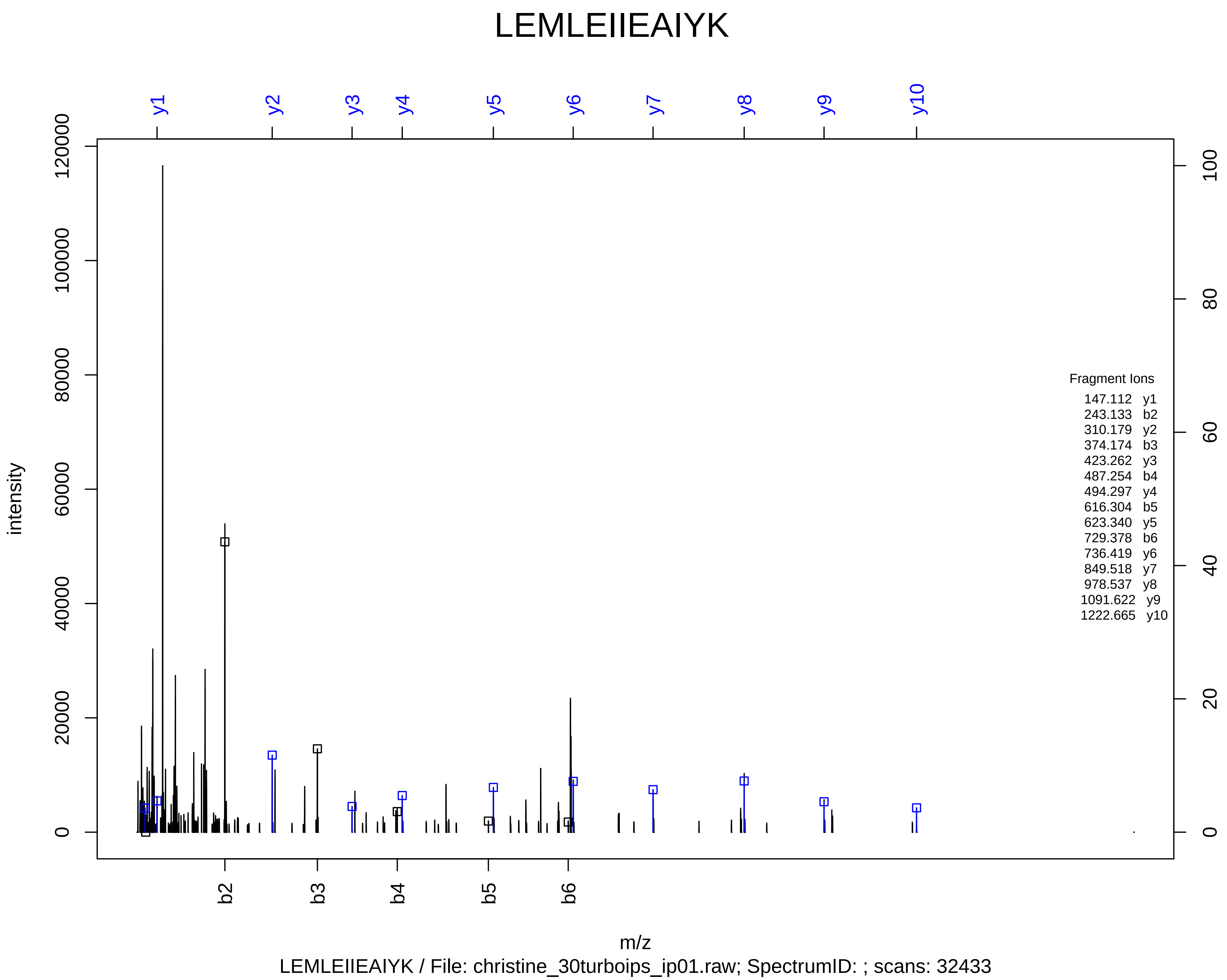

LEMLEIIEAIYK
y1
y2
y3
y4
y5
y6
y7
y8
y9
y10
b2
b3
b4
b5
b6
intensity
m/z
Fragment Ions
147.112 y1
243.133 b2
310.179 y2
374.174 b3
423.262 y3
487.254 b4
494.297 y4
616.304 b5
623.340 y5
729.378 b6
736.419 y6
849.518 y7
978.537 y8
1091.622 y9
1222.665 y10
LEMLEIIEAIYK / File: christine_30turboips_ip01.raw; SpectrumID: ; scans: 32433

ETLGTGAFSEVVLAEEK

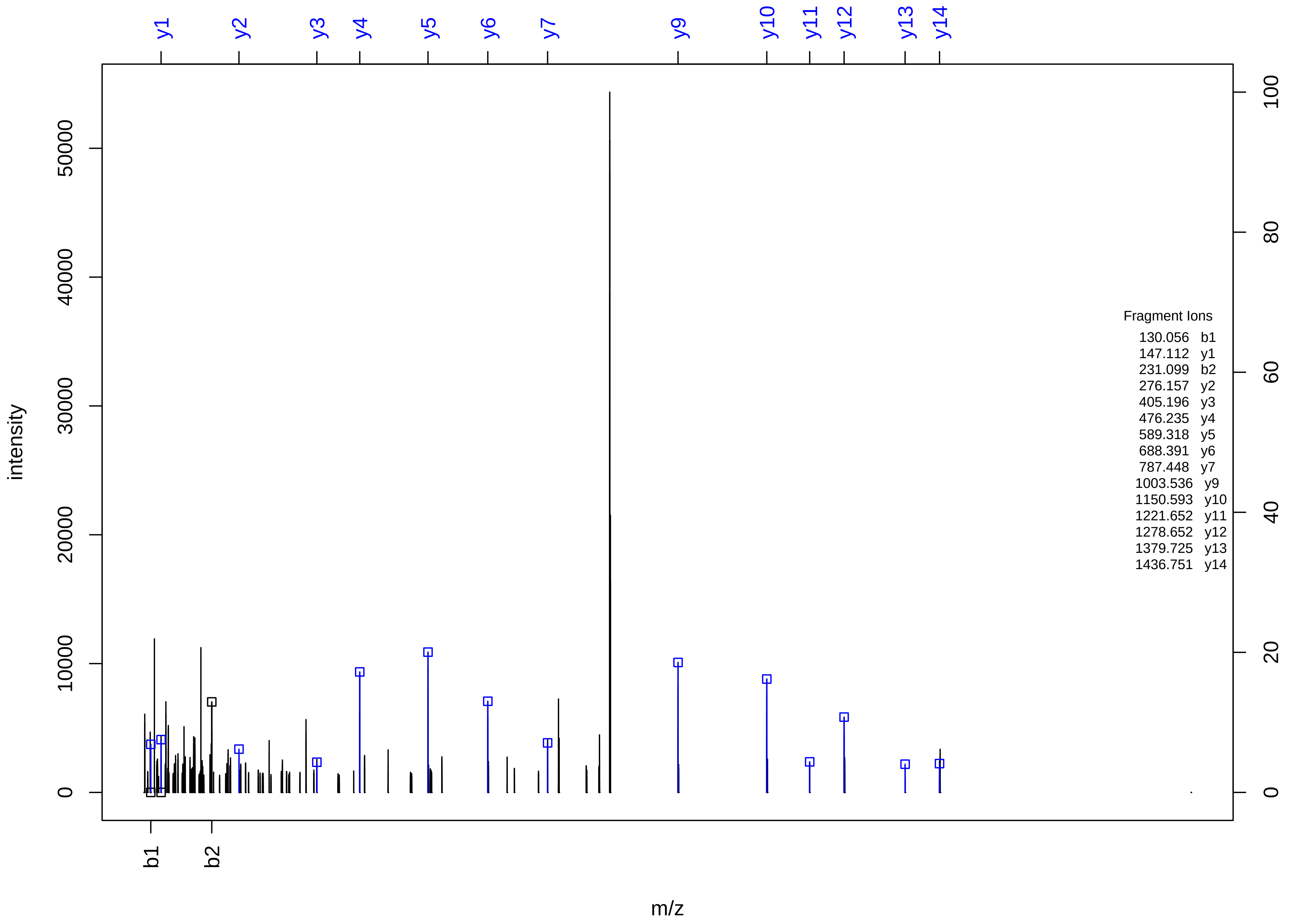

ETLGTGAFSEVVLAEEK / File: christine_30turboips_ip01.raw; SpectrumID: ; scans: 17957

RVSMELGGLAPFIVFDSANVDQAVAGAMASK

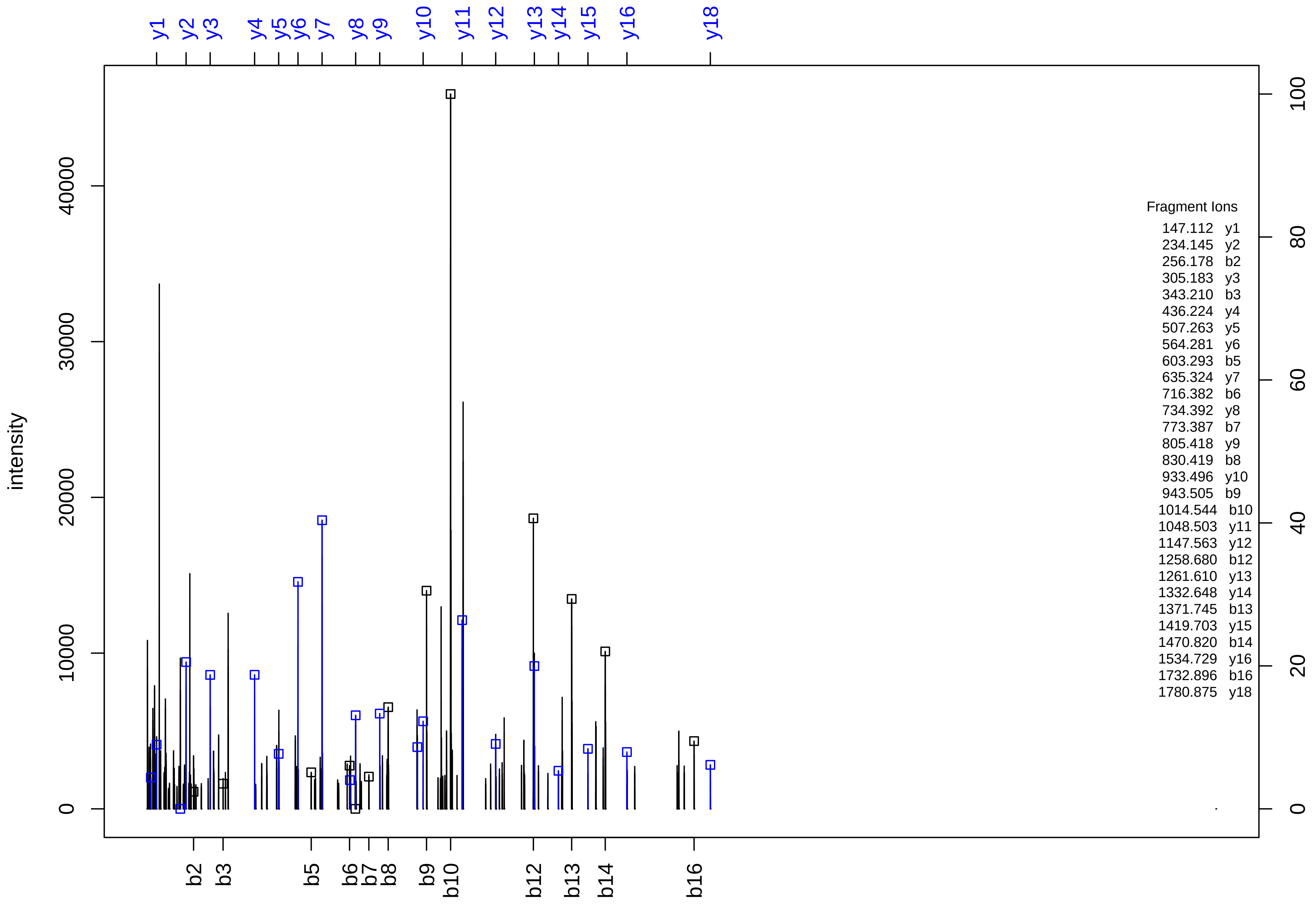

RVSMELGGLAPFIVFDSANVDQAVAGAMASK / File: christine_30turboips_ip01.raw; SpectrumID: ; scans: 32117

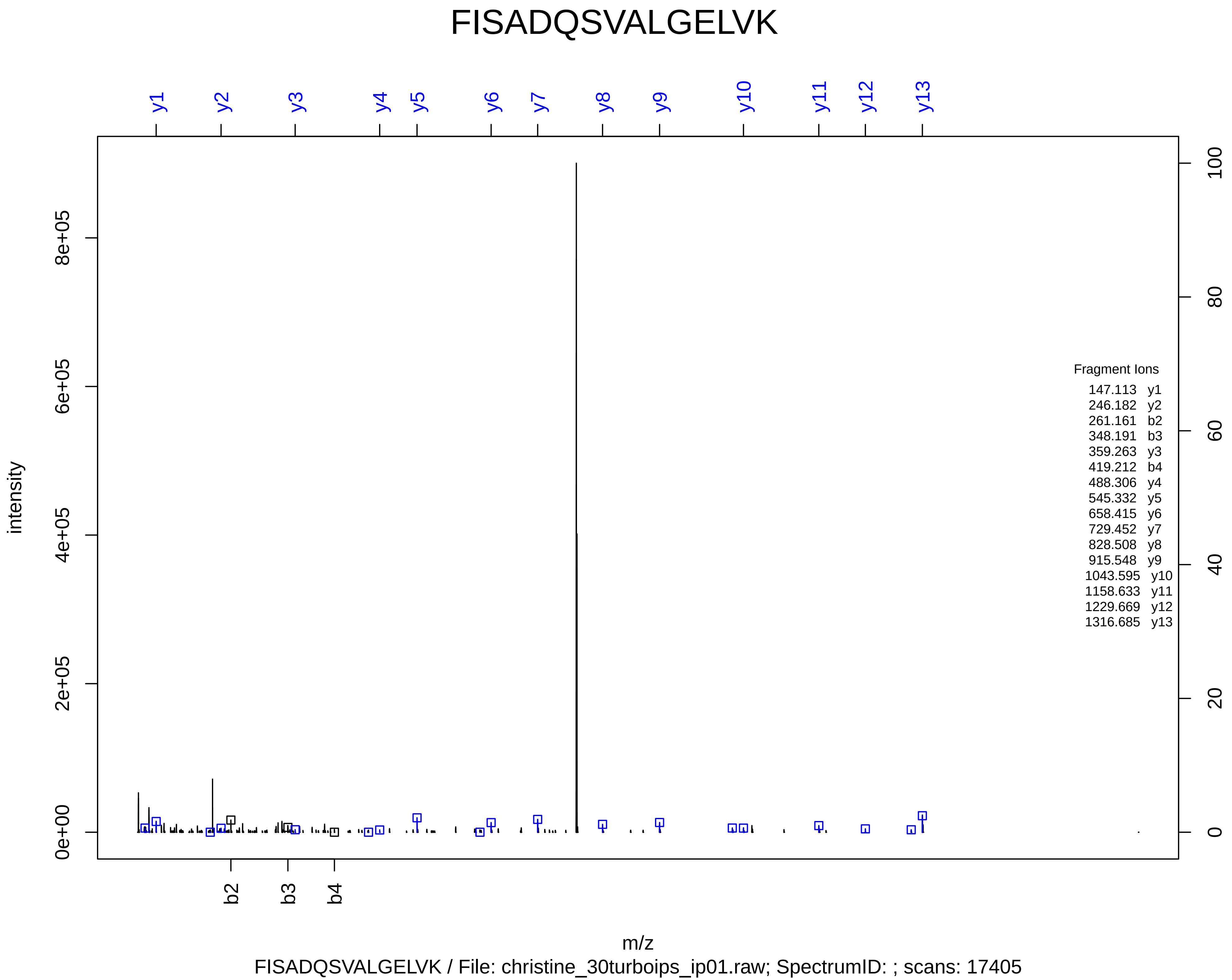
FISADQSVALGELVK
y1
y2
y3
y4
y5
y6
y7
y8
y9
y10
y11
y12
y13
b2
b3
b4
intensity
0e+00
2e+05
4e+05
6e+05
8e+05
0
20
40
60
80
100
m/z
Fragment Ions
147.113 y1
246.182 y2
261.161 b2
348.191 b3
359.263 y3
419.212 b4
488.306 y4
545.332 y5
658.415 y6
729.452 y7
828.508 y8
915.548 y9
1043.595 y10
1158.633 y11
1229.669 y12
1316.685 y13
FISADQSVALGELVK / File: christine_30turboips_ip01.raw; SpectrumID: ; scans: 17405

LCEAICPAQAITIEAEPR

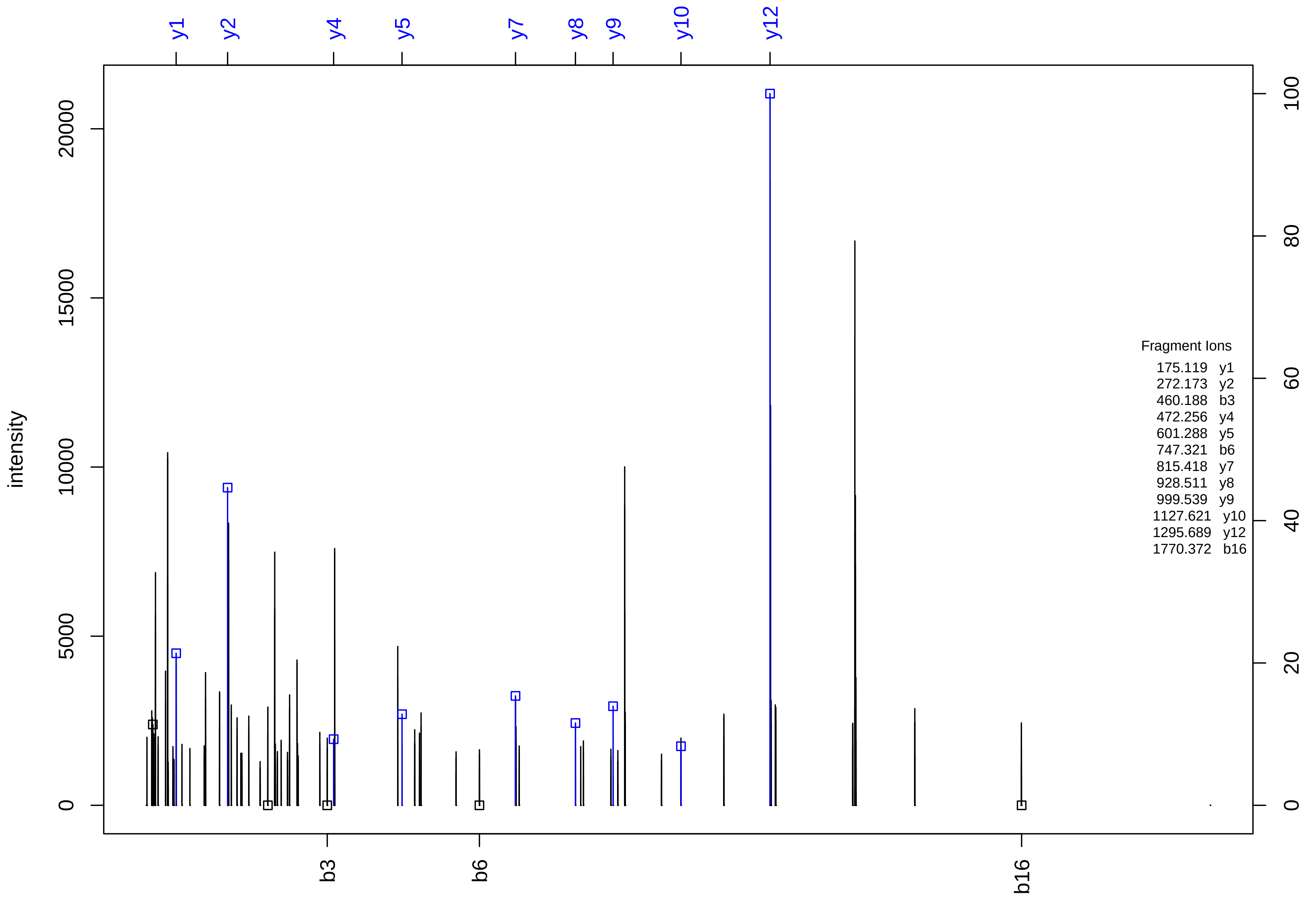

LCEAICPAQAITIEAEPR / File: christine_30turboips_ip01.raw; SpectrumID: ; scans: 14965

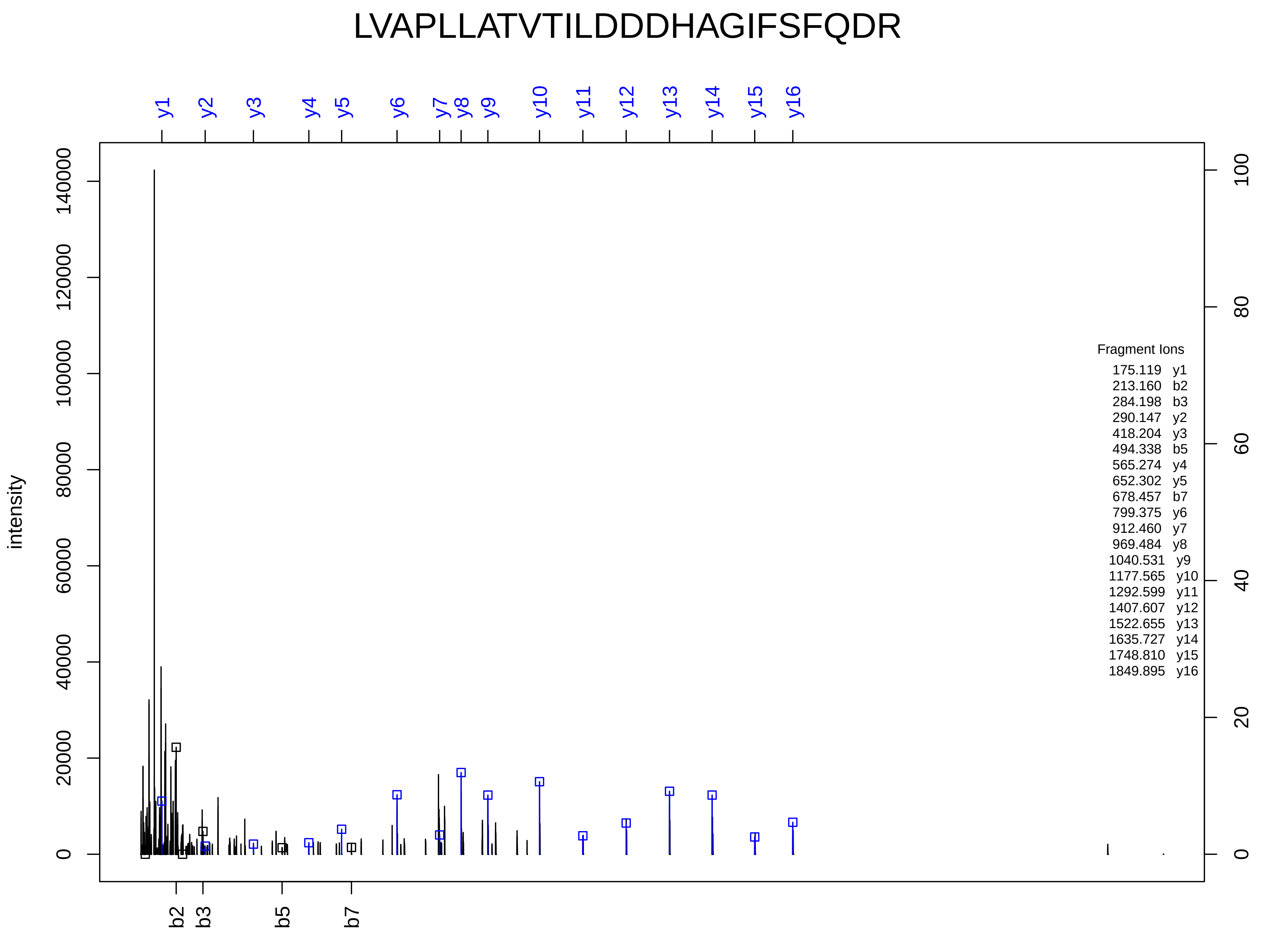

LVAPLLATVTILDDDHAGIFSFQDR / File: christine_30turboips_ip01.raw; SpectrumID: ; scans: 32412

IQHPSNVLHFFNAPLEVTEENFFEICDELGVK

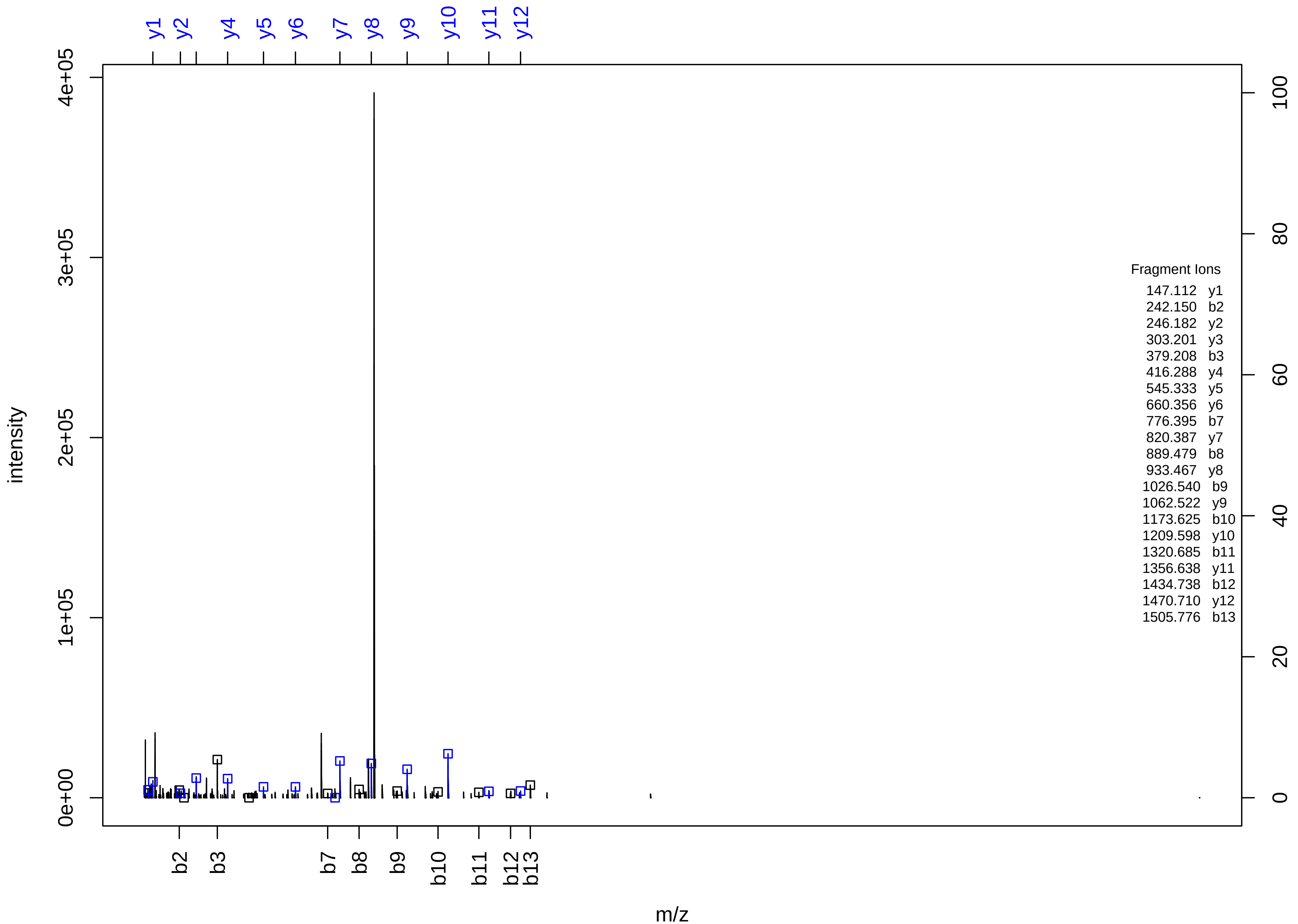

IQHPSNVLHFFNAPLEVTEENFFEICDELGVK / File: christine_30turboips_ip01.raw; SpectrumID: ; scans: 32134

VICLVGAGISTSAGIPDFR

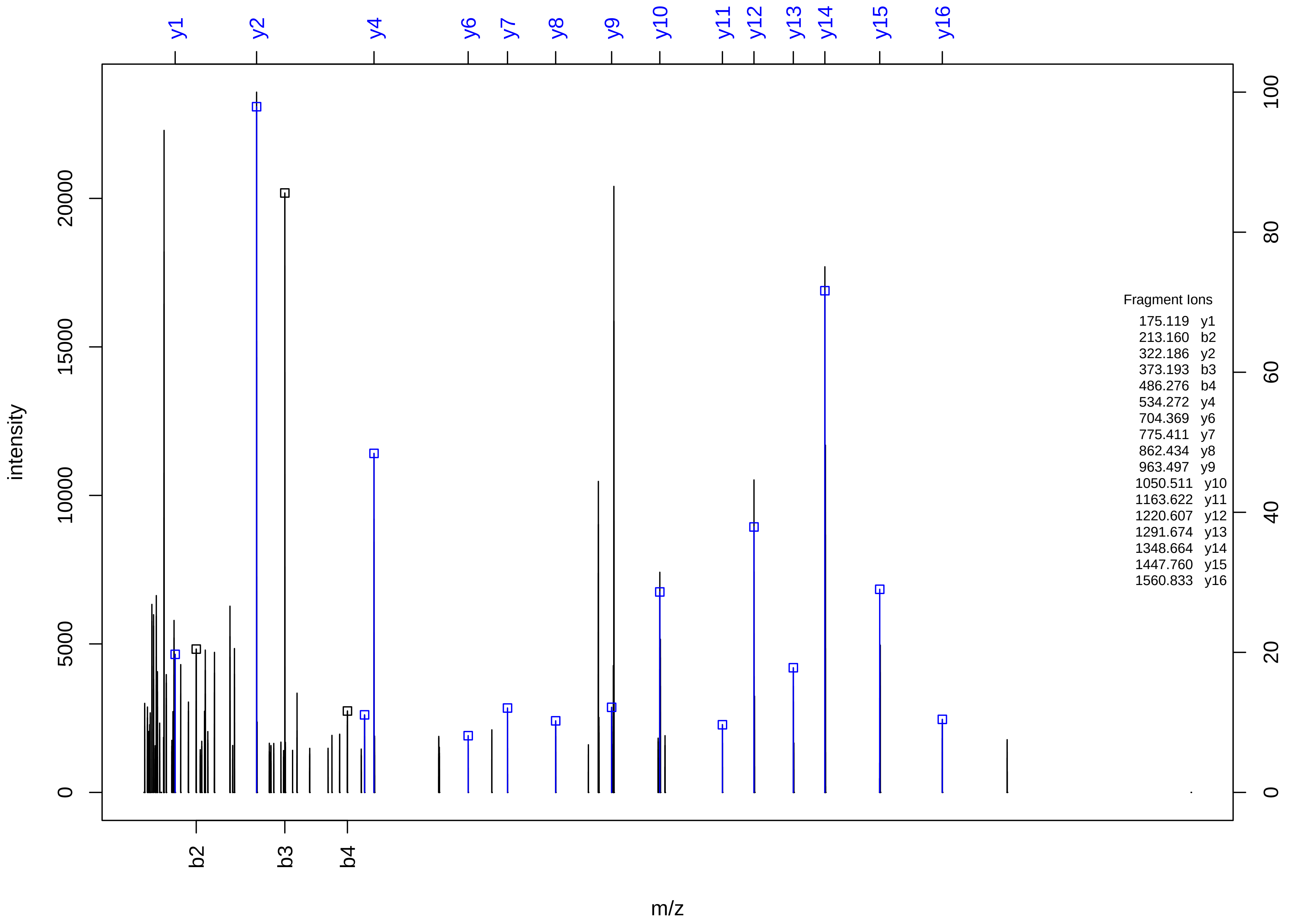

VICLVGAGISTSAGIPDFR / File: christine_30turboips_ip01.raw; SpectrumID: ; scans: 21828

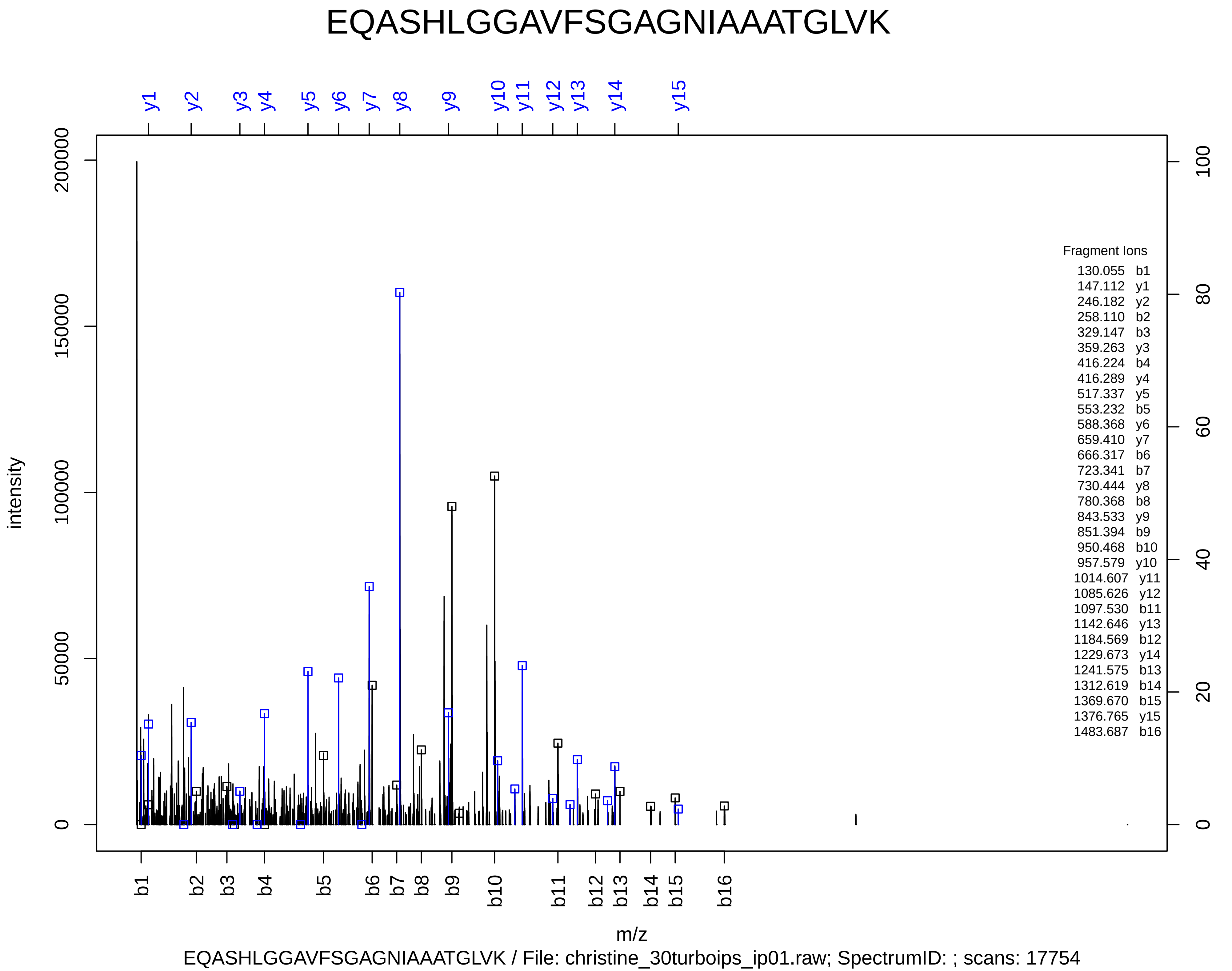
EQASHLGGAVFSGAGNIAAATGLVK
y1 y2 y3 y4 y5 y6 y7 y8 y9 y10 y11 y12 y13 y14 y15
intensity
0
50000
100000
150000
200000
0
20
40
60
80
100
Fragment Ions
130.055 b1
147.112 y1
246.182 y2
258.110 b2
329.147 b3
359.263 y3
416.224 b4
416.289 y4
517.337 y5
553.232 b5
588.368 y6
659.410 y7
666.317 b6
723.341 b7
730.444 y8
780.368 b8
843.533 y9
851.394 b9
950.468 b10
957.579 y10
1014.607 y11
1085.626 y12
1097.530 b11
1142.646 y13
1184.569 b12
1229.673 y14
1241.575 b13
1312.619 b14
1369.670 b15
1376.765 y15
1483.687 b16
b1 b2 b3 b4 b5 b6 b7 b8 b9 b10 b11 b12 b13 b14 b15 b16
m/z
EQASHLGGAVFSGAGNIAAATGLVK / File: christine_30turboips_ip01.raw; SpectrumID: ; scans: 17754

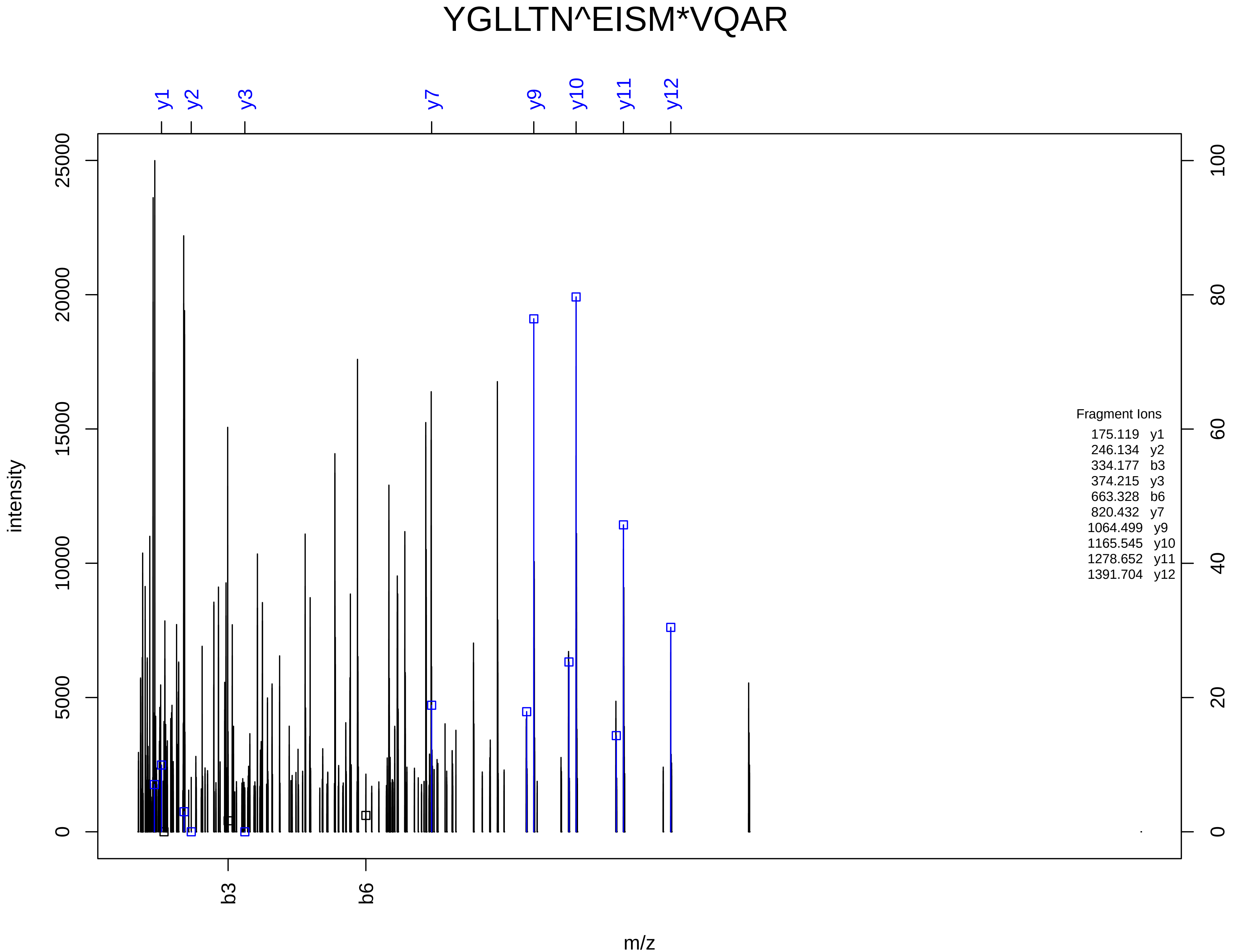
YGLLTN^EISM*VQAR
y1
y2
y3
y7
y9
y10
y11
y12
intensity
0
5000
10000
15000
20000
25000
0
20
40
60
80
100
b3
b6
m/z
Fragment Ions
175.119 y1
246.134 y2
334.177 b3
374.215 y3
663.328 b6
820.432 y7
1064.499 y9
1165.545 y10
1278.652 y11
1391.704 y12
YGLLTNEISMVQAR / File: christine_30turboips_ip01.raw; SpectrumID: ; scans: 19578

AVGFVSEDEYLEIQGITR

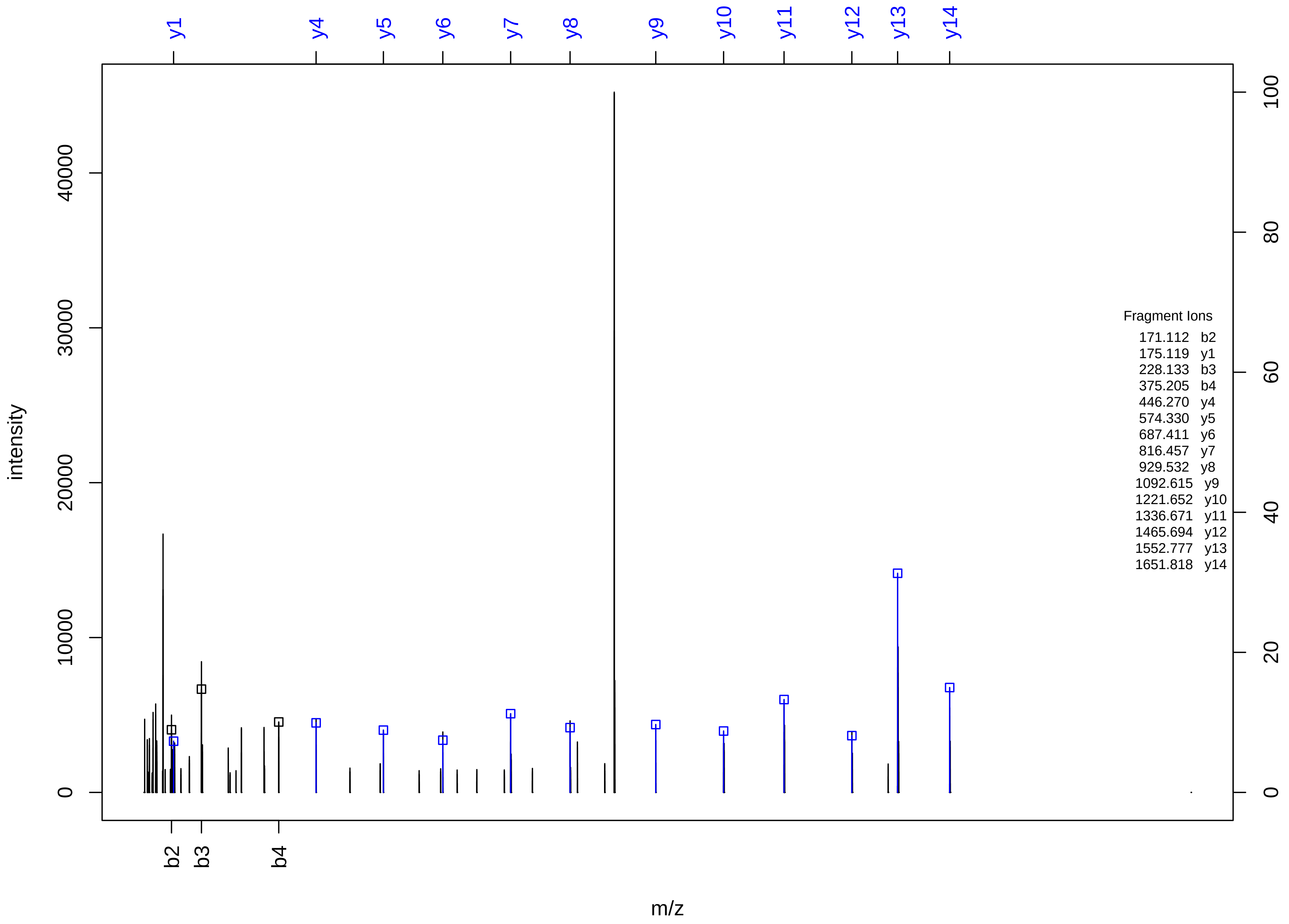

AVGFVSEDEYLEIQGITR / File: christine_30turboips_ip01.raw; SpectrumID: ; scans: 20673

(Ac)SESLVVCDVAEDLVEK

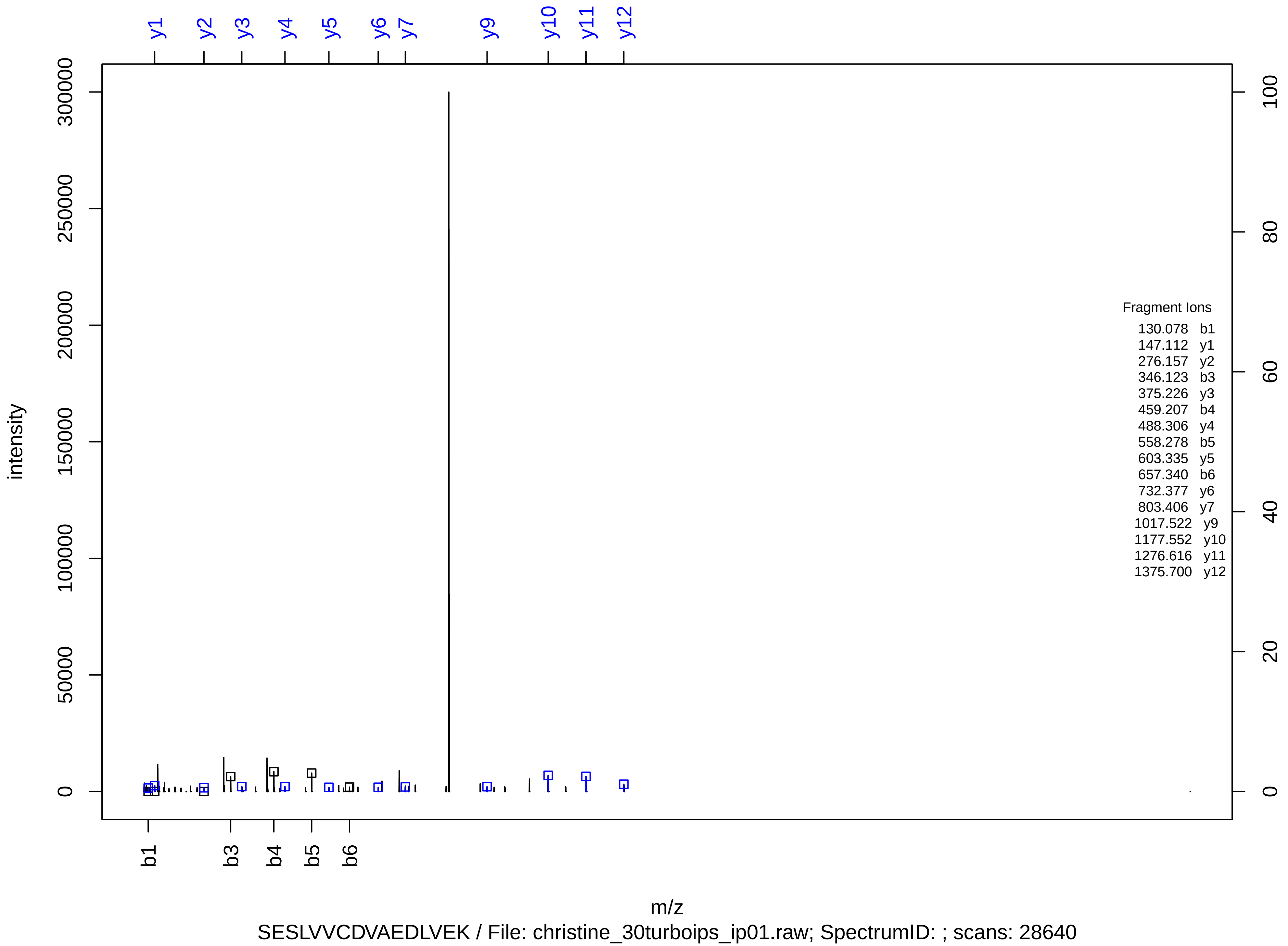

SESLVVCDVAEDLVEK / File: christine_30turboips_ip01.raw; SpectrumID: ; scans: 28640

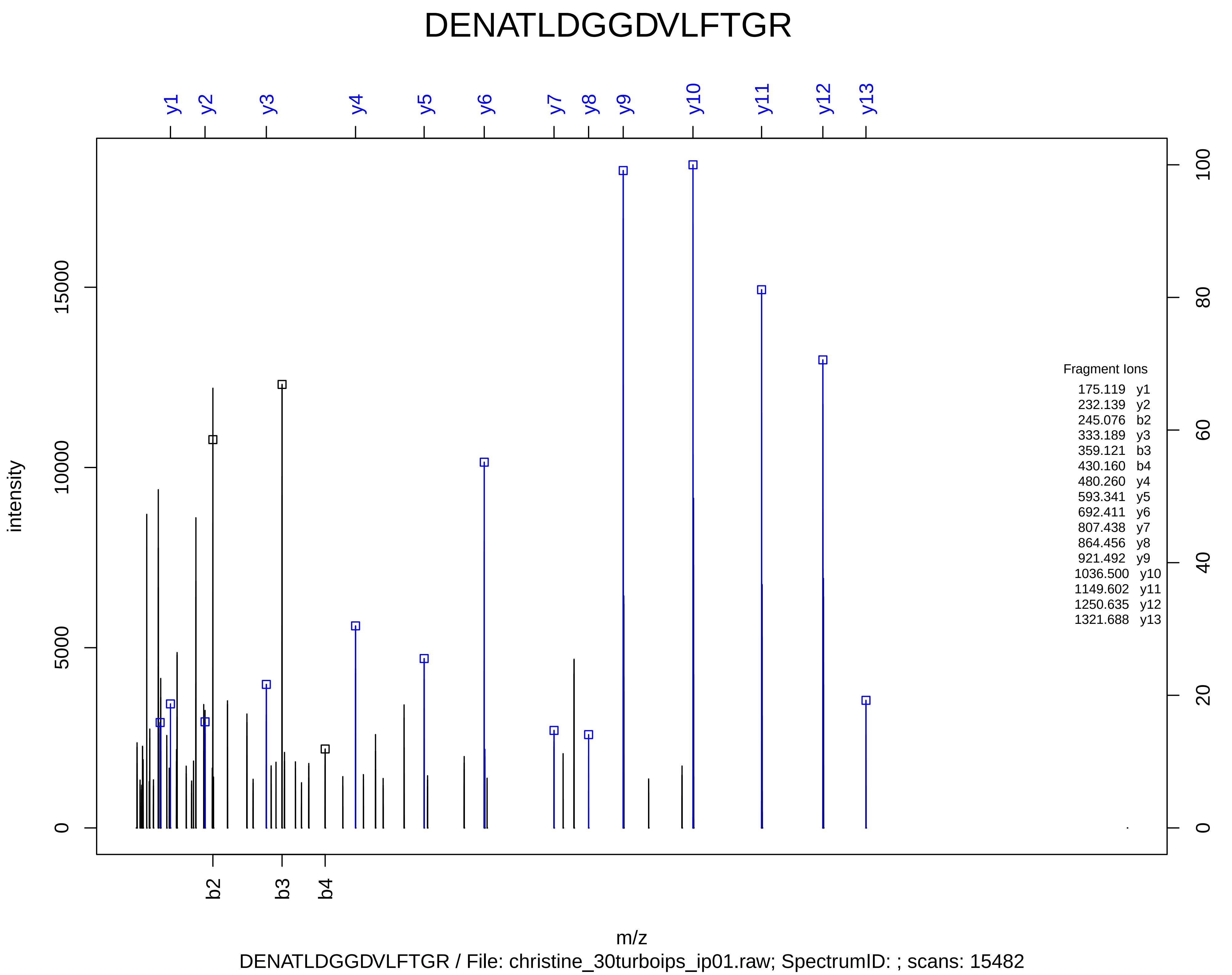

DENATLDGGDVLFTGR
y1
y2
y3
y4
y5
y6
y7
y8
y9
y10
y11
y12
y13
intensity
0
5000
10000
15000
0
20
40
60
80
100
b2
b3
b4
m/z
Fragment Ions
175.119 y1
232.139 y2
245.076 b2
333.189 y3
359.121 b3
430.160 b4
480.260 y4
593.341 y5
692.411 y6
807.438 y7
864.456 y8
921.492 y9
1036.500 y10
1149.602 y11
1250.635 y12
1321.688 y13
DENATLDGGDVLFTGR / File: christine_30turboips_ip01.raw; SpectrumID: ; scans: 15482

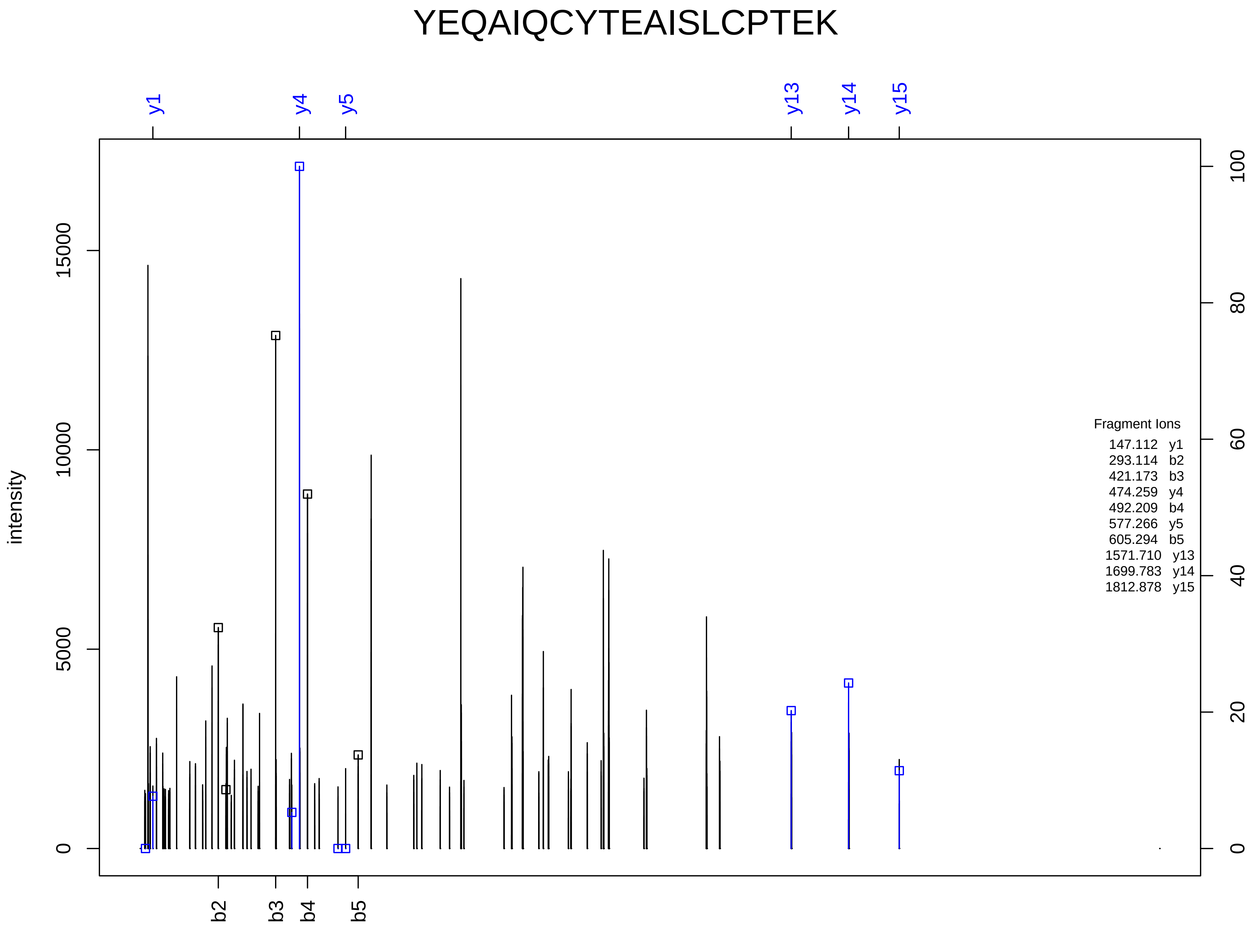

YEQAIQCYTEAISLCPTEK / File: christine_30turboips_ip01.raw; SpectrumID: ; scans: 15943

VFLLGEEVAQYDGAYK

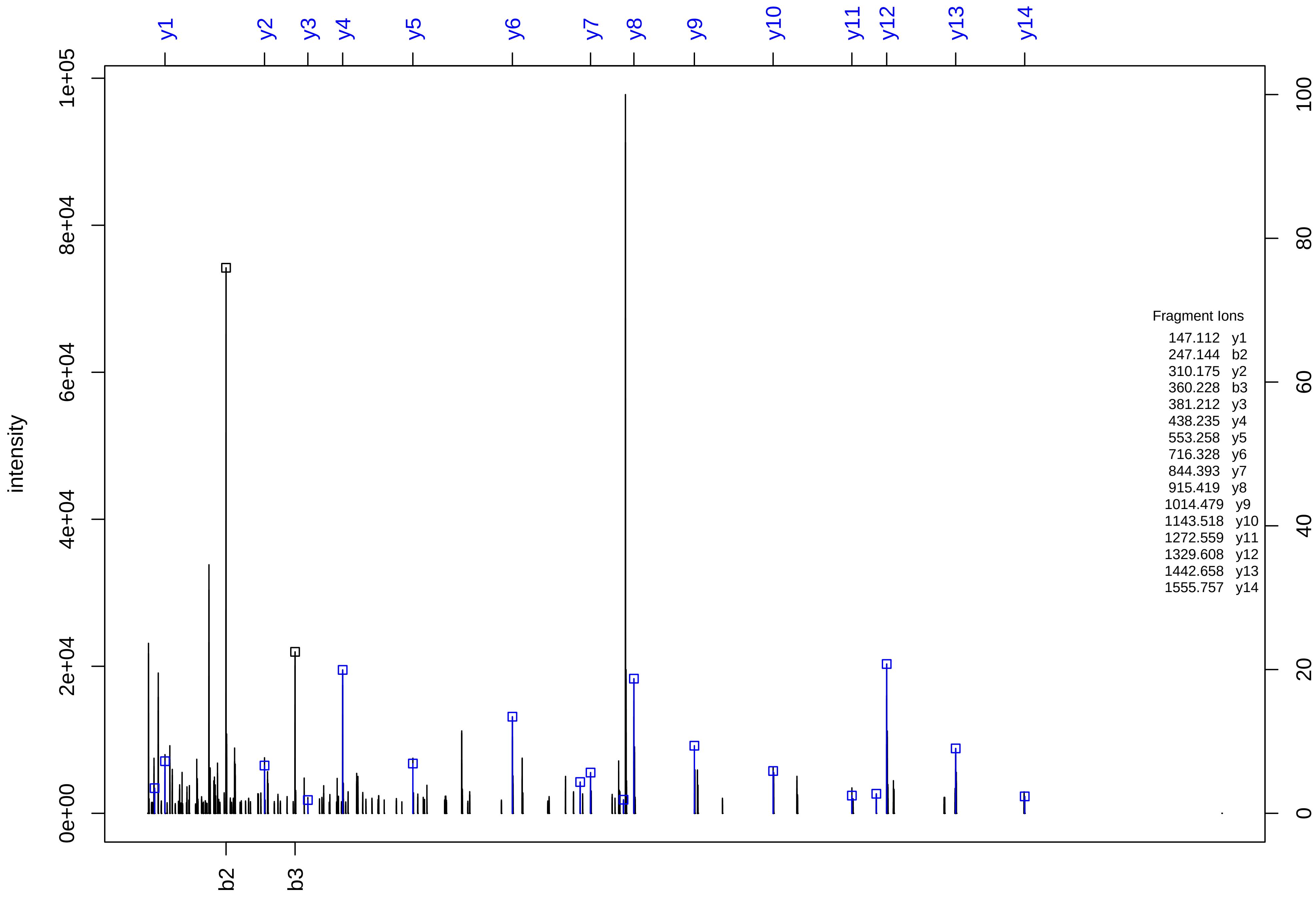

VFLLGEEVAQYDGAYK / File: christine_30turboips_ip01.raw; SpectrumID: ; scans: 19952

LIAVIGDEDTVTGFLLGGIGELNK

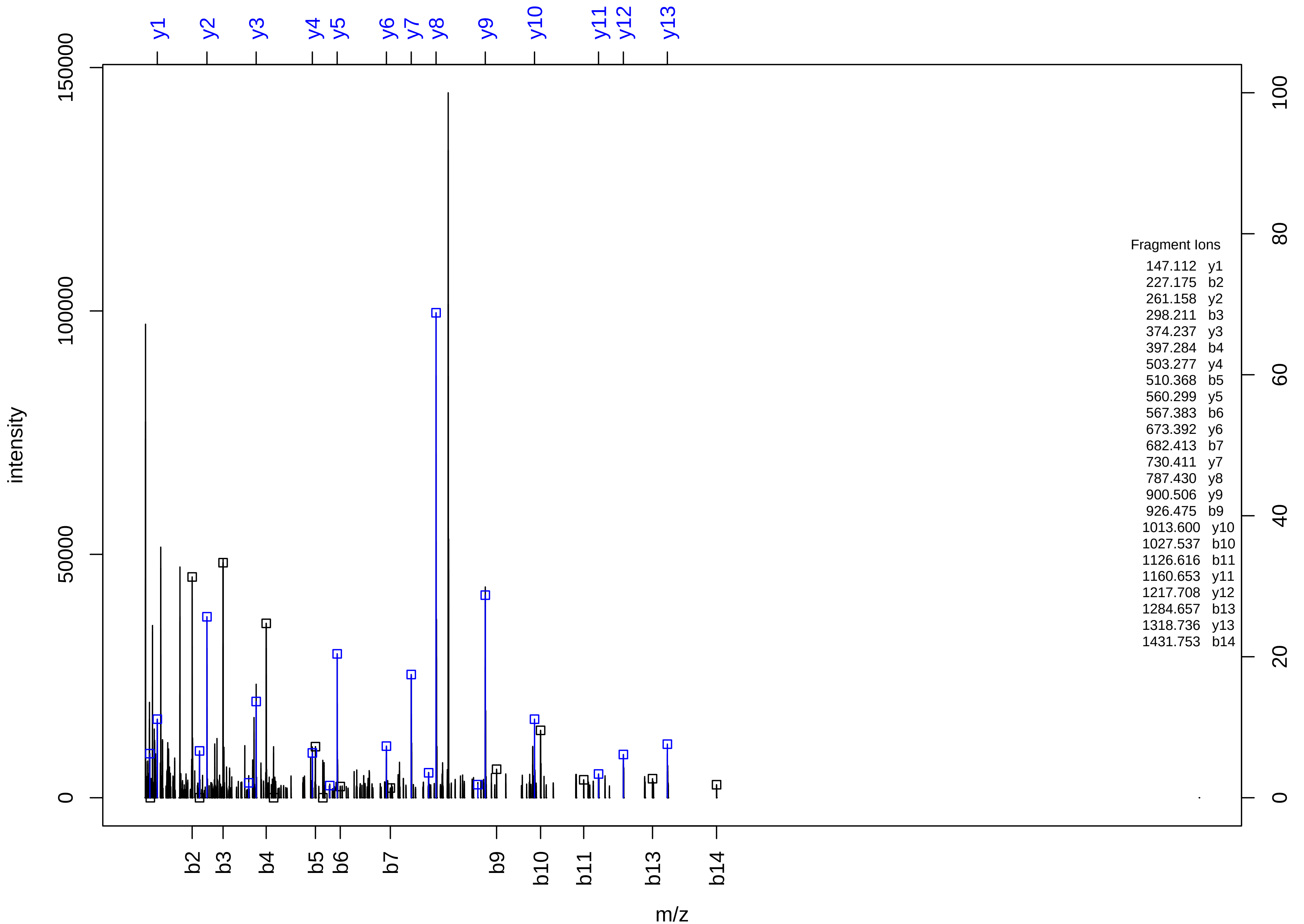

LIAVIGDEDTVTGFLLGGIGELNK / File: christine_30turboips_ip01.raw; SpectrumID: ; scans: 32544

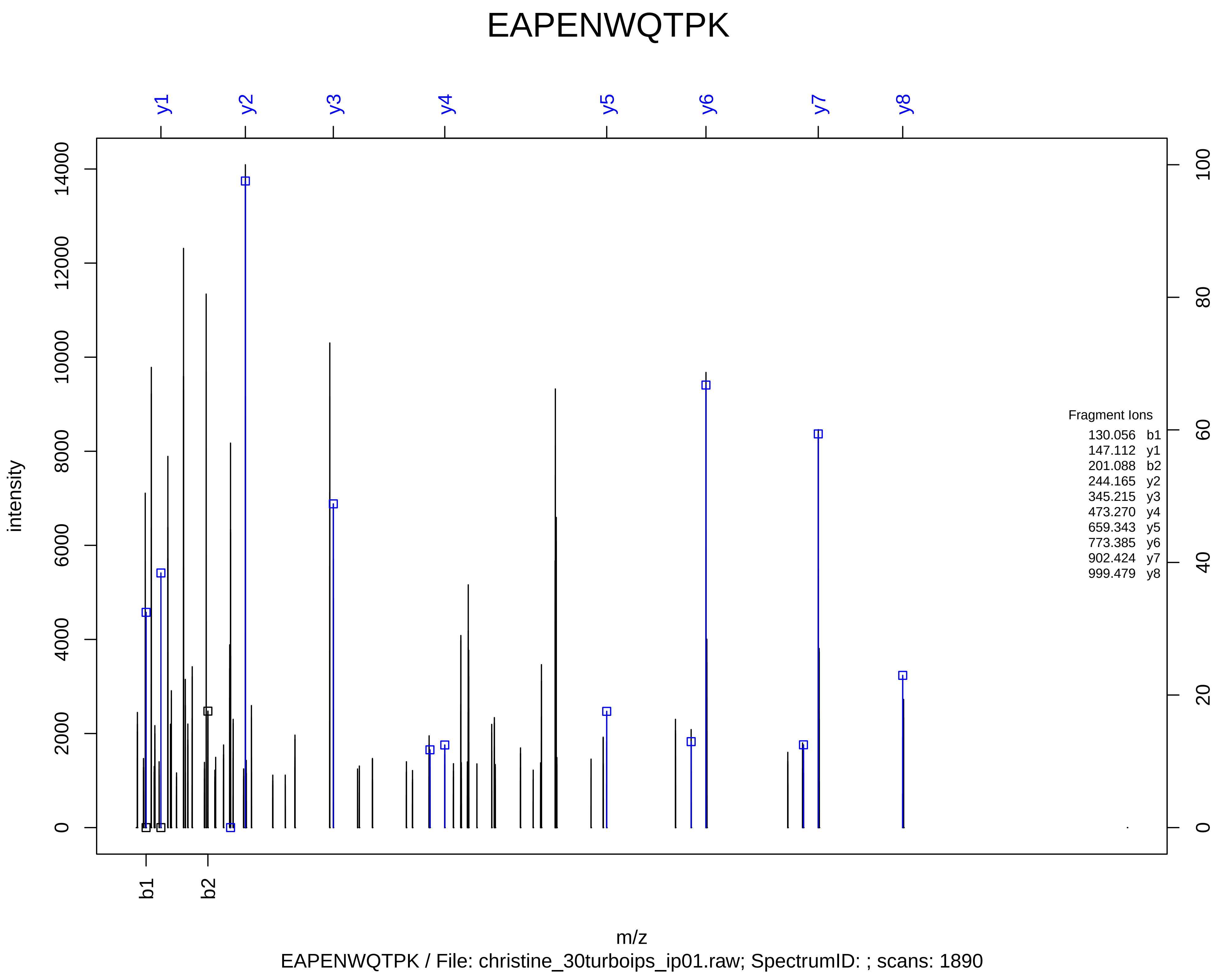
EAPENWQTPK
y1
y2
y3
y4
y5
y6
y7
y8
b1
b2
intensity
m/z
0
2000
4000
6000
8000
10000
12000
14000
0
20
40
60
80
100
Fragment Ions
130.056 b1
147.112 y1
201.088 b2
244.165 y2
345.215 y3
473.270 y4
659.343 y5
773.385 y6
902.424 y7
999.479 y8
EAPENWQTPK / File: christine_30turboips_ip01.raw; SpectrumID: ; scans: 1890

THCPIYLVNVSSISAGDVIAAAK

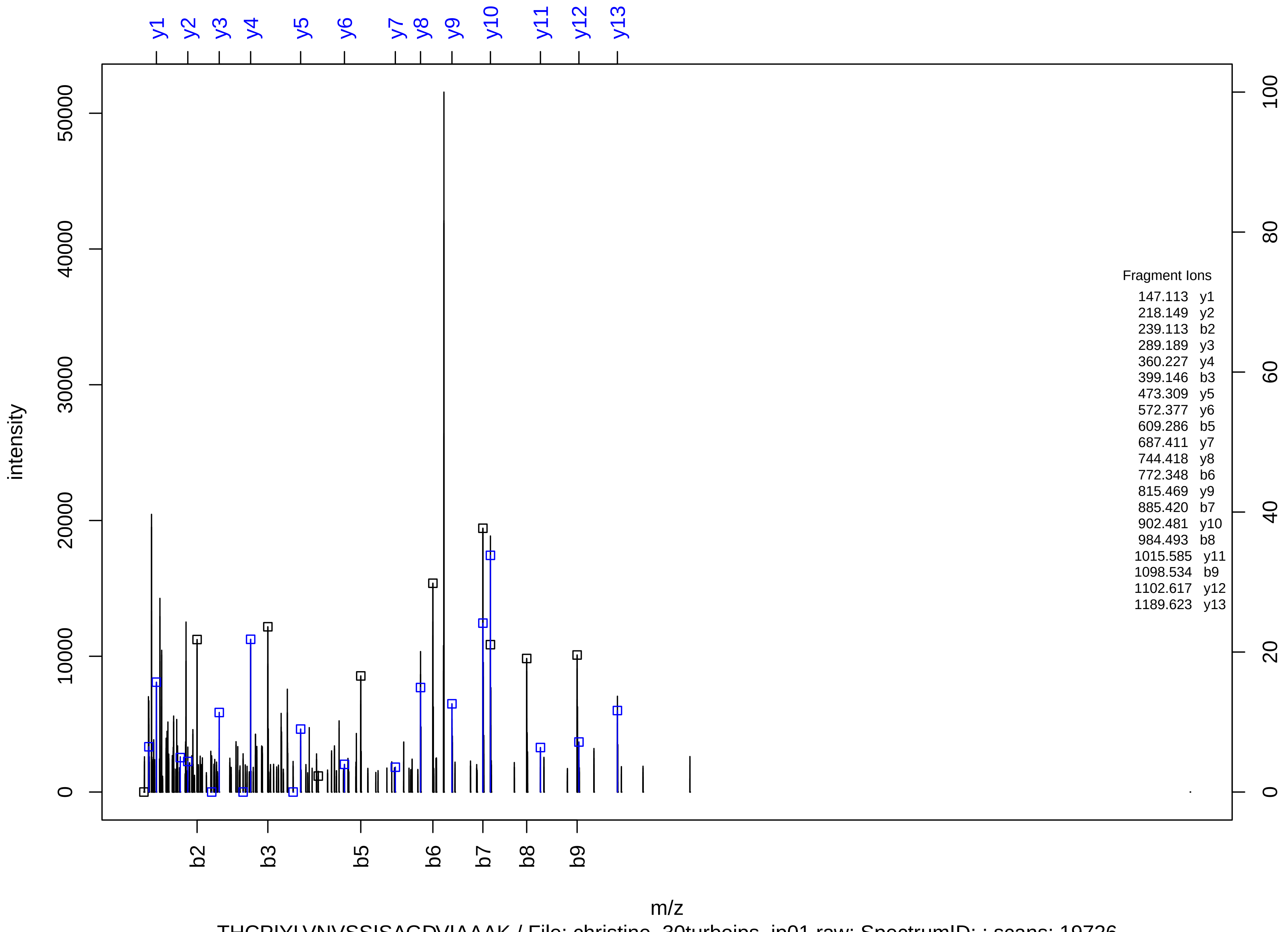

THCPIYLVNVSSISAGDVIAAAK / File: christine_30turboips_ip01.raw; SpectrumID: ; scans: 19726

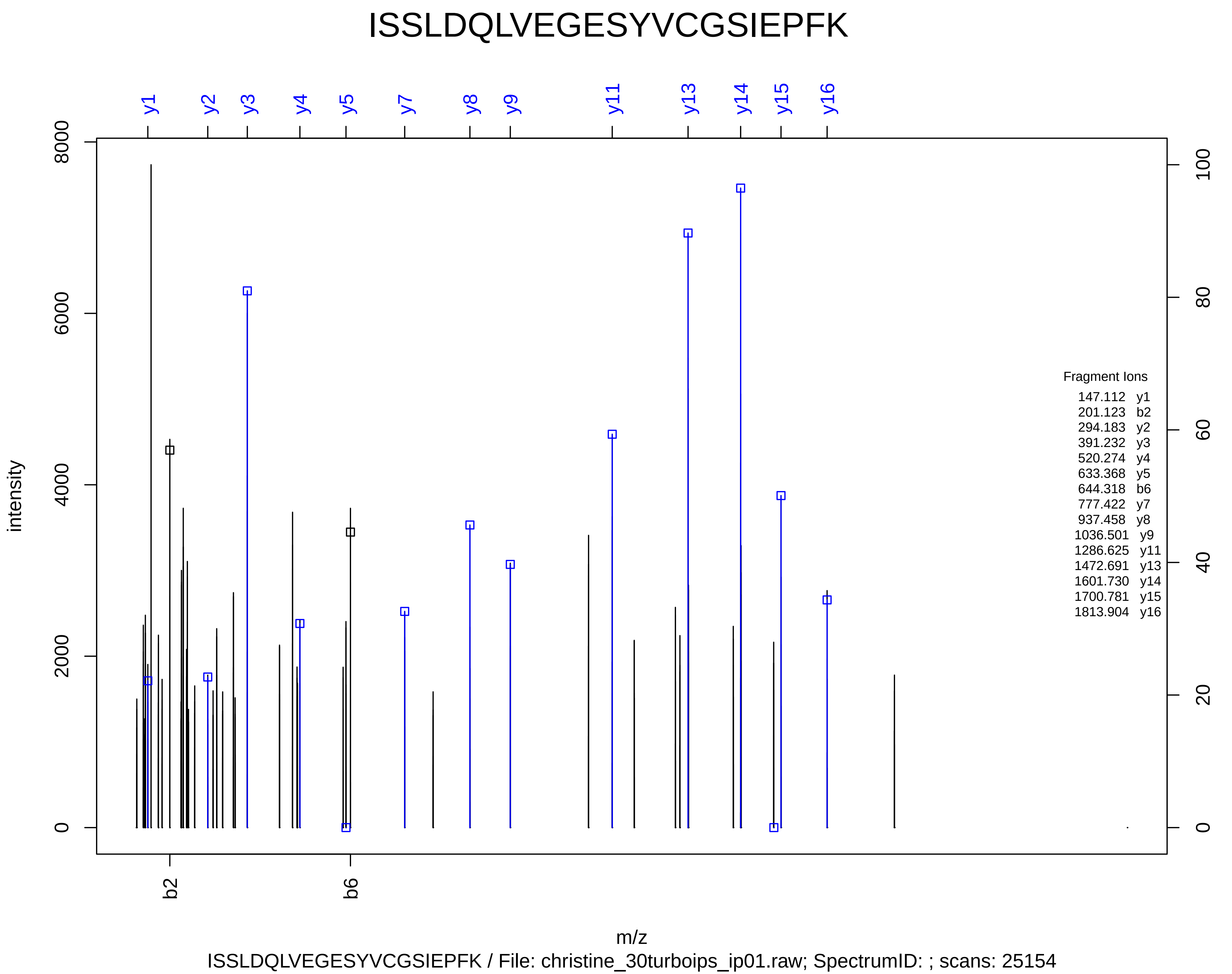
ISSLDQLVEGESYVCGSIEPFK
y1
y2
y3
y4
y5
y7
y8
y9
y11
y13
y14
y15
y16
b2
b6
intensity
m/z
0
2000
4000
6000
8000
20
40
60
80
100
Fragment Ions
147.112 y1
201.123 b2
294.183 y2
391.232 y3
520.274 y4
633.368 y5
644.318 b6
777.422 y7
937.458 y8
1036.501 y9
1286.625 y11
1472.691 y13
1601.730 y14
1700.781 y15
1813.904 y16
ISSLDQLVEGESYVCGSIEPFK / File: christine_30turboips_ip01.raw; SpectrumID: ; scans: 25154

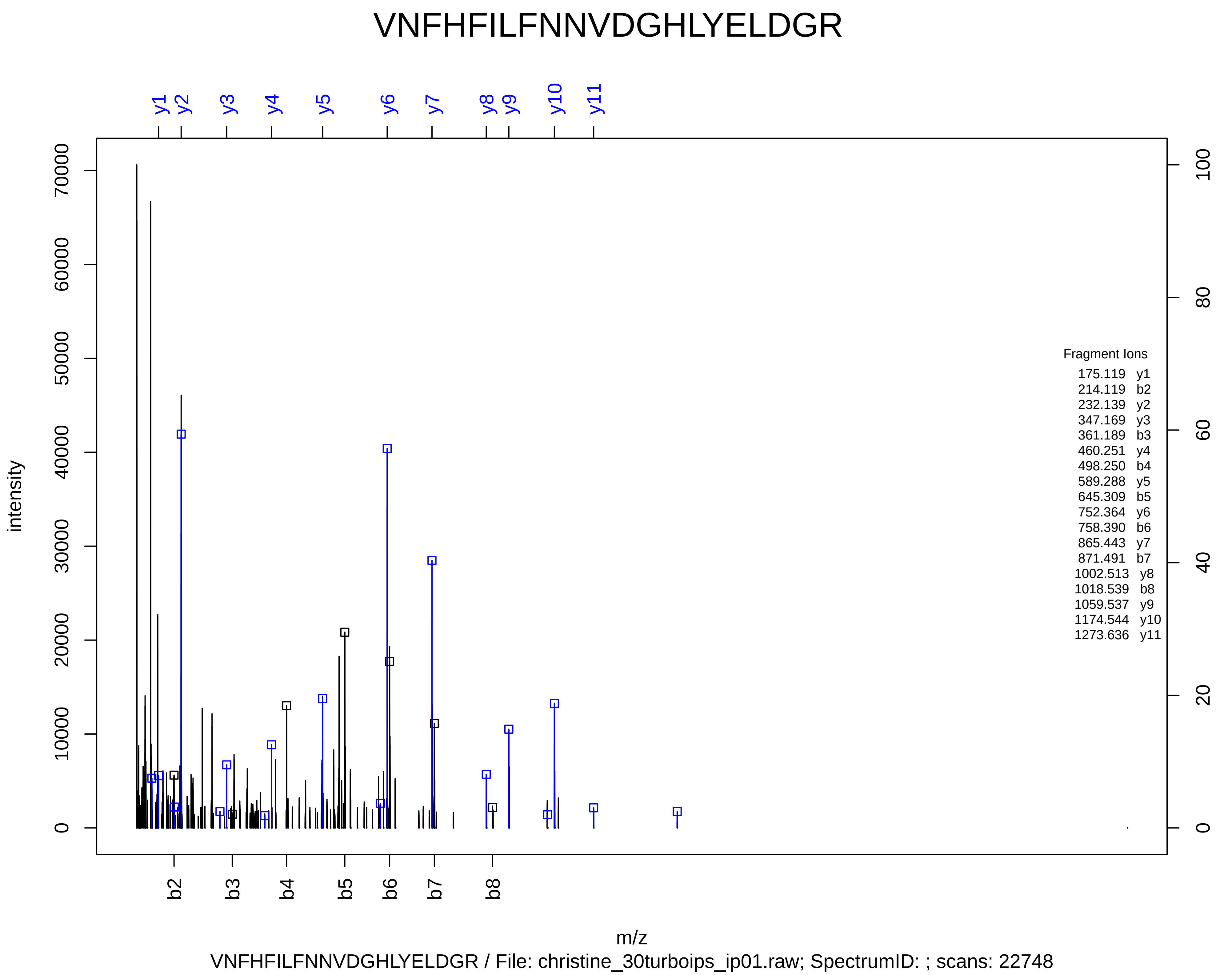
VNFHFILFNNVDGHLYELDGR
y1 y2 y3 y4 y5 y6 y7 y8 y9 y10 y11
b2 b3 b4 b5 b6 b7 b8
intensity
m/z
Fragment Ions
175.119 y1
214.119 b2
232.139 y2
347.169 y3
361.189 b3
460.251 y4
498.250 b4
589.288 y5
645.309 b5
752.364 y6
758.390 b6
865.443 y7
871.491 b7
1002.513 y8
1018.539 b8
1059.537 y9
1174.544 y10
1273.636 y11
VNFHFILFNNVDGHLYELDGR / File: christine_30turboips_ip01.raw; SpectrumID: ; scans: 22748

NDFTEEEEAQVR

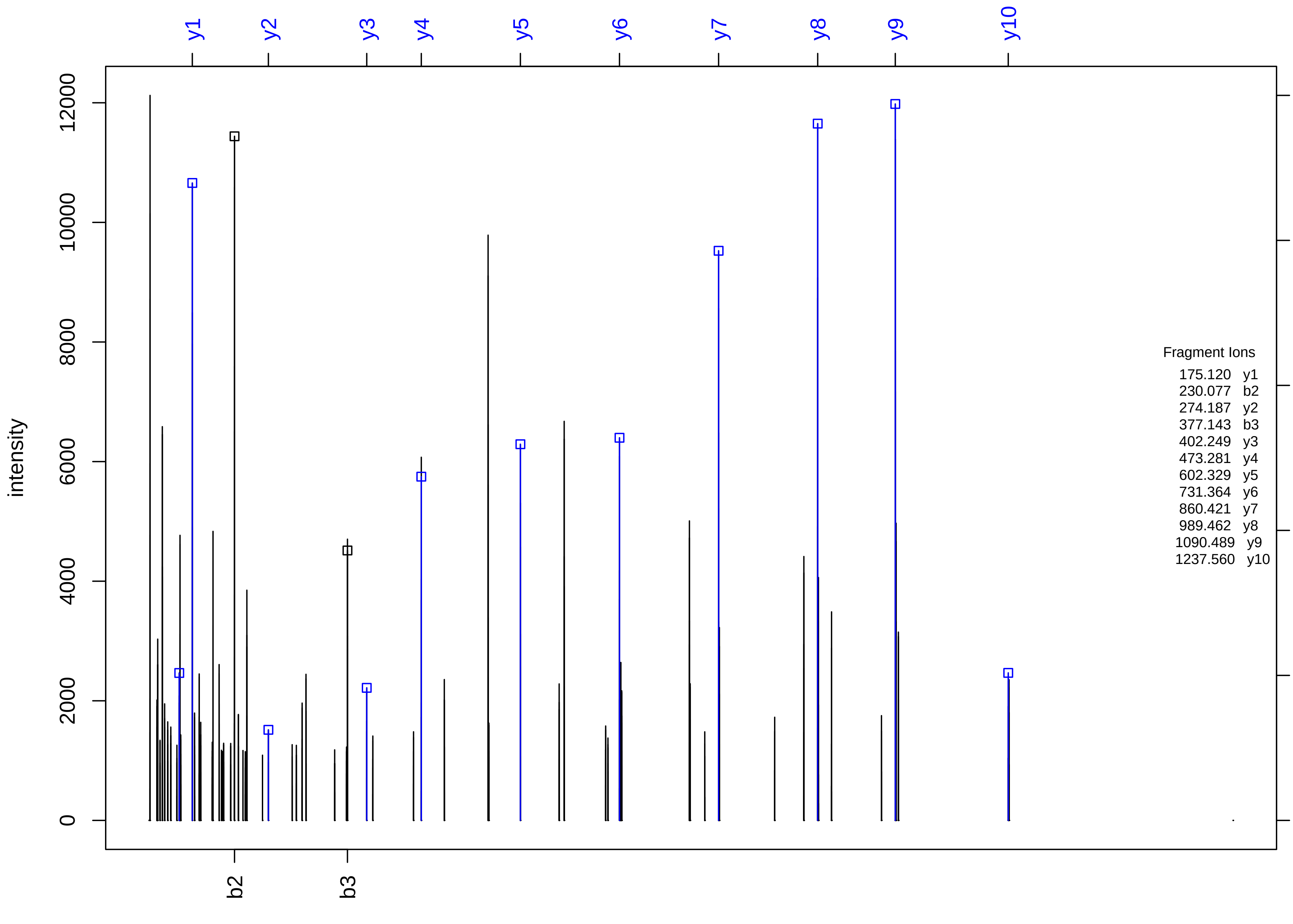

NDFTEEEEAQVR / File: christine_30turboips_ip01.raw; SpectrumID: ; scans: 3322

AIFLADGNVFTTGFSR

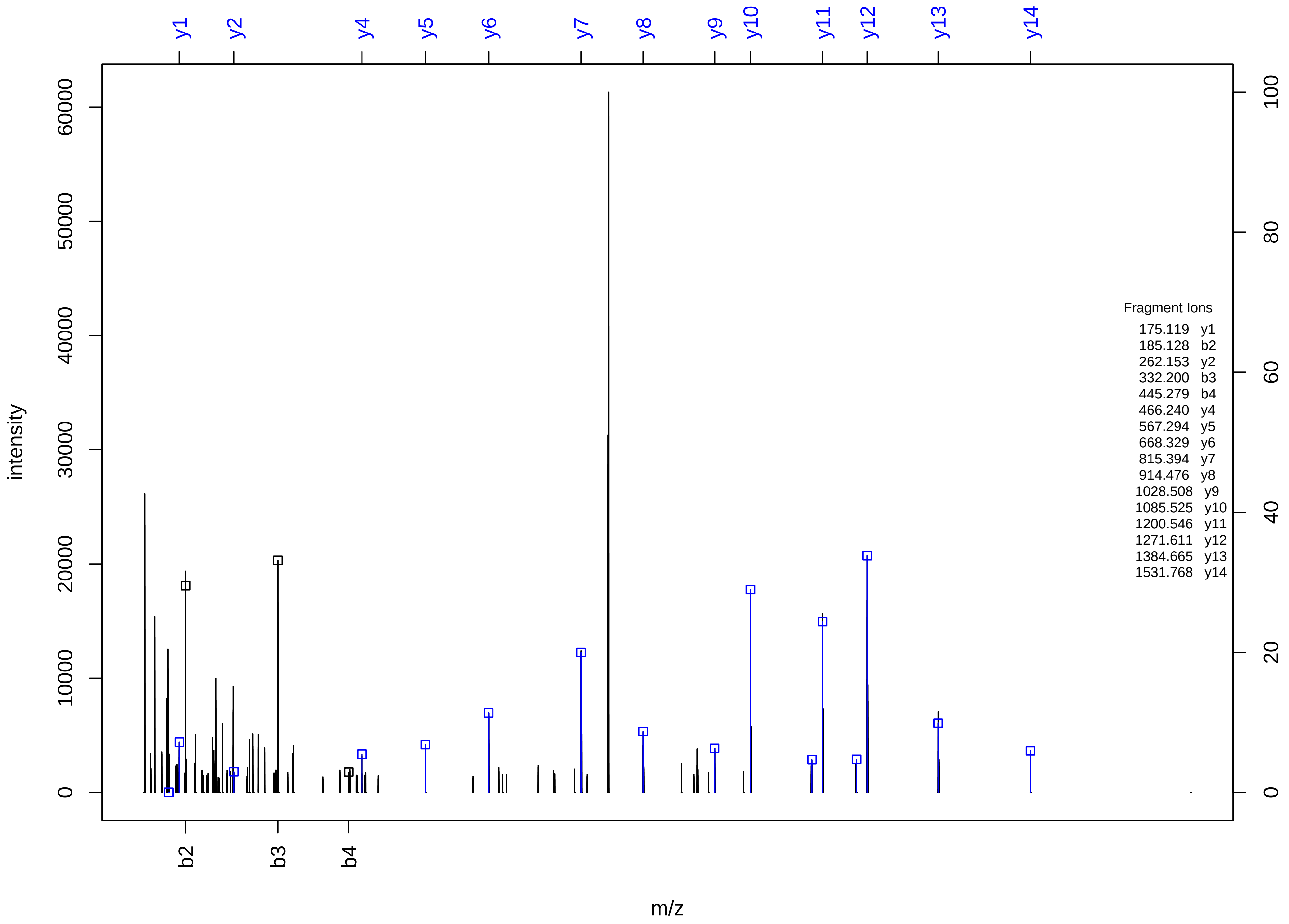

AIFLADGNVFTTGFSR / File: christine_30turboips_ip01.raw; SpectrumID: ; scans: 22749

KM*DLDM*R

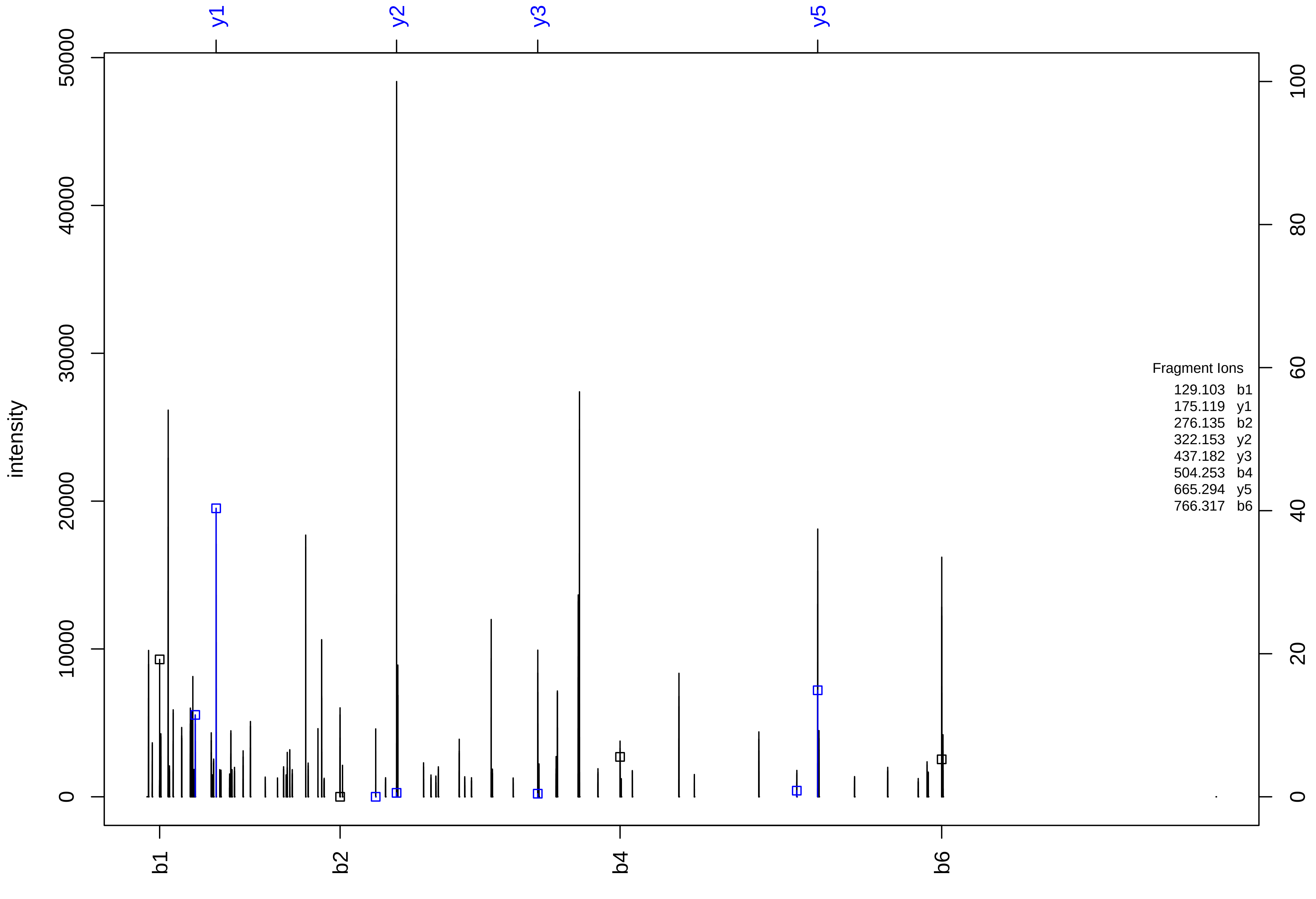

KMDLDMR / File: christine_30turboips_ip01.raw; SpectrumID: ; scans: 4028

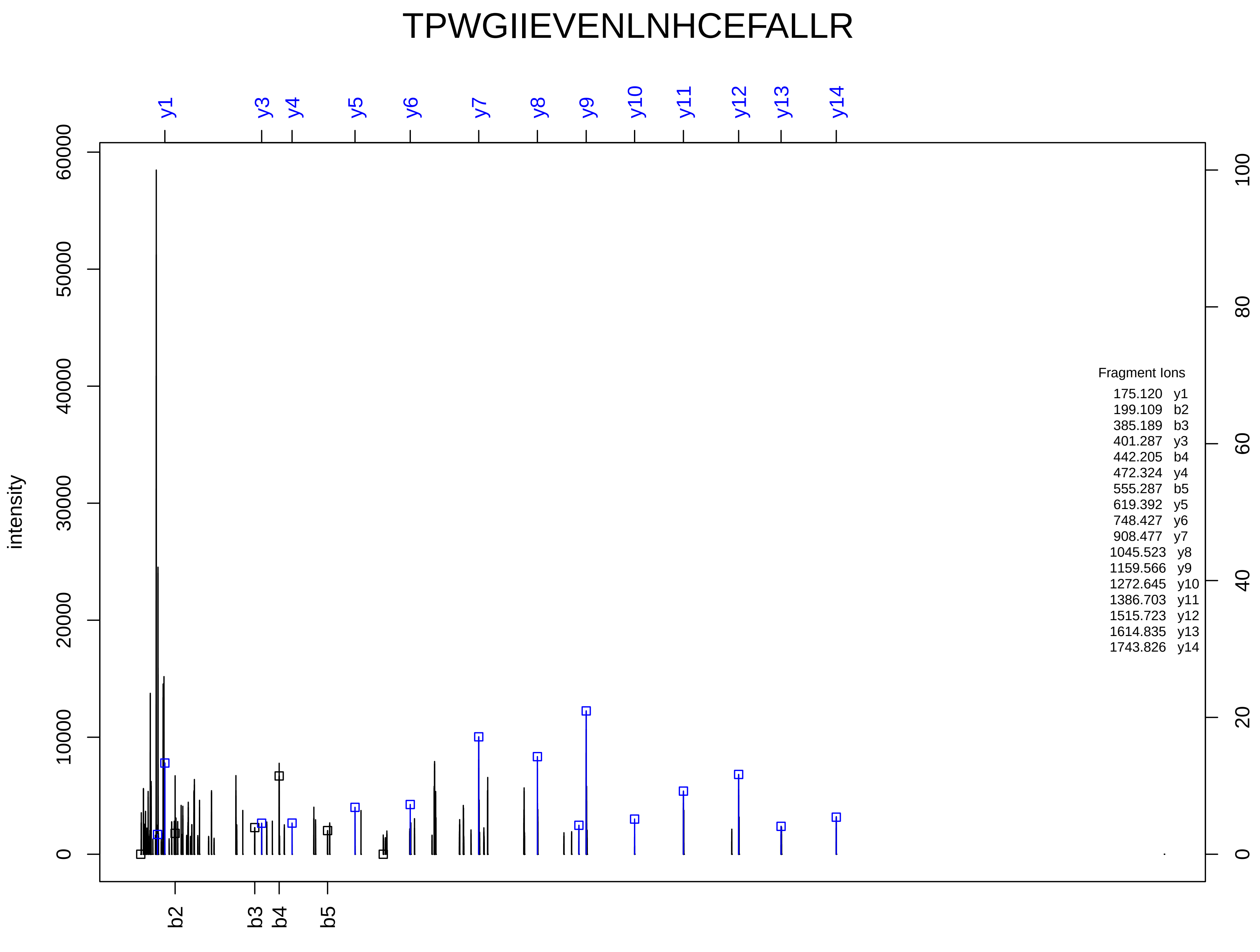

TPWGIIEVENLNHCEFALLR / File: christine_30turboips_ip01.raw; SpectrumID: ; scans: 26159

SFQGPVLIGSAQGGVNIEDVAAENPEAIVK

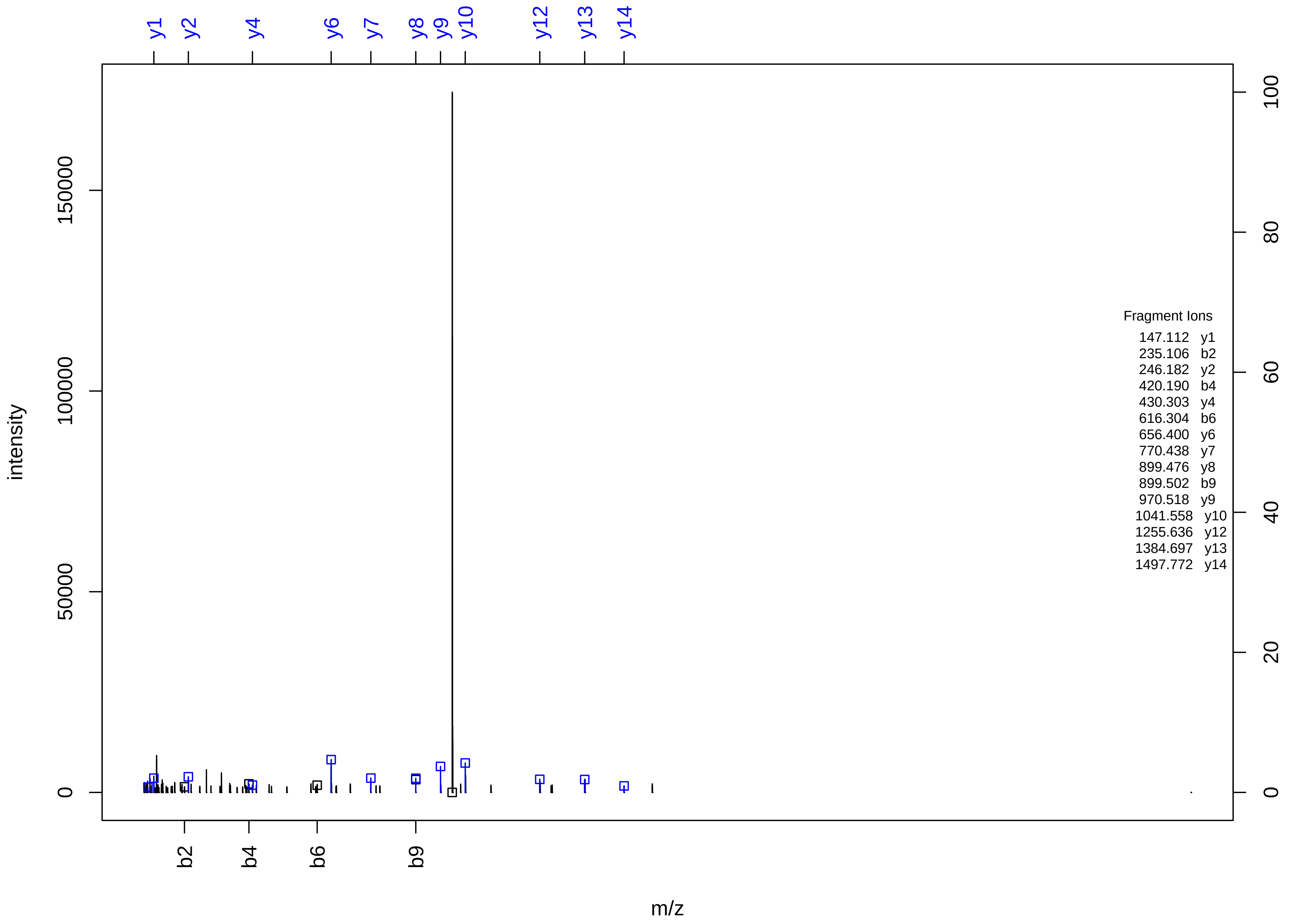

SFQGPVLIGSAQGGVNIEDVAAENPEAIVK / File: christine_30turboips_ip01.raw; SpectrumID: ; scans: 23557

ESAPFAVIGSNTVVEAK

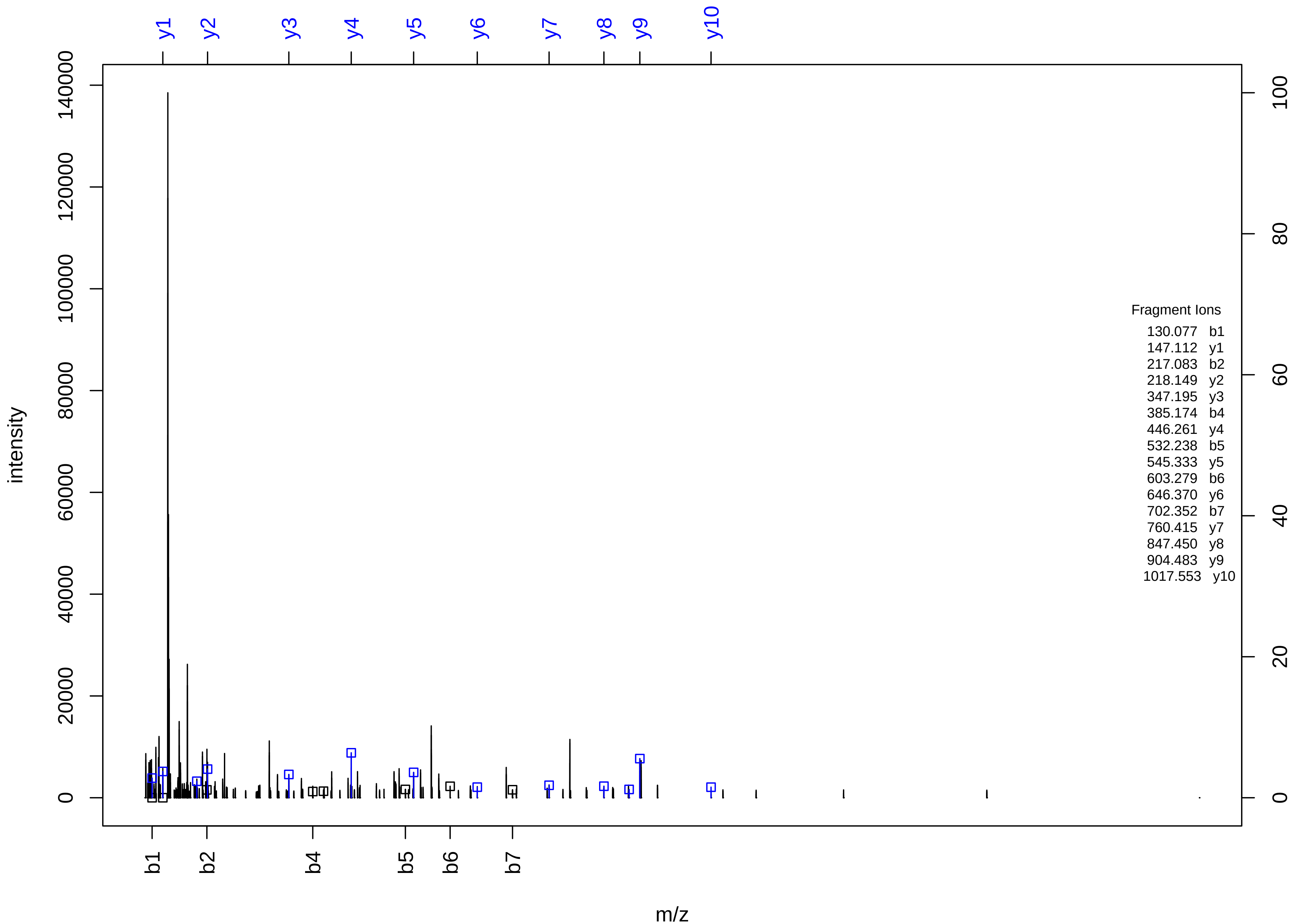

ESAPFAVIGSNTVVEAK / File: christine_30turboips_ip01.raw; SpectrumID: ; scans: 14556

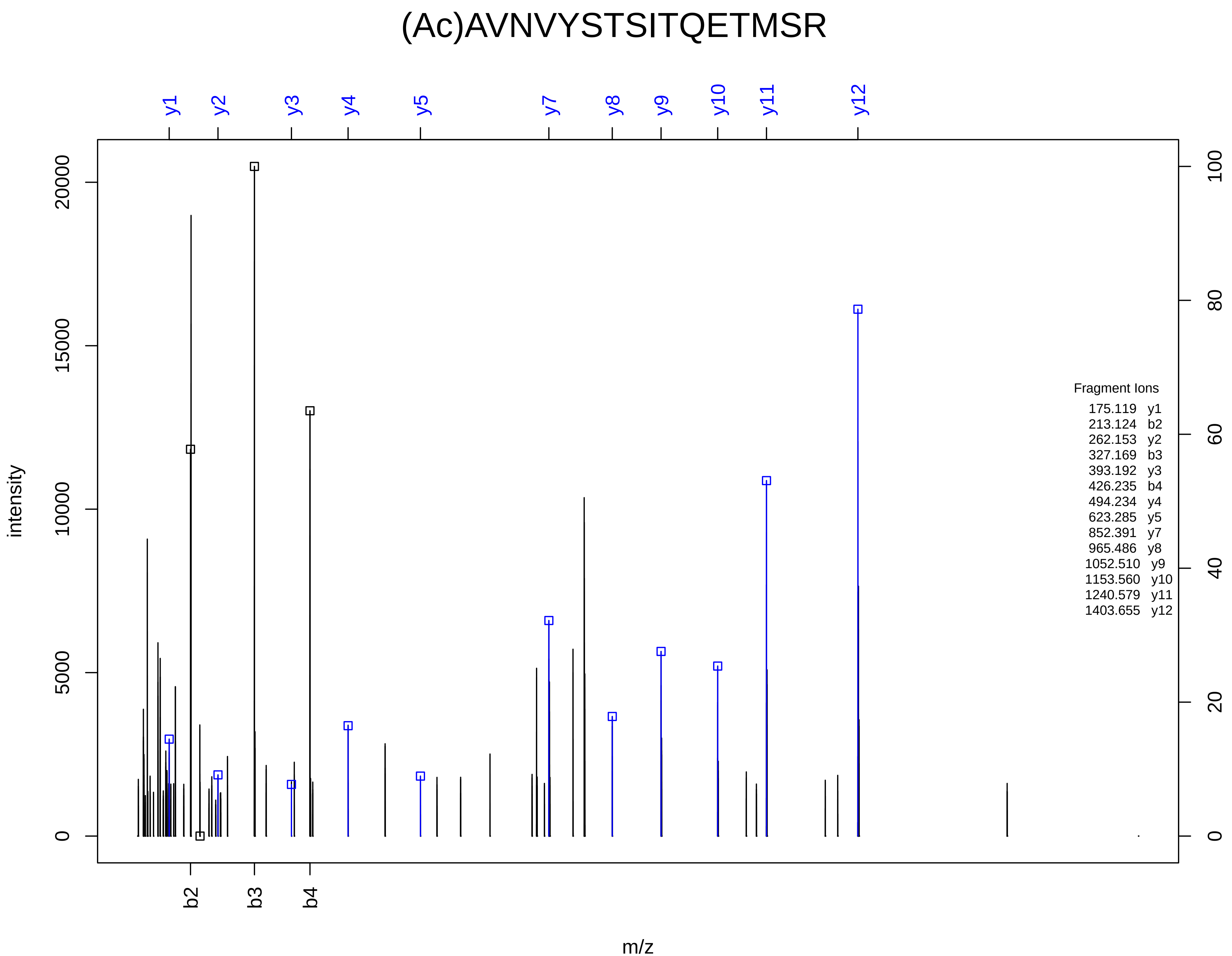
(Ac)AVNVYSTSITQETMSR
y1
y2
y3
y4
y5
y7
y8
y9
y10
y11
y12
b2
b3
b4
intensity
m/z
0
5000
10000
15000
20000
0
20
40
60
80
100
Fragment Ions
175.119 y1
213.124 b2
262.153 y2
327.169 b3
393.192 y3
426.235 b4
494.234 y4
623.285 y5
852.391 y7
965.486 y8
1052.510 y9
1153.560 y10
1240.579 y11
1403.655 y12
AVNVYSTSITQETMSR / File: christine_30turboips_ip01.raw; SpectrumID: ; scans: 16745

IFQANNDATEVVLNK

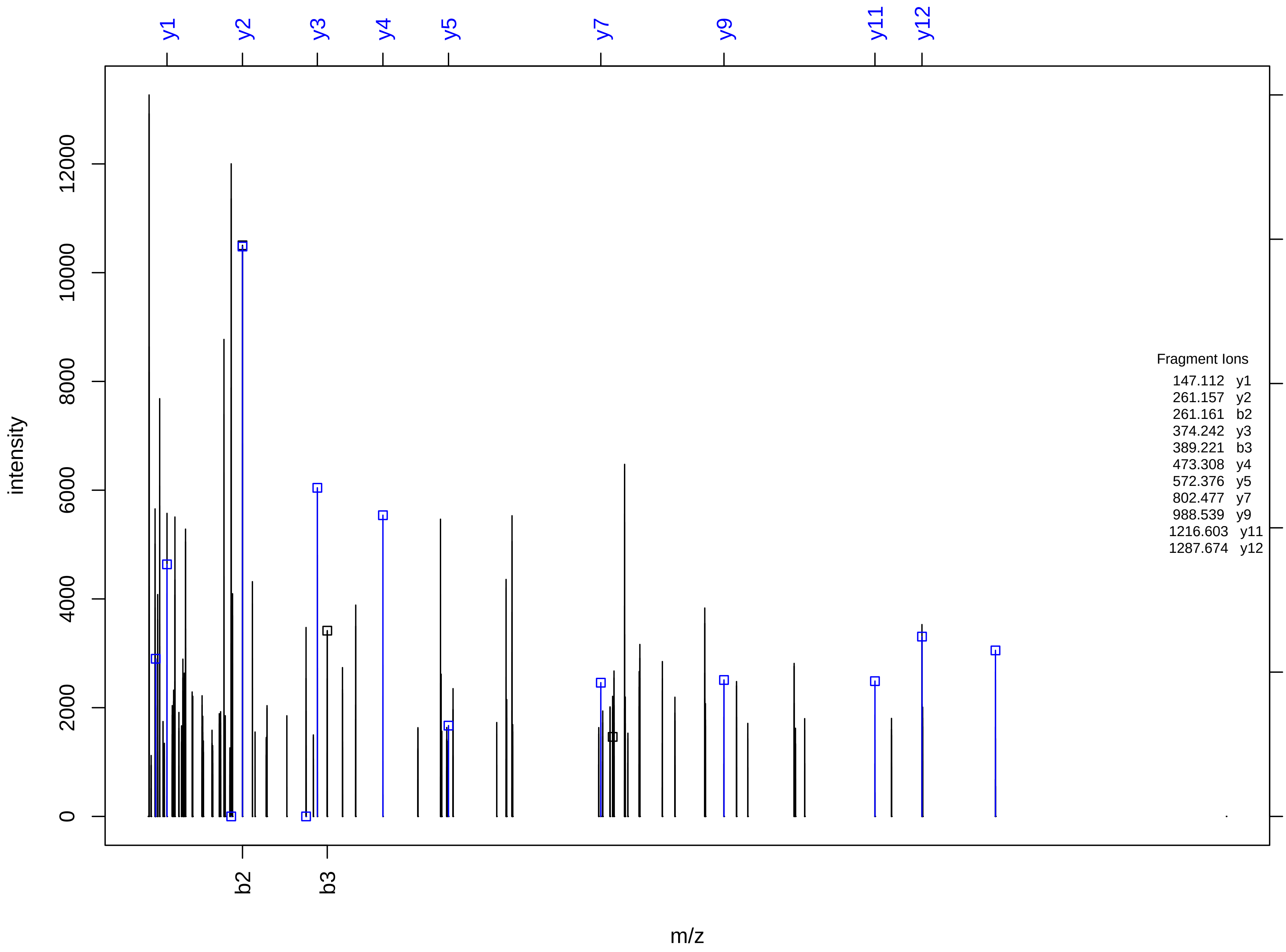

IFQANNDATEVVLNK / File: christine_30turboips_ip30.raw; SpectrumID: ; scans: 9820

(Ac)M*DPIQLLFYVNGQK

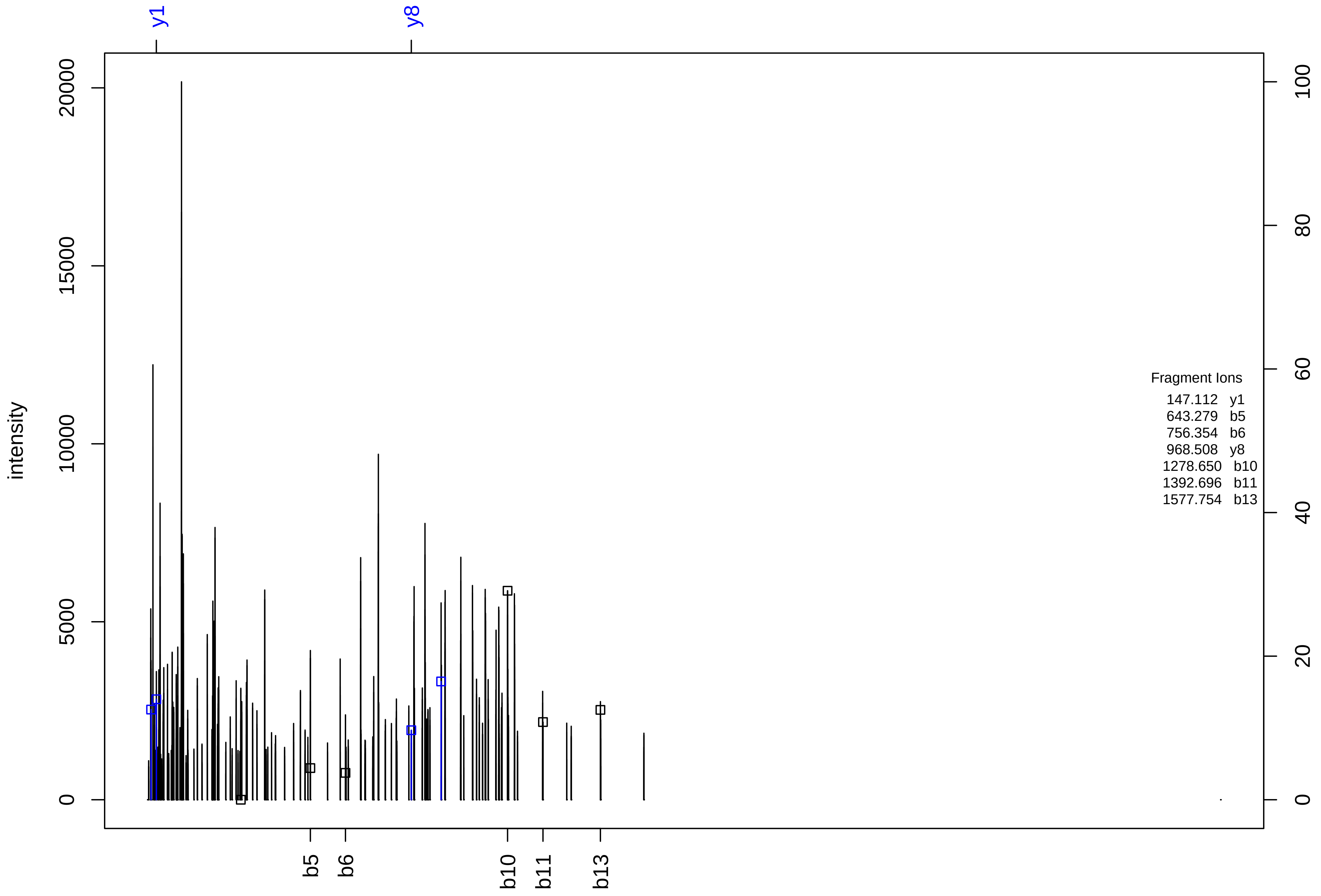

MDPIQLLFYVNGQK / File: christine_30turboips_ip30.raw; SpectrumID: ; scans: 20411

DDVILNEPSADAPAAR

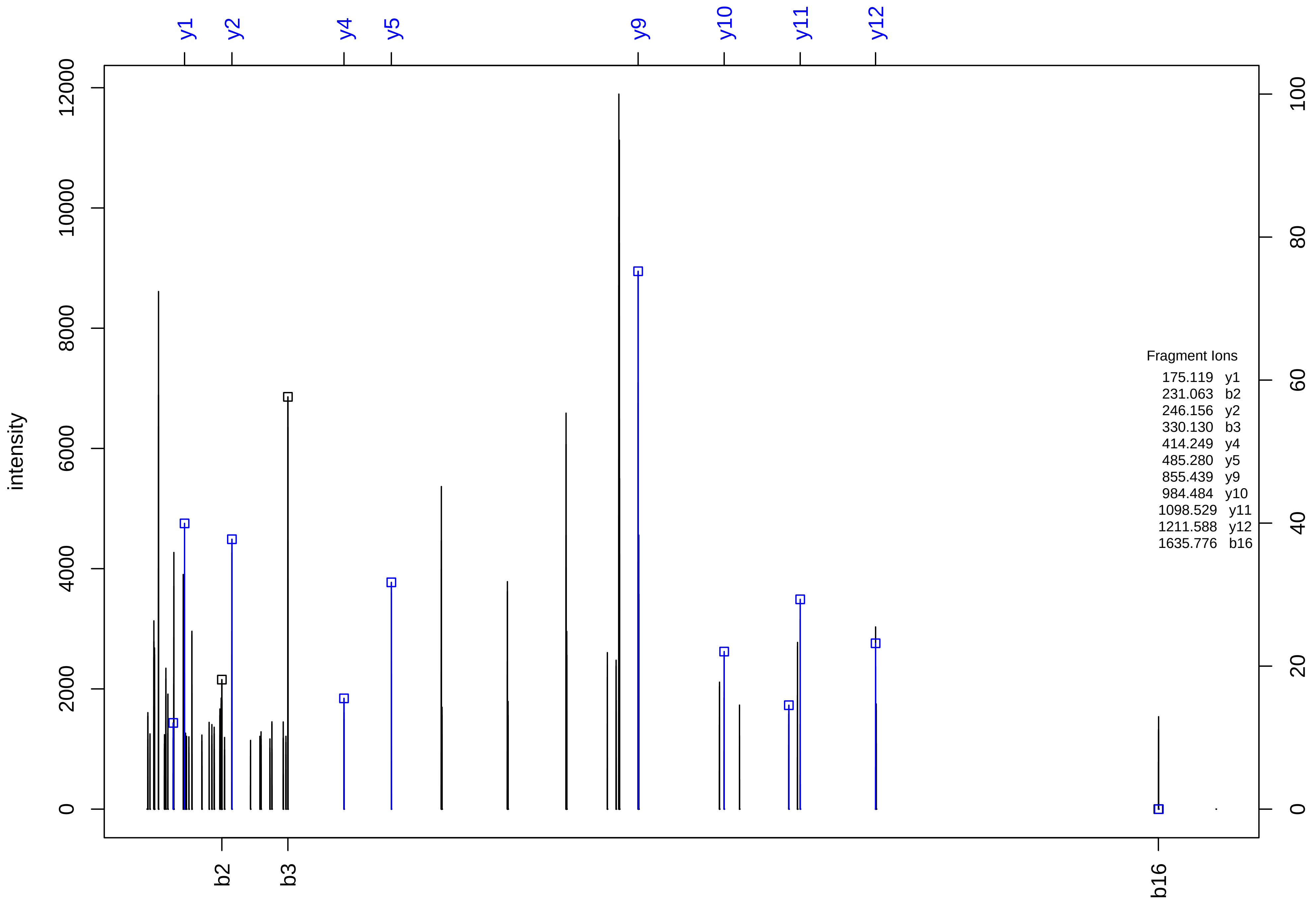

DDVILNEPSADAPAAR / File: christine_30turboips_ip30.raw; SpectrumID: ; scans: 8308

DIVQFVPFR

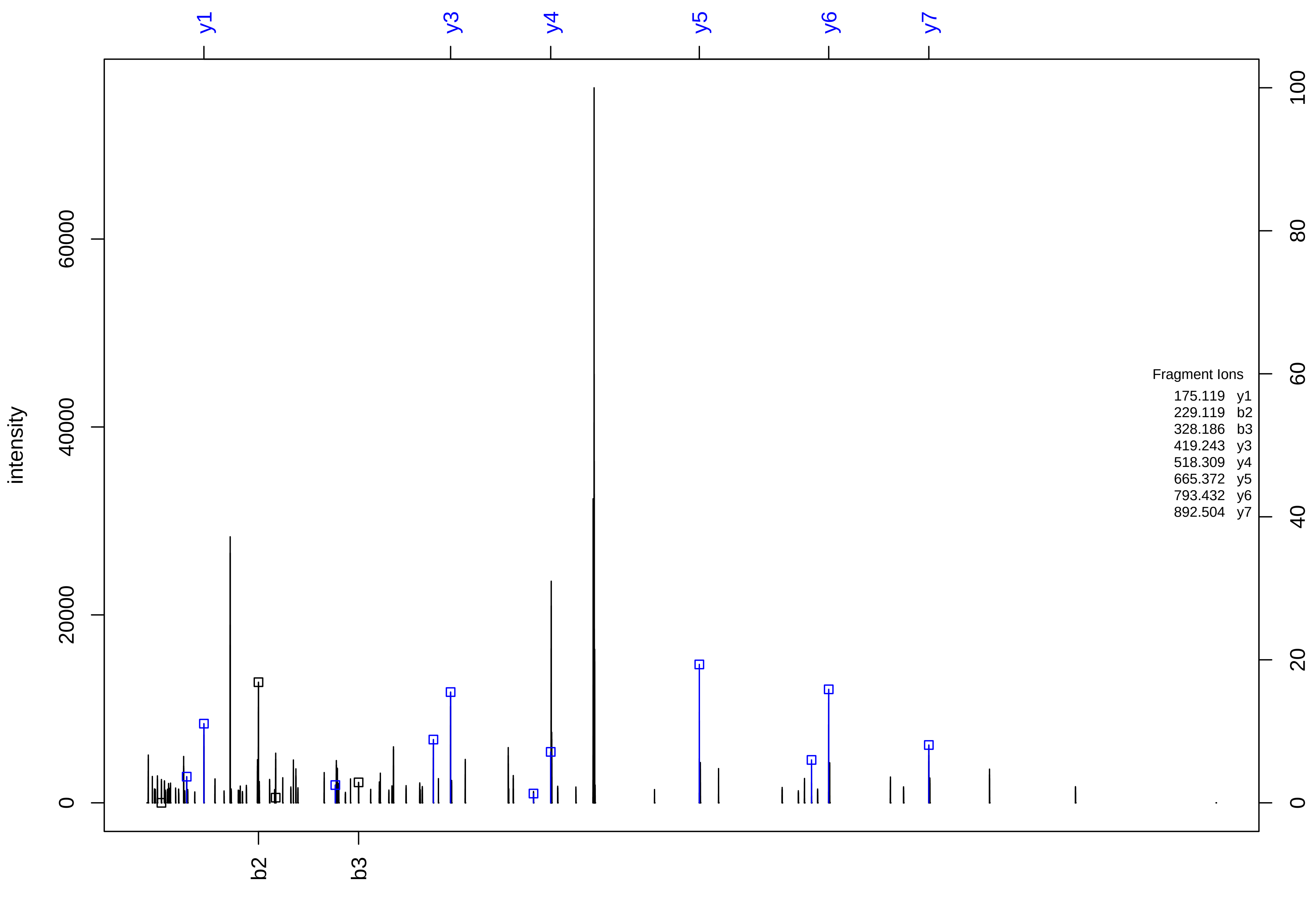

DIVQFVPFR / File: christine_30turboips_ip30.raw; SpectrumID: ; scans: 21777

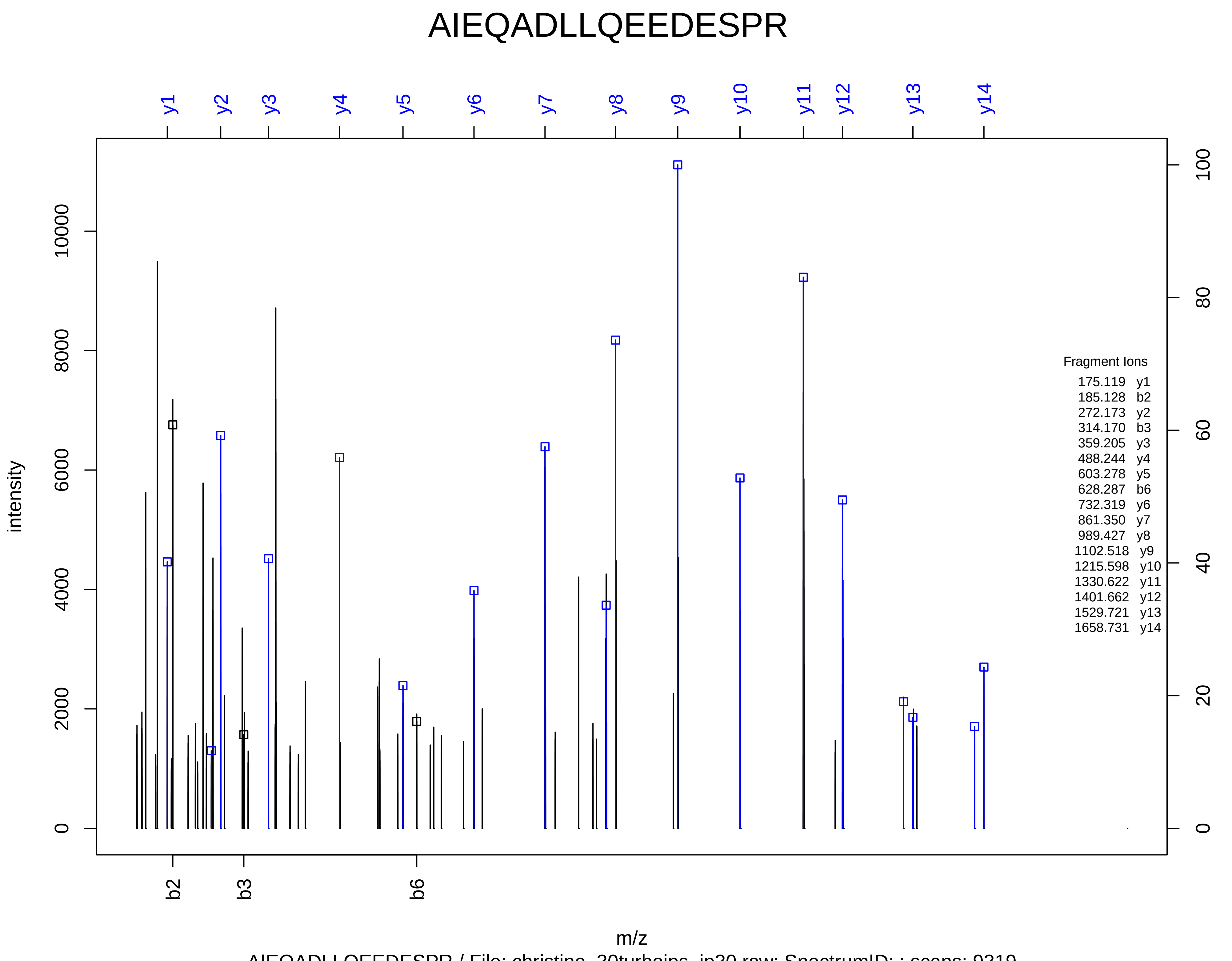
AIEQADLLQEEDESPR
y1
y2
y3
y4
y5
y6
y7
y8
y9
y10
y11
y12
y13
y14
b2
b3
b6
intensity
0
2000
4000
6000
8000
10000
0
20
40
60
80
100
m/z
Fragment Ions
175.119 y1
185.128 b2
272.173 y2
314.170 b3
359.205 y3
488.244 y4
603.278 y5
628.287 b6
732.319 y6
861.350 y7
989.427 y8
1102.518 y9
1215.598 y10
1330.622 y11
1401.662 y12
1529.721 y13
1658.731 y14
AIEQADLLQEEDESPR / File: christine_30turboips_ip30.raw; SpectrumID: ; scans: 9319

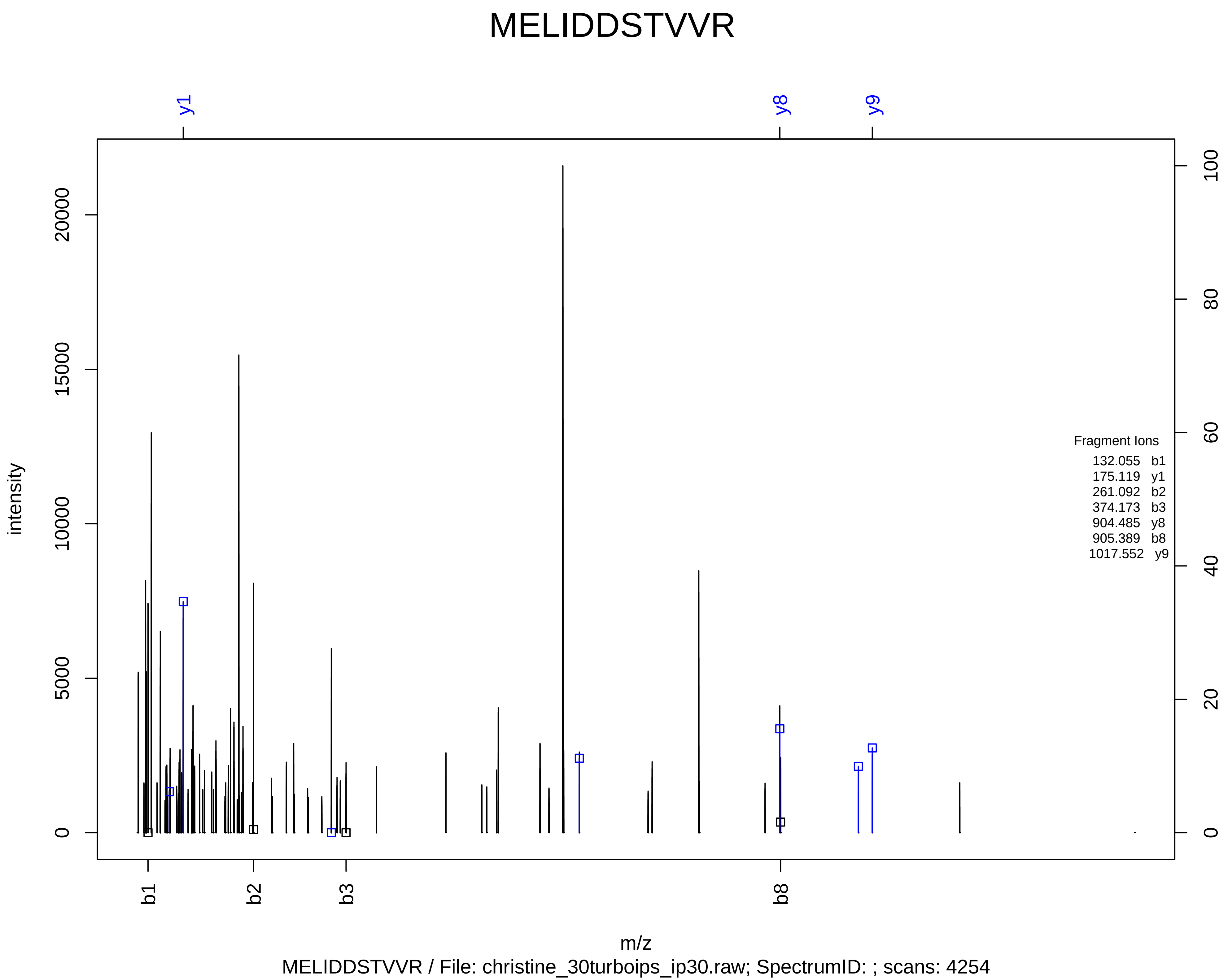

MELIDDSTVVR
y1
y8
y9
intensity
0
5000
10000
15000
20000
0
20
40
60
80
100
Fragment Ions
132.055 b1
175.119 y1
261.092 b2
374.173 b3
904.485 y8
905.389 b8
1017.552 y9
b1
b2
b3
b8
m/z
MELIDDSTVVR / File: christine_30turboips_ip30.raw; SpectrumID: ; scans: 4254

LYVYNTDTDNCR

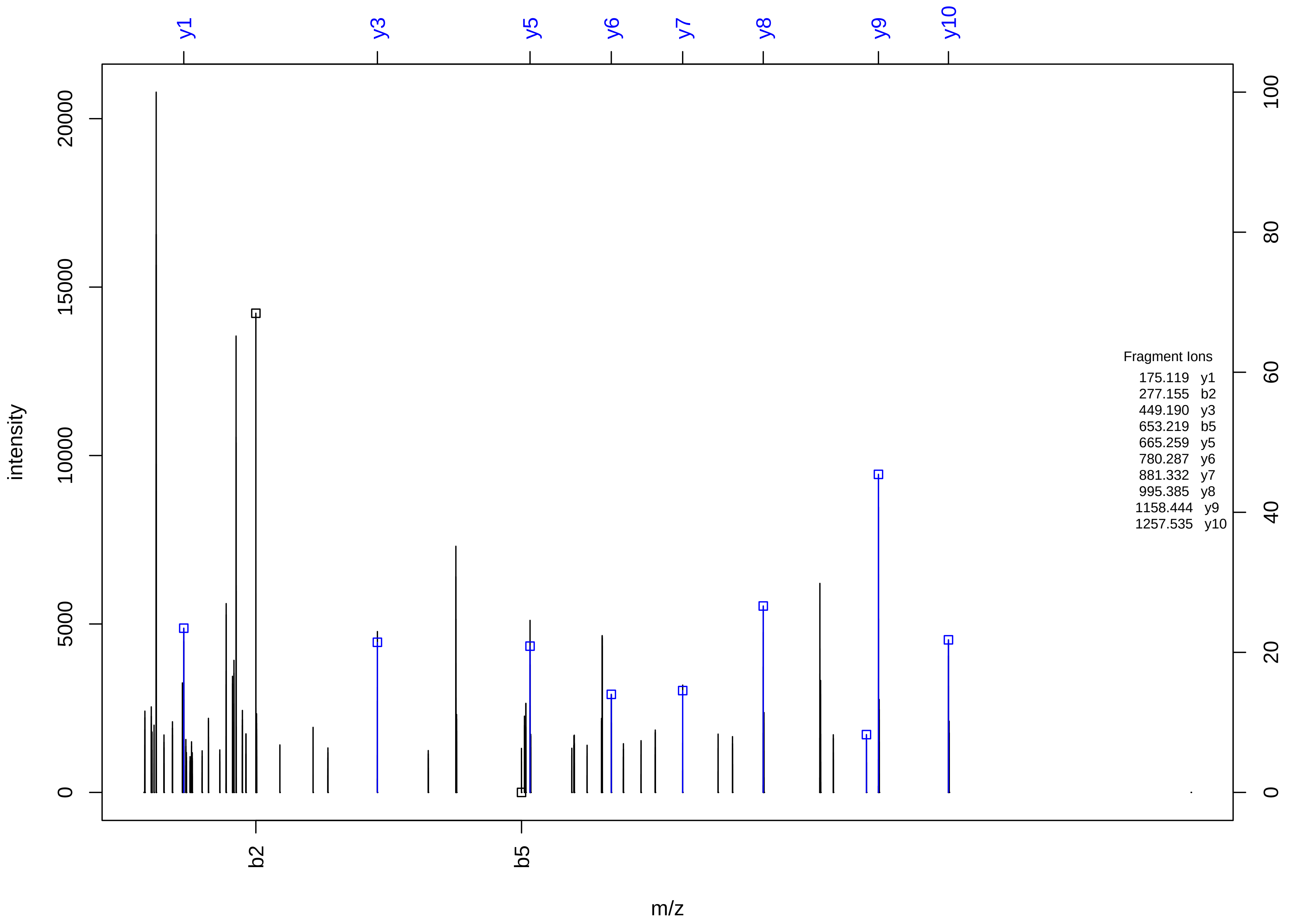

LYVYNTDTDNCR / File: christine_30turboips_ip30.raw; SpectrumID: ; scans: 3438

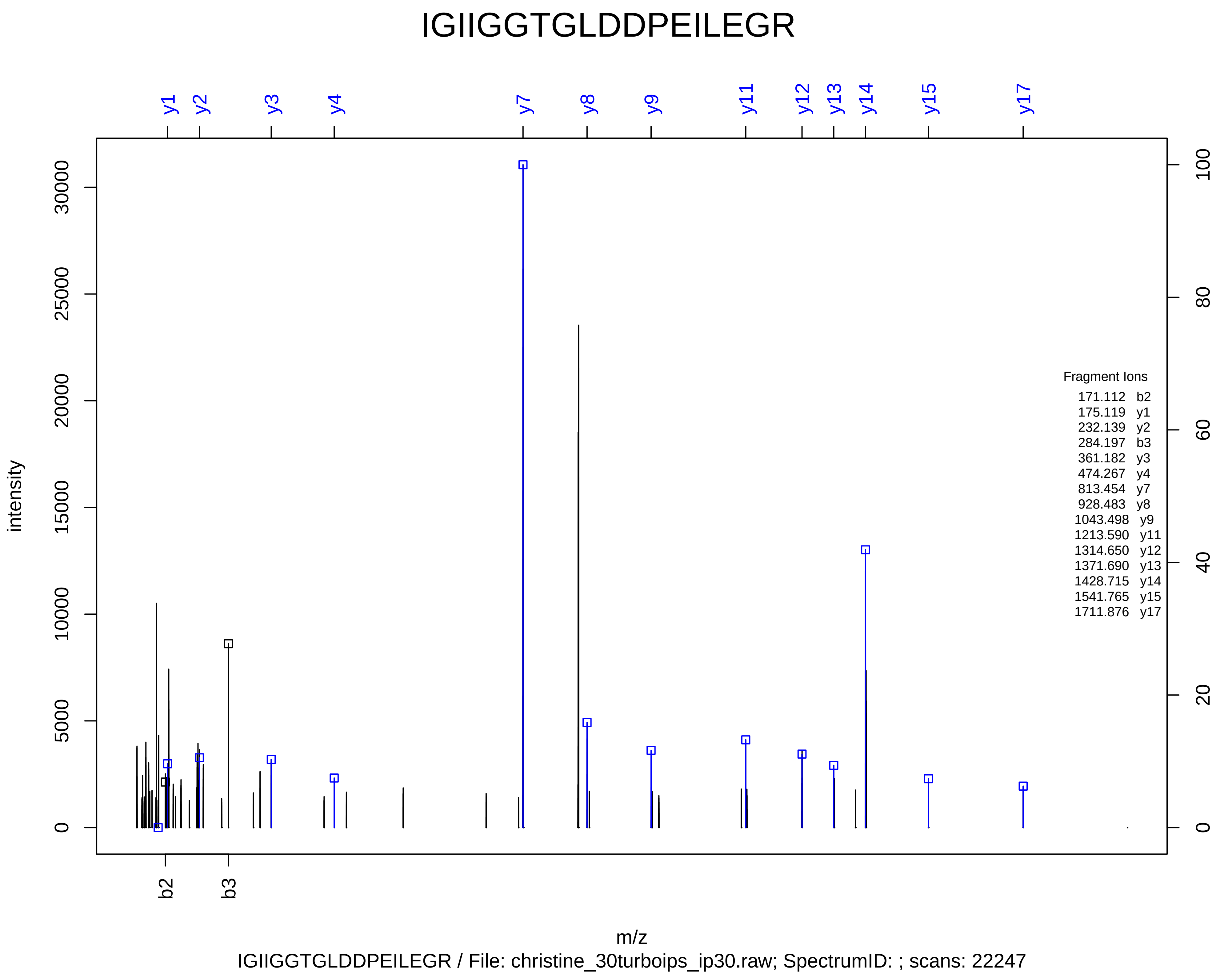
IGIIGGTGLDDPEILEGR
y1
y2
y3
y4
y7
y8
y9
y11
y12
y13
y14
y15
y17
intensity
0
5000
10000
15000
20000
25000
30000
0
20
40
60
80
100
b2
b3
m/z
Fragment Ions
171.112 b2
175.119 y1
232.139 y2
284.197 b3
361.182 y3
474.267 y4
813.454 y7
928.483 y8
1043.498 y9
1213.590 y11
1314.650 y12
1371.690 y13
1428.715 y14
1541.765 y15
1711.876 y17
IGIIGGTGLDDPEILEGR / File: christine_30turboips_ip30.raw; SpectrumID: ; scans: 22247

DLPDGPDAPIDR

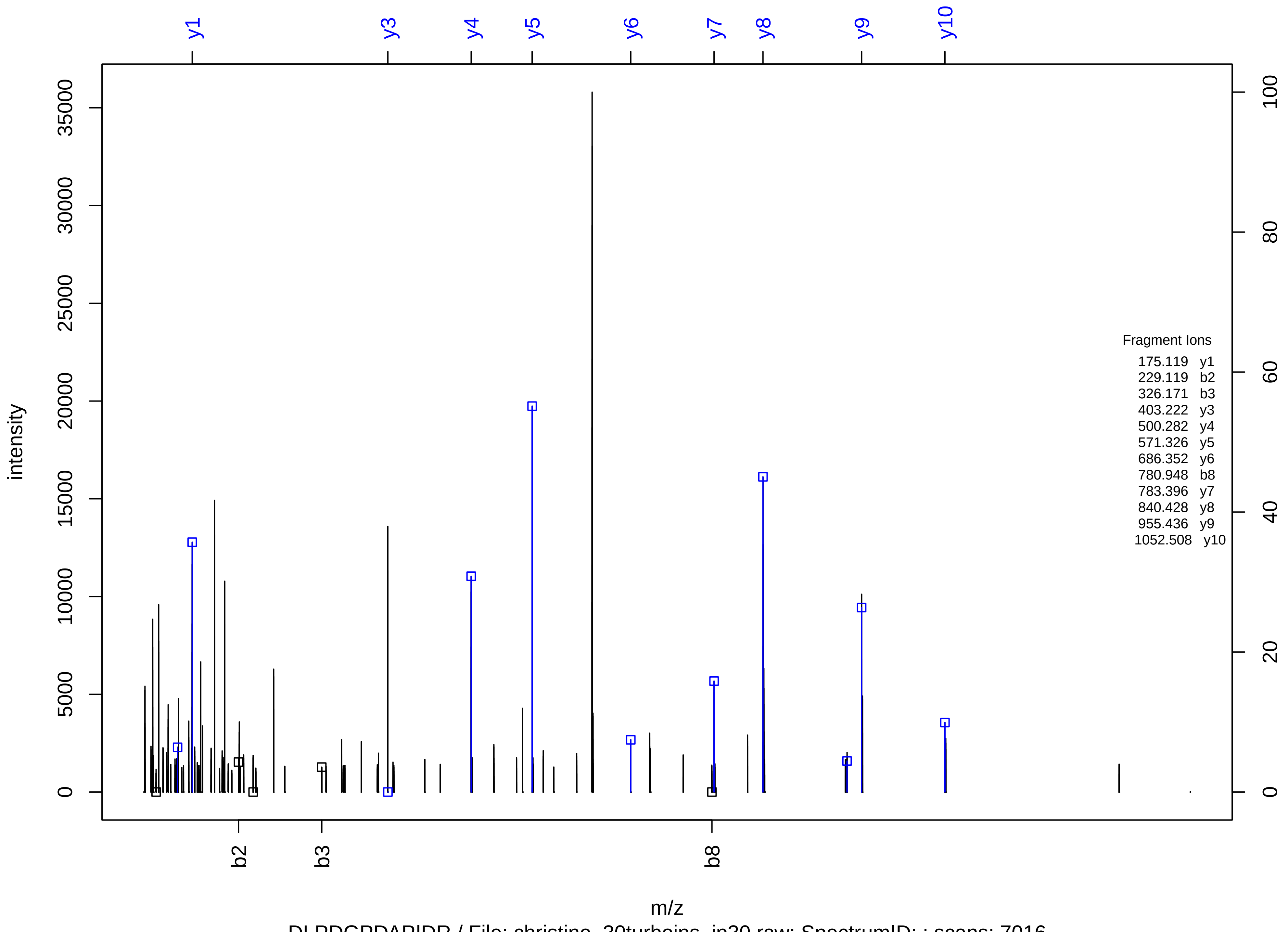

DLPDGPDAPIDR / File: christine_30turboips_ip30.raw; SpectrumID: ; scans: 7016

LIDAAGTLGTDELR

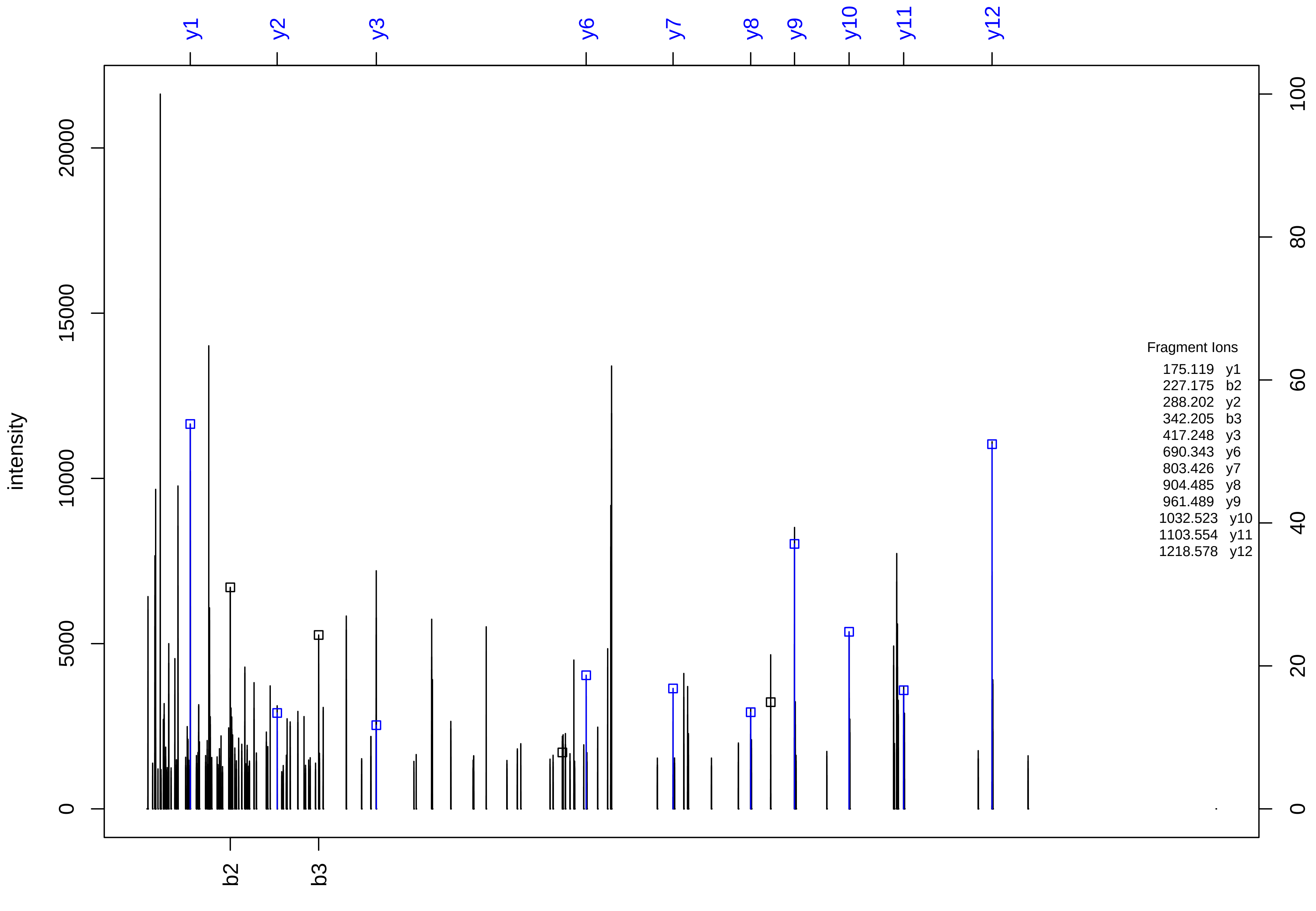

LIDAAGTLGTDELR / File: christine_30turboips_ip24.raw; SpectrumID: ; scans: 12695

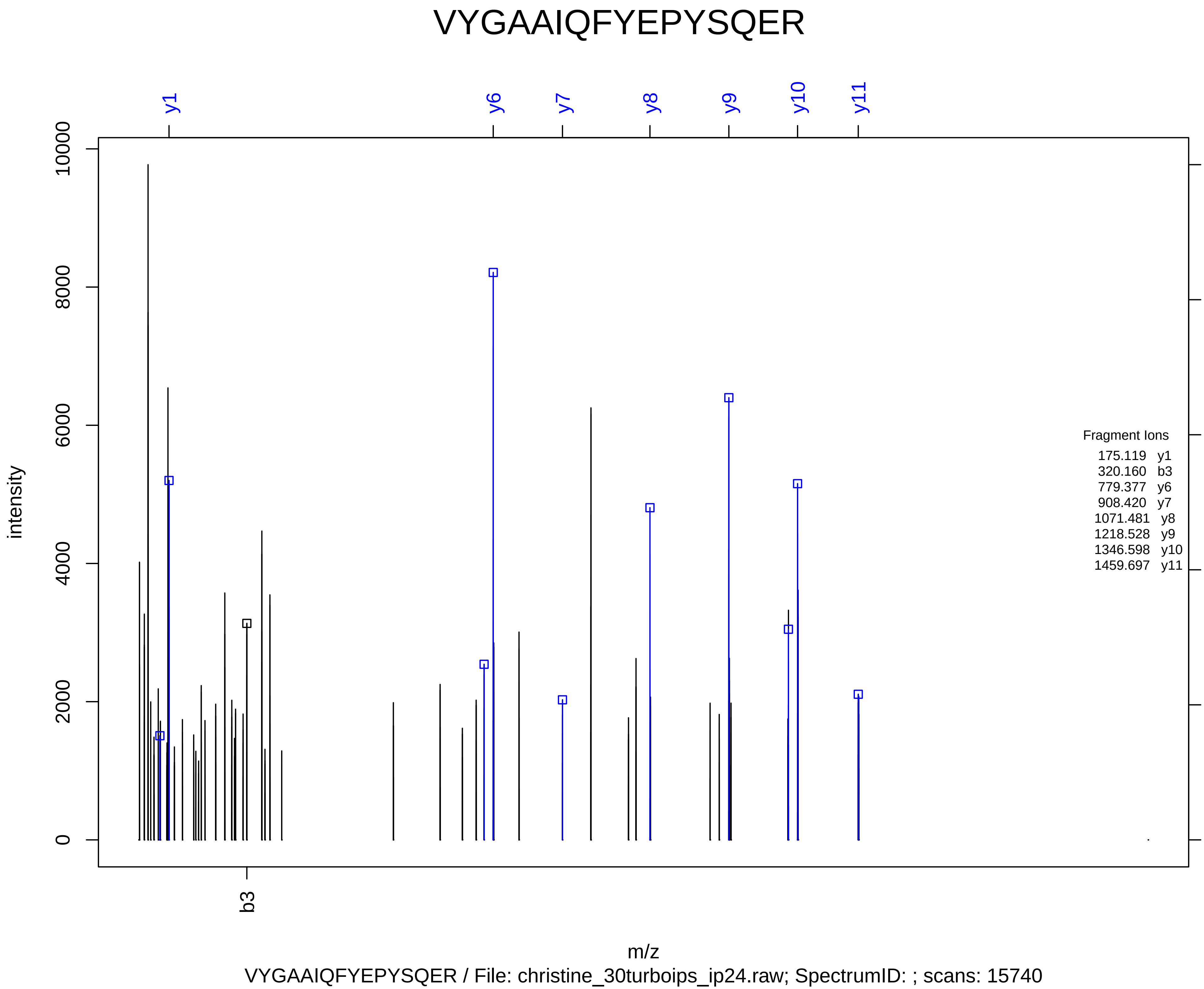
VYGAAIQFYEPYSQER
y1
y6
y7
y8
y9
y10
y11
b3
intensity
m/z
0
2000
4000
6000
8000
10000
0
20
40
60
80
100
Fragment Ions
175.119 y1
320.160 b3
779.377 y6
908.420 y7
1071.481 y8
1218.528 y9
1346.598 y10
1459.697 y11
VYGAAIQFYEPYSQER / File: christine_30turboips_ip24.raw; SpectrumID: ; scans: 15740

AVVVEEPVGELR

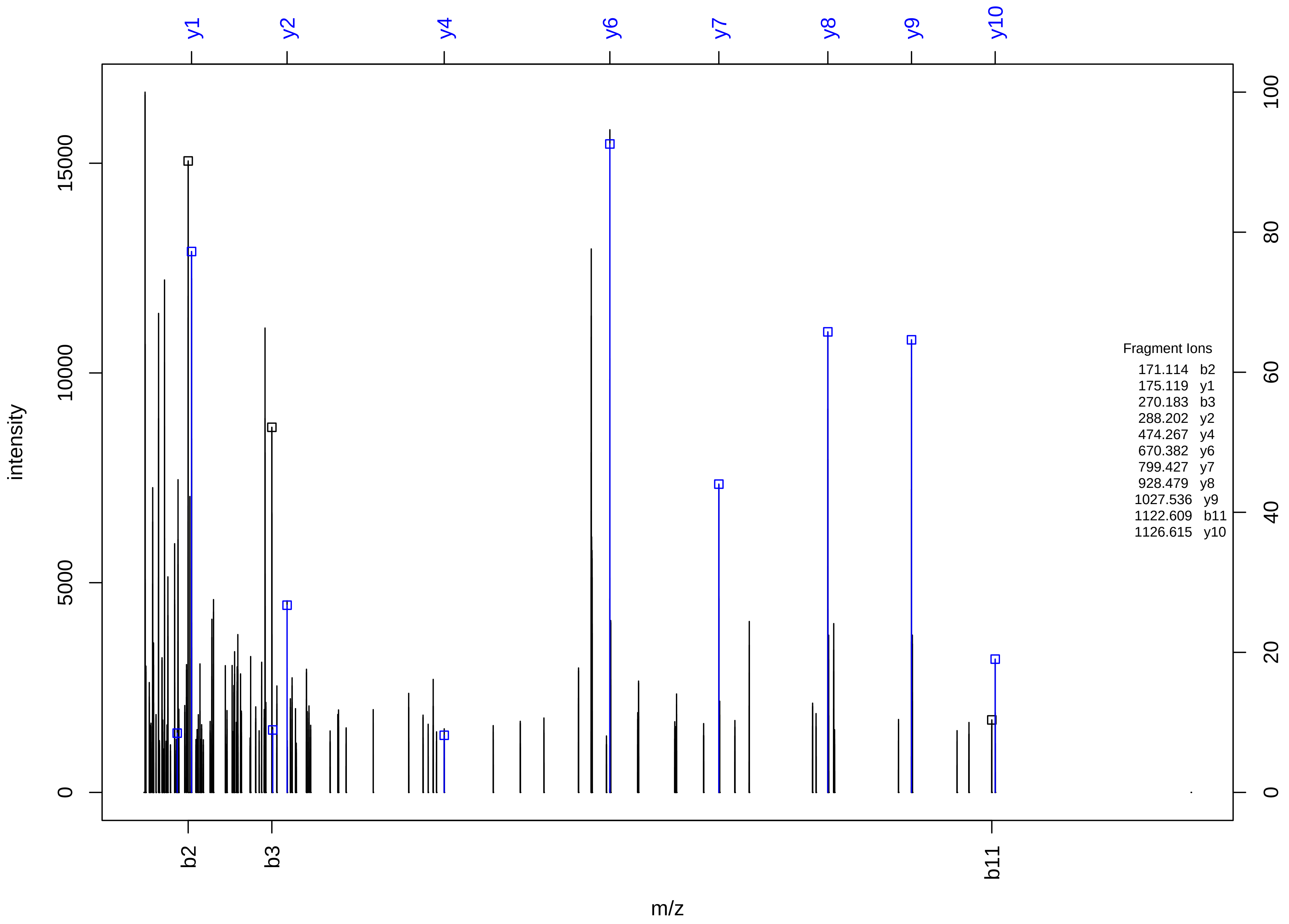

AVVVEEPVGELR / File: christine_30turboips_ip24.raw; SpectrumID: ; scans: 9519

FQQAADLIDAEQR

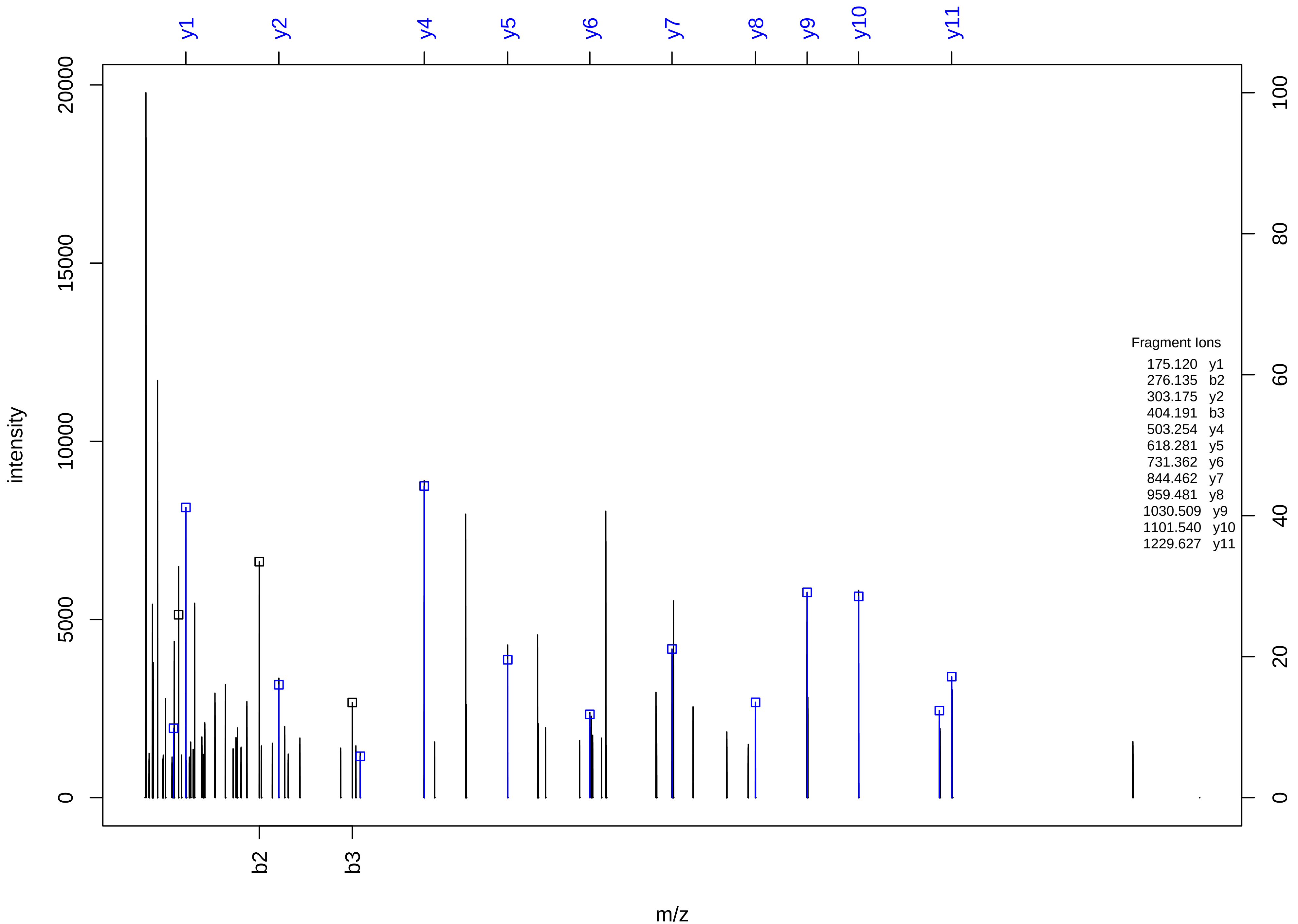

FQQAADLIDAEQR / File: christine_30turboips_ip24.raw; SpectrumID: ; scans: 9121

VPAPEVASGPDPEEEIR

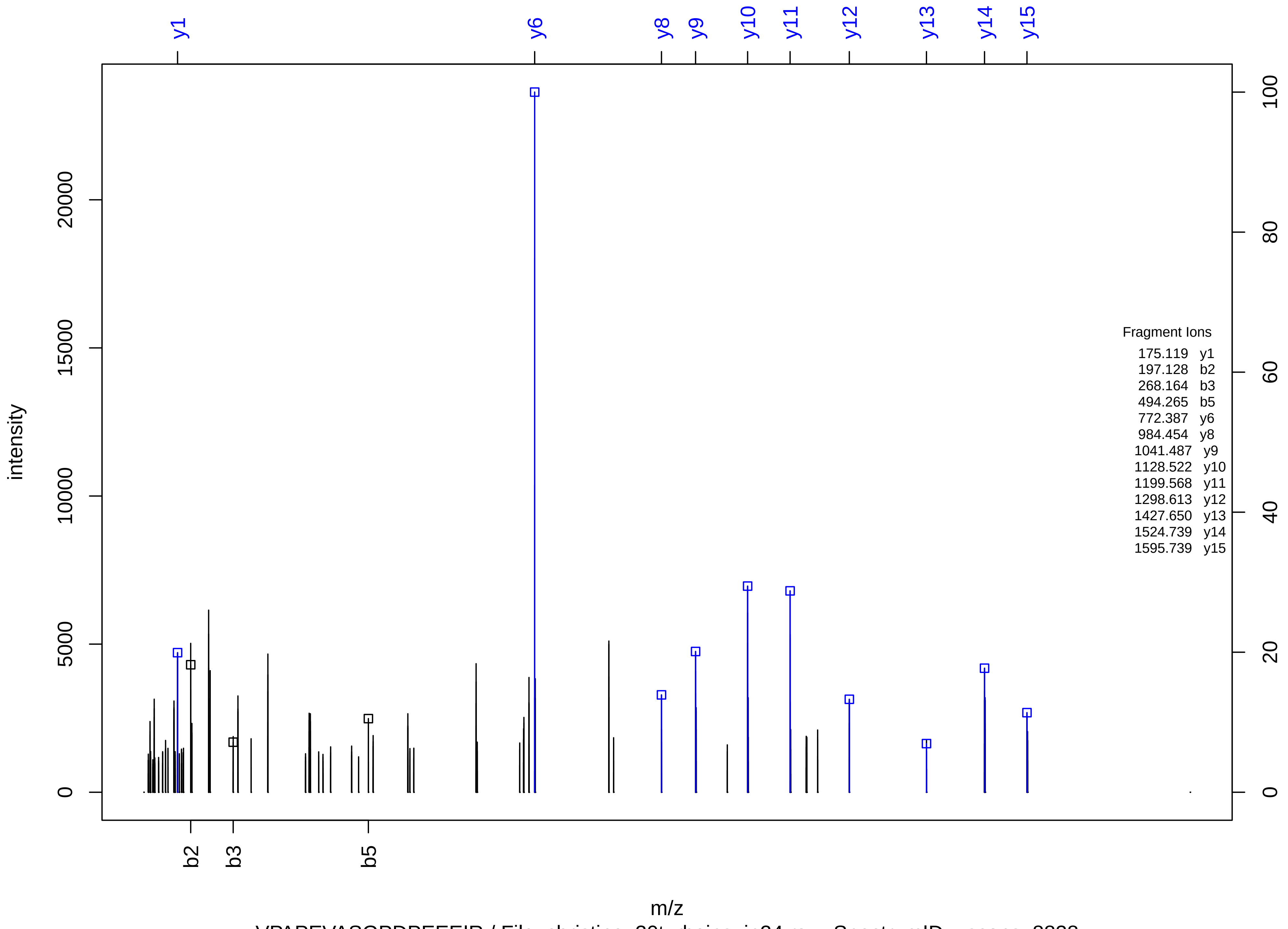

VPAPEVASGPDPEEEIR / File: christine_30turboips_ip24.raw; SpectrumID: ; scans: 8838

(Ac)AAAVVEEAAAGDVQR

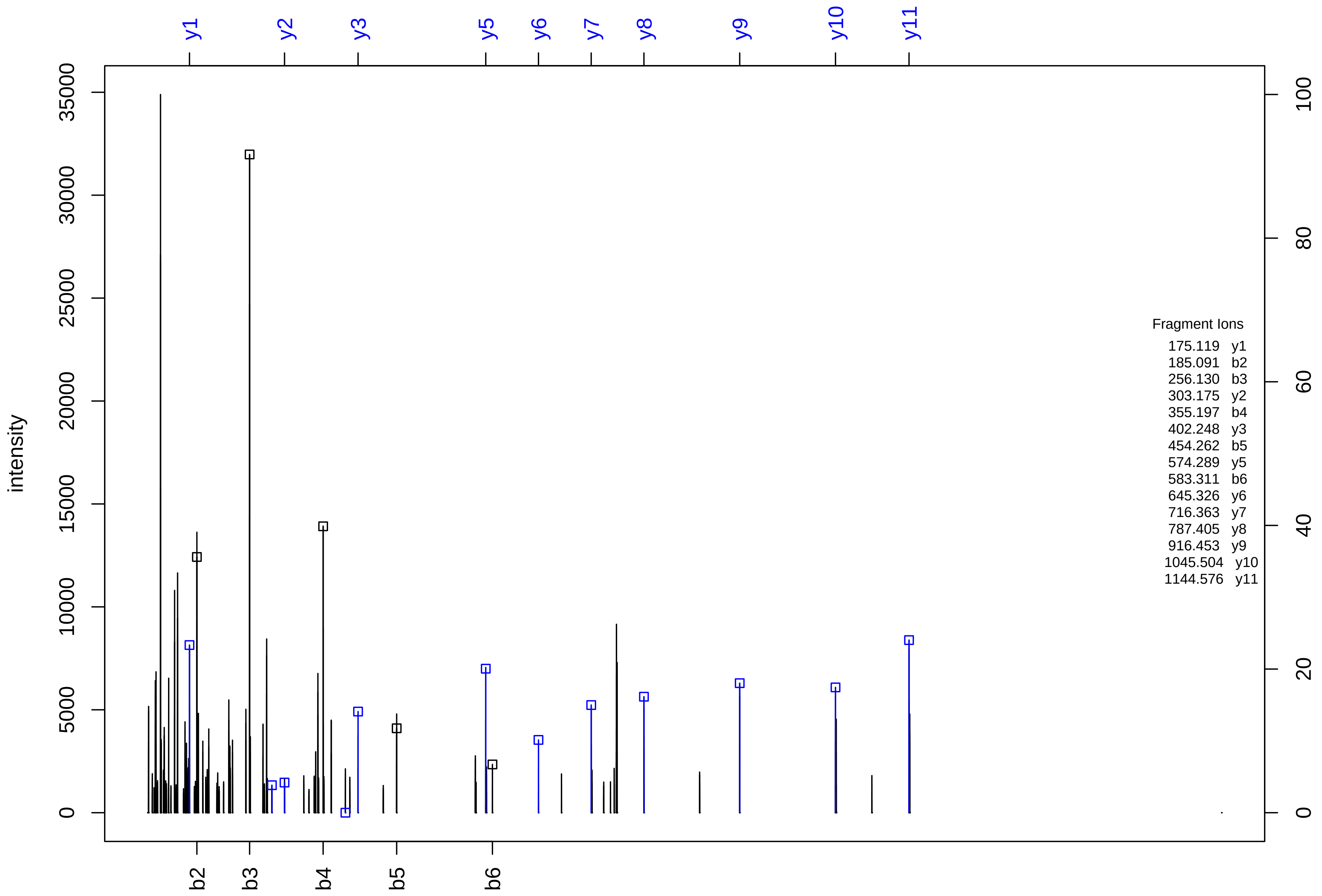

AAAVVEEAAAGDVQR / File: christine_30turboips_ip24.raw; SpectrumID: ; scans: 18603

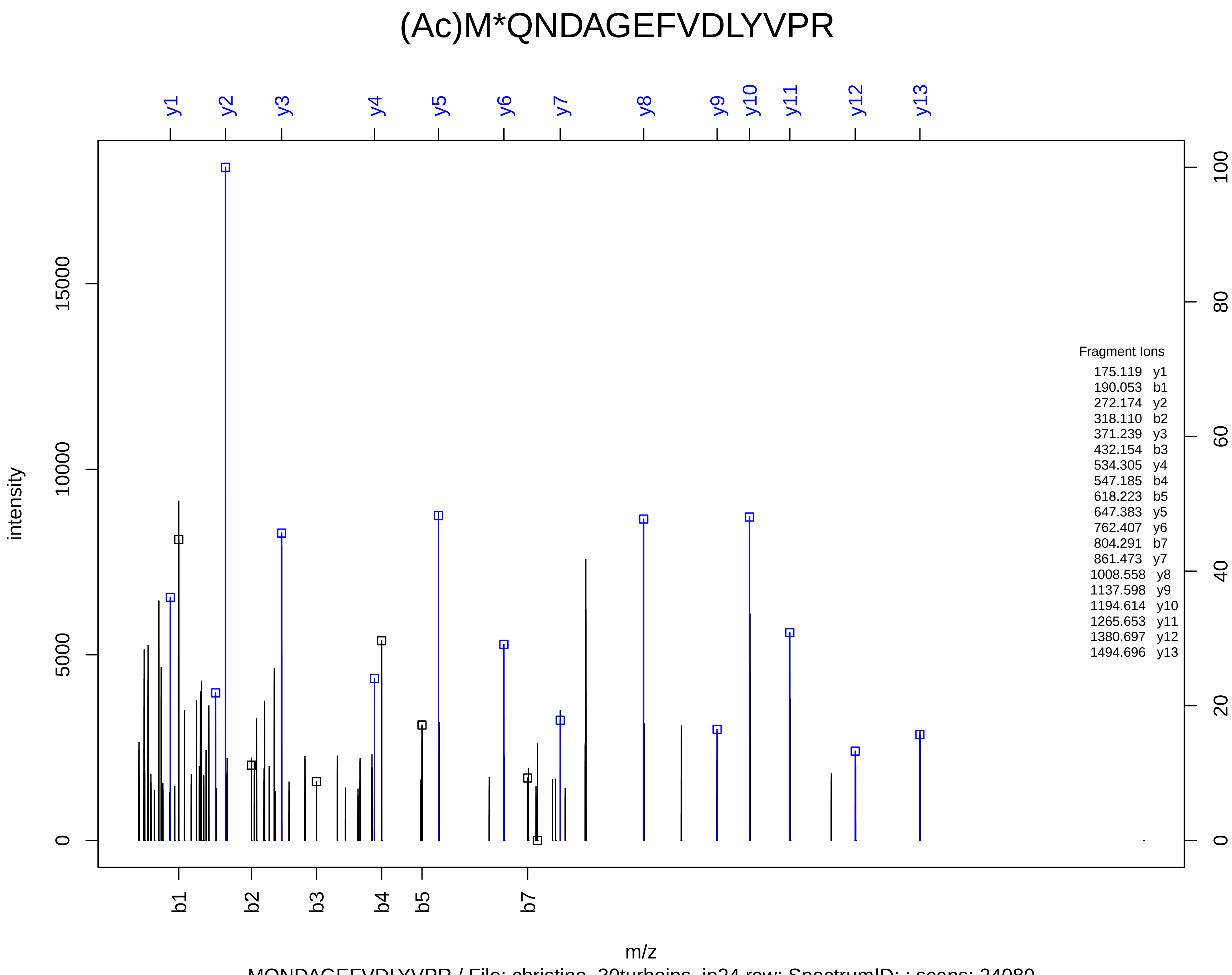
(Ac)M*QNDAGEFVDLYVPR
y1 y2 y3 y4 y5 y6 y7 y8 y9 y10 y11 y12 y13
b1 b2 b3 b4 b5 b7
intensity
0
5000
10000
15000
0
20
40
60
80
100
m/z
Fragment Ions
175.119 y1
190.053 b1
272.174 y2
318.110 b2
371.239 y3
432.154 b3
534.305 y4
547.185 b4
618.223 b5
647.383 y5
762.407 y6
804.291 b7
861.473 y7
1008.558 y8
1137.598 y9
1194.614 y10
1265.653 y11
1380.697 y12
1494.696 y13
MQNDAGEFVDLYVPR / File: christine_30turboips_ip24.raw; SpectrumID: ; scans: 24080

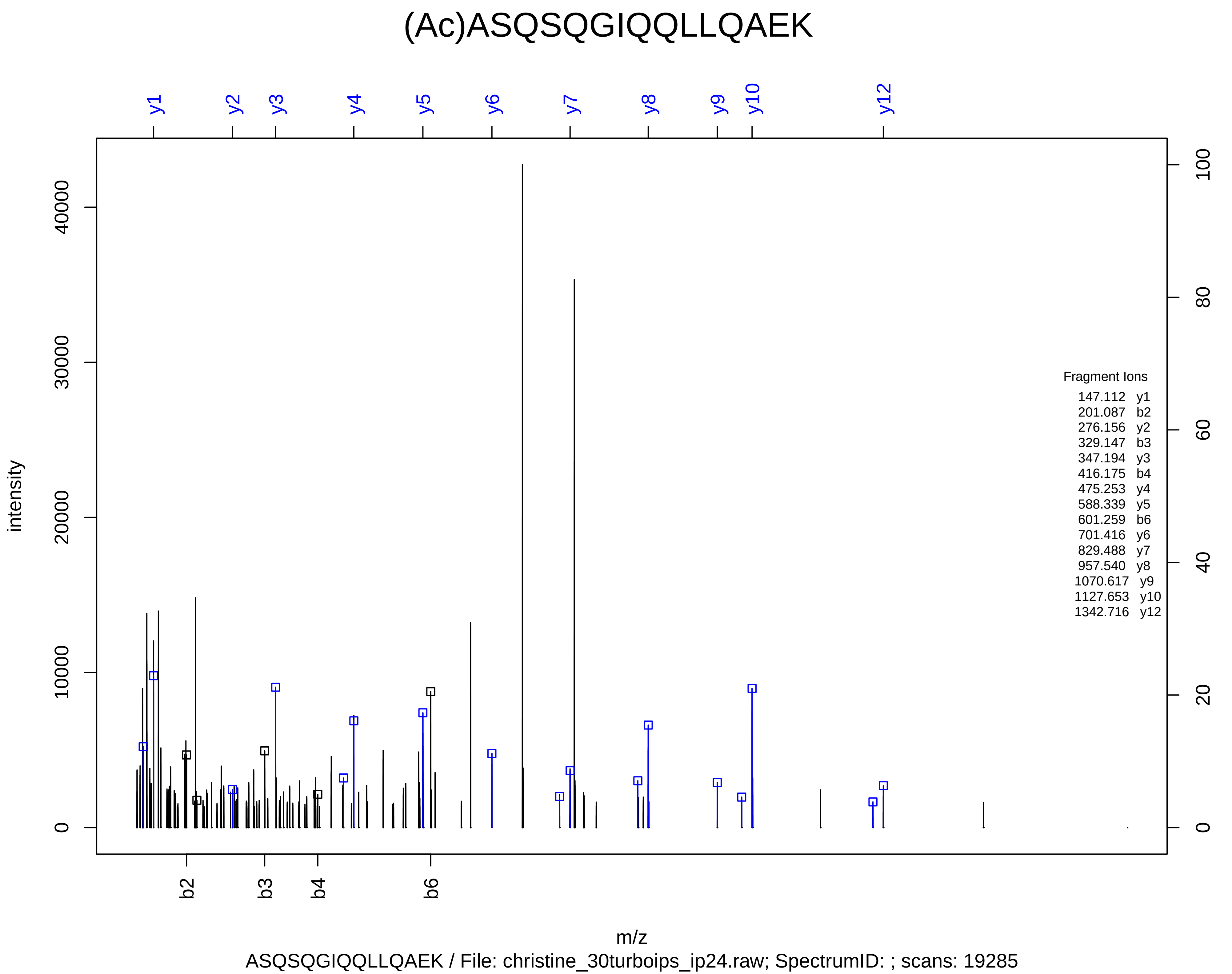

(Ac)ASQSQGIQQLLQAEK
y1
y2
y3
y4
y5
y6
y7
y8
y9
y10
y12
intensity
0
10000
20000
30000
40000
0
20
40
60
80
100
b2
b3
b4
b6
m/z
Fragment Ions
147.112 y1
201.087 b2
276.156 y2
329.147 b3
347.194 y3
416.175 b4
475.253 y4
588.339 y5
601.259 b6
701.416 y6
829.488 y7
957.540 y8
1070.617 y9
1127.653 y10
1342.716 y12
ASQSQGIQQLLQAEK / File: christine_30turboips_ip24.raw; SpectrumID: ; scans: 19285

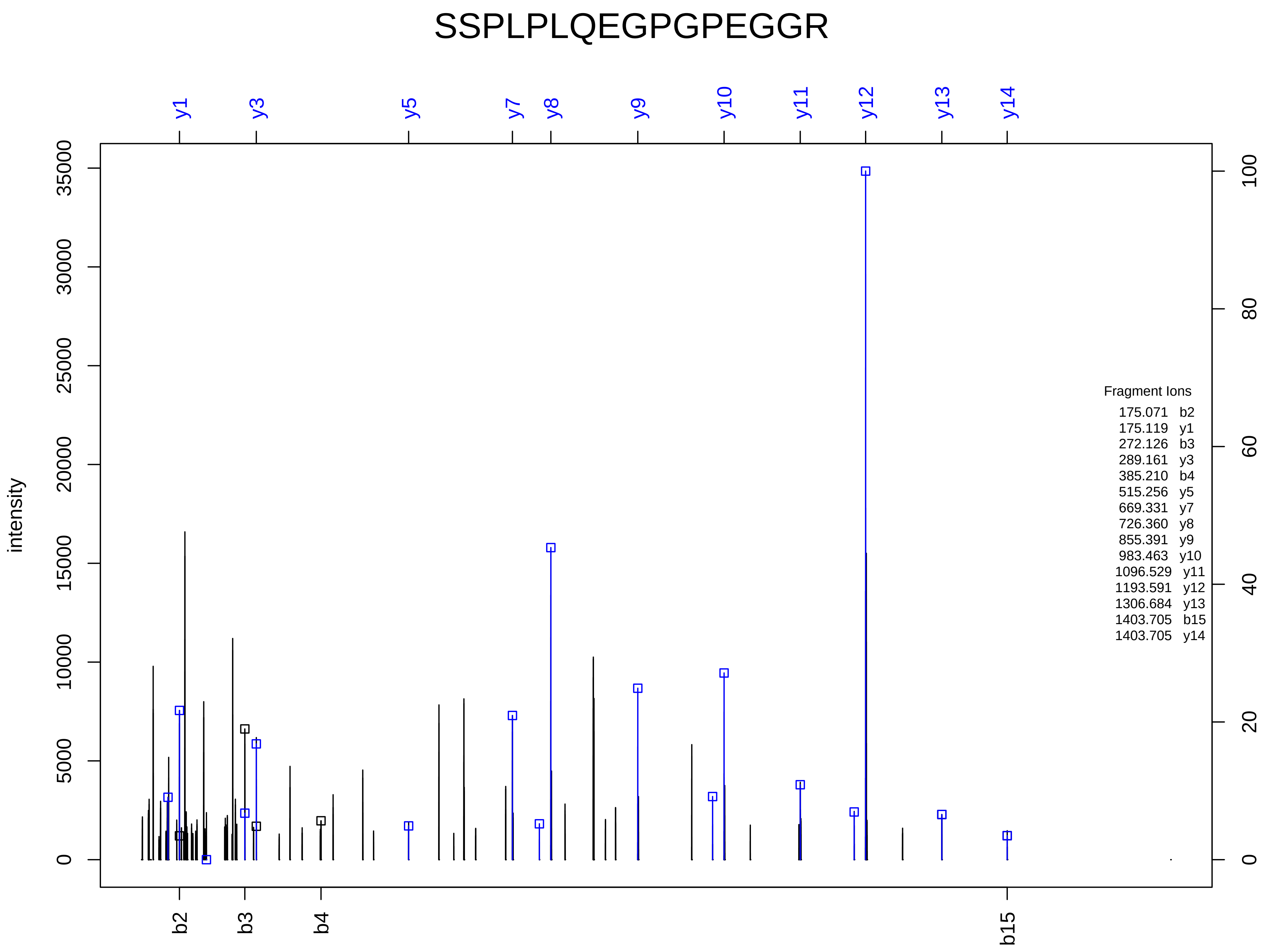

SSPLPLQEGPGPEGGR / File: christine_30turboips_ip24.raw; SpectrumID: ; scans: 7136

AAQEQELESLR

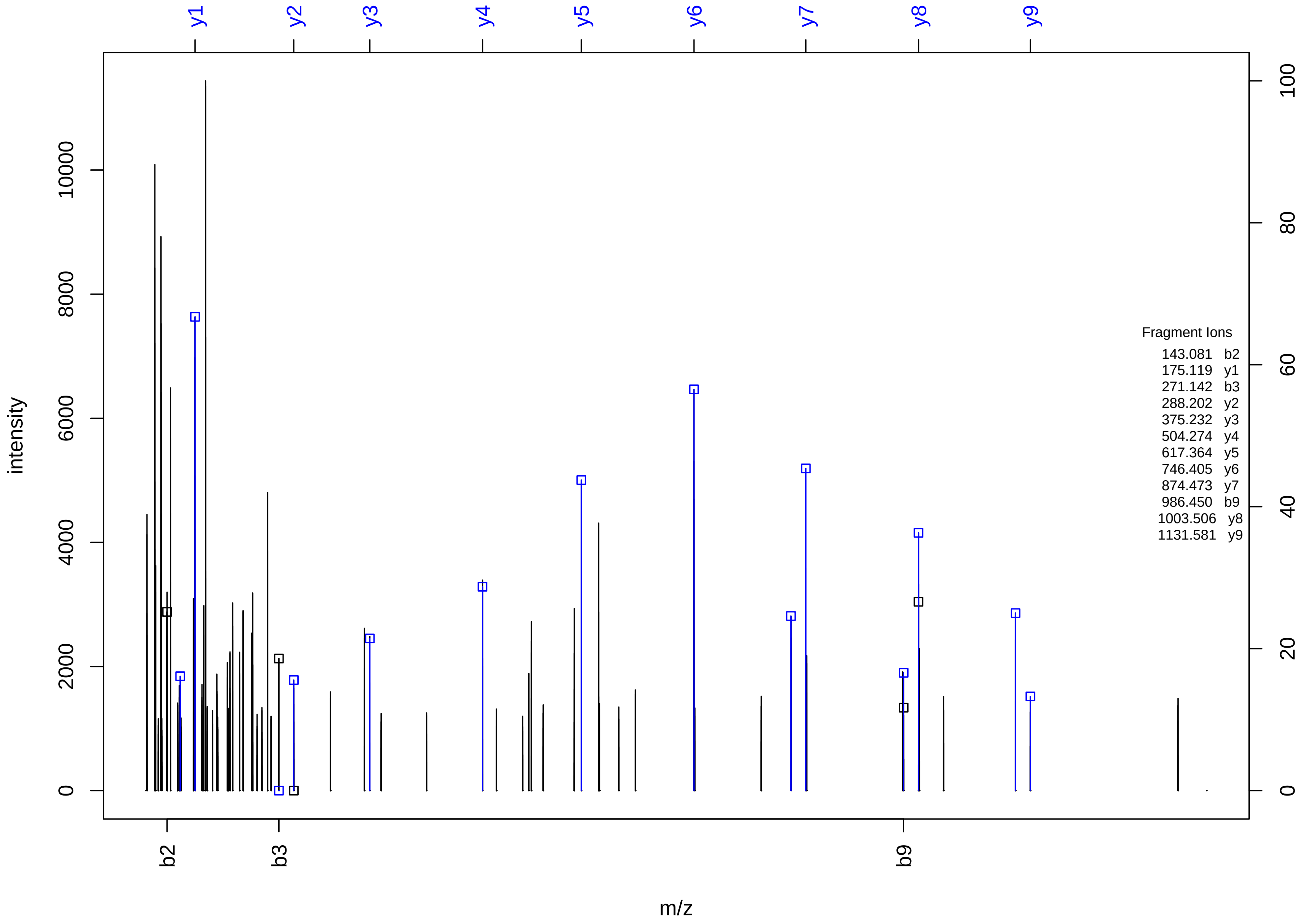

AAQEQELESLR / File: christine_30turboips_ip24.raw; SpectrumID: ; scans: 3806

DTDIVDEAIYYFK

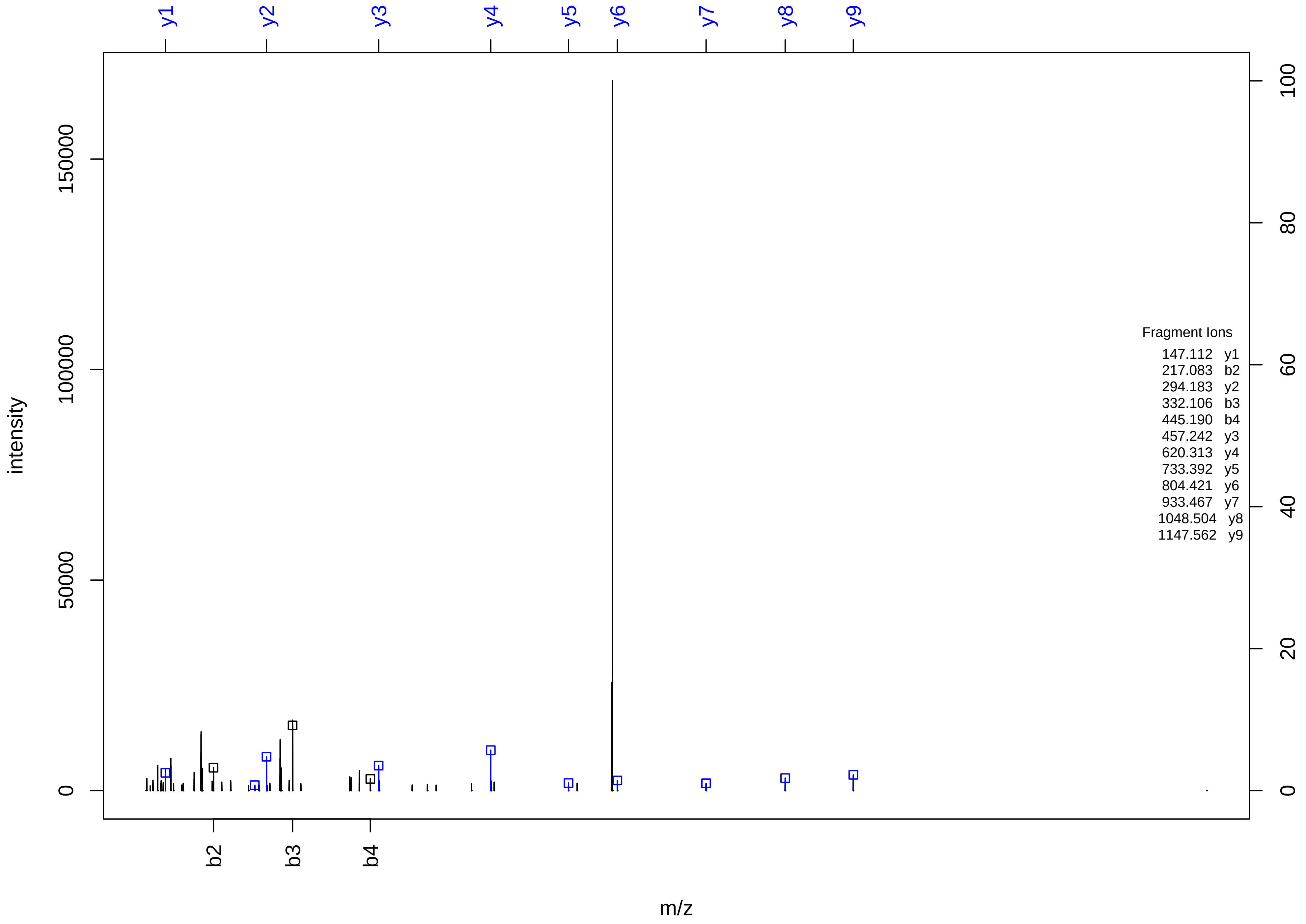

DTDIVDEAIYYFK / File: christine_30turboips_ip24.raw; SpectrumID: ; scans: 28802

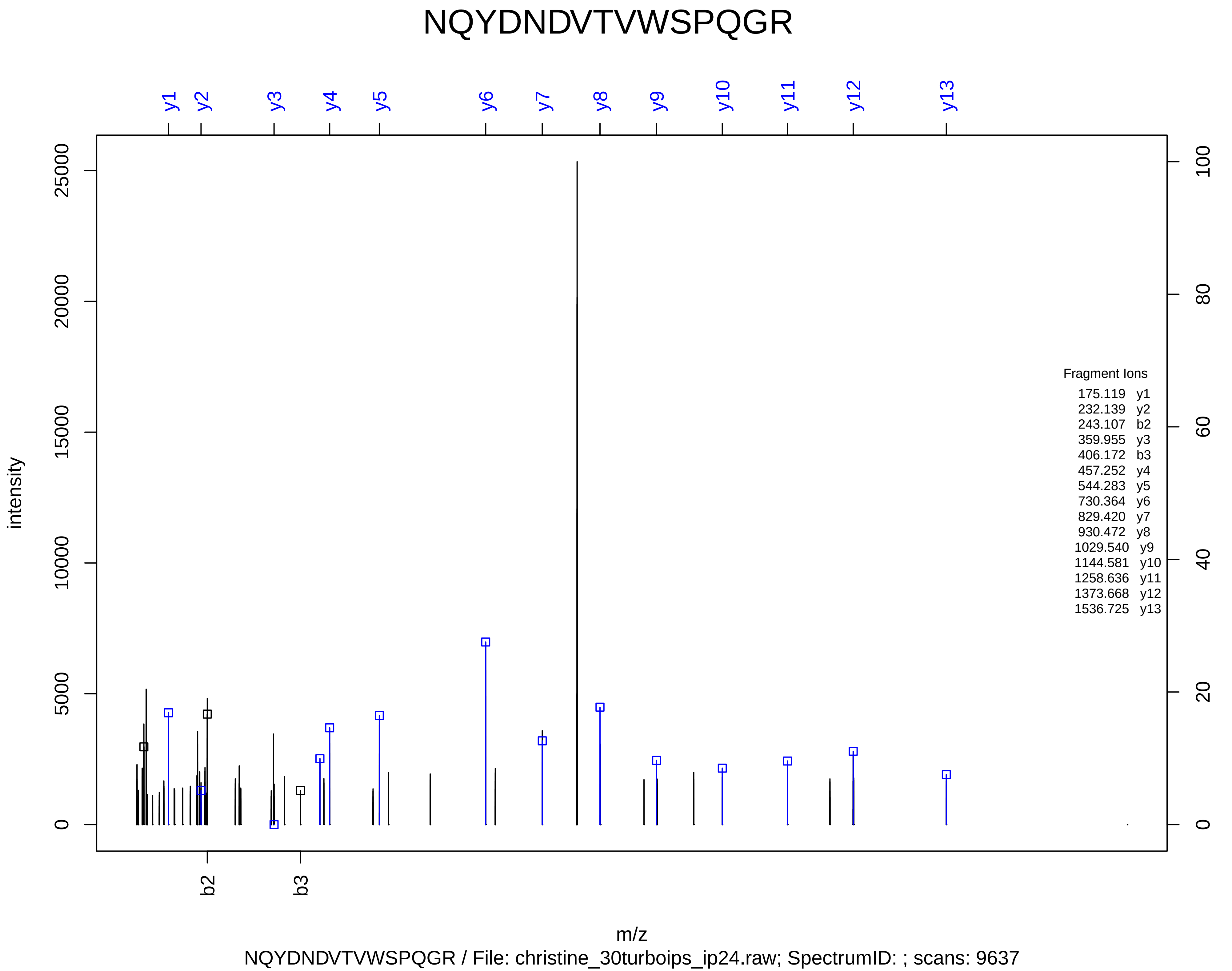
NQYDNDVTVWSPQGR
y1 y2 y3 y4 y5 y6 y7 y8 y9 y10 y11 y12 y13
intensity
0
5000
10000
15000
20000
25000
0
20
40
60
80
100
b2 b3
m/z
Fragment Ions
175.119 y1
232.139 y2
243.107 b2
359.955 y3
406.172 b3
457.252 y4
544.283 y5
730.364 y6
829.420 y7
930.472 y8
1029.540 y9
1144.581 y10
1258.636 y11
1373.668 y12
1536.725 y13
NQYDNDVTVWSPQGR / File: christine_30turboips_ip24.raw; SpectrumID: ; scans: 9637

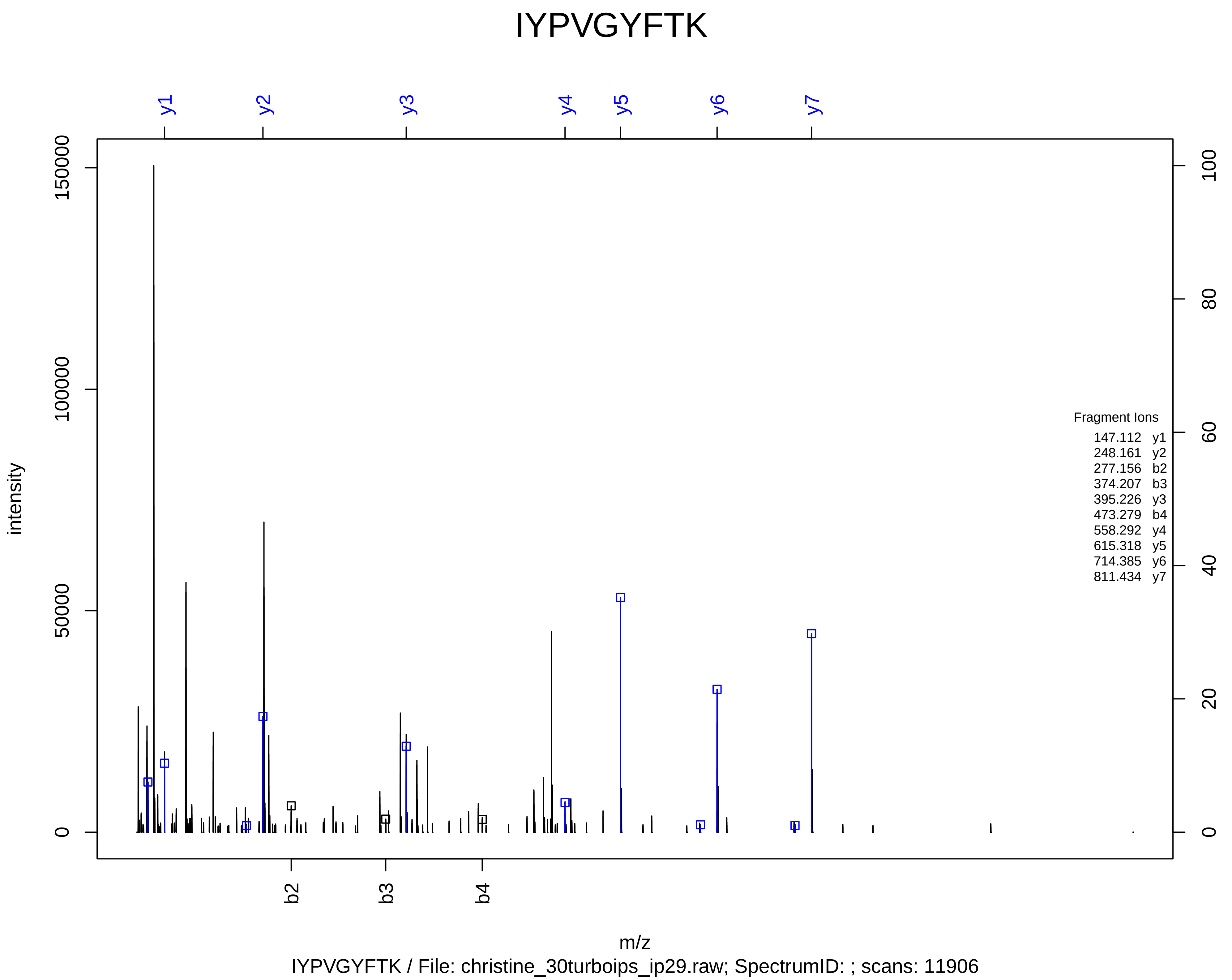
IYPVGYFTK
y1
y2
y3
y4
y5
y6
y7
b2
b3
b4
intensity
m/z
Fragment Ions
147.112 y1
248.161 y2
277.156 b2
374.207 b3
395.226 y3
473.279 b4
558.292 y4
615.318 y5
714.385 y6
811.434 y7
IYPVGYFTK / File: christine_30turboips_ip29.raw; SpectrumID: ; scans: 11906

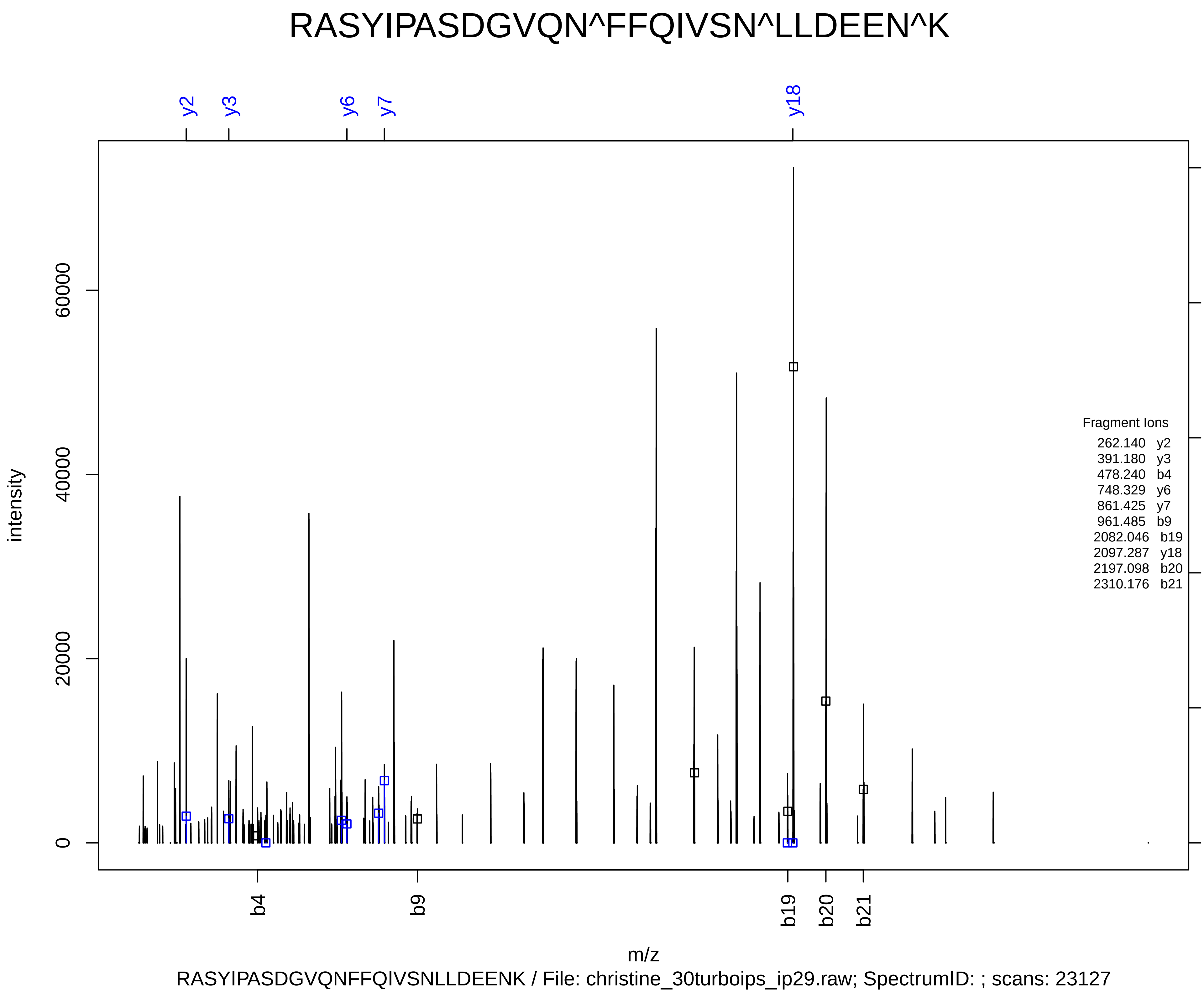
RASYIPASDGVQN^FFQIVSN^LLDEEN^K
y2
y3
y6
y7
y18
intensity
60000
40000
20000
0
100
80
60
40
20
0
Fragment Ions
262.140 y2
391.180 y3
478.240 b4
748.329 y6
861.425 y7
961.485 b9
2082.046 b19
2097.287 y18
2197.098 b20
2310.176 b21
b4
b9
b19
b20
b21
m/z
RASYIPASDGVQNFFQIVSNLLDEENK / File: christine_30turboips_ip29.raw; SpectrumID: ; scans: 23127

(Ac)APSADPGM*VR

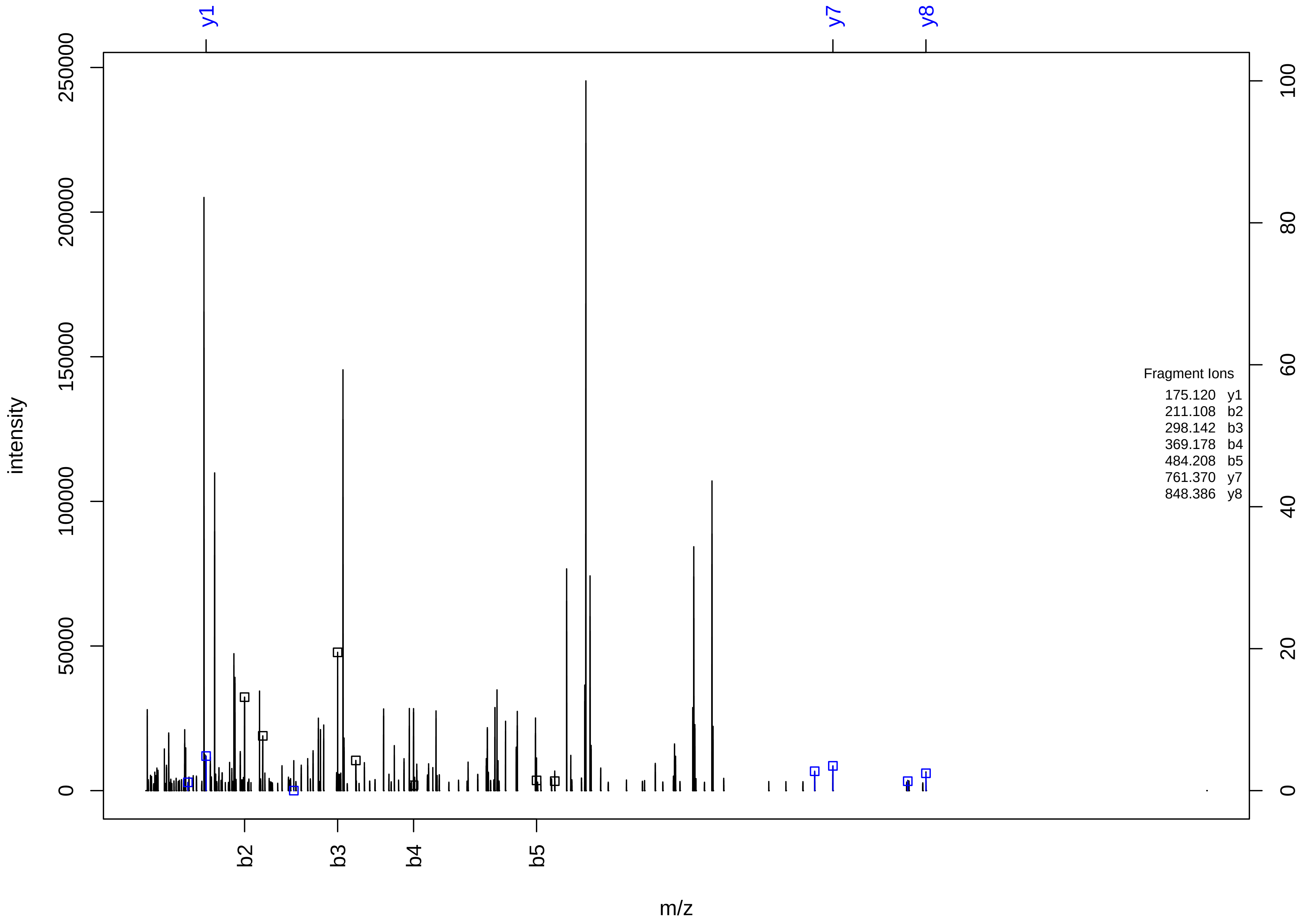

APSADPGMVR / File: christine_30turboips_ip29.raw; SpectrumID: ; scans: 12371

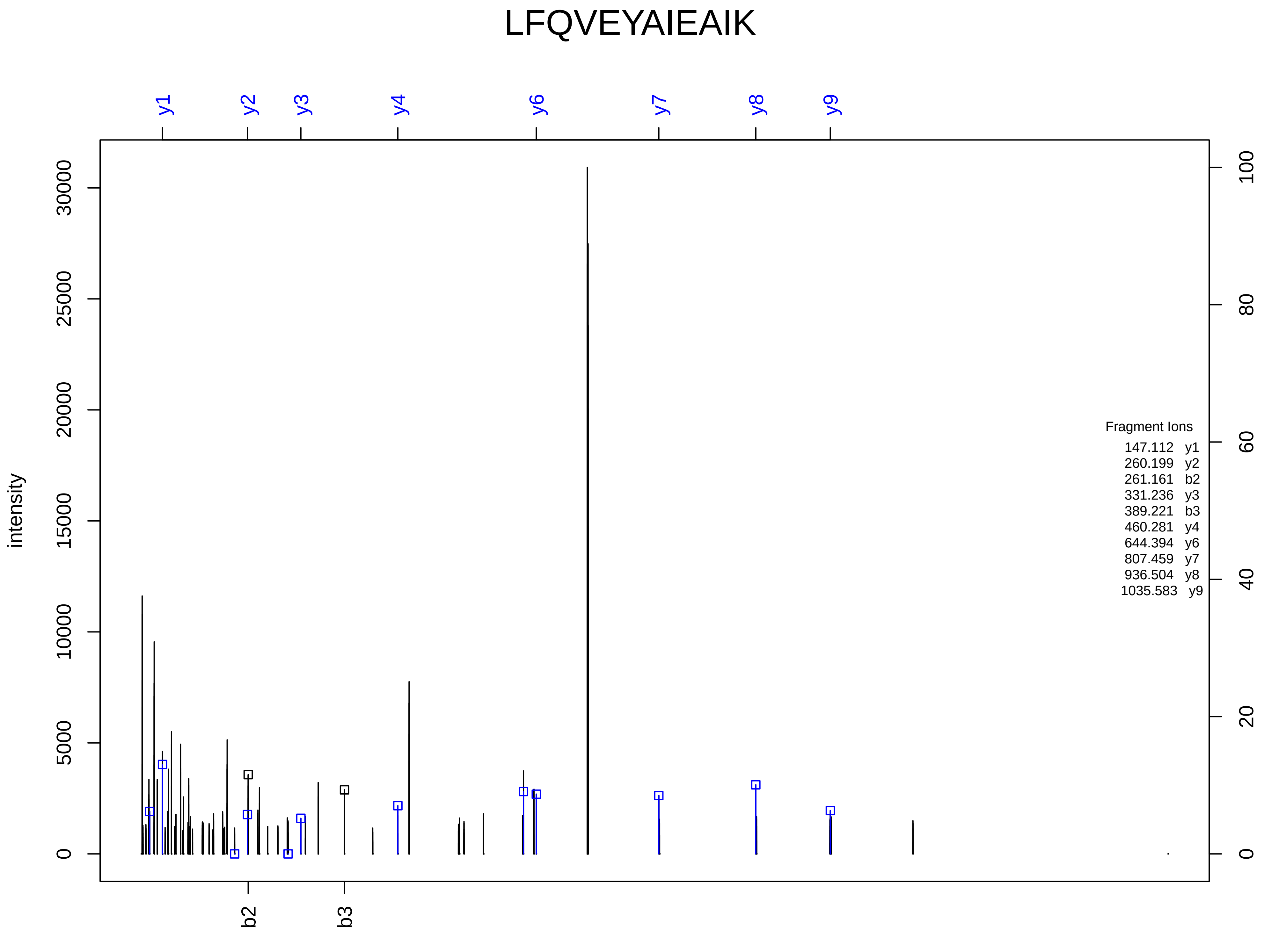

LFQVEYAIEAIK / File: christine_30turboips_ip29.raw; SpectrumID: ; scans: 22433

(Ac)DQENQTSISEFILLGLSN^QAEK

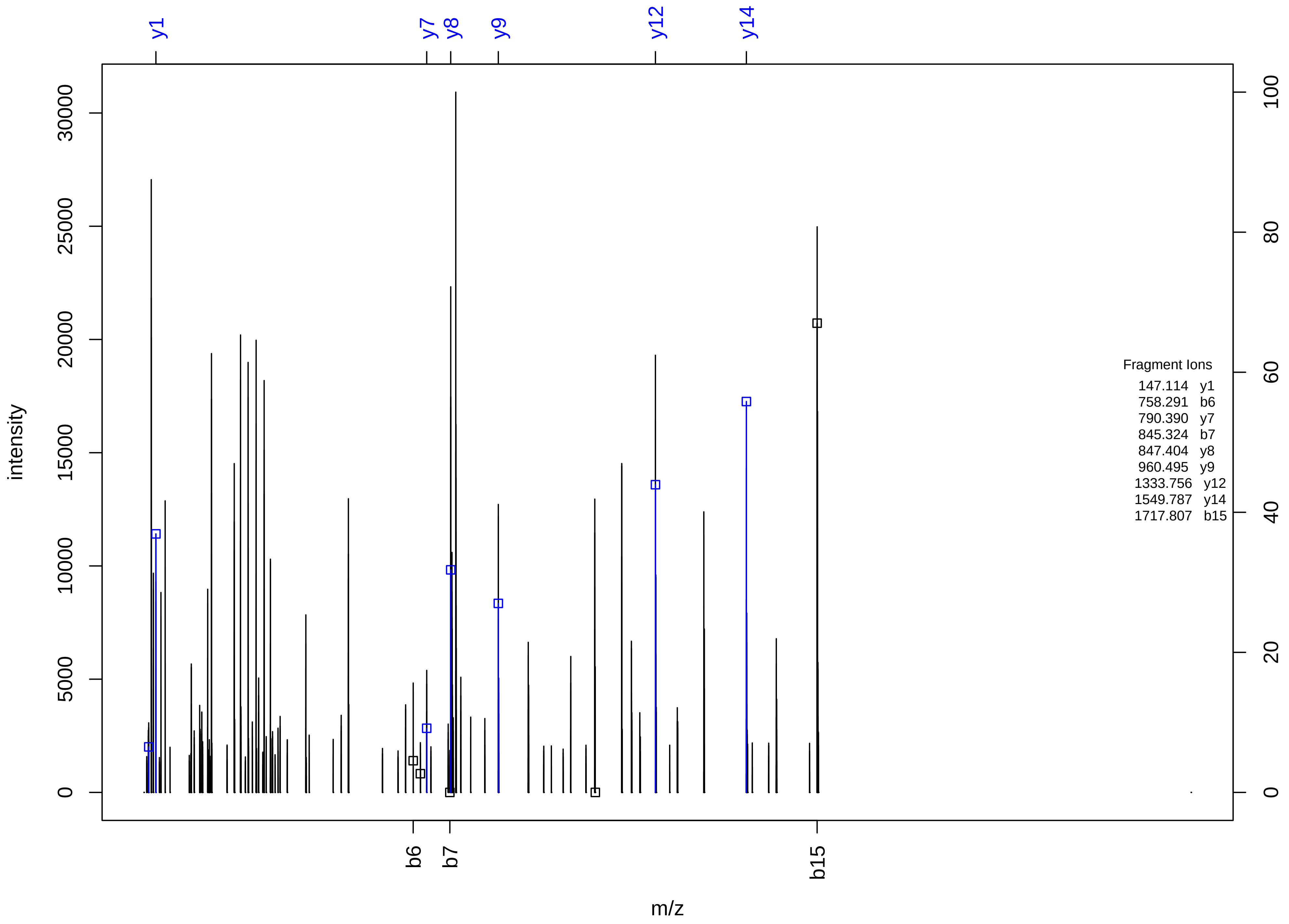

DQENQTSISEFILLGLSNQAEK / File: christine_30turboips_ip29.raw; SpectrumID: ; scans: 20418

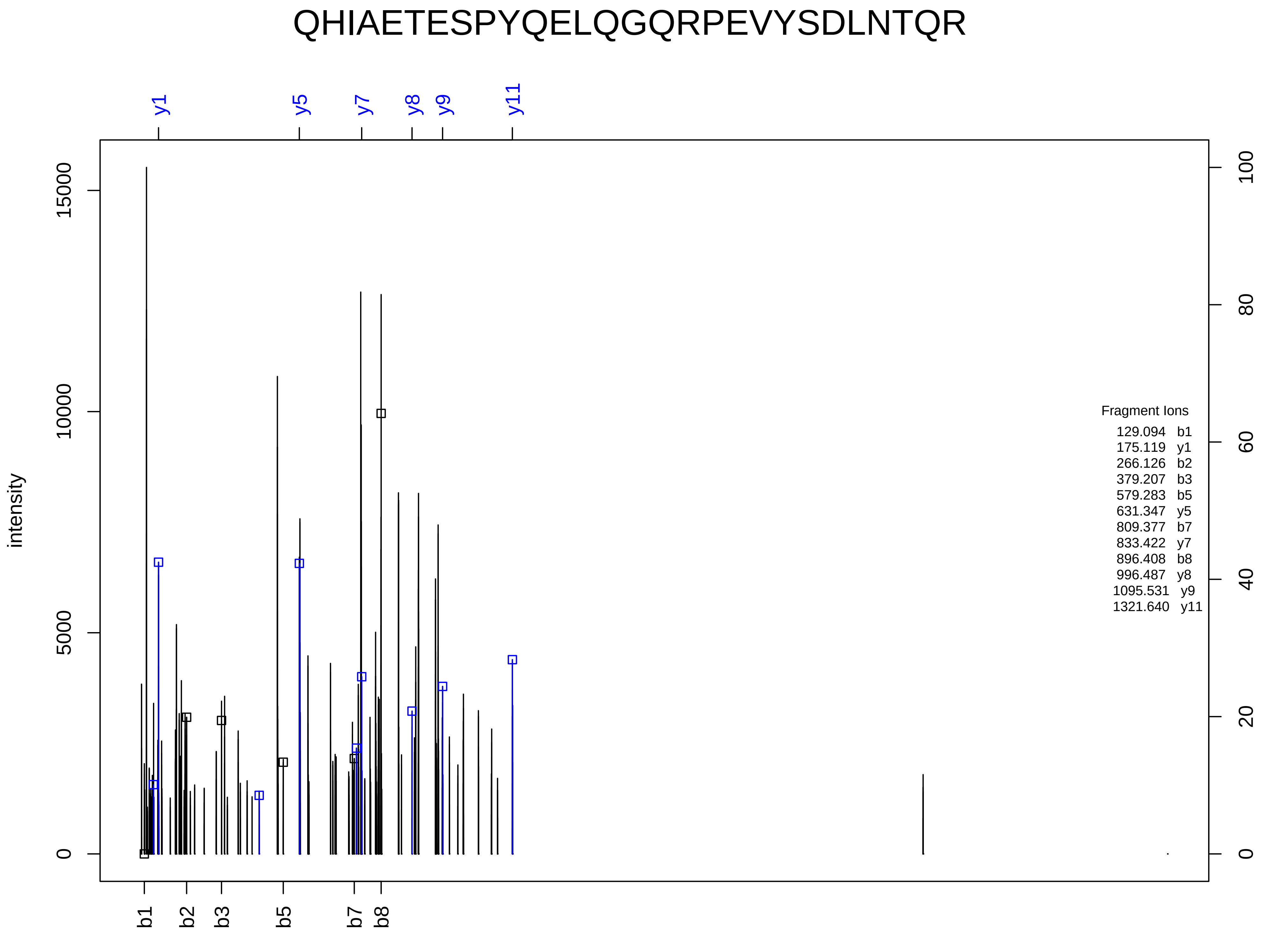

QHIAETESPYQELQGQRPEVYSDLNTQR / File: christine_30turboips_ip27.raw; SpectrumID: ; scans: 9532

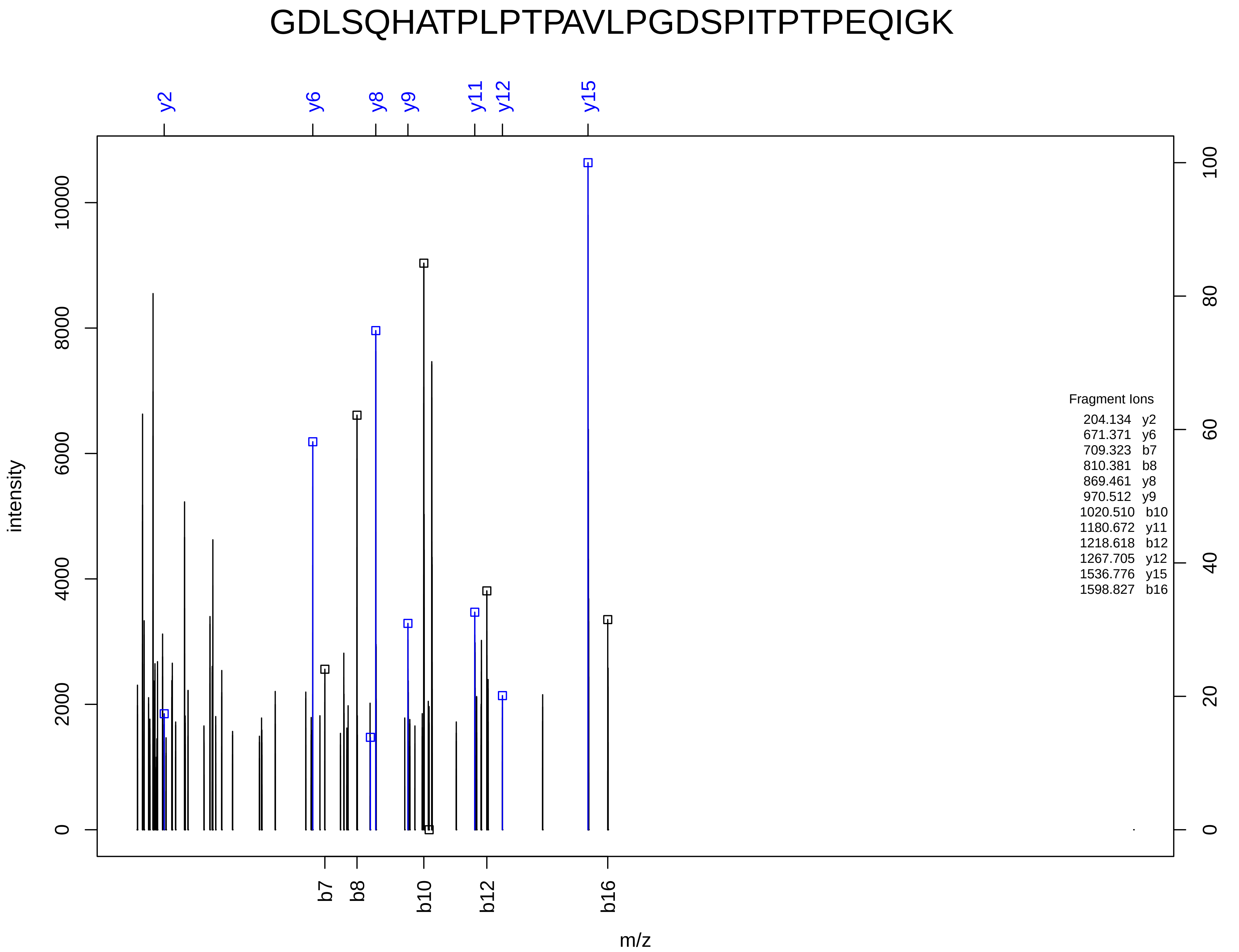

GDLSQHATPLPTPAVLPGDSPITPTPEQIGK / File: christine_30turboips_ip27.raw; SpectrumID: ; scans: 18996

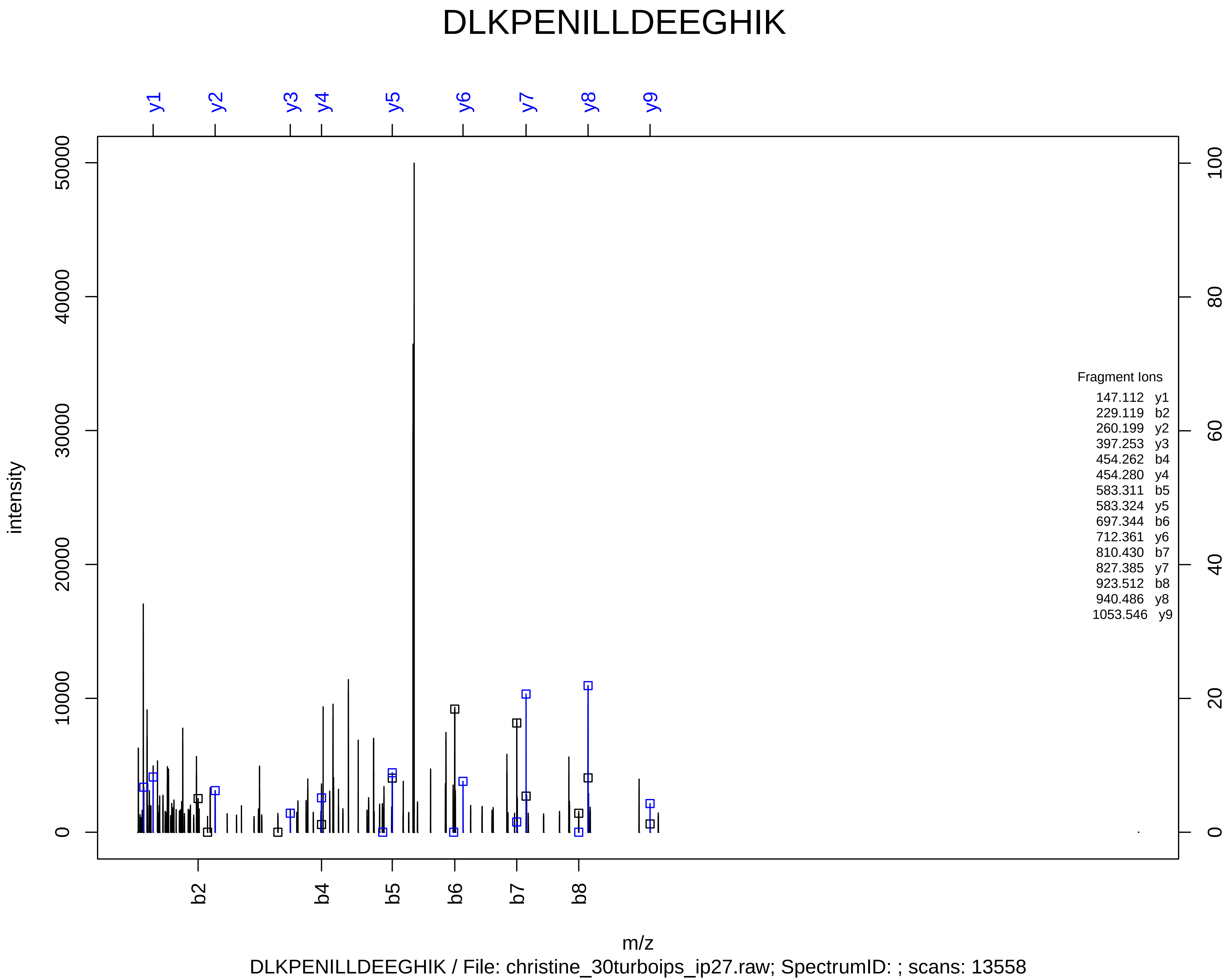
DLKPENILLDEEGHIK
y1
y2
y3
y4
y5
y6
y7
y8
y9
intensity
0
10000
20000
30000
40000
50000
0
20
40
60
80
100
b2
b4
b5
b6
b7
b8
m/z
Fragment Ions
147.112 y1
229.119 b2
260.199 y2
397.253 y3
454.262 b4
454.280 y4
583.311 b5
583.324 y5
697.344 b6
712.361 y6
810.430 b7
827.385 y7
923.512 b8
940.486 y8
1053.546 y9
DLKPENILLDEEGHIK / File: christine_30turboips_ip27.raw; SpectrumID: ; scans: 13558

TVSTTISPLLLIP

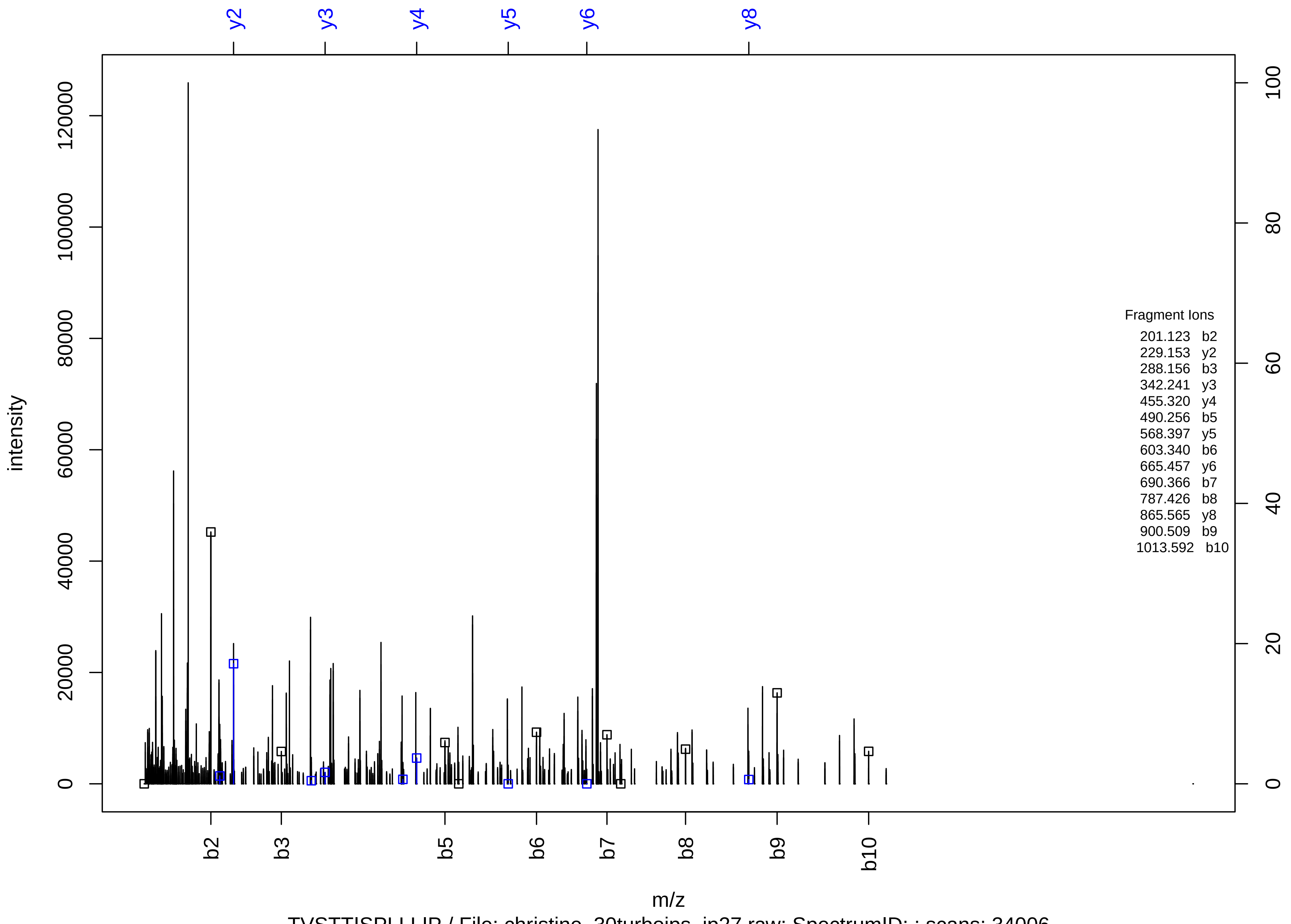

TVSTTISPLLLIP / File: christine_30turboips_ip27.raw; SpectrumID: ; scans: 34006

VAQLEQVYIR

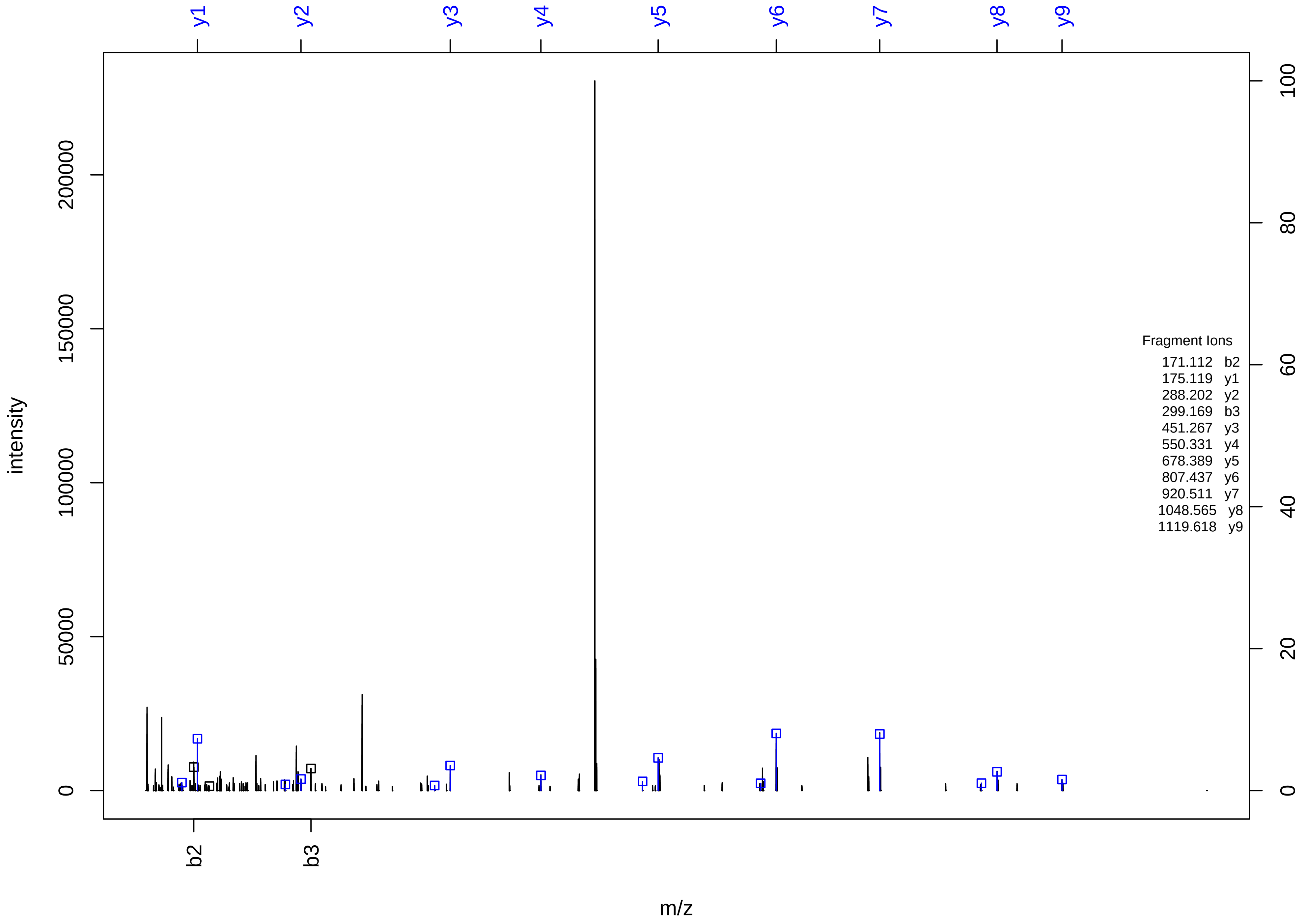

VAQLEQVYIR / File: christine_30turboips_ip27.raw; SpectrumID: ; scans: 8823

AGITGETEFAEADQDFSDENR

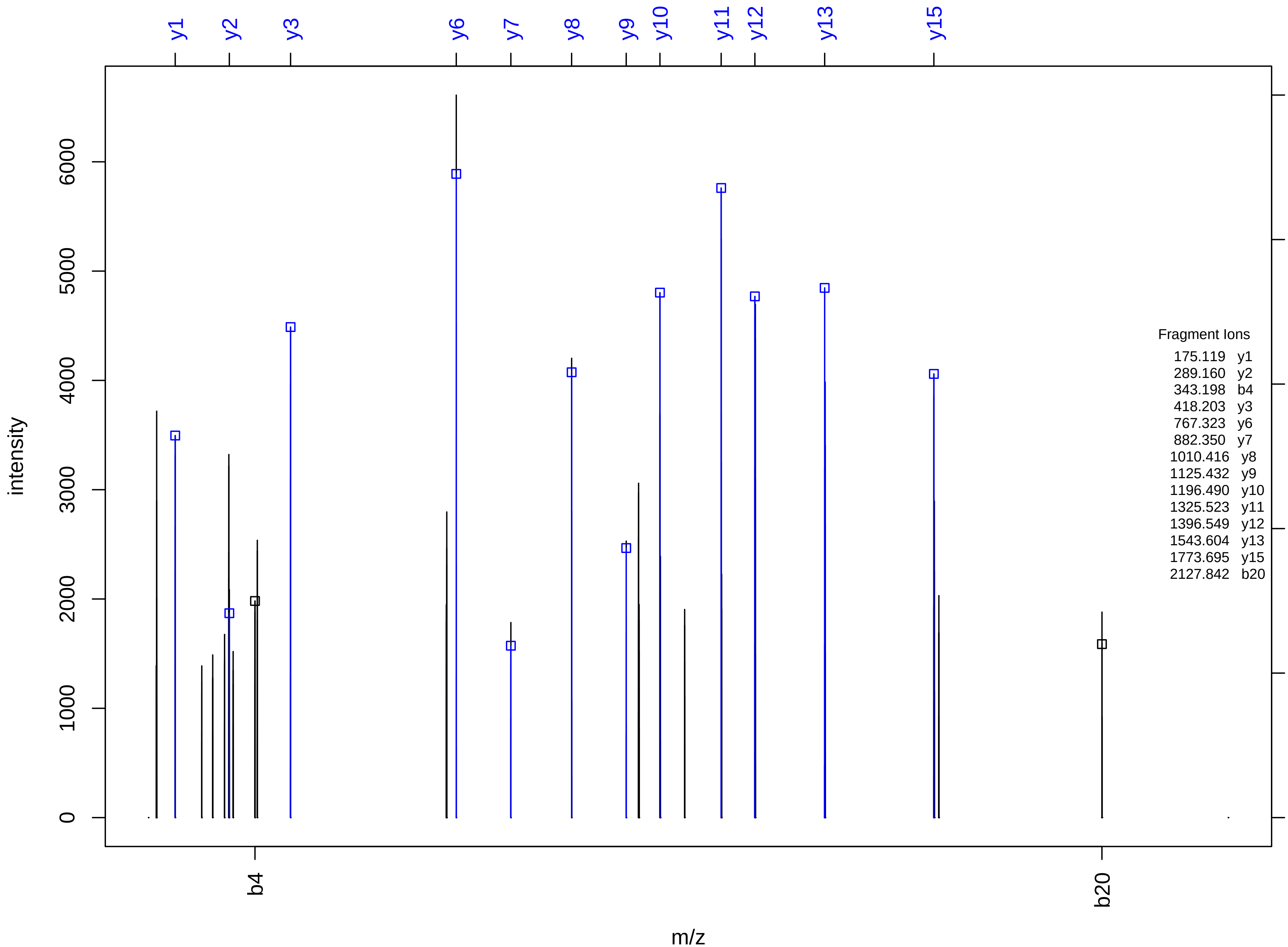

AGITGETEFAEADQDFSDENR / File: christine_30turboips_ip27.raw; SpectrumID: ; scans: 13497

DVAQIFNNILR

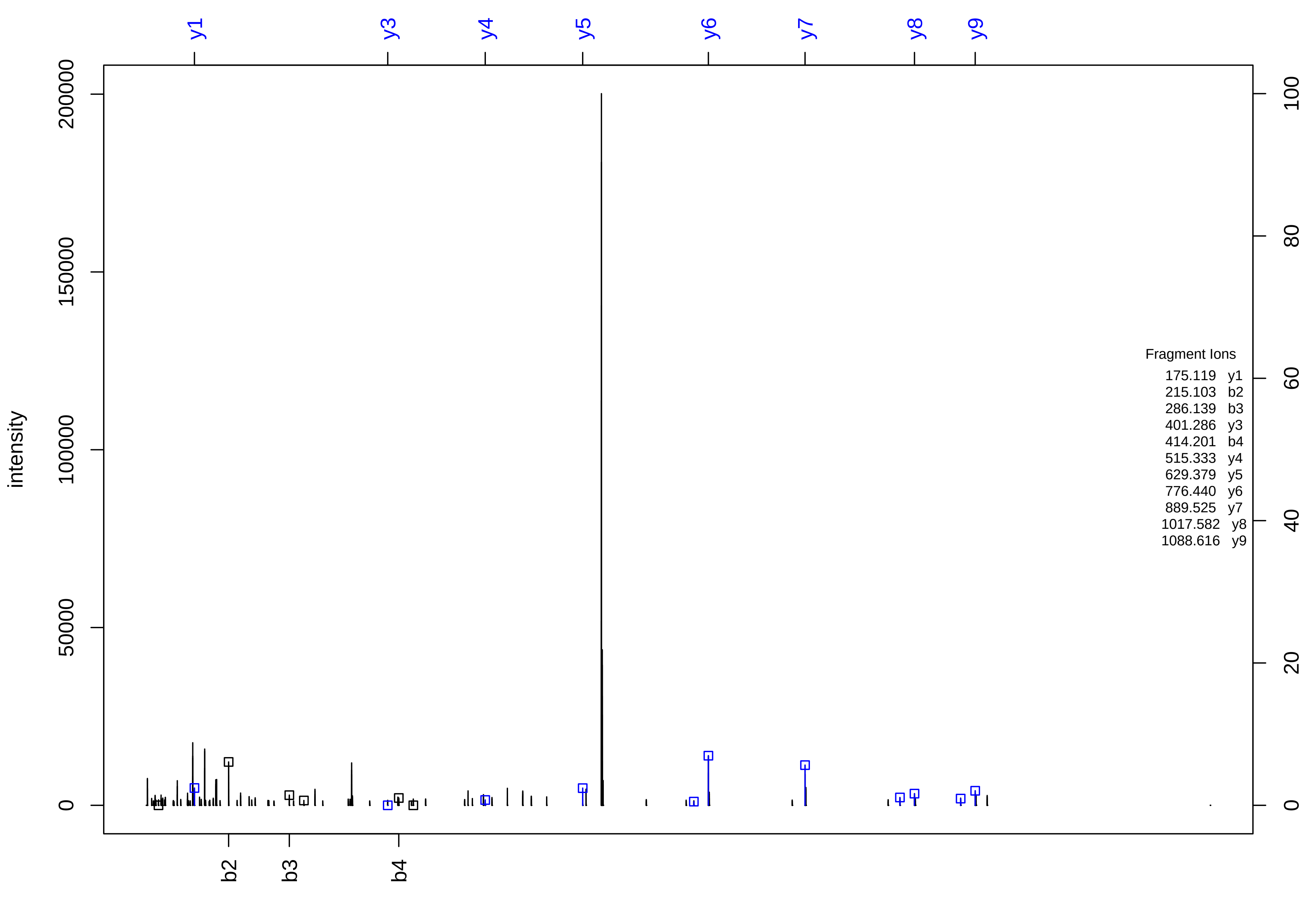

DVAQIFNNILR / File: christine_30turboips_ip27.raw; SpectrumID: ; scans: 27895

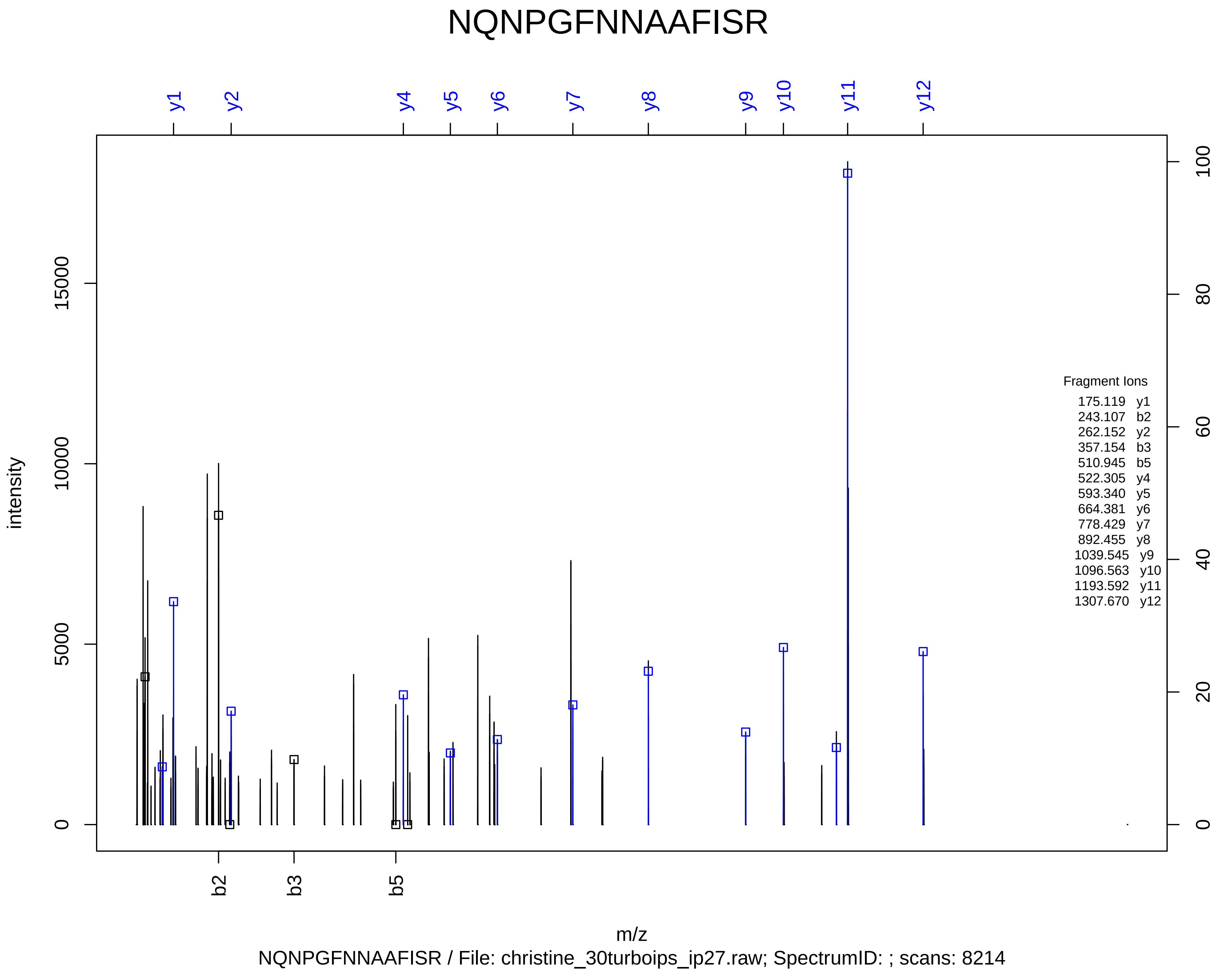
NQNPGFNNAAFISR
y1
y2
y4
y5
y6
y7
y8
y9
y10
y11
y12
intensity
0
5000
10000
15000
0
20
40
60
80
100
b2
b3
b5
m/z
Fragment Ions
175.119 y1
243.107 b2
262.152 y2
357.154 b3
510.945 b5
522.305 y4
593.340 y5
664.381 y6
778.429 y7
892.455 y8
1039.545 y9
1096.563 y10
1193.592 y11
1307.670 y12
NQNPGFNNAAFISR / File: christine_30turboips_ip27.raw; SpectrumID: ; scans: 8214

IELVVVGPEAPLAAGIVGDLTSAGVR

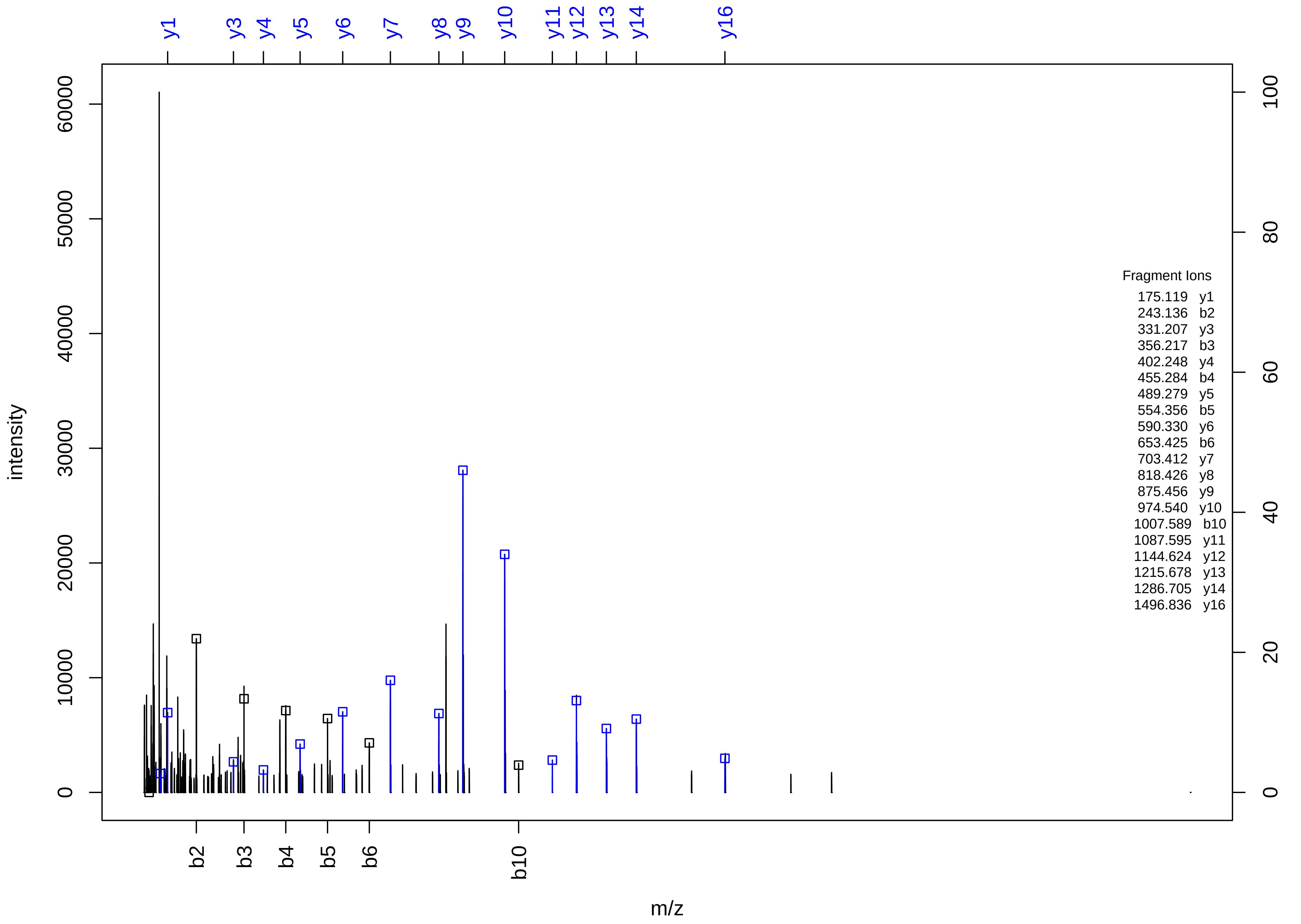

IELVVVGPEAPLAAGIVGDLTSAGVR / File: christine_30turboips_ip27.raw; SpectrumID: ; scans: 33594

YVILDIPLLFETK

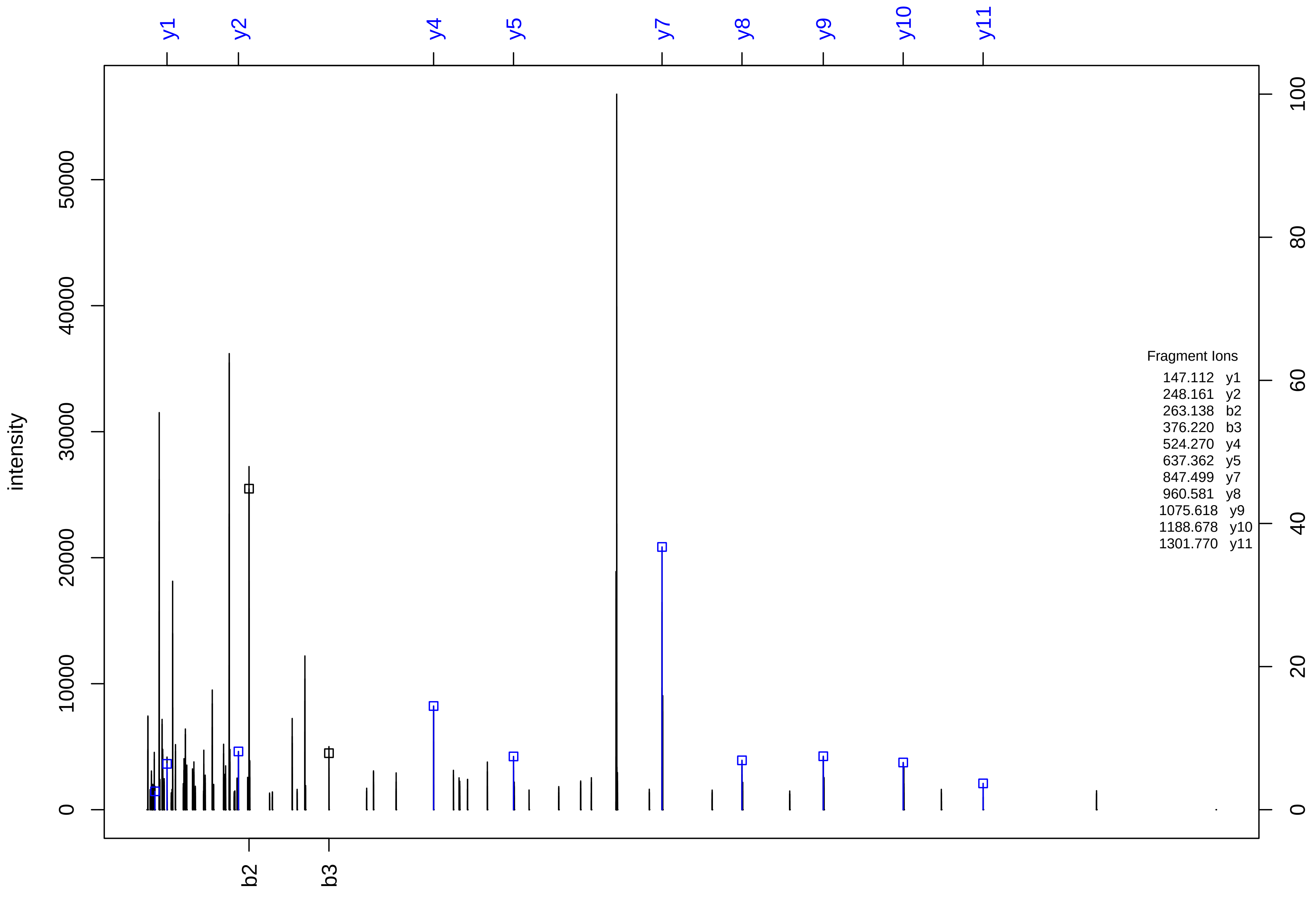

YVILDIPLLFETK / File: christine_30turboips_ip27.raw; SpectrumID: ; scans: 33694

TIFDTPDEDPNYNPLPEERPGGFAWGEGQR

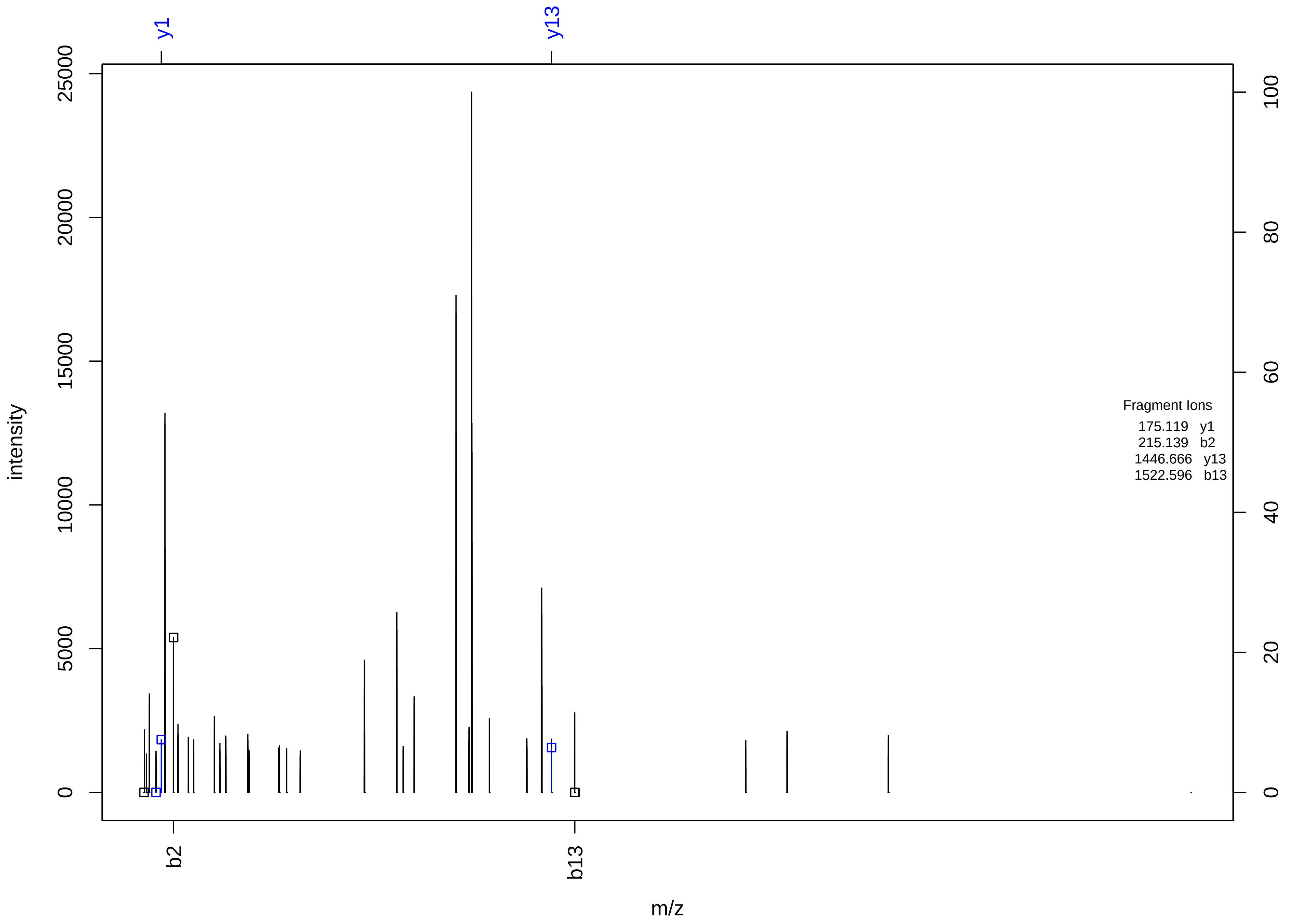

TIFDTPDEDPNYNPLPEERPGGFAWGEGQR / File: christine_30turboips_ip27.raw; SpectrumID: ; scans: 20321

VDVIQEPGLSGR

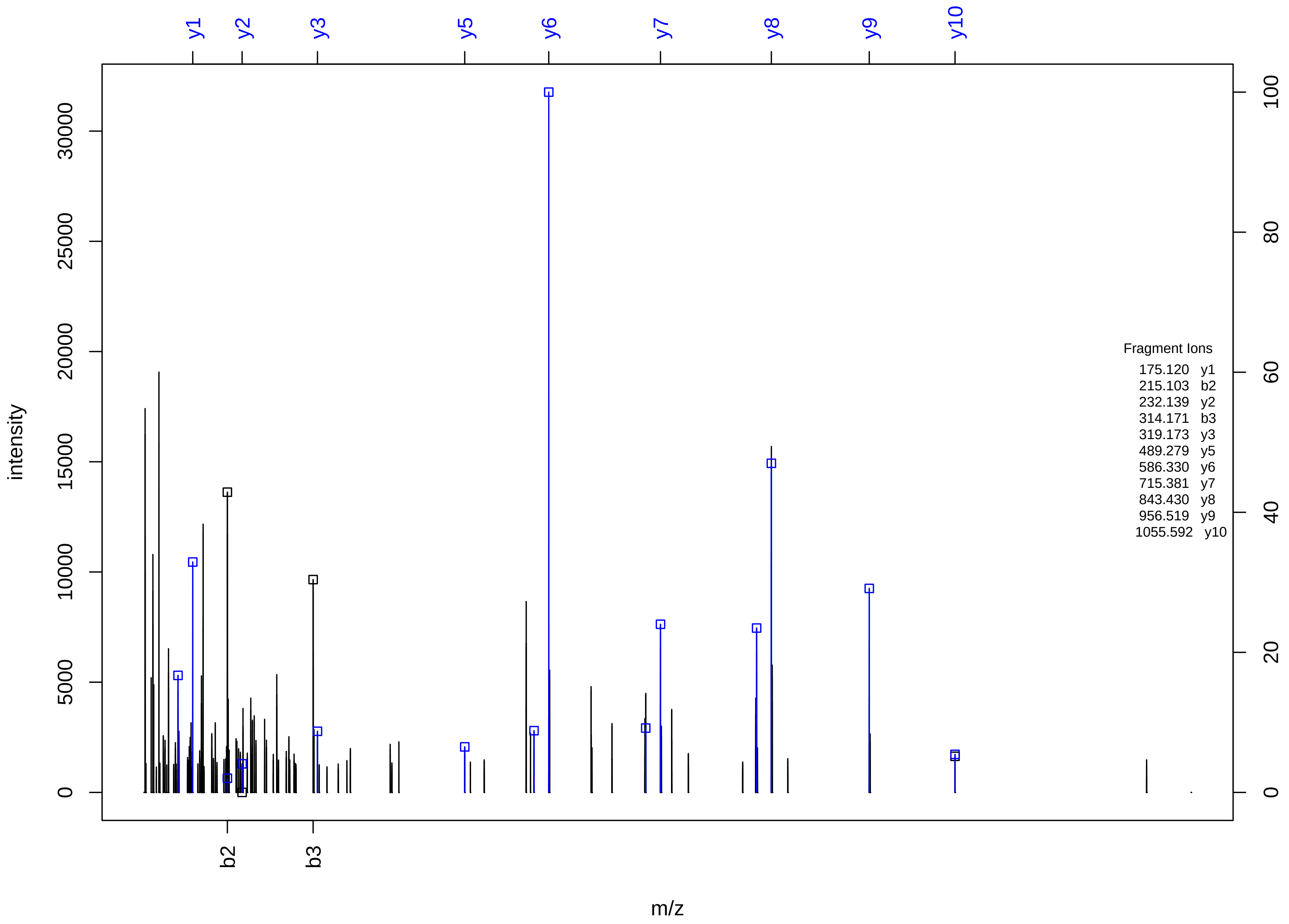

VDVIQEPGLSGR / File: christine_30turboips_ip27.raw; SpectrumID: ; scans: 7581

TASTSFTNIAYDLCAK

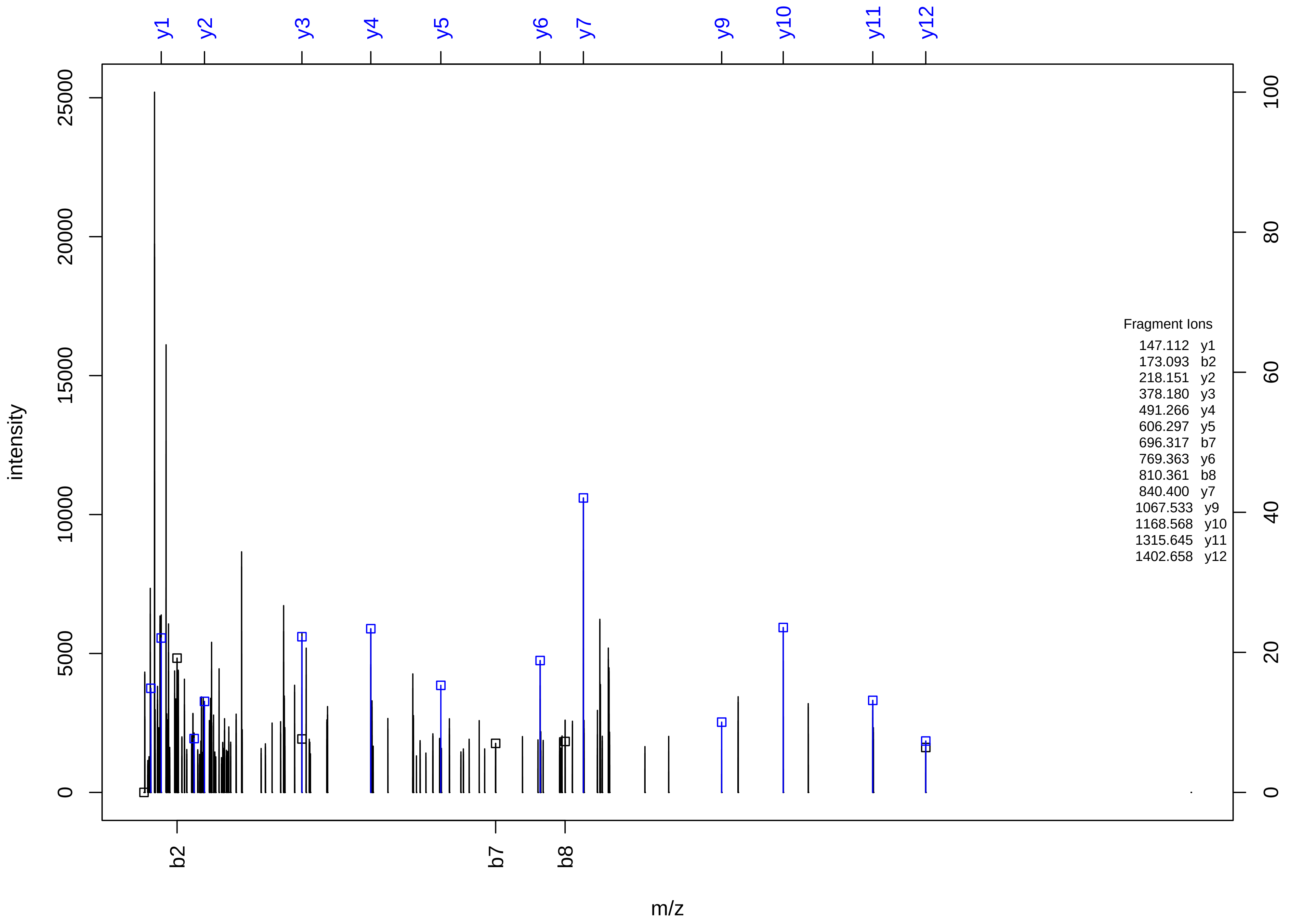

TASTSFTNIAYDLCAK / File: christine_30turboips_ip27.raw; SpectrumID: ; scans: 16928

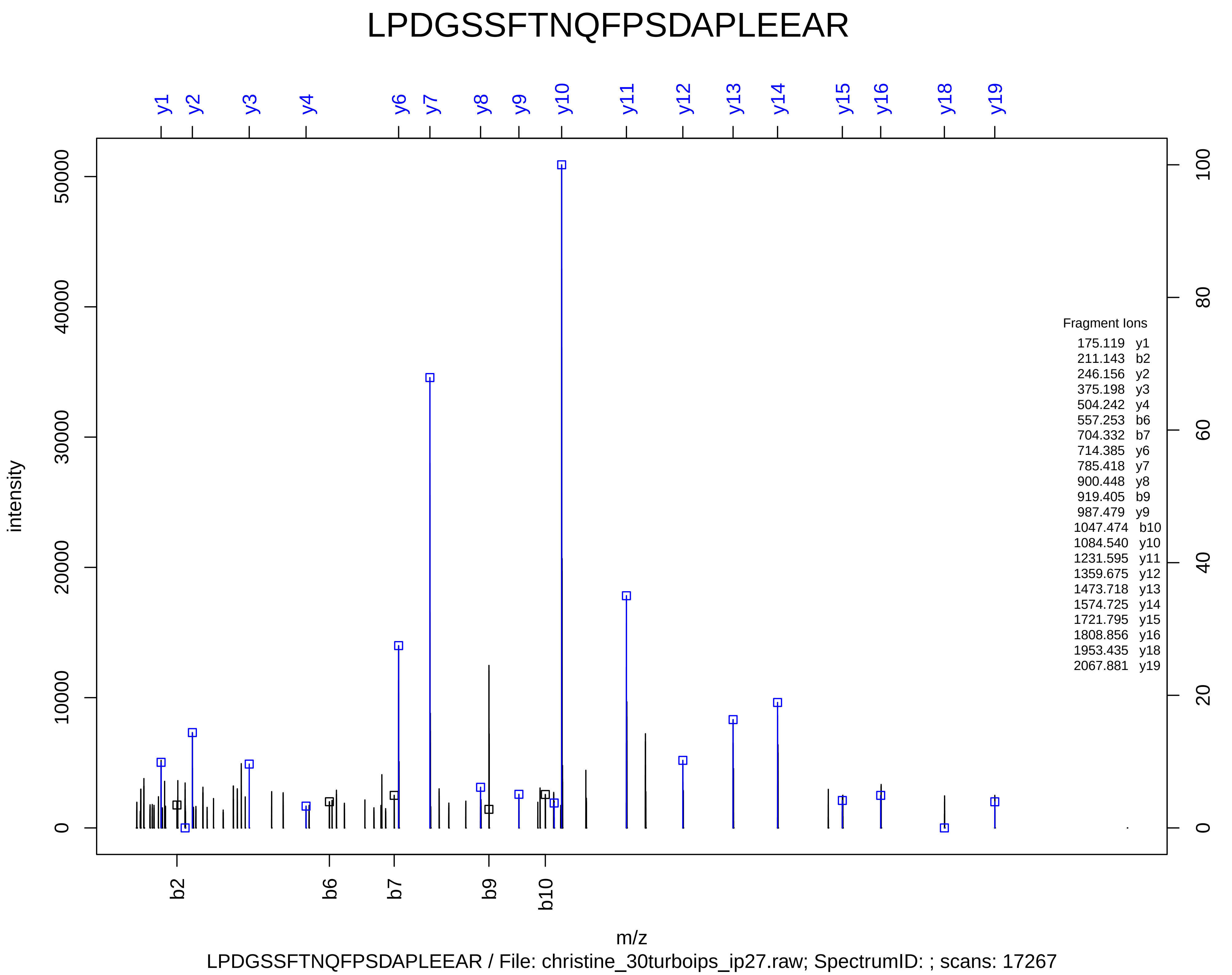
LPDGSSFTNQFPSDAPLEEAR
y1
y2
y3
y4
y6
y7
y8
y9
y10
y11
y12
y13
y14
y15
y16
y18
y19
intensity
0
10000
20000
30000
40000
50000
0
20
40
60
80
100
Fragment Ions
175.119 y1
211.143 b2
246.156 y2
375.198 y3
504.242 y4
557.253 b6
704.332 b7
714.385 y6
785.418 y7
900.448 y8
919.405 b9
987.479 y9
1047.474 b10
1084.540 y10
1231.595 y11
1359.675 y12
1473.718 y13
1574.725 y14
1721.795 y15
1808.856 y16
1953.435 y18
2067.881 y19
b2
b6
b7
b9
b10
m/z
LPDGSSFTNQFPSDAPLEEAR / File: christine_30turboips_ip27.raw; SpectrumID: ; scans: 17267

LFISPGLLPEAPTQSGPPKPDPAGTPK

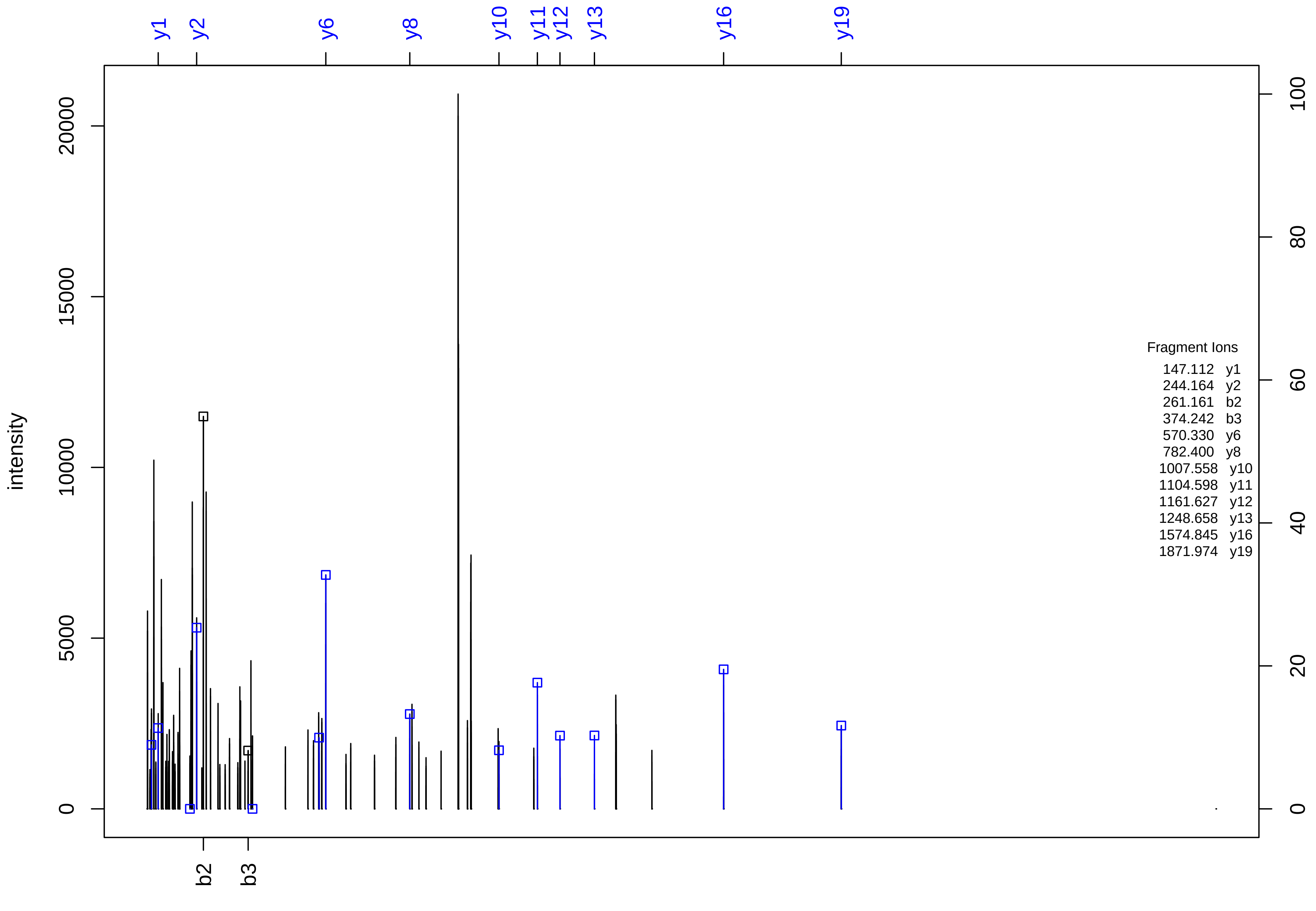

LFISPGLLPEAPTQSGPPKPDPAGTPK / File: christine_30turboips_ip27.raw; SpectrumID: ; scans: 18215

VSLYYESLCGACR

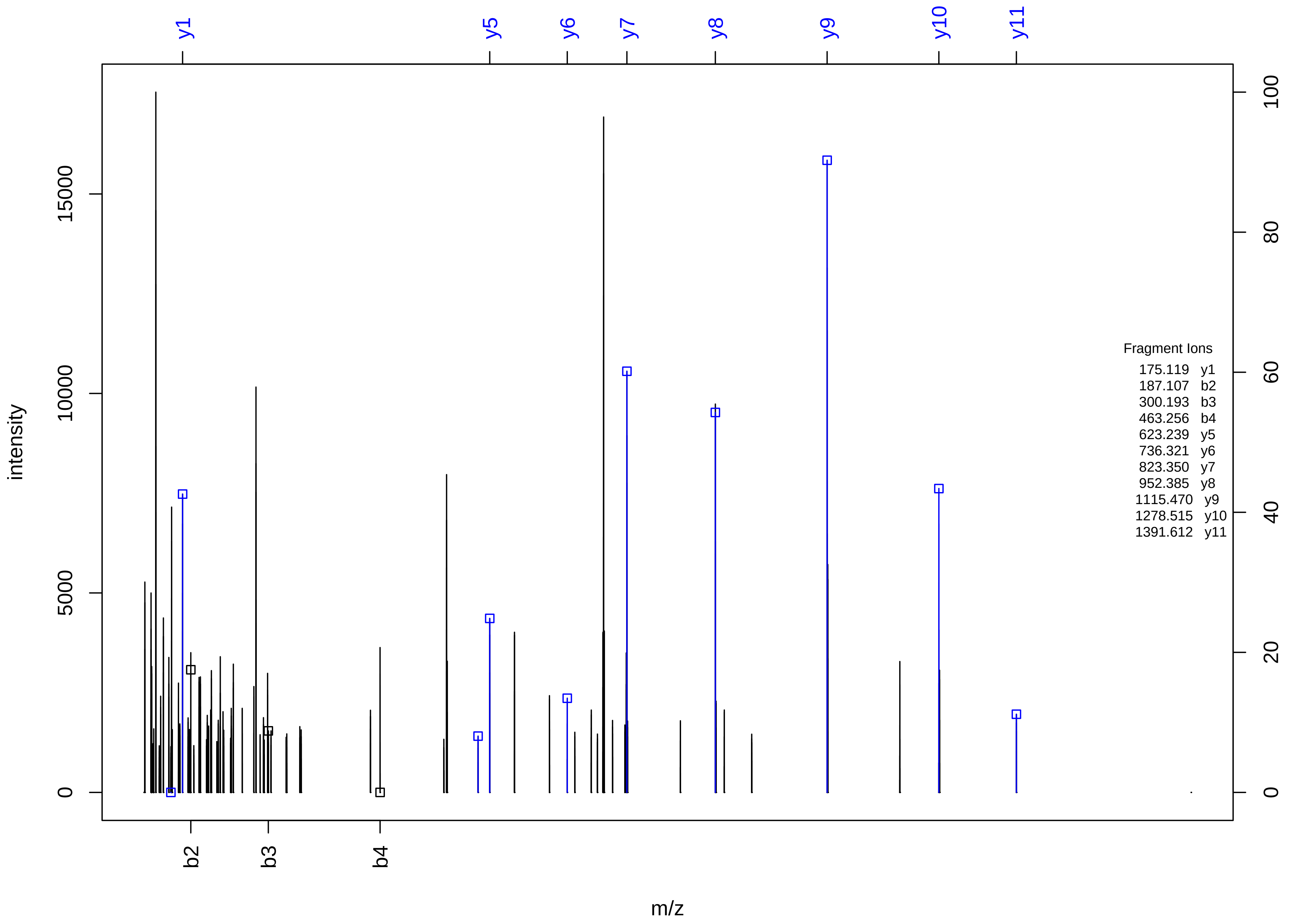

VSLYYESLCGACR / File: christine_30turboips_ip27.raw; SpectrumID: ; scans: 11306

AVAVVVDPIQSVK

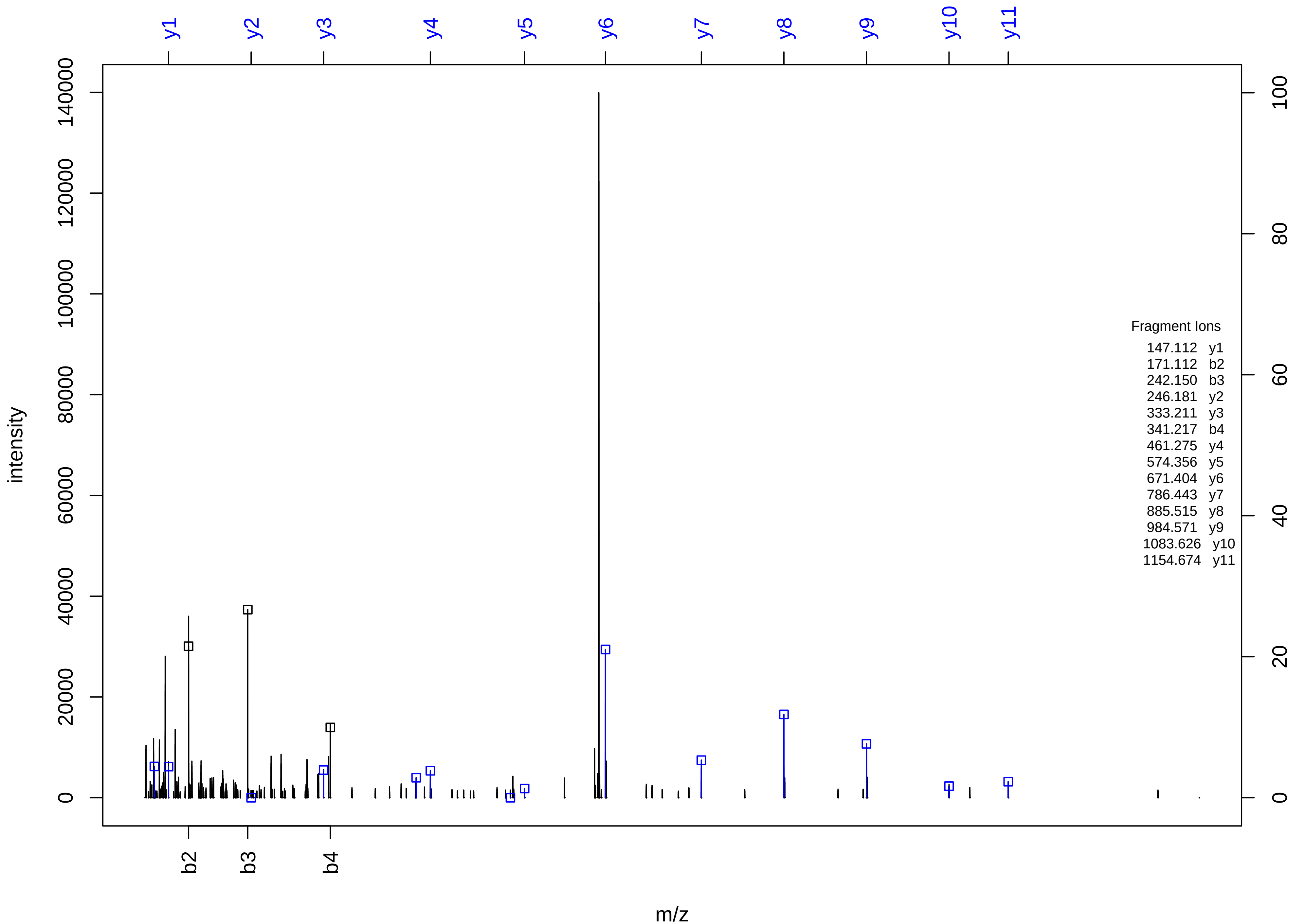

AVAVVVDPIQSVK / File: christine_30turboips_ip28.raw; SpectrumID: ; scans: 13321

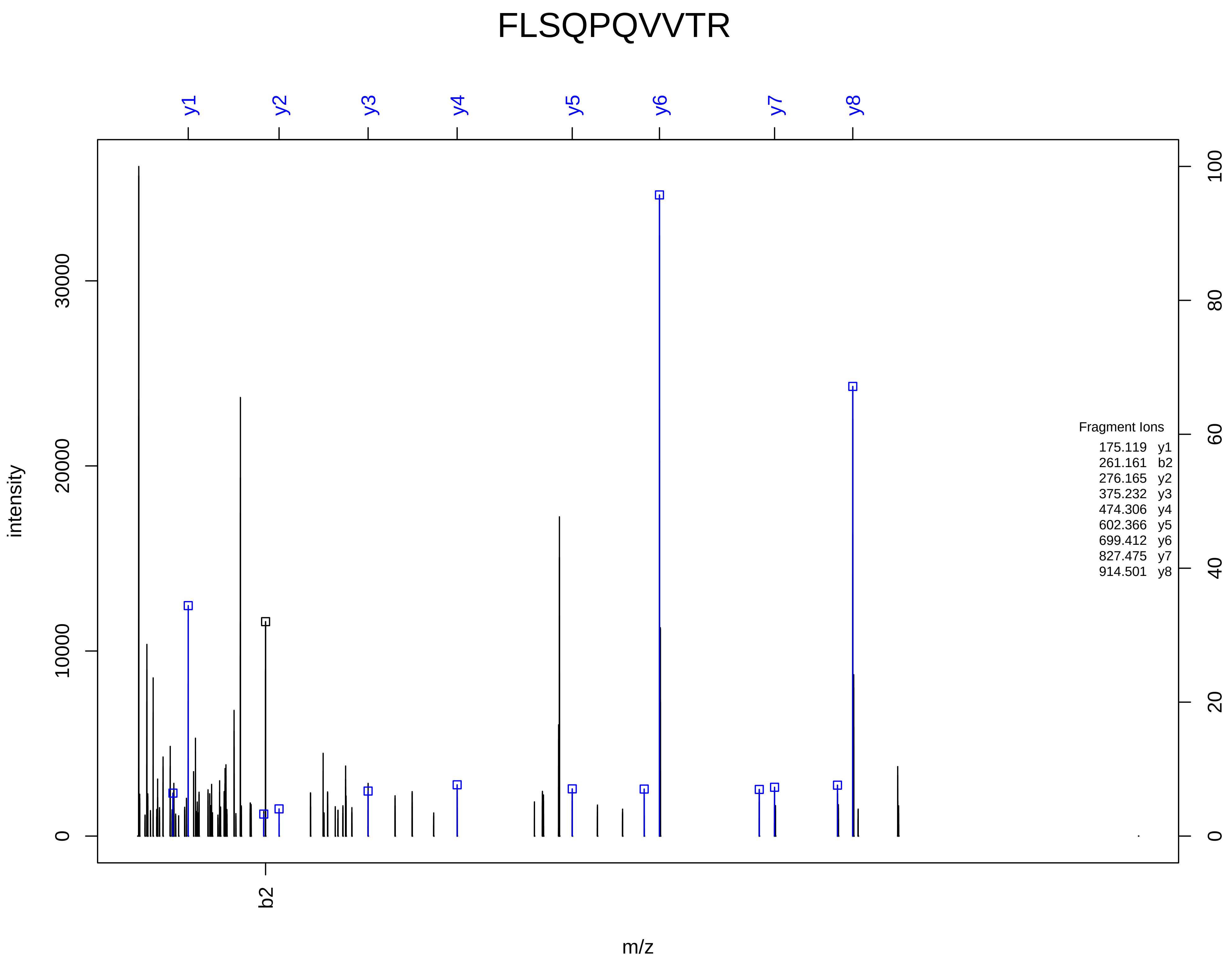

FLSQPQVVTR / File: christine_30turboips_ip28.raw; SpectrumID: ; scans: 5218

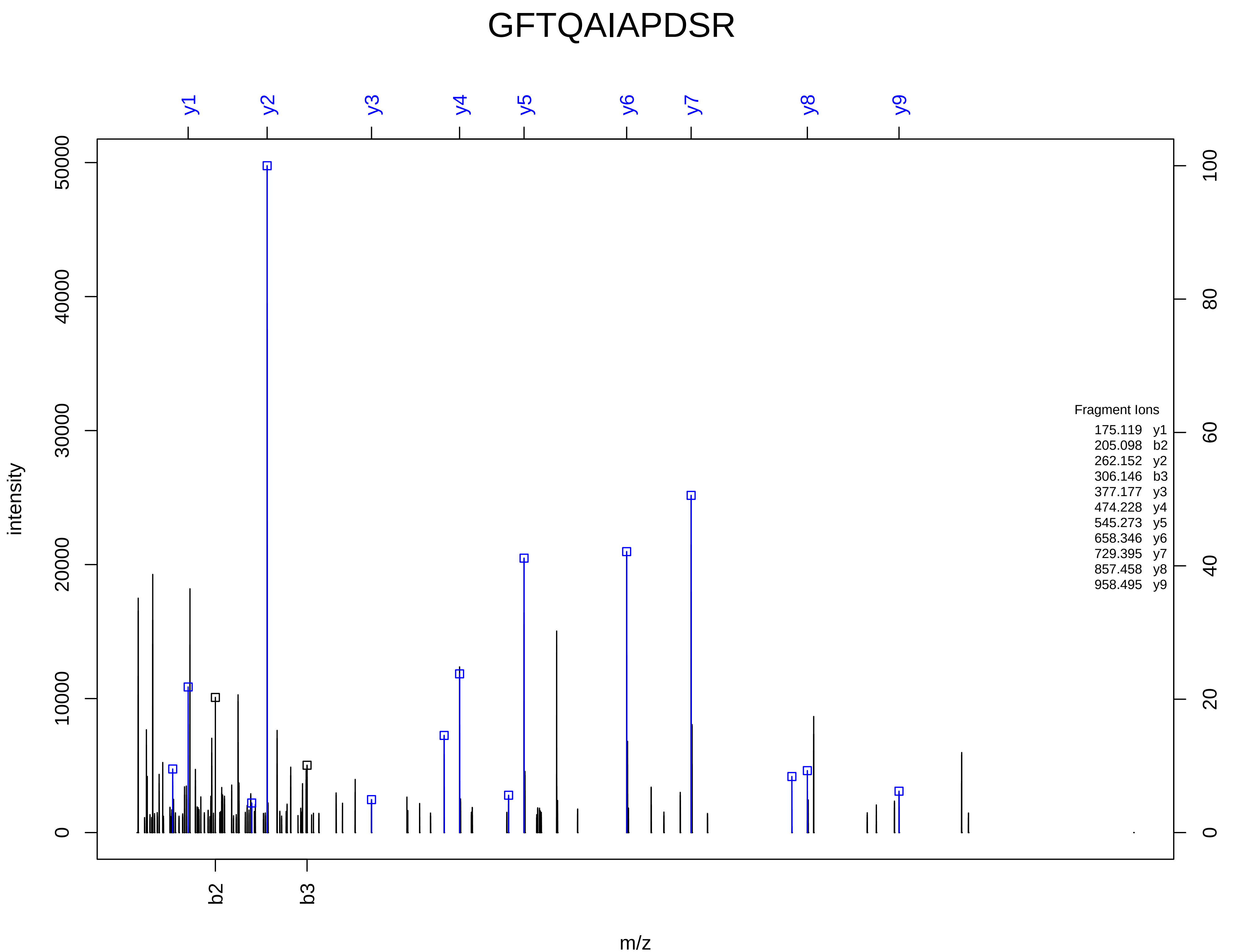
GFTQAIAPDSR
y1
y2
y3
y4
y5
y6
y7
y8
y9
b2
b3
intensity
m/z
Fragment Ions
175.119 y1
205.098 b2
262.152 y2
306.146 b3
377.177 y3
474.228 y4
545.273 y5
658.346 y6
729.395 y7
857.458 y8
958.495 y9
GFTQAIAPDSR / File: christine_30turboips_ip28.raw; SpectrumID: ; scans: 3923

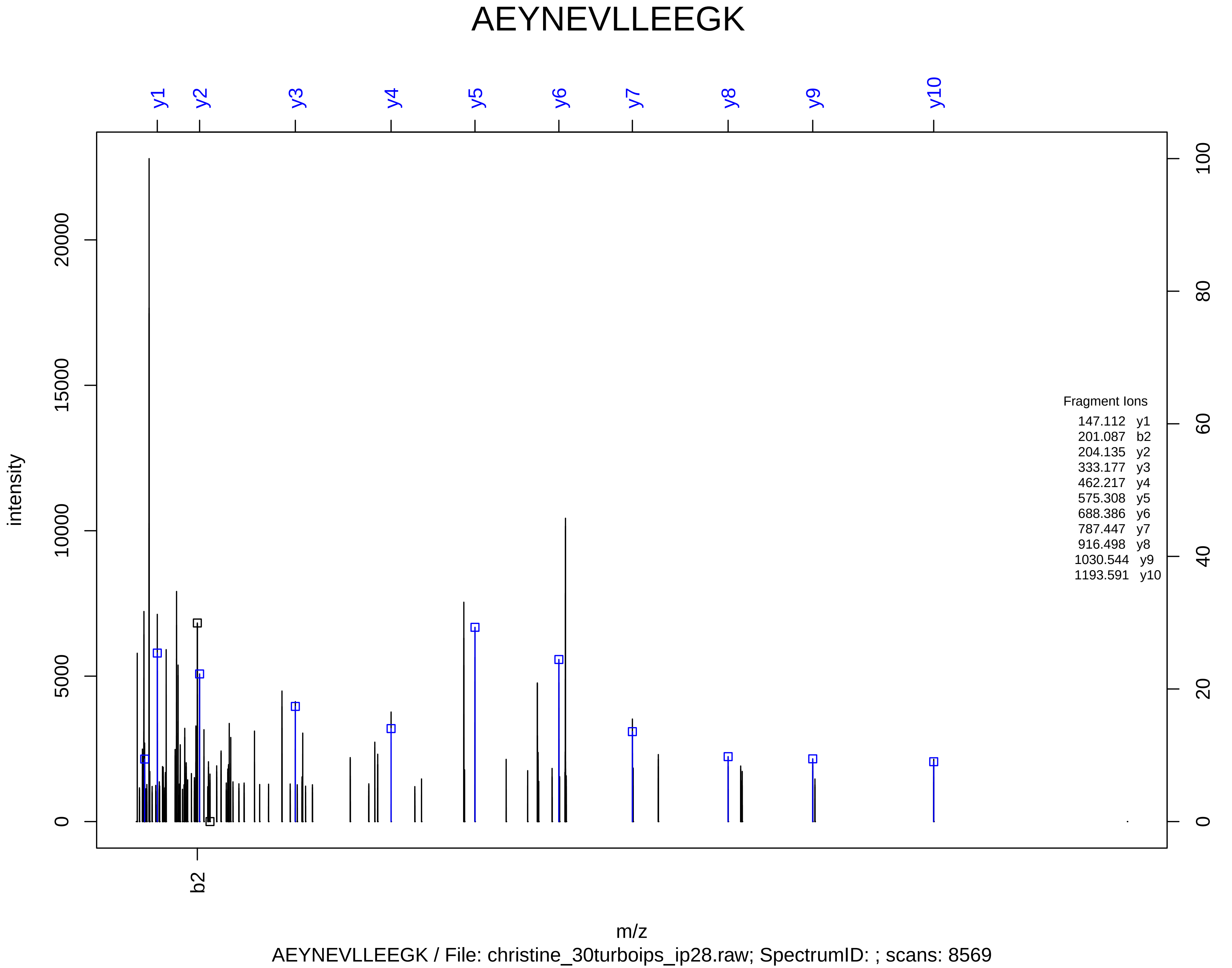
AEYNEVLLEEGK
y1
y2
y3
y4
y5
y6
y7
y8
y9
y10
intensity
0
5000
10000
15000
20000
0
20
40
60
80
100
b2
m/z
Fragment Ions
147.112 y1
201.087 b2
204.135 y2
333.177 y3
462.217 y4
575.308 y5
688.386 y6
787.447 y7
916.498 y8
1030.544 y9
1193.591 y10
AEYNEVLLEEGK / File: christine_30turboips_ip28.raw; SpectrumID: ; scans: 8569

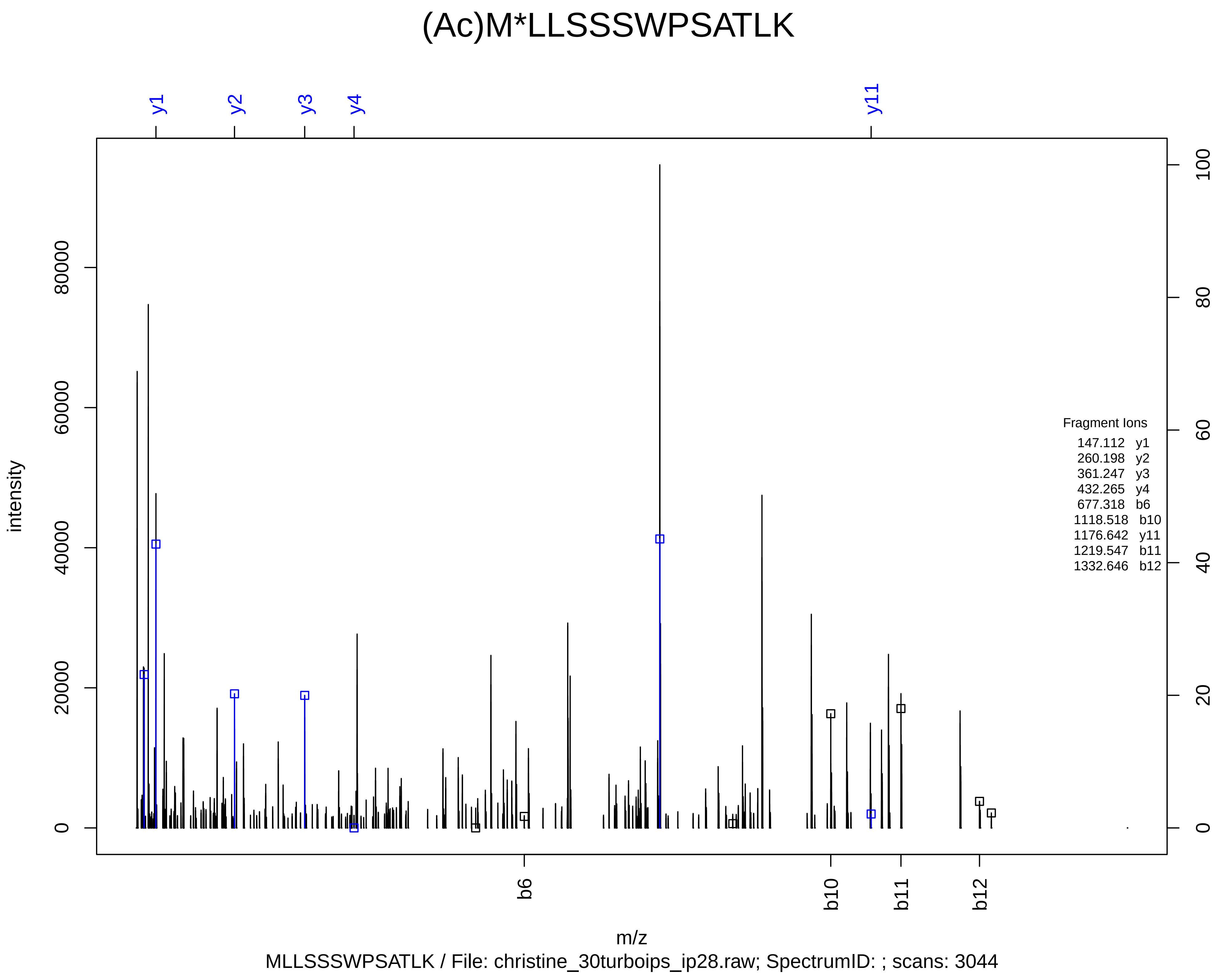
(Ac)M*LLSSSWPSATLK
y1
y2
y3
y4
y11
intensity
0
20000
40000
60000
80000
0
20
40
60
80
100
Fragment Ions
147.112 y1
260.198 y2
361.247 y3
432.265 y4
677.318 b6
1118.518 b10
1176.642 y11
1219.547 b11
1332.646 b12
b6
b10
b11
b12
m/z
MLLSSSWPSATLK / File: christine_30turboips_ip28.raw; SpectrumID: ; scans: 3044

SLEDEINR

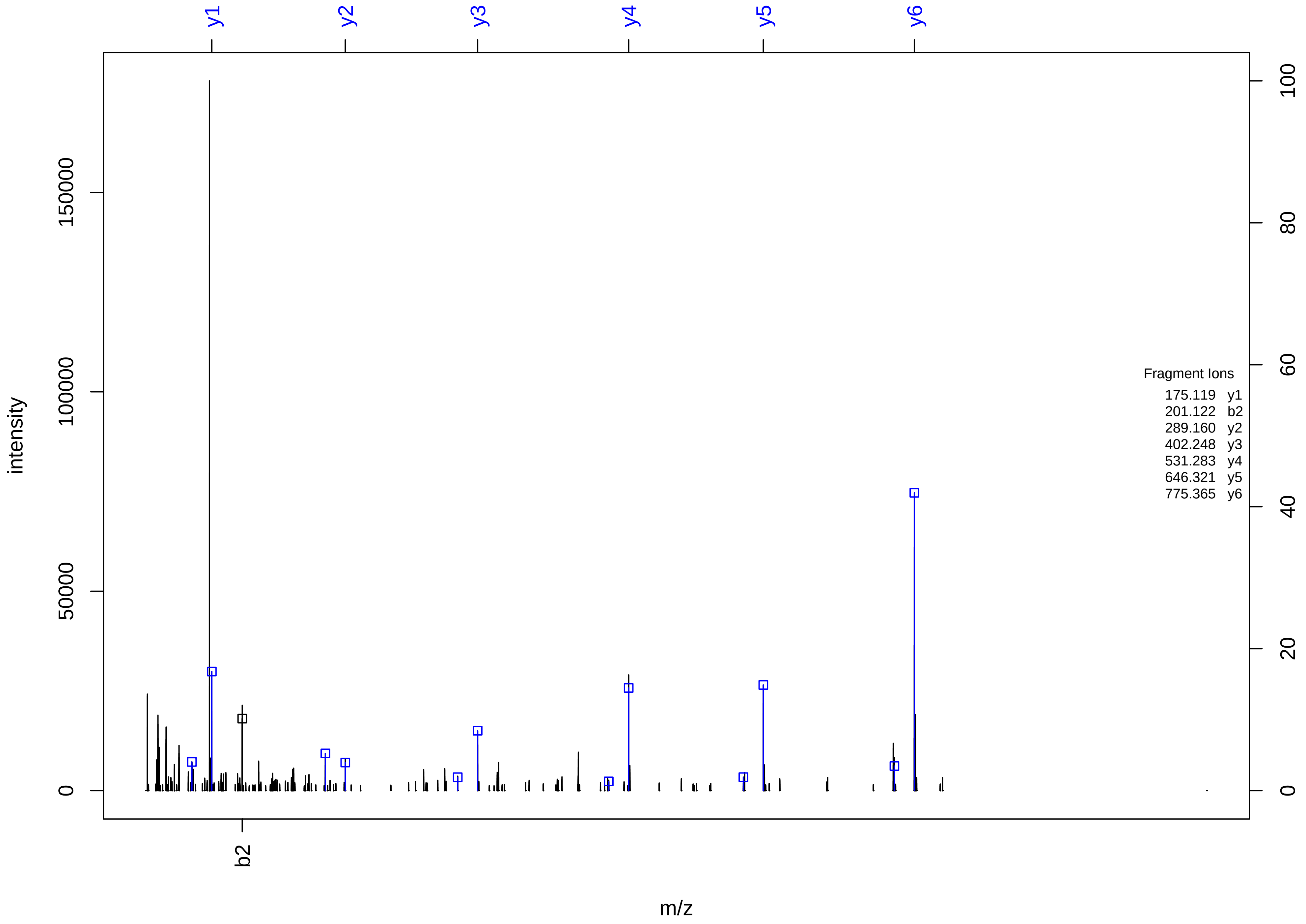

SLEDEINR / File: christine_30turboips_ip28.raw; SpectrumID: ; scans: 3189

LQDDTSCSAVCK

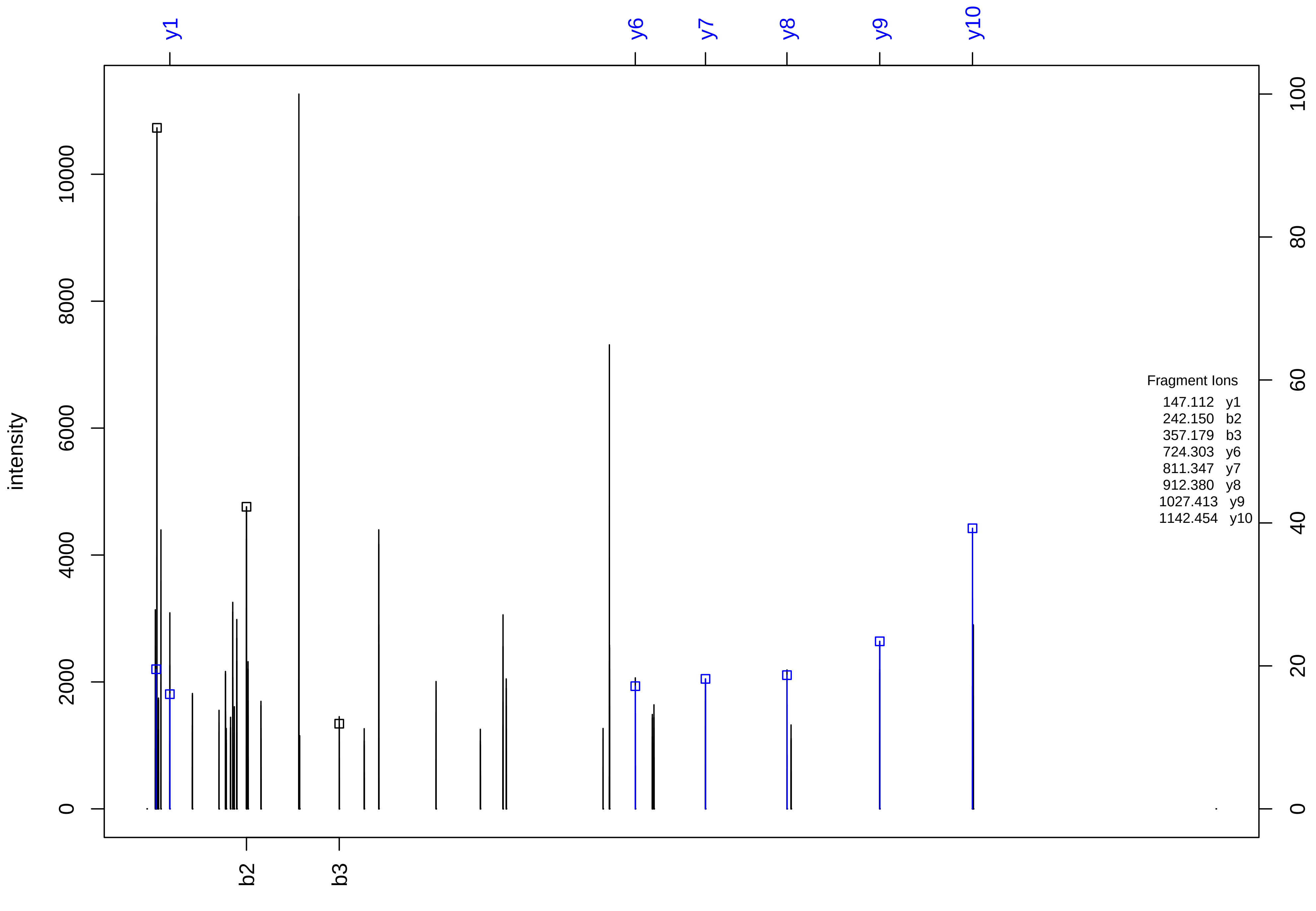

LQDDTSCSAVCK / File: christine_30turboips_ip28.raw; SpectrumID: ; scans: 1103

EQTSPPHPEETR

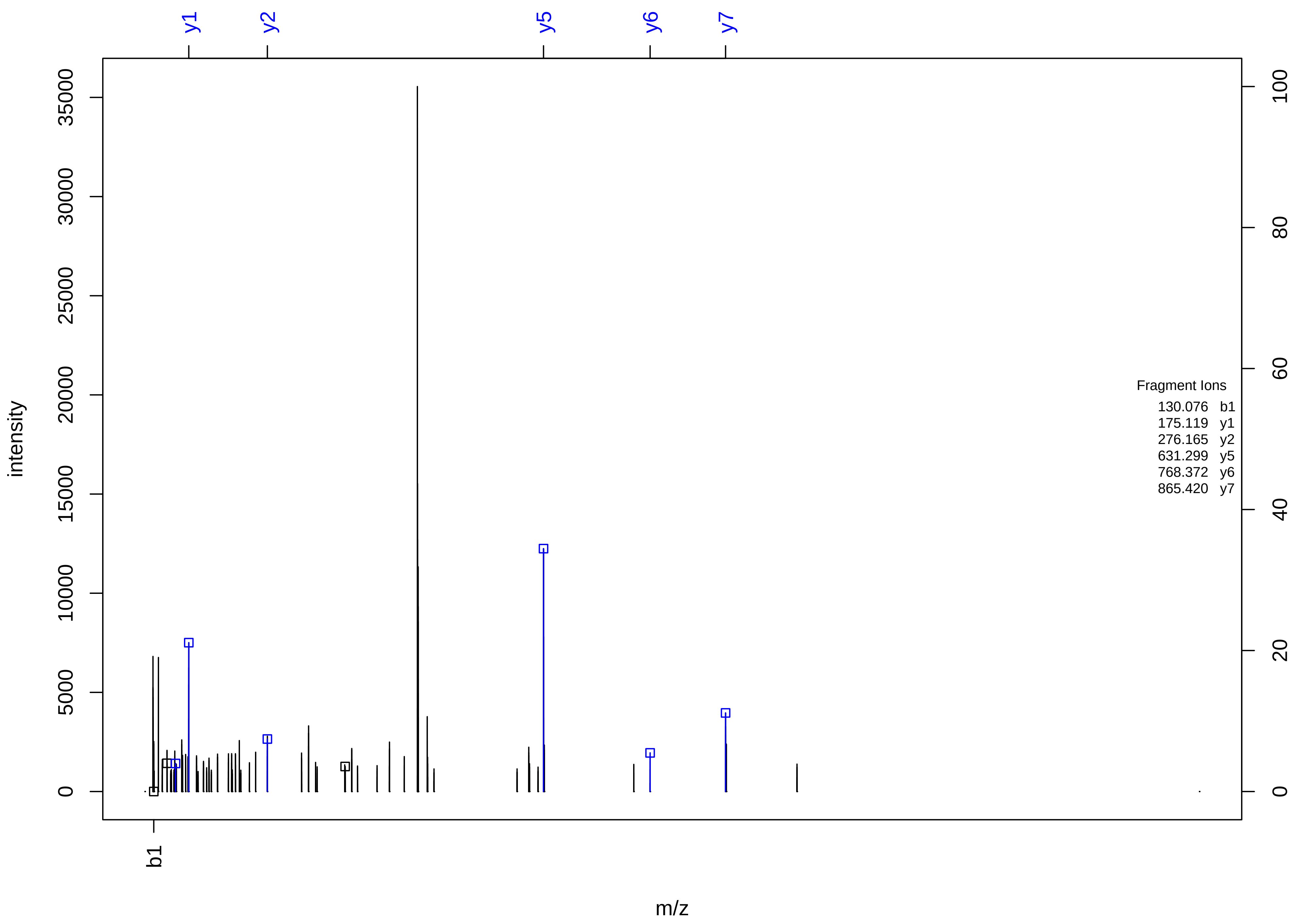

EQTSPPHPEETR / File: christine_30turboips_ip28.raw; SpectrumID: ; scans: 762

LLDEDN^TGTVEFK

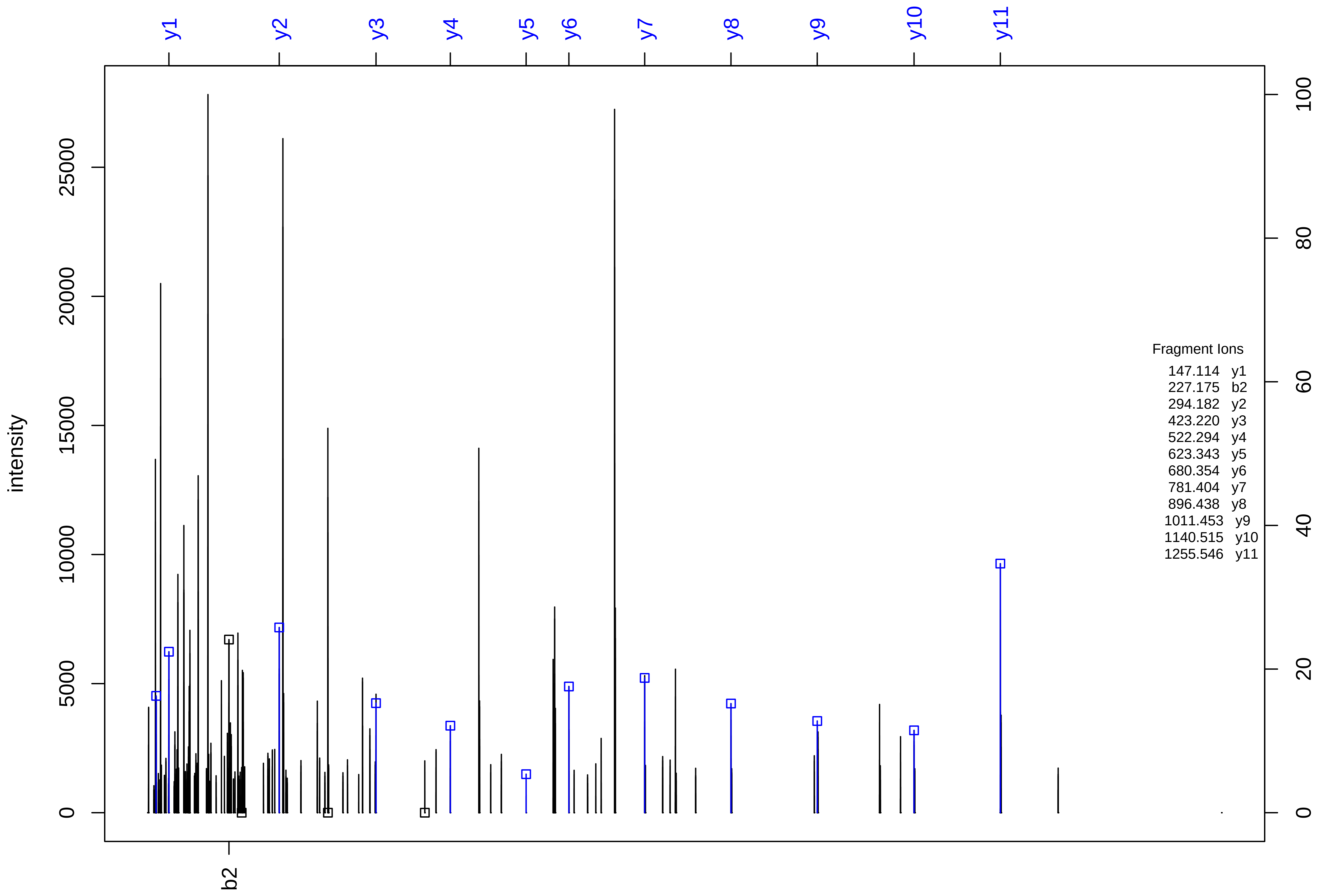

LLDEDNTGTVEFK / File: christine_30turboips_ip28.raw; SpectrumID: ; scans: 10842

(Ac)M*IN^FSAFLGAATM*YTRYK

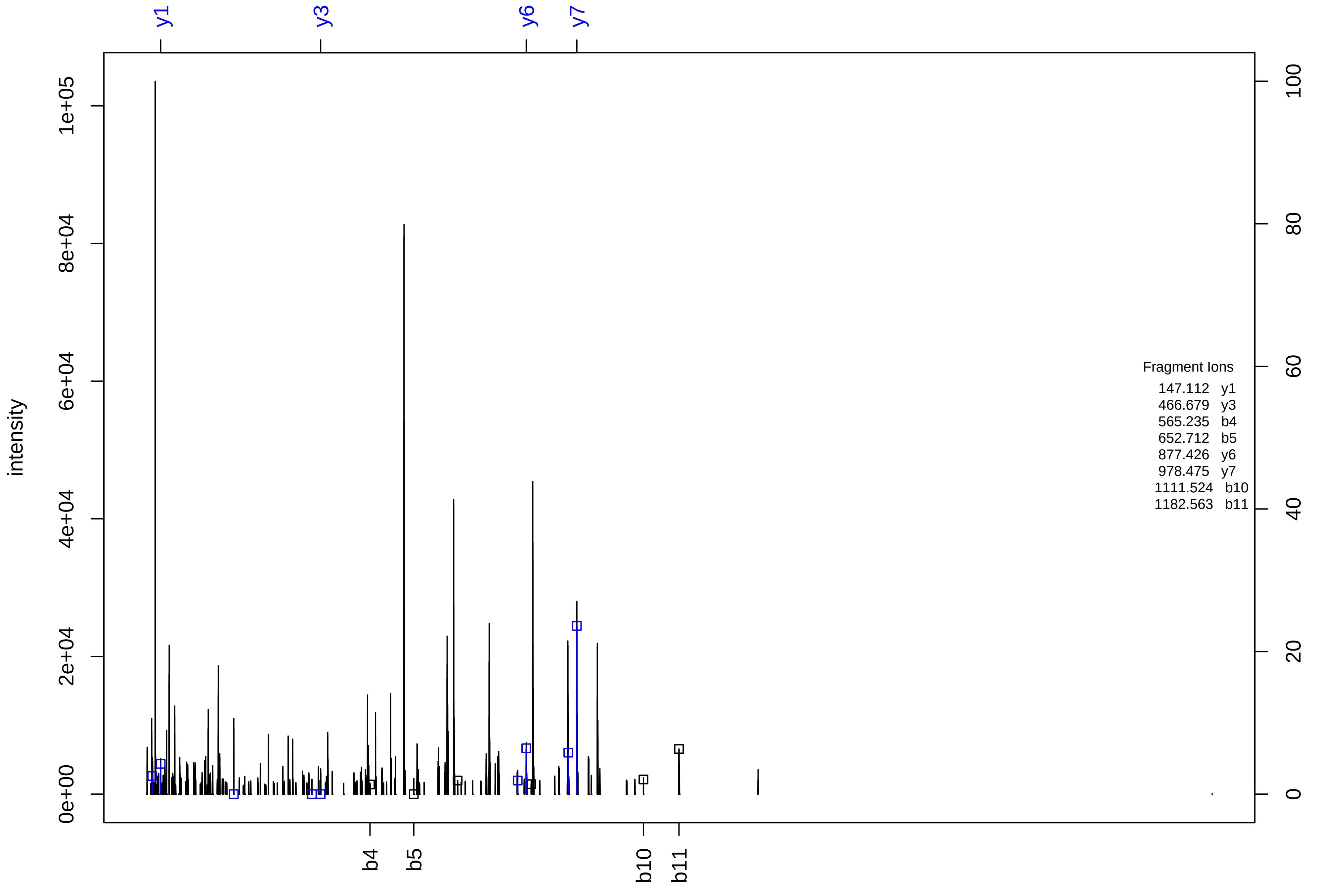

MINFSAFLGAATMYTRYK / File: christine_30turboips_ip28.raw; SpectrumID: ; scans: 10957

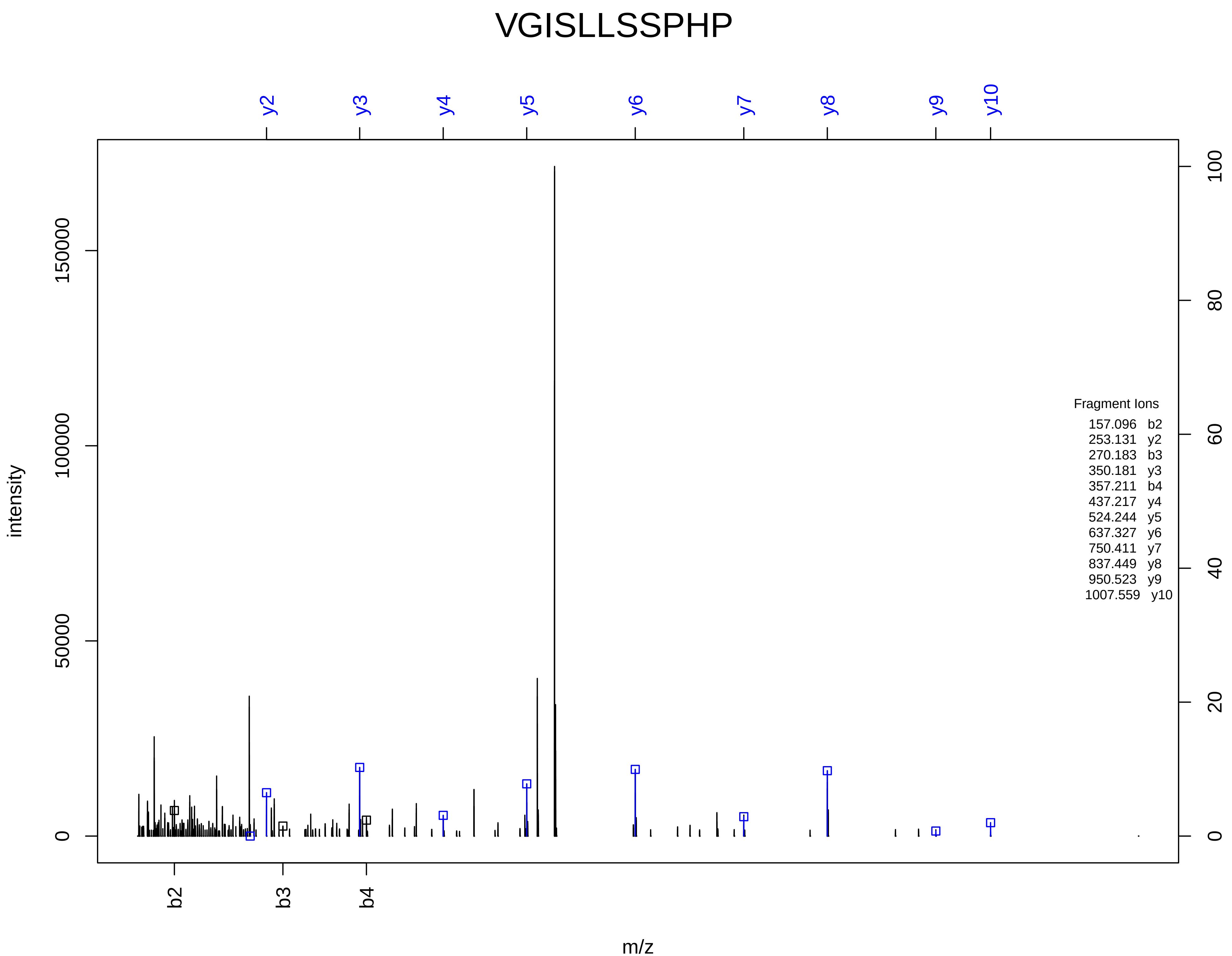
VGISLLSSPHP
y2
y3
y4
y5
y6
y7
y8
y9
y10
intensity
0
50000
100000
150000
0
20
40
60
80
100
Fragment Ions
157.096 b2
253.131 y2
270.183 b3
350.181 y3
357.211 b4
437.217 y4
524.244 y5
637.327 y6
750.411 y7
837.449 y8
950.523 y9
1007.559 y10
b2
b3
b4
m/z
VGISLLSSPHP / File: christine_30turboips_ip26.raw; SpectrumID: ; scans: 13645

DTAVTLTPFEDTLTR

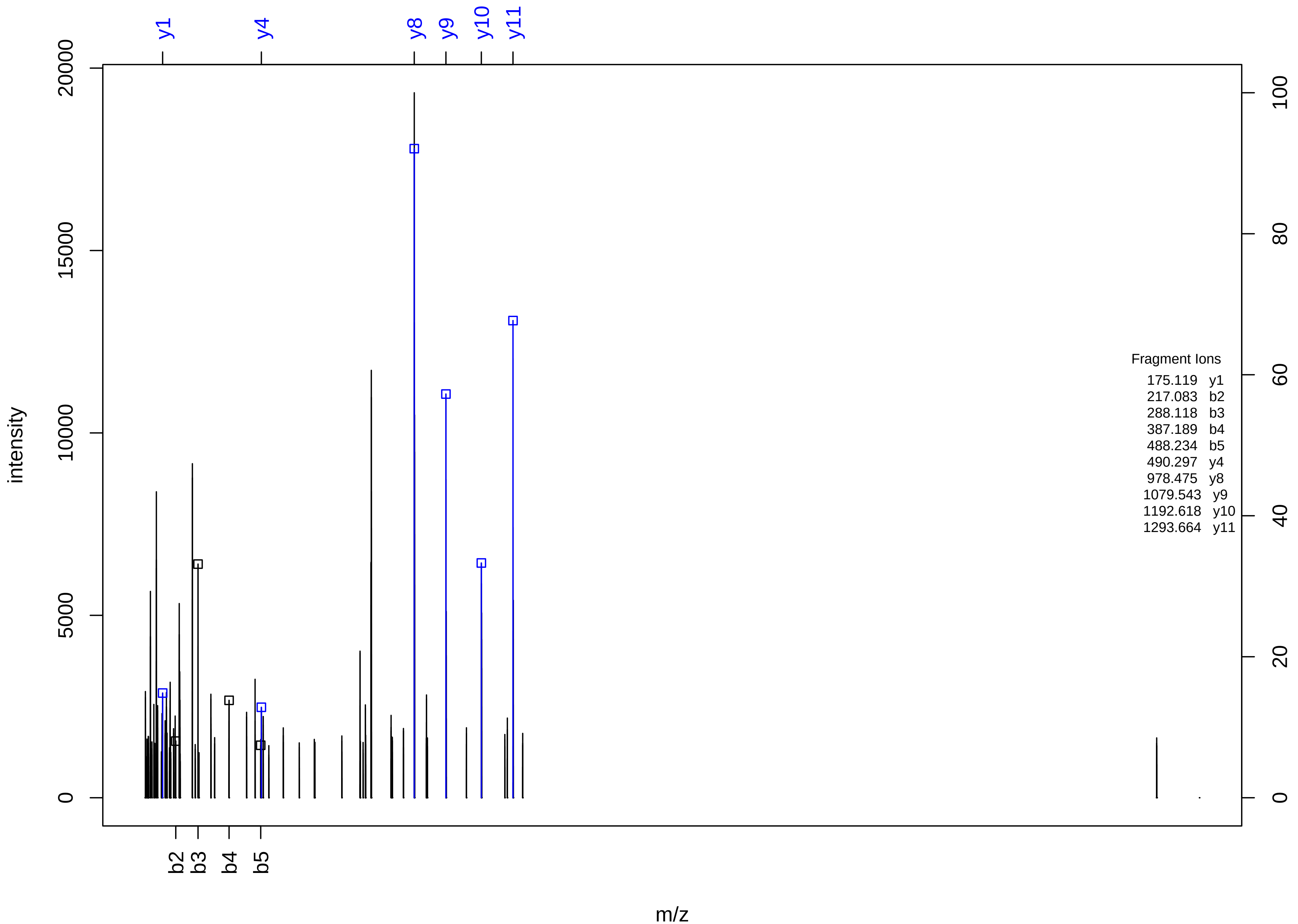

DTAVTLTPFEDTLTR / File: christine_30turboips_ip26.raw; SpectrumID: ; scans: 20543

VPTAHLEDVLPLAEDITTILSK

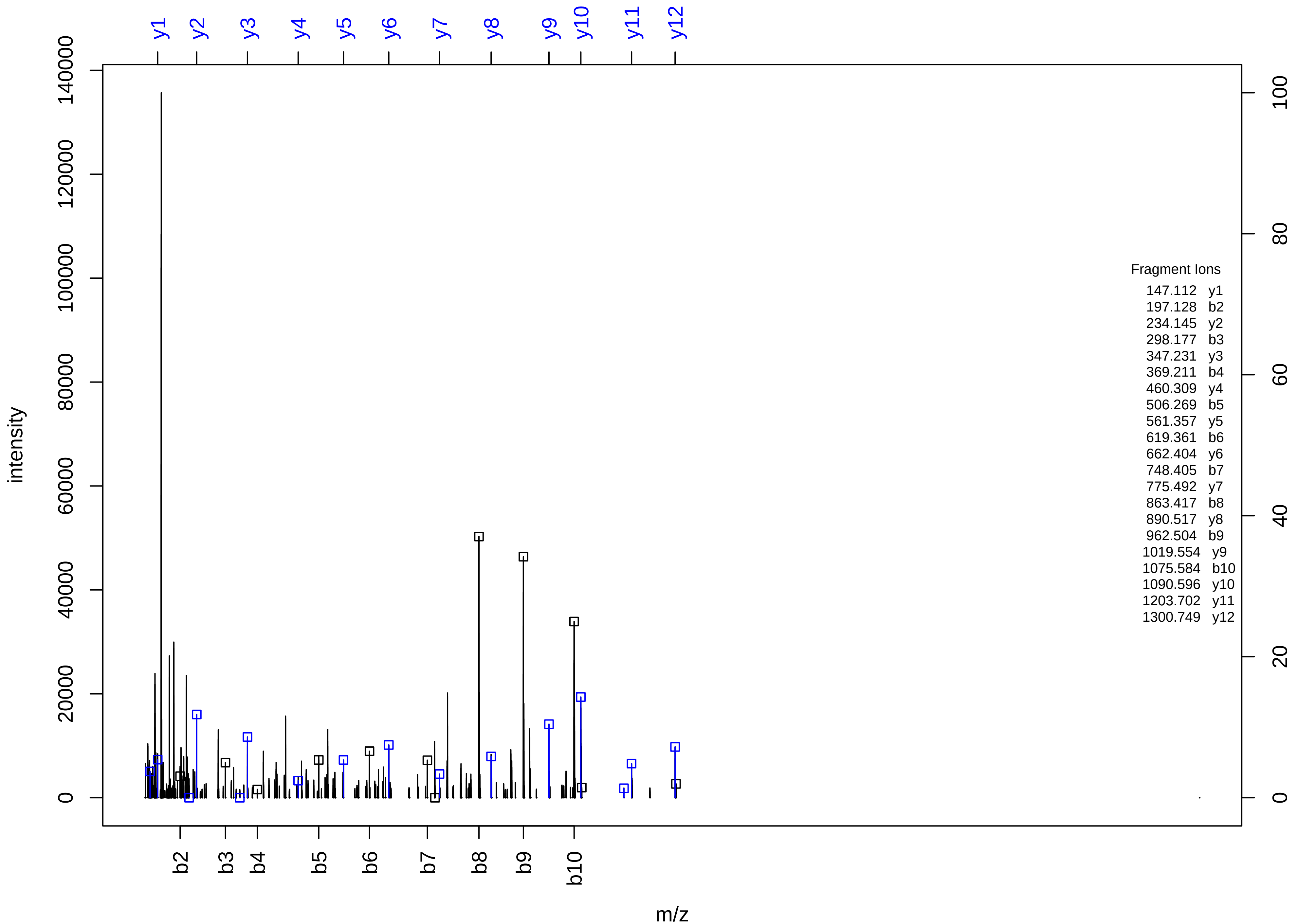

VPTAHLEDVLPLAEDITTILSK / File: christine_30turboips_ip25.raw; SpectrumID: ; scans: 32147

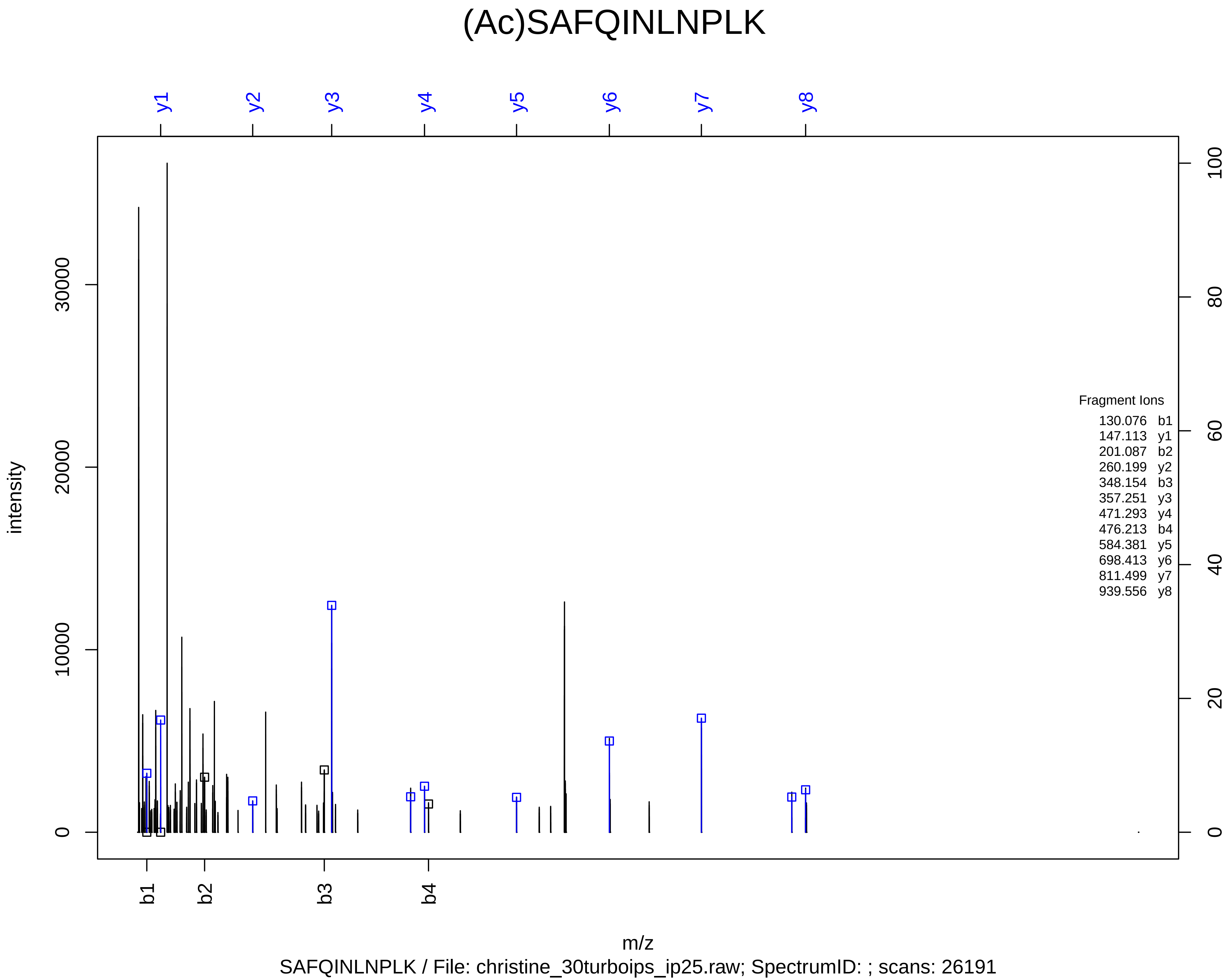

(Ac)SAFQINLNPLK
y1
y2
y3
y4
y5
y6
y7
y8
intensity
0
10000
20000
30000
0
20
40
60
80
100
Fragment Ions
130.076 b1
147.113 y1
201.087 b2
260.199 y2
348.154 b3
357.251 y3
471.293 y4
476.213 b4
584.381 y5
698.413 y6
811.499 y7
939.556 y8
b1
b2
b3
b4
m/z
SAFQINLNPLK / File: christine_30turboips_ip25.raw; SpectrumID: ; scans: 26191

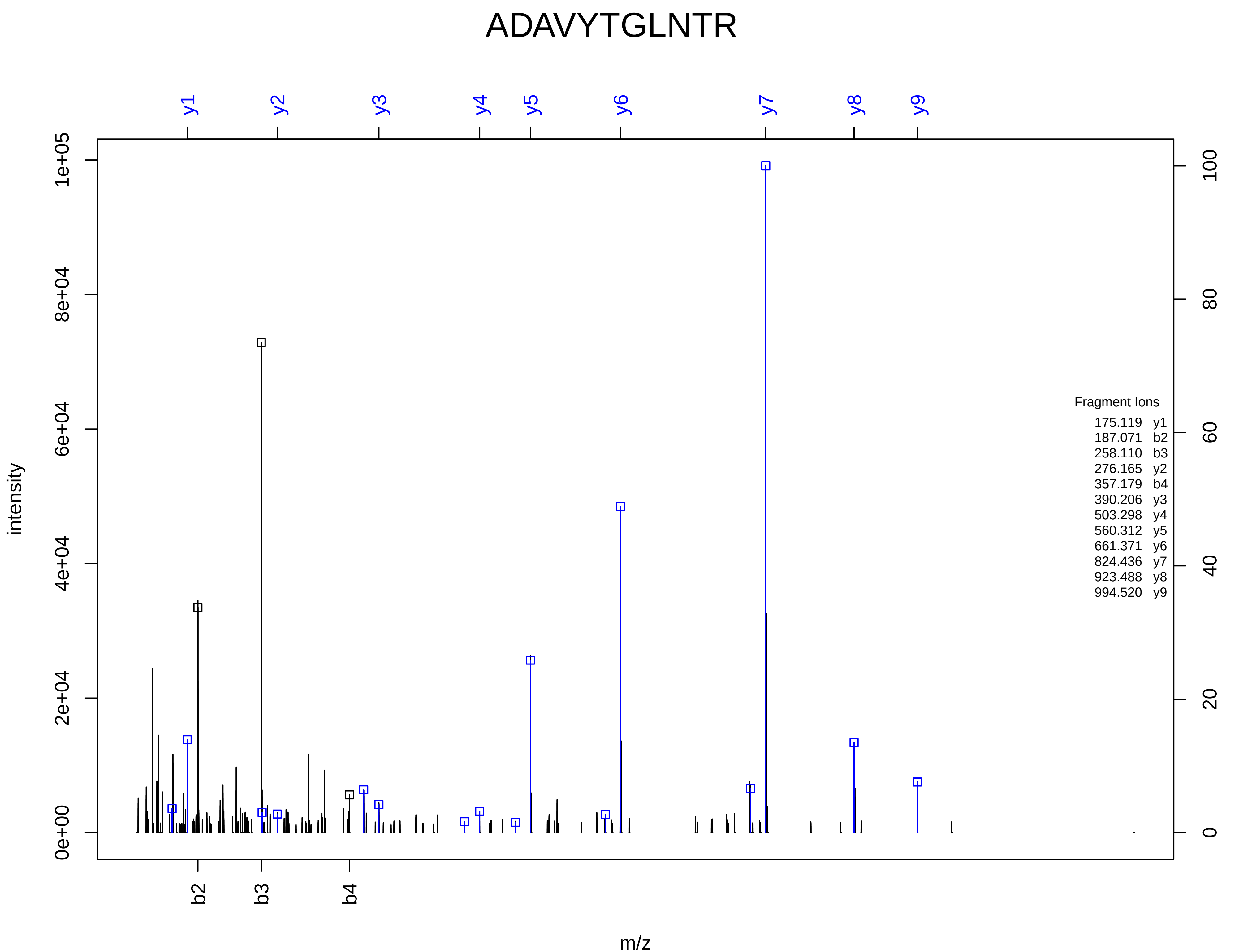
ADAVYTGLNTR
y1
y2
y3
y4
y5
y6
y7
y8
y9
b2
b3
b4
intensity
0e+00
2e+04
4e+04
6e+04
8e+04
1e+05
0
20
40
60
80
100
Fragment Ions
175.119 y1
187.071 b2
258.110 b3
276.165 y2
357.179 b4
390.206 y3
503.298 y4
560.312 y5
661.371 y6
824.436 y7
923.488 y8
994.520 y9
m/z
ADAVYTGLNTR / File: christine_30turboips_ip25.raw; SpectrumID: ; scans: 3175

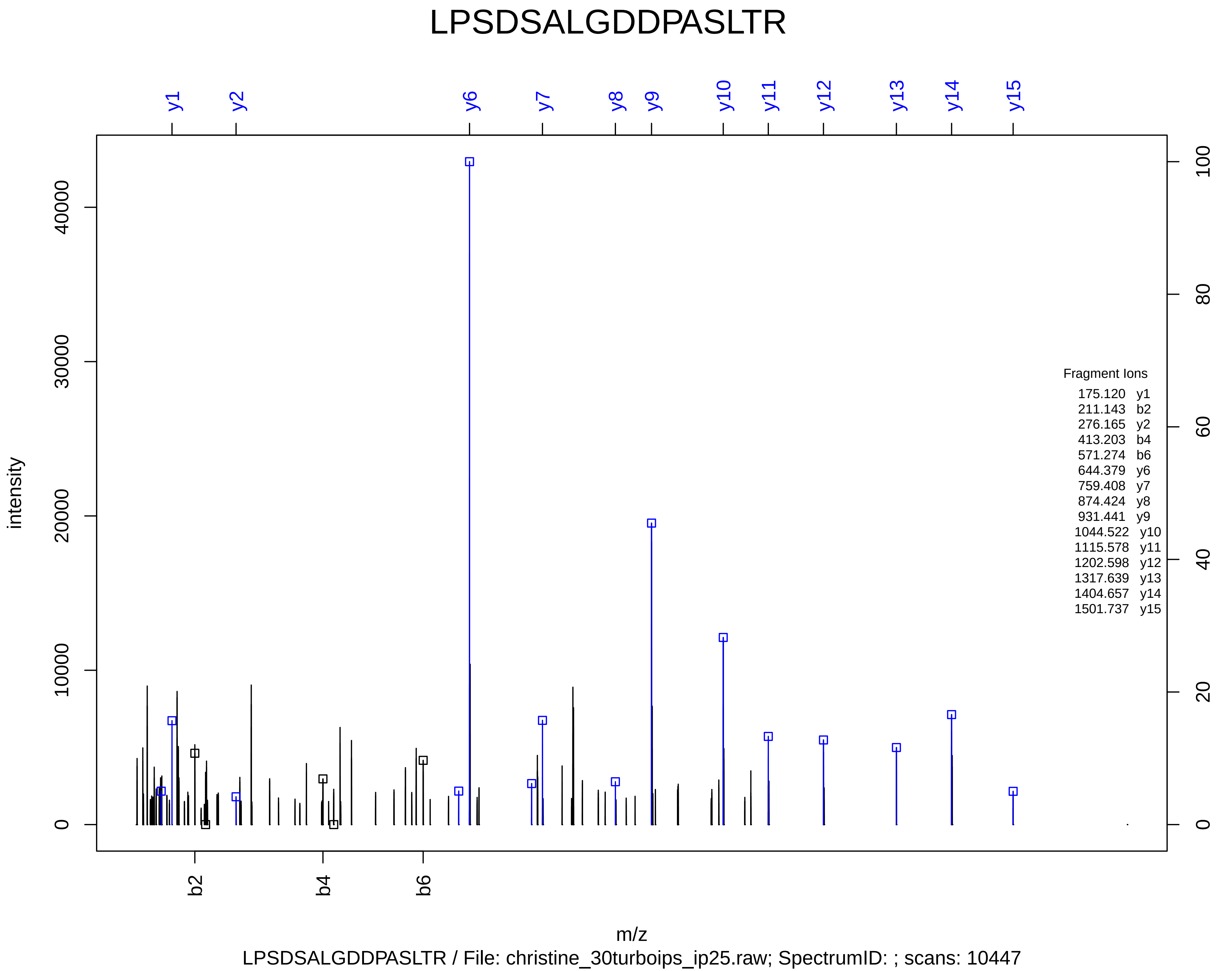
LPSDSALGDDPASLTR
y1
y2
y6
y7
y8
y9
y10
y11
y12
y13
y14
y15
intensity
40000
30000
20000
10000
0
100
80
60
40
20
0
Fragment Ions
175.120 y1
211.143 b2
276.165 y2
413.203 b4
571.274 b6
644.379 y6
759.408 y7
874.424 y8
931.441 y9
1044.522 y10
1115.578 y11
1202.598 y12
1317.639 y13
1404.657 y14
1501.737 y15
b2
b4
b6
m/z
LPSDSALGDDPASLTR / File: christine_30turboips_ip25.raw; SpectrumID: ; scans: 10447

LEPAPLDSSPAVSTHEGSK

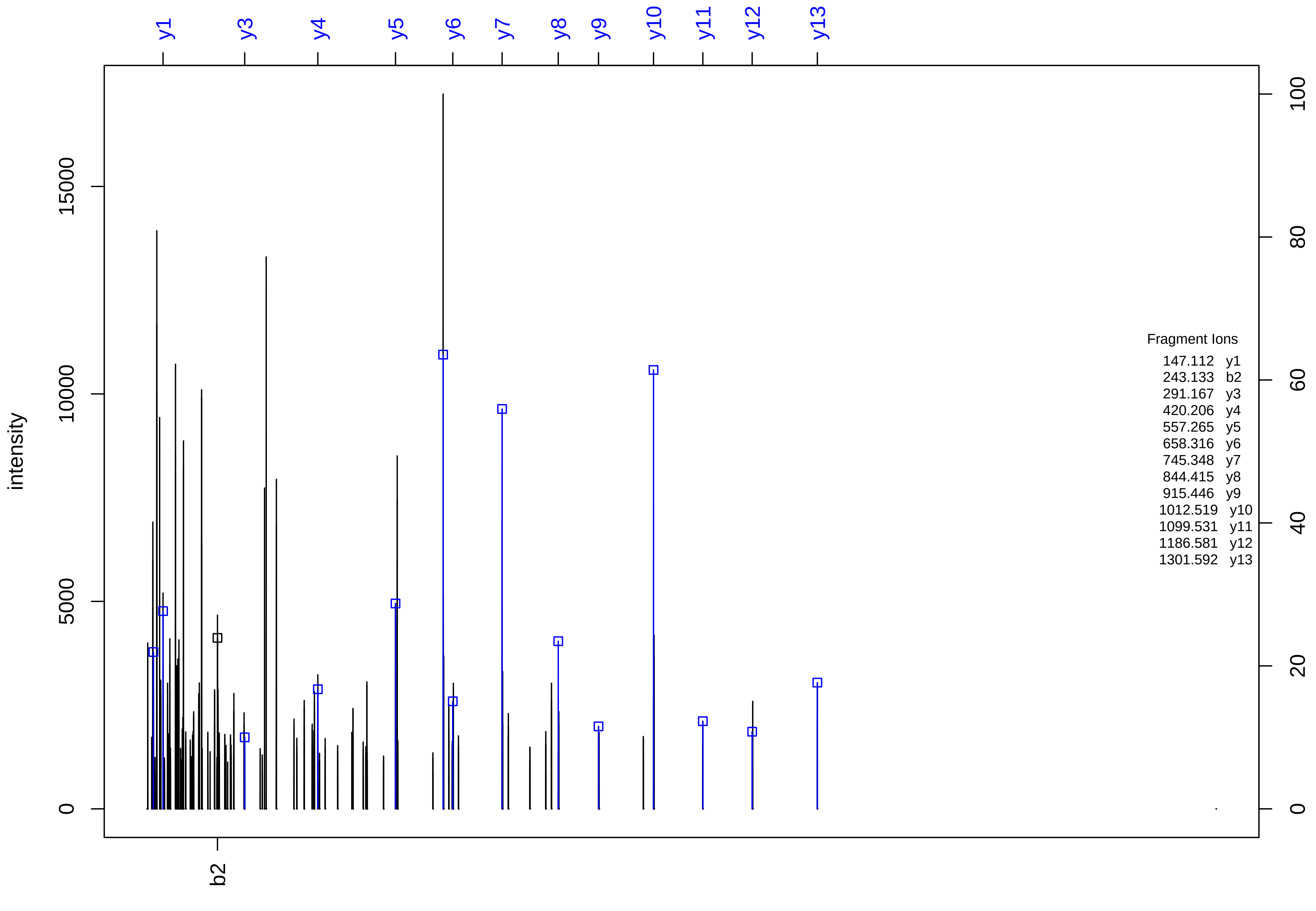

LEPAPLDSSPAVSTHEGSK / File: christine_30turboips_ip25.raw; SpectrumID: ; scans: 3626

(Ac)SHTILLVQPTK

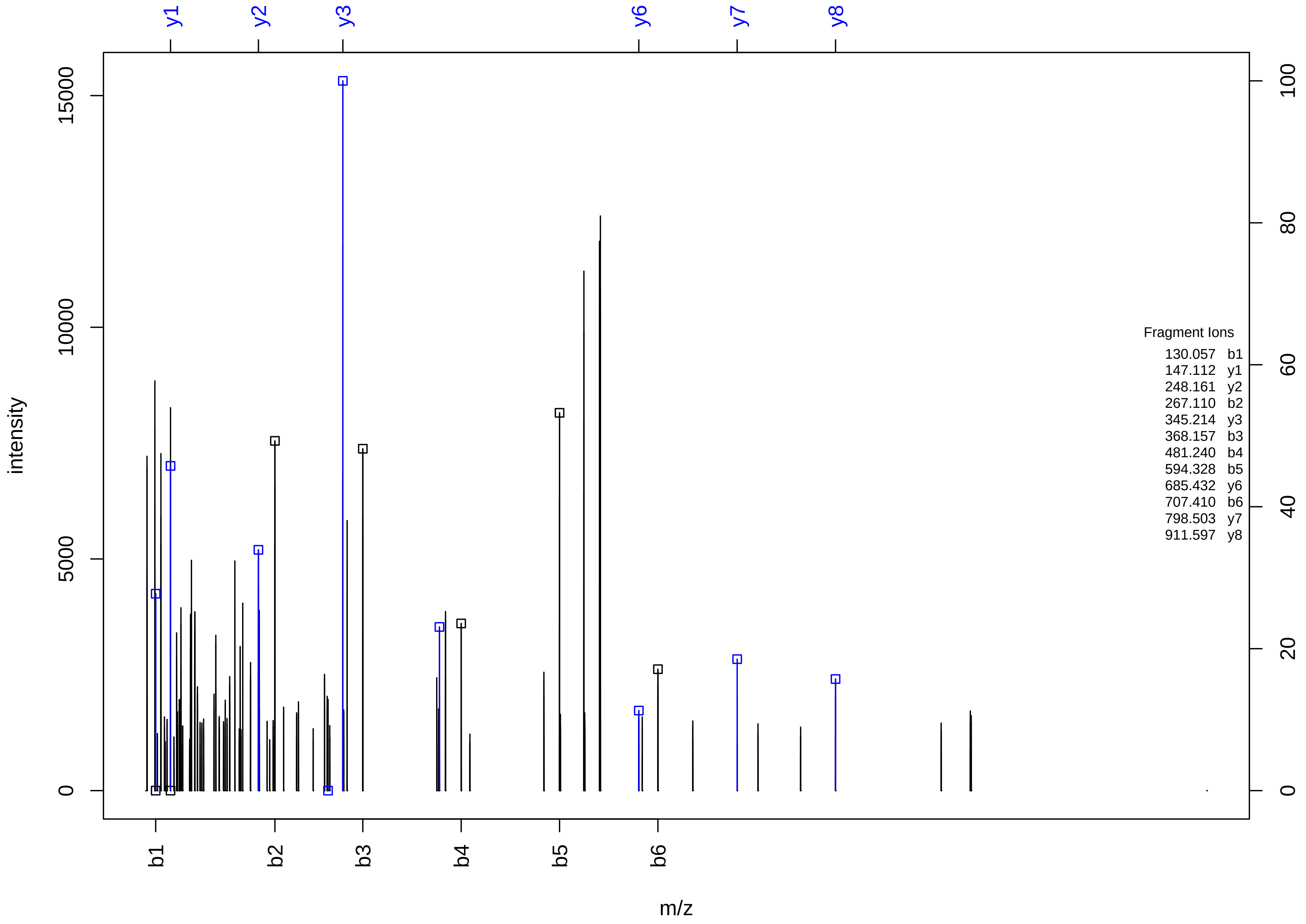

SHTILLVQPTK / File: christine_30turboips_ip25.raw; SpectrumID: ; scans: 10492

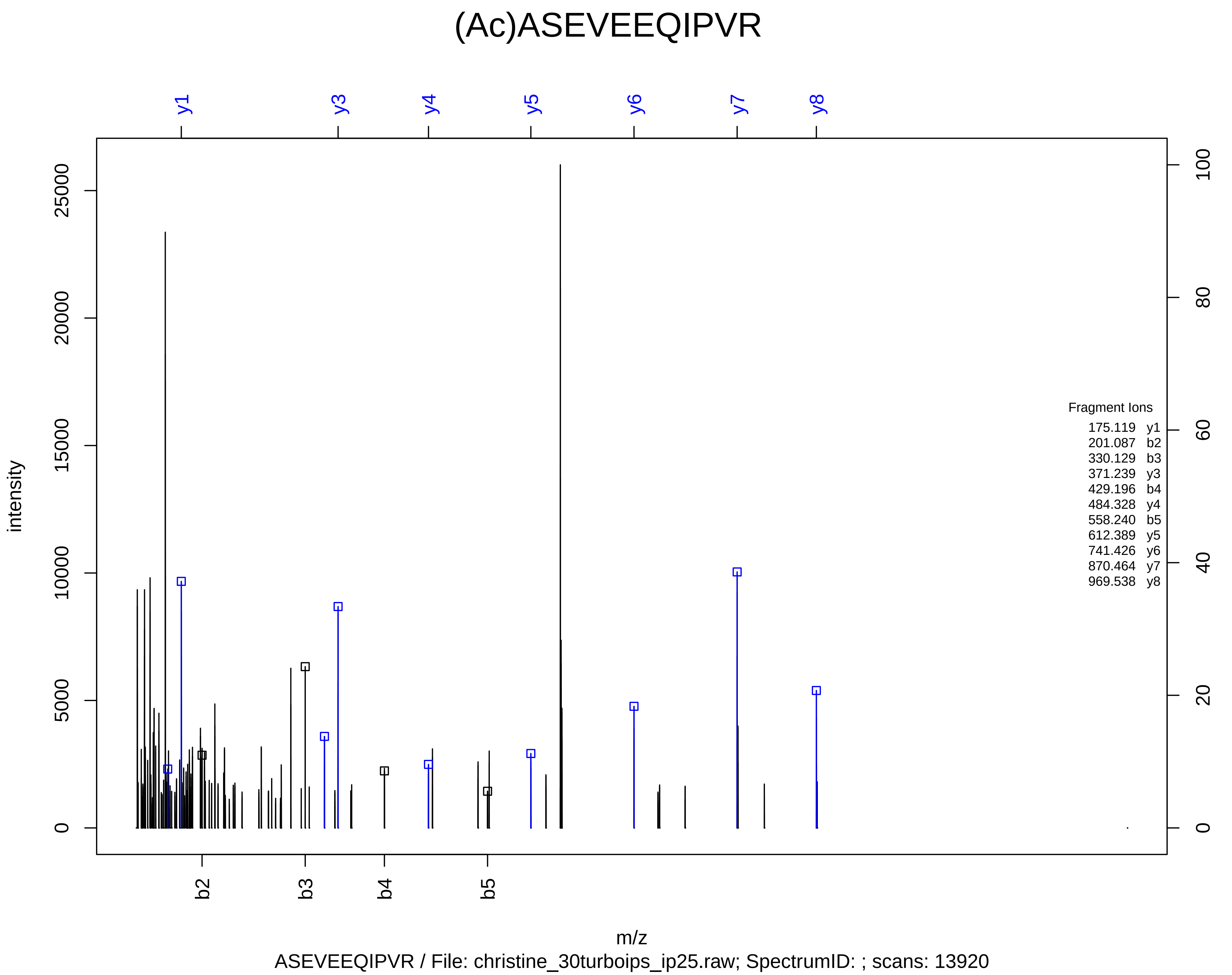
(Ac)ASEVEEQIPVR
y1
y3
y4
y5
y6
y7
y8
intensity
0
5000
10000
15000
20000
25000
0
20
40
60
80
100
b2
b3
b4
b5
m/z
Fragment Ions
175.119 y1
201.087 b2
330.129 b3
371.239 y3
429.196 b4
484.328 y4
558.240 b5
612.389 y5
741.426 y6
870.464 y7
969.538 y8
ASEVEEQIPVR / File: christine_30turboips_ip25.raw; SpectrumID: ; scans: 13920

EVVCTENLTPWK

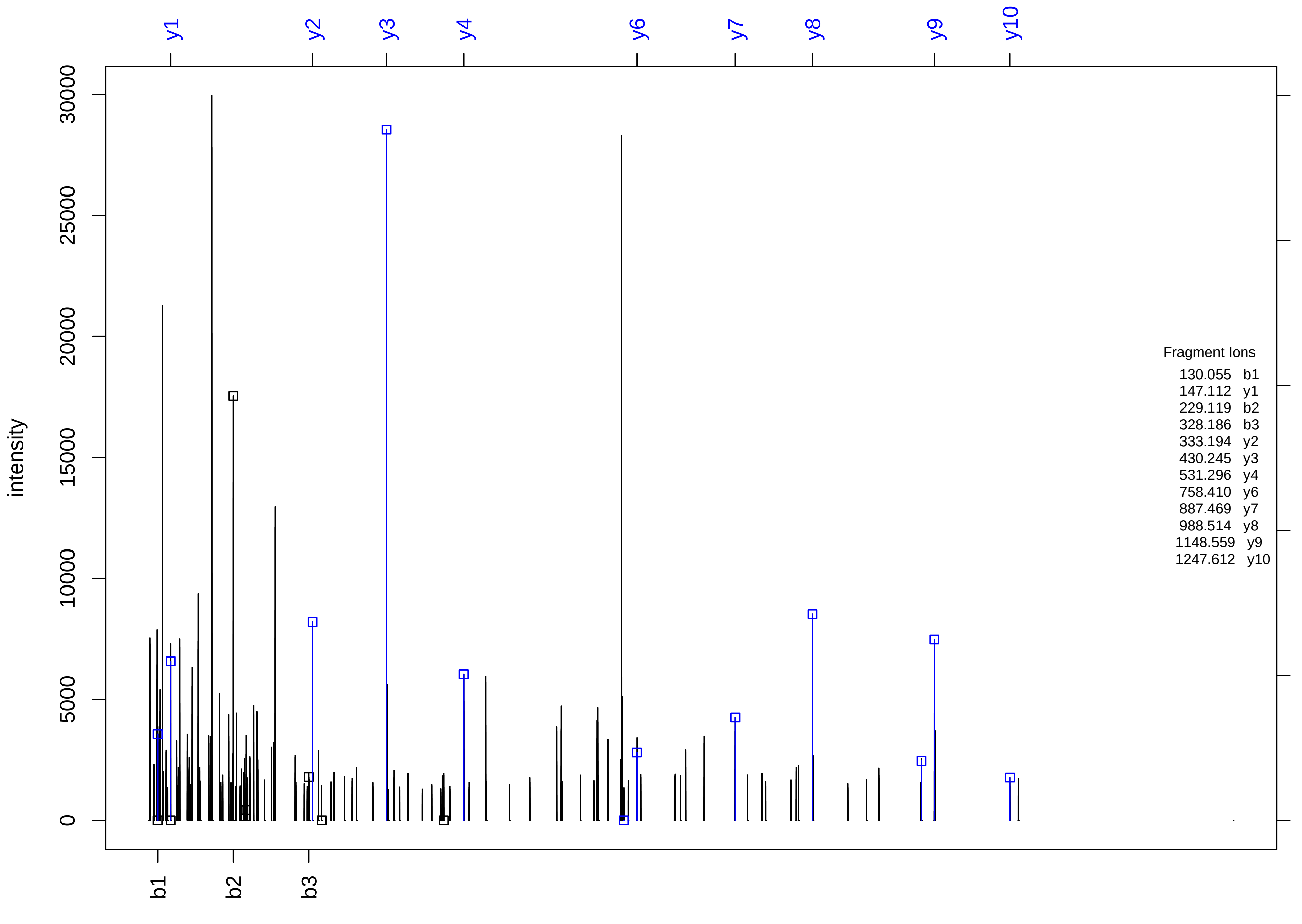

EVVCTENLTPWK / File: christine_30turboips_ip25.raw; SpectrumID: ; scans: 12456

SAIAVQGGLTELPWDGGLK

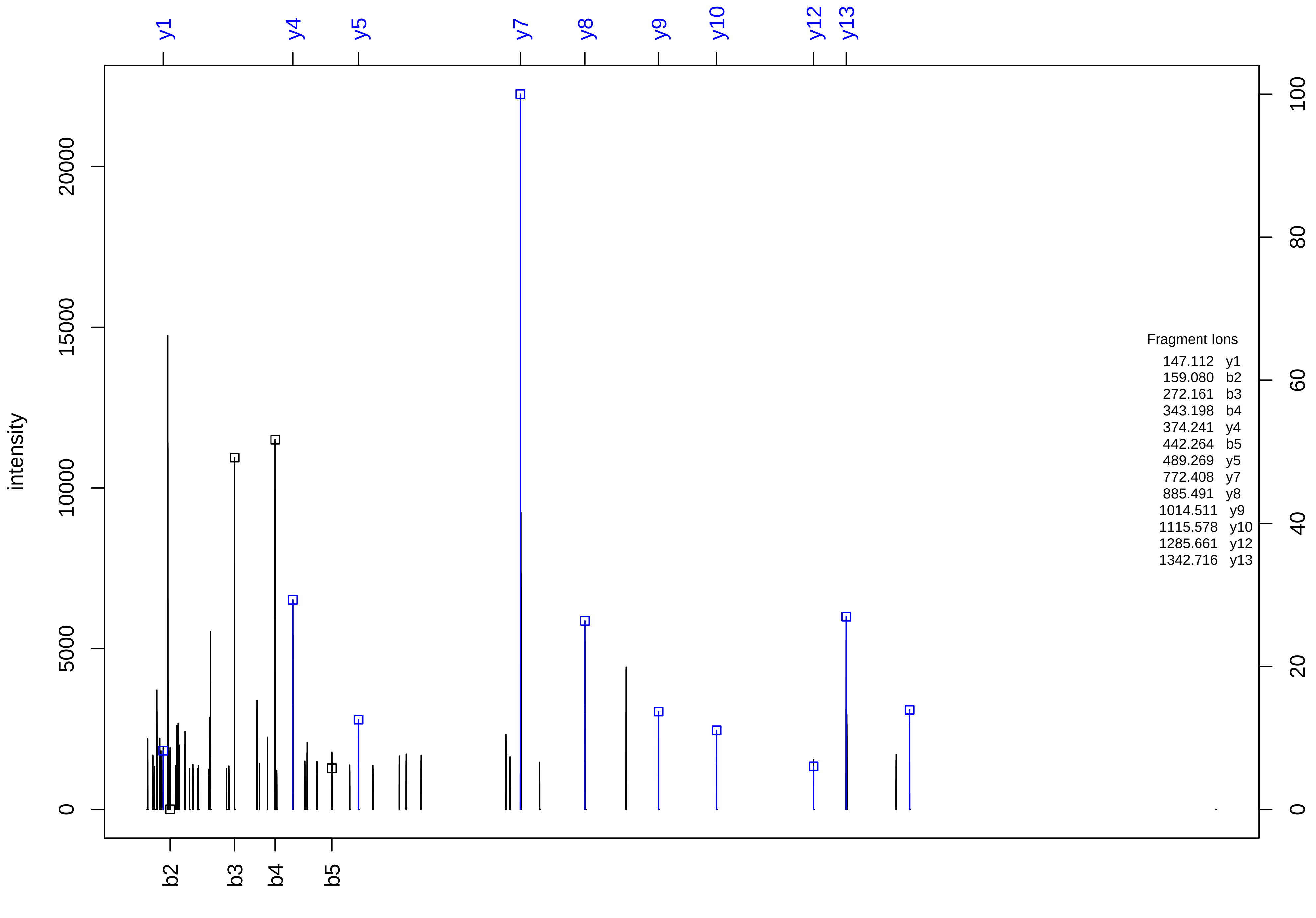

SAIAVQGGLTELPWDGGLK / File: christine_30turboips_ip25.raw; SpectrumID: ; scans: 22781

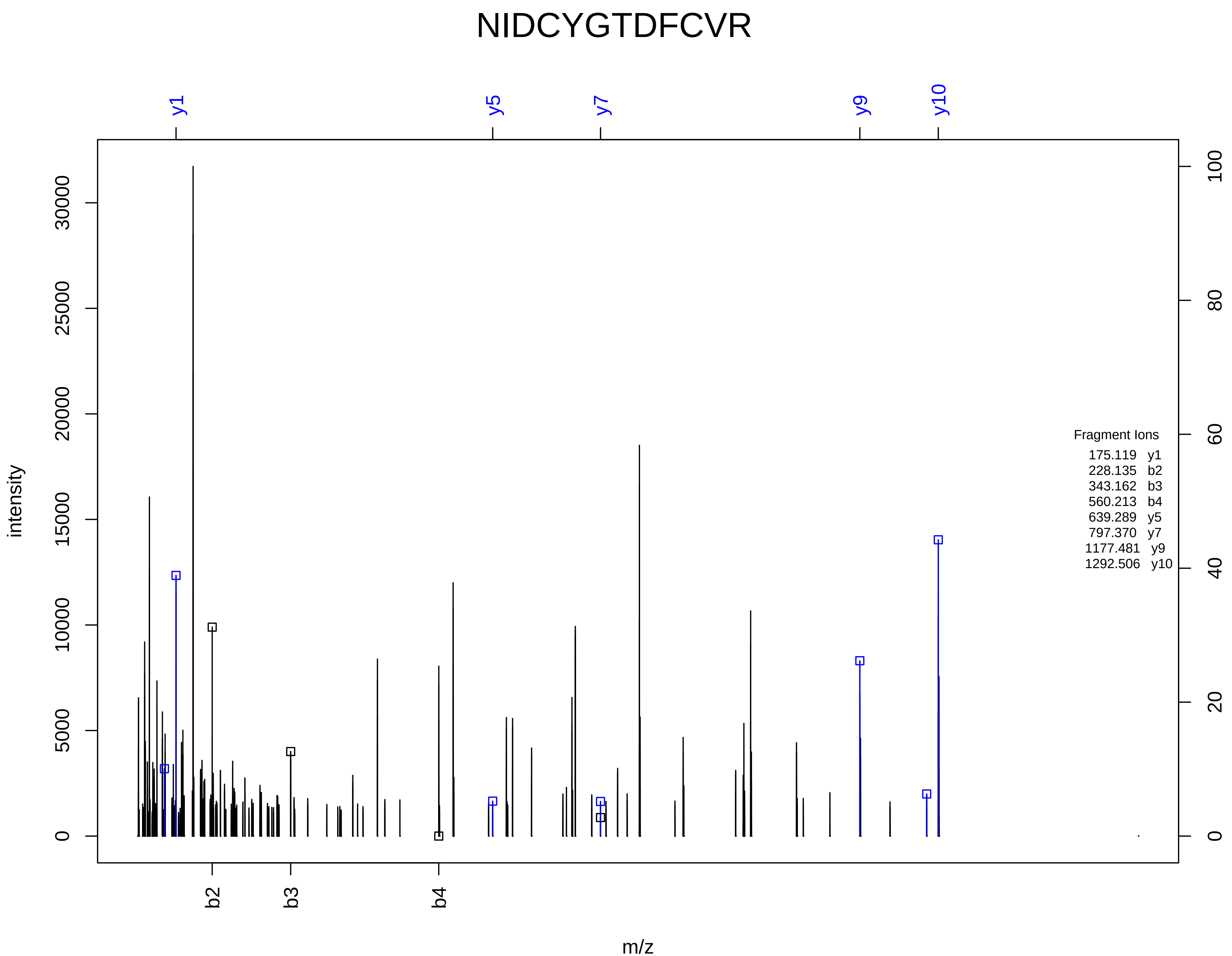

NIDCYGTDFCVR / File: christine_30turboips_ip25.raw; SpectrumID: ; scans: 10037

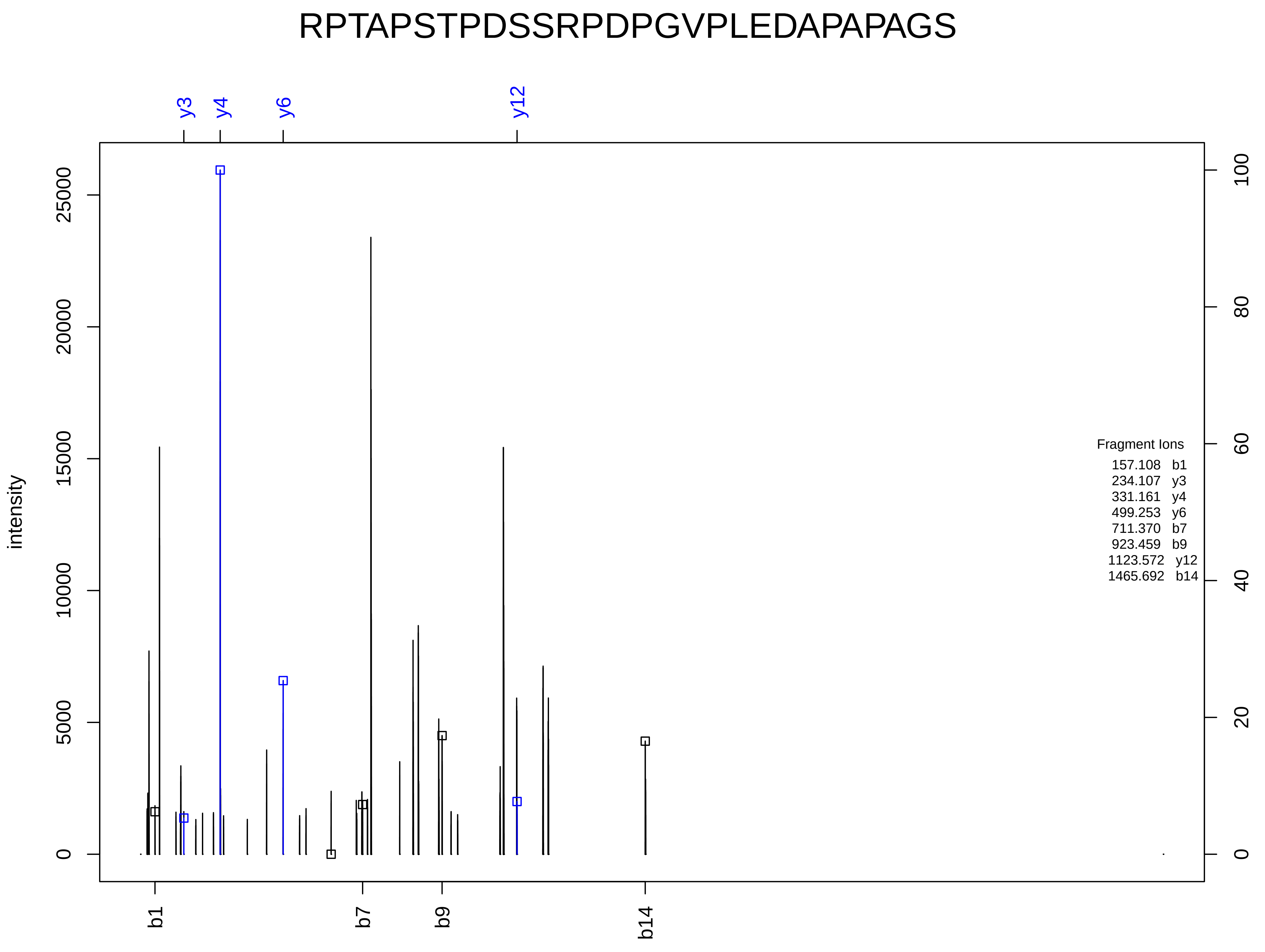

RPTAPSTPDSSRPDPGVPLEDAPAPAGS / File: christine_30turboips_ip25.raw; SpectrumID: ; scans: 7104

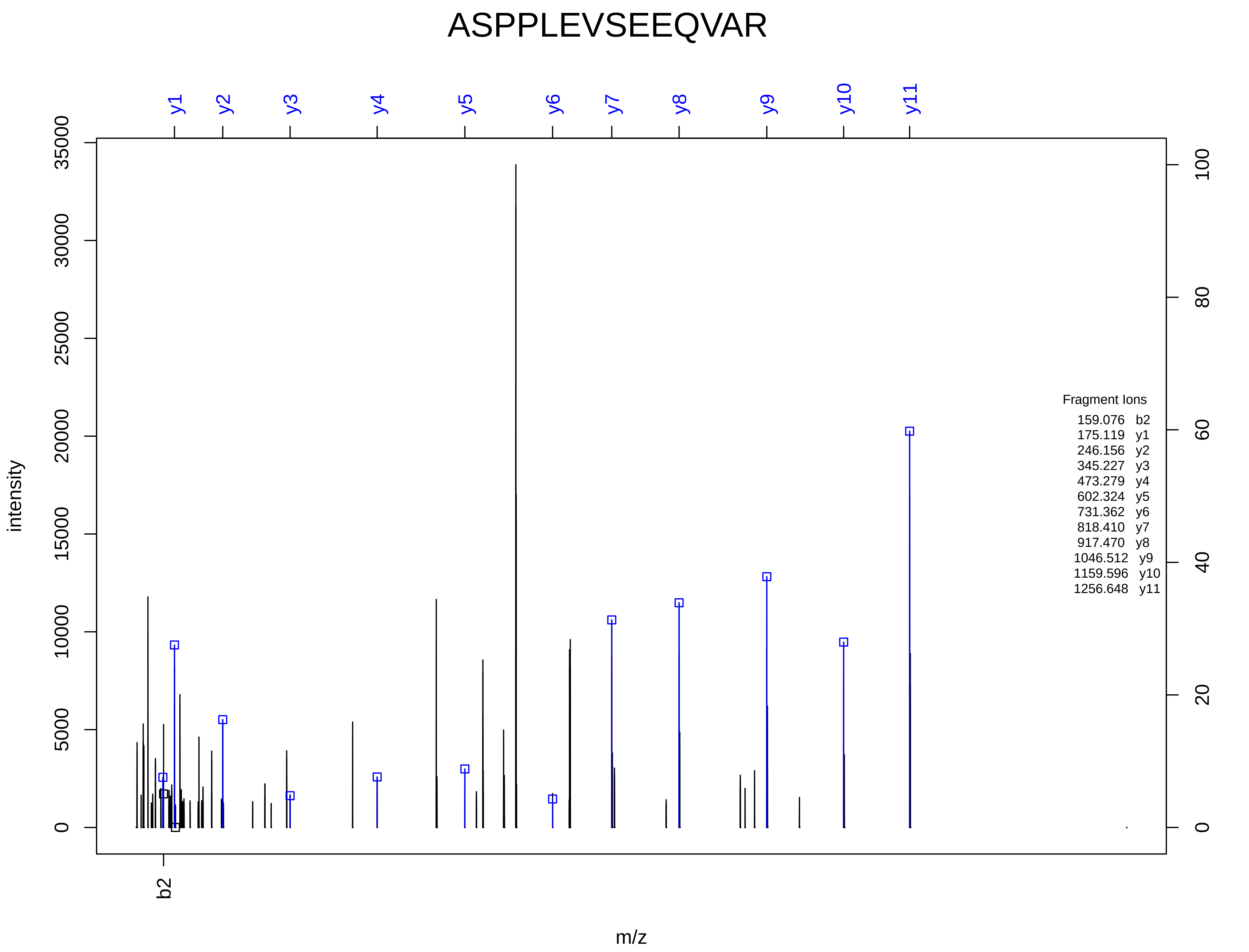
ASPPLEVSEEQVAR
y1
y2
y3
y4
y5
y6
y7
y8
y9
y10
y11
intensity
0
5000
10000
15000
20000
25000
30000
35000
0
20
40
60
80
100
b2
m/z
Fragment Ions
159.076 b2
175.119 y1
246.156 y2
345.227 y3
473.279 y4
602.324 y5
731.362 y6
818.410 y7
917.470 y8
1046.512 y9
1159.596 y10
1256.648 y11
ASPPLEVSEEQVAR / File: christine_30turboips_ip25.raw; SpectrumID: ; scans: 6336

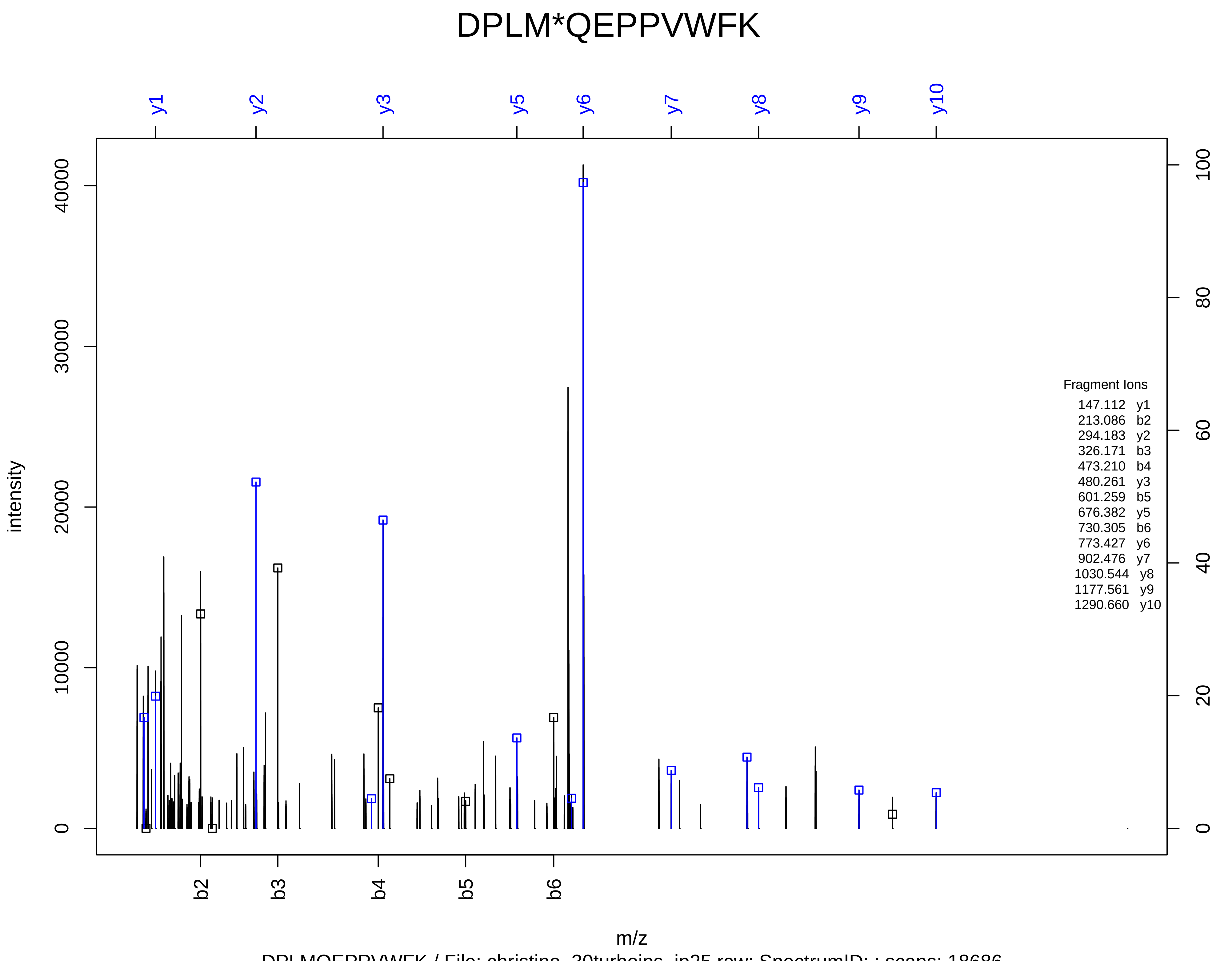
DPLM*QEPPVWFK
y1
y2
y3
y5
y6
y7
y8
y9
y10
intensity
0
10000
20000
30000
40000
0
20
40
60
80
100
b2
b3
b4
b5
b6
m/z
Fragment Ions
147.112 y1
213.086 b2
294.183 y2
326.171 b3
473.210 b4
480.261 y3
601.259 b5
676.382 y5
730.305 b6
773.427 y6
902.476 y7
1030.544 y8
1177.561 y9
1290.660 y10
DPLMQEPPVWFK / File: christine_30turboips_ip25.raw; SpectrumID: ; scans: 18686

APLCTEQFGSGAPR

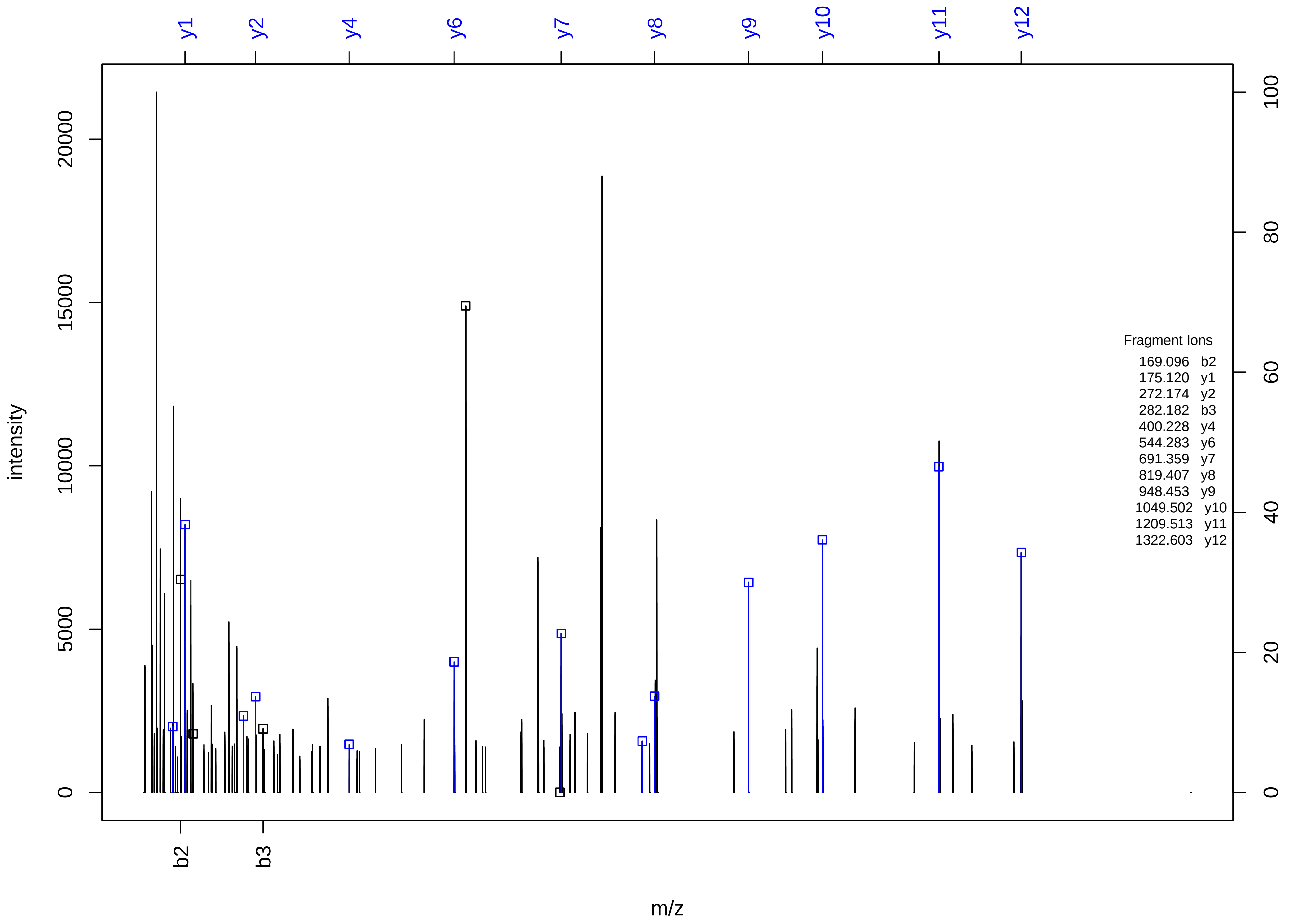

APLCTEQFGSGAPR / File: christine_30turboips_ip25.raw; SpectrumID: ; scans: 4808

SAAEVAQDVFDAVGK

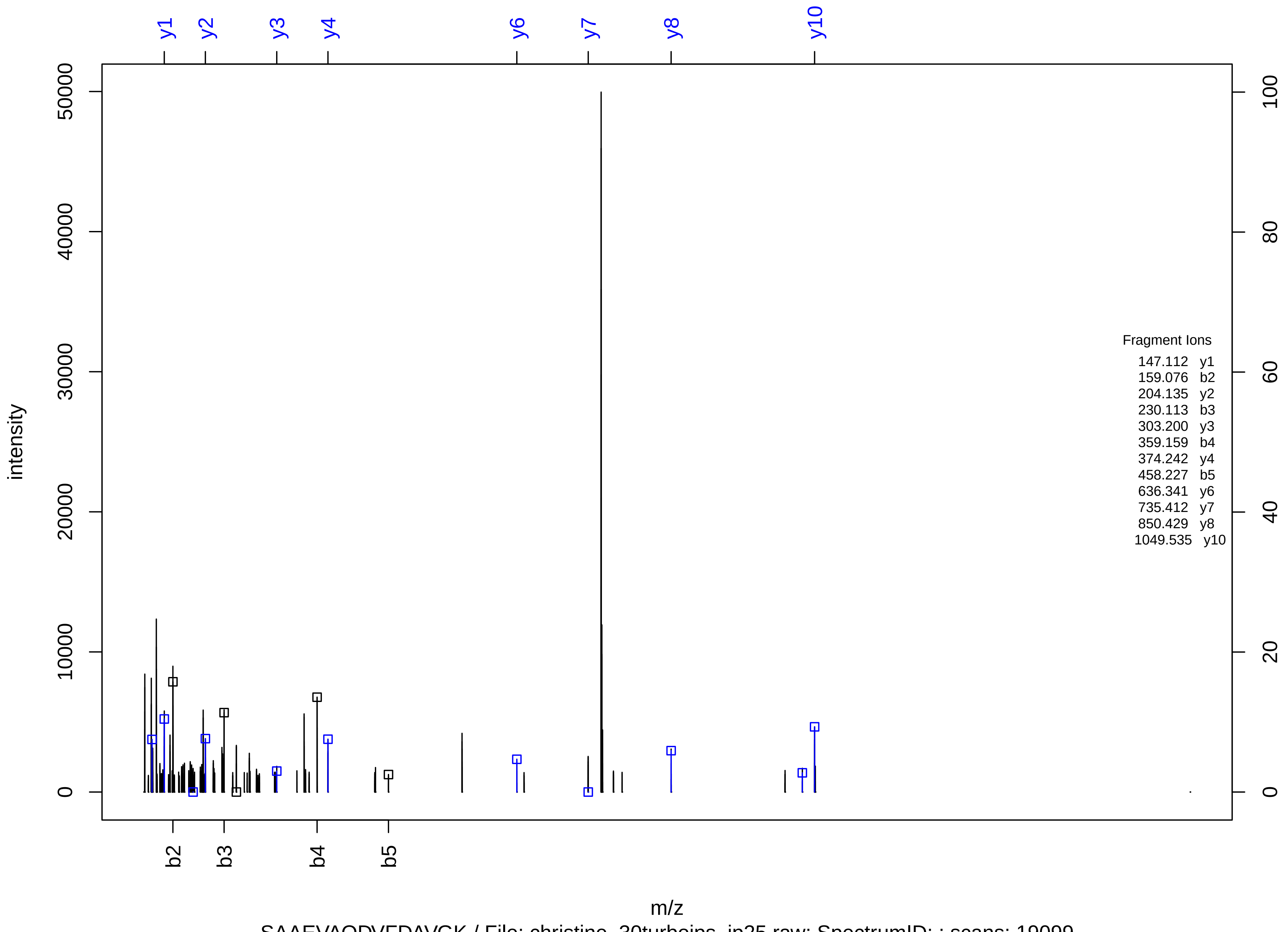

SAAEVAQDVFDAVGK / File: christine_30turboips_ip25.raw; SpectrumID: ; scans: 19099

(Ac)AGAEDAPGRQPELDEDETAEGR

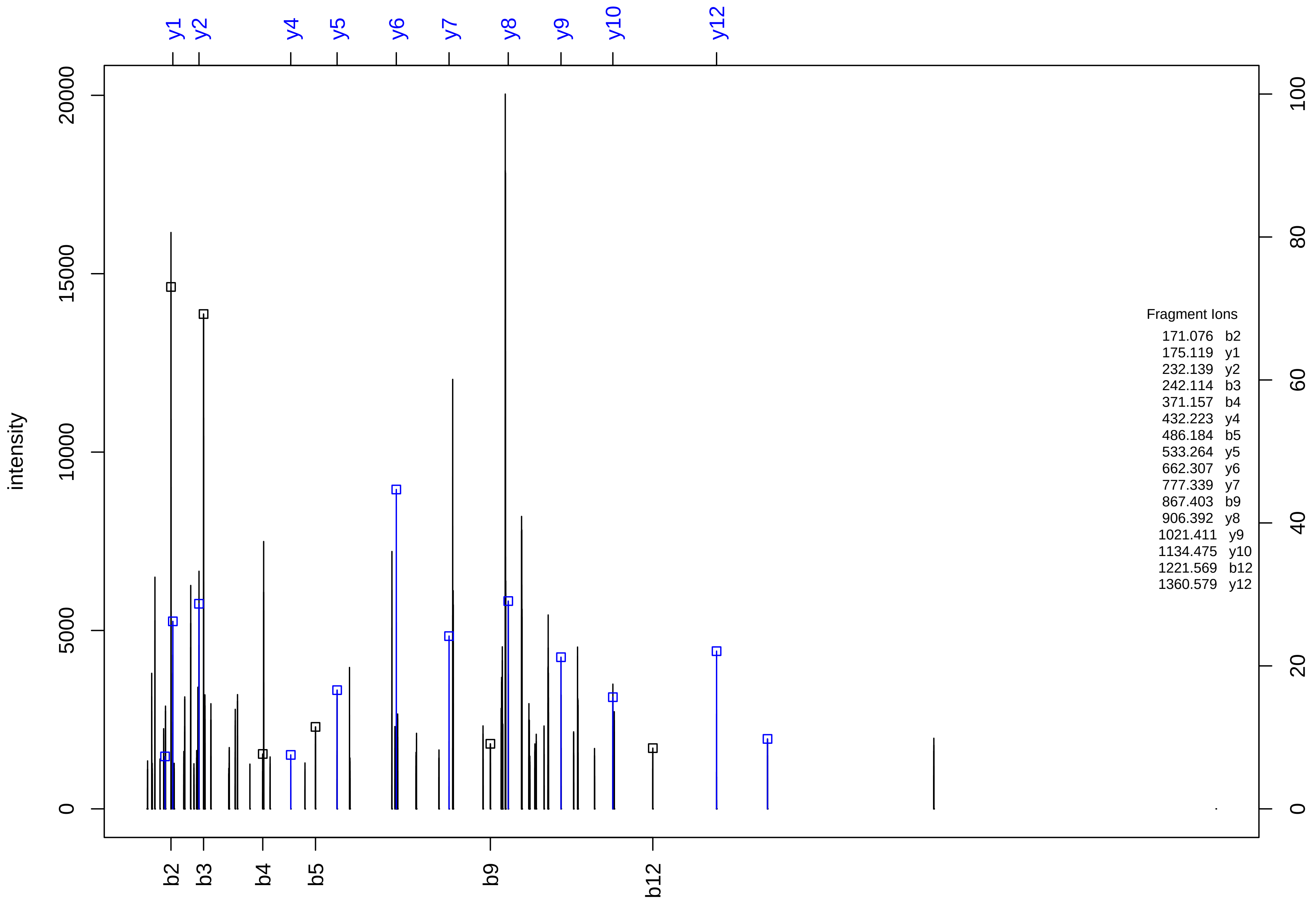

AGAEDAPGRQPELDEDETAEGR / File: christine_30turboips_ip25.raw; SpectrumID: ; scans: 5065

(Ac)AFASEDNVYHSSNAVYR

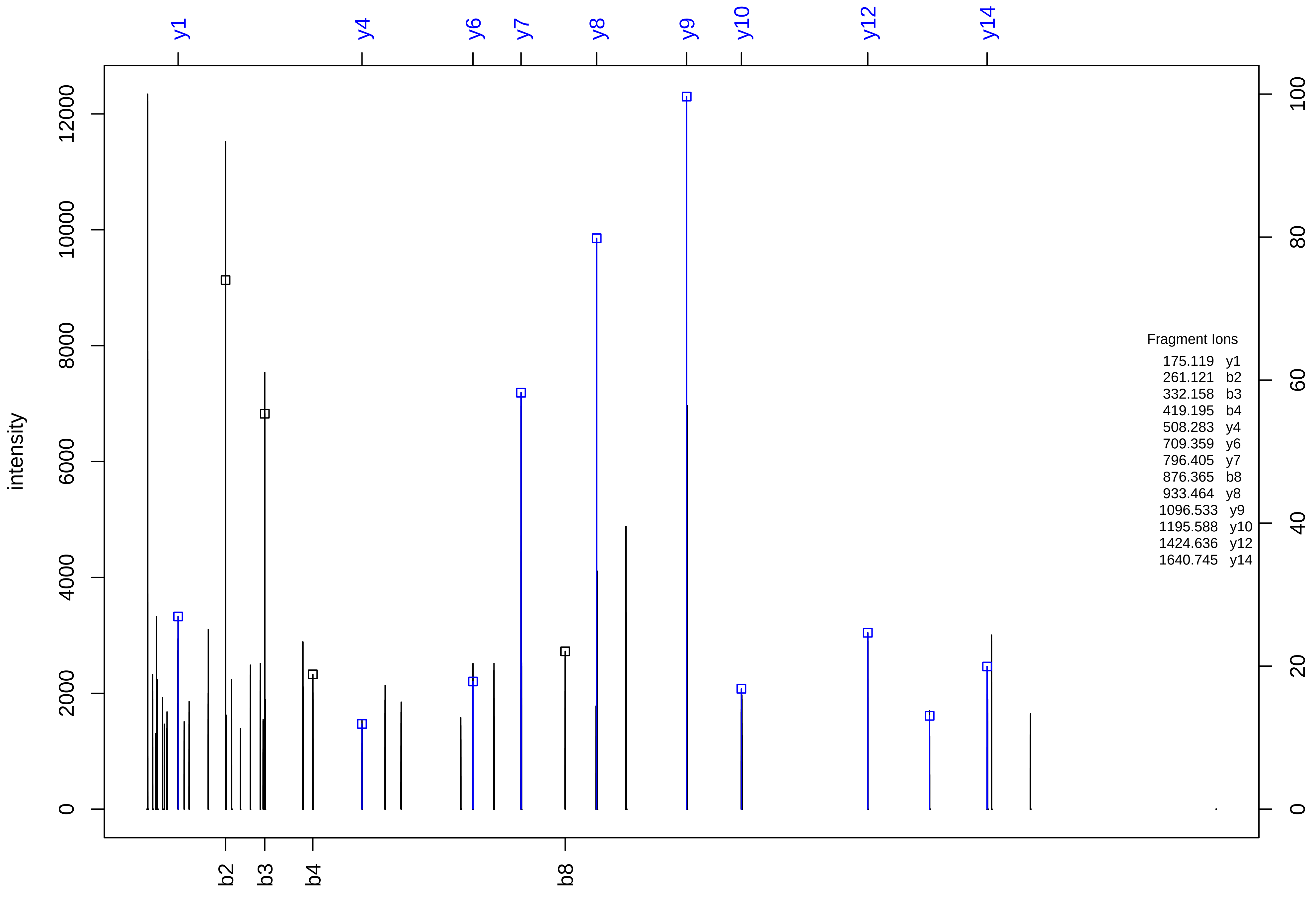

AFASEDNVYHSSNAVYR / File: christine_30turboips_ip25.raw; SpectrumID: ; scans: 10156

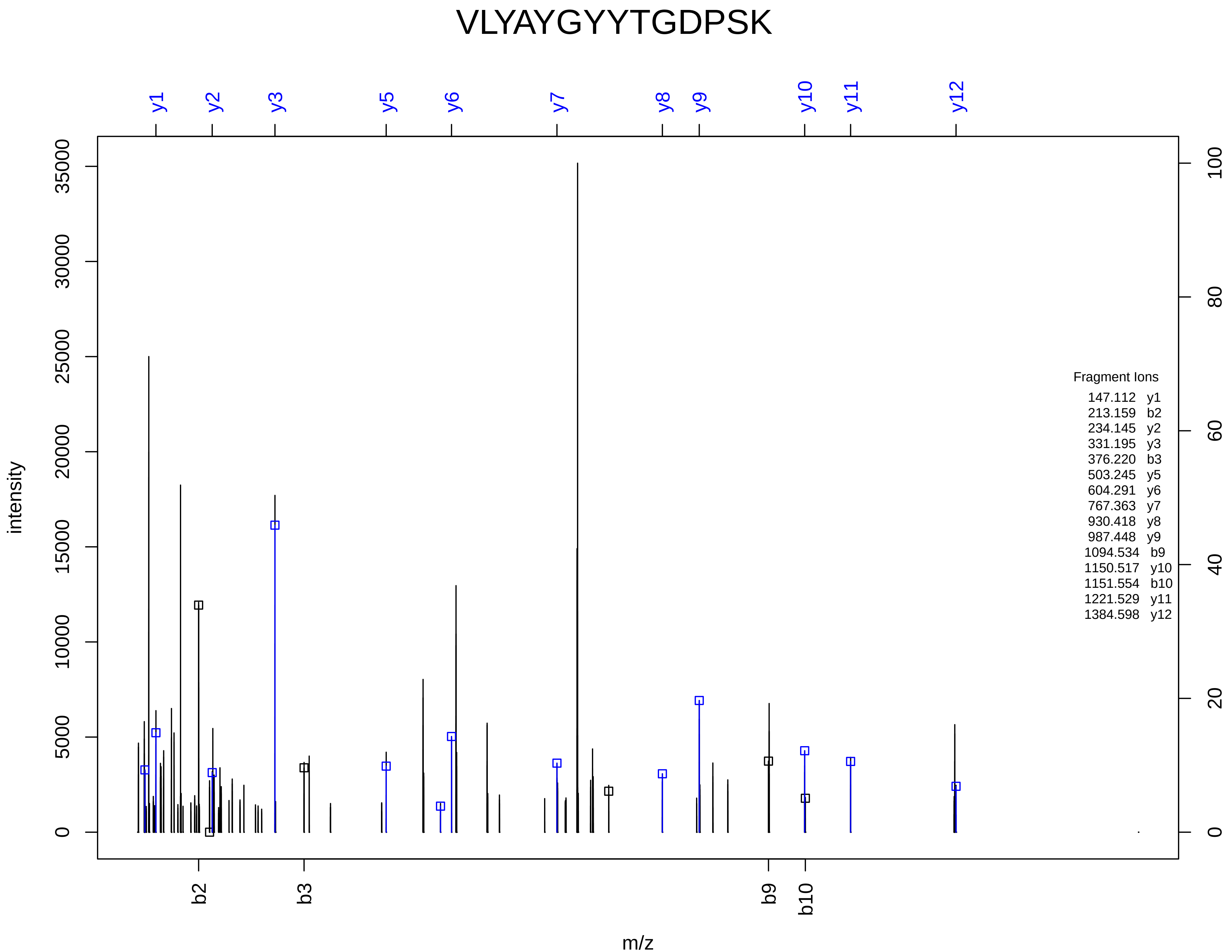

VLYAYGYYTGDPSK / File: christine_30turboips_ip25.raw; SpectrumID: ; scans: 10369

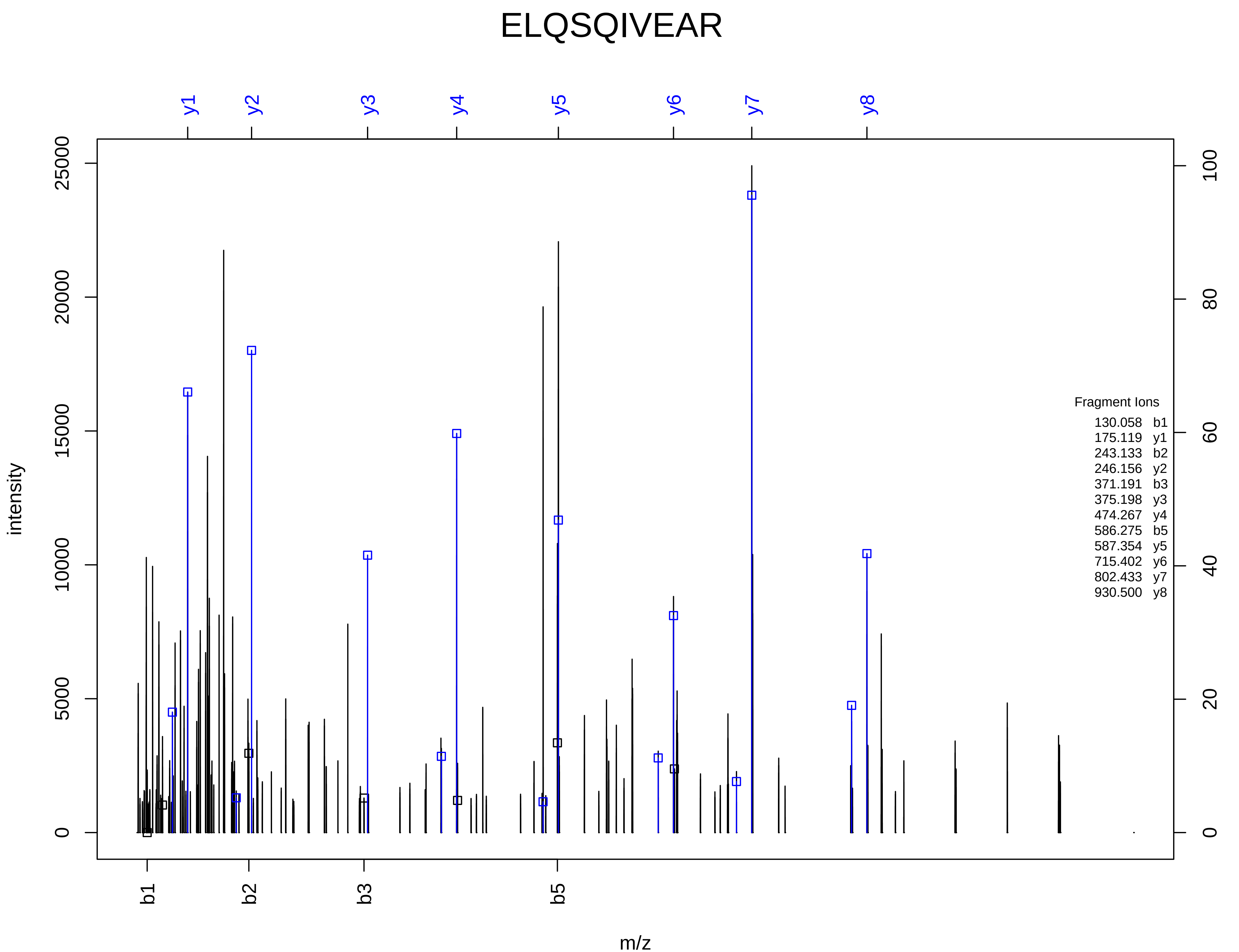

ELQSQIVEAR / File: christine_30turboips_ip25.raw; SpectrumID: ; scans: 2618

(Ac)AFSDLTSR

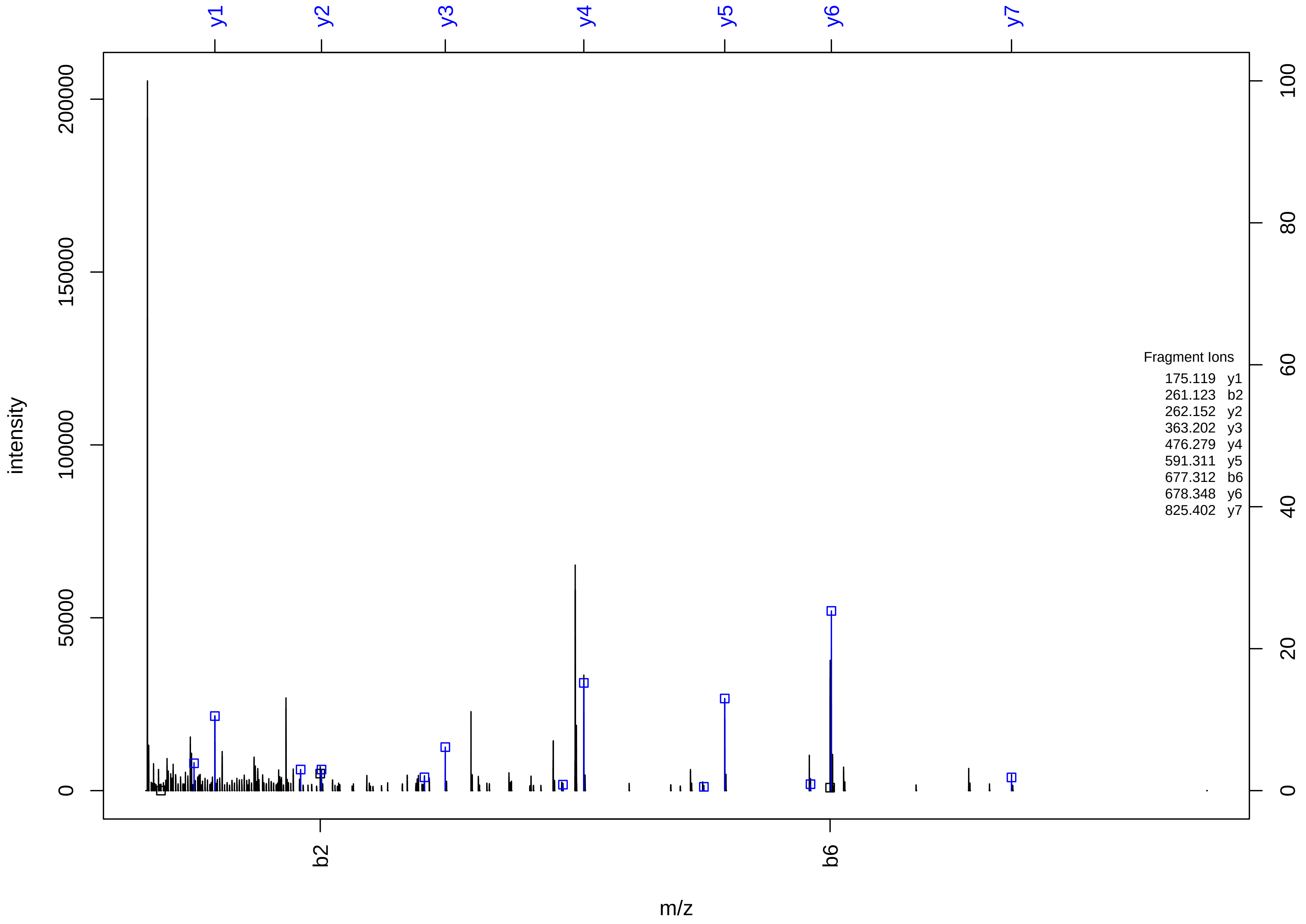

AFSDLTSR / File: christine_30turboips_ip25.raw; SpectrumID: ; scans: 13994

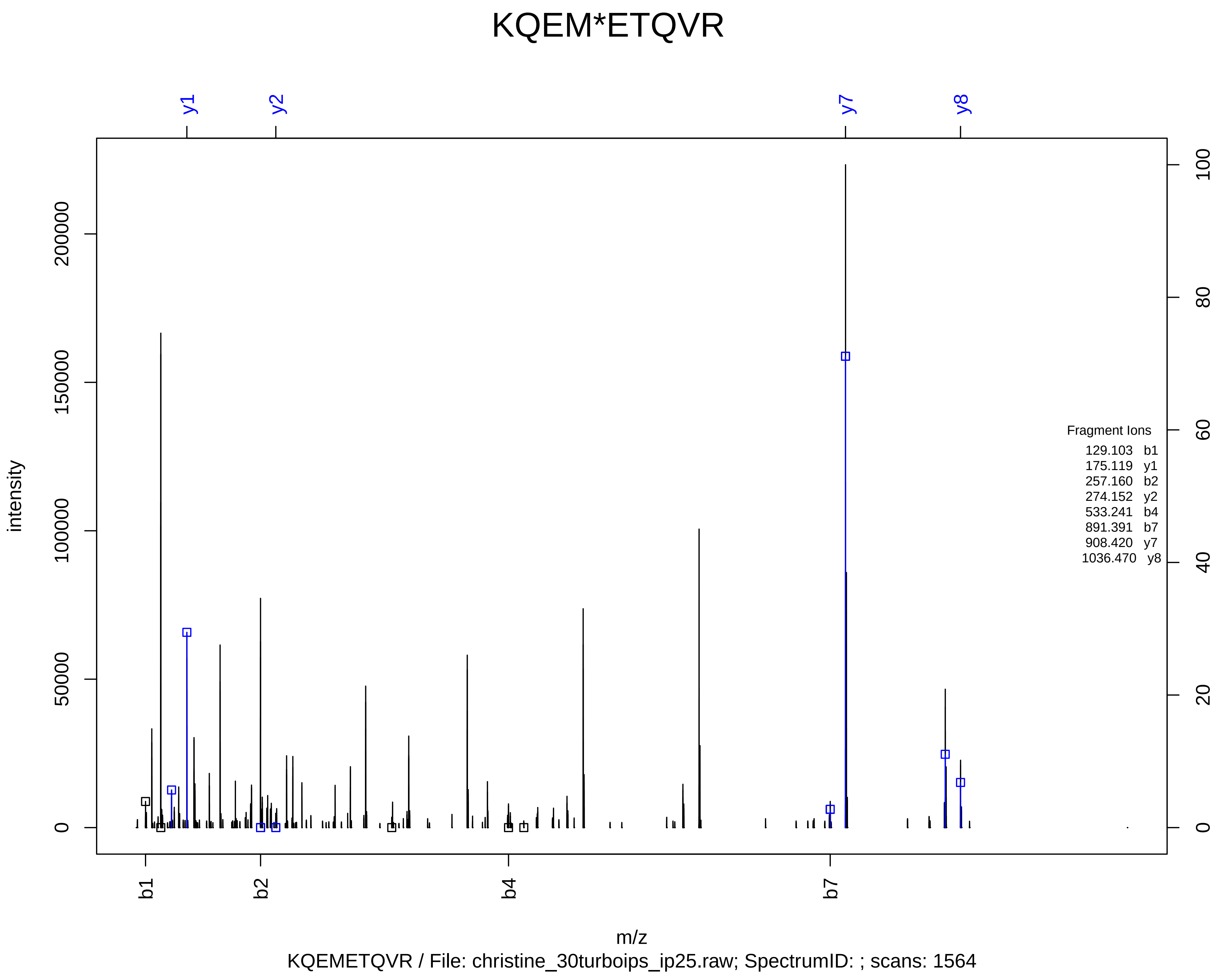

KQEM*ETQVR
y1
y2
y7
y8
intensity
0
50000
100000
150000
200000
0
20
40
60
80
100
Fragment Ions
129.103 b1
175.119 y1
257.160 b2
274.152 y2
533.241 b4
891.391 b7
908.420 y7
1036.470 y8
b1
b2
b4
b7
m/z
KQEMETQVR / File: christine_30turboips_ip25.raw; SpectrumID: ; scans: 1564

(Ac)M*ASN^VTNKTDPRSM*NSR

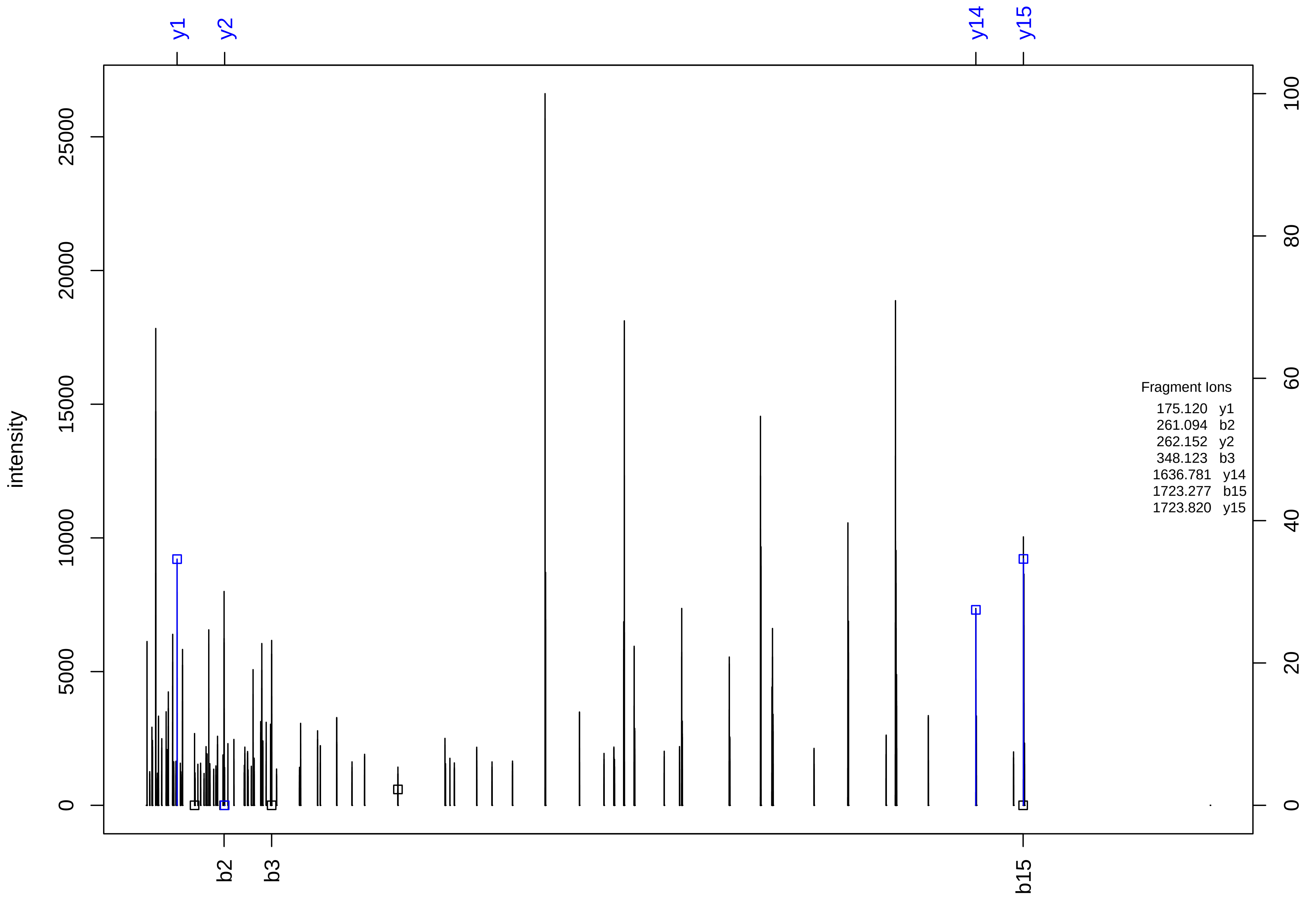

MASNVTNKTDPRSMNSR / File: christine_30turboips_ip25.raw; SpectrumID: ; scans: 14055

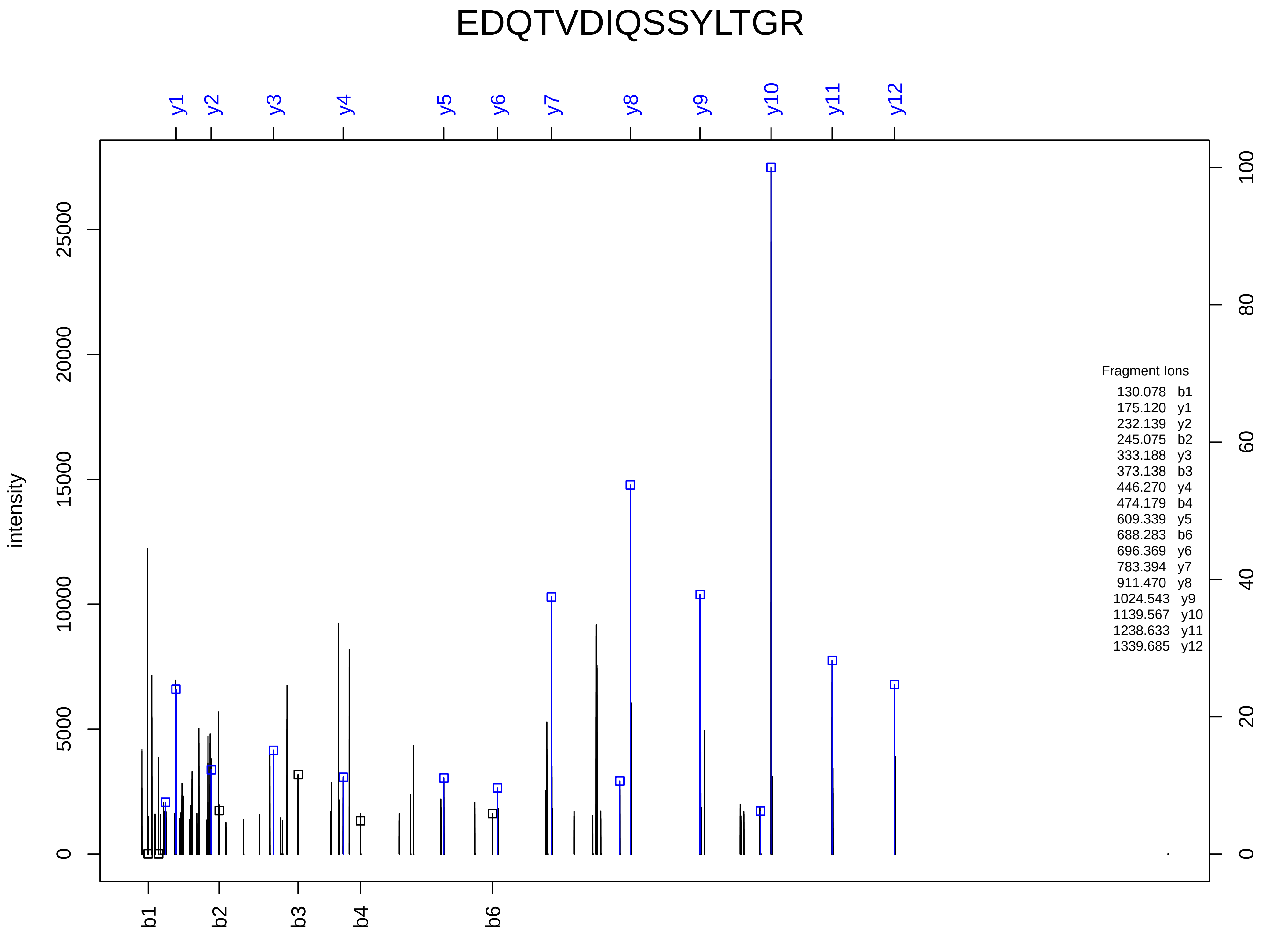

EDQTVDIQSSYLTGR / File: christine_30turboips_ip25.raw; SpectrumID: ; scans: 10304

KTPN^PANQYQFDKV

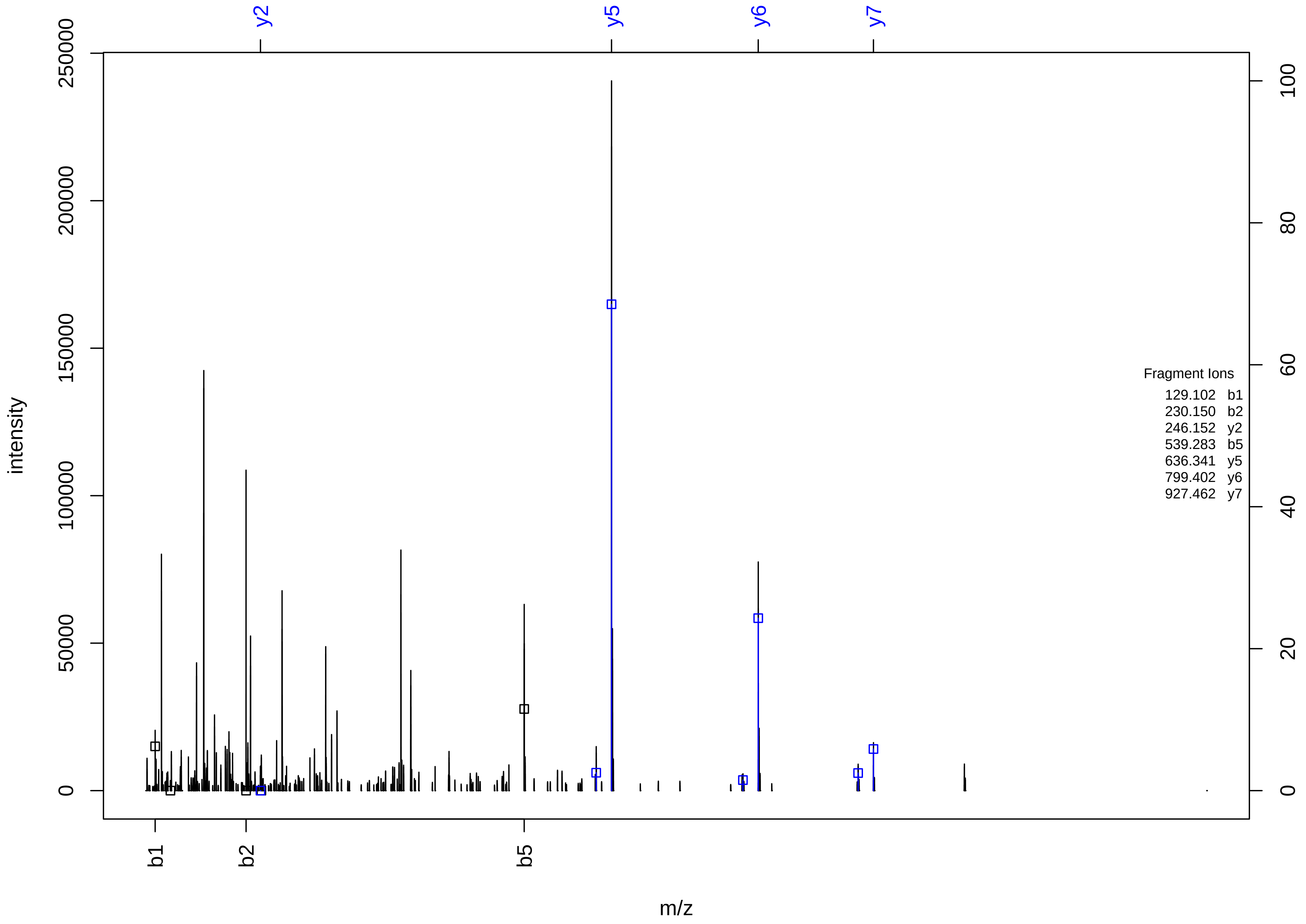

KTPNPANQYQFDKV / File: christine_30turboips_ip25.raw; SpectrumID: ; scans: 1501

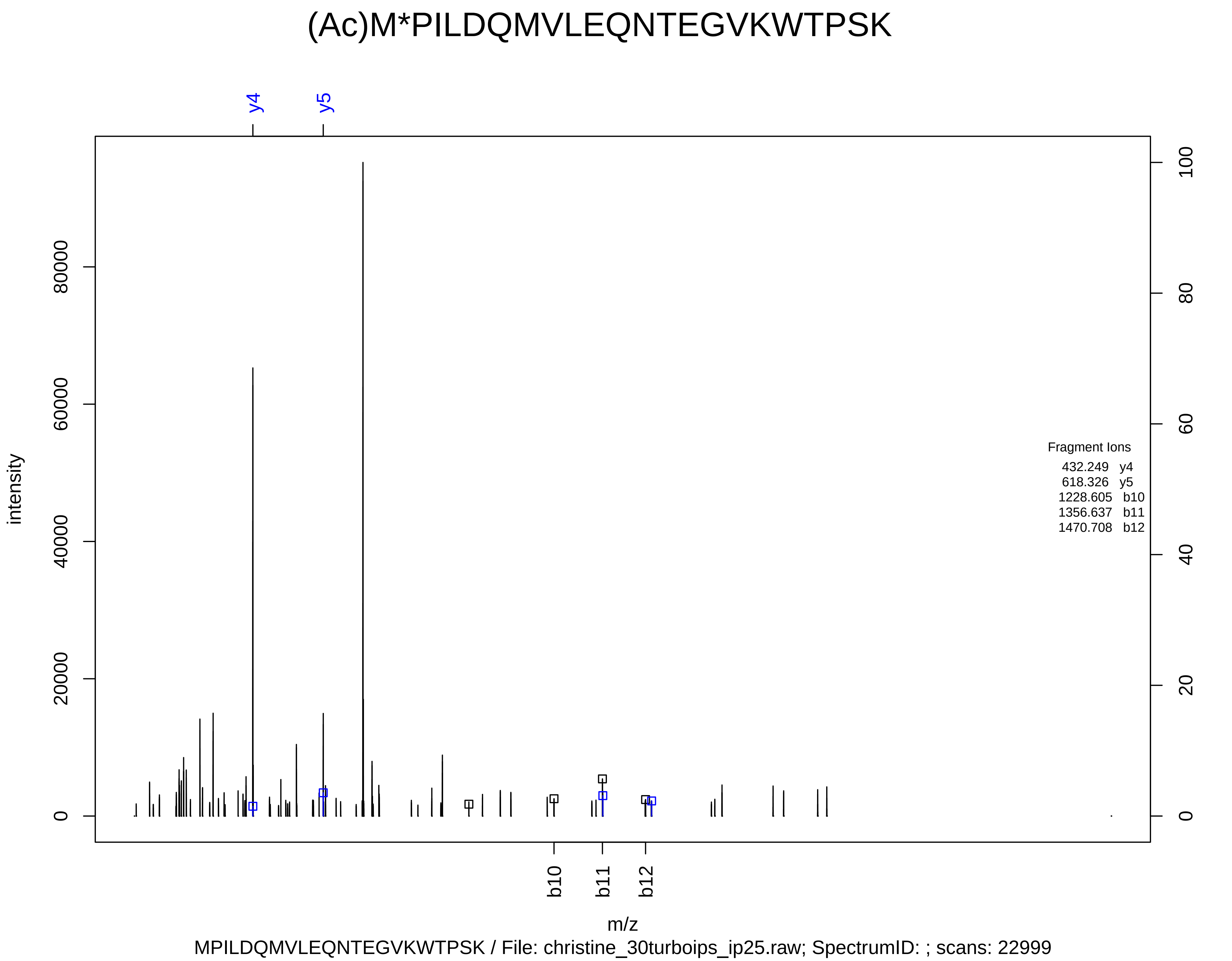

(Ac)M*PILDQMVLEQNTEGVKWTPSK
y4
y5
intensity
0
20000
40000
60000
80000
0
20
40
60
80
100
Fragment Ions
432.249 y4
618.326 y5
1228.605 b10
1356.637 b11
1470.708 b12
b10
b11
b12
m/z
MPILDQMVLEQNTEGVKWTPSK / File: christine_30turboips_ip25.raw; SpectrumID: ; scans: 22999
