# Supplementary material for: Proximity Labeling Proteomics Reveals Kv1.3 Potassium Channel Immune Interactors in Microglia
Source: Mol Cell Proteomics. 2024 Jun 25;23(8):100809. doi: 10.1016/j.mcpro.2024.100809 (PMC11780389; doi:10.1016/j.mcpro.2024.100809)
Supplement: OHW_HEK_AP [file mmc10.pdf]

ENVLEN^LQKER

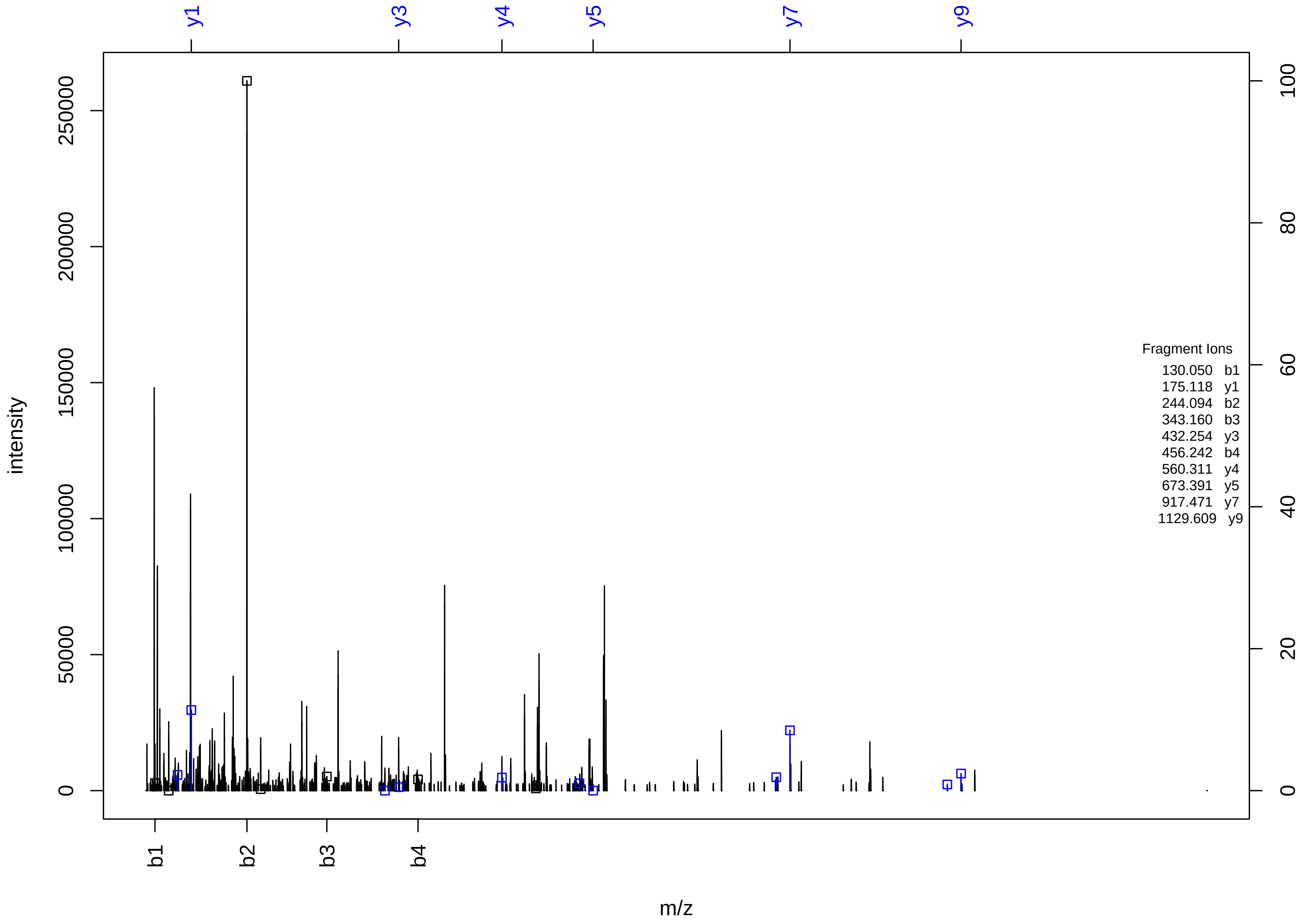

ENVLENLQKER / File: christine_ip_01.raw; SpectrumID: ; scans: 8440

ILYNPLQGQK

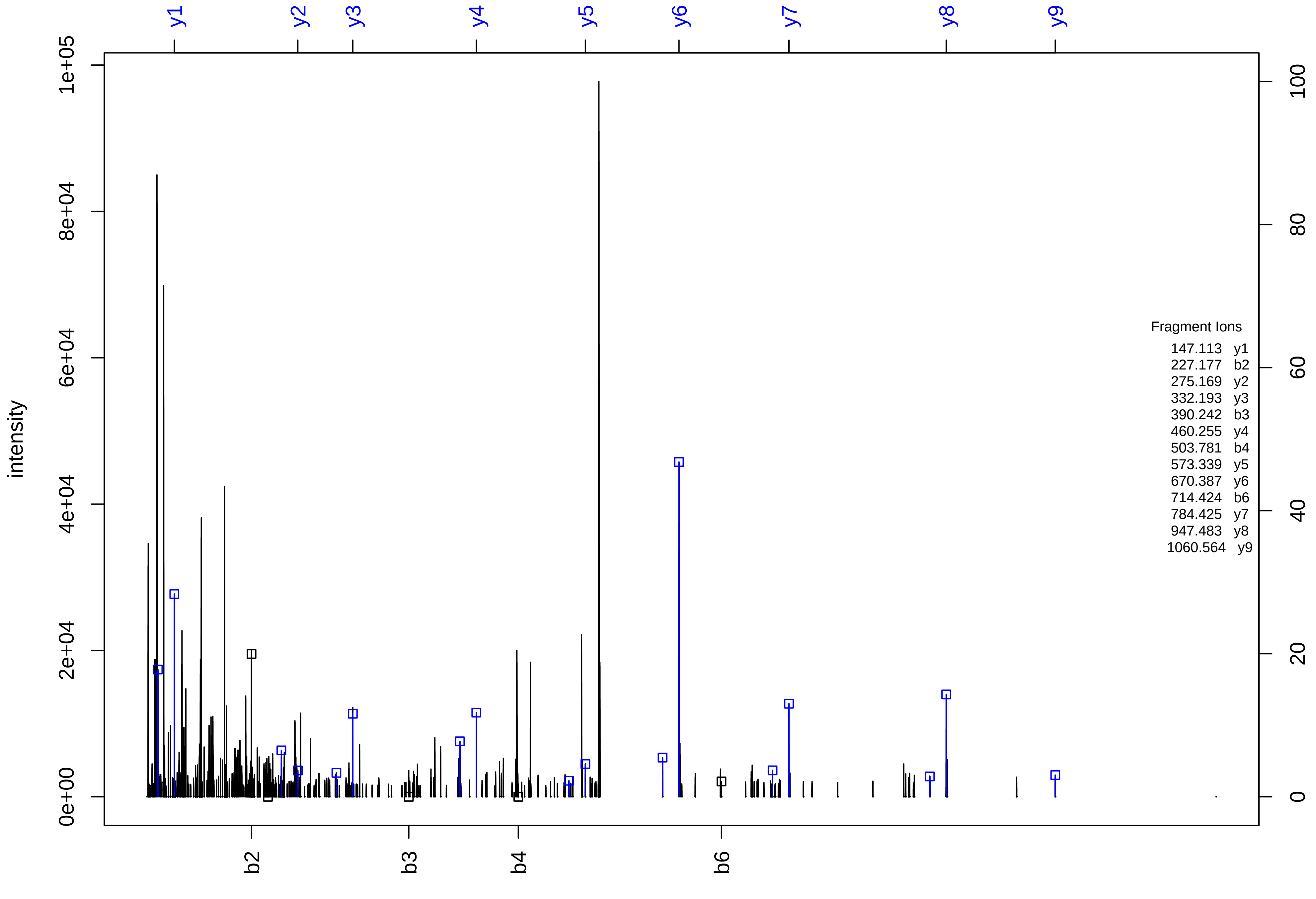

ILYNPLQGQK / File: christine_ip_01.raw; SpectrumID: ; scans: 8632

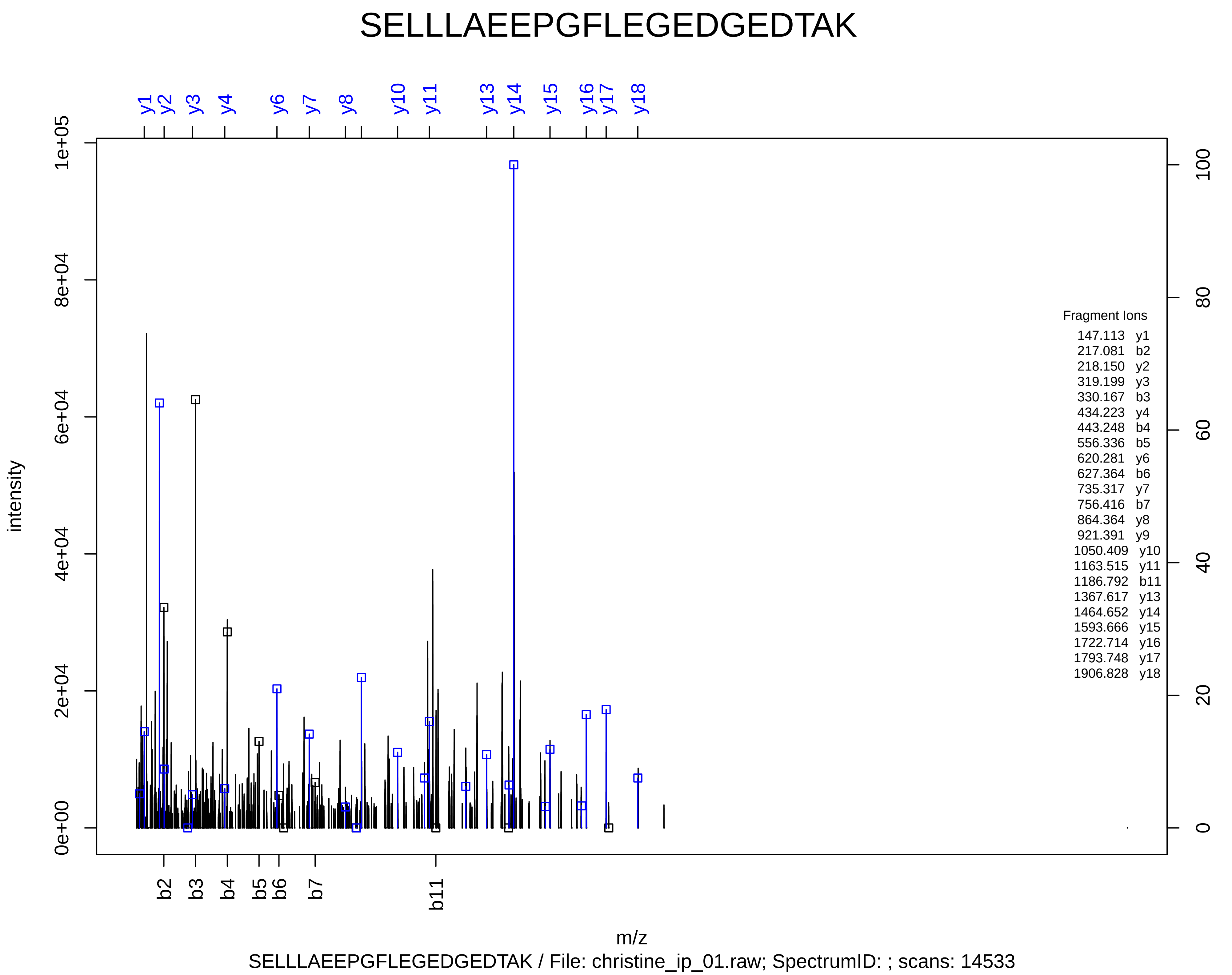
SELLLAEEPGFLEGEDGEDTAK
y1
y2
y3
y4
y6
y7
y8
y10
y11
y13
y14
y15
y16
y17
y18
intensity
0e+00
2e+04
4e+04
6e+04
8e+04
1e+05
0
20
40
60
80
100
Fragment Ions
147.113 y1
217.081 b2
218.150 y2
319.199 y3
330.167 b3
434.223 y4
443.248 b4
556.336 b5
620.281 y6
627.364 b6
735.317 y7
756.416 b7
864.364 y8
921.391 y9
1050.409 y10
1163.515 y11
1186.792 b11
1367.617 y13
1464.652 y14
1593.666 y15
1722.714 y16
1793.748 y17
1906.828 y18
b2
b3
b4
b5
b6
b7
b11
m/z
SELLLAEEPGFLEGEDGEDTAK / File: christine_ip_01.raw; SpectrumID: ; scans: 14533

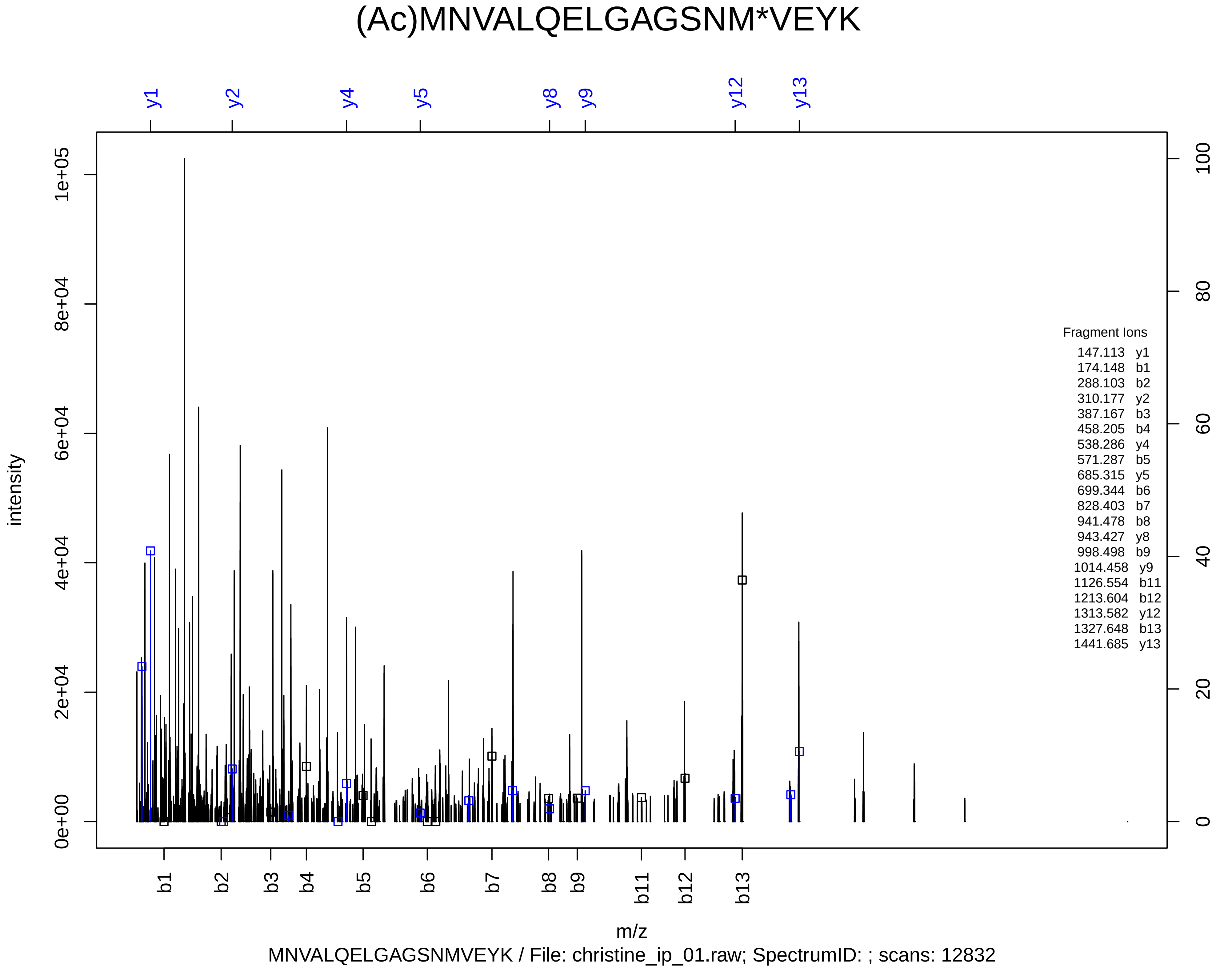

(Ac)MNVALQELGAGSNM*VEYK
y1
y2
y4
y5
y8
y9
y12
y13
intensity
0e+00
2e+04
4e+04
6e+04
8e+04
1e+05
0
20
40
60
80
100
Fragment Ions
147.113 y1
174.148 b1
288.103 b2
310.177 y2
387.167 b3
458.205 b4
538.286 y4
571.287 b5
685.315 y5
699.344 b6
828.403 b7
941.478 b8
943.427 y8
998.498 b9
1014.458 y9
1126.554 b11
1213.604 b12
1313.582 y12
1327.648 b13
1441.685 y13
b1
b2
b3
b4
b5
b6
b7
b8
b9
b11
b12
b13
m/z
MNVALQELGAGSNMVEYK / File: christine_ip_01.raw; SpectrumID: ; scans: 12832

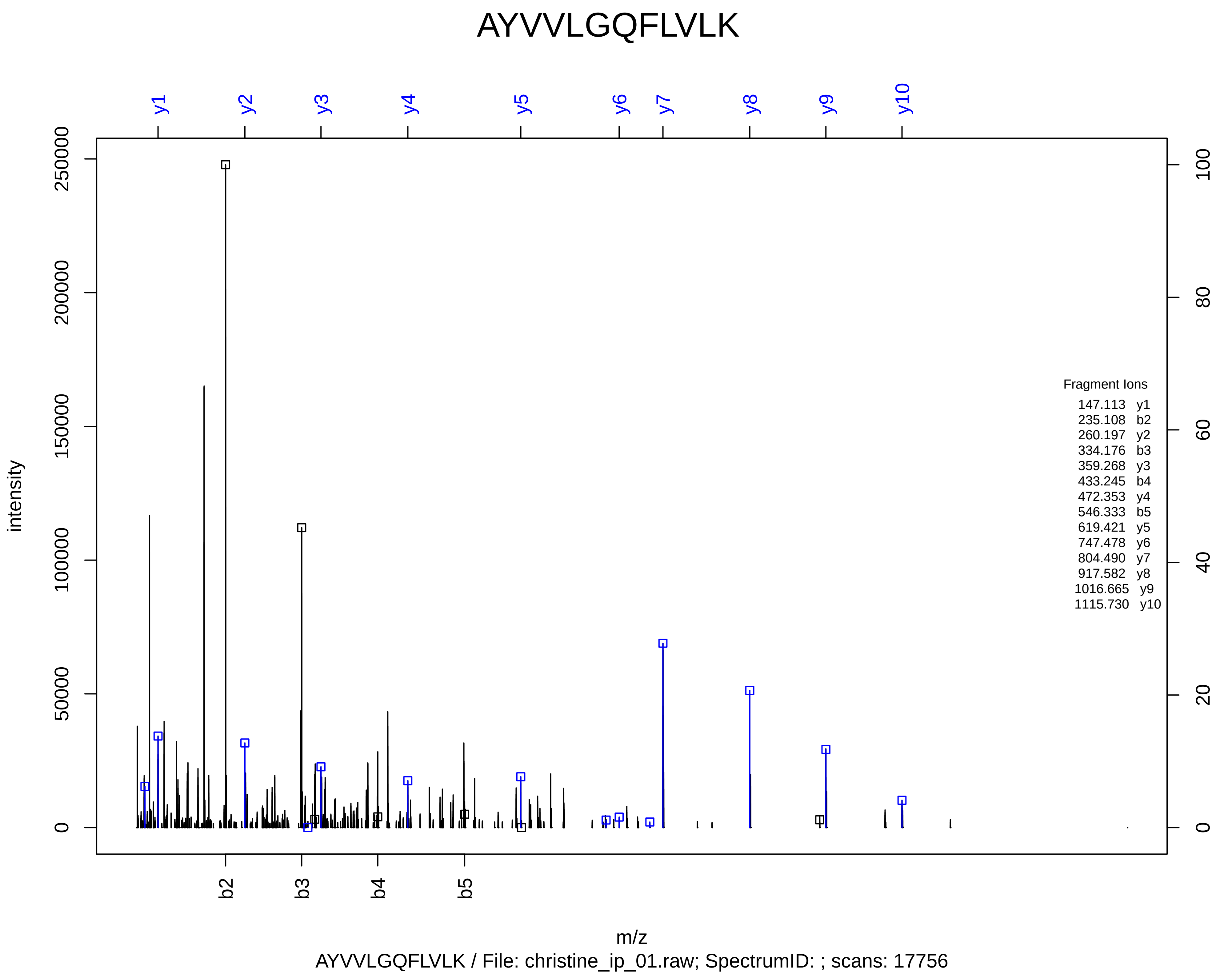
AYVVLGQFLVLK
y1
y2
y3
y4
y5
y6
y7
y8
y9
y10
intensity
0
50000
100000
150000
200000
250000
0
20
40
60
80
100
b2
b3
b4
b5
m/z
Fragment Ions
147.113 y1
235.108 b2
260.197 y2
334.176 b3
359.268 y3
433.245 b4
472.353 y4
546.333 b5
619.421 y5
747.478 y6
804.490 y7
917.582 y8
1016.665 y9
1115.730 y10
AYVVLGQFLVLK / File: christine_ip_01.raw; SpectrumID: ; scans: 17756

YSDMIVAAIQAEK

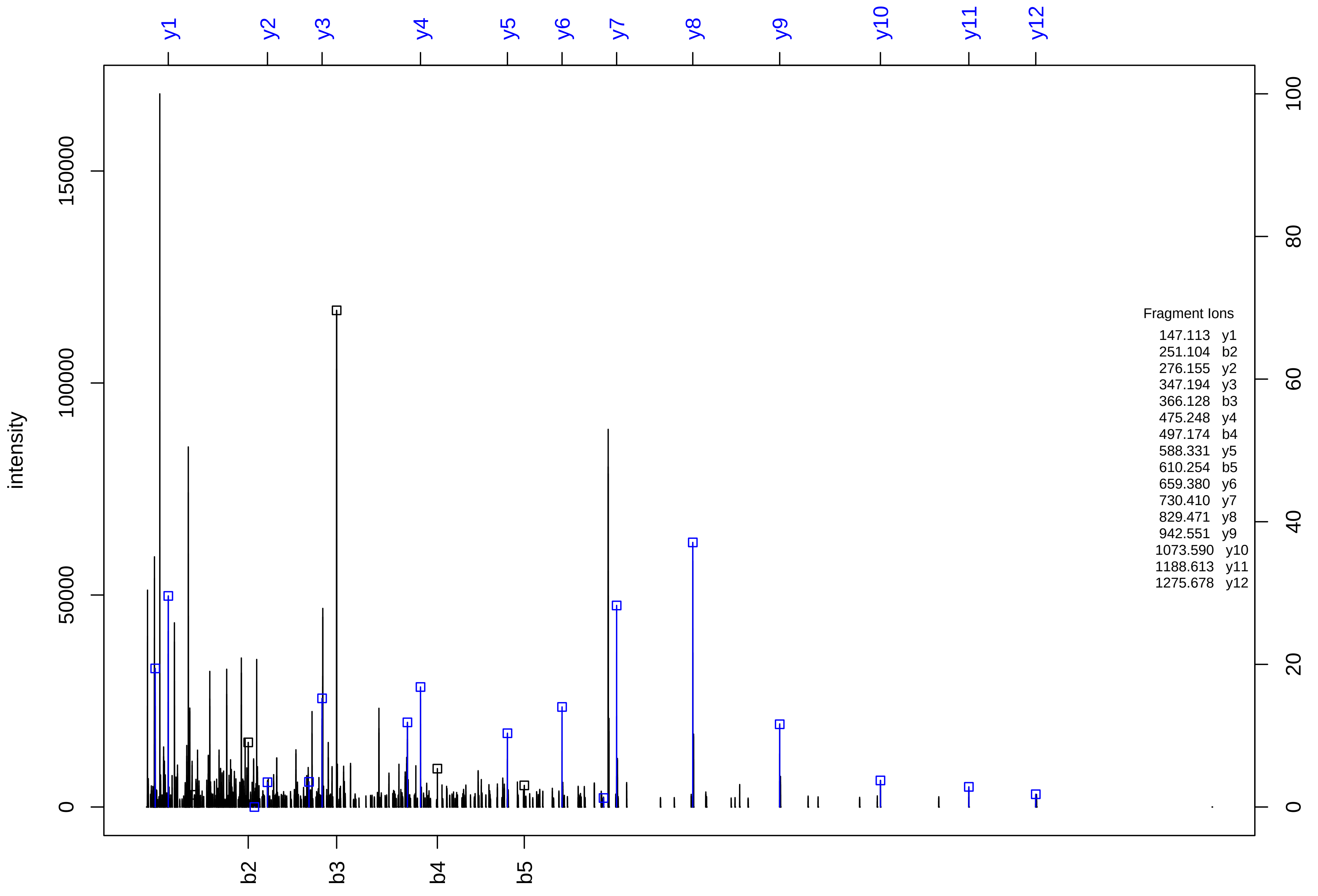

YSDMIVAAIQAEK / File: christine_ip_01.raw; SpectrumID: ; scans: 11695

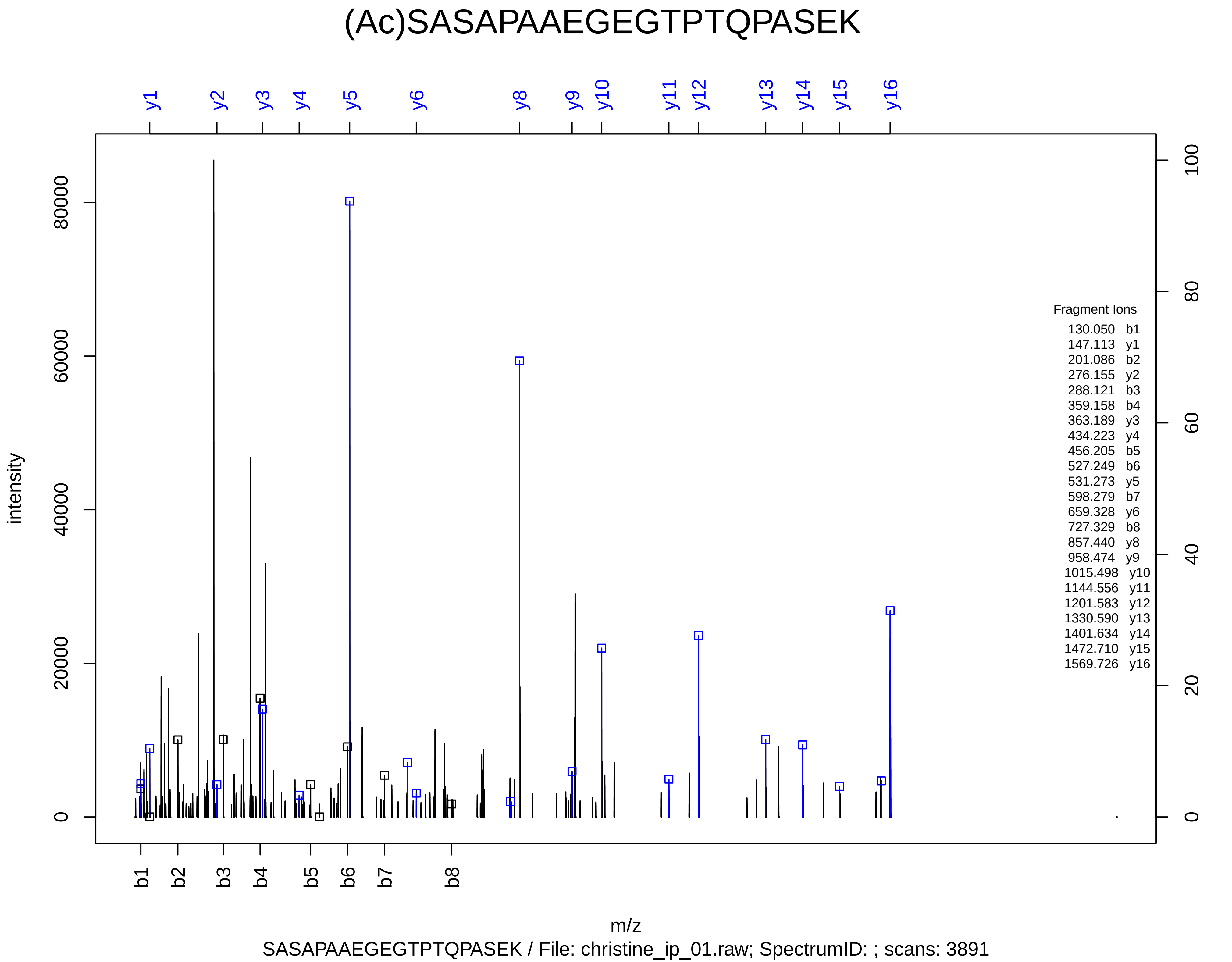
(Ac)SASAPAAEGEGTPTQPASEK
y1 y2 y3 y4 y5 y6 y8 y9 y10 y11 y12 y13 y14 y15 y16
b1 b2 b3 b4 b5 b6 b7 b8
intensity
0
20000
40000
60000
80000
0
20
40
60
80
100
m/z
Fragment Ions
130.050 b1
147.113 y1
201.086 b2
276.155 y2
288.121 b3
359.158 b4
363.189 y3
434.223 y4
456.205 b5
527.249 b6
531.273 y5
598.279 b7
659.328 y6
727.329 b8
857.440 y8
958.474 y9
1015.498 y10
1144.556 y11
1201.583 y12
1330.590 y13
1401.634 y14
1472.710 y15
1569.726 y16
SASAPAAEGEGTPTQPASEK / File: christine_ip_01.raw; SpectrumID: ; scans: 3891

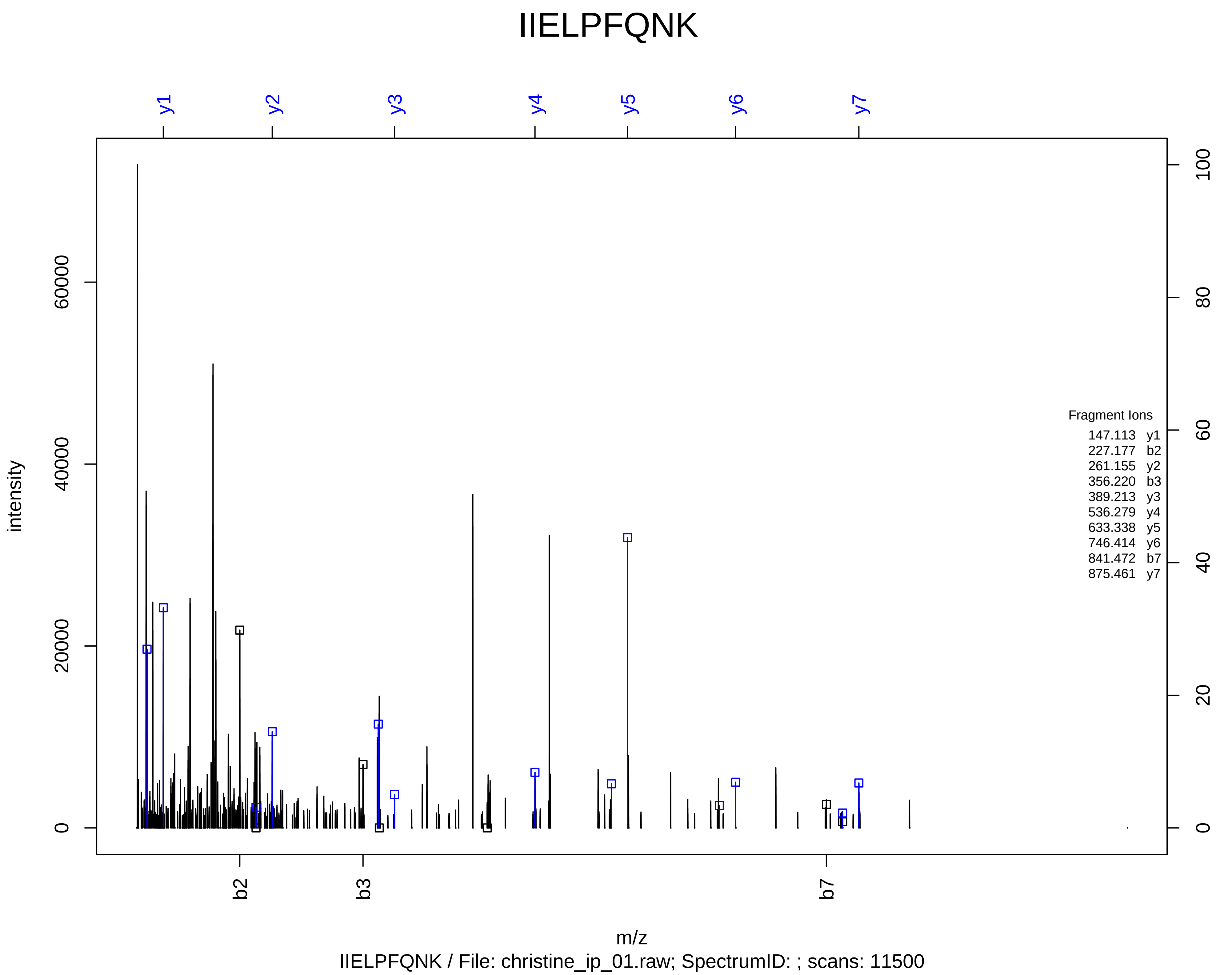
IIELPFQNK
y1
y2
y3
y4
y5
y6
y7
intensity
0
20000
40000
60000
0
20
40
60
80
100
Fragment Ions
147.113 y1
227.177 b2
261.155 y2
356.220 b3
389.213 y3
536.279 y4
633.338 y5
746.414 y6
841.472 b7
875.461 y7
b2
b3
b7
m/z
IIELPFQNK / File: christine_ip_01.raw; SpectrumID: ; scans: 11500

HPEAPLQNTN^AN^LN^MRKR

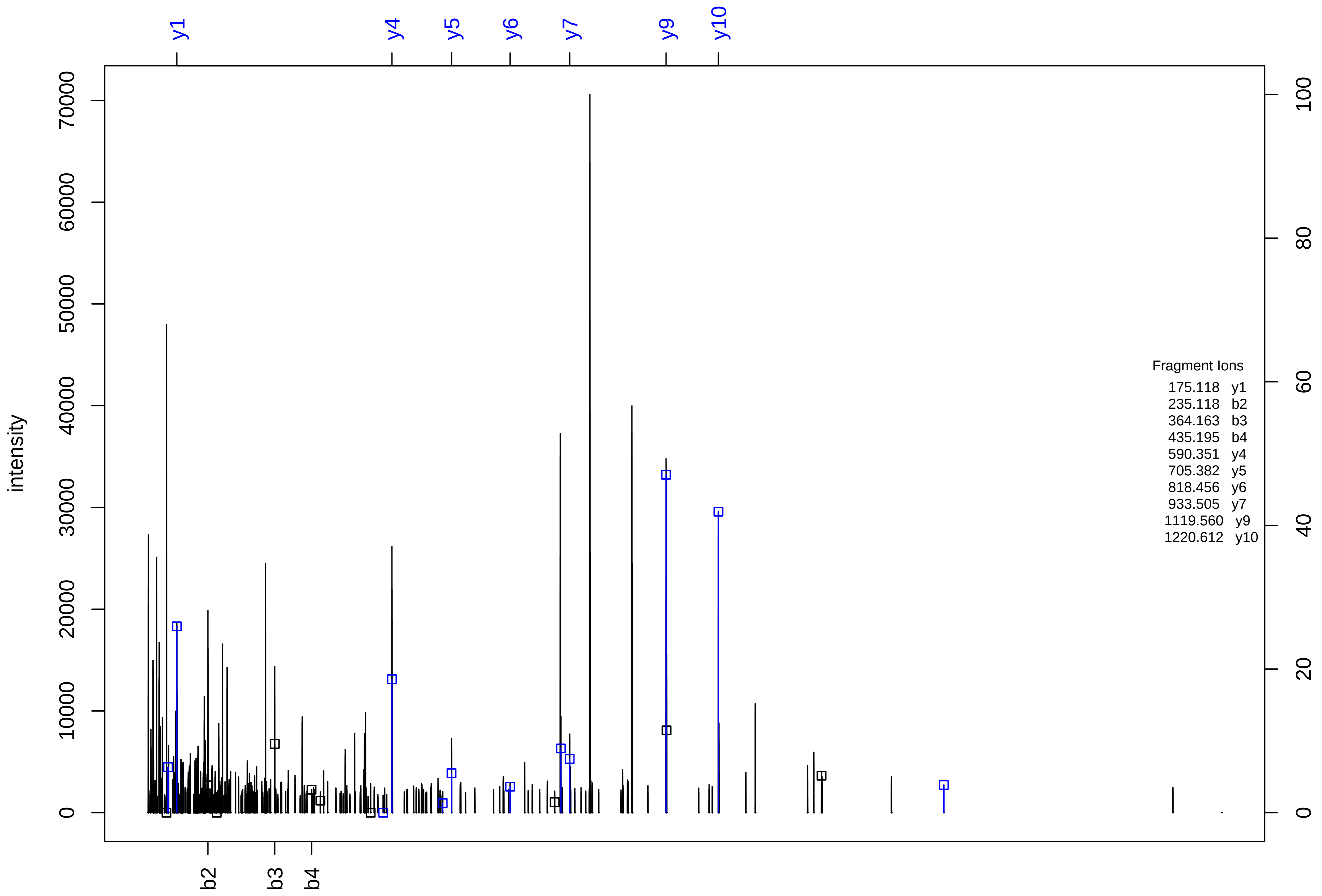

HPEAPLQNTNANLNMRKR / File: christine_ip_01.raw; SpectrumID: ; scans: 14909

VQLPPEIQLAQR

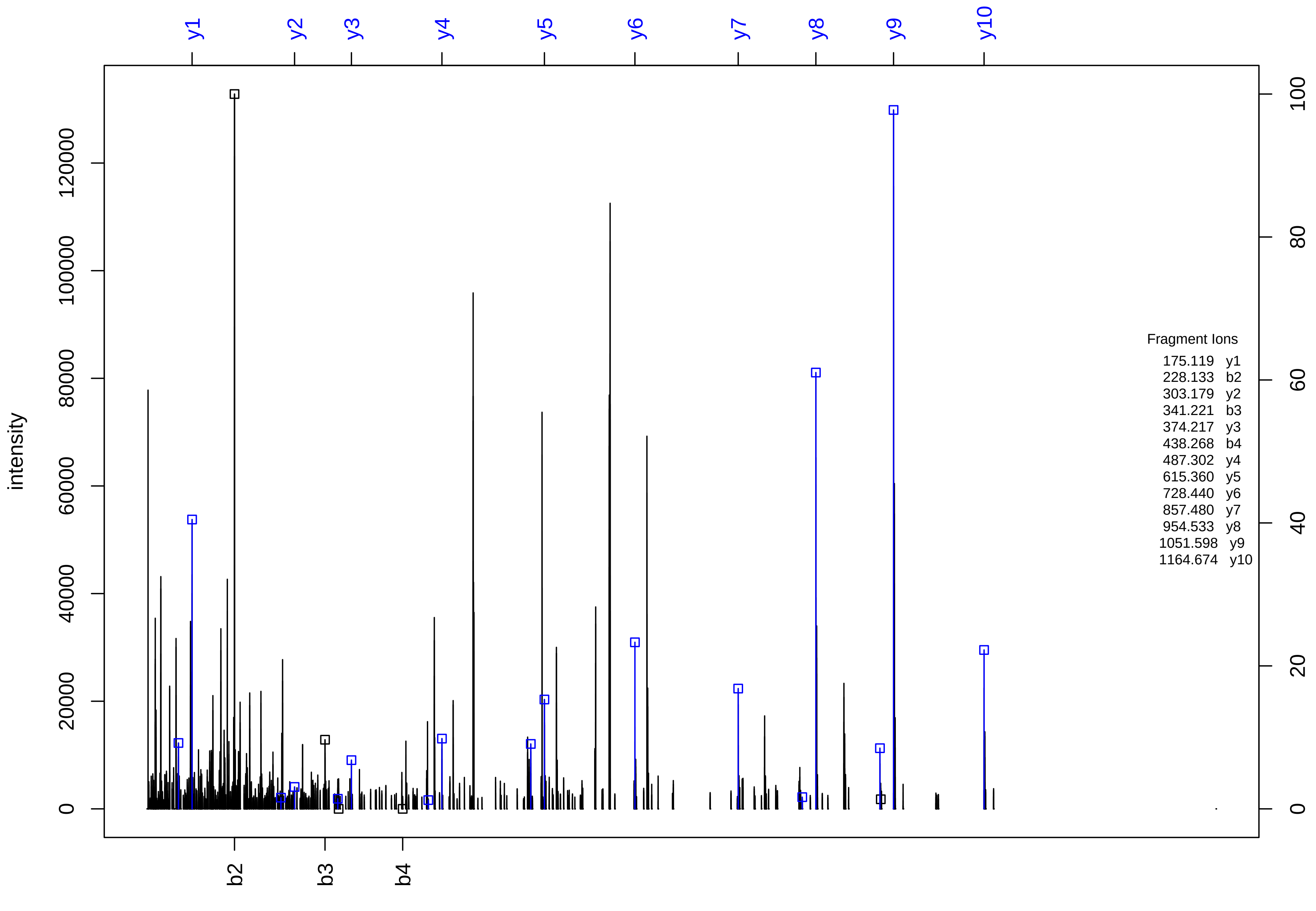

VQLPPEIQLAQR / File: christine_ip_01.raw; SpectrumID: ; scans: 11064

GFAFVYFENVDDAK

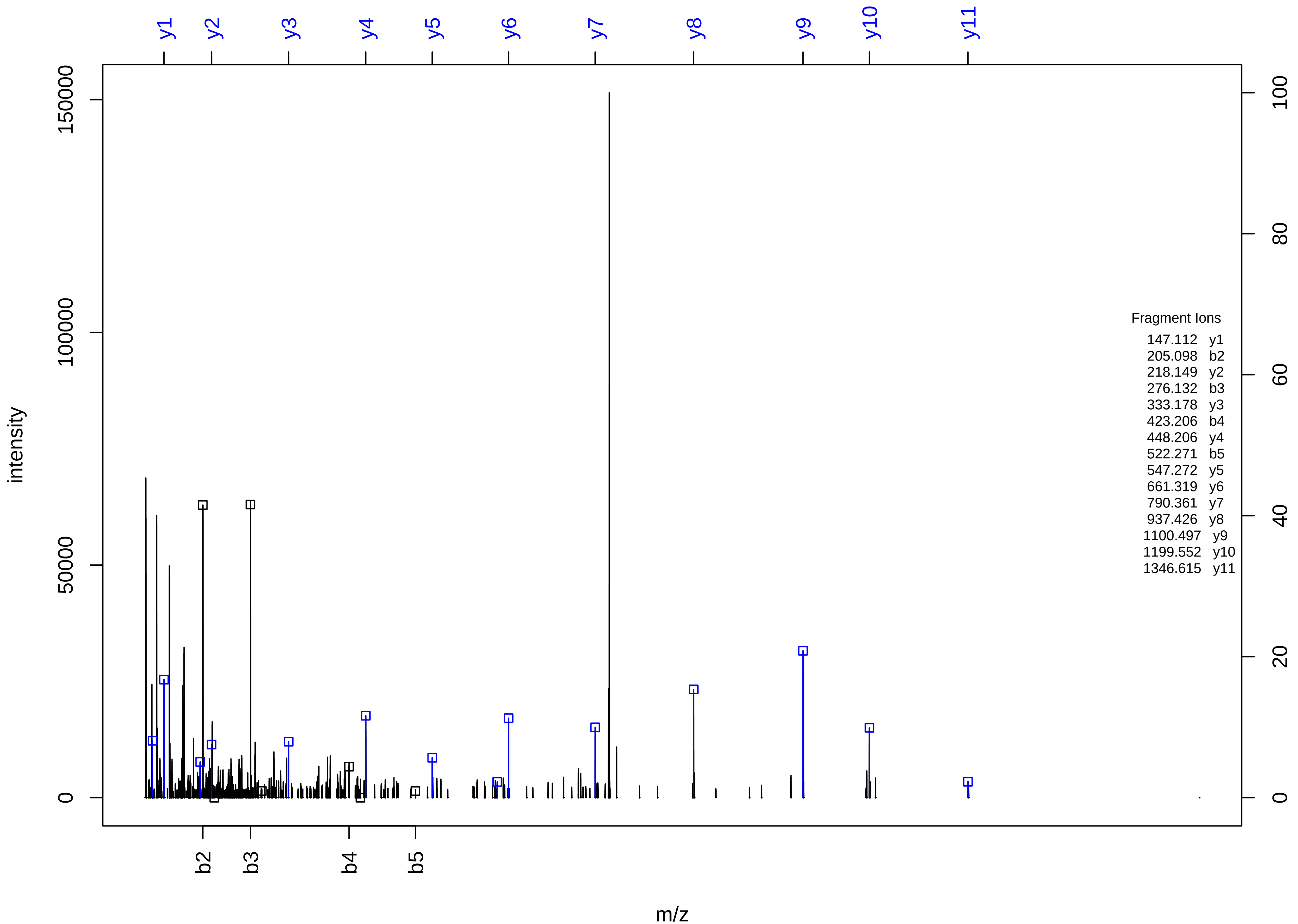

GFAFVYFENVDDAK / File: christine_ip_01.raw; SpectrumID: ; scans: 14980

ASGAGSEFQDQTR

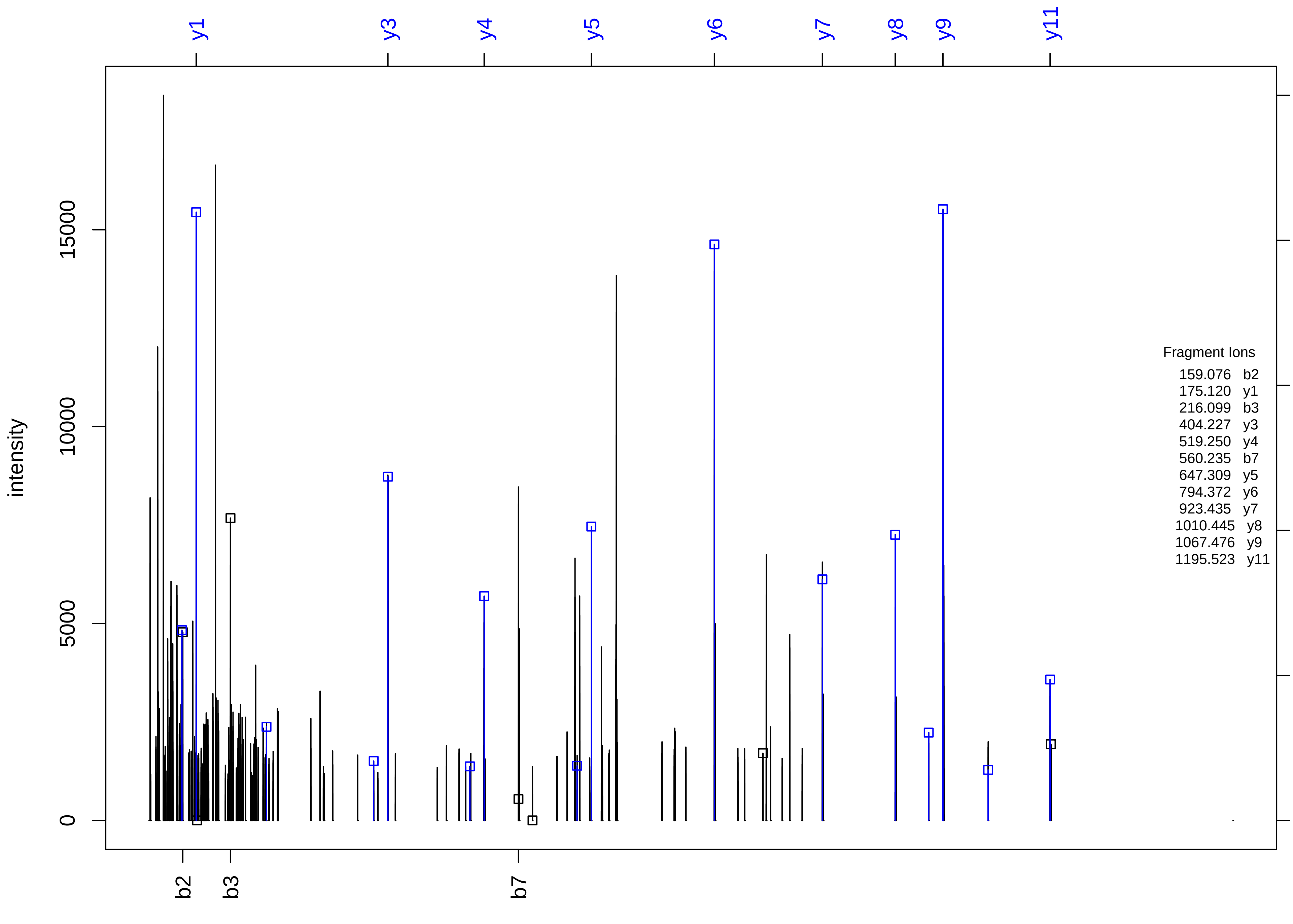

ASGAGSEFQDQTR / File: christine_ip_01.raw; SpectrumID: ; scans: 2270

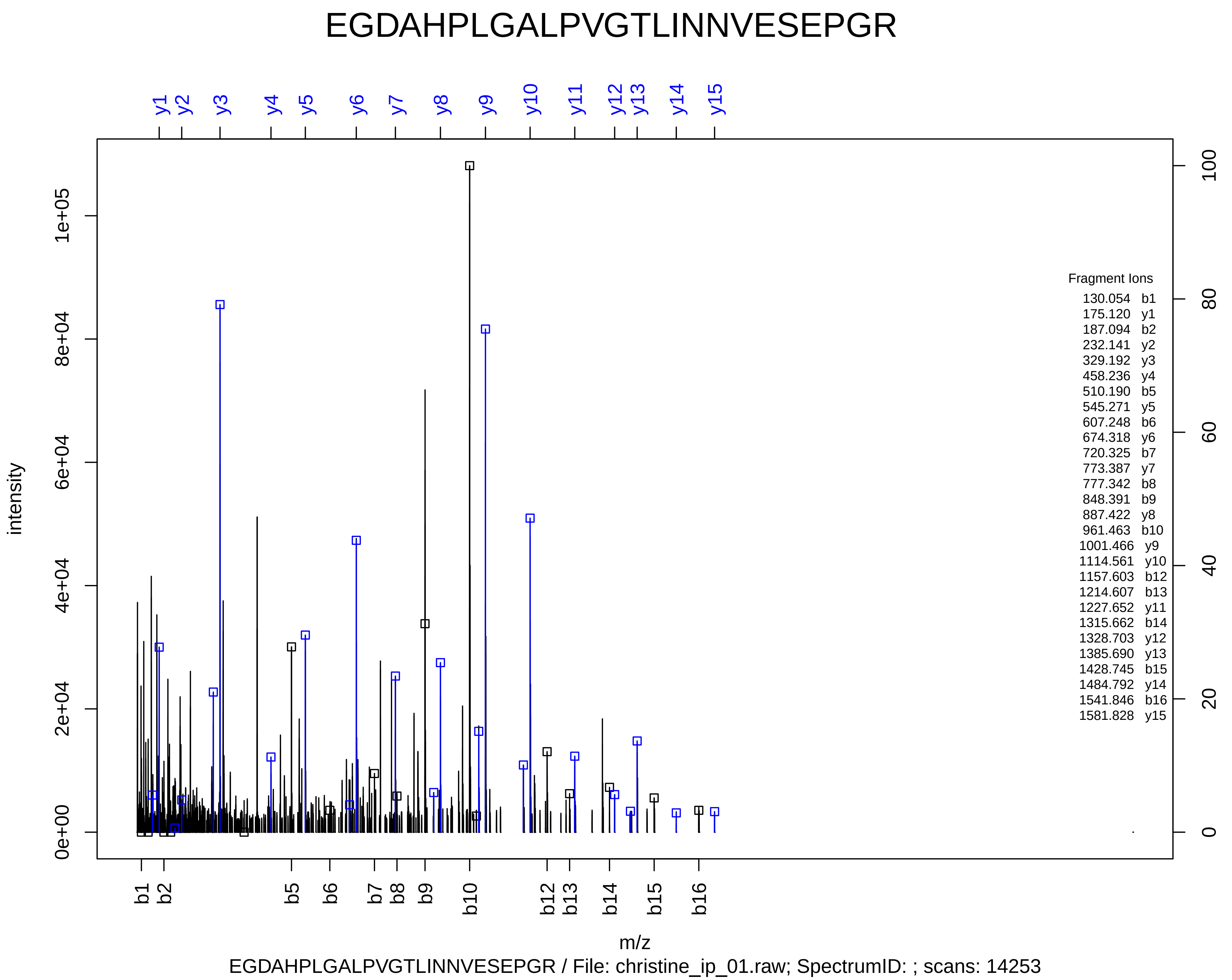

EGDAHPLGALPVGTLINNVESEPGR
y1
y2
y3
y4
y5
y6
y7
y8
y9
y10
y11
y12
y13
y14
y15
intensity
0e+00
2e+04
4e+04
6e+04
8e+04
1e+05
0
20
40
60
80
100
b1
b2
b5
b6
b7
b8
b9
b10
b12
b13
b14
b15
b16
m/z
Fragment Ions
130.054 b1
175.120 y1
187.094 b2
232.141 y2
329.192 y3
458.236 y4
510.190 b5
545.271 y5
607.248 b6
674.318 y6
720.325 b7
773.387 y7
777.342 b8
848.391 b9
887.422 y8
961.463 b10
1001.466 y9
1114.561 y10
1157.603 b12
1214.607 b13
1227.652 y11
1315.662 b14
1328.703 y12
1385.690 y13
1428.745 b15
1484.792 y14
1541.846 b16
1581.828 y15
EGDAHPLGALPVGTLINNVESEPGR / File: christine_ip_01.raw; SpectrumID: ; scans: 14253

LSDSLLTN^EPVK

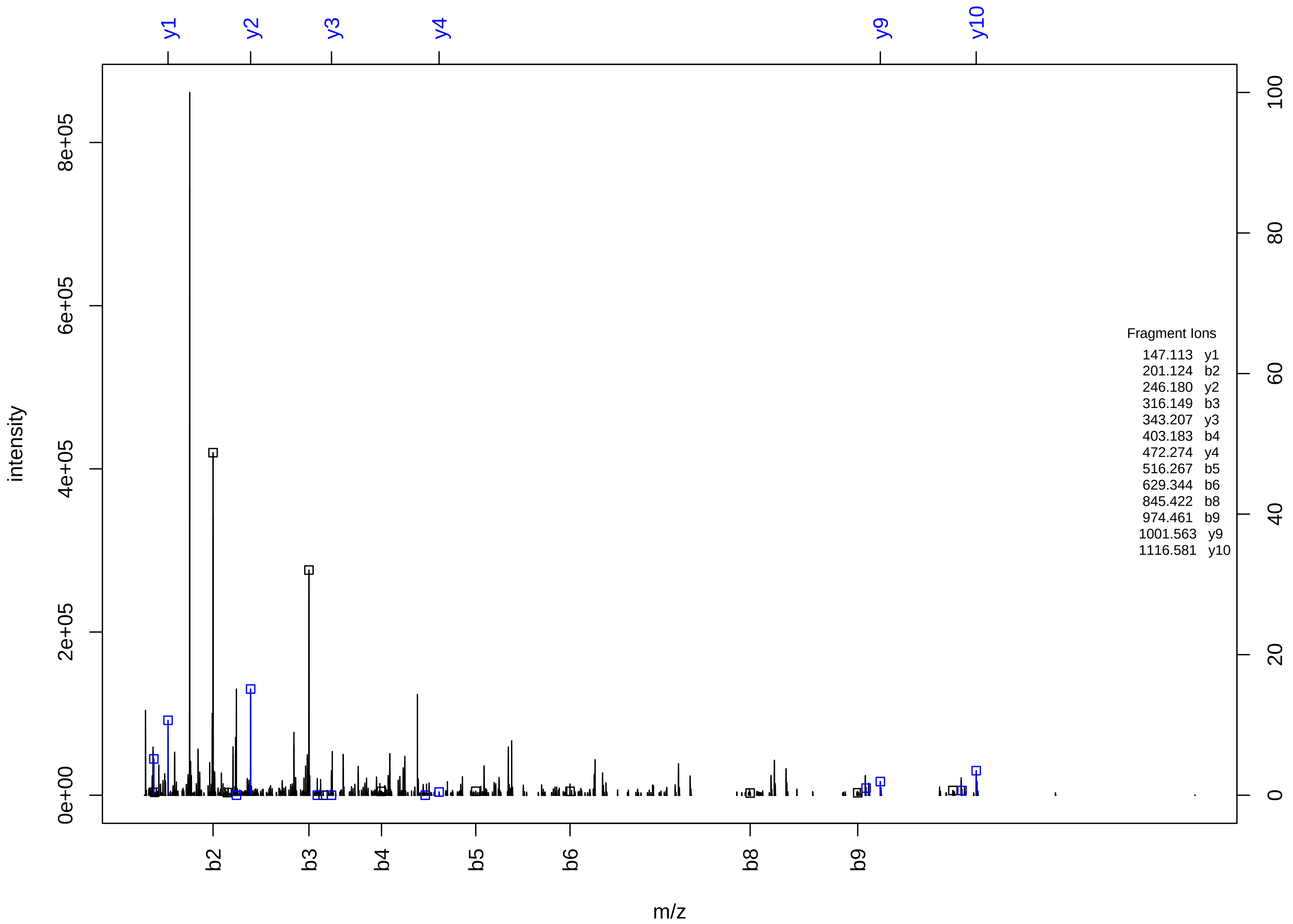

LSDSLLTNEPVK / File: christine_ip_01.raw; SpectrumID: ; scans: 16475

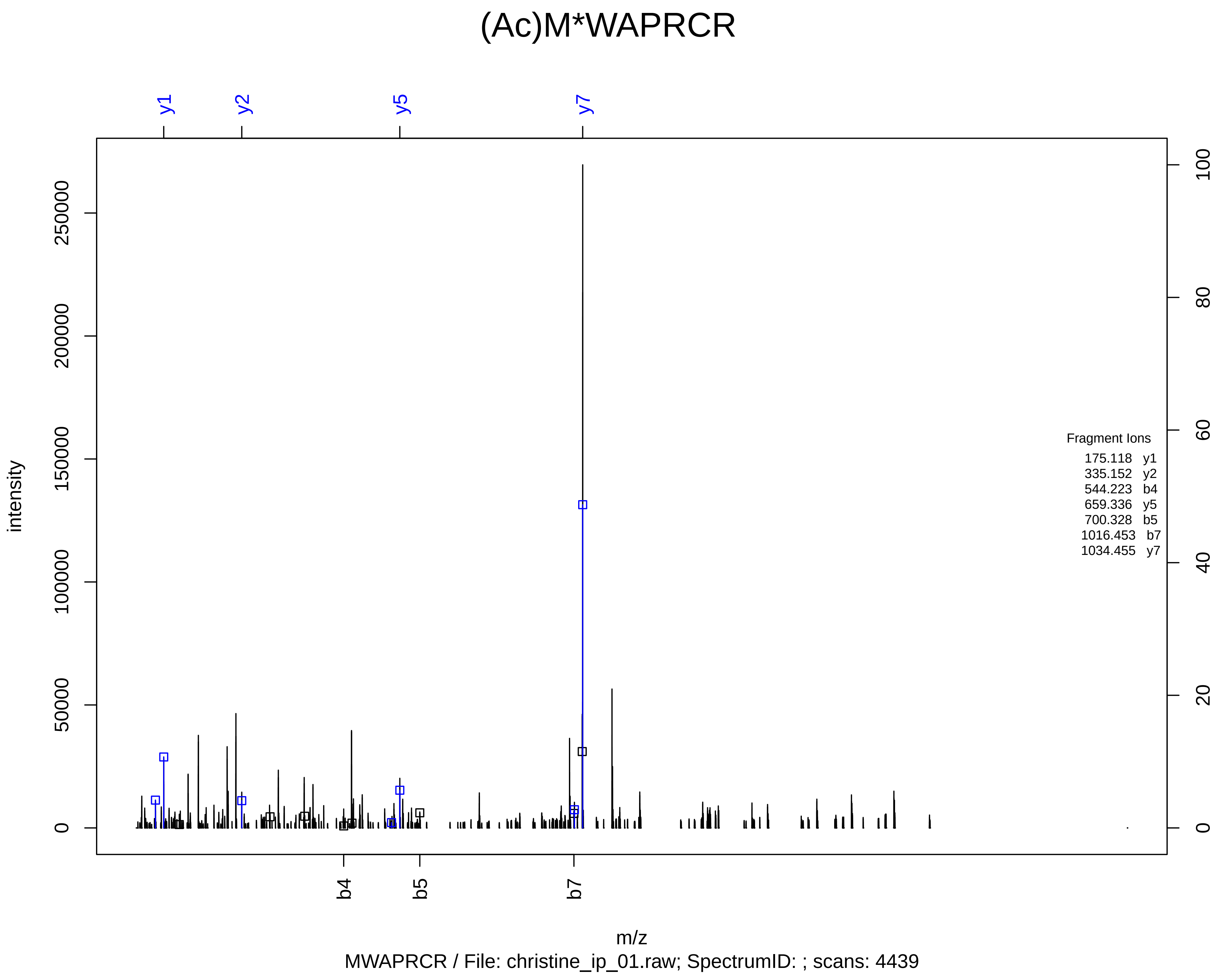

(Ac)M*WAPRCR
y1
y2
y5
y7
intensity
0
50000
100000
150000
200000
250000
0
20
40
60
80
100
Fragment Ions
175.118 y1
335.152 y2
544.223 b4
659.336 y5
700.328 b5
1016.453 b7
1034.455 y7
b4
b5
b7
m/z
MWAPRCR / File: christine_ip_01.raw; SpectrumID: ; scans: 4439

VEQLGAEGNVEESQK

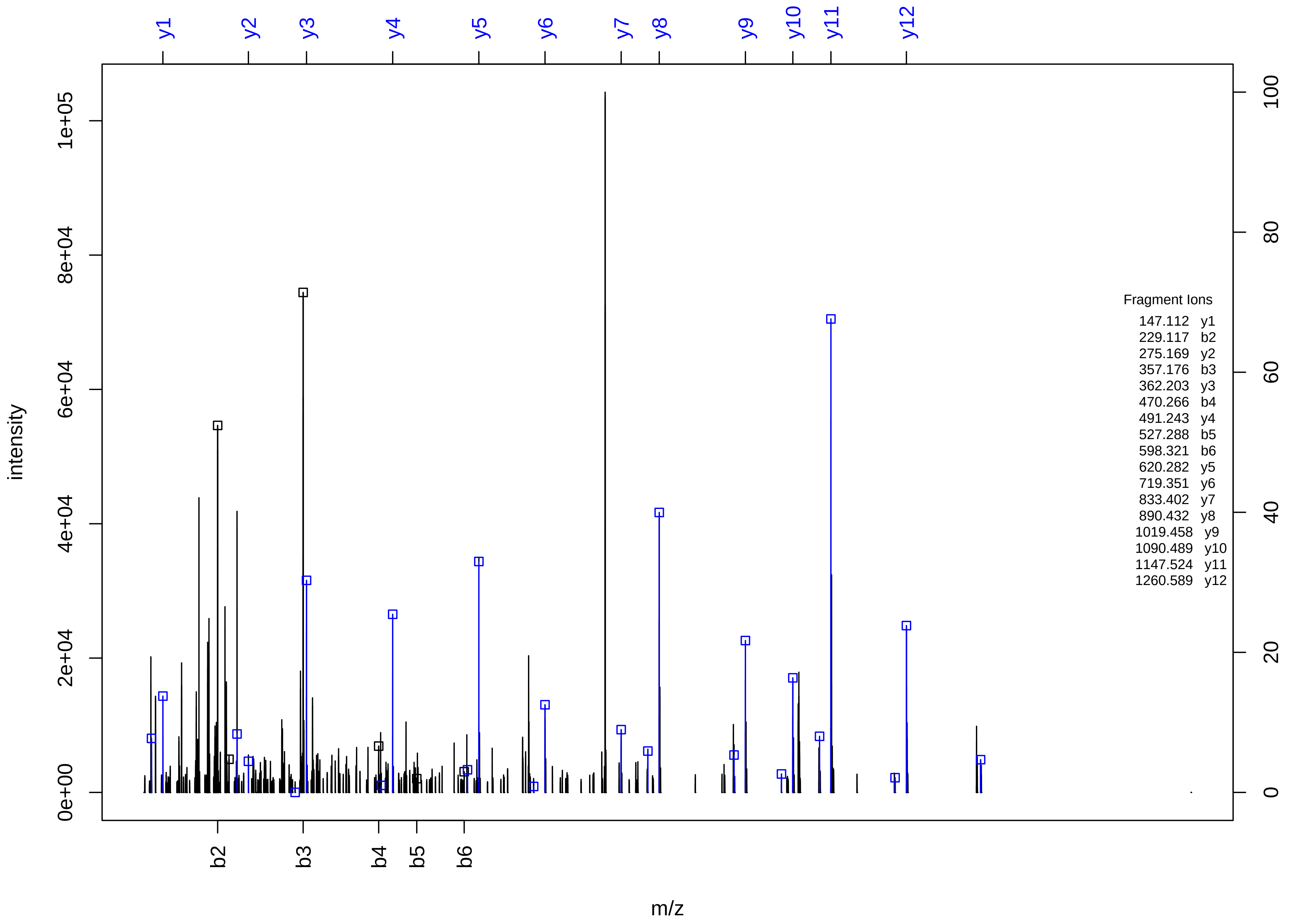

VEQLGAEGNVEESQK / File: christine_ip_01.raw; SpectrumID: ; scans: 3309

EENSTEEQALEDQNAK

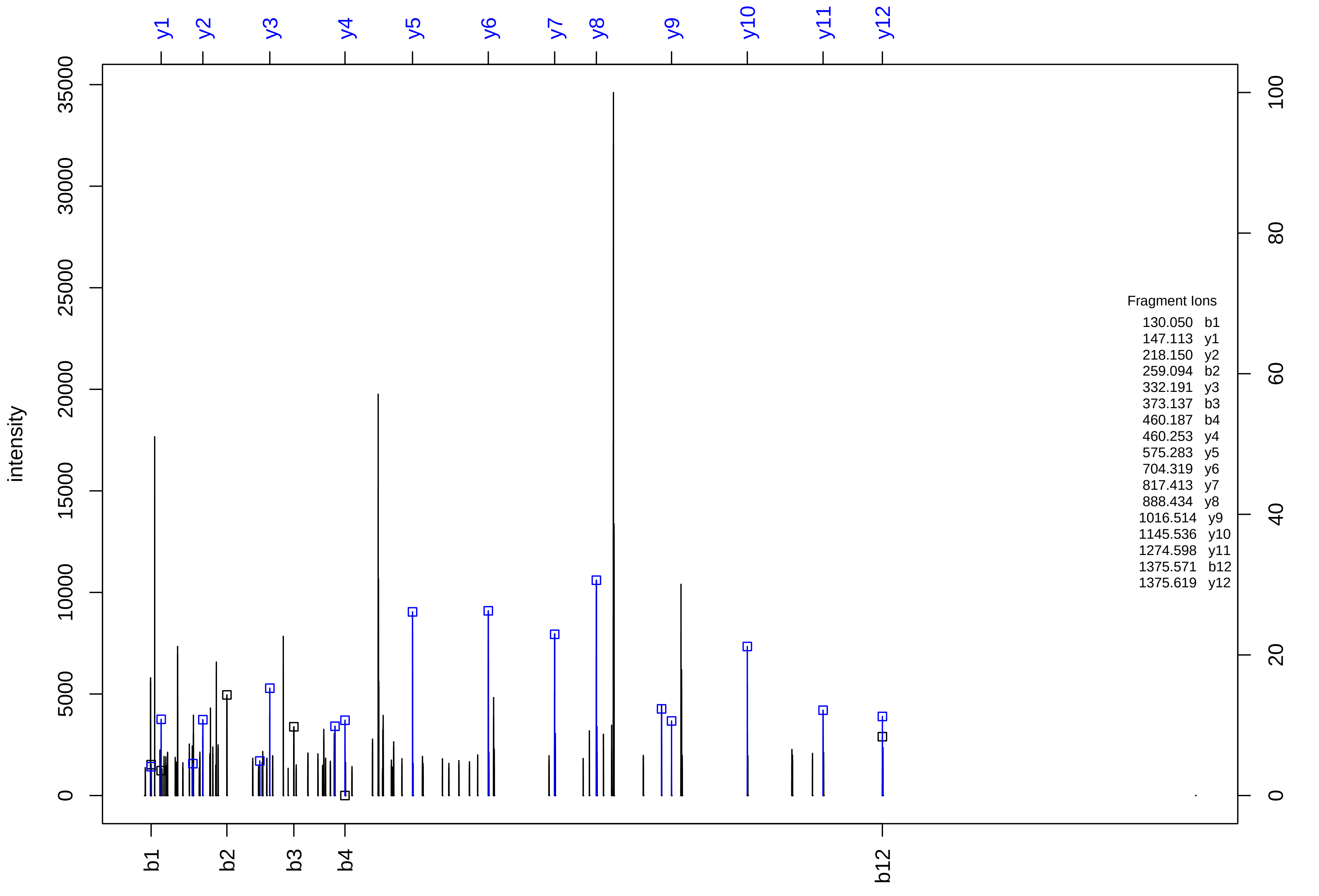

EENSTEEQALEDQNAK / File: christine_ip_01.raw; SpectrumID: ; scans: 3063

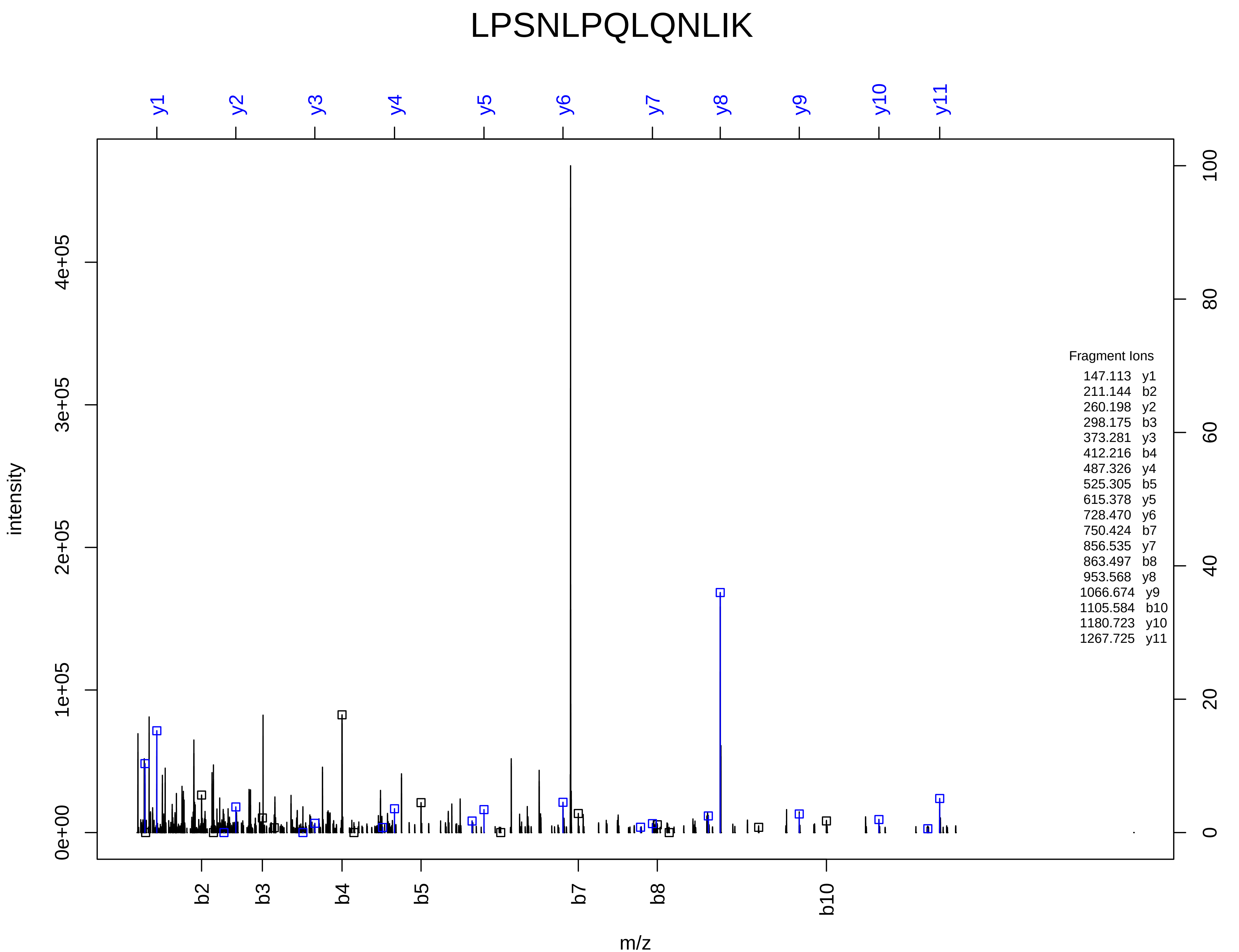

LPSNLPQLQNLIK / File: christine_ip_01.raw; SpectrumID: ; scans: 14422

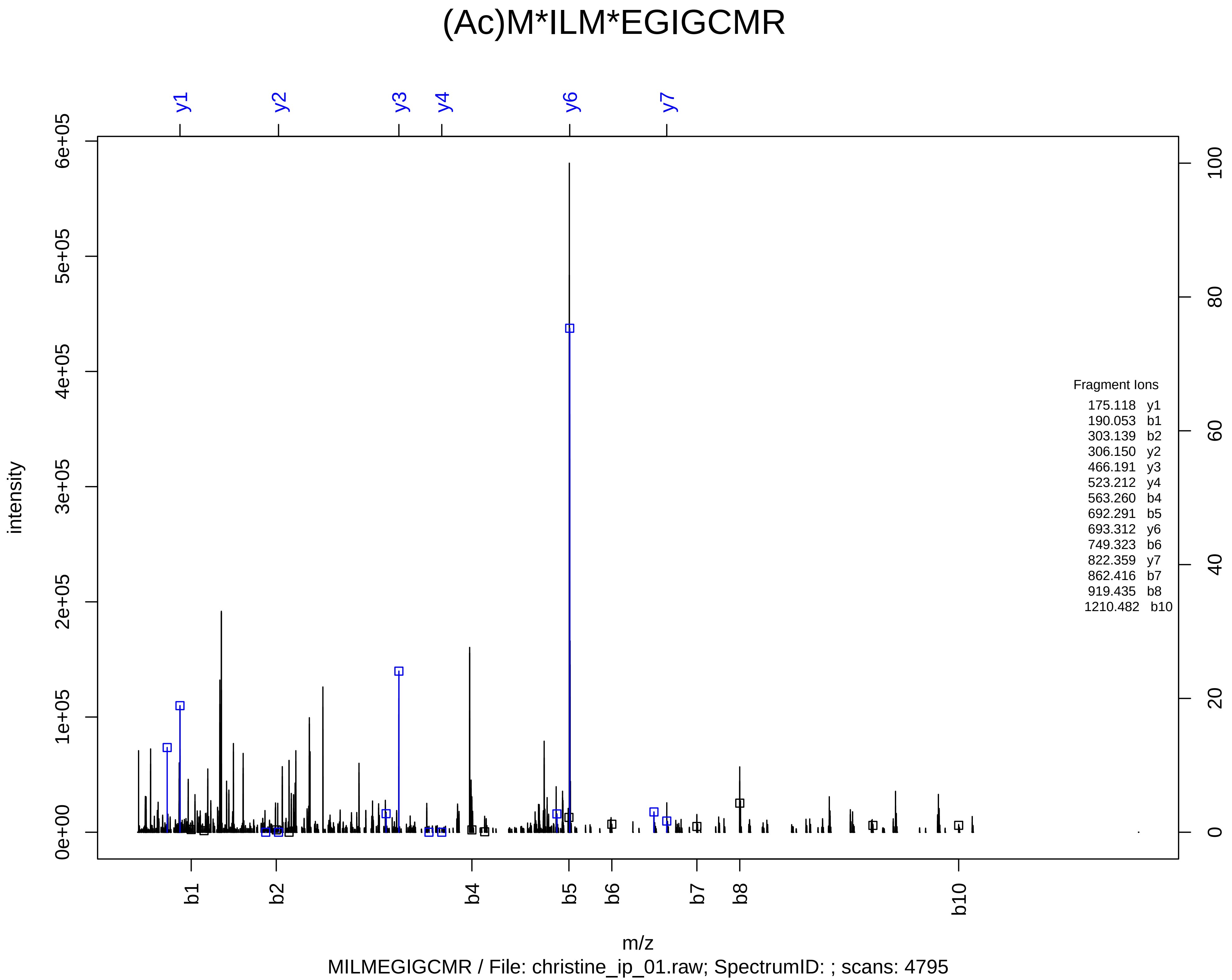

(Ac)M*ILM*EGIGCMR
y1
y2
y3
y4
y6
y7
intensity
0e+00
1e+05
2e+05
3e+05
4e+05
5e+05
6e+05
0
20
40
60
80
100
Fragment Ions
175.118 y1
190.053 b1
303.139 b2
306.150 y2
466.191 y3
523.212 y4
563.260 b4
692.291 b5
693.312 y6
749.323 b6
822.359 y7
862.416 b7
919.435 b8
1210.482 b10
b1
b2
b4
b5
b6
b7
b8
b10
m/z
MILMEGIGCMR / File: christine_ip_01.raw; SpectrumID: ; scans: 4795

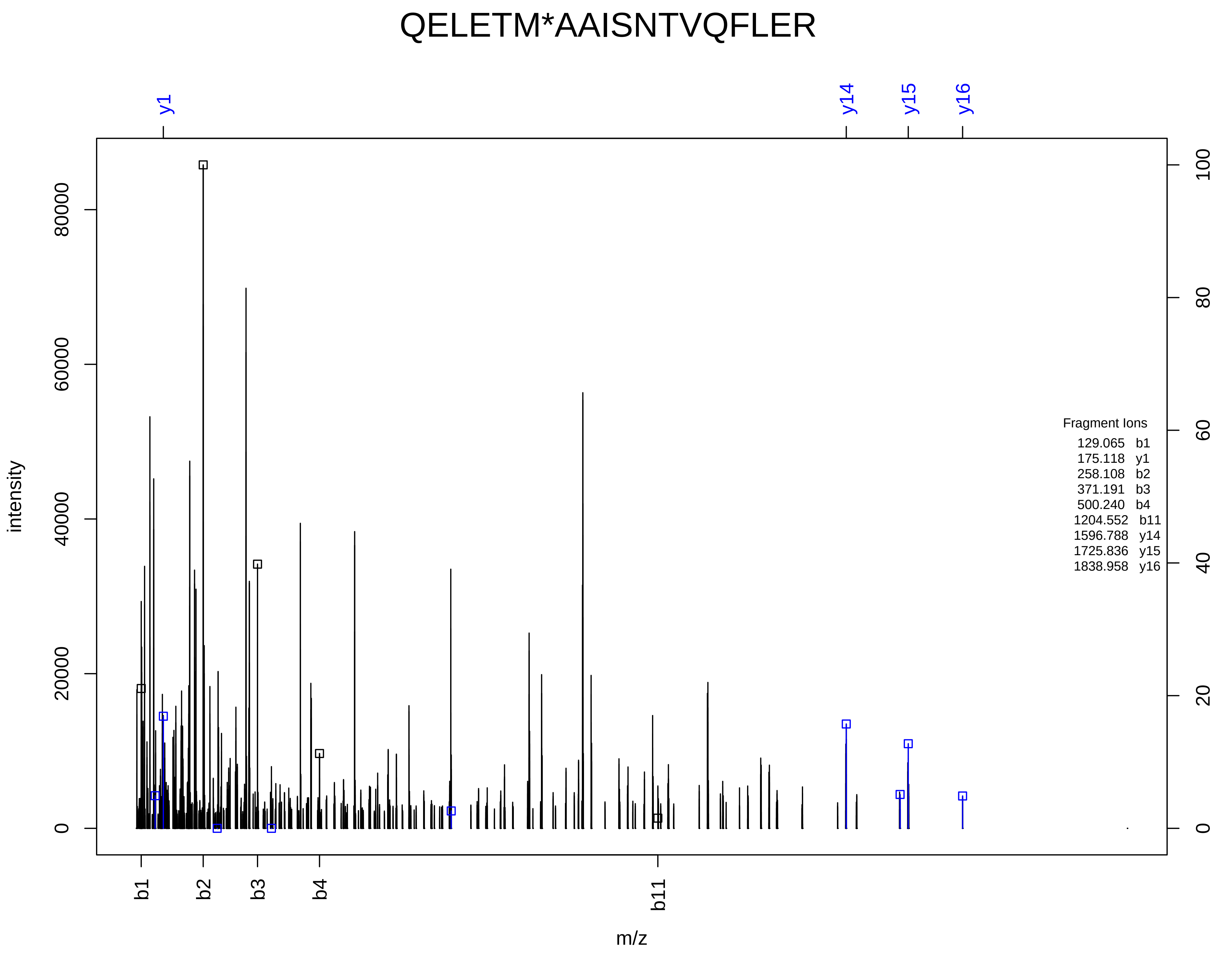
QELETM*AAISNTVQFLER
y1
y14
y15
y16
intensity
0
20000
40000
60000
80000
0
20
40
60
80
100
Fragment Ions
129.065 b1
175.118 y1
258.108 b2
371.191 b3
500.240 b4
1204.552 b11
1596.788 y14
1725.836 y15
1838.958 y16
b1
b2
b3
b4
b11
m/z
QELETMAAISNTVQFLER / File: christine_ip_01.raw; SpectrumID: ; scans: 14266

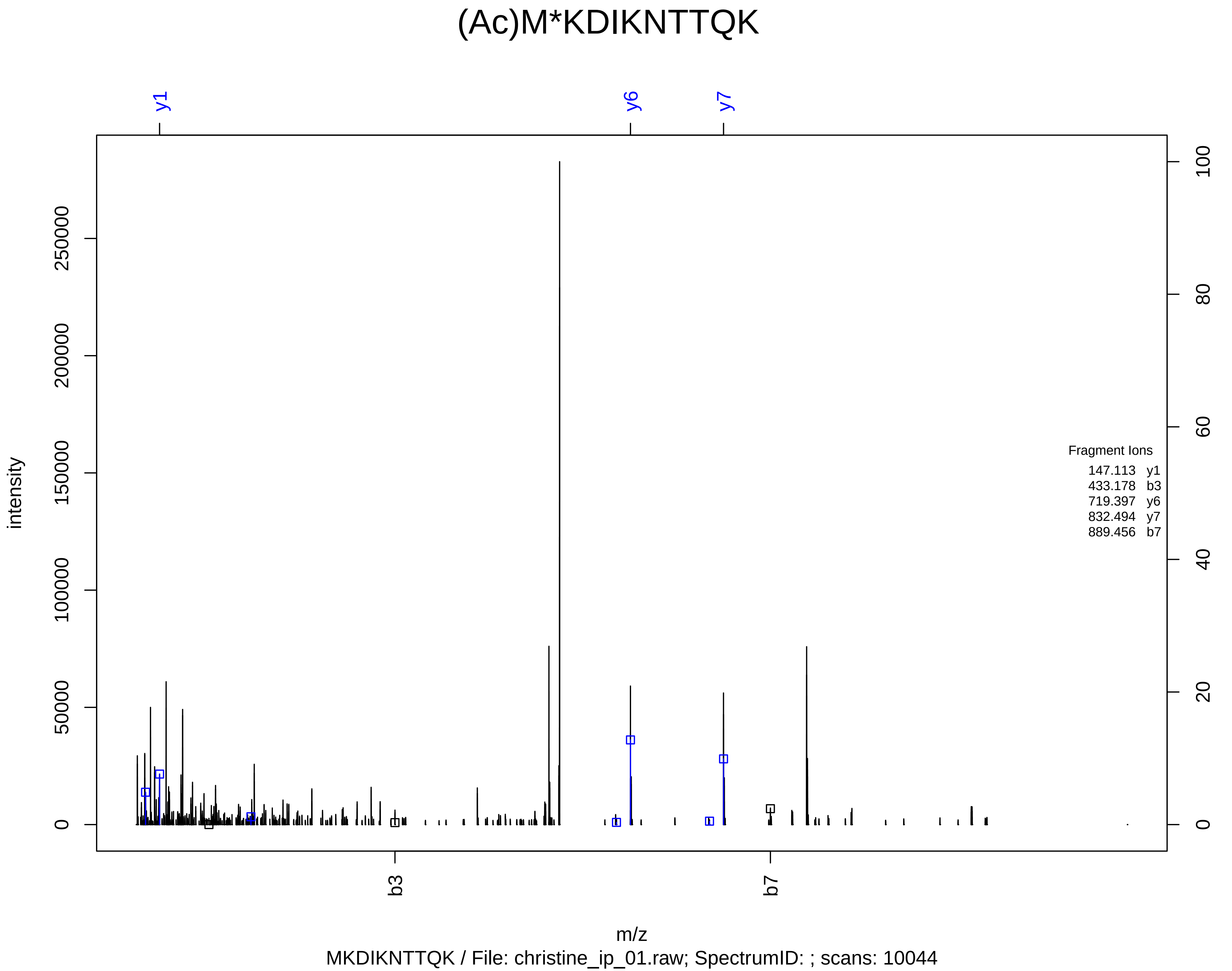

(Ac)M*KDIKNTTQK
y1
y6
y7
intensity
0
50000
100000
150000
200000
250000
0
20
40
60
80
100
Fragment Ions
147.113 y1
433.178 b3
719.397 y6
832.494 y7
889.456 b7
b3
b7
m/z
MKDIKNTTQK / File: christine_ip_01.raw; SpectrumID: ; scans: 10044

HSARTGGMAGRASASSPAFEN^

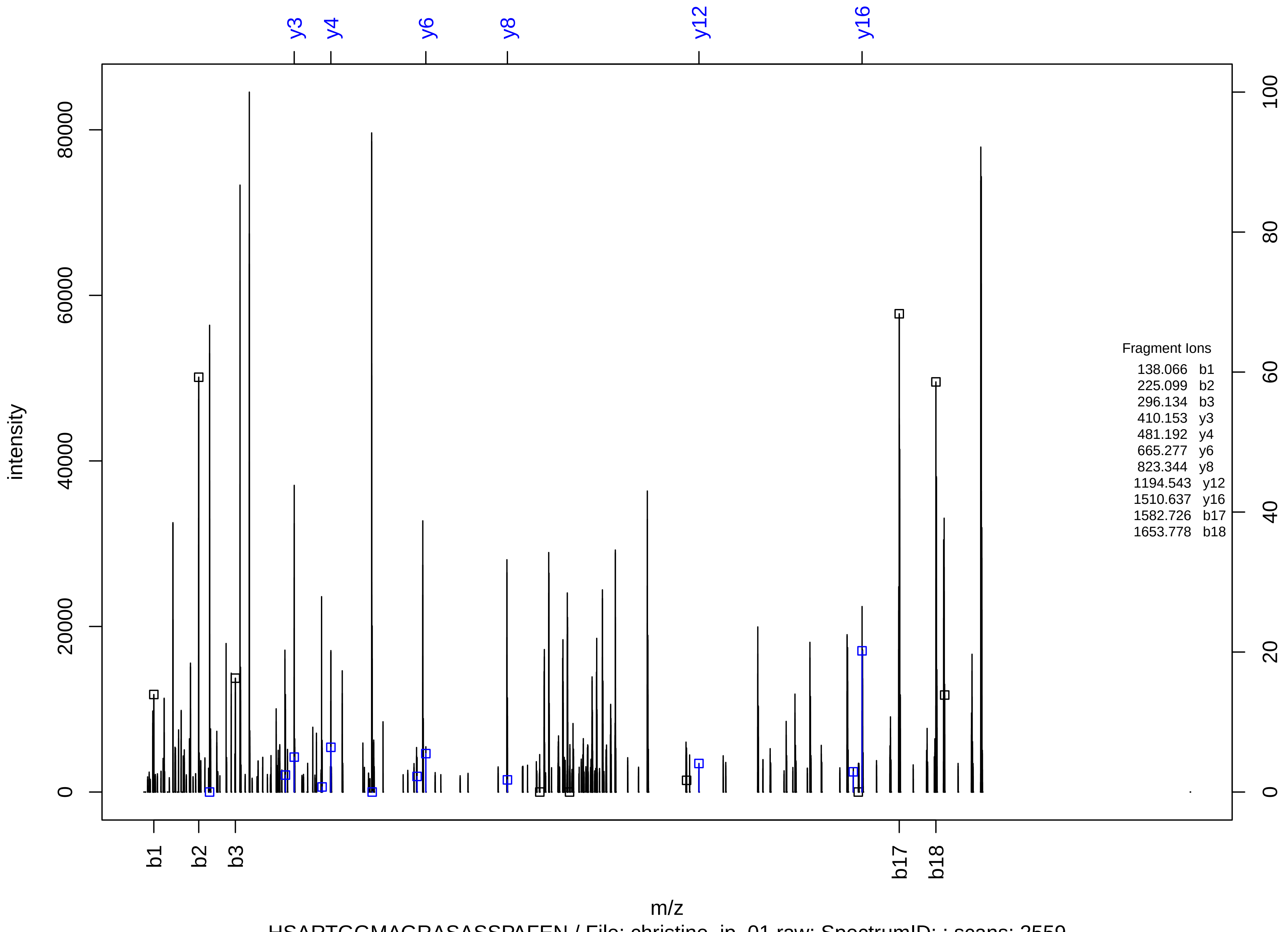

HSARTGGMAGRASASSPAFEN / File: christine_ip_01.raw; SpectrumID: ; scans: 2559

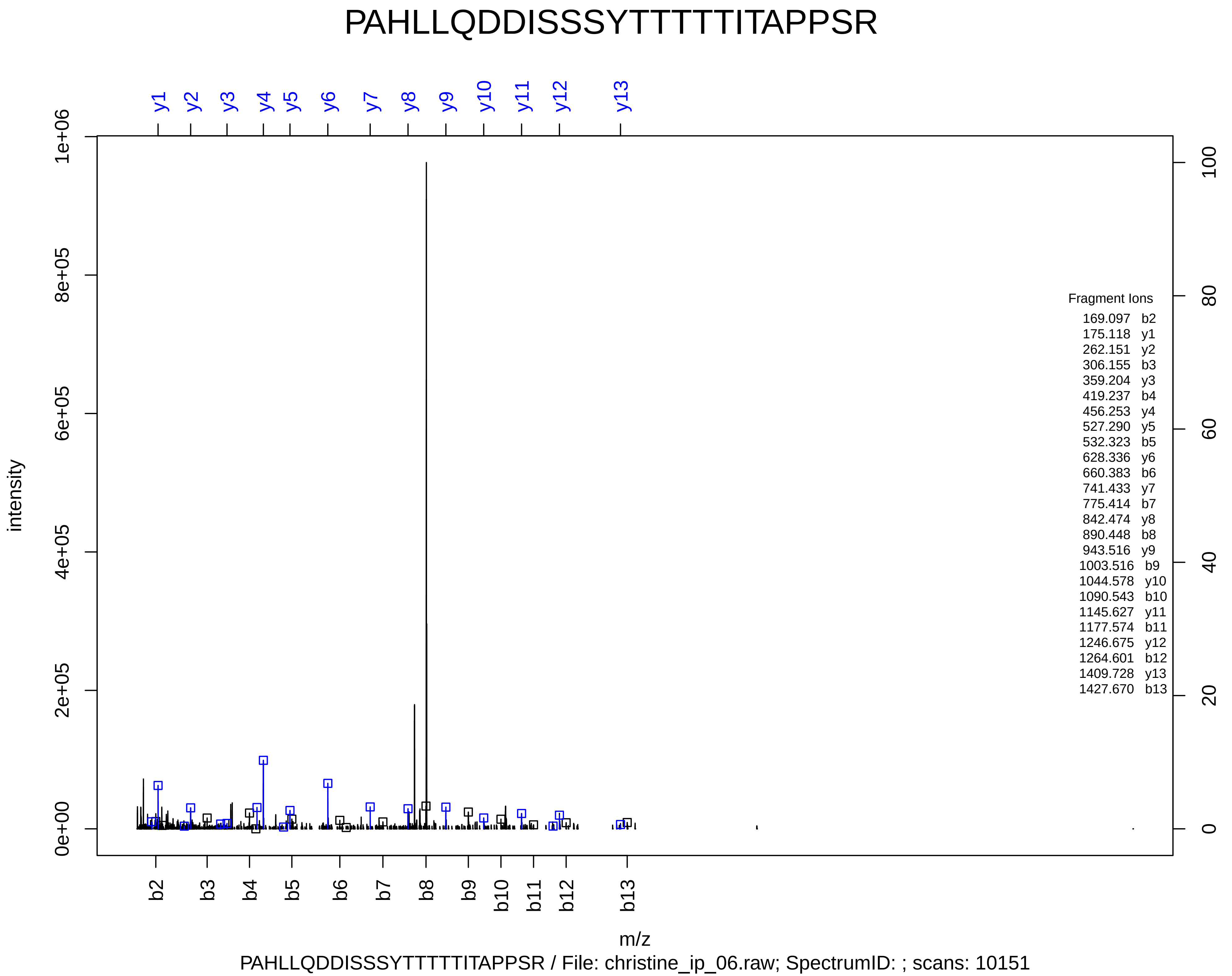
PAHLLQDDISSSYTTTTTTITAPPSR
y1 y2 y3 y4 y5 y6 y7 y8 y9 y10 y11 y12 y13
b2 b3 b4 b5 b6 b7 b8 b9 b10 b11 b12 b13
intensity
0e+00 2e+05 4e+05 6e+05 8e+05 1e+06
0 20 40 60 80 100
m/z
Fragment Ions
169.097 b2
175.118 y1
262.151 y2
306.155 b3
359.204 y3
419.237 b4
456.253 y4
527.290 y5
532.323 b5
628.336 y6
660.383 b6
741.433 y7
775.414 b7
842.474 y8
890.448 b8
943.516 y9
1003.516 b9
1044.578 y10
1090.543 b10
1145.627 y11
1177.574 b11
1246.675 y12
1264.601 b12
1409.728 y13
1427.670 b13
PAHLLQDDISSSYTTTTTTITAPPSR / File: christine_ip_06.raw; SpectrumID: ; scans: 10151

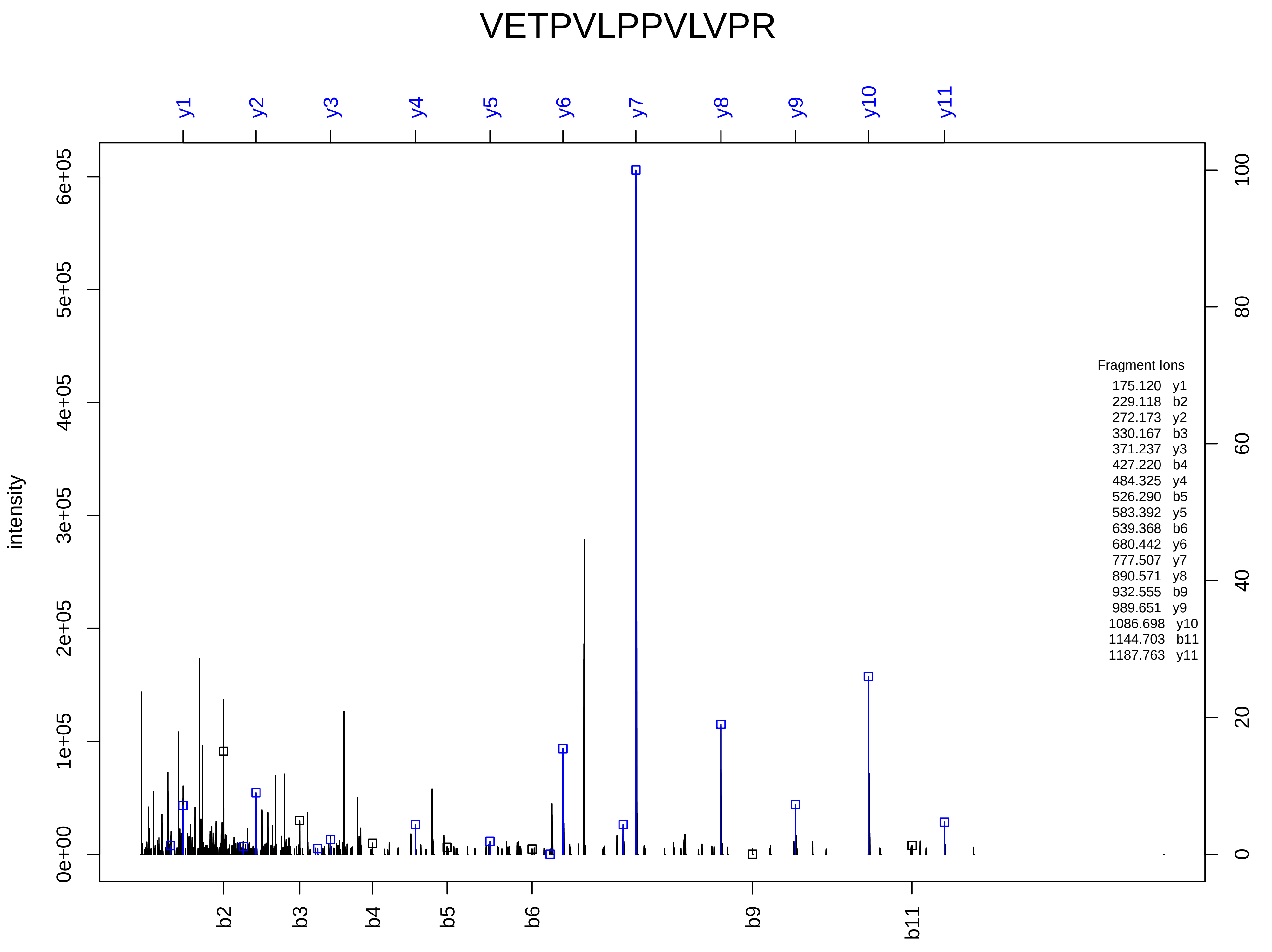

VETPVLPPVLVPR
y1
y2
y3
y4
y5
y6
y7
y8
y9
y10
y11
intensity
6e+05
5e+05
4e+05
3e+05
2e+05
1e+05
0e+00
100
80
60
40
20
0
Fragment Ions
175.120 y1
229.118 b2
272.173 y2
330.167 b3
371.237 y3
427.220 b4
484.325 y4
526.290 b5
583.392 y5
639.368 b6
680.442 y6
777.507 y7
890.571 y8
932.555 b9
989.651 y9
1086.698 y10
1144.703 b11
1187.763 y11
b2
b3
b4
b5
b6
b9
b11
m/z
VETPVLPPVLVPR / File: christine_ip_06.raw; SpectrumID: ; scans: 15718

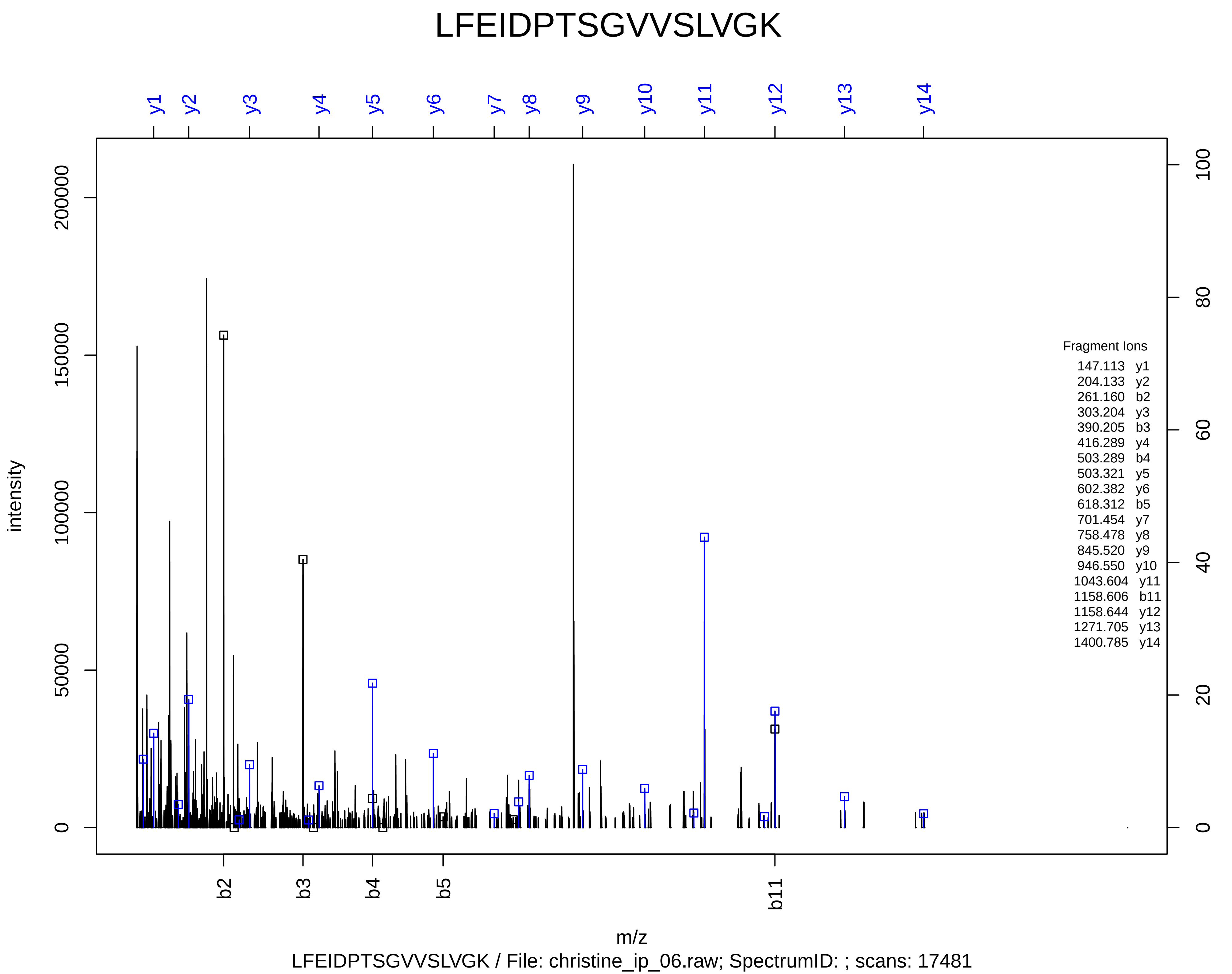
LFEIDPTSGVVSLVGK
y1
y2
y3
y4
y5
y6
y7
y8
y9
y10
y11
y12
y13
y14
intensity
0
50000
100000
150000
200000
0
20
40
60
80
100
b2
b3
b4
b5
b11
m/z
Fragment Ions
147.113 y1
204.133 y2
261.160 b2
303.204 y3
390.205 b3
416.289 y4
503.289 b4
503.321 y5
602.382 y6
618.312 b5
701.454 y7
758.478 y8
845.520 y9
946.550 y10
1043.604 y11
1158.606 b11
1158.644 y12
1271.705 y13
1400.785 y14
LFEIDPTSGVVSLVGK / File: christine_ip_06.raw; SpectrumID: ; scans: 17481

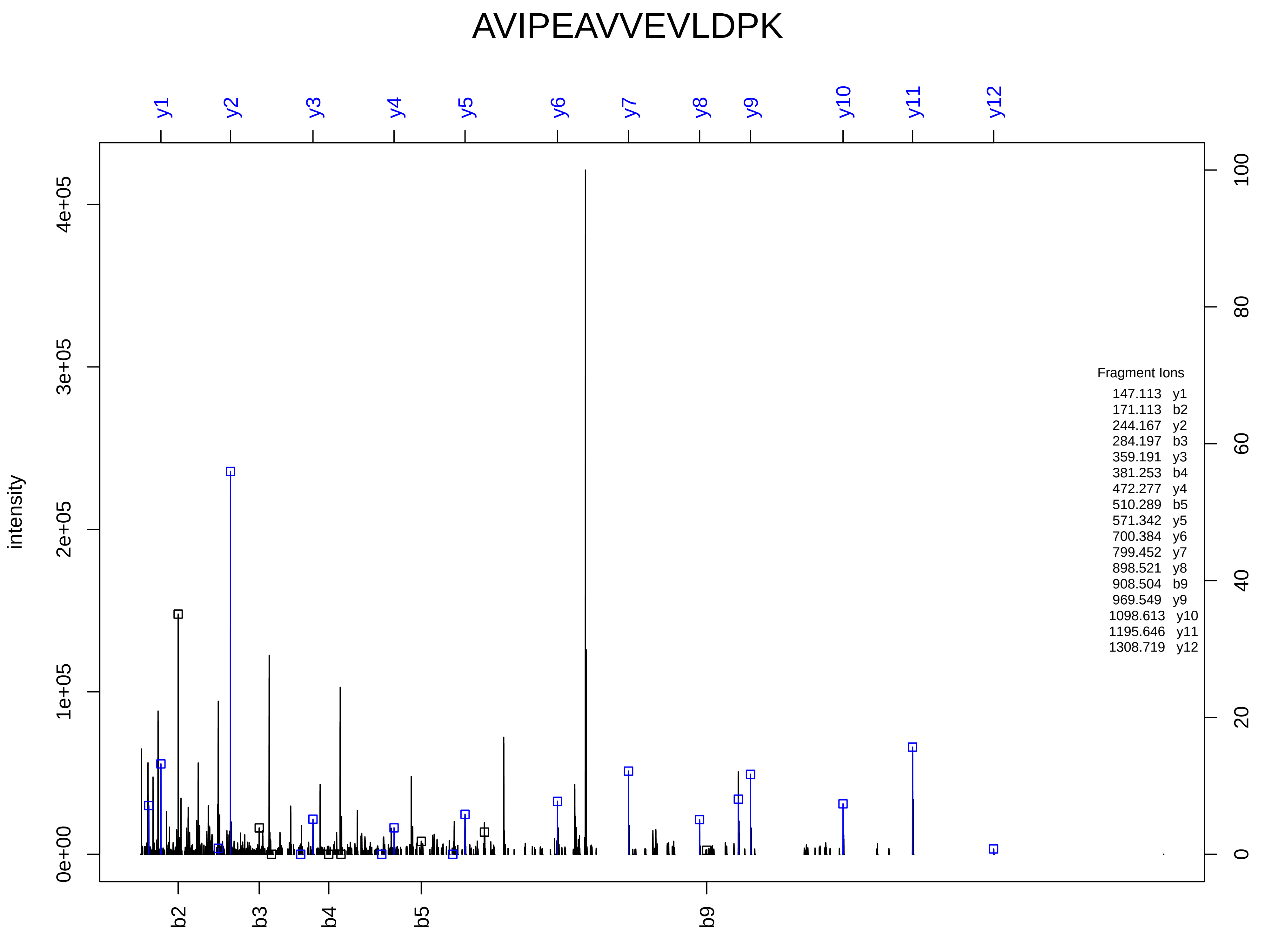

AVIPEAVVEVLDPK / File: christine_ip_06.raw; SpectrumID: ; scans: 17859

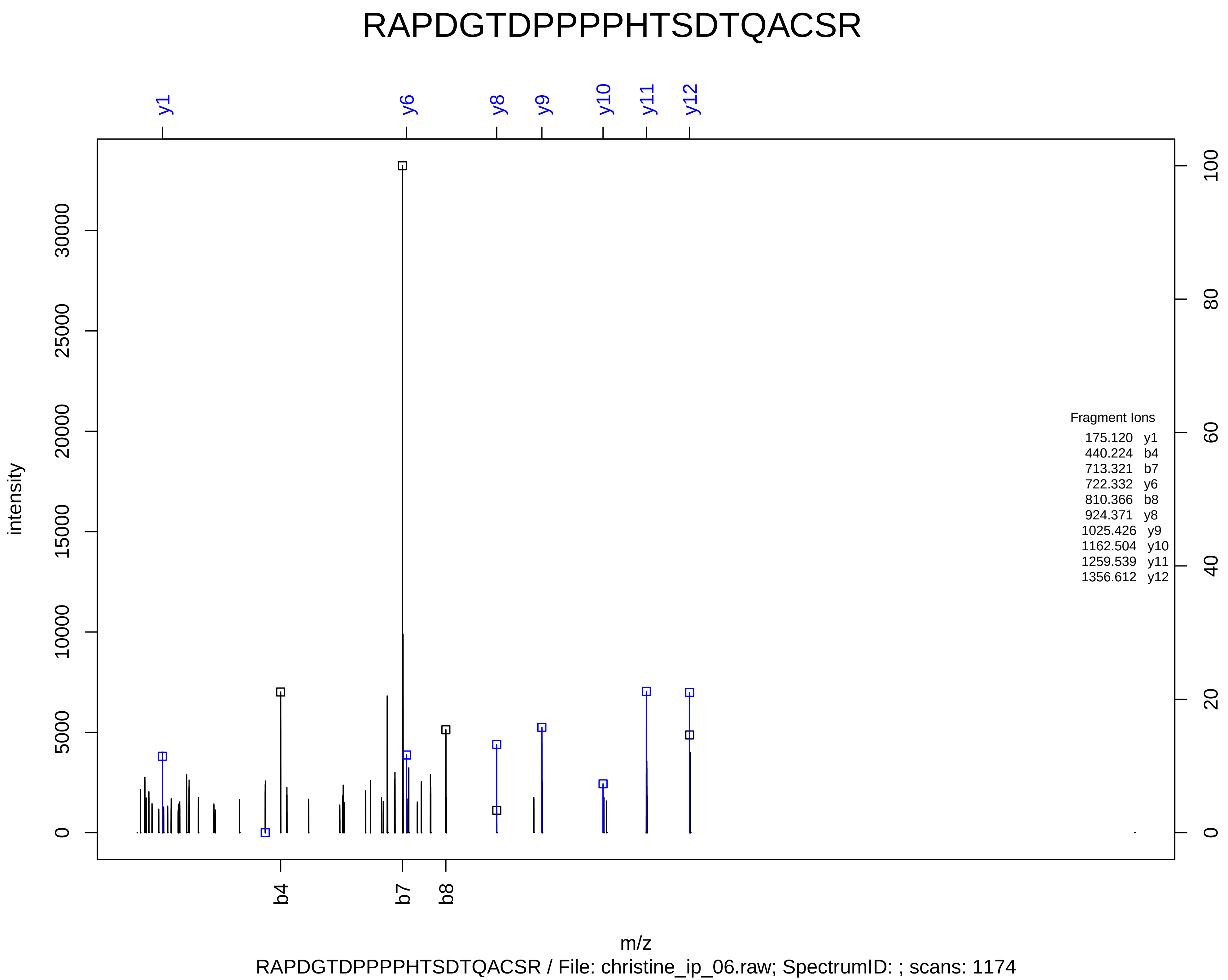

RAPDGTDPPPPHTSDTQACSR
y1
y6
y8
y9
y10
y11
y12
intensity
0
5000
10000
15000
20000
25000
30000
0
20
40
60
80
100
Fragment Ions
175.120 y1
440.224 b4
713.321 b7
722.332 y6
810.366 b8
924.371 y8
1025.426 y9
1162.504 y10
1259.539 y11
1356.612 y12
b4
b7
b8
m/z
RAPDGTDPPPPHTSDTQACSR / File: christine_ip_06.raw; SpectrumID: ; scans: 1174

LDDLVNWAR

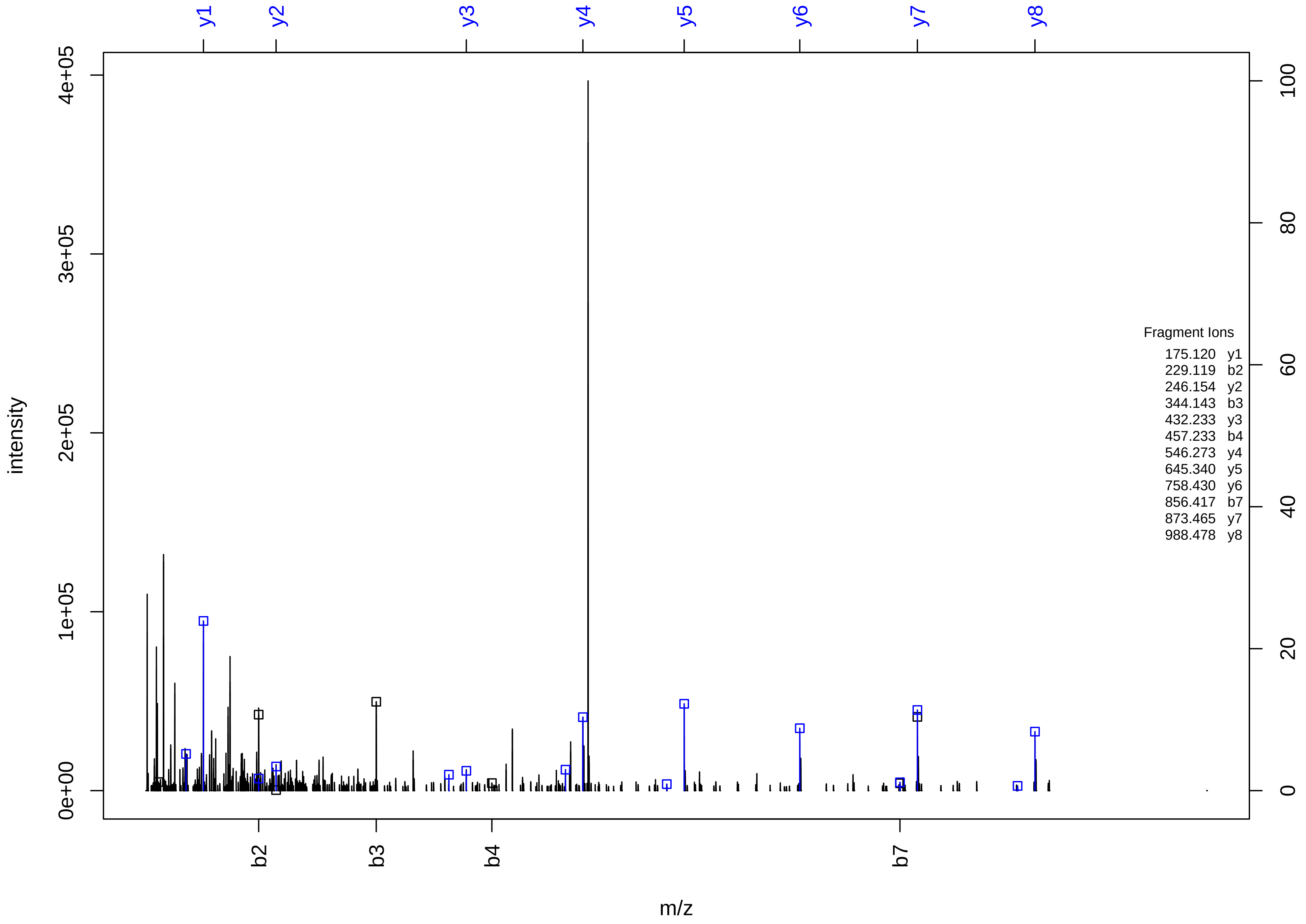

LDDLVNWAR / File: christine_ip_06.raw; SpectrumID: ; scans: 11153

DDNEECGDICPGTAK

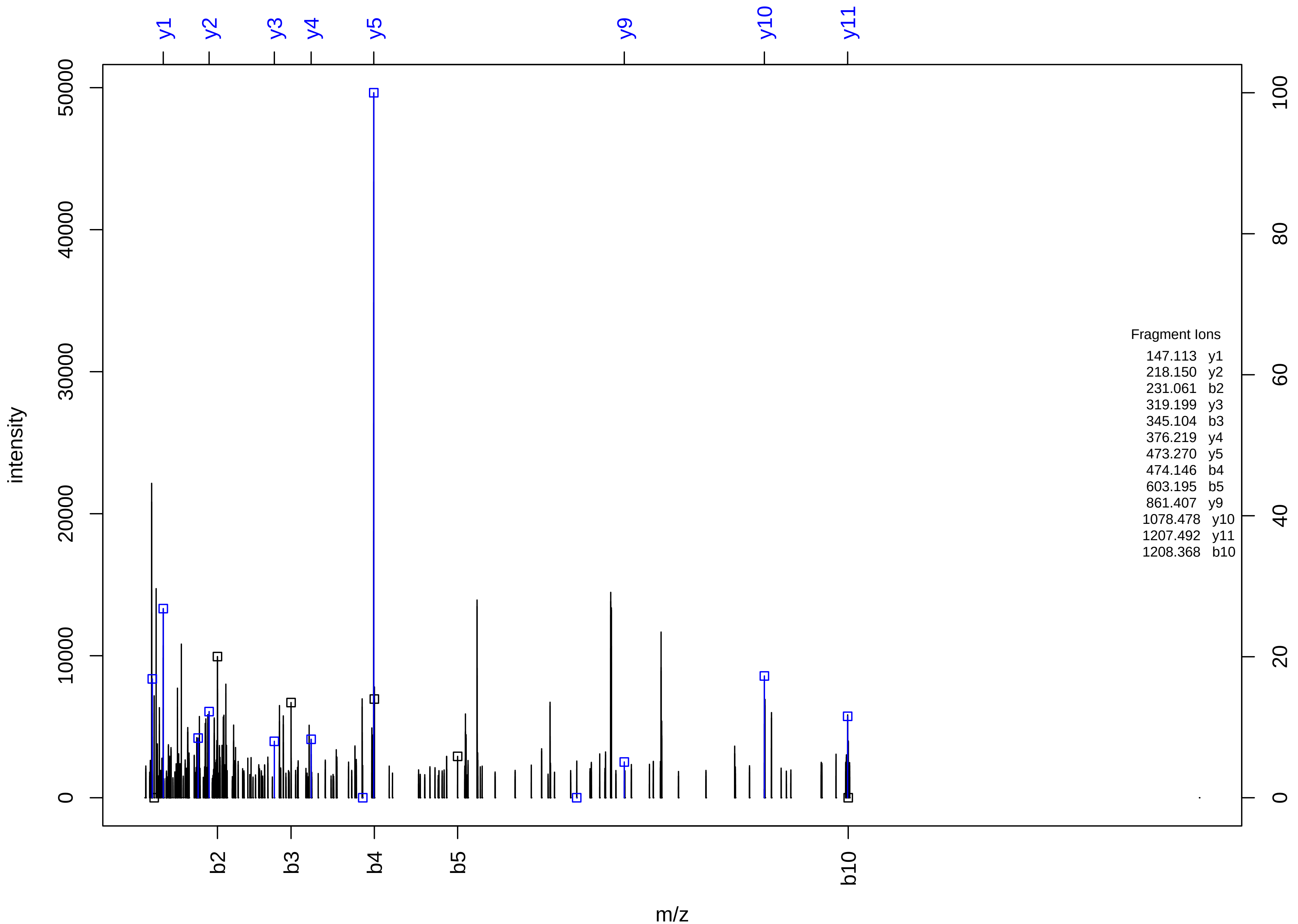

DDNEECGDICPGTAK / File: christine_ip_06.raw; SpectrumID: ; scans: 2826

TQVELLEAPTPALK

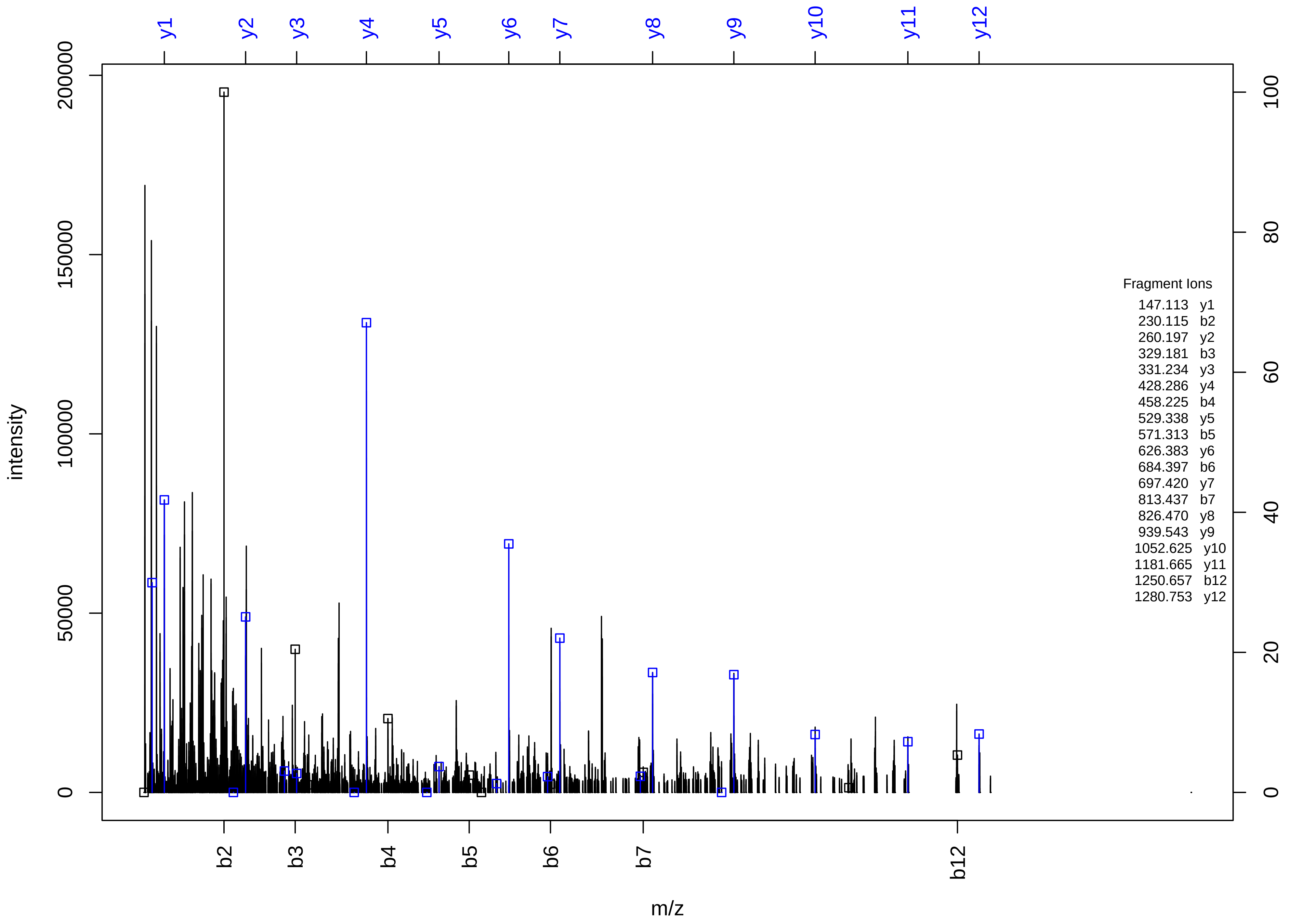

TQVELLEAPTPALK / File: christine_ip_06.raw; SpectrumID: ; scans: 12071

ISTLPQLNSALVQDLAK

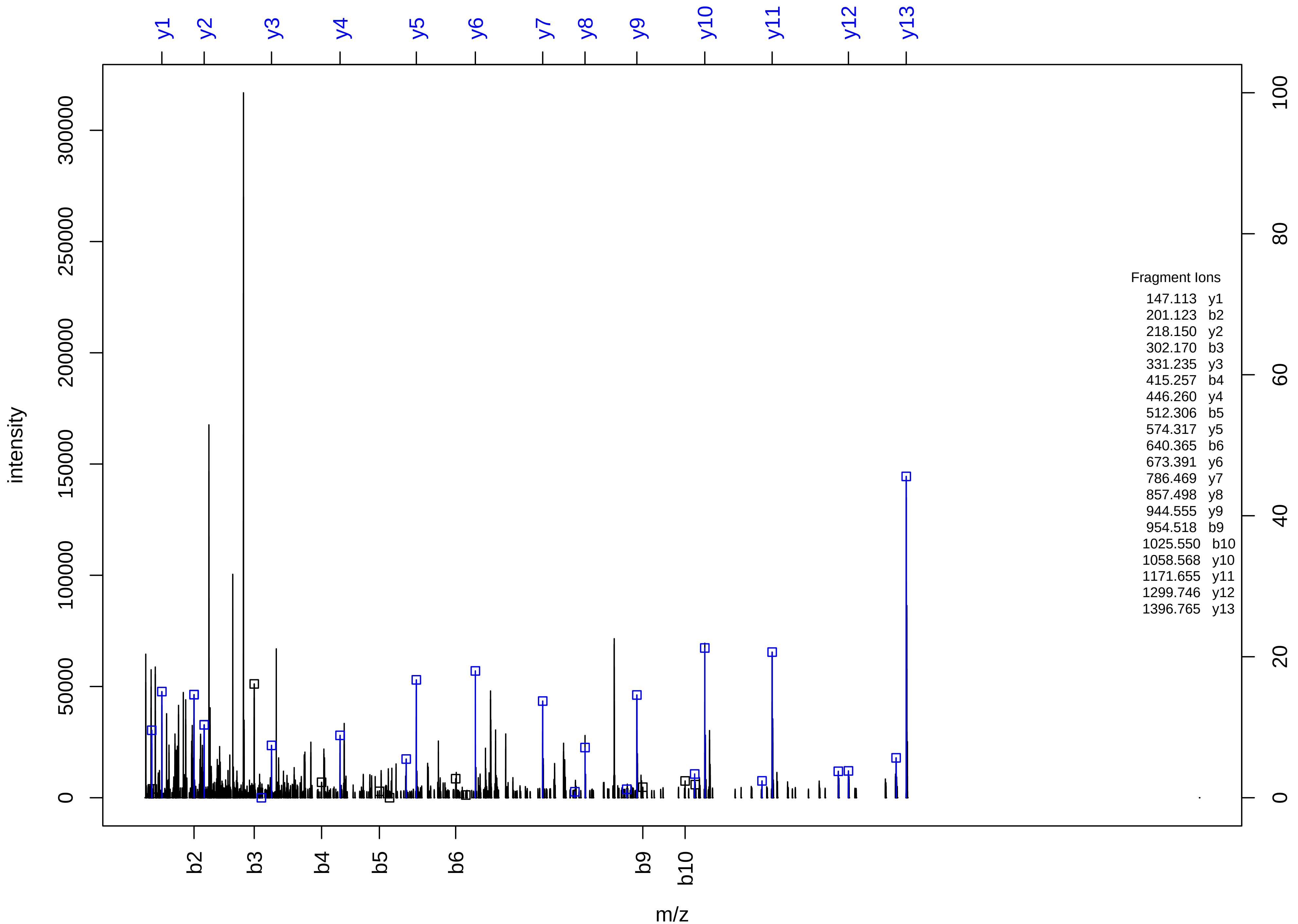

ISTLPQLNSALVQDLAK / File: christine_ip_06.raw; SpectrumID: ; scans: 16372

HTLADNFNPVSEER

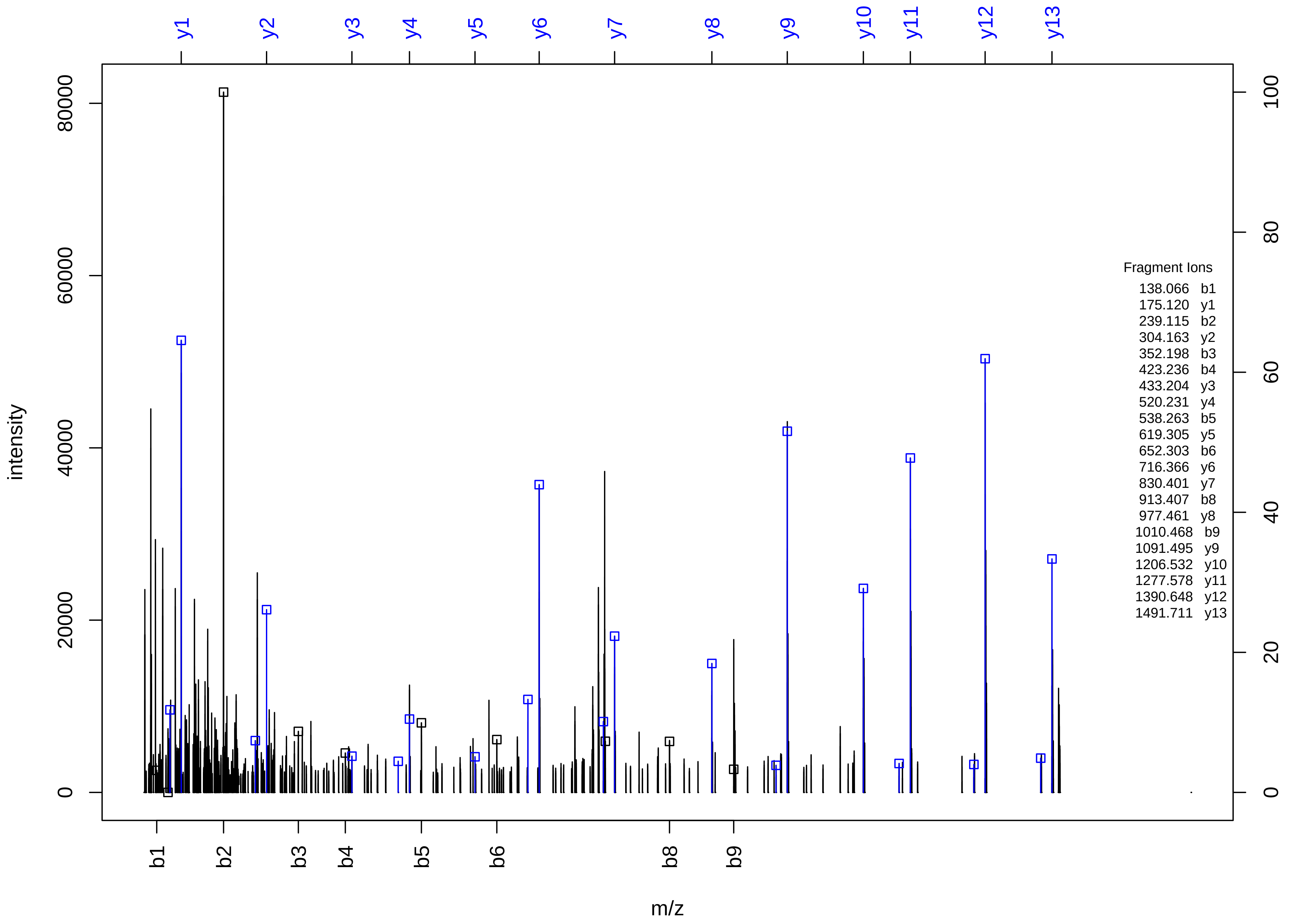

HTLADNFNPVSEER / File: christine_ip_06.raw; SpectrumID: ; scans: 7039

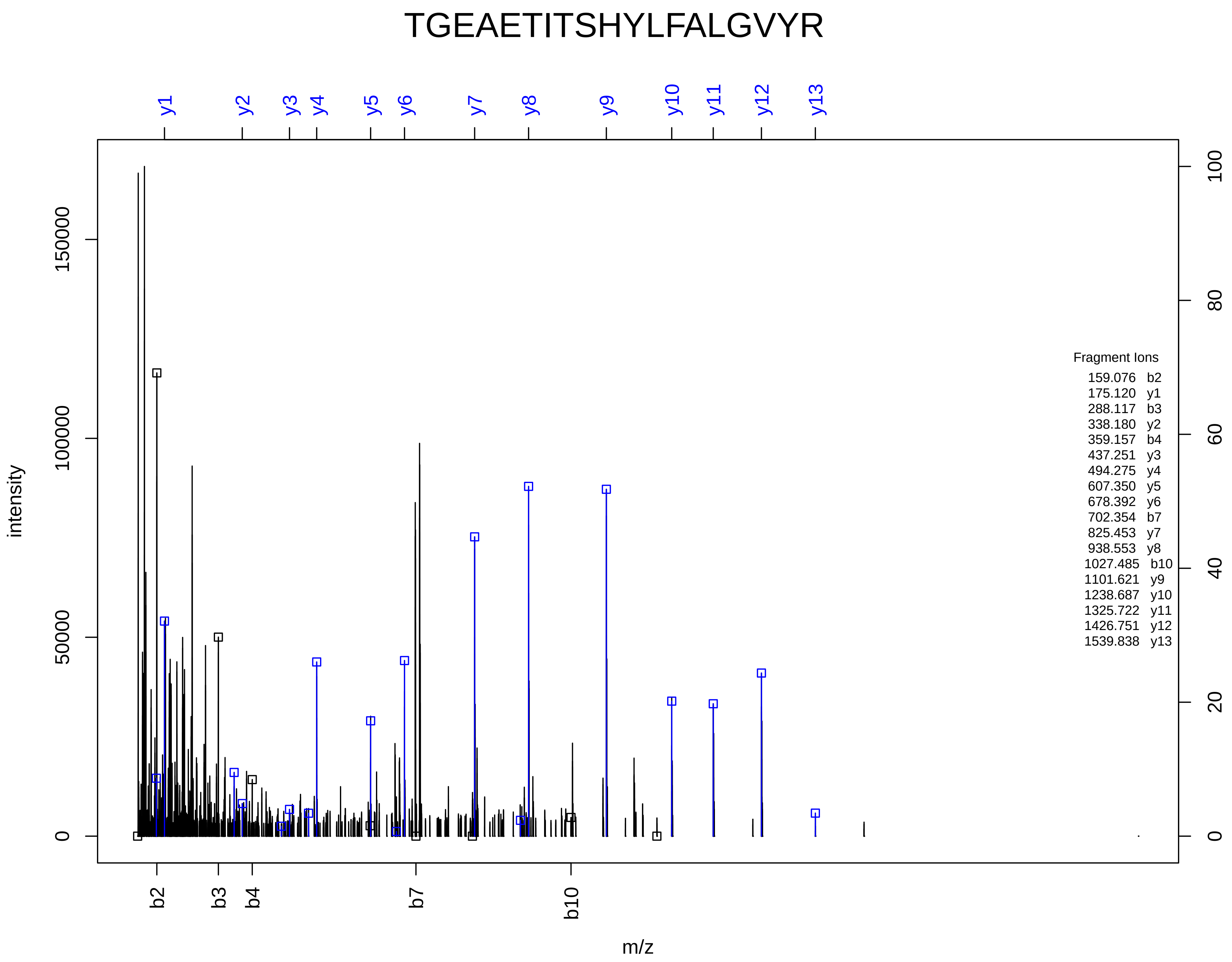

TGEAETITSHYLFALGVYR
y1
y2
y3
y4
y5
y6
y7
y8
y9
y10
y11
y12
y13
intensity
0
50000
100000
150000
0
20
40
60
80
100
Fragment Ions
159.076 b2
175.120 y1
288.117 b3
338.180 y2
359.157 b4
437.251 y3
494.275 y4
607.350 y5
678.392 y6
702.354 b7
825.453 y7
938.553 y8
1027.485 b10
1101.621 y9
1238.687 y10
1325.722 y11
1426.751 y12
1539.838 y13
b2
b3
b4
b7
b10
m/z
TGEAETITSHYLFALGVYR / File: christine_ip_06.raw; SpectrumID: ; scans: 15845

LAAIAESGVER

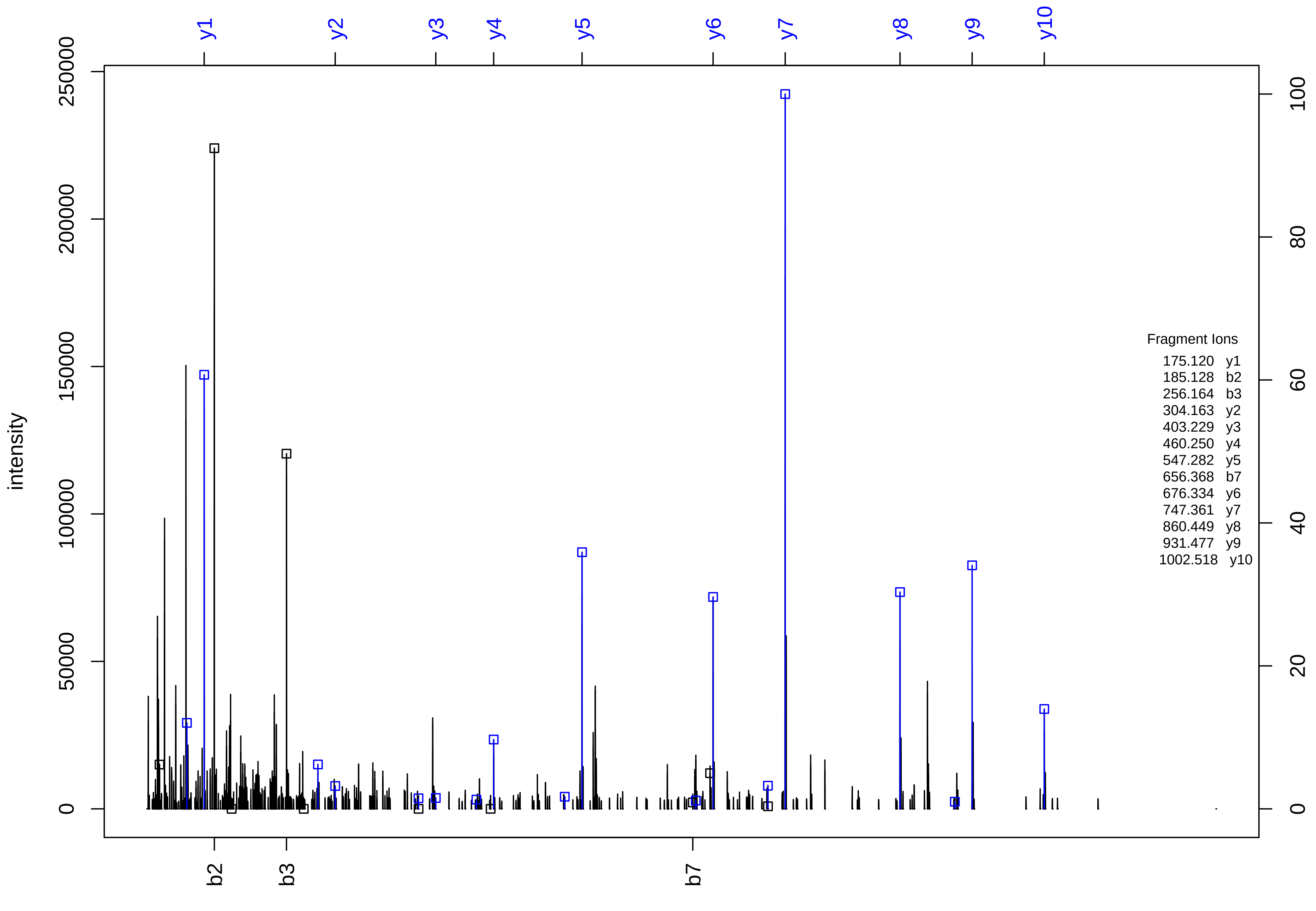

LAAIAESGVER / File: christine_ip_06.raw; SpectrumID: ; scans: 4511

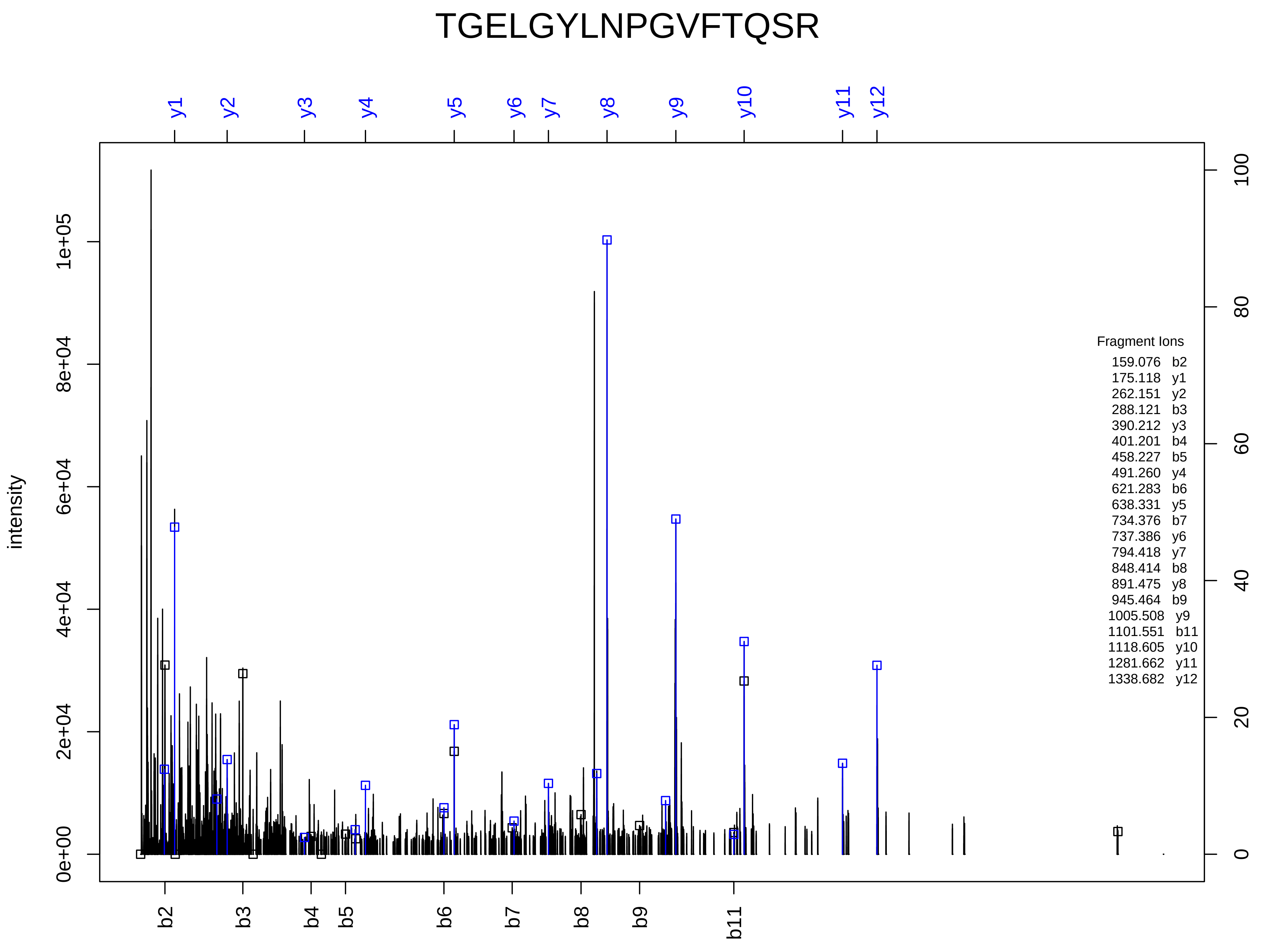

TGELGYLNPGVFTQSR / File: christine_ip_06.raw; SpectrumID: ; scans: 13605

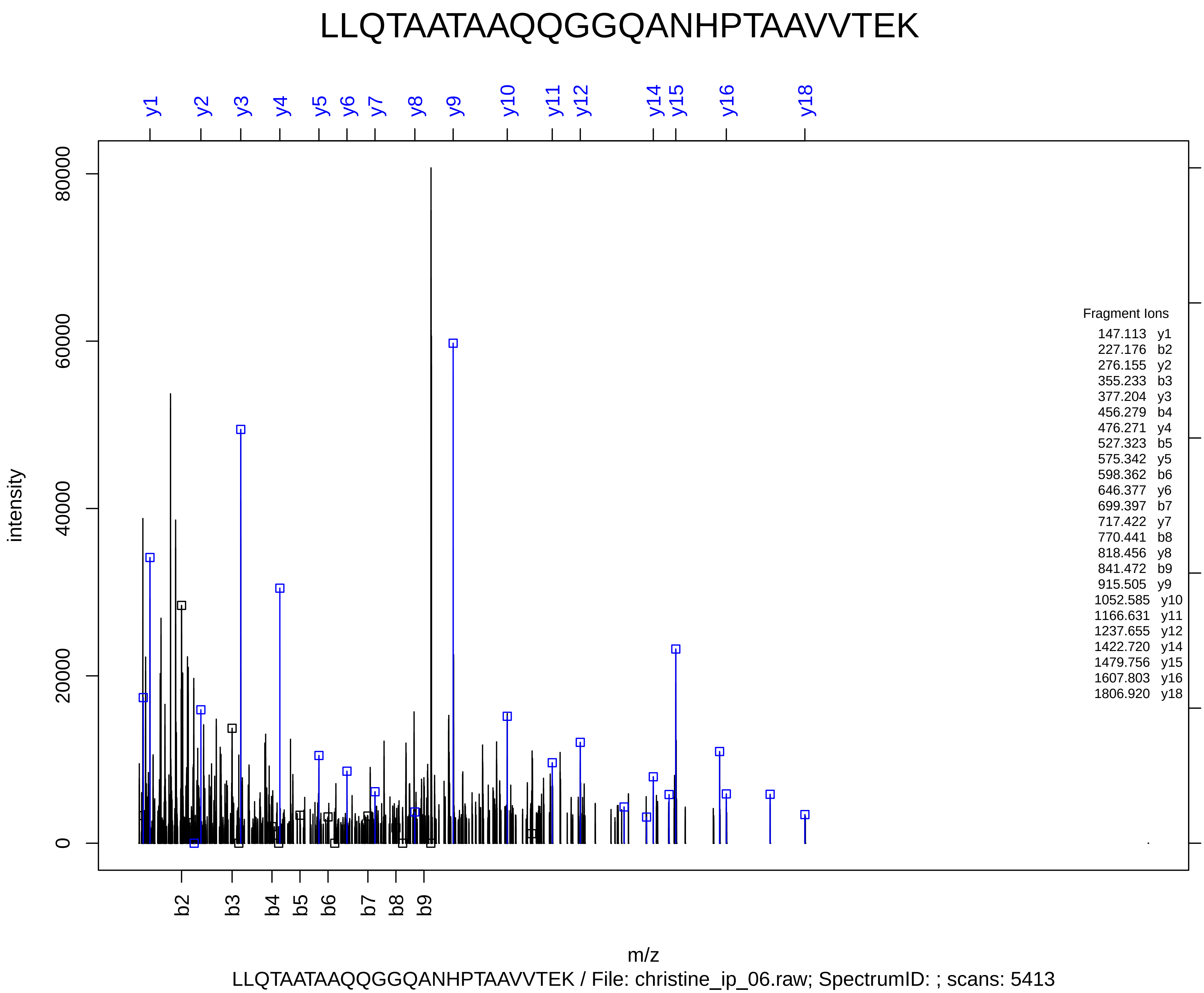
LLQTAATAAQQGGQANHPTAAVVTEK
y1 y2 y3 y4 y5 y6 y7 y8 y9 y10 y11 y12 y14 y15 y16 y18
b2 b3 b4 b5 b6 b7 b8 b9
intensity
m/z
Fragment Ions
147.113 y1
227.176 b2
276.155 y2
355.233 b3
377.204 y3
456.279 b4
476.271 y4
527.323 b5
575.342 y5
598.362 b6
646.377 y6
699.397 b7
717.422 y7
770.441 b8
818.456 y8
841.472 b9
915.505 y9
1052.585 y10
1166.631 y11
1237.655 y12
1422.720 y14
1479.756 y15
1607.803 y16
1806.920 y18
LLQTAATAAQQGGQANHPTAAVVTEK / File: christine_ip_06.raw; SpectrumID: ; scans: 5413

EAEEEETTN^DN^GVLVLEPAR

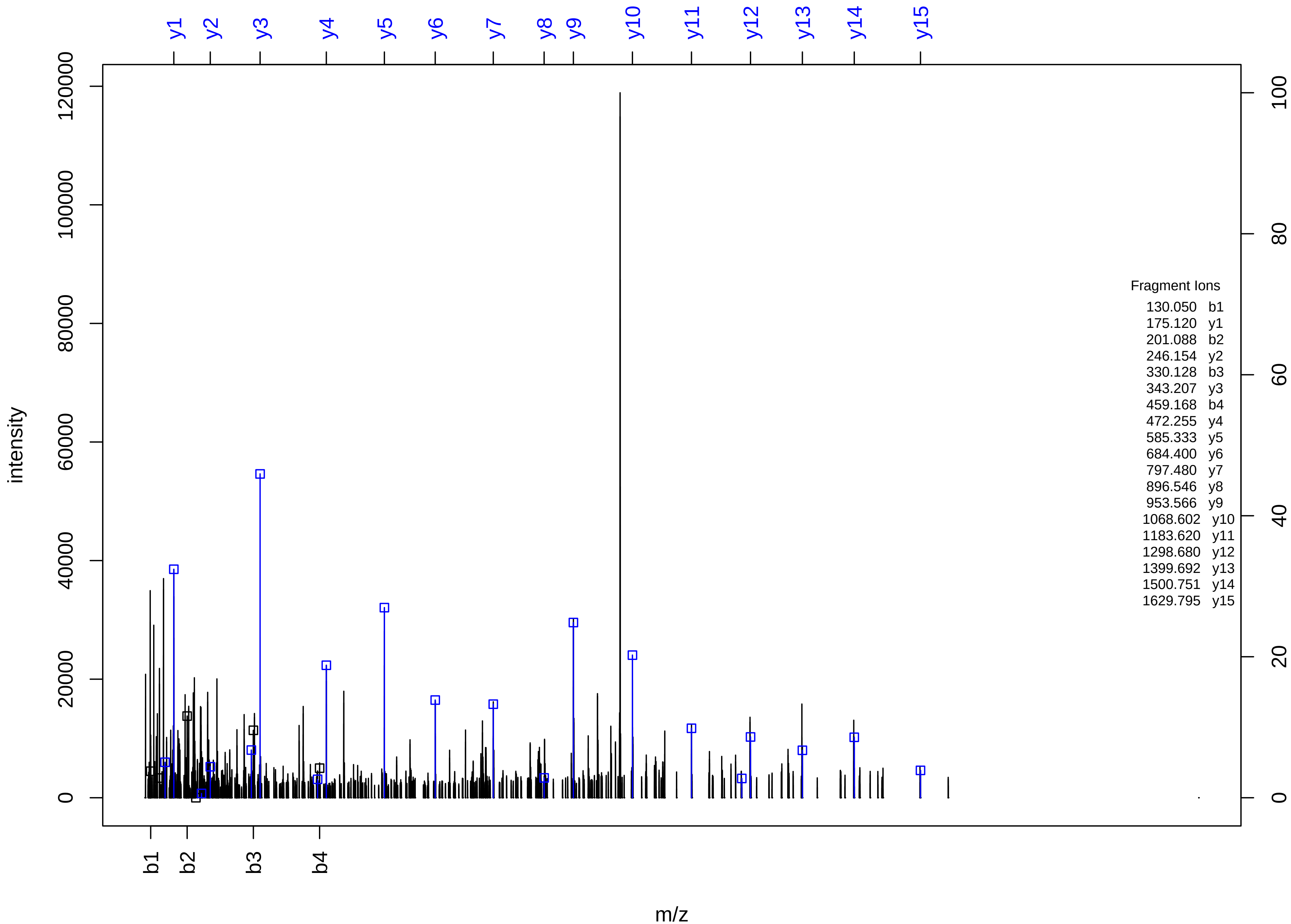

EAEEEETTNDNGVLVLEPAR / File: christine_ip_06.raw; SpectrumID: ; scans: 10247

LDFLIPLSK

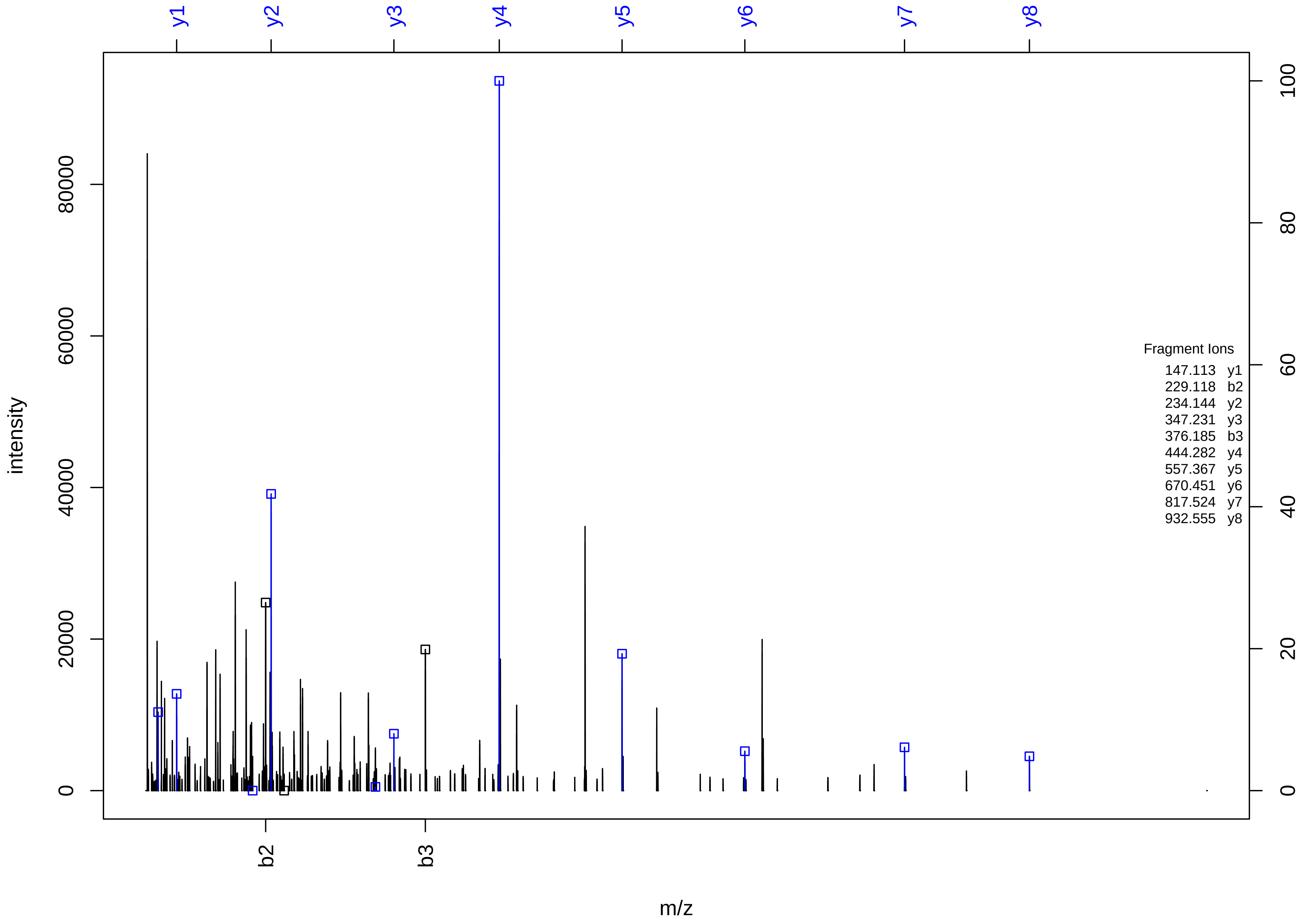

LDFLIPLSK / File: christine_ip_06.raw; SpectrumID: ; scans: 16453

ILFIGGITAPTVR

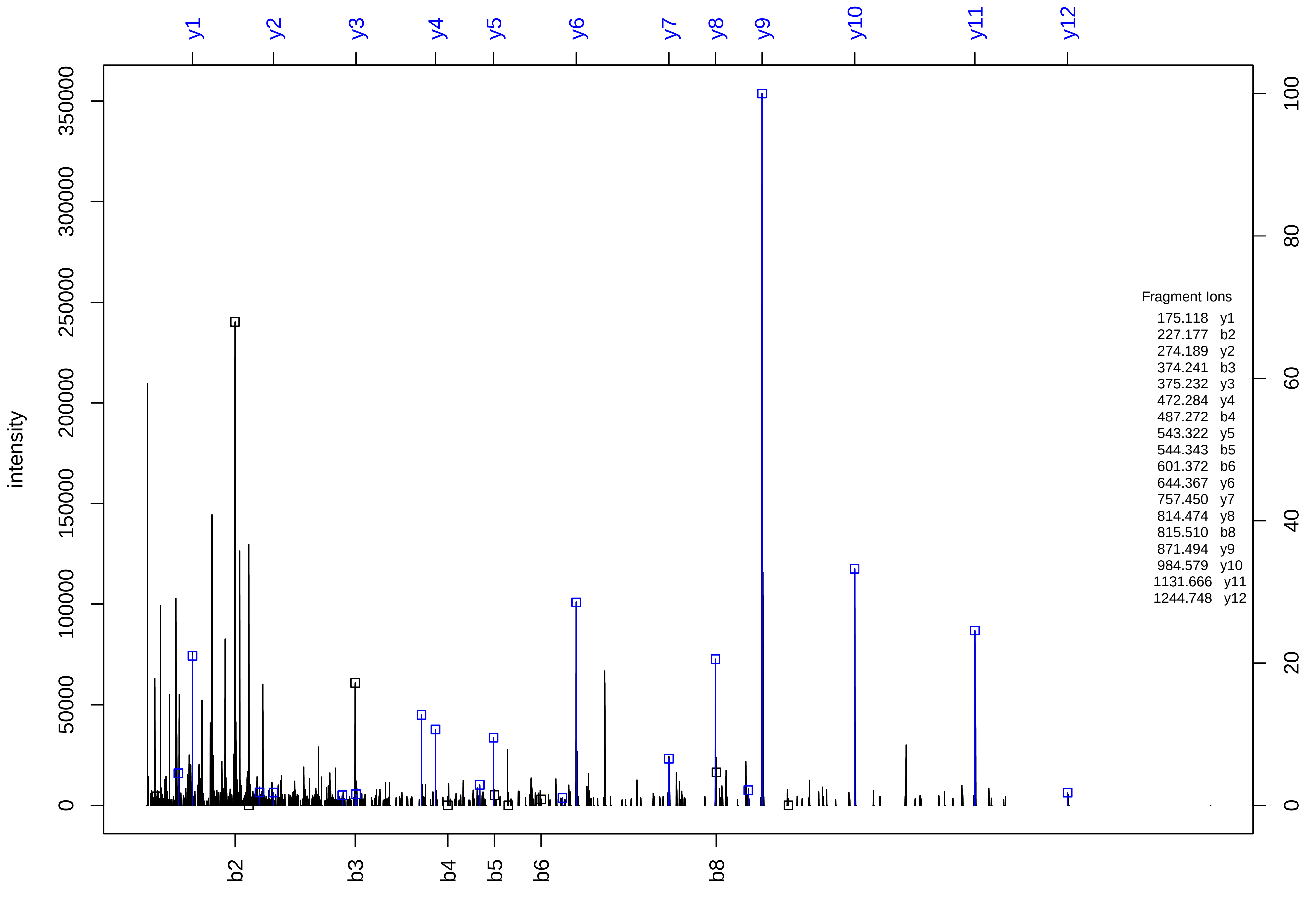

ILFIGGITAPTVR / File: christine_ip_06.raw; SpectrumID: ; scans: 15843

DFSPSGIFGAFQR

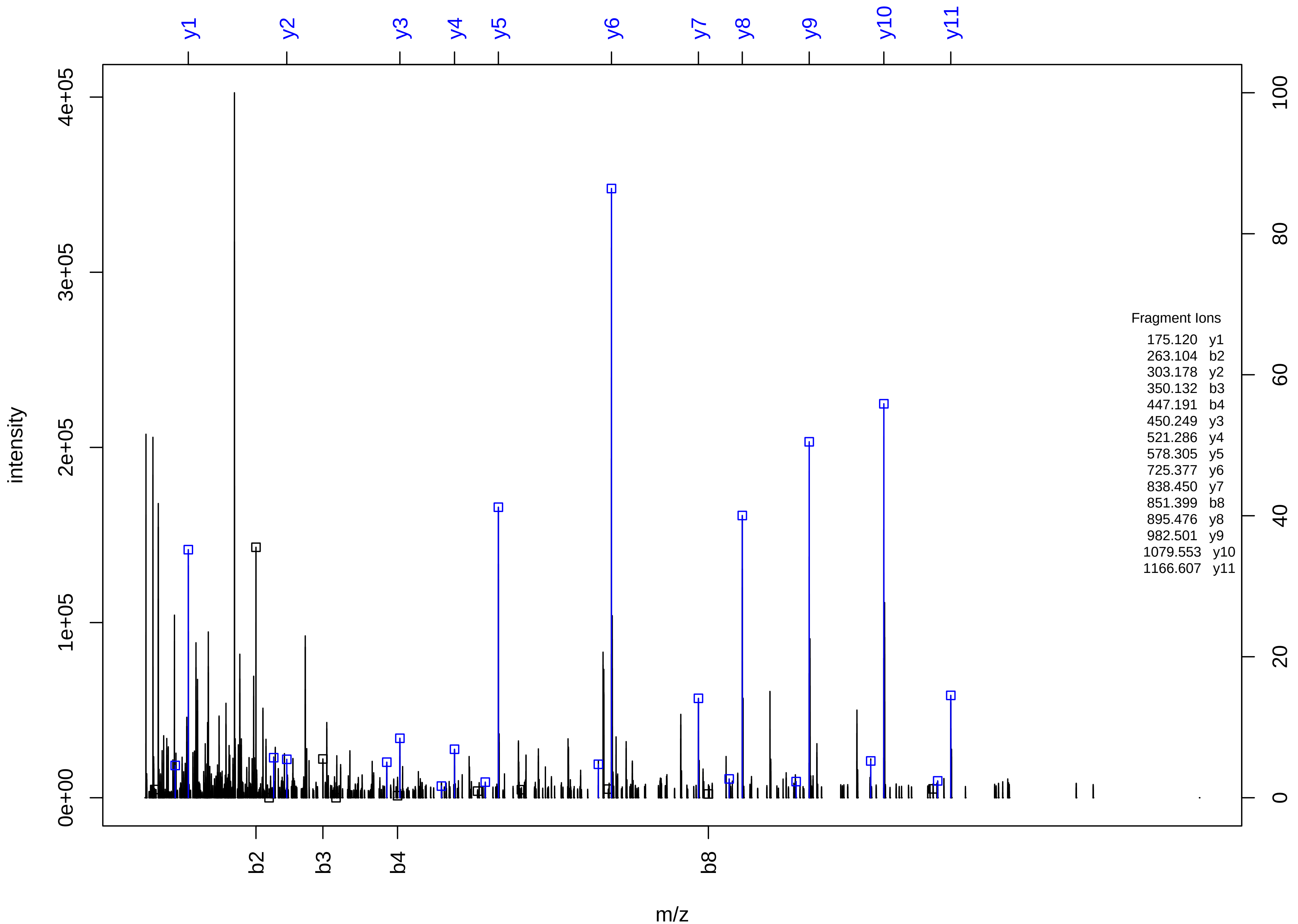

DFSPSGIFGAFQR / File: christine_ip_06.raw; SpectrumID: ; scans: 17269

QNRPIPQWIR

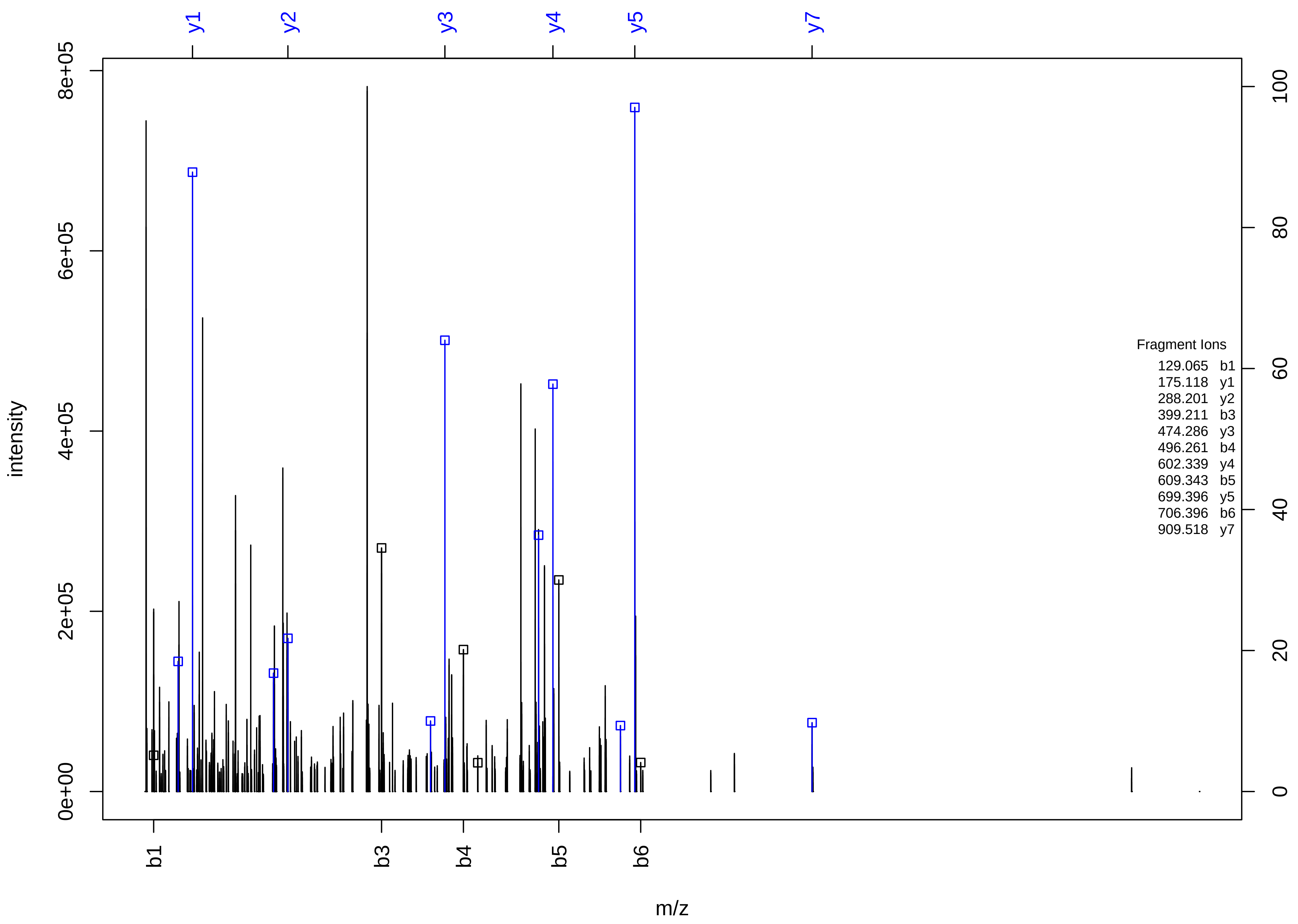

QNRPIPQWIR / File: christine_ip_06.raw; SpectrumID: ; scans: 6754

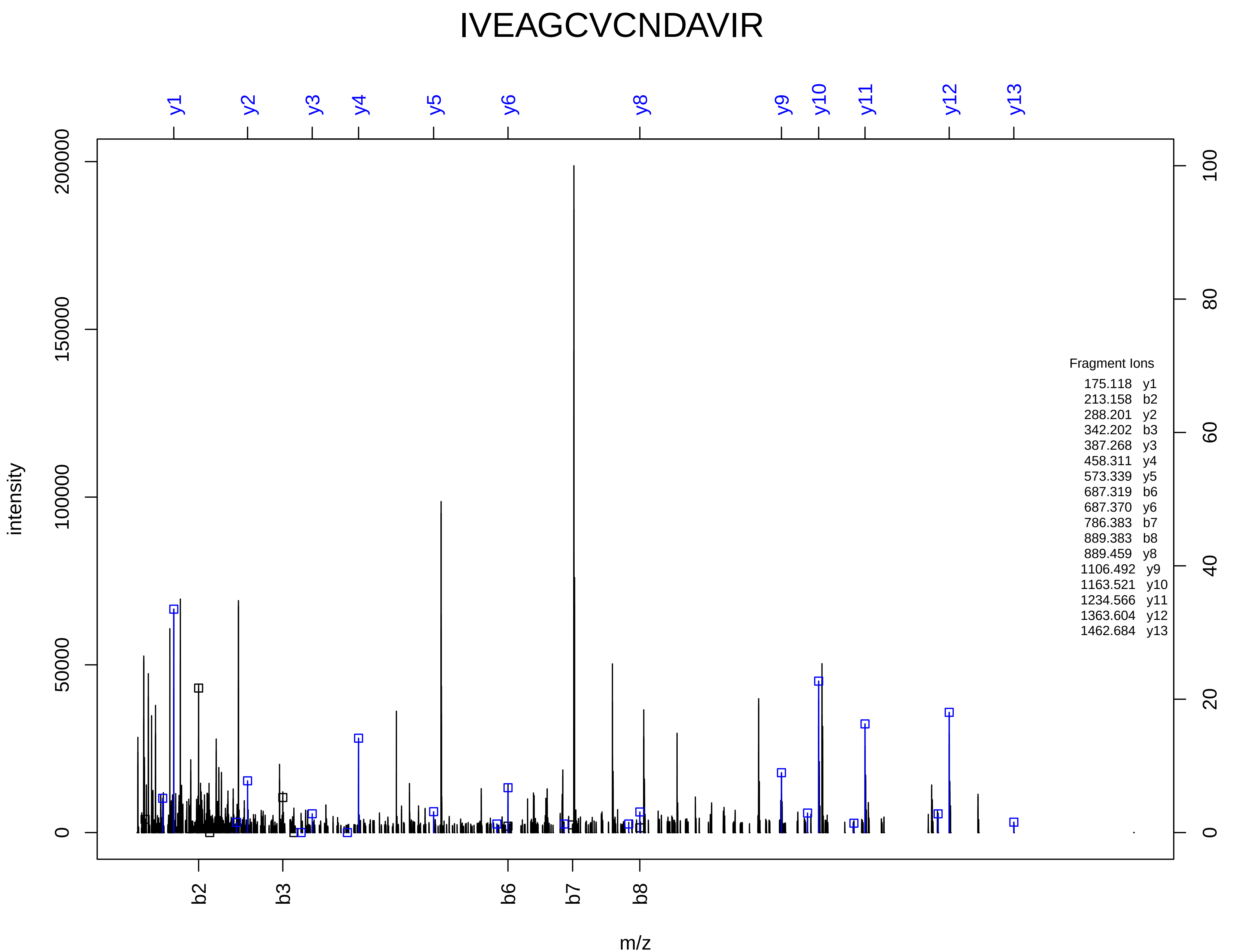

IVEAGCVCNDAVIR / File: christine_ip_06.raw; SpectrumID: ; scans: 6682

QVAAVGQEPQVFGR

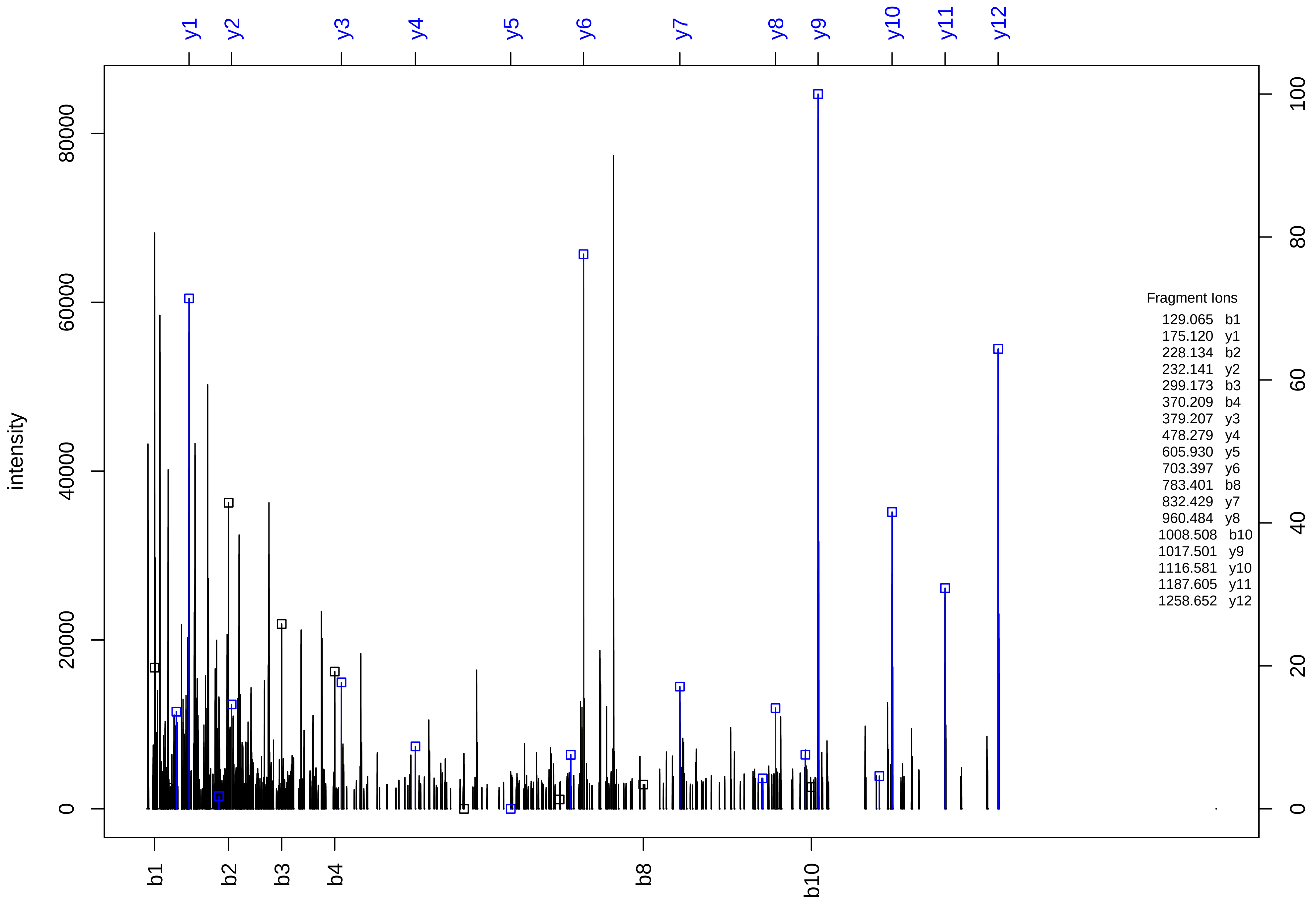

QVAAVGQEPQVFGR / File: christine_ip_06.raw; SpectrumID: ; scans: 7650

WEESGPQFITNSEEVR

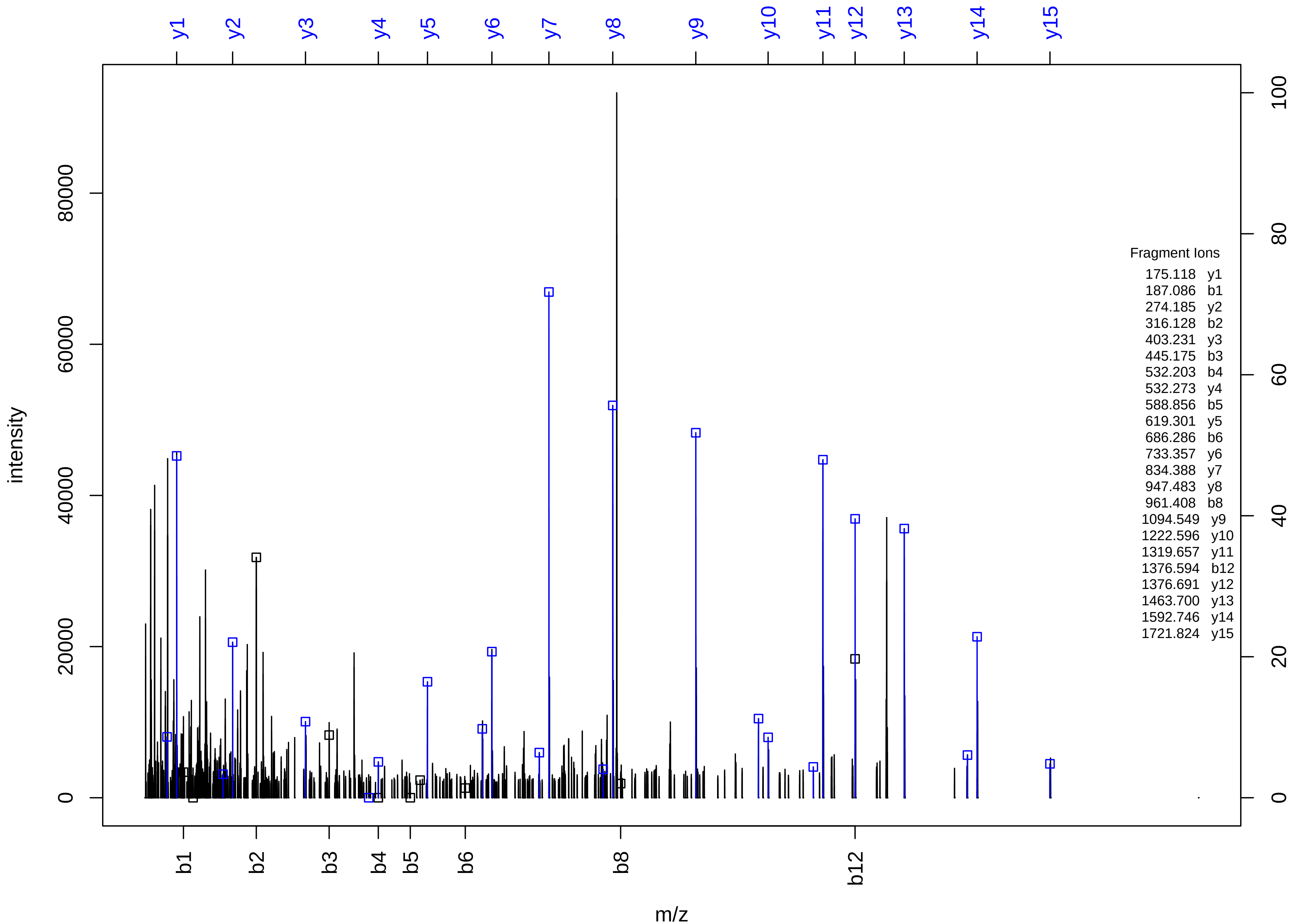

WEESGPQFITNSEEVR / File: christine_ip_06.raw; SpectrumID: ; scans: 10116

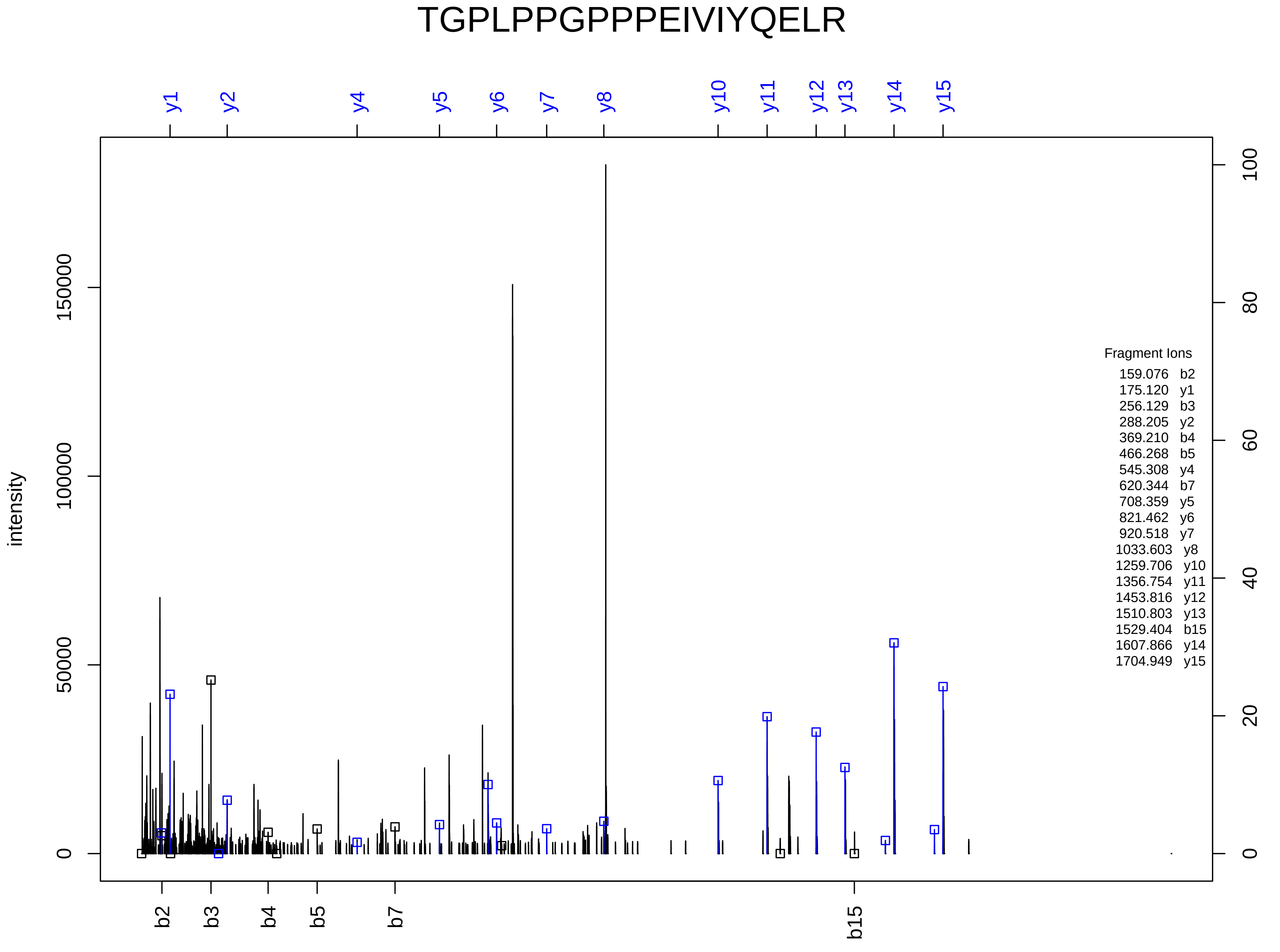

TGPLPPGPPPEIVIYQELR / File: christine_ip_06.raw; SpectrumID: ; scans: 17103

LLSTDAEAVSTR

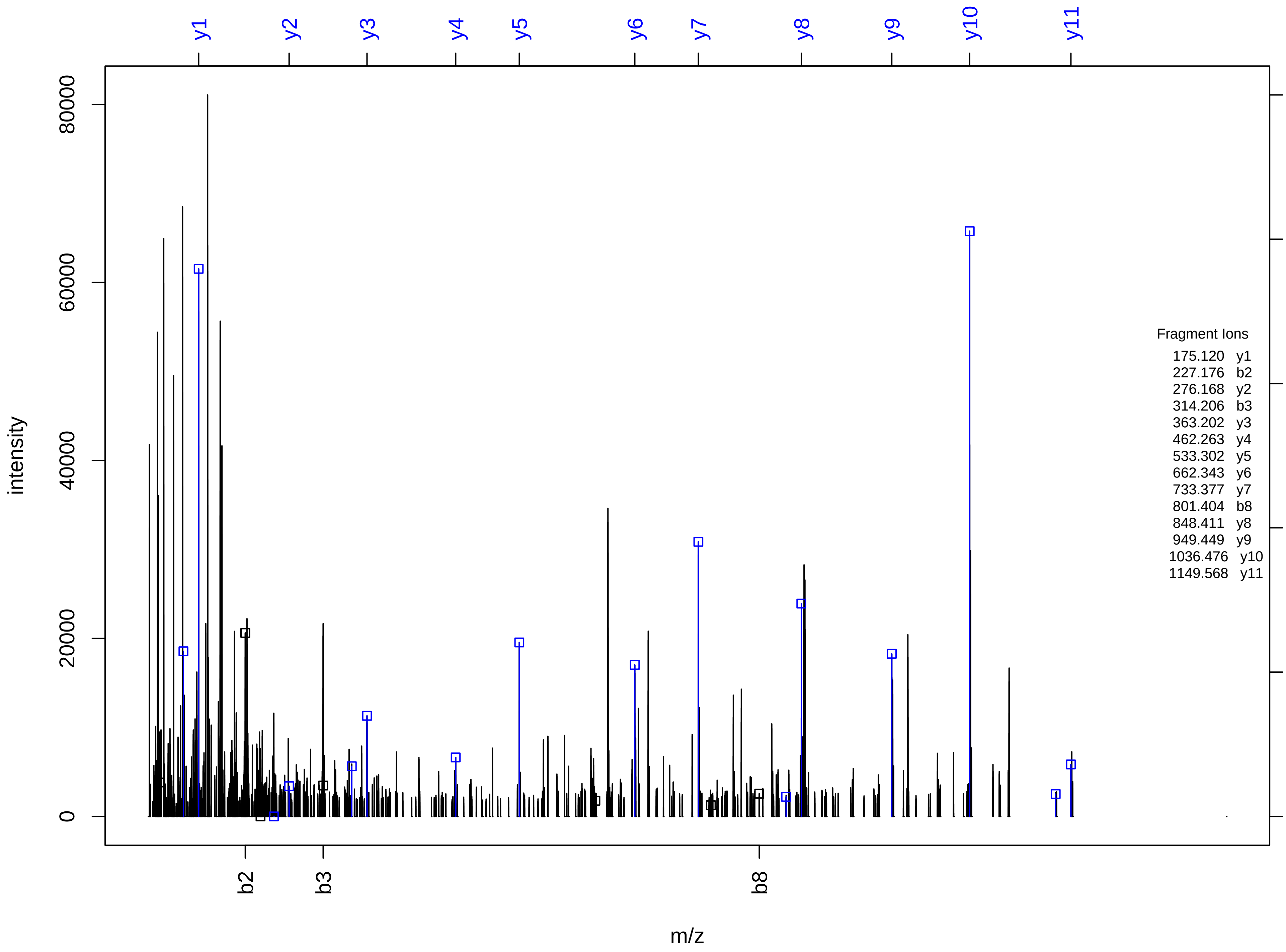

LLSTDAEAVSTR / File: christine_ip_06.raw; SpectrumID: ; scans: 4839

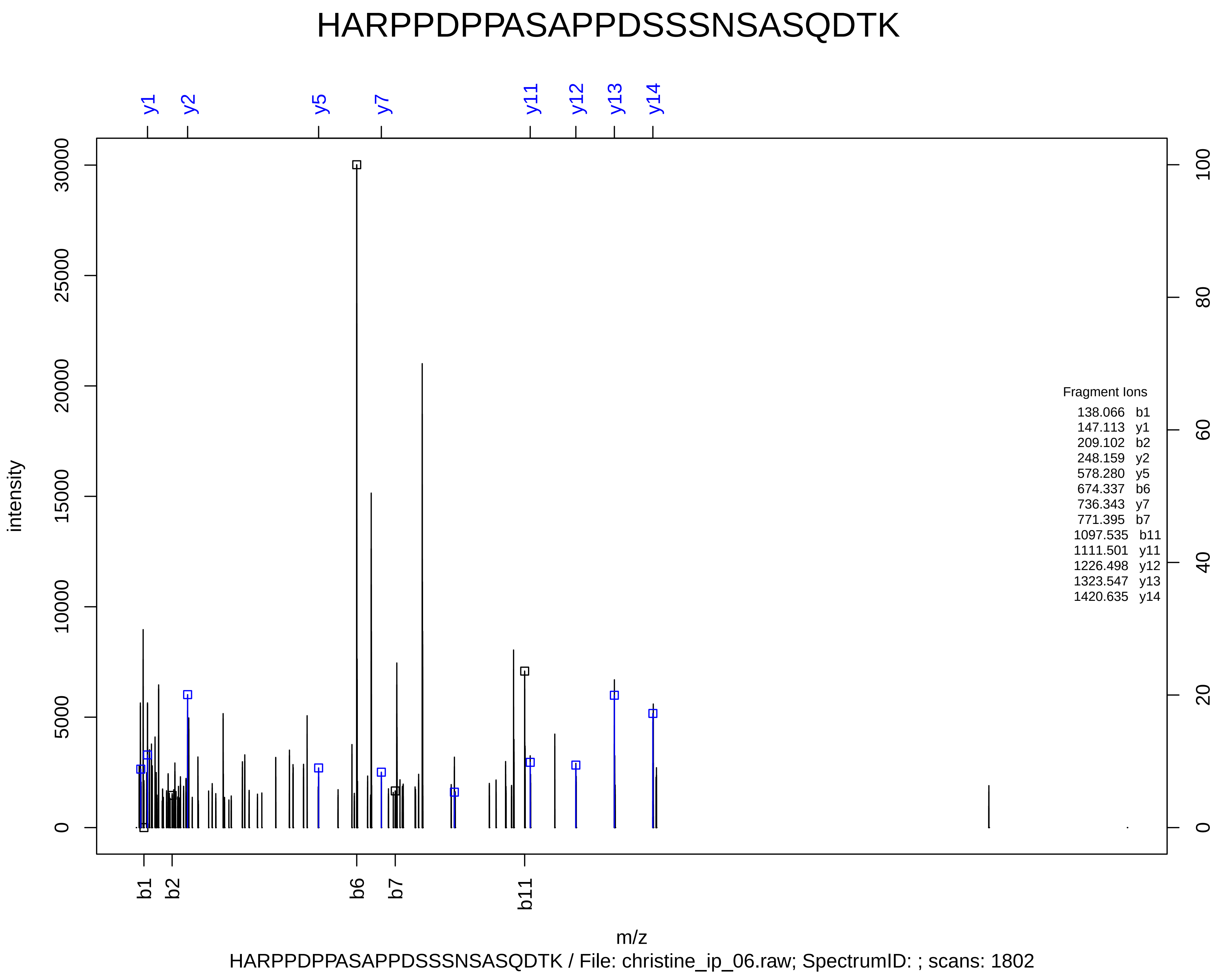
HARPPDPPASAPPDSSSNSASQDTK
y1
y2
y5
y7
y11
y12
y13
y14
intensity
0
5000
10000
15000
20000
25000
30000
0
20
40
60
80
100
Fragment Ions
138.066 b1
147.113 y1
209.102 b2
248.159 y2
578.280 y5
674.337 b6
736.343 y7
771.395 b7
1097.535 b11
1111.501 y11
1226.498 y12
1323.547 y13
1420.635 y14
b1
b2
b6
b7
b11
m/z
HARPPDPPASAPPDSSSNSASQDTK / File: christine_ip_06.raw; SpectrumID: ; scans: 1802

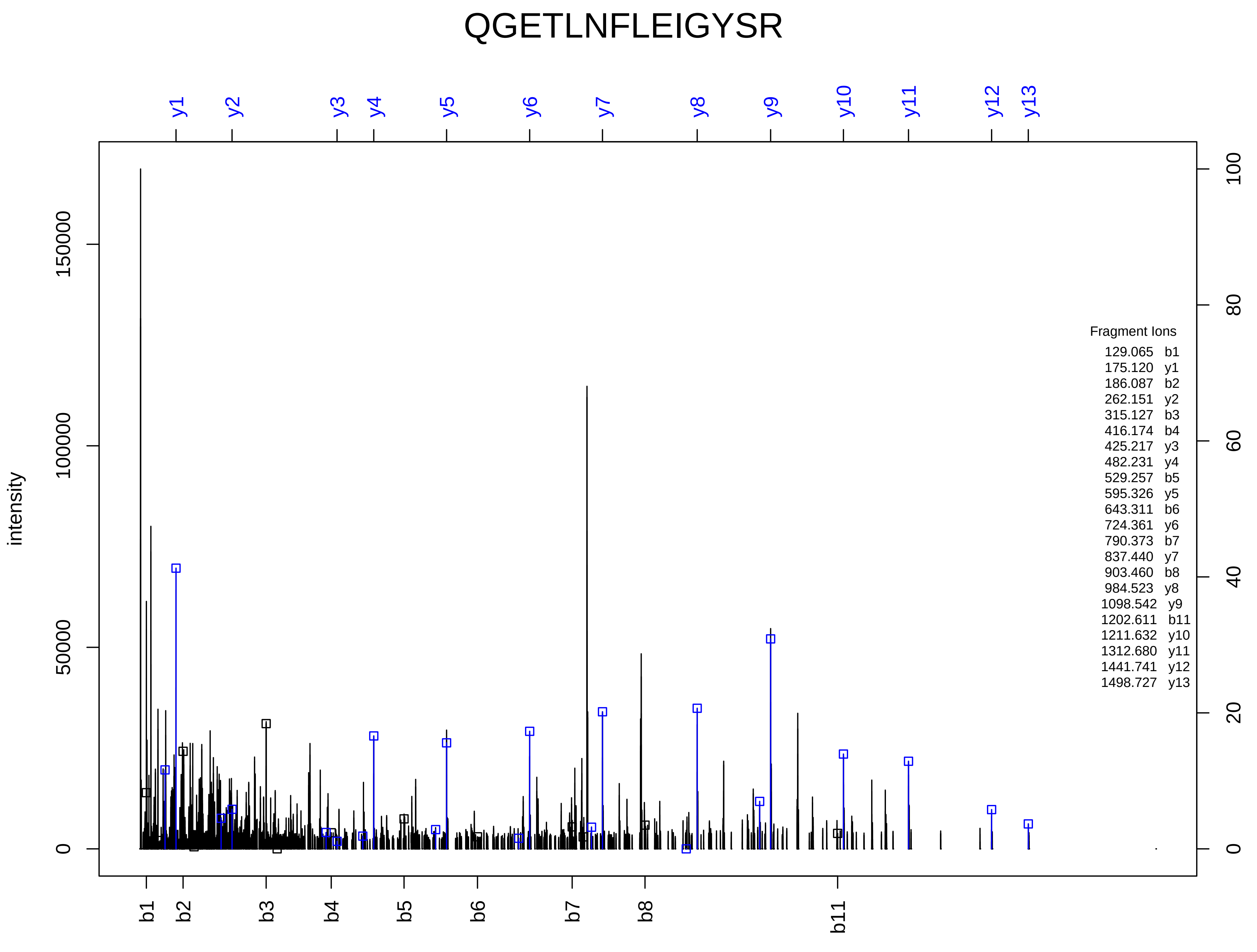

QGETLNFLEIGYSR / File: christine_ip_06.raw; SpectrumID: ; scans: 14905

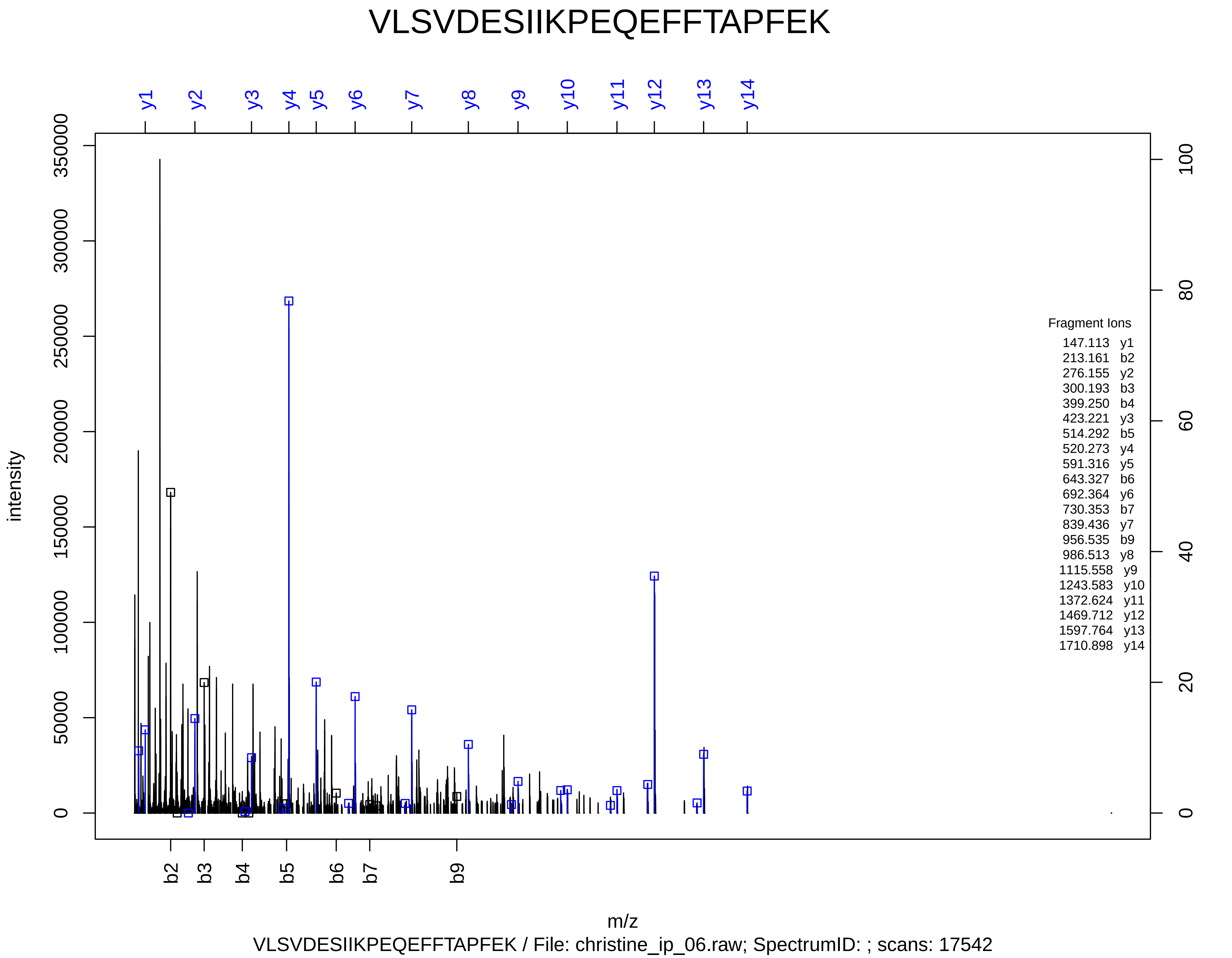
VLSVDESIIKPEQEFFTAPFEK
y1 y2 y3 y4 y5 y6 y7 y8 y9 y10 y11 y12 y13 y14
intensity
0
50000
100000
150000
200000
250000
300000
350000
0
20
40
60
80
100
b2 b3 b4 b5 b6 b7 b9
m/z
Fragment Ions
147.113 y1
213.161 b2
276.155 y2
300.193 b3
399.250 b4
423.221 y3
514.292 b5
520.273 y4
591.316 y5
643.327 b6
692.364 y6
730.353 b7
839.436 y7
956.535 b9
986.513 y8
1115.558 y9
1243.583 y10
1372.624 y11
1469.712 y12
1597.764 y13
1710.898 y14
VLSVDESIIKPEQEFFTAPFEK / File: christine_ip_06.raw; SpectrumID: ; scans: 17542

FTEDLVGSVVHVLSHR

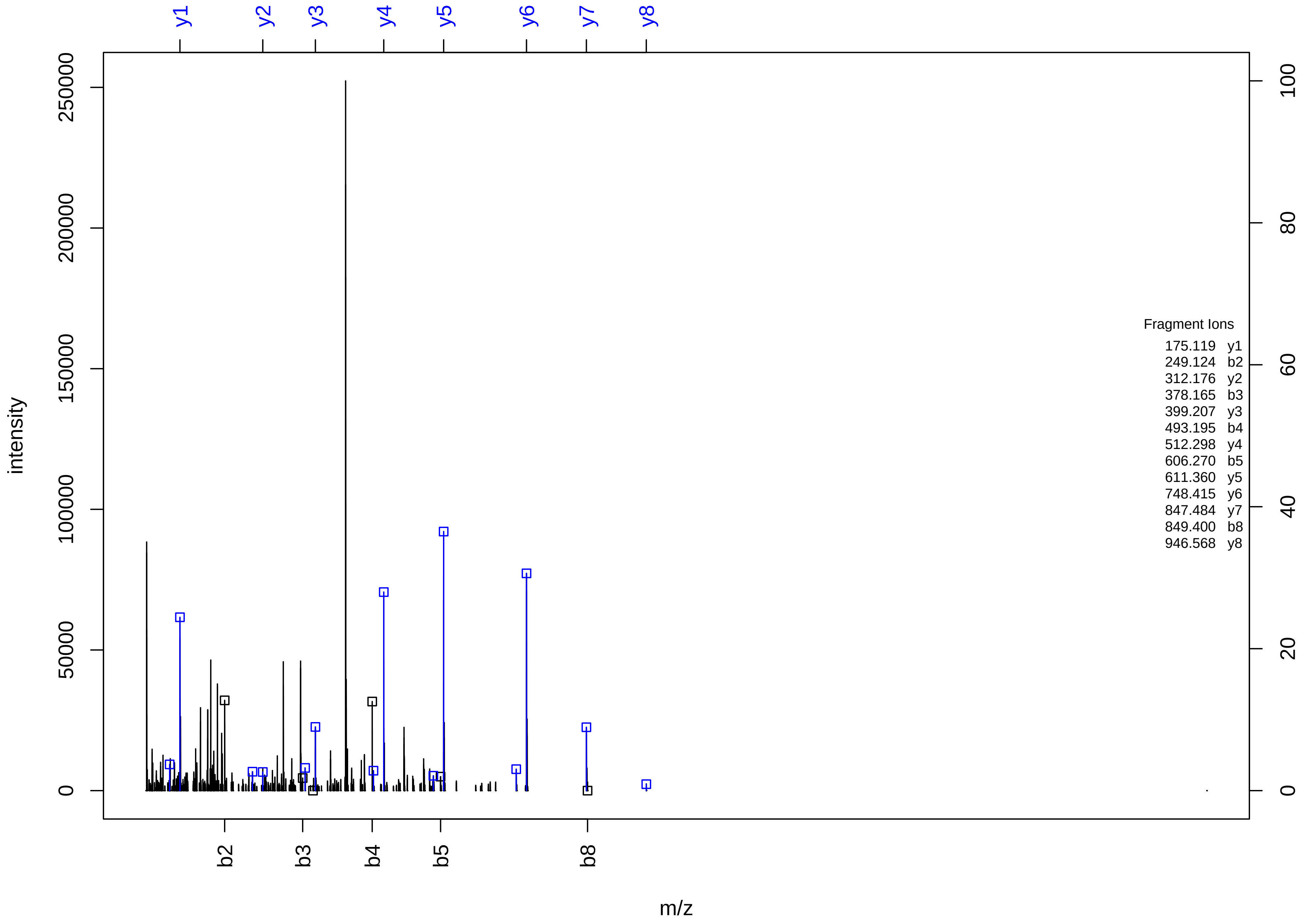

FTEDLVGSVVHVLSHR / File: christine_ip_06.raw; SpectrumID: ; scans: 15113

(Ac)SQVLFHQLVPLQVK

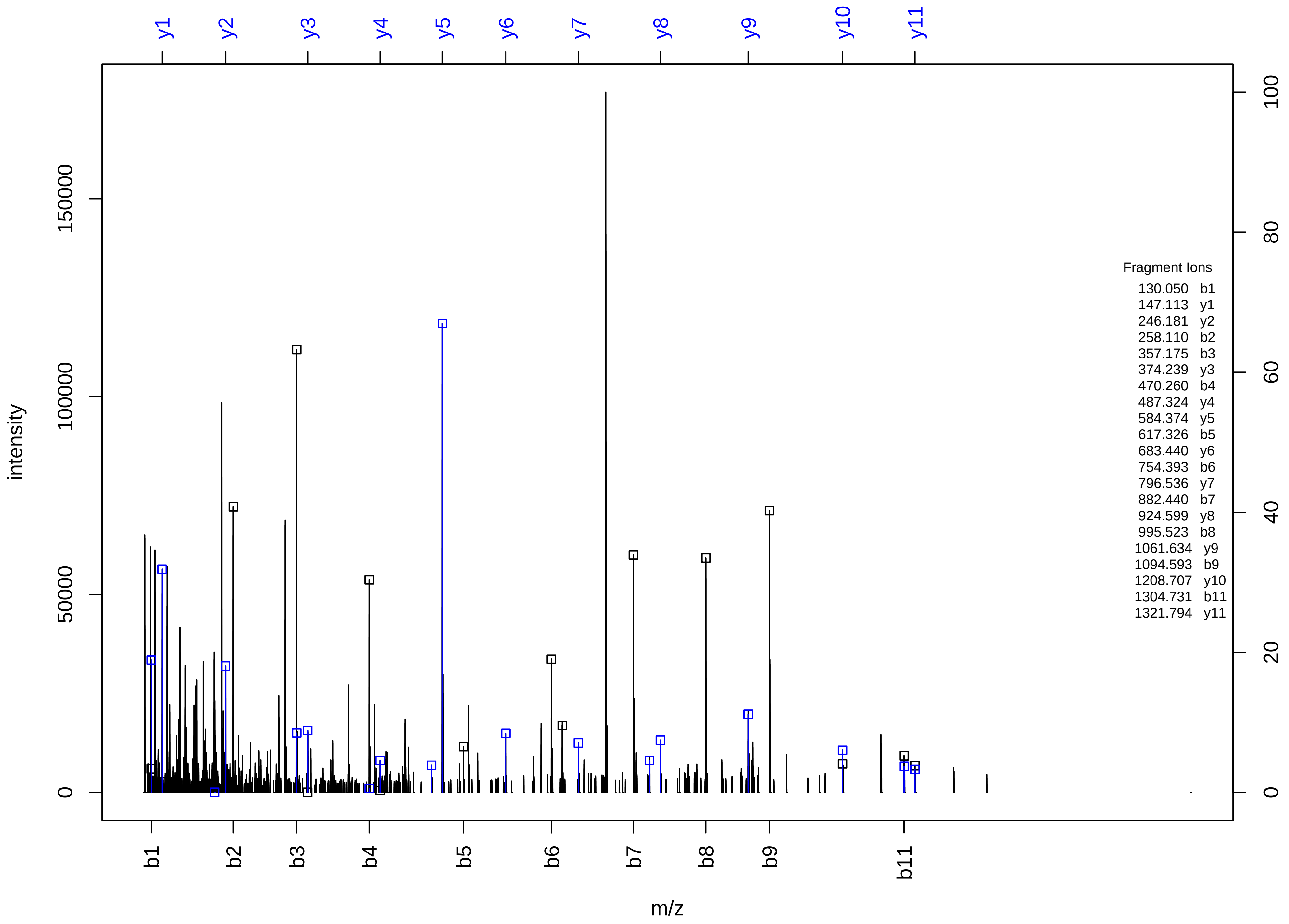

SQVLFHQLVPLQVK / File: christine_ip_06.raw; SpectrumID: ; scans: 17904

DLLFGSIVAVDEPTRPIYR

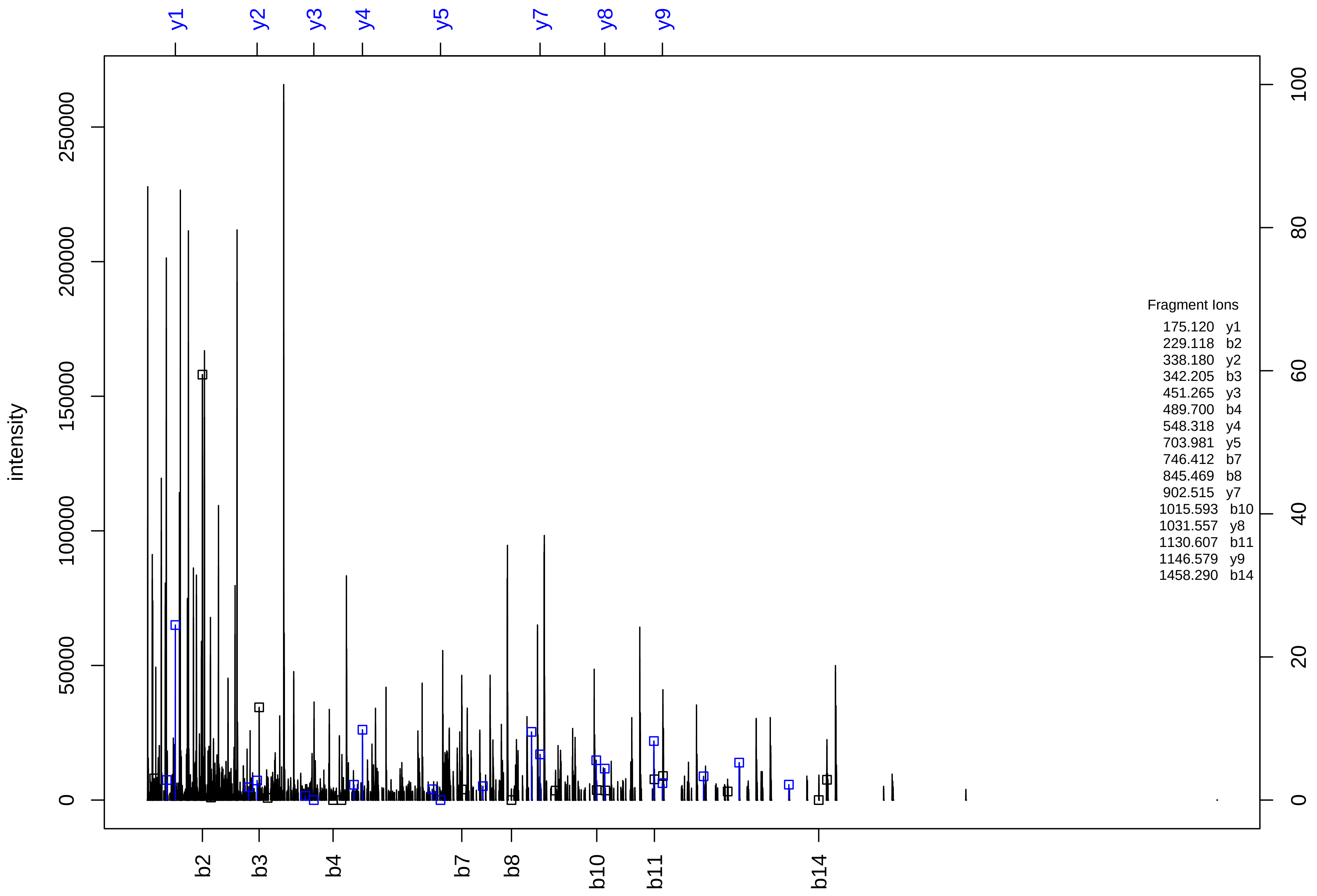

DLLFGSIVAVDEPTRPIYR / File: christine_ip_06.raw; SpectrumID: ; scans: 16711

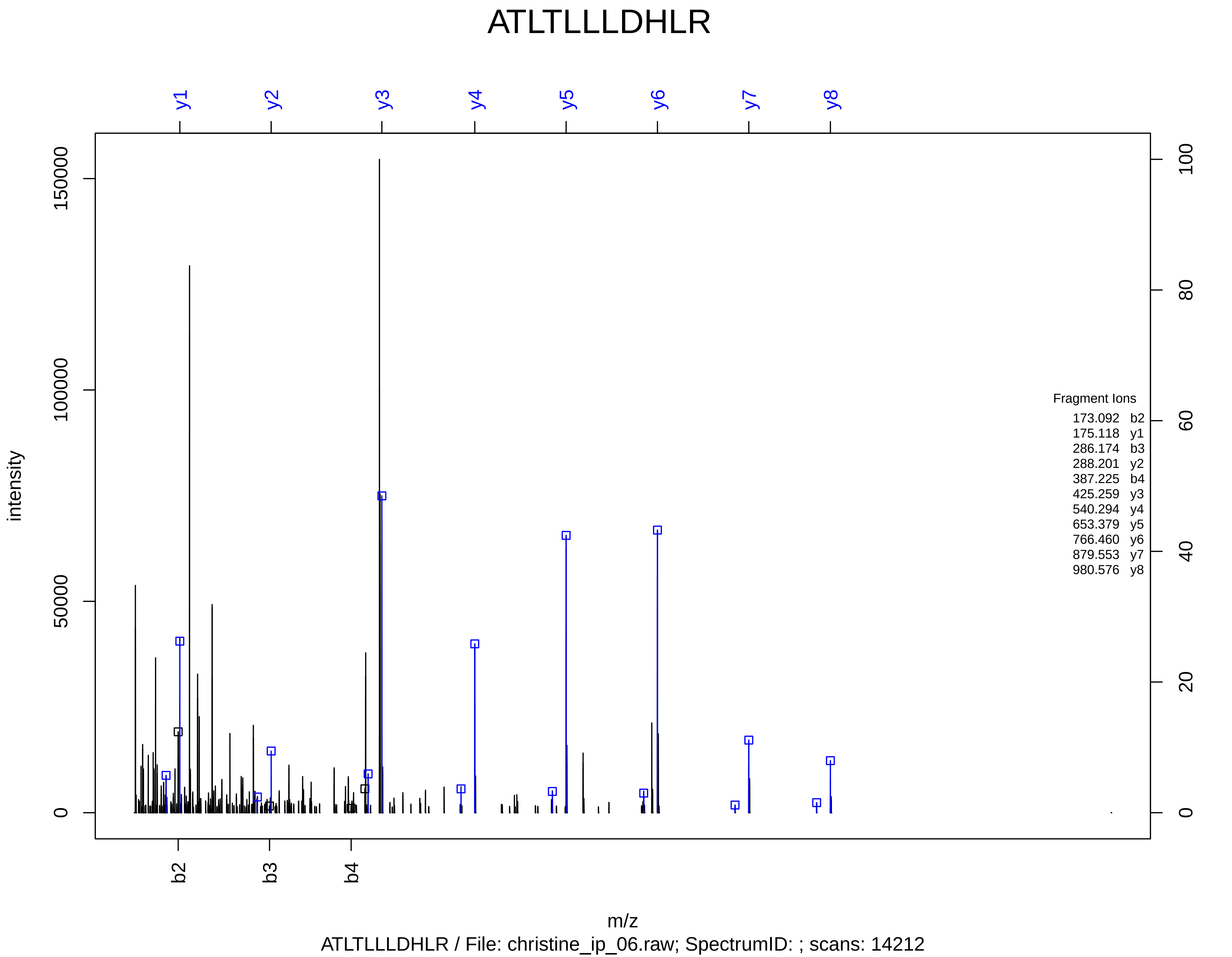

ATLTLLLDHLR
y1
y2
y3
y4
y5
y6
y7
y8
intensity
0
50000
100000
150000
0
20
40
60
80
100
Fragment Ions
173.092 b2
175.118 y1
286.174 b3
288.201 y2
387.225 b4
425.259 y3
540.294 y4
653.379 y5
766.460 y6
879.553 y7
980.576 y8
b2
b3
b4
m/z
ATLTLLLDHLR / File: christine_ip_06.raw; SpectrumID: ; scans: 14212

AGRPEPSPDPEQPAGR

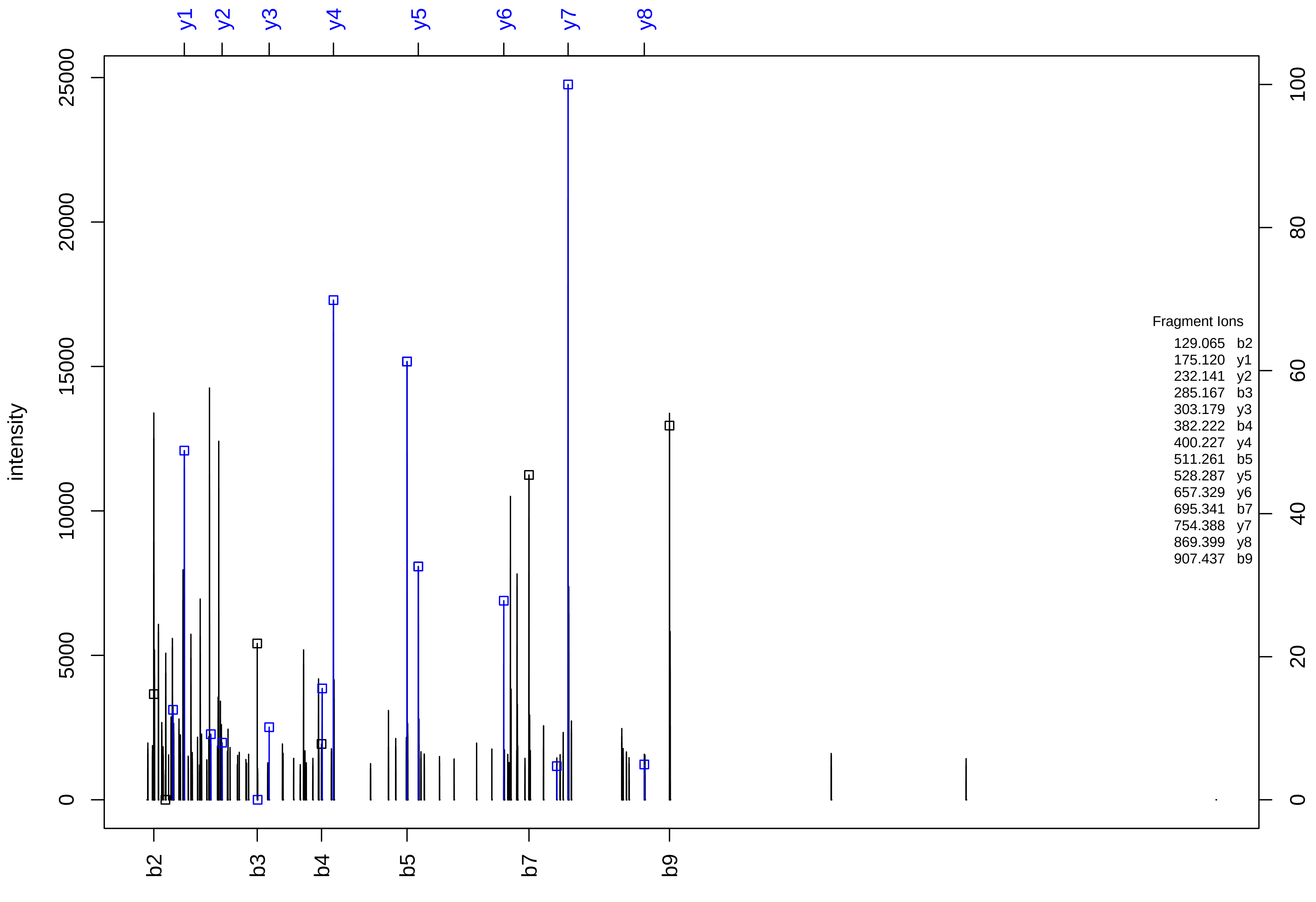

AGRPEPSPDPEQPAGR / File: christine_ip_06.raw; SpectrumID: ; scans: 1659

SGSSSSSIPESQSNHSNQSDSGVSDTQPAGHVR

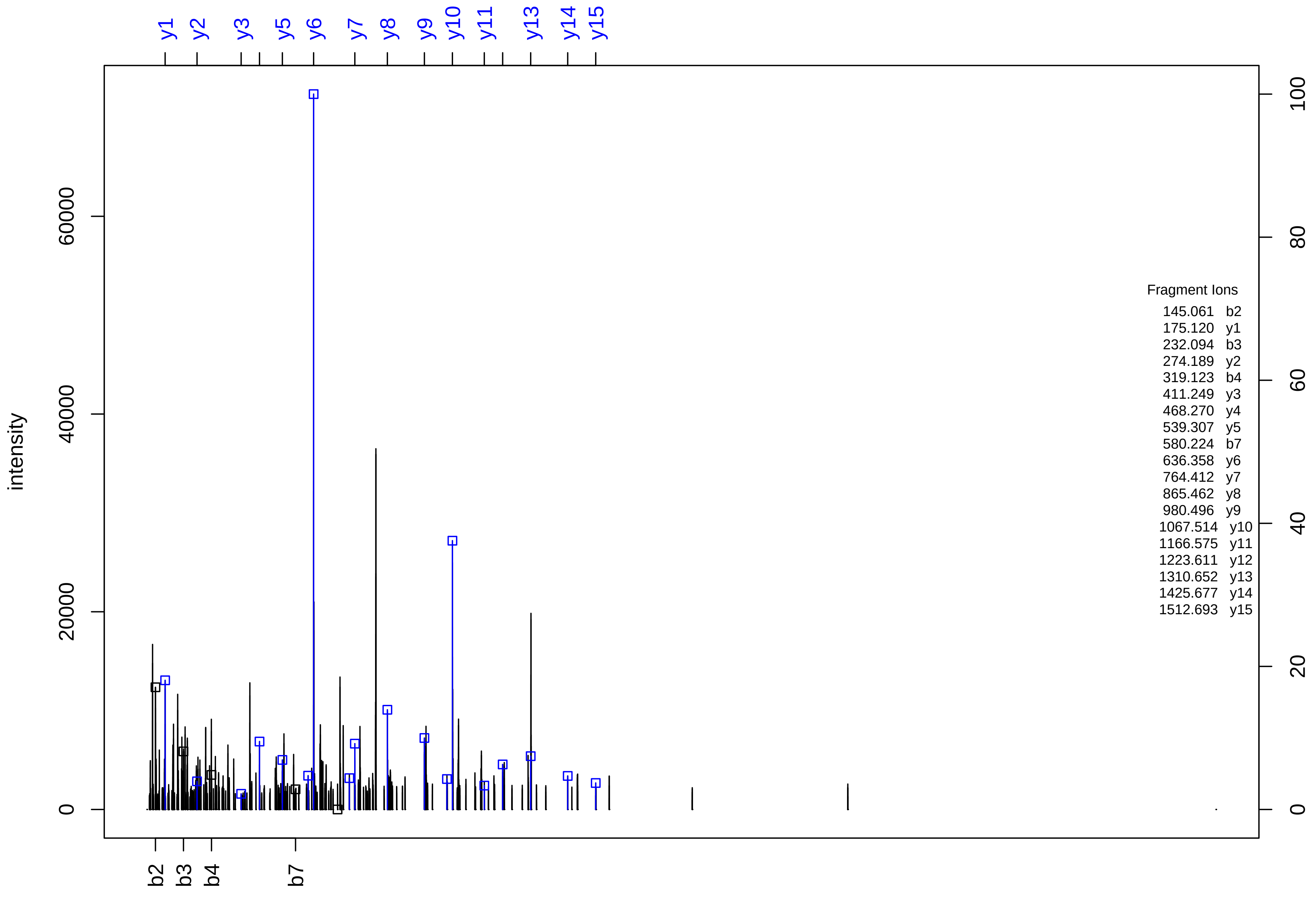

SGSSSSSIPESQSNHSNQSDSGVSDTQPAGHVR / File: christine_ip_06.raw; SpectrumID: ; scans: 2281

(Ac)AAAAAAVGPGAGGAGSAVPGGAGPCATVSVFPGAR

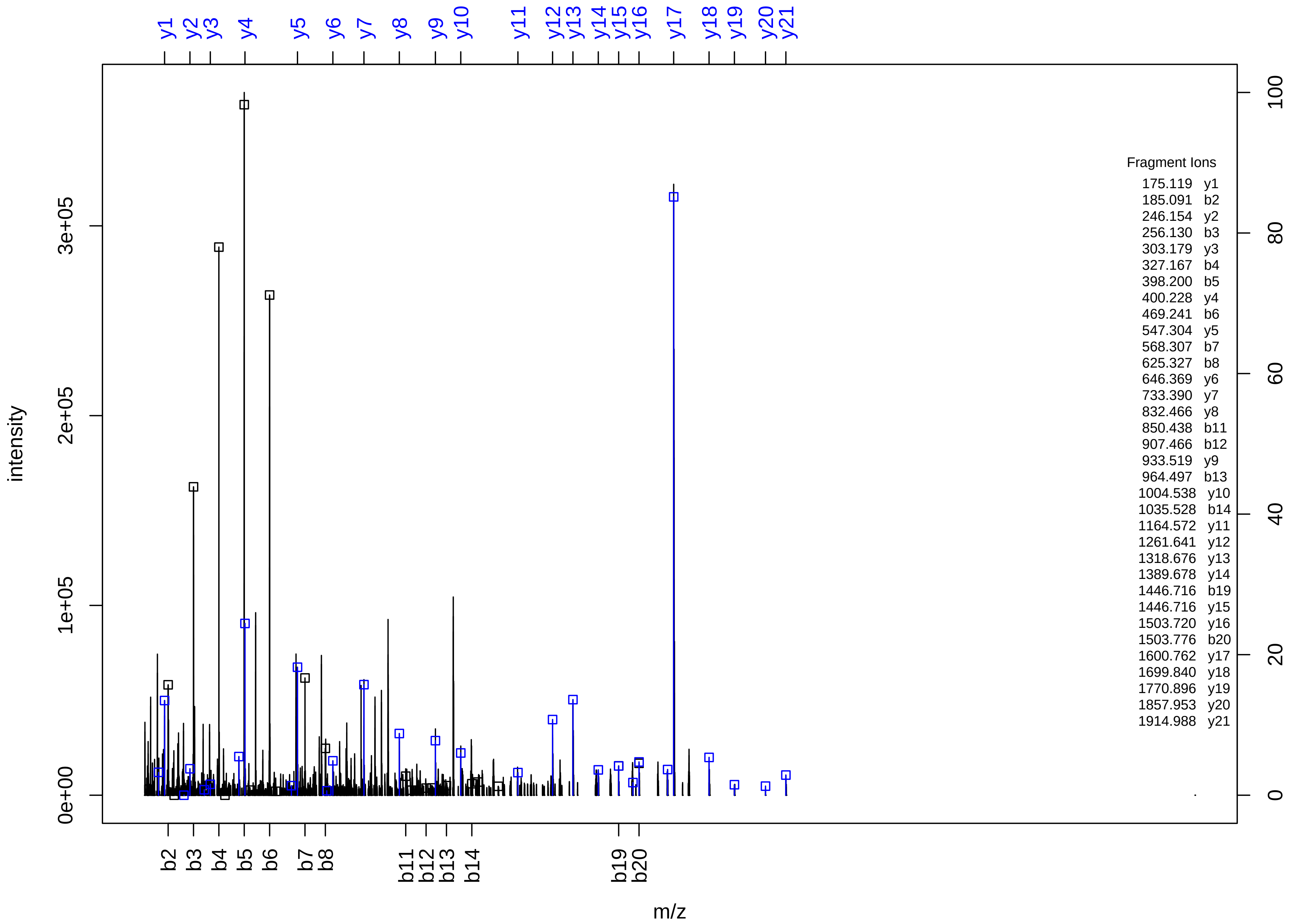

AAAAAAVGPGAGGAGSAVPGGAGPCATVSVFPGAR / File: christine_ip_06.raw; SpectrumID: ; scans: 15218

DLAFVDPEDCTPLSTITR

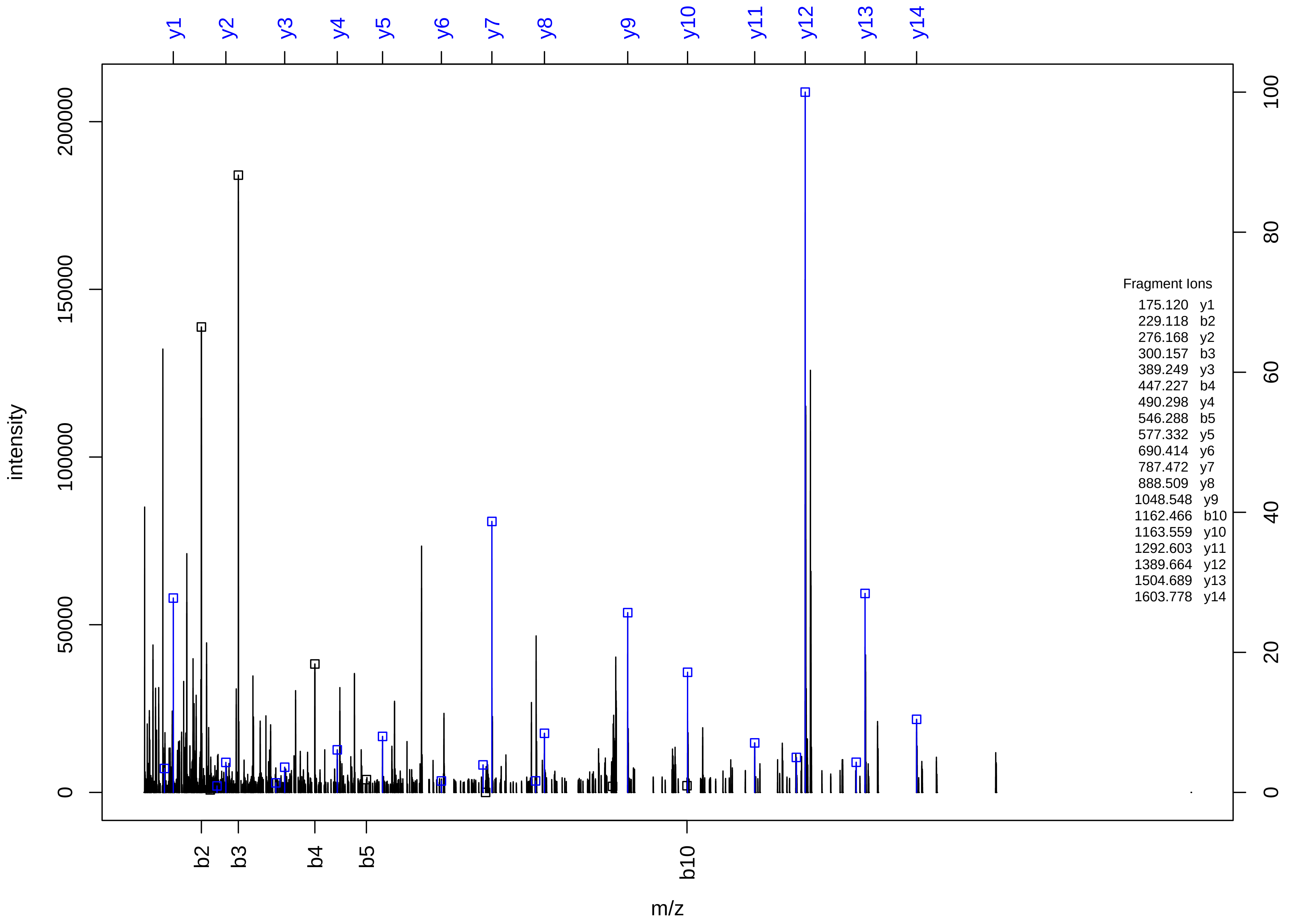

DLAFVDPEDCTPLSTITR / File: christine_ip_06.raw; SpectrumID: ; scans: 16773

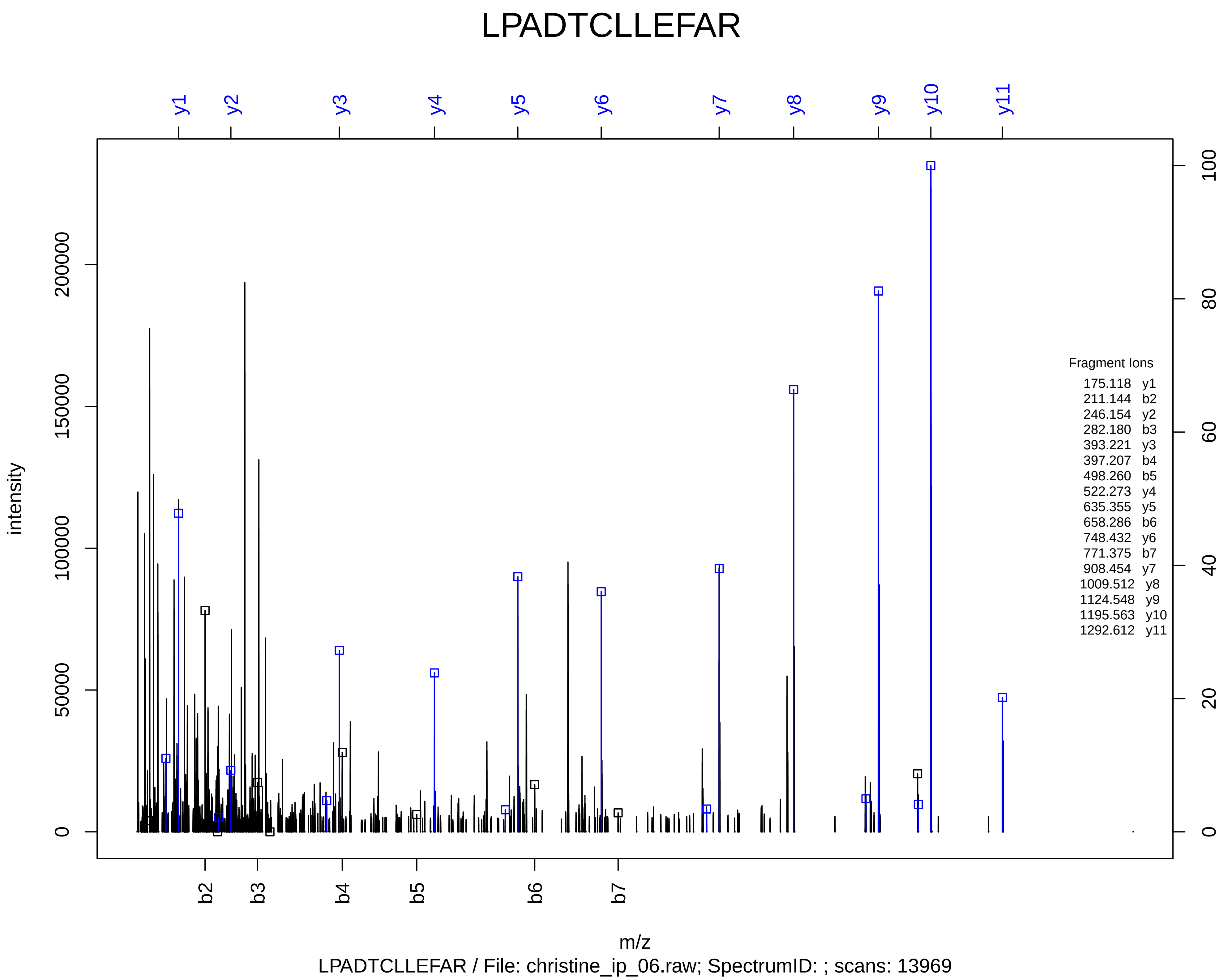
LPADTCLLEFAR
y1
y2
y3
y4
y5
y6
y7
y8
y9
y10
y11
intensity
0
50000
100000
150000
200000
0
20
40
60
80
100
Fragment Ions
175.118 y1
211.144 b2
246.154 y2
282.180 b3
393.221 y3
397.207 b4
498.260 b5
522.273 y4
635.355 y5
658.286 b6
748.432 y6
771.375 b7
908.454 y7
1009.512 y8
1124.548 y9
1195.563 y10
1292.612 y11
b2
b3
b4
b5
b6
b7
m/z
LPADTCLLEFAR / File: christine_ip_06.raw; SpectrumID: ; scans: 13969

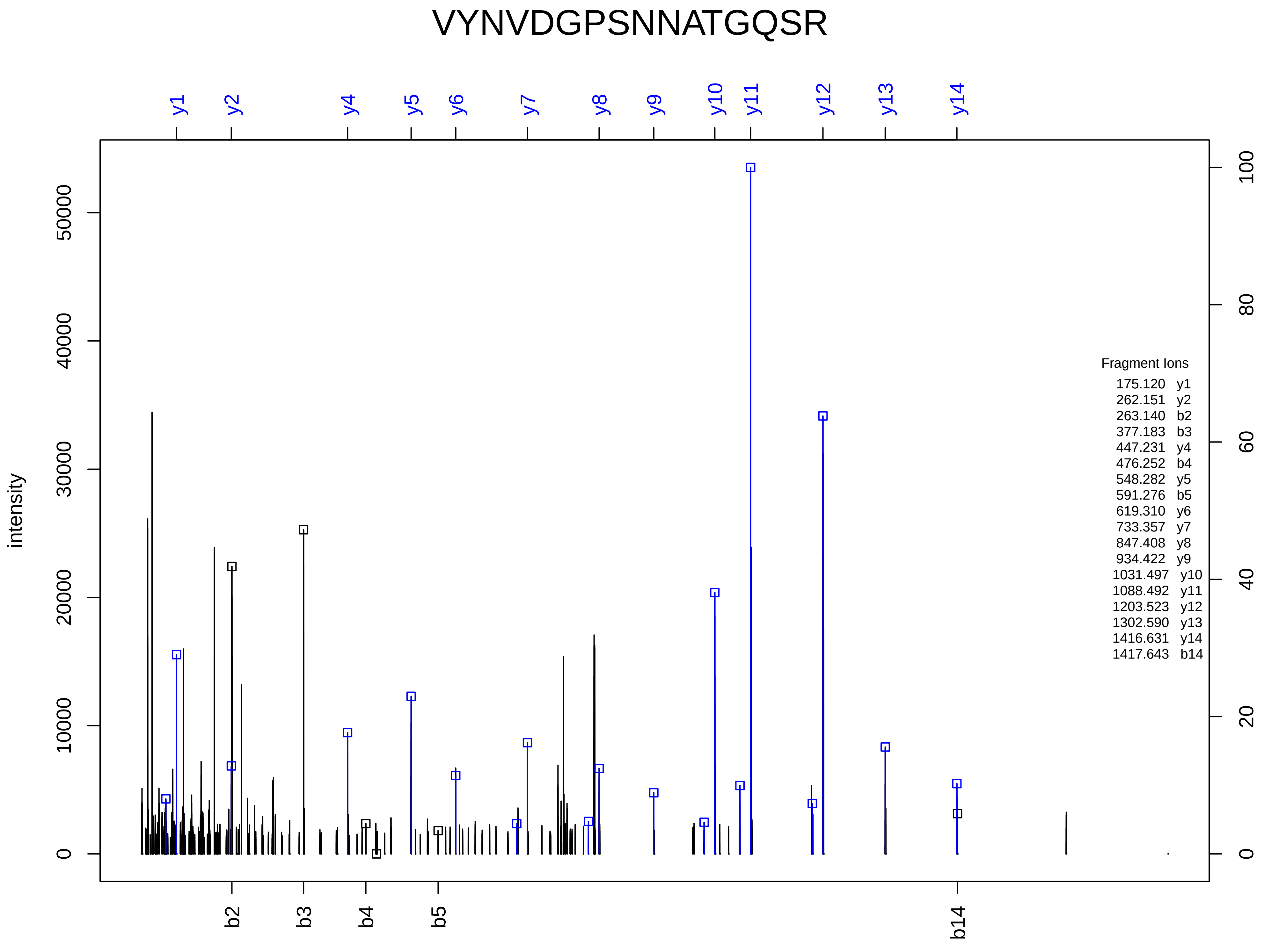

VYNVDGPSNNATGQSR / File: christine_ip_06.raw; SpectrumID: ; scans: 2596

(Ac)AENSESLGTVPEHER

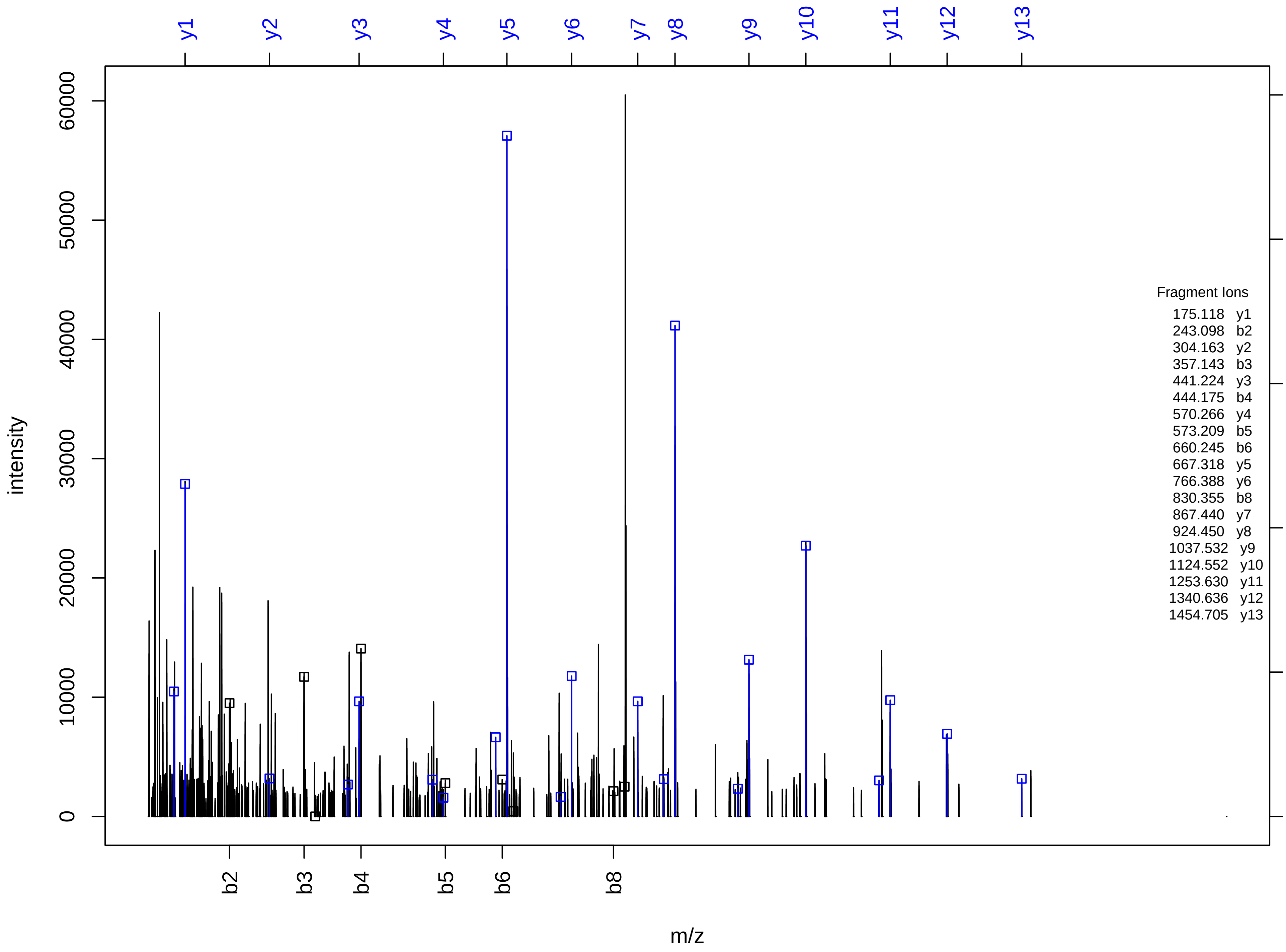

AENSESLGTVPEHER / File: christine_ip_06.raw; SpectrumID: ; scans: 4563

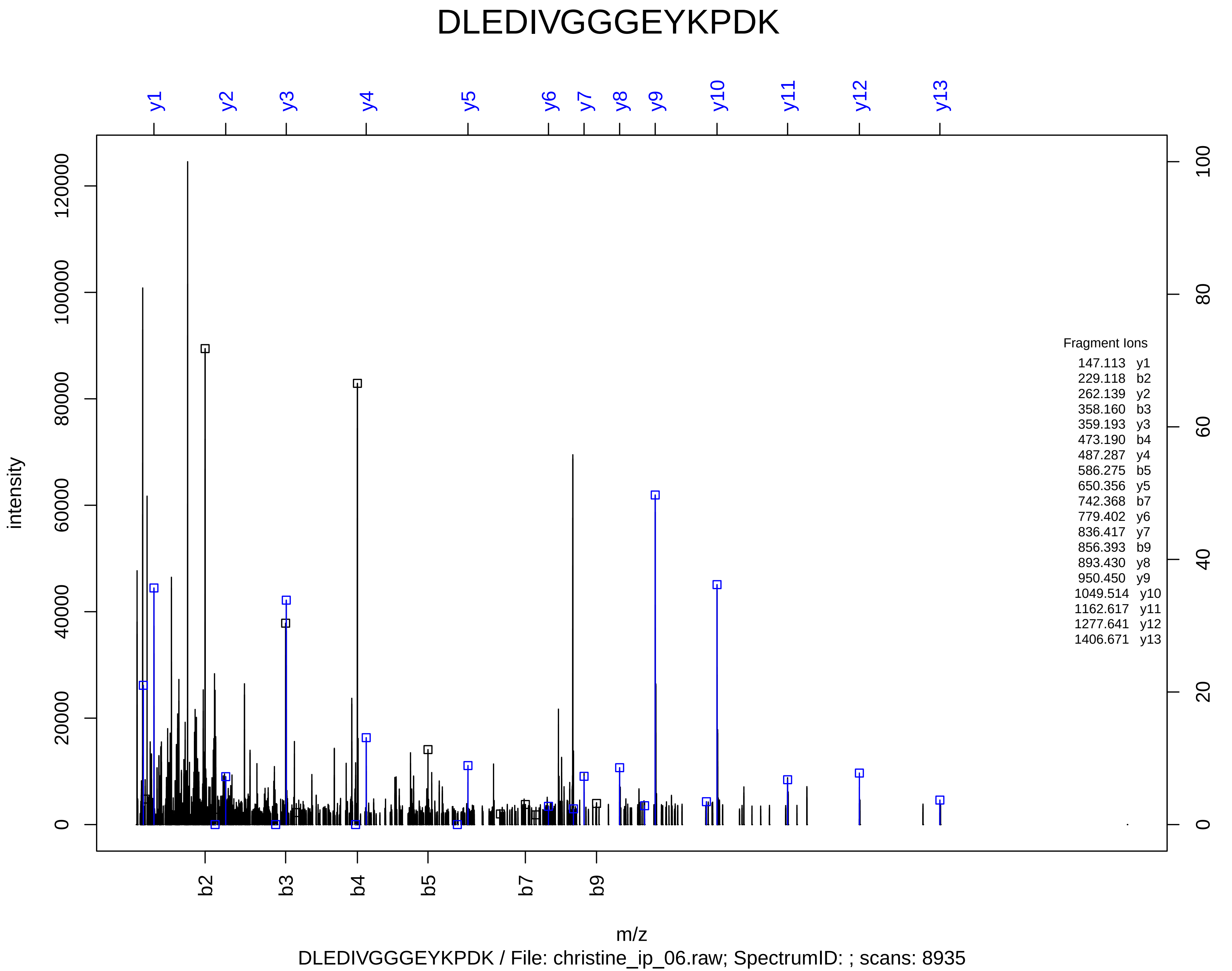
DLEDIVGGGEYKPDK
y1
y2
y3
y4
y5
y6
y7
y8
y9
y10
y11
y12
y13
intensity
0
20000
40000
60000
80000
100000
120000
0
20
40
60
80
100
b2
b3
b4
b5
b7
b9
m/z
Fragment Ions
147.113 y1
229.118 b2
262.139 y2
358.160 b3
359.193 y3
473.190 b4
487.287 y4
586.275 b5
650.356 y5
742.368 b7
779.402 y6
836.417 y7
856.393 b9
893.430 y8
950.450 y9
1049.514 y10
1162.617 y11
1277.641 y12
1406.671 y13
DLEDIVGGGEYKPDK / File: christine_ip_06.raw; SpectrumID: ; scans: 8935

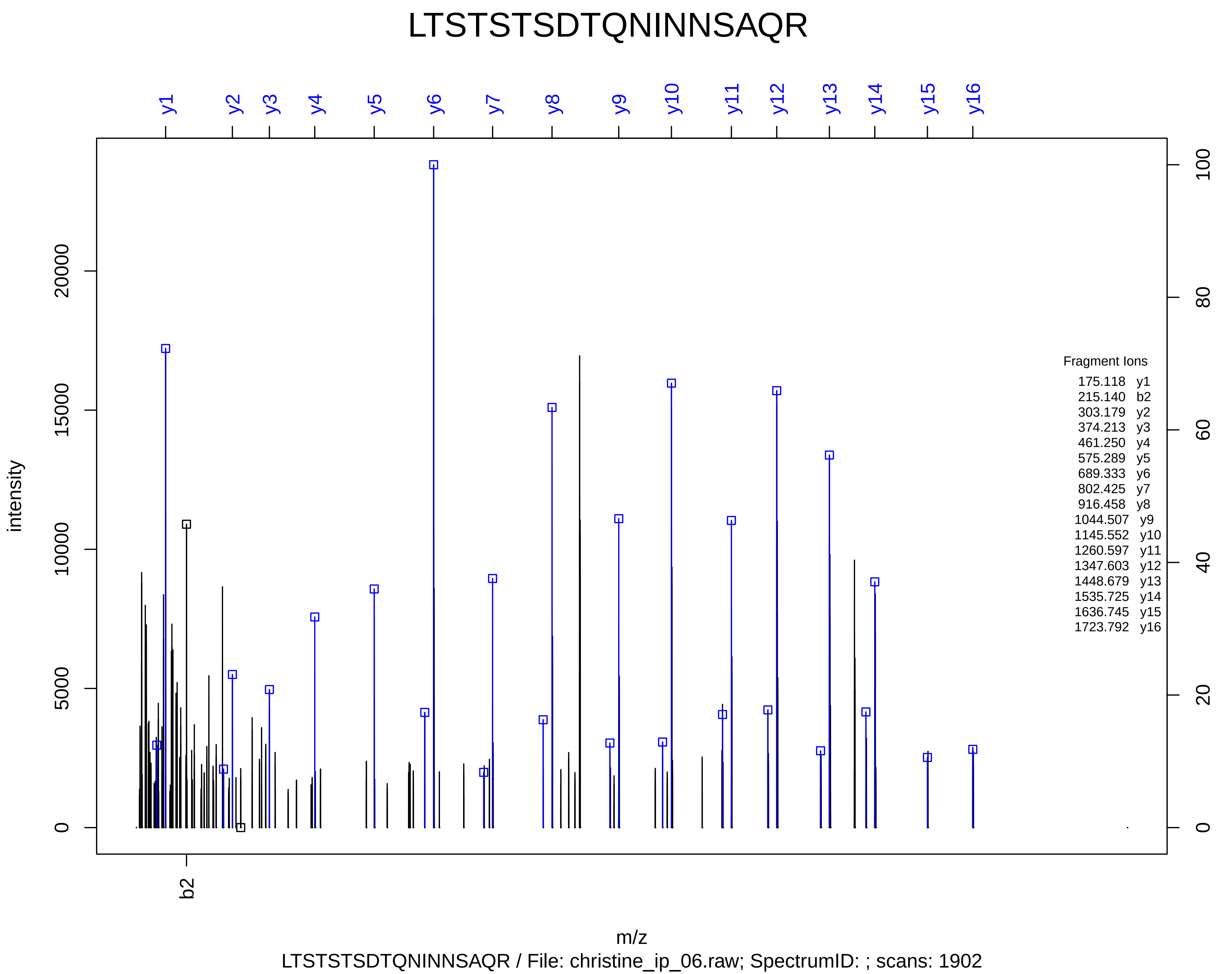

LTSTSTSDTQNINNSAQR
y1
y2
y3
y4
y5
y6
y7
y8
y9
y10
y11
y12
y13
y14
y15
y16
b2
intensity
0
5000
10000
15000
20000
0
20
40
60
80
100
Fragment Ions
175.118 y1
215.140 b2
303.179 y2
374.213 y3
461.250 y4
575.289 y5
689.333 y6
802.425 y7
916.458 y8
1044.507 y9
1145.552 y10
1260.597 y11
1347.603 y12
1448.679 y13
1535.725 y14
1636.745 y15
1723.792 y16
m/z
LTSTSTSDTQNINNSAQR / File: christine_ip_06.raw; SpectrumID: ; scans: 1902

QYAGYDYSQQGR

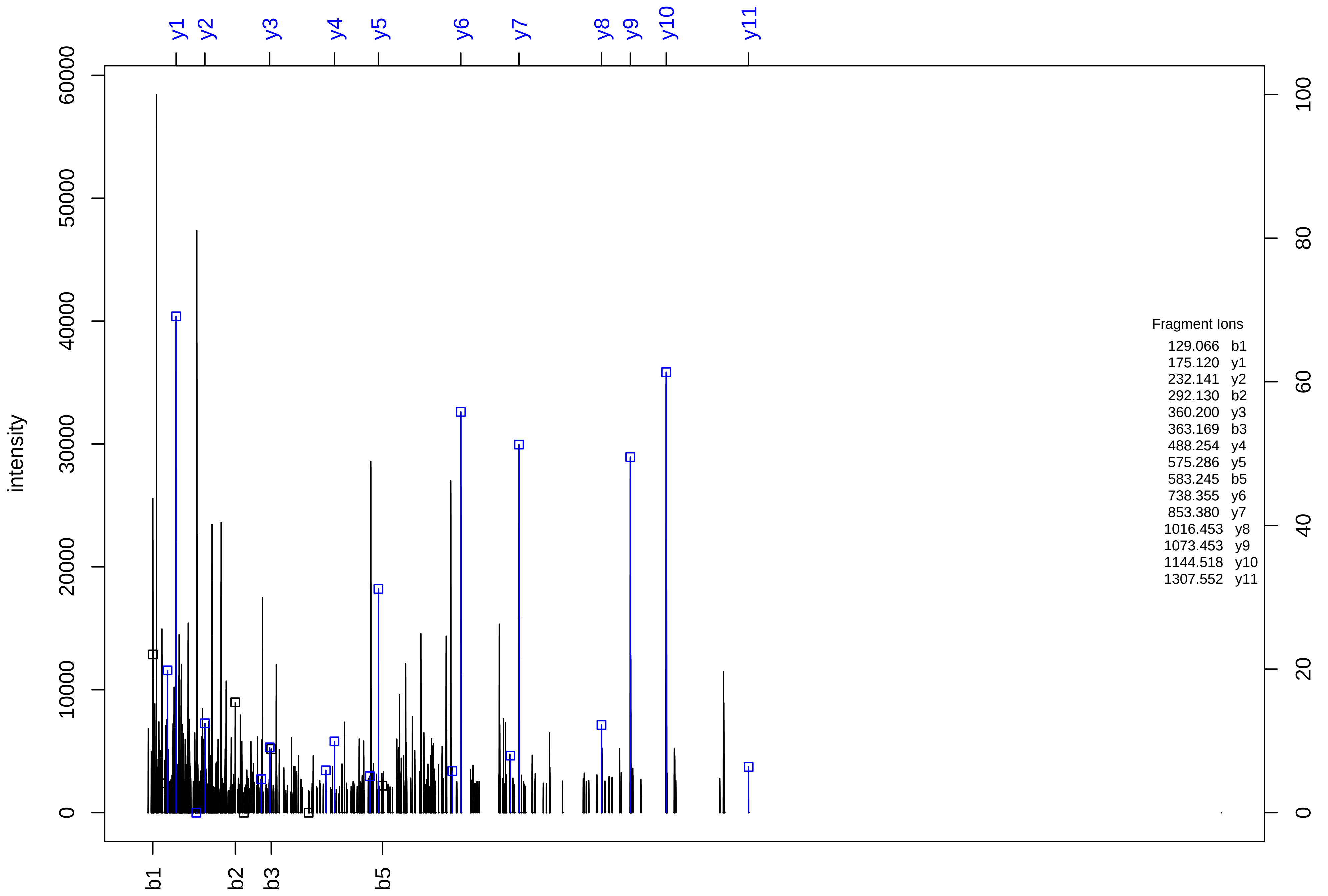

QYAGYDYSQQGR / File: christine_ip_06.raw; SpectrumID: ; scans: 2977

DGPEPEAEREPVR

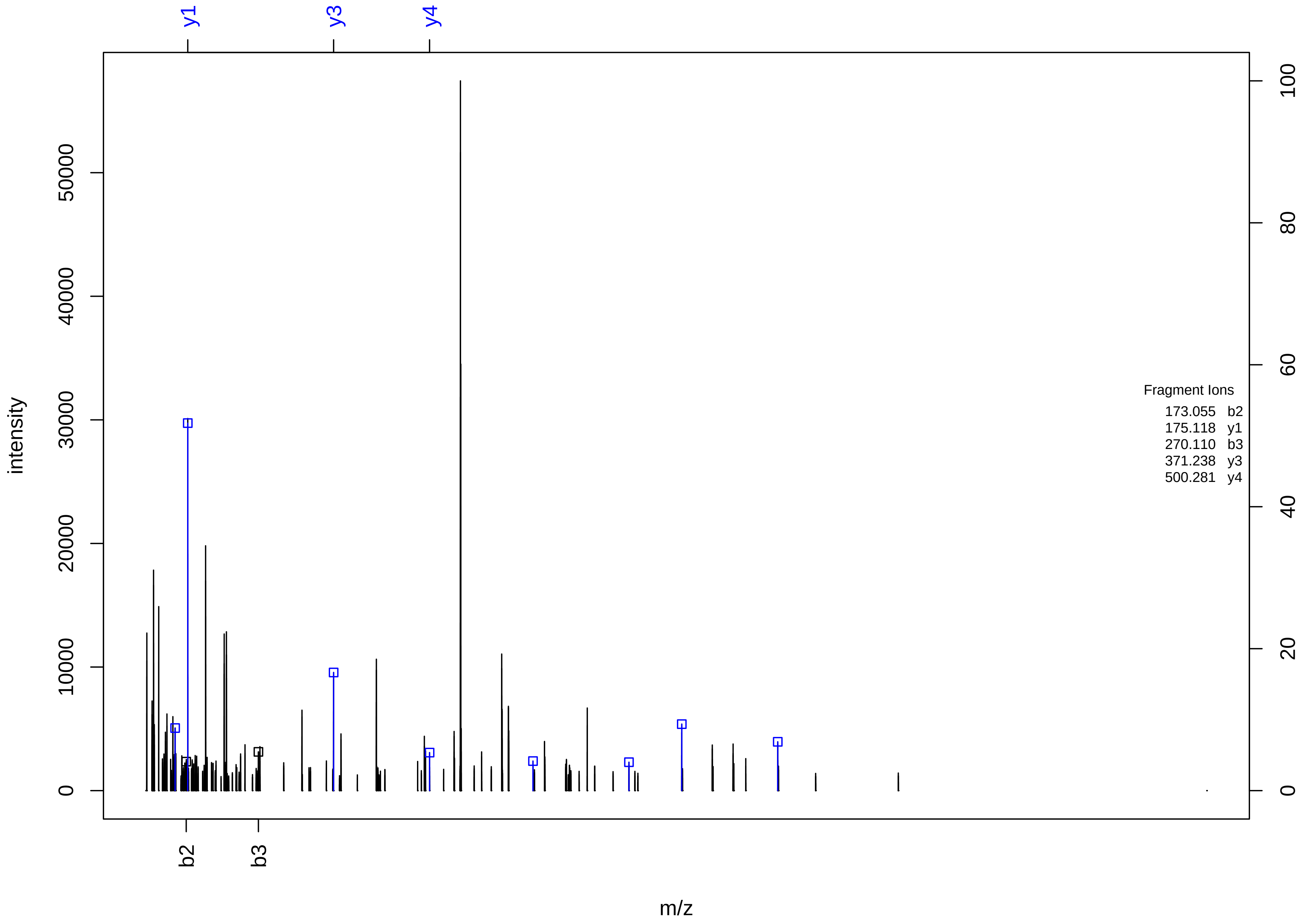

DGPEPEAEREPVR / File: christine_ip_06.raw; SpectrumID: ; scans: 1999

LAPQAYLTHFFLR

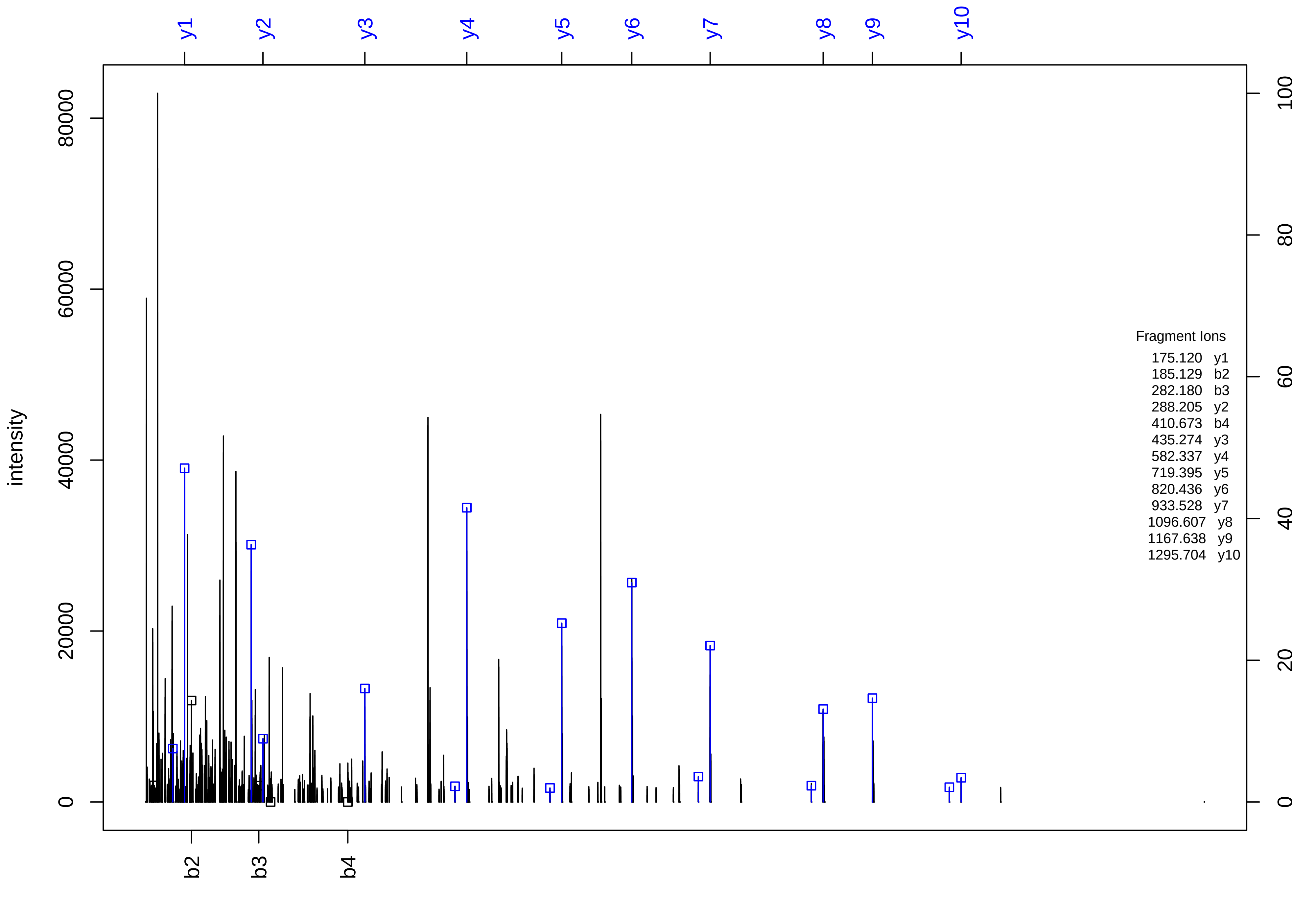

LAPQAYLTHFFLR / File: christine_ip_06.raw; SpectrumID: ; scans: 16038

(Ac)ANEAYPCPCDIGHR

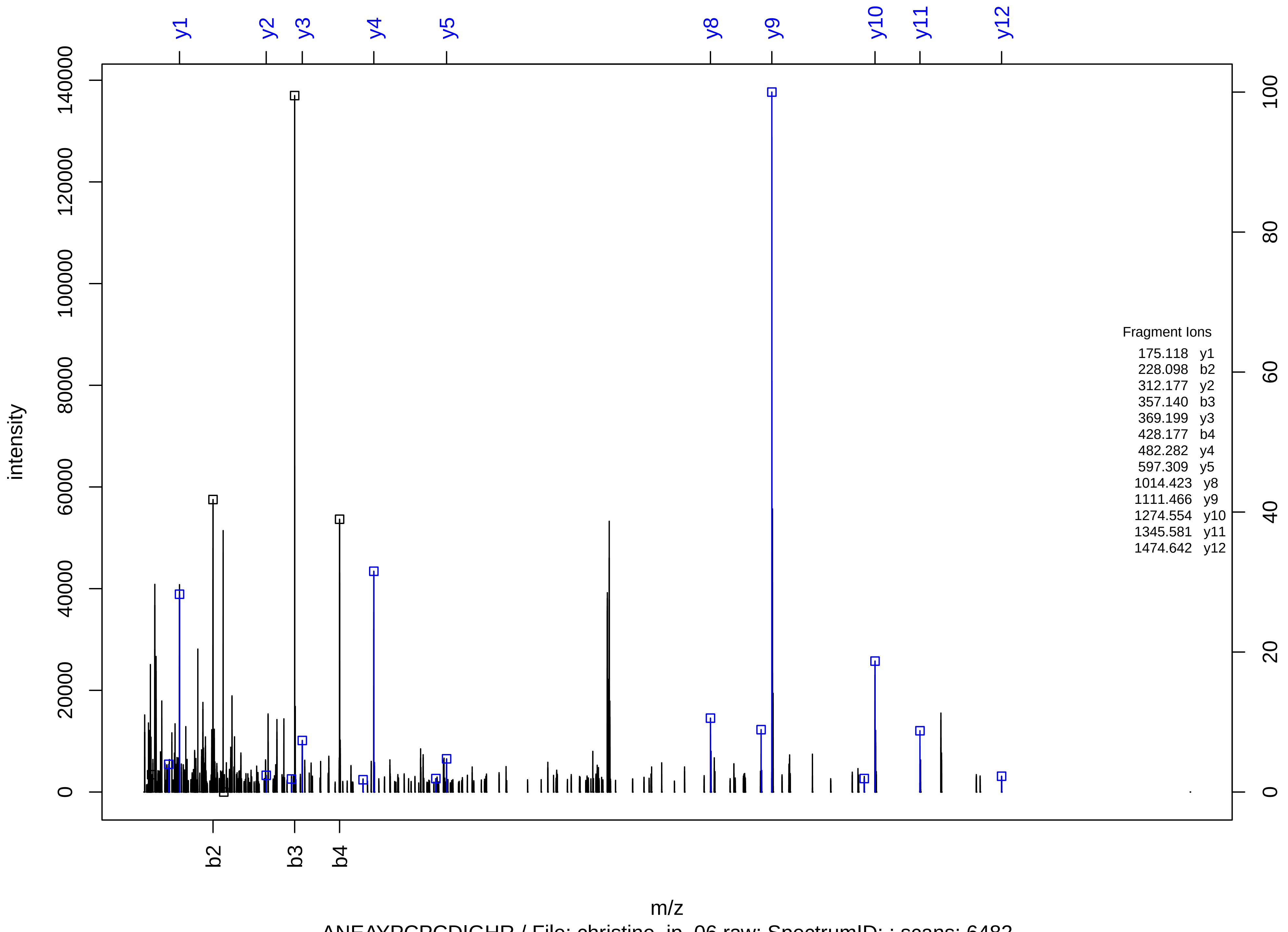

ANEAYPCPCDIGHR / File: christine_ip_06.raw; SpectrumID: ; scans: 6482

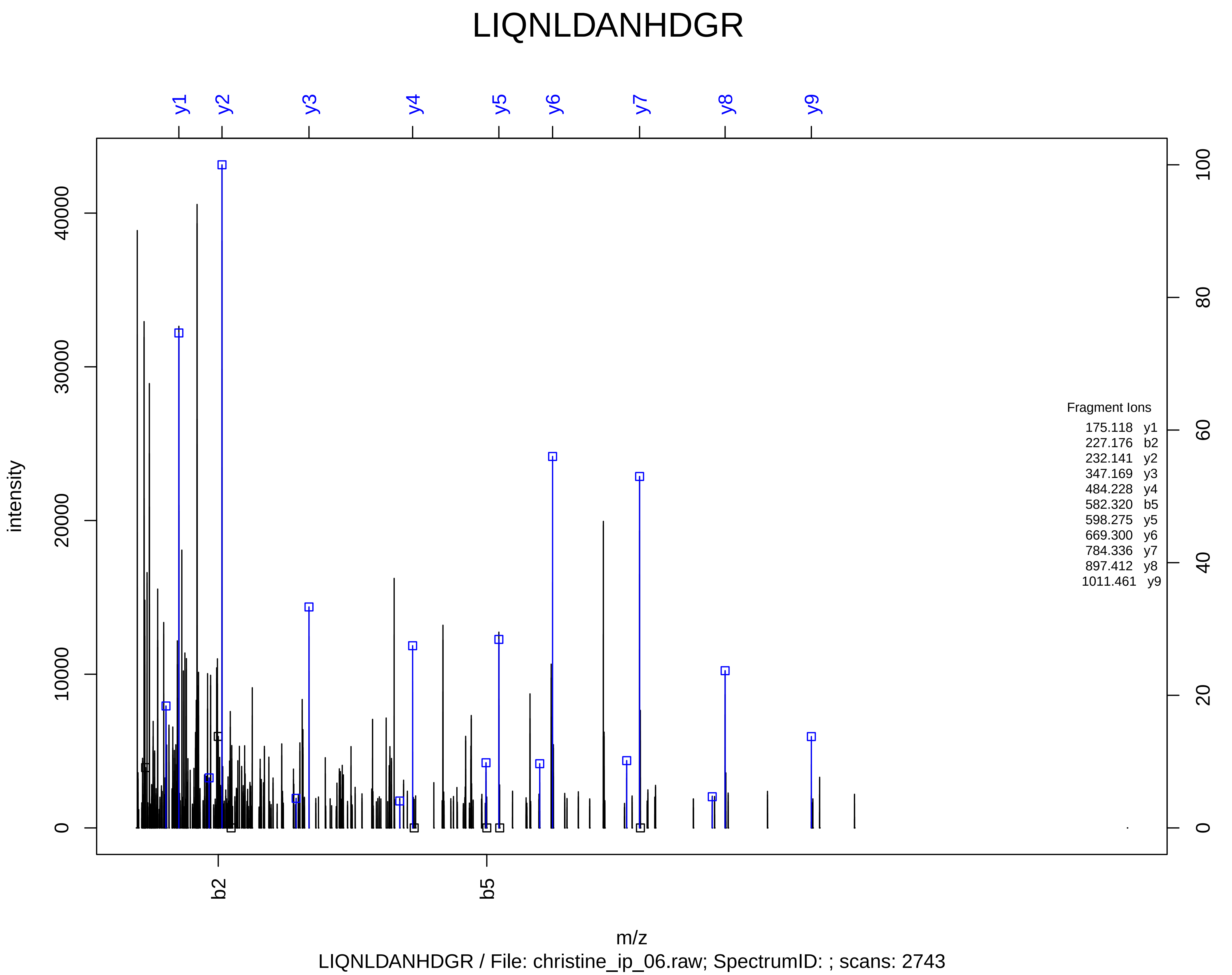
LIQNLDANHDGR
y1
y2
y3
y4
y5
y6
y7
y8
y9
intensity
0
10000
20000
30000
40000
0
20
40
60
80
100
b2
b5
Fragment Ions
175.118 y1
227.176 b2
232.141 y2
347.169 y3
484.228 y4
582.320 b5
598.275 y5
669.300 y6
784.336 y7
897.412 y8
1011.461 y9
m/z
LIQNLDANHDGR / File: christine_ip_06.raw; SpectrumID: ; scans: 2743

HLQLNETSTANHIHSR

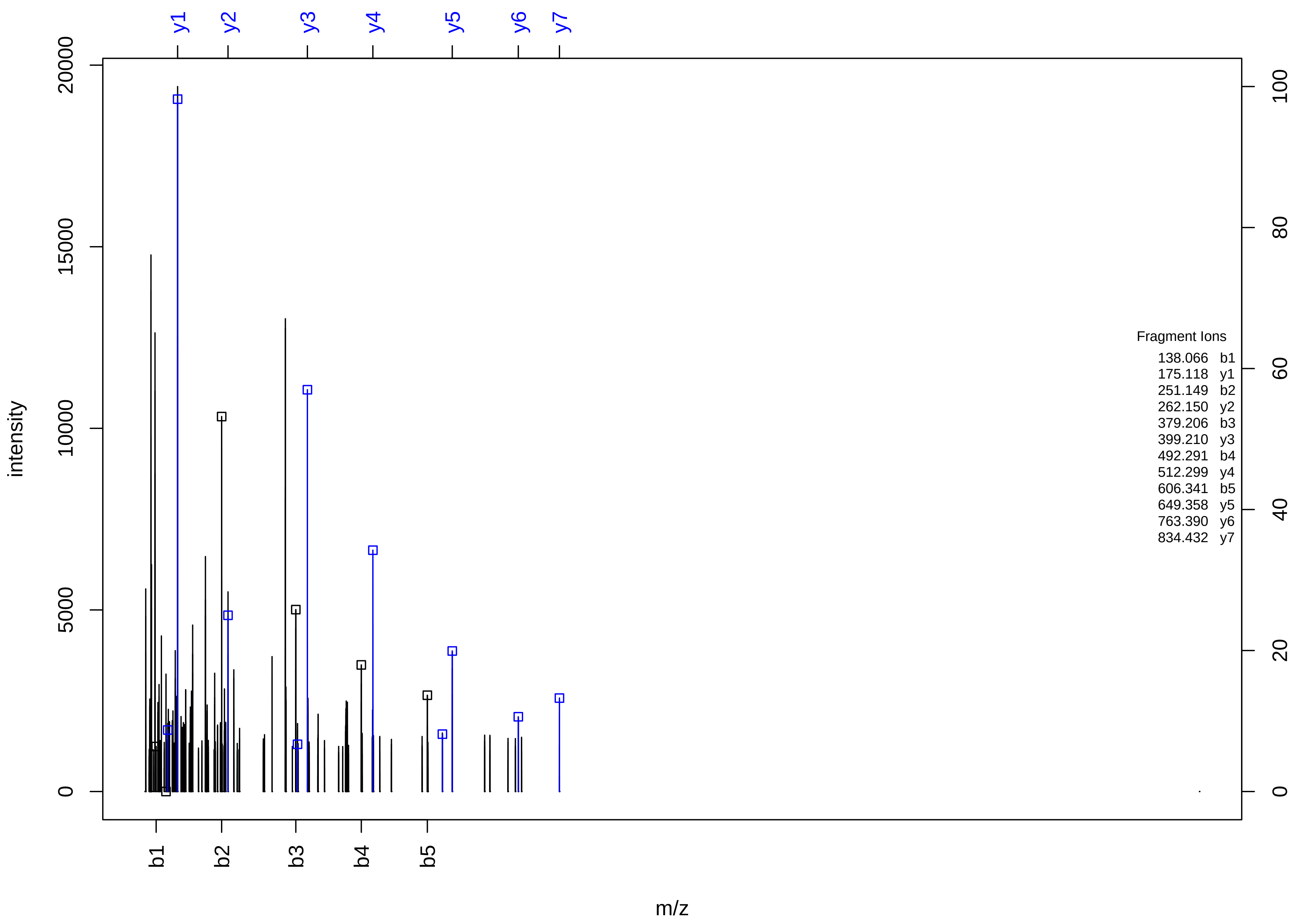

HLQLNETSTANHIHSR / File: christine_ip_06.raw; SpectrumID: ; scans: 2204

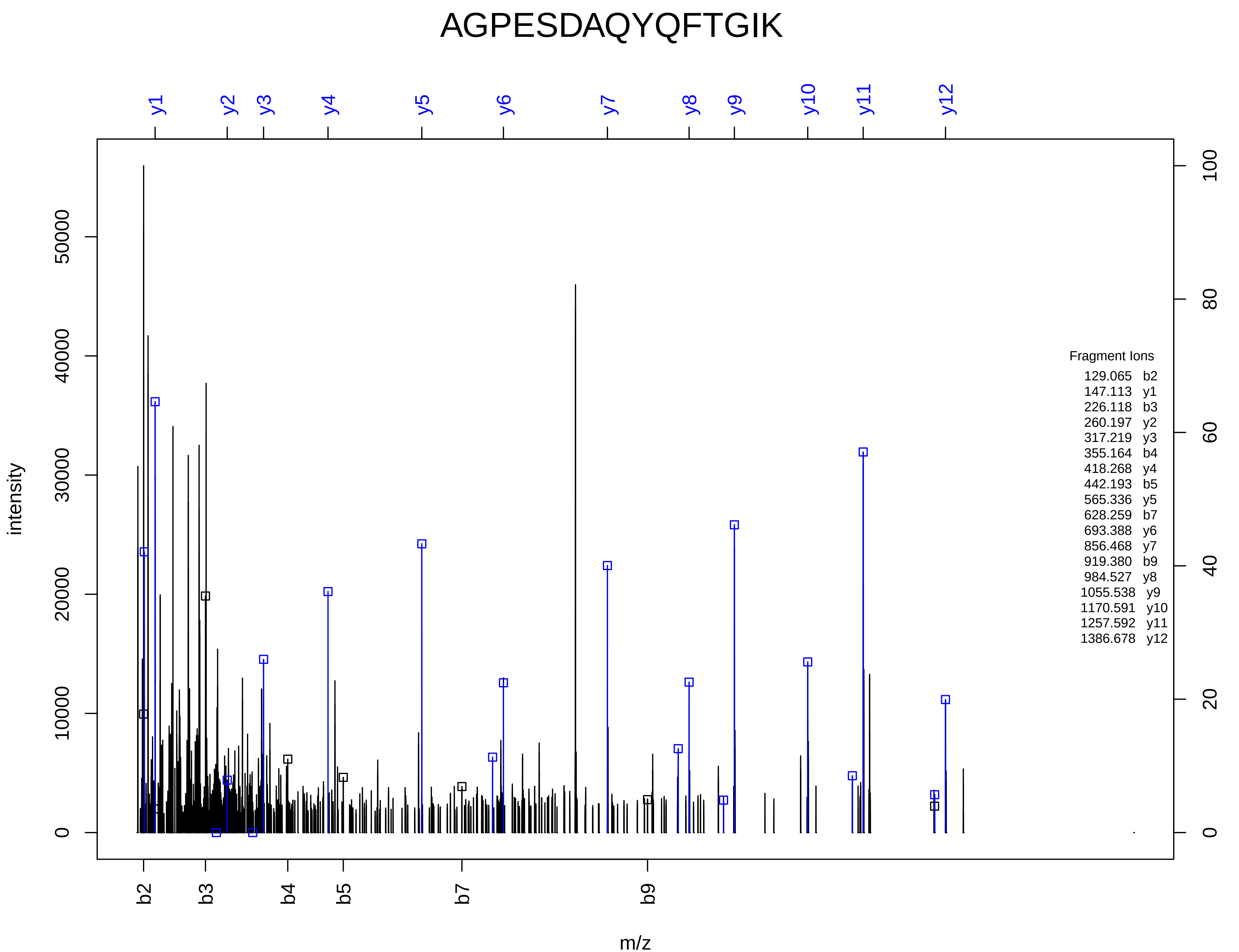

AGPESDAQYQFTGIK
y1
y2
y3
y4
y5
y6
y7
y8
y9
y10
y11
y12
intensity
0
10000
20000
30000
40000
50000
0
20
40
60
80
100
Fragment Ions
129.065 b2
147.113 y1
226.118 b3
260.197 y2
317.219 y3
355.164 b4
418.268 y4
442.193 b5
565.336 y5
628.259 b7
693.388 y6
856.468 y7
919.380 b9
984.527 y8
1055.538 y9
1170.591 y10
1257.592 y11
1386.678 y12
b2
b3
b4
b5
b7
b9
m/z
AGPESDAQYQFTGIK / File: christine_ip_06.raw; SpectrumID: ; scans: 7866

NIPVATN^N^PAR

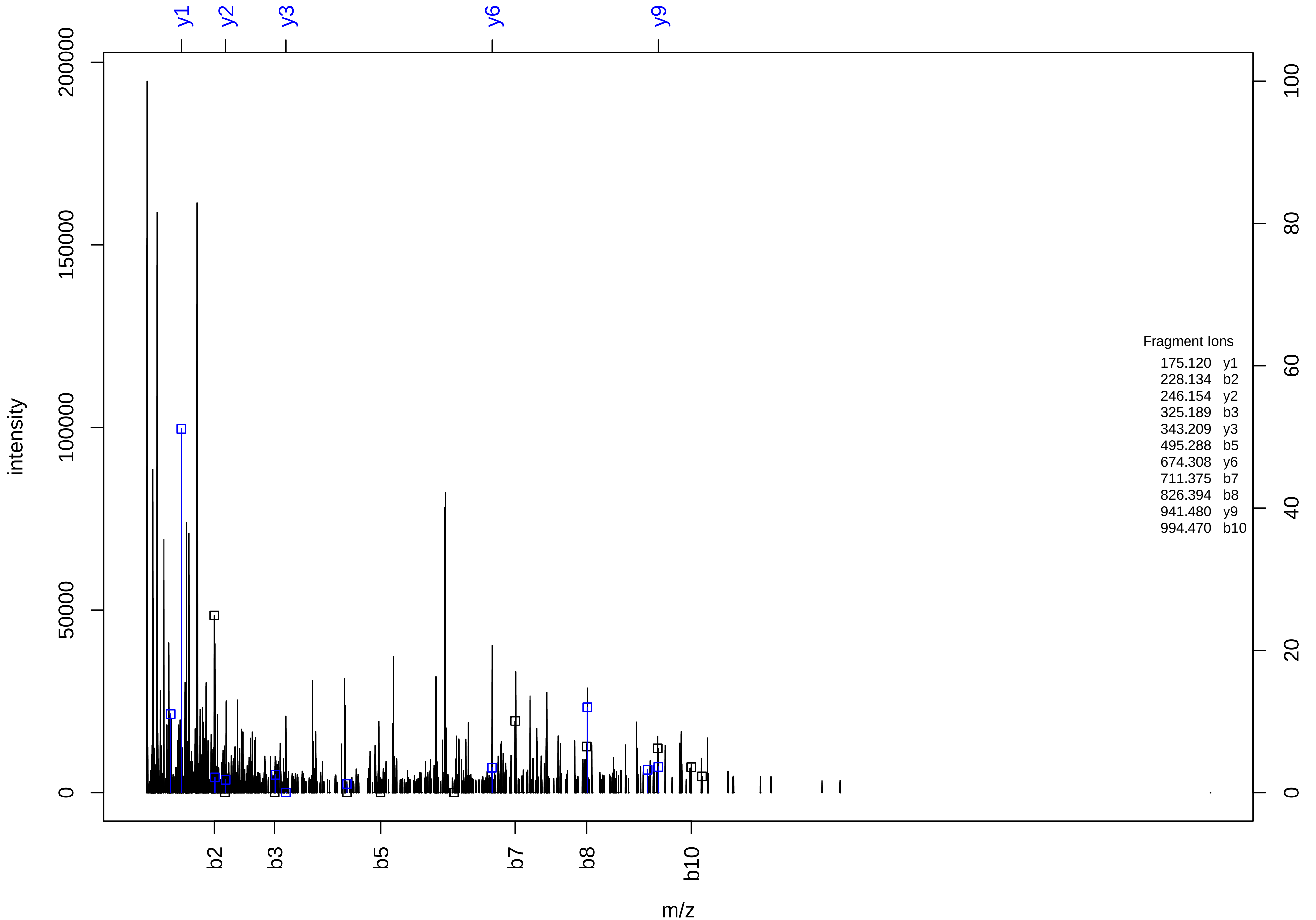

NIPVATNNPAR / File: christine_ip_06.raw; SpectrumID: ; scans: 6977

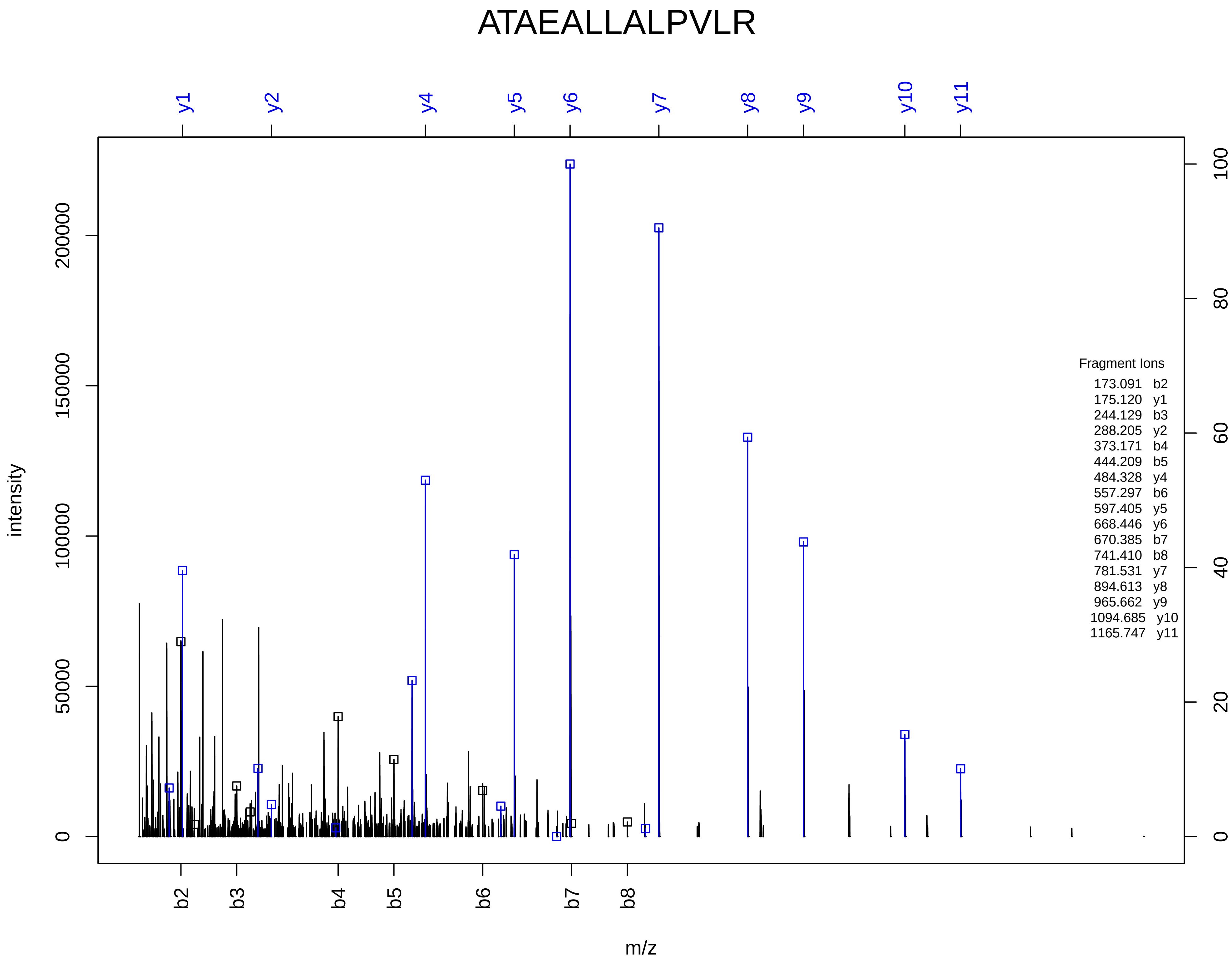

ATAEALLALPVLR / File: christine_ip_06.raw; SpectrumID: ; scans: 17649

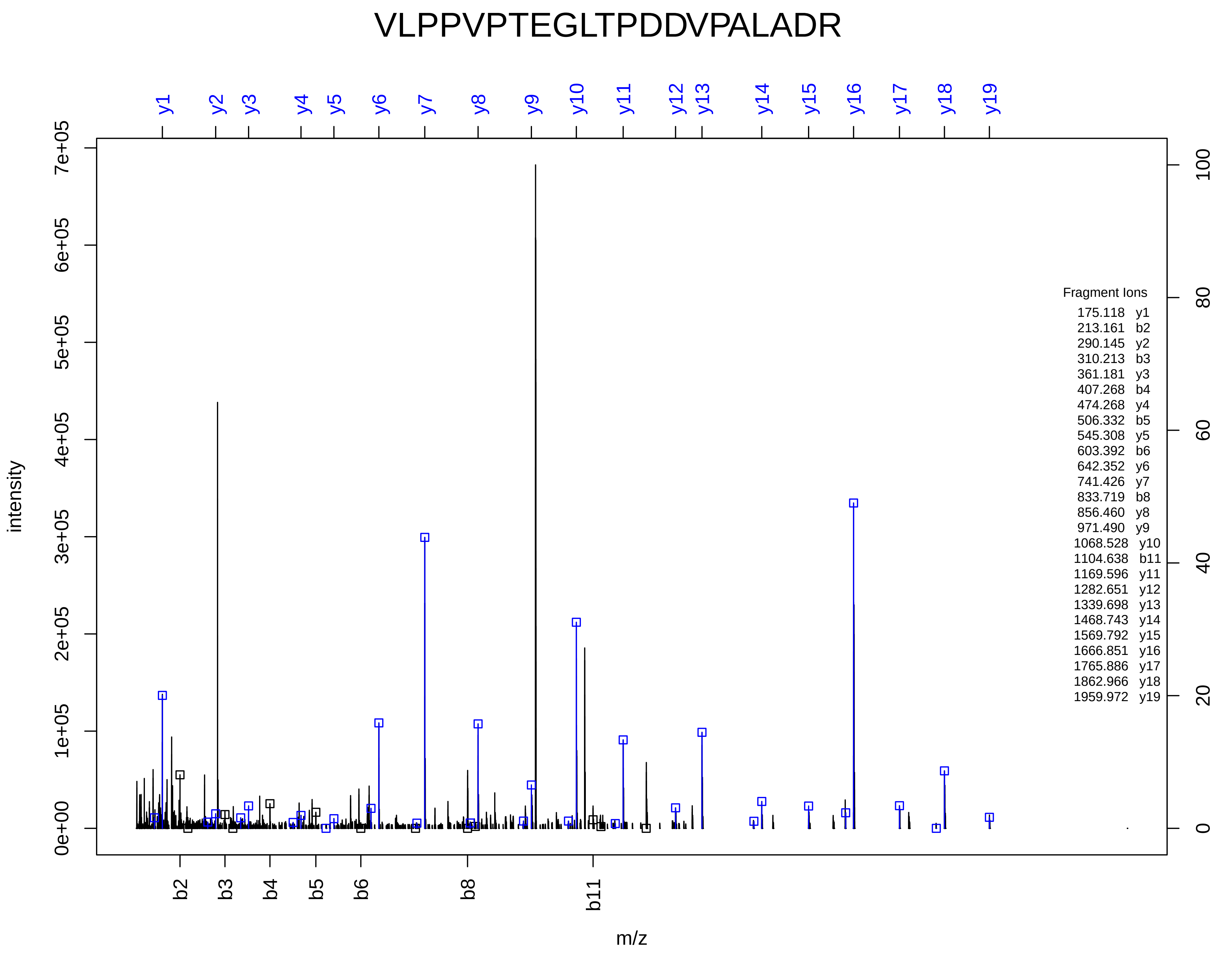

VLPPVPTEGLTPDDVPALADR / File: christine_ip_06.raw; SpectrumID: ; scans: 15637

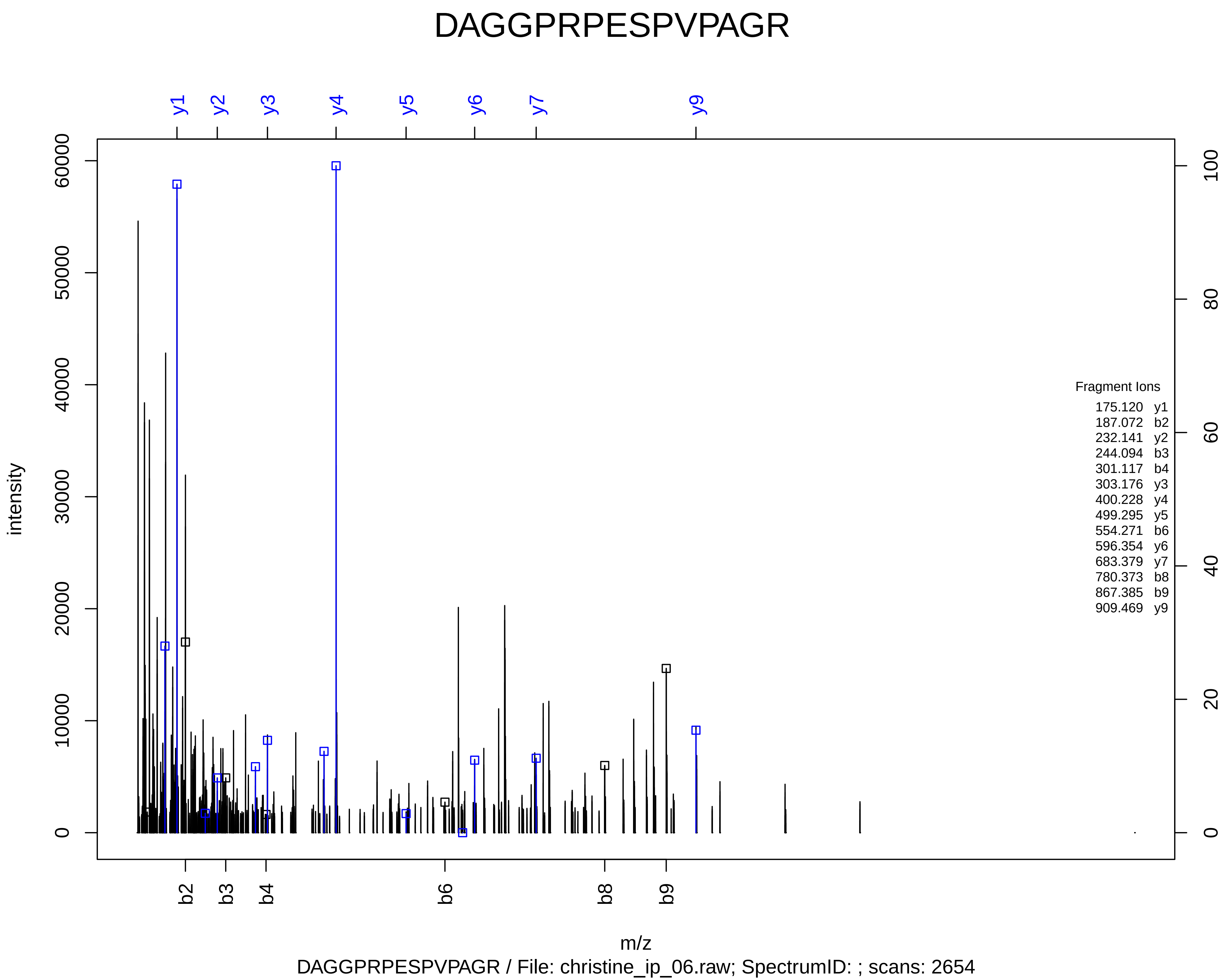
DAGGPRPESPVPAGR
y1
y2
y3
y4
y5
y6
y7
y9
intensity
0
10000
20000
30000
40000
50000
60000
0
20
40
60
80
100
b2
b3
b4
b6
b8
b9
m/z
Fragment Ions
175.120 y1
187.072 b2
232.141 y2
244.094 b3
301.117 b4
303.176 y3
400.228 y4
499.295 y5
554.271 b6
596.354 y6
683.379 y7
780.373 b8
867.385 b9
909.469 y9
DAGGPRPESPVPAGR / File: christine_ip_06.raw; SpectrumID: ; scans: 2654

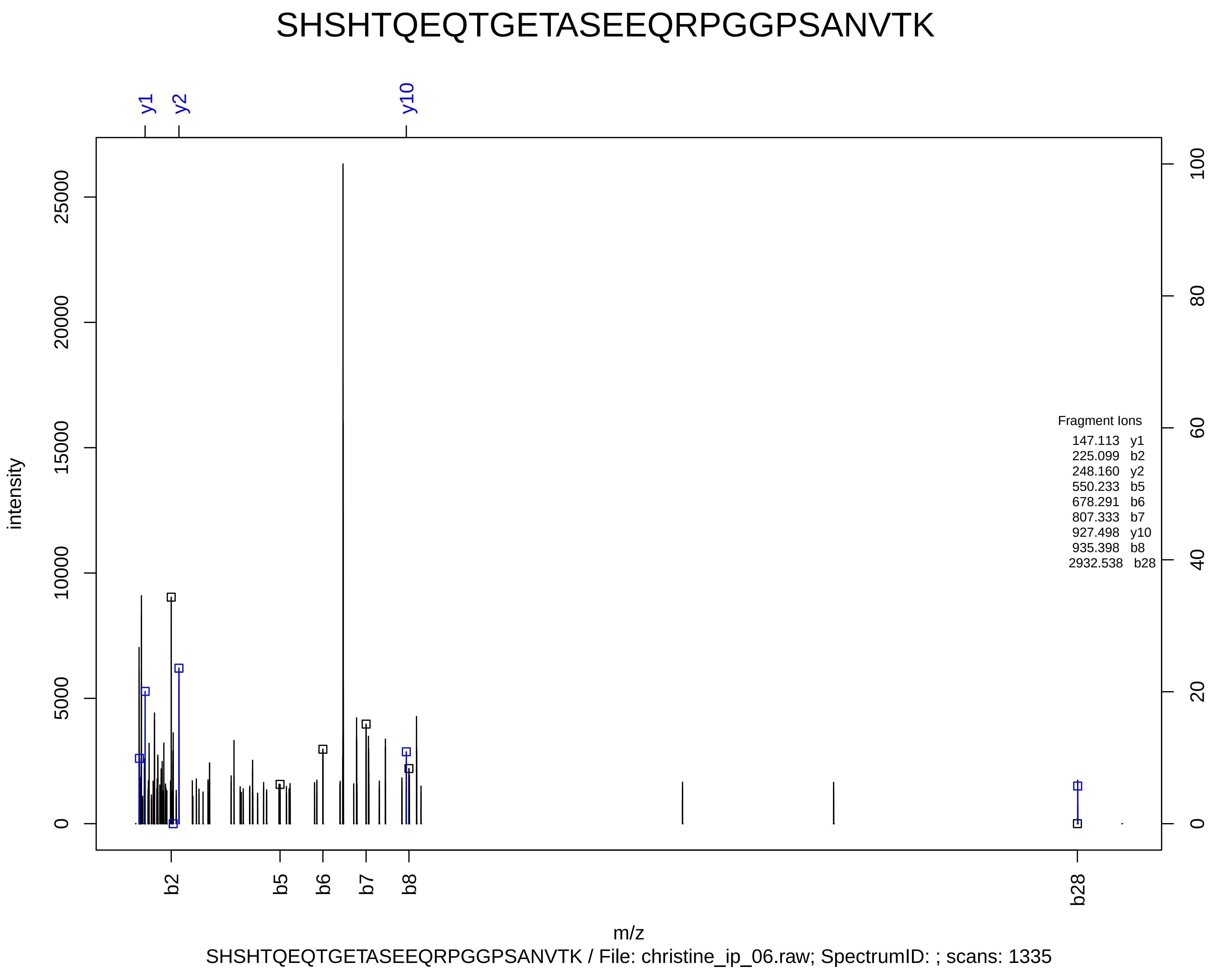

SHSHTQEQTGETASEEQRPGGPSANVTK
y1
y2
y10
intensity
0
5000
10000
15000
20000
25000
0
20
40
60
80
100
Fragment Ions
147.113 y1
225.099 b2
248.160 y2
550.233 b5
678.291 b6
807.333 b7
927.498 y10
935.398 b8
2932.538 b28
b2
b5
b6
b7
b8
b28
m/z
SHSHTQEQTGETASEEQRPGGPSANVTK / File: christine_ip_06.raw; SpectrumID: ; scans: 1335

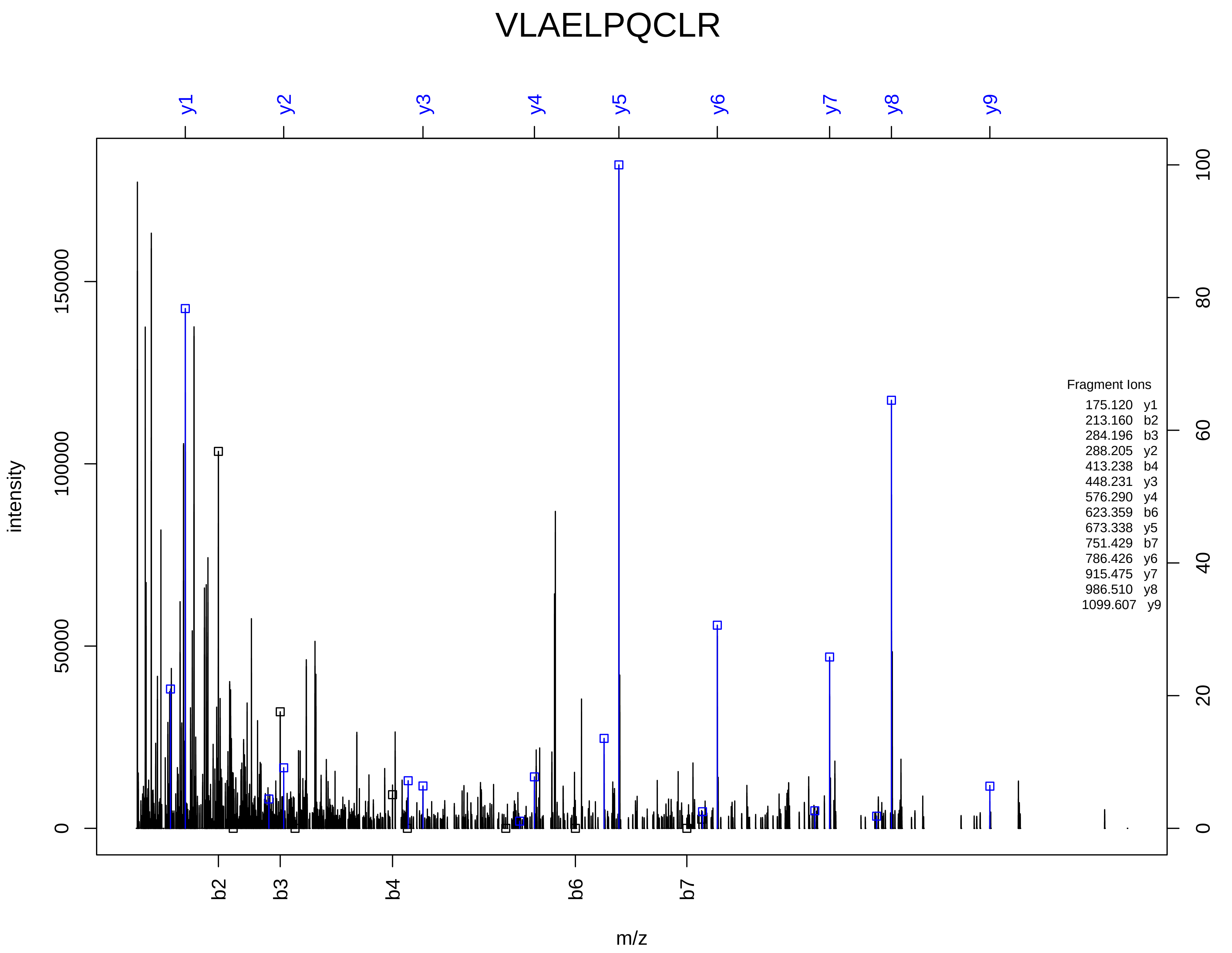
VLAELPQCLR
y1
y2
y3
y4
y5
y6
y7
y8
y9
intensity
0
50000
100000
150000
0
20
40
60
80
100
b2
b3
b4
b6
b7
m/z
Fragment Ions
175.120 y1
213.160 b2
284.196 b3
288.205 y2
413.238 b4
448.231 y3
576.290 y4
623.359 b6
673.338 y5
751.429 b7
786.426 y6
915.475 y7
986.510 y8
1099.607 y9
VLAELPQCLR / File: christine_ip_06.raw; SpectrumID: ; scans: 9994

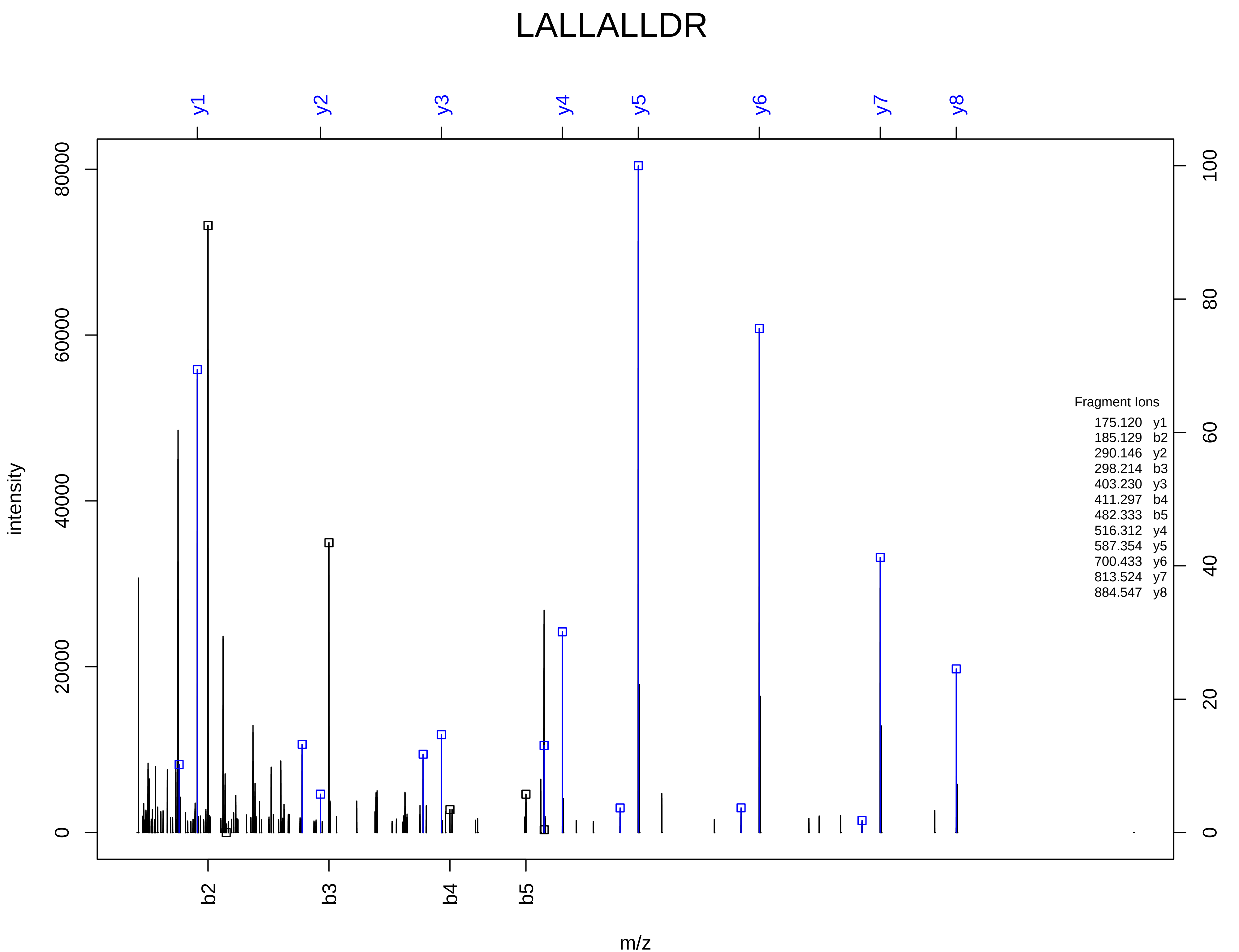

LALLALLDR
y1
y2
y3
y4
y5
y6
y7
y8
b2
b3
b4
b5
intensity
m/z
Fragment Ions
175.120 y1
185.129 b2
290.146 y2
298.214 b3
403.230 y3
411.297 b4
482.333 b5
516.312 y4
587.354 y5
700.433 y6
813.524 y7
884.547 y8
LALLALLDR / File: christine_ip_06.raw; SpectrumID: ; scans: 18215

DNSSNEVFPQGAEER

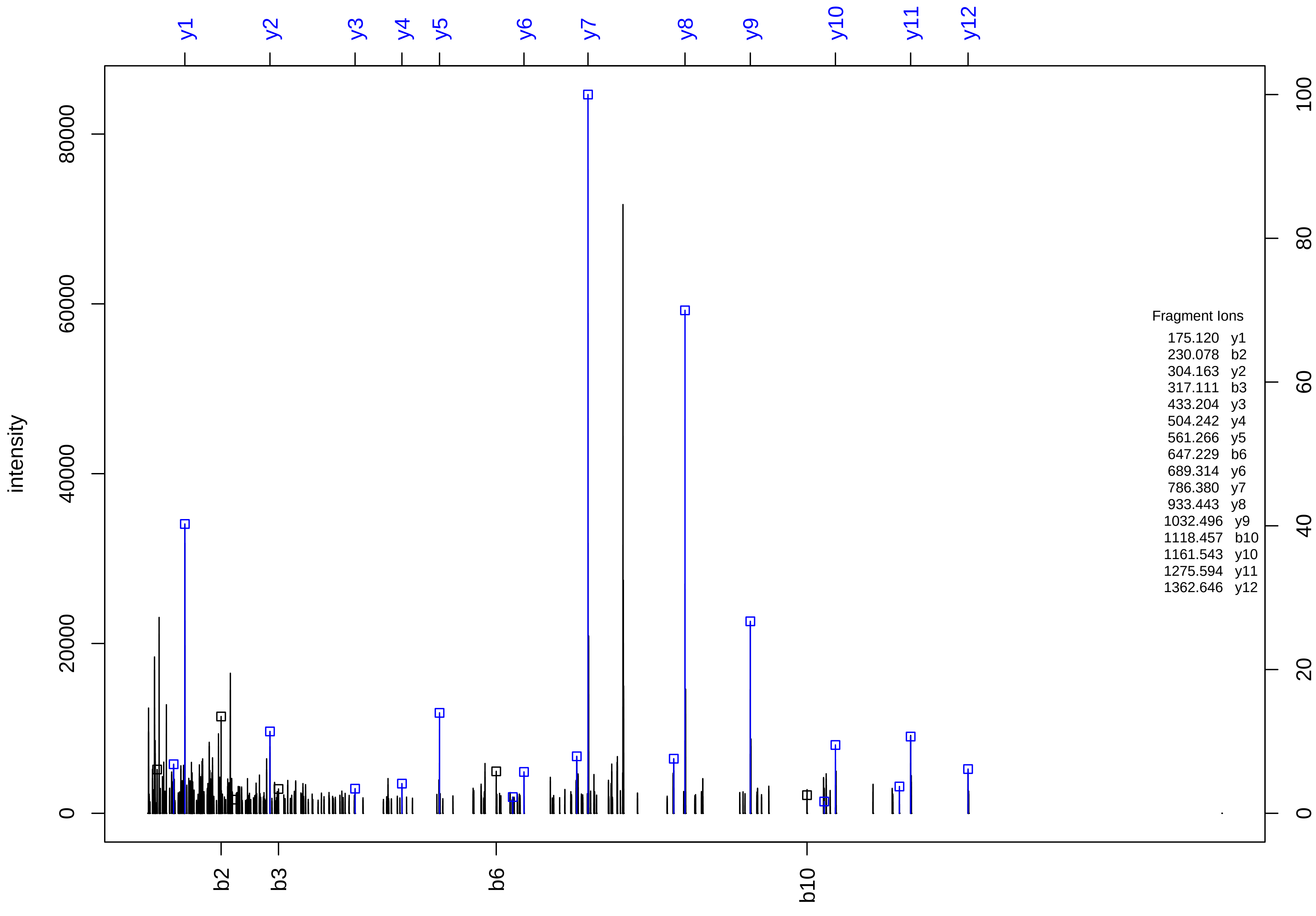

DNSSNEVFPQGAEER / File: christine_ip_06.raw; SpectrumID: ; scans: 5171

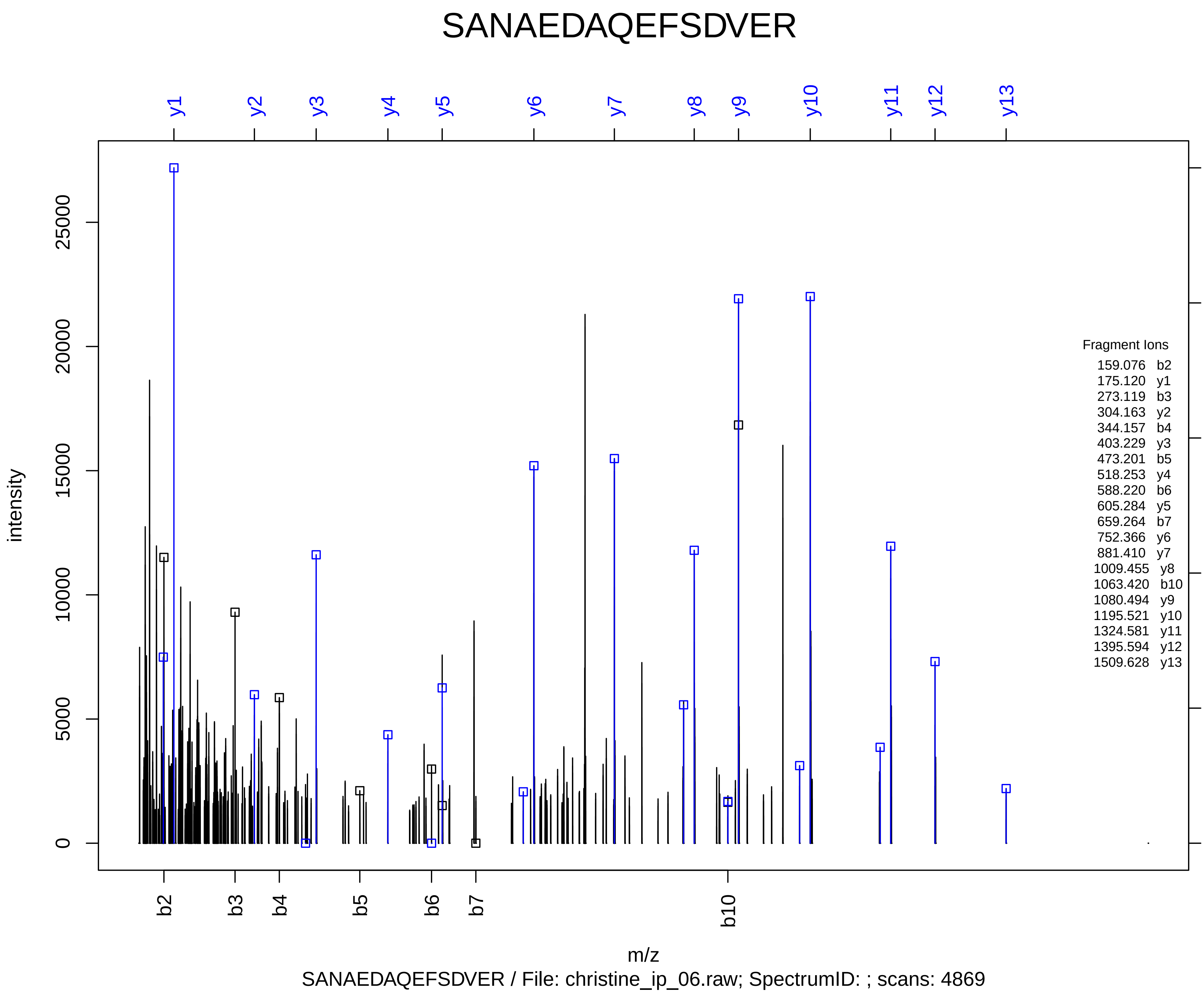
SANAEDAQEFSDVER
intensity
m/z
y1
y2
y3
y4
y5
y6
y7
y8
y9
y10
y11
y12
y13
b2
b3
b4
b5
b6
b7
b10
0
5000
10000
15000
20000
25000
0
20
40
60
80
100
Fragment Ions
159.076 b2
175.120 y1
273.119 b3
304.163 y2
344.157 b4
403.229 y3
473.201 b5
518.253 y4
588.220 b6
605.284 y5
659.264 b7
752.366 y6
881.410 y7
1009.455 y8
1063.420 b10
1080.494 y9
1195.521 y10
1324.581 y11
1395.594 y12
1509.628 y13
SANAEDAQEFSDVER / File: christine_ip_06.raw; SpectrumID: ; scans: 4869

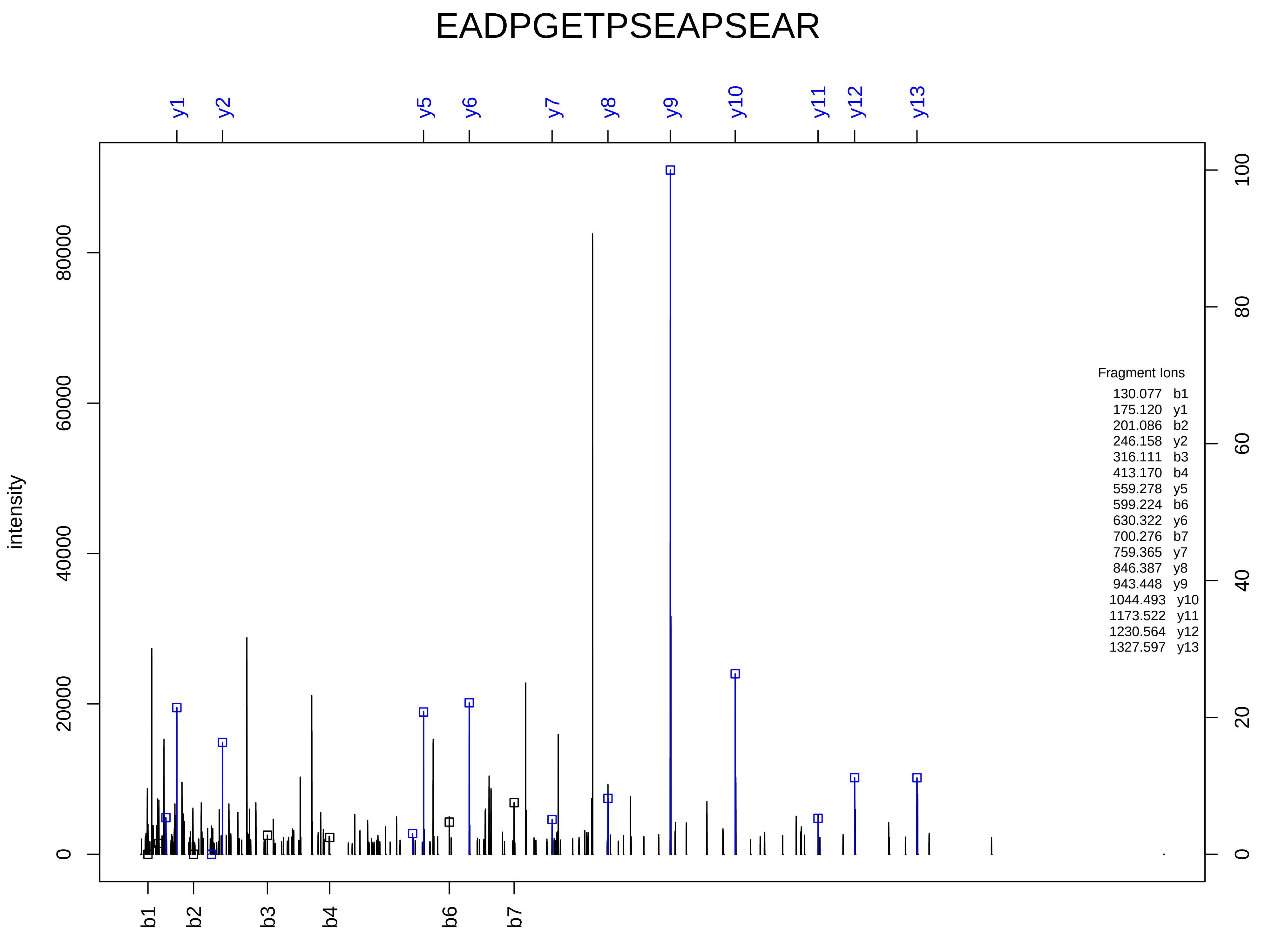

EADPGETPSEAPSEAR / File: christine_ip_06.raw; SpectrumID: ; scans: 2254

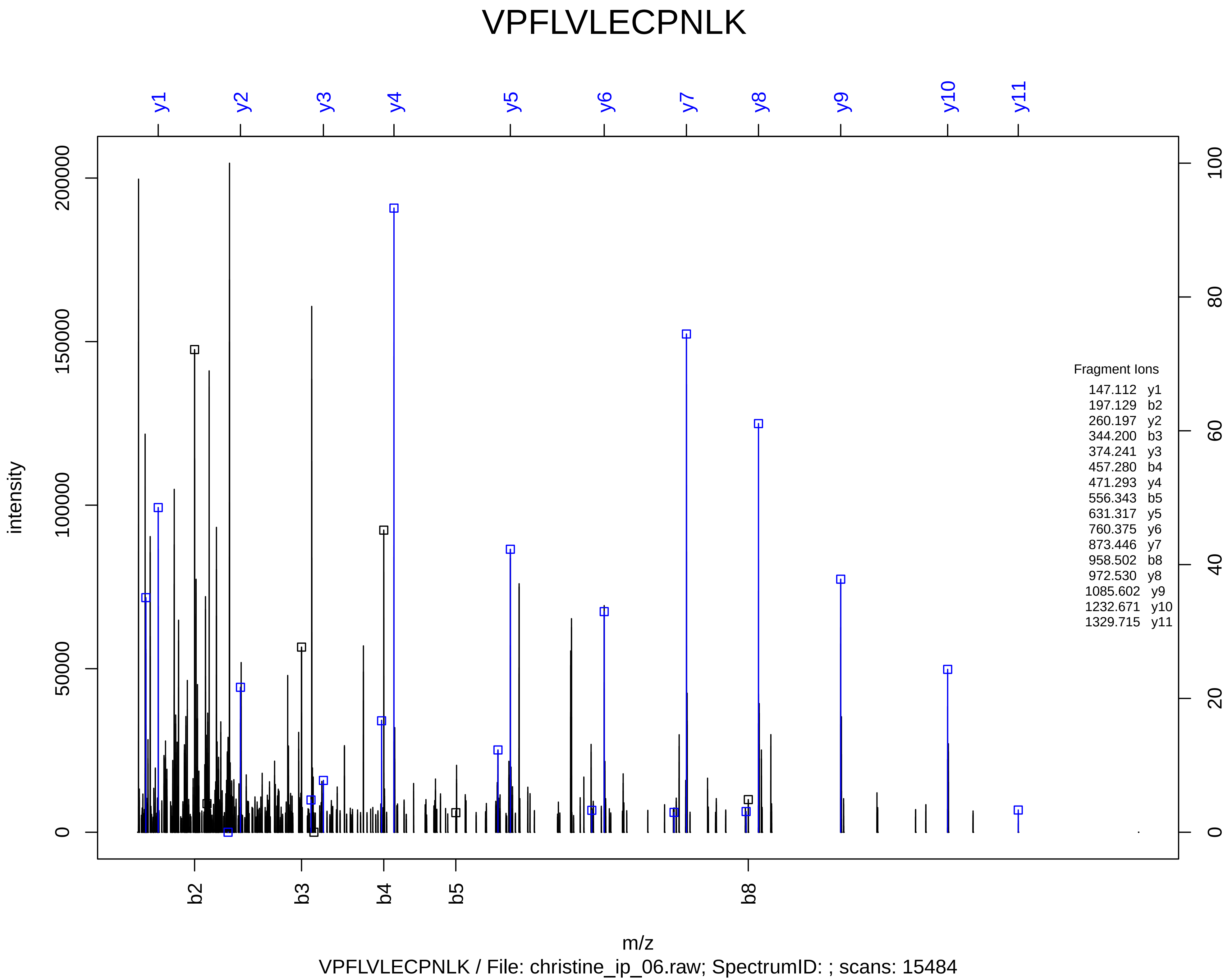
VPFLVLECPNLK
y1
y2
y3
y4
y5
y6
y7
y8
y9
y10
y11
intensity
0
50000
100000
150000
200000
0
20
40
60
80
100
b2
b3
b4
b5
b8
m/z
Fragment Ions
147.112 y1
197.129 b2
260.197 y2
344.200 b3
374.241 y3
457.280 b4
471.293 y4
556.343 b5
631.317 y5
760.375 y6
873.446 y7
958.502 b8
972.530 y8
1085.602 y9
1232.671 y10
1329.715 y11
VPFLVLECPNLK / File: christine_ip_06.raw; SpectrumID: ; scans: 15484

VLDIIYQPQAIFR

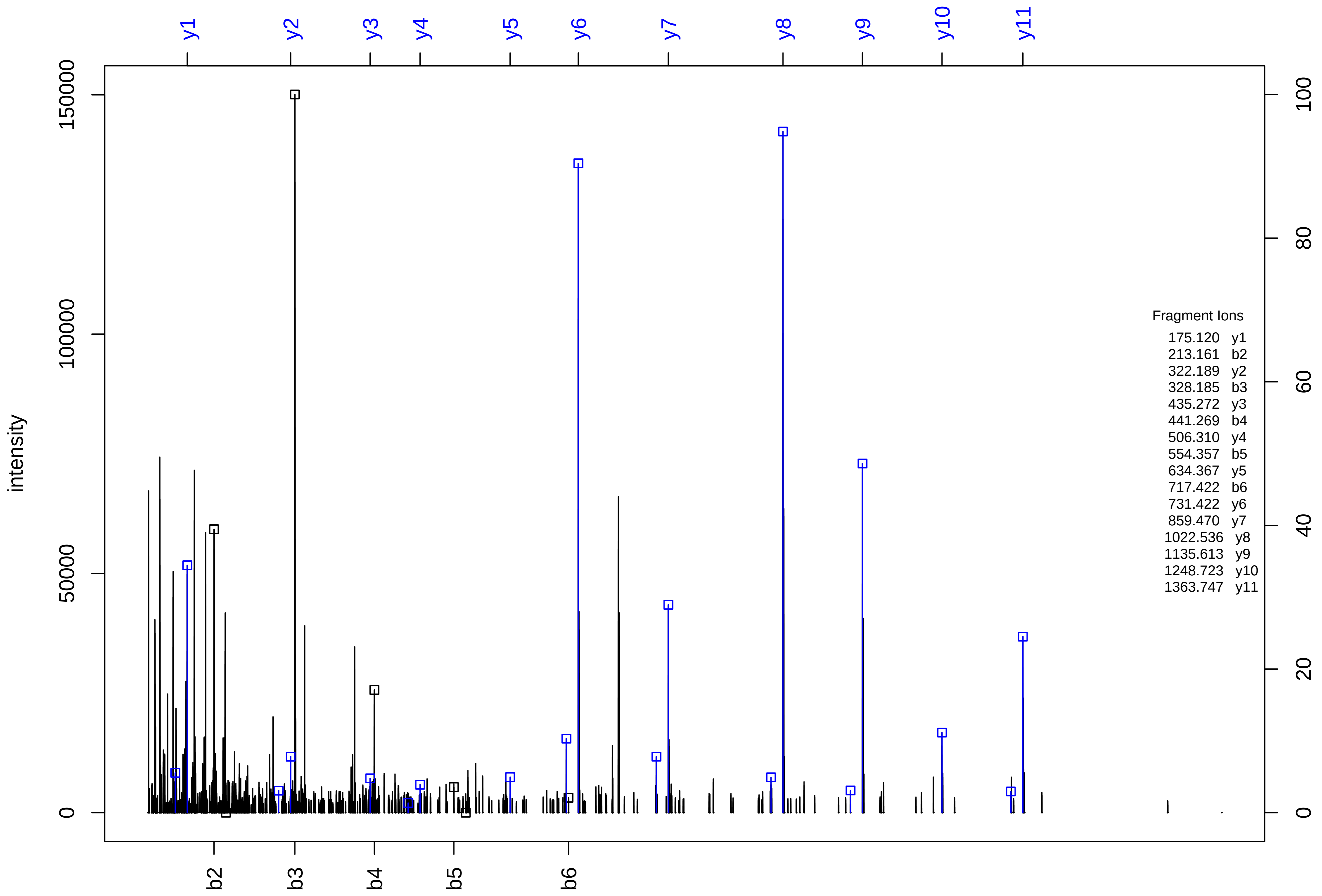

VLDIIYQPQAIFR / File: christine_ip_06.raw; SpectrumID: ; scans: 17153

VLALLDVPDK

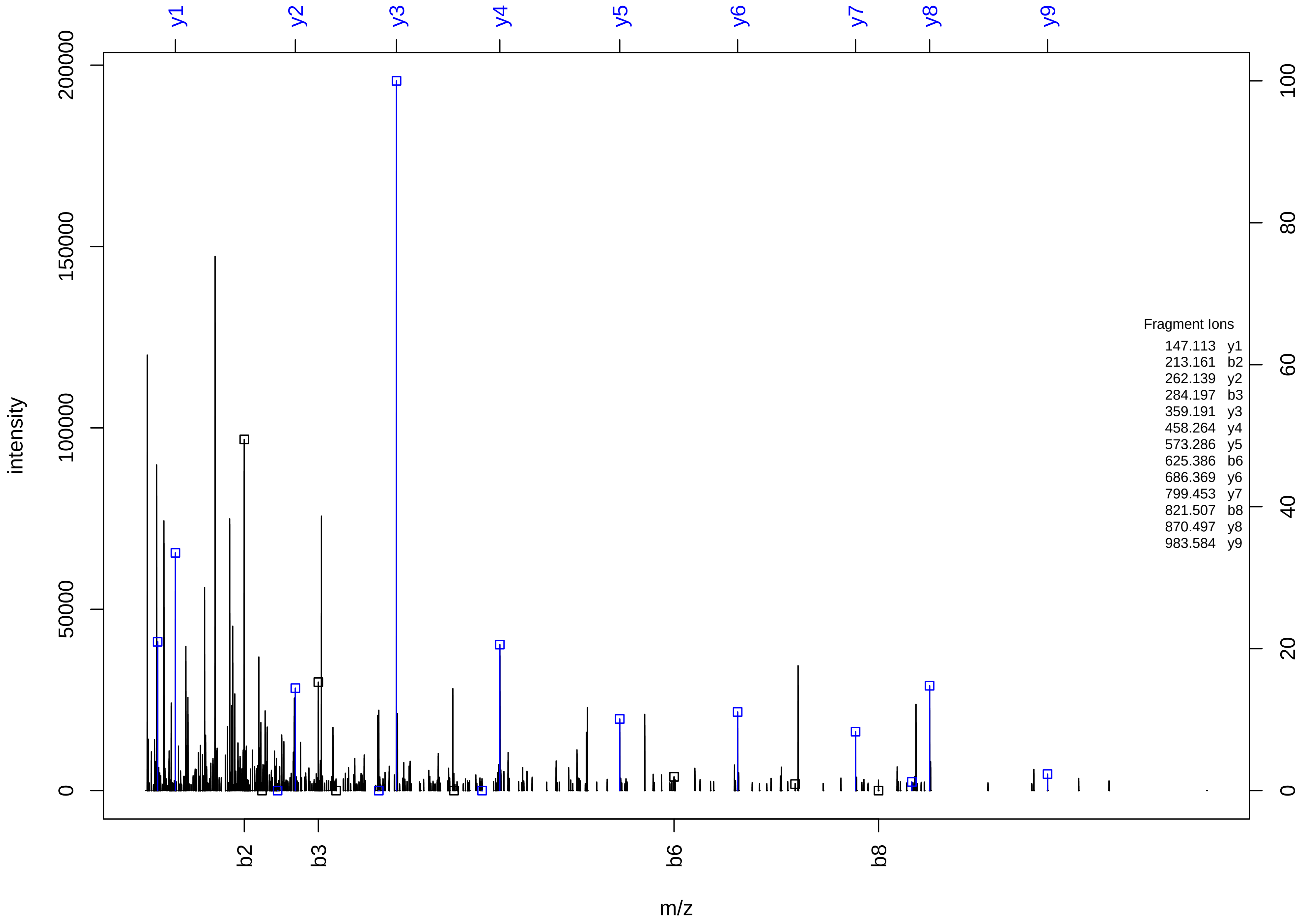

VLALLDVPDK / File: christine_ip_06.raw; SpectrumID: ; scans: 12698

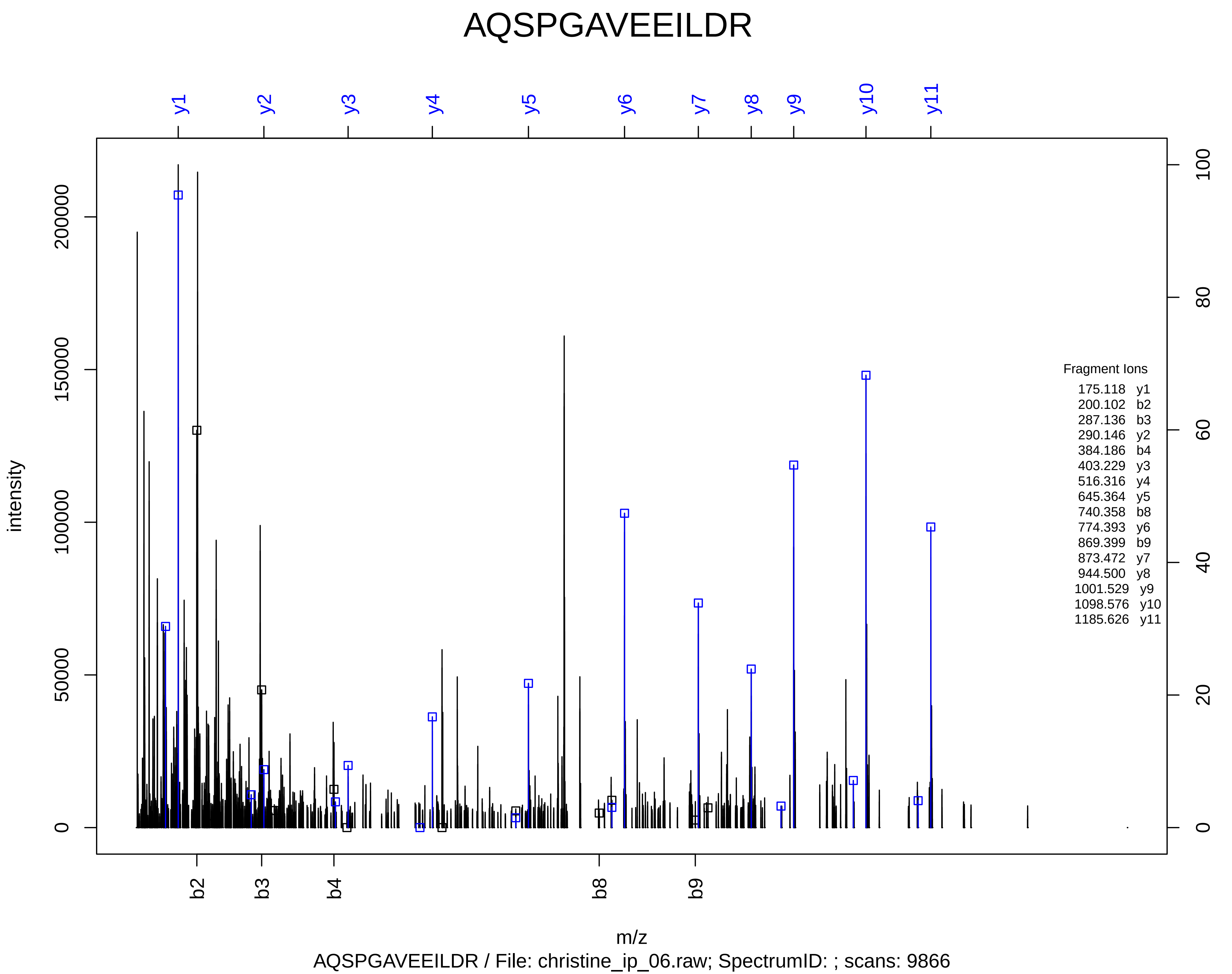
AQSPGAVEEILDR
y1
y2
y3
y4
y5
y6
y7
y8
y9
y10
y11
intensity
0
50000
100000
150000
200000
0
20
40
60
80
100
b2
b3
b4
b8
b9
m/z
Fragment Ions
175.118 y1
200.102 b2
287.136 b3
290.146 y2
384.186 b4
403.229 y3
516.316 y4
645.364 y5
740.358 b8
774.393 y6
869.399 b9
873.472 y7
944.500 y8
1001.529 y9
1098.576 y10
1185.626 y11
AQSPGAVEEILDR / File: christine_ip_06.raw; SpectrumID: ; scans: 9866

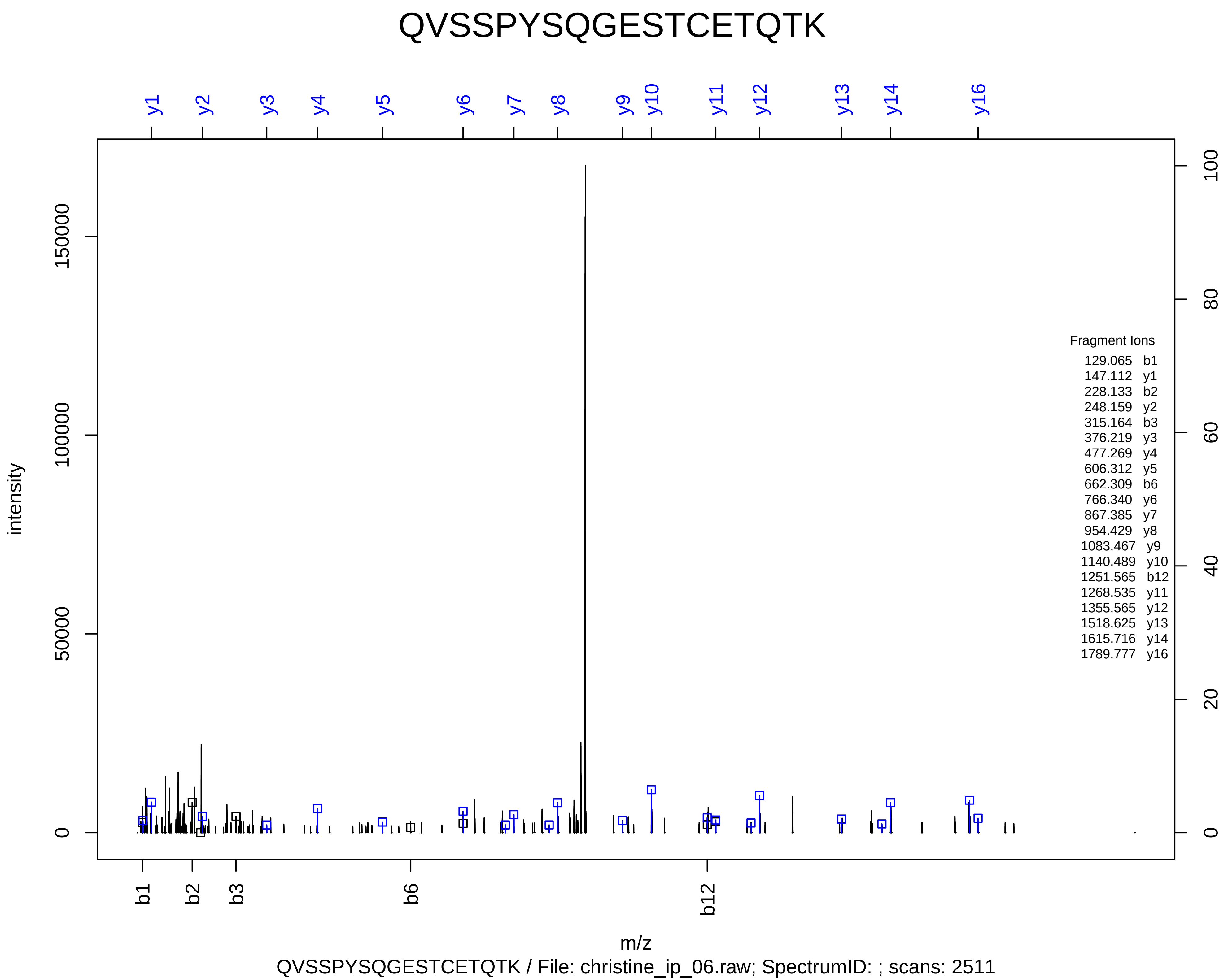
QVSSPYSQGESTCETQTK
y1
y2
y3
y4
y5
y6
y7
y8
y9
y10
y11
y12
y13
y14
y16
intensity
0
50000
100000
150000
0
20
40
60
80
100
Fragment Ions
129.065 b1
147.112 y1
228.133 b2
248.159 y2
315.164 b3
376.219 y3
477.269 y4
606.312 y5
662.309 b6
766.340 y6
867.385 y7
954.429 y8
1083.467 y9
1140.489 y10
1251.565 b12
1268.535 y11
1355.565 y12
1518.625 y13
1615.716 y14
1789.777 y16
b1
b2
b3
b6
b12
m/z
QVSSPYSQGESTCETQTK / File: christine_ip_06.raw; SpectrumID: ; scans: 2511

EIPVEGGQEQQTDSTK

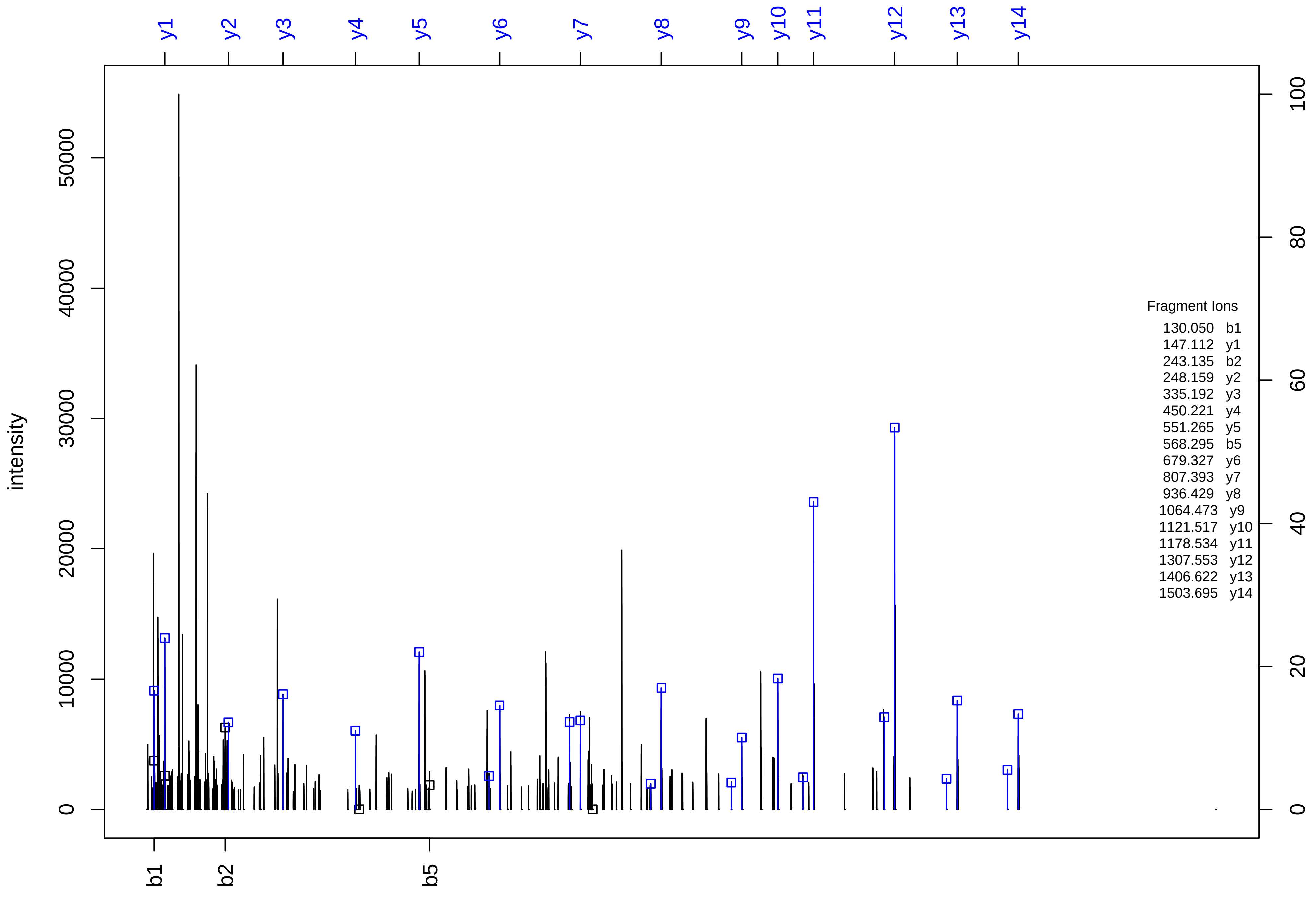

EIPVEGGQEQQTDSTK / File: christine_ip_06.raw; SpectrumID: ; scans: 2848

EGQHDVFGDISFLQGNYELLPGNQEK

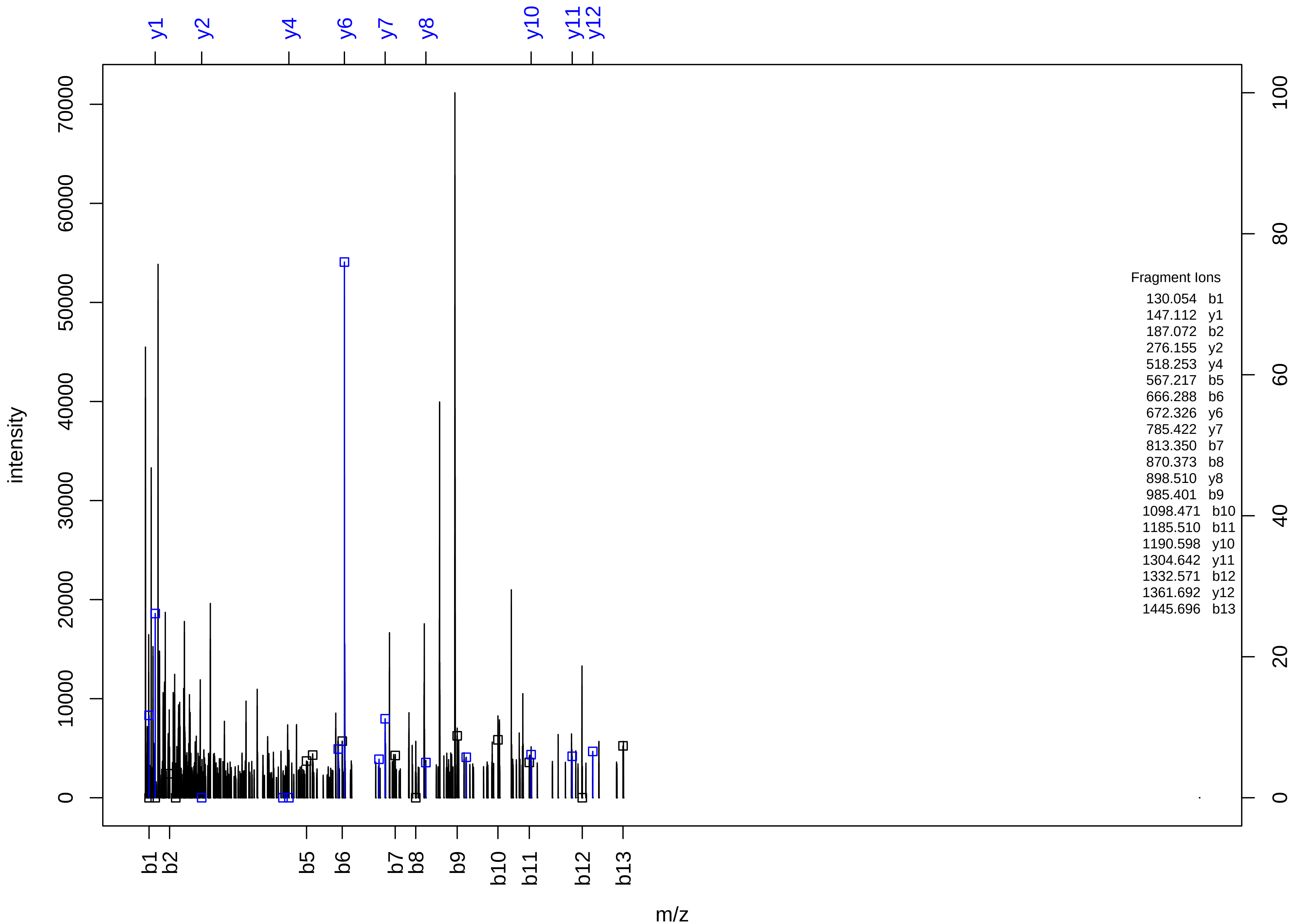

EGQHDVFGDISFLQGNYELLPGNQEK / File: christine_ip_06.raw; SpectrumID: ; scans: 17596

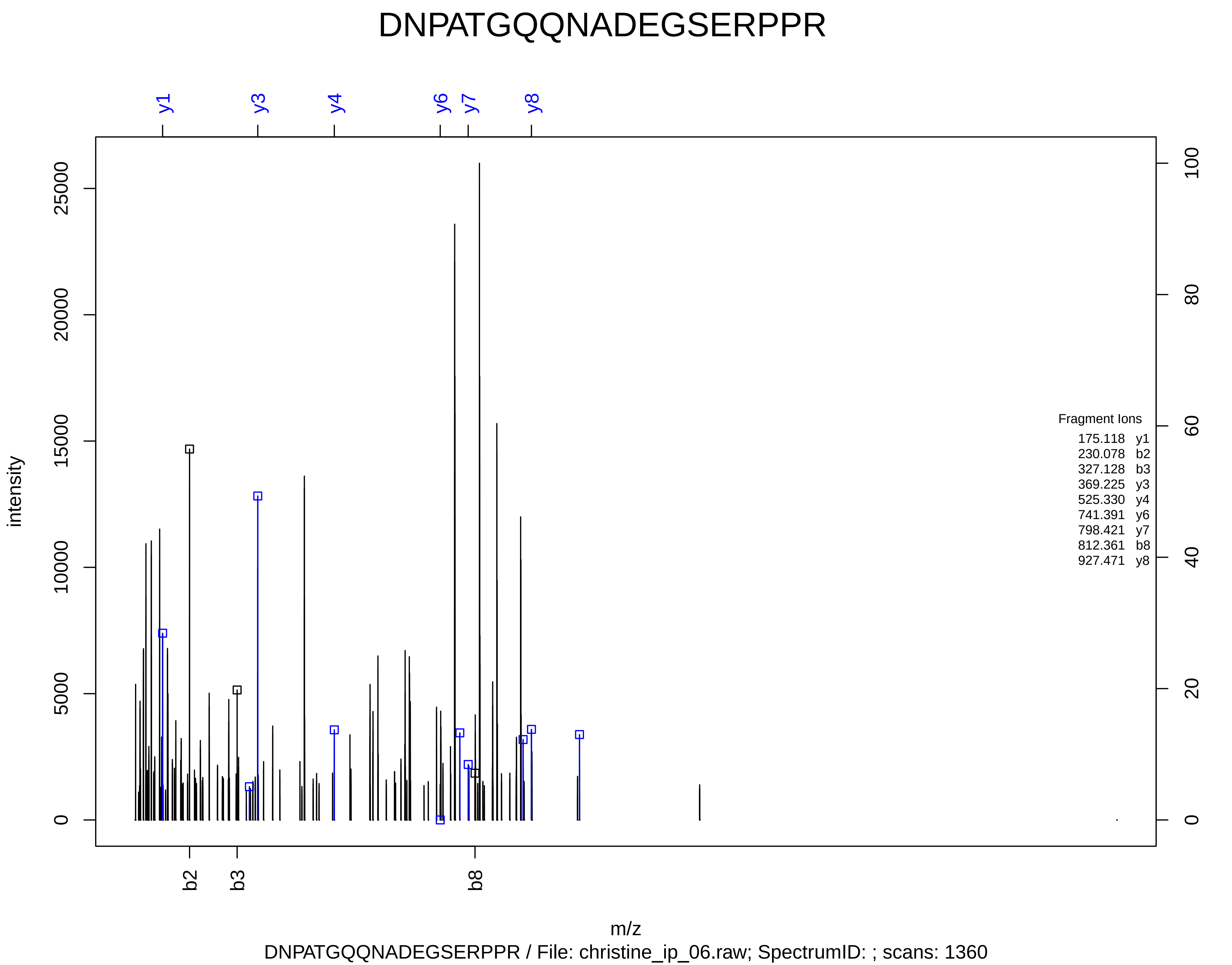
DNPATGQQNADEGSERPPR
y1
y3
y4
y6
y7
y8
intensity
0
5000
10000
15000
20000
25000
0
20
40
60
80
100
b2
b3
b8
m/z
Fragment Ions
175.118 y1
230.078 b2
327.128 b3
369.225 y3
525.330 y4
741.391 y6
798.421 y7
812.361 b8
927.471 y8
DNPATGQQNADEGSERPPR / File: christine_ip_06.raw; SpectrumID: ; scans: 1360

QQSEEDLLLQDFSR

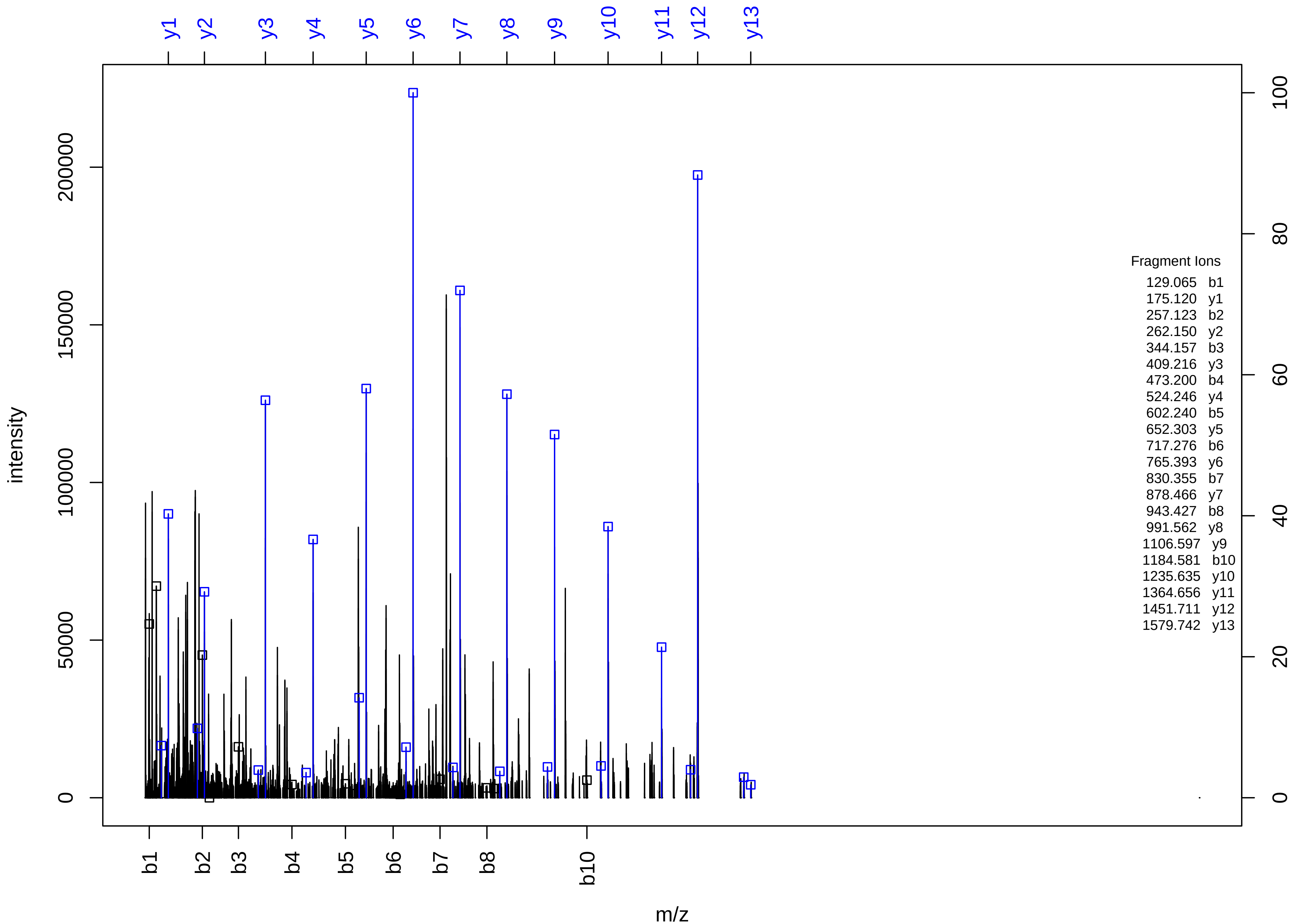

QQSEEDLLLQDFSR / File: christine_ip_06.raw; SpectrumID: ; scans: 13666

LLDPSSPLALALSAR

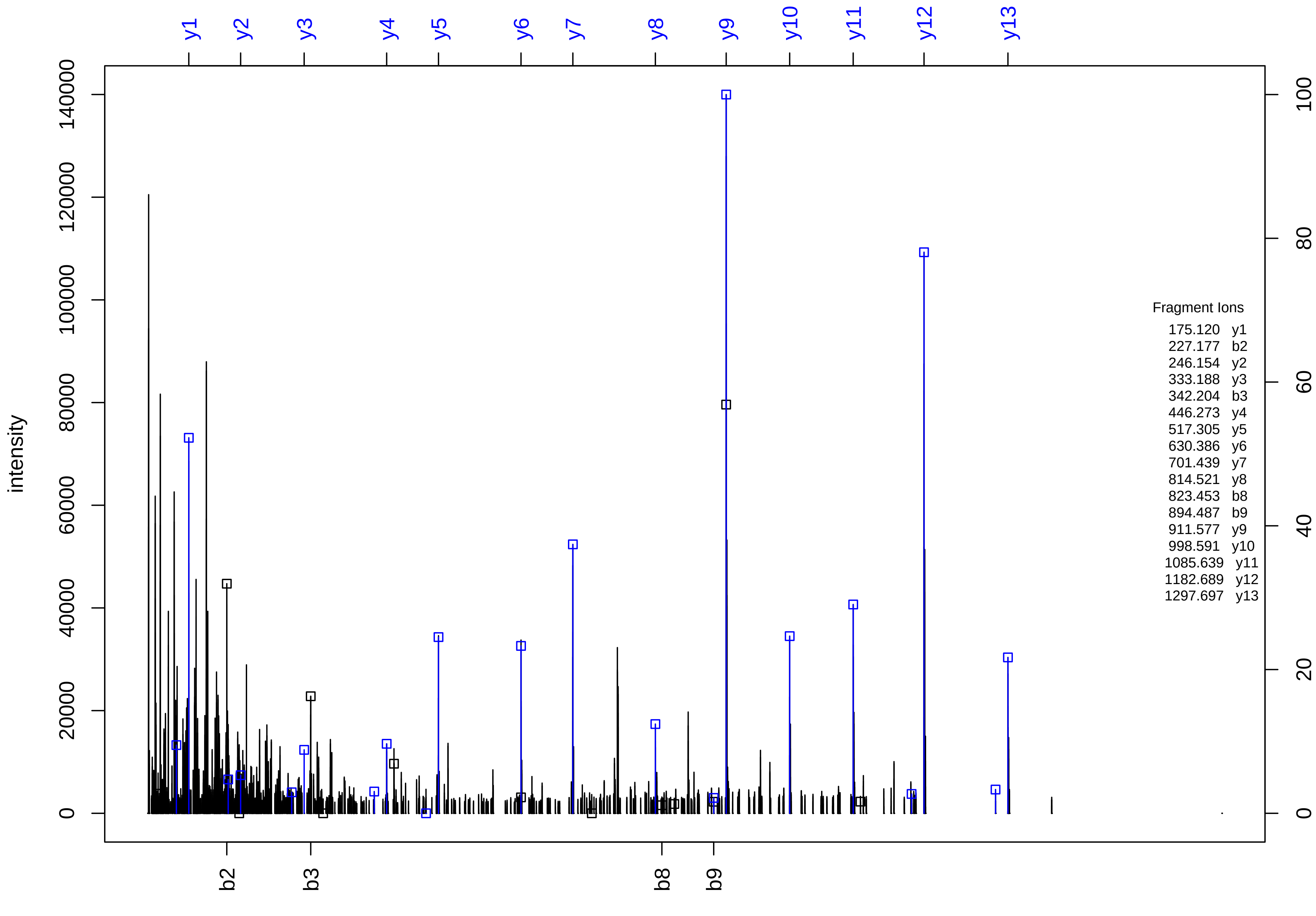

LLDPSSPLALALSAR / File: christine_ip_06.raw; SpectrumID: ; scans: 17253

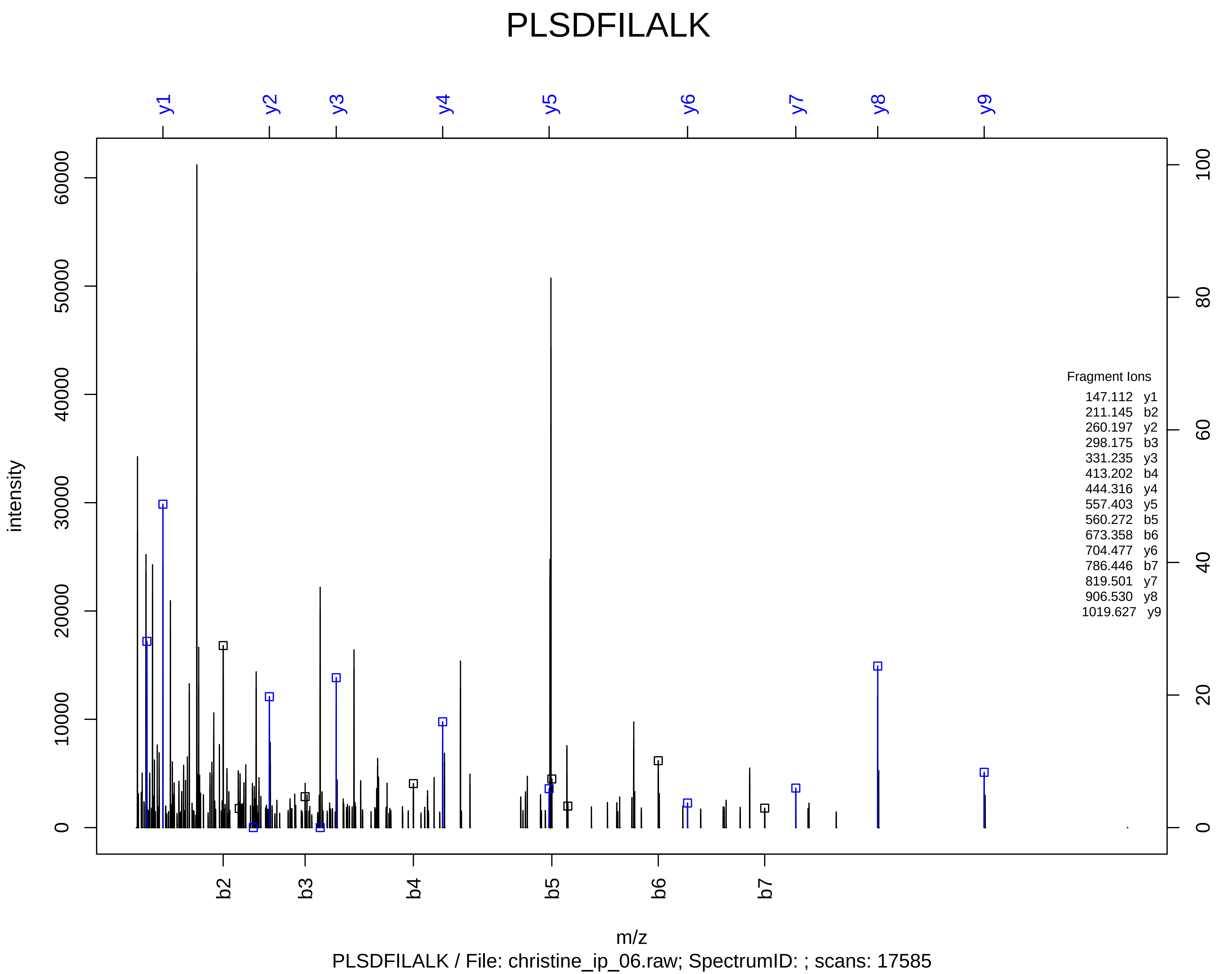

PLSDFILALK
y1
y2
y3
y4
y5
y6
y7
y8
y9
b2
b3
b4
b5
b6
b7
intensity
m/z
Fragment Ions
147.112 y1
211.145 b2
260.197 y2
298.175 b3
331.235 y3
413.202 b4
444.316 y4
557.403 y5
560.272 b5
673.358 b6
704.477 y6
786.446 b7
819.501 y7
906.530 y8
1019.627 y9
PLSDFILALK / File: christine_ip_06.raw; SpectrumID: ; scans: 17585

QSLLFQLN^EN^TGEISTAK

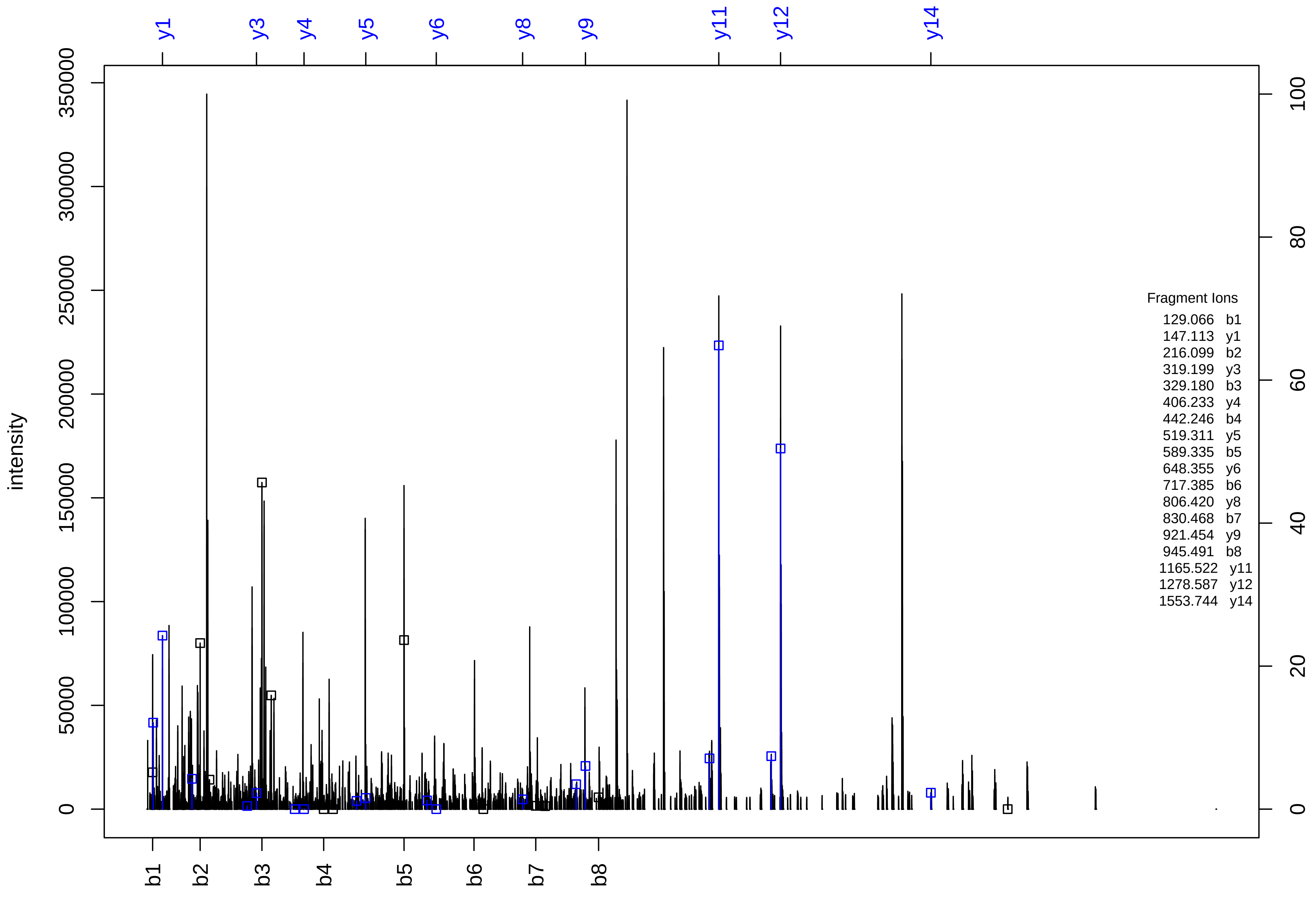

QSLLFQLNENTGEISTAK / File: christine_ip_06.raw; SpectrumID: ; scans: 9998

(Ac)EAELTPLESAIN^VLEM*IK

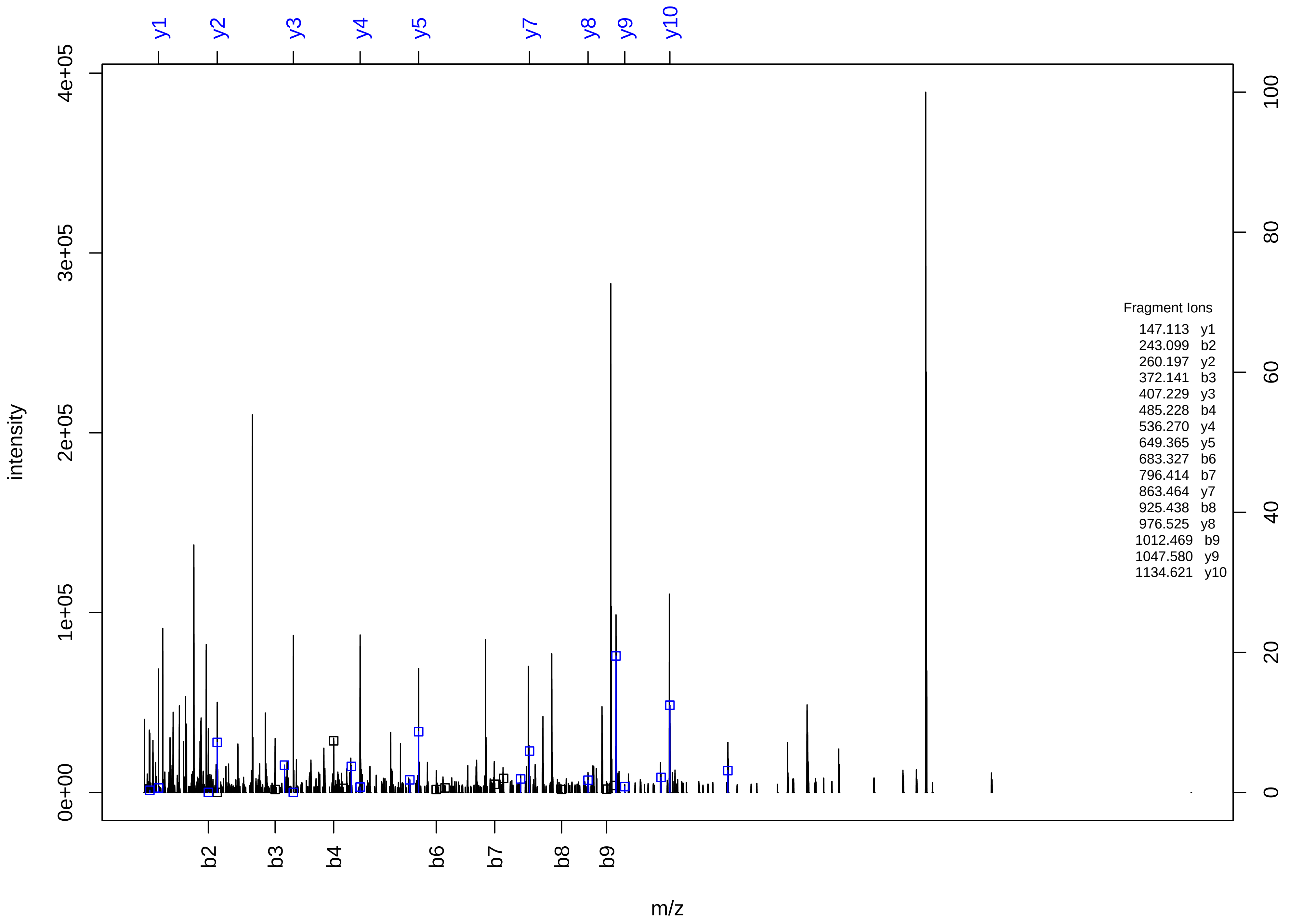

EAELTPLESAINVLEMIK / File: christine_ip_06.raw; SpectrumID: ; scans: 16820

DGGFDLSDALPDNENK

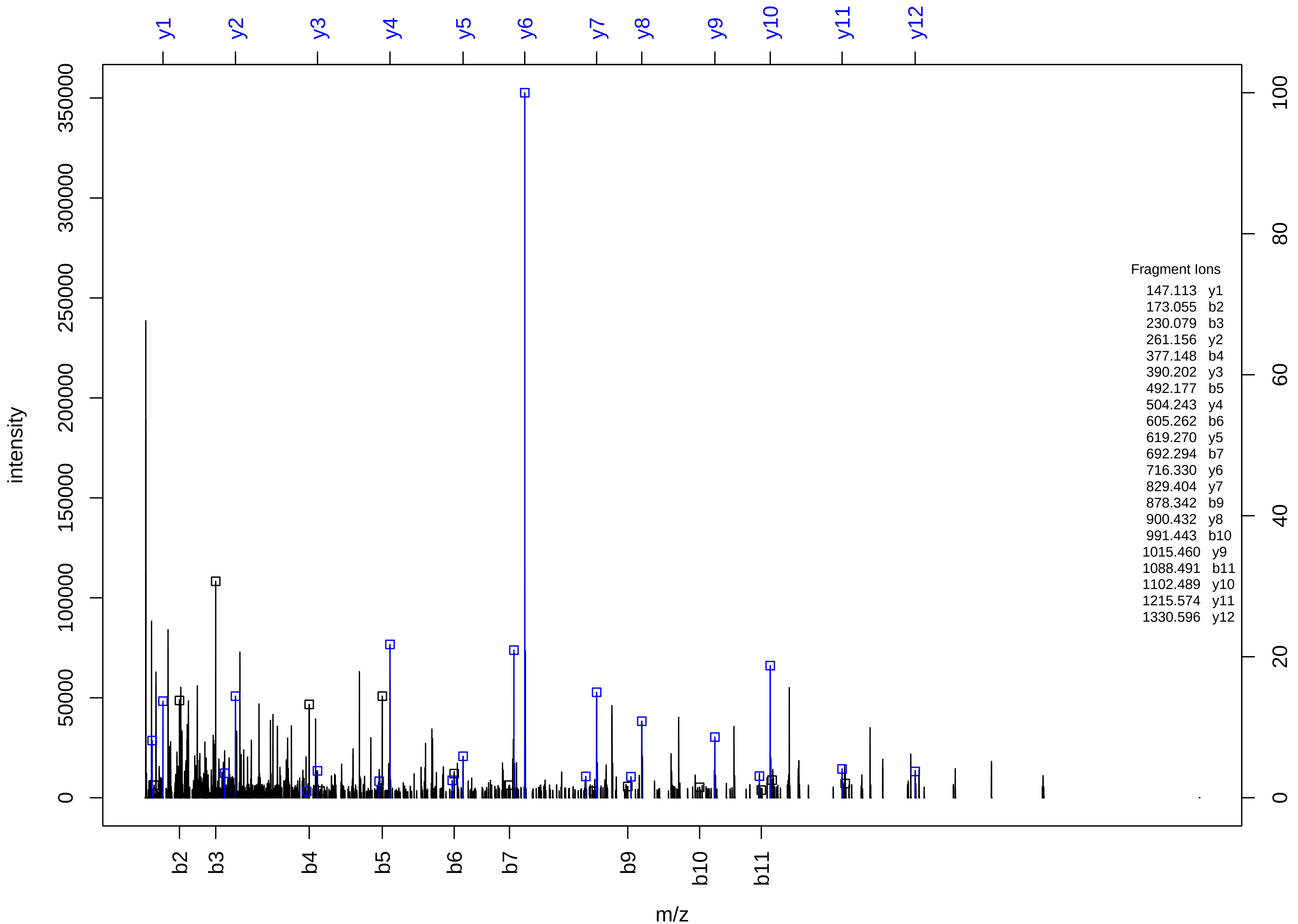

DGGFDLSDALPDNENK / File: christine_ip_06.raw; SpectrumID: ; scans: 12030

DRHKLVSSFLTTAMLK

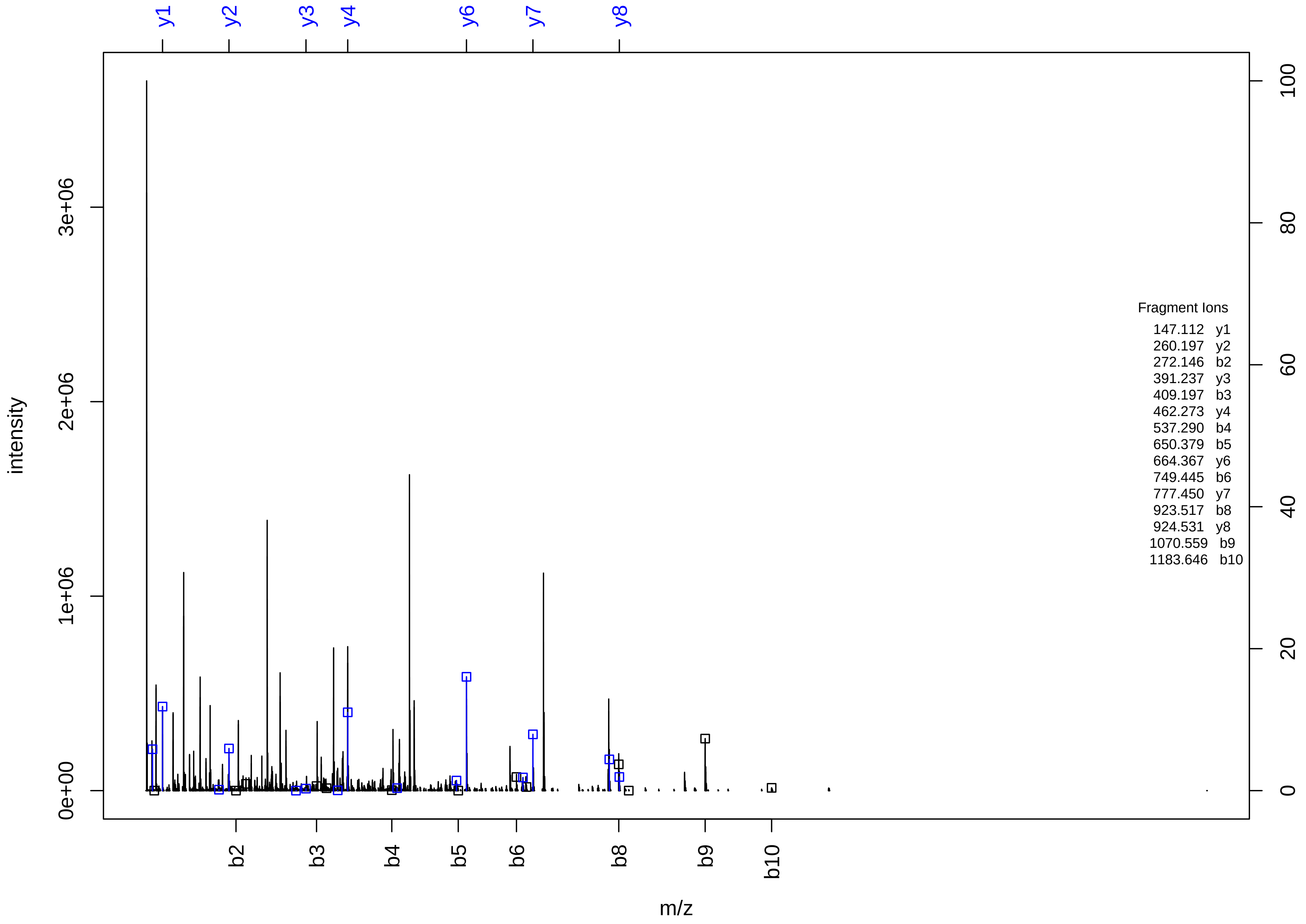

DRHKLVSSFLTTAMLK / File: christine_ip_06.raw; SpectrumID: ; scans: 19213

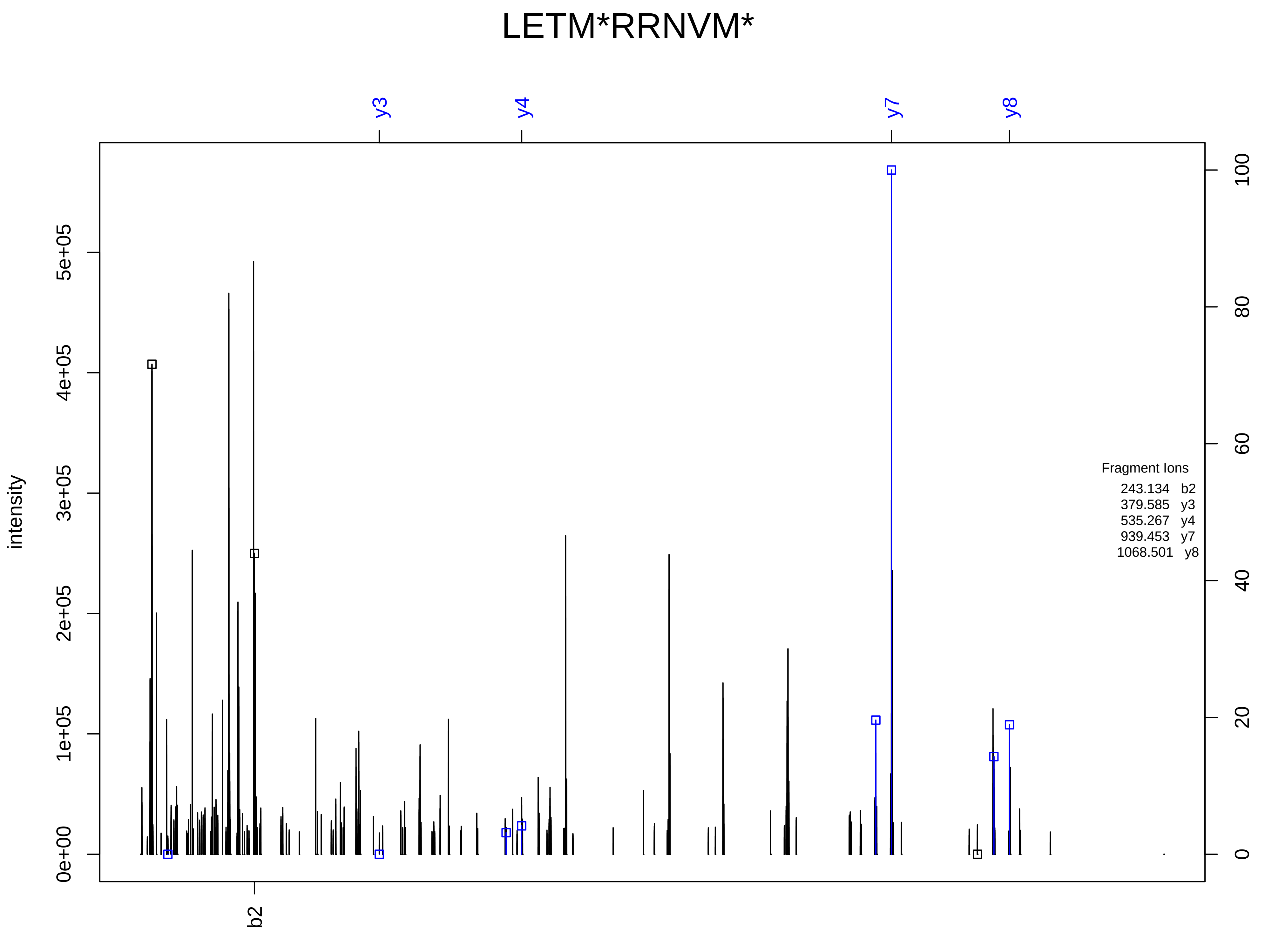
LETM*RRNVM*
intensity
0e+00
1e+05
2e+05
3e+05
4e+05
5e+05
0
20
40
60
80
100
y3
y4
y7
y8
b2
Fragment Ions
243.134 b2
379.585 y3
535.267 y4
939.453 y7
1068.501 y8
m/z
LETMRRNVM / File: christine_ip_06.raw; SpectrumID: ; scans: 3994

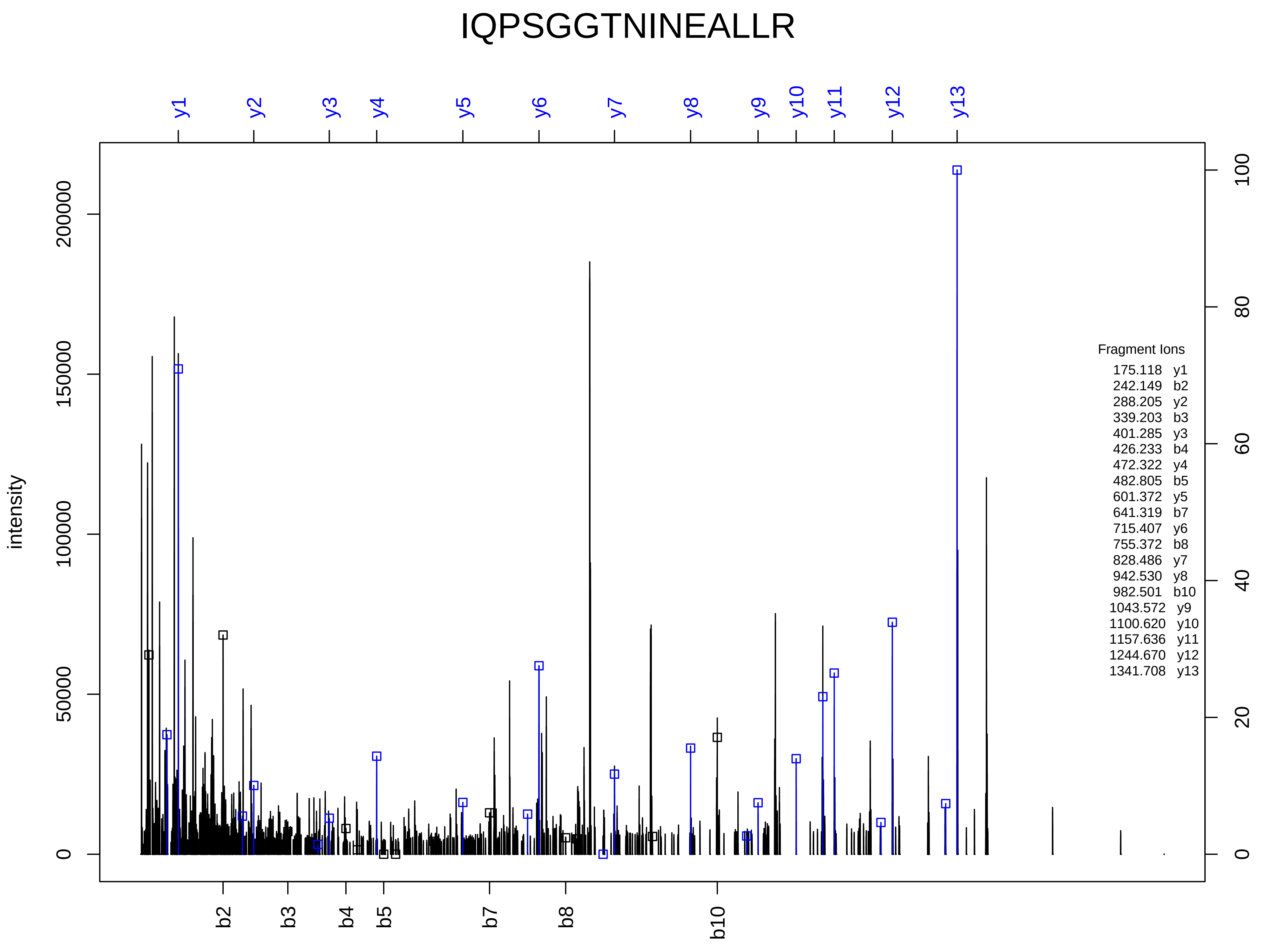

IQPSGGTNINEALLR / File: christine_ip_08.raw; SpectrumID: ; scans: 9920

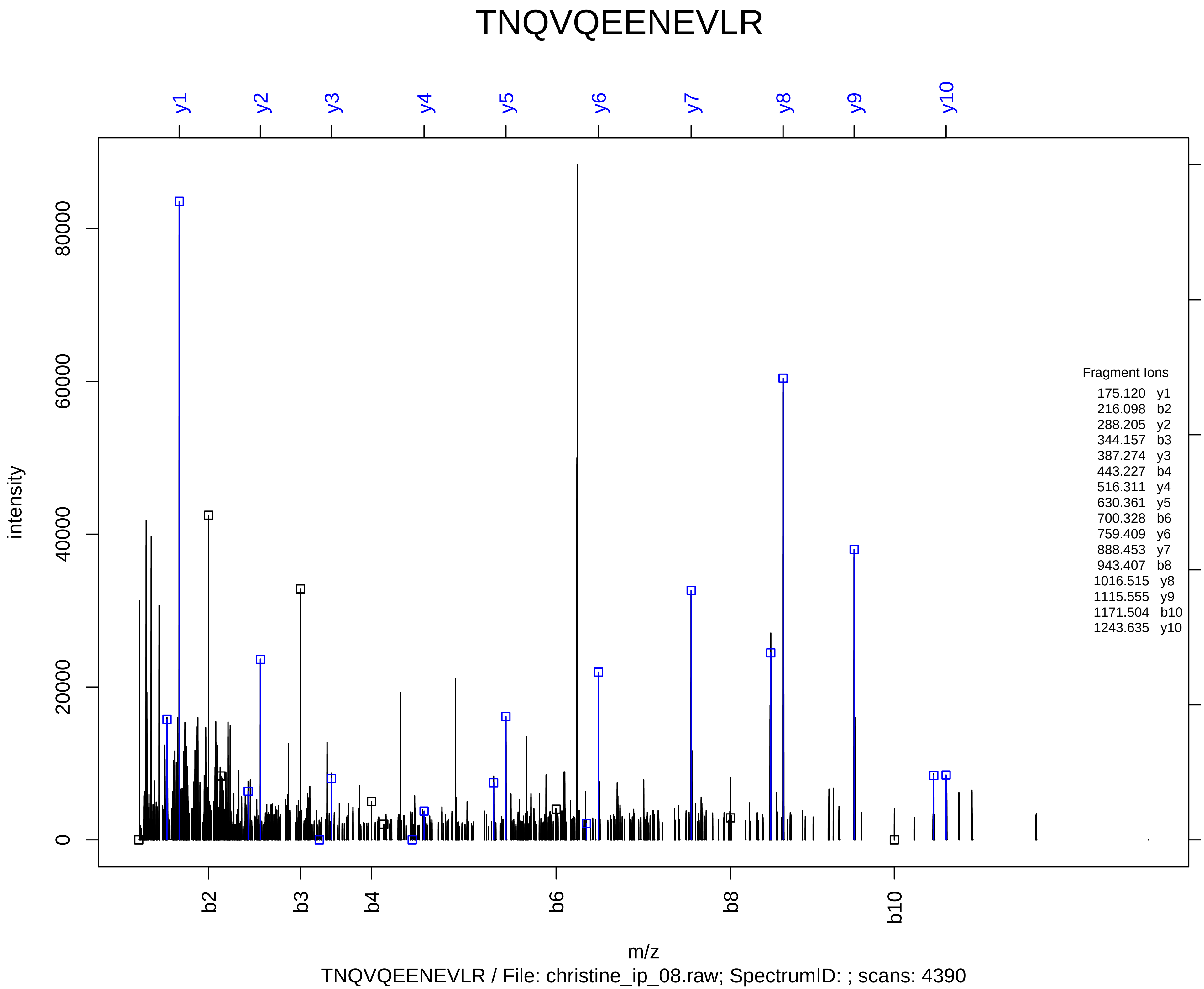
TNQVQEENEVLR
y1
y2
y3
y4
y5
y6
y7
y8
y9
y10
intensity
0
20000
40000
60000
80000
0
20
40
60
80
100
b2
b3
b4
b6
b8
b10
m/z
Fragment Ions
175.120 y1
216.098 b2
288.205 y2
344.157 b3
387.274 y3
443.227 b4
516.311 y4
630.361 y5
700.328 b6
759.409 y6
888.453 y7
943.407 b8
1016.515 y8
1115.555 y9
1171.504 b10
1243.635 y10
TNQVQEENEVLR / File: christine_ip_08.raw; SpectrumID: ; scans: 4390

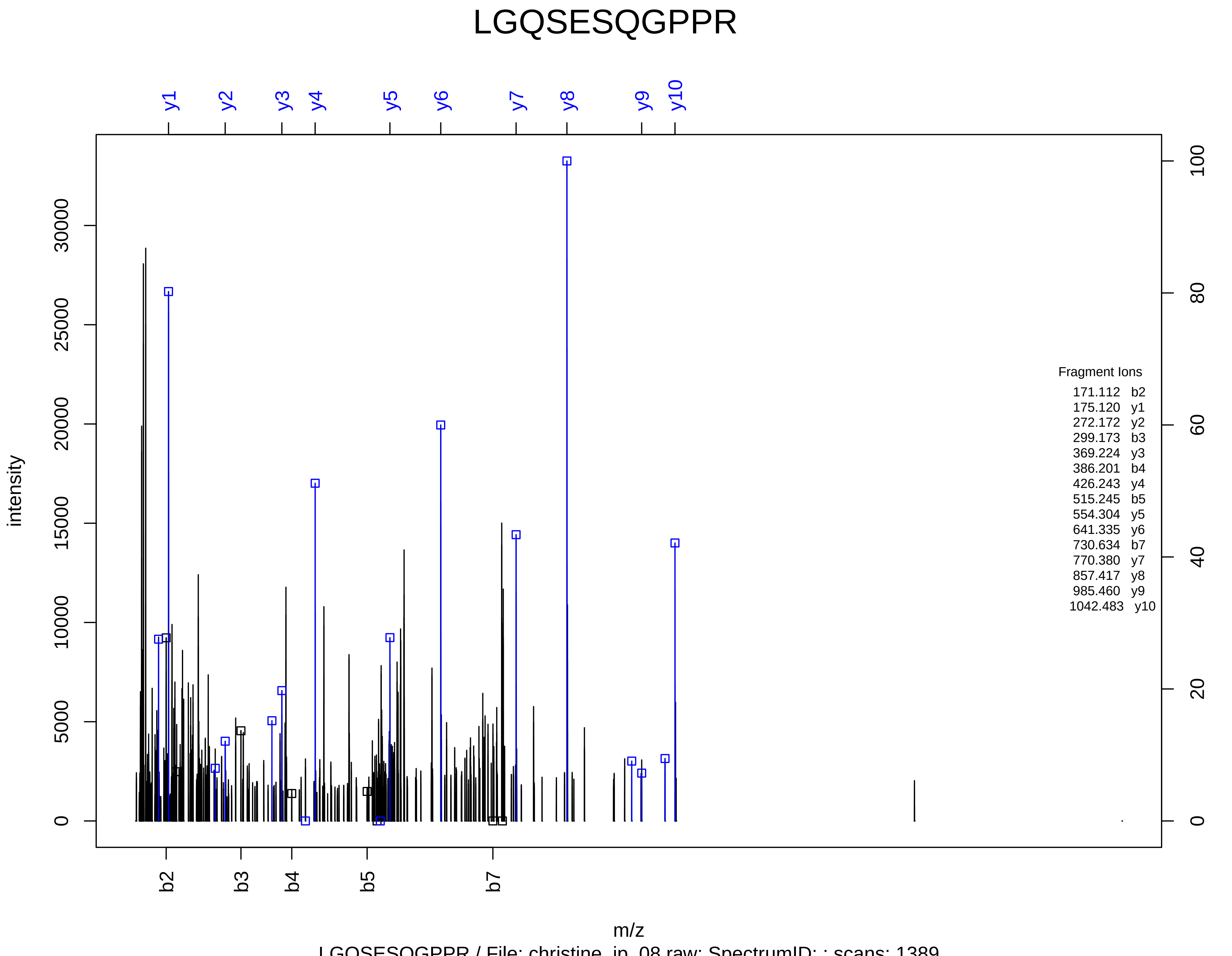

LGQSESQGPPR / File: christine_ip_08.raw; SpectrumID: ; scans: 1389

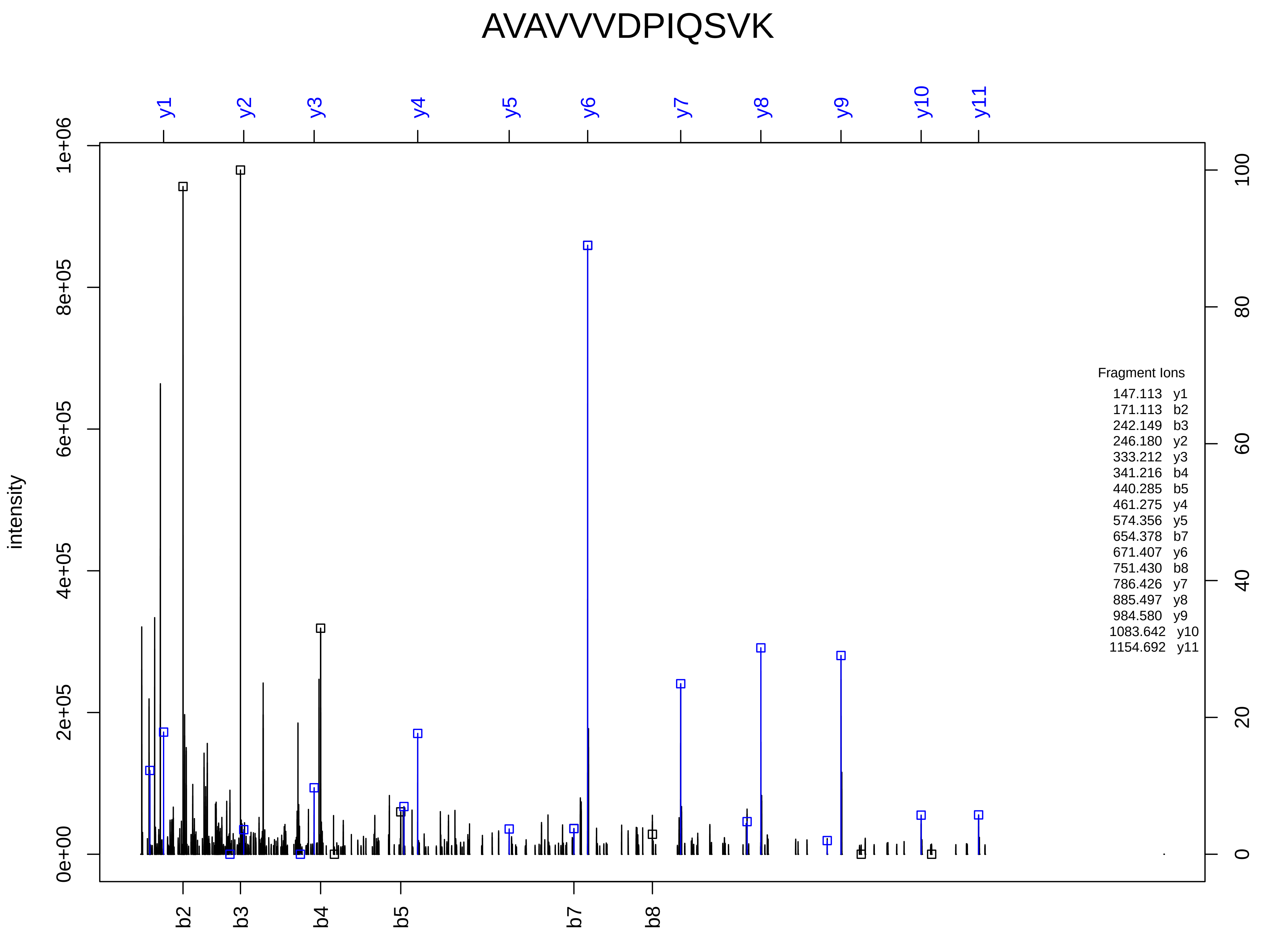

AVAVVVDPIQSVK / File: christine_ip_08.raw; SpectrumID: ; scans: 12477

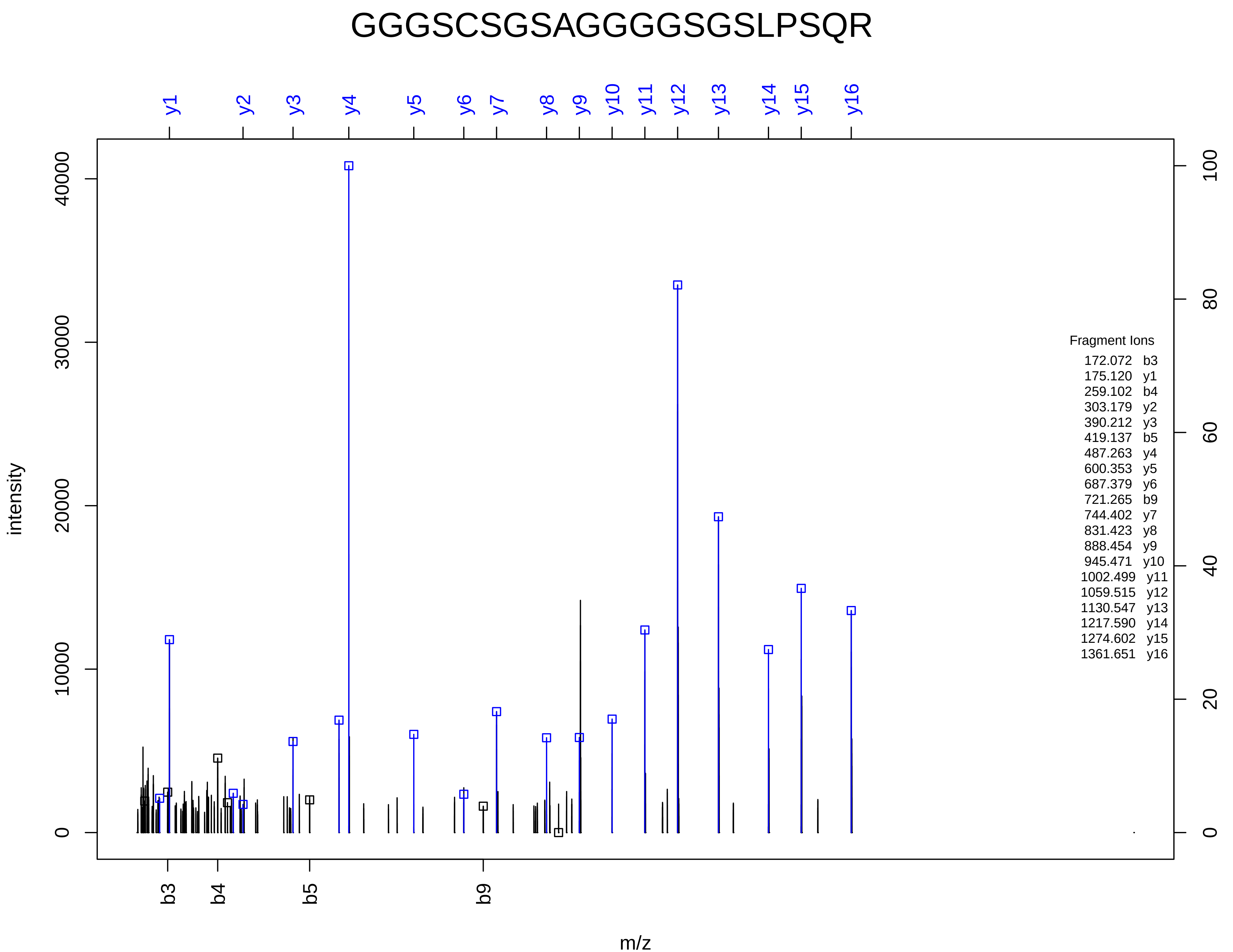

GGGSCSGSAGGGGSGSLPSQR / File: christine_ip_08.raw; SpectrumID: ; scans: 1670

VDVDEYDENK

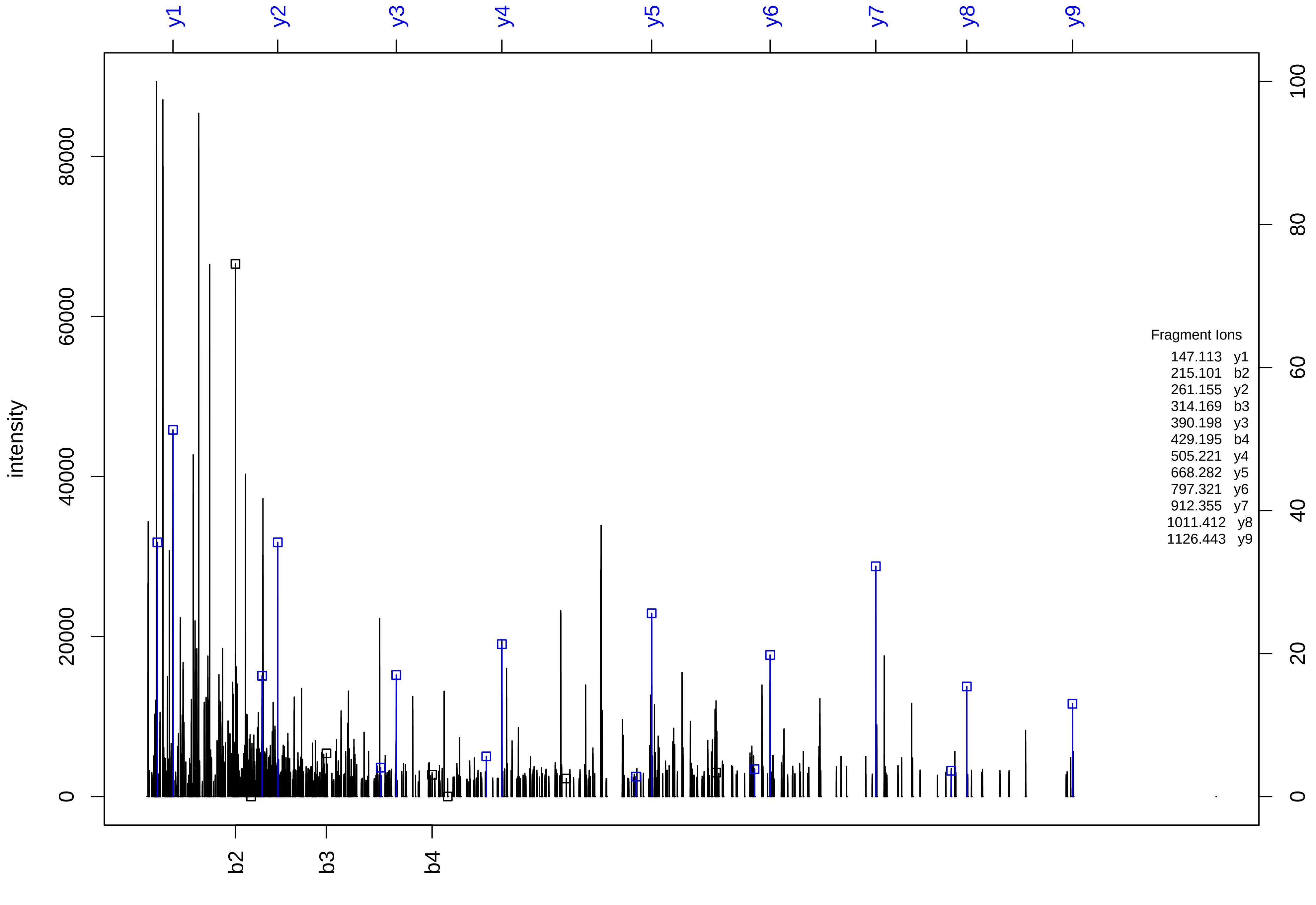

VDVDEYDENK / File: christine_ip_08.raw; SpectrumID: ; scans: 3418

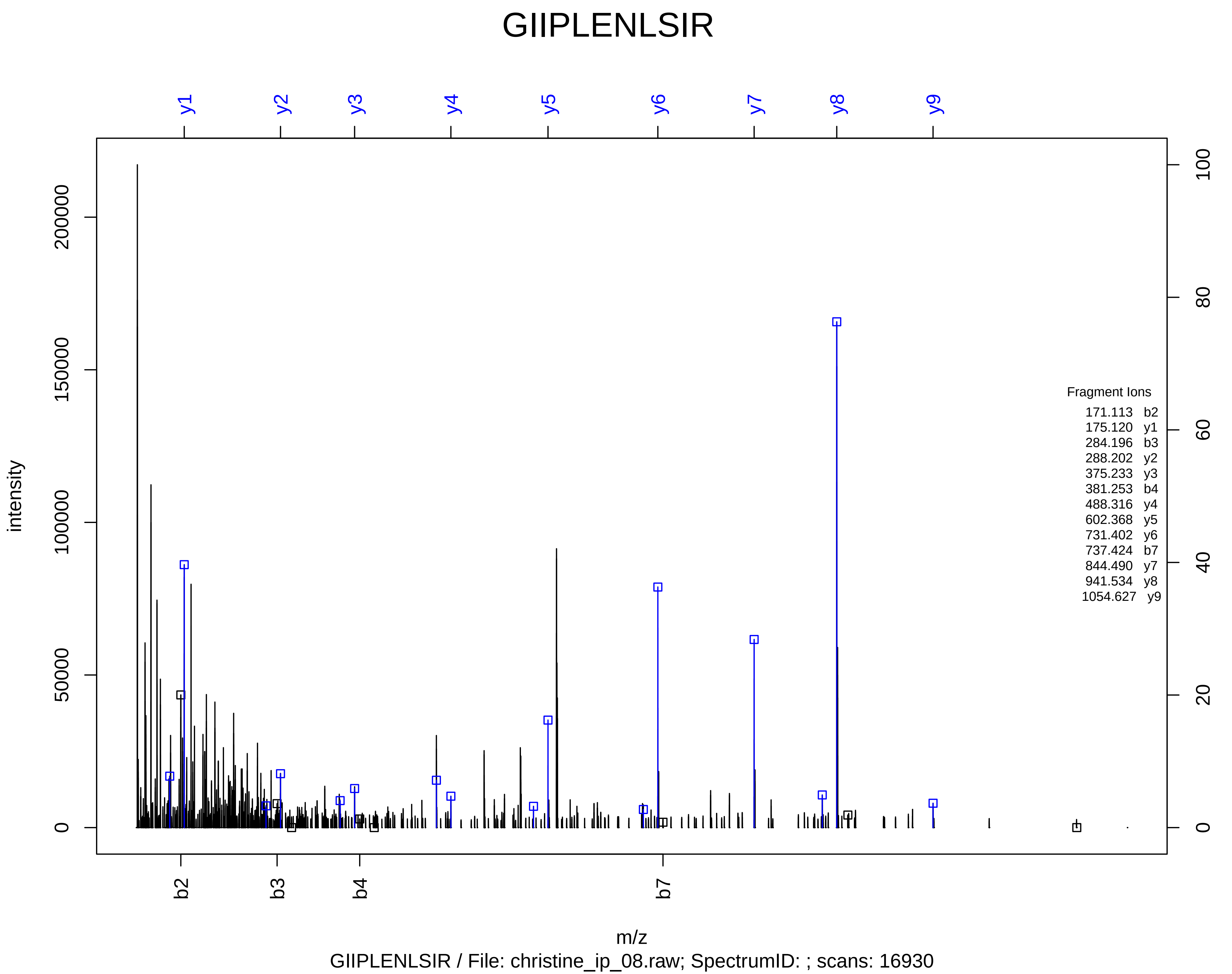
GIIPLENLSIR
y1
y2
y3
y4
y5
y6
y7
y8
y9
b2
b3
b4
b7
intensity
m/z
Fragment Ions
171.113 b2
175.120 y1
284.196 b3
288.202 y2
375.233 y3
381.253 b4
488.316 y4
602.368 y5
731.402 y6
737.424 b7
844.490 y7
941.534 y8
1054.627 y9
GIIPLENLSIR / File: christine_ip_08.raw; SpectrumID: ; scans: 16930

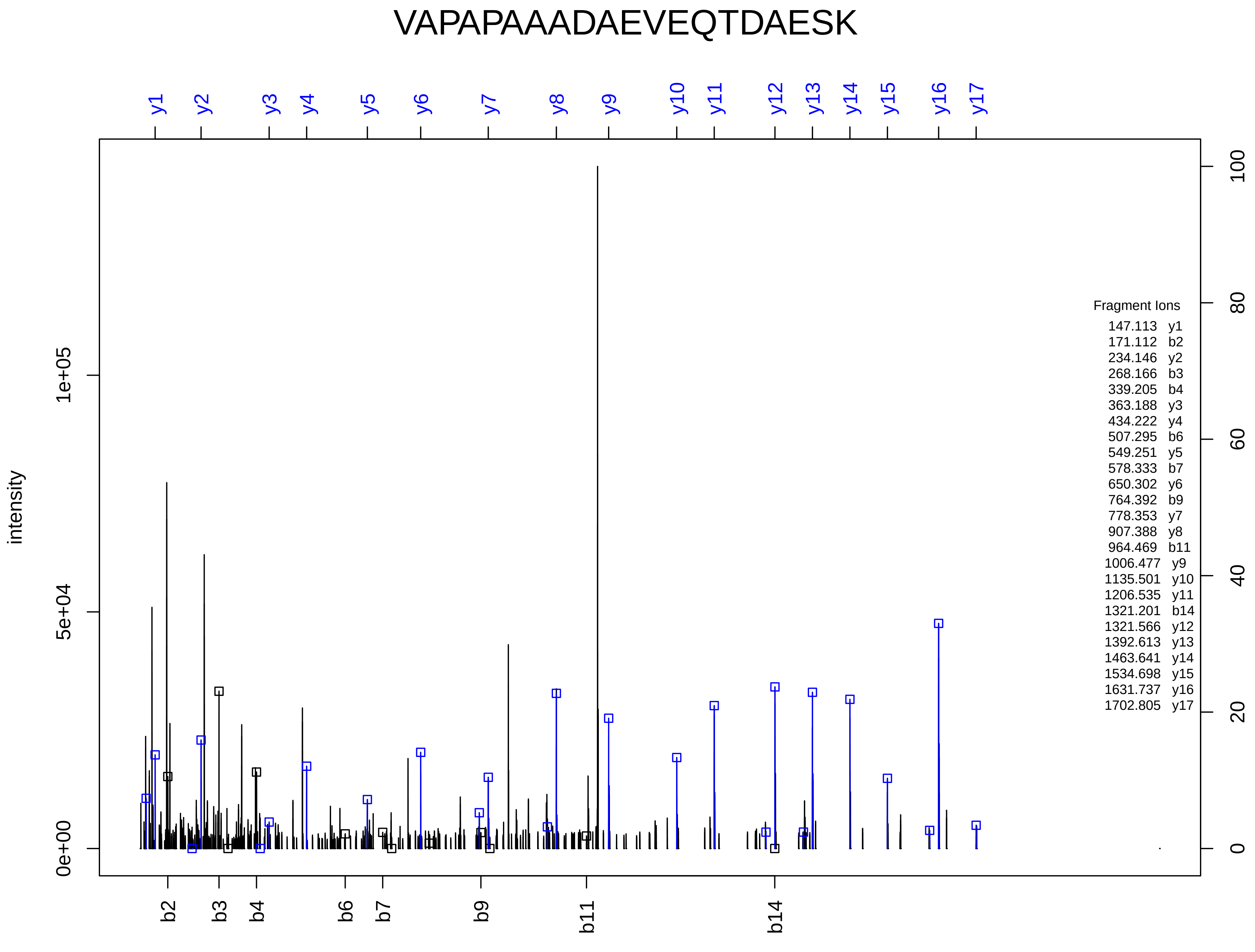

VAPAPAAADAEVEQTDAESK / File: christine_ip_08.raw; SpectrumID: ; scans: 5546

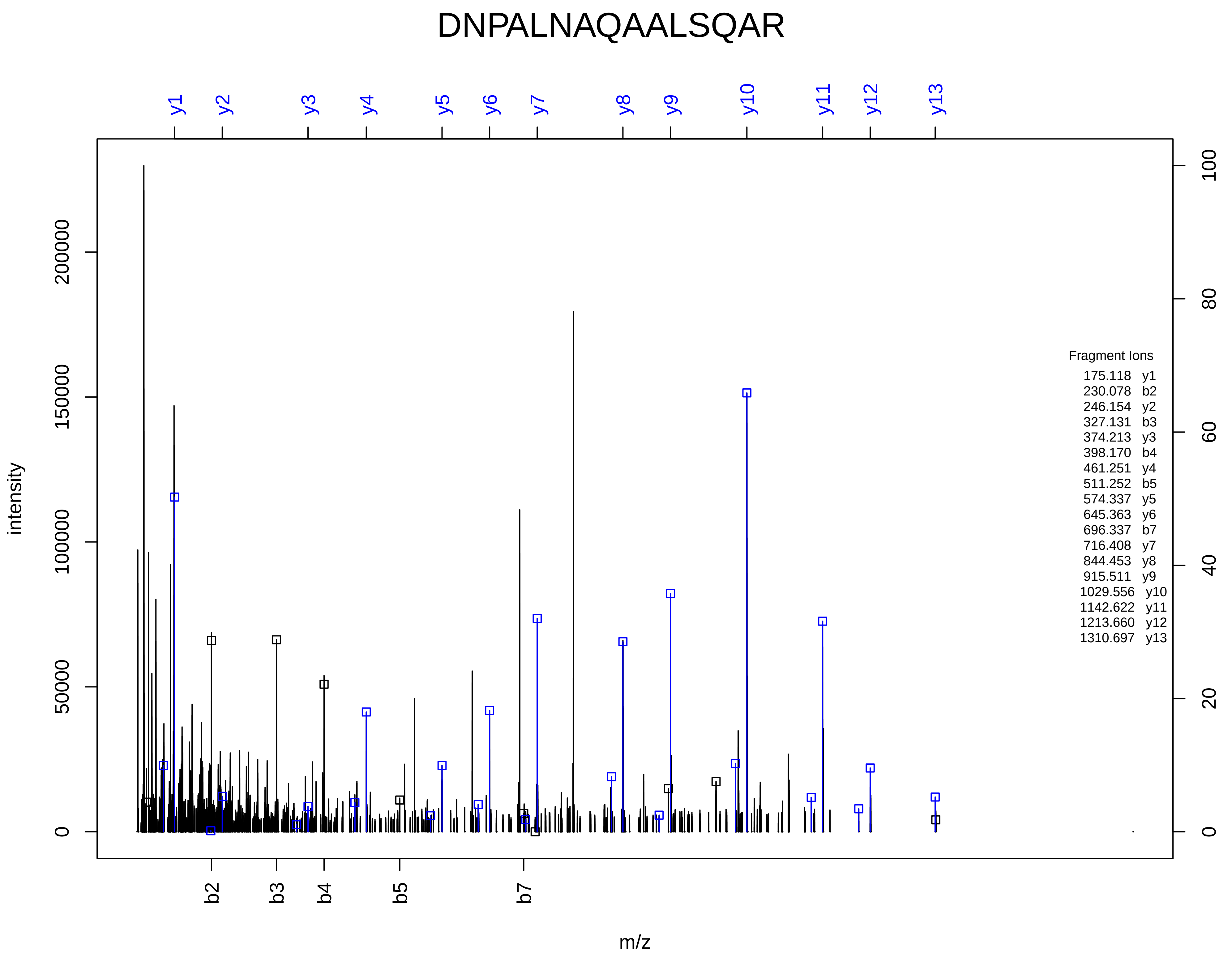

DNPALNAQAALSQAR / File: christine_ip_08.raw; SpectrumID: ; scans: 9420

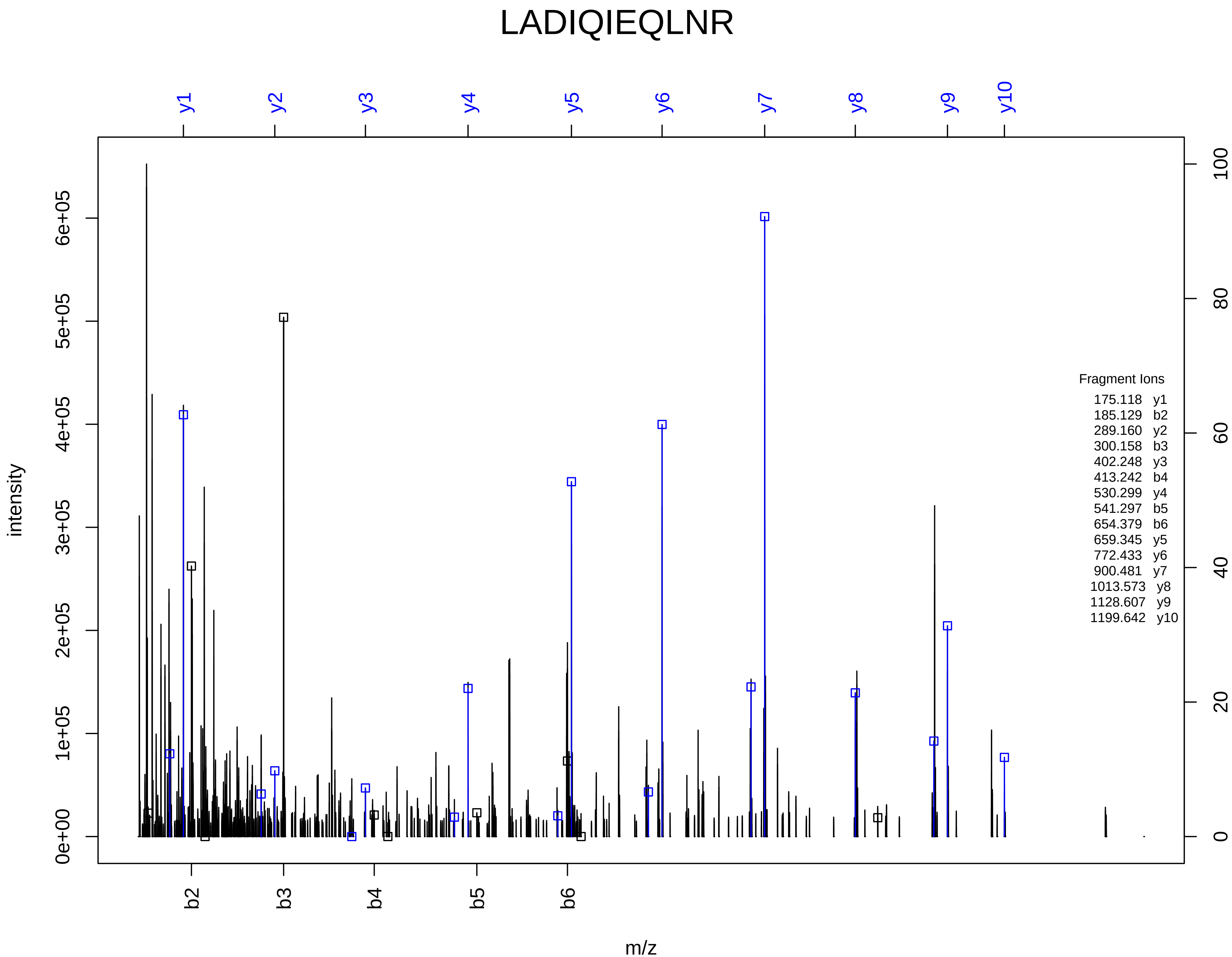

LADIQIEQLNR
y1
y2
y3
y4
y5
y6
y7
y8
y9
y10
intensity
0e+00
1e+05
2e+05
3e+05
4e+05
5e+05
6e+05
0
20
40
60
80
100
b2
b3
b4
b5
b6
m/z
Fragment Ions
175.118 y1
185.129 b2
289.160 y2
300.158 b3
402.248 y3
413.242 b4
530.299 y4
541.297 b5
654.379 b6
659.345 y5
772.433 y6
900.481 y7
1013.573 y8
1128.607 y9
1199.642 y10
LADIQIEQLNR / File: christine_ip_08.raw; SpectrumID: ; scans: 10492

YLLPLILGGR

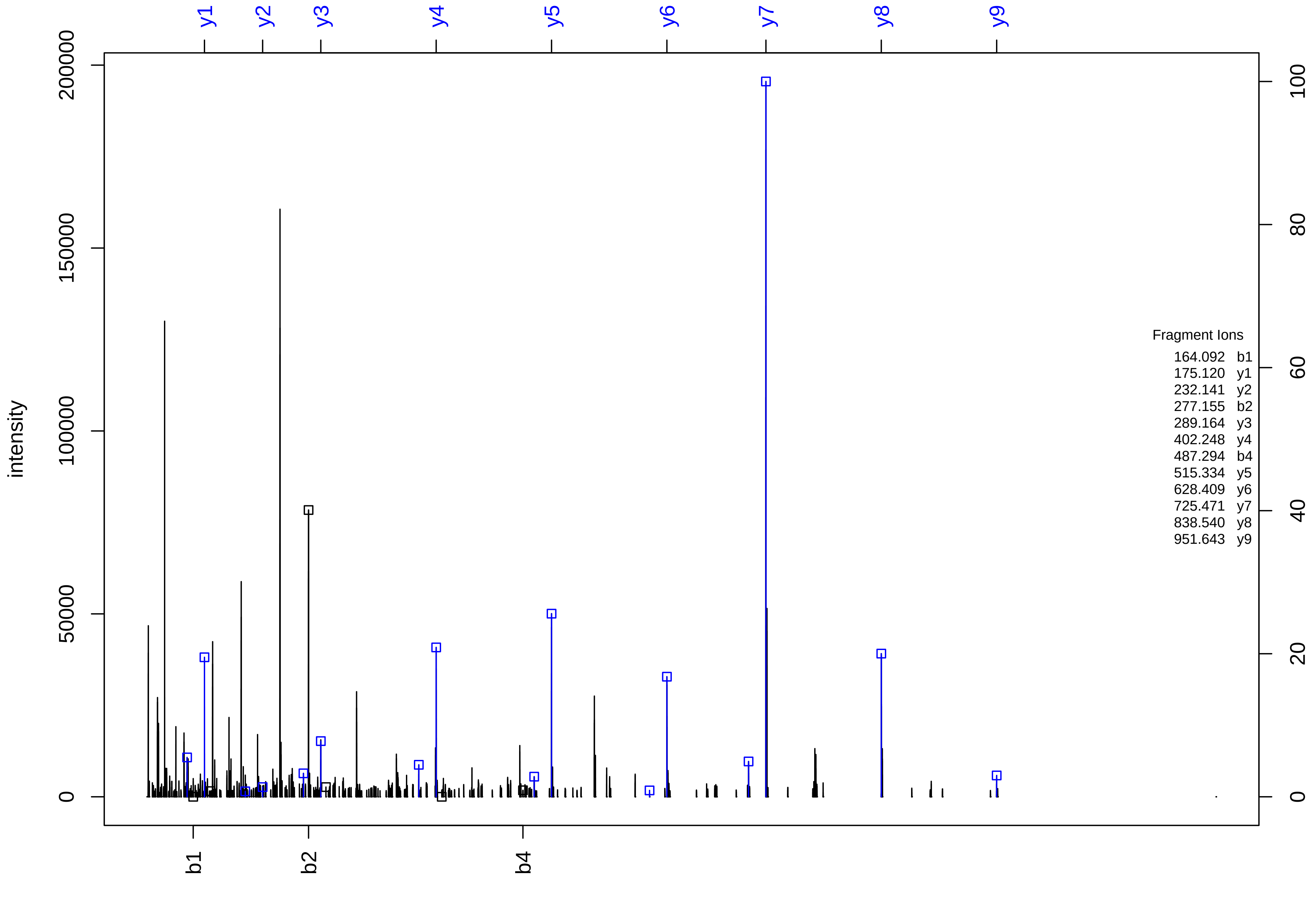

YLLPLILGGR / File: christine_ip_08.raw; SpectrumID: ; scans: 20041

TIVQLENEIYQIK

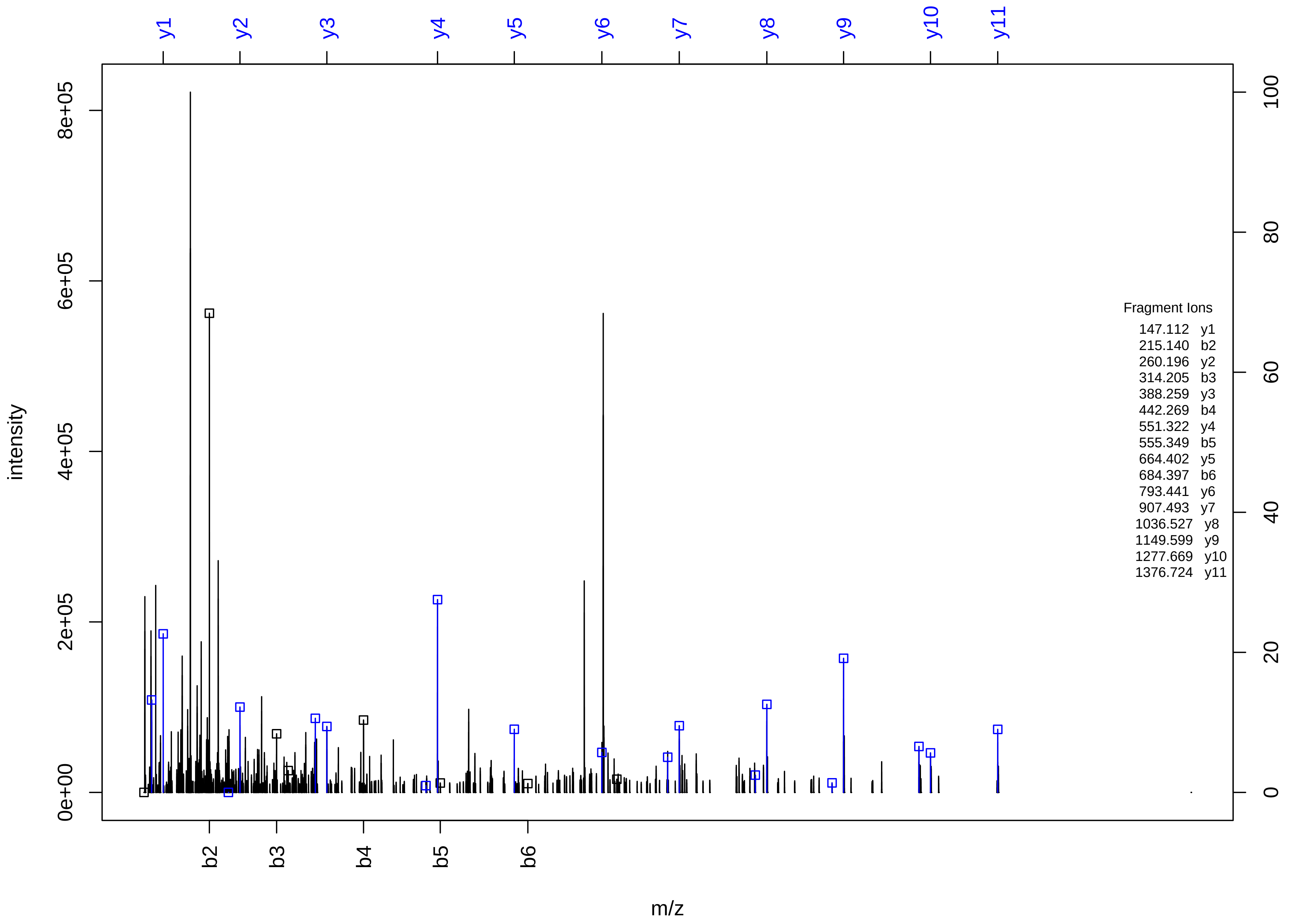

TIVQLENEIYQIK / File: christine_ip_08.raw; SpectrumID: ; scans: 15892

ADTSLNNCEGAAGSTSEK

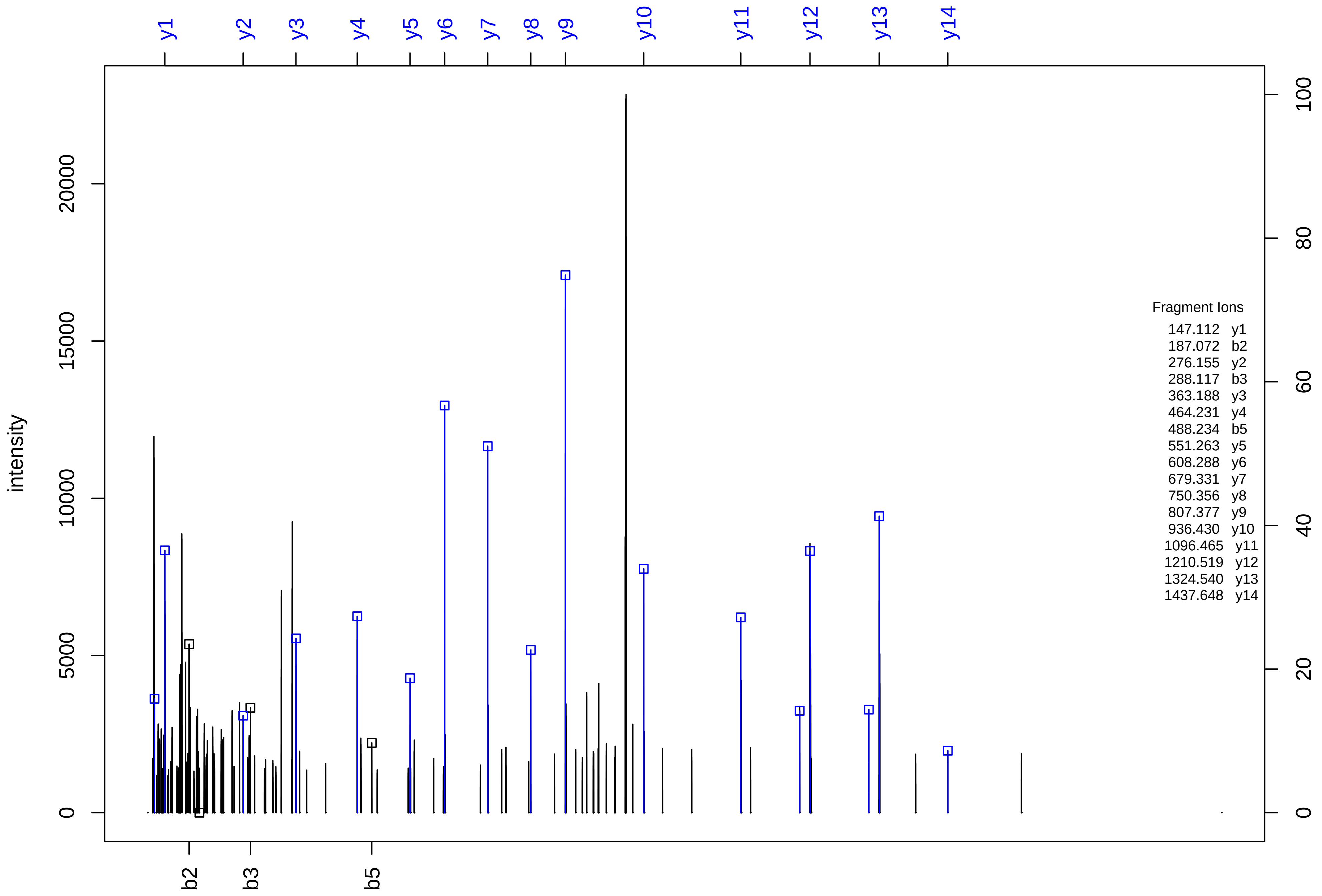

ADTSLNNCEGAAGSTSEK / File: christine_ip_08.raw; SpectrumID: ; scans: 2101

AFVDVVN^GEYVPR

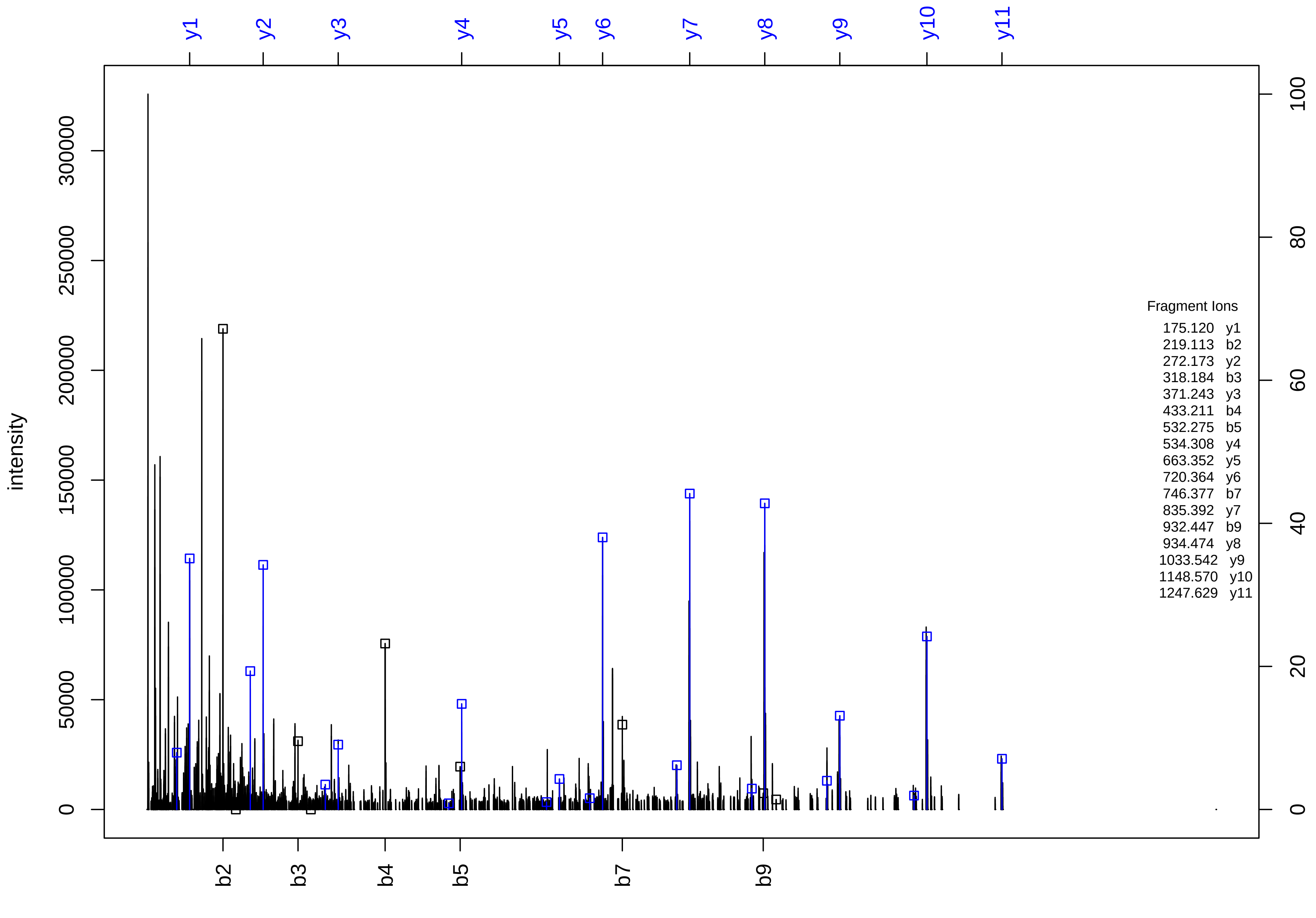

AFVDVVNGEYVPR / File: christine_ip_08.raw; SpectrumID: ; scans: 12898

LNIPAILR

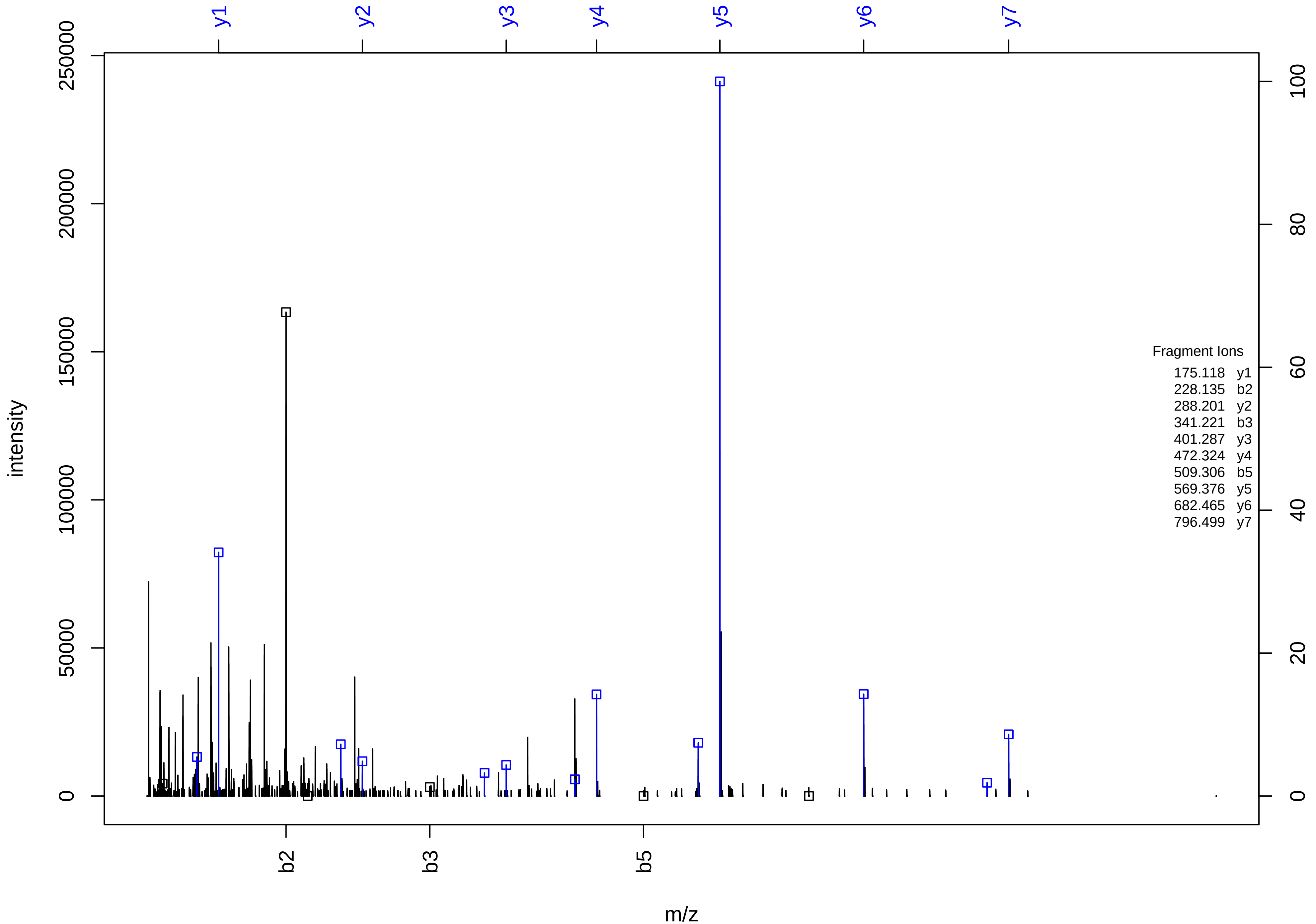

LNIPAILR / File: christine_ip_08.raw; SpectrumID: ; scans: 14832

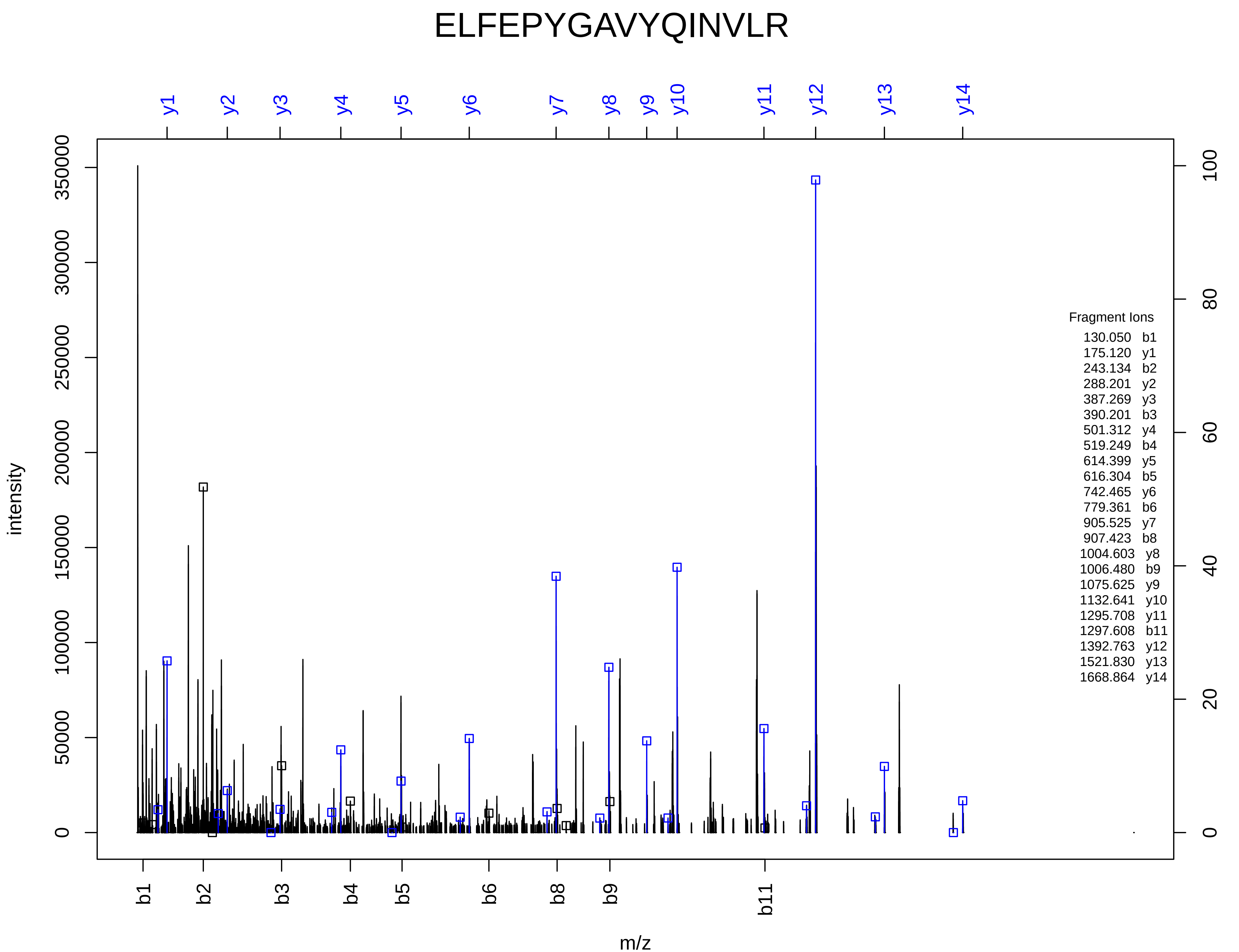
ELFEPYGAVYQINVLR
y1 y2 y3 y4 y5 y6 y7 y8 y9 y10 y11 y12 y13 y14
b1 b2 b3 b4 b5 b6 b8 b9 b11
intensity
m/z
Fragment Ions
130.050 b1
175.120 y1
243.134 b2
288.201 y2
387.269 y3
390.201 b3
501.312 y4
519.249 b4
614.399 y5
616.304 b5
742.465 y6
779.361 b6
905.525 y7
907.423 b8
1004.603 y8
1006.480 b9
1075.625 y9
1132.641 y10
1295.708 y11
1297.608 b11
1392.763 y12
1521.830 y13
1668.864 y14
ELFEPYGAVYQINVLR / File: christine_ip_08.raw; SpectrumID: ; scans: 19533

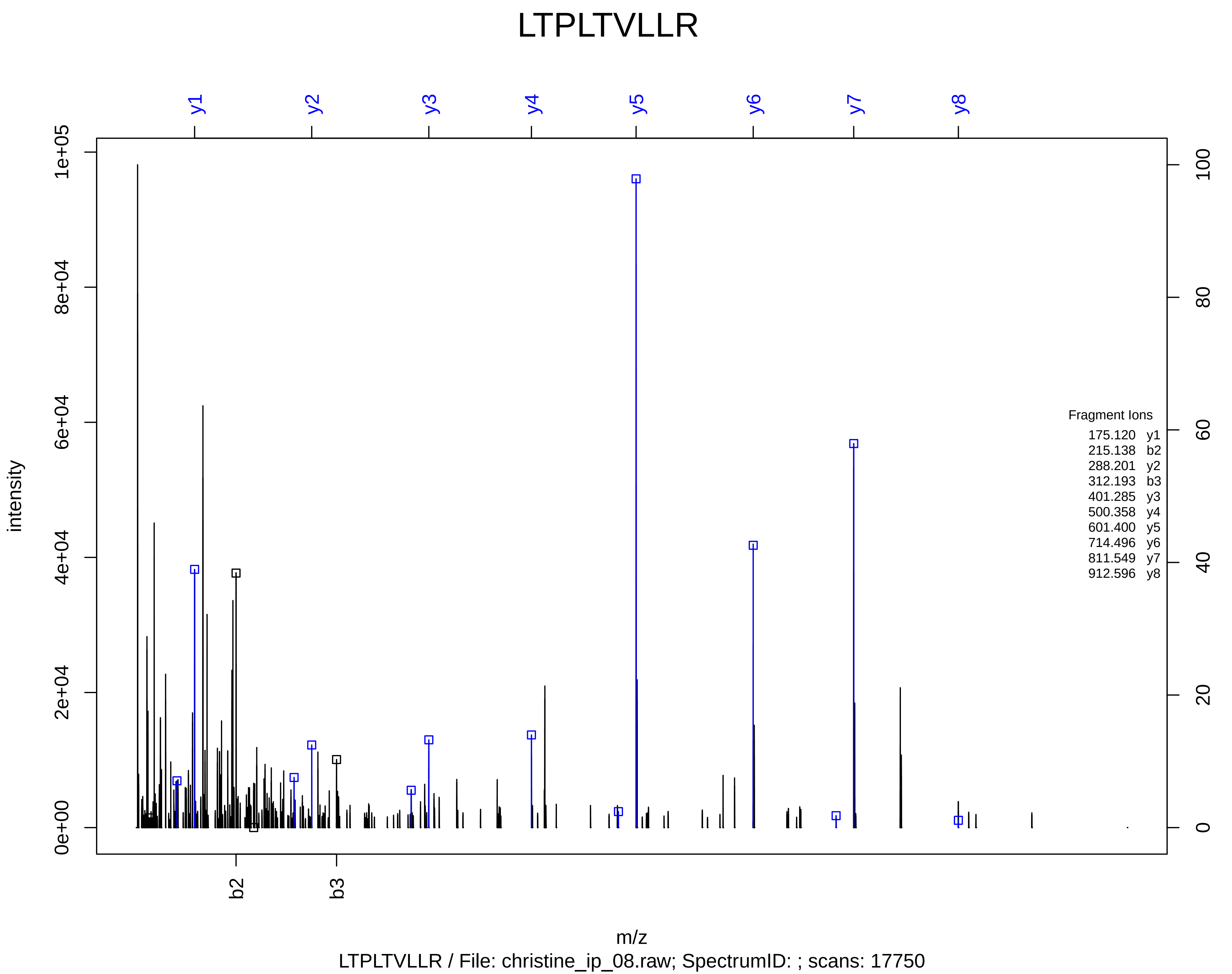
LTPLTVLLR
y1
y2
y3
y4
y5
y6
y7
y8
b2
b3
intensity
0e+00
2e+04
4e+04
6e+04
8e+04
1e+05
0
20
40
60
80
100
Fragment Ions
175.120 y1
215.138 b2
288.201 y2
312.193 b3
401.285 y3
500.358 y4
601.400 y5
714.496 y6
811.549 y7
912.596 y8
m/z
LTPLTVLLR / File: christine_ip_08.raw; SpectrumID: ; scans: 17750

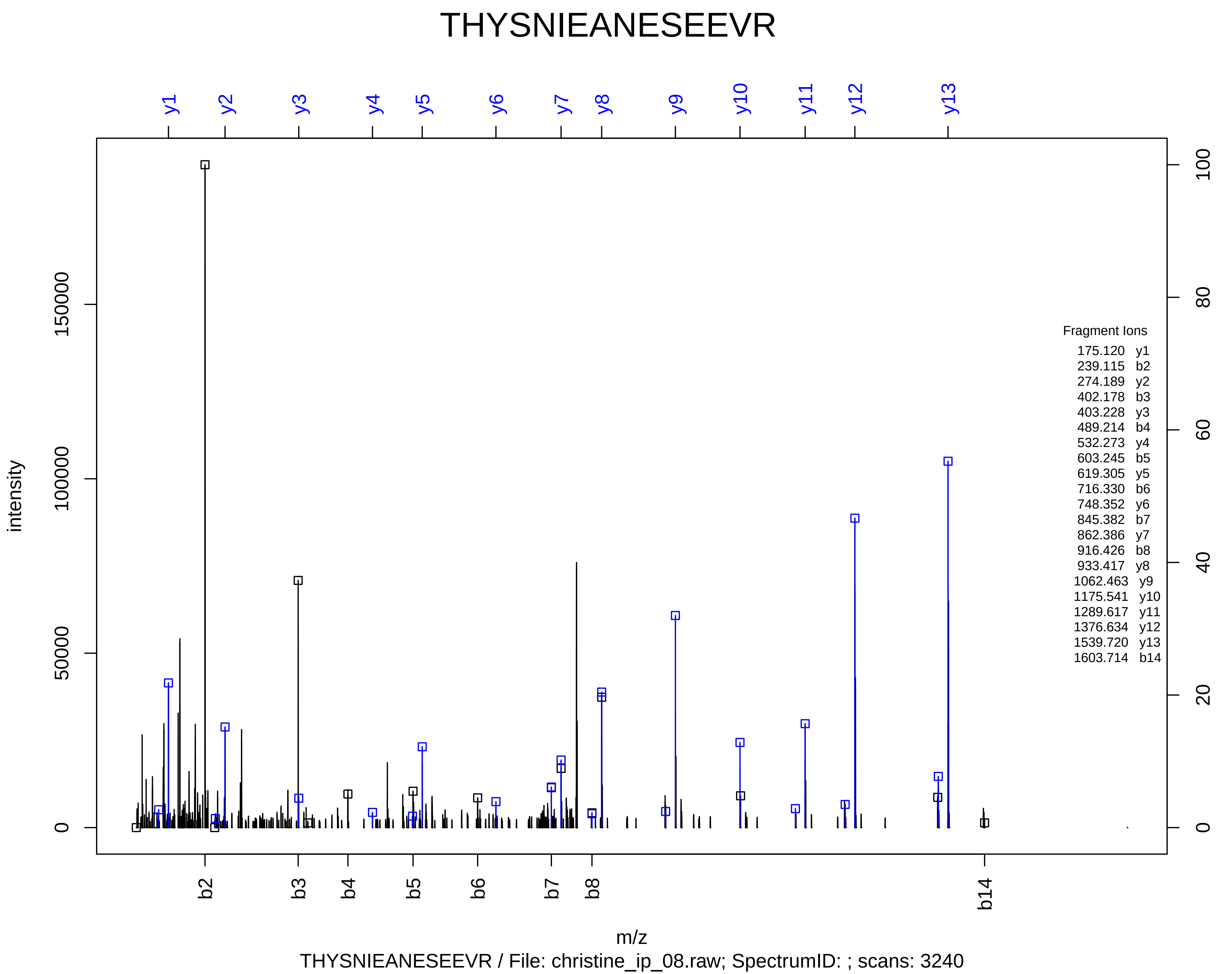
THYSNIEANESEEVR
y1
y2
y3
y4
y5
y6
y7
y8
y9
y10
y11
y12
y13
intensity
0
50000
100000
150000
0
20
40
60
80
100
Fragment Ions
175.120 y1
239.115 b2
274.189 y2
402.178 b3
403.228 y3
489.214 b4
532.273 y4
603.245 b5
619.305 y5
716.330 b6
748.352 y6
845.382 b7
862.386 y7
916.426 b8
933.417 y8
1062.463 y9
1175.541 y10
1289.617 y11
1376.634 y12
1539.720 y13
1603.714 b14
b2
b3
b4
b5
b6
b7
b8
b14
m/z
THYSNIEANESEEVR / File: christine_ip_08.raw; SpectrumID: ; scans: 3240

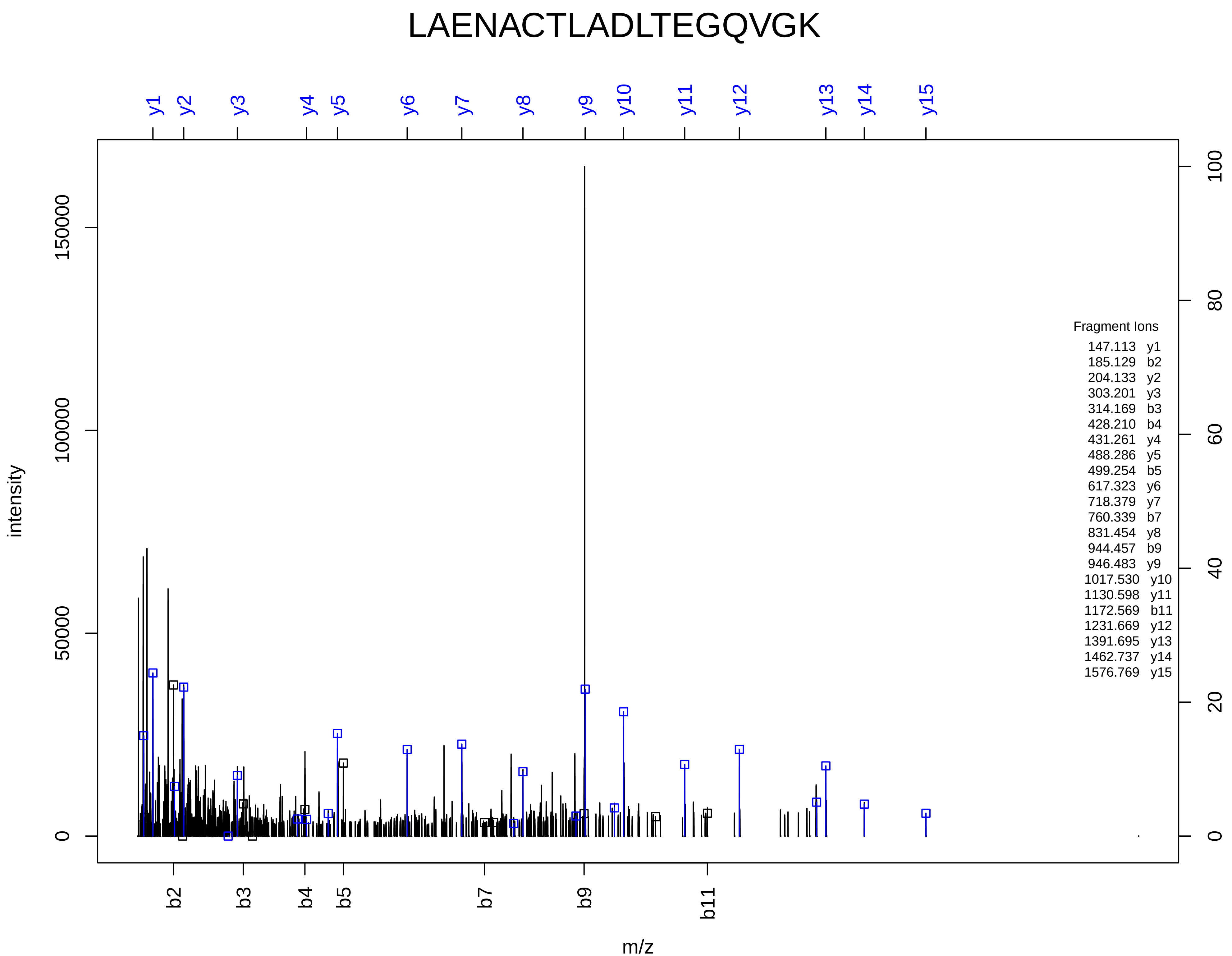

LAENACTLADLTEGQVGK / File: christine_ip_08.raw; SpectrumID: ; scans: 12389

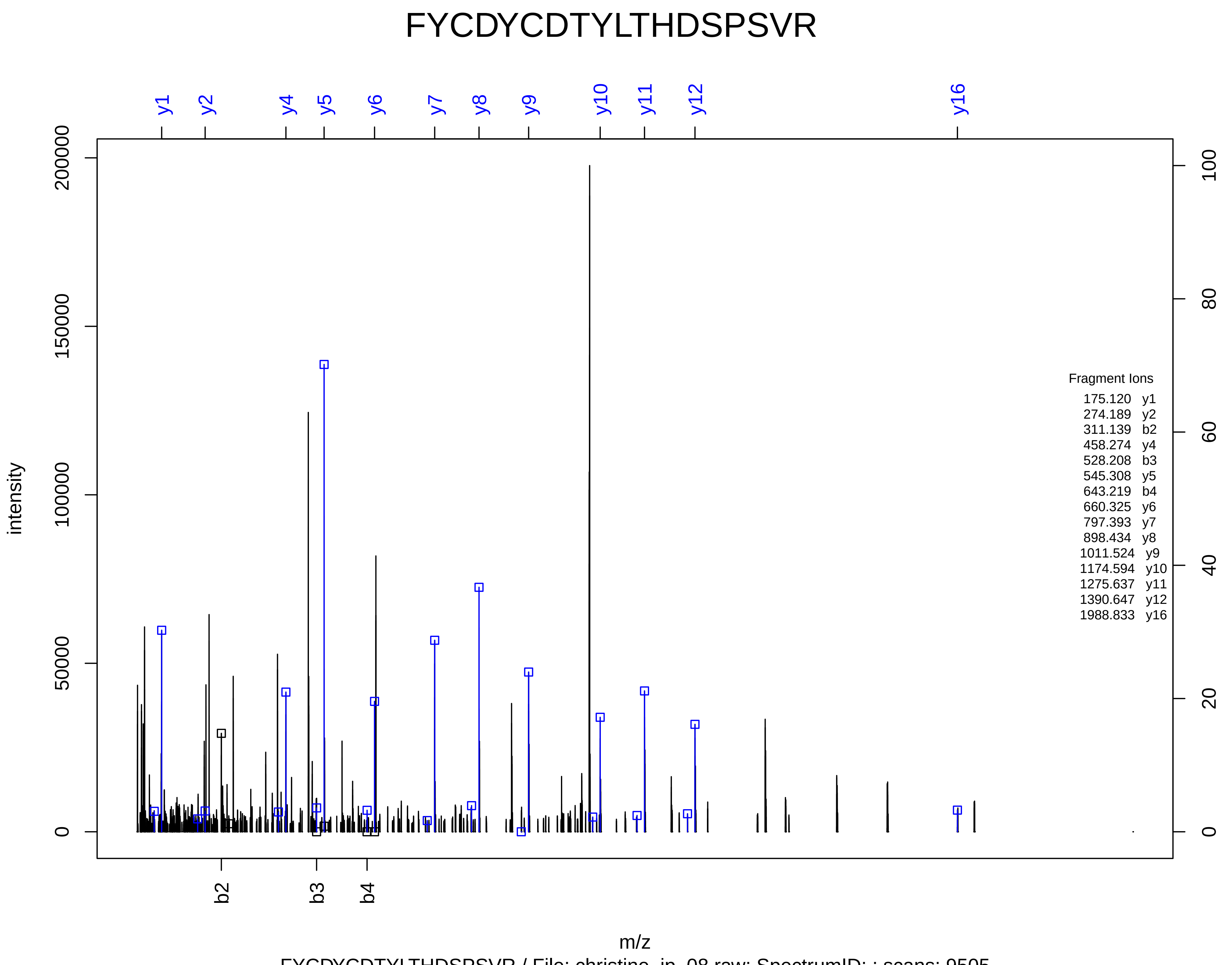
FYCDYCDTYLTHDSPSVR
y1 y2 y4 y5 y6 y7 y8 y9 y10 y11 y12 y16
b2 b3 b4
intensity
0
50000
100000
150000
200000
0
20
40
60
80
100
m/z
Fragment Ions
175.120 y1
274.189 y2
311.139 b2
458.274 y4
528.208 b3
545.308 y5
643.219 b4
660.325 y6
797.393 y7
898.434 y8
1011.524 y9
1174.594 y10
1275.637 y11
1390.647 y12
1988.833 y16
FYCDYCDTYLTHDSPSVR / File: christine_ip_08.raw; SpectrumID: ; scans: 9505

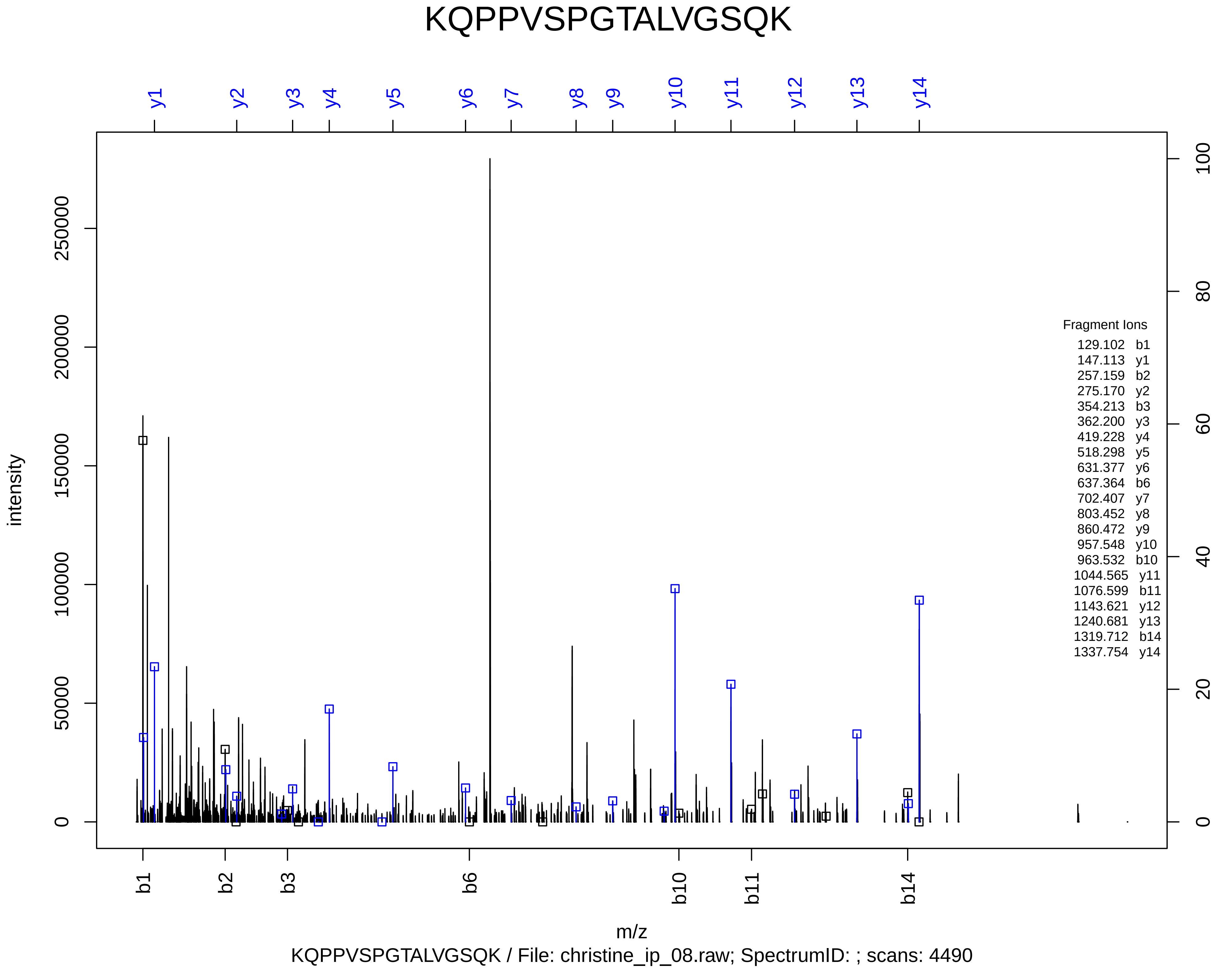
KQPPVSPGTALVGSQK
y1
y2
y3
y4
y5
y6
y7
y8
y9
y10
y11
y12
y13
y14
intensity
0
50000
100000
150000
200000
250000
0
20
40
60
80
100
Fragment Ions
129.102 b1
147.113 y1
257.159 b2
275.170 y2
354.213 b3
362.200 y3
419.228 y4
518.298 y5
631.377 y6
637.364 b6
702.407 y7
803.452 y8
860.472 y9
957.548 y10
963.532 b10
1044.565 y11
1076.599 b11
1143.621 y12
1240.681 y13
1319.712 b14
1337.754 y14
b1
b2
b3
b6
b10
b11
b14
m/z
KQPPVSPGTALVGSQK / File: christine_ip_08.raw; SpectrumID: ; scans: 4490

GYLVTQDELDQTLEEFK

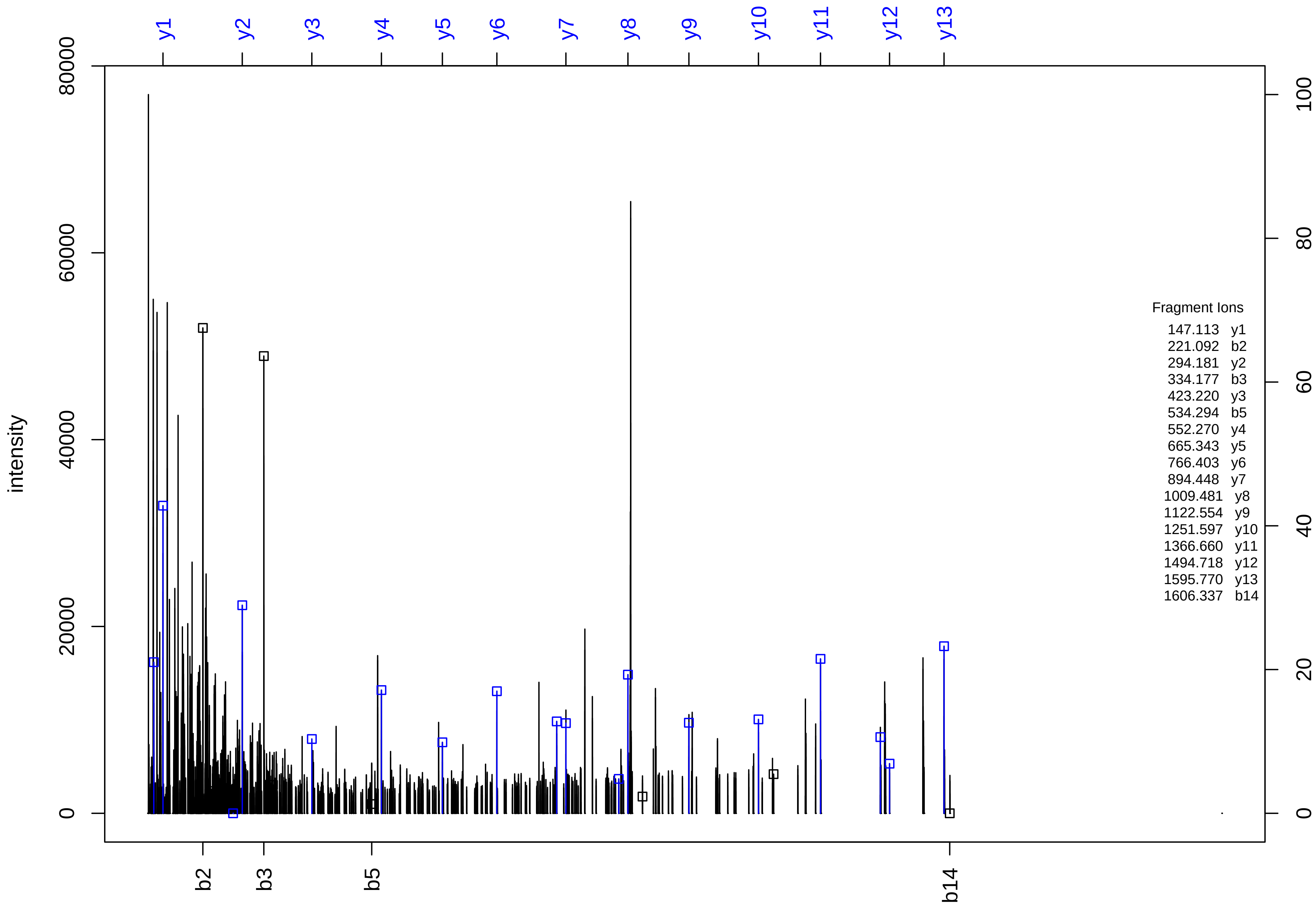

GYLVTQDELDQTLEEFK / File: christine_ip_08.raw; SpectrumID: ; scans: 19250

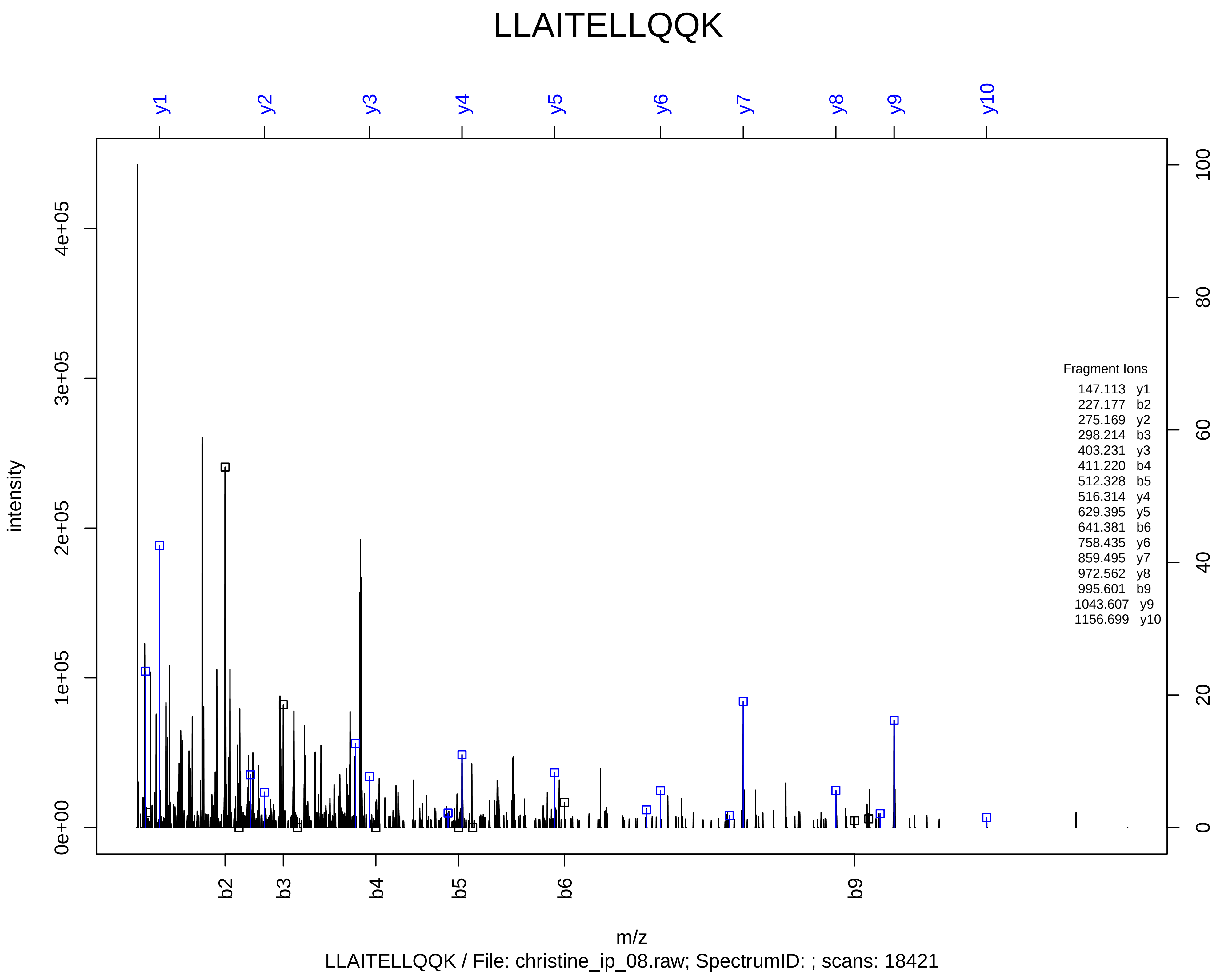

LLAITELLQQK
y1
y2
y3
y4
y5
y6
y7
y8
y9
y10
b2
b3
b4
b5
b6
b9
intensity
0e+00
1e+05
2e+05
3e+05
4e+05
0
20
40
60
80
100
Fragment Ions
147.113 y1
227.177 b2
275.169 y2
298.214 b3
403.231 y3
411.220 b4
512.328 b5
516.314 y4
629.395 y5
641.381 b6
758.435 y6
859.495 y7
972.562 y8
995.601 b9
1043.607 y9
1156.699 y10
m/z
LLAITELLQQK / File: christine_ip_08.raw; SpectrumID: ; scans: 18421

LAEKEETGM*AM*R

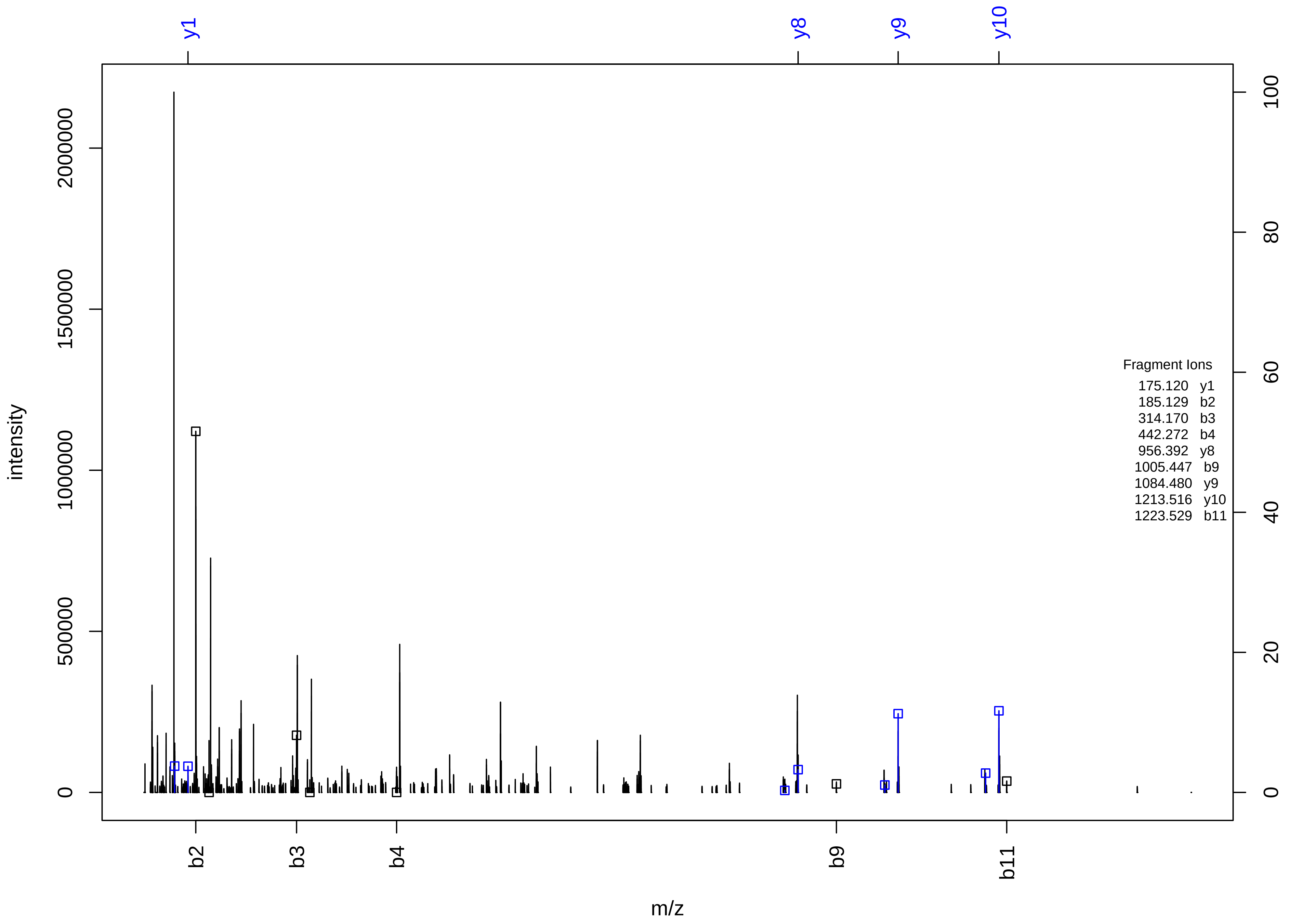

LAEKEETGMAMR / File: christine_ip_08.raw; SpectrumID: ; scans: 5428

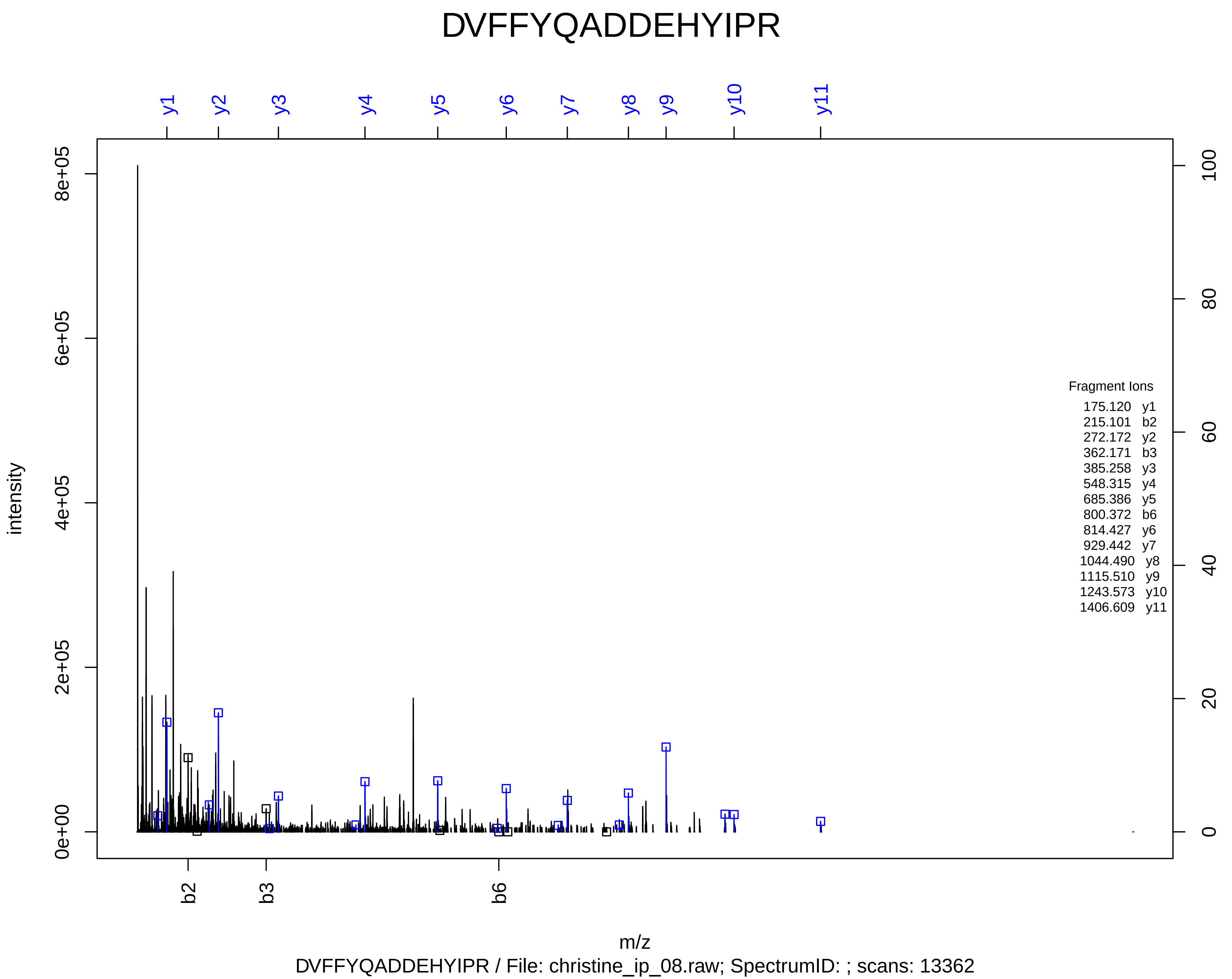

DVFFYQADDEHYIPR
y1
y2
y3
y4
y5
y6
y7
y8
y9
y10
y11
intensity
0e+00
2e+05
4e+05
6e+05
8e+05
0
20
40
60
80
100
b2
b3
b6
Fragment Ions
175.120 y1
215.101 b2
272.172 y2
362.171 b3
385.258 y3
548.315 y4
685.386 y5
800.372 b6
814.427 y6
929.442 y7
1044.490 y8
1115.510 y9
1243.573 y10
1406.609 y11
m/z
DVFFYQADDEHYIPR / File: christine_ip_08.raw; SpectrumID: ; scans: 13362

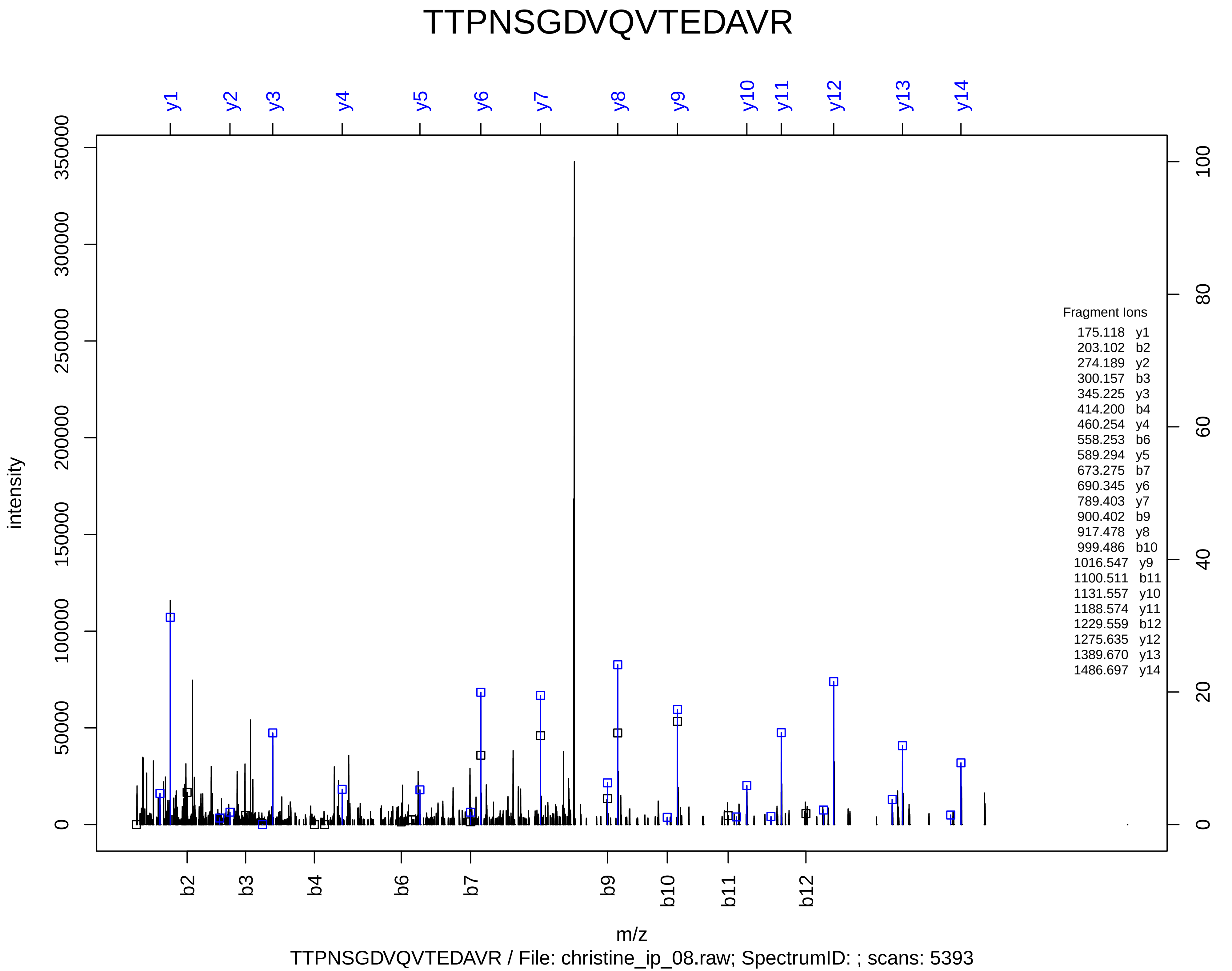
TTPNSGDVQVTEDAVR
intensity
m/z
y1
y2
y3
y4
y5
y6
y7
y8
y9
y10
y11
y12
y13
y14
b2
b3
b4
b6
b7
b9
b10
b11
b12
Fragment Ions
175.118 y1
203.102 b2
274.189 y2
300.157 b3
345.225 y3
414.200 b4
460.254 y4
558.253 b6
589.294 y5
673.275 b7
690.345 y6
789.403 y7
900.402 b9
917.478 y8
999.486 b10
1016.547 y9
1100.511 b11
1131.557 y10
1188.574 y11
1229.559 b12
1275.635 y12
1389.670 y13
1486.697 y14
TTPNSGDVQVTEDAVR / File: christine_ip_08.raw; SpectrumID: ; scans: 5393

VVTDLISLIR

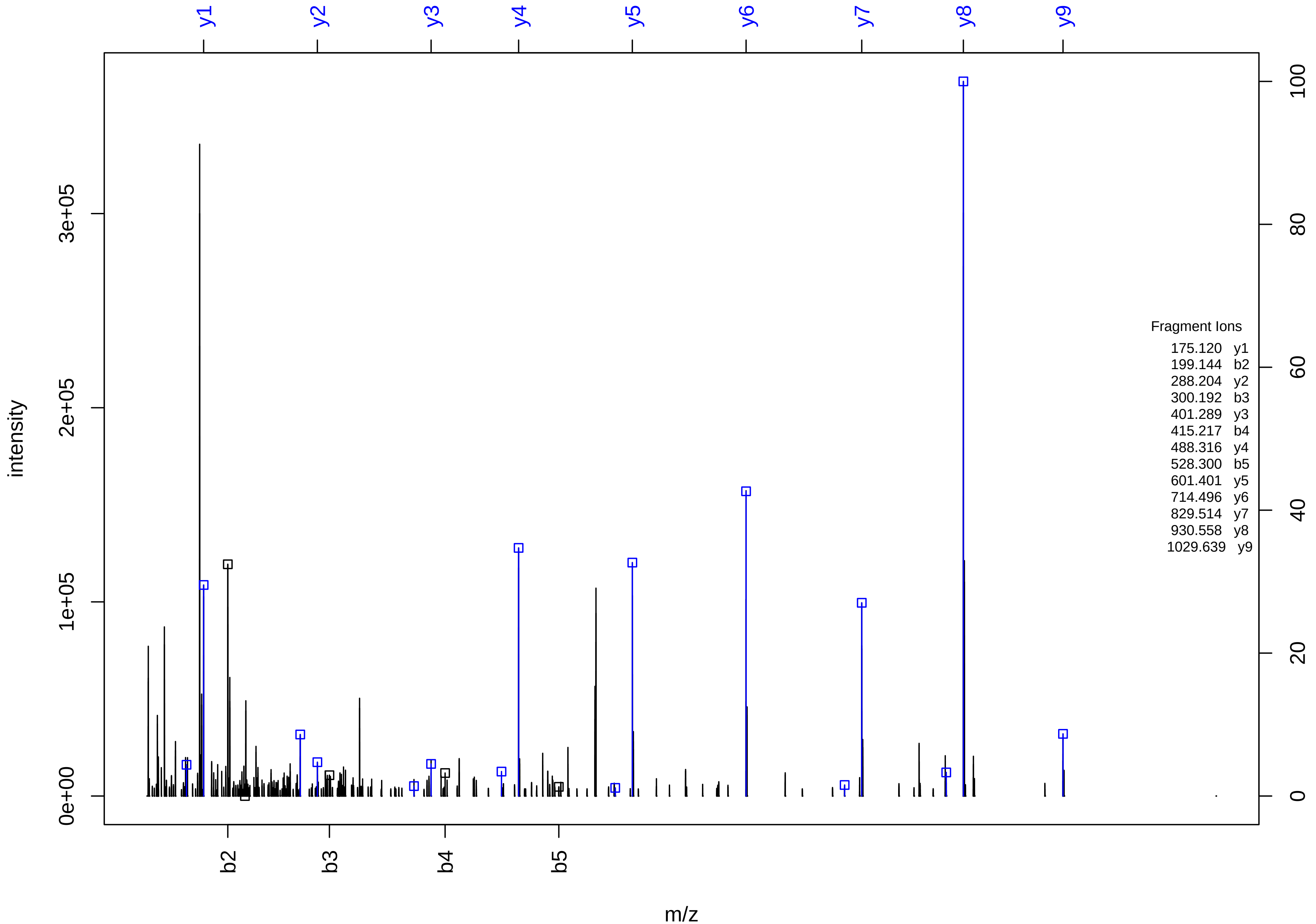

VVTDLISLIR / File: christine_ip_08.raw; SpectrumID: ; scans: 18481

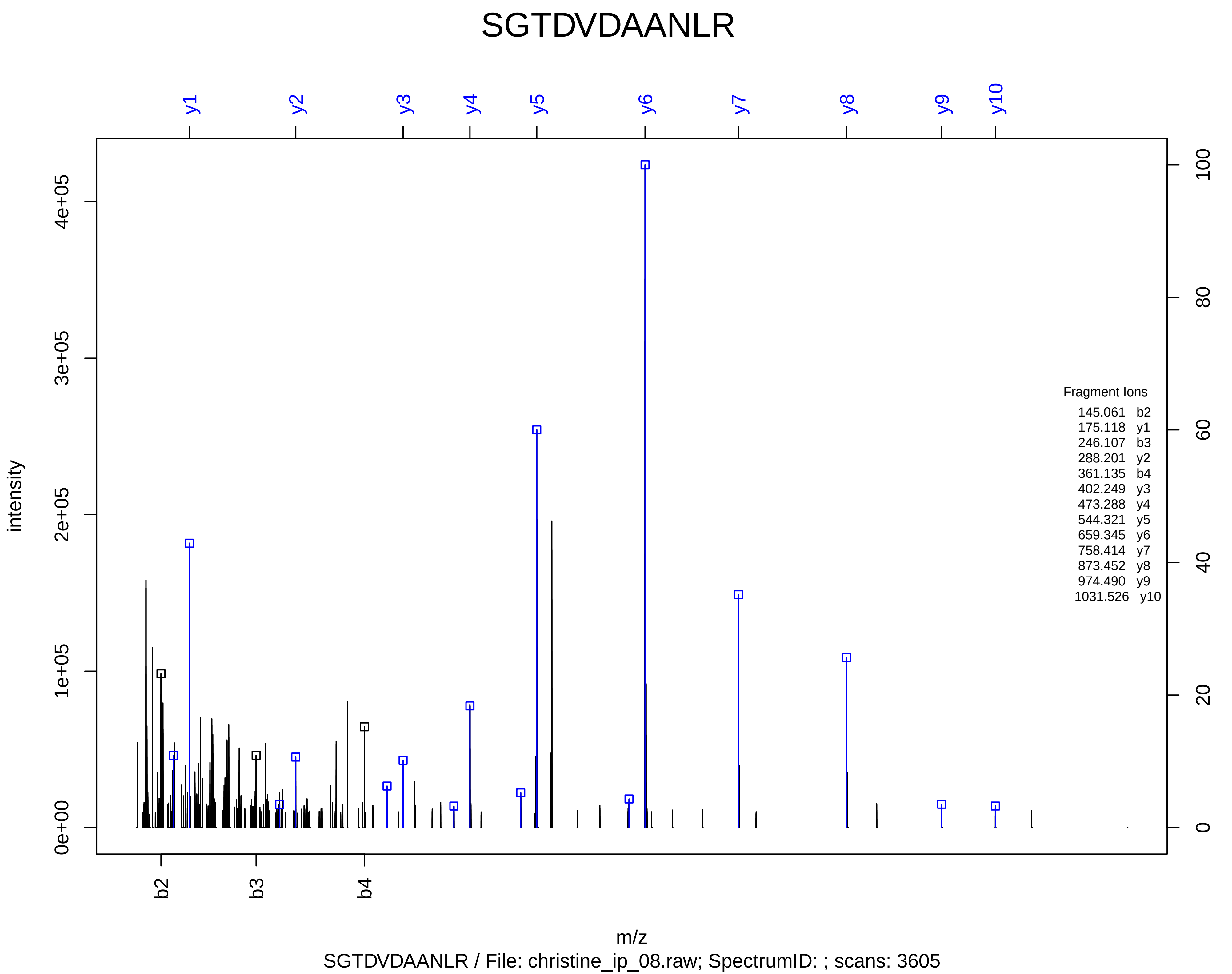
SGTDVDAANLR
y1
y2
y3
y4
y5
y6
y7
y8
y9
y10
intensity
0e+00
1e+05
2e+05
3e+05
4e+05
0
20
40
60
80
100
b2
b3
b4
m/z
Fragment Ions
145.061 b2
175.118 y1
246.107 b3
288.201 y2
361.135 b4
402.249 y3
473.288 y4
544.321 y5
659.345 y6
758.414 y7
873.452 y8
974.490 y9
1031.526 y10
SGTDVDAANLR / File: christine_ip_08.raw; SpectrumID: ; scans: 3605

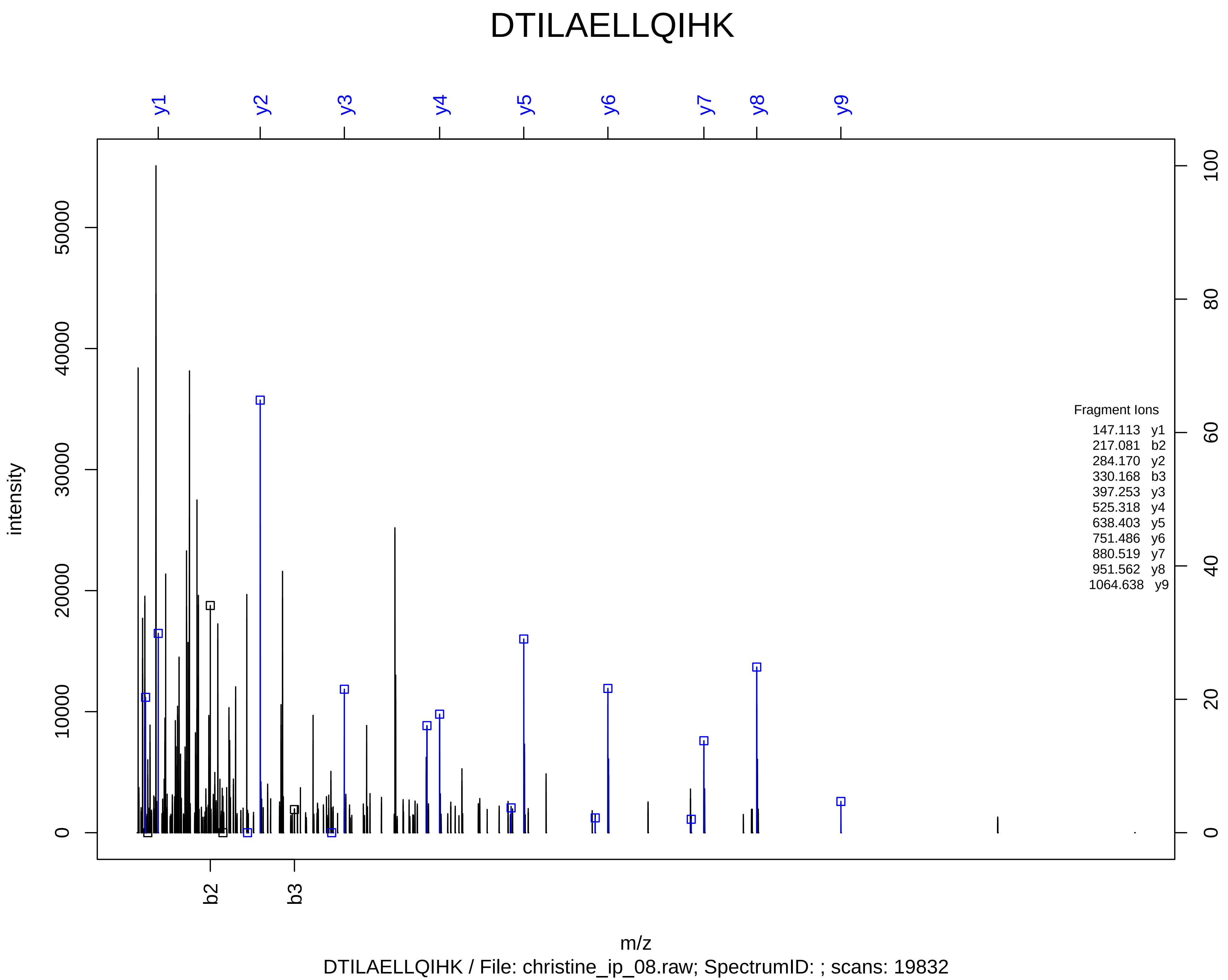
DTILAELLQIHK
y1
y2
y3
y4
y5
y6
y7
y8
y9
intensity
0
10000
20000
30000
40000
50000
0
20
40
60
80
100
Fragment Ions
147.113 y1
217.081 b2
284.170 y2
330.168 b3
397.253 y3
525.318 y4
638.403 y5
751.486 y6
880.519 y7
951.562 y8
1064.638 y9
b2
b3
m/z
DTILAELLQIHK / File: christine_ip_08.raw; SpectrumID: ; scans: 19832

NEVSFVIHNLPVLAK

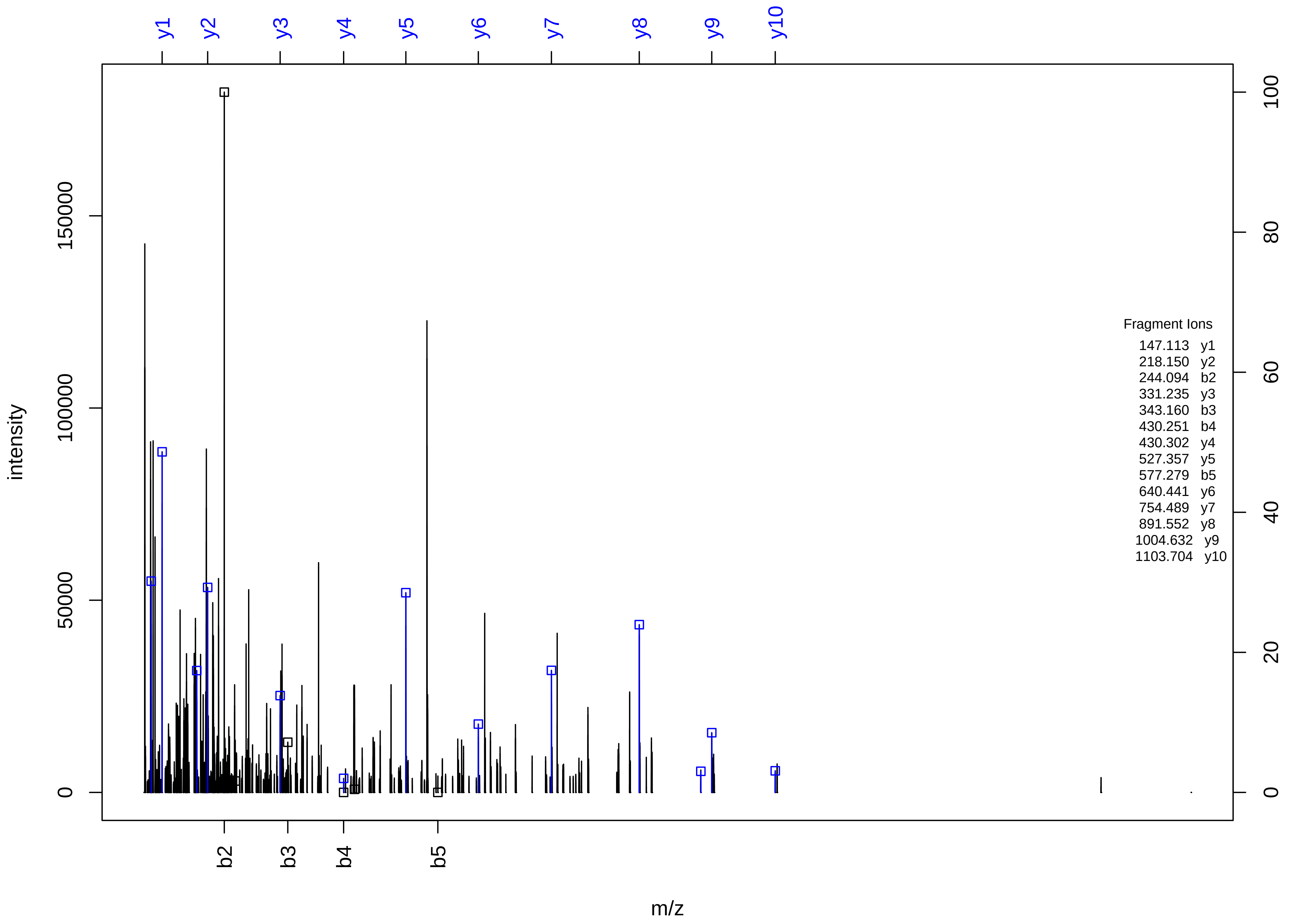

NEVSFVIHNLPVLAK / File: christine_ip_08.raw; SpectrumID: ; scans: 16633

SDSYVELSQYR

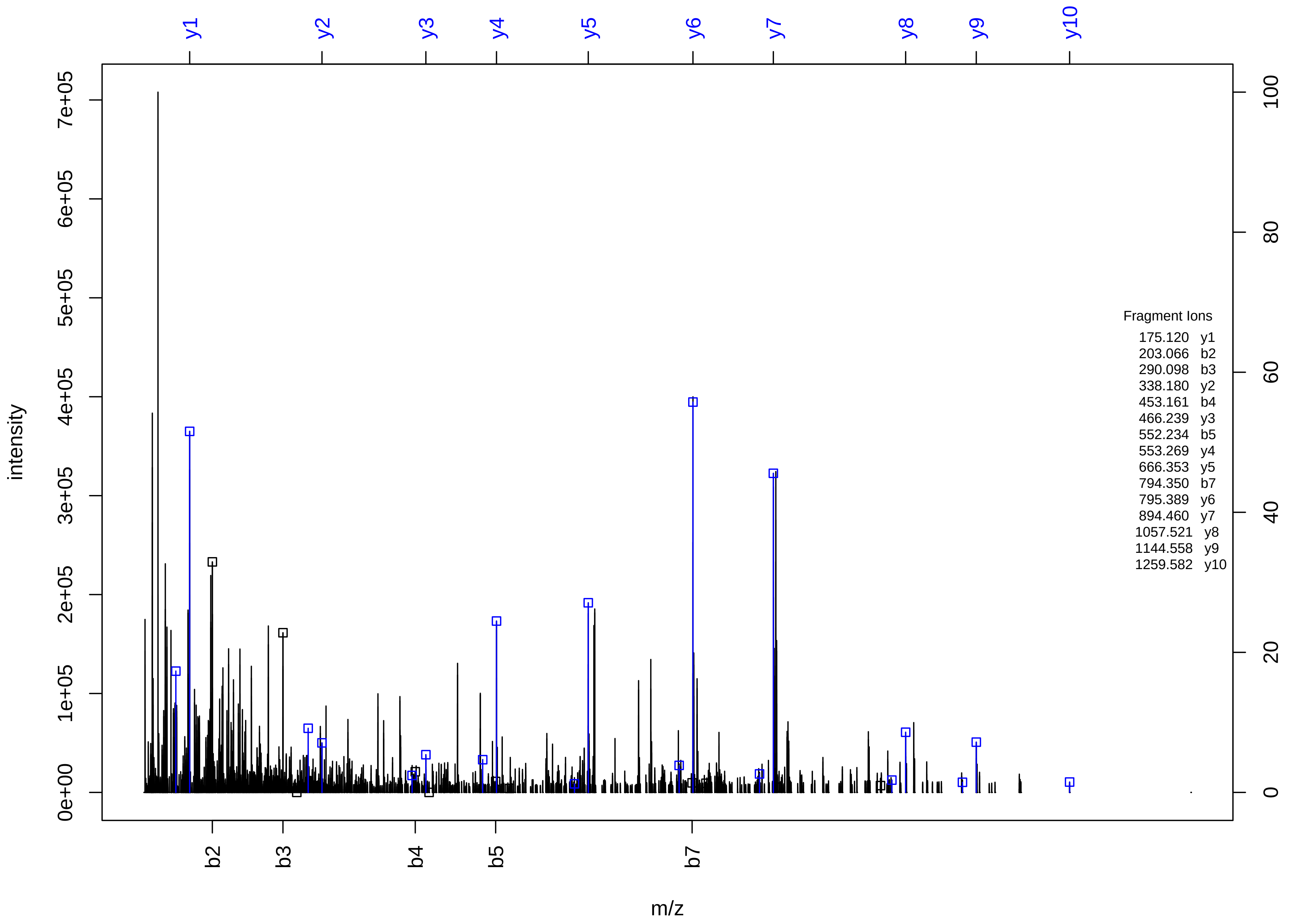

SDSYVELSQYR / File: christine_ip_08.raw; SpectrumID: ; scans: 7982

GEGAGQPSTSAQGQPAAPAPQK

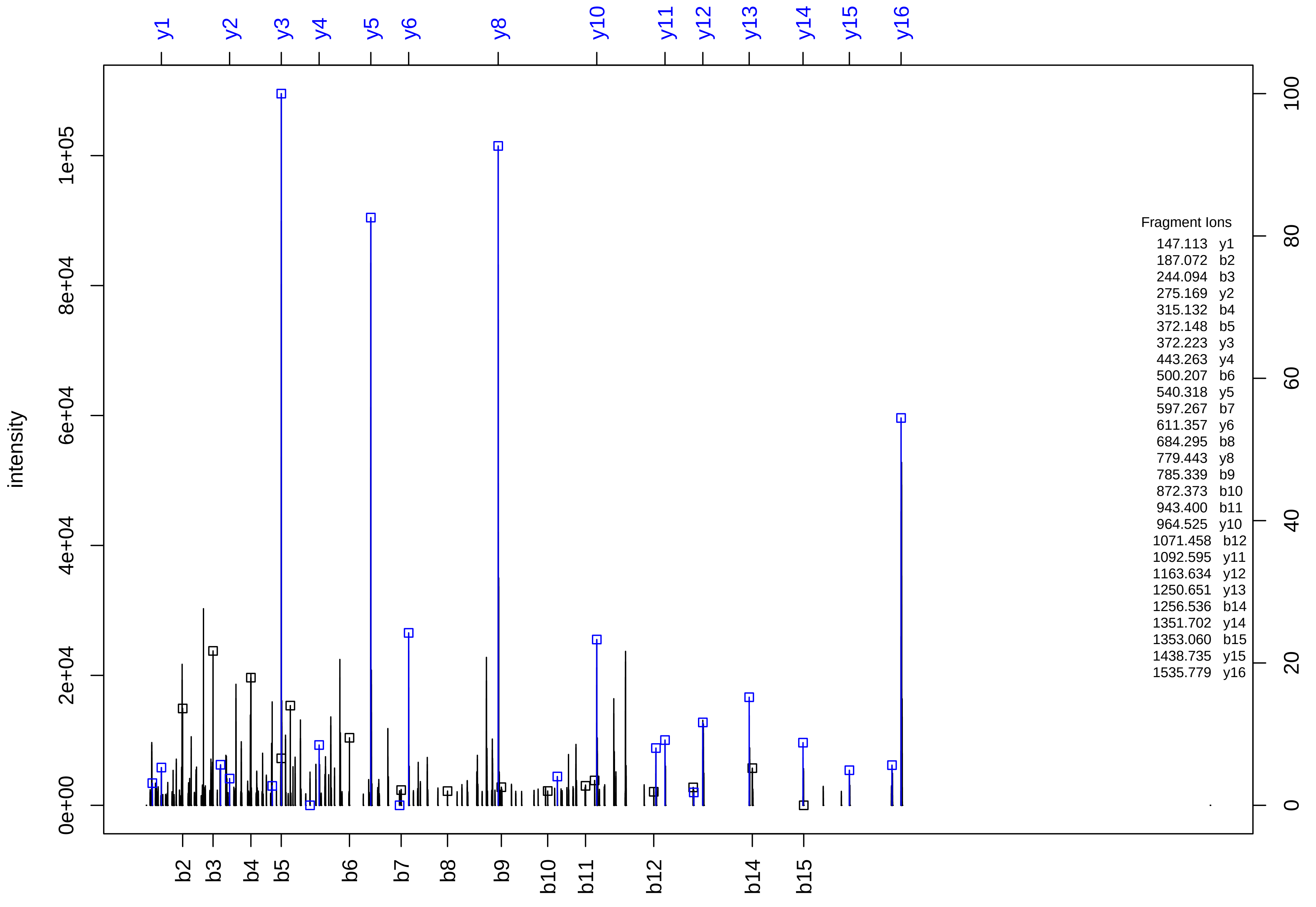

GEGAGQPSTSAQGQPAAPAPQK / File: christine_ip_08.raw; SpectrumID: ; scans: 2276

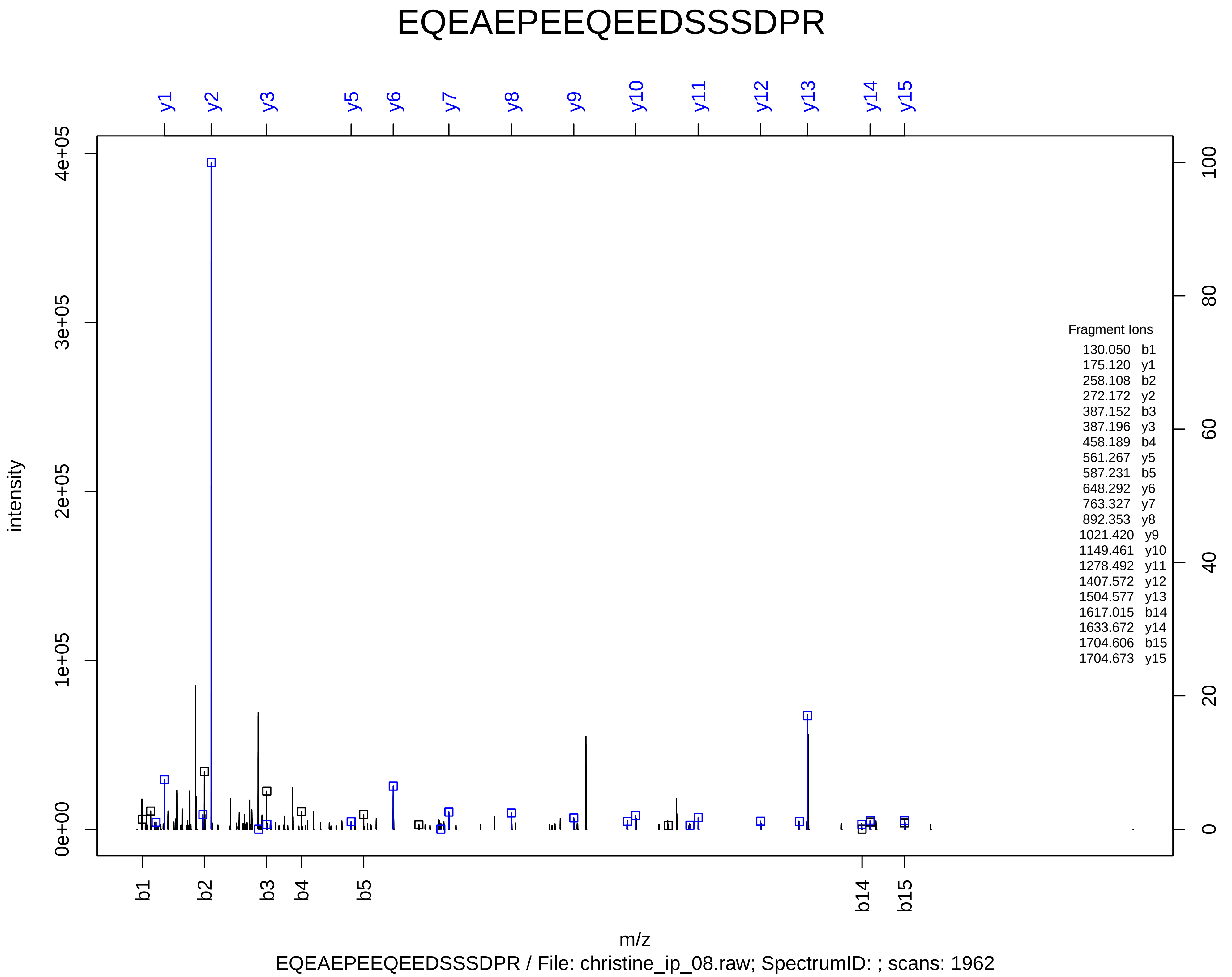
EQEAEPEEQEEDSSSDPR
y1 y2 y3 y5 y6 y7 y8 y9 y10 y11 y12 y13 y14 y15
b1 b2 b3 b4 b5 b14 b15
intensity
m/z
Fragment Ions
130.050 b1
175.120 y1
258.108 b2
272.172 y2
387.152 b3
387.196 y3
458.189 b4
561.267 y5
587.231 b5
648.292 y6
763.327 y7
892.353 y8
1021.420 y9
1149.461 y10
1278.492 y11
1407.572 y12
1504.577 y13
1617.015 b14
1633.672 y14
1704.606 b15
1704.673 y15
EQEAEPEEQEEDSSSDPR / File: christine_ip_08.raw; SpectrumID: ; scans: 1962

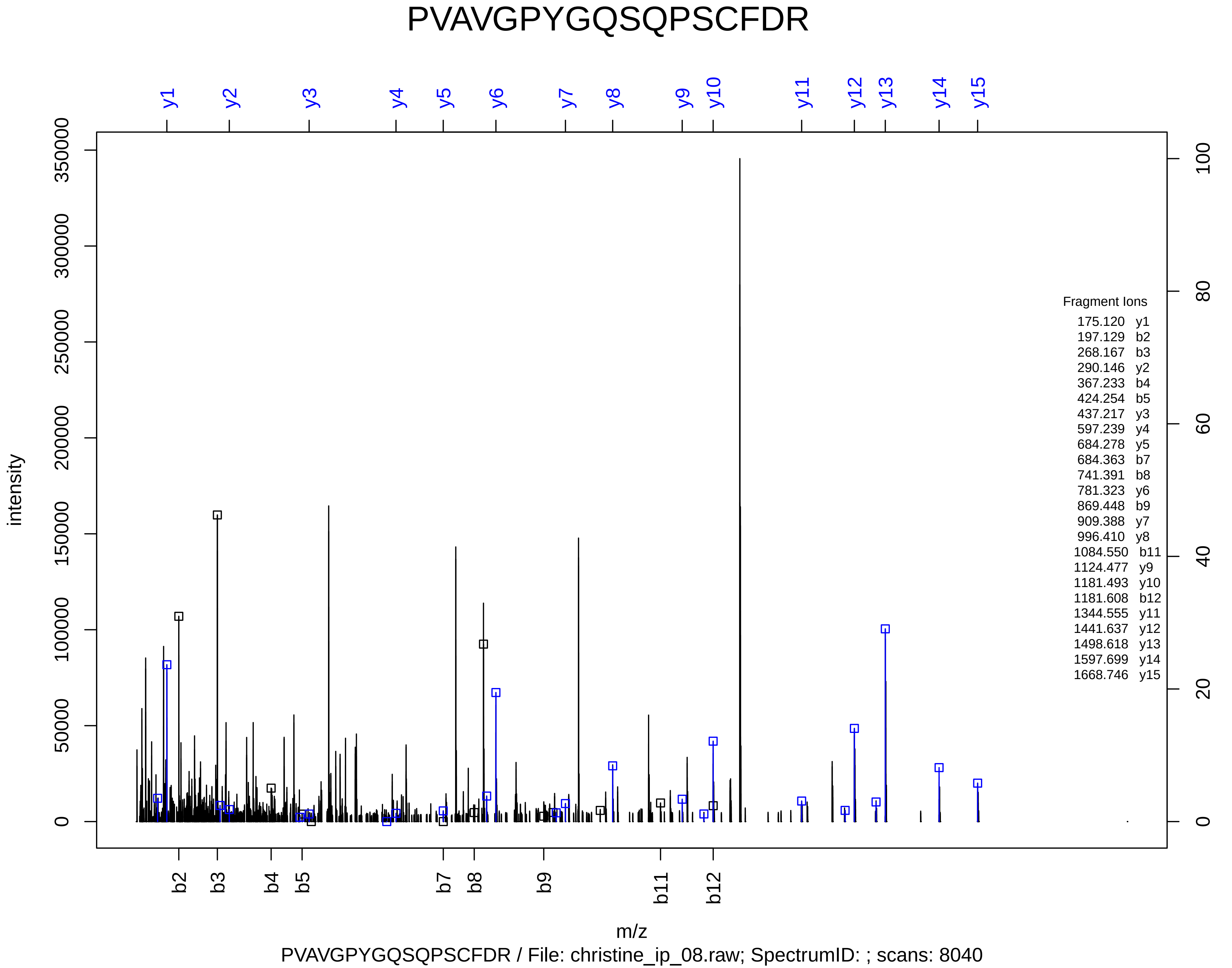

PVAVGPYGQSQPSCFDR
y1
y2
y3
y4
y5
y6
y7
y8
y9
y10
y11
y12
y13
y14
y15
intensity
0
50000
100000
150000
200000
250000
300000
350000
0
20
40
60
80
100
b2
b3
b4
b5
b7
b8
b9
b11
b12
m/z
Fragment Ions
175.120 y1
197.129 b2
268.167 b3
290.146 y2
367.233 b4
424.254 b5
437.217 y3
597.239 y4
684.278 y5
684.363 b7
741.391 b8
781.323 y6
869.448 b9
909.388 y7
996.410 y8
1084.550 b11
1124.477 y9
1181.493 y10
1181.608 b12
1344.555 y11
1441.637 y12
1498.618 y13
1597.699 y14
1668.746 y15
PVAVGPYGQSQPSCFDR / File: christine_ip_08.raw; SpectrumID: ; scans: 8040

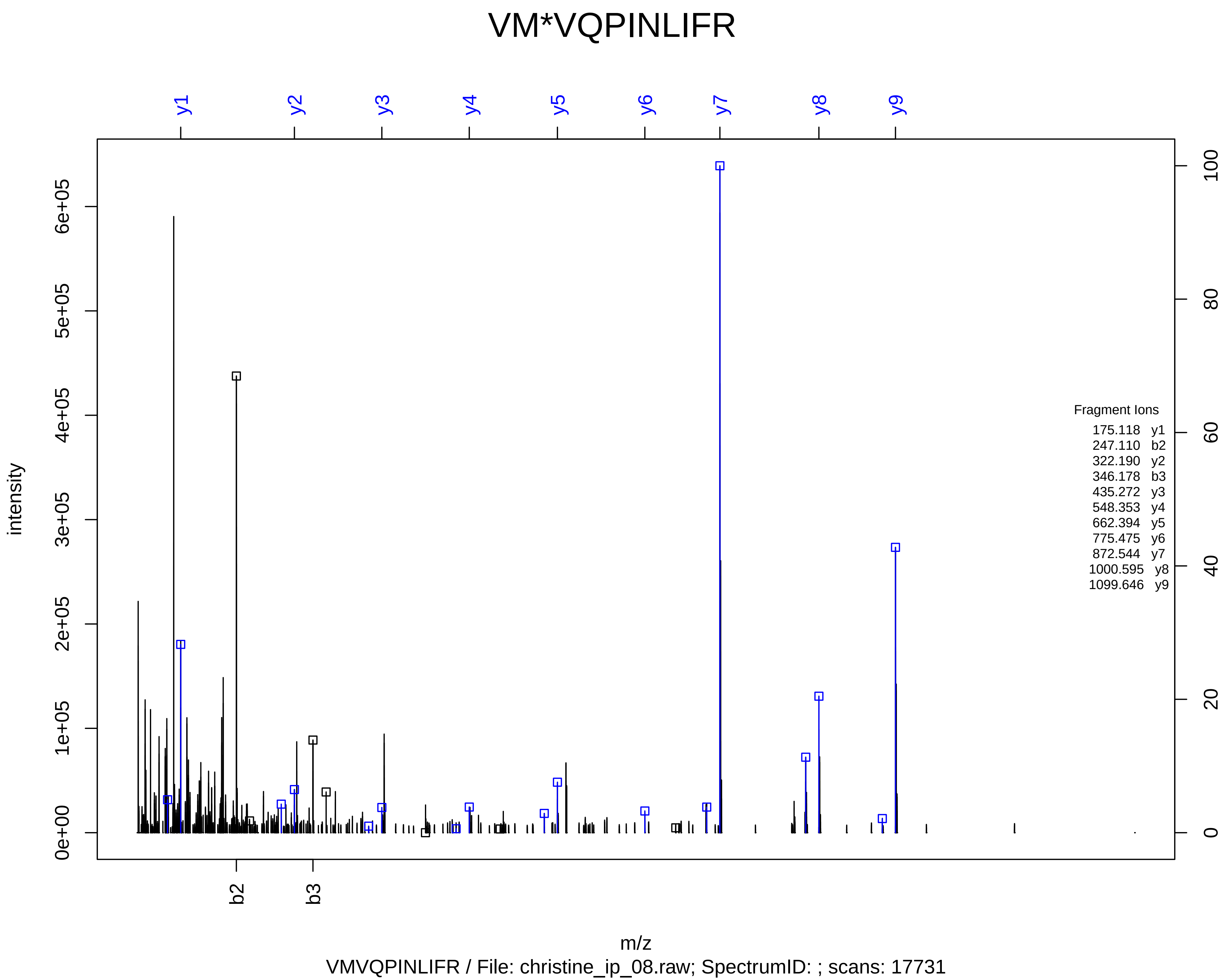
VM*VQPINLIFR
y1
y2
y3
y4
y5
y6
y7
y8
y9
b2
b3
intensity
m/z
Fragment Ions
175.118 y1
247.110 b2
322.190 y2
346.178 b3
435.272 y3
548.353 y4
662.394 y5
775.475 y6
872.544 y7
1000.595 y8
1099.646 y9
VMVQPINLIFR / File: christine_ip_08.raw; SpectrumID: ; scans: 17731

EGDVLTLLESER

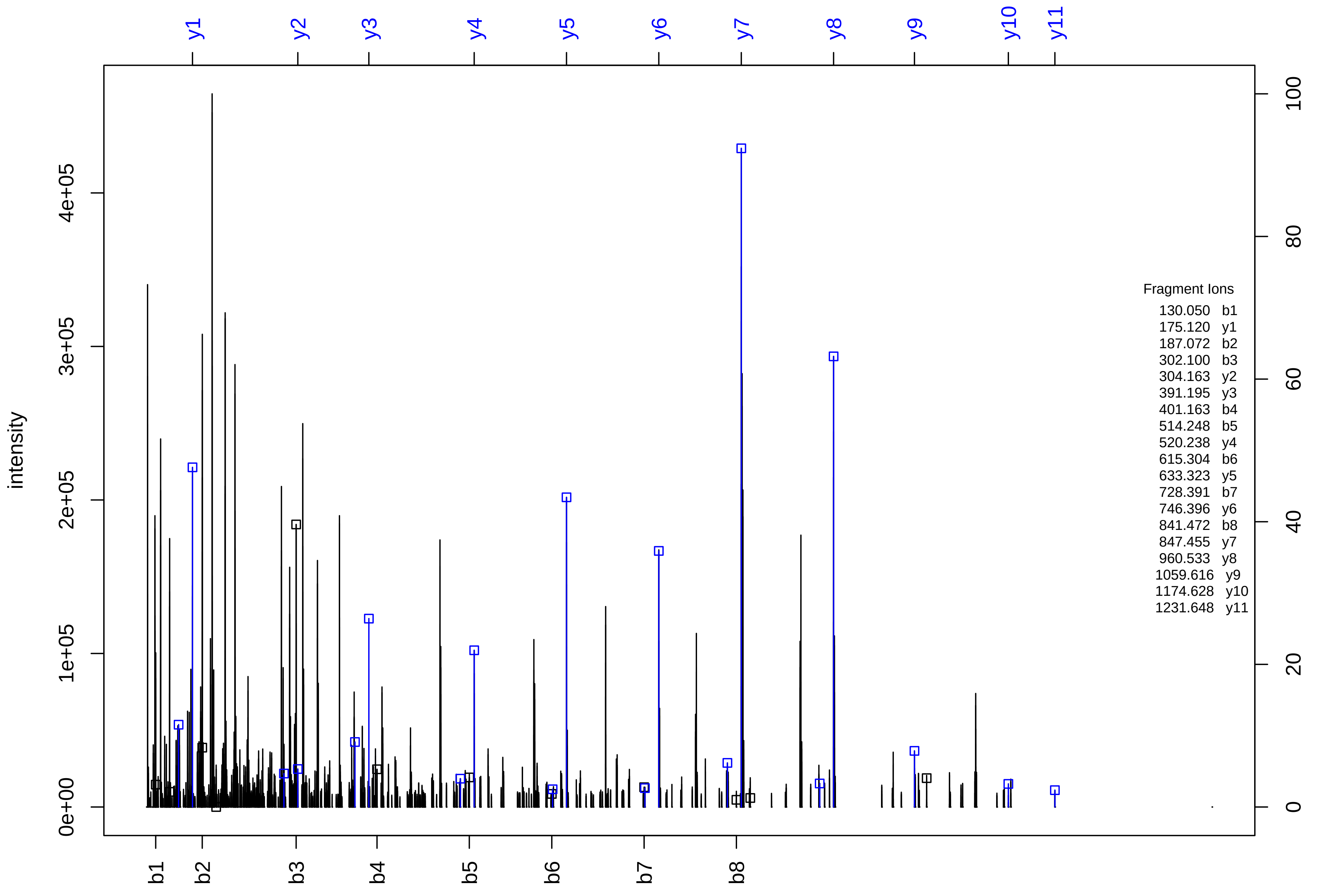

EGDVLTLLESER / File: christine_ip_08.raw; SpectrumID: ; scans: 16285

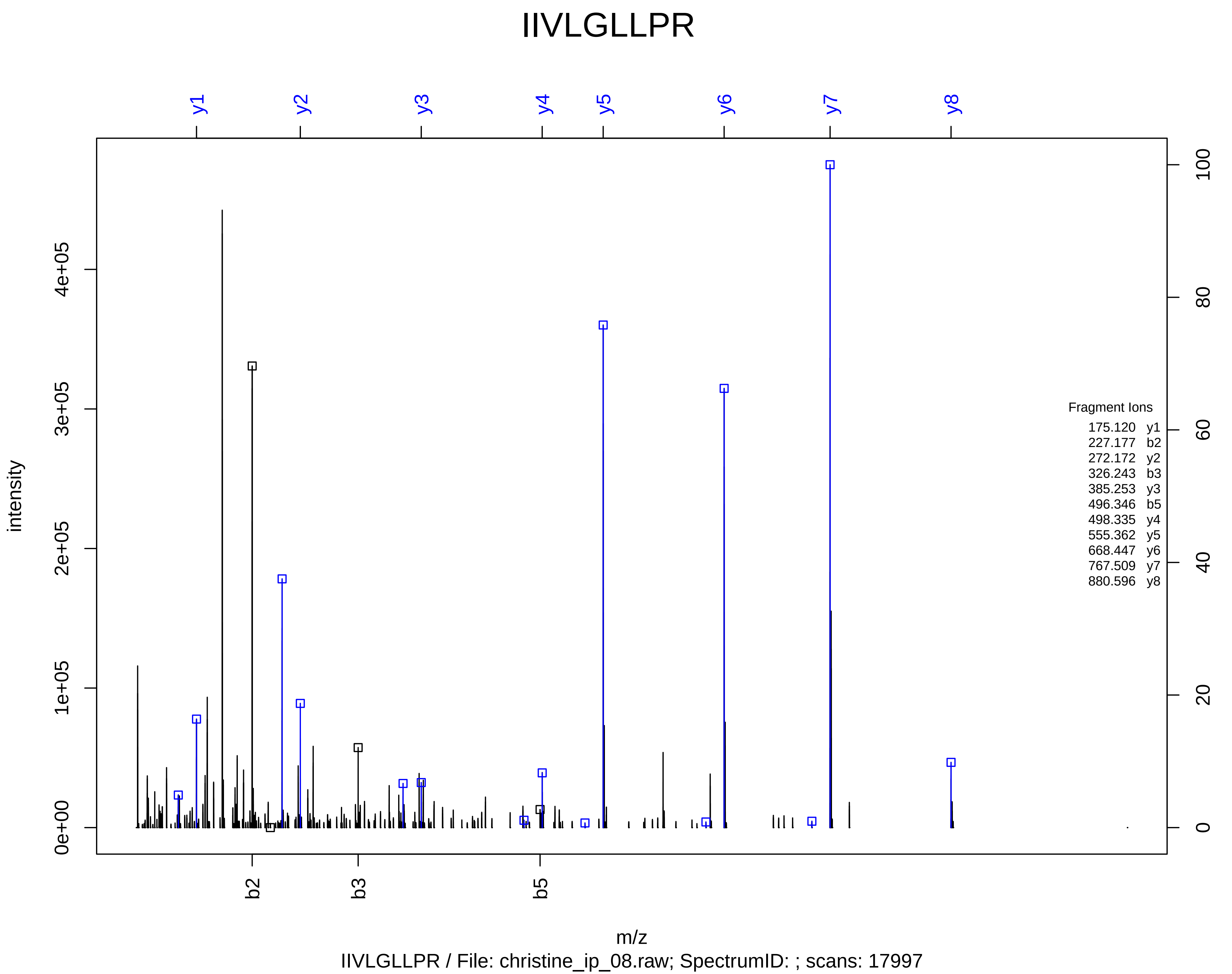
IIVLGLLPR
y1
y2
y3
y4
y5
y6
y7
y8
b2
b3
b5
intensity
0e+00
1e+05
2e+05
3e+05
4e+05
0
20
40
60
80
100
Fragment Ions
175.120 y1
227.177 b2
272.172 y2
326.243 b3
385.253 y3
496.346 b5
498.335 y4
555.362 y5
668.447 y6
767.509 y7
880.596 y8
m/z
IIVLGLLPR / File: christine_ip_08.raw; SpectrumID: ; scans: 17997

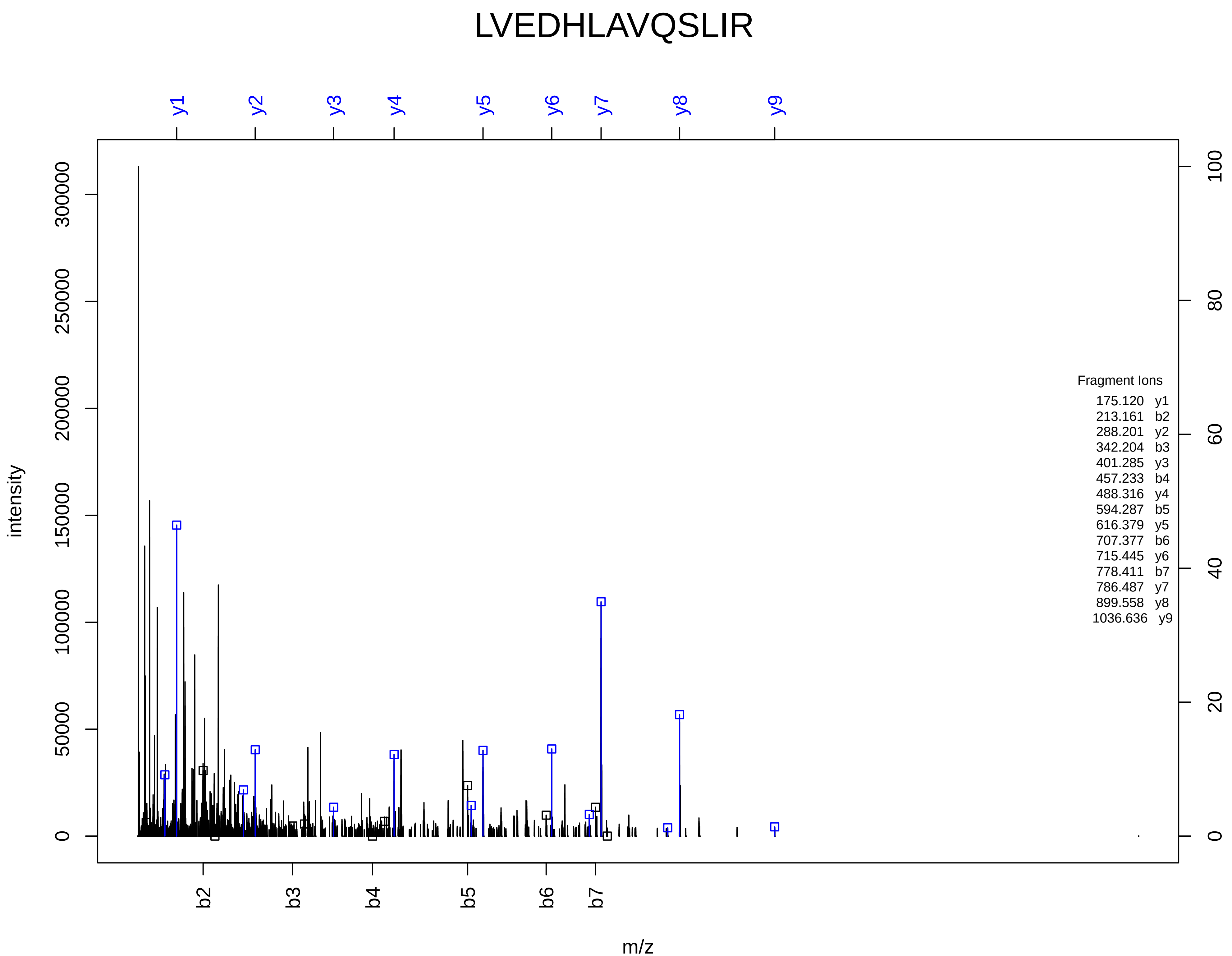
LVEDHLAVQSLIR
y1
y2
y3
y4
y5
y6
y7
y8
y9
intensity
0
50000
100000
150000
200000
250000
300000
0
20
40
60
80
100
Fragment Ions
175.120 y1
213.161 b2
288.201 y2
342.204 b3
401.285 y3
457.233 b4
488.316 y4
594.287 b5
616.379 y5
707.377 b6
715.445 y6
778.411 b7
786.487 y7
899.558 y8
1036.636 y9
b2
b3
b4
b5
b6
b7
m/z
LVEDHLAVQSLIR / File: christine_ip_08.raw; SpectrumID: ; scans: 11884

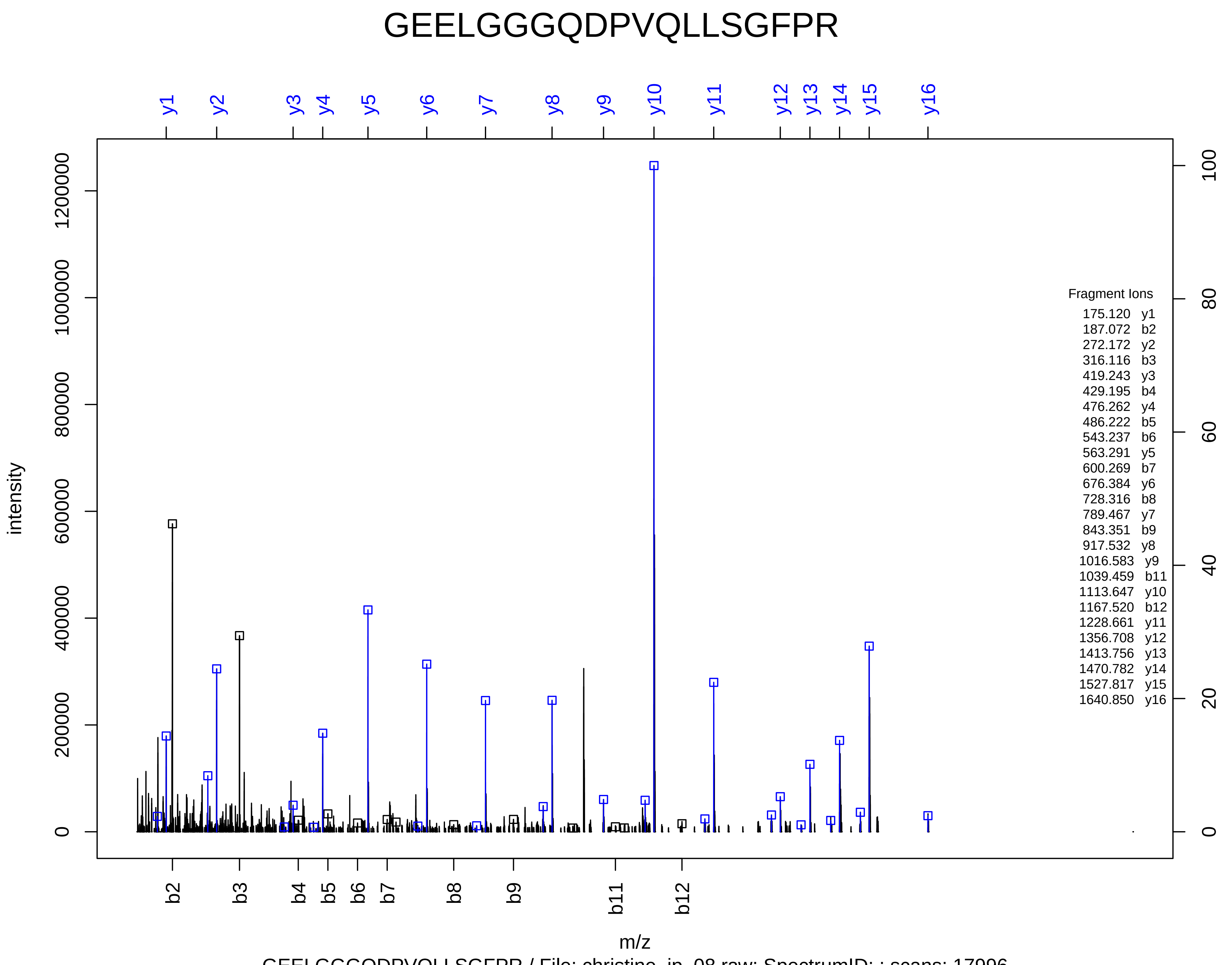
GEELGGGQDPVQLLSGFPR
y1 y2 y3 y4 y5 y6 y7 y8 y9 y10 y11 y12 y13 y14 y15 y16
intensity
0
200000
400000
600000
800000
1000000
1200000
0
20
40
60
80
100
b2 b3 b4 b5 b6 b7 b8 b9 b11 b12
Fragment Ions
175.120 y1
187.072 b2
272.172 y2
316.116 b3
419.243 y3
429.195 b4
476.262 y4
486.222 b5
543.237 b6
563.291 y5
600.269 b7
676.384 y6
728.316 b8
789.467 y7
843.351 b9
917.532 y8
1016.583 y9
1039.459 b11
1113.647 y10
1167.520 b12
1228.661 y11
1356.708 y12
1413.756 y13
1470.782 y14
1527.817 y15
1640.850 y16
m/z
GEELGGGQDPVQLLSGFPR / File: christine_ip_08.raw; SpectrumID: ; scans: 17996

LLAEALNQVTQR

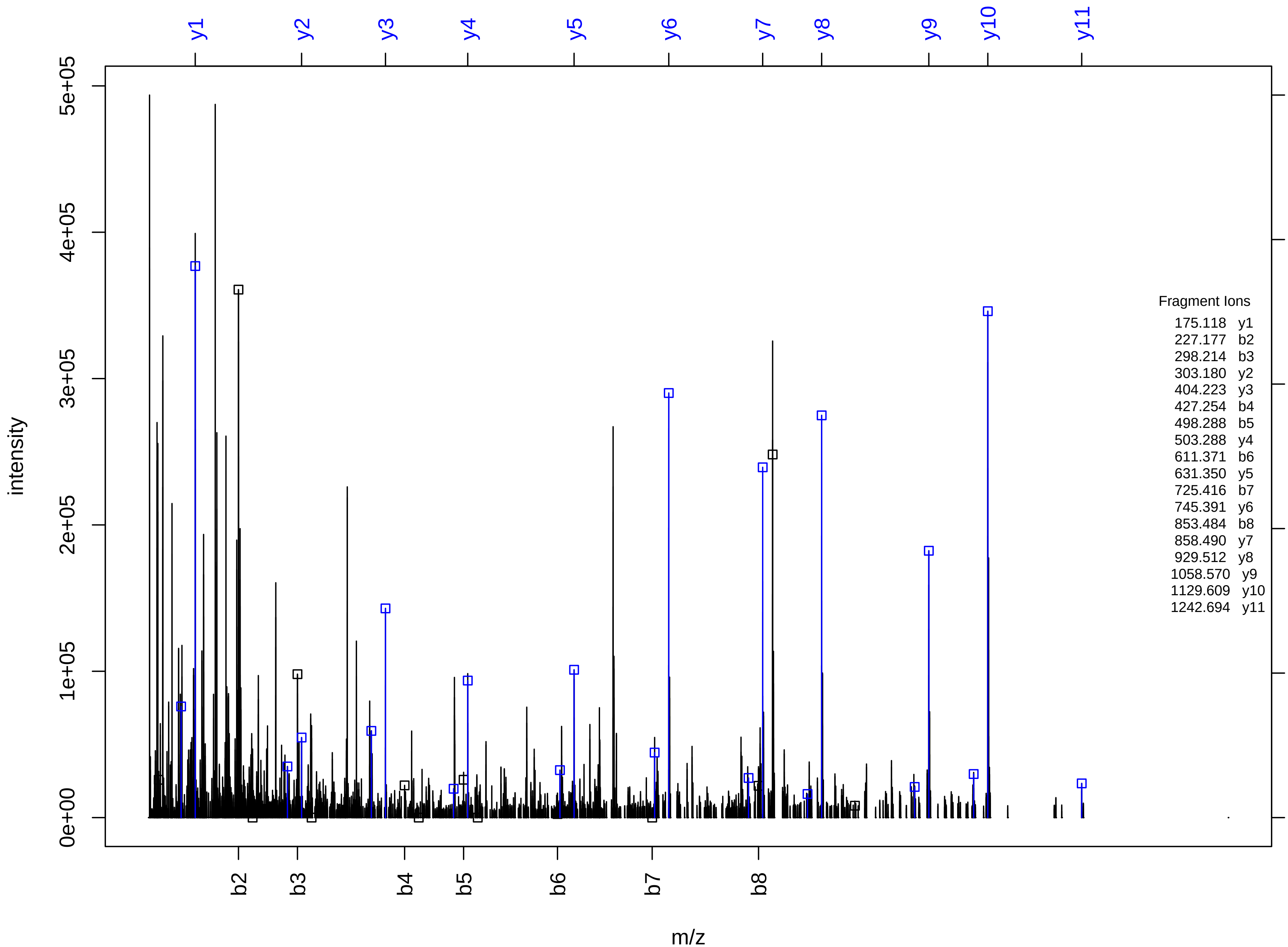

LLAEALNQVTQR / File: christine_ip_08.raw; SpectrumID: ; scans: 11330

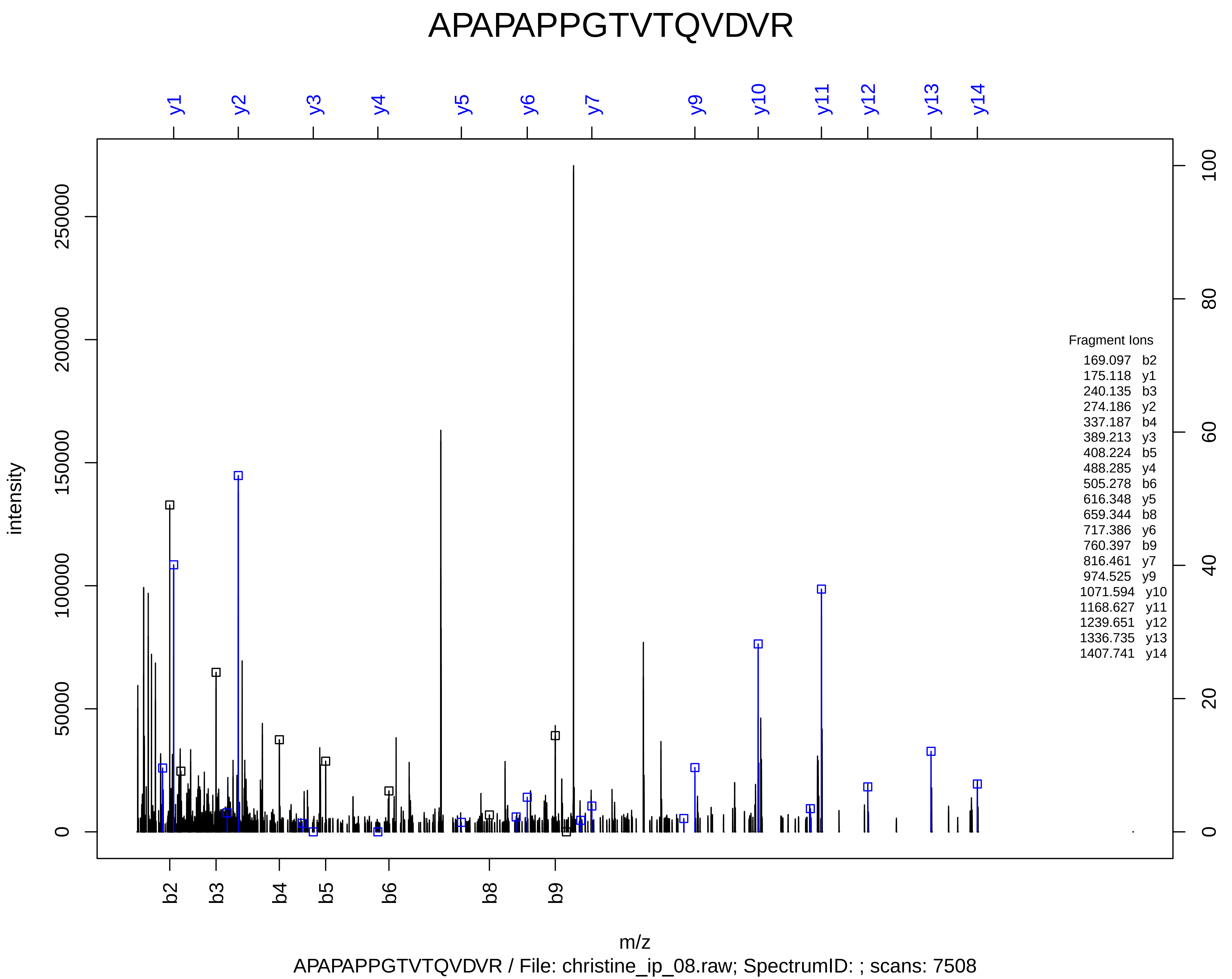
APAPAPPGTVTQVDVR
y1
y2
y3
y4
y5
y6
y7
y9
y10
y11
y12
y13
y14
intensity
0
50000
100000
150000
200000
250000
0
20
40
60
80
100
b2
b3
b4
b5
b6
b8
b9
Fragment Ions
169.097 b2
175.118 y1
240.135 b3
274.186 y2
337.187 b4
389.213 y3
408.224 b5
488.285 y4
505.278 b6
616.348 y5
659.344 b8
717.386 y6
760.397 b9
816.461 y7
974.525 y9
1071.594 y10
1168.627 y11
1239.651 y12
1336.735 y13
1407.741 y14
m/z
APAPAPPGTVTQVDVR / File: christine_ip_08.raw; SpectrumID: ; scans: 7508

IGIIGGTGLDDPEILEGR

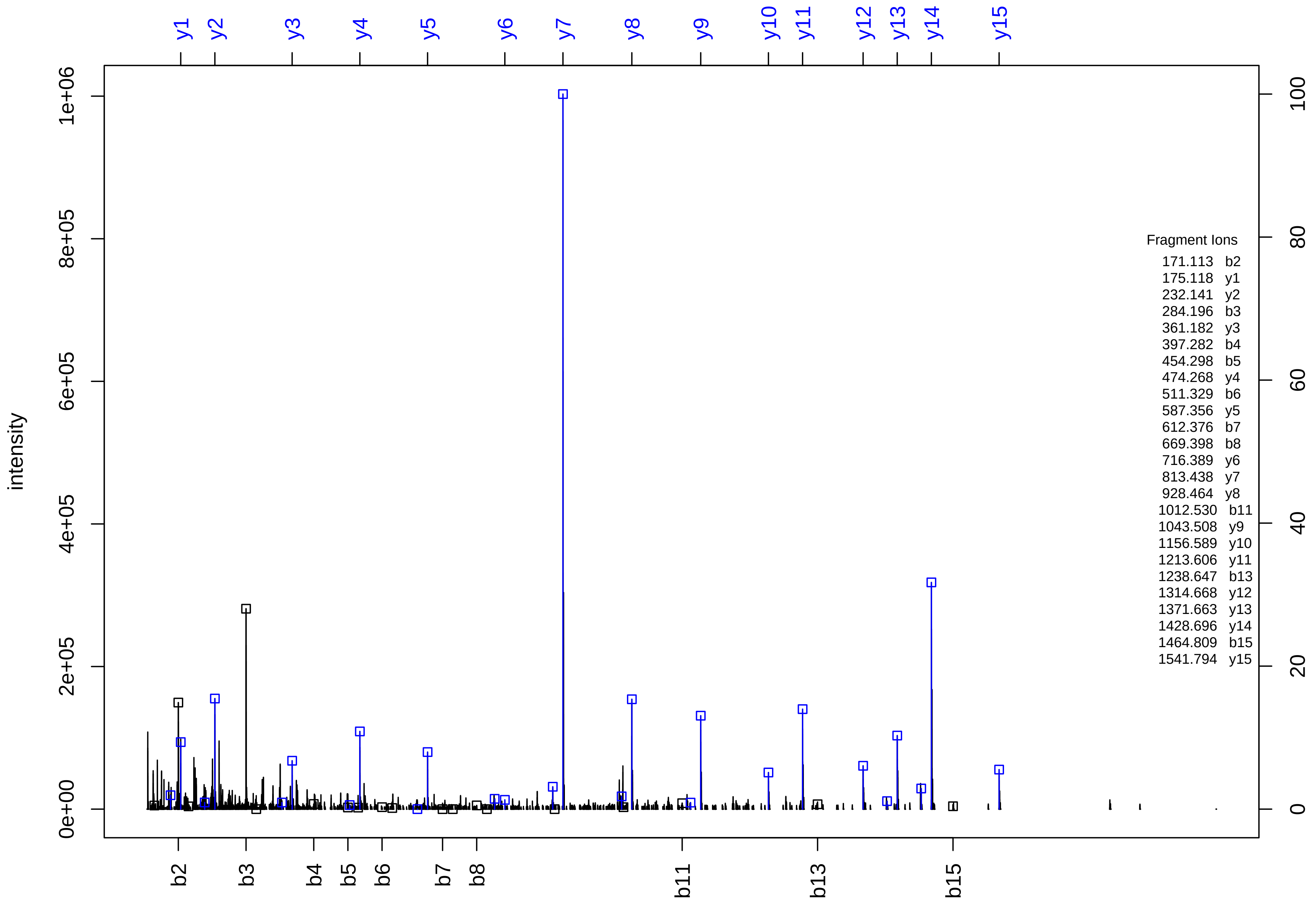

IGIIGGTGLDDPEILEGR / File: christine_ip_08.raw; SpectrumID: ; scans: 17596

(Ac)SGGSSCSQTPSR

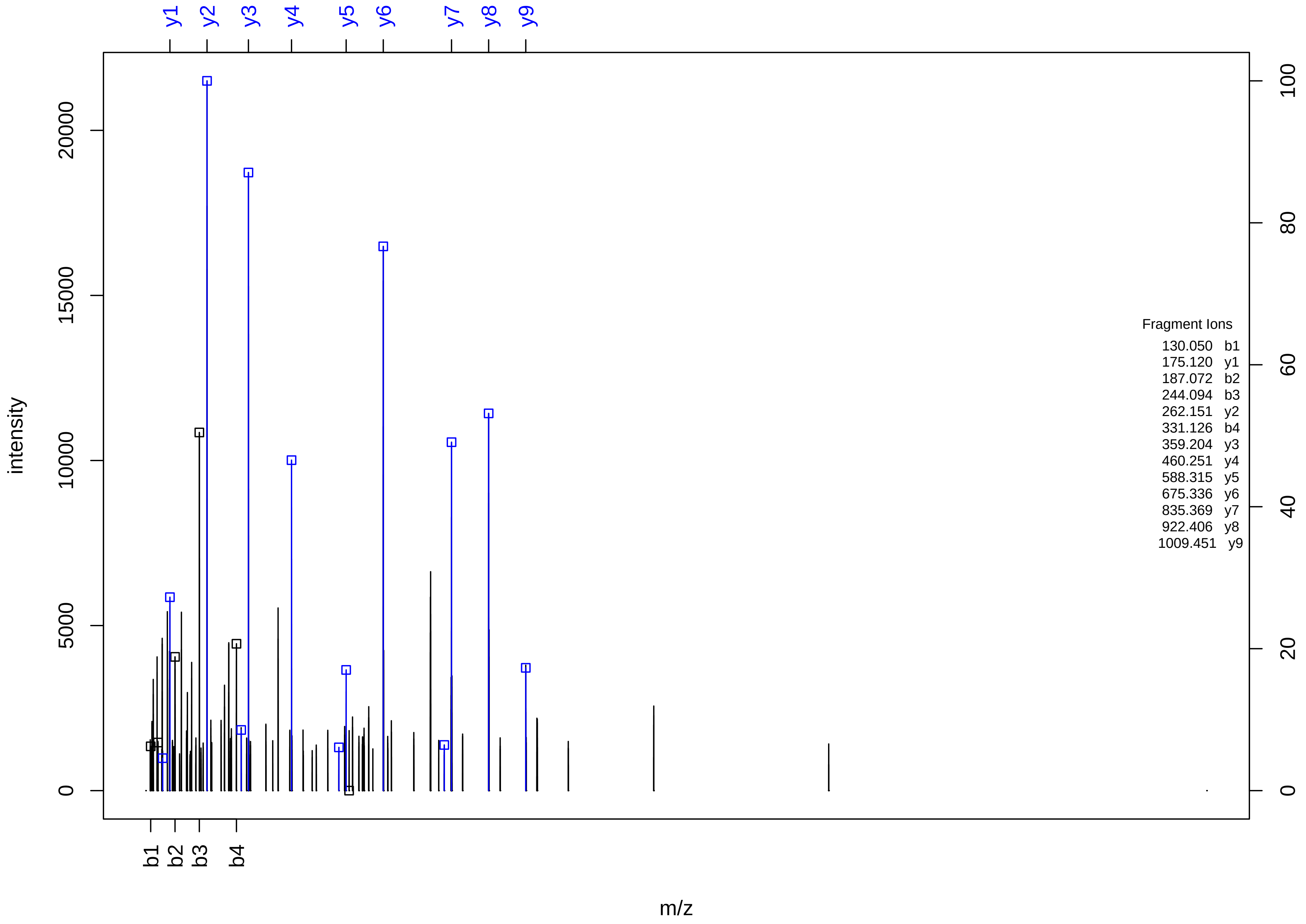

SGGSSCSQTPSR / File: christine_ip_08.raw; SpectrumID: ; scans: 772

VEEATVEER

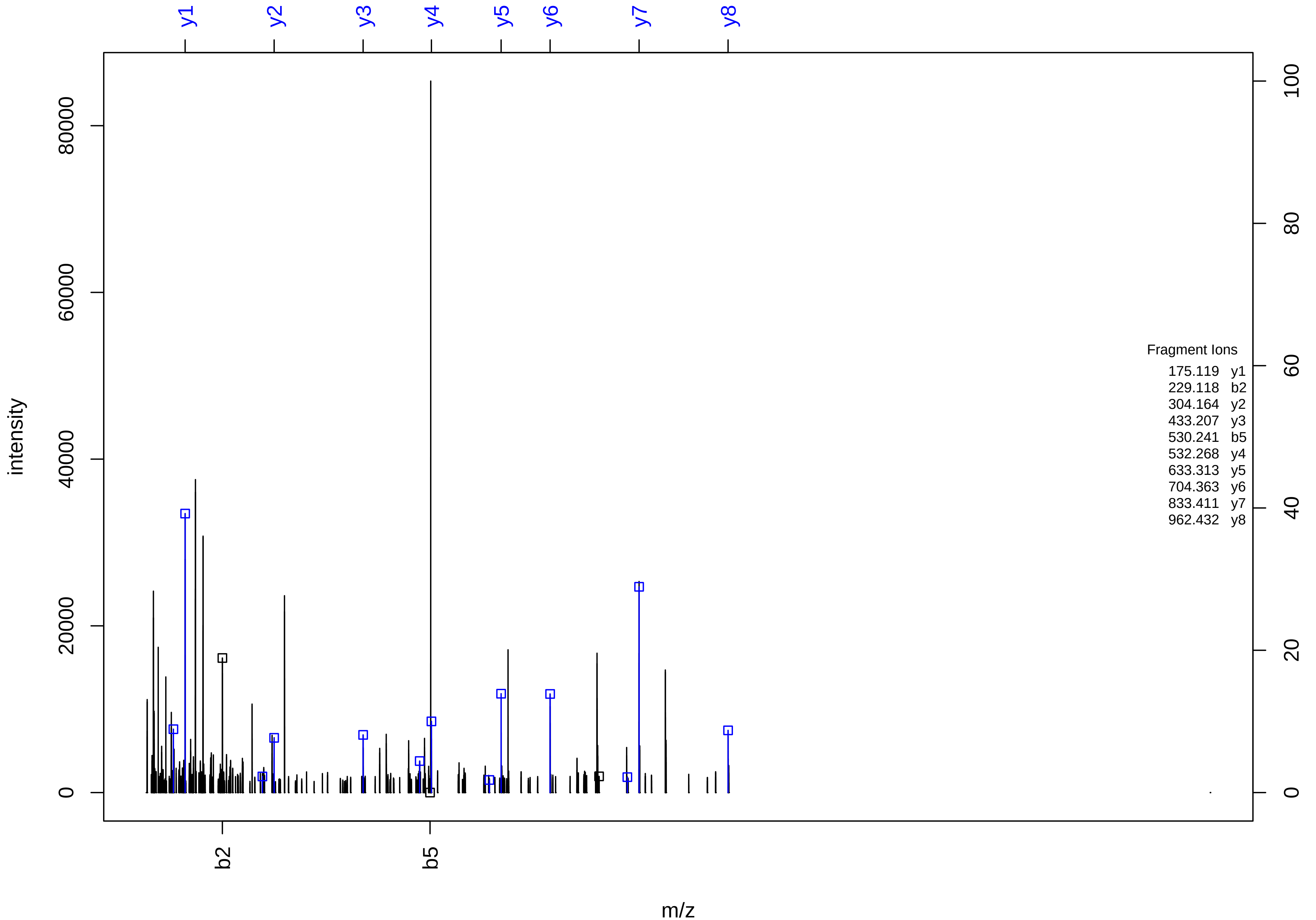

VEEATVEER / File: christine_ip_08.raw; SpectrumID: ; scans: 1630

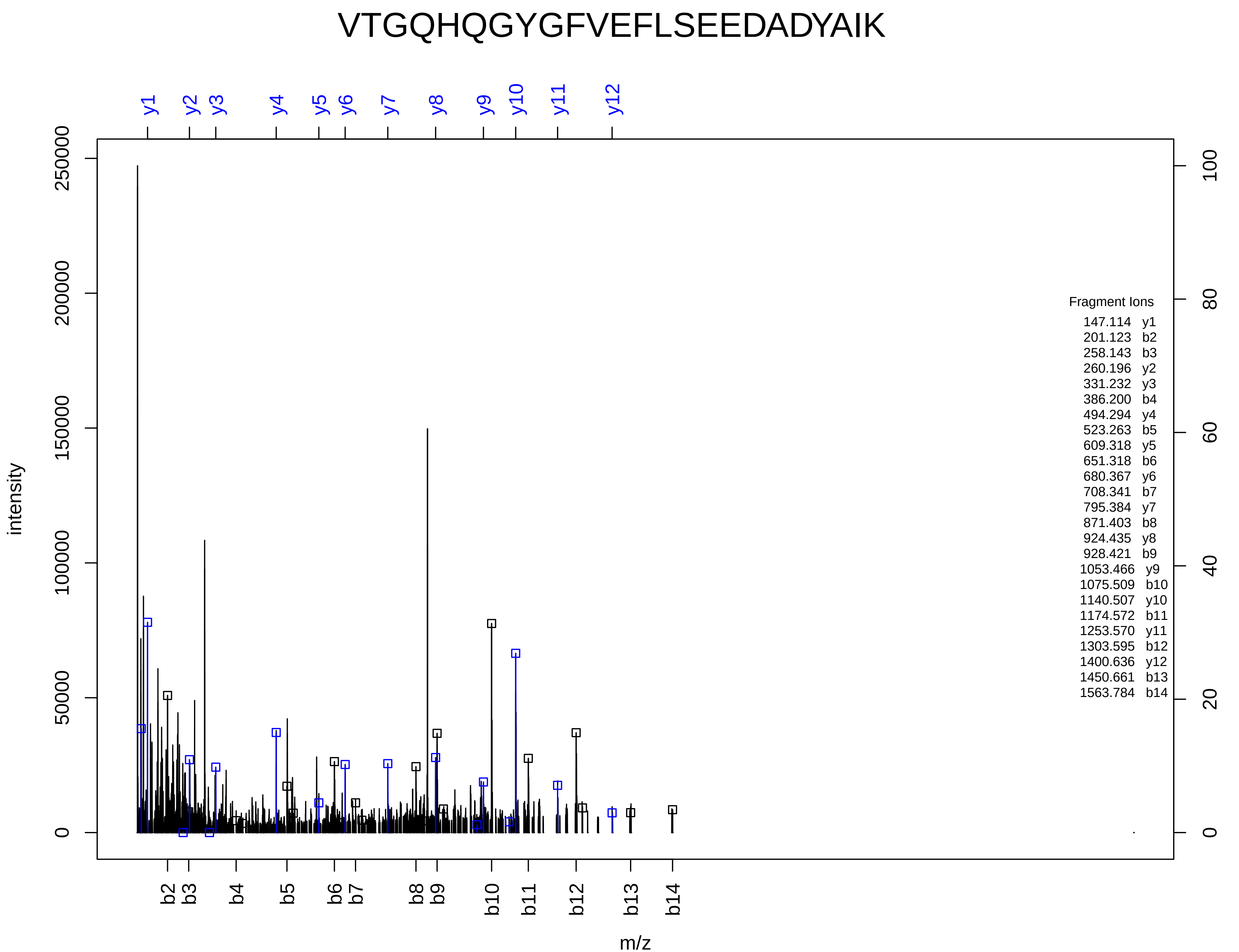
VTGQHQGYGFVEFLSEEDADYAIK
y1
y2
y3
y4
y5
y6
y7
y8
y9
y10
y11
y12
intensity
250000
200000
150000
100000
50000
0
100
80
60
40
20
0
Fragment Ions
147.114 y1
201.123 b2
258.143 b3
260.196 y2
331.232 y3
386.200 b4
494.294 y4
523.263 b5
609.318 y5
651.318 b6
680.367 y6
708.341 b7
795.384 y7
871.403 b8
924.435 y8
928.421 b9
1053.466 y9
1075.509 b10
1140.507 y10
1174.572 b11
1253.570 y11
1303.595 b12
1400.636 y12
1450.661 b13
1563.784 b14
b2
b3
b4
b5
b6
b7
b8
b9
b10
b11
b12
b13
b14
m/z
VTGQHQGYGFVEFLSEEDADYAIK / File: christine_ip_08.raw; SpectrumID: ; scans: 17195

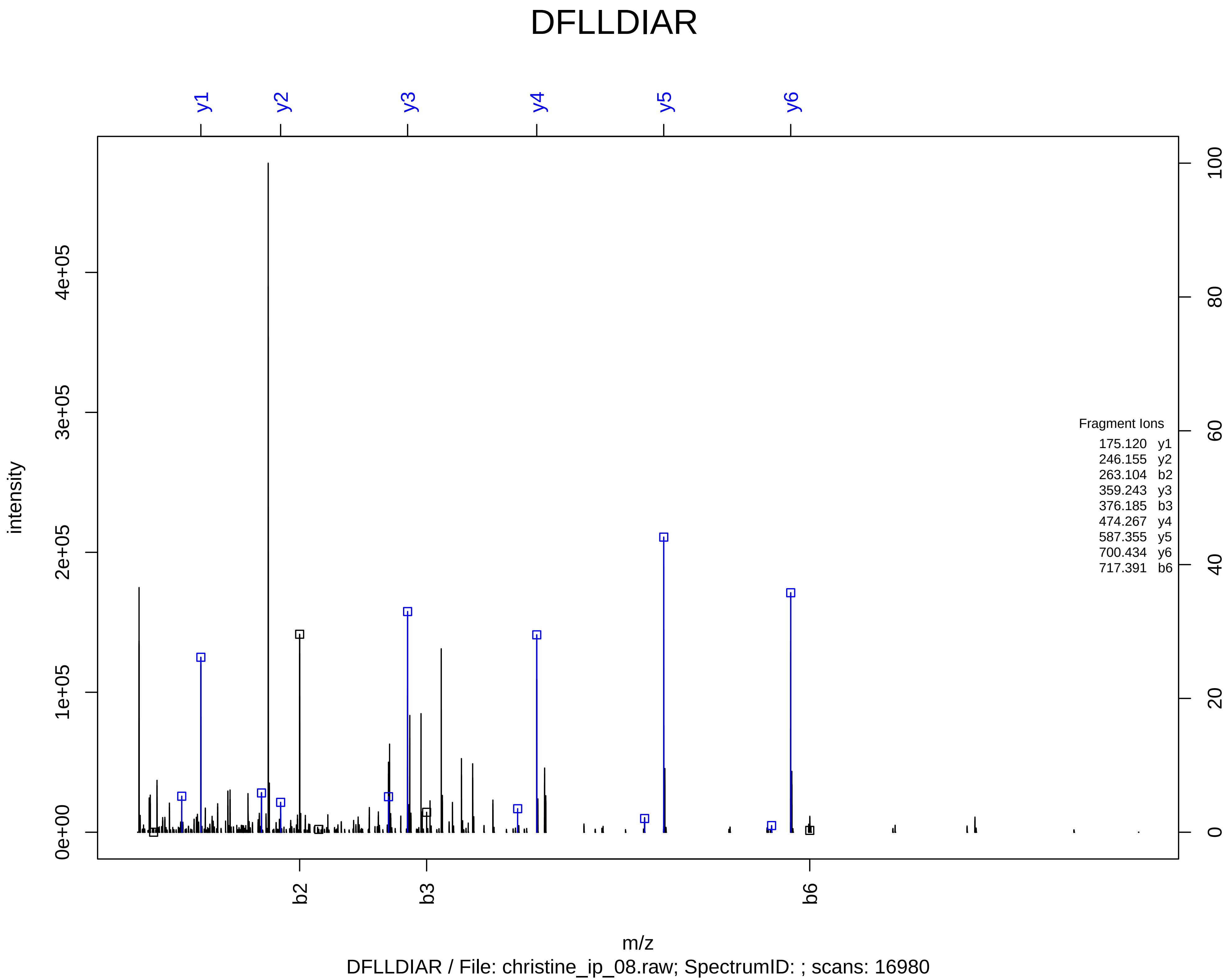
DFLLDIAR
y1
y2
y3
y4
y5
y6
intensity
0e+00
1e+05
2e+05
3e+05
4e+05
0
20
40
60
80
100
Fragment Ions
175.120 y1
246.155 y2
263.104 b2
359.243 y3
376.185 b3
474.267 y4
587.355 y5
700.434 y6
717.391 b6
b2
b3
b6
m/z
DFLLDIAR / File: christine_ip_08.raw; SpectrumID: ; scans: 16980

YIHLENLLAR

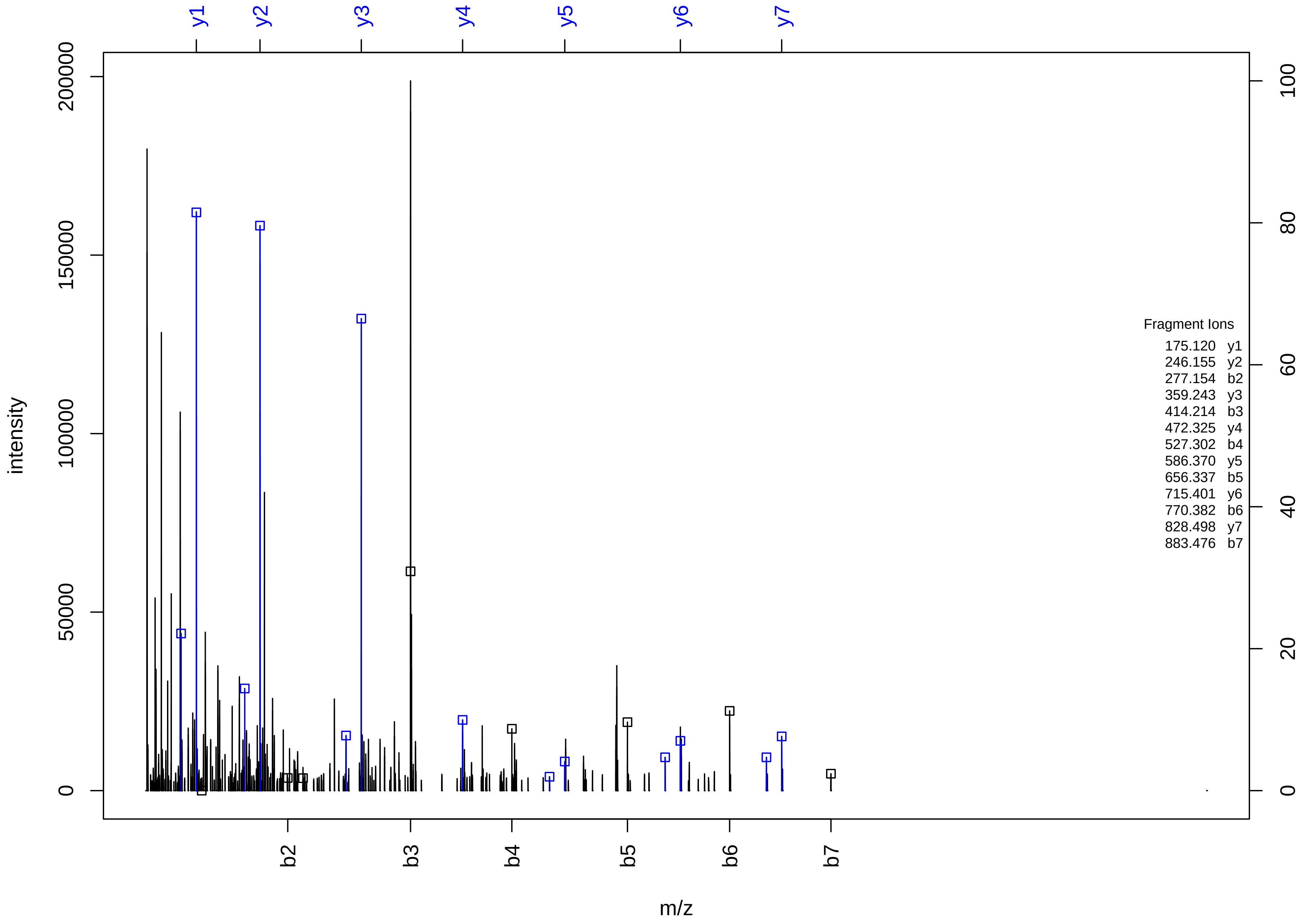

YIHLENLLAR / File: christine_ip_08.raw; SpectrumID: ; scans: 12561

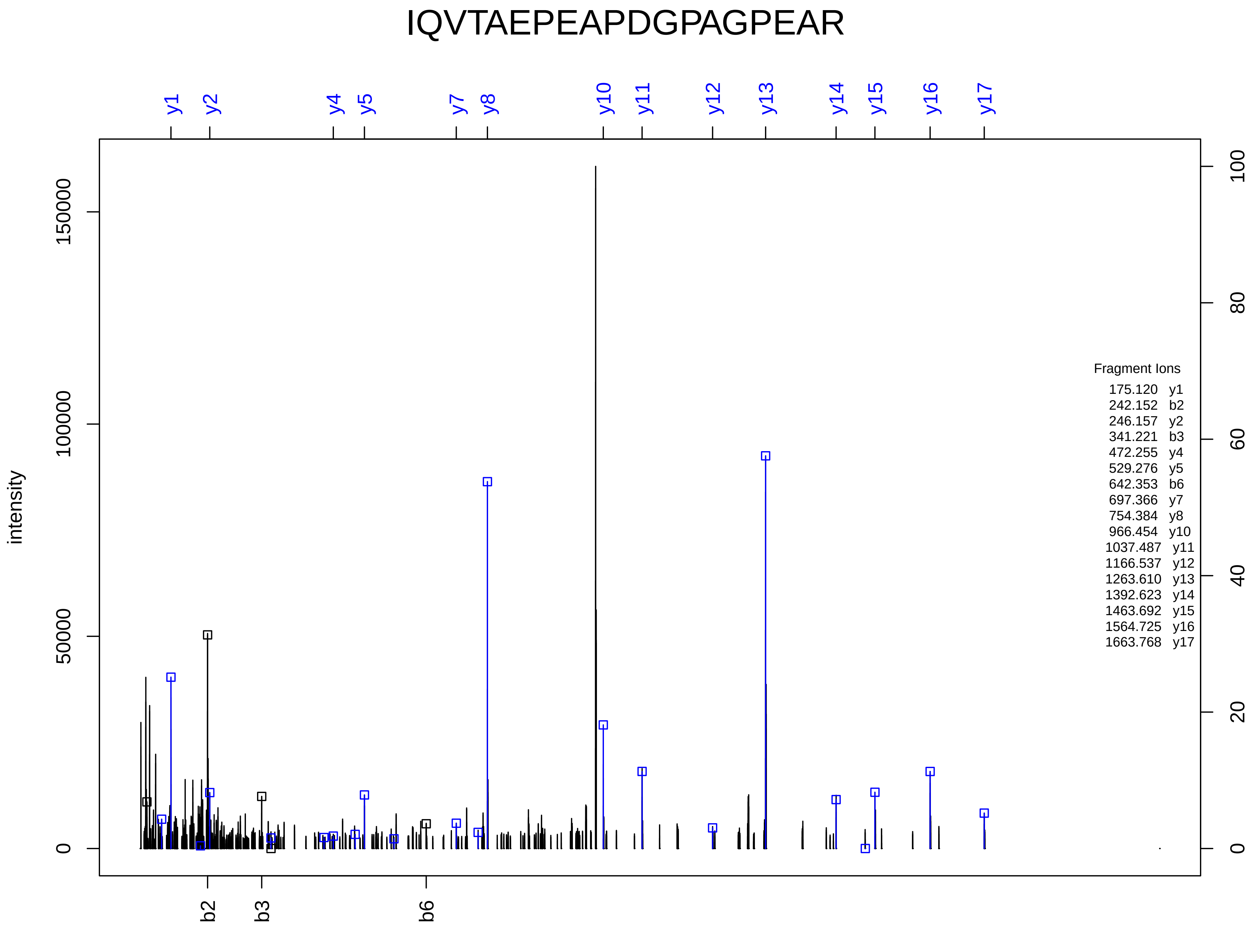
IQVTAEPEAPDGPAGPEAR
y1
y2
y4
y5
y7
y8
y10
y11
y12
y13
y14
y15
y16
y17
intensity
150000
100000
50000
0
100
80
60
40
20
0
b2
b3
b6
m/z
Fragment Ions
175.120 y1
242.152 b2
246.157 y2
341.221 b3
472.255 y4
529.276 y5
642.353 b6
697.366 y7
754.384 y8
966.454 y10
1037.487 y11
1166.537 y12
1263.610 y13
1392.623 y14
1463.692 y15
1564.725 y16
1663.768 y17
IQVTAEPEAPDGPAGPEAR / File: christine_ip_08.raw; SpectrumID: ; scans: 7138

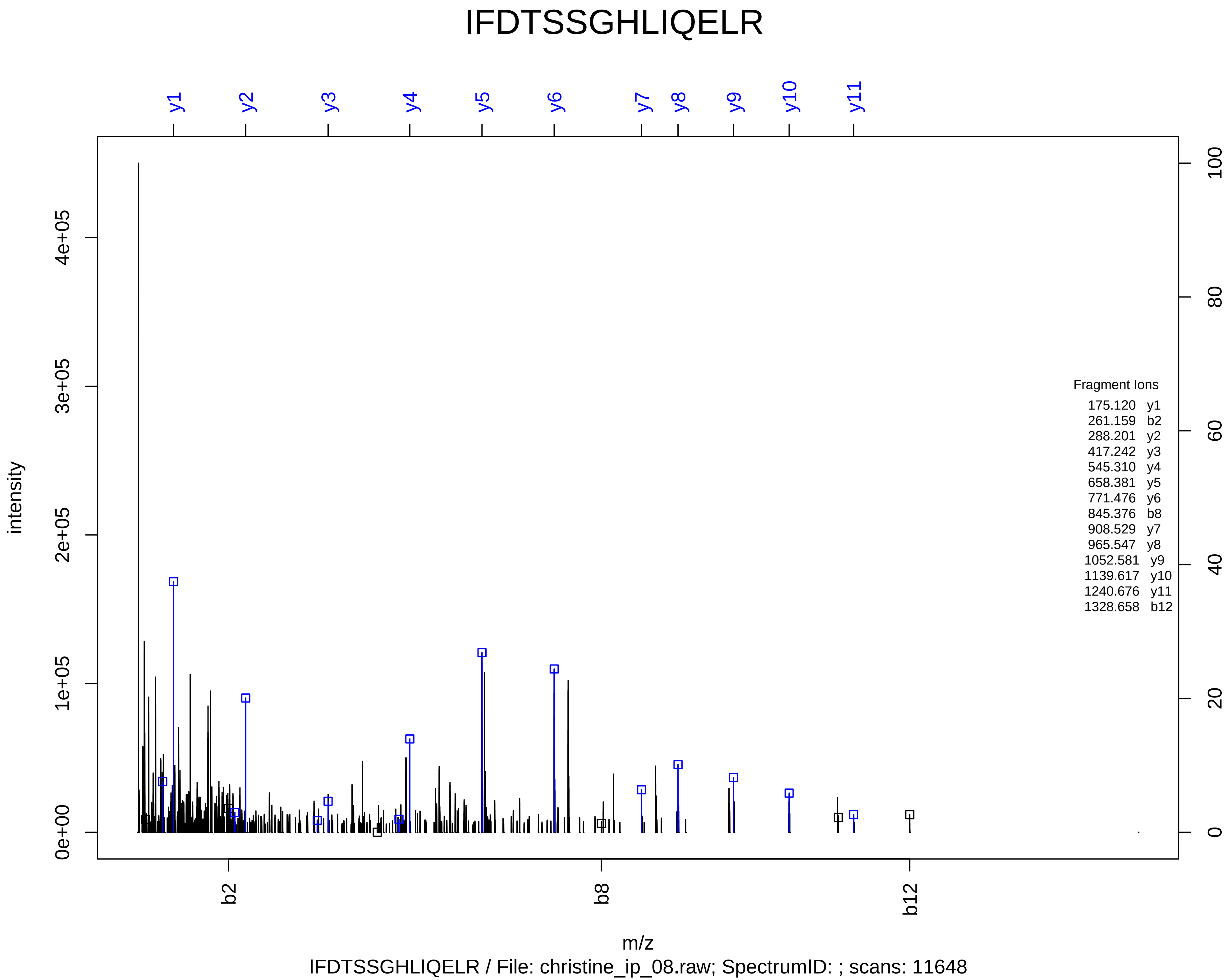

IFDTSSGHLIQELR
y1
y2
y3
y4
y5
y6
y7
y8
y9
y10
y11
intensity
0e+00
1e+05
2e+05
3e+05
4e+05
0
20
40
60
80
100
Fragment Ions
175.120 y1
261.159 b2
288.201 y2
417.242 y3
545.310 y4
658.381 y5
771.476 y6
845.376 b8
908.529 y7
965.547 y8
1052.581 y9
1139.617 y10
1240.676 y11
1328.658 b12
b2
b8
b12
m/z
IFDTSSGHLIQELR / File: christine_ip_08.raw; SpectrumID: ; scans: 11648

VLELLLPIR

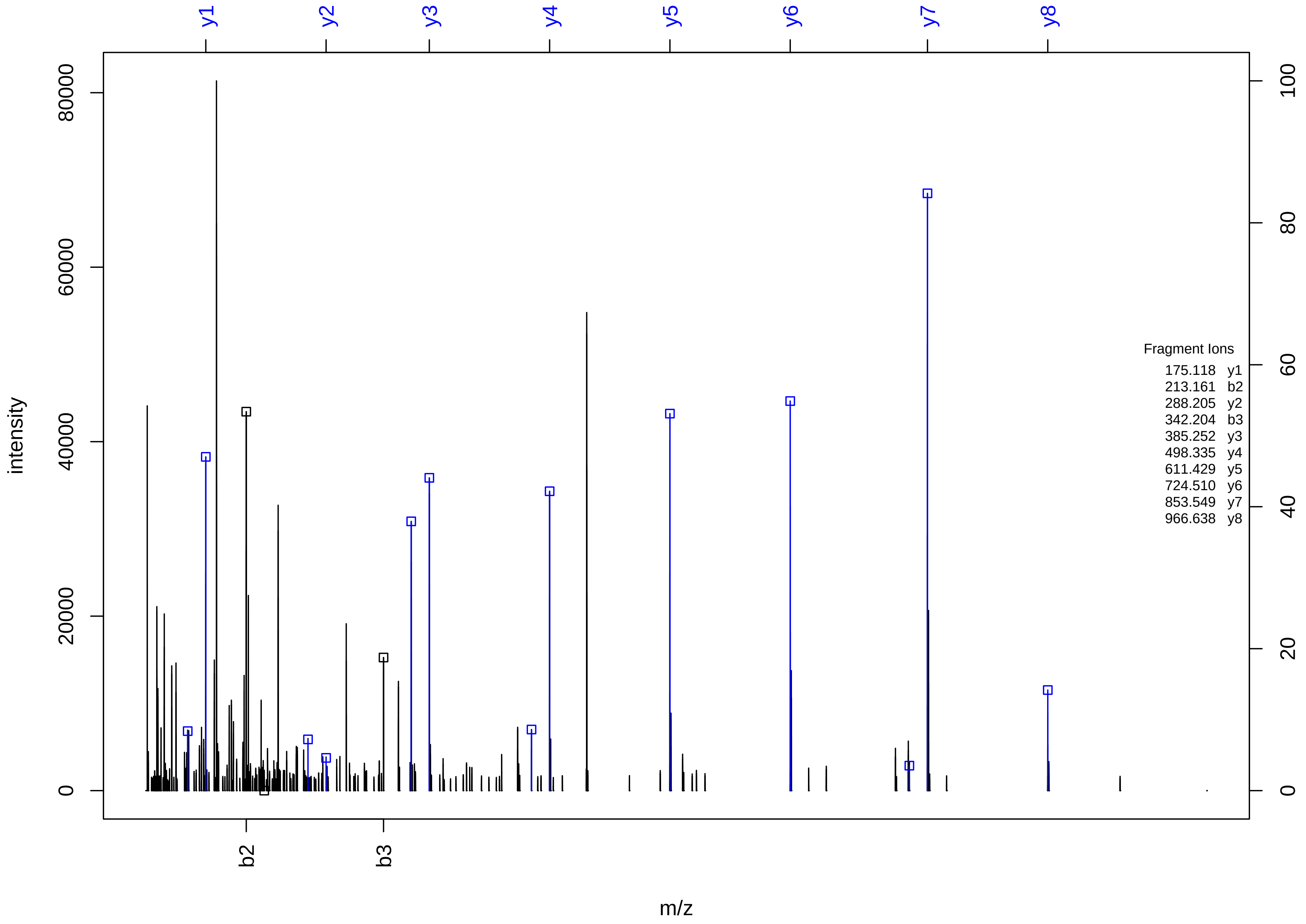

VLELLLPIR / File: christine_ip_08.raw; SpectrumID: ; scans: 19934

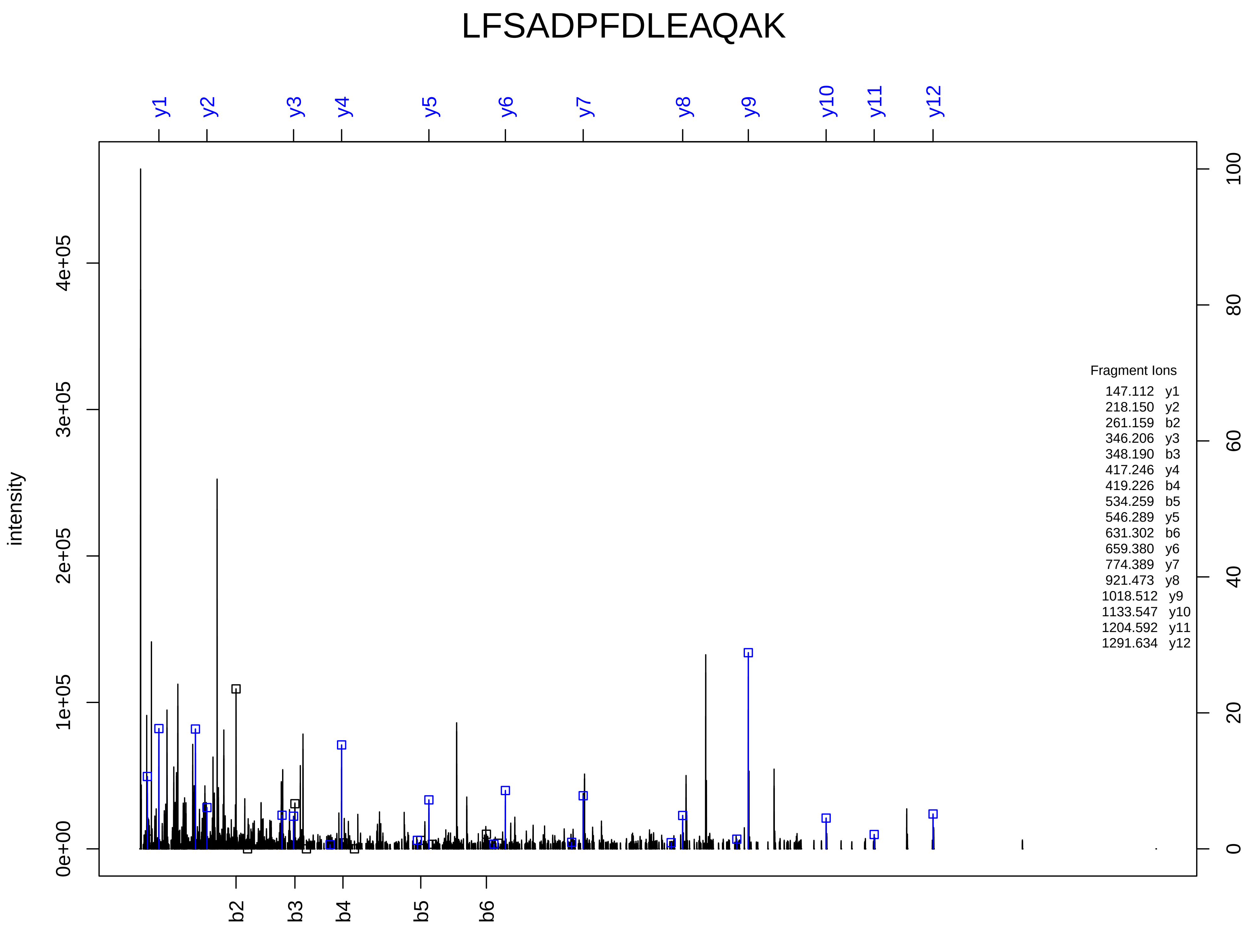

LFSADPFDLEAQAK / File: christine_ip_08.raw; SpectrumID: ; scans: 16413

LLALLDALASHK

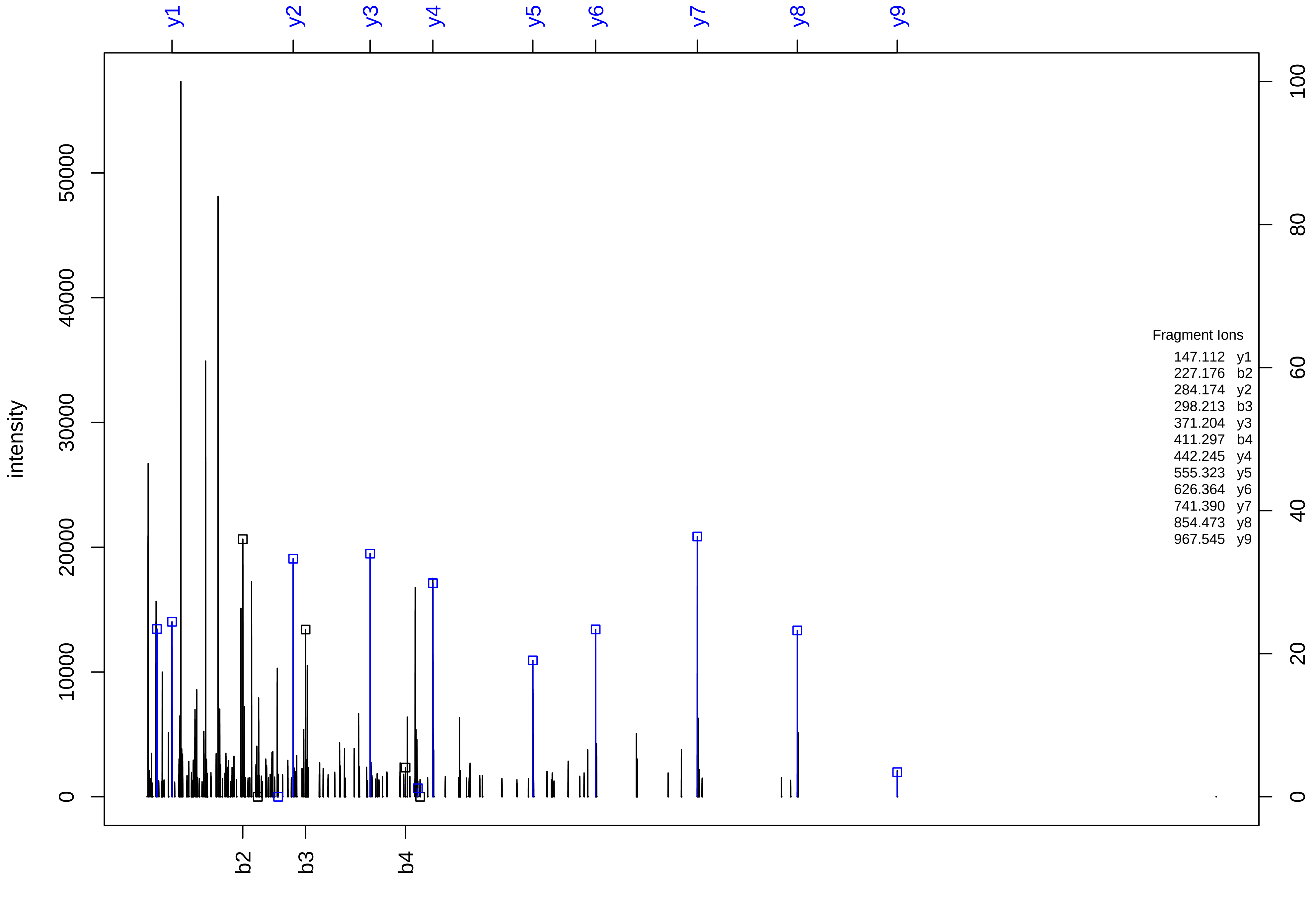

LLALLDALASHK / File: christine_ip_08.raw; SpectrumID: ; scans: 17417

QDLMQSLAKLQERFHLDQN^IGR

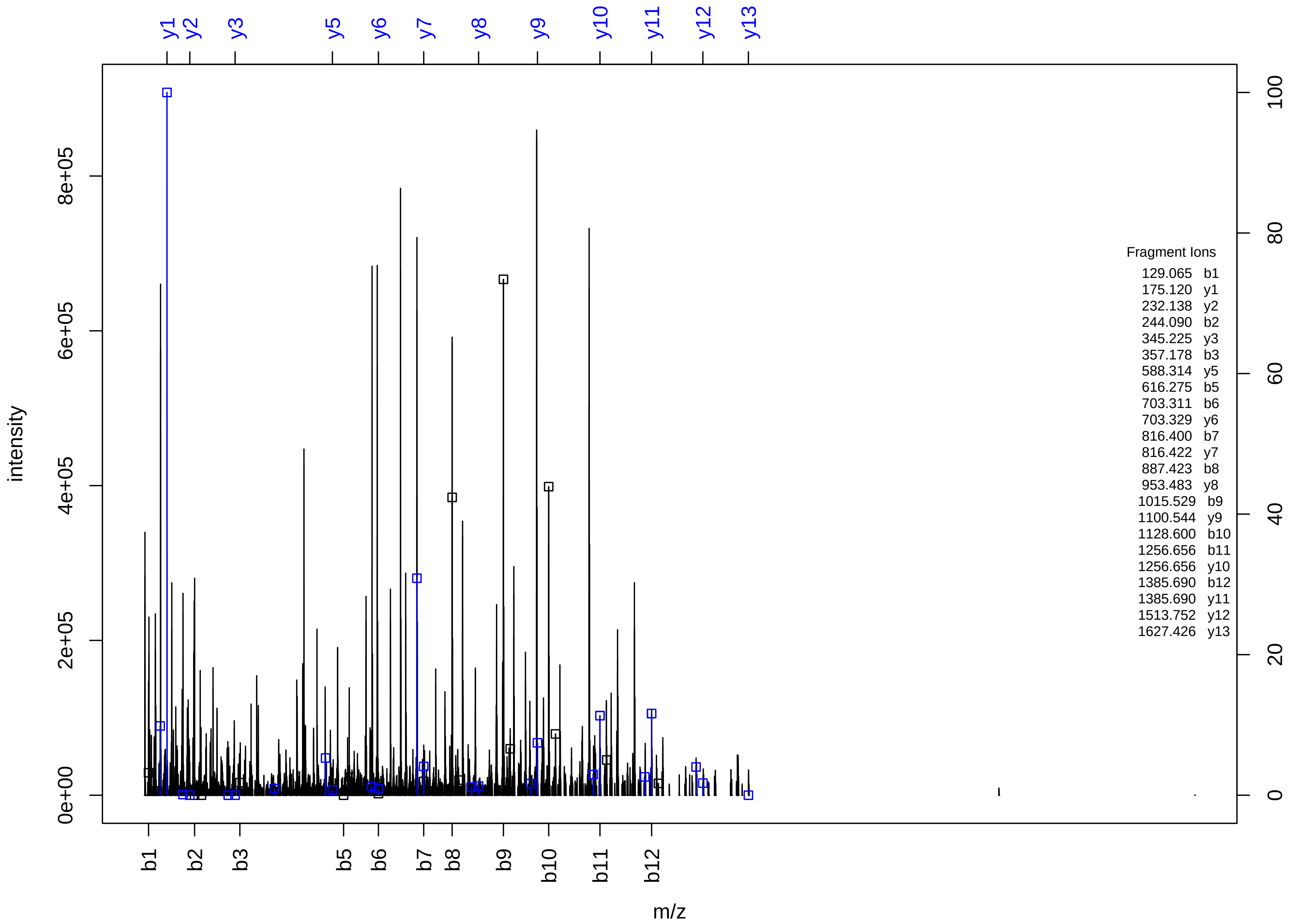

QDLMQSLAKLQERFHLDQNIGR / File: christine_ip_08.raw; SpectrumID: ; scans: 17944

LNLLQGQVSELPLR

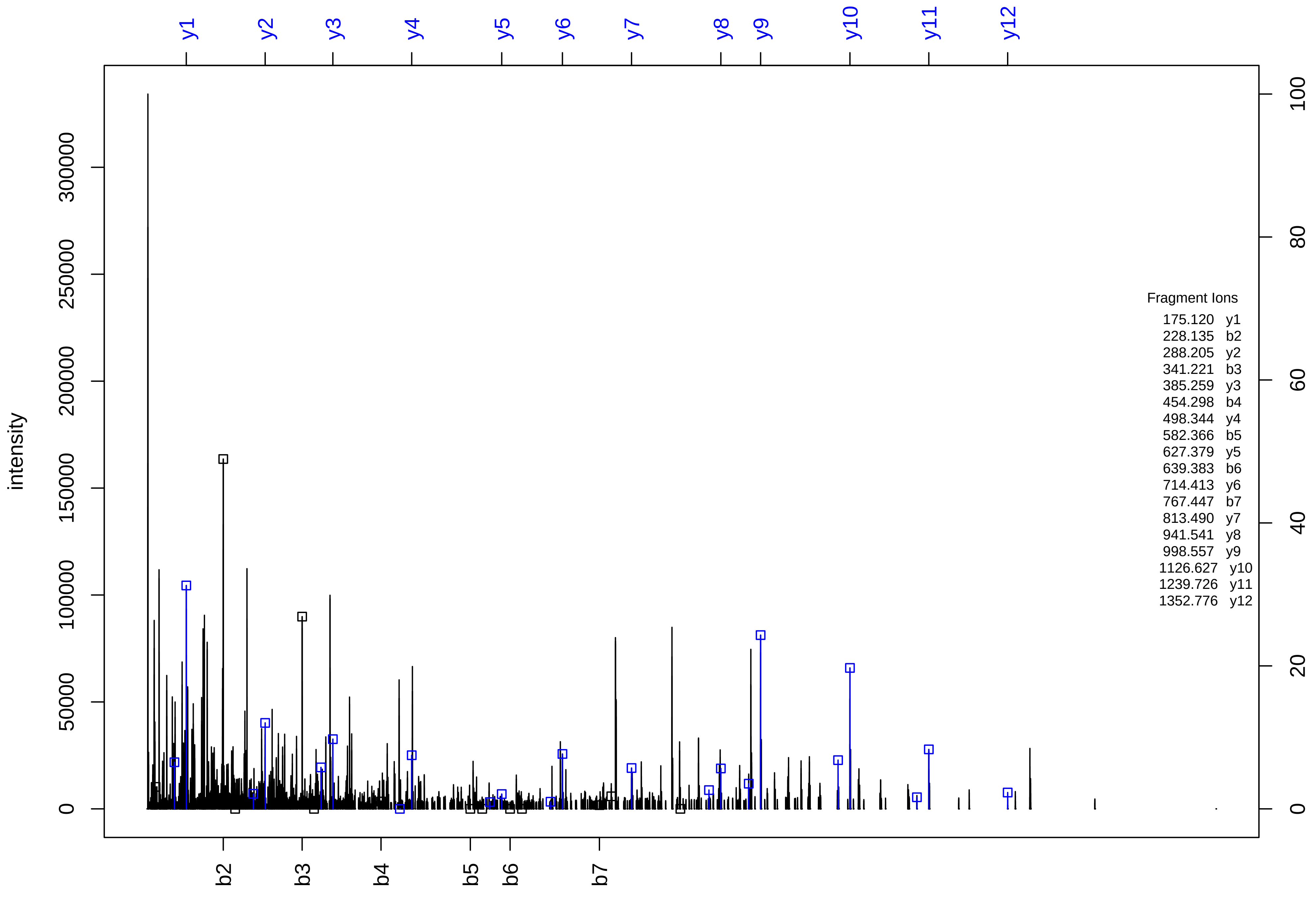

LNLLQGQVSELPLR / File: christine_ip_08.raw; SpectrumID: ; scans: 16662

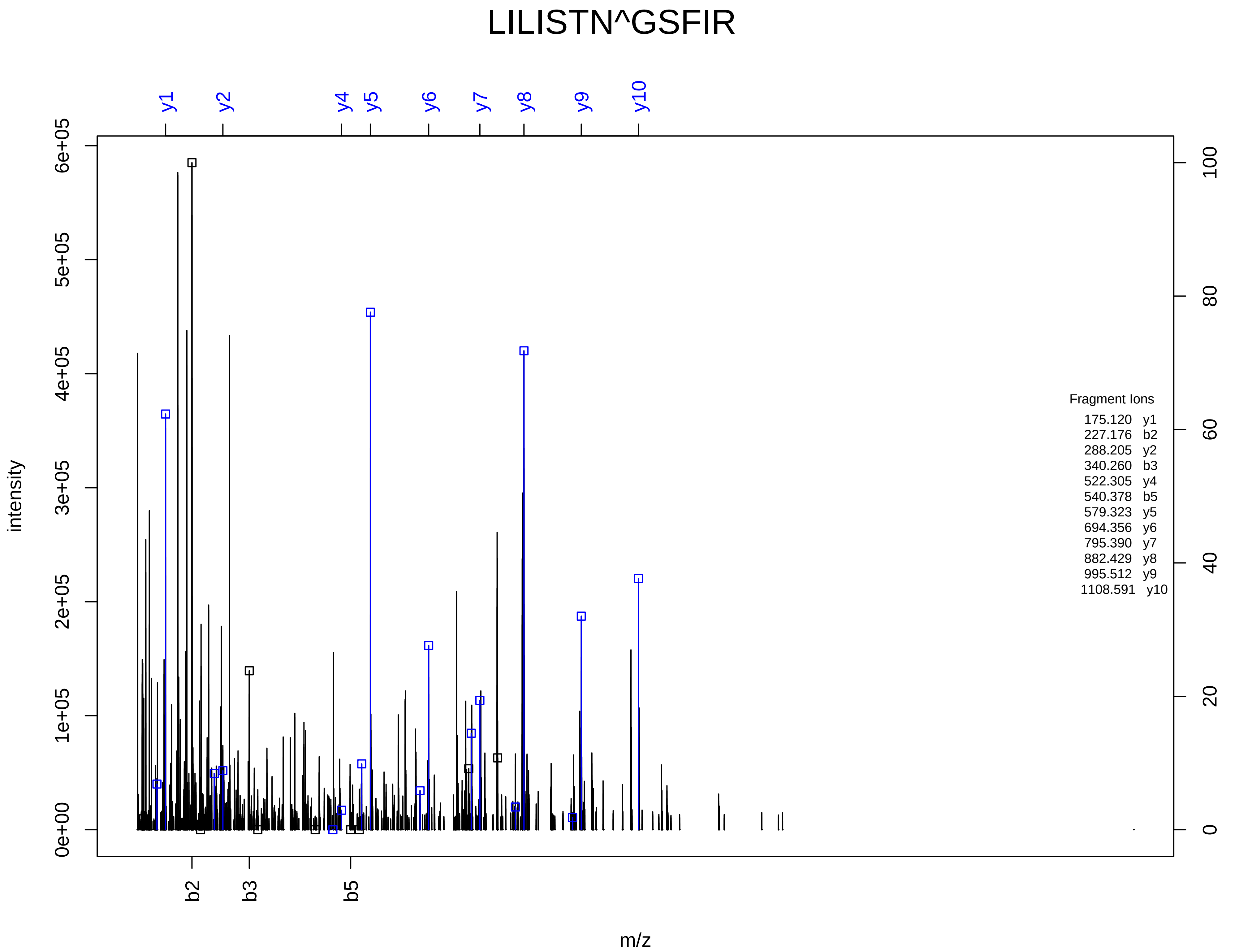

LILISTNGSFIR / File: christine_ip_08.raw; SpectrumID: ; scans: 16975

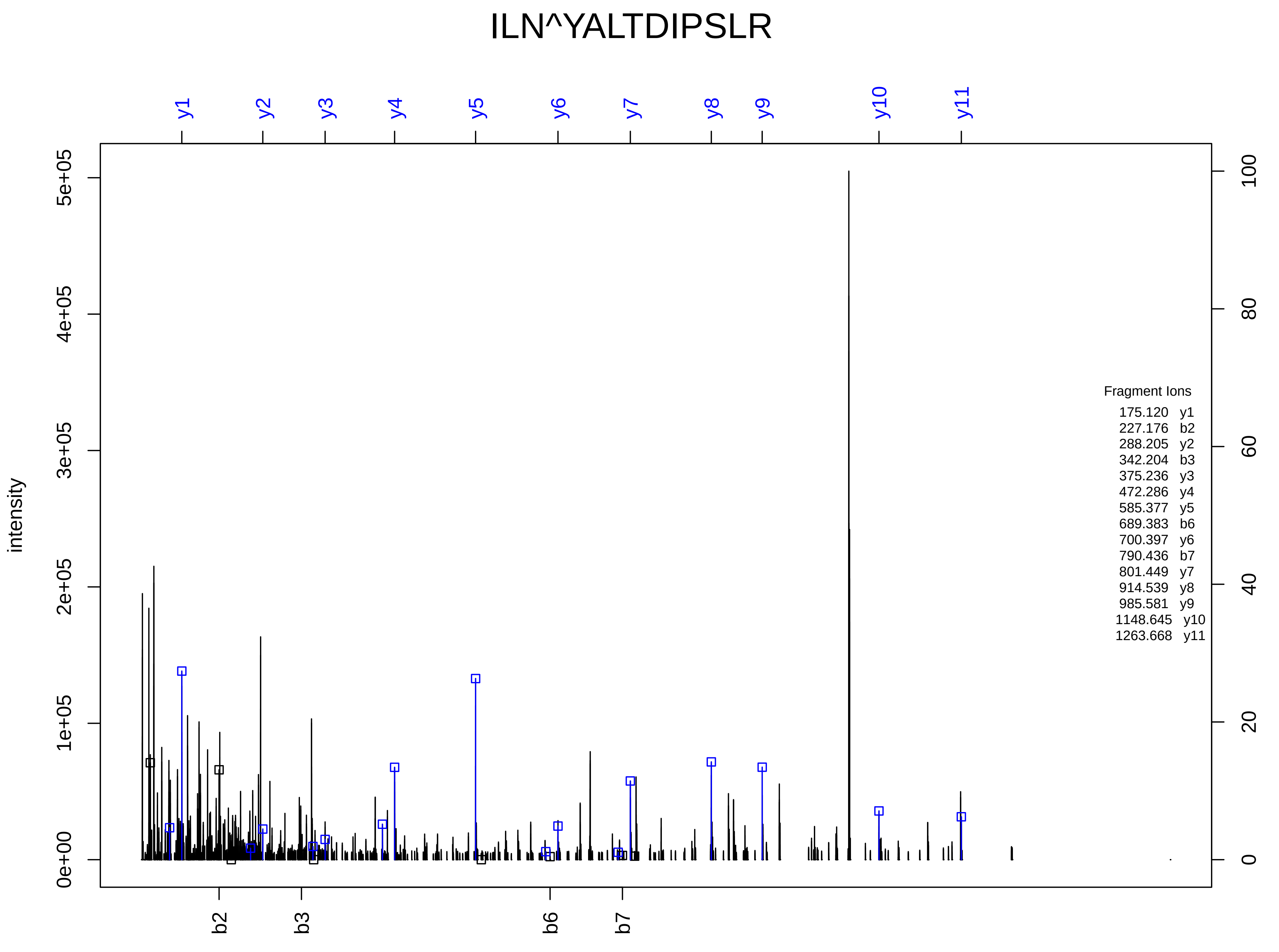

ILNYALTDIPSLR / File: christine_ip_08.raw; SpectrumID: ; scans: 17023

ESPGAAATSSSGPQAQQHR

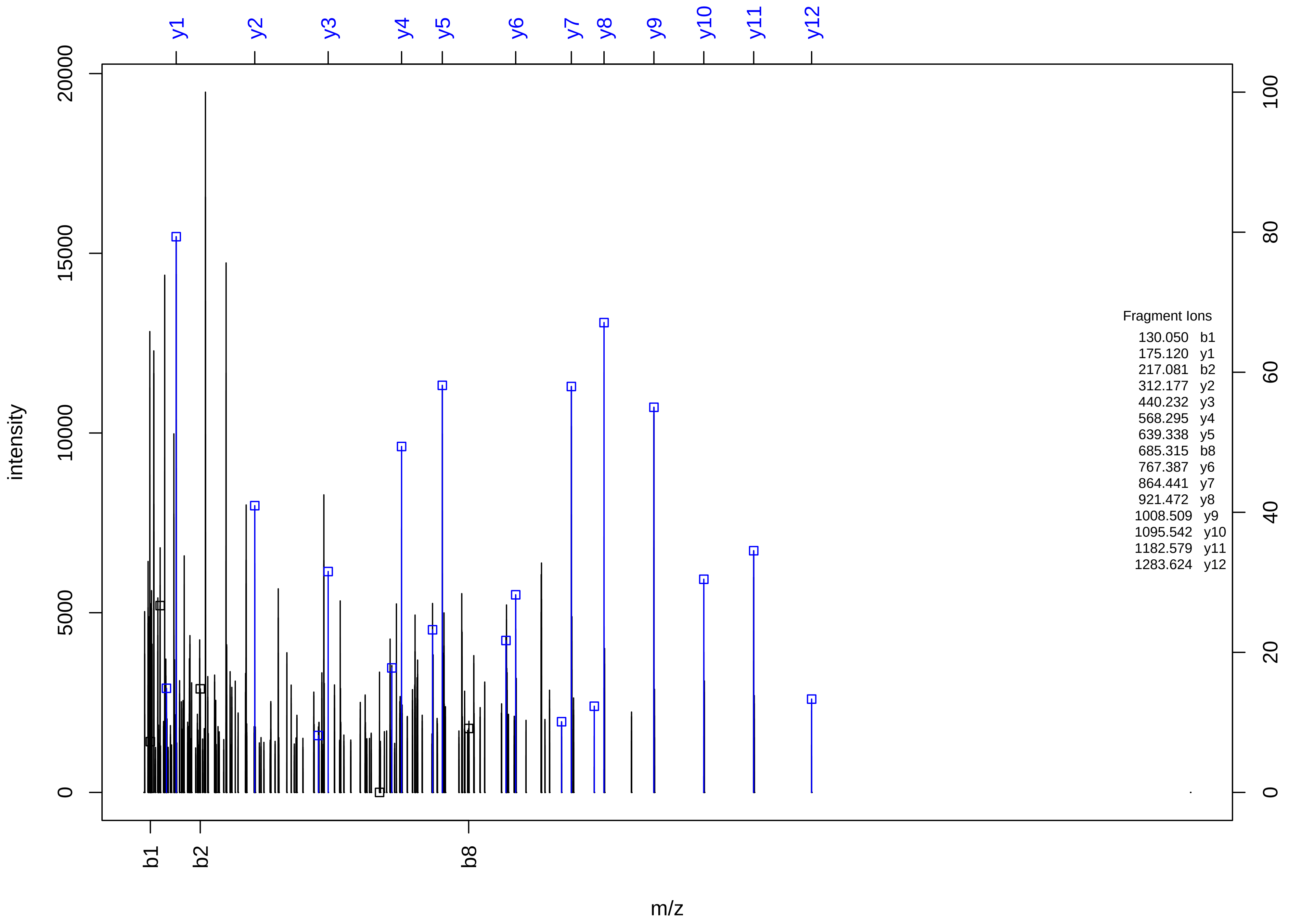

ESPGAAATSSSGPQAQQHR / File: christine_ip_08.raw; SpectrumID: ; scans: 1183

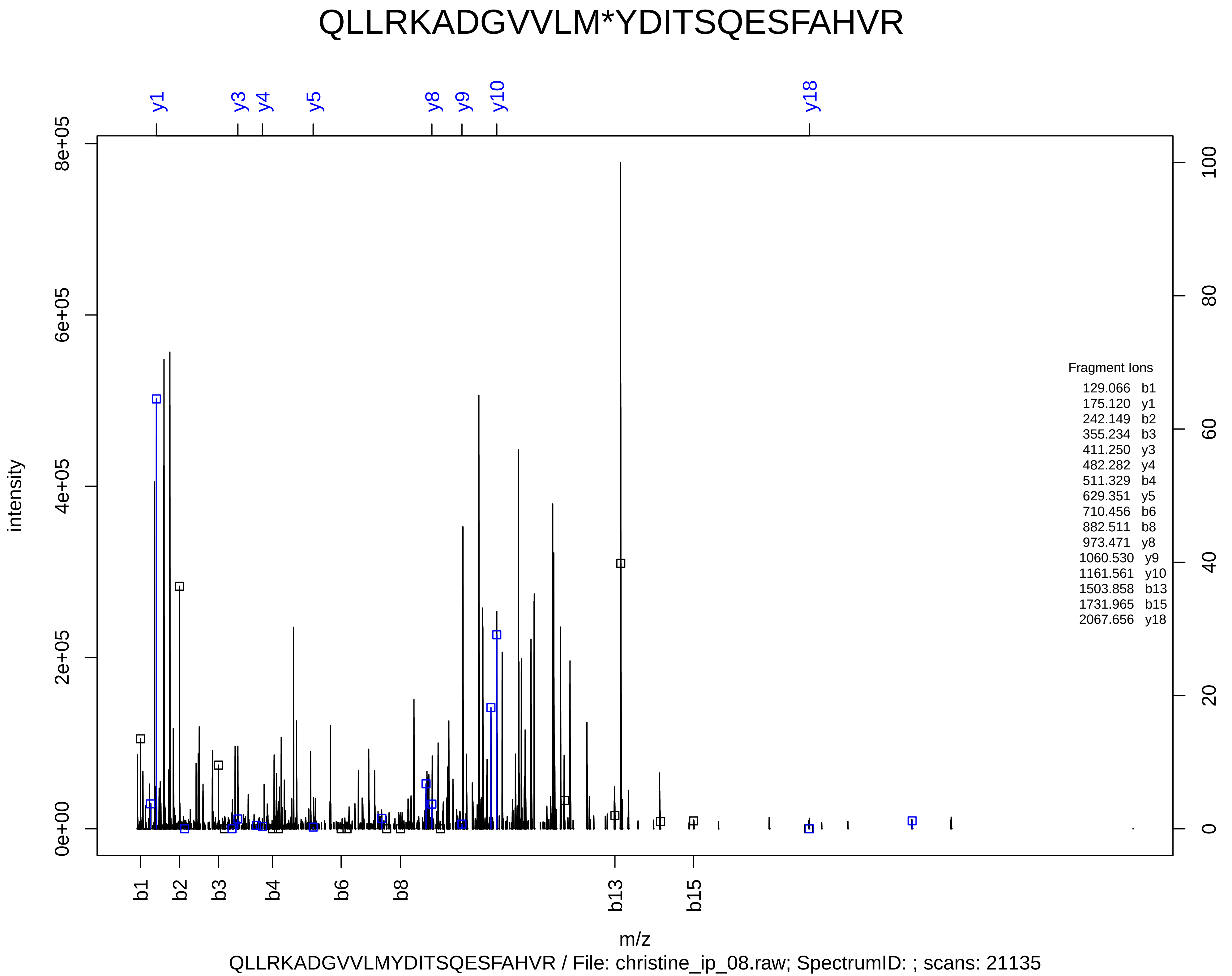
QLLRKADGVVLM*YDITSQESFAHVR
y1
y3
y4
y5
y8
y9
y10
y18
intensity
0e+00
2e+05
4e+05
6e+05
8e+05
0
20
40
60
80
100
Fragment Ions
129.066 b1
175.120 y1
242.149 b2
355.234 b3
411.250 y3
482.282 y4
511.329 b4
629.351 y5
710.456 b6
882.511 b8
973.471 y8
1060.530 y9
1161.561 y10
1503.858 b13
1731.965 b15
2067.656 y18
b1
b2
b3
b4
b6
b8
b13
b15
m/z
QLLRKADGVVLMYDITSQESFAHVR / File: christine_ip_08.raw; SpectrumID: ; scans: 21135

IFELTELR

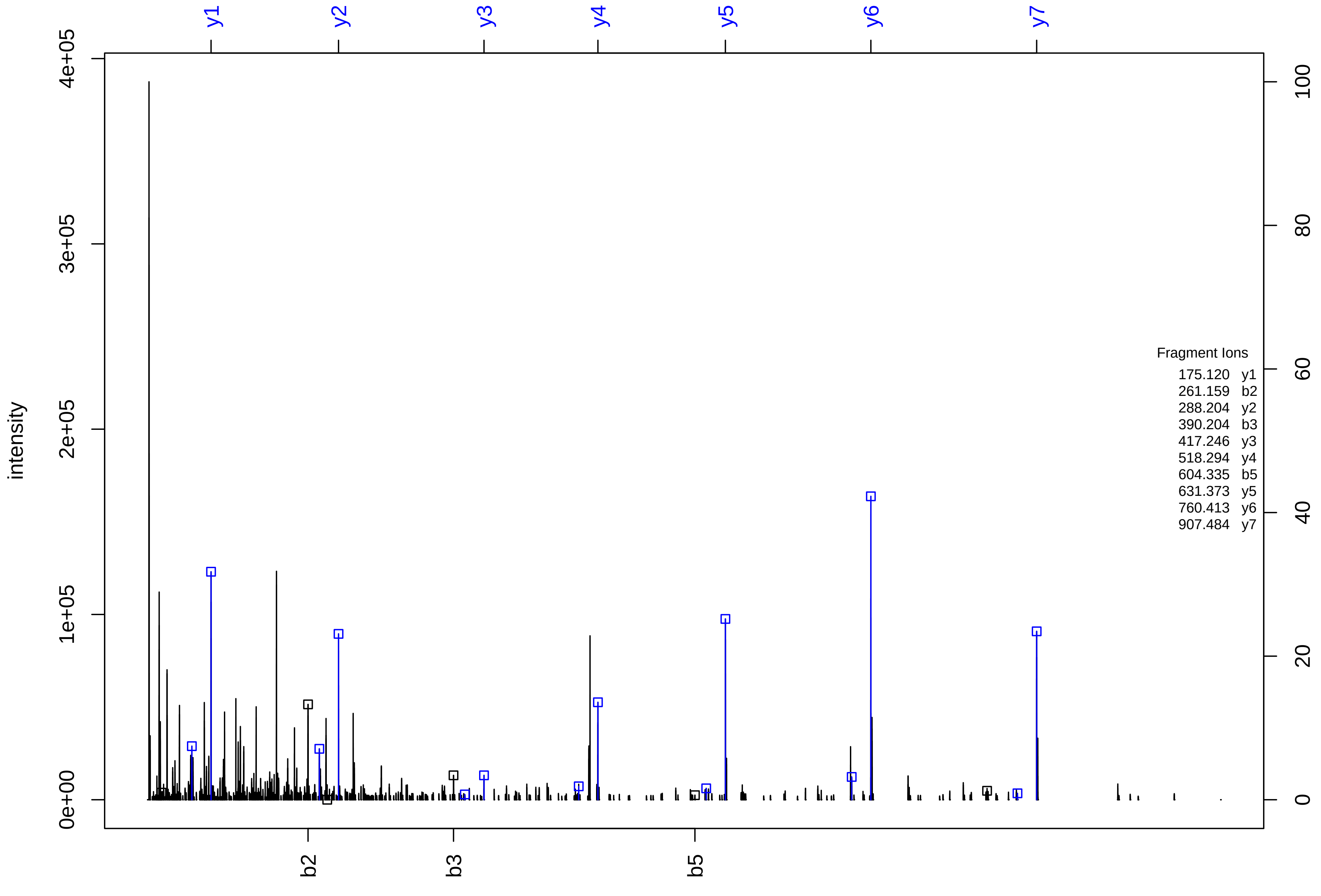

IFELTELR / File: christine_ip_08.raw; SpectrumID: ; scans: 13996

VADYCENNYIQATDK

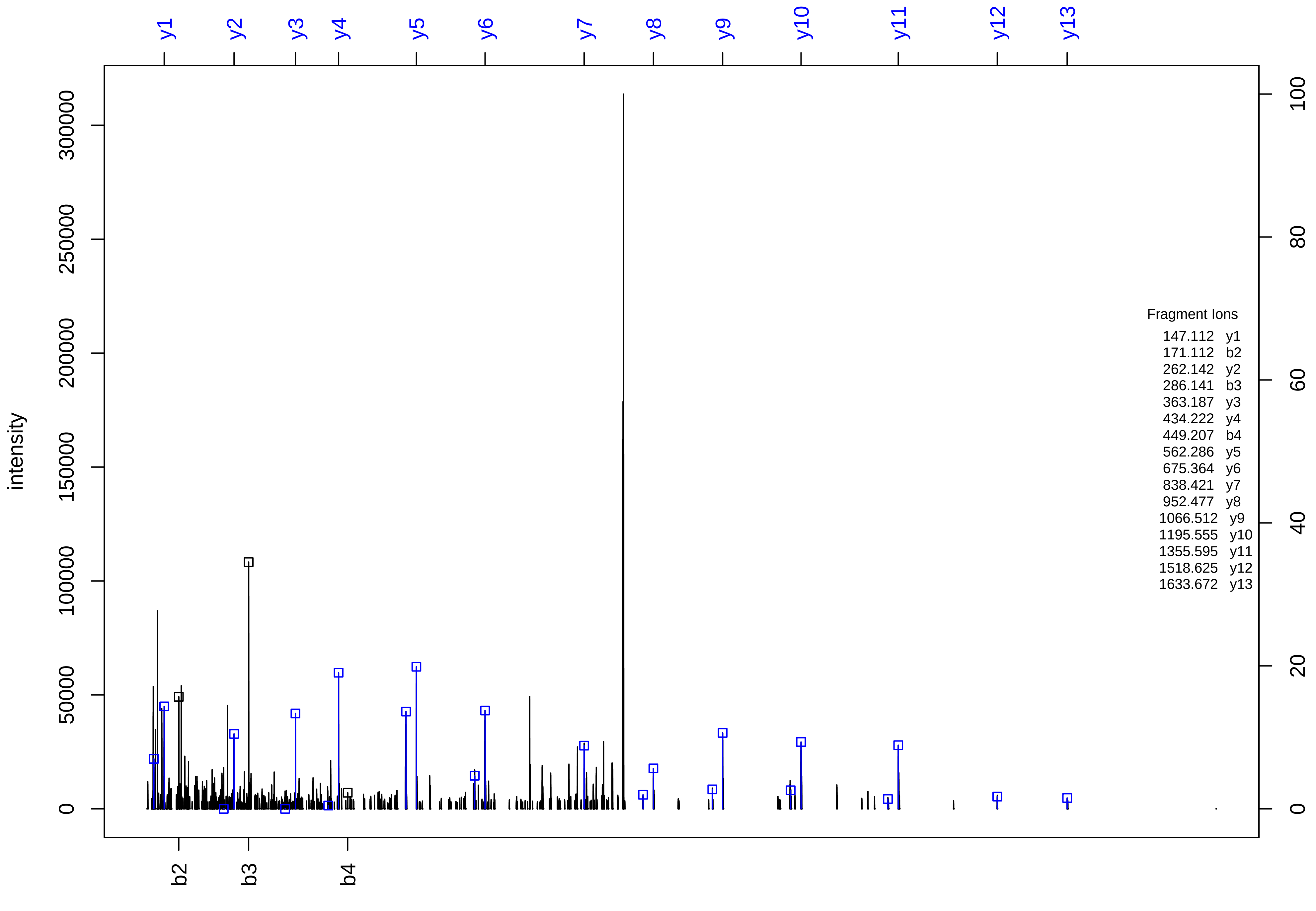

VADYCENNYIQATDK / File: christine_ip_08.raw; SpectrumID: ; scans: 6261

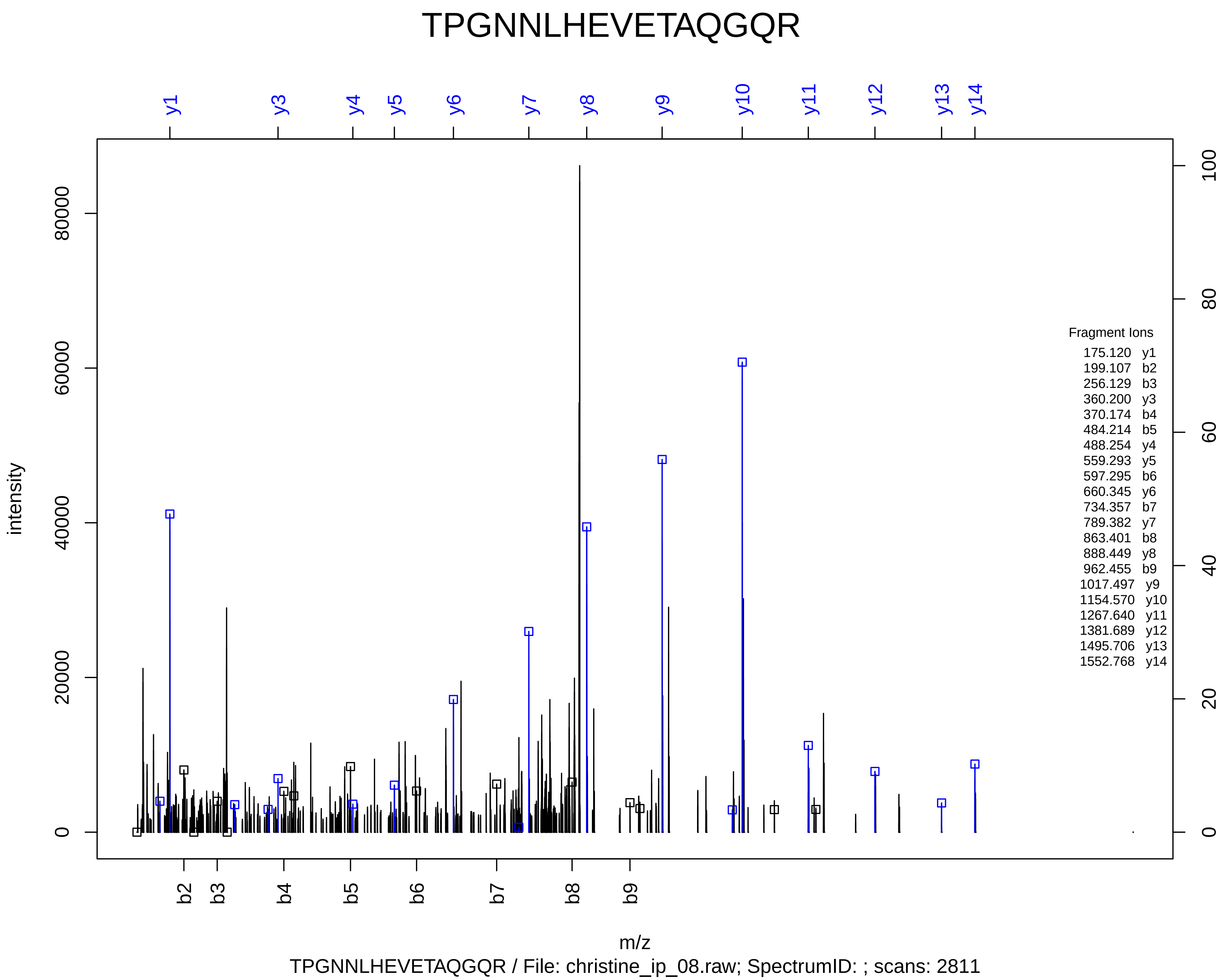
TPGNNLHEVETAQGQR
y1
y3
y4
y5
y6
y7
y8
y9
y10
y11
y12
y13
y14
intensity
0
20000
40000
60000
80000
0
20
40
60
80
100
b2
b3
b4
b5
b6
b7
b8
b9
m/z
Fragment Ions
175.120 y1
199.107 b2
256.129 b3
360.200 y3
370.174 b4
484.214 b5
488.254 y4
559.293 y5
597.295 b6
660.345 y6
734.357 b7
789.382 y7
863.401 b8
888.449 y8
962.455 b9
1017.497 y9
1154.570 y10
1267.640 y11
1381.689 y12
1495.706 y13
1552.768 y14
TPGNNLHEVETAQGQR / File: christine_ip_08.raw; SpectrumID: ; scans: 2811

DPESLLVPAPLSGPR

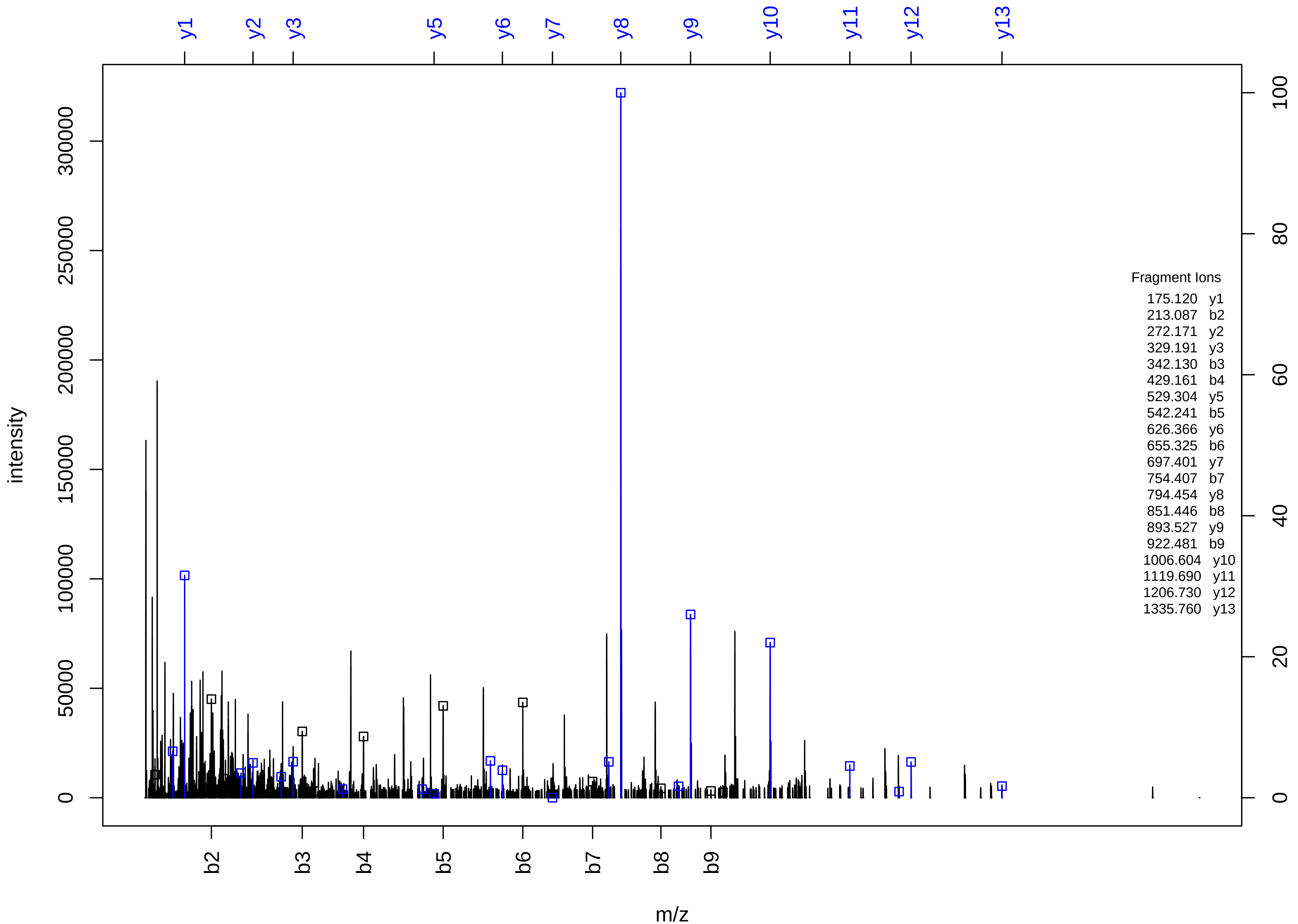

DPESLLVPAPLSGPR / File: christine_ip_08.raw; SpectrumID: ; scans: 16612

KEEEDLEALIAHFQTLDAK

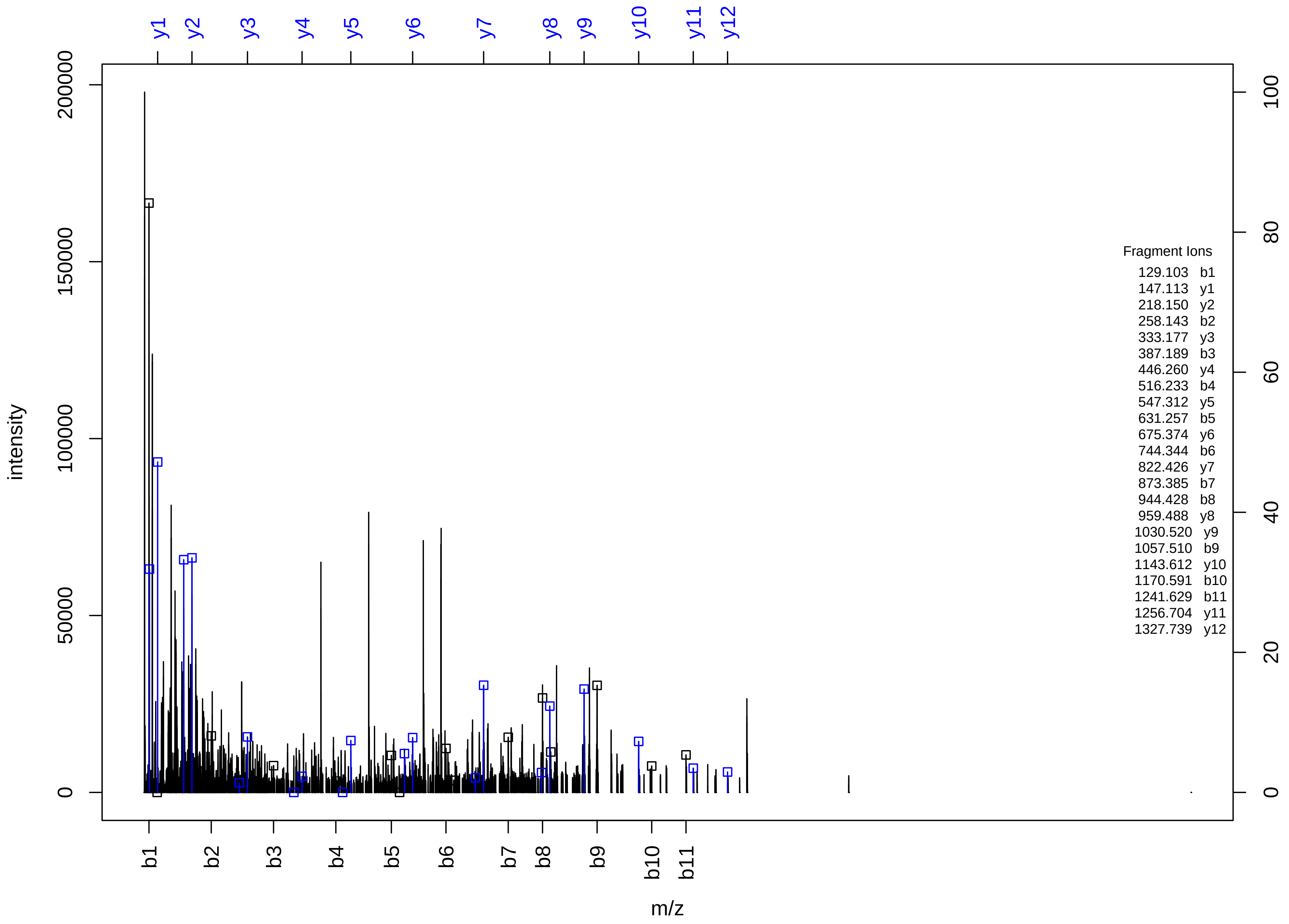

KEEEDLEALIAHFQTLDAK / File: christine_ip_08.raw; SpectrumID: ; scans: 17862

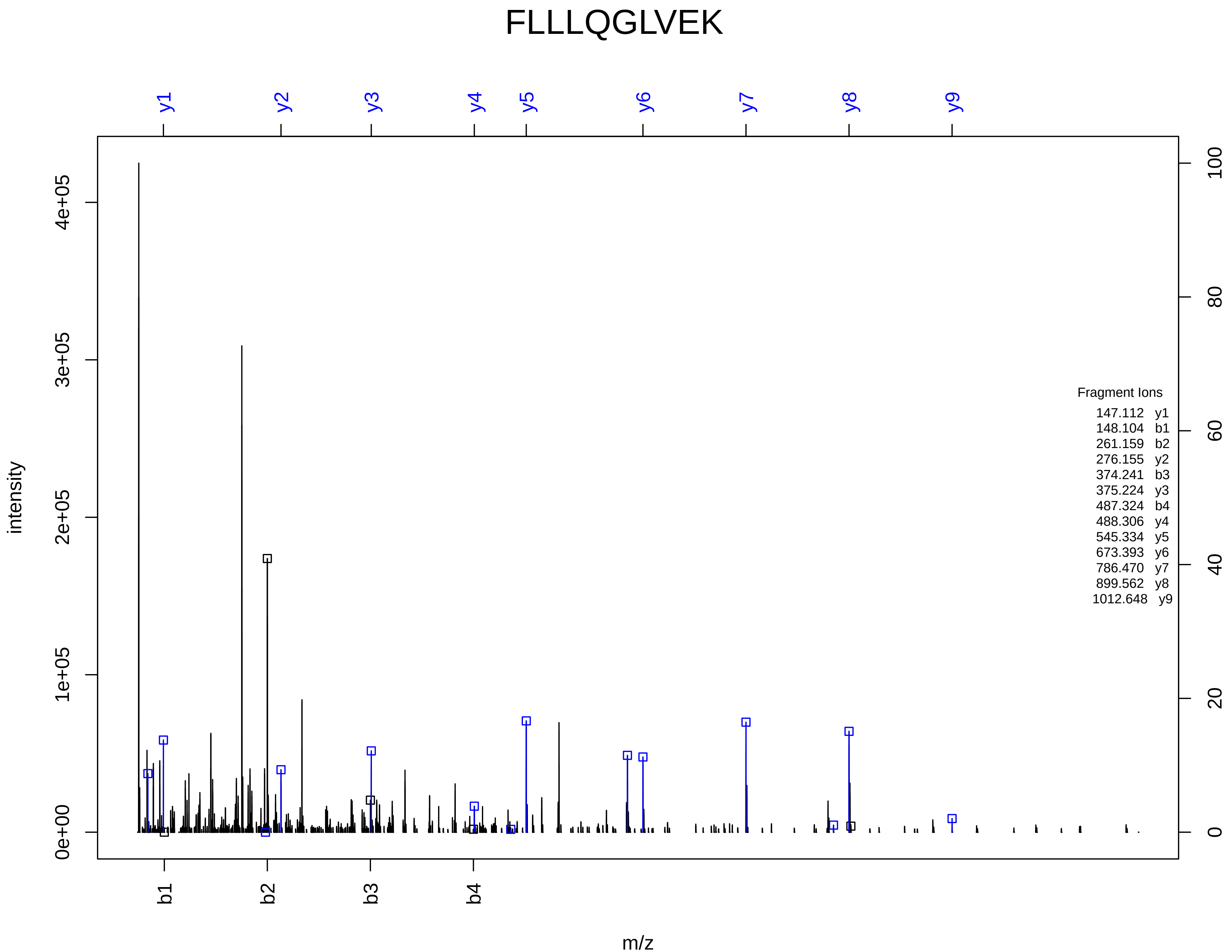

FLLLQGLVEK / File: christine_ip_08.raw; SpectrumID: ; scans: 18441

DIDPQNDLTFLR

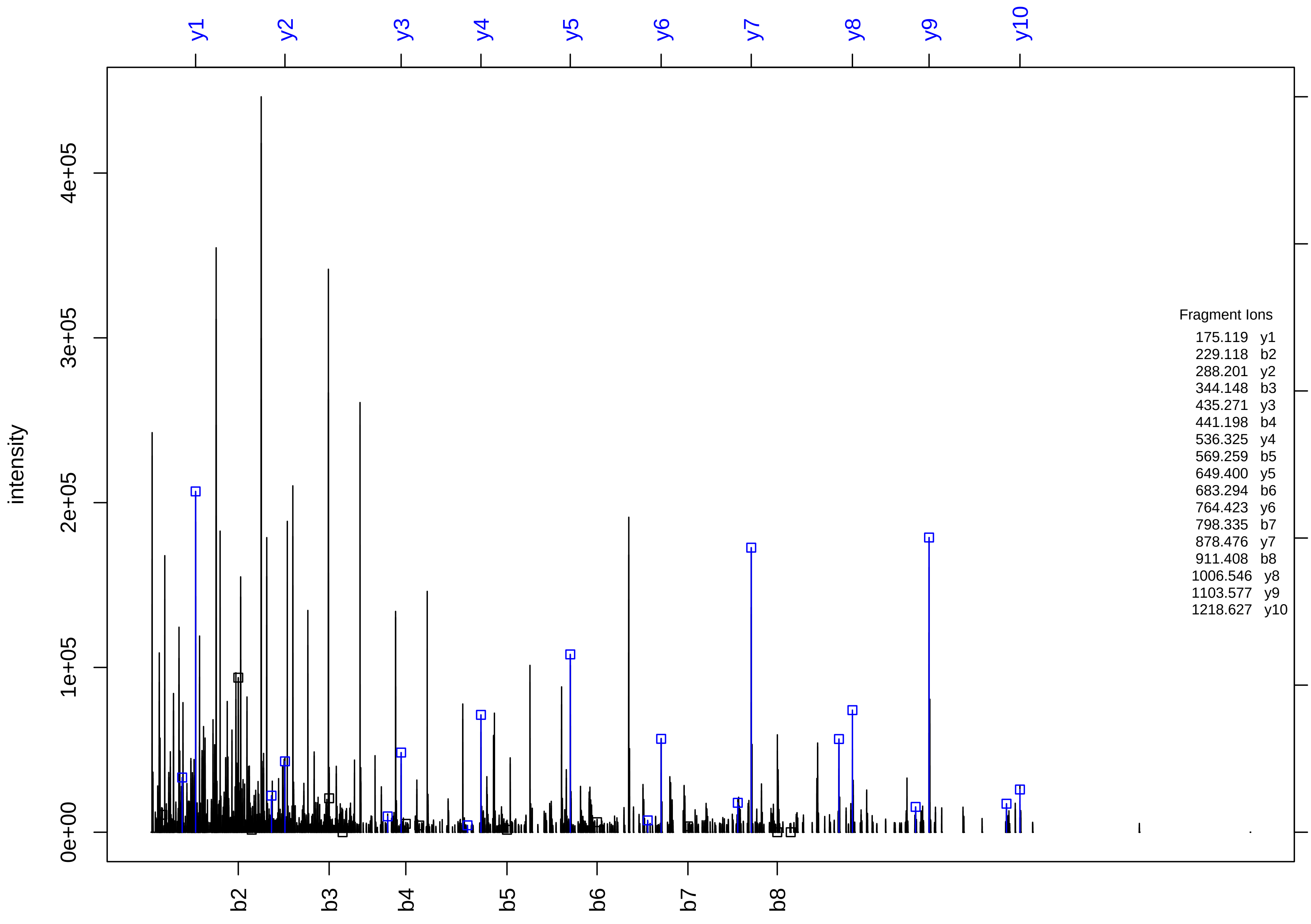

DIDPQNDLTFLR / File: christine_ip_08.raw; SpectrumID: ; scans: 15859

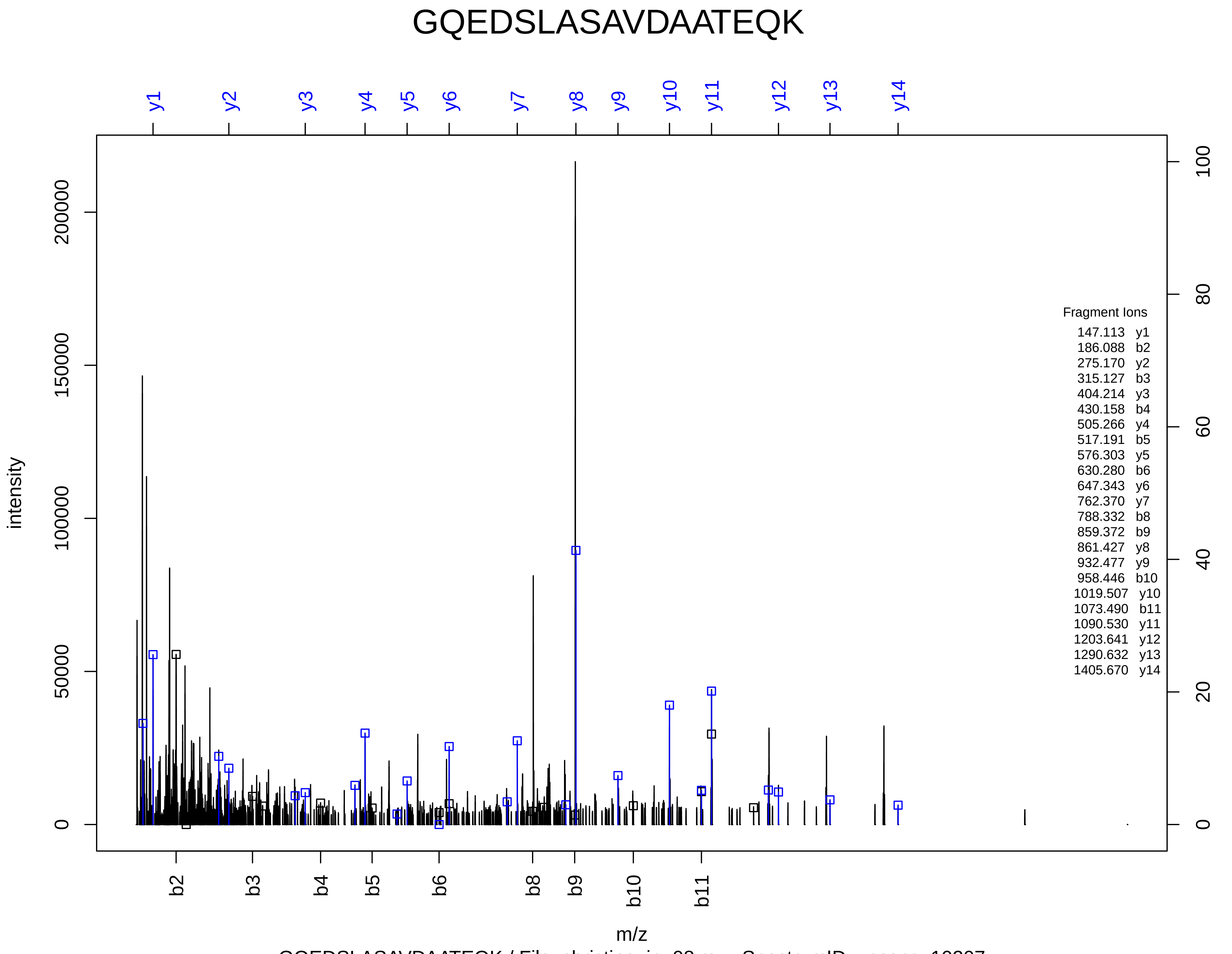

GQEDSLASAVDAATEQK / File: christine_ip_08.raw; SpectrumID: ; scans: 10207

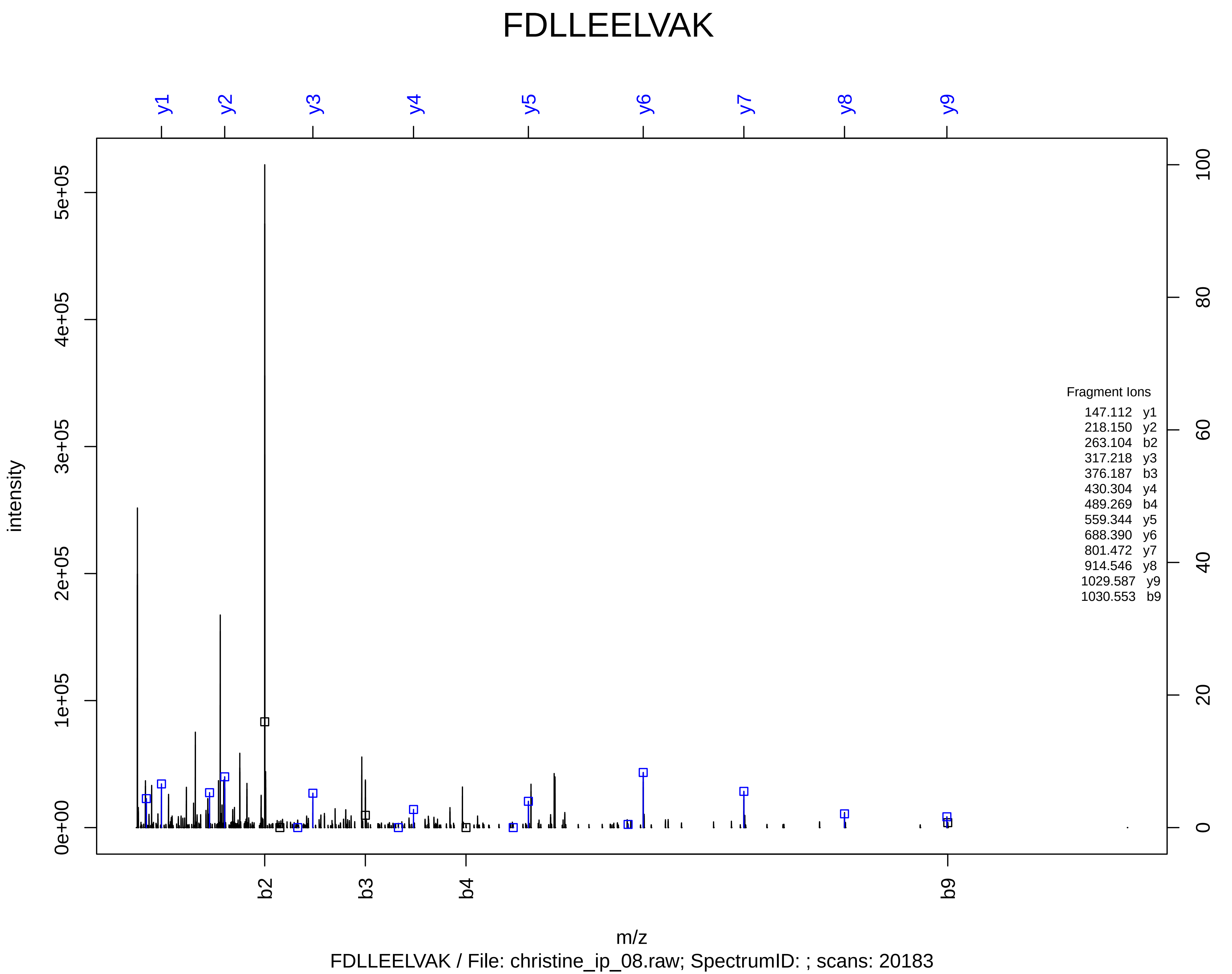

FDLLEELVAK
y1
y2
y3
y4
y5
y6
y7
y8
y9
intensity
0e+00
1e+05
2e+05
3e+05
4e+05
5e+05
0
20
40
60
80
100
Fragment Ions
147.112 y1
218.150 y2
263.104 b2
317.218 y3
376.187 b3
430.304 y4
489.269 b4
559.344 y5
688.390 y6
801.472 y7
914.546 y8
1029.587 y9
1030.553 b9
b2
b3
b4
b9
m/z
FDLLEELVAK / File: christine_ip_08.raw; SpectrumID: ; scans: 20183

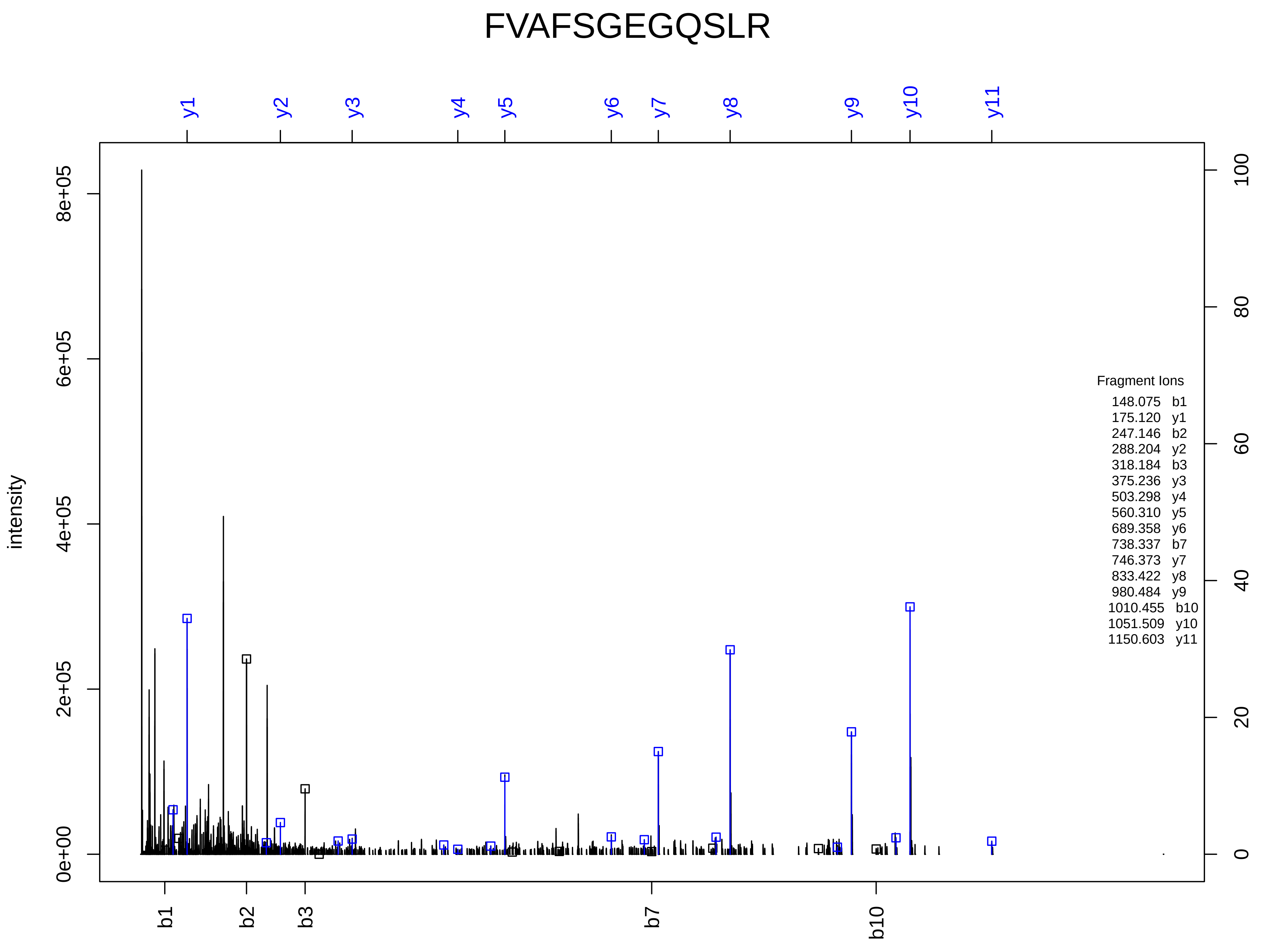

FVAFSGEGQSLR / File: christine_ip_08.raw; SpectrumID: ; scans: 9109

TPASGLQTPTSTPAPGSATR

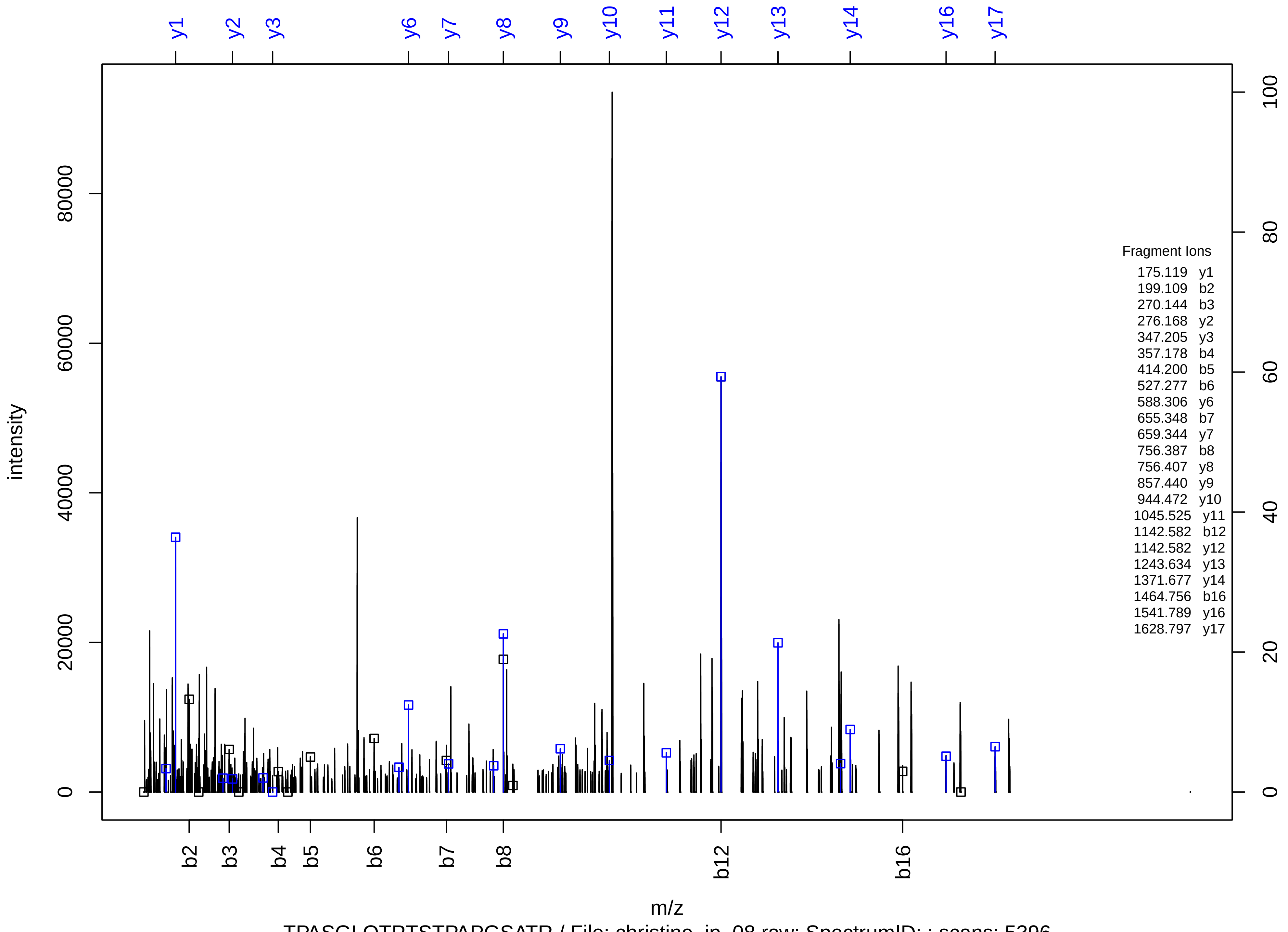

TPASGLQTPTSTPAPGSATR / File: christine_ip_08.raw; SpectrumID: ; scans: 5396

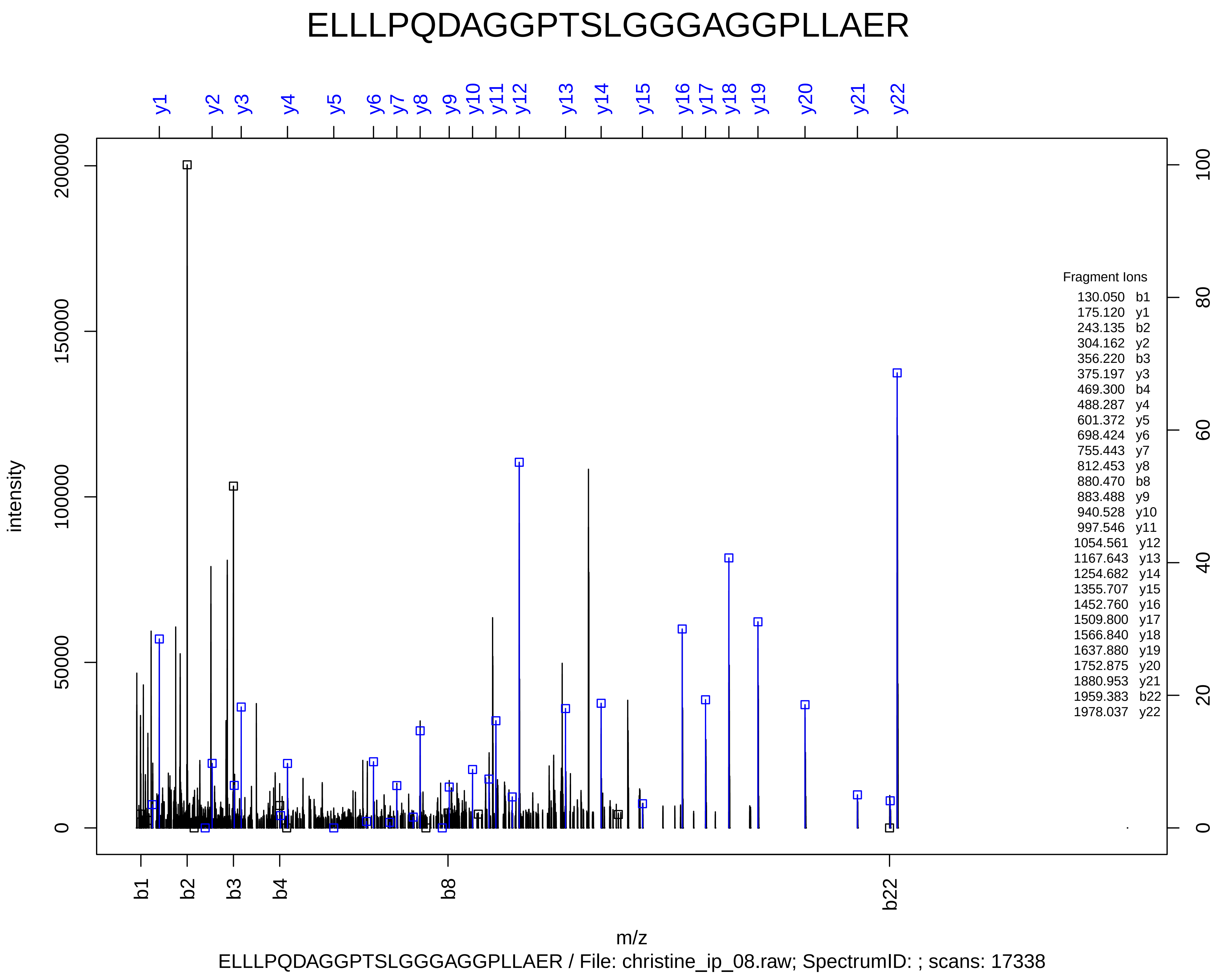
ELLLPQDAGGPTSLGGGAGGPLLAER
y1 y2 y3 y4 y5 y6 y7 y8 y9 y10 y11 y12 y13 y14 y15 y16 y17 y18 y19 y20 y21 y22
b1 b2 b3 b4 b8 b22
intensity
m/z
Fragment Ions
130.050 b1
175.120 y1
243.135 b2
304.162 y2
356.220 b3
375.197 y3
469.300 b4
488.287 y4
601.372 y5
698.424 y6
755.443 y7
812.453 y8
880.470 b8
883.488 y9
940.528 y10
997.546 y11
1054.561 y12
1167.643 y13
1254.682 y14
1355.707 y15
1452.760 y16
1509.800 y17
1566.840 y18
1637.880 y19
1752.875 y20
1880.953 y21
1959.383 b22
1978.037 y22
ELLLPQDAGGPTSLGGGAGGPLLAER / File: christine_ip_08.raw; SpectrumID: ; scans: 17338

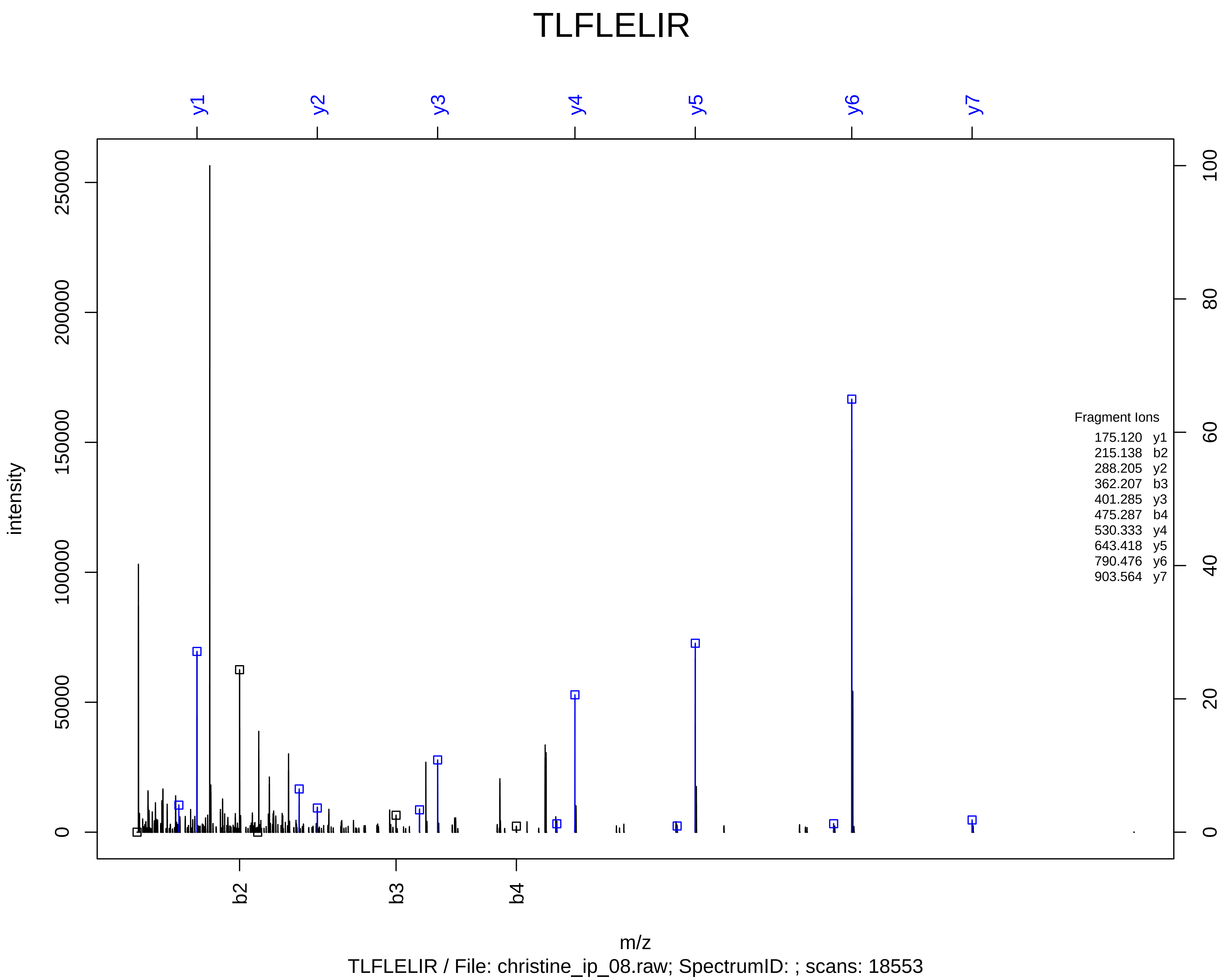
TLFLELIR
y1
y2
y3
y4
y5
y6
y7
intensity
250000
200000
150000
100000
50000
0
100
80
60
40
20
0
Fragment Ions
175.120 y1
215.138 b2
288.205 y2
362.207 b3
401.285 y3
475.287 b4
530.333 y4
643.418 y5
790.476 y6
903.564 y7
b2
b3
b4
m/z
TLFLELIR / File: christine_ip_08.raw; SpectrumID: ; scans: 18553

IASLLGLLSK

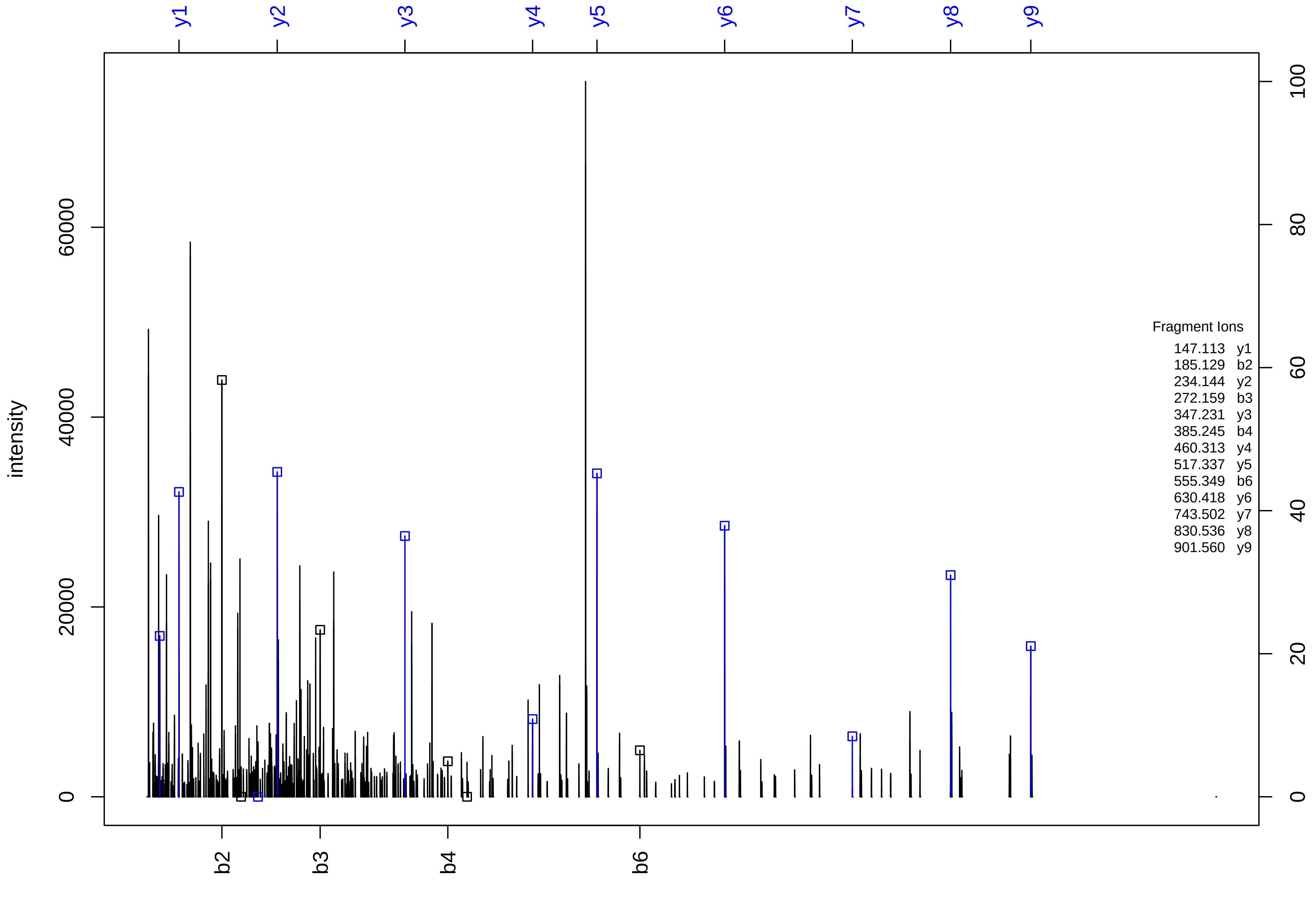

IASLLGLLSK / File: christine_ip_08.raw; SpectrumID: ; scans: 18528

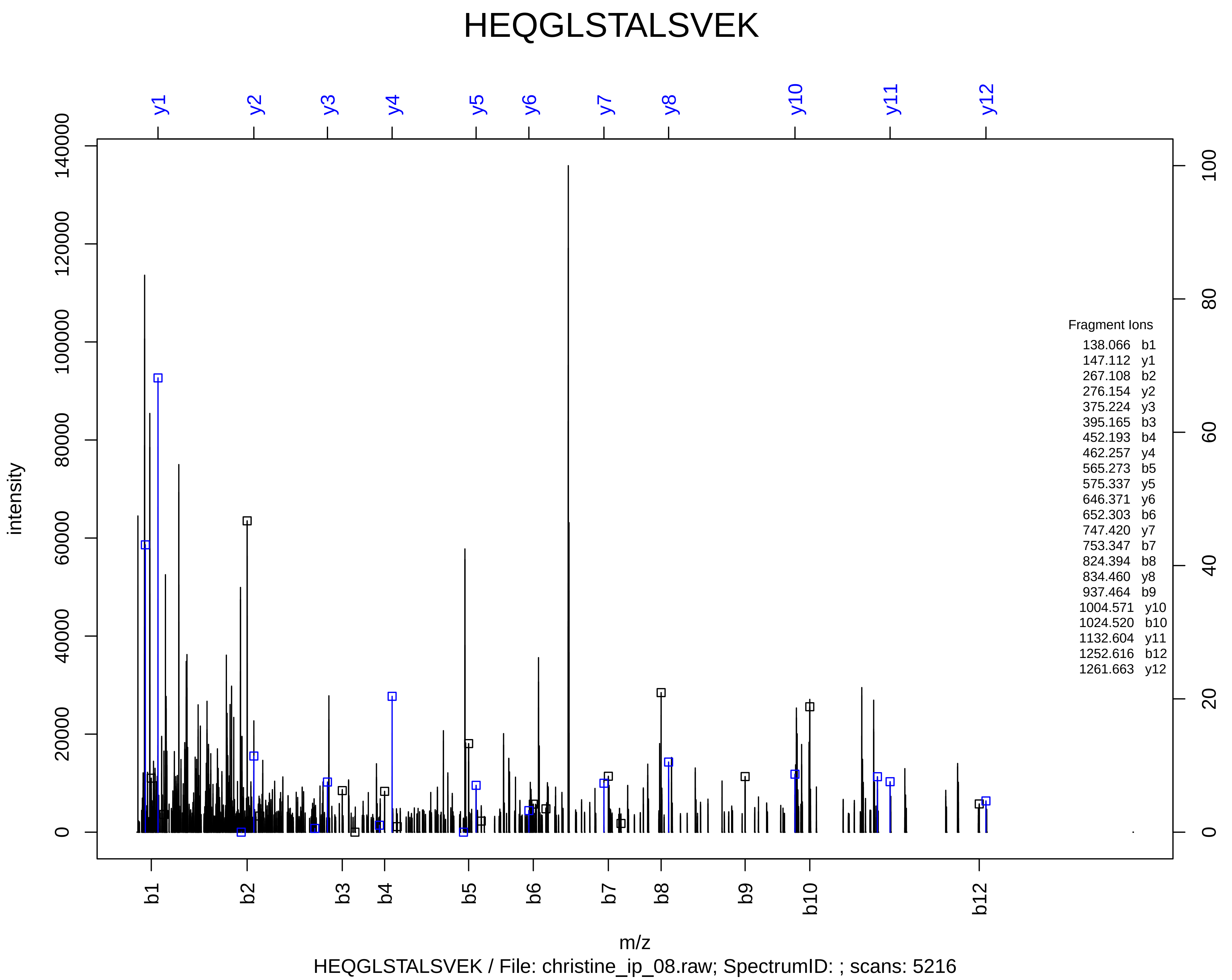
HEQGLSTALSVEK
intensity
m/z
y1 y2 y3 y4 y5 y6 y7 y8 y10 y11 y12
b1 b2 b3 b4 b5 b6 b7 b8 b9 b10 b12
Fragment Ions
138.066 b1
147.112 y1
267.108 b2
276.154 y2
375.224 y3
395.165 b3
452.193 b4
462.257 y4
565.273 b5
575.337 y5
646.371 y6
652.303 b6
747.420 y7
753.347 b7
824.394 b8
834.460 y8
937.464 b9
1004.571 y10
1024.520 b10
1132.604 y11
1252.616 b12
1261.663 y12
HEQGLSTALSVEK / File: christine_ip_08.raw; SpectrumID: ; scans: 5216

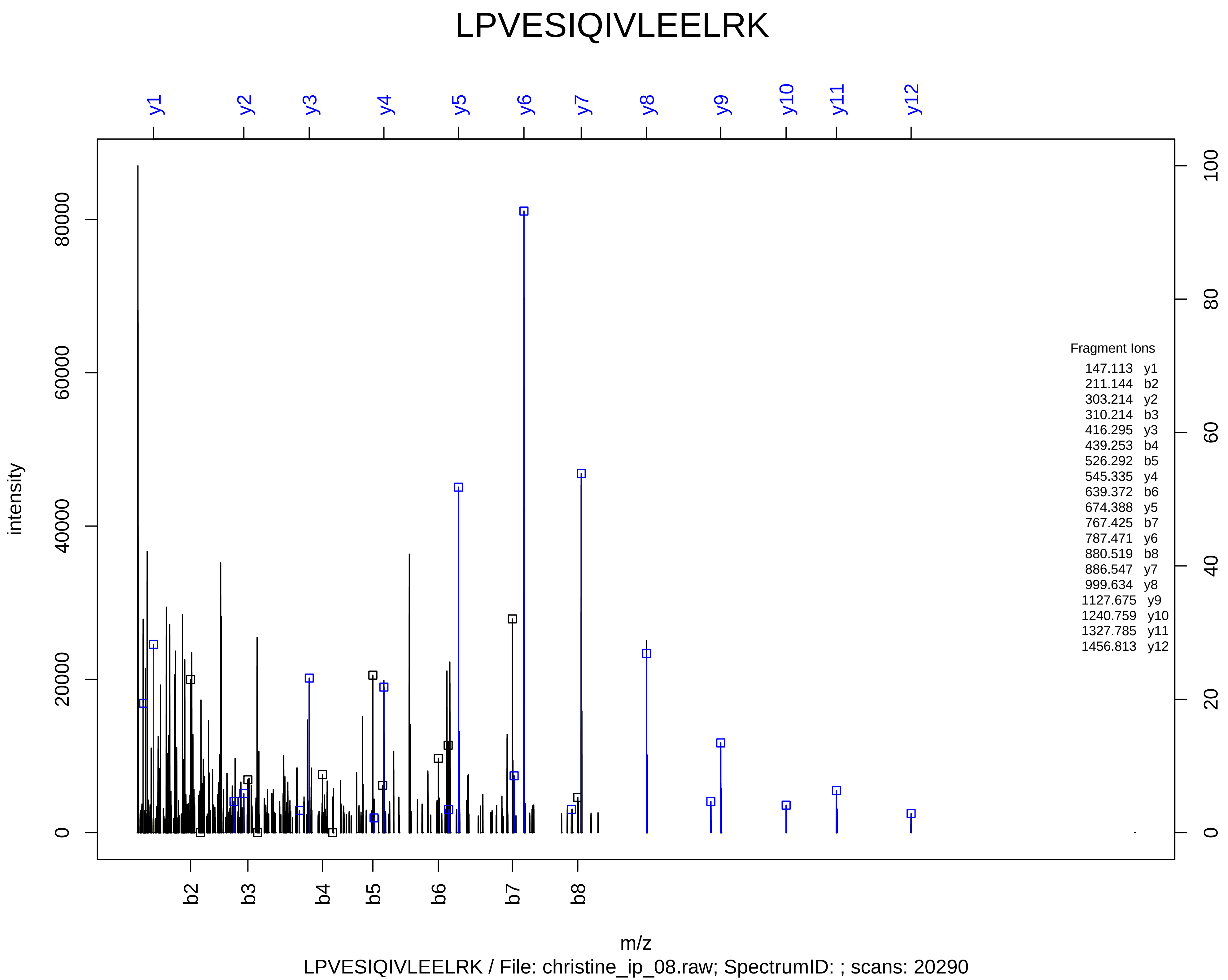

LPVESIQIVLEELRK
y1
y2
y3
y4
y5
y6
y7
y8
y9
y10
y11
y12
b2
b3
b4
b5
b6
b7
b8
intensity
m/z
Fragment Ions
147.113 y1
211.144 b2
303.214 y2
310.214 b3
416.295 y3
439.253 b4
526.292 b5
545.335 y4
639.372 b6
674.388 y5
767.425 b7
787.471 y6
880.519 b8
886.547 y7
999.634 y8
1127.675 y9
1240.759 y10
1327.785 y11
1456.813 y12
LPVESIQIVLEELRK / File: christine_ip_08.raw; SpectrumID: ; scans: 20290

DSLTDLYVQHAIPLPQR

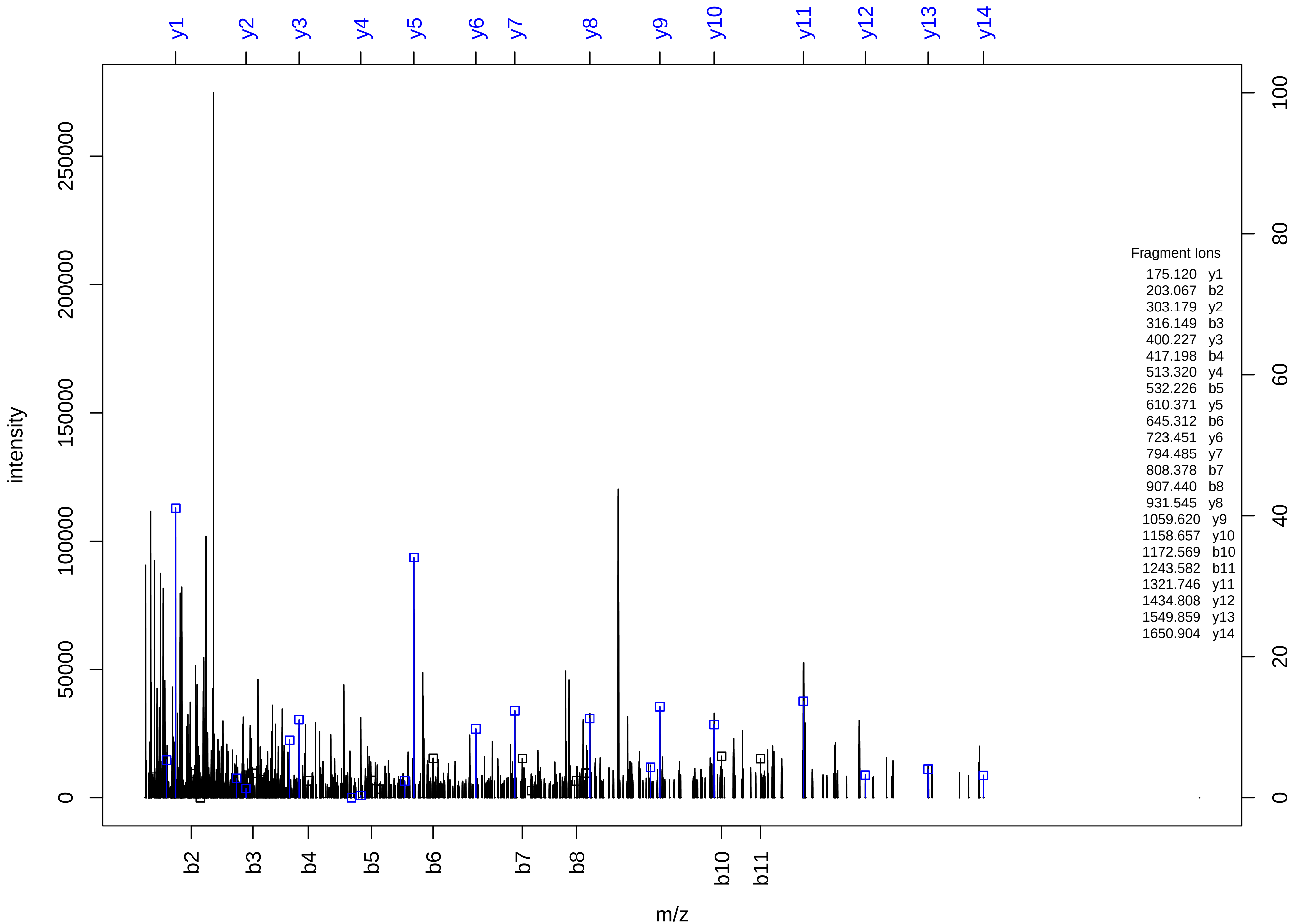

DSLTDLYVQHAIPLPQR / File: christine_ip_08.raw; SpectrumID: ; scans: 15584

ETVQNEVYLGLPIFR

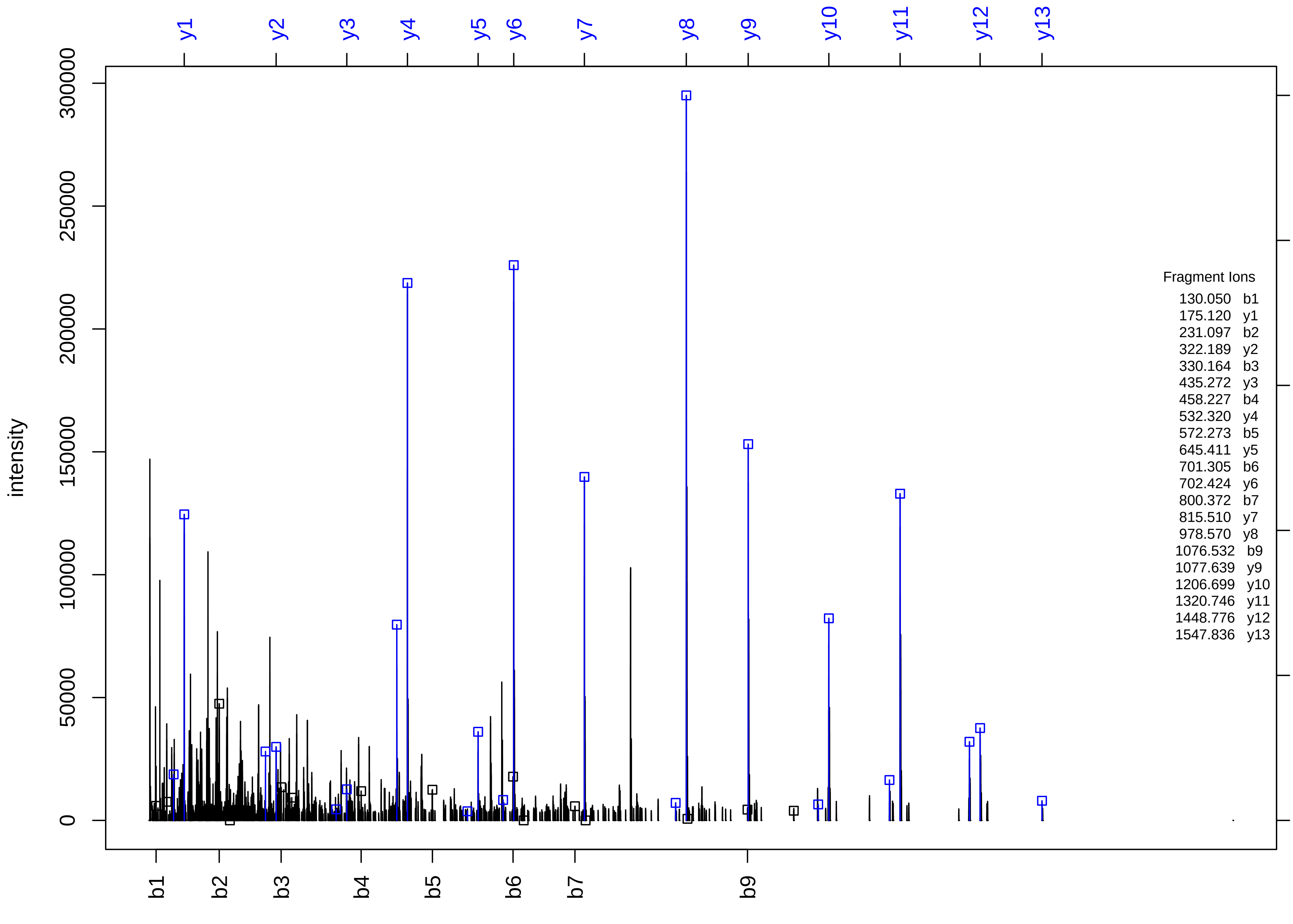

ETVQNEVYLGLPIFR / File: christine_ip_08.raw; SpectrumID: ; scans: 20443

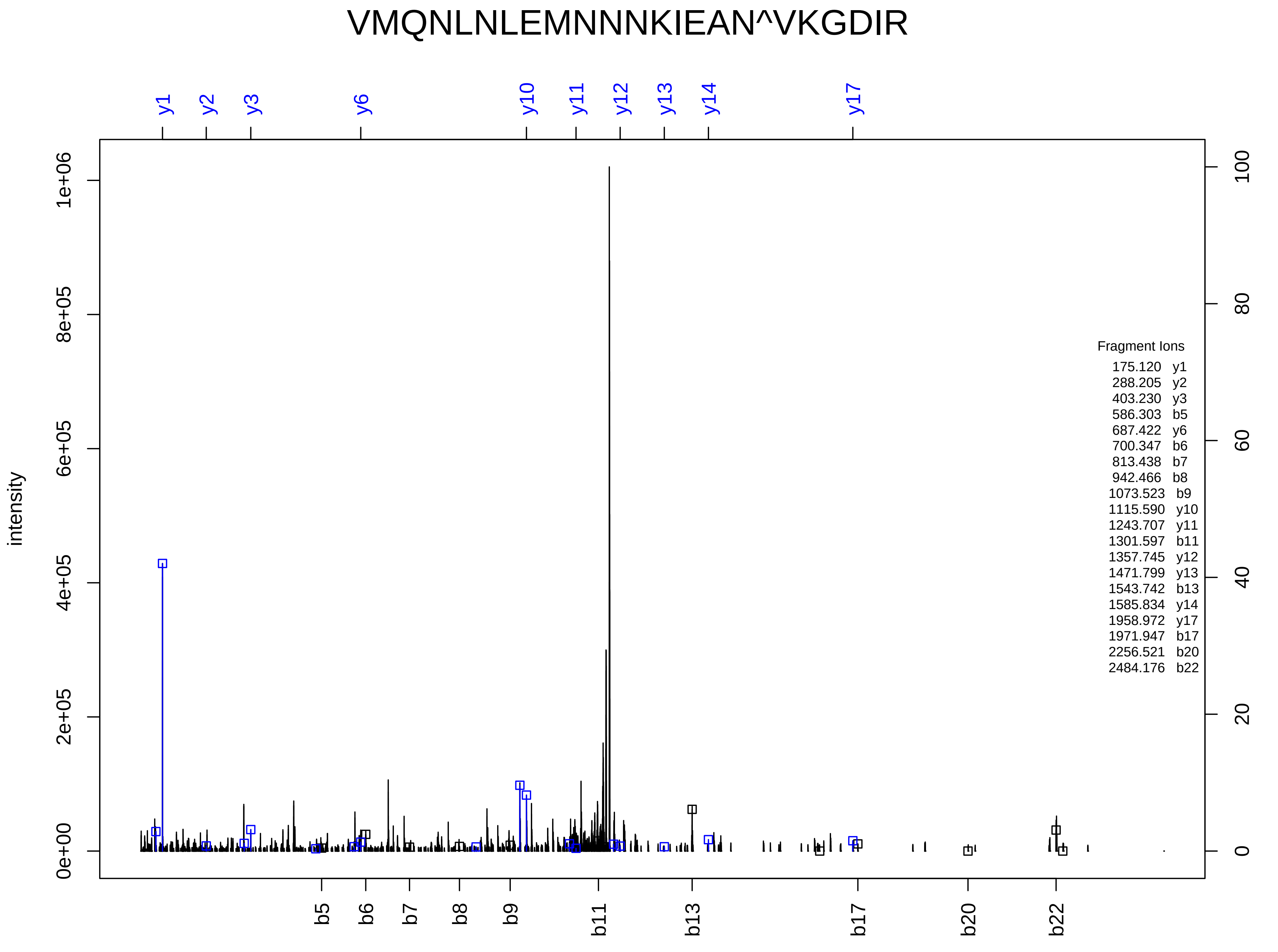

VMQNLNLEMNNNKIEANVKGDIR / File: christine_ip_08.raw; SpectrumID: ; scans: 17900

LLAIDN^IRLDN^CPM*EDAVQILR

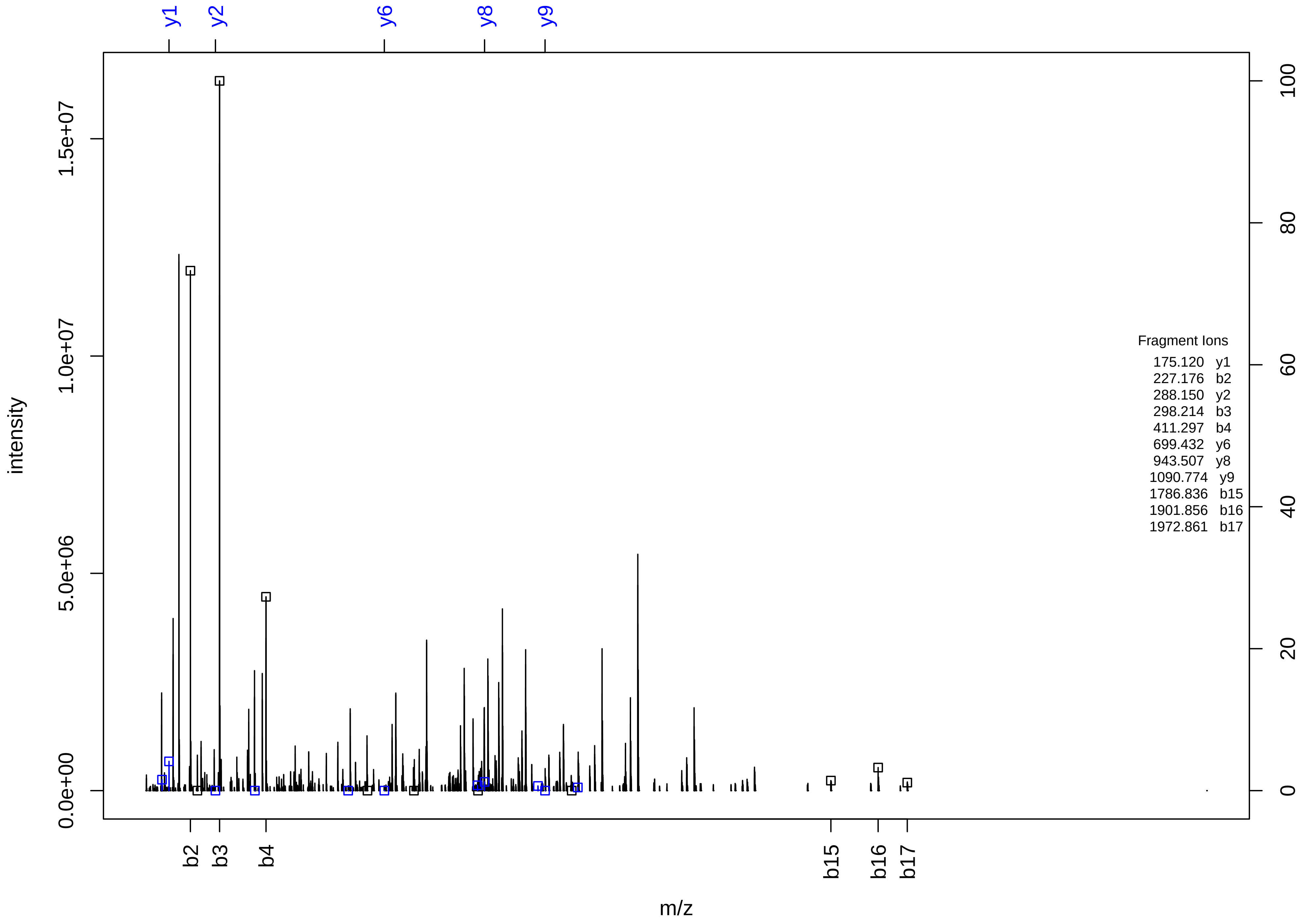

LLAIDNIRLDNCPMEDAVQILR / File: christine_ip_08.raw; SpectrumID: ; scans: 14695

APAPAPPPSGSTSCGDR

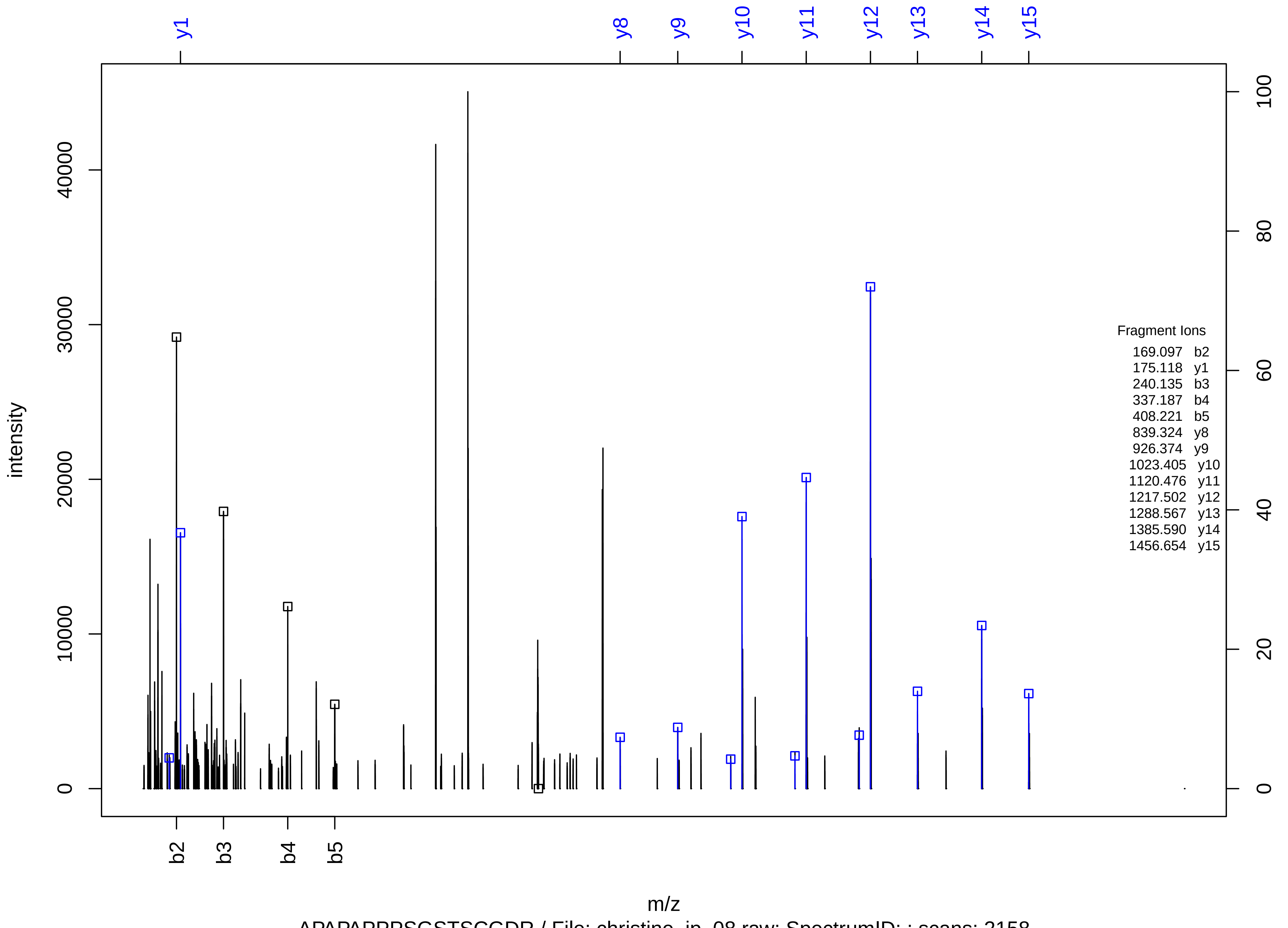

APAPAPPPSGSTSCGDR / File: christine_ip_08.raw; SpectrumID: ; scans: 2158

ALLSN^IEKYKPN^M*IK

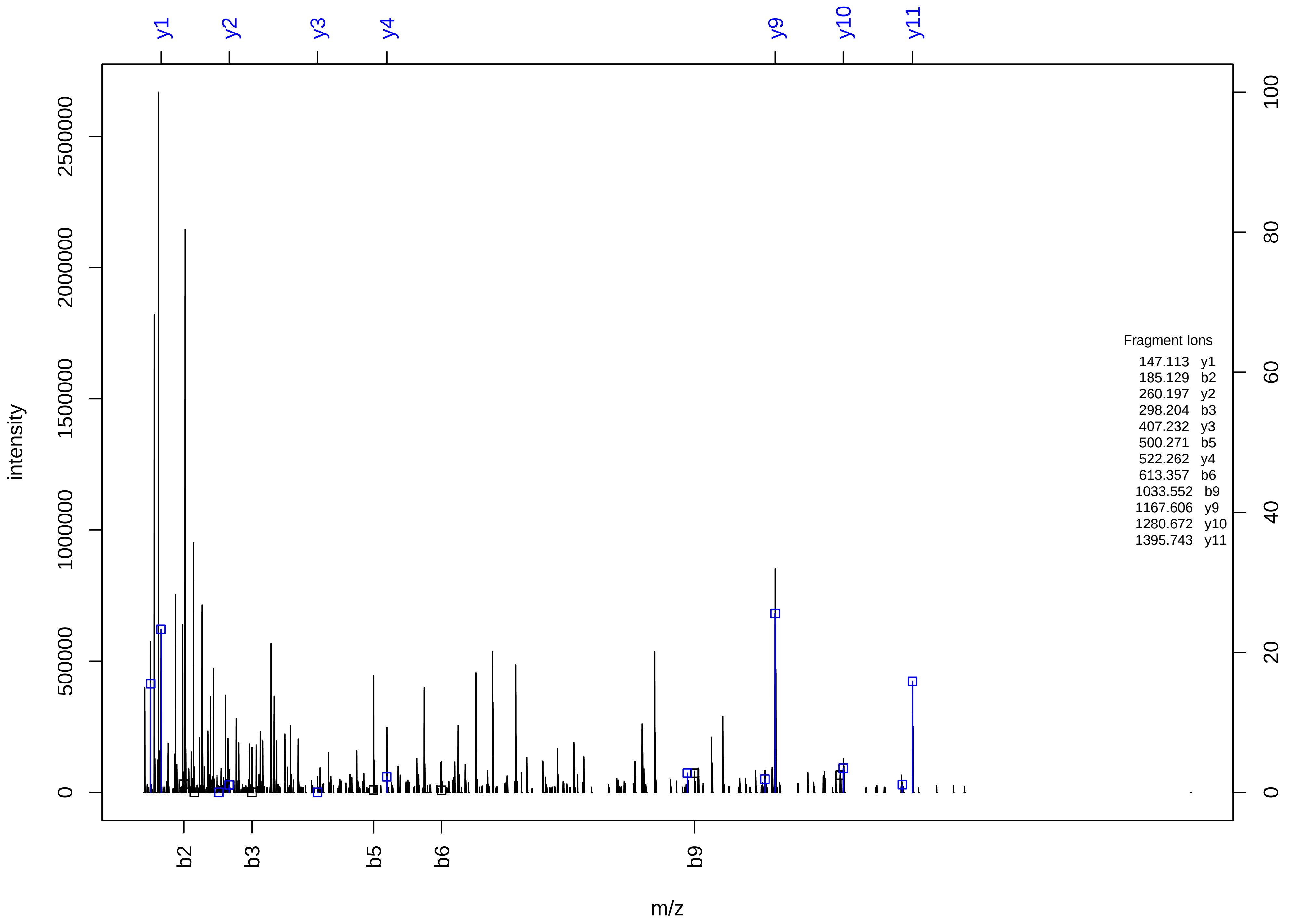

ALLSNIEKYKPNMIK / File: christine_ip_08.raw; SpectrumID: ; scans: 17808

NFVLPEEIIQEVR

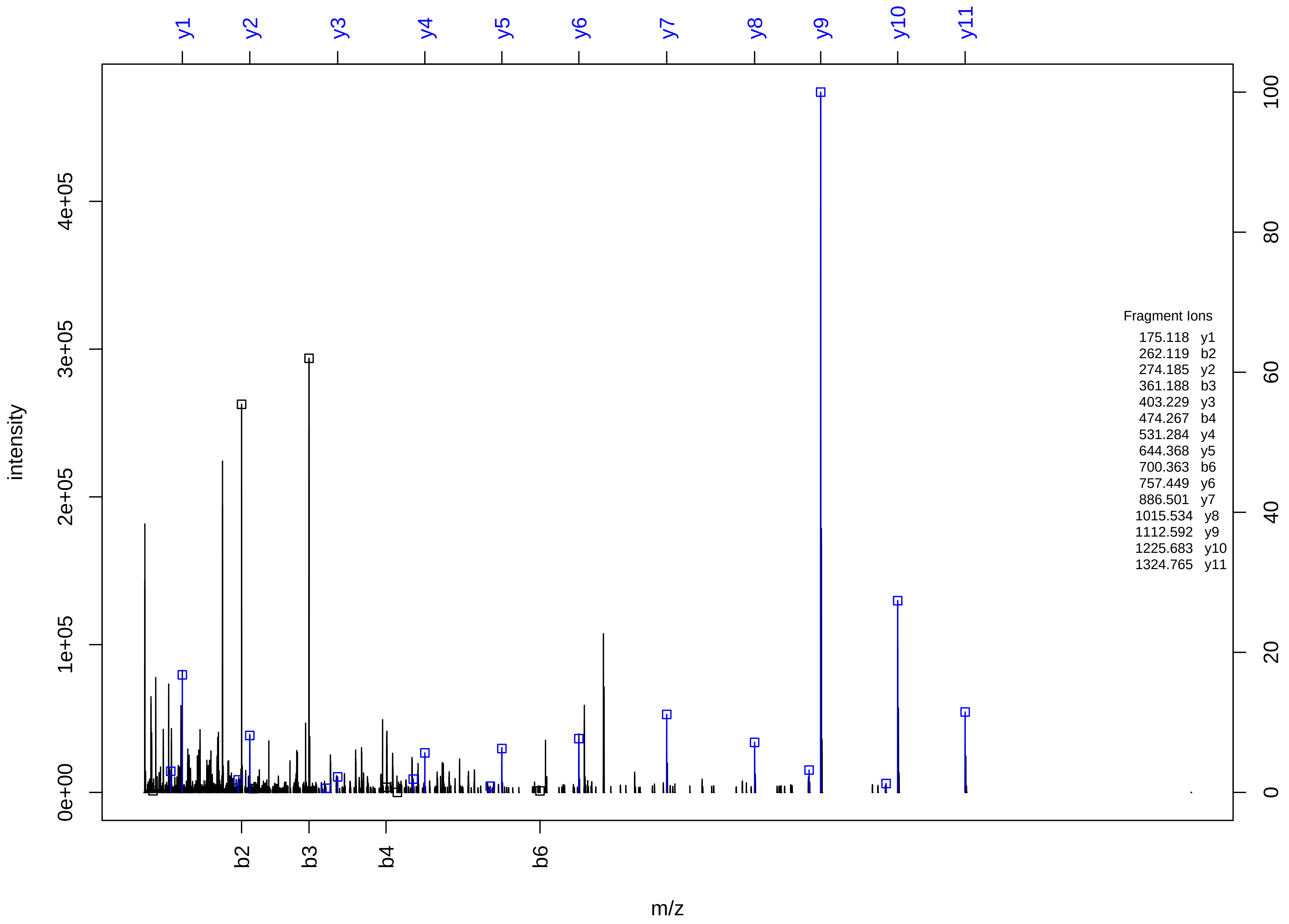

NFVLPEEIIQEVR / File: christine_ip_08.raw; SpectrumID: ; scans: 19818

QAQAQESEEEEESR

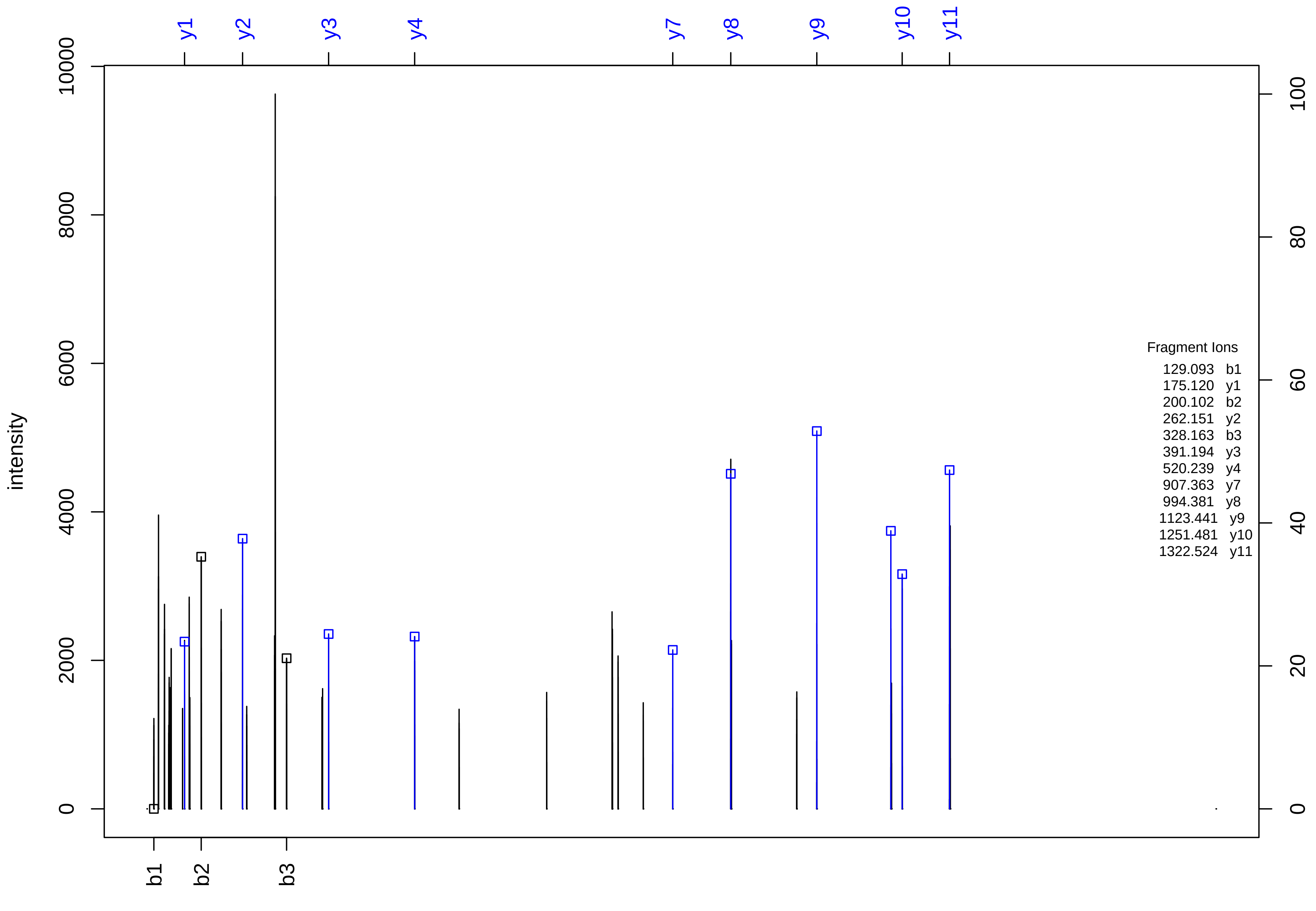

QAQAQESEEEEESR / File: christine_ip_08.raw; SpectrumID: ; scans: 898

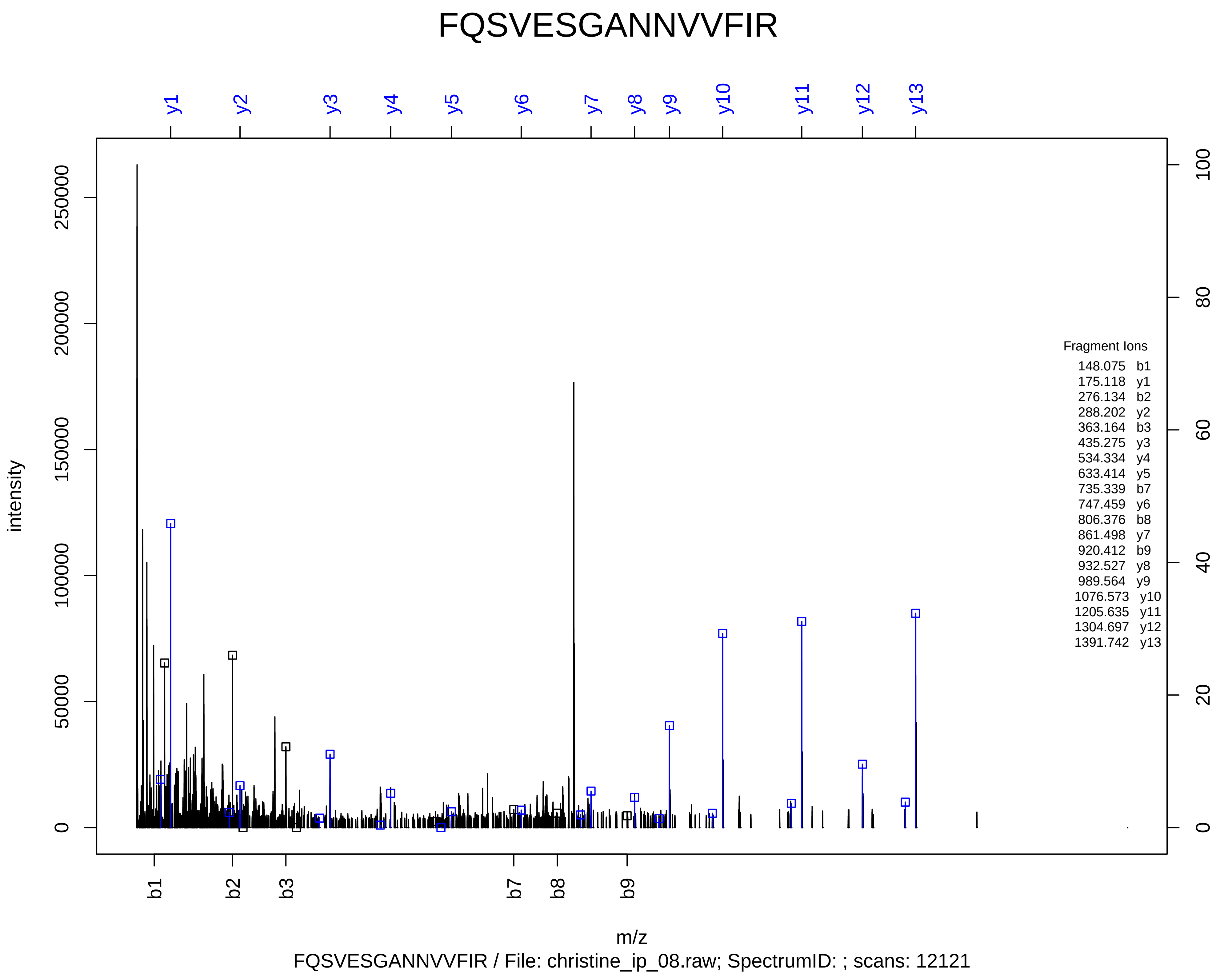
FQSVESGANNVVFIR
intensity
m/z
y1
y2
y3
y4
y5
y6
y7
y8
y9
y10
y11
y12
y13
b1
b2
b3
b7
b8
b9
Fragment Ions
148.075 b1
175.118 y1
276.134 b2
288.202 y2
363.164 b3
435.275 y3
534.334 y4
633.414 y5
735.339 b7
747.459 y6
806.376 b8
861.498 y7
920.412 b9
932.527 y8
989.564 y9
1076.573 y10
1205.635 y11
1304.697 y12
1391.742 y13
FQSVESGANNVVFIR / File: christine_ip_08.raw; SpectrumID: ; scans: 12121

LPVDFSNIPTYLLK

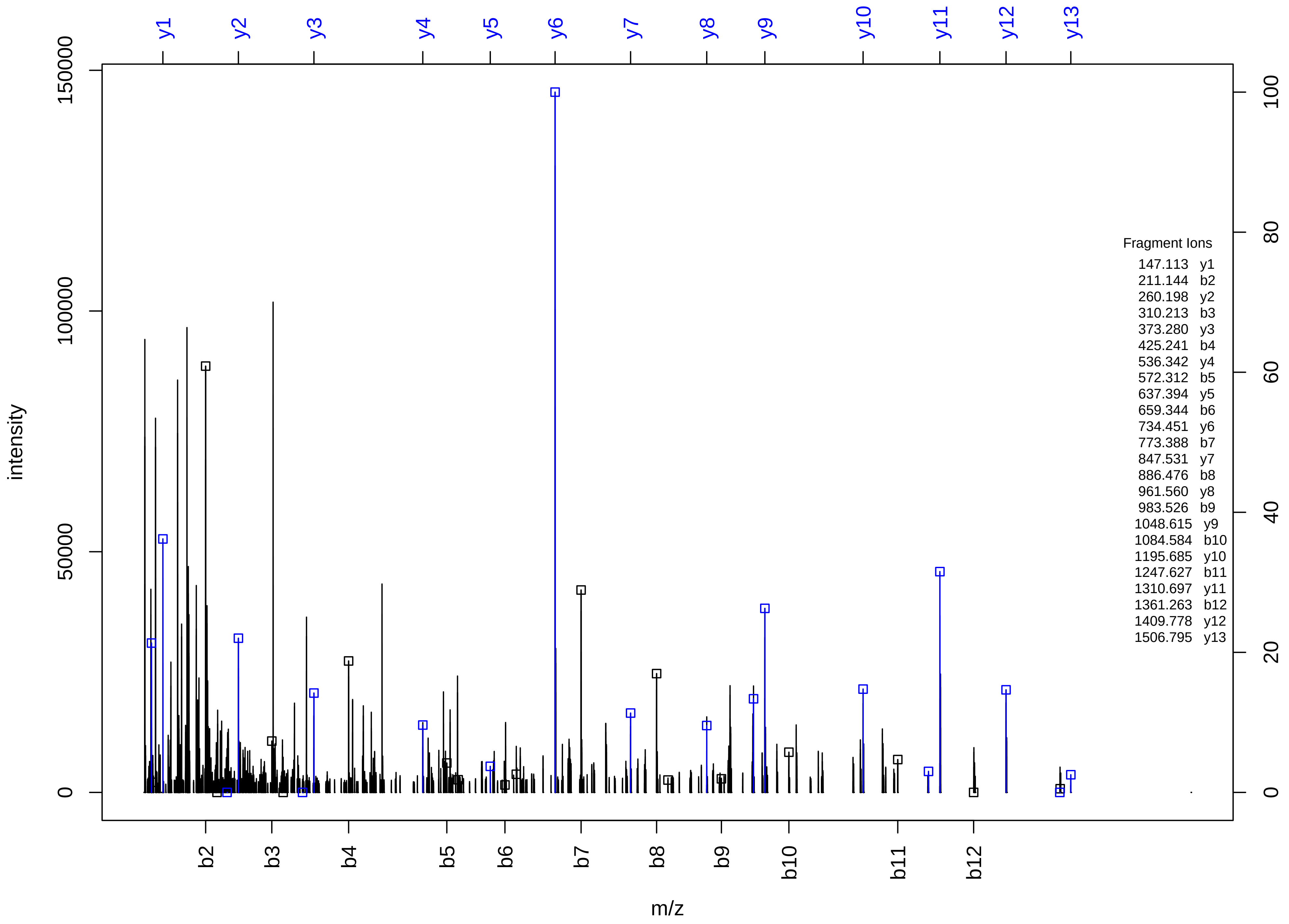

LPVDFSNIPTYLLK / File: christine_ip_08.raw; SpectrumID: ; scans: 20914

AVEAVVNDTSGENK

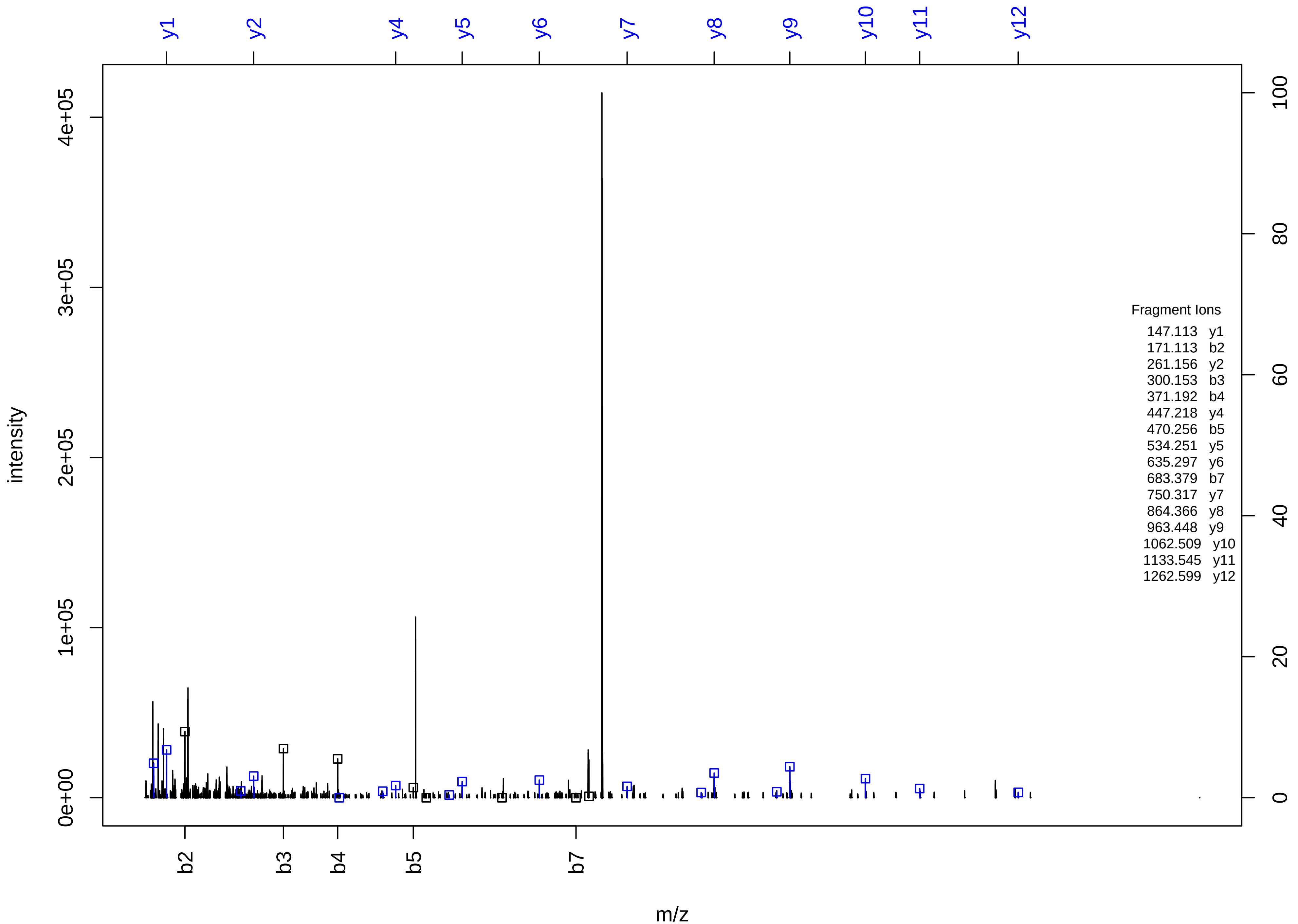

AVEAVVNDTSGENK / File: christine_ip_08.raw; SpectrumID: ; scans: 3114

VAIFTLINEINK

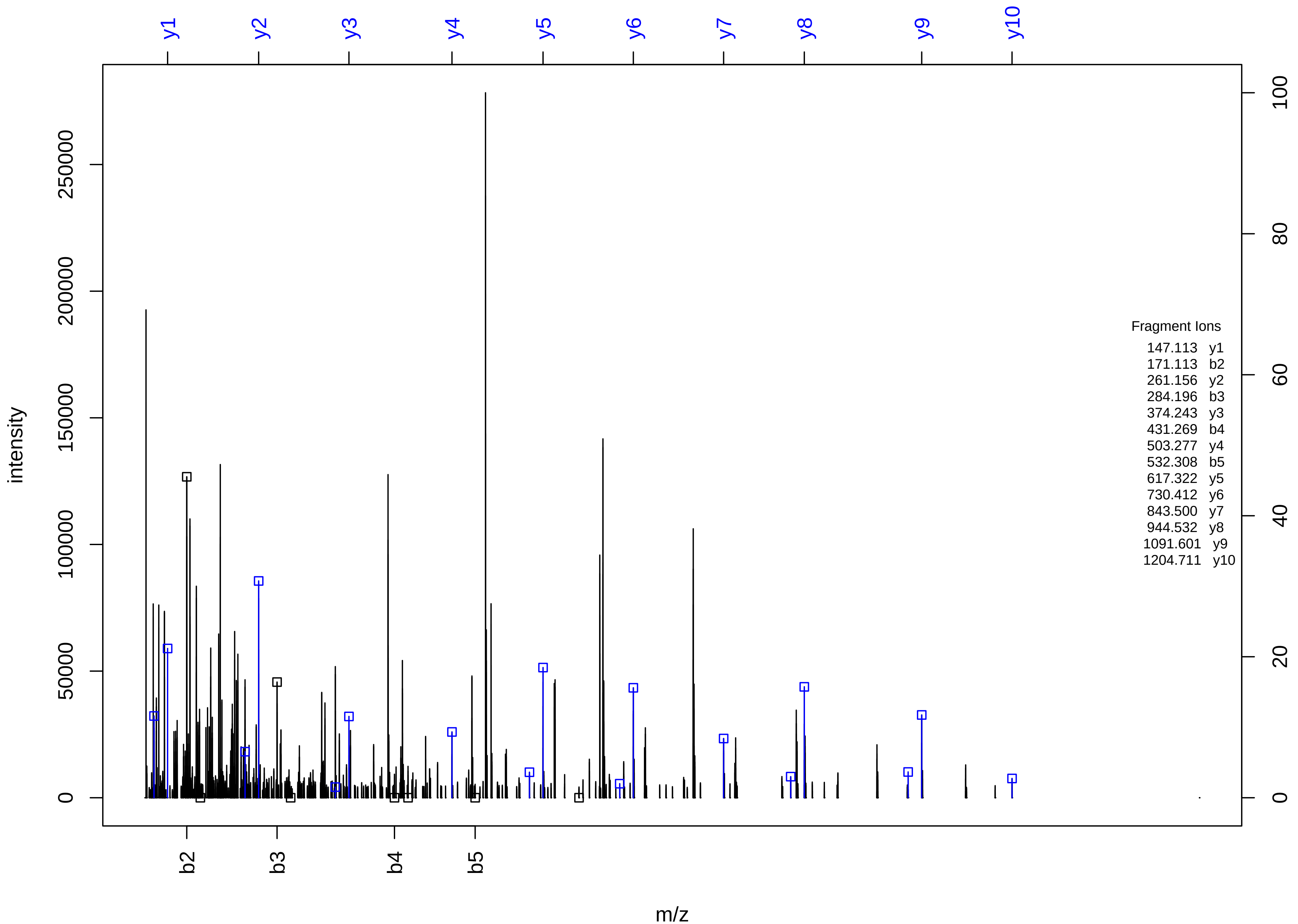

VAIFTLINEINK / File: christine_ip_08.raw; SpectrumID: ; scans: 19901

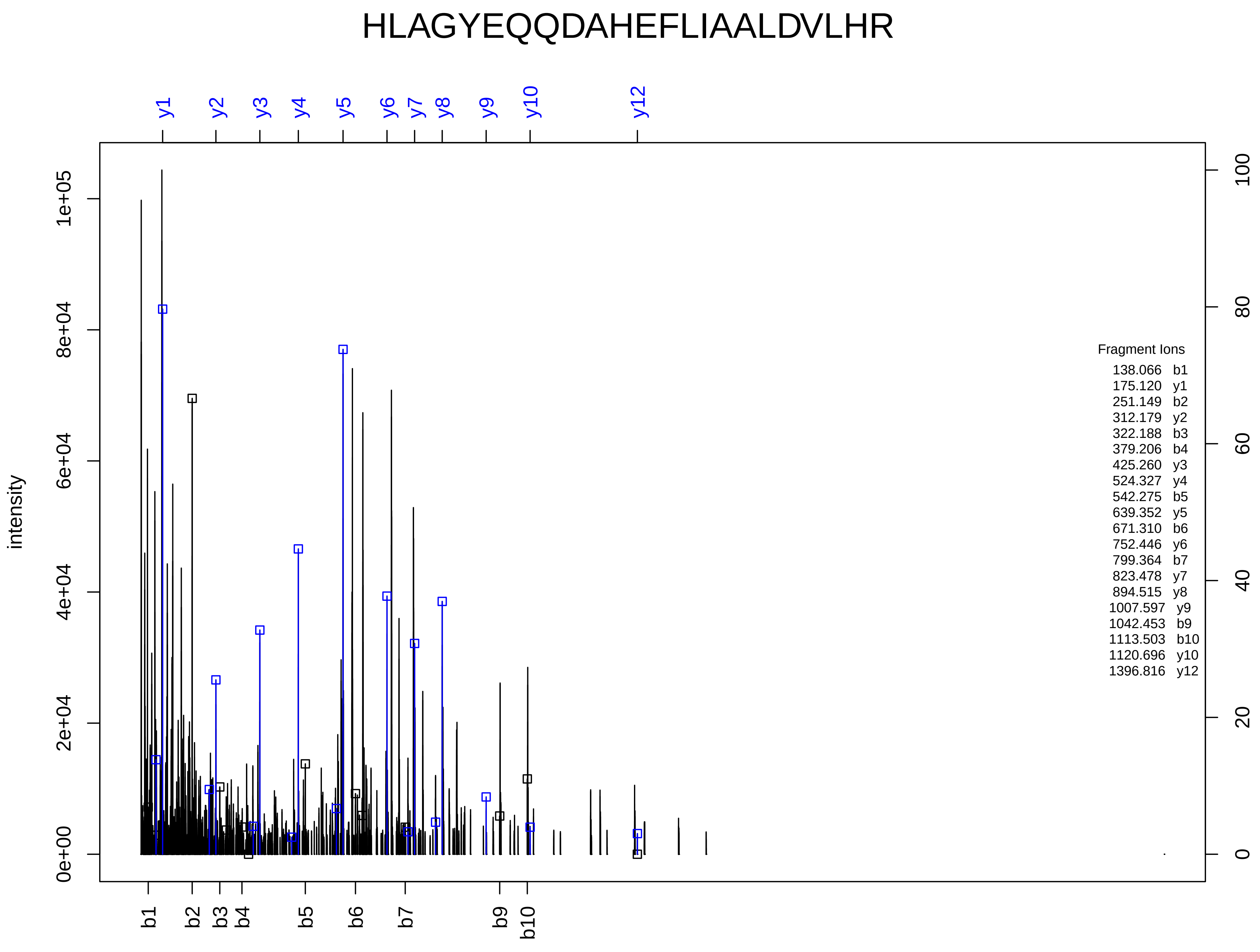

HLAGYEQQDAHEFLIAALDVLHR / File: christine_ip_08.raw; SpectrumID: ; scans: 19224

HPIGVLLNAITELQK

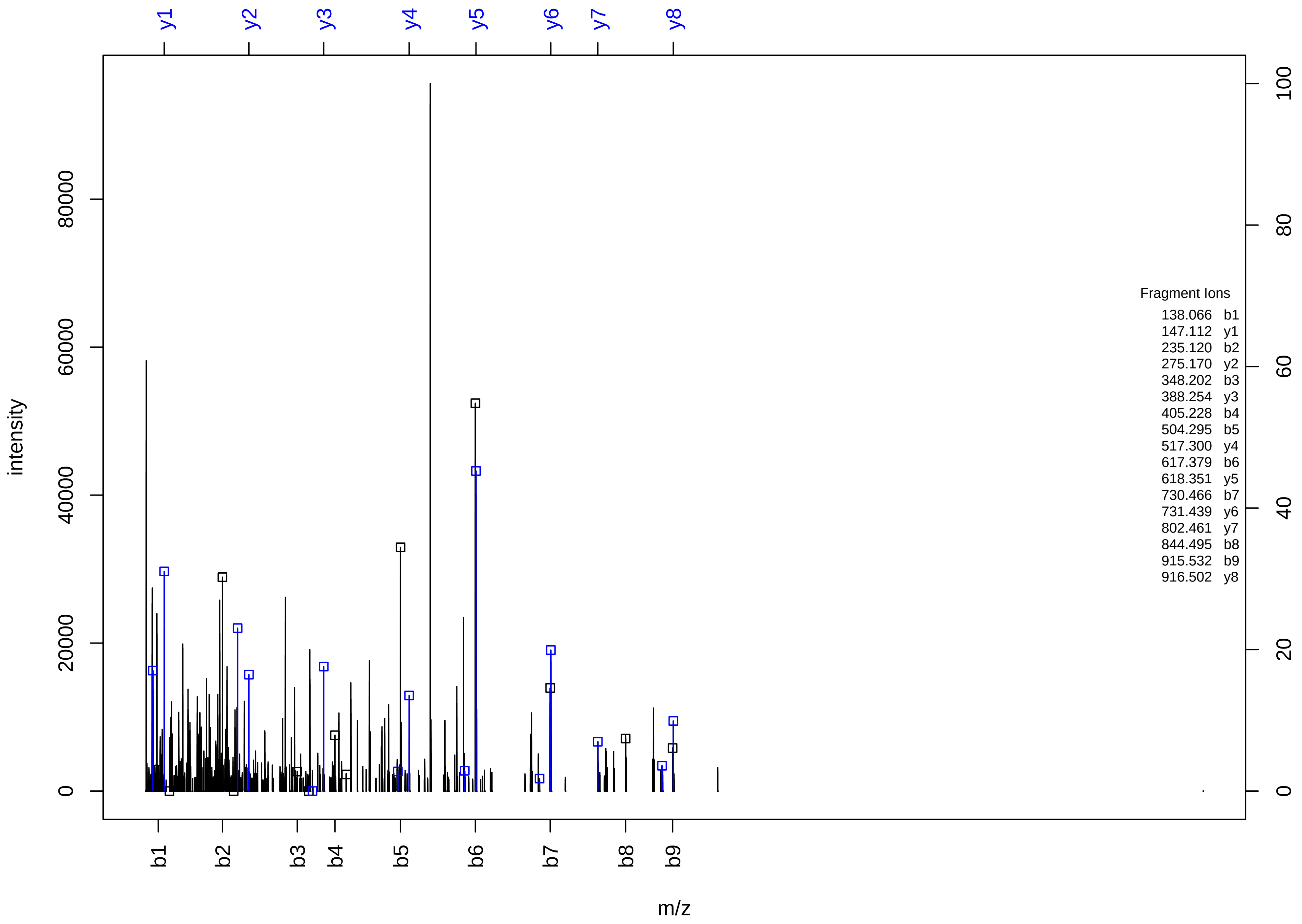

HPIGVLLNAITELQK / File: christine_ip_08.raw; SpectrumID: ; scans: 19643

GFGFVDFLTK

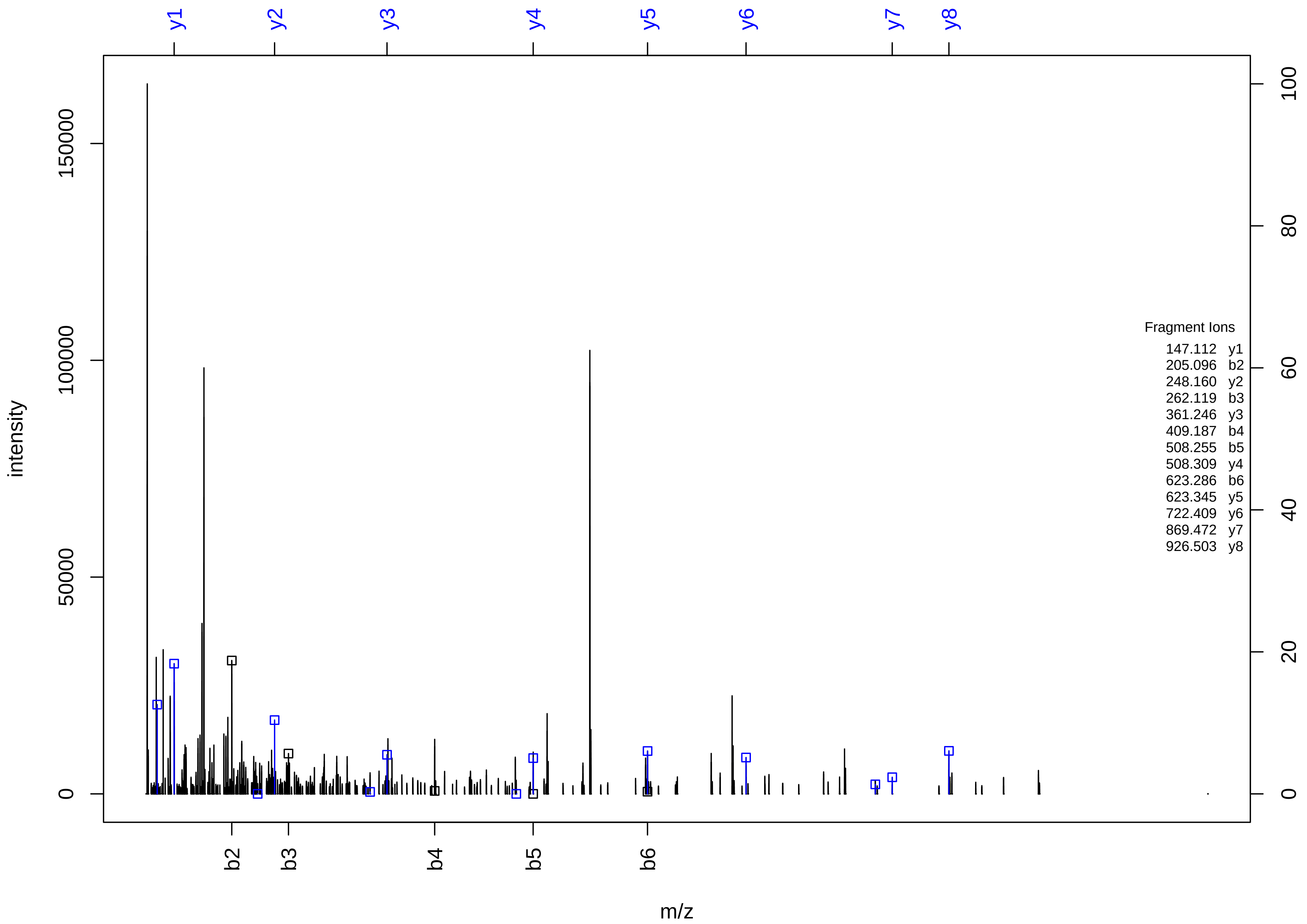

GFGFVDFLTK / File: christine_ip_08.raw; SpectrumID: ; scans: 19772

AIDNAADLLIFGK

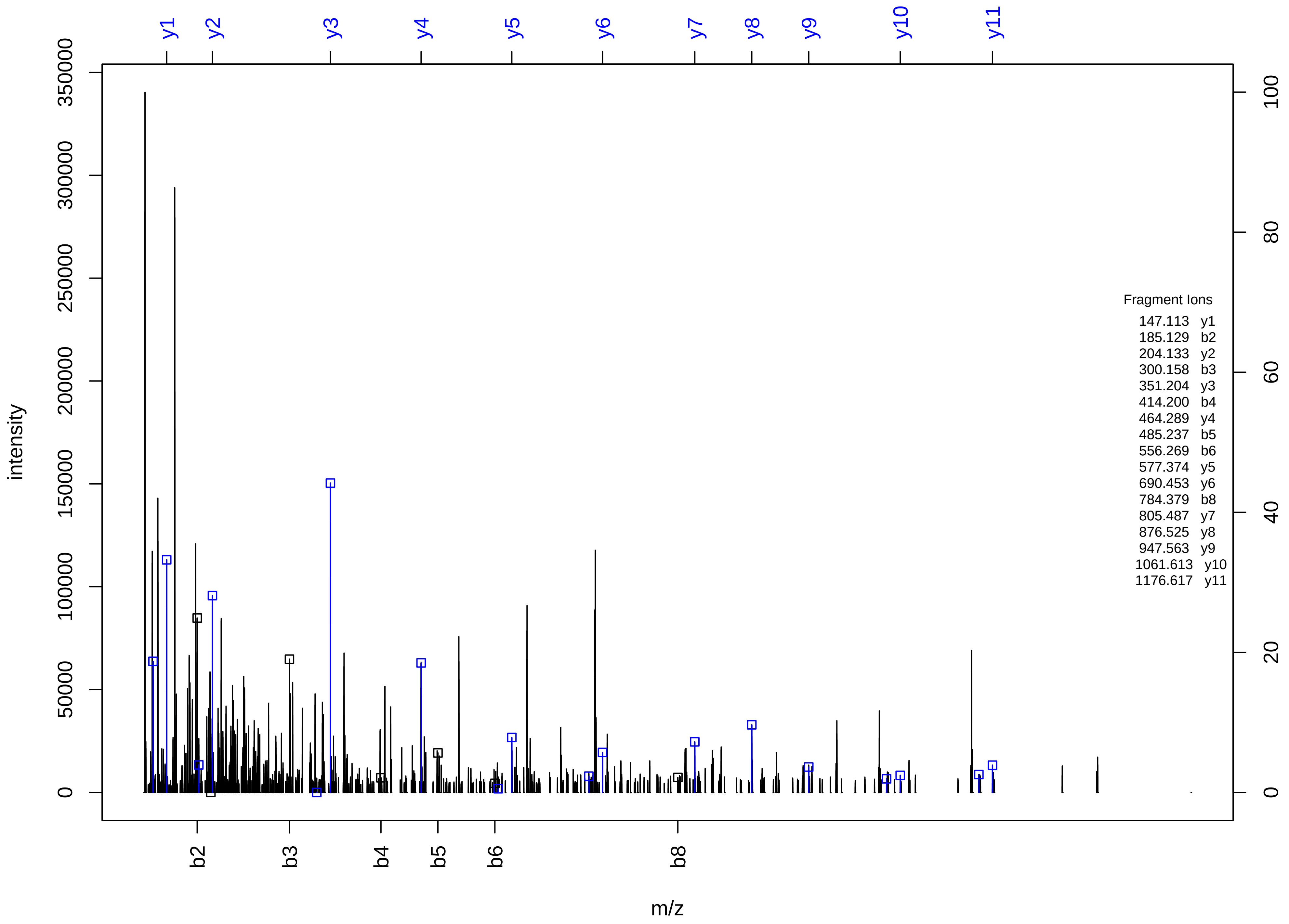

AIDNAADLLIFGK / File: christine_ip_08.raw; SpectrumID: ; scans: 18050

AGGEEEDDDDEAAGGR

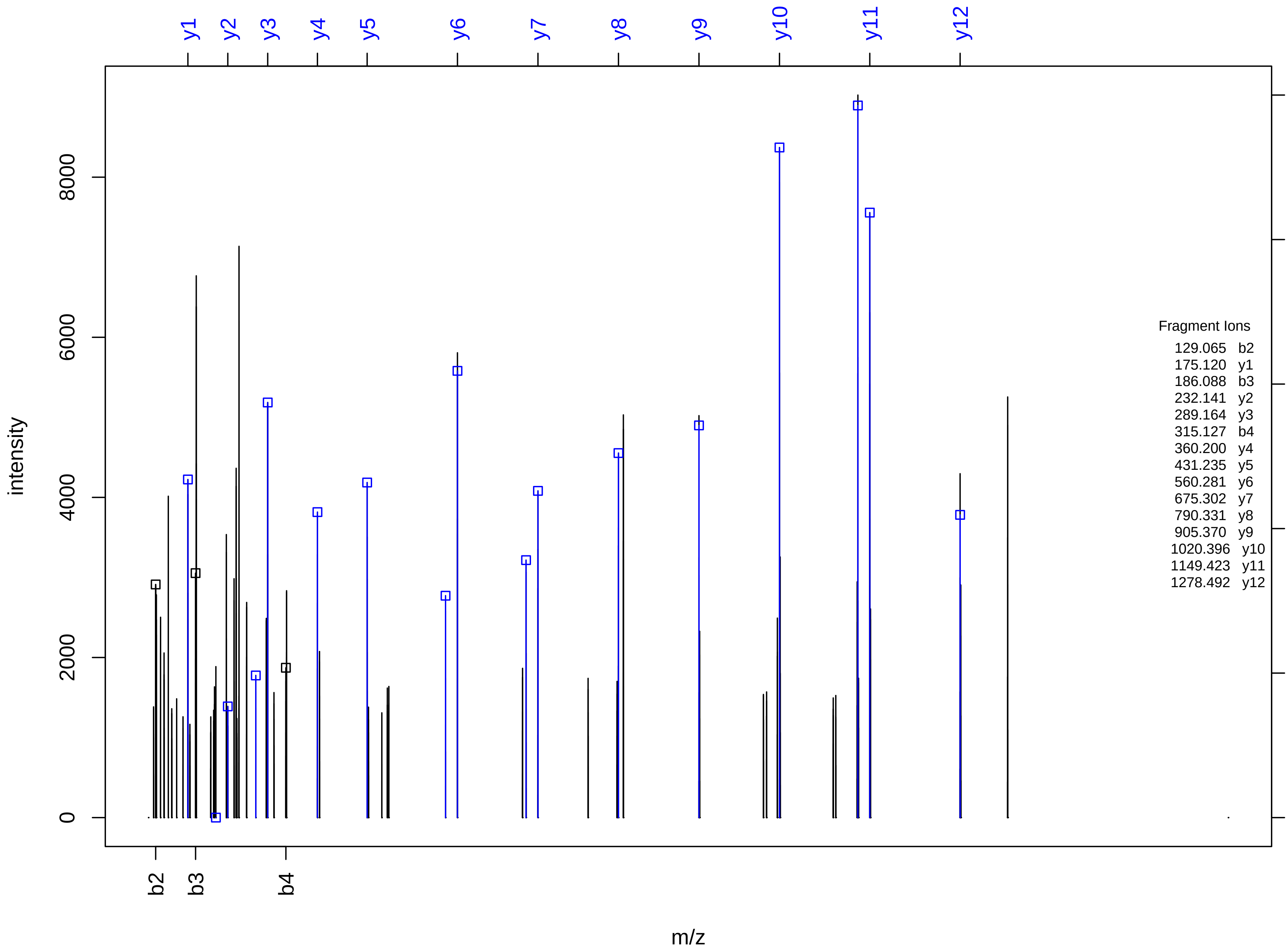

AGGEEEDDDDEAAGGR / File: christine_ip_08.raw; SpectrumID: ; scans: 867

MVEN^FKN^LVAVGSKHQN^K

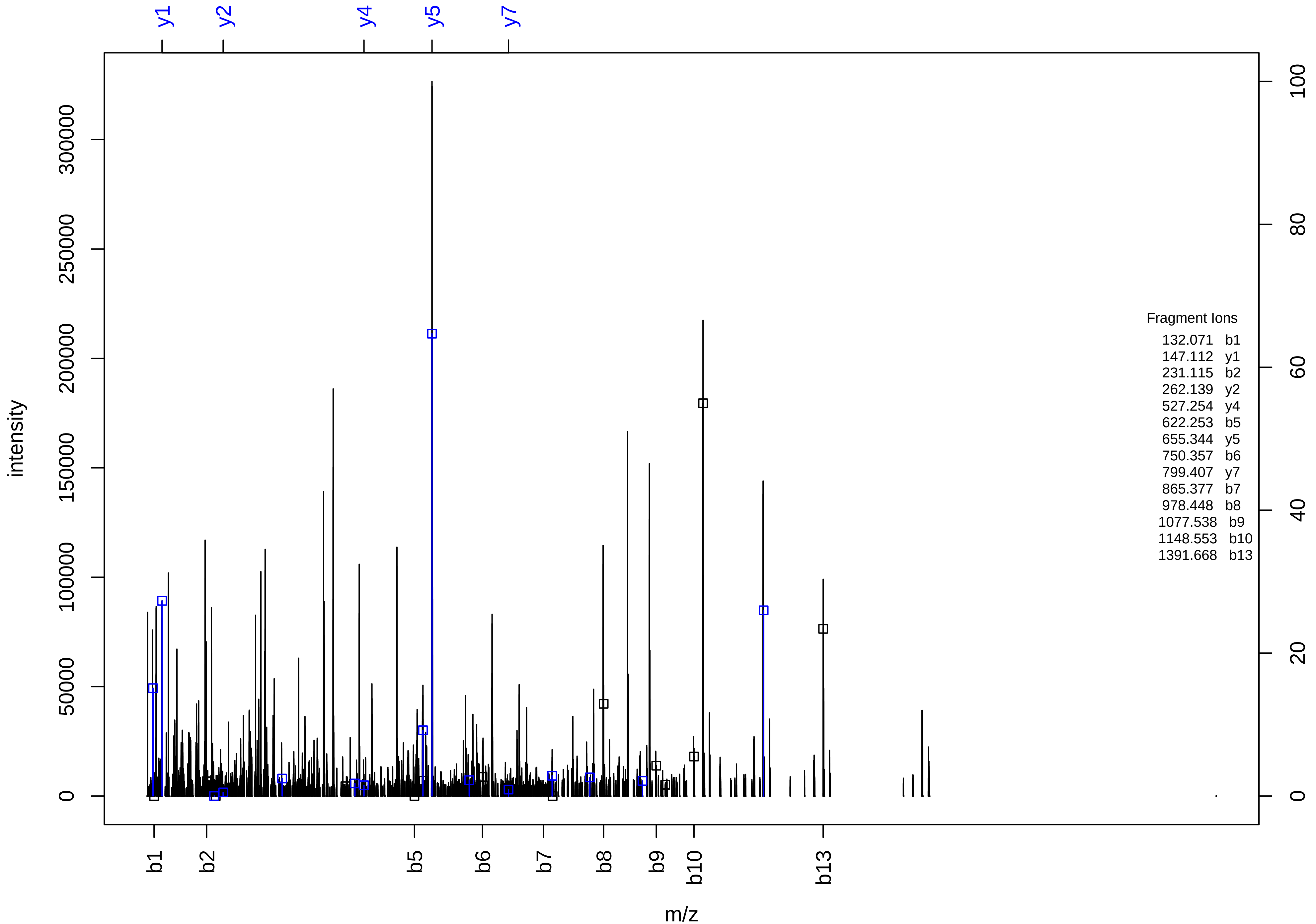

MVENFKNLVAVGSKHQNK / File: christine_ip_08.raw; SpectrumID: ; scans: 15935

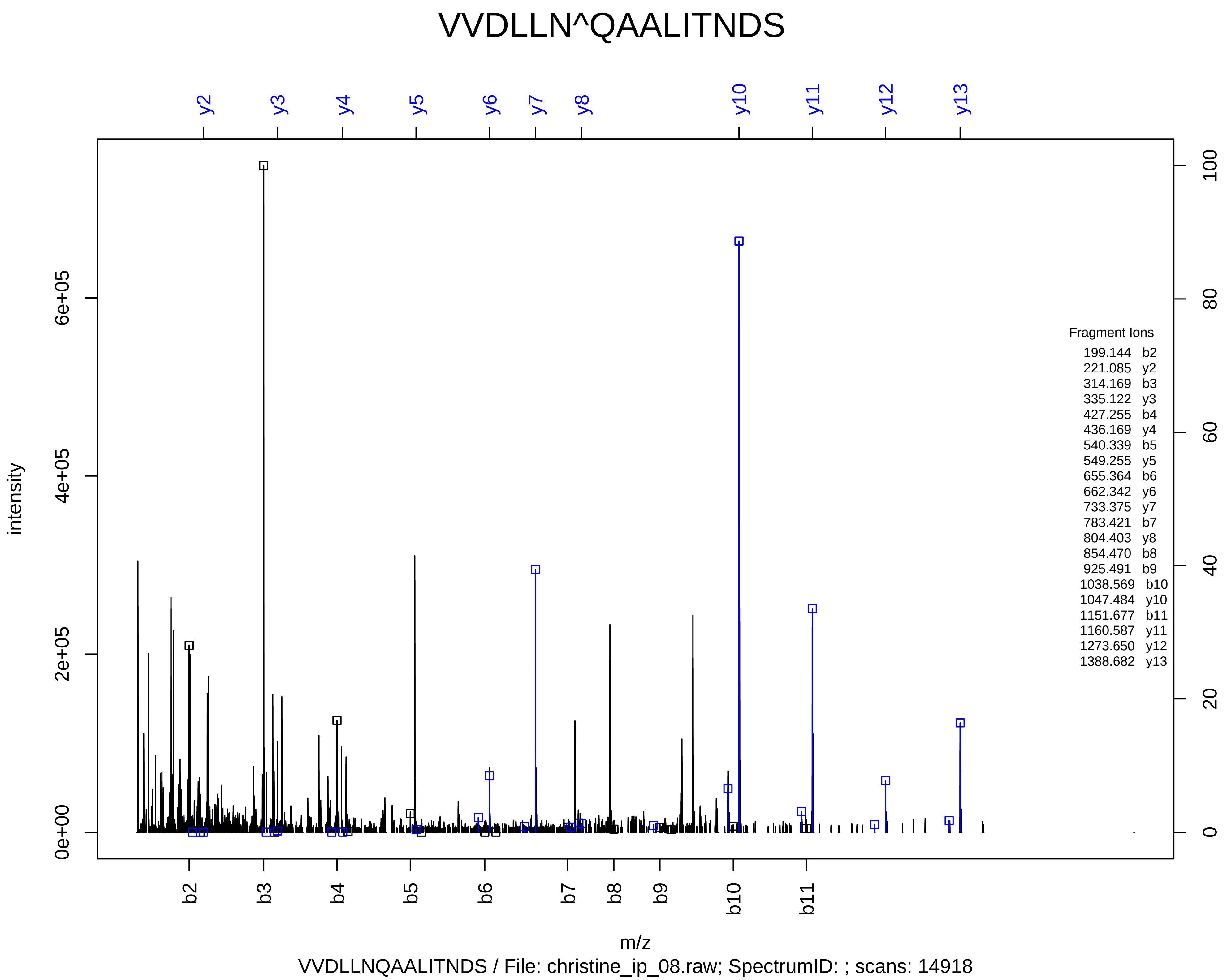
VVDLLN^QAALITNDS
y2
y3
y4
y5
y6
y7
y8
y10
y11
y12
y13
intensity
0e+00
2e+05
4e+05
6e+05
0
20
40
60
80
100
b2
b3
b4
b5
b6
b7
b8
b9
b10
b11
m/z
Fragment Ions
199.144 b2
221.085 y2
314.169 b3
335.122 y3
427.255 b4
436.169 y4
540.339 b5
549.255 y5
655.364 b6
662.342 y6
733.375 y7
783.421 b7
804.403 y8
854.470 b8
925.491 b9
1038.569 b10
1047.484 y10
1151.677 b11
1160.587 y11
1273.650 y12
1388.682 y13
VVDLLNQAALITNDS / File: christine_ip_08.raw; SpectrumID: ; scans: 14918

DELEEEEELQELLSK

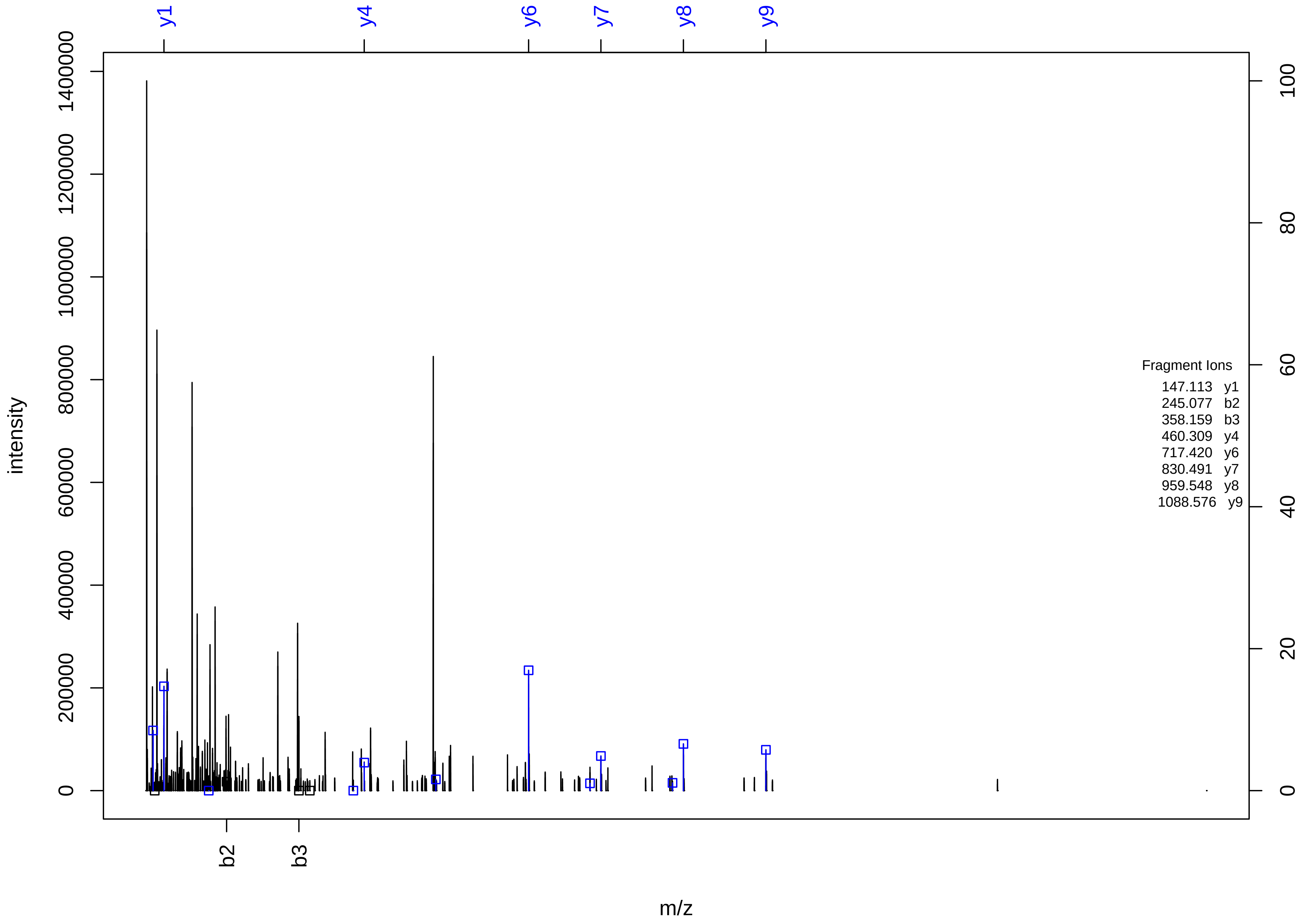

DELEEEEELQELLSK / File: christine_ip_09.raw; SpectrumID: ; scans: 10583

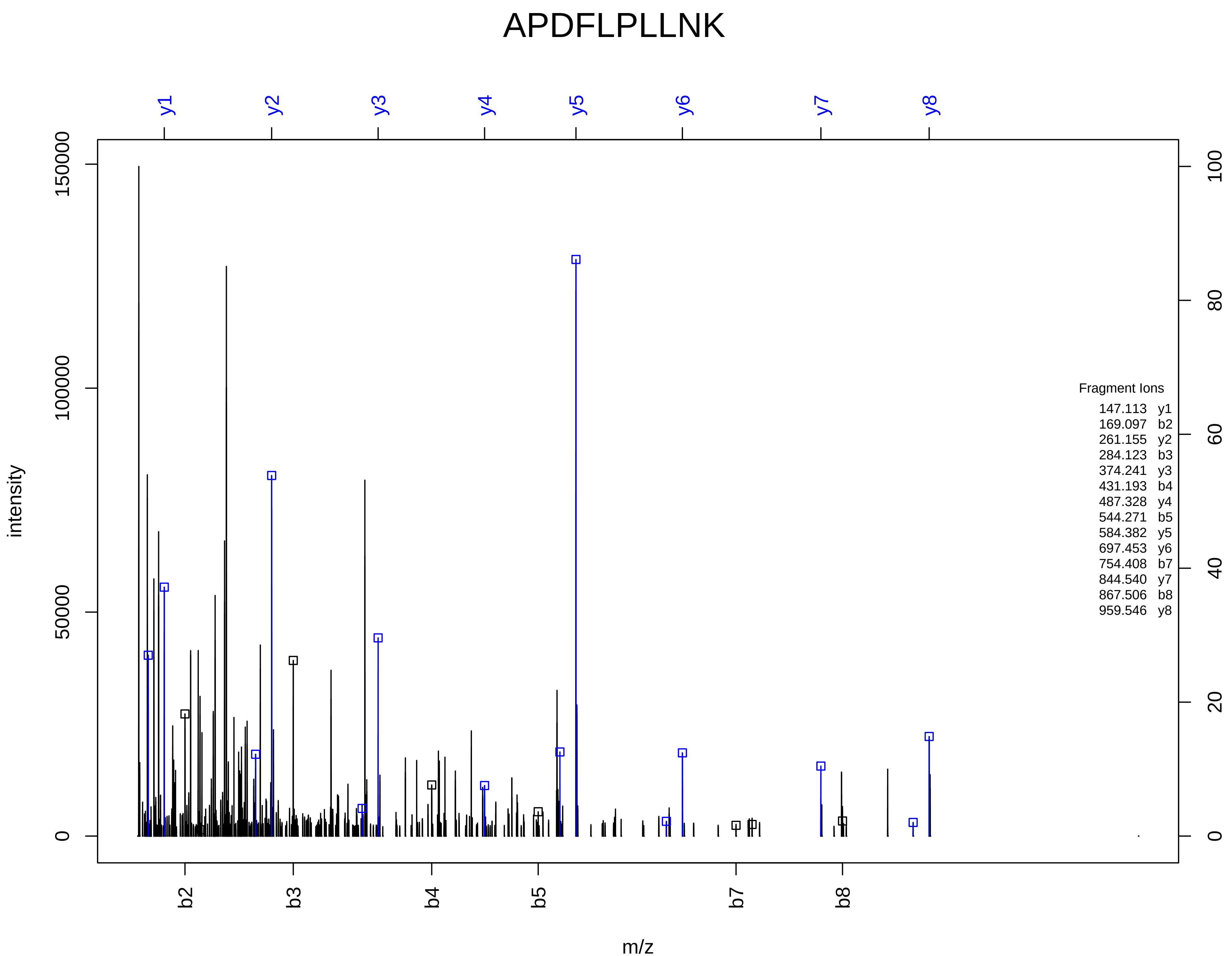

APDFLPLLNK / File: christine_ip_09.raw; SpectrumID: ; scans: 16260

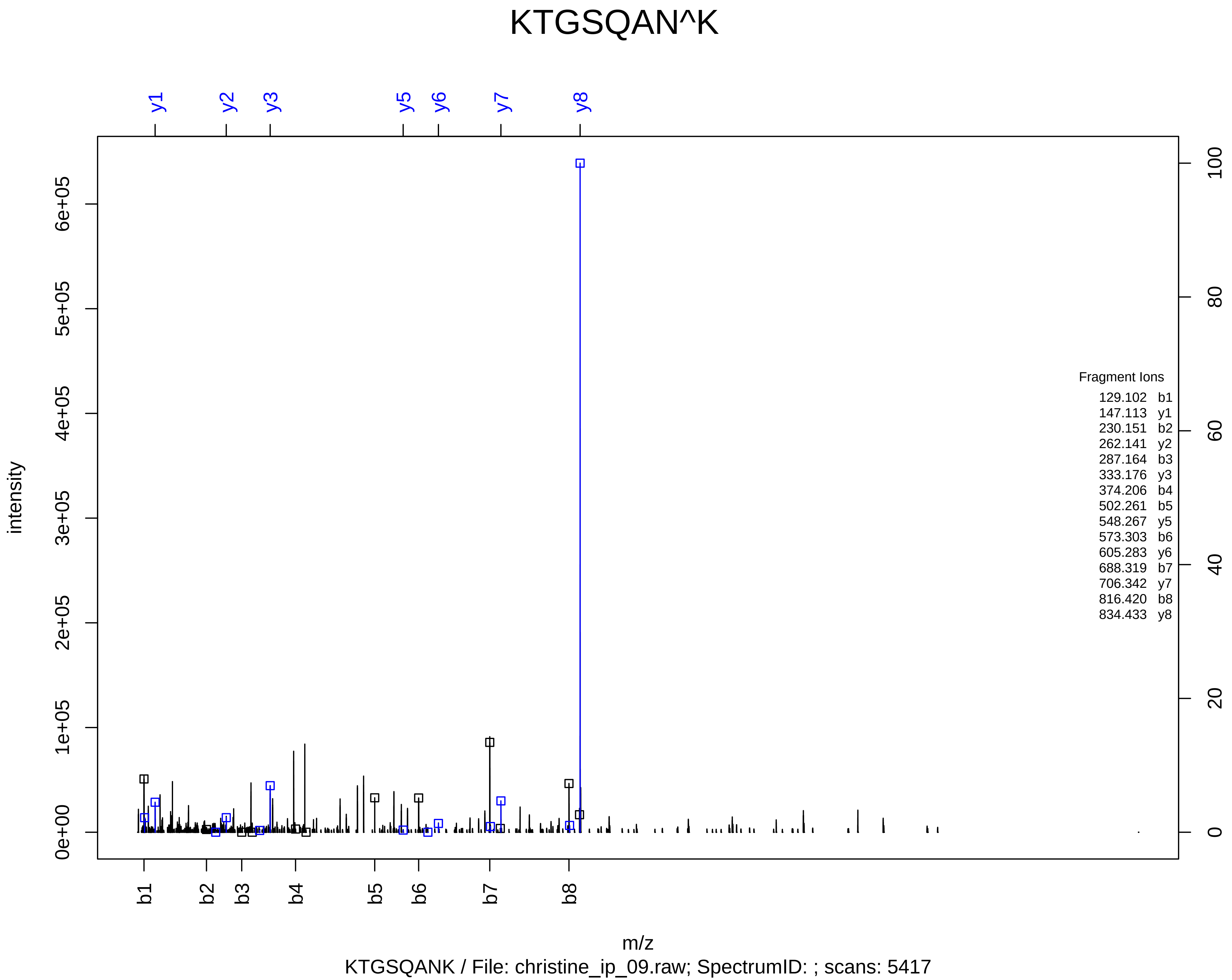
KTGSQAN^K
y1
y2
y3
y5
y6
y7
y8
intensity
0e+00
1e+05
2e+05
3e+05
4e+05
5e+05
6e+05
0
20
40
60
80
100
b1
b2
b3
b4
b5
b6
b7
b8
m/z
Fragment Ions
129.102 b1
147.113 y1
230.151 b2
262.141 y2
287.164 b3
333.176 y3
374.206 b4
502.261 b5
548.267 y5
573.303 b6
605.283 y6
688.319 b7
706.342 y7
816.420 b8
834.433 y8
KTGSQANK / File: christine_ip_09.raw; SpectrumID: ; scans: 5417

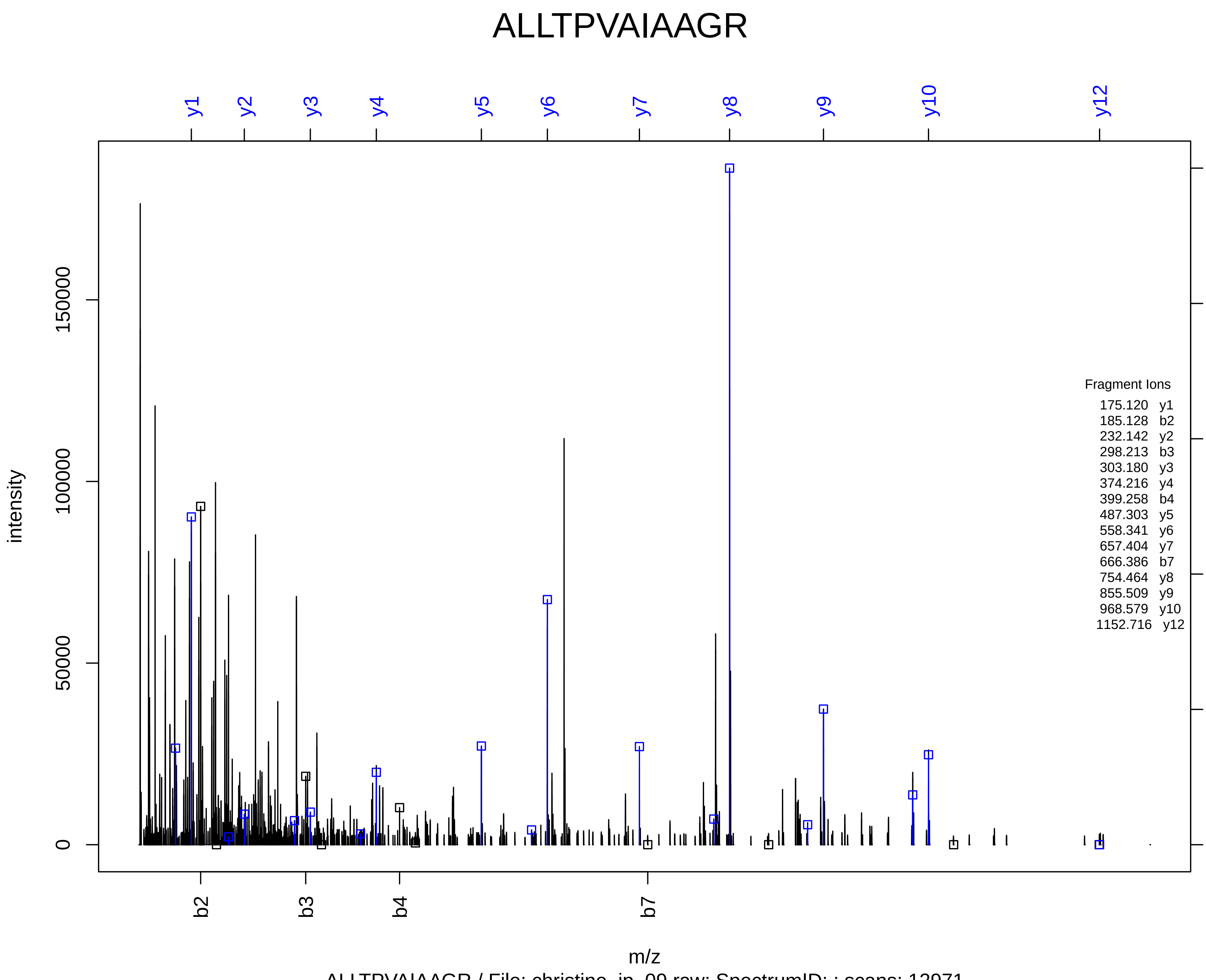

ALLLTPVAIAAGR
y1
y2
y3
y4
y5
y6
y7
y8
y9
y10
y12
intensity
0
50000
100000
150000
0
20
40
60
80
100
b2
b3
b4
b7
m/z
Fragment Ions
175.120 y1
185.128 b2
232.142 y2
298.213 b3
303.180 y3
374.216 y4
399.258 b4
487.303 y5
558.341 y6
657.404 y7
666.386 b7
754.464 y8
855.509 y9
968.579 y10
1152.716 y12
ALLLTPVAIAAGR / File: christine_ip_09.raw; SpectrumID: ; scans: 12971

LEEGPPVTTVLTR

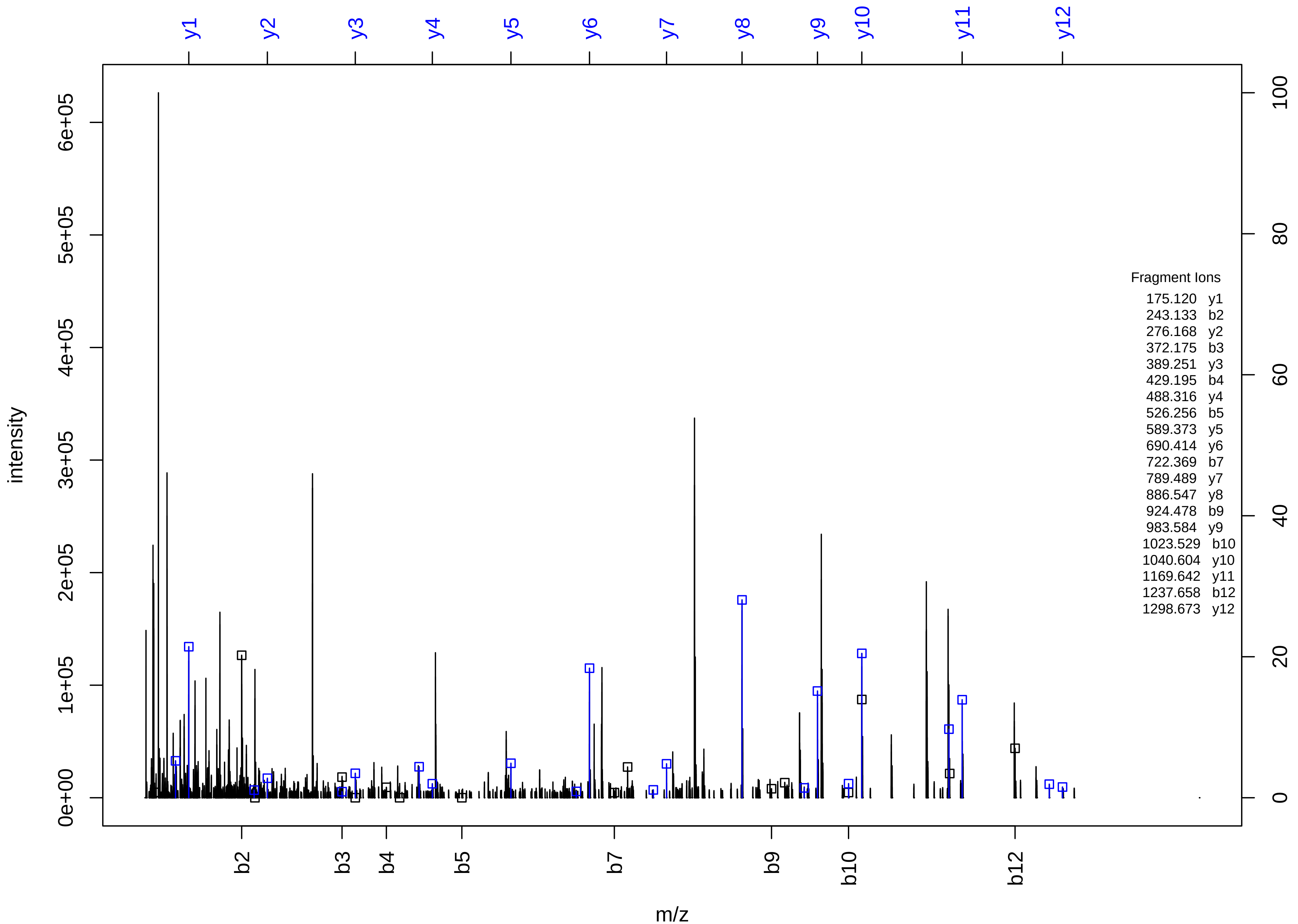

LEEGPPVTTVLTR / File: christine_ip_09.raw; SpectrumID: ; scans: 10862

ENAEQGEVDMESHR

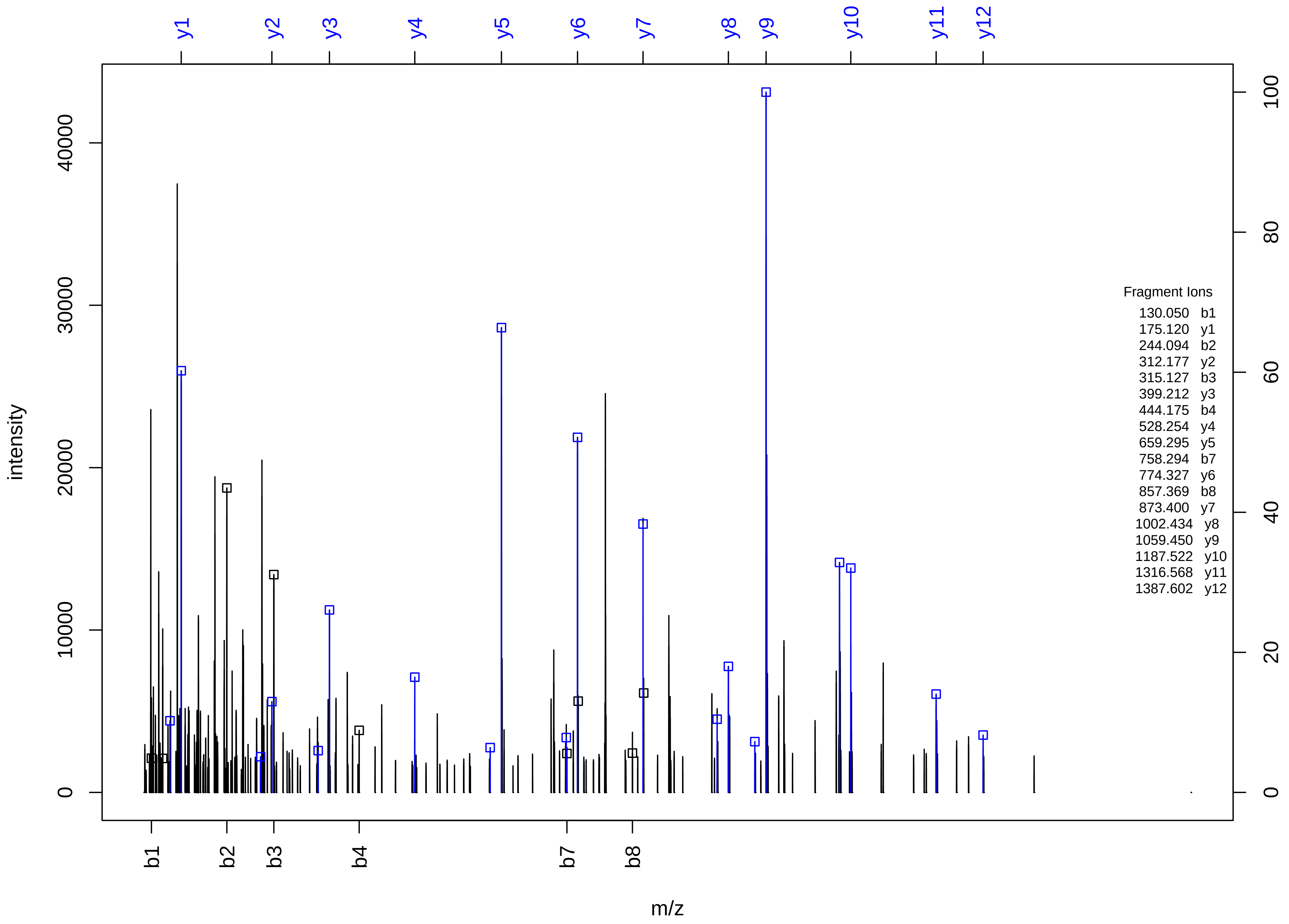

ENAEQGEVDMESHR / File: christine_ip_09.raw; SpectrumID: ; scans: 2309

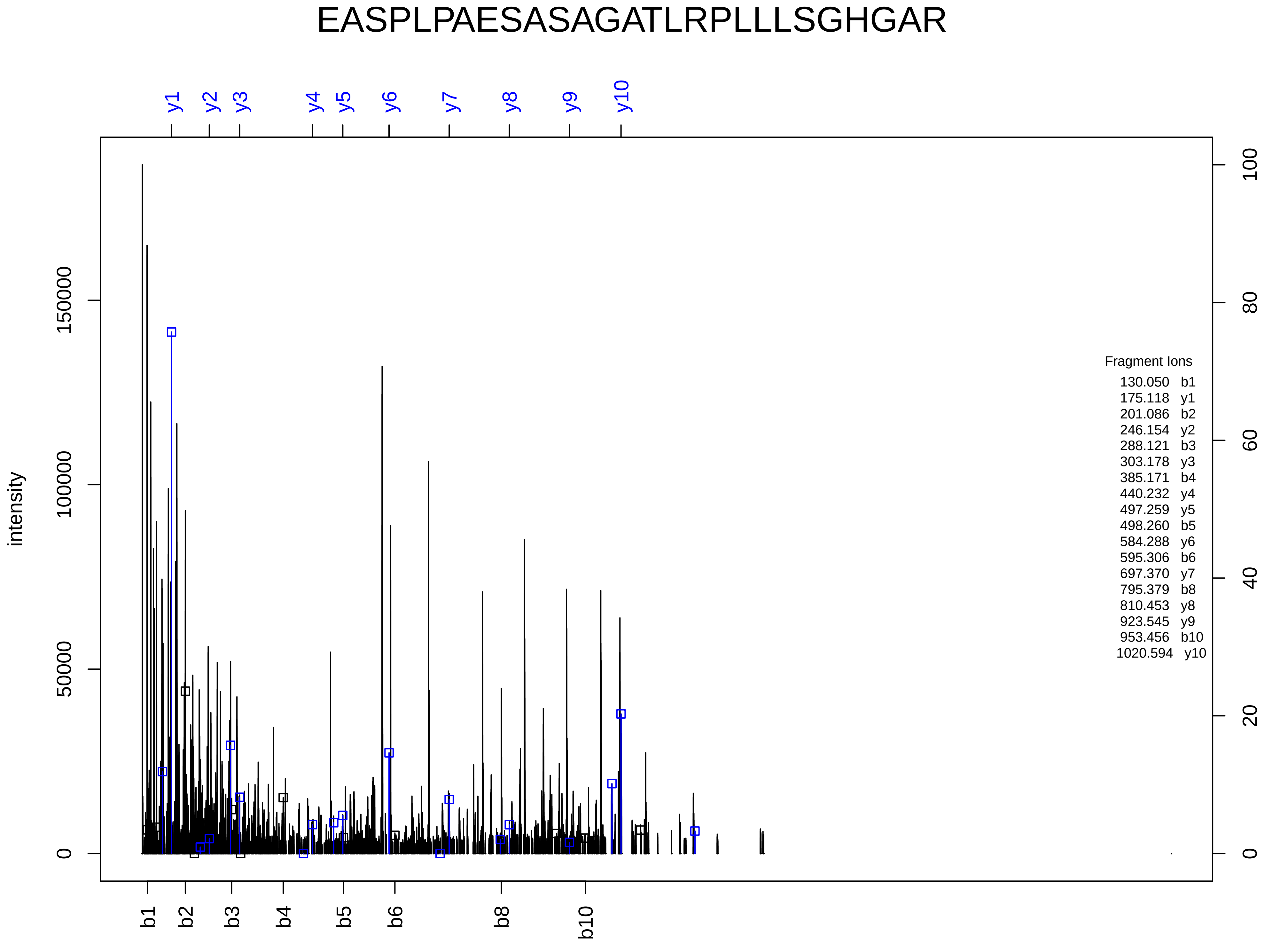

EASPLPAESASAGATLRPLLLSGHGAR / File: christine_ip_09.raw; SpectrumID: ; scans: 12325

(Ac)SAEVPEAASAEEQK

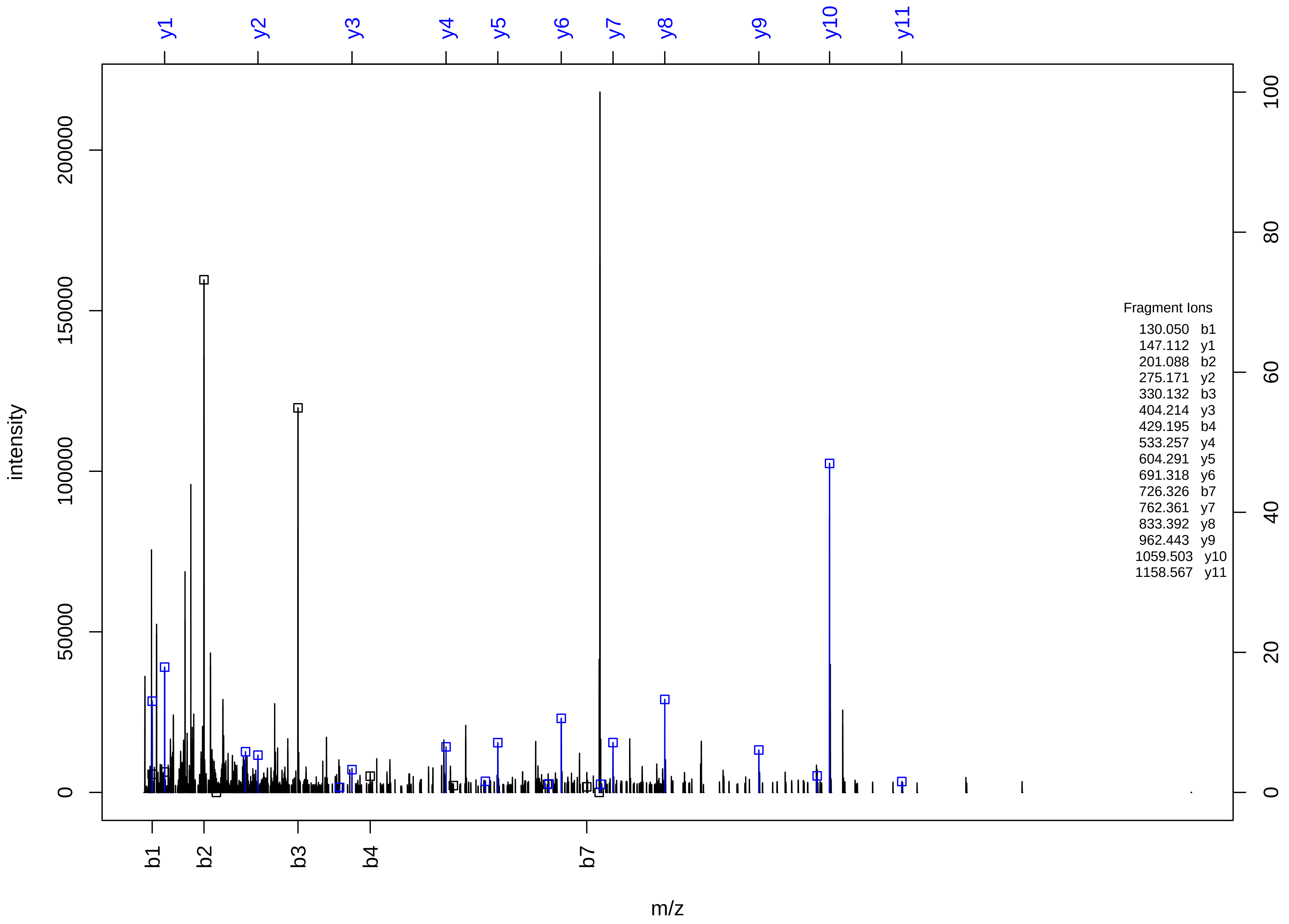

SAEVPEAASAEEQK / File: christine_ip_09.raw; SpectrumID: ; scans: 5915

PGPTPSGTNVGSSGR

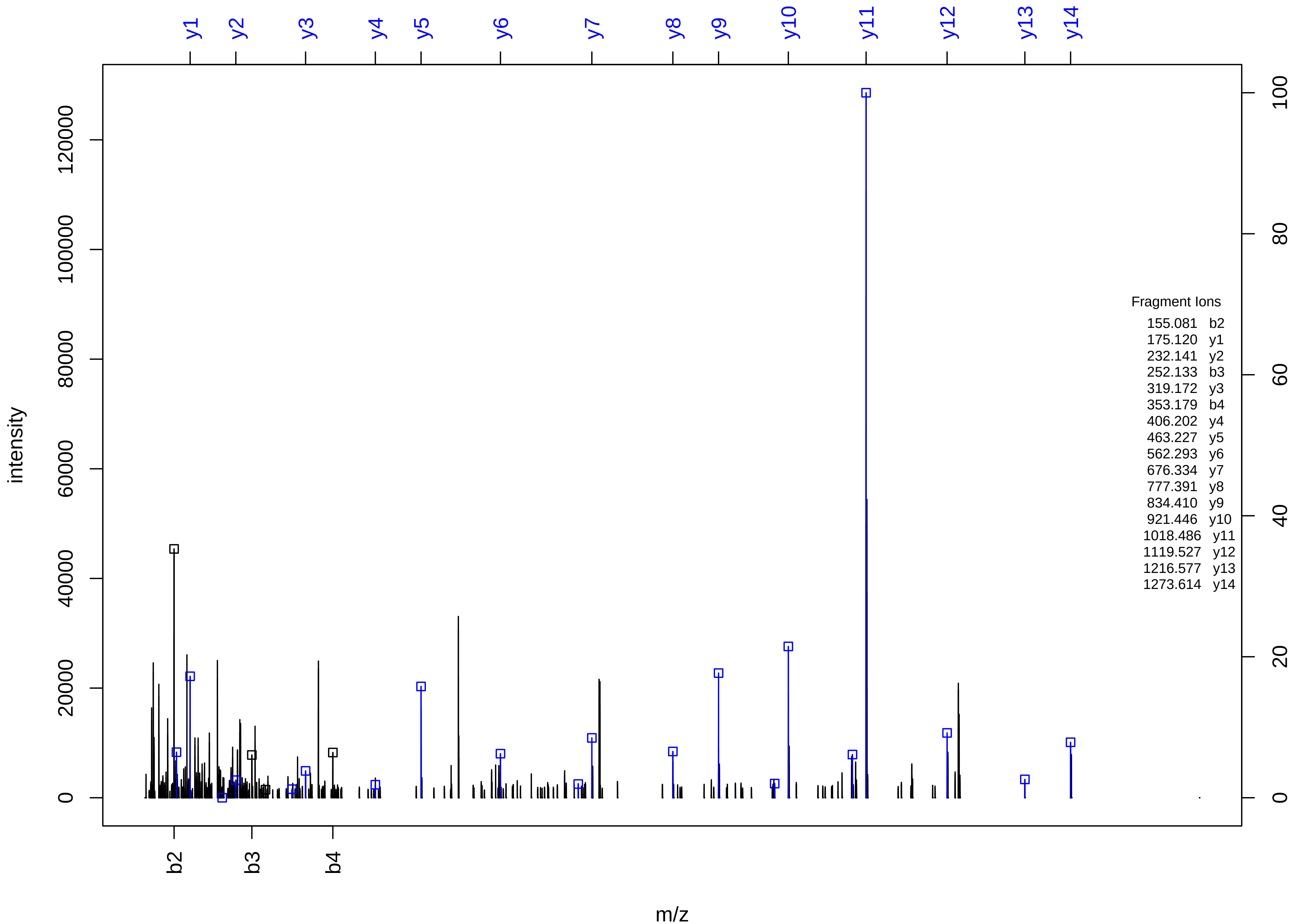

PGPTPSGTNVGSSGR / File: christine_ip_09.raw; SpectrumID: ; scans: 1915

AIEQADLLQEEDESPR

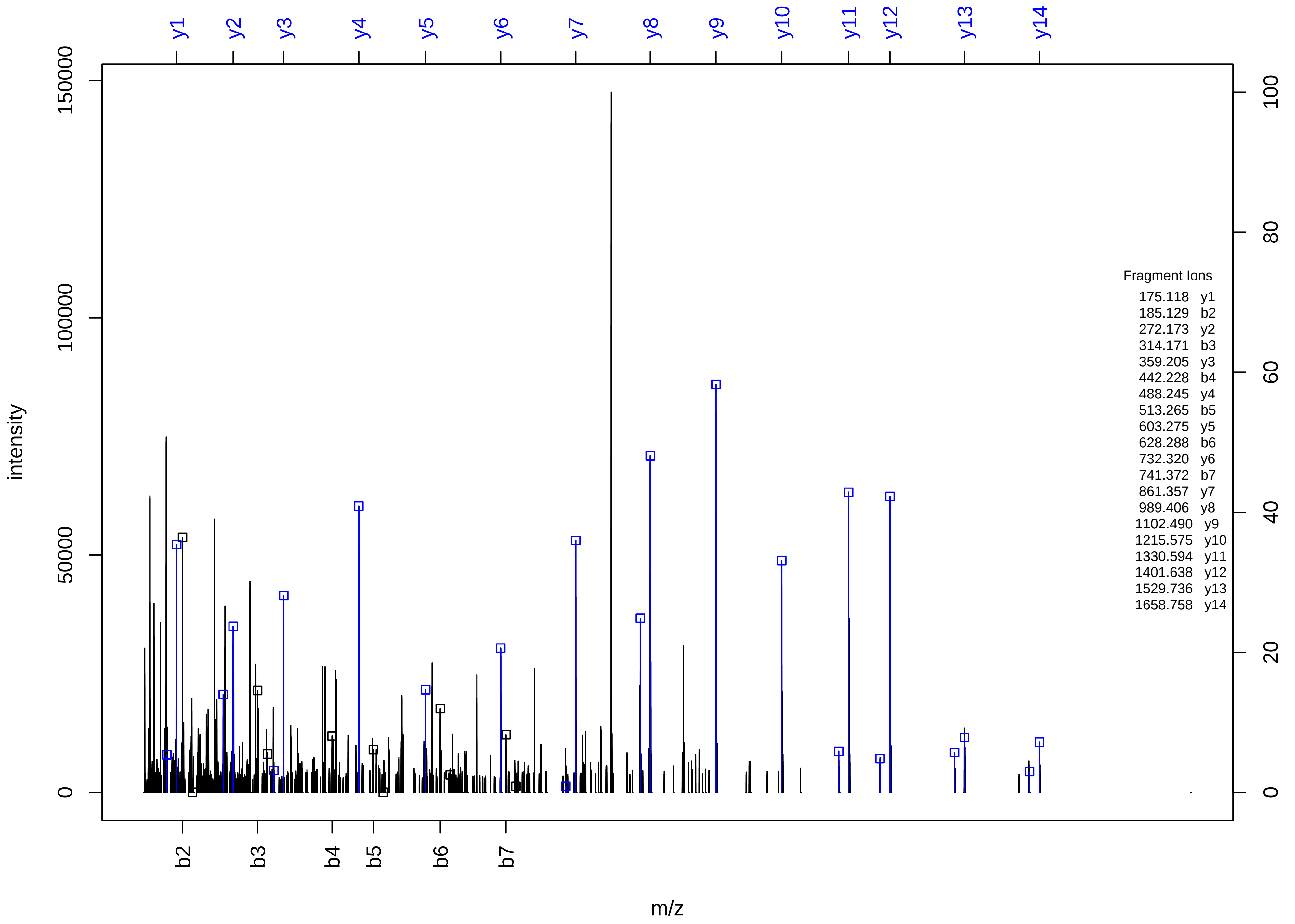

AIEQADLLQEEDESPR / File: christine_ip_09.raw; SpectrumID: ; scans: 8890

YSFLQFDPAPR

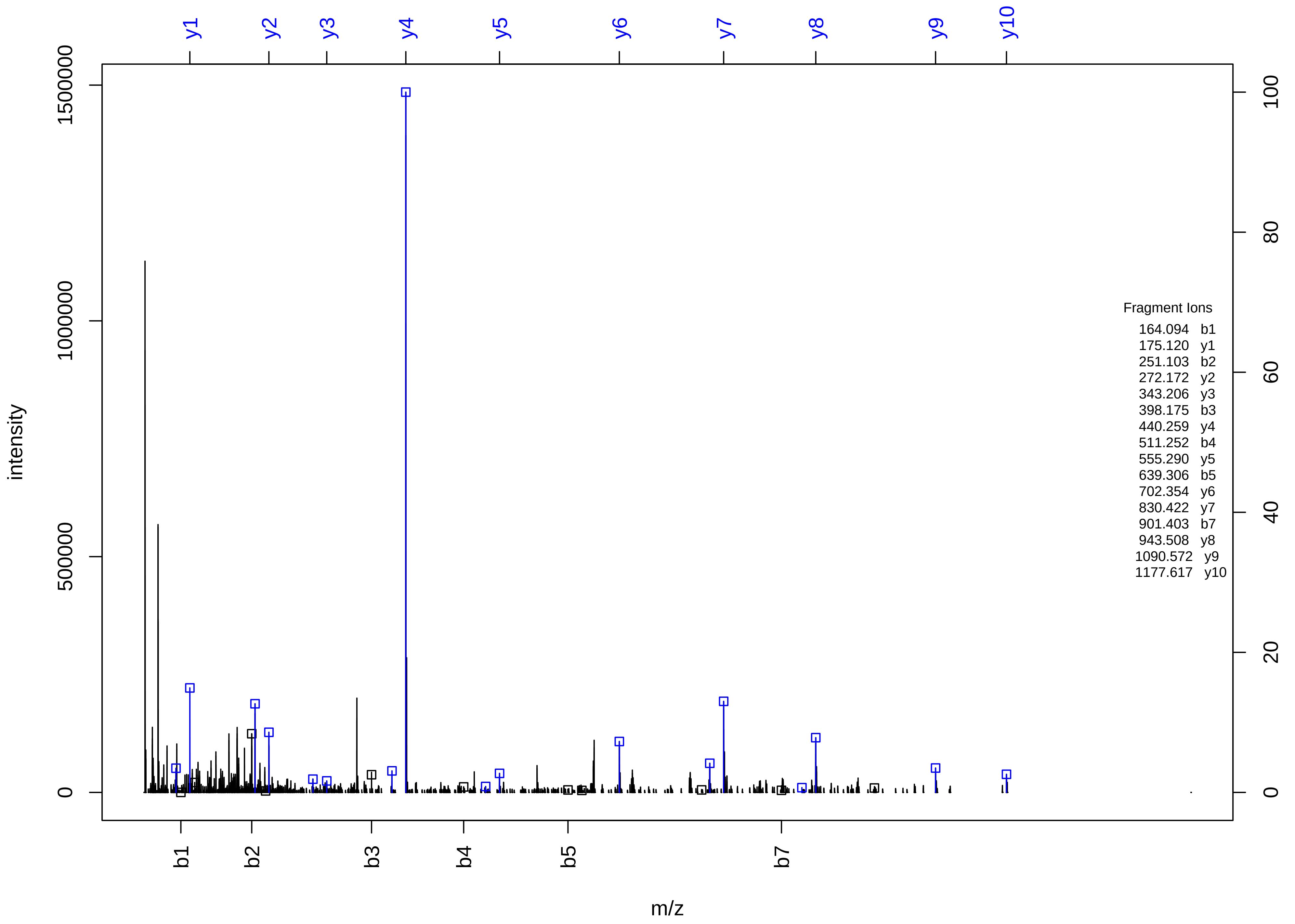

YSFLQFDPAPR / File: christine_ip_09.raw; SpectrumID: ; scans: 14670

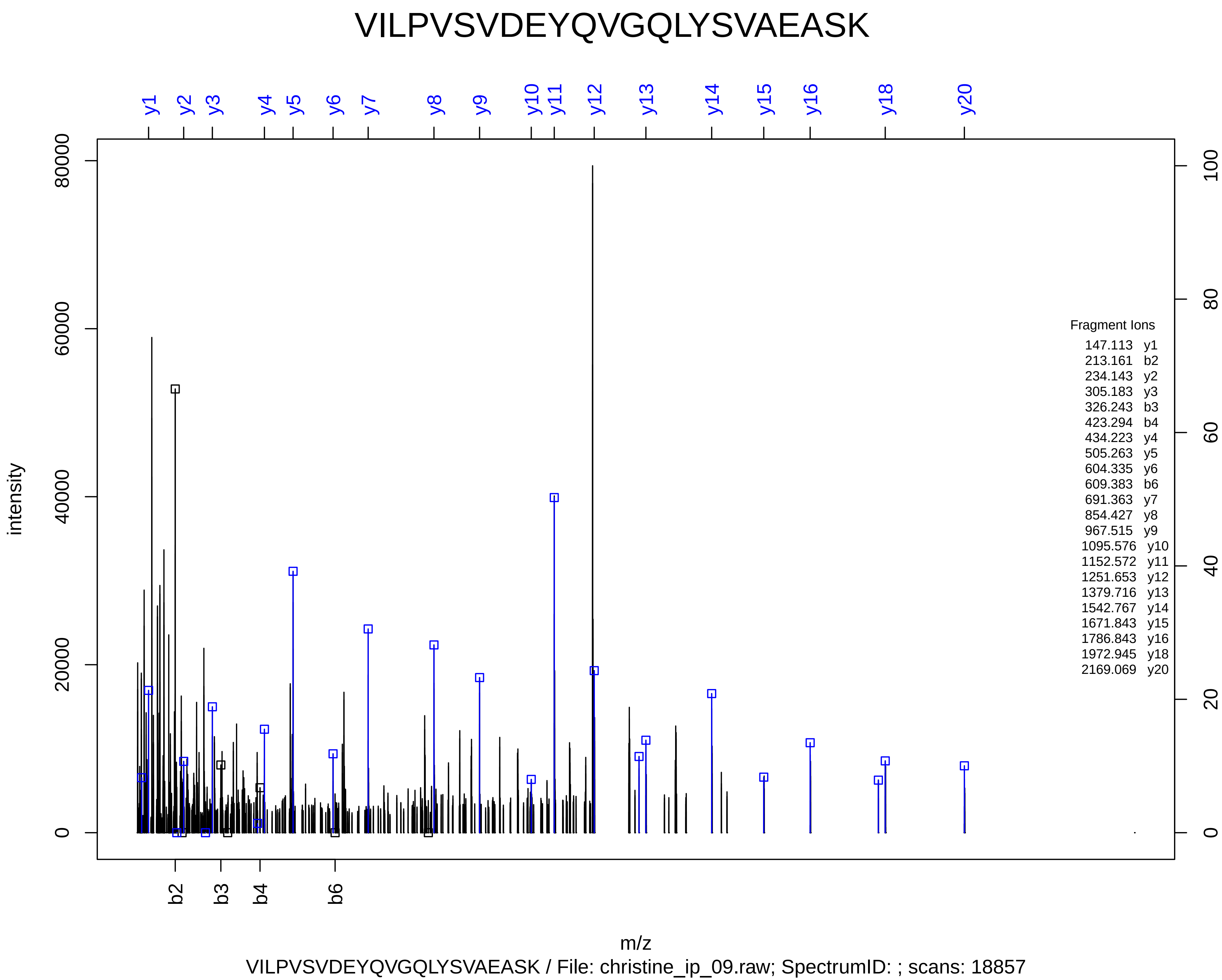
VILPVSVDEYQVGQLYSVAEASK
y1 y2 y3 y4 y5 y6 y7 y8 y9 y10 y11 y12 y13 y14 y15 y16 y18 y20
b2 b3 b4 b6
intensity
0
20000
40000
60000
80000
0
20
40
60
80
100
m/z
Fragment Ions
147.113 y1
213.161 b2
234.143 y2
305.183 y3
326.243 b3
423.294 b4
434.223 y4
505.263 y5
604.335 y6
609.383 b6
691.363 y7
854.427 y8
967.515 y9
1095.576 y10
1152.572 y11
1251.653 y12
1379.716 y13
1542.767 y14
1671.843 y15
1786.843 y16
1972.945 y18
2169.069 y20
VILPVSVDEYQVGQLYSVAEASK / File: christine_ip_09.raw; SpectrumID: ; scans: 18857

SPGIISQASAPR

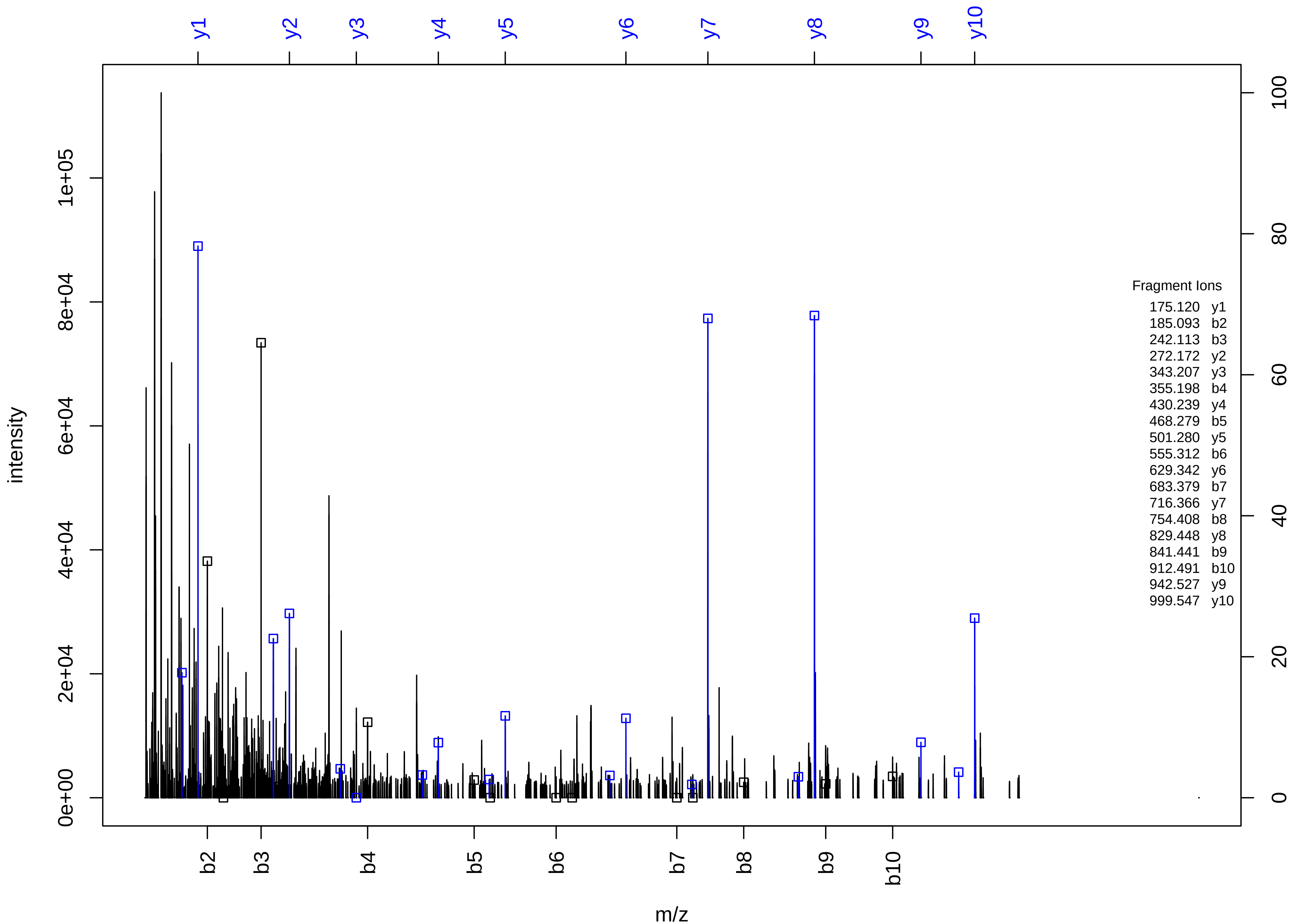

SPGIISQASAPR / File: christine_ip_09.raw; SpectrumID: ; scans: 5012

ILQEDPTNTAAR

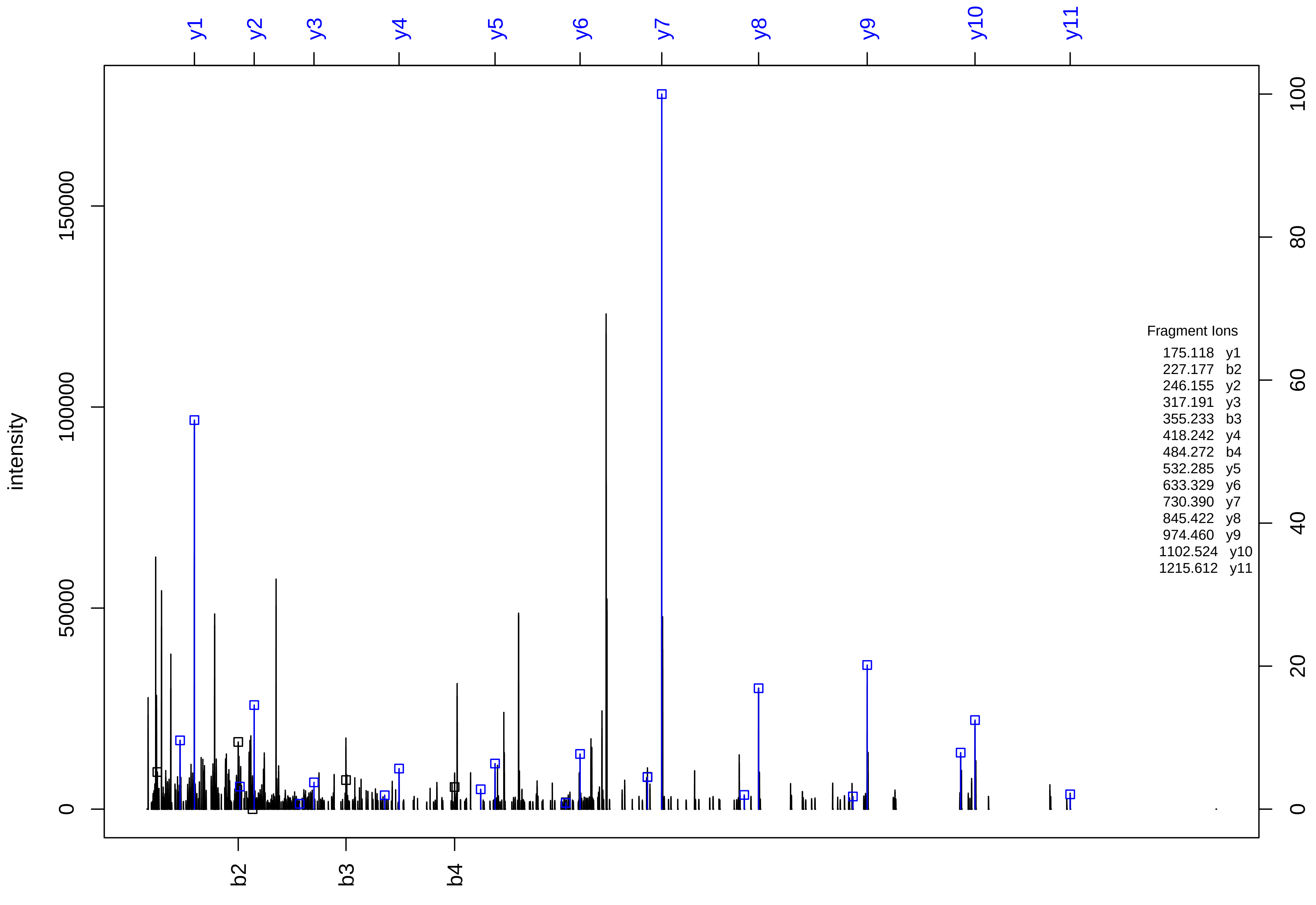

ILQEDPTNTAAR / File: christine_ip_09.raw; SpectrumID: ; scans: 3452

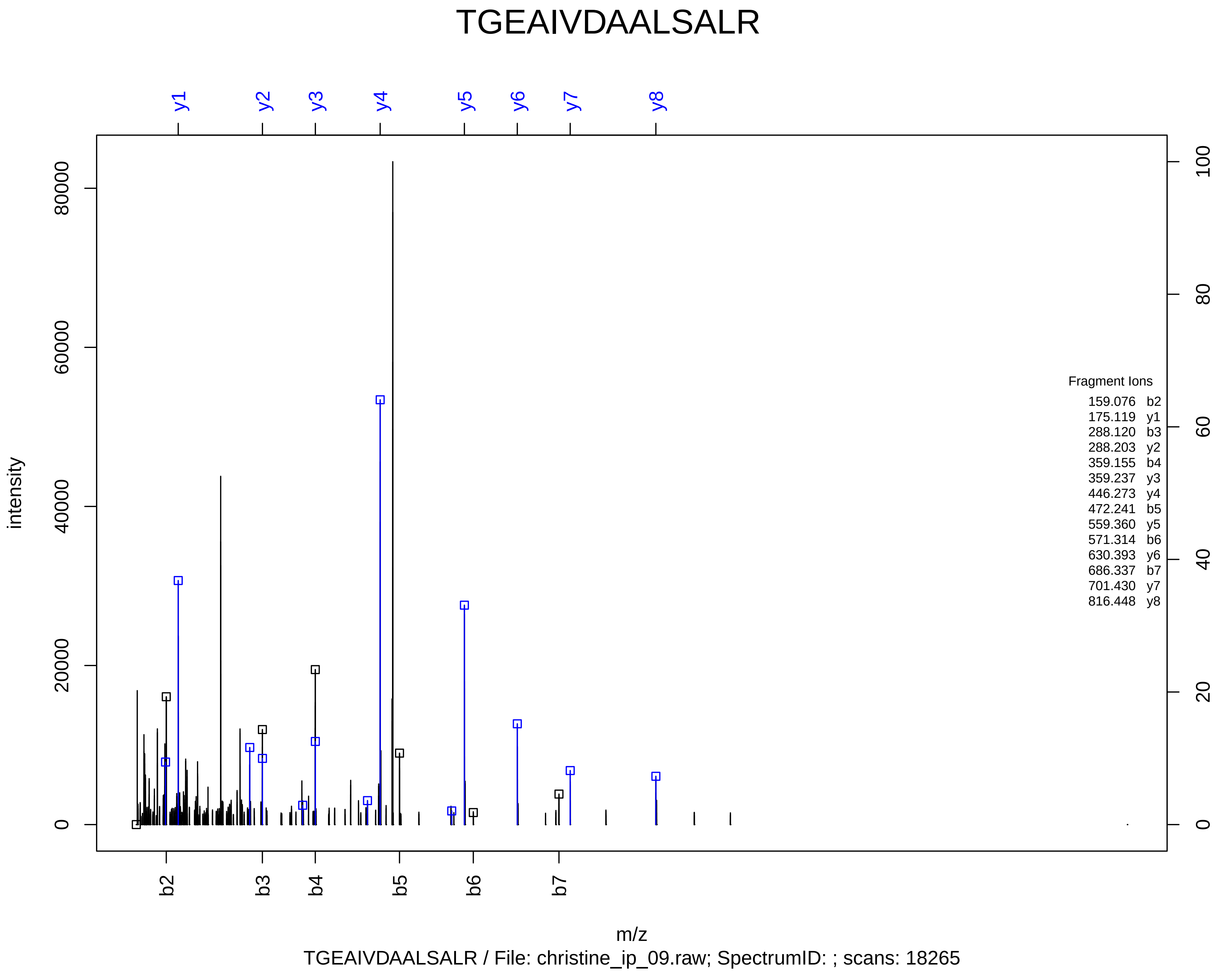
TGEAIVDAALSALR
y1
y2
y3
y4
y5
y6
y7
y8
intensity
0
20000
40000
60000
80000
0
20
40
60
80
100
Fragment Ions
159.076 b2
175.119 y1
288.120 b3
288.203 y2
359.155 b4
359.237 y3
446.273 y4
472.241 b5
559.360 y5
571.314 b6
630.393 y6
686.337 b7
701.430 y7
816.448 y8
b2
b3
b4
b5
b6
b7
m/z
TGEAIVDAALSALR / File: christine_ip_09.raw; SpectrumID: ; scans: 18265

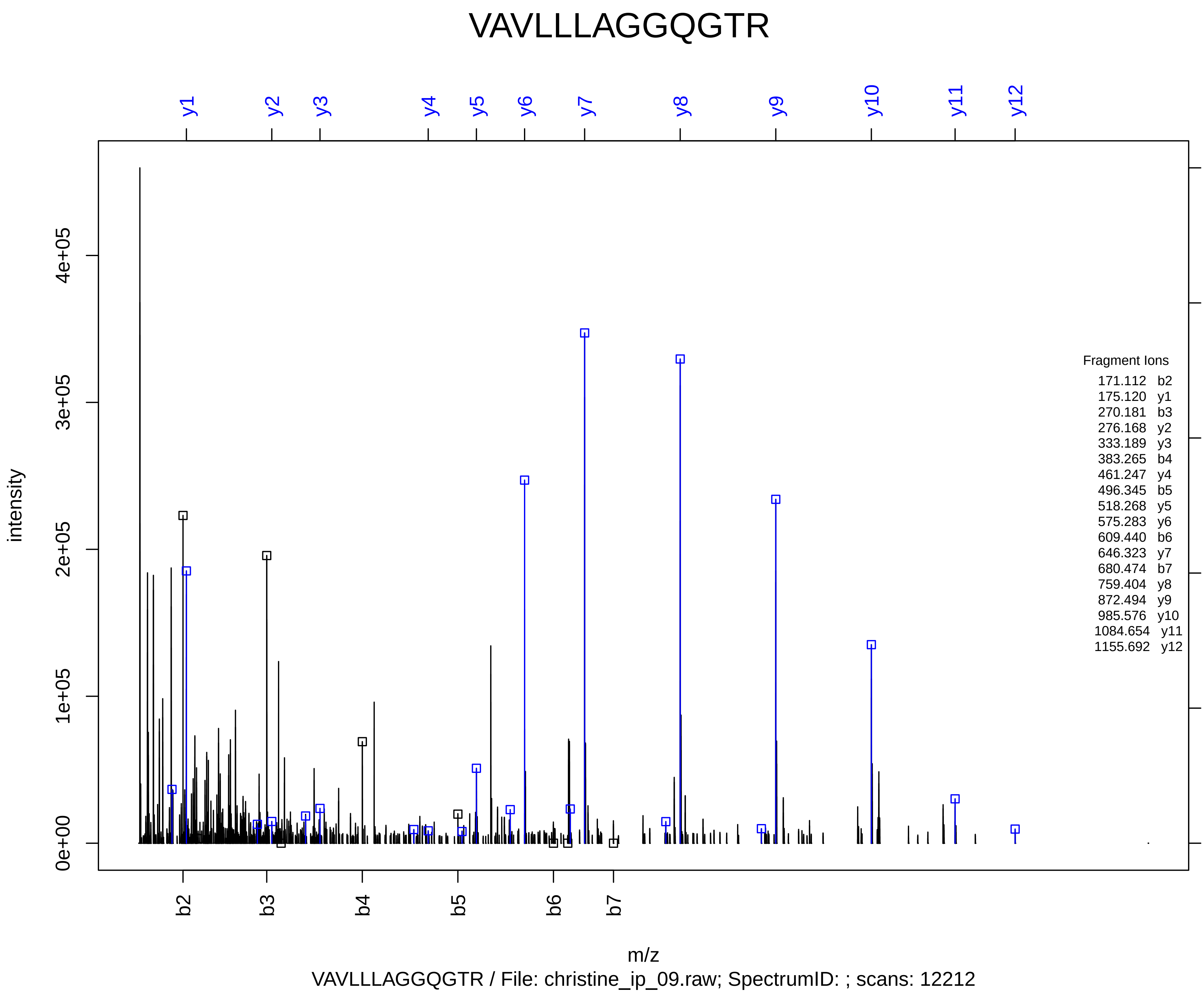
VAVLLLAGGQGTR
y1
y2
y3
y4
y5
y6
y7
y8
y9
y10
y11
y12
intensity
0e+00
1e+05
2e+05
3e+05
4e+05
0
20
40
60
80
100
Fragment Ions
171.112 b2
175.120 y1
270.181 b3
276.168 y2
333.189 y3
383.265 b4
461.247 y4
496.345 b5
518.268 y5
575.283 y6
609.440 b6
646.323 y7
680.474 b7
759.404 y8
872.494 y9
985.576 y10
1084.654 y11
1155.692 y12
b2
b3
b4
b5
b6
b7
m/z
VAVLLLAGGQGTR / File: christine_ip_09.raw; SpectrumID: ; scans: 12212

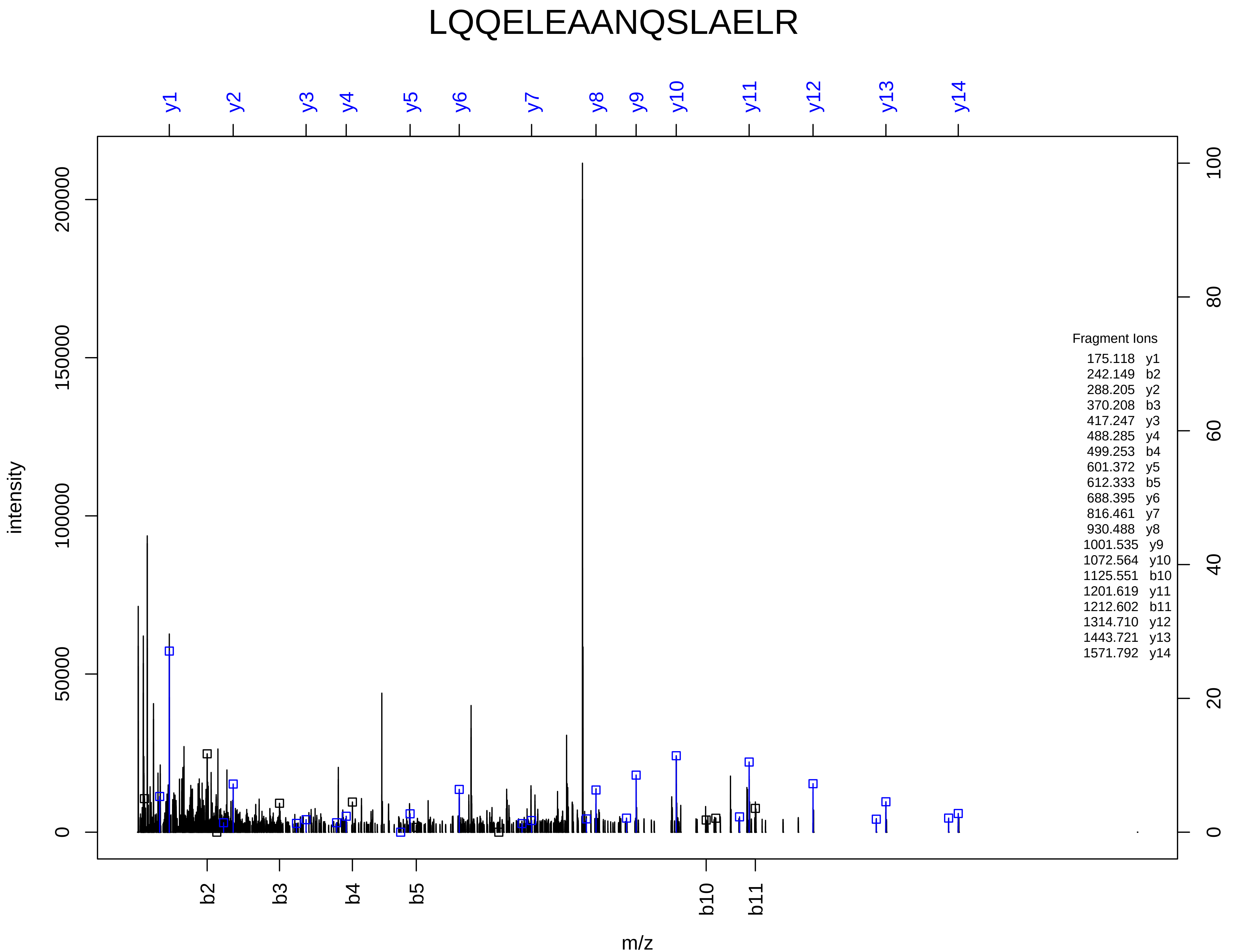

LQQELEAANQSLAELR / File: christine_ip_09.raw; SpectrumID: ; scans: 11991

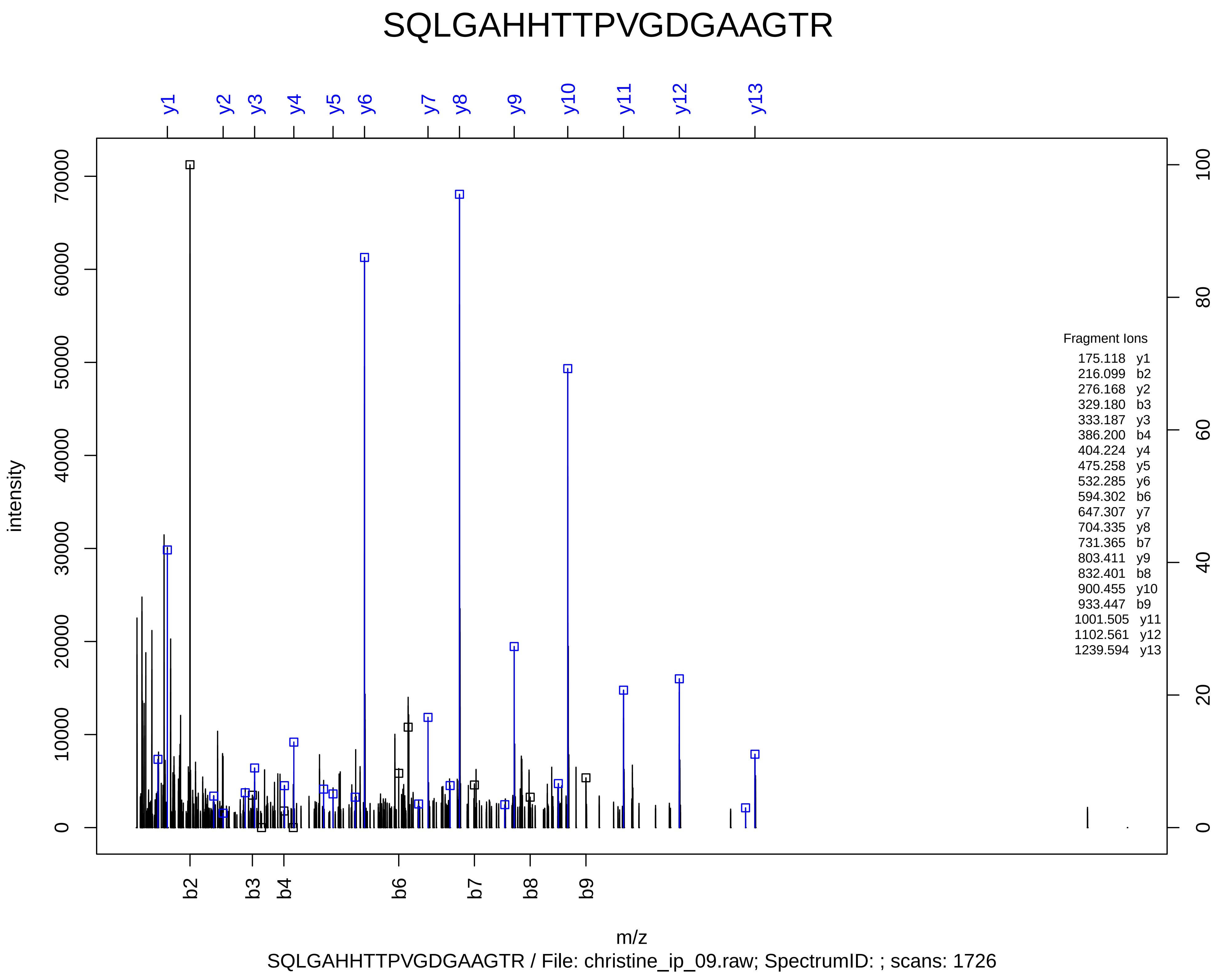
SQLGAHHTTPVGDGAAGTR
y1
y2
y3
y4
y5
y6
y7
y8
y9
y10
y11
y12
y13
intensity
0
10000
20000
30000
40000
50000
60000
70000
0
20
40
60
80
100
b2
b3
b4
b6
b7
b8
b9
m/z
Fragment Ions
175.118 y1
216.099 b2
276.168 y2
329.180 b3
333.187 y3
386.200 b4
404.224 y4
475.258 y5
532.285 y6
594.302 b6
647.307 y7
704.335 y8
731.365 b7
803.411 y9
832.401 b8
900.455 y10
933.447 b9
1001.505 y11
1102.561 y12
1239.594 y13
SQLGAHHTTPVGDGAAGTR / File: christine_ip_09.raw; SpectrumID: ; scans: 1726

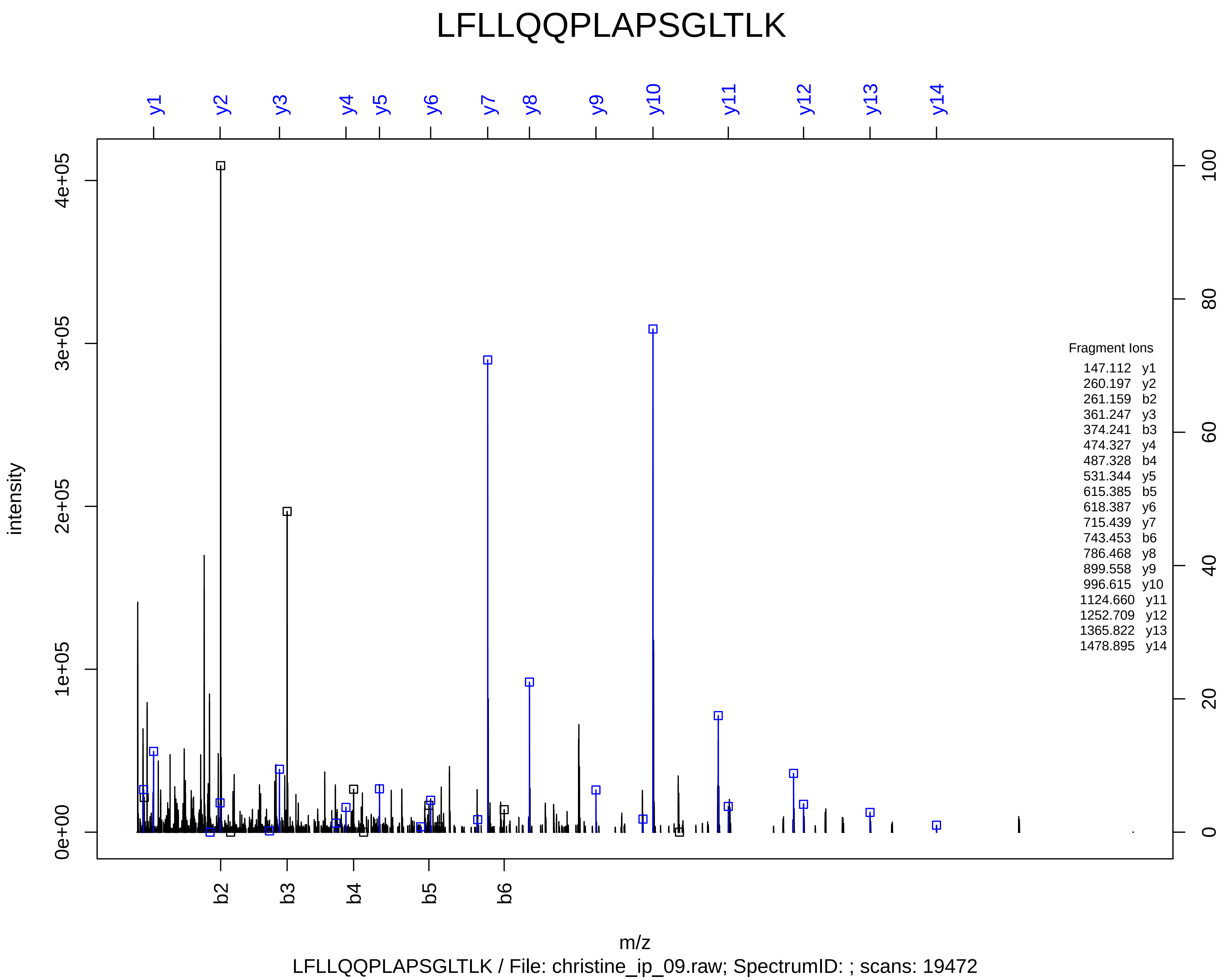
LFLLQQPLAPSGLTLK
y1
y2
y3
y4
y5
y6
y7
y8
y9
y10
y11
y12
y13
y14
b2
b3
b4
b5
b6
intensity
0e+00
1e+05
2e+05
3e+05
4e+05
0
20
40
60
80
100
Fragment Ions
147.112 y1
260.197 y2
261.159 b2
361.247 y3
374.241 b3
474.327 y4
487.328 b4
531.344 y5
615.385 b5
618.387 y6
715.439 y7
743.453 b6
786.468 y8
899.558 y9
996.615 y10
1124.660 y11
1252.709 y12
1365.822 y13
1478.895 y14
m/z
LFLLQQPLAPSGLTLK / File: christine_ip_09.raw; SpectrumID: ; scans: 19472

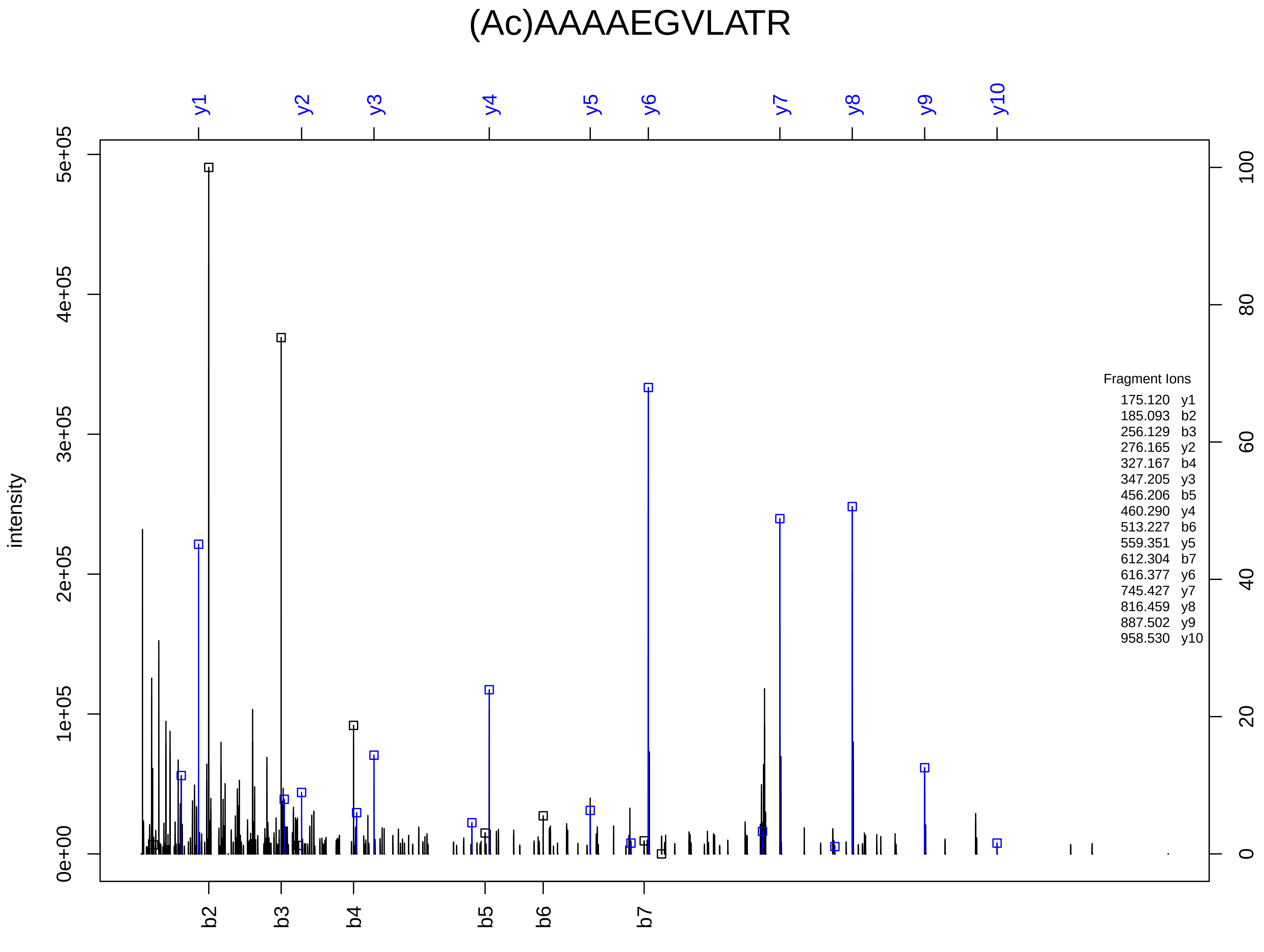

AAAAEGVLATR / File: christine_ip_09.raw; SpectrumID: ; scans: 11562

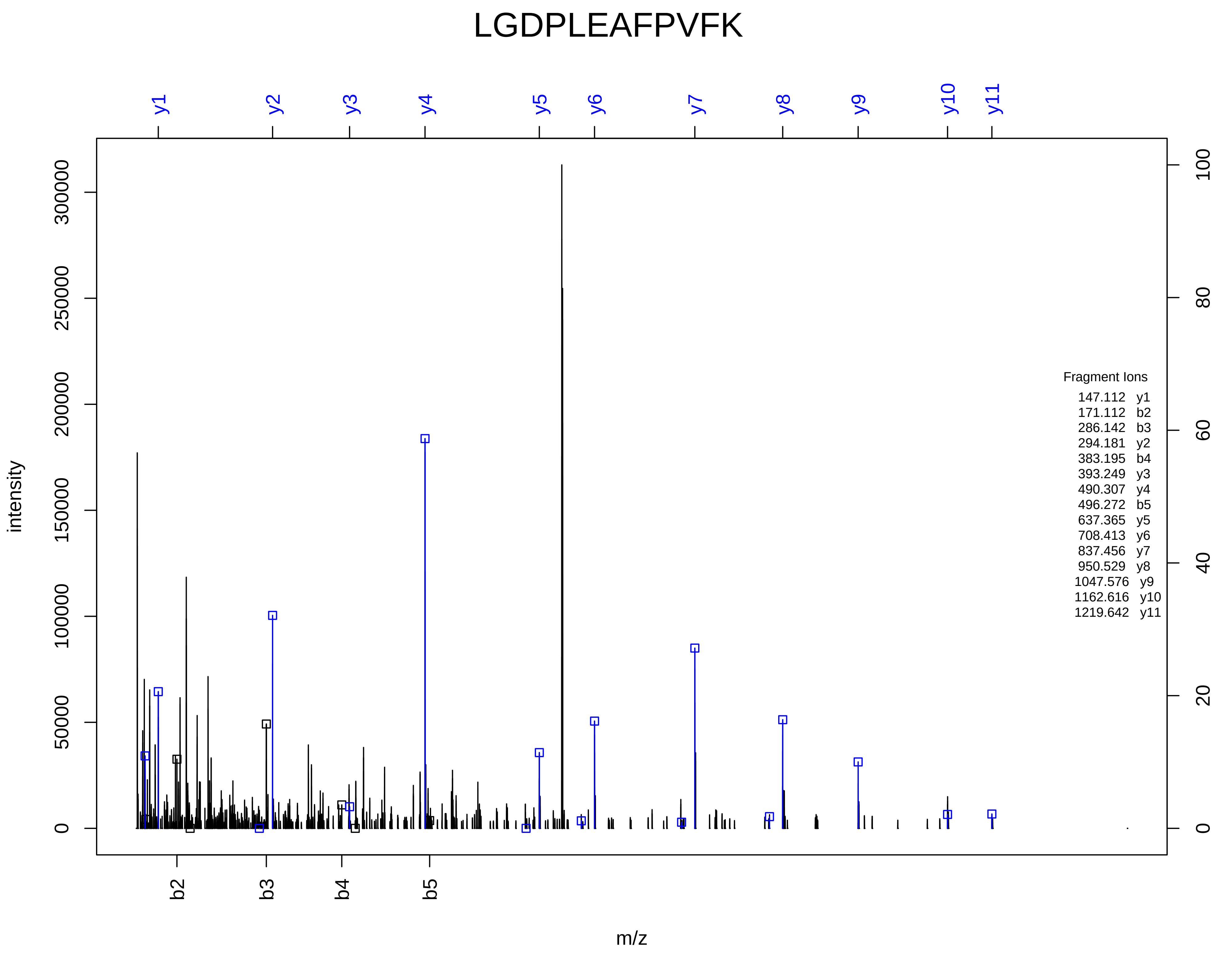
LGDPLEAFPVFK
y1
y2
y3
y4
y5
y6
y7
y8
y9
y10
y11
b2
b3
b4
b5
intensity
m/z
Fragment Ions
147.112 y1
171.112 b2
286.142 b3
294.181 y2
383.195 b4
393.249 y3
490.307 y4
496.272 b5
637.365 y5
708.413 y6
837.456 y7
950.529 y8
1047.576 y9
1162.616 y10
1219.642 y11
LGDPLEAFPVFK / File: christine_ip_09.raw; SpectrumID: ; scans: 18802

ELENEENQEEQGLEEK

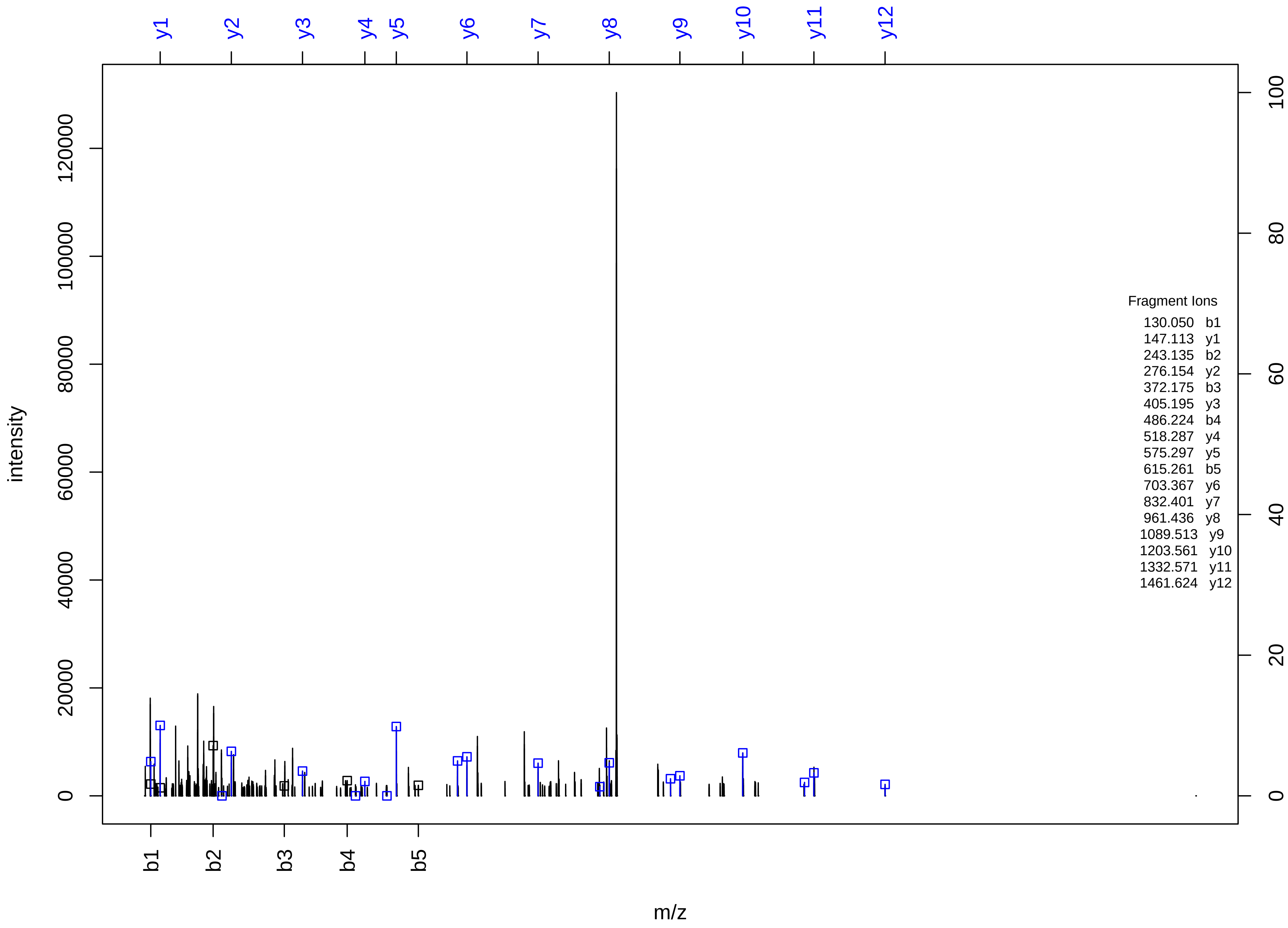

ELENEENQEEQGLEEK / File: christine_ip_09.raw; SpectrumID: ; scans: 4370

(Ac)KLSVN^EAQLGFYLGSLSHLSACPGIDPR

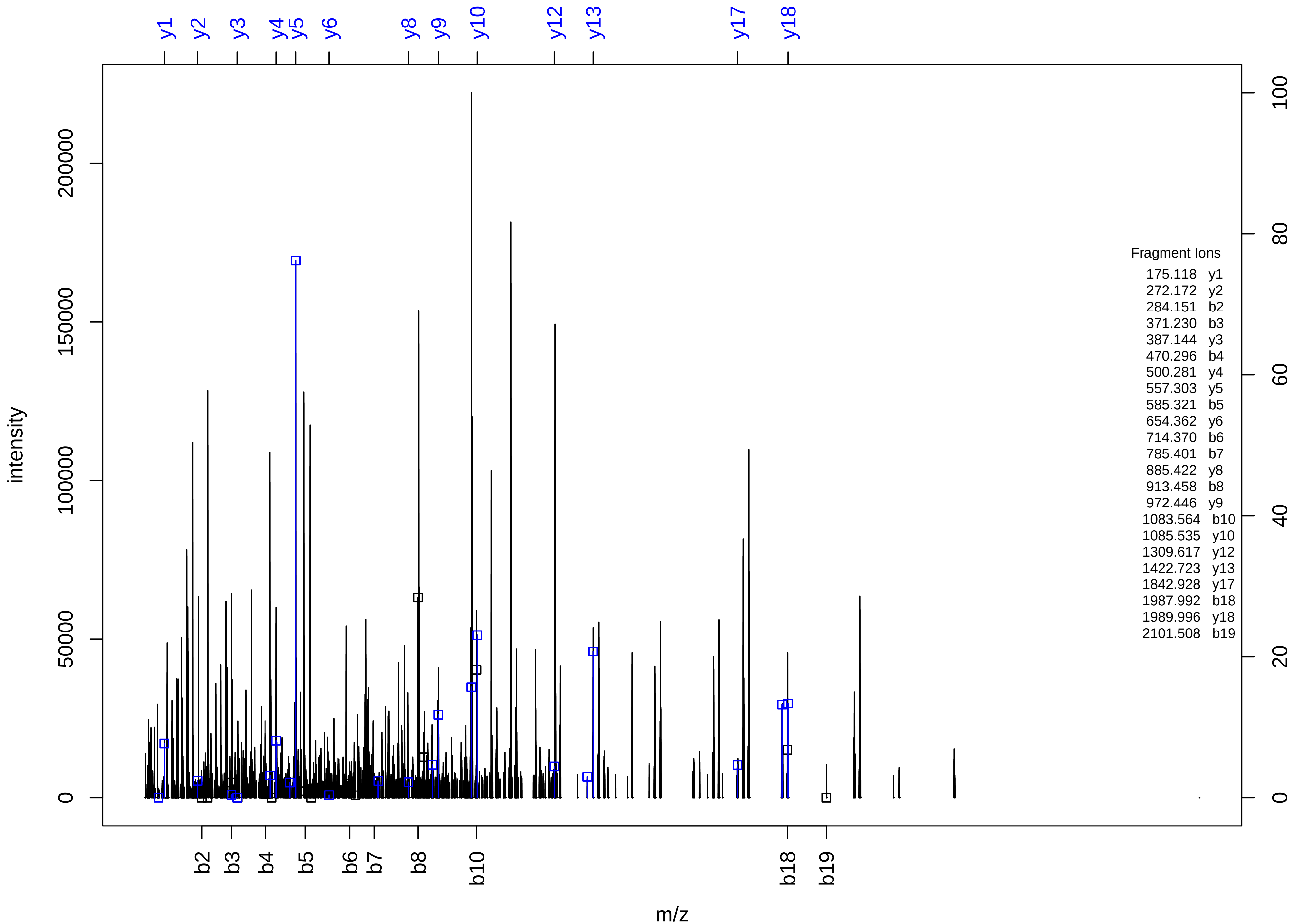

KLSVNEAQLGFYLGSLSHLSACPGIDPR / File: christine_ip_09.raw; SpectrumID: ; scans: 16862

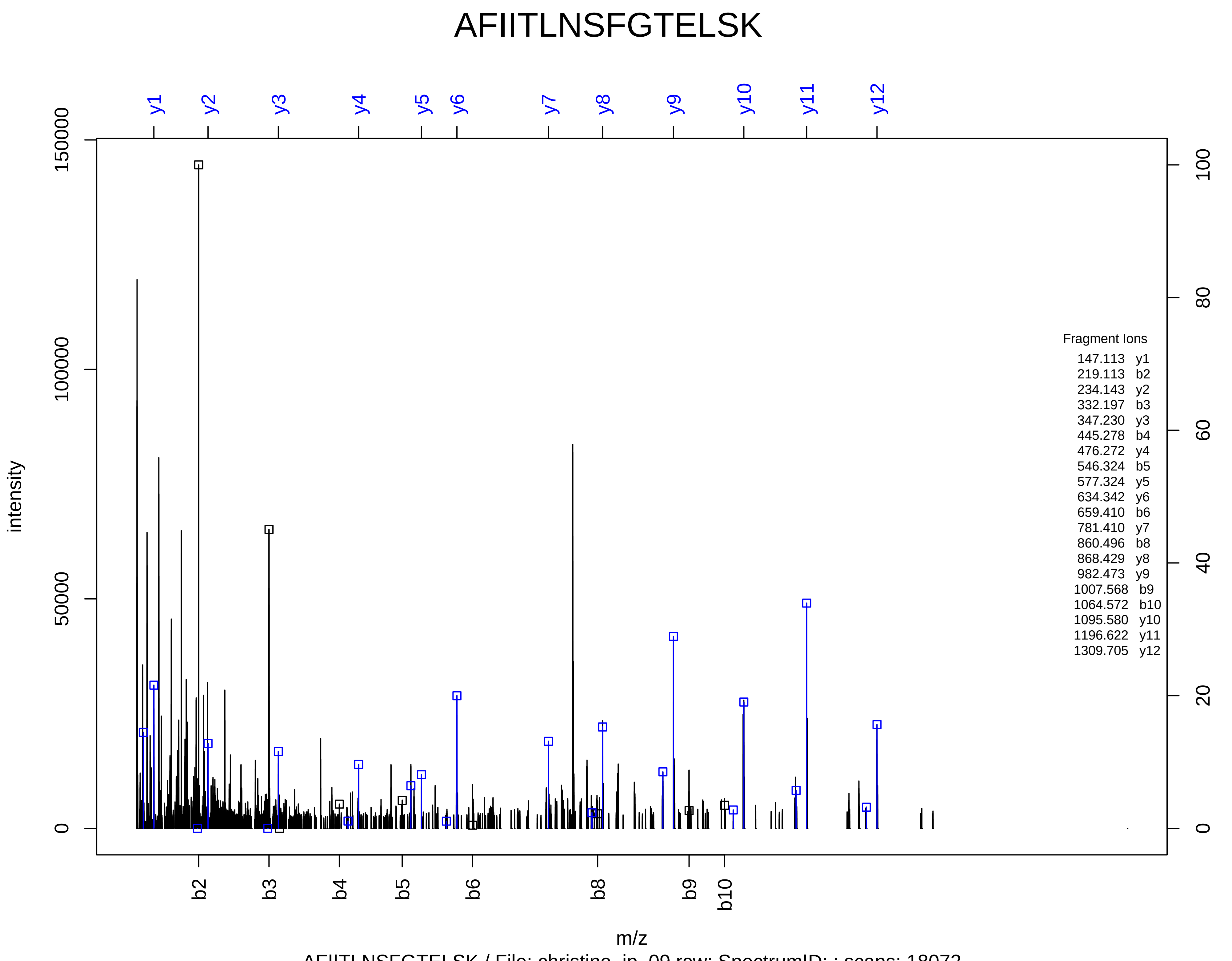

AFIITLNSFGTELSK
y1
y2
y3
y4
y5
y6
y7
y8
y9
y10
y11
y12
intensity
0
50000
100000
150000
0
20
40
60
80
100
Fragment Ions
147.113 y1
219.113 b2
234.143 y2
332.197 b3
347.230 y3
445.278 b4
476.272 y4
546.324 b5
577.324 y5
634.342 y6
659.410 b6
781.410 y7
860.496 b8
868.429 y8
982.473 y9
1007.568 b9
1064.572 b10
1095.580 y10
1196.622 y11
1309.705 y12
b2
b3
b4
b5
b6
b8
b9
b10
m/z
AFIITLNSFGTELSK / File: christine_ip_09.raw; SpectrumID: ; scans: 18072

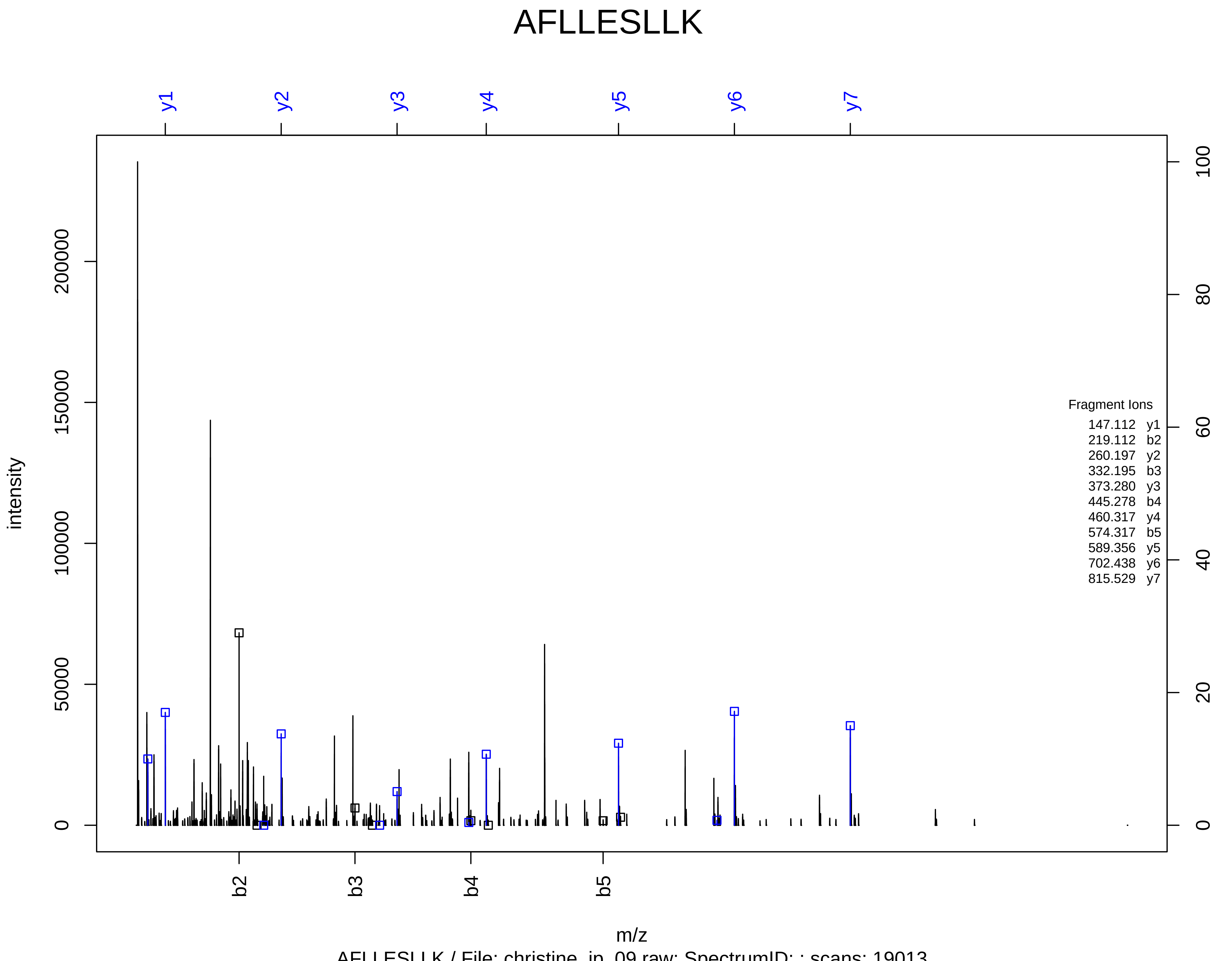

AFLLESLLK
y1
y2
y3
y4
y5
y6
y7
intensity
0
50000
100000
150000
200000
0
20
40
60
80
100
b2
b3
b4
b5
m/z
Fragment Ions
147.112 y1
219.112 b2
260.197 y2
332.195 b3
373.280 y3
445.278 b4
460.317 y4
574.317 b5
589.356 y5
702.438 y6
815.529 y7
AFLLESLLK / File: christine_ip_09.raw; SpectrumID: ; scans: 19013

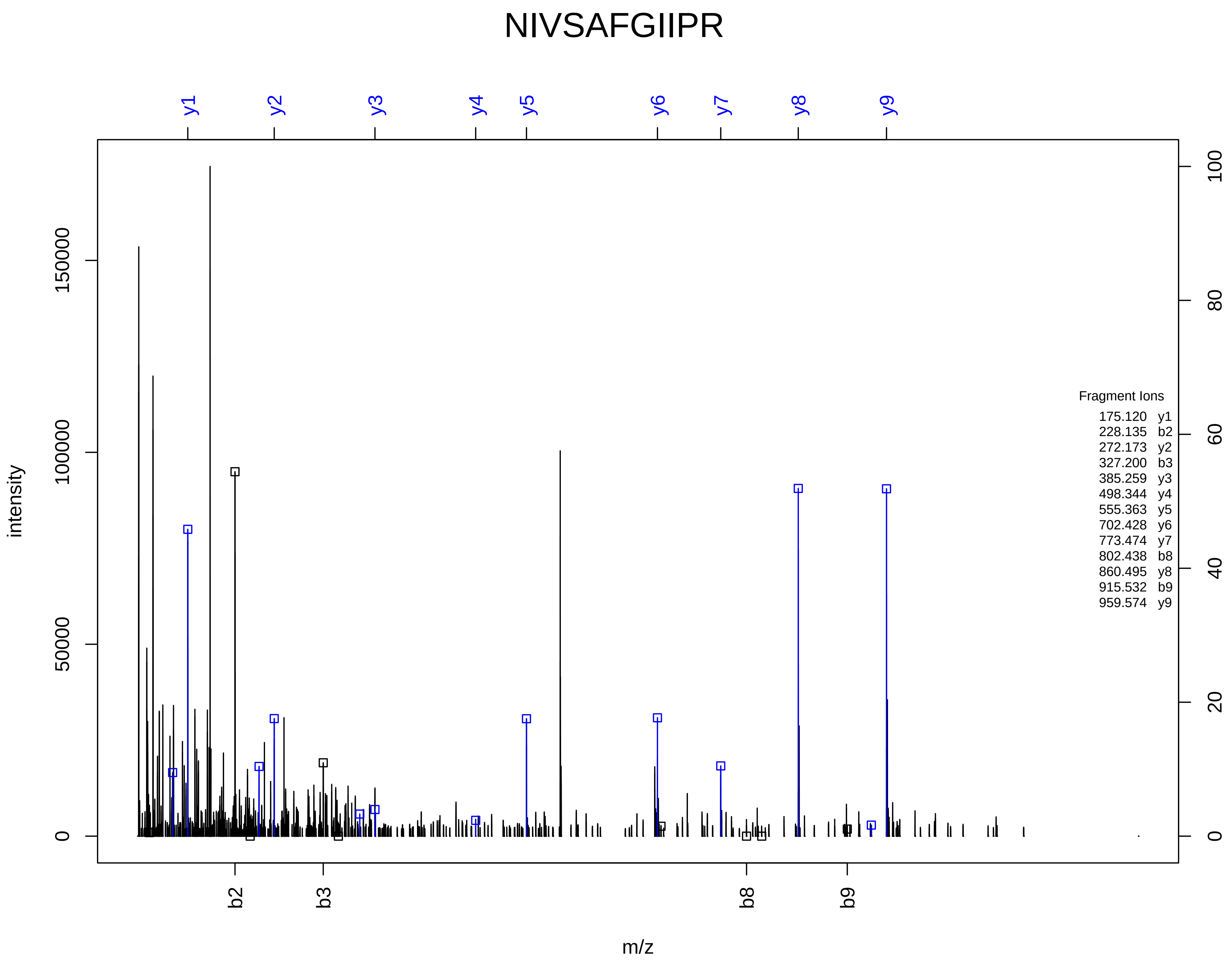

NIVSAFGIIPR
y1
y2
y3
y4
y5
y6
y7
y8
y9
b2
b3
b8
b9
intensity
m/z
0
50000
100000
150000
0
20
40
60
80
100
Fragment Ions
175.120 y1
228.135 b2
272.173 y2
327.200 b3
385.259 y3
498.344 y4
555.363 y5
702.428 y6
773.474 y7
802.438 b8
860.495 y8
915.532 b9
959.574 y9
NIVSAFGIIPR / File: christine_ip_09.raw; SpectrumID: ; scans: 15850

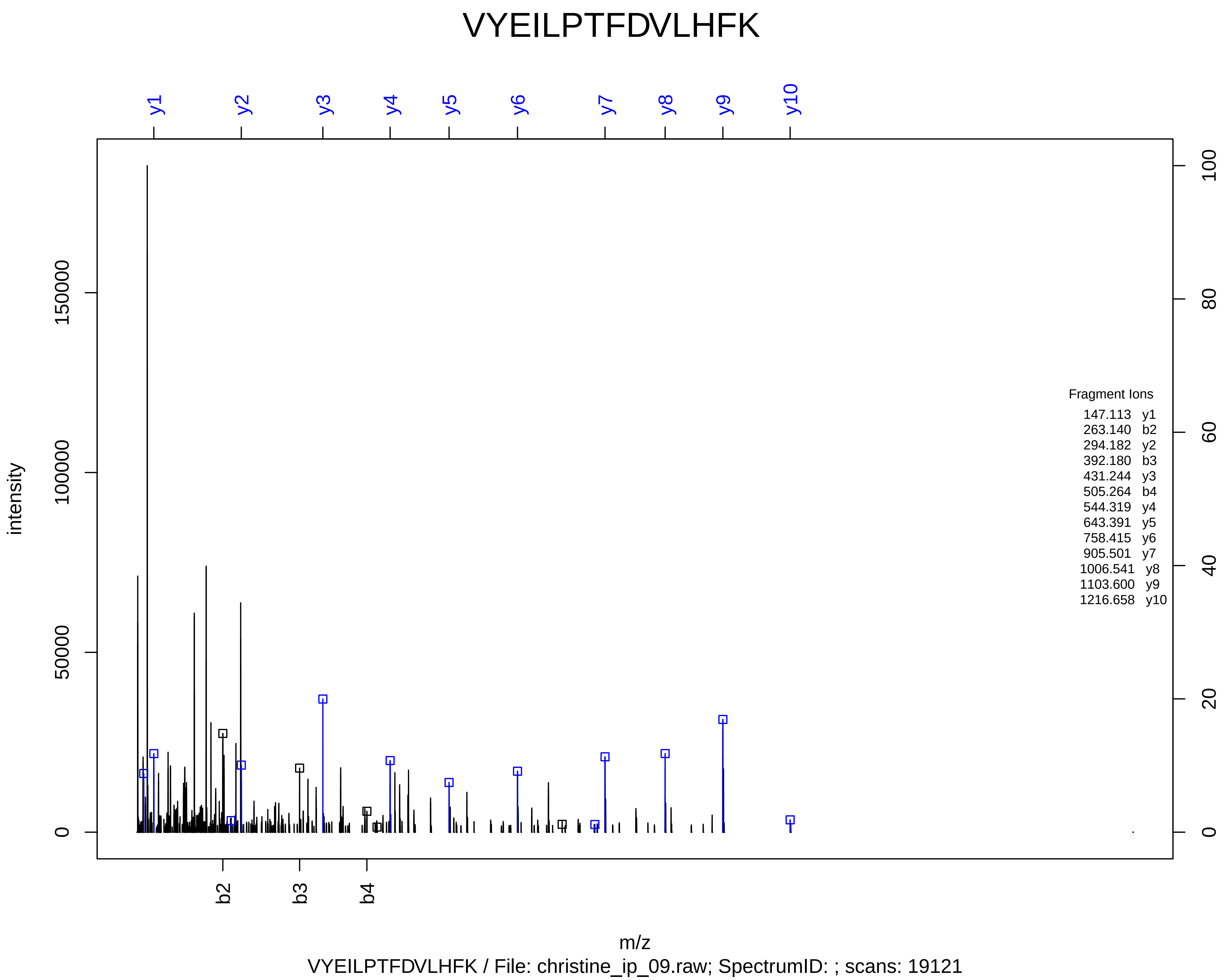
VYEILPTFDVLHFK
y1
y2
y3
y4
y5
y6
y7
y8
y9
y10
intensity
150000
100000
50000
0
100
80
60
40
20
0
b2
b3
b4
m/z
Fragment Ions
147.113 y1
263.140 b2
294.182 y2
392.180 b3
431.244 y3
505.264 b4
544.319 y4
643.391 y5
758.415 y6
905.501 y7
1006.541 y8
1103.600 y9
1216.658 y10
VYEILPTFDVLHFK / File: christine_ip_09.raw; SpectrumID: ; scans: 19121

VLATDFDDEFDDEEPLPAIGTCK

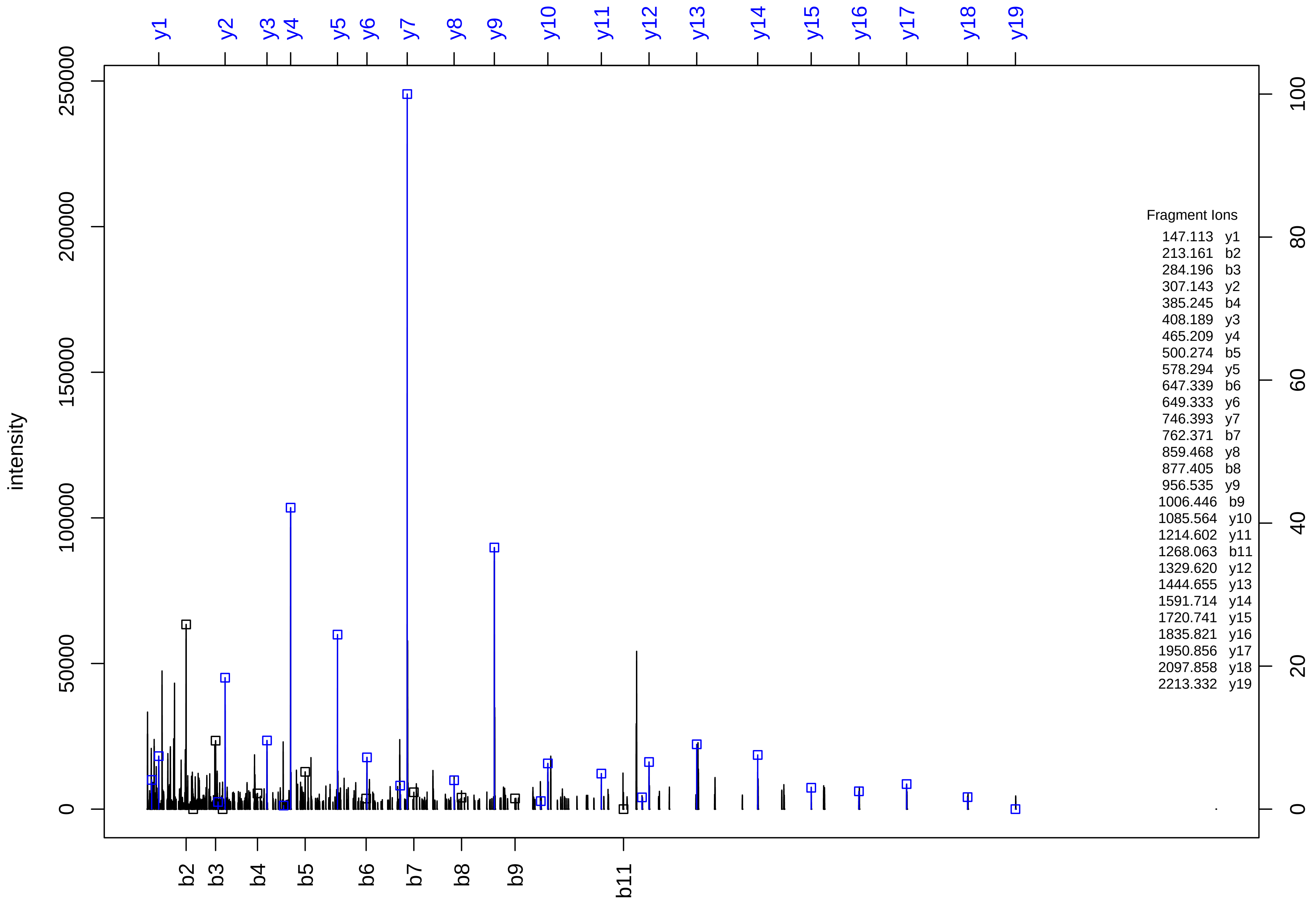

VLATDFDDEFDDEEPLPAIGTCK / File: christine_ip_09.raw; SpectrumID: ; scans: 16753

NFLSTPQFLYR

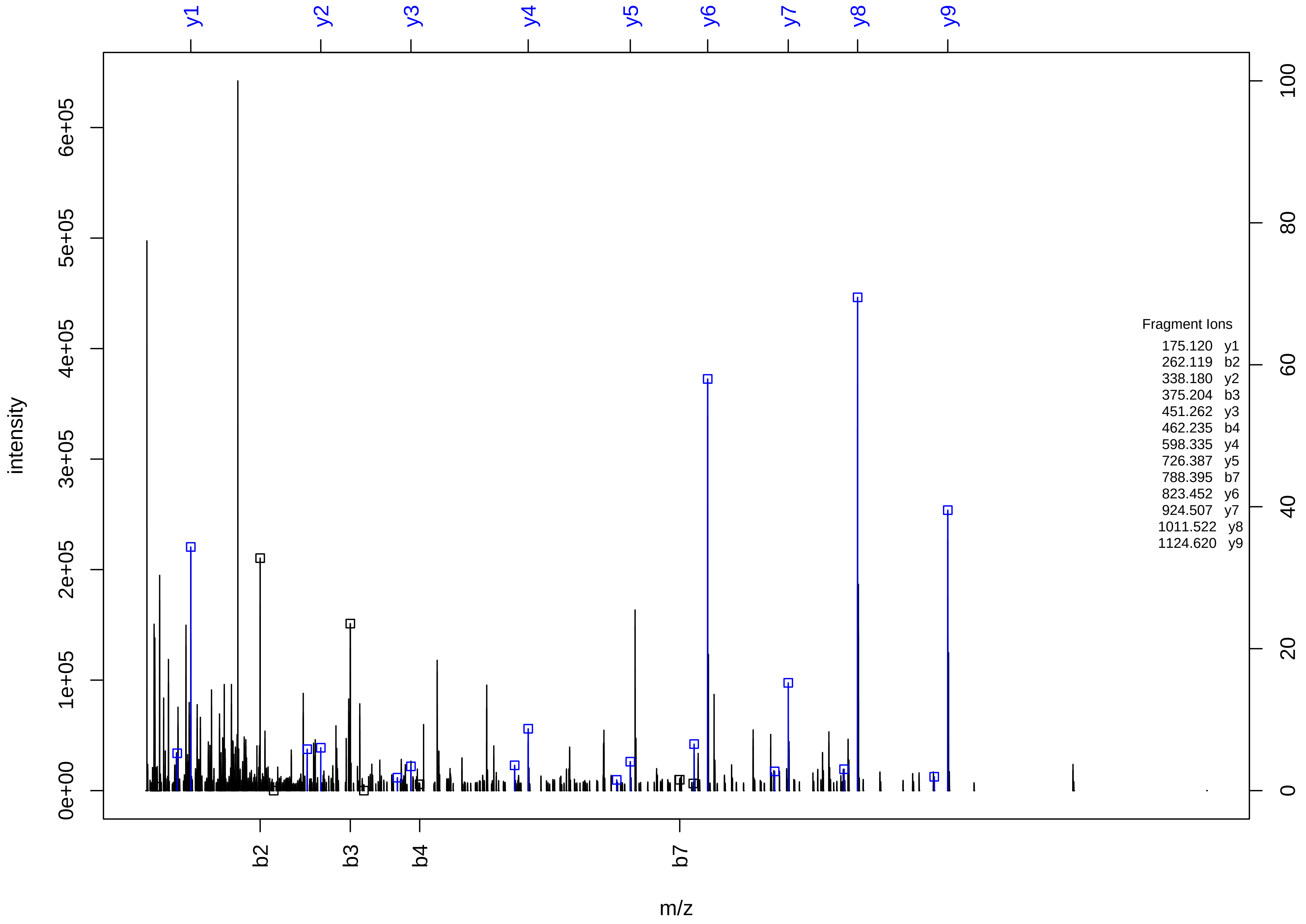

NFLSTPQFLYR / File: christine_ip_09.raw; SpectrumID: ; scans: 15108

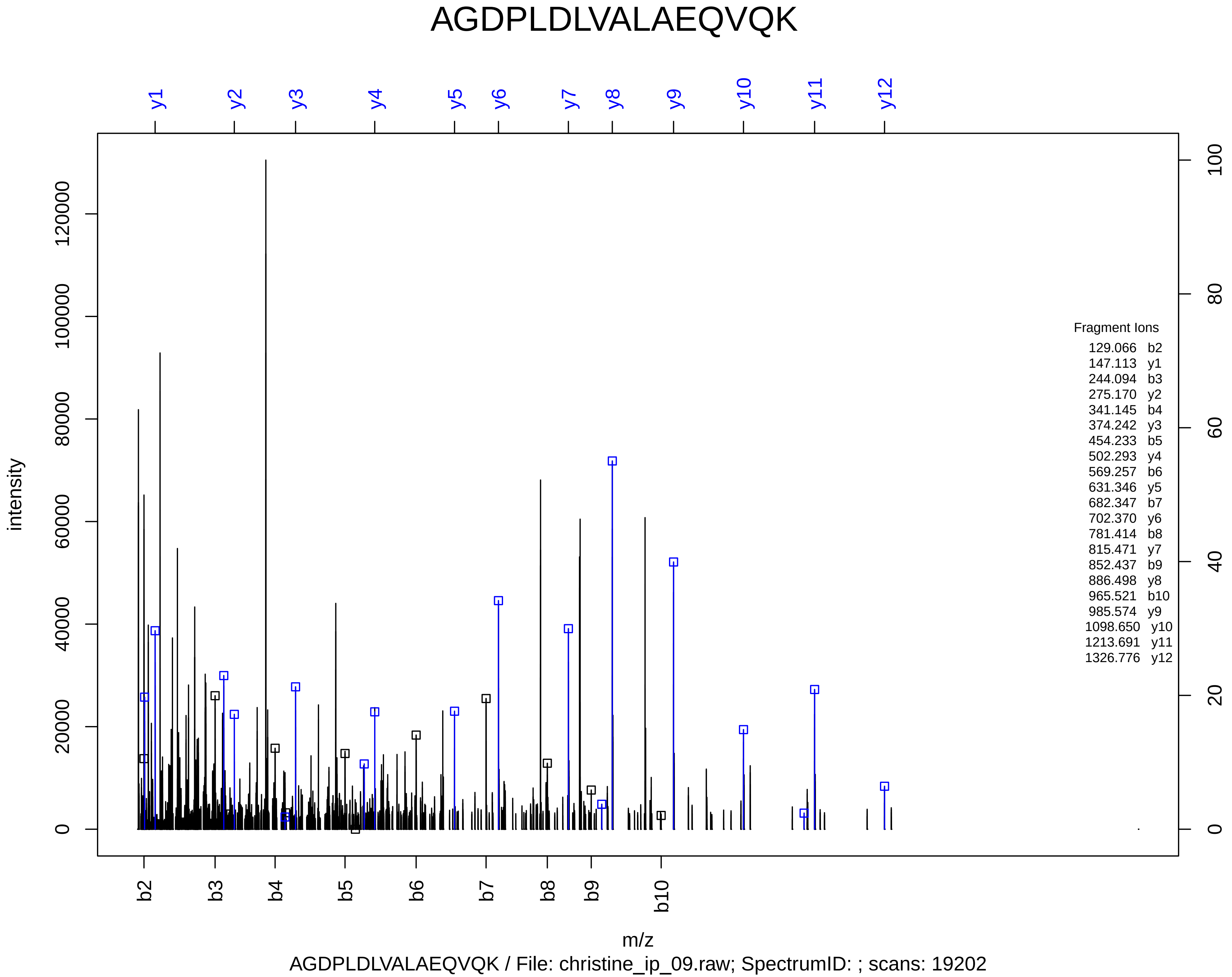
AGDPLDLVALAEQVQK
y1
y2
y3
y4
y5
y6
y7
y8
y9
y10
y11
y12
intensity
0
20000
40000
60000
80000
100000
120000
0
20
40
60
80
100
Fragment Ions
129.066 b2
147.113 y1
244.094 b3
275.170 y2
341.145 b4
374.242 y3
454.233 b5
502.293 y4
569.257 b6
631.346 y5
682.347 b7
702.370 y6
781.414 b8
815.471 y7
852.437 b9
886.498 y8
965.521 b10
985.574 y9
1098.650 y10
1213.691 y11
1326.776 y12
b2
b3
b4
b5
b6
b7
b8
b9
b10
m/z
AGDPLDLVALAEQVQK / File: christine_ip_09.raw; SpectrumID: ; scans: 19202

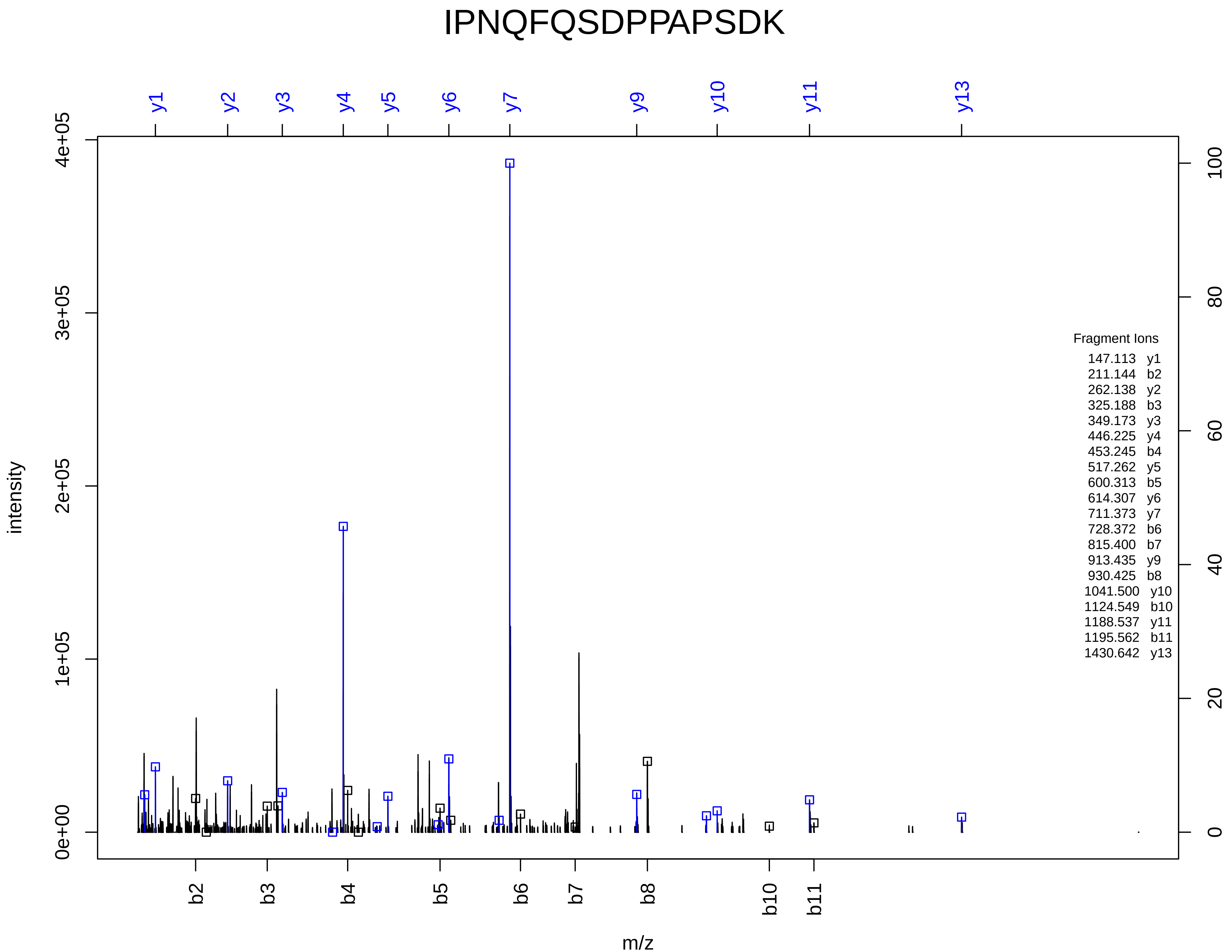

IPNQFQSDPPAPSDK / File: christine_ip_09.raw; SpectrumID: ; scans: 5269

LPAELGPQLLHK

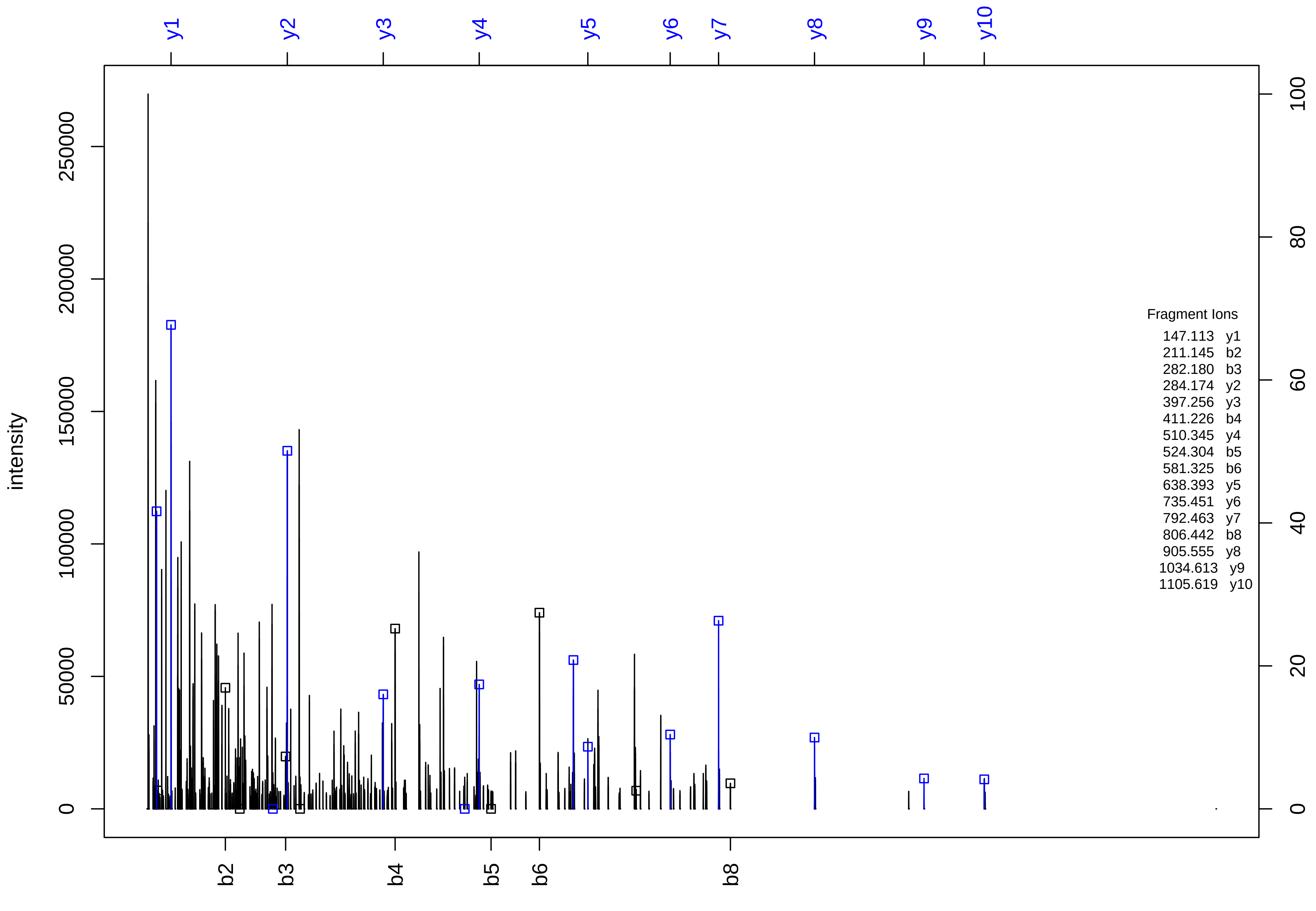

LPAELGPQLLHK / File: christine_ip_09.raw; SpectrumID: ; scans: 10042

SLSTPQFN^SLDVIADVNPK

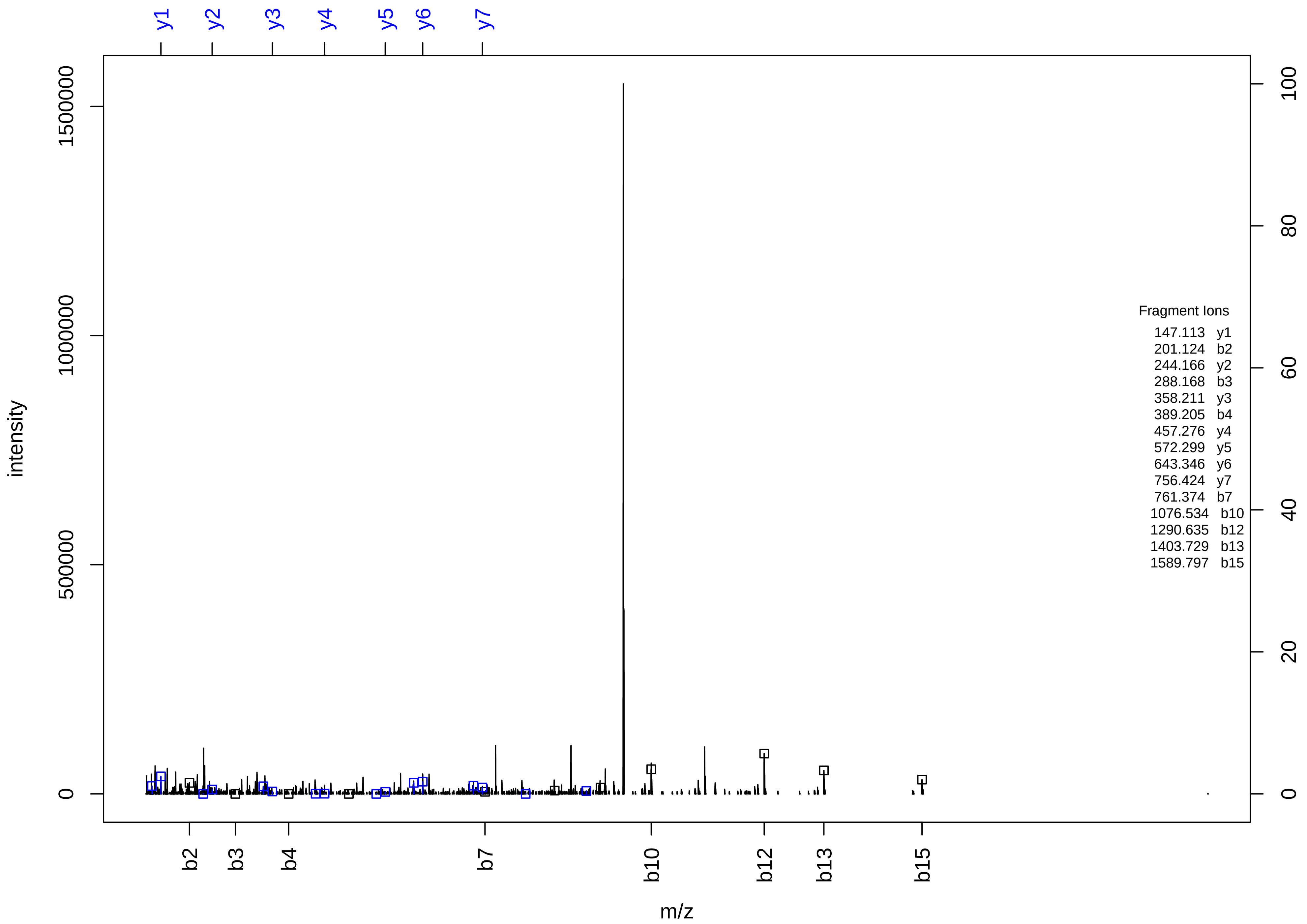

SLSTPQFNSLDVIADVNPK / File: christine_ip_09.raw; SpectrumID: ; scans: 15269

DPLVIELGQK

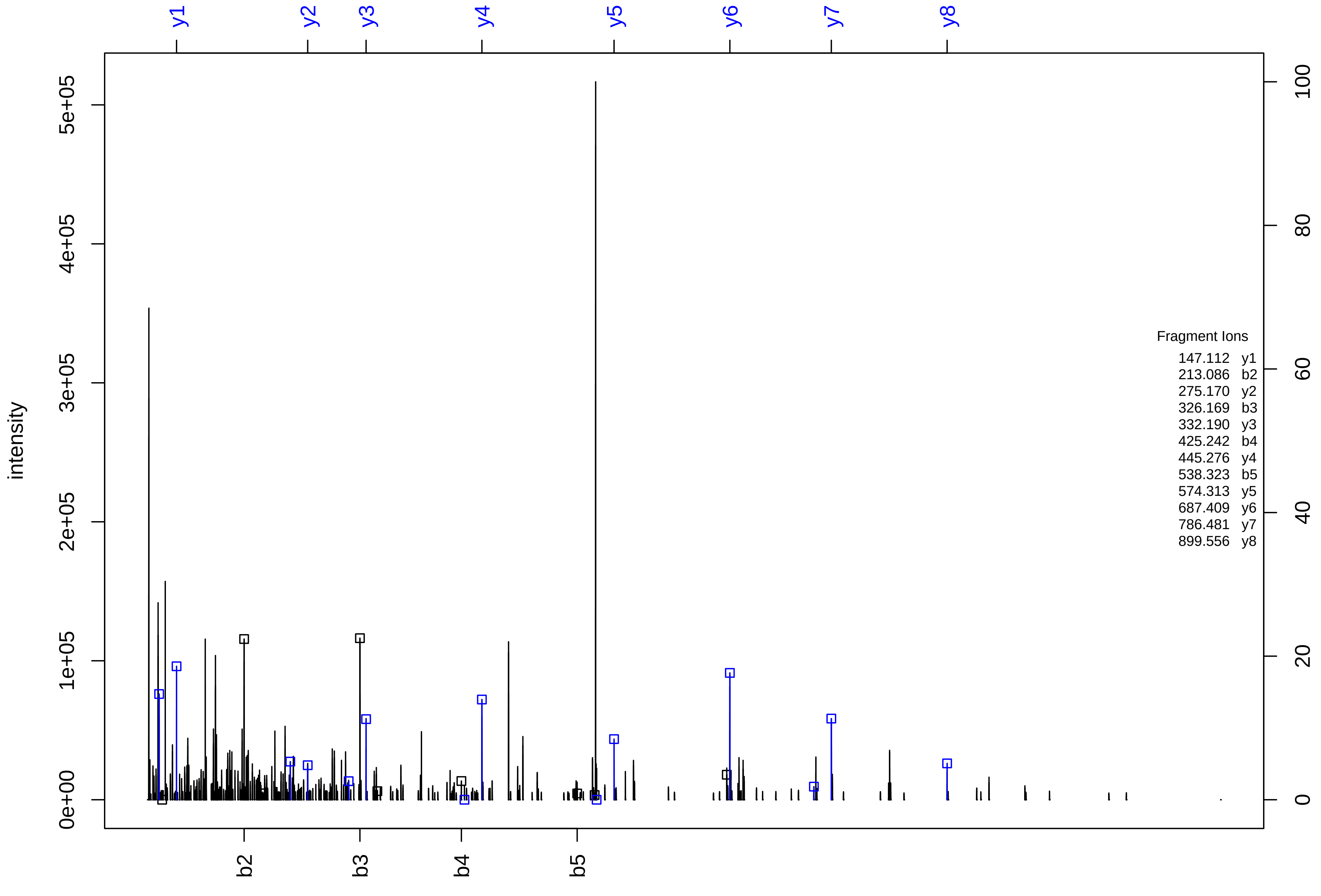

DPLVIELGQK / File: christine_ip_09.raw; SpectrumID: ; scans: 12167

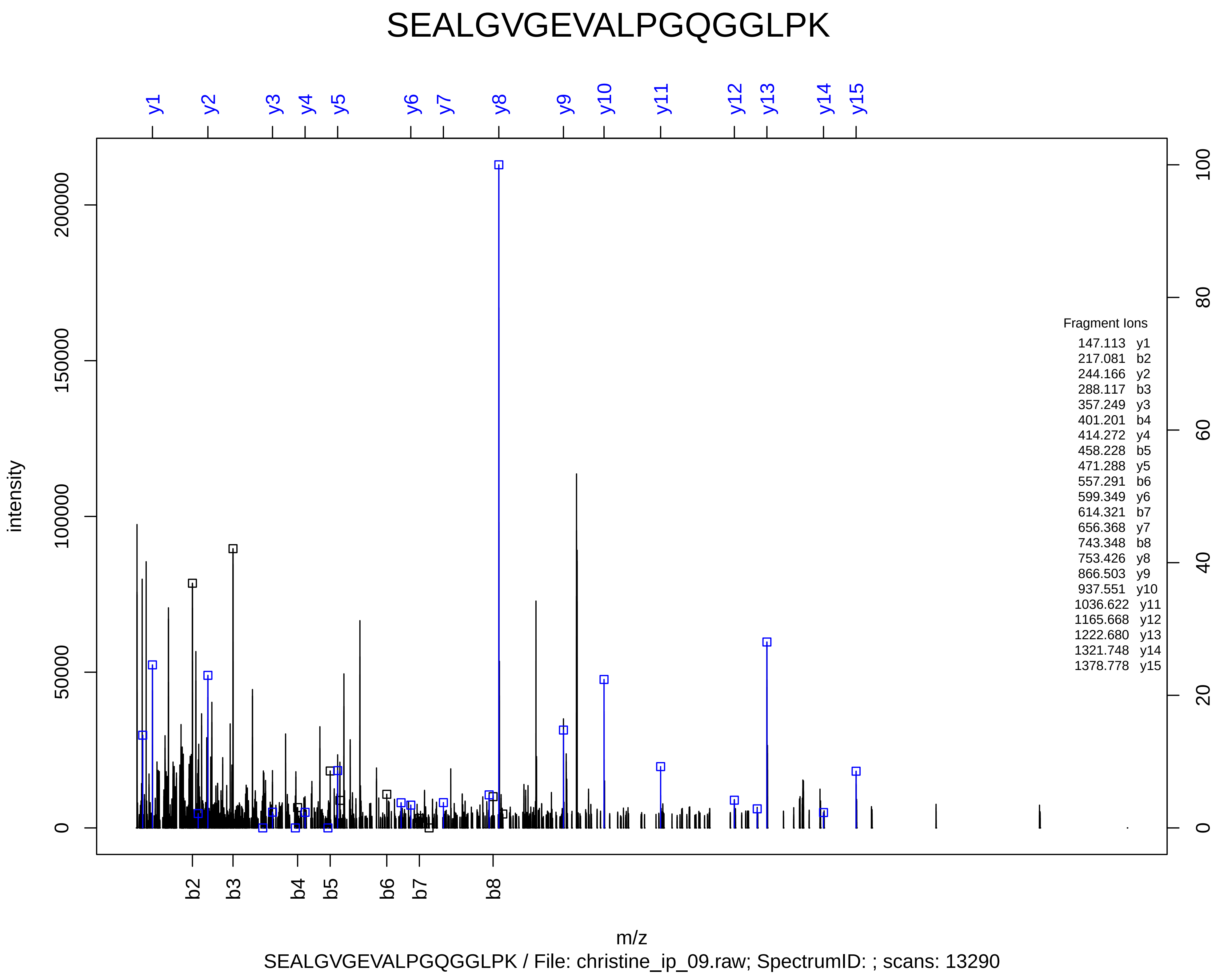
SEALGVGEVALPGQGGLPK
y1 y2 y3 y4 y5 y6 y7 y8 y9 y10 y11 y12 y13 y14 y15
intensity
0
50000
100000
150000
200000
0
20
40
60
80
100
b2 b3 b4 b5 b6 b7 b8
m/z
Fragment Ions
147.113 y1
217.081 b2
244.166 y2
288.117 b3
357.249 y3
401.201 b4
414.272 y4
458.228 b5
471.288 y5
557.291 b6
599.349 y6
614.321 b7
656.368 y7
743.348 b8
753.426 y8
866.503 y9
937.551 y10
1036.622 y11
1165.668 y12
1222.680 y13
1321.748 y14
1378.778 y15
SEALGVGEVALPGQGGLPK / File: christine_ip_09.raw; SpectrumID: ; scans: 13290

APPPSLTDCIGTVDSR

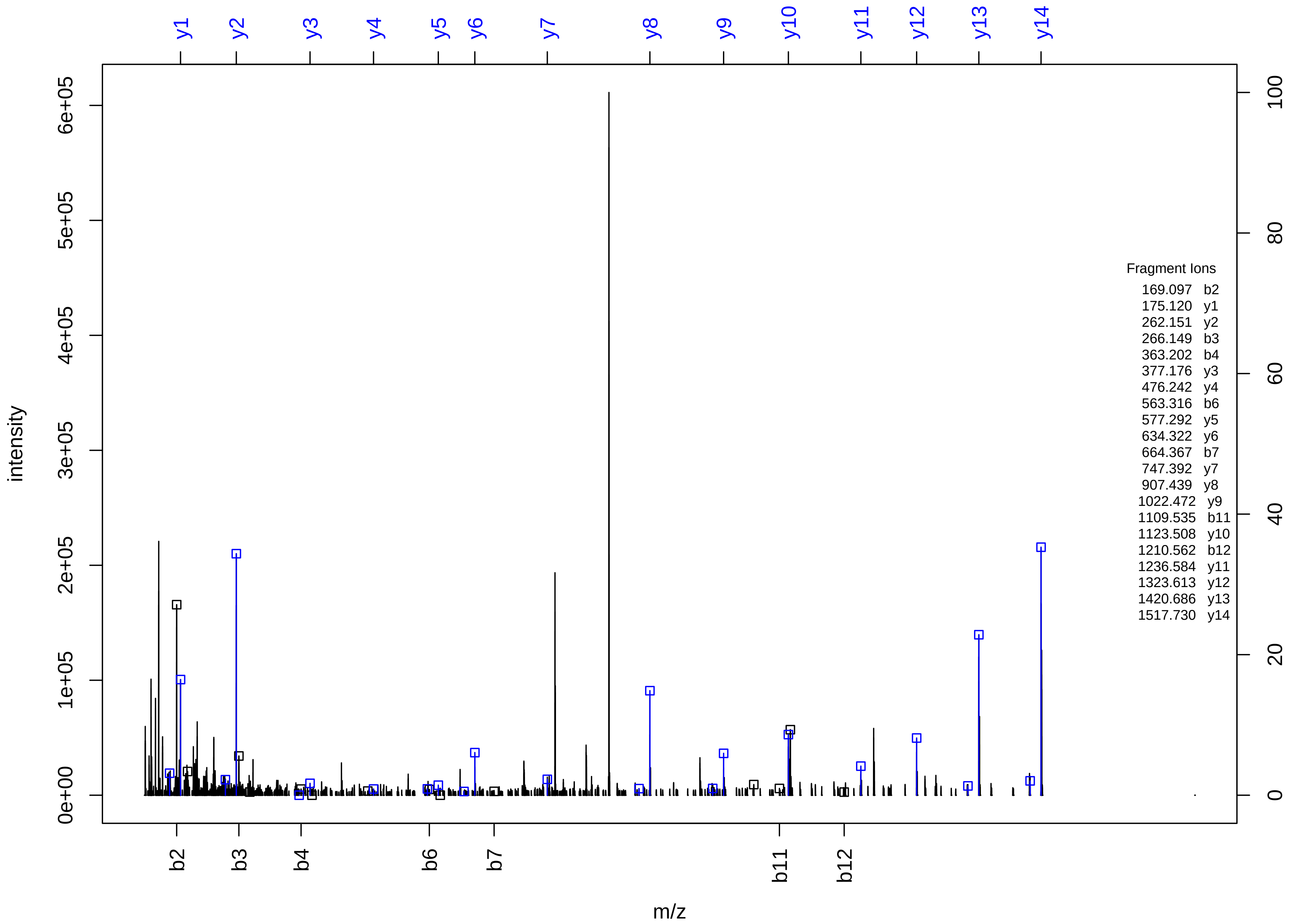

APPPSLTDCIGTVDSR / File: christine_ip_09.raw; SpectrumID: ; scans: 9573

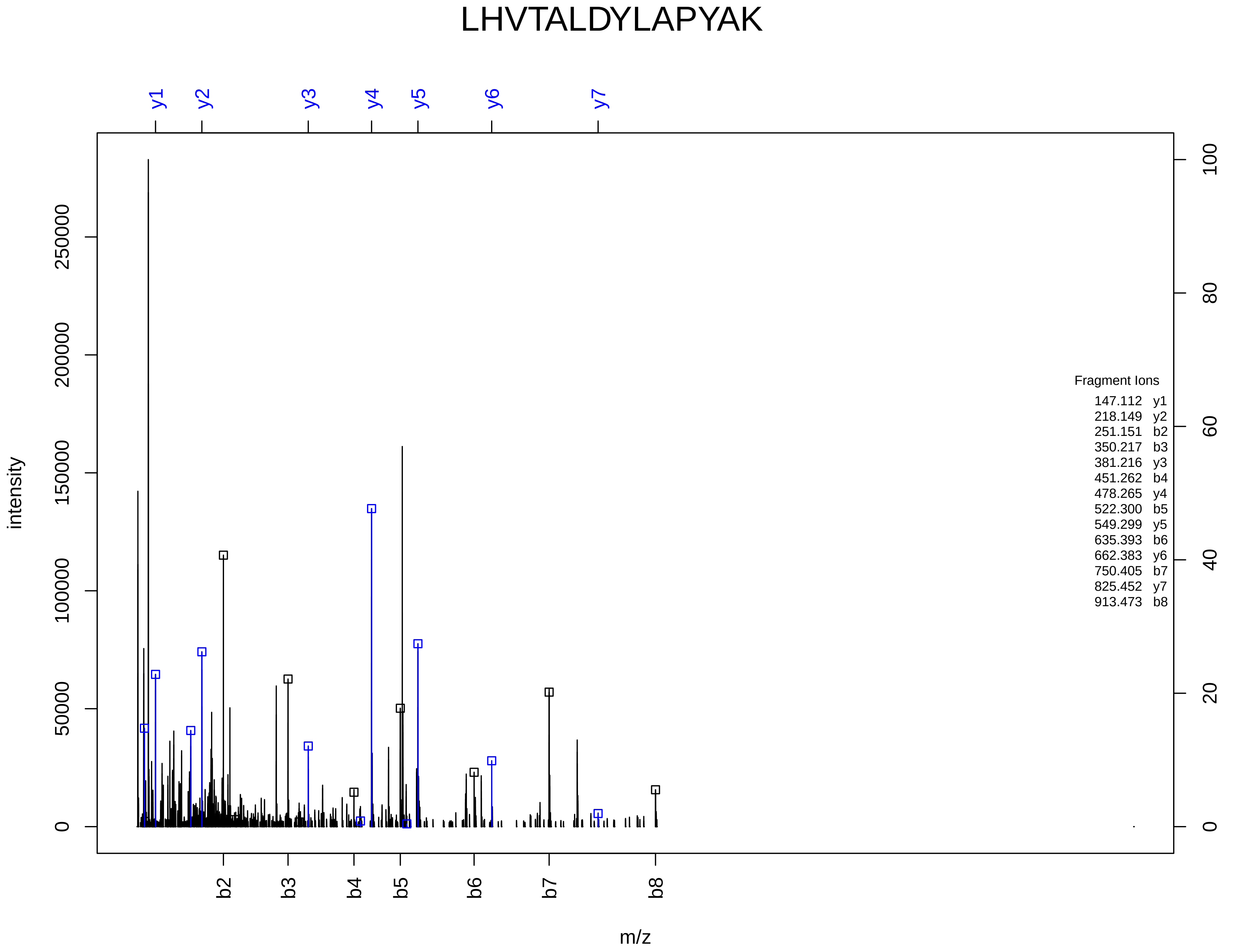

LHVTALDYLAPYAK / File: christine_ip_09.raw; SpectrumID: ; scans: 13959

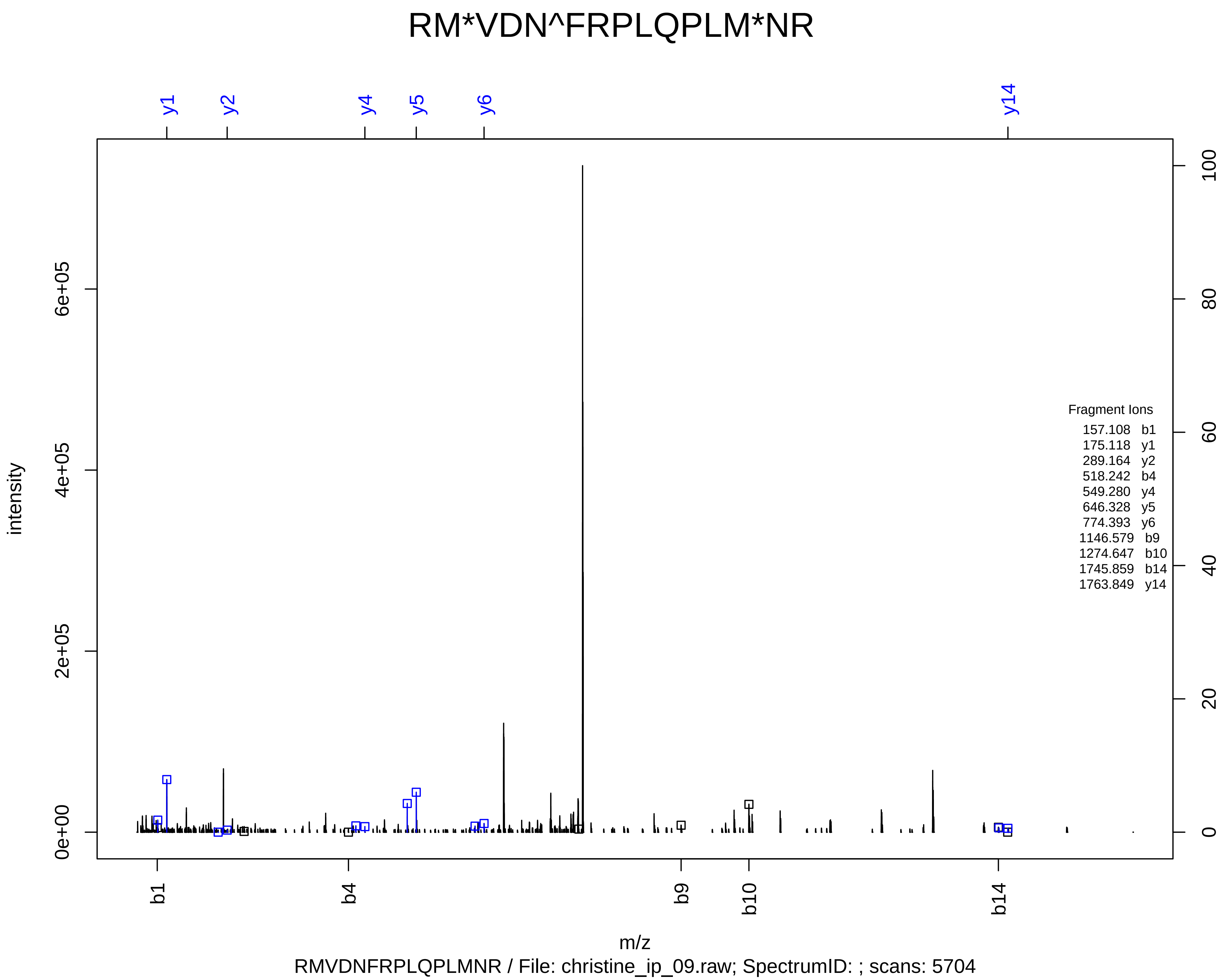
RM*VDN^FRPLQPLM*NR
y1
y2
y4
y5
y6
y14
intensity
6e+05
4e+05
2e+05
0e+00
100
80
60
40
20
0
Fragment Ions
157.108 b1
175.118 y1
289.164 y2
518.242 b4
549.280 y4
646.328 y5
774.393 y6
1146.579 b9
1274.647 b10
1745.859 b14
1763.849 y14
b1
b4
b9
b10
b14
m/z
RMVDNFRPLQPLMNR / File: christine_ip_09.raw; SpectrumID: ; scans: 5704

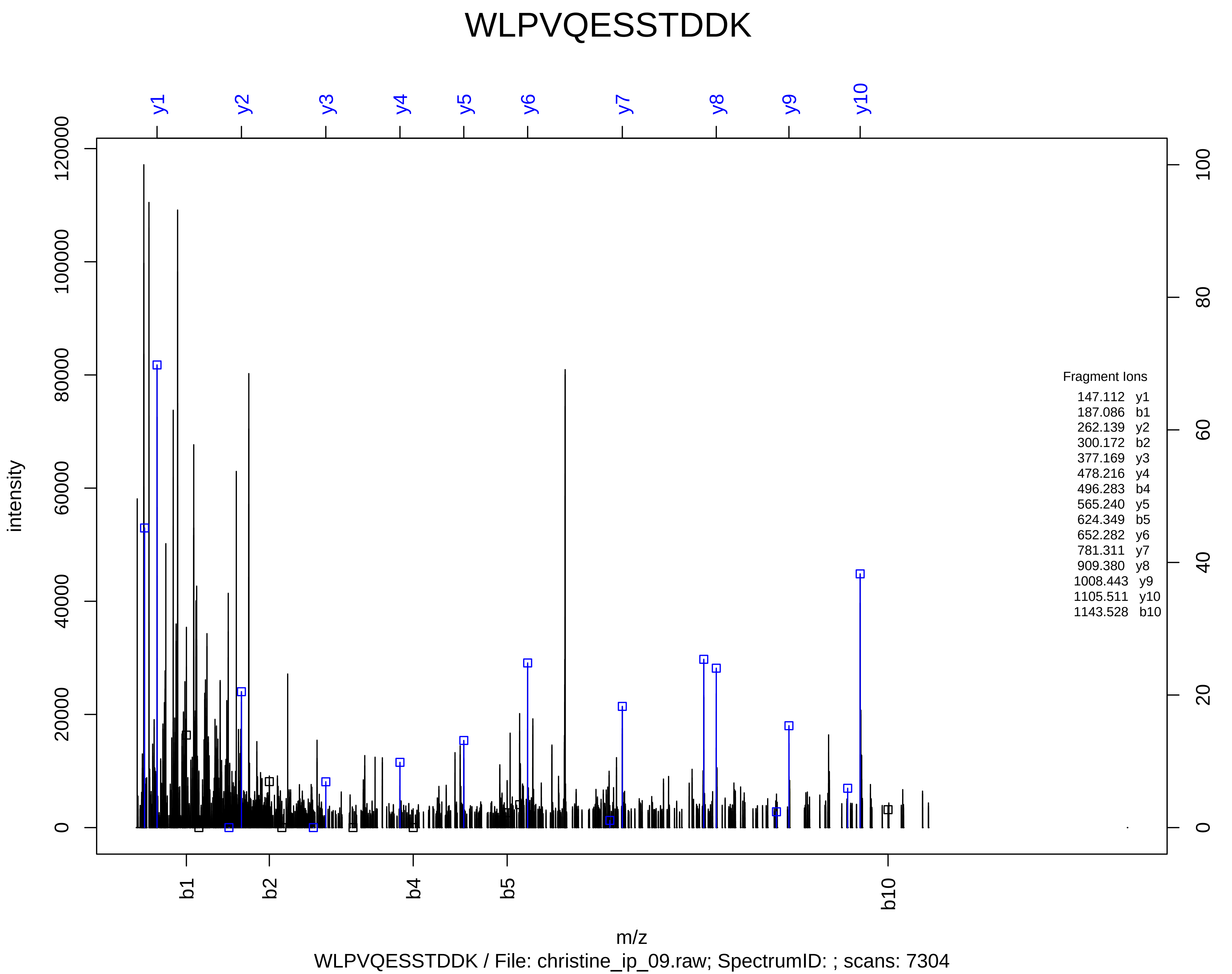
WLPVQESSTDDK
y1
y2
y3
y4
y5
y6
y7
y8
y9
y10
intensity
0
20000
40000
60000
80000
100000
120000
0
20
40
60
80
100
Fragment Ions
147.112 y1
187.086 b1
262.139 y2
300.172 b2
377.169 y3
478.216 y4
496.283 b4
565.240 y5
624.349 b5
652.282 y6
781.311 y7
909.380 y8
1008.443 y9
1105.511 y10
1143.528 b10
b1
b2
b4
b5
b10
m/z
WLPVQESSTDDK / File: christine_ip_09.raw; SpectrumID: ; scans: 7304

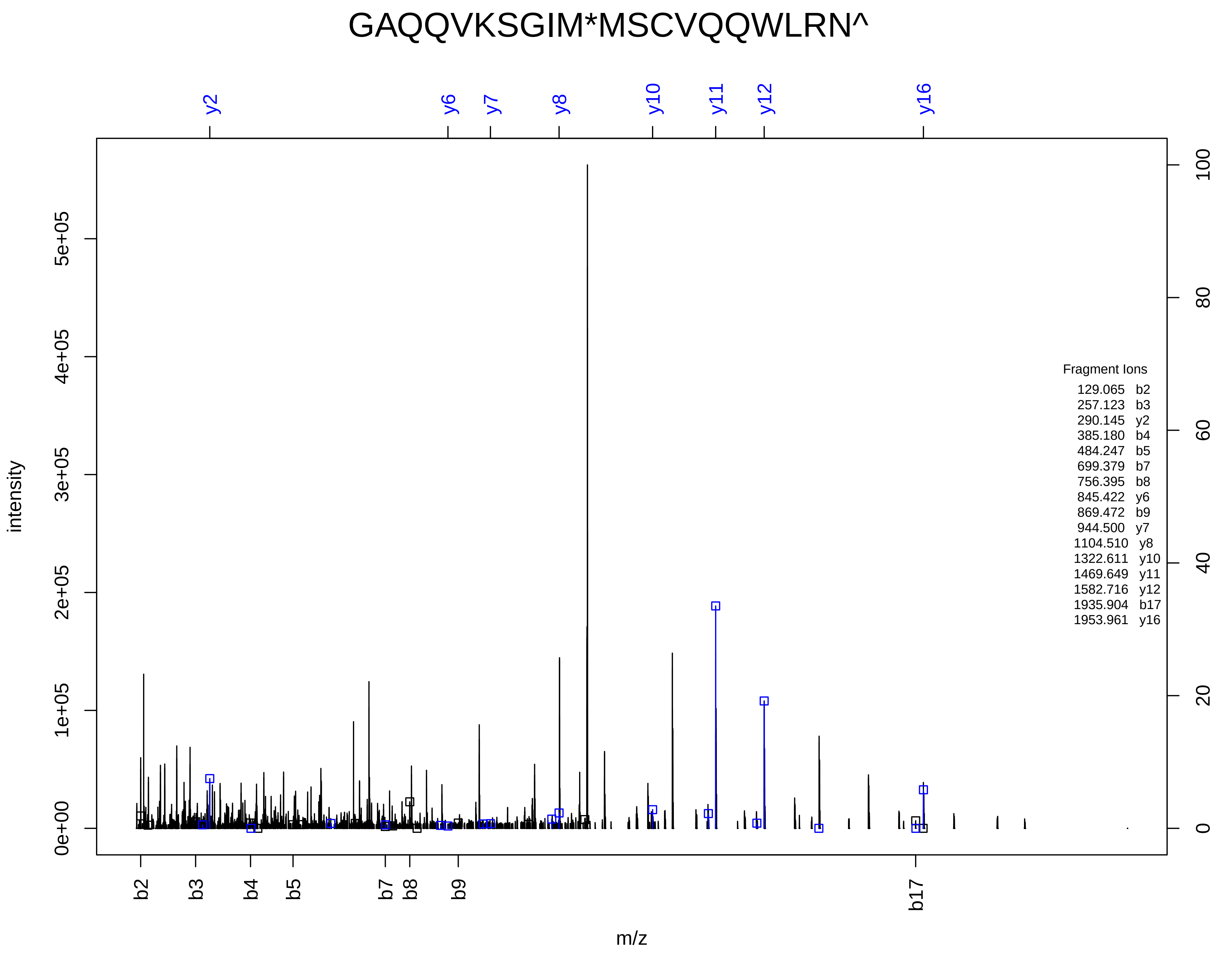
GAQQVKSGIM*MSCVQQWLRN^
y2
y6
y7
y8
y10
y11
y12
y16
intensity
0e+00
1e+05
2e+05
3e+05
4e+05
5e+05
0
20
40
60
80
100
Fragment Ions
129.065 b2
257.123 b3
290.145 y2
385.180 b4
484.247 b5
699.379 b7
756.395 b8
845.422 y6
869.472 b9
944.500 y7
1104.510 y8
1322.611 y10
1469.649 y11
1582.716 y12
1935.904 b17
1953.961 y16
b2
b3
b4
b5
b7
b8
b9
b17
m/z
GAQQVKSGIMMSCVQQWLRN / File: christine_ip_09.raw; SpectrumID: ; scans: 11231

FIYEAWQGVER

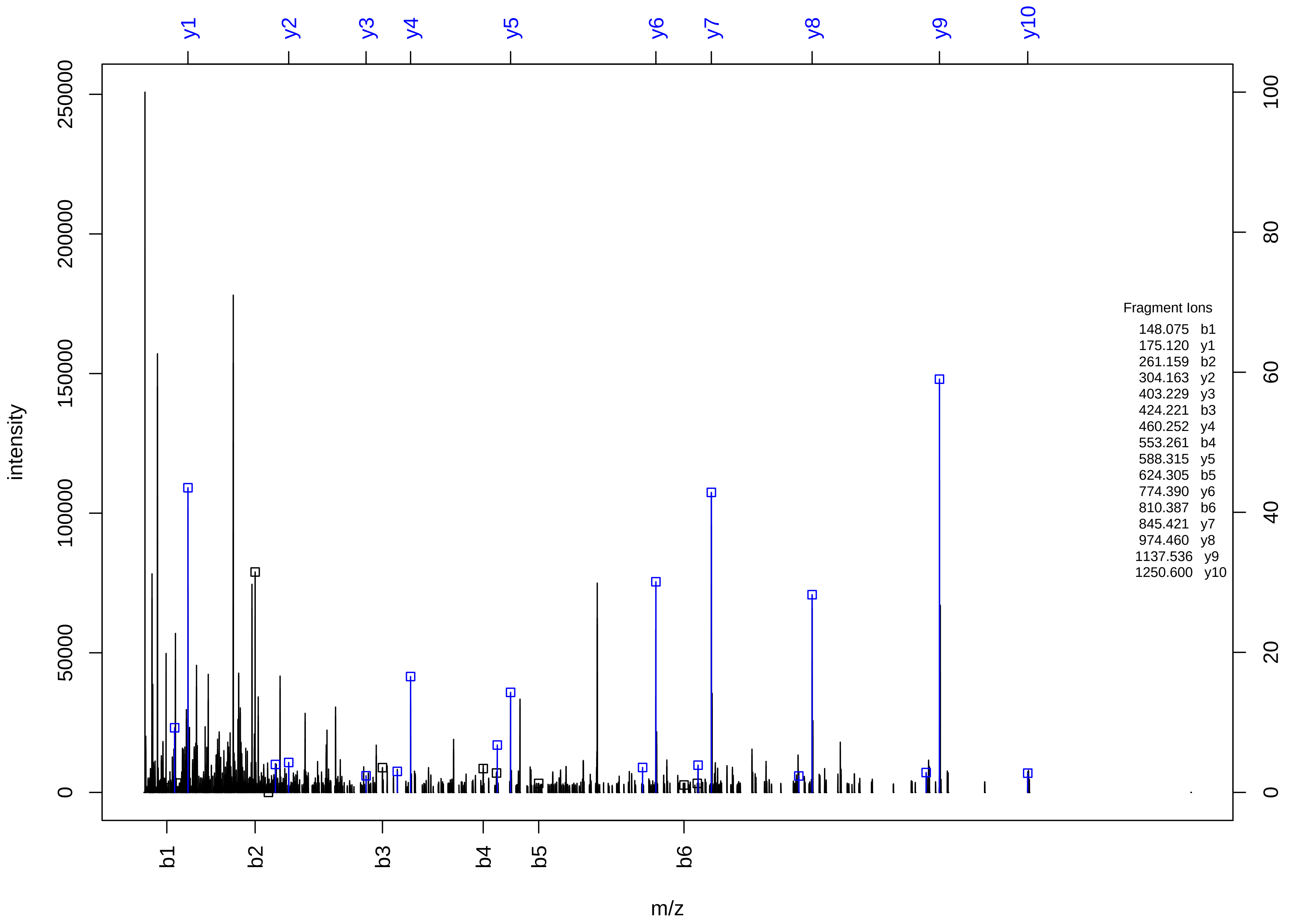

FIYEAWQGVER / File: christine_ip_10.raw; SpectrumID: ; scans: 11468

AYTVVGGPPGGPPVR

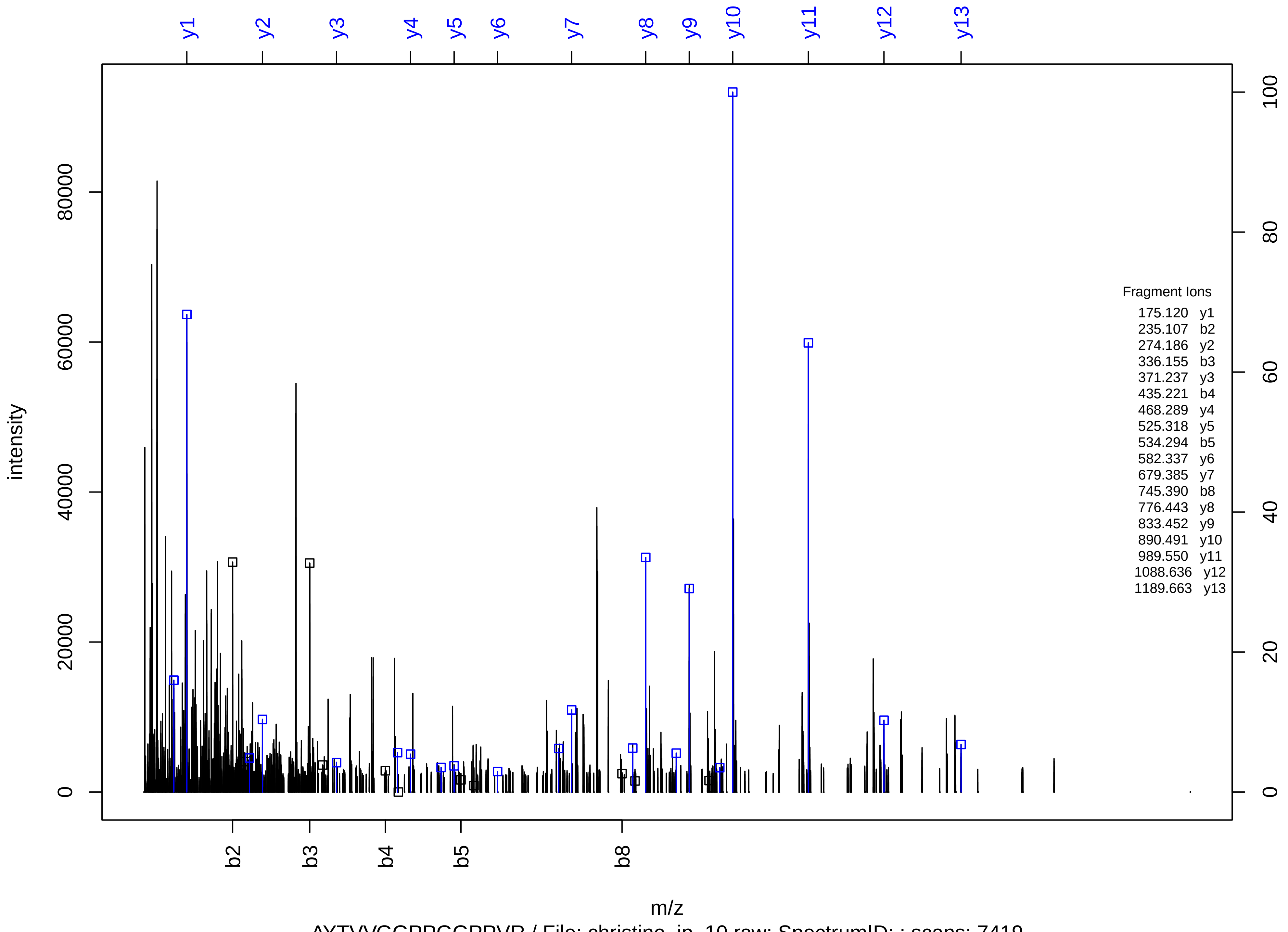

AYTVVGGPPGGPPVR / File: christine_ip_10.raw; SpectrumID: ; scans: 7419

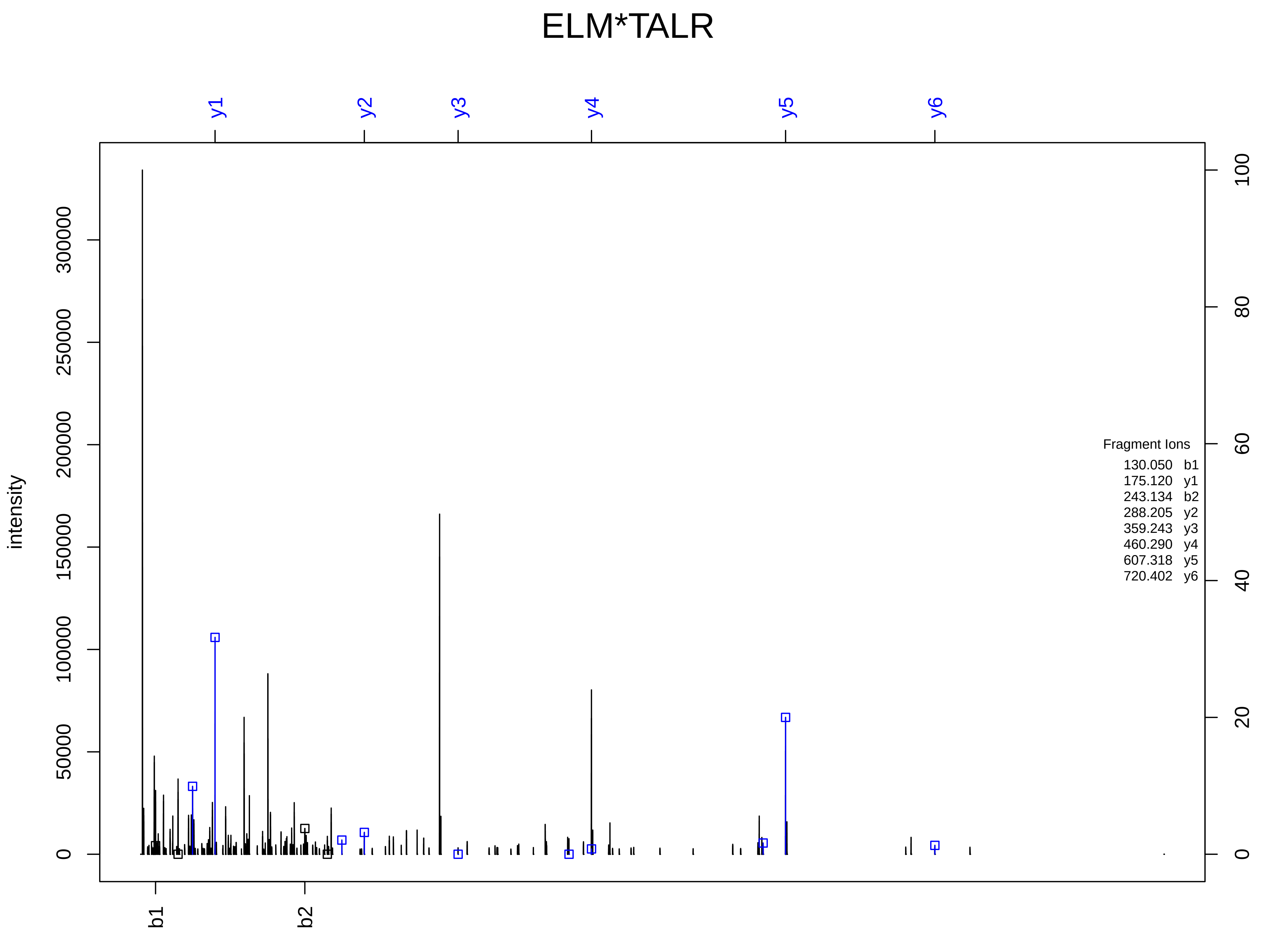
ELM*TALR
y1
y2
y3
y4
y5
y6
intensity
0
50000
100000
150000
200000
250000
300000
0
20
40
60
80
100
Fragment Ions
130.050 b1
175.120 y1
243.134 b2
288.205 y2
359.243 y3
460.290 y4
607.318 y5
720.402 y6
b1
b2
m/z
ELMTALR / File: christine_ip_10.raw; SpectrumID: ; scans: 9623

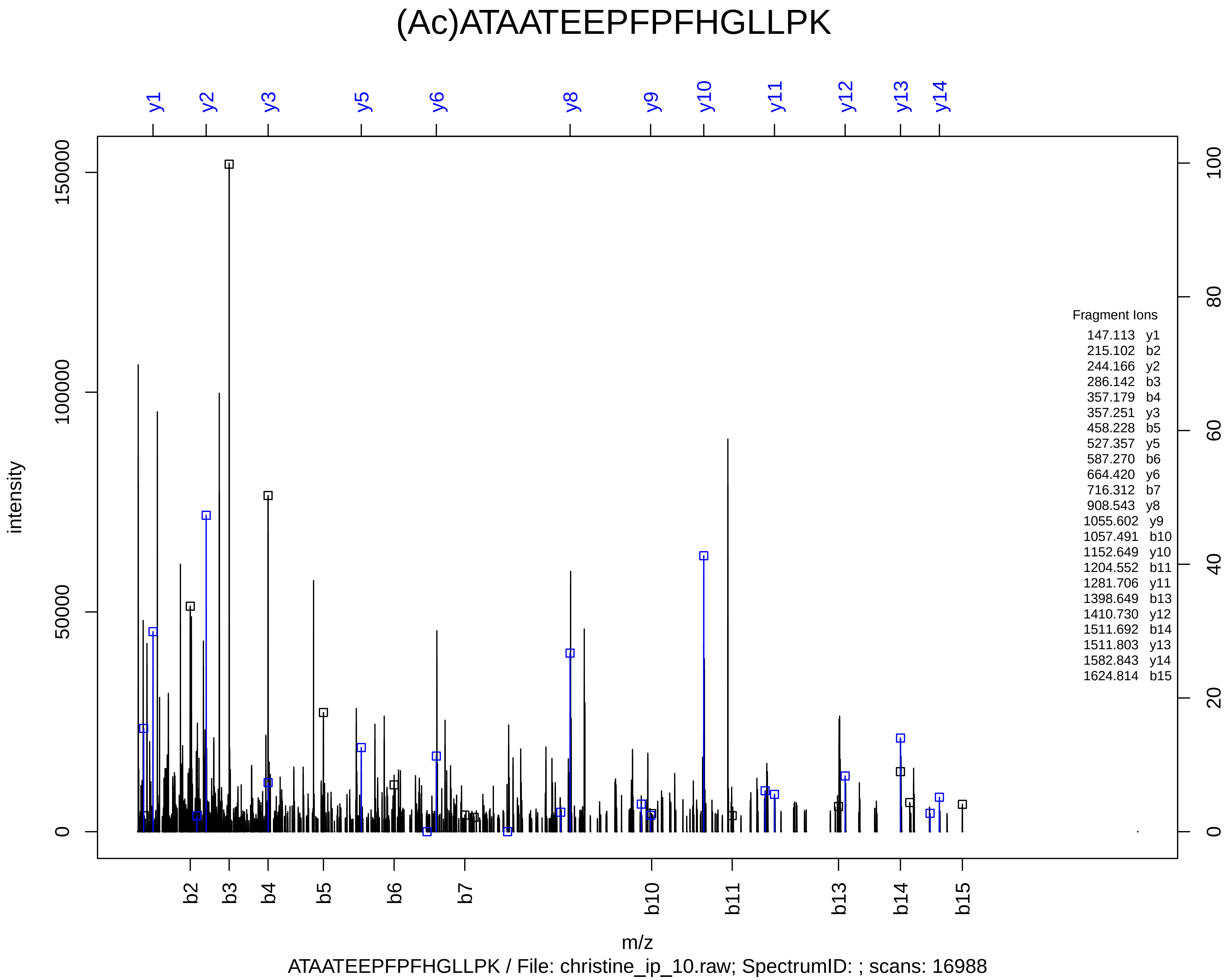
(Ac)ATAATEEPFPFHGLLPK
y1
y2
y3
y5
y6
y8
y9
y10
y11
y12
y13
y14
intensity
150000
100000
50000
0
100
80
60
40
20
0
Fragment Ions
147.113 y1
215.102 b2
244.166 y2
286.142 b3
357.179 b4
357.251 y3
458.228 b5
527.357 y5
587.270 b6
664.420 y6
716.312 b7
908.543 y8
1055.602 y9
1057.491 b10
1152.649 y10
1204.552 b11
1281.706 y11
1398.649 b13
1410.730 y12
1511.692 b14
1511.803 y13
1582.843 y14
1624.814 b15
b2
b3
b4
b5
b6
b7
b10
b11
b13
b14
b15
m/z
ATAATEEPFPFHGLLPK / File: christine_ip_10.raw; SpectrumID: ; scans: 16988

VSHQGYSTEAEFEEPR

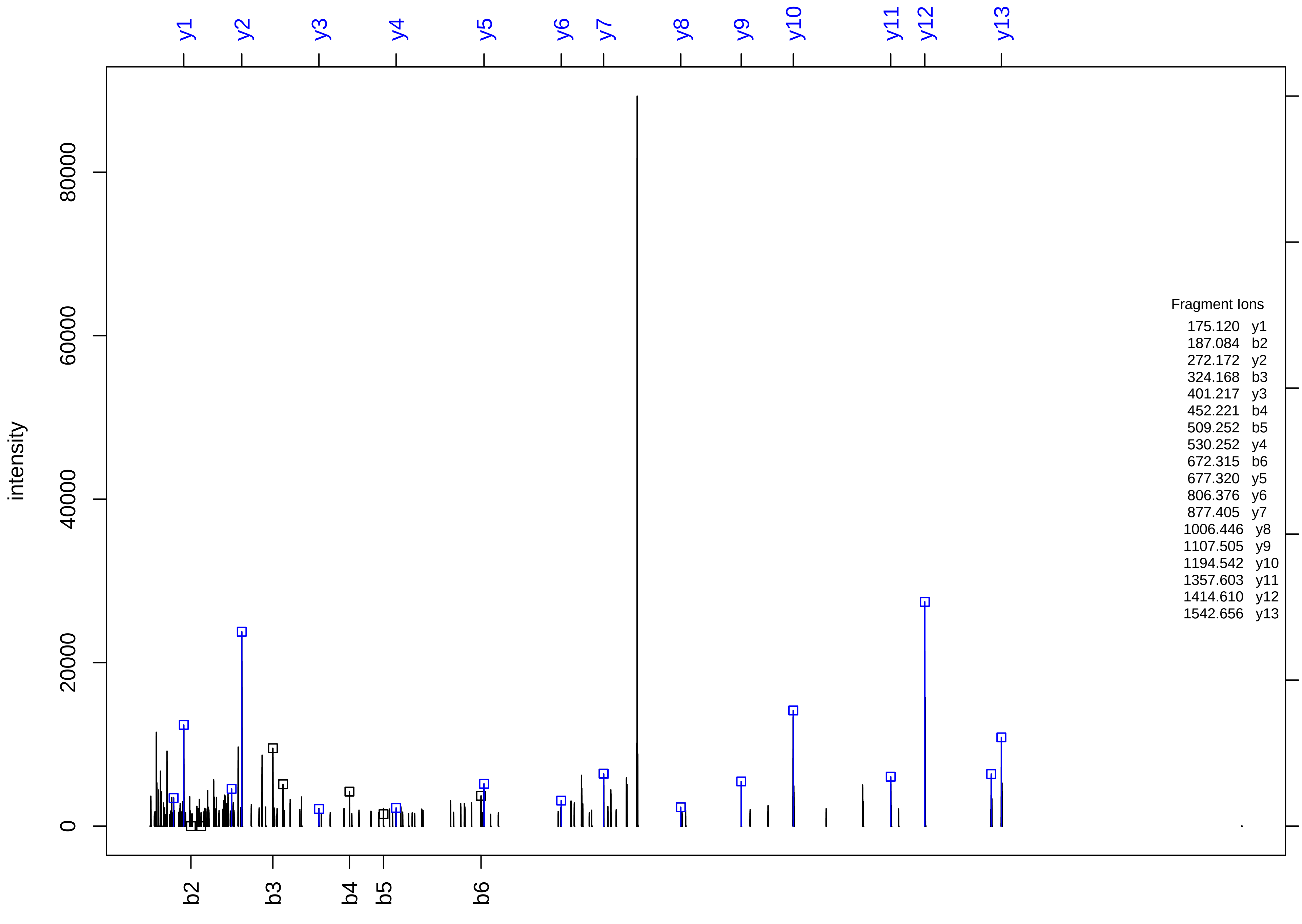

VSHQGYSTEAEFEEPR / File: christine_ip_10.raw; SpectrumID: ; scans: 4353

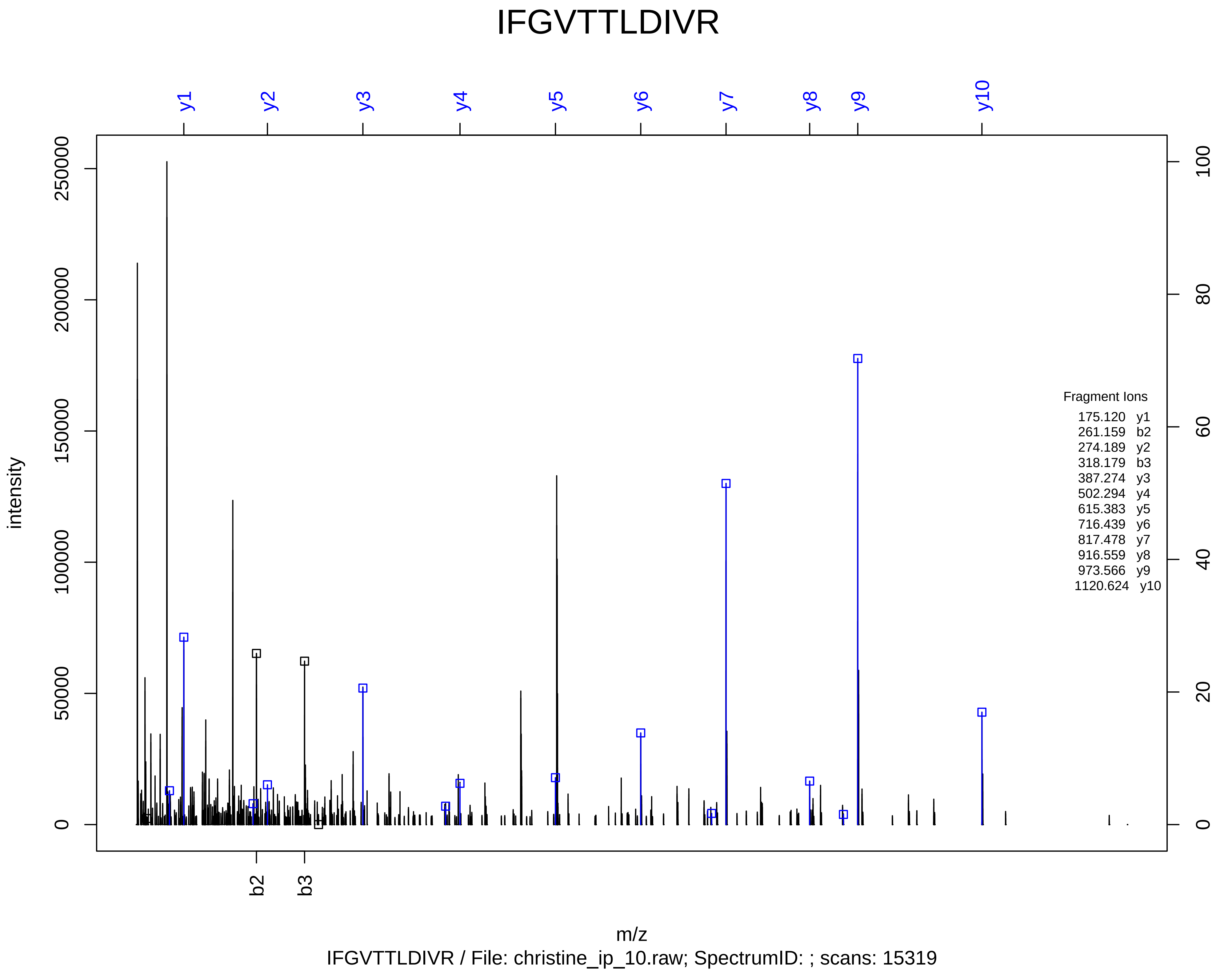
IFGVTTLDIVR
y1
y2
y3
y4
y5
y6
y7
y8
y9
y10
intensity
0
50000
100000
150000
200000
250000
0
20
40
60
80
100
b2
b3
m/z
Fragment Ions
175.120 y1
261.159 b2
274.189 y2
318.179 b3
387.274 y3
502.294 y4
615.383 y5
716.439 y6
817.478 y7
916.559 y8
973.566 y9
1120.624 y10
IFGVTTLDIVR / File: christine_ip_10.raw; SpectrumID: ; scans: 15319

FVAPEEVLPFTEGDILEK

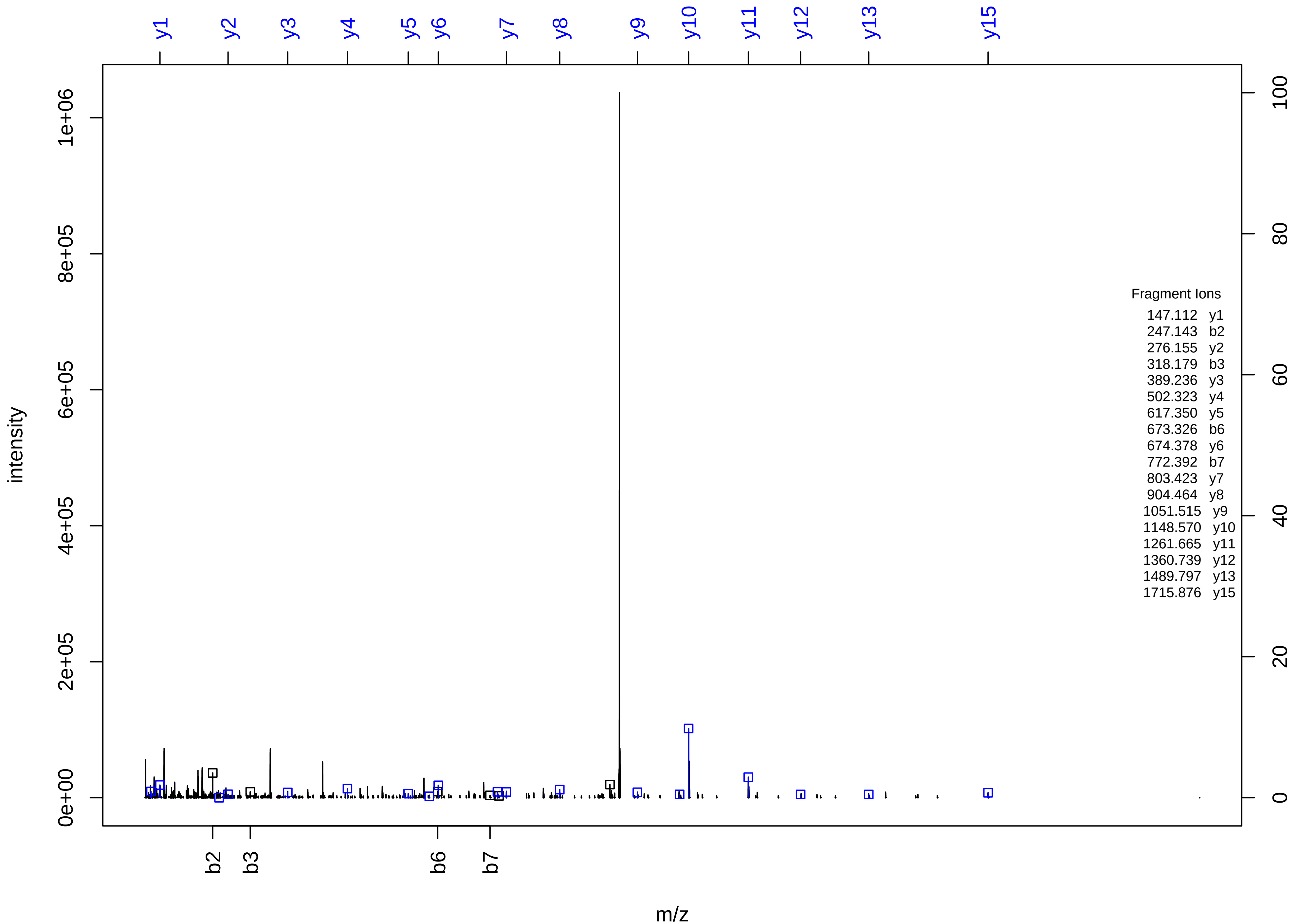

FVAPEEVLPFTEGDILEK / File: christine_ip_10.raw; SpectrumID: ; scans: 18477

EFPGFLENQK

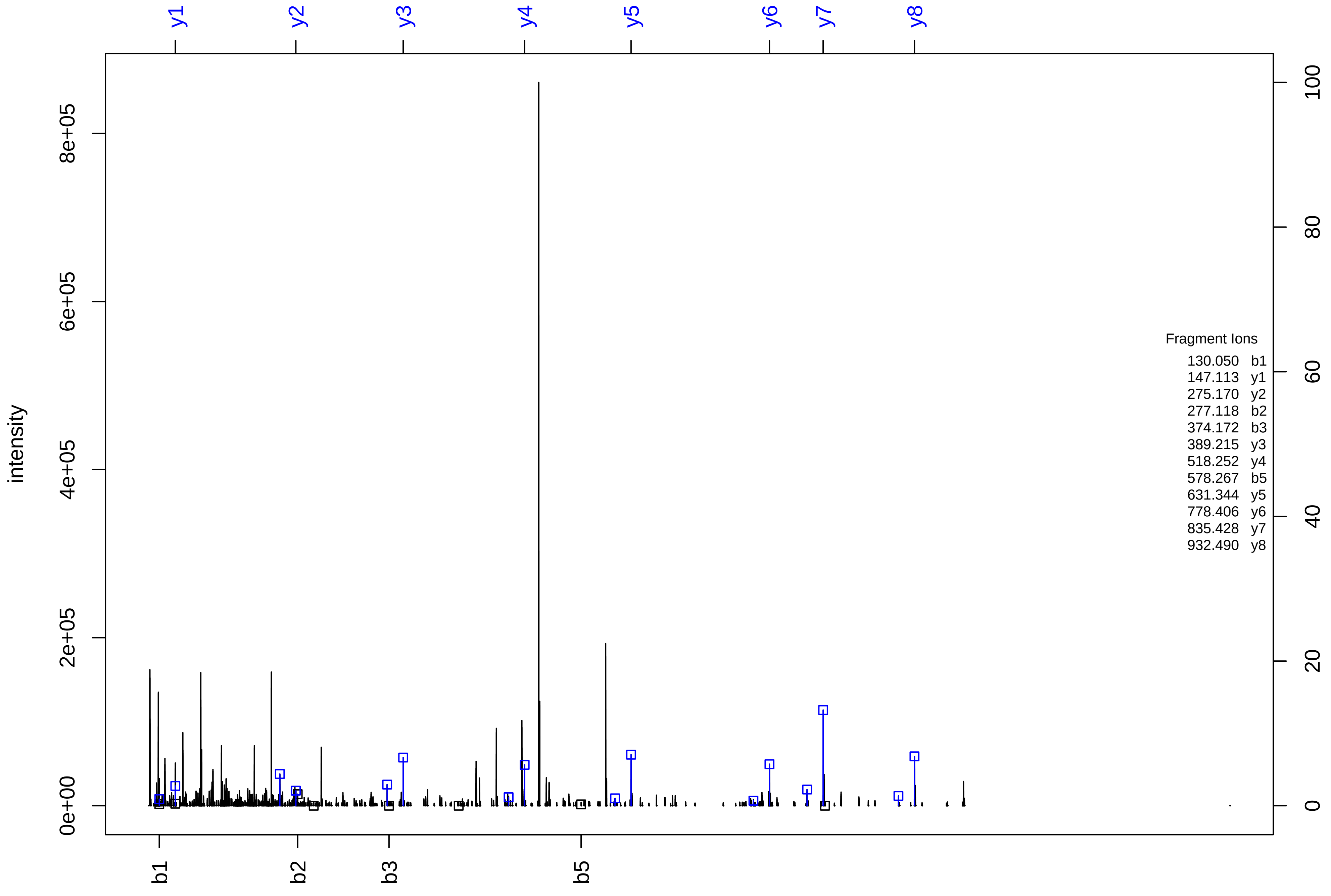

EFPGFLENQK / File: christine_ip_10.raw; SpectrumID: ; scans: 10596

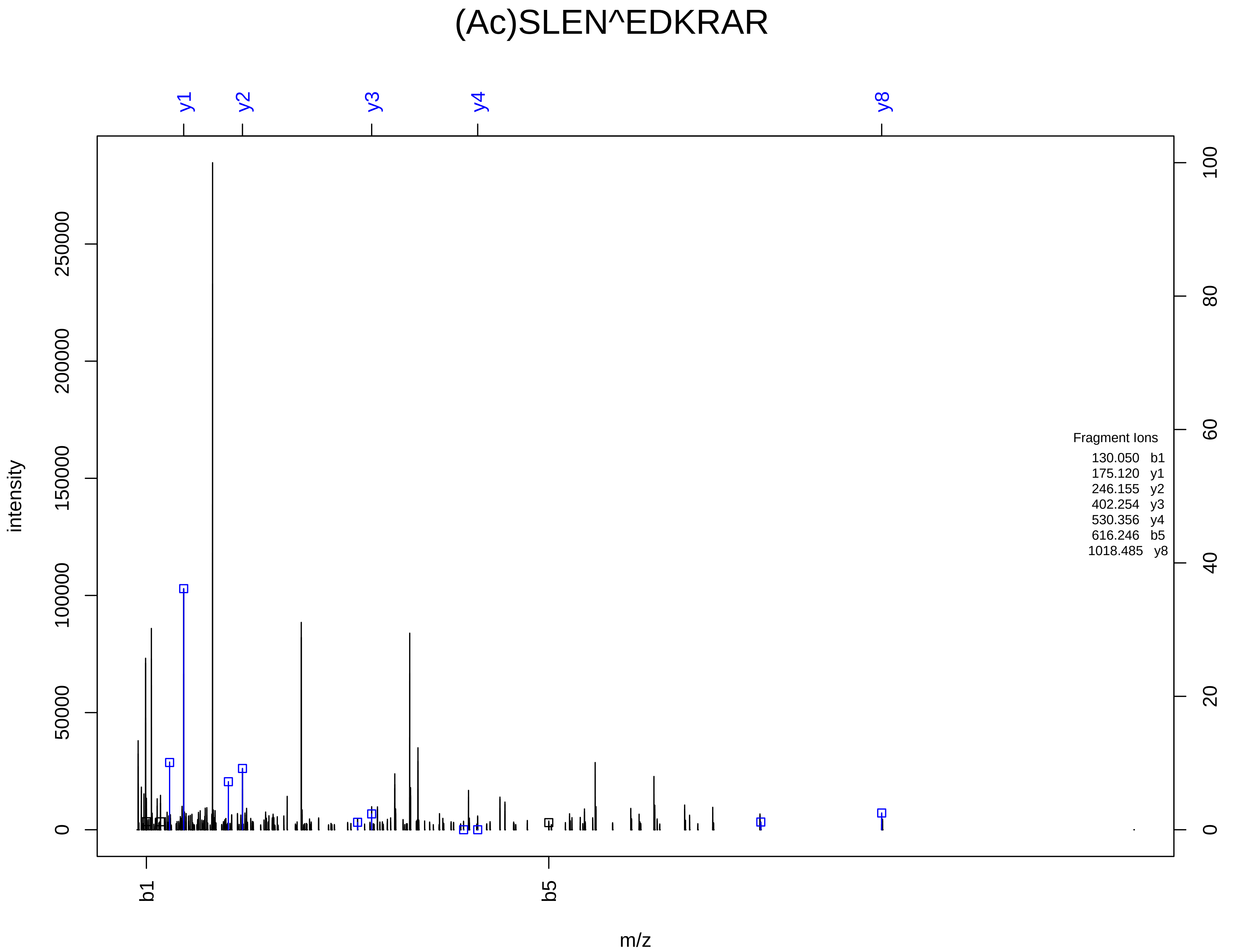

SLENEDKRAR / File: christine_ip_10.raw; SpectrumID: ; scans: 2303

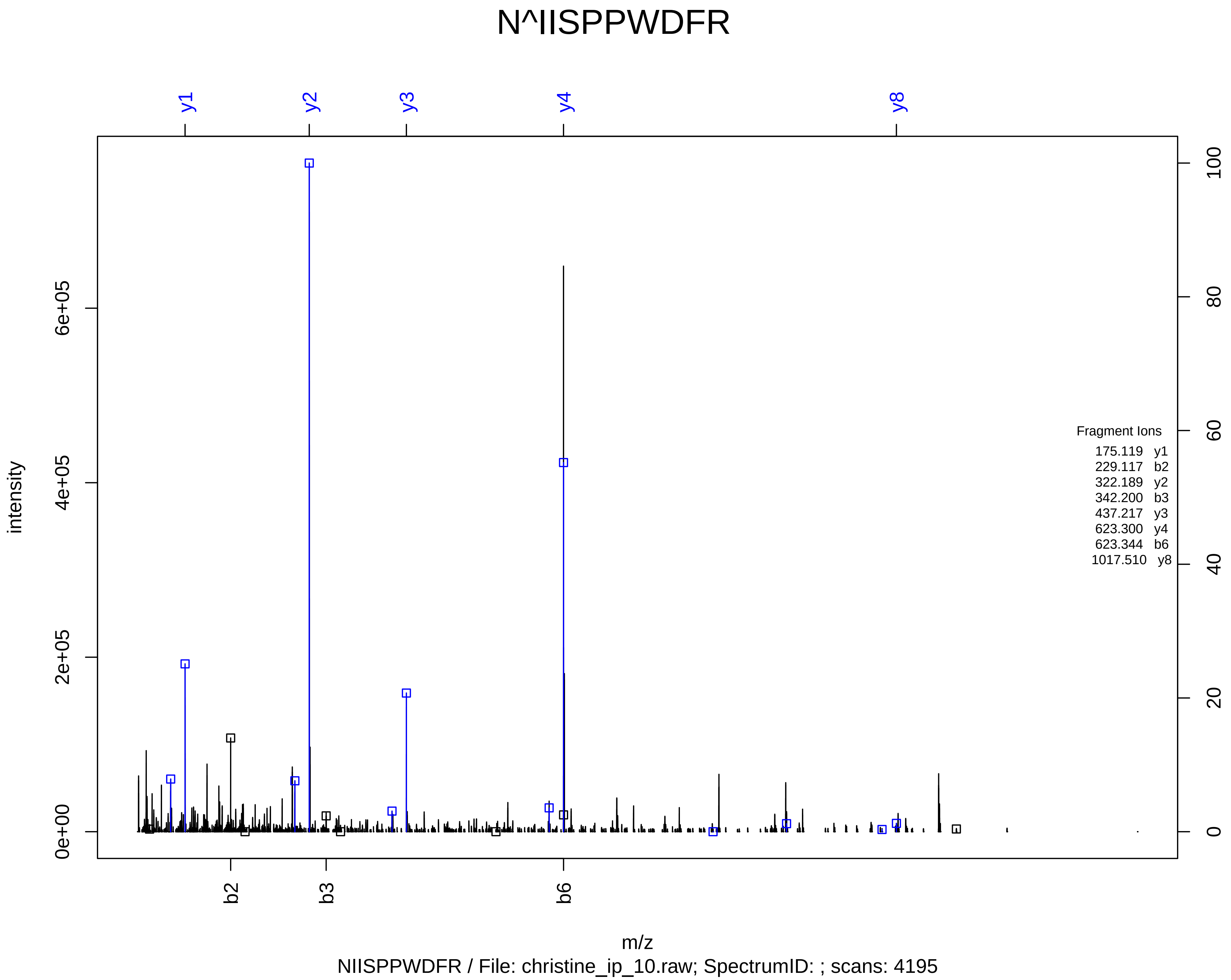
N^IISPPWDFR
y1
y2
y3
y4
y8
intensity
0e+00
2e+05
4e+05
6e+05
0
20
40
60
80
100
b2
b3
b6
m/z
Fragment Ions
175.119 y1
229.117 b2
322.189 y2
342.200 b3
437.217 y3
623.300 y4
623.344 b6
1017.510 y8
NIISPPWDFR / File: christine_ip_10.raw; SpectrumID: ; scans: 4195

DDDIEEGDLPEHK

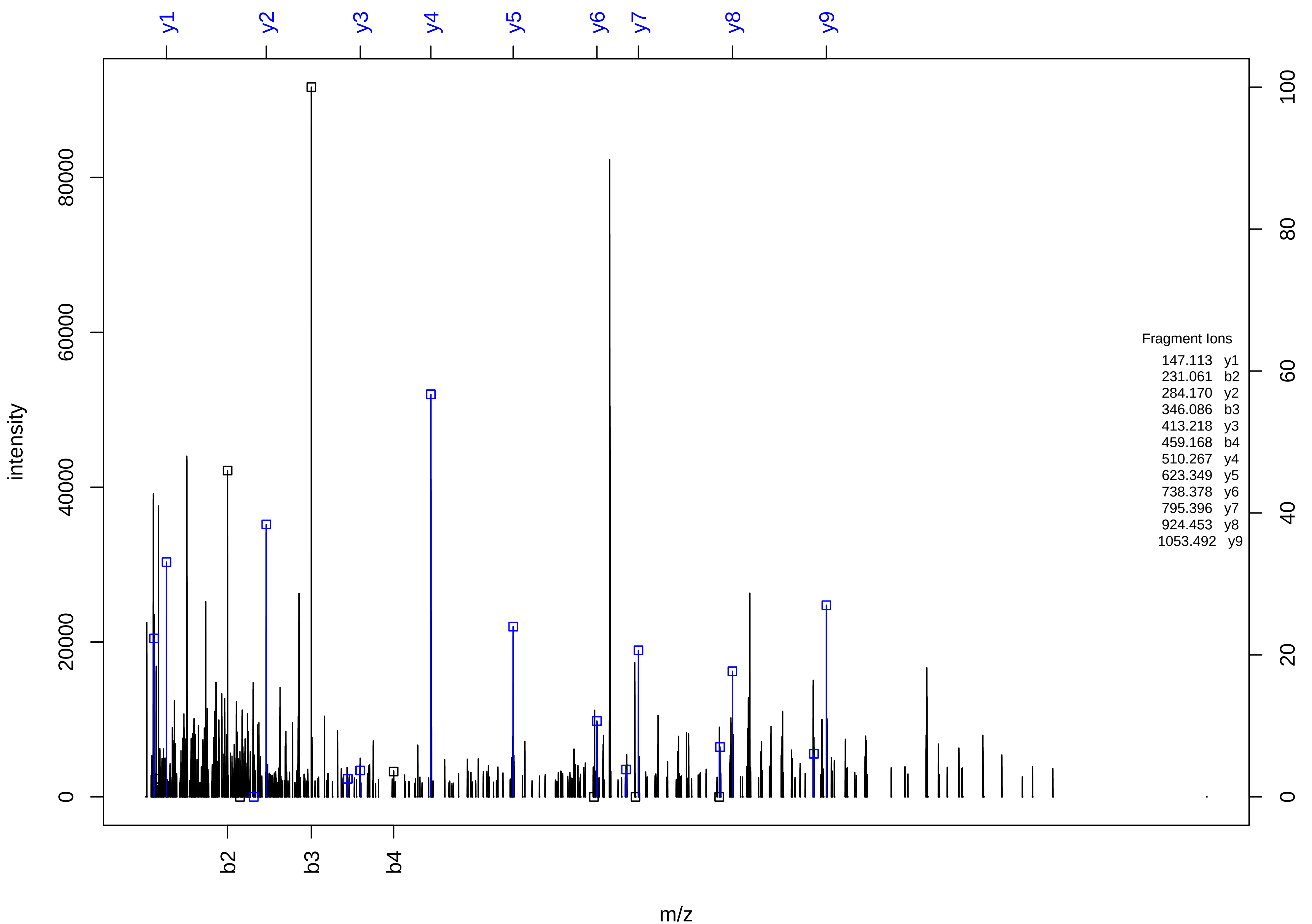

DDDIEEGDLPEHK / File: christine_ip_10.raw; SpectrumID: ; scans: 4009

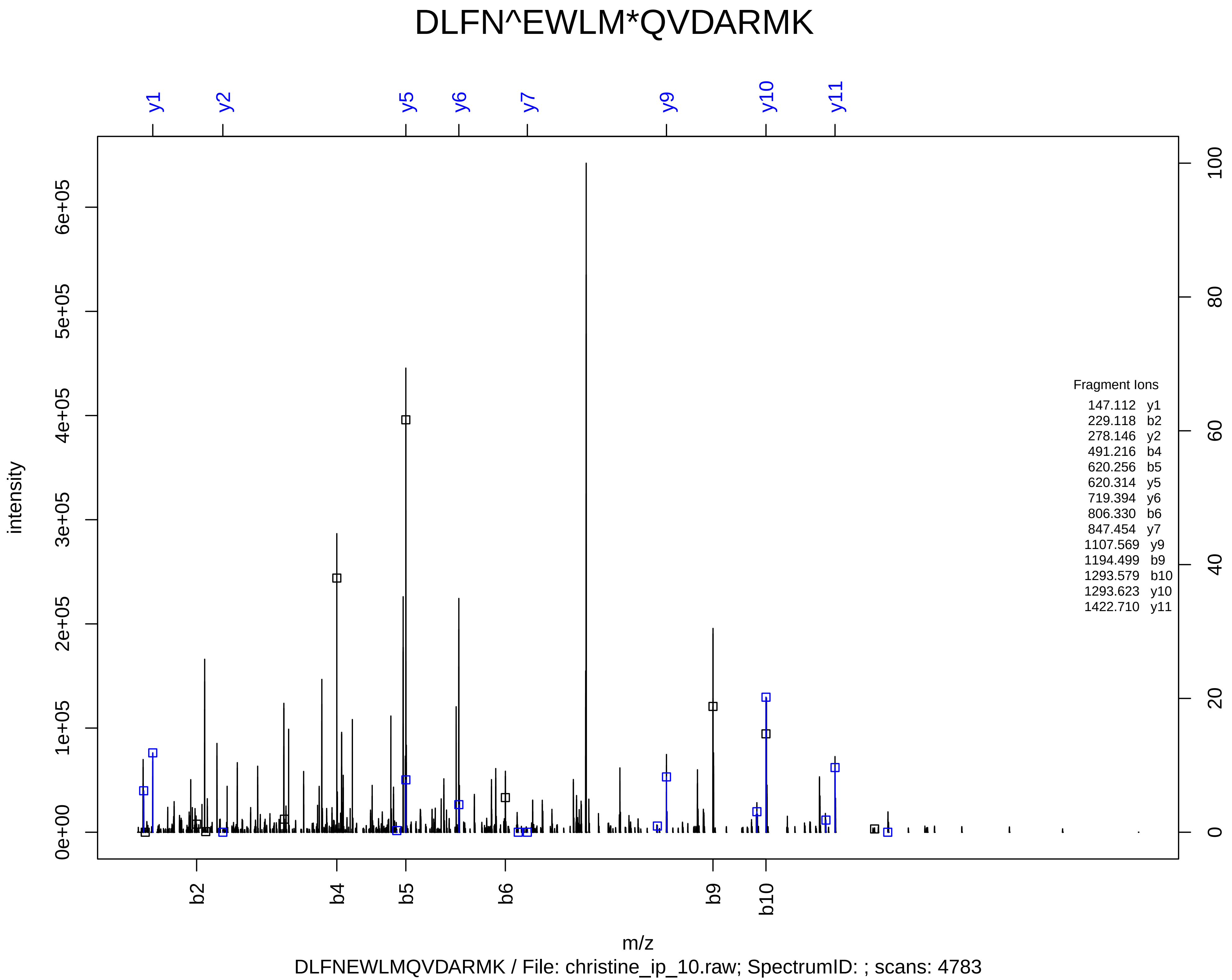
DLFN^EWLM*QVDARMK
y1
y2
y5
y6
y7
y9
y10
y11
intensity
0e+00
1e+05
2e+05
3e+05
4e+05
5e+05
6e+05
0
20
40
60
80
100
Fragment Ions
147.112 y1
229.118 b2
278.146 y2
491.216 b4
620.256 b5
620.314 y5
719.394 y6
806.330 b6
847.454 y7
1107.569 y9
1194.499 b9
1293.579 b10
1293.623 y10
1422.710 y11
b2
b4
b5
b6
b9
b10
m/z
DLFNEWLMQVDARMK / File: christine_ip_10.raw; SpectrumID: ; scans: 4783

(Ac)SYNCCSGNFSSR

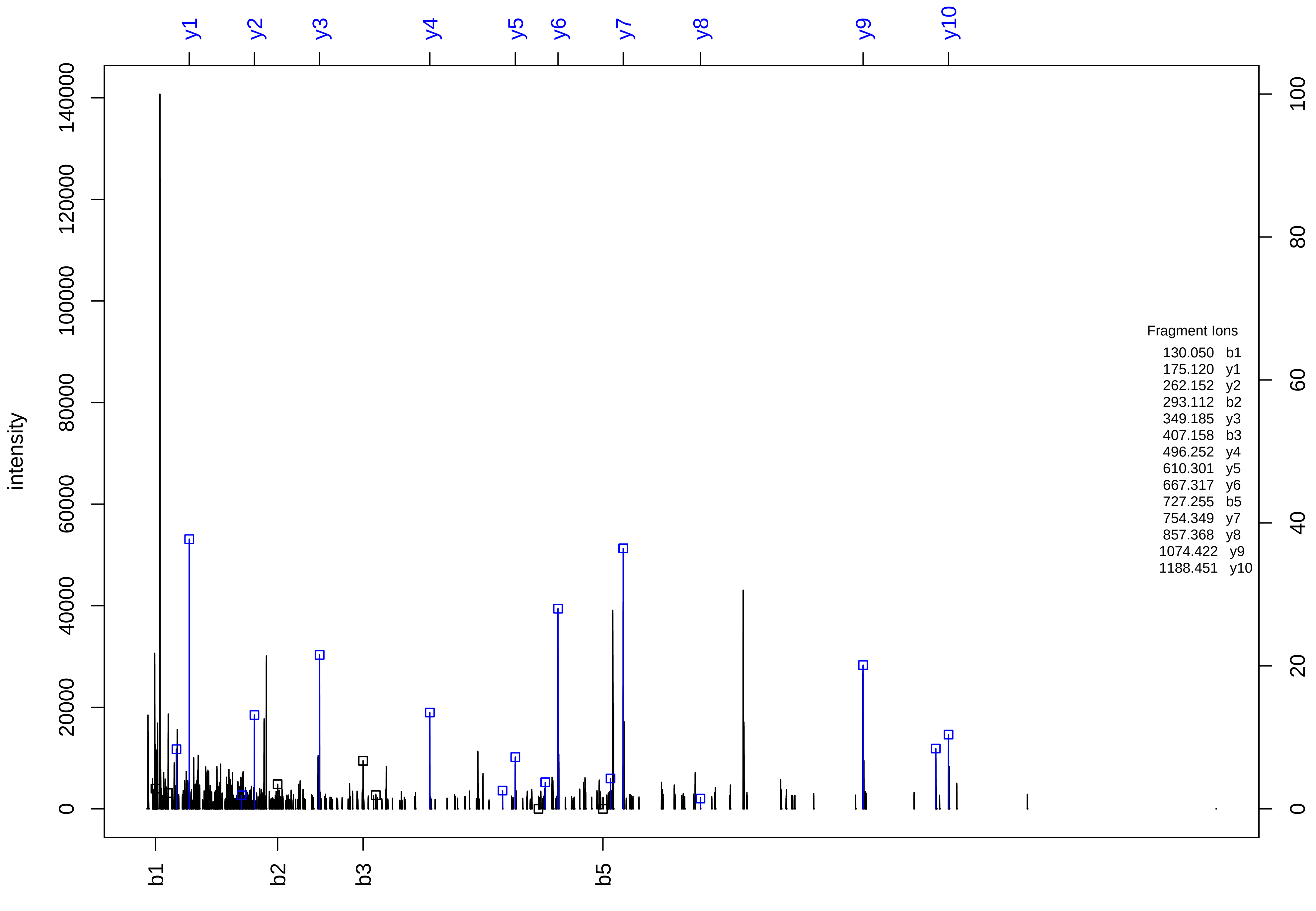

SYNCCSGNFSSR / File: christine_ip_10.raw; SpectrumID: ; scans: 4388

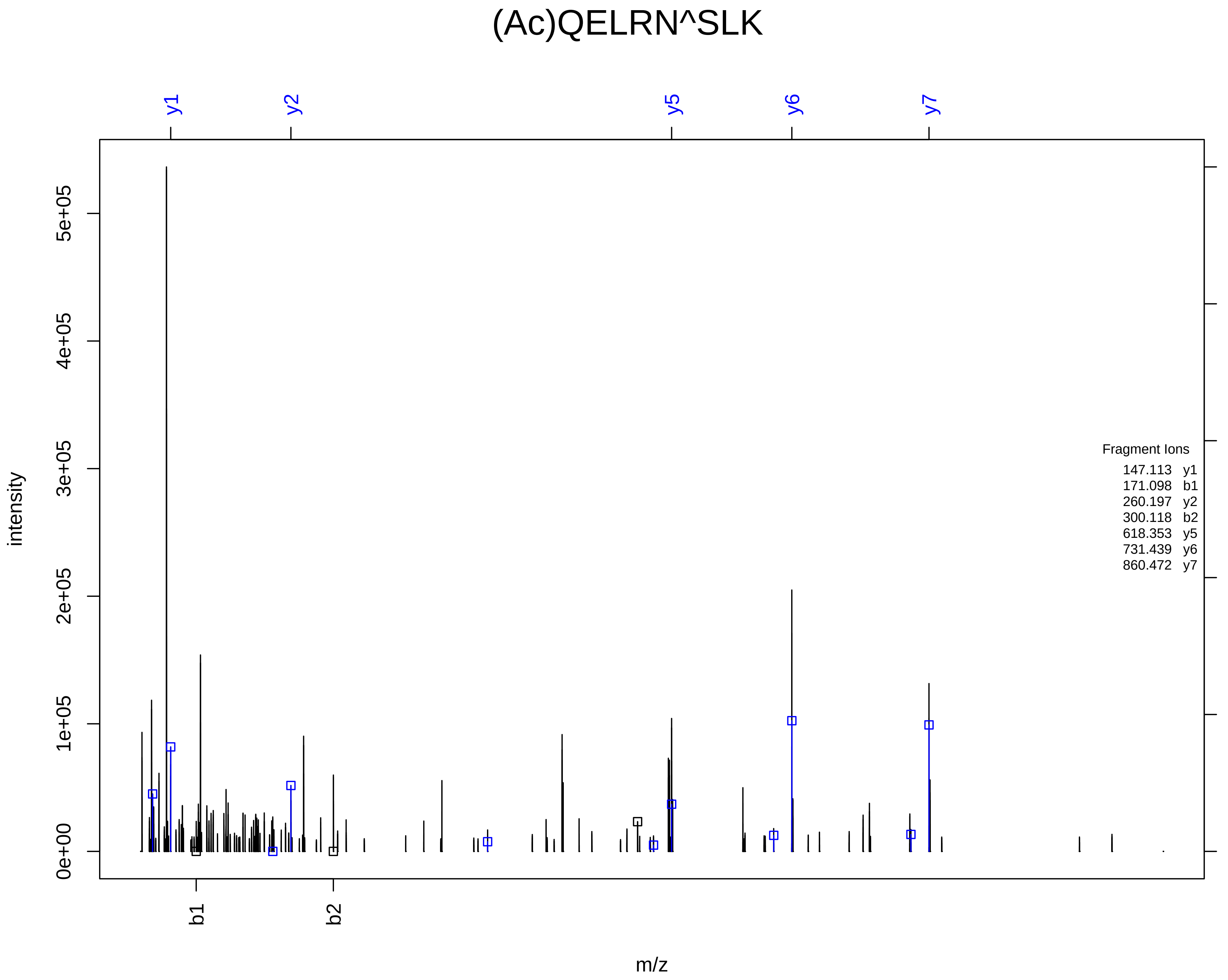

(Ac)QELRN^SLK
y1
y2
y5
y6
y7
intensity
0e+00
1e+05
2e+05
3e+05
4e+05
5e+05
0
20
40
60
80
100
Fragment Ions
147.113 y1
171.098 b1
260.197 y2
300.118 b2
618.353 y5
731.439 y6
860.472 y7
b1
b2
m/z
QELRNSLK / File: christine_ip_10.raw; SpectrumID: ; scans: 6895

TYQQSCVSSCR

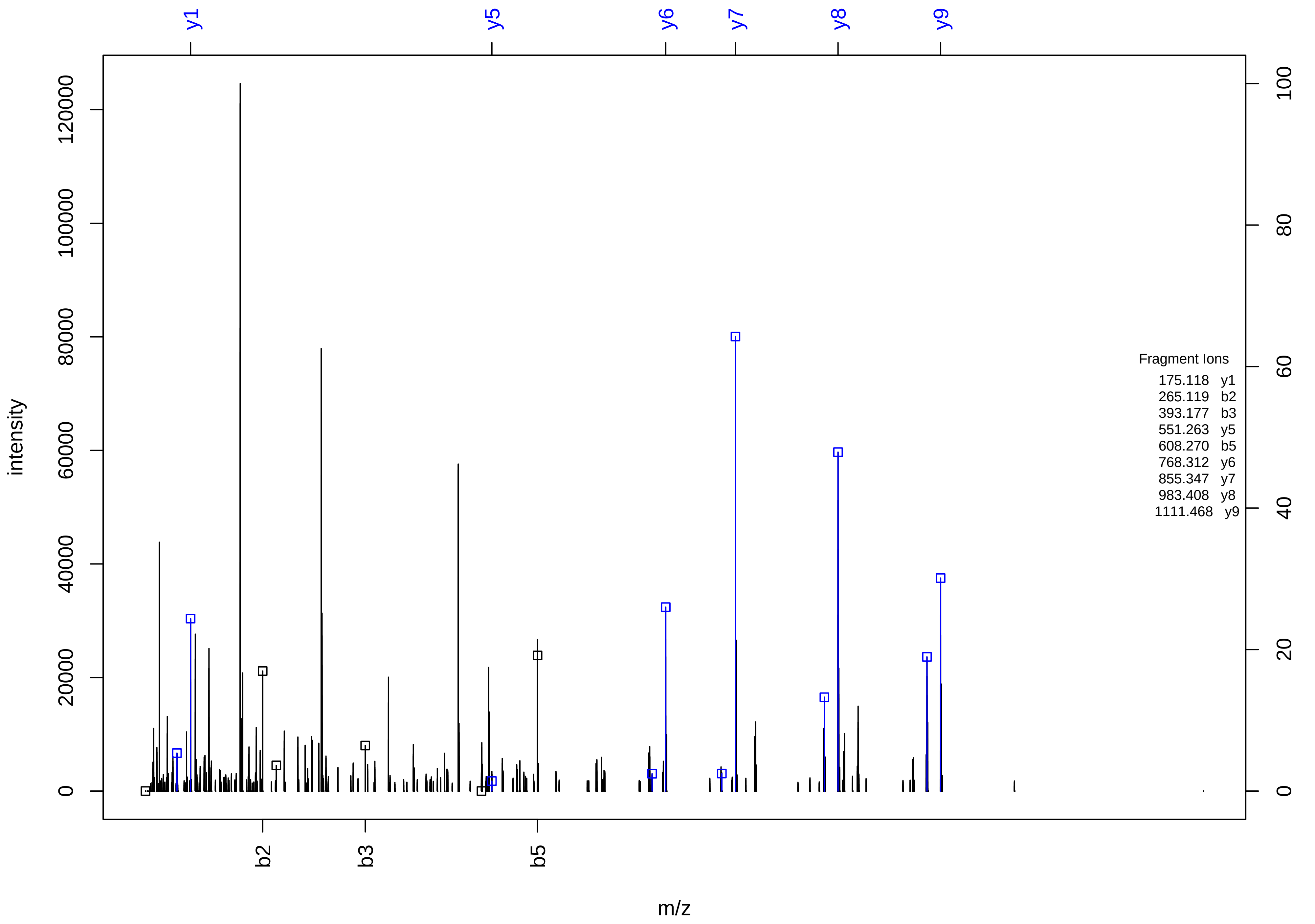

TYQQSCVSSCR / File: christine_ip_10.raw; SpectrumID: ; scans: 1274

(Ac)TEFHLQSQM*PSIR

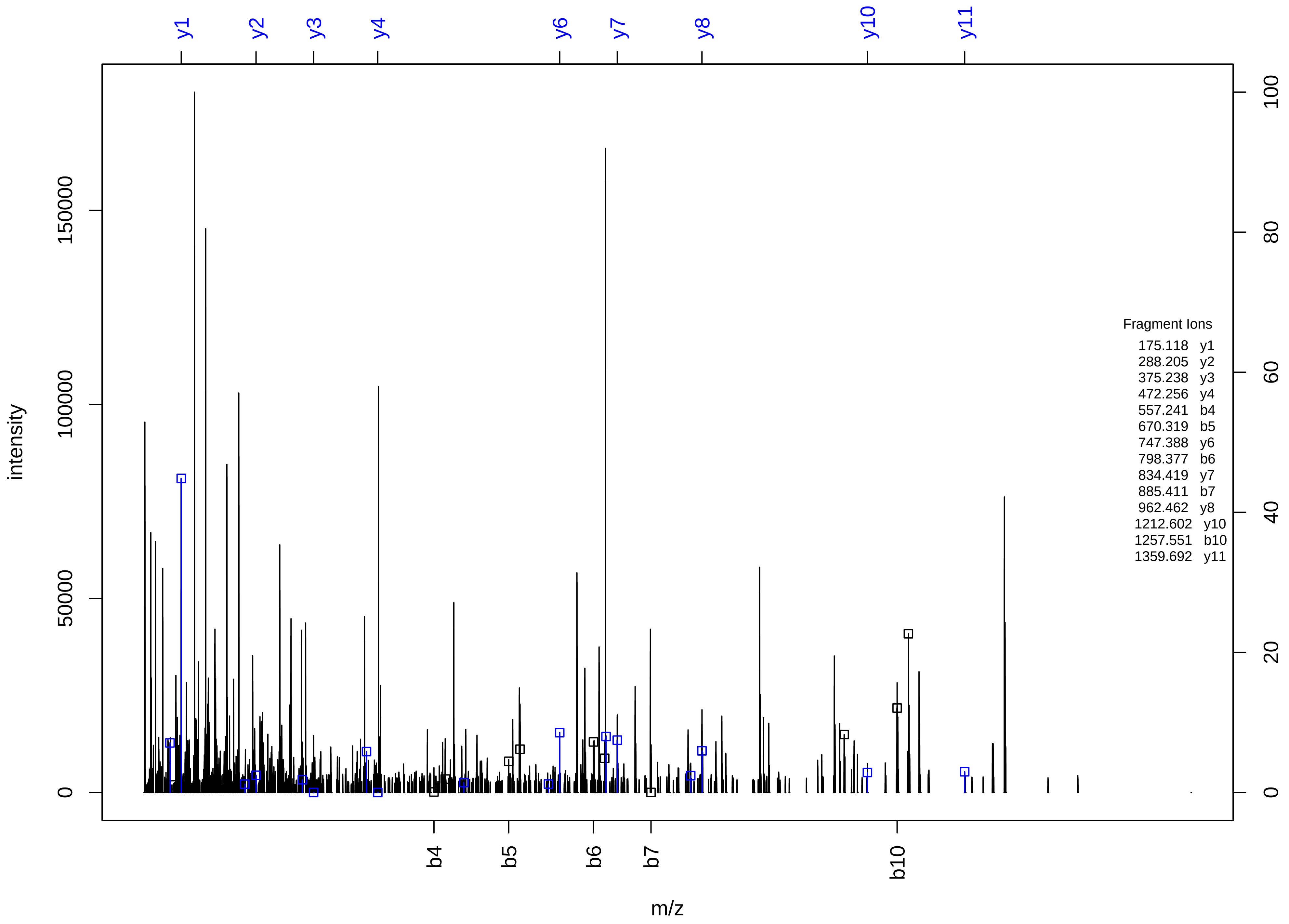

TEFHLQSQMPSIR / File: christine_ip_10.raw; SpectrumID: ; scans: 8964

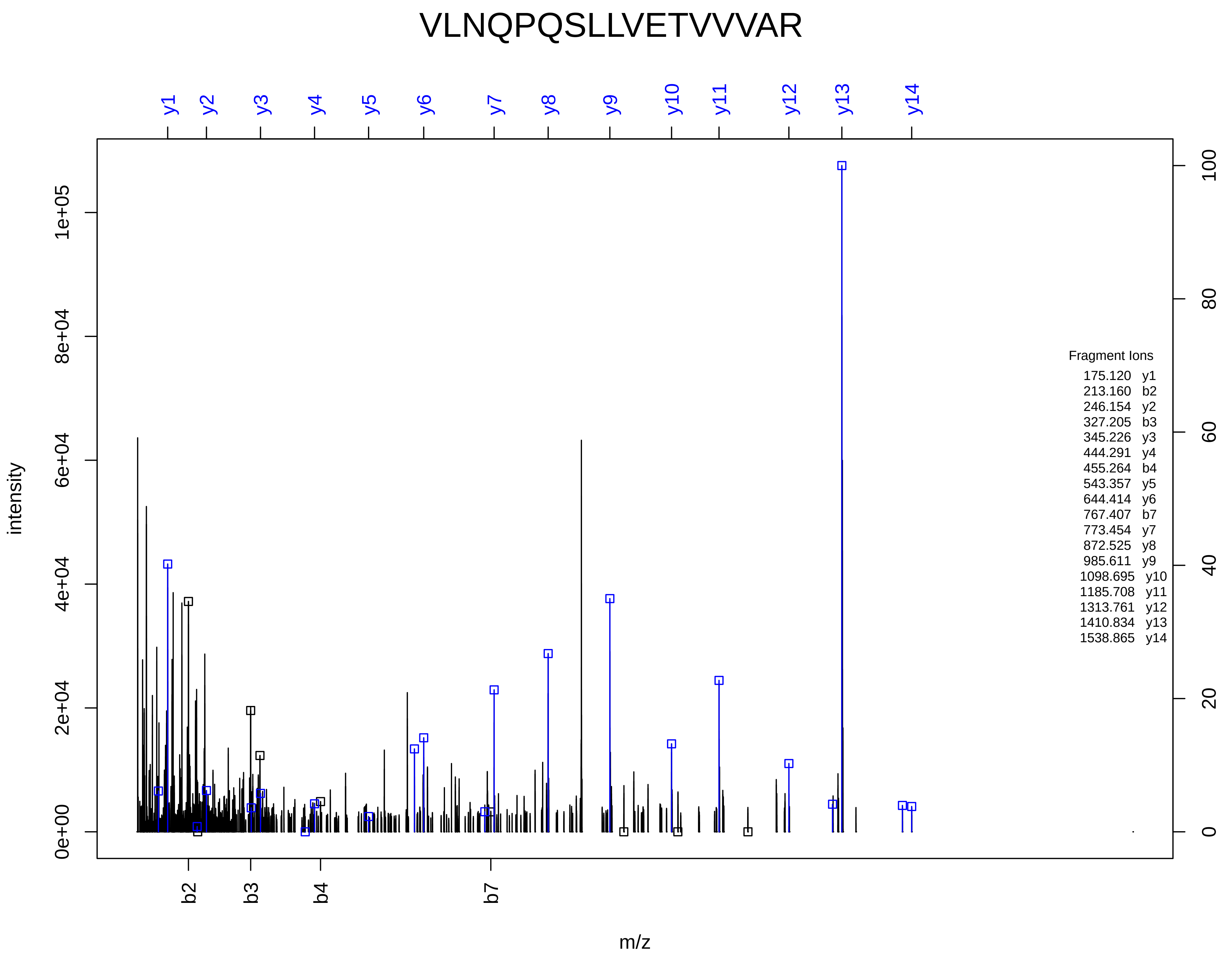

VLNQPQSLLVETVVVAR / File: christine_ip_10.raw; SpectrumID: ; scans: 15692

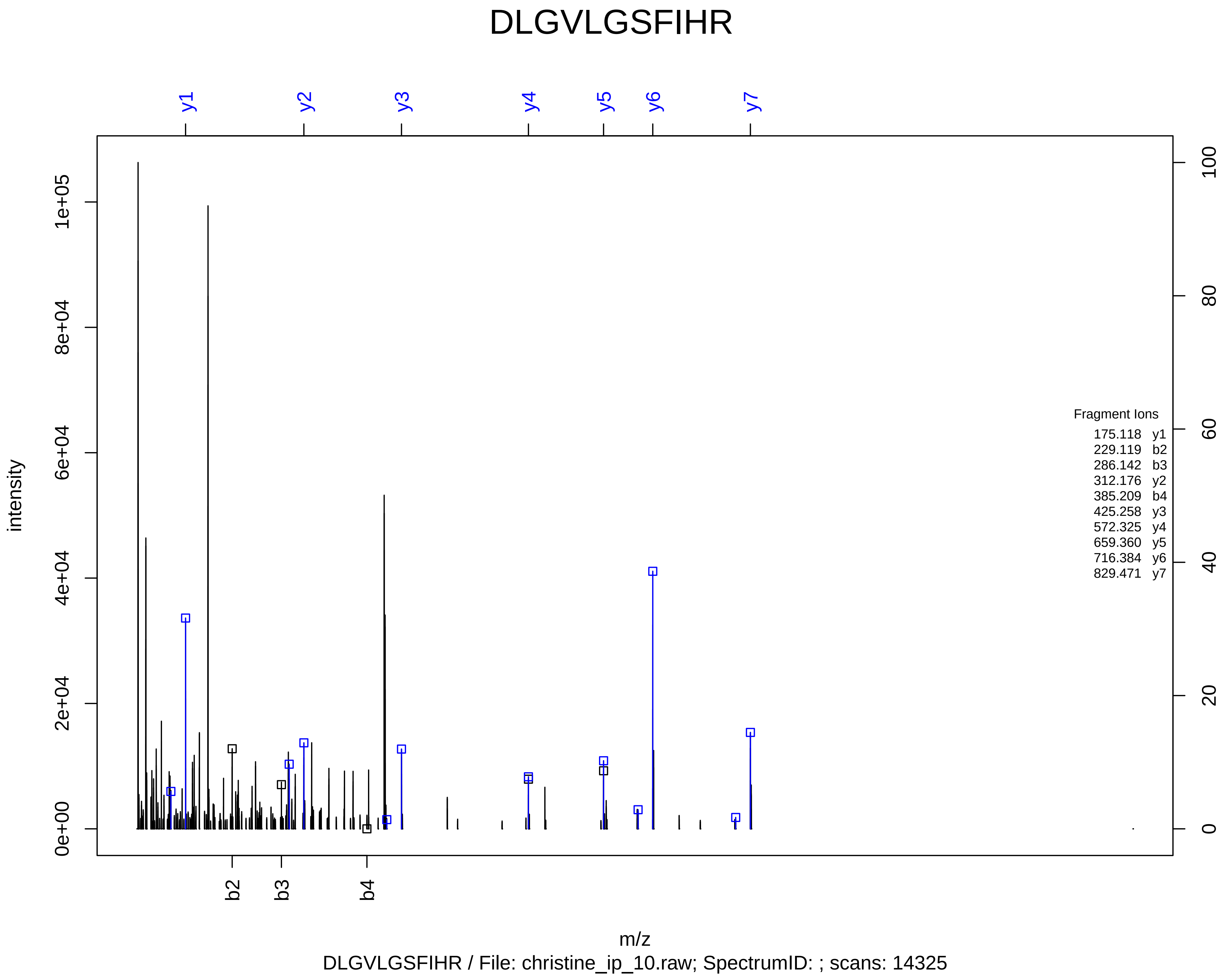
DLGVLGSFIHR
y1
y2
y3
y4
y5
y6
y7
b2
b3
b4
intensity
m/z
0e+00
2e+04
4e+04
6e+04
8e+04
1e+05
0
20
40
60
80
100
Fragment Ions
175.118 y1
229.119 b2
286.142 b3
312.176 y2
385.209 b4
425.258 y3
572.325 y4
659.360 y5
716.384 y6
829.471 y7
DLGVLGSFIHR / File: christine_ip_10.raw; SpectrumID: ; scans: 14325

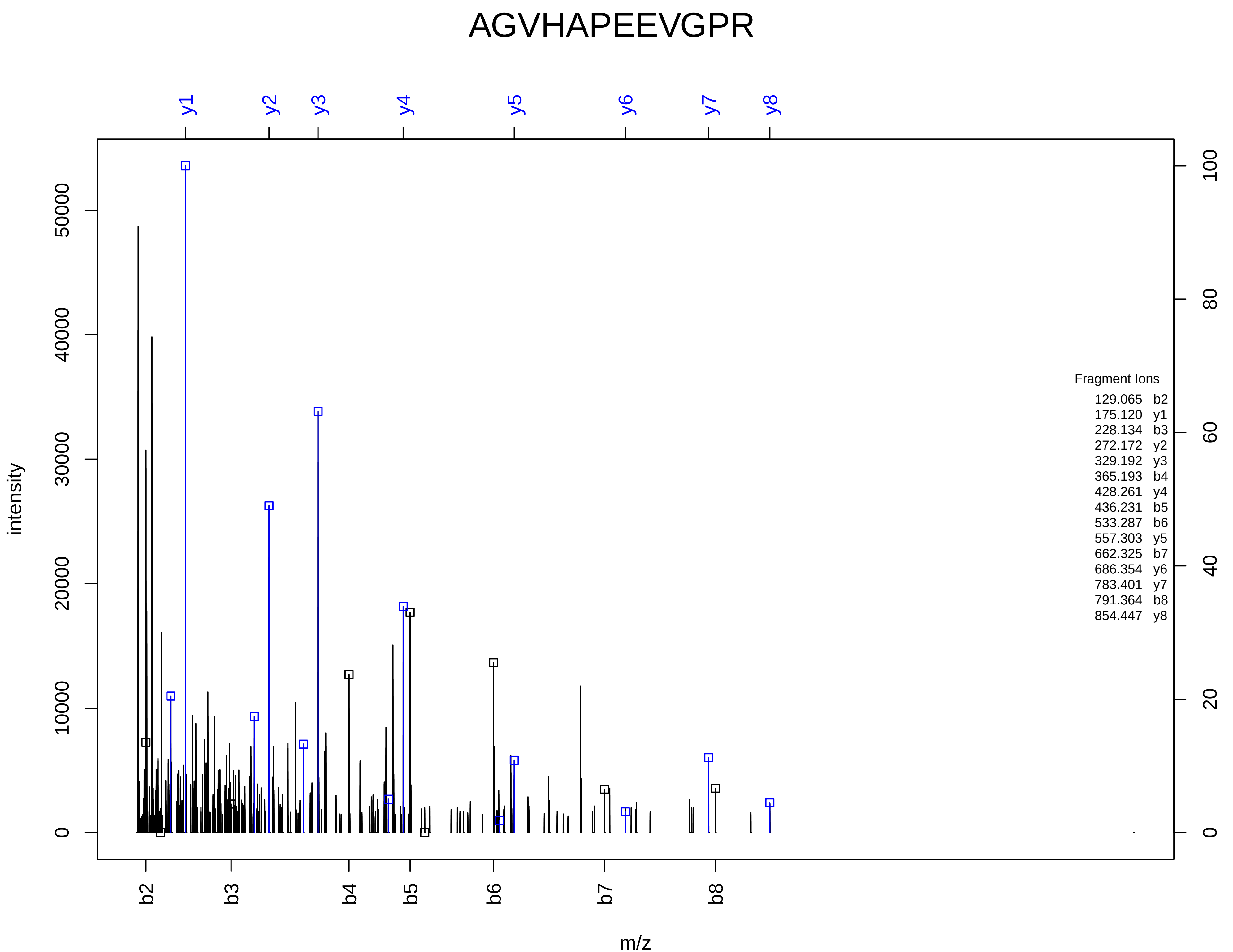
AGVHAPEEVGPR
y1
y2
y3
y4
y5
y6
y7
y8
b2
b3
b4
b5
b6
b7
b8
intensity
m/z
Fragment Ions
129.065 b2
175.120 y1
228.134 b3
272.172 y2
329.192 y3
365.193 b4
428.261 y4
436.231 b5
533.287 b6
557.303 y5
662.325 b7
686.354 y6
783.401 y7
791.364 b8
854.447 y8
AGVHAPEEVGPR / File: christine_ip_10.raw; SpectrumID: ; scans: 2520

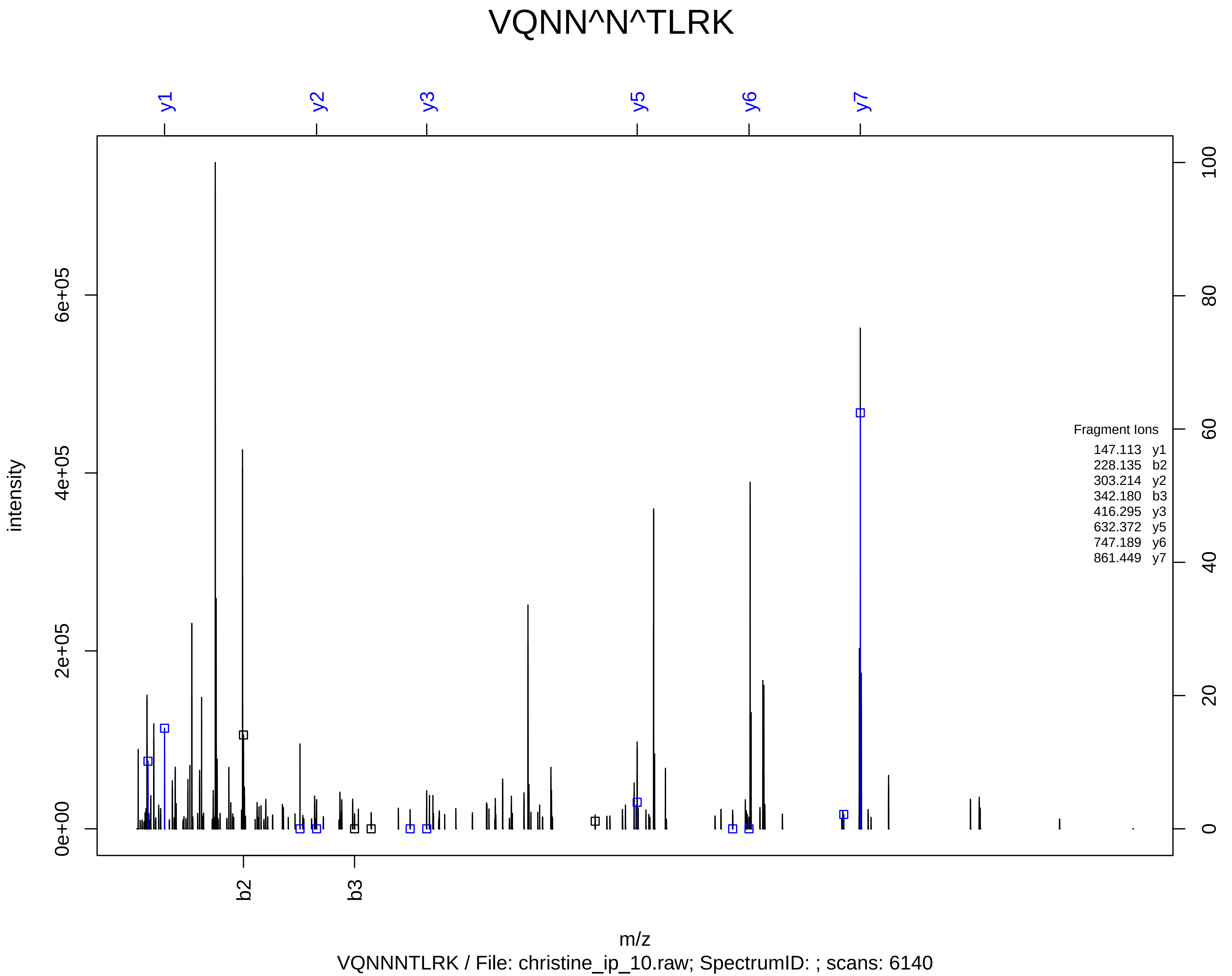
VQNN^N^TLRK
y1
y2
y3
y5
y6
y7
b2
b3
intensity
0e+00
2e+05
4e+05
6e+05
0
20
40
60
80
100
Fragment Ions
147.113 y1
228.135 b2
303.214 y2
342.180 b3
416.295 y3
632.372 y5
747.189 y6
861.449 y7
m/z
VQNNNTLRK / File: christine_ip_10.raw; SpectrumID: ; scans: 6140

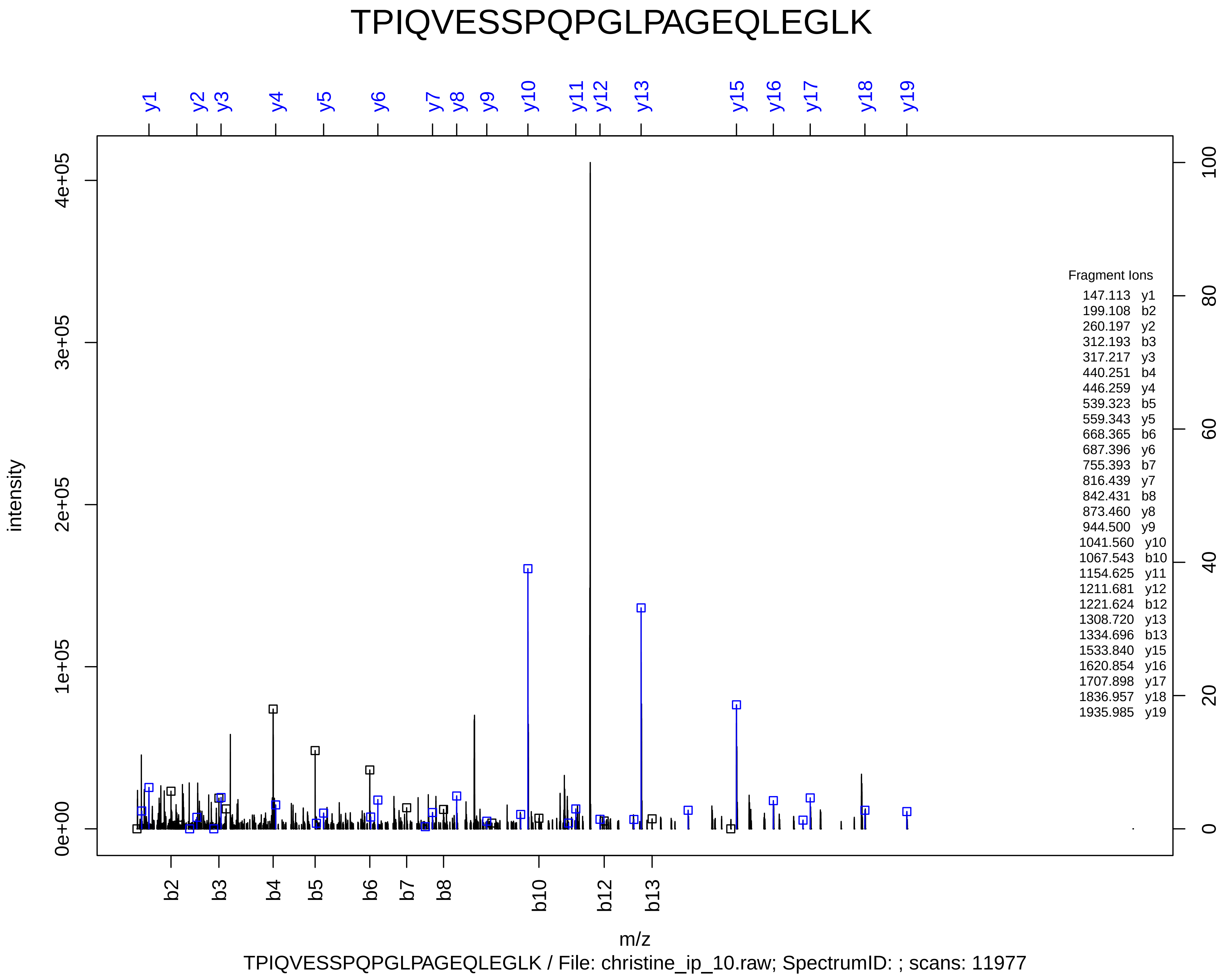
TPIQVESSPQPGLPAGEQLEGLK
y1 y2 y3 y4 y5 y6 y7 y8 y9 y10 y11 y12 y13 y15 y16 y17 y18 y19
b2 b3 b4 b5 b6 b7 b8 b10 b12 b13
intensity
0e+00
1e+05
2e+05
3e+05
4e+05
0
20
40
60
80
100
m/z
Fragment Ions
147.113 y1
199.108 b2
260.197 y2
312.193 b3
317.217 y3
440.251 b4
446.259 y4
539.323 b5
559.343 y5
668.365 b6
687.396 y6
755.393 b7
816.439 y7
842.431 b8
873.460 y8
944.500 y9
1041.560 y10
1067.543 b10
1154.625 y11
1211.681 y12
1221.624 b12
1308.720 y13
1334.696 b13
1533.840 y15
1620.854 y16
1707.898 y17
1836.957 y18
1935.985 y19
TPIQVESSPQPGLPAGEQLEGLK / File: christine_ip_10.raw; SpectrumID: ; scans: 11977

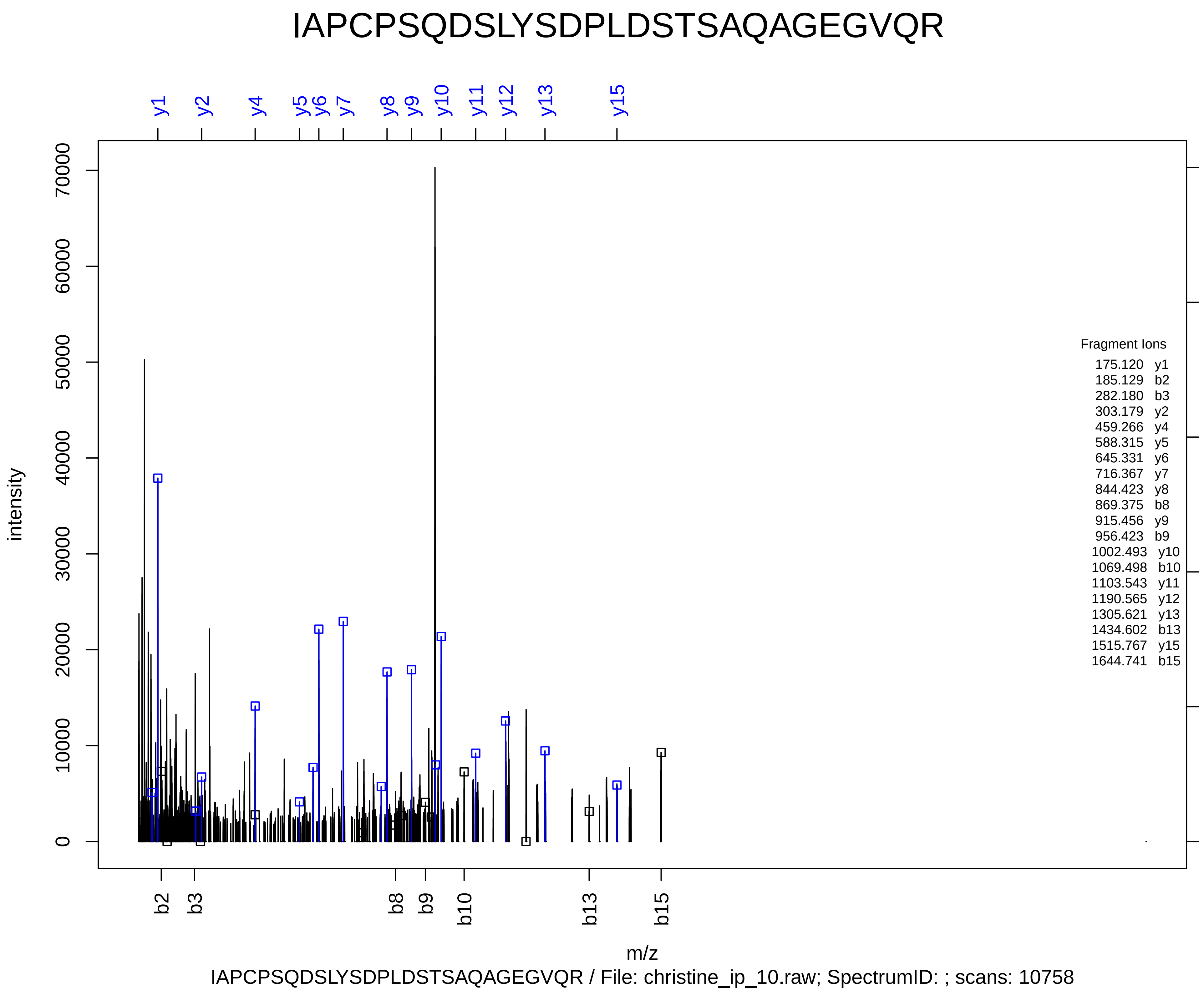
IAPCPSQDSLYSDPLDSTSAQAGEGVQR
y1
y2
y4
y5
y6
y7
y8
y9
y10
y11
y12
y13
y15
intensity
m/z
b2
b3
b8
b9
b10
b13
b15
Fragment Ions
175.120 y1
185.129 b2
282.180 b3
303.179 y2
459.266 y4
588.315 y5
645.331 y6
716.367 y7
844.423 y8
869.375 b8
915.456 y9
956.423 b9
1002.493 y10
1069.498 b10
1103.543 y11
1190.565 y12
1305.621 y13
1434.602 b13
1515.767 y15
1644.741 b15
IAPCPSQDSLYSDPLDSTSAQAGEGVQR / File: christine_ip_10.raw; SpectrumID: ; scans: 10758

ARKM*EFFN^PVLN^EN^QK

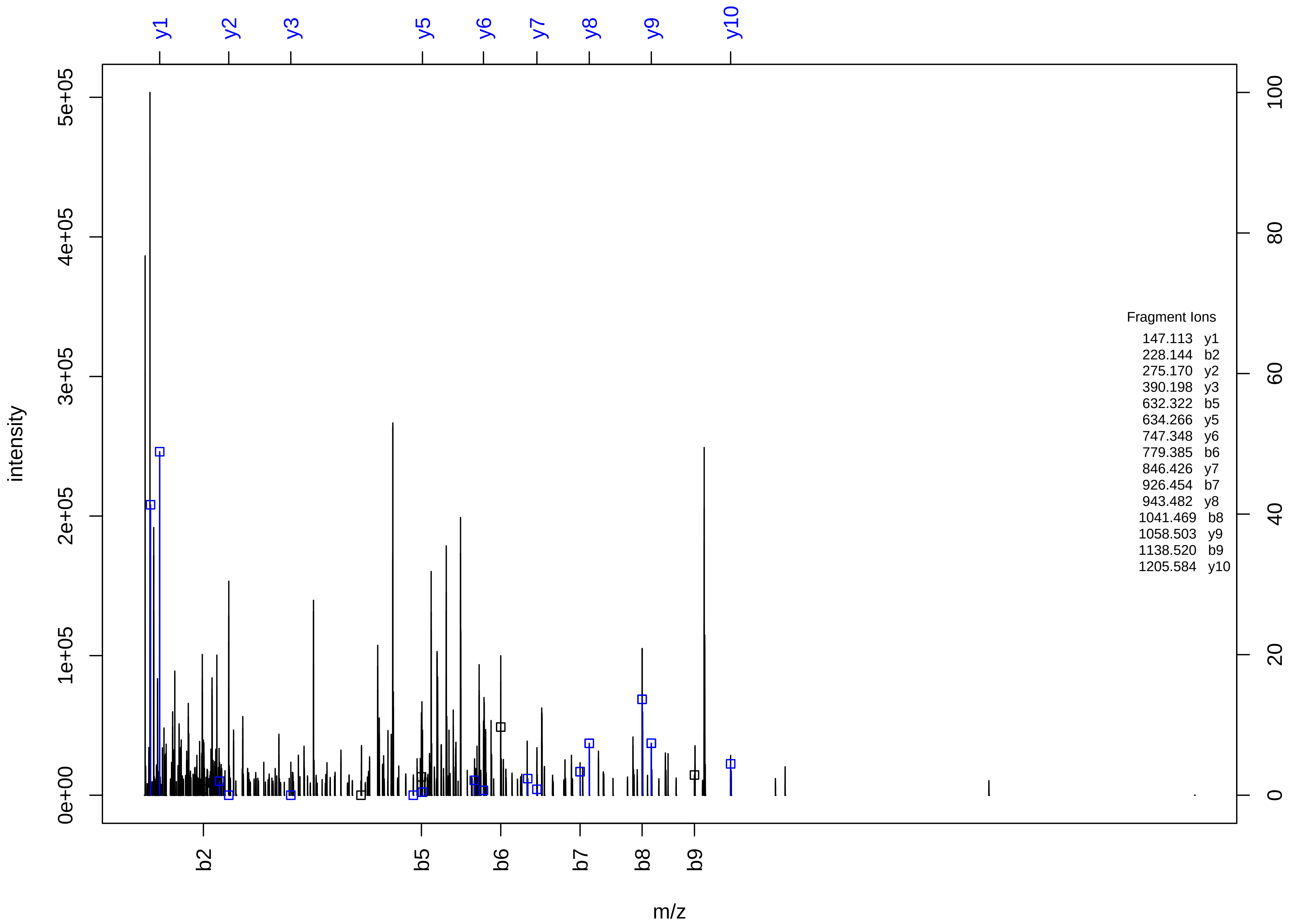

ARKMEFFNPVLNENQK / File: christine_ip_10.raw; SpectrumID: ; scans: 8104

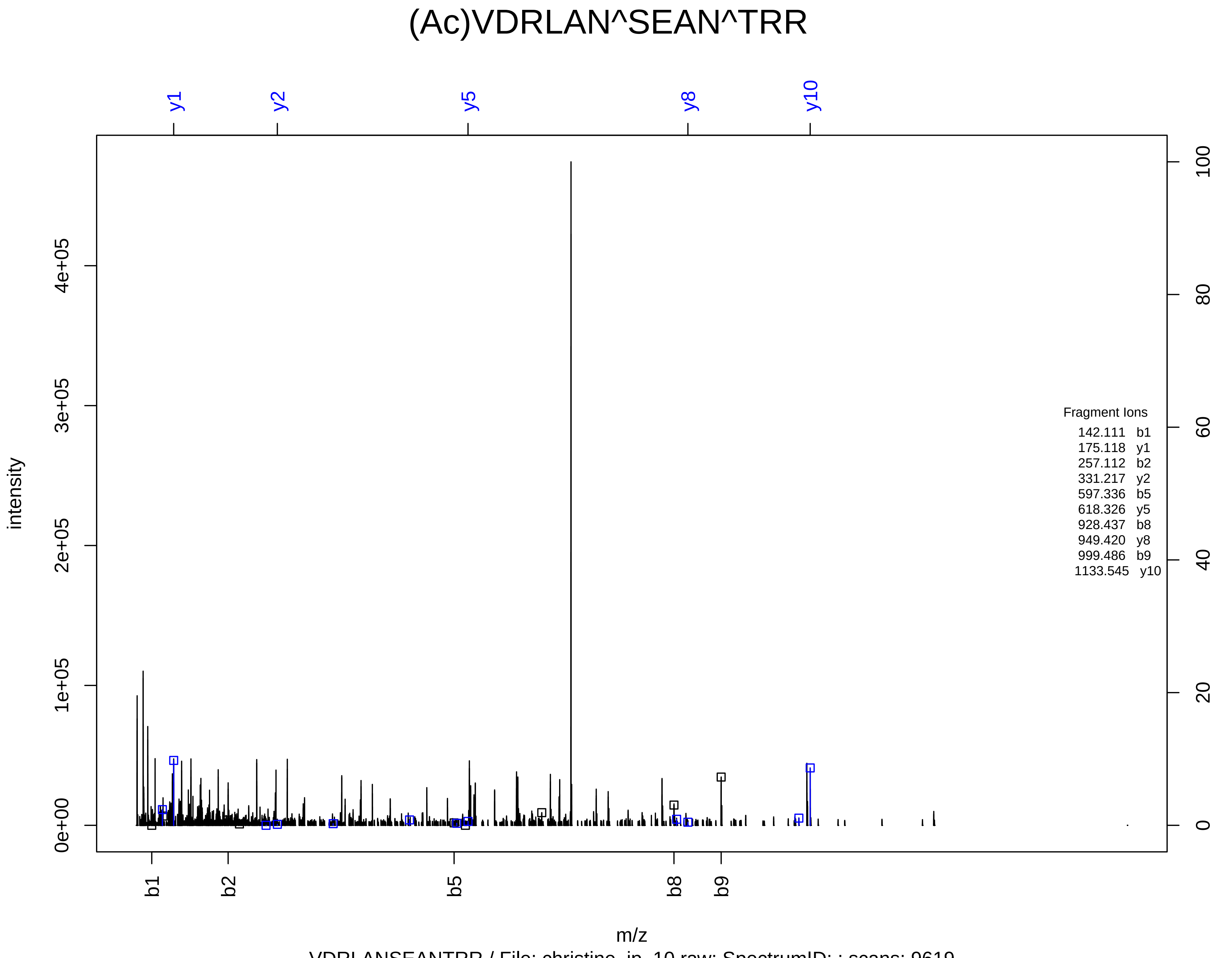
(Ac)VDRLAN^SEAN^TRR
y1
y2
y5
y8
y10
intensity
0e+00
1e+05
2e+05
3e+05
4e+05
0
20
40
60
80
100
b1
b2
b5
b8
b9
m/z
Fragment Ions
142.111 b1
175.118 y1
257.112 b2
331.217 y2
597.336 b5
618.326 y5
928.437 b8
949.420 y8
999.486 b9
1133.545 y10
VDRLANSEANTRR / File: christine_ip_10.raw; SpectrumID: ; scans: 9619

ITDTIGPTETSIAPR

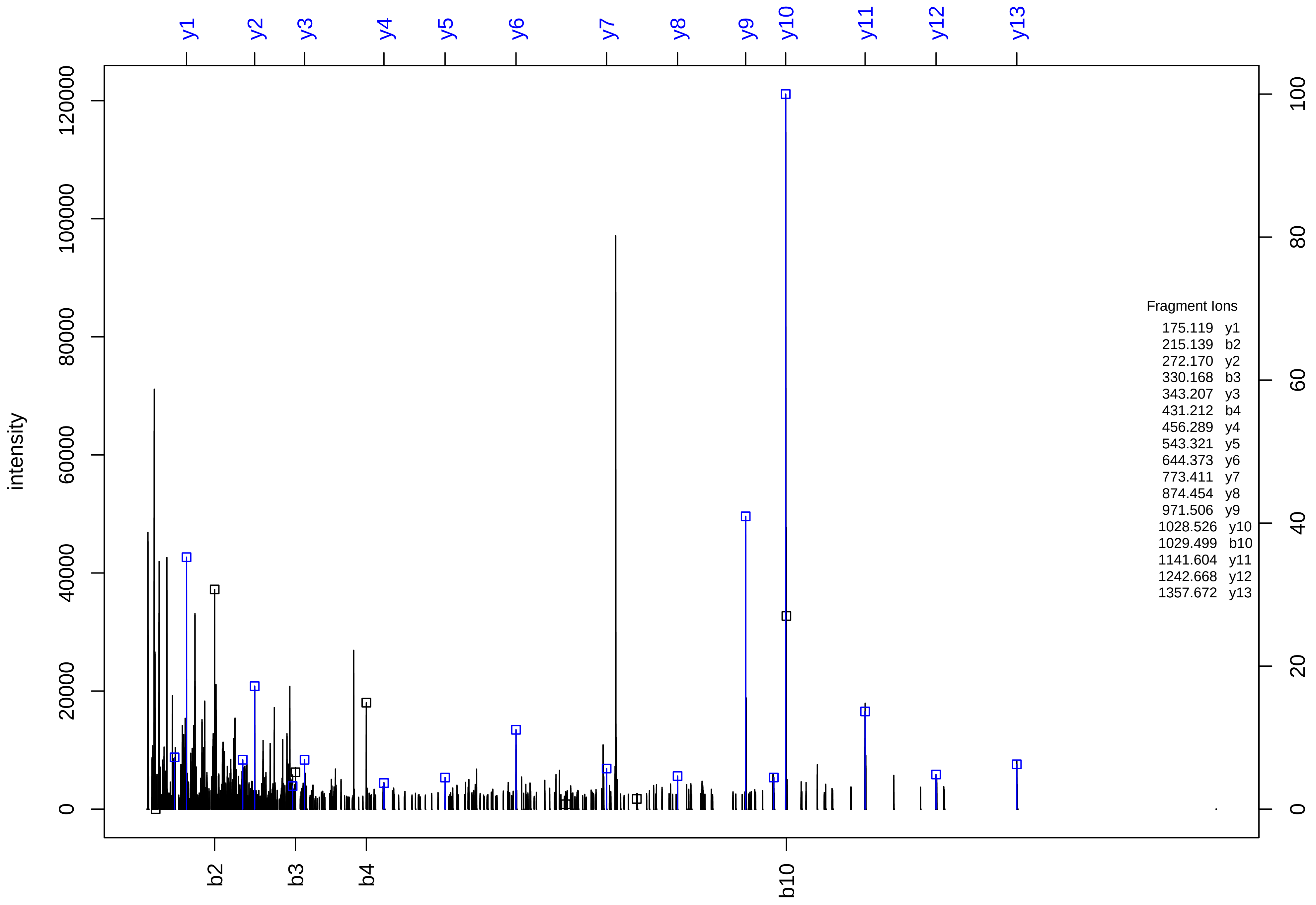

ITDTIGPTETSIAPR / File: christine_ip_10.raw; SpectrumID: ; scans: 8038

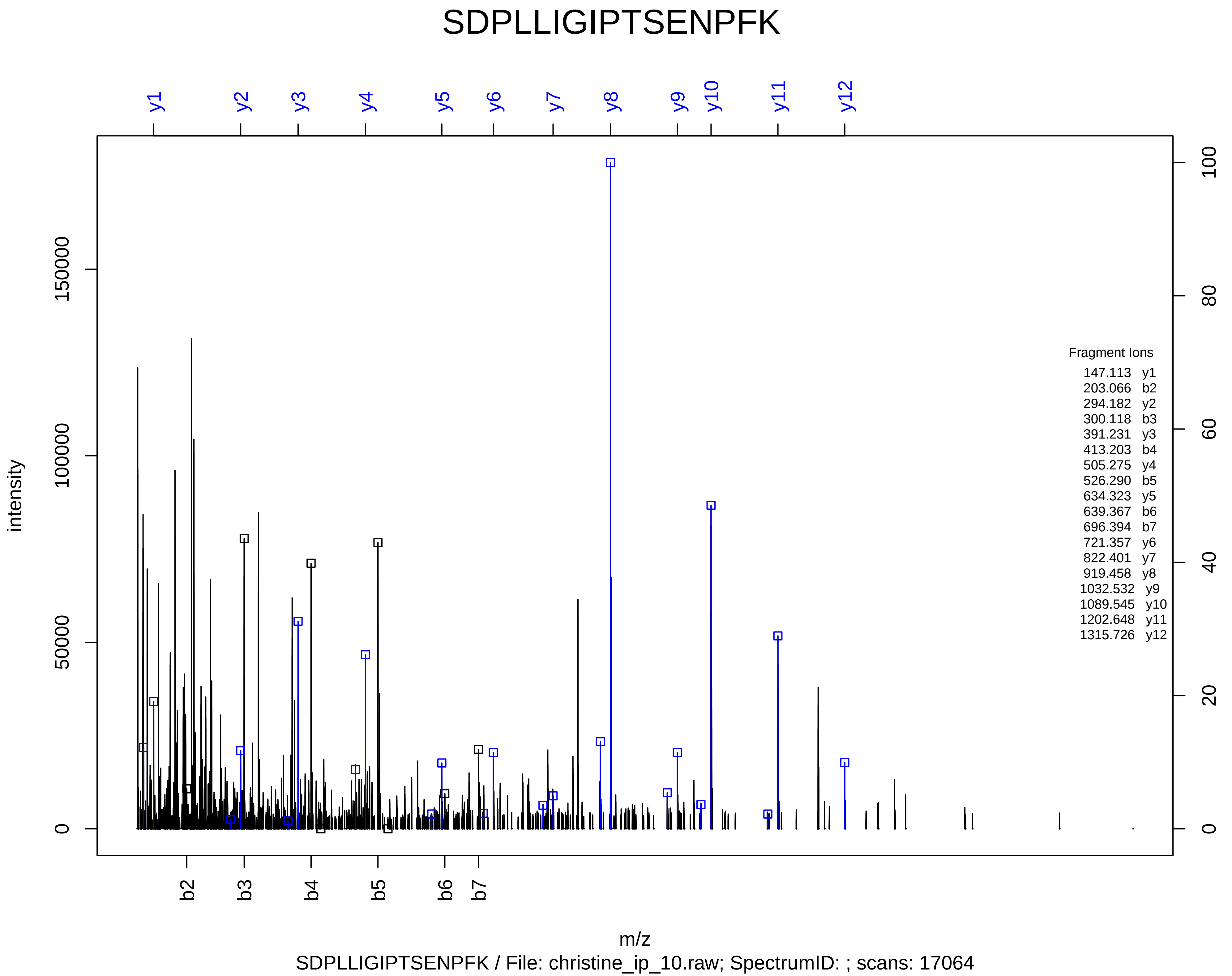
SDPLLIGIPTSENPFK
y1
y2
y3
y4
y5
y6
y7
y8
y9
y10
y11
y12
b2
b3
b4
b5
b6
b7
intensity
m/z
Fragment Ions
147.113 y1
203.066 b2
294.182 y2
300.118 b3
391.231 y3
413.203 b4
505.275 y4
526.290 b5
634.323 y5
639.367 b6
696.394 b7
721.357 y6
822.401 y7
919.458 y8
1032.532 y9
1089.545 y10
1202.648 y11
1315.726 y12
SDPLLIGIPTSENPFK / File: christine_ip_10.raw; SpectrumID: ; scans: 17064

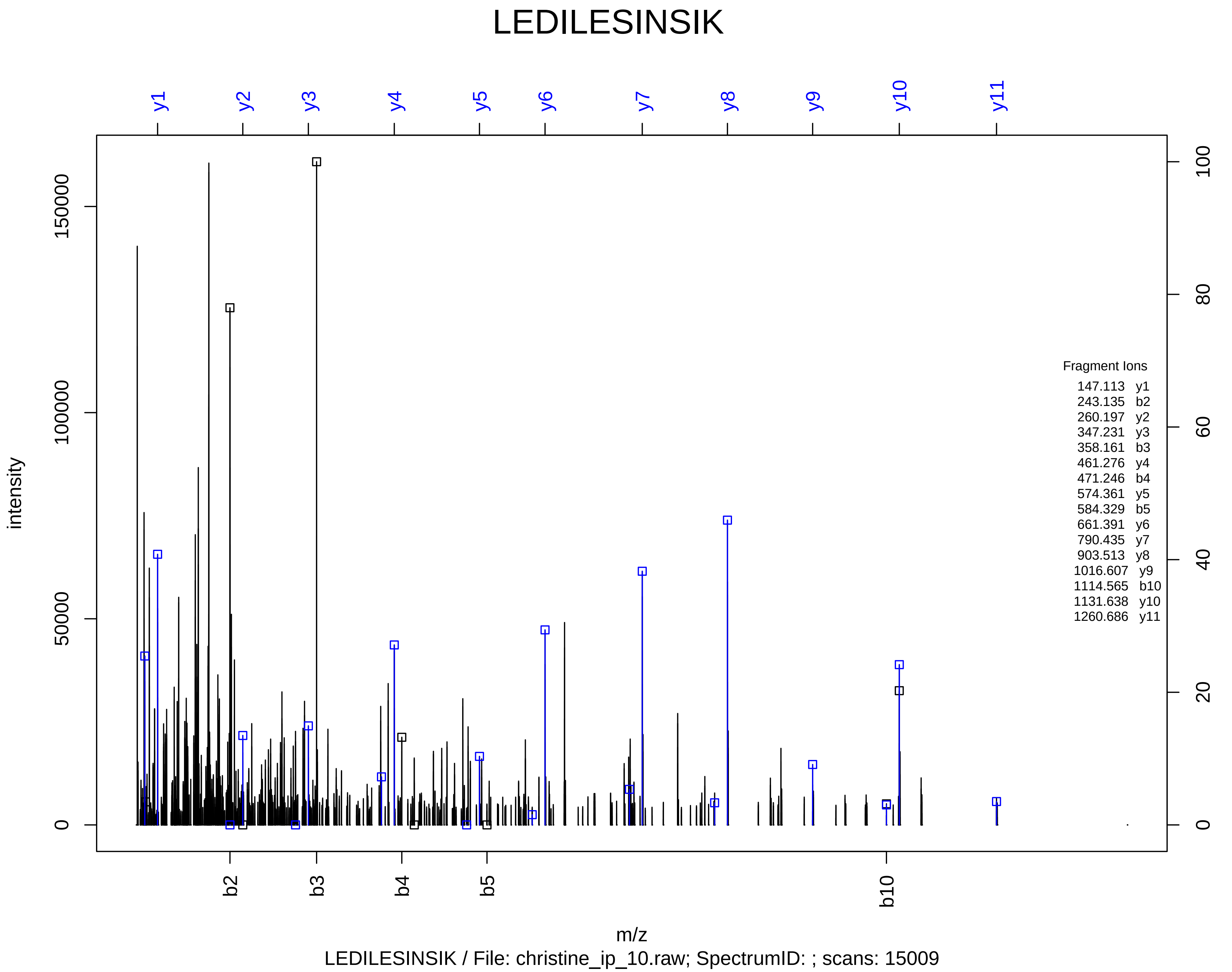

LEDILESINSIK
y1
y2
y3
y4
y5
y6
y7
y8
y9
y10
y11
intensity
0
50000
100000
150000
0
20
40
60
80
100
Fragment Ions
147.113 y1
243.135 b2
260.197 y2
347.231 y3
358.161 b3
461.276 y4
471.246 b4
574.361 y5
584.329 b5
661.391 y6
790.435 y7
903.513 y8
1016.607 y9
1114.565 b10
1131.638 y10
1260.686 y11
b2
b3
b4
b5
b10
m/z
LEDILESINSIK / File: christine_ip_10.raw; SpectrumID: ; scans: 15009

SYQDAVLEDIFK

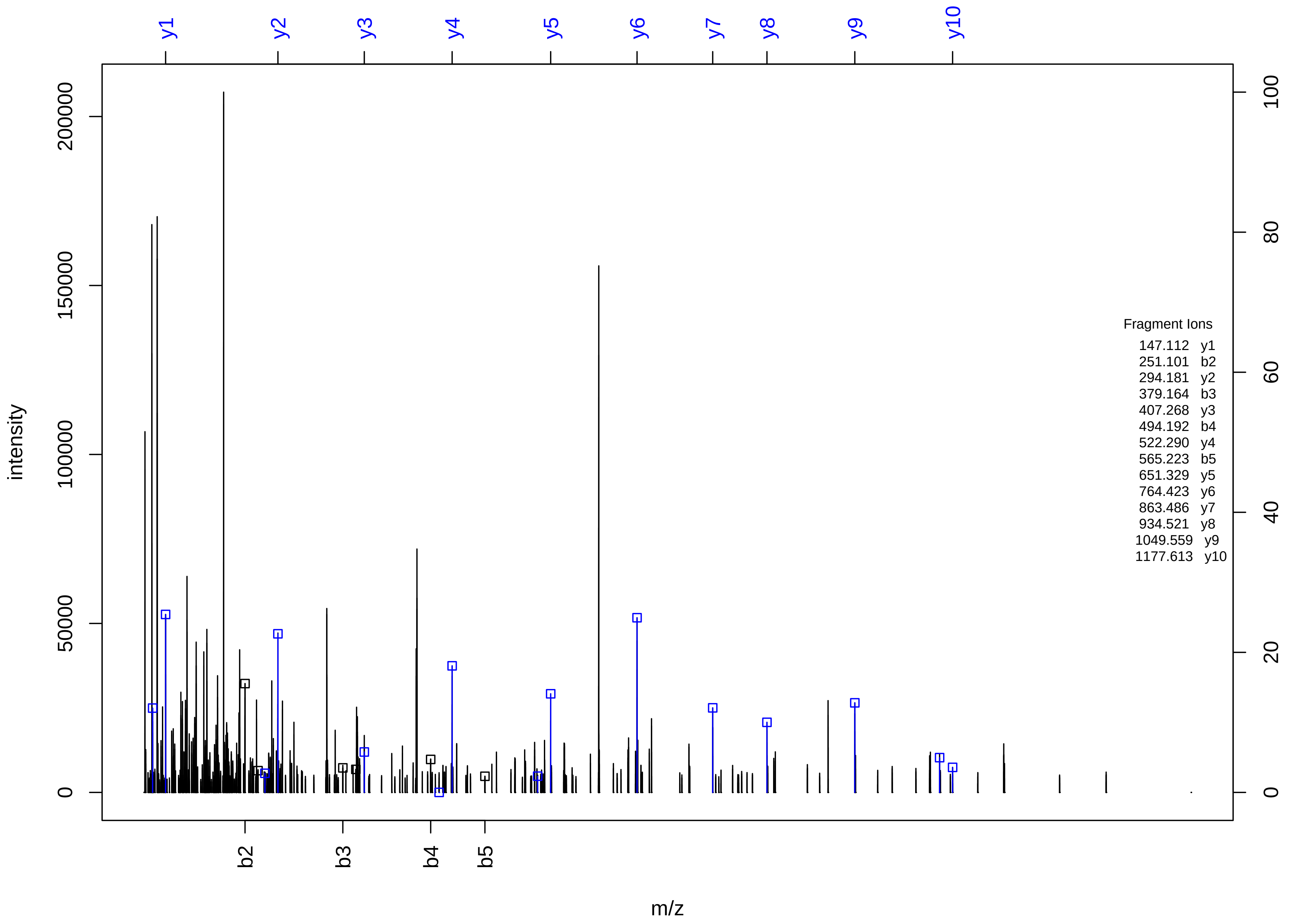

SYQDAVLEDIFK / File: christine_ip_10.raw; SpectrumID: ; scans: 16169

VINSLSM*GIN^K

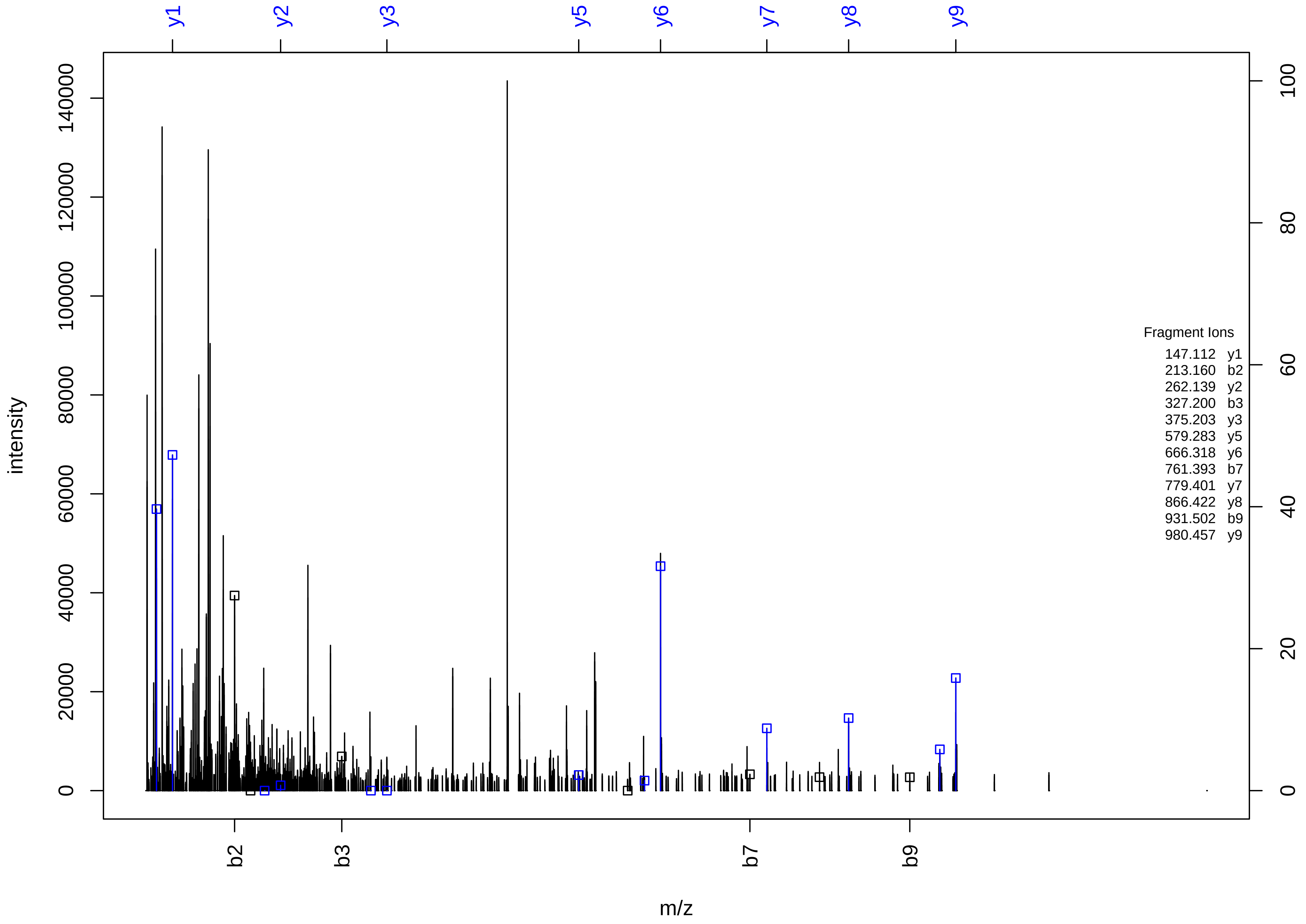

VINSLSMGINK / File: christine_ip_14.raw; SpectrumID: ; scans: 5985

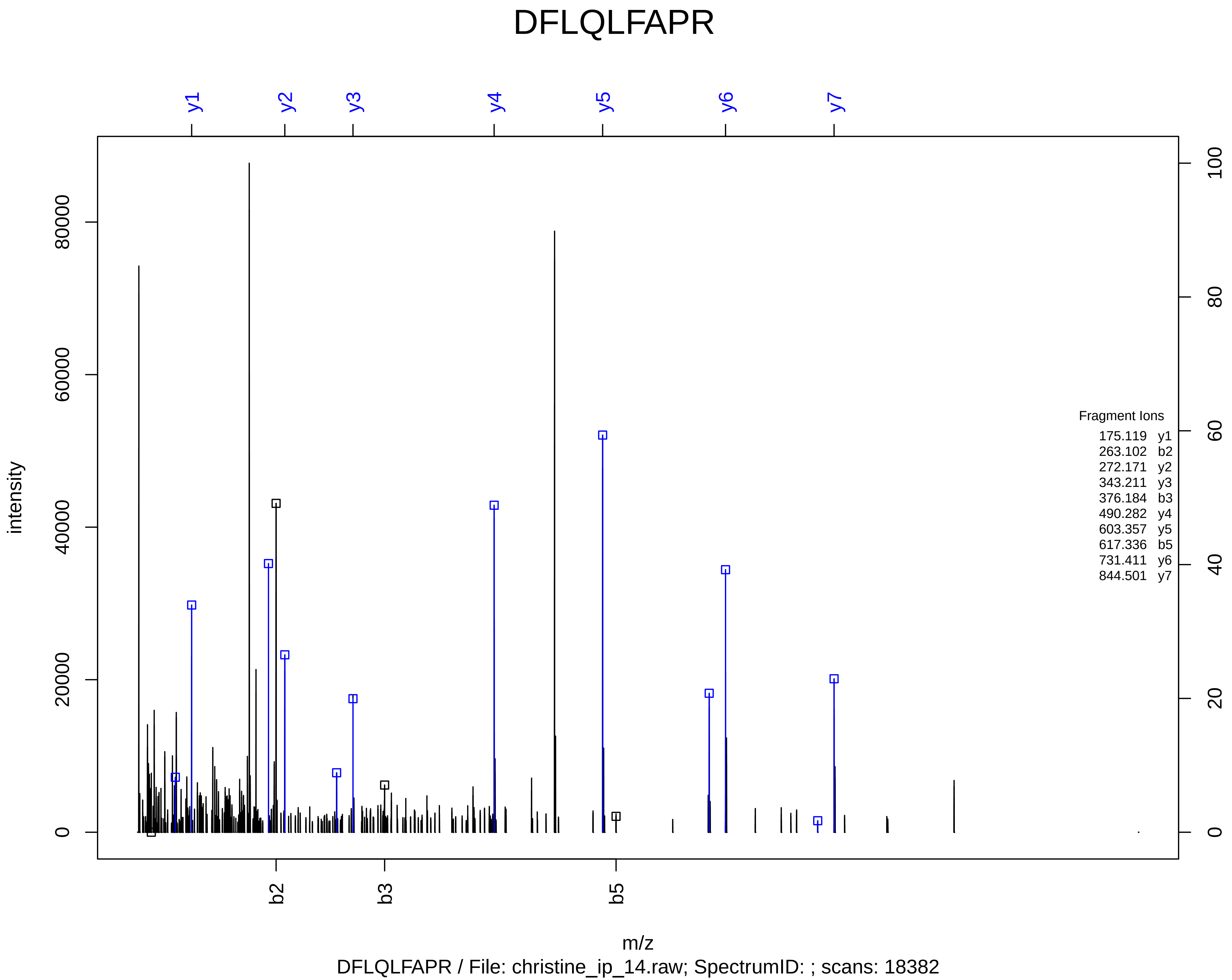
DFLQLFAPR
y1
y2
y3
y4
y5
y6
y7
b2
b3
b5
intensity
0
20000
40000
60000
80000
0
20
40
60
80
100
m/z
Fragment Ions
175.119 y1
263.102 b2
272.171 y2
343.211 y3
376.184 b3
490.282 y4
603.357 y5
617.336 b5
731.411 y6
844.501 y7
DFLQLFAPR / File: christine_ip_14.raw; SpectrumID: ; scans: 18382

VPGTQAASTSHQPPTR

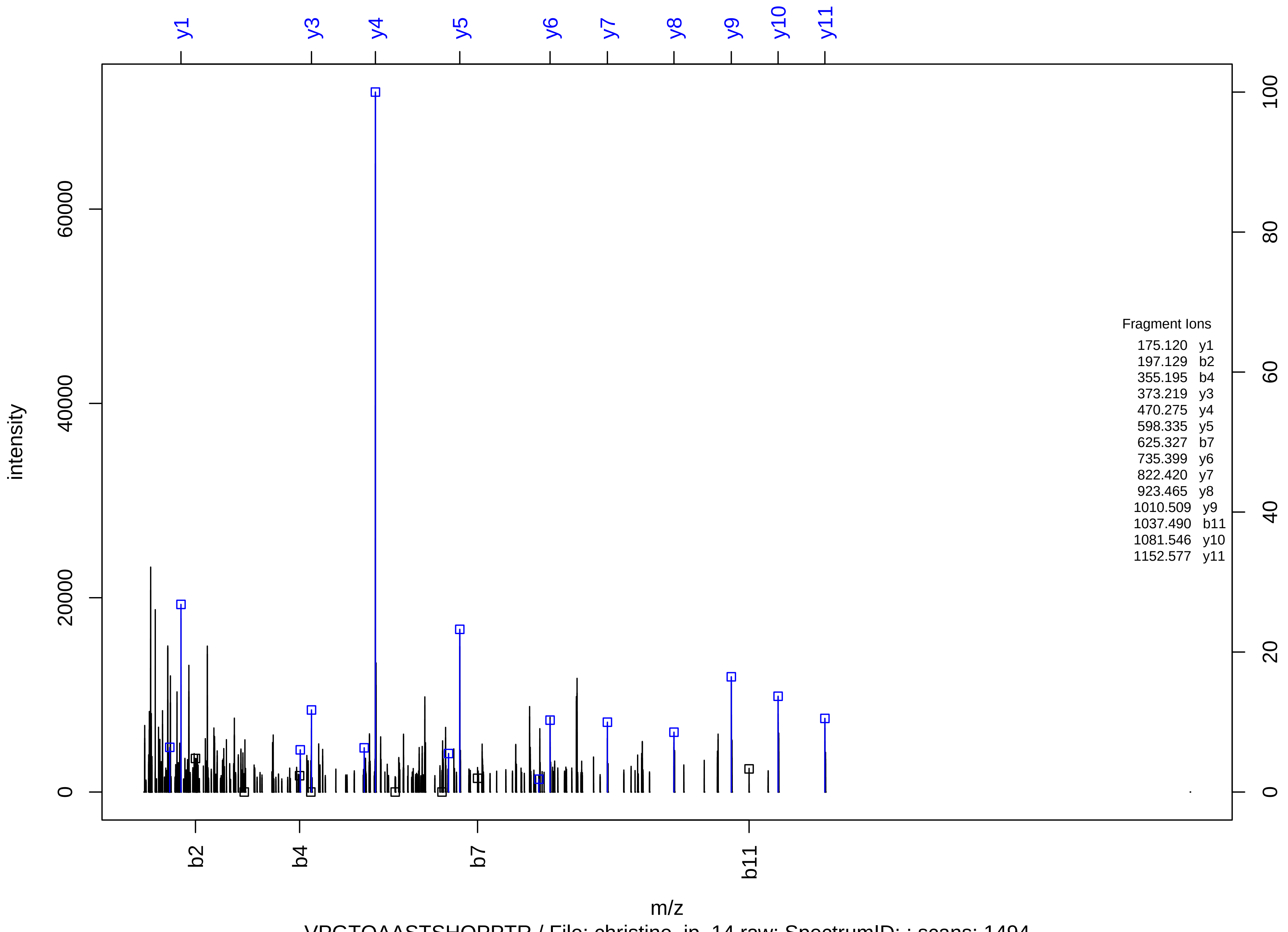

VPGTQAASTSHQPPTR / File: christine_ip_14.raw; SpectrumID: ; scans: 1494

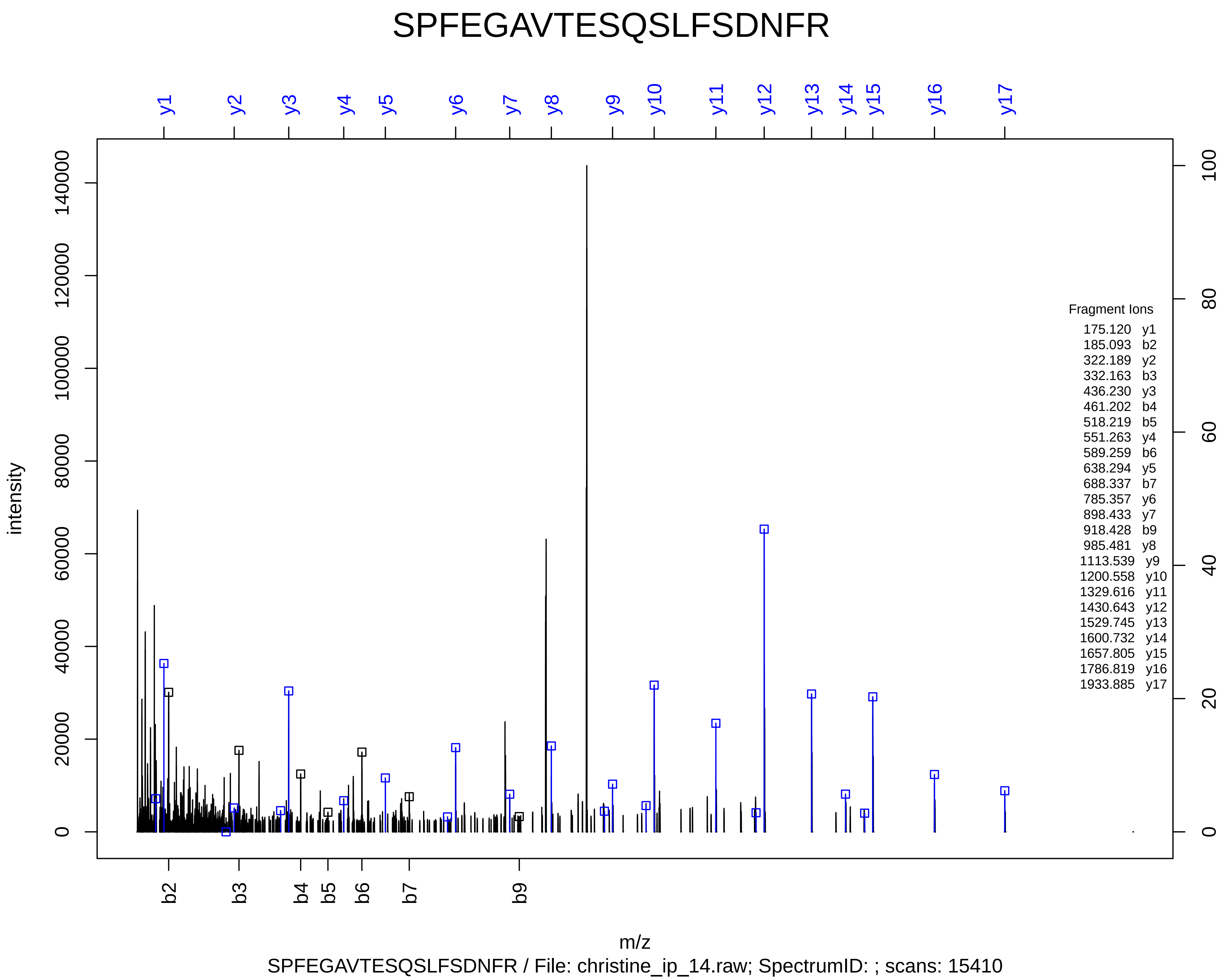

SPFEGAVTESQSLFSDNFR
y1
y2
y3
y4
y5
y6
y7
y8
y9
y10
y11
y12
y13
y14
y15
y16
y17
intensity
0
20000
40000
60000
80000
100000
120000
140000
0
20
40
60
80
100
Fragment Ions
175.120 y1
185.093 b2
322.189 y2
332.163 b3
436.230 y3
461.202 b4
518.219 b5
551.263 y4
589.259 b6
638.294 y5
688.337 b7
785.357 y6
898.433 y7
918.428 b9
985.481 y8
1113.539 y9
1200.558 y10
1329.616 y11
1430.643 y12
1529.745 y13
1600.732 y14
1657.805 y15
1786.819 y16
1933.885 y17
b2
b3
b4
b5
b6
b7
b9
m/z
SPFEGAVTESQSLFSDNFR / File: christine_ip_14.raw; SpectrumID: ; scans: 15410

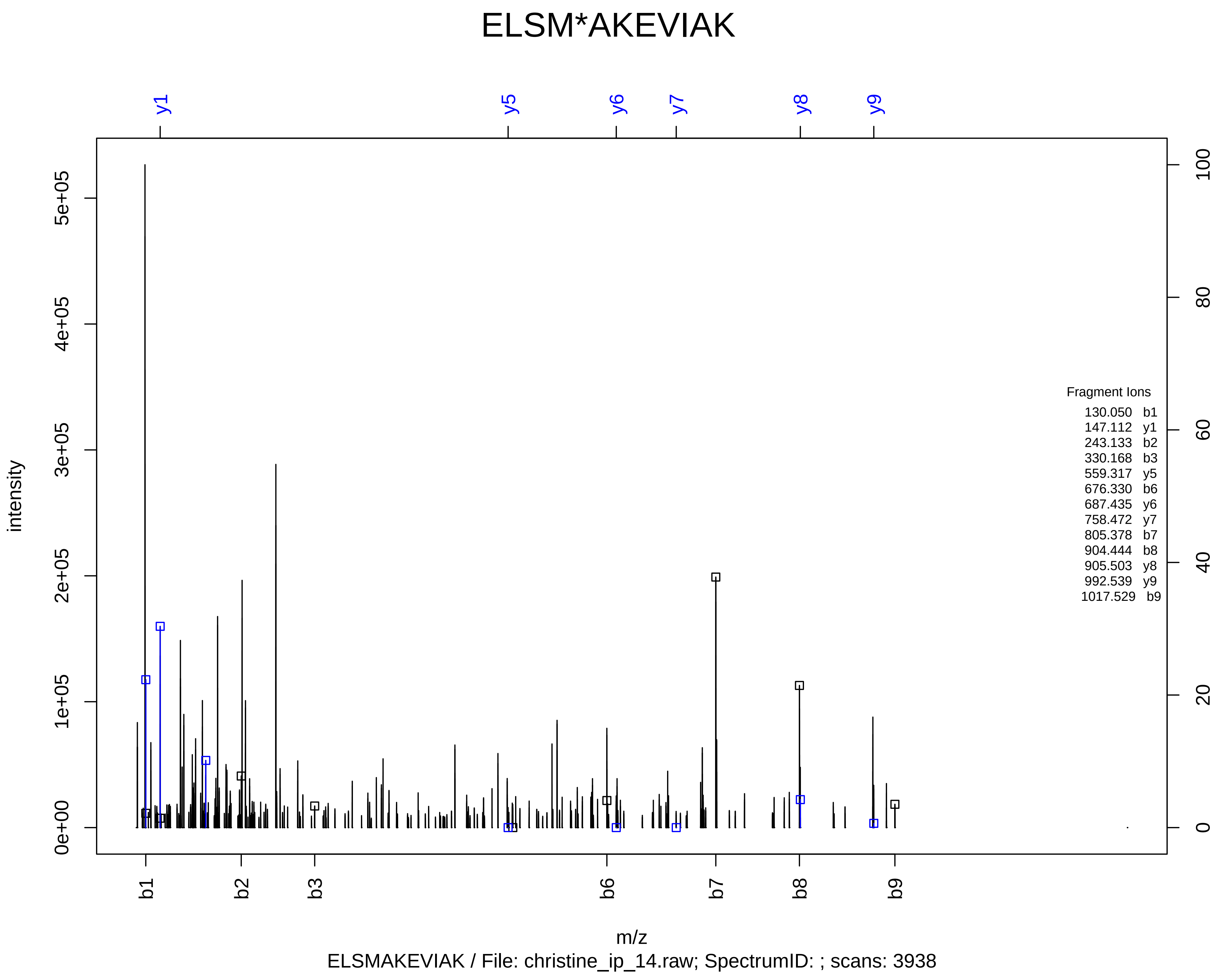
ELSM*AKEVIAK
y1
y5
y6
y7
y8
y9
intensity
0e+00
1e+05
2e+05
3e+05
4e+05
5e+05
0
20
40
60
80
100
Fragment Ions
130.050 b1
147.112 y1
243.133 b2
330.168 b3
559.317 y5
676.330 b6
687.435 y6
758.472 y7
805.378 b7
904.444 b8
905.503 y8
992.539 y9
1017.529 b9
b1
b2
b3
b6
b7
b8
b9
m/z
ELSMAKEVIAK / File: christine_ip_14.raw; SpectrumID: ; scans: 3938

GRPQGPPQQGGHQQGPPPPPPGKPQGPPPQGGRPQGPPQGQSPQ

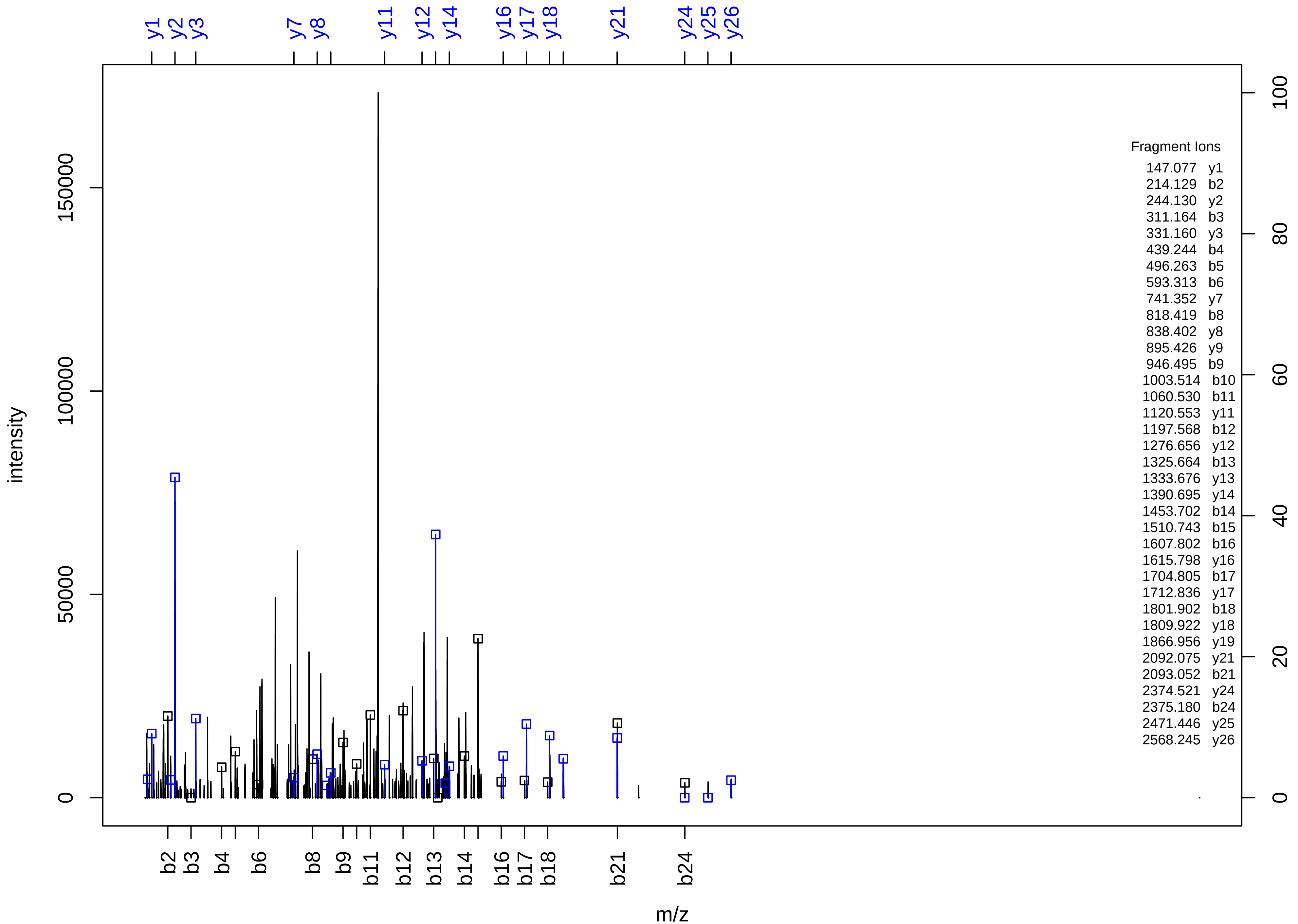

GRPQGPPQQGGHQQGPPPPPPGKPQGPPPQGGRPQGPPQGQSPQ / File: christine_ip_14.raw; SpectrumID: ; scans: 2921

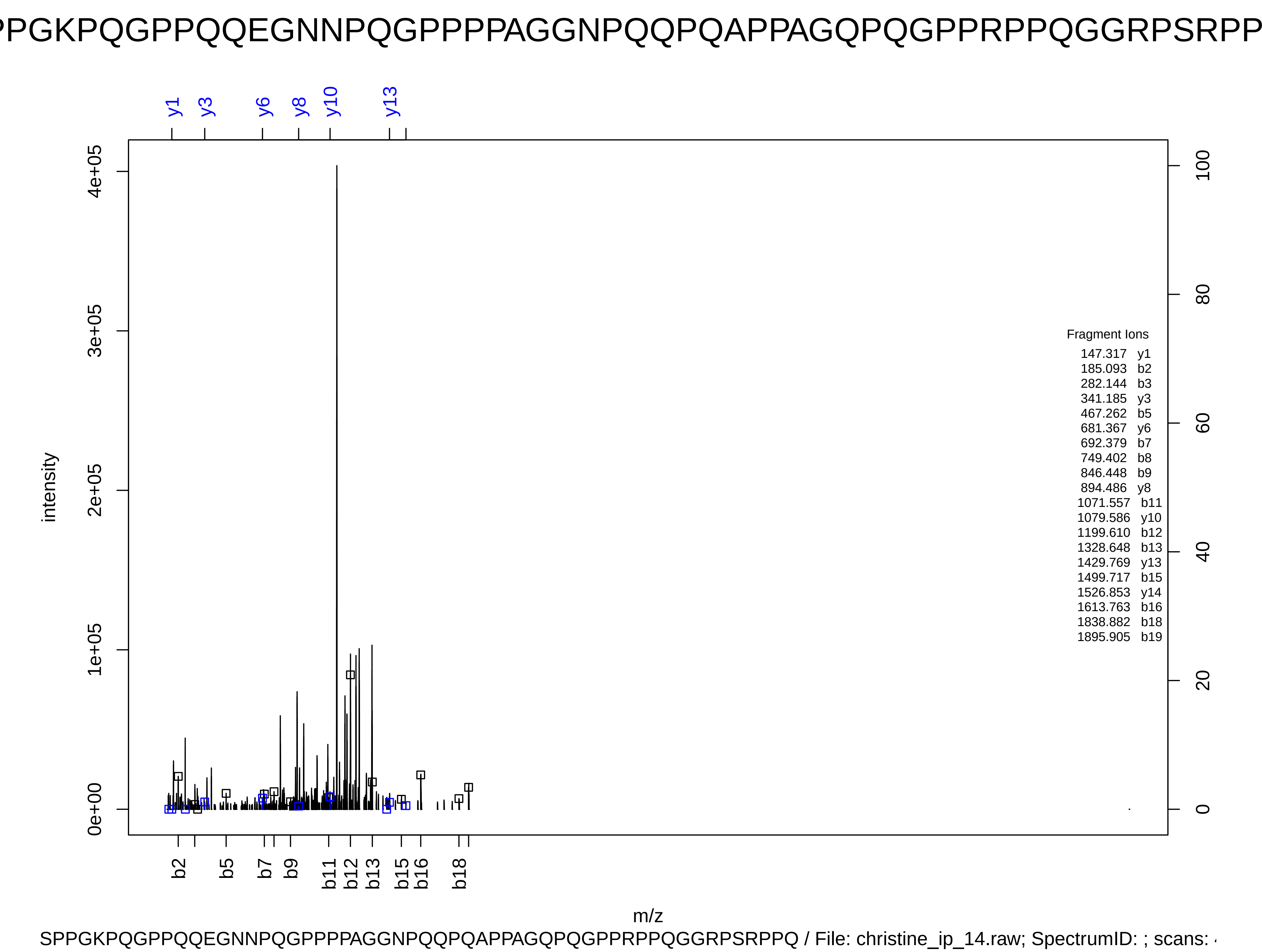
PPGKPQGPPQQEGNNPQGPPPPAGGNPQQPQAPPAGQPQGPPRPPQGGRPSRPP
y1
y3
y6
y8
y10
y13
intensity
0e+00
1e+05
2e+05
3e+05
4e+05
0
20
40
60
80
100
b2
b5
b7
b9
b11
b12
b13
b15
b16
b18
m/z
Fragment Ions
147.317 y1
185.093 b2
282.144 b3
341.185 y3
467.262 b5
681.367 y6
692.379 b7
749.402 b8
846.448 b9
894.486 y8
1071.557 b11
1079.586 y10
1199.610 b12
1328.648 b13
1429.769 y13
1499.717 b15
1526.853 y14
1613.763 b16
1838.882 b18
1895.905 b19
SPPGKPQGPPQQEGNNPQGPPPPAGGNPQQPQAPPAGQPQGPPRPPQGGRPSRPPQ / File: christine_ip_14.raw; SpectrumID: ; scans:

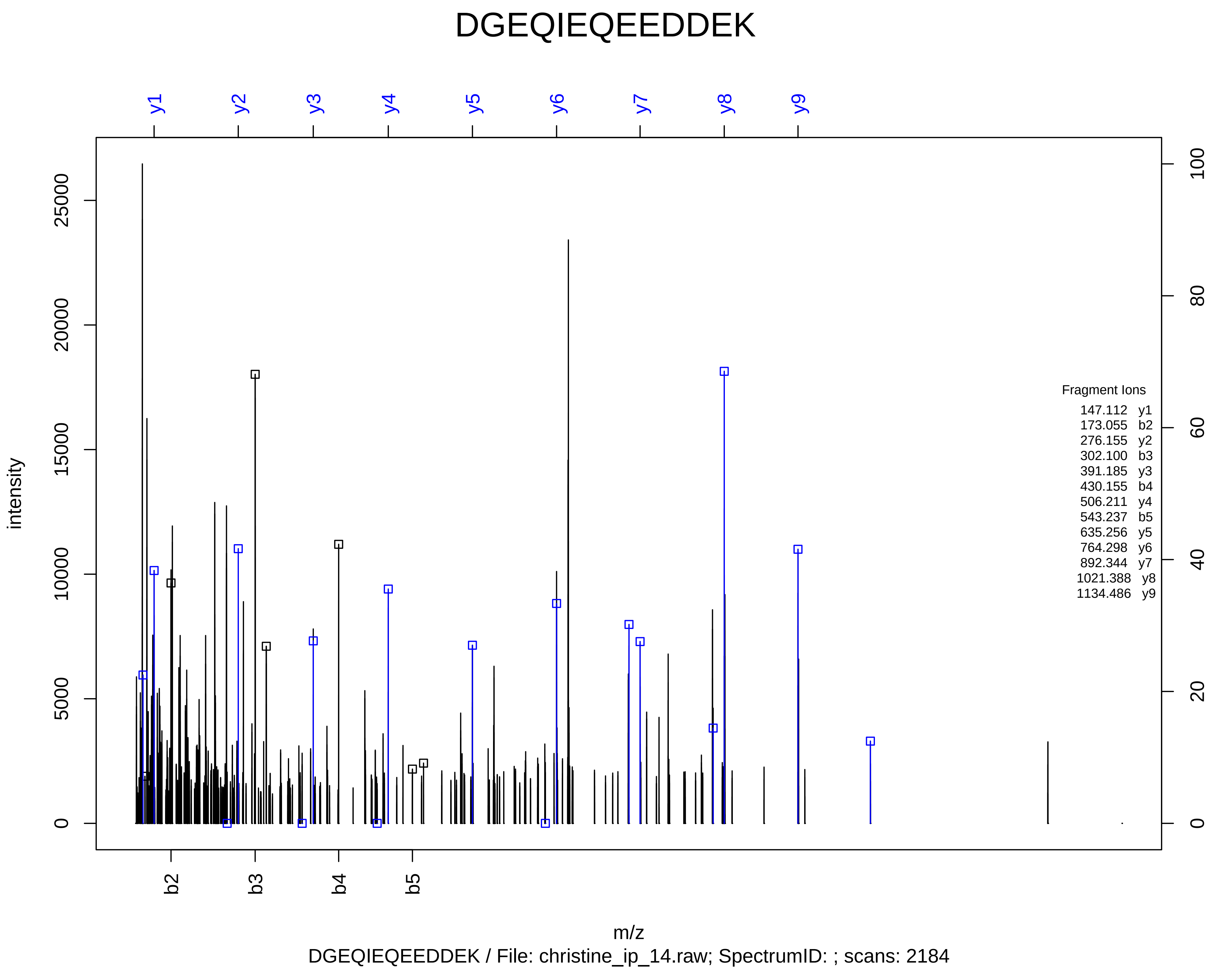
DGEQIEQEEDDEK
intensity
m/z
y1 y2 y3 y4 y5 y6 y7 y8 y9
b2 b3 b4 b5
0
5000
10000
15000
20000
25000
0
20
40
60
80
100
Fragment Ions
147.112 y1
173.055 b2
276.155 y2
302.100 b3
391.185 y3
430.155 b4
506.211 y4
543.237 b5
635.256 y5
764.298 y6
892.344 y7
1021.388 y8
1134.486 y9
DGEQIEQEEDDEK / File: christine_ip_14.raw; SpectrumID: ; scans: 2184

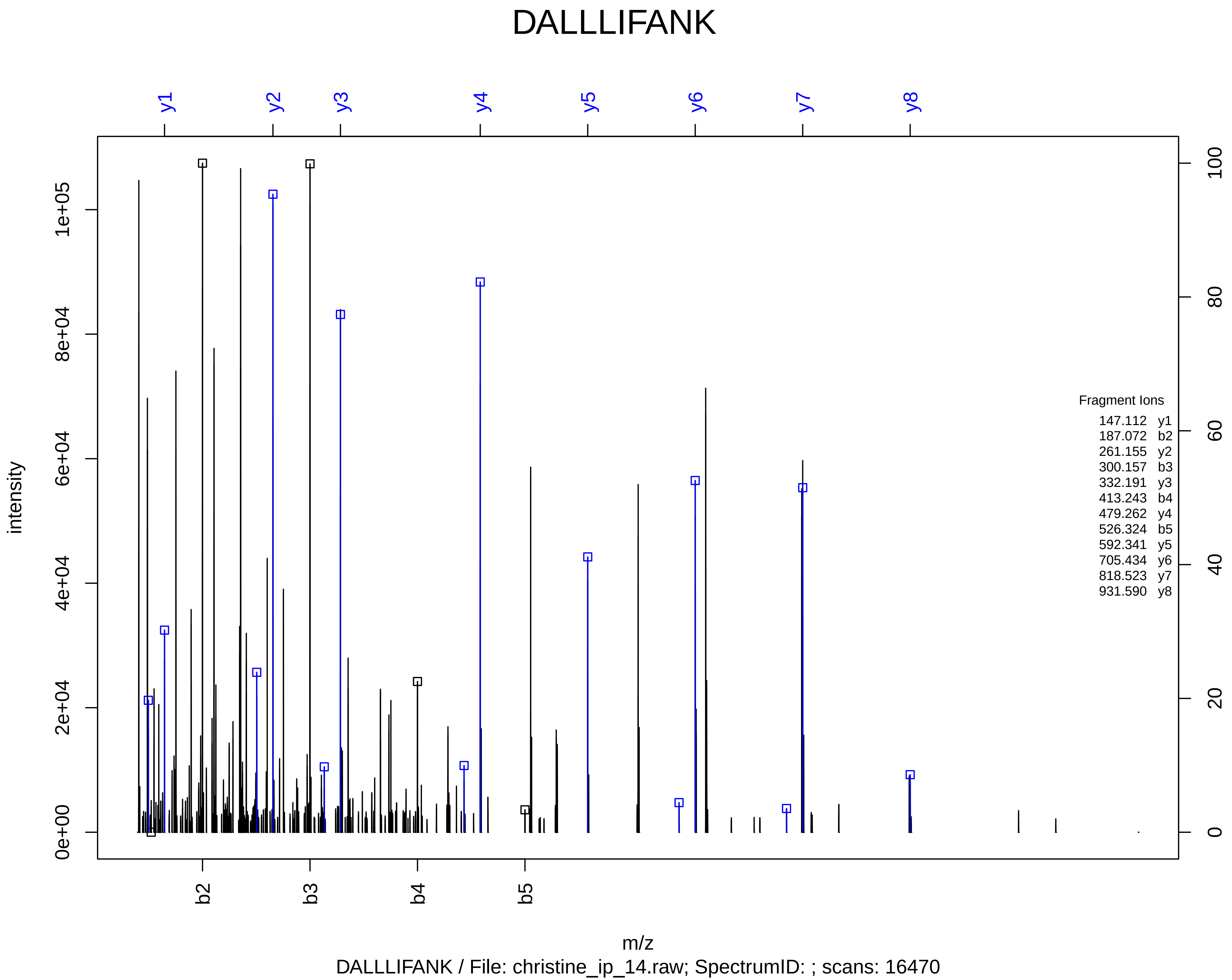

DALLLIFANK
y1
y2
y3
y4
y5
y6
y7
y8
b2
b3
b4
b5
intensity
0e+00
2e+04
4e+04
6e+04
8e+04
1e+05
0
20
40
60
80
100
m/z
Fragment Ions
147.112 y1
187.072 b2
261.155 y2
300.157 b3
332.191 y3
413.243 b4
479.262 y4
526.324 b5
592.341 y5
705.434 y6
818.523 y7
931.590 y8
DALLLIFANK / File: christine_ip_14.raw; SpectrumID: ; scans: 16470

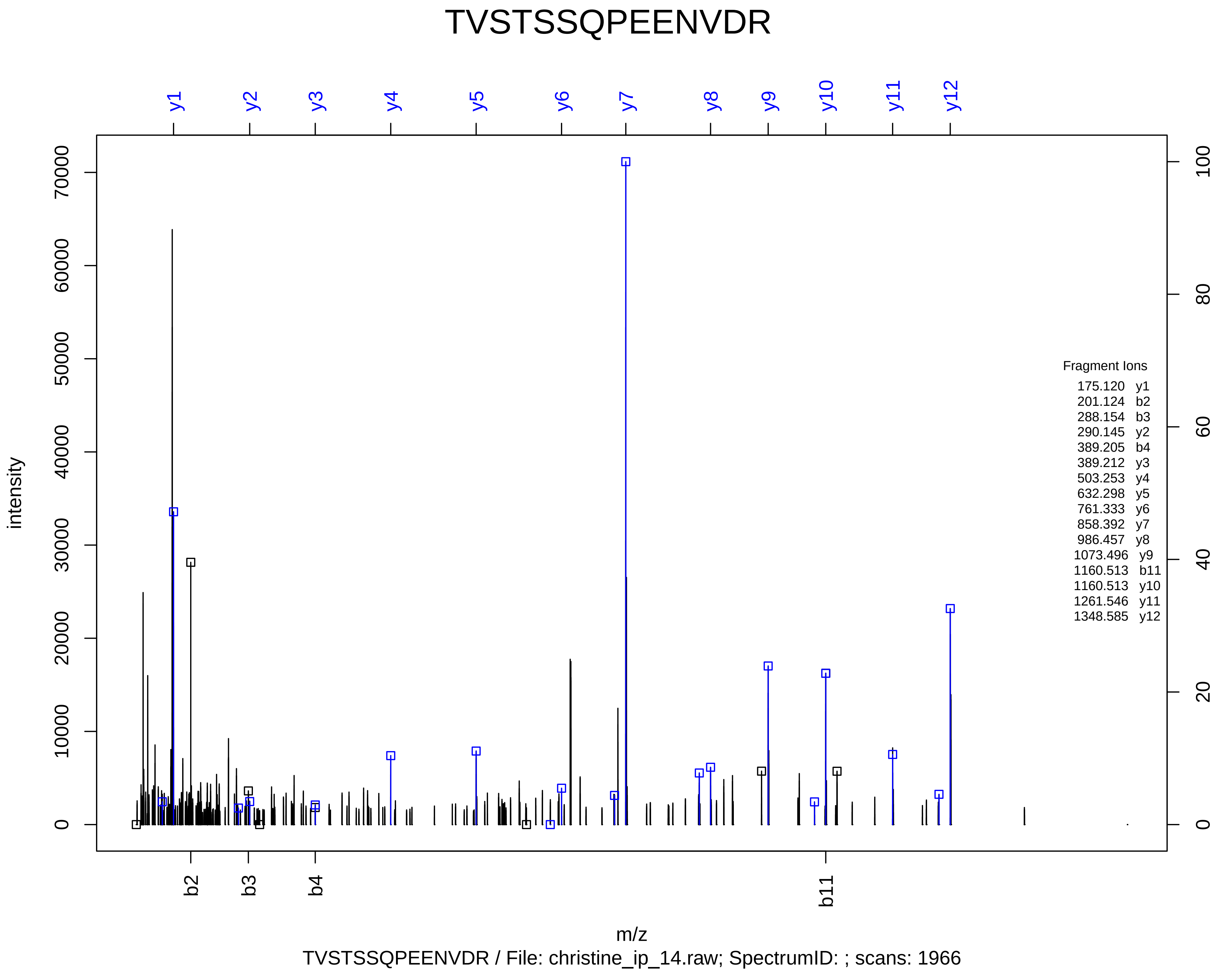

TVSTSSQPEENVDR
y1
y2
y3
y4
y5
y6
y7
y8
y9
y10
y11
y12
intensity
0
10000
20000
30000
40000
50000
60000
70000
0
20
40
60
80
100
b2
b3
b4
b11
Fragment Ions
175.120 y1
201.124 b2
288.154 b3
290.145 y2
389.205 b4
389.212 y3
503.253 y4
632.298 y5
761.333 y6
858.392 y7
986.457 y8
1073.496 y9
1160.513 b11
1160.513 y10
1261.546 y11
1348.585 y12
m/z
TVSTSSQPEENVDR / File: christine_ip_14.raw; SpectrumID: ; scans: 1966

ADALQAGASQFETSAAK

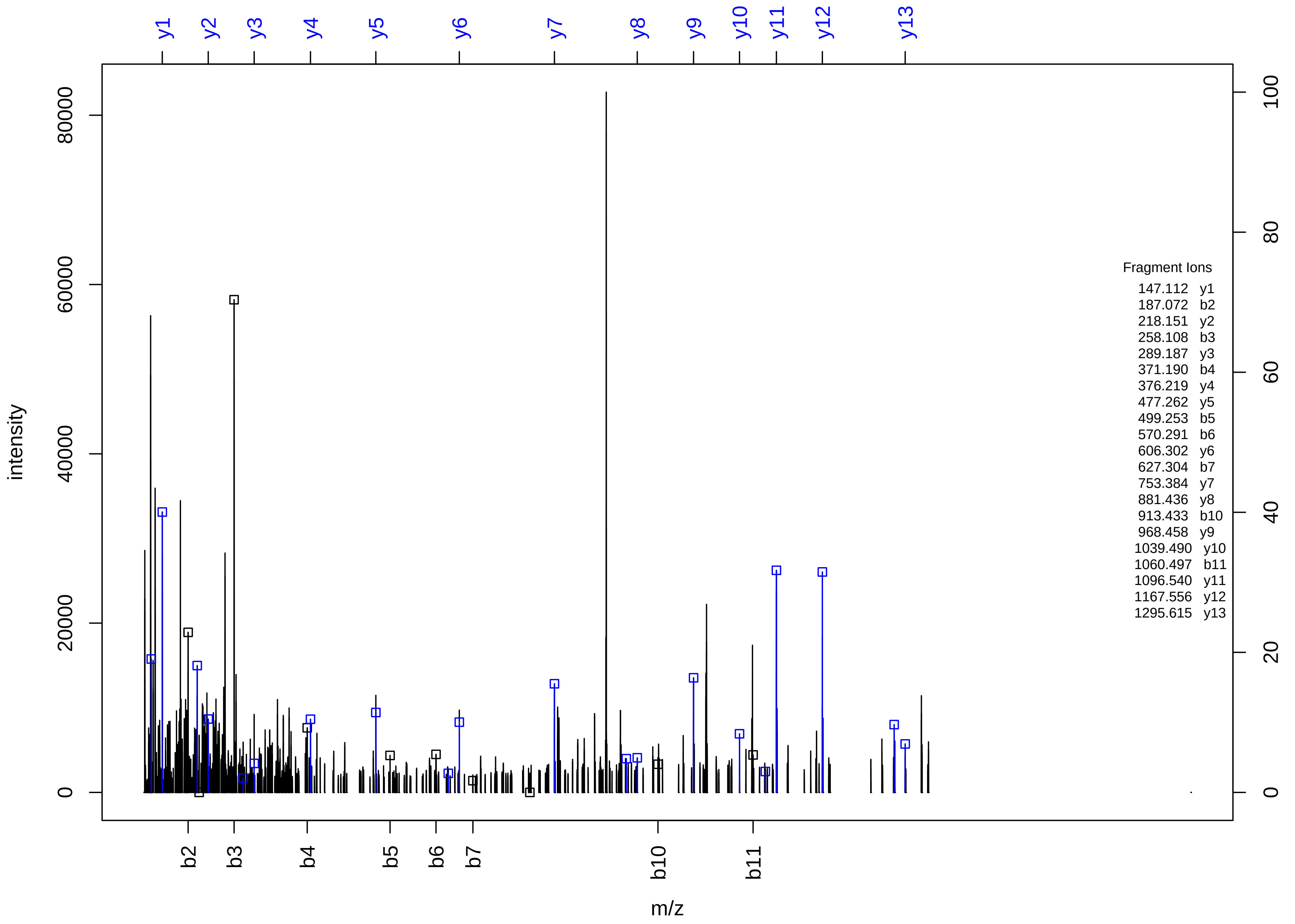

ADALQAGASQFETSAAK / File: christine_ip_14.raw; SpectrumID: ; scans: 7454

HLQLSFQEPHFYLR

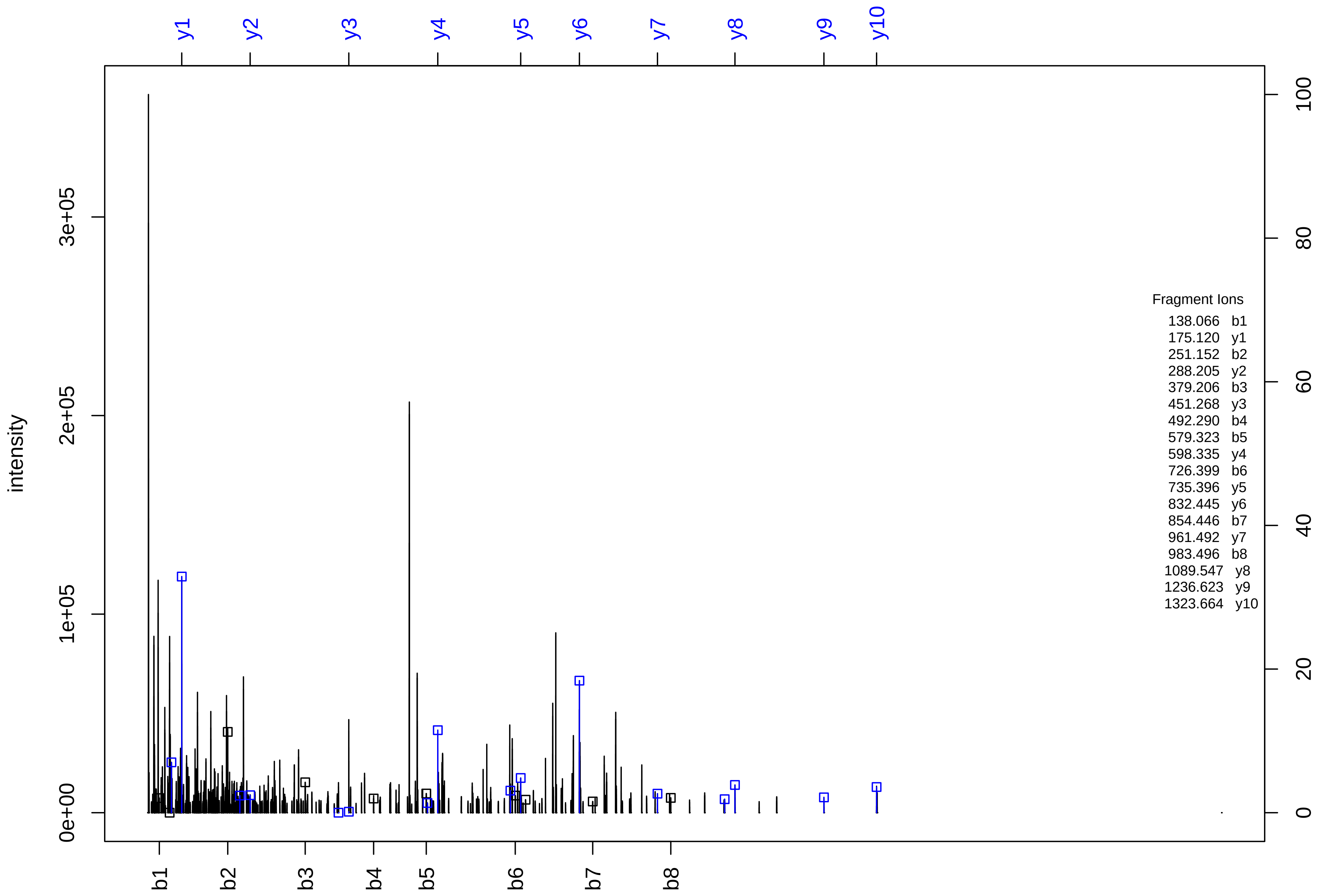

HLQLSFQEPHFYLR / File: christine_ip_14.raw; SpectrumID: ; scans: 12255

HHLPQPEGPPDAR

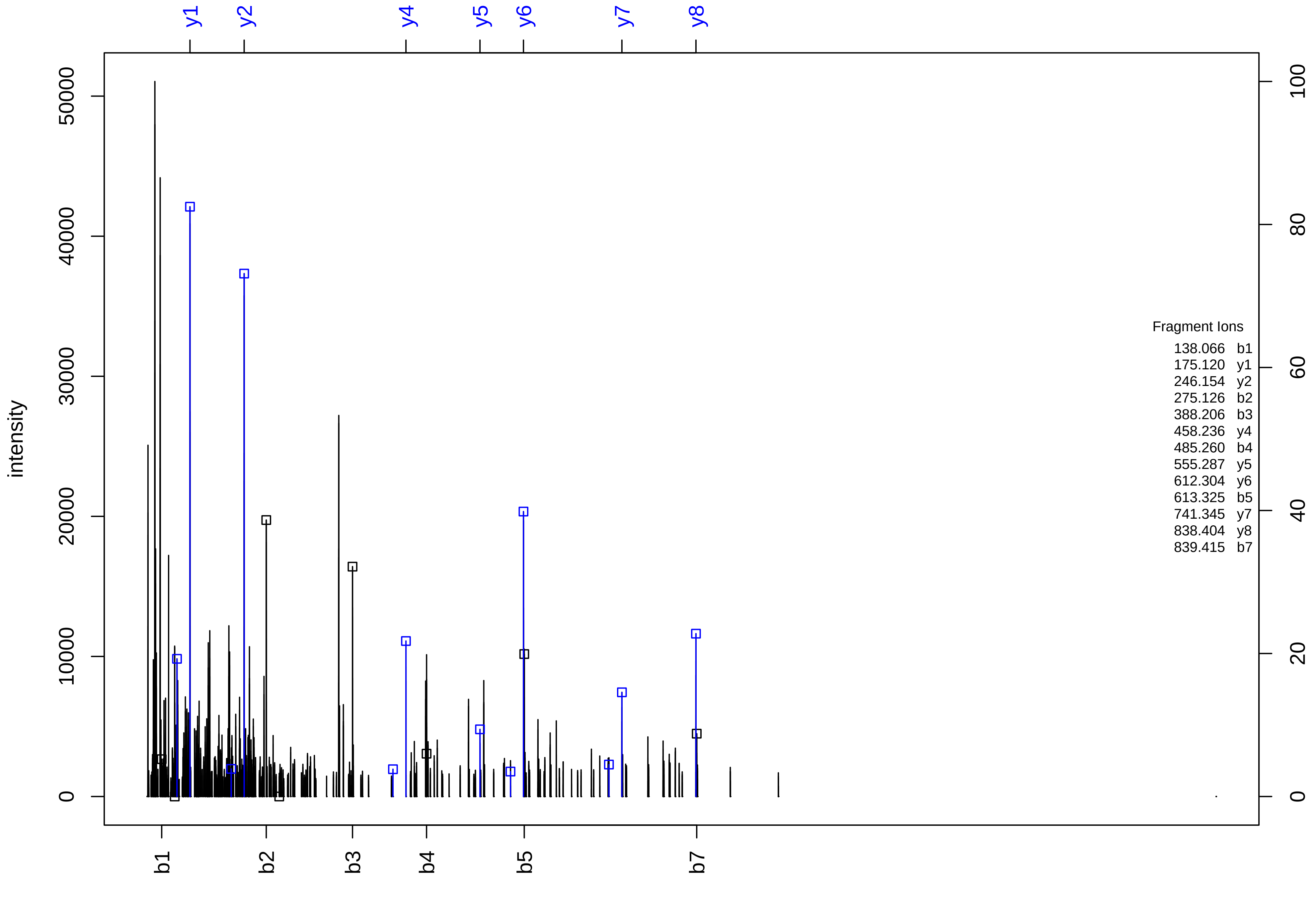

HHLPQPEGPPDAR / File: christine_ip_14.raw; SpectrumID: ; scans: 2150

SAIAIQGGLTELPWDGGLK

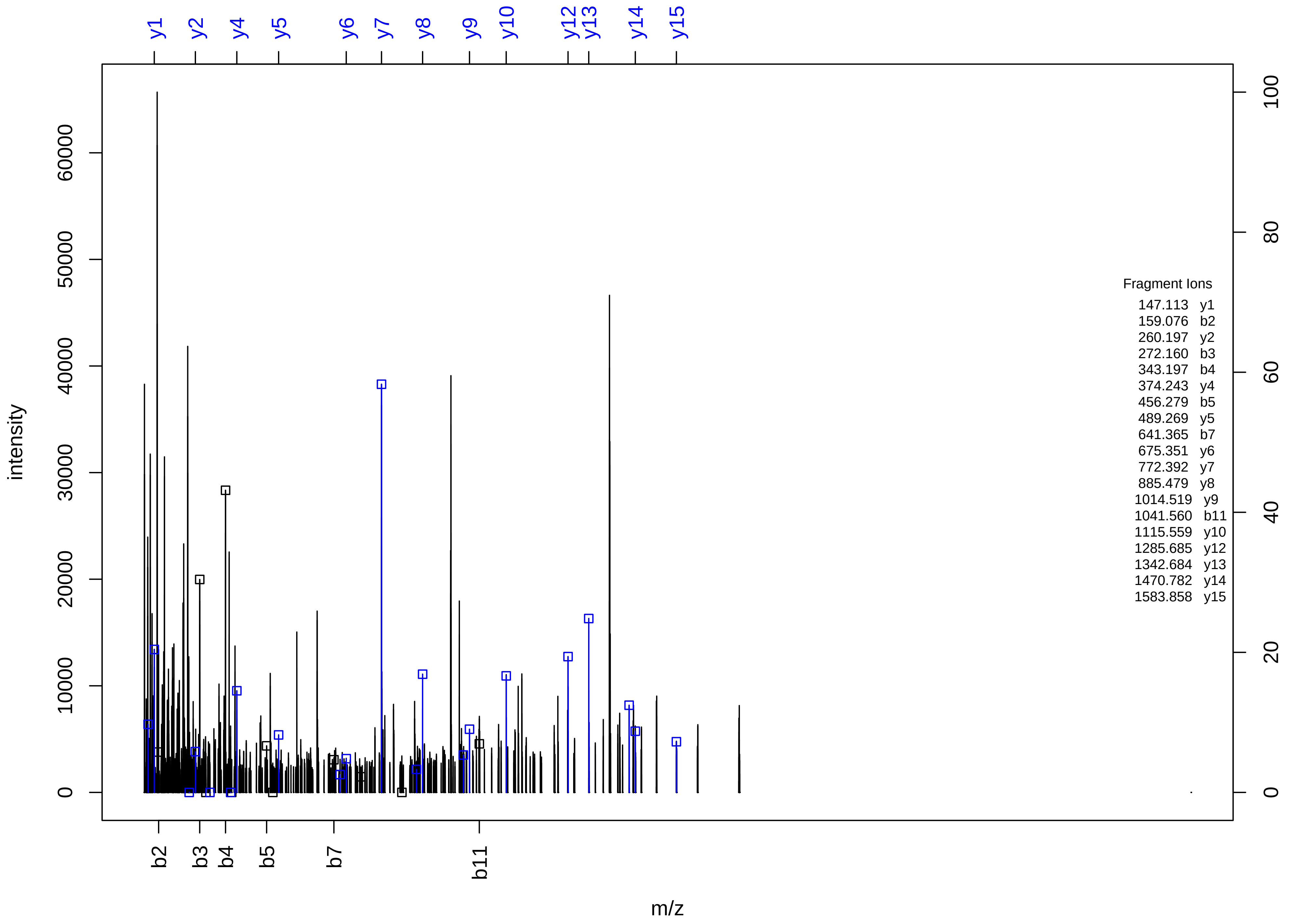

SAIAIQGGLTELPWDGGLK / File: christine_ip_14.raw; SpectrumID: ; scans: 17207

FLATEVSQFSPSLISEK

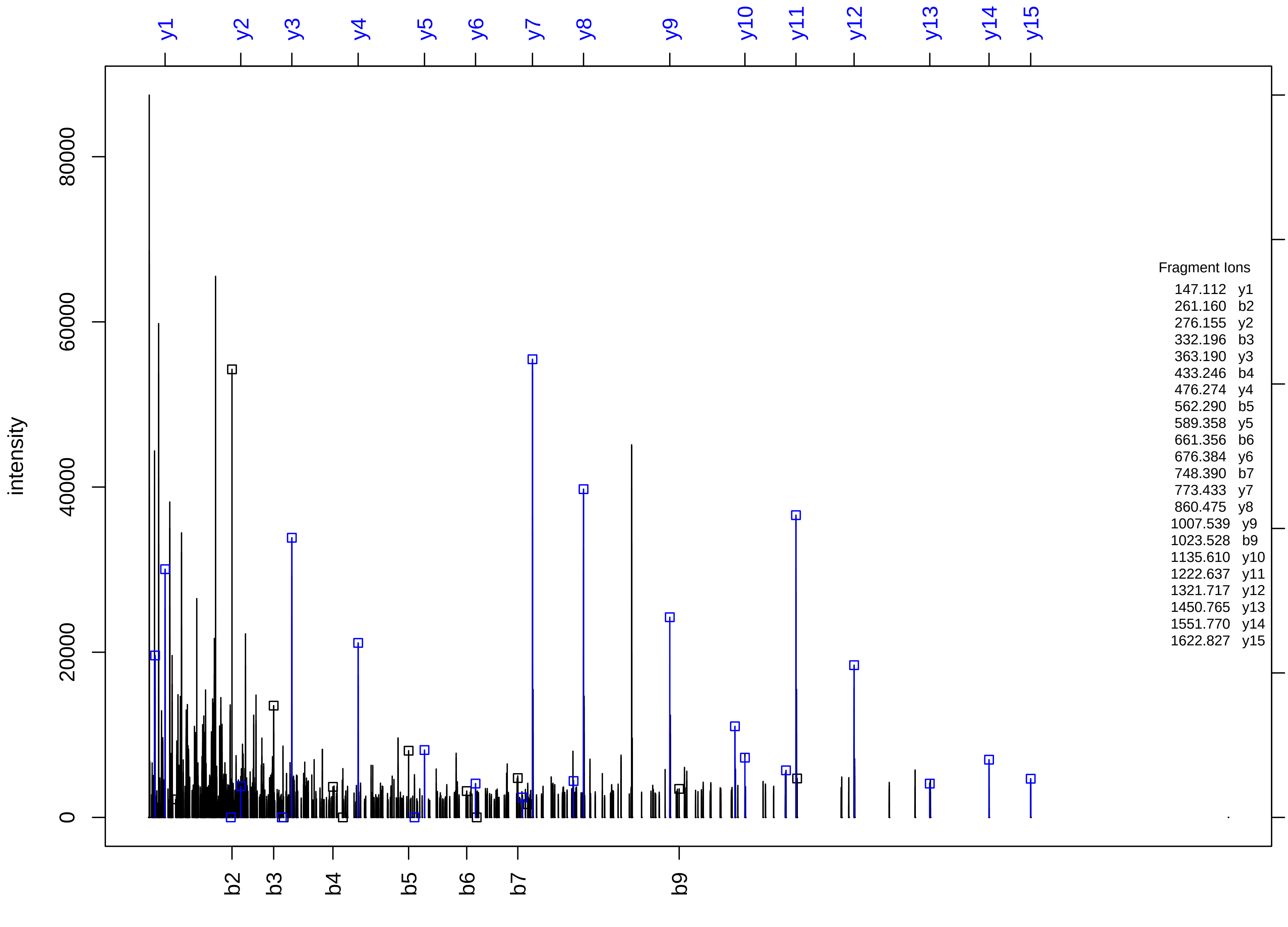

FLATEVSQFSPSLISEK / File: christine_ip_14.raw; SpectrumID: ; scans: 14807

DTEPGPASGGTLGPSGEEAPRPR

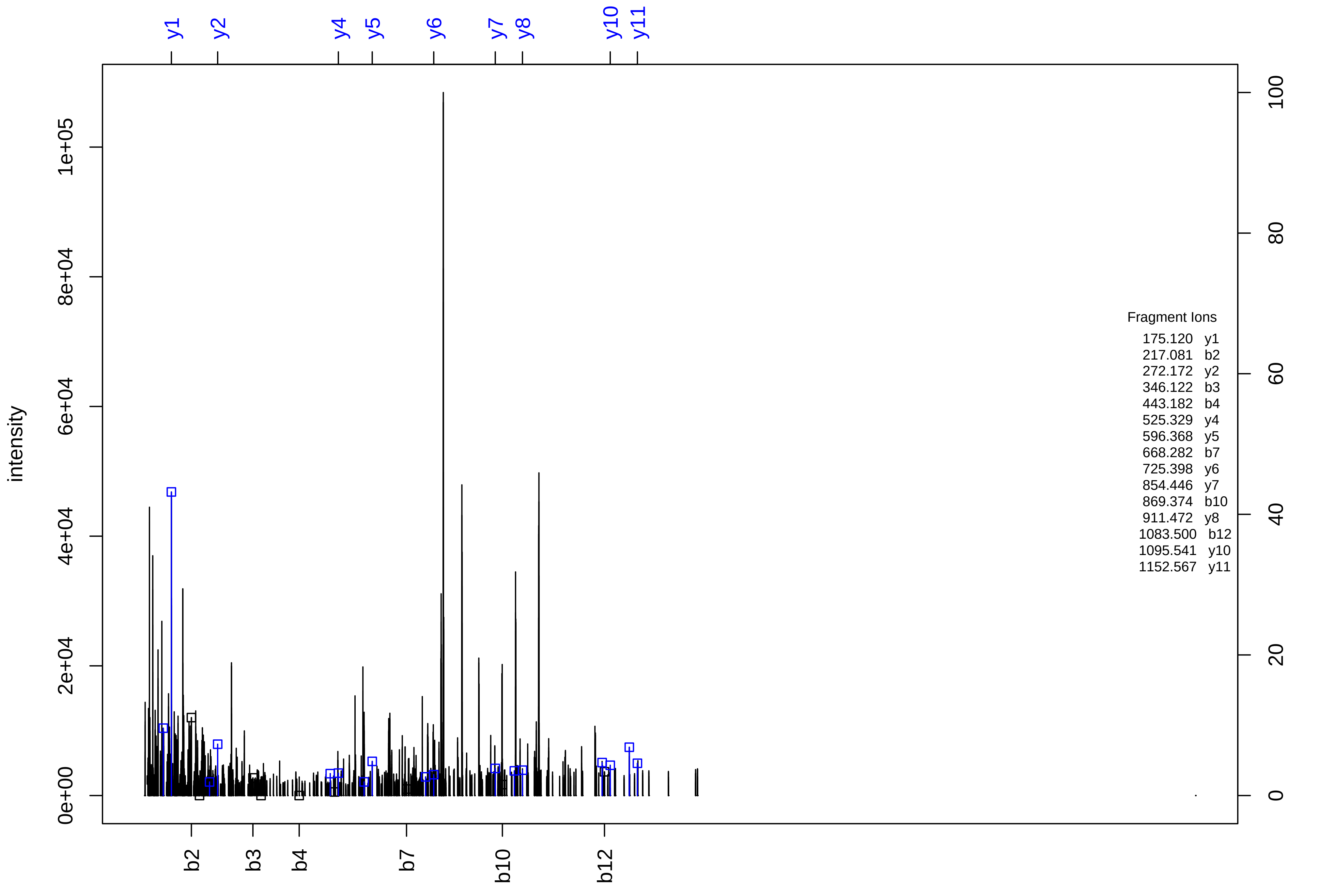

DTEPGPASGGTLGPSGEEAPRPR / File: christine_ip_14.raw; SpectrumID: ; scans: 4479

VYELPFLVALDHR

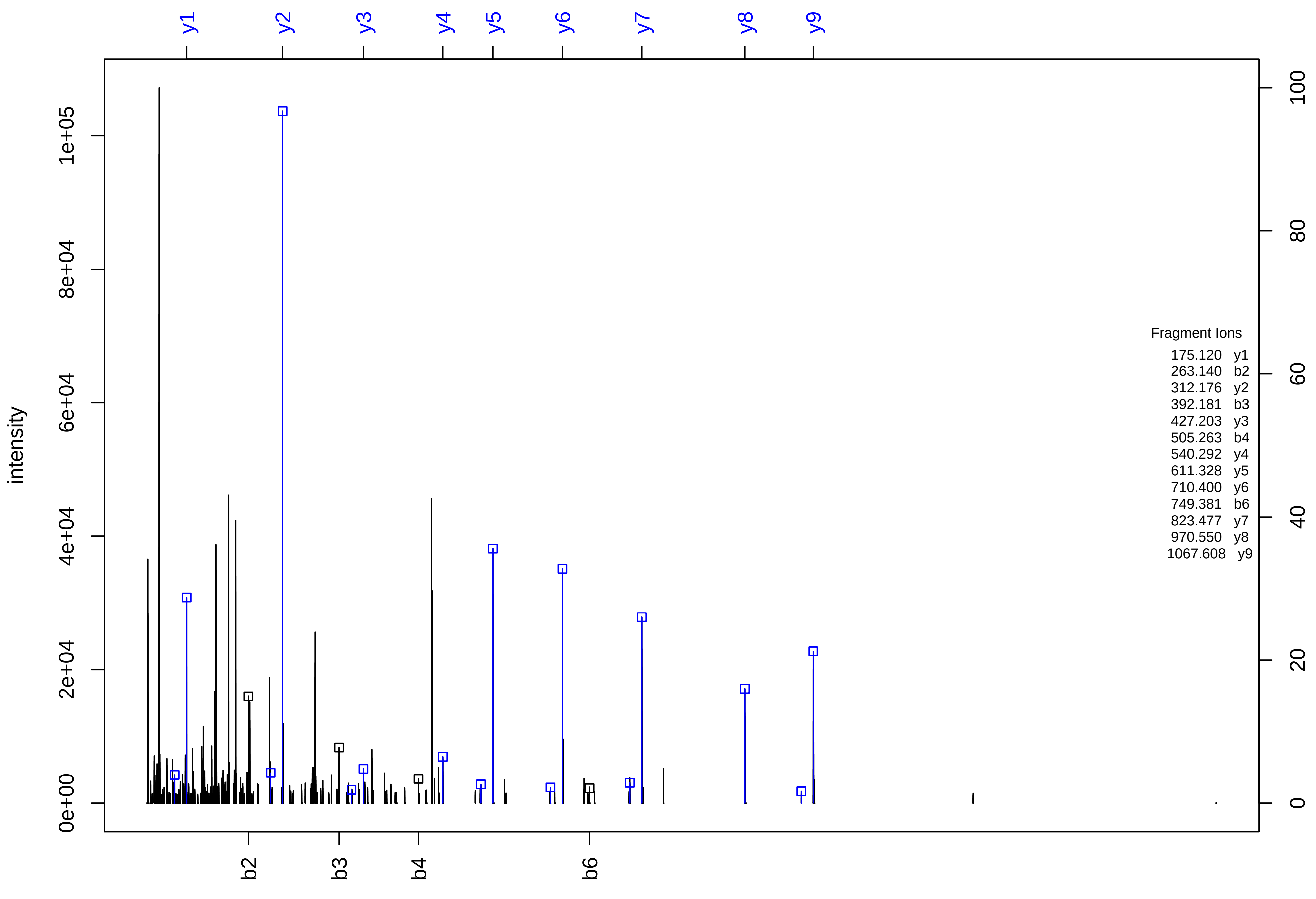

VYELPFLVALDHR / File: christine_ip_14.raw; SpectrumID: ; scans: 18166

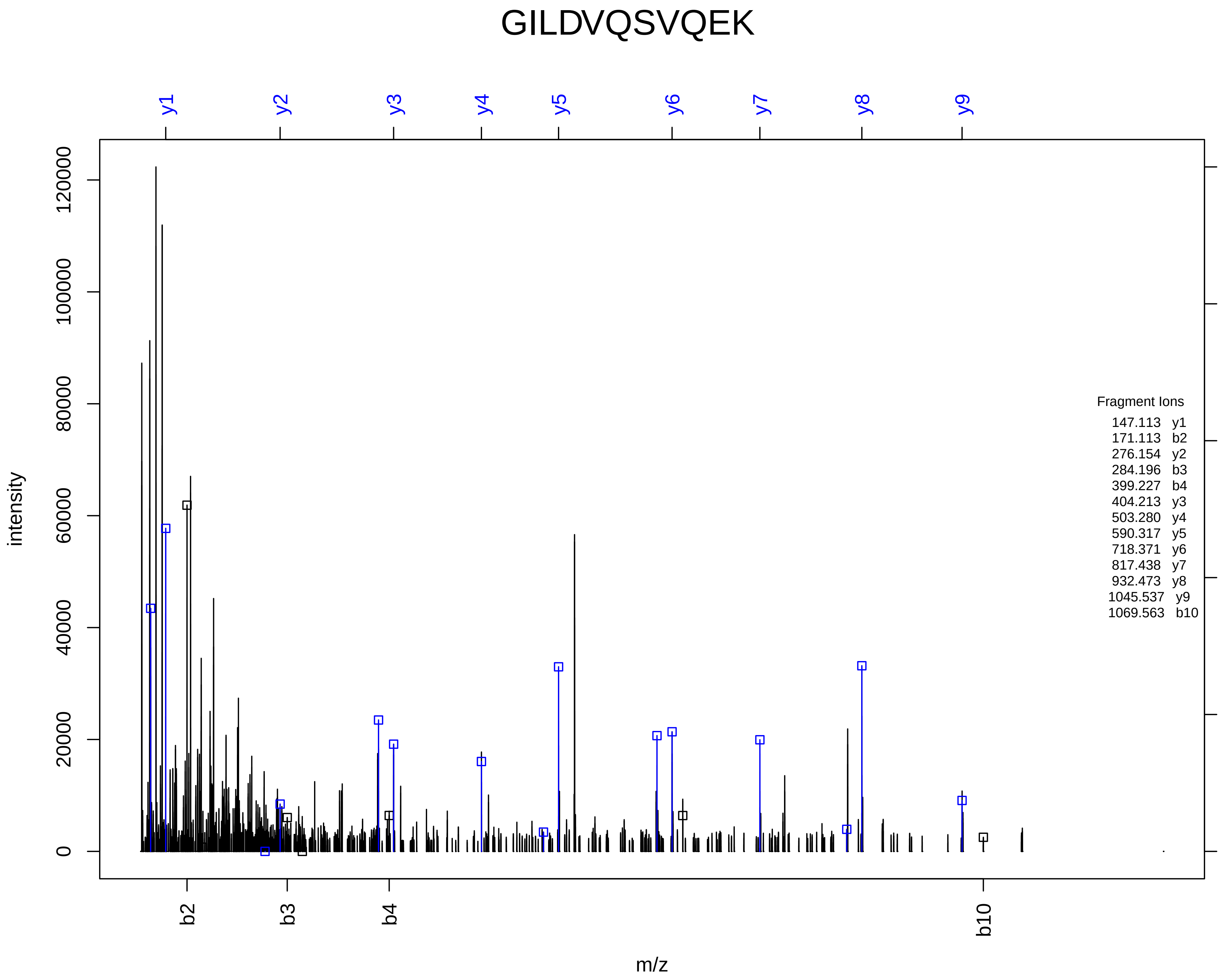

GILDVQSVQEK / File: christine_ip_14.raw; SpectrumID: ; scans: 8011

ILDETQEAVEYQR

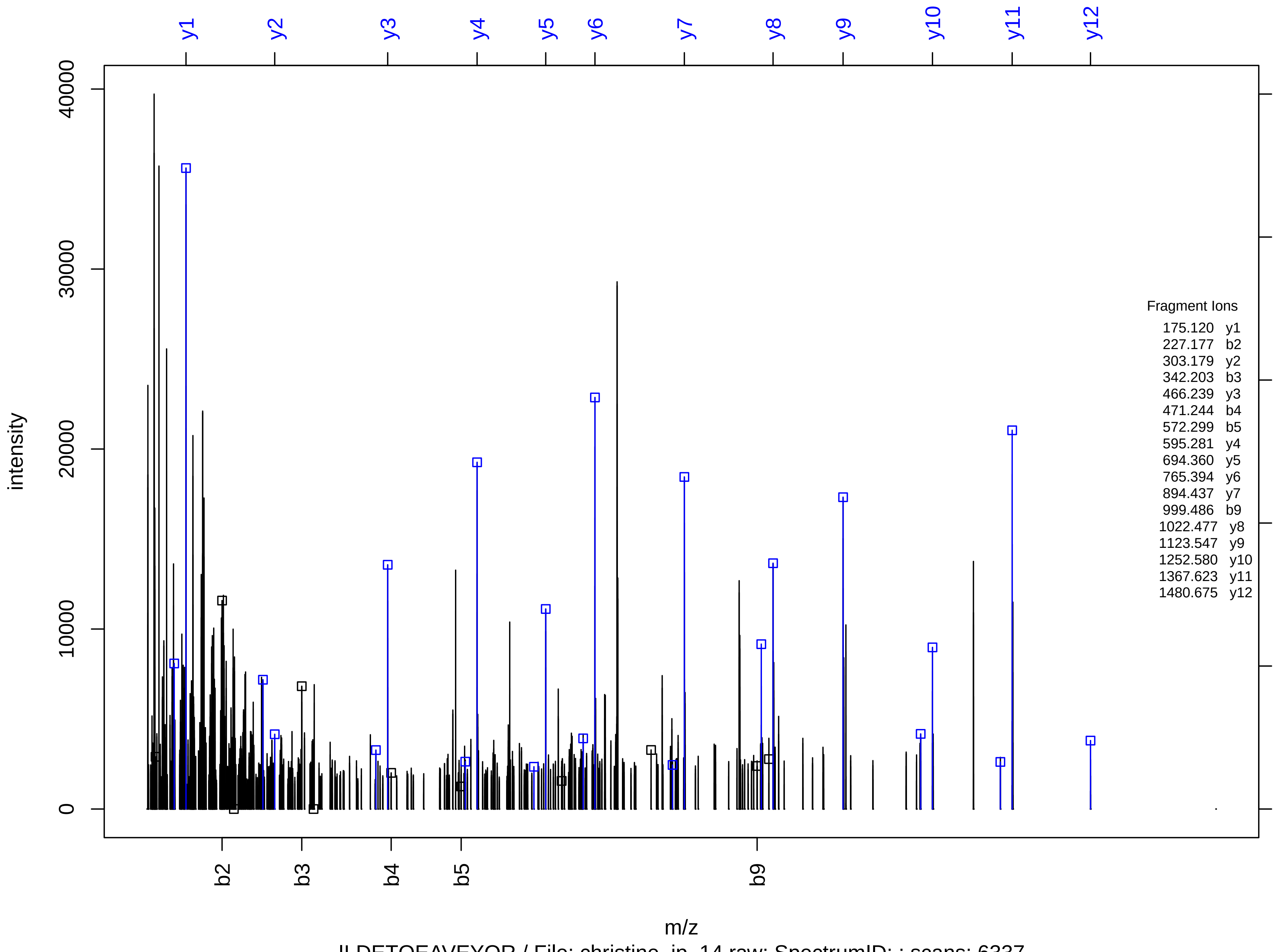

ILDETQEAVEYQR / File: christine_ip_14.raw; SpectrumID: ; scans: 6337

SHYADVDPENQNFLLESNLGK

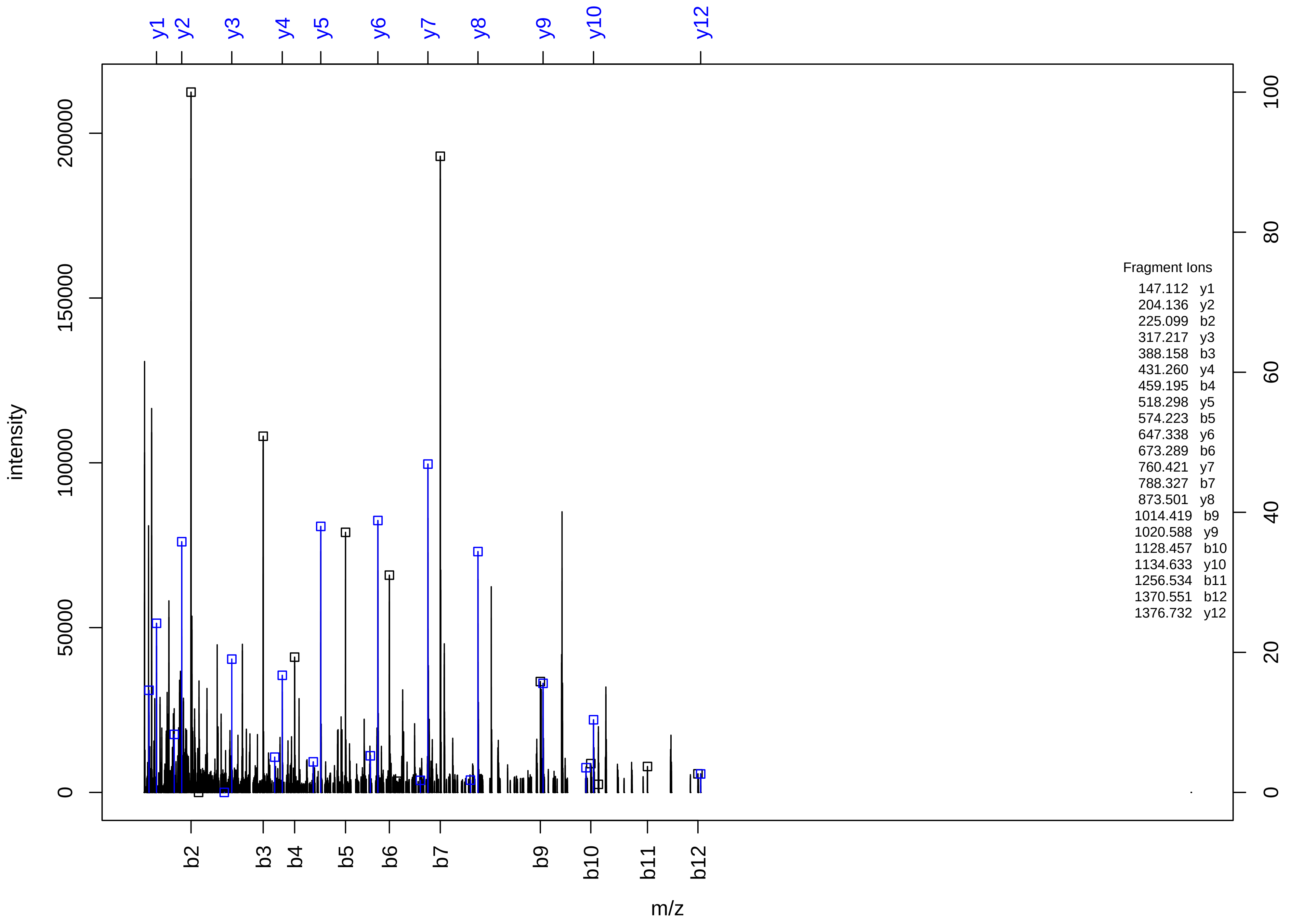

SHYADVDPENQNFLLESNLGK / File: christine_ip_14.raw; SpectrumID: ; scans: 11303

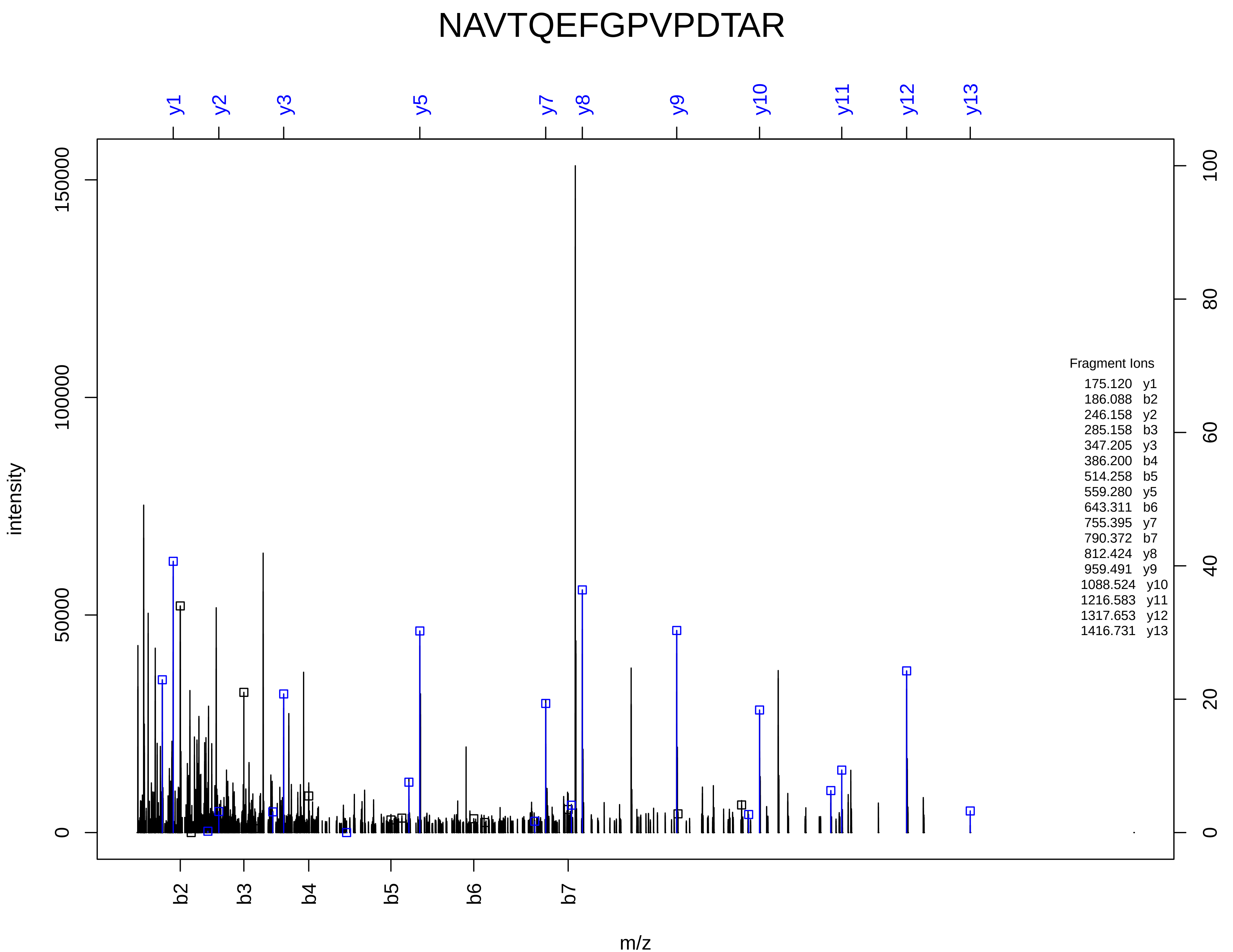

NAVTQEFGPVPDTAR
y1
y2
y3
y5
y7
y8
y9
y10
y11
y12
y13
intensity
150000
100000
50000
0
100
80
60
40
20
0
b2
b3
b4
b5
b6
b7
m/z
Fragment Ions
175.120 y1
186.088 b2
246.158 y2
285.158 b3
347.205 y3
386.200 b4
514.258 b5
559.280 y5
643.311 b6
755.395 y7
790.372 b7
812.424 y8
959.491 y9
1088.524 y10
1216.583 y11
1317.653 y12
1416.731 y13
NAVTQEFGPVPDTAR / File: christine_ip_14.raw; SpectrumID: ; scans: 8393

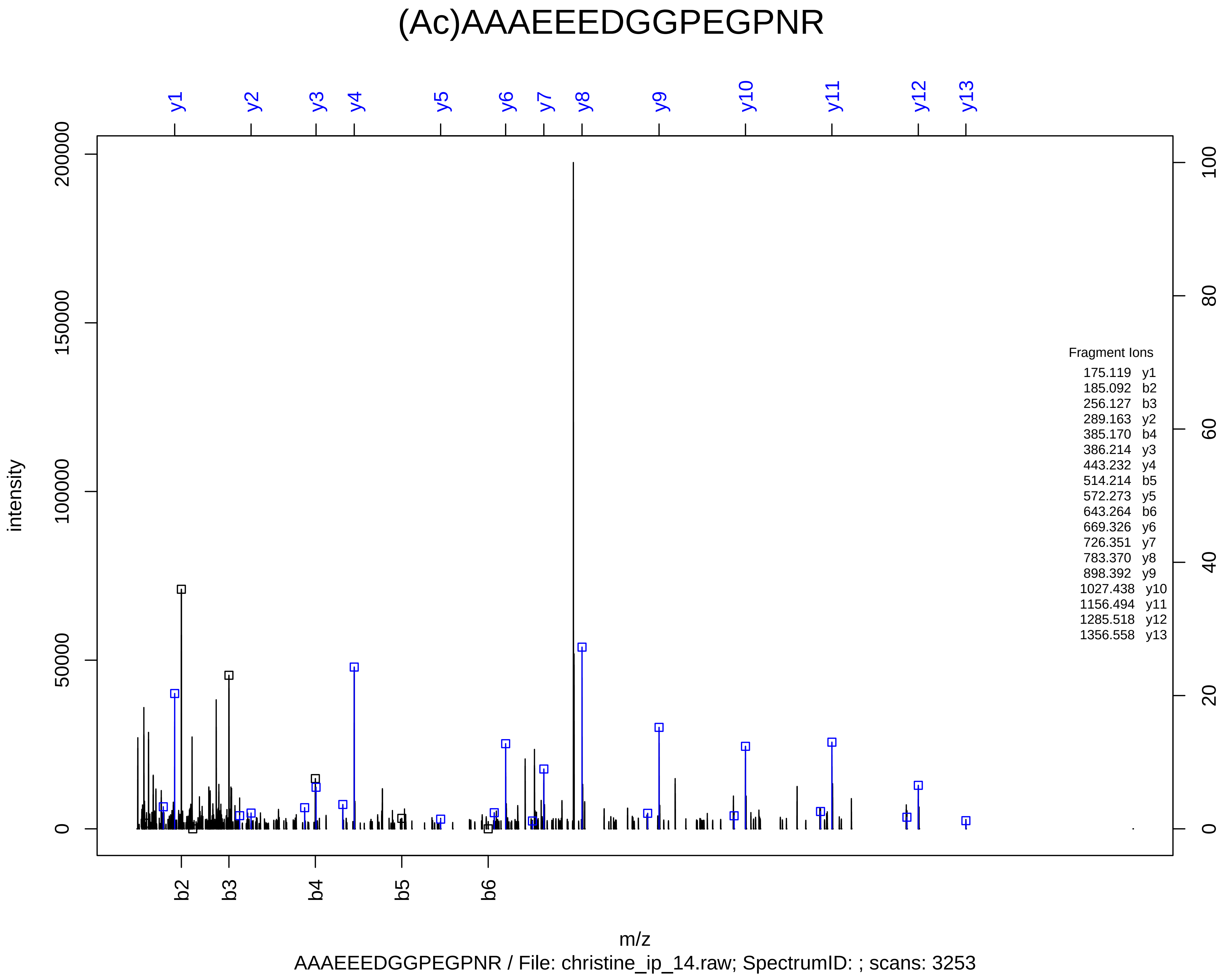
(Ac)AAAEEEDGGPEGPNR
y1
y2
y3
y4
y5
y6
y7
y8
y9
y10
y11
y12
y13
intensity
0
50000
100000
150000
200000
0
20
40
60
80
100
b2
b3
b4
b5
b6
m/z
Fragment Ions
175.119 y1
185.092 b2
256.127 b3
289.163 y2
385.170 b4
386.214 y3
443.232 y4
514.214 b5
572.273 y5
643.264 b6
669.326 y6
726.351 y7
783.370 y8
898.392 y9
1027.438 y10
1156.494 y11
1285.518 y12
1356.558 y13
AAAEEEDGGPEGPNR / File: christine_ip_14.raw; SpectrumID: ; scans: 3253

FVGLGVFVDTYPNEEK

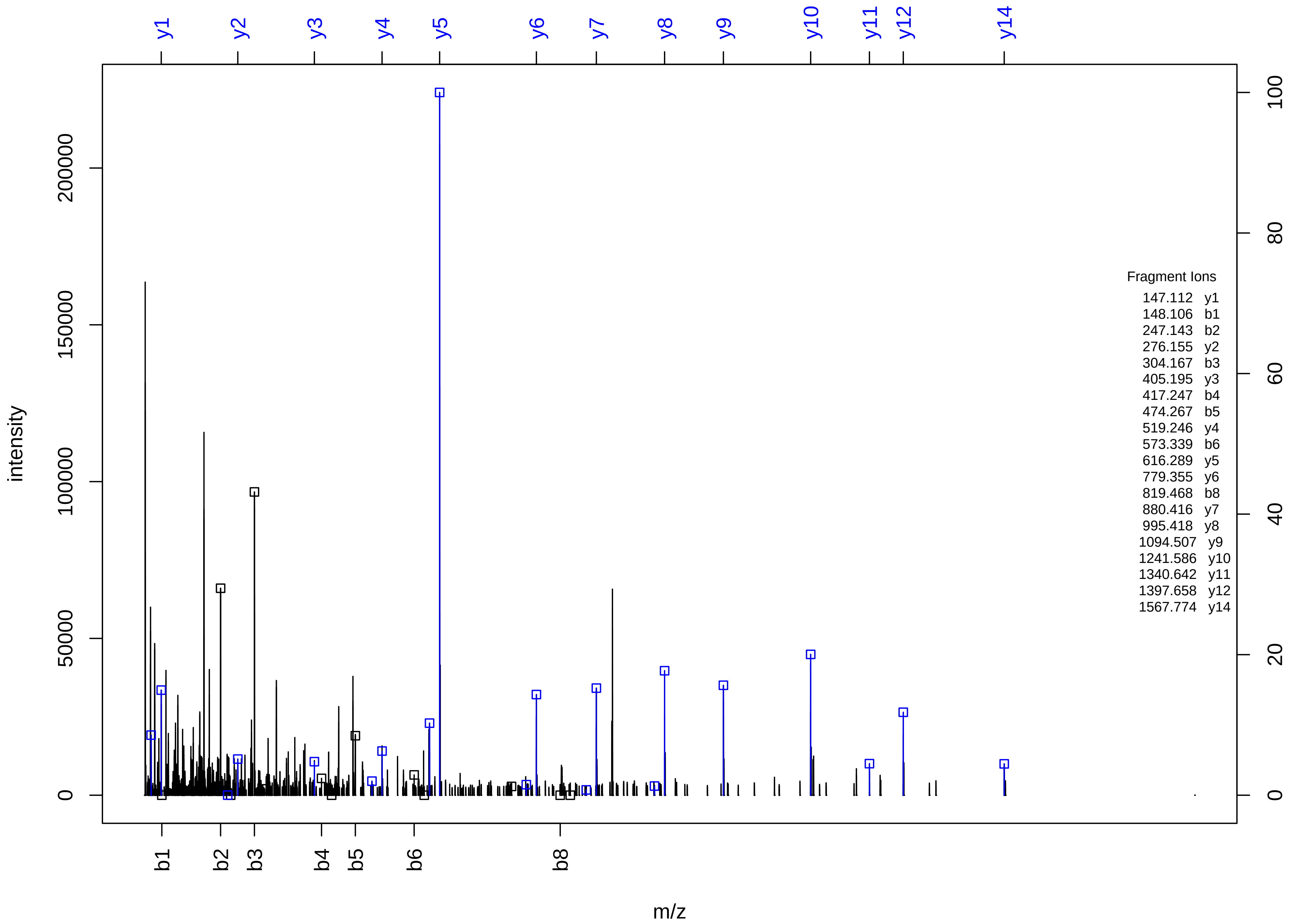

FVGLGVFVDTYPNEEK / File: christine_ip_14.raw; SpectrumID: ; scans: 15932

SEEPEVPDQEGLQR

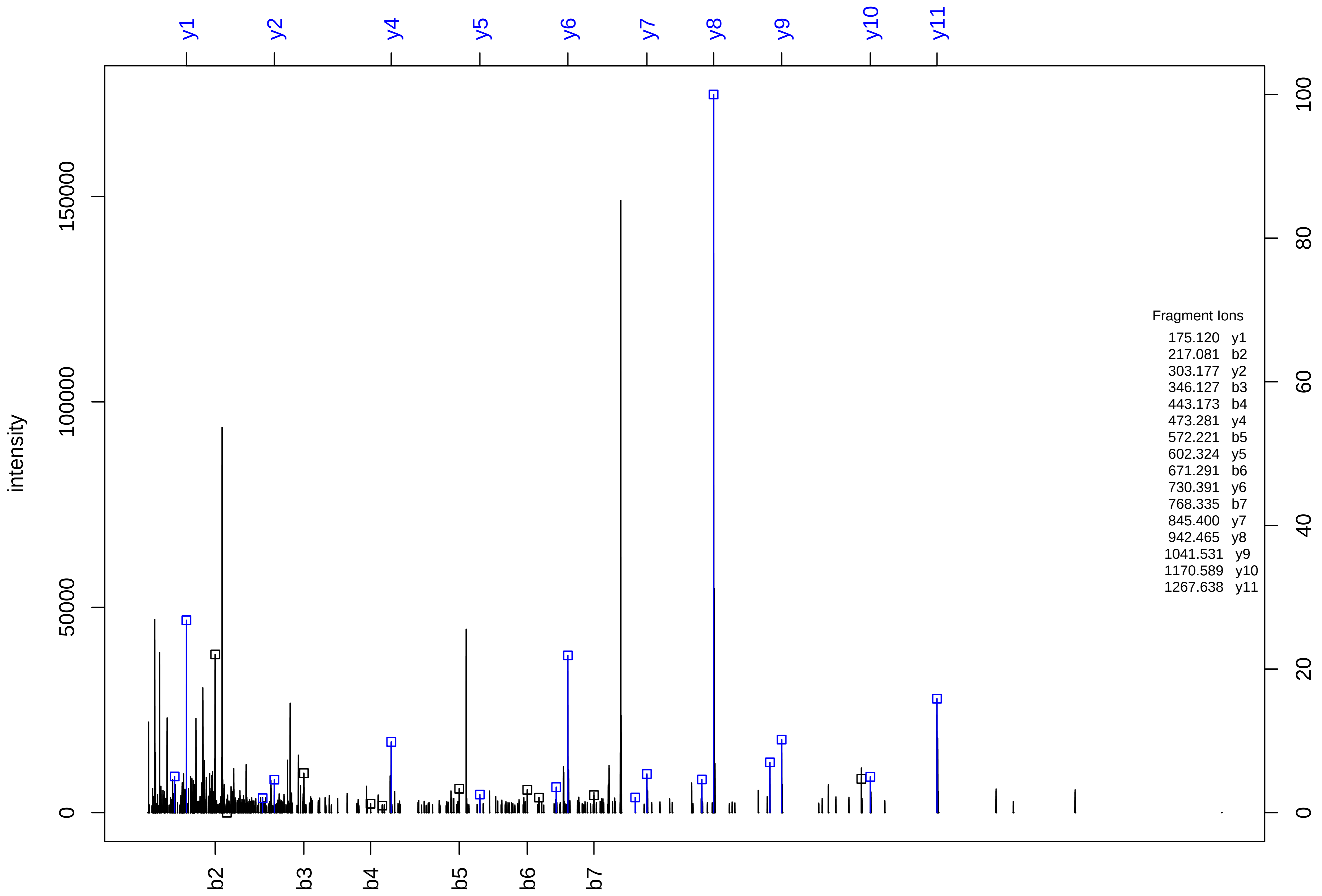

SEEPEVPDQEGLQR / File: christine_ip_14.raw; SpectrumID: ; scans: 5261

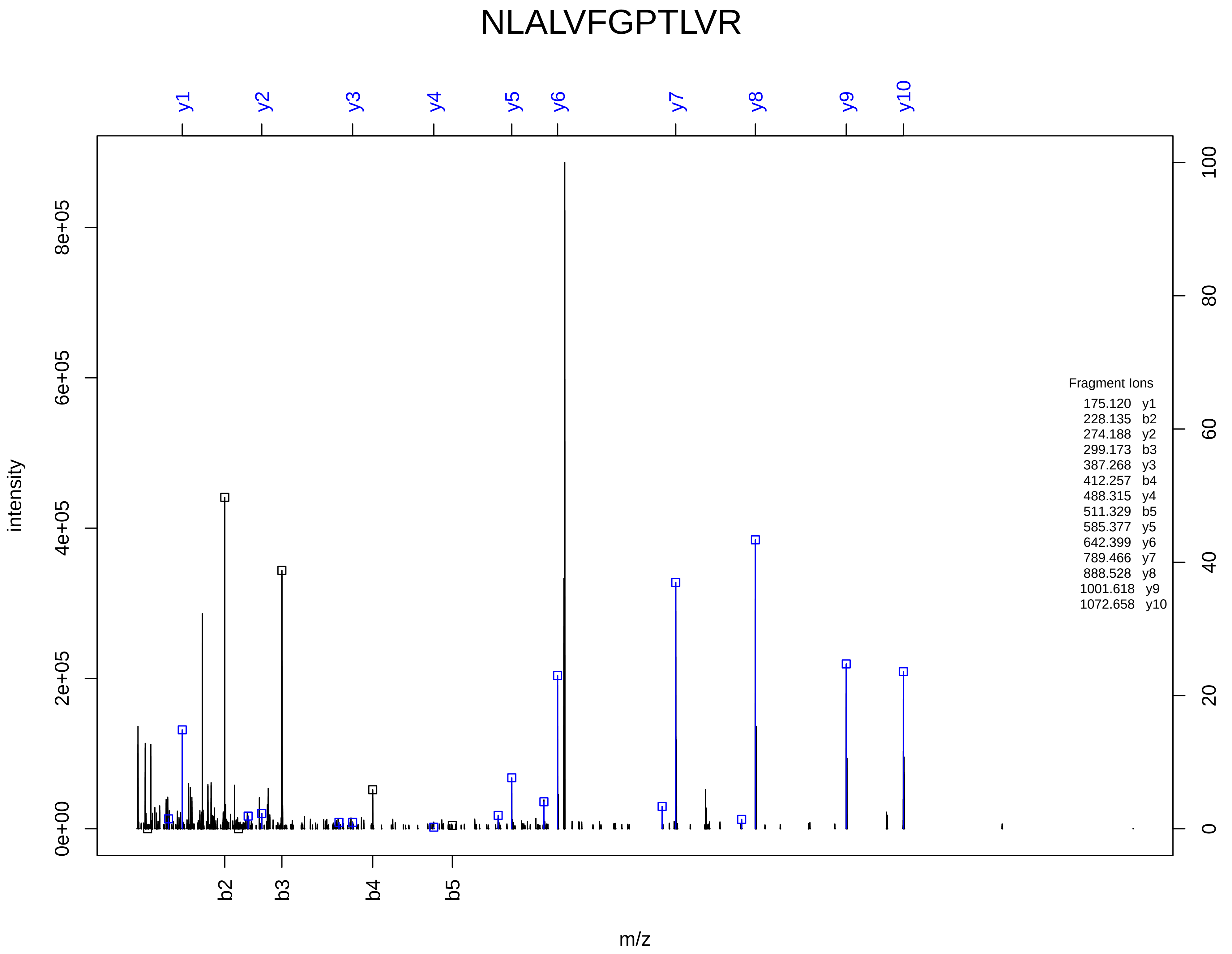

NLALVFGPTLVR / File: christine_ip_14.raw; SpectrumID: ; scans: 16196

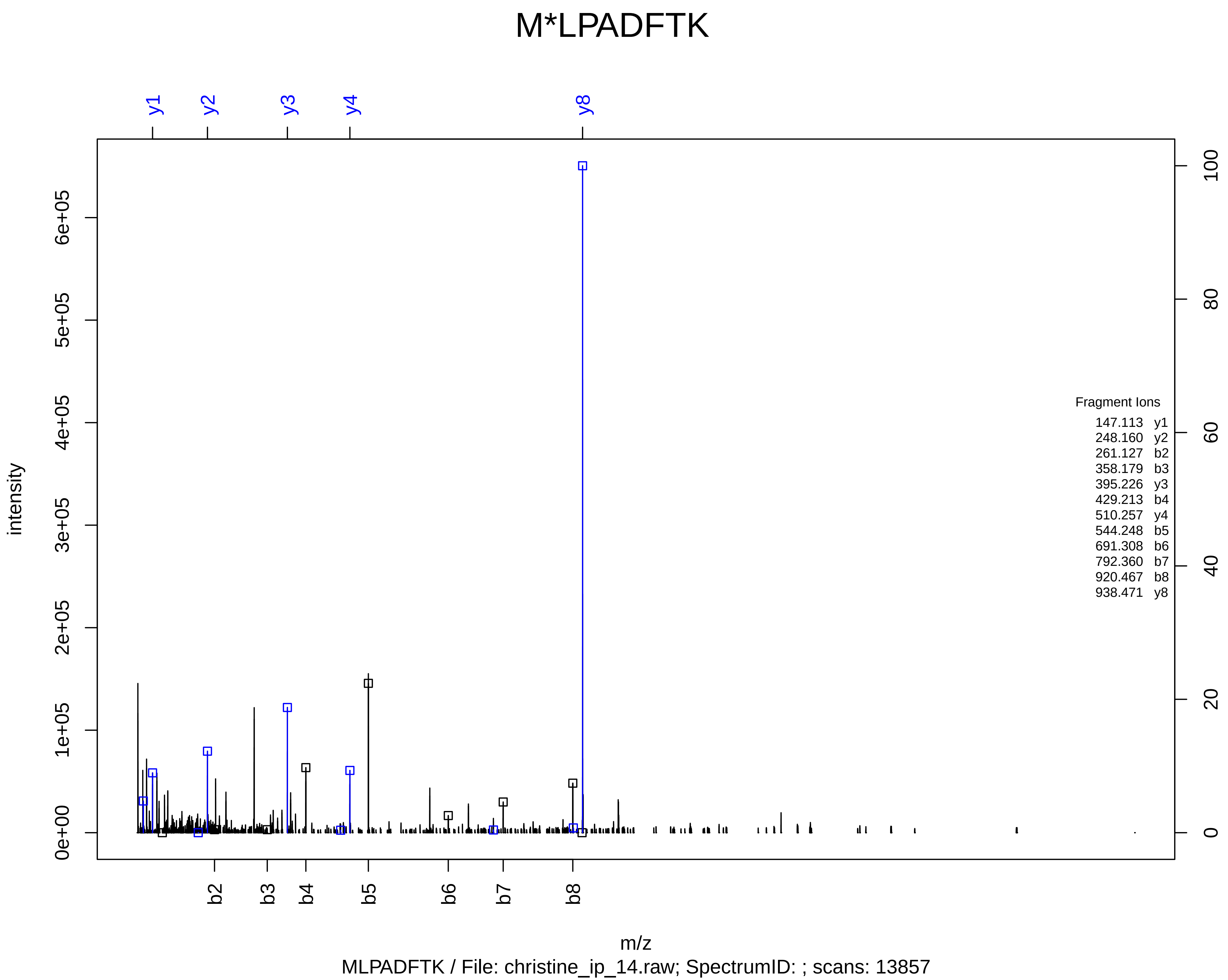

M*LPADFTK
y1
y2
y3
y4
y8
intensity
0e+00
1e+05
2e+05
3e+05
4e+05
5e+05
6e+05
0
20
40
60
80
100
b2
b3
b4
b5
b6
b7
b8
m/z
Fragment Ions
147.113 y1
248.160 y2
261.127 b2
358.179 b3
395.226 y3
429.213 b4
510.257 y4
544.248 b5
691.308 b6
792.360 b7
920.467 b8
938.471 y8
MLPADFTK / File: christine_ip_14.raw; SpectrumID: ; scans: 13857

TTQTSNAPDVNDAIVK

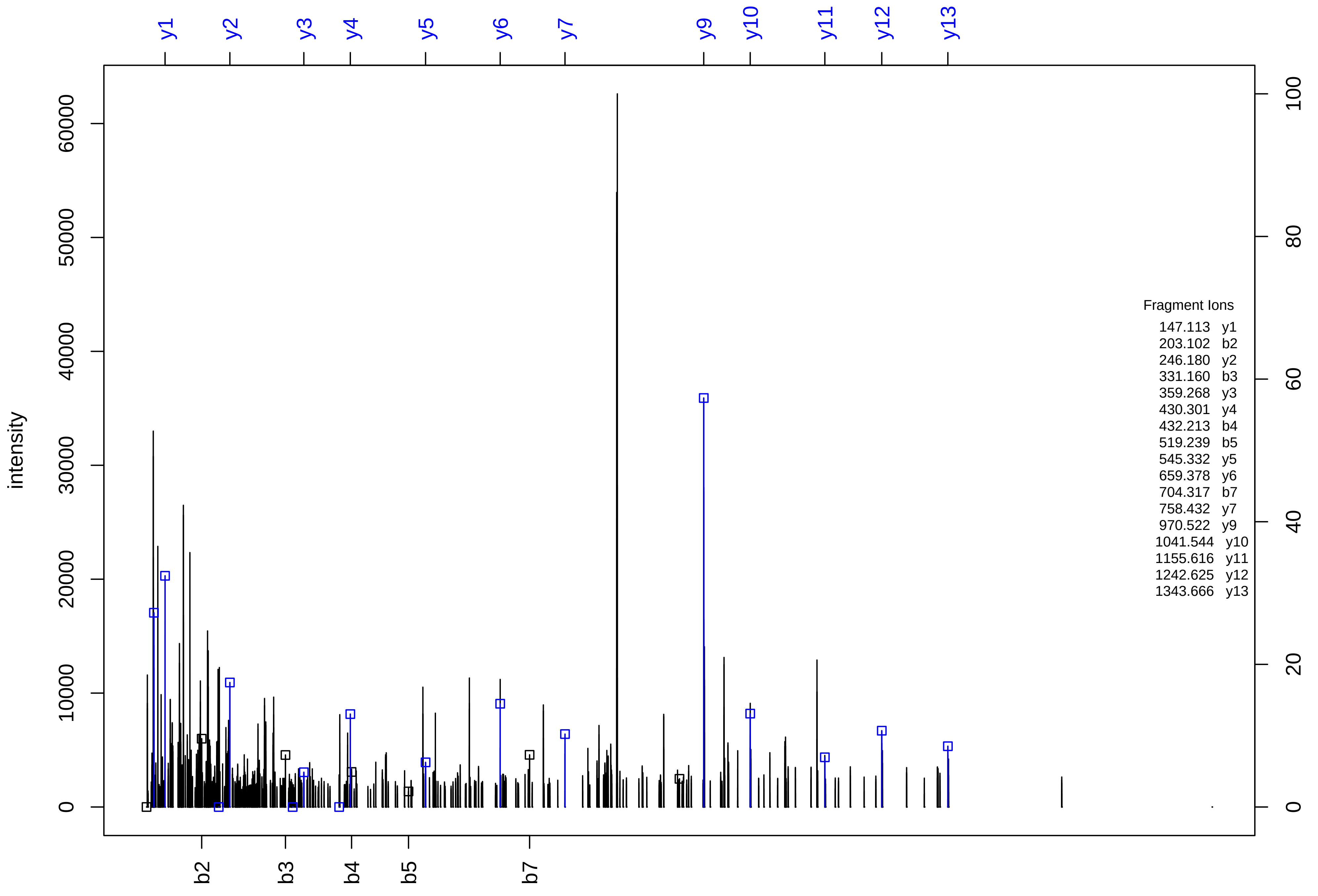

TTQTSNAPDVNDAIVK / File: christine_ip_13.raw; SpectrumID: ; scans: 5351

LLSHHAN^IEALNK

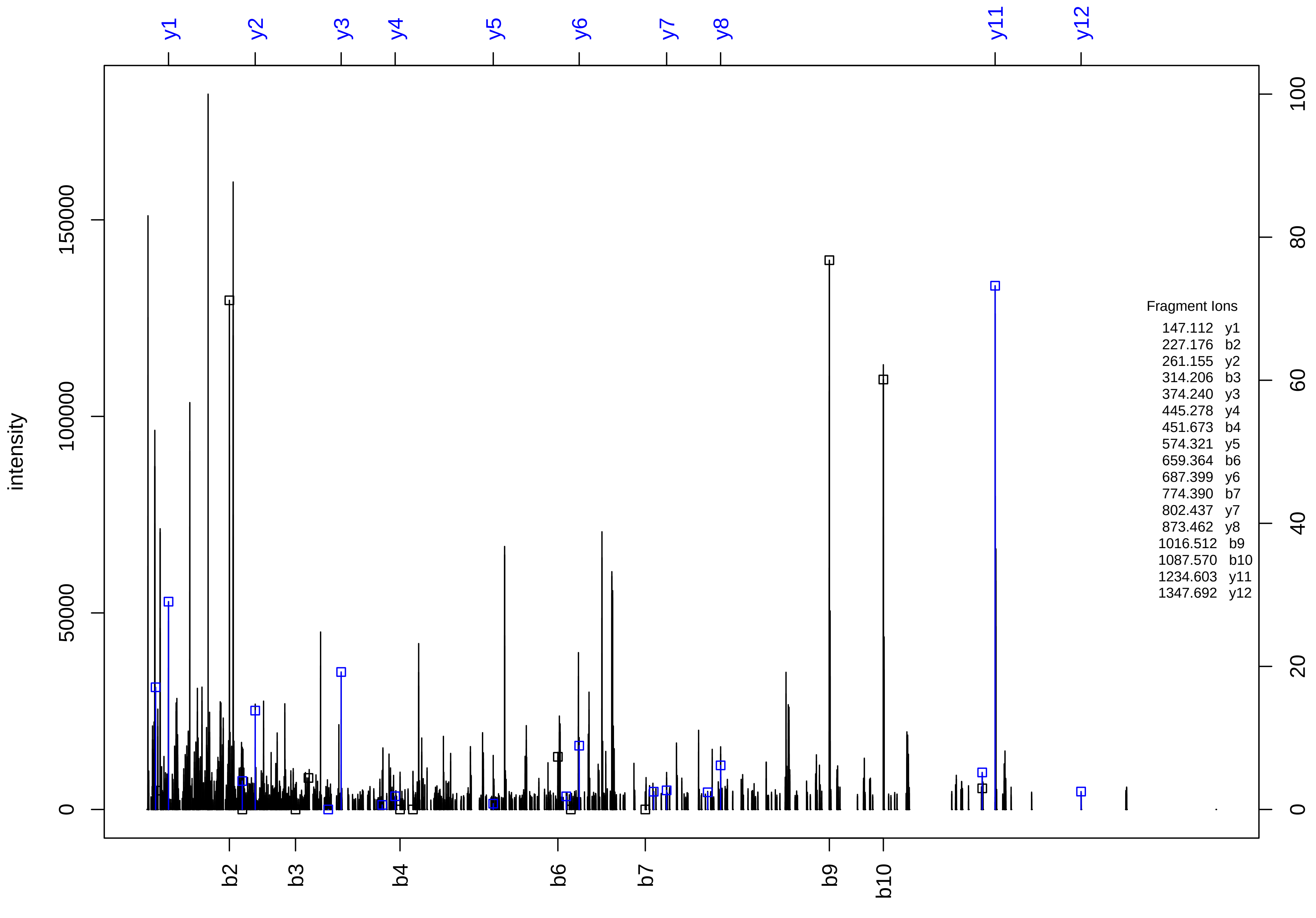

LLSHHANIEALNK / File: christine_ip_13.raw; SpectrumID: ; scans: 7105

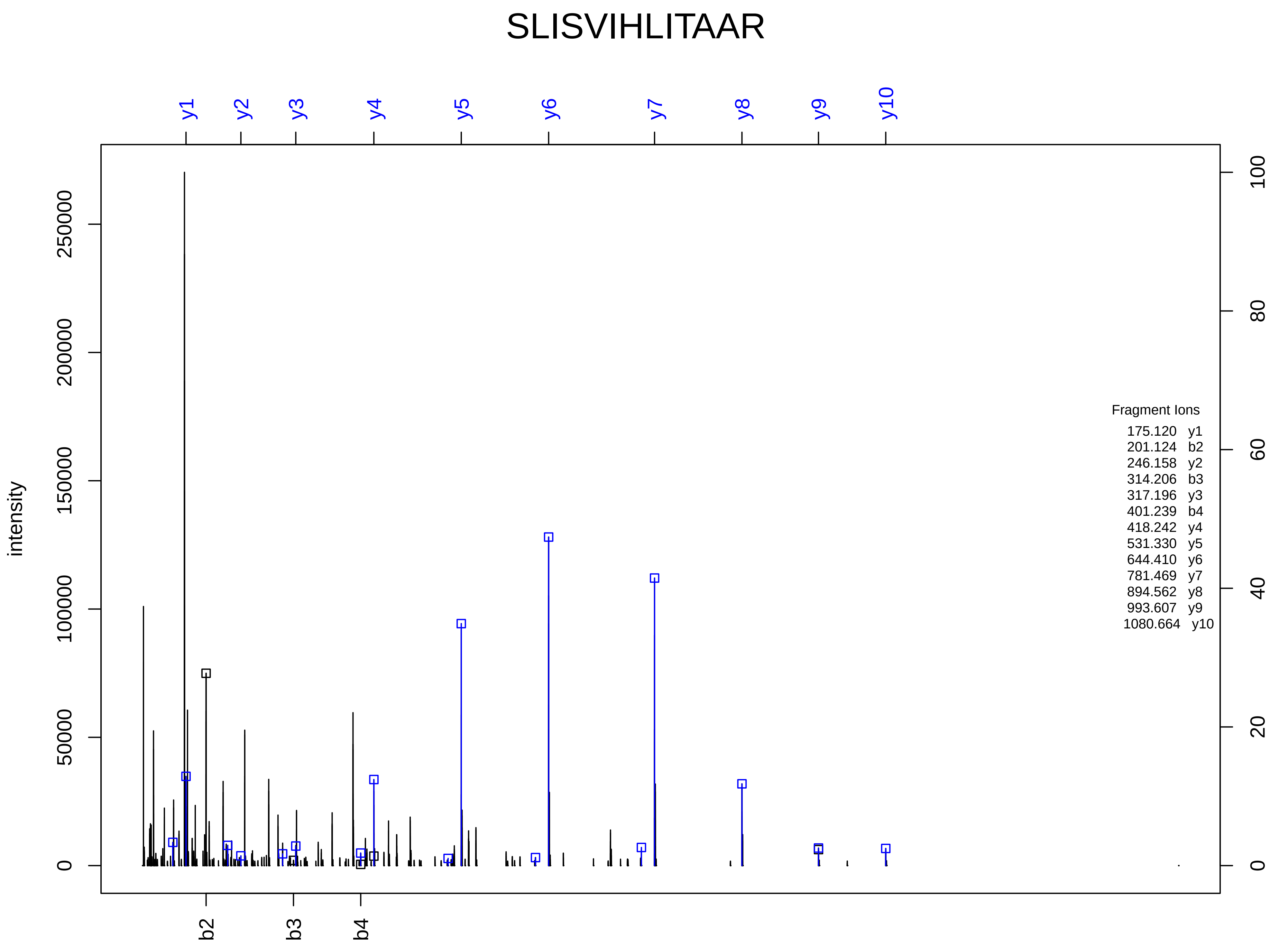

SLISVIHLITAAR / File: christine_ip_13.raw; SpectrumID: ; scans: 17829

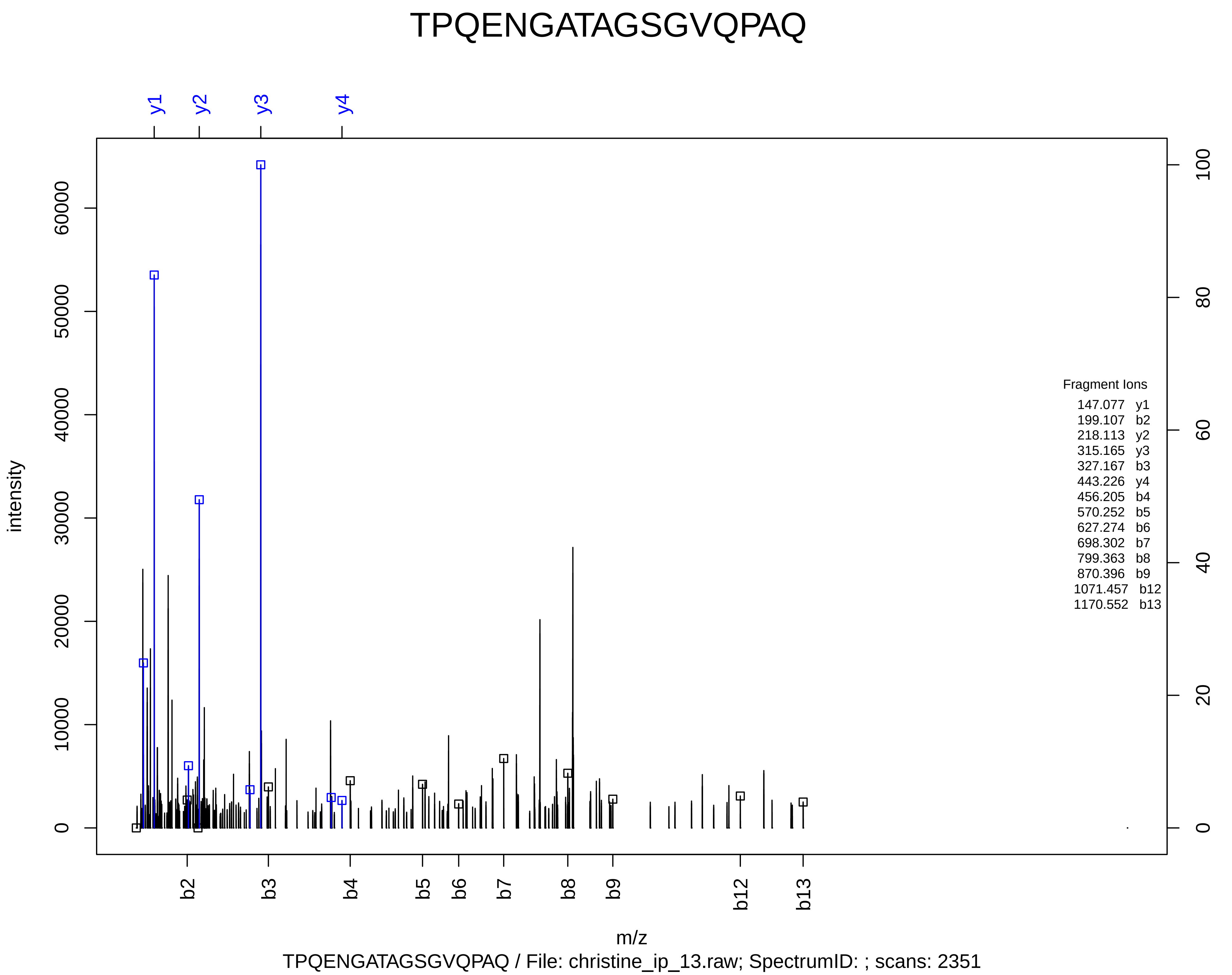
TPQENGATAGSGVQPAQ
y1
y2
y3
y4
intensity
0
10000
20000
30000
40000
50000
60000
0
20
40
60
80
100
Fragment Ions
147.077 y1
199.107 b2
218.113 y2
315.165 y3
327.167 b3
443.226 y4
456.205 b4
570.252 b5
627.274 b6
698.302 b7
799.363 b8
870.396 b9
1071.457 b12
1170.552 b13
b2
b3
b4
b5
b6
b7
b8
b9
b12
b13
m/z
TPQENGATAGSGVQPAQ / File: christine_ip_13.raw; SpectrumID: ; scans: 2351

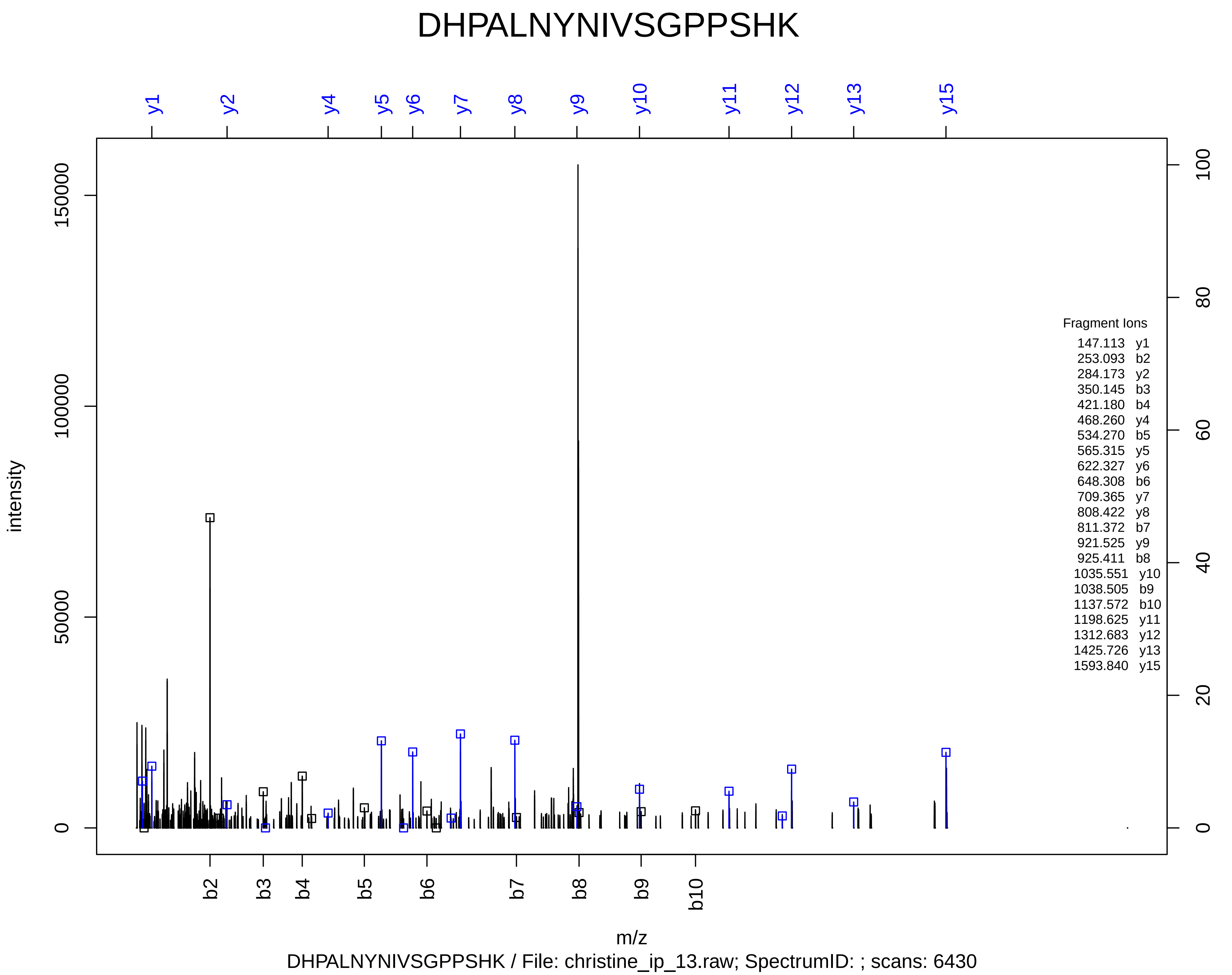
DHPALNYNIVSGPPSHK
y1 y2 y4 y5 y6 y7 y8 y9 y10 y11 y12 y13 y15
b2 b3 b4 b5 b6 b7 b8 b9 b10
intensity
0
50000
100000
150000
0
20
40
60
80
100
m/z
Fragment Ions
147.113 y1
253.093 b2
284.173 y2
350.145 b3
421.180 b4
468.260 y4
534.270 b5
565.315 y5
622.327 y6
648.308 b6
709.365 y7
808.422 y8
811.372 b7
921.525 y9
925.411 b8
1035.551 y10
1038.505 b9
1137.572 b10
1198.625 y11
1312.683 y12
1425.726 y13
1593.840 y15
DHPALNYNIVSGPPSHK / File: christine_ip_13.raw; SpectrumID: ; scans: 6430

EPVGAGVSSTSENNVSK

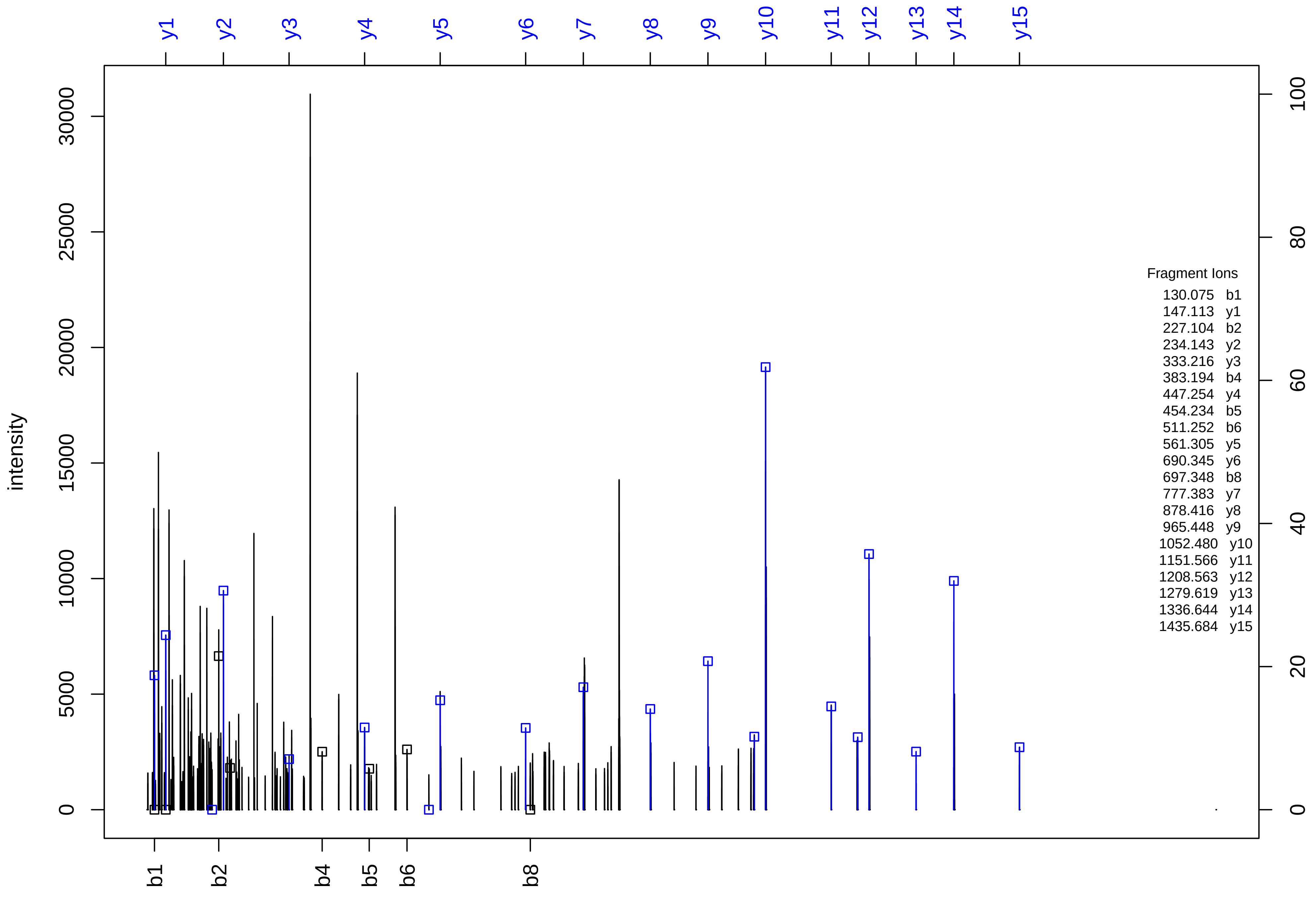

EPVGAGVSSTSENNVSK / File: christine_ip_13.raw; SpectrumID: ; scans: 2420

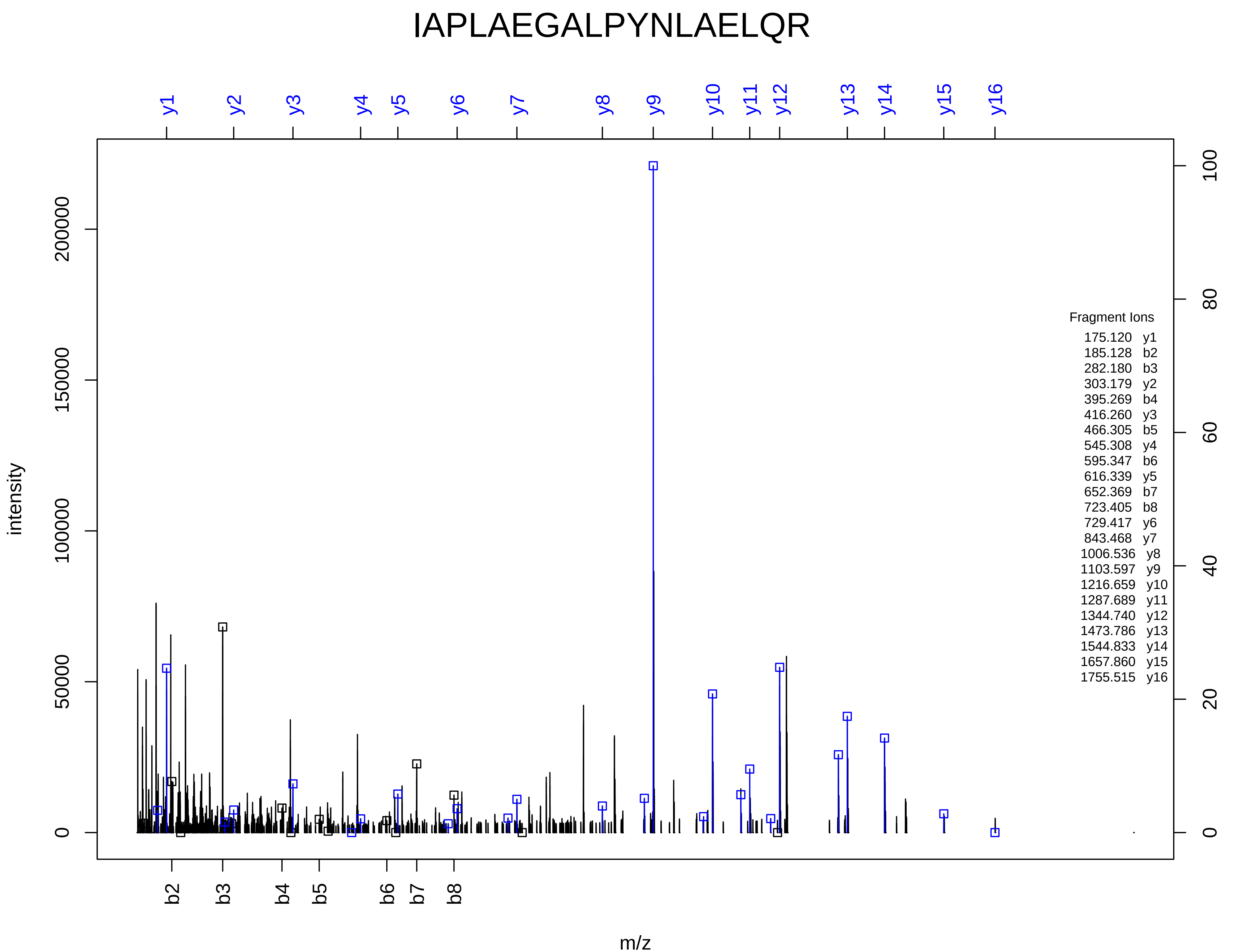
IAPLAEGALPYNLAELQR
y1 y2 y3 y4 y5 y6 y7 y8 y9 y10 y11 y12 y13 y14 y15 y16
b2 b3 b4 b5 b6 b7 b8
intensity
0
50000
100000
150000
200000
0
20
40
60
80
100
Fragment Ions
175.120 y1
185.128 b2
282.180 b3
303.179 y2
395.269 b4
416.260 y3
466.305 b5
545.308 y4
595.347 b6
616.339 y5
652.369 b7
723.405 b8
729.417 y6
843.468 y7
1006.536 y8
1103.597 y9
1216.659 y10
1287.689 y11
1344.740 y12
1473.786 y13
1544.833 y14
1657.860 y15
1755.515 y16
m/z
IAPLAEGALPYNLAELQR / File: christine_ip_13.raw; SpectrumID: ; scans: 16611

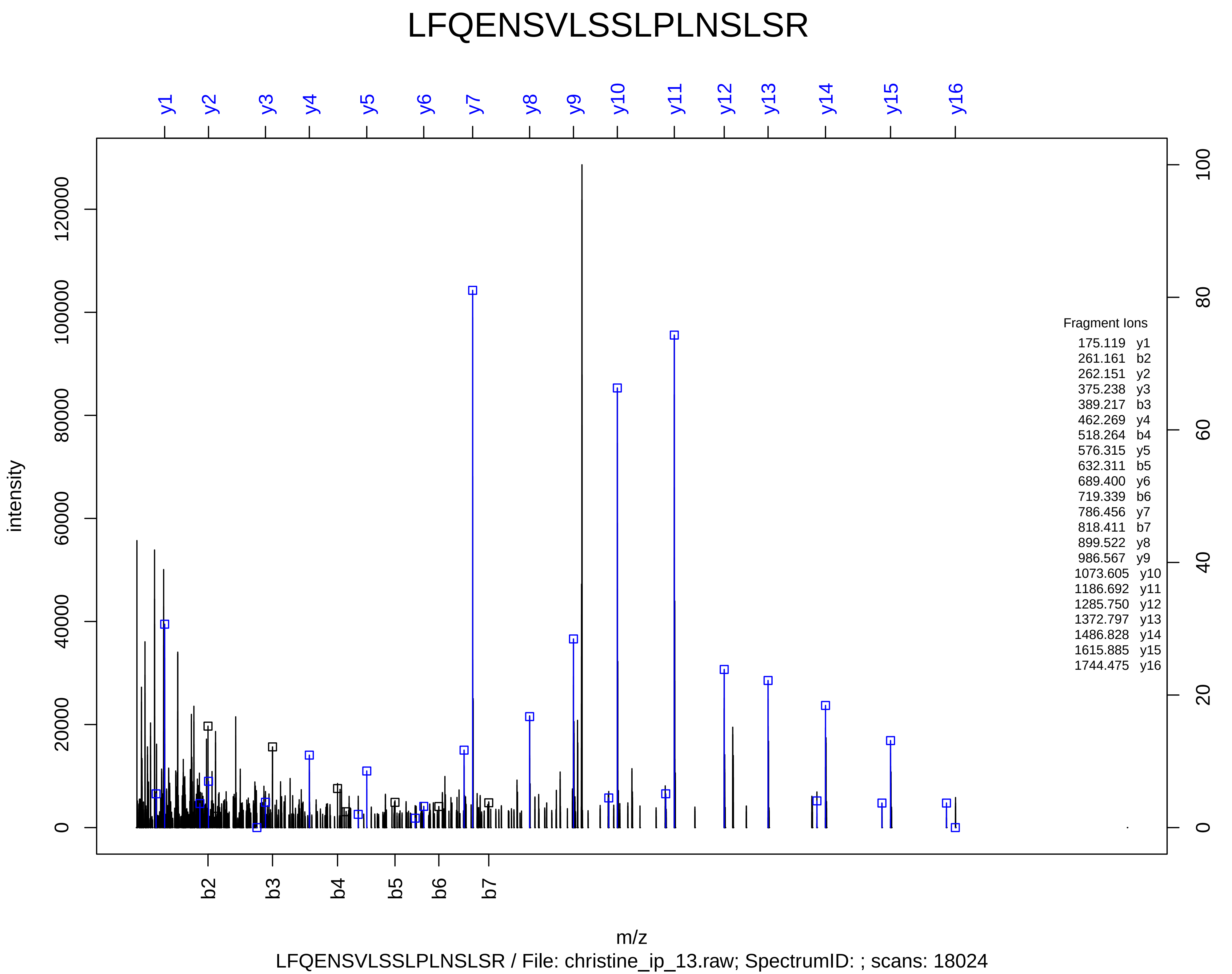
LFQENSVLSSLPLNSLSR
y1 y2 y3 y4 y5 y6 y7 y8 y9 y10 y11 y12 y13 y14 y15 y16
b2 b3 b4 b5 b6 b7
intensity
m/z
Fragment Ions
175.119 y1
261.161 b2
262.151 y2
375.238 y3
389.217 b3
462.269 y4
518.264 b4
576.315 y5
632.311 b5
689.400 y6
719.339 b6
786.456 y7
818.411 b7
899.522 y8
986.567 y9
1073.605 y10
1186.692 y11
1285.750 y12
1372.797 y13
1486.828 y14
1615.885 y15
1744.475 y16
LFQENSVLSSLPLNSLSR / File: christine_ip_13.raw; SpectrumID: ; scans: 18024

AWDDFFPGSDR

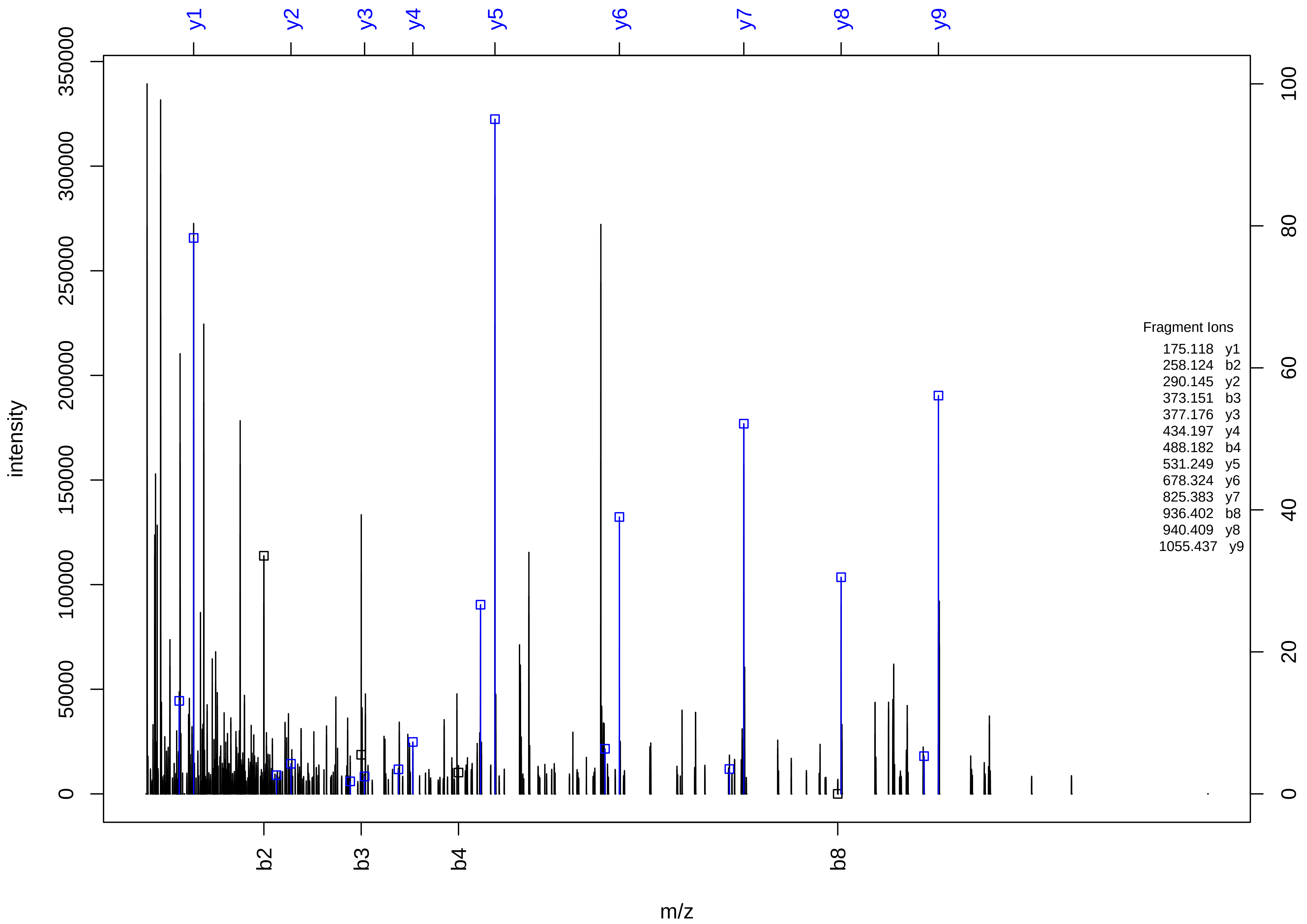

AWDDFFPGSDR / File: christine_ip_13.raw; SpectrumID: ; scans: 13763

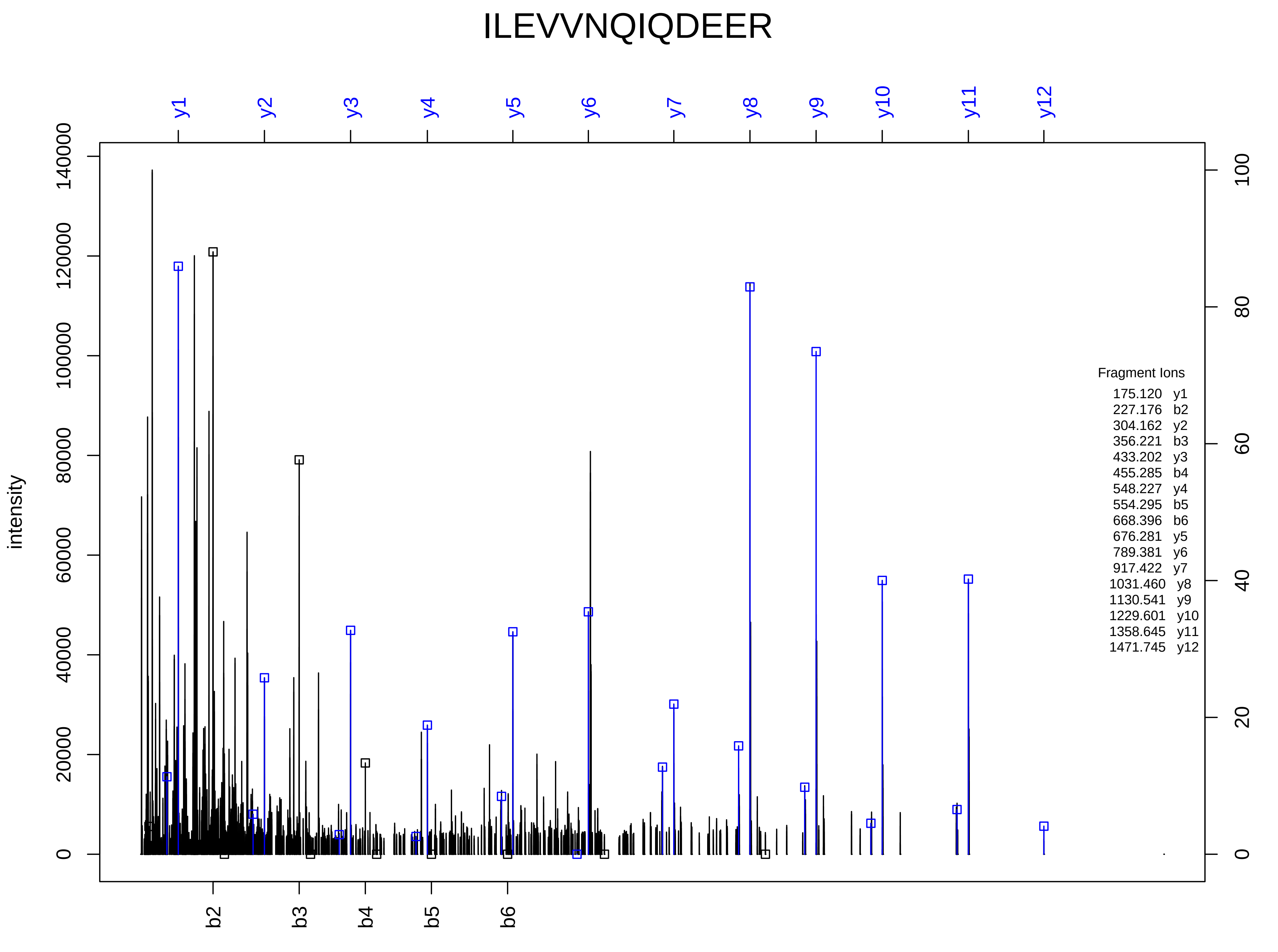

ILEVVNQIQDEER / File: christine_ip_13.raw; SpectrumID: ; scans: 10586

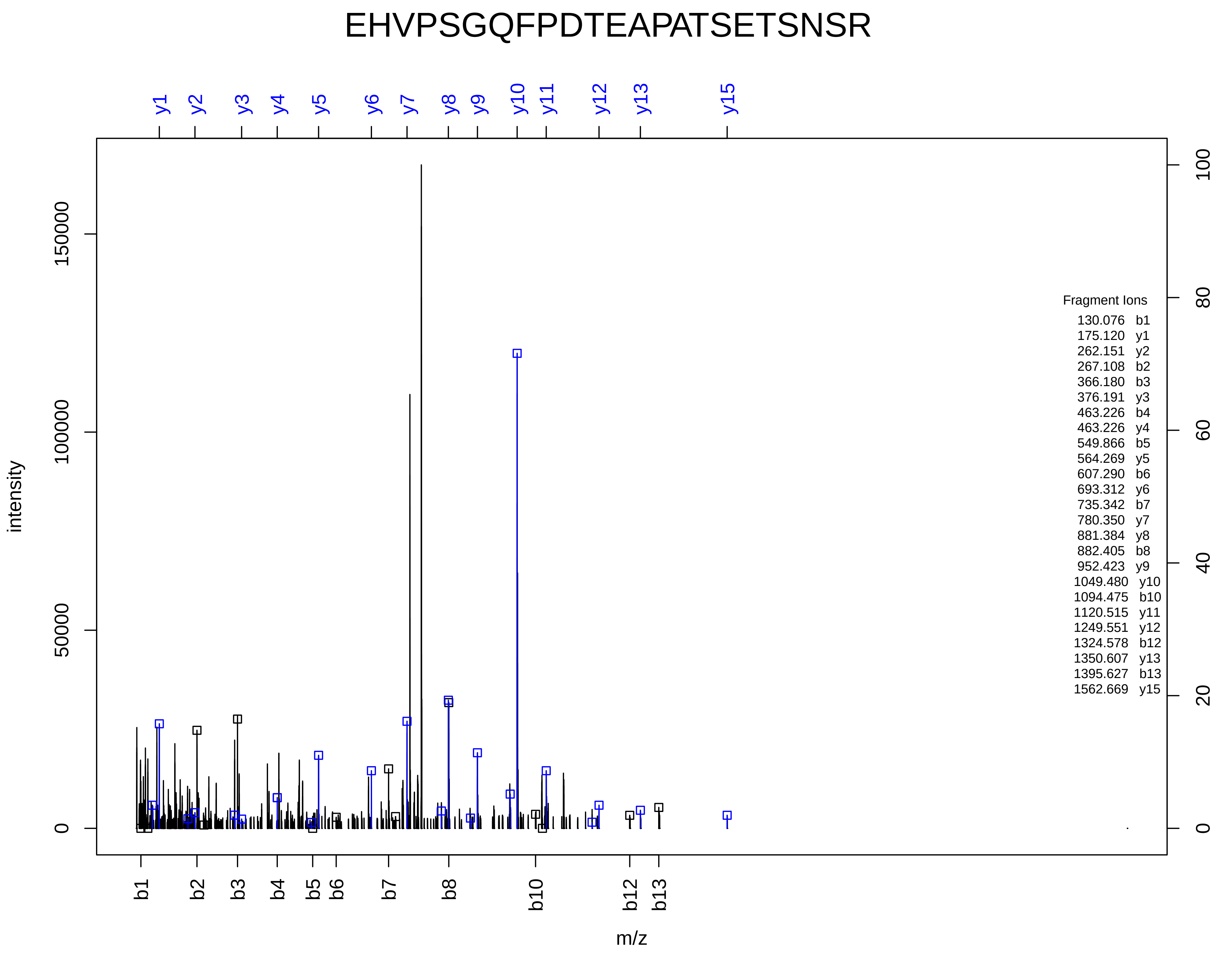

EHVPSGQFPDTEAPATSETSNSR / File: christine_ip_13.raw; SpectrumID: ; scans: 4826

NPPDIVVQPGHIR

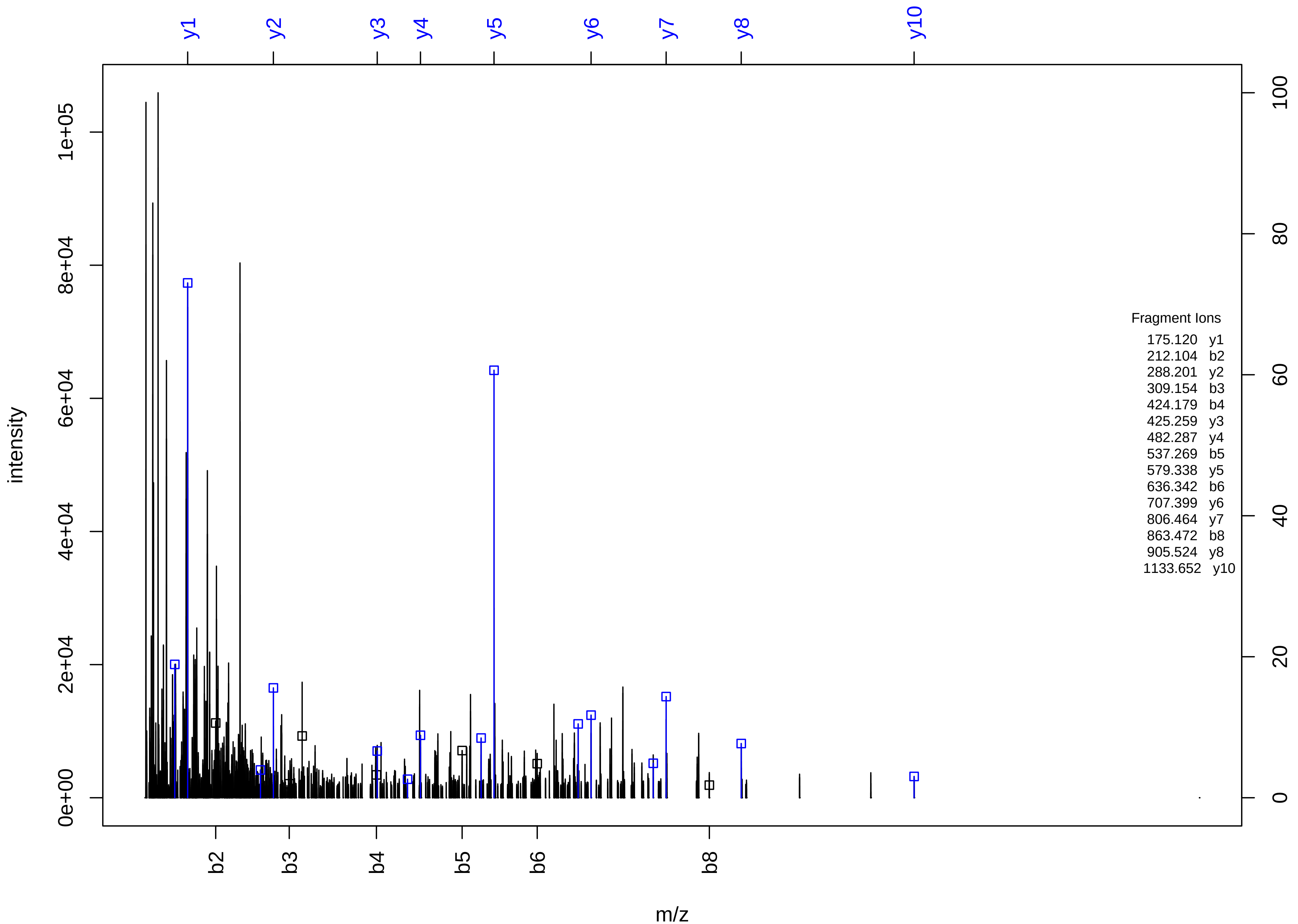

NPPDIVVQPGHIR / File: christine_ip_13.raw; SpectrumID: ; scans: 6505

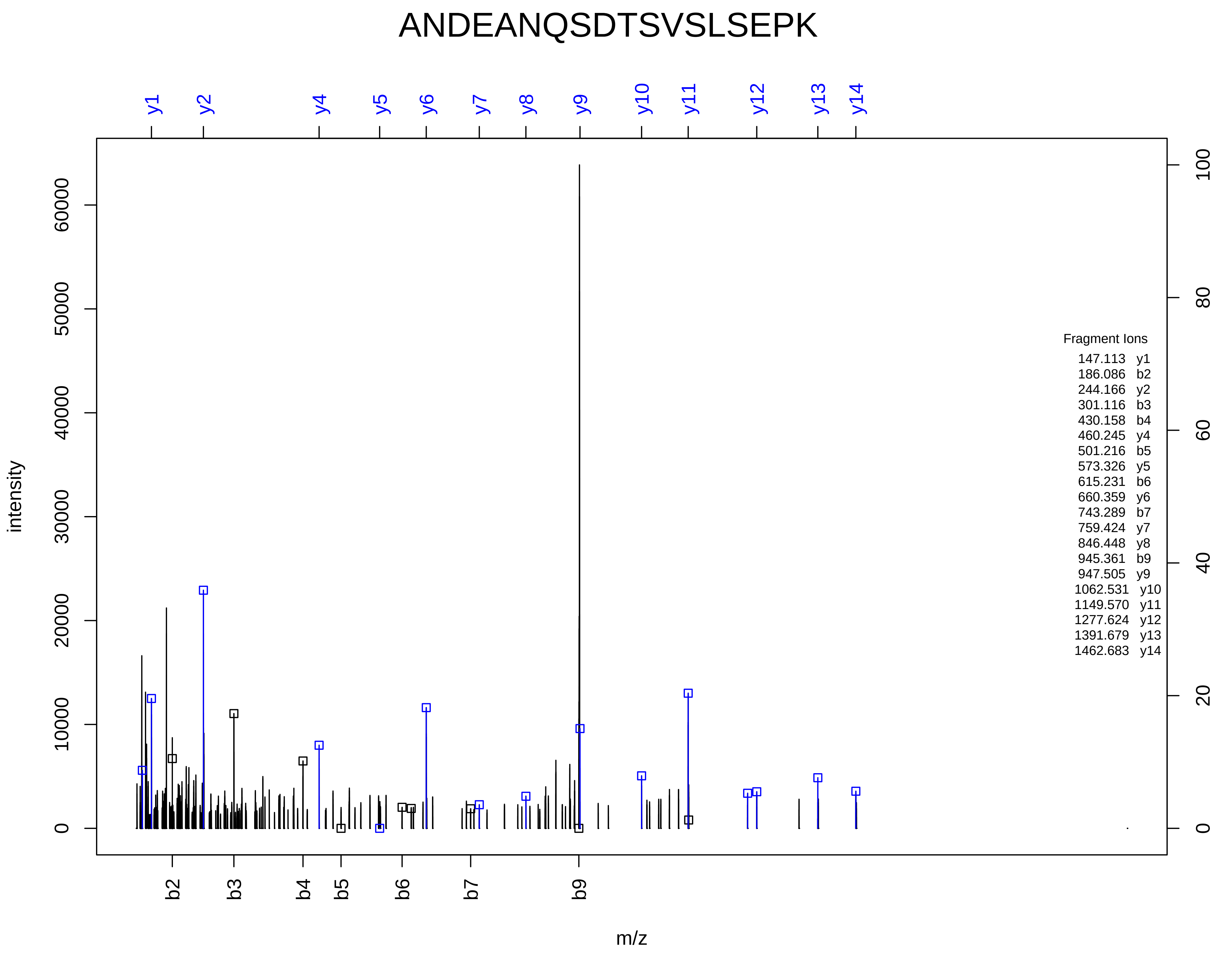
ANDEANQSDTSVSLSEPK
y1
y2
y4
y5
y6
y7
y8
y9
y10
y11
y12
y13
y14
b2
b3
b4
b5
b6
b7
b9
intensity
m/z
Fragment Ions
147.113 y1
186.086 b2
244.166 y2
301.116 b3
430.158 b4
460.245 y4
501.216 b5
573.326 y5
615.231 b6
660.359 y6
743.289 b7
759.424 y7
846.448 y8
945.361 b9
947.505 y9
1062.531 y10
1149.570 y11
1277.624 y12
1391.679 y13
1462.683 y14
ANDEANQSDTSVSLSEPK / File: christine_ip_13.raw; SpectrumID: ; scans: 4129

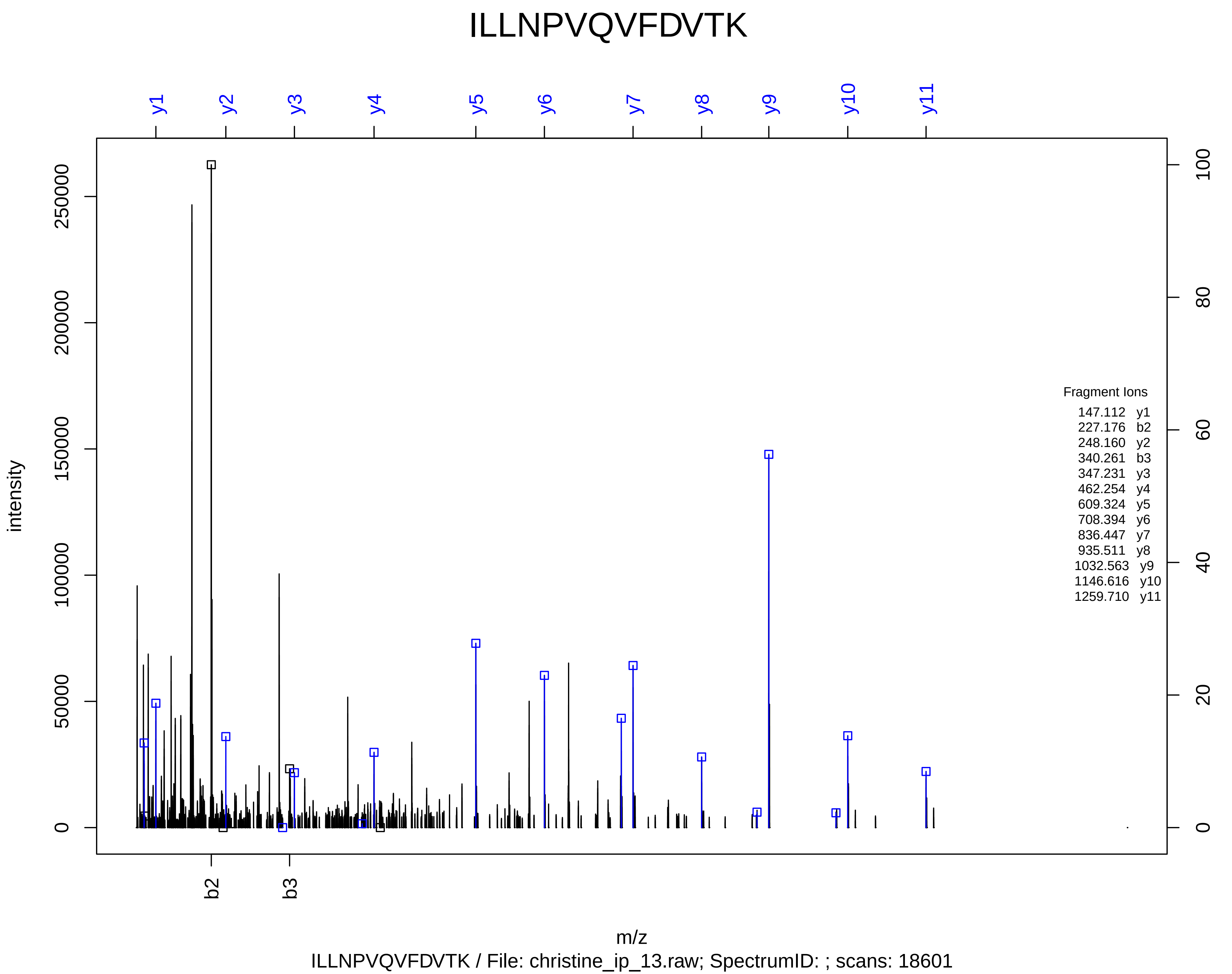
ILLNPVQVFDVTK
y1
y2
y3
y4
y5
y6
y7
y8
y9
y10
y11
intensity
0
50000
100000
150000
200000
250000
0
20
40
60
80
100
Fragment Ions
147.112 y1
227.176 b2
248.160 y2
340.261 b3
347.231 y3
462.254 y4
609.324 y5
708.394 y6
836.447 y7
935.511 y8
1032.563 y9
1146.616 y10
1259.710 y11
b2
b3
m/z
ILLNPVQVFDVTK / File: christine_ip_13.raw; SpectrumID: ; scans: 18601

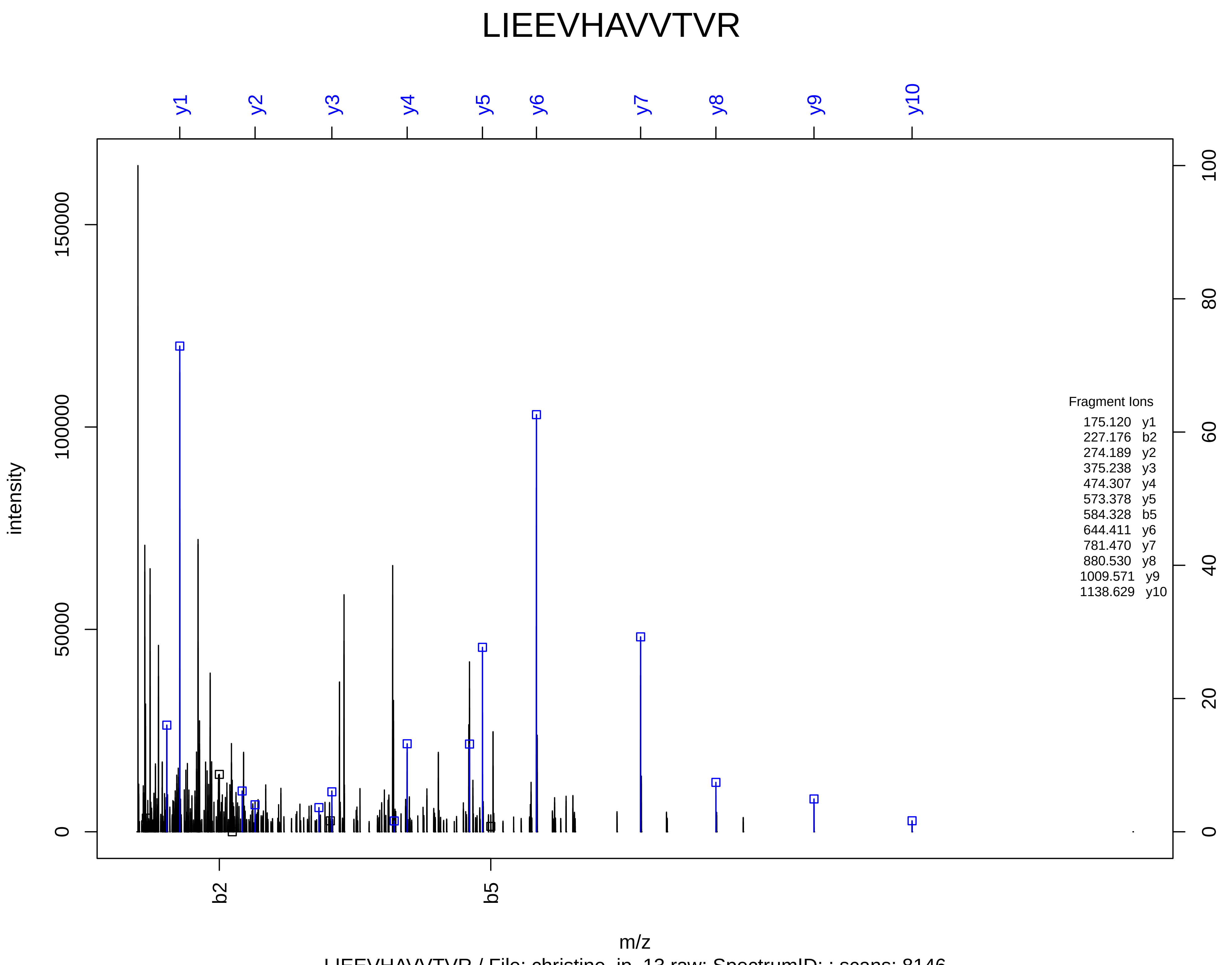
LIEEVHAVVTVR
y1
y2
y3
y4
y5
y6
y7
y8
y9
y10
intensity
0
50000
100000
150000
0
20
40
60
80
100
Fragment Ions
175.120 y1
227.176 b2
274.189 y2
375.238 y3
474.307 y4
573.378 y5
584.328 b5
644.411 y6
781.470 y7
880.530 y8
1009.571 y9
1138.629 y10
b2
b5
m/z
LIEEVHAVVTVR / File: christine_ip_13.raw; SpectrumID: ; scans: 8146

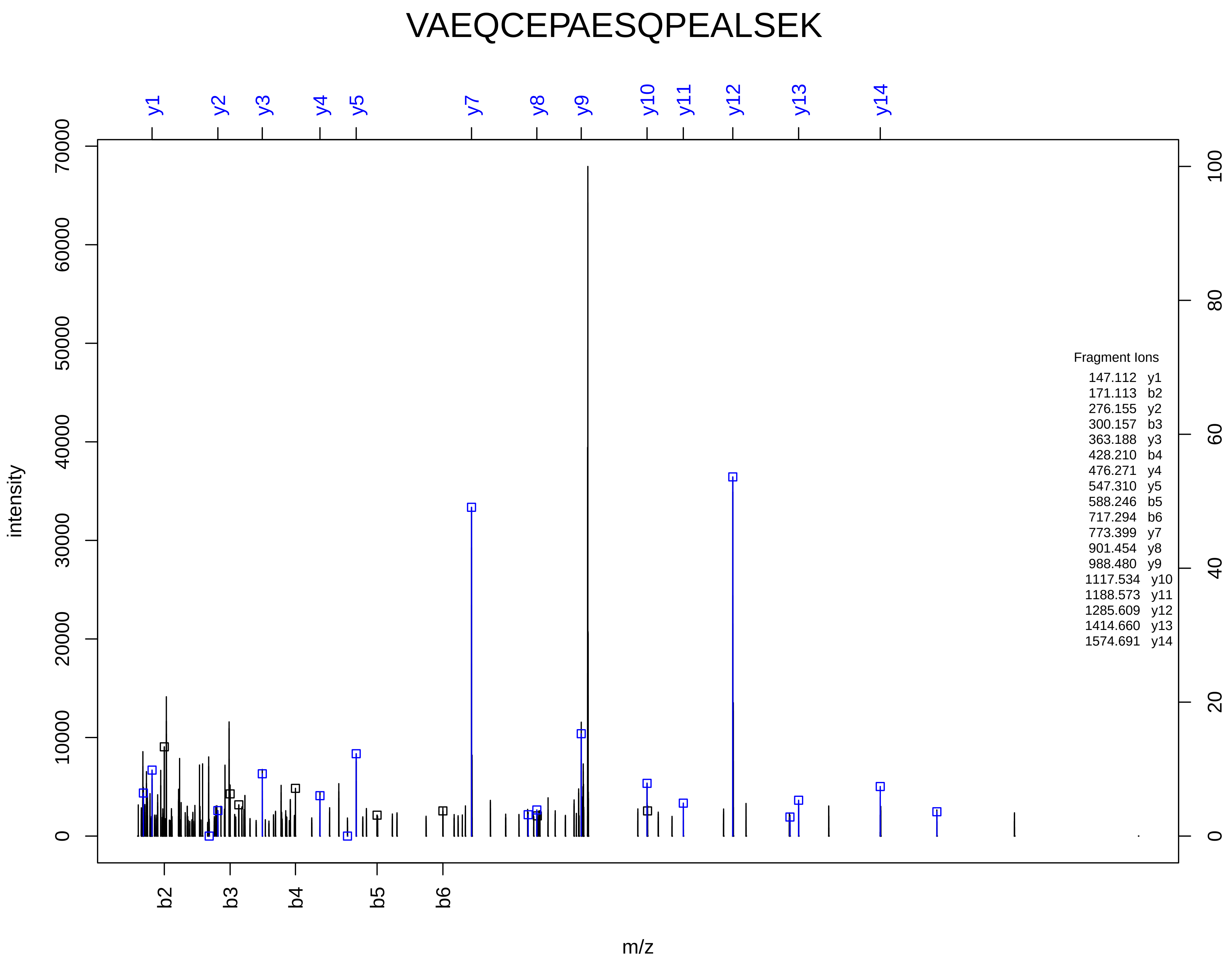

VAEQCEPAESQPEALSEK / File: christine_ip_13.raw; SpectrumID: ; scans: 3933

FEDLEVSTFER

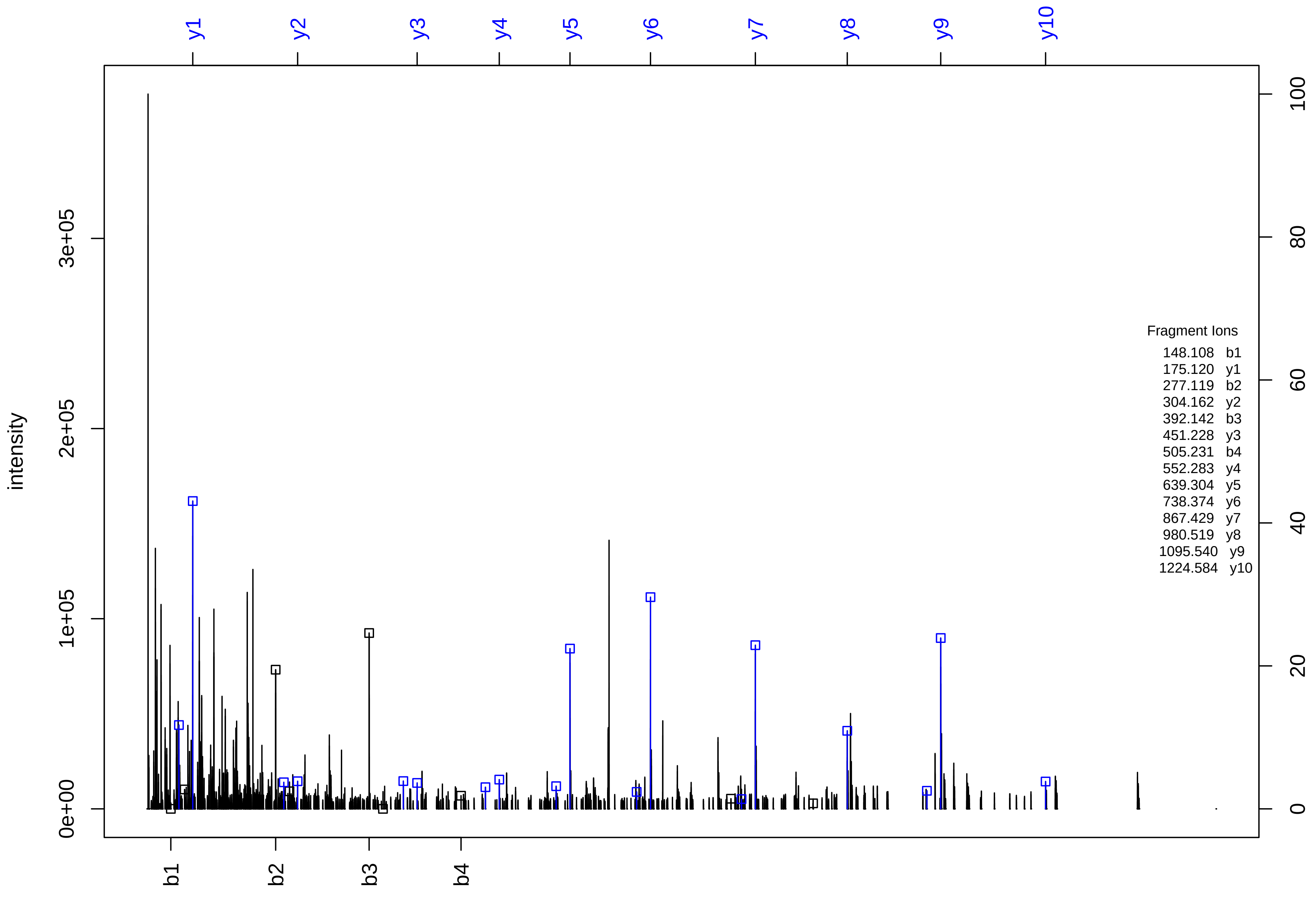

FEDLEVSTFER / File: christine_ip_13.raw; SpectrumID: ; scans: 11012

DCSGPLSALTELNTK

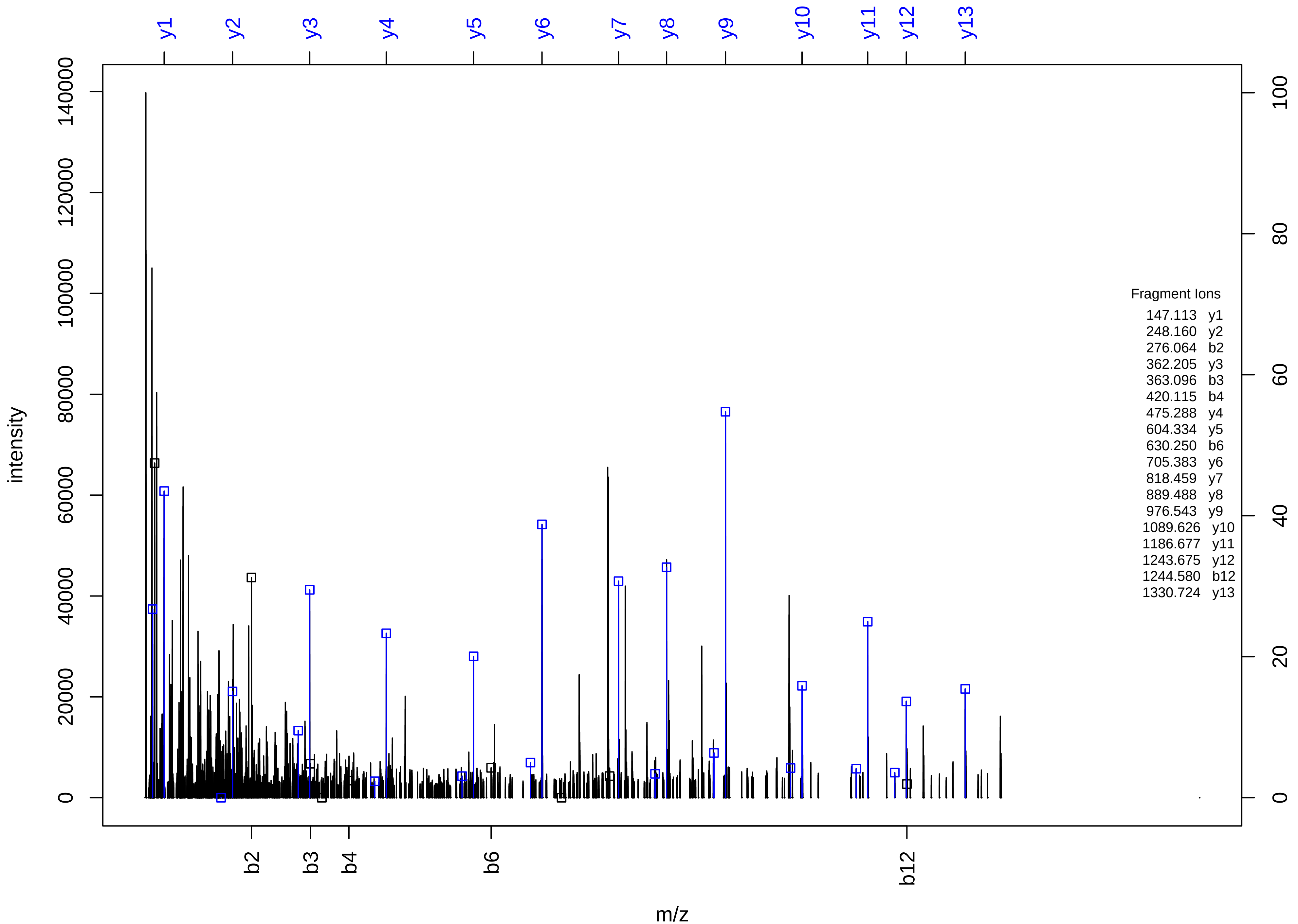

DCSGPLSALTELNTK / File: christine_ip_13.raw; SpectrumID: ; scans: 14731

ELTLQDVELLK

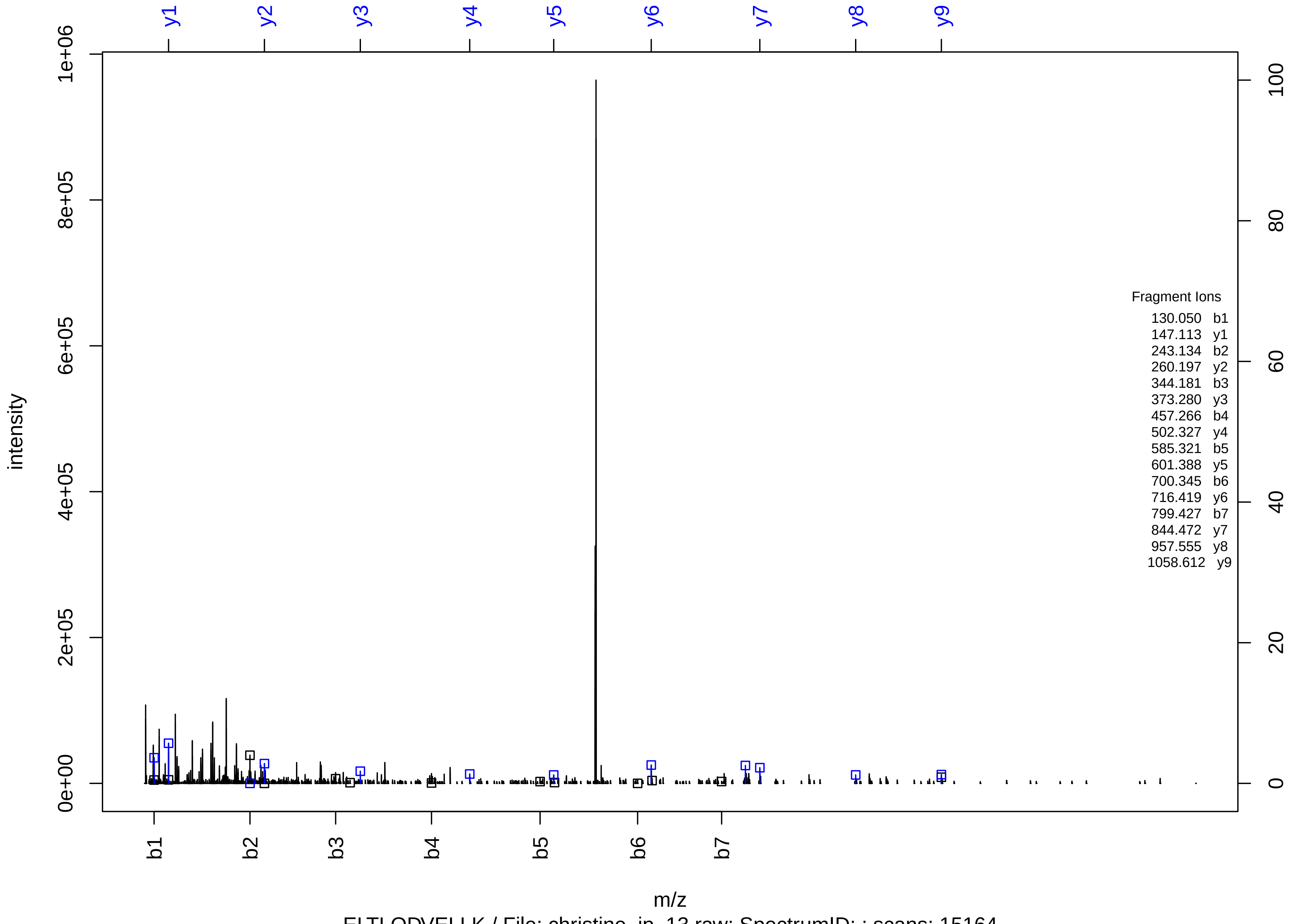

ELTLQDVELLK / File: christine_ip_13.raw; SpectrumID: ; scans: 15164

VLAQQGEYSEAIPILR

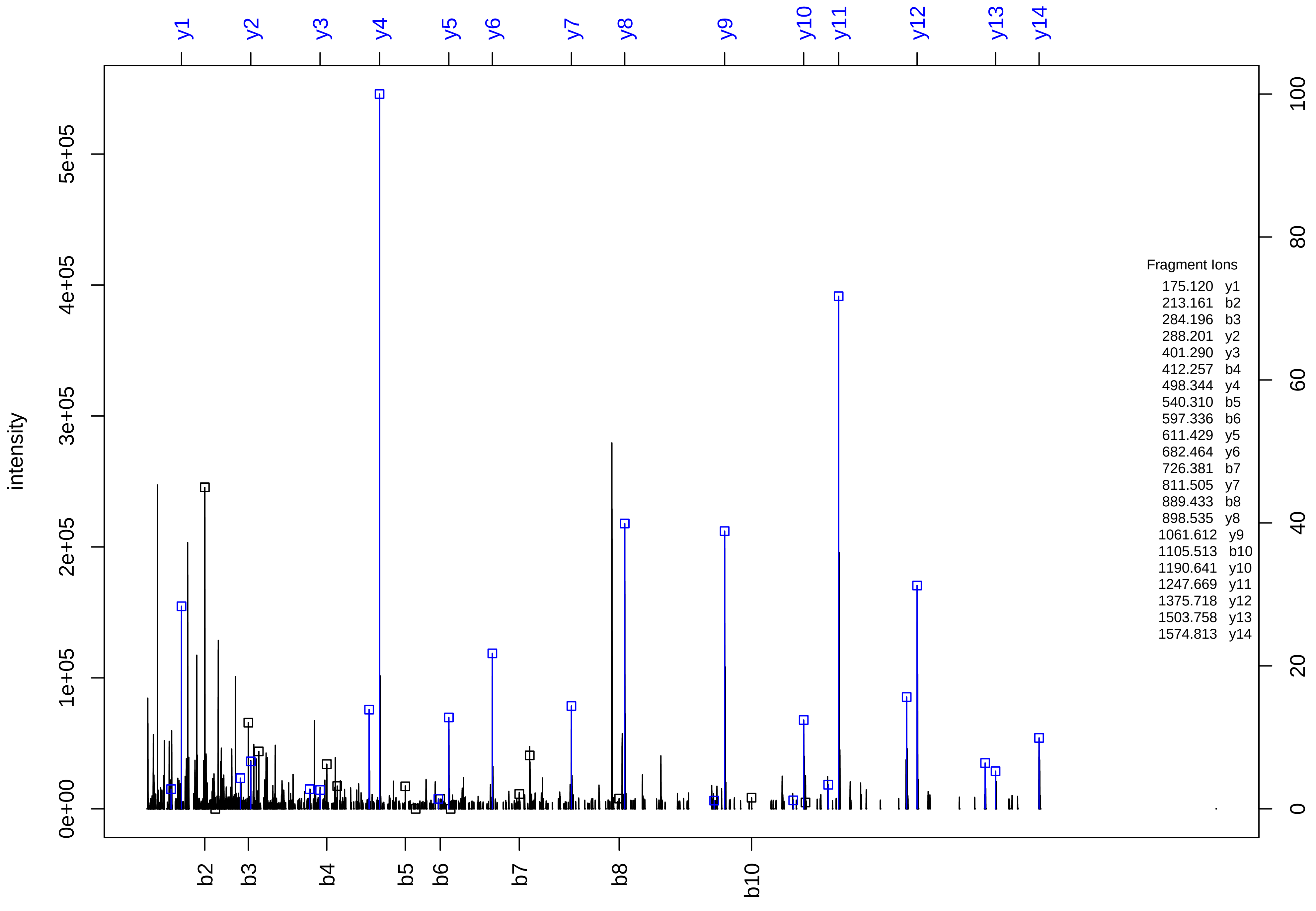

VLAQQGEYSEAIPILR / File: christine_ip_13.raw; SpectrumID: ; scans: 13547

VPVSDLLLSYESPK

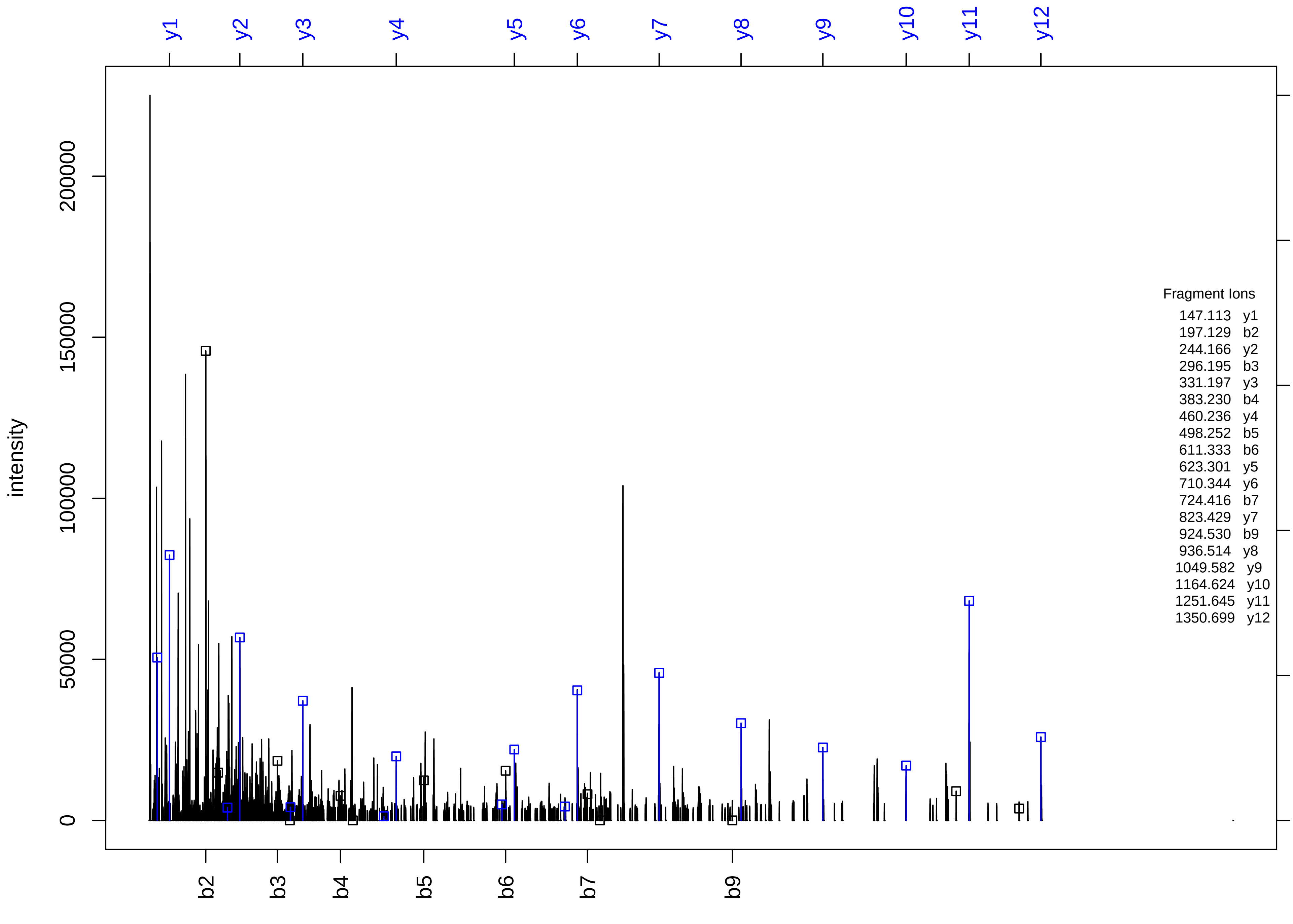

VPVSDLLLSYESPK / File: christine_ip_13.raw; SpectrumID: ; scans: 14574

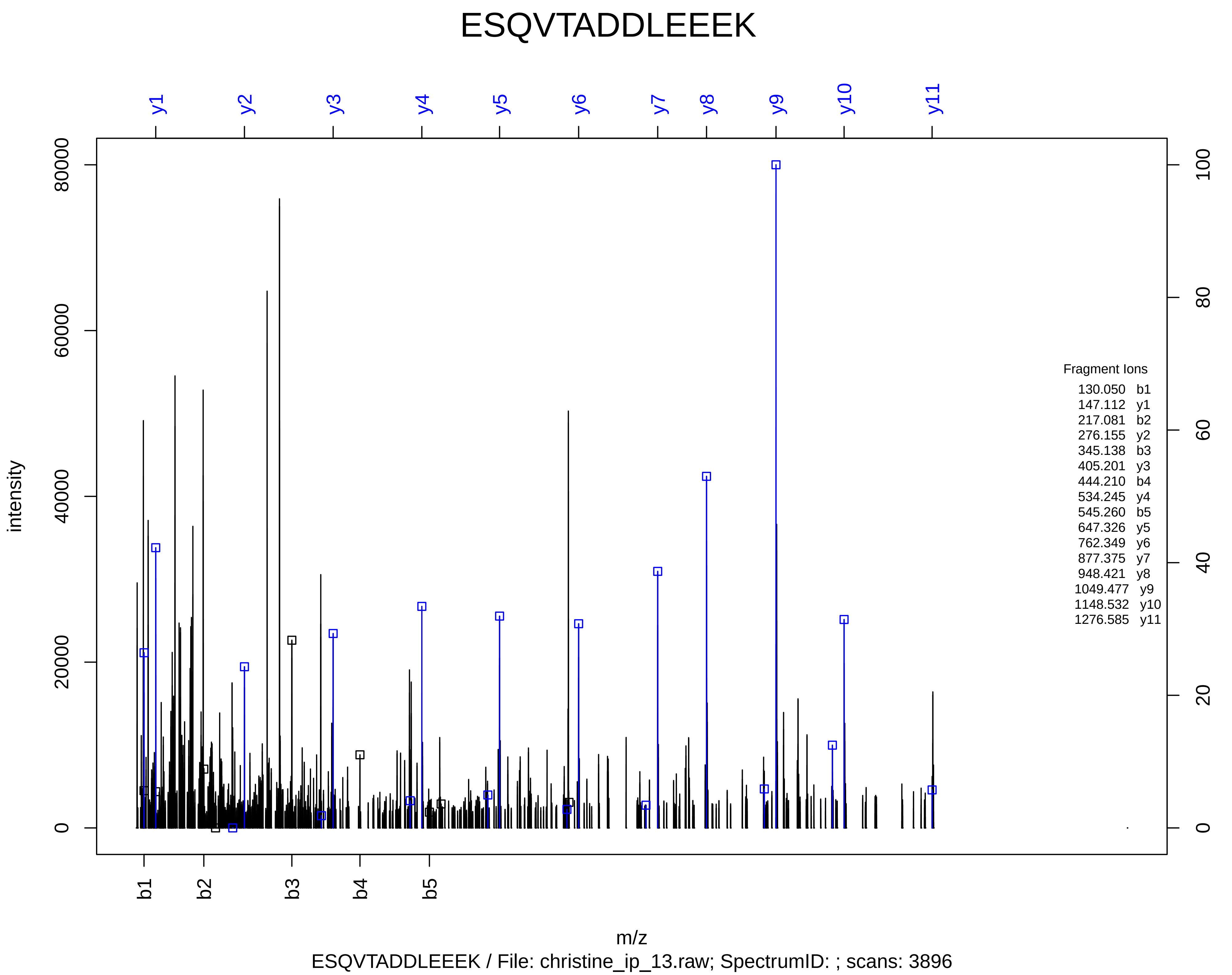

ESQVTADDLEEEK
intensity
m/z
y1 y2 y3 y4 y5 y6 y7 y8 y9 y10 y11
b1 b2 b3 b4 b5
0 20000 40000 60000 80000
0 20 40 60 80 100
Fragment Ions
130.050 b1
147.112 y1
217.081 b2
276.155 y2
345.138 b3
405.201 y3
444.210 b4
534.245 y4
545.260 b5
647.326 y5
762.349 y6
877.375 y7
948.421 y8
1049.477 y9
1148.532 y10
1276.585 y11
ESQVTADDLEEEK / File: christine_ip_13.raw; SpectrumID: ; scans: 3896

APGAEEDDSELQR

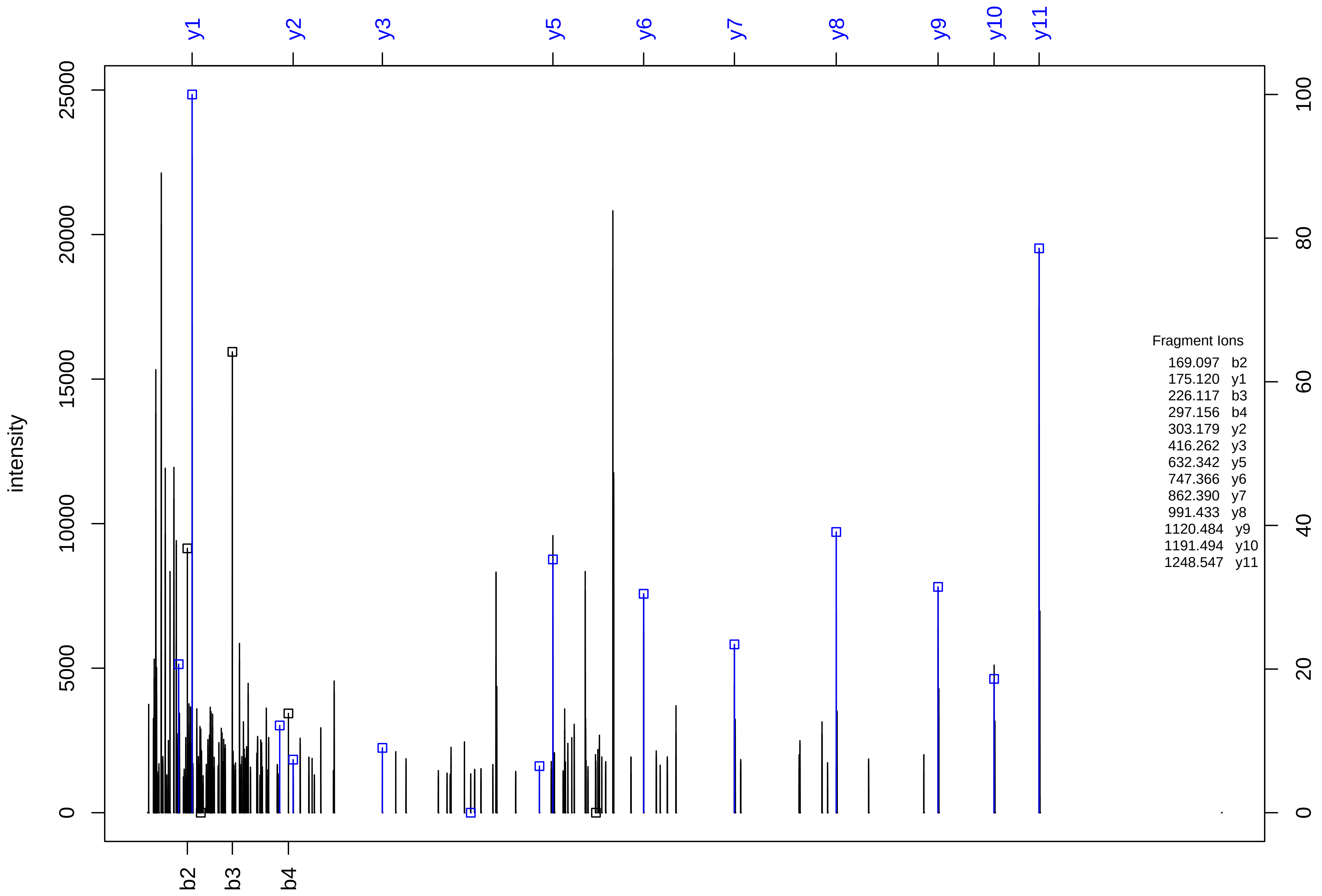

APGAEEDDSELQR / File: christine_ip_13.raw; SpectrumID: ; scans: 2264

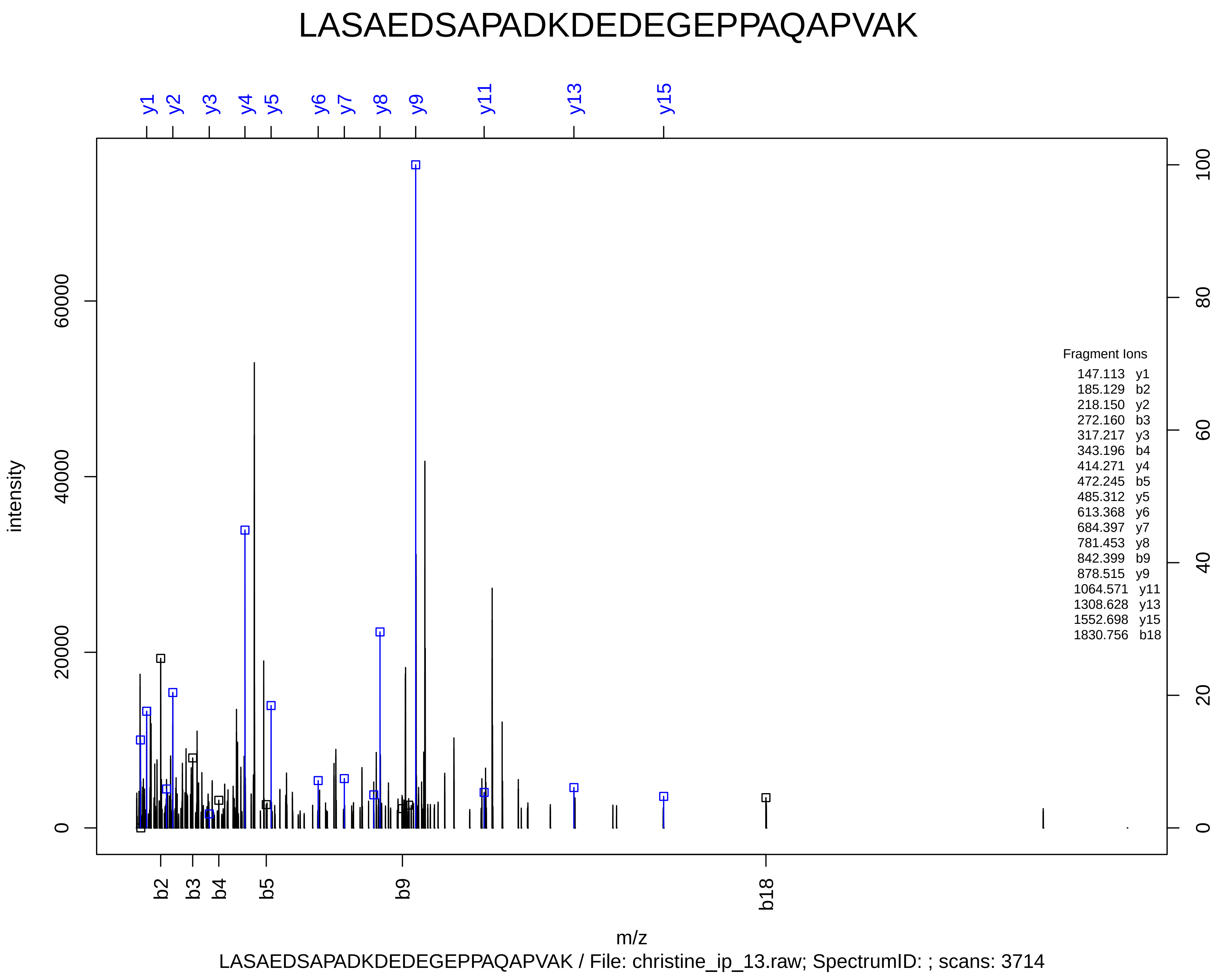
LASAEDSAPADKDEDEGEPPAQAPVAK
y1 y2 y3 y4 y5 y6 y7 y8 y9 y11 y13 y15
b2 b3 b4 b5 b9 b18
intensity
m/z
Fragment Ions
147.113 y1
185.129 b2
218.150 y2
272.160 b3
317.217 y3
343.196 b4
414.271 y4
472.245 b5
485.312 y5
613.368 y6
684.397 y7
781.453 y8
842.399 b9
878.515 y9
1064.571 y11
1308.628 y13
1552.698 y15
1830.756 b18
LASAEDSAPADKDEDEGEPPAQAPVAK / File: christine_ip_13.raw; SpectrumID: ; scans: 3714

LALLEEAR

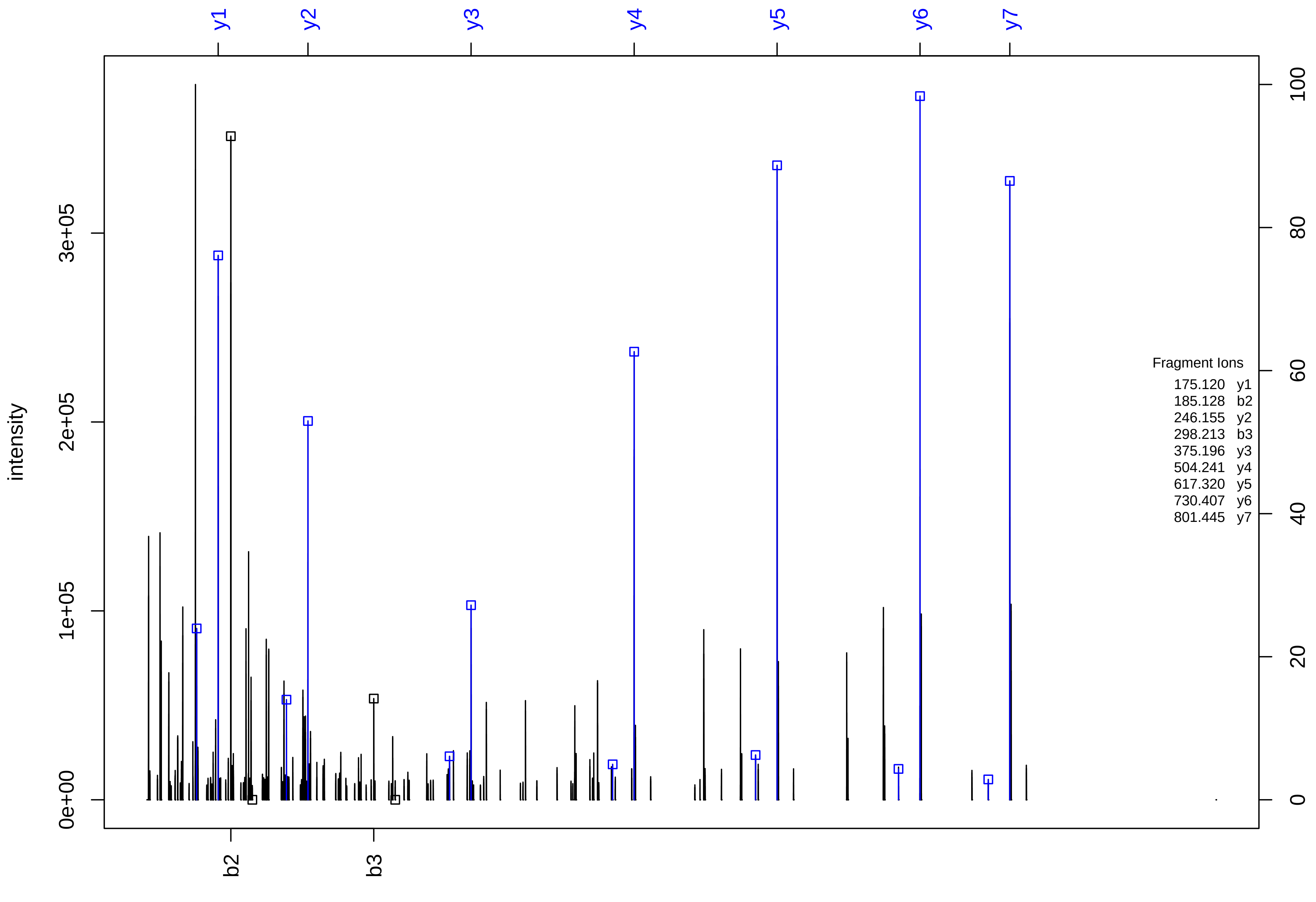

LALLEEAR / File: christine_ip_13.raw; SpectrumID: ; scans: 7921

EKVM*VAN^SFRN^K

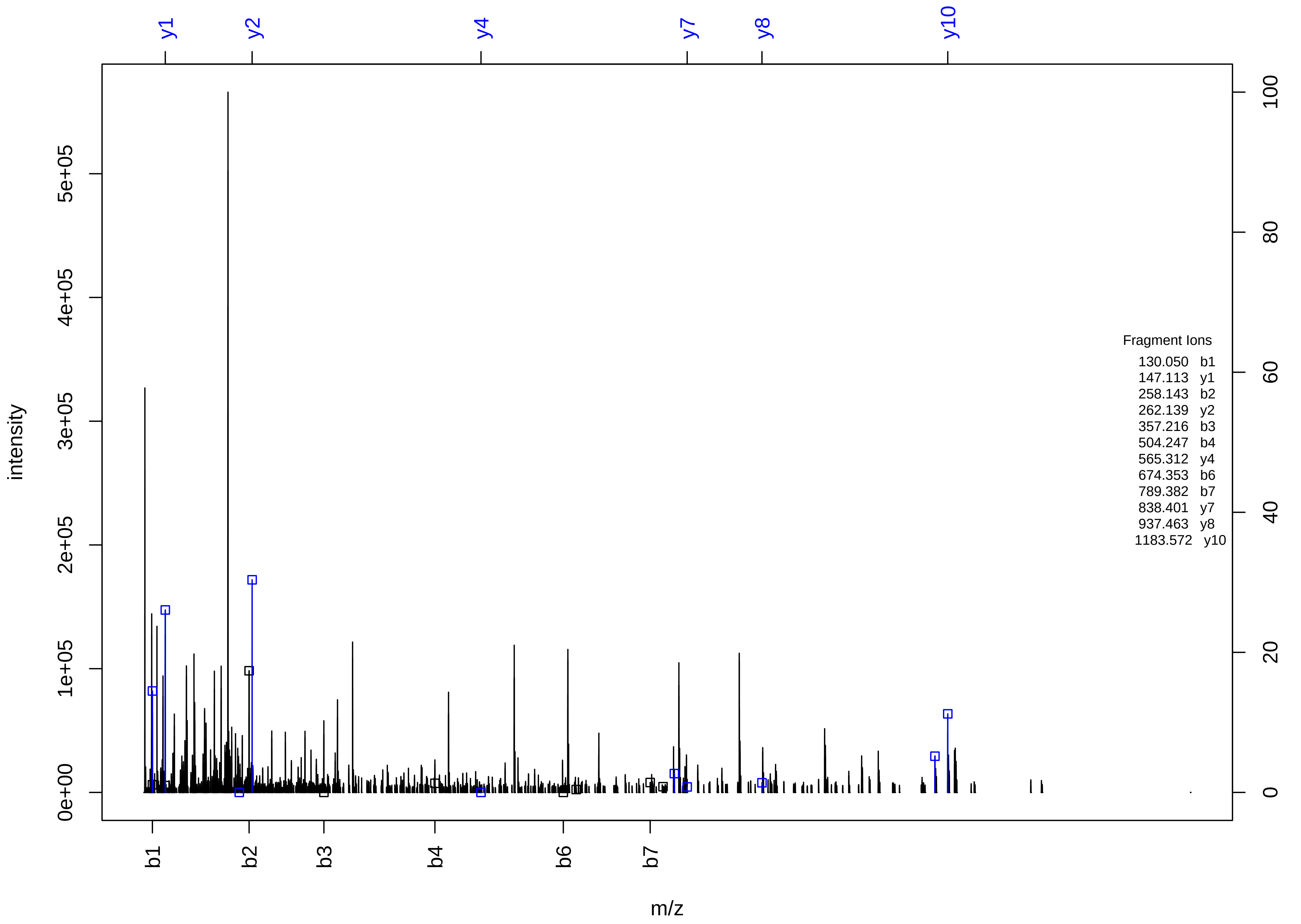

EKVMVANSFRNK / File: christine_ip_13.raw; SpectrumID: ; scans: 12981

TPDSFEESQGEEIGK

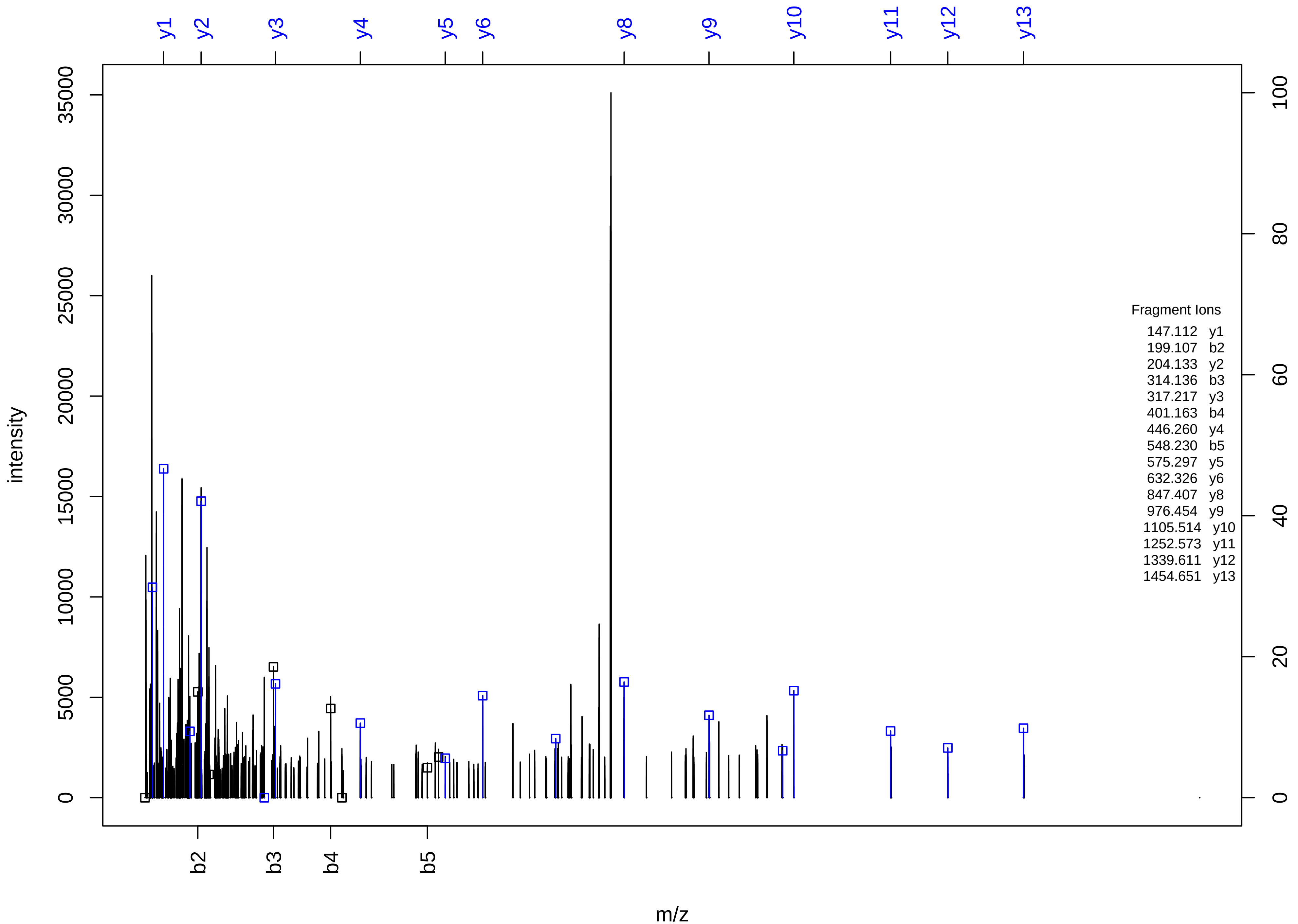

TPDSFEESQGEEIGK / File: christine_ip_13.raw; SpectrumID: ; scans: 4841

SLSPFAITYLDR

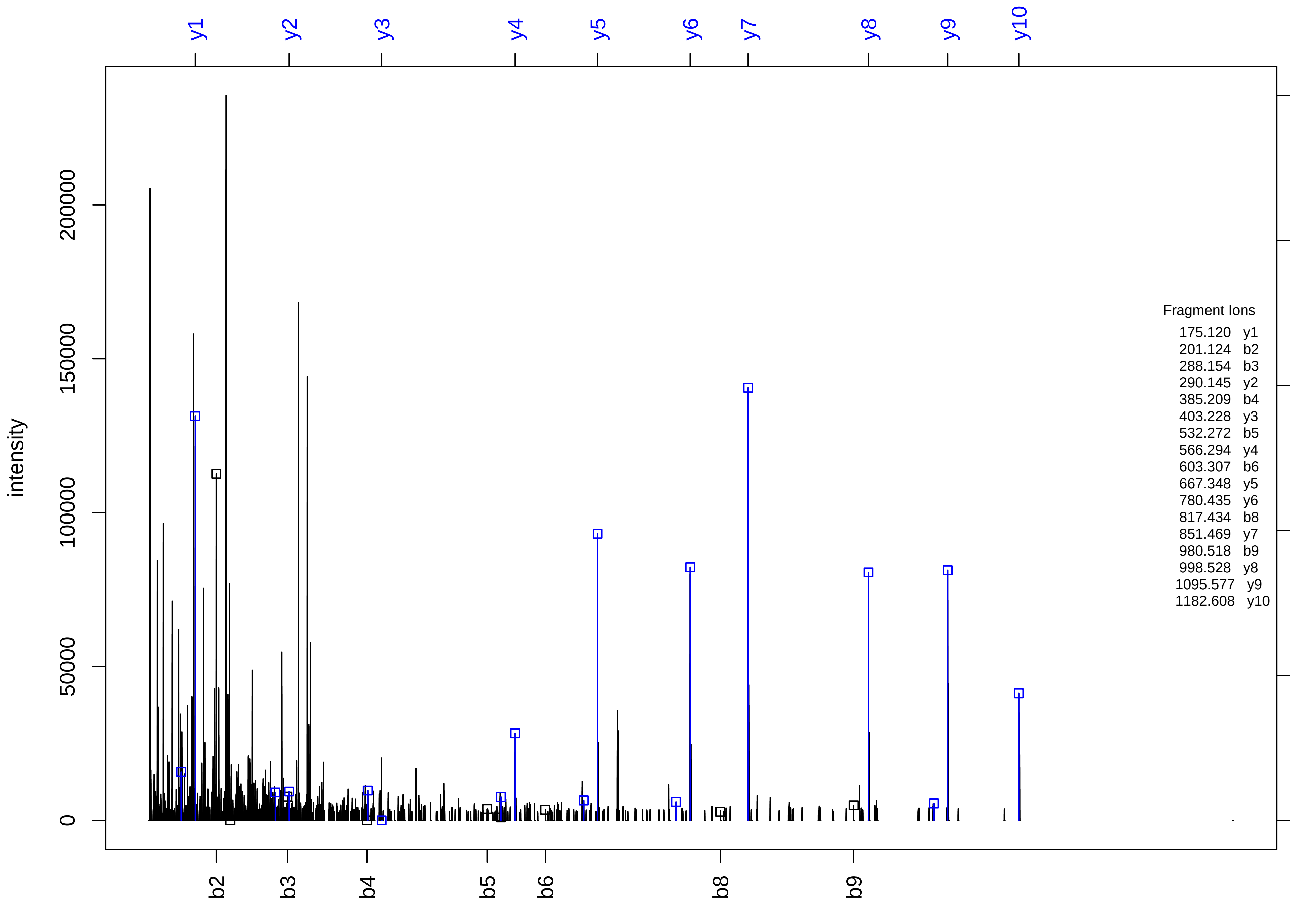

SLSPFAITYLDR / File: christine_ip_13.raw; SpectrumID: ; scans: 15753

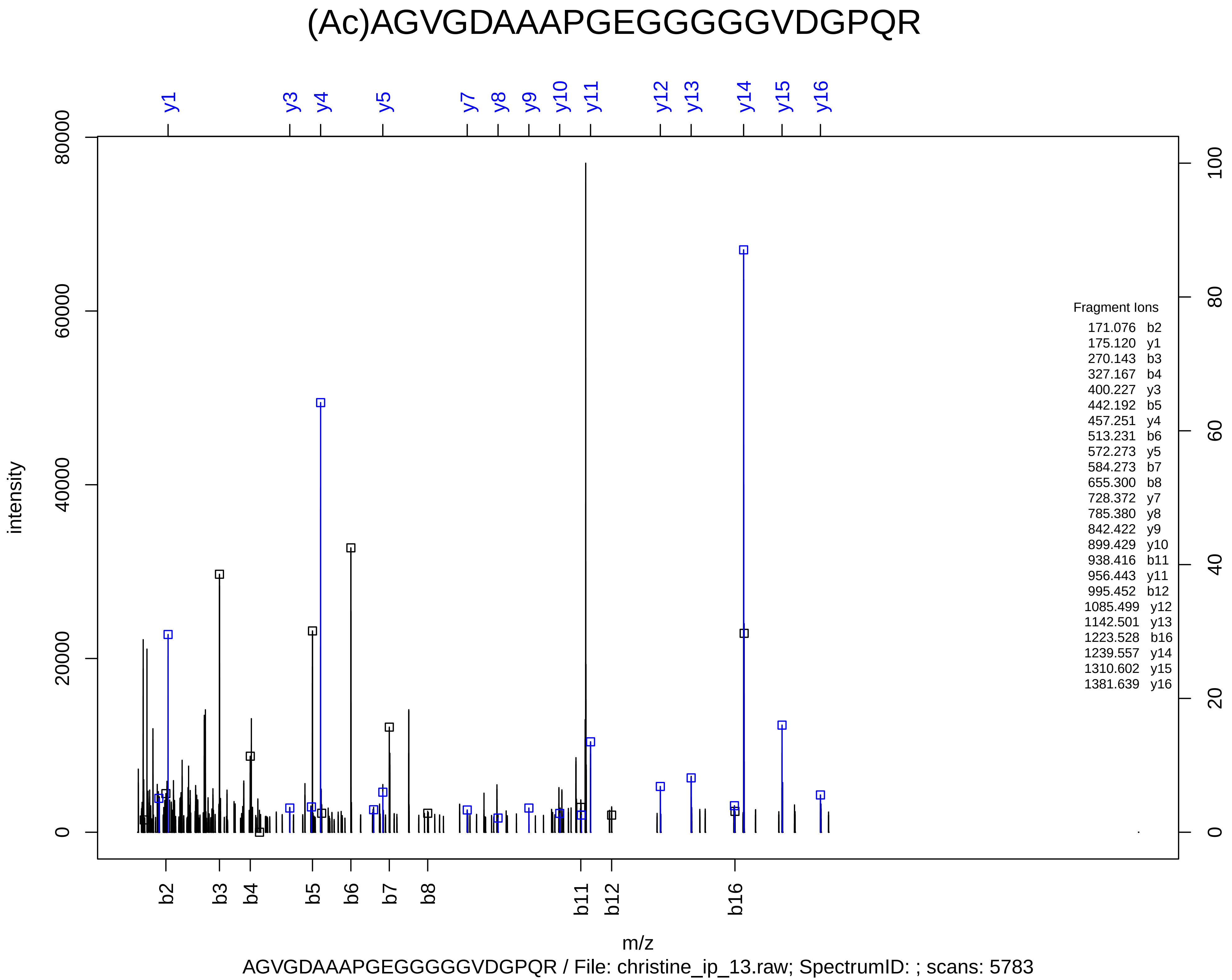

(Ac)AGVGDAAAPGEGGGGGVDGPQR
y1
y3
y4
y5
y7
y8
y9
y10
y11
y12
y13
y14
y15
y16
intensity
0
20000
40000
60000
80000
0
20
40
60
80
100
b2
b3
b4
b5
b6
b7
b8
b11
b12
b16
m/z
Fragment Ions
171.076 b2
175.120 y1
270.143 b3
327.167 b4
400.227 y3
442.192 b5
457.251 y4
513.231 b6
572.273 y5
584.273 b7
655.300 b8
728.372 y7
785.380 y8
842.422 y9
899.429 y10
938.416 b11
956.443 y11
995.452 b12
1085.499 y12
1142.501 y13
1223.528 b16
1239.557 y14
1310.602 y15
1381.639 y16
AGVGDAAAPGEGGGGGVDGPQR / File: christine_ip_13.raw; SpectrumID: ; scans: 5783

IPEDILQLLSK

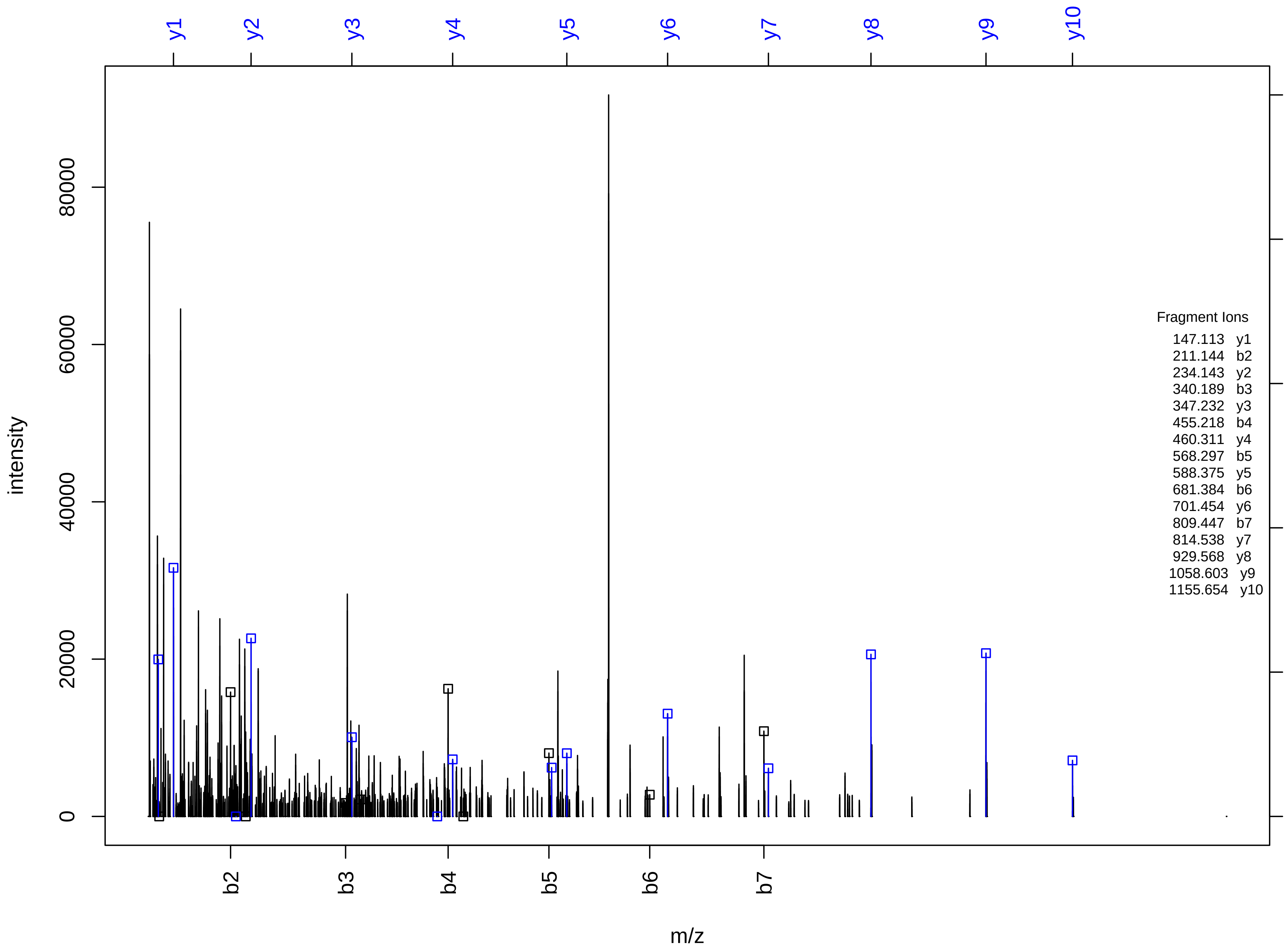

IPEDILQLLSK / File: christine_ip_13.raw; SpectrumID: ; scans: 18523

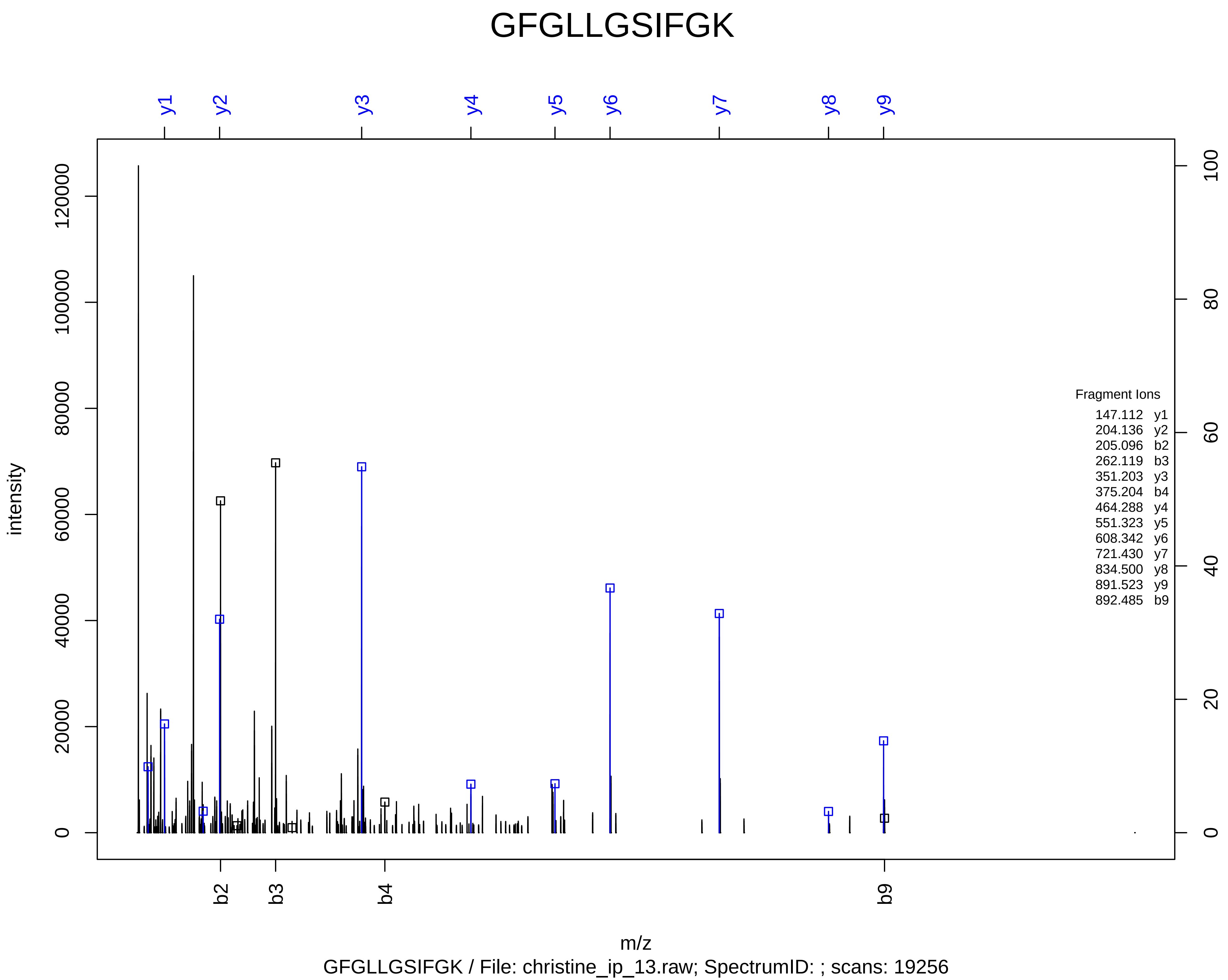
GFGLLGSIFGK
y1
y2
y3
y4
y5
y6
y7
y8
y9
b2
b3
b4
b9
intensity
m/z
Fragment Ions
147.112 y1
204.136 y2
205.096 b2
262.119 b3
351.203 y3
375.204 b4
464.288 y4
551.323 y5
608.342 y6
721.430 y7
834.500 y8
891.523 y9
892.485 b9
GFGLLGSIFGK / File: christine_ip_13.raw; SpectrumID: ; scans: 19256

VVSSTSEEEEAFTEK

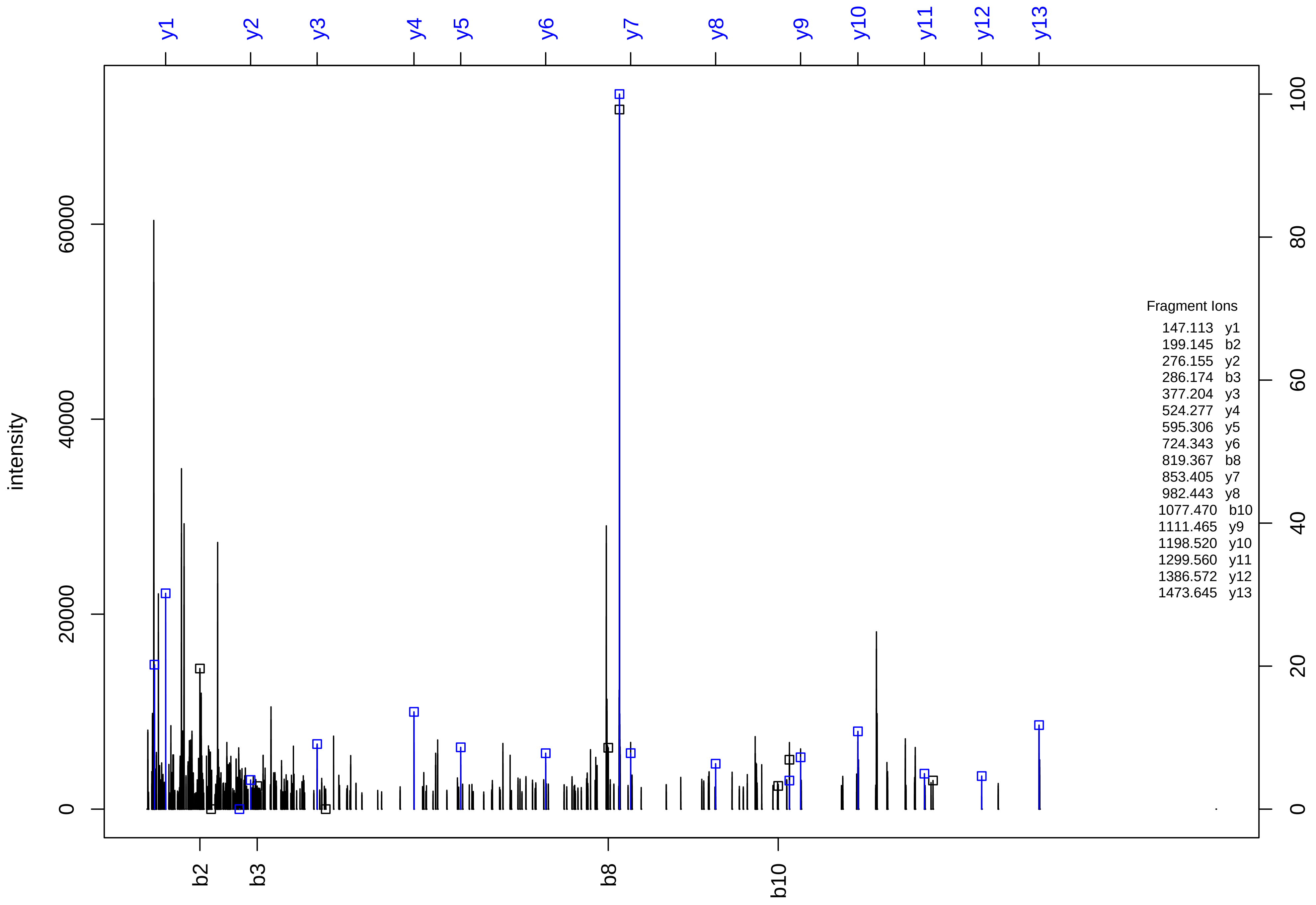

VVSSTSEEEEAFTEK / File: christine_ip_13.raw; SpectrumID: ; scans: 5056

IYHGNCNTPIPED

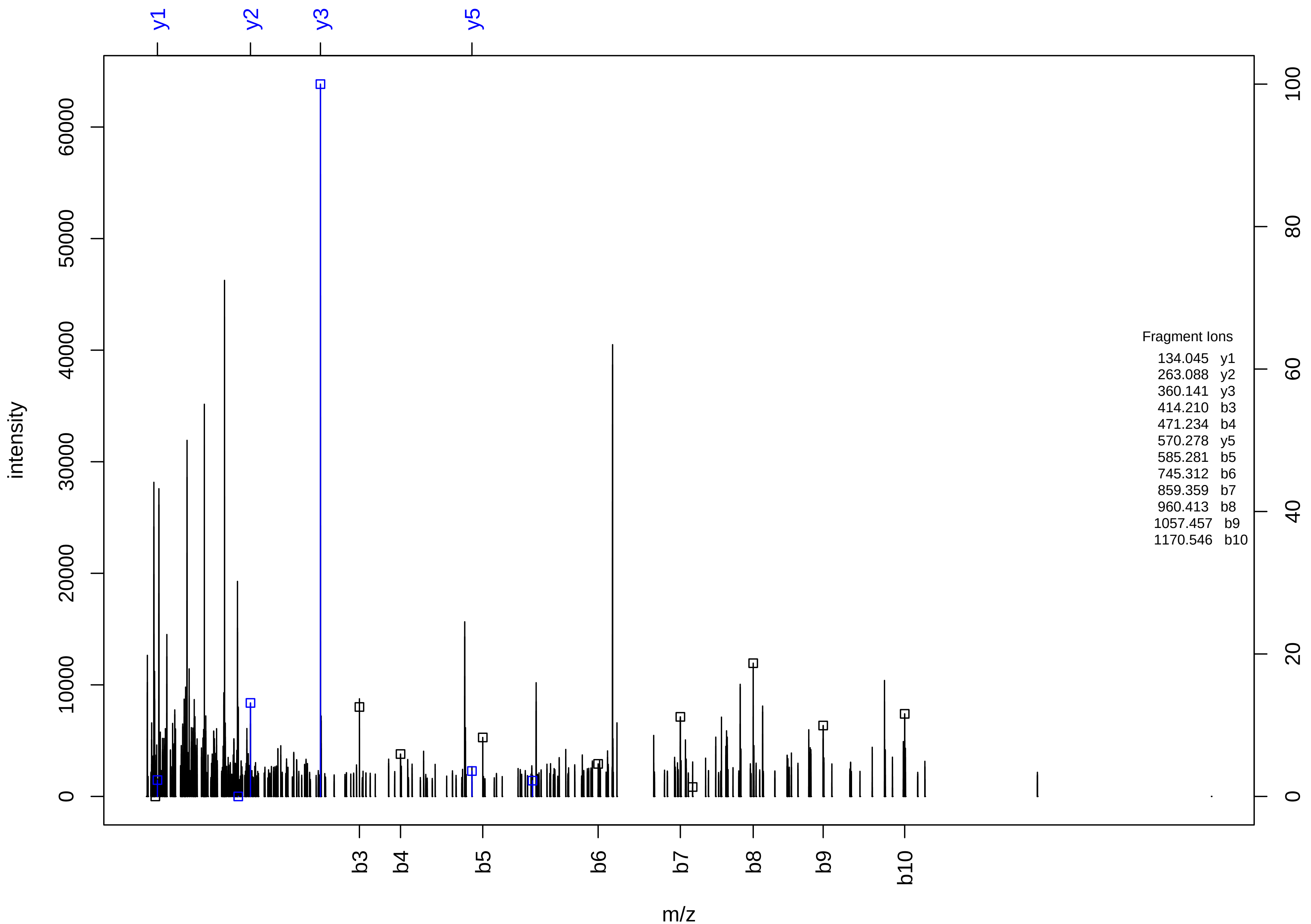

IYHGNCNTPIPED / File: christine_ip_13.raw; SpectrumID: ; scans: 4650

TLEVLLK

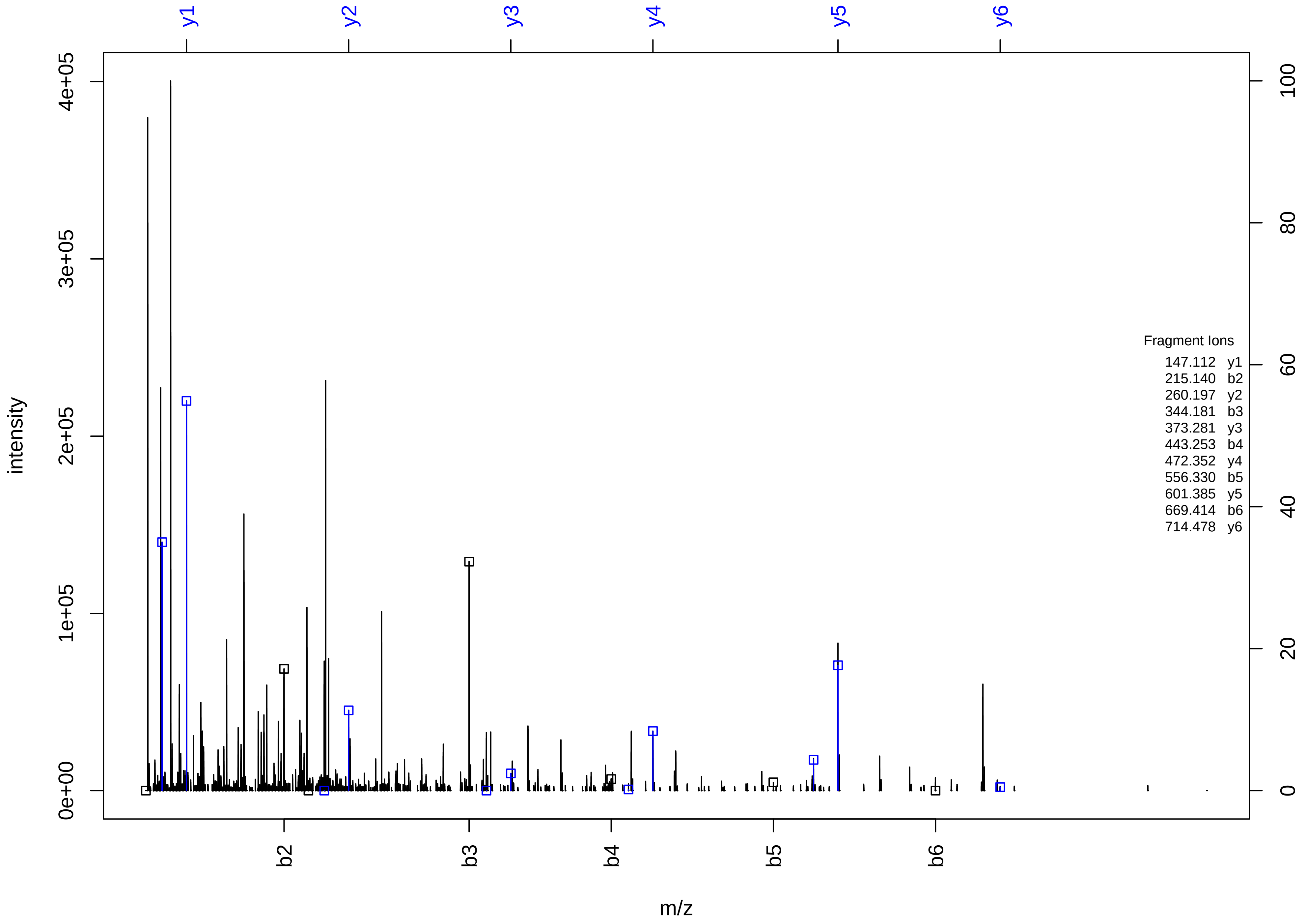

TLEVLLK / File: christine_ip_13.raw; SpectrumID: ; scans: 7632

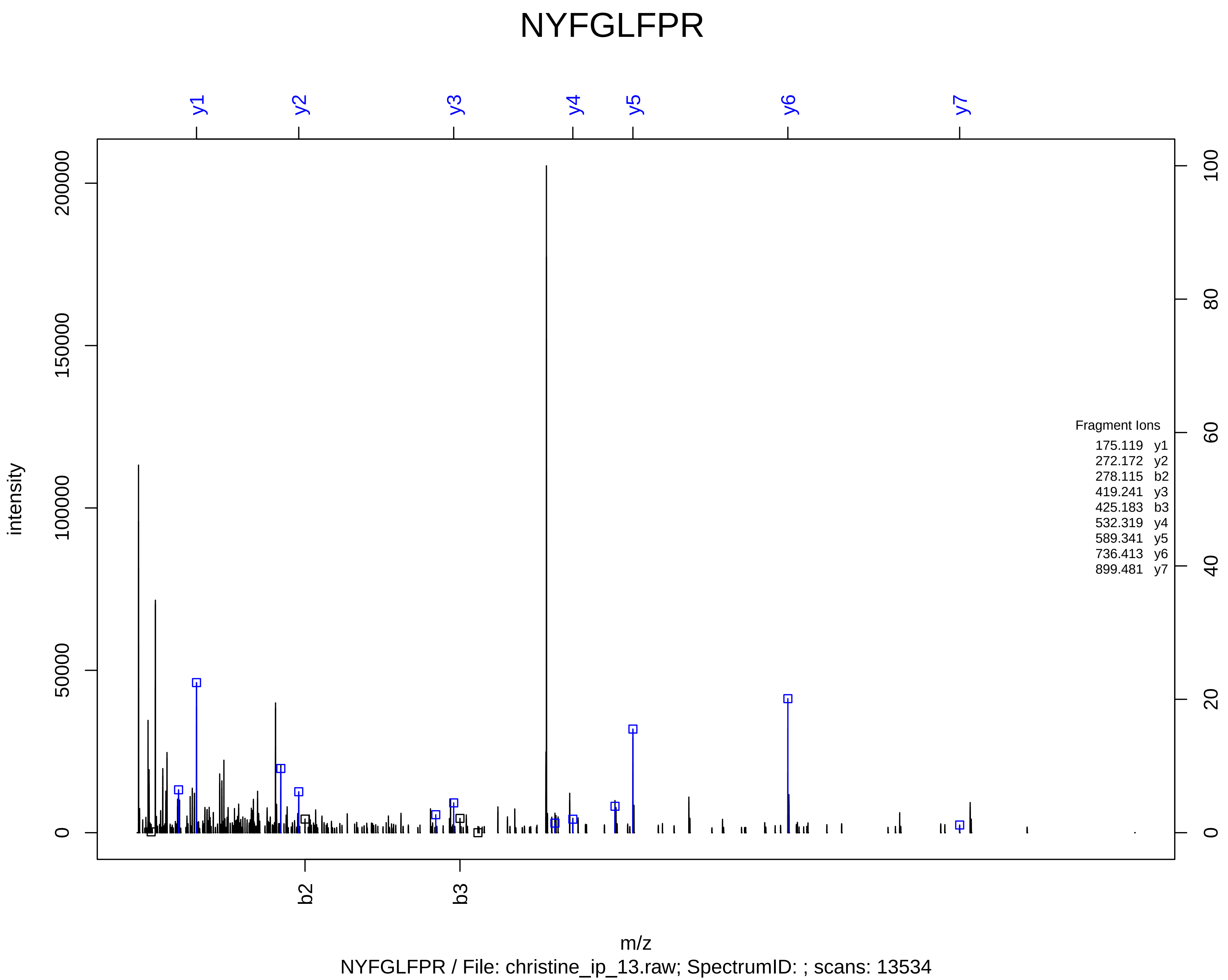

NYFGLFPR
y1
y2
y3
y4
y5
y6
y7
intensity
200000
150000
100000
50000
0
100
80
60
40
20
0
Fragment Ions
175.119 y1
272.172 y2
278.115 b2
419.241 y3
425.183 b3
532.319 y4
589.341 y5
736.413 y6
899.481 y7
b2
b3
m/z
NYFGLFPR / File: christine_ip_13.raw; SpectrumID: ; scans: 13534

SSPSGPSNPSNPSVEEK

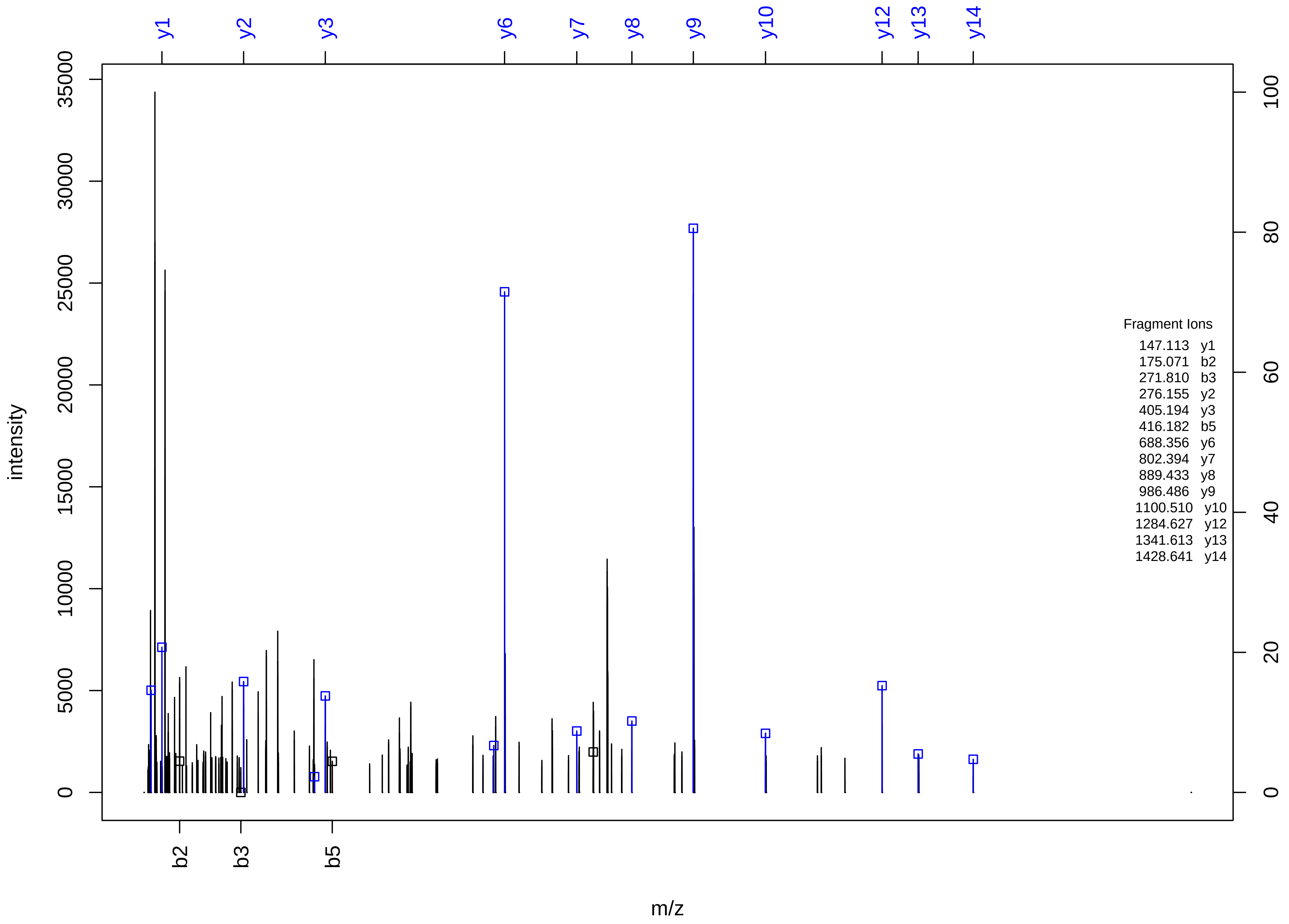

SSPSGPSNPSNPSVEEK / File: christine_ip_13.raw; SpectrumID: ; scans: 1806

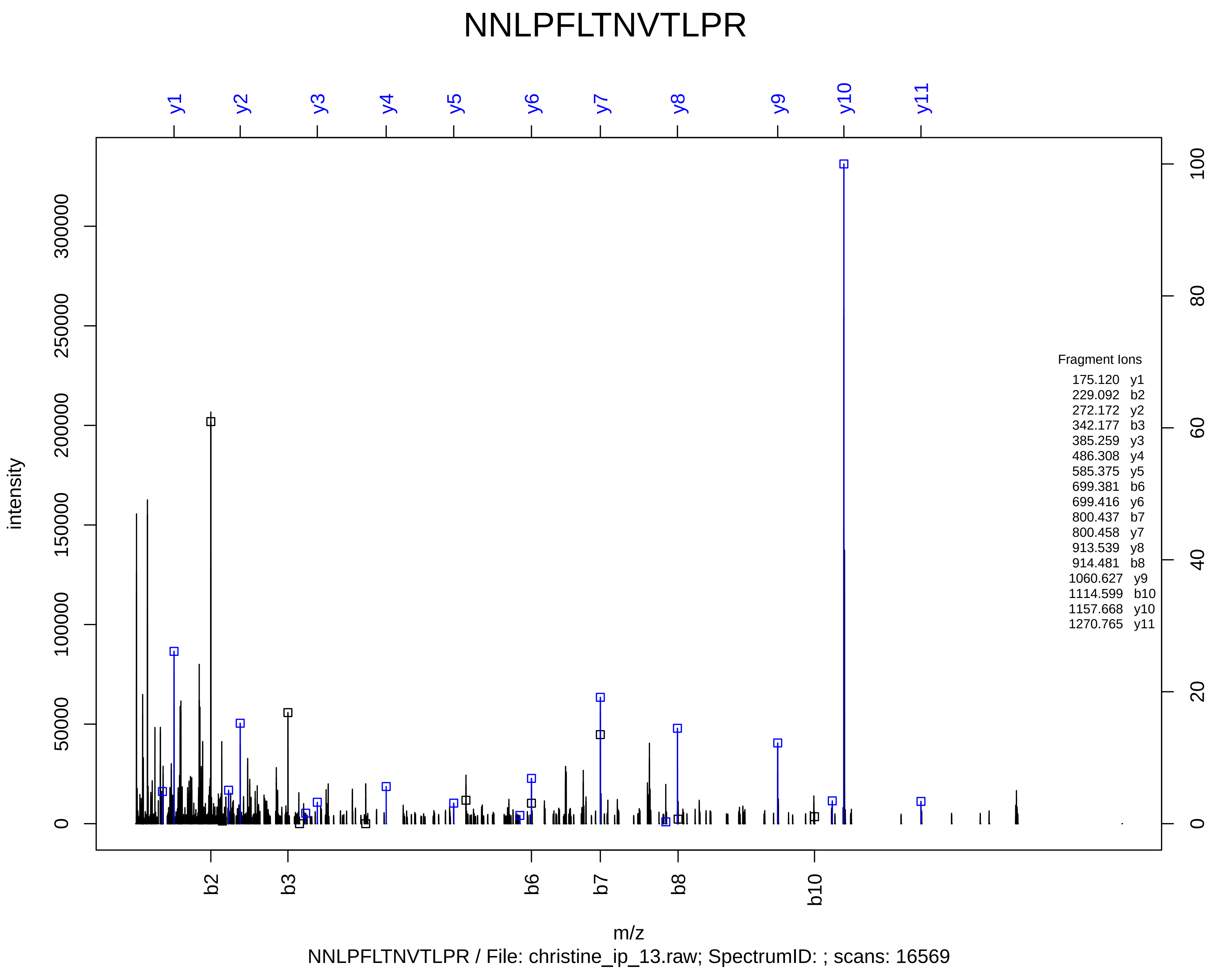
NNLPFLTNVTLPR
y1
y2
y3
y4
y5
y6
y7
y8
y9
y10
y11
intensity
0
50000
100000
150000
200000
250000
300000
0
20
40
60
80
100
b2
b3
b6
b7
b8
b10
m/z
Fragment Ions
175.120 y1
229.092 b2
272.172 y2
342.177 b3
385.259 y3
486.308 y4
585.375 y5
699.381 b6
699.416 y6
800.437 b7
800.458 y7
913.539 y8
914.481 b8
1060.627 y9
1114.599 b10
1157.668 y10
1270.765 y11
NNLPFLTNVTLPR / File: christine_ip_13.raw; SpectrumID: ; scans: 16569

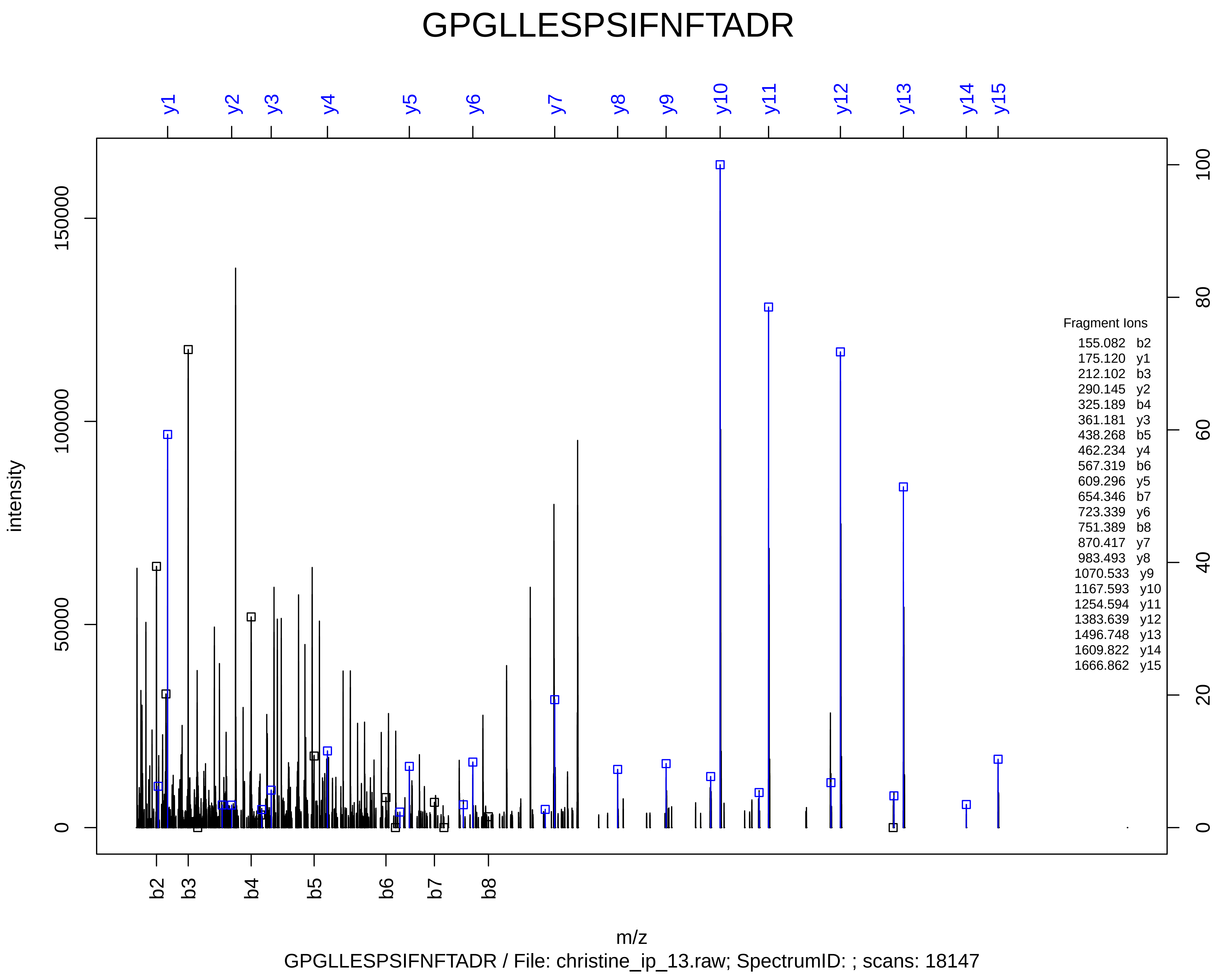
GPGLLESPSIFNFTADR
y1
y2
y3
y4
y5
y6
y7
y8
y9
y10
y11
y12
y13
y14
y15
intensity
0
50000
100000
150000
0
20
40
60
80
100
b2
b3
b4
b5
b6
b7
b8
Fragment Ions
155.082 b2
175.120 y1
212.102 b3
290.145 y2
325.189 b4
361.181 y3
438.268 b5
462.234 y4
567.319 b6
609.296 y5
654.346 b7
723.339 y6
751.389 b8
870.417 y7
983.493 y8
1070.533 y9
1167.593 y10
1254.594 y11
1383.639 y12
1496.748 y13
1609.822 y14
1666.862 y15
m/z
GPGLLESPSIFNFTADR / File: christine_ip_13.raw; SpectrumID: ; scans: 18147

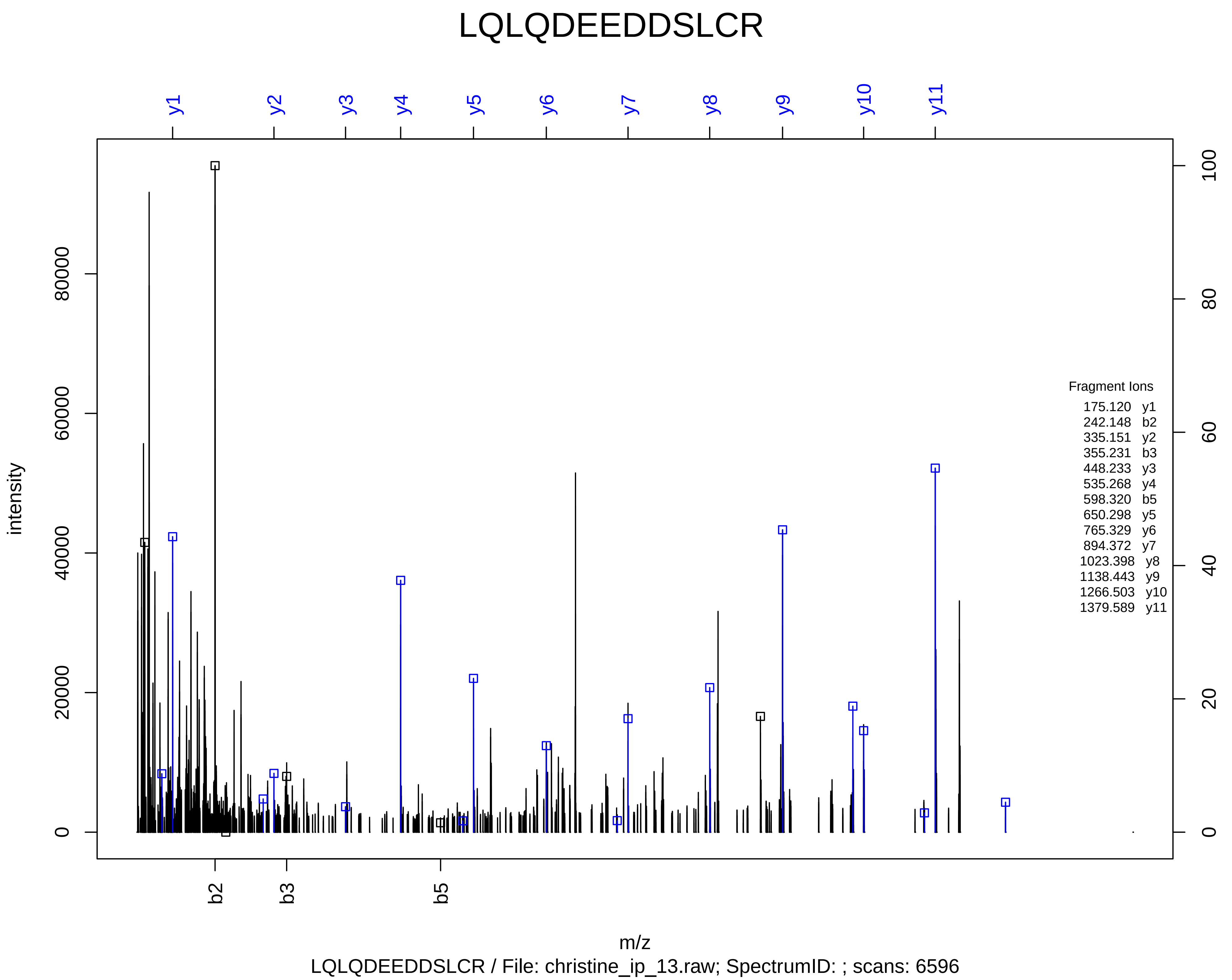

LQLQDEEDDSLCR
y1
y2
y3
y4
y5
y6
y7
y8
y9
y10
y11
b2
b3
b5
intensity
0
20000
40000
60000
80000
0
20
40
60
80
100
Fragment Ions
175.120 y1
242.148 b2
335.151 y2
355.231 b3
448.233 y3
535.268 y4
598.320 b5
650.298 y5
765.329 y6
894.372 y7
1023.398 y8
1138.443 y9
1266.503 y10
1379.589 y11
m/z
LQLQDEEDDSLCR / File: christine_ip_13.raw; SpectrumID: ; scans: 6596

APPNATLEHFYLTSGK

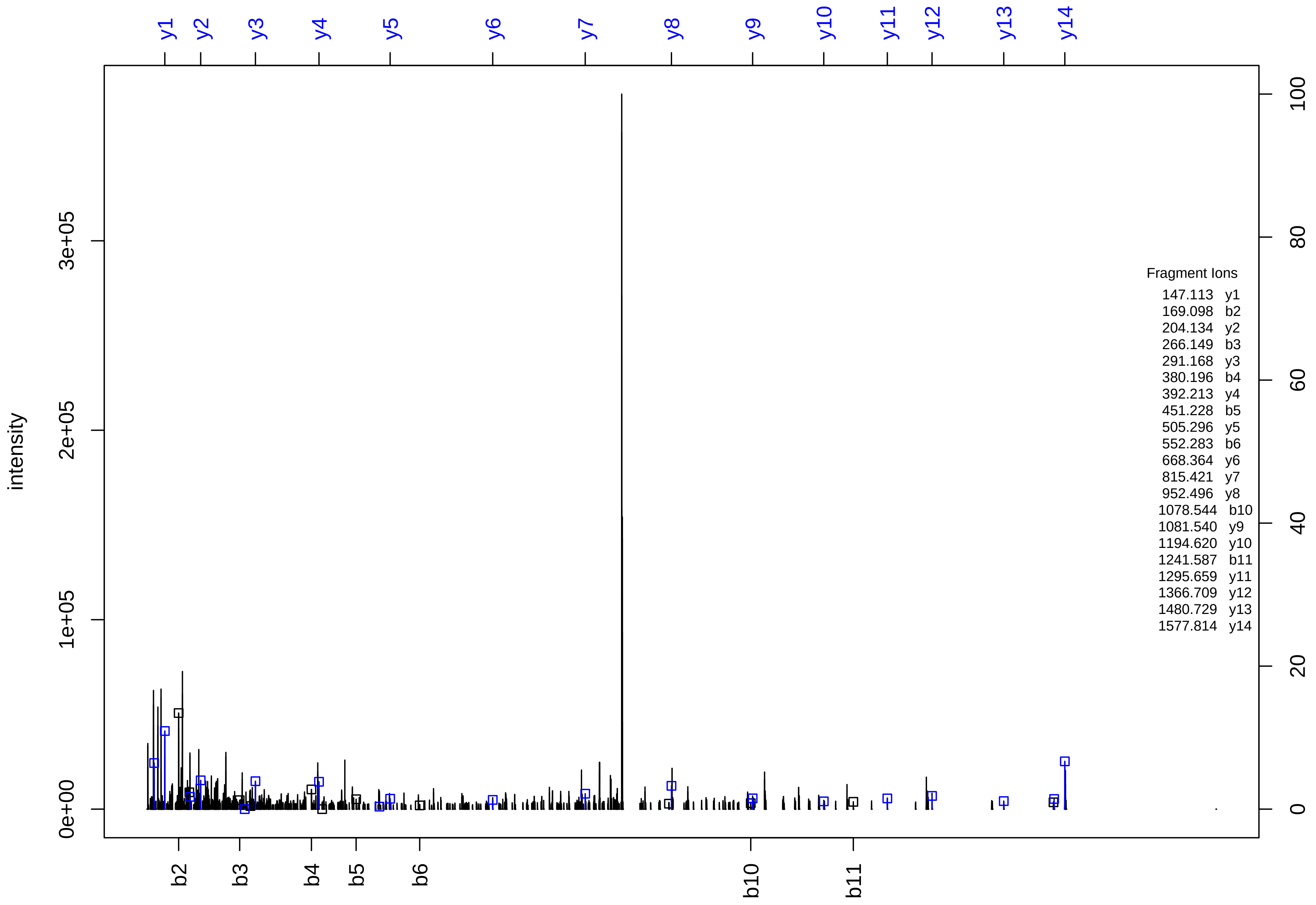

APPNATLEHFYLTSGK / File: christine_ip_13.raw; SpectrumID: ; scans: 9563

EHHN^GNFTDPSSVNEK

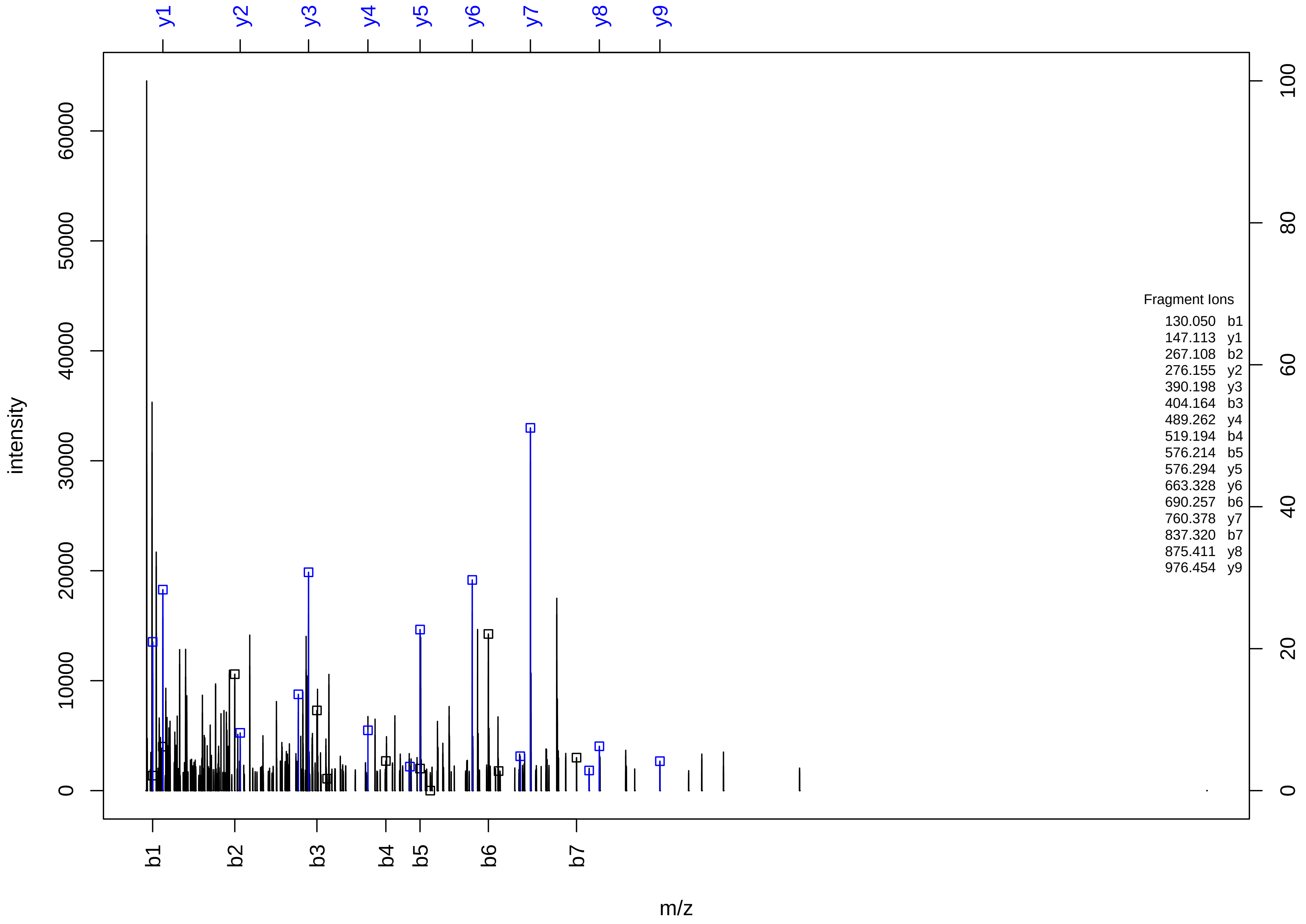

EHHNGNFTDPSSVNEK / File: christine_ip_13.raw; SpectrumID: ; scans: 1928

LLEATAKPEPEEK

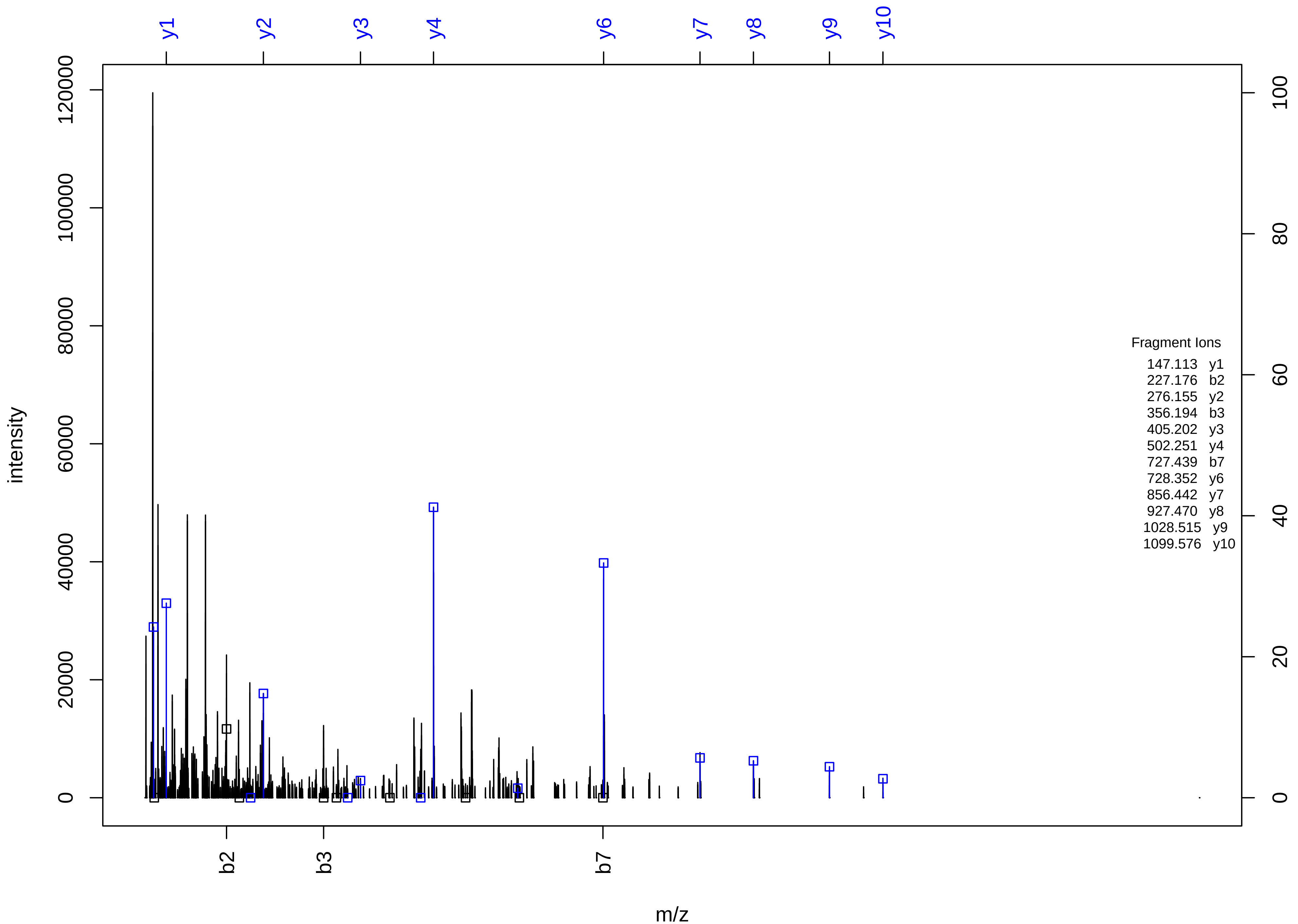

LLEATAKPEPEEK / File: christine_ip_13.raw; SpectrumID: ; scans: 2746

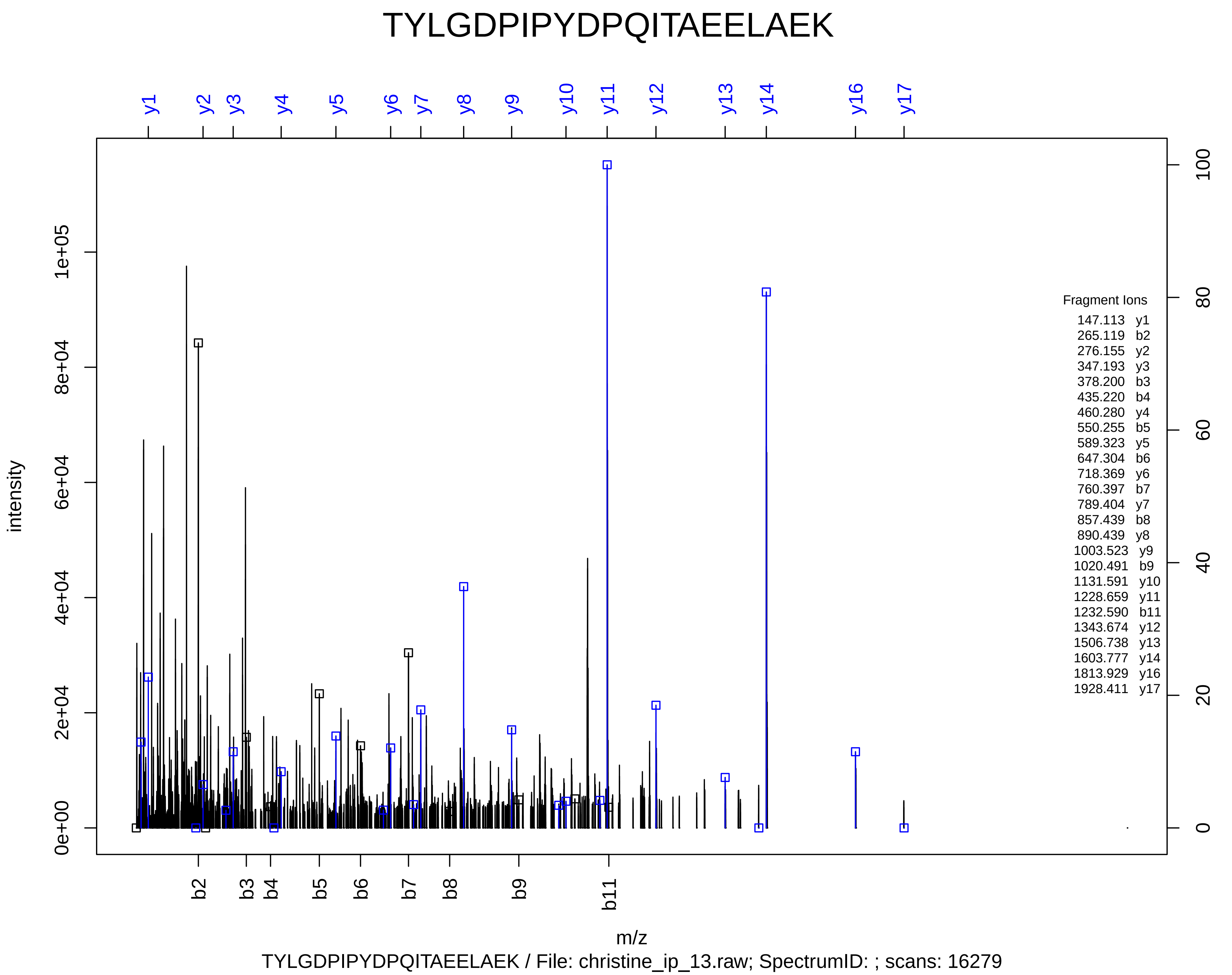

TYLGDPIPYDPQITAEELAEK
y1
y2
y3
y4
y5
y6
y7
y8
y9
y10
y11
y12
y13
y14
y16
y17
intensity
0e+00
2e+04
4e+04
6e+04
8e+04
1e+05
0
20
40
60
80
100
Fragment Ions
147.113 y1
265.119 b2
276.155 y2
347.193 y3
378.200 b3
435.220 b4
460.280 y4
550.255 b5
589.323 y5
647.304 b6
718.369 y6
760.397 b7
789.404 y7
857.439 b8
890.439 y8
1003.523 y9
1020.491 b9
1131.591 y10
1228.659 y11
1232.590 b11
1343.674 y12
1506.738 y13
1603.777 y14
1813.929 y16
1928.411 y17
b2
b3
b4
b5
b6
b7
b8
b9
b11
m/z
TYLGDPIPYDPQITAEELAEK / File: christine_ip_13.raw; SpectrumID: ; scans: 16279

YLTGTYVQEESPEGGR

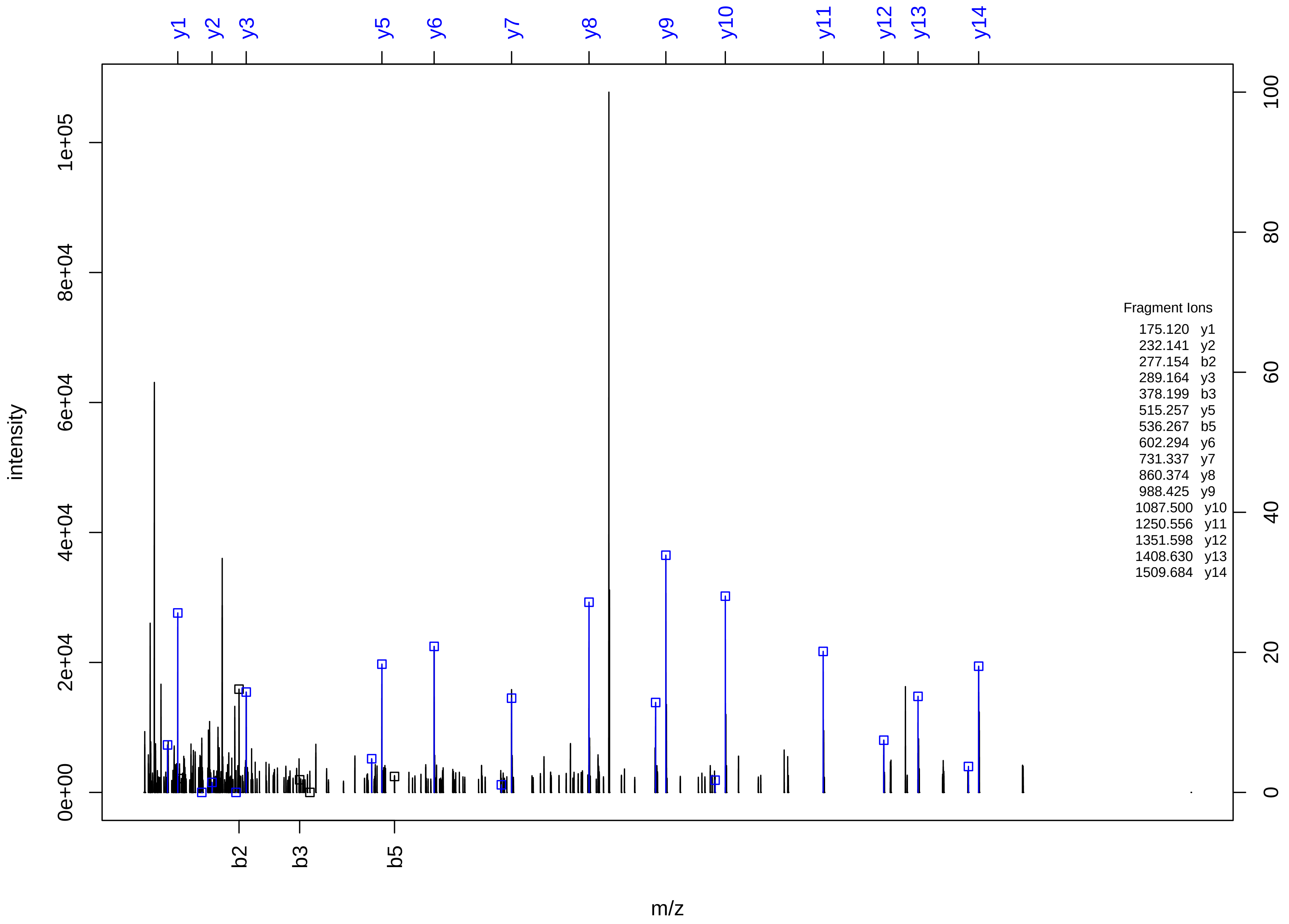

YLTGTYVQEESPEGGR / File: christine_ip_13.raw; SpectrumID: ; scans: 6130

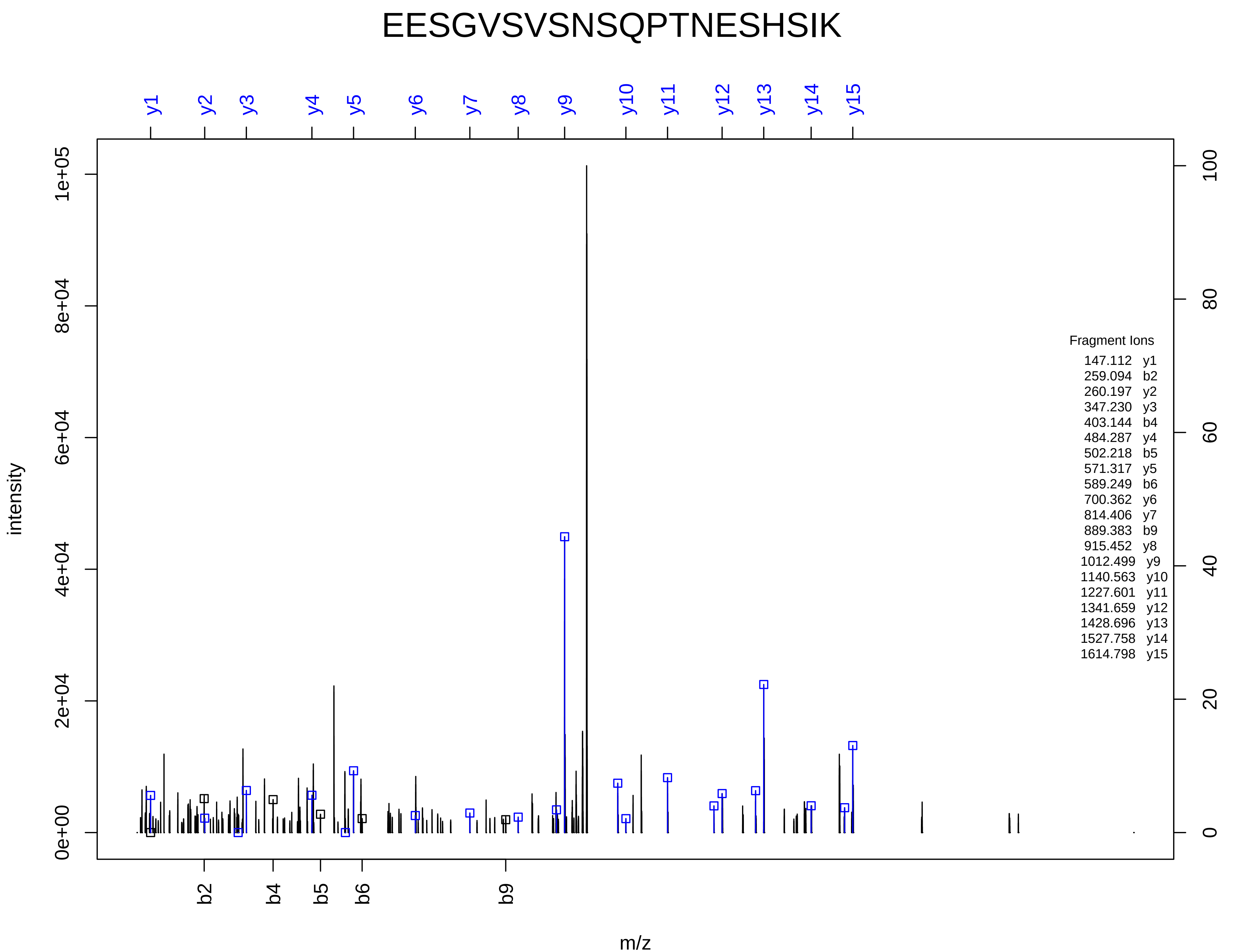

EESGVSVSNSQPTNESHSIK / File: christine_ip_13.raw; SpectrumID: ; scans: 2481

NVQVLYSEQSPLSHDLILNLTQDGIK

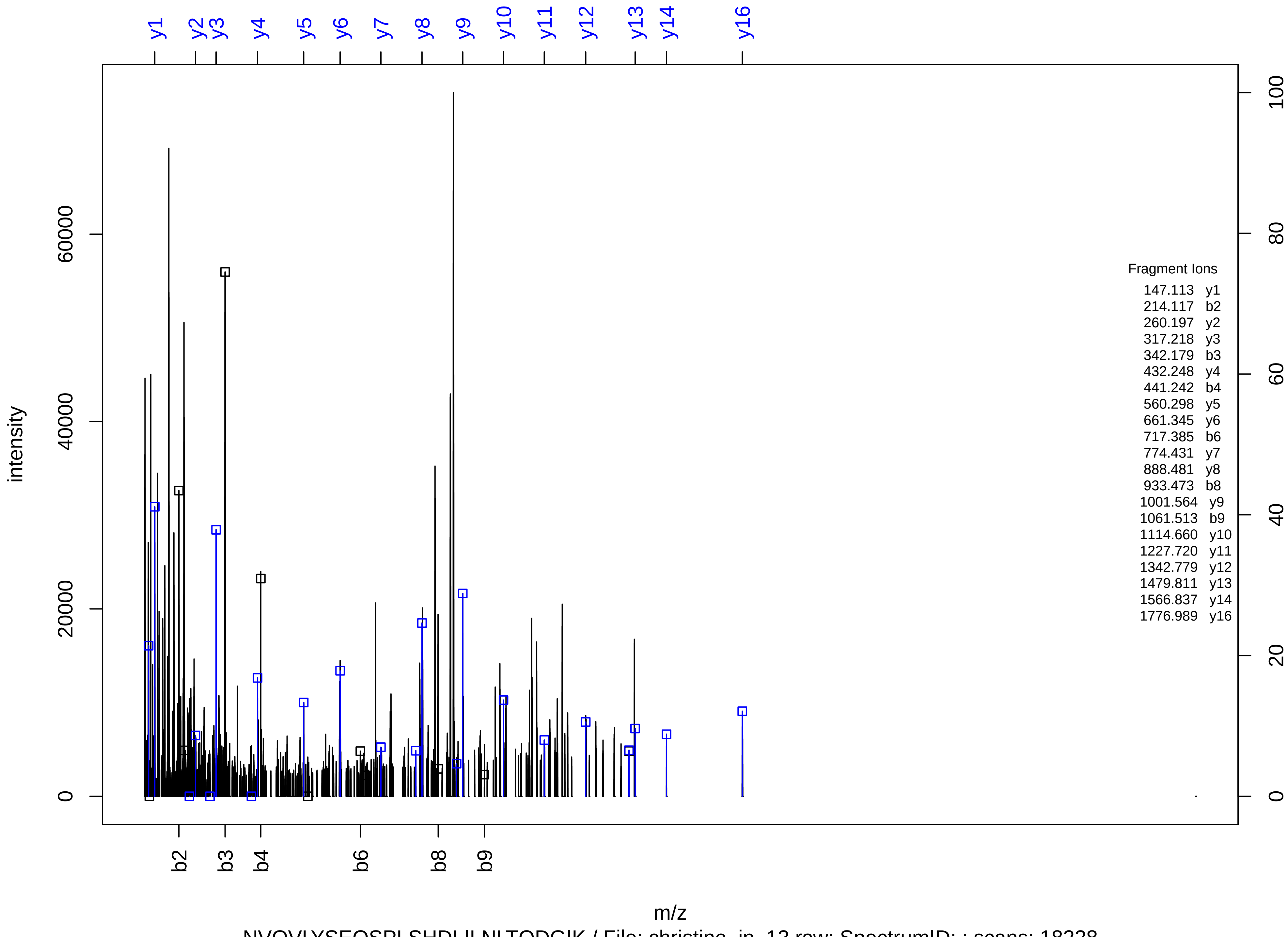

NVQVLYSEQSPLSHDLILNLTQDGIK / File: christine_ip_13.raw; SpectrumID: ; scans: 18228

REAPVDVLTQIGR

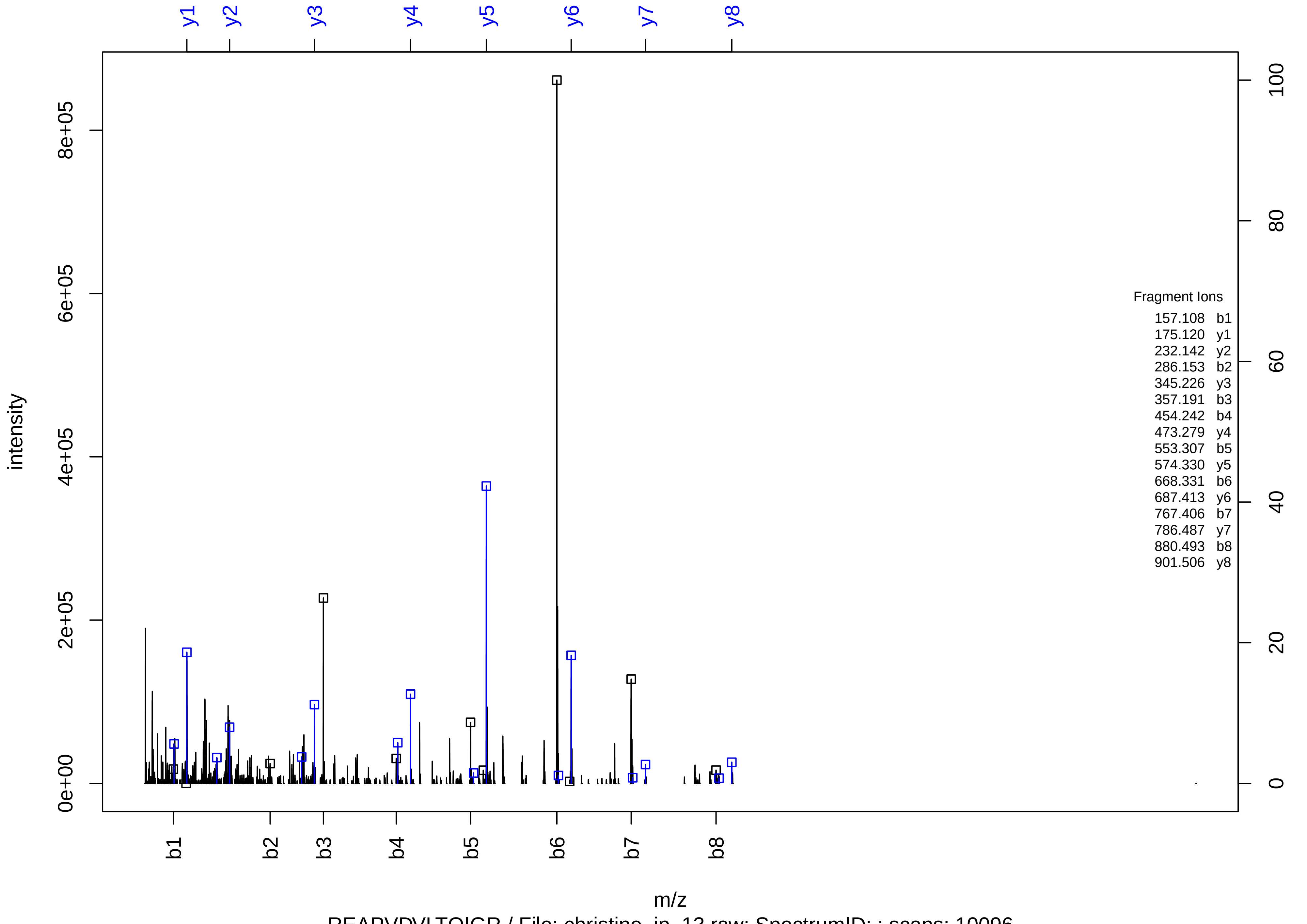

REAPVDVLTQIGR / File: christine_ip_13.raw; SpectrumID: ; scans: 10096

LLADVTHLLLK

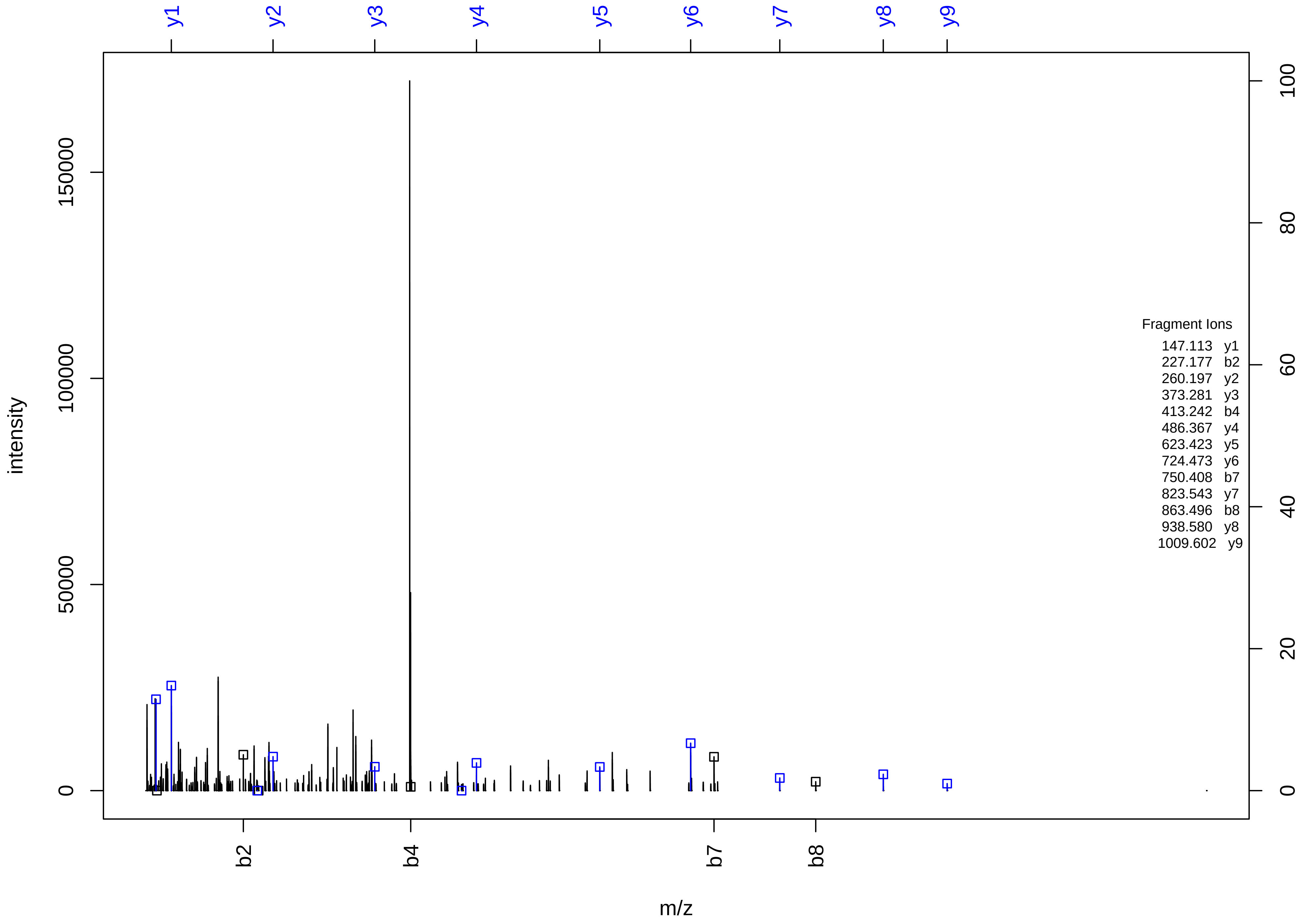

LLADVTHLLLK / File: christine_ip_13.raw; SpectrumID: ; scans: 15167

EPPAPAQQLQPQPVAVQGPEPAR

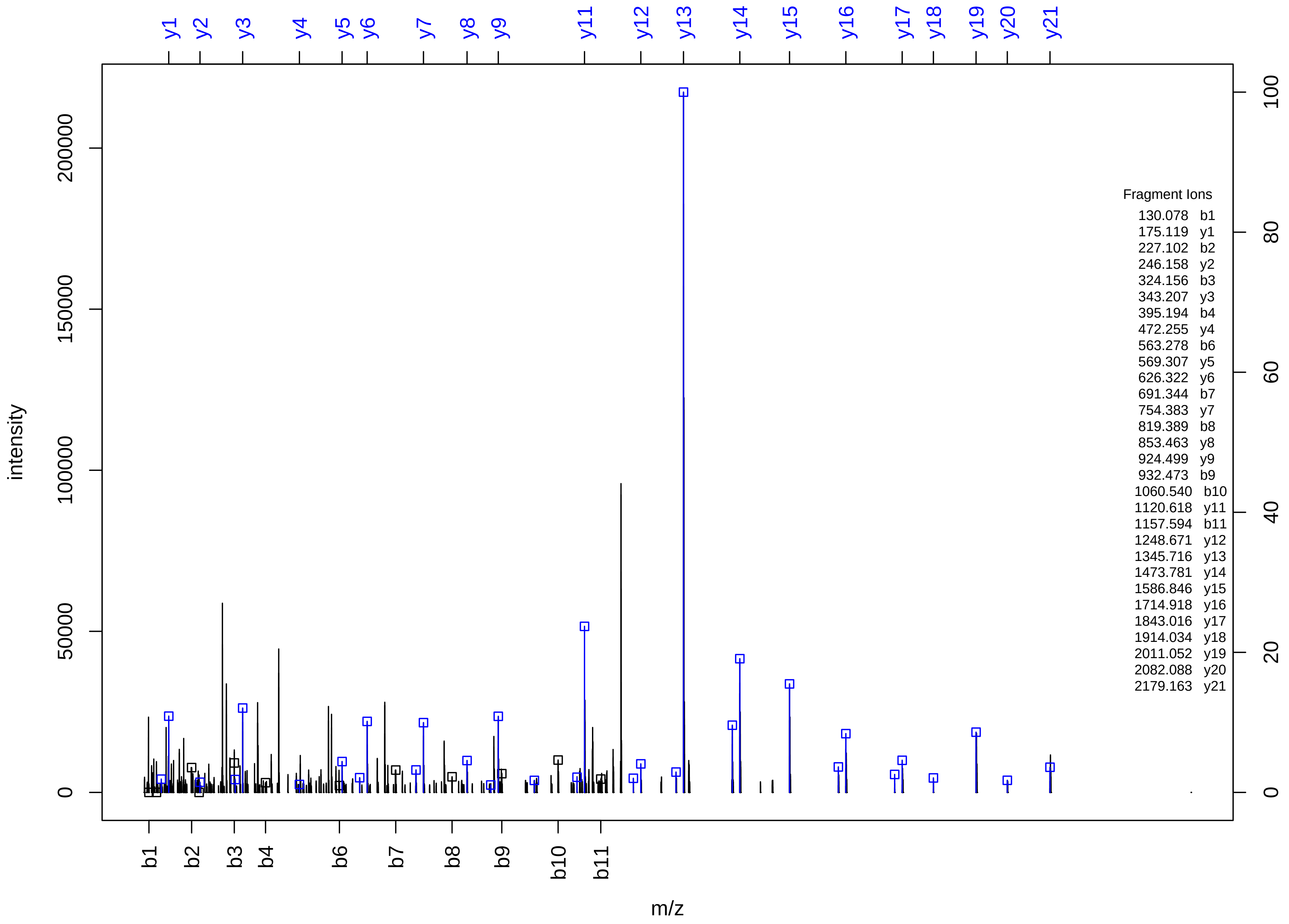

EPPAPAQQLQPQPVAVQGPEPAR / File: christine_ip_13.raw; SpectrumID: ; scans: 8549

GLIPVFALGR

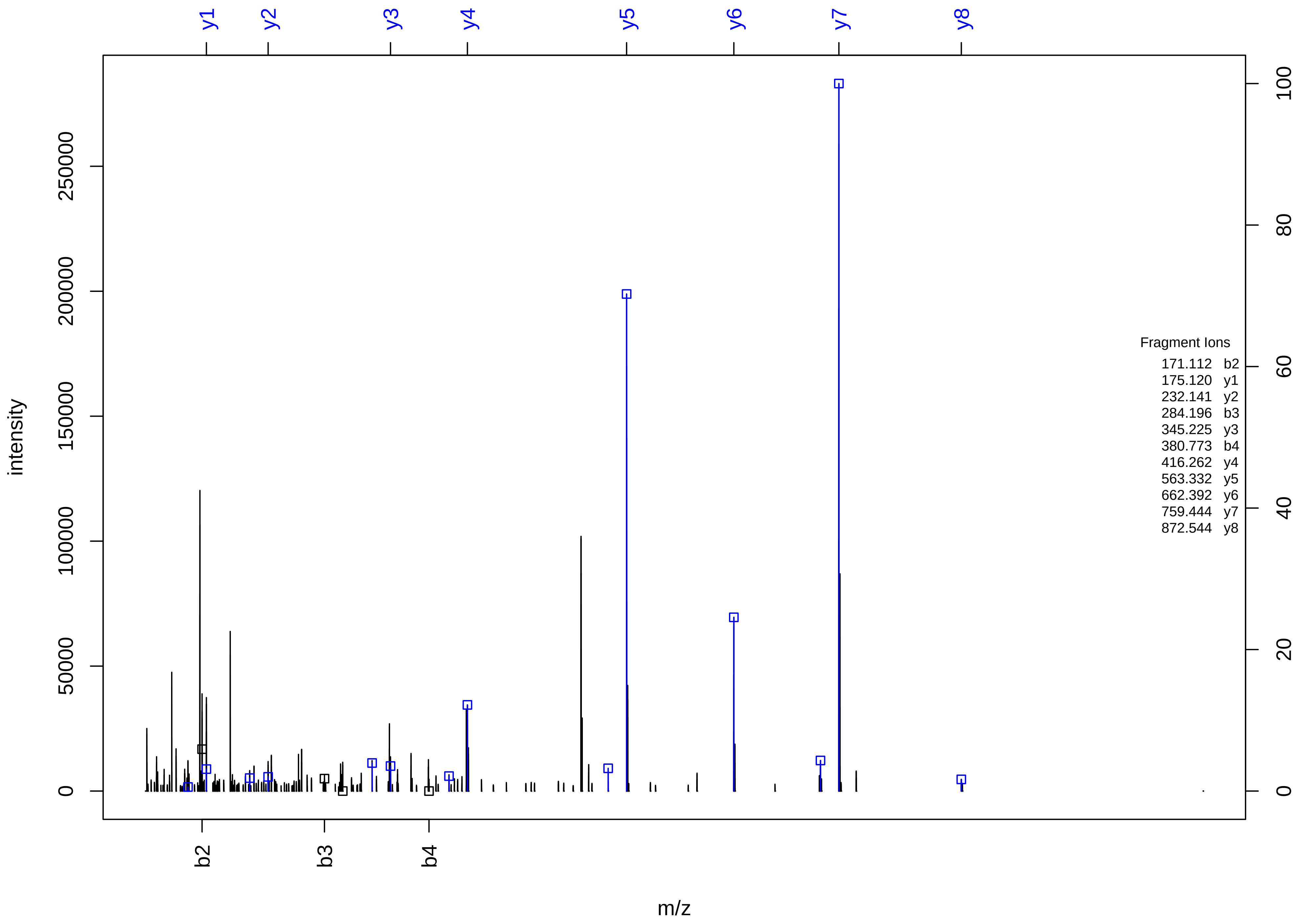

GLIPVFALGR / File: christine_ip_13.raw; SpectrumID: ; scans: 17173

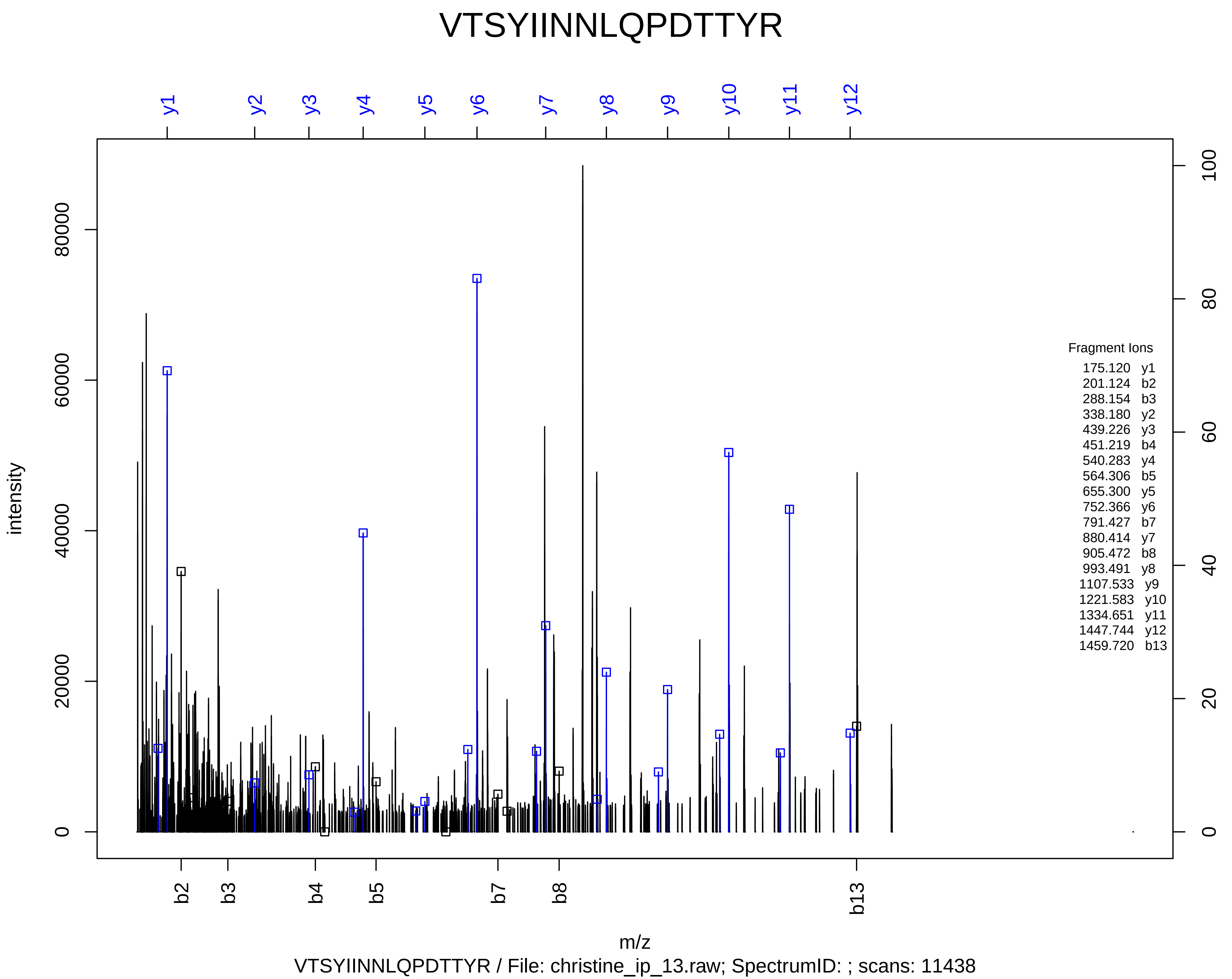
VTSYIINNLQPDTTYR
y1
y2
y3
y4
y5
y6
y7
y8
y9
y10
y11
y12
intensity
0
20000
40000
60000
80000
0
20
40
60
80
100
Fragment Ions
175.120 y1
201.124 b2
288.154 b3
338.180 y2
439.226 y3
451.219 b4
540.283 y4
564.306 b5
655.300 y5
752.366 y6
791.427 b7
880.414 y7
905.472 b8
993.491 y8
1107.533 y9
1221.583 y10
1334.651 y11
1447.744 y12
1459.720 b13
b2
b3
b4
b5
b7
b8
b13
m/z
VTSYIINNLQPDTTYR / File: christine_ip_13.raw; SpectrumID: ; scans: 11438

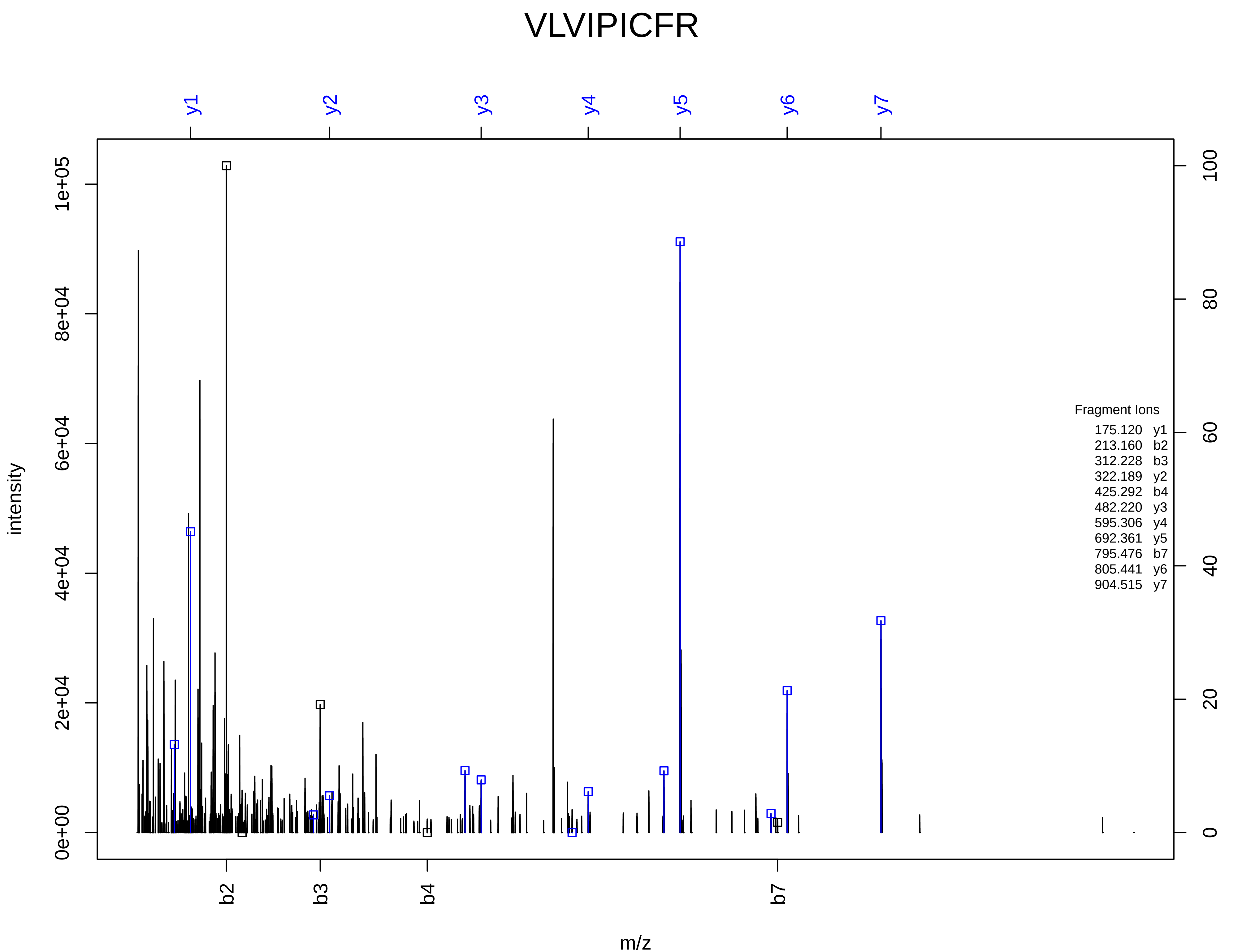

VLVIPICFR
y1
y2
y3
y4
y5
y6
y7
intensity
0e+00
2e+04
4e+04
6e+04
8e+04
1e+05
0
20
40
60
80
100
Fragment Ions
175.120 y1
213.160 b2
312.228 b3
322.189 y2
425.292 b4
482.220 y3
595.306 y4
692.361 y5
795.476 b7
805.441 y6
904.515 y7
b2
b3
b4
b7
m/z
VLVIPICFR / File: christine_ip_13.raw; SpectrumID: ; scans: 16699

AGDNIPEEQPVASTPTTVSDGENKK

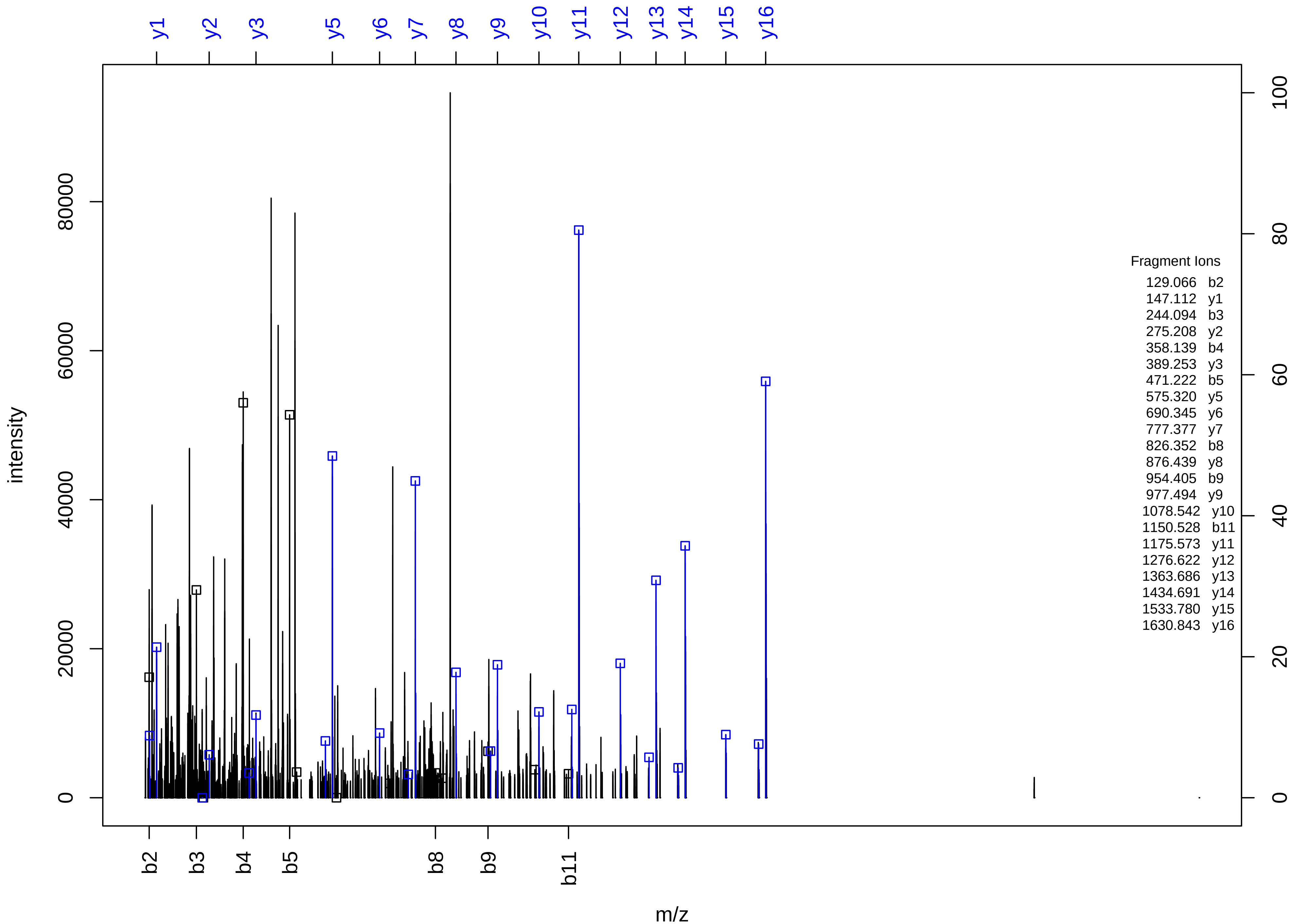

AGDNIPEEQPVASTPTTVSDGENKK / File: christine_ip_13.raw; SpectrumID: ; scans: 4996

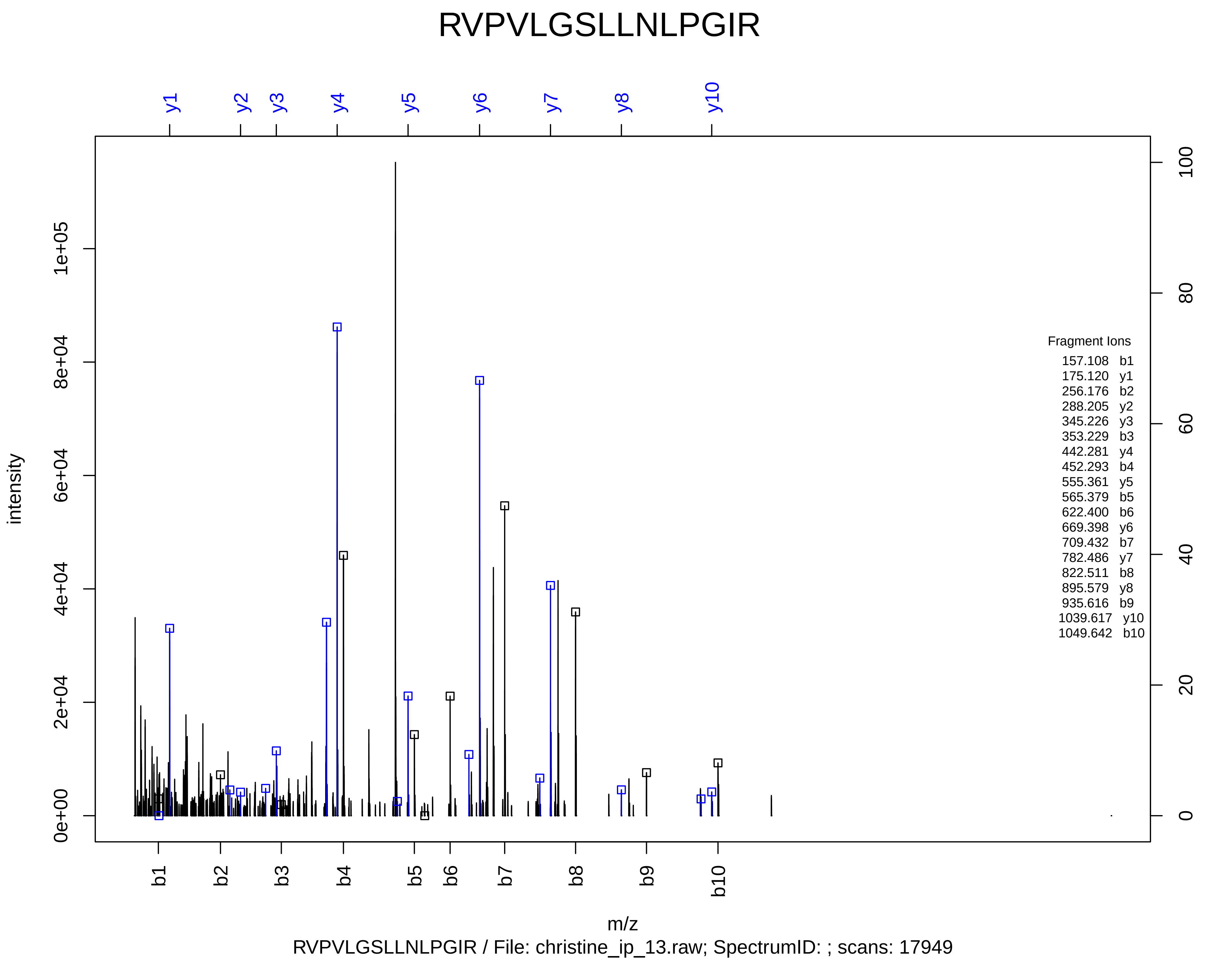

RVPVLGSLLNLPGIR
y1
y2
y3
y4
y5
y6
y7
y8
y10
intensity
0e+00
2e+04
4e+04
6e+04
8e+04
1e+05
0
20
40
60
80
100
Fragment Ions
157.108 b1
175.120 y1
256.176 b2
288.205 y2
345.226 y3
353.229 b3
442.281 y4
452.293 b4
555.361 y5
565.379 b5
622.400 b6
669.398 y6
709.432 b7
782.486 y7
822.511 b8
895.579 y8
935.616 b9
1039.617 y10
1049.642 b10
b1
b2
b3
b4
b5
b6
b7
b8
b9
b10
m/z
RVPVLGSLLNLPGIR / File: christine_ip_13.raw; SpectrumID: ; scans: 17949

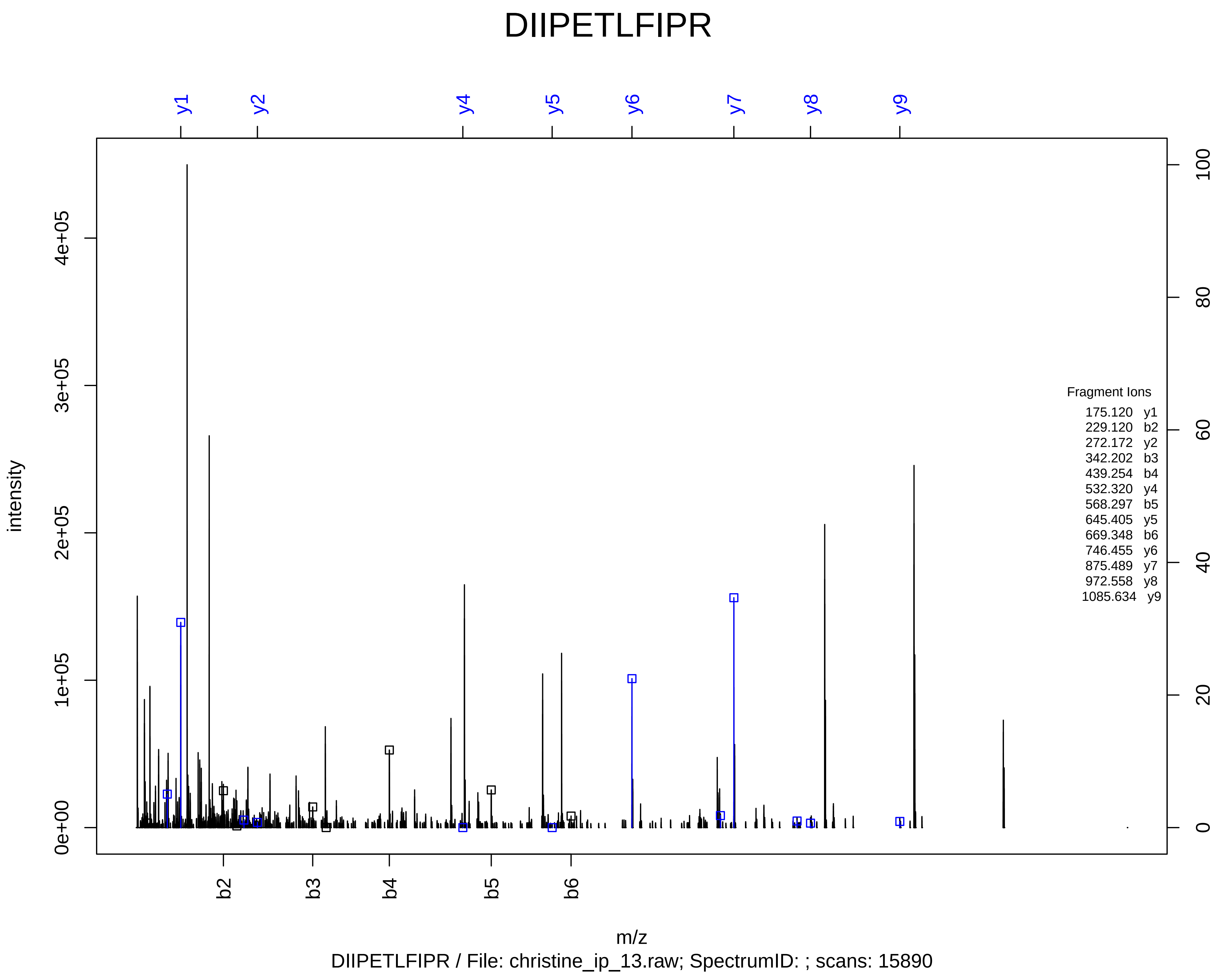
DIIPETLFIPR
y1
y2
y4
y5
y6
y7
y8
y9
b2
b3
b4
b5
b6
intensity
0e+00
1e+05
2e+05
3e+05
4e+05
0
20
40
60
80
100
m/z
Fragment Ions
175.120 y1
229.120 b2
272.172 y2
342.202 b3
439.254 b4
532.320 y4
568.297 b5
645.405 y5
669.348 b6
746.455 y6
875.489 y7
972.558 y8
1085.634 y9
DIIPETLFIPR / File: christine_ip_13.raw; SpectrumID: ; scans: 15890

ESLCDSPHQNLSR

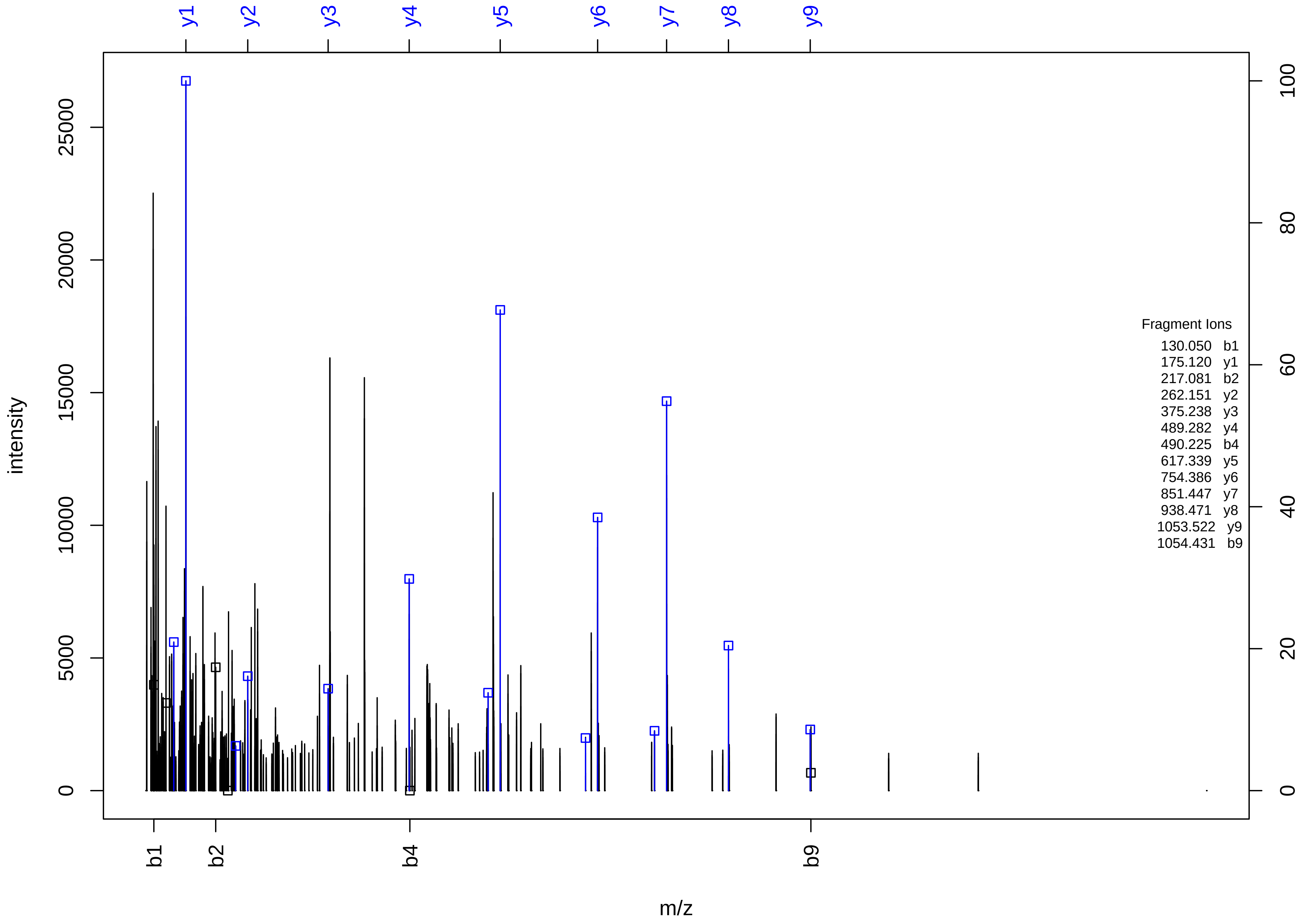

ESLCDSPHQNLSR / File: christine_ip_13.raw; SpectrumID: ; scans: 2216

IAPGAVVCVESEIR

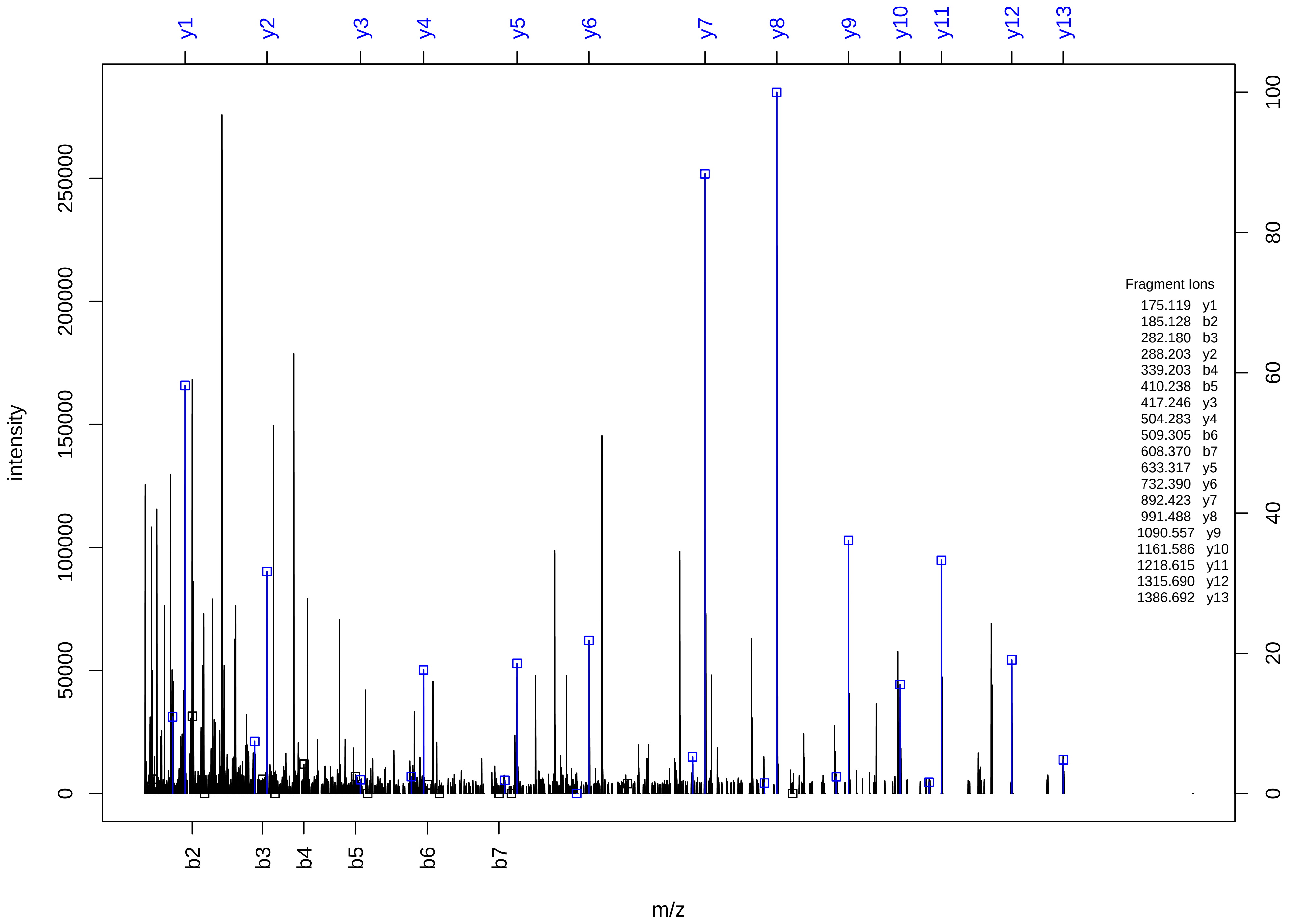

IAPGAVVCVESEIR / File: christine_ip_12.raw; SpectrumID: ; scans: 12016

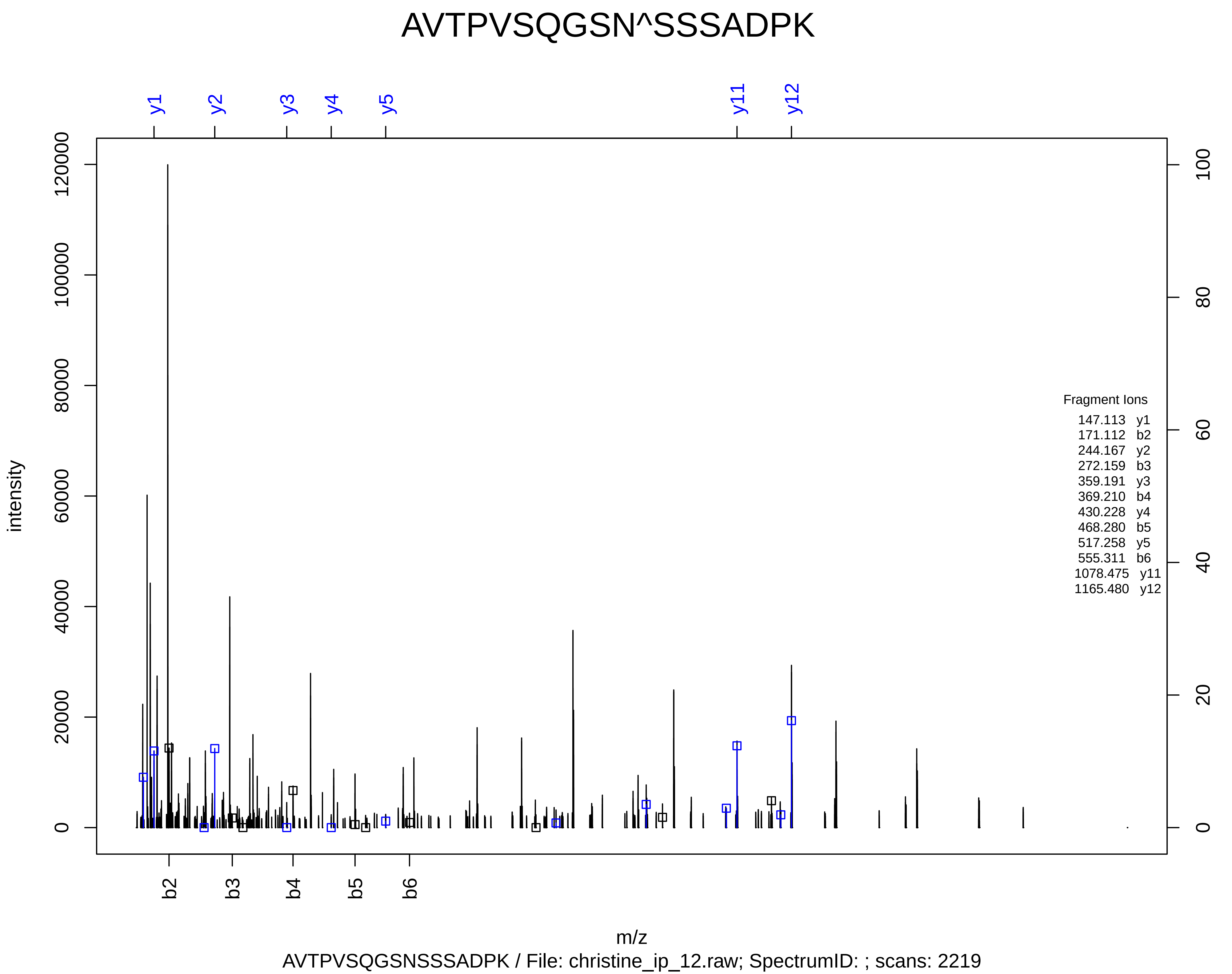

AVTPVSQGSN^SSSADPK
y1
y2
y3
y4
y5
y11
y12
intensity
0
20000
40000
60000
80000
100000
120000
0
20
40
60
80
100
b2
b3
b4
b5
b6
Fragment Ions
147.113 y1
171.112 b2
244.167 y2
272.159 b3
359.191 y3
369.210 b4
430.228 y4
468.280 b5
517.258 y5
555.311 b6
1078.475 y11
1165.480 y12
m/z
AVTPVSQGSNSSSADPK / File: christine_ip_12.raw; SpectrumID: ; scans: 2219

AQQEFAAGVFSNPAVR

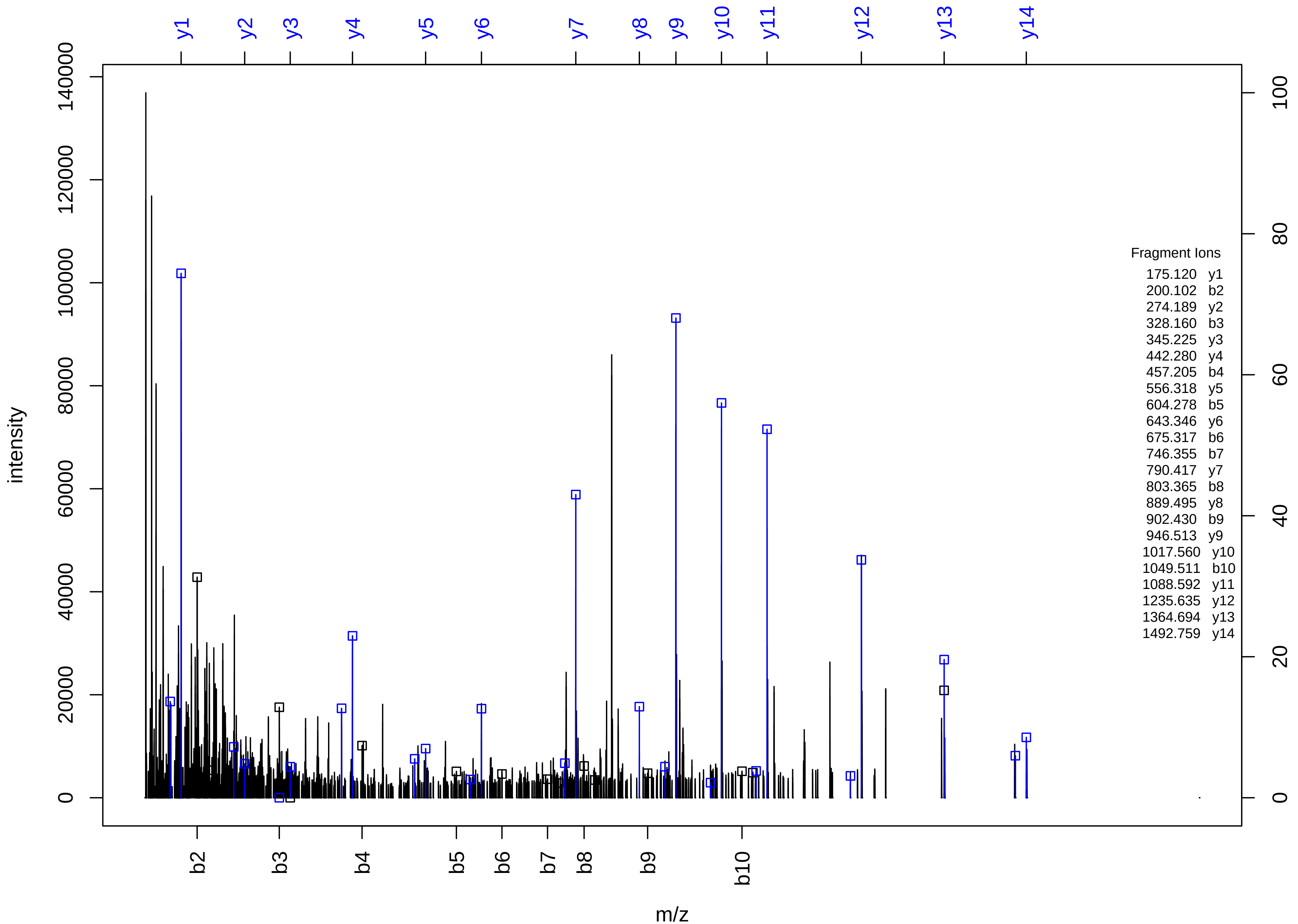

AQQEFAAGVFSNPAVR / File: christine_ip_12.raw; SpectrumID: ; scans: 12296

DTDIVDEAIYYFK

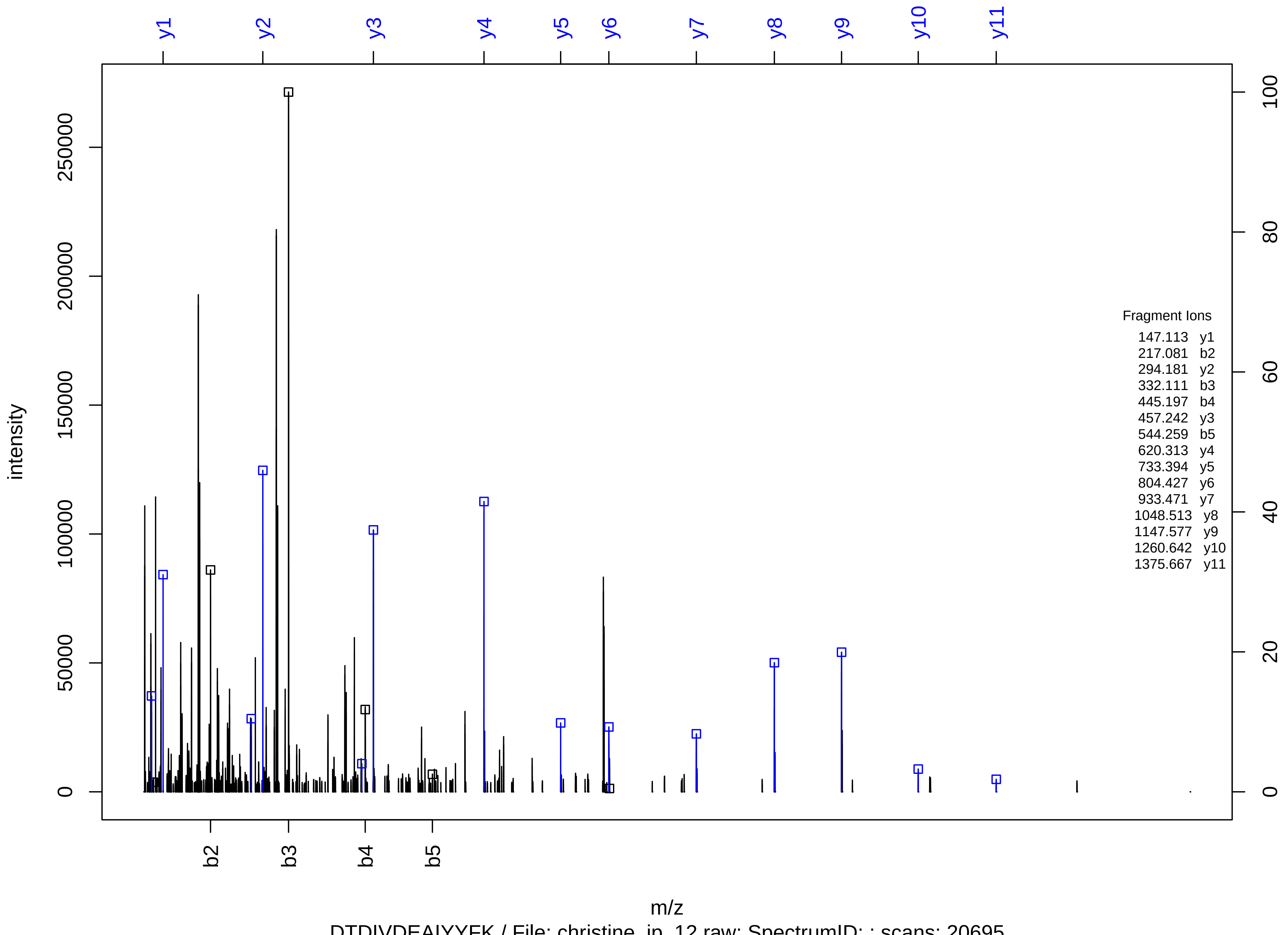

DTDIVDEAIYYFK / File: christine_ip_12.raw; SpectrumID: ; scans: 20695

LISSSQVDQETGFNR

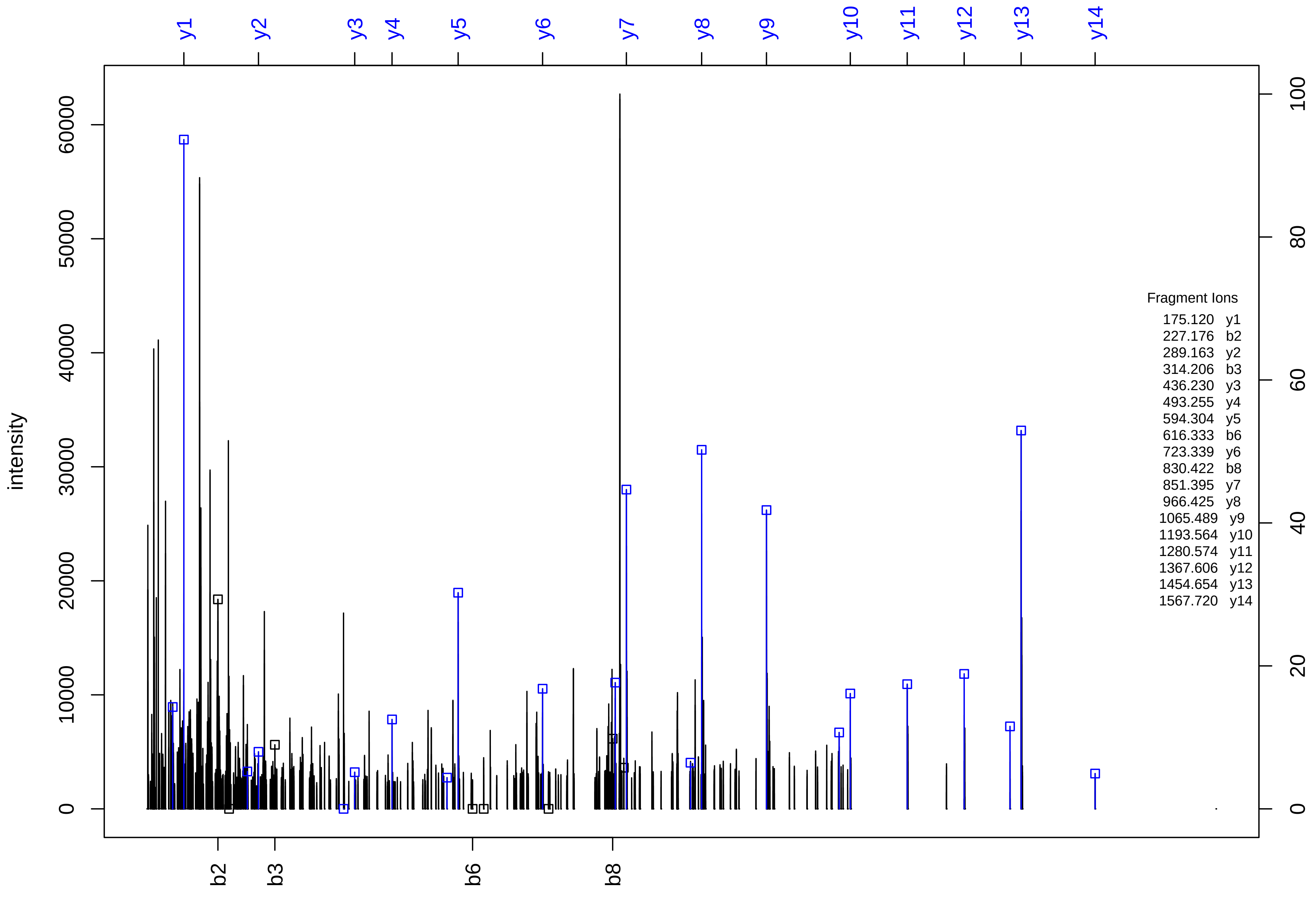

LISSSQVDQETGFNR / File: christine_ip_12.raw; SpectrumID: ; scans: 7391

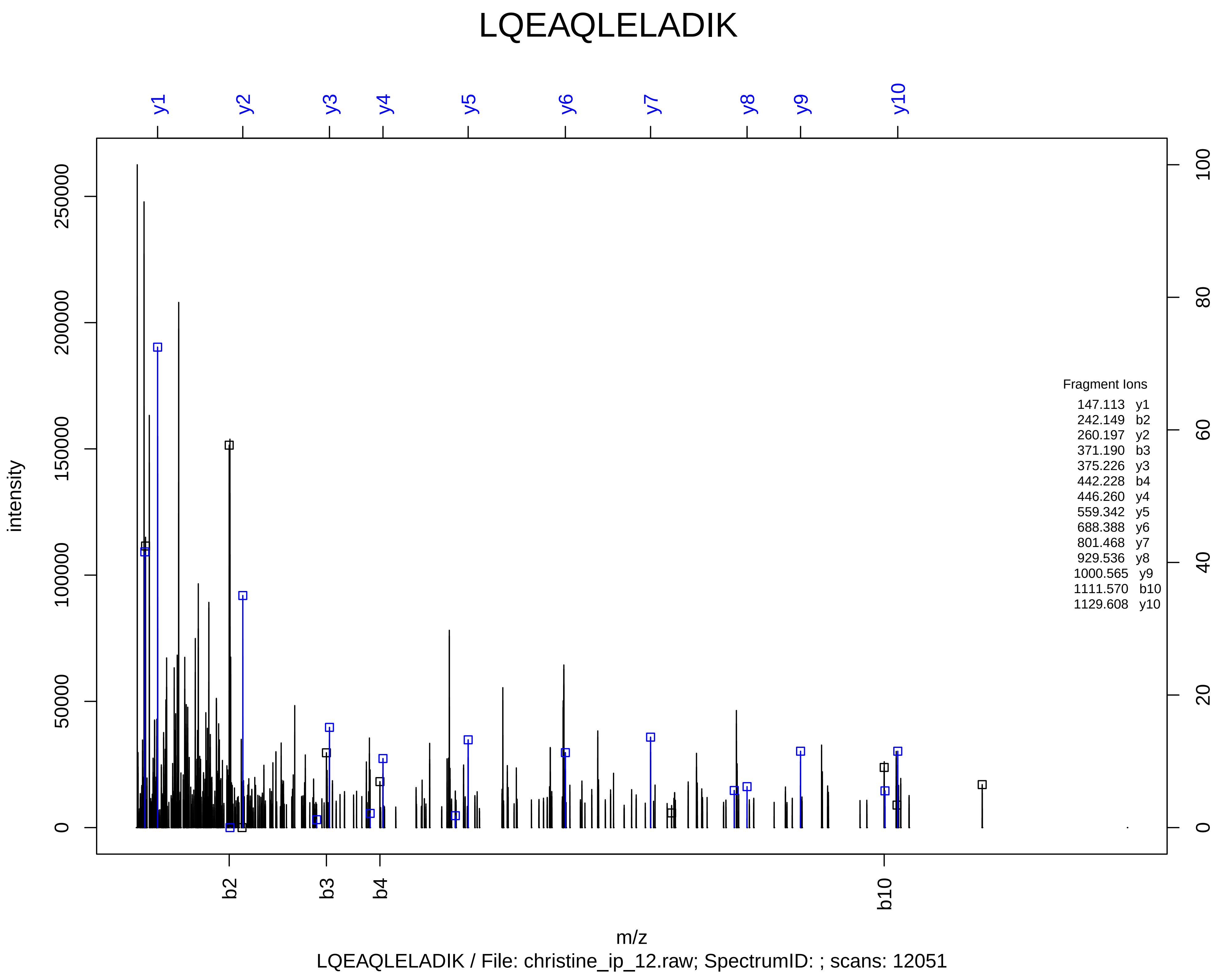
LQEAQLELADIK
y1
y2
y3
y4
y5
y6
y7
y8
y9
y10
intensity
0
50000
100000
150000
200000
250000
0
20
40
60
80
100
Fragment Ions
147.113 y1
242.149 b2
260.197 y2
371.190 b3
375.226 y3
442.228 b4
446.260 y4
559.342 y5
688.388 y6
801.468 y7
929.536 y8
1000.565 y9
1111.570 b10
1129.608 y10
b2
b3
b4
b10
m/z
LQEAQLELADIK / File: christine_ip_12.raw; SpectrumID: ; scans: 12051

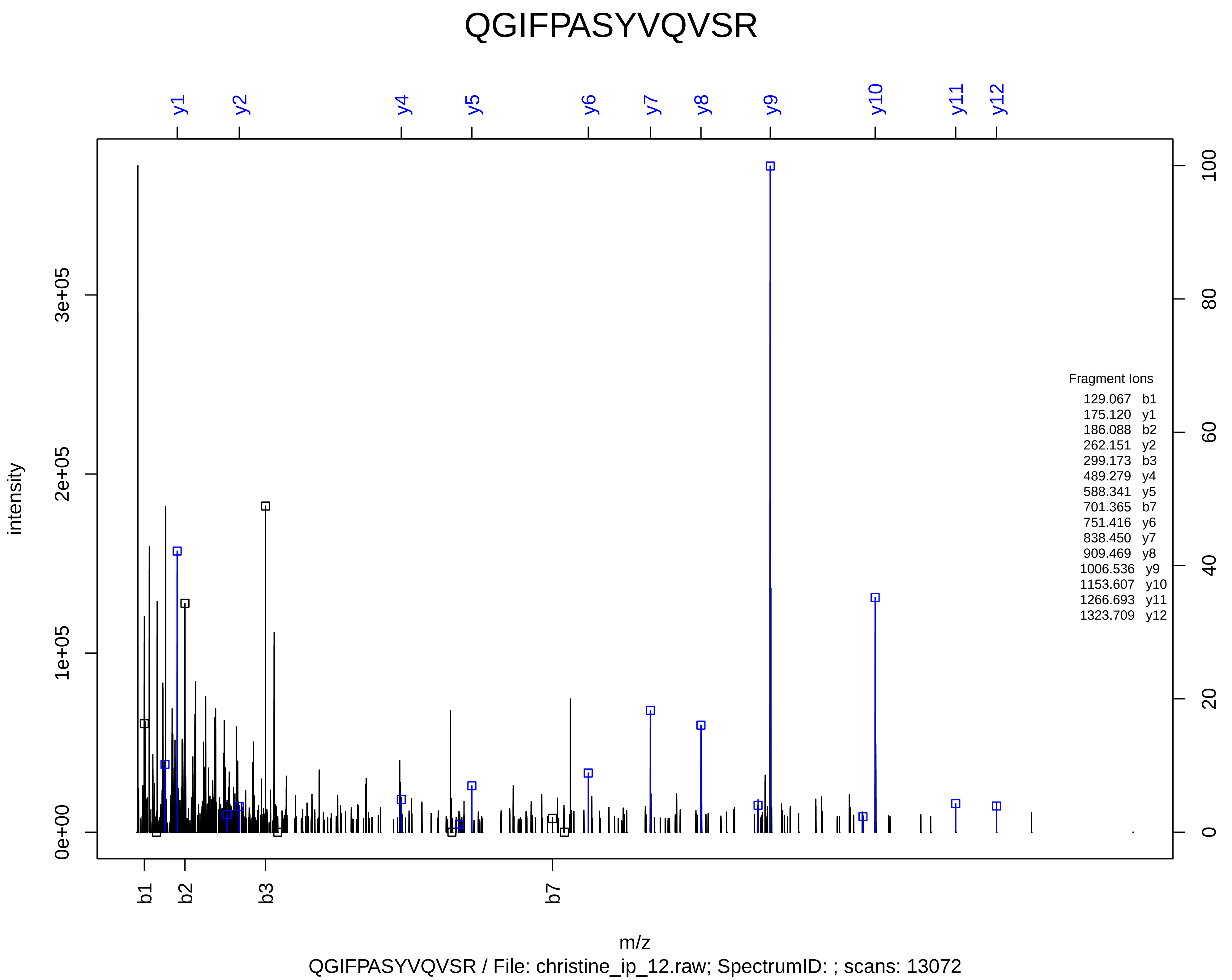
QGIFPASYVQVSR
y1
y2
y4
y5
y6
y7
y8
y9
y10
y11
y12
b1
b2
b3
b7
intensity
m/z
Fragment Ions
129.067 b1
175.120 y1
186.088 b2
262.151 y2
299.173 b3
489.279 y4
588.341 y5
701.365 b7
751.416 y6
838.450 y7
909.469 y8
1006.536 y9
1153.607 y10
1266.693 y11
1323.709 y12
QGIFPASYVQVSR / File: christine_ip_12.raw; SpectrumID: ; scans: 13072

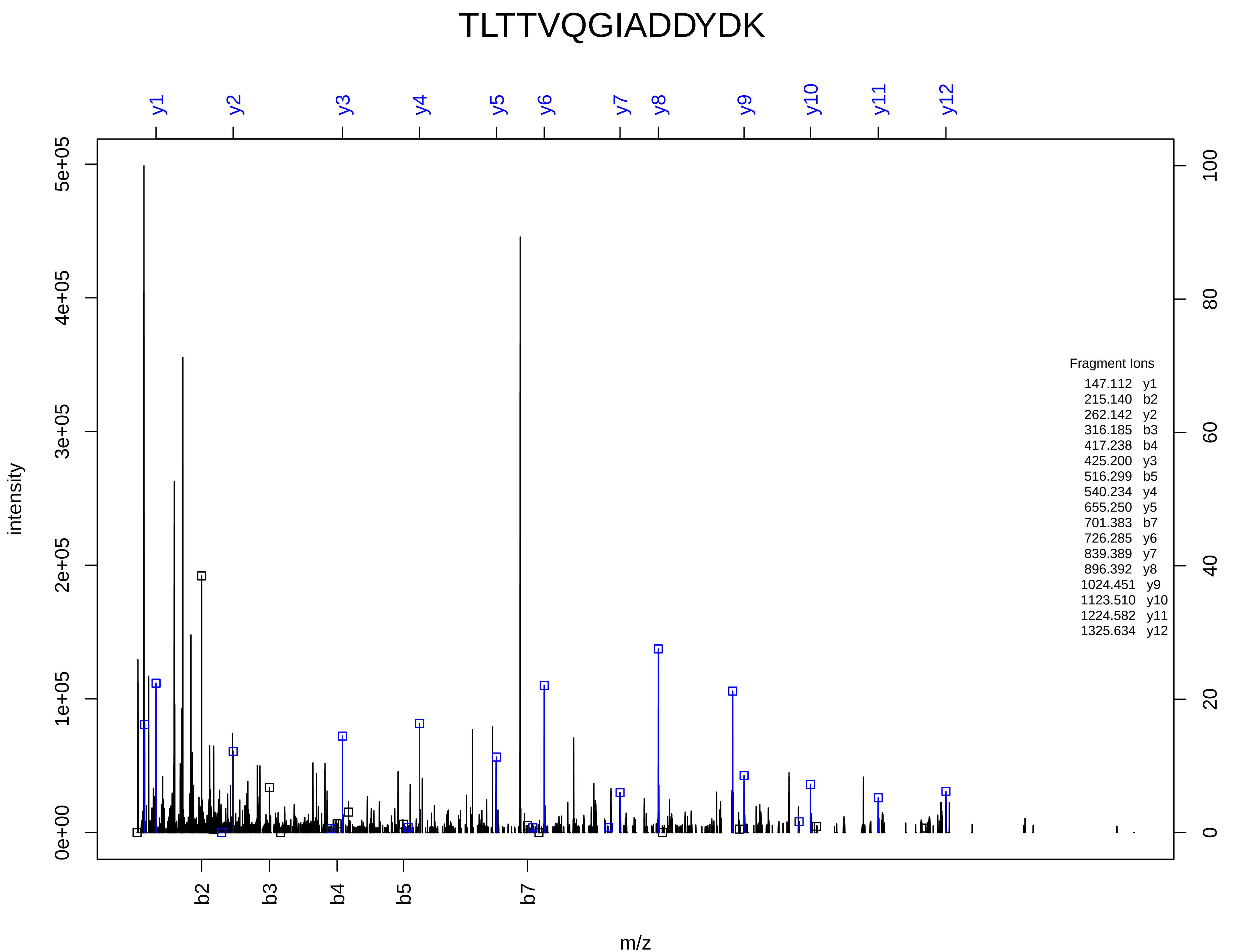

TLTTVQGIADDYDK / File: christine_ip_12.raw; SpectrumID: ; scans: 9905

HLEPEPEEEIIAEDYDDDPVDYEATR

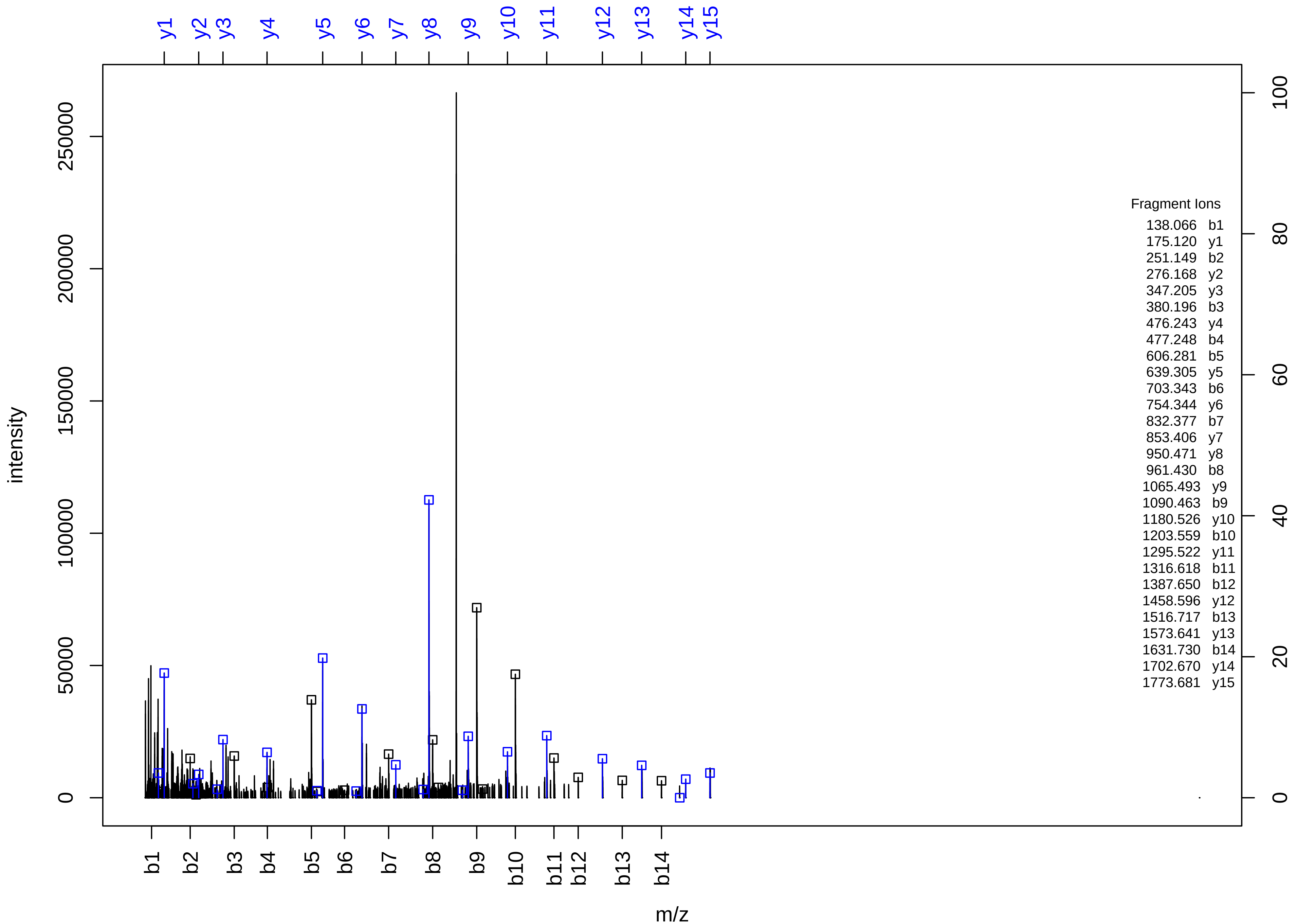

HLEPEPEEEIIAEDYDDDPVDYEATR / File: christine_ip_12.raw; SpectrumID: ; scans: 13653

NDFLELLR

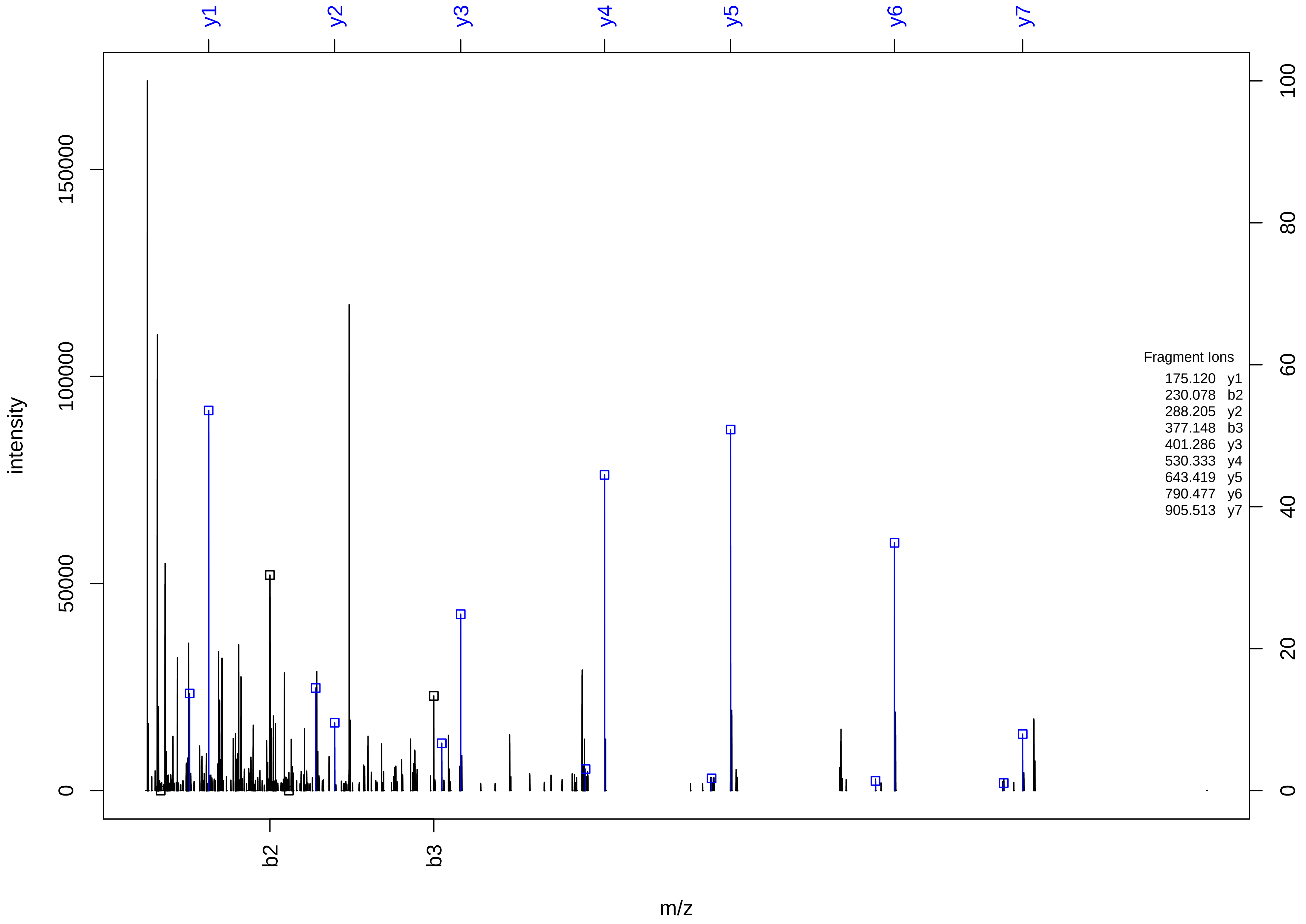

NDFLELLR / File: christine_ip_12.raw; SpectrumID: ; scans: 17066

(Ac)AAGTSSYWEDLRK

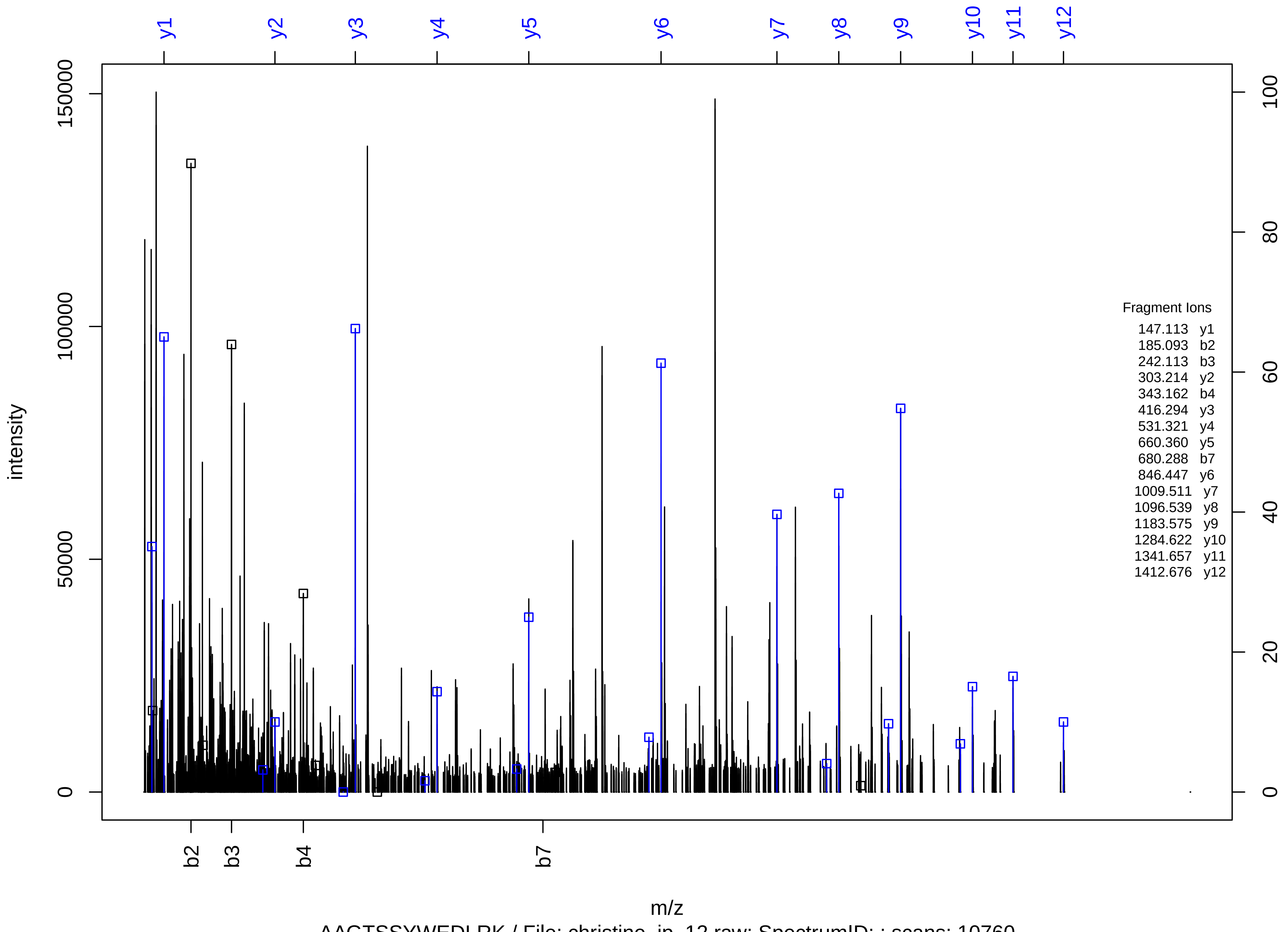

AAGTSSYWEDLRK / File: christine_ip_12.raw; SpectrumID: ; scans: 10760

VVLVNNILQNAQER

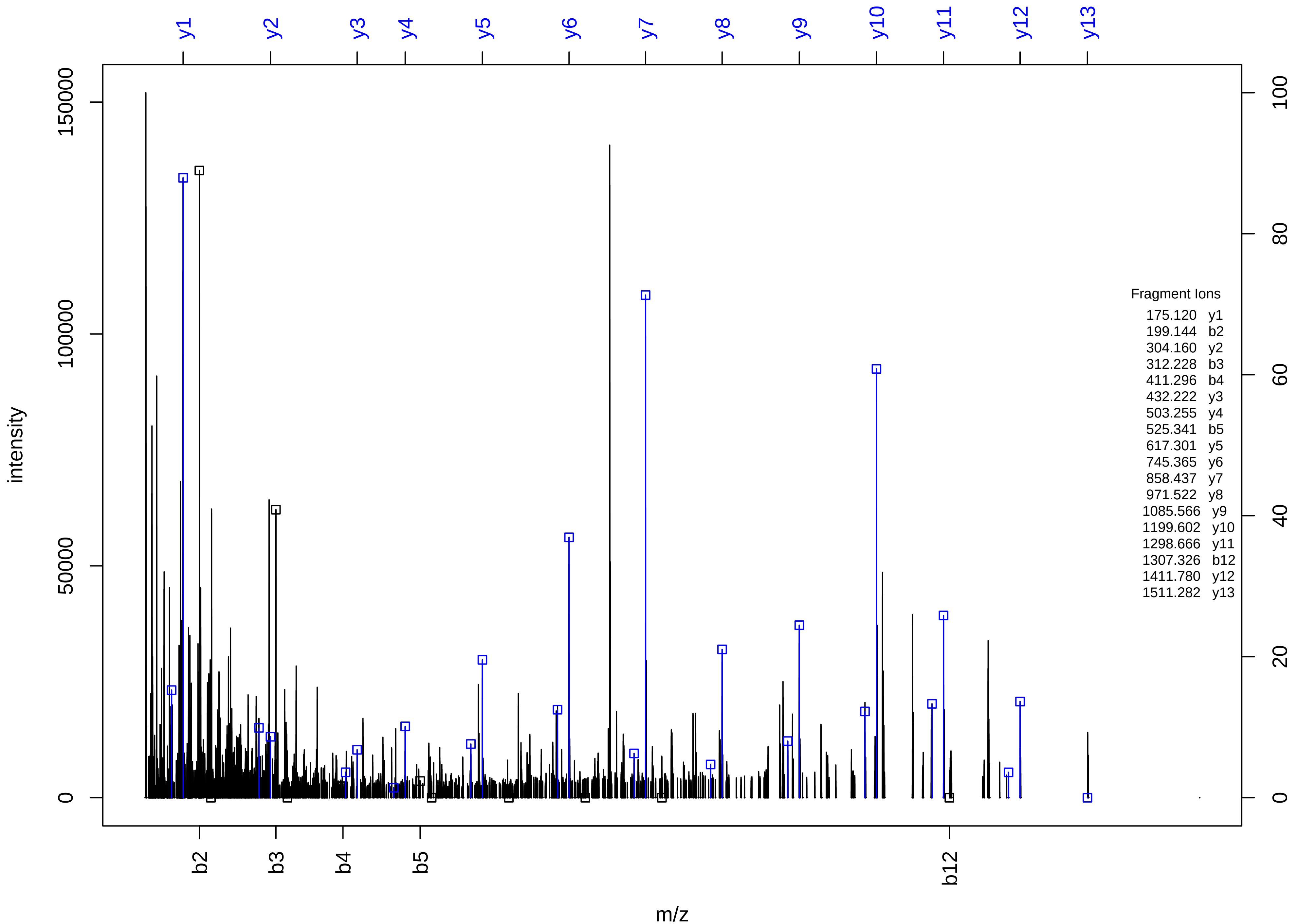

VVLVNNILQNAQER / File: christine_ip_12.raw; SpectrumID: ; scans: 13624

(Ac)MQEIIASVDHIK

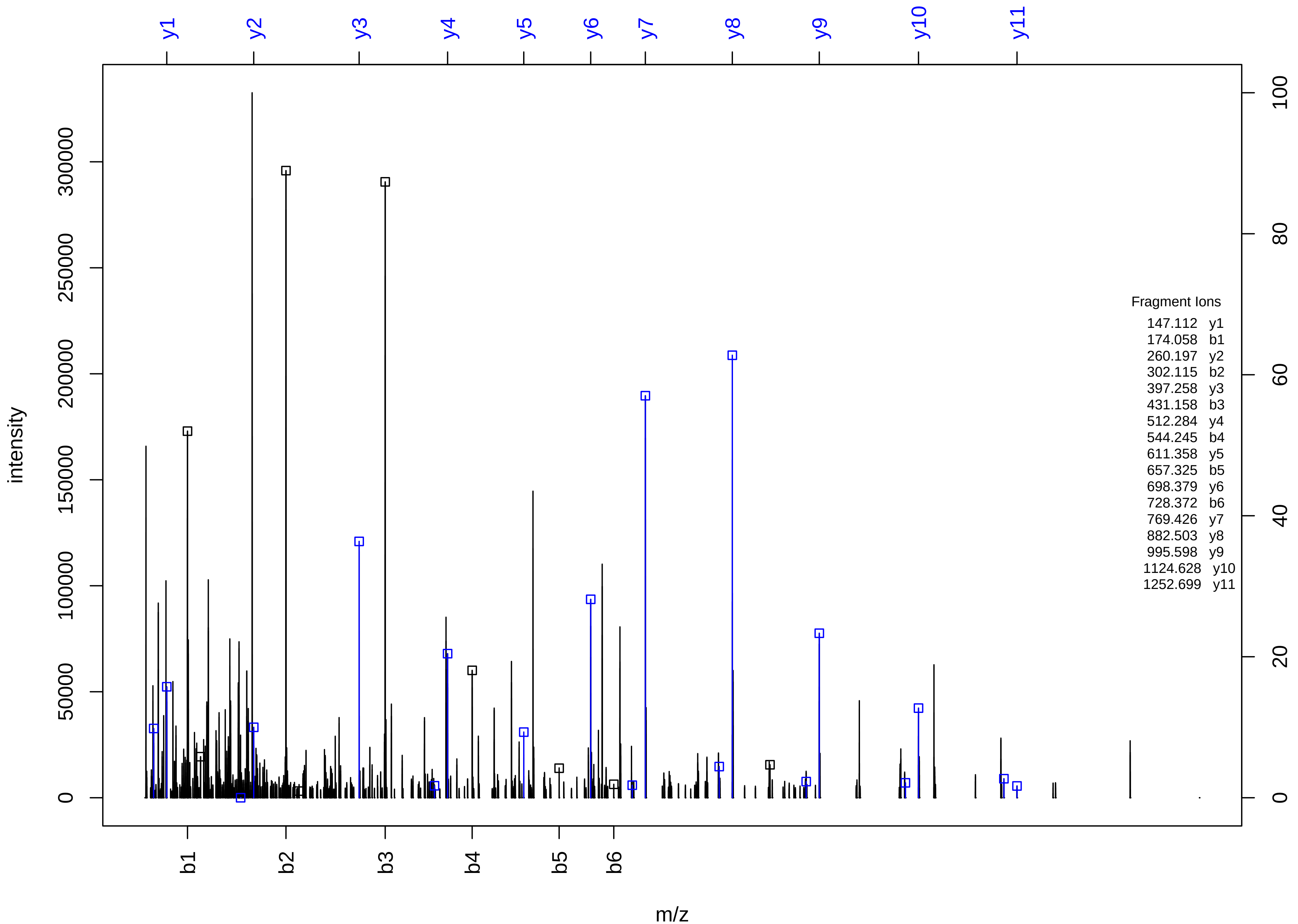

MQEIIASVDHIK / File: christine_ip_12.raw; SpectrumID: ; scans: 20431

GKPDLNTALPVR

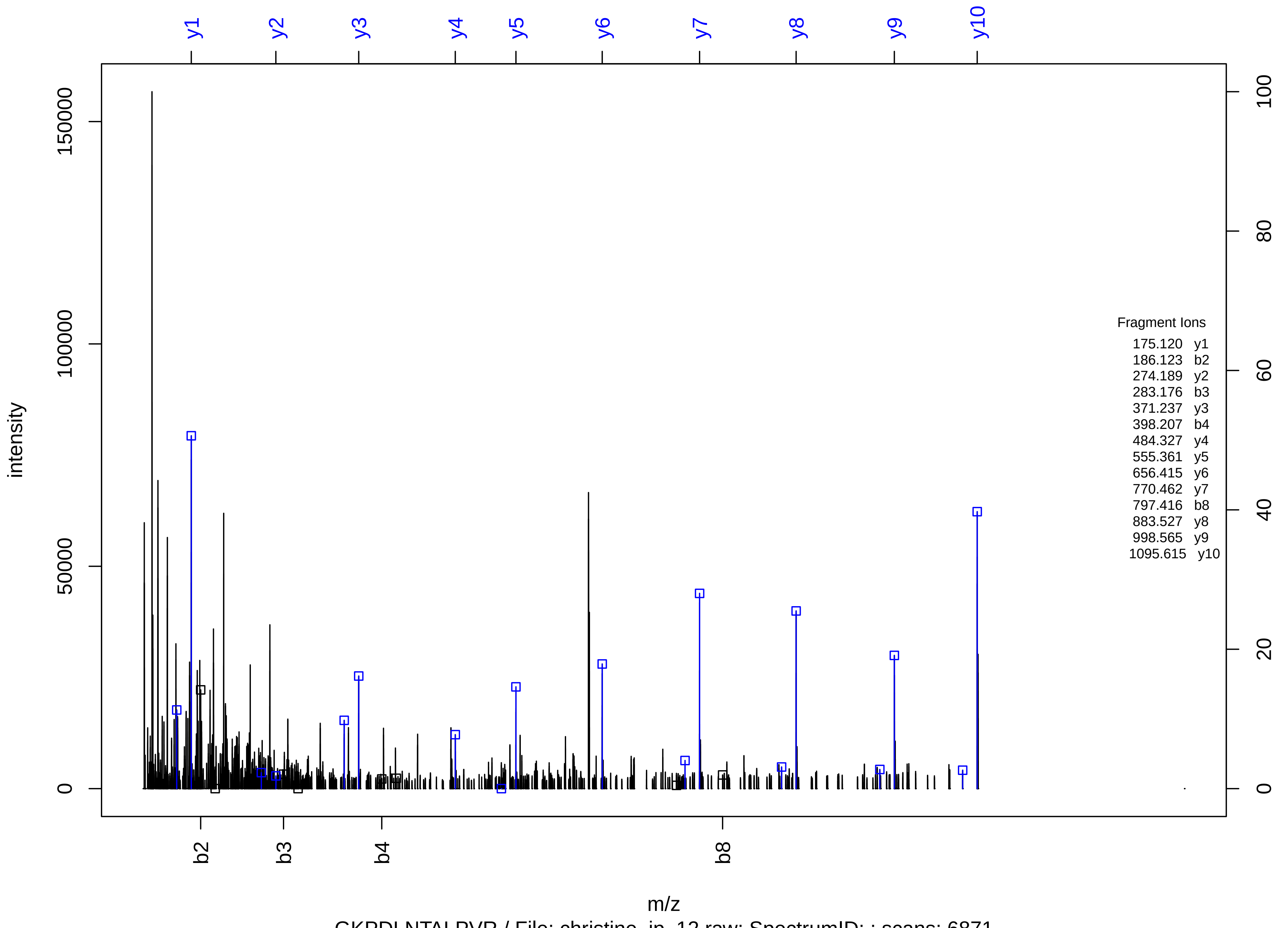

GKPDLNTALPVR / File: christine_ip_12.raw; SpectrumID: ; scans: 6871

ISQTYQQQYGR

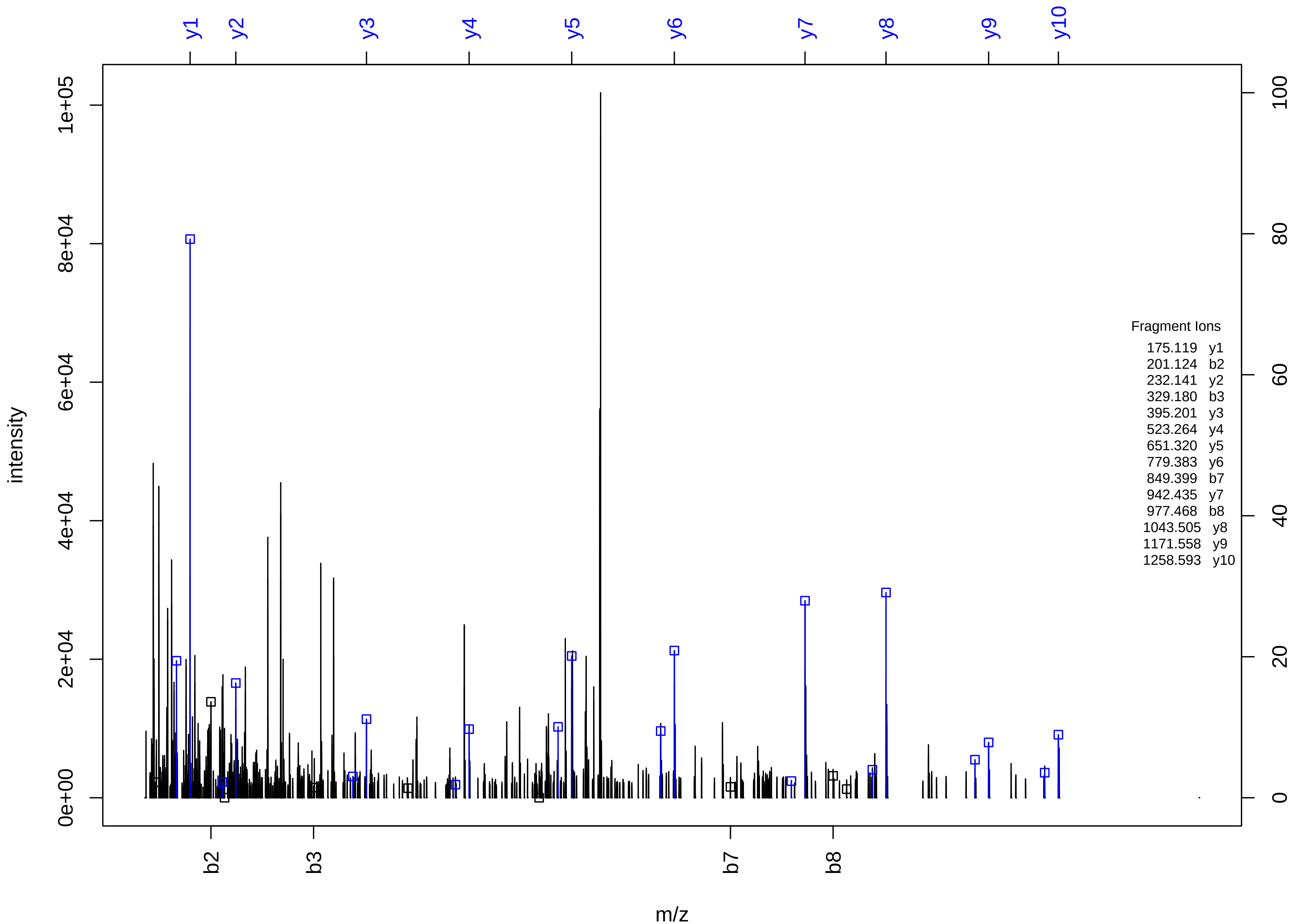

ISQTYQQQYGR / File: christine_ip_12.raw; SpectrumID: ; scans: 2595

TALLDAAGVASLLTTAEVVVTEIPK

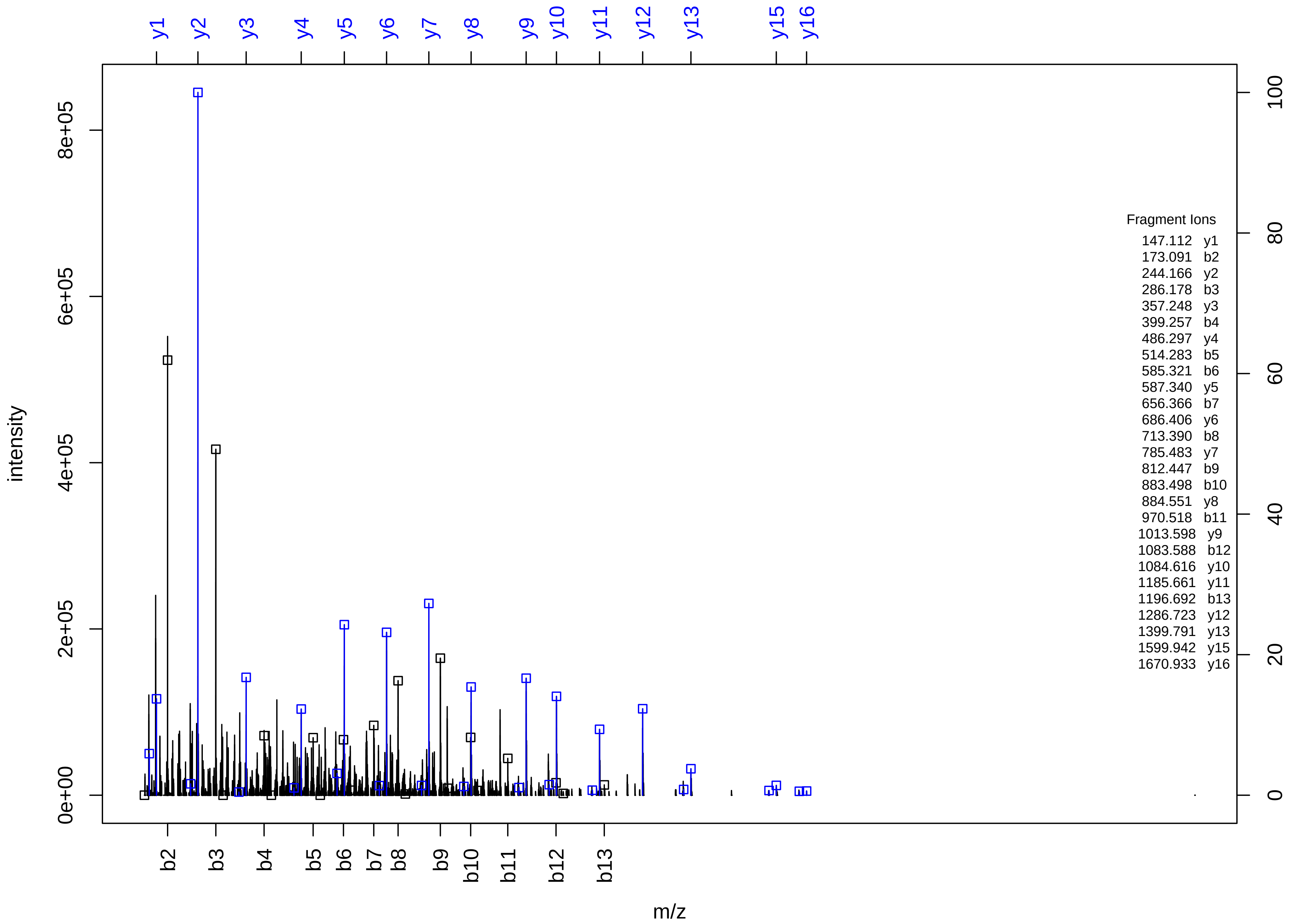

TALLDAAGVASLLTTAEVVVTEIPK / File: christine_ip_12.raw; SpectrumID: ; scans: 21296

FGEVISYQQLAALAGNPK

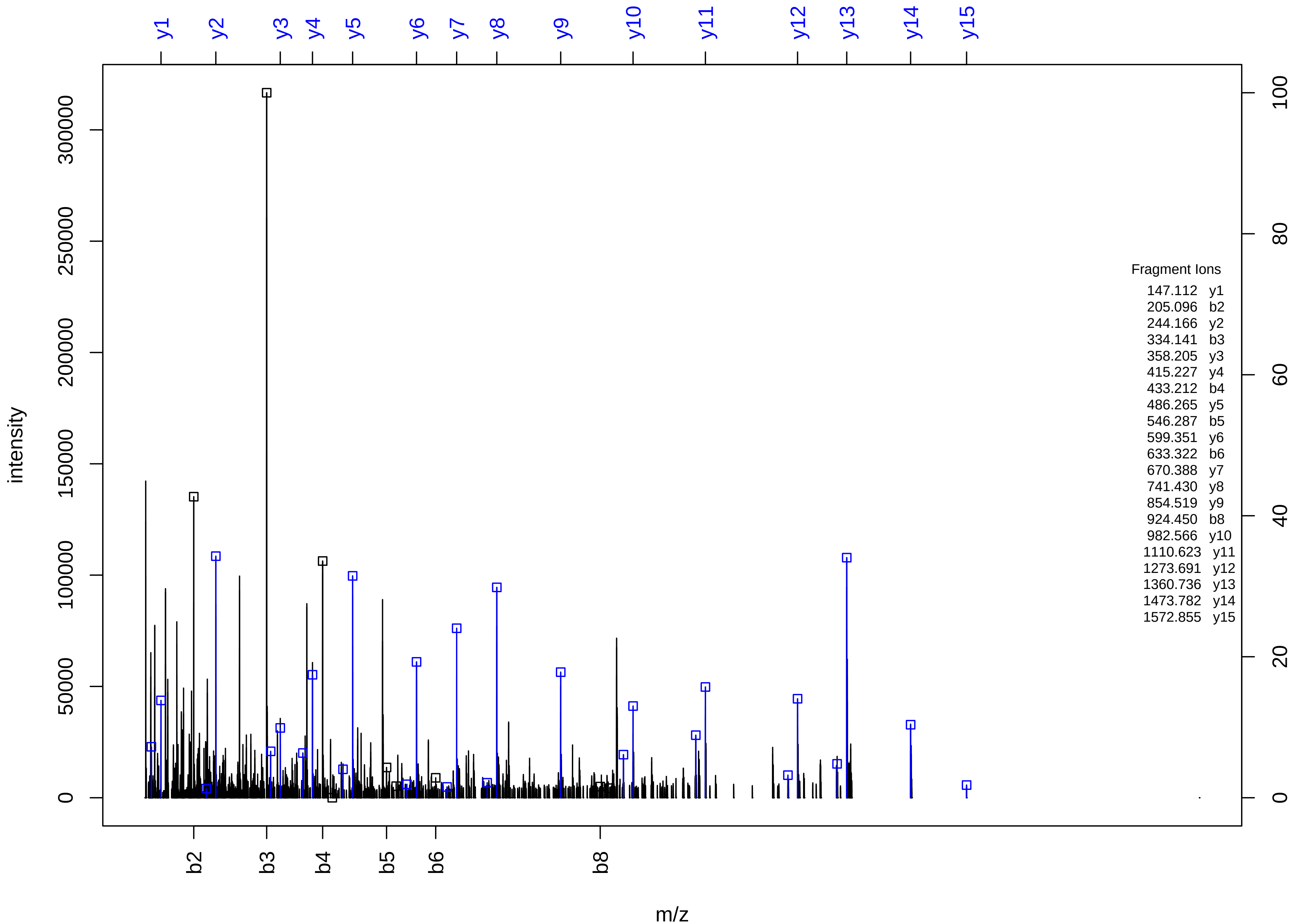

FGEVISYQQLAALAGNPK / File: christine_ip_12.raw; SpectrumID: ; scans: 17712

FYGAEIVSALDYLHSEK

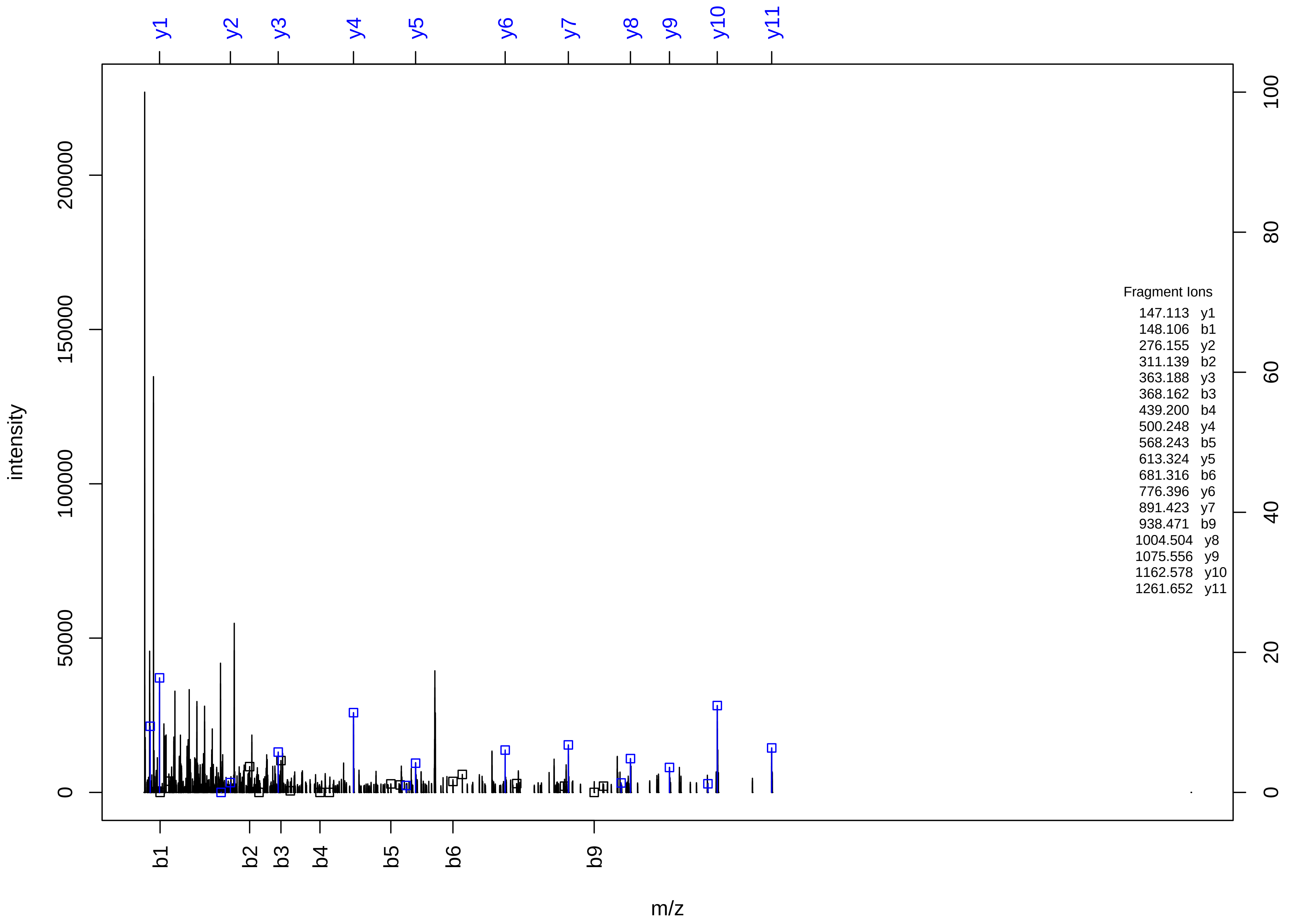

FYGAEIVSALDYLHSEK / File: christine_ip_12.raw; SpectrumID: ; scans: 18417

VLLESEQFLTELTR

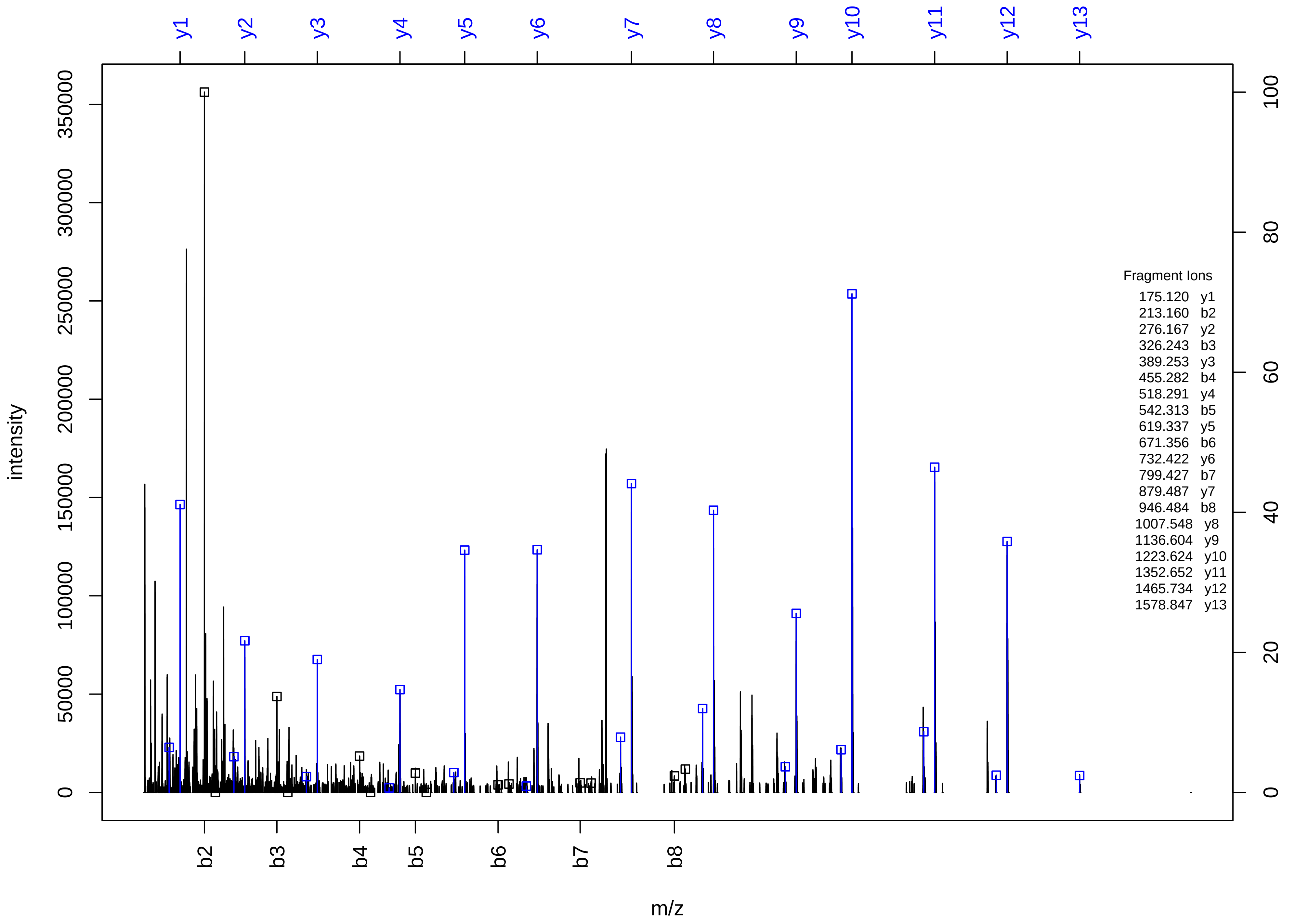

VLLESEQFLTELTR / File: christine_ip_12.raw; SpectrumID: ; scans: 19162

LTWHAYPEDAENK

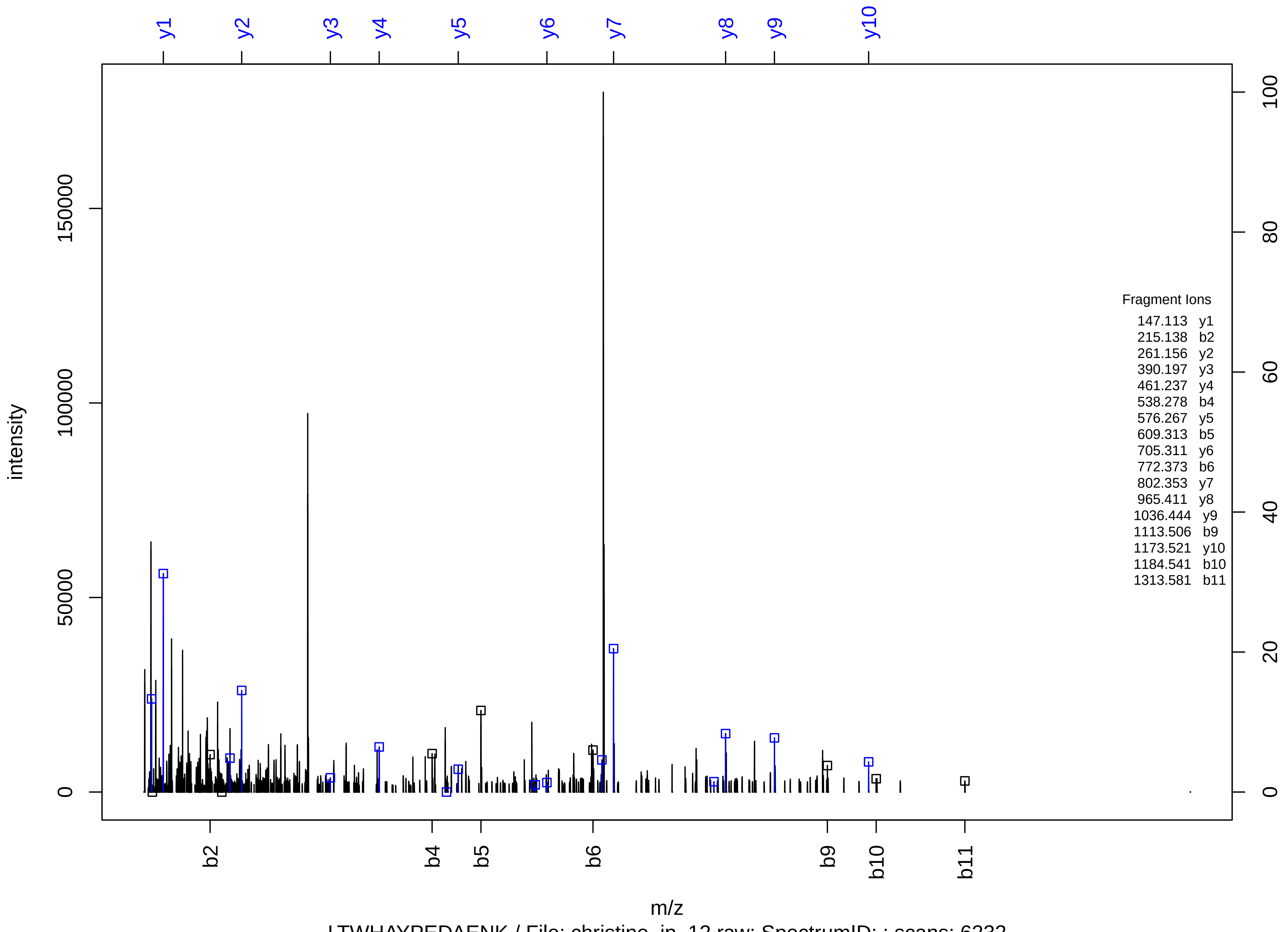

LTWHAYPEDAENK / File: christine_ip_12.raw; SpectrumID: ; scans: 6232

IYHPNINSN^GSICLDILR

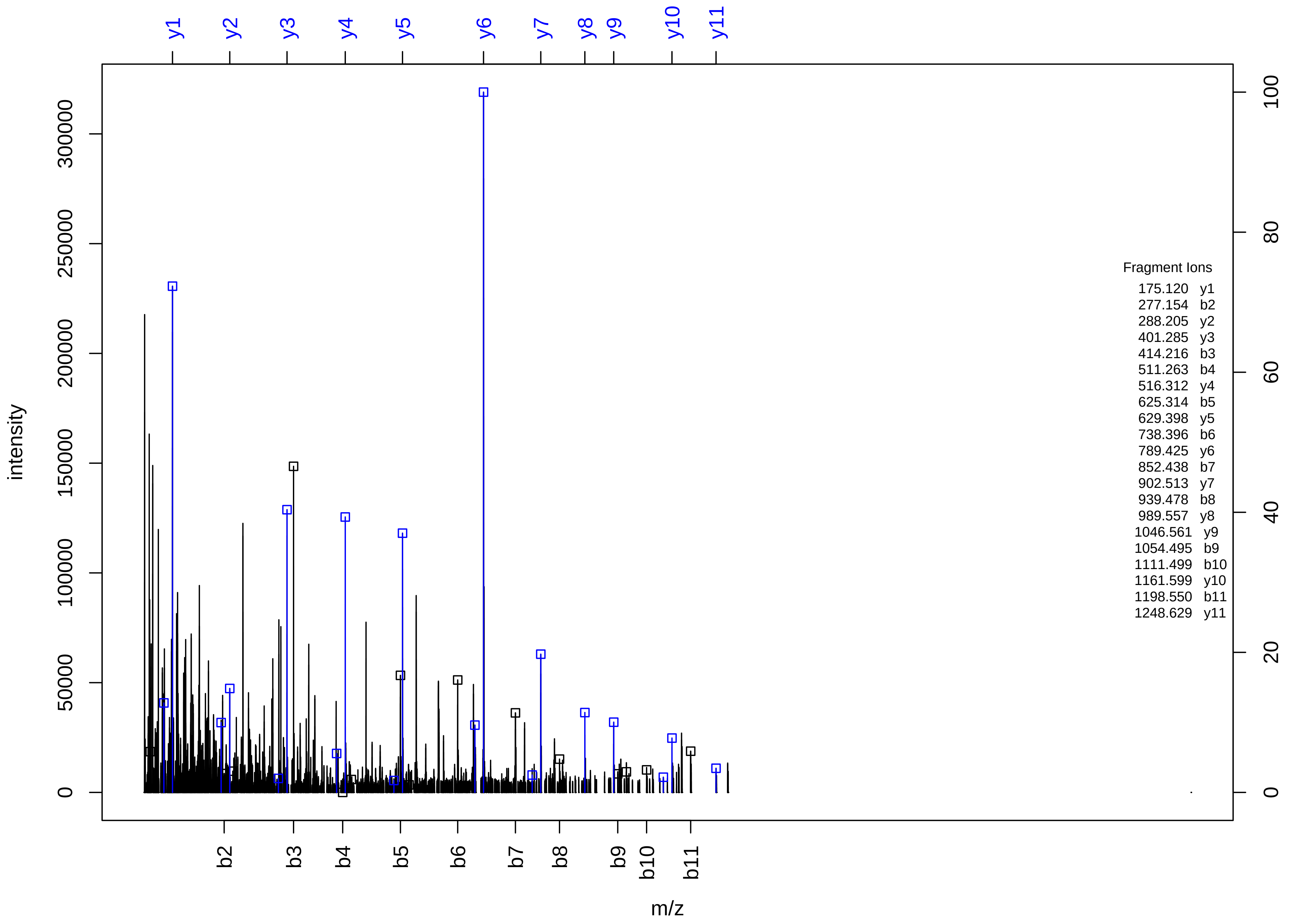

IYHPNINSNGSICLDILR / File: christine_ip_12.raw; SpectrumID: ; scans: 14884

ELADITLDPPPNCSAGPK

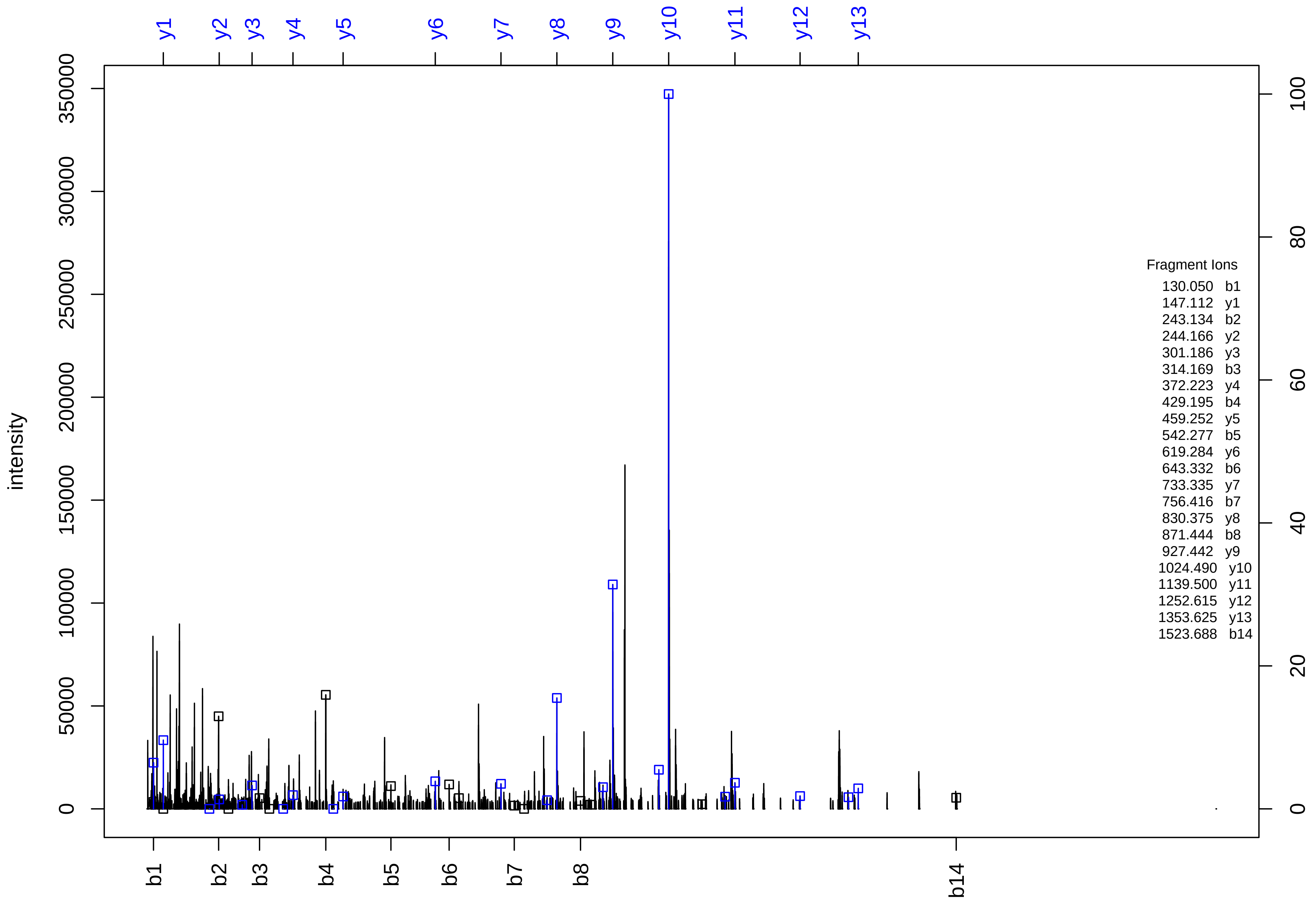

ELADITLDPPPNCSAGPK / File: christine_ip_12.raw; SpectrumID: ; scans: 11713

PGIVELPTLEELK

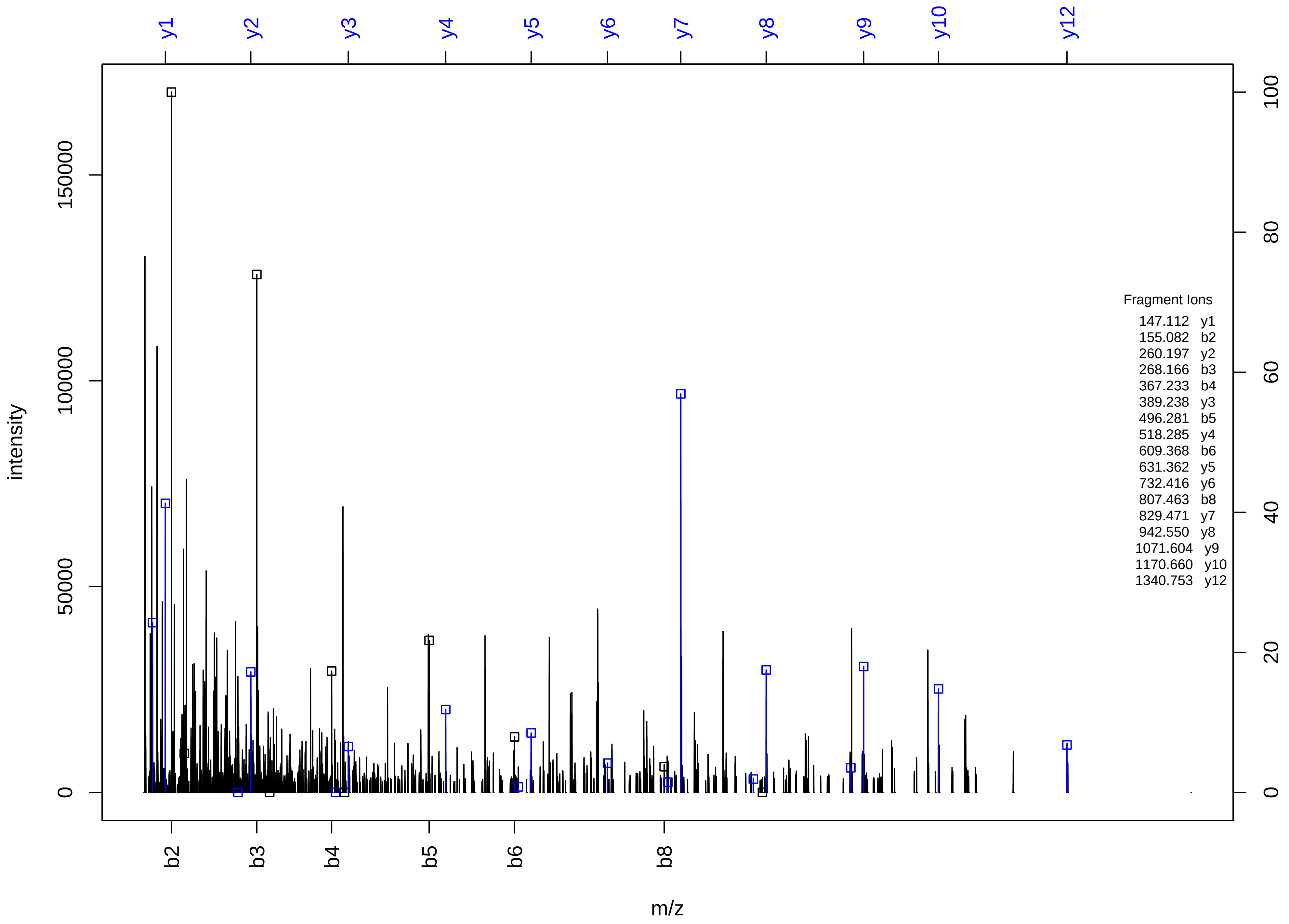

PGIVELPTLEELK / File: christine_ip_12.raw; SpectrumID: ; scans: 17891

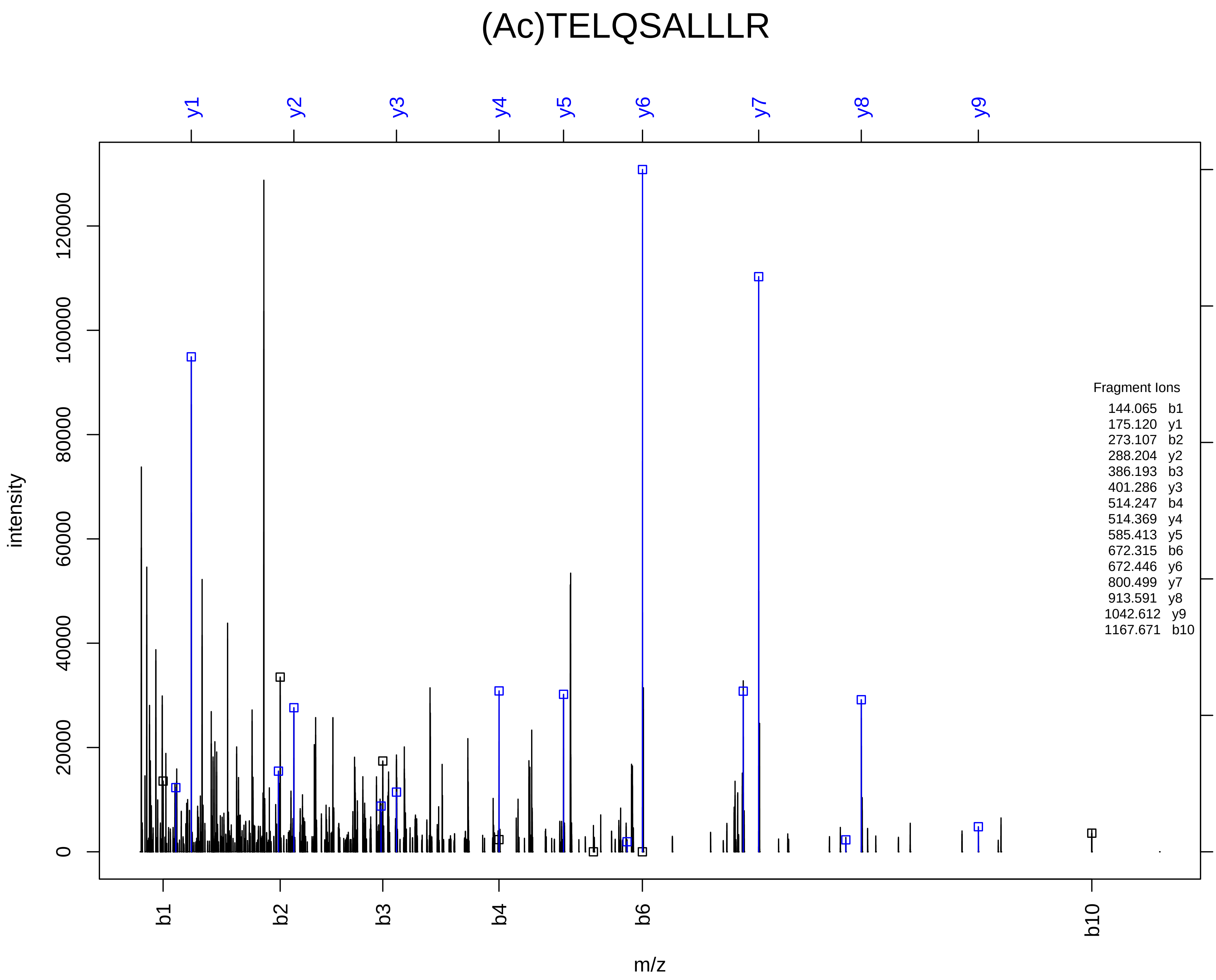
(Ac)TELQSALLLR
y1
y2
y3
y4
y5
y6
y7
y8
y9
intensity
0
20000
40000
60000
80000
100000
120000
0
20
40
60
80
100
b1
b2
b3
b4
b6
b10
m/z
Fragment Ions
144.065 b1
175.120 y1
273.107 b2
288.204 y2
386.193 b3
401.286 y3
514.247 b4
514.369 y4
585.413 y5
672.315 b6
672.446 y6
800.499 y7
913.591 y8
1042.612 y9
1167.671 b10
TELQSALLLR / File: christine_ip_12.raw; SpectrumID: ; scans: 19306

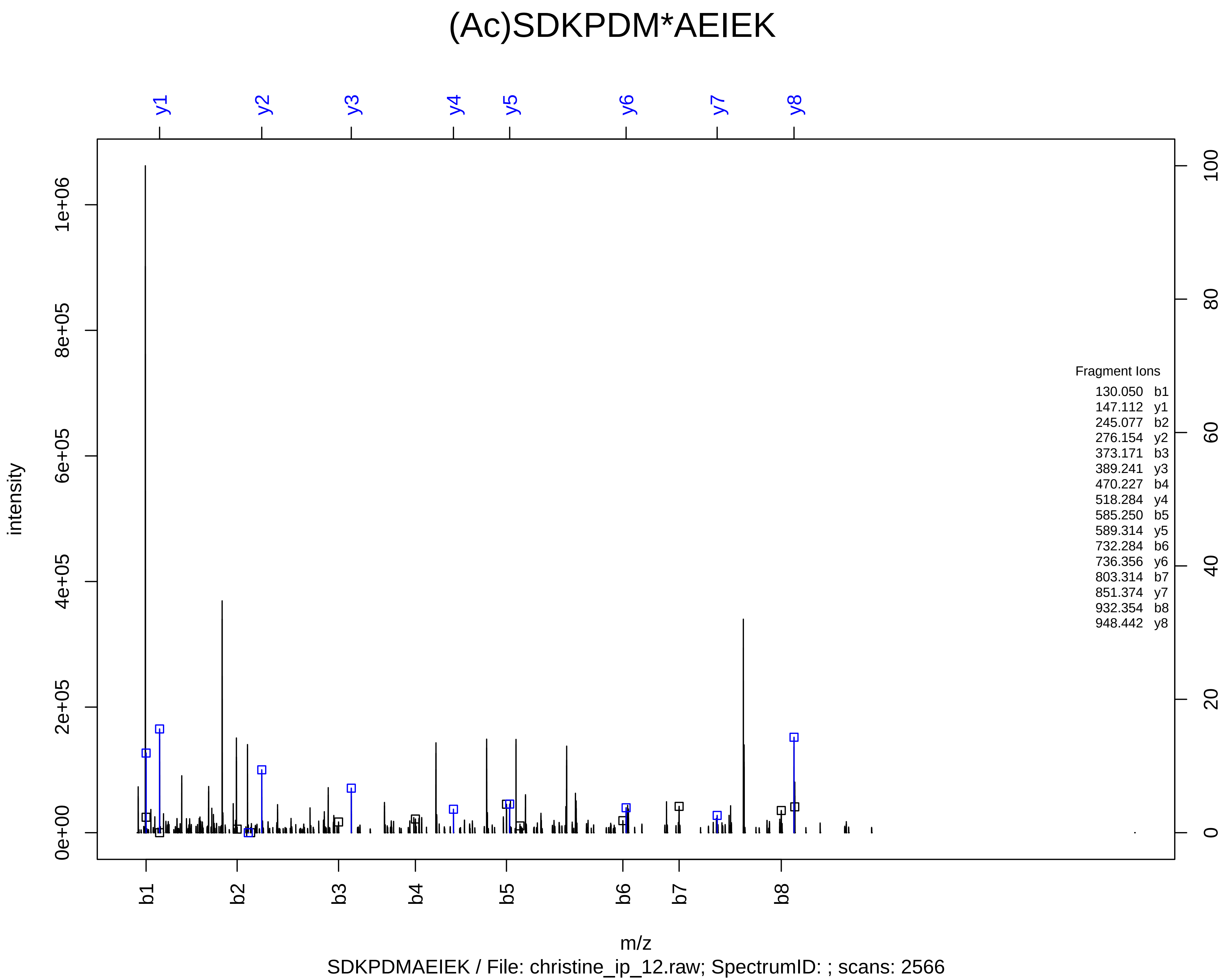
(Ac)SDKPDM*AEIEK
y1
y2
y3
y4
y5
y6
y7
y8
intensity
0e+00
2e+05
4e+05
6e+05
8e+05
1e+06
0
20
40
60
80
100
Fragment Ions
130.050 b1
147.112 y1
245.077 b2
276.154 y2
373.171 b3
389.241 y3
470.227 b4
518.284 y4
585.250 b5
589.314 y5
732.284 b6
736.356 y6
803.314 b7
851.374 y7
932.354 b8
948.442 y8
b1
b2
b3
b4
b5
b6
b7
b8
m/z
SDKPDMAEIEK / File: christine_ip_12.raw; SpectrumID: ; scans: 2566

(Ac)ADKPDM*GEIASFDK

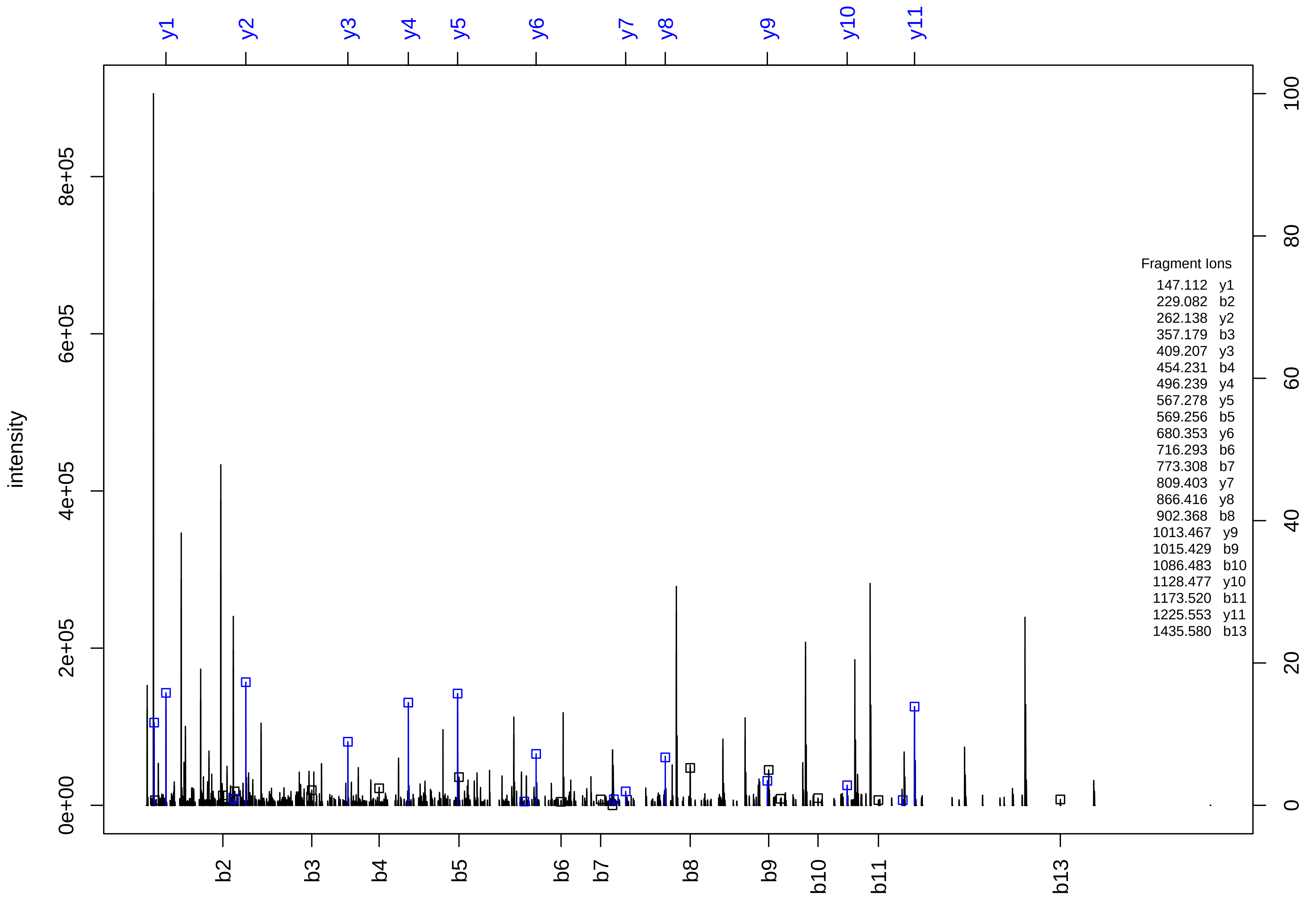

ADKPDMGEIASFDK / File: christine_ip_12.raw; SpectrumID: ; scans: 7454

GPEDSGAGGTGCGGADDPAK

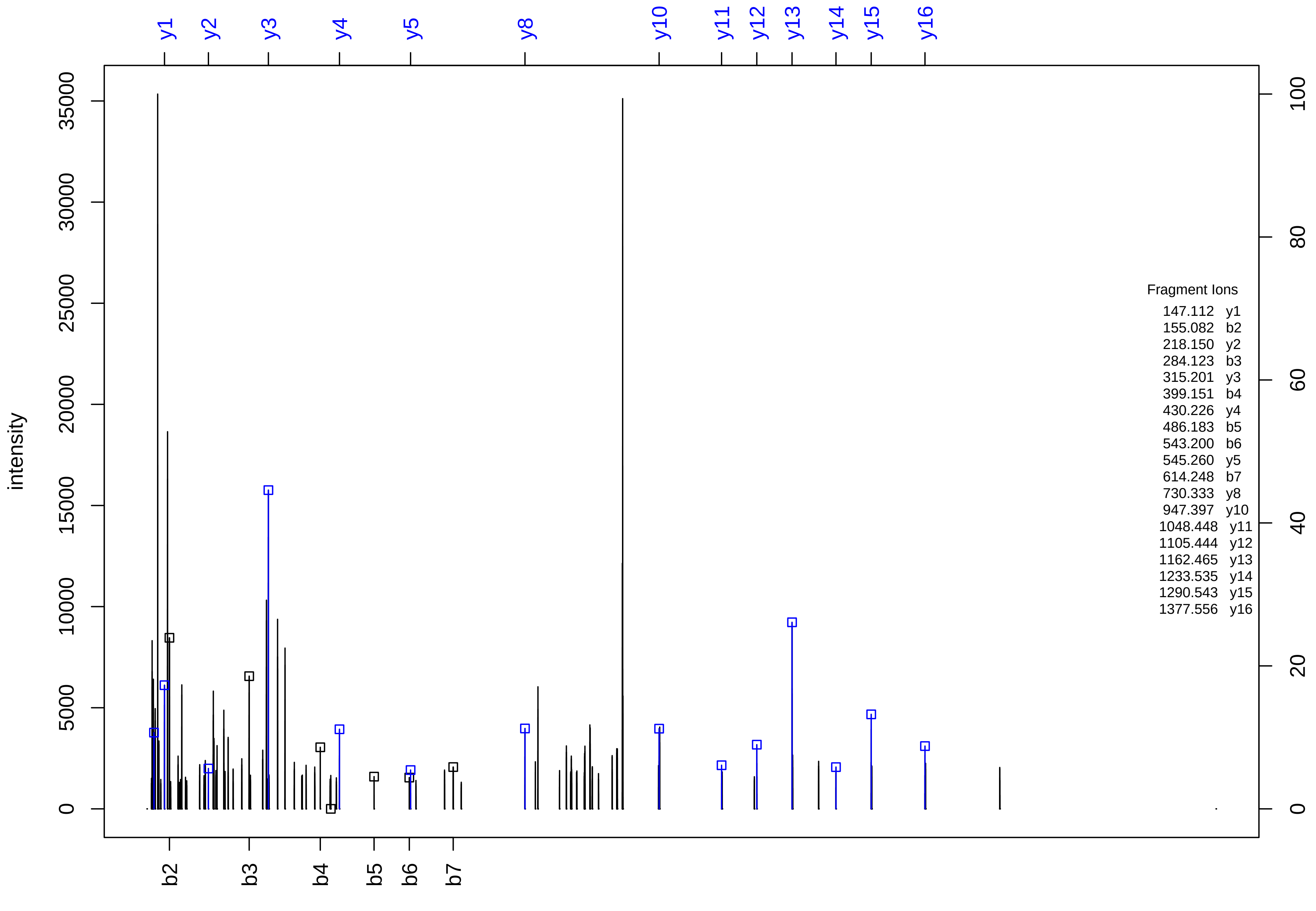

GPEDSGAGGTGCGGADDPAK / File: christine_ip_12.raw; SpectrumID: ; scans: 1678

NEEEDQKEDEEDQNEEK

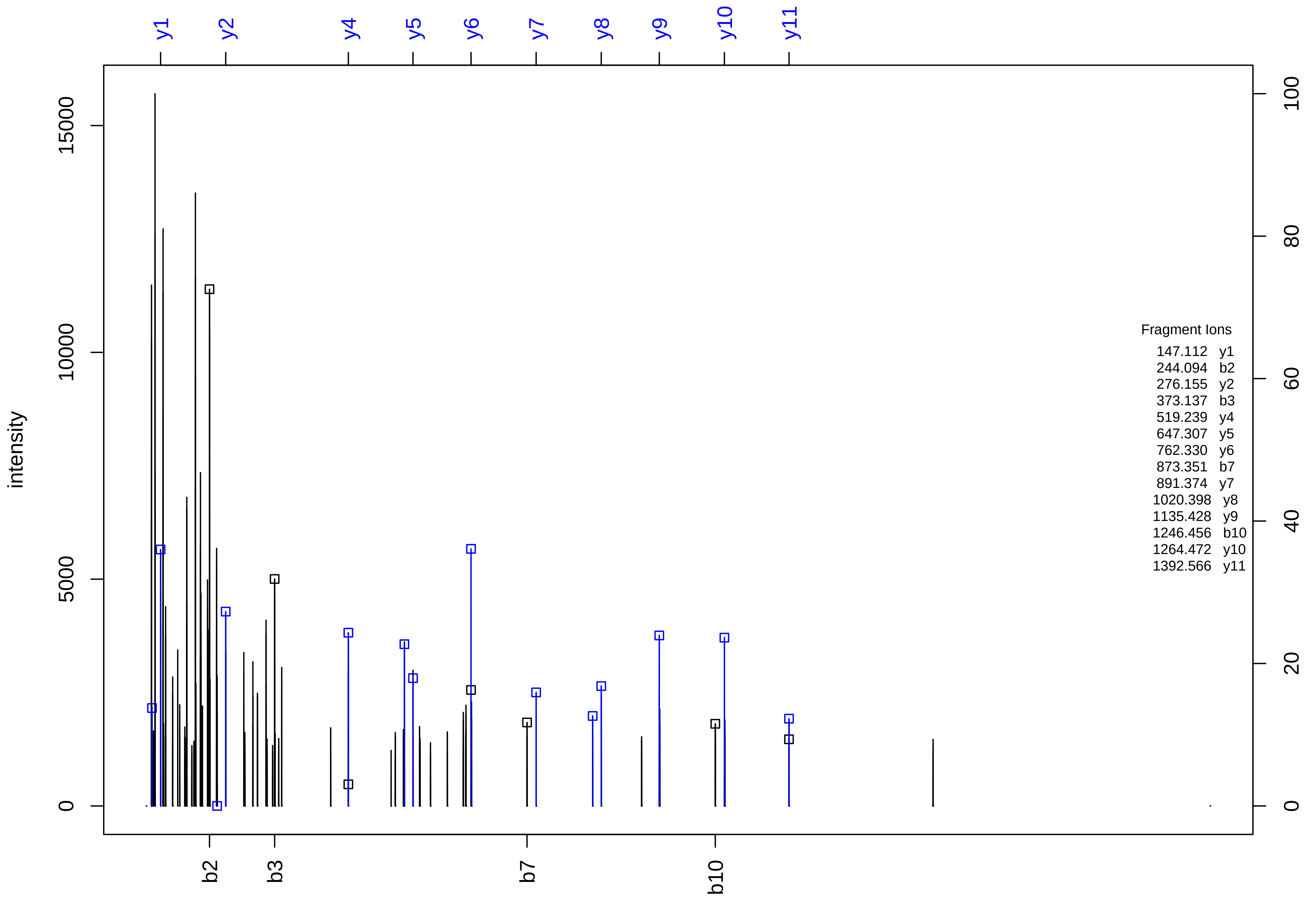

NEEEDQKEDEEDQNEEK / File: christine_ip_12.raw; SpectrumID: ; scans: 776

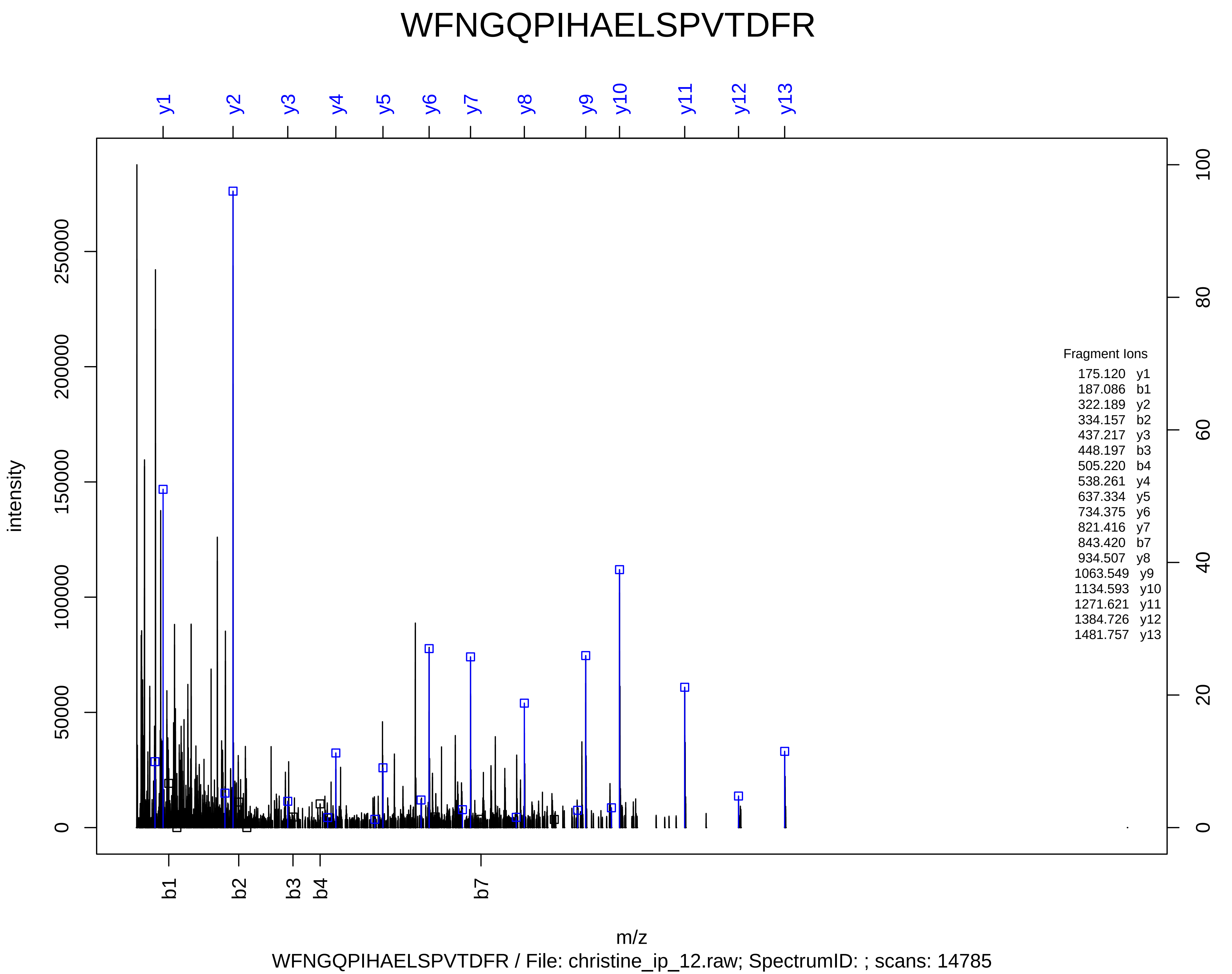

WFNGQPIHAELSPVTDFR
y1
y2
y3
y4
y5
y6
y7
y8
y9
y10
y11
y12
y13
b1
b2
b3
b4
b7
intensity
0
50000
100000
150000
200000
250000
0
20
40
60
80
100
m/z
Fragment Ions
175.120 y1
187.086 b1
322.189 y2
334.157 b2
437.217 y3
448.197 b3
505.220 b4
538.261 y4
637.334 y5
734.375 y6
821.416 y7
843.420 b7
934.507 y8
1063.549 y9
1134.593 y10
1271.621 y11
1384.726 y12
1481.757 y13
WFNGQPIHAELSPVTDFR / File: christine_ip_12.raw; SpectrumID: ; scans: 14785

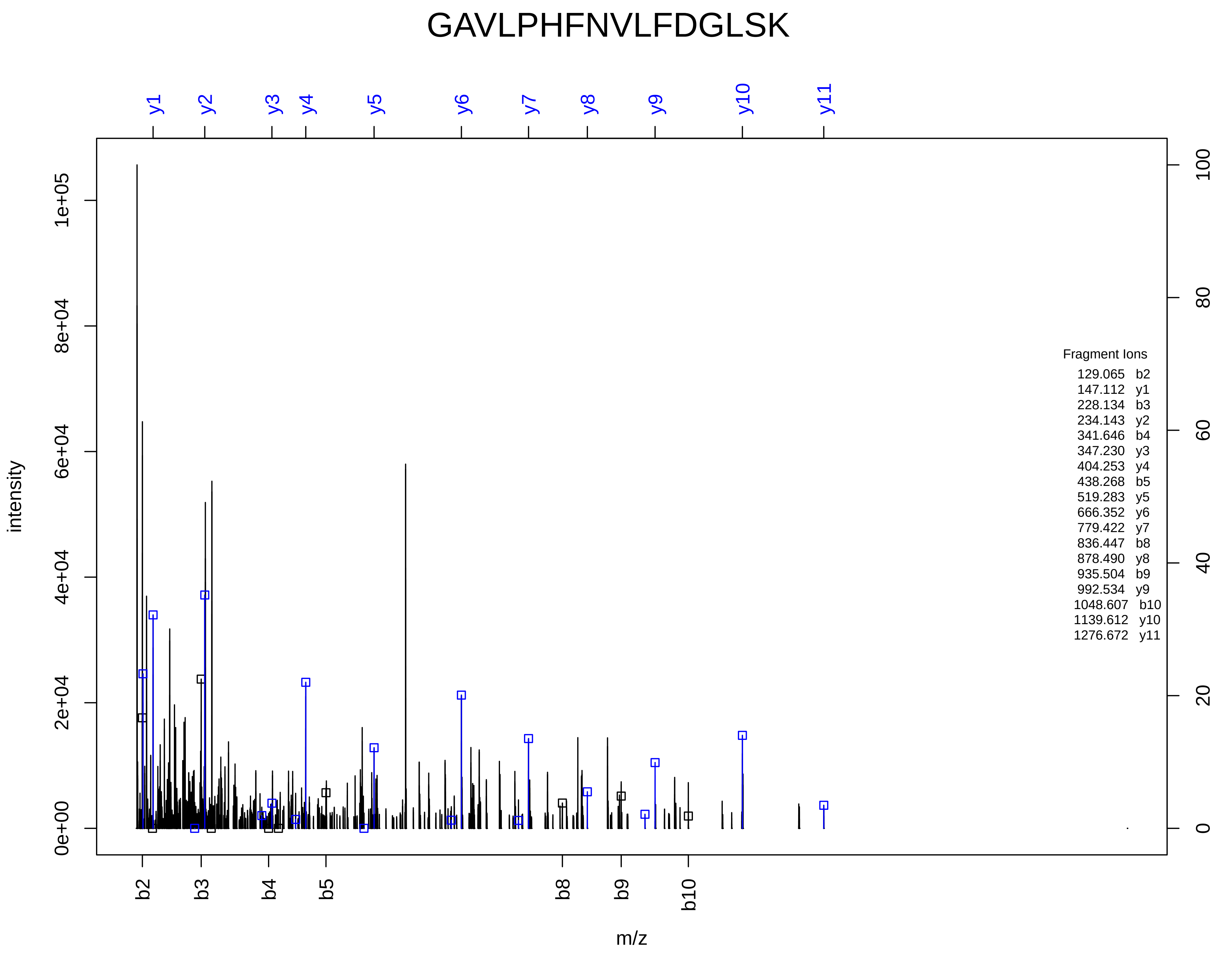
GAVLPHFNVLFDGLSK
y1
y2
y3
y4
y5
y6
y7
y8
y9
y10
y11
intensity
0e+00
2e+04
4e+04
6e+04
8e+04
1e+05
0
20
40
60
80
100
Fragment Ions
129.065 b2
147.112 y1
228.134 b3
234.143 y2
341.646 b4
347.230 y3
404.253 y4
438.268 b5
519.283 y5
666.352 y6
779.422 y7
836.447 b8
878.490 y8
935.504 b9
992.534 y9
1048.607 b10
1139.612 y10
1276.672 y11
b2
b3
b4
b5
b8
b9
b10
m/z
GAVLPHFNVLFDGLSK / File: christine_ip_12.raw; SpectrumID: ; scans: 17946

LVLPSLISSR

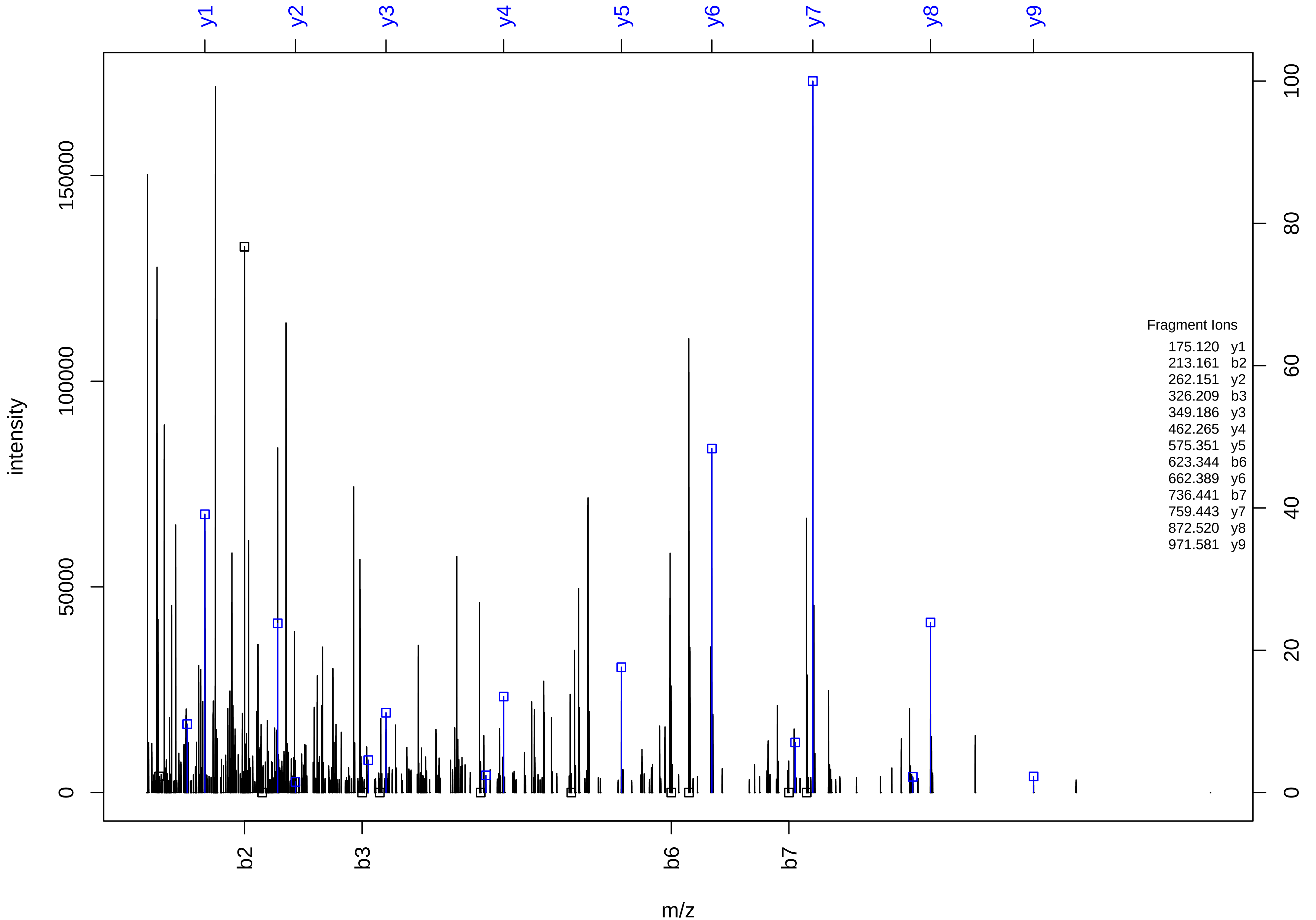

LVLPSLISSR / File: christine_ip_12.raw; SpectrumID: ; scans: 15336

AQQEDALAQQAFEEAR

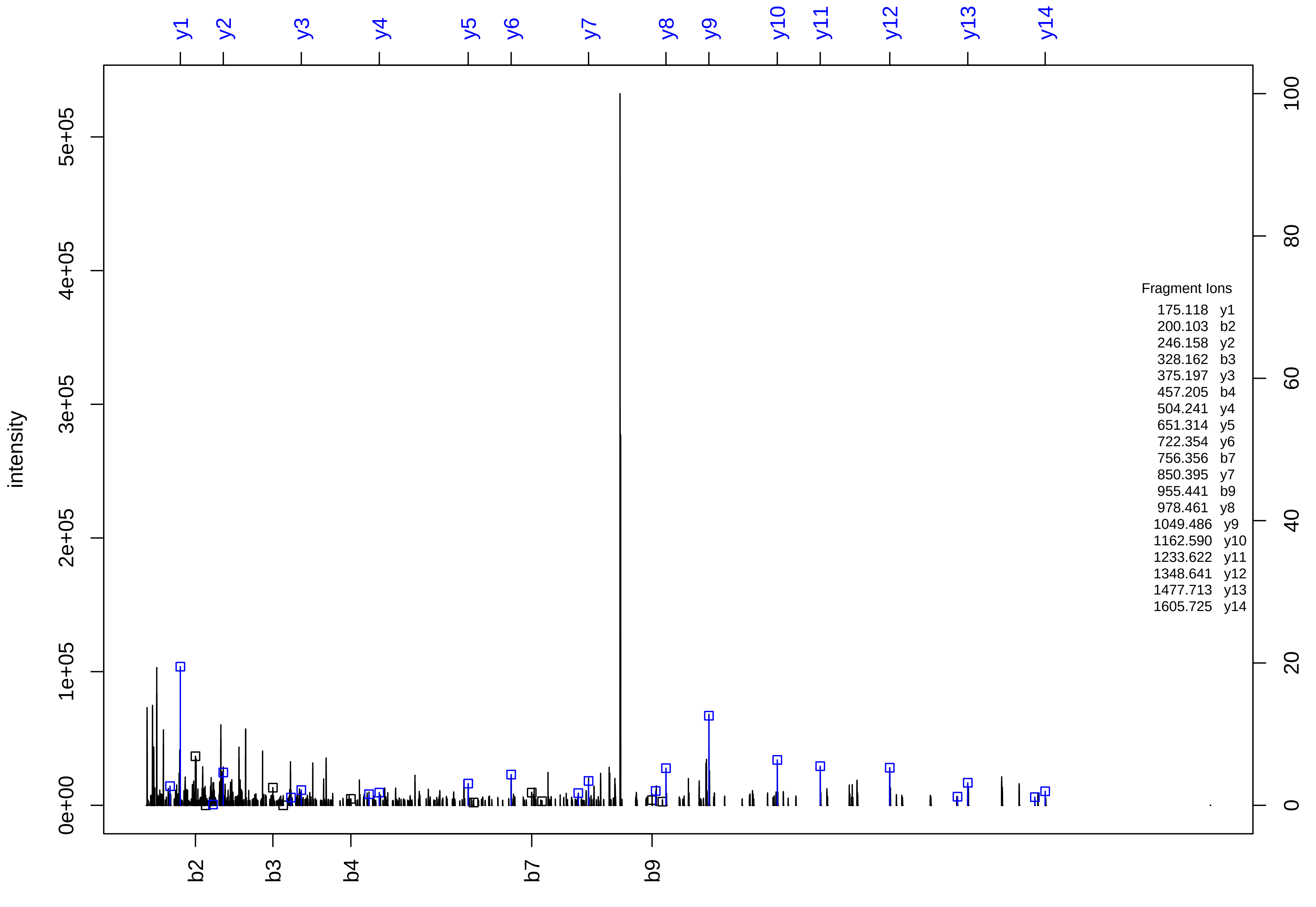

AQQEDALAQQAFEEAR / File: christine_ip_12.raw; SpectrumID: ; scans: 9955

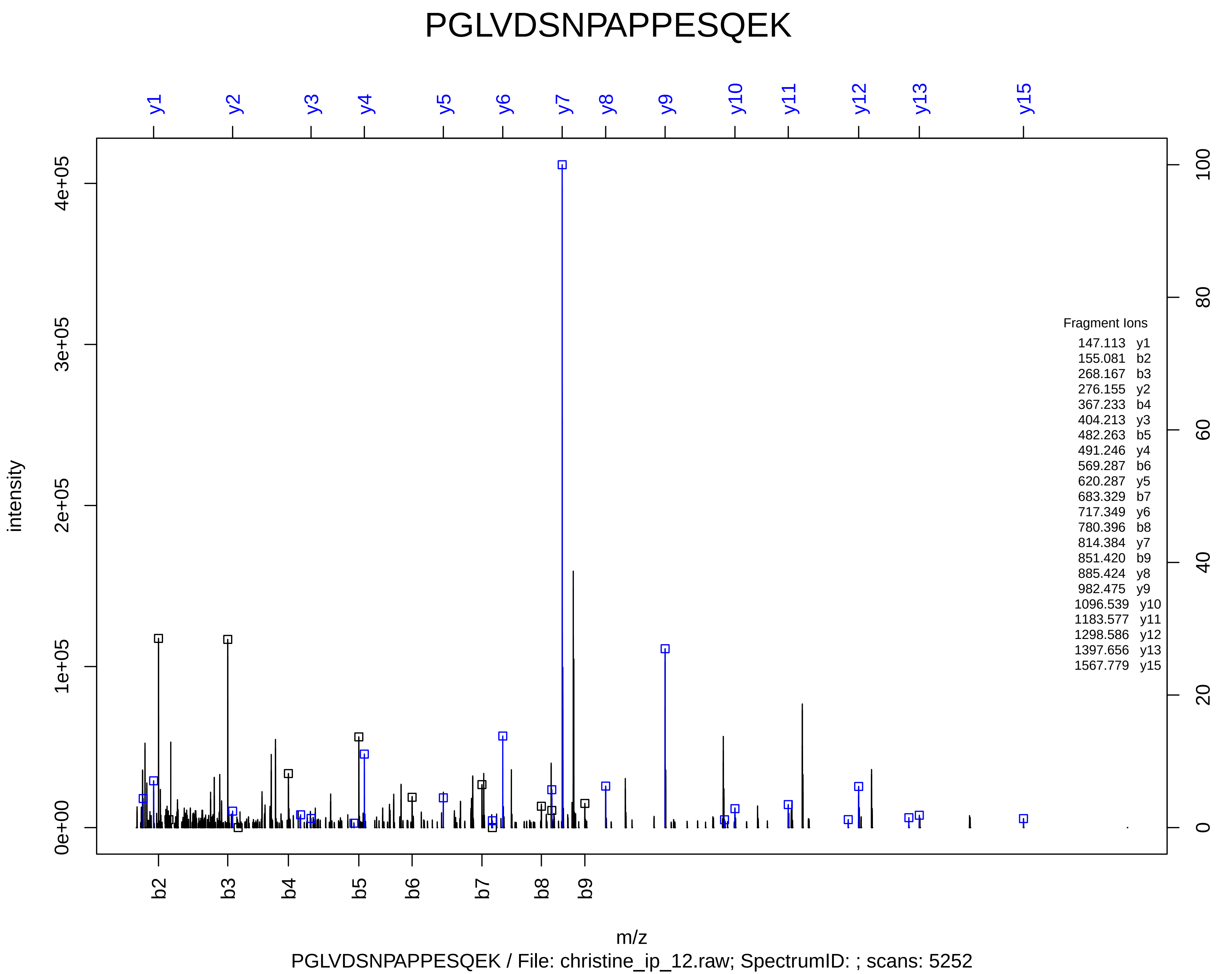
PGLVDSNPAPPESQEK
y1 y2 y3 y4 y5 y6 y7 y8 y9 y10 y11 y12 y13 y15
b2 b3 b4 b5 b6 b7 b8 b9
intensity
0e+00
1e+05
2e+05
3e+05
4e+05
0
20
40
60
80
100
m/z
Fragment Ions
147.113 y1
155.081 b2
268.167 b3
276.155 y2
367.233 b4
404.213 y3
482.263 b5
491.246 y4
569.287 b6
620.287 y5
683.329 b7
717.349 y6
780.396 b8
814.384 y7
851.420 b9
885.424 y8
982.475 y9
1096.539 y10
1183.577 y11
1298.586 y12
1397.656 y13
1567.779 y15
PGLVDSNPAPPESQEK / File: christine_ip_12.raw; SpectrumID: ; scans: 5252

EVDEGPSPPEQFTAVK

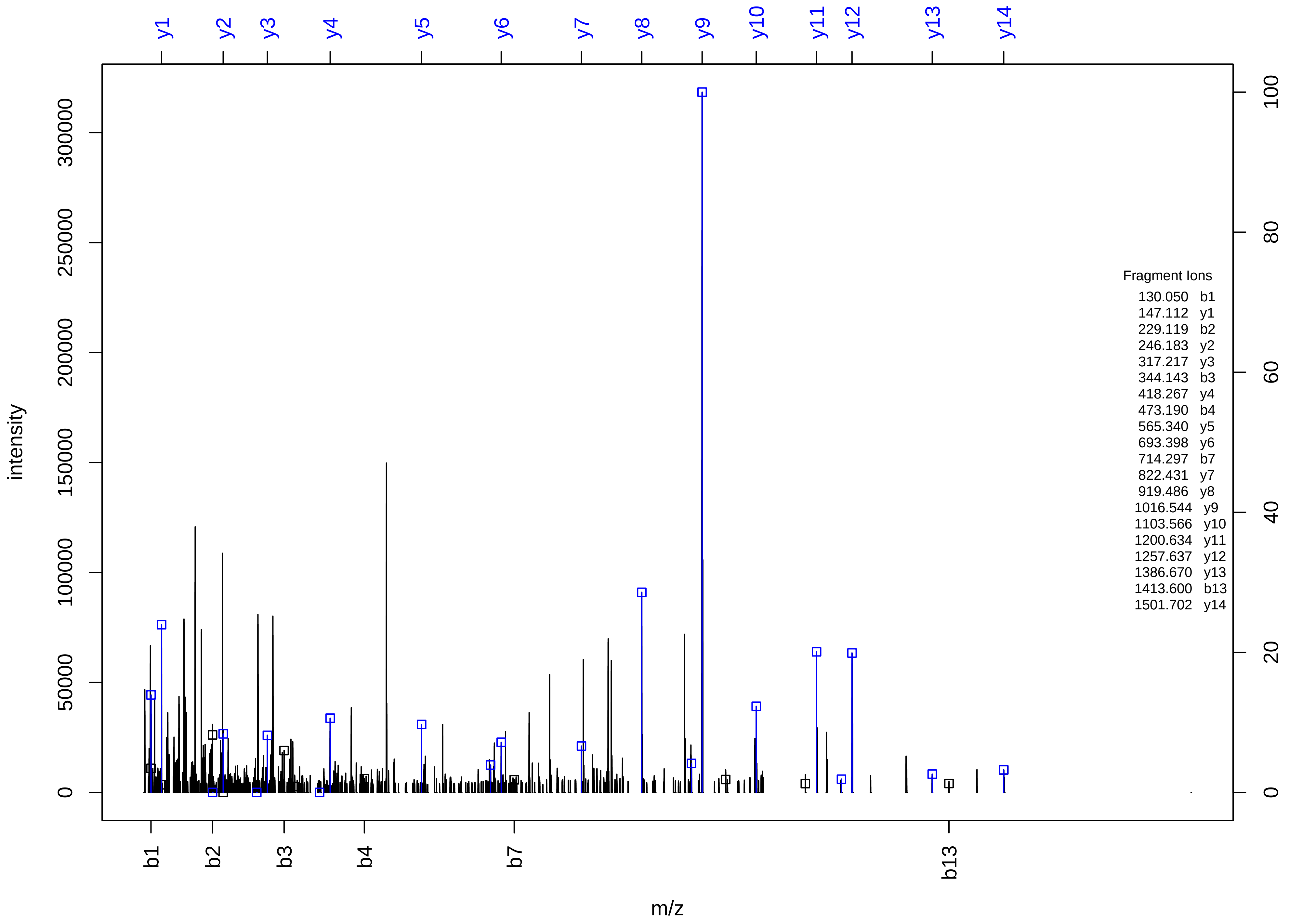

EVDEGPSPPEQFTAVK / File: christine_ip_12.raw; SpectrumID: ; scans: 8949

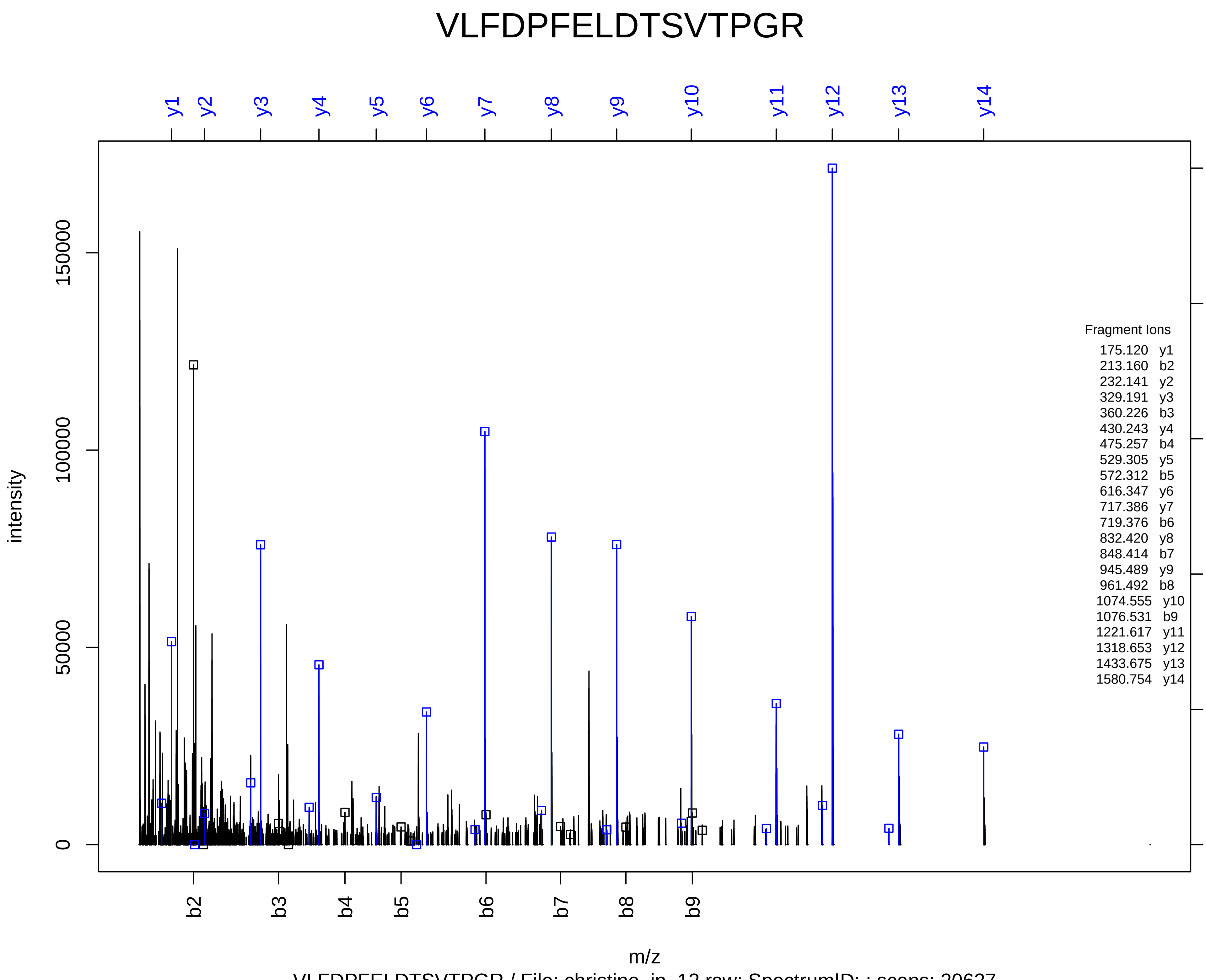
VLFDPFELDTSVTPGR
y1 y2 y3 y4 y5 y6 y7 y8 y9 y10 y11 y12 y13 y14
intensity
0
50000
100000
150000
0
20
40
60
80
100
b2 b3 b4 b5 b6 b7 b8 b9
m/z
Fragment Ions
175.120 y1
213.160 b2
232.141 y2
329.191 y3
360.226 b3
430.243 y4
475.257 b4
529.305 y5
572.312 b5
616.347 y6
717.386 y7
719.376 b6
832.420 y8
848.414 b7
945.489 y9
961.492 b8
1074.555 y10
1076.531 b9
1221.617 y11
1318.653 y12
1433.675 y13
1580.754 y14
VLFDPFELDTSVTPGR / File: christine_ip_12.raw; SpectrumID: ; scans: 20627

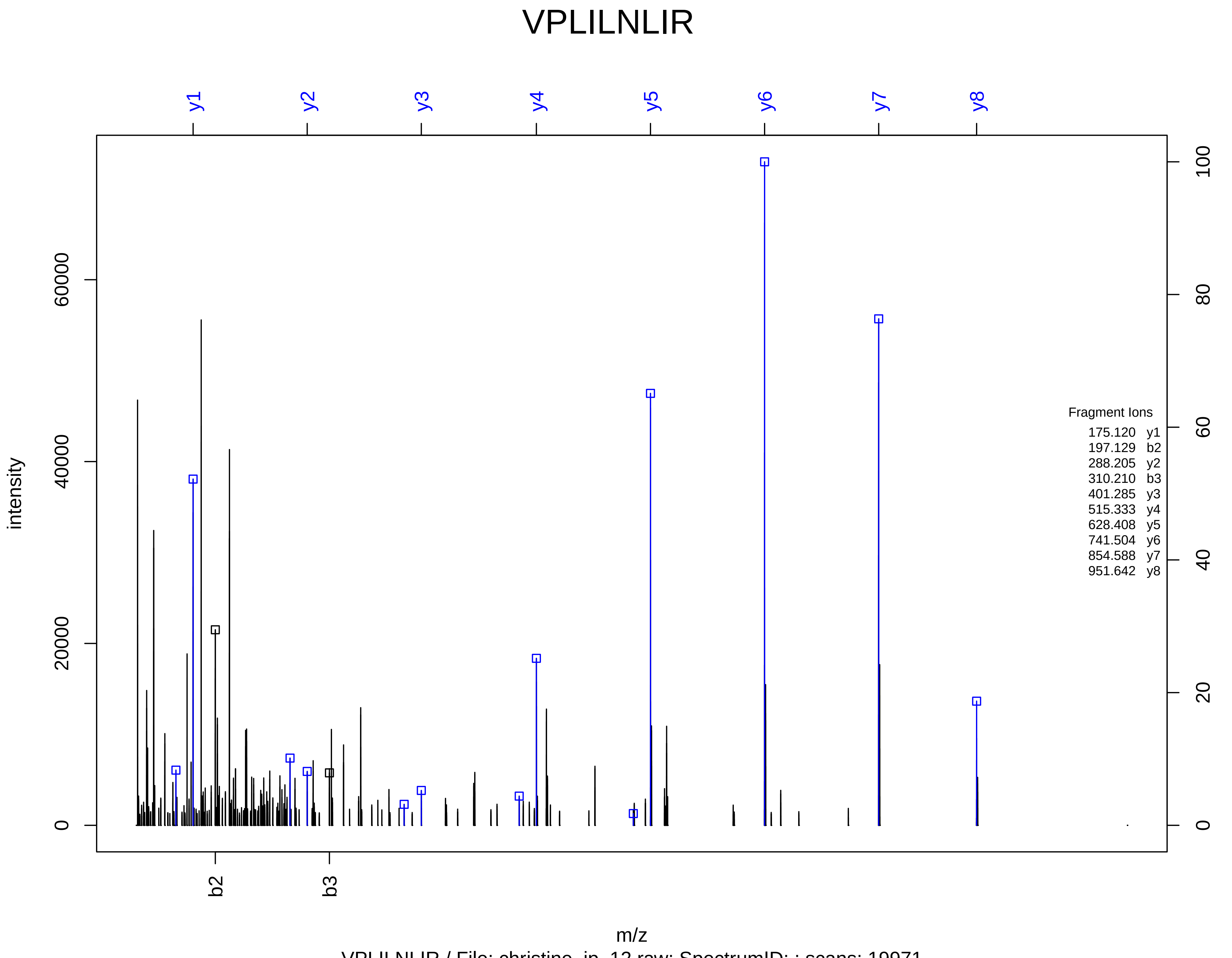
VPLILNLIR
y1
y2
y3
y4
y5
y6
y7
y8
intensity
0
20000
40000
60000
0
20
40
60
80
100
b2
b3
m/z
Fragment Ions
175.120 y1
197.129 b2
288.205 y2
310.210 b3
401.285 y3
515.333 y4
628.408 y5
741.504 y6
854.588 y7
951.642 y8
VPLILNLIR / File: christine_ip_12.raw; SpectrumID: ; scans: 19971

AELVQLEDEITTLR

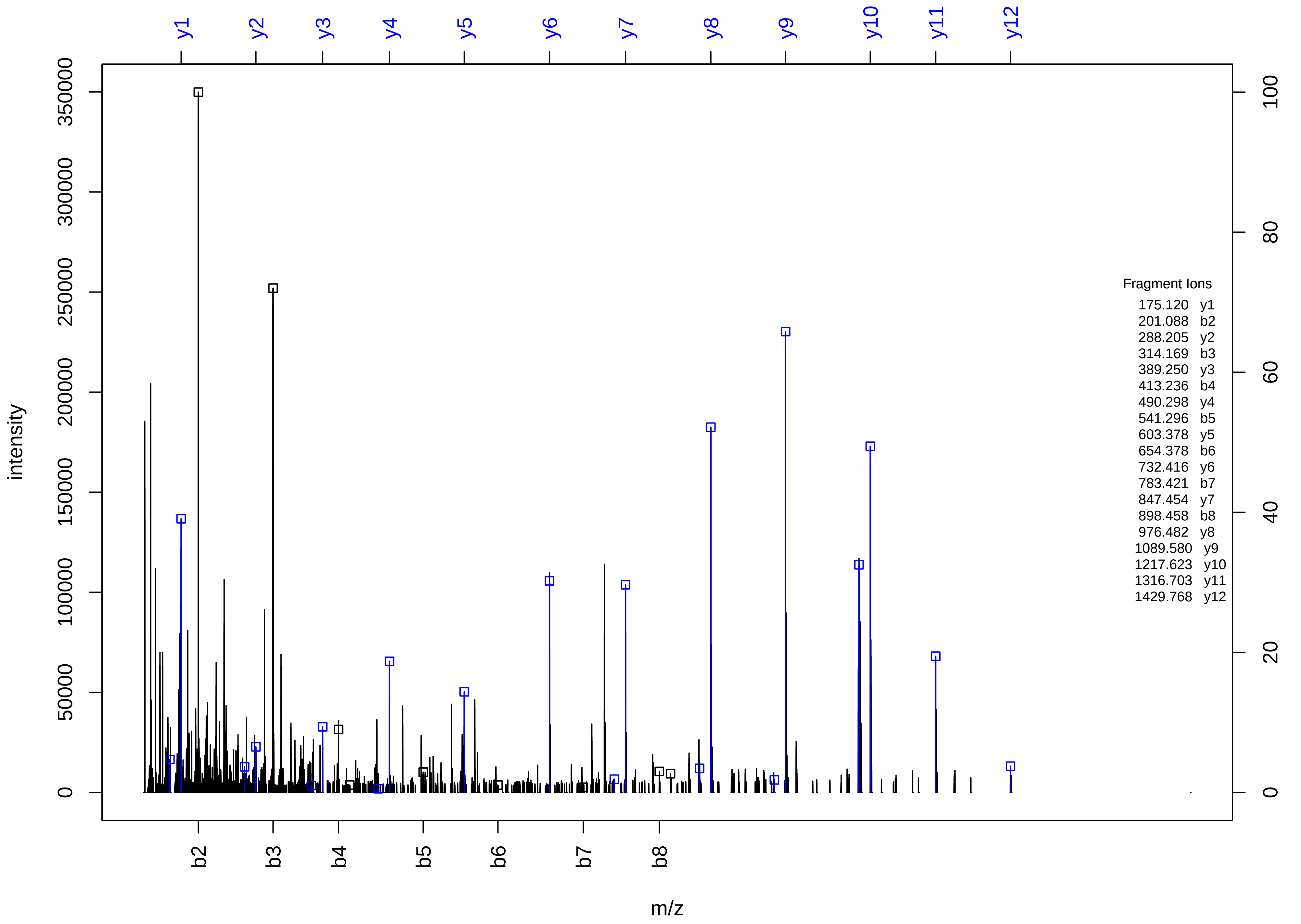

AELVQLEDEITTLR / File: christine_ip_12.raw; SpectrumID: ; scans: 17013

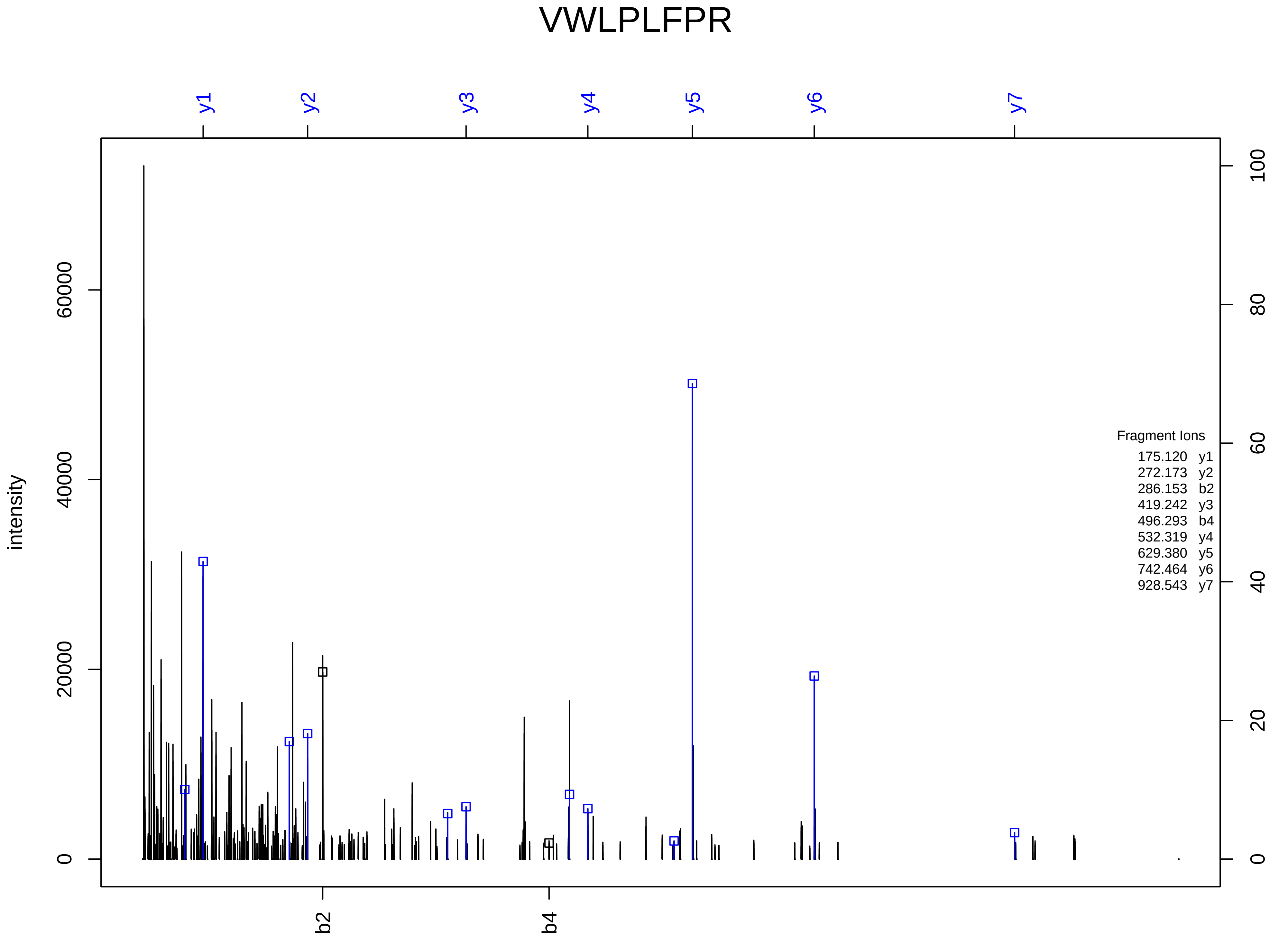
VWLPLFPR
y1
y2
y3
y4
y5
y6
y7
b2
b4
intensity
m/z
Fragment Ions
175.120 y1
272.173 y2
286.153 b2
419.242 y3
496.293 b4
532.319 y4
629.380 y5
742.464 y6
928.543 y7
0
20000
40000
60000
0
20
40
60
80
100
VWLPLFPR / File: christine_ip_12.raw; SpectrumID: ; scans: 19645

SLPAPVAQRPDSPGGGLQAPGQK

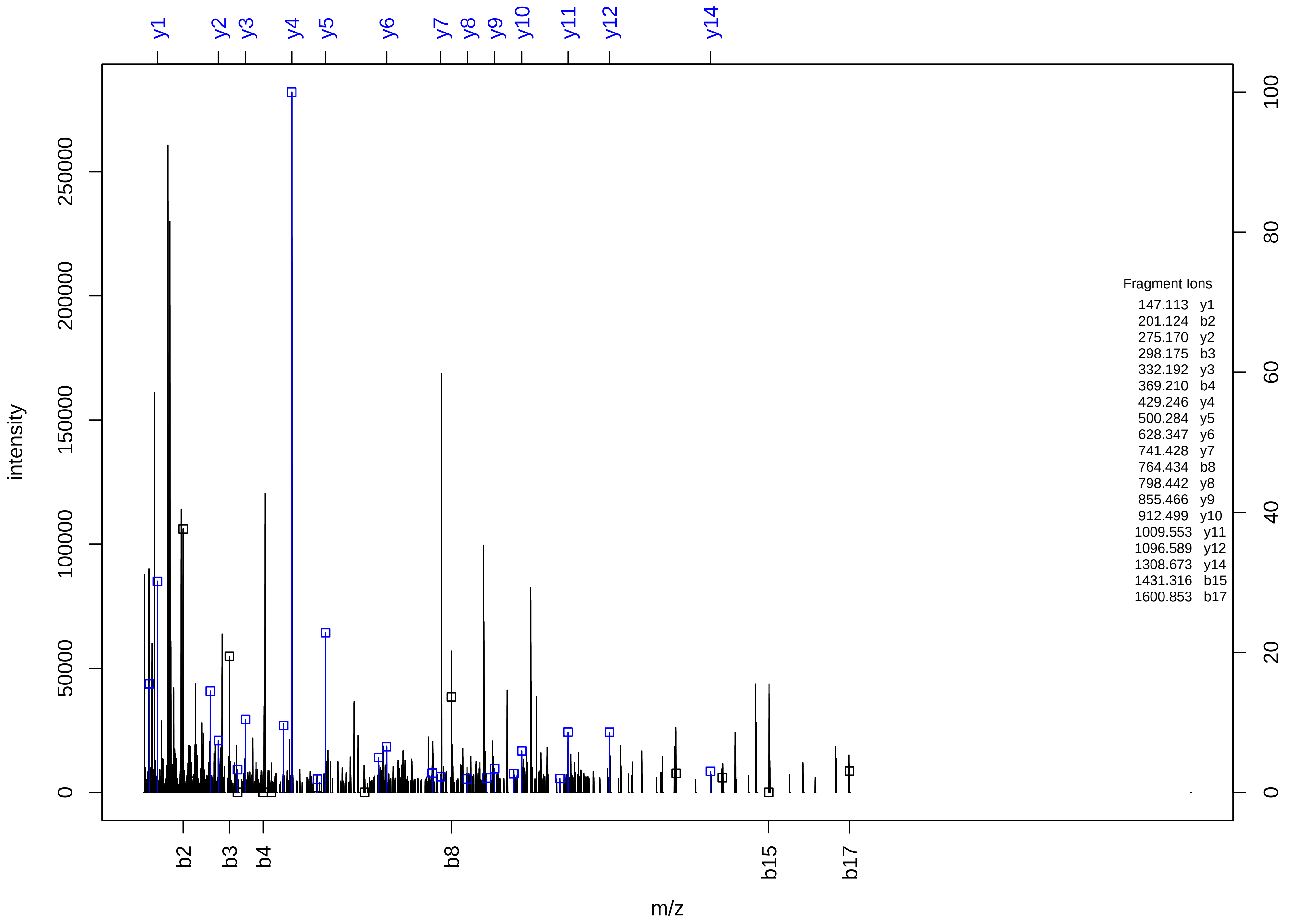

SLPAPVAQRPDSPGGGLQAPGQK / File: christine_ip_12.raw; SpectrumID: ; scans: 7436

LLGIPLLQPYTTEER

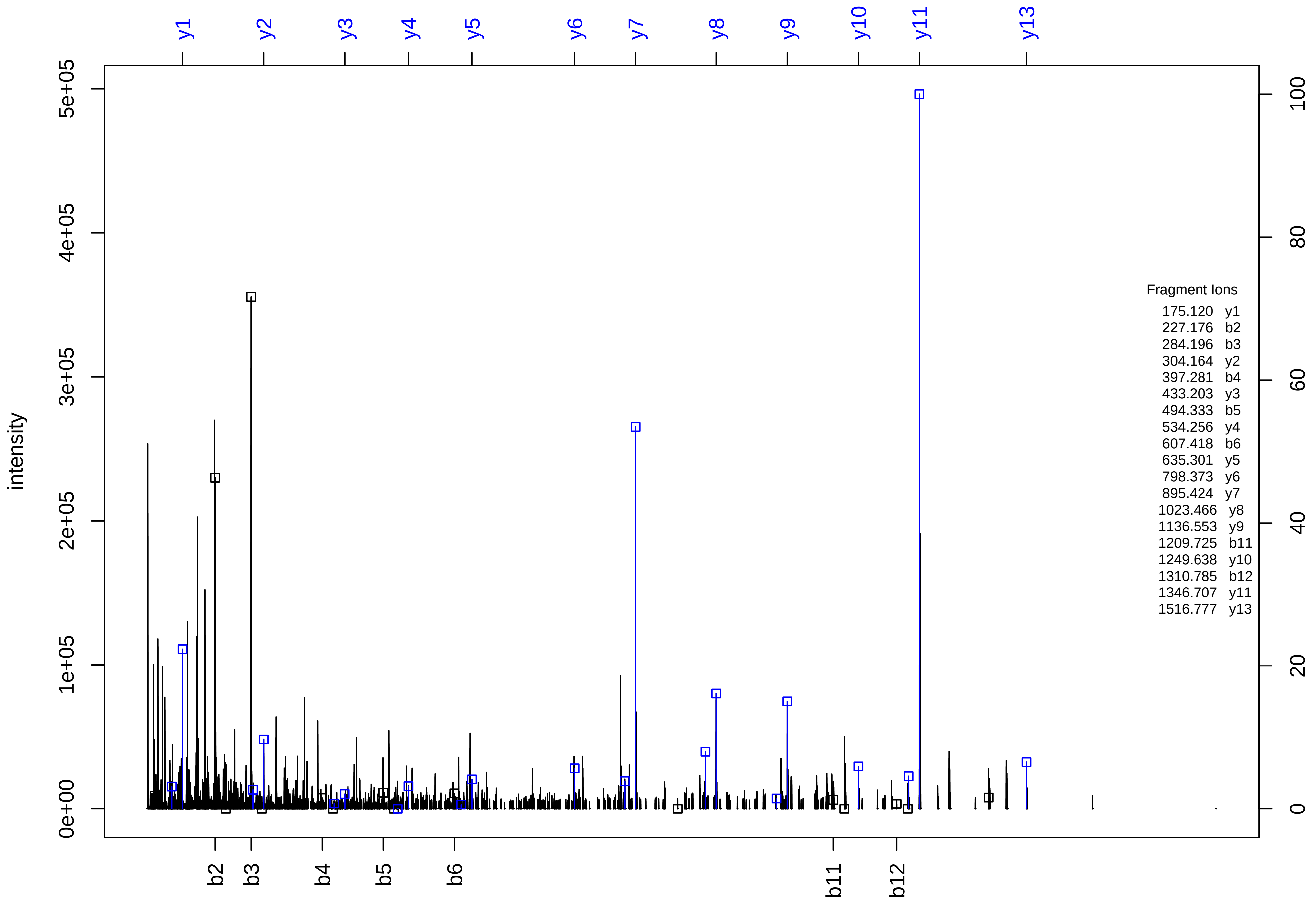

LLGIPLLQPYTTEER / File: christine_ip_12.raw; SpectrumID: ; scans: 20661

LGPSTGSTAETQCR

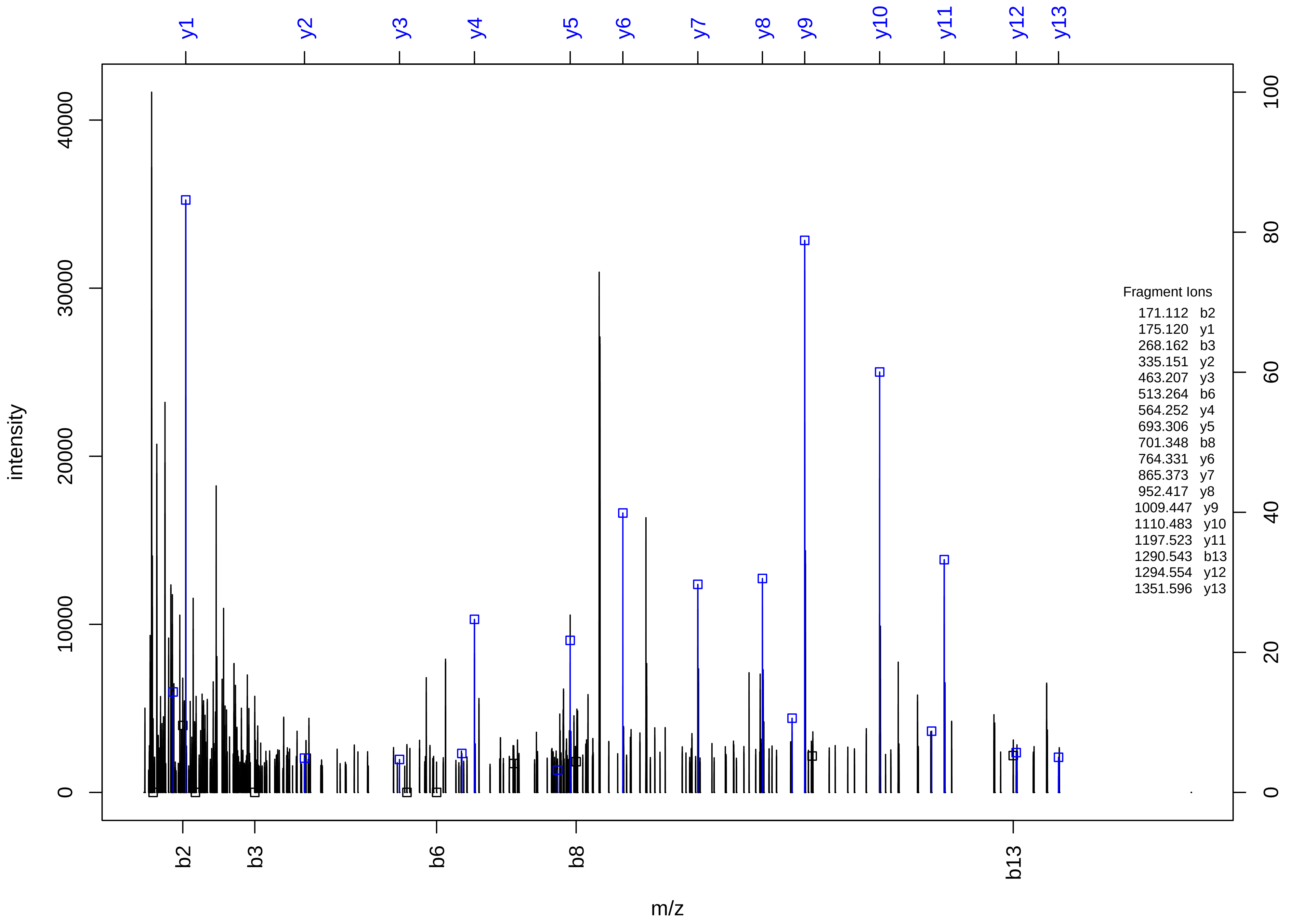

LGPSTGSTAETQCR / File: christine_ip_12.raw; SpectrumID: ; scans: 2446

VVPGFIVQGGDPTGTGSGGESIYGAPFK

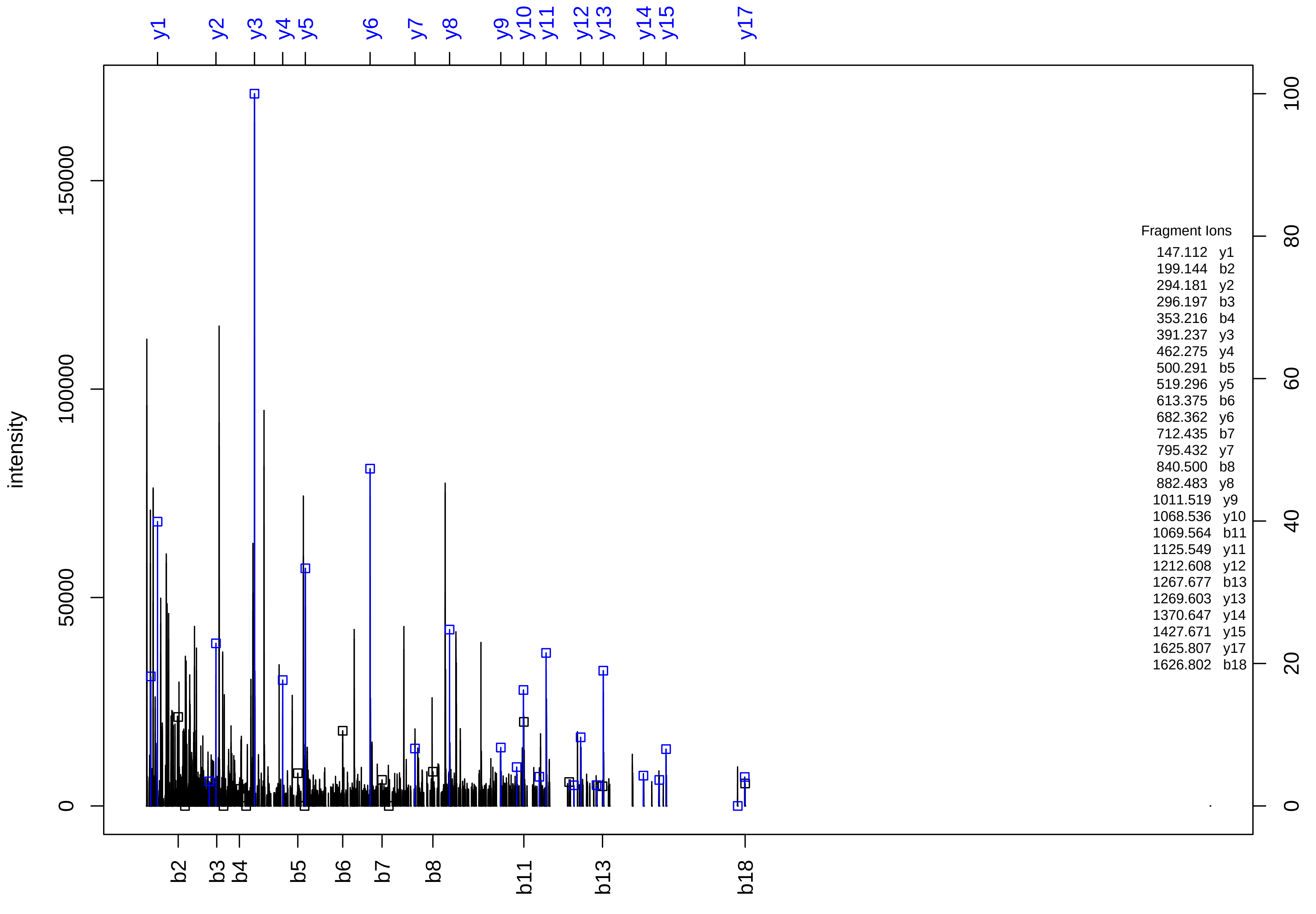

VVPGFIVQGGDPTGTGSGGESIYGAPFK / File: christine_ip_12.raw; SpectrumID: ; scans: 16858

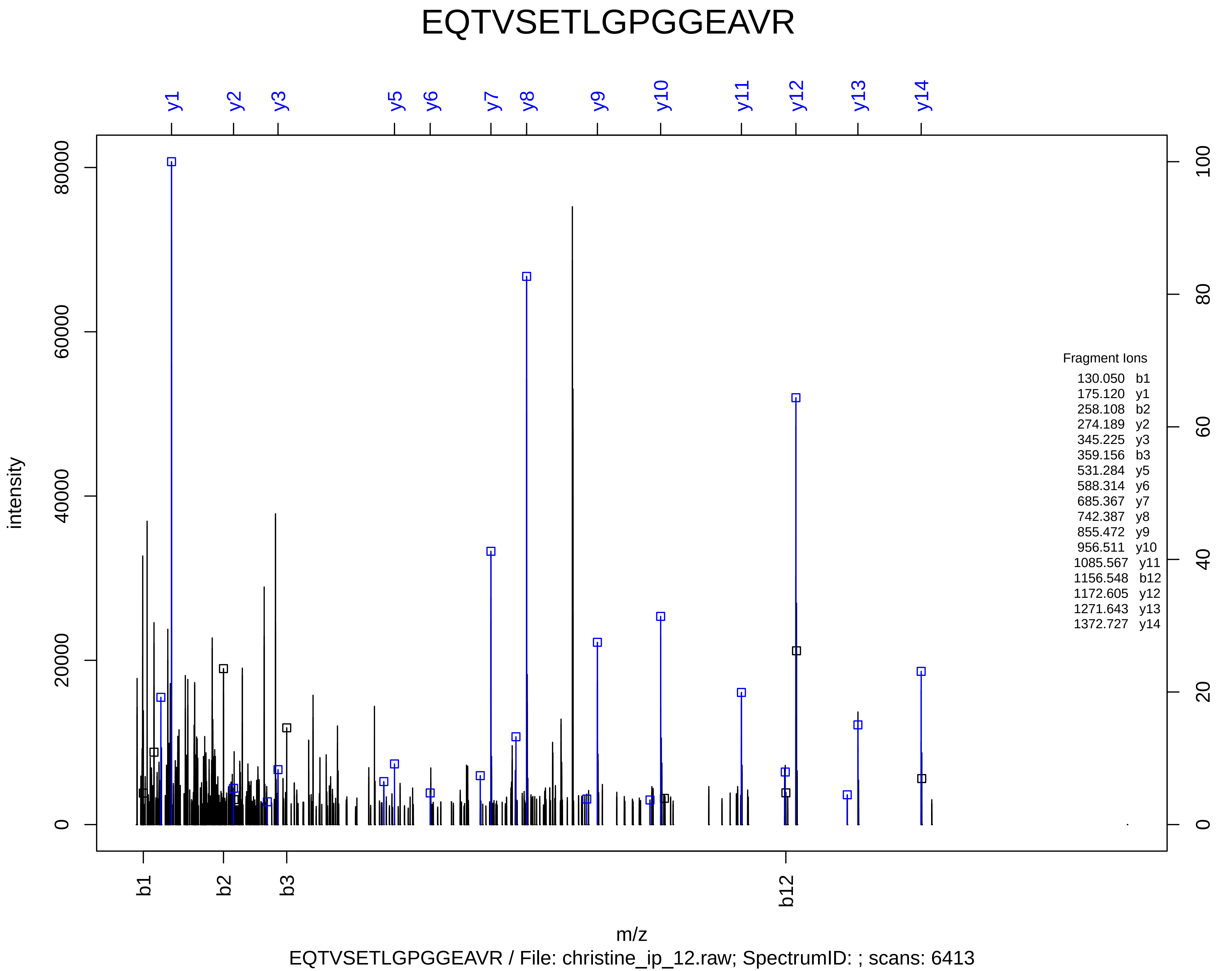

EQTVSETLGPGGEAVR
y1
y2
y3
y5
y6
y7
y8
y9
y10
y11
y12
y13
y14
b1
b2
b3
b12
intensity
0
20000
40000
60000
80000
0
20
40
60
80
100
m/z
Fragment Ions
130.050 b1
175.120 y1
258.108 b2
274.189 y2
345.225 y3
359.156 b3
531.284 y5
588.314 y6
685.367 y7
742.387 y8
855.472 y9
956.511 y10
1085.567 y11
1156.548 b12
1172.605 y12
1271.643 y13
1372.727 y14
EQTVSETLGPGGEAVR / File: christine_ip_12.raw; SpectrumID: ; scans: 6413

AQQLLDAVEQR

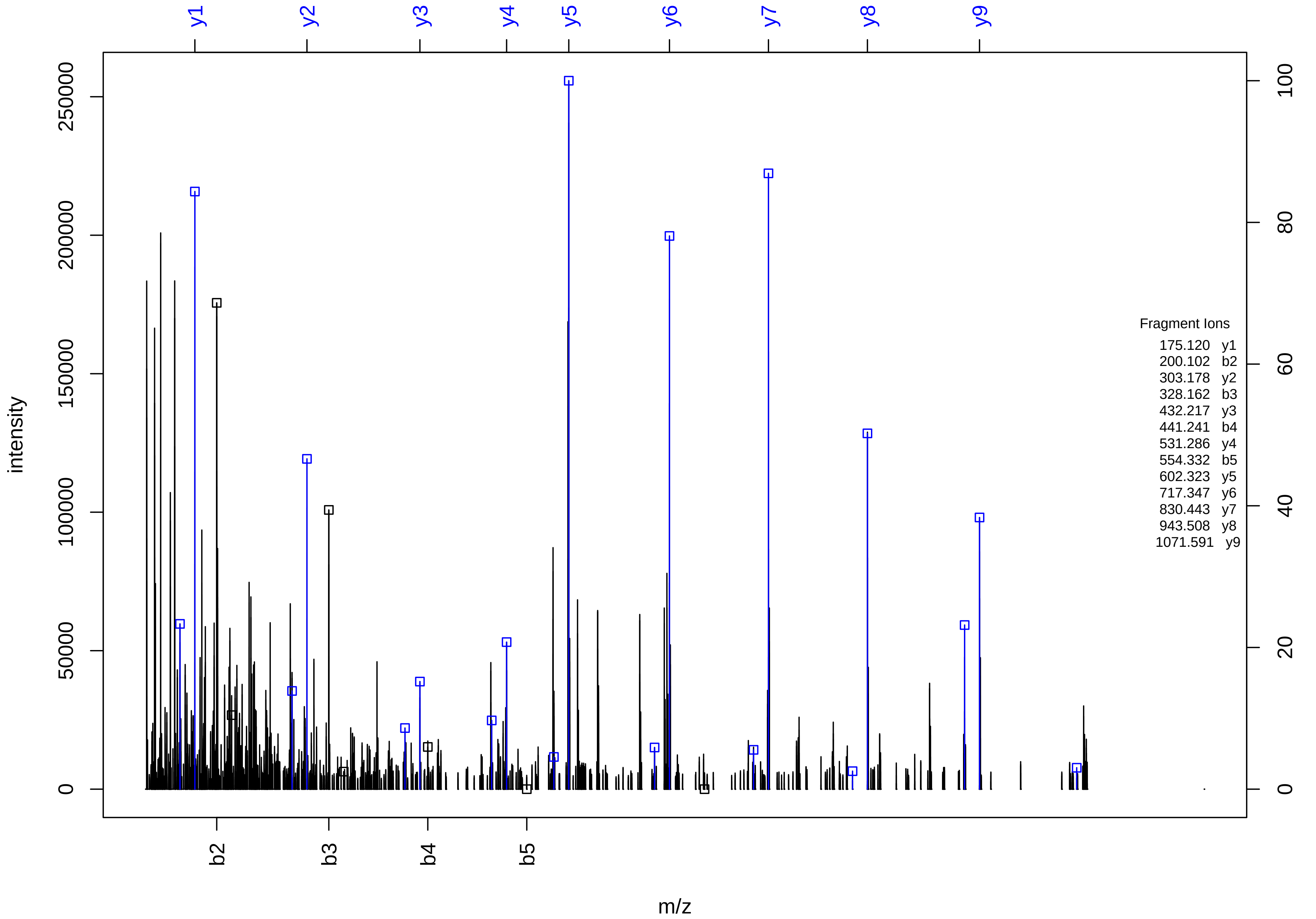

AQQLLDAVEQR / File: christine_ip_12.raw; SpectrumID: ; scans: 8607

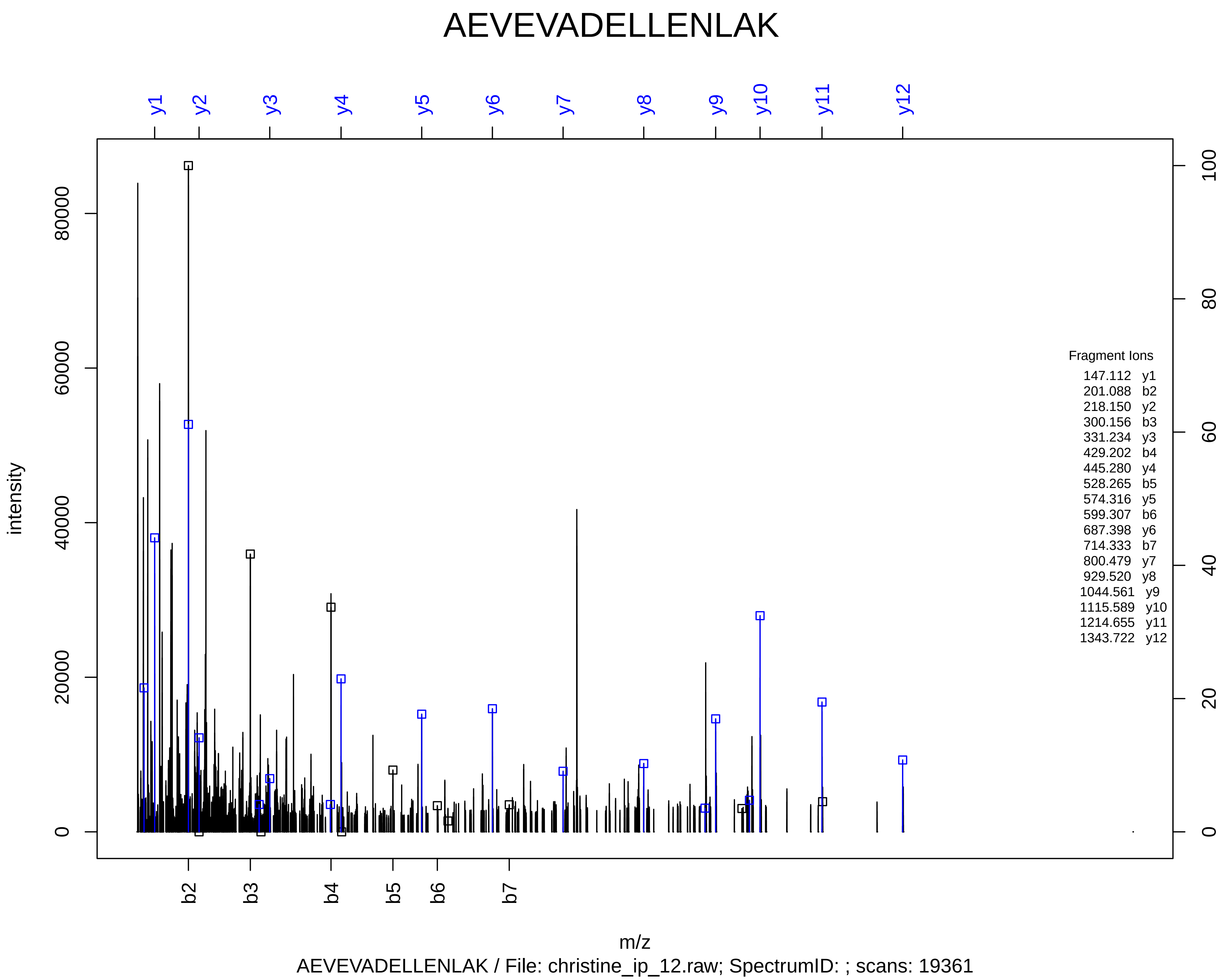
AEVEVADELLENLAK
y1
y2
y3
y4
y5
y6
y7
y8
y9
y10
y11
y12
intensity
0
20000
40000
60000
80000
0
20
40
60
80
100
Fragment Ions
147.112 y1
201.088 b2
218.150 y2
300.156 b3
331.234 y3
429.202 b4
445.280 y4
528.265 b5
574.316 y5
599.307 b6
687.398 y6
714.333 b7
800.479 y7
929.520 y8
1044.561 y9
1115.589 y10
1214.655 y11
1343.722 y12
b2
b3
b4
b5
b6
b7
m/z
AEVEVADELLENLAK / File: christine_ip_12.raw; SpectrumID: ; scans: 19361

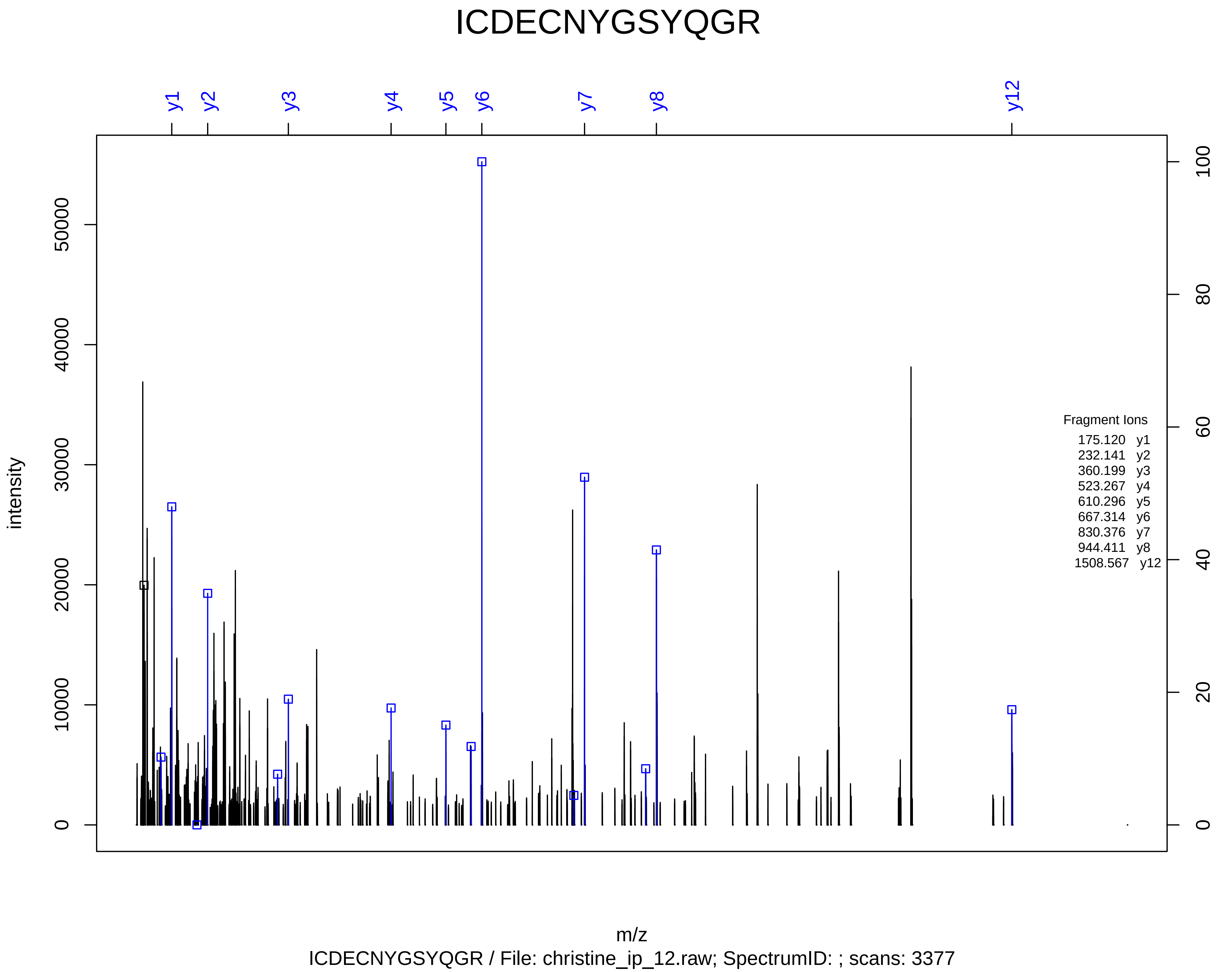

ICDECNYGSYQGR
y1
y2
y3
y4
y5
y6
y7
y8
y12
intensity
0
10000
20000
30000
40000
50000
0
20
40
60
80
100
Fragment Ions
175.120 y1
232.141 y2
360.199 y3
523.267 y4
610.296 y5
667.314 y6
830.376 y7
944.411 y8
1508.567 y12
m/z
ICDECNYGSYQGR / File: christine_ip_12.raw; SpectrumID: ; scans: 3377

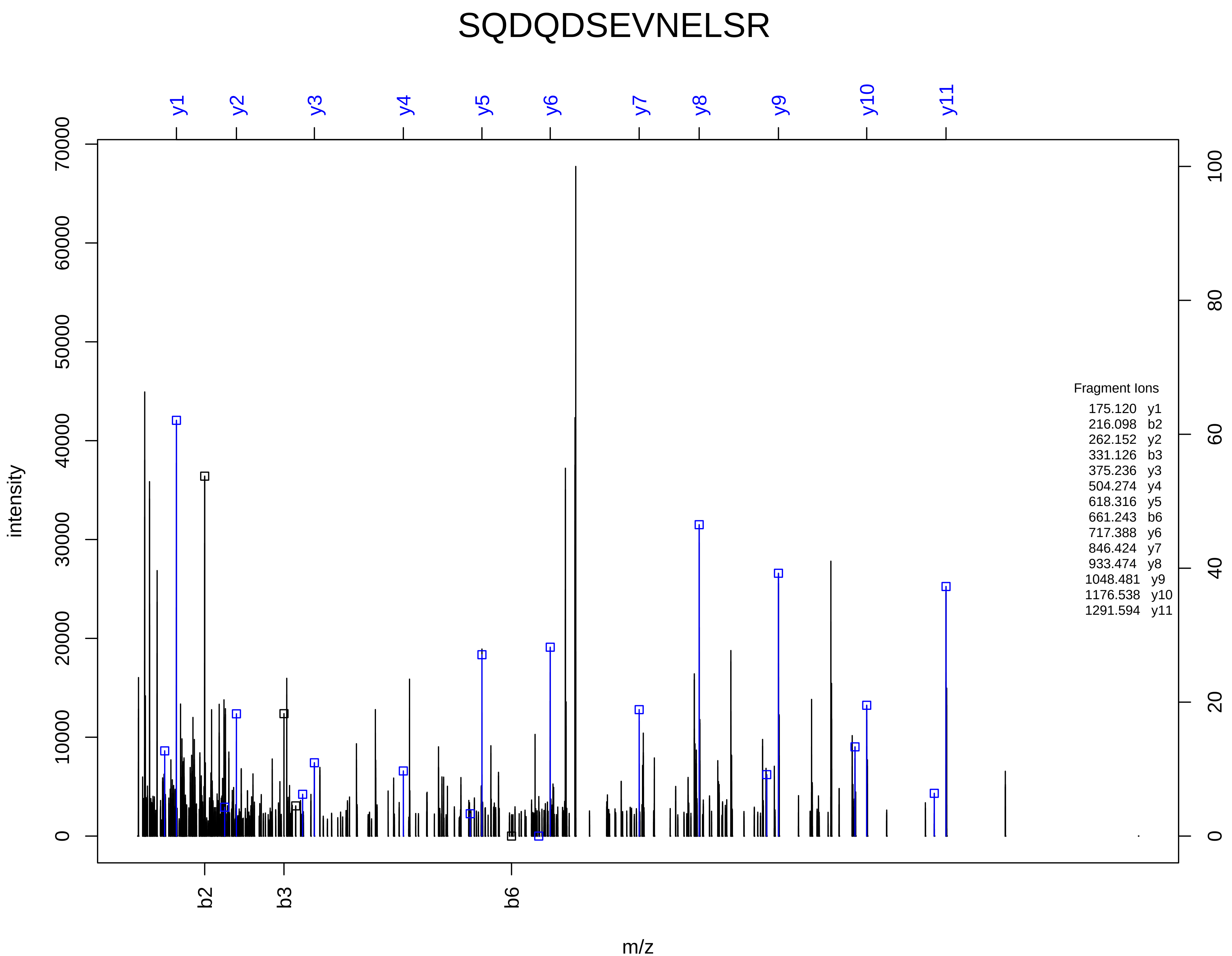

SQDQDSEVNELSR / File: christine_ip_12.raw; SpectrumID: ; scans: 3692

ISLLLHLLEDELDHR

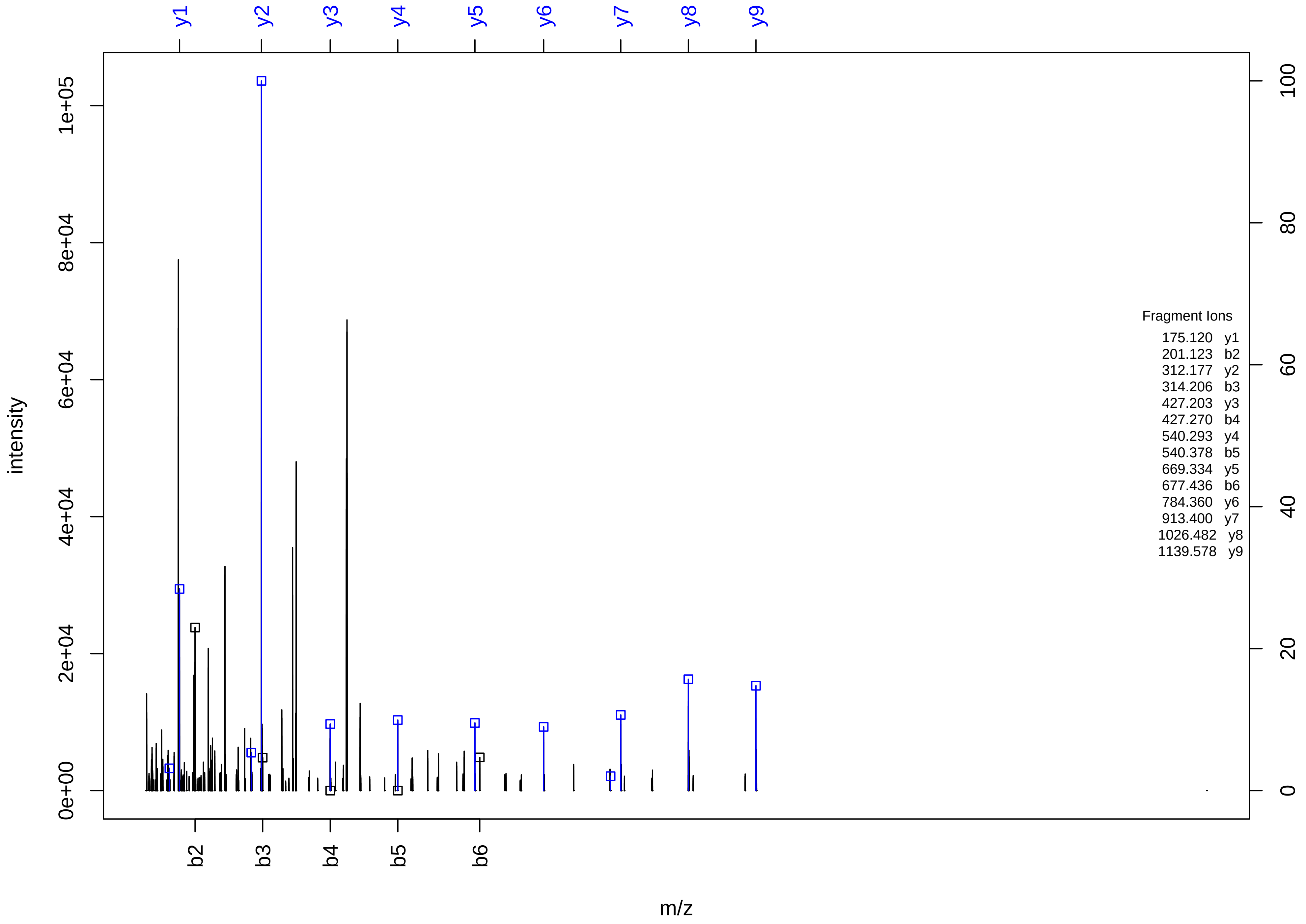

ISLLLHLLEDELDHR / File: christine_ip_12.raw; SpectrumID: ; scans: 20683

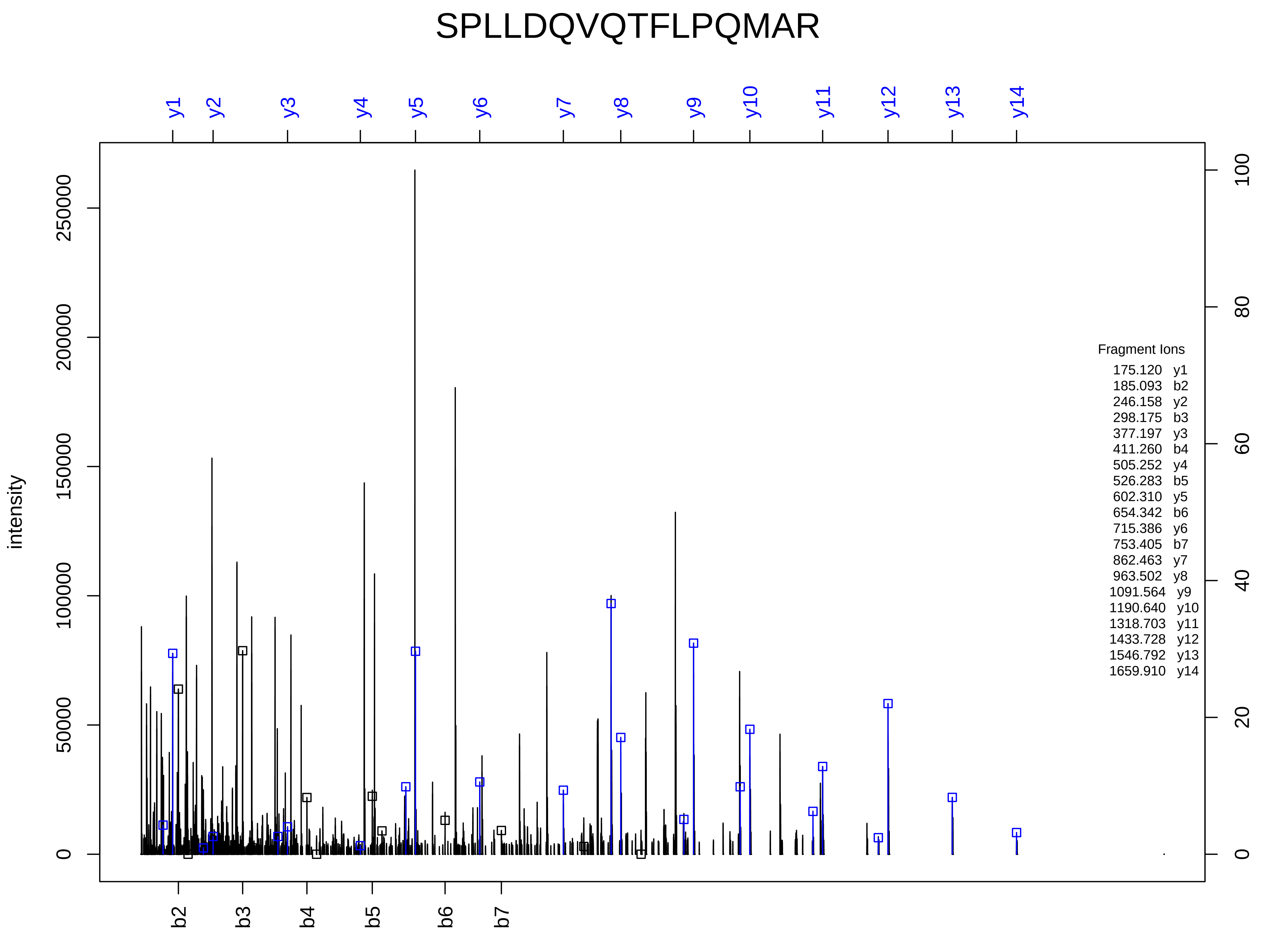

SPLLDQVQTFLPQMAR / File: christine_ip_12.raw; SpectrumID: ; scans: 20240

LNPQEYLTSTACR

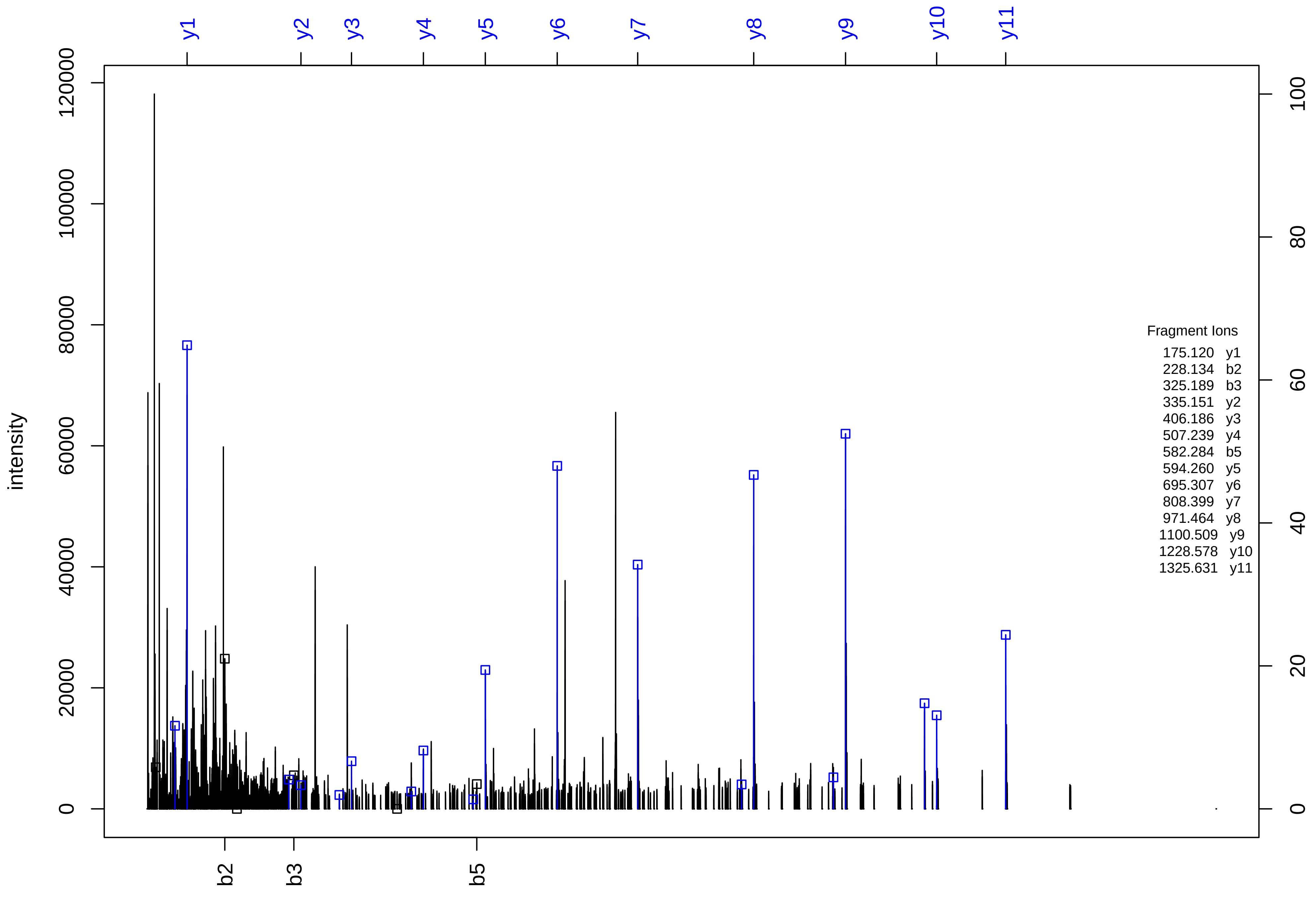

LNPQEYLTSTACR / File: christine_ip_12.raw; SpectrumID: ; scans: 10042

VASTLTEEGGGGGGGGGSVAPKPPR

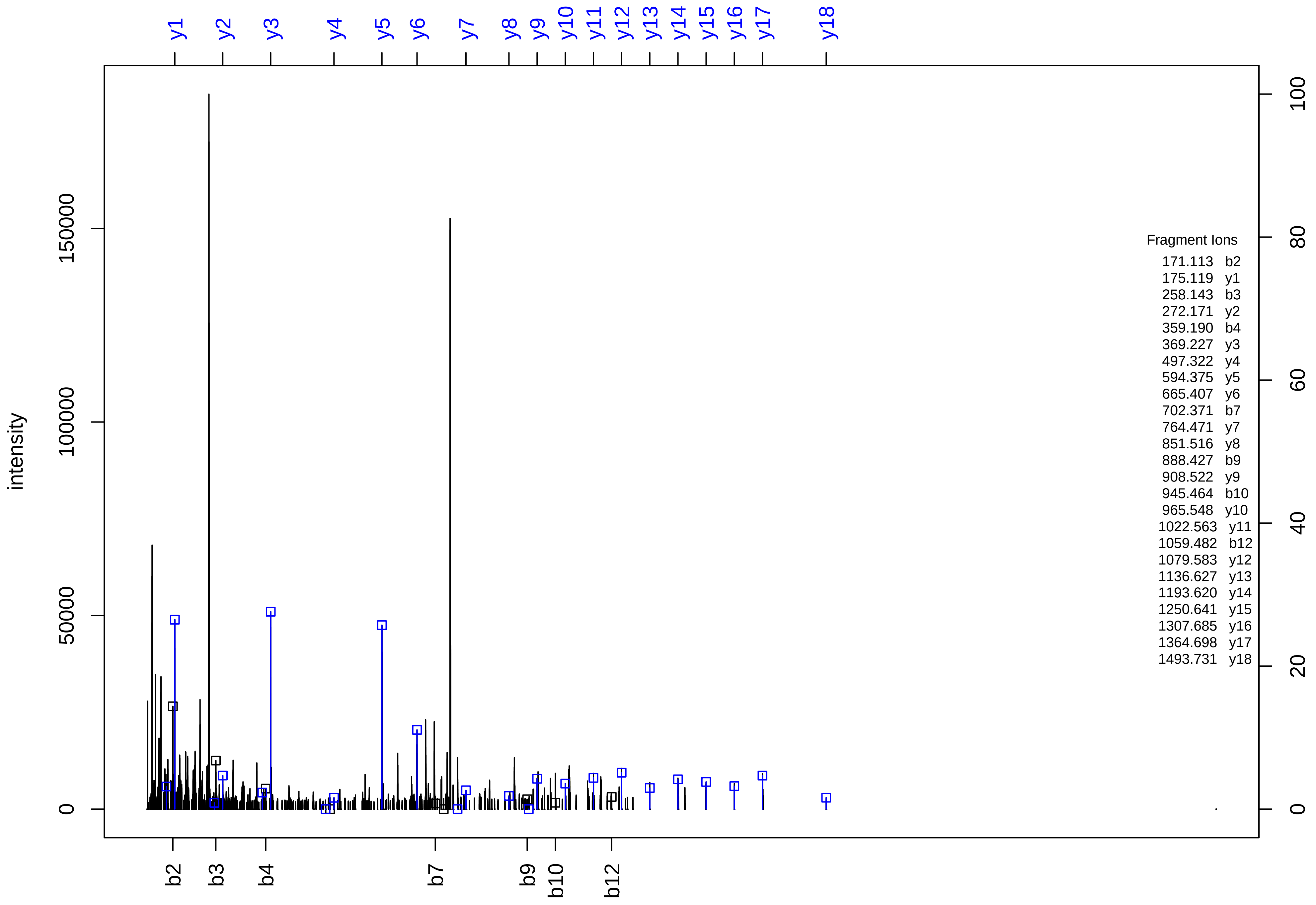

VASTLTEEGGGGGGGGGSVAPKPPR / File: christine_ip_12.raw; SpectrumID: ; scans: 4505

LLM*M*PSVNK

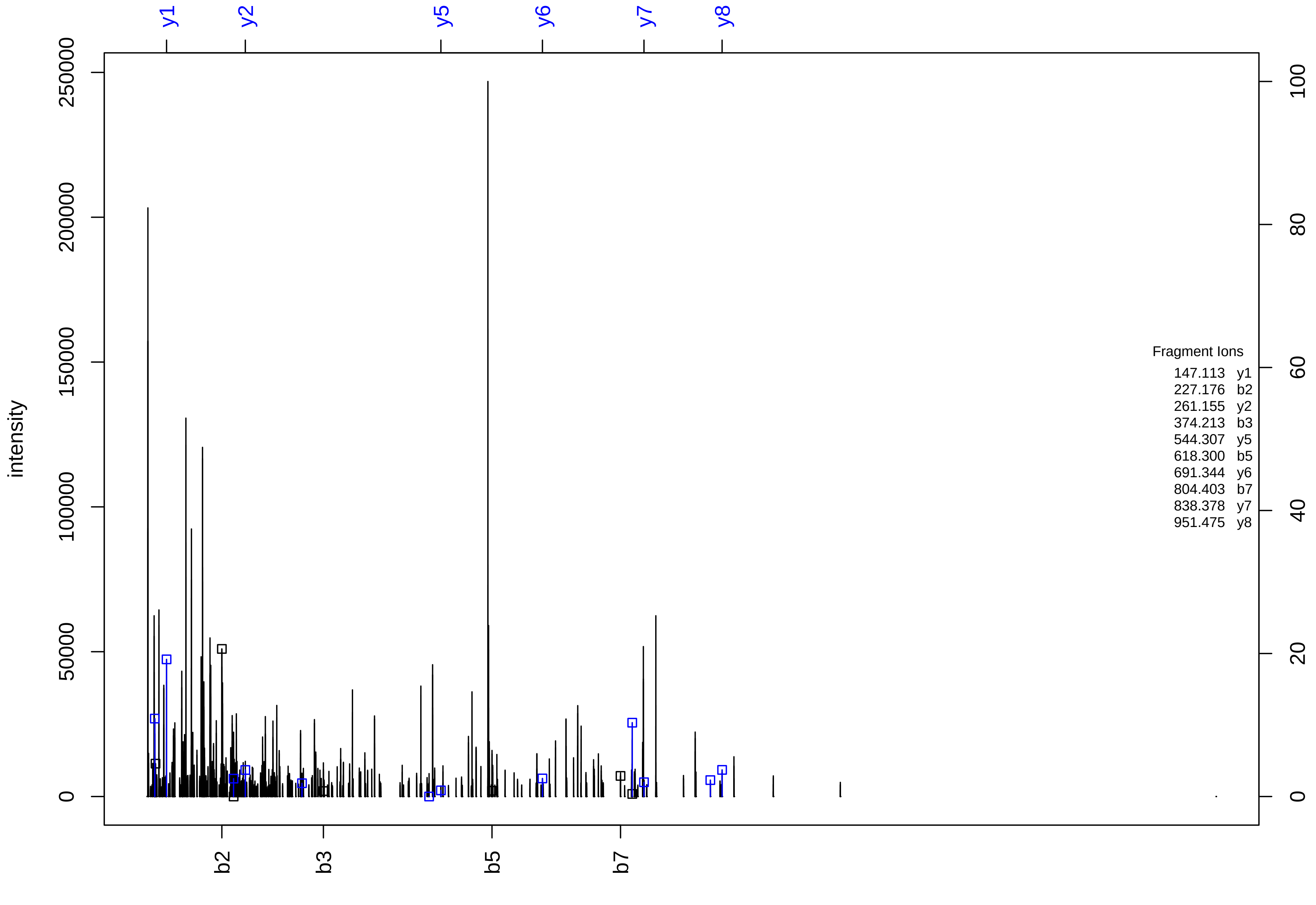

LLMMPSVNK / File: christine_ip_12.raw; SpectrumID: ; scans: 14422

TILQGSSEGTGLSALLPQPK

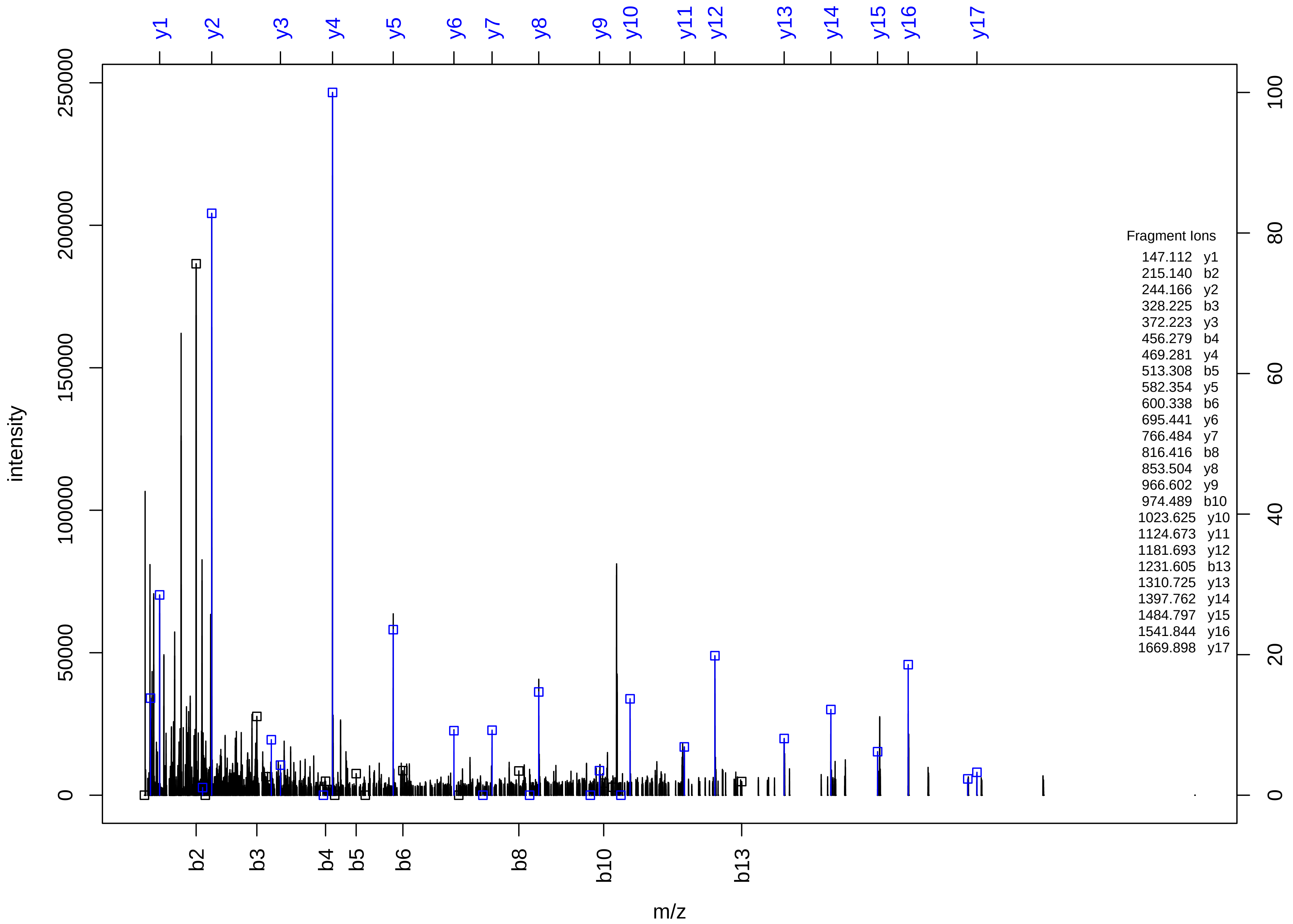

TILQGSSEGTGLSALLPQPK / File: christine_ip_12.raw; SpectrumID: ; scans: 16529

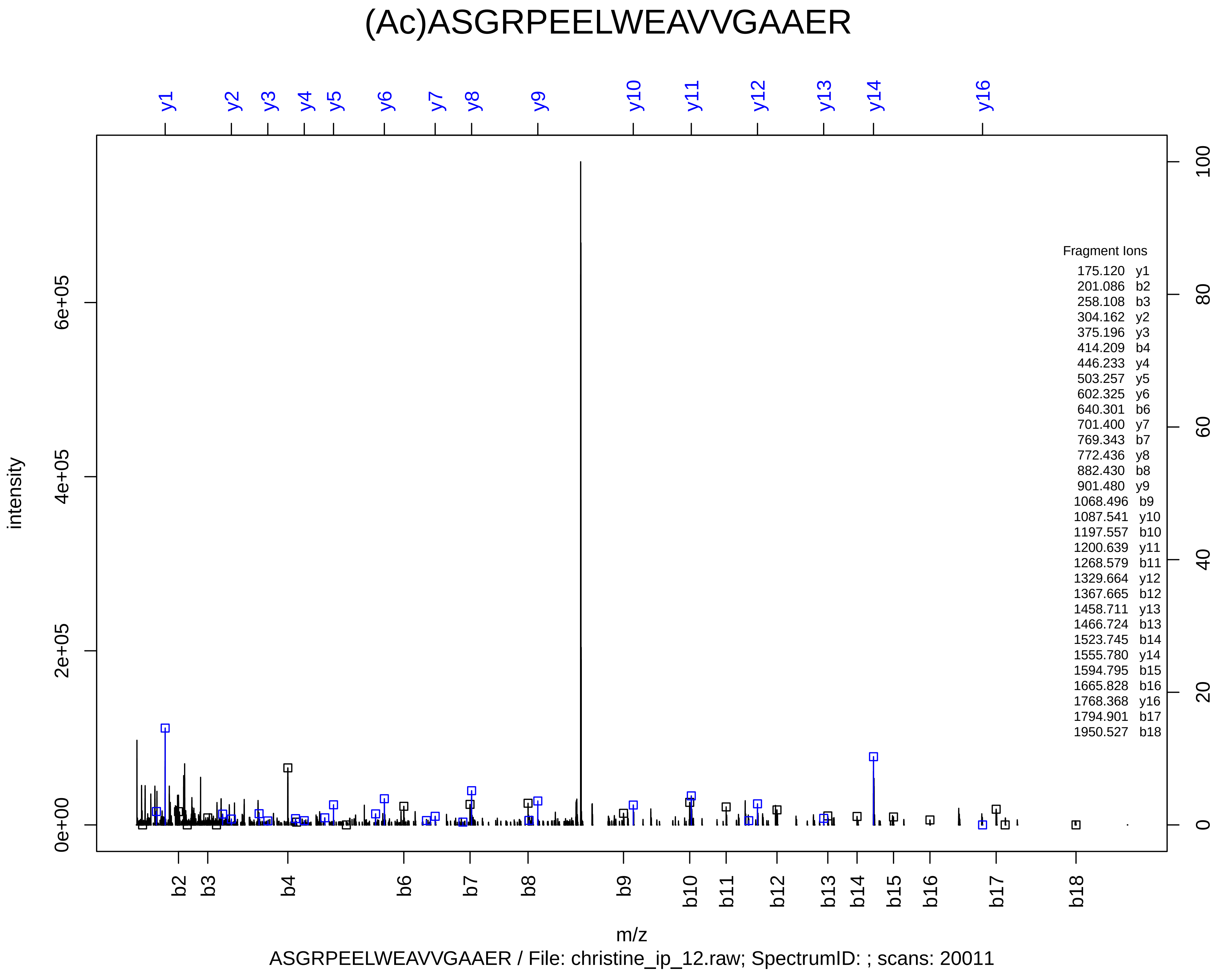
(Ac)ASGRPEELWEAVVGAAER
y1
y2
y3
y4
y5
y6
y7
y8
y9
y10
y11
y12
y13
y14
y16
b2
b3
b4
b6
b7
b8
b9
b10
b11
b12
b13
b14
b15
b16
b17
b18
intensity
0e+00
2e+05
4e+05
6e+05
0
20
40
60
80
100
m/z
Fragment Ions
175.120 y1
201.086 b2
258.108 b3
304.162 y2
375.196 y3
414.209 b4
446.233 y4
503.257 y5
602.325 y6
640.301 b6
701.400 y7
769.343 b7
772.436 y8
882.430 b8
901.480 y9
1068.496 b9
1087.541 y10
1197.557 b10
1200.639 y11
1268.579 b11
1329.664 y12
1367.665 b12
1458.711 y13
1466.724 b13
1523.745 b14
1555.780 y14
1594.795 b15
1665.828 b16
1768.368 y16
1794.901 b17
1950.527 b18
ASGRPEELWEAVVGAAER / File: christine_ip_12.raw; SpectrumID: ; scans: 20011

LFFTSVPGGR

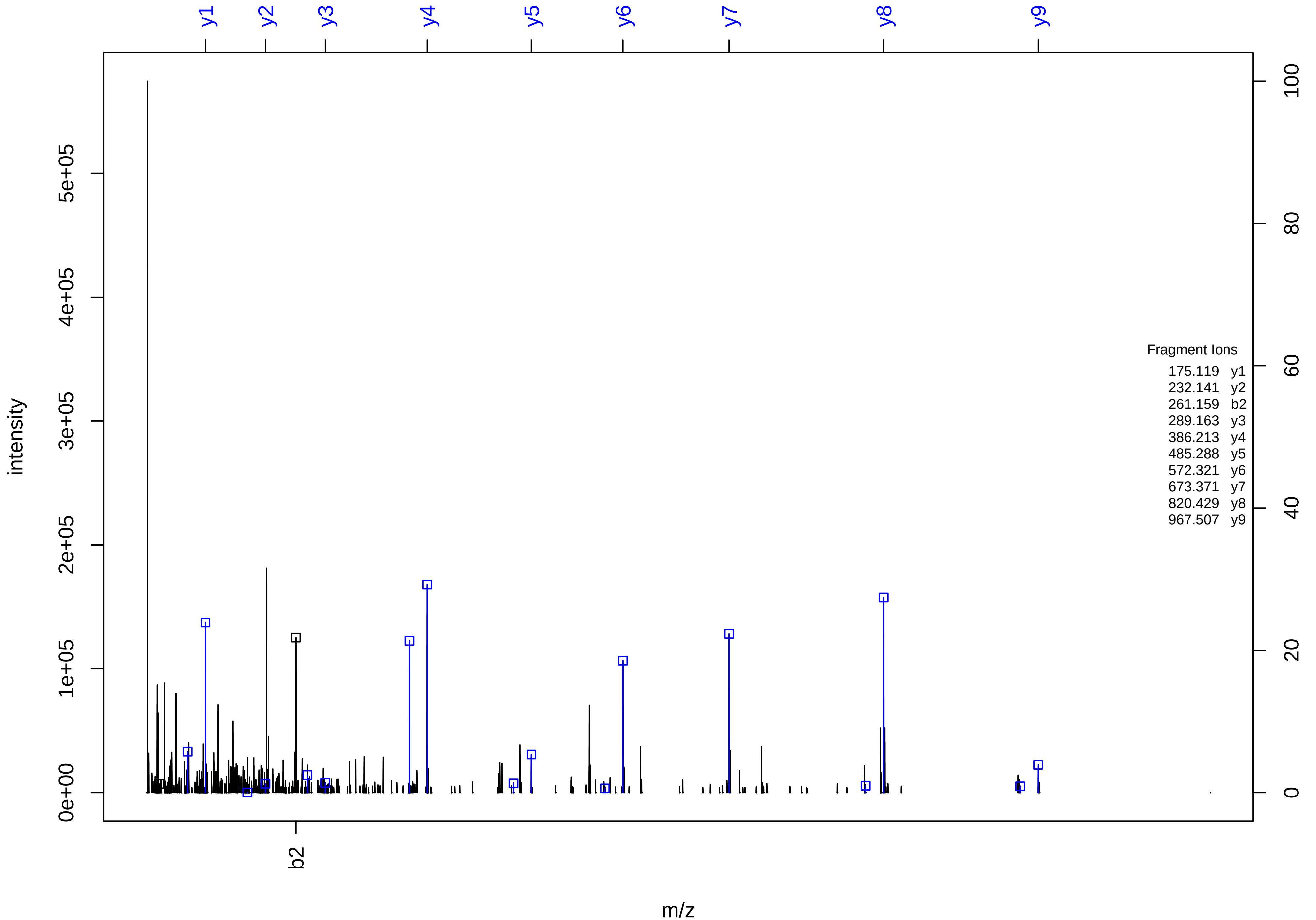

LFFTSVPGGR / File: christine_ip_12.raw; SpectrumID: ; scans: 12427

DLIHDVSFDFHGR

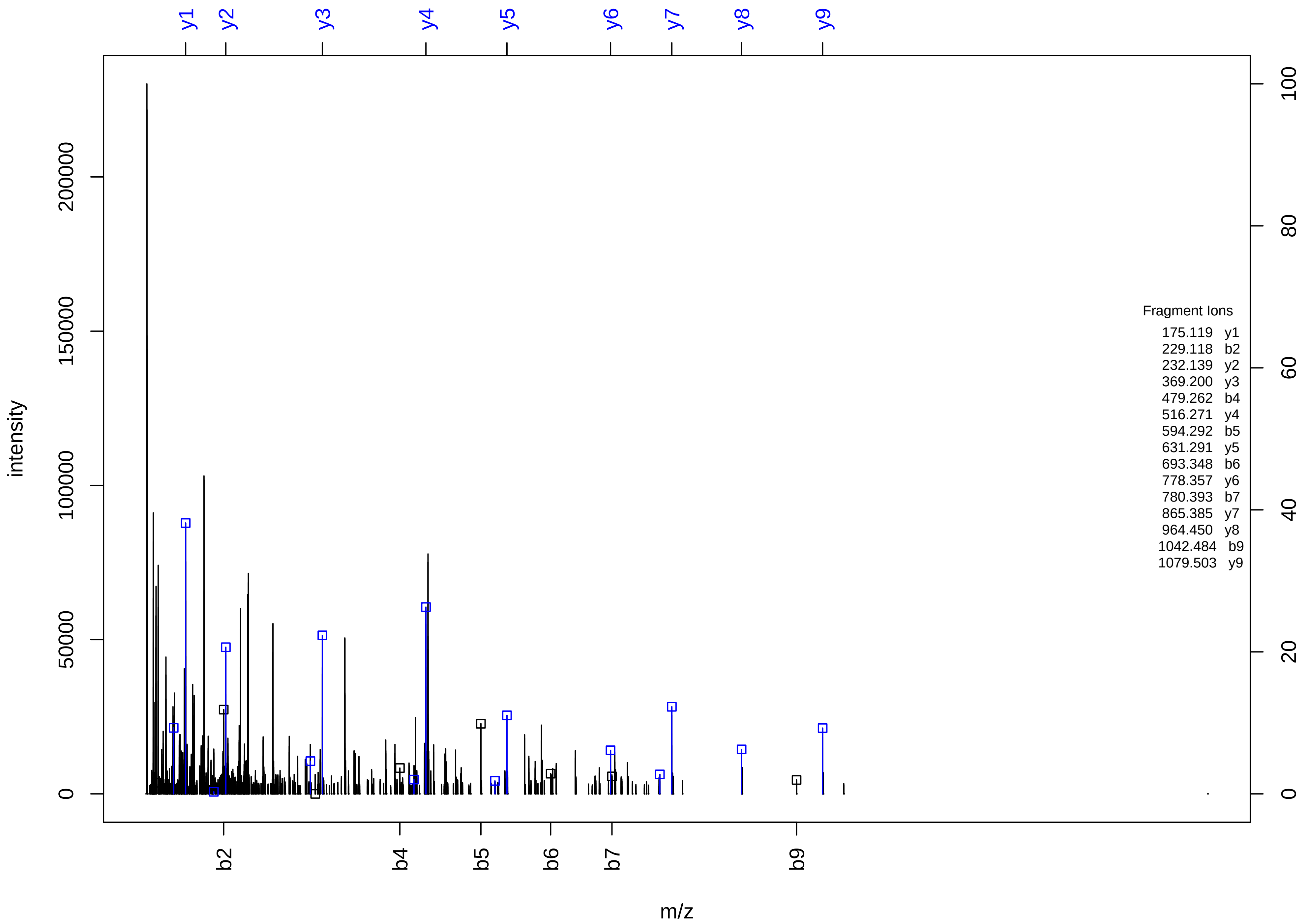

DLIHDVSFDFHGR / File: christine_ip_12.raw; SpectrumID: ; scans: 13382

TILFIDEIHR

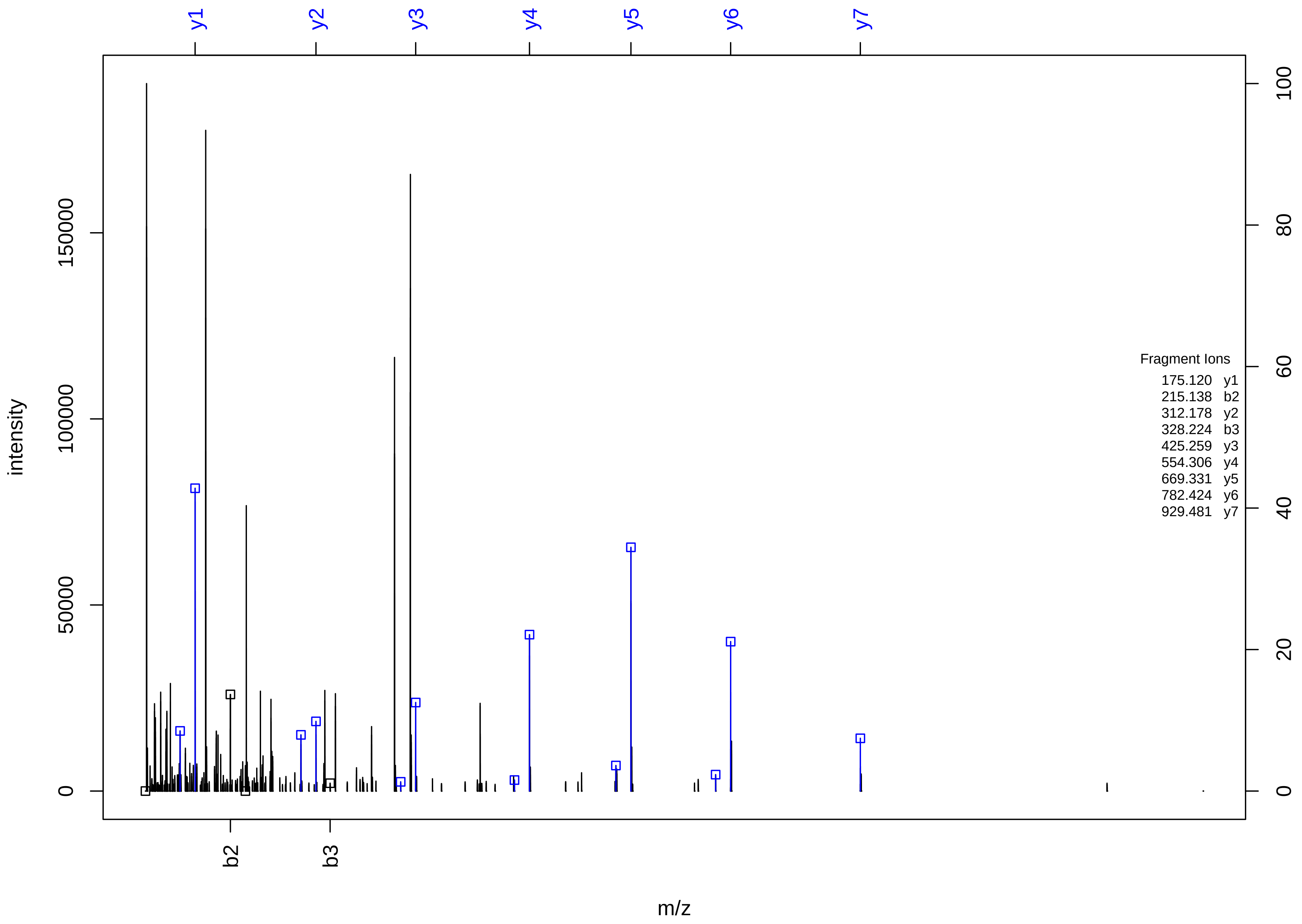

TILFIDEIHR / File: christine_ip_12.raw; SpectrumID: ; scans: 13447

YSDEENLPEK

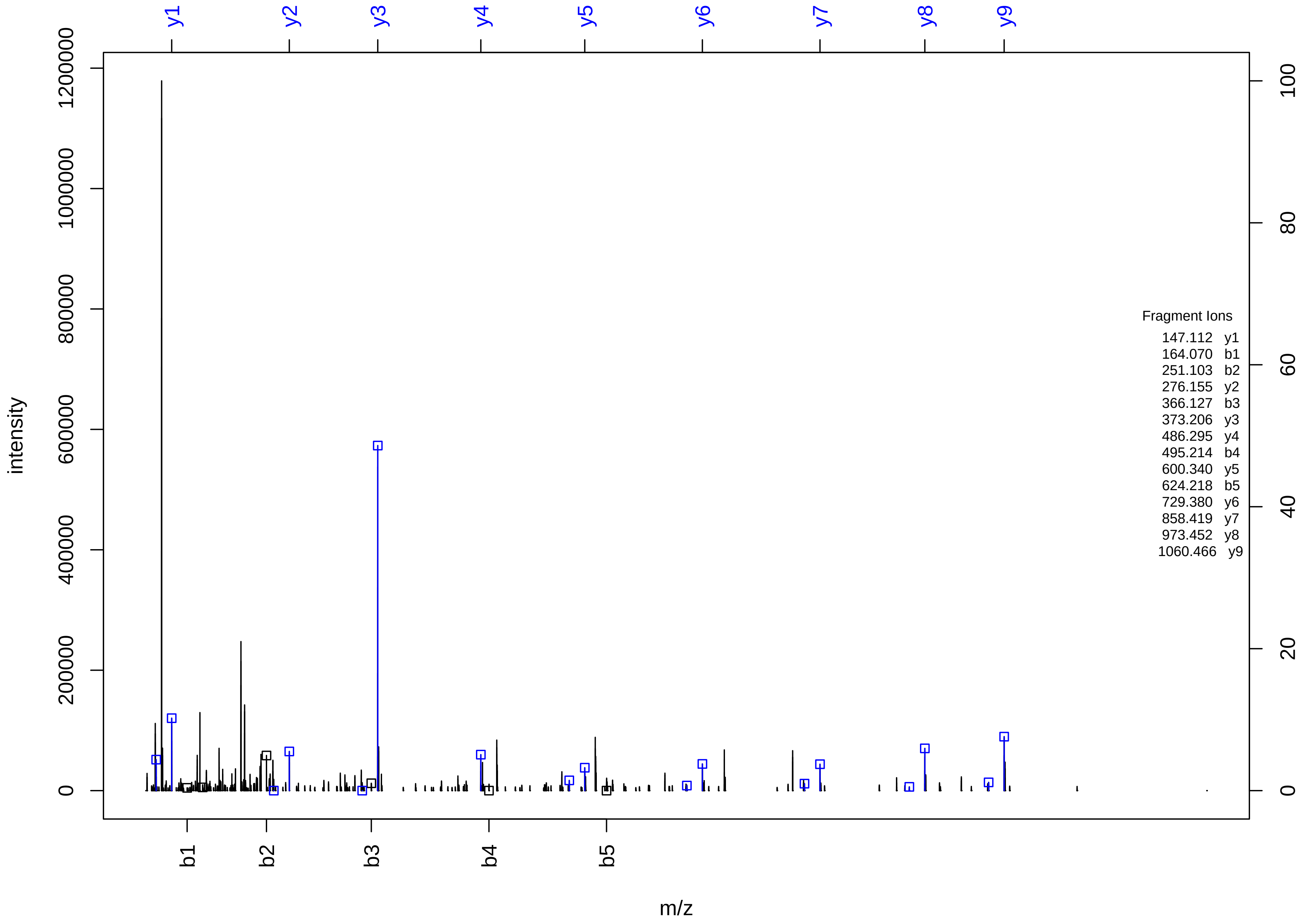

YSDEENLPEK / File: christine_ip_12.raw; SpectrumID: ; scans: 3008

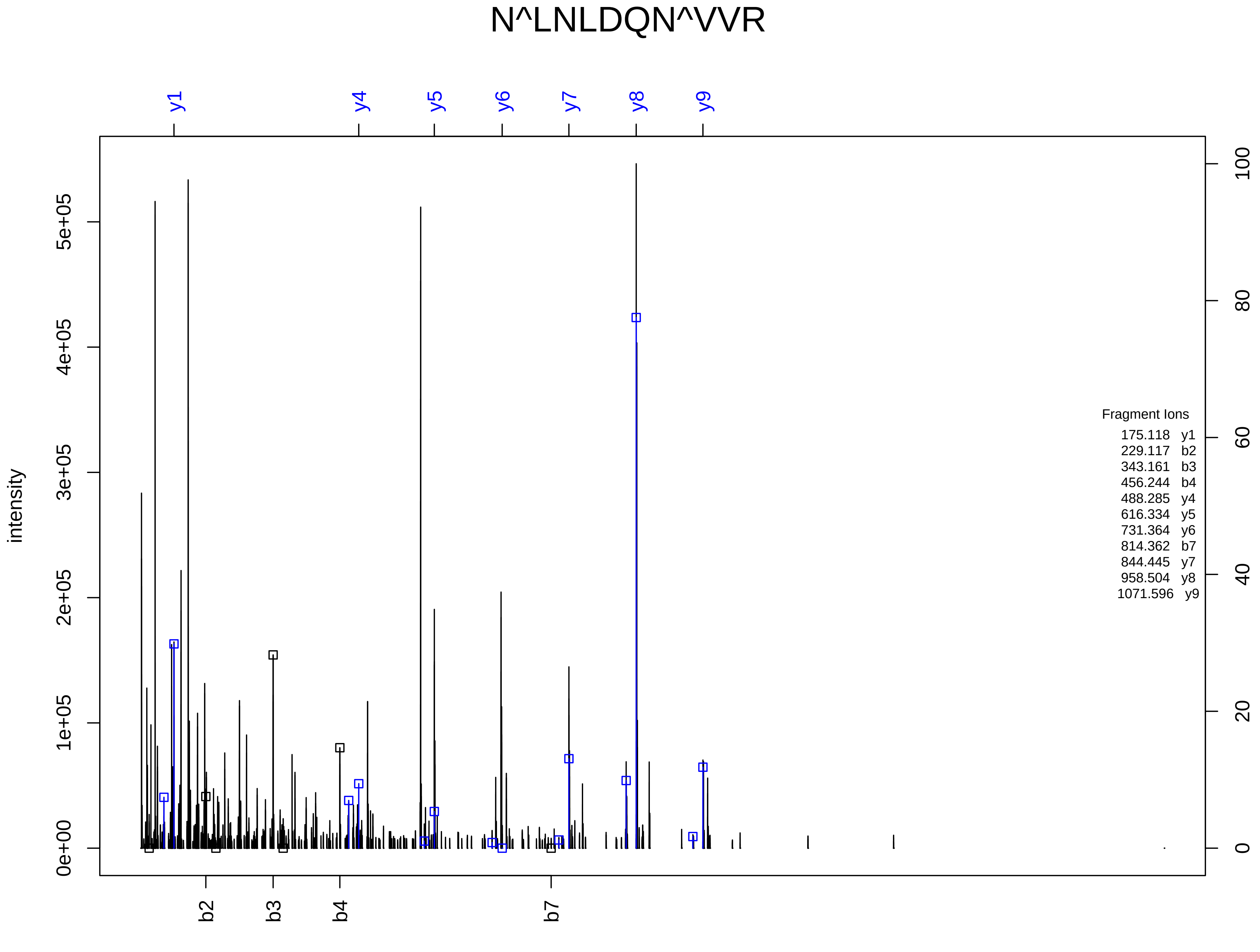
N^LNLDQN^VVR
y1
y4
y5
y6
y7
y8
y9
b2
b3
b4
b7
intensity
m/z
Fragment Ions
175.118 y1
229.117 b2
343.161 b3
456.244 b4
488.285 y4
616.334 y5
731.364 y6
814.362 b7
844.445 y7
958.504 y8
1071.596 y9
0e+00
1e+05
2e+05
3e+05
4e+05
5e+05
0
20
40
60
80
100
NLNLDQNVVR / File: christine_ip_12.raw; SpectrumID: ; scans: 15450

SIHLEGDGQQLLDALQHELVTTQR

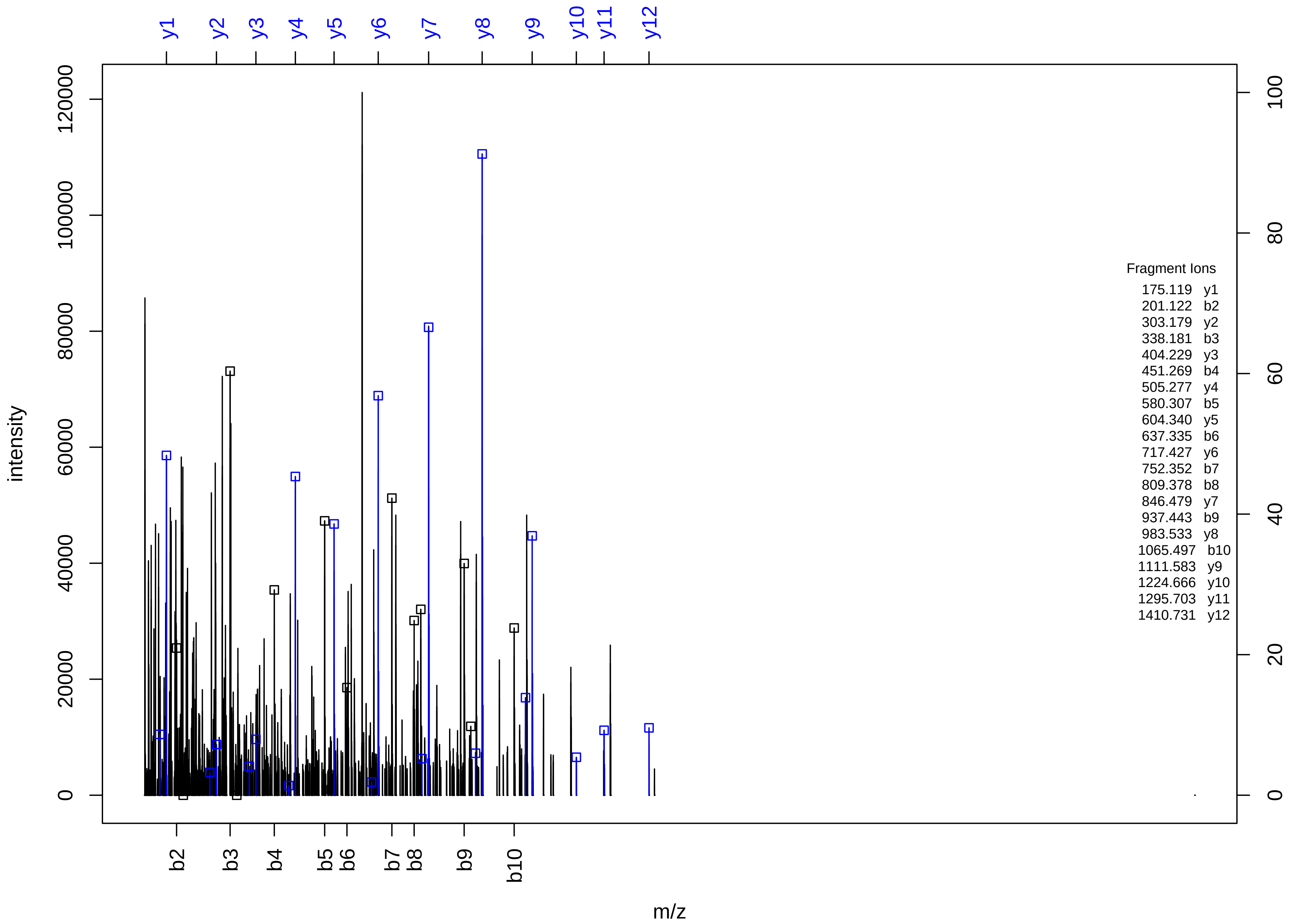

SIHLEGDGQQLLDALQHELVTTQR / File: christine_ip_12.raw; SpectrumID: ; scans: 19043

QGPVSQSATQQPVTADK

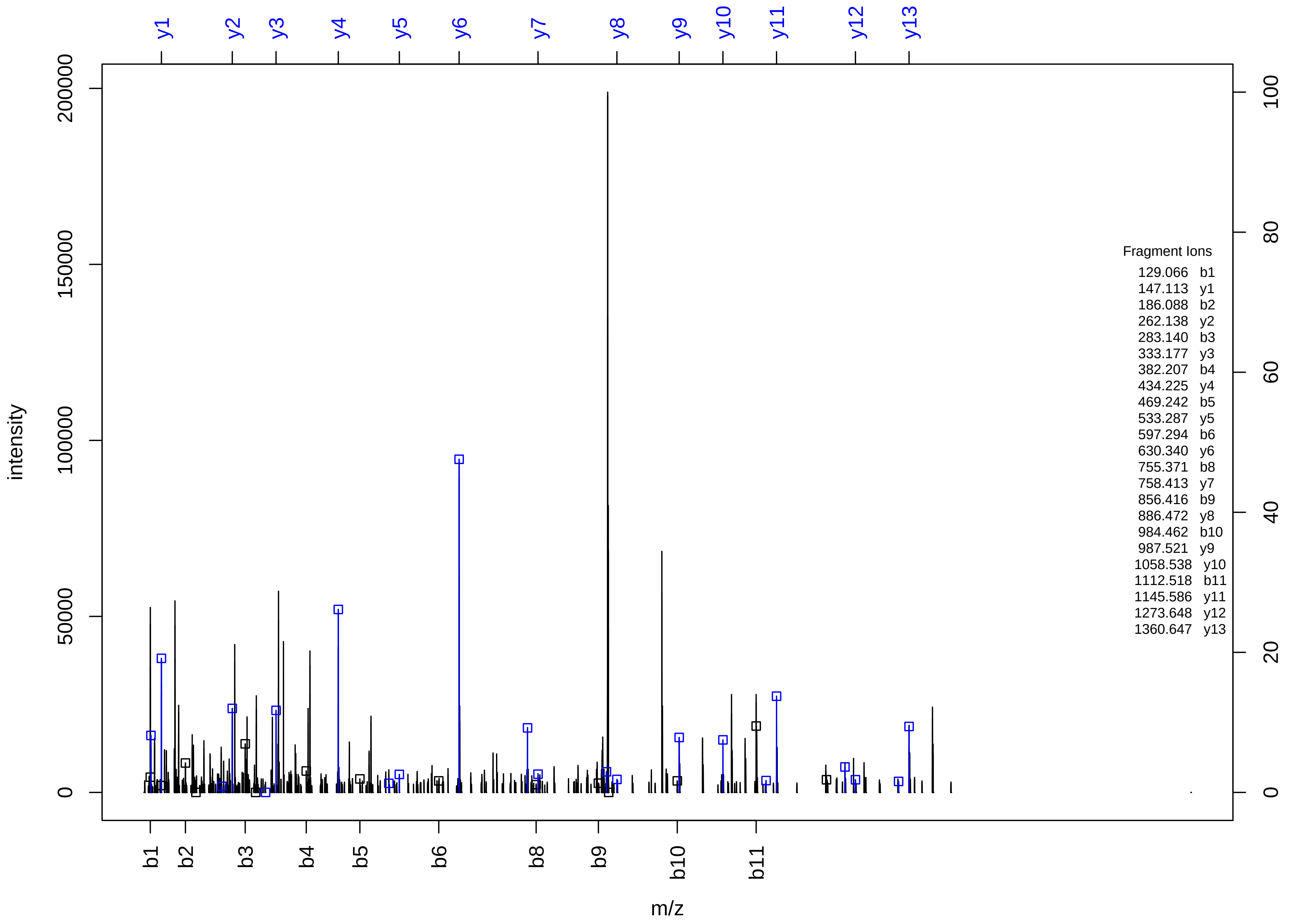

QGPVSQSATQQPVTADK / File: christine_ip_12.raw; SpectrumID: ; scans: 3068

TGSVDIIVTDLPFGK

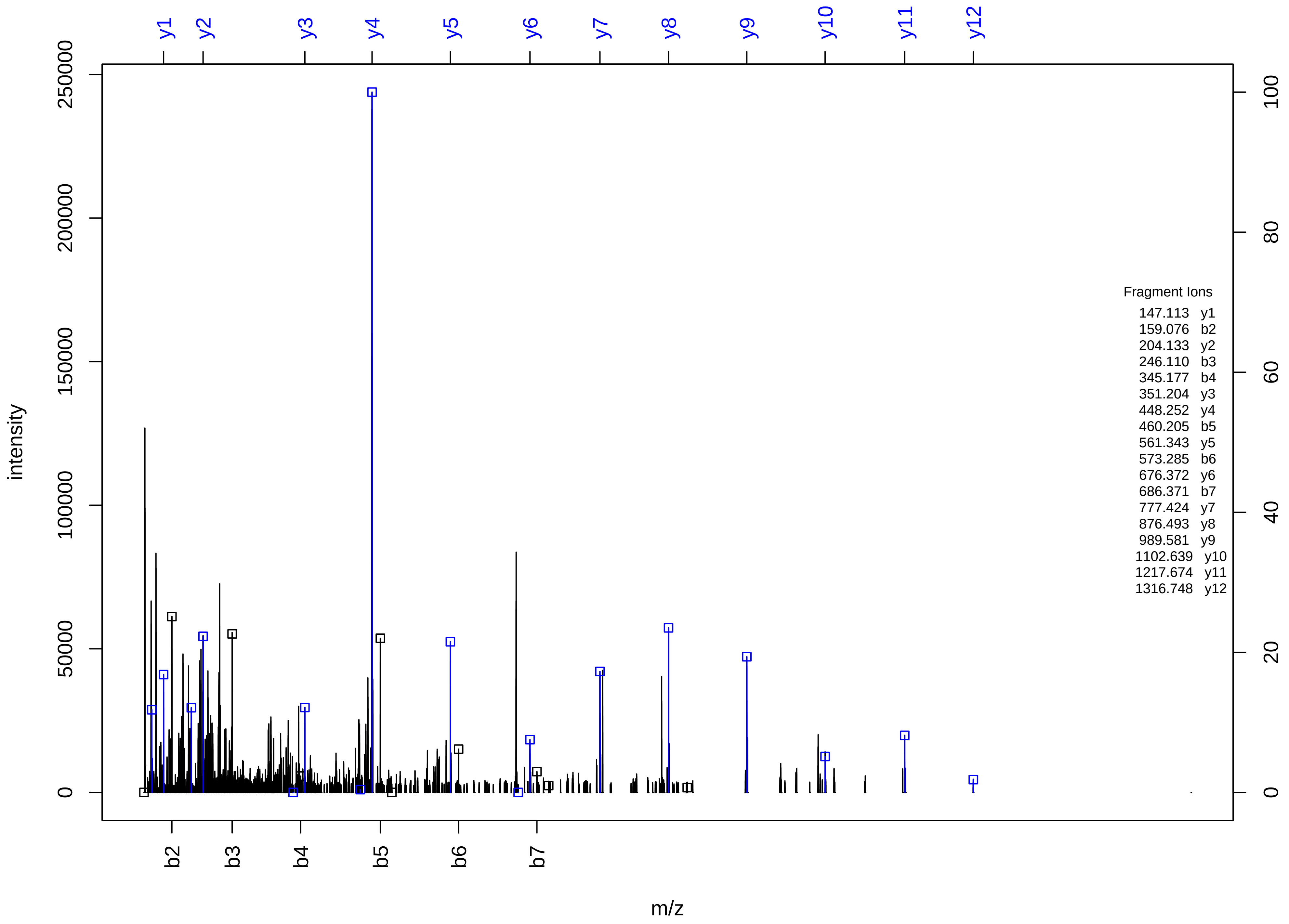

TGSVDIIVTDLPFGK / File: christine_ip_12.raw; SpectrumID: ; scans: 18834

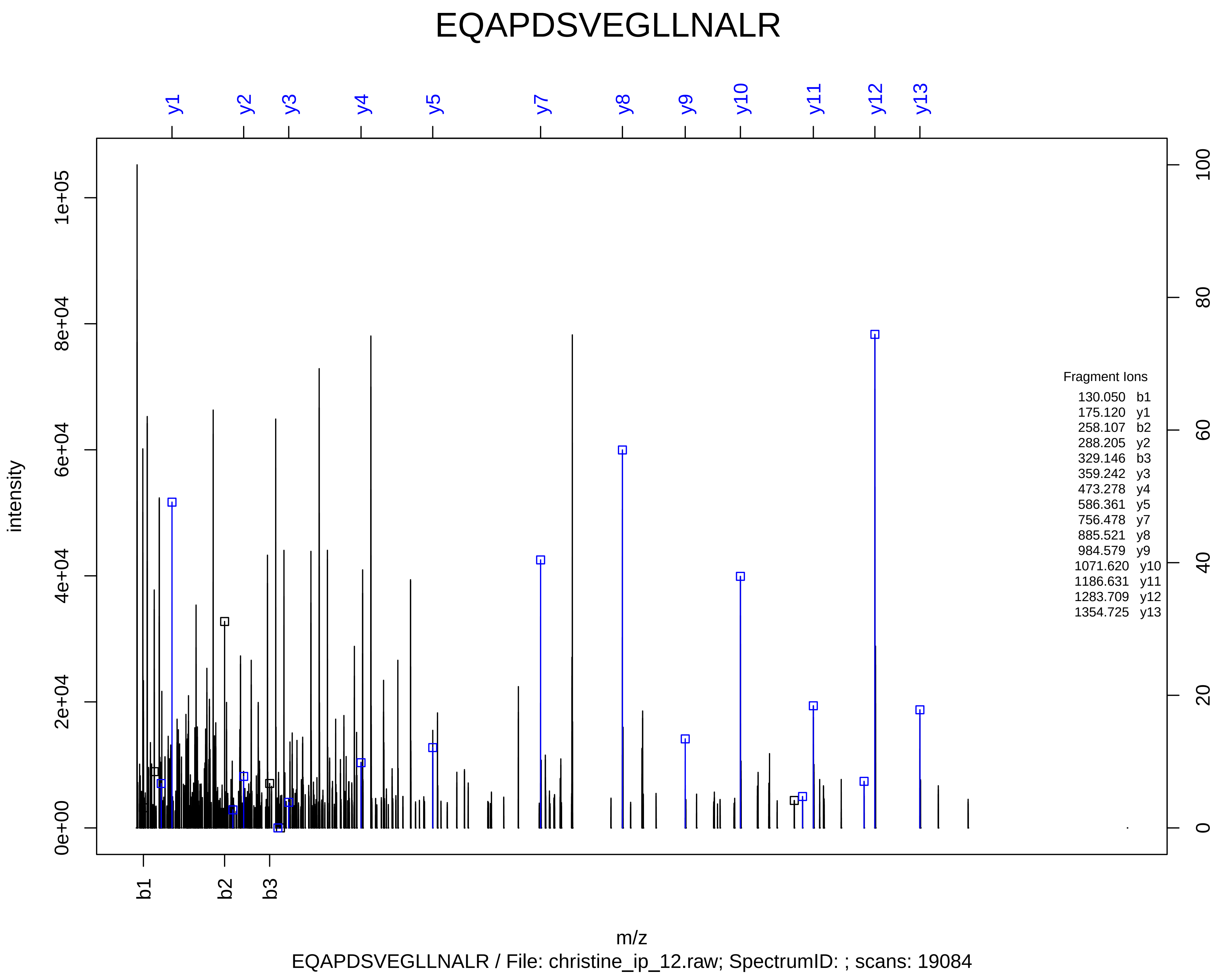
EQAPDSVEGLLNALR
y1
y2
y3
y4
y5
y7
y8
y9
y10
y11
y12
y13
intensity
0e+00
2e+04
4e+04
6e+04
8e+04
1e+05
0
20
40
60
80
100
b1
b2
b3
m/z
Fragment Ions
130.050 b1
175.120 y1
258.107 b2
288.205 y2
329.146 b3
359.242 y3
473.278 y4
586.361 y5
756.478 y7
885.521 y8
984.579 y9
1071.620 y10
1186.631 y11
1283.709 y12
1354.725 y13
EQAPDSVEGLLNALR / File: christine_ip_12.raw; SpectrumID: ; scans: 19084

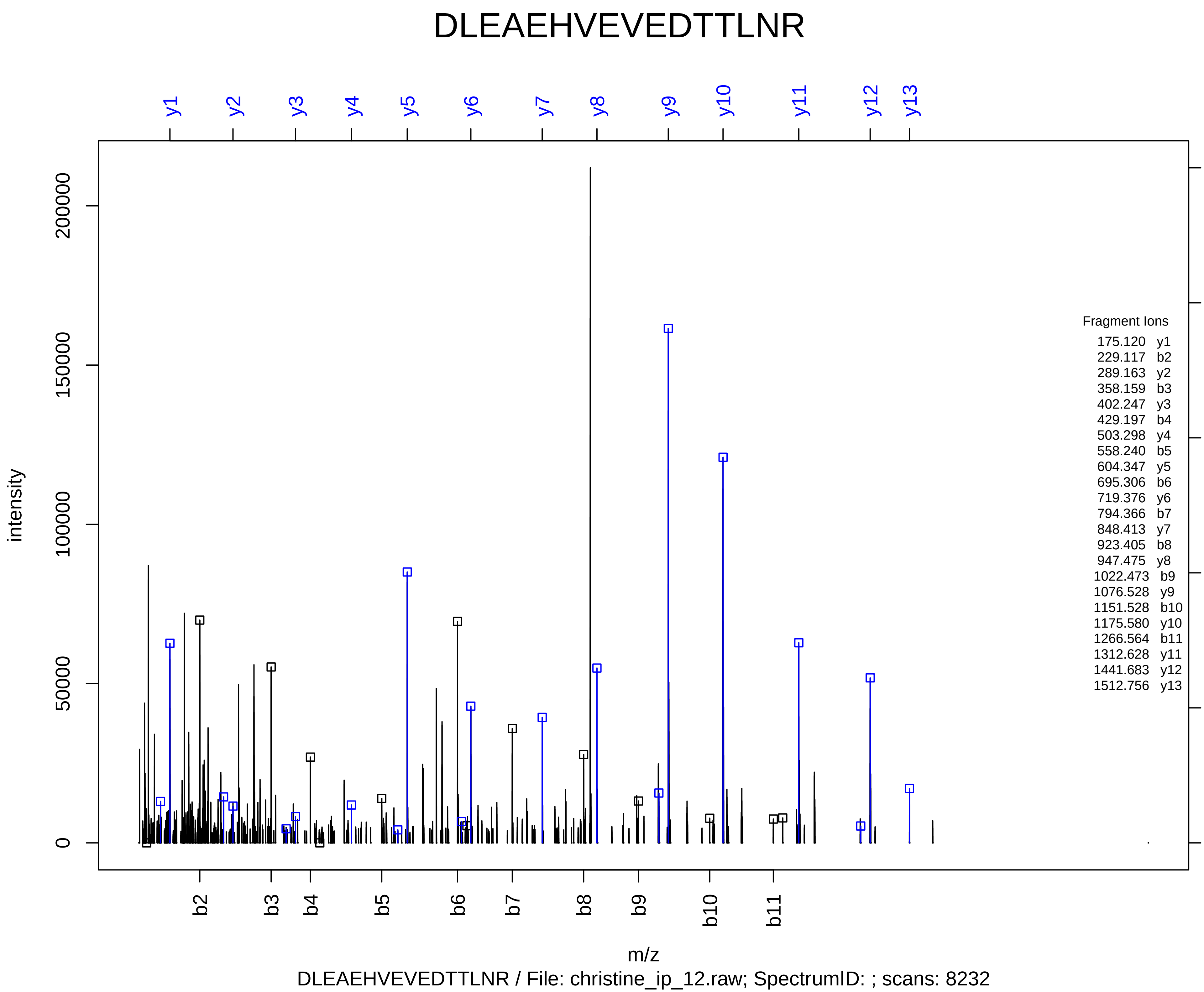
DLEAEHVEVEDTTLNR
y1
y2
y3
y4
y5
y6
y7
y8
y9
y10
y11
y12
y13
intensity
0
50000
100000
150000
200000
0
20
40
60
80
100
b2
b3
b4
b5
b6
b7
b8
b9
b10
b11
m/z
Fragment Ions
175.120 y1
229.117 b2
289.163 y2
358.159 b3
402.247 y3
429.197 b4
503.298 y4
558.240 b5
604.347 y5
695.306 b6
719.376 y6
794.366 b7
848.413 y7
923.405 b8
947.475 y8
1022.473 b9
1076.528 y9
1151.528 b10
1175.580 y10
1266.564 b11
1312.628 y11
1441.683 y12
1512.756 y13
DLEAEHVEVEDTTLNR / File: christine_ip_12.raw; SpectrumID: ; scans: 8232

FIPDDITFDDEPK

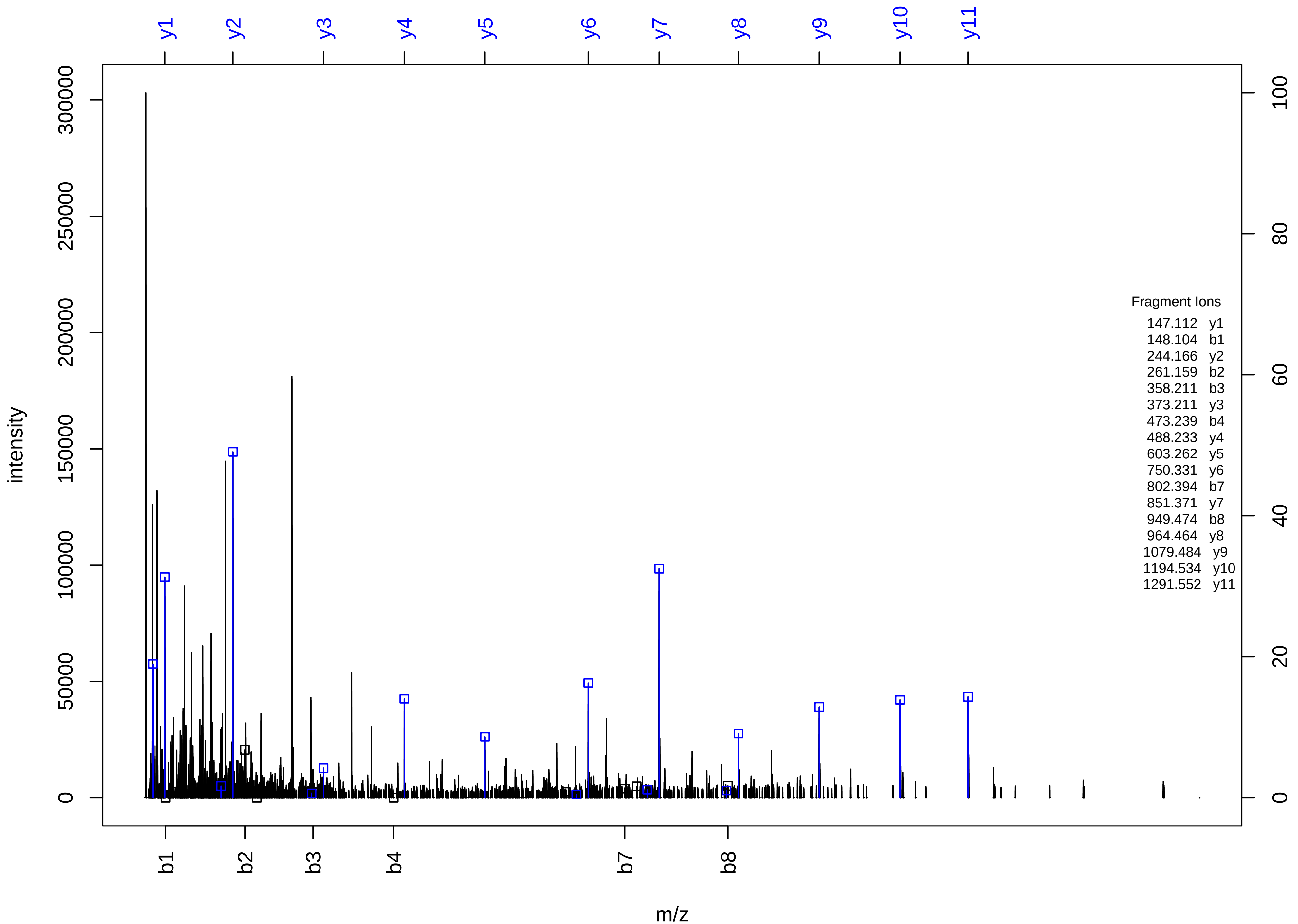

FIPDDITFDDEPK / File: christine_ip_12.raw; SpectrumID: ; scans: 13804

GGHPPAIQSLINLLADNR

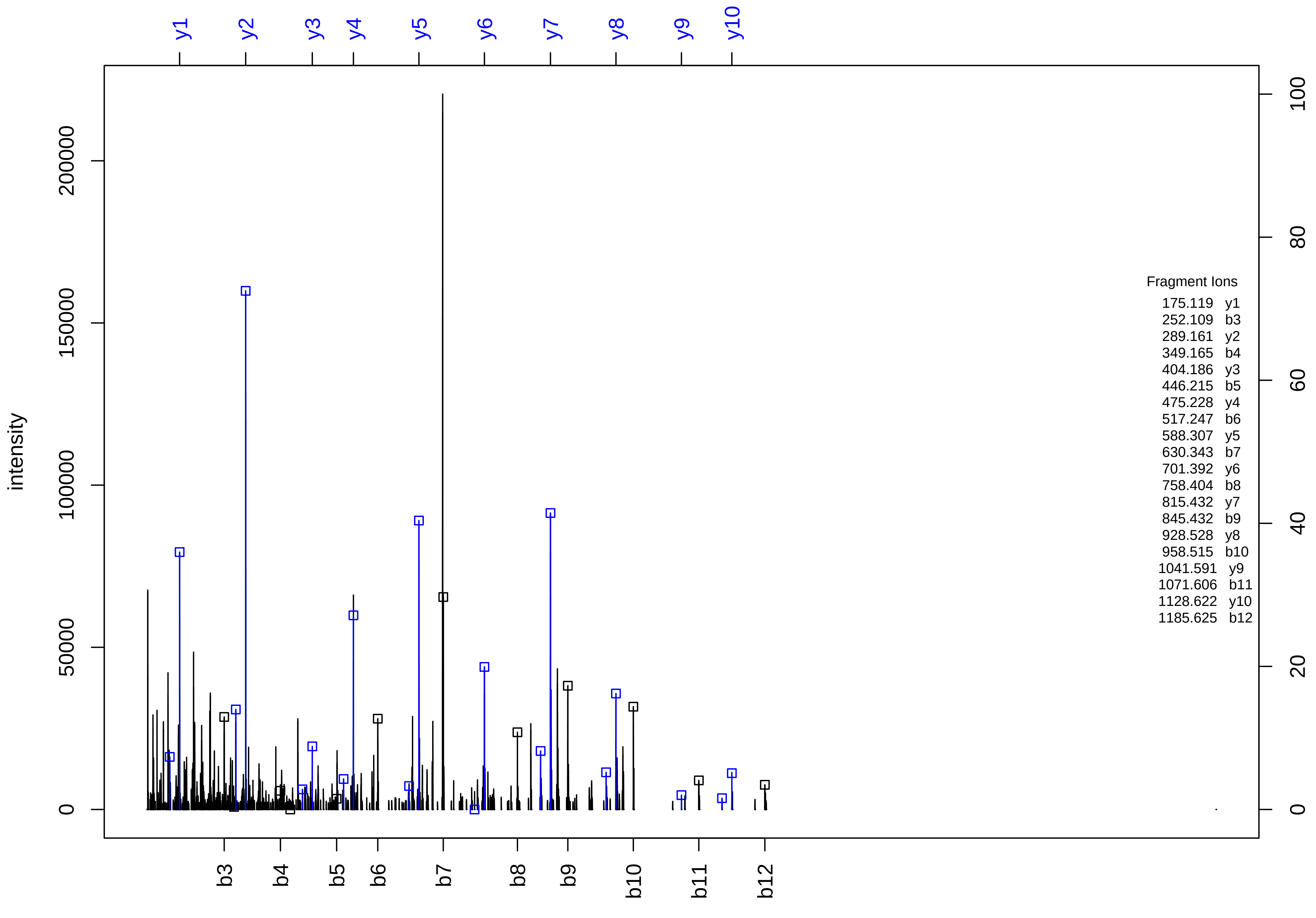

GGHPPAIQSLINLLADNR / File: christine_ip_12.raw; SpectrumID: ; scans: 19899

AQILGGANTPYEK

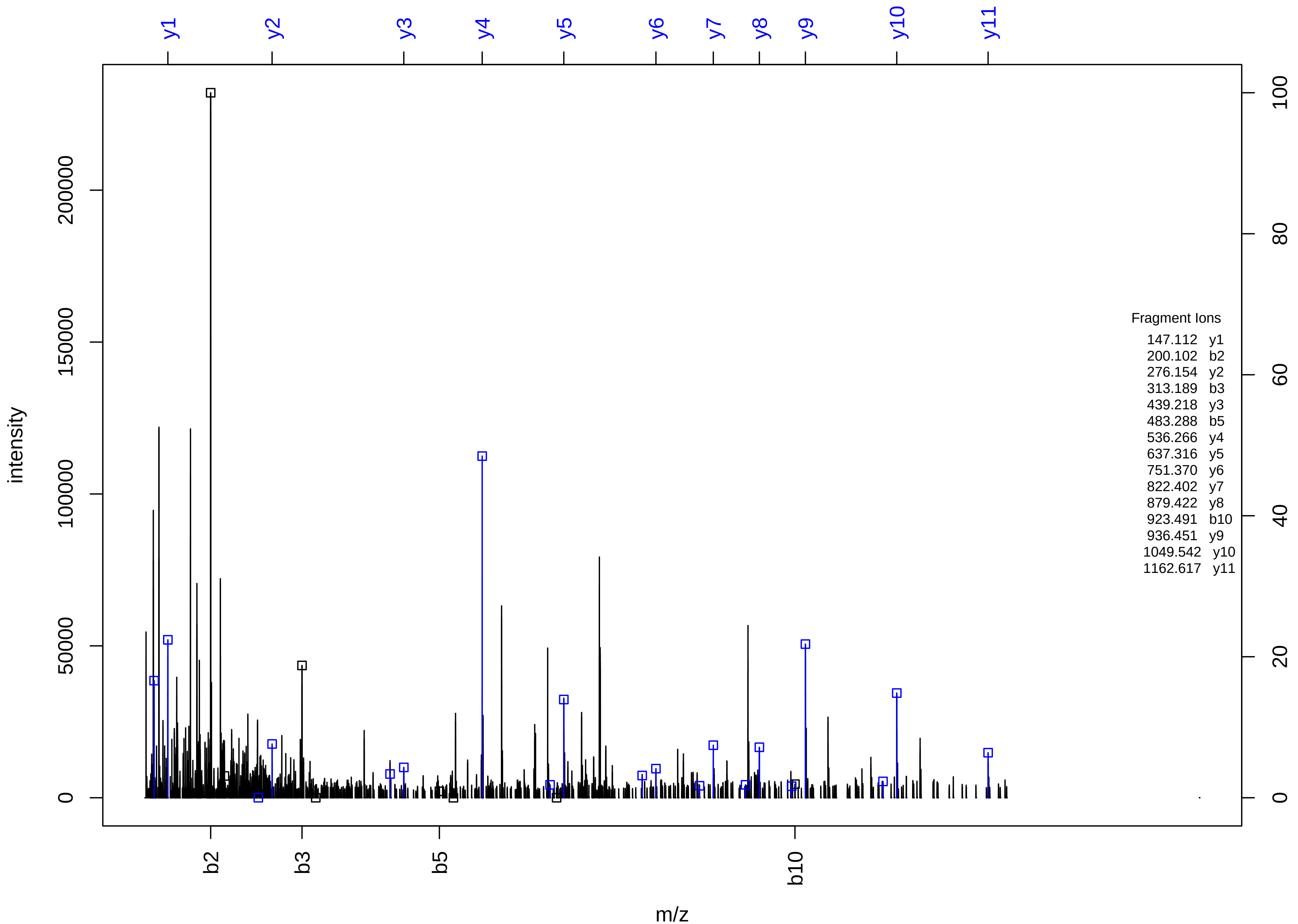

AQILGGANTPYEK / File: christine_ip_12.raw; SpectrumID: ; scans: 6410

ALQDLQLDQGNQK

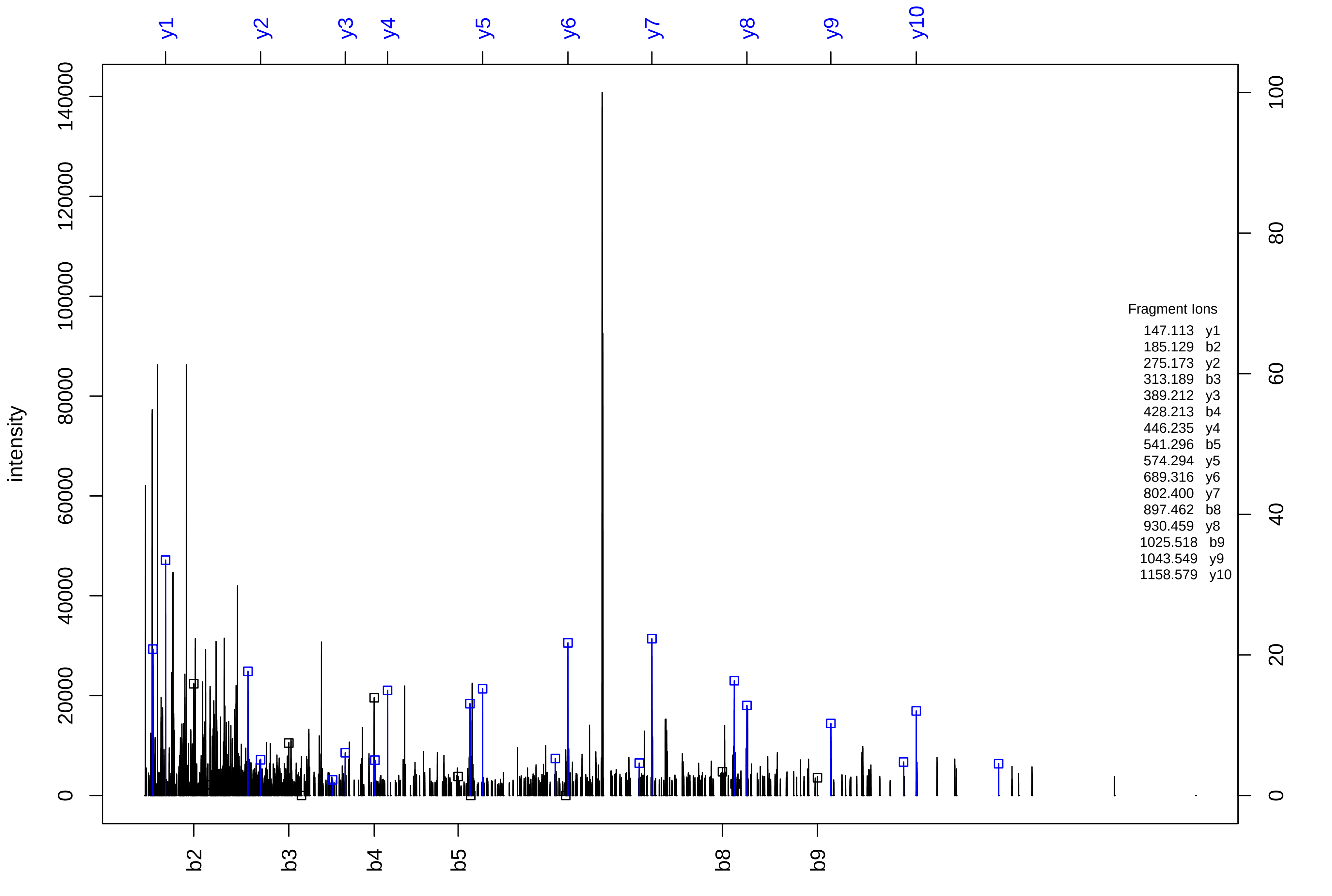

ALQDLQLDQGNQK / File: christine_ip_12.raw; SpectrumID: ; scans: 7160

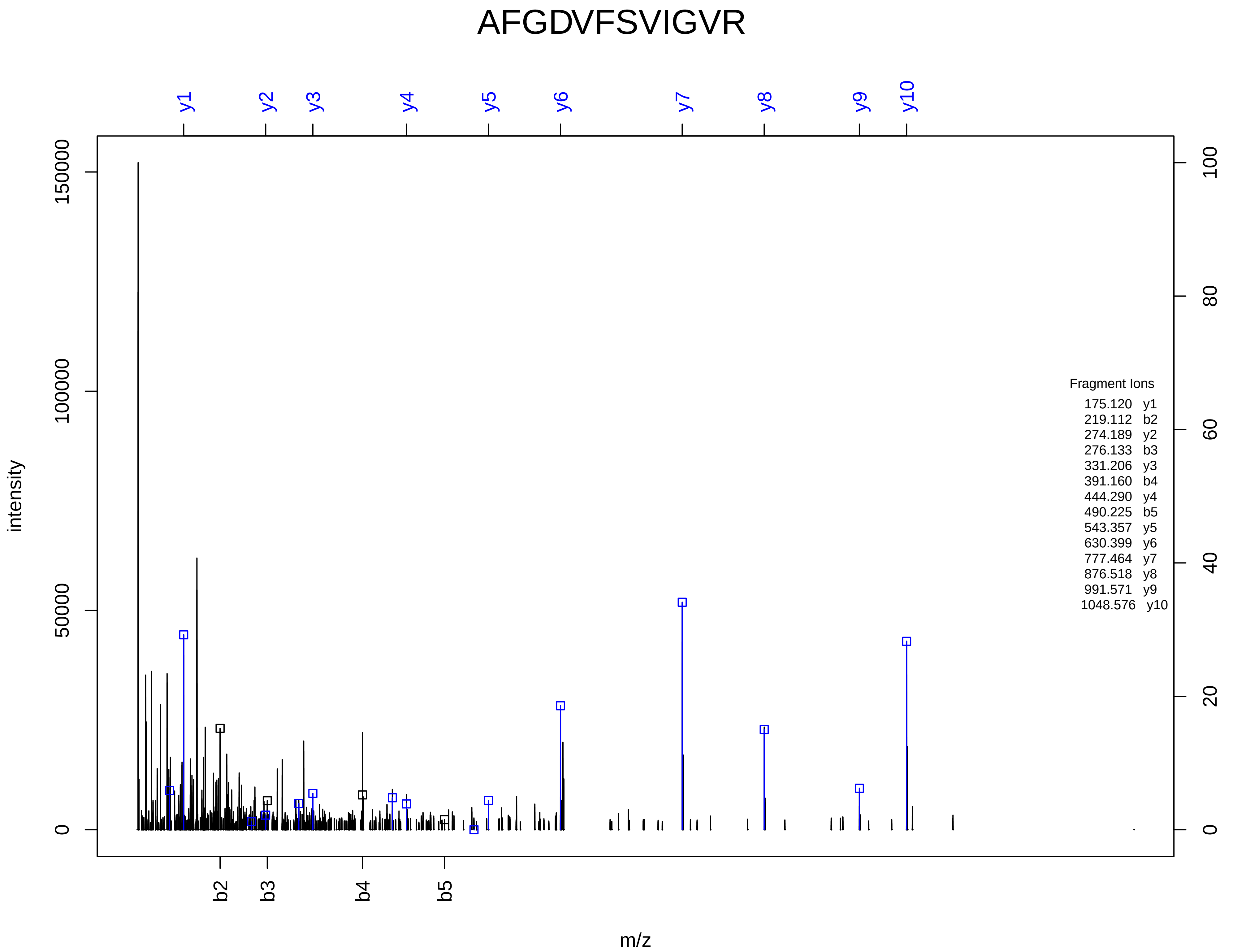

AFGDVFSVIGVR / File: christine_ip_12.raw; SpectrumID: ; scans: 19371

TDFEEFFLR

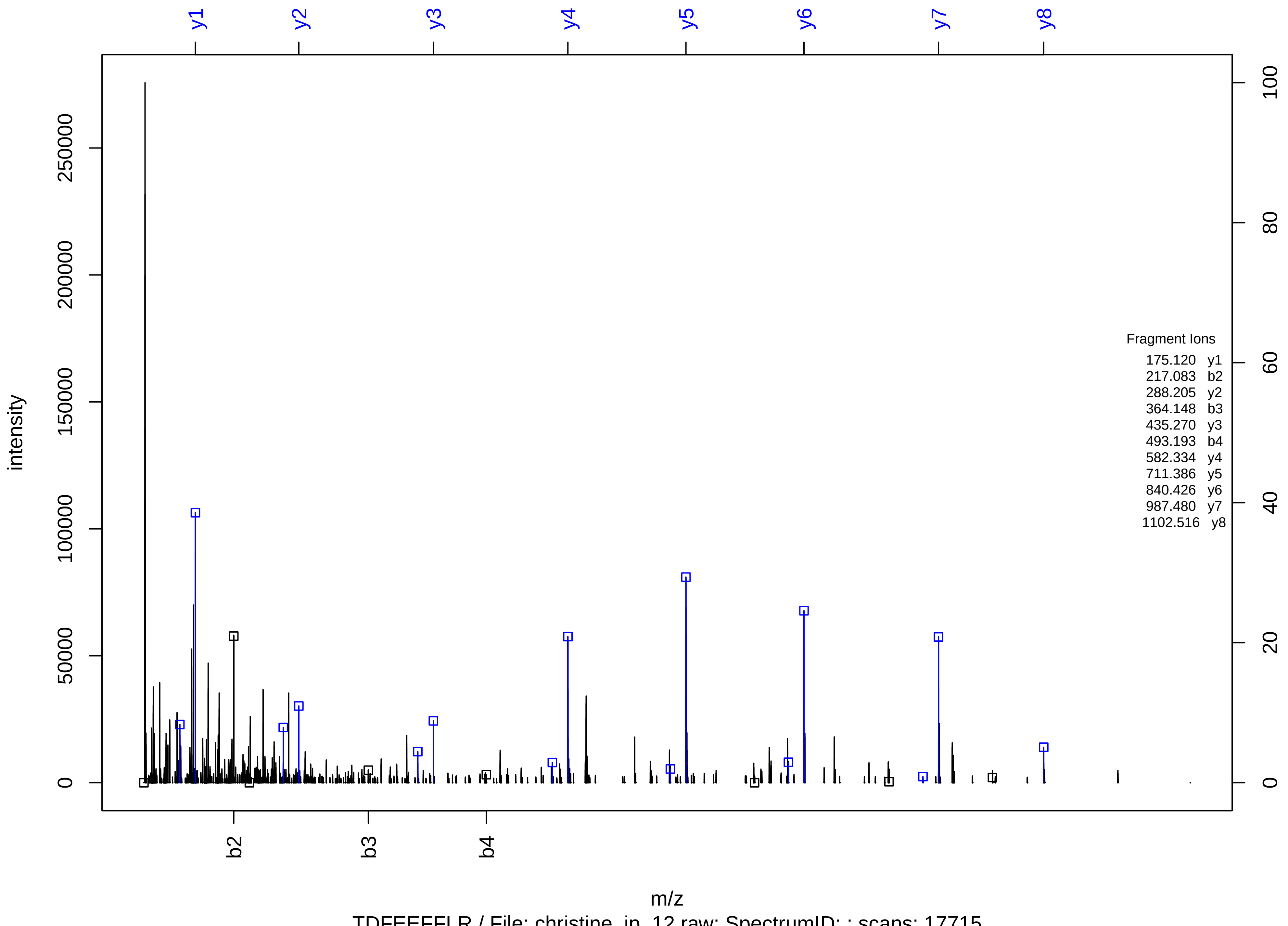

TDFEEFFLR / File: christine_ip_12.raw; SpectrumID: ; scans: 17715

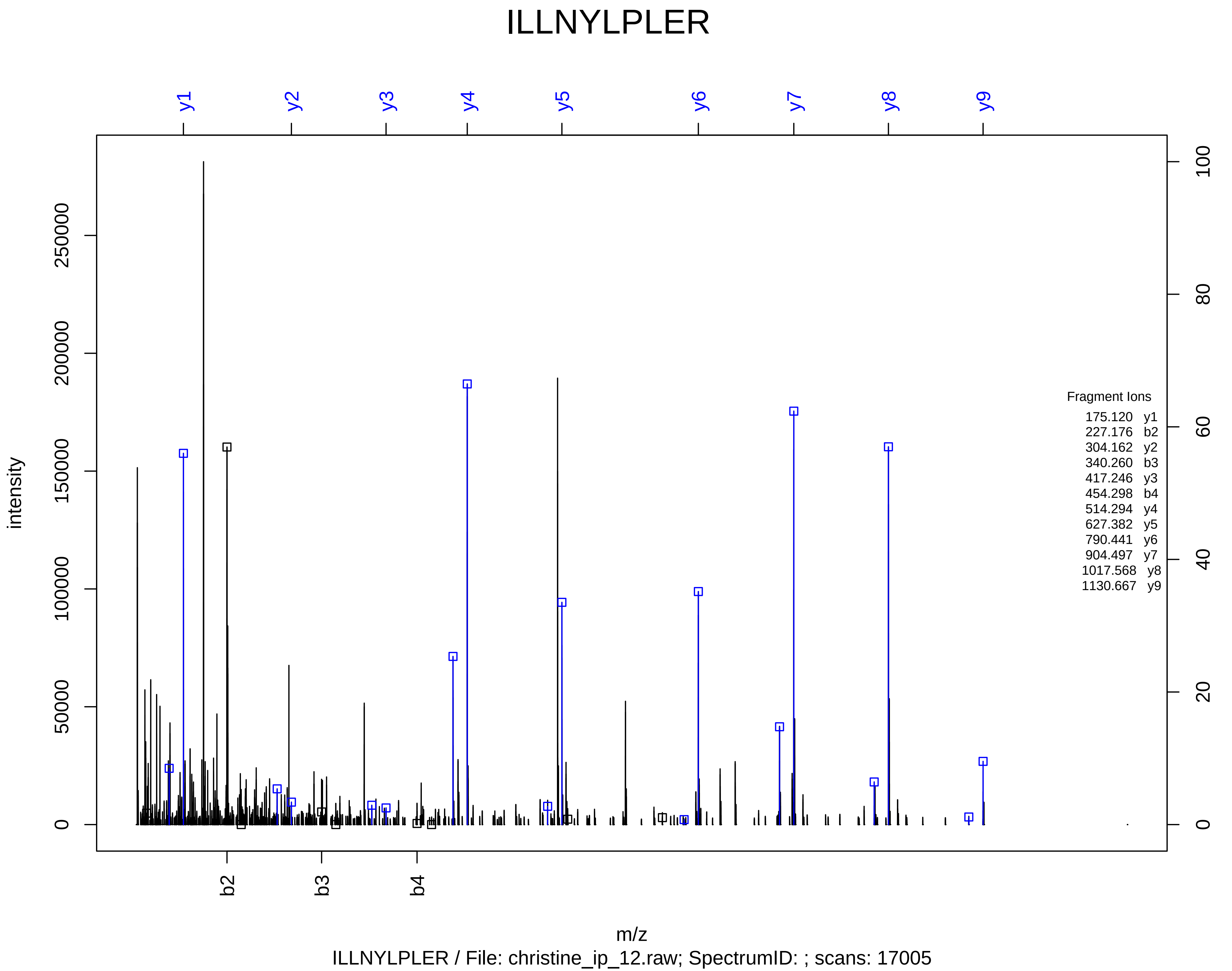

ILLNYLPLER
y1
y2
y3
y4
y5
y6
y7
y8
y9
b2
b3
b4
intensity
m/z
0
50000
100000
150000
200000
250000
0
20
40
60
80
100
Fragment Ions
175.120 y1
227.176 b2
304.162 y2
340.260 b3
417.246 y3
454.298 b4
514.294 y4
627.382 y5
790.441 y6
904.497 y7
1017.568 y8
1130.667 y9
ILLNYLPLER / File: christine_ip_12.raw; SpectrumID: ; scans: 17005

TDTAVQATGSVPSTPIAHR

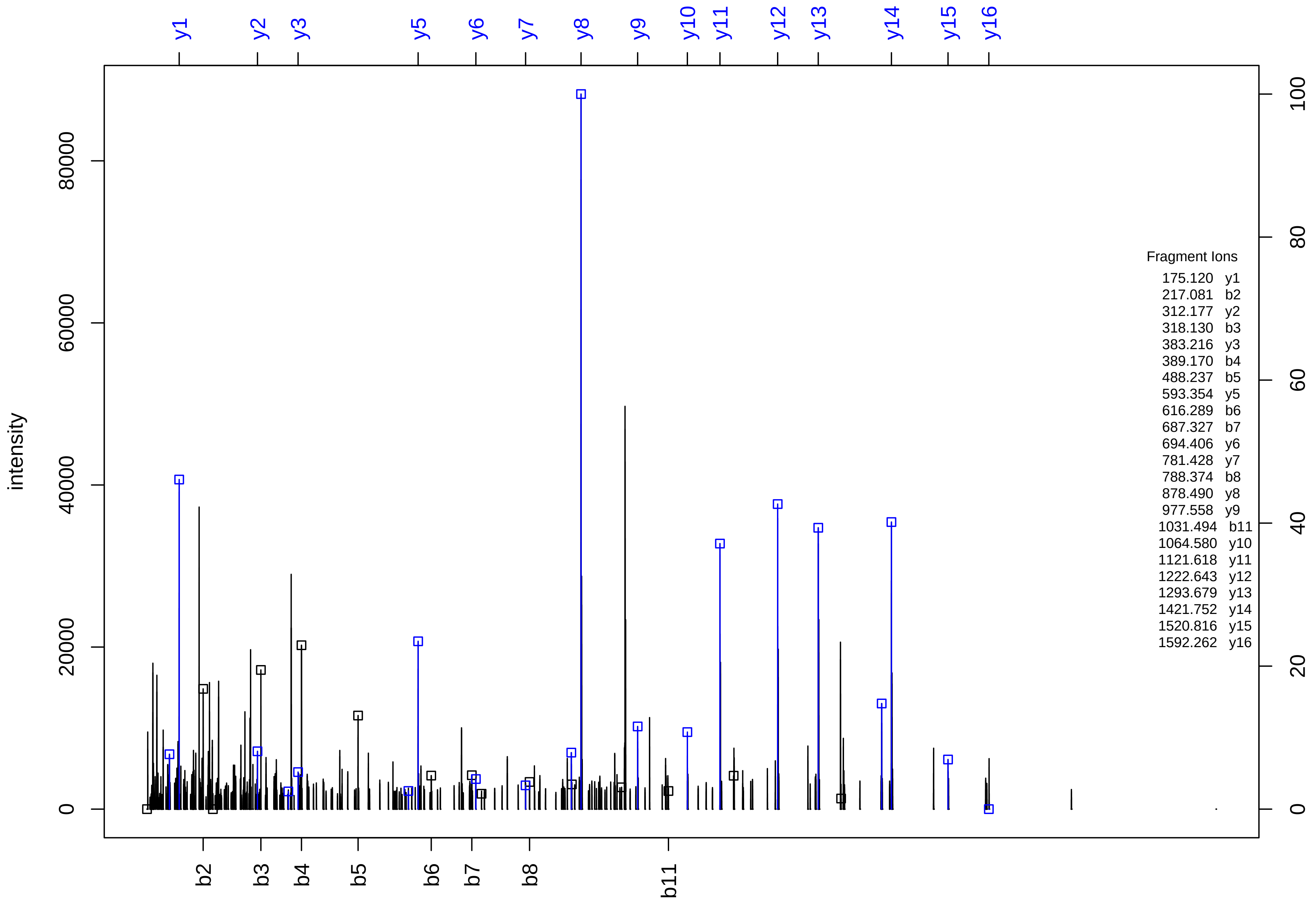

TDTAVQATGSVPSTPIAHR / File: christine_ip_12.raw; SpectrumID: ; scans: 6068

NAEDCLYELPENIR

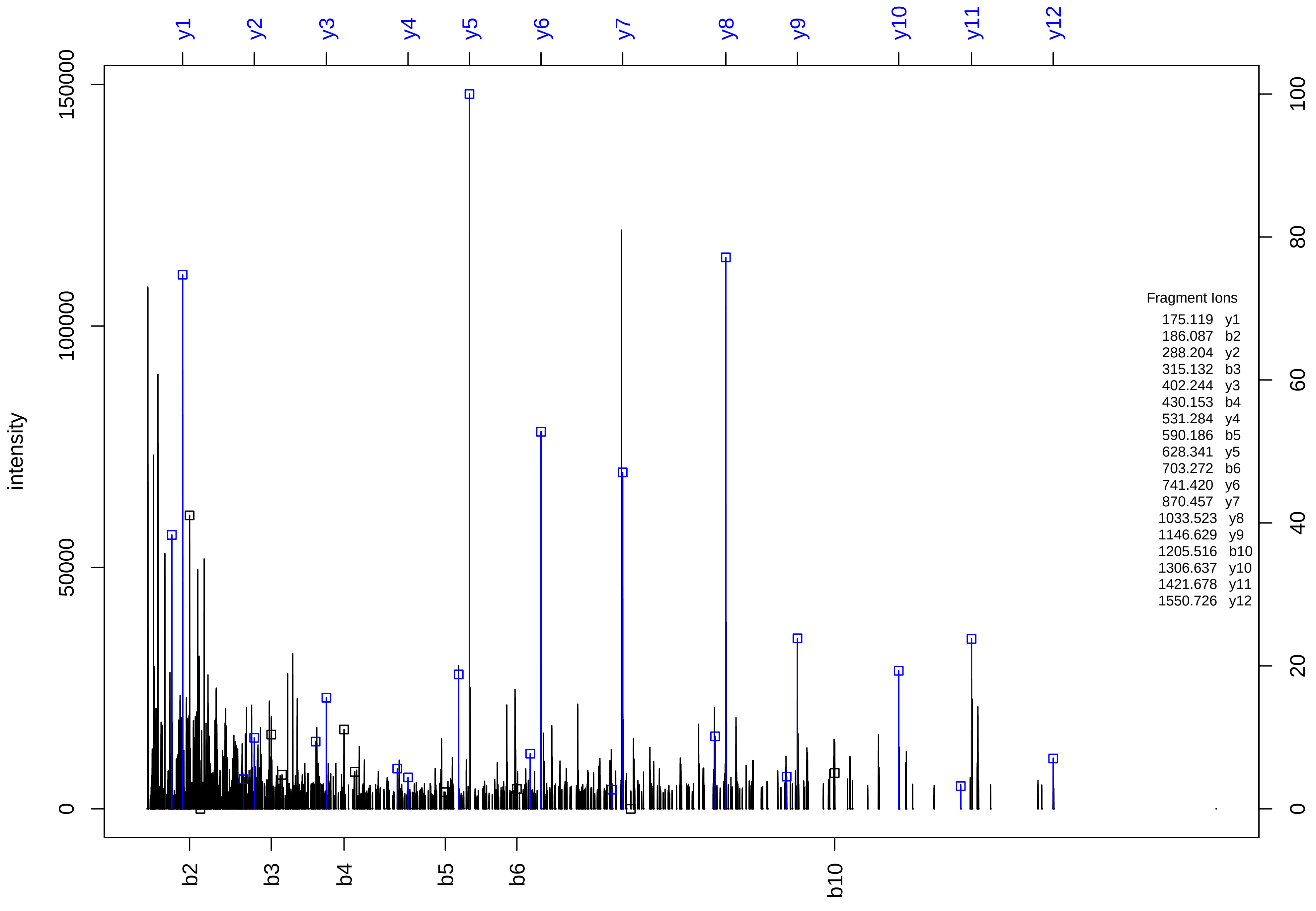

NAEDCLYELPENIR / File: christine_ip_12.raw; SpectrumID: ; scans: 13405

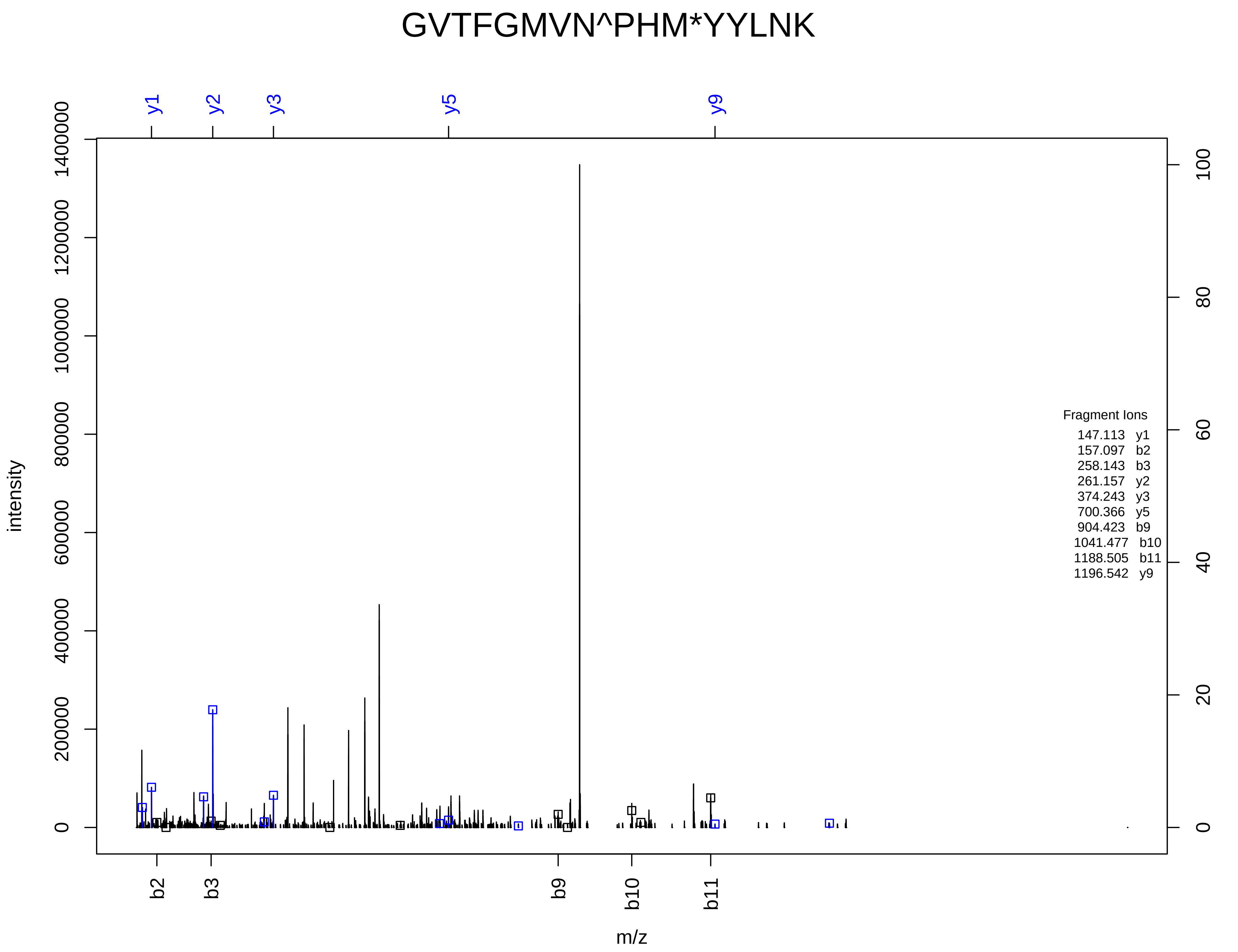
GVTFGMVN^PHM*YYLNK
y1
y2
y3
y5
y9
intensity
0
200000
400000
600000
800000
1000000
1200000
1400000
0
20
40
60
80
100
Fragment Ions
147.113 y1
157.097 b2
258.143 b3
261.157 y2
374.243 y3
700.366 y5
904.423 b9
1041.477 b10
1188.505 b11
1196.542 y9
b2
b3
b9
b10
b11
m/z
GVTFGMVNPHMYYLNK / File: christine_ip_12.raw; SpectrumID: ; scans: 10857

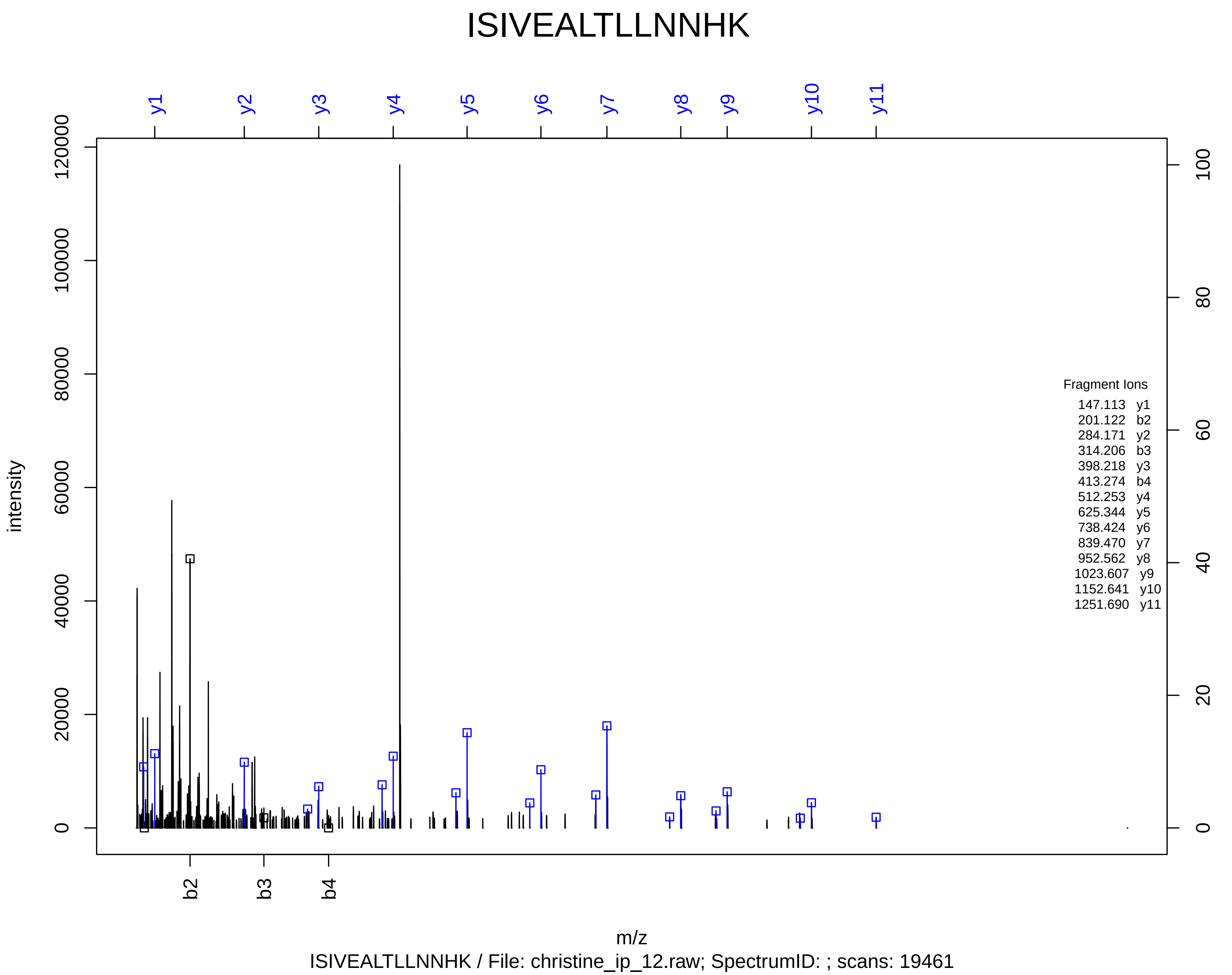
ISIVEALTLLNNHK
intensity
m/z
y1
y2
y3
y4
y5
y6
y7
y8
y9
y10
y11
b2
b3
b4
Fragment Ions
147.113 y1
201.122 b2
284.171 y2
314.206 b3
398.218 y3
413.274 b4
512.253 y4
625.344 y5
738.424 y6
839.470 y7
952.562 y8
1023.607 y9
1152.641 y10
1251.690 y11
ISIVEALTLLNNHK / File: christine_ip_12.raw; SpectrumID: ; scans: 19461

ELTVDNSFEFEK

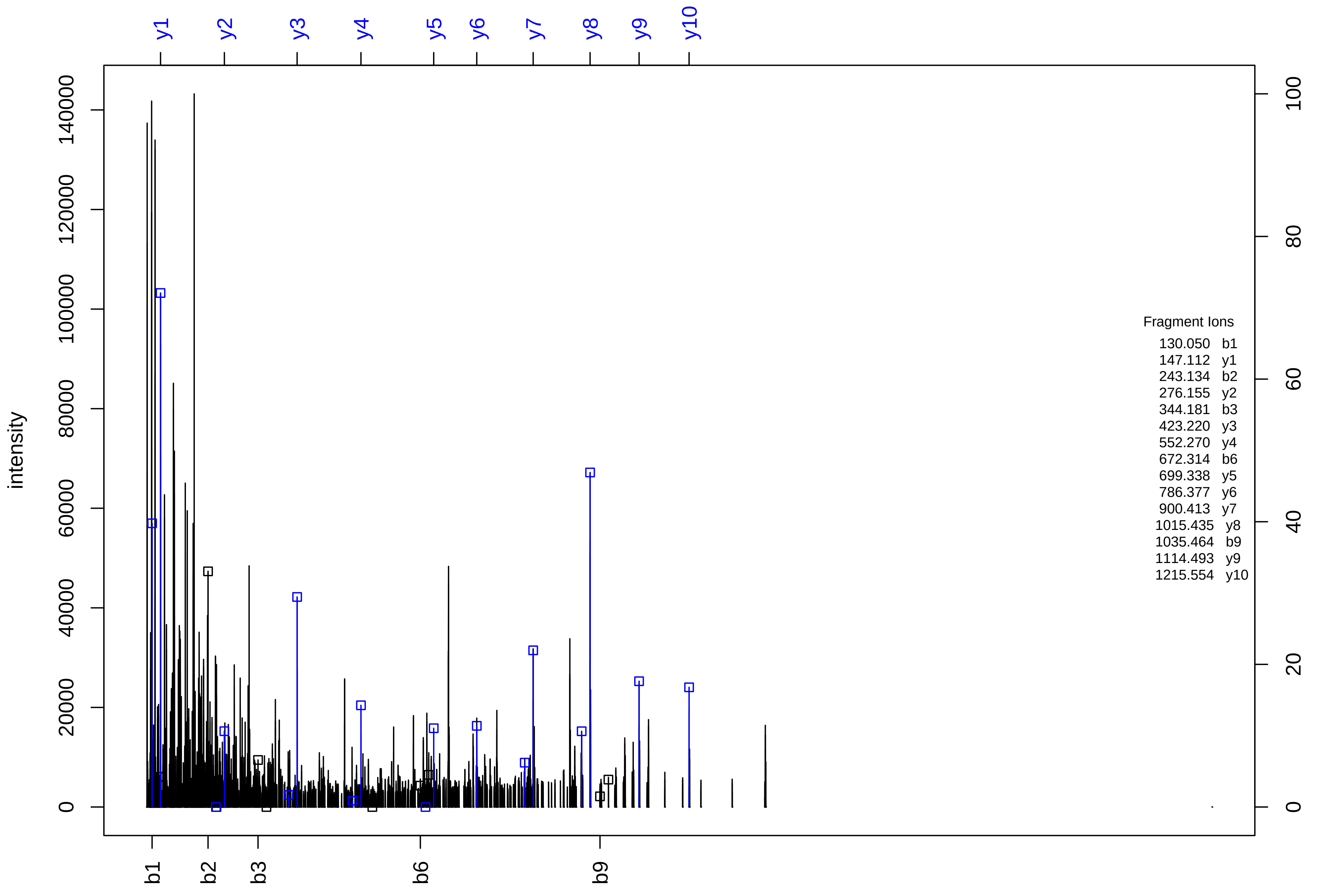

ELTVDNSFEFEK / File: christine_ip_12.raw; SpectrumID: ; scans: 11993

ELNESNSQM*EADM*IKLR

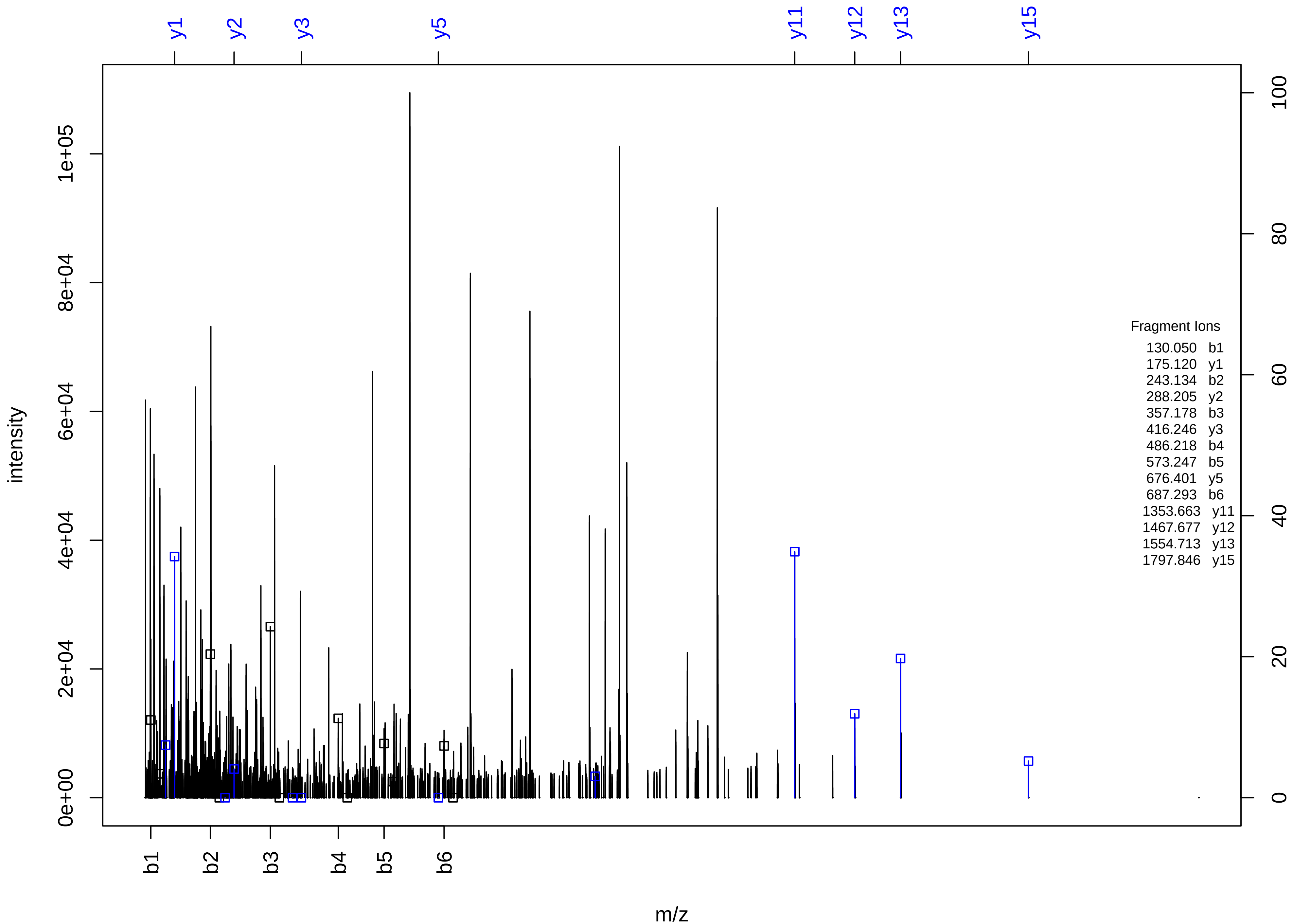

ELNESNSQMEADMIKLR / File: christine_ip_12.raw; SpectrumID: ; scans: 16552

YPFILPHQQVDK

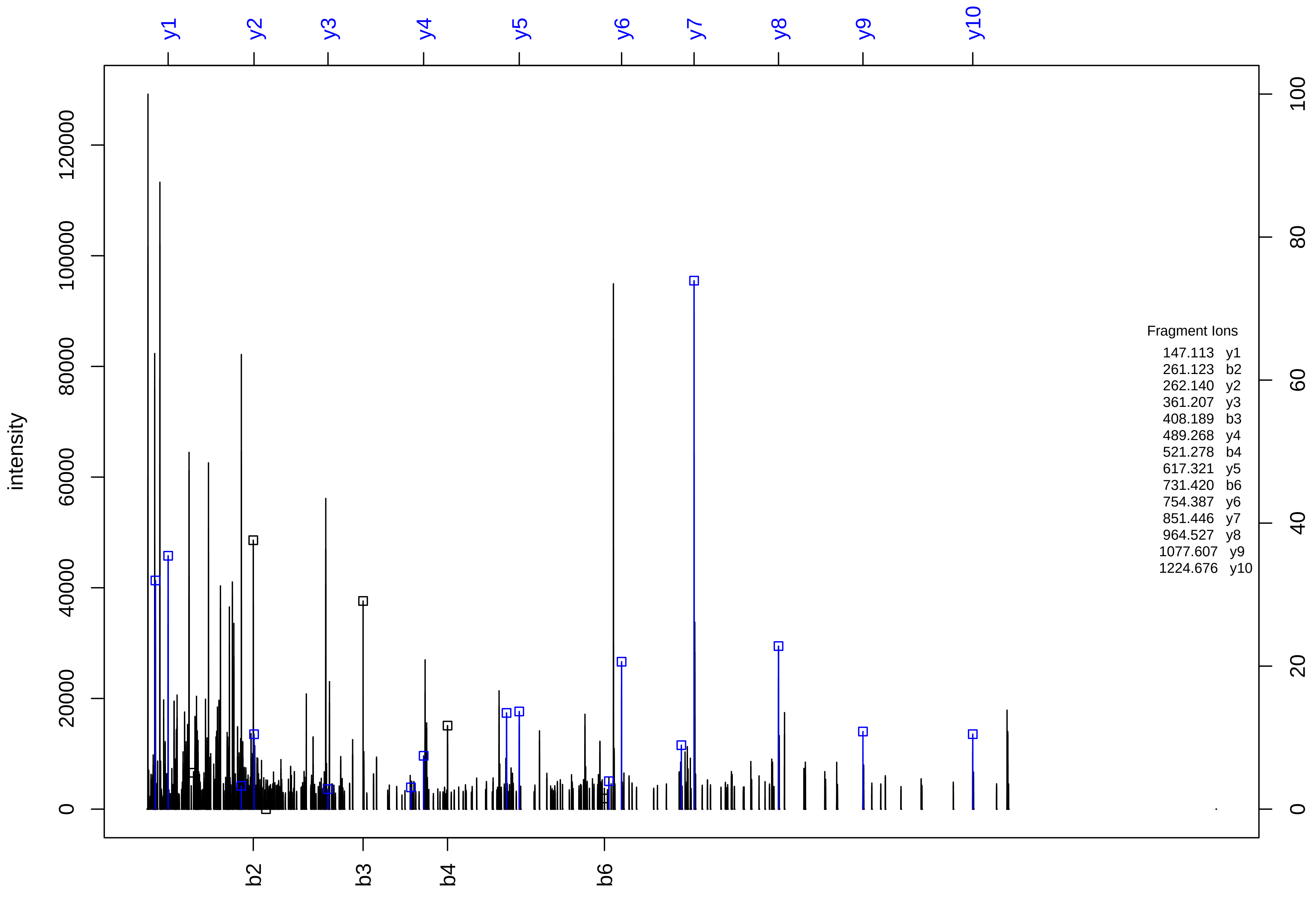

YPFILPHQQVDK / File: christine_ip_12.raw; SpectrumID: ; scans: 9801

VAQIQNAGLGEFR

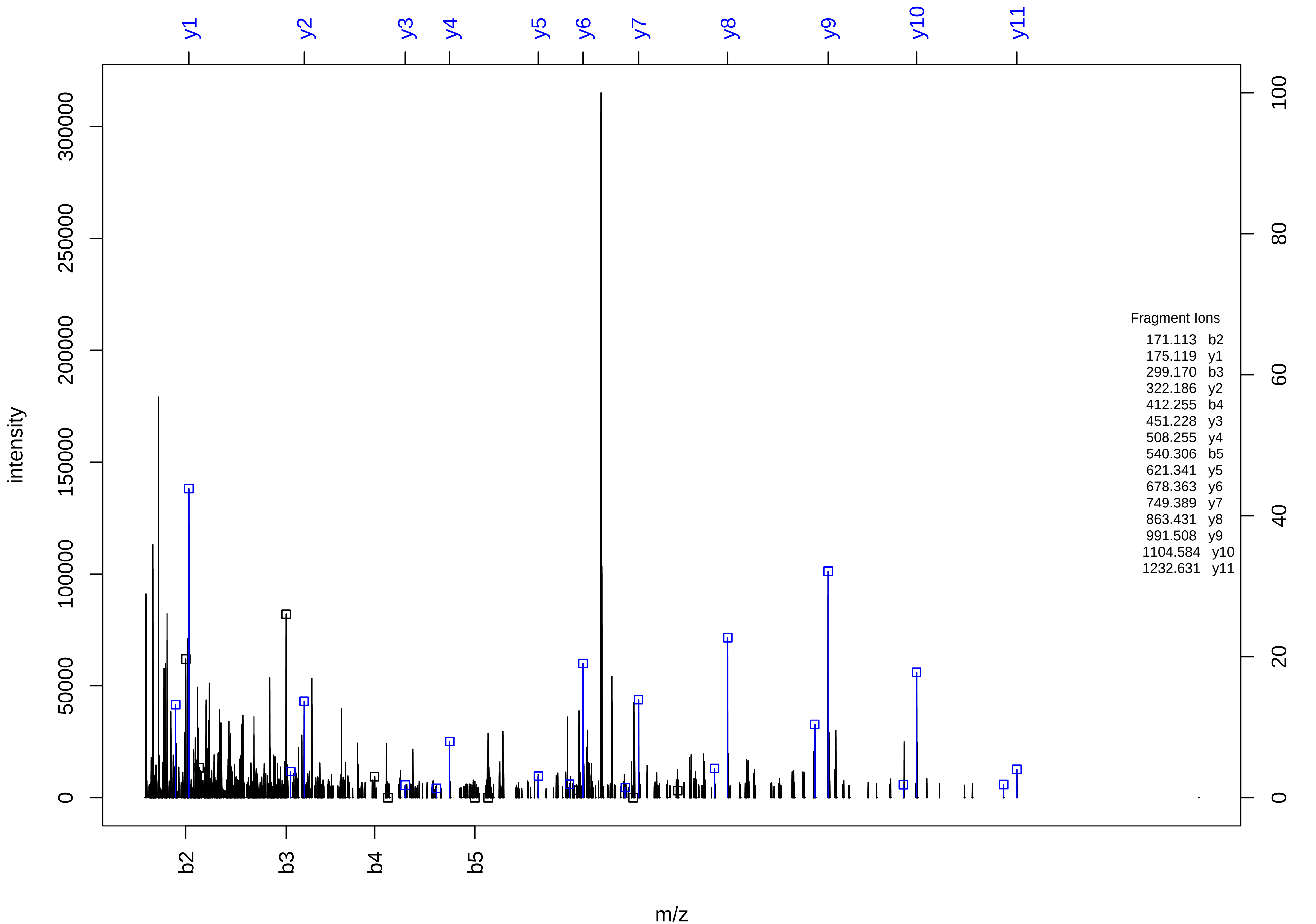

VAQIQNAGLGEFR / File: christine_ip_12.raw; SpectrumID: ; scans: 9727

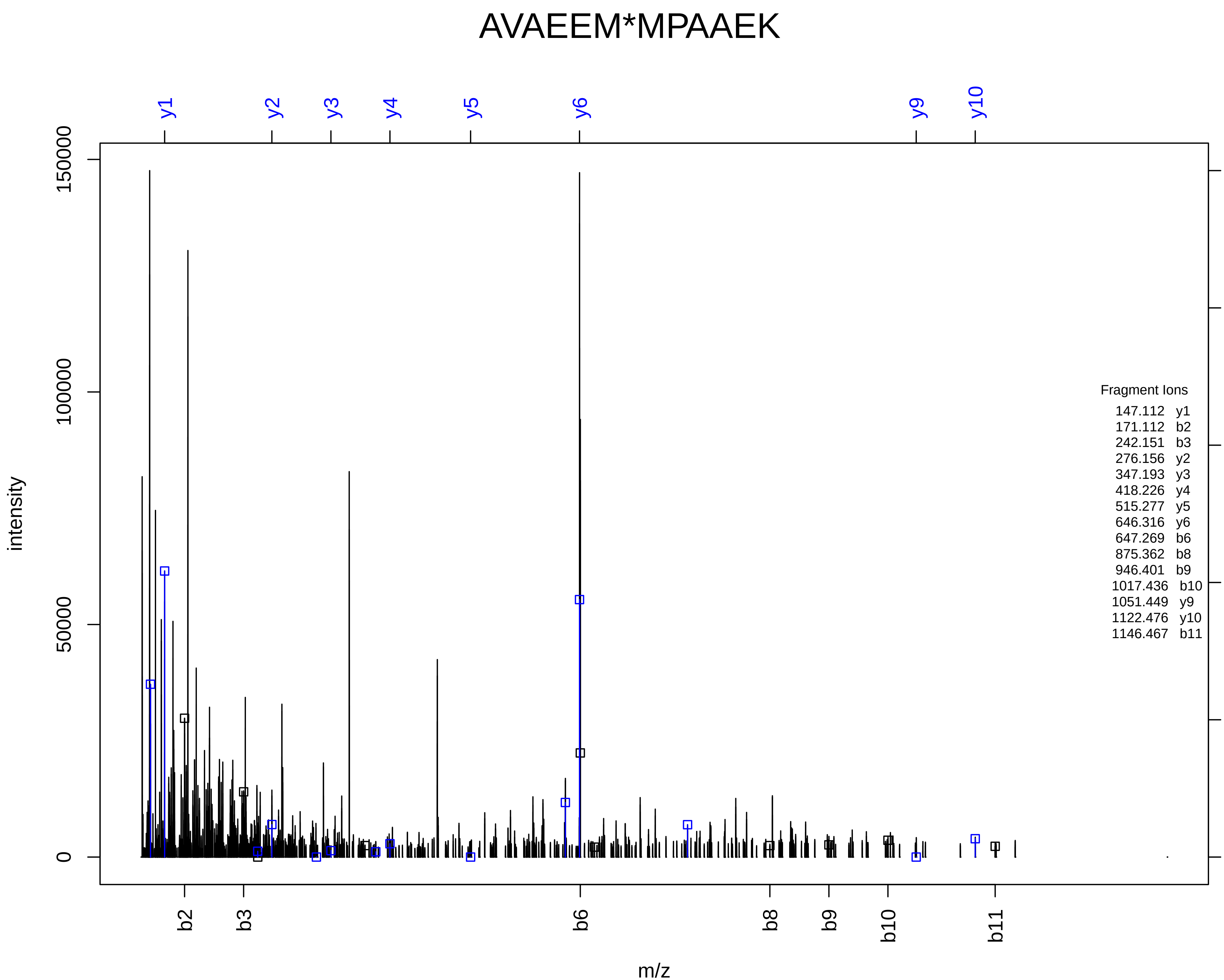

AVAEEMMPAAEK / File: christine_ip_12.raw; SpectrumID: ; scans: 4738

HVVFGEVTEGLDVLR

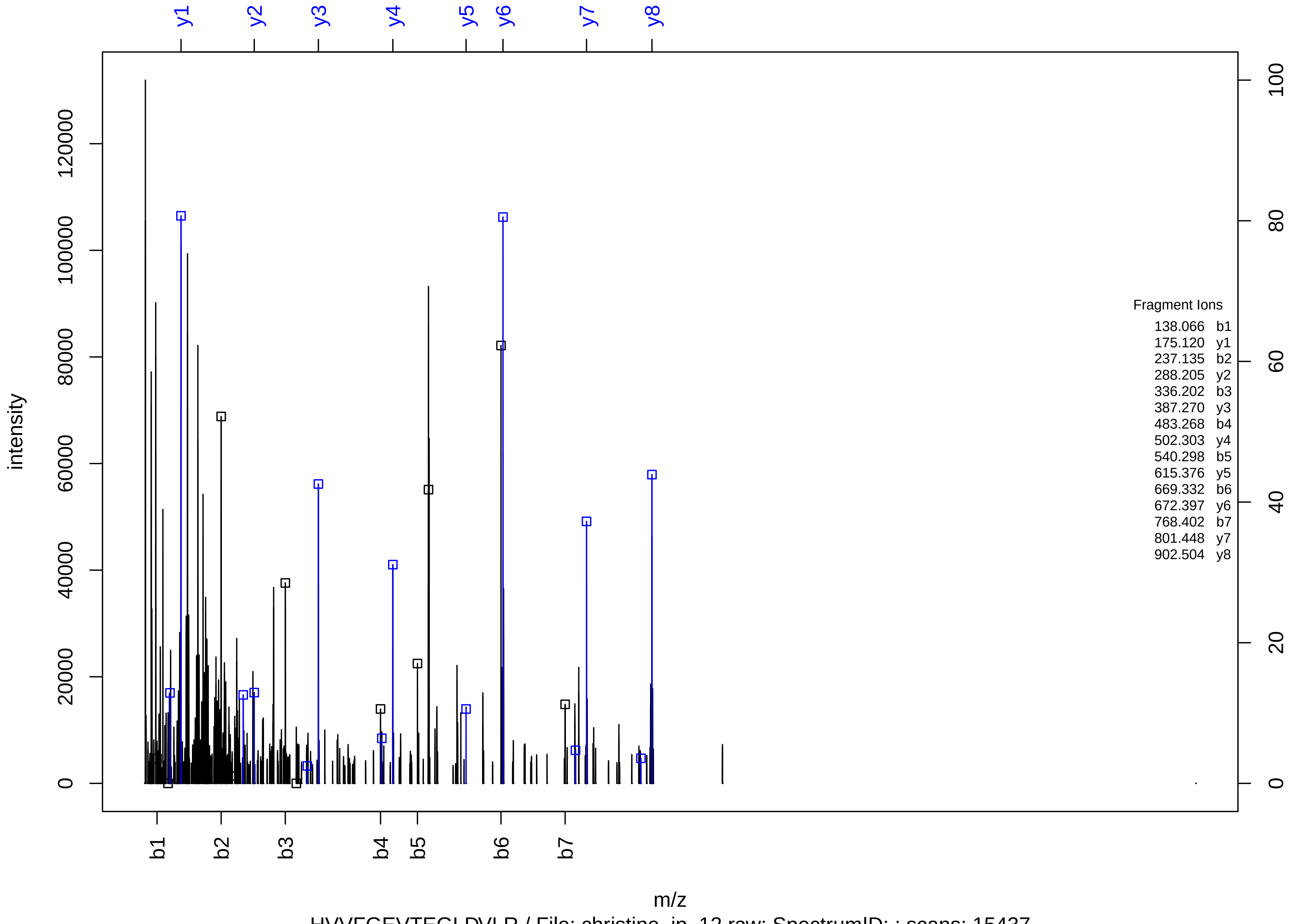

HVVFGEVTEGLDVLR / File: christine_ip_12.raw; SpectrumID: ; scans: 15437

LPFLSSSNLSLDVLR

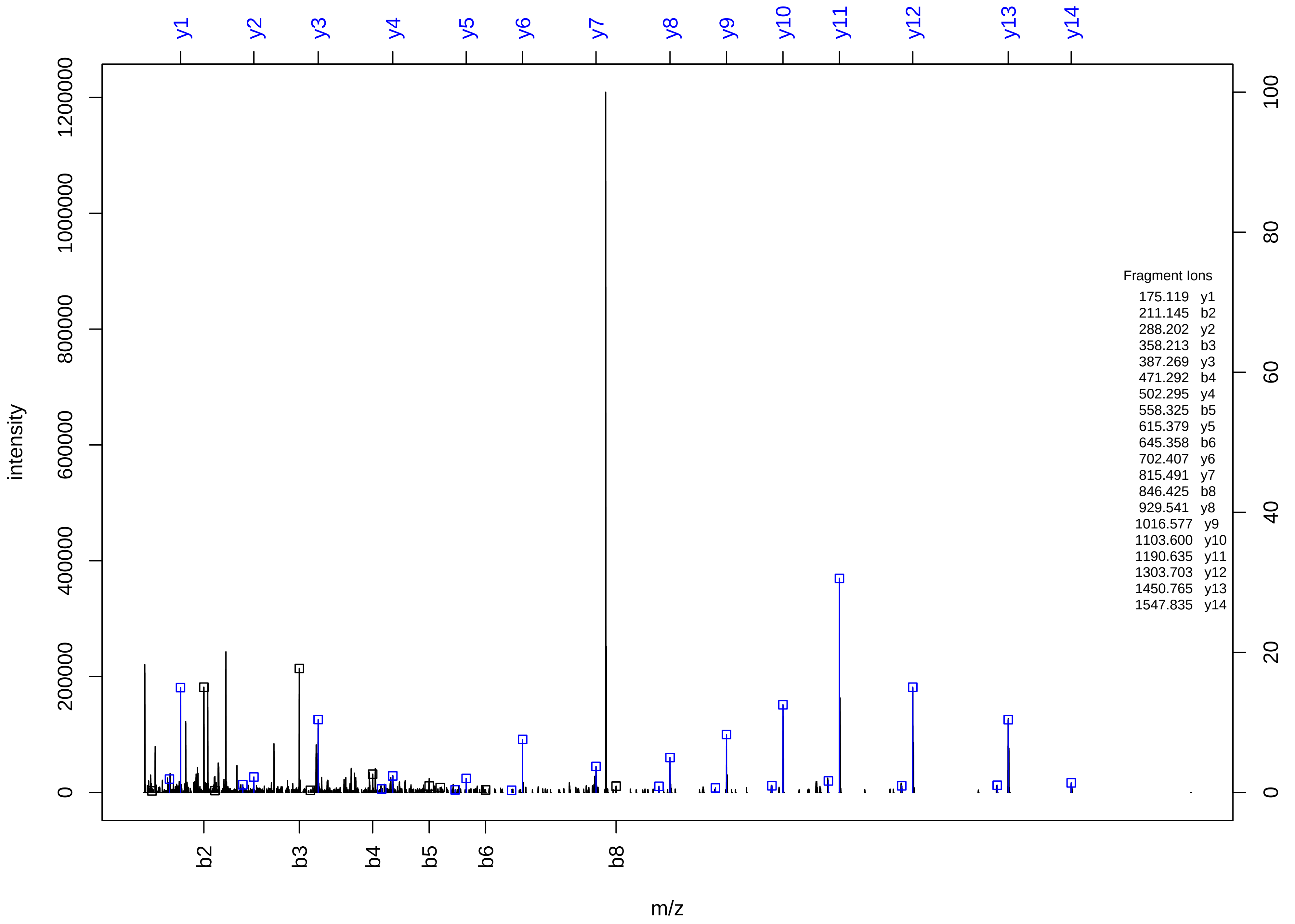

LPFLSSSNLSLDVLR / File: christine_ip_12.raw; SpectrumID: ; scans: 20755

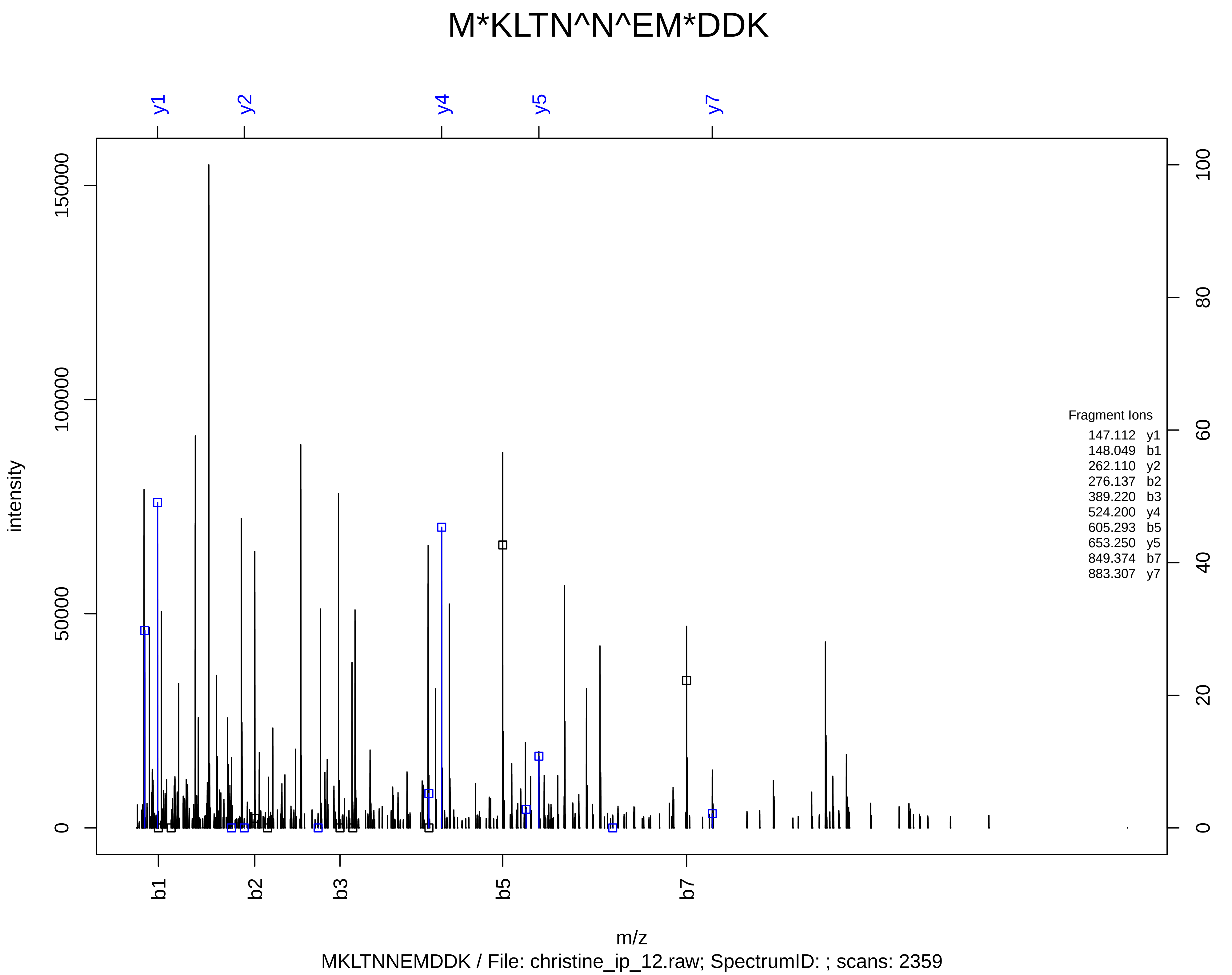
M*KLTN^N^EM*DDK
y1
y2
y4
y5
y7
intensity
0
50000
100000
150000
0
20
40
60
80
100
b1
b2
b3
b5
b7
m/z
Fragment Ions
147.112 y1
148.049 b1
262.110 y2
276.137 b2
389.220 b3
524.200 y4
605.293 b5
653.250 y5
849.374 b7
883.307 y7
MKLTNNEMDDK / File: christine_ip_12.raw; SpectrumID: ; scans: 2359

IPDEIIDMVK

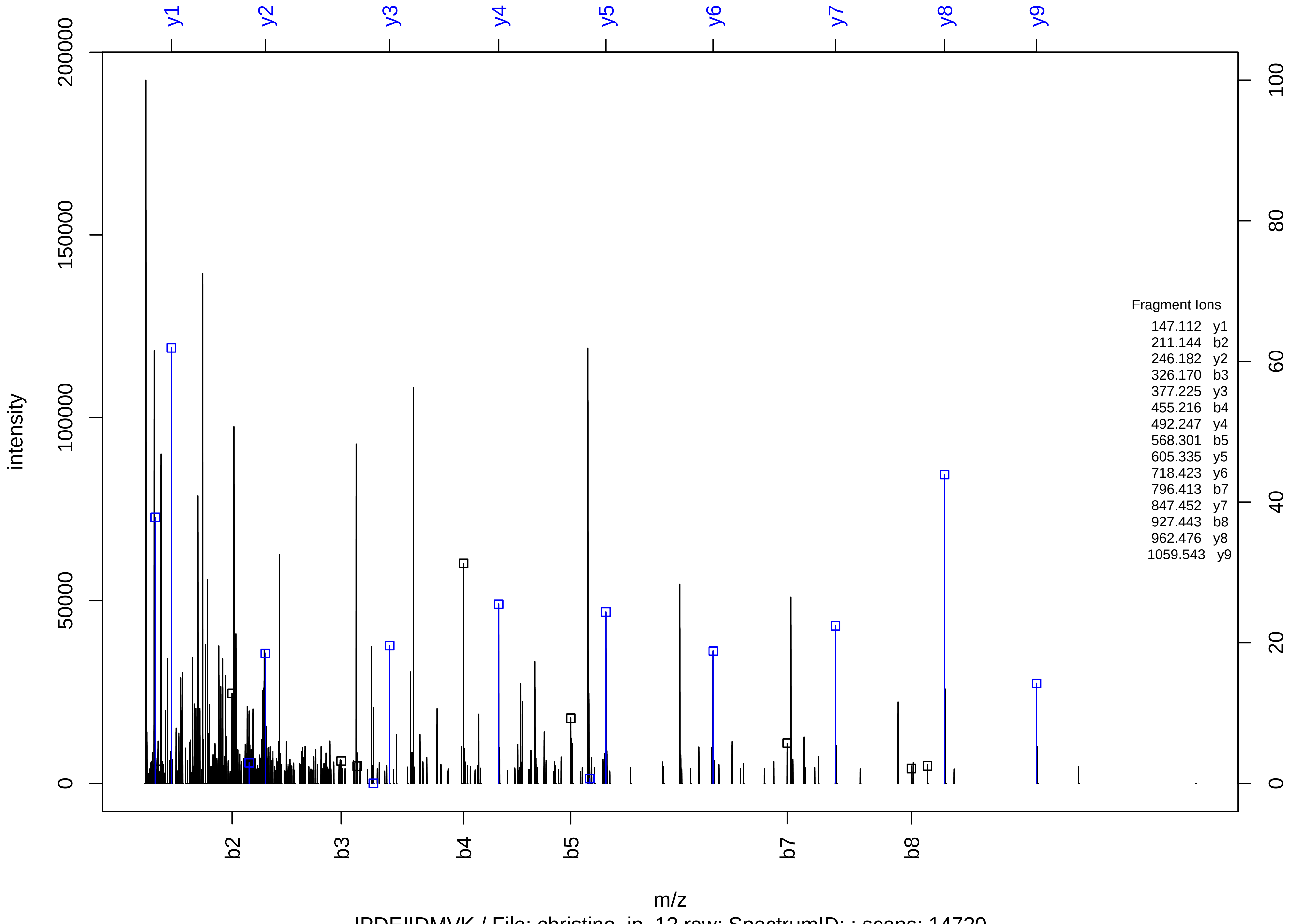

IPDEIIDMVK / File: christine_ip_12.raw; SpectrumID: ; scans: 14720

RVEELGDLAQAHIQQLSEAAGEDDHFLIR

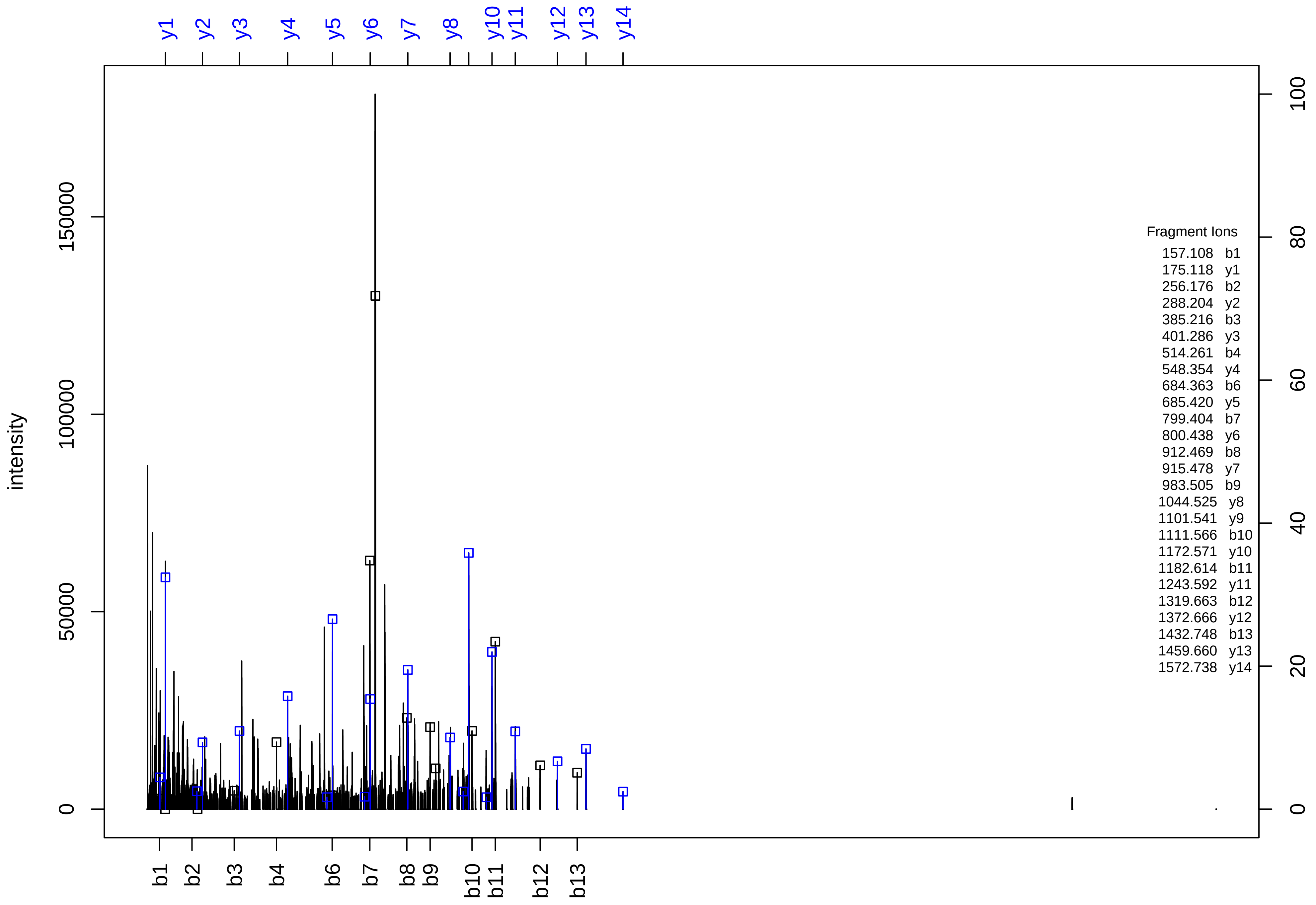

RVEELGDLAQAHIQQLSEAAGEDDHFLIR / File: christine_ip_04.raw; SpectrumID: ; scans: 15454

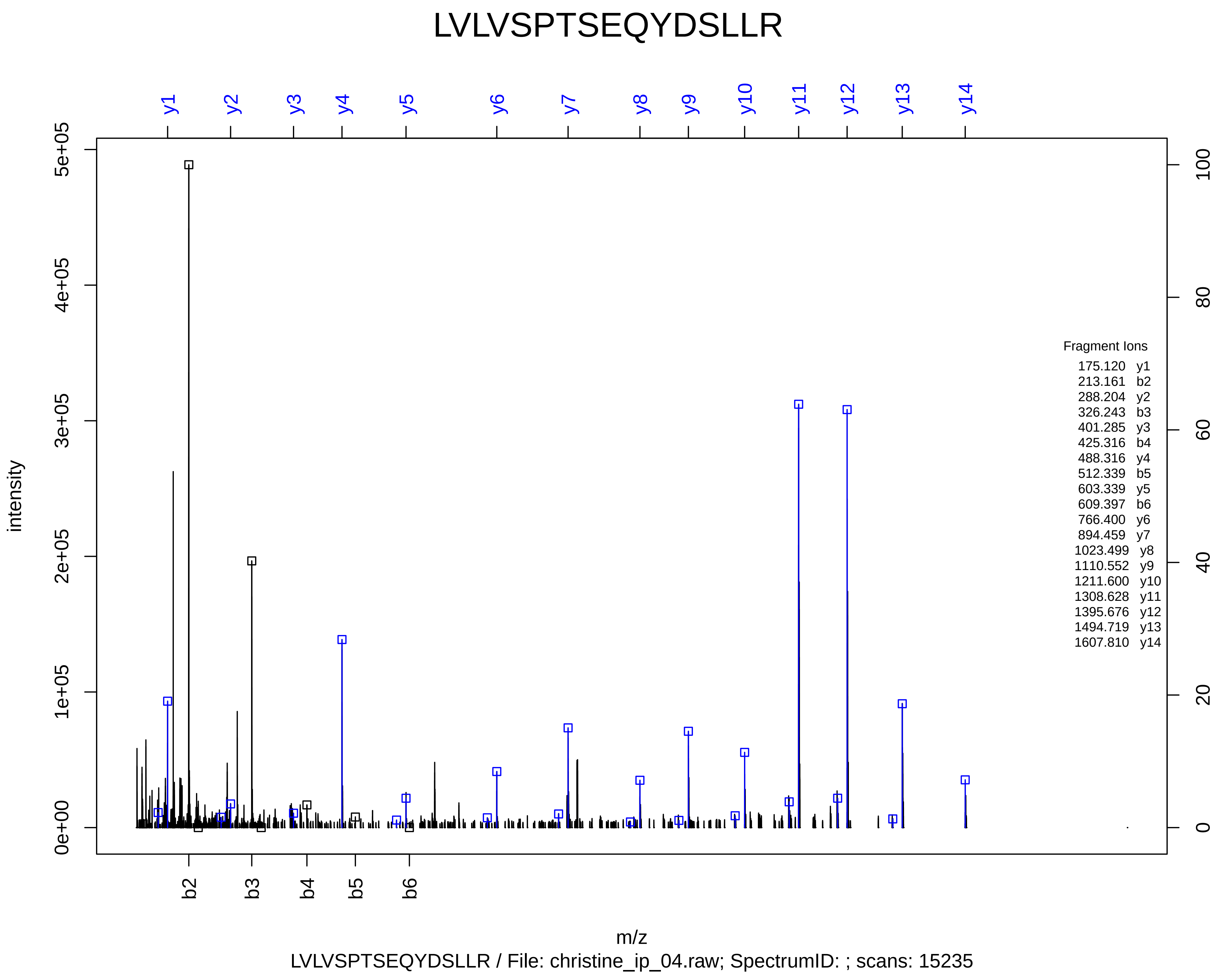
LVLVSPTSEQYDSLLR
y1
y2
y3
y4
y5
y6
y7
y8
y9
y10
y11
y12
y13
y14
b2
b3
b4
b5
b6
intensity
0e+00
1e+05
2e+05
3e+05
4e+05
5e+05
0
20
40
60
80
100
Fragment Ions
175.120 y1
213.161 b2
288.204 y2
326.243 b3
401.285 y3
425.316 b4
488.316 y4
512.339 b5
603.339 y5
609.397 b6
766.400 y6
894.459 y7
1023.499 y8
1110.552 y9
1211.600 y10
1308.628 y11
1395.676 y12
1494.719 y13
1607.810 y14
m/z
LVLVSPTSEQYDSLLR / File: christine_ip_04.raw; SpectrumID: ; scans: 15235

(Ac)AAAAELSLLEK

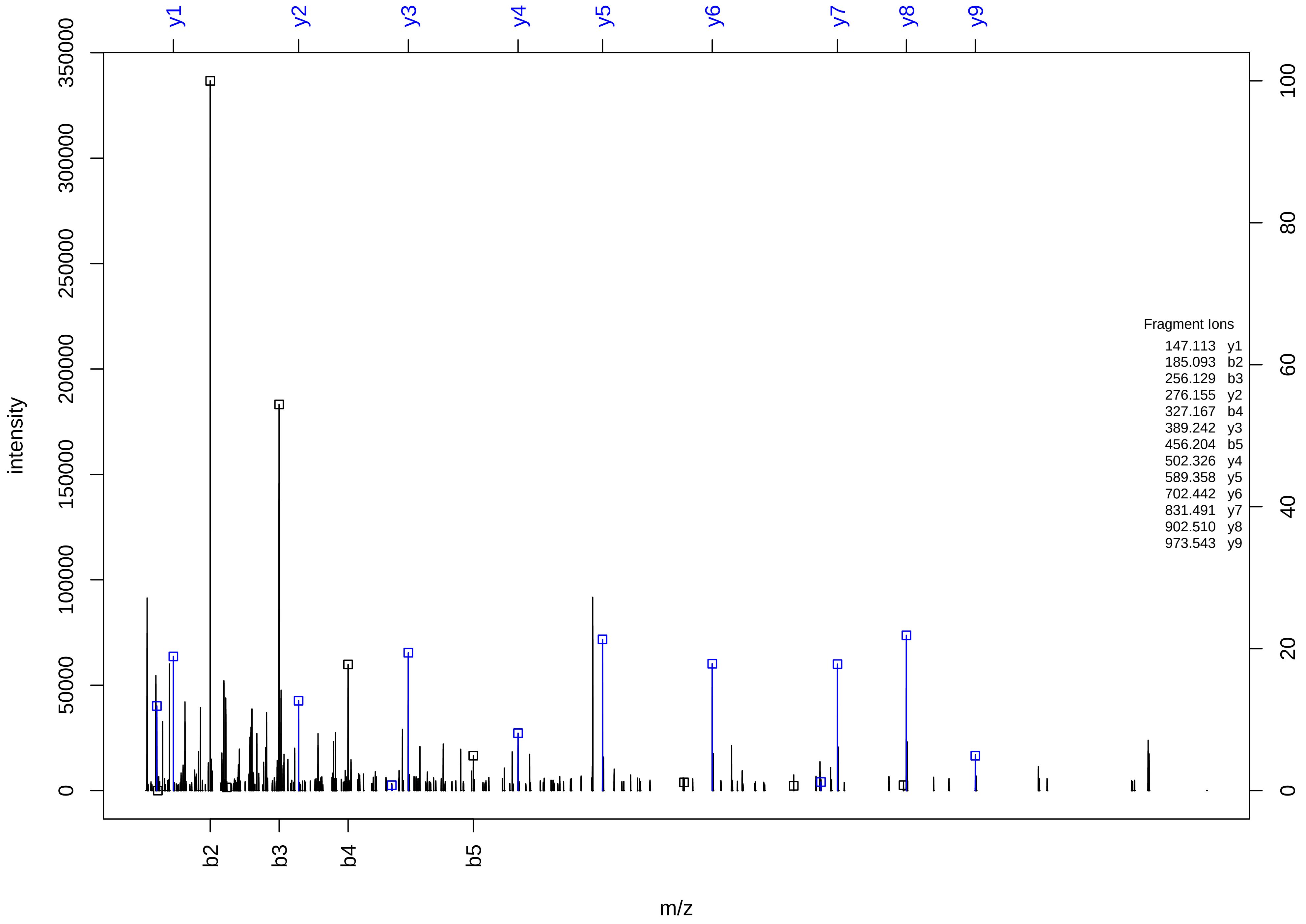

AAAAELSLLEK / File: christine_ip_04.raw; SpectrumID: ; scans: 17343

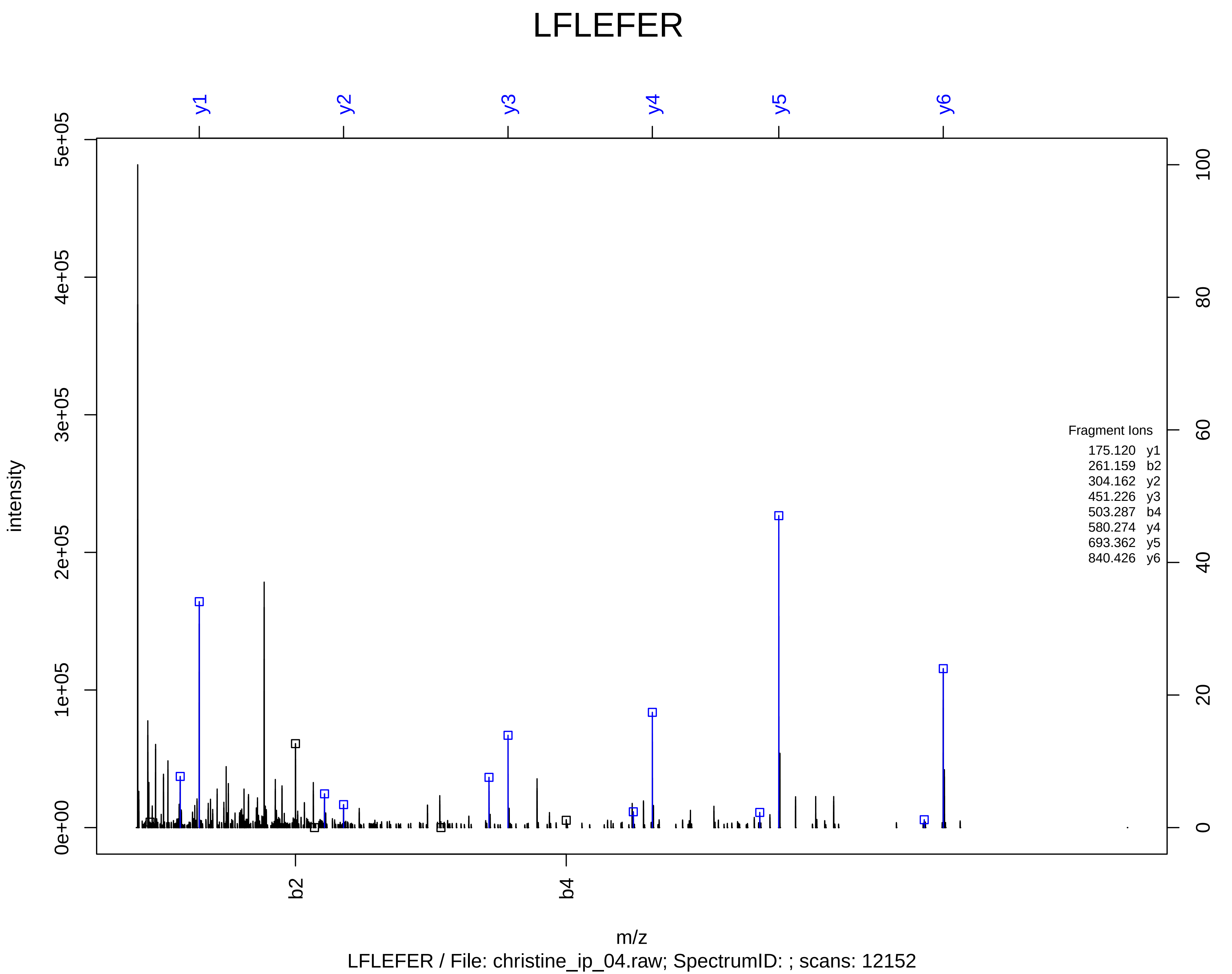
LFLEFER
y1
y2
y3
y4
y5
y6
intensity
0e+00
1e+05
2e+05
3e+05
4e+05
5e+05
0
20
40
60
80
100
Fragment Ions
175.120 y1
261.159 b2
304.162 y2
451.226 y3
503.287 b4
580.274 y4
693.362 y5
840.426 y6
b2
b4
m/z
LFLEFER / File: christine_ip_04.raw; SpectrumID: ; scans: 12152

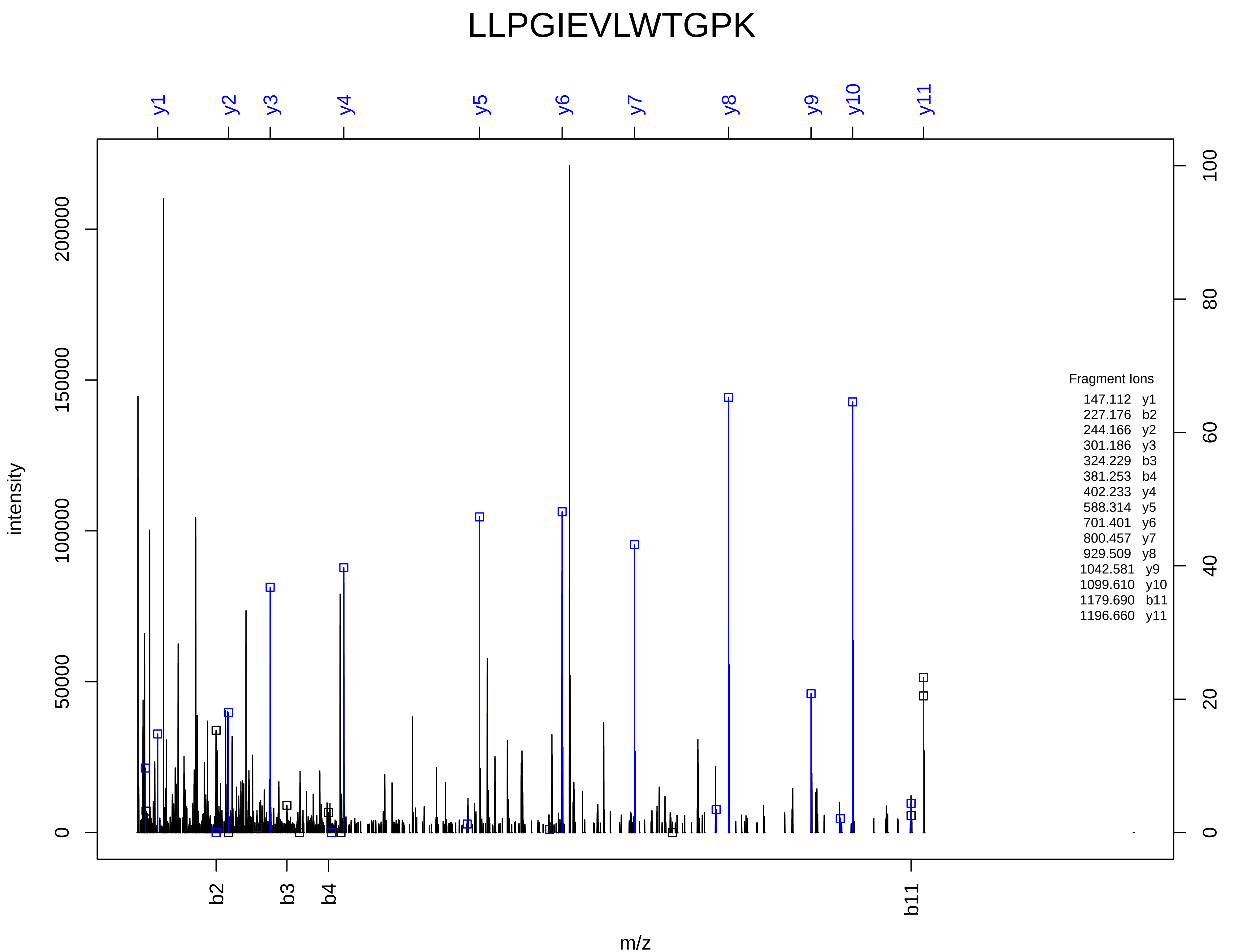
LLPGIEVLWTGPK
y1
y2
y3
y4
y5
y6
y7
y8
y9
y10
y11
intensity
0
50000
100000
150000
200000
0
20
40
60
80
100
Fragment Ions
147.112 y1
227.176 b2
244.166 y2
301.186 y3
324.229 b3
381.253 b4
402.233 y4
588.314 y5
701.401 y6
800.457 y7
929.509 y8
1042.581 y9
1099.610 y10
1179.690 b11
1196.660 y11
b2
b3
b4
b11
m/z
LLPGIEVLWTGPK / File: christine_ip_04.raw; SpectrumID: ; scans: 19349

EQYEEETEAK

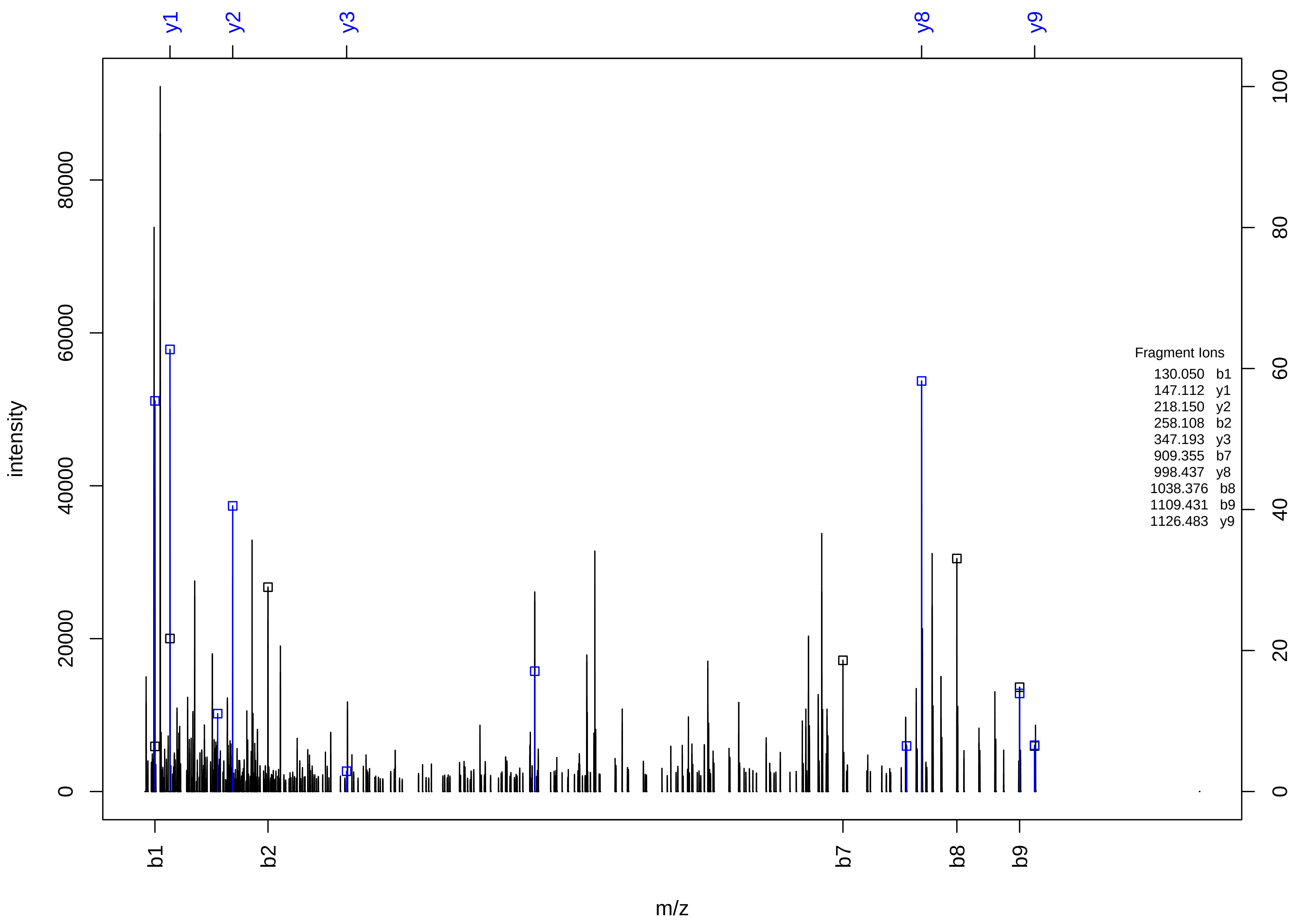

EQYEEETEAK / File: christine_ip_04.raw; SpectrumID: ; scans: 1916

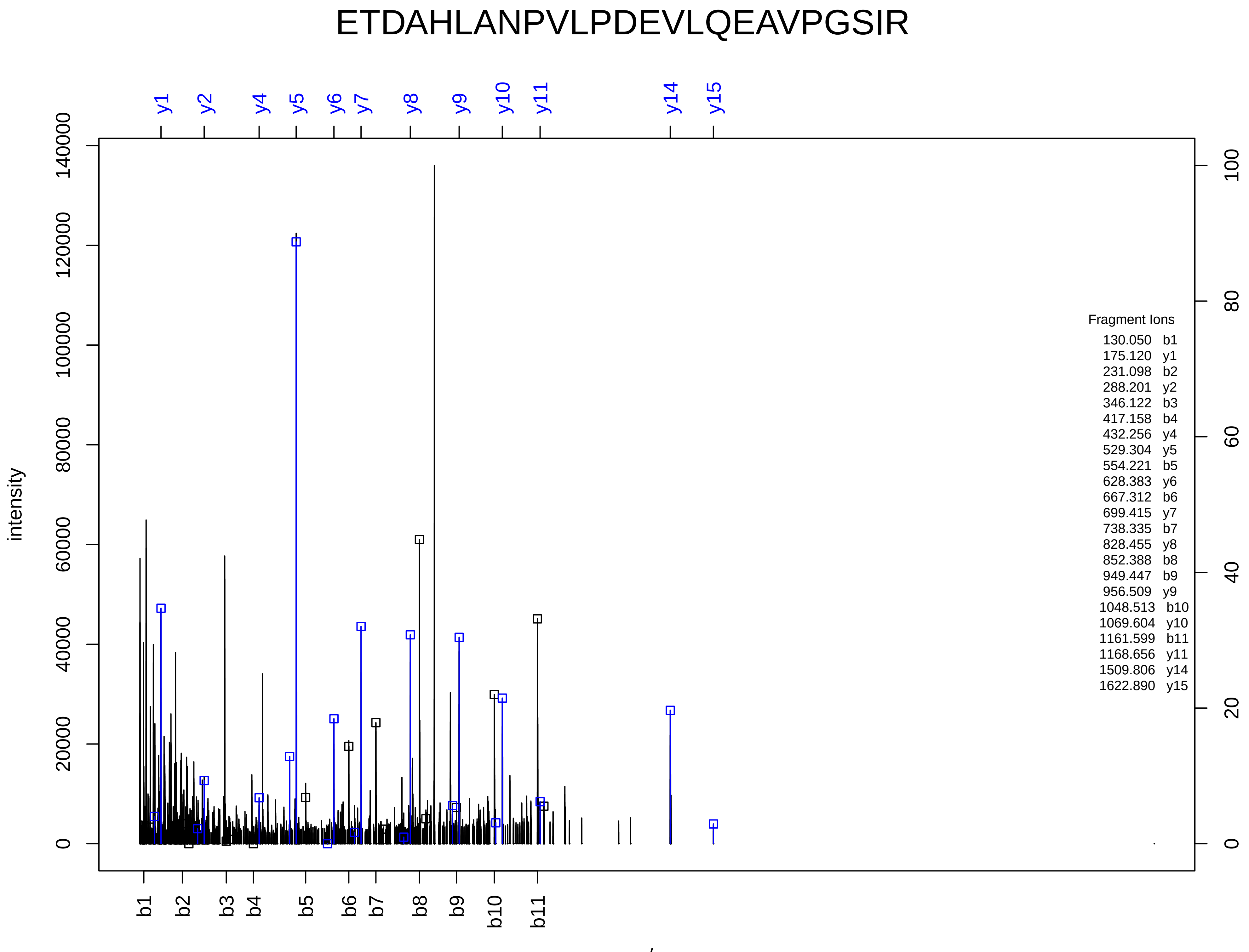

ETDAHLANPVLPDEVLQEAVPGSIR / File: christine_ip_04.raw; SpectrumID: ; scans: 16395

TTIPEEEEEEEEAAGVVVEEELFHQQGTPR

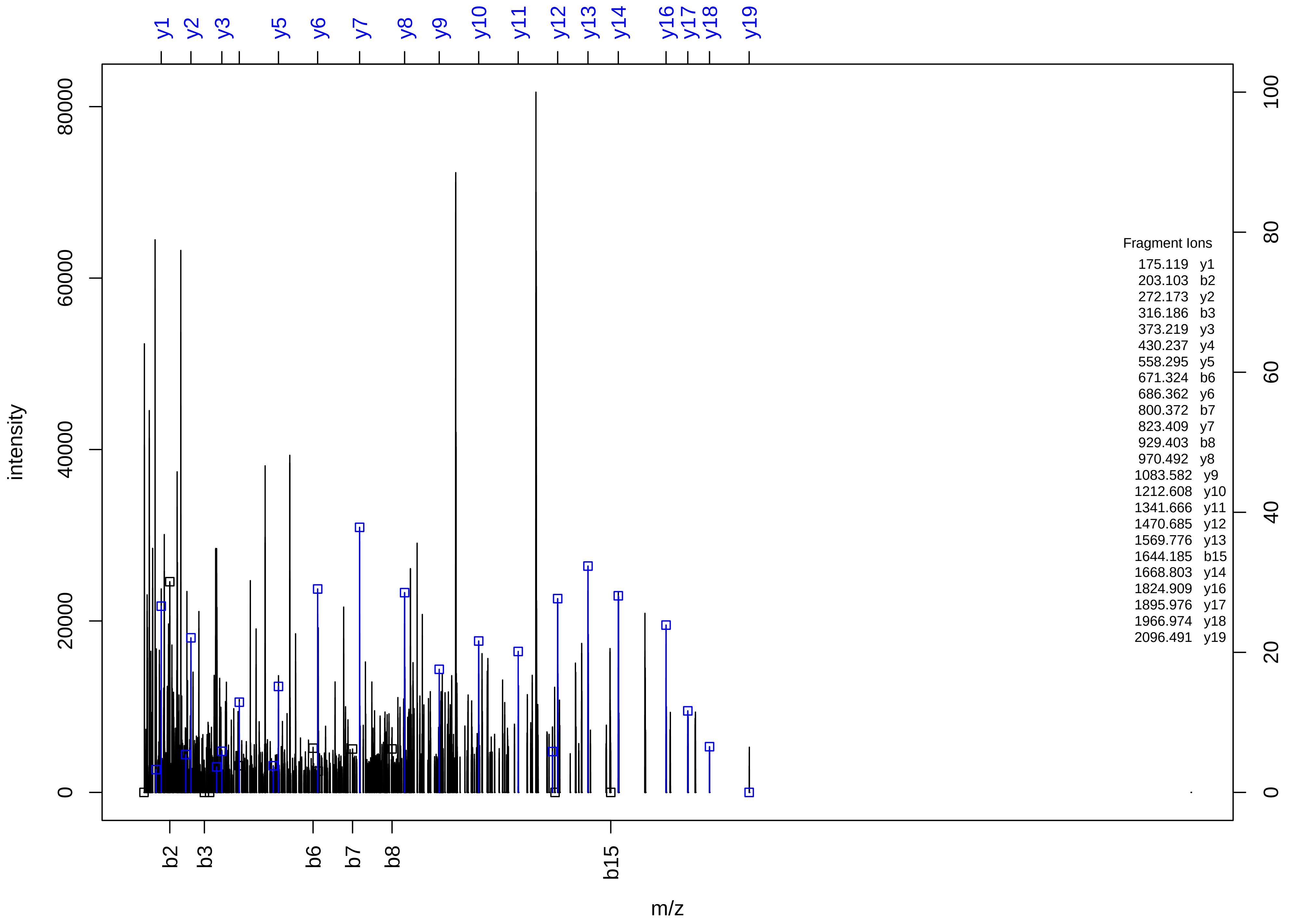

TTIPEEEEEEEEAAGVVVEEELFHQQGTPR / File: christine_ip_04.raw; SpectrumID: ; scans: 17722

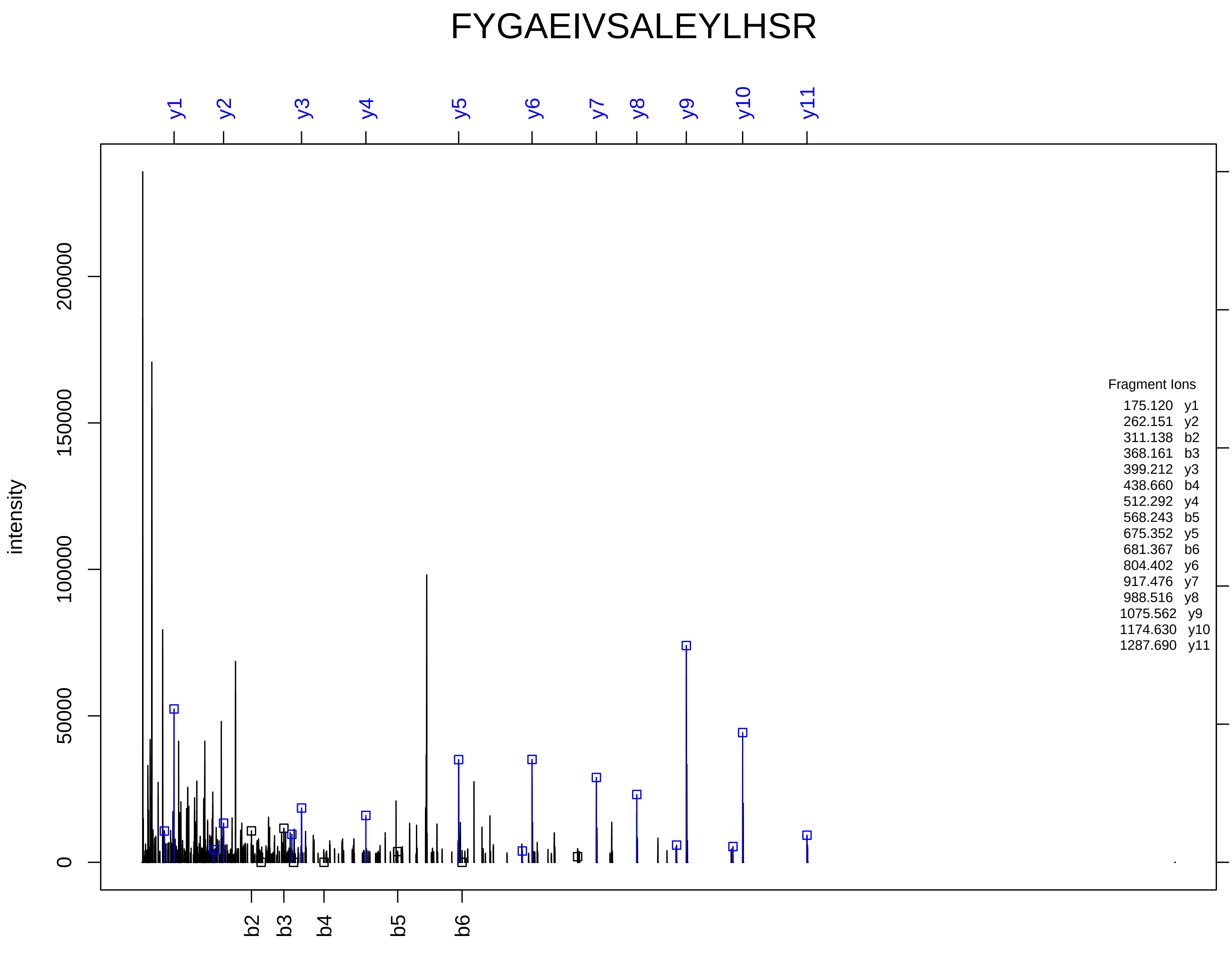
FYGAEIVSALEYLHSR
y1 y2 y3 y4 y5 y6 y7 y8 y9 y10 y11
b2 b3 b4 b5 b6
intensity
m/z
0
50000
100000
150000
200000
0
20
40
60
80
100
Fragment Ions
175.120 y1
262.151 y2
311.138 b2
368.161 b3
399.212 y3
438.660 b4
512.292 y4
568.243 b5
675.352 y5
681.367 b6
804.402 y6
917.476 y7
988.516 y8
1075.562 y9
1174.630 y10
1287.690 y11
FYGAEIVSALEYLHSR / File: christine_ip_04.raw; SpectrumID: ; scans: 18542

DVVFNYLHATAFQGTPLAQAVEGPSENVR

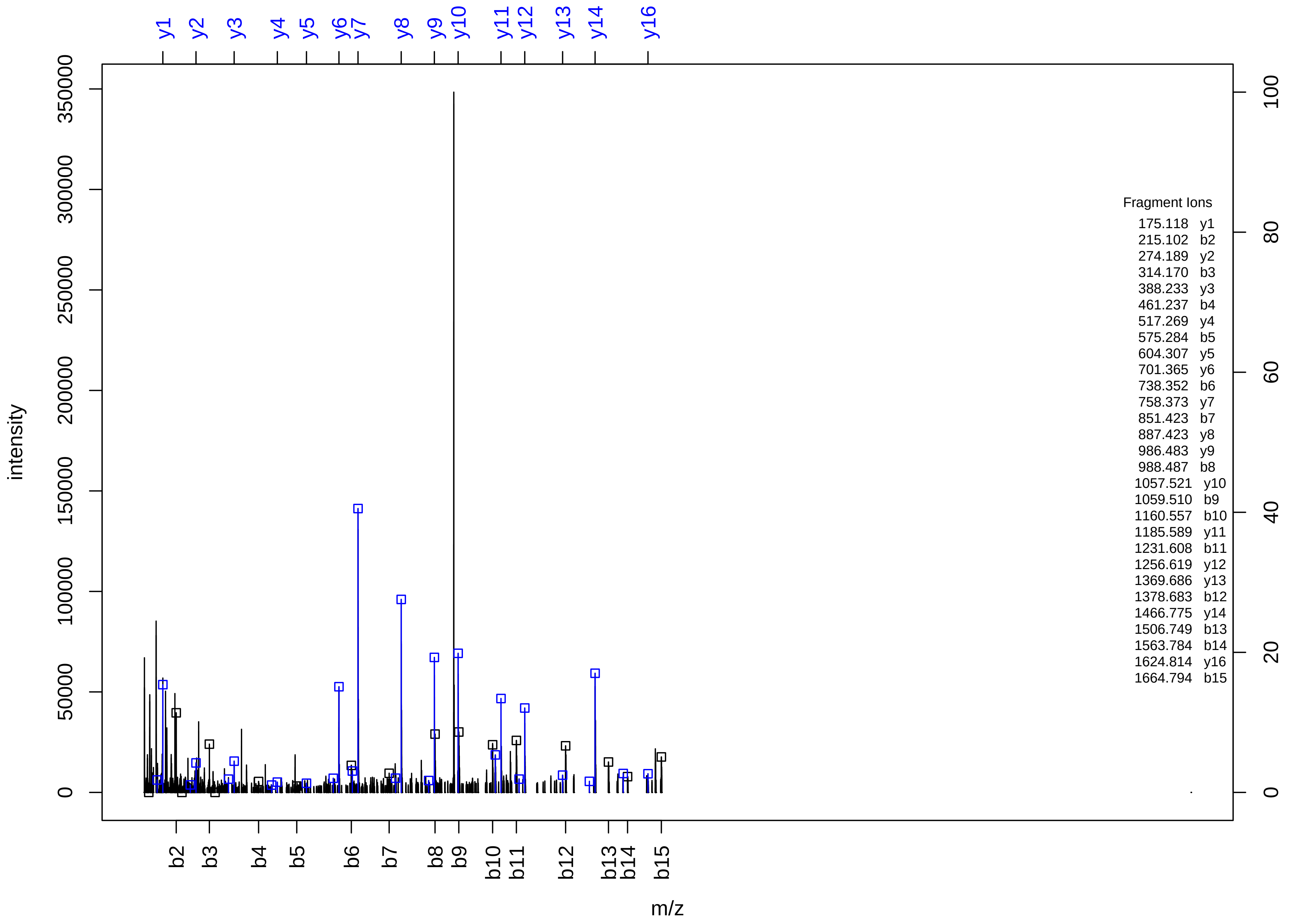

DVVFNYLHATAFQGTPLAQAVEGPSENVR / File: christine_ip_04.raw; SpectrumID: ; scans: 19002

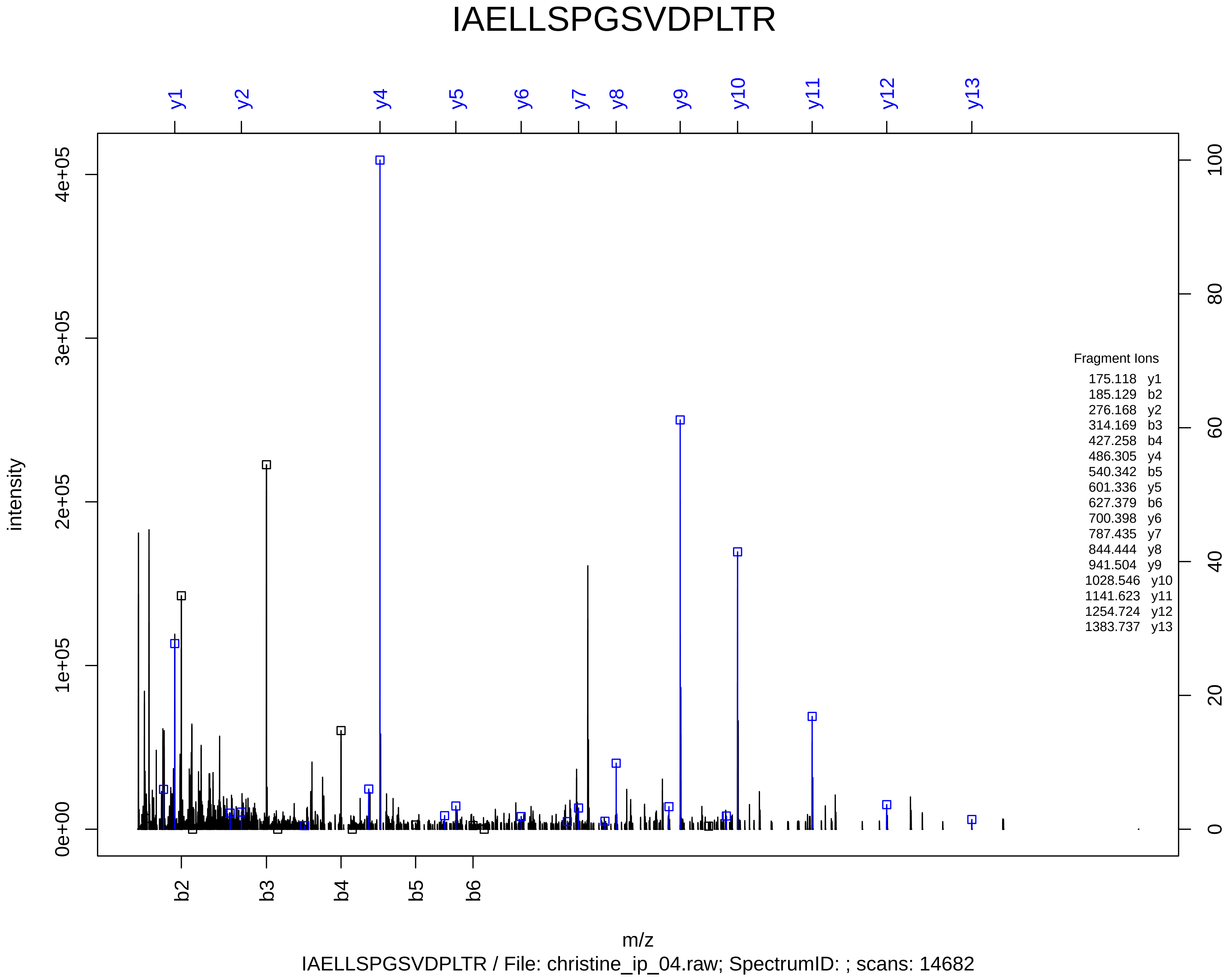
IAELLSPGSVDPLTR
y1 y2 y4 y5 y6 y7 y8 y9 y10 y11 y12 y13
b2 b3 b4 b5 b6
intensity
m/z
Fragment Ions
175.118 y1
185.129 b2
276.168 y2
314.169 b3
427.258 b4
486.305 y4
540.342 b5
601.336 y5
627.379 b6
700.398 y6
787.435 y7
844.444 y8
941.504 y9
1028.546 y10
1141.623 y11
1254.724 y12
1383.737 y13
IAELLSPGSVDPLTR / File: christine_ip_04.raw; SpectrumID: ; scans: 14682

IYGLGSLALYEK

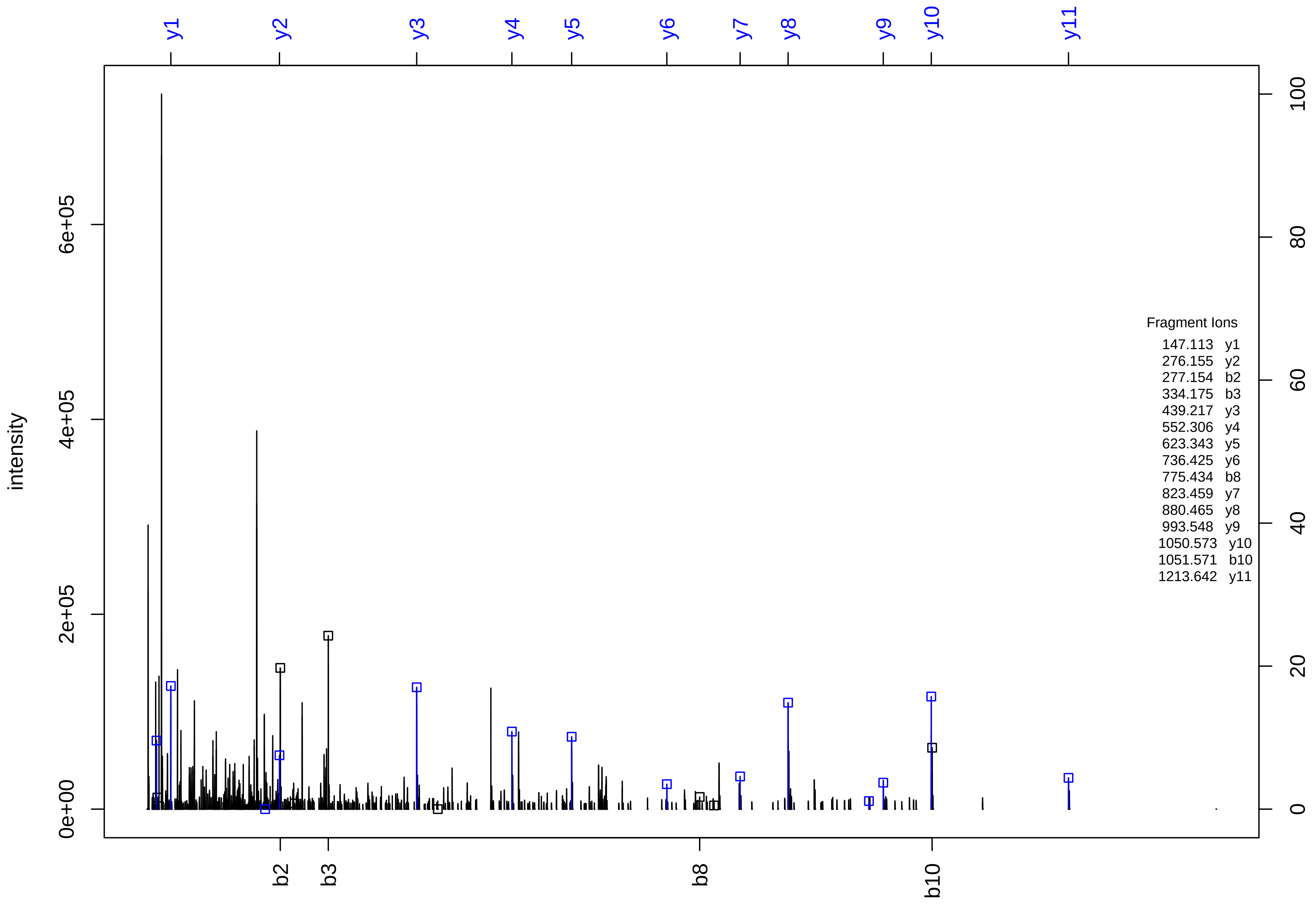

IYGLGSLALYEK / File: christine_ip_04.raw; SpectrumID: ; scans: 14485

TVLGTPEVLLGALPGAGGTQR

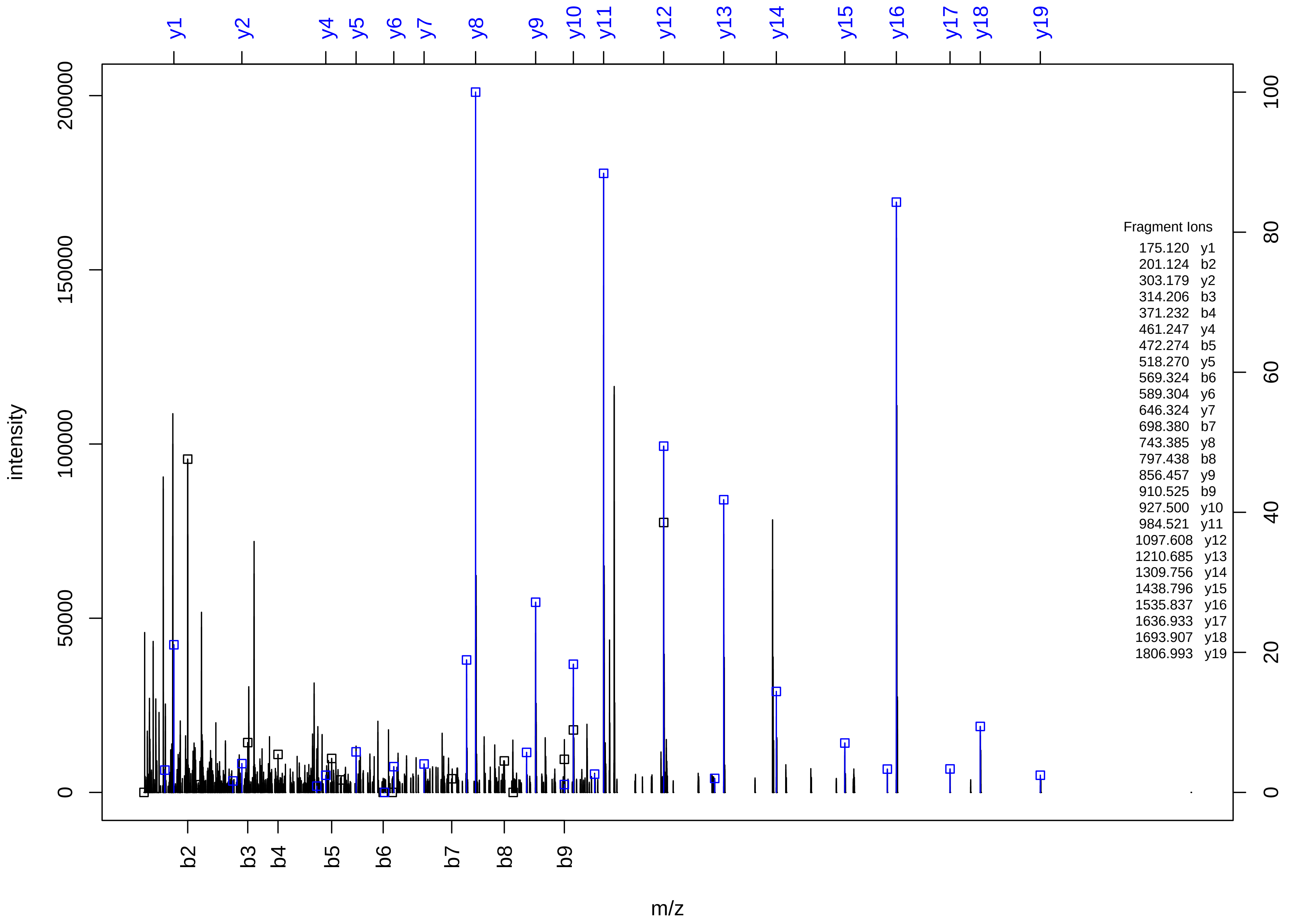

TVLGTPEVLLGALPGAGGTQR / File: christine_ip_04.raw; SpectrumID: ; scans: 18310

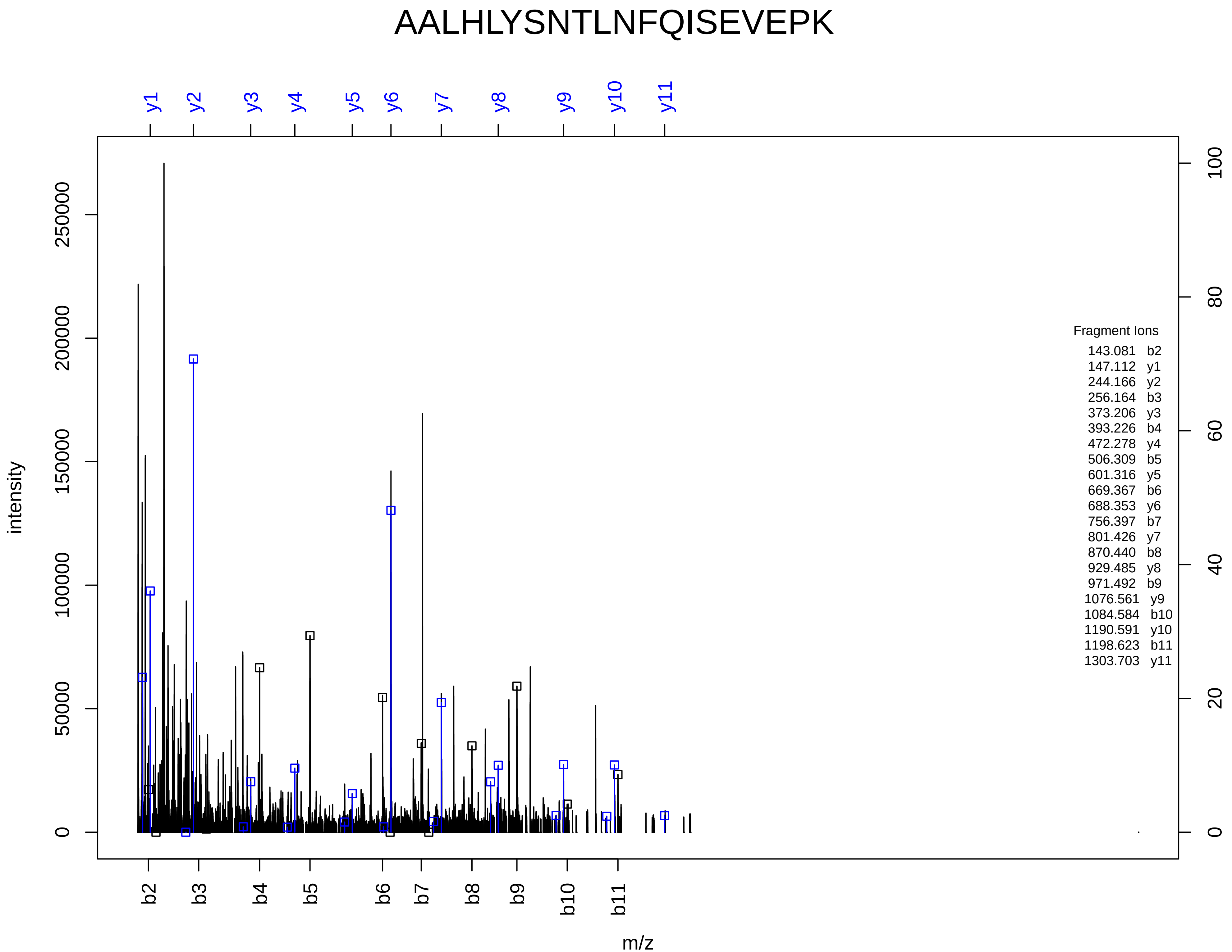

AALHLYSNTLNFQISEVEPK / File: christine_ip_04.raw; SpectrumID: ; scans: 14319

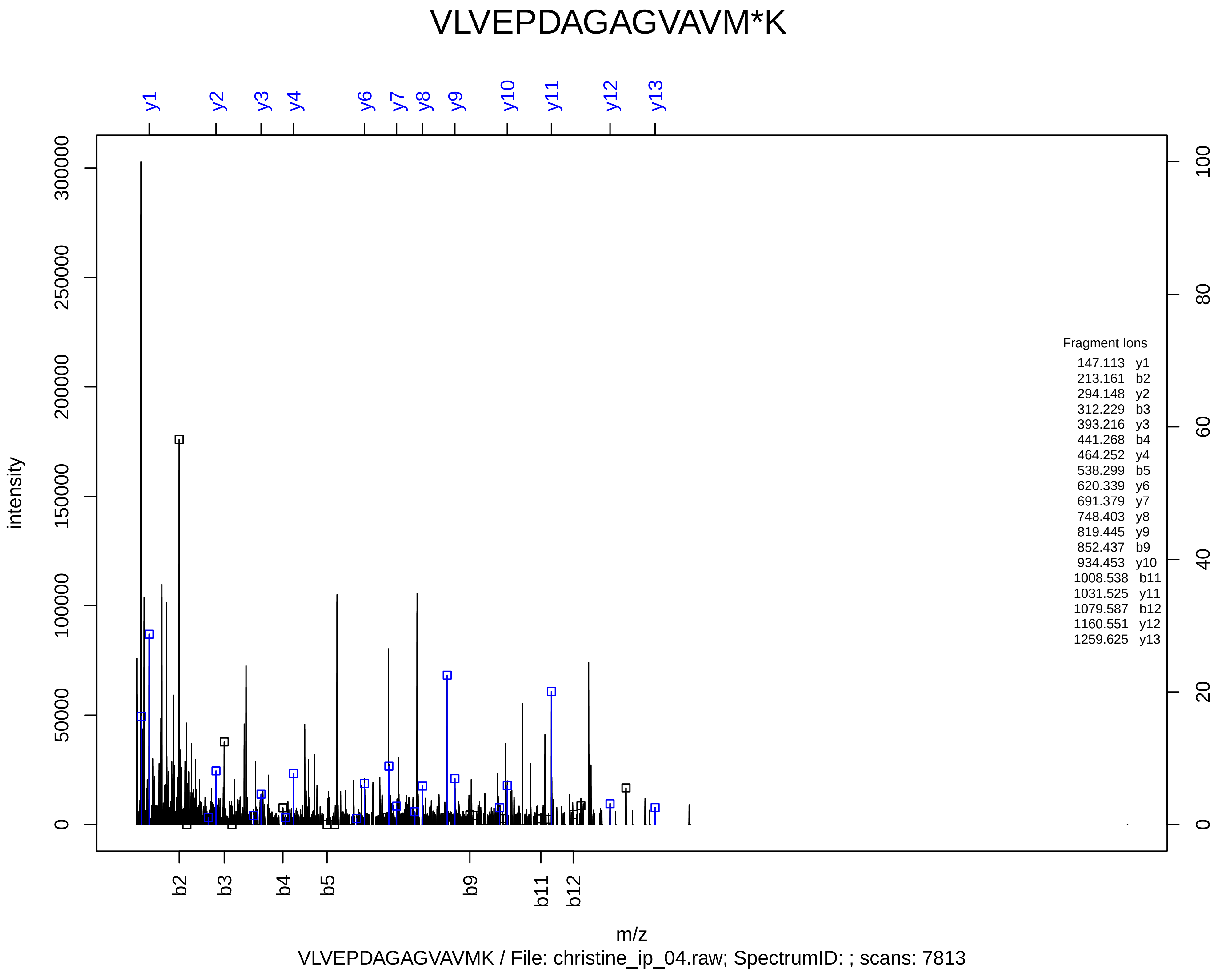
VLVEPDAGAGVAVM*K
y1
y2
y3
y4
y6
y7
y8
y9
y10
y11
y12
y13
intensity
300000
250000
200000
150000
100000
50000
0
100
80
60
40
20
0
Fragment Ions
147.113 y1
213.161 b2
294.148 y2
312.229 b3
393.216 y3
441.268 b4
464.252 y4
538.299 b5
620.339 y6
691.379 y7
748.403 y8
819.445 y9
852.437 b9
934.453 y10
1008.538 b11
1031.525 y11
1079.587 b12
1160.551 y12
1259.625 y13
b2
b3
b4
b5
b9
b11
b12
m/z
VLVEPDAGAGVAVMK / File: christine_ip_04.raw; SpectrumID: ; scans: 7813

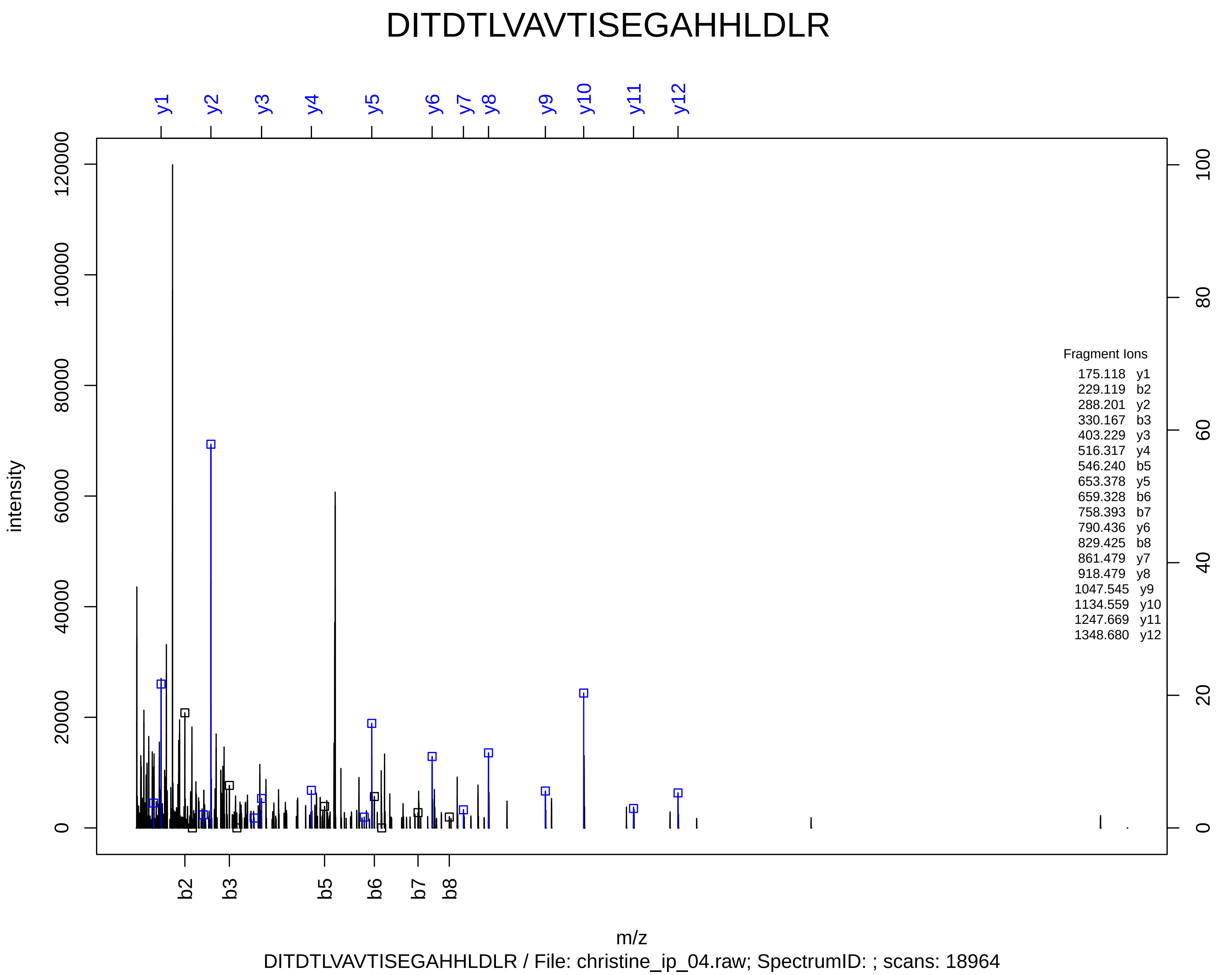
DITDTLVAVTISEGAHHLDLR
y1
y2
y3
y4
y5
y6
y7
y8
y9
y10
y11
y12
intensity
0
20000
40000
60000
80000
100000
120000
0
20
40
60
80
100
b2
b3
b5
b6
b7
b8
m/z
Fragment Ions
175.118 y1
229.119 b2
288.201 y2
330.167 b3
403.229 y3
516.317 y4
546.240 b5
653.378 y5
659.328 b6
758.393 b7
790.436 y6
829.425 b8
861.479 y7
918.479 y8
1047.545 y9
1134.559 y10
1247.669 y11
1348.680 y12
DITDTLVAVTISEGAHHLDLR / File: christine_ip_04.raw; SpectrumID: ; scans: 18964

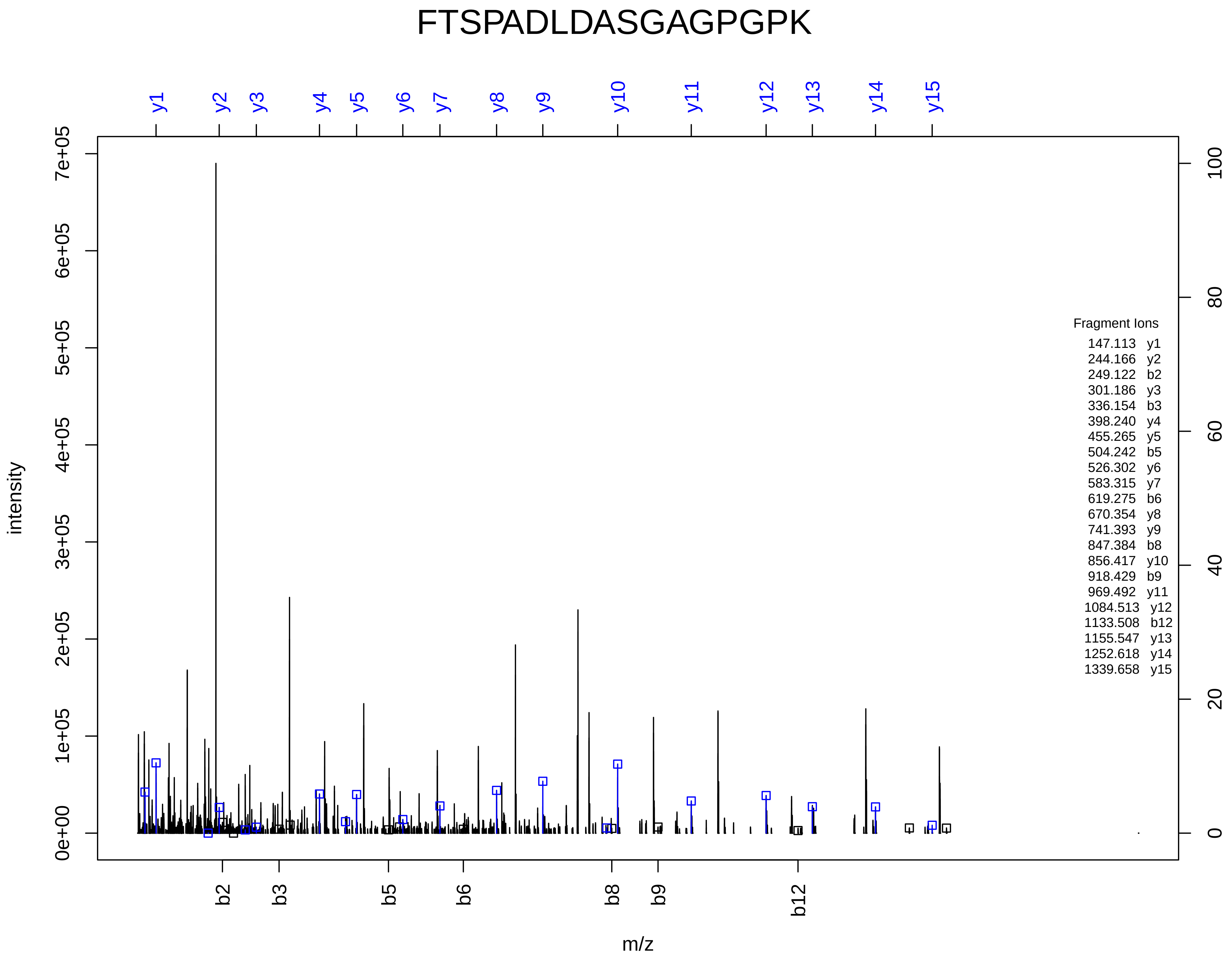

FTSPADLDASGAGPGPK / File: christine_ip_04.raw; SpectrumID: ; scans: 7656

GVEEEEEEDGEMRE

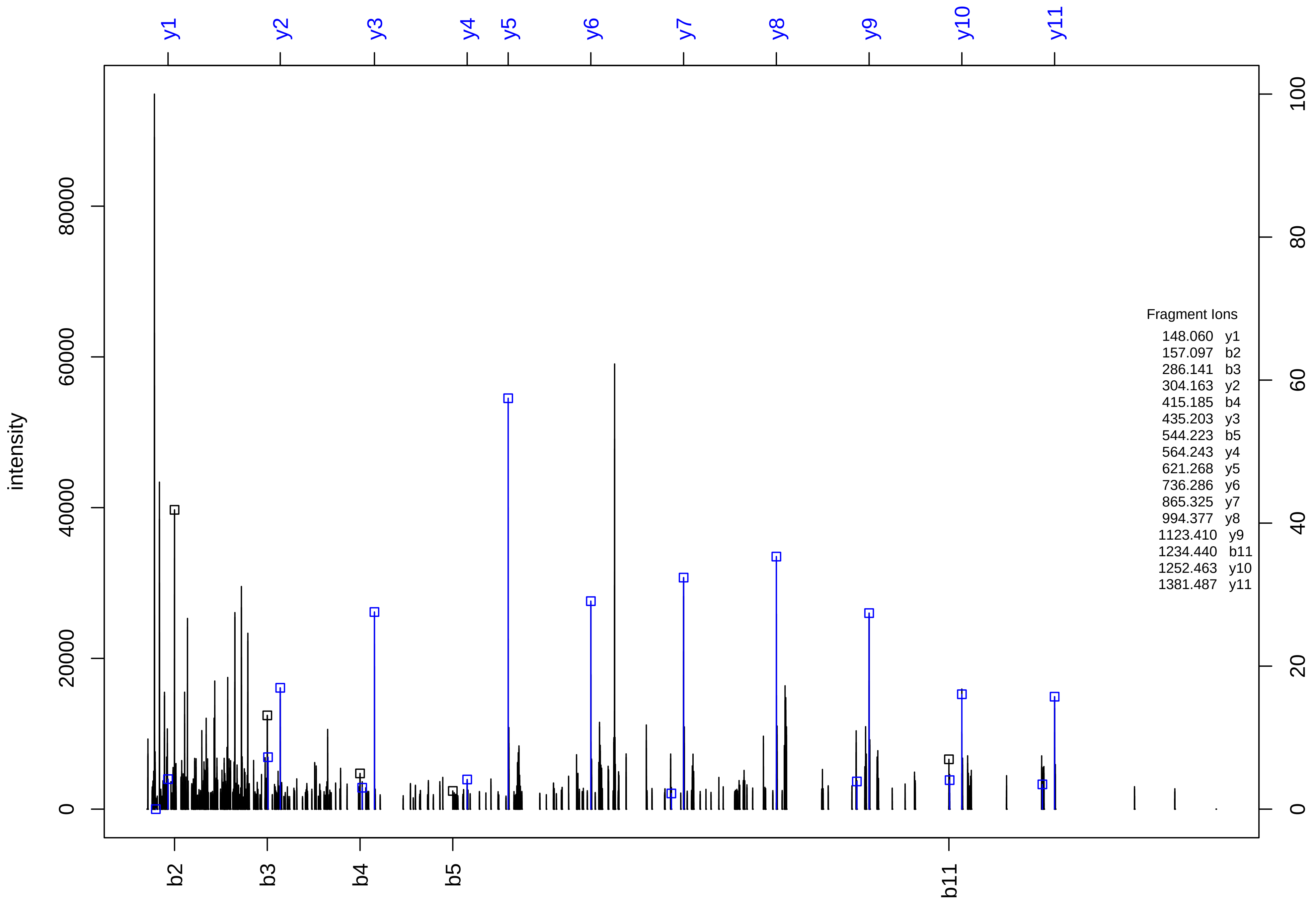

GVEEEEEEDGEMRE / File: christine_ip_04.raw; SpectrumID: ; scans: 3102

AVATAVEQIYPFVFESR

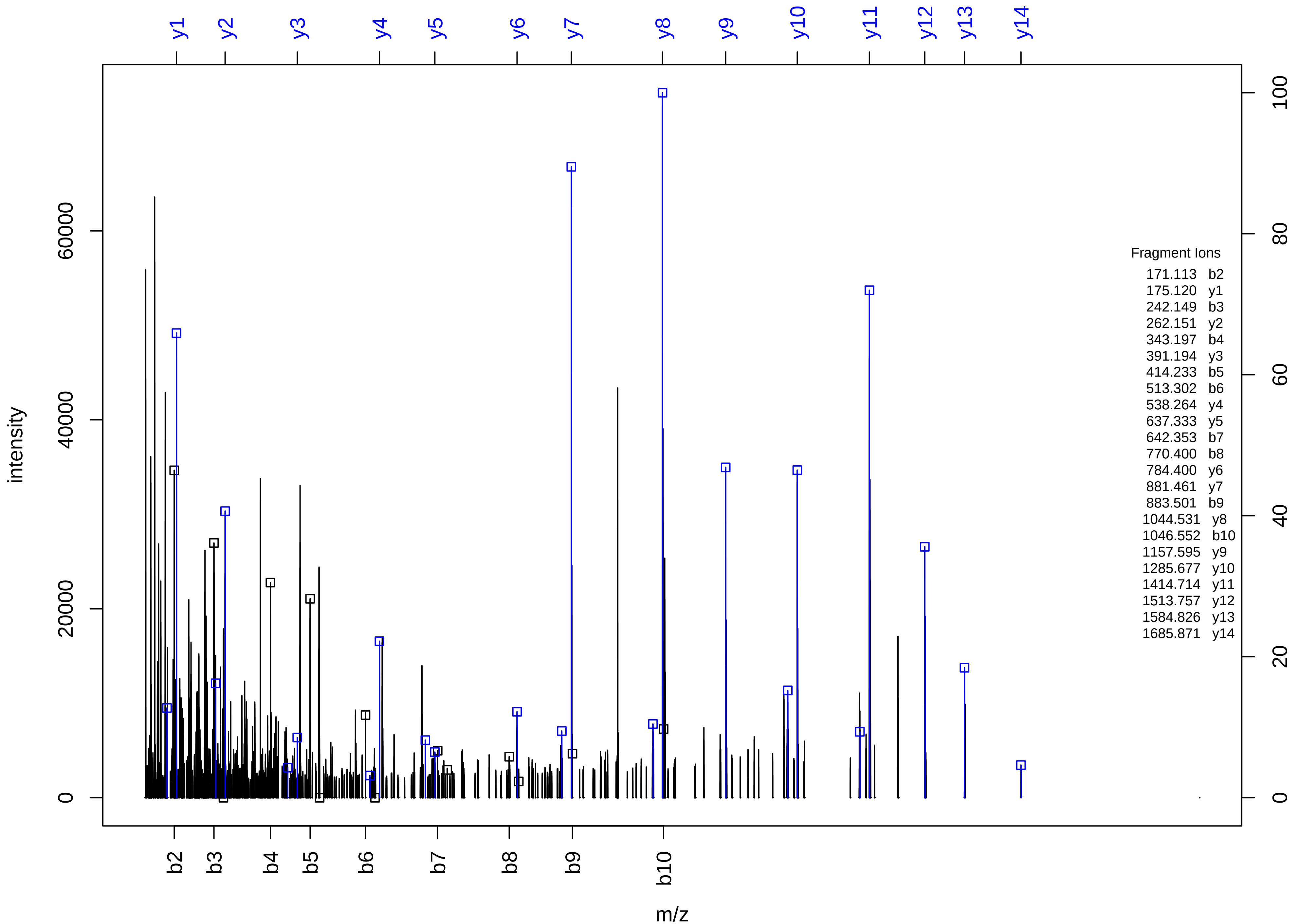

AVATAVEQIYPFVFESR / File: christine_ip_04.raw; SpectrumID: ; scans: 18541

NFGSYVTHETK

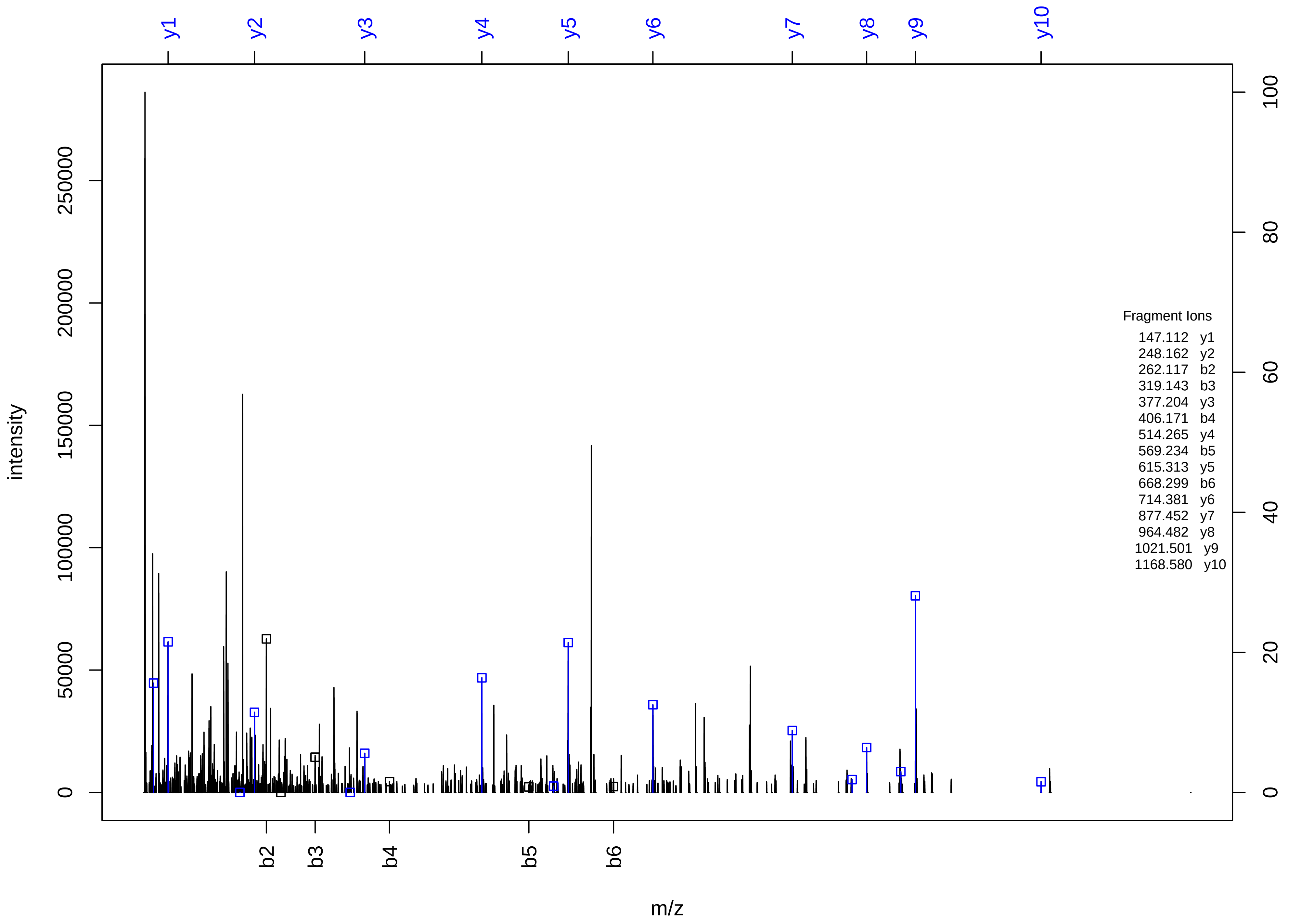

NFGSYVTHETK / File: christine_ip_04.raw; SpectrumID: ; scans: 3589

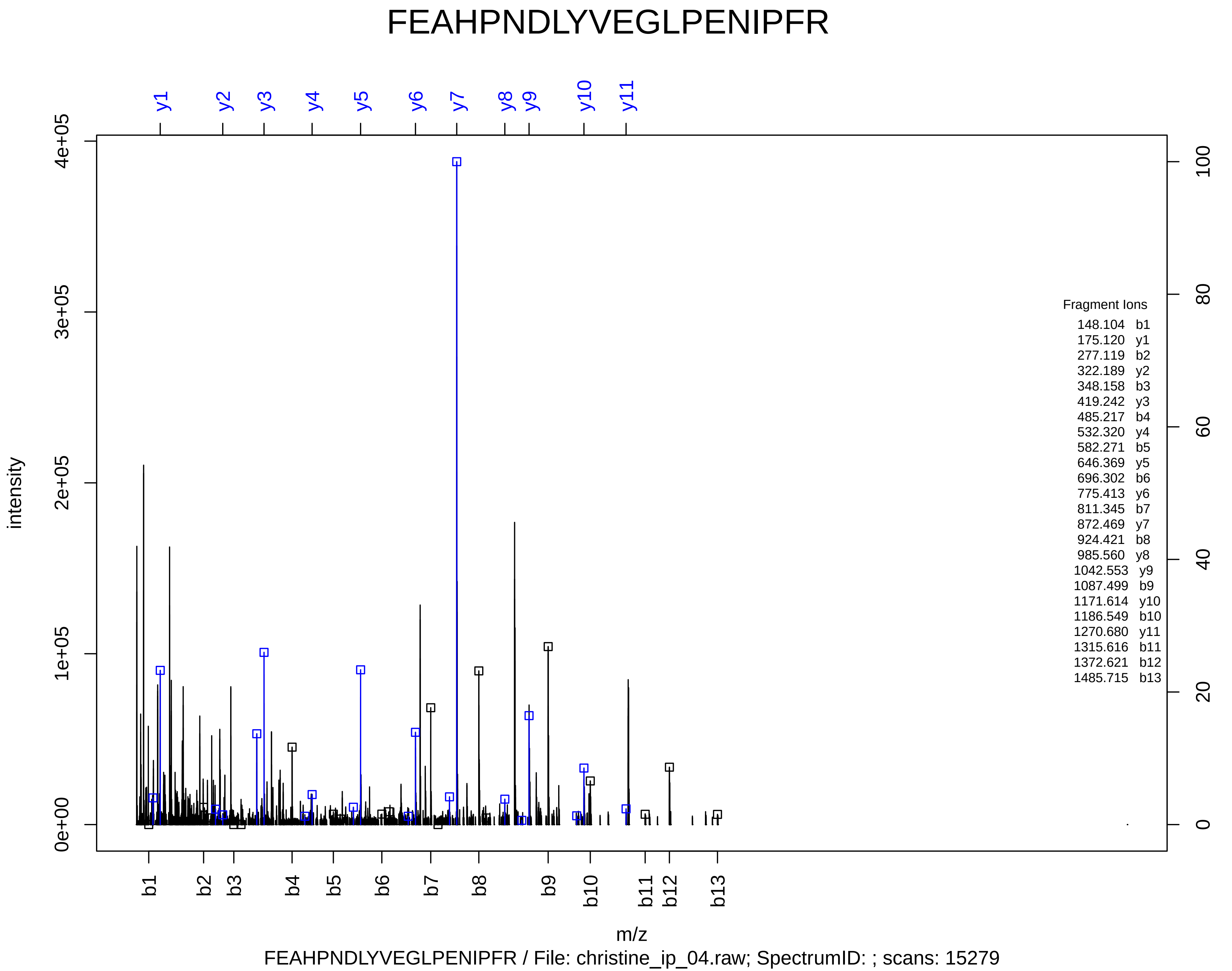
FEAHPNDLYVEGLPENIPFR
y1
y2
y3
y4
y5
y6
y7
y8
y9
y10
y11
intensity
0e+00
1e+05
2e+05
3e+05
4e+05
0
20
40
60
80
100
Fragment Ions
148.104 b1
175.120 y1
277.119 b2
322.189 y2
348.158 b3
419.242 y3
485.217 b4
532.320 y4
582.271 b5
646.369 y5
696.302 b6
775.413 y6
811.345 b7
872.469 y7
924.421 b8
985.560 y8
1042.553 y9
1087.499 b9
1171.614 y10
1186.549 b10
1270.680 y11
1315.616 b11
1372.621 b12
1485.715 b13
b1
b2
b3
b4
b5
b6
b7
b8
b9
b10
b11
b12
b13
m/z
FEAHPNDLYVEGLPENIPFR / File: christine_ip_04.raw; SpectrumID: ; scans: 15279

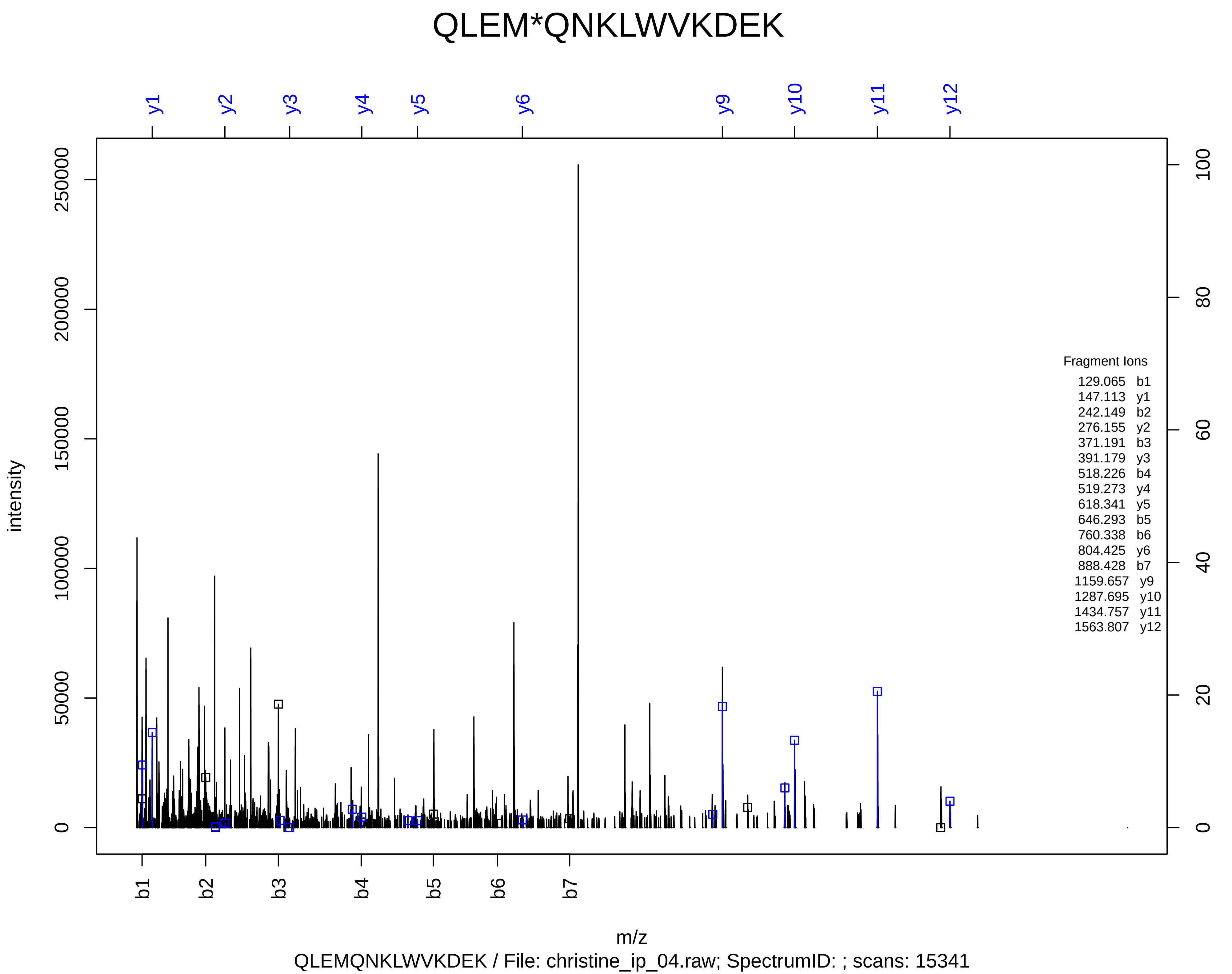
QLEM*QNKLWVKDEK
y1
y2
y3
y4
y5
y6
y9
y10
y11
y12
intensity
0
50000
100000
150000
200000
250000
0
20
40
60
80
100
b1
b2
b3
b4
b5
b6
b7
m/z
Fragment Ions
129.065 b1
147.113 y1
242.149 b2
276.155 y2
371.191 b3
391.179 y3
518.226 b4
519.273 y4
618.341 y5
646.293 b5
760.338 b6
804.425 y6
888.428 b7
1159.657 y9
1287.695 y10
1434.757 y11
1563.807 y12
QLEMQNKLWVKDEK / File: christine_ip_04.raw; SpectrumID: ; scans: 15341

TGALLLQGFIQDR

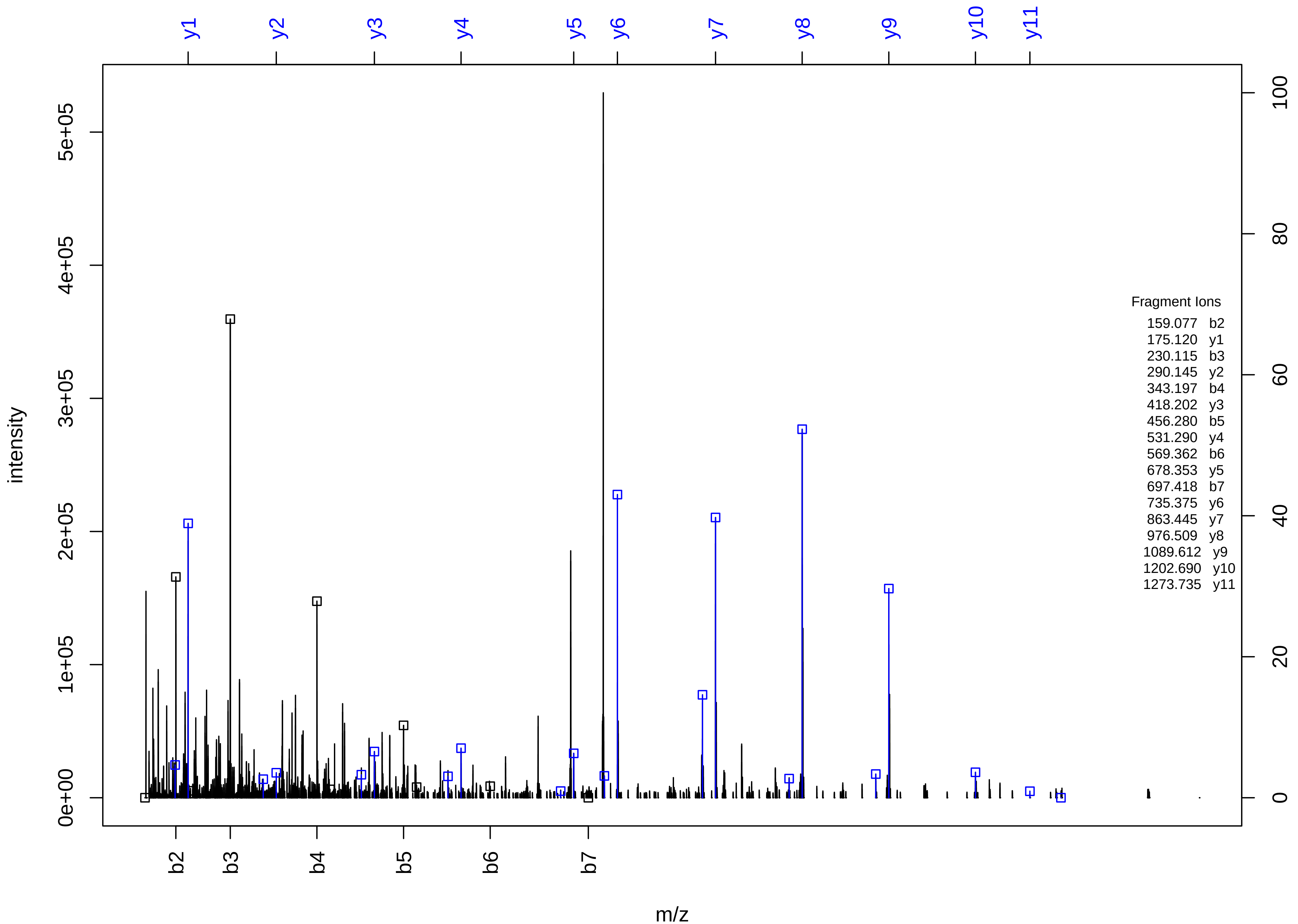

TGALLLQGFIQDR / File: christine_ip_04.raw; SpectrumID: ; scans: 16393

VVVLGLLPR

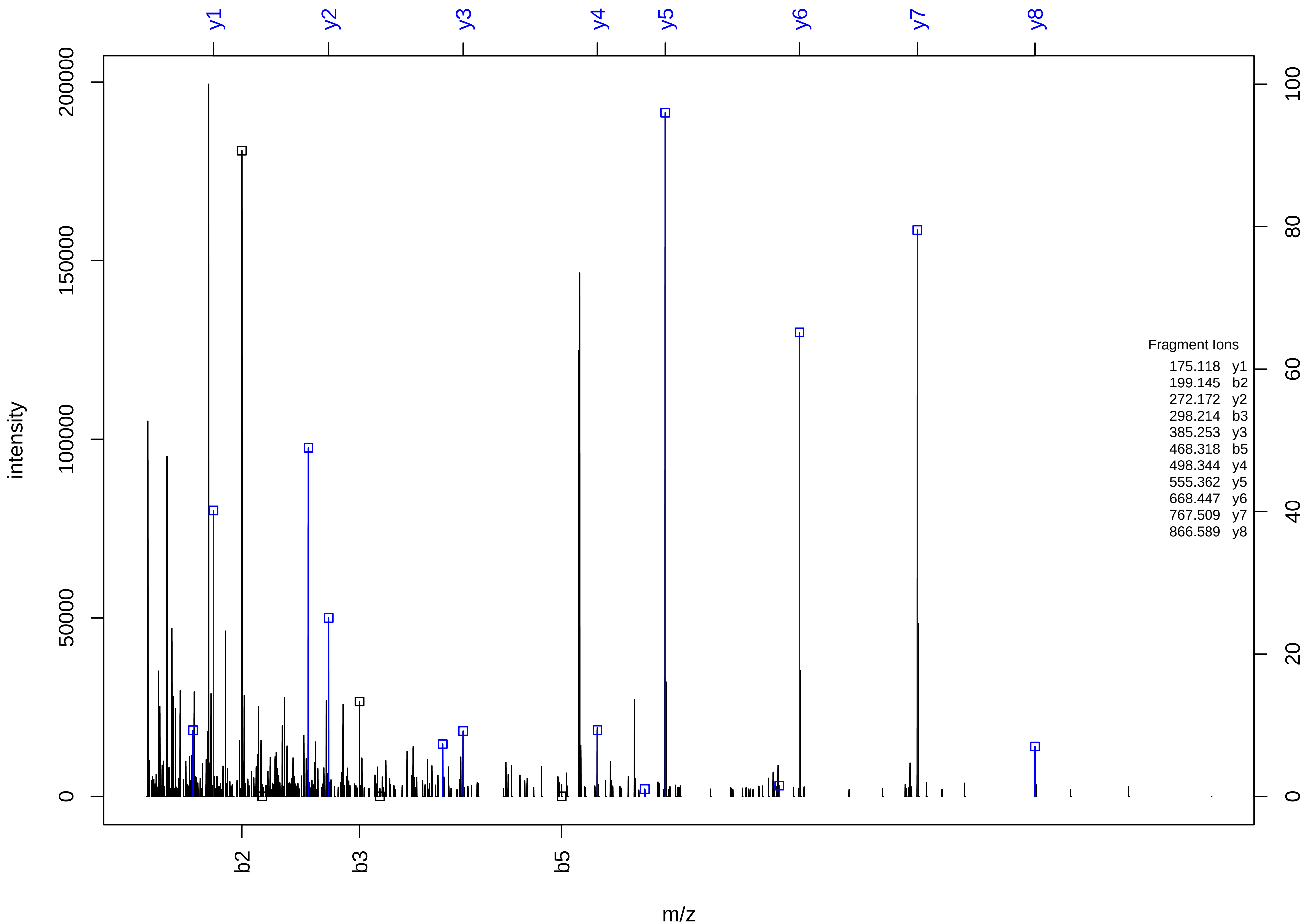

VVVLGLLPR / File: christine_ip_04.raw; SpectrumID: ; scans: 14839

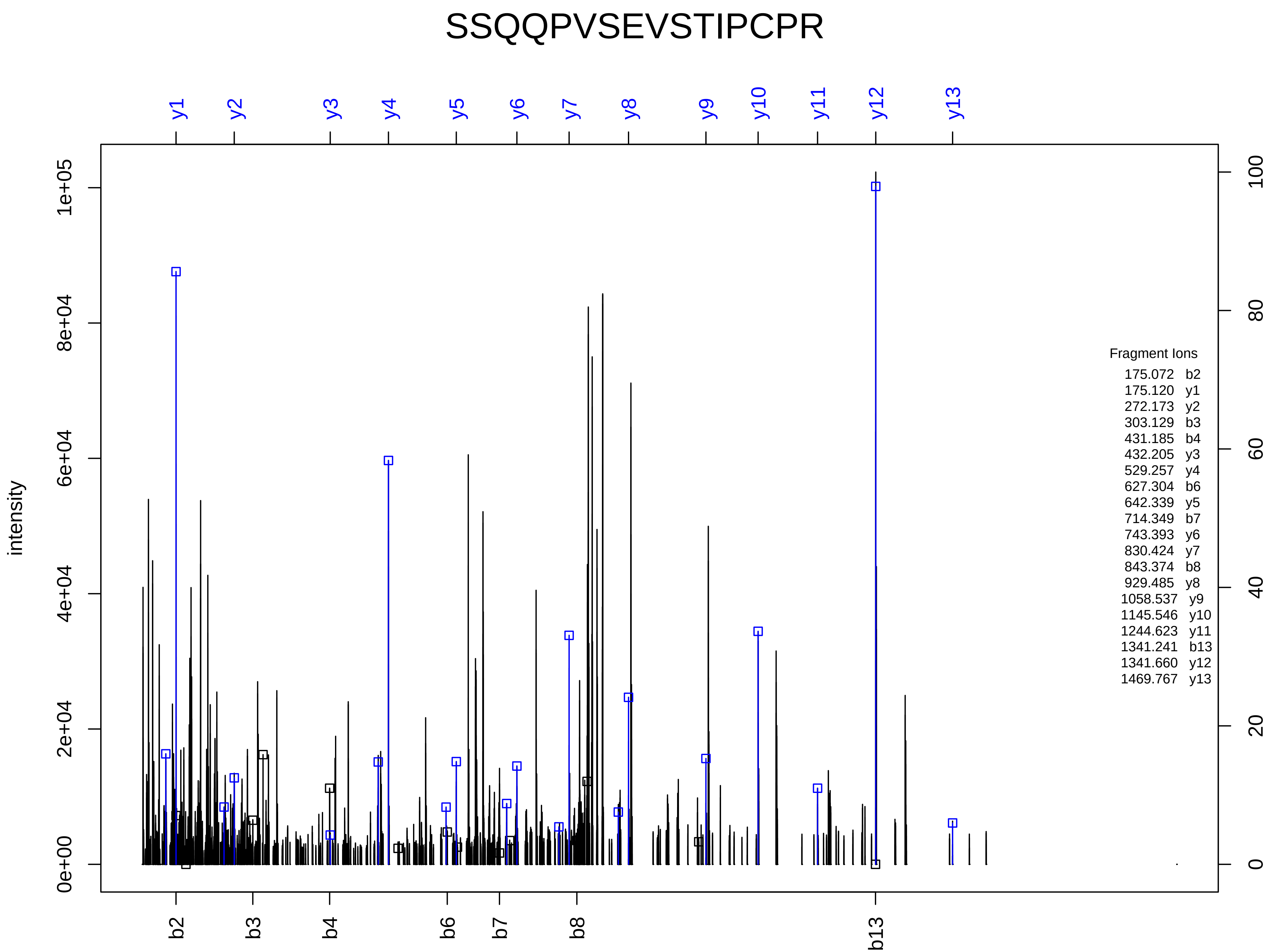

SSQQPVSEVSTIPCPR / File: christine_ip_04.raw; SpectrumID: ; scans: 7429

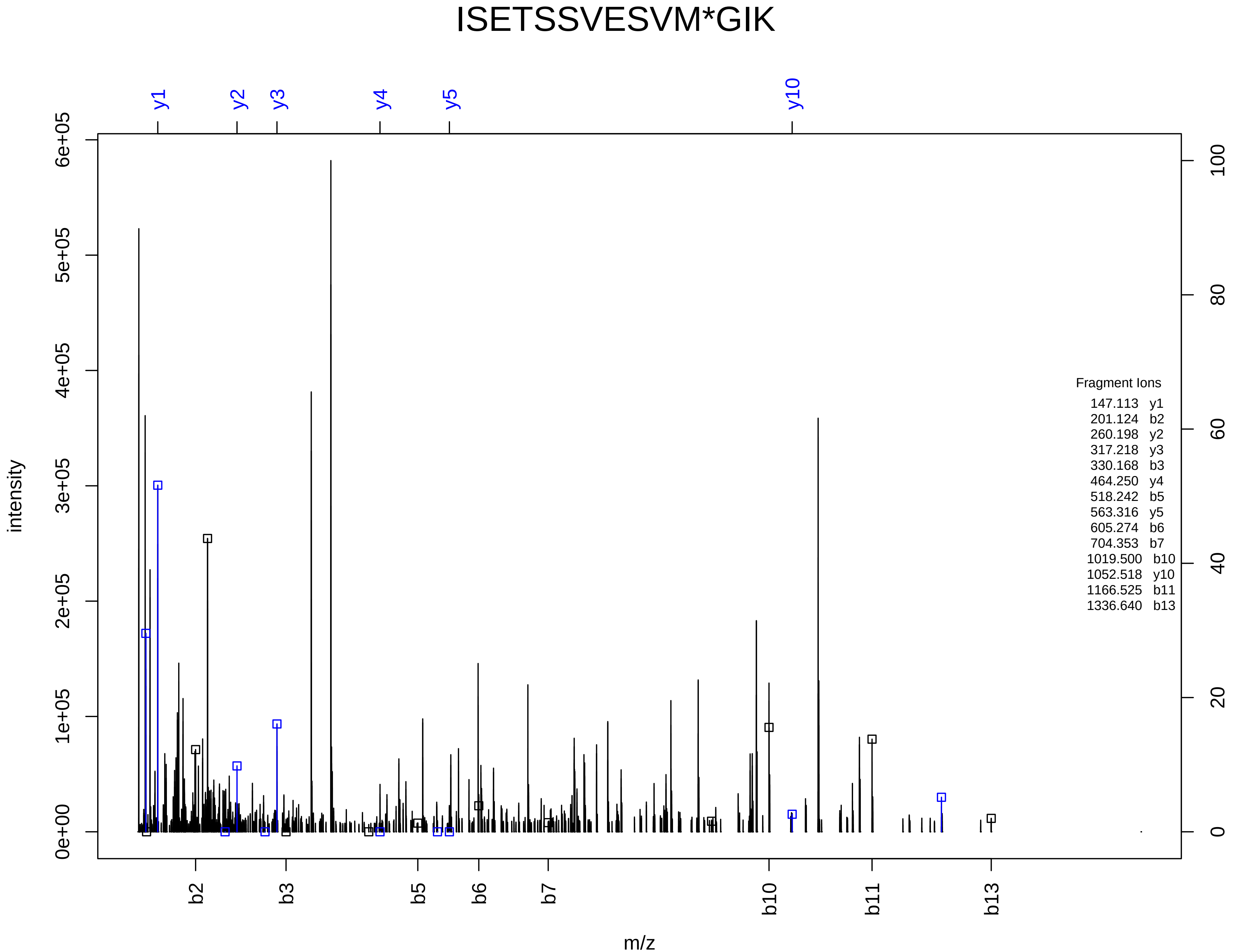
ISETSSVESVM*GIK
y1
y2
y3
y4
y5
y10
intensity
0e+00
1e+05
2e+05
3e+05
4e+05
5e+05
6e+05
0
20
40
60
80
100
b2
b3
b5
b6
b7
b10
b11
b13
m/z
Fragment Ions
147.113 y1
201.124 b2
260.198 y2
317.218 y3
330.168 b3
464.250 y4
518.242 b5
563.316 y5
605.274 b6
704.353 b7
1019.500 b10
1052.518 y10
1166.525 b11
1336.640 b13
ISETSSVESVMGIK / File: christine_ip_04.raw; SpectrumID: ; scans: 12504

SEVVESTTESQDK

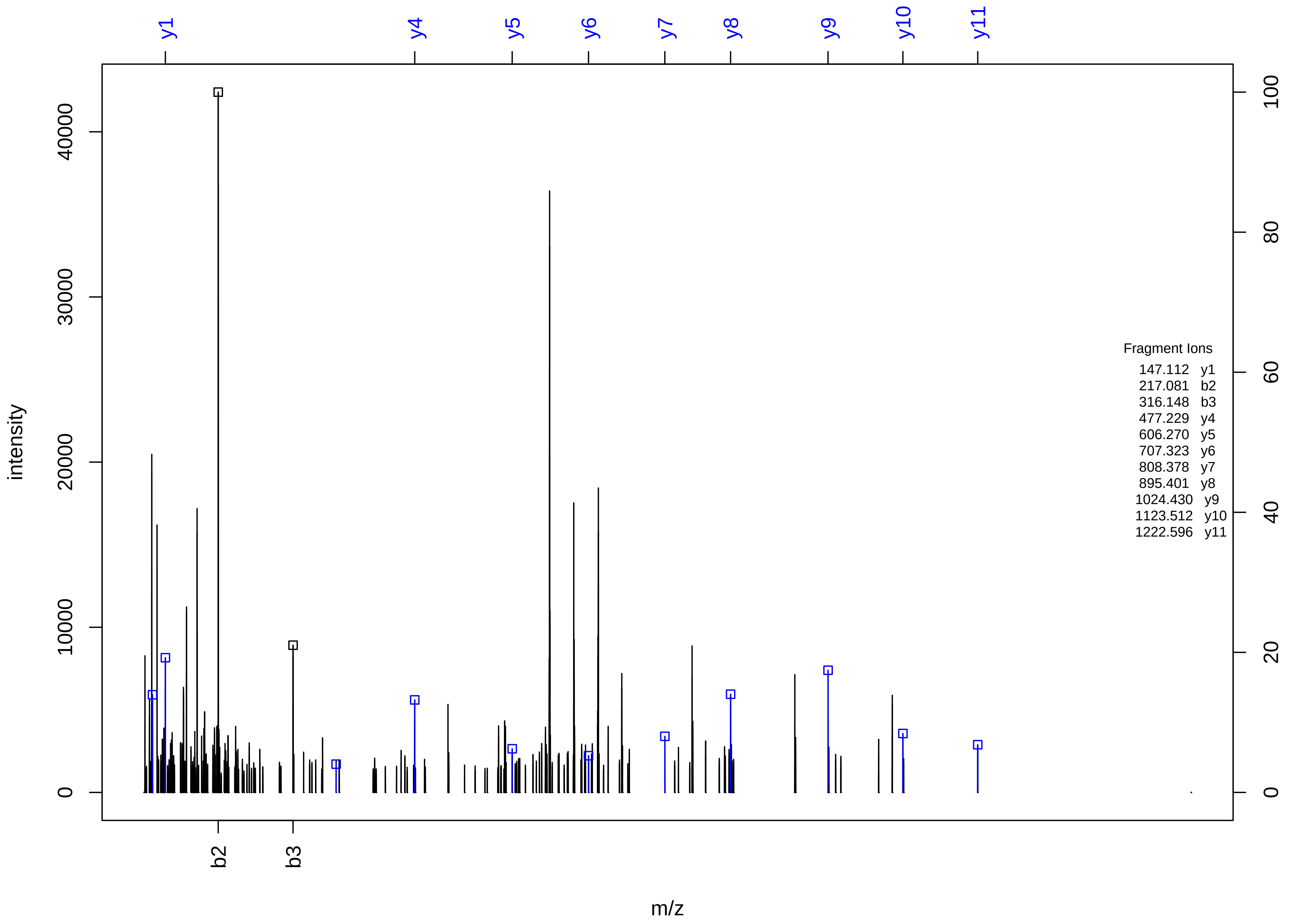

SEVVESTTESQDK / File: christine_ip_04.raw; SpectrumID: ; scans: 1564

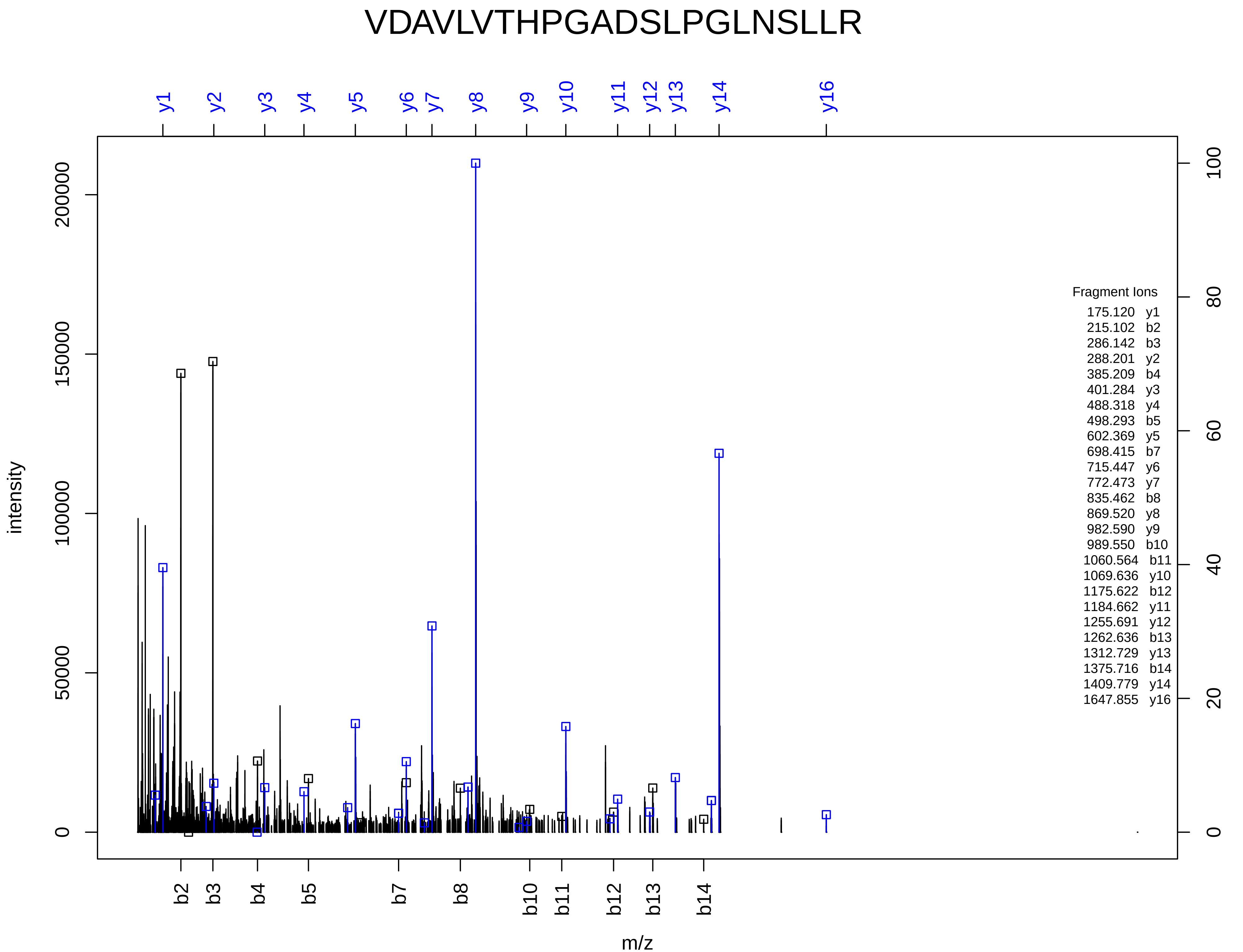

VDAVLVTHPGADSLPGLNSLLR / File: christine_ip_04.raw; SpectrumID: ; scans: 16779

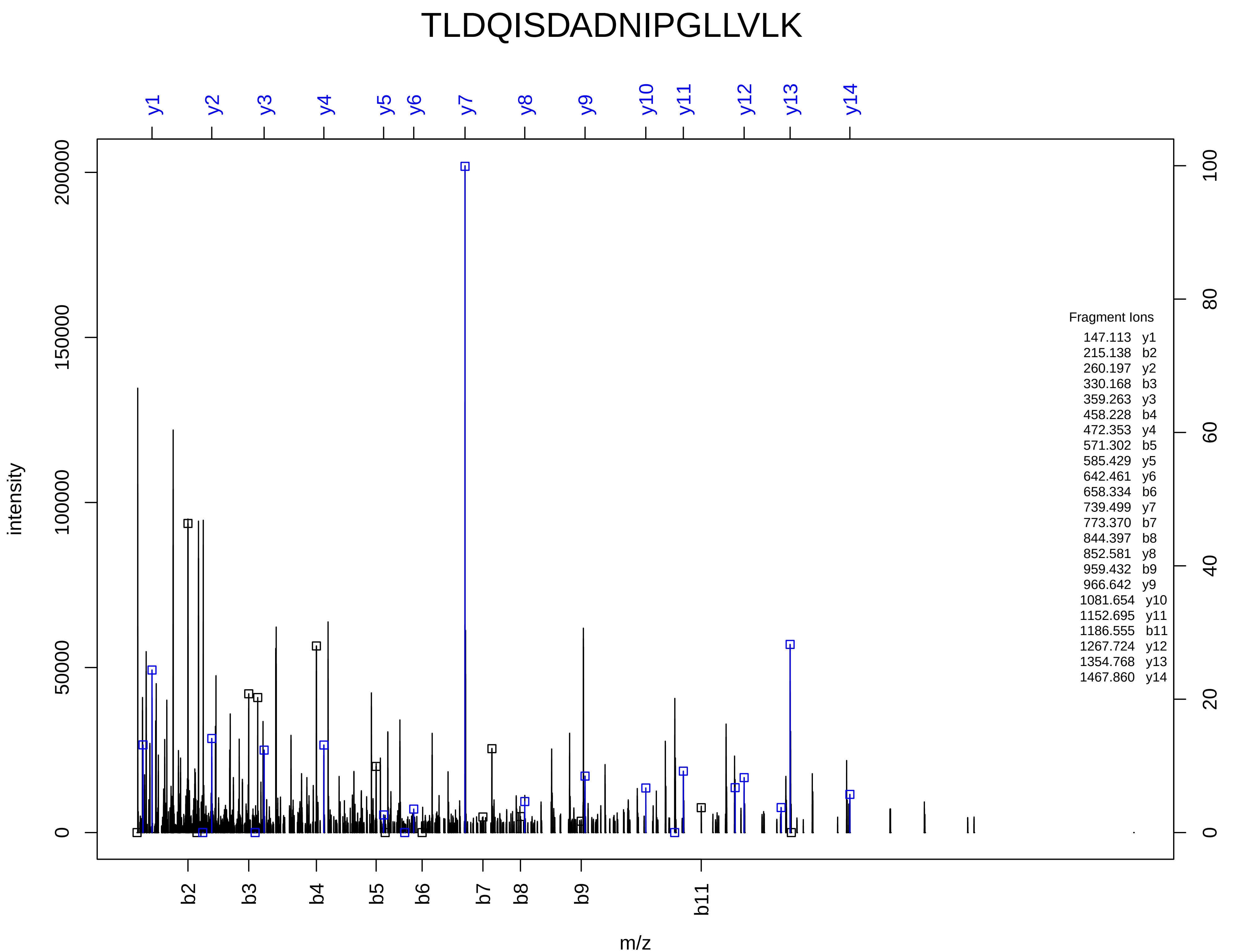

TLDQISDADNIPGLLVLK / File: christine_ip_04.raw; SpectrumID: ; scans: 18868

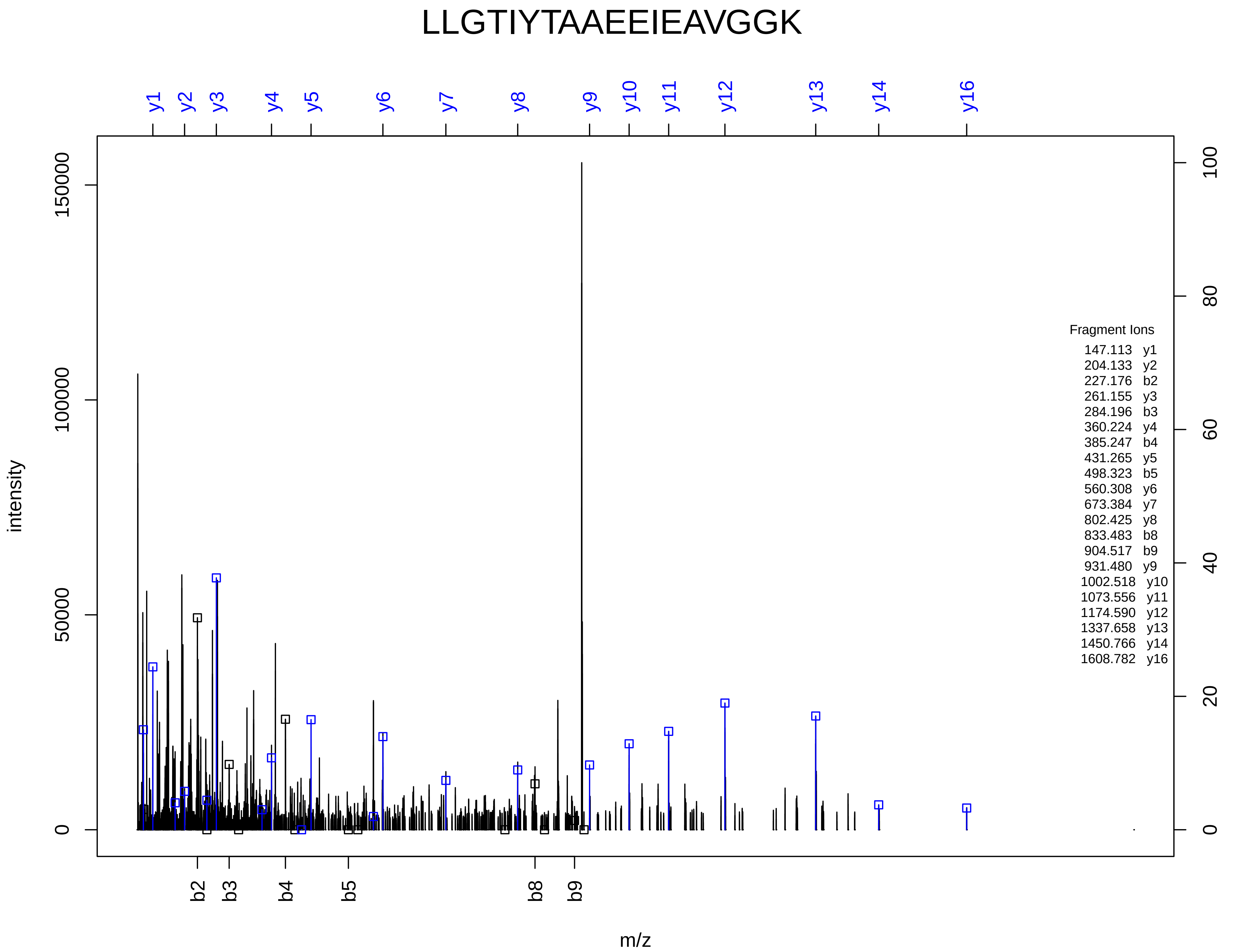

LLGTIYTAAEEIEAVGGK / File: christine_ip_04.raw; SpectrumID: ; scans: 18124

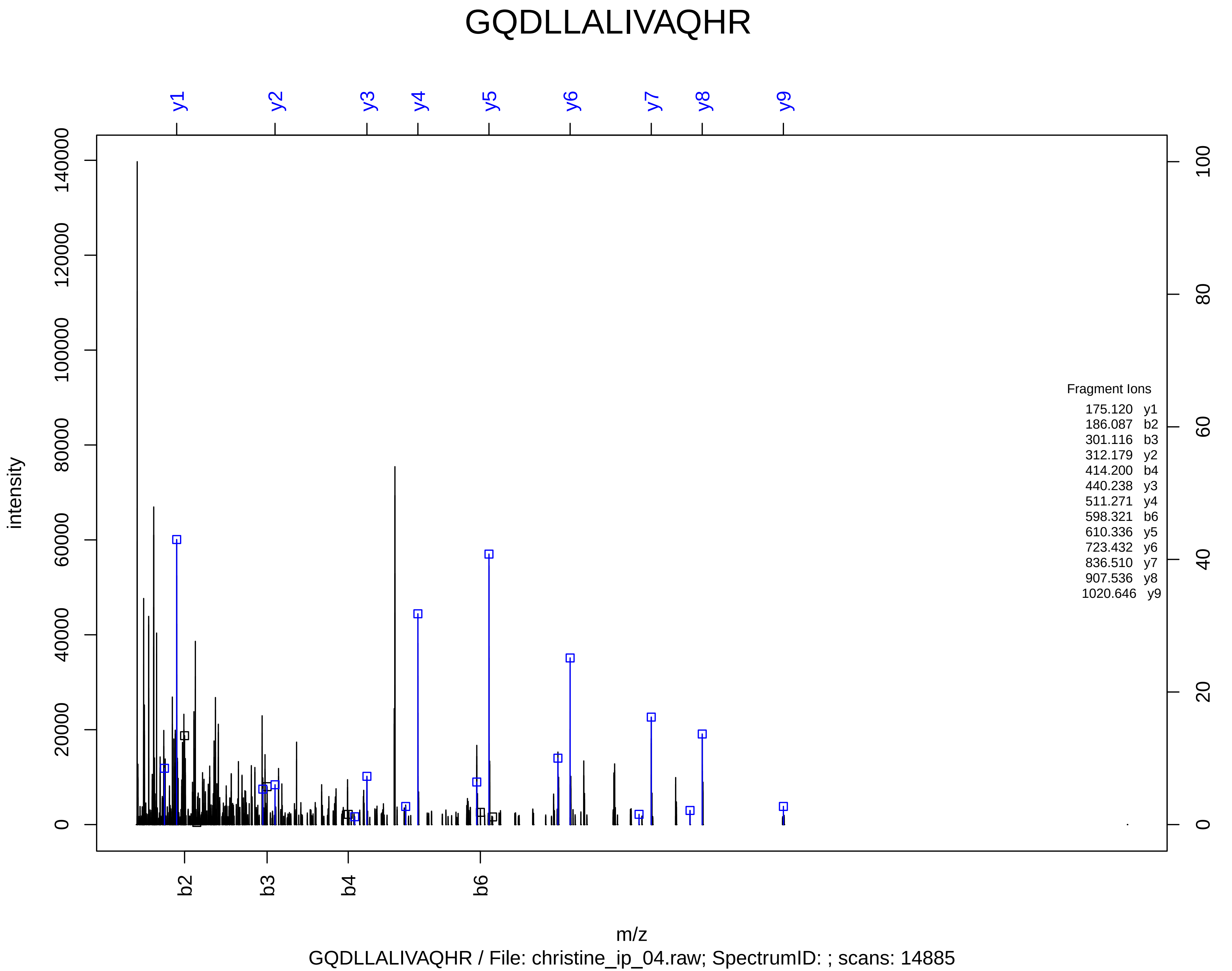
GQDLLALIVAQHR
y1
y2
y3
y4
y5
y6
y7
y8
y9
intensity
0
20000
40000
60000
80000
100000
120000
140000
0
20
40
60
80
100
b2
b3
b4
b6
m/z
Fragment Ions
175.120 y1
186.087 b2
301.116 b3
312.179 y2
414.200 b4
440.238 y3
511.271 y4
598.321 b6
610.336 y5
723.432 y6
836.510 y7
907.536 y8
1020.646 y9
GQDLLALIVAQHR / File: christine_ip_04.raw; SpectrumID: ; scans: 14885

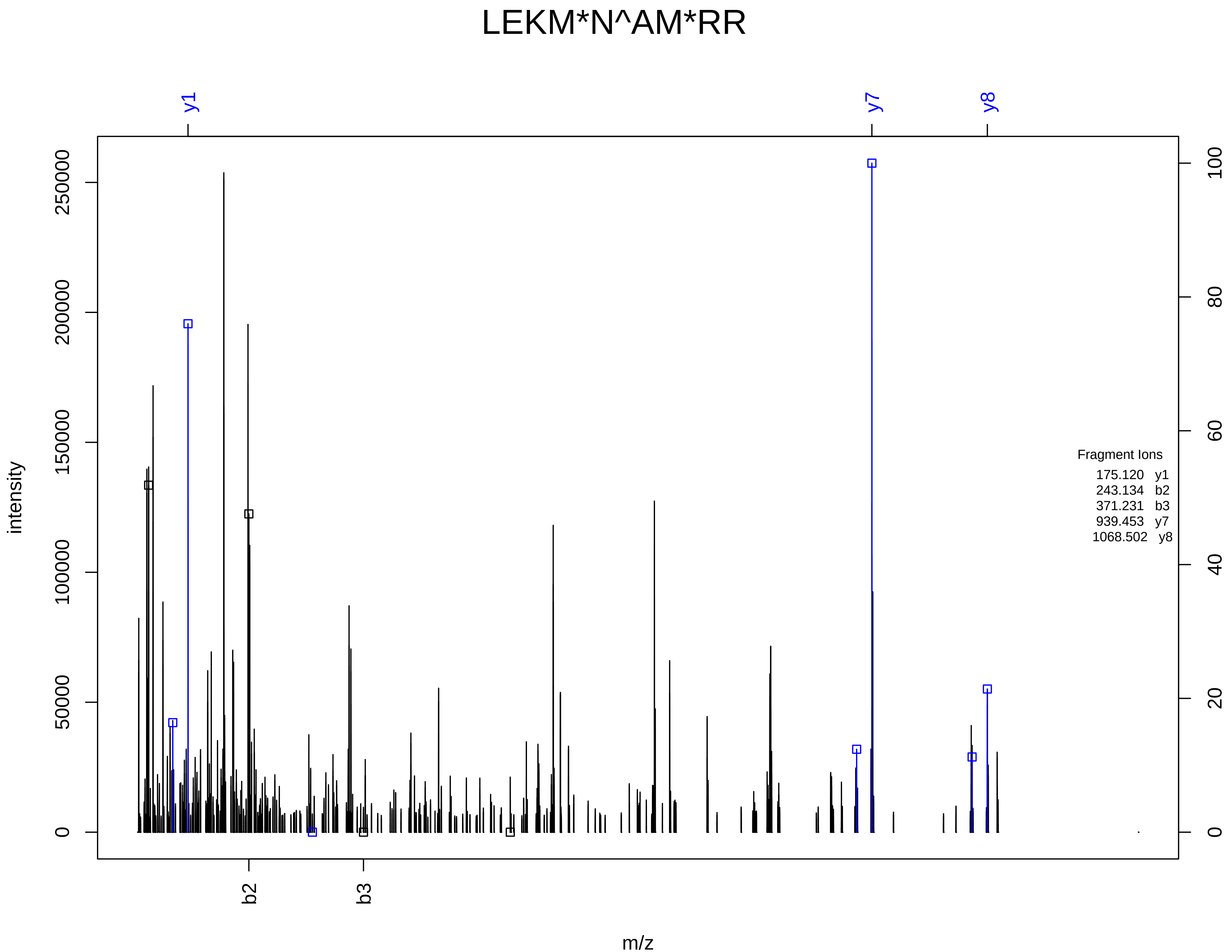

LEKMNAMRR / File: christine_ip_04.raw; SpectrumID: ; scans: 4395

HLAGVPLIGWVLR

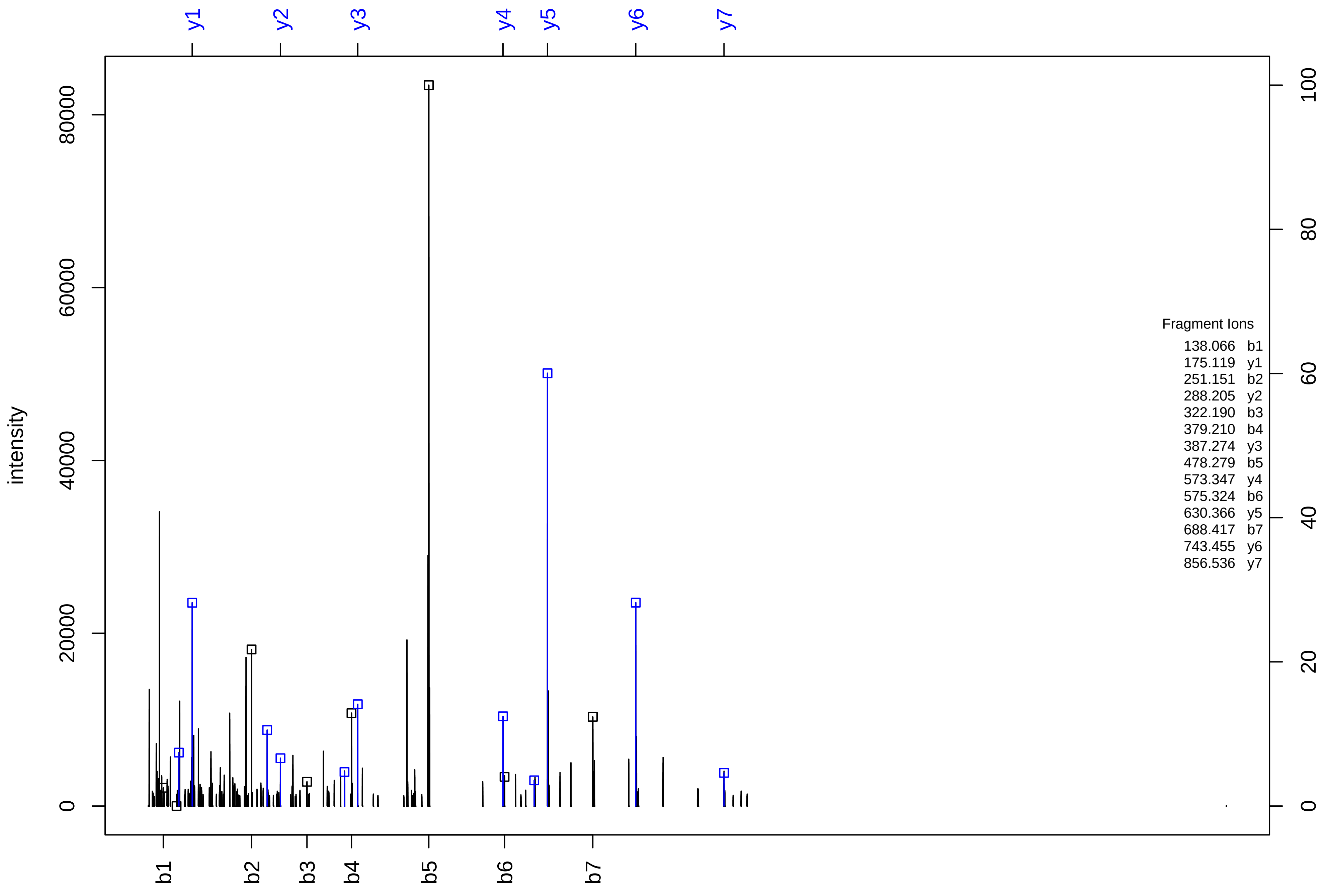

HLAGVPLIGWVLR / File: christine_ip_04.raw; SpectrumID: ; scans: 19187

DIPAAILHAFLR

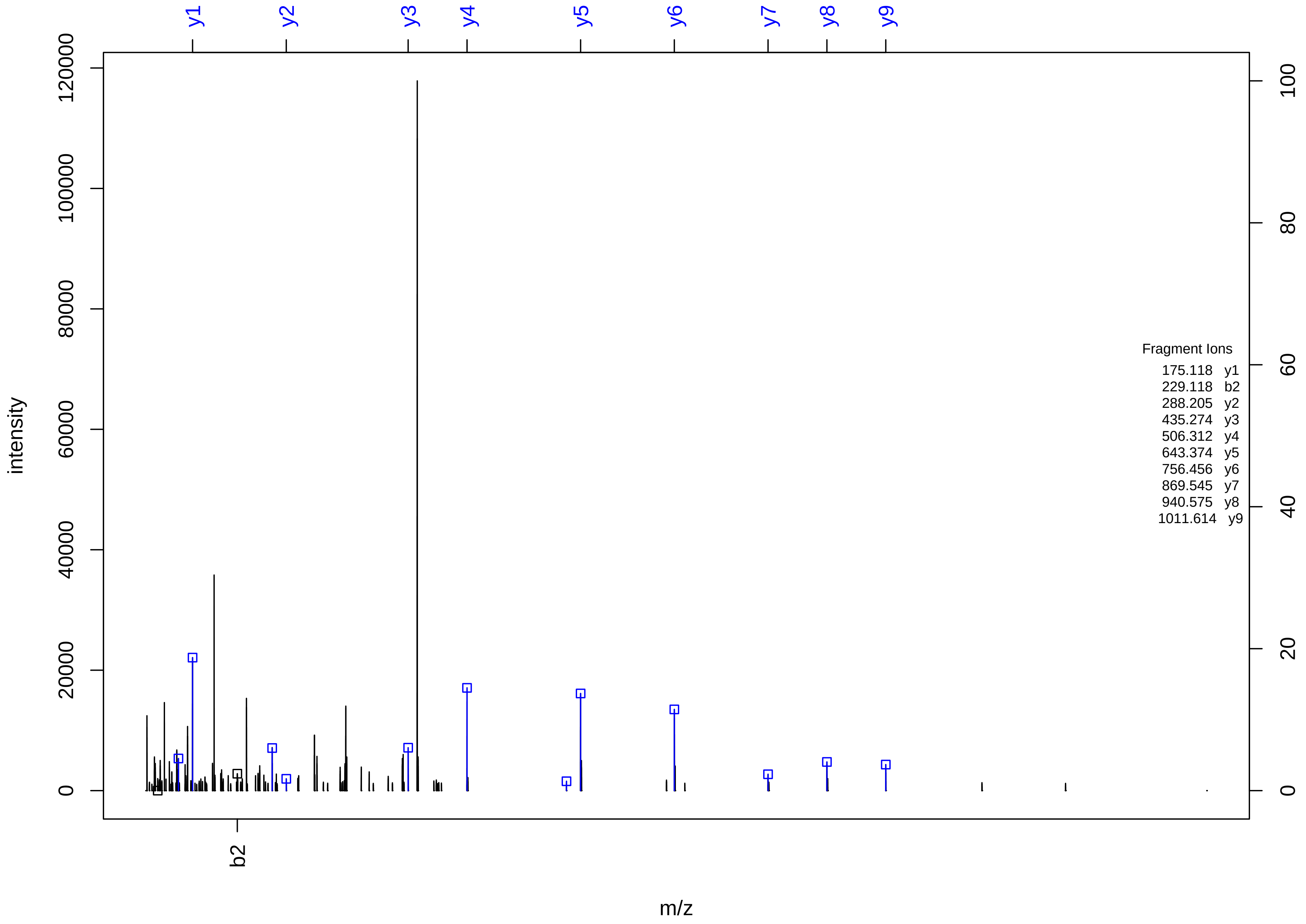

DIPAAILHAFLR / File: christine_ip_04.raw; SpectrumID: ; scans: 19732

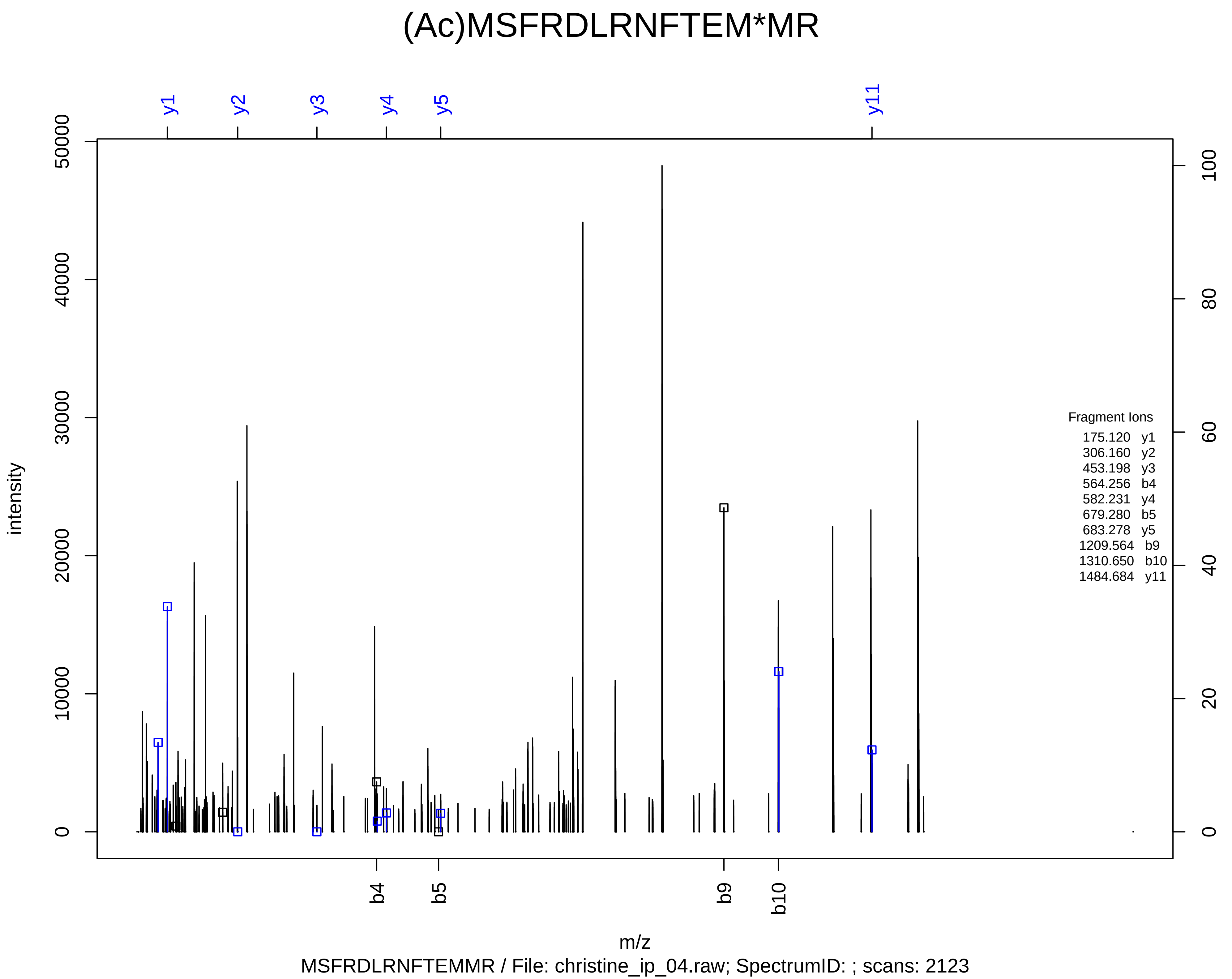
(Ac)MSFRDLRNFTEM*MR
y1
y2
y3
y4
y5
y11
intensity
0
10000
20000
30000
40000
50000
0
20
40
60
80
100
b4
b5
b9
b10
m/z
Fragment Ions
175.120 y1
306.160 y2
453.198 y3
564.256 b4
582.231 y4
679.280 b5
683.278 y5
1209.564 b9
1310.650 b10
1484.684 y11
MSFRDLRNFTEMMR / File: christine_ip_04.raw; SpectrumID: ; scans: 2123

LLALNPDAVELFK

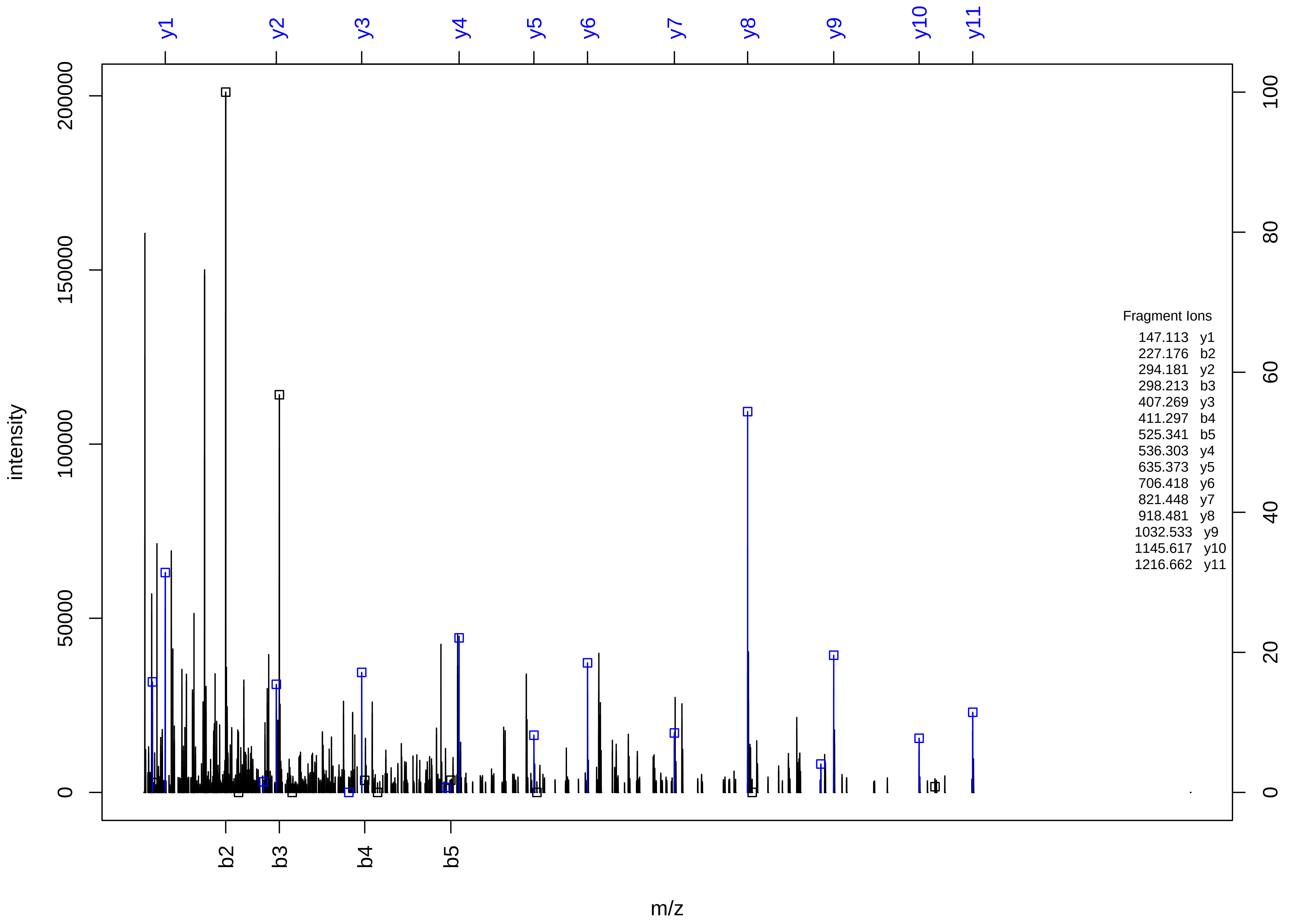

LLALNPDAVELFK / File: christine_ip_04.raw; SpectrumID: ; scans: 18532

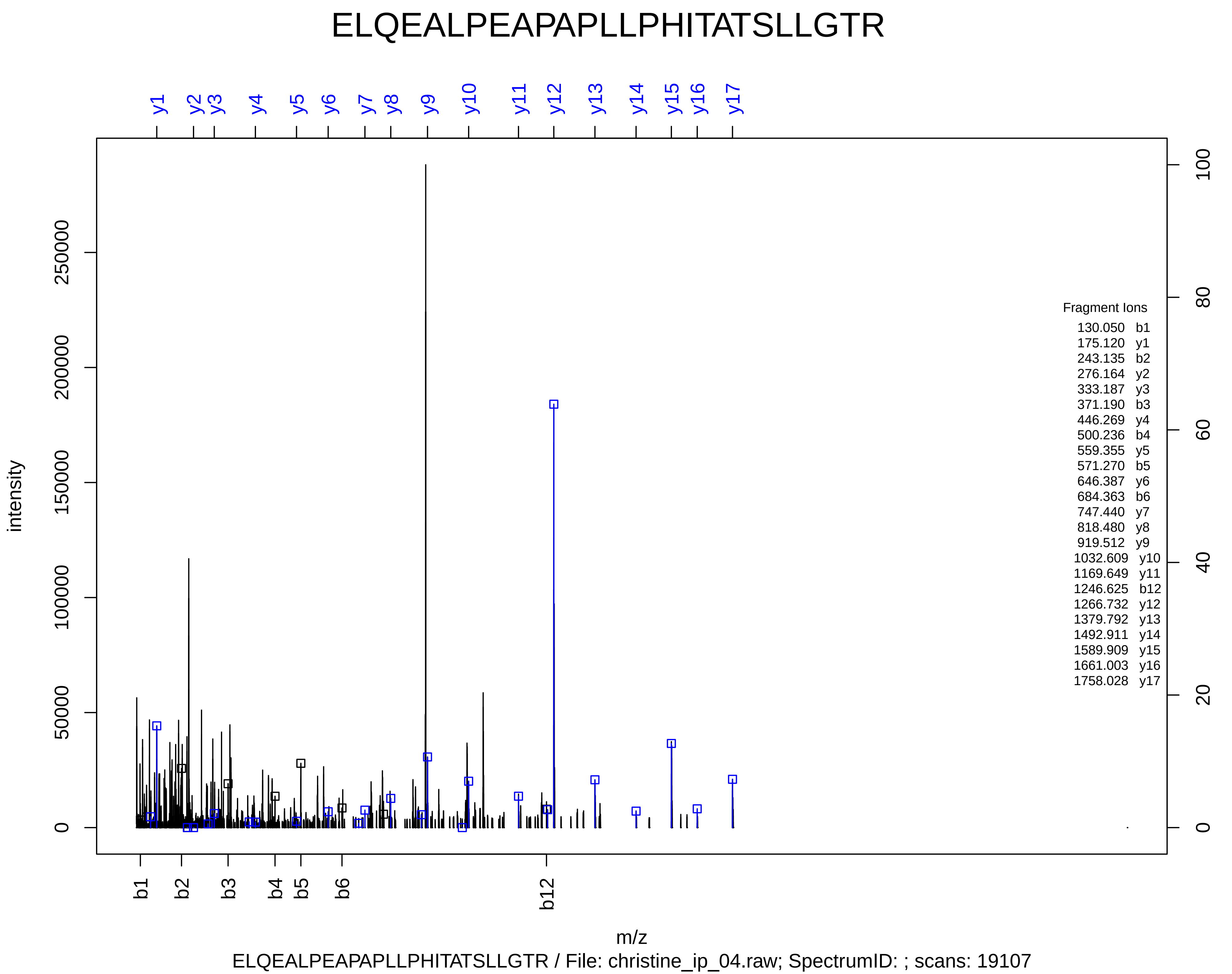
ELQEALPEAPAPLLPHITATSLLGTR
y1
y2
y3
y4
y5
y6
y7
y8
y9
y10
y11
y12
y13
y14
y15
y16
y17
intensity
0
50000
100000
150000
200000
250000
0
20
40
60
80
100
b1
b2
b3
b4
b5
b6
b12
m/z
Fragment Ions
130.050 b1
175.120 y1
243.135 b2
276.164 y2
333.187 y3
371.190 b3
446.269 y4
500.236 b4
559.355 y5
571.270 b5
646.387 y6
684.363 b6
747.440 y7
818.480 y8
919.512 y9
1032.609 y10
1169.649 y11
1246.625 b12
1266.732 y12
1379.792 y13
1492.911 y14
1589.909 y15
1661.003 y16
1758.028 y17
ELQEALPEAPAPLLPHITATSLLGTR / File: christine_ip_04.raw; SpectrumID: ; scans: 19107

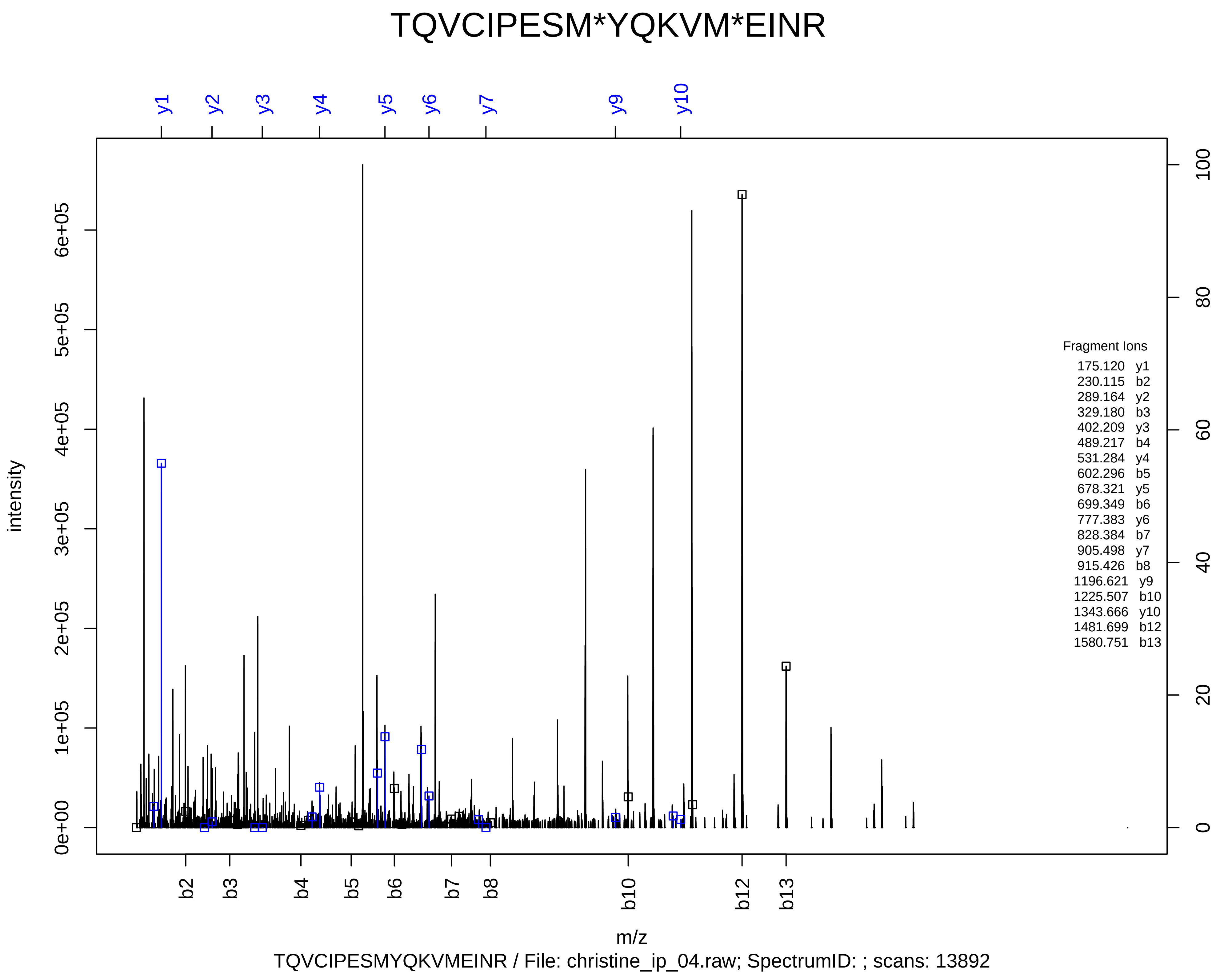

TQVCIPESM*YQKVM*EINR
y1
y2
y3
y4
y5
y6
y7
y9
y10
intensity
0e+00
1e+05
2e+05
3e+05
4e+05
5e+05
6e+05
0
20
40
60
80
100
b2
b3
b4
b5
b6
b7
b8
b10
b12
b13
Fragment Ions
175.120 y1
230.115 b2
289.164 y2
329.180 b3
402.209 y3
489.217 b4
531.284 y4
602.296 b5
678.321 y5
699.349 b6
777.383 y6
828.384 b7
905.498 y7
915.426 b8
1196.621 y9
1225.507 b10
1343.666 y10
1481.699 b12
1580.751 b13
m/z
TQVCIPESMYQKVMEINR / File: christine_ip_04.raw; SpectrumID: ; scans: 13892

HQTLVLTLPTVAFLEDK

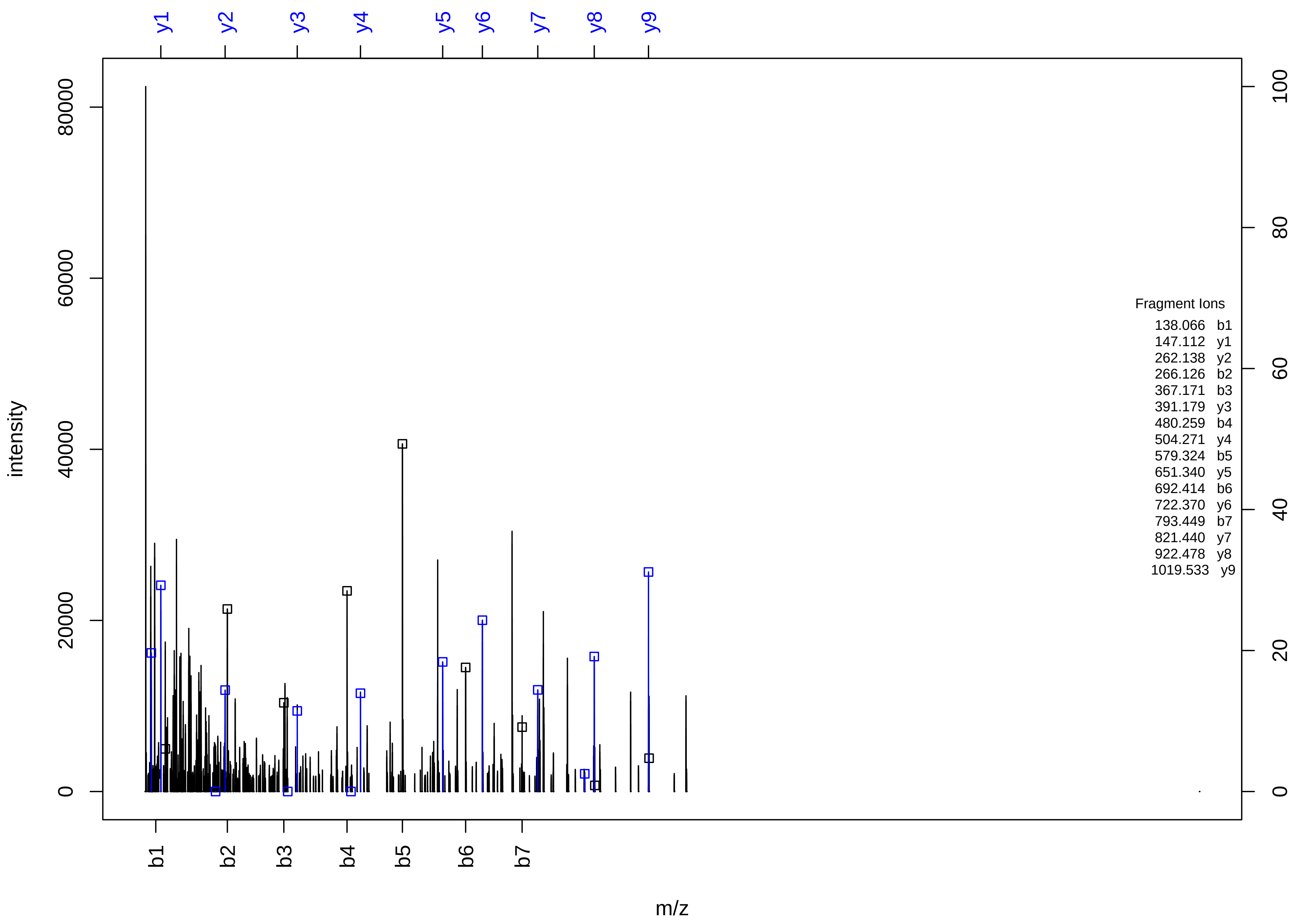

HQTLVLTLPTVAFLEDK / File: christine_ip_04.raw; SpectrumID: ; scans: 19082

GEVFSVLEFAPSNHSFK

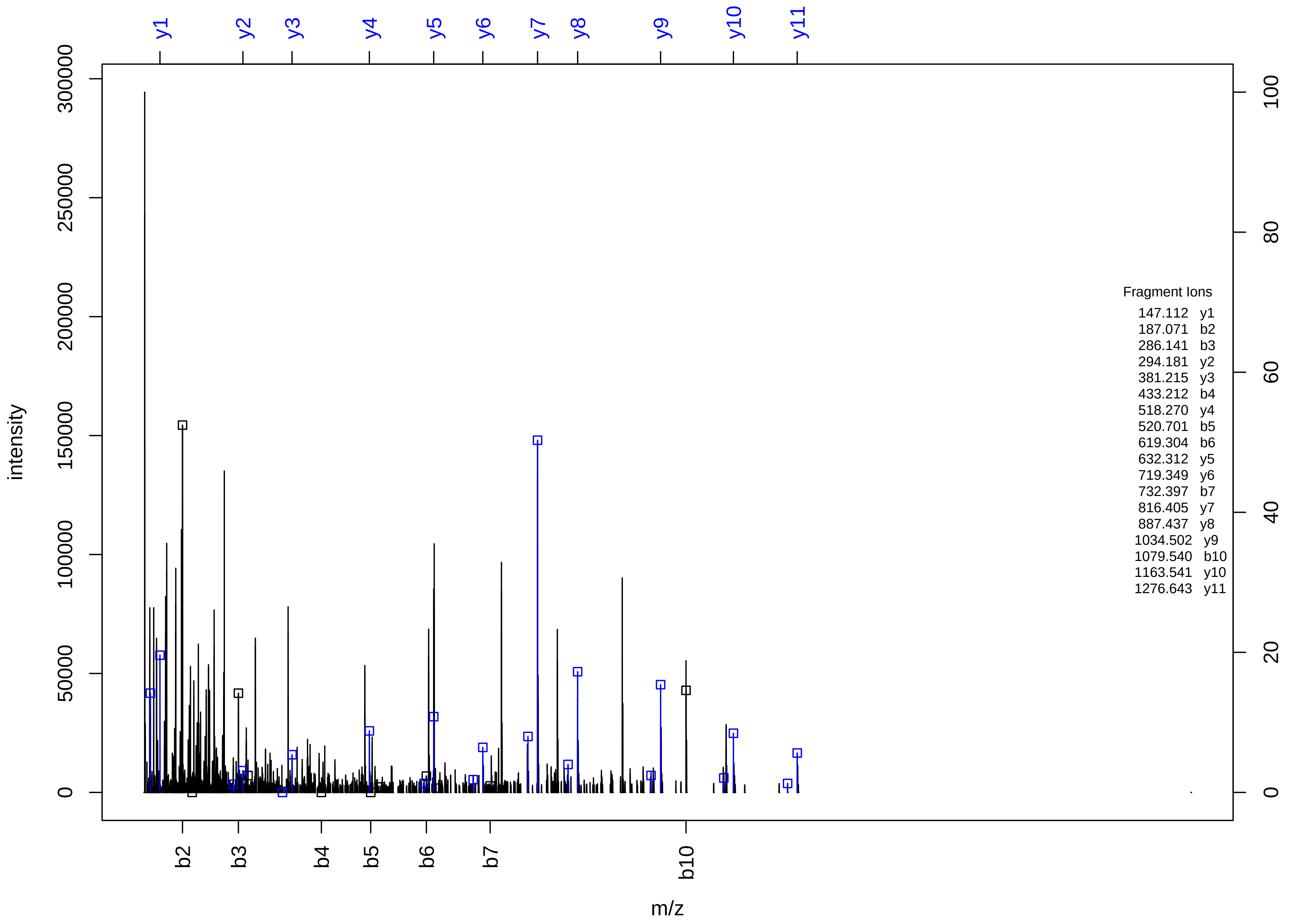

GEVFSVLEFAPSNHSFK / File: christine_ip_04.raw; SpectrumID: ; scans: 15013

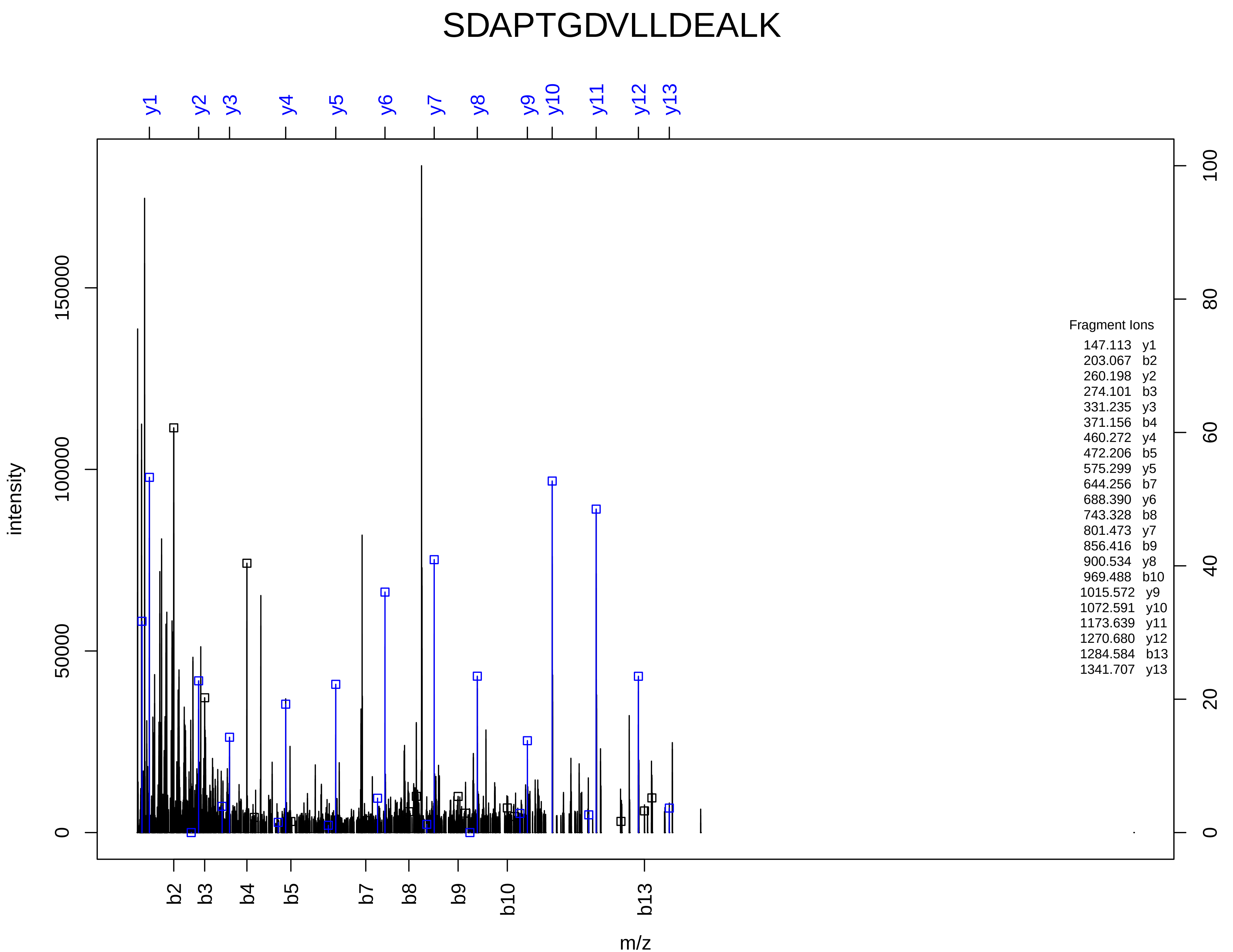

SDAPTGDVLLDEALK / File: christine_ip_04.raw; SpectrumID: ; scans: 13882

(Ac)M*VNLAAM*VWR

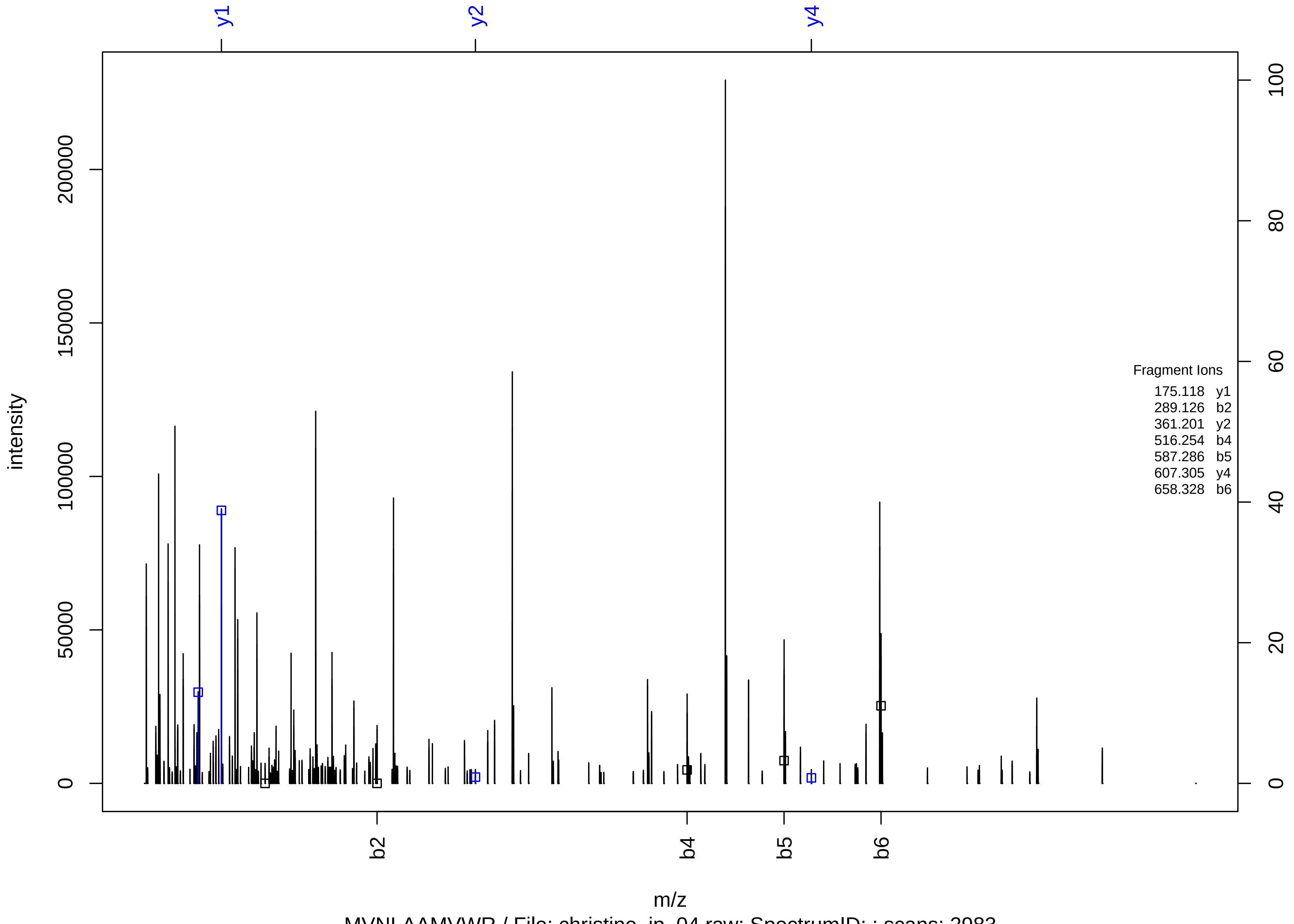

MVNLAAMVWR / File: christine_ip_04.raw; SpectrumID: ; scans: 2983

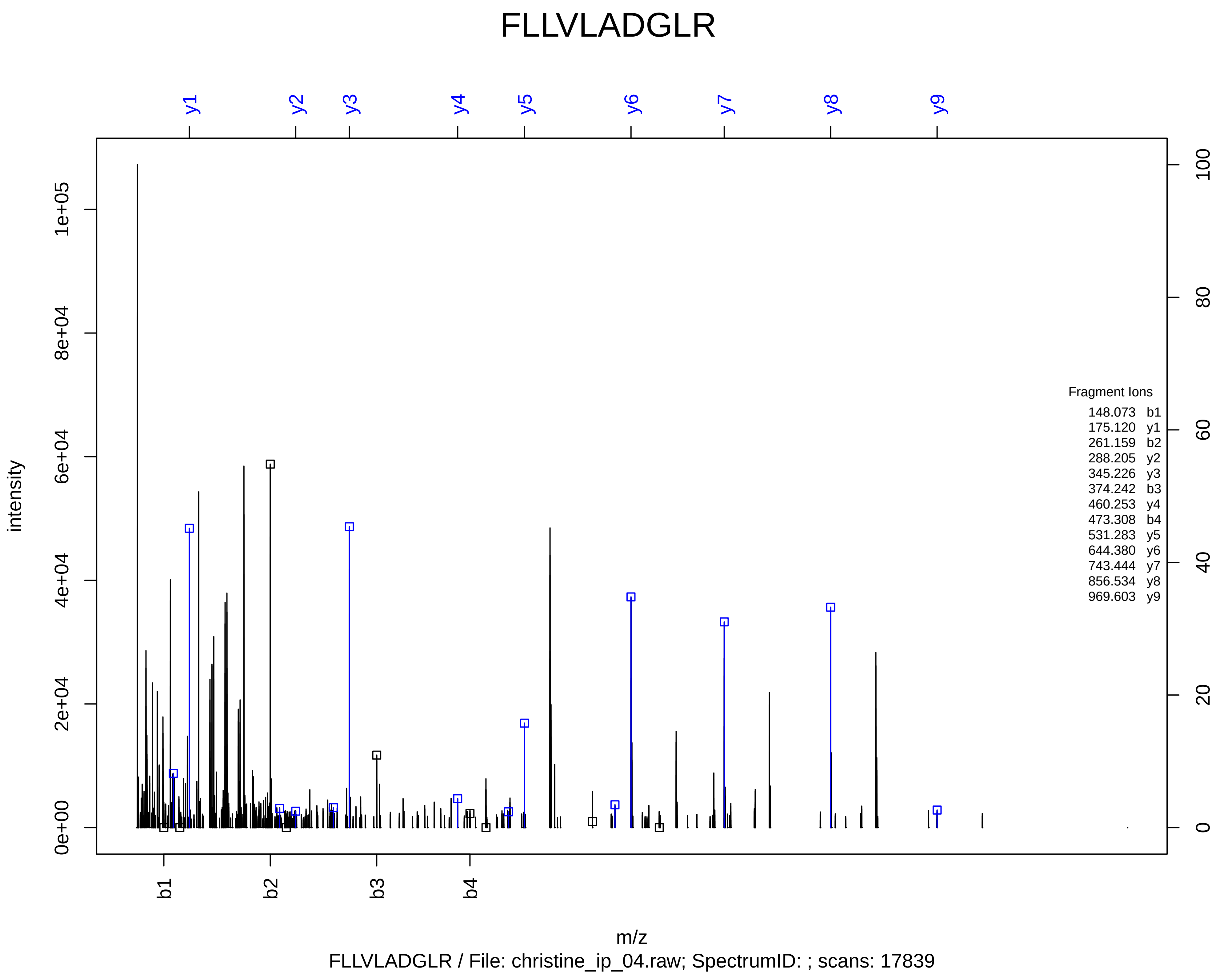

FLLVLADGLR
y1
y2
y3
y4
y5
y6
y7
y8
y9
intensity
0e+00
2e+04
4e+04
6e+04
8e+04
1e+05
0
20
40
60
80
100
b1
b2
b3
b4
m/z
Fragment Ions
148.073 b1
175.120 y1
261.159 b2
288.205 y2
345.226 y3
374.242 b3
460.253 y4
473.308 b4
531.283 y5
644.380 y6
743.444 y7
856.534 y8
969.603 y9
FLLVLADGLR / File: christine_ip_04.raw; SpectrumID: ; scans: 17839

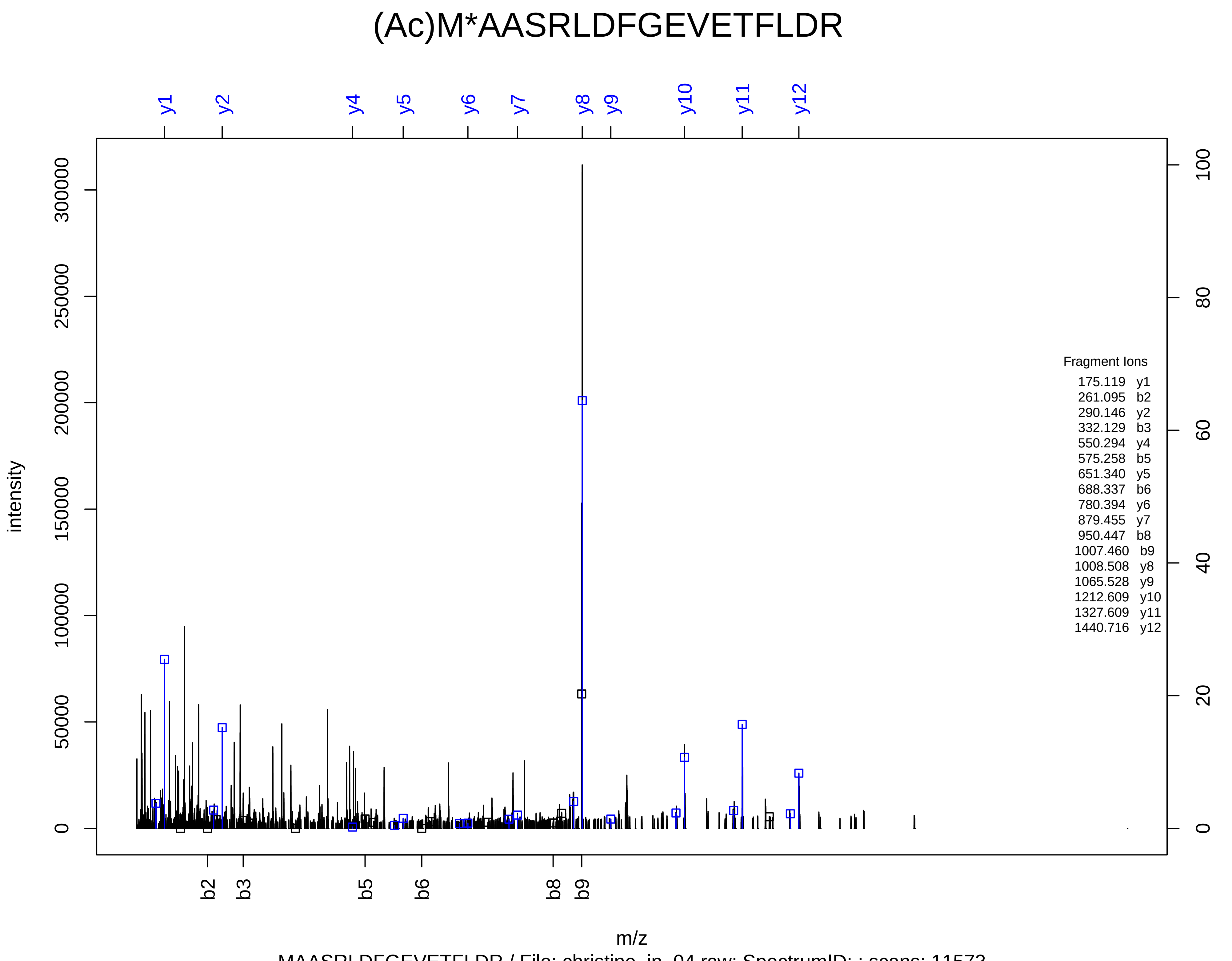
(Ac)M*AASRLDFGEVETFLDR
y1
y2
y4
y5
y6
y7
y8
y9
y10
y11
y12
intensity
0
50000
100000
150000
200000
250000
300000
0
20
40
60
80
100
b2
b3
b5
b6
b8
b9
m/z
Fragment Ions
175.119 y1
261.095 b2
290.146 y2
332.129 b3
550.294 y4
575.258 b5
651.340 y5
688.337 b6
780.394 y6
879.455 y7
950.447 b8
1007.460 b9
1008.508 y8
1065.528 y9
1212.609 y10
1327.609 y11
1440.716 y12
MAASRLDFGEVETFLDR / File: christine_ip_04.raw; SpectrumID: ; scans: 11573

EECPVFTPPGGETLDQVK

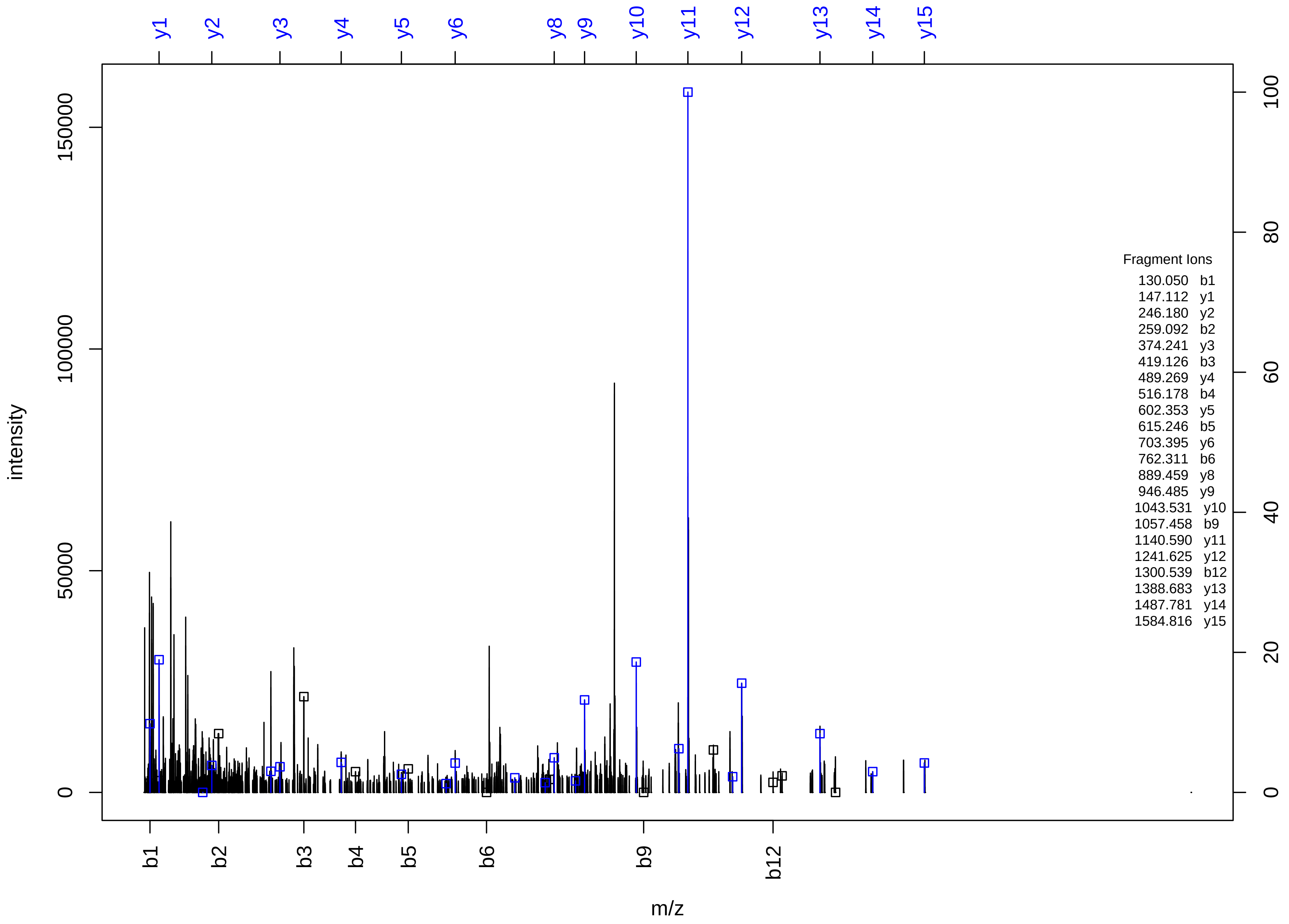

EECPVFTPPGGETLDQVK / File: christine_ip_04.raw; SpectrumID: ; scans: 11748

RNLFAEIIEEHLANR

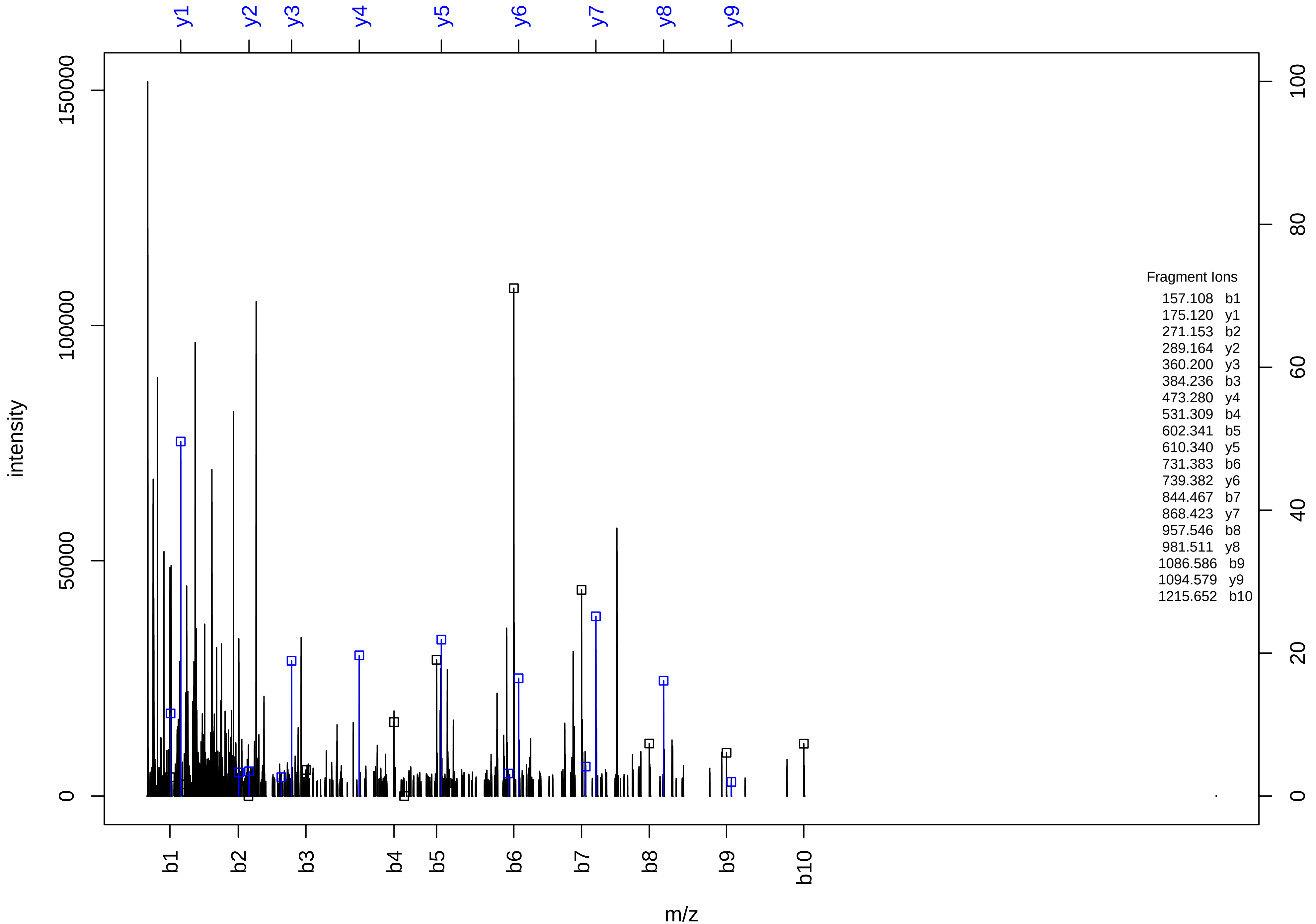

RNLFAEIIEEHLANR / File: christine_ip_04.raw; SpectrumID: ; scans: 15144

IALLPLLQAETDRR

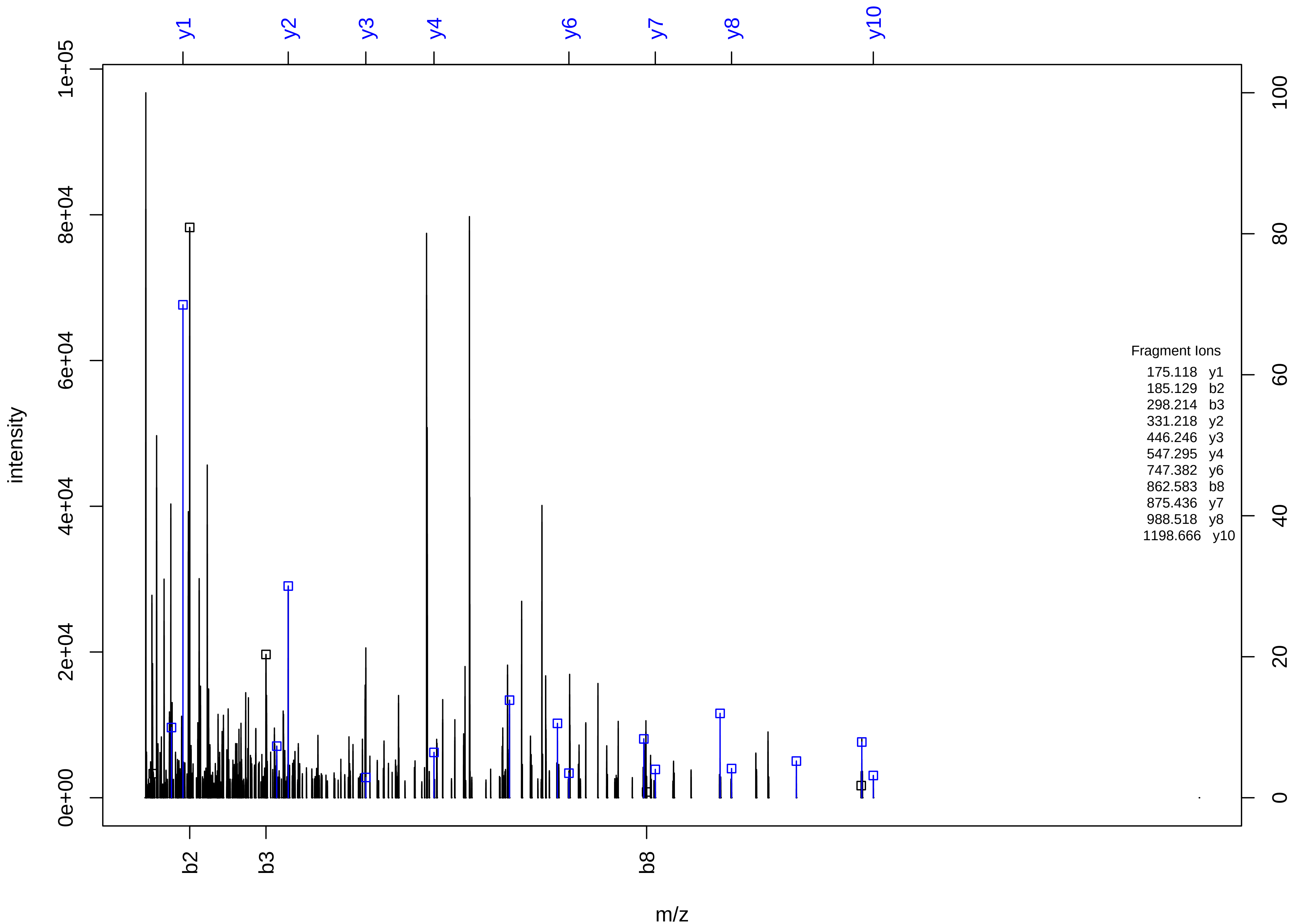

IALLPLLQAETDRR / File: christine_ip_04.raw; SpectrumID: ; scans: 16045

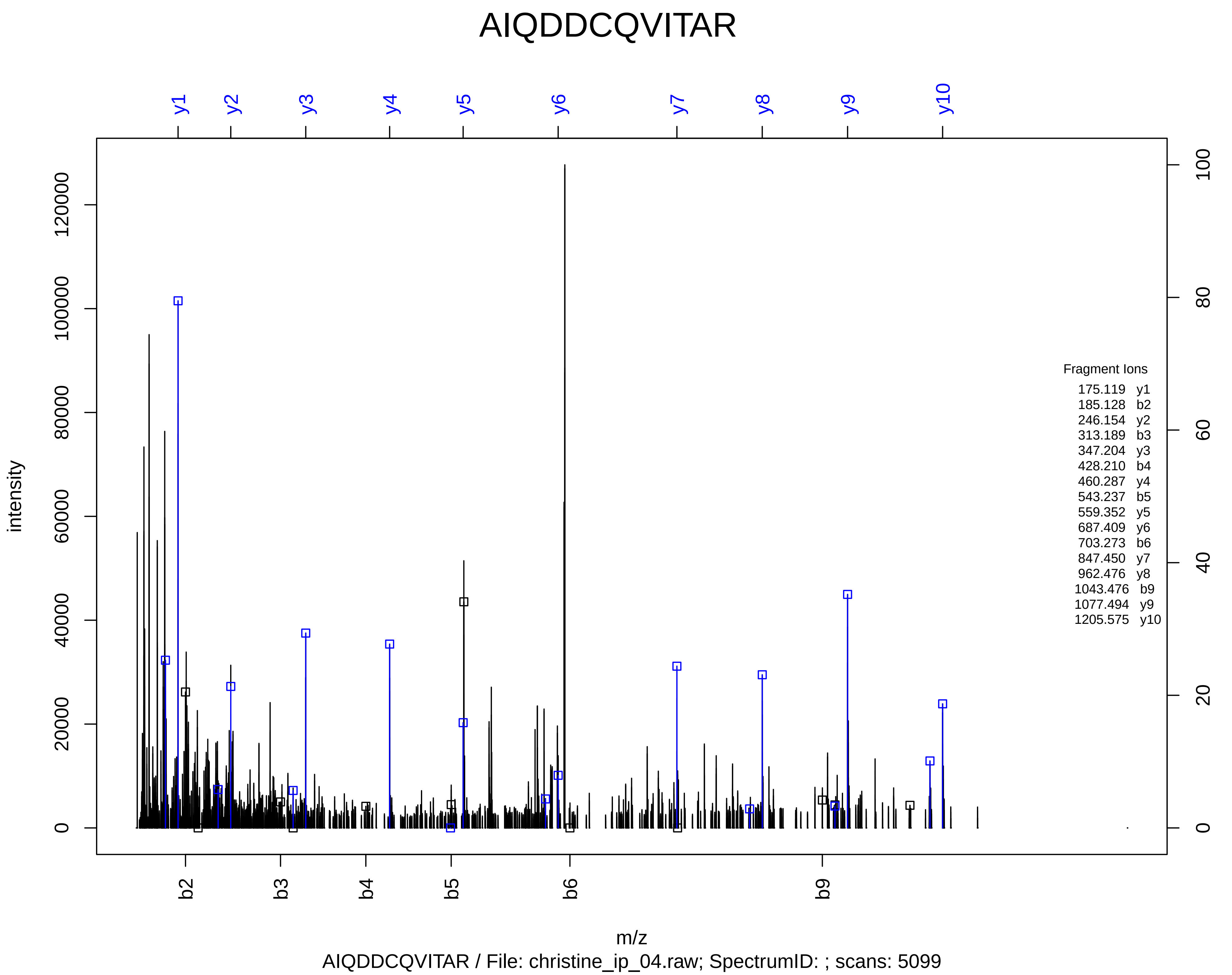
AIQDDCQVITAR
y1
y2
y3
y4
y5
y6
y7
y8
y9
y10
intensity
0
20000
40000
60000
80000
100000
120000
0
20
40
60
80
100
b2
b3
b4
b5
b6
b9
m/z
Fragment Ions
175.119 y1
185.128 b2
246.154 y2
313.189 b3
347.204 y3
428.210 b4
460.287 y4
543.237 b5
559.352 y5
687.409 y6
703.273 b6
847.450 y7
962.476 y8
1043.476 b9
1077.494 y9
1205.575 y10
AIQDDCQVITAR / File: christine_ip_04.raw; SpectrumID: ; scans: 5099

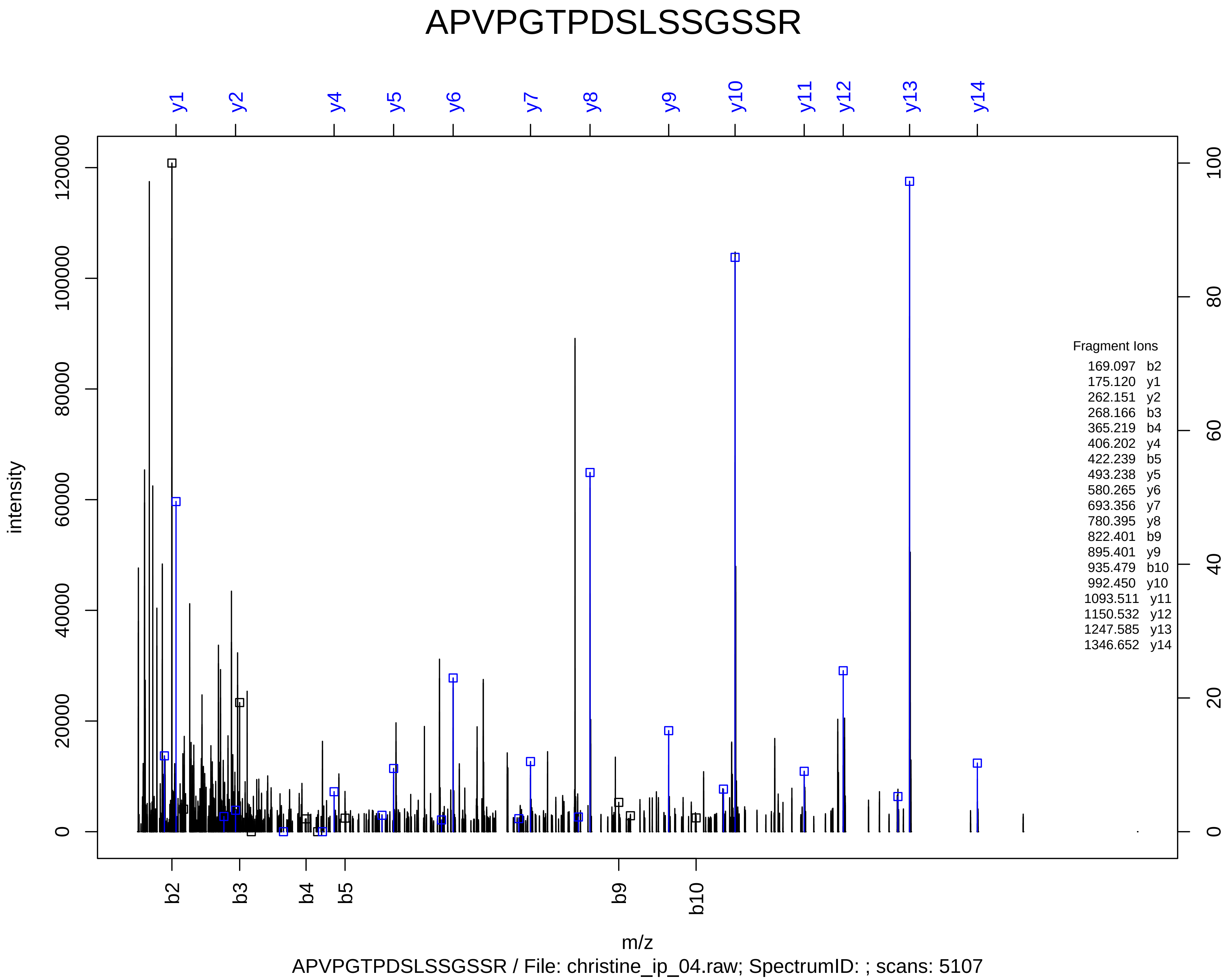
APVPGTPDSLSSGSSR
y1
y2
y4
y5
y6
y7
y8
y9
y10
y11
y12
y13
y14
intensity
0
20000
40000
60000
80000
100000
120000
0
20
40
60
80
100
b2
b3
b4
b5
b9
b10
m/z
Fragment Ions
169.097 b2
175.120 y1
262.151 y2
268.166 b3
365.219 b4
406.202 y4
422.239 b5
493.238 y5
580.265 y6
693.356 y7
780.395 y8
822.401 b9
895.401 y9
935.479 b10
992.450 y10
1093.511 y11
1150.532 y12
1247.585 y13
1346.652 y14
APVPGTPDSLSSGSSR / File: christine_ip_04.raw; SpectrumID: ; scans: 5107

EEIIYECDK

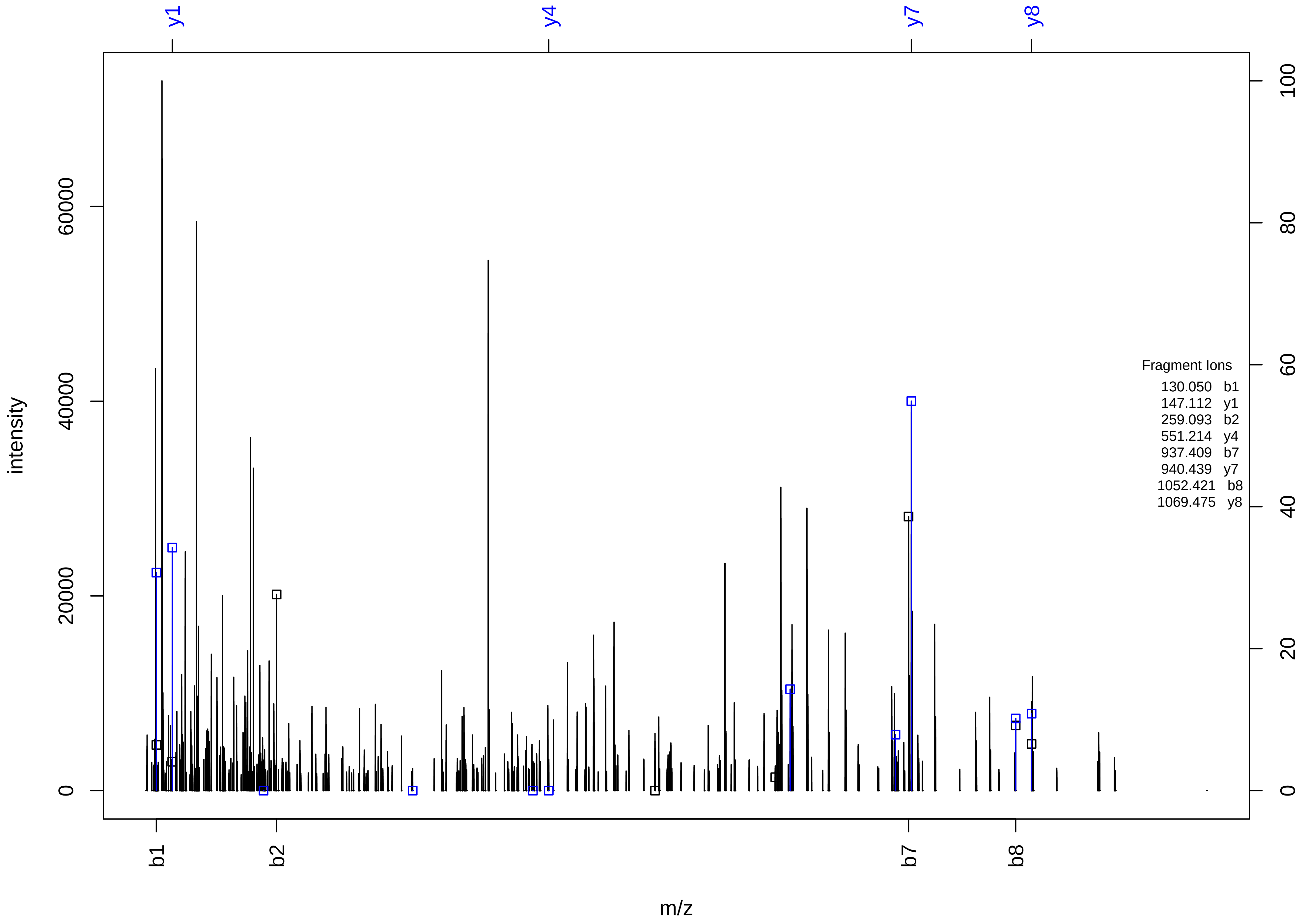

EEIIYECDK / File: christine_ip_03.raw; SpectrumID: ; scans: 2095

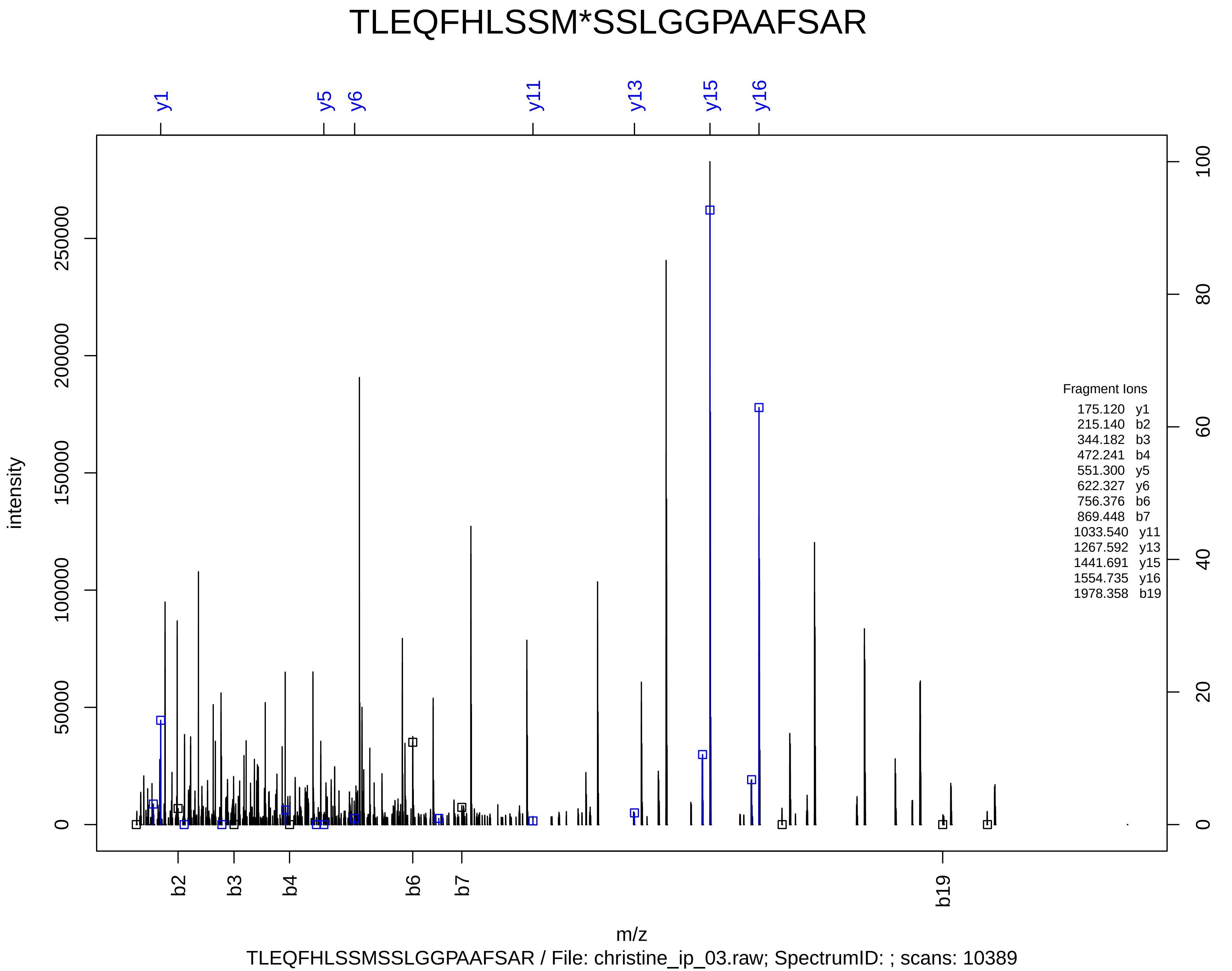
TLEQFHLSSM*SSLGGPAAFSAR
y1
y5
y6
y11
y13
y15
y16
b2
b3
b4
b6
b7
b19
intensity
m/z
0
50000
100000
150000
200000
250000
0
20
40
60
80
100
Fragment Ions
175.120 y1
215.140 b2
344.182 b3
472.241 b4
551.300 y5
622.327 y6
756.376 b6
869.448 b7
1033.540 y11
1267.592 y13
1441.691 y15
1554.735 y16
1978.358 b19
TLEQFHLSSMSSLGGPAAFSAR / File: christine_ip_03.raw; SpectrumID: ; scans: 10389

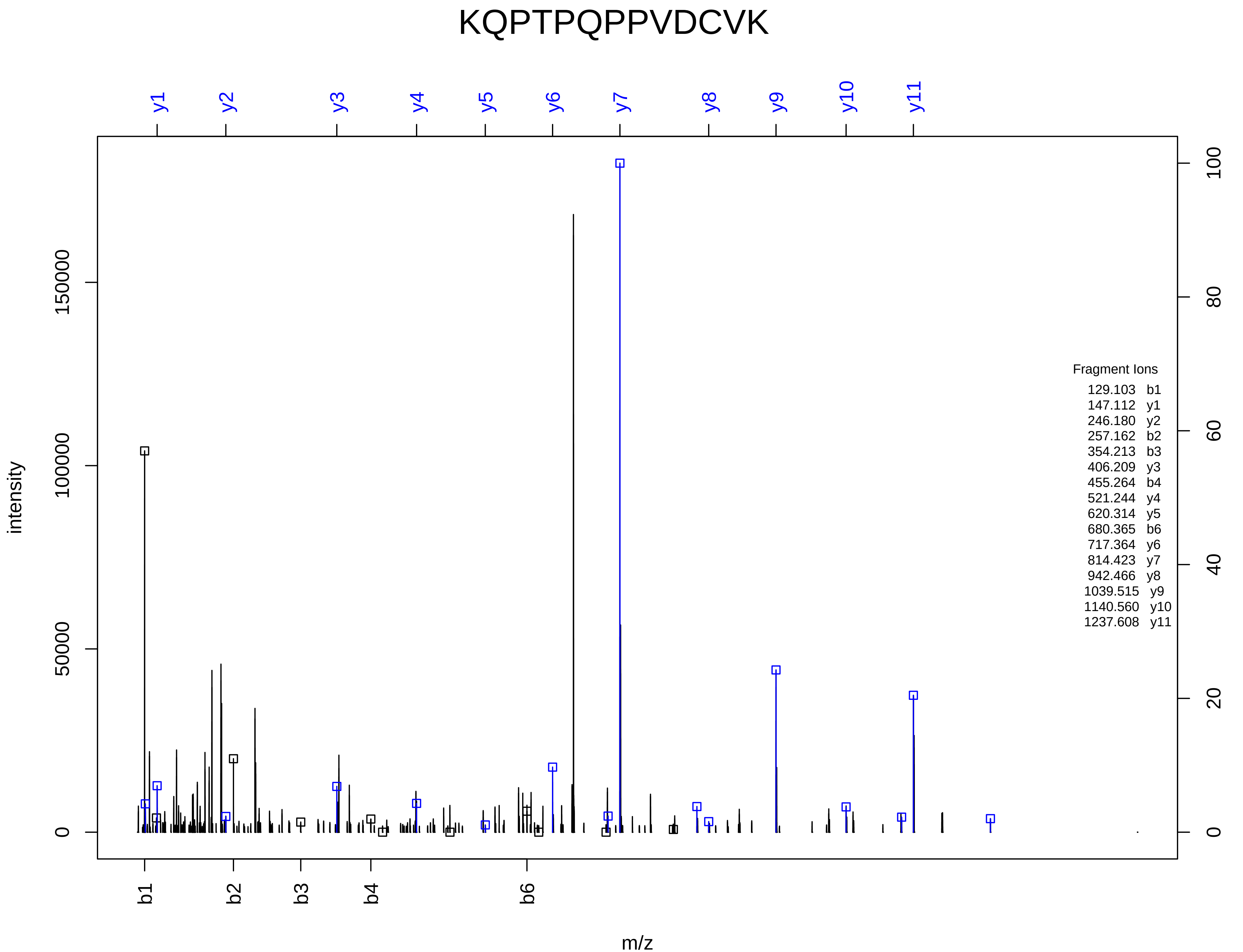

KQPTPQPPVDCVK / File: christine_ip_03.raw; SpectrumID: ; scans: 3086

ENFPNFLSACDK

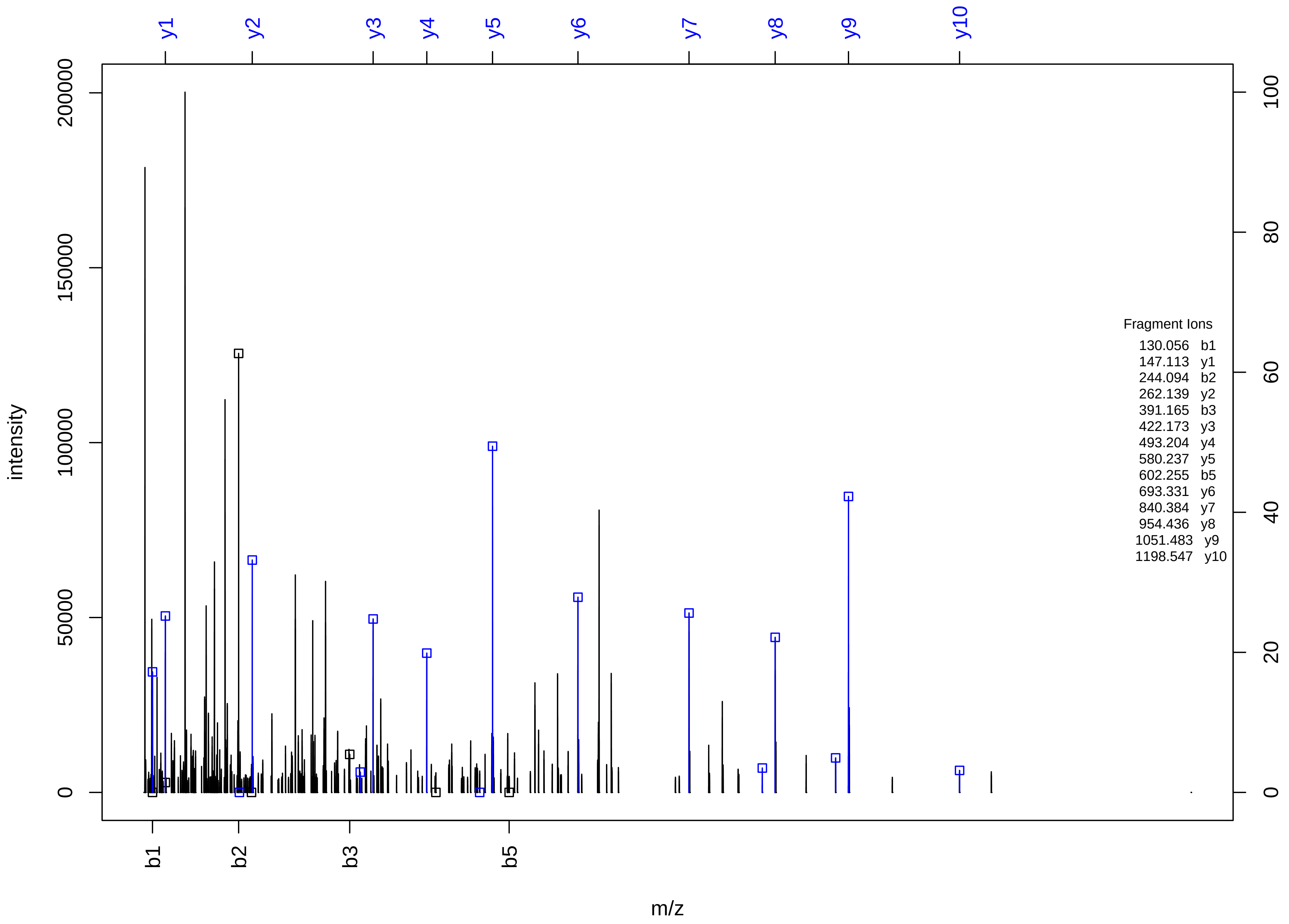

ENFPNFLSACDK / File: christine_ip_03.raw; SpectrumID: ; scans: 11163

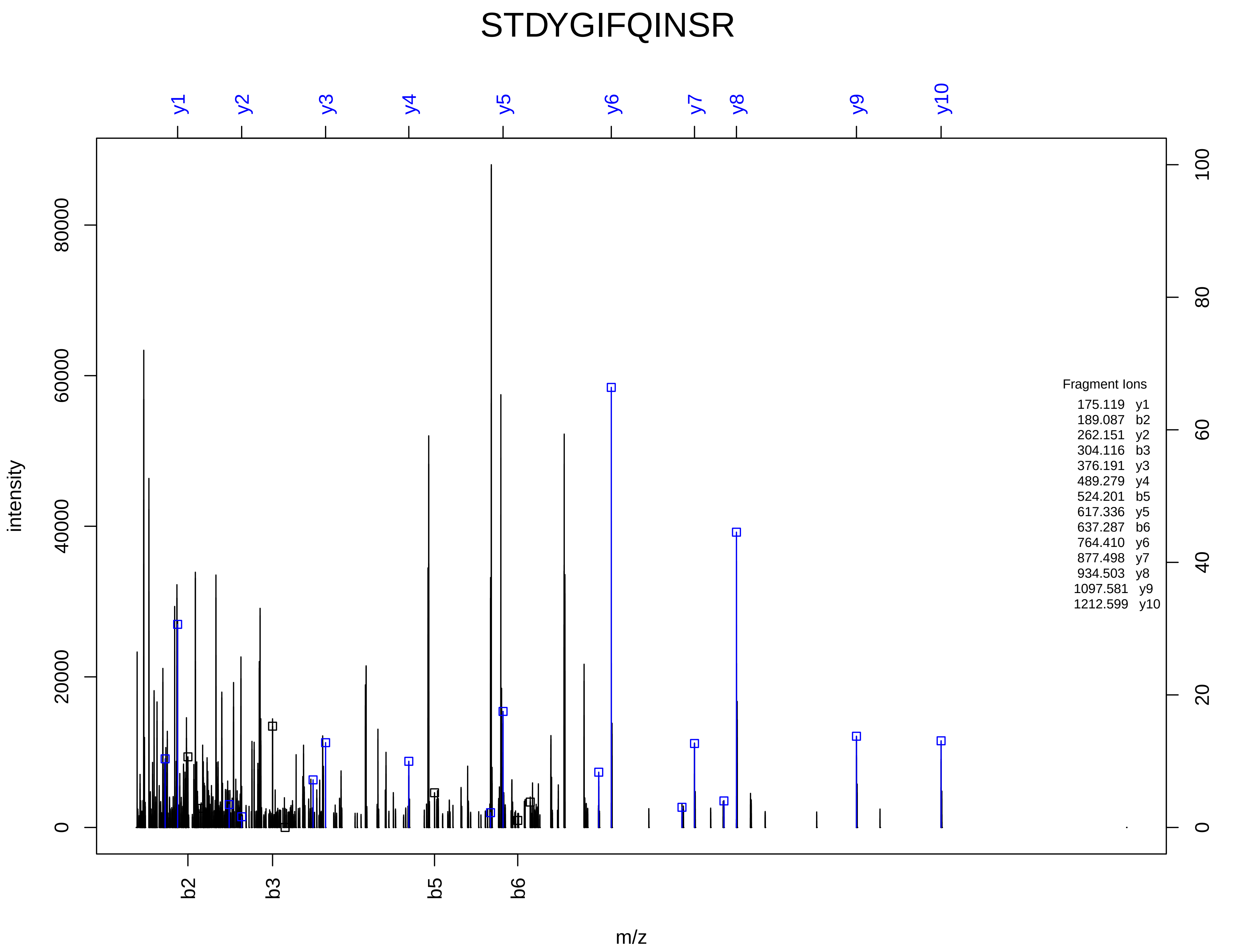
STDYGIFQINSR
y1
y2
y3
y4
y5
y6
y7
y8
y9
y10
intensity
0
20000
40000
60000
80000
0
20
40
60
80
100
Fragment Ions
175.119 y1
189.087 b2
262.151 y2
304.116 b3
376.191 y3
489.279 y4
524.201 b5
617.336 y5
637.287 b6
764.410 y6
877.498 y7
934.503 y8
1097.581 y9
1212.599 y10
b2
b3
b5
b6
m/z
STDYGIFQINSR / File: christine_ip_03.raw; SpectrumID: ; scans: 10257

(Ac)M*EEQQPEPK

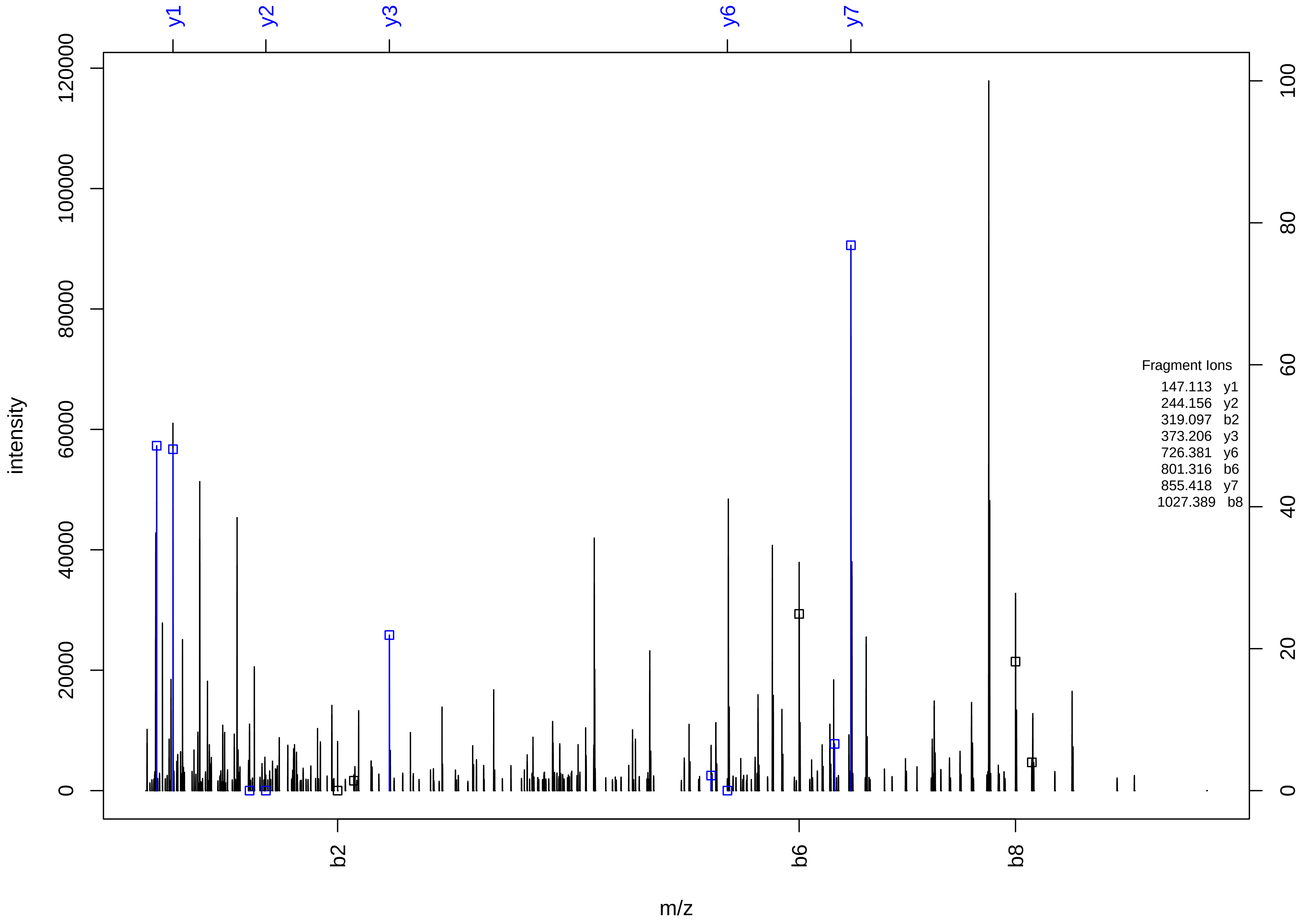

MEEQQPEPK / File: christine_ip_03.raw; SpectrumID: ; scans: 3187

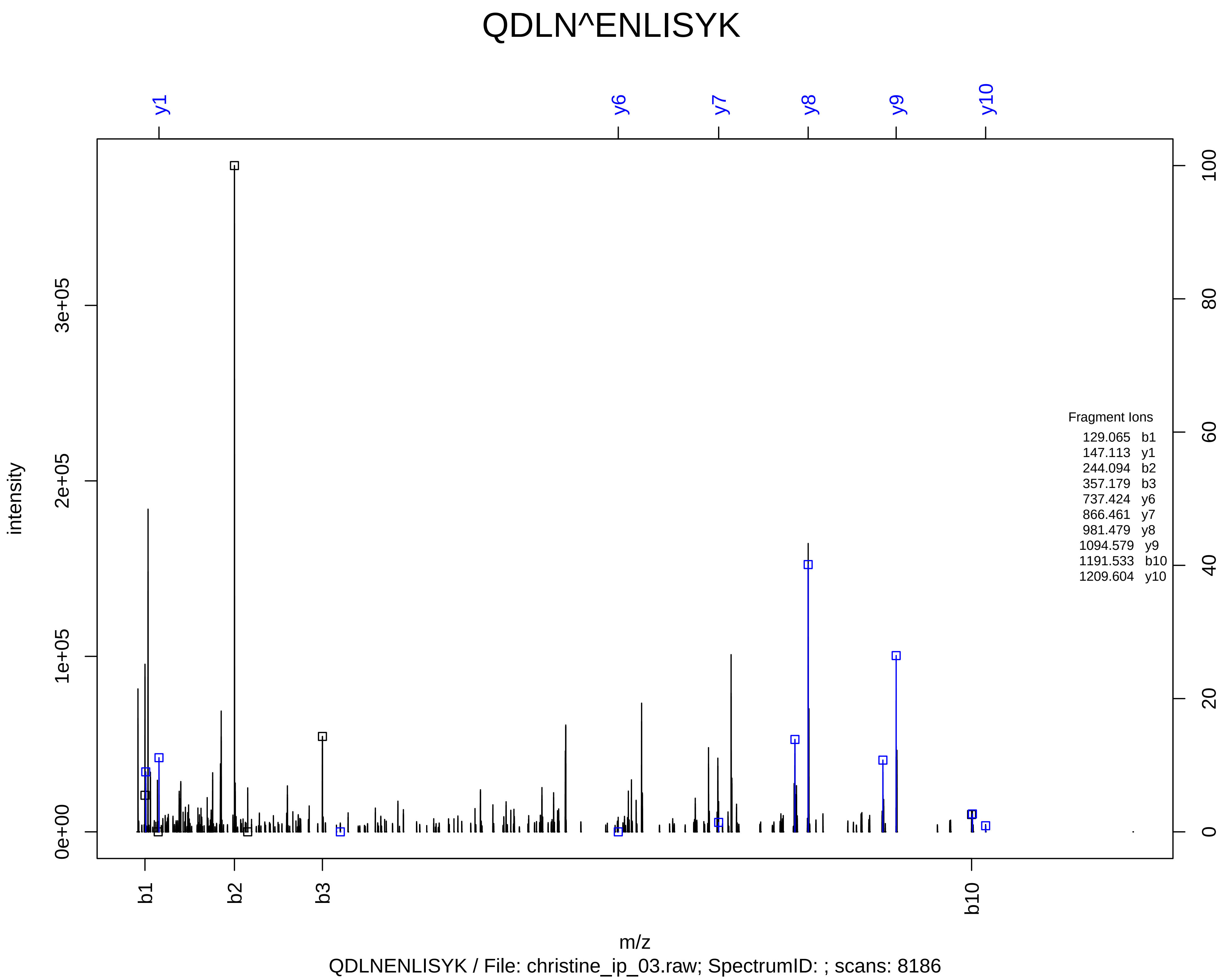
QDLN^ENLISYK
y1
y6
y7
y8
y9
y10
intensity
3e+05
2e+05
1e+05
0e+00
100
80
60
40
20
0
Fragment Ions
129.065 b1
147.113 y1
244.094 b2
357.179 b3
737.424 y6
866.461 y7
981.479 y8
1094.579 y9
1191.533 b10
1209.604 y10
b1
b2
b3
b10
m/z
QDLNENLISYK / File: christine_ip_03.raw; SpectrumID: ; scans: 8186

LLQIGNELR

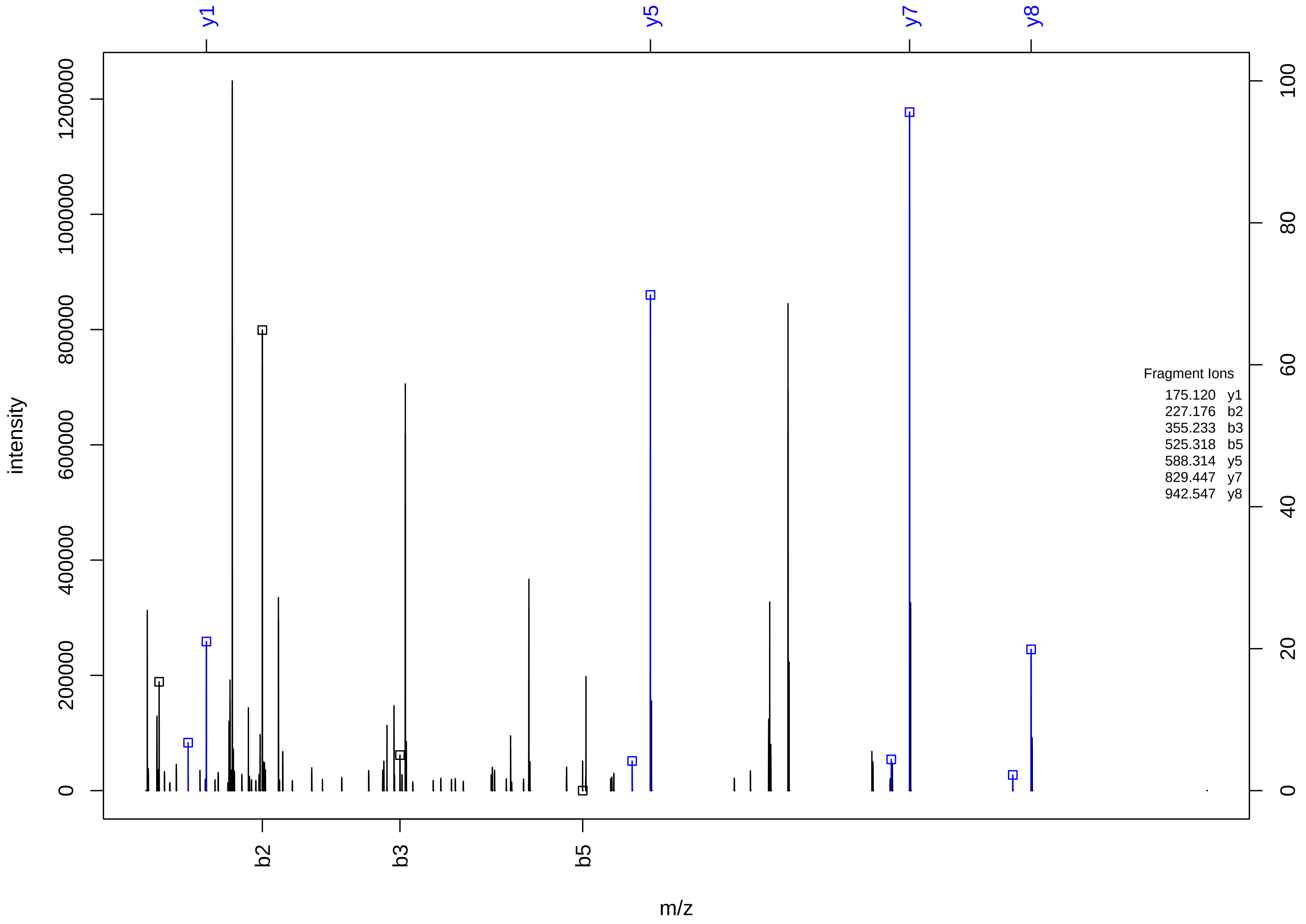

LLQIGNELR / File: christine_ip_03.raw; SpectrumID: ; scans: 8405

NQNYM*NILR

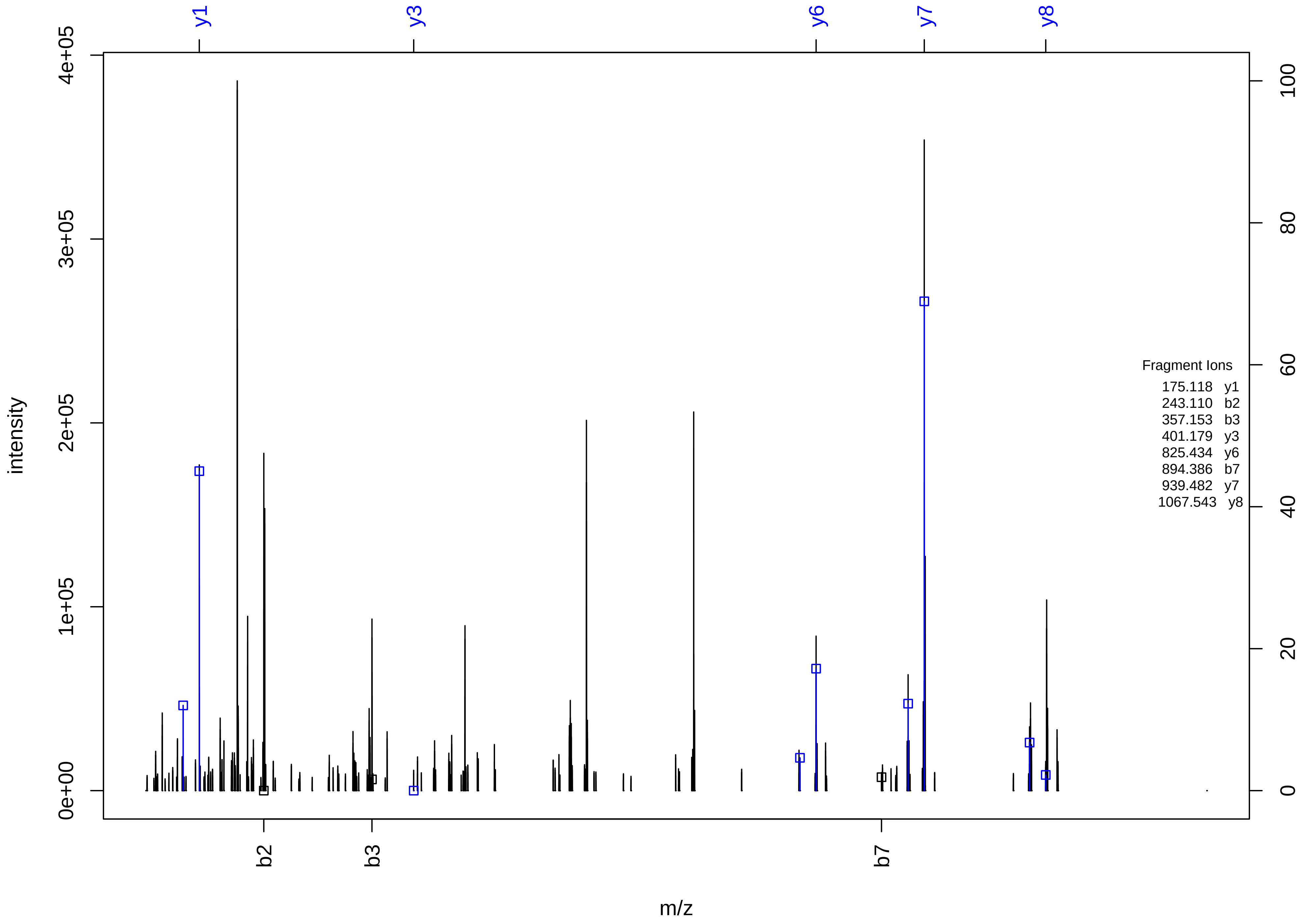

NQNYMNILR / File: christine_ip_03.raw; SpectrumID: ; scans: 4023

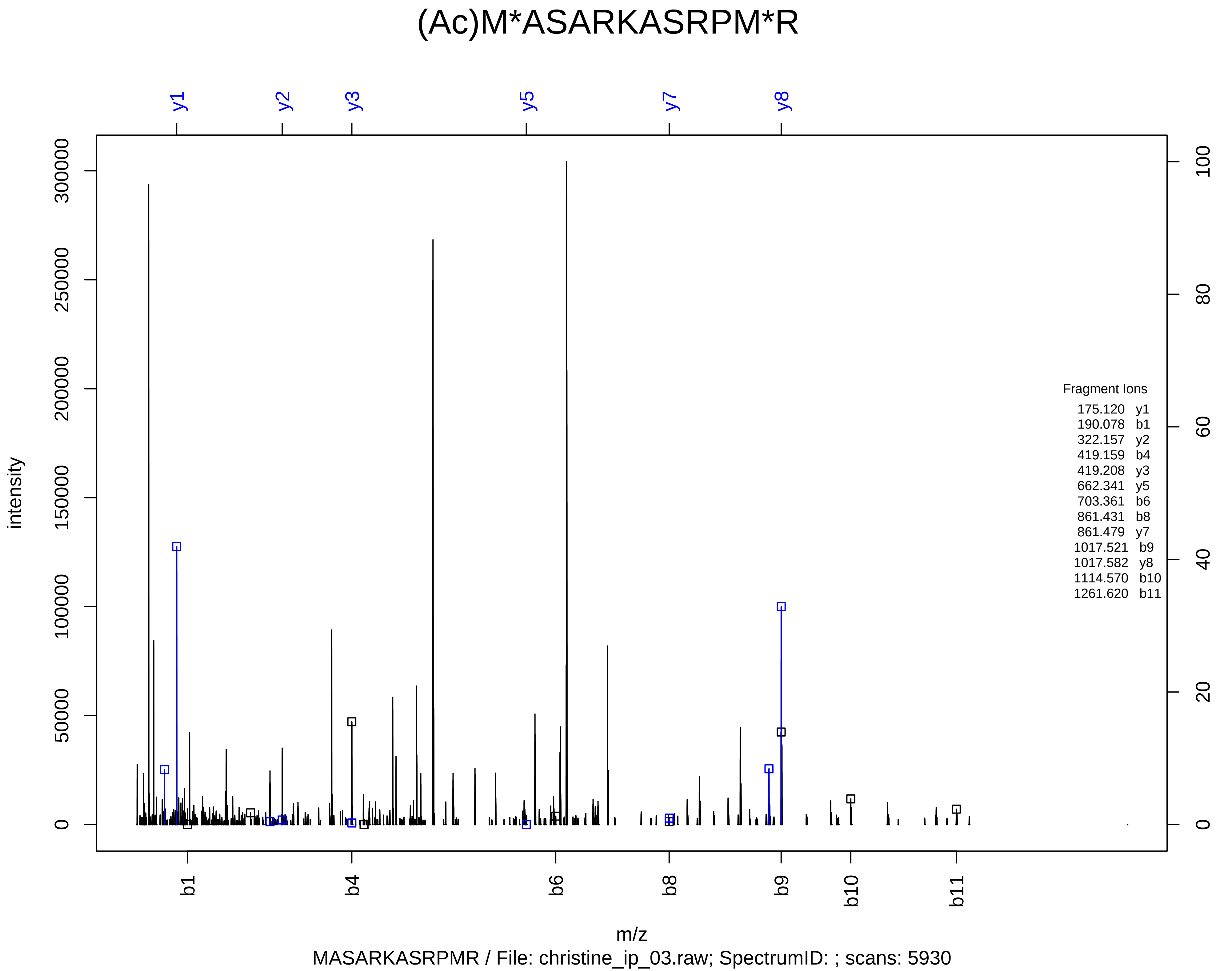
(Ac)M*ASARKASRPM*R
y1
y2
y3
y5
y7
y8
intensity
0
50000
100000
150000
200000
250000
300000
0
20
40
60
80
100
Fragment Ions
175.120 y1
190.078 b1
322.157 y2
419.159 b4
419.208 y3
662.341 y5
703.361 b6
861.431 b8
861.479 y7
1017.521 b9
1017.582 y8
1114.570 b10
1261.620 b11
b1
b4
b6
b8
b9
b10
b11
m/z
MASARKASRPMR / File: christine_ip_03.raw; SpectrumID: ; scans: 5930

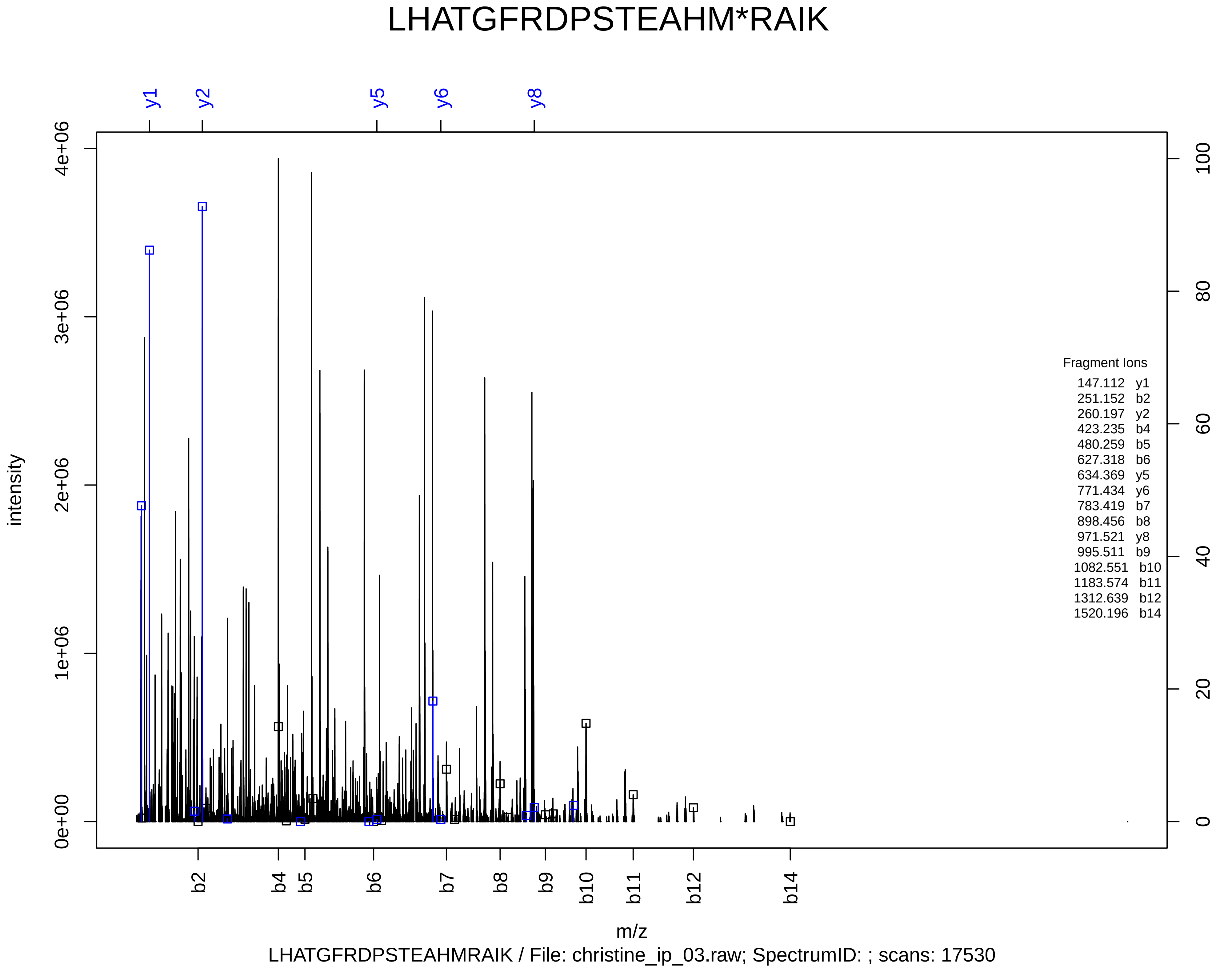
LHATGFRDPSTEAHM*RAIK
y1
y2
y5
y6
y8
intensity
0e+00
1e+06
2e+06
3e+06
4e+06
0
20
40
60
80
100
b2
b4
b5
b6
b7
b8
b9
b10
b11
b12
b14
m/z
Fragment Ions
147.112 y1
251.152 b2
260.197 y2
423.235 b4
480.259 b5
627.318 b6
634.369 y5
771.434 y6
783.419 b7
898.456 b8
971.521 y8
995.511 b9
1082.551 b10
1183.574 b11
1312.639 b12
1520.196 b14
LHATGFRDPSTEAHMRAIK / File: christine_ip_03.raw; SpectrumID: ; scans: 17530

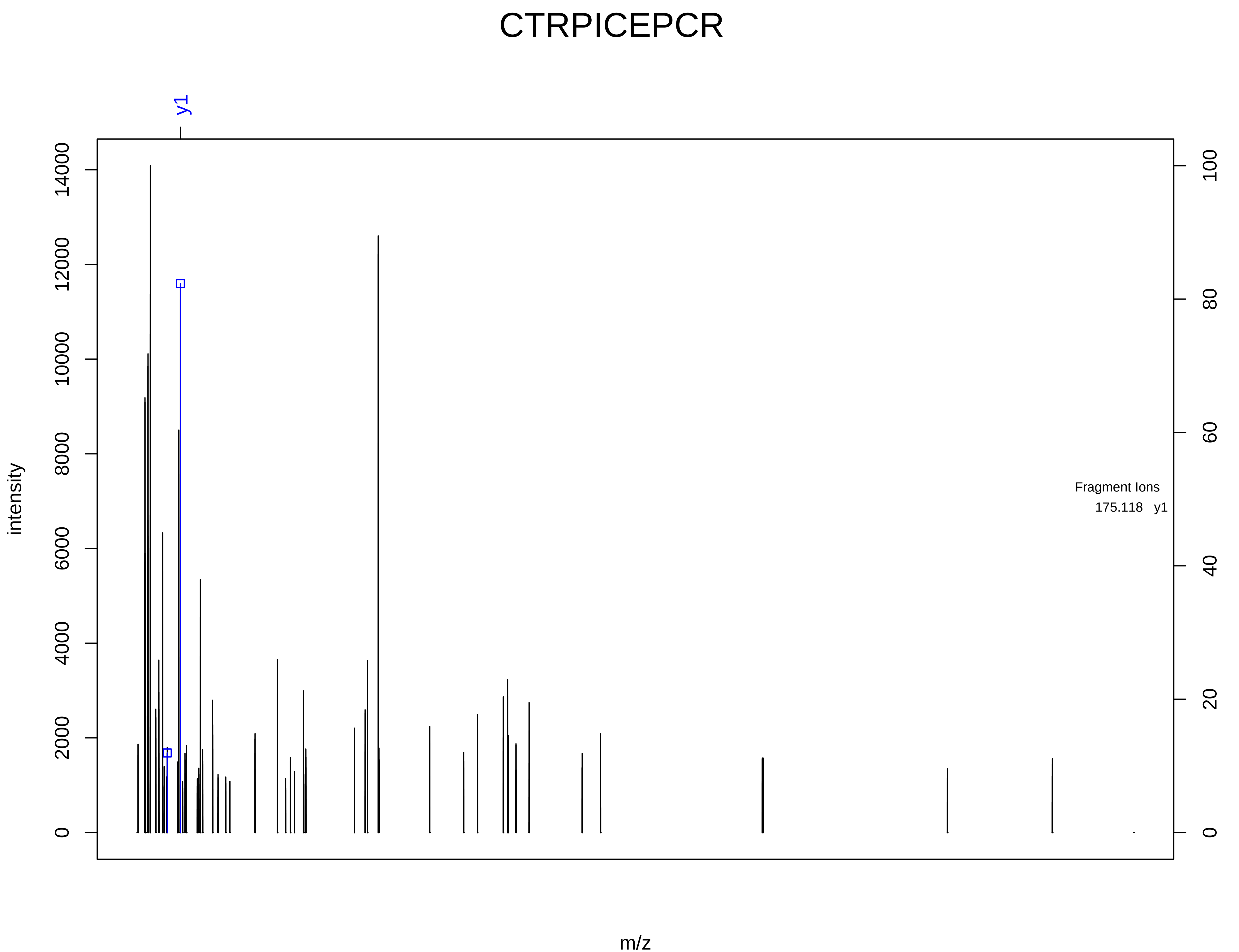
CTRPICEPCR
y1
intensity
14000
12000
10000
8000
6000
4000
2000
0
100
80
60
40
20
0
Fragment Ions
175.118 y1
m/z
CTRPICEPCR / File: christine_ip_02.raw; SpectrumID: ; scans: 1425

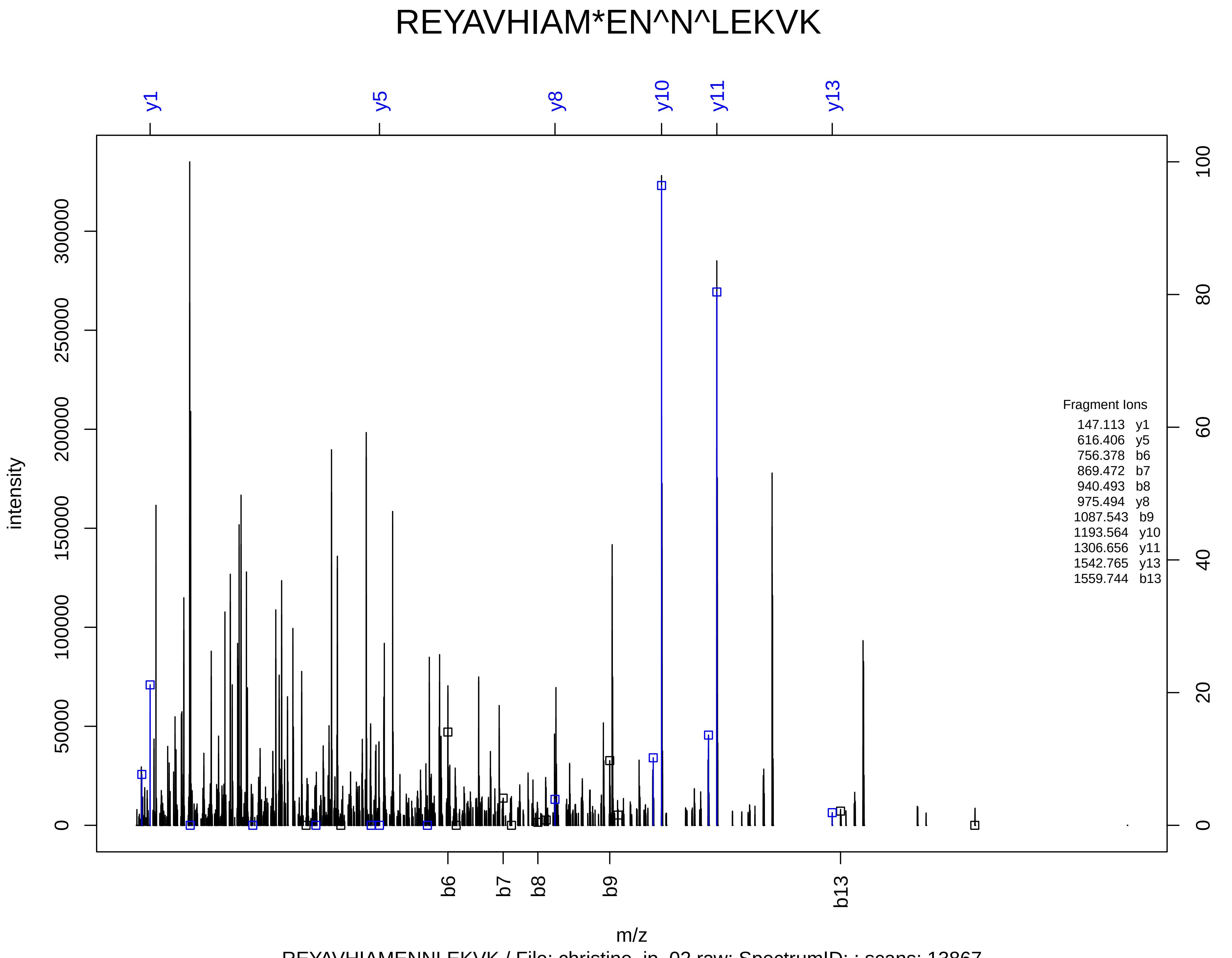

REYAVHIAM*EN^N^LEKVK
y1
y5
y8
y10
y11
y13
intensity
0
50000
100000
150000
200000
250000
300000
0
20
40
60
80
100
Fragment Ions
147.113 y1
616.406 y5
756.378 b6
869.472 b7
940.493 b8
975.494 y8
1087.543 b9
1193.564 y10
1306.656 y11
1542.765 y13
1559.744 b13
b6
b7
b8
b9
b13
m/z
REYAVHIAMENNLEKVK / File: christine_ip_02.raw; SpectrumID: ; scans: 13867

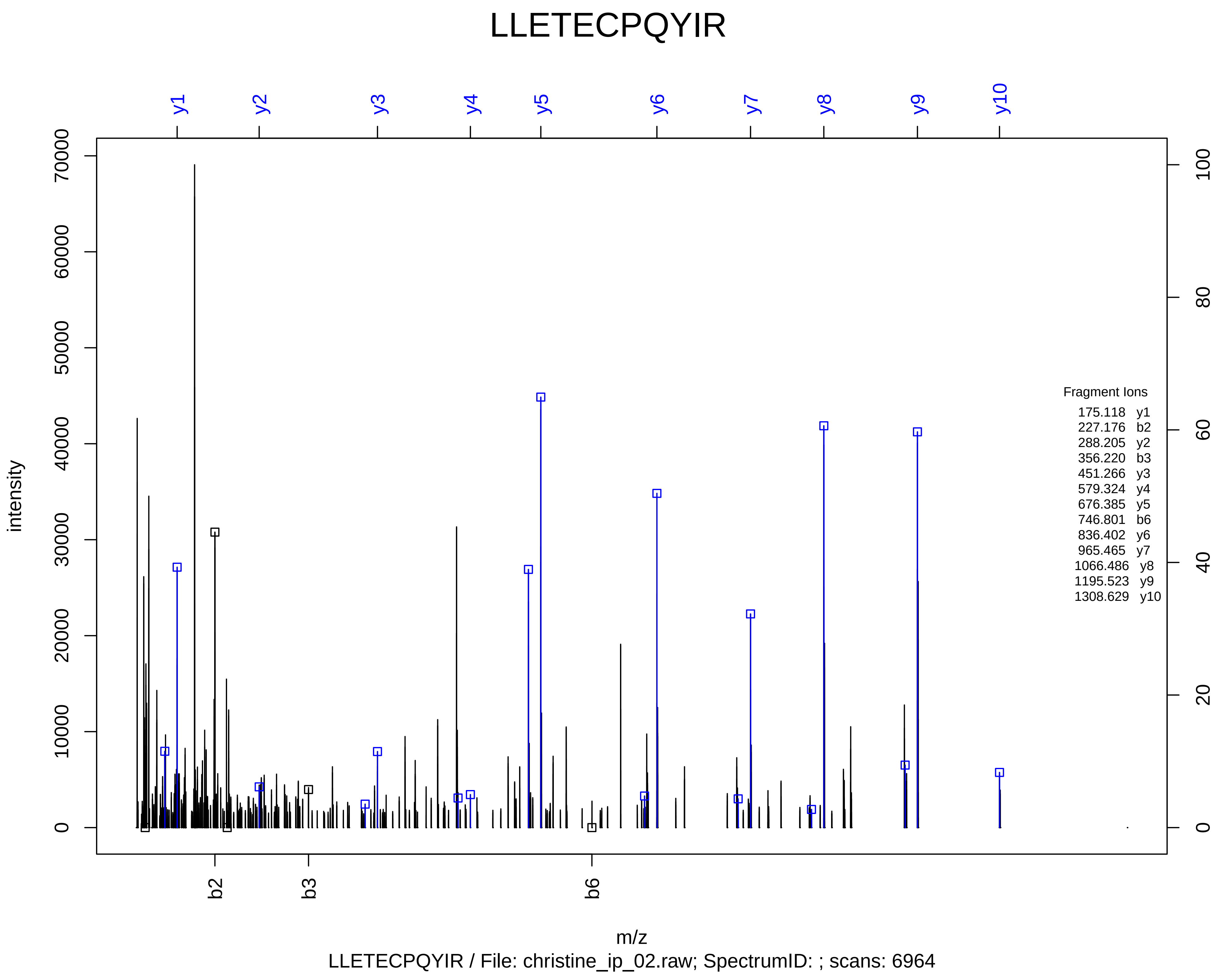
LLETECPQYIR
y1
y2
y3
y4
y5
y6
y7
y8
y9
y10
b2
b3
b6
intensity
m/z
Fragment Ions
175.118 y1
227.176 b2
288.205 y2
356.220 b3
451.266 y3
579.324 y4
676.385 y5
746.801 b6
836.402 y6
965.465 y7
1066.486 y8
1195.523 y9
1308.629 y10
LLETECPQYIR / File: christine_ip_02.raw; SpectrumID: ; scans: 6964

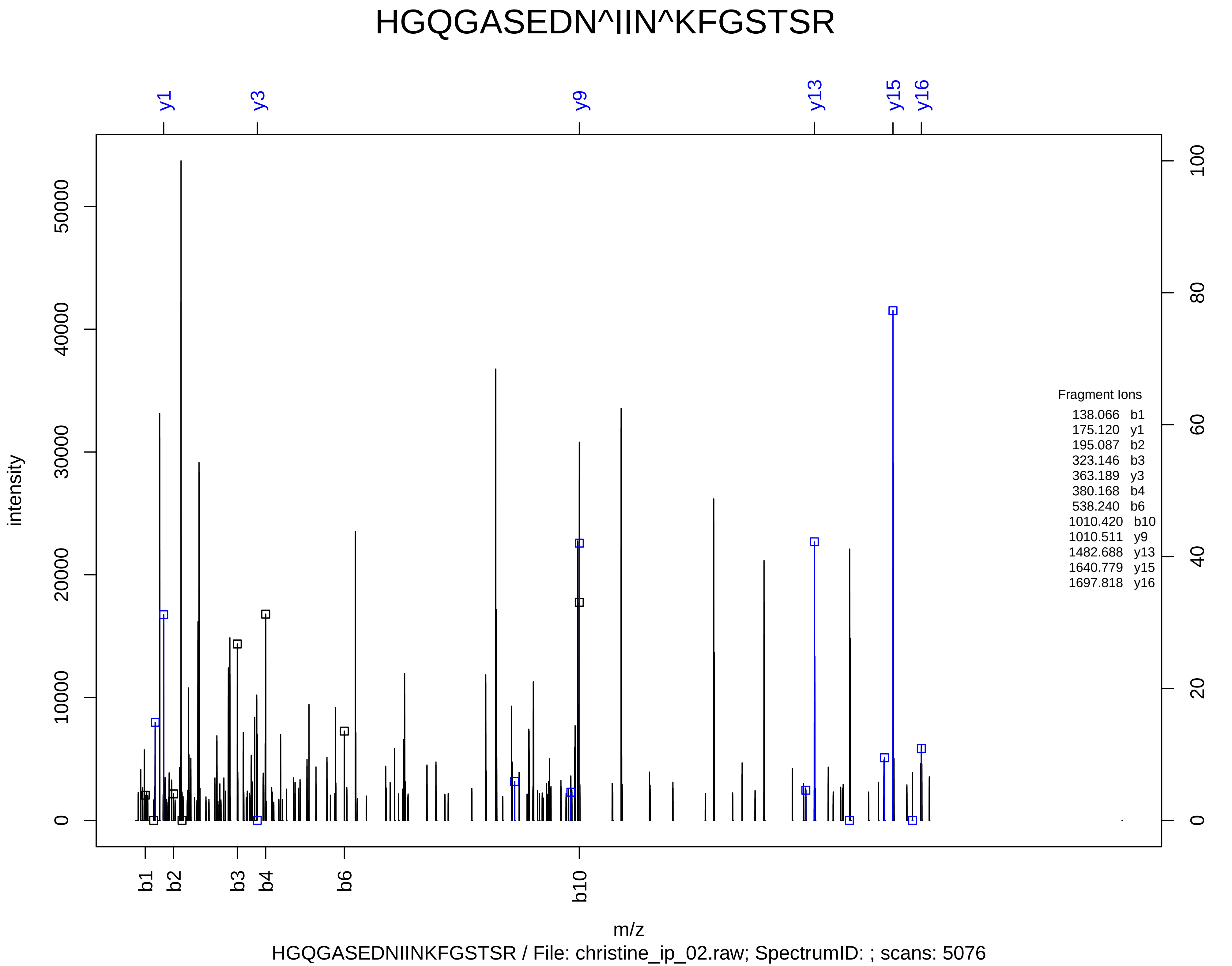
HGQGASEDN^IIN^KFGSTSR
y1
y3
y9
y13
y15
y16
intensity
m/z
b1
b2
b3
b4
b6
b10
Fragment Ions
138.066 b1
175.120 y1
195.087 b2
323.146 b3
363.189 y3
380.168 b4
538.240 b6
1010.420 b10
1010.511 y9
1482.688 y13
1640.779 y15
1697.818 y16
HGQGASEDNIINKFGSTSR / File: christine_ip_02.raw; SpectrumID: ; scans: 5076

ELEQQLAAVR

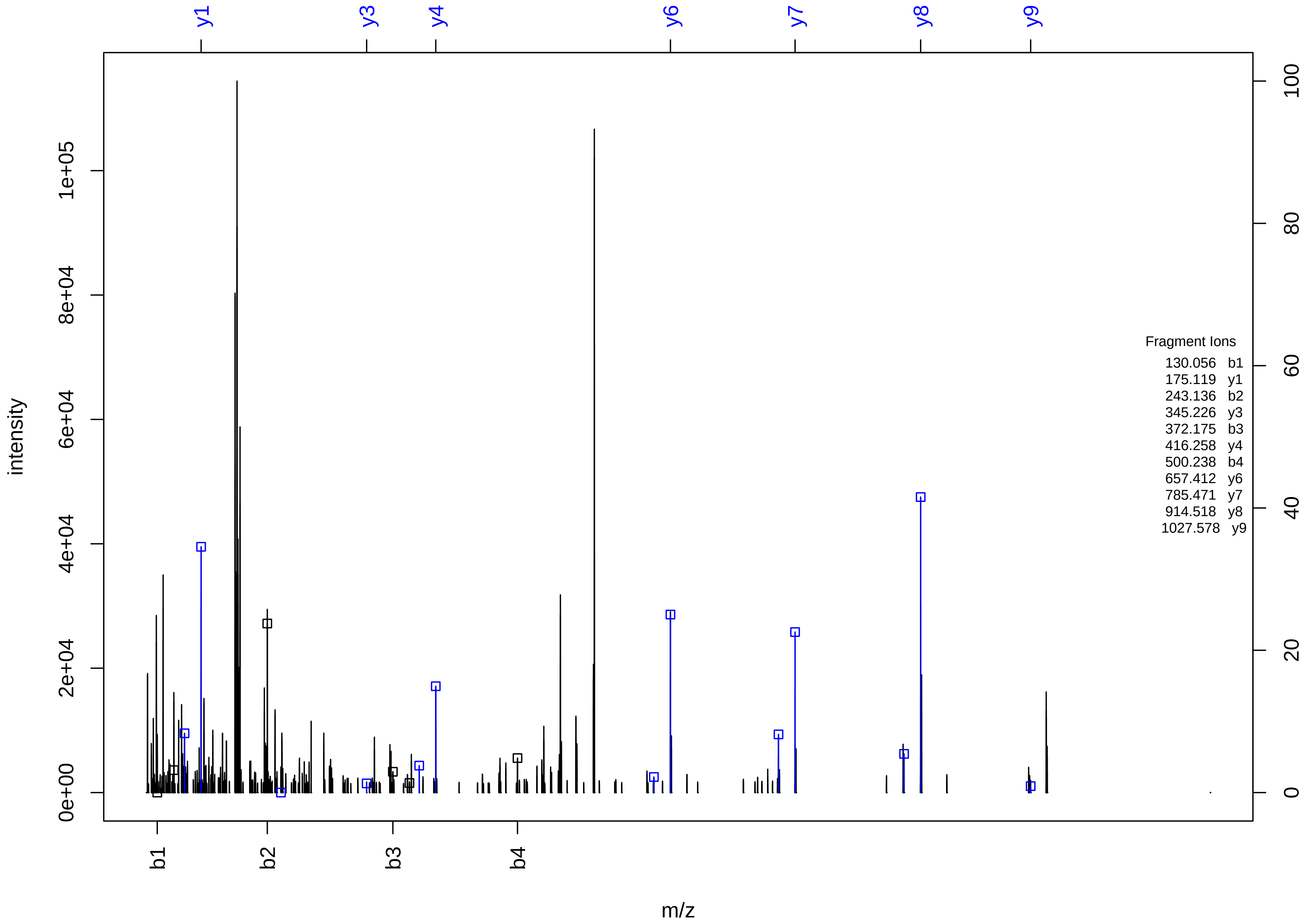

ELEQQLAAVR / File: christine_ip_02.raw; SpectrumID: ; scans: 4959

EAPEGWQTPK

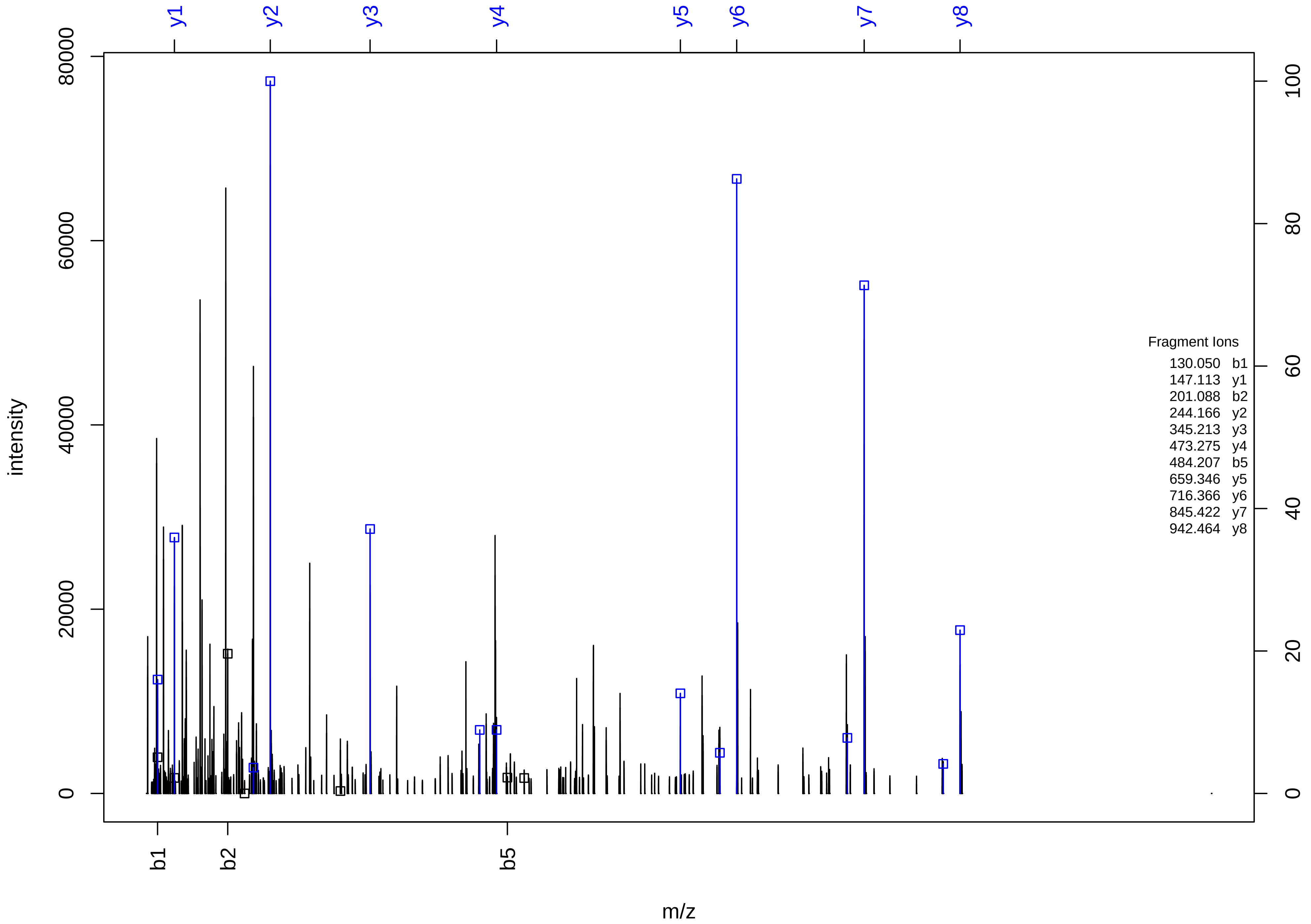

EAPEGWQTPK / File: christine_ip_02.raw; SpectrumID: ; scans: 3796

LGAETLPR

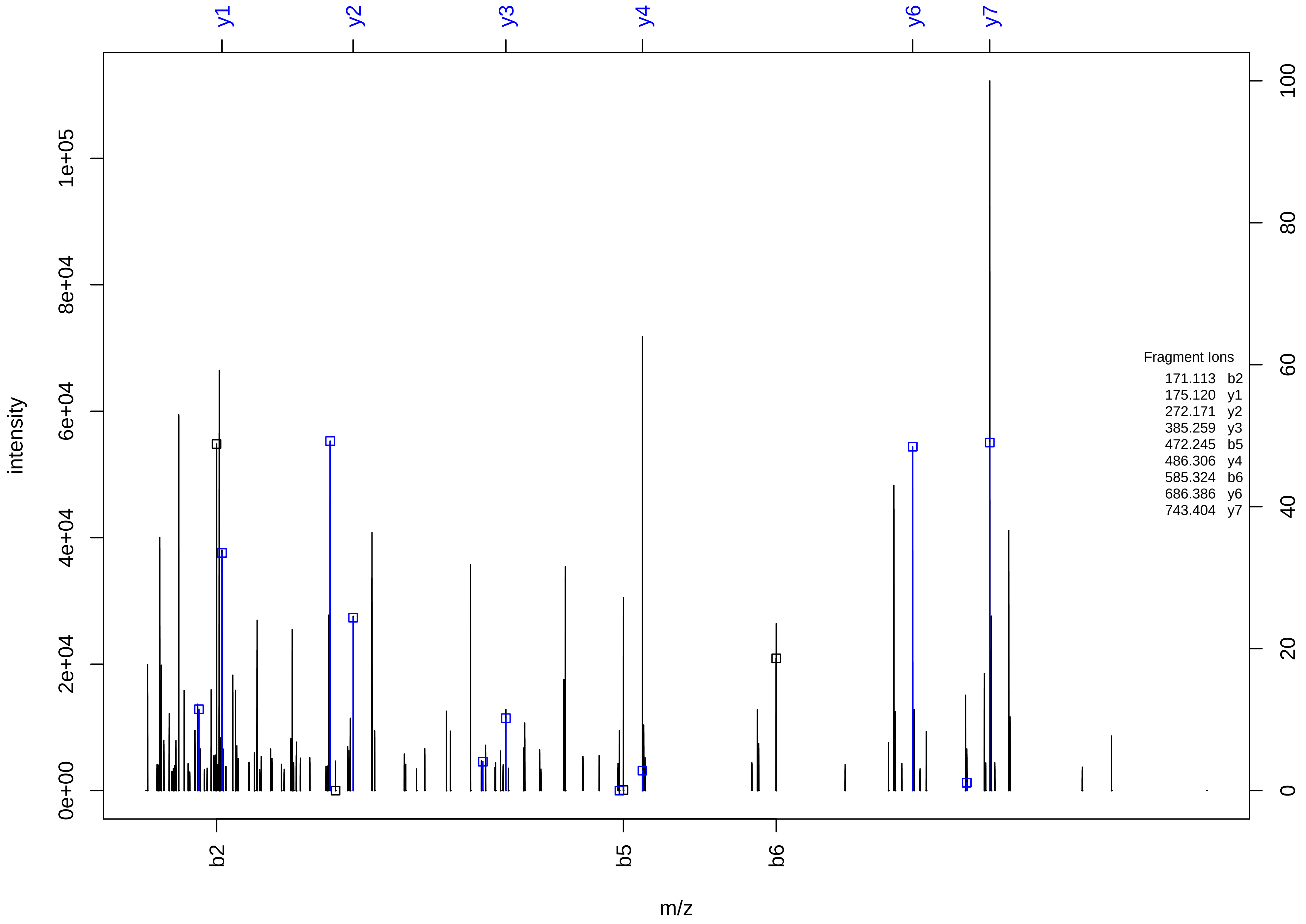

LGAETLPR / File: christine_ip_02.raw; SpectrumID: ; scans: 5292

EVLLQTFLDDTSPGDK

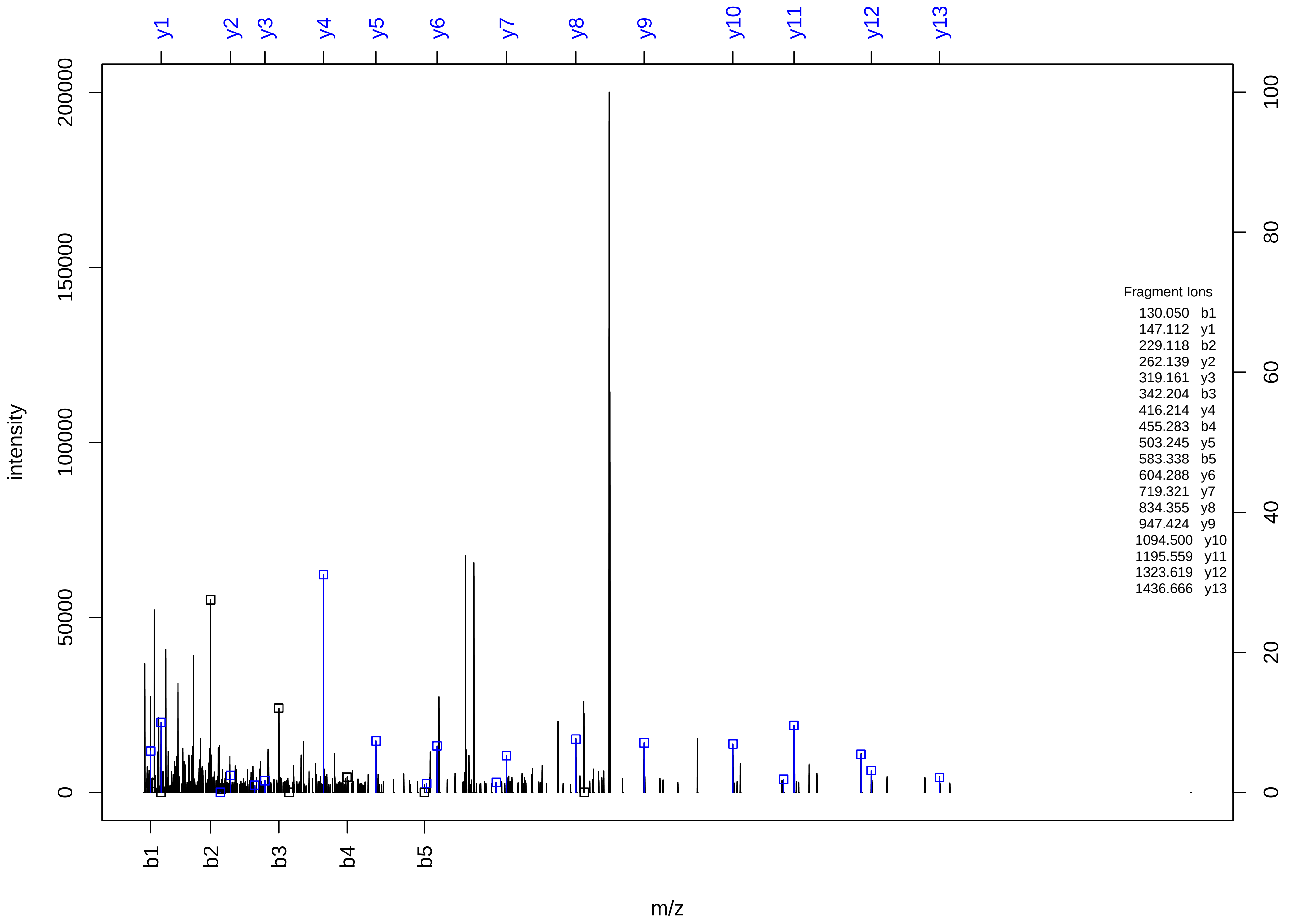

EVLLQTFLDDTSPGDK / File: christine_ip_05.raw; SpectrumID: ; scans: 16498

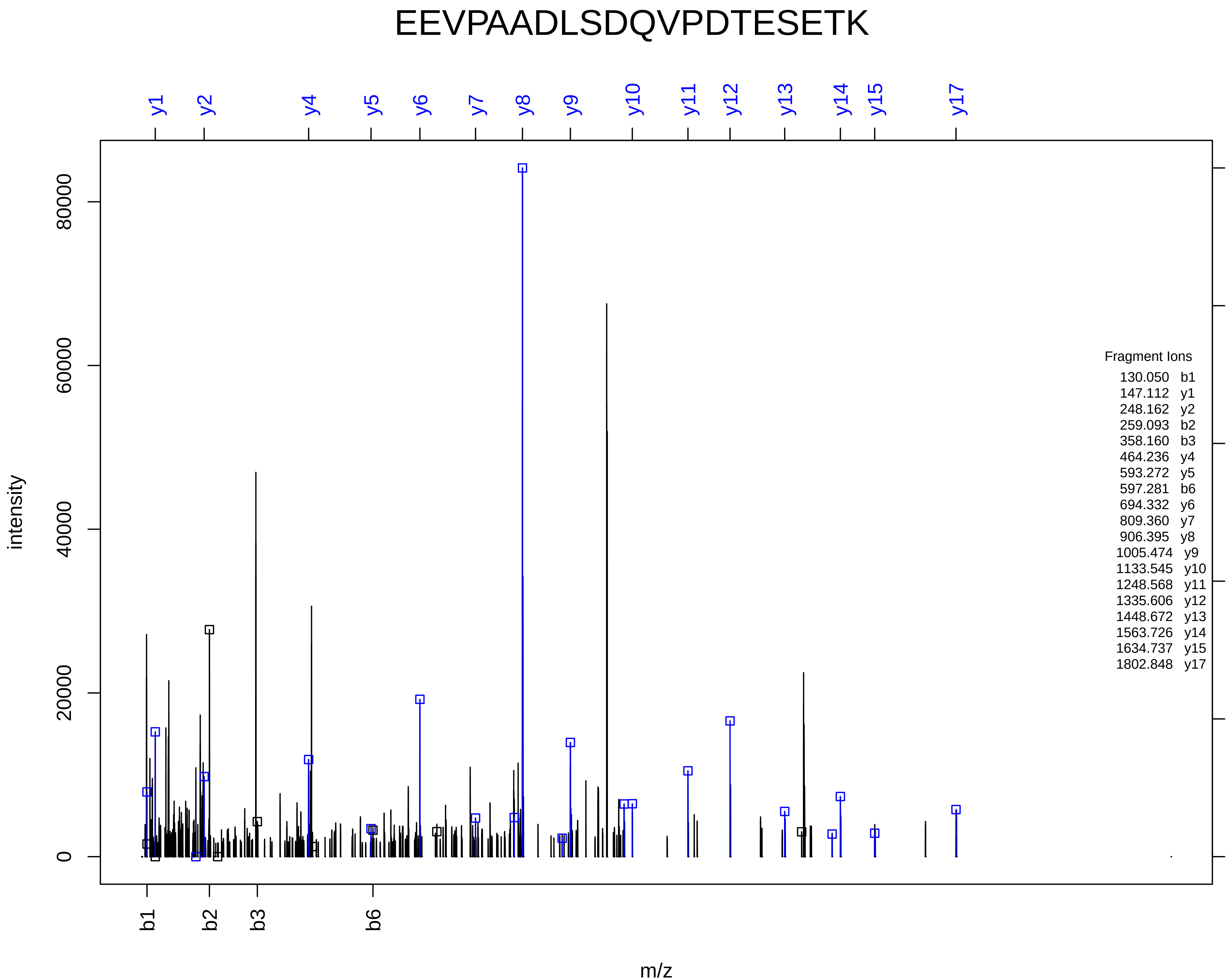

EEVPAADLSDQVPDTESETK / File: christine_ip_05.raw; SpectrumID: ; scans: 8321

LALFNPDVCWDR

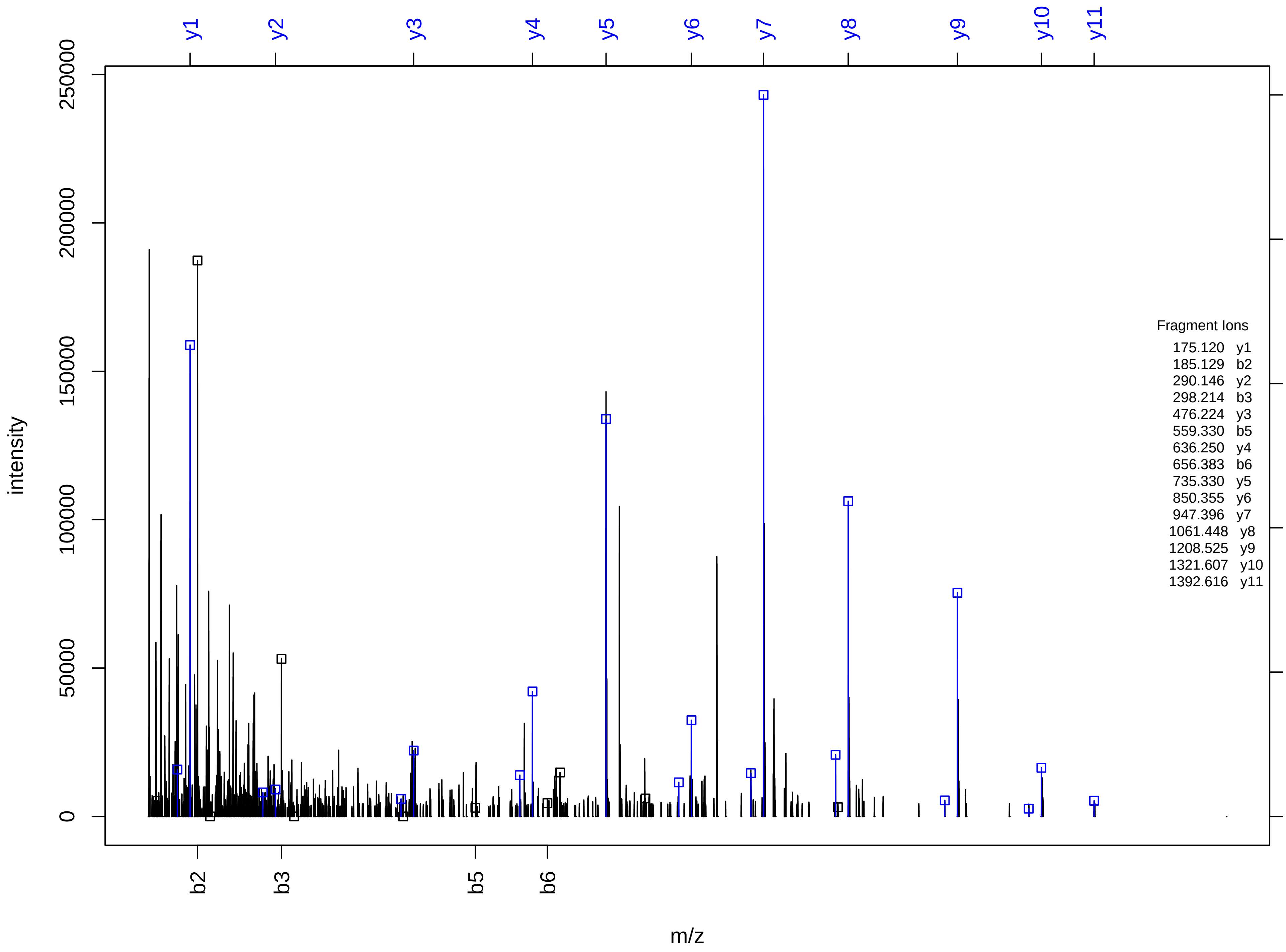

LALFNPDVCWDR / File: christine_ip_05.raw; SpectrumID: ; scans: 15026

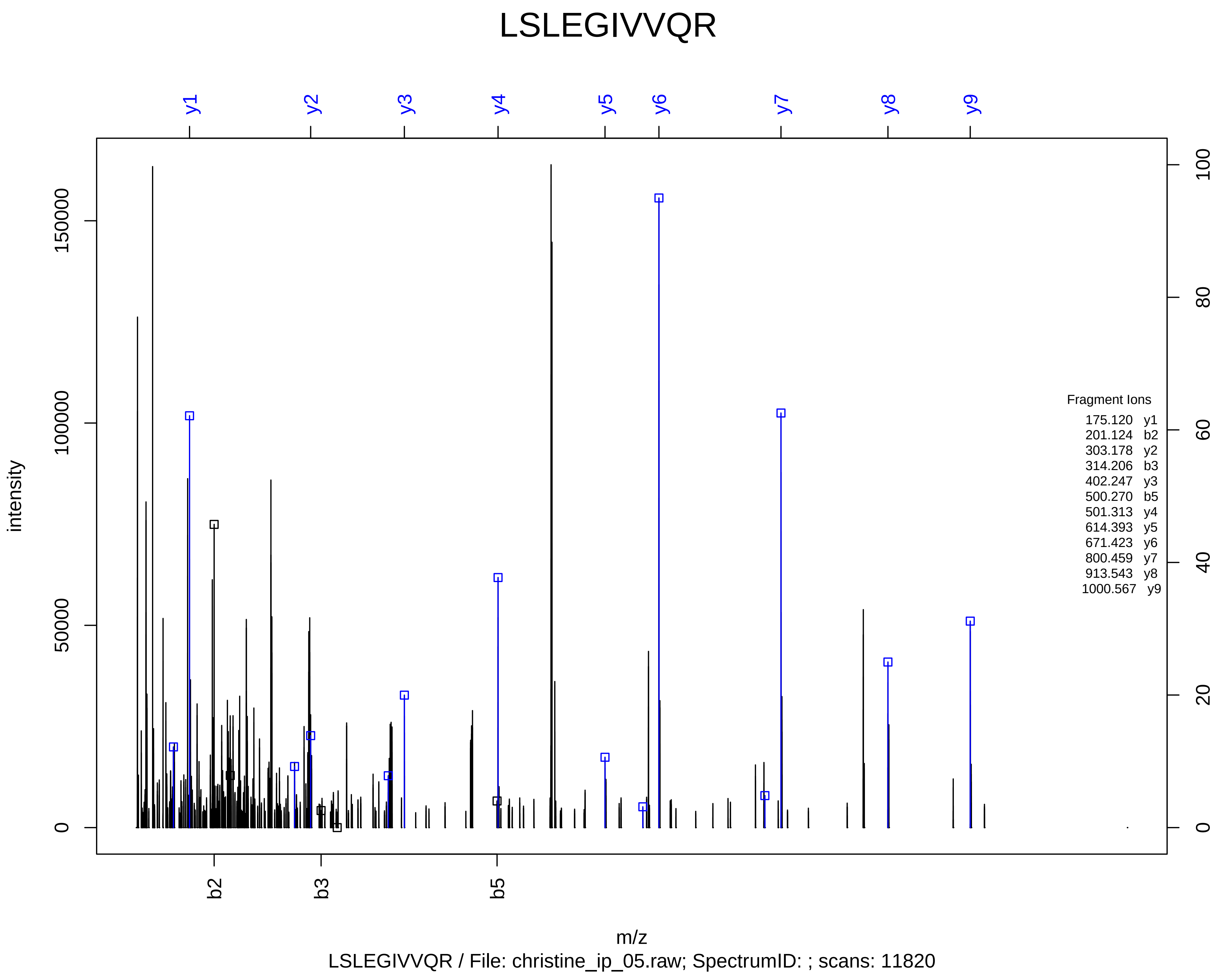
LSLEGIVVQR
y1
y2
y3
y4
y5
y6
y7
y8
y9
150000
100000
50000
0
intensity
100
80
60
40
20
0
Fragment Ions
175.120 y1
201.124 b2
303.178 y2
314.206 b3
402.247 y3
500.270 b5
501.313 y4
614.393 y5
671.423 y6
800.459 y7
913.543 y8
1000.567 y9
b2
b3
b5
m/z
LSLEGIVVQR / File: christine_ip_05.raw; SpectrumID: ; scans: 11820

DEVLYVFPSDFCR

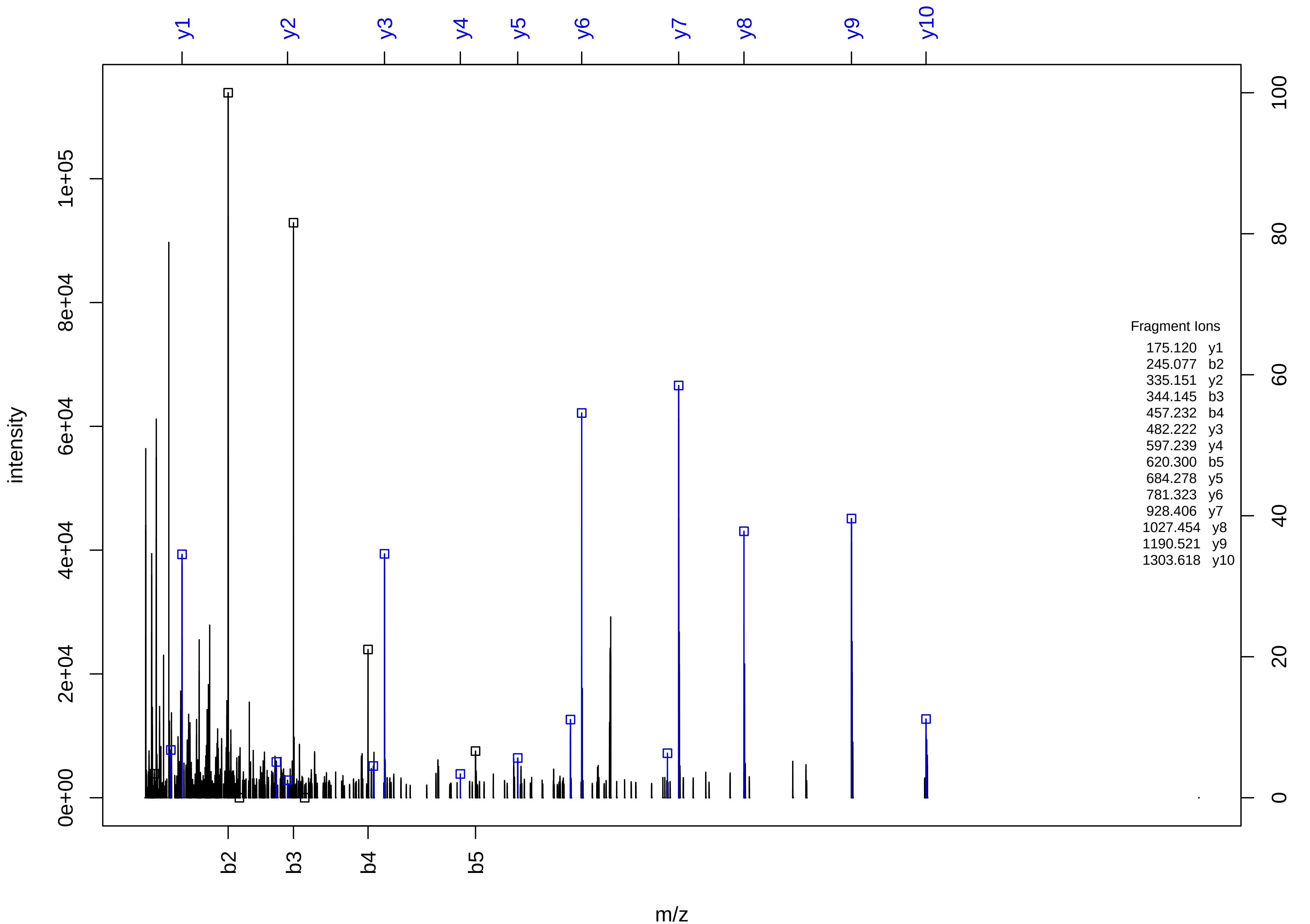

DEVLYVFPSDFCR / File: christine_ip_05.raw; SpectrumID: ; scans: 15934

DLEEGIQM*LMGR

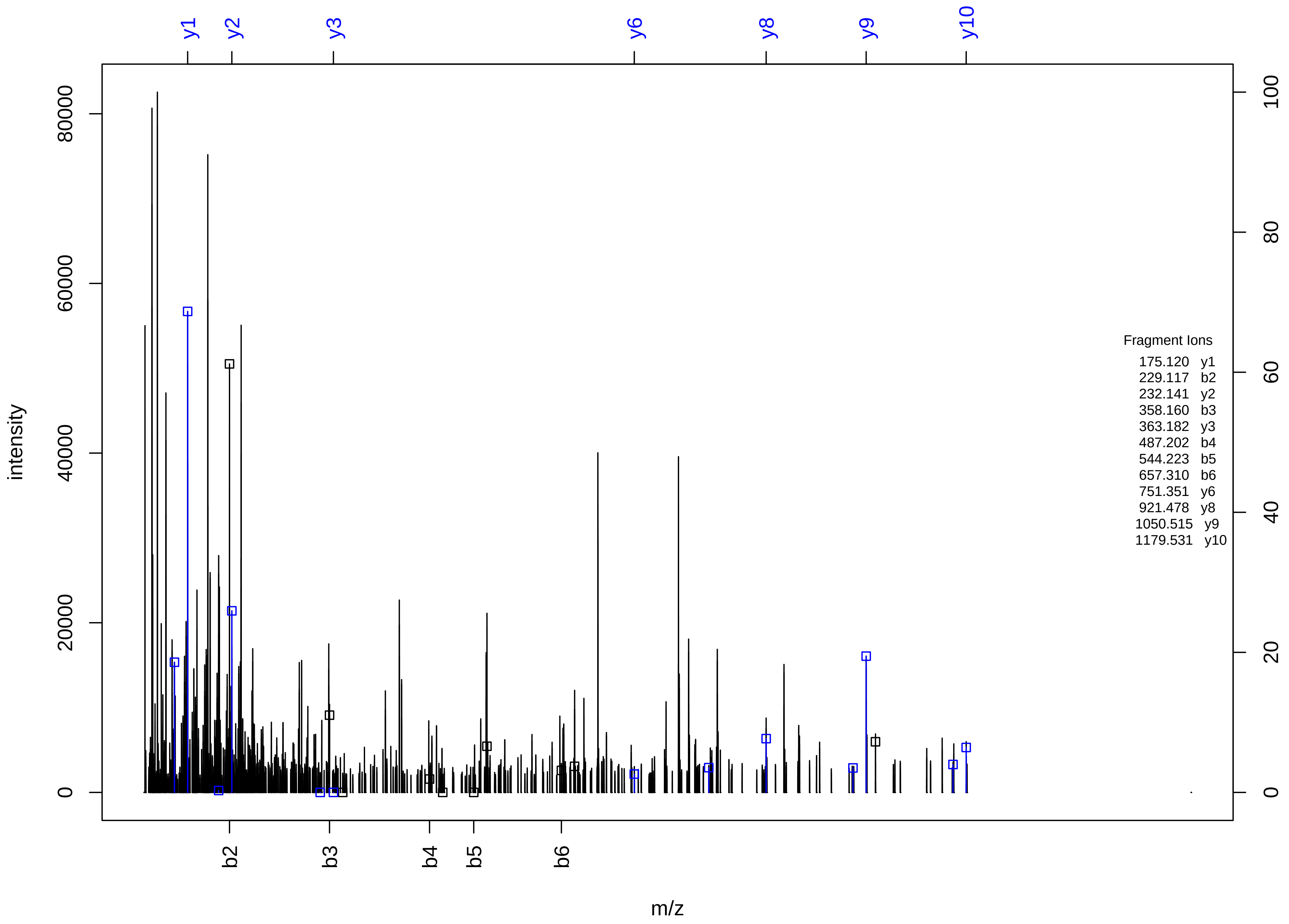

DLEEGIQMLMGR / File: christine_ip_05.raw; SpectrumID: ; scans: 8048

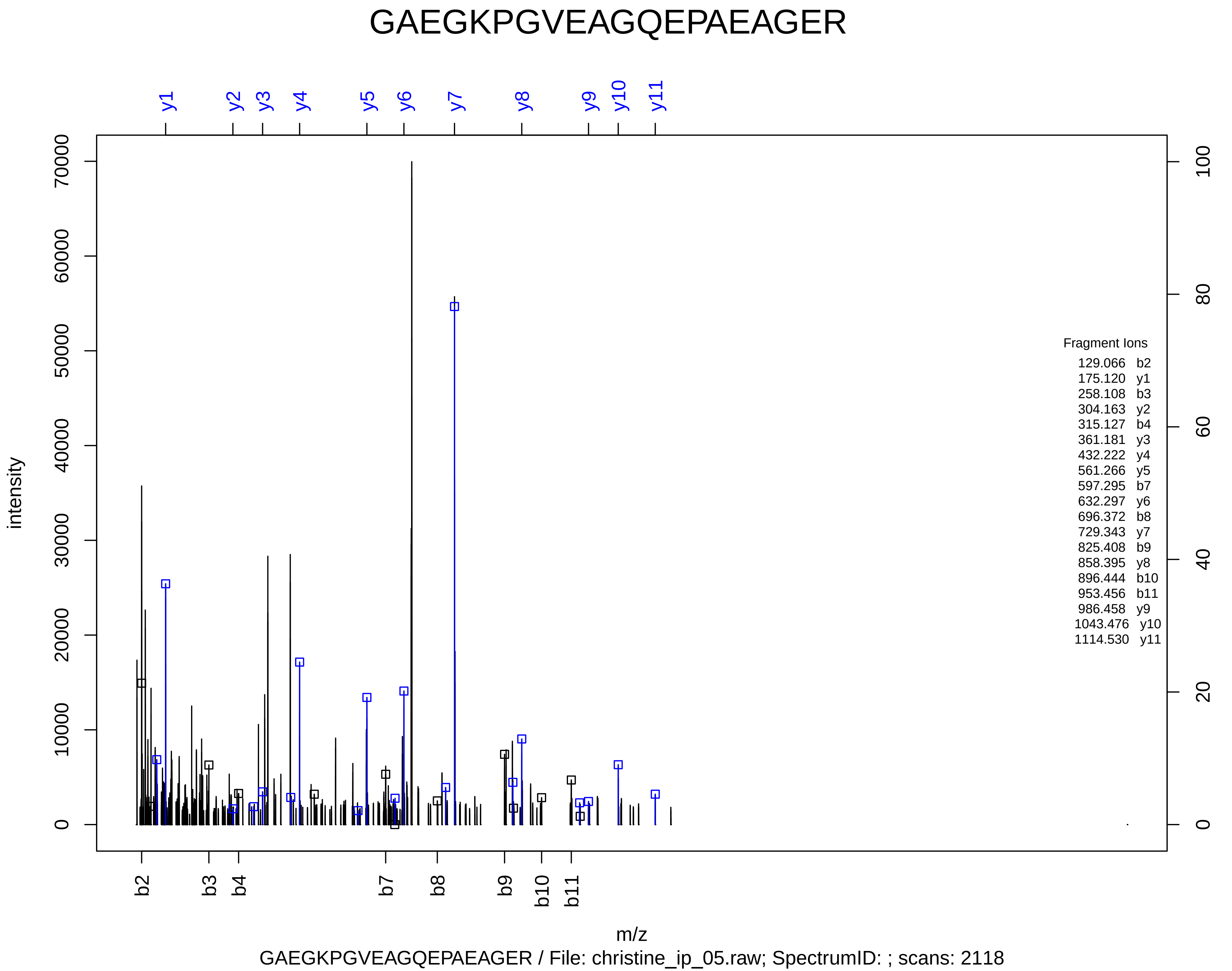
GAEGKPGVEAGQEPAEAGER
y1 y2 y3 y4 y5 y6 y7 y8 y9 y10 y11
intensity
0 10000 20000 30000 40000 50000 60000 70000
0 20 40 60 80 100
Fragment Ions
129.066 b2
175.120 y1
258.108 b3
304.163 y2
315.127 b4
361.181 y3
432.222 y4
561.266 y5
597.295 b7
632.297 y6
696.372 b8
729.343 y7
825.408 b9
858.395 y8
896.444 b10
953.456 b11
986.458 y9
1043.476 y10
1114.530 y11
b2 b3 b4 b7 b8 b9 b10 b11
m/z
GAEGKPGVEAGQEPAEAGER / File: christine_ip_05.raw; SpectrumID: ; scans: 2118

EPEPEIITEPVDVPTFLK

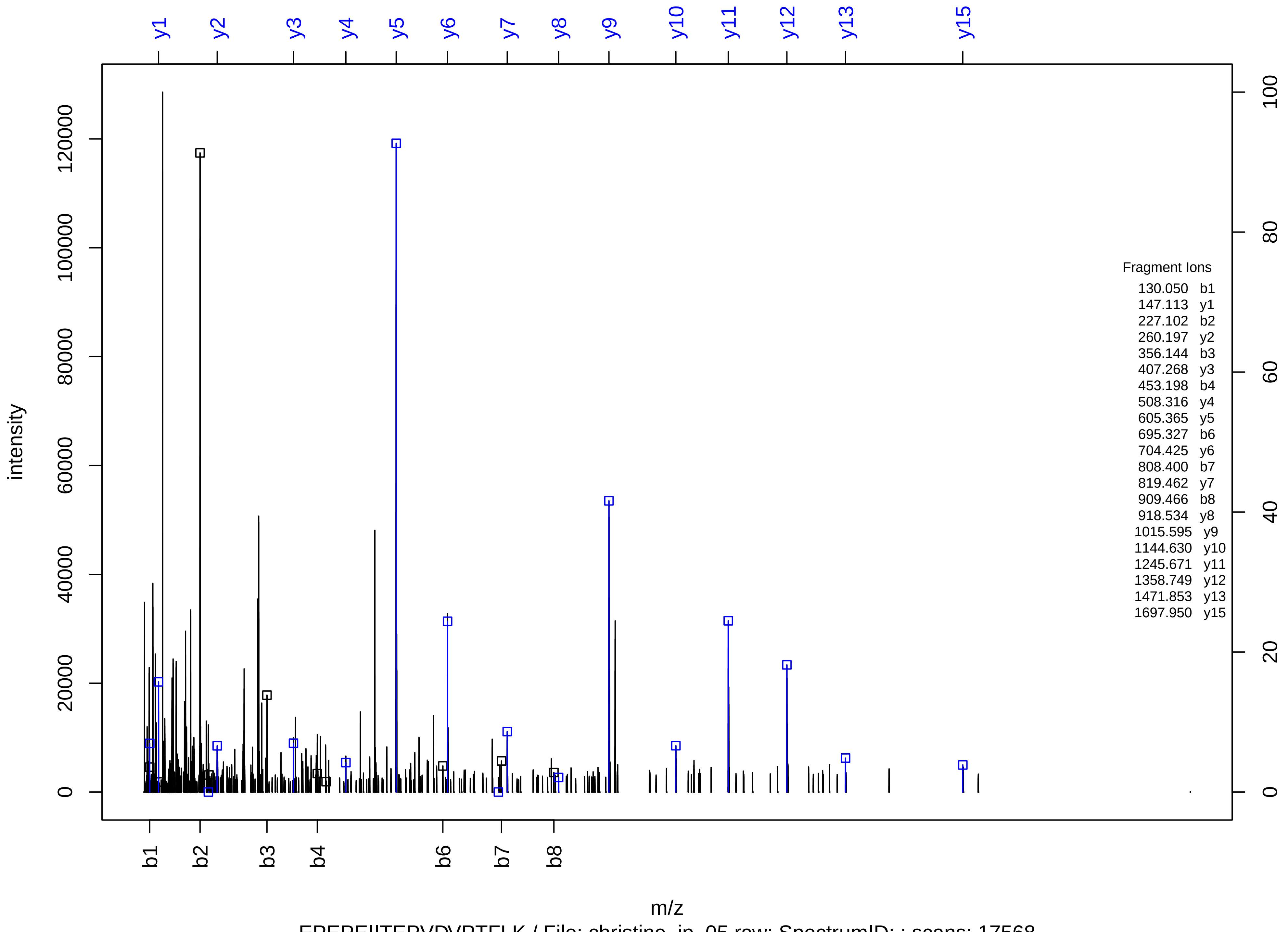

EPEPEIITEPVDVPTFLK / File: christine_ip_05.raw; SpectrumID: ; scans: 17568

LVQTAAQQVAEDK

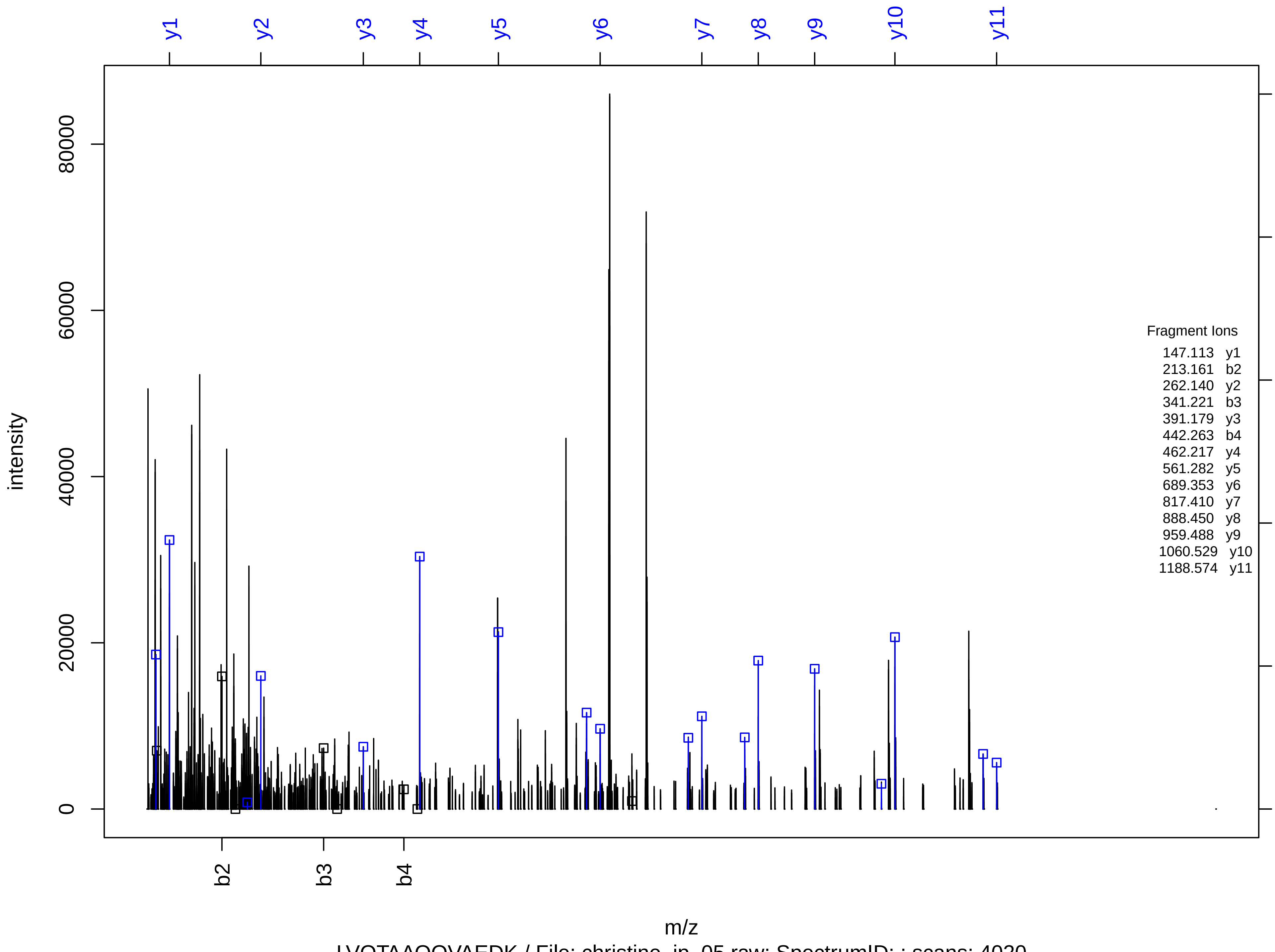

LVQTAAQQVAEDK / File: christine_ip_05.raw; SpectrumID: ; scans: 4020

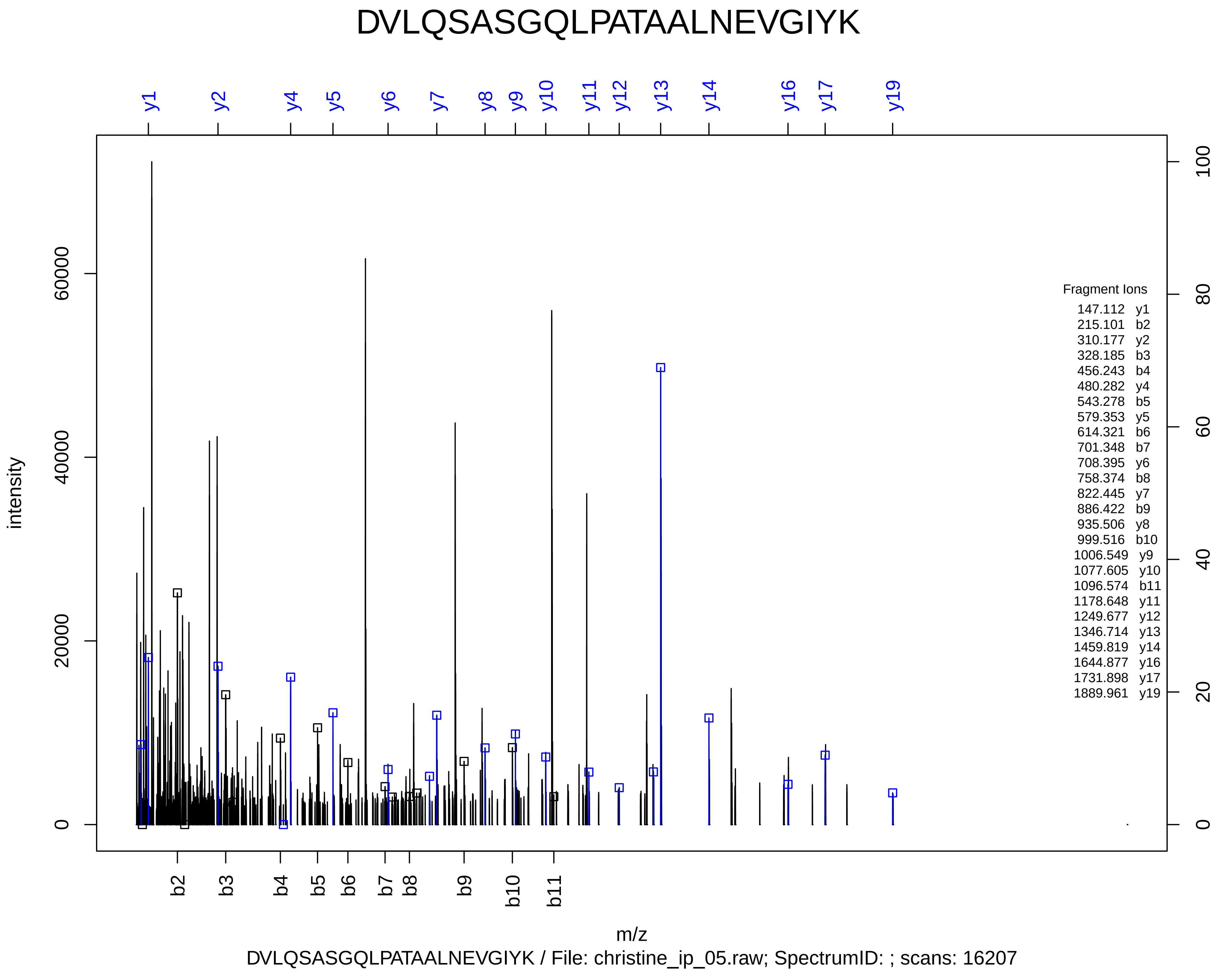
DVLQSASGQLPATAALNEVGIYK
y1
y2
y4
y5
y6
y7
y8
y9
y10
y11
y12
y13
y14
y16
y17
y19
intensity
0
20000
40000
60000
0
20
40
60
80
100
Fragment Ions
147.112 y1
215.101 b2
310.177 y2
328.185 b3
456.243 b4
480.282 y4
543.278 b5
579.353 y5
614.321 b6
701.348 b7
708.395 y6
758.374 b8
822.445 y7
886.422 b9
935.506 y8
999.516 b10
1006.549 y9
1077.605 y10
1096.574 b11
1178.648 y11
1249.677 y12
1346.714 y13
1459.819 y14
1644.877 y16
1731.898 y17
1889.961 y19
b2
b3
b4
b5
b6
b7
b8
b9
b10
b11
m/z
DVLQSASGQLPATAALNEVGIYK / File: christine_ip_05.raw; SpectrumID: ; scans: 16207

GLLLYGPPGTGK

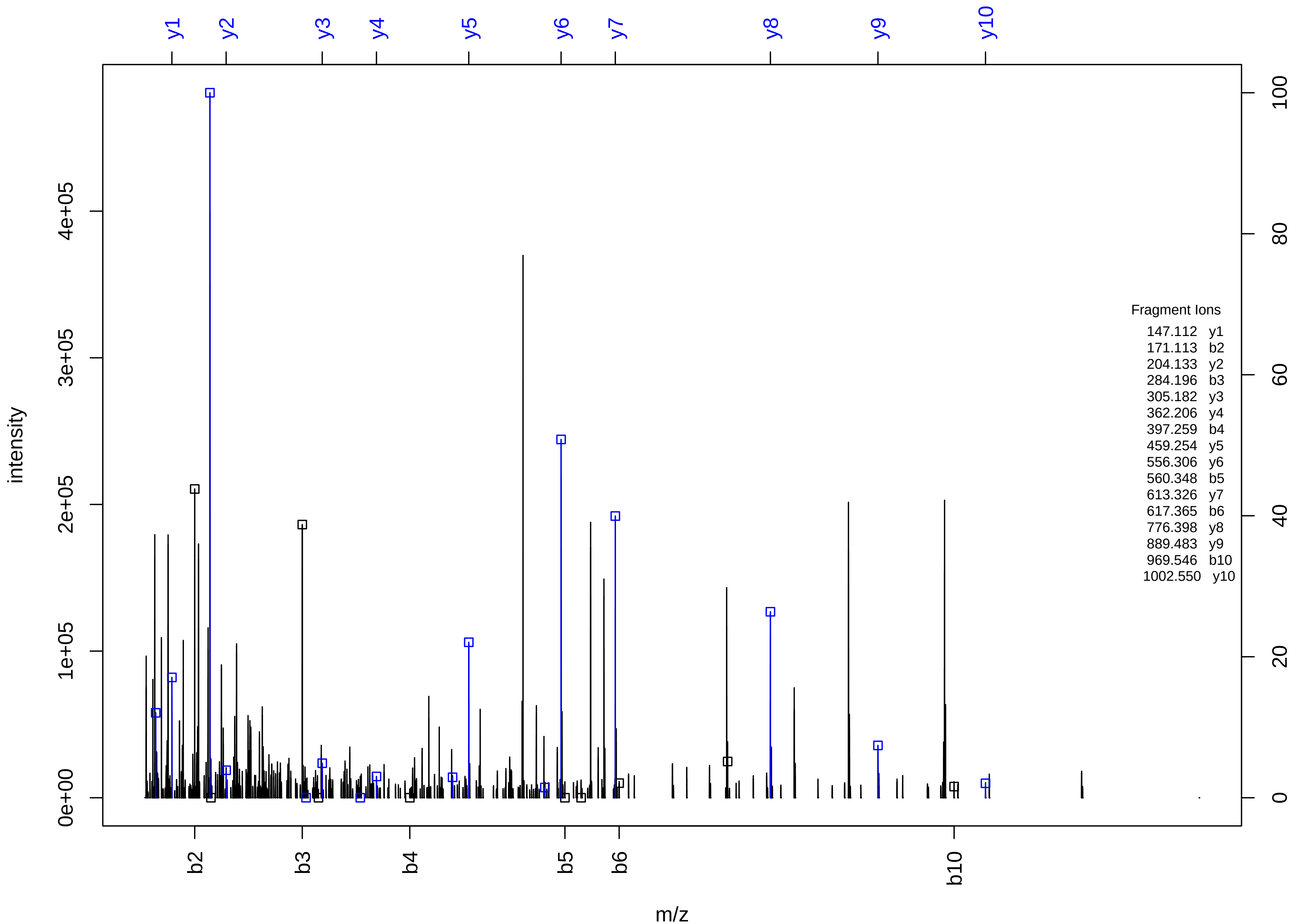

GLLLYGPPGTGK / File: christine_ip_05.raw; SpectrumID: ; scans: 10326

DGPVRPQNAEEEK

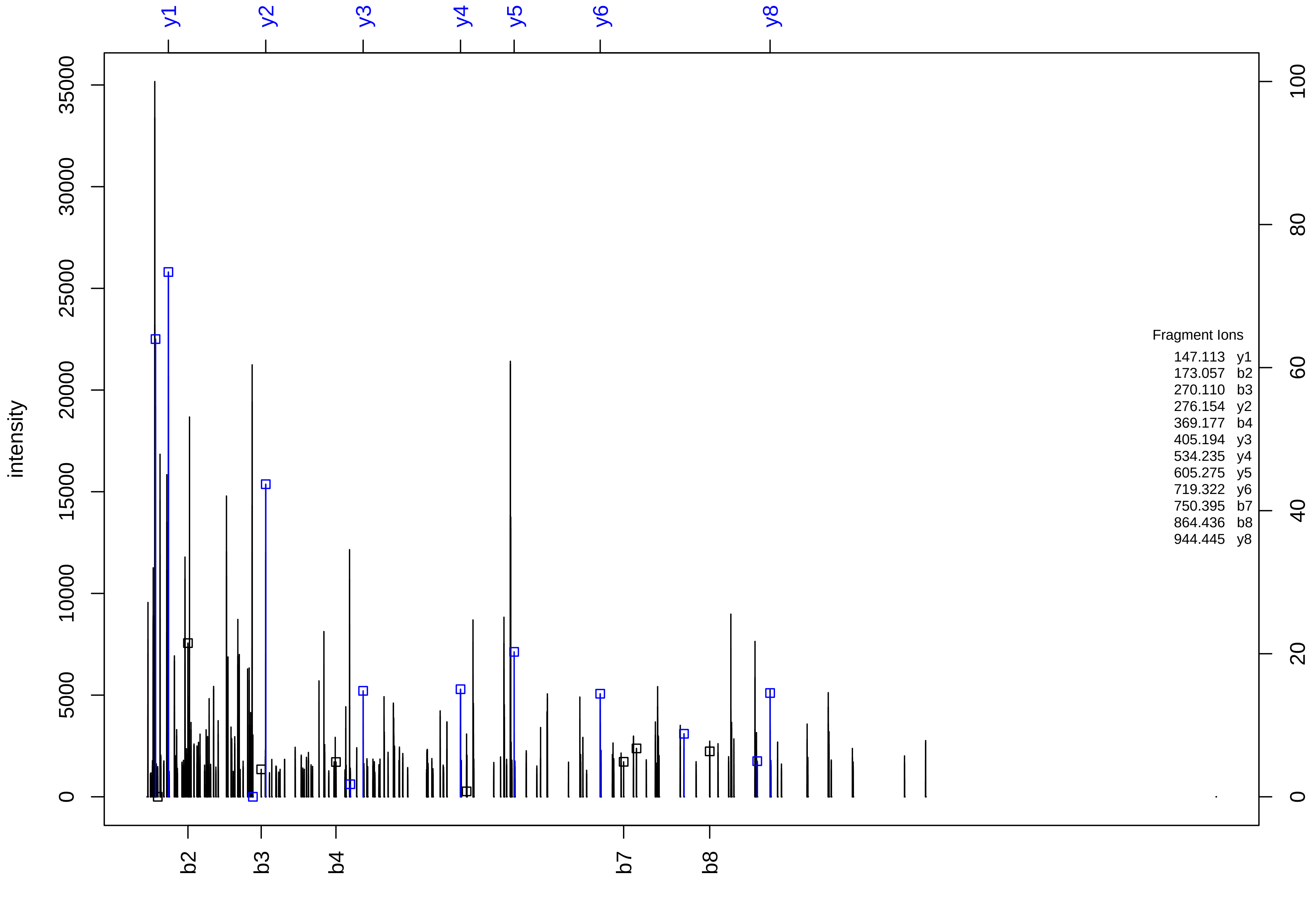

DGPVRPQNAEEEK / File: christine_ip_05.raw; SpectrumID: ; scans: 1345

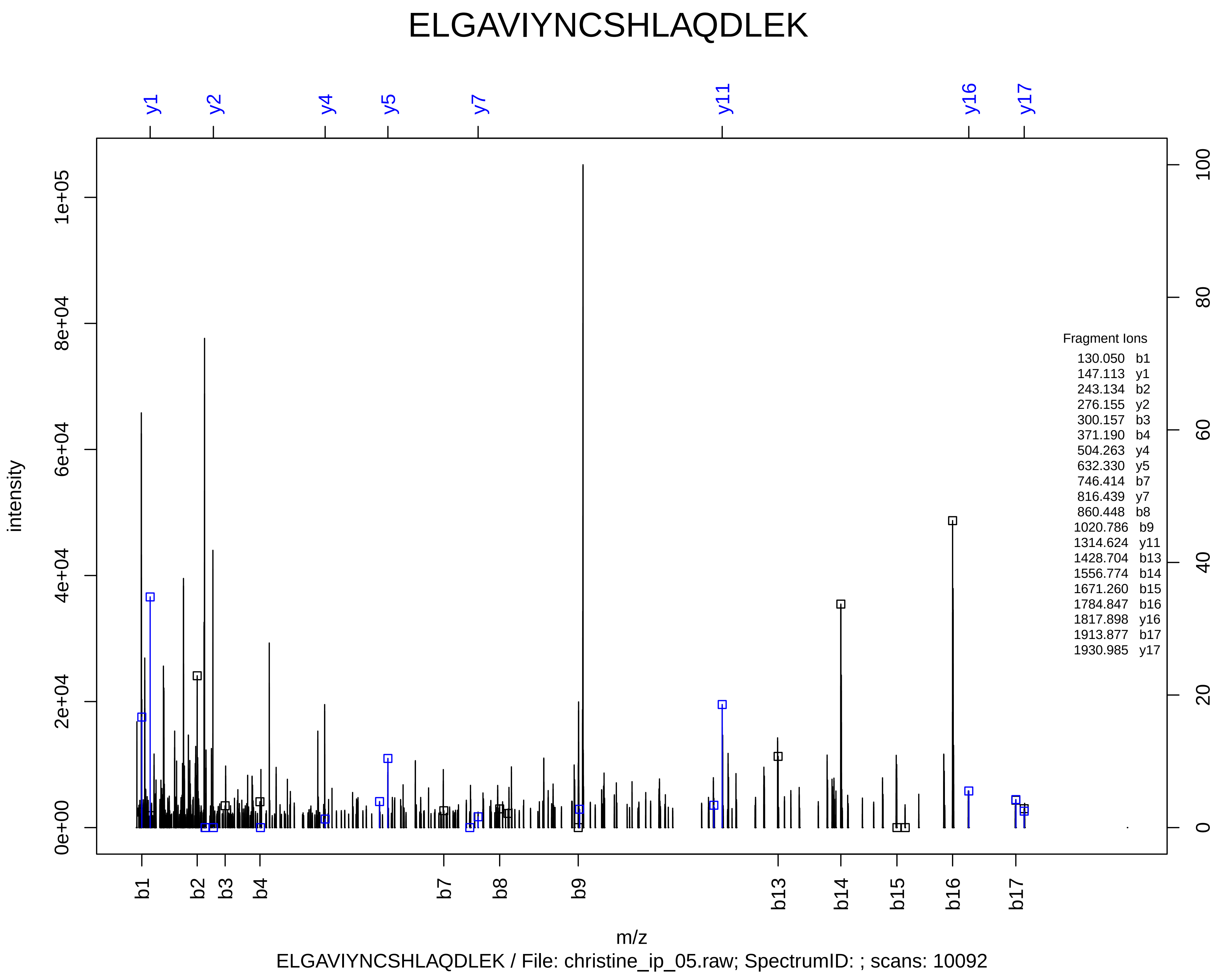
ELGAVIYNCSHLAQDLEK
y1
y2
y4
y5
y7
y11
y16
y17
intensity
0e+00
2e+04
4e+04
6e+04
8e+04
1e+05
0
20
40
60
80
100
Fragment Ions
130.050 b1
147.113 y1
243.134 b2
276.155 y2
300.157 b3
371.190 b4
504.263 y4
632.330 y5
746.414 b7
816.439 y7
860.448 b8
1020.786 b9
1314.624 y11
1428.704 b13
1556.774 b14
1671.260 b15
1784.847 b16
1817.898 y16
1913.877 b17
1930.985 y17
b1
b2
b3
b4
b7
b8
b9
b13
b14
b15
b16
b17
m/z
ELGAVIYNCSHLAQDLEK / File: christine_ip_05.raw; SpectrumID: ; scans: 10092

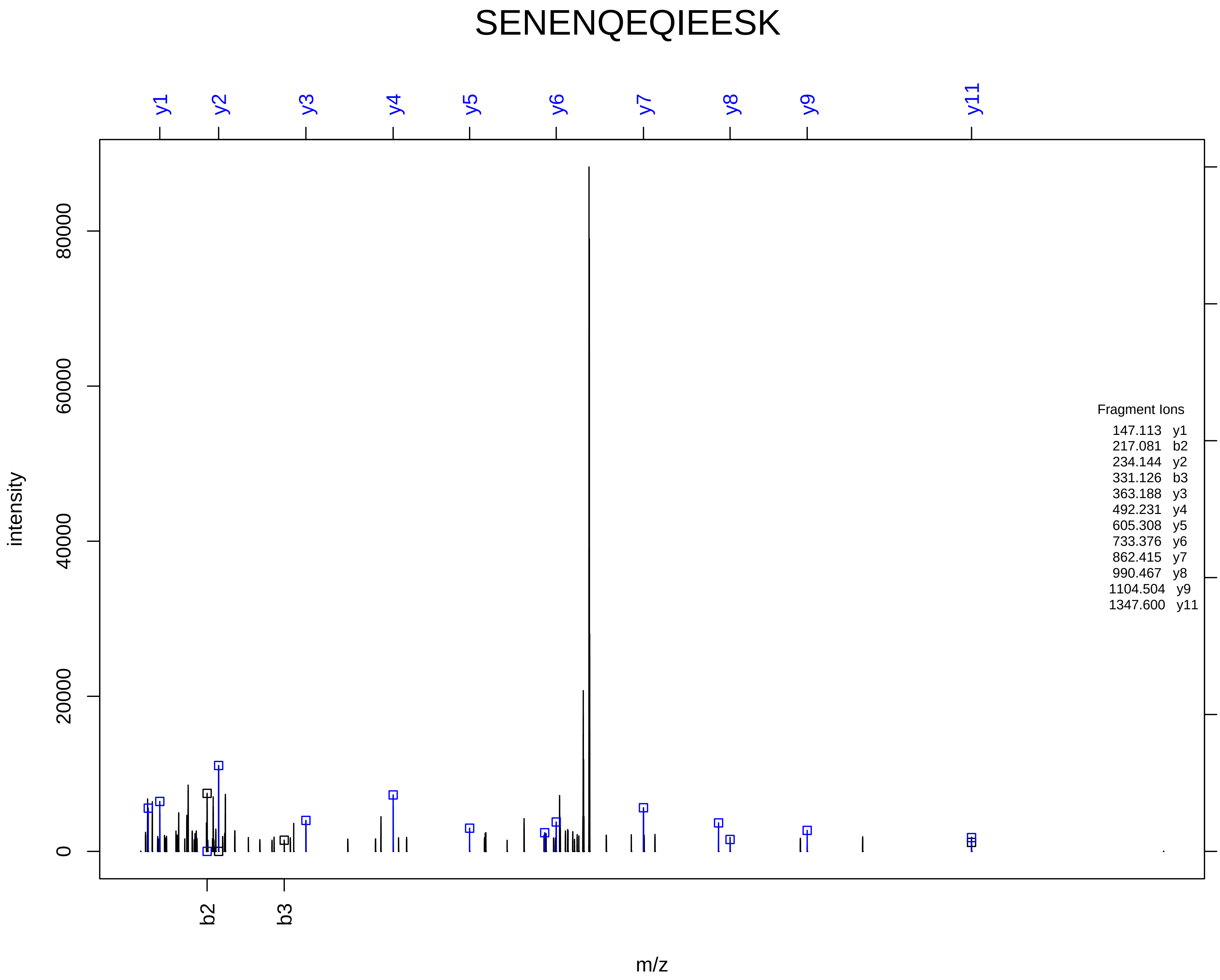
SENENQEQIEESK
y1
y2
y3
y4
y5
y6
y7
y8
y9
y11
intensity
0
20000
40000
60000
80000
0
20
40
60
80
100
b2
b3
m/z
Fragment Ions
147.113 y1
217.081 b2
234.144 y2
331.126 b3
363.188 y3
492.231 y4
605.308 y5
733.376 y6
862.415 y7
990.467 y8
1104.504 y9
1347.600 y11
SENENQEQIEESK / File: christine_ip_05.raw; SpectrumID: ; scans: 1441

IIN^IVEVM*N^R

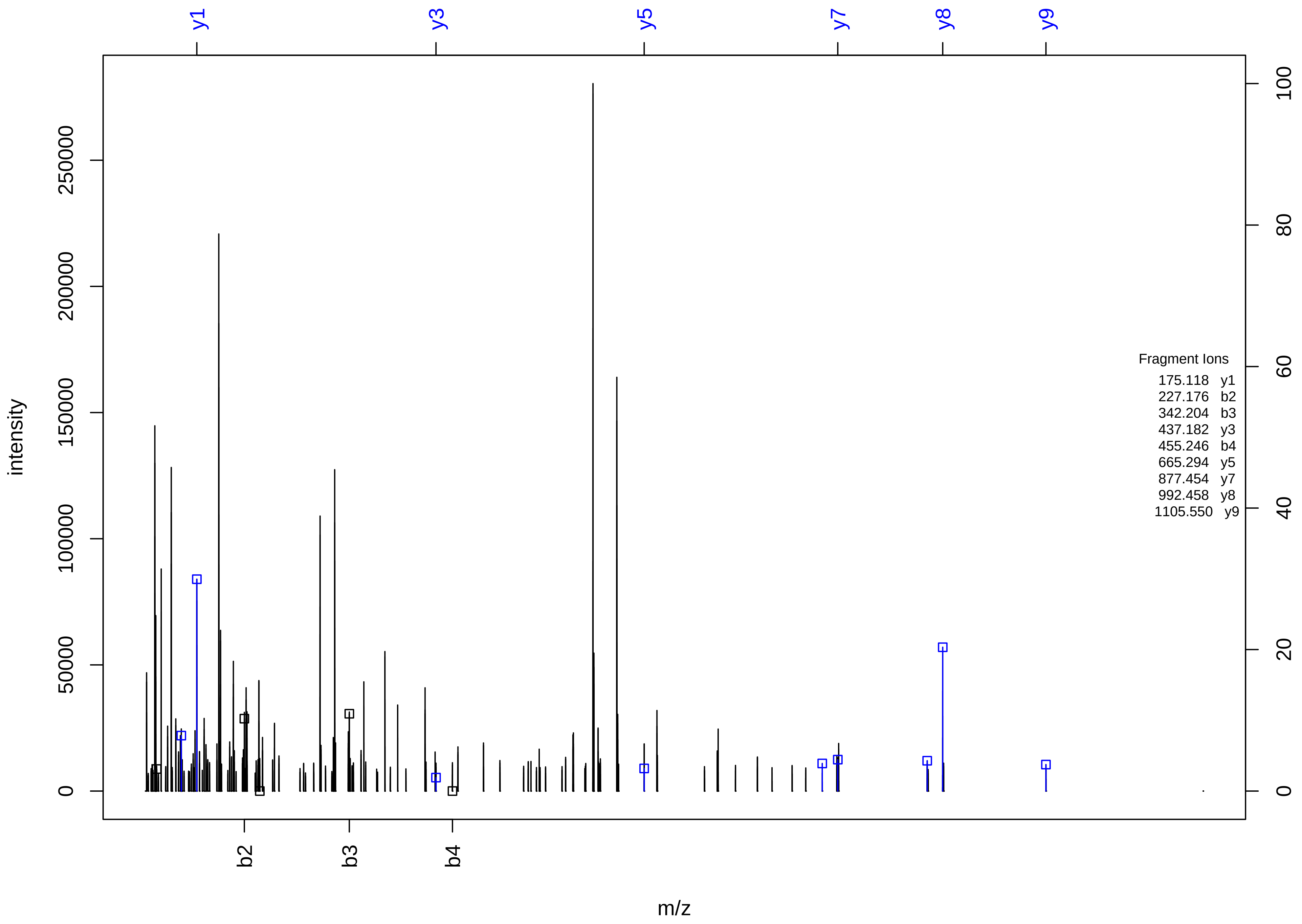

IINIVEVMNR / File: christine_ip_05.raw; SpectrumID: ; scans: 6823

NVLDSEDEIEELSK

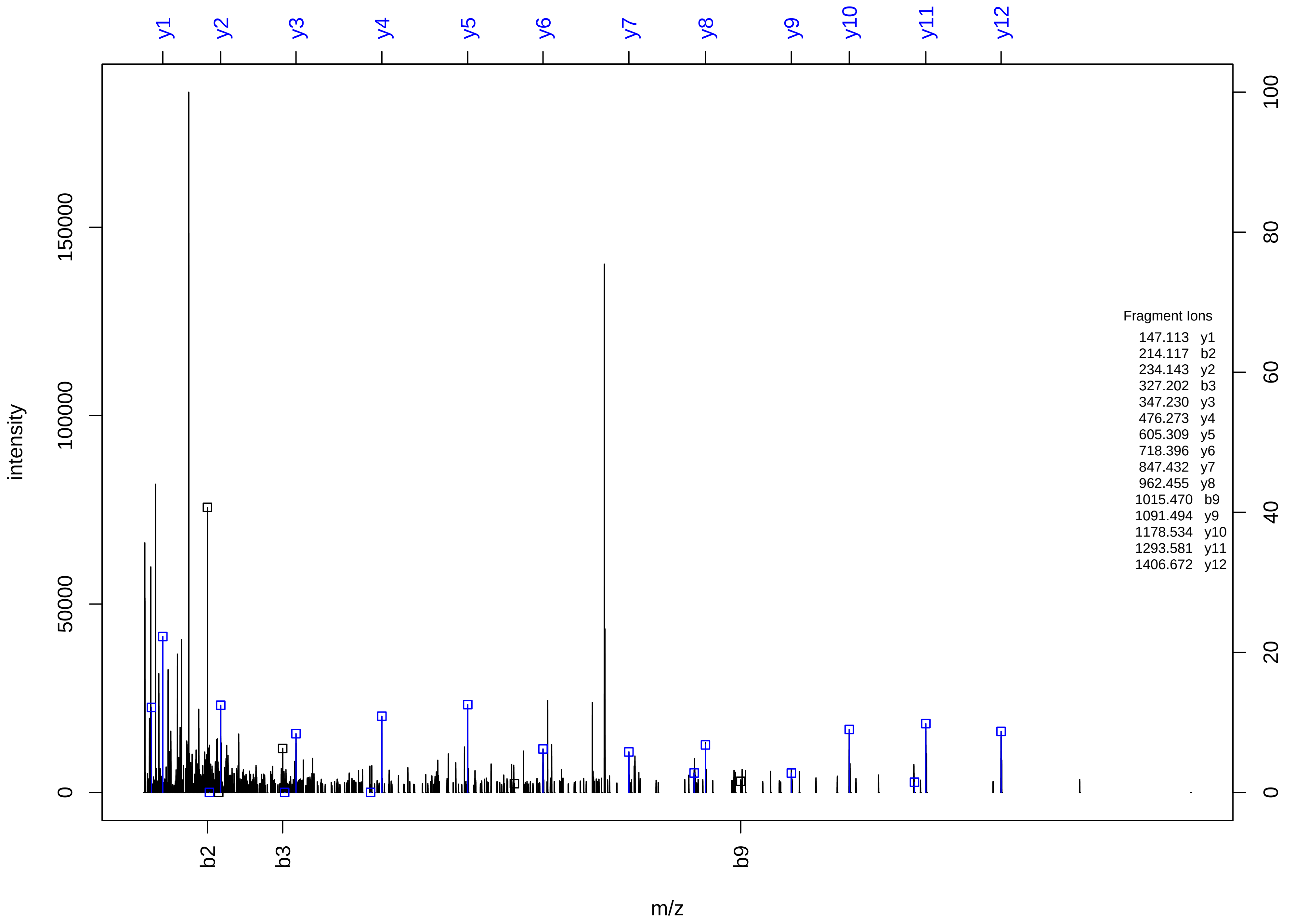

NVLDSEDEIEELSK / File: christine_ip_05.raw; SpectrumID: ; scans: 11365

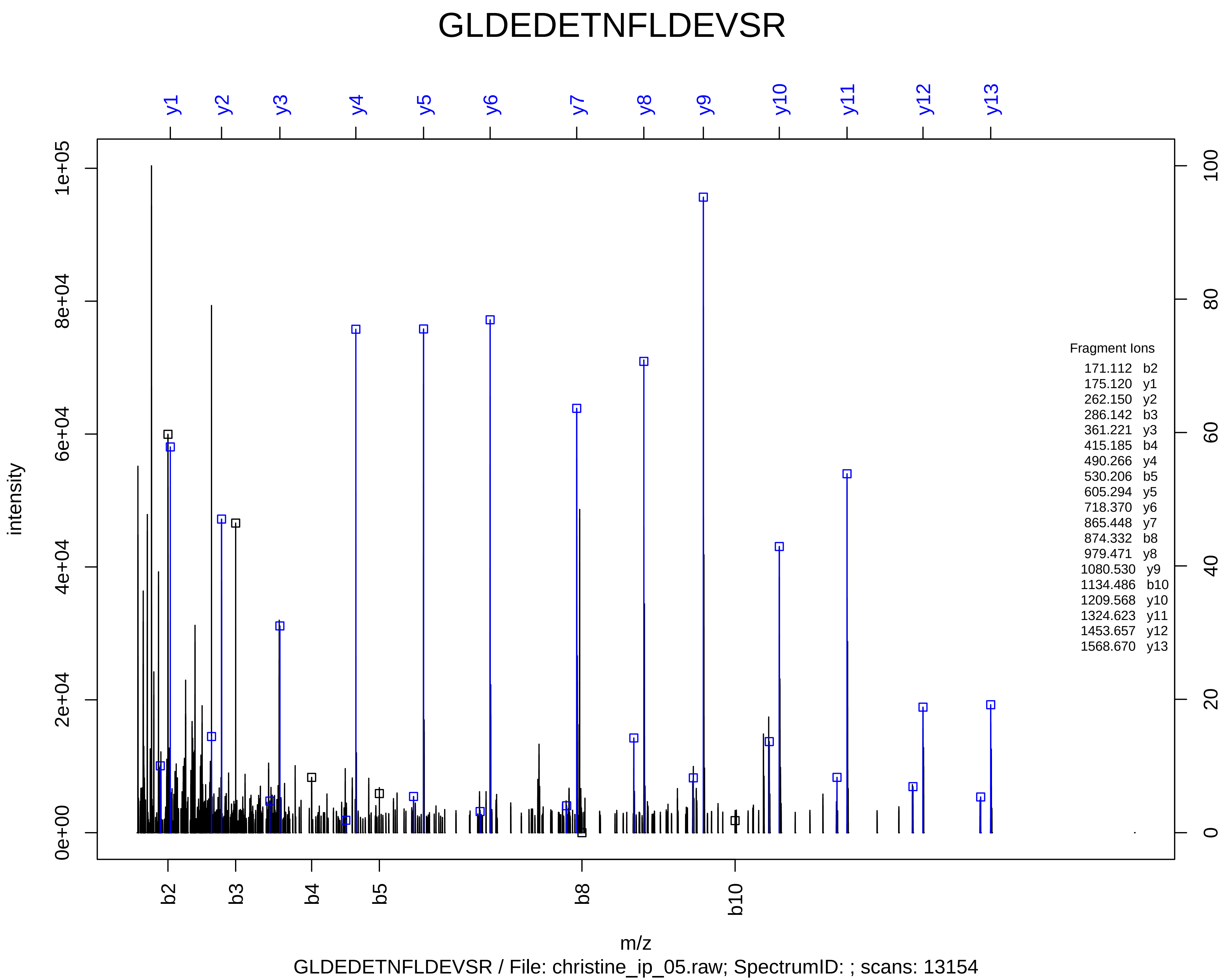
GLDEDETNFLDEVSR
y1
y2
y3
y4
y5
y6
y7
y8
y9
y10
y11
y12
y13
b2
b3
b4
b5
b8
b10
intensity
0e+00
2e+04
4e+04
6e+04
8e+04
1e+05
0
20
40
60
80
100
m/z
Fragment Ions
171.112 b2
175.120 y1
262.150 y2
286.142 b3
361.221 y3
415.185 b4
490.266 y4
530.206 b5
605.294 y5
718.370 y6
865.448 y7
874.332 b8
979.471 y8
1080.530 y9
1134.486 b10
1209.568 y10
1324.623 y11
1453.657 y12
1568.670 y13
GLDEDETNFLDEVSR / File: christine_ip_05.raw; SpectrumID: ; scans: 13154

ELQSQIQEAR

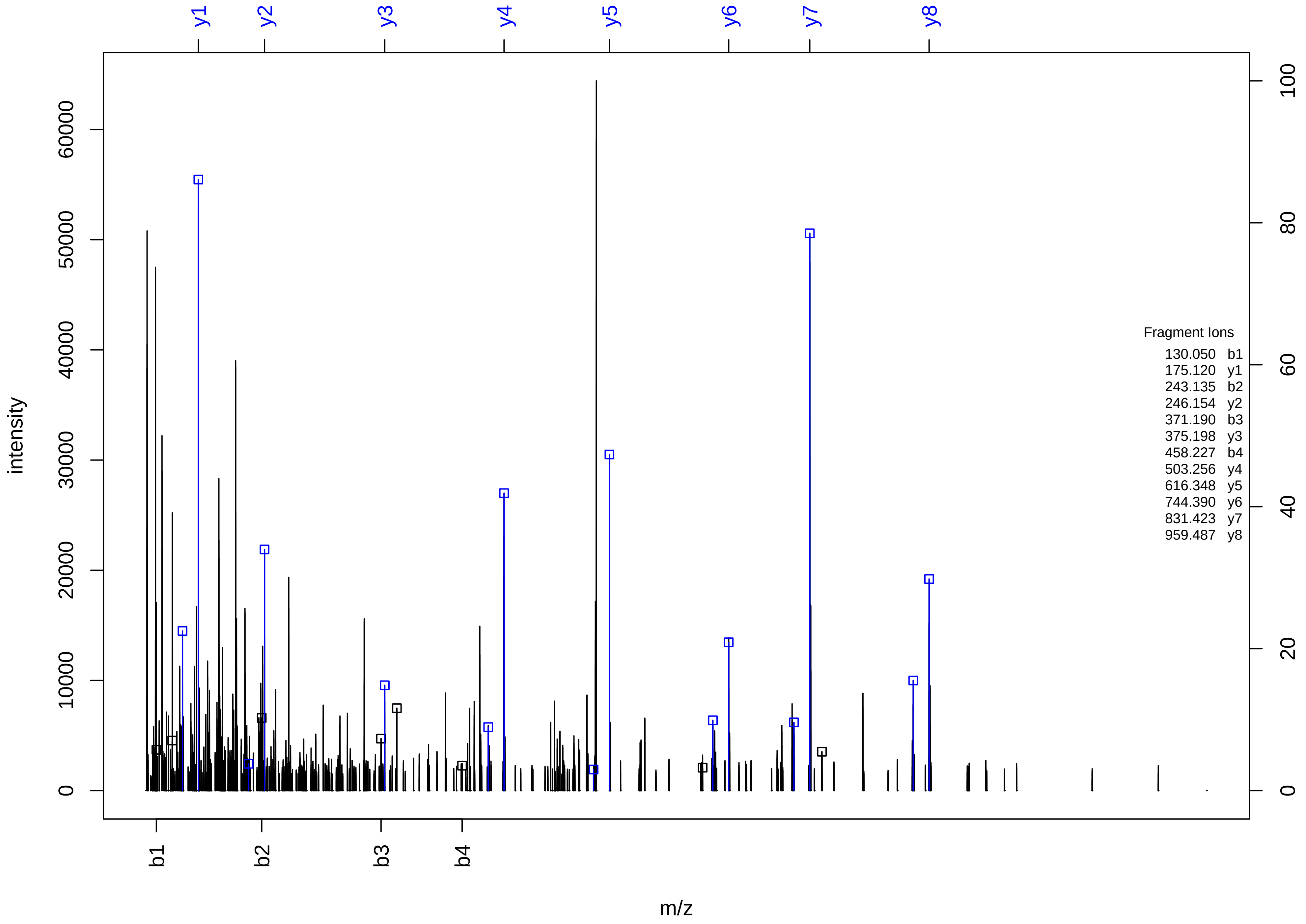

ELQSQIQEAR / File: christine_ip_05.raw; SpectrumID: ; scans: 3218

GVTNDQVDPSVDVLK

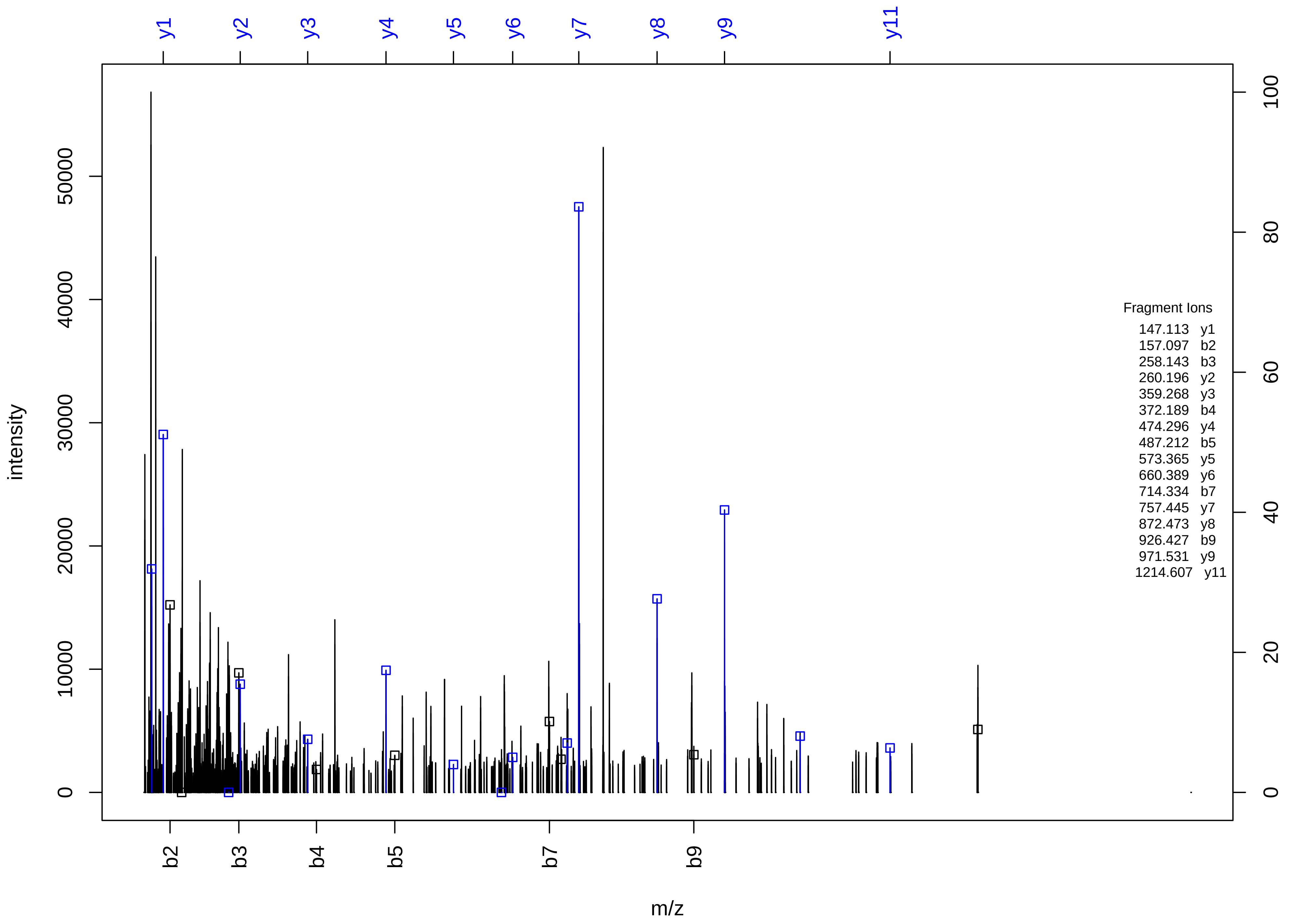

GVTNDQVDPSVDVLK / File: christine_ip_05.raw; SpectrumID: ; scans: 9181

LFTESCSISPK

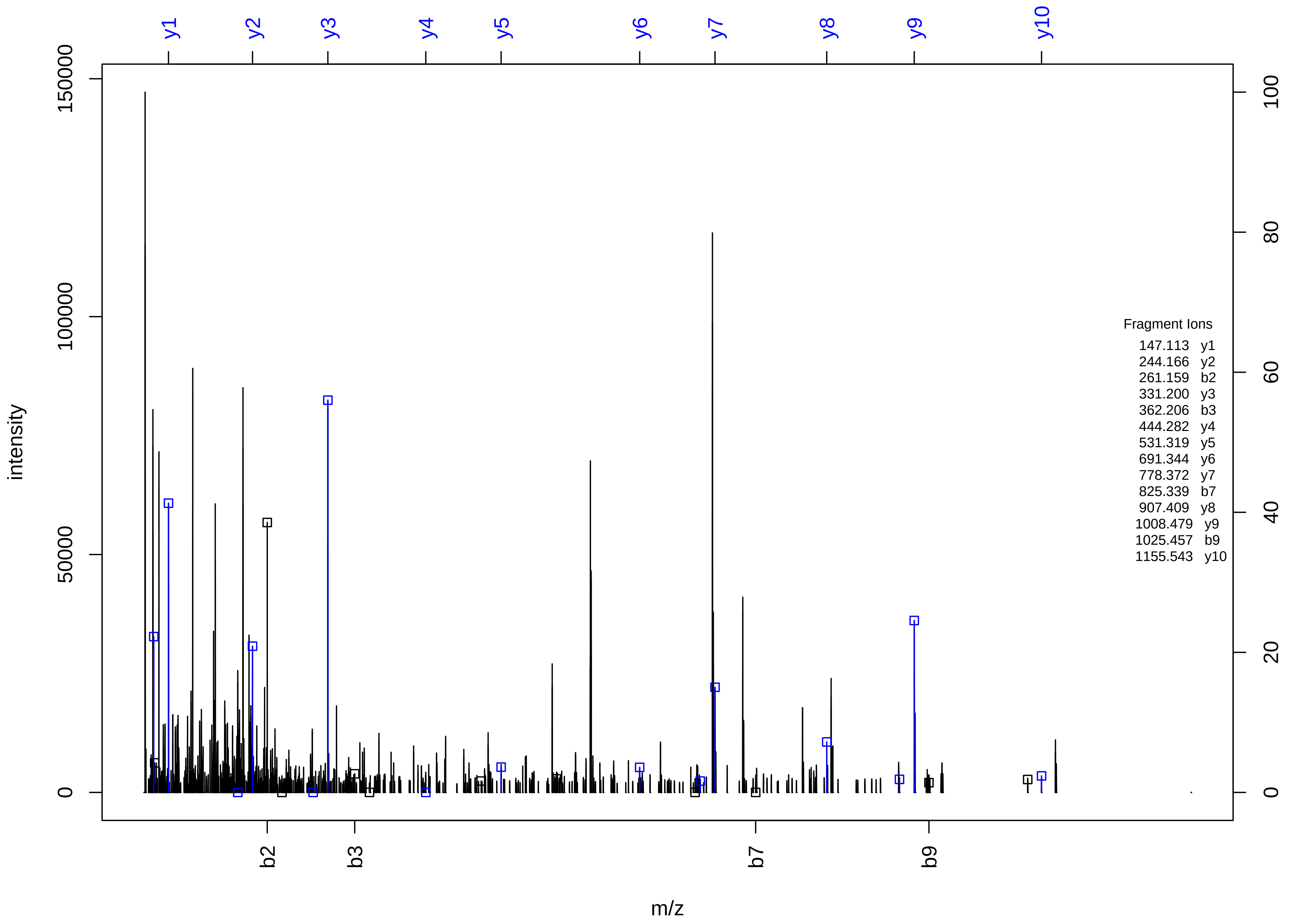

LFTESCSISPK / File: christine_ip_05.raw; SpectrumID: ; scans: 6627
