# Supplementary material for: Proximity Labeling Proteomics Reveals Kv1.3 Potassium Channel Immune Interactors in Microglia
Source: Mol Cell Proteomics. 2024 Jun 25;23(8):100809. doi: 10.1016/j.mcpro.2024.100809 (PMC11780389; doi:10.1016/j.mcpro.2024.100809)
Supplement: OHW_HEK_Lysate [file mmc8.pdf]

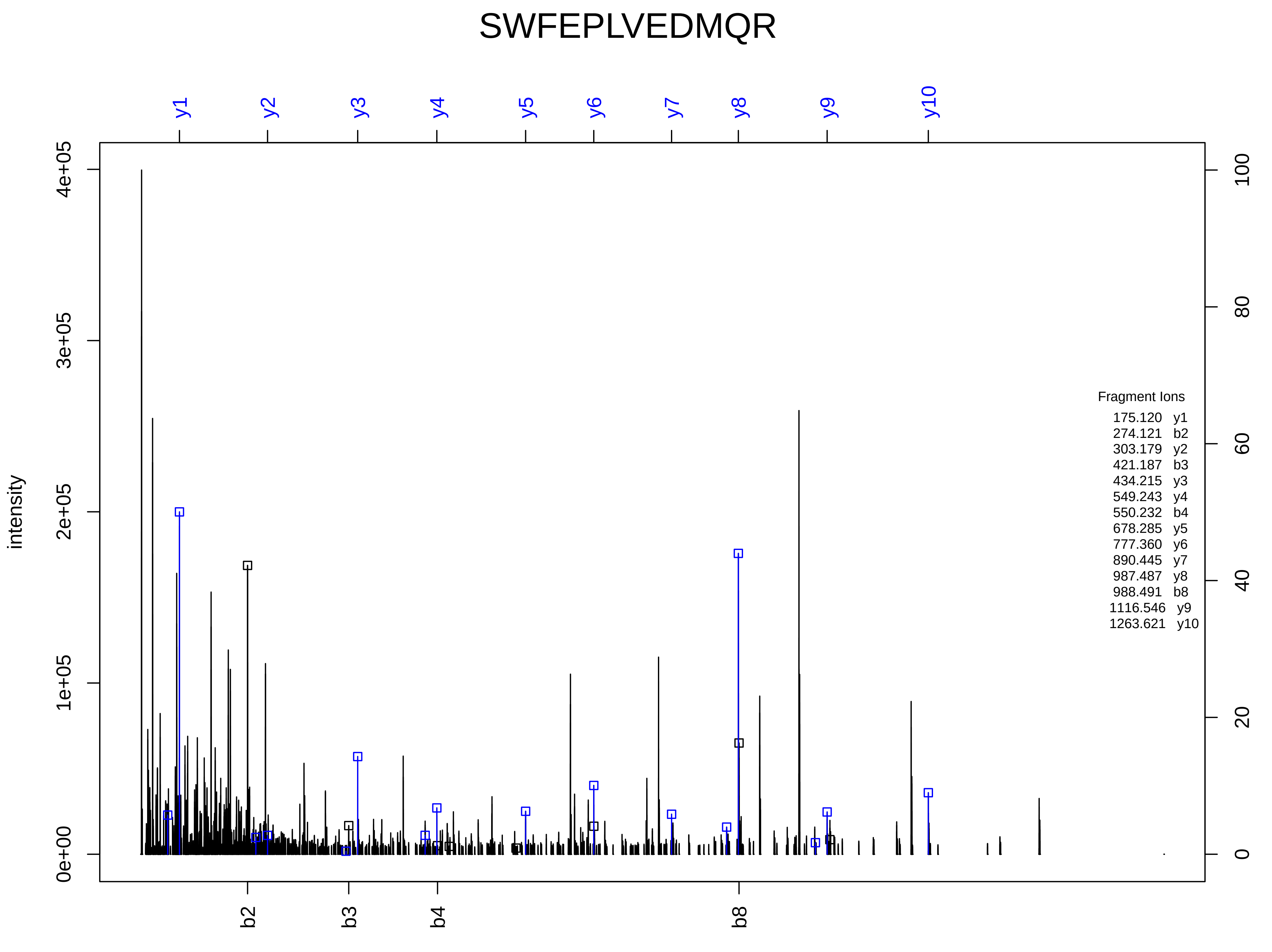

SWFEPLVEDMQR / File: christine_pool_04.raw; SpectrumID: ; scans: 19334

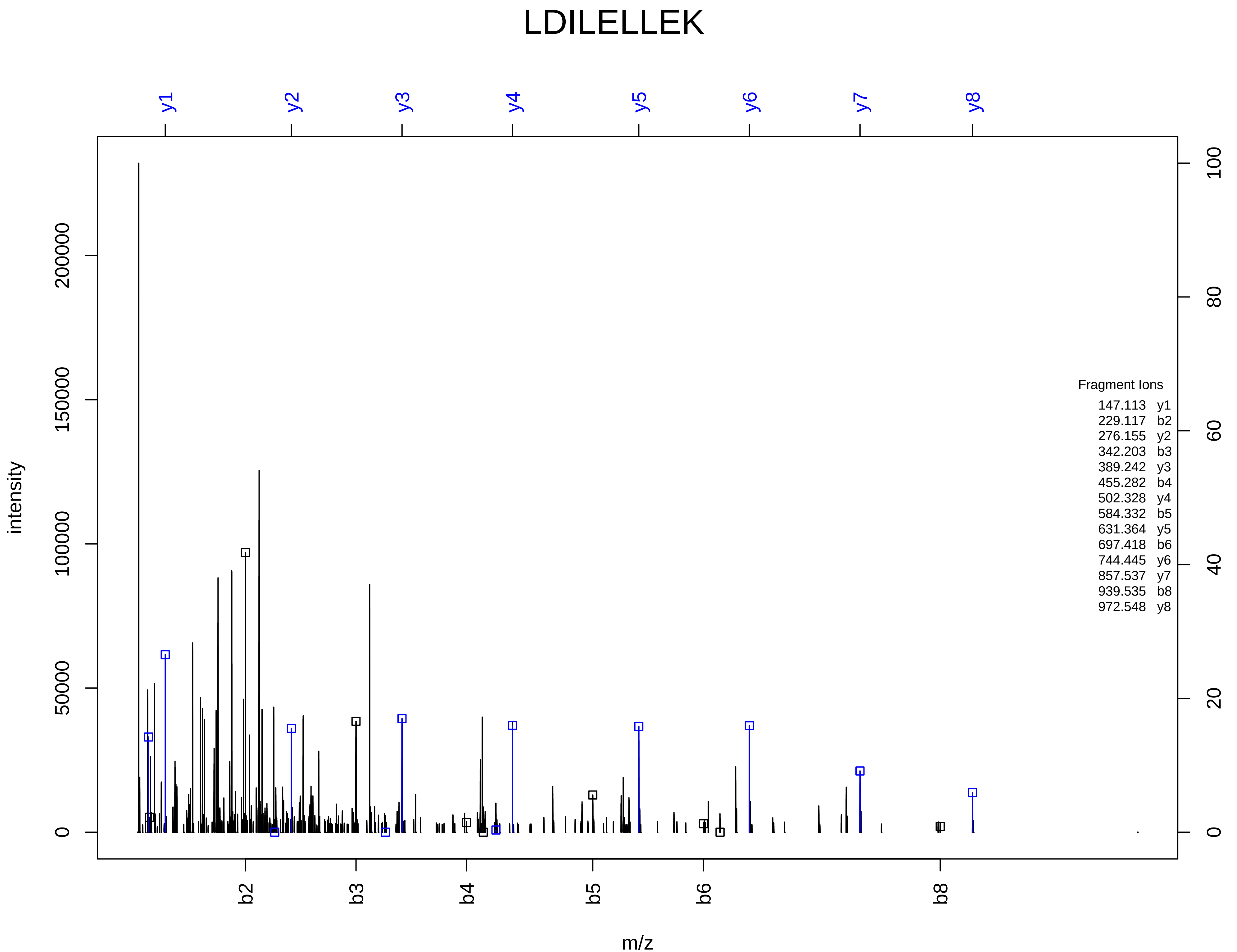
LDILELLEK
y1
y2
y3
y4
y5
y6
y7
y8
intensity
0
50000
100000
150000
200000
0
20
40
60
80
100
Fragment Ions
147.113 y1
229.117 b2
276.155 y2
342.203 b3
389.242 y3
455.282 b4
502.328 y4
584.332 b5
631.364 y5
697.418 b6
744.445 y6
857.537 y7
939.535 b8
972.548 y8
b2
b3
b4
b5
b6
b8
m/z
LDILELLEK / File: christine_pool_04.raw; SpectrumID: ; scans: 22934

YDGCCEGDVVQCIR

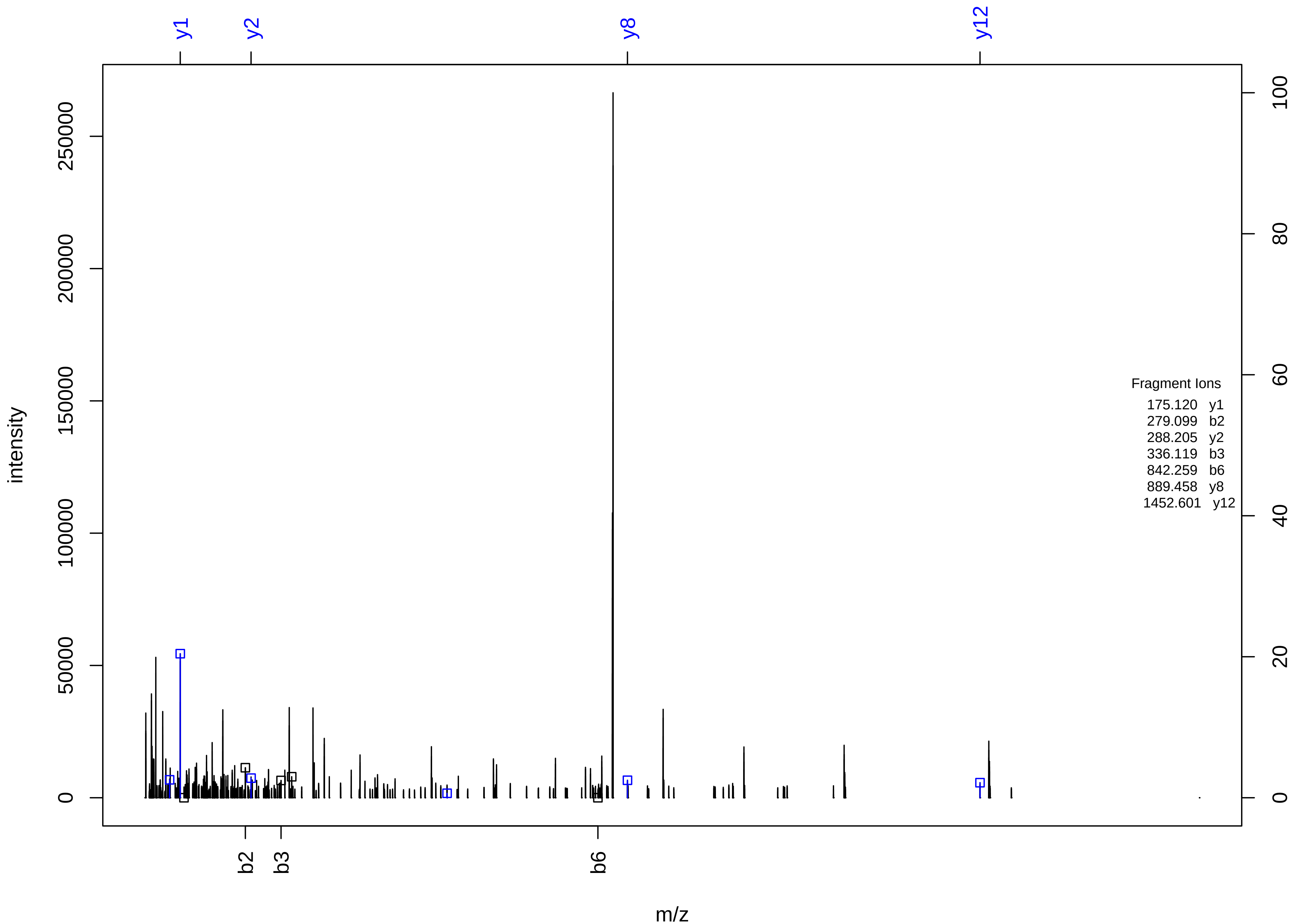

YDGCCEGDVVQCIR / File: christine_pool_04.raw; SpectrumID: ; scans: 7385

LFCDFGDEFEVLDTTGEEPK

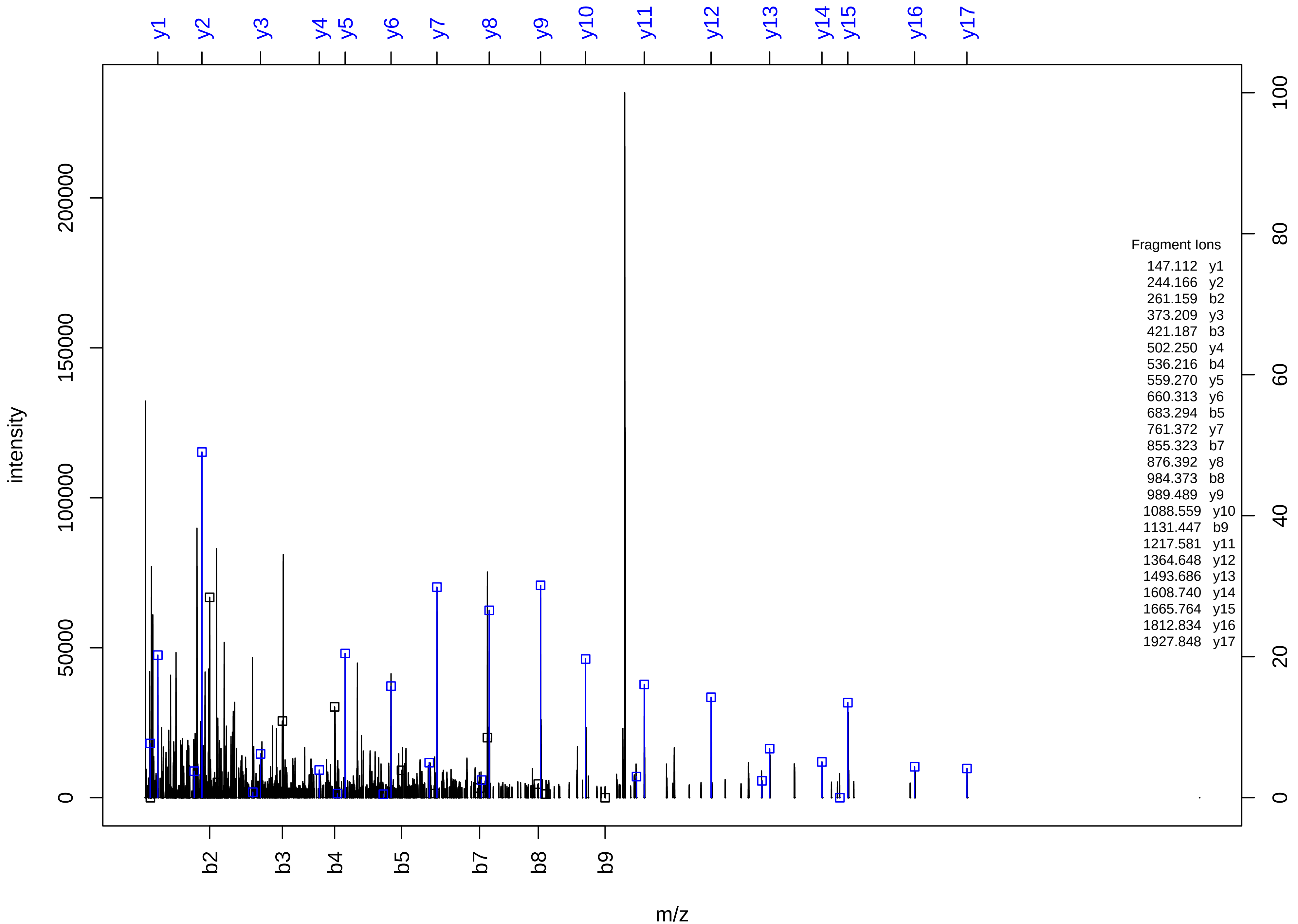

LFCDFGDEFEVLDTTGEEPK / File: christine_pool_04.raw; SpectrumID: ; scans: 21230

ILAIELENLK

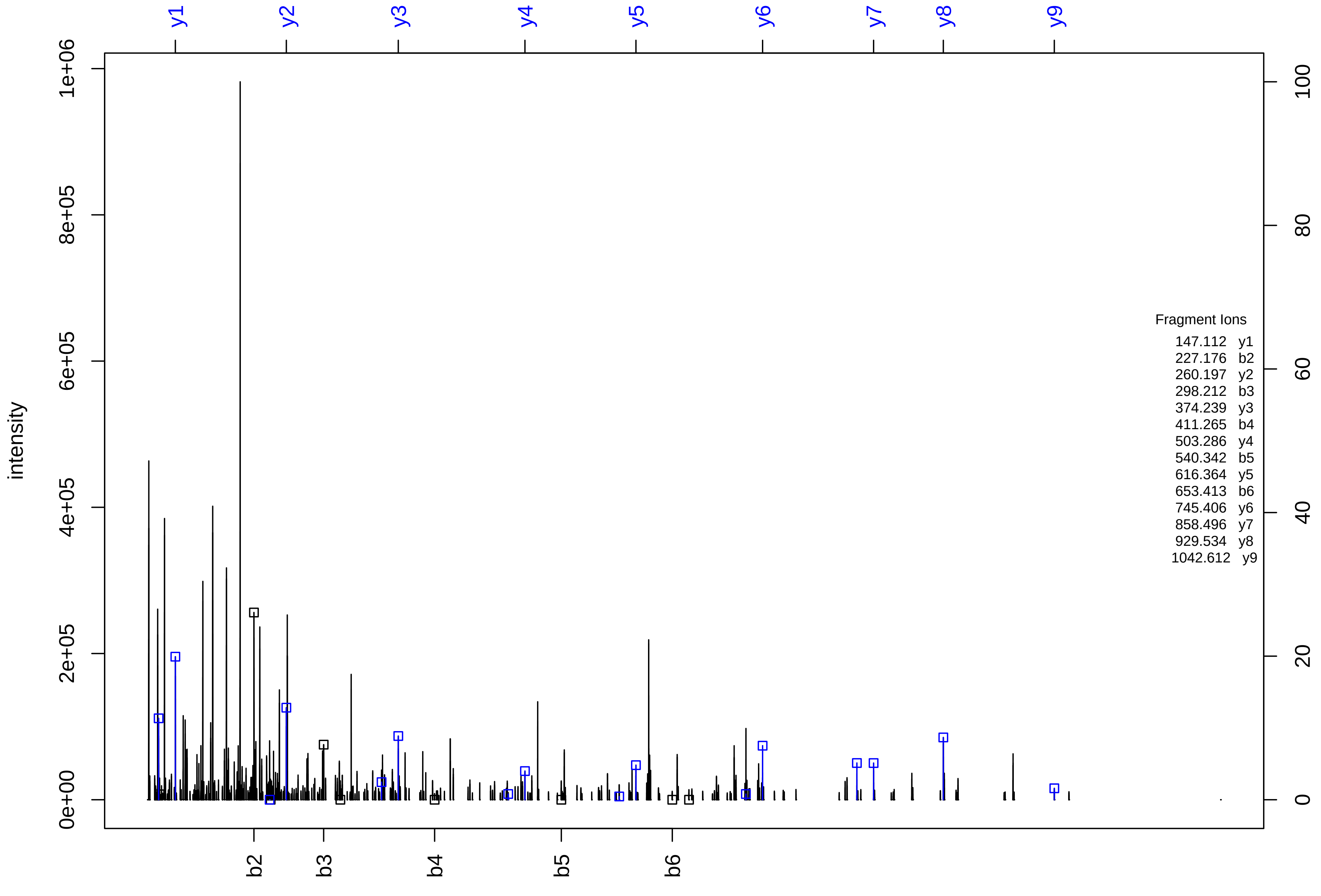

ILAIELENLK / File: christine_pool_04.raw; SpectrumID: ; scans: 16090

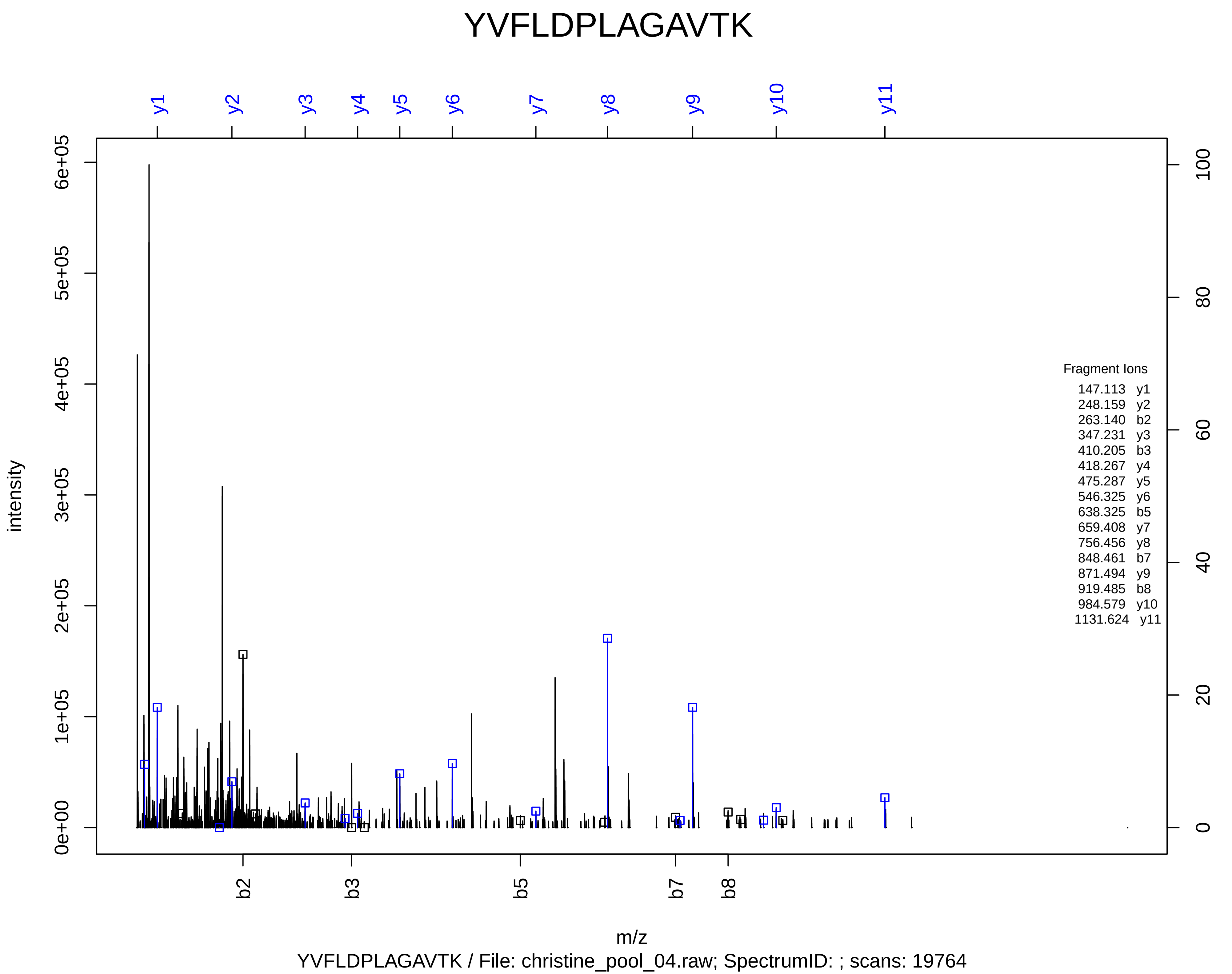

YVFLDPLAGAVTK
y1
y2
y3
y4
y5
y6
y7
y8
y9
y10
y11
intensity
0e+00
1e+05
2e+05
3e+05
4e+05
5e+05
6e+05
0
20
40
60
80
100
Fragment Ions
147.113 y1
248.159 y2
263.140 b2
347.231 y3
410.205 b3
418.267 y4
475.287 y5
546.325 y6
638.325 b5
659.408 y7
756.456 y8
848.461 b7
871.494 y9
919.485 b8
984.579 y10
1131.624 y11
b2
b3
b5
b7
b8
m/z
YVFLDPLAGAVTK / File: christine_pool_04.raw; SpectrumID: ; scans: 19764

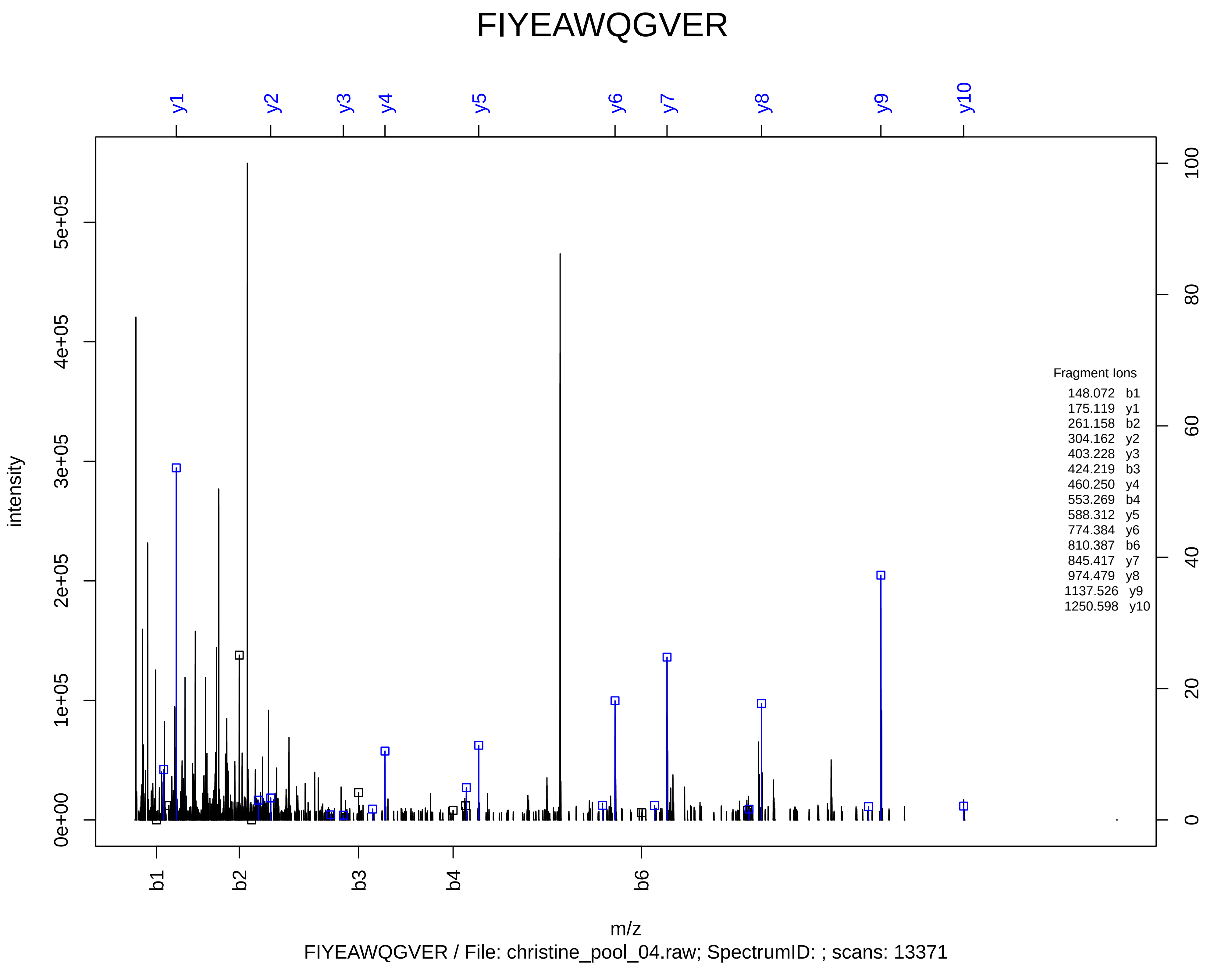
FIYEAWQGVER
y1
y2
y3
y4
y5
y6
y7
y8
y9
y10
intensity
0e+00
1e+05
2e+05
3e+05
4e+05
5e+05
0
20
40
60
80
100
b1
b2
b3
b4
b6
m/z
Fragment Ions
148.072 b1
175.119 y1
261.158 b2
304.162 y2
403.228 y3
424.219 b3
460.250 y4
553.269 b4
588.312 y5
774.384 y6
810.387 b6
845.417 y7
974.479 y8
1137.526 y9
1250.598 y10
FIYEAWQGVER / File: christine_pool_04.raw; SpectrumID: ; scans: 13371

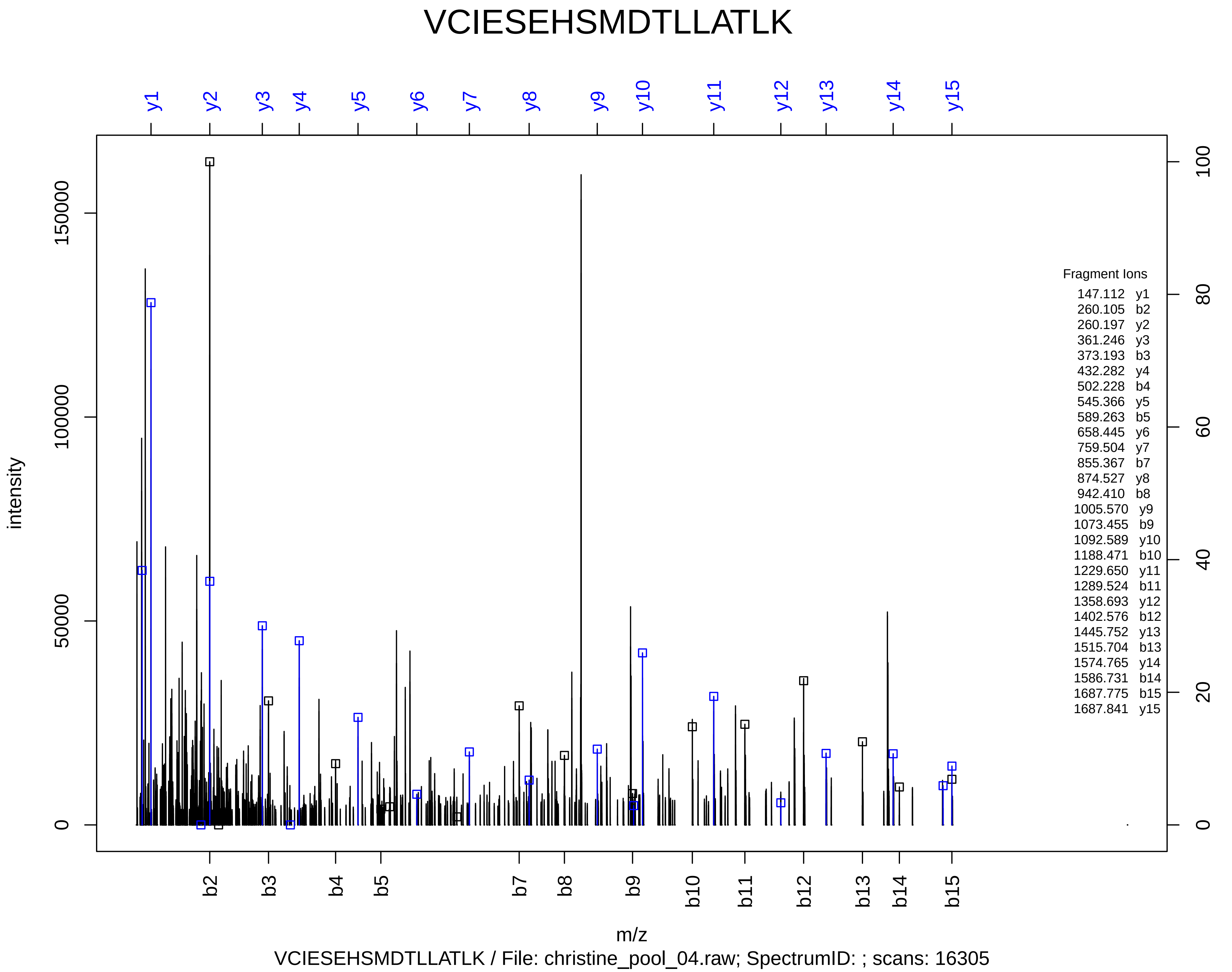
VCIESEHSMDTLLATLK
y1 y2 y3 y4 y5 y6 y7 y8 y9 y10 y11 y12 y13 y14 y15
b2 b3 b4 b5 b7 b8 b9 b10 b11 b12 b13 b14 b15
intensity
0
50000
100000
150000
0
20
40
60
80
100
m/z
Fragment Ions
147.112 y1
260.105 b2
260.197 y2
361.246 y3
373.193 b3
432.282 y4
502.228 b4
545.366 y5
589.263 b5
658.445 y6
759.504 y7
855.367 b7
874.527 y8
942.410 b8
1005.570 y9
1073.455 b9
1092.589 y10
1188.471 b10
1229.650 y11
1289.524 b11
1358.693 y12
1402.576 b12
1445.752 y13
1515.704 b13
1574.765 y14
1586.731 b14
1687.775 b15
1687.841 y15
VCIESEHSMDTLLATLK / File: christine_pool_04.raw; SpectrumID: ; scans: 16305

FQIGDYLDIAITPPNR

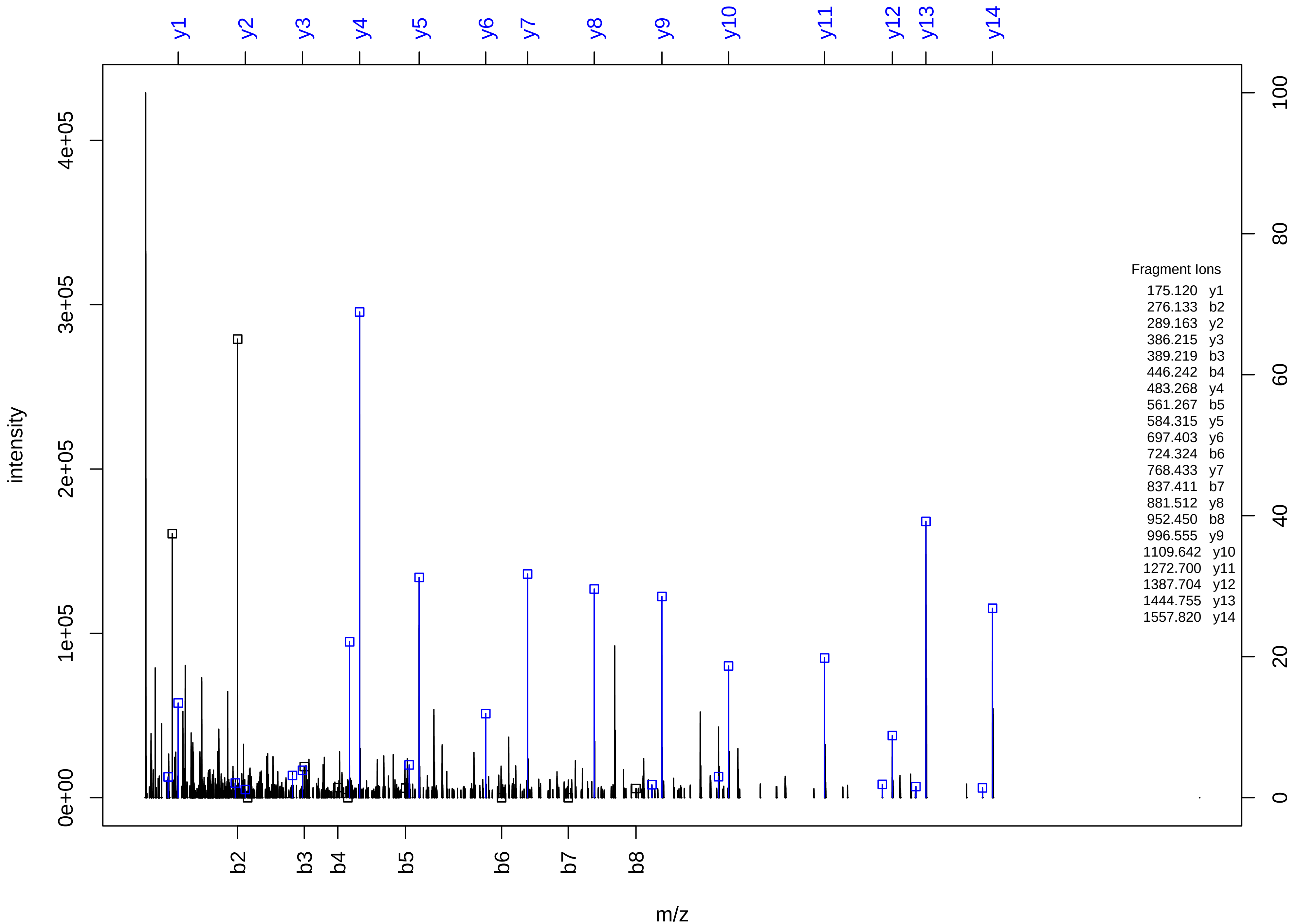

FQIGDYLDIAITPPNR / File: christine_pool_04.raw; SpectrumID: ; scans: 22388

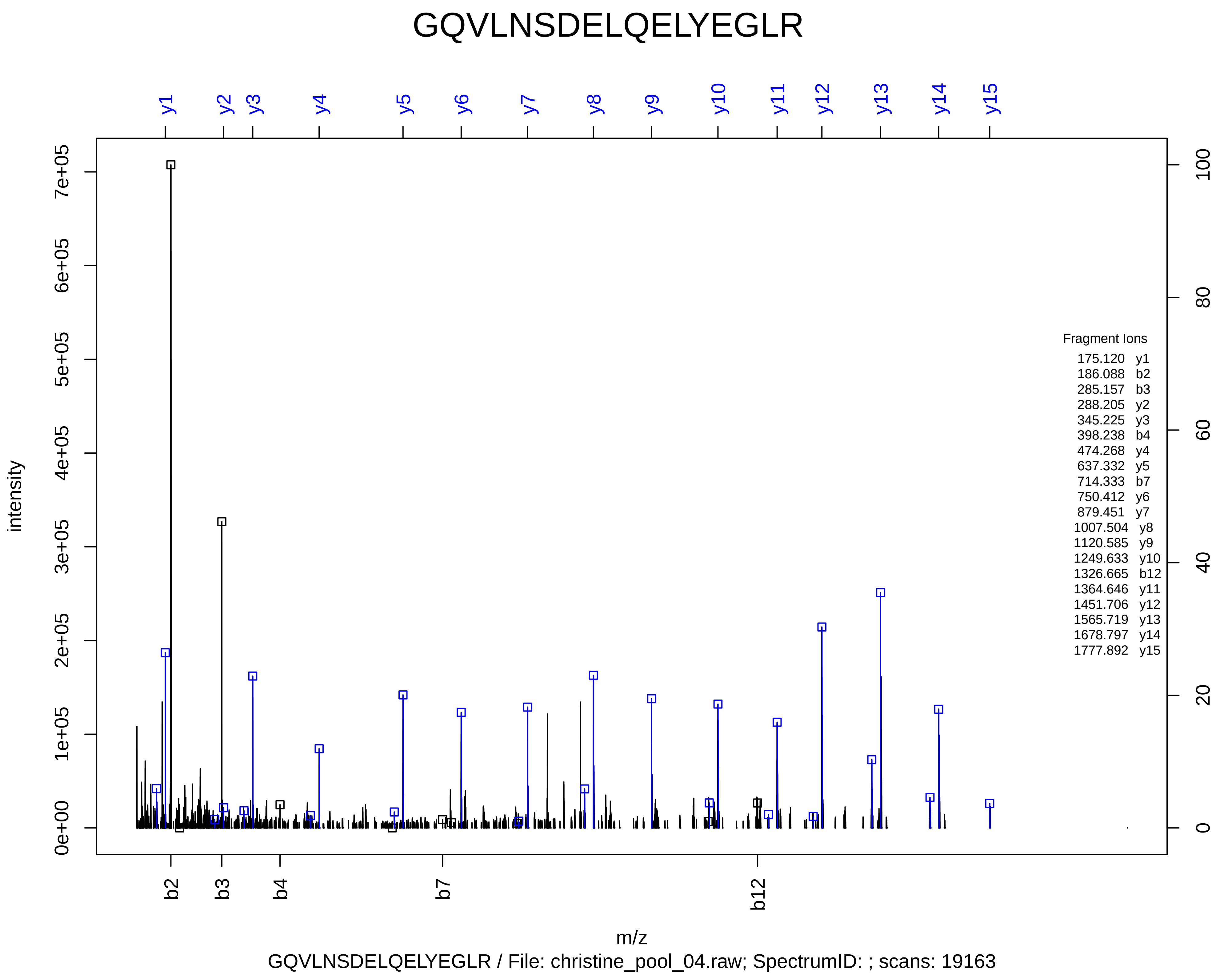
GQVLNSDELQELYEGLR
y1 y2 y3 y4 y5 y6 y7 y8 y9 y10 y11 y12 y13 y14 y15
b2 b3 b4 b7 b12
intensity
m/z
Fragment Ions
175.120 y1
186.088 b2
285.157 b3
288.205 y2
345.225 y3
398.238 b4
474.268 y4
637.332 y5
714.333 b7
750.412 y6
879.451 y7
1007.504 y8
1120.585 y9
1249.633 y10
1326.665 b12
1364.646 y11
1451.706 y12
1565.719 y13
1678.797 y14
1777.892 y15
GQVLNSDELQELYEGLR / File: christine_pool_04.raw; SpectrumID: ; scans: 19163

HVTTAEGTPGTTDQEGPPPDGPPEK

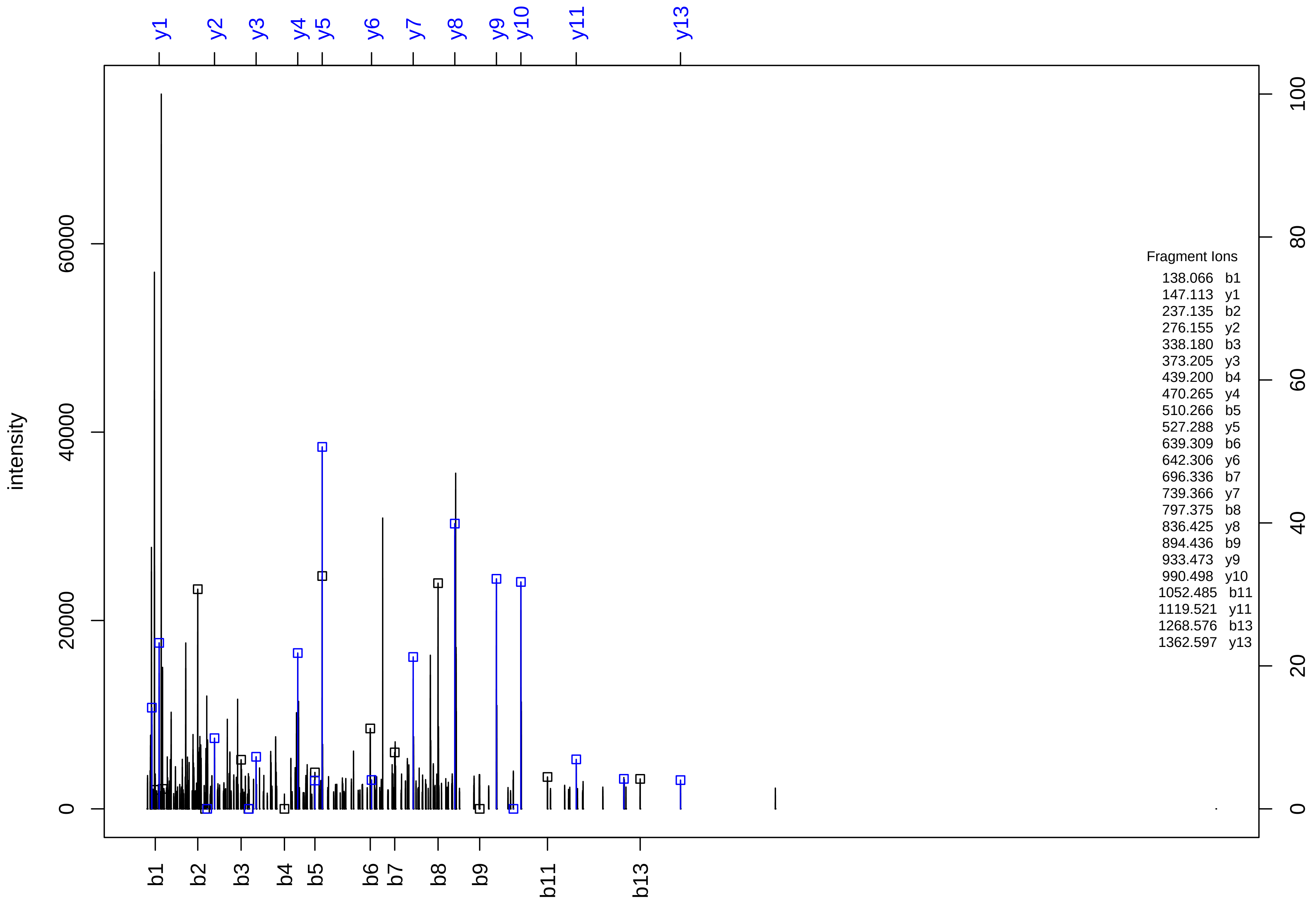

HVTTAEGTPGTTDQEGPPPDGPPEK / File: christine_pool_04.raw; SpectrumID: ; scans: 3962

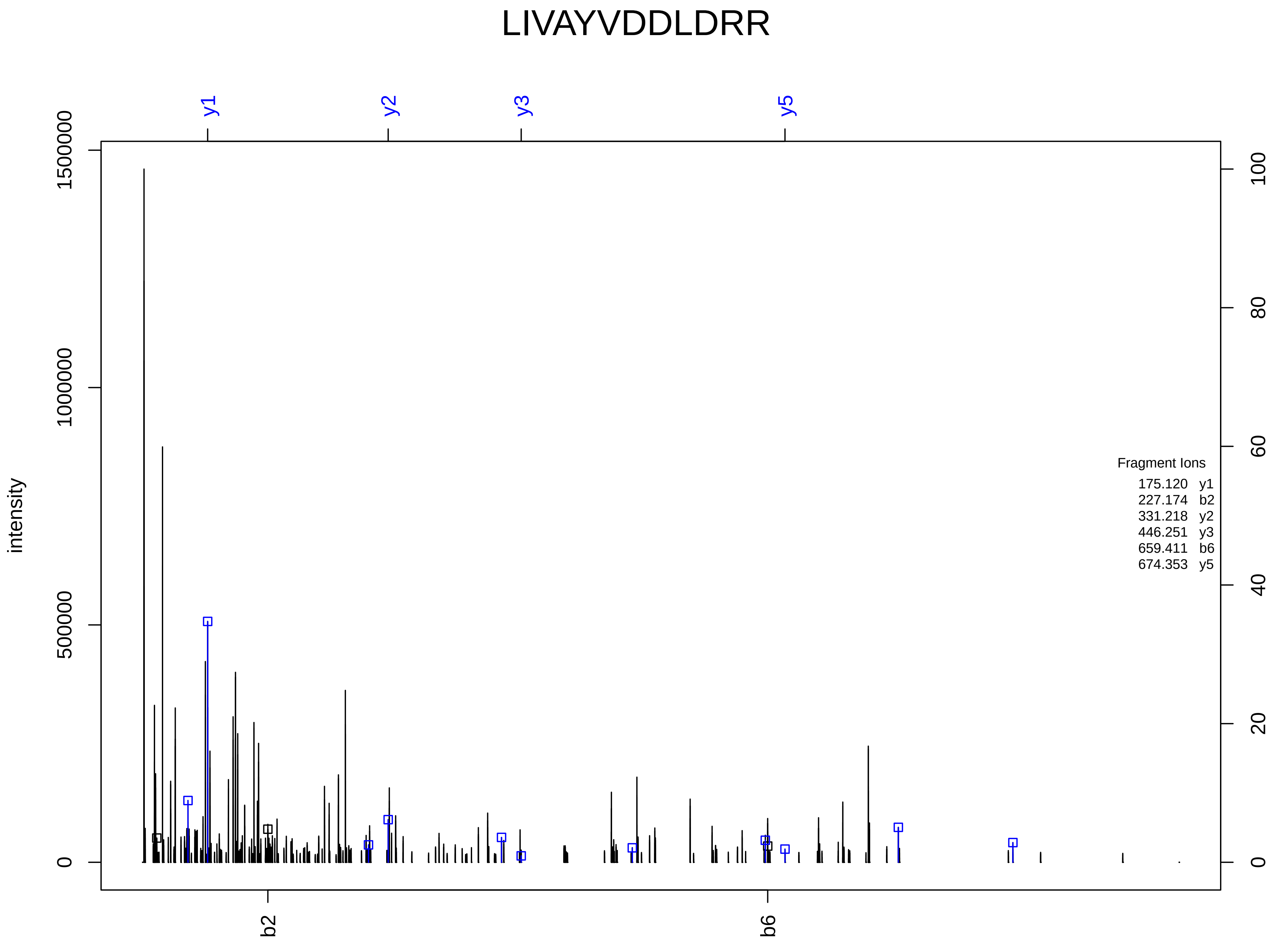

LIVAYVDDLDRR / File: christine_pool_04.raw; SpectrumID: ; scans: 12074

YLVQDTDEFILPTGANK

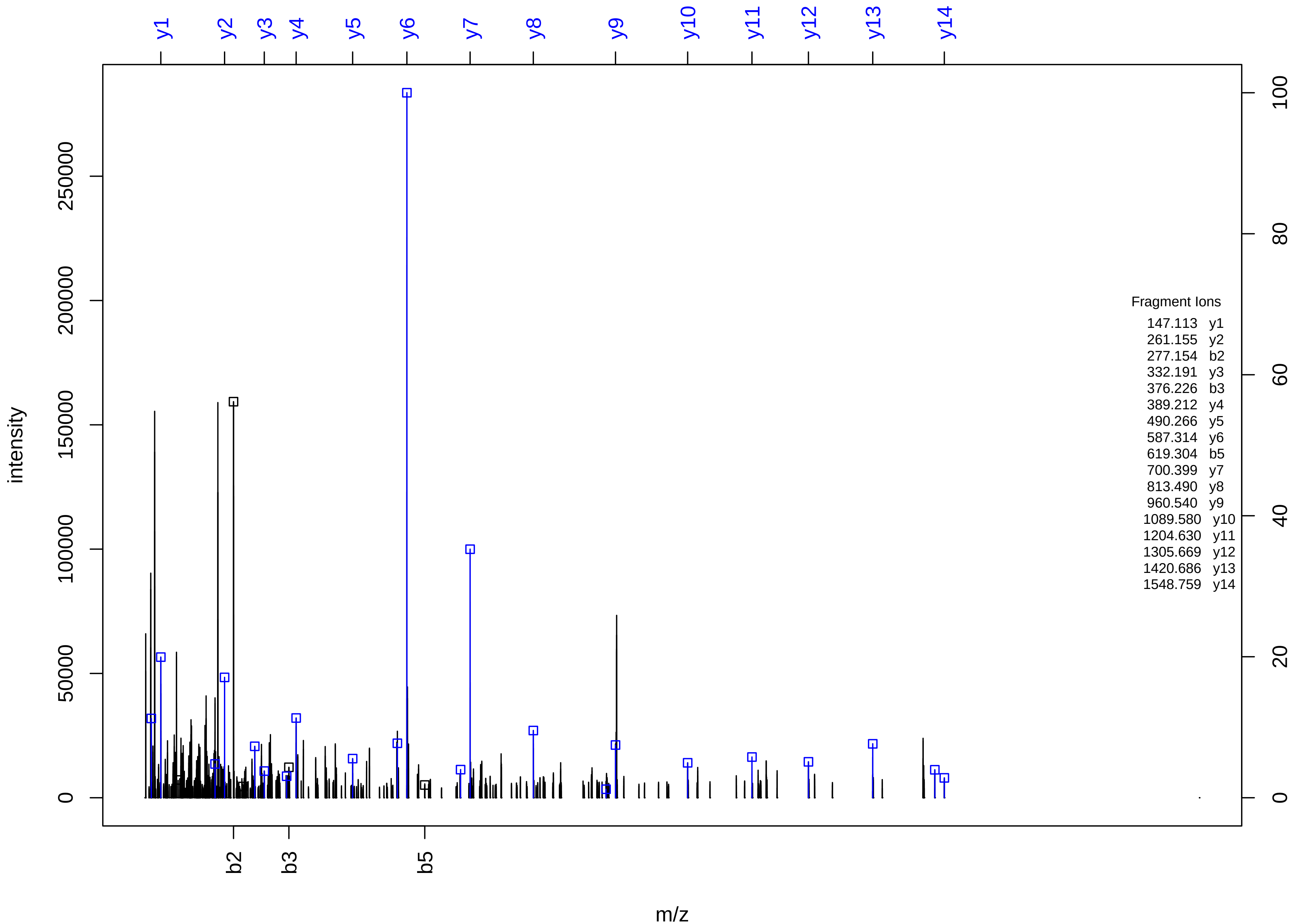

YLVQDTDEFILPTGANK / File: christine_pool_04.raw; SpectrumID: ; scans: 16576

HVISYSLSPFEQR

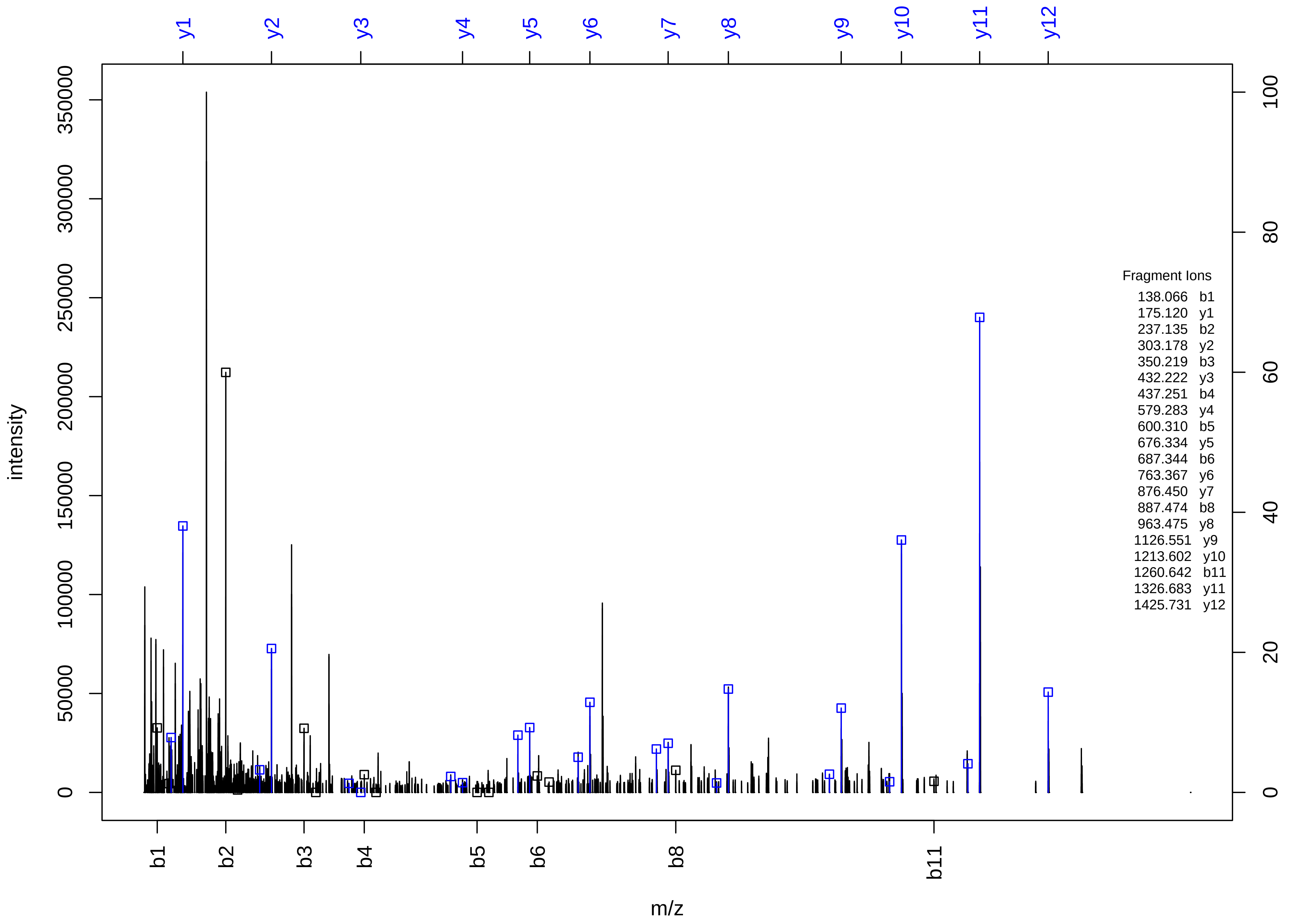

HVISYSLSPFEQR / File: christine_pool_04.raw; SpectrumID: ; scans: 10934

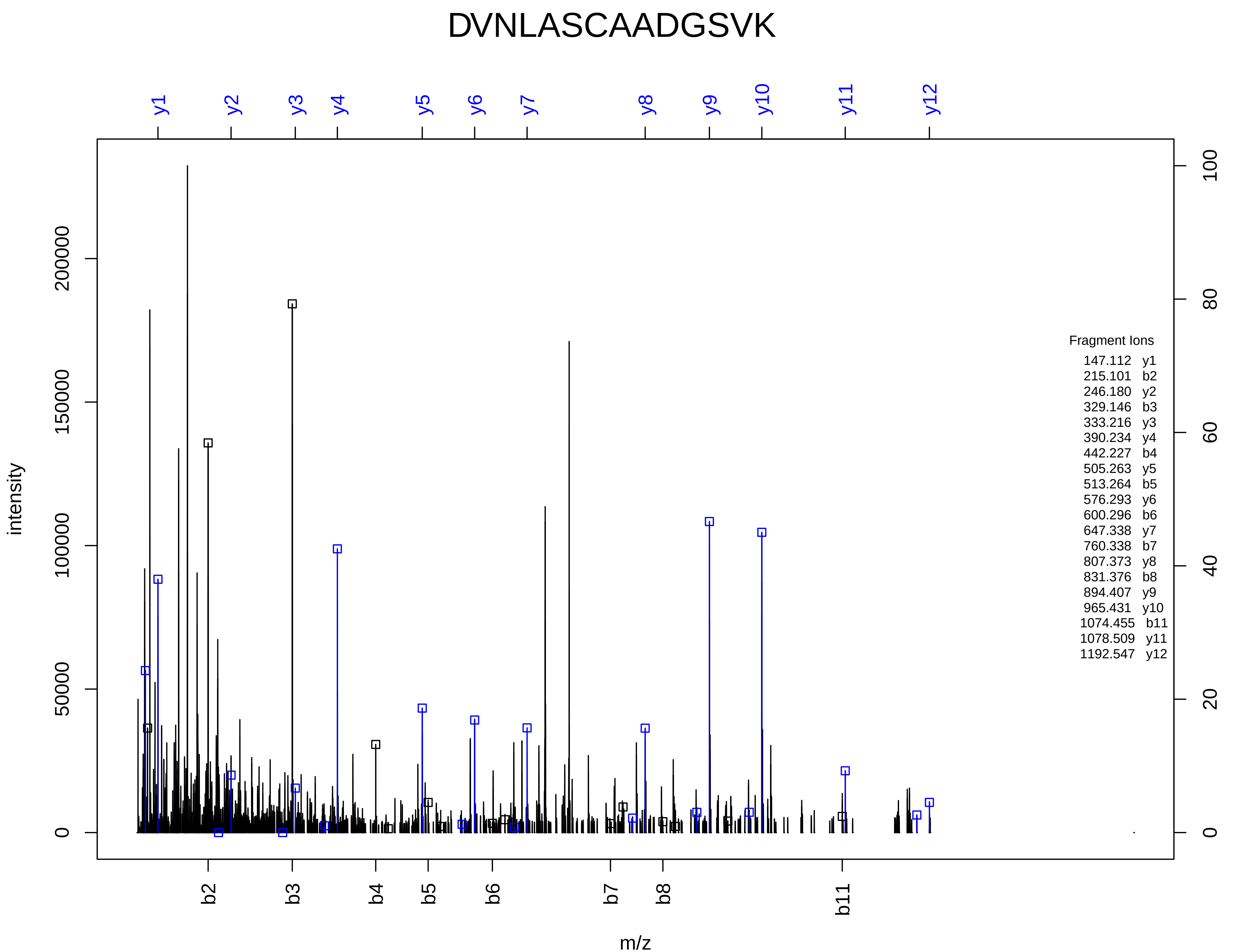

DVNLASCAADGSVK / File: christine_pool_04.raw; SpectrumID: ; scans: 6580

LIEDFLAR

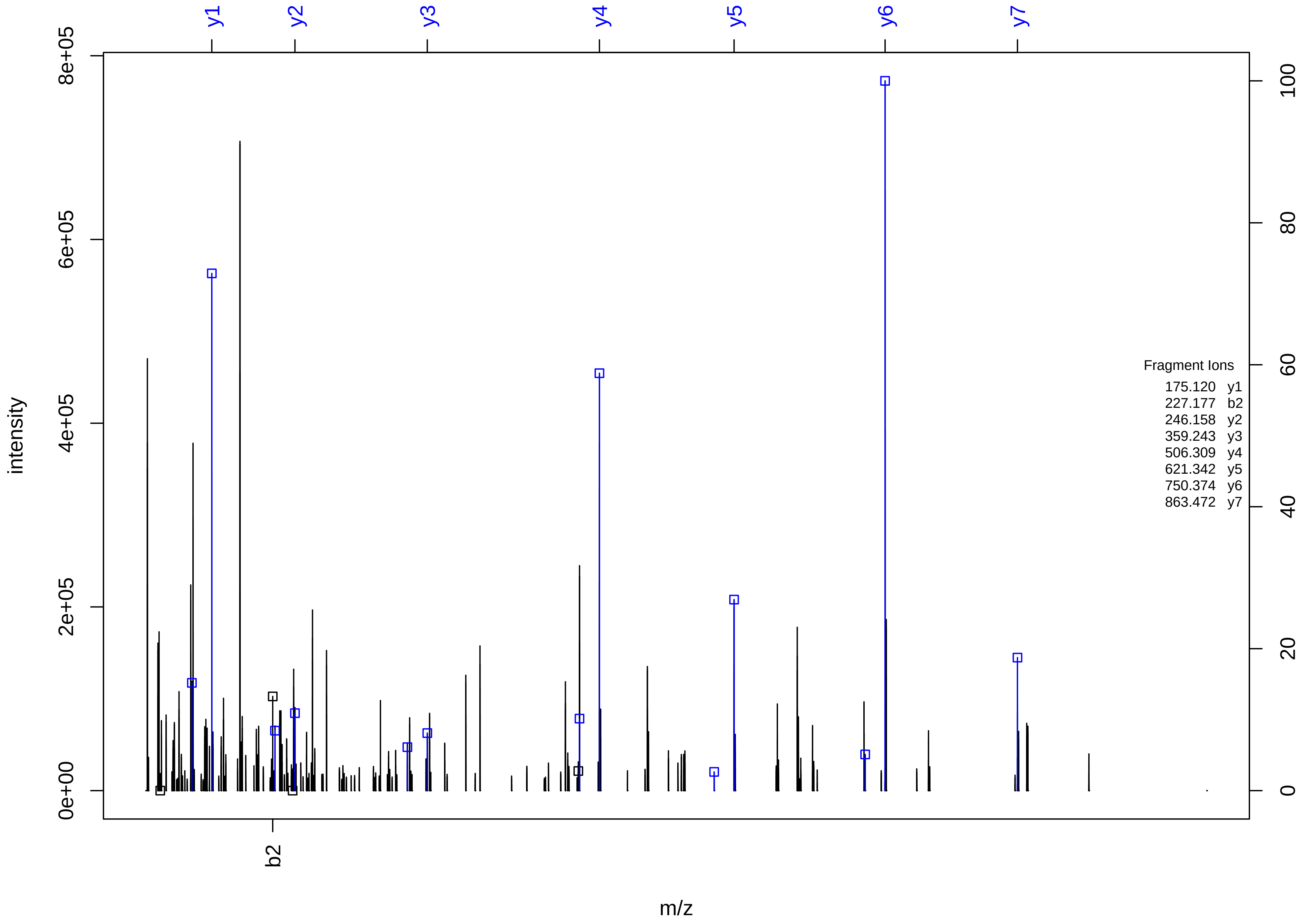

LIEDFLAR / File: christine_pool_04.raw; SpectrumID: ; scans: 13894

LFLEFER

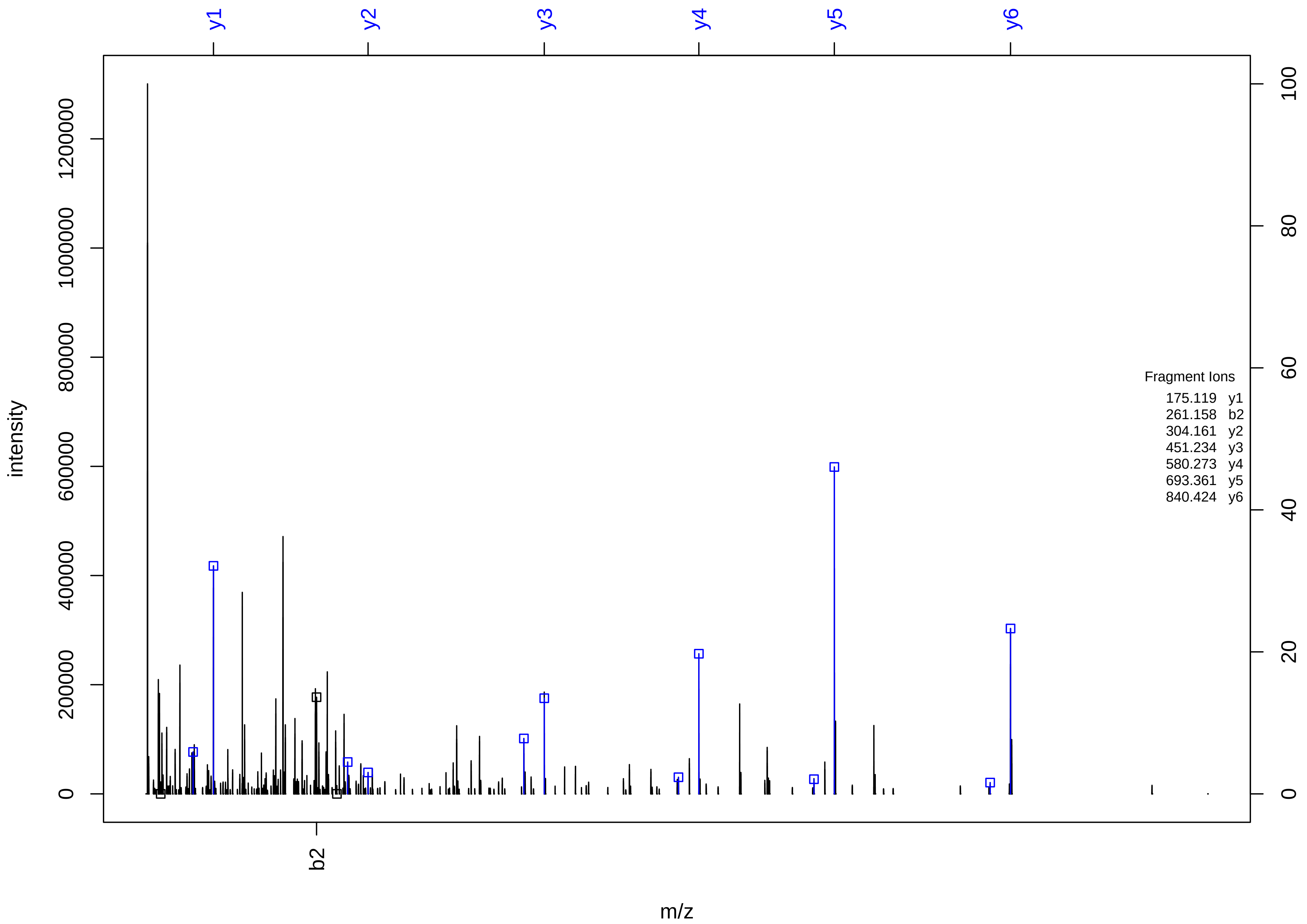

LFLEFER / File: christine_pool_04.raw; SpectrumID: ; scans: 13616

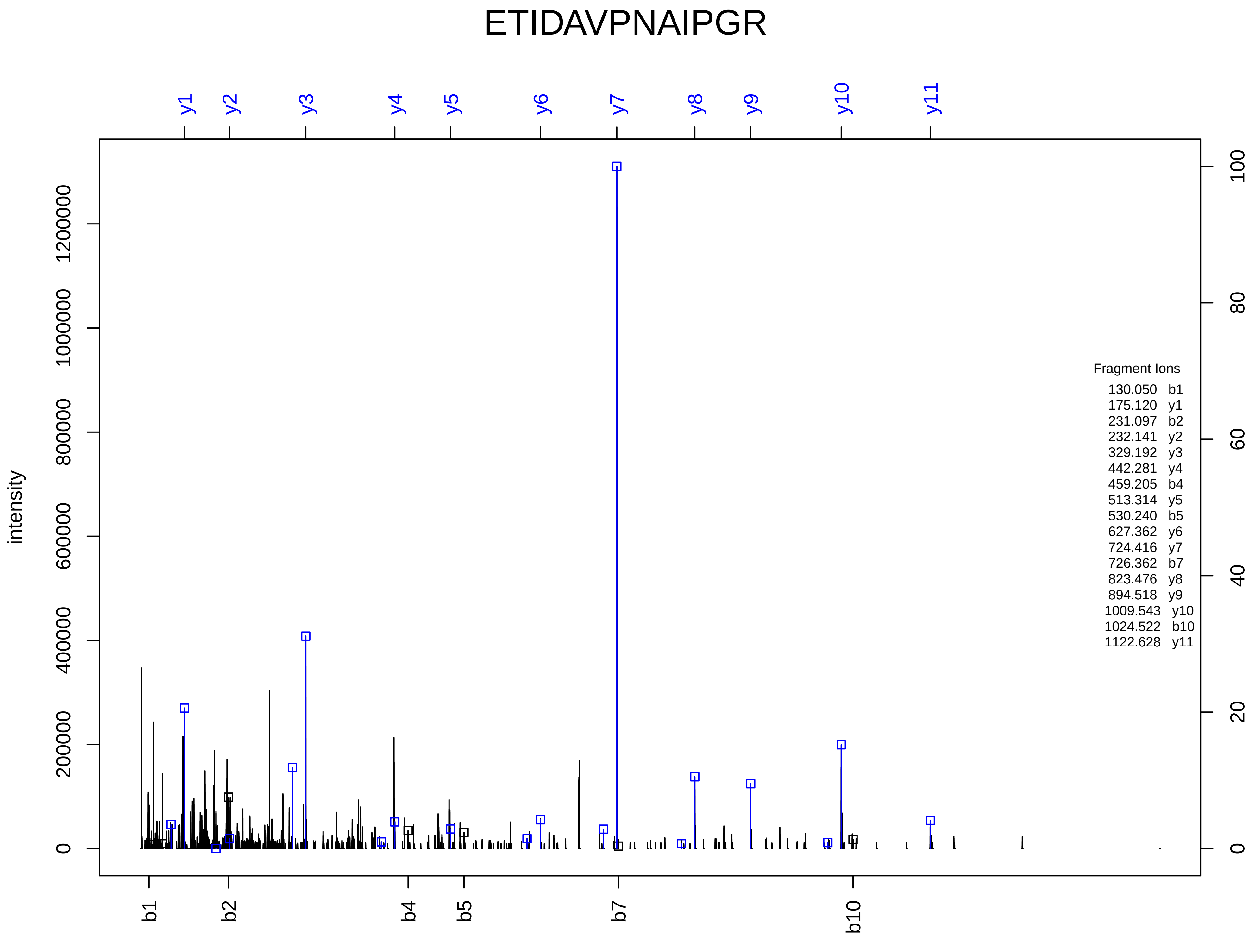

ETIDAVPNAIPGR / File: christine_pool_04.raw; SpectrumID: ; scans: 9655

SVSFSDVFFK

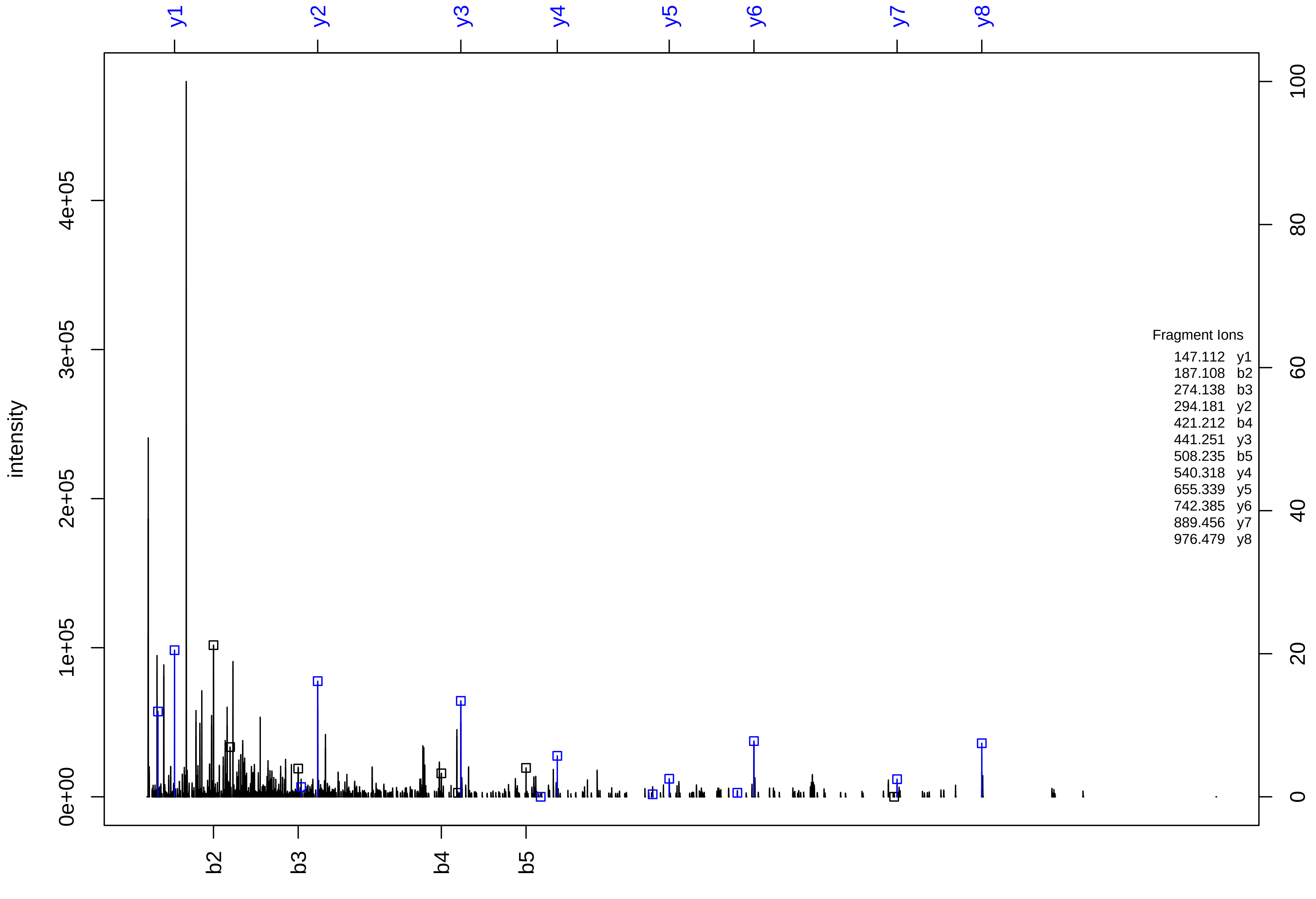

SVSFSDVFFK / File: christine_pool_04.raw; SpectrumID: ; scans: 17498

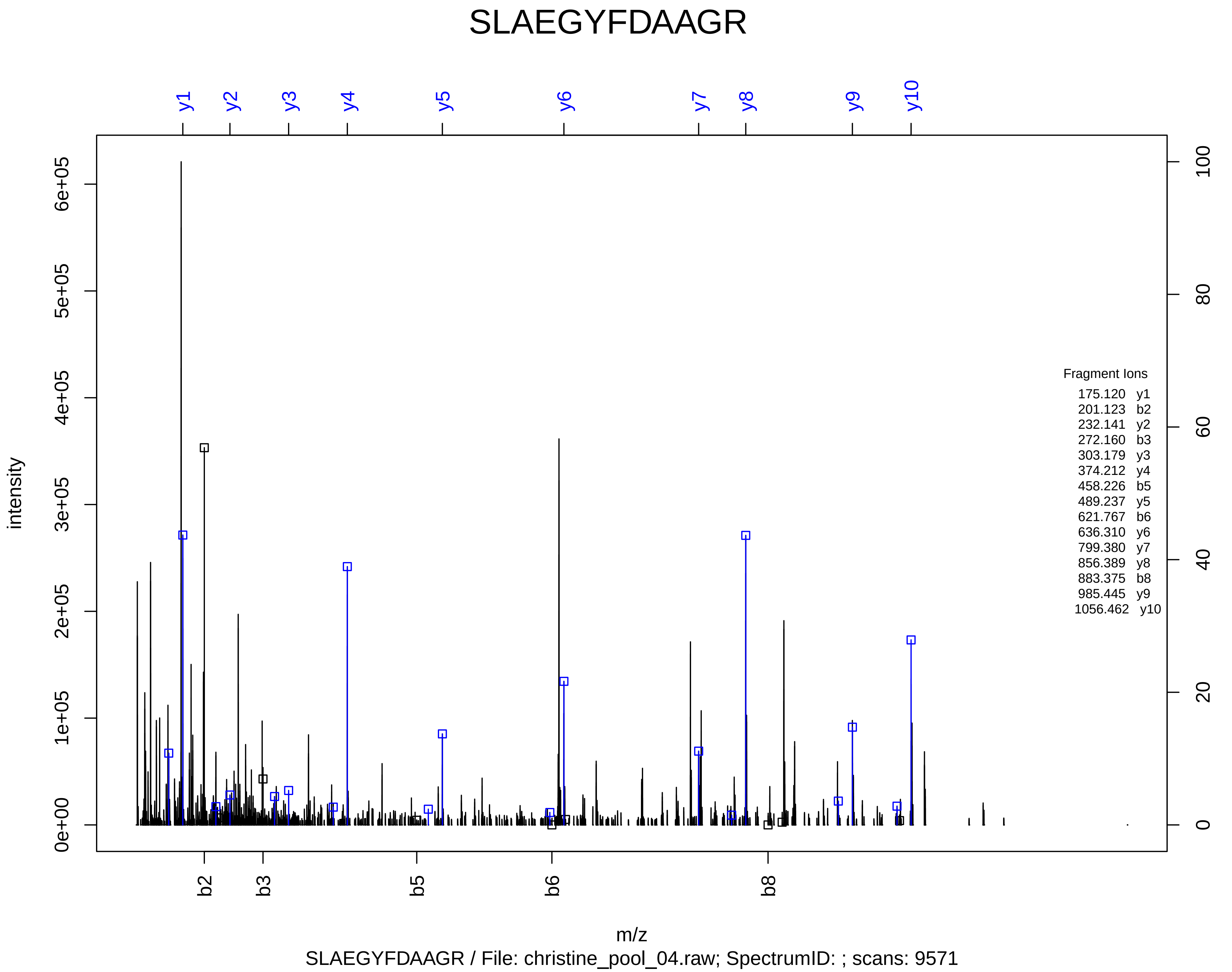
SLAEGYFDAAGR
intensity
m/z
y1 y2 y3 y4 y5 y6 y7 y8 y9 y10
b2 b3 b5 b6 b8
0e+00 1e+05 2e+05 3e+05 4e+05 5e+05 6e+05
0 20 40 60 80 100
Fragment Ions
175.120 y1
201.123 b2
232.141 y2
272.160 b3
303.179 y3
374.212 y4
458.226 b5
489.237 y5
621.767 b6
636.310 y6
799.380 y7
856.389 y8
883.375 b8
985.445 y9
1056.462 y10
SLAEGYFDAAGR / File: christine_pool_04.raw; SpectrumID: ; scans: 9571

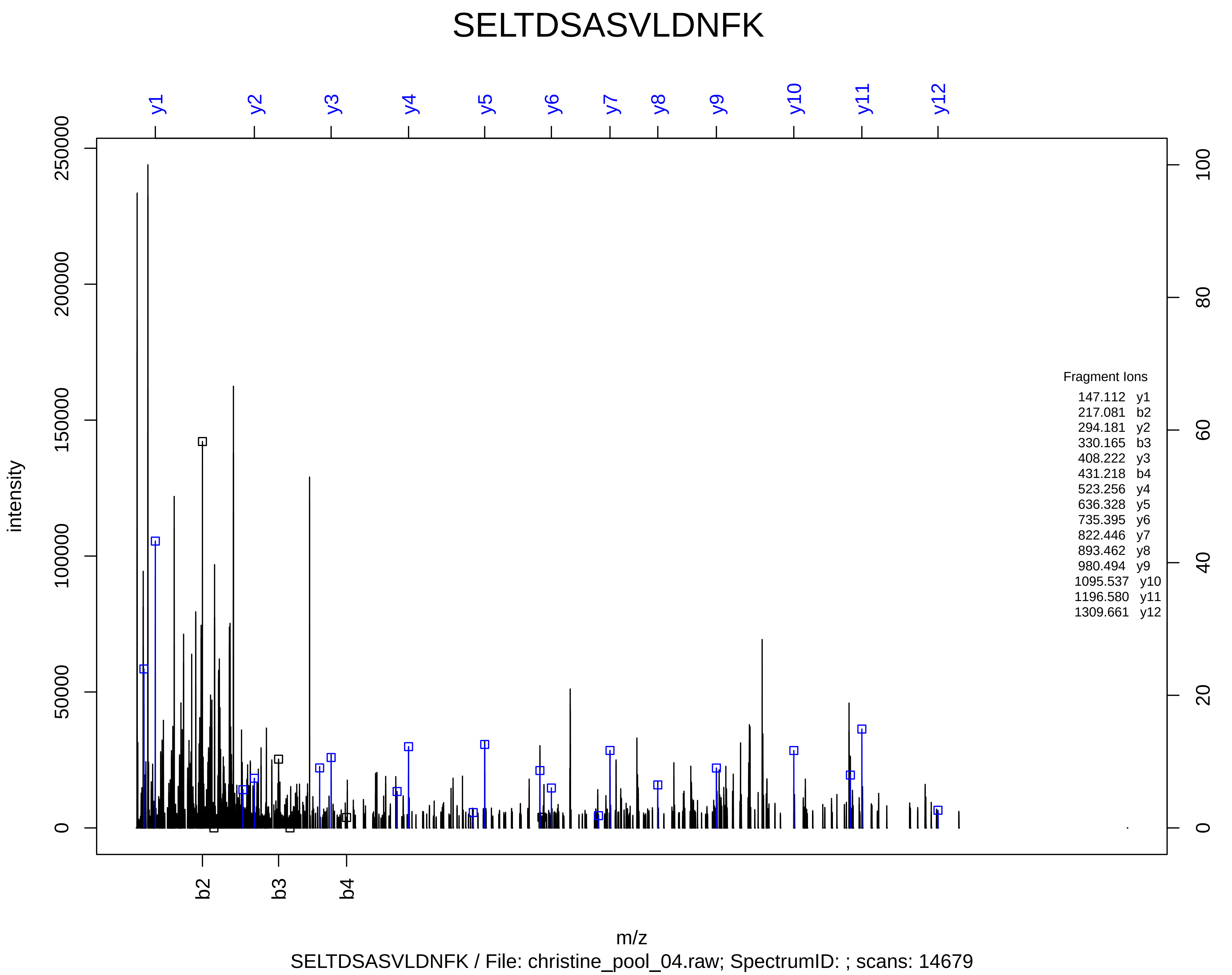
SELTDSASVLDNFK
y1
y2
y3
y4
y5
y6
y7
y8
y9
y10
y11
y12
intensity
250000
200000
150000
100000
50000
0
100
80
60
40
20
0
b2
b3
b4
m/z
Fragment Ions
147.112 y1
217.081 b2
294.181 y2
330.165 b3
408.222 y3
431.218 b4
523.256 y4
636.328 y5
735.395 y6
822.446 y7
893.462 y8
980.494 y9
1095.537 y10
1196.580 y11
1309.661 y12
SELTDSASVLDNFK / File: christine_pool_04.raw; SpectrumID: ; scans: 14679

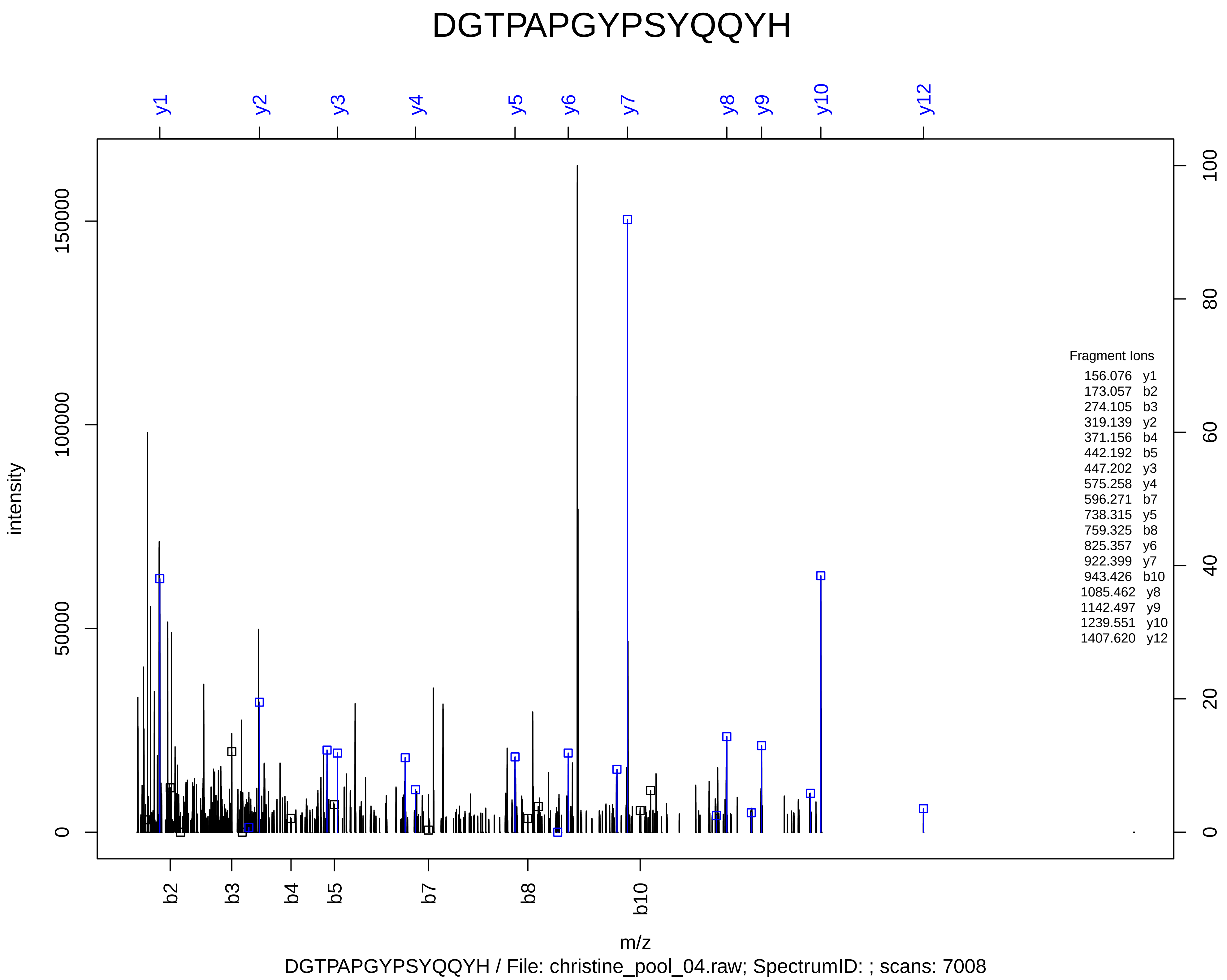
DGTPAPGYPSYQQYH
y1
y2
y3
y4
y5
y6
y7
y8
y9
y10
y12
b2
b3
b4
b5
b7
b8
b10
intensity
m/z
Fragment Ions
156.076 y1
173.057 b2
274.105 b3
319.139 y2
371.156 b4
442.192 b5
447.202 y3
575.258 y4
596.271 b7
738.315 y5
759.325 b8
825.357 y6
922.399 y7
943.426 b10
1085.462 y8
1142.497 y9
1239.551 y10
1407.620 y12
DGTPAPGYPSYQQYH / File: christine_pool_04.raw; SpectrumID: ; scans: 7008

EDAGEVTFSLGN^SR

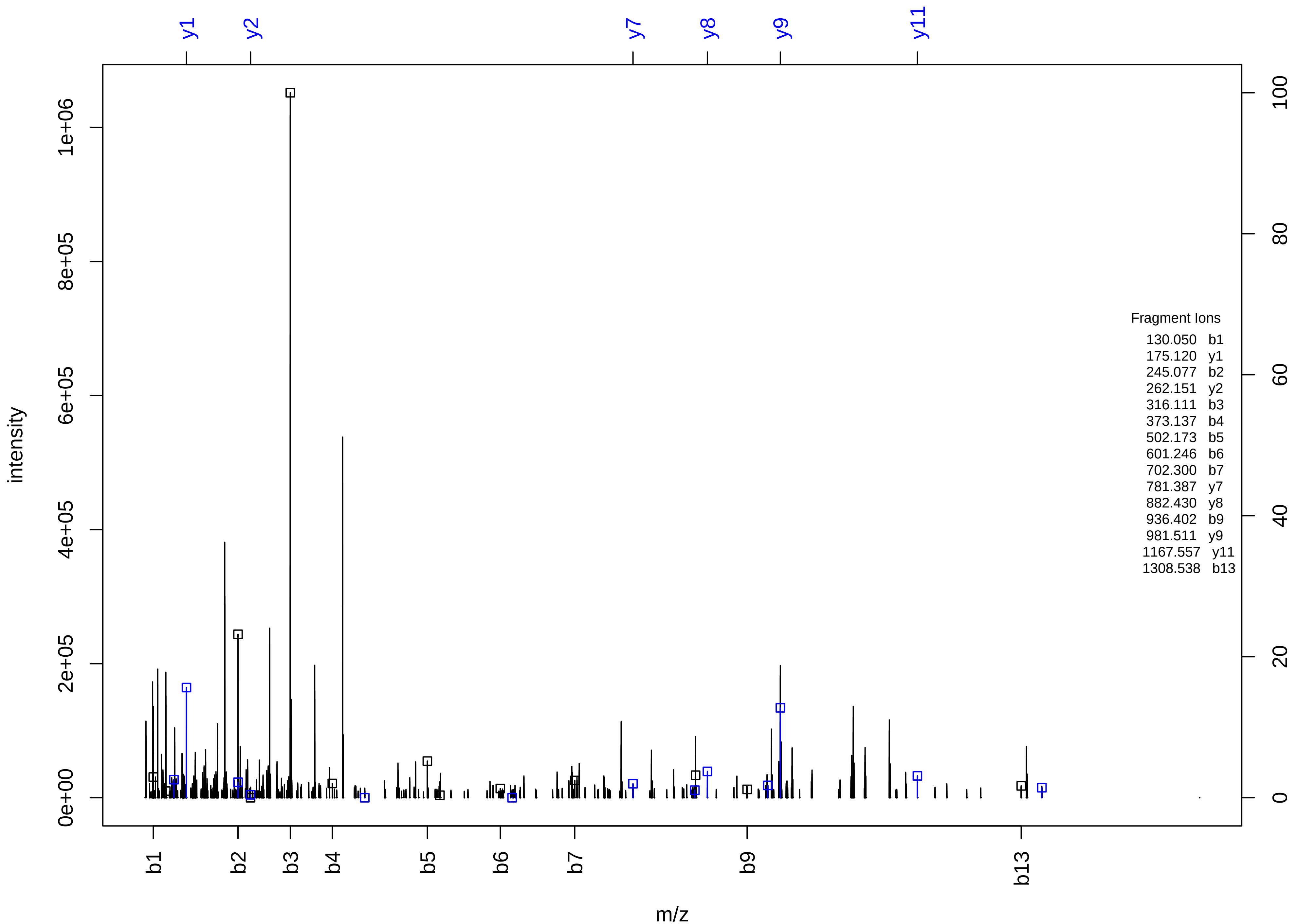

EDAGEVTFSLGNSR / File: christine_pool_04.raw; SpectrumID: ; scans: 7376

YTDQGGEEEEDYESEEQLQHR

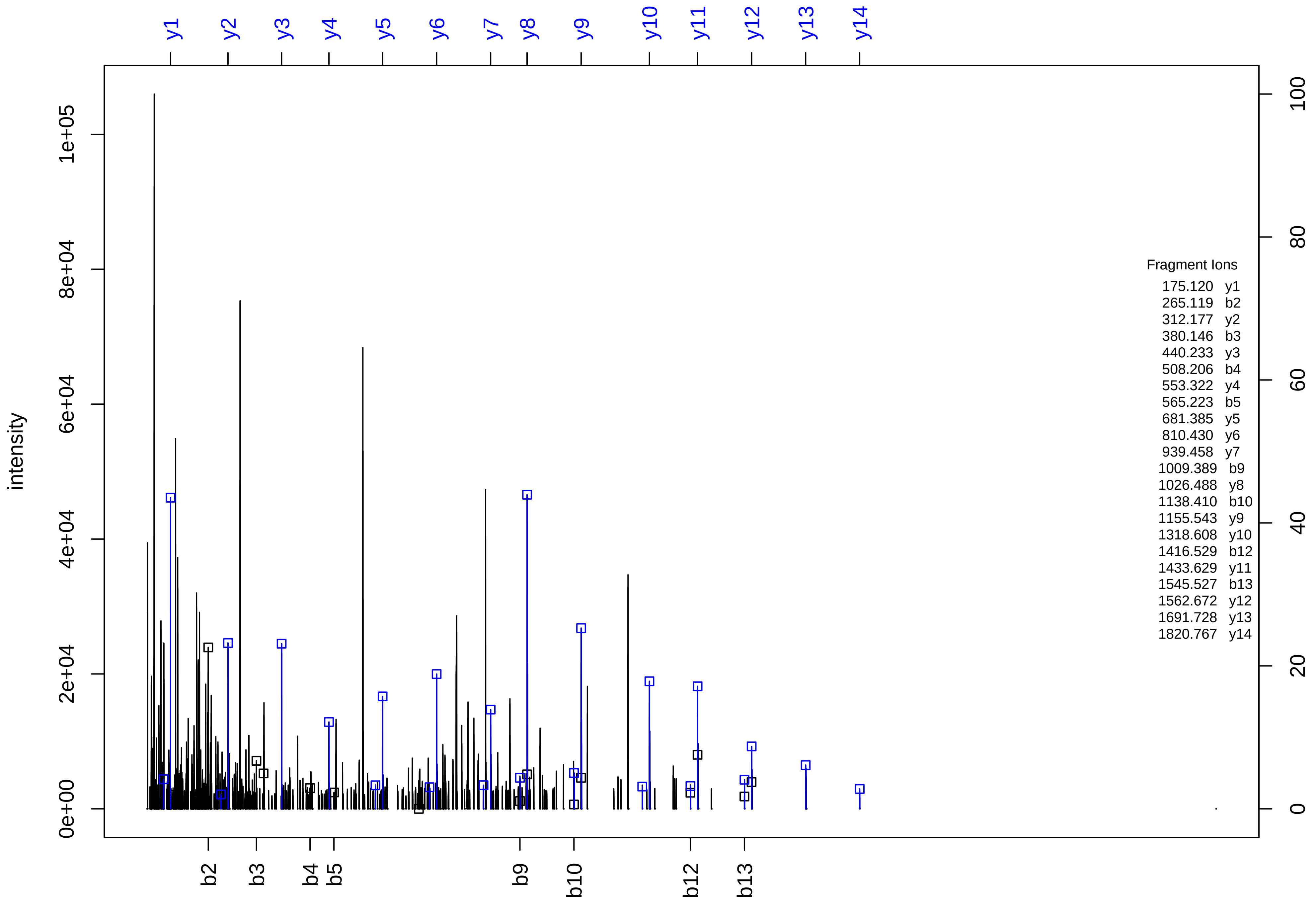

YTDQGGEEEEDYESEEQLQHR / File: christine_pool_04.raw; SpectrumID: ; scans: 4800

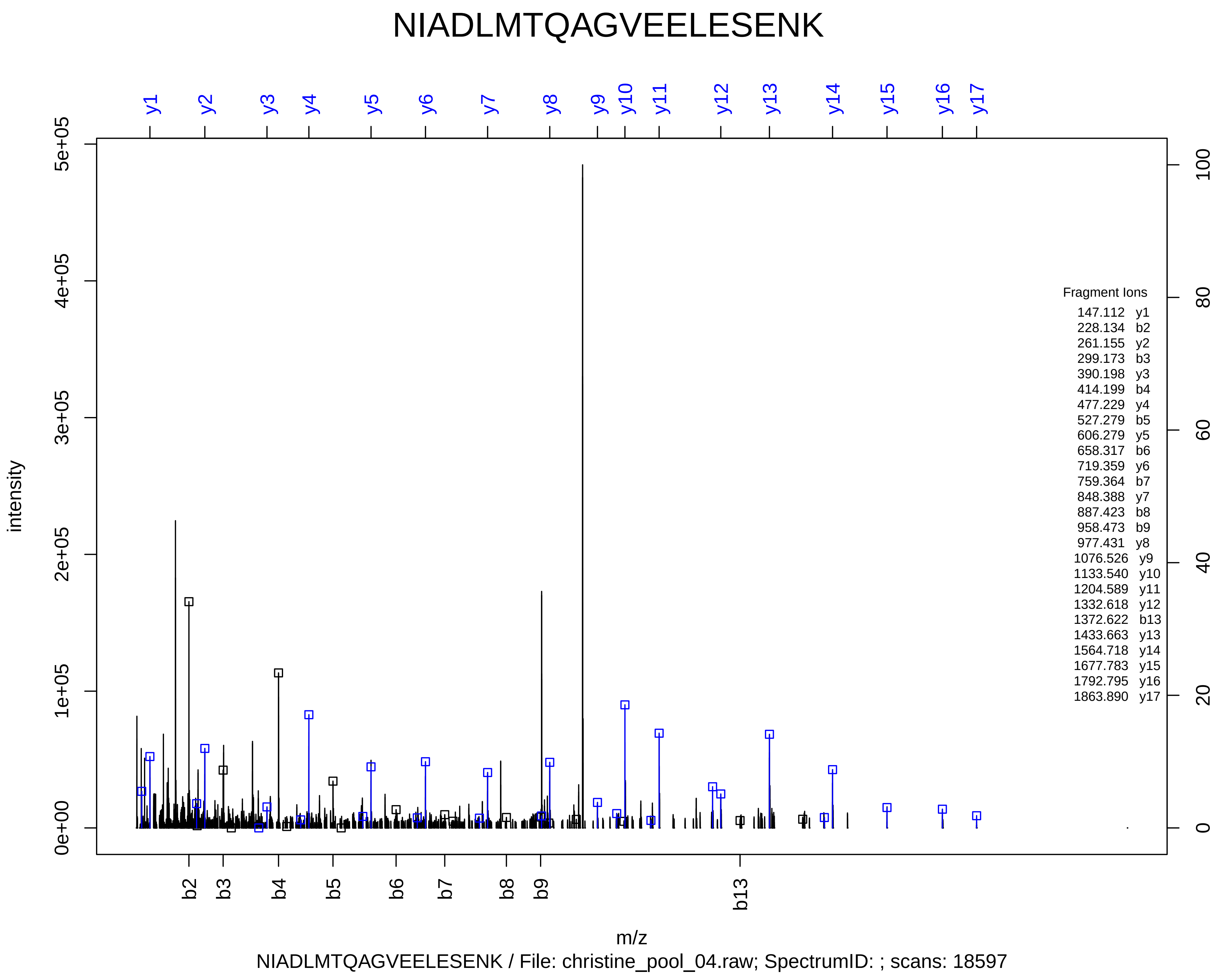
NIADLMTQAGVEELESENK
y1 y2 y3 y4 y5 y6 y7 y8 y9 y10 y11 y12 y13 y14 y15 y16 y17
b2 b3 b4 b5 b6 b7 b8 b9 b13
intensity
m/z
0e+00 1e+05 2e+05 3e+05 4e+05 5e+05
0 20 40 60 80 100
Fragment Ions
147.112 y1
228.134 b2
261.155 y2
299.173 b3
390.198 y3
414.199 b4
477.229 y4
527.279 b5
606.279 y5
658.317 b6
719.359 y6
759.364 b7
848.388 y7
887.423 b8
958.473 b9
977.431 y8
1076.526 y9
1133.540 y10
1204.589 y11
1332.618 y12
1372.622 b13
1433.663 y13
1564.718 y14
1677.783 y15
1792.795 y16
1863.890 y17
NIADLMTQAGVEELESENK / File: christine_pool_04.raw; SpectrumID: ; scans: 18597

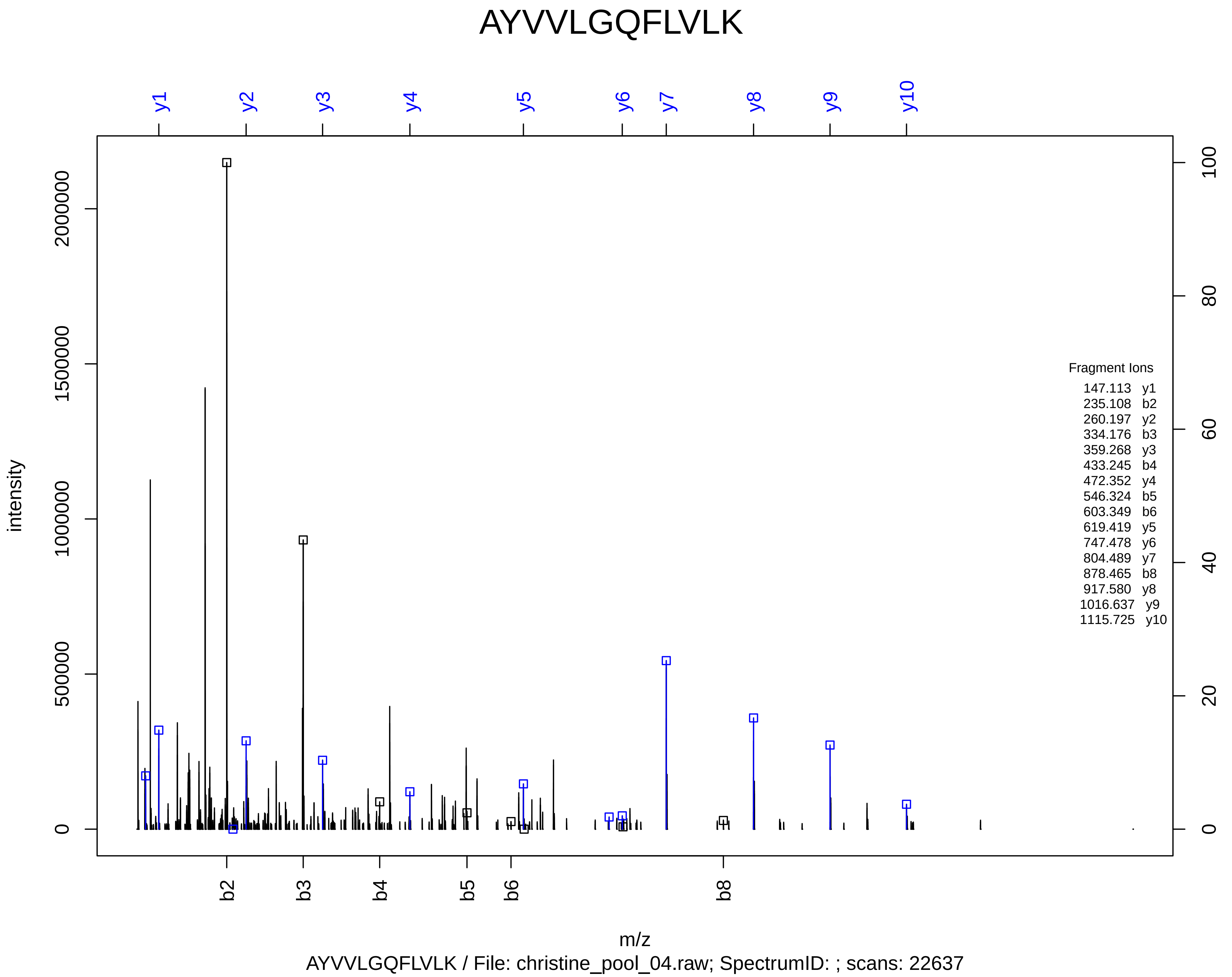
AYVVLGQFLVLK
y1
y2
y3
y4
y5
y6
y7
y8
y9
y10
b2
b3
b4
b5
b6
b8
intensity
m/z
Fragment Ions
147.113 y1
235.108 b2
260.197 y2
334.176 b3
359.268 y3
433.245 b4
472.352 y4
546.324 b5
603.349 b6
619.419 y5
747.478 y6
804.489 y7
878.465 b8
917.580 y8
1016.637 y9
1115.725 y10
AYVVLGQFLVLK / File: christine_pool_04.raw; SpectrumID: ; scans: 22637

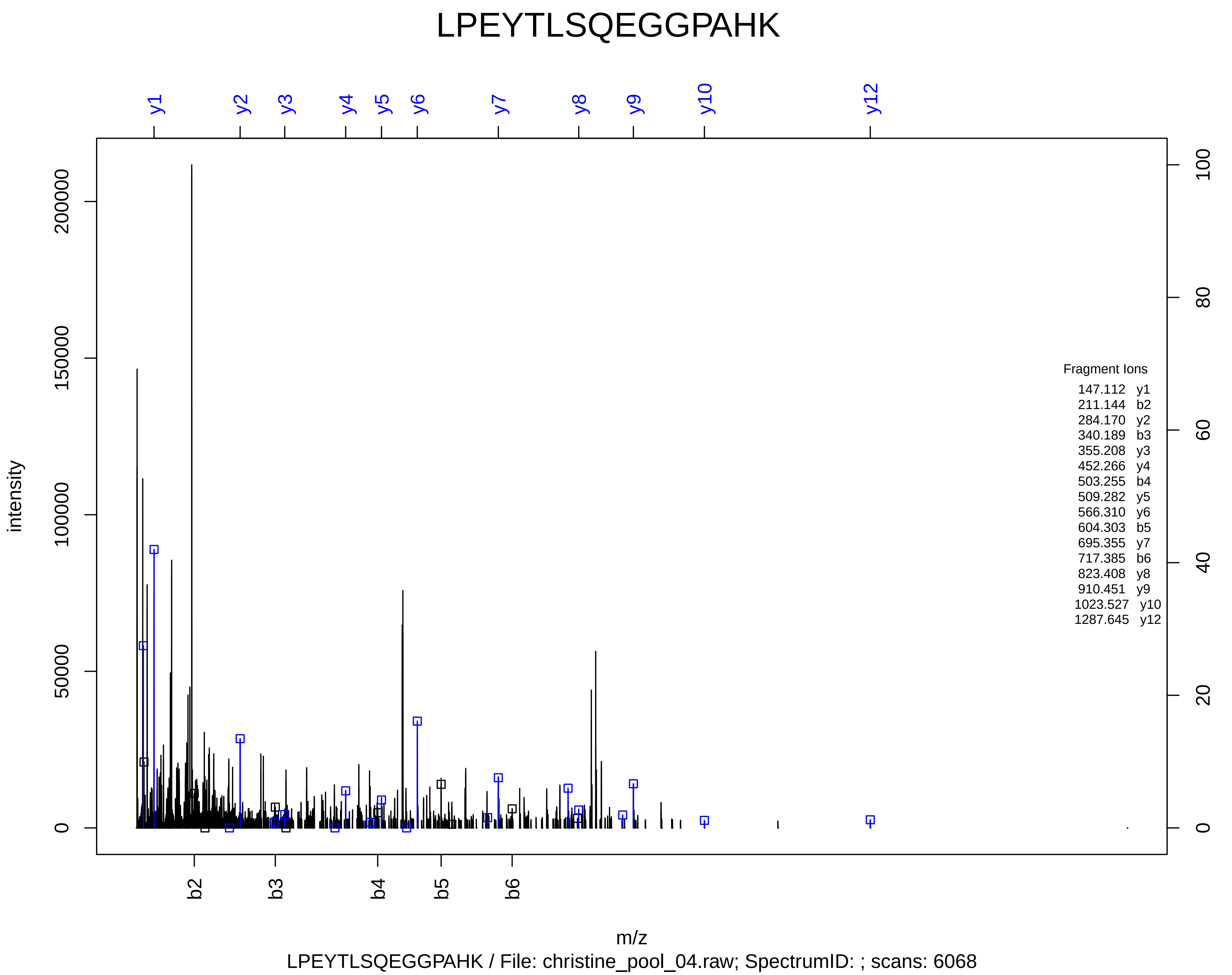

LPEYTLSQEGGPAHK
y1
y2
y3
y4
y5
y6
y7
y8
y9
y10
y12
intensity
0
50000
100000
150000
200000
0
20
40
60
80
100
b2
b3
b4
b5
b6
m/z
Fragment Ions
147.112 y1
211.144 b2
284.170 y2
340.189 b3
355.208 y3
452.266 y4
503.255 b4
509.282 y5
566.310 y6
604.303 b5
695.355 y7
717.385 b6
823.408 y8
910.451 y9
1023.527 y10
1287.645 y12
LPEYTLSQEGGPAHK / File: christine_pool_04.raw; SpectrumID: ; scans: 6068

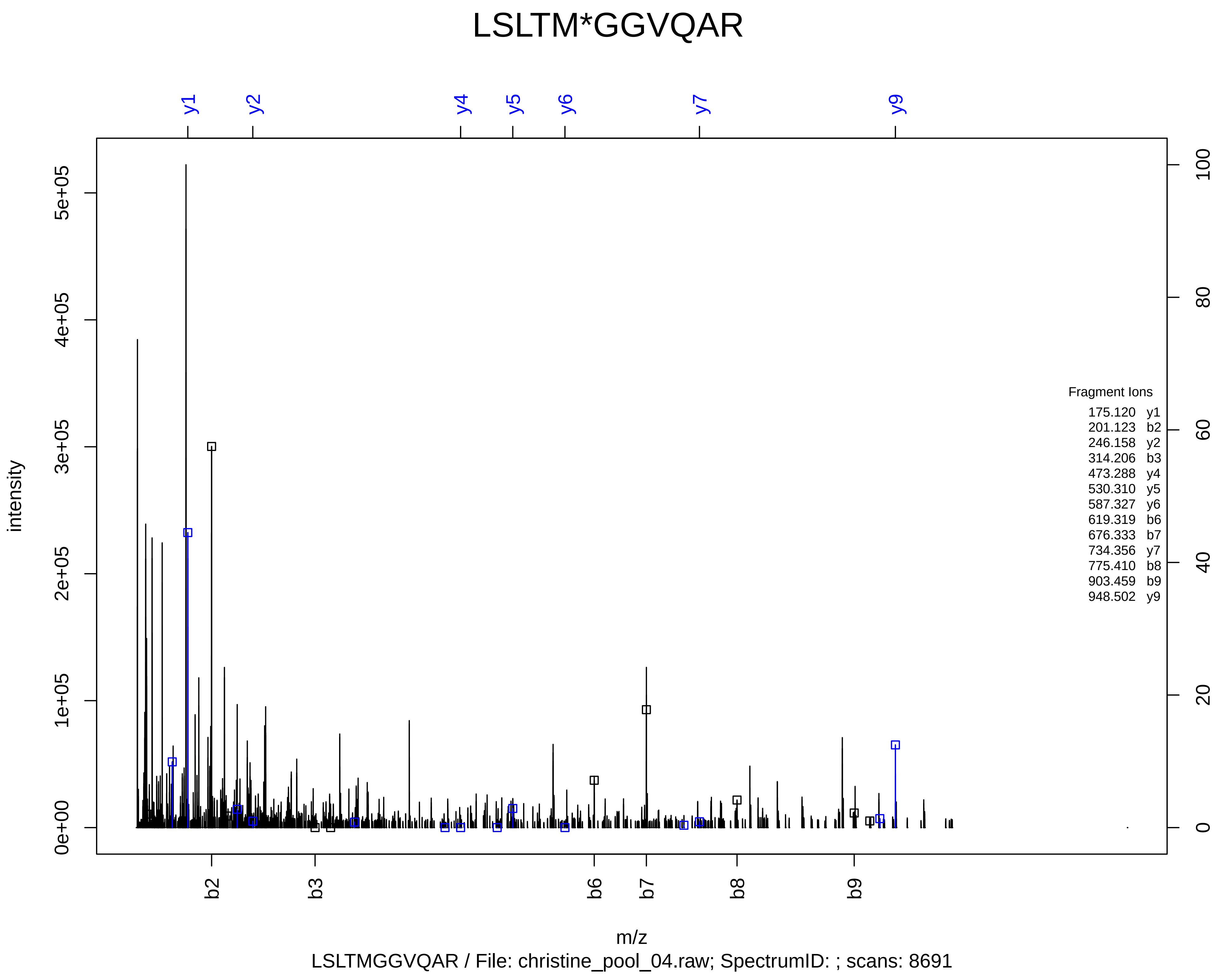

LSLTM*GGVQAR
y1
y2
y4
y5
y6
y7
y9
intensity
0e+00
1e+05
2e+05
3e+05
4e+05
5e+05
0
20
40
60
80
100
b2
b3
b6
b7
b8
b9
m/z
Fragment Ions
175.120 y1
201.123 b2
246.158 y2
314.206 b3
473.288 y4
530.310 y5
587.327 y6
619.319 b6
676.333 b7
734.356 y7
775.410 b8
903.459 b9
948.502 y9
LSLTMGGVQAR / File: christine_pool_04.raw; SpectrumID: ; scans: 8691

LVGNMHGDETVSR

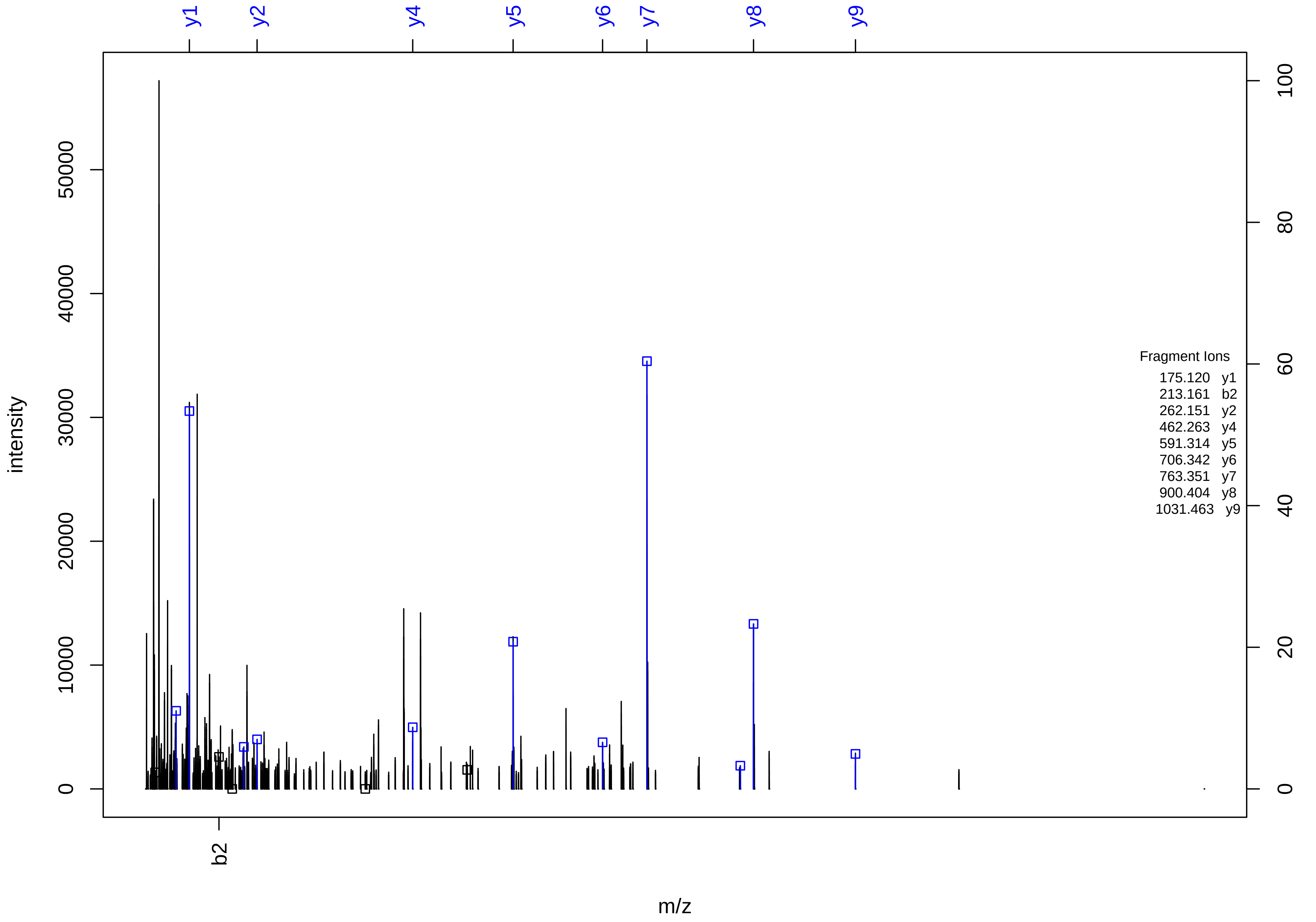

LVGNMHGDETVSR / File: christine_pool_04.raw; SpectrumID: ; scans: 3204

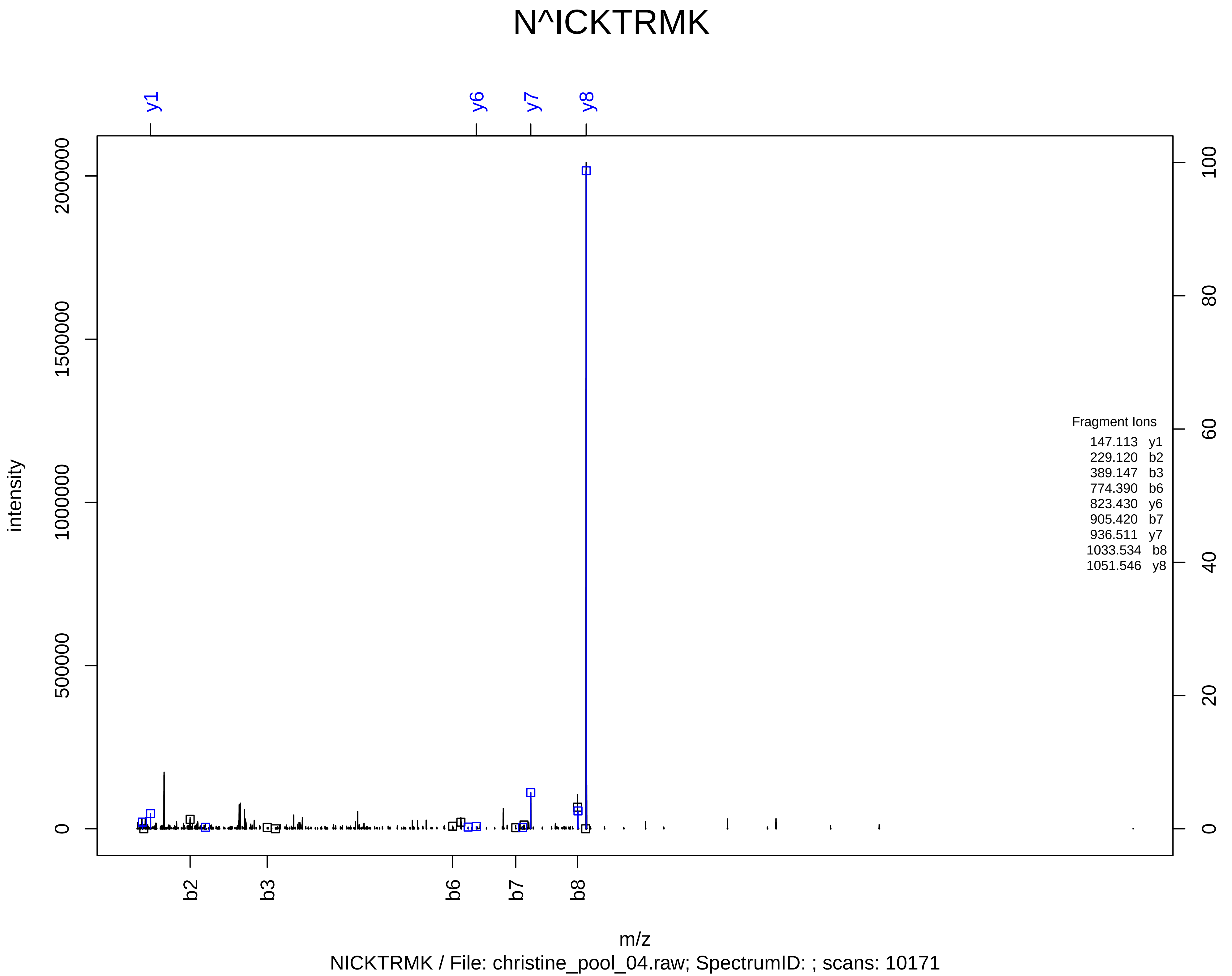
N^ICKTRMK
y1
y6
y7
y8
intensity
2000000
1500000
1000000
500000
0
100
80
60
40
20
0
Fragment Ions
147.113 y1
229.120 b2
389.147 b3
774.390 b6
823.430 y6
905.420 b7
936.511 y7
1033.534 b8
1051.546 y8
b2
b3
b6
b7
b8
m/z
NICKTRMK / File: christine_pool_04.raw; SpectrumID: ; scans: 10171

TYDLPGNFLTQALTQR

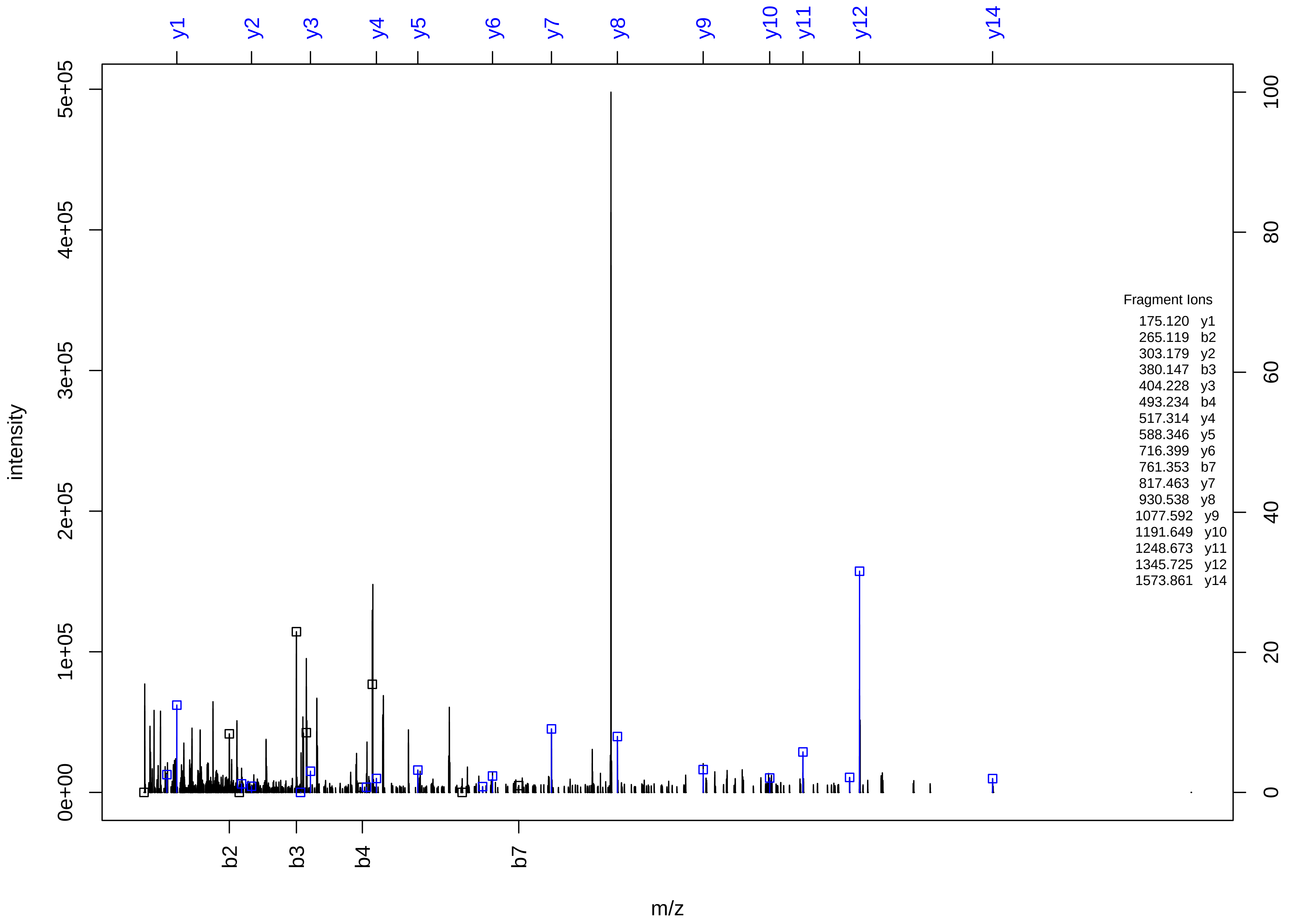

TYDLPGNFLTQALTQR / File: christine_pool_04.raw; SpectrumID: ; scans: 21325

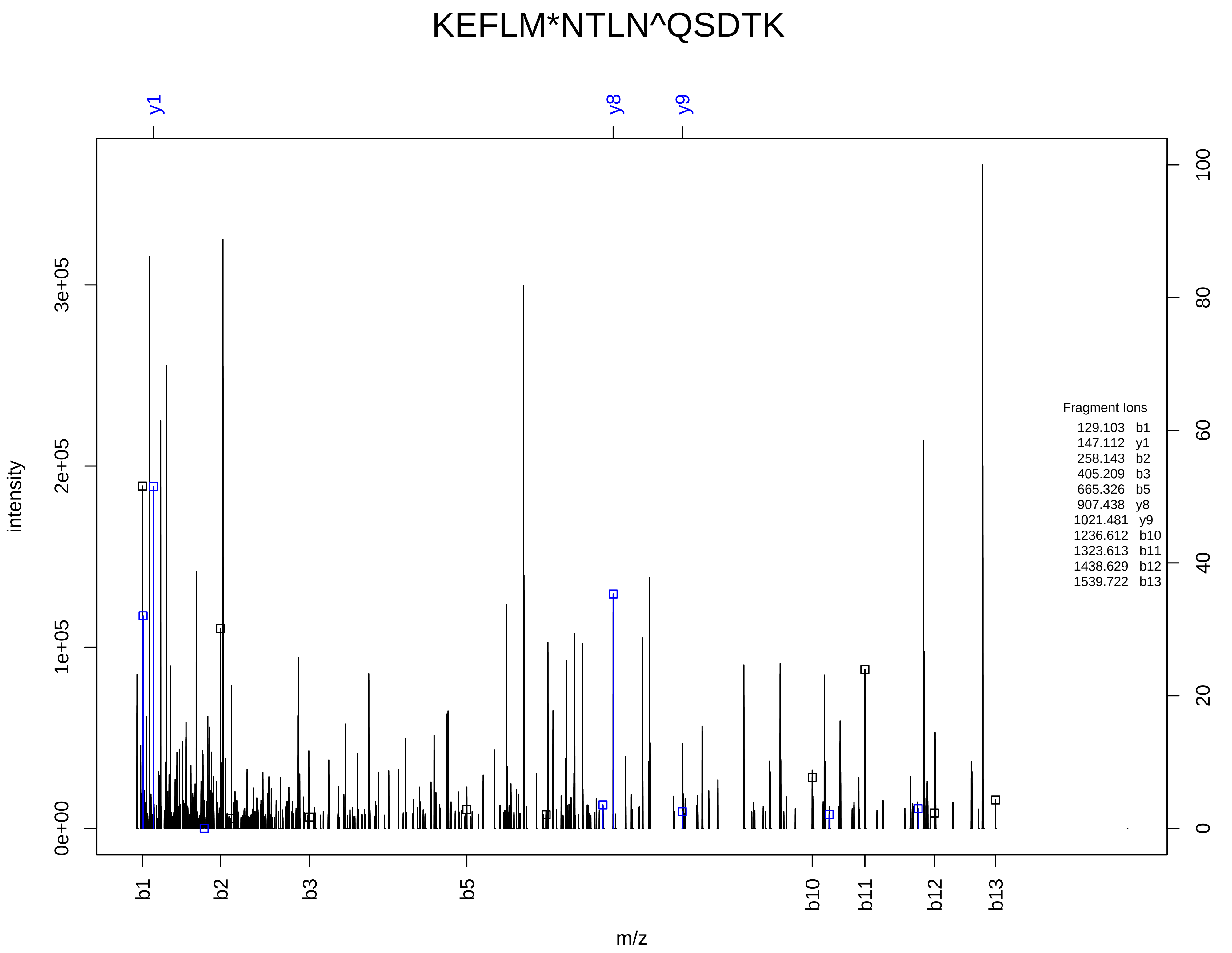

KEFLM*NTLN^QSDTK
y1
y8
y9
intensity
0e+00
1e+05
2e+05
3e+05
0
20
40
60
80
100
b1
b2
b3
b5
b10
b11
b12
b13
m/z
Fragment Ions
129.103 b1
147.112 y1
258.143 b2
405.209 b3
665.326 b5
907.438 y8
1021.481 y9
1236.612 b10
1323.613 b11
1438.629 b12
1539.722 b13
KEFLMNTLNQSDTK / File: christine_pool_04.raw; SpectrumID: ; scans: 10183

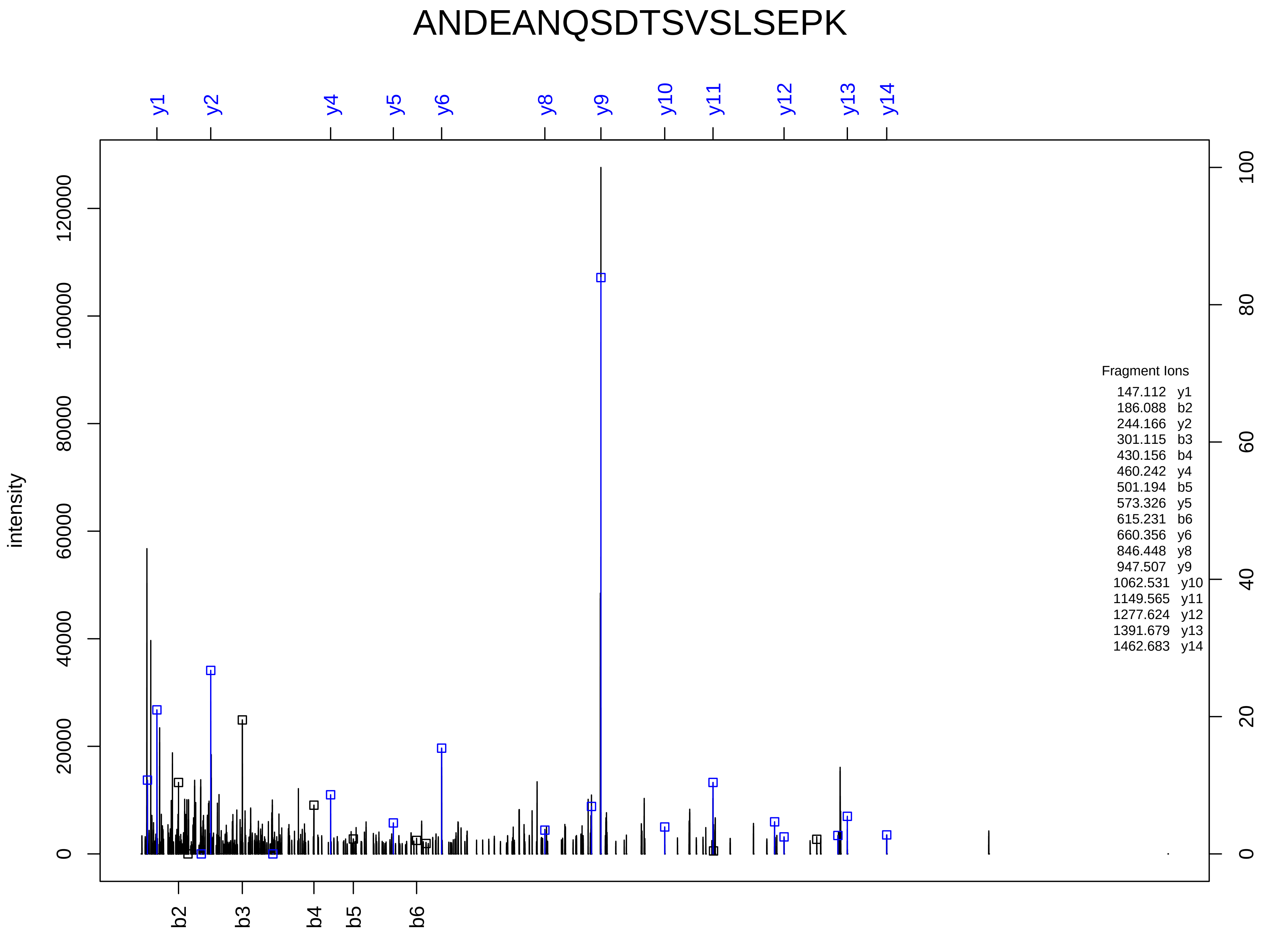

ANDEANQSDTSVSLSEPK / File: christine_pool_04.raw; SpectrumID: ; scans: 5032

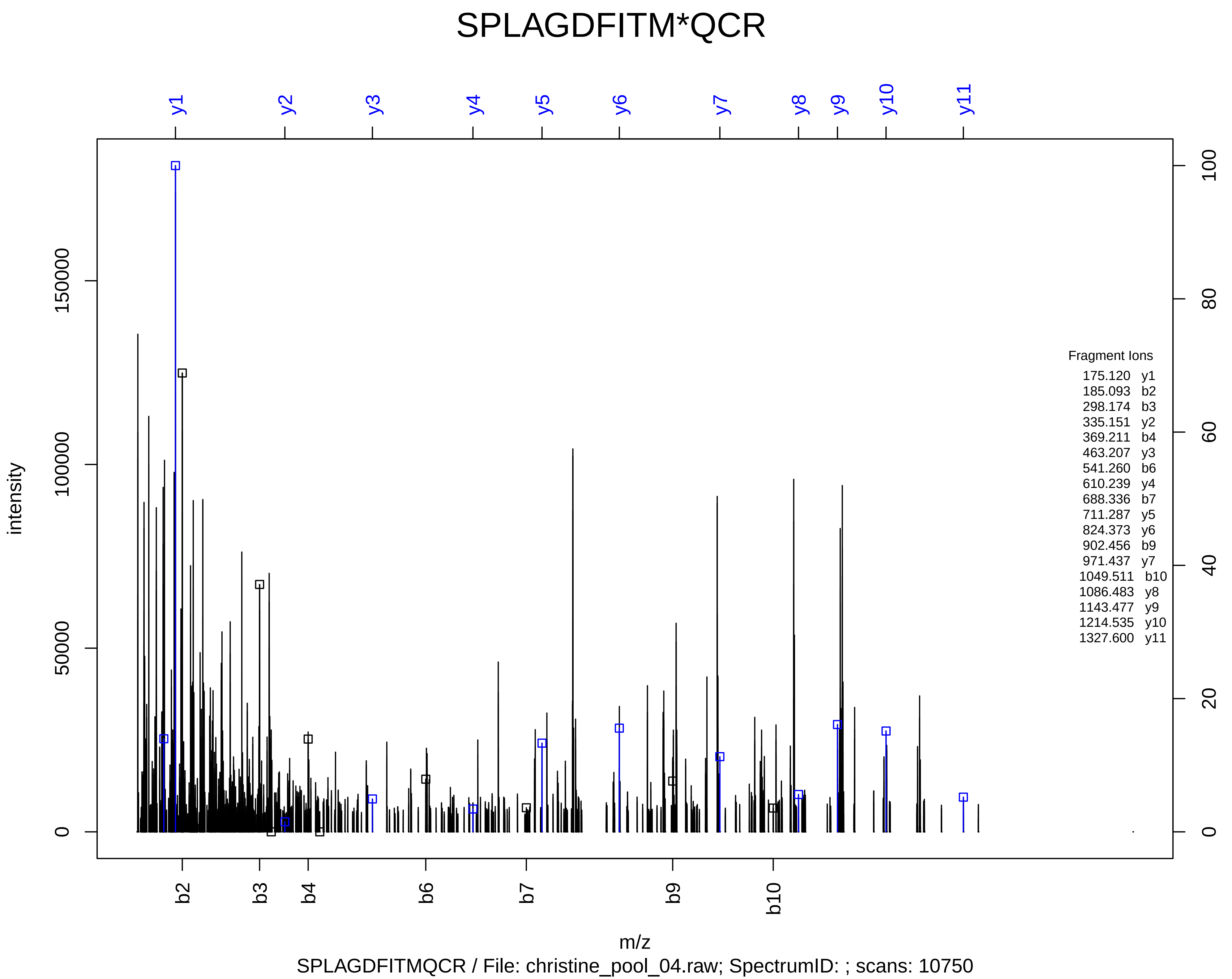

SPLAGDFITM*QCR
y1
y2
y3
y4
y5
y6
y7
y8
y9
y10
y11
intensity
0
50000
100000
150000
0
20
40
60
80
100
Fragment Ions
175.120 y1
185.093 b2
298.174 b3
335.151 y2
369.211 b4
463.207 y3
541.260 b6
610.239 y4
688.336 b7
711.287 y5
824.373 y6
902.456 b9
971.437 y7
1049.511 b10
1086.483 y8
1143.477 y9
1214.535 y10
1327.600 y11
b2
b3
b4
b6
b7
b9
b10
m/z
SPLAGDFITMQCR / File: christine_pool_04.raw; SpectrumID: ; scans: 10750

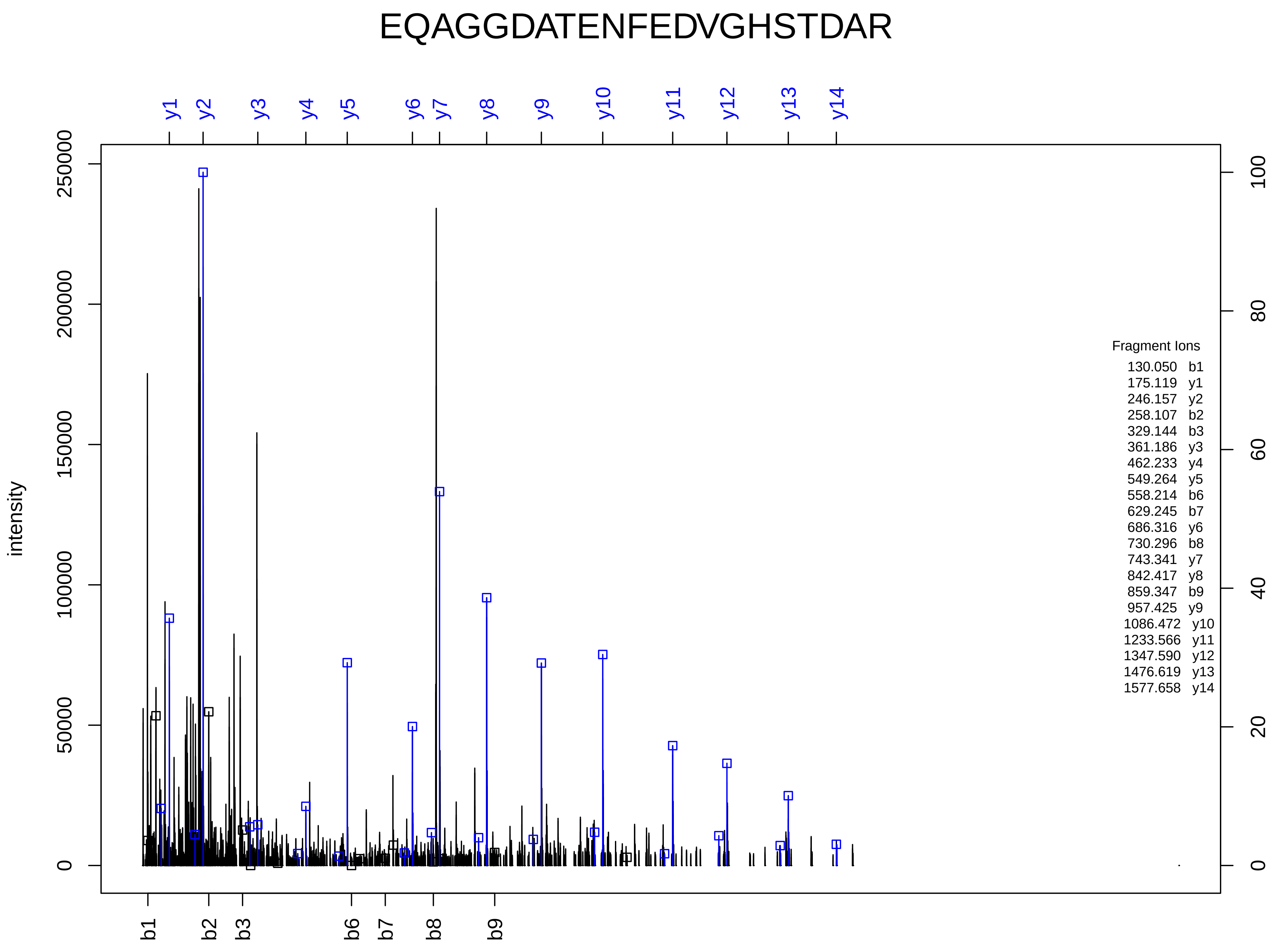

EQAGGDATENFEDVGHSTDAR / File: christine_pool_04.raw; SpectrumID: ; scans: 5357

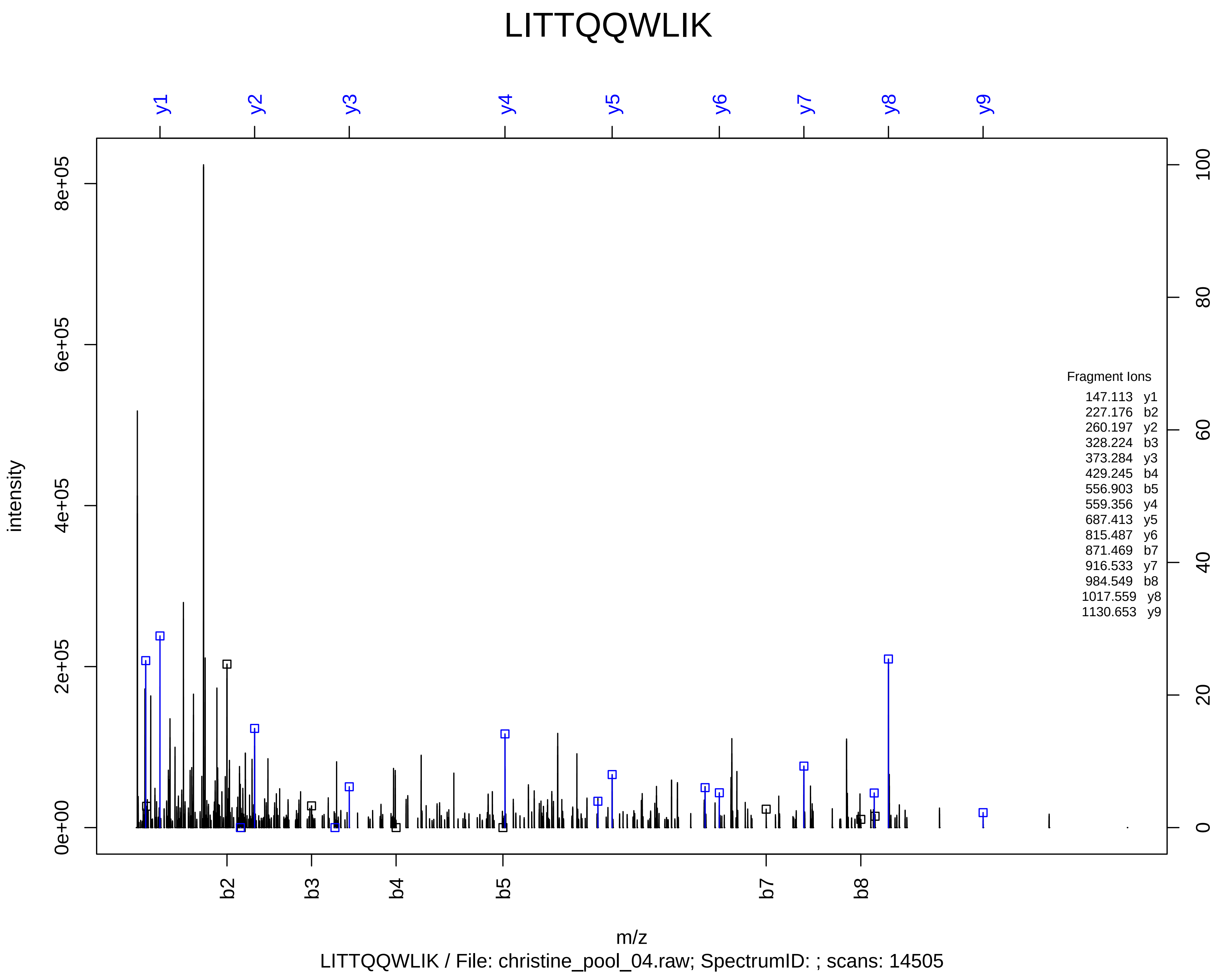
LITTQQWLIK
y1
y2
y3
y4
y5
y6
y7
y8
y9
intensity
0e+00
2e+05
4e+05
6e+05
8e+05
0
20
40
60
80
100
b2
b3
b4
b5
b7
b8
m/z
Fragment Ions
147.113 y1
227.176 b2
260.197 y2
328.224 b3
373.284 y3
429.245 b4
556.903 b5
559.356 y4
687.413 y5
815.487 y6
871.469 b7
916.533 y7
984.549 b8
1017.559 y8
1130.653 y9
LITTQQWLIK / File: christine_pool_04.raw; SpectrumID: ; scans: 14505

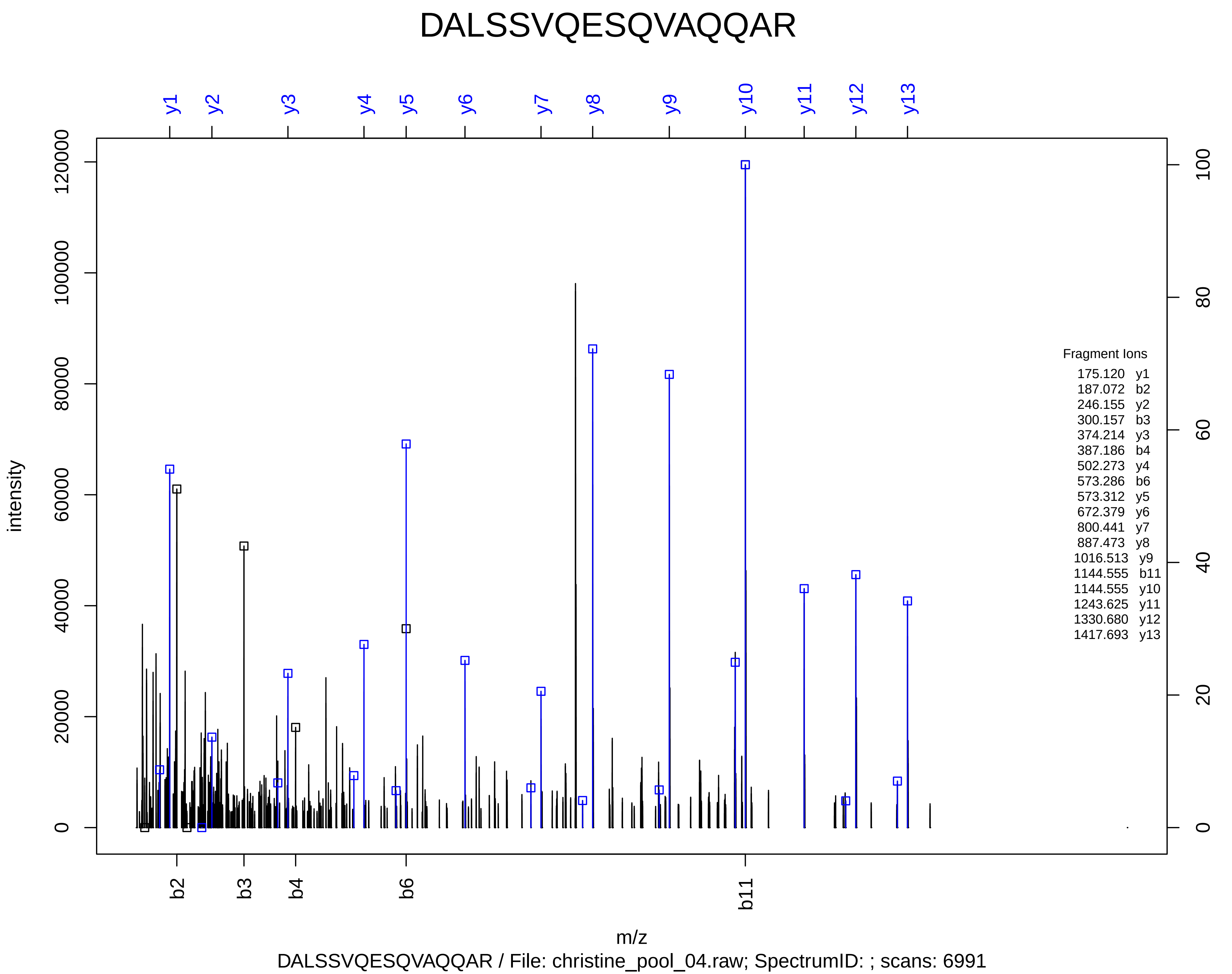

DALSSVQESQVAQQAR
y1
y2
y3
y4
y5
y6
y7
y8
y9
y10
y11
y12
y13
intensity
0
20000
40000
60000
80000
100000
120000
0
20
40
60
80
100
b2
b3
b4
b6
b11
m/z
Fragment Ions
175.120 y1
187.072 b2
246.155 y2
300.157 b3
374.214 y3
387.186 b4
502.273 y4
573.286 b6
573.312 y5
672.379 y6
800.441 y7
887.473 y8
1016.513 y9
1144.555 b11
1144.555 y10
1243.625 y11
1330.680 y12
1417.693 y13
DALSSVQESQVAQQAR / File: christine_pool_04.raw; SpectrumID: ; scans: 6991

FEEDVEEETER

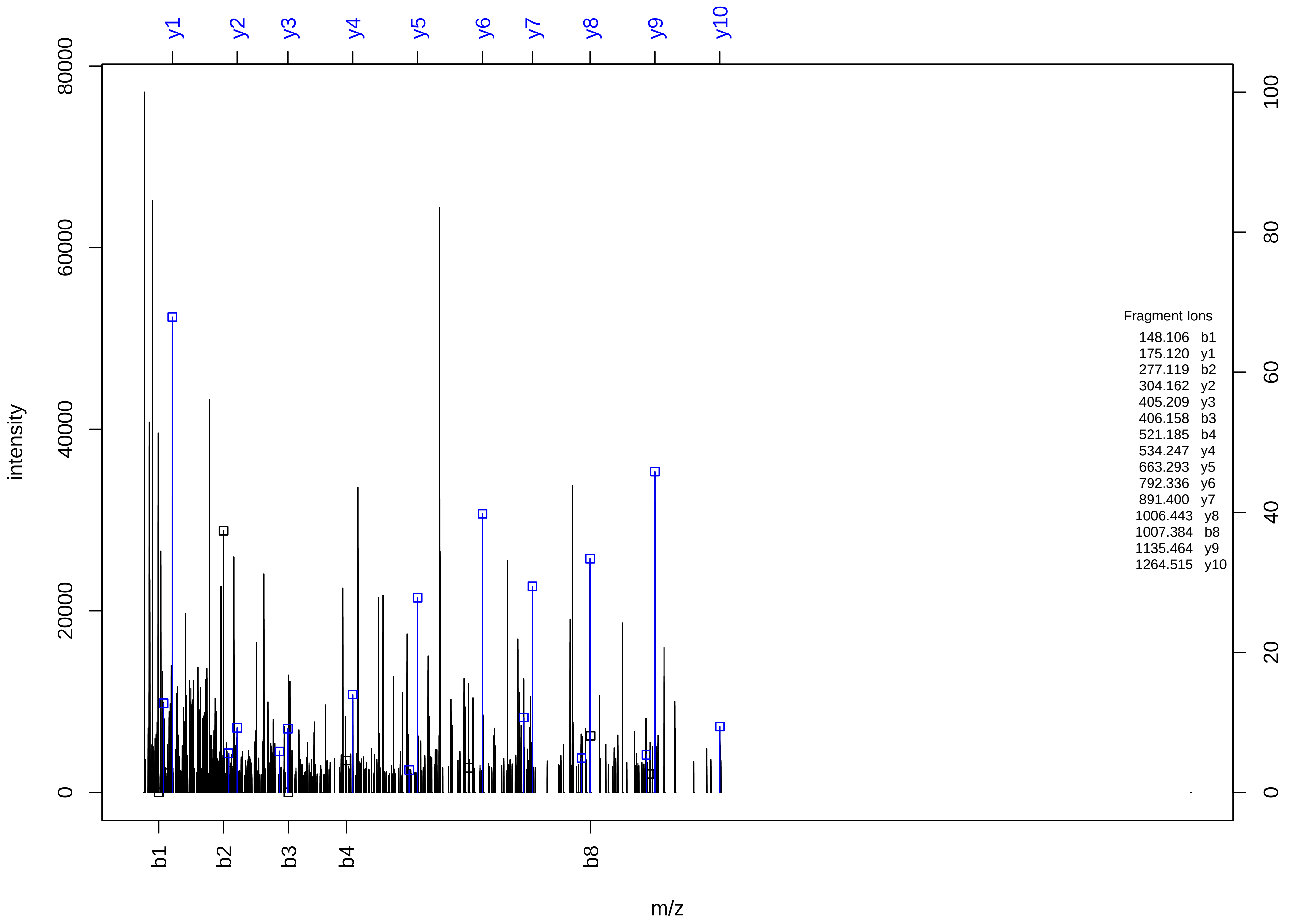

FEEDVEEETER / File: christine_pool_04.raw; SpectrumID: ; scans: 4727

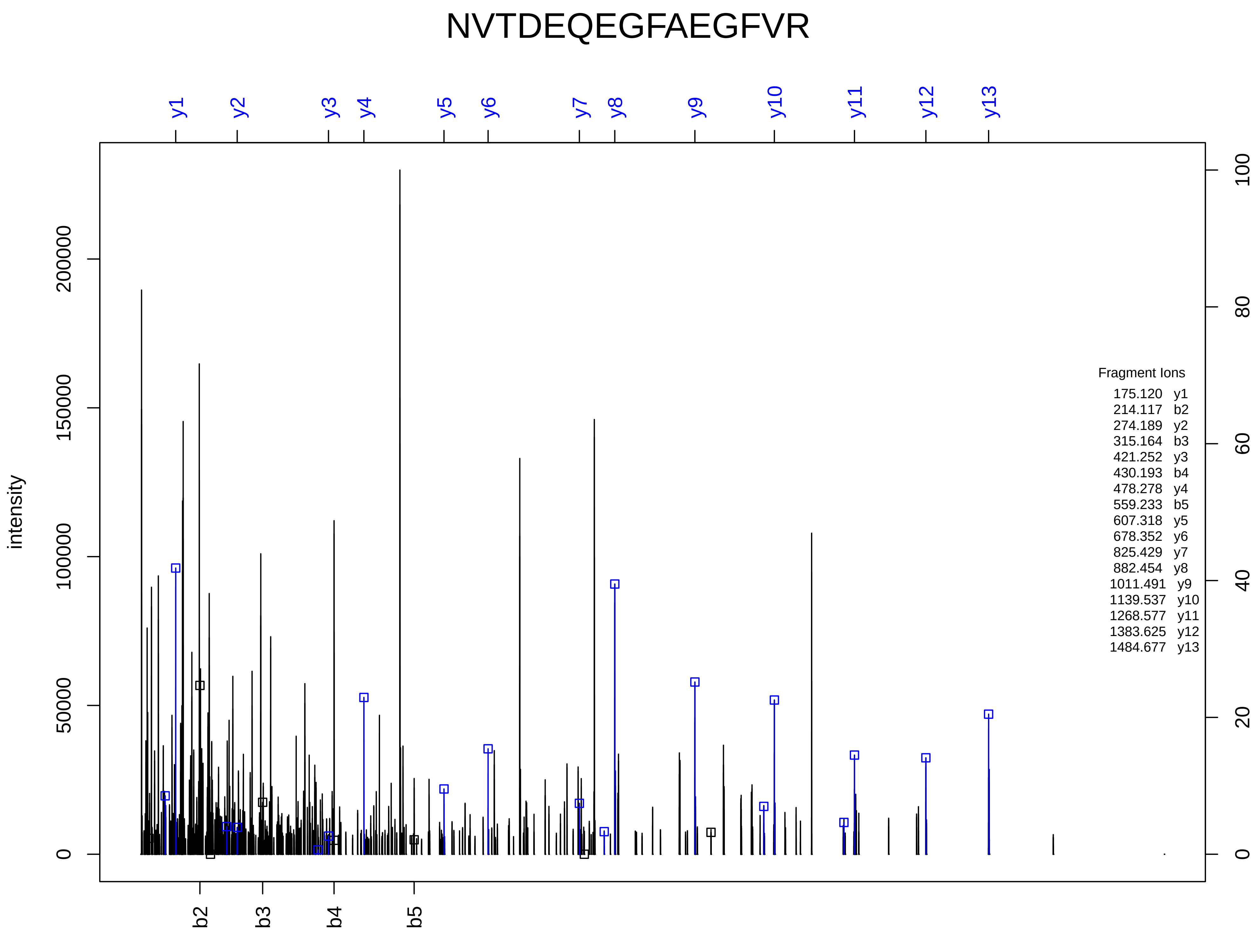

NVTDEQEGFAEGFVR / File: christine_pool_04.raw; SpectrumID: ; scans: 11951

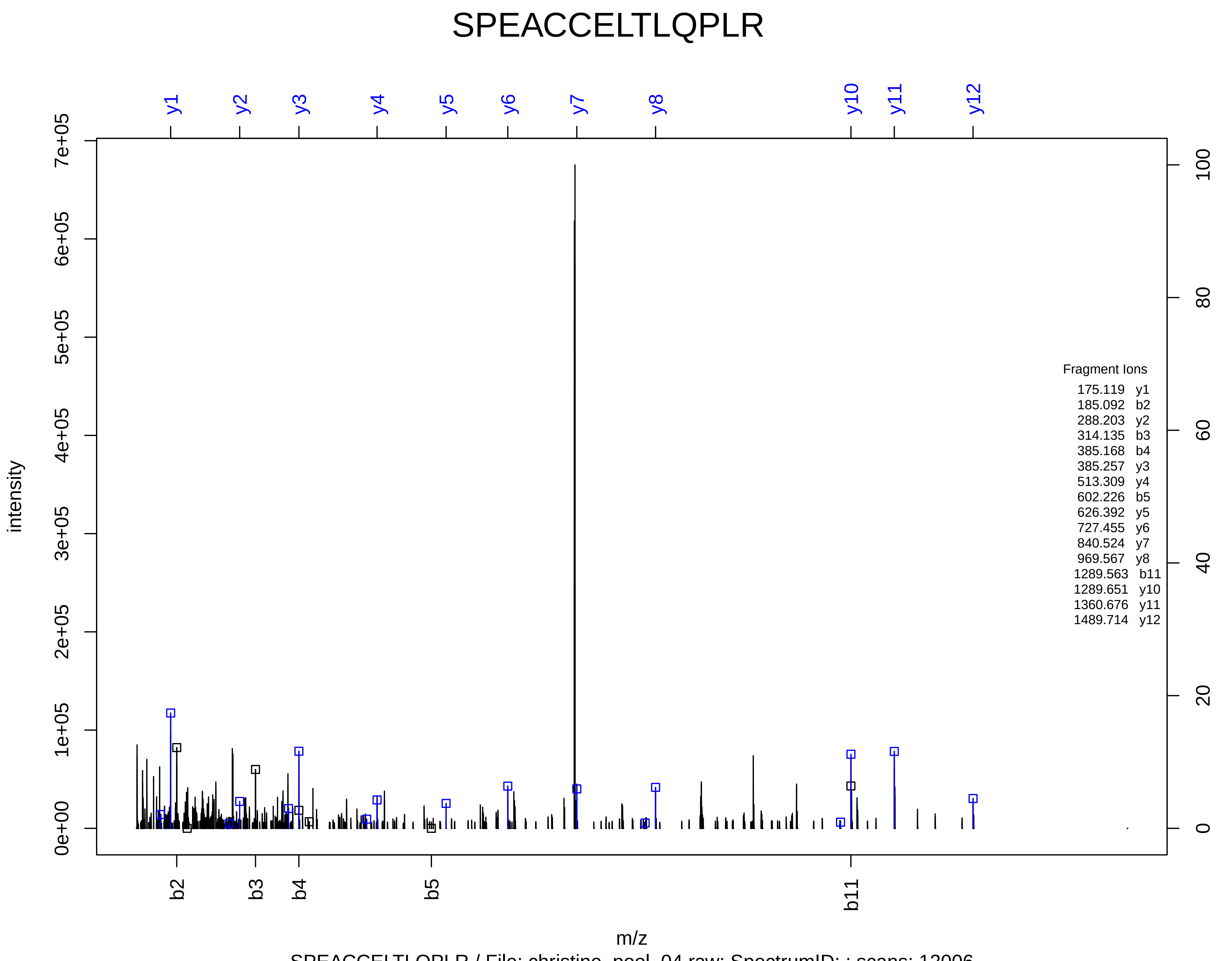
SPEACCELTLQPLR
y1 y2 y3 y4 y5 y6 y7 y8 y10 y11 y12
b2 b3 b4 b5 b11
intensity
m/z
Fragment Ions
175.119 y1
185.092 b2
288.203 y2
314.135 b3
385.168 b4
385.257 y3
513.309 y4
602.226 b5
626.392 y5
727.455 y6
840.524 y7
969.567 y8
1289.563 b11
1289.651 y10
1360.676 y11
1489.714 y12
SPEACCELTLQPLR / File: christine_pool_04.raw; SpectrumID: ; scans: 12006

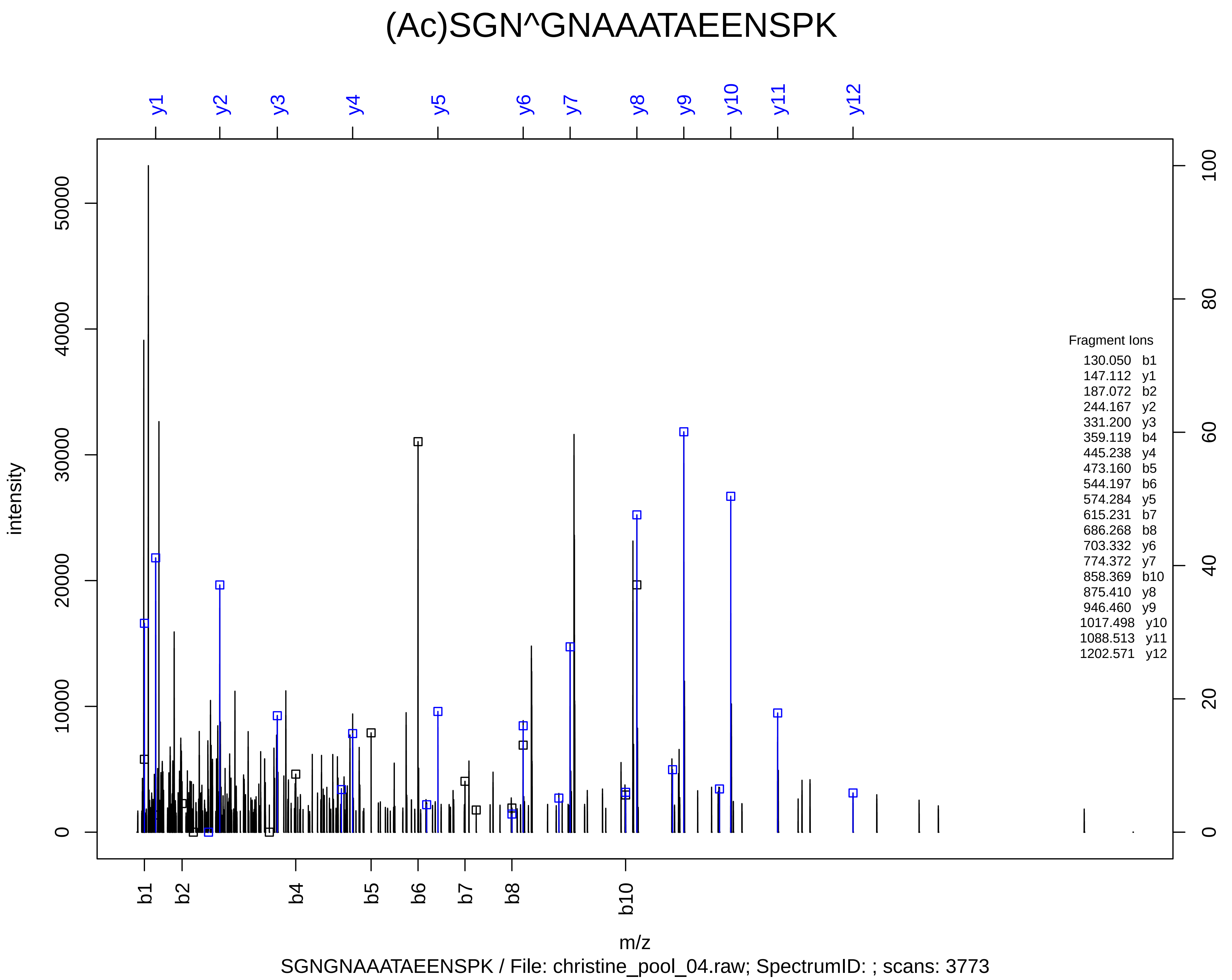
(Ac)SGN^GNAAATAEENSPK
intensity
m/z
Fragment Ions
130.050 b1
147.112 y1
187.072 b2
244.167 y2
331.200 y3
359.119 b4
445.238 y4
473.160 b5
544.197 b6
574.284 y5
615.231 b7
686.268 b8
703.332 y6
774.372 y7
858.369 b10
875.410 y8
946.460 y9
1017.498 y10
1088.513 y11
1202.571 y12
SGNGNAAATAEENSPK / File: christine_pool_04.raw; SpectrumID: ; scans: 3773

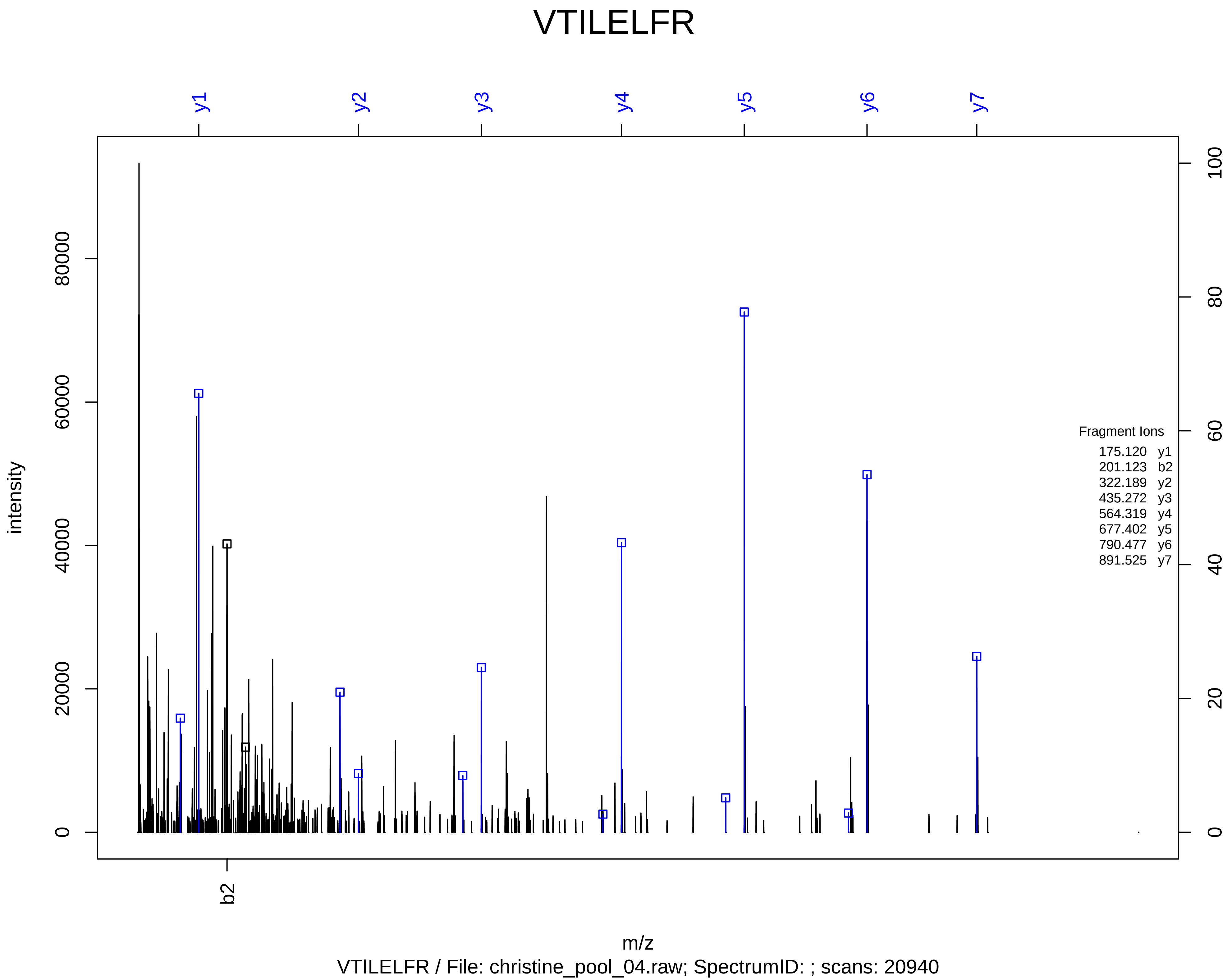
VTILELFR
y1
y2
y3
y4
y5
y6
y7
b2
intensity
m/z
0
20000
40000
60000
80000
100
80
60
40
20
0
Fragment Ions
175.120 y1
201.123 b2
322.189 y2
435.272 y3
564.319 y4
677.402 y5
790.477 y6
891.525 y7
VTILELFR / File: christine_pool_04.raw; SpectrumID: ; scans: 20940

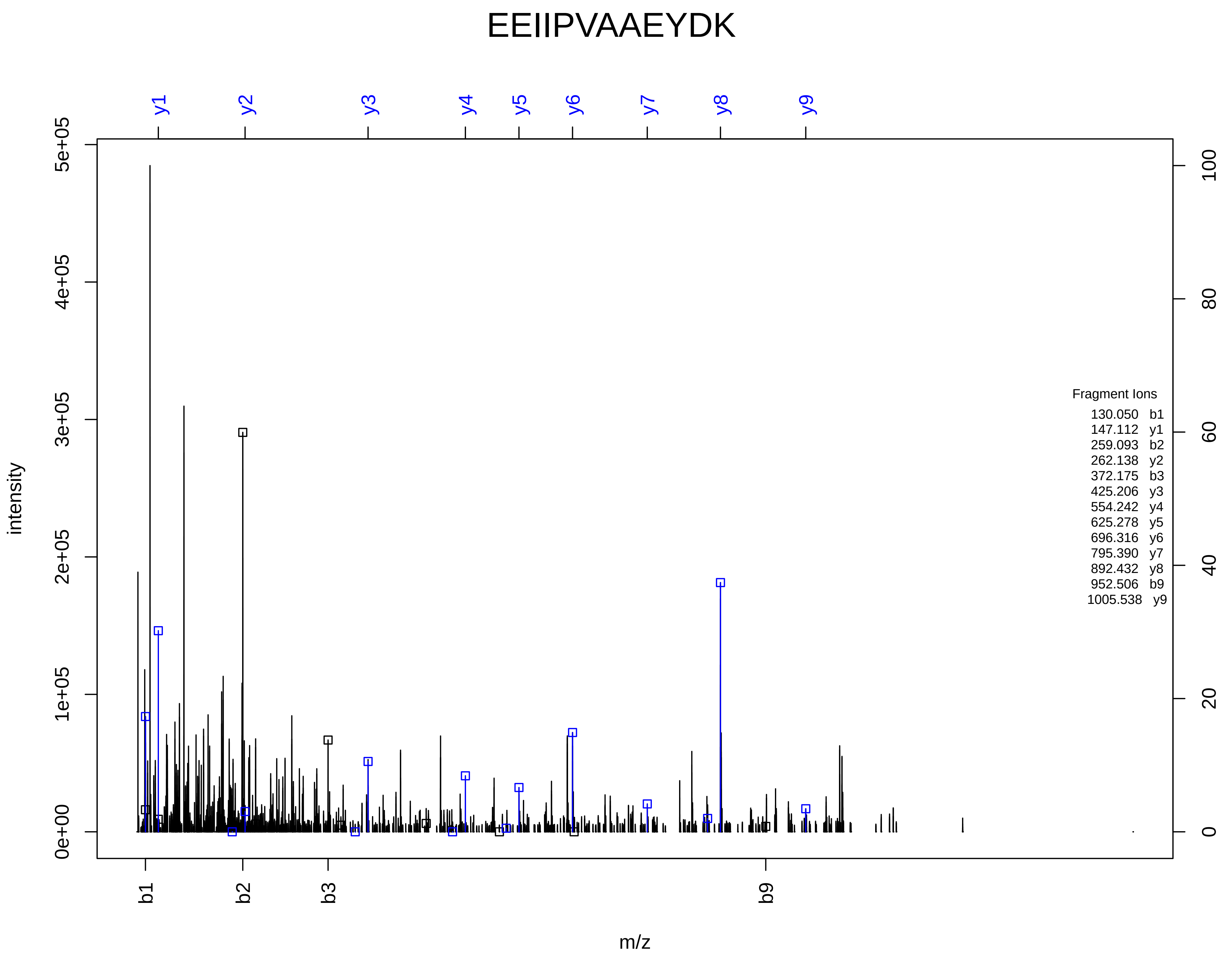

EEIIPVAAEYDK / File: christine_pool_04.raw; SpectrumID: ; scans: 11712

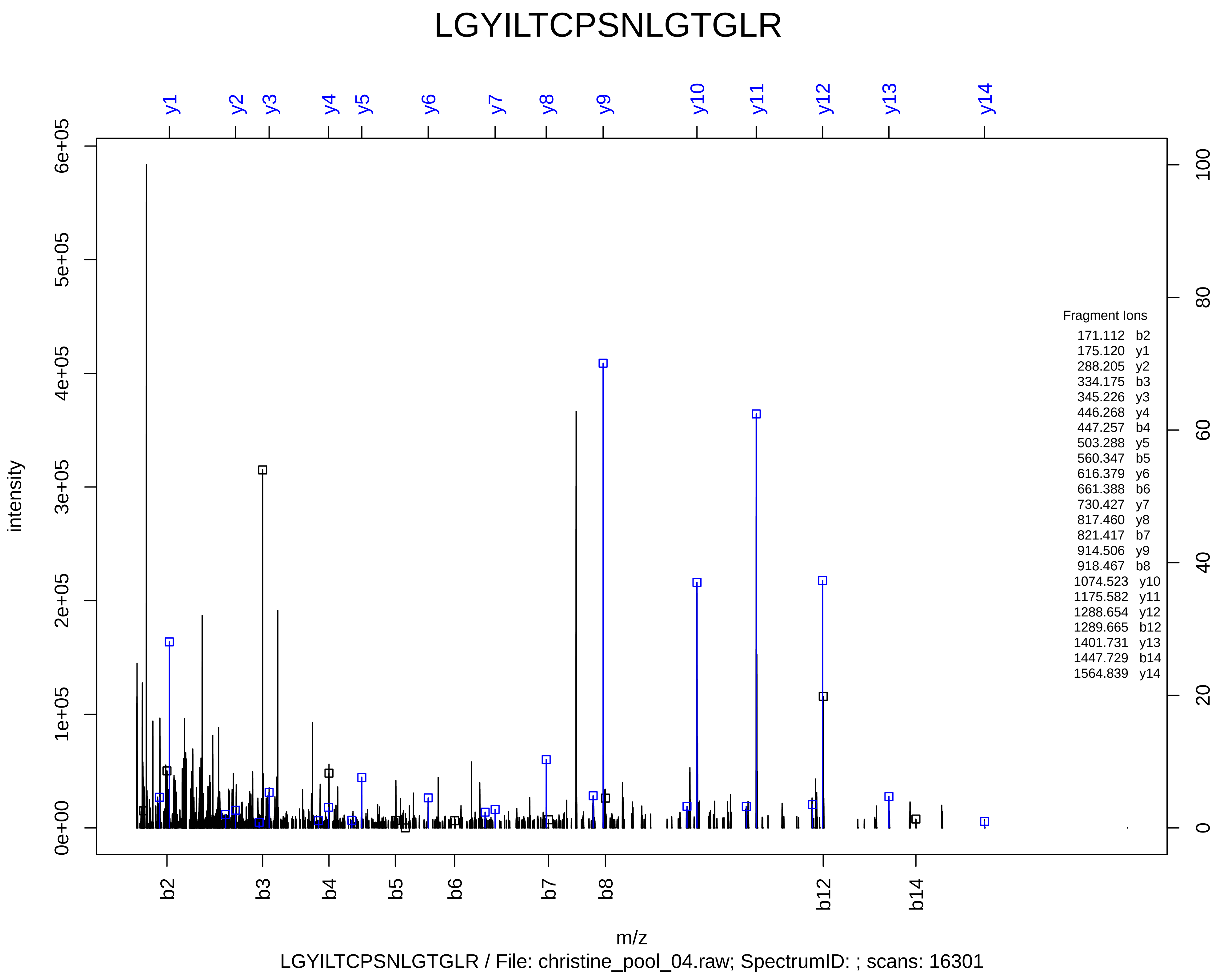
LGYILTCPSNLGTGLR
intensity
m/z
Fragment Ions
171.112 b2
175.120 y1
288.205 y2
334.175 b3
345.226 y3
446.268 y4
447.257 b4
503.288 y5
560.347 b5
616.379 y6
661.388 b6
730.427 y7
817.460 y8
821.417 b7
914.506 y9
918.467 b8
1074.523 y10
1175.582 y11
1288.654 y12
1289.665 b12
1401.731 y13
1447.729 b14
1564.839 y14
LGYILTCPSNLGTGLR / File: christine_pool_04.raw; SpectrumID: ; scans: 16301

DETEFYLGK

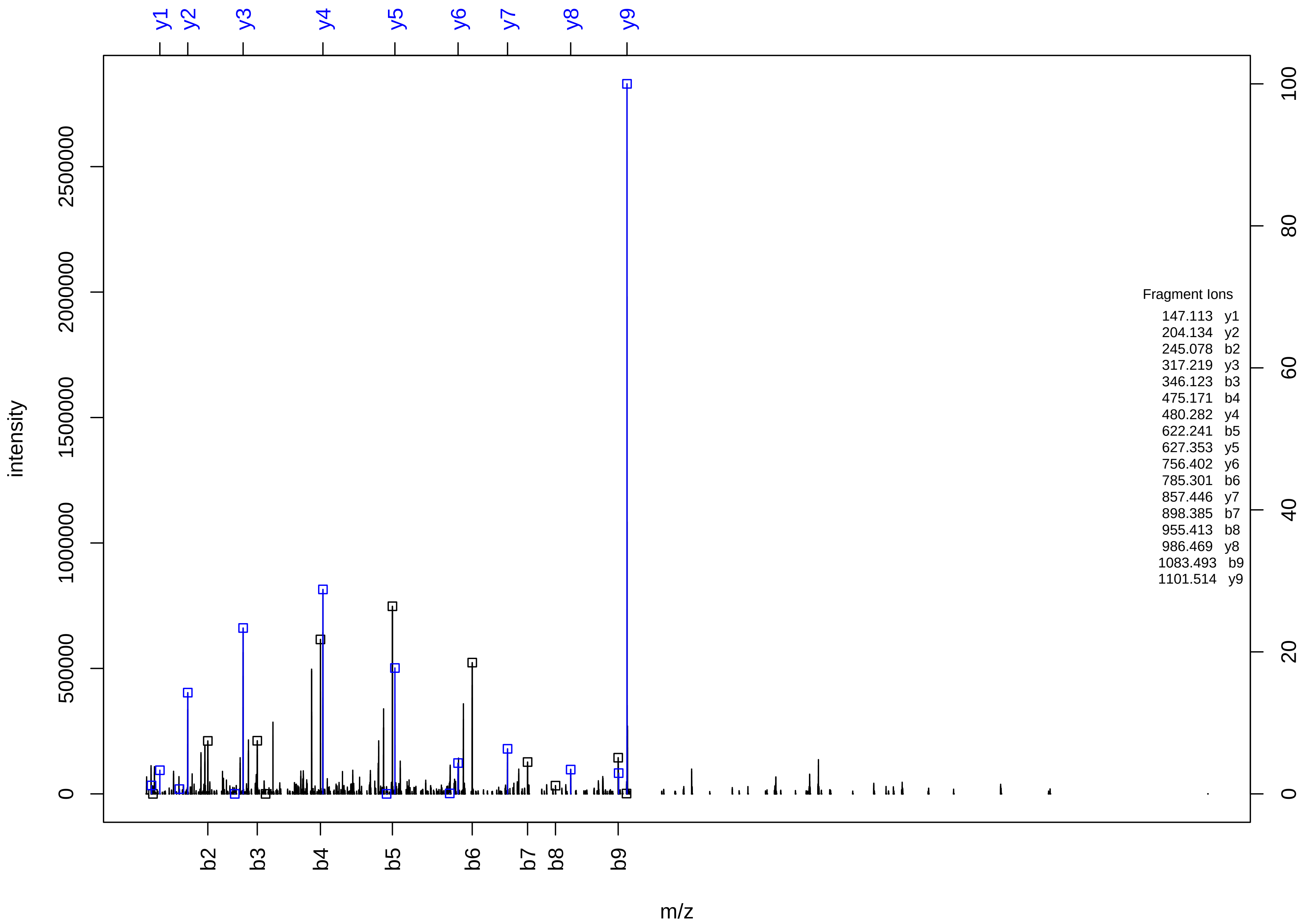

DETEFYLGK / File: christine_pool_04.raw; SpectrumID: ; scans: 9819

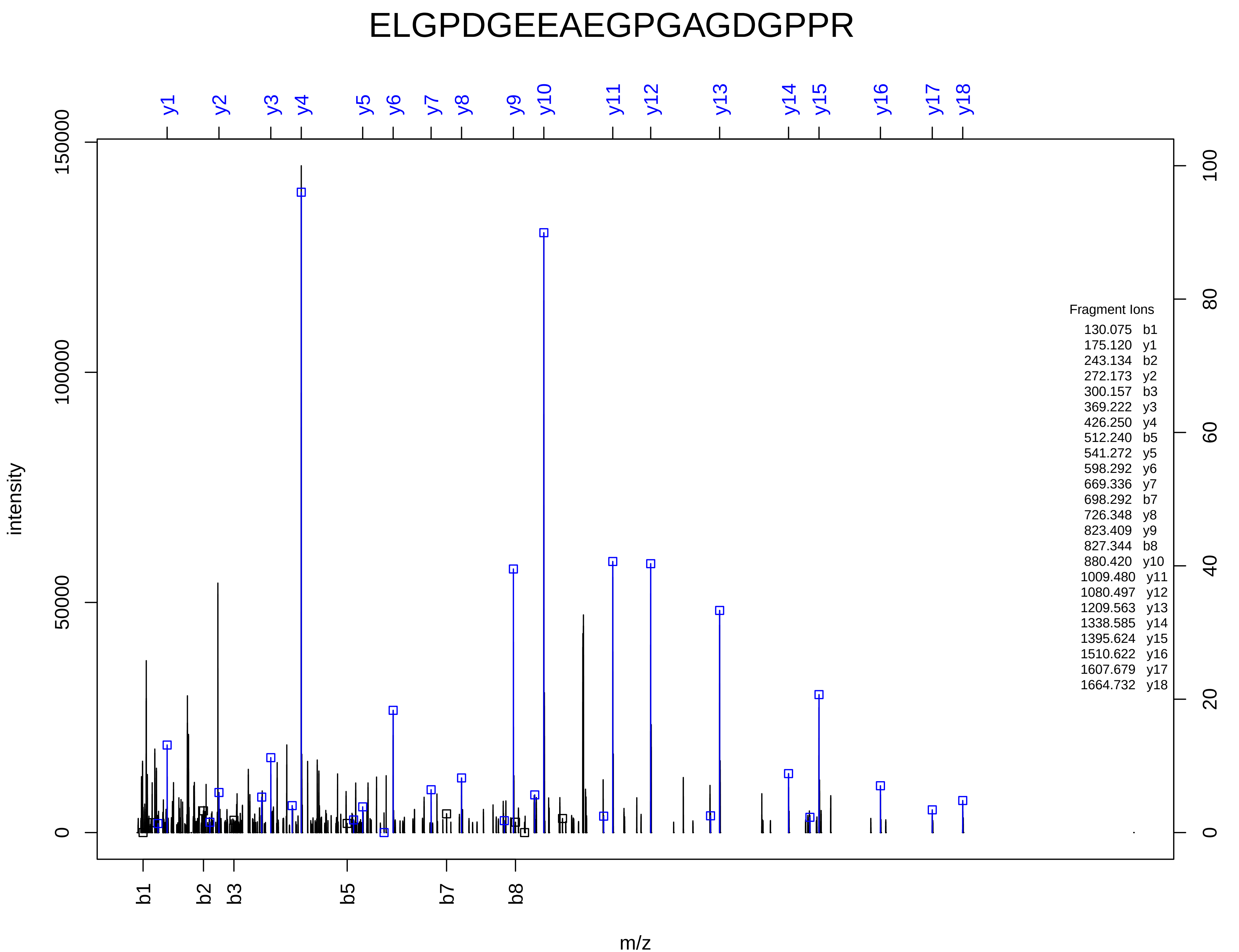

ELGPDGEEAEGPGAGDGPPR / File: christine_pool_04.raw; SpectrumID: ; scans: 5438

RQTSGGPVDASSEYQQELER

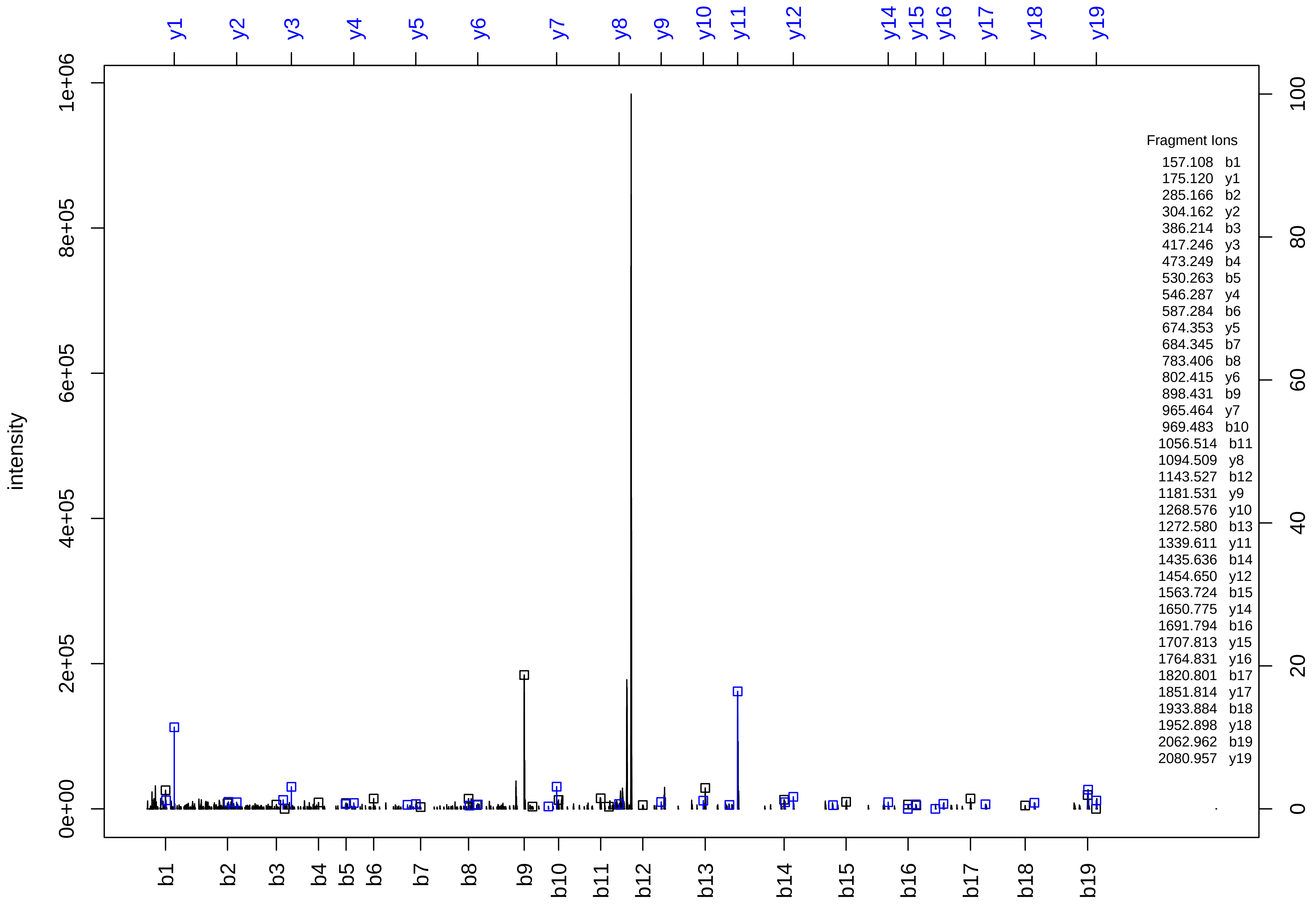

RQTSGGPVDASSEYQQELER / File: christine_pool_04.raw; SpectrumID: ; scans: 6304

YGDLLPADGILIQGNDLK

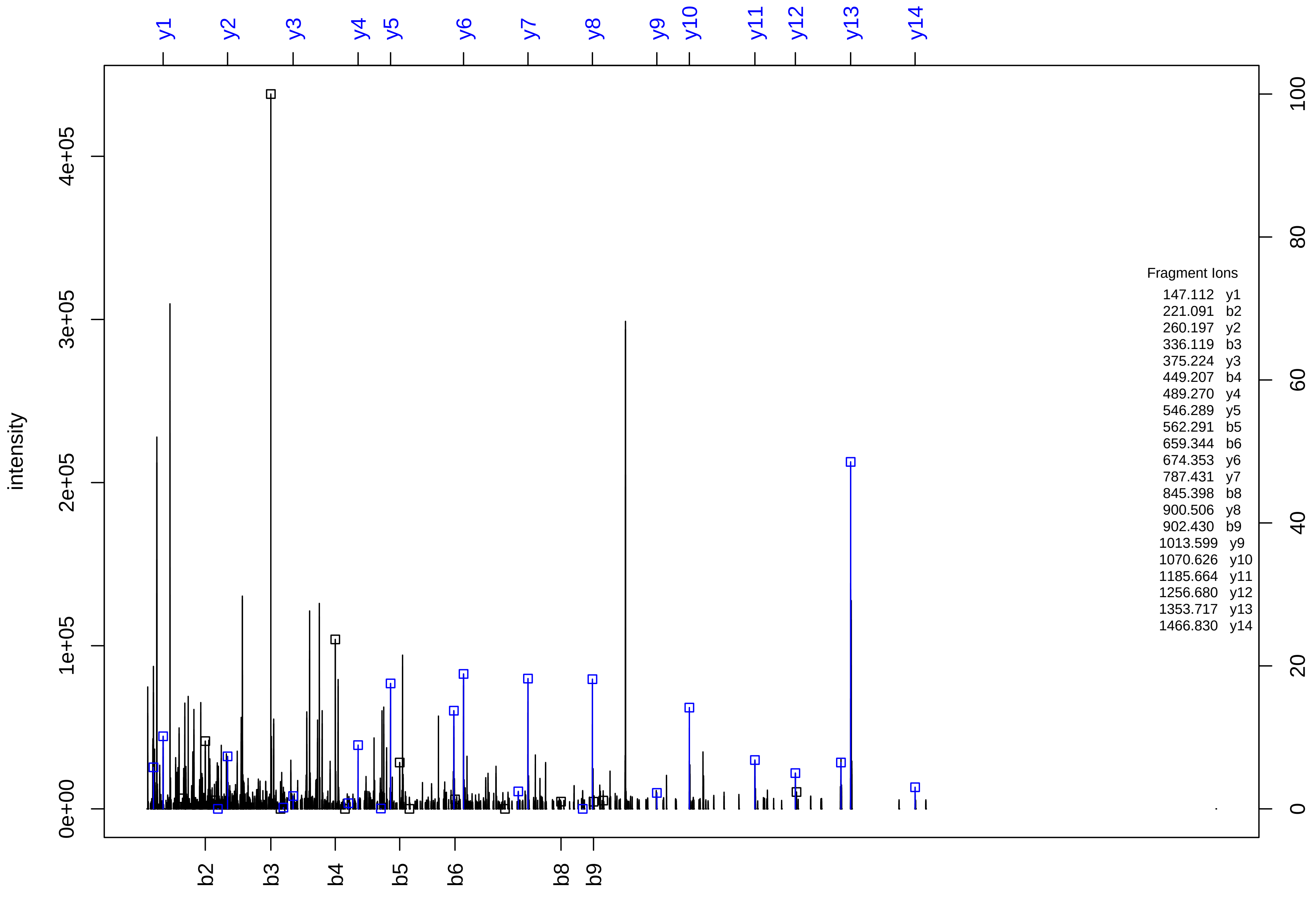

YGDLLPADGILIQGNDLK / File: christine_pool_04.raw; SpectrumID: ; scans: 19702

DVLETFTVK

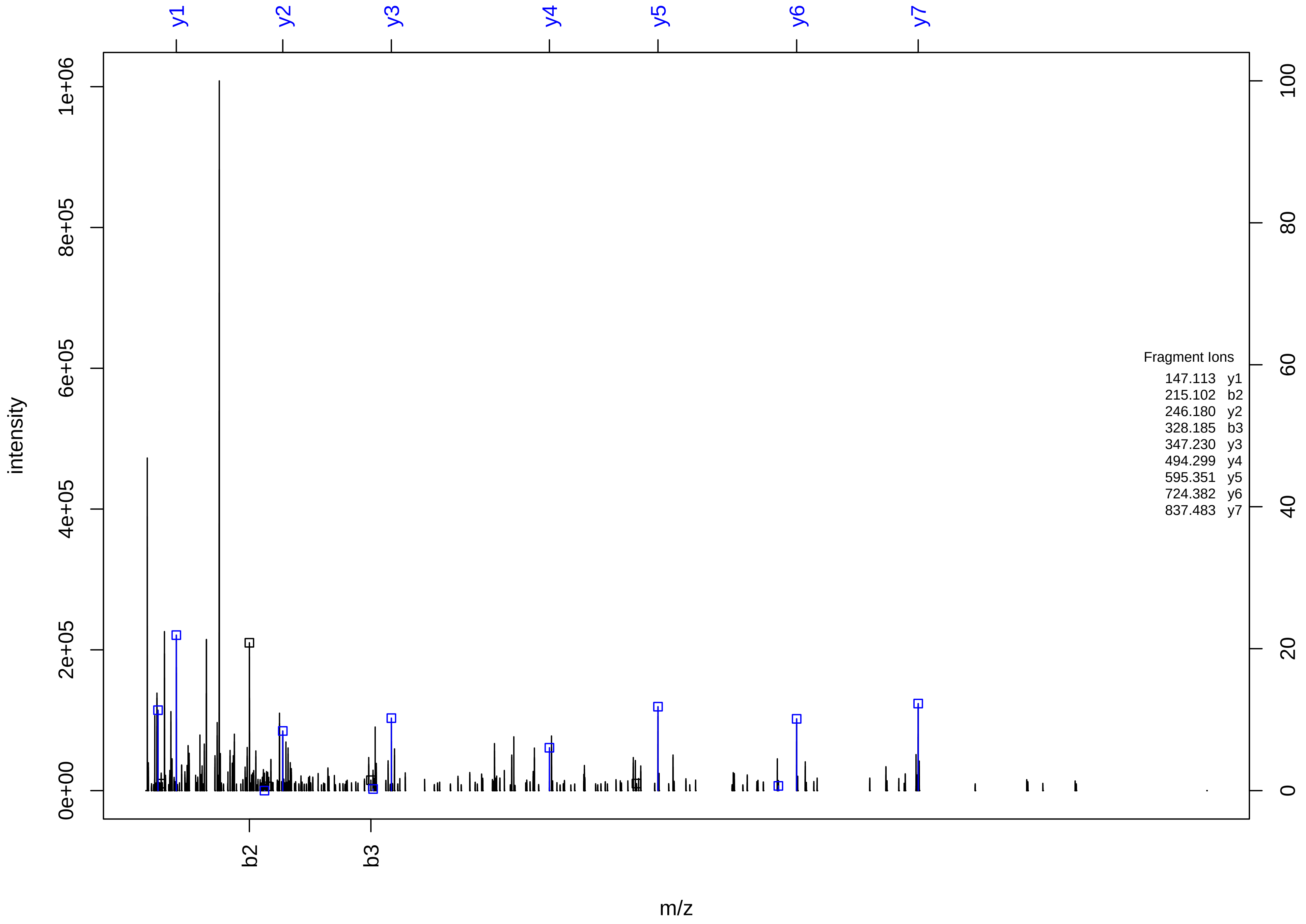

DVLETFTVK / File: christine_pool_04.raw; SpectrumID: ; scans: 13362

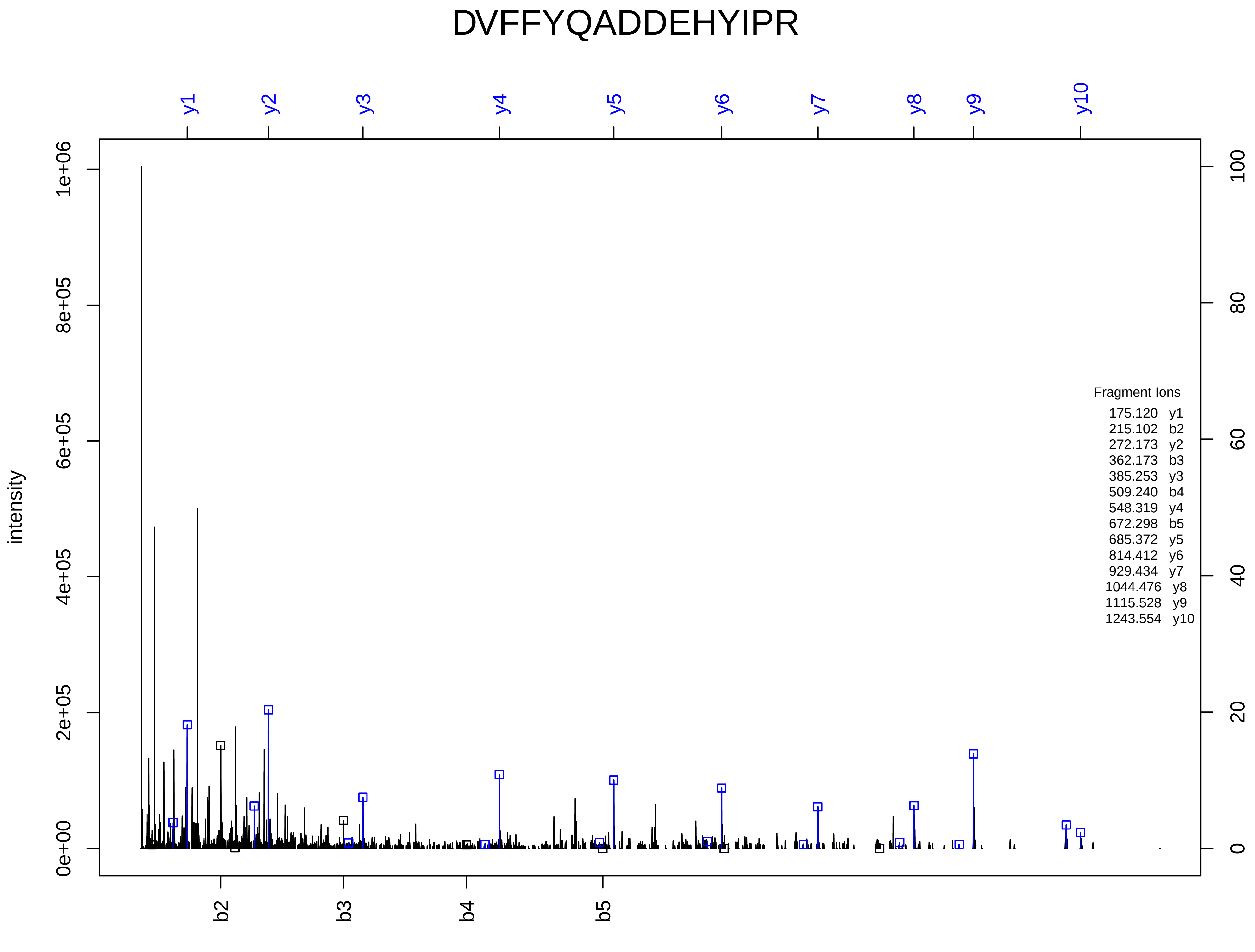

DVFFYQADDEHYIPR / File: christine_pool_04.raw; SpectrumID: ; scans: 14073

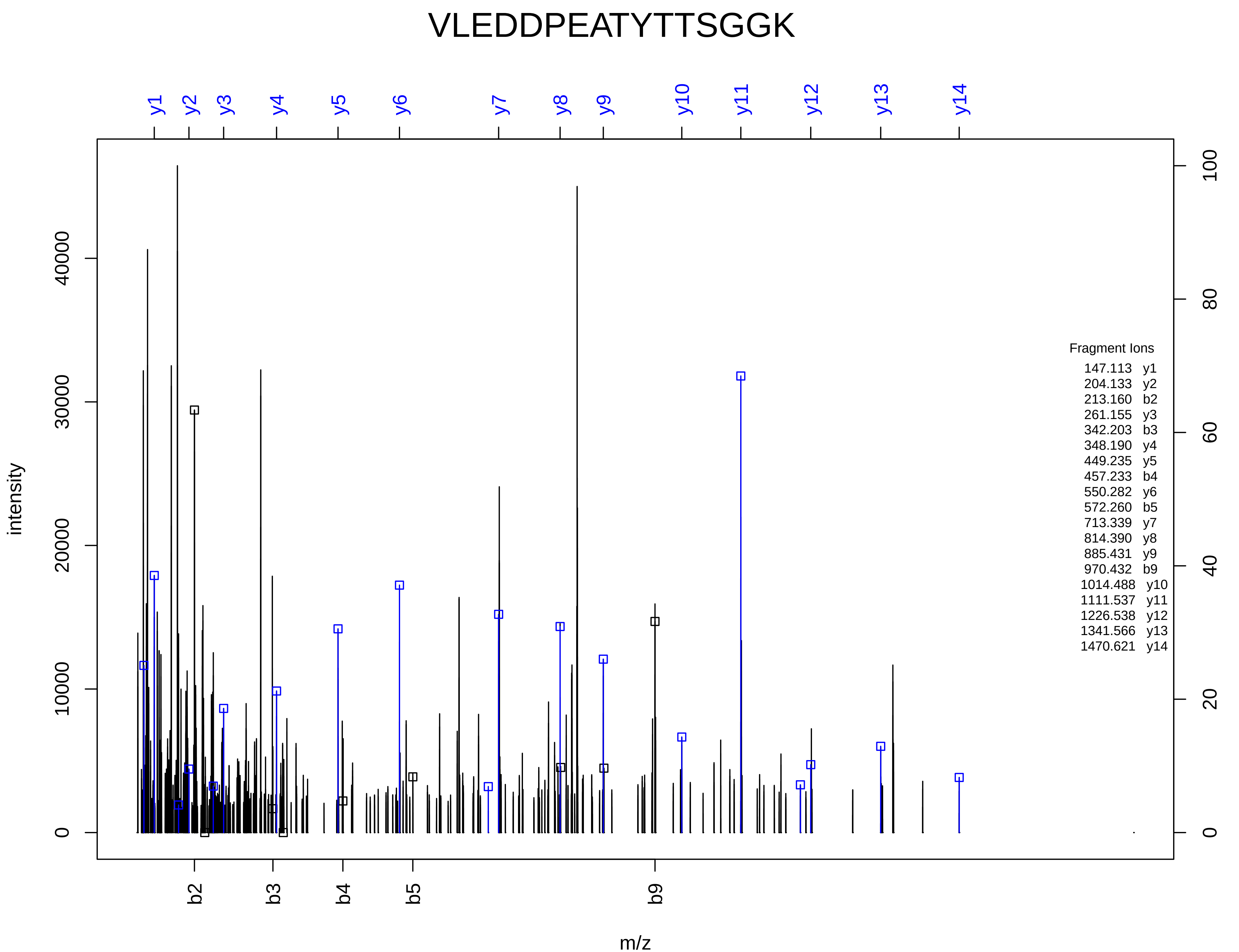
VLEDDPEATYTTSGGK
y1
y2
y3
y4
y5
y6
y7
y8
y9
y10
y11
y12
y13
y14
intensity
0
10000
20000
30000
40000
0
20
40
60
80
100
Fragment Ions
147.113 y1
204.133 y2
213.160 b2
261.155 y3
342.203 b3
348.190 y4
449.235 y5
457.233 b4
550.282 y6
572.260 b5
713.339 y7
814.390 y8
885.431 y9
970.432 b9
1014.488 y10
1111.537 y11
1226.538 y12
1341.566 y13
1470.621 y14
b2
b3
b4
b5
b9
m/z
VLEDDPEATYTTSGGK / File: christine_pool_04.raw; SpectrumID: ; scans: 5882

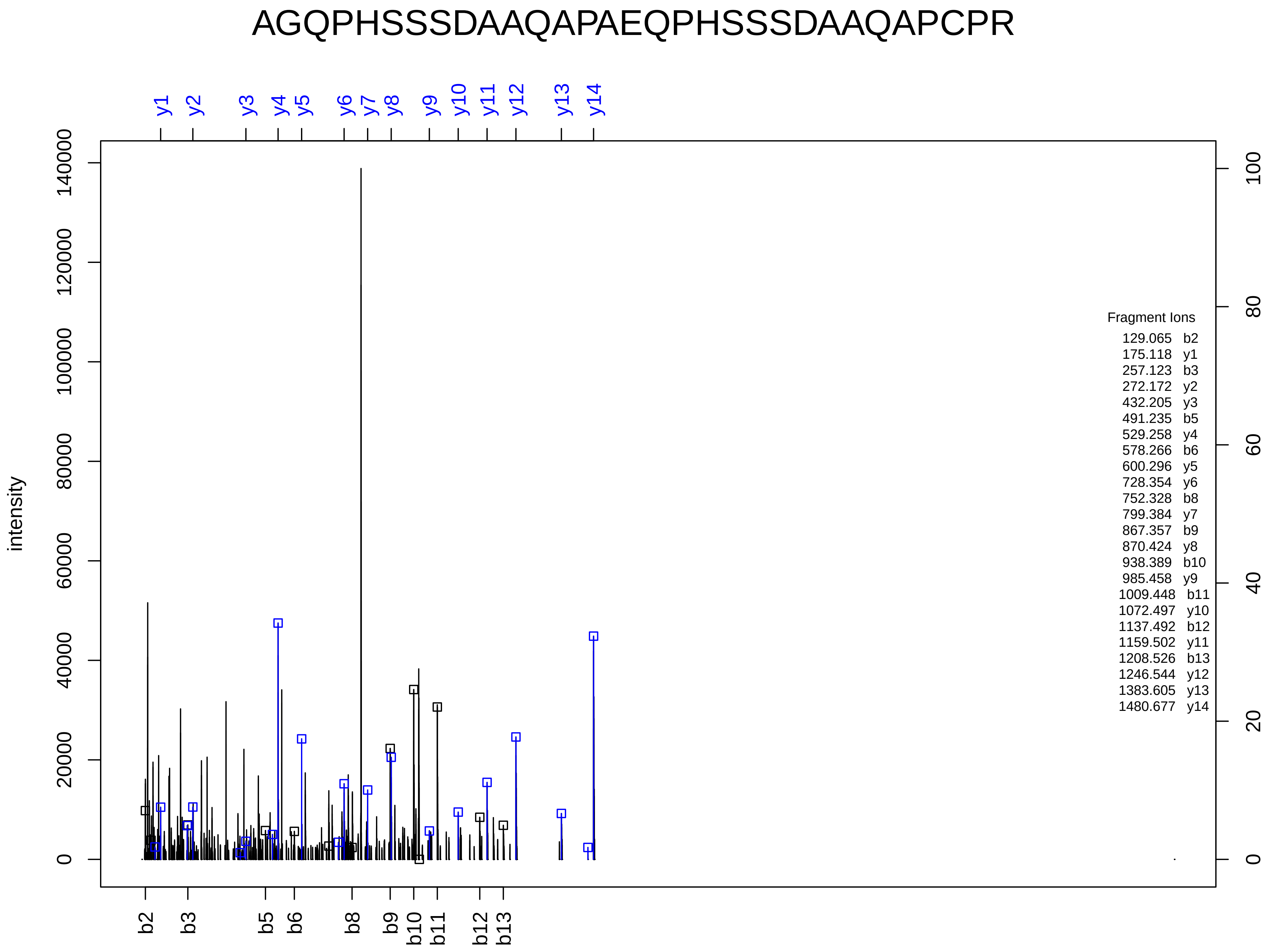

AGQPHSSSDAAQAPAEQPHSSSDAAQAPCPR / File: christine_pool_04.raw; SpectrumID: ; scans: 2850

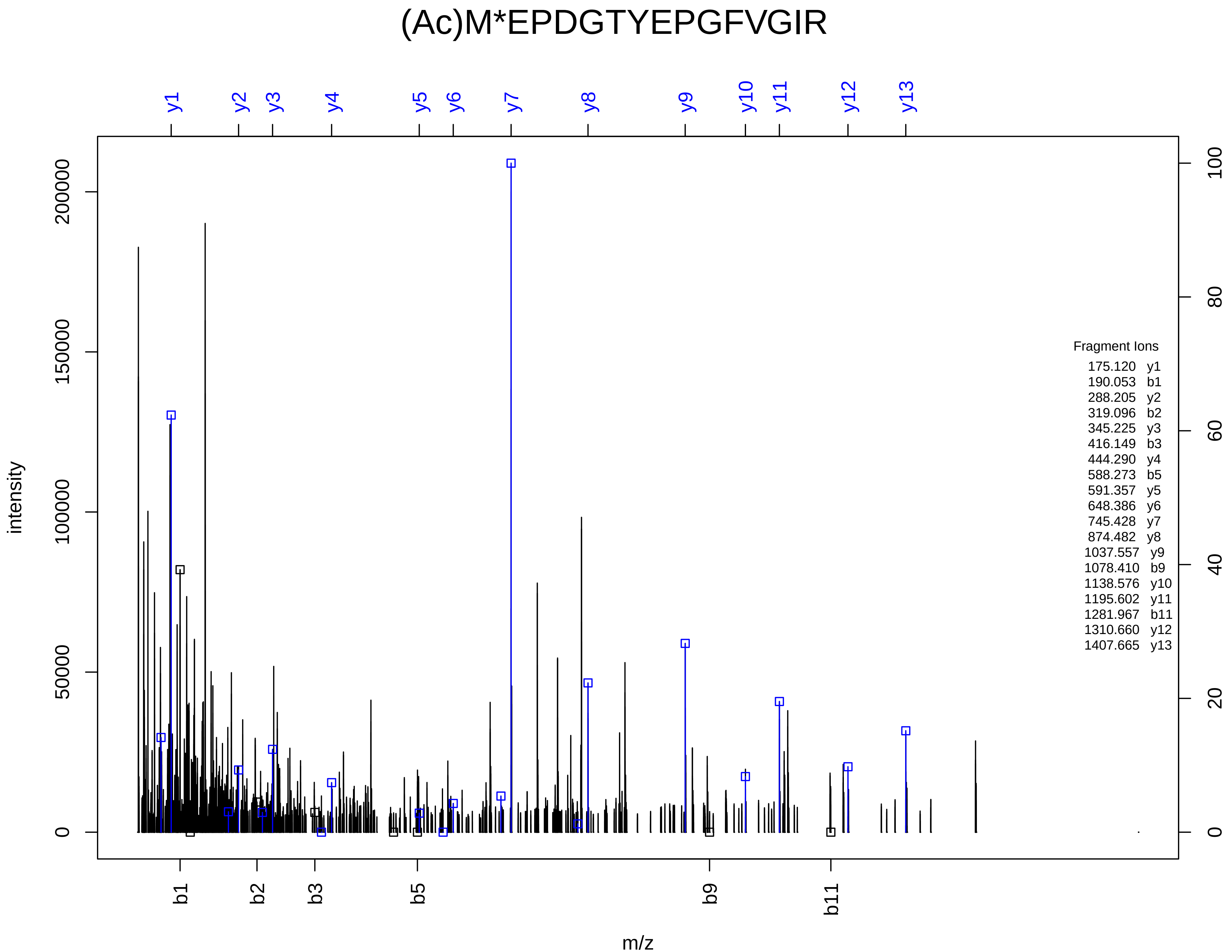

MEPDGTYEPGFVGIR / File: christine_pool_04.raw; SpectrumID: ; scans: 15791

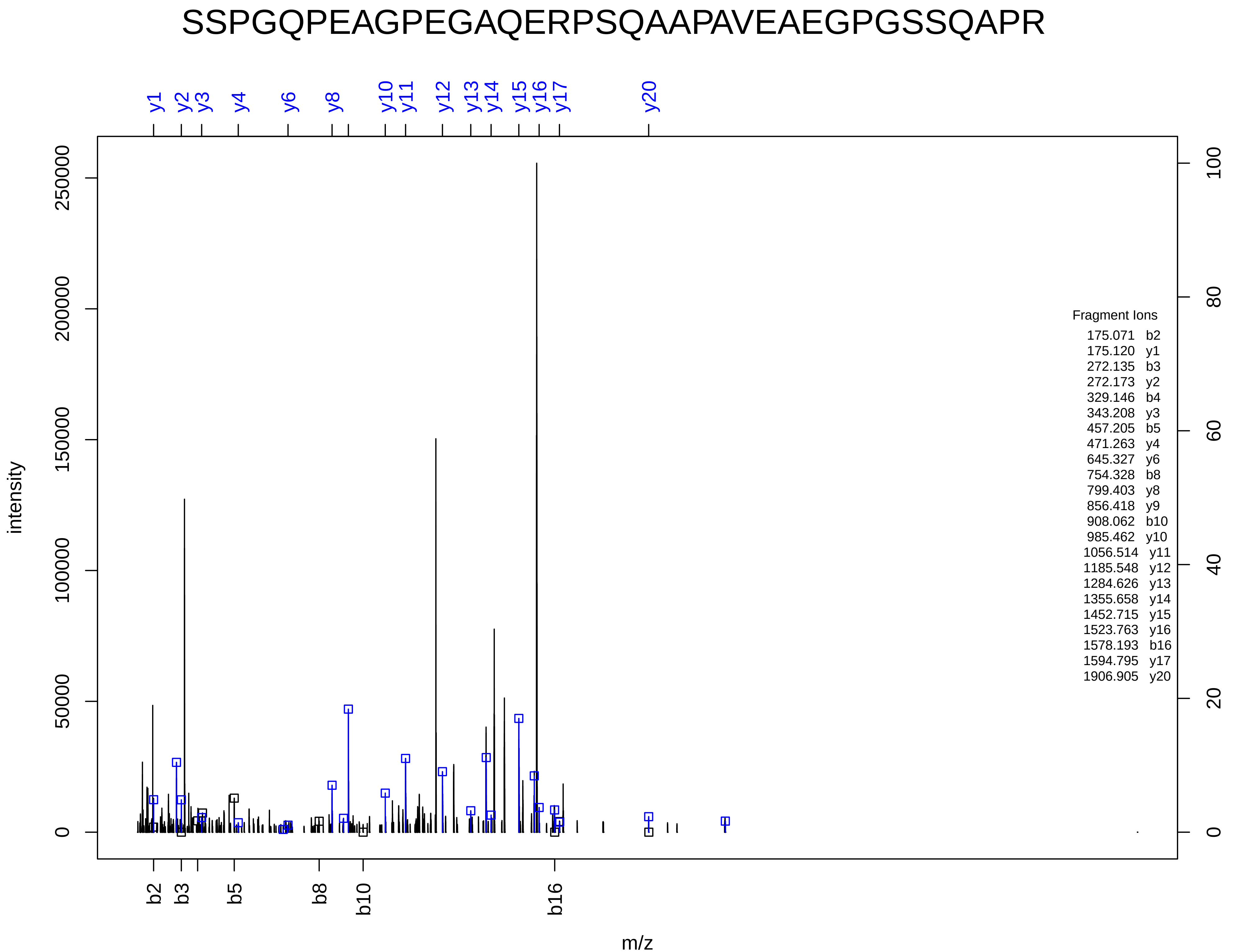

SSPGQPEAGPEGAQERPSQAAPAVEAEGPGSSQAPR / File: christine_pool_04.raw; SpectrumID: ; scans: 5661

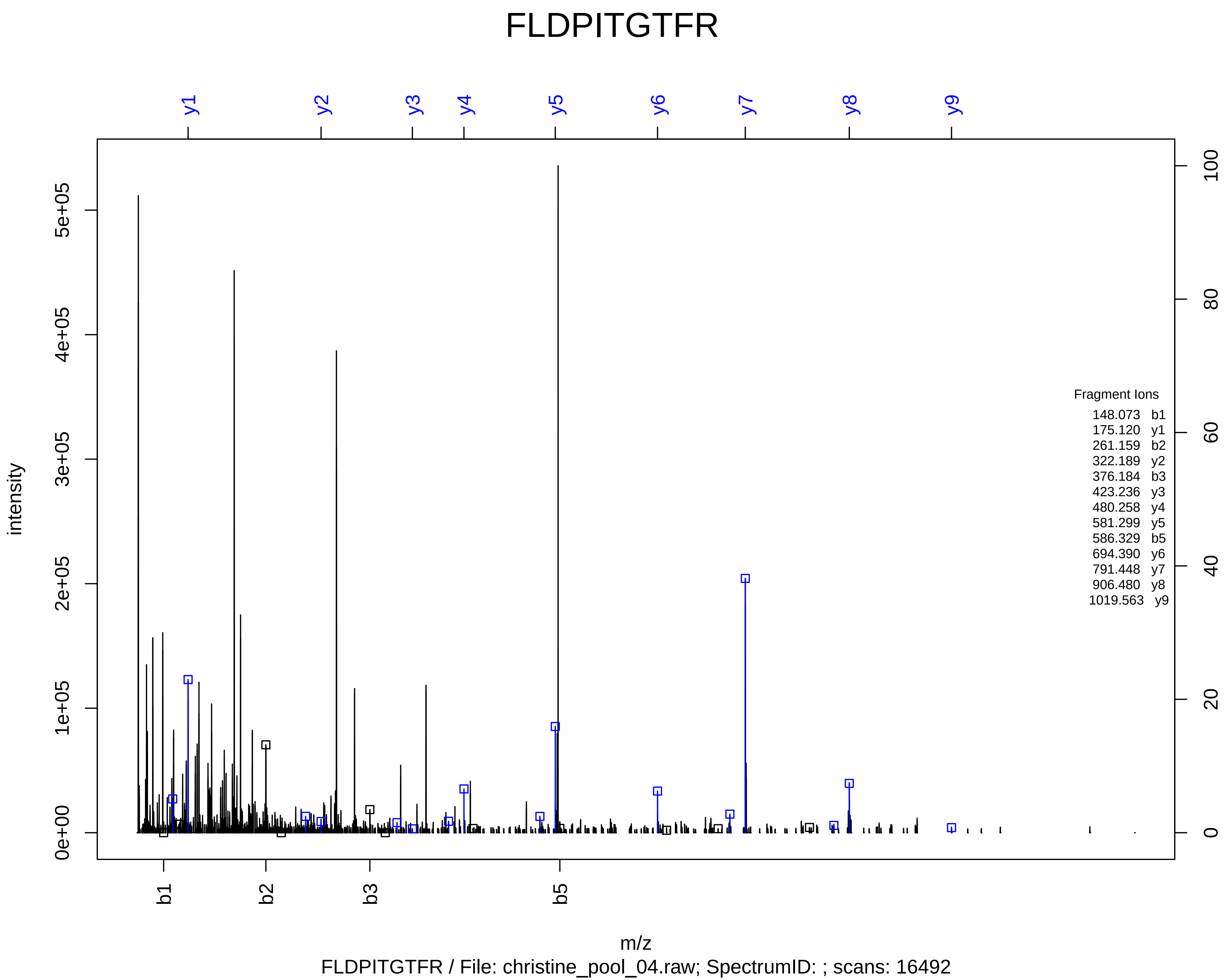
FLDPITGTFR
y1
y2
y3
y4
y5
y6
y7
y8
y9
b1
b2
b3
b5
intensity
m/z
0e+00
1e+05
2e+05
3e+05
4e+05
5e+05
0
20
40
60
80
100
Fragment Ions
148.073 b1
175.120 y1
261.159 b2
322.189 y2
376.184 b3
423.236 y3
480.258 y4
581.299 y5
586.329 b5
694.390 y6
791.448 y7
906.480 y8
1019.563 y9
FLDPITGTFR / File: christine_pool_04.raw; SpectrumID: ; scans: 16492

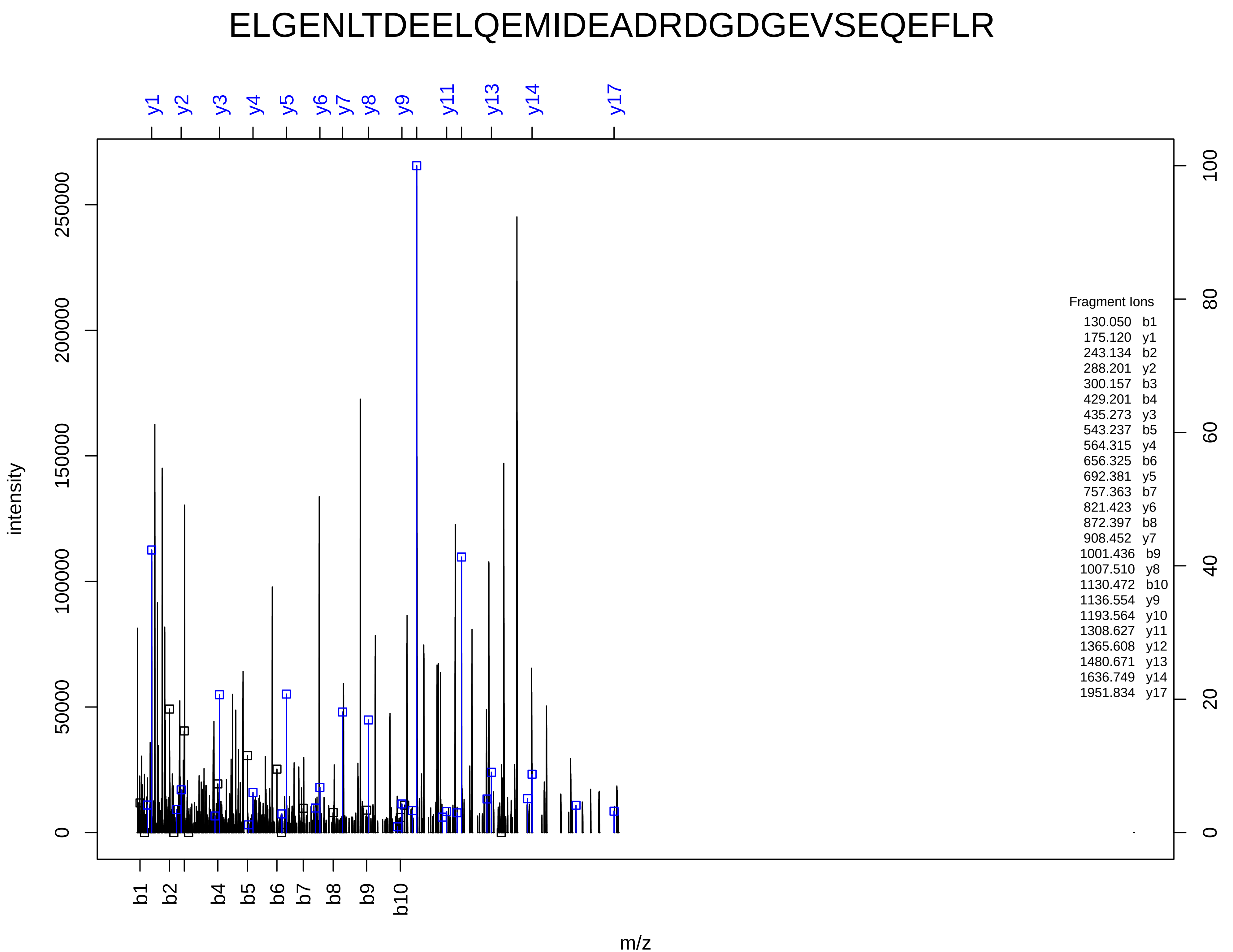

ELGENLTDEELQEMIDEADRDGDGEVSEQEFLR / File: christine_pool_04.raw; SpectrumID: ; scans: 21491

TSTTSSMVASAEQPR

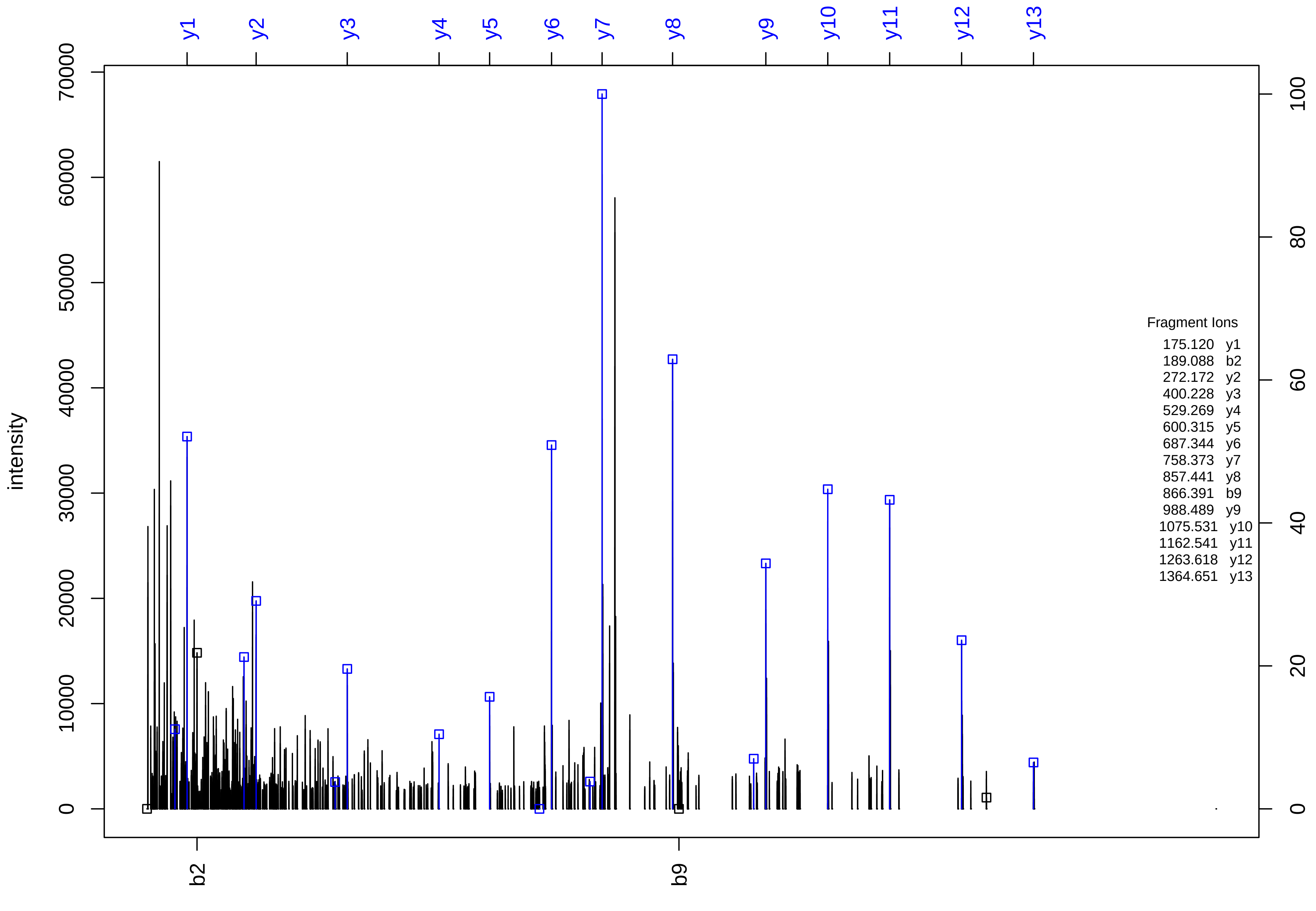

TSTTSSMVASAEQPR / File: christine_pool_04.raw; SpectrumID: ; scans: 4587

ELVNDDEDIDWVQTEK

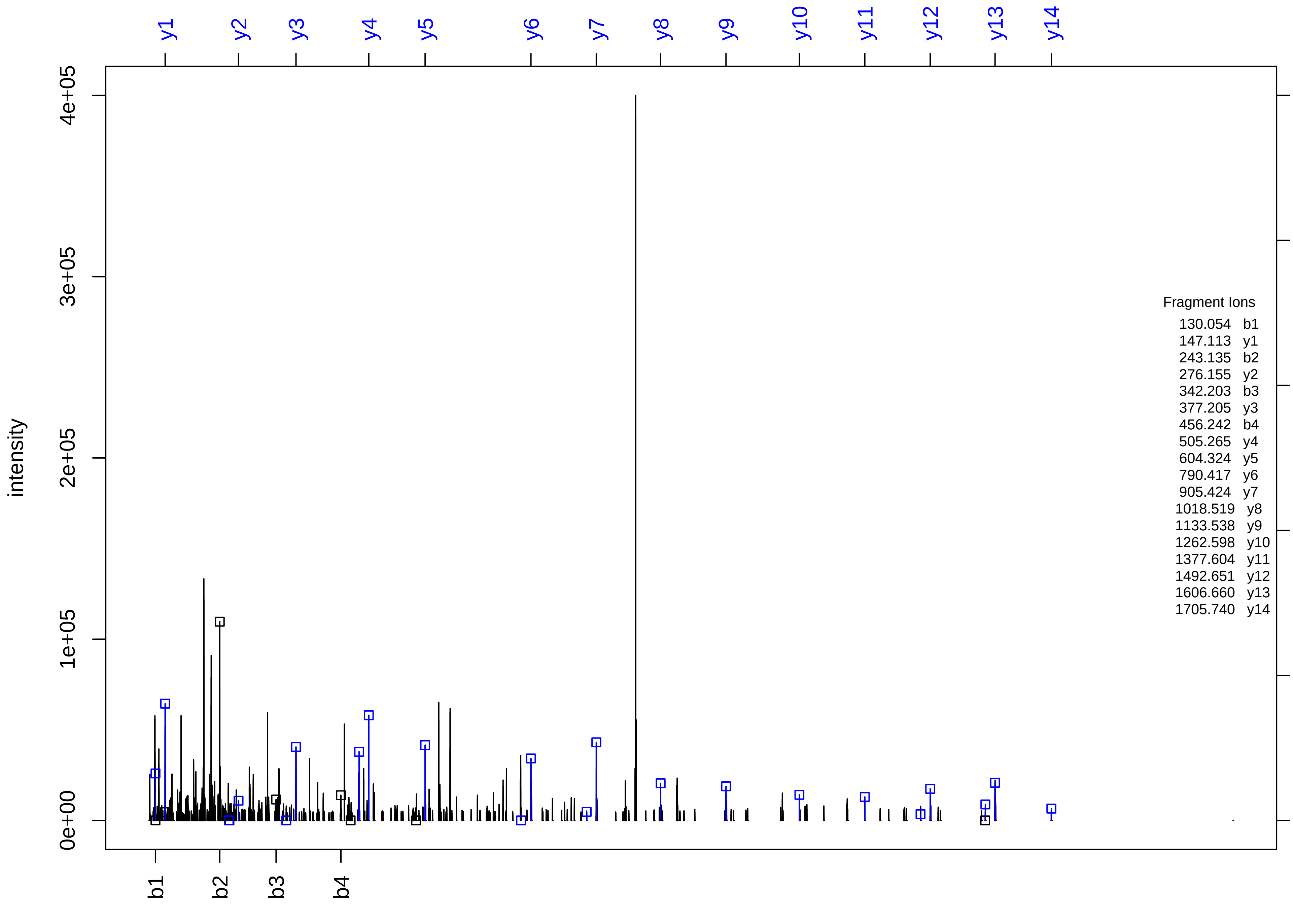

ELVNDDEDIDWVQTEK / File: christine_pool_04.raw; SpectrumID: ; scans: 13111

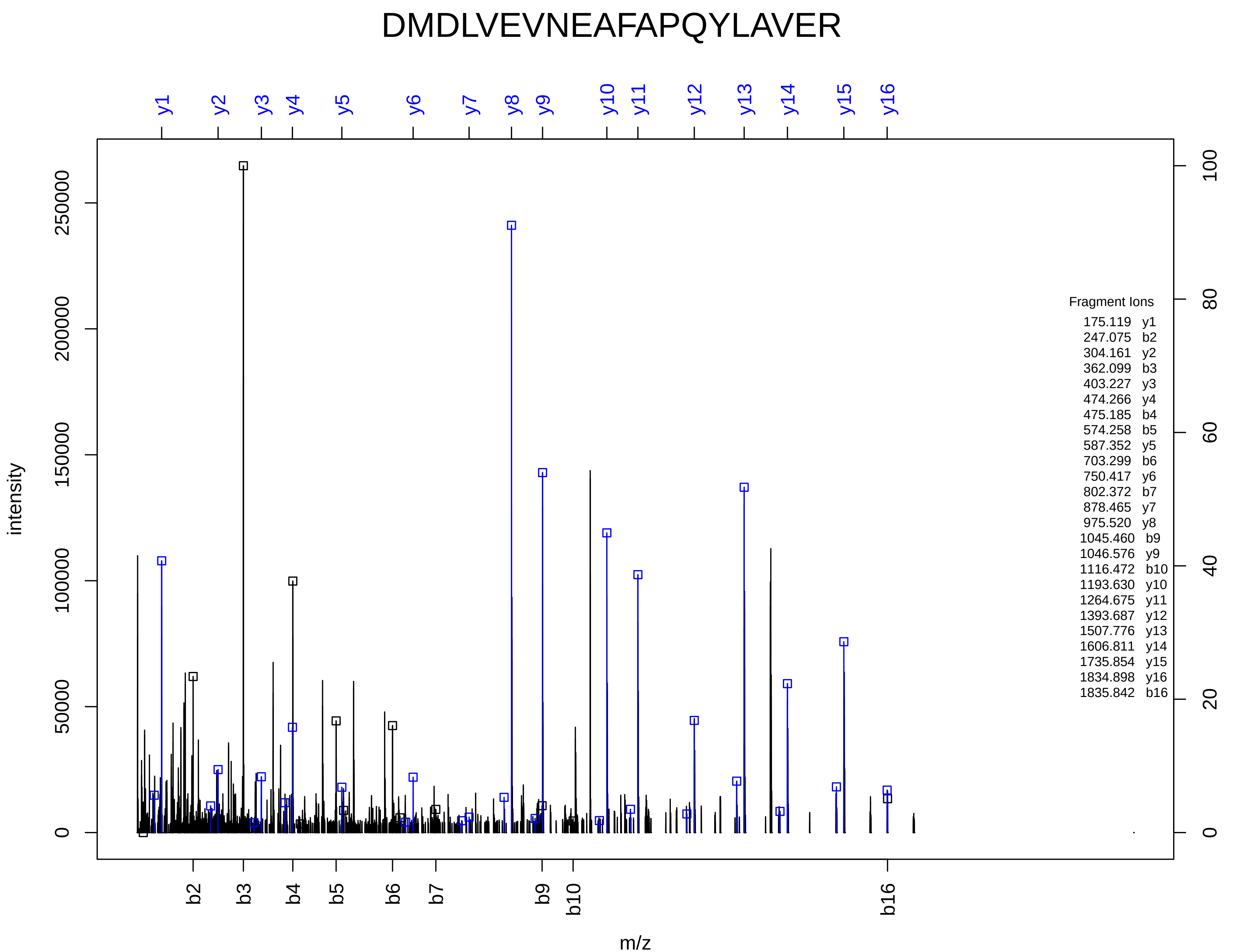

DMDLVEVNEAFAPQYLAVER / File: christine_pool_04.raw; SpectrumID: ; scans: 22265

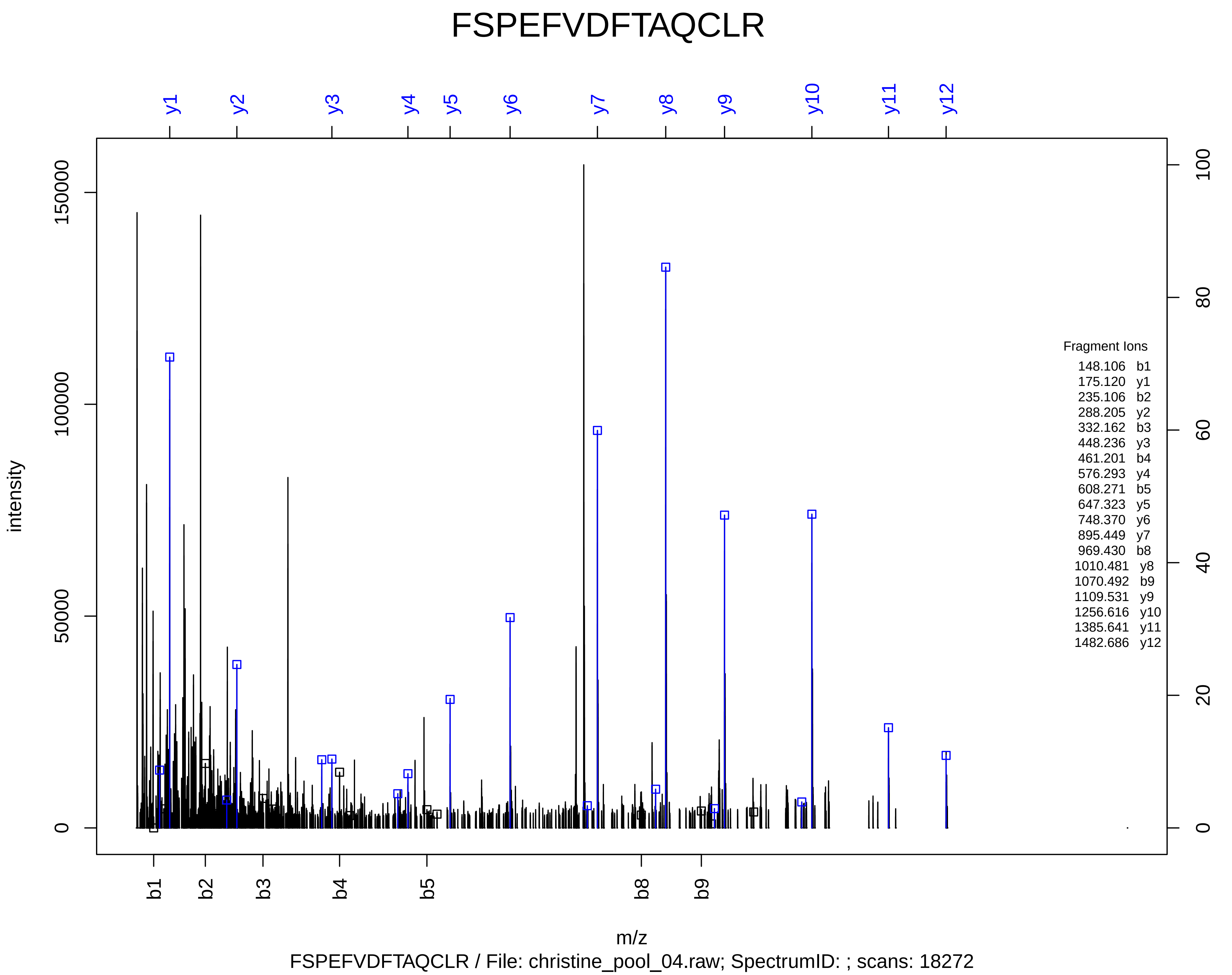

FSPEFVDFTAQCLR
y1
y2
y3
y4
y5
y6
y7
y8
y9
y10
y11
y12
b1
b2
b3
b4
b5
b8
b9
intensity
m/z
Fragment Ions
148.106 b1
175.120 y1
235.106 b2
288.205 y2
332.162 b3
448.236 y3
461.201 b4
576.293 y4
608.271 b5
647.323 y5
748.370 y6
895.449 y7
969.430 b8
1010.481 y8
1070.492 b9
1109.531 y9
1256.616 y10
1385.641 y11
1482.686 y12
FSPEFVDFTAQCLR / File: christine_pool_04.raw; SpectrumID: ; scans: 18272

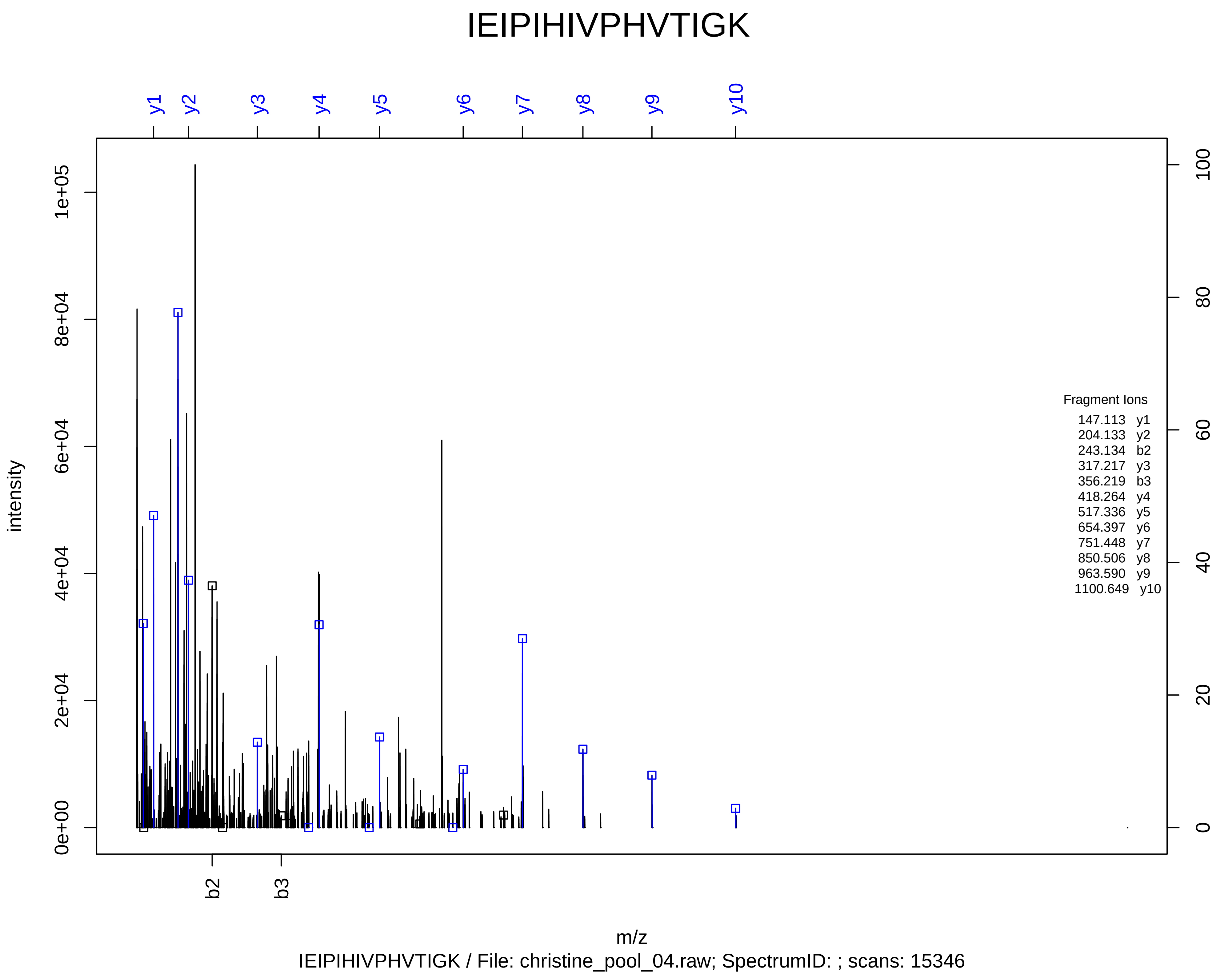
IEIPIHIVPHVTIGK
y1
y2
y3
y4
y5
y6
y7
y8
y9
y10
intensity
0e+00
2e+04
4e+04
6e+04
8e+04
1e+05
0
20
40
60
80
100
b2
b3
m/z
Fragment Ions
147.113 y1
204.133 y2
243.134 b2
317.217 y3
356.219 b3
418.264 y4
517.336 y5
654.397 y6
751.448 y7
850.506 y8
963.590 y9
1100.649 y10
IEIPIHIVPHVTIGK / File: christine_pool_04.raw; SpectrumID: ; scans: 15346

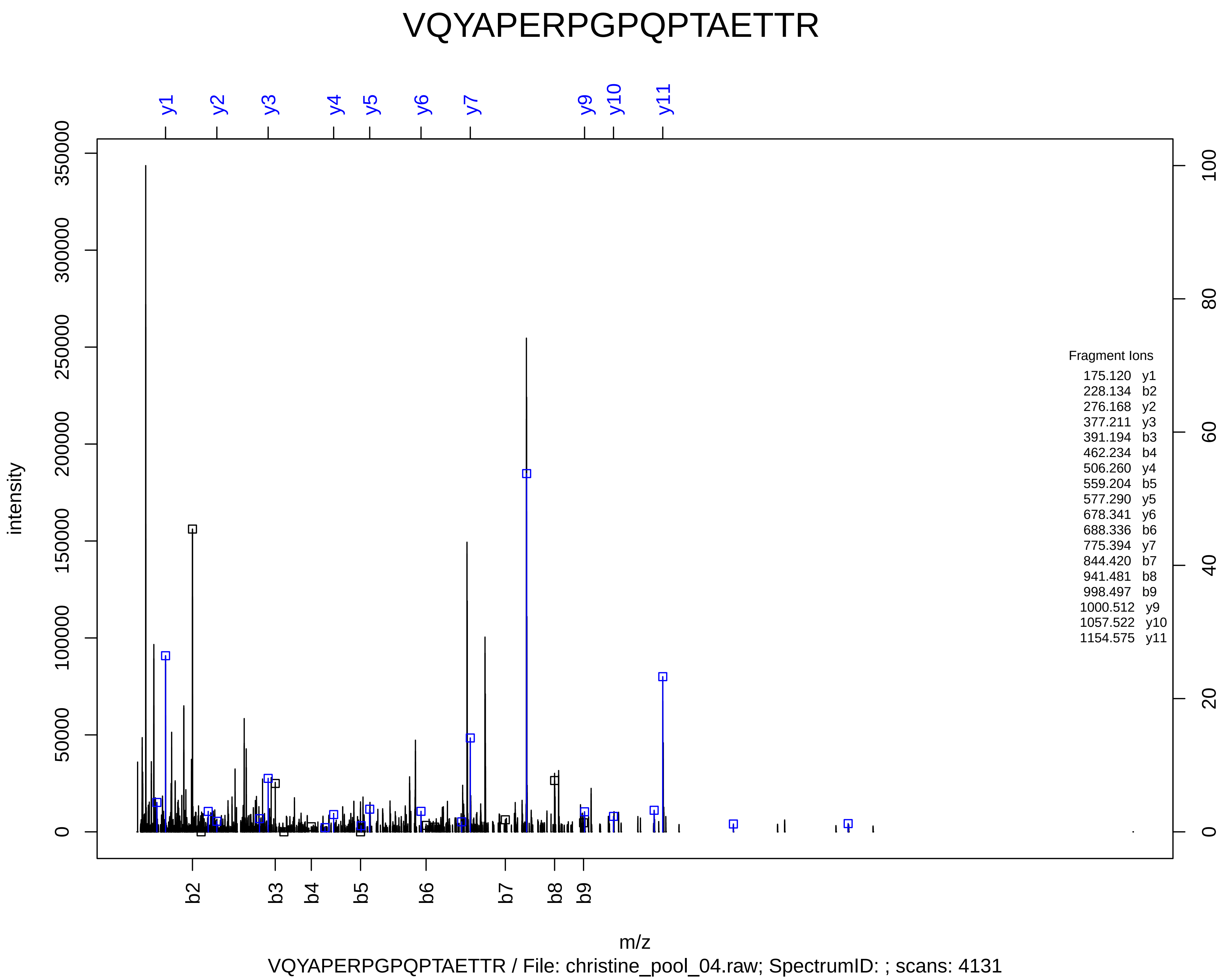
VQYAPERPGPQPTAETTR
y1
y2
y3
y4
y5
y6
y7
y9
y10
y11
intensity
0
50000
100000
150000
200000
250000
300000
350000
0
20
40
60
80
100
b2
b3
b4
b5
b6
b7
b8
b9
Fragment Ions
175.120 y1
228.134 b2
276.168 y2
377.211 y3
391.194 b3
462.234 b4
506.260 y4
559.204 b5
577.290 y5
678.341 y6
688.336 b6
775.394 y7
844.420 b7
941.481 b8
998.497 b9
1000.512 y9
1057.522 y10
1154.575 y11
m/z
VQYAPERPGPQPTAETTR / File: christine_pool_04.raw; SpectrumID: ; scans: 4131

VPFLPGDSDLDQLTR

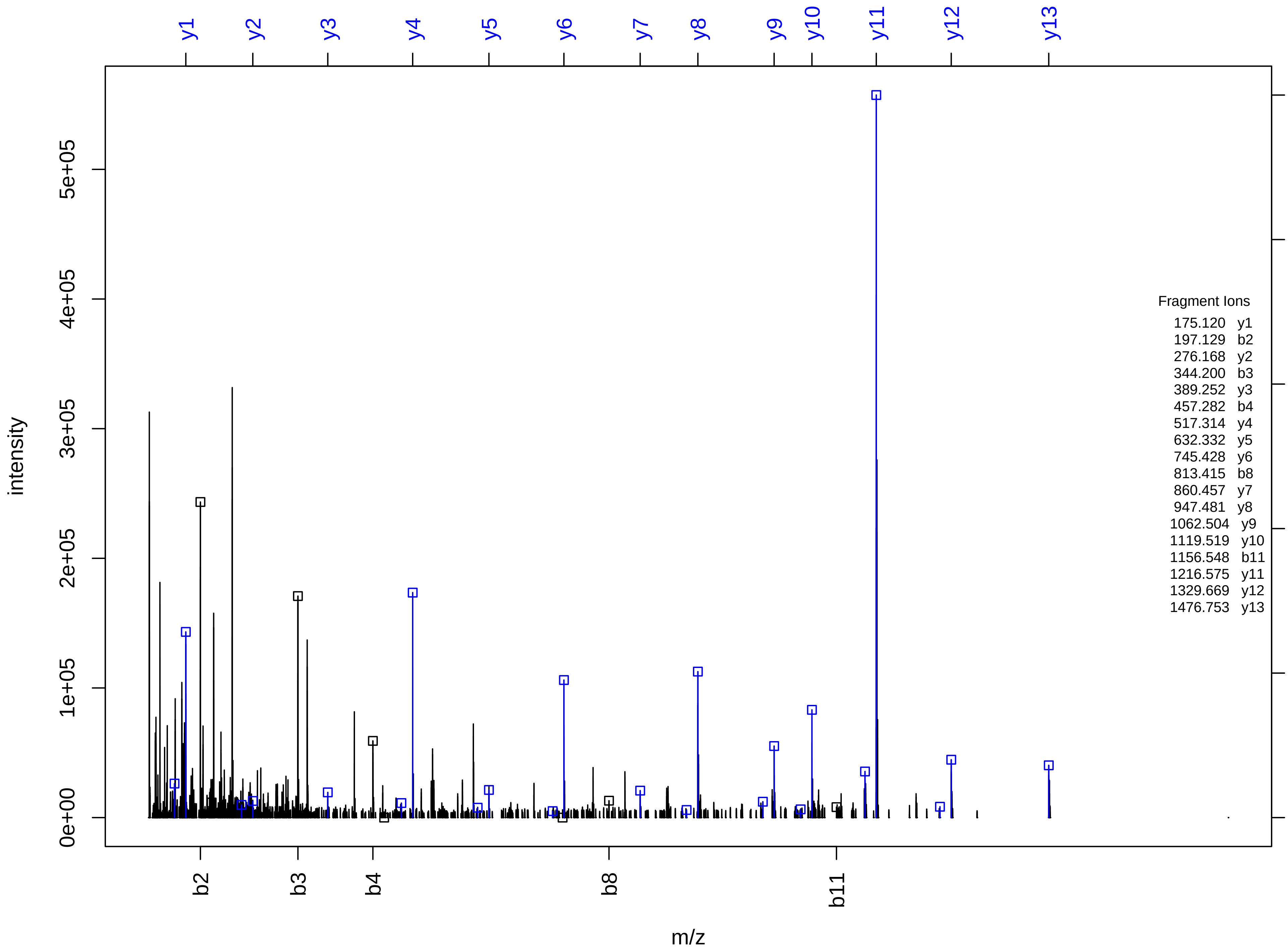

VPFLPGDSDLDQLTR / File: christine_pool_04.raw; SpectrumID: ; scans: 17943

SFPPPGPAEGLLR

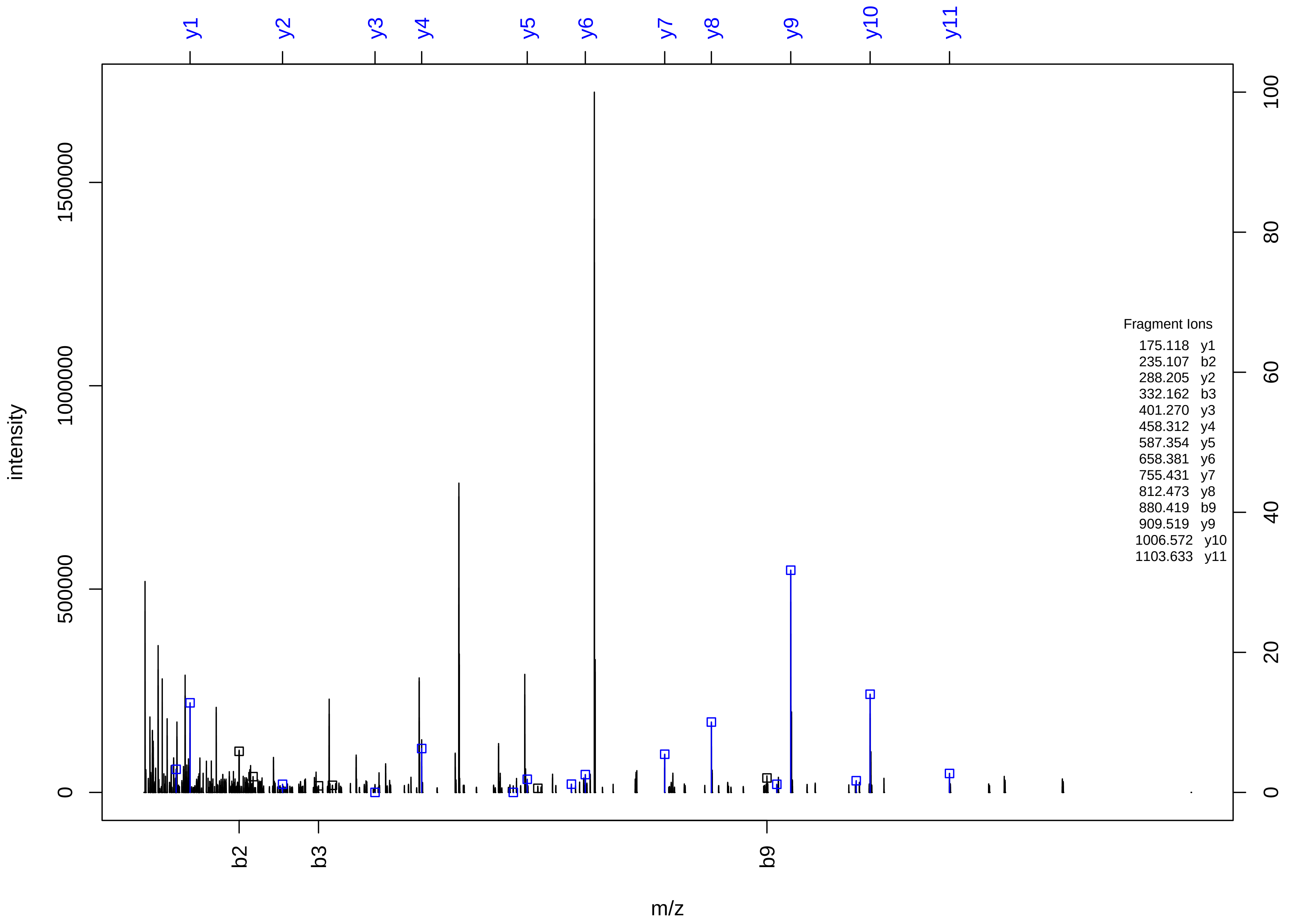

SFPPPGPAEGLLR / File: christine_pool_04.raw; SpectrumID: ; scans: 14176

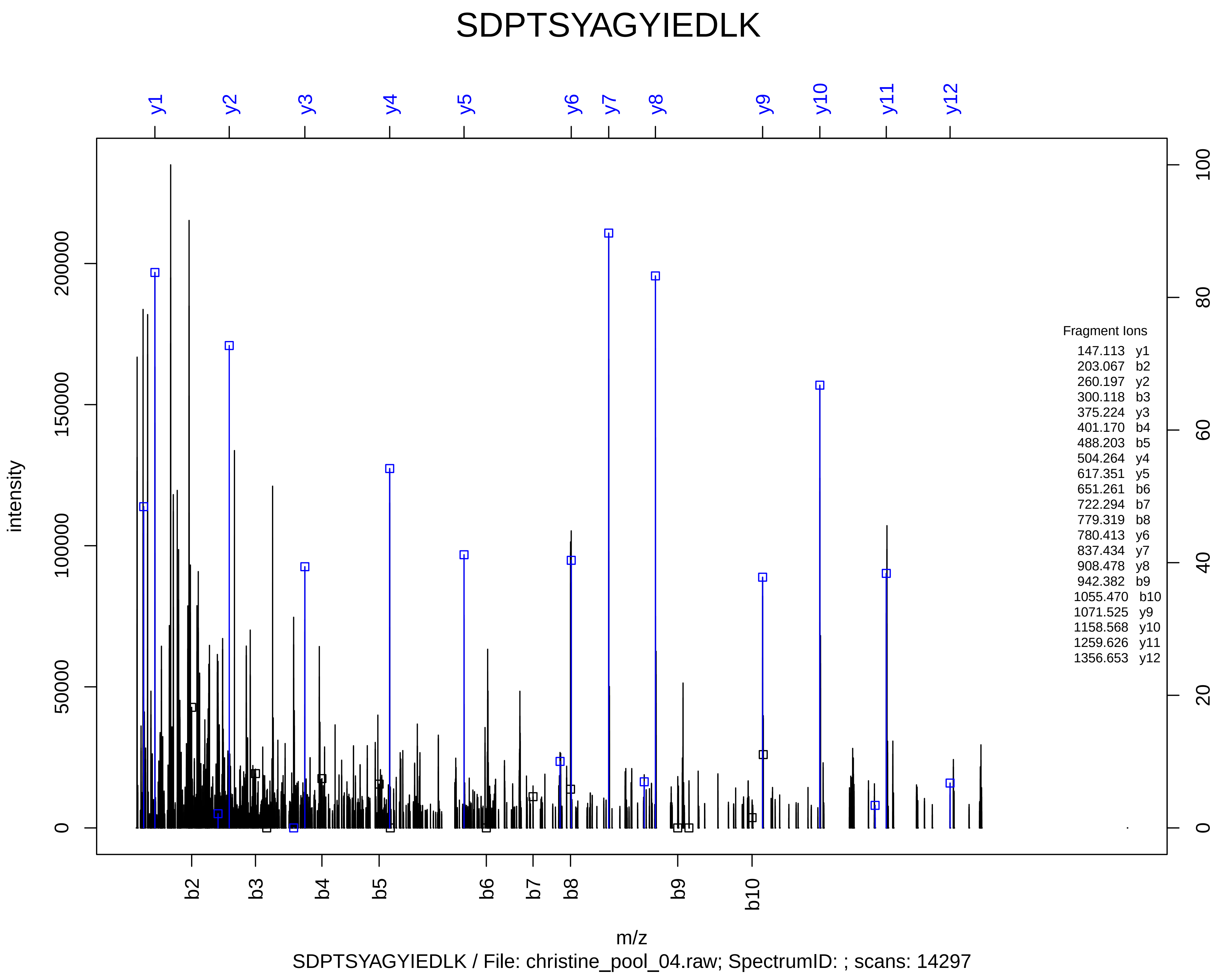
SDPTSYAGYIEDLK
y1
y2
y3
y4
y5
y6
y7
y8
y9
y10
y11
y12
b2
b3
b4
b5
b6
b7
b8
b9
b10
intensity
0
50000
100000
150000
200000
0
20
40
60
80
100
m/z
Fragment Ions
147.113 y1
203.067 b2
260.197 y2
300.118 b3
375.224 y3
401.170 b4
488.203 b5
504.264 y4
617.351 y5
651.261 b6
722.294 b7
779.319 b8
780.413 y6
837.434 y7
908.478 y8
942.382 b9
1055.470 b10
1071.525 y9
1158.568 y10
1259.626 y11
1356.653 y12
SDPTSYAGYIEDLK / File: christine_pool_04.raw; SpectrumID: ; scans: 14297

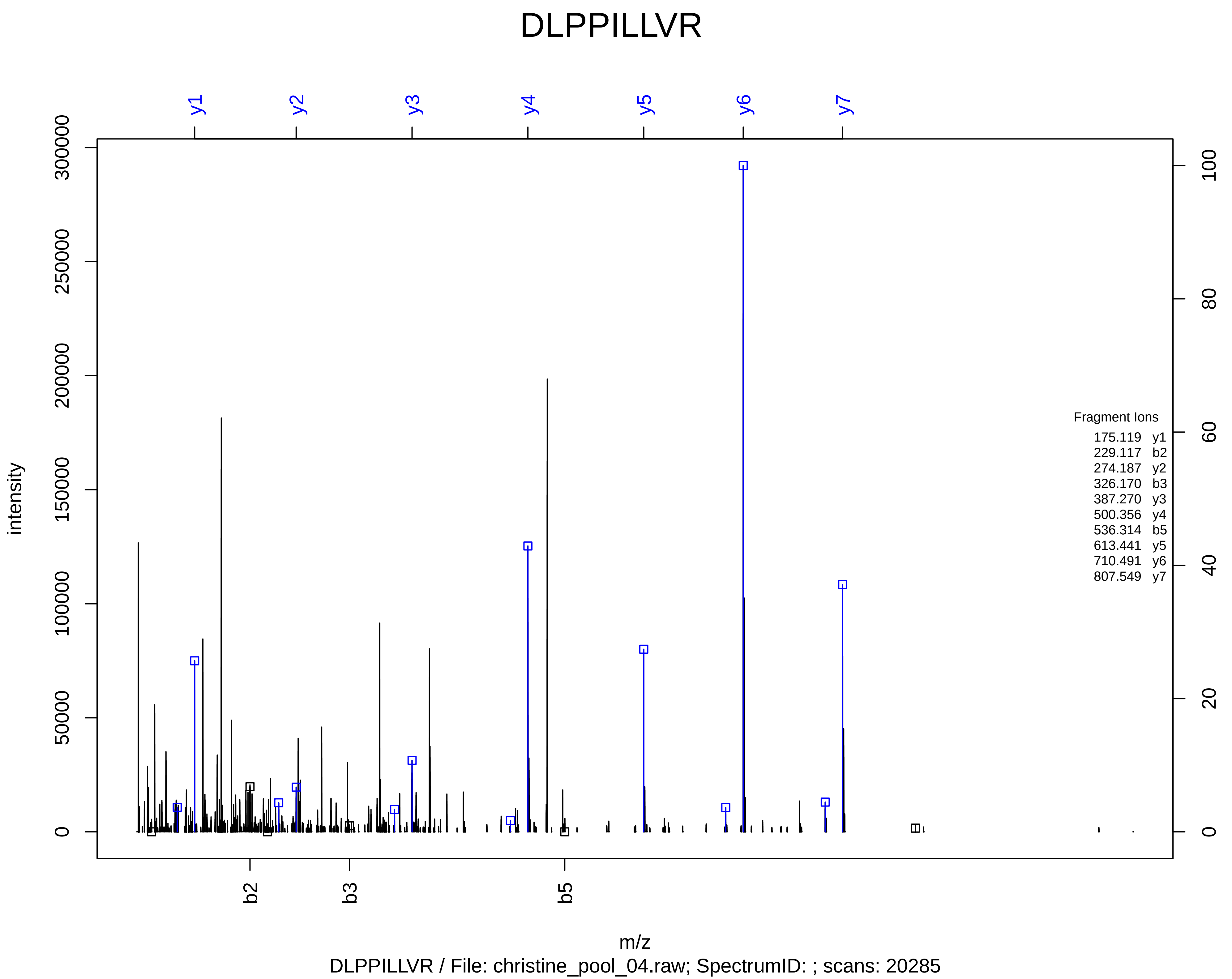
DLPPILLVR
y1
y2
y3
y4
y5
y6
y7
b2
b3
b5
intensity
0
50000
100000
150000
200000
250000
300000
0
20
40
60
80
100
Fragment Ions
175.119 y1
229.117 b2
274.187 y2
326.170 b3
387.270 y3
500.356 y4
536.314 b5
613.441 y5
710.491 y6
807.549 y7
m/z
DLPPILLVR / File: christine_pool_04.raw; SpectrumID: ; scans: 20285

SAAASNLSGLSLQEAQQILNVSK

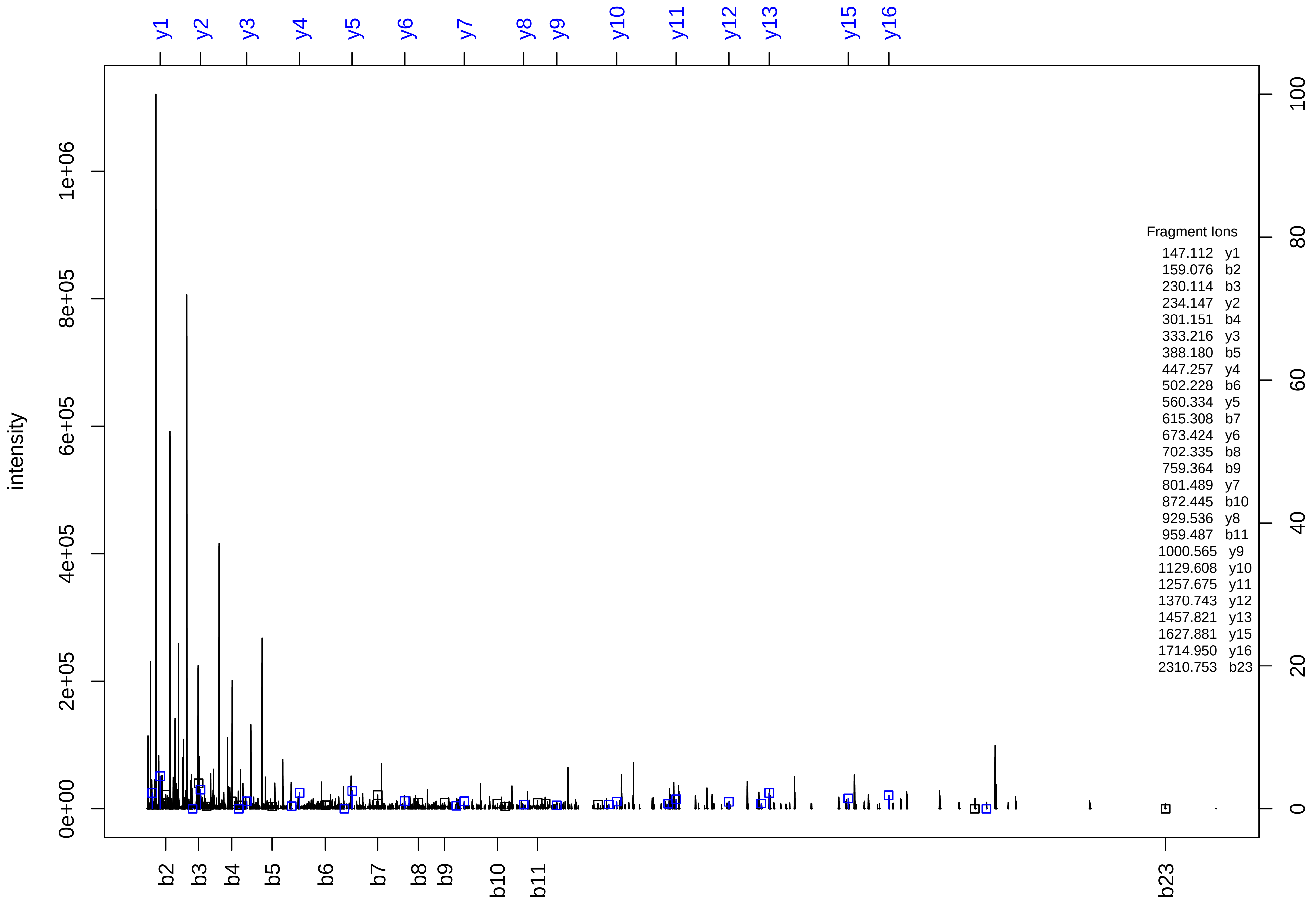

SAAASNLSGLSLQEAQQILNVSK / File: christine_pool_04.raw; SpectrumID: ; scans: 20310

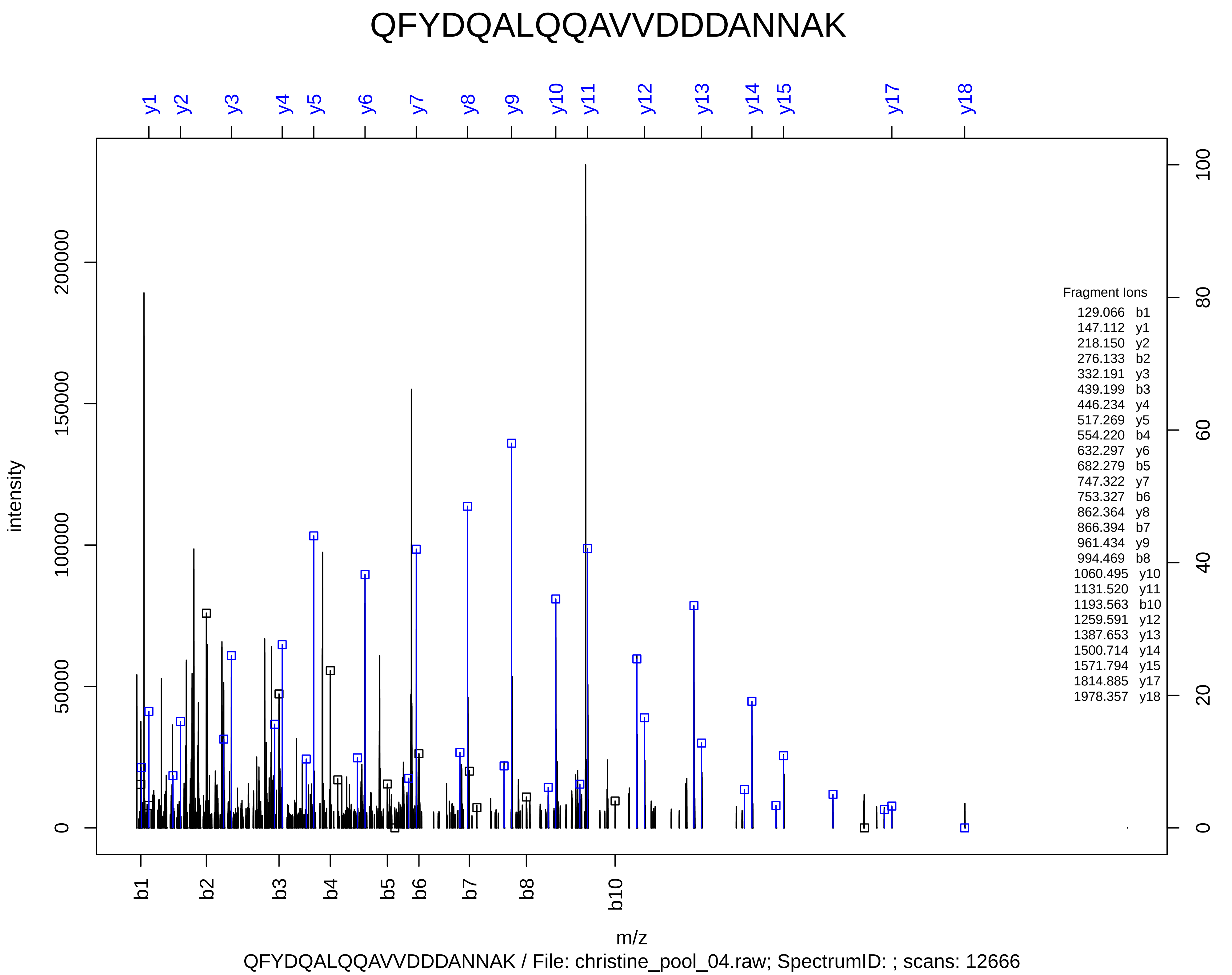
QFYDQALQQAVVDDDANNAK
intensity
m/z
Fragment Ions
129.066 b1
147.112 y1
218.150 y2
276.133 b2
332.191 y3
439.199 b3
446.234 y4
517.269 y5
554.220 b4
632.297 y6
682.279 b5
747.322 y7
753.327 b6
862.364 y8
866.394 b7
961.434 y9
994.469 b8
1060.495 y10
1131.520 y11
1193.563 b10
1259.591 y12
1387.653 y13
1500.714 y14
1571.794 y15
1814.885 y17
1978.357 y18
QFYDQALQQAVVDDDANNAK / File: christine_pool_04.raw; SpectrumID: ; scans: 12666

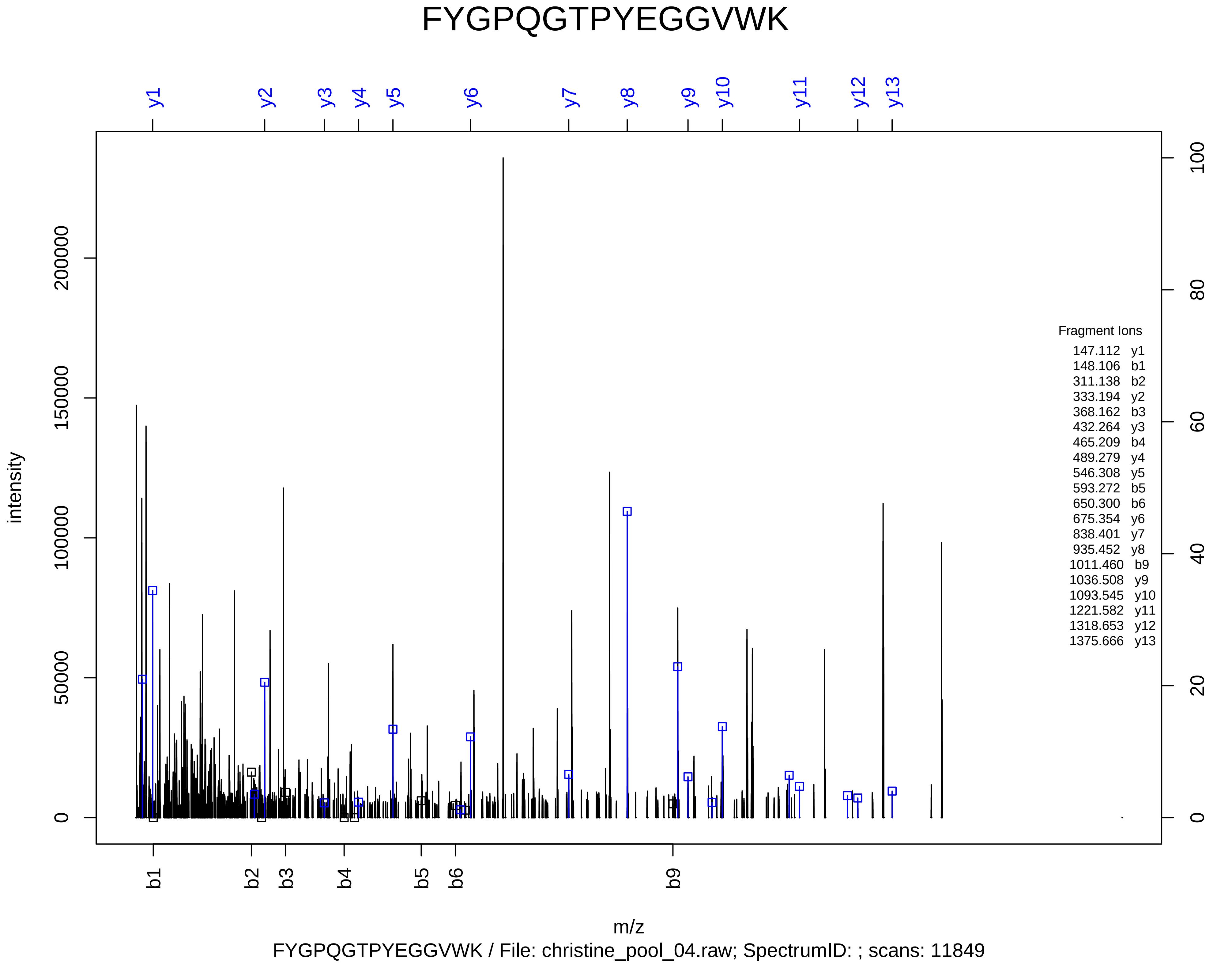
FYGPQGTPYEGGVWK
y1 y2 y3 y4 y5 y6 y7 y8 y9 y10 y11 y12 y13
b1 b2 b3 b4 b5 b6 b9
intensity
0
50000
100000
150000
200000
0
20
40
60
80
100
Fragment Ions
147.112 y1
148.106 b1
311.138 b2
333.194 y2
368.162 b3
432.264 y3
465.209 b4
489.279 y4
546.308 y5
593.272 b5
650.300 b6
675.354 y6
838.401 y7
935.452 y8
1011.460 b9
1036.508 y9
1093.545 y10
1221.582 y11
1318.653 y12
1375.666 y13
m/z
FYGPQGTPYEGGVWK / File: christine_pool_04.raw; SpectrumID: ; scans: 11849

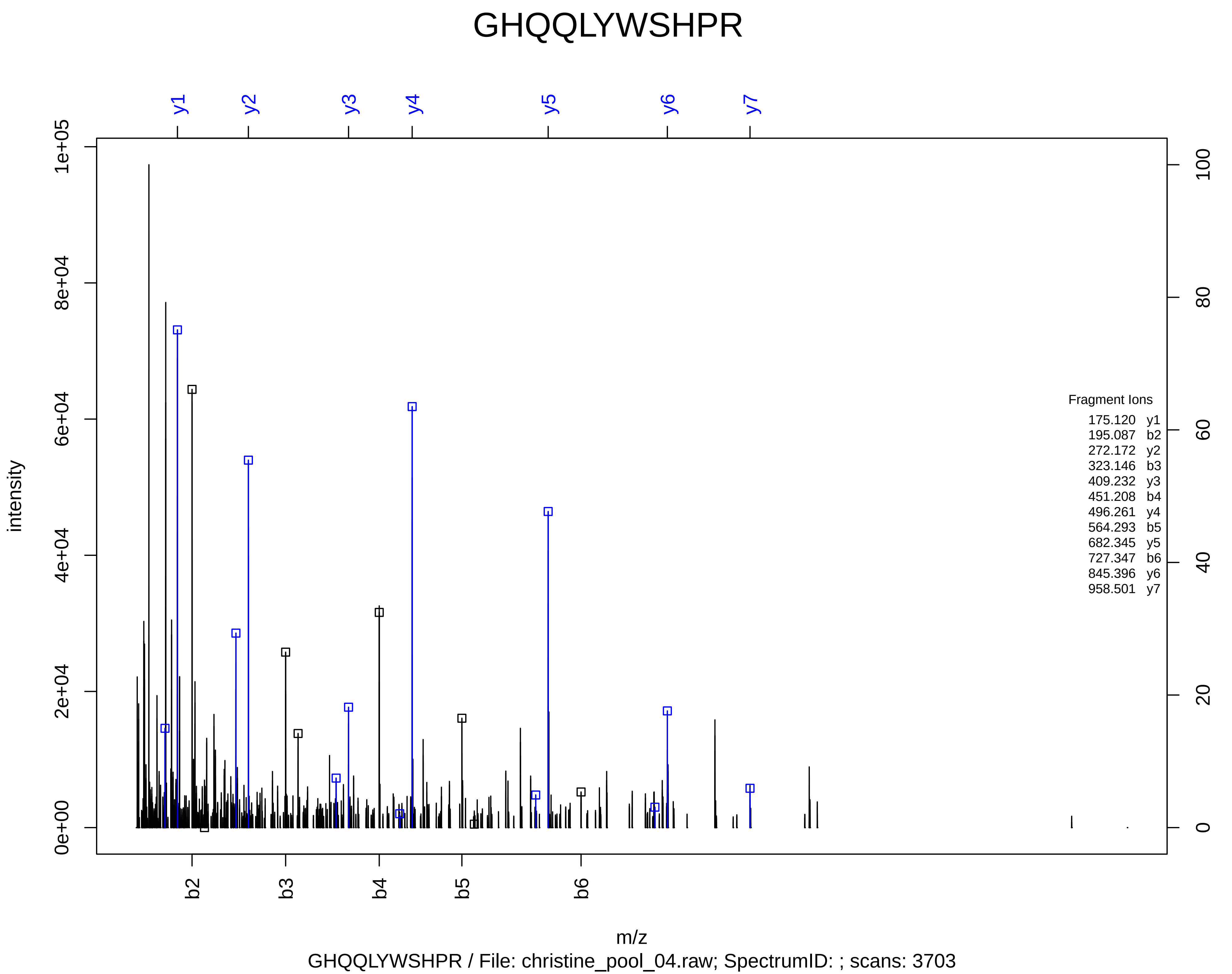

GHQQLYWSHPR
y1
y2
y3
y4
y5
y6
y7
intensity
0e+00
2e+04
4e+04
6e+04
8e+04
1e+05
0
20
40
60
80
100
b2
b3
b4
b5
b6
Fragment Ions
175.120 y1
195.087 b2
272.172 y2
323.146 b3
409.232 y3
451.208 b4
496.261 y4
564.293 b5
682.345 y5
727.347 b6
845.396 y6
958.501 y7
m/z
GHQQLYWSHPR / File: christine_pool_04.raw; SpectrumID: ; scans: 3703

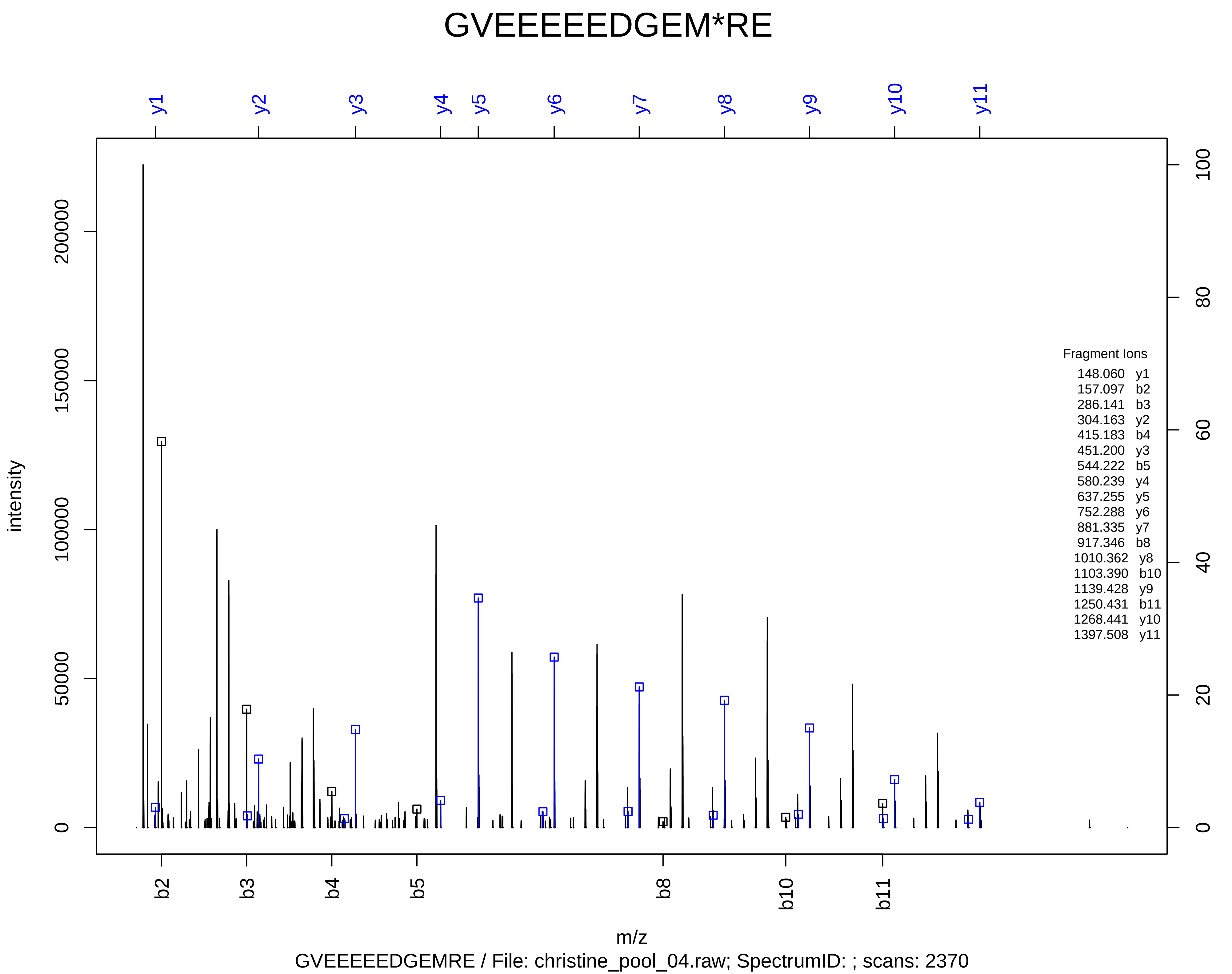

GVEEEEEEDGEM*RE
y1
y2
y3
y4
y5
y6
y7
y8
y9
y10
y11
intensity
0
50000
100000
150000
200000
0
20
40
60
80
100
Fragment Ions
148.060 y1
157.097 b2
286.141 b3
304.163 y2
415.183 b4
451.200 y3
544.222 b5
580.239 y4
637.255 y5
752.288 y6
881.335 y7
917.346 b8
1010.362 y8
1103.390 b10
1139.428 y9
1250.431 b11
1268.441 y10
1397.508 y11
b2
b3
b4
b5
b8
b10
b11
m/z
GVEEEEEEDGEMRE / File: christine_pool_04.raw; SpectrumID: ; scans: 2370

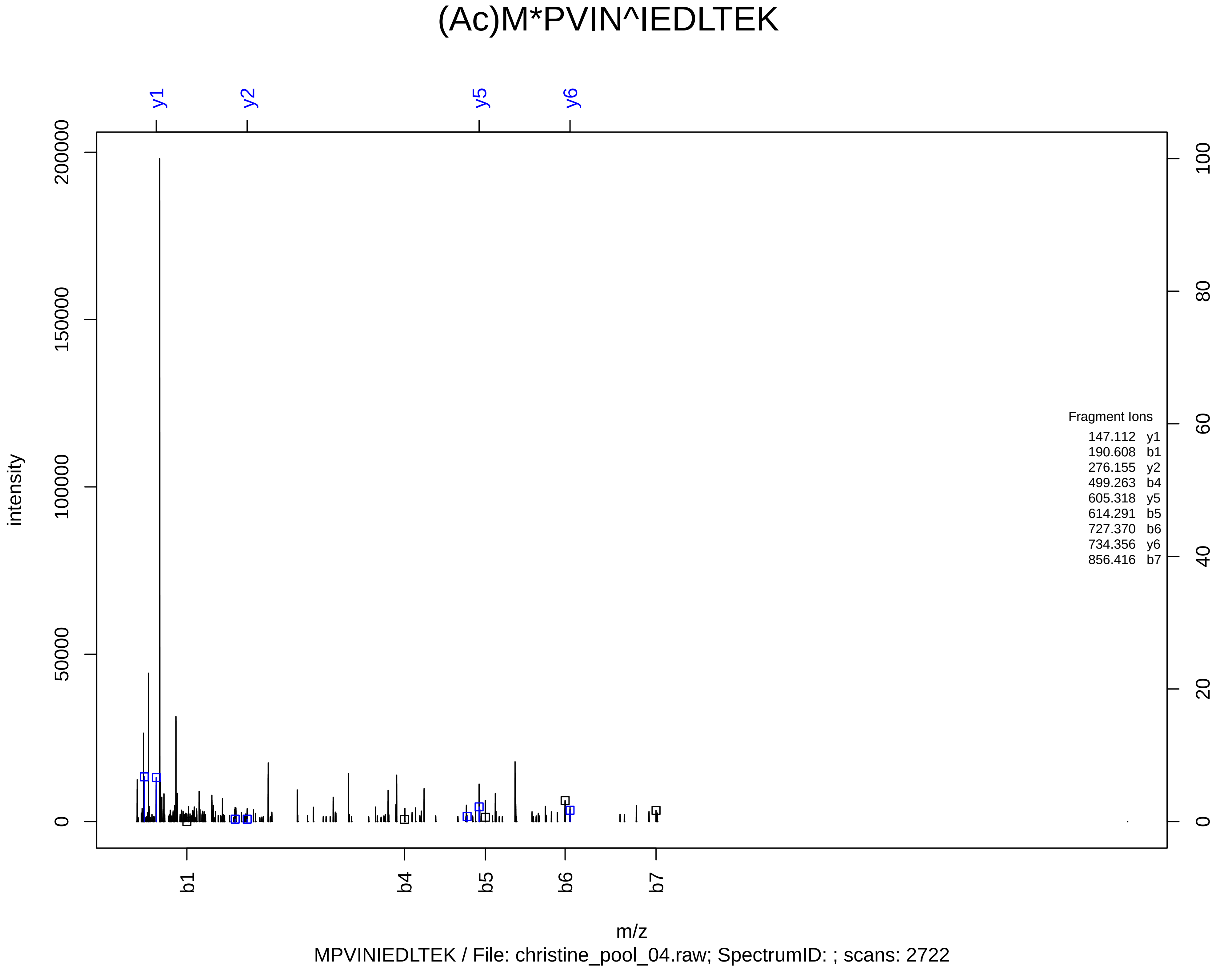
(Ac)M*PVIN^IEDLTEK
y1
y2
y5
y6
intensity
0
50000
100000
150000
200000
0
20
40
60
80
100
Fragment Ions
147.112 y1
190.608 b1
276.155 y2
499.263 b4
605.318 y5
614.291 b5
727.370 b6
734.356 y6
856.416 b7
b1
b4
b5
b6
b7
m/z
MPVINIEDLTEK / File: christine_pool_04.raw; SpectrumID: ; scans: 2722

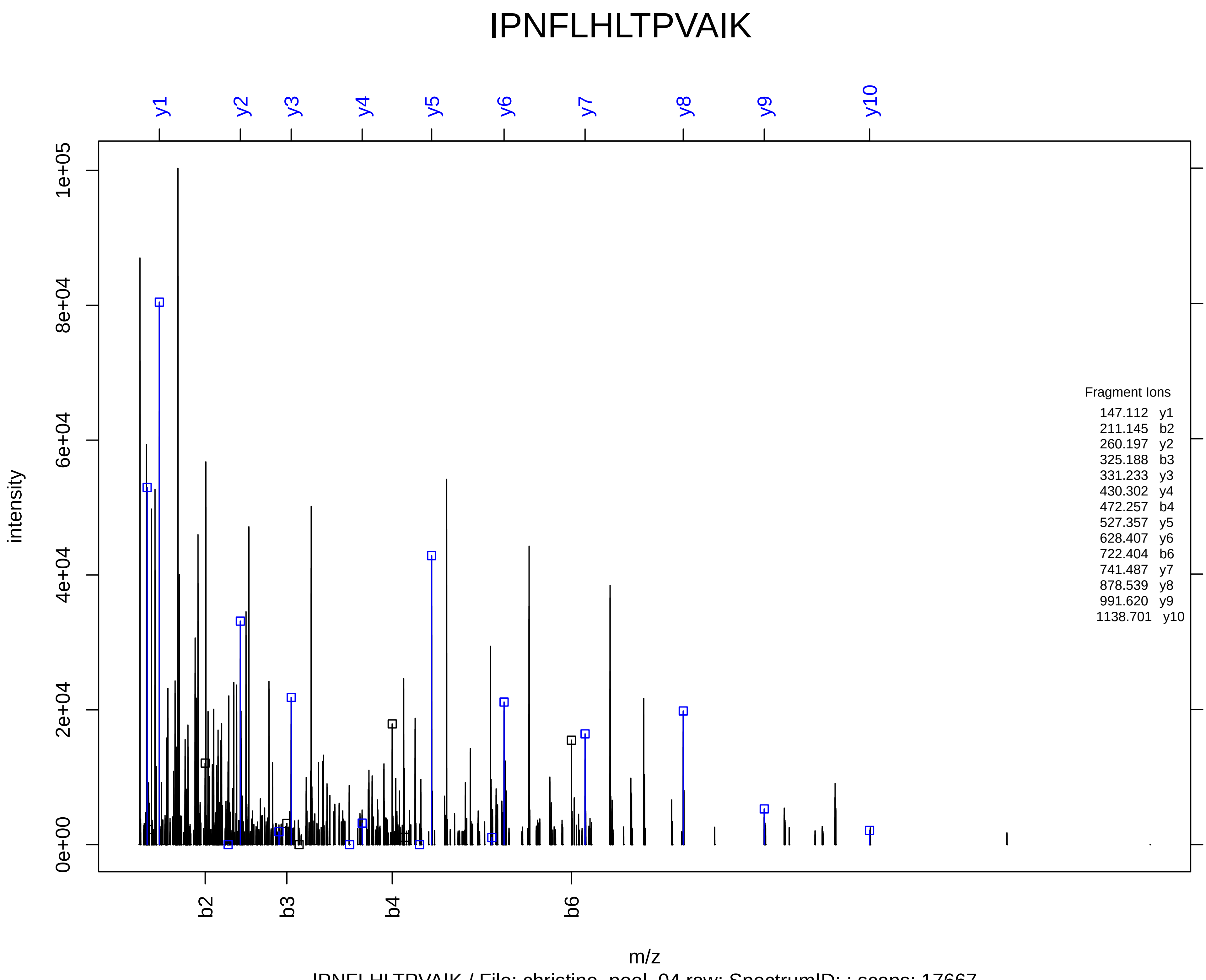

IPNFLHLTPVAIK
y1
y2
y3
y4
y5
y6
y7
y8
y9
y10
intensity
0e+00
2e+04
4e+04
6e+04
8e+04
1e+05
0
20
40
60
80
100
b2
b3
b4
b6
m/z
Fragment Ions
147.112 y1
211.145 b2
260.197 y2
325.188 b3
331.233 y3
430.302 y4
472.257 b4
527.357 y5
628.407 y6
722.404 b6
741.487 y7
878.539 y8
991.620 y9
1138.701 y10
IPNFLHLTPVAIK / File: christine_pool_04.raw; SpectrumID: ; scans: 17667

LLTSQCGAAEEEFVQR

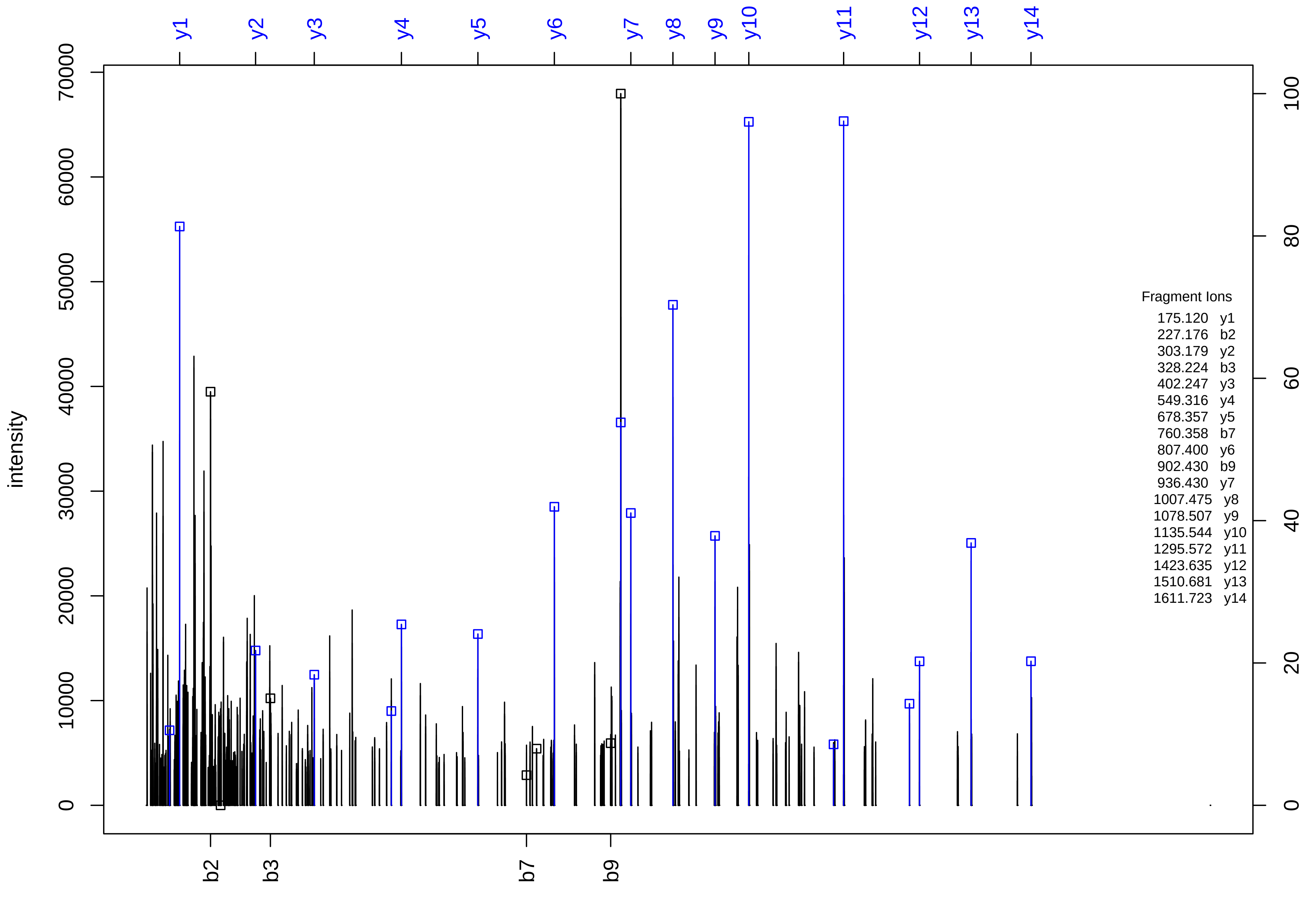

LLTSQCGAAEEEFVQR / File: christine_pool_04.raw; SpectrumID: ; scans: 9417

SADGSAPAGEGEGVTLQR

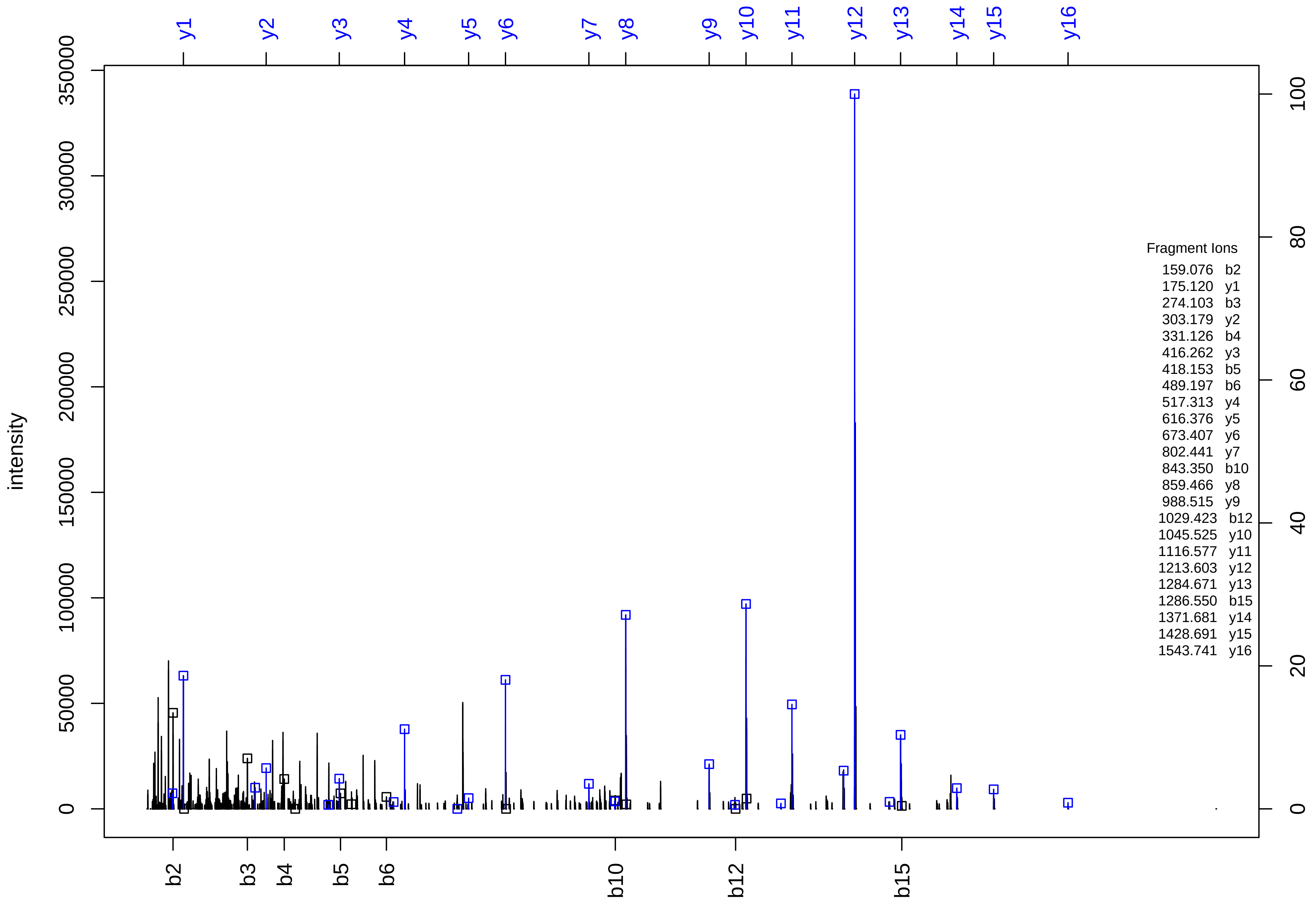

SADGSAPAGEGEGVTLQR / File: christine_pool_04.raw; SpectrumID: ; scans: 4911

TEDFIIDTLELR

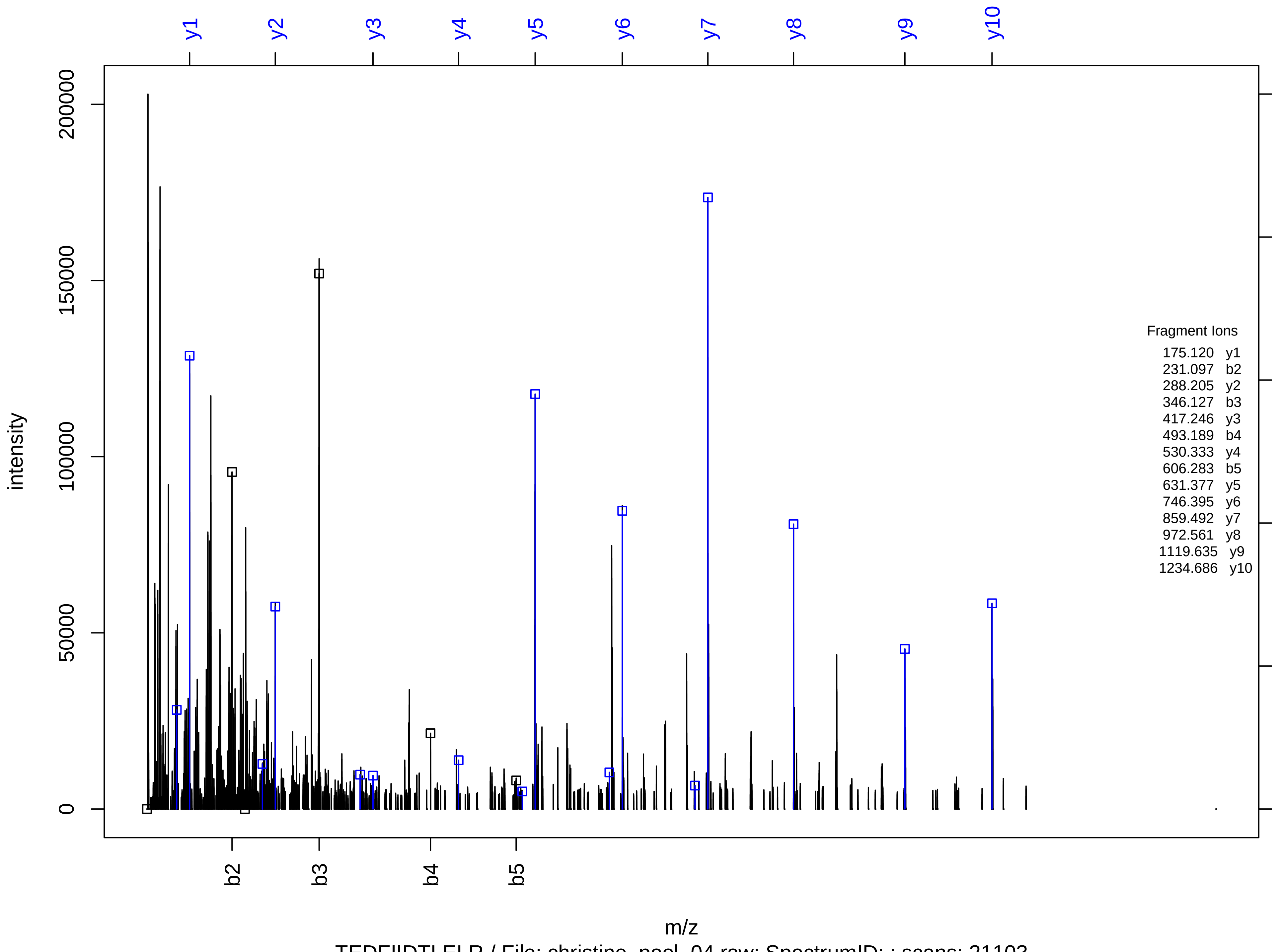

TEDFIIDTLELR / File: christine_pool_04.raw; SpectrumID: ; scans: 21103

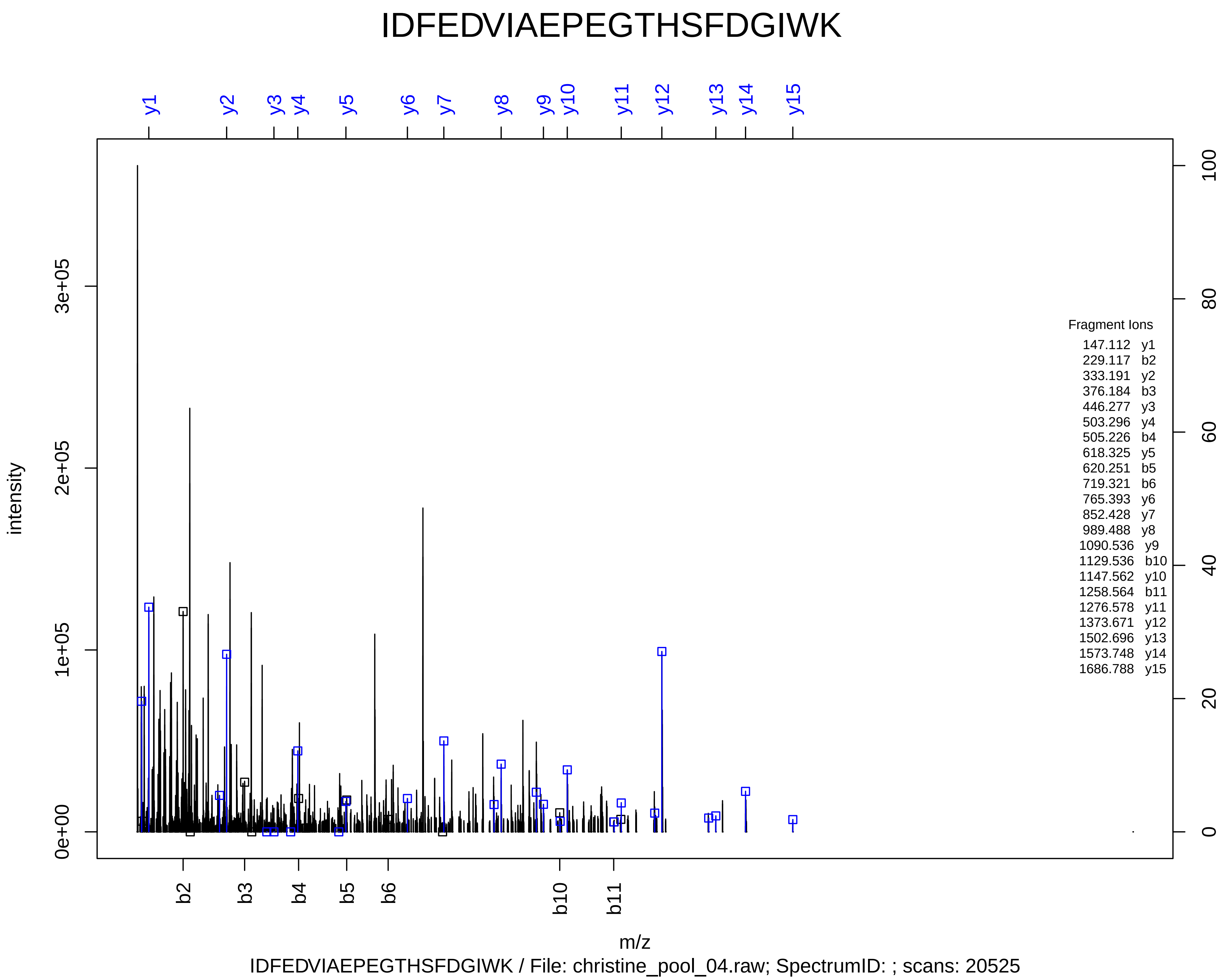

IDFEDVIAEPEGTHSFDGIWK
y1
y2
y3
y4
y5
y6
y7
y8
y9
y10
y11
y12
y13
y14
y15
intensity
0e+00
1e+05
2e+05
3e+05
0
20
40
60
80
100
b2
b3
b4
b5
b6
b10
b11
m/z
Fragment Ions
147.112 y1
229.117 b2
333.191 y2
376.184 b3
446.277 y3
503.296 y4
505.226 b4
618.325 y5
620.251 b5
719.321 b6
765.393 y6
852.428 y7
989.488 y8
1090.536 y9
1129.536 b10
1147.562 y10
1258.564 b11
1276.578 y11
1373.671 y12
1502.696 y13
1573.748 y14
1686.788 y15
IDFEDVIAEPEGTHSFDGIWK / File: christine_pool_04.raw; SpectrumID: ; scans: 20525

KLETEETVPETDVETK

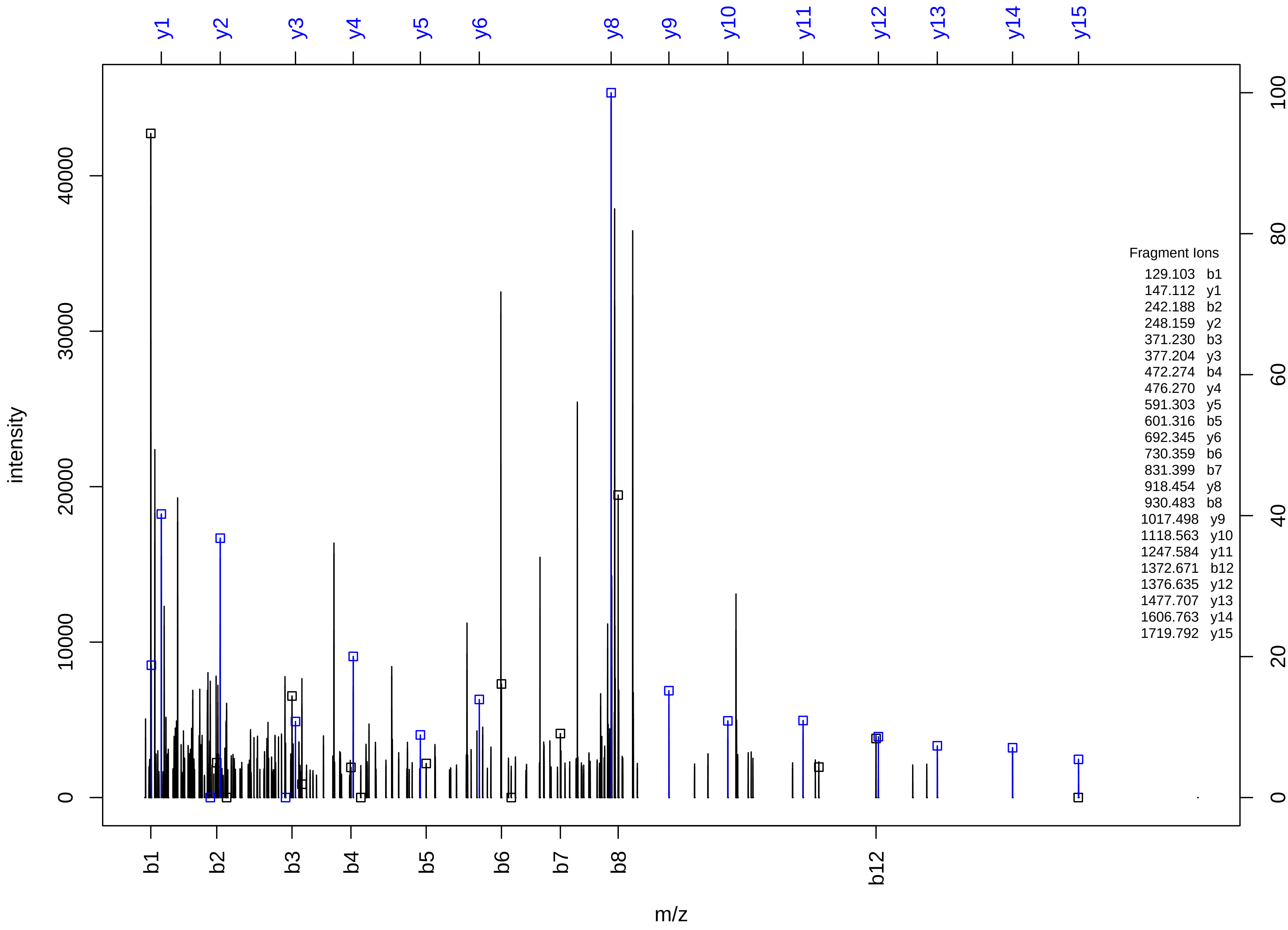

KLETEETVPETDVETK / File: christine_pool_04.raw; SpectrumID: ; scans: 5300

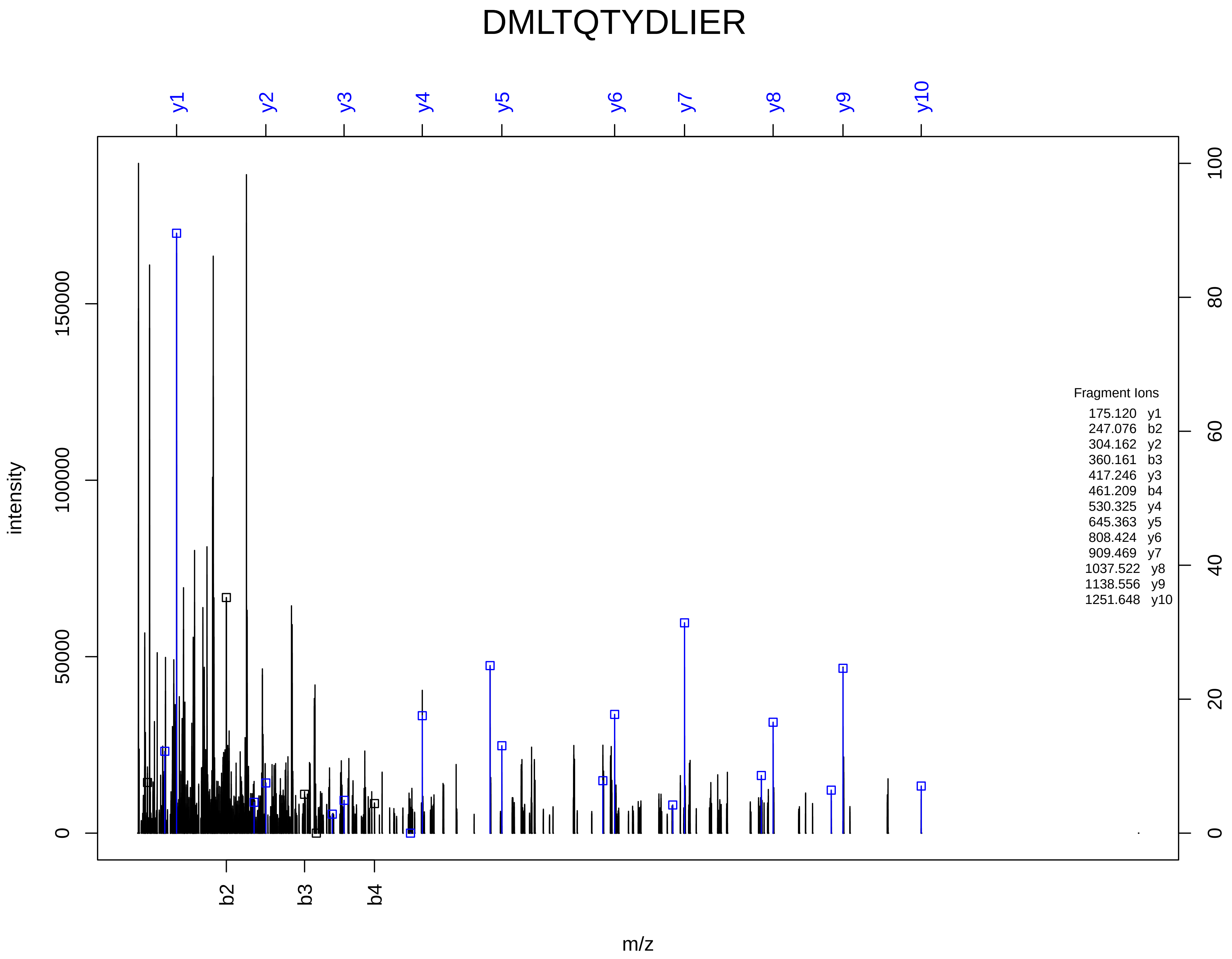
DMLTQTYDLIER
y1
y2
y3
y4
y5
y6
y7
y8
y9
y10
intensity
0
50000
100000
150000
0
20
40
60
80
100
b2
b3
b4
m/z
Fragment Ions
175.120 y1
247.076 b2
304.162 y2
360.161 b3
417.246 y3
461.209 b4
530.325 y4
645.363 y5
808.424 y6
909.469 y7
1037.522 y8
1138.556 y9
1251.648 y10
DMLTQTYDLIER / File: christine_pool_04.raw; SpectrumID: ; scans: 19047

LVQASEELLR

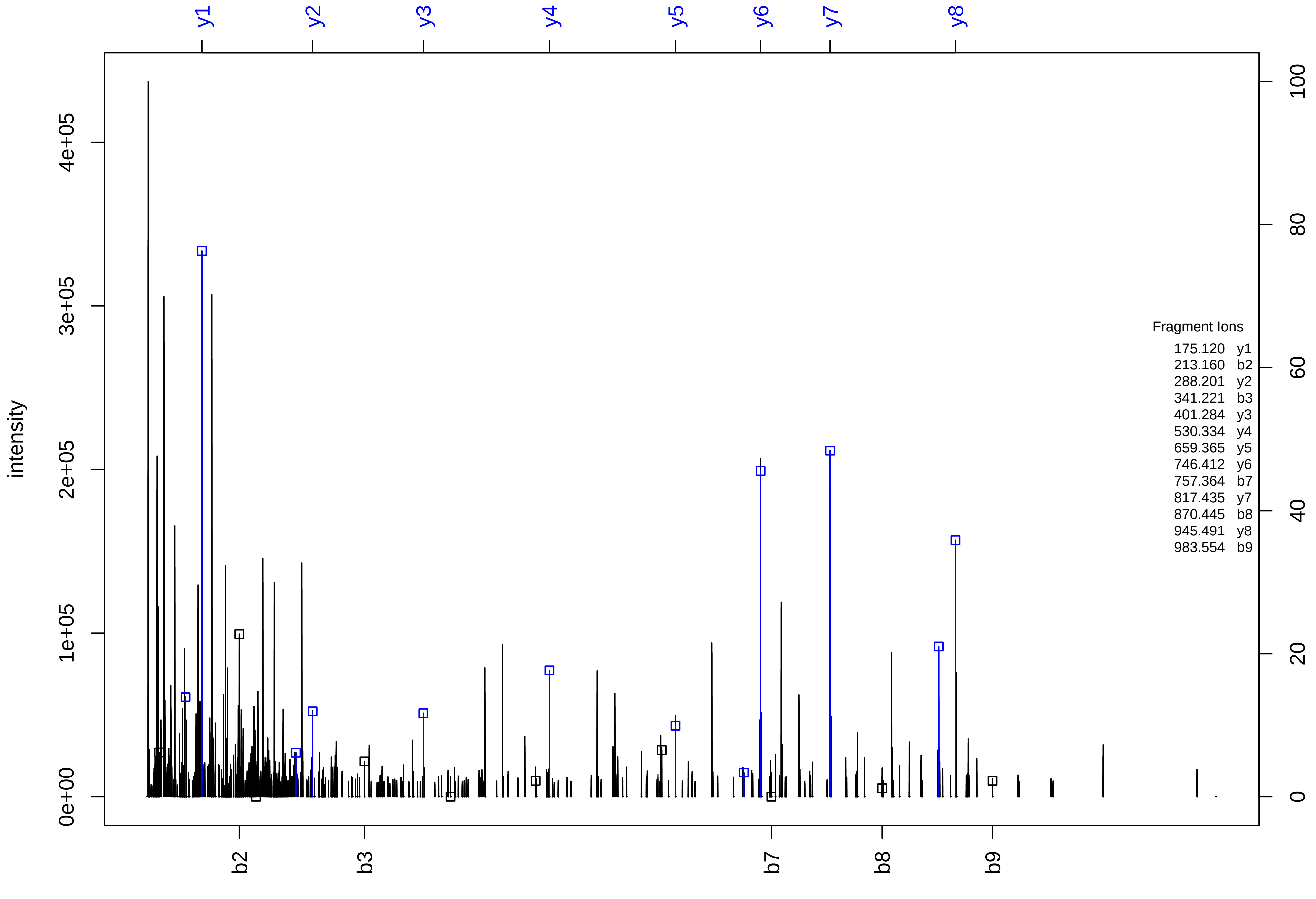

LVQASEELLR / File: christine_pool_04.raw; SpectrumID: ; scans: 8968

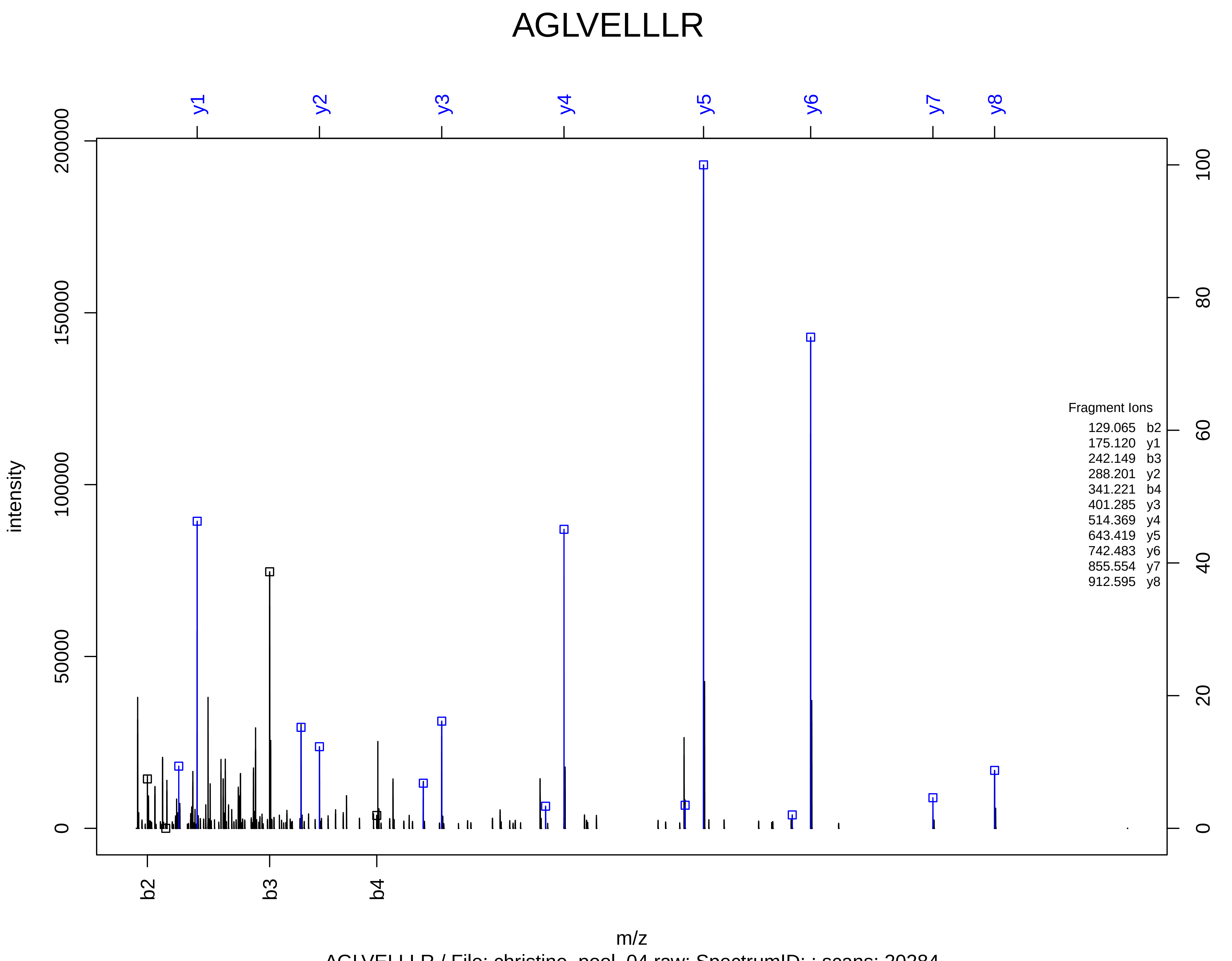
AGLVELLLR
y1
y2
y3
y4
y5
y6
y7
y8
b2
b3
b4
intensity
0
50000
100000
150000
200000
0
20
40
60
80
100
Fragment Ions
129.065 b2
175.120 y1
242.149 b3
288.201 y2
341.221 b4
401.285 y3
514.369 y4
643.419 y5
742.483 y6
855.554 y7
912.595 y8
m/z
AGLVELLLR / File: christine_pool_04.raw; SpectrumID: ; scans: 20284

DEVLYVFPSDFCR

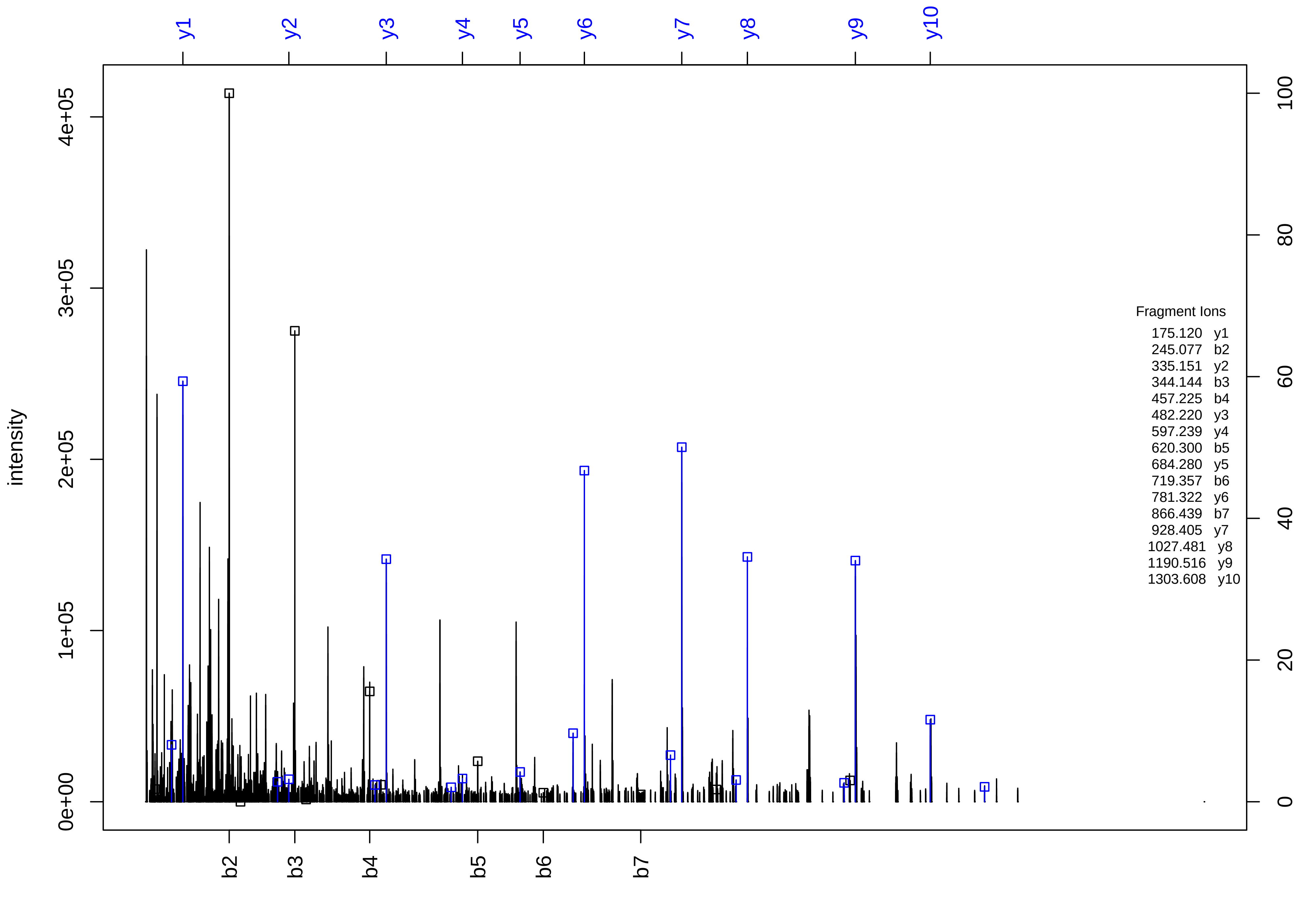

DEVLYVFPSDFCR / File: christine_pool_04.raw; SpectrumID: ; scans: 19368

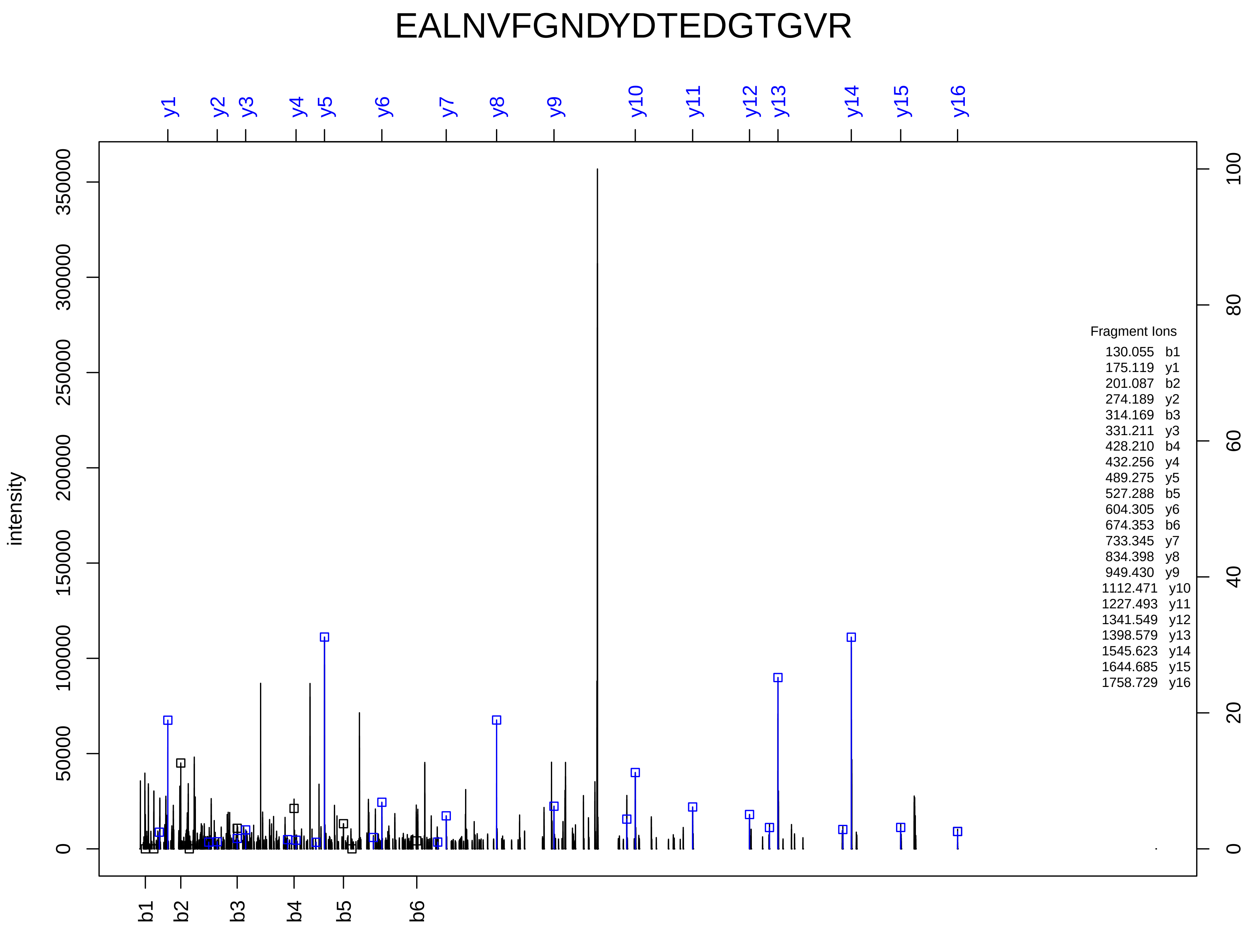

EALNVFGNDYDTEDGTGVR / File: christine_pool_04.raw; SpectrumID: ; scans: 13185

EWLIEVPGNADPLEDQFAK

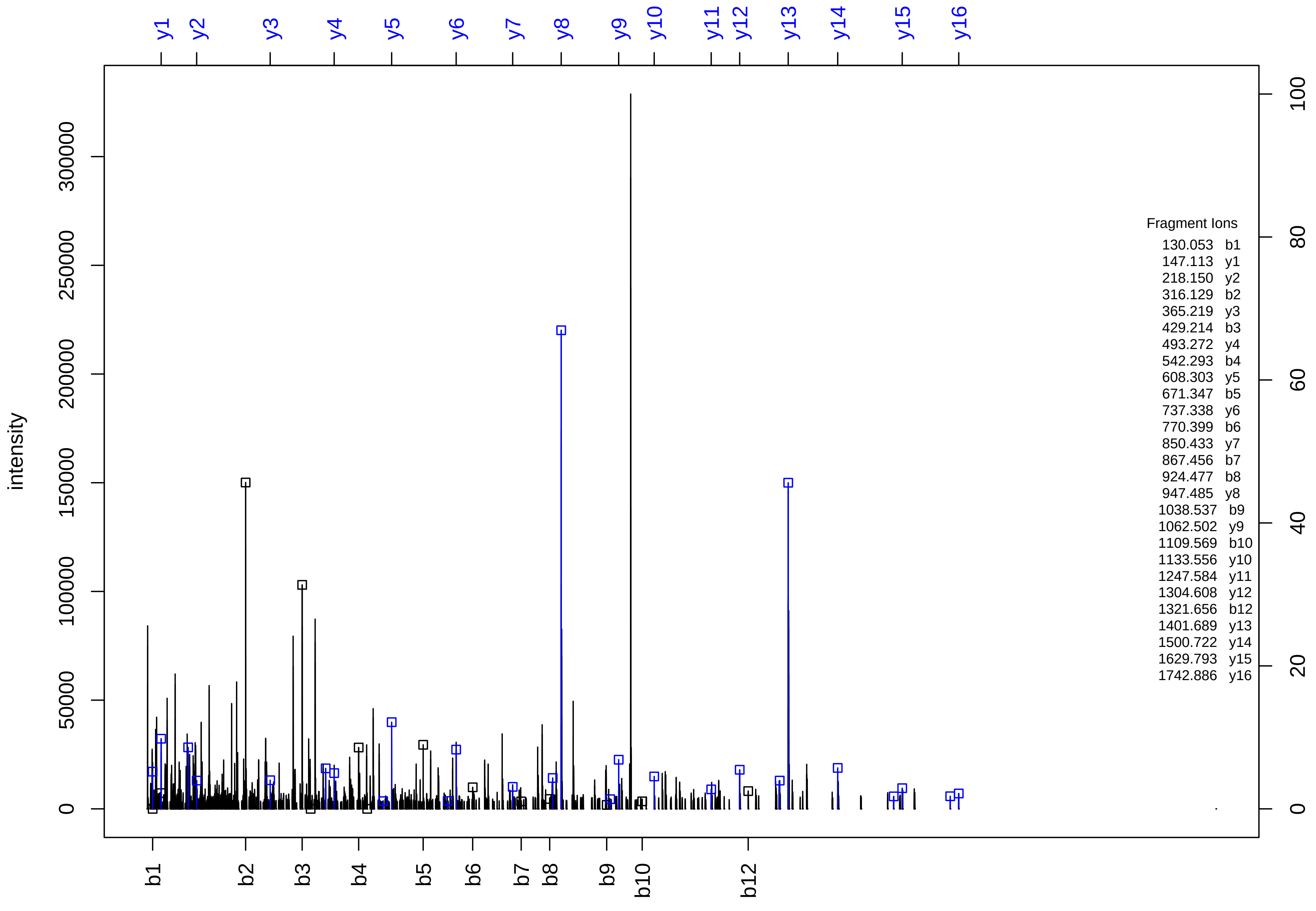

EWLIEVPGNADPLEDQFAK / File: christine_pool_04.raw; SpectrumID: ; scans: 21953

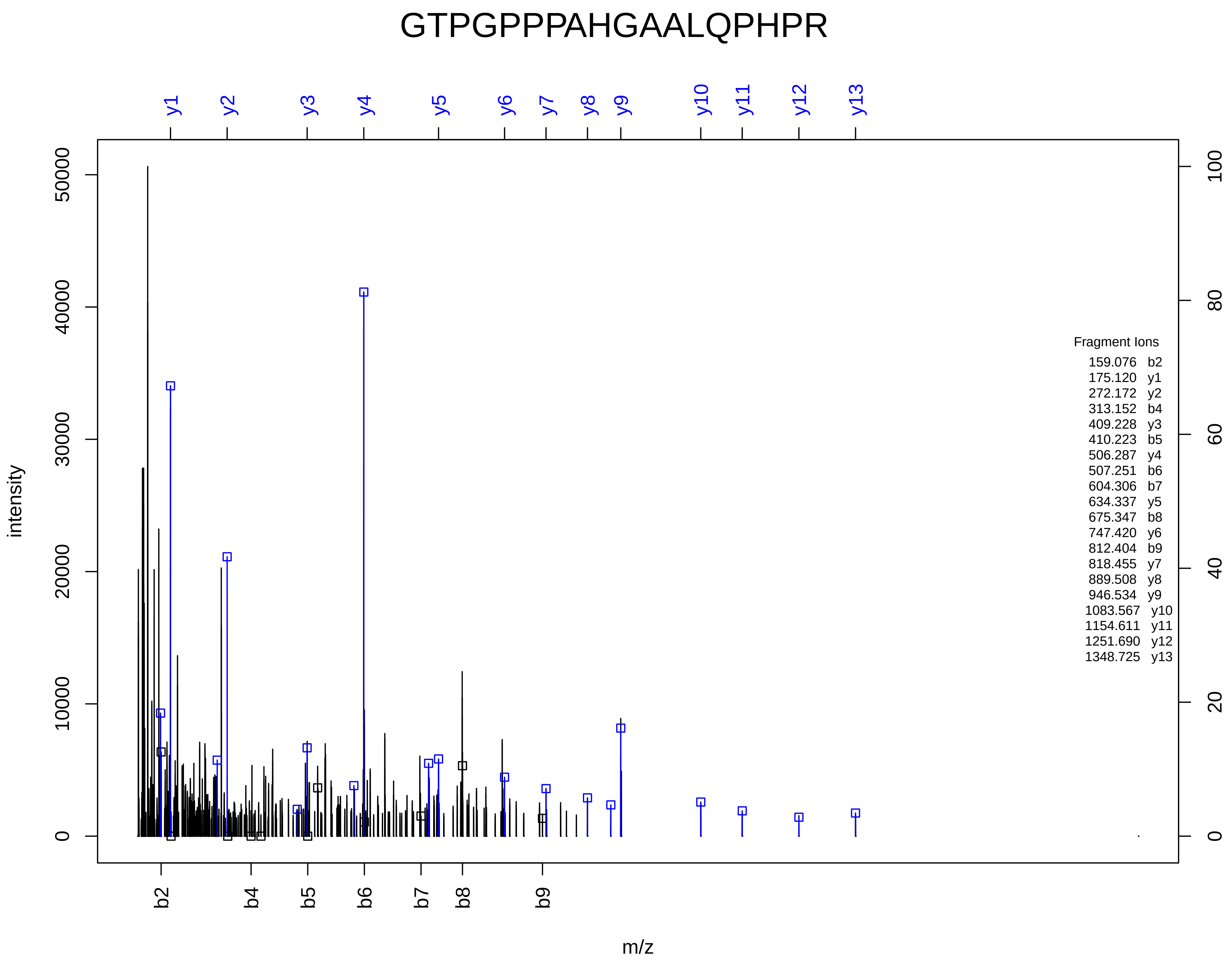

GTPGPPPAHGAALQPHPR
y1
y2
y3
y4
y5
y6
y7
y8
y9
y10
y11
y12
y13
intensity
0
10000
20000
30000
40000
50000
0
20
40
60
80
100
b2
b4
b5
b6
b7
b8
b9
m/z
Fragment Ions
159.076 b2
175.120 y1
272.172 y2
313.152 b4
409.228 y3
410.223 b5
506.287 y4
507.251 b6
604.306 b7
634.337 y5
675.347 b8
747.420 y6
812.404 b9
818.455 y7
889.508 y8
946.534 y9
1083.567 y10
1154.611 y11
1251.690 y12
1348.725 y13
GTPGPPPAHGAALQPHPR / File: christine_pool_04.raw; SpectrumID: ; scans: 3372

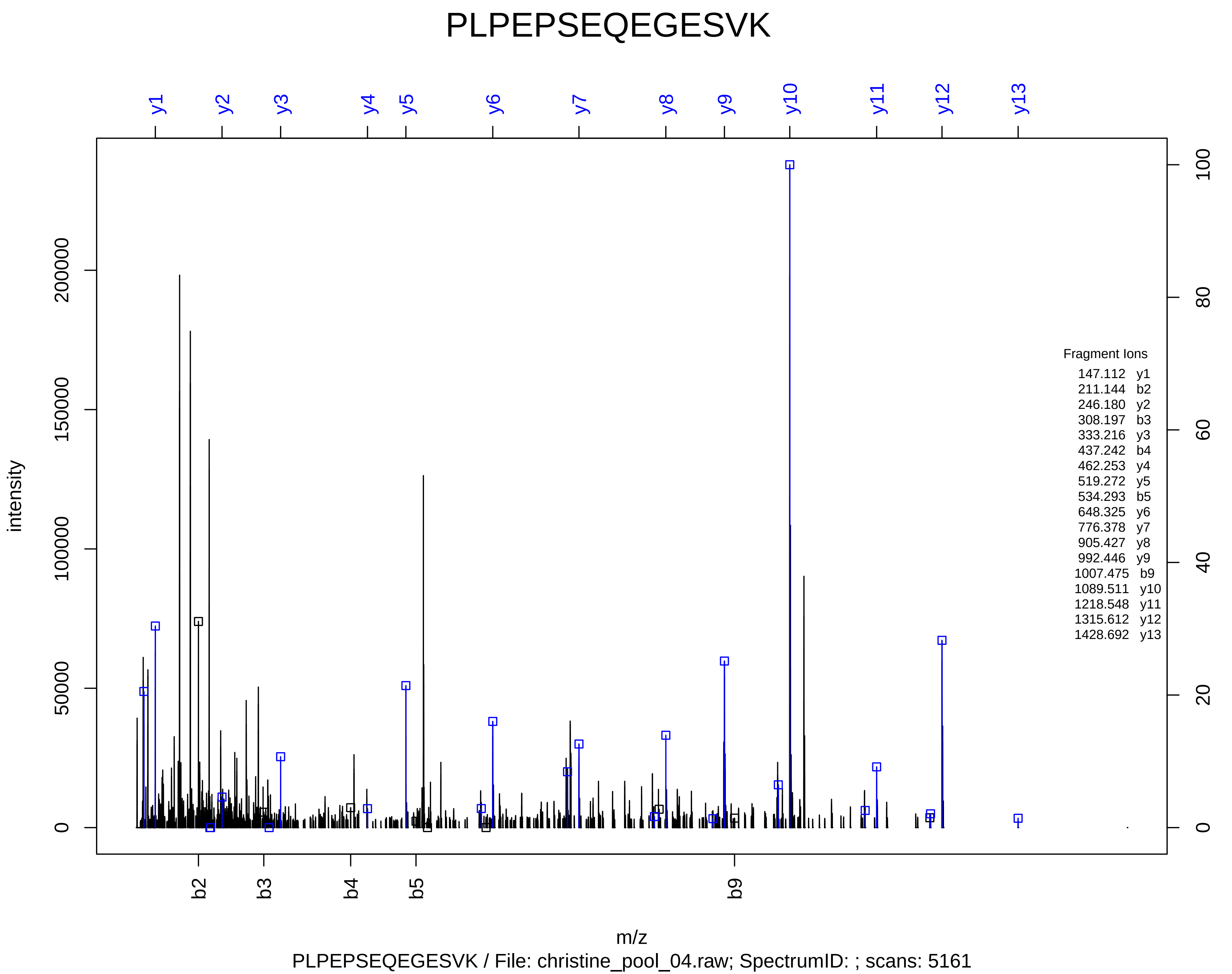

PLPEPSEQEGESVK
y1 y2 y3 y4 y5 y6 y7 y8 y9 y10 y11 y12 y13
b2 b3 b4 b5 b9
intensity
m/z
0 50000 100000 150000 200000
0 20 40 60 80 100
Fragment Ions
147.112 y1
211.144 b2
246.180 y2
308.197 b3
333.216 y3
437.242 b4
462.253 y4
519.272 y5
534.293 b5
648.325 y6
776.378 y7
905.427 y8
992.446 y9
1007.475 b9
1089.511 y10
1218.548 y11
1315.612 y12
1428.692 y13
PLPEPSEQEGESVK / File: christine_pool_04.raw; SpectrumID: ; scans: 5161

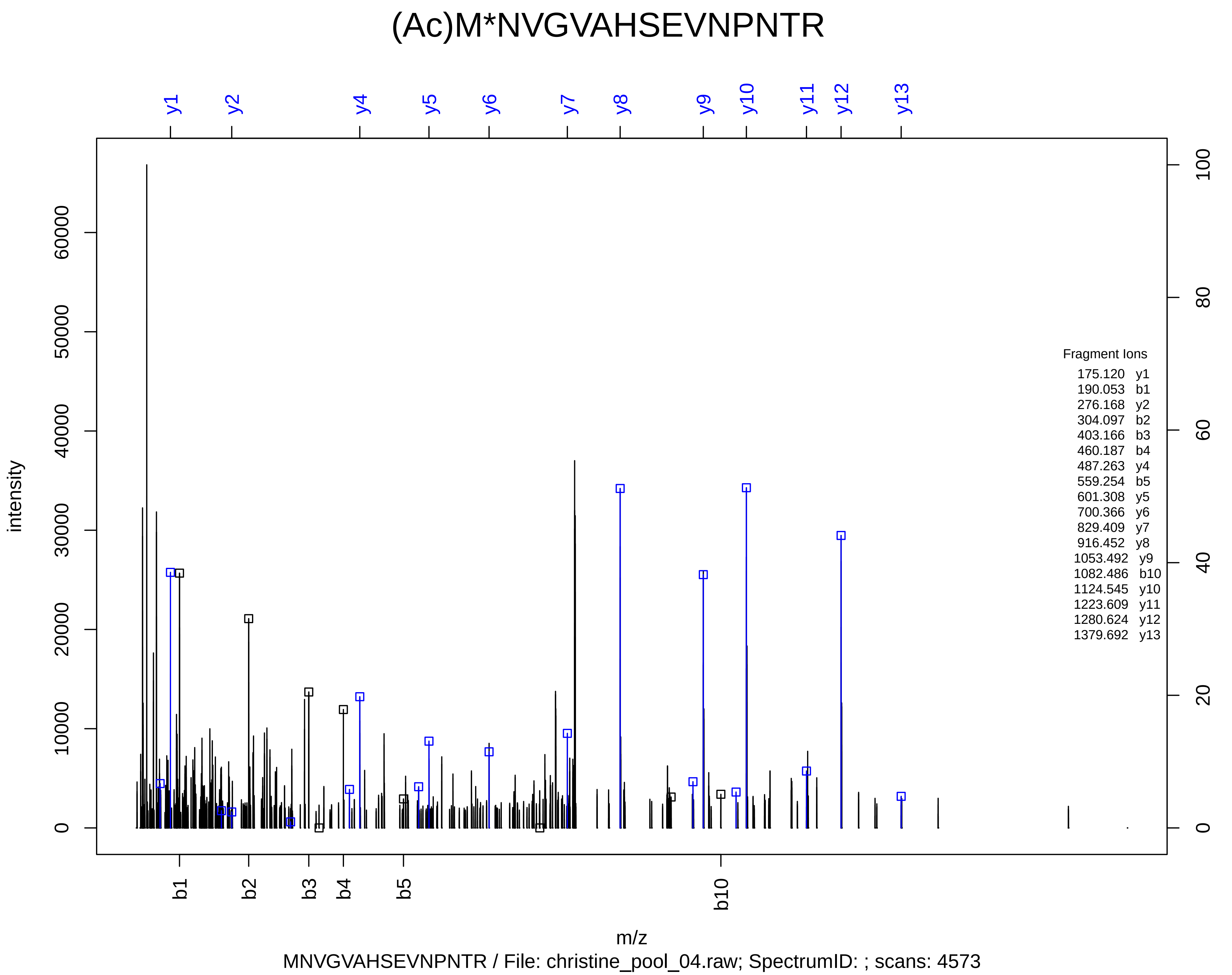

(Ac)M*NVGVAHSEVNPNTR
y1
y2
y4
y5
y6
y7
y8
y9
y10
y11
y12
y13
intensity
0
10000
20000
30000
40000
50000
60000
0
20
40
60
80
100
b1
b2
b3
b4
b5
b10
m/z
Fragment Ions
175.120 y1
190.053 b1
276.168 y2
304.097 b2
403.166 b3
460.187 b4
487.263 y4
559.254 b5
601.308 y5
700.366 y6
829.409 y7
916.452 y8
1053.492 y9
1082.486 b10
1124.545 y10
1223.609 y11
1280.624 y12
1379.692 y13
MNVGVAHSEVNPNTR / File: christine_pool_04.raw; SpectrumID: ; scans: 4573

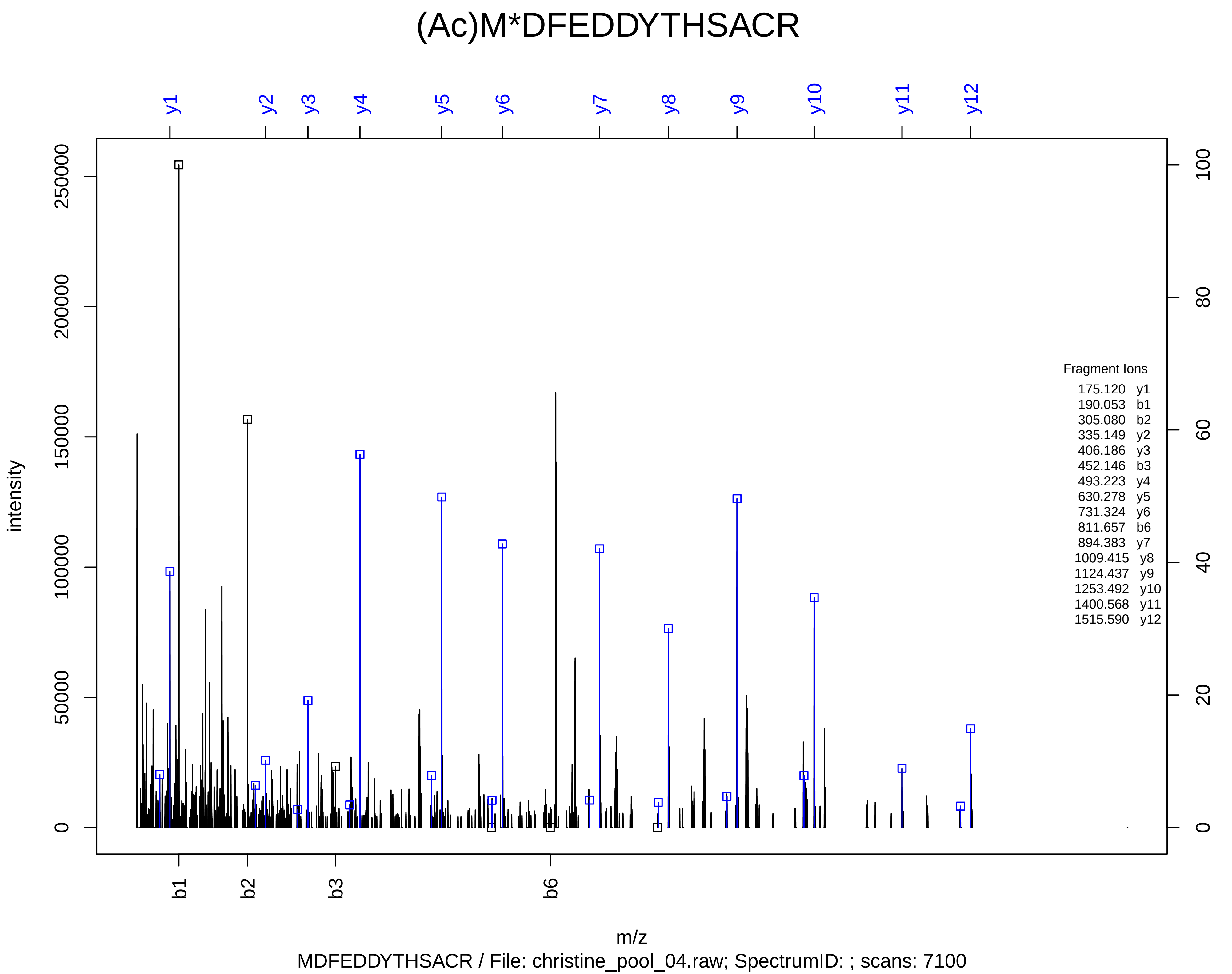

(Ac)M*DFEDDYTHSACR
y1 y2 y3 y4 y5 y6 y7 y8 y9 y10 y11 y12
b1 b2 b3 b6
intensity
0
50000
100000
150000
200000
250000
0
20
40
60
80
100
m/z
Fragment Ions
175.120 y1
190.053 b1
305.080 b2
335.149 y2
406.186 y3
452.146 b3
493.223 y4
630.278 y5
731.324 y6
811.657 b6
894.383 y7
1009.415 y8
1124.437 y9
1253.492 y10
1400.568 y11
1515.590 y12
MDFEDDYTHSACR / File: christine_pool_04.raw; SpectrumID: ; scans: 7100

NLDNAAEAAEQFK

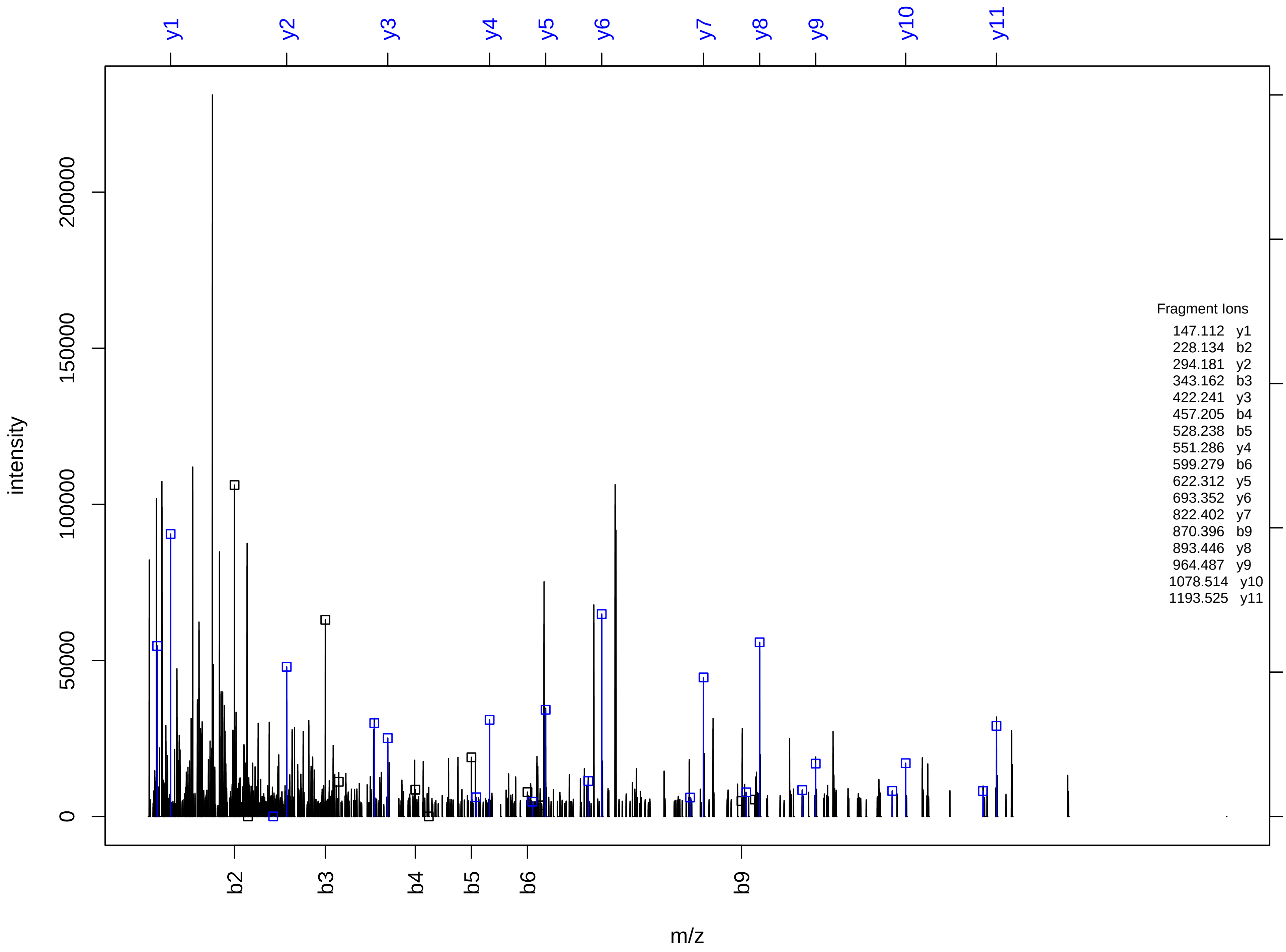

NLDNAAEAAEQFK / File: christine_pool_04.raw; SpectrumID: ; scans: 8863

ELDFWQEGWR

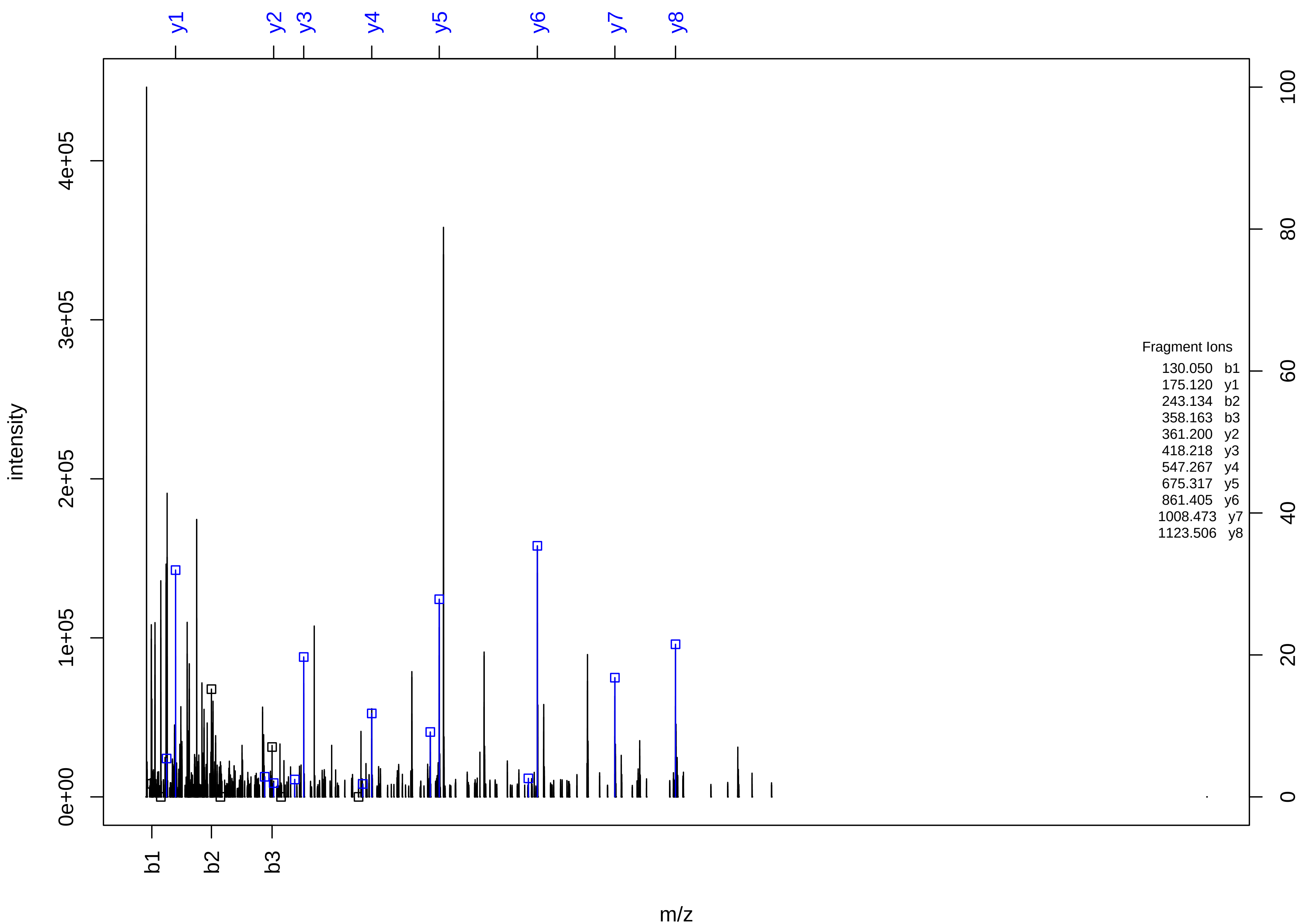

ELDFWQEGWR / File: christine_pool_04.raw; SpectrumID: ; scans: 17715

VLELLLPIR

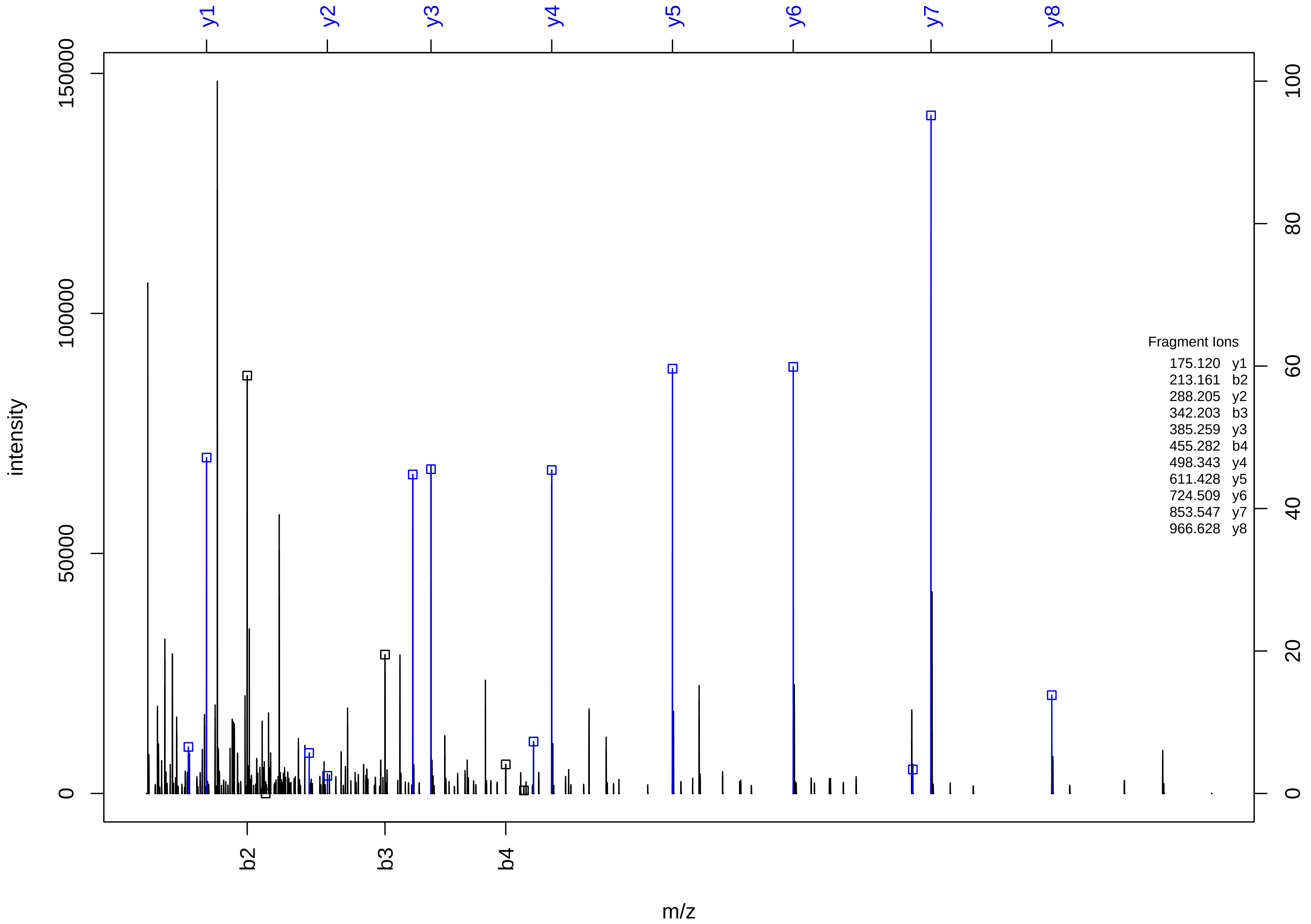

VLELLLPIR / File: christine_pool_04.raw; SpectrumID: ; scans: 21780

FLAEAAIR

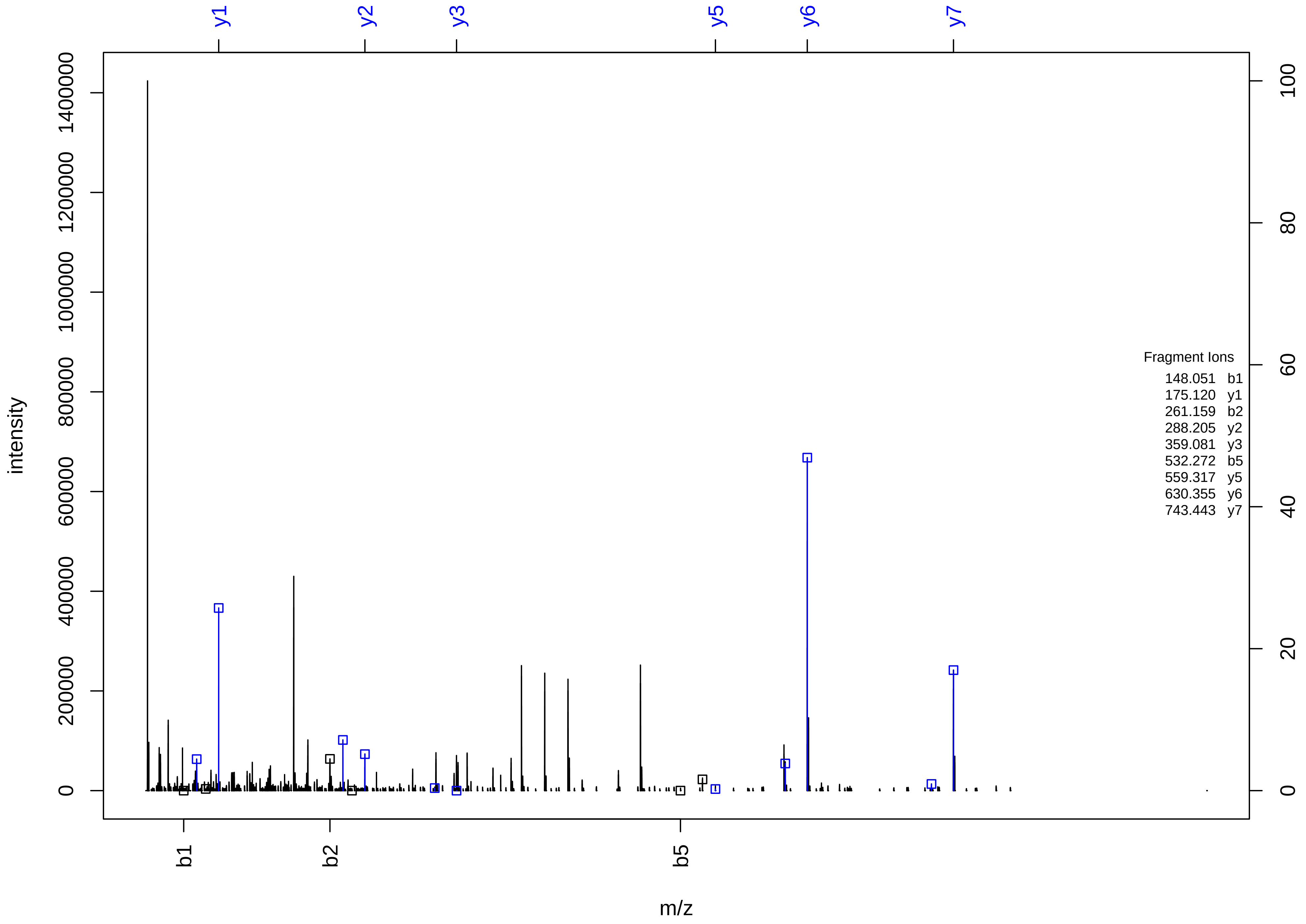

FLAEAAIR / File: christine_pool_04.raw; SpectrumID: ; scans: 10611

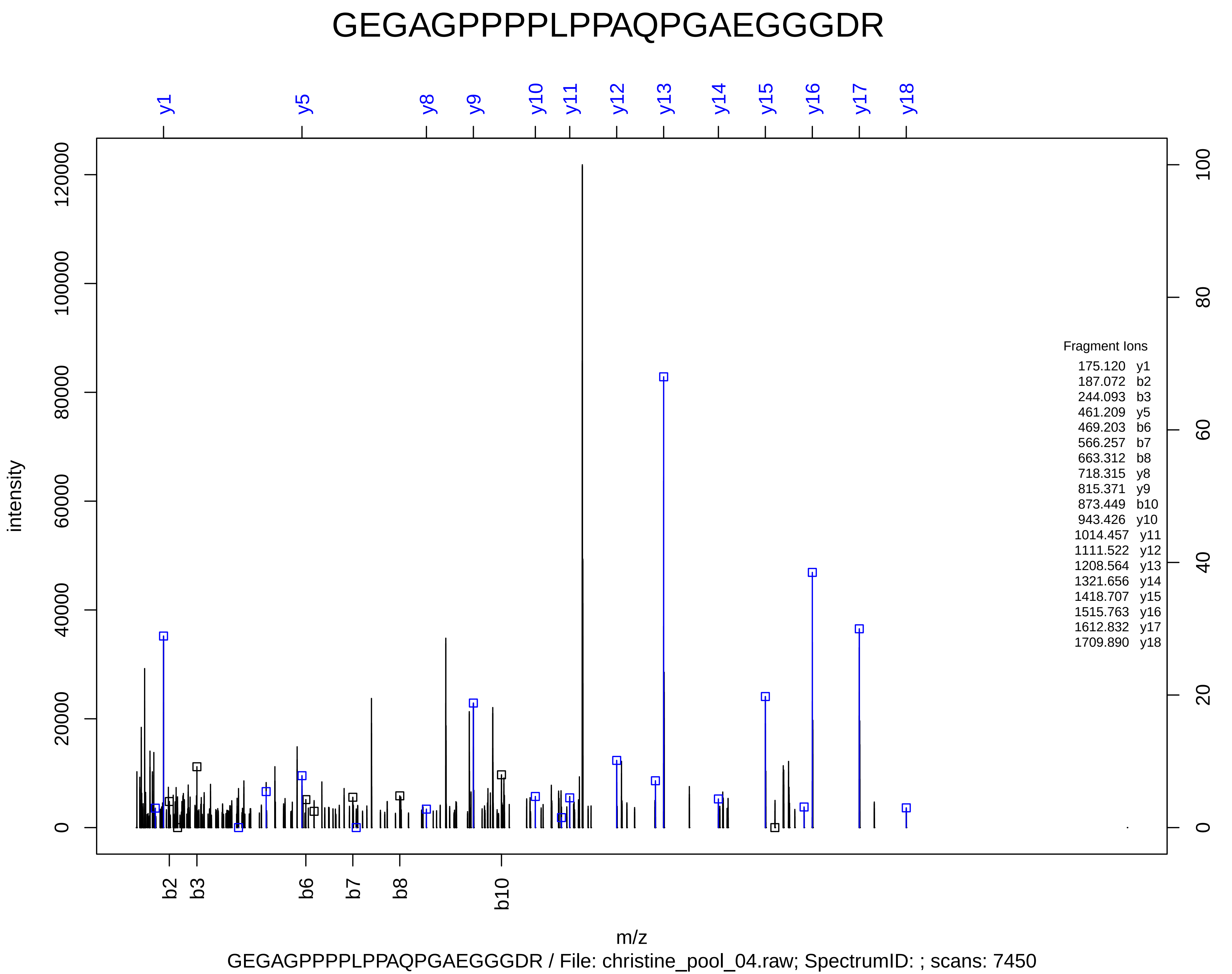
GEGAGPPPPLPPAQPGAEGGGDR
y1
y5
y8
y9
y10
y11
y12
y13
y14
y15
y16
y17
y18
b2
b3
b6
b7
b8
b10
intensity
m/z
0
20000
40000
60000
80000
100000
120000
0
20
40
60
80
100
Fragment Ions
175.120 y1
187.072 b2
244.093 b3
461.209 y5
469.203 b6
566.257 b7
663.312 b8
718.315 y8
815.371 y9
873.449 b10
943.426 y10
1014.457 y11
1111.522 y12
1208.564 y13
1321.656 y14
1418.707 y15
1515.763 y16
1612.832 y17
1709.890 y18
GEGAGPPPPLPPAQPGAEGGGDR / File: christine_pool_04.raw; SpectrumID: ; scans: 7450

LPEPSASLPNPPSK

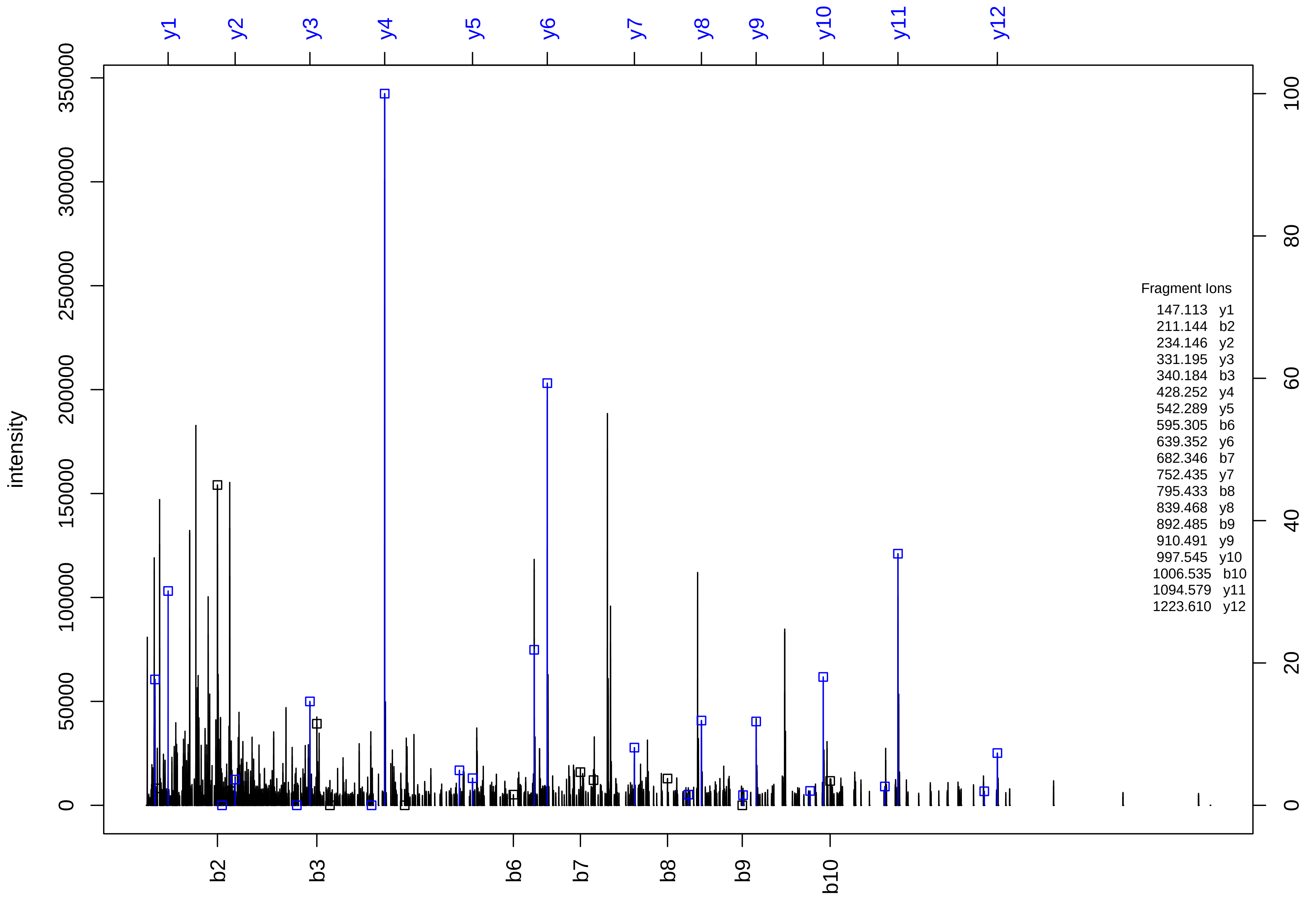

LPEPSASLPNPPSK / File: christine_pool_04.raw; SpectrumID: ; scans: 8798

FDLIWNR

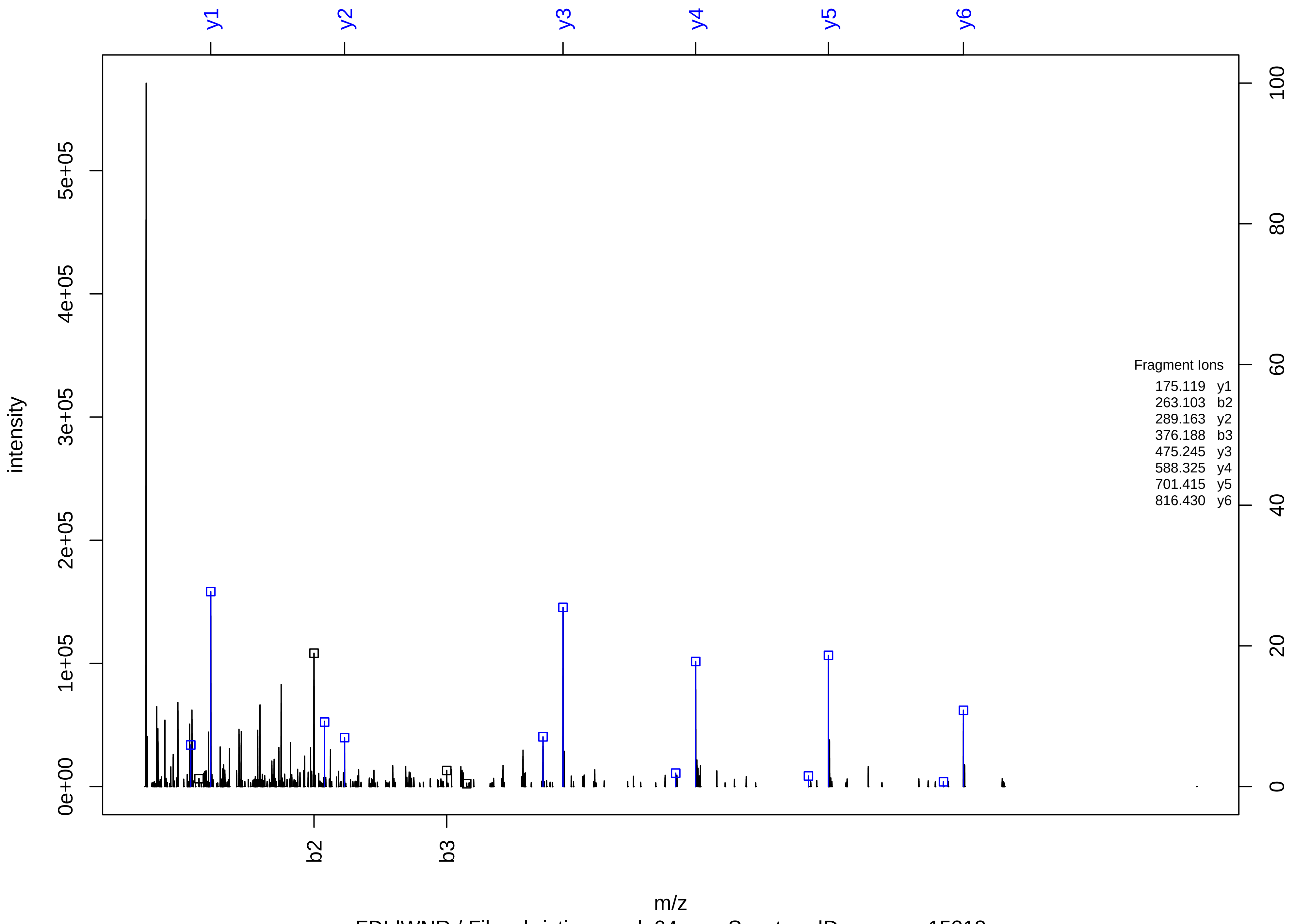

FDLIWNR / File: christine_pool_04.raw; SpectrumID: ; scans: 15318

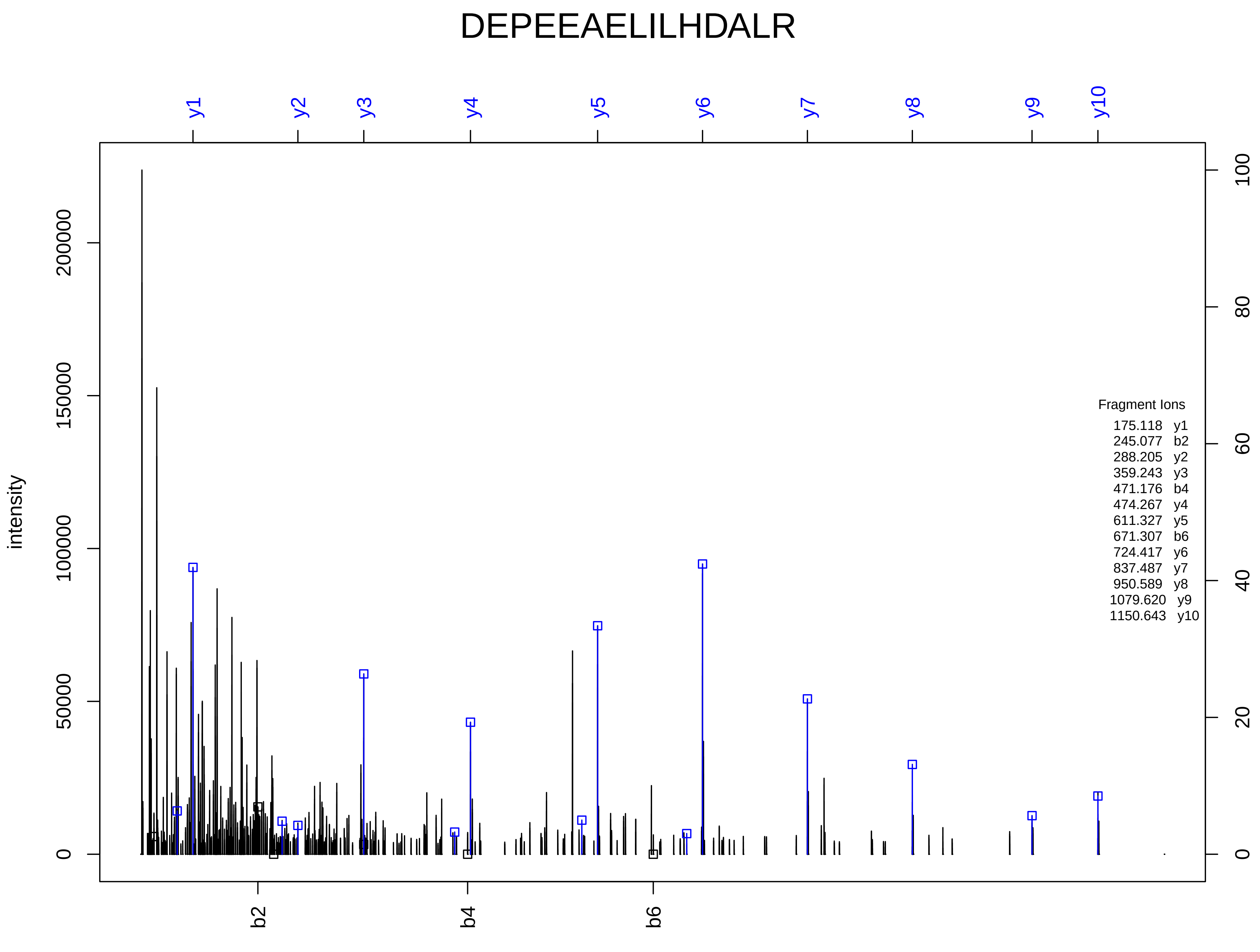

DEPEEEAELILHDALR / File: christine_pool_04.raw; SpectrumID: ; scans: 17235

EAVEQFESQGVDLSNIVK

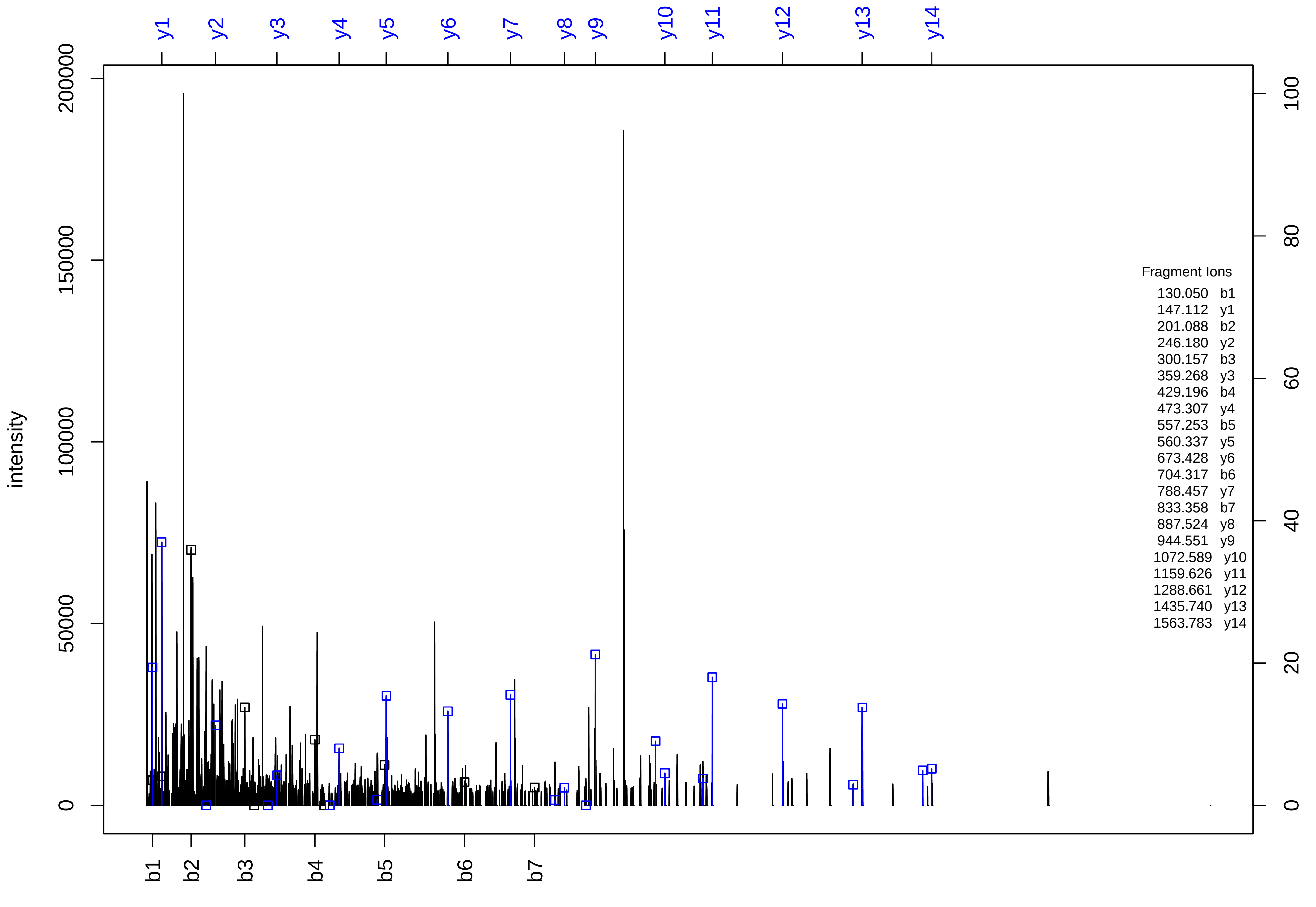

EAVEQFESQGVDLSNIVK / File: christine_pool_04.raw; SpectrumID: ; scans: 16158

GPASTPTYSPAPTQPAPR

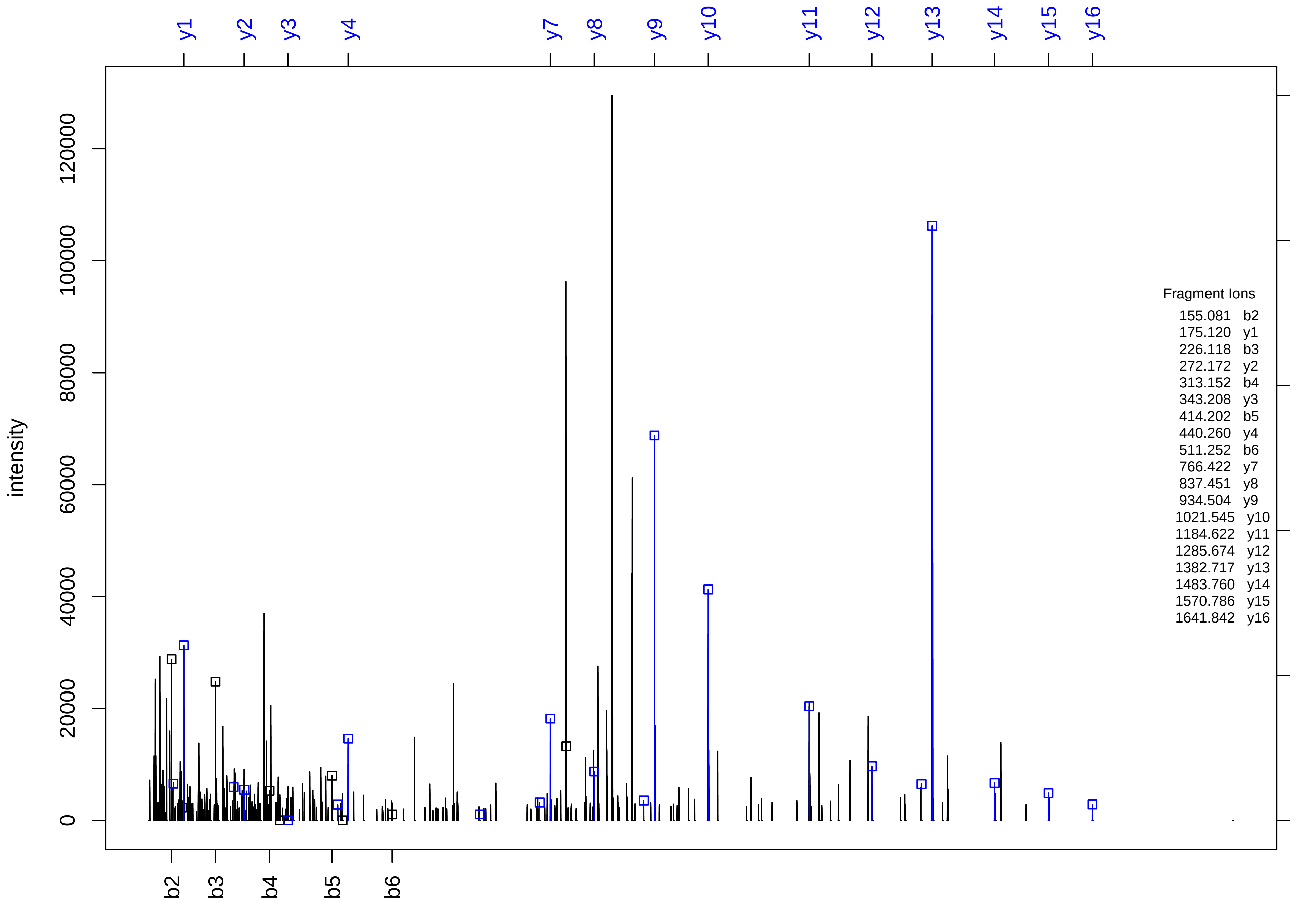

GPASTPTYSPAPTQPAPR / File: christine_pool_04.raw; SpectrumID: ; scans: 5288

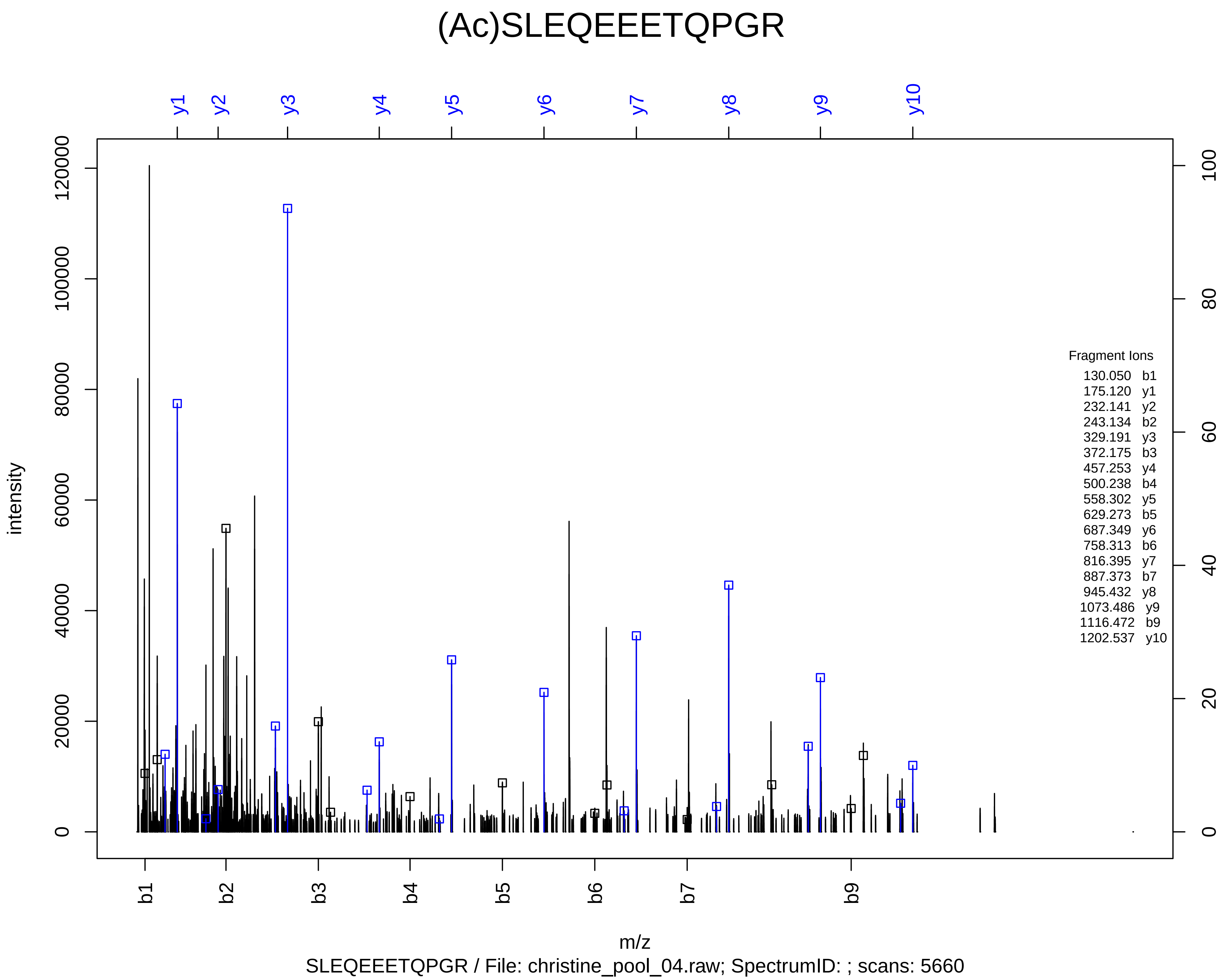
(Ac)SLEQEEETQPGR
intensity
m/z
Fragment Ions
130.050 b1
175.120 y1
232.141 y2
243.134 b2
329.191 y3
372.175 b3
457.253 y4
500.238 b4
558.302 y5
629.273 b5
687.349 y6
758.313 b6
816.395 y7
887.373 b7
945.432 y8
1073.486 y9
1116.472 b9
1202.537 y10
SLEQEEETQPGR / File: christine_pool_04.raw; SpectrumID: ; scans: 5660

GVPHPEDDHSQVEGPESLR

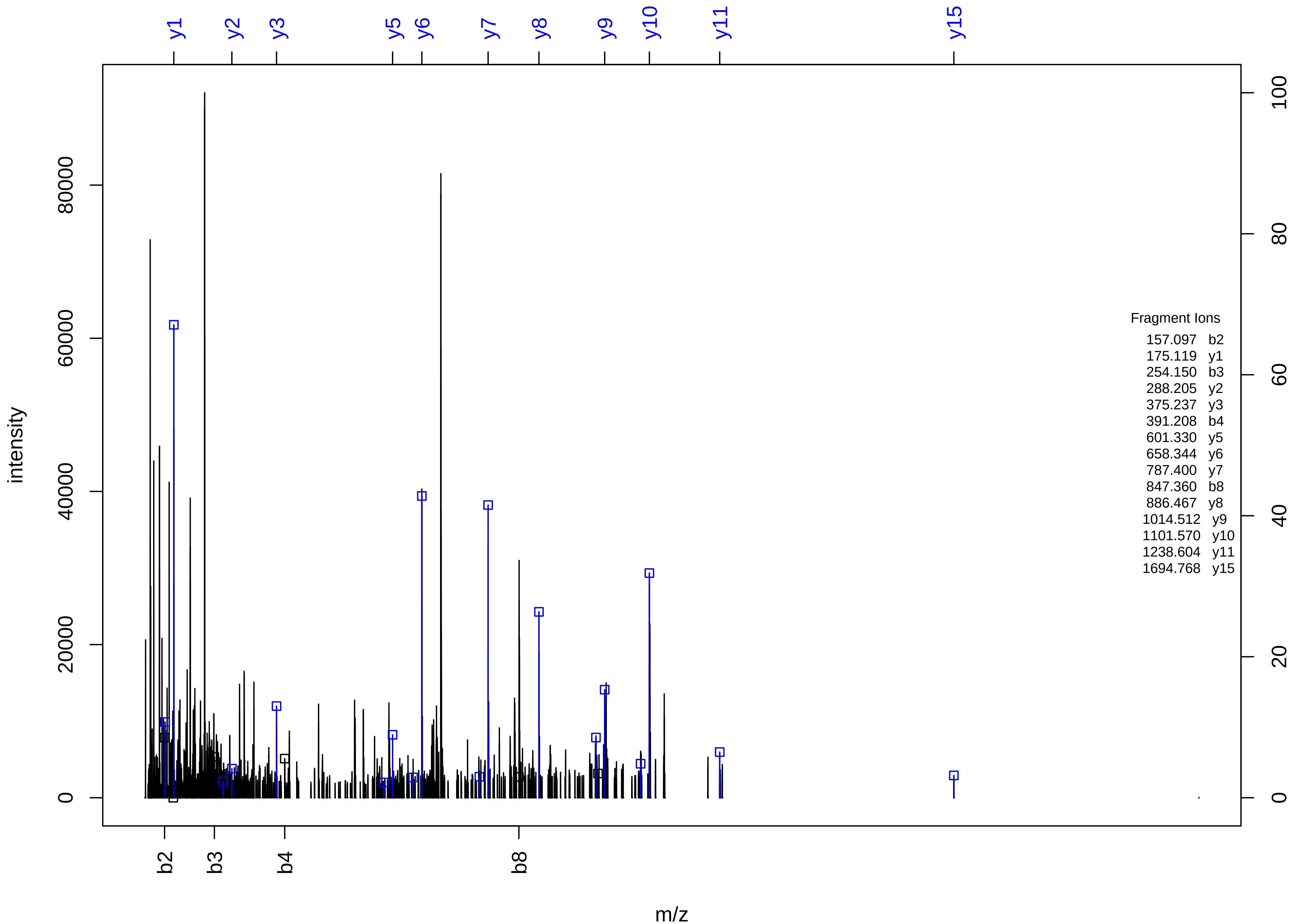

GVPHPEDDHSQVEGPESLR / File: christine_pool_04.raw; SpectrumID: ; scans: 4995

DFDGLVQVIIPQDESAASVK

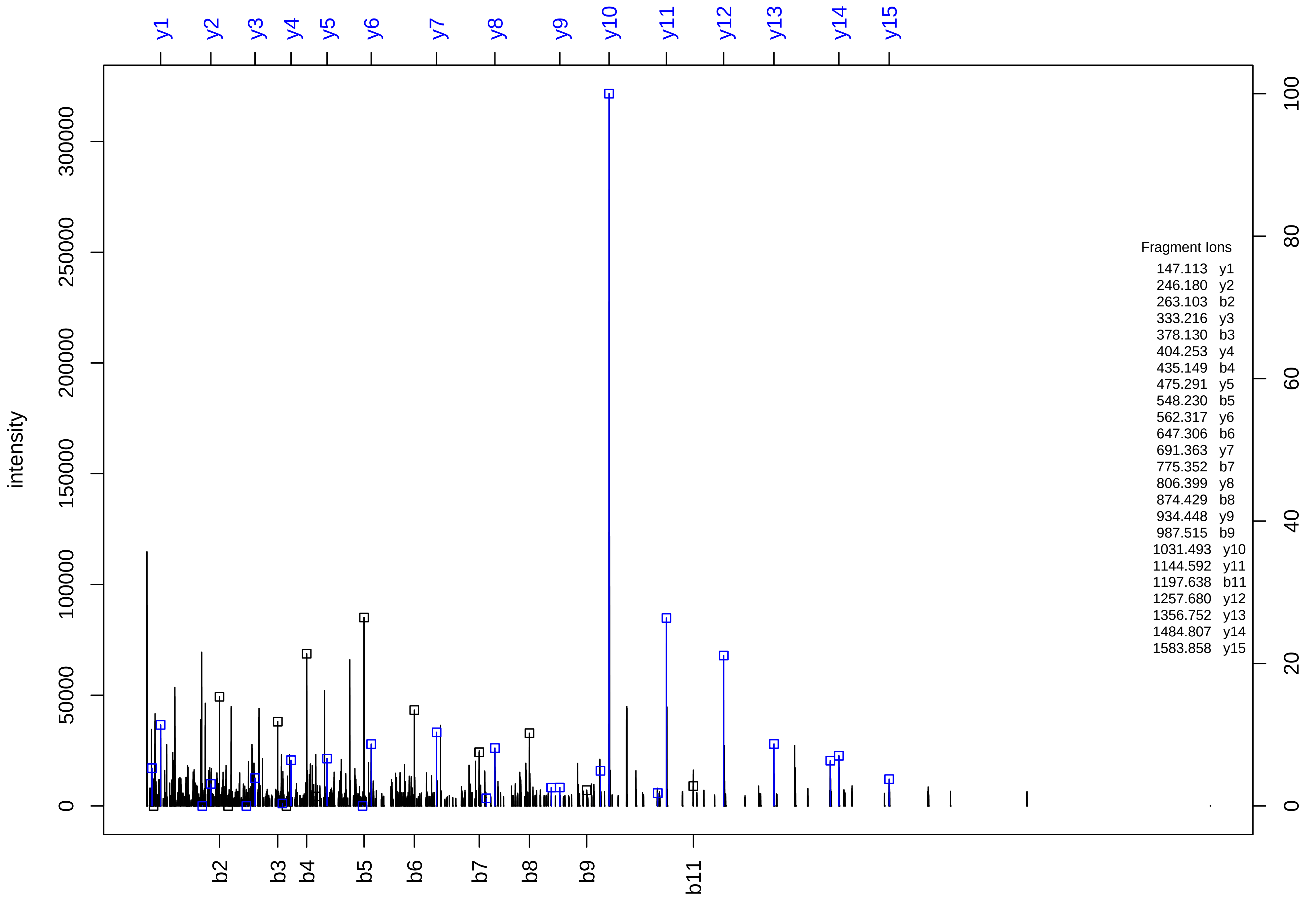

DFDGLVQVIIPQDESAASVK / File: christine_pool_04.raw; SpectrumID: ; scans: 21870

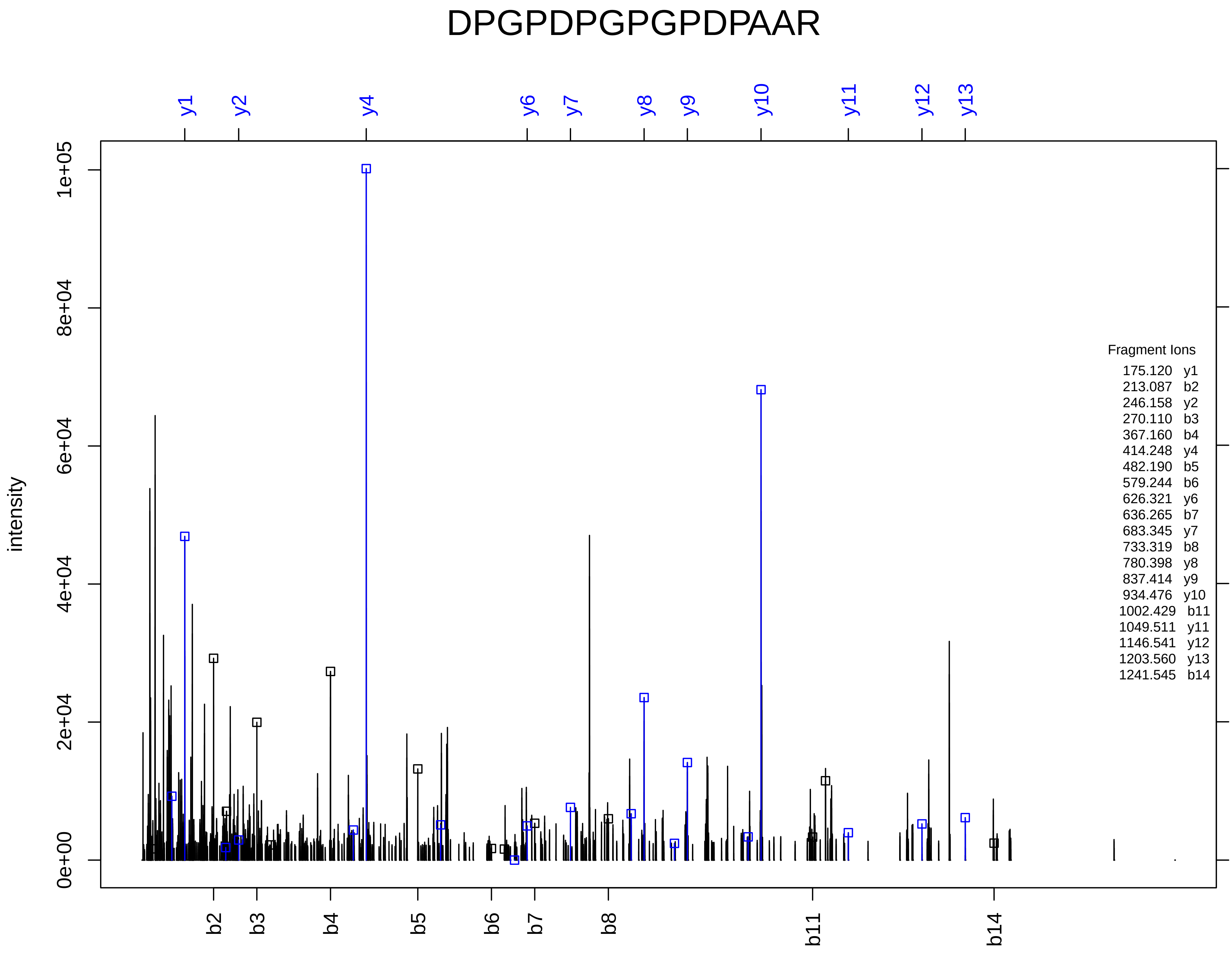

DPGPDPGPGPDPAAR
y1
y2
y4
y6
y7
y8
y9
y10
y11
y12
y13
intensity
0e+00
2e+04
4e+04
6e+04
8e+04
1e+05
0
20
40
60
80
100
Fragment Ions
175.120 y1
213.087 b2
246.158 y2
270.110 b3
367.160 b4
414.248 y4
482.190 b5
579.244 b6
626.321 y6
636.265 b7
683.345 y7
733.319 b8
780.398 y8
837.414 y9
934.476 y10
1002.429 b11
1049.511 y11
1146.541 y12
1203.560 y13
1241.545 b14
b2
b3
b4
b5
b6
b7
b8
b11
b14
m/z
DPGPDPGPGPDPAAR / File: christine_pool_04.raw; SpectrumID: ; scans: 4326

GLGGEVPGSHQGPDPYR

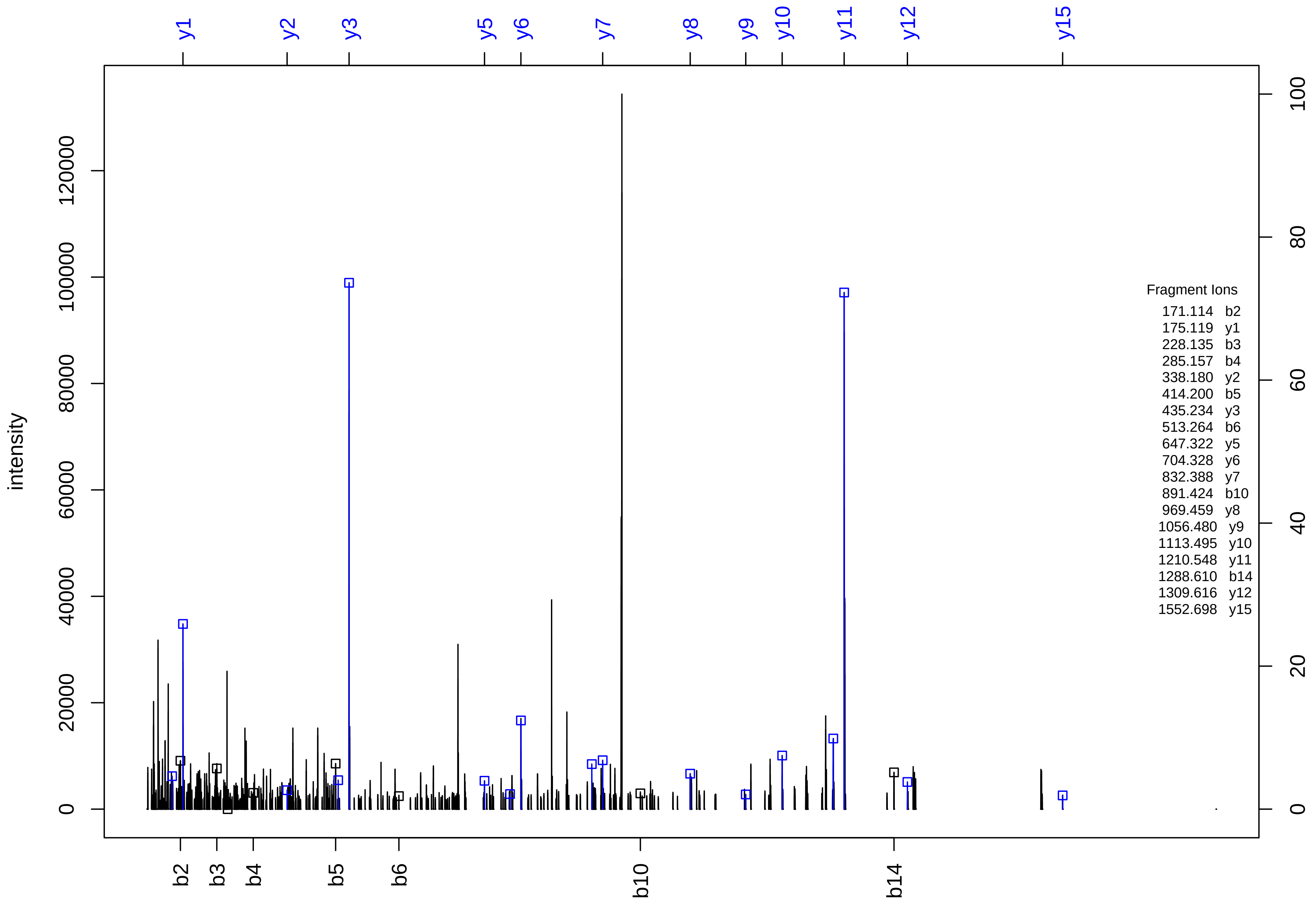

GLGGEVPGSHQGPDPYR / File: christine_pool_04.raw; SpectrumID: ; scans: 5325

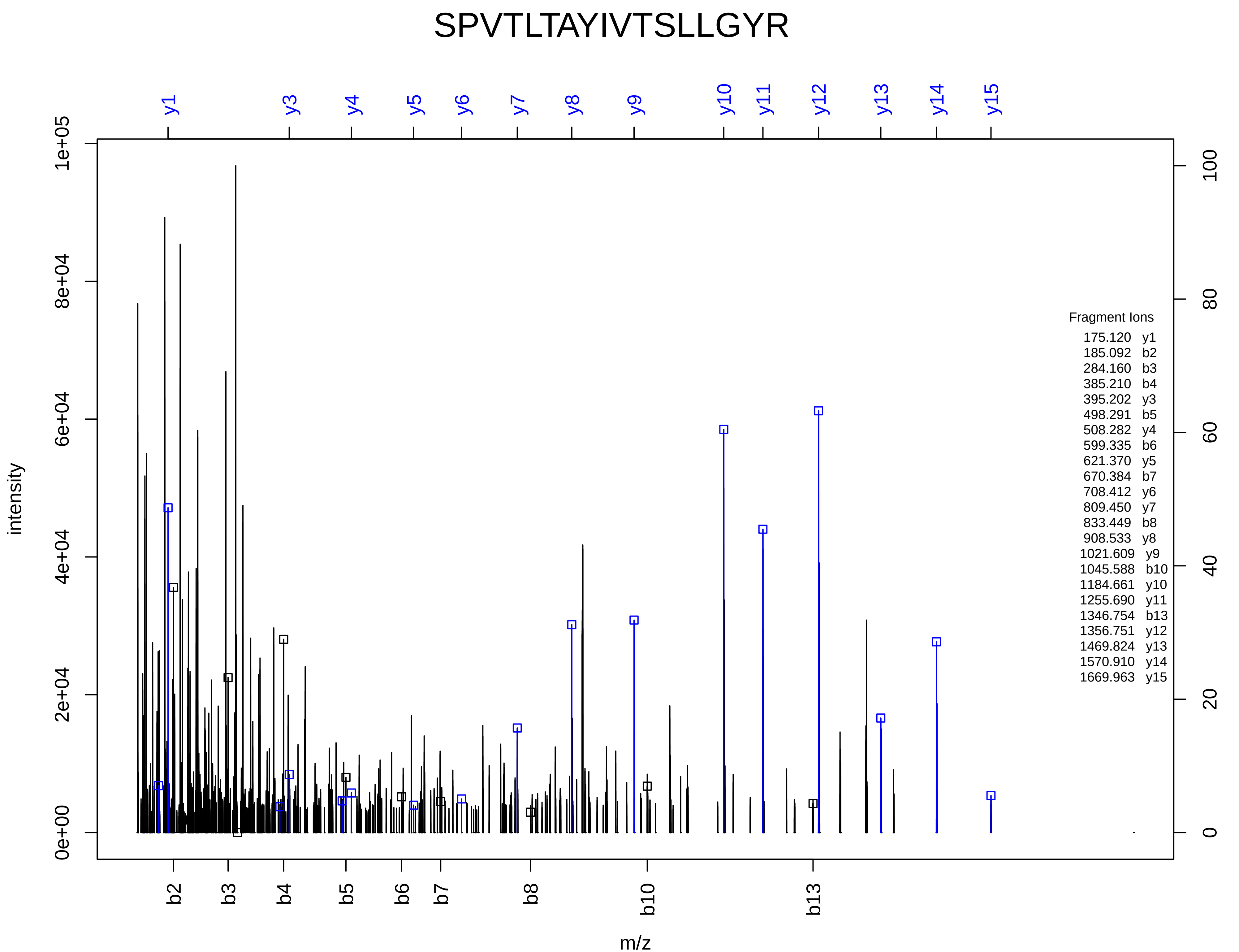

SPVTLTAYIVTSLLGYR / File: christine_pool_04.raw; SpectrumID: ; scans: 23388

IAAIDSLNQYTR

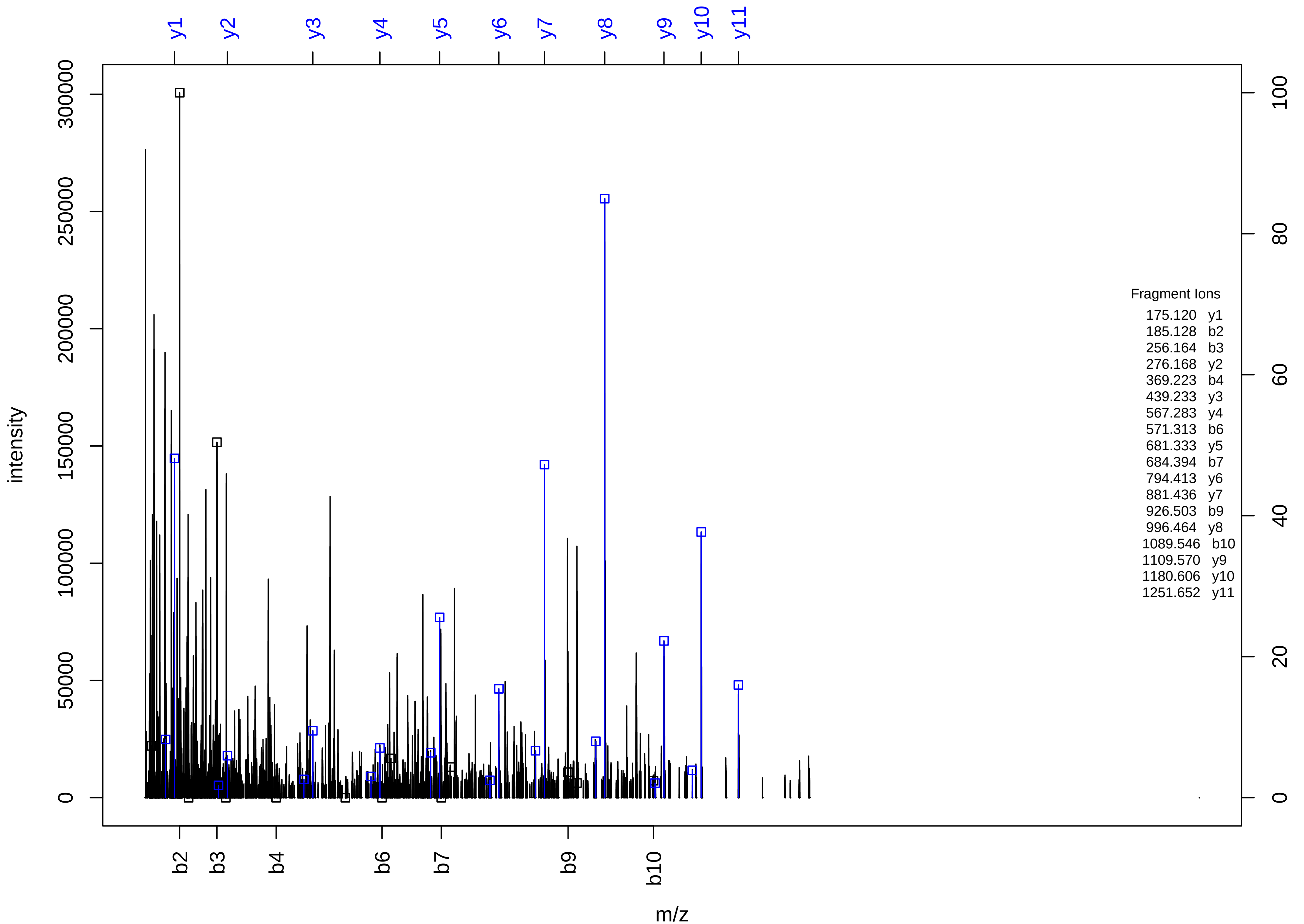

IAAIDSLNQYTR / File: christine_pool_04.raw; SpectrumID: ; scans: 10788

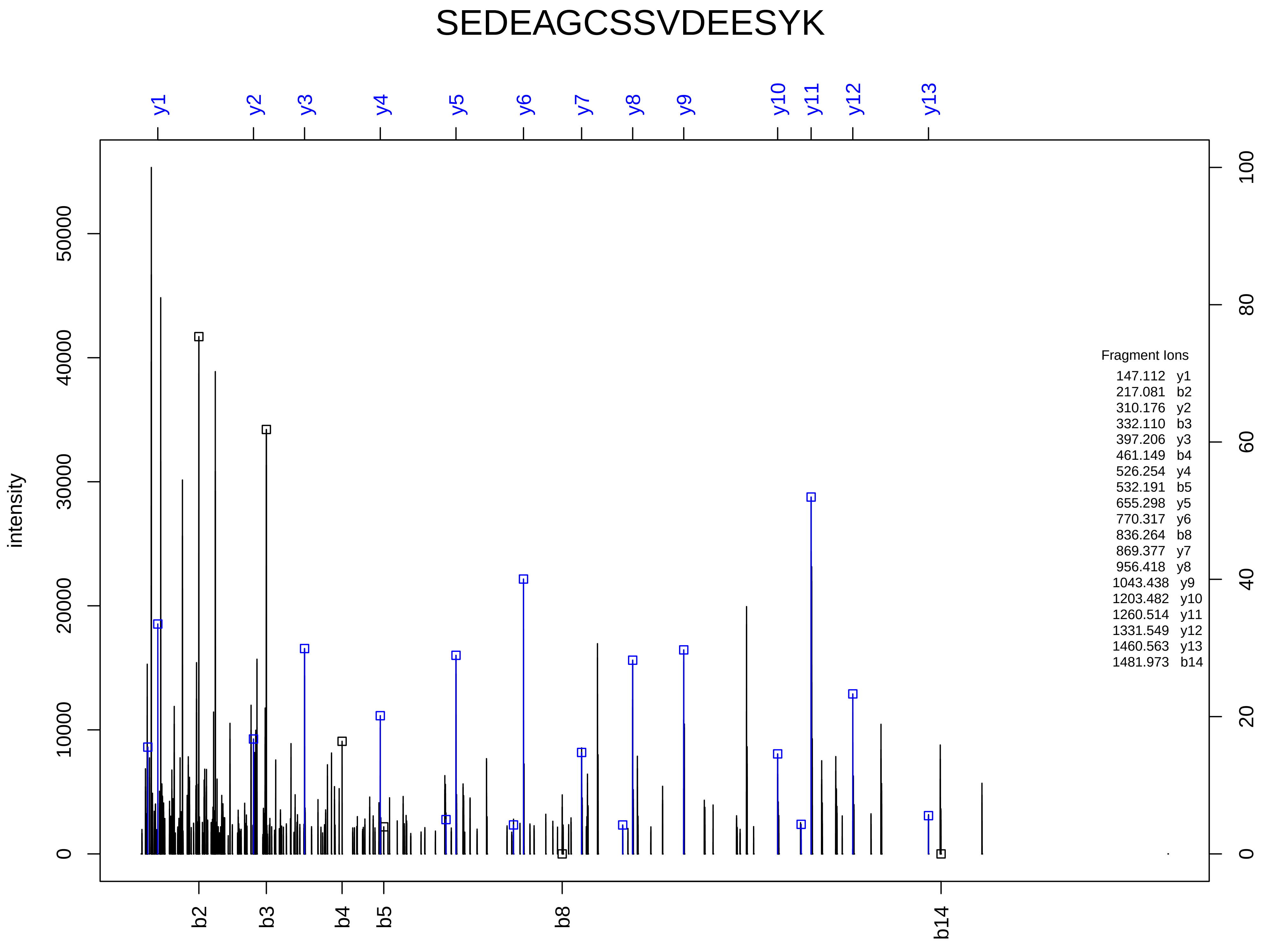

SEDEAGCSSVDEESYK / File: christine_pool_04.raw; SpectrumID: ; scans: 3262

HFSQAEGSQLDEVR

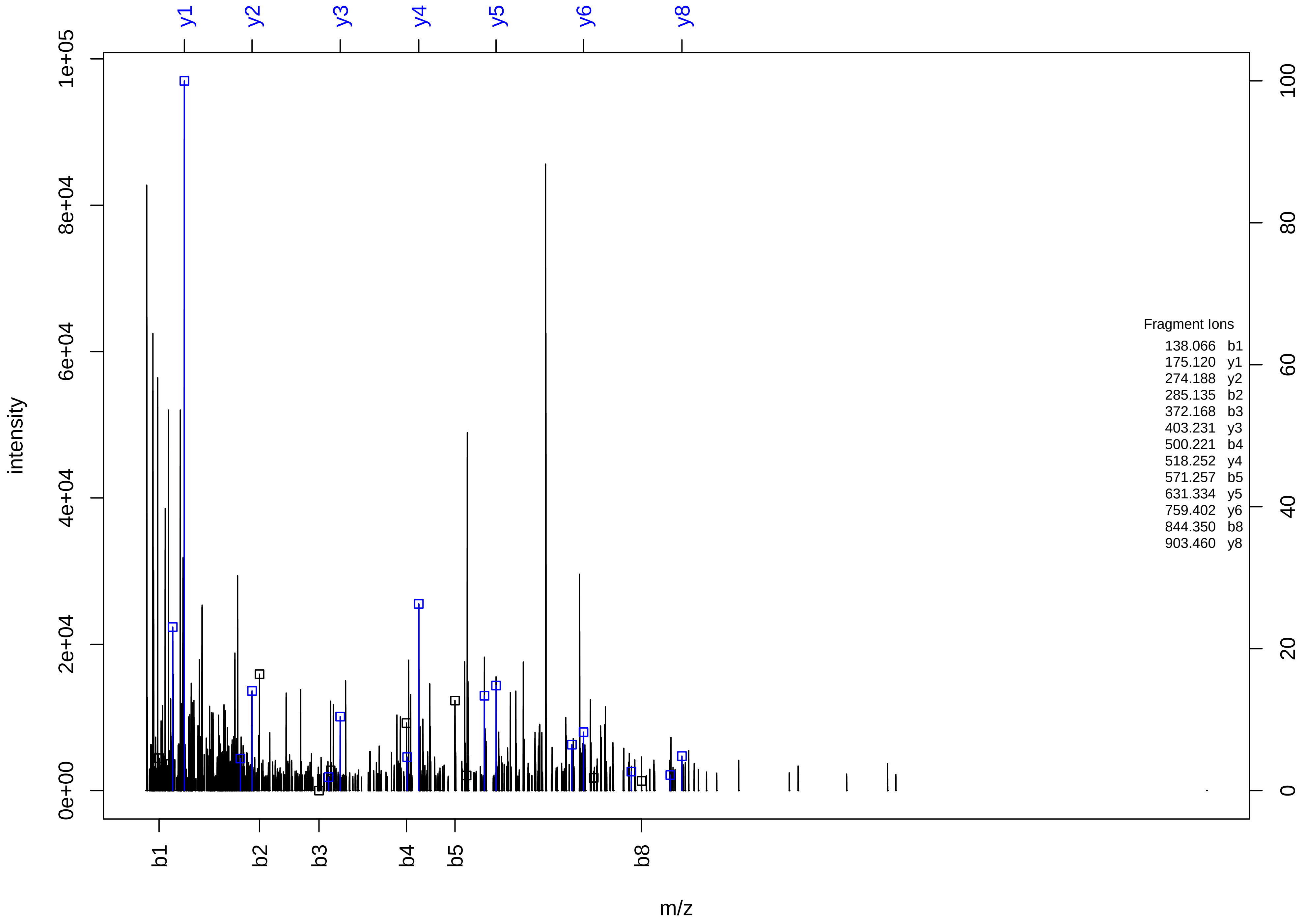

HFSQAEGSQLDEVR / File: christine_pool_04.raw; SpectrumID: ; scans: 4595

DPEIAELFFK

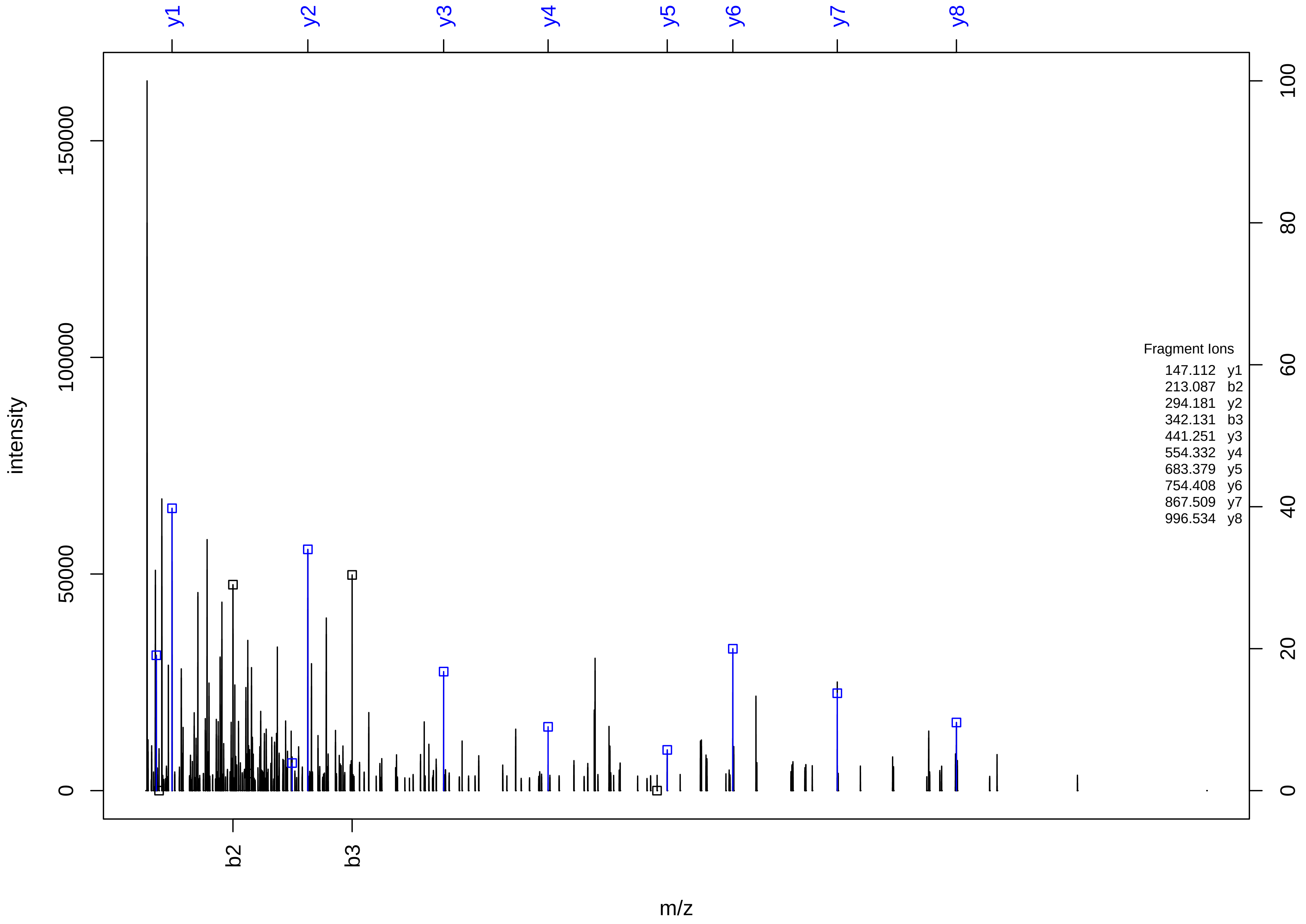

DPEIAELFFK / File: christine_pool_04.raw; SpectrumID: ; scans: 21671

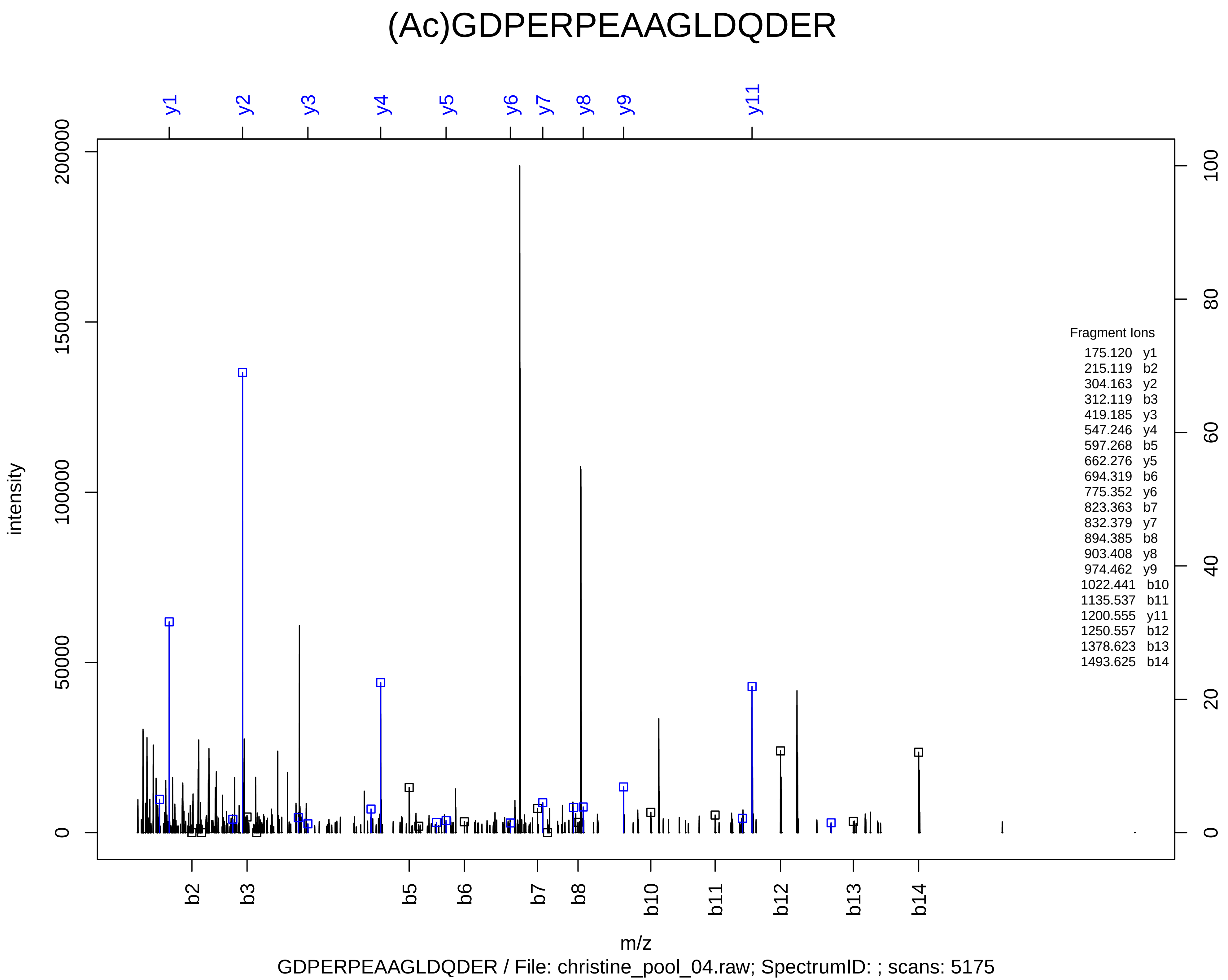
(Ac)GDPERPEAAGLDQDER
y1
y2
y3
y4
y5
y6
y7
y8
y9
y11
intensity
0
50000
100000
150000
200000
0
20
40
60
80
100
b2
b3
b5
b6
b7
b8
b10
b11
b12
b13
b14
m/z
Fragment Ions
175.120 y1
215.119 b2
304.163 y2
312.119 b3
419.185 y3
547.246 y4
597.268 b5
662.276 y5
694.319 b6
775.352 y6
823.363 b7
832.379 y7
894.385 b8
903.408 y8
974.462 y9
1022.441 b10
1135.537 b11
1200.555 y11
1250.557 b12
1378.623 b13
1493.625 b14
GDPERPEAAGLDQDER / File: christine_pool_04.raw; SpectrumID: ; scans: 5175

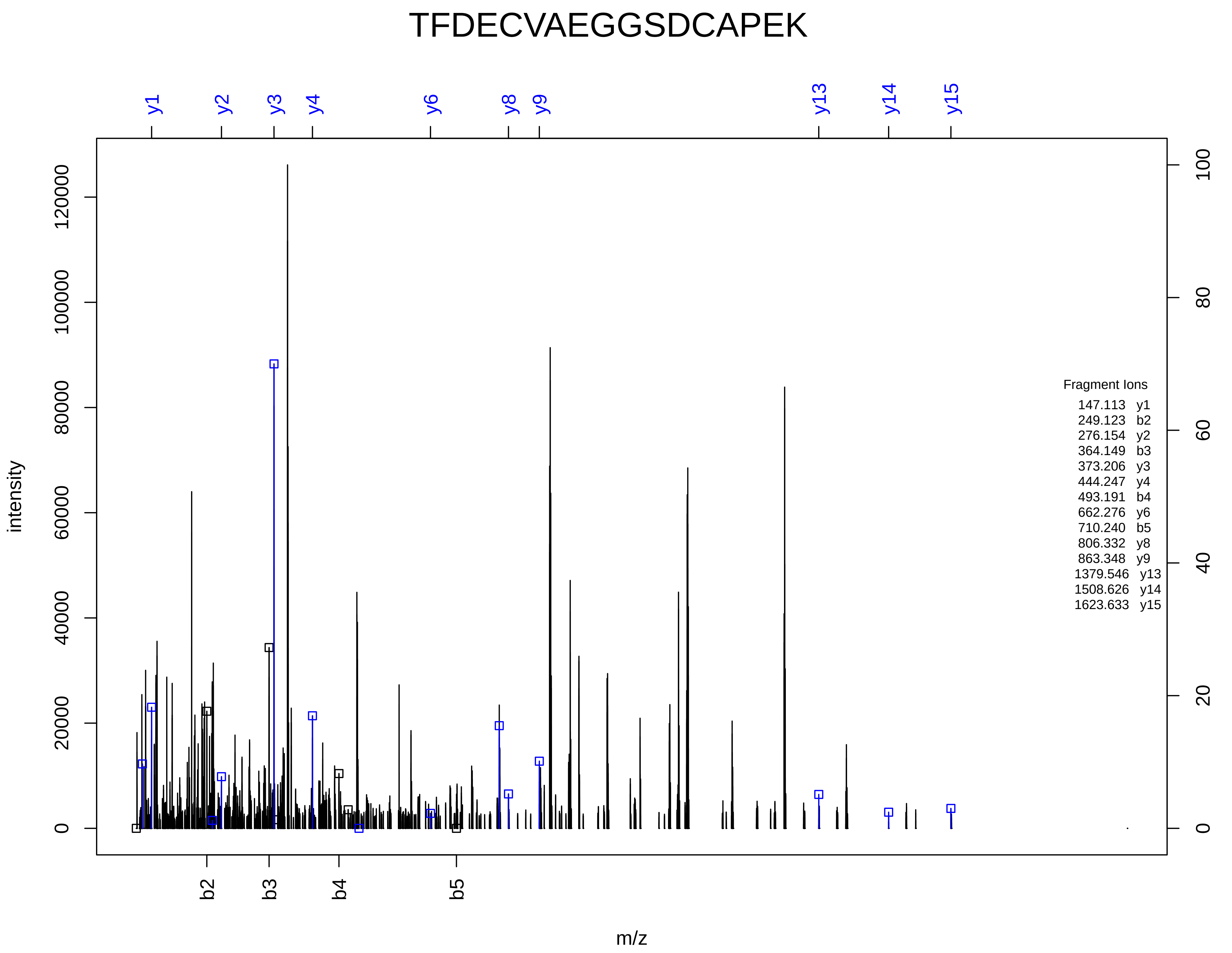

TFDECVAEGGSDCAPEK / File: christine_pool_04.raw; SpectrumID: ; scans: 5306

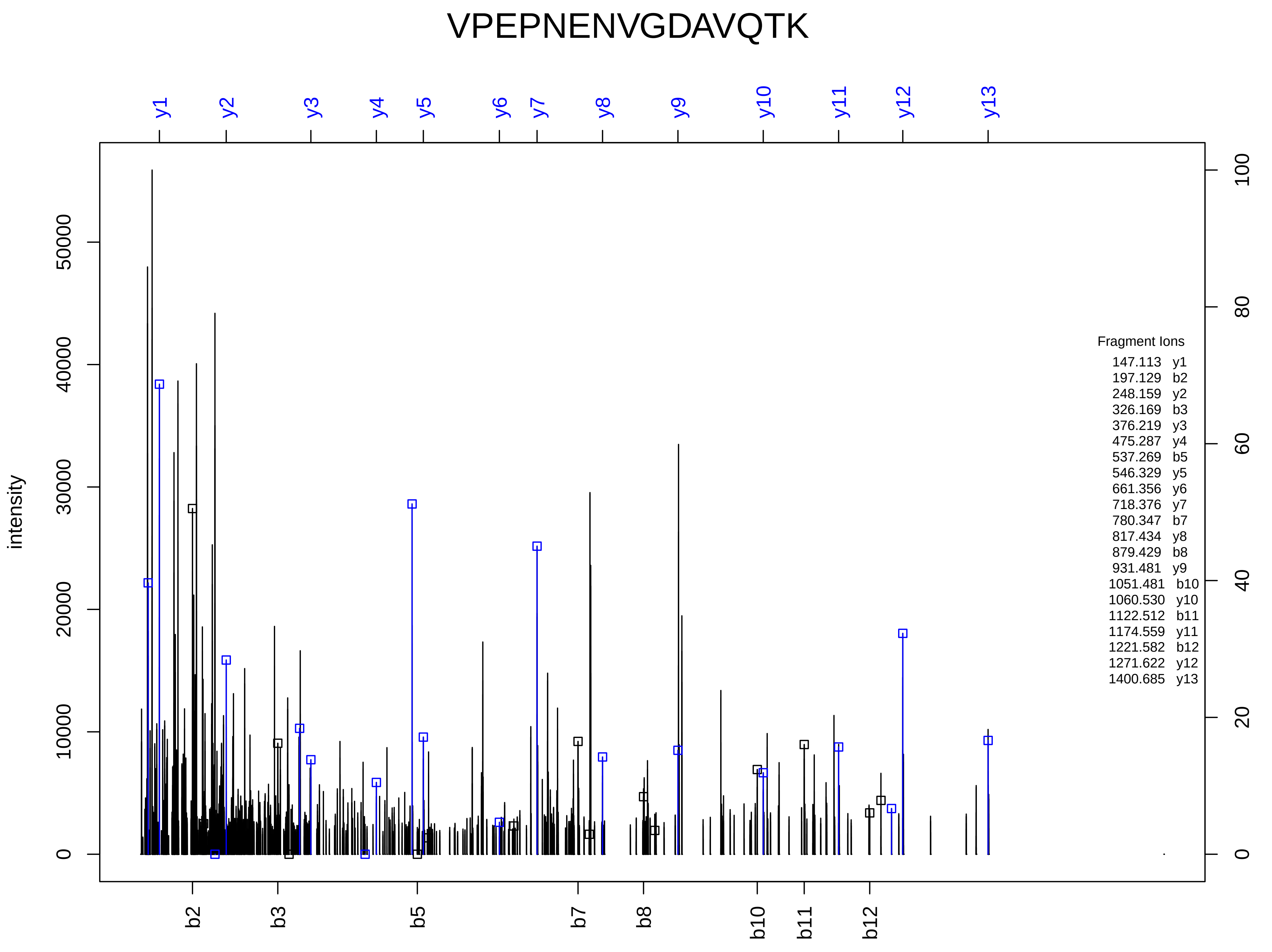

VPEPNENVGDAVQTK / File: christine_pool_04.raw; SpectrumID: ; scans: 5447

NLSSDEATNPISR

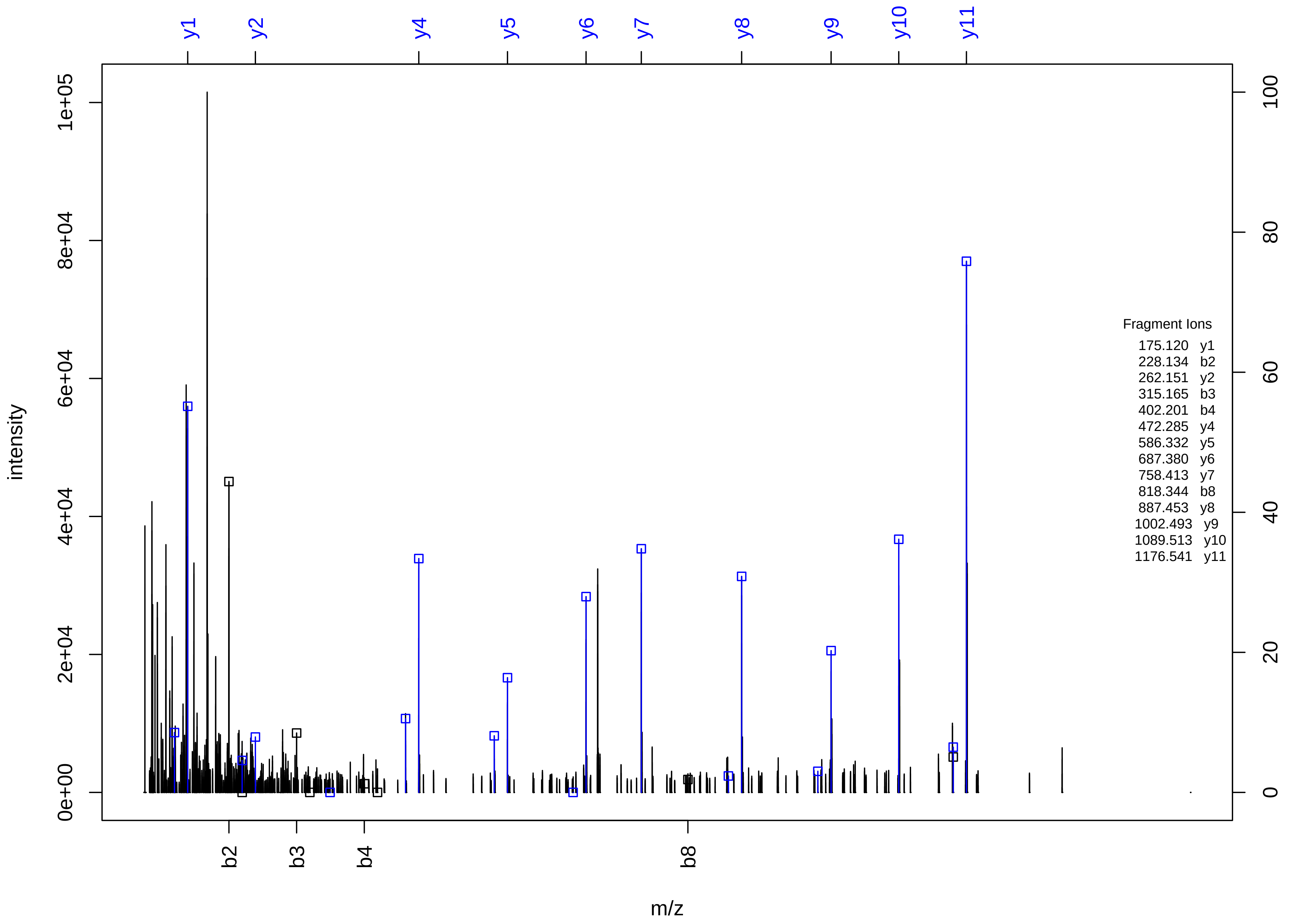

NLSSDEATNPISR / File: christine_pool_04.raw; SpectrumID: ; scans: 5216

RSGASAAPAASAAAALAPSATR

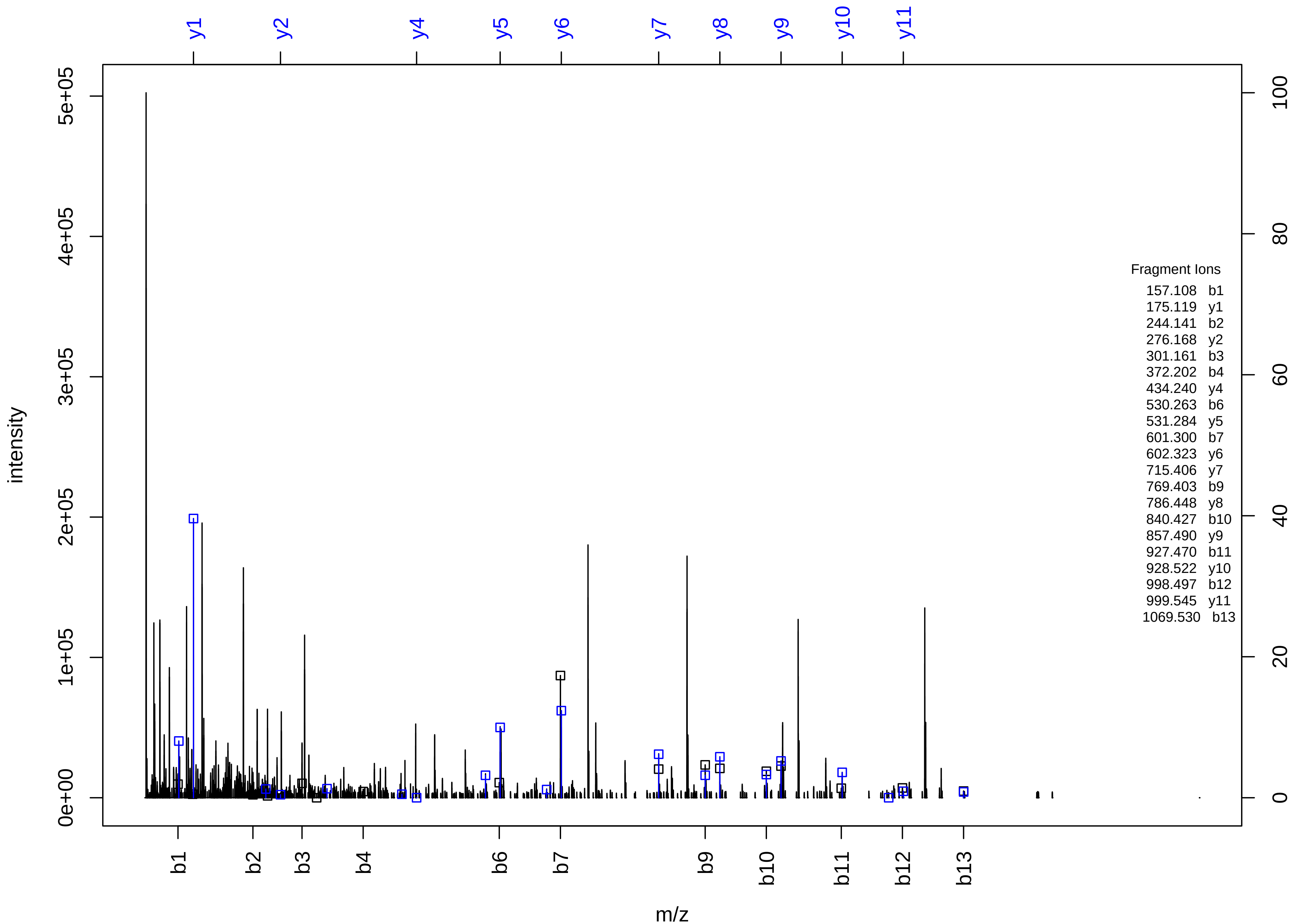

RSGASAAPAASAAAALAPSATR / File: christine_pool_04.raw; SpectrumID: ; scans: 7145

(Ac)ATTGTPTADRGDAAATDDPAAR

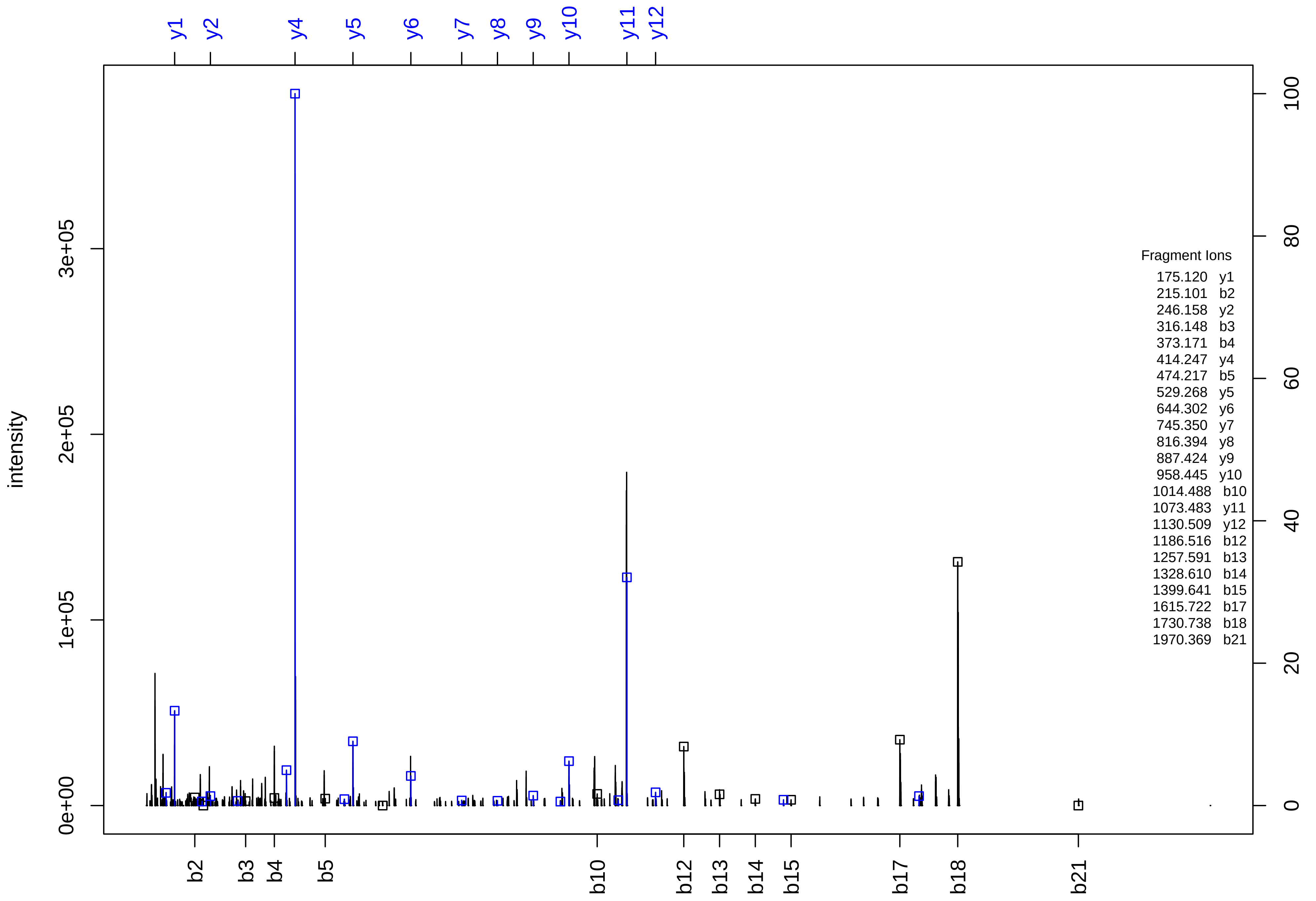

ATTGTPTADRGDAAATDDPAAR / File: christine_pool_04.raw; SpectrumID: ; scans: 4468

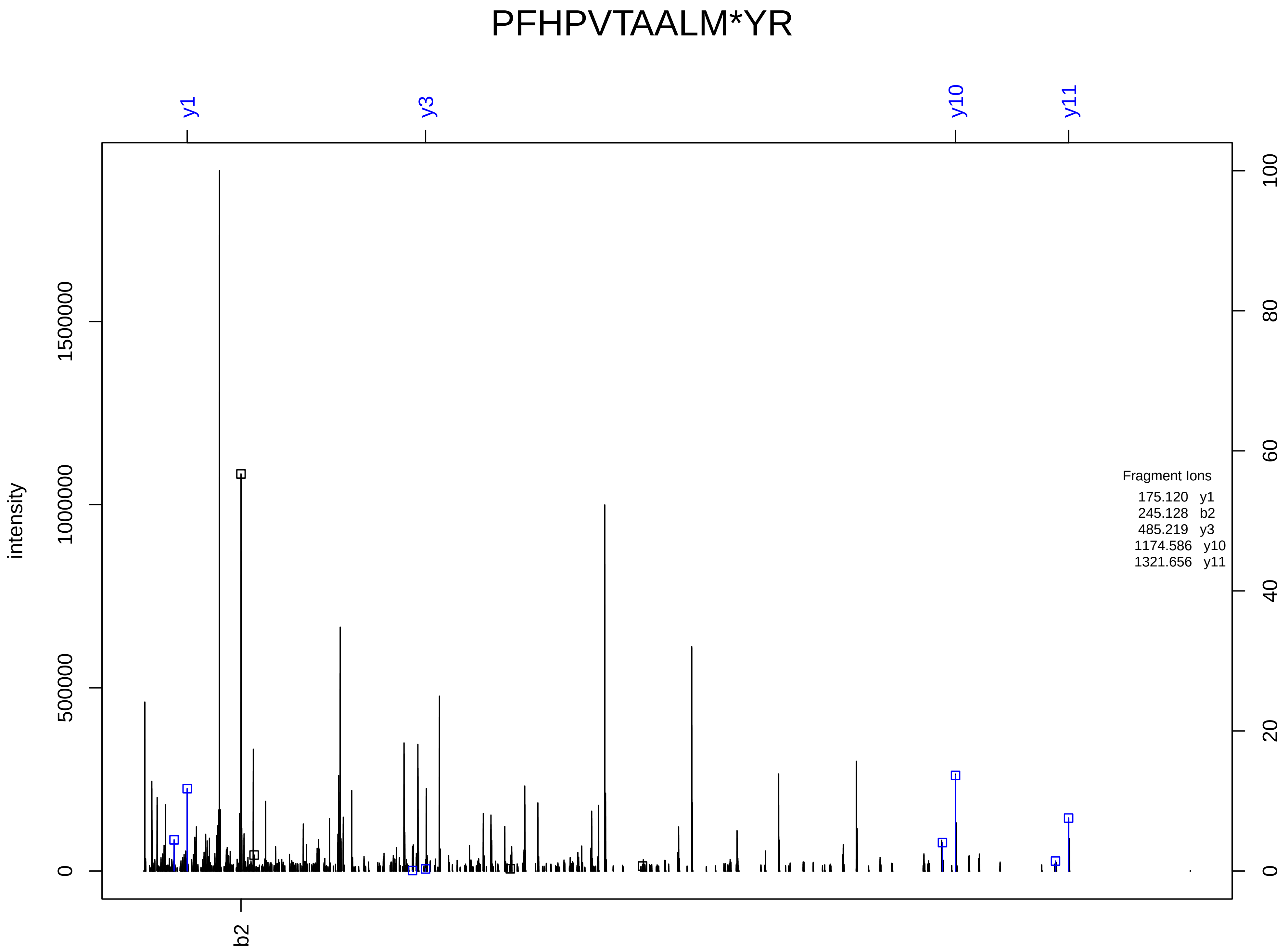

PFHPVTAALMYR / File: christine_pool_04.raw; SpectrumID: ; scans: 9834

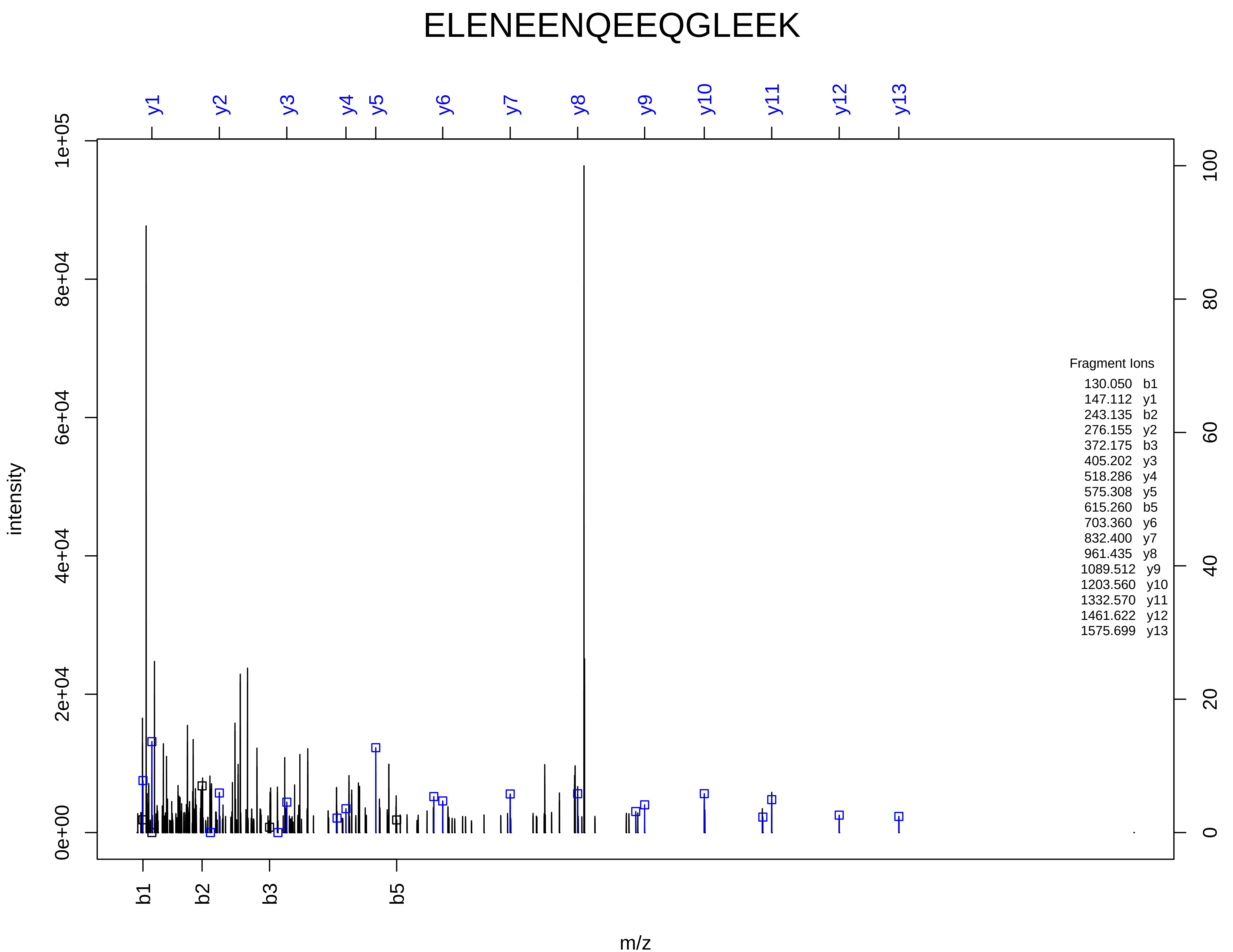
ELENEENQEEQGLEEK
y1 y2 y3 y4 y5 y6 y7 y8 y9 y10 y11 y12 y13
b1 b2 b3 b5
intensity
0e+00 2e+04 4e+04 6e+04 8e+04 1e+05
0 20 40 60 80 100
m/z
Fragment Ions
130.050 b1
147.112 y1
243.135 b2
276.155 y2
372.175 b3
405.202 y3
518.286 y4
575.308 y5
615.260 b5
703.360 y6
832.400 y7
961.435 y8
1089.512 y9
1203.560 y10
1332.570 y11
1461.622 y12
1575.699 y13
ELENEENQEEQGLEEK / File: christine_pool_04.raw; SpectrumID: ; scans: 4677

ITADGAHFELR

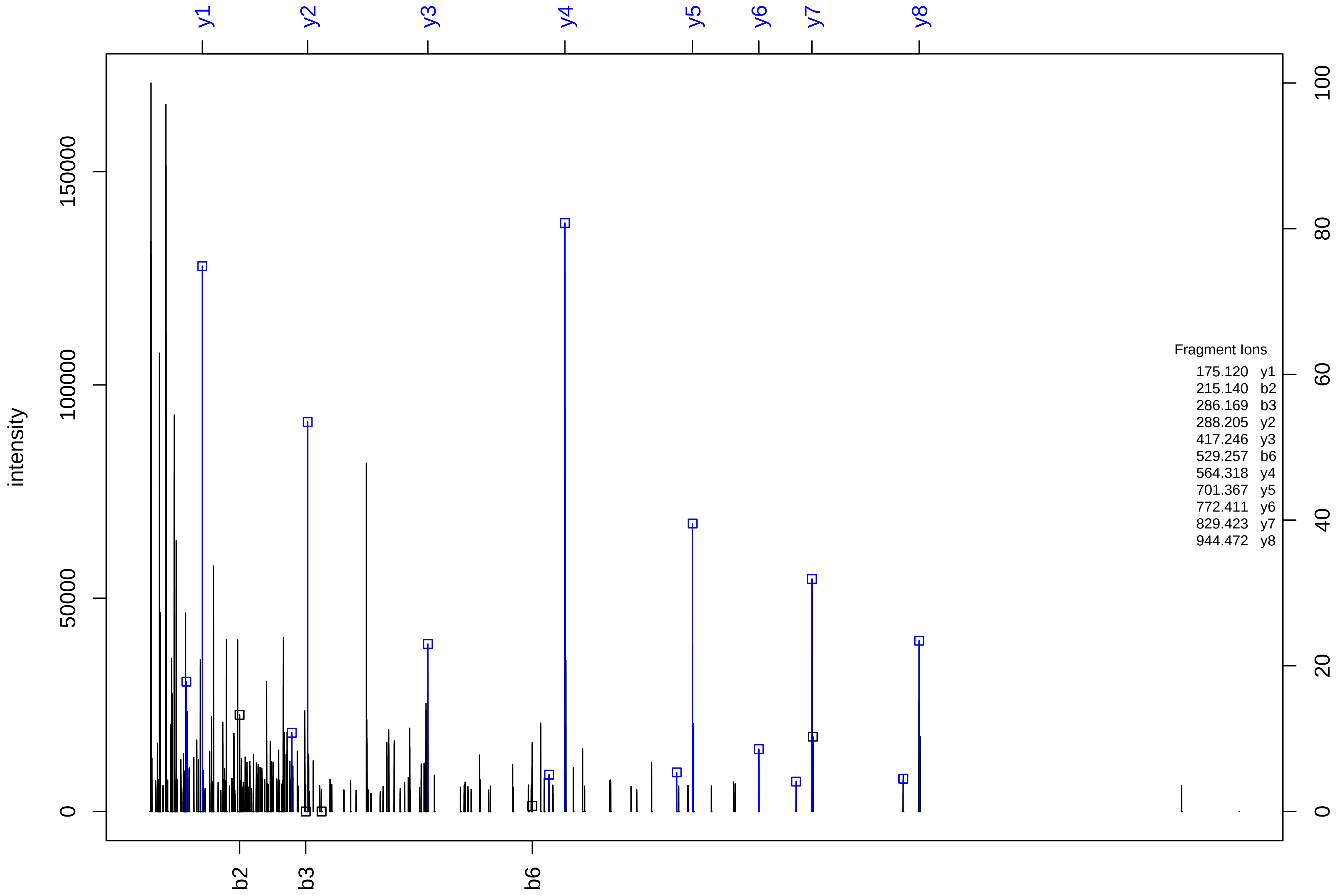

ITADGAHFELR / File: christine_pool_04.raw; SpectrumID: ; scans: 7118

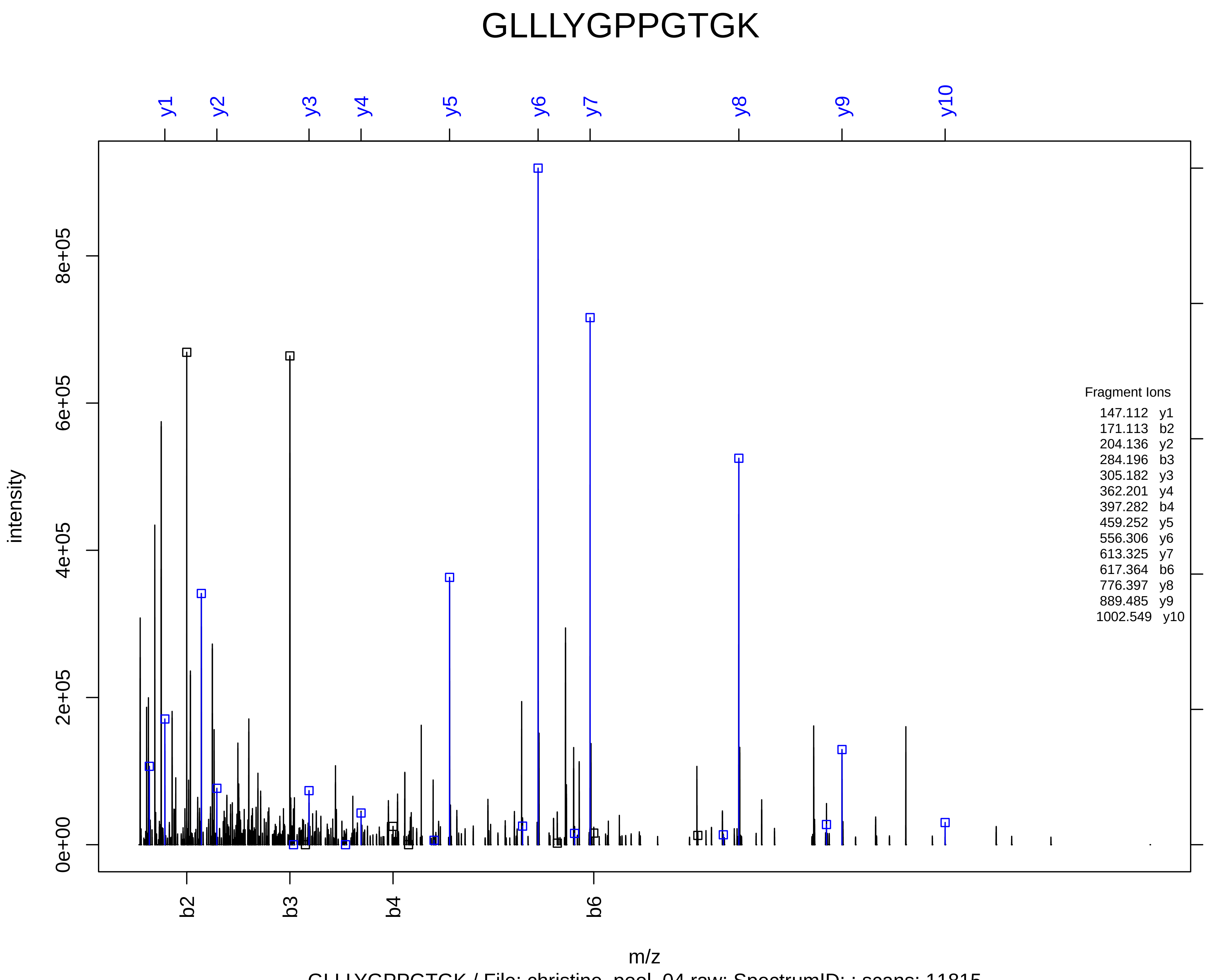
GLLLYGPPGTGK
y1
y2
y3
y4
y5
y6
y7
y8
y9
y10
intensity
0e+00
2e+05
4e+05
6e+05
8e+05
0
20
40
60
80
100
b2
b3
b4
b6
m/z
Fragment Ions
147.112 y1
171.113 b2
204.136 y2
284.196 b3
305.182 y3
362.201 y4
397.282 b4
459.252 y5
556.306 y6
613.325 y7
617.364 b6
776.397 y8
889.485 y9
1002.549 y10
GLLLYGPPGTGK / File: christine_pool_04.raw; SpectrumID: ; scans: 11815

VADYCENNYIQATDK

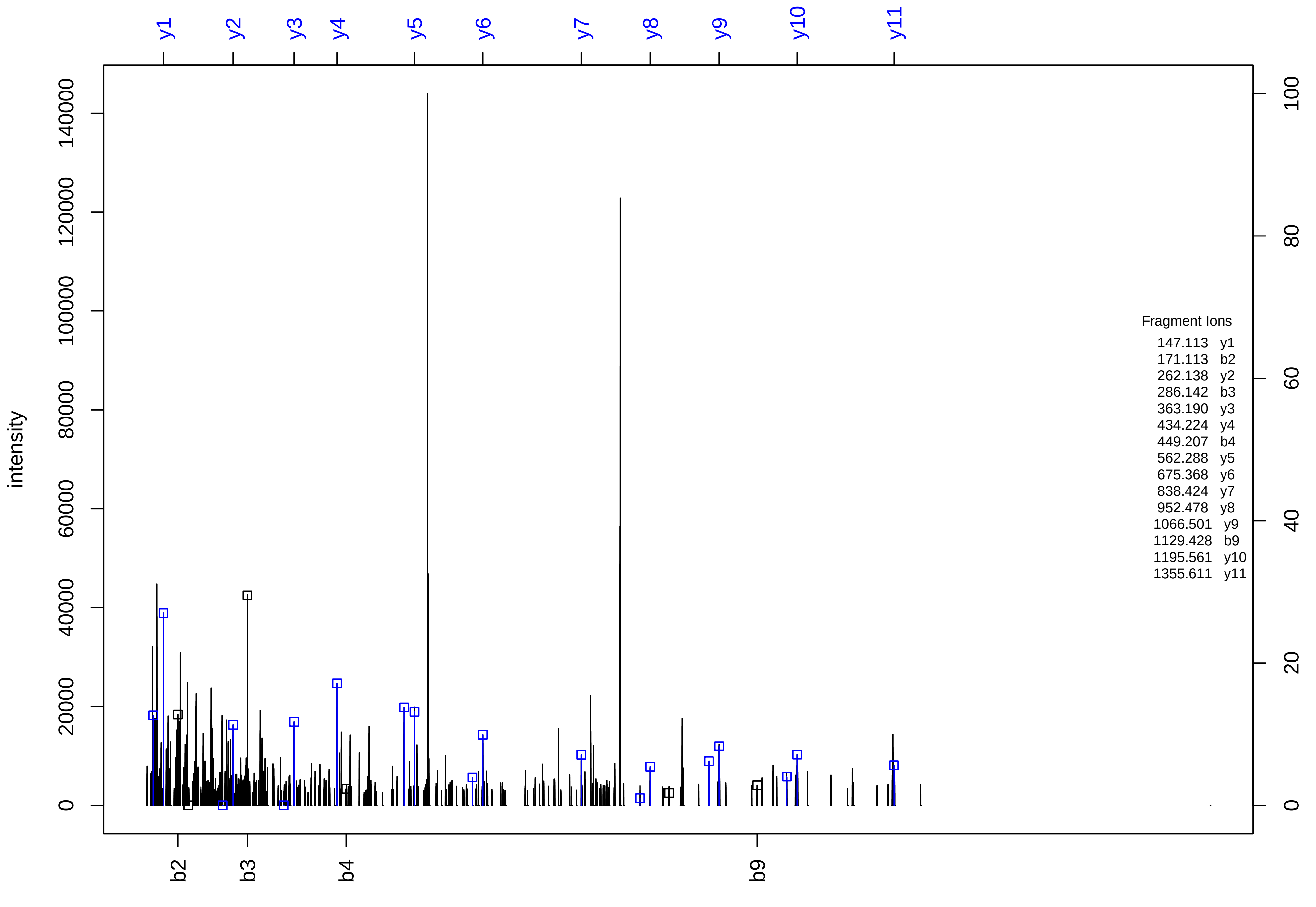

VADYCENNYIQATDK / File: christine_pool_04.raw; SpectrumID: ; scans: 6538

QDNYNEEVADLK

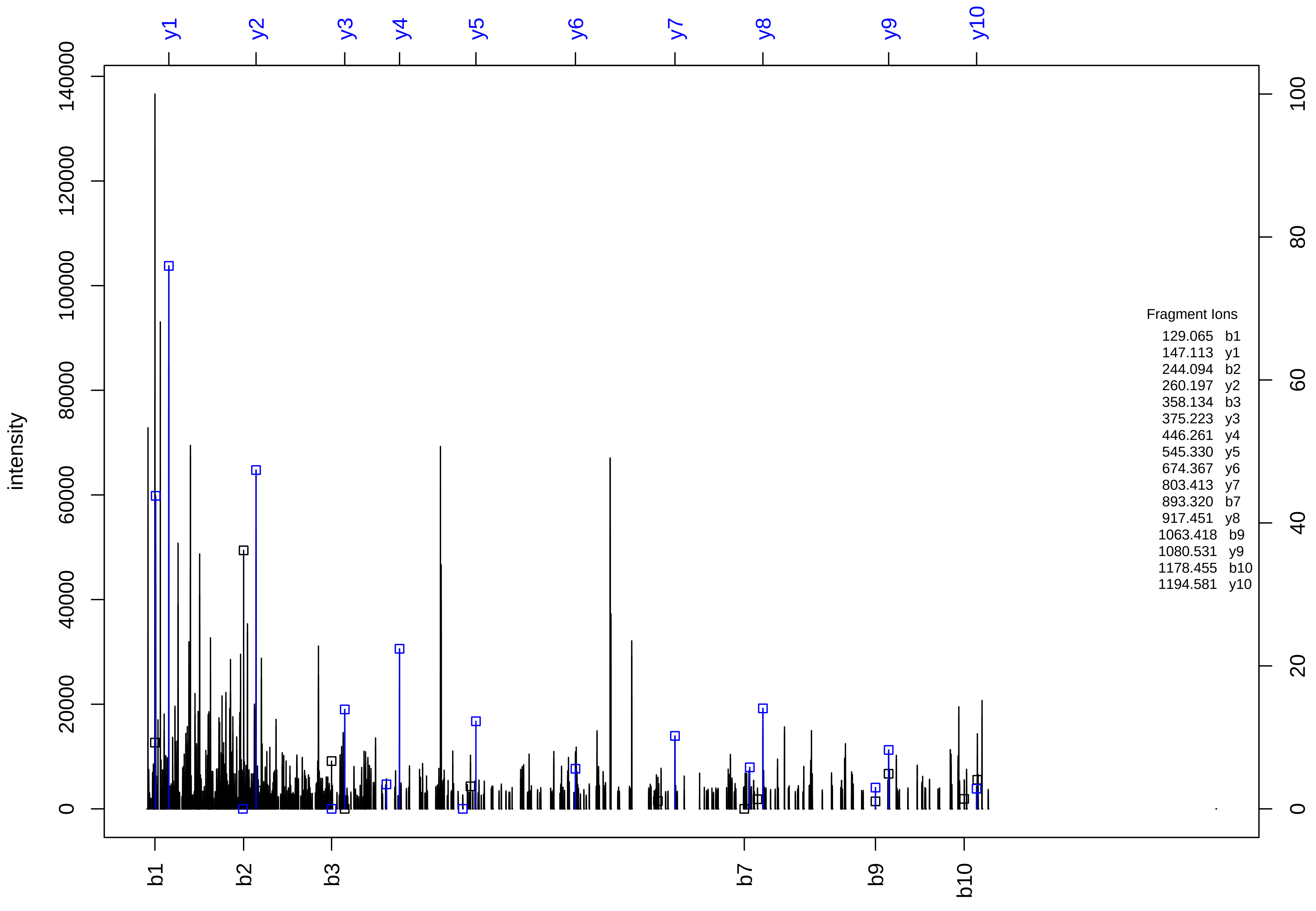

QDNYNEEVADLK / File: christine_pool_04.raw; SpectrumID: ; scans: 6508

DGIEFAFK

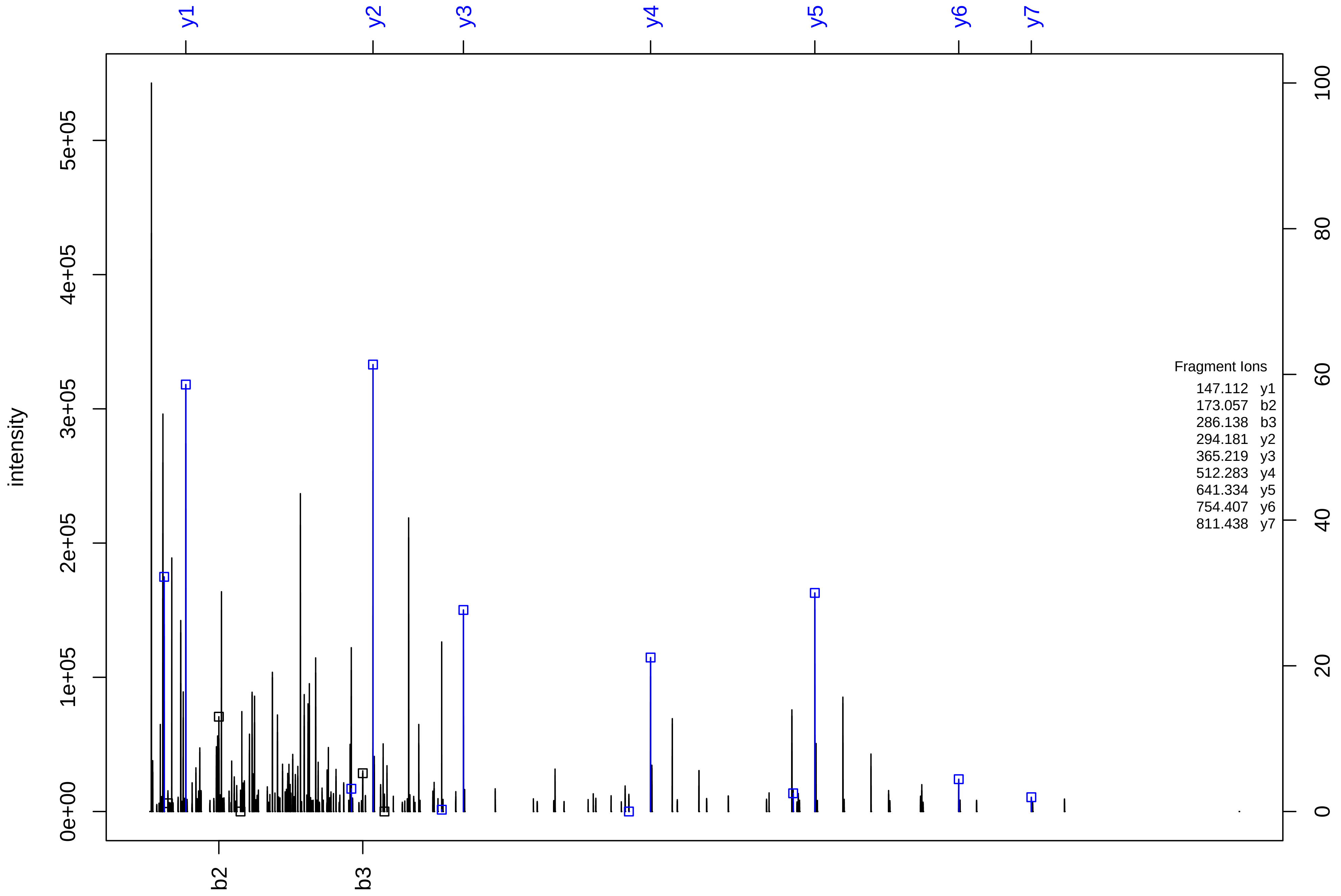

DGIEFAFK / File: christine_pool_04.raw; SpectrumID: ; scans: 14007

NLLGLALGR

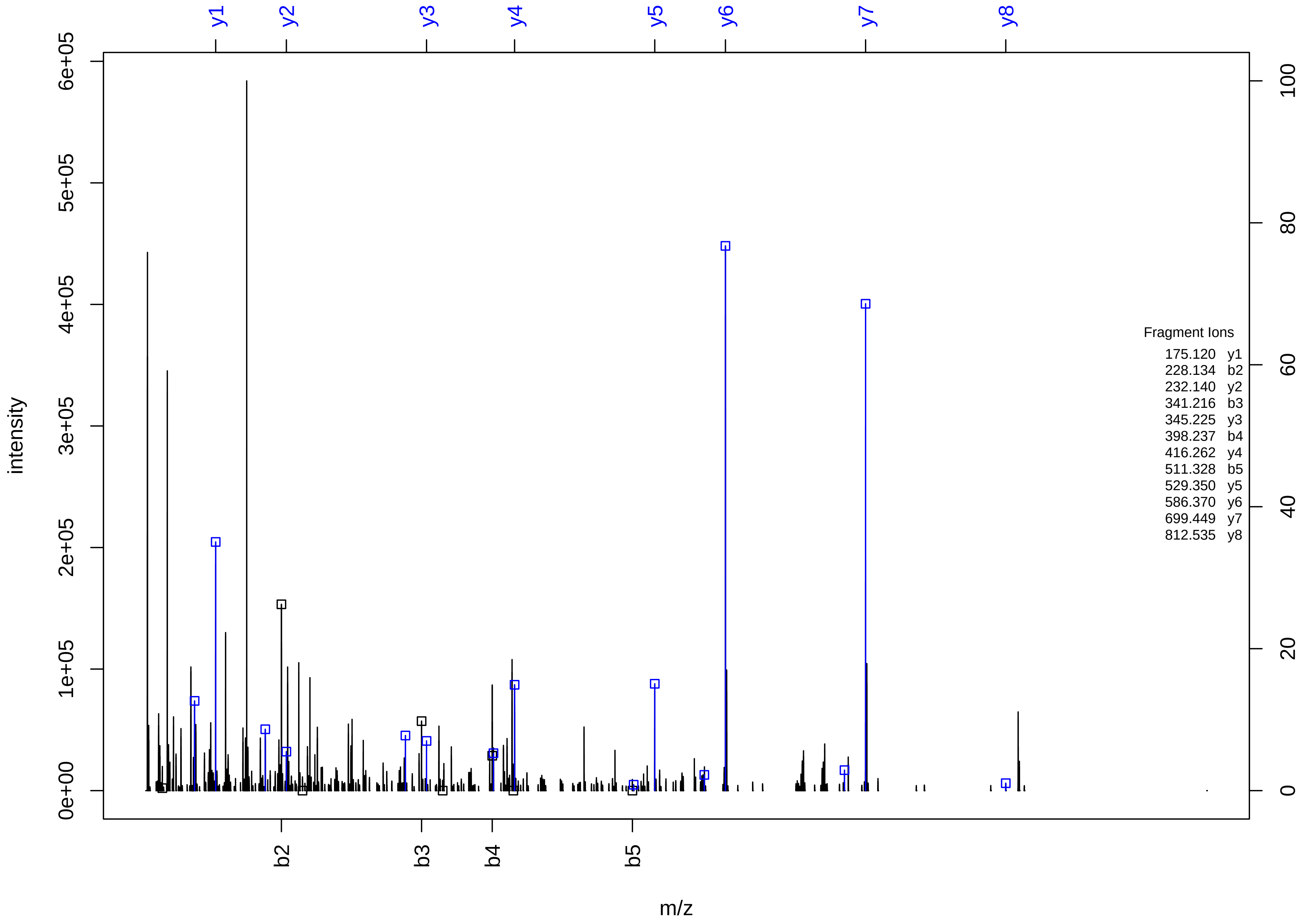

NLLGLALGR / File: christine_pool_04.raw; SpectrumID: ; scans: 16496

EYVNSTSEESHDEDEIRPVQQQDLHR

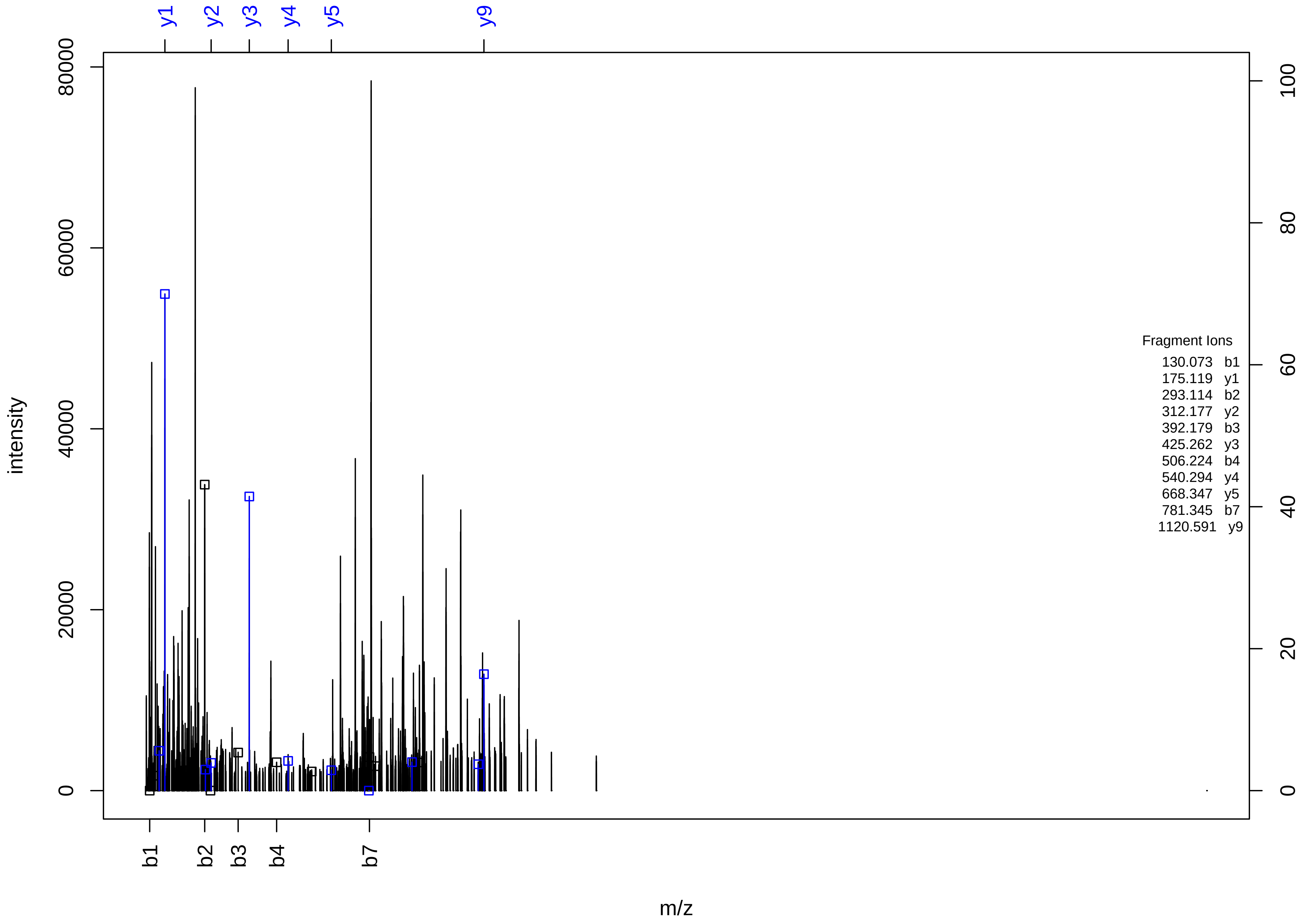

EYVNSTSEESHDEDEIRPVQQQDLHR / File: christine_pool_04.raw; SpectrumID: ; scans: 5389

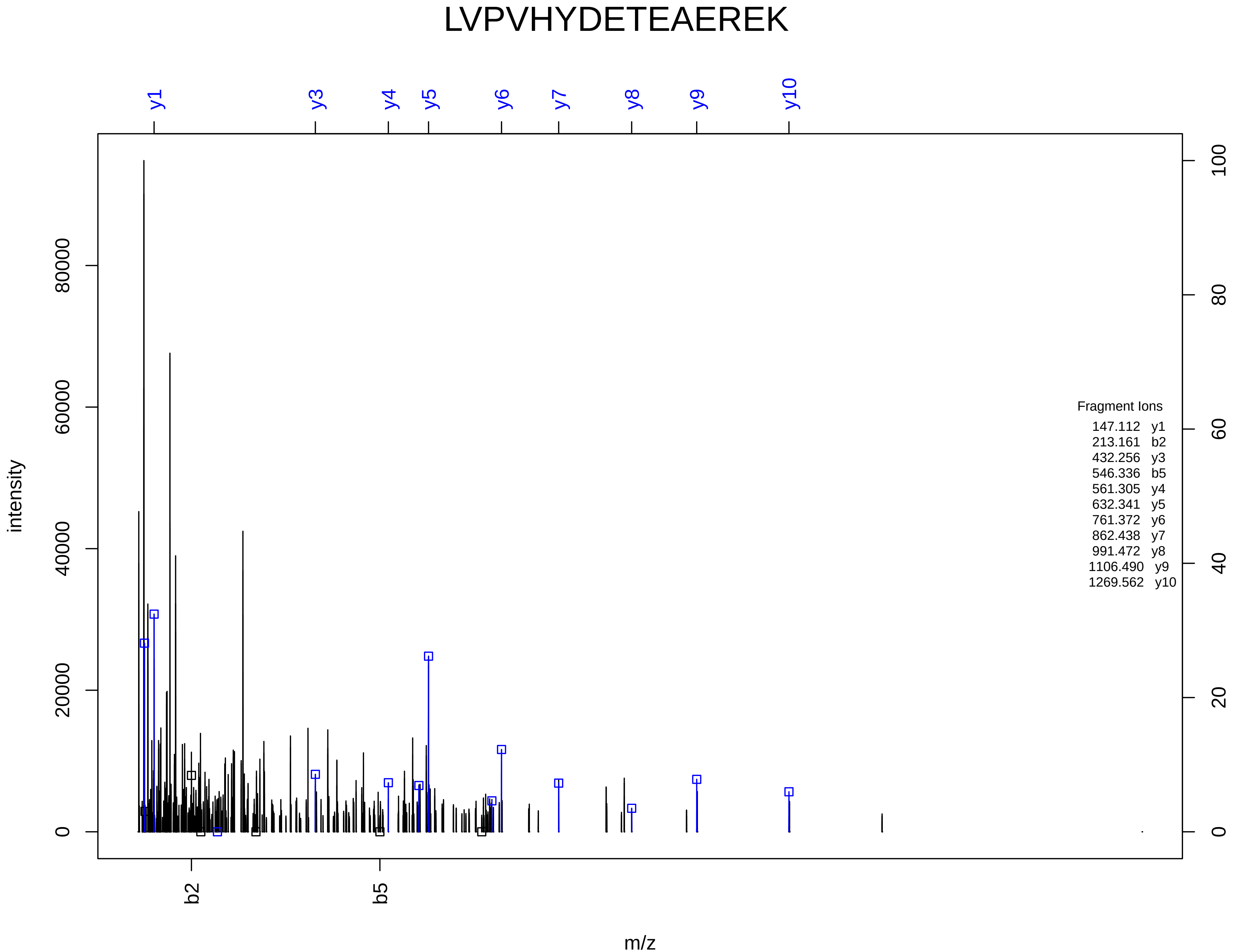

LVPVHYDETEAEREK / File: christine_pool_04.raw; SpectrumID: ; scans: 4461

LPADTCLLEFAR

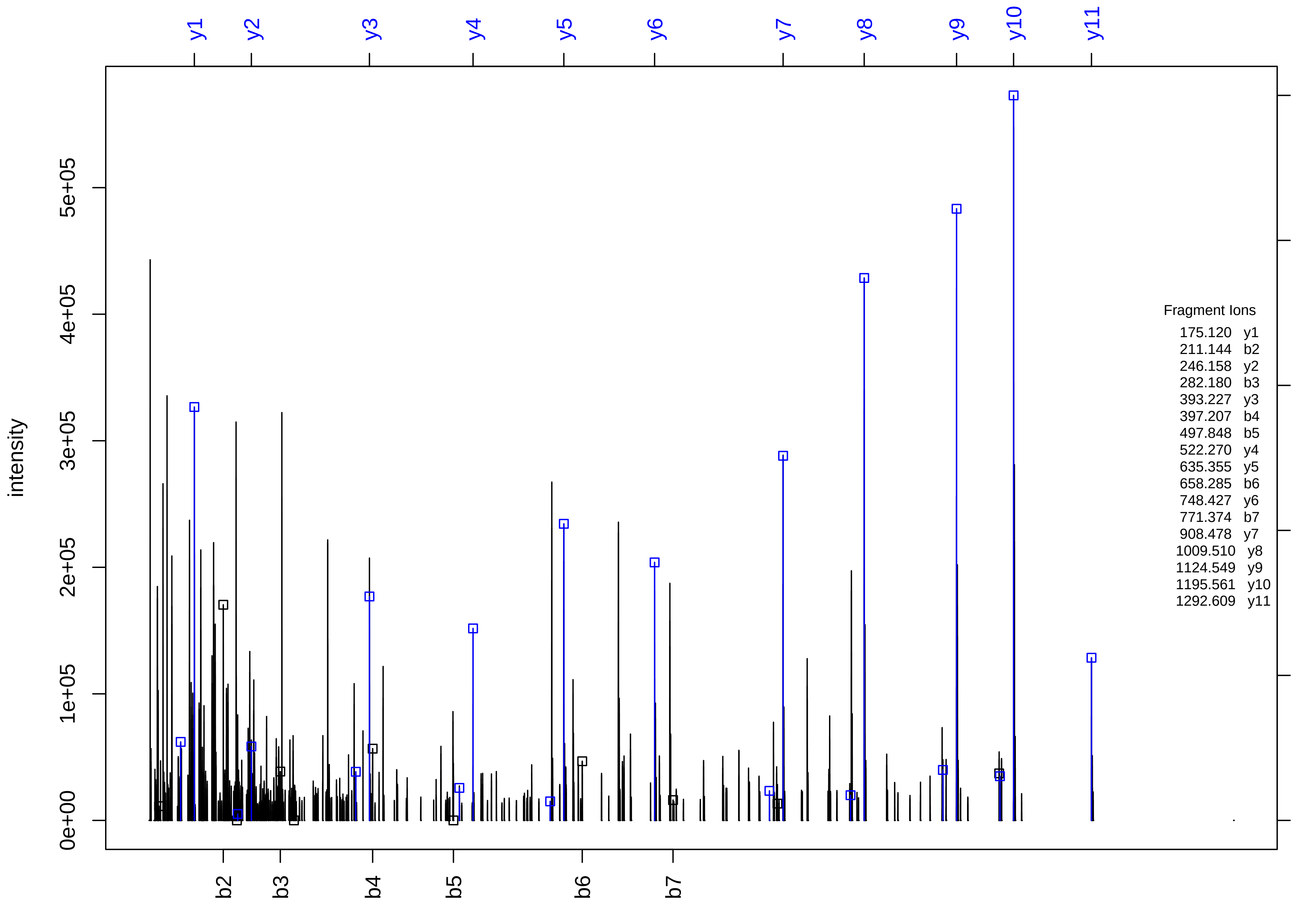

LPADTCLLEFAR / File: christine_pool_04.raw; SpectrumID: ; scans: 16762

MLM*TLVSPSGR

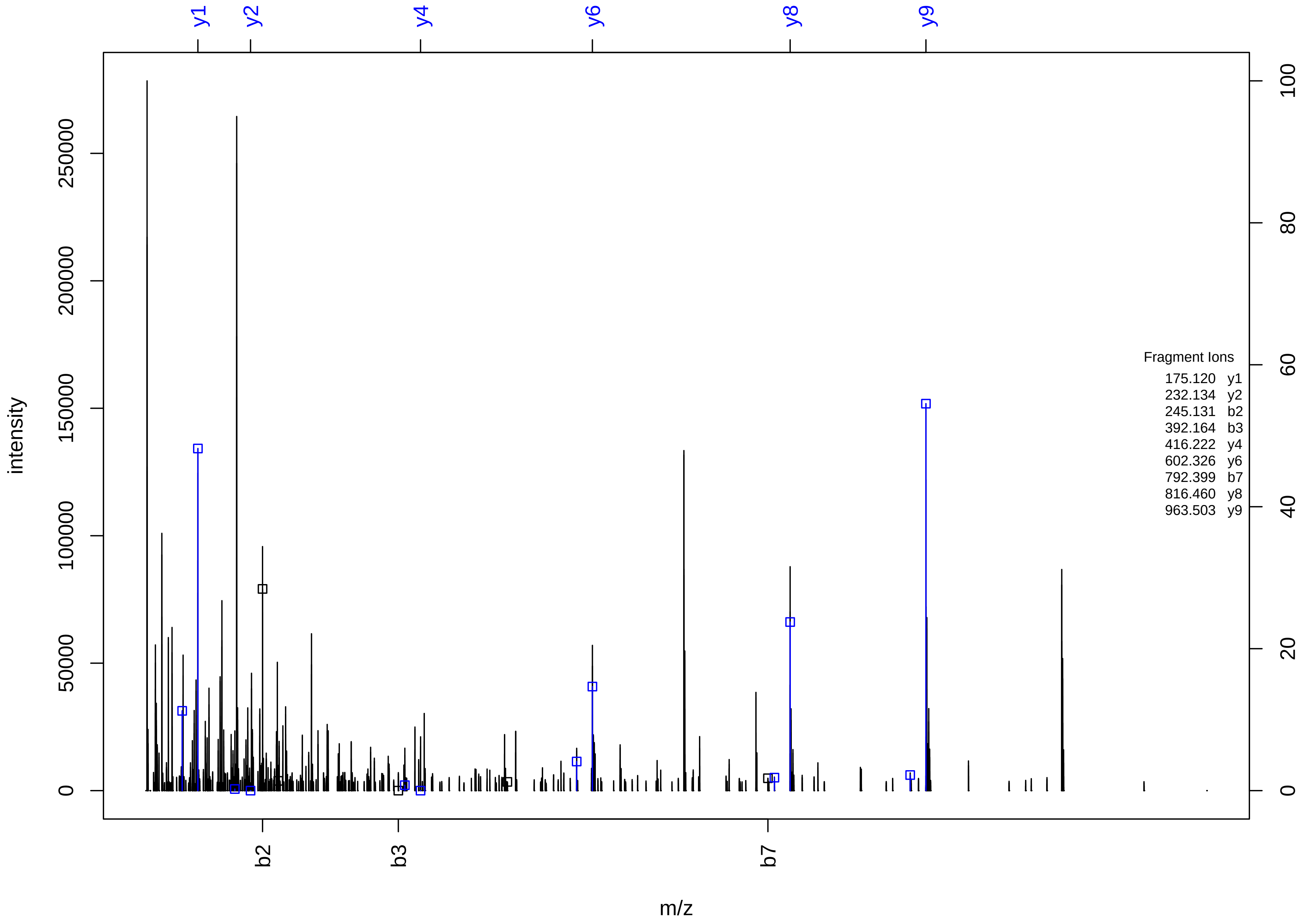

MLMTLVSPSGR / File: christine_pool_04.raw; SpectrumID: ; scans: 20687

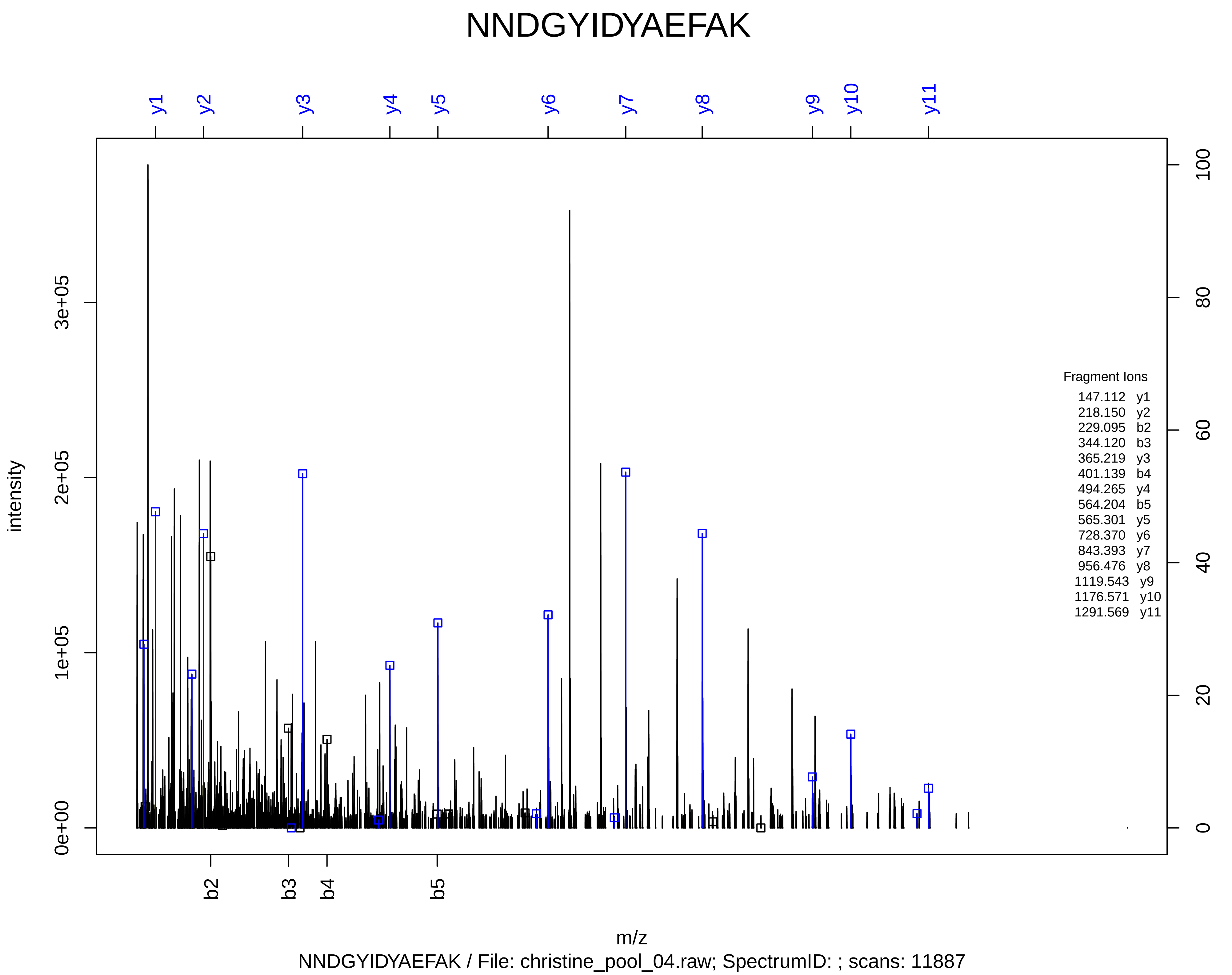
NNDGYIDYAEFAK
y1
y2
y3
y4
y5
y6
y7
y8
y9
y10
y11
b2
b3
b4
b5
intensity
0e+00
1e+05
2e+05
3e+05
0
20
40
60
80
100
m/z
Fragment Ions
147.112 y1
218.150 y2
229.095 b2
344.120 b3
365.219 y3
401.139 b4
494.265 y4
564.204 b5
565.301 y5
728.370 y6
843.393 y7
956.476 y8
1119.543 y9
1176.571 y10
1291.569 y11
NNDGYIDYAEFAK / File: christine_pool_04.raw; SpectrumID: ; scans: 11887

HGAGAEISTVHPEQYAK

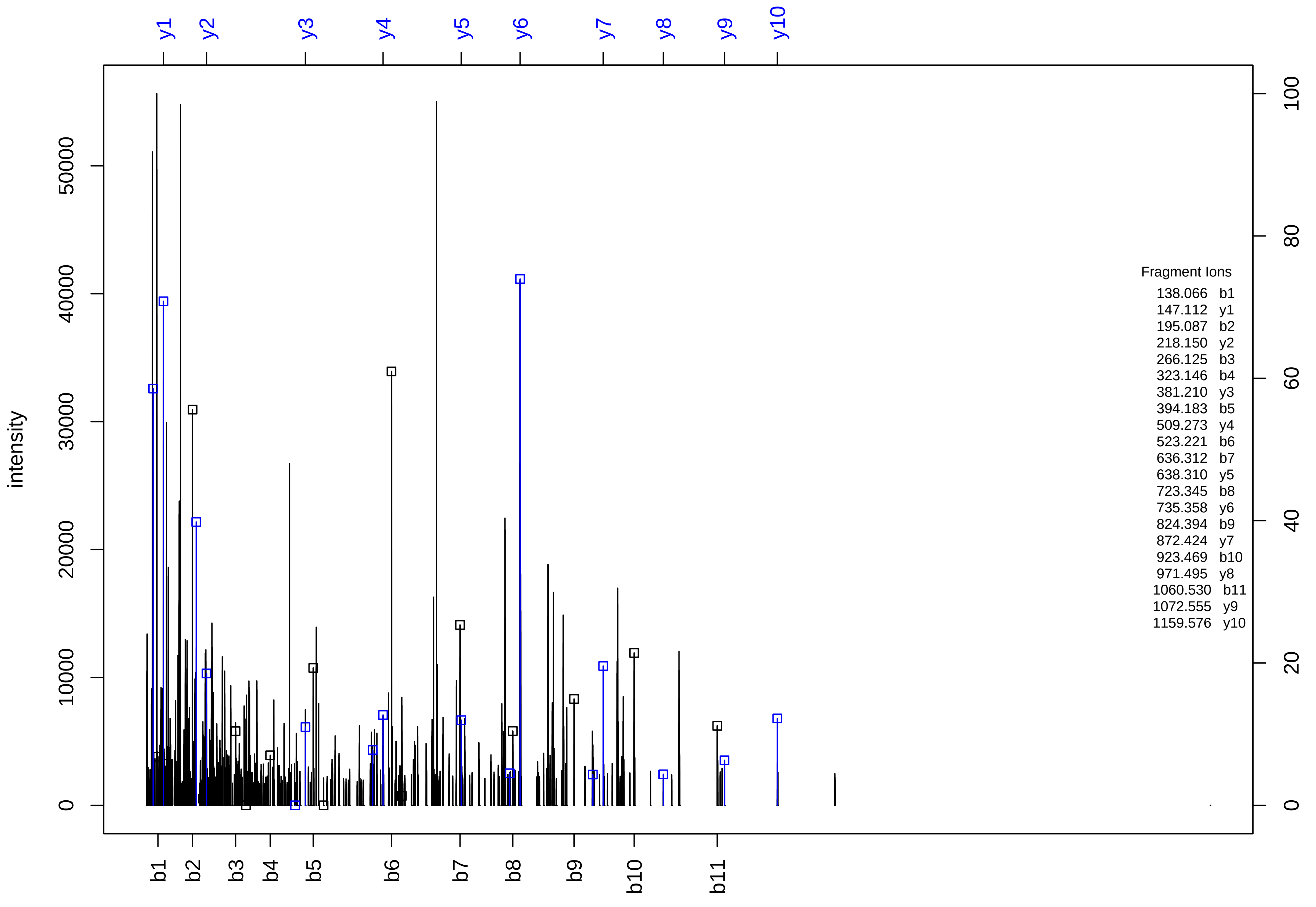

HGAGAEISTVHPEQYAK / File: christine_pool_04.raw; SpectrumID: ; scans: 4406

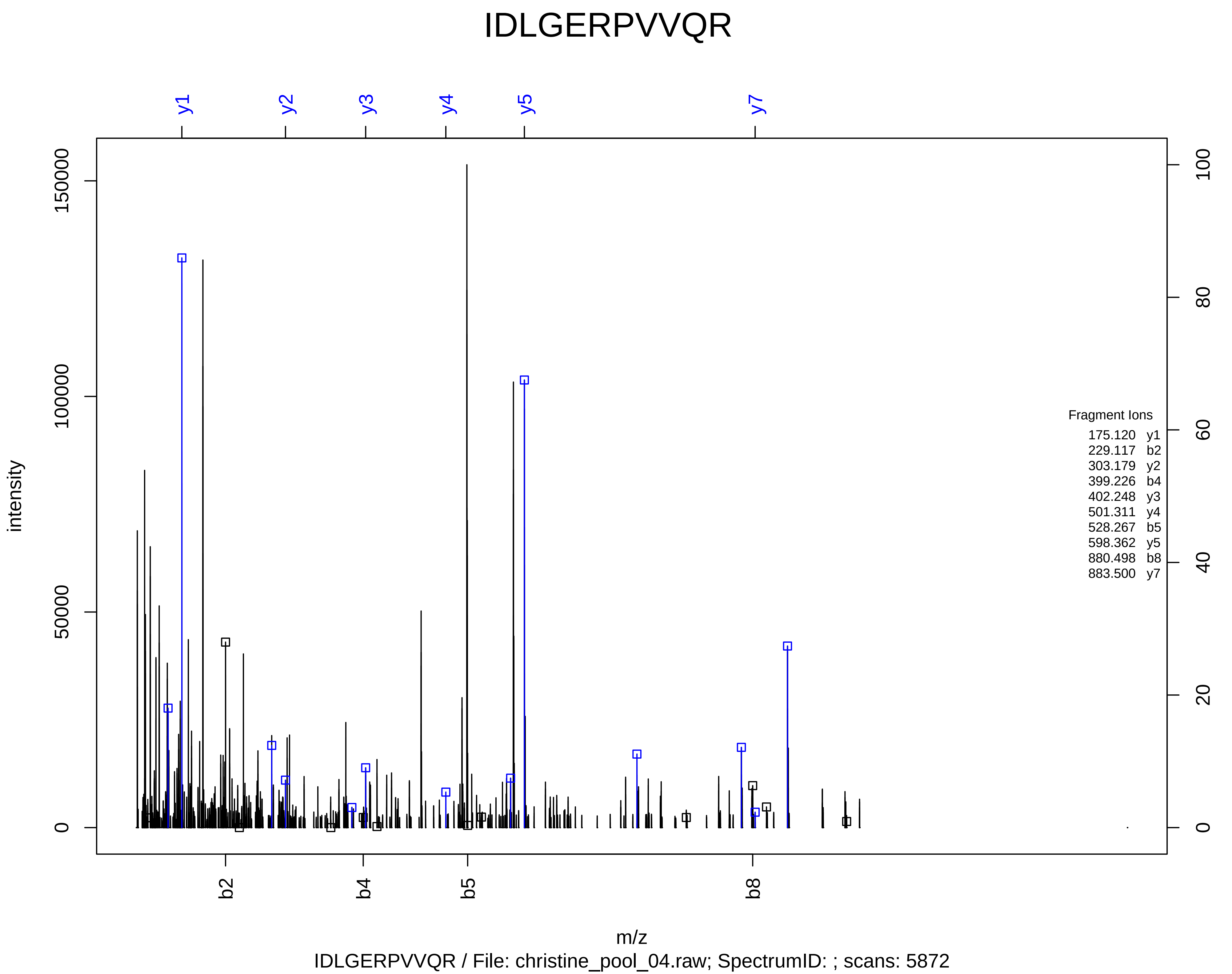
IDLGERPVVQR
y1
y2
y3
y4
y5
y7
intensity
150000
100000
50000
0
100
80
60
40
20
0
Fragment Ions
175.120 y1
229.117 b2
303.179 y2
399.226 b4
402.248 y3
501.311 y4
528.267 b5
598.362 y5
880.498 b8
883.500 y7
b2
b4
b5
b8
m/z
IDLGERPVVQR / File: christine_pool_04.raw; SpectrumID: ; scans: 5872

DGVPGLNIEELIEK

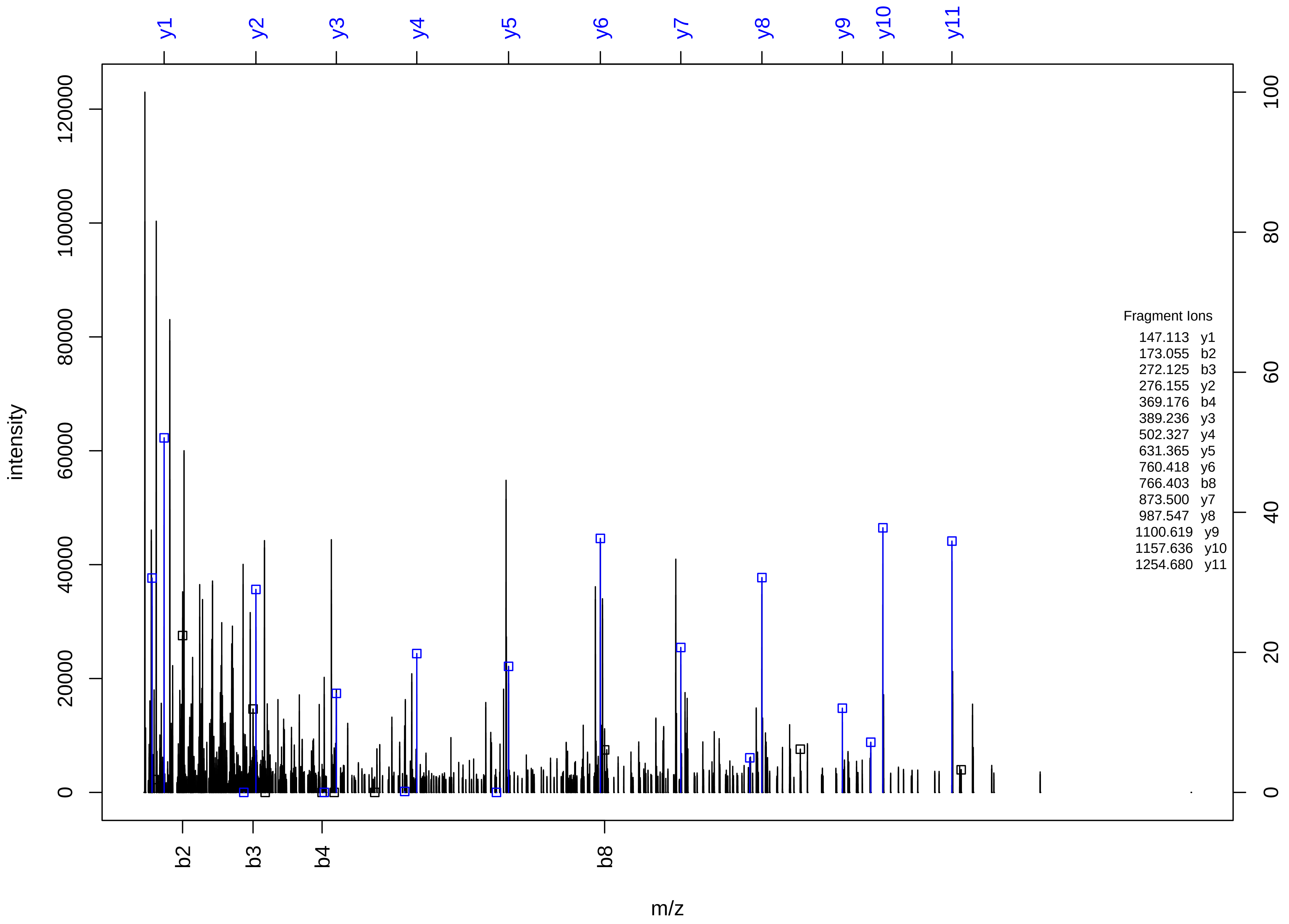

DGVPGLNIEELIEK / File: christine_pool_04.raw; SpectrumID: ; scans: 21777

VLFPLLTK

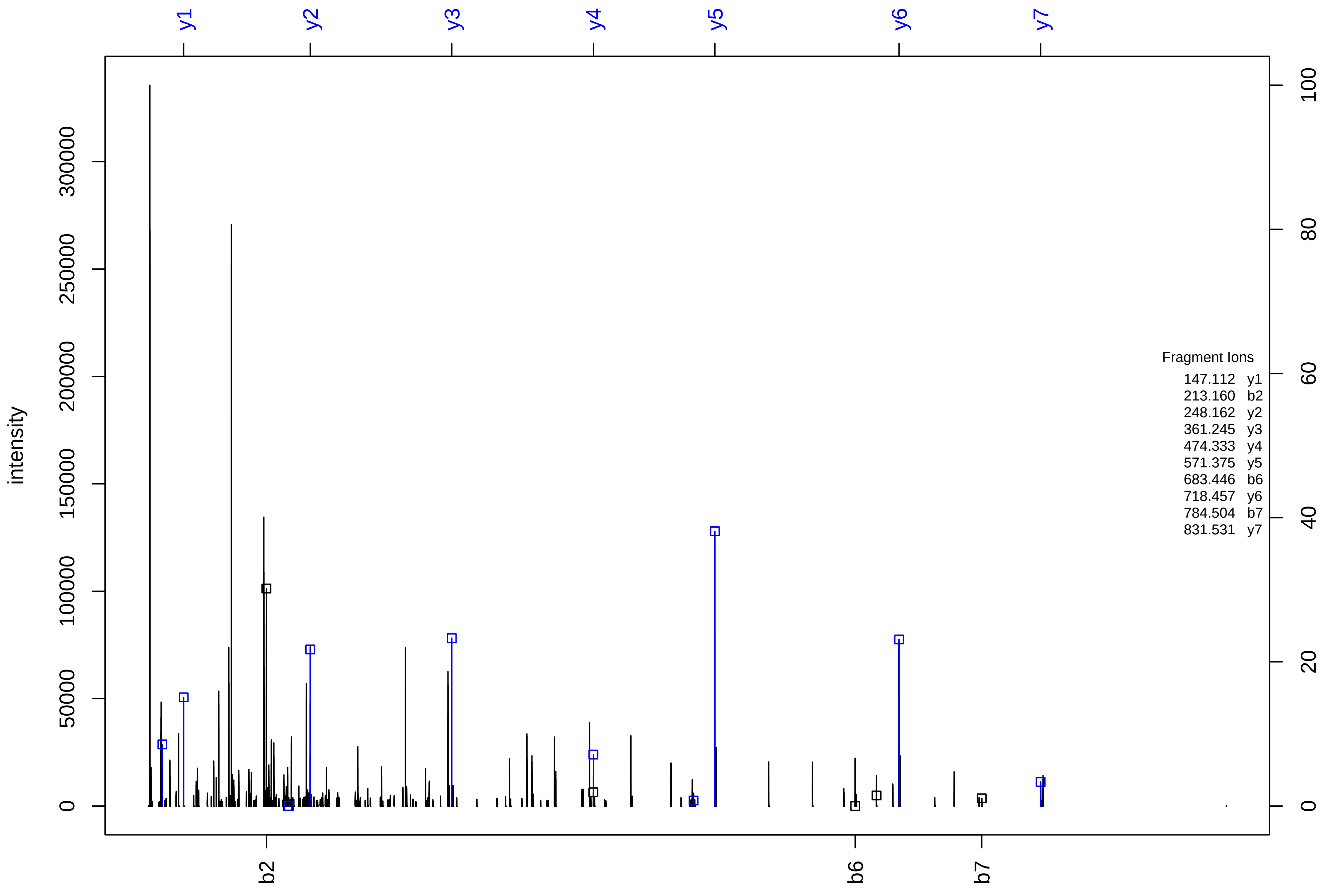

VLFPLLTK / File: christine_pool_04.raw; SpectrumID: ; scans: 18504

ELFSFFR

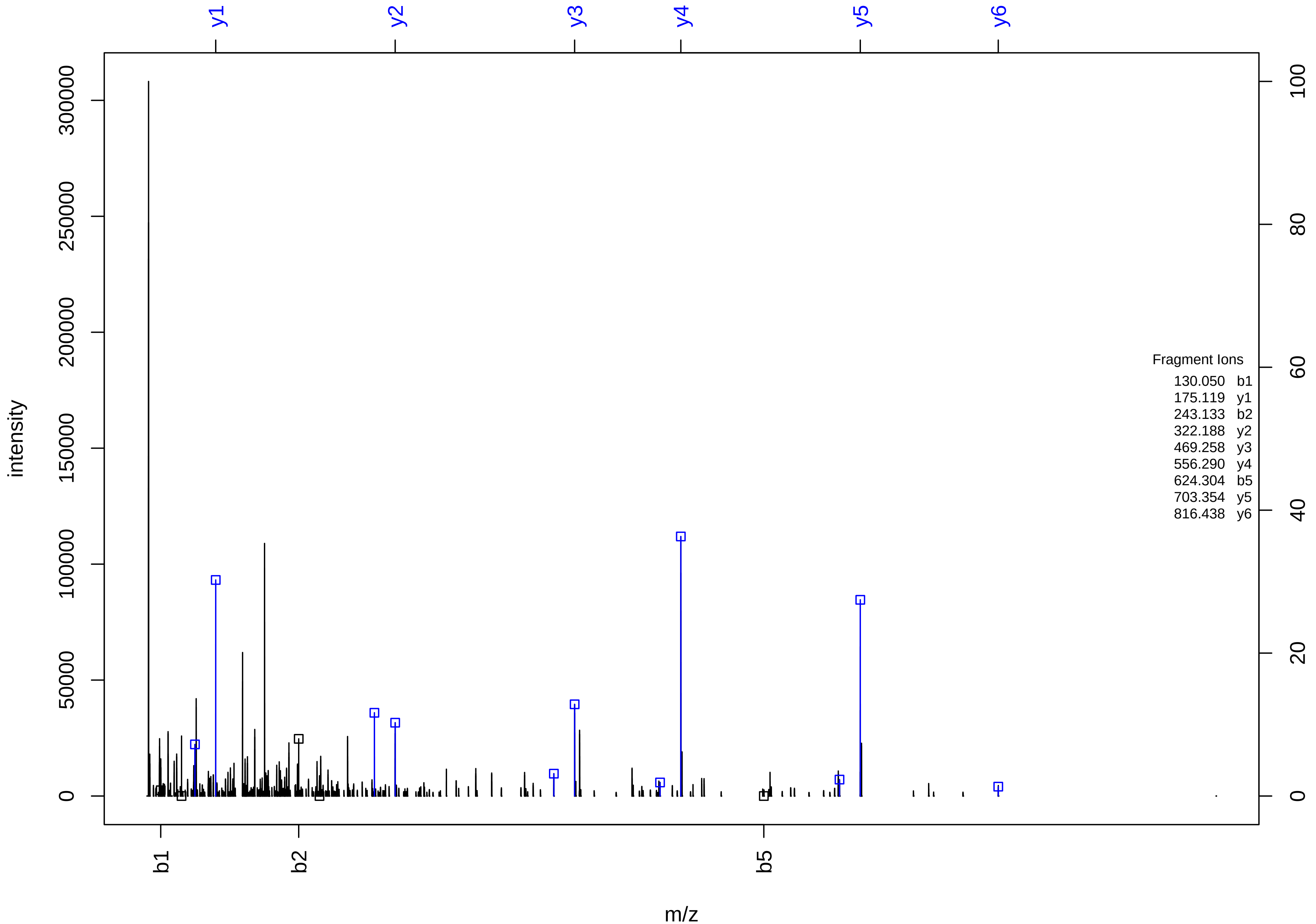

ELFSFFR / File: christine_pool_04.raw; SpectrumID: ; scans: 18756

EAPETDTSPSLWDVEFAK

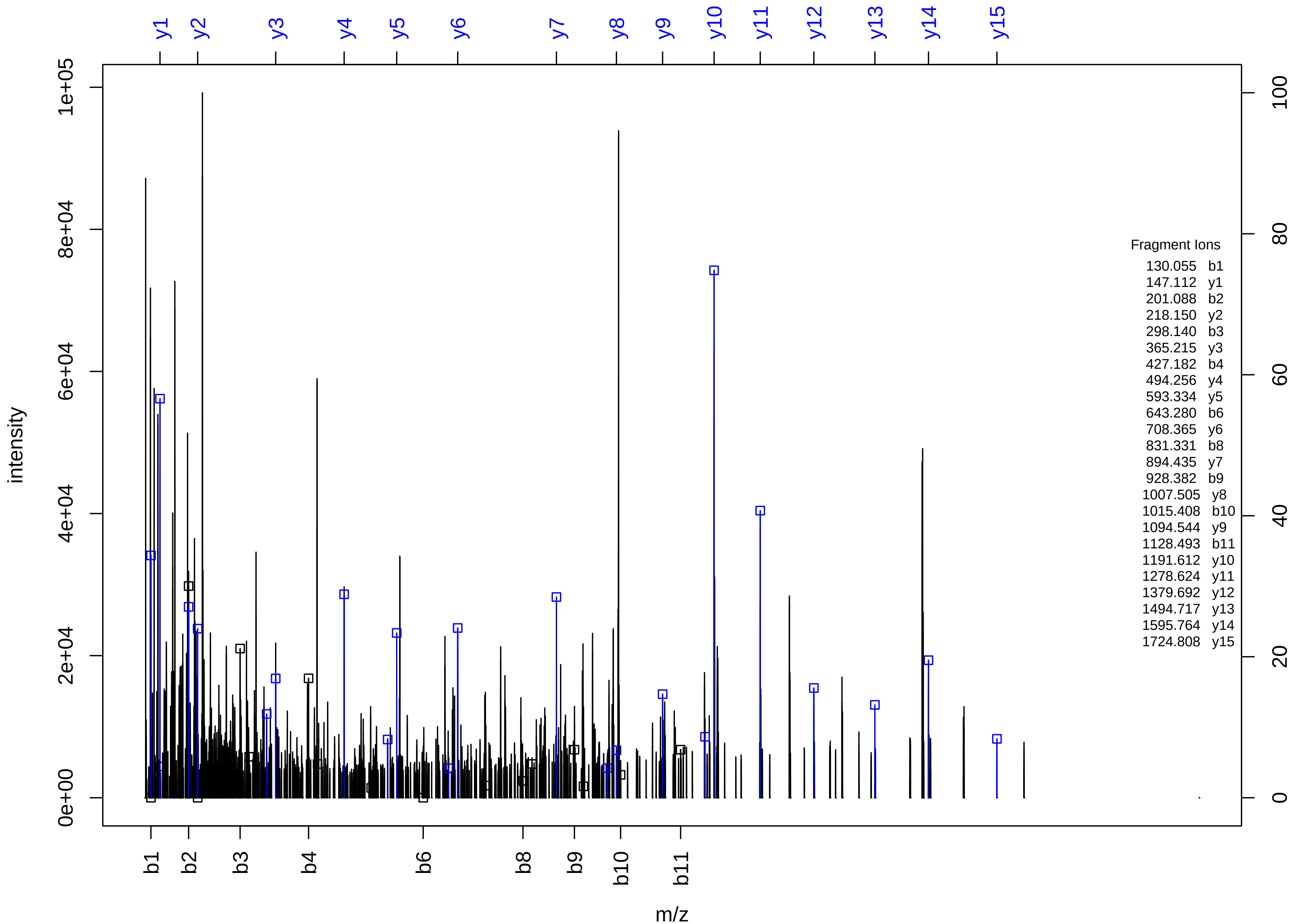

EAPETDTSPSLWDVEFAK / File: christine_pool_04.raw; SpectrumID: ; scans: 17127

(Ac)AAVVENVVK

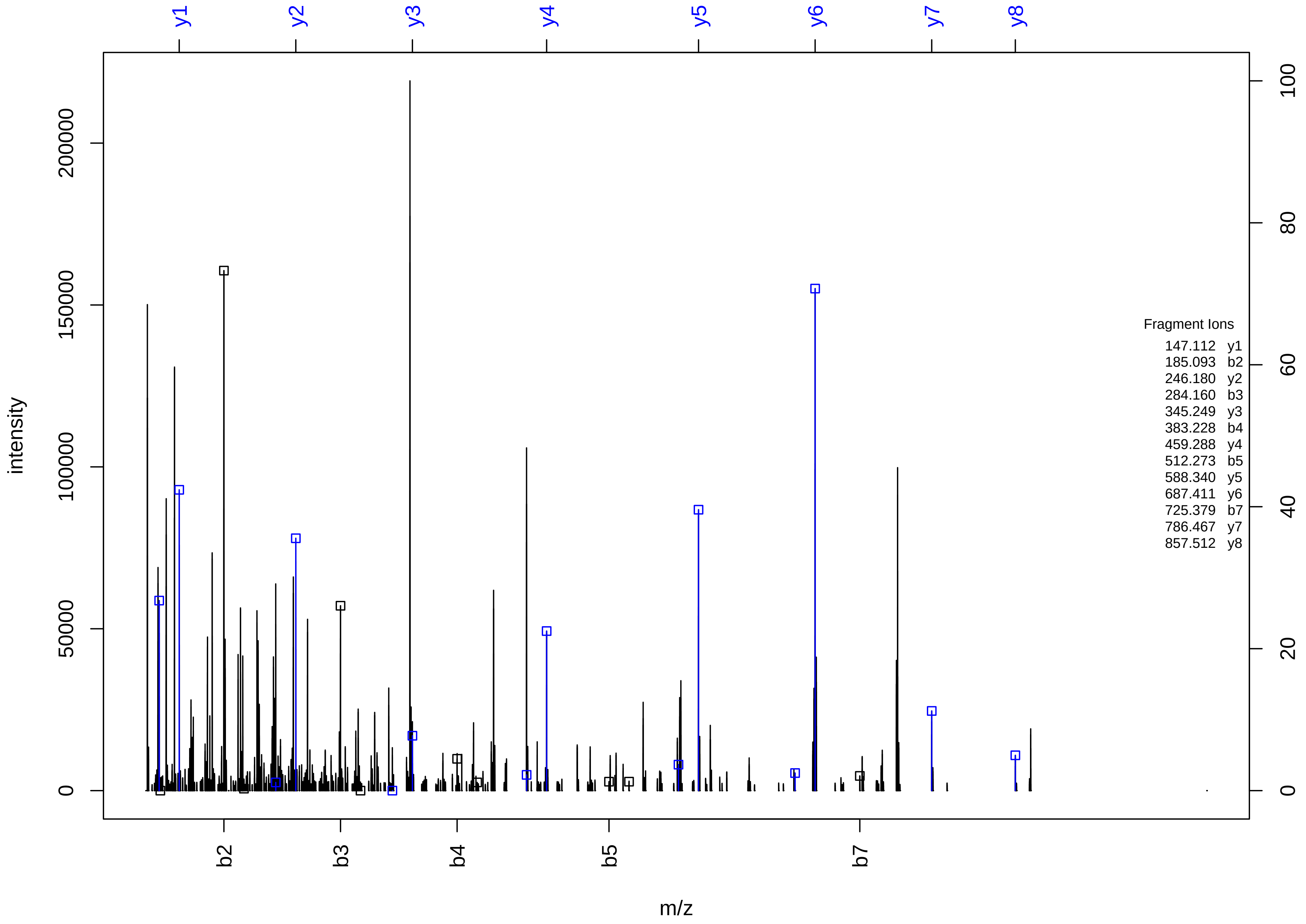

AAVVENVVK / File: christine_pool_04.raw; SpectrumID: ; scans: 15986

SINGILFPGGSVDLR

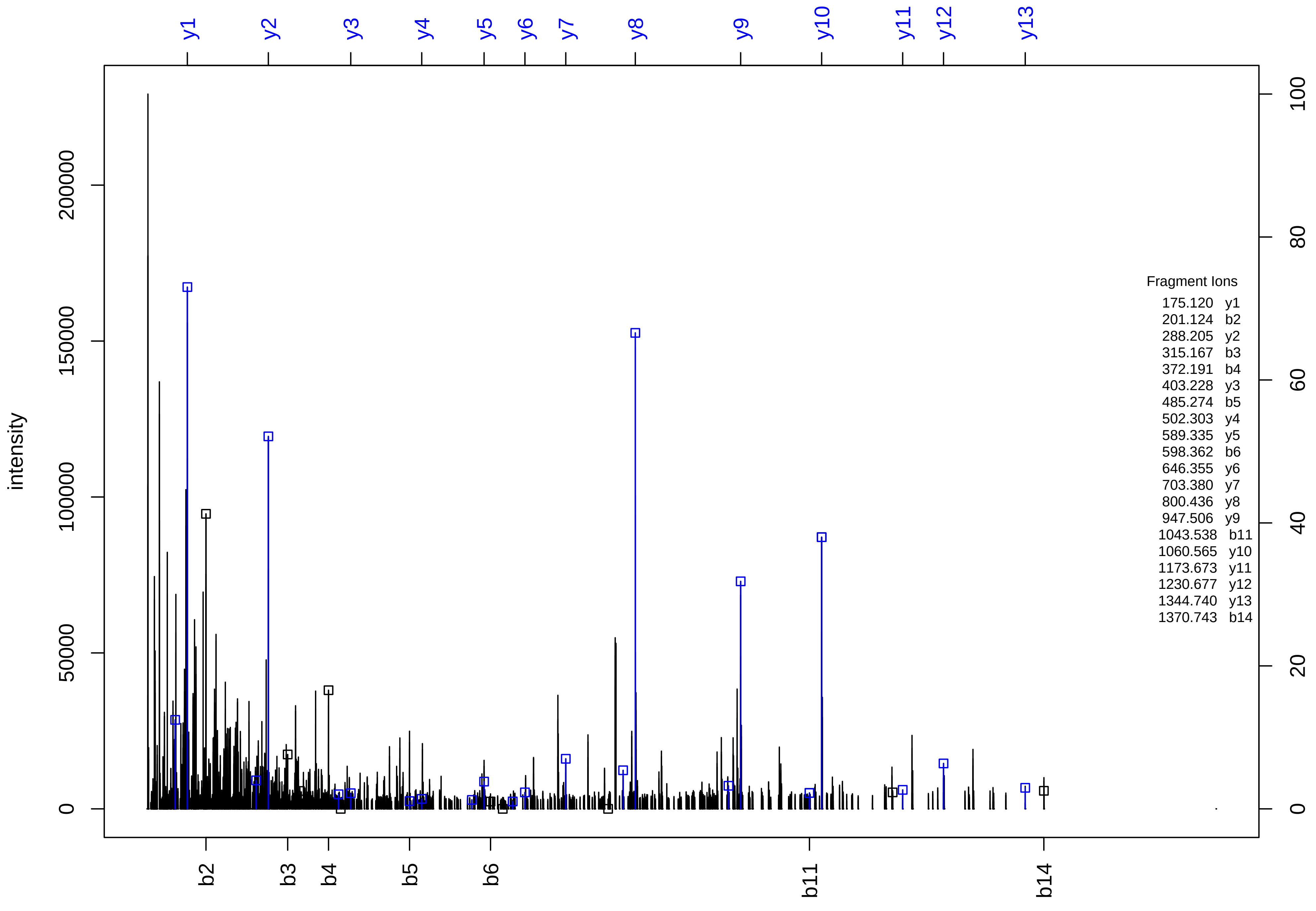

SINGILFPGGSVDLR / File: christine_pool_04.raw; SpectrumID: ; scans: 18589

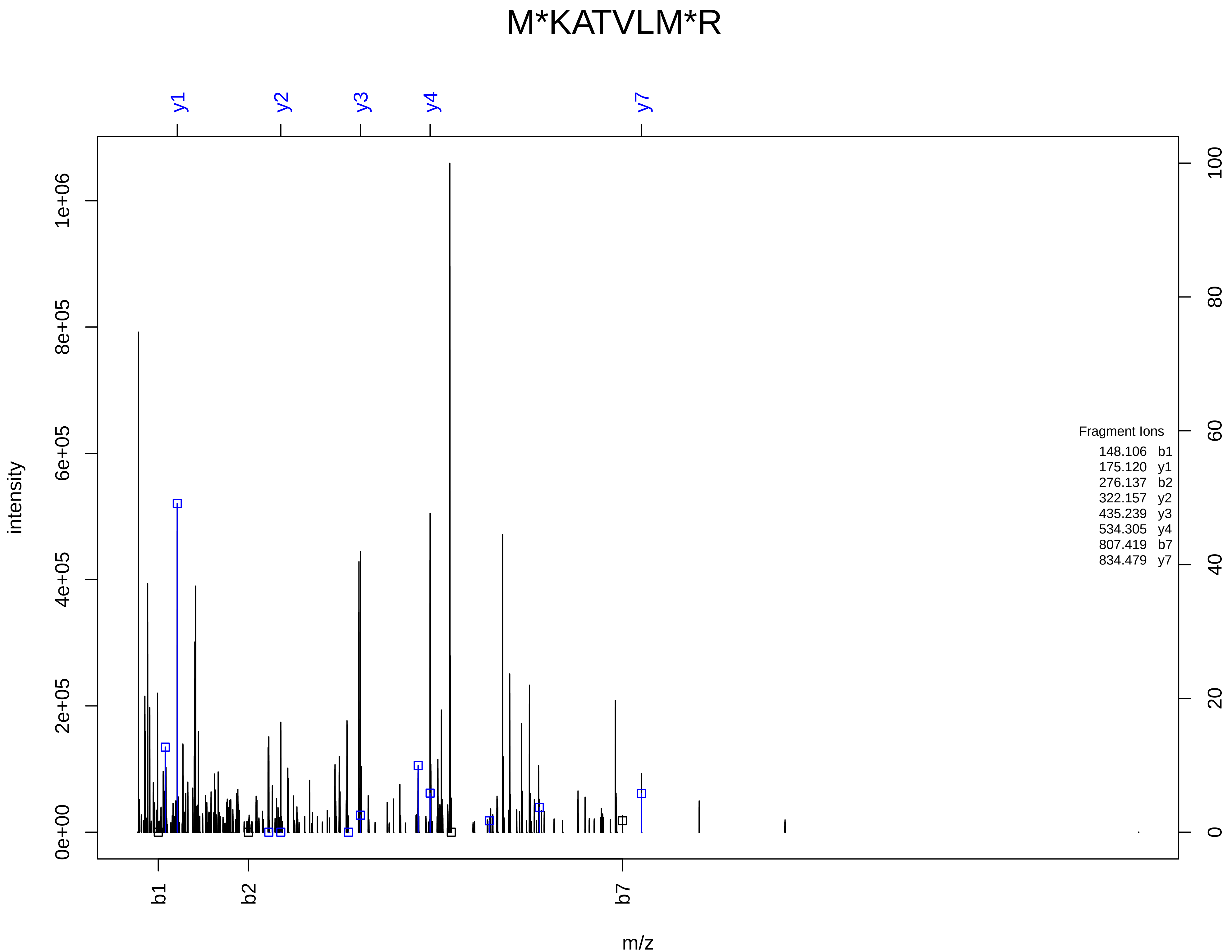

MKATVLMR / File: christine_pool_04.raw; SpectrumID: ; scans: 12269

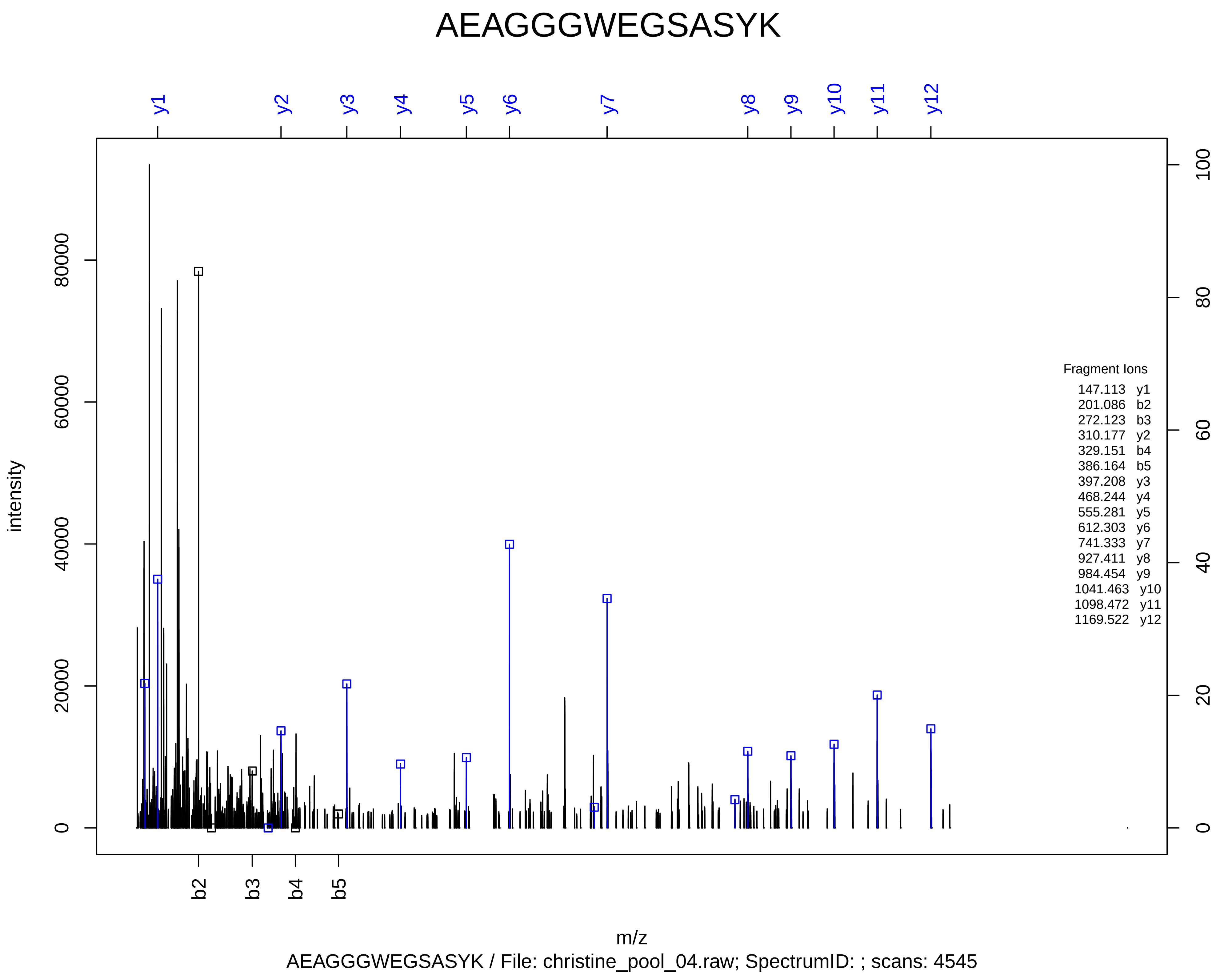
AEAGGGWEGSASYK
y1
y2
y3
y4
y5
y6
y7
y8
y9
y10
y11
y12
b2
b3
b4
b5
intensity
m/z
Fragment Ions
147.113 y1
201.086 b2
272.123 b3
310.177 y2
329.151 b4
386.164 b5
397.208 y3
468.244 y4
555.281 y5
612.303 y6
741.333 y7
927.411 y8
984.454 y9
1041.463 y10
1098.472 y11
1169.522 y12
AEAGGGWEGSASYK / File: christine_pool_04.raw; SpectrumID: ; scans: 4545

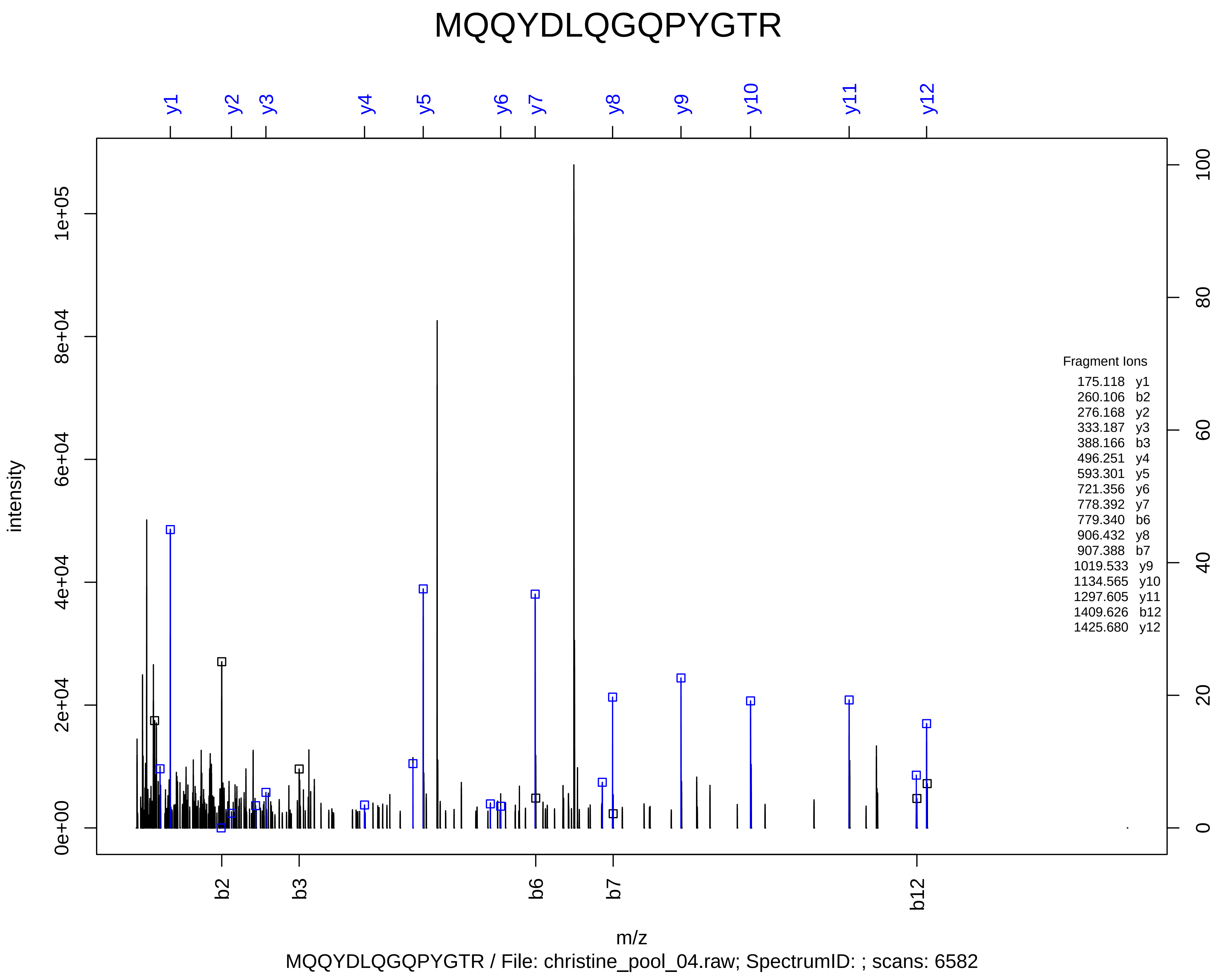

MQQYDLQGQPYGTR
y1 y2 y3 y4 y5 y6 y7 y8 y9 y10 y11 y12
b2 b3 b6 b7 b12
intensity
0e+00
2e+04
4e+04
6e+04
8e+04
1e+05
0
20
40
60
80
100
m/z
Fragment Ions
175.118 y1
260.106 b2
276.168 y2
333.187 y3
388.166 b3
496.251 y4
593.301 y5
721.356 y6
778.392 y7
779.340 b6
906.432 y8
907.388 b7
1019.533 y9
1134.565 y10
1297.605 y11
1409.626 b12
1425.680 y12
MQQYDLQGQPYGTR / File: christine_pool_04.raw; SpectrumID: ; scans: 6582

ELQQELQEYEVVTESEK

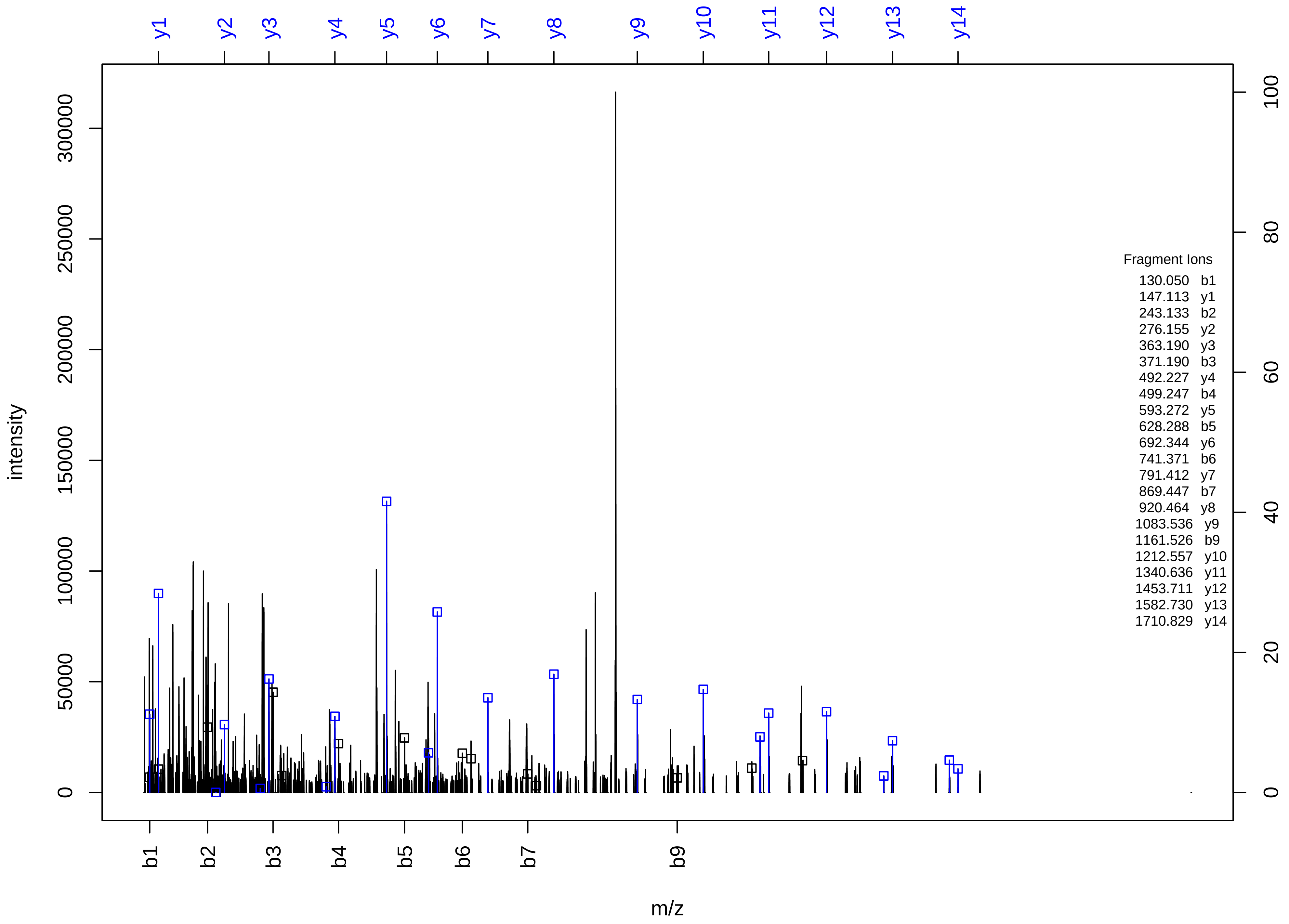

ELQQELQEYEVVTESEK / File: christine_pool_04.raw; SpectrumID: ; scans: 13947

DTFYDNFENEPILYGR

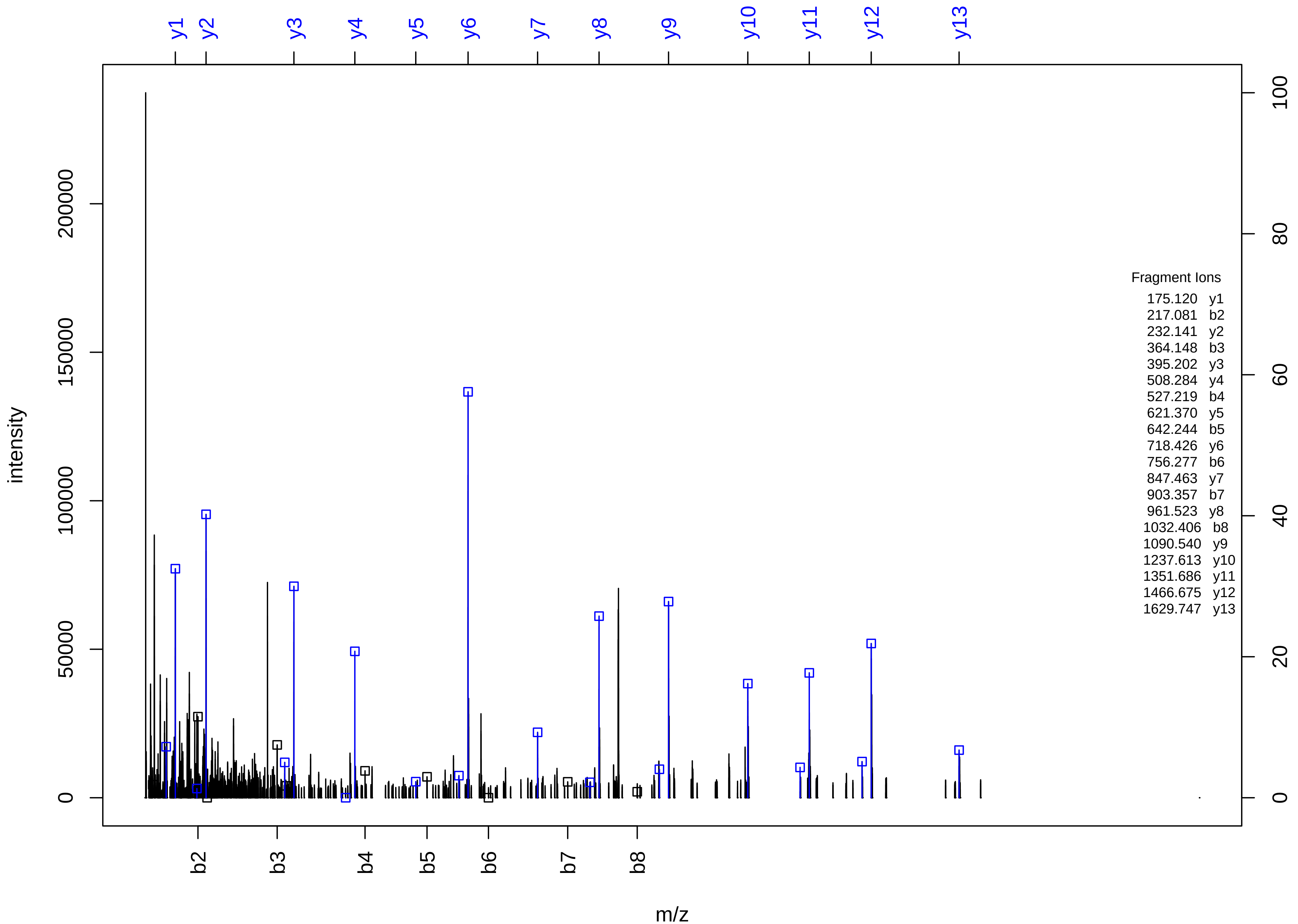

DTFYDNFENEPILYGR / File: christine_pool_04.raw; SpectrumID: ; scans: 18229

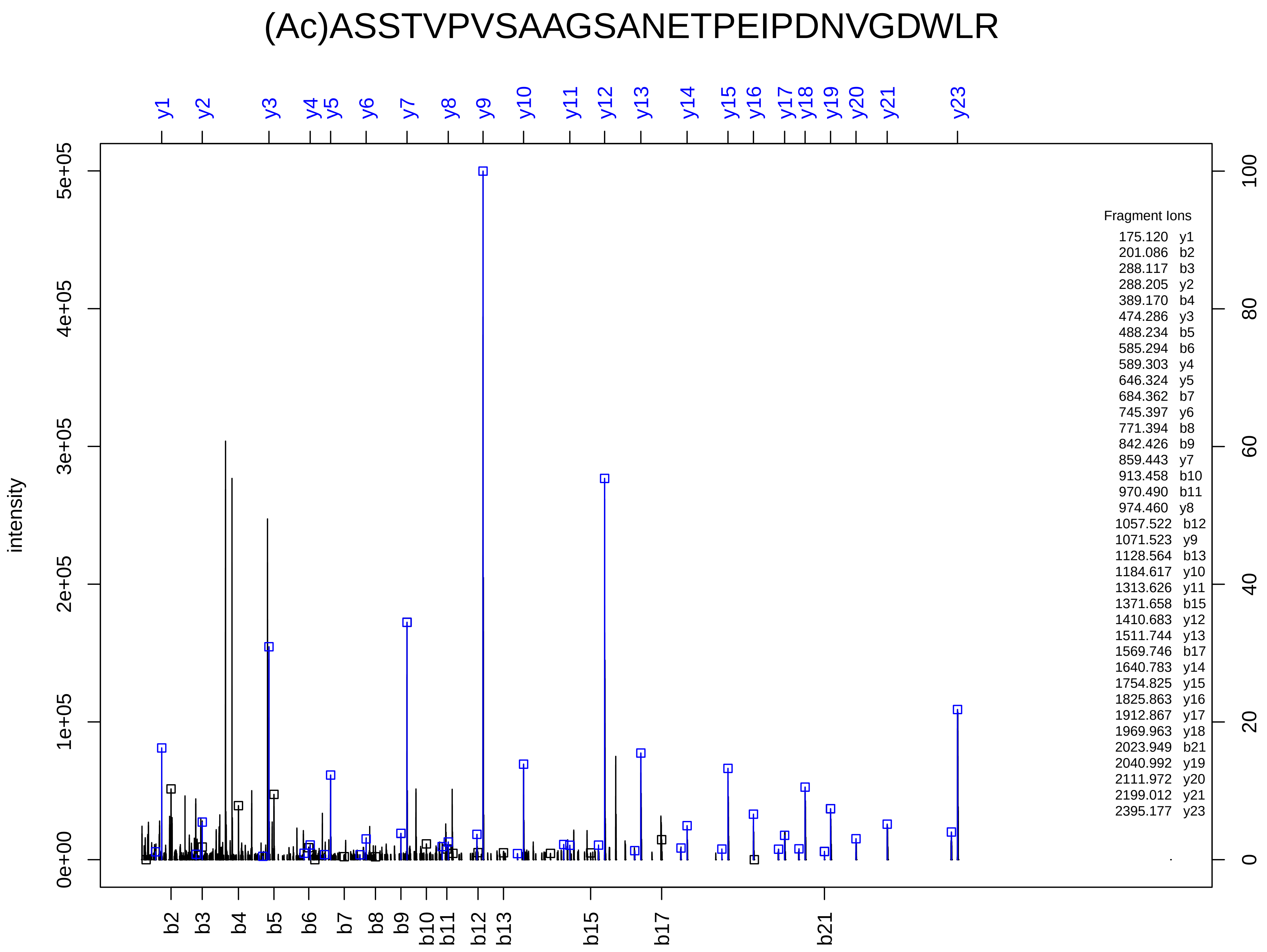

ASSTVPVSAAGSANETPEIPDNVGDWLR / File: christine_pool_04.raw; SpectrumID: ; scans: 22393

GFGFVTFADPASVDK

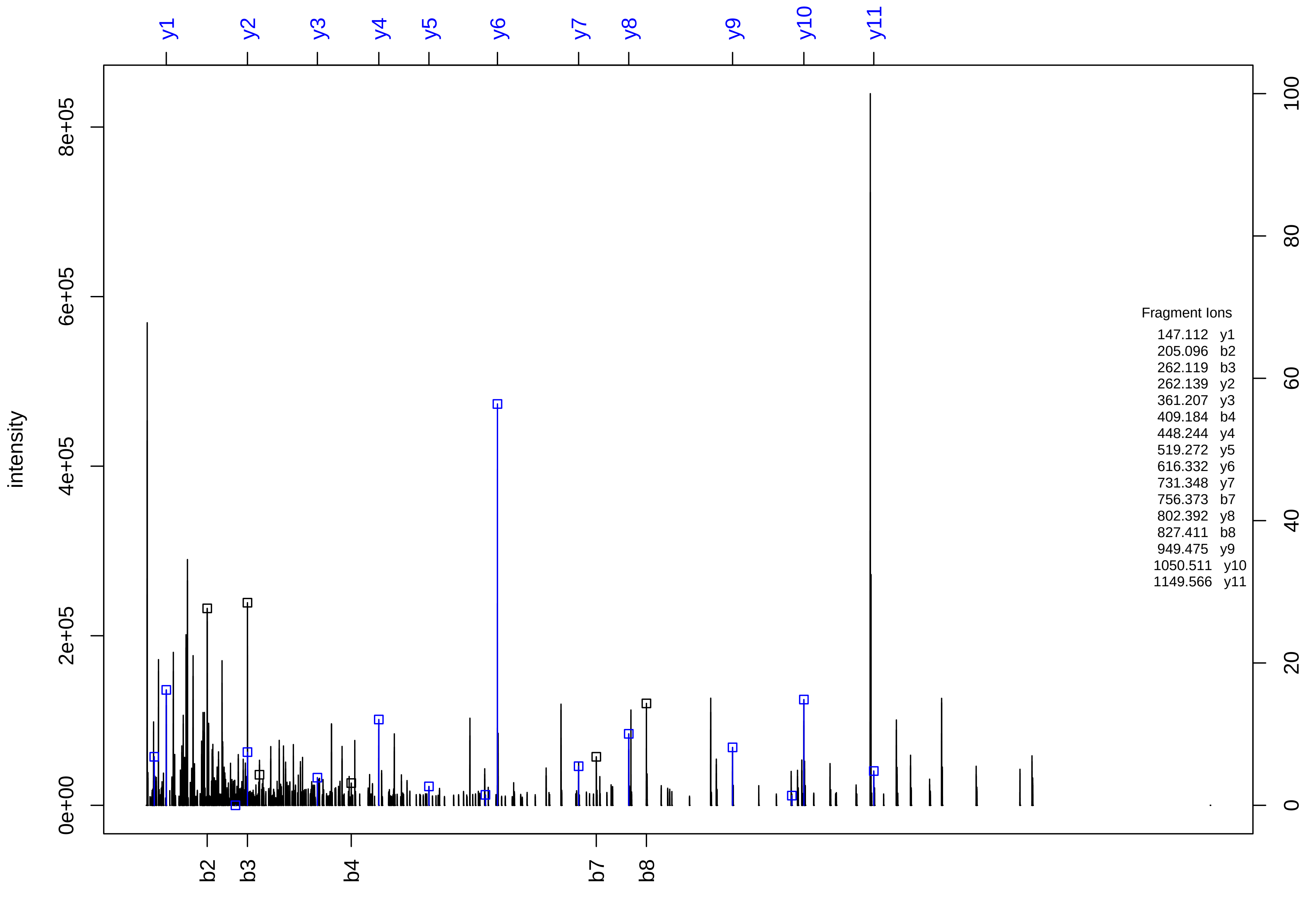

GFGFVTFADPASVDK / File: christine_pool_04.raw; SpectrumID: ; scans: 18523

FALDFGLVPAPPAGTR

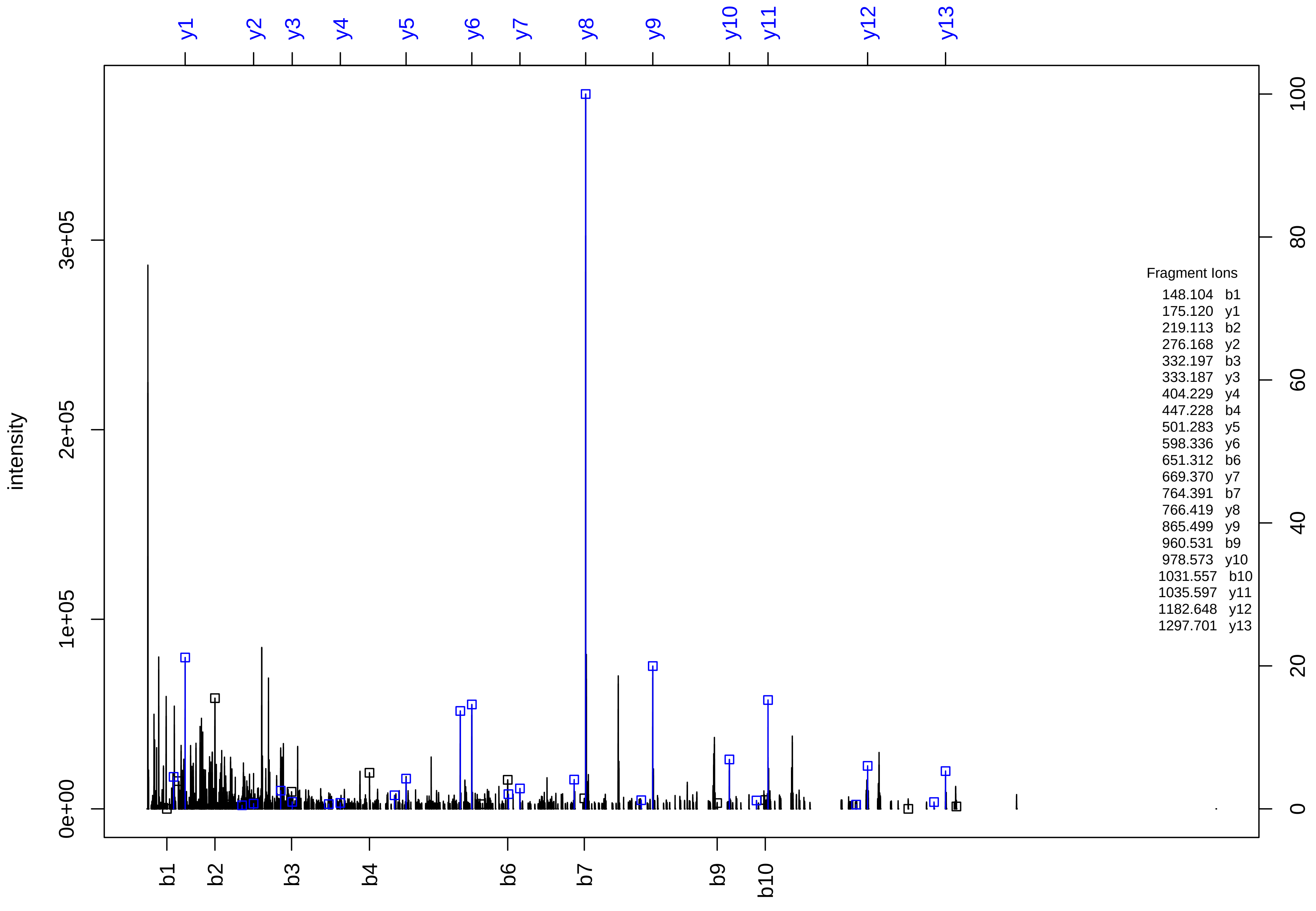

FALDFGLVPAPPAGTR / File: christine_pool_04.raw; SpectrumID: ; scans: 21554

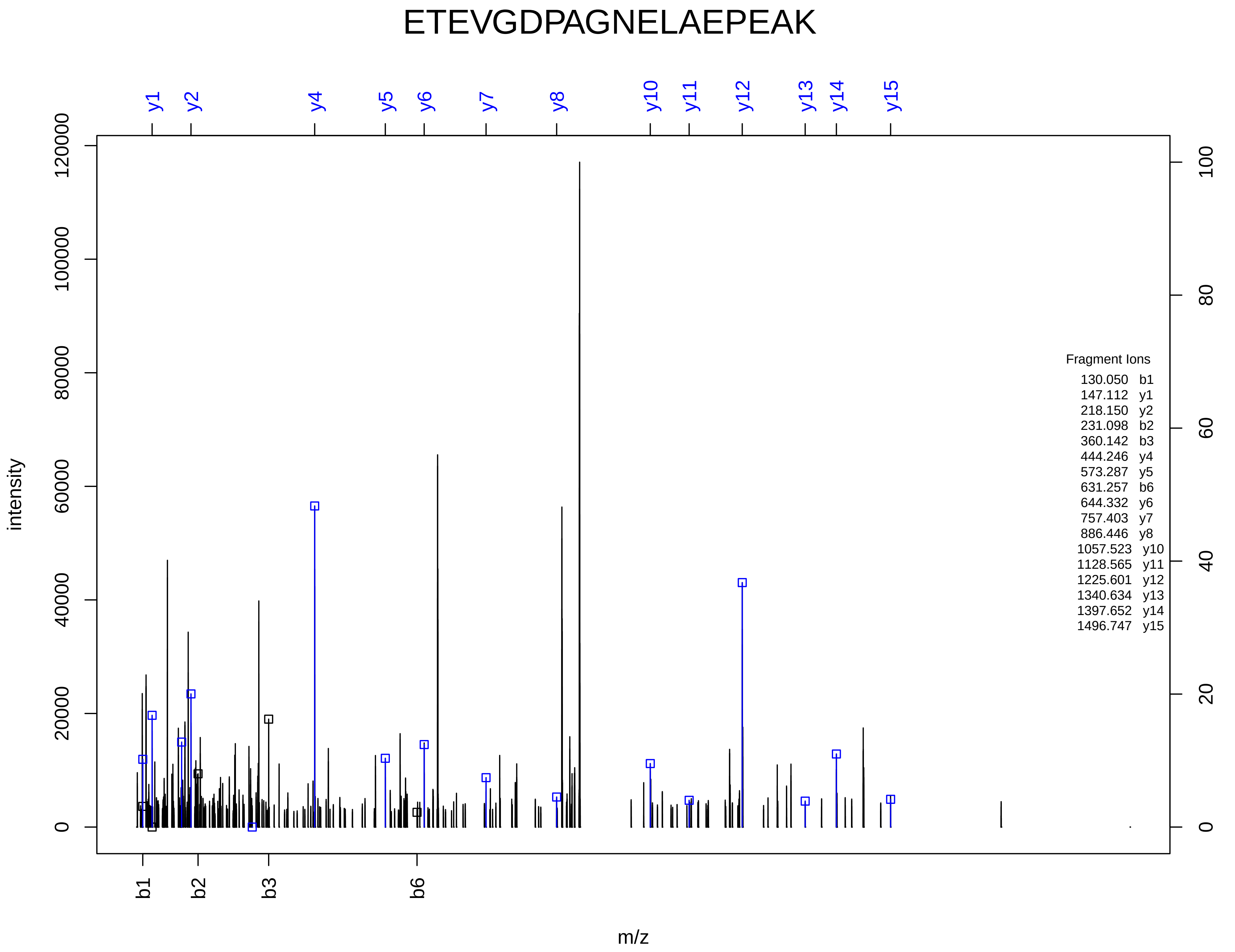

ETEVGDPAGNELAEPEAK / File: christine_pool_04.raw; SpectrumID: ; scans: 6865

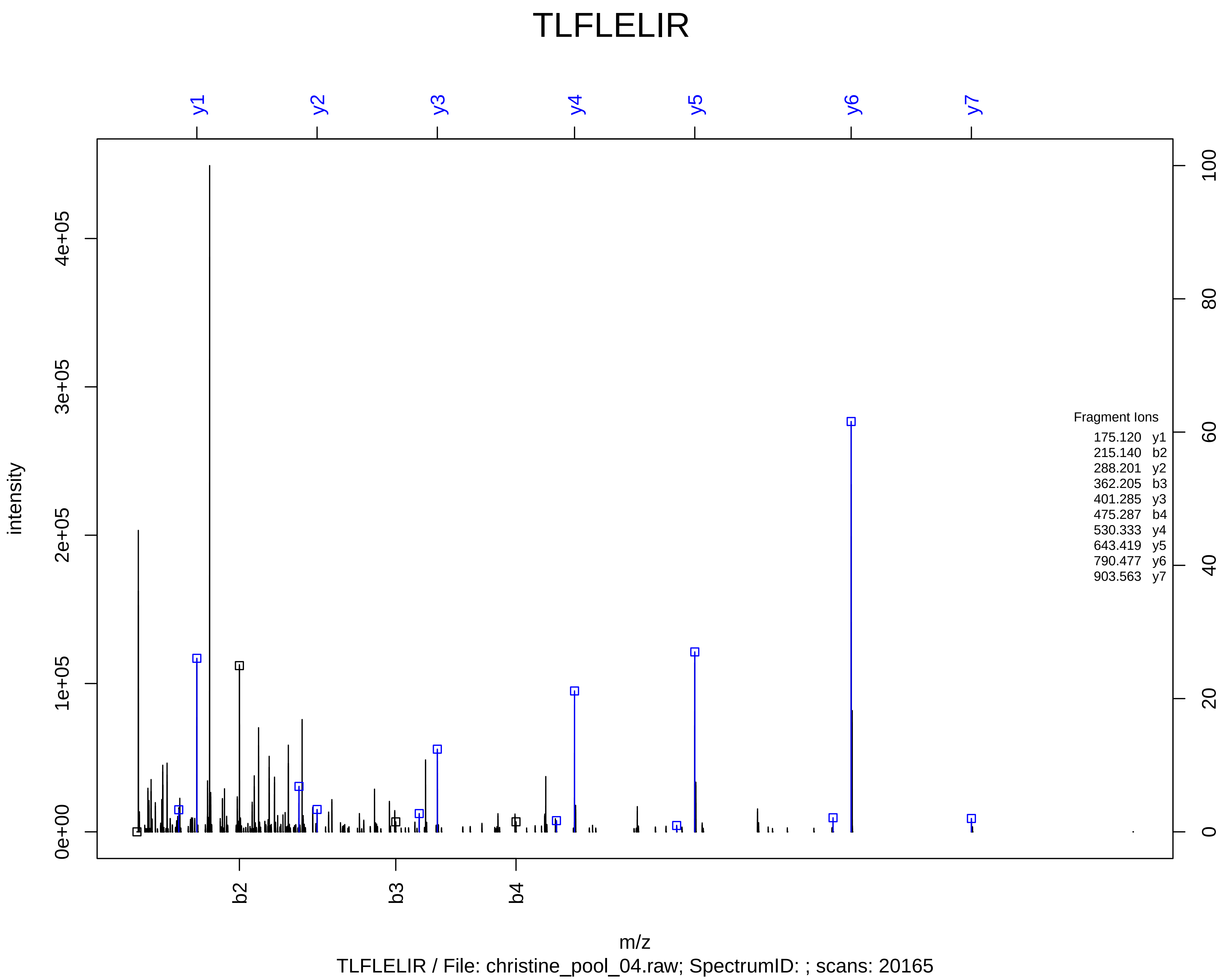
TLFLELIR
y1
y2
y3
y4
y5
y6
y7
b2
b3
b4
intensity
m/z
0e+00
1e+05
2e+05
3e+05
4e+05
0
20
40
60
80
100
Fragment Ions
175.120 y1
215.140 b2
288.201 y2
362.205 b3
401.285 y3
475.287 b4
530.333 y4
643.419 y5
790.477 y6
903.563 y7
TLFLELIR / File: christine_pool_04.raw; SpectrumID: ; scans: 20165

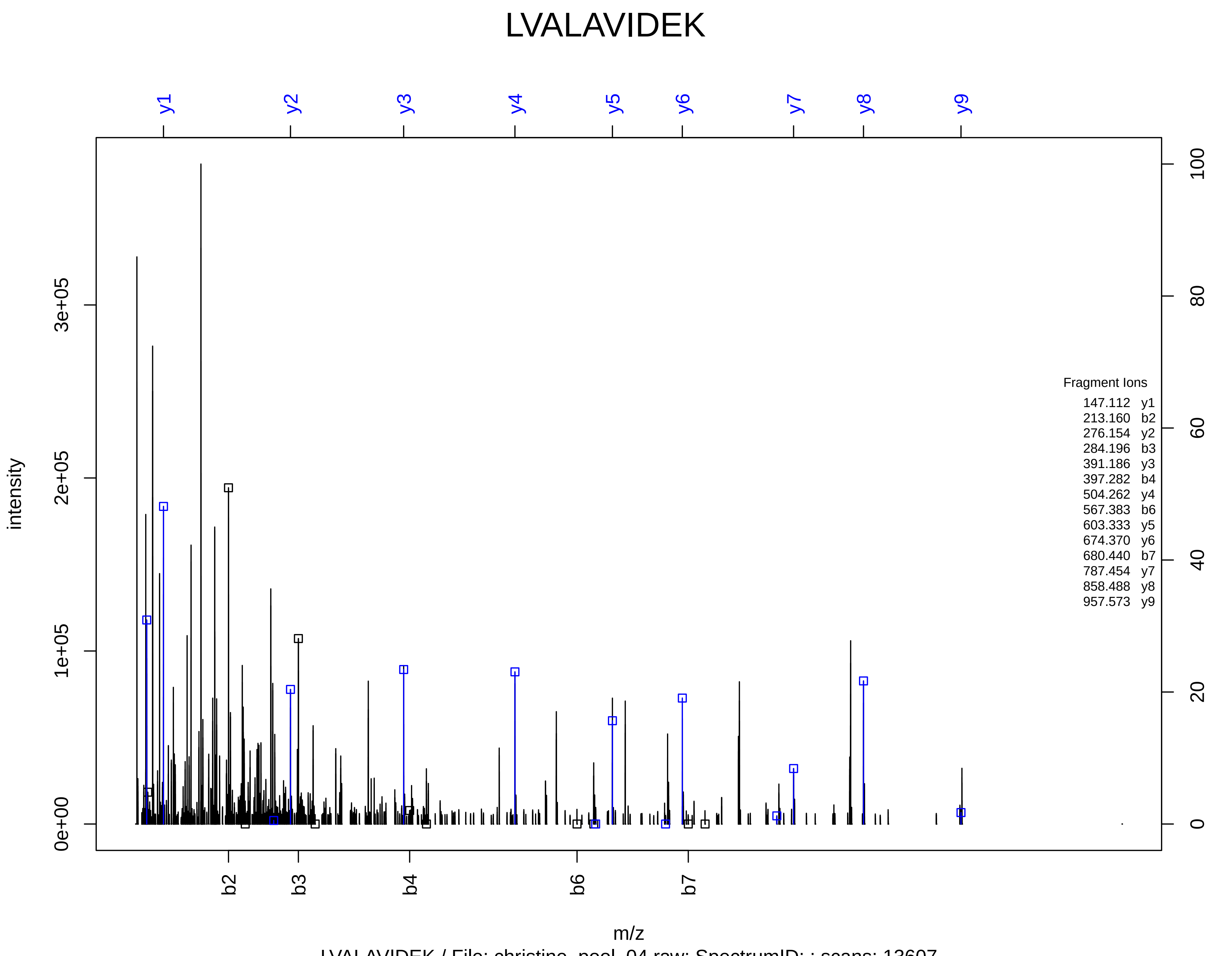
LVALAVIDEK
y1
y2
y3
y4
y5
y6
y7
y8
y9
b2
b3
b4
b6
b7
intensity
0e+00
1e+05
2e+05
3e+05
0
20
40
60
80
100
m/z
Fragment Ions
147.112 y1
213.160 b2
276.154 y2
284.196 b3
391.186 y3
397.282 b4
504.262 y4
567.383 b6
603.333 y5
674.370 y6
680.440 b7
787.454 y7
858.488 y8
957.573 y9
LVALAVIDEK / File: christine_pool_04.raw; SpectrumID: ; scans: 13607

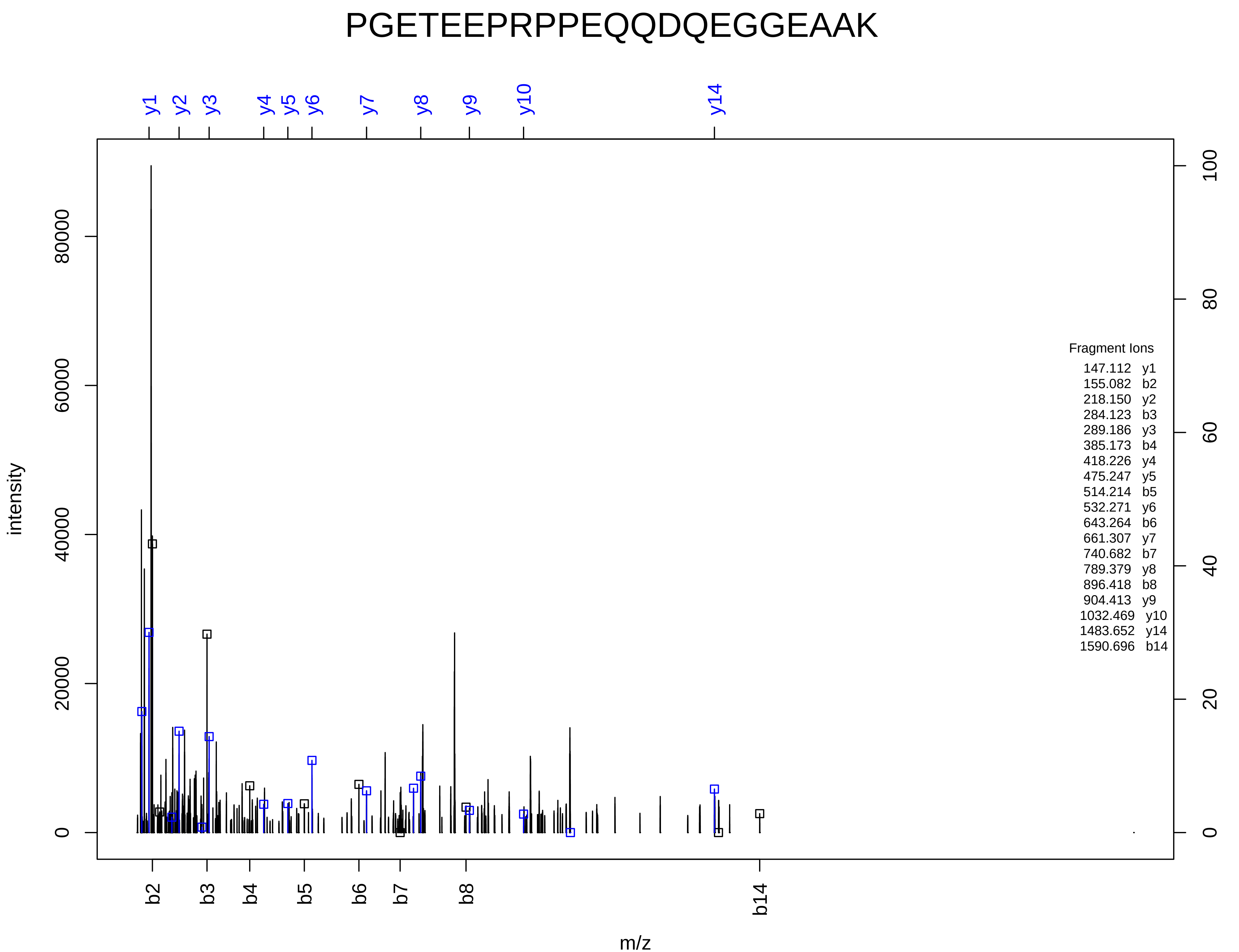

PGETEEPRPPEQQDQEGGEAAK / File: christine_pool_04.raw; SpectrumID: ; scans: 2881

TVHYLPILFIDQLSNR

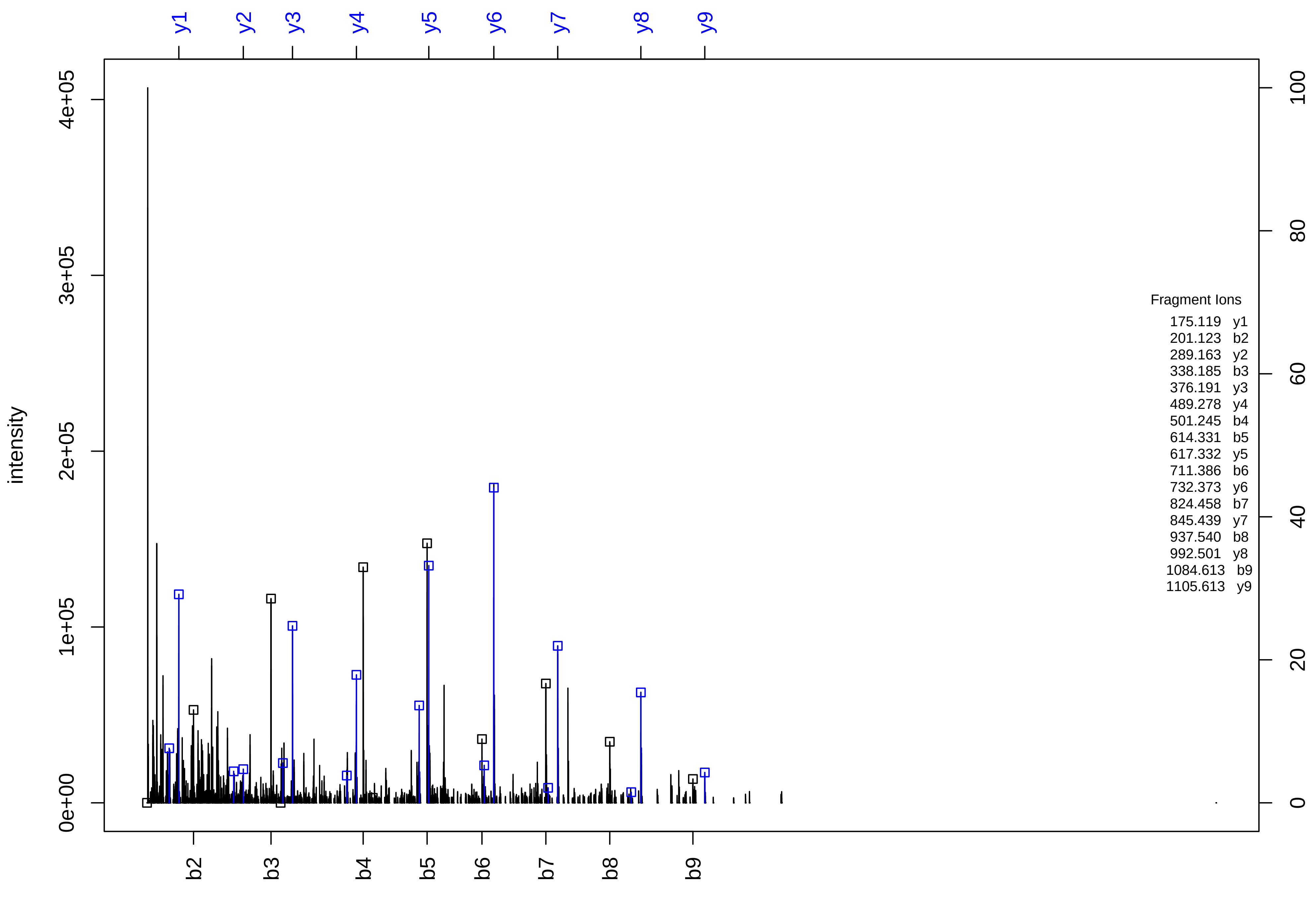

TVHYLPILFIDQLSNR / File: christine_pool_04.raw; SpectrumID: ; scans: 22454

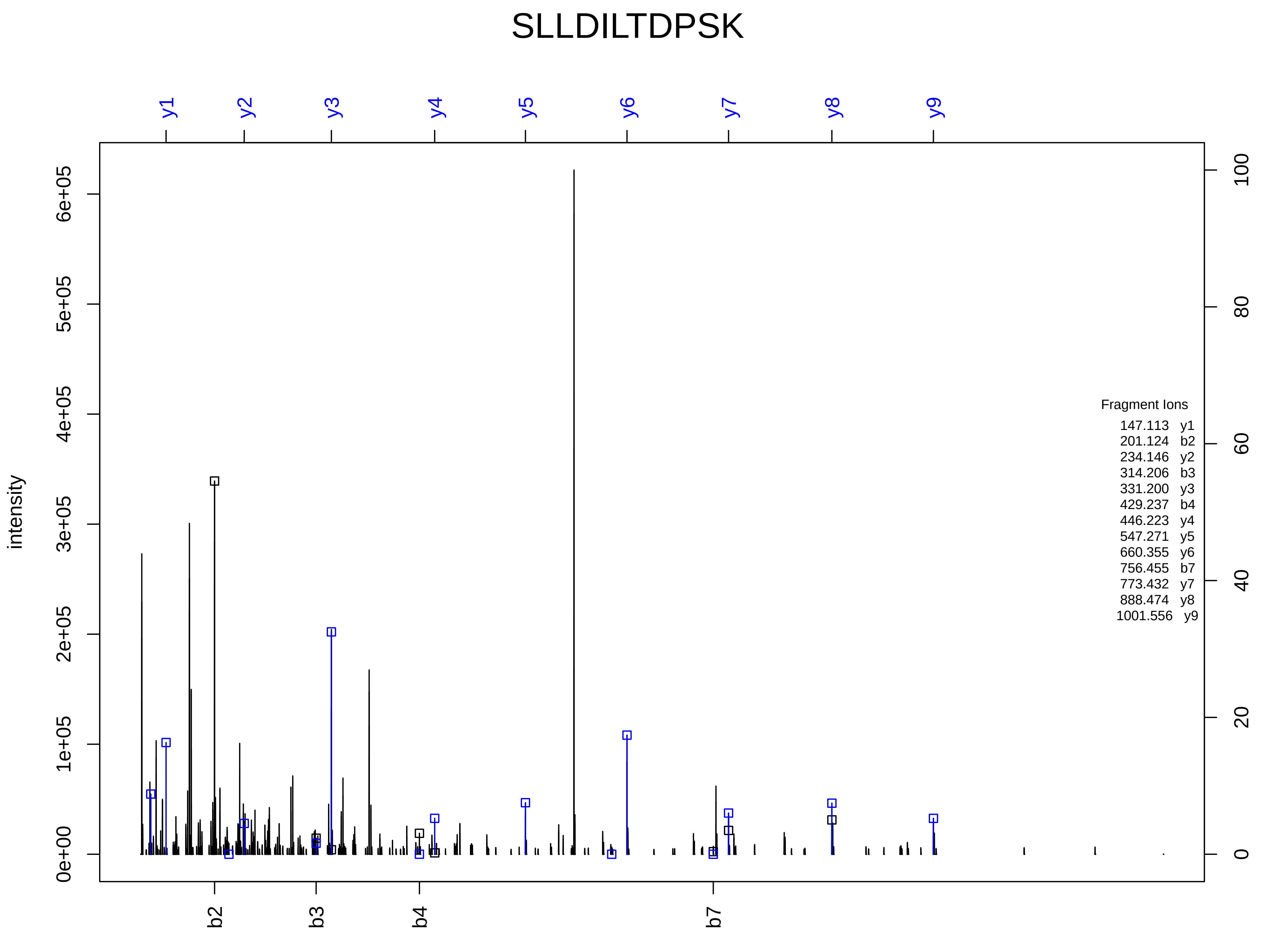

SLLDILTDPSK / File: christine_pool_04.raw; SpectrumID: ; scans: 20269

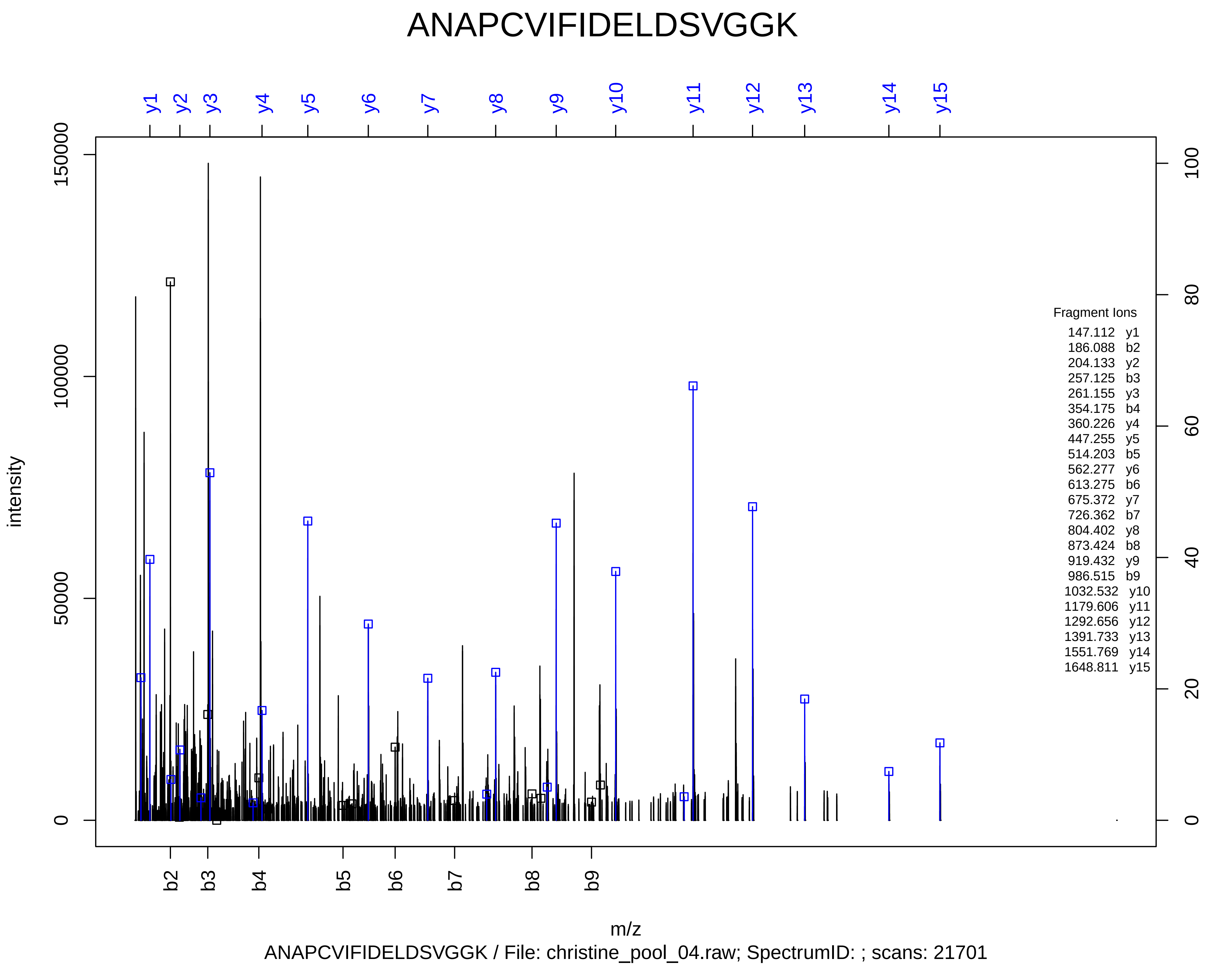

ANAPCVIFIDELDSVGGK
y1 y2 y3 y4 y5 y6 y7 y8 y9 y10 y11 y12 y13 y14 y15
b2 b3 b4 b5 b6 b7 b8 b9
intensity
m/z
0
50000
100000
150000
0
20
40
60
80
100
Fragment Ions
147.112 y1
186.088 b2
204.133 y2
257.125 b3
261.155 y3
354.175 b4
360.226 y4
447.255 y5
514.203 b5
562.277 y6
613.275 b6
675.372 y7
726.362 b7
804.402 y8
873.424 b8
919.432 y9
986.515 b9
1032.532 y10
1179.606 y11
1292.656 y12
1391.733 y13
1551.769 y14
1648.811 y15
ANAPCVIFIDELDSVGGK / File: christine_pool_04.raw; SpectrumID: ; scans: 21701

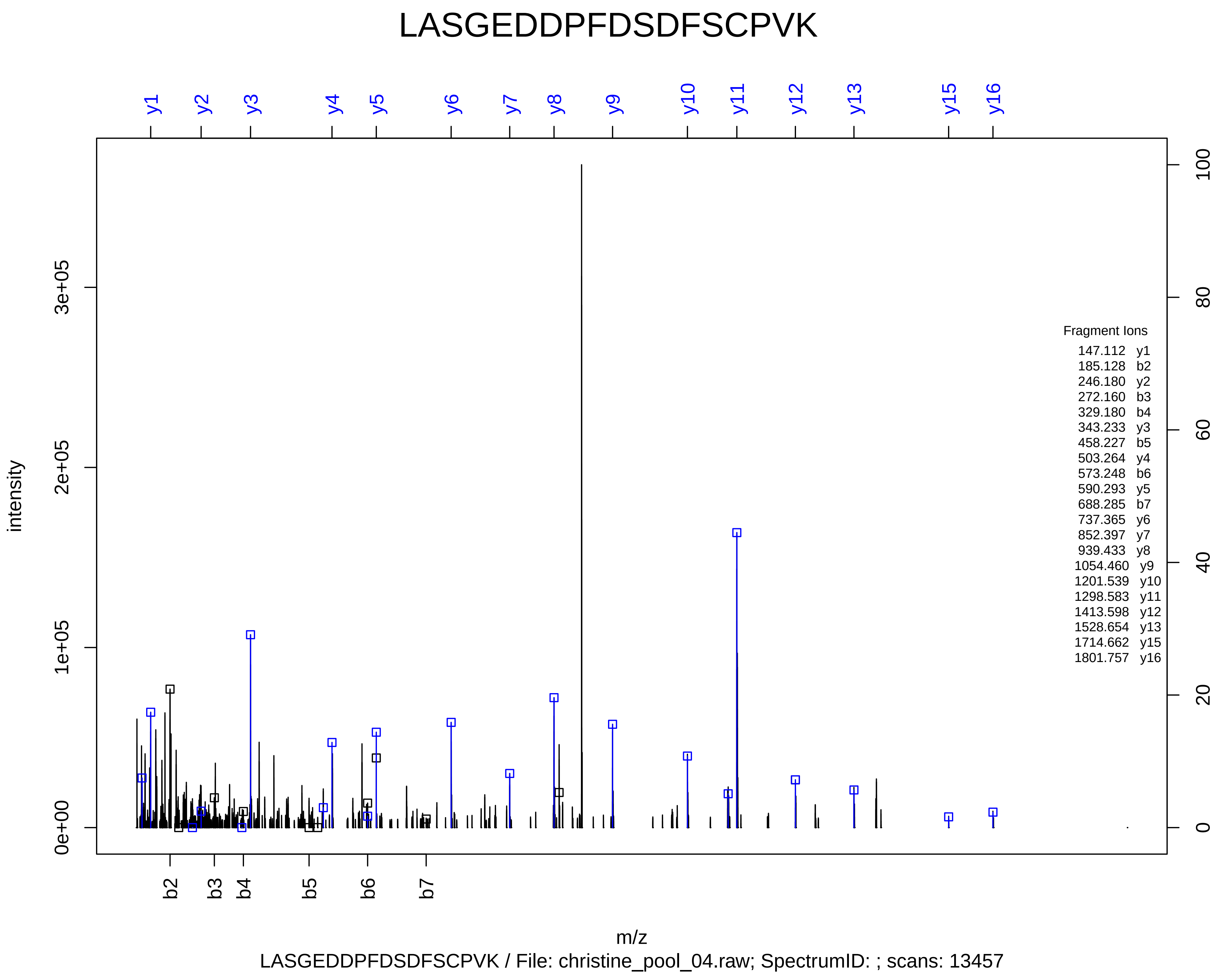

LASGEDDPFDSDFSCPVK
y1
y2
y3
y4
y5
y6
y7
y8
y9
y10
y11
y12
y13
y15
y16
intensity
0e+00
1e+05
2e+05
3e+05
0
20
40
60
80
100
b2
b3
b4
b5
b6
b7
m/z
Fragment Ions
147.112 y1
185.128 b2
246.180 y2
272.160 b3
329.180 b4
343.233 y3
458.227 b5
503.264 y4
573.248 b6
590.293 y5
688.285 b7
737.365 y6
852.397 y7
939.433 y8
1054.460 y9
1201.539 y10
1298.583 y11
1413.598 y12
1528.654 y13
1714.662 y15
1801.757 y16
LASGEDDPFDSDFSCPVK / File: christine_pool_04.raw; SpectrumID: ; scans: 13457

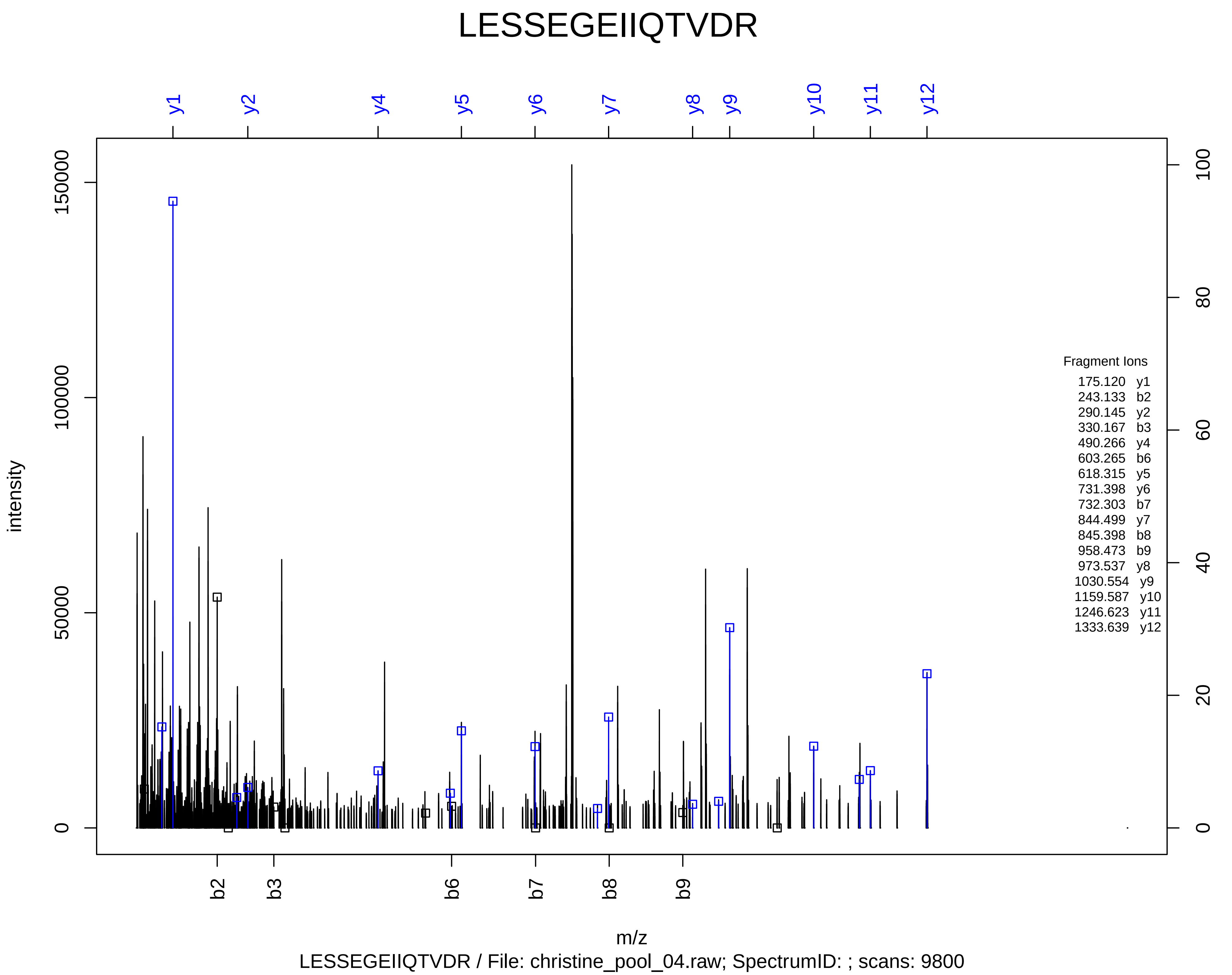

LESSEGEIIQTVDR
y1
y2
y4
y5
y6
y7
y8
y9
y10
y11
y12
intensity
150000
100000
50000
0
100
80
60
40
20
0
b2
b3
b6
b7
b8
b9
m/z
Fragment Ions
175.120 y1
243.133 b2
290.145 y2
330.167 b3
490.266 y4
603.265 b6
618.315 y5
731.398 y6
732.303 b7
844.499 y7
845.398 b8
958.473 b9
973.537 y8
1030.554 y9
1159.587 y10
1246.623 y11
1333.639 y12
LESSEGEIIQTVDR / File: christine_pool_04.raw; SpectrumID: ; scans: 9800

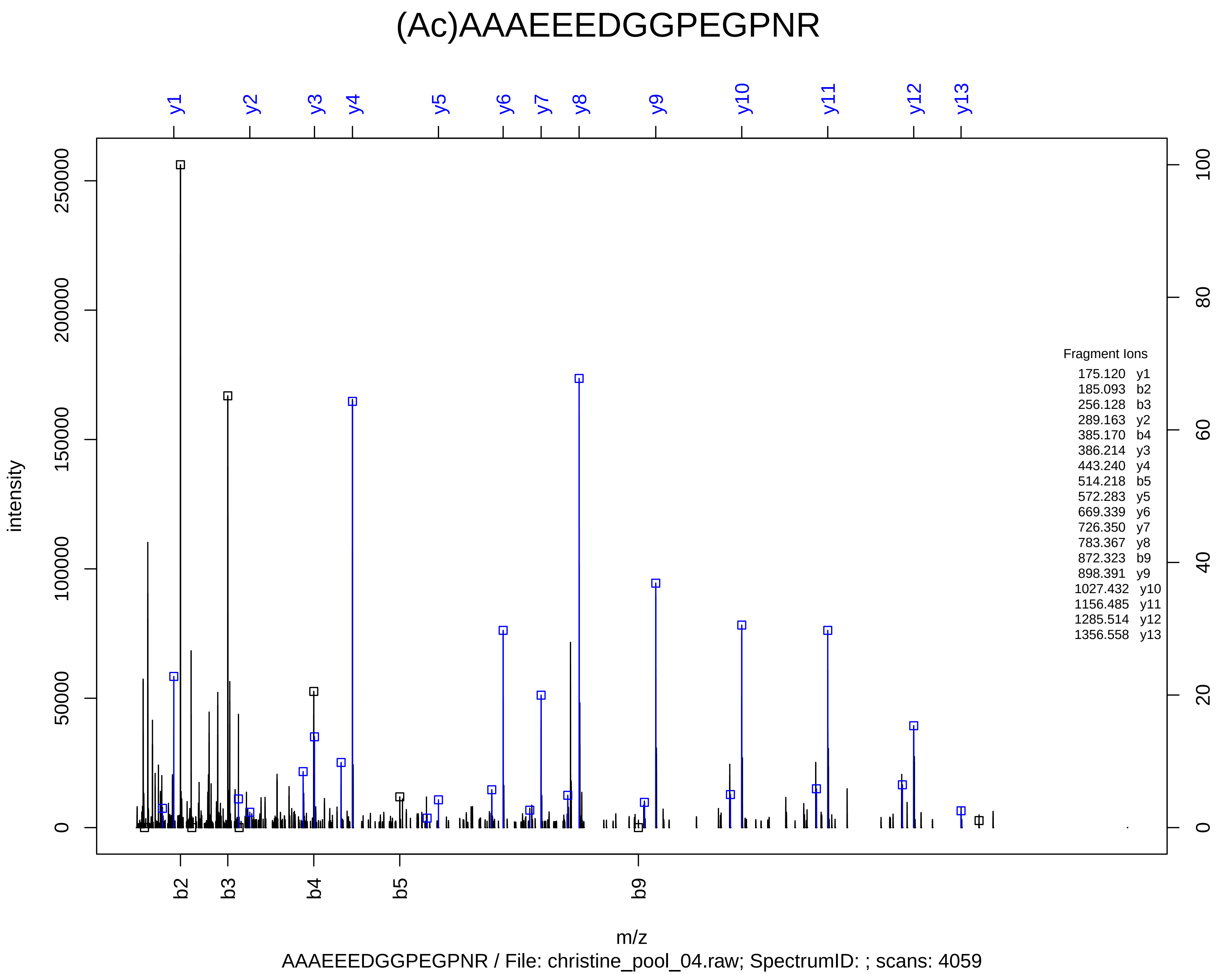
(Ac)AAAEEEDGGPEGPNR
y1
y2
y3
y4
y5
y6
y7
y8
y9
y10
y11
y12
y13
intensity
0
50000
100000
150000
200000
250000
0
20
40
60
80
100
b2
b3
b4
b5
b9
m/z
Fragment Ions
175.120 y1
185.093 b2
256.128 b3
289.163 y2
385.170 b4
386.214 y3
443.240 y4
514.218 b5
572.283 y5
669.339 y6
726.350 y7
783.367 y8
872.323 b9
898.391 y9
1027.432 y10
1156.485 y11
1285.514 y12
1356.558 y13
AAAEEEDGGPEGPNR / File: christine_pool_04.raw; SpectrumID: ; scans: 4059

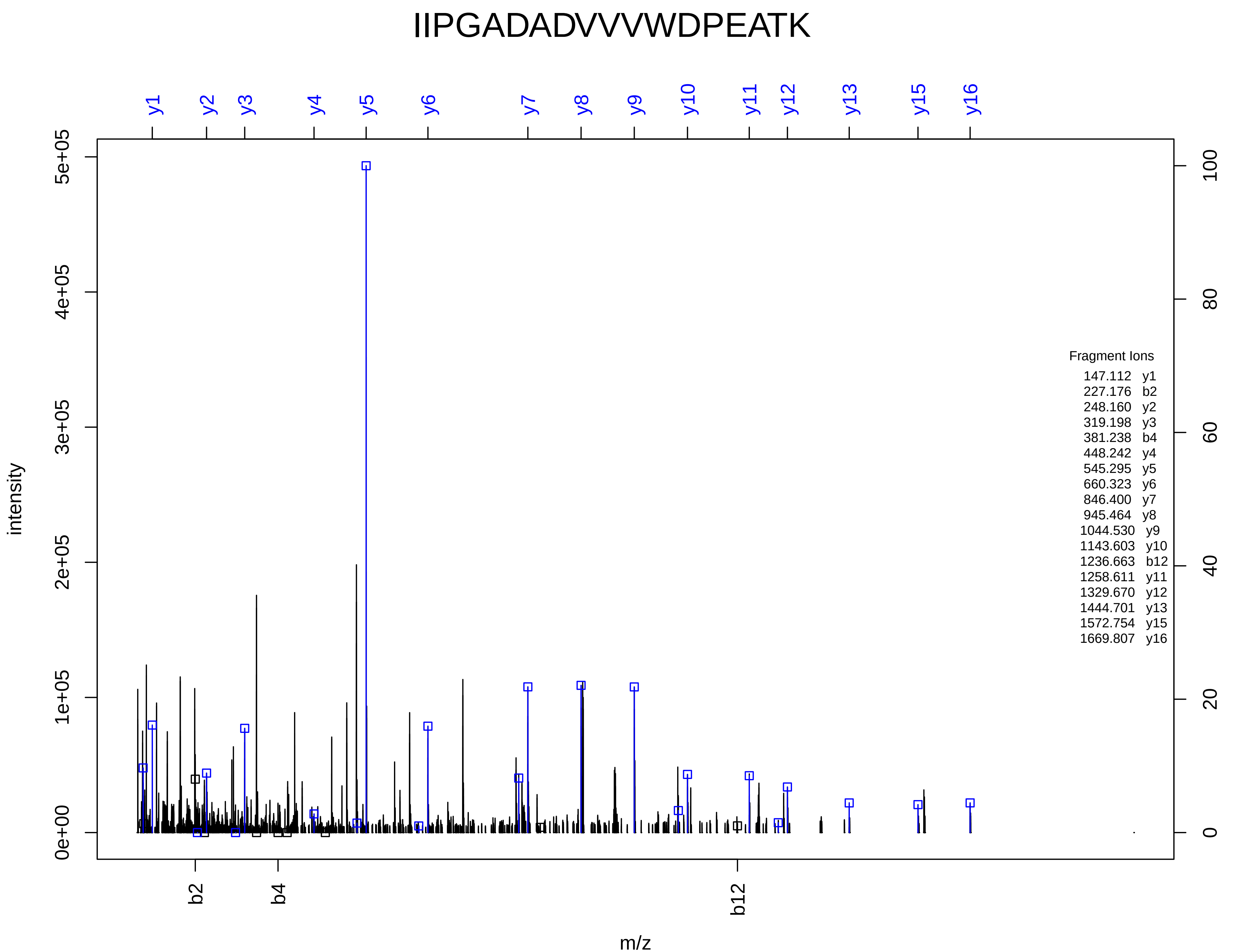
IIPGADADVVVWDPEATK
y1
y2
y3
y4
y5
y6
y7
y8
y9
y10
y11
y12
y13
y15
y16
intensity
0e+00
1e+05
2e+05
3e+05
4e+05
5e+05
0
20
40
60
80
100
Fragment Ions
147.112 y1
227.176 b2
248.160 y2
319.198 y3
381.238 b4
448.242 y4
545.295 y5
660.323 y6
846.400 y7
945.464 y8
1044.530 y9
1143.603 y10
1236.663 b12
1258.611 y11
1329.670 y12
1444.701 y13
1572.754 y15
1669.807 y16
b2
b4
b12
m/z
IIPGADADVVVWDPEATK / File: christine_pool_04.raw; SpectrumID: ; scans: 17105

LHLDEDYPCSLVGNWNTWYGEQDQAVHLWR

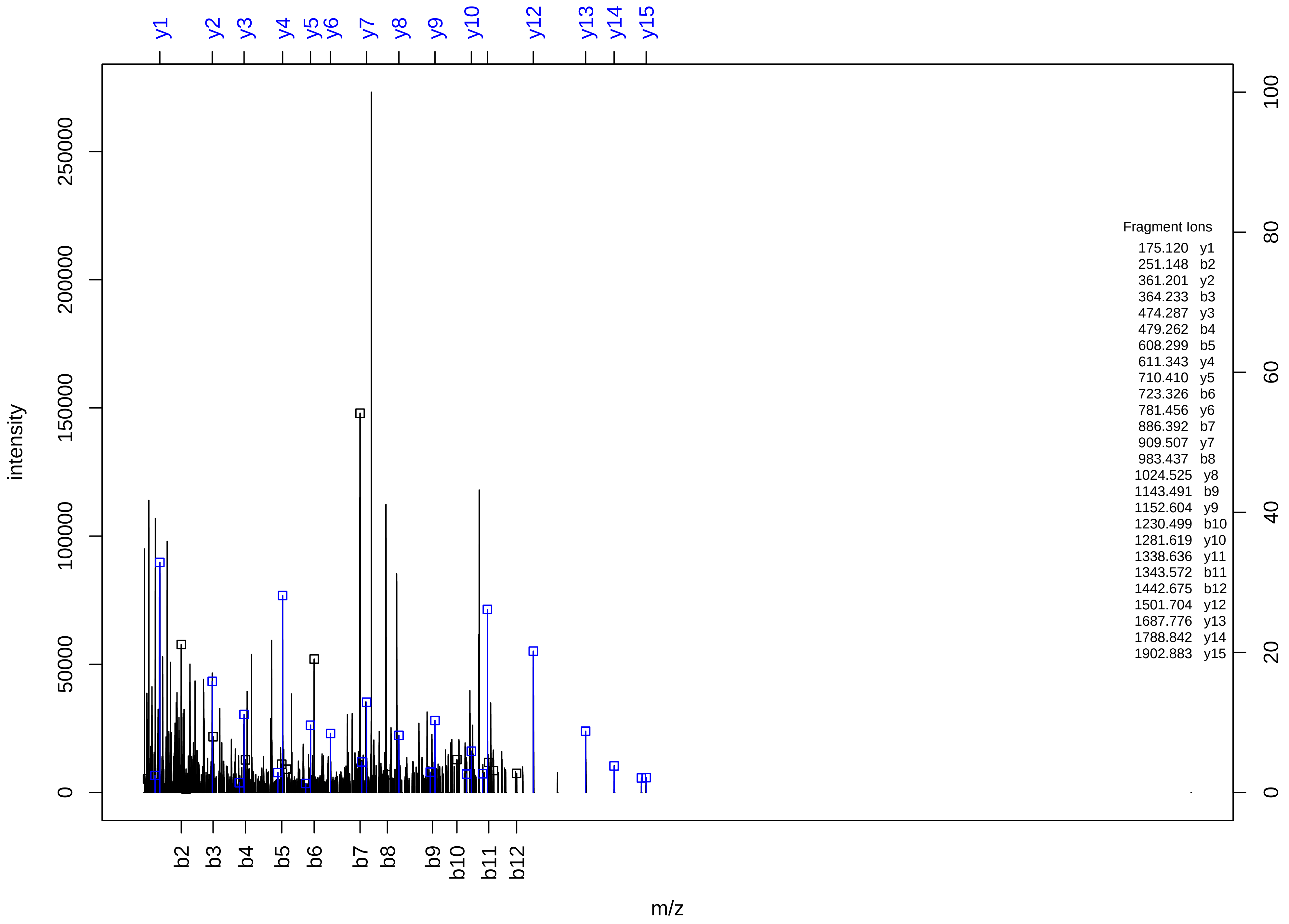

LHLDEDYPCSLVGNWNTWYGEQDQAVHLWR / File: christine_pool_04.raw; SpectrumID: ; scans: 21071

FLVFDWNWR

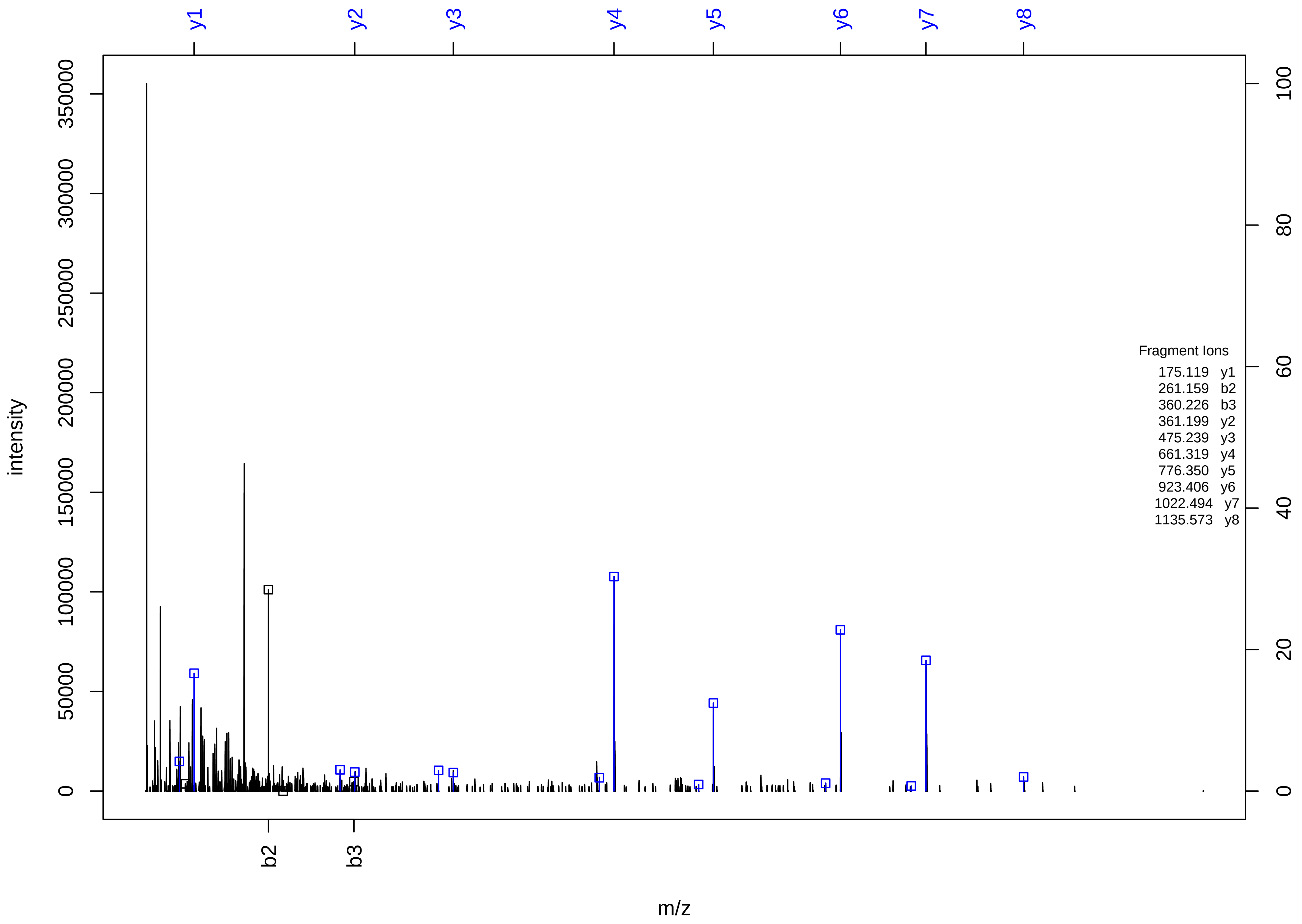

FLVFDWNWR / File: christine_pool_04.raw; SpectrumID: ; scans: 23027

AHAAQVTCVAASPHK

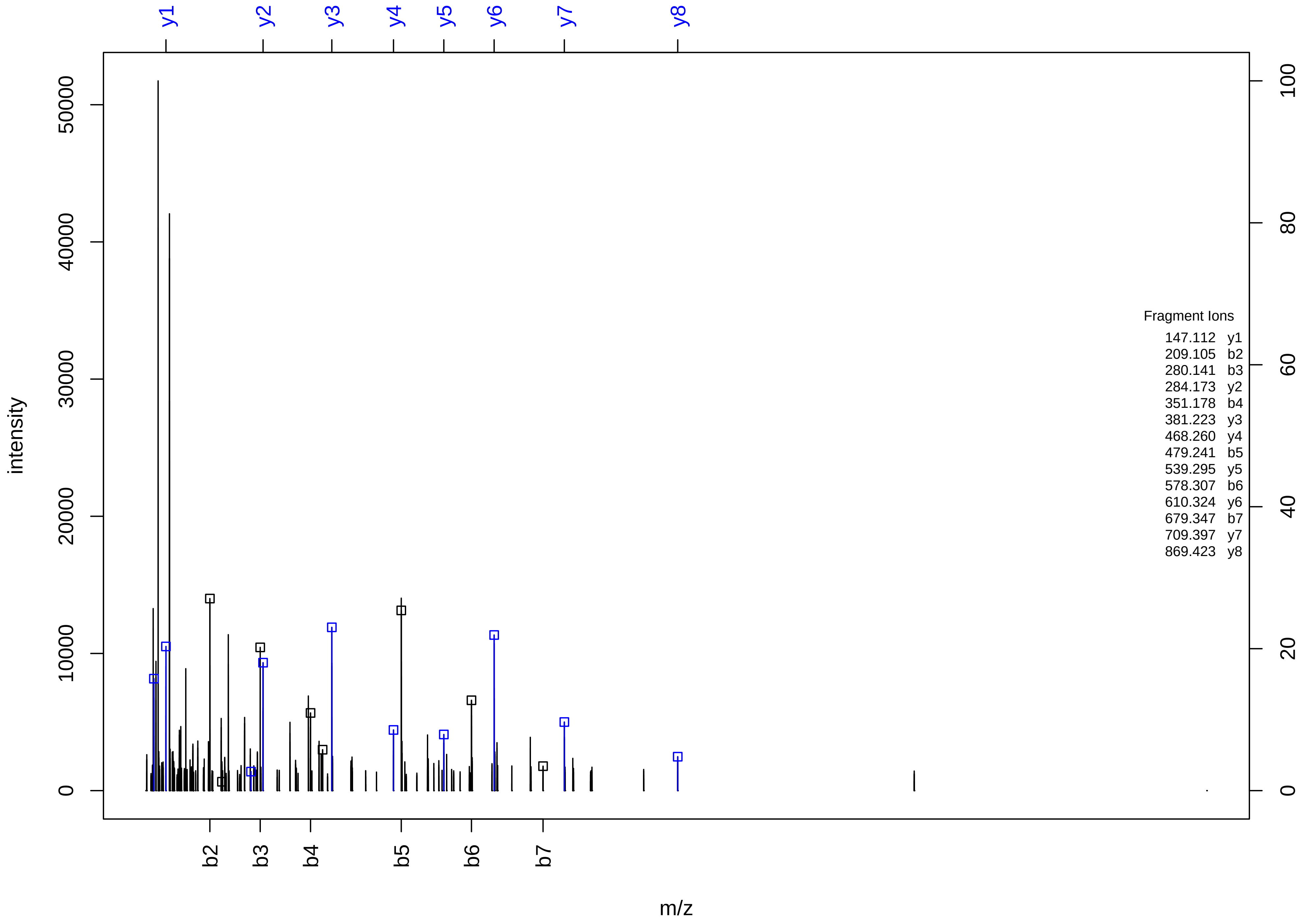

AHAAQVTCVAASPHK / File: christine_pool_04.raw; SpectrumID: ; scans: 2161

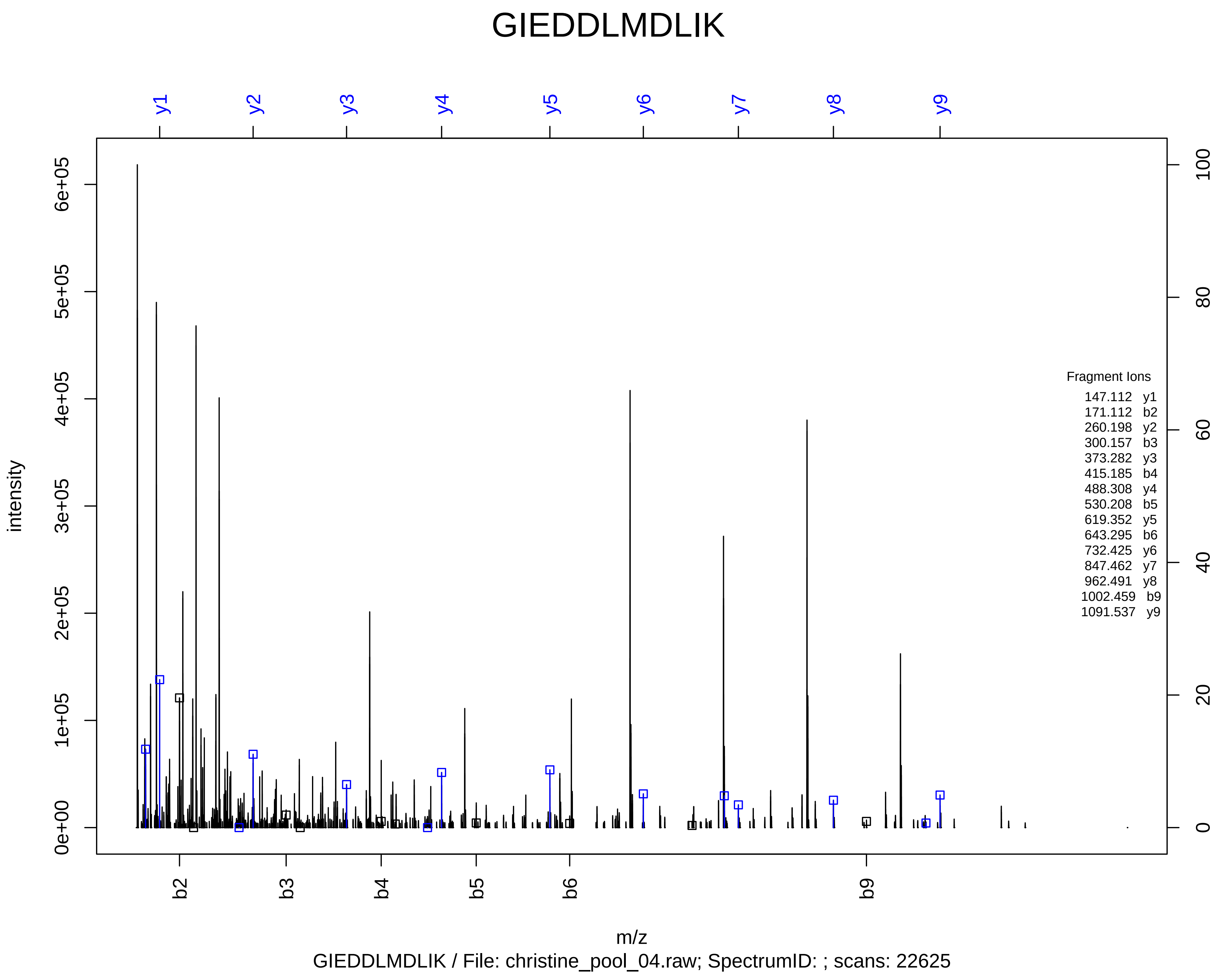
GIEDDLMDLIK
y1
y2
y3
y4
y5
y6
y7
y8
y9
intensity
0e+00
1e+05
2e+05
3e+05
4e+05
5e+05
6e+05
0
20
40
60
80
100
Fragment Ions
147.112 y1
171.112 b2
260.198 y2
300.157 b3
373.282 y3
415.185 b4
488.308 y4
530.208 b5
619.352 y5
643.295 b6
732.425 y6
847.462 y7
962.491 y8
1002.459 b9
1091.537 y9
b2
b3
b4
b5
b6
b9
m/z
GIEDDLMDLIK / File: christine_pool_04.raw; SpectrumID: ; scans: 22625

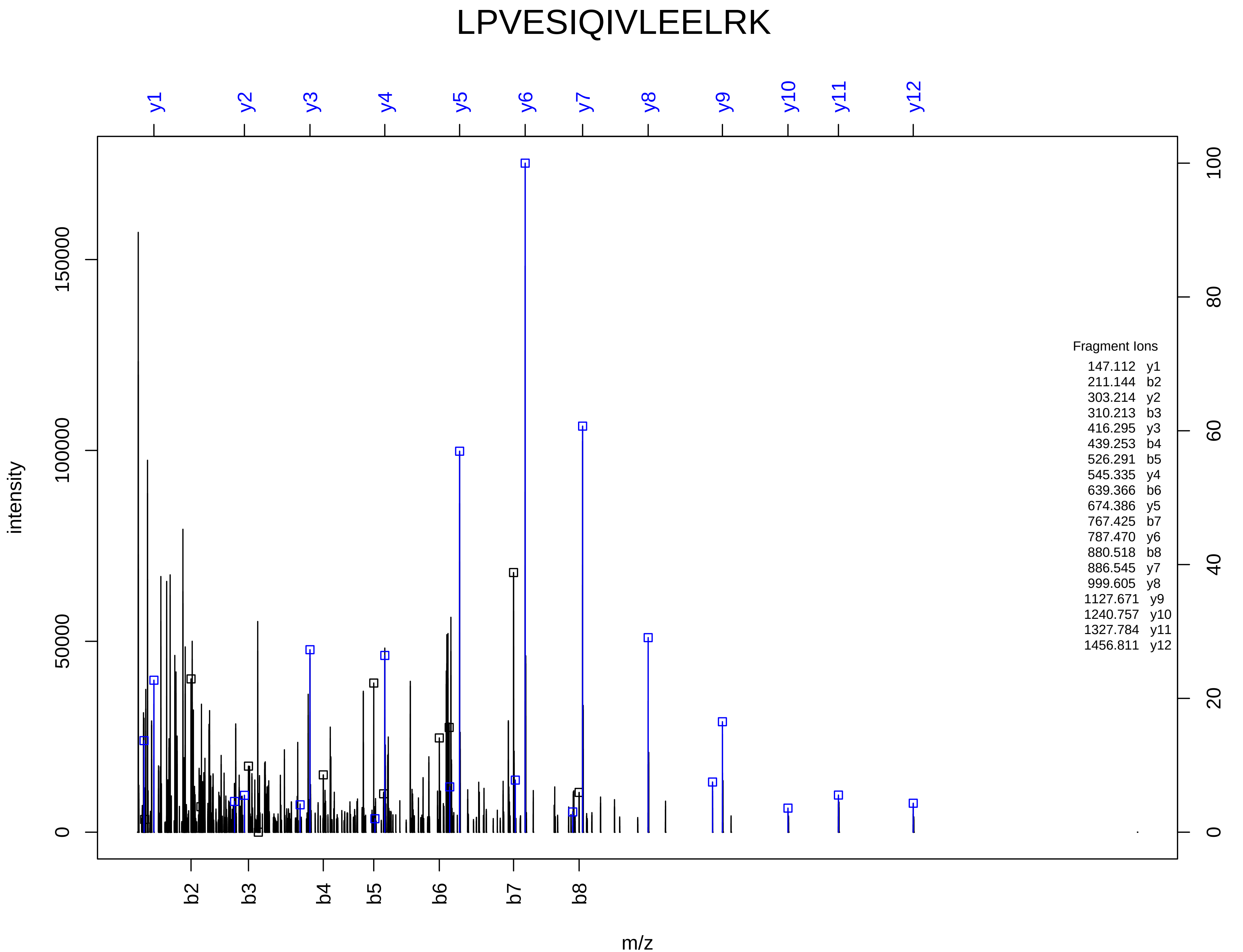

LPVESIQIVLEELRK
y1
y2
y3
y4
y5
y6
y7
y8
y9
y10
y11
y12
intensity
0
50000
100000
150000
0
20
40
60
80
100
b2
b3
b4
b5
b6
b7
b8
Fragment Ions
147.112 y1
211.144 b2
303.214 y2
310.213 b3
416.295 y3
439.253 b4
526.291 b5
545.335 y4
639.366 b6
674.386 y5
767.425 b7
787.470 y6
880.518 b8
886.545 y7
999.605 y8
1127.671 y9
1240.757 y10
1327.784 y11
1456.811 y12
m/z
LPVESIQIVLEELRK / File: christine_pool_04.raw; SpectrumID: ; scans: 22156

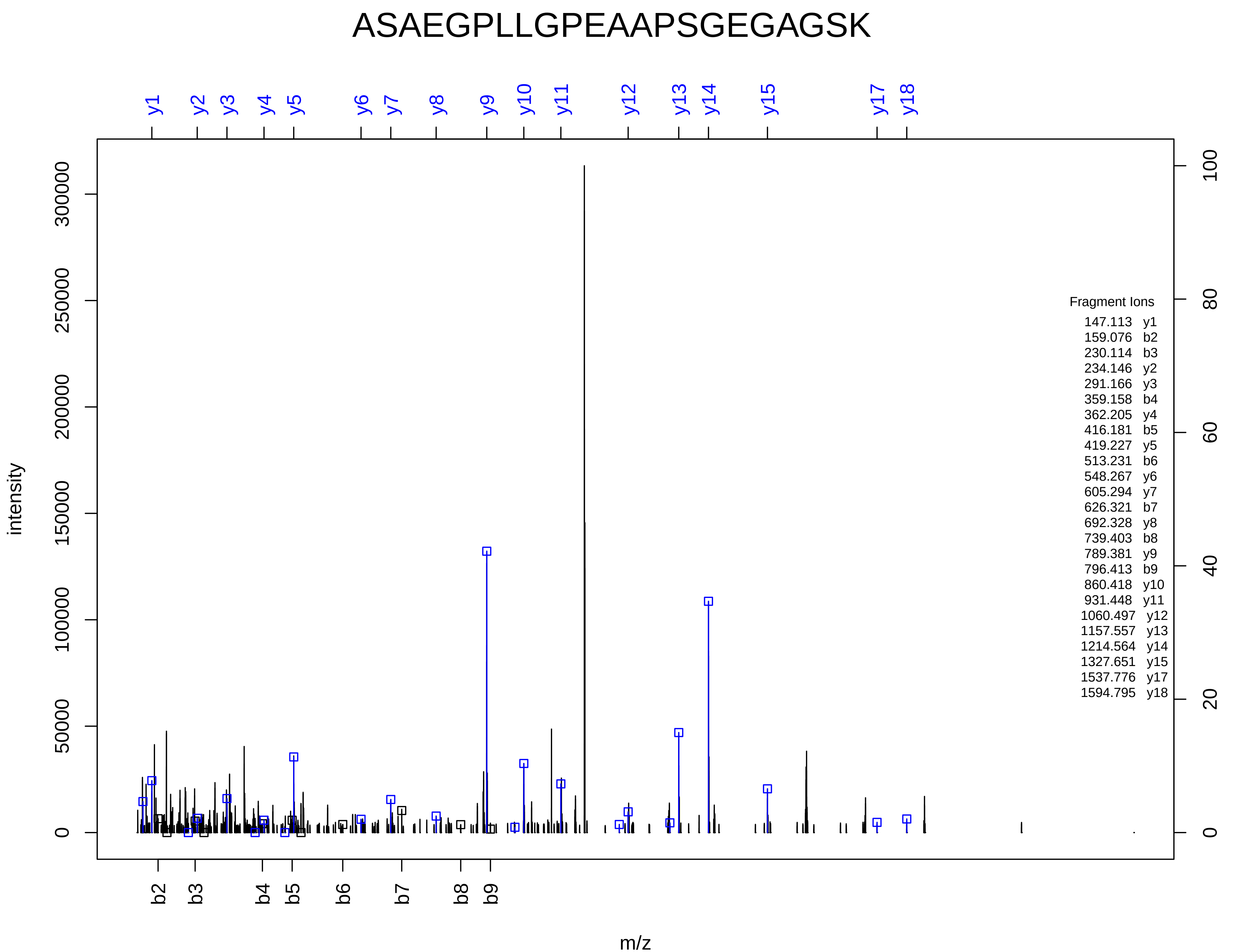

ASAEGPLLGPEAAPSGEGAGSK / File: christine_pool_04.raw; SpectrumID: ; scans: 8226

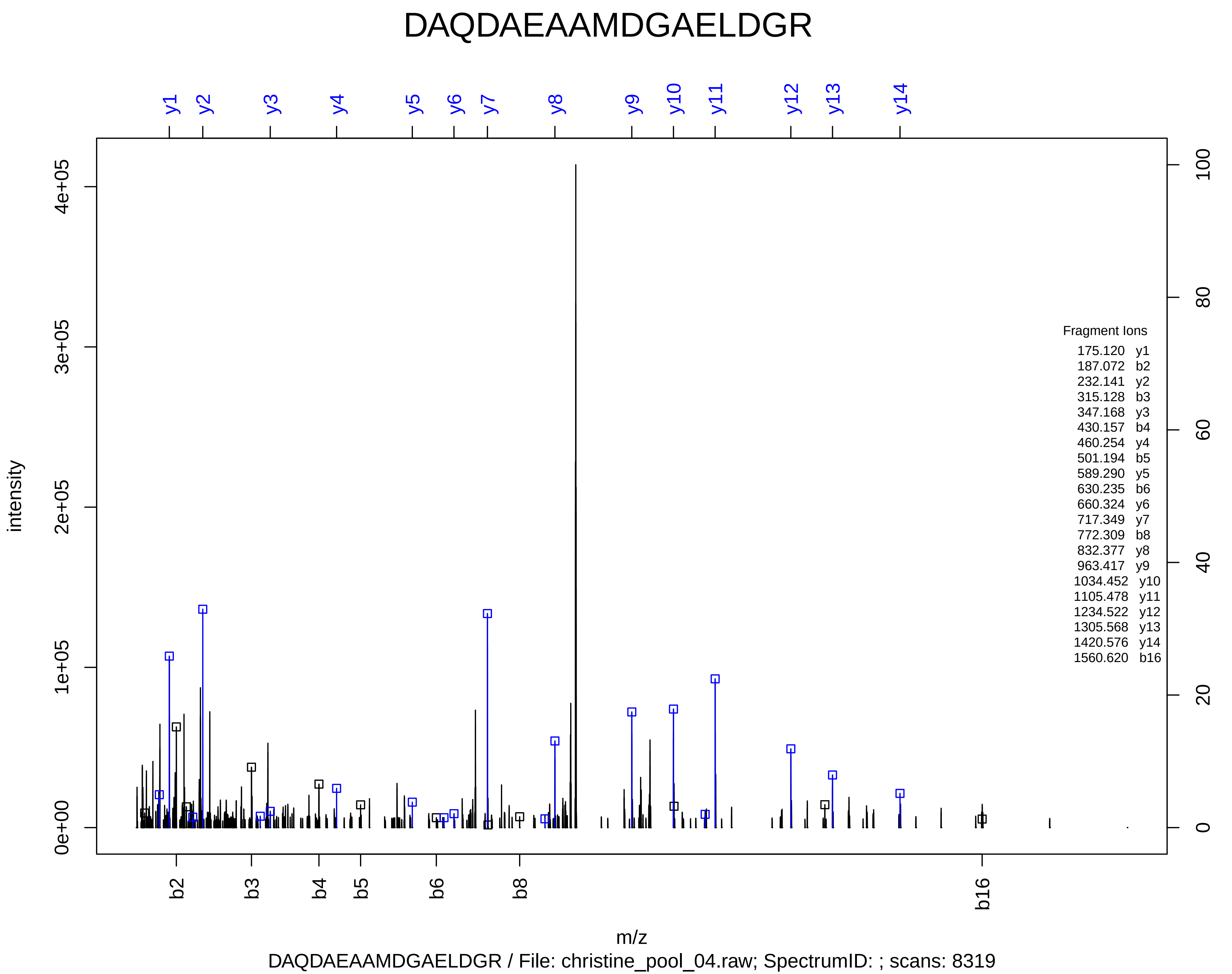
DAQDAEAAMDGAELDGR
y1
y2
y3
y4
y5
y6
y7
y8
y9
y10
y11
y12
y13
y14
intensity
0e+00
1e+05
2e+05
3e+05
4e+05
0
20
40
60
80
100
b2
b3
b4
b5
b6
b8
b16
m/z
Fragment Ions
175.120 y1
187.072 b2
232.141 y2
315.128 b3
347.168 y3
430.157 b4
460.254 y4
501.194 b5
589.290 y5
630.235 b6
660.324 y6
717.349 y7
772.309 b8
832.377 y8
963.417 y9
1034.452 y10
1105.478 y11
1234.522 y12
1305.568 y13
1420.576 y14
1560.620 b16
DAQDAEAAMDGAELDGR / File: christine_pool_04.raw; SpectrumID: ; scans: 8319

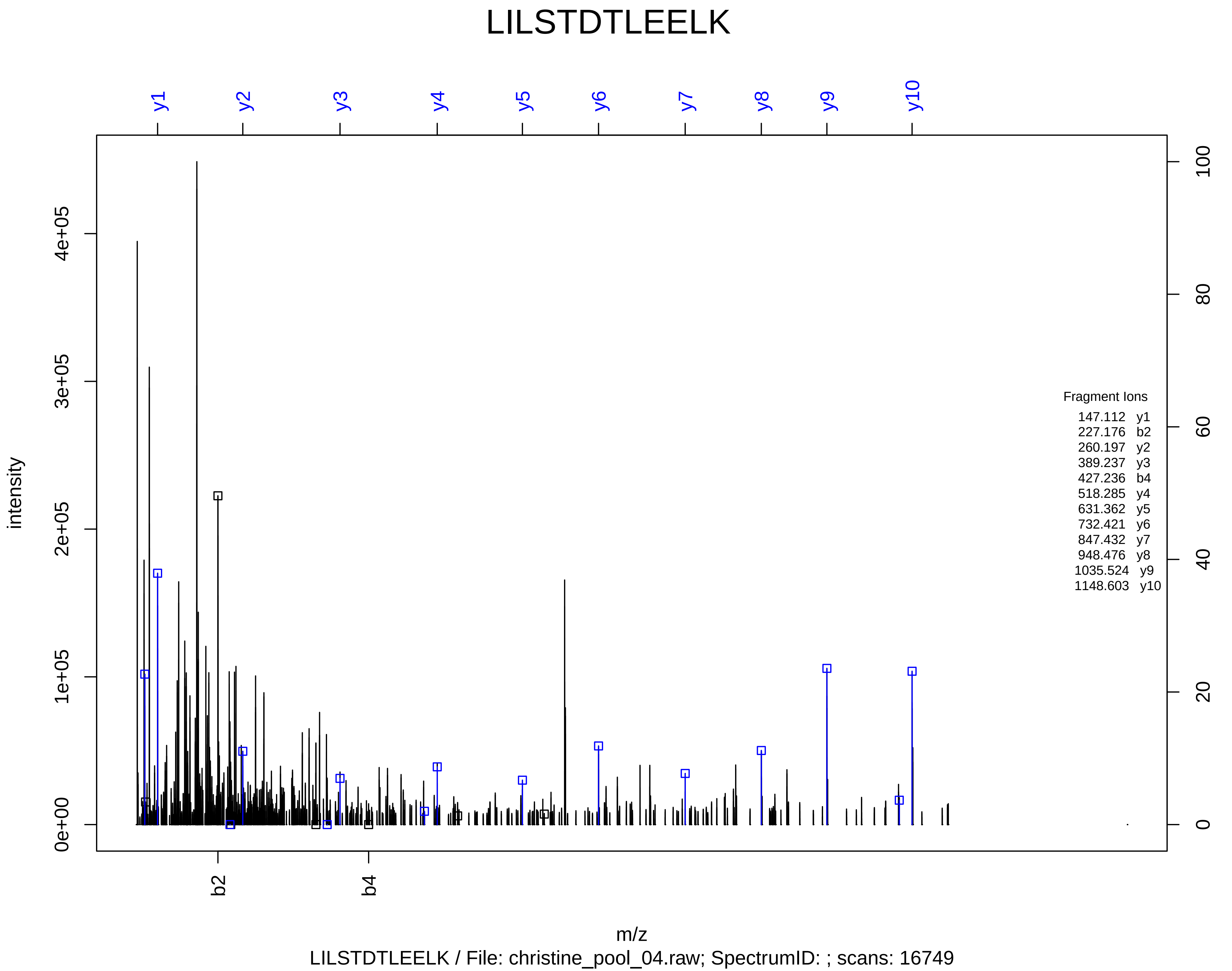

LILSTDTLEELK
y1
y2
y3
y4
y5
y6
y7
y8
y9
y10
intensity
0e+00
1e+05
2e+05
3e+05
4e+05
0
20
40
60
80
100
b2
b4
m/z
Fragment Ions
147.112 y1
227.176 b2
260.197 y2
389.237 y3
427.236 b4
518.285 y4
631.362 y5
732.421 y6
847.432 y7
948.476 y8
1035.524 y9
1148.603 y10
LILSTDTLEELK / File: christine_pool_04.raw; SpectrumID: ; scans: 16749

LGAFFLER

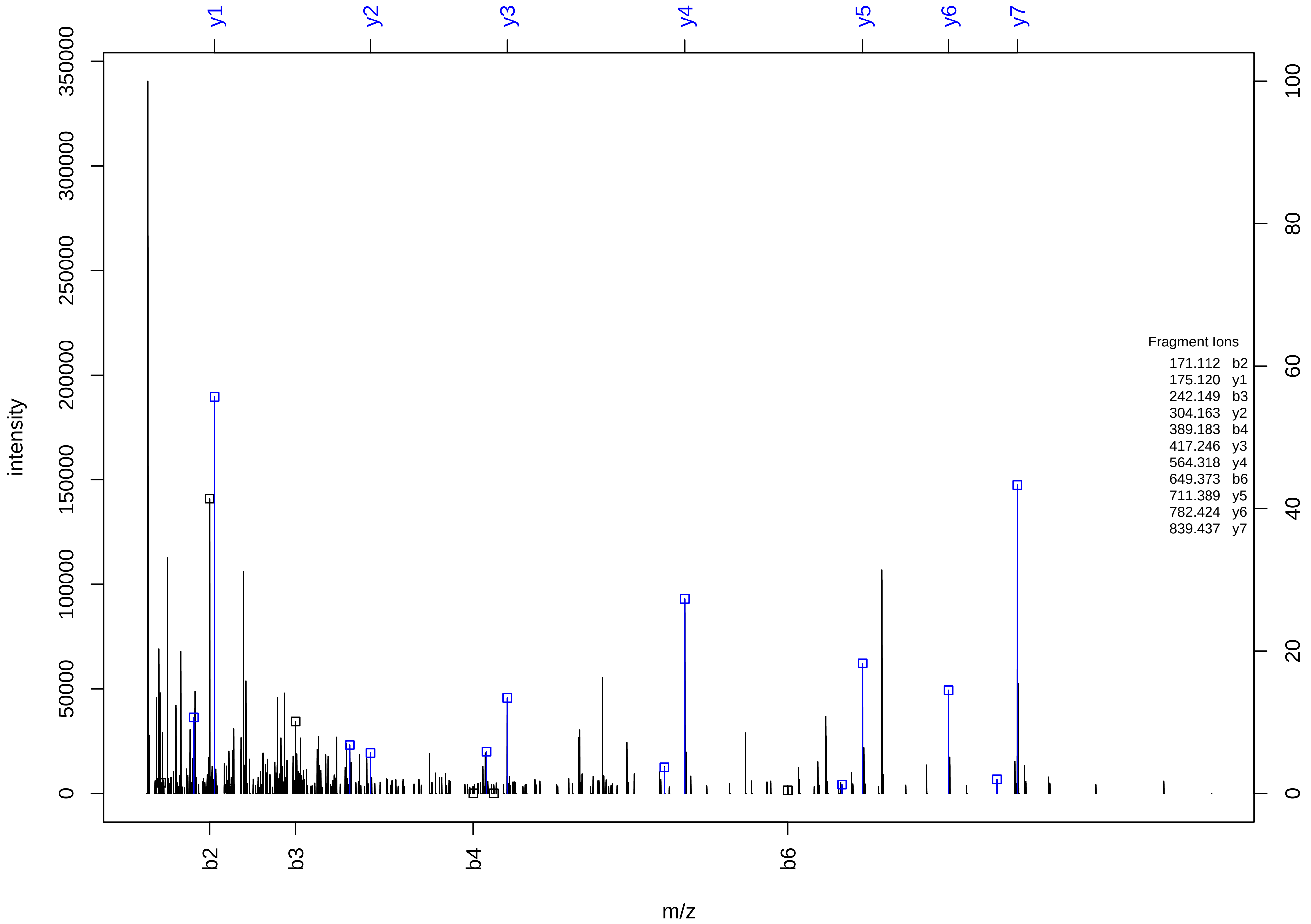

LGAFFLER / File: christine_pool_04.raw; SpectrumID: ; scans: 14897

VNLLSVLEAAK

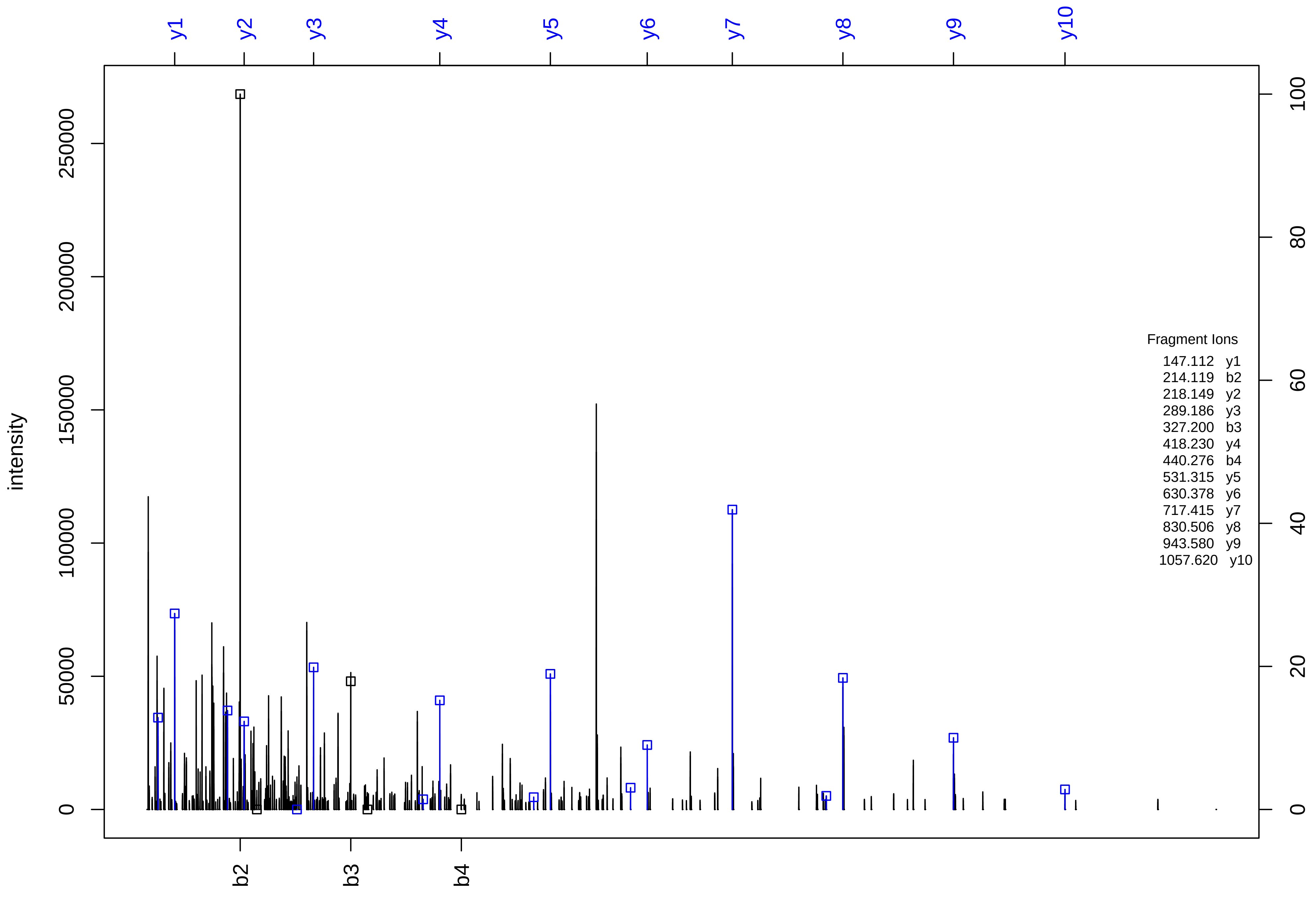

VNLLSVLEAAK / File: christine_pool_04.raw; SpectrumID: ; scans: 20823

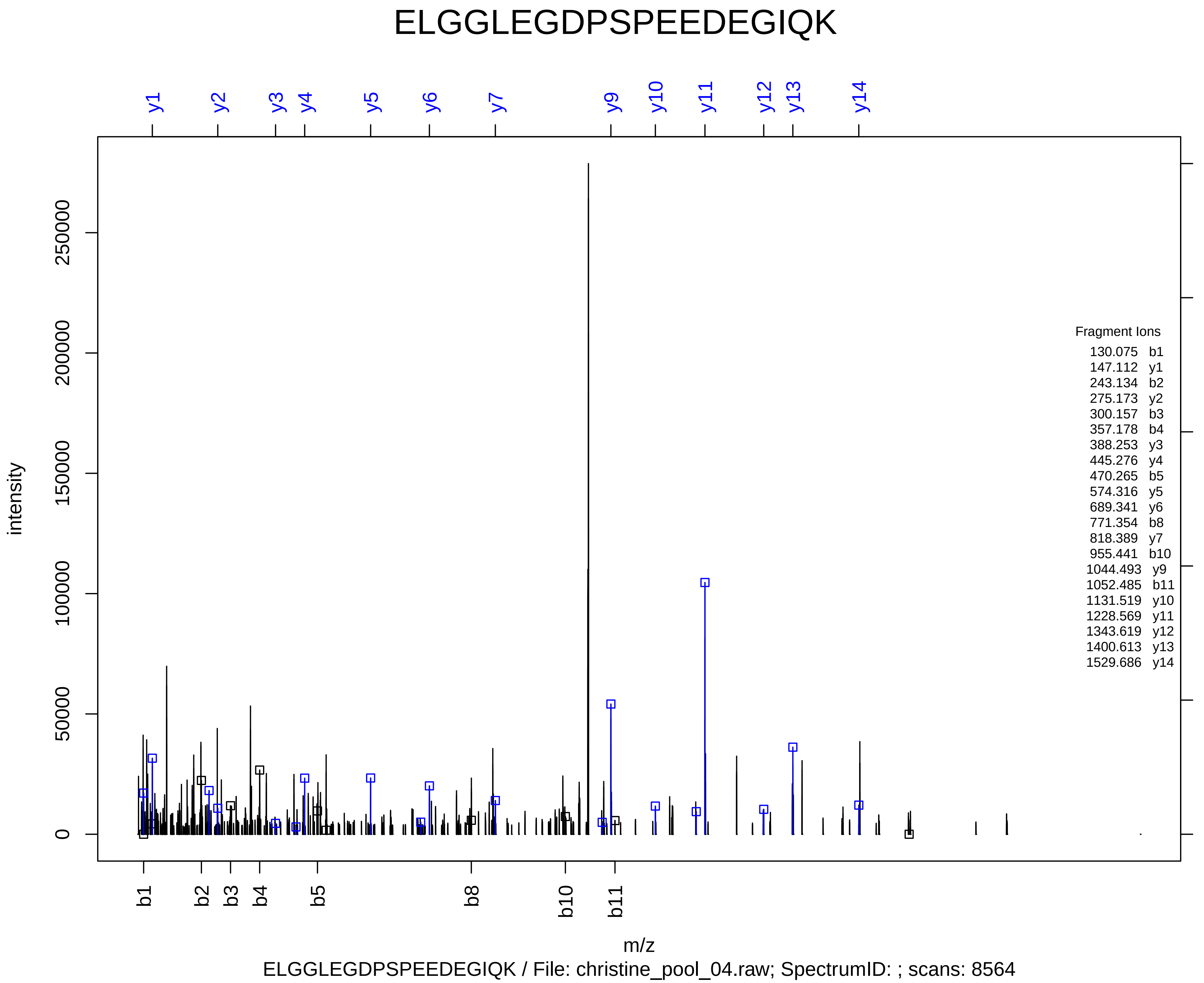

ELGGLEGDPSPEEDEGIQK
y1 y2 y3 y4 y5 y6 y7 y9 y10 y11 y12 y13 y14
intensity
0
50000
100000
150000
200000
250000
0
20
40
60
80
100
b1 b2 b3 b4 b5 b8 b10 b11
m/z
Fragment Ions
130.075 b1
147.112 y1
243.134 b2
275.173 y2
300.157 b3
357.178 b4
388.253 y3
445.276 y4
470.265 b5
574.316 y5
689.341 y6
771.354 b8
818.389 y7
955.441 b10
1044.493 y9
1052.485 b11
1131.519 y10
1228.569 y11
1343.619 y12
1400.613 y13
1529.686 y14
ELGGLEGDPSPEEDEGIQK / File: christine_pool_04.raw; SpectrumID: ; scans: 8564

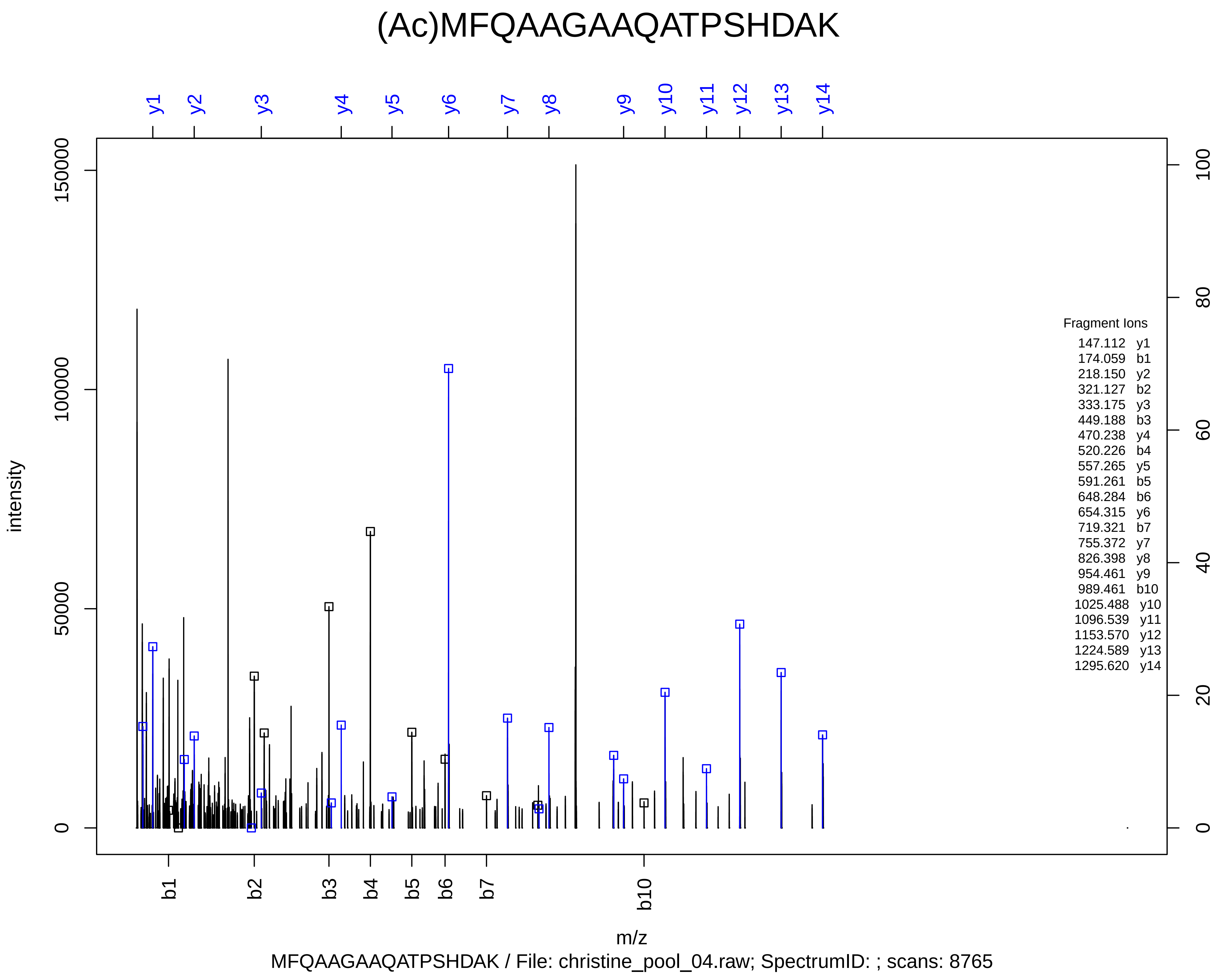

(Ac)MFQAAGAAQATPSHDAK
y1
y2
y3
y4
y5
y6
y7
y8
y9
y10
y11
y12
y13
y14
intensity
0
50000
100000
150000
0
20
40
60
80
100
Fragment Ions
147.112 y1
174.059 b1
218.150 y2
321.127 b2
333.175 y3
449.188 b3
470.238 y4
520.226 b4
557.265 y5
591.261 b5
648.284 b6
654.315 y6
719.321 b7
755.372 y7
826.398 y8
954.461 y9
989.461 b10
1025.488 y10
1096.539 y11
1153.570 y12
1224.589 y13
1295.620 y14
b1
b2
b3
b4
b5
b6
b7
b10
m/z
MFQAAGAAQATPSHDAK / File: christine_pool_04.raw; SpectrumID: ; scans: 8765

VDELSLYSVPEGQSK

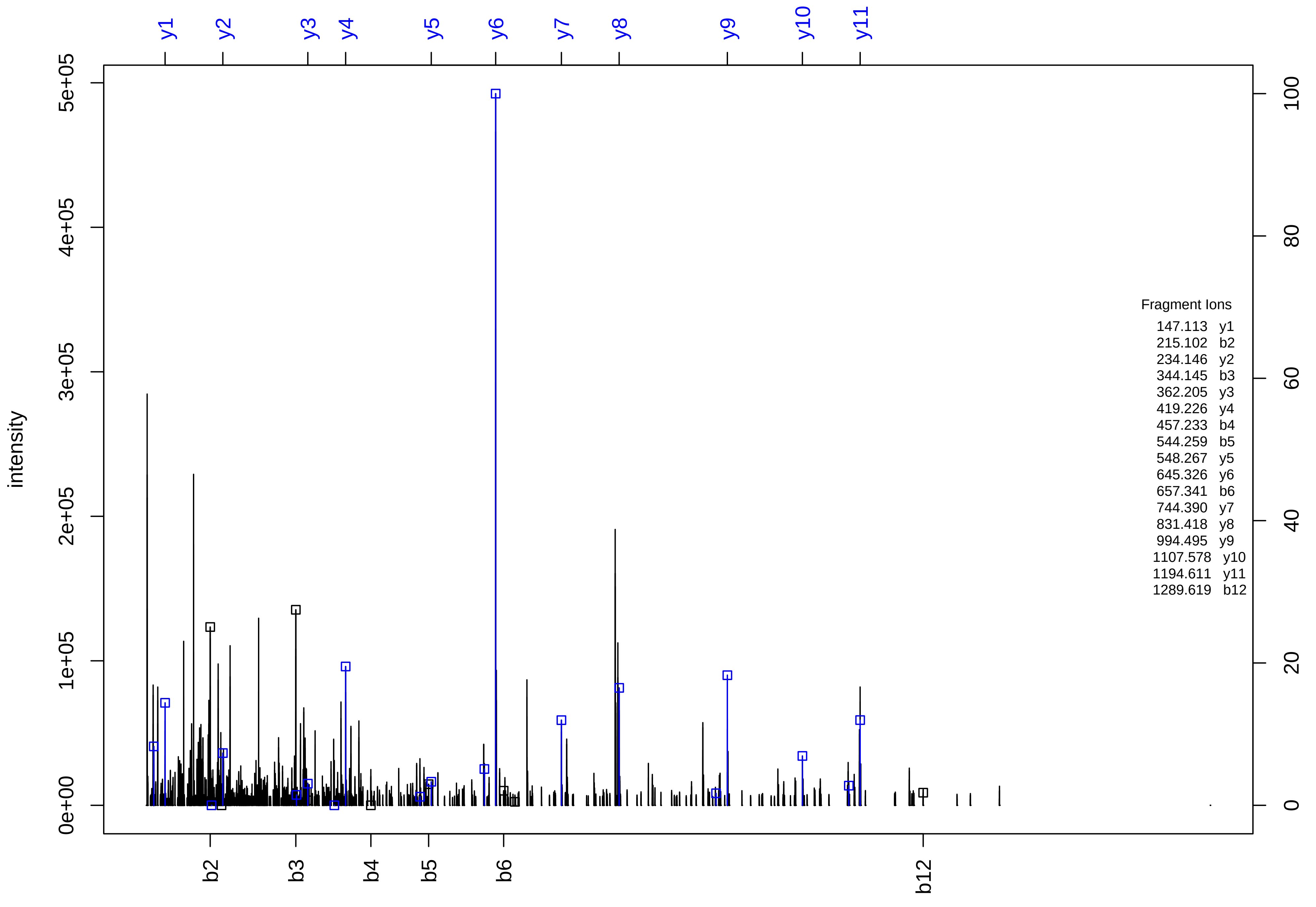

VDELSLYSVPEGQSK / File: christine_pool_04.raw; SpectrumID: ; scans: 11785

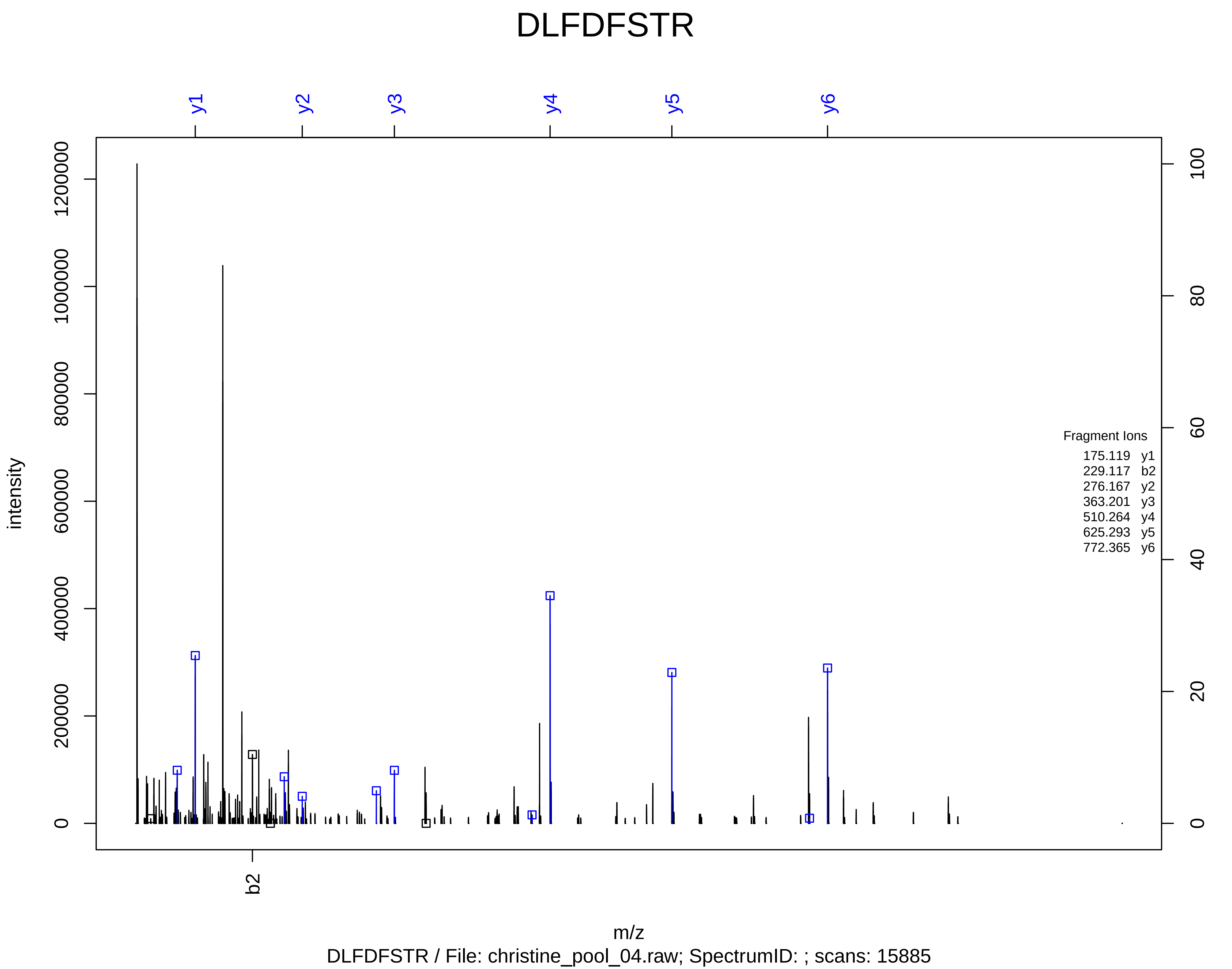
DLFDFSTR
y1
y2
y3
y4
y5
y6
b2
intensity
m/z
0
200000
400000
600000
800000
1000000
1200000
0
20
40
60
80
100
Fragment Ions
175.119 y1
229.117 b2
276.167 y2
363.201 y3
510.264 y4
625.293 y5
772.365 y6
DLFDFSTR / File: christine_pool_04.raw; SpectrumID: ; scans: 15885

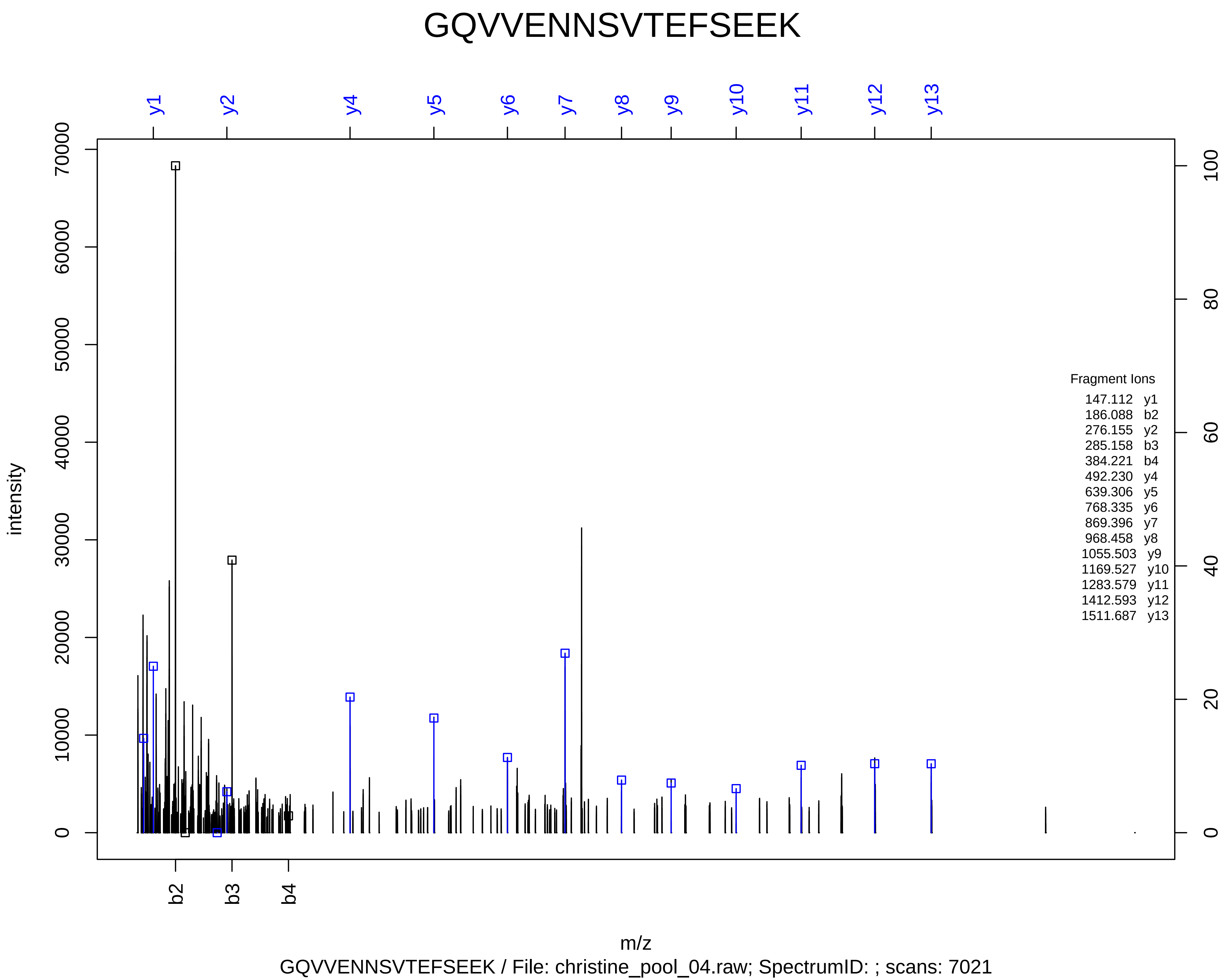
GQVVENNSVTEFSEEK
y1
y2
y4
y5
y6
y7
y8
y9
y10
y11
y12
y13
intensity
0
10000
20000
30000
40000
50000
60000
70000
0
20
40
60
80
100
b2
b3
b4
m/z
Fragment Ions
147.112 y1
186.088 b2
276.155 y2
285.158 b3
384.221 b4
492.230 y4
639.306 y5
768.335 y6
869.396 y7
968.458 y8
1055.503 y9
1169.527 y10
1283.579 y11
1412.593 y12
1511.687 y13
GQVVENNSVTEFSEEK / File: christine_pool_04.raw; SpectrumID: ; scans: 7021

RPVHGESDTEQLQDDDIETTK

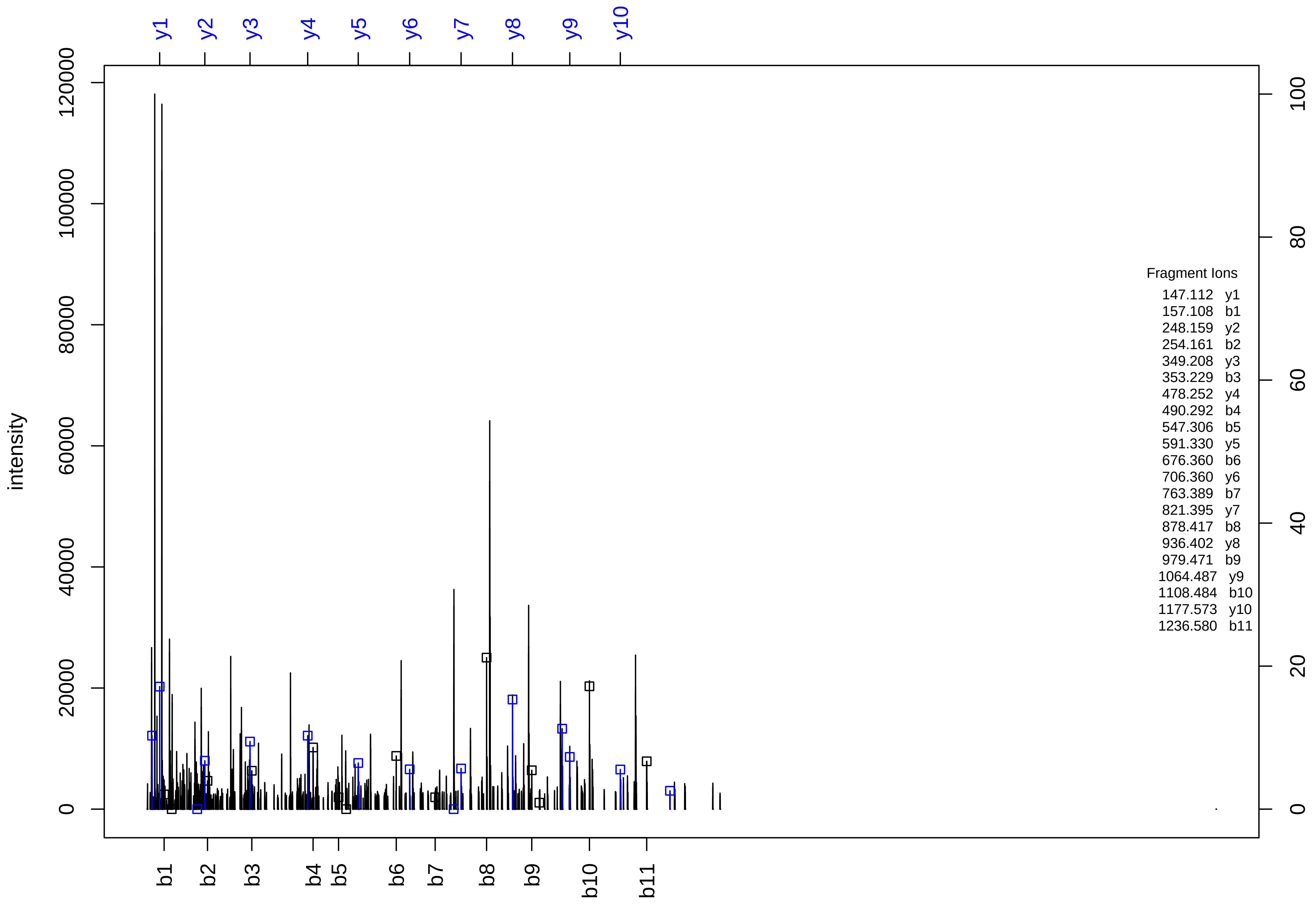

RPVHGESDTEQLQDDDIETTK / File: christine_pool_04.raw; SpectrumID: ; scans: 3987

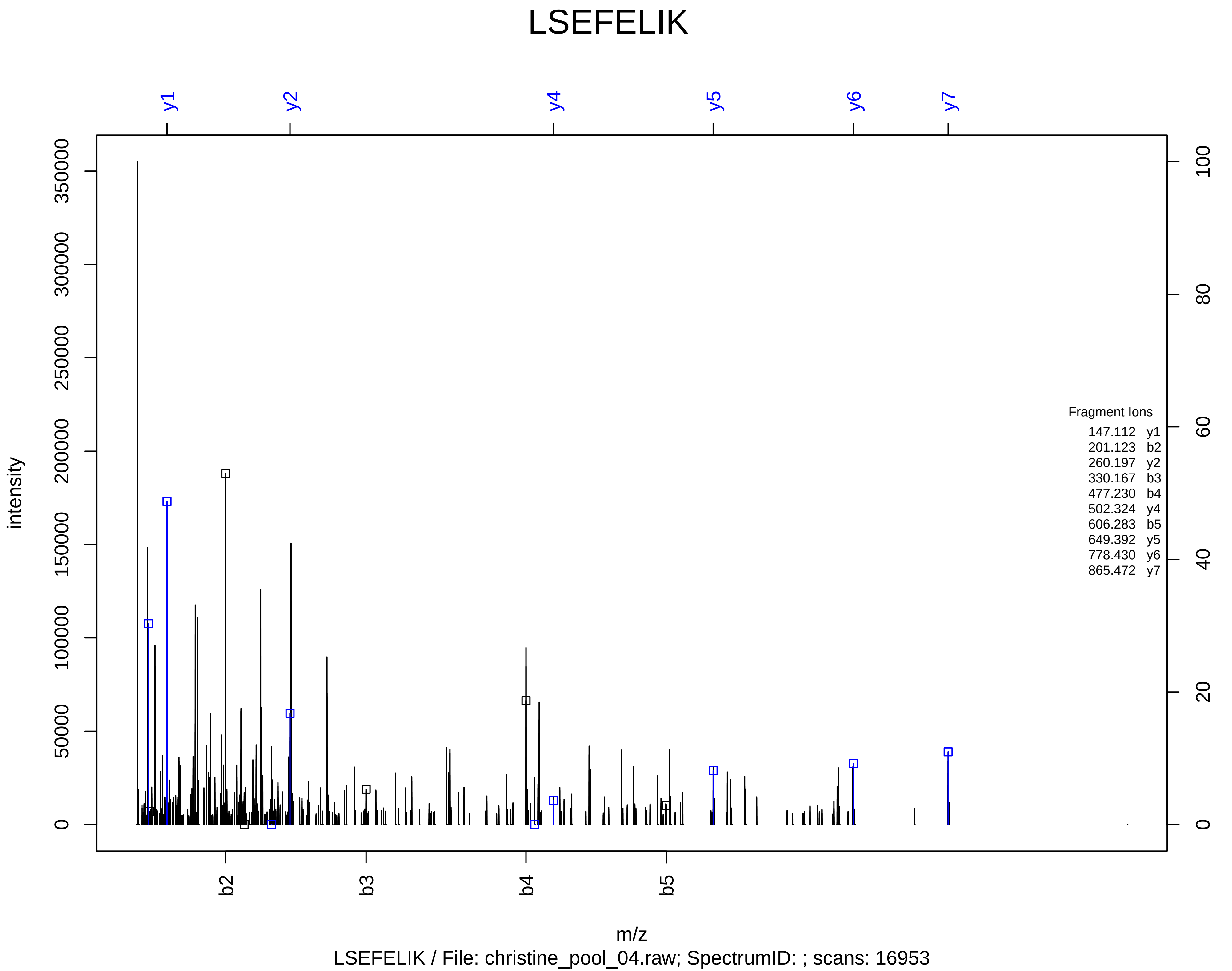

LSEFELIK
y1
y2
y4
y5
y6
y7
intensity
0
50000
100000
150000
200000
250000
300000
350000
0
20
40
60
80
100
b2
b3
b4
b5
m/z
Fragment Ions
147.112 y1
201.123 b2
260.197 y2
330.167 b3
477.230 b4
502.324 y4
606.283 b5
649.392 y5
778.430 y6
865.472 y7
LSEFELIK / File: christine_pool_04.raw; SpectrumID: ; scans: 16953

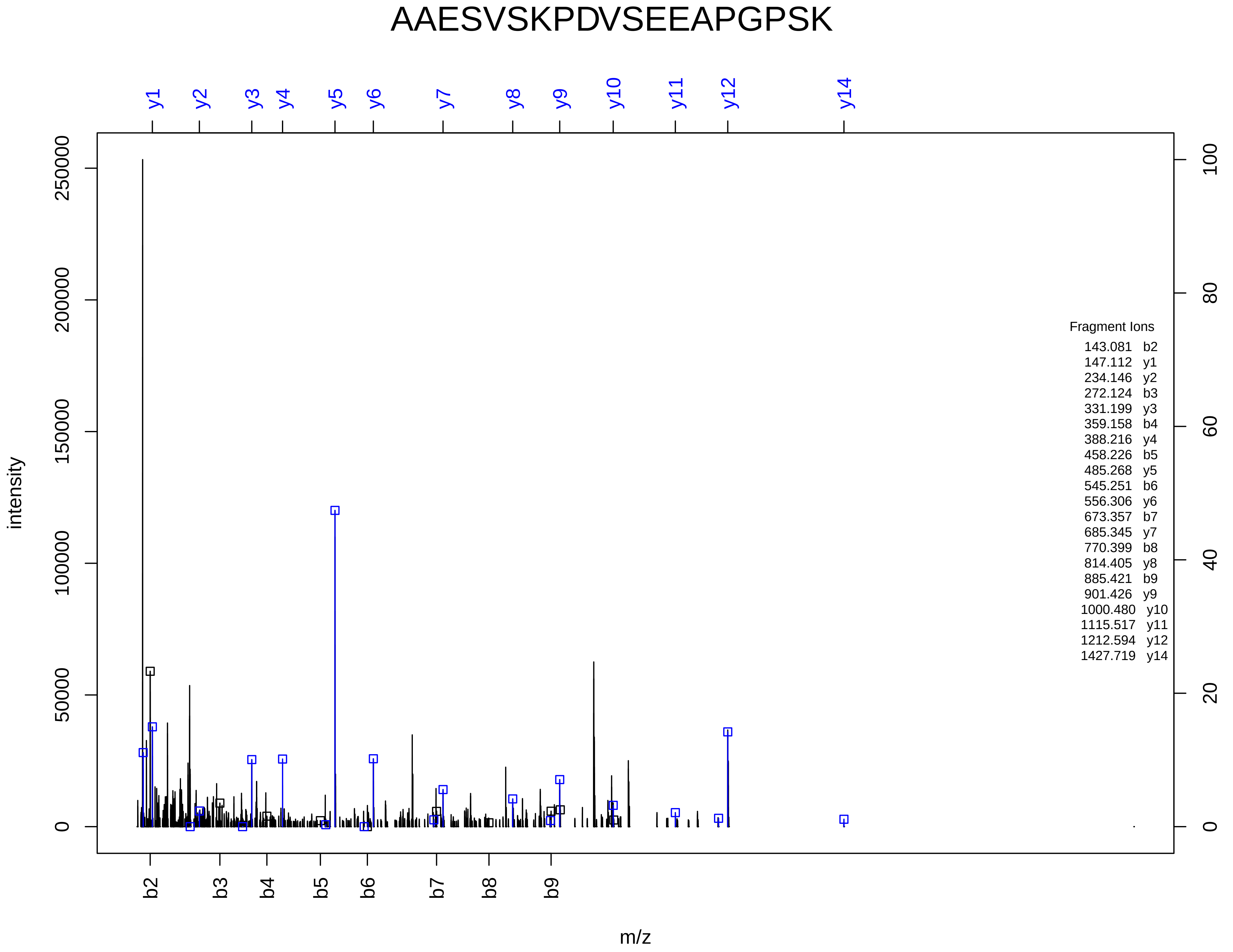

AAESVSKPDVSEEAPGPSK
y1
y2
y3
y4
y5
y6
y7
y8
y9
y10
y11
y12
y14
intensity
0
50000
100000
150000
200000
250000
0
20
40
60
80
100
Fragment Ions
143.081 b2
147.112 y1
234.146 y2
272.124 b3
331.199 y3
359.158 b4
388.216 y4
458.226 b5
485.268 y5
545.251 b6
556.306 y6
673.357 b7
685.345 y7
770.399 b8
814.405 y8
885.421 b9
901.426 y9
1000.480 y10
1115.517 y11
1212.594 y12
1427.719 y14
b2
b3
b4
b5
b6
b7
b8
b9
m/z
AAESVSKPDVSEEAPGPSK / File: christine_pool_04.raw; SpectrumID: ; scans: 3788

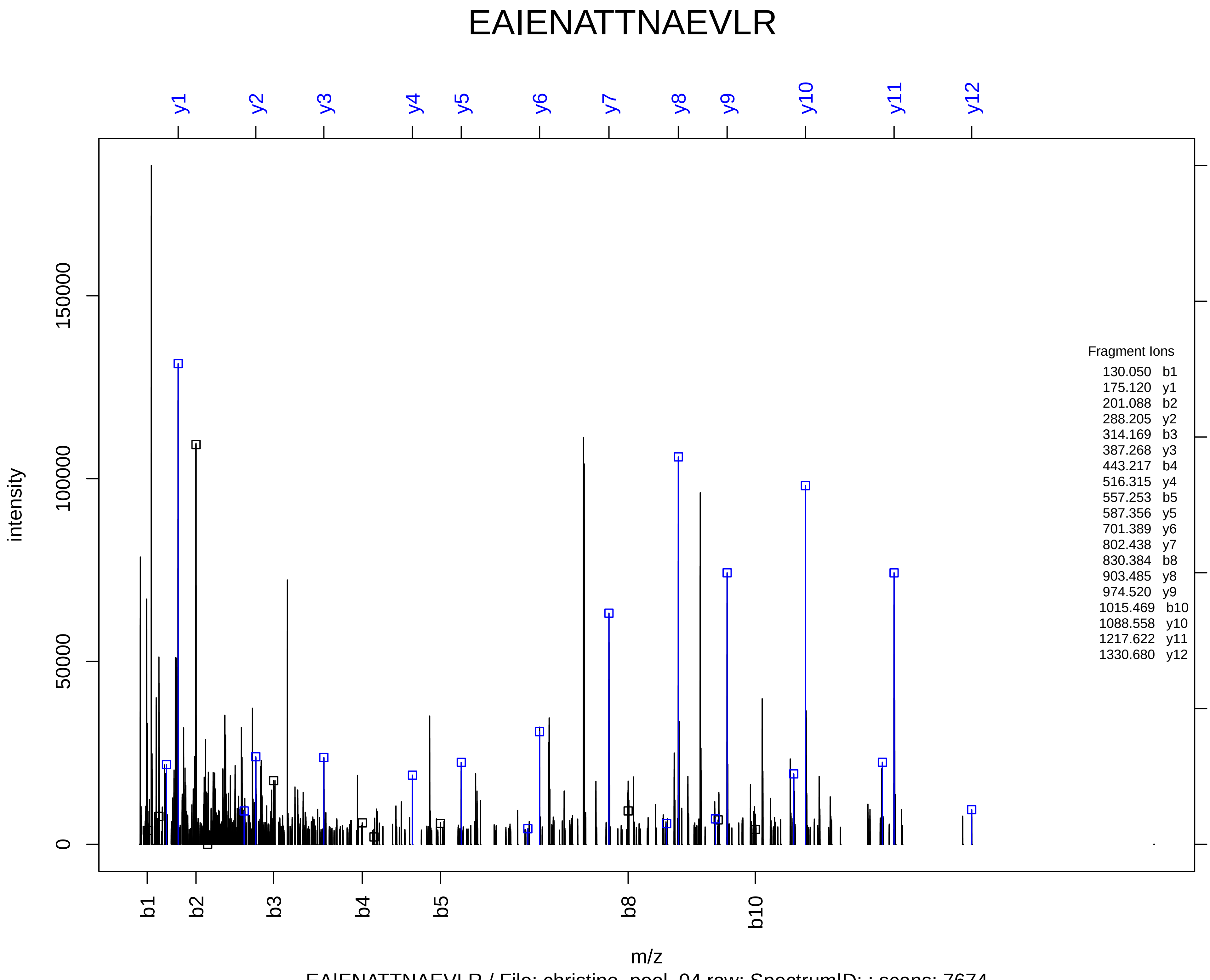

EAIENATTNAEVLR / File: christine_pool_04.raw; SpectrumID: ; scans: 7674

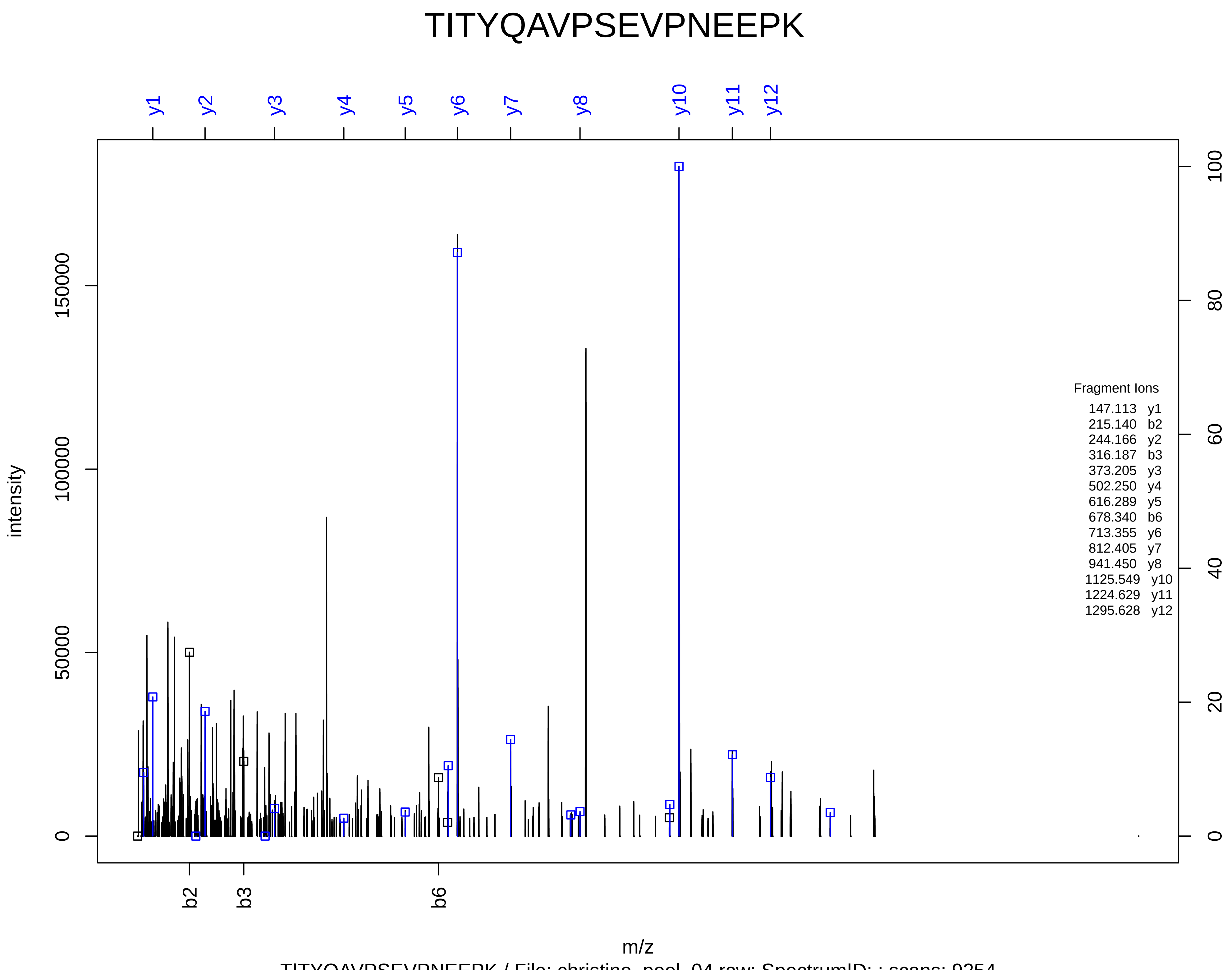

TITYQAVPSEVPNEEPK
y1
y2
y3
y4
y5
y6
y7
y8
y10
y11
y12
b2
b3
b6
intensity
0
50000
100000
150000
0
20
40
60
80
100
Fragment Ions
147.113 y1
215.140 b2
244.166 y2
316.187 b3
373.205 y3
502.250 y4
616.289 y5
678.340 b6
713.355 y6
812.405 y7
941.450 y8
1125.549 y10
1224.629 y11
1295.628 y12
m/z
TITYQAVPSEVPNEEPK / File: christine_pool_04.raw; SpectrumID: ; scans: 9254

M*MGLGLM*AKDKDK

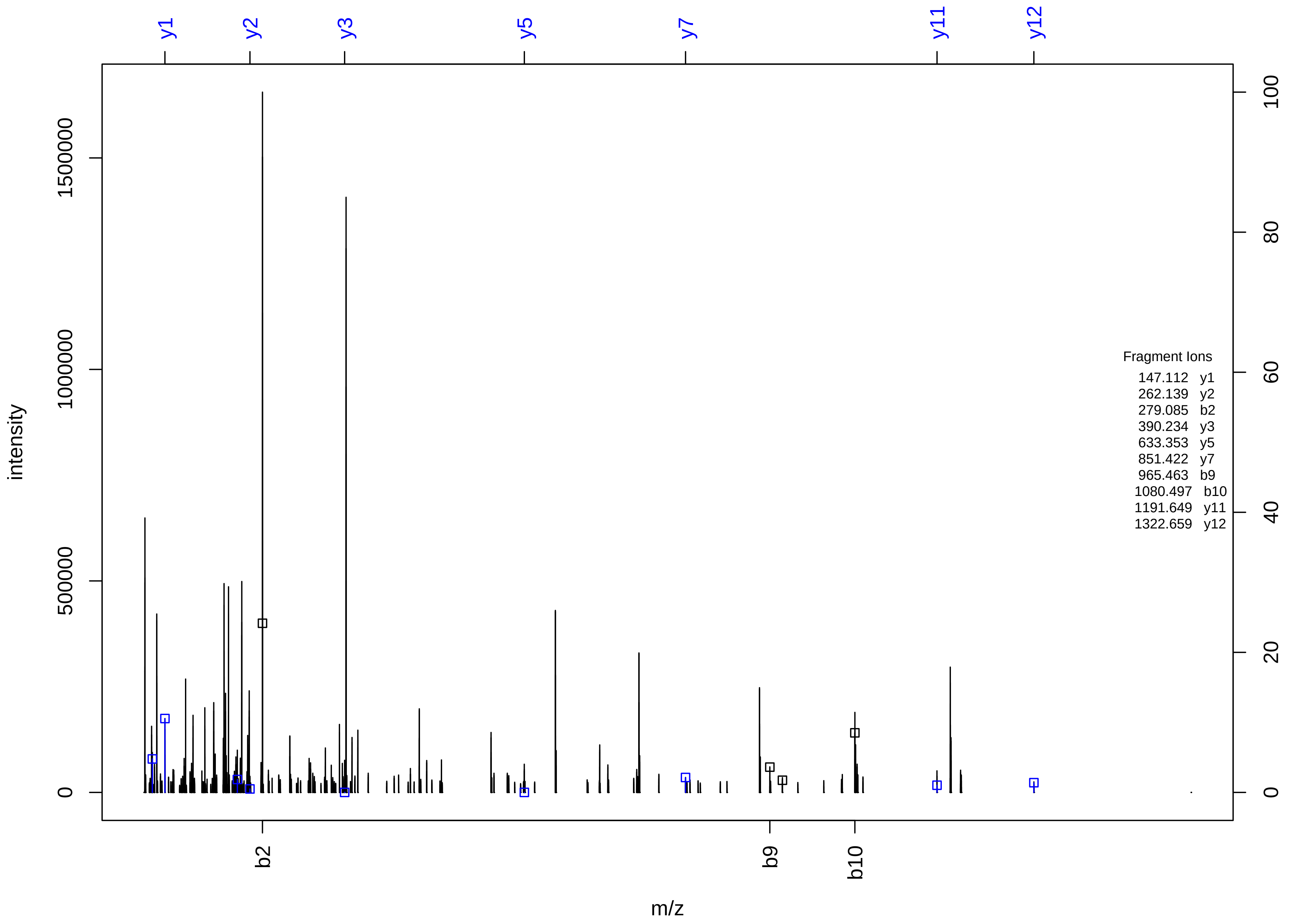

MMGLGLMAKDKDK / File: christine_pool_04.raw; SpectrumID: ; scans: 12011

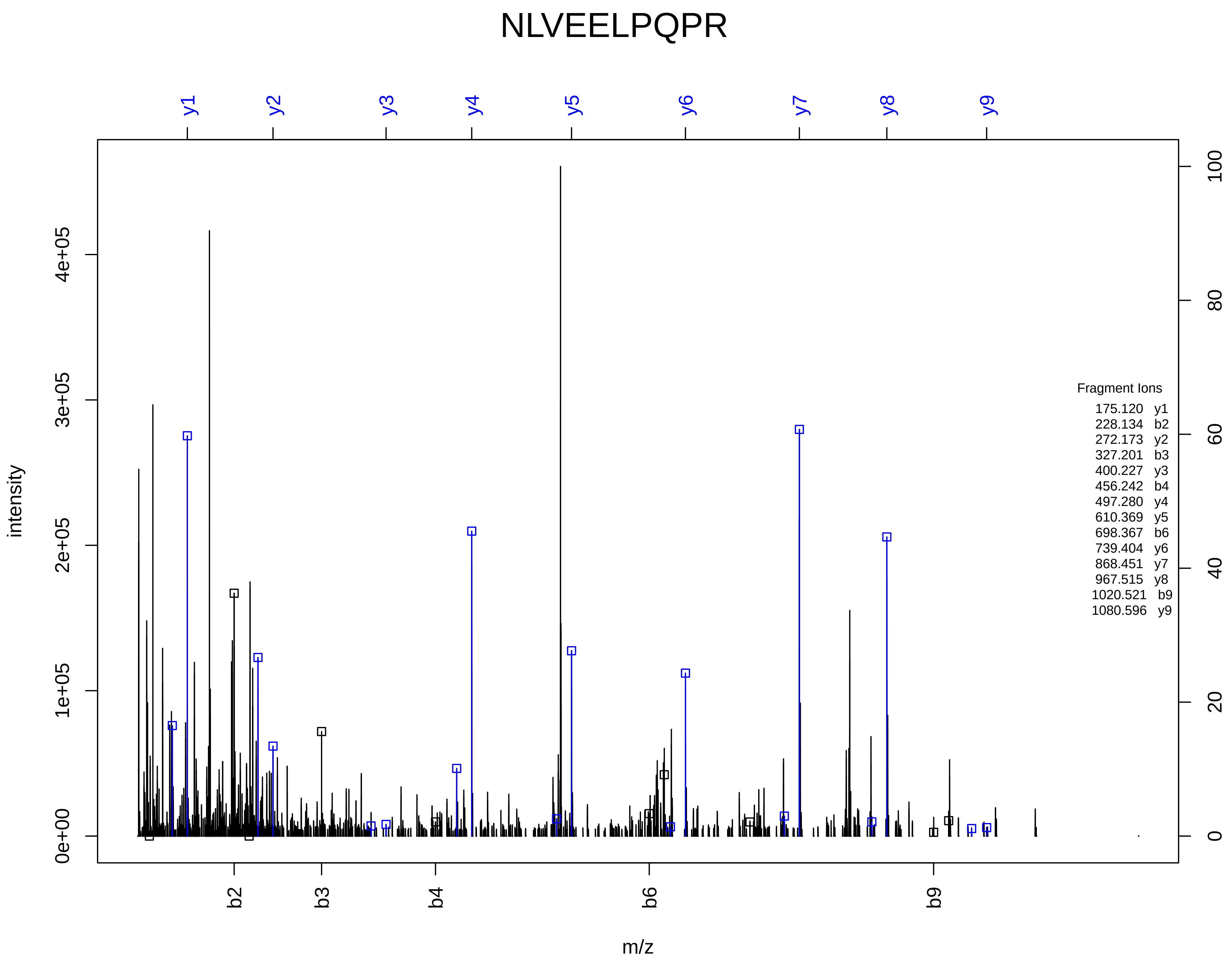

NLVEELPQPR
y1
y2
y3
y4
y5
y6
y7
y8
y9
intensity
0e+00
1e+05
2e+05
3e+05
4e+05
0
20
40
60
80
100
b2
b3
b4
b6
b9
m/z
Fragment Ions
175.120 y1
228.134 b2
272.173 y2
327.201 b3
400.227 y3
456.242 b4
497.280 y4
610.369 y5
698.367 b6
739.404 y6
868.451 y7
967.515 y8
1020.521 b9
1080.596 y9
NLVEELPQPR / File: christine_pool_04.raw; SpectrumID: ; scans: 10170

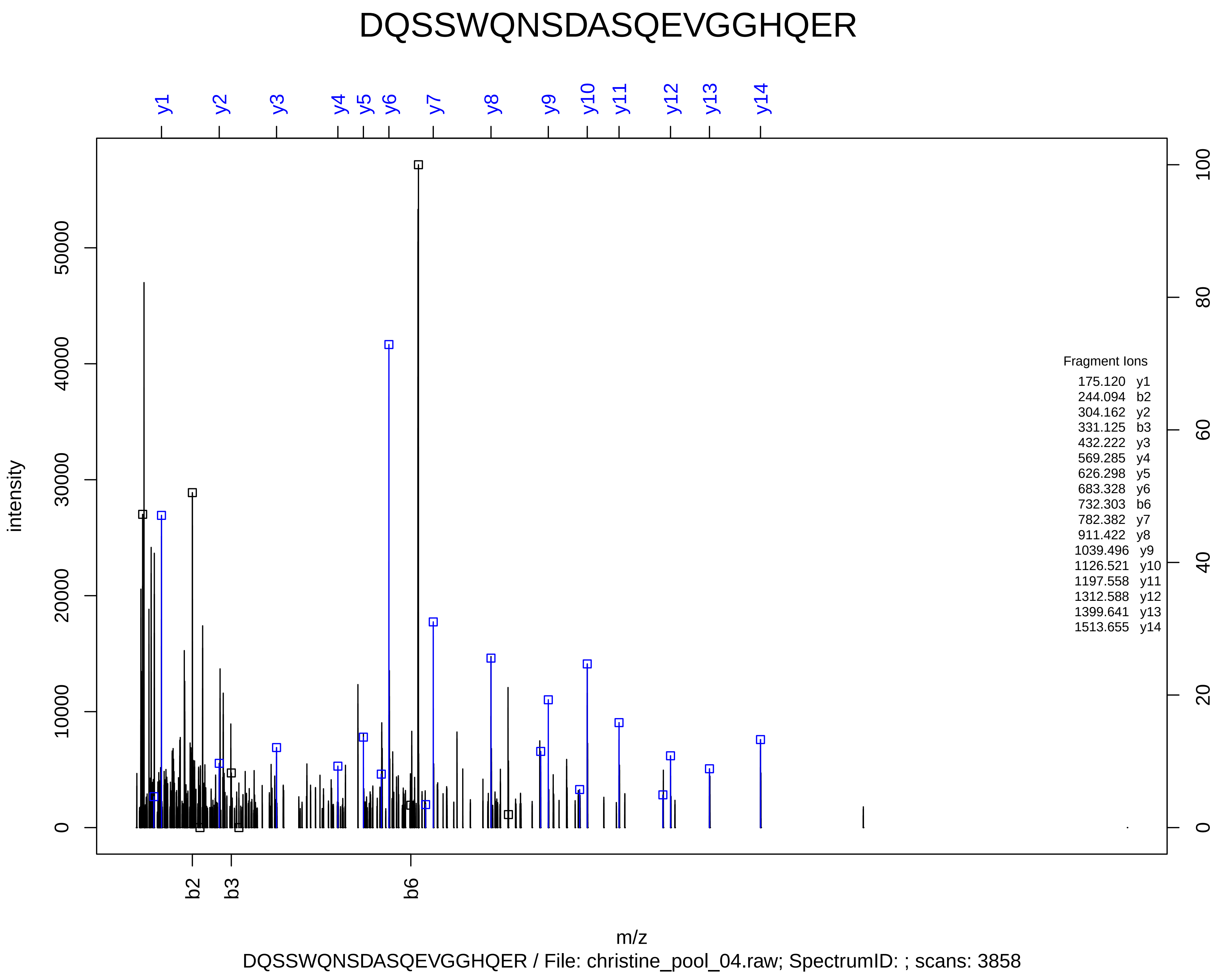
DQSSWQNSDASQEVGGHQER
y1
y2
y3
y4
y5
y6
y7
y8
y9
y10
y11
y12
y13
y14
b2
b3
b6
intensity
0
10000
20000
30000
40000
50000
0
20
40
60
80
100
Fragment Ions
175.120 y1
244.094 b2
304.162 y2
331.125 b3
432.222 y3
569.285 y4
626.298 y5
683.328 y6
732.303 b6
782.382 y7
911.422 y8
1039.496 y9
1126.521 y10
1197.558 y11
1312.588 y12
1399.641 y13
1513.655 y14
m/z
DQSSWQNSDASQEVGGHQER / File: christine_pool_04.raw; SpectrumID: ; scans: 3858

LLADVTHLLLK

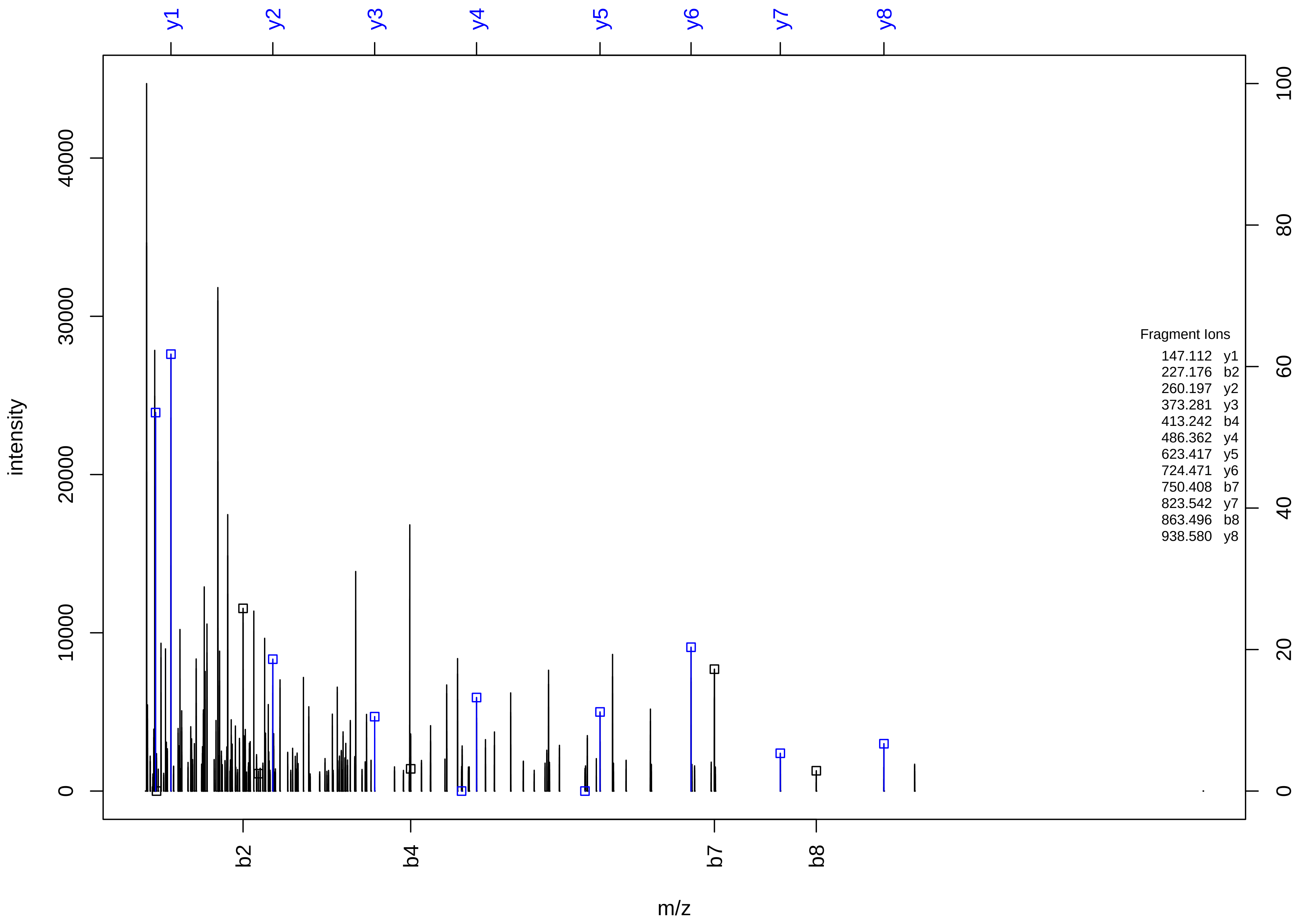

LLADVTHLLLK / File: christine_pool_04.raw; SpectrumID: ; scans: 17880

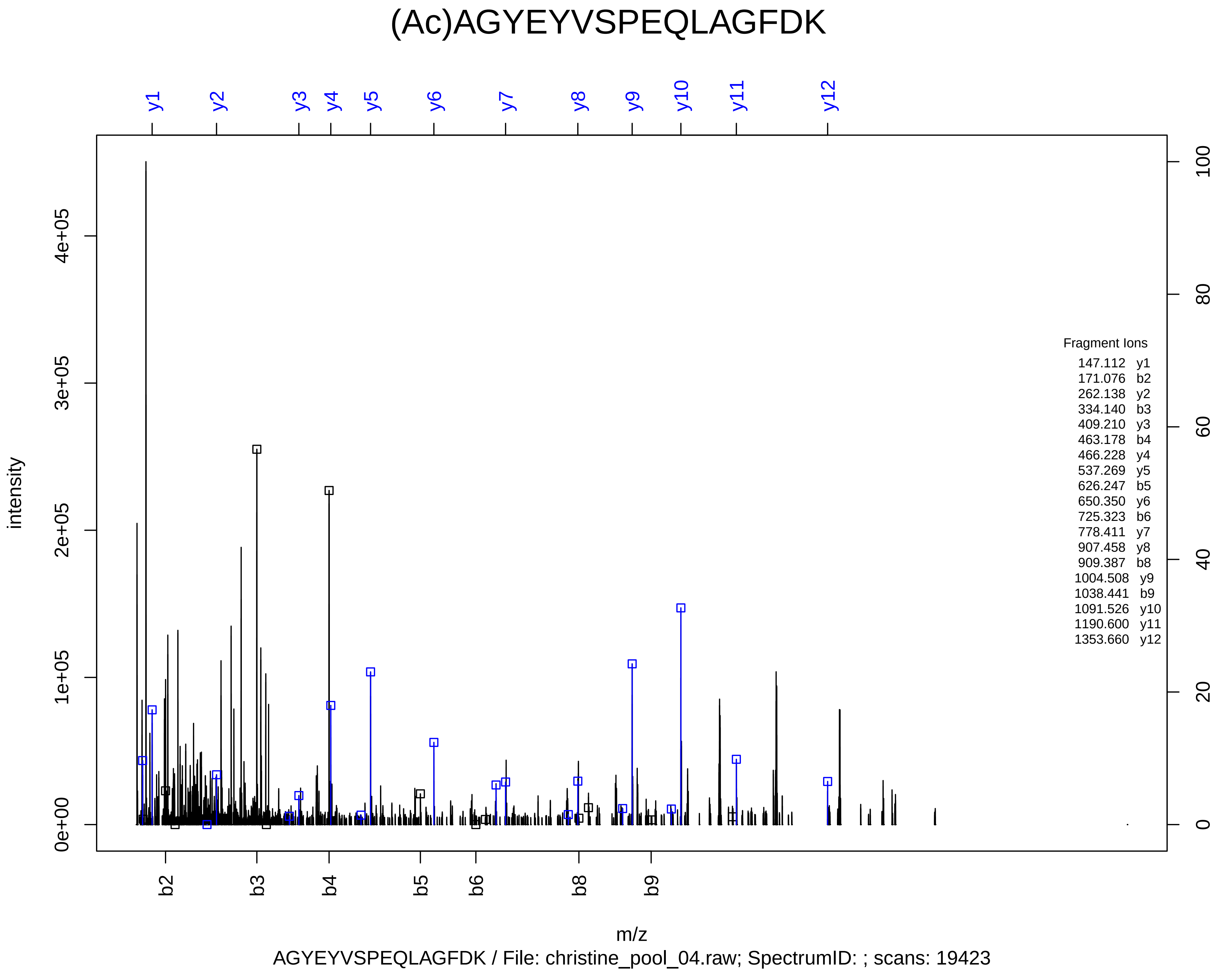

(Ac)AGYEYVSPEQLAGFDK
intensity
m/z
Fragment Ions
147.112 y1
171.076 b2
262.138 y2
334.140 b3
409.210 y3
463.178 b4
466.228 y4
537.269 y5
626.247 b5
650.350 y6
725.323 b6
778.411 y7
907.458 y8
909.387 b8
1004.508 y9
1038.441 b9
1091.526 y10
1190.600 y11
1353.660 y12
AGYEYVSPEQLAGFDK / File: christine_pool_04.raw; SpectrumID: ; scans: 19423

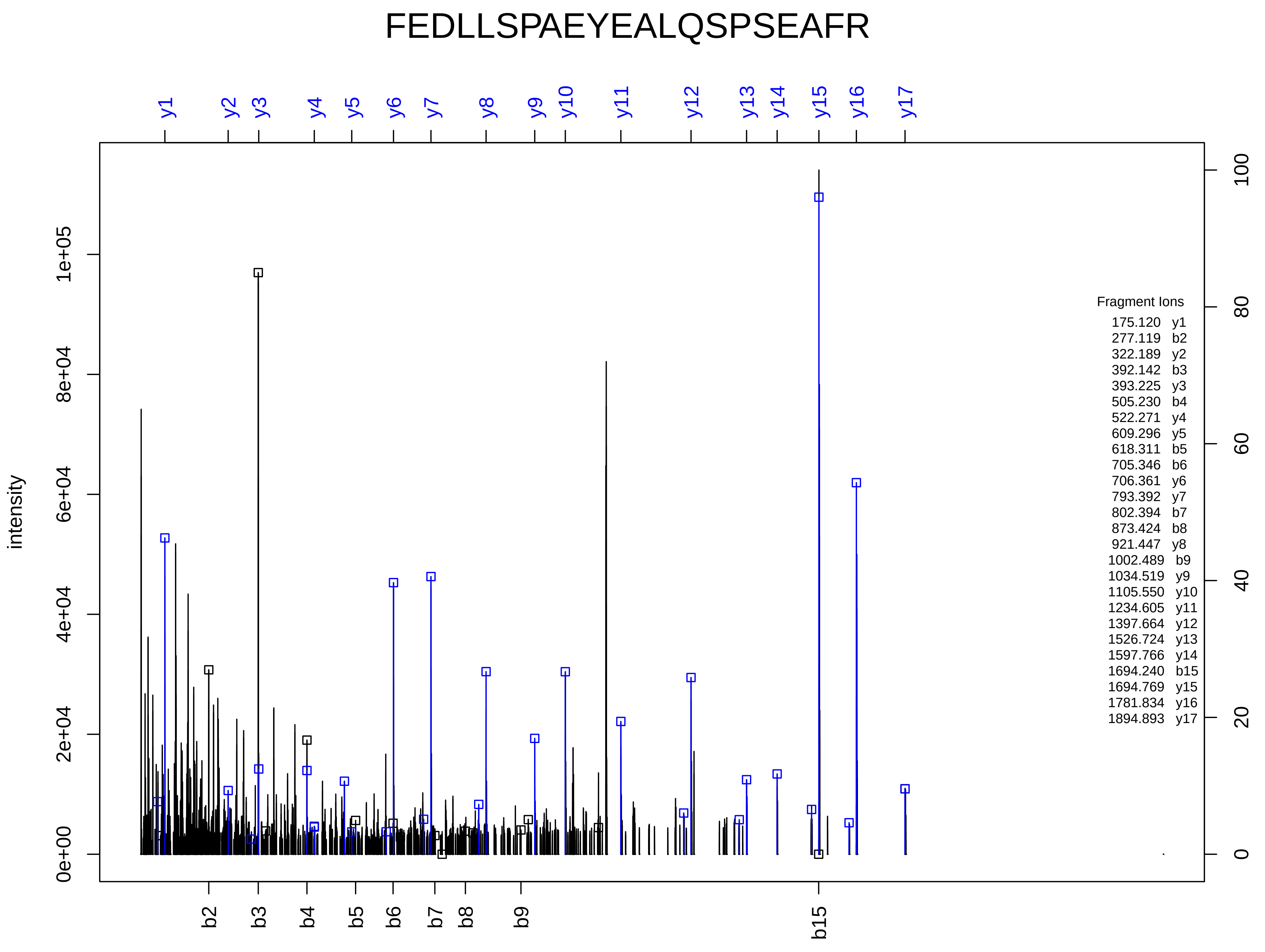

FEDLLSPAEYEALQSPSEAFR / File: christine_pool_04.raw; SpectrumID: ; scans: 20904

AGAPADQGAEAADNQREEAADNQR

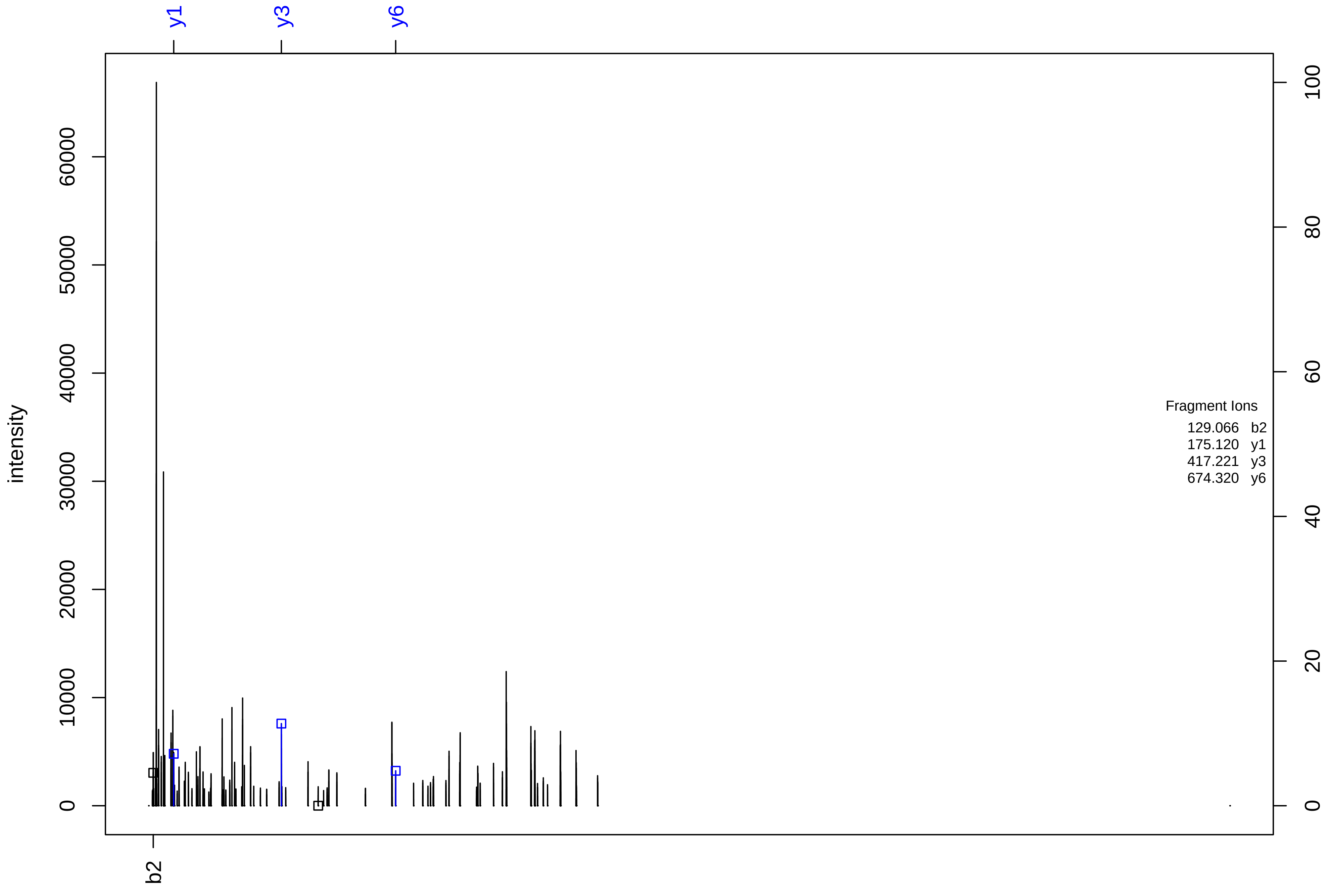

AGAPADQGAEAADNQREEAADNQR / File: christine_pool_04.raw; SpectrumID: ; scans: 2556

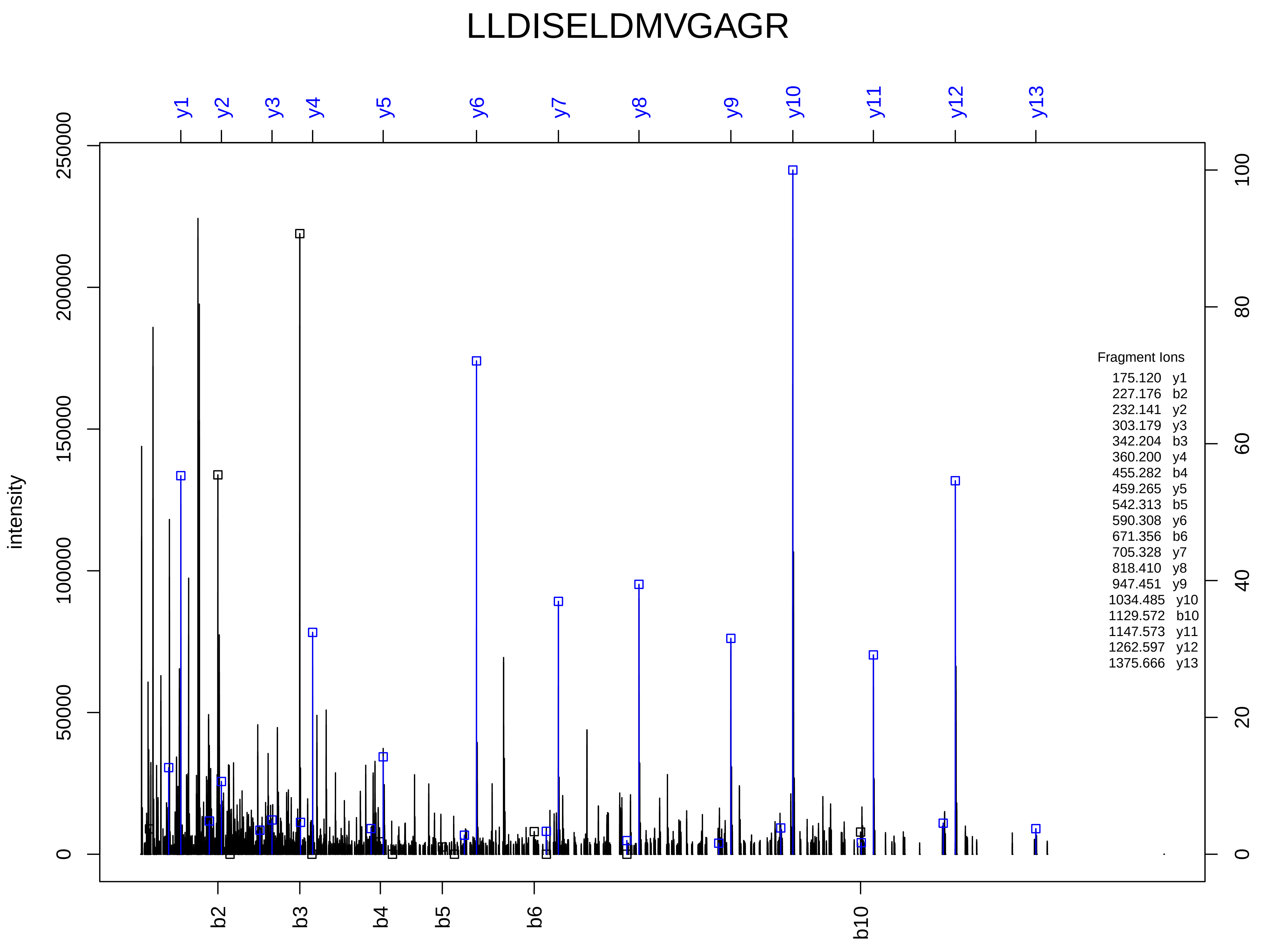
LLDISELDMVGAGR
y1 y2 y3 y4 y5 y6 y7 y8 y9 y10 y11 y12 y13
b2 b3 b4 b5 b6 b10
intensity
m/z
Fragment Ions
175.120 y1
227.176 b2
232.141 y2
303.179 y3
342.204 b3
360.200 y4
455.282 b4
459.265 y5
542.313 b5
590.308 y6
671.356 b6
705.328 y7
818.410 y8
947.451 y9
1034.485 y10
1129.572 b10
1147.573 y11
1262.597 y12
1375.666 y13
LLDISELDMVGAGR / File: christine_pool_04.raw; SpectrumID: ; scans: 18923

AGGPPQVAGAQAACSEDR

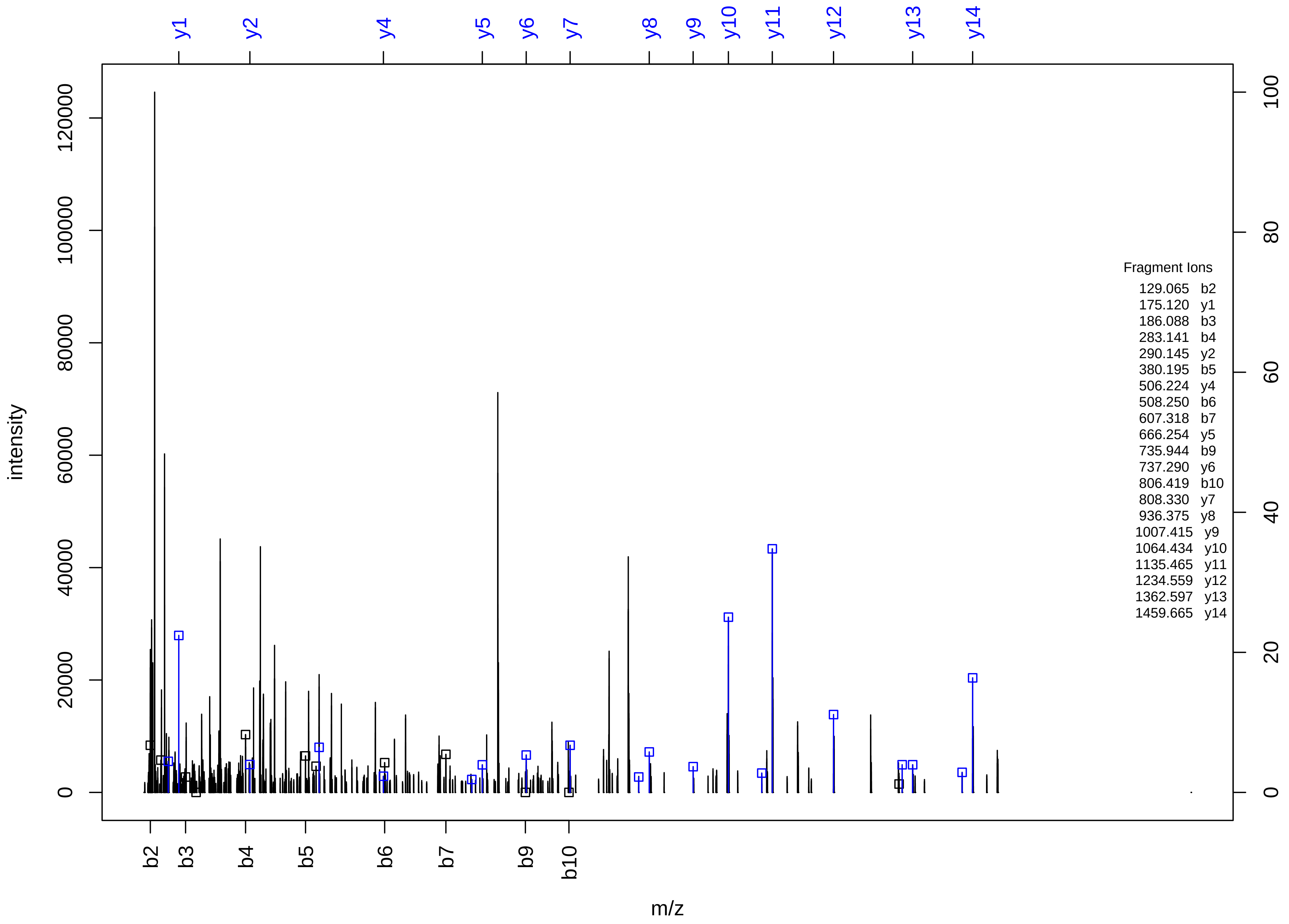

AGGPPQVAGAQAACSEDR / File: christine_pool_04.raw; SpectrumID: ; scans: 4046

FDAELLEIK

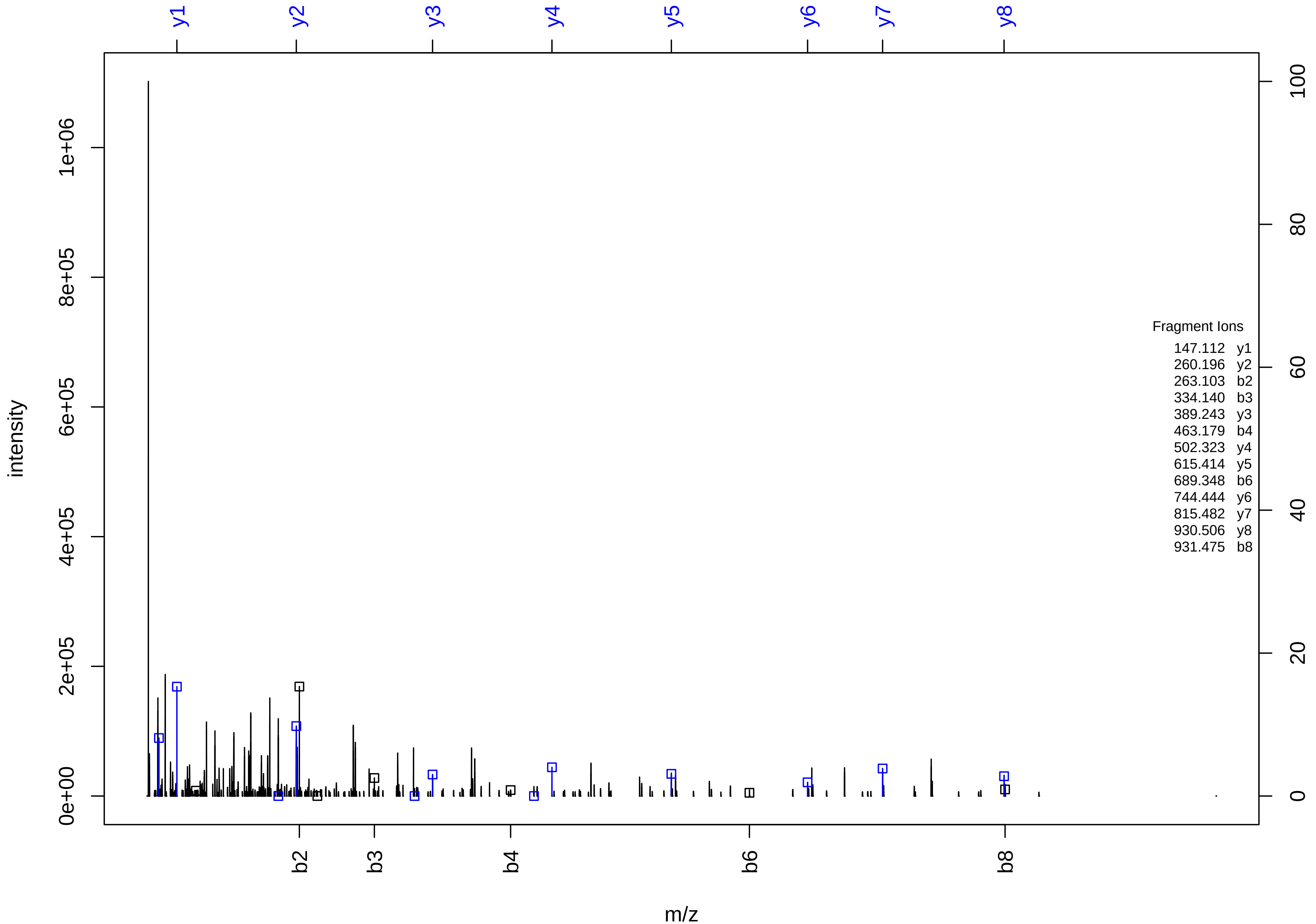

FDAELLEIK / File: christine_pool_04.raw; SpectrumID: ; scans: 15331

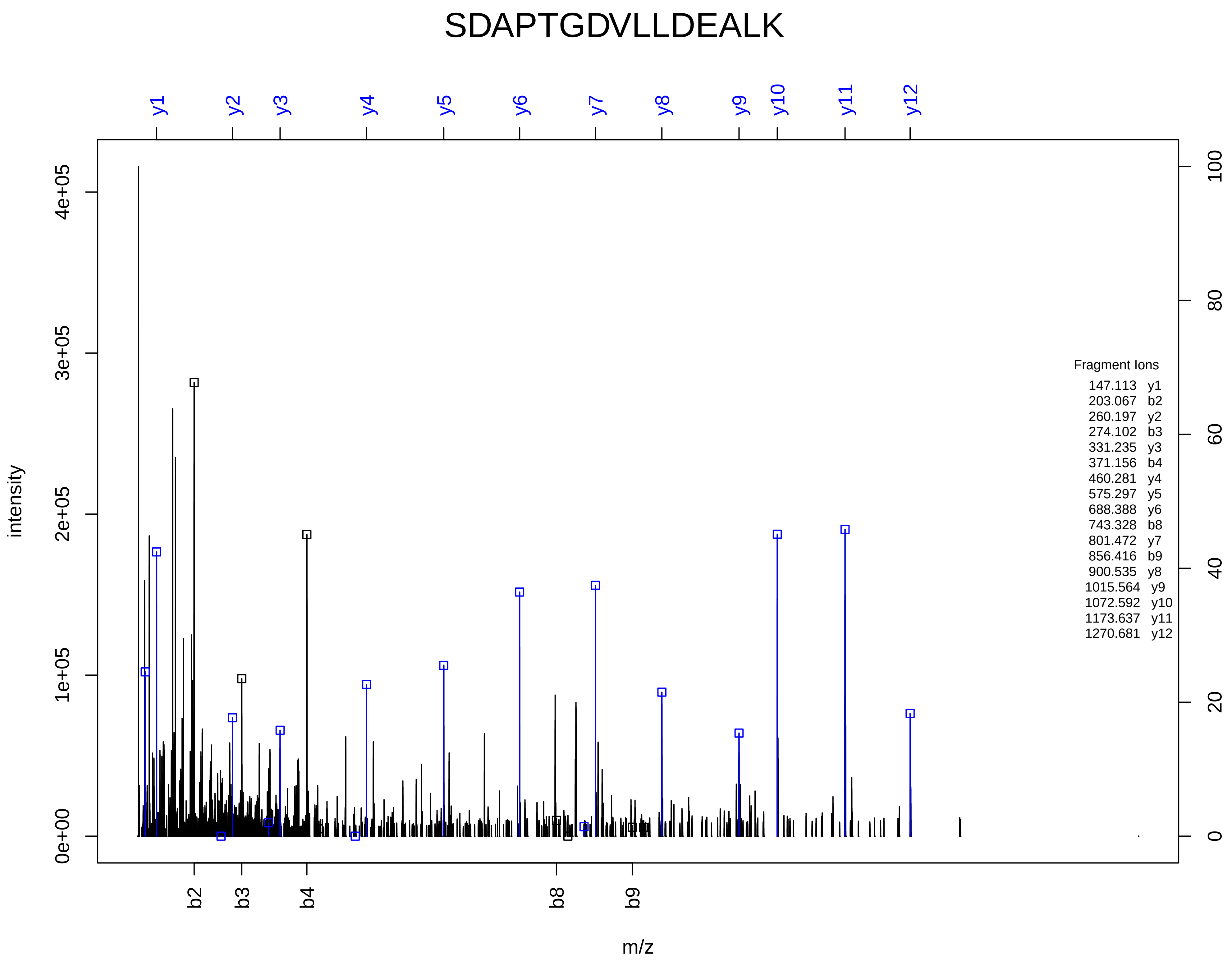

SDAPTGDVLLDEALK / File: christine_pool_04.raw; SpectrumID: ; scans: 15801

EAQQYSEALASTR

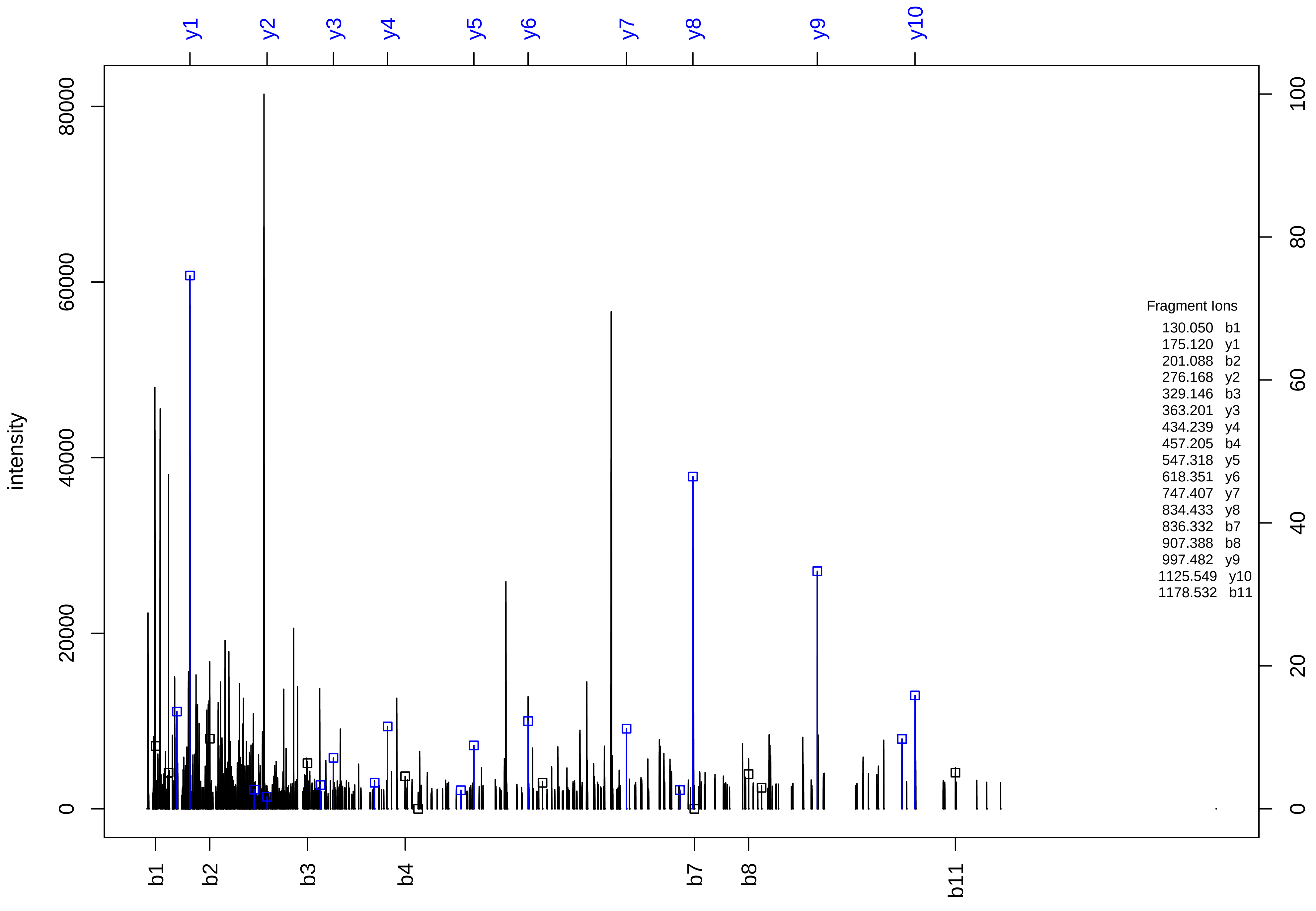

EAQQYSEALASTR / File: christine_pool_04.raw; SpectrumID: ; scans: 5247

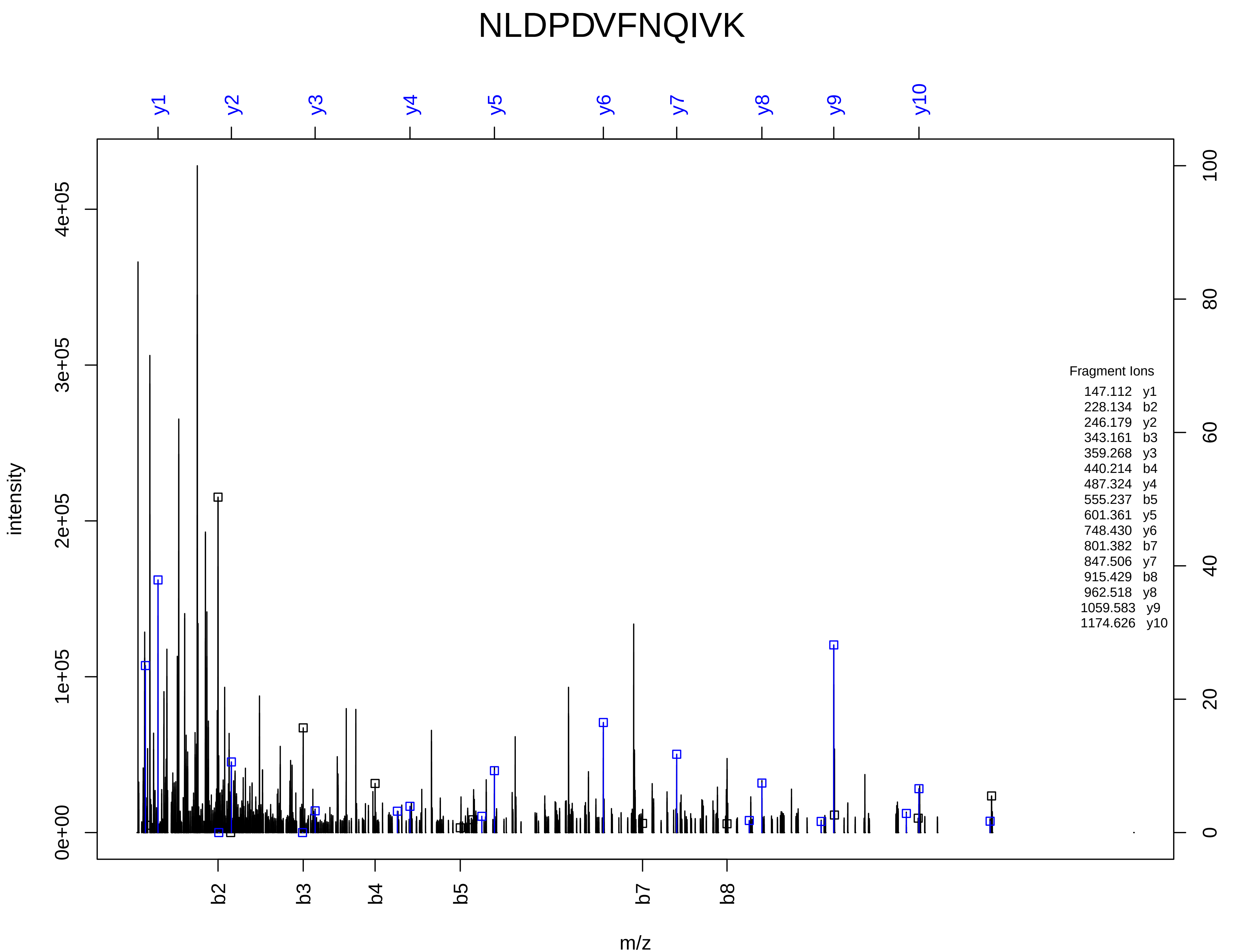

NLDPDVFNQIVK / File: christine_pool_04.raw; SpectrumID: ; scans: 16657

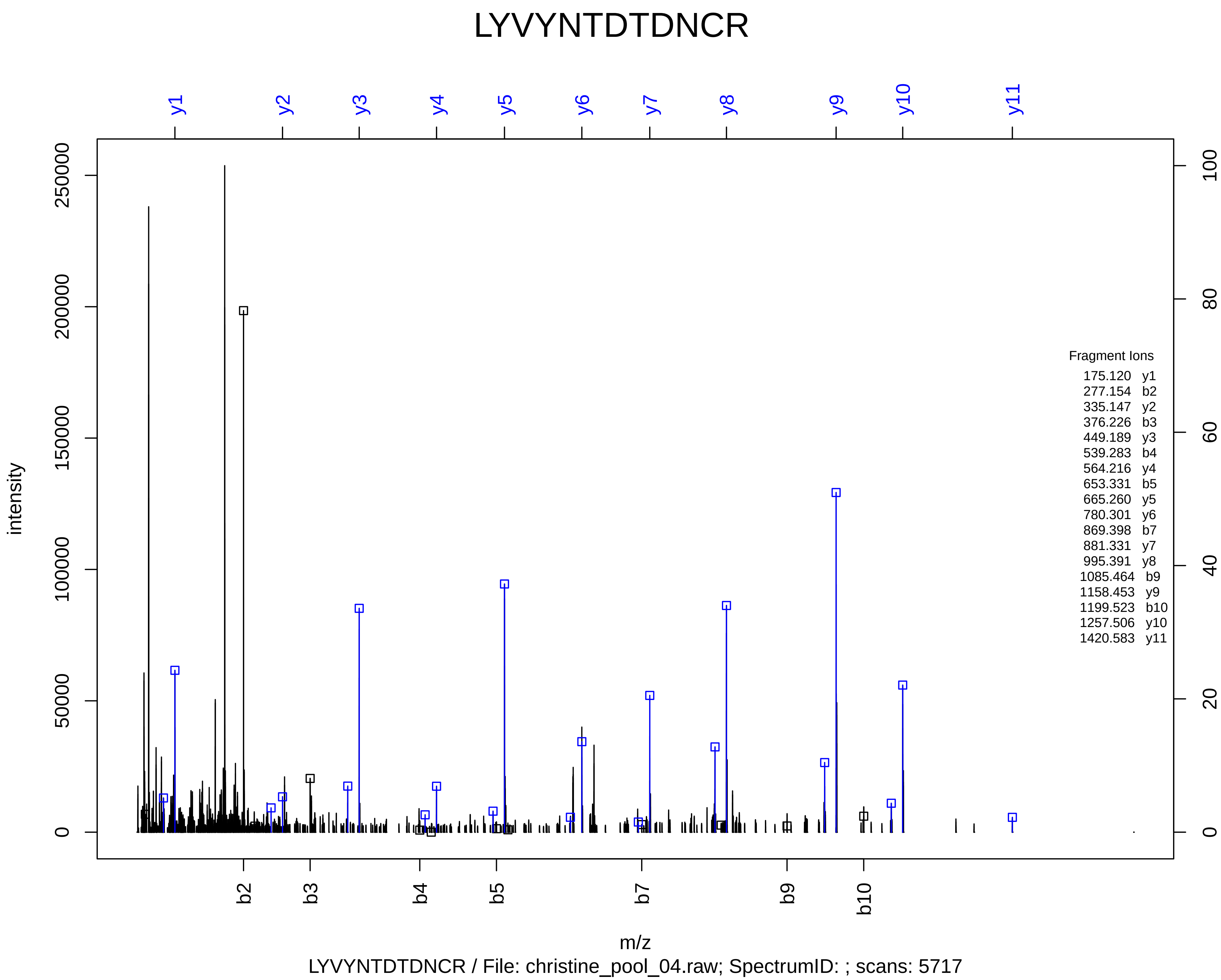

LYVYNTDTDNCR
y1
y2
y3
y4
y5
y6
y7
y8
y9
y10
y11
b2
b3
b4
b5
b7
b9
b10
intensity
0
50000
100000
150000
200000
250000
0
20
40
60
80
100
Fragment Ions
175.120 y1
277.154 b2
335.147 y2
376.226 b3
449.189 y3
539.283 b4
564.216 y4
653.331 b5
665.260 y5
780.301 y6
869.398 b7
881.331 y7
995.391 y8
1085.464 b9
1158.453 y9
1199.523 b10
1257.506 y10
1420.583 y11
m/z
LYVYNTDTDNCR / File: christine_pool_04.raw; SpectrumID: ; scans: 5717

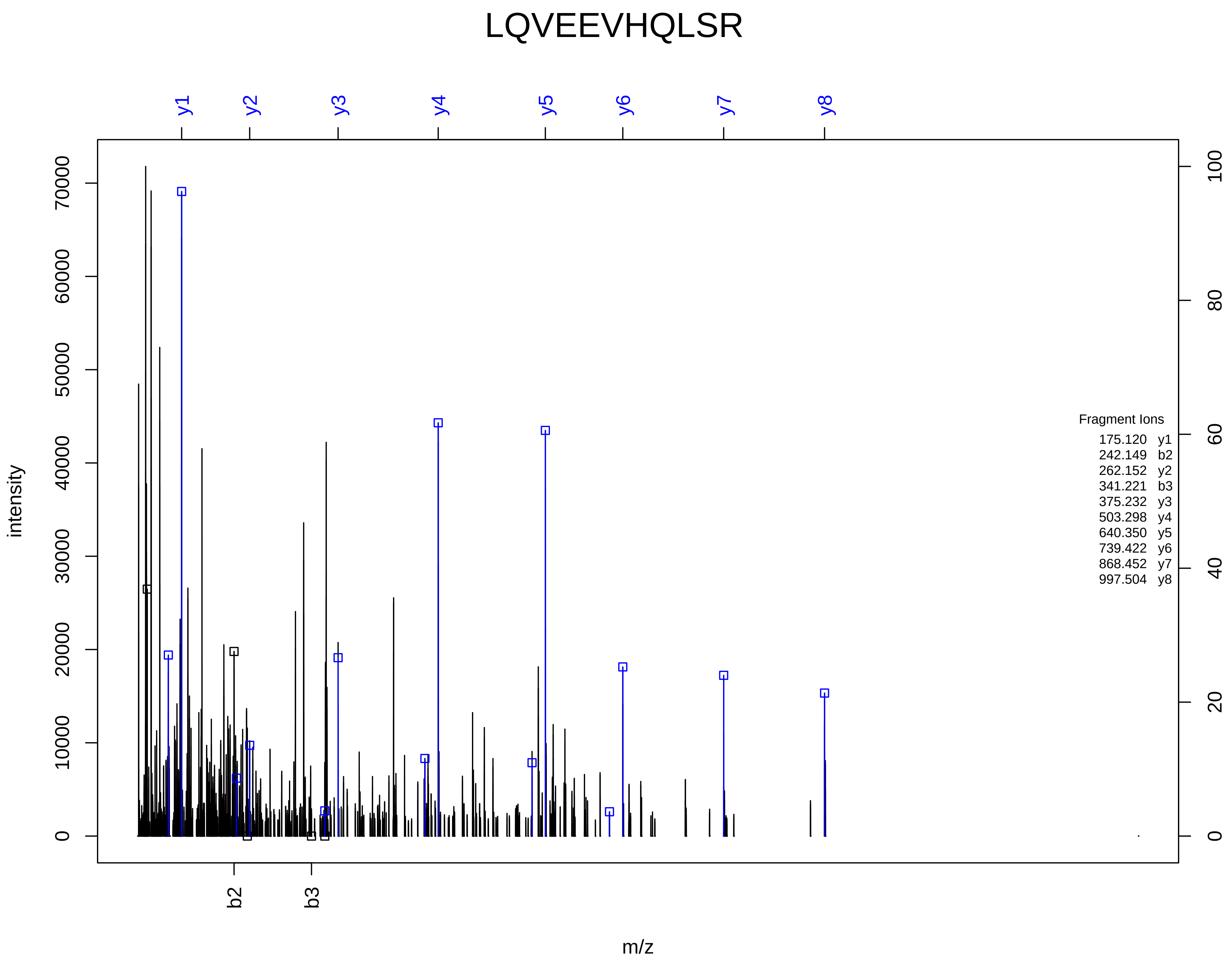

LQVEEVHQLSR / File: christine_pool_04.raw; SpectrumID: ; scans: 5434

FVELHFDELPSSELETILHK

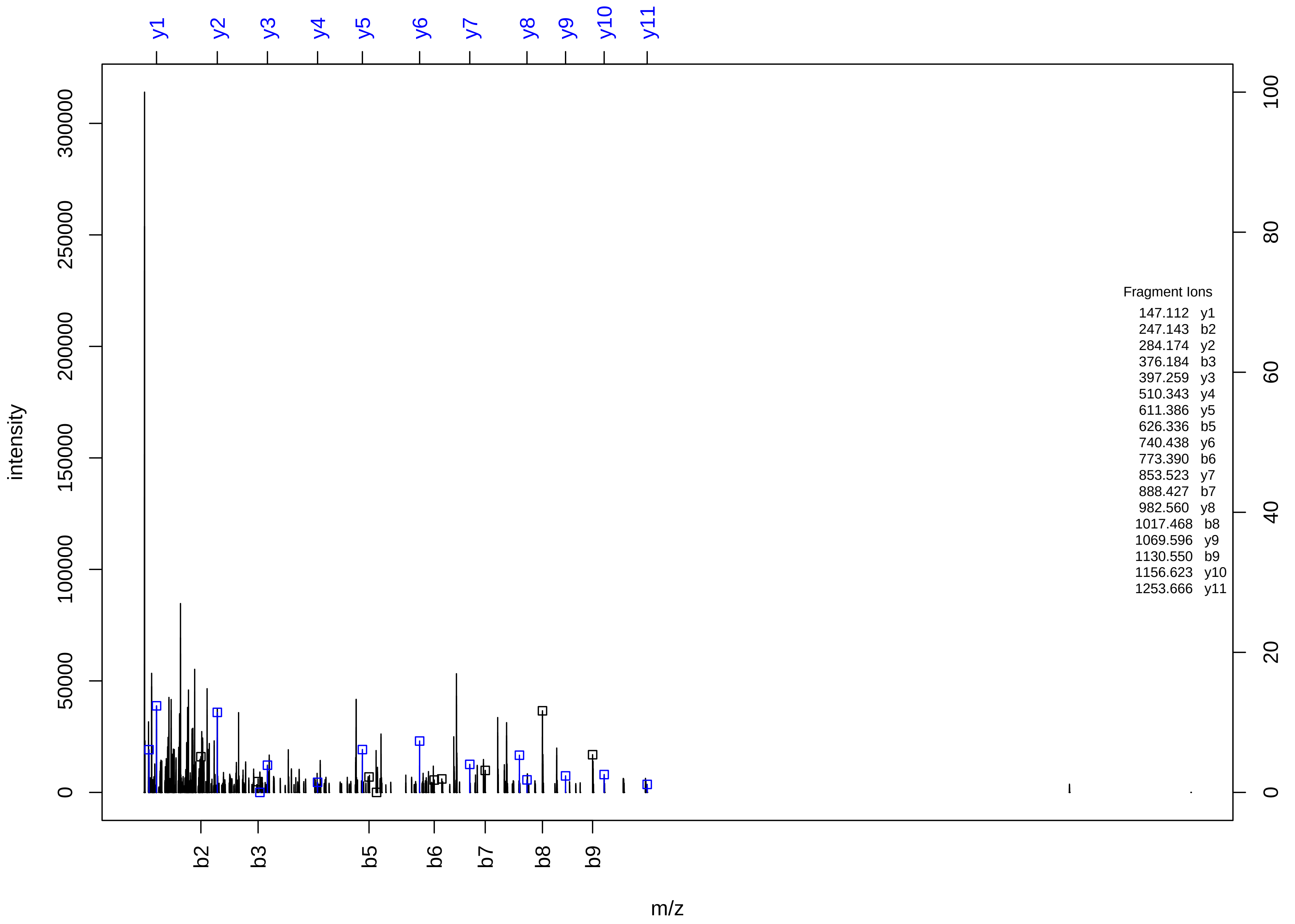

FVELHFDELPSSELETILHK / File: christine_pool_04.raw; SpectrumID: ; scans: 21092

FDYSGVGSSDGNSEESTLGK

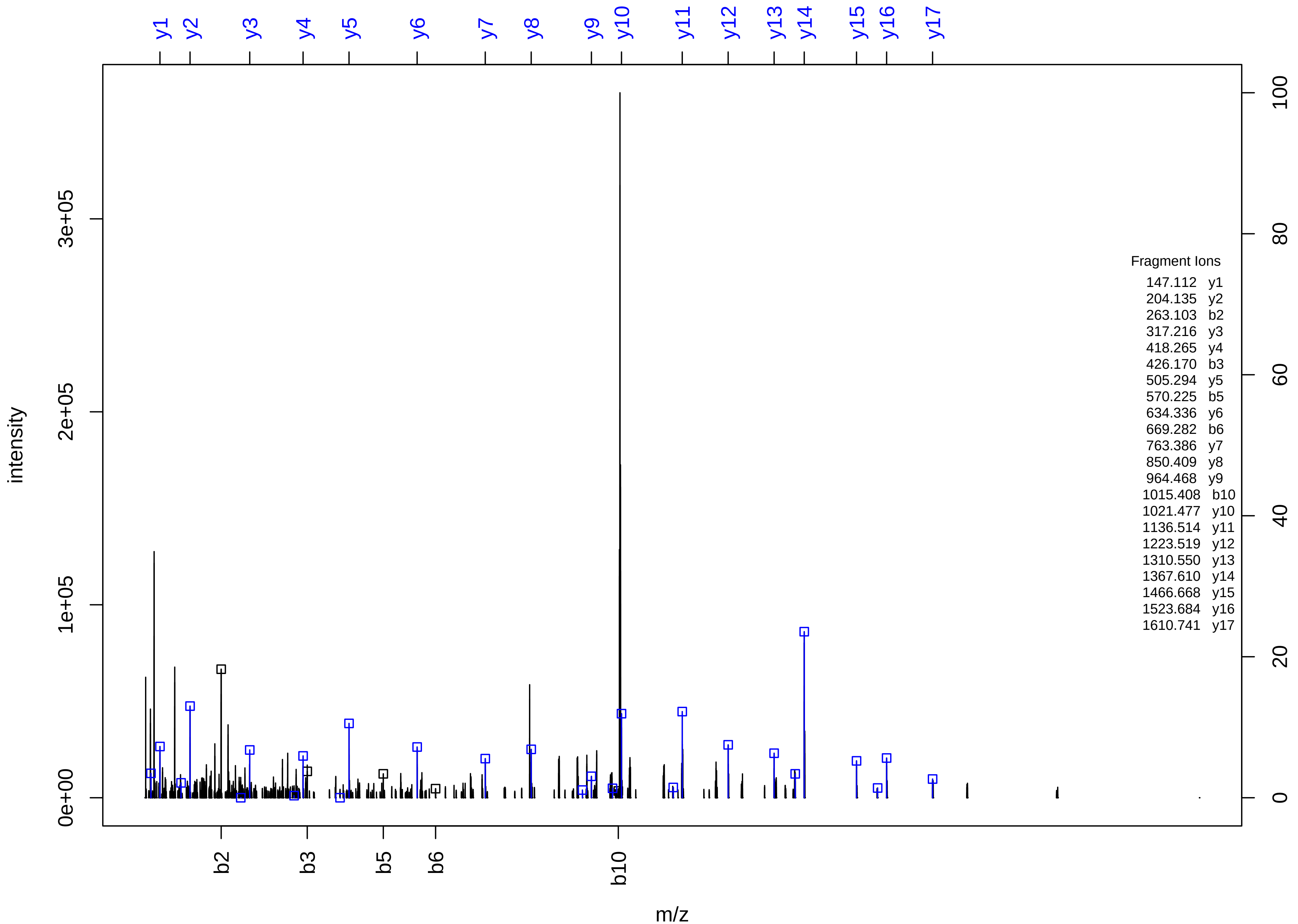

FDYSGVGSSDGNSEESTLGK / File: christine_pool_04.raw; SpectrumID: ; scans: 7798

TSN^PLVLEEASASQAGSR

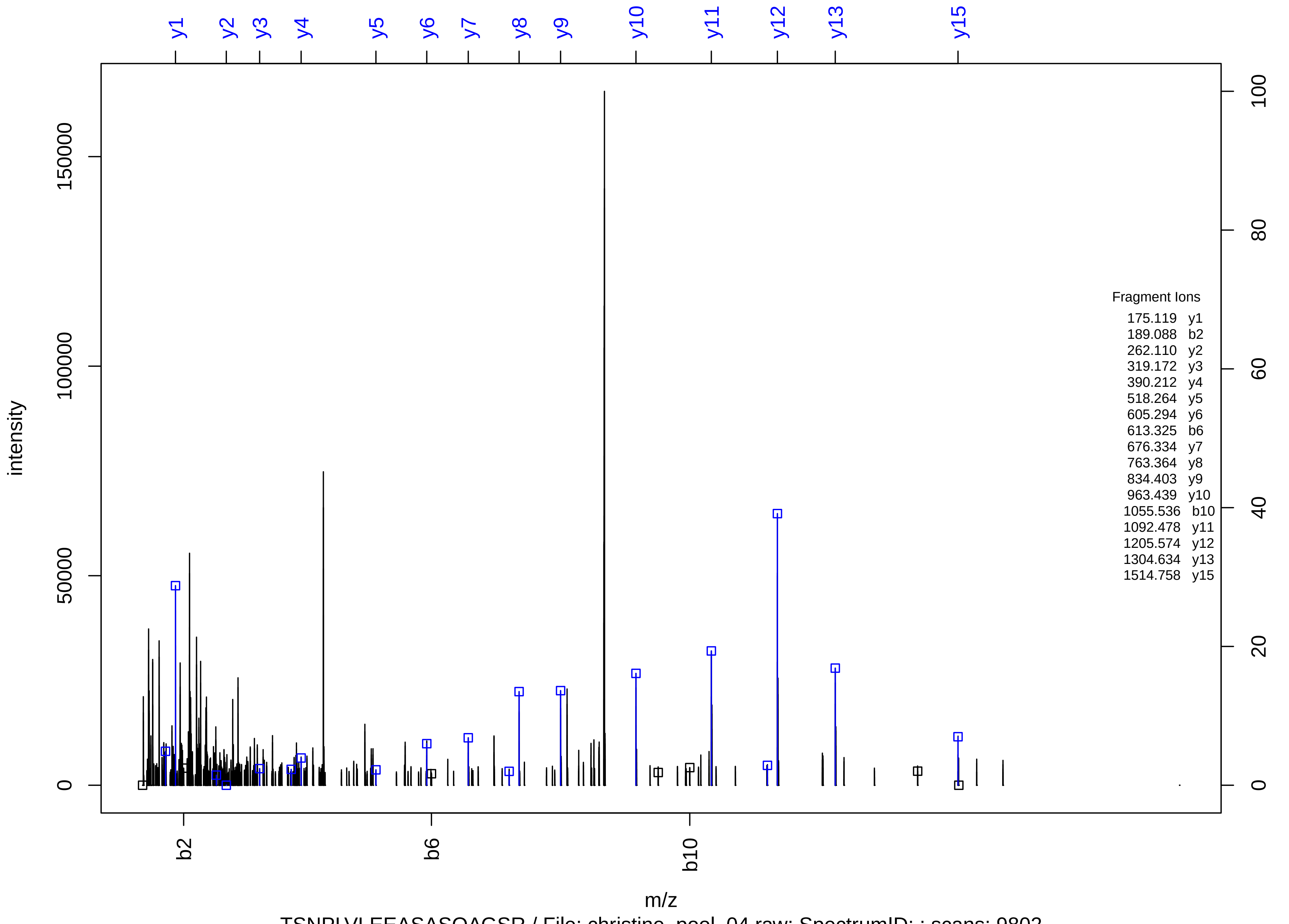

TSNPLVLEEASASQAGSR / File: christine_pool_04.raw; SpectrumID: ; scans: 9802

EGEERE DADVSVTQR

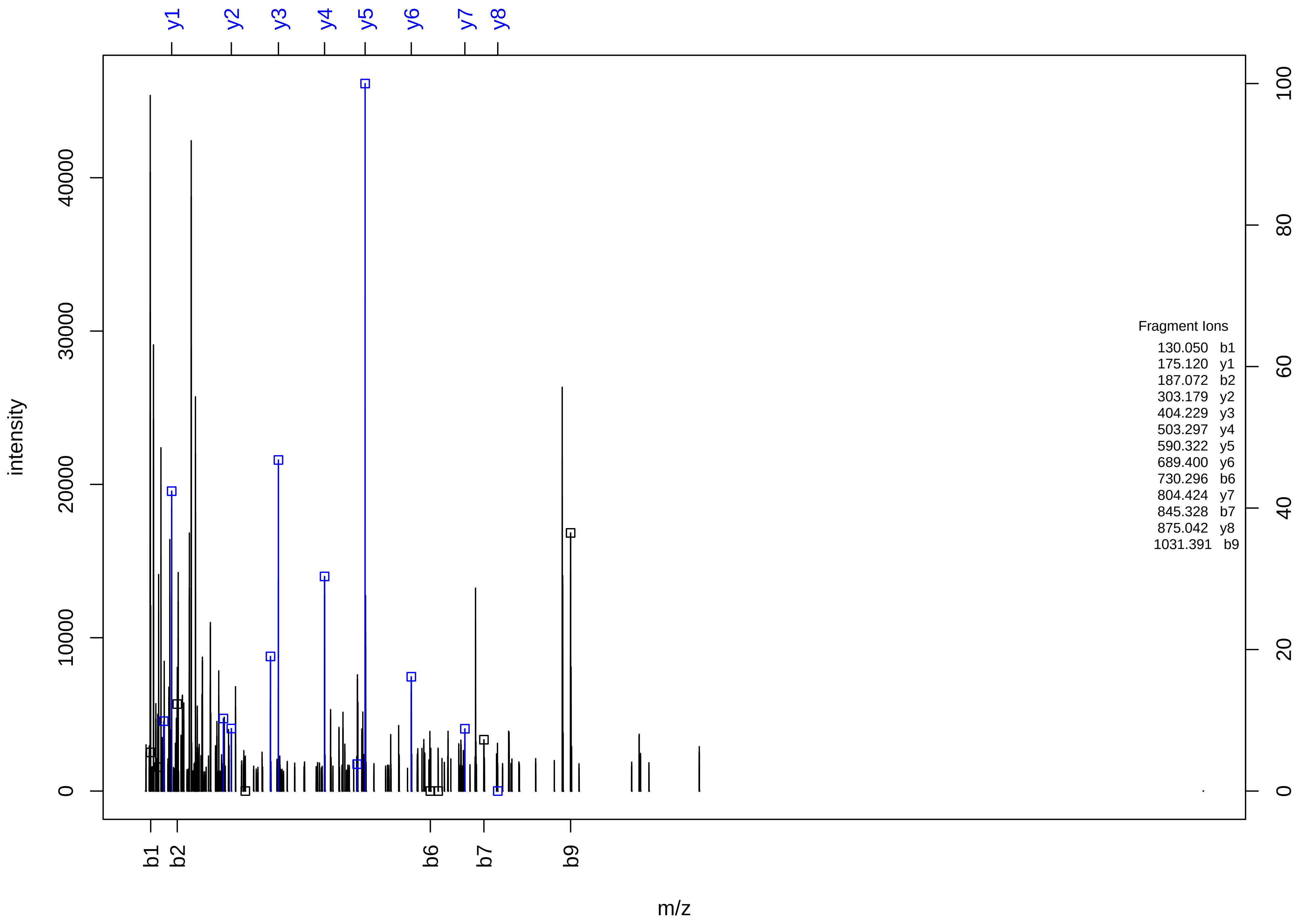

EGEEREDADVSVTQR / File: christine_pool_04.raw; SpectrumID: ; scans: 2847

ESPNPPNPSGQCPICR

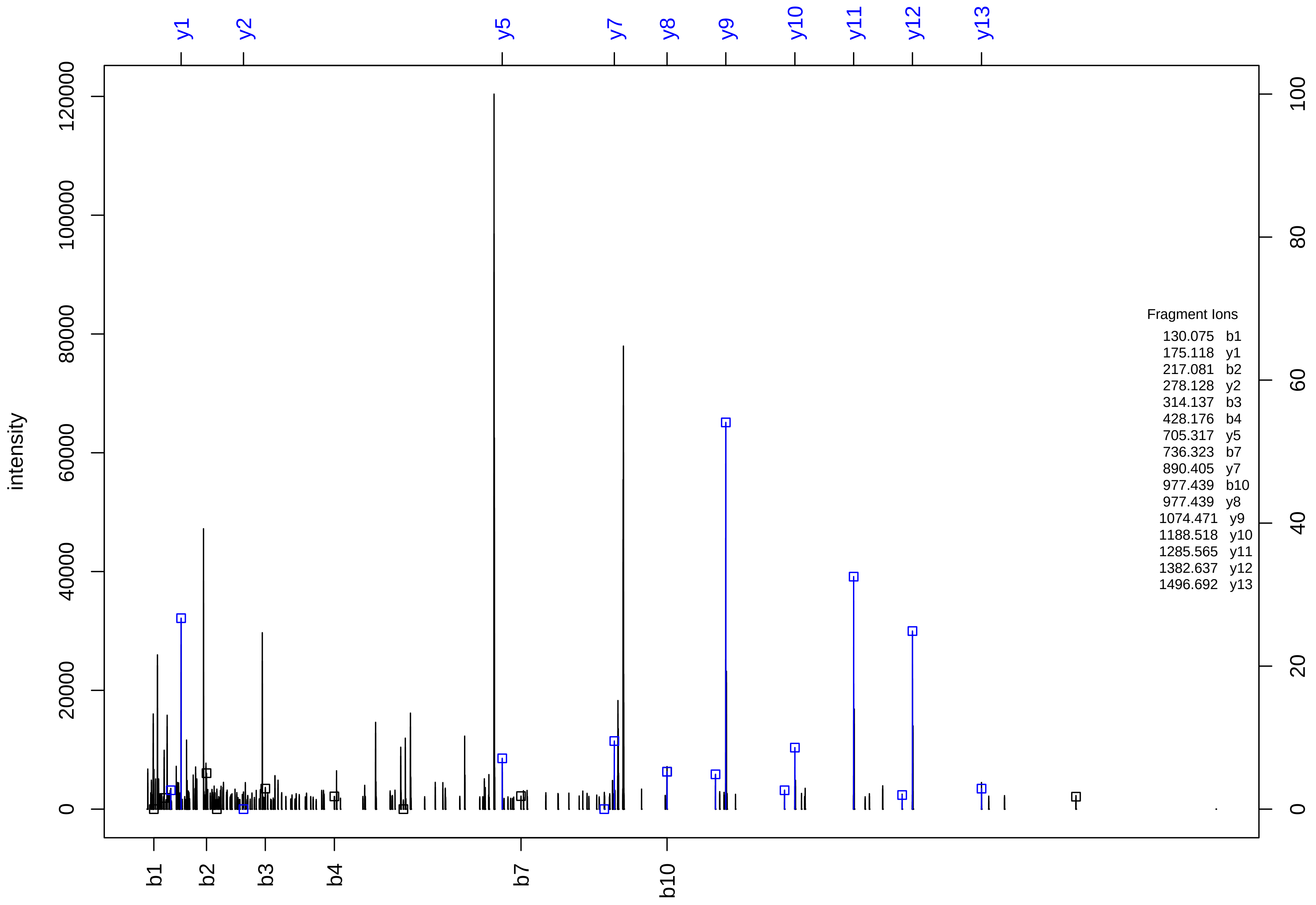

ESPNPPNPSGQCPICR / File: christine_pool_04.raw; SpectrumID: ; scans: 4795

GPPNPNVEYIPFDEMK

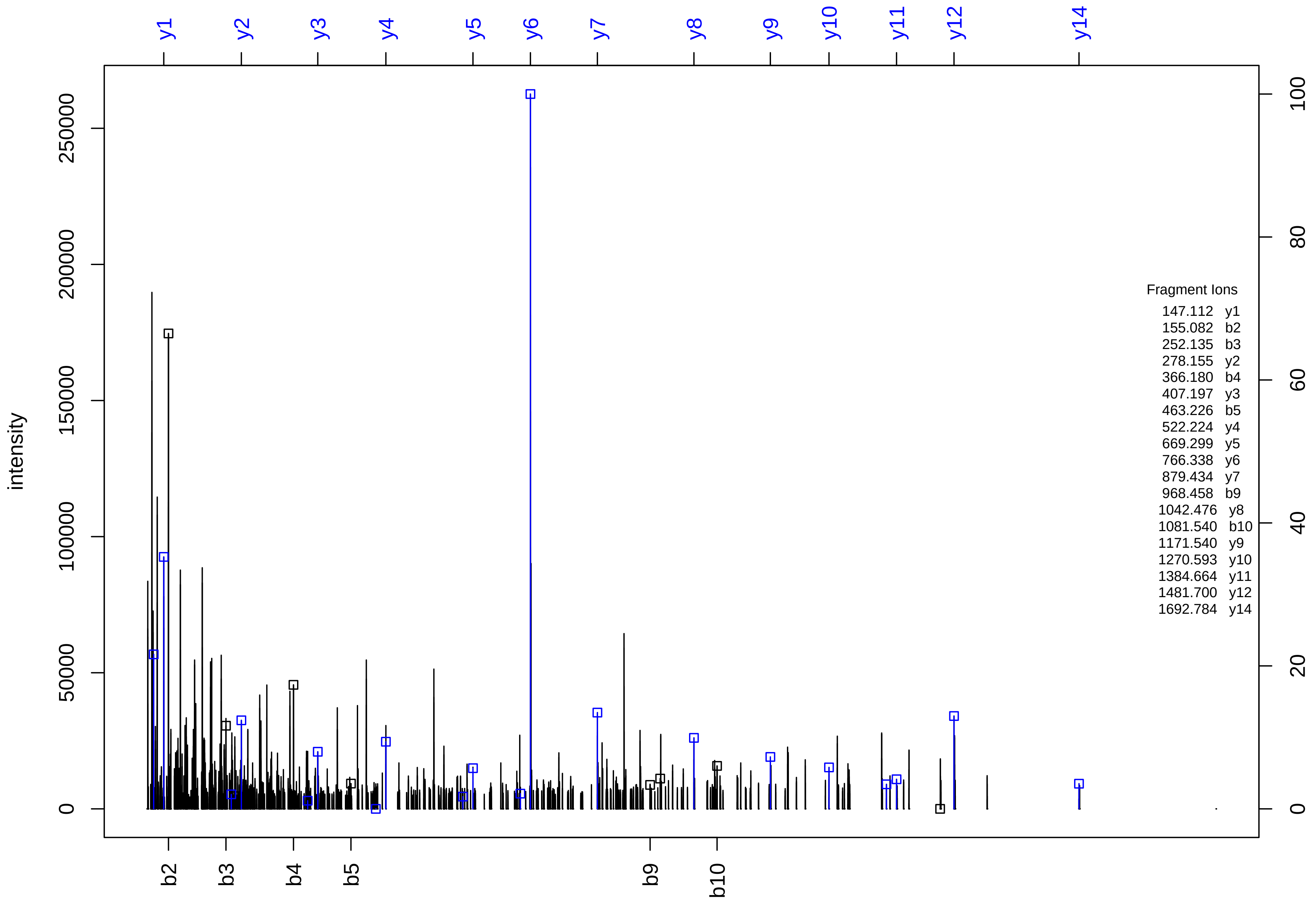

GPPNPNVEYIPFDEMK / File: christine_pool_04.raw; SpectrumID: ; scans: 15735

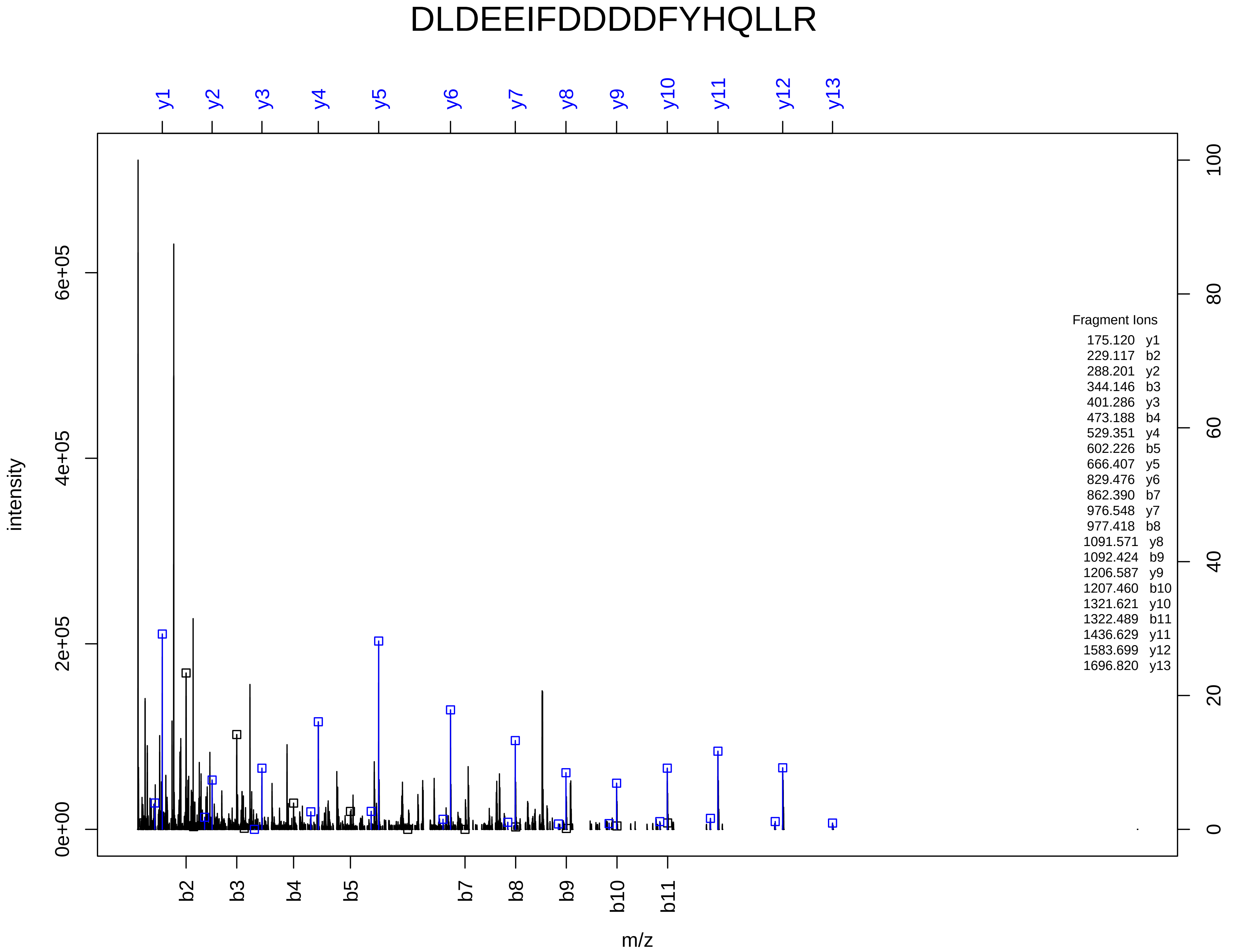

DLDEEIFDDDDFYHQLLR / File: christine_pool_04.raw; SpectrumID: ; scans: 22158

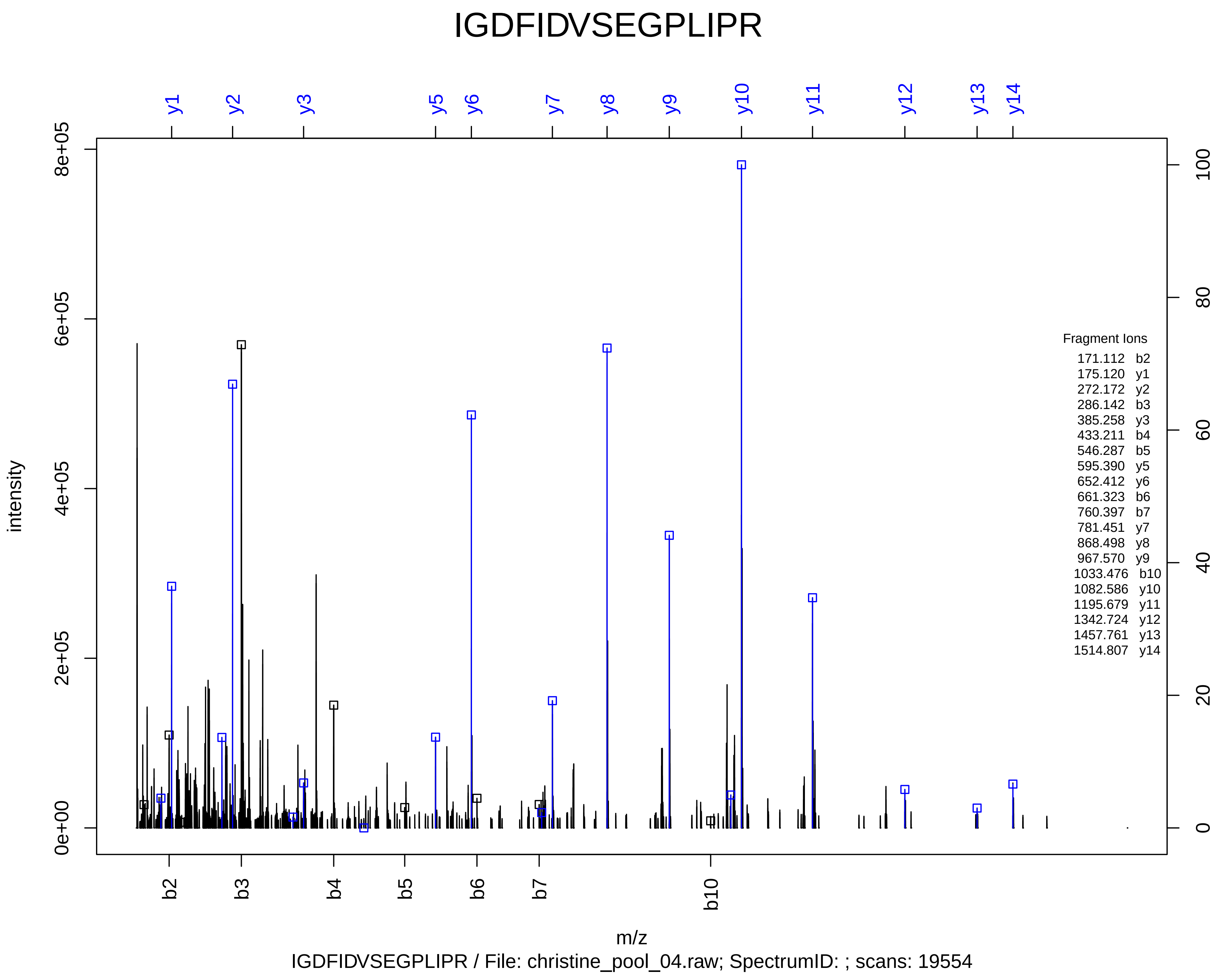
IGDFIDVSEGPLIPR
y1
y2
y3
y5
y6
y7
y8
y9
y10
y11
y12
y13
y14
b2
b3
b4
b5
b6
b7
b10
intensity
0e+00
2e+05
4e+05
6e+05
8e+05
0
20
40
60
80
100
m/z
Fragment Ions
171.112 b2
175.120 y1
272.172 y2
286.142 b3
385.258 y3
433.211 b4
546.287 b5
595.390 y5
652.412 y6
661.323 b6
760.397 b7
781.451 y7
868.498 y8
967.570 y9
1033.476 b10
1082.586 y10
1195.679 y11
1342.724 y12
1457.761 y13
1514.807 y14
IGDFIDVSEGPLIPR / File: christine_pool_04.raw; SpectrumID: ; scans: 19554

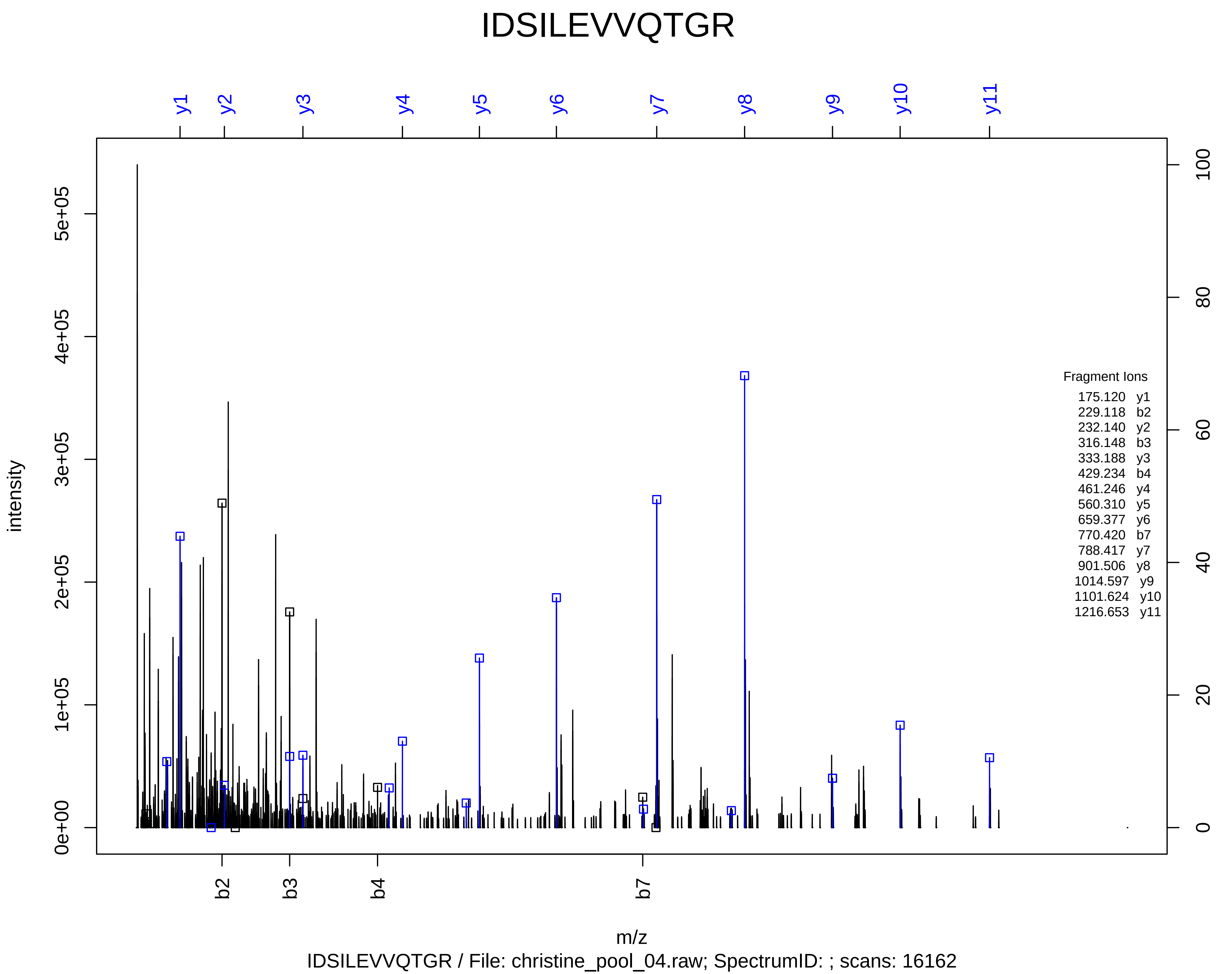
IDSILEVVQTGR
y1 y2 y3 y4 y5 y6 y7 y8 y9 y10 y11
b2 b3 b4 b7
intensity
0e+00
1e+05
2e+05
3e+05
4e+05
5e+05
0
20
40
60
80
100
m/z
Fragment Ions
175.120 y1
229.118 b2
232.140 y2
316.148 b3
333.188 y3
429.234 b4
461.246 y4
560.310 y5
659.377 y6
770.420 b7
788.417 y7
901.506 y8
1014.597 y9
1101.624 y10
1216.653 y11
IDSILEVVQTGR / File: christine_pool_04.raw; SpectrumID: ; scans: 16162

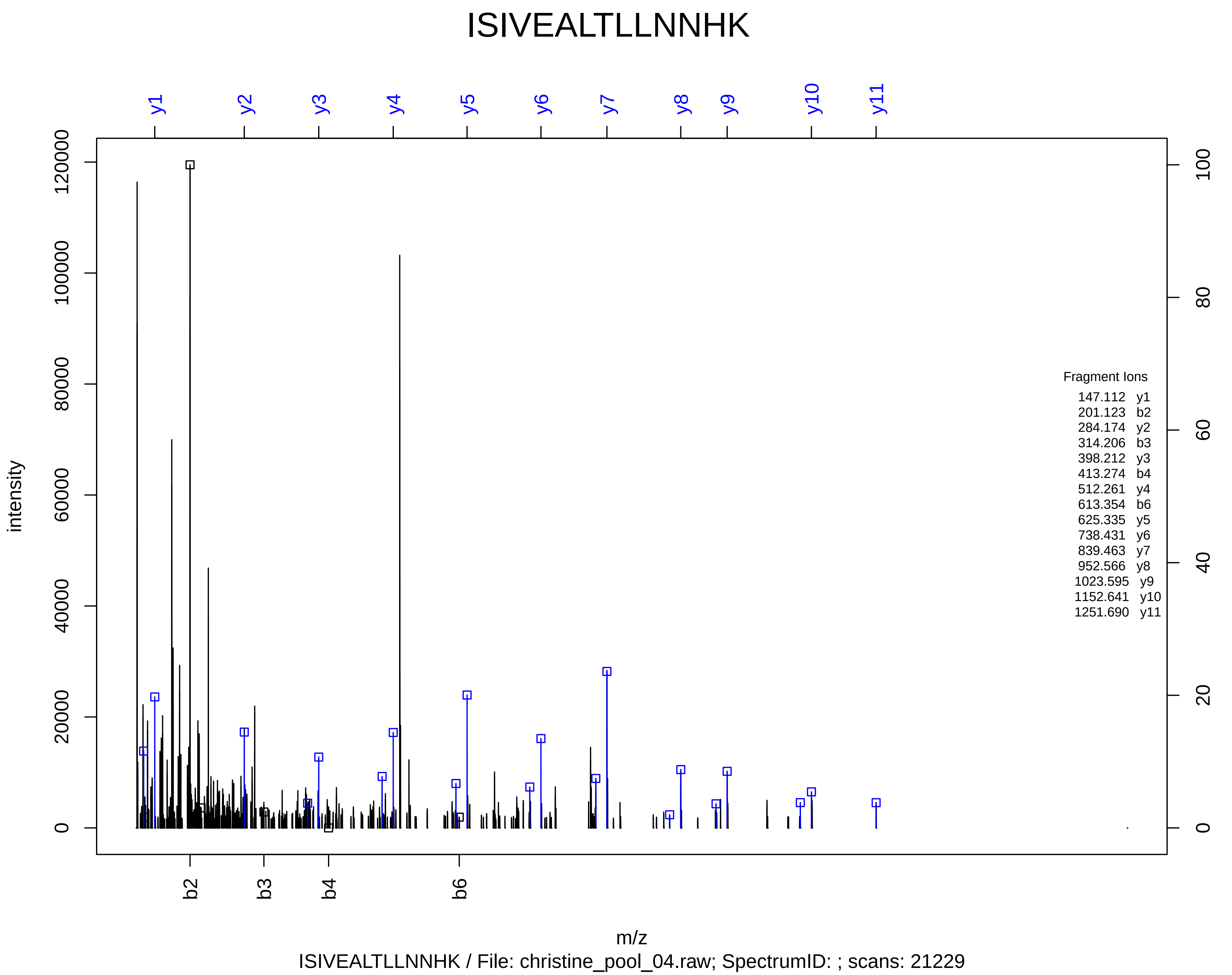

ISIVEALTLLNNHK
y1
y2
y3
y4
y5
y6
y7
y8
y9
y10
y11
intensity
0
20000
40000
60000
80000
100000
120000
0
20
40
60
80
100
b2
b3
b4
b6
Fragment Ions
147.112 y1
201.123 b2
284.174 y2
314.206 b3
398.212 y3
413.274 b4
512.261 y4
613.354 b6
625.335 y5
738.431 y6
839.463 y7
952.566 y8
1023.595 y9
1152.641 y10
1251.690 y11
m/z
ISIVEALTLLNNHK / File: christine_pool_04.raw; SpectrumID: ; scans: 21229

QDEDGYITLNIK

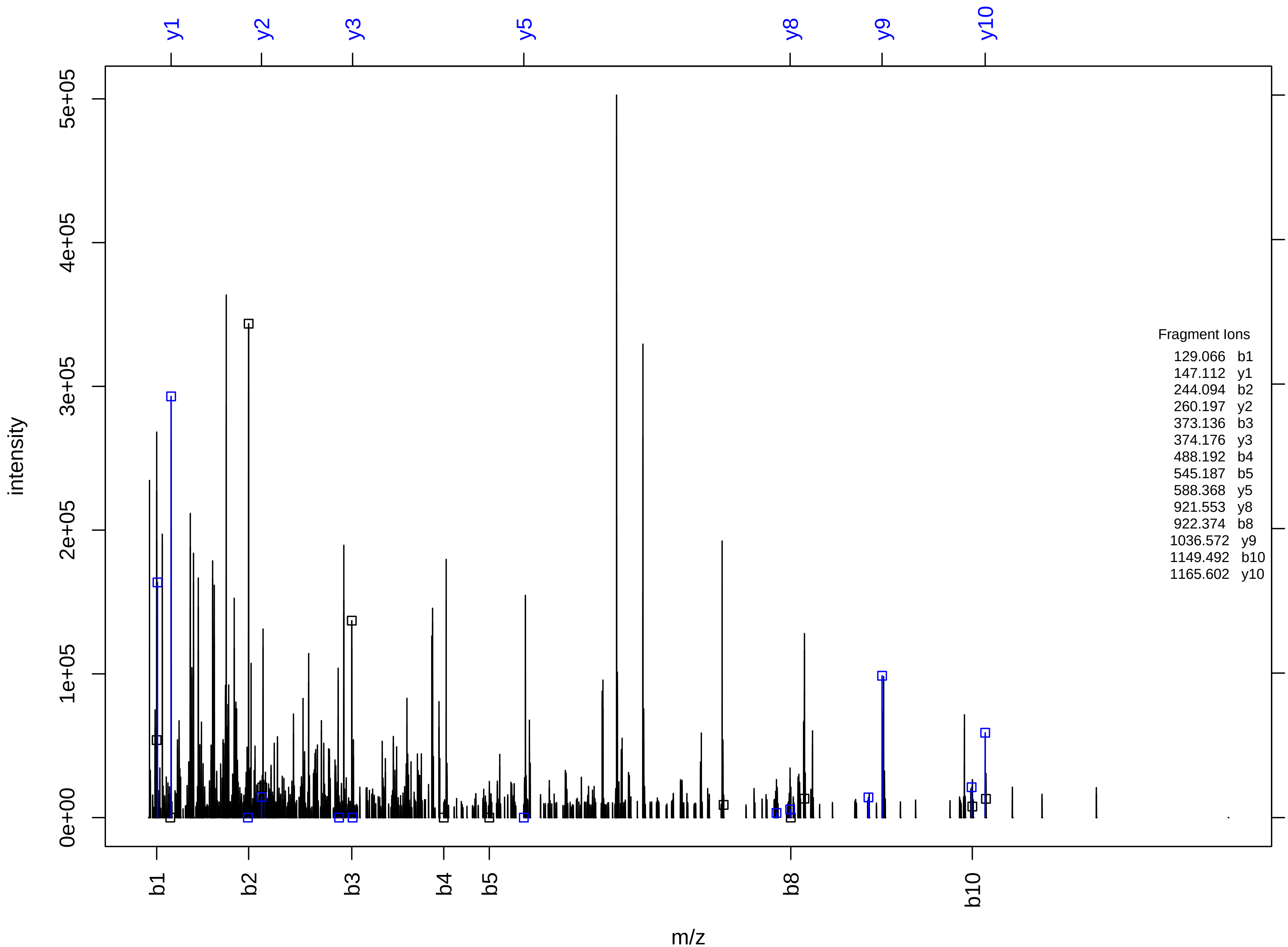

QDEDGYITLNIK / File: christine_pool_04.raw; SpectrumID: ; scans: 10310

(Ac)MNVDHEVNLLVEEIHR

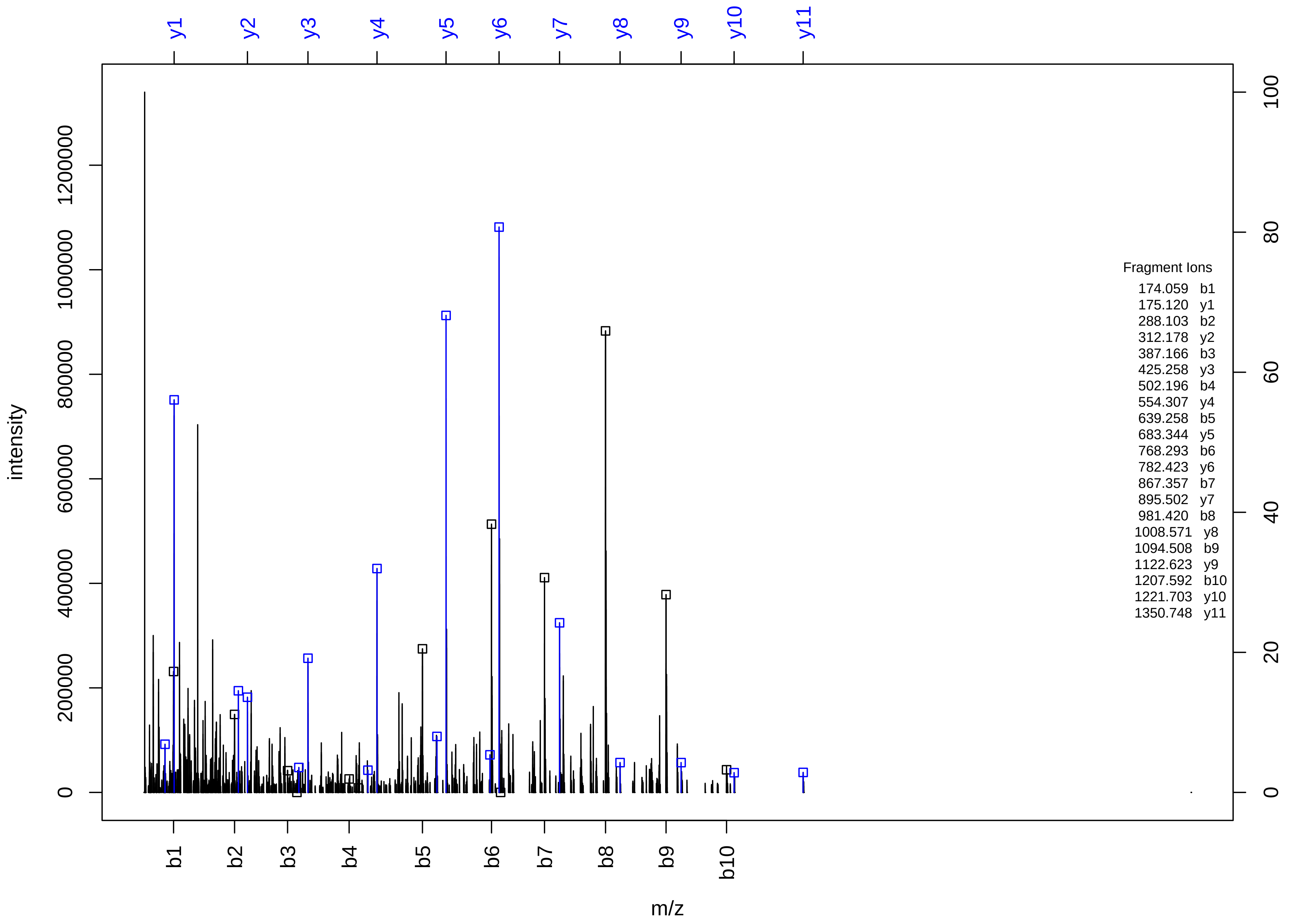

MNVDHEVNLLVEEIHR / File: christine_pool_04.raw; SpectrumID: ; scans: 20154

TPTRHLLN^GSLVDGEPPM*KR

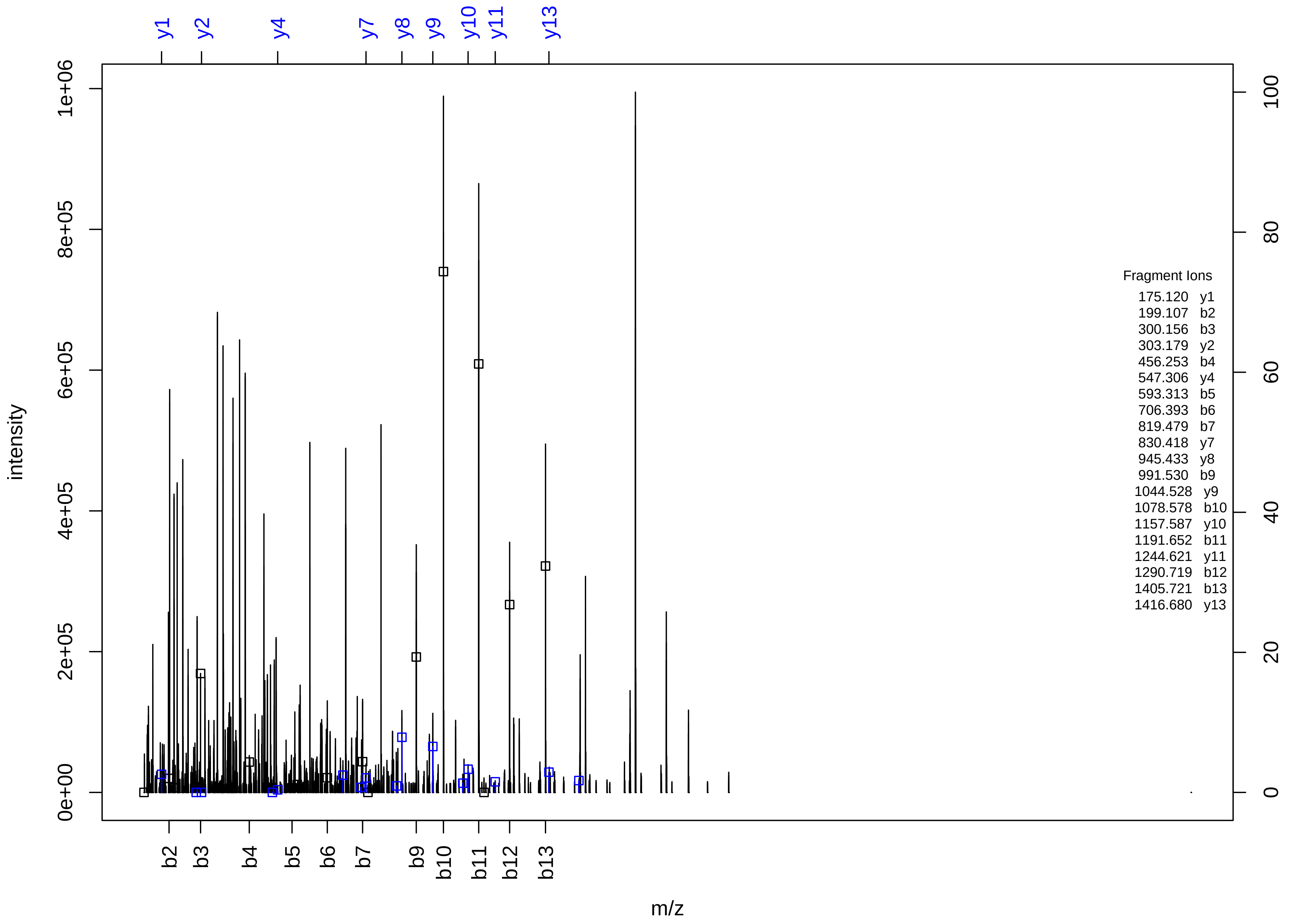

TPTRHLLNGSLVDGEPPMKR / File: christine_pool_04.raw; SpectrumID: ; scans: 23324

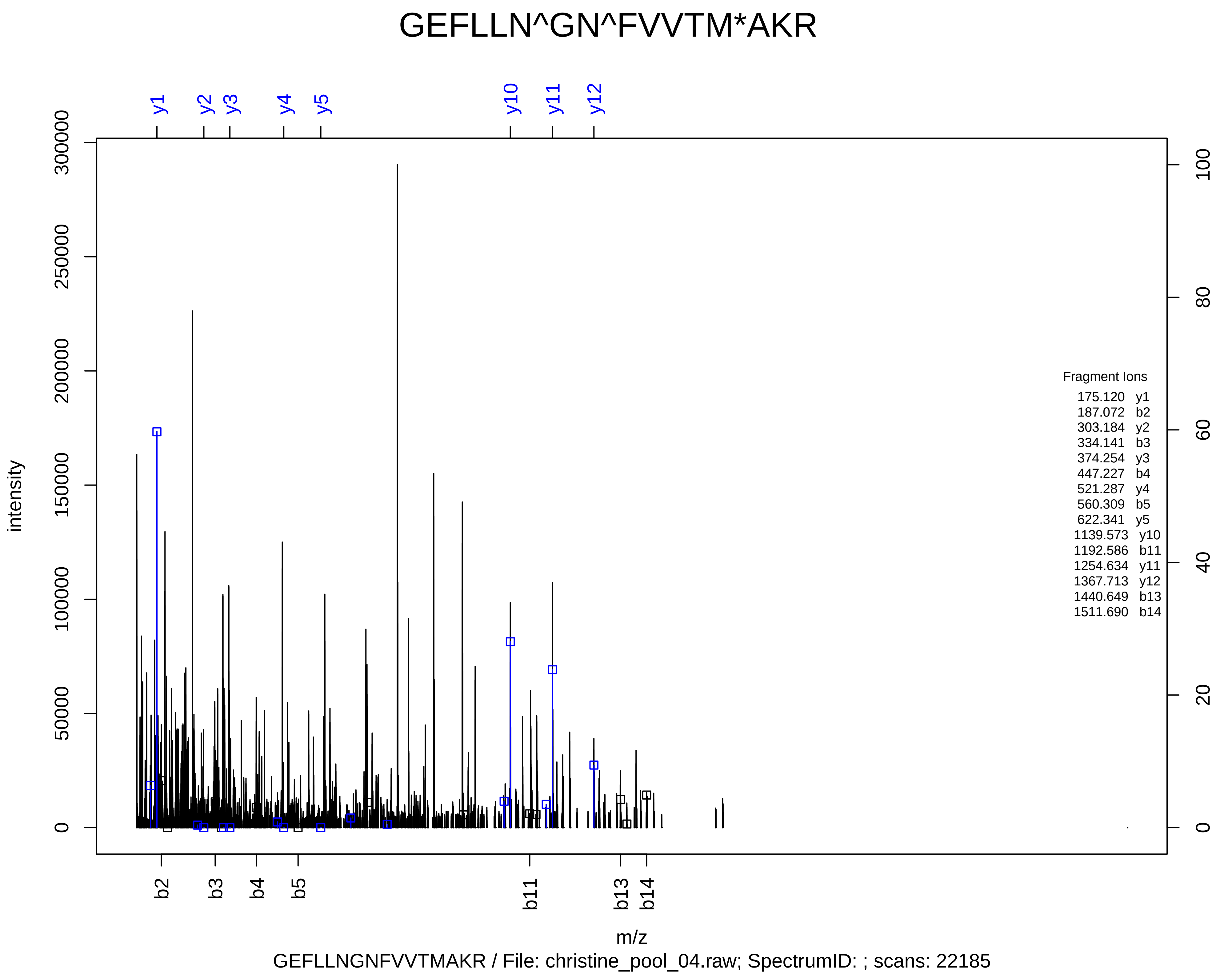

GEFLLN^GN^FVVTM*AKR
y1
y2
y3
y4
y5
y10
y11
y12
intensity
300000
250000
200000
150000
100000
50000
0
100
80
60
40
20
0
Fragment Ions
175.120 y1
187.072 b2
303.184 y2
334.141 b3
374.254 y3
447.227 b4
521.287 y4
560.309 b5
622.341 y5
1139.573 y10
1192.586 b11
1254.634 y11
1367.713 y12
1440.649 b13
1511.690 b14
b2
b3
b4
b5
b11
b13
b14
m/z
GEFLLNGNFVVTMAKR / File: christine_pool_04.raw; SpectrumID: ; scans: 22185

IDVYGFSALWK

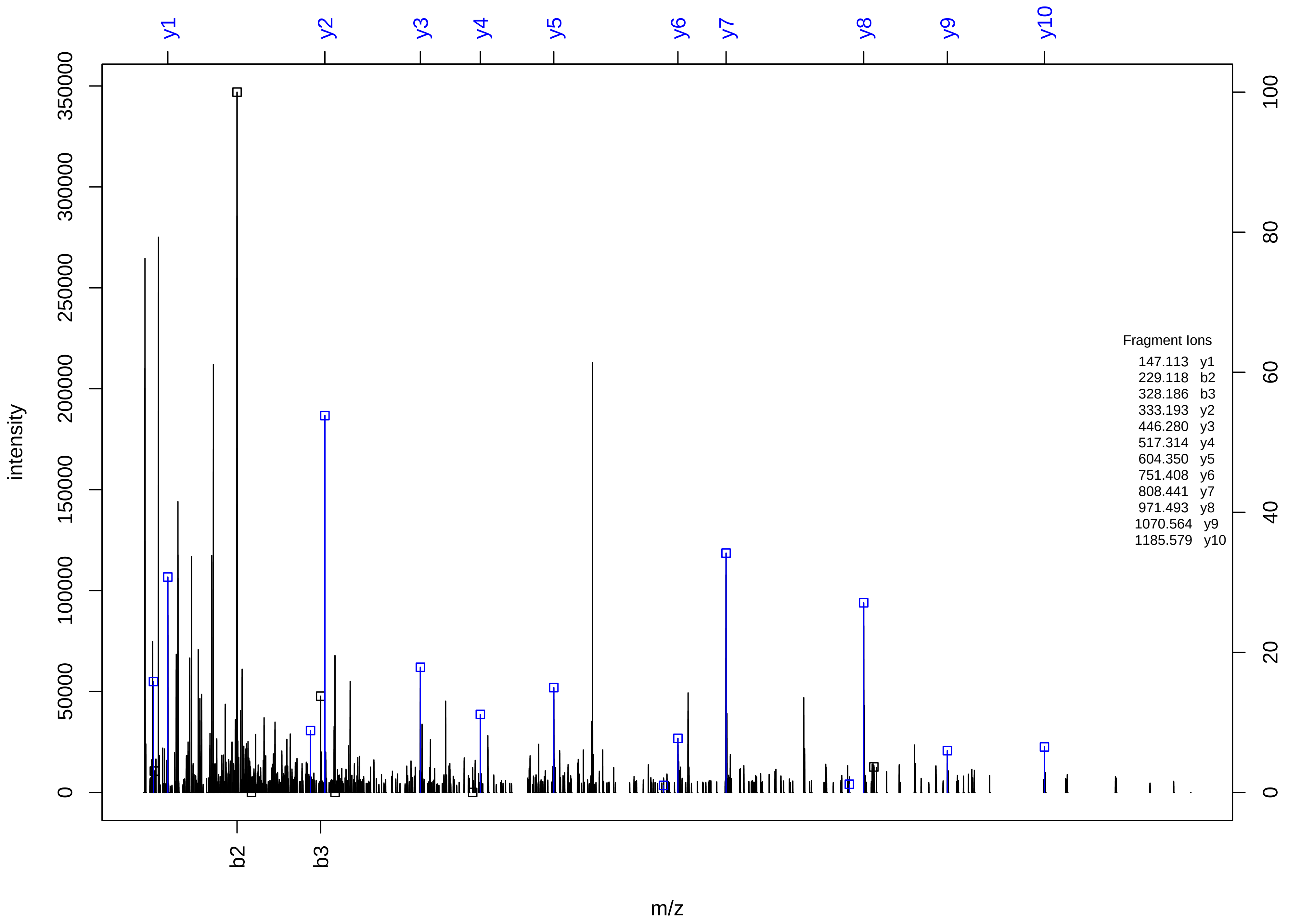

IDVYGFSALWK / File: christine_pool_04.raw; SpectrumID: ; scans: 20871

DLHQGIEAASDEEDLR

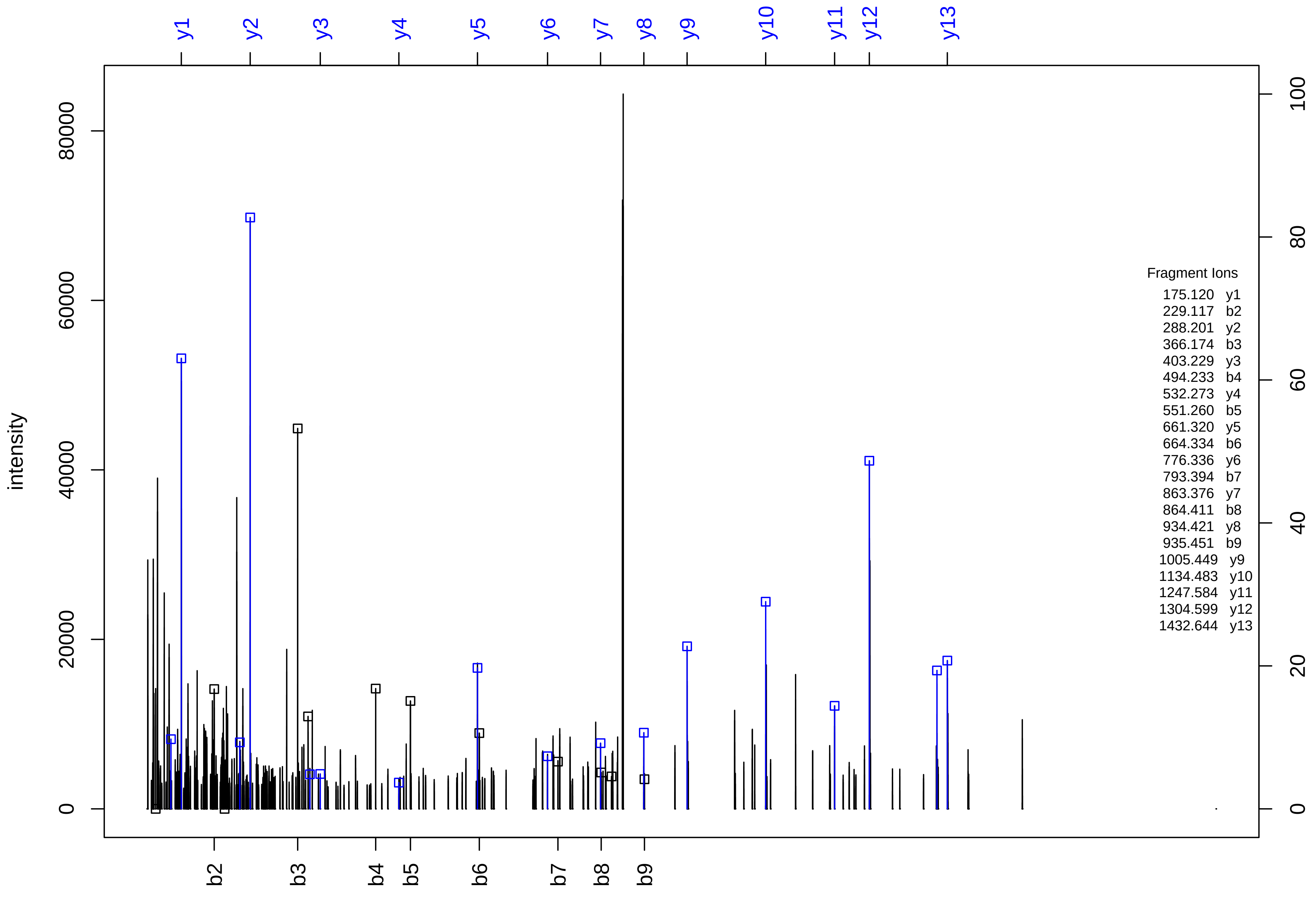

DLHQGIEAASDEEDLR / File: christine_pool_04.raw; SpectrumID: ; scans: 7737

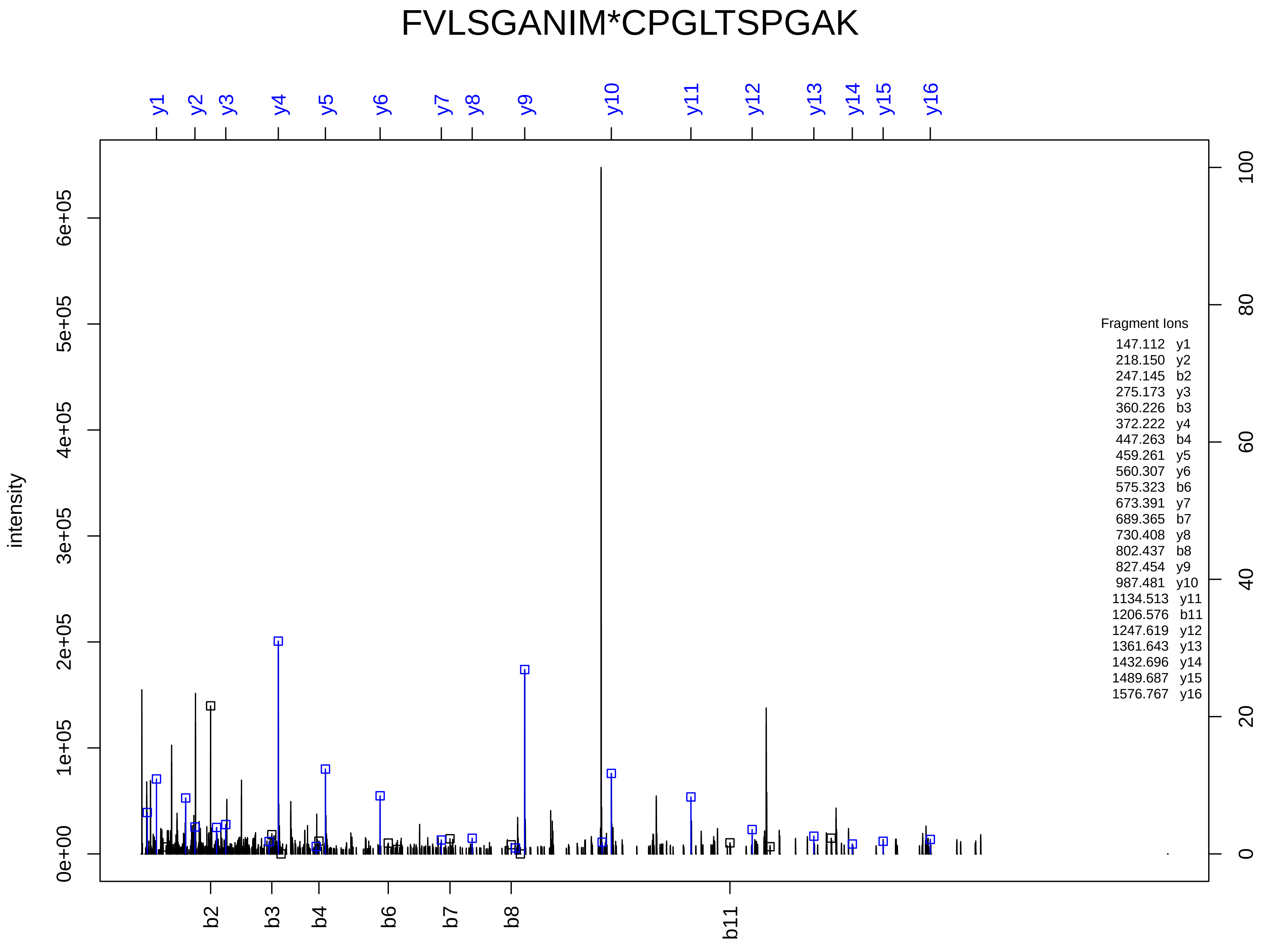

FVLSGANIMCPGLTSPGAK / File: christine_pool_04.raw; SpectrumID: ; scans: 13952

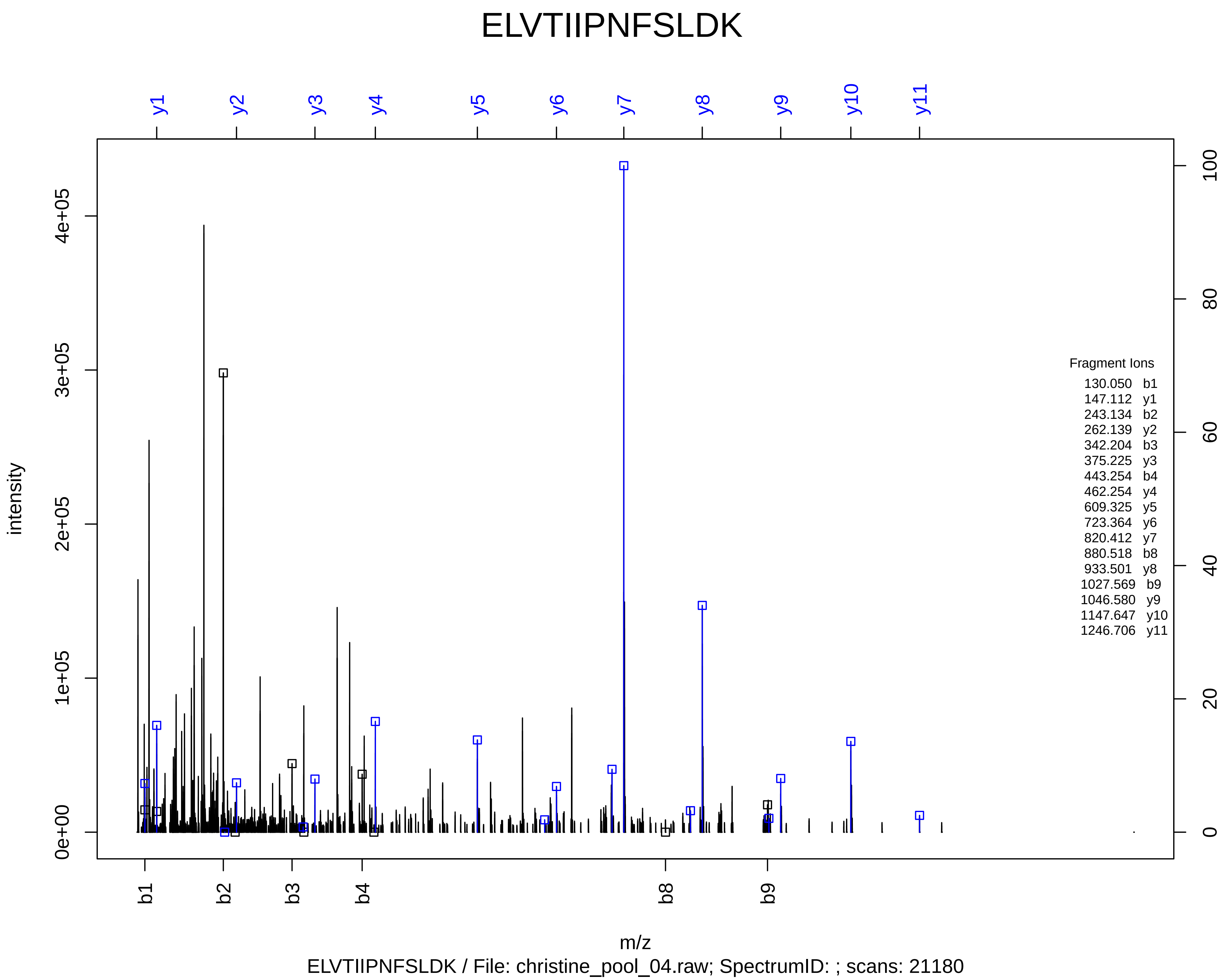
ELVTIIPNFSLDK
y1 y2 y3 y4 y5 y6 y7 y8 y9 y10 y11
b1 b2 b3 b4 b8 b9
intensity
0e+00 1e+05 2e+05 3e+05 4e+05
0 20 40 60 80 100
Fragment Ions
130.050 b1
147.112 y1
243.134 b2
262.139 y2
342.204 b3
375.225 y3
443.254 b4
462.254 y4
609.325 y5
723.364 y6
820.412 y7
880.518 b8
933.501 y8
1027.569 b9
1046.580 y9
1147.647 y10
1246.706 y11
m/z
ELVTIIPNFSLDK / File: christine_pool_04.raw; SpectrumID: ; scans: 21180

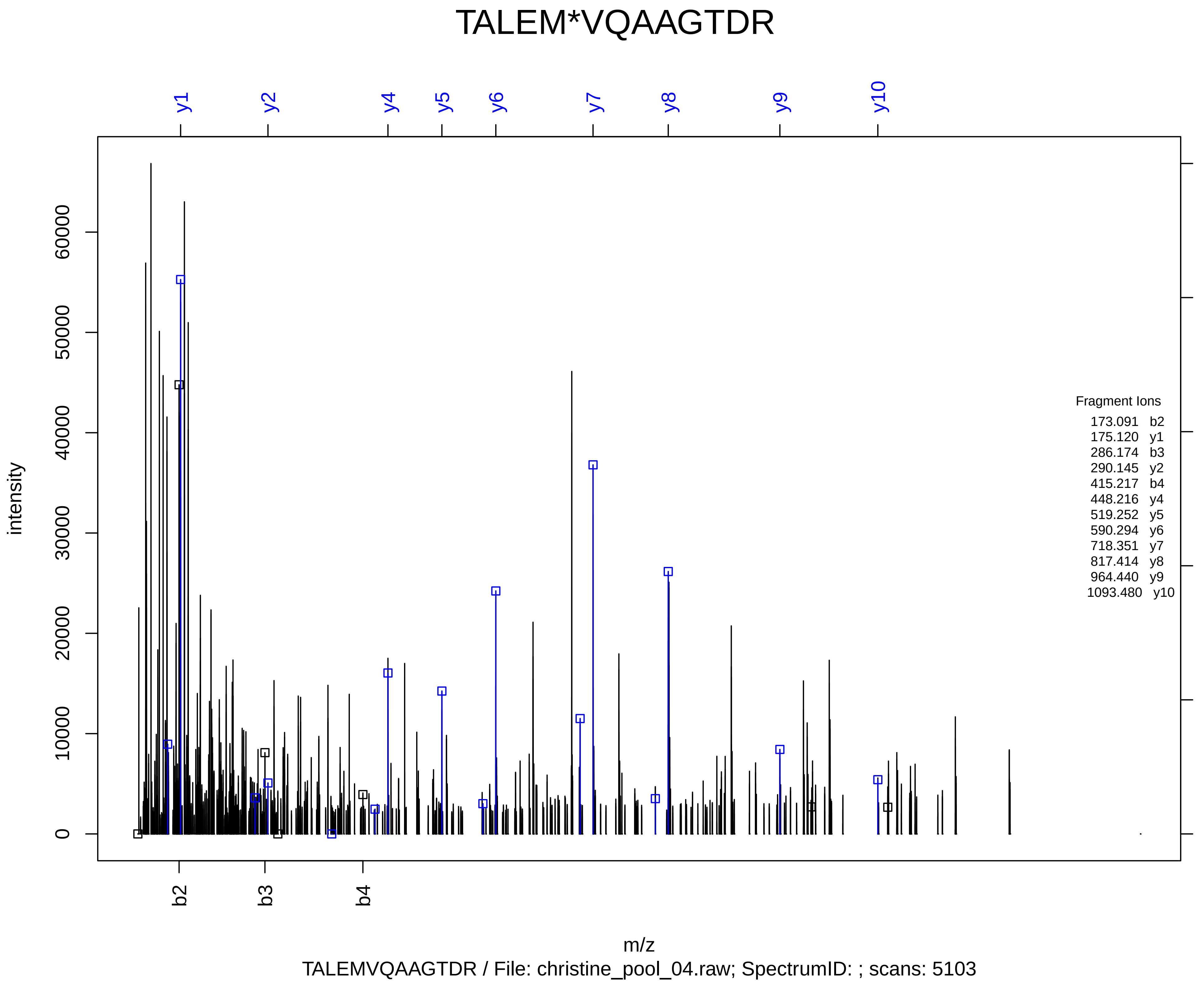
TALEM*VQAAGTDR
y1
y2
y4
y5
y6
y7
y8
y9
y10
intensity
0
10000
20000
30000
40000
50000
60000
0
20
40
60
80
100
b2
b3
b4
m/z
Fragment Ions
173.091 b2
175.120 y1
286.174 b3
290.145 y2
415.217 b4
448.216 y4
519.252 y5
590.294 y6
718.351 y7
817.414 y8
964.440 y9
1093.480 y10
TALEMVQAAGTDR / File: christine_pool_04.raw; SpectrumID: ; scans: 5103

IELPELFDR

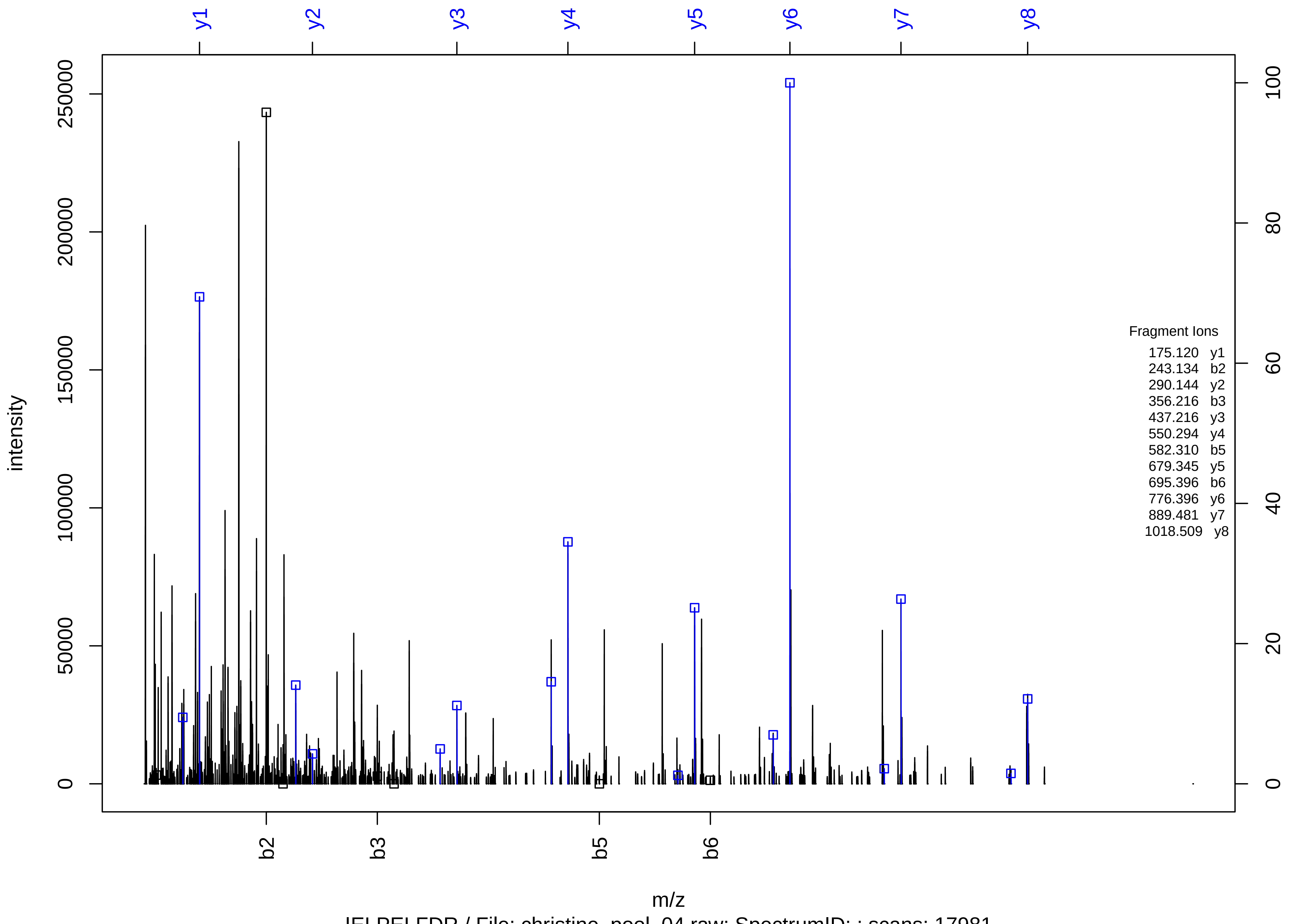

IELPELFDR / File: christine_pool_04.raw; SpectrumID: ; scans: 17981

ALSGTSPDDVQPGPSVGPPSK

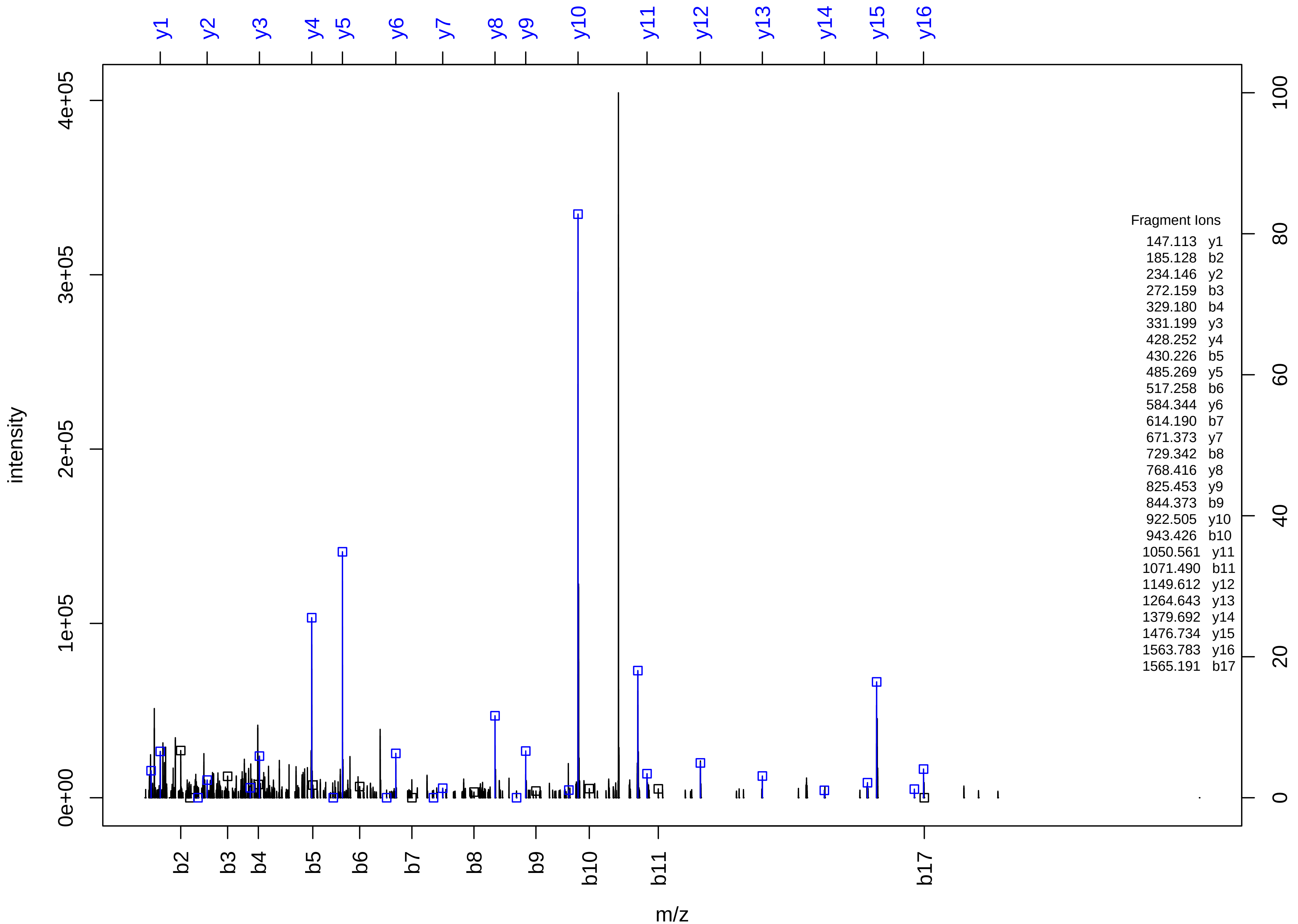

ALSGTSPDDVQPGPSVGPPSK / File: christine_pool_04.raw; SpectrumID: ; scans: 7108

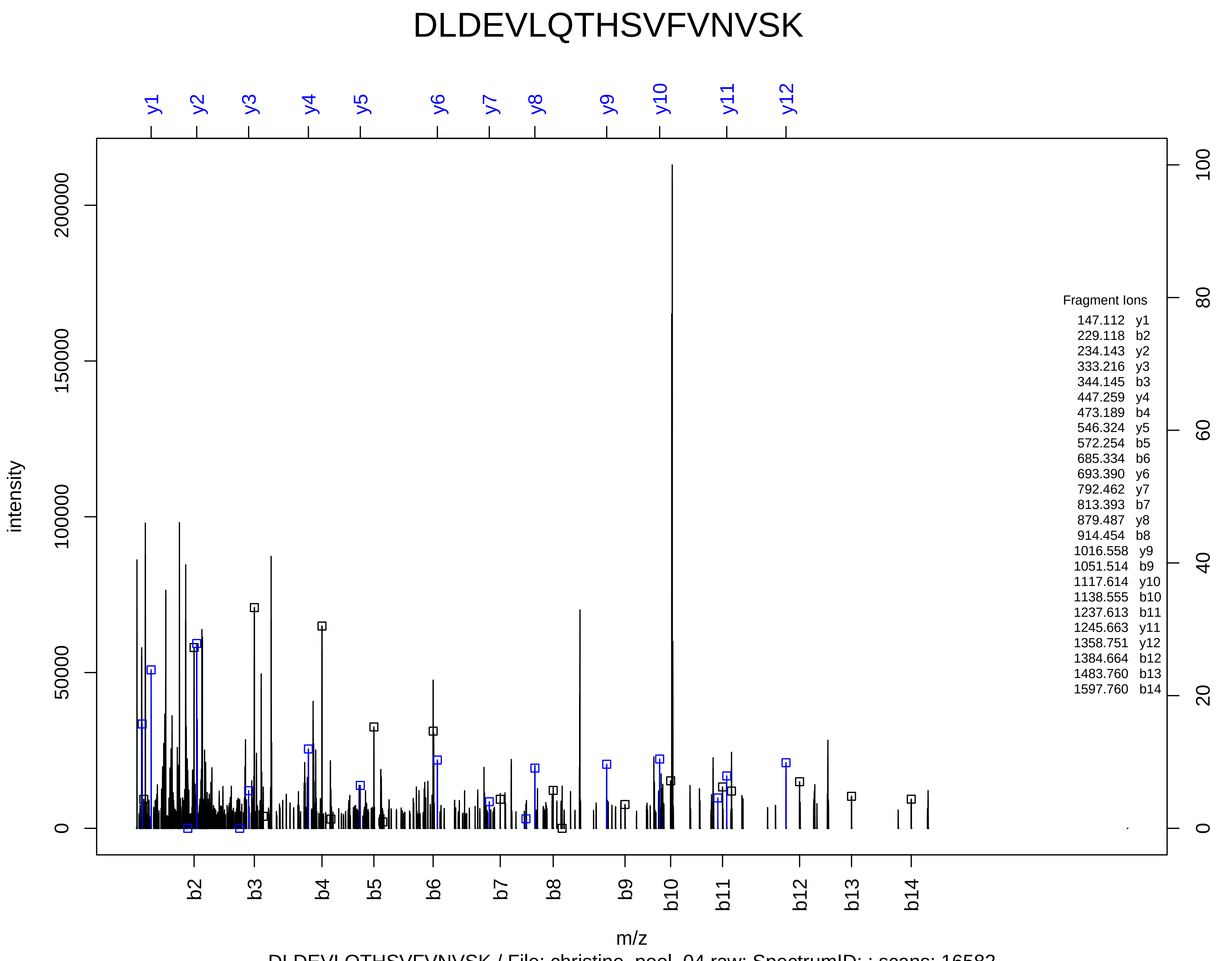
DLDEVLQTHSVFVNVSK
y1 y2 y3 y4 y5 y6 y7 y8 y9 y10 y11 y12
b2 b3 b4 b5 b6 b7 b8 b9 b10 b11 b12 b13 b14
intensity
m/z
0
50000
100000
150000
200000
0
20
40
60
80
100
Fragment Ions
147.112 y1
229.118 b2
234.143 y2
333.216 y3
344.145 b3
447.259 y4
473.189 b4
546.324 y5
572.254 b5
685.334 b6
693.390 y6
792.462 y7
813.393 b7
879.487 y8
914.454 b8
1016.558 y9
1051.514 b9
1117.614 y10
1138.555 b10
1237.613 b11
1245.663 y11
1358.751 y12
1384.664 b12
1483.760 b13
1597.760 b14
DLDEVLQTHSVFVNVSK / File: christine_pool_04.raw; SpectrumID: ; scans: 16582

LCNLEEGSPGSGTYTR

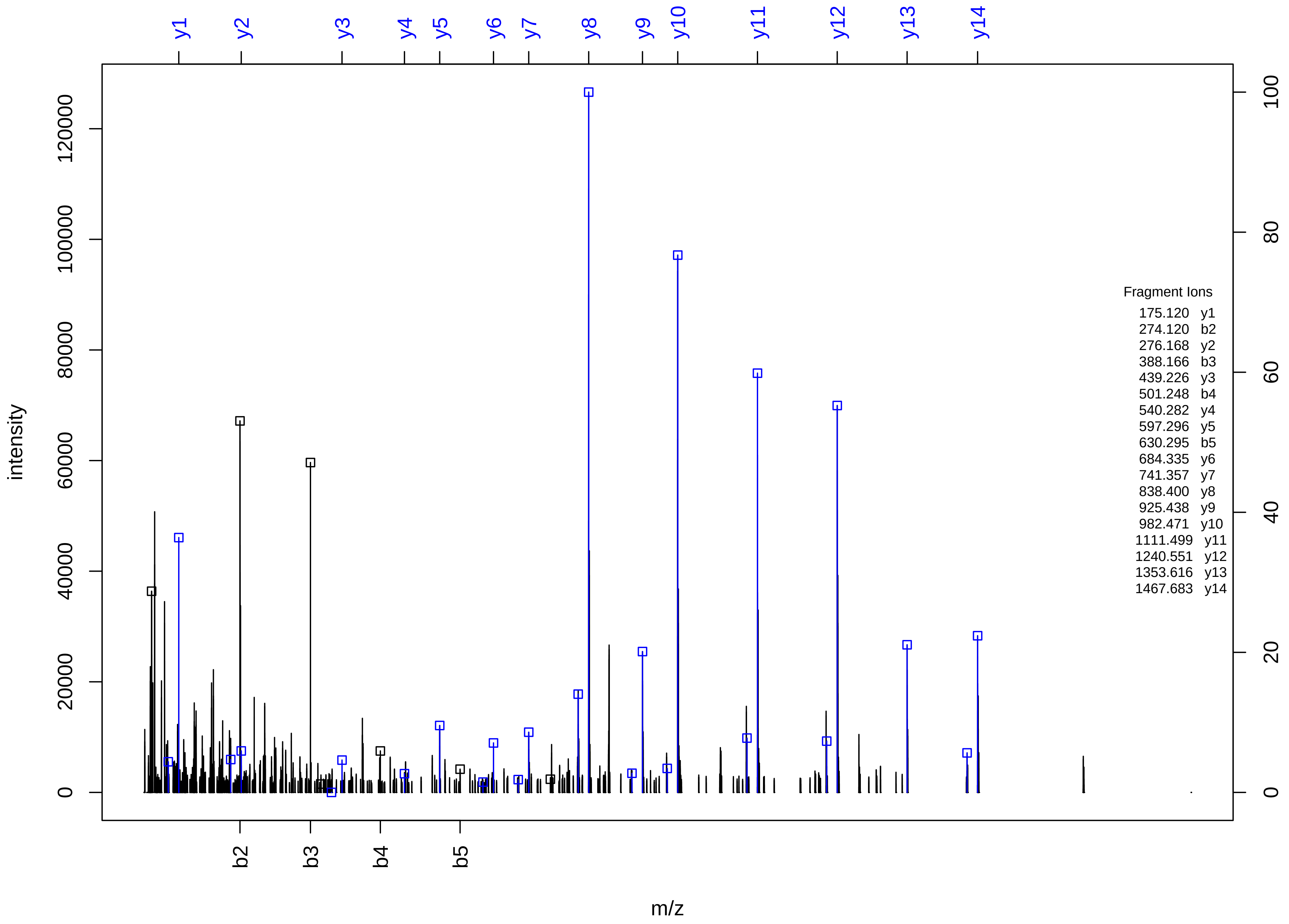

LCNLEEGSPGSGTYTR / File: christine_pool_04.raw; SpectrumID: ; scans: 5542

ELLLLIR

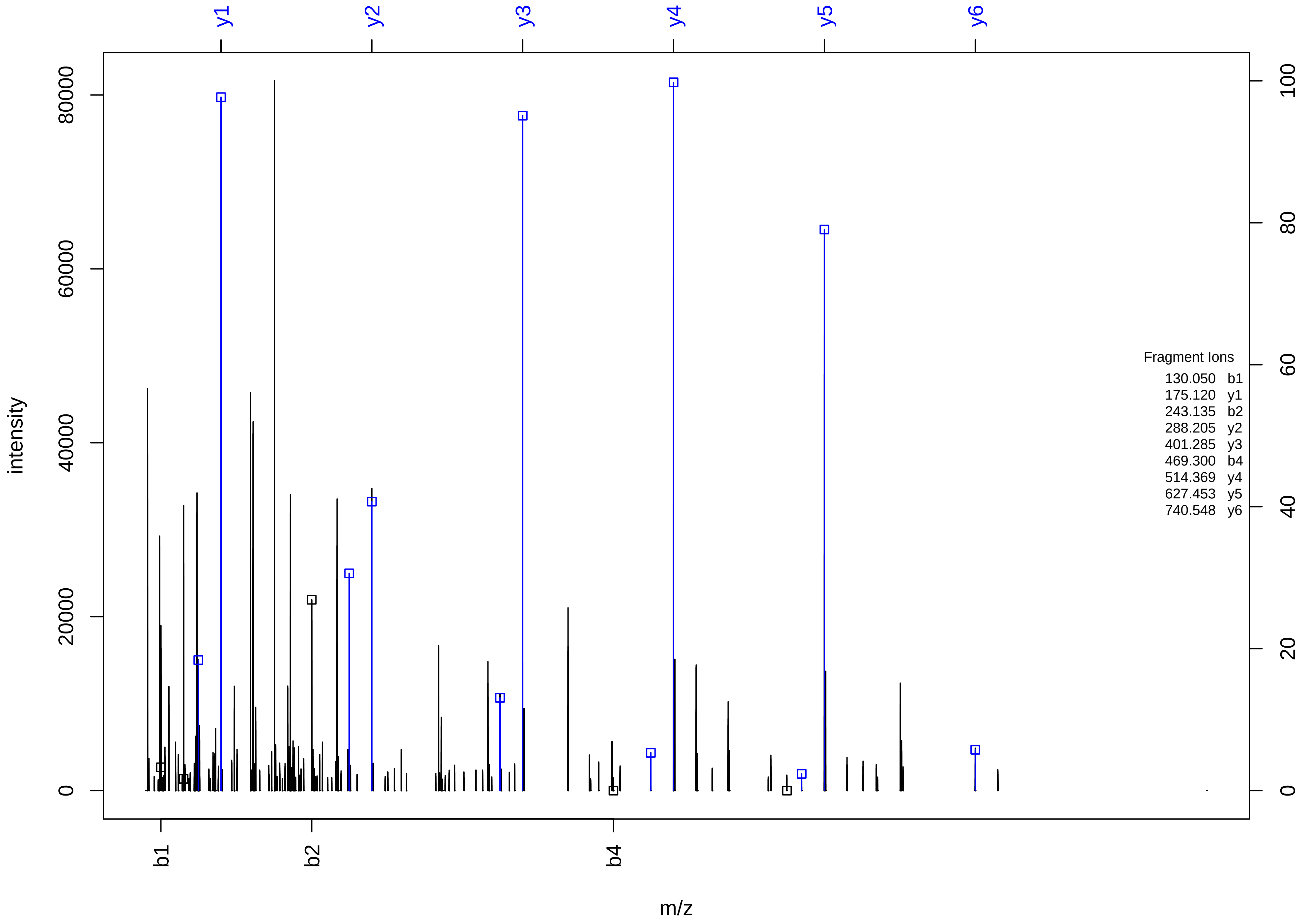

ELLLLIR / File: christine_pool_04.raw; SpectrumID: ; scans: 17812

DLFALAR

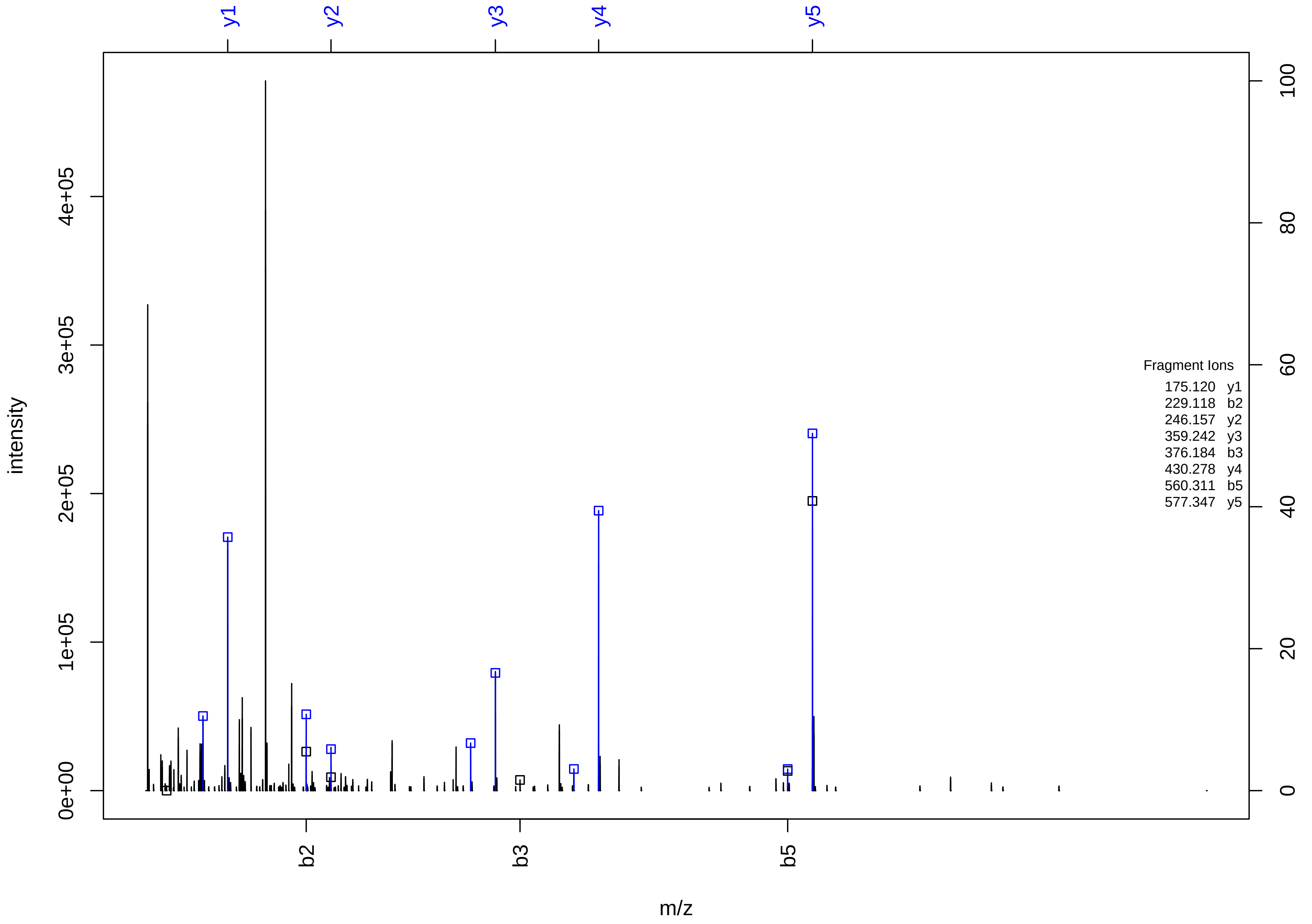

DLFALAR / File: christine_pool_04.raw; SpectrumID: ; scans: 14607

(Ac)AANATTNPSQLLPLELVDK

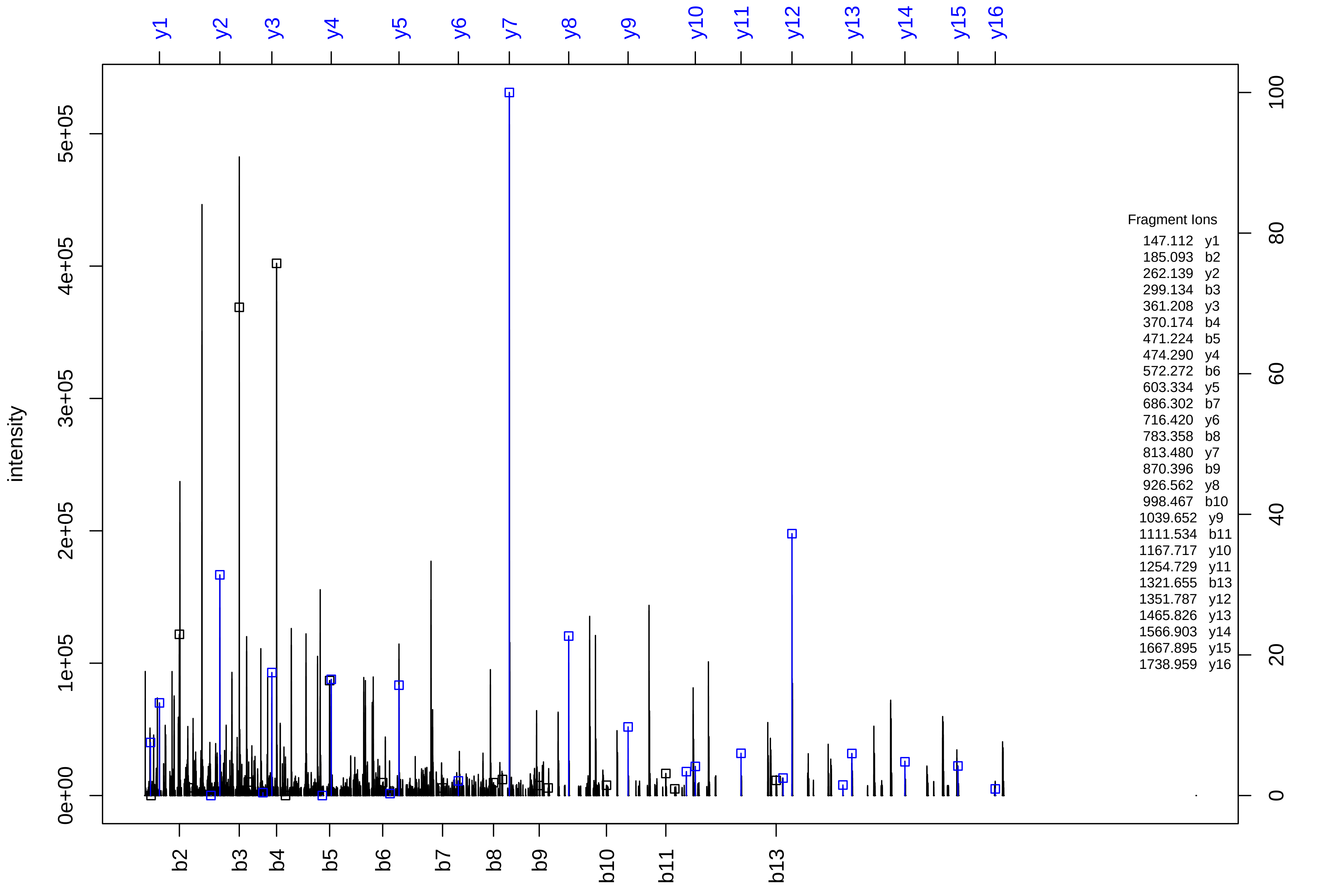

AANATTNPSQLLPLELVDK / File: christine_pool_04.raw; SpectrumID: ; scans: 23149

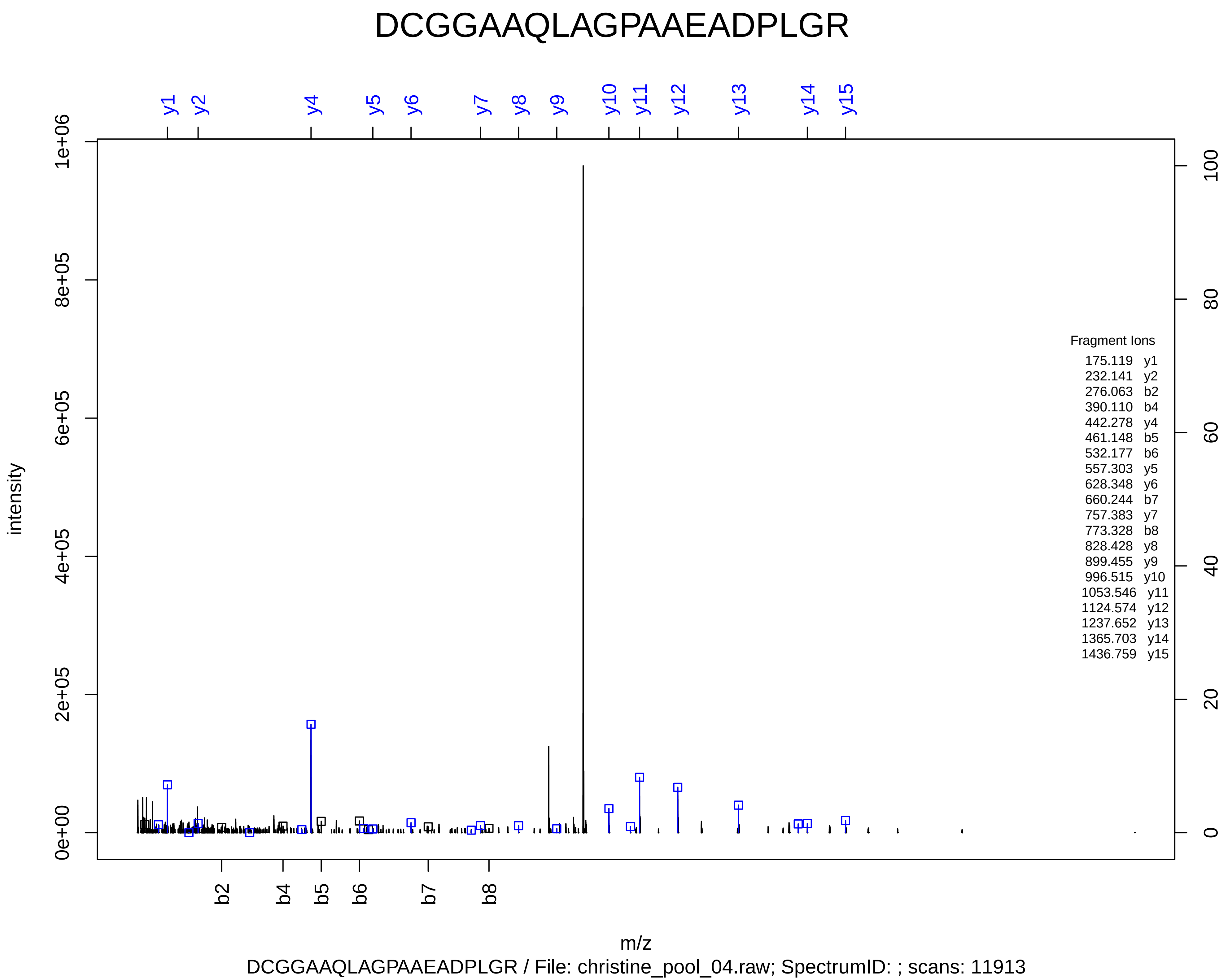
DCGGAAQLAGPAAEADPLGR
y1
y2
y4
y5
y6
y7
y8
y9
y10
y11
y12
y13
y14
y15
intensity
0e+00
2e+05
4e+05
6e+05
8e+05
1e+06
0
20
40
60
80
100
b2
b4
b5
b6
b7
b8
m/z
Fragment Ions
175.119 y1
232.141 y2
276.063 b2
390.110 b4
442.278 y4
461.148 b5
532.177 b6
557.303 y5
628.348 y6
660.244 b7
757.383 y7
773.328 b8
828.428 y8
899.455 y9
996.515 y10
1053.546 y11
1124.574 y12
1237.652 y13
1365.703 y14
1436.759 y15
DCGGAAQLAGPAAEADPLGR / File: christine_pool_04.raw; SpectrumID: ; scans: 11913

DLVHTEGQLQNEEIVAHDGSATYLR

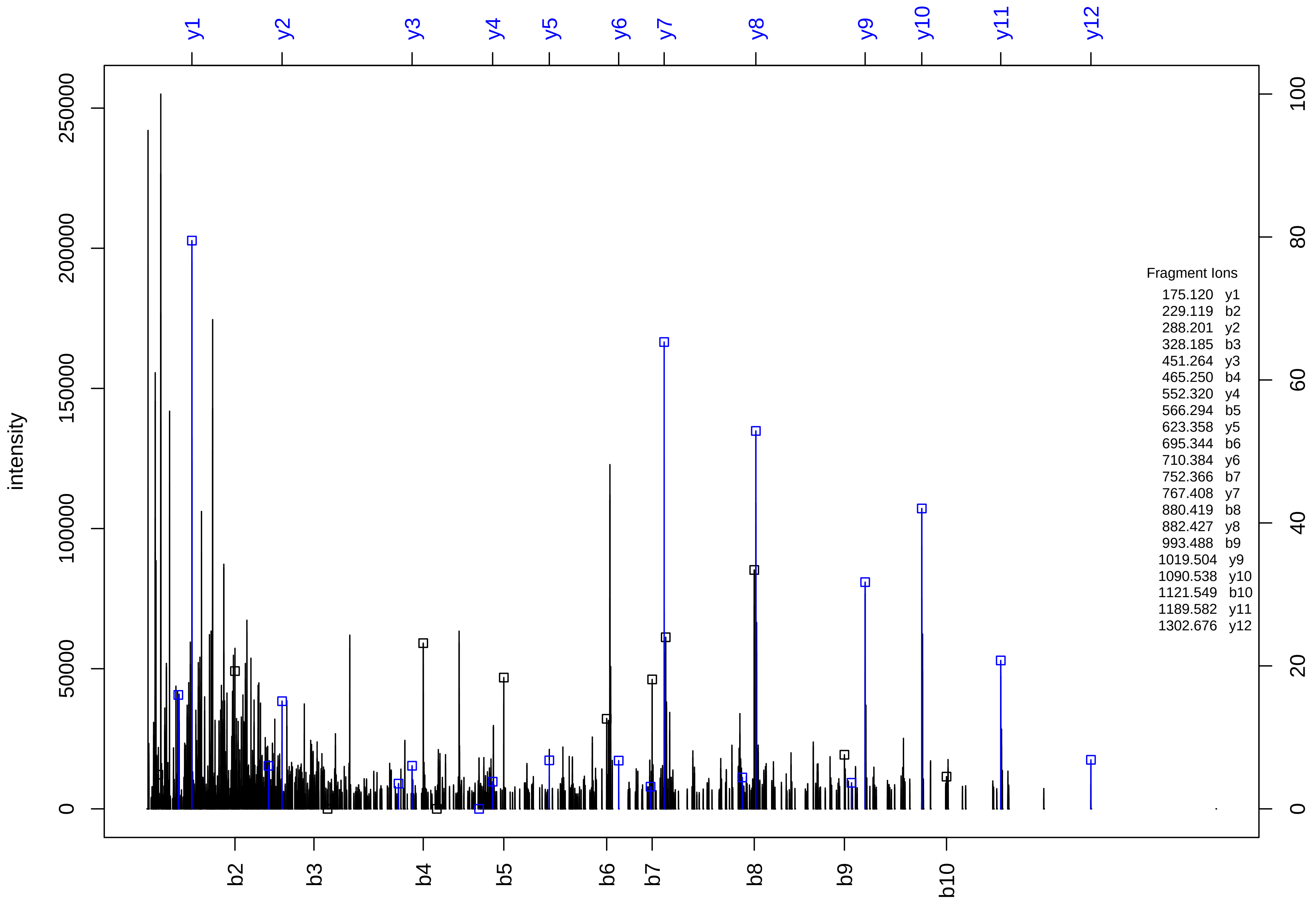

DLVHTEGQLQNEEIVAHDGSATYLR / File: christine_pool_04.raw; SpectrumID: ; scans: 11588

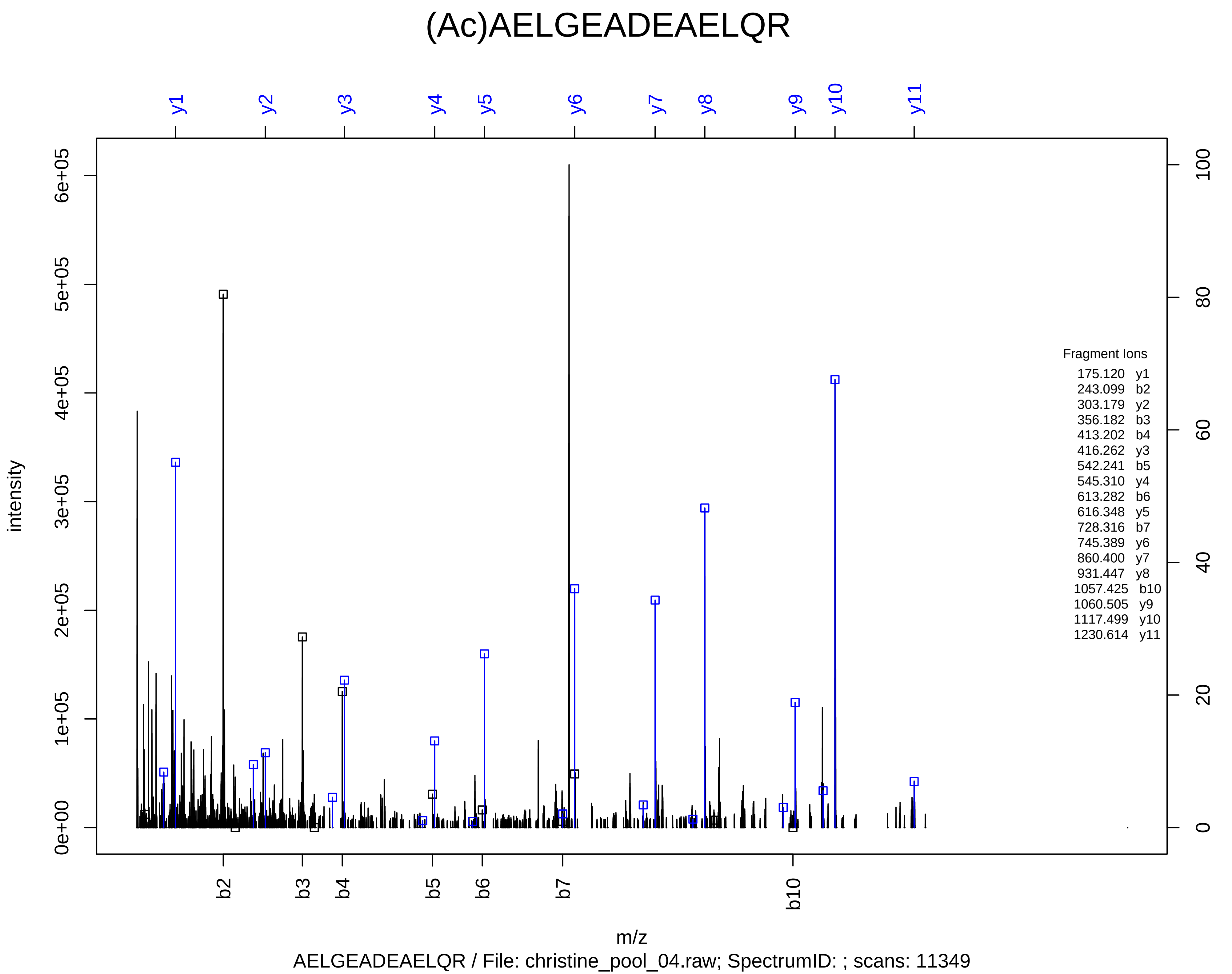

(Ac)AELGEADEAELQR
y1
y2
y3
y4
y5
y6
y7
y8
y9
y10
y11
intensity
0e+00
1e+05
2e+05
3e+05
4e+05
5e+05
6e+05
0
20
40
60
80
100
b2
b3
b4
b5
b6
b7
b10
m/z
Fragment Ions
175.120 y1
243.099 b2
303.179 y2
356.182 b3
413.202 b4
416.262 y3
542.241 b5
545.310 y4
613.282 b6
616.348 y5
728.316 b7
745.389 y6
860.400 y7
931.447 y8
1057.425 b10
1060.505 y9
1117.499 y10
1230.614 y11
AELGEADEAELQR / File: christine_pool_04.raw; SpectrumID: ; scans: 11349

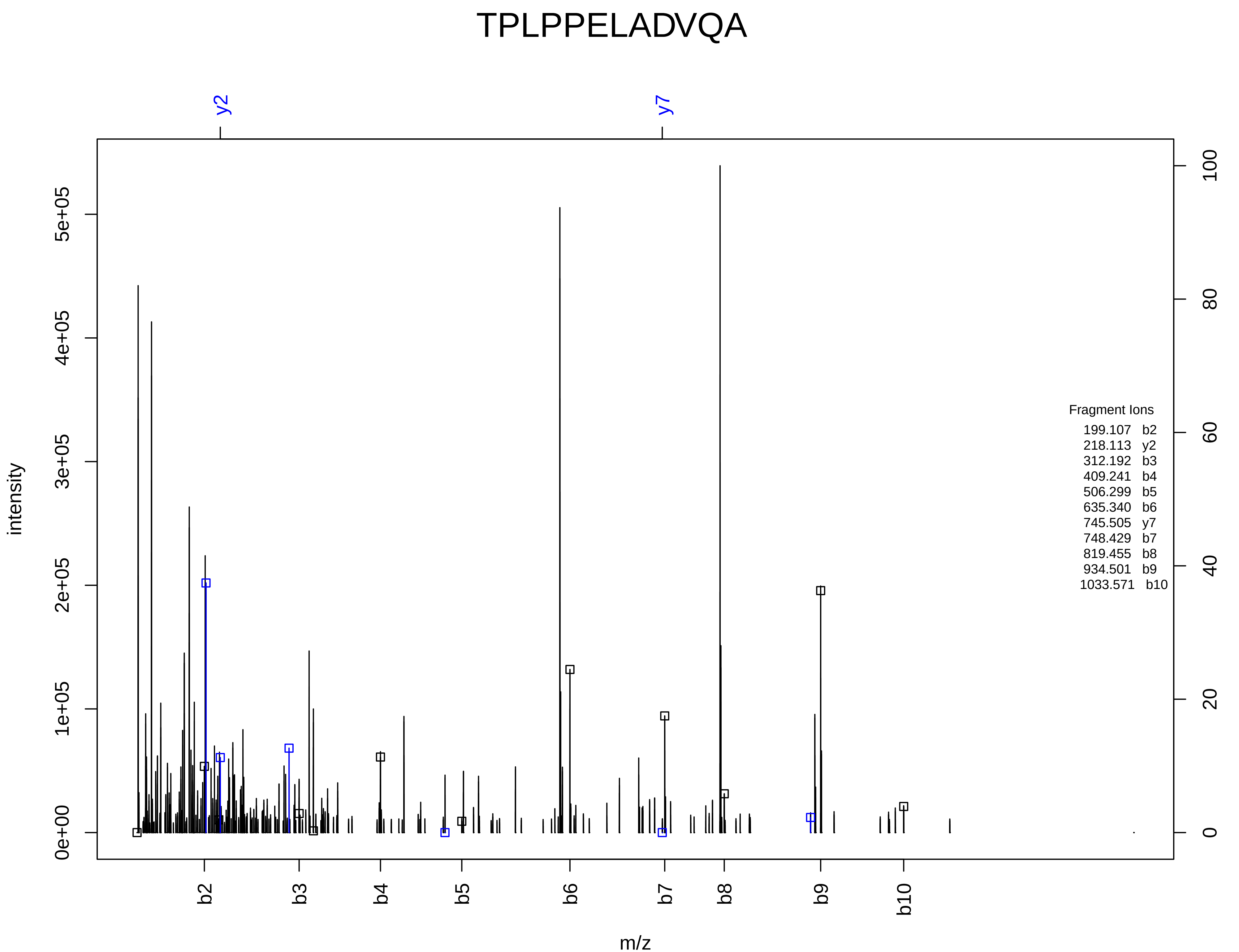

TPLPPELADVQA / File: christine_pool_04.raw; SpectrumID: ; scans: 16669

RPDEVPDDDEPAGPM*K

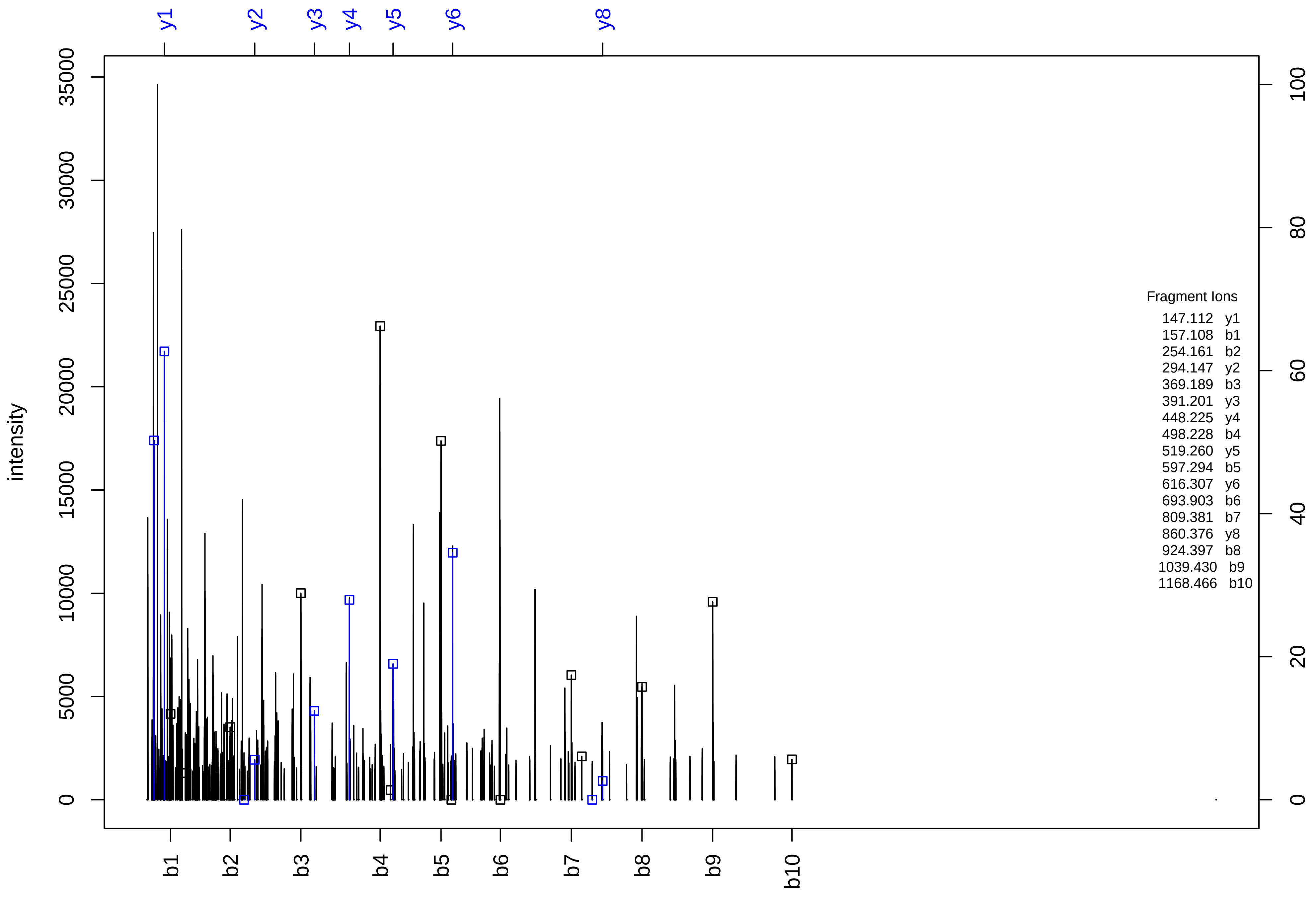

RPDEVPDDDEPAGPMK / File: christine_pool_04.raw; SpectrumID: ; scans: 3264

N^FLKMWMK

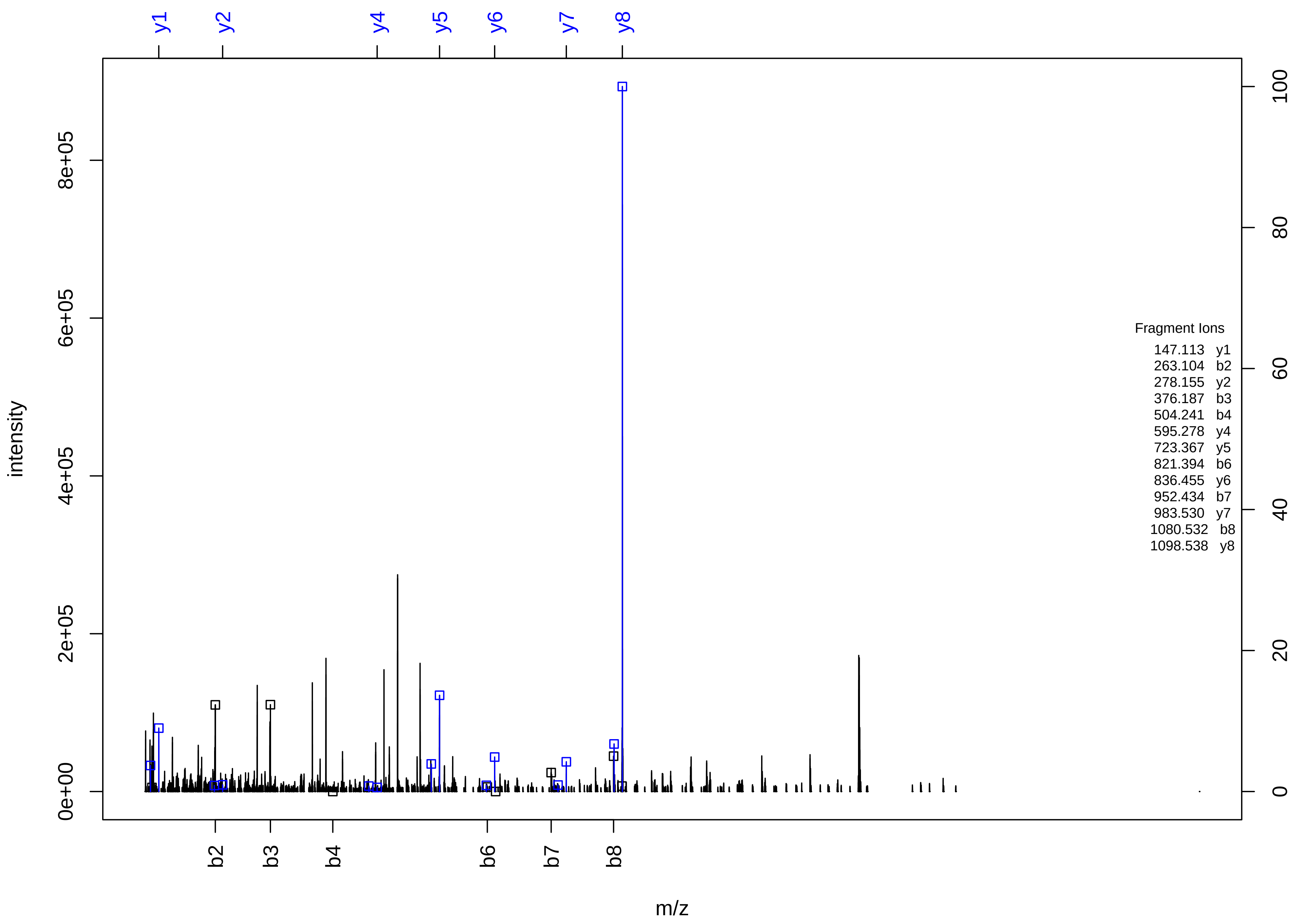

NFLKMWMK / File: christine_pool_04.raw; SpectrumID: ; scans: 17129

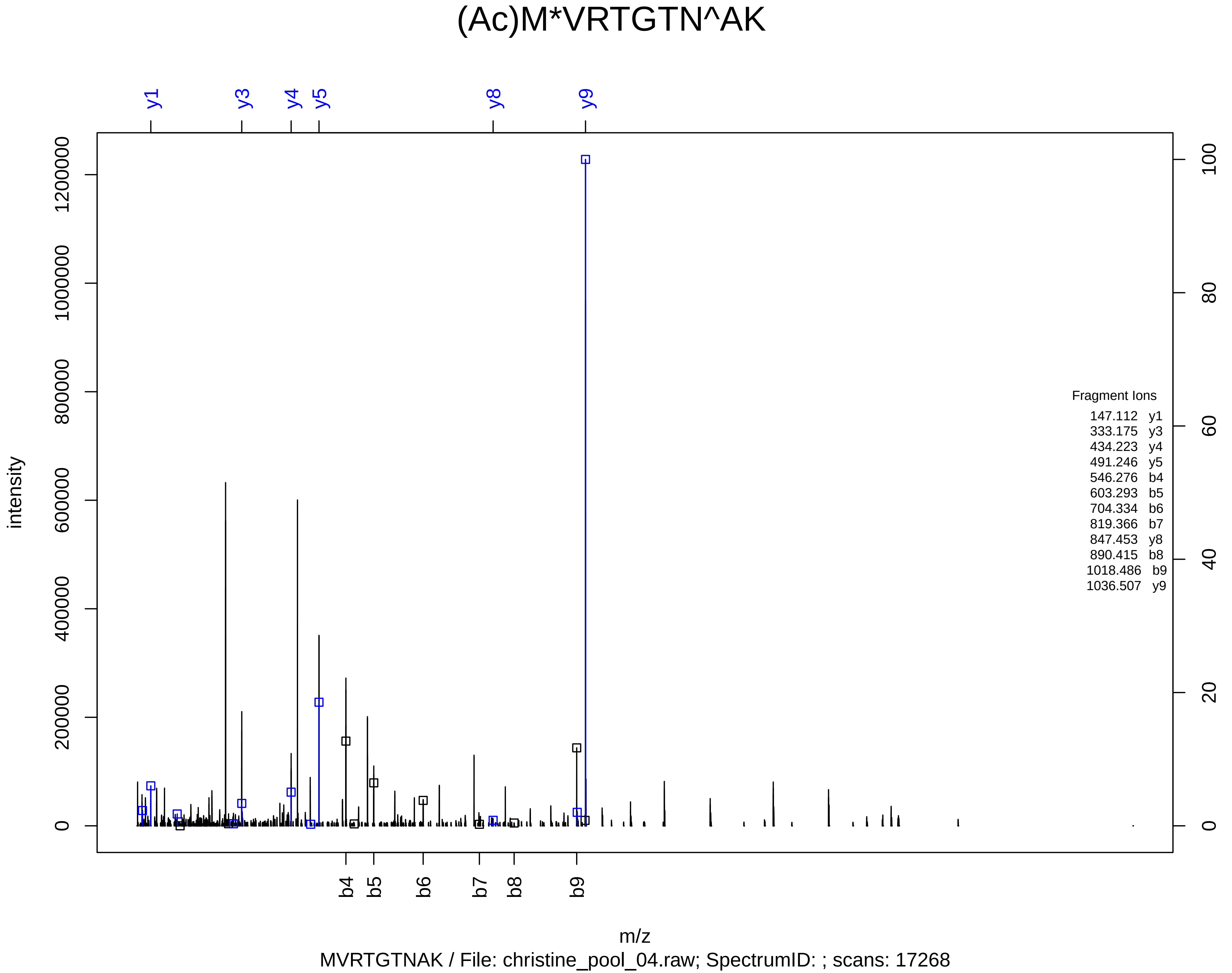

(Ac)M*VRTGTN^AK
y1
y3
y4
y5
y8
y9
intensity
0
200000
400000
600000
800000
1000000
1200000
0
20
40
60
80
100
Fragment Ions
147.112 y1
333.175 y3
434.223 y4
491.246 y5
546.276 b4
603.293 b5
704.334 b6
819.366 b7
847.453 y8
890.415 b8
1018.486 b9
1036.507 y9
b4
b5
b6
b7
b8
b9
m/z
MVRTGTNAK / File: christine_pool_04.raw; SpectrumID: ; scans: 17268

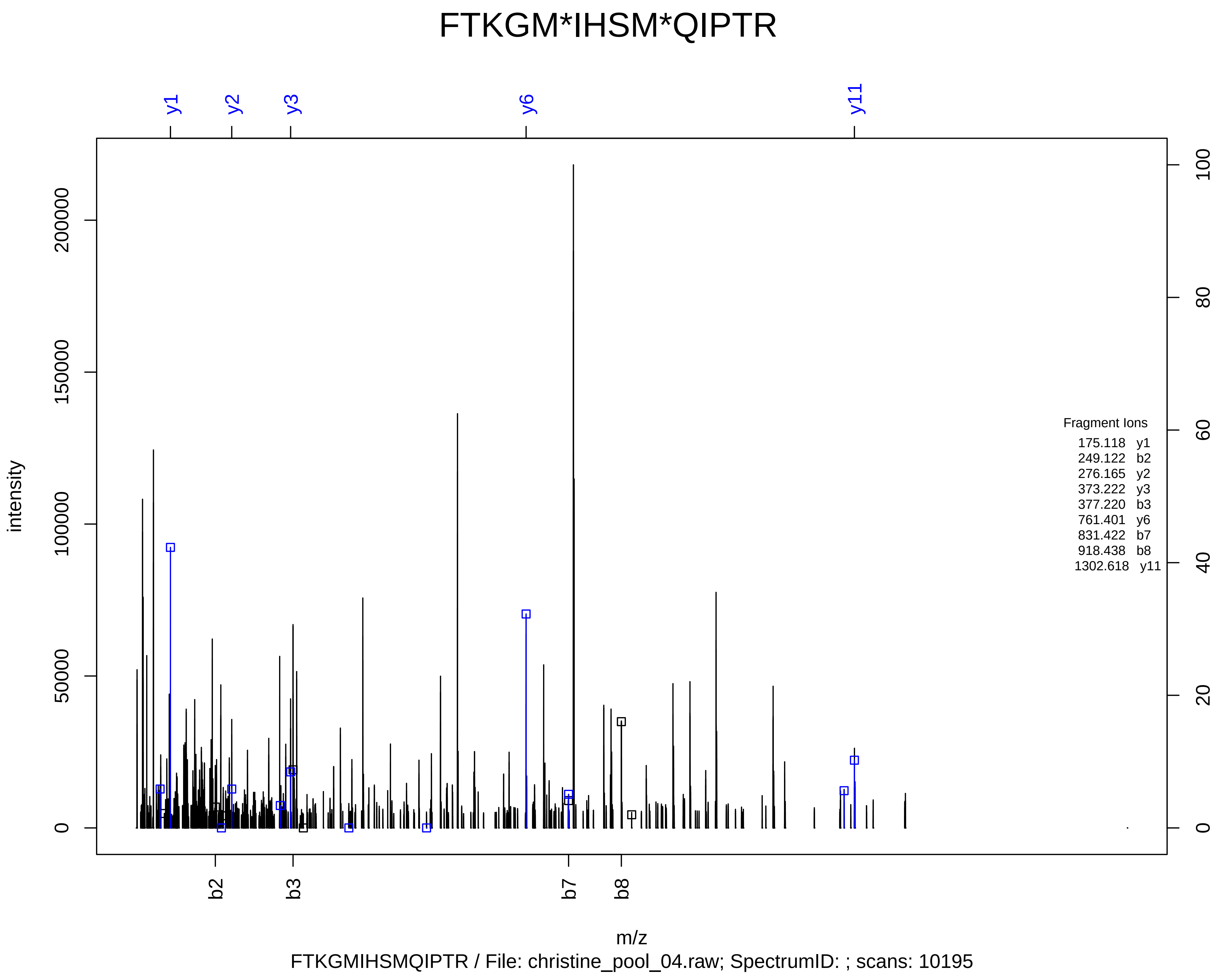
FTKGM*IHSM*QIPTR
y1
y2
y3
y6
y11
intensity
0
50000
100000
150000
200000
0
20
40
60
80
100
b2
b3
b7
b8
m/z
Fragment Ions
175.118 y1
249.122 b2
276.165 y2
373.222 y3
377.220 b3
761.401 y6
831.422 b7
918.438 b8
1302.618 y11
FTKGMIHSMQIPTR / File: christine_pool_04.raw; SpectrumID: ; scans: 10195

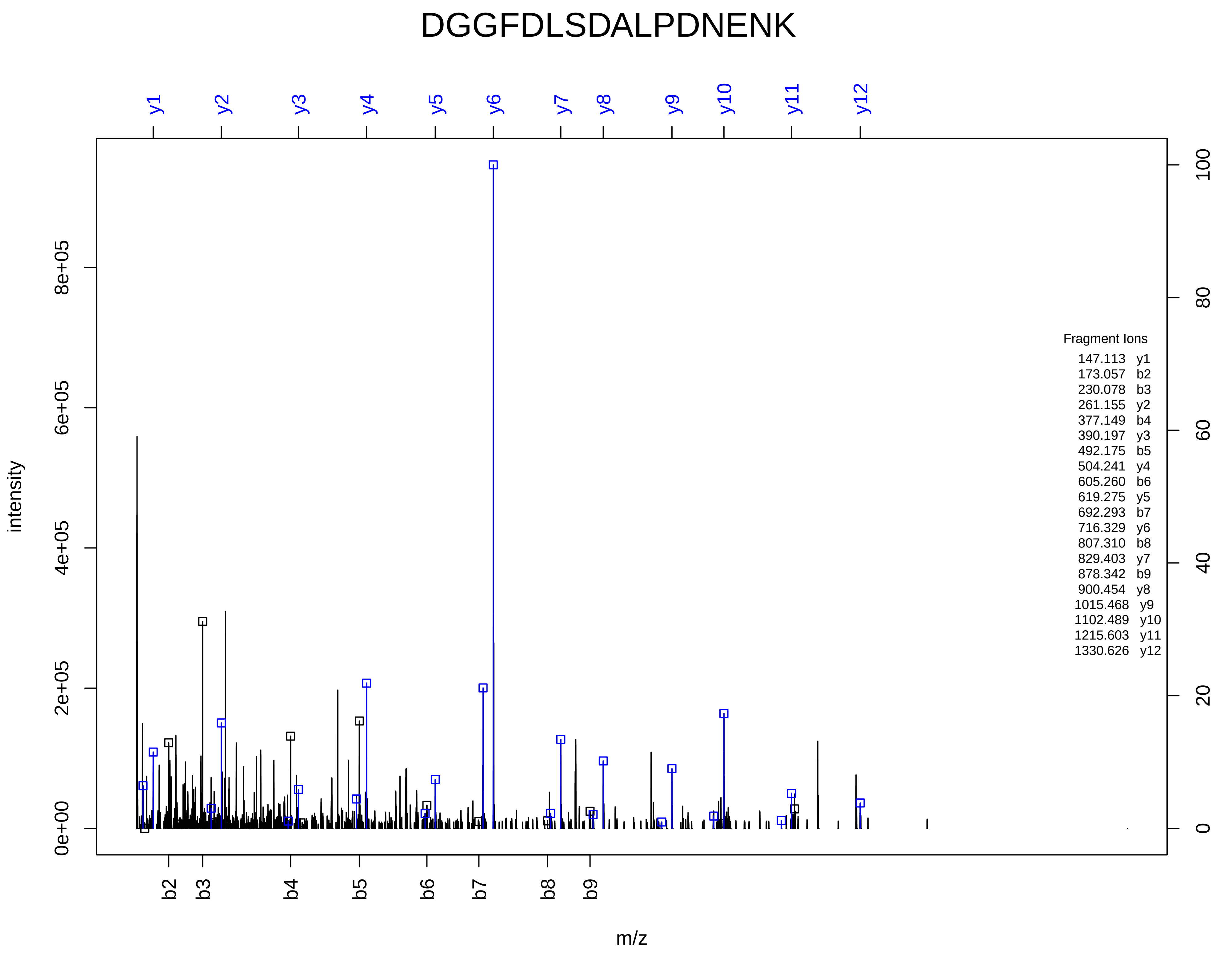

DGGFDLSDALPDNENK
y1 y2 y3 y4 y5 y6 y7 y8 y9 y10 y11 y12
b2 b3 b4 b5 b6 b7 b8 b9
intensity
0e+00 2e+05 4e+05 6e+05 8e+05
0 20 40 60 80 100
m/z
Fragment Ions
147.113 y1
173.057 b2
230.078 b3
261.155 y2
377.149 b4
390.197 y3
492.175 b5
504.241 y4
605.260 b6
619.275 y5
692.293 b7
716.329 y6
807.310 b8
829.403 y7
878.342 b9
900.454 y8
1015.468 y9
1102.489 y10
1215.603 y11
1330.626 y12
DGGFDLSDALPDNENK / File: christine_pool_04.raw; SpectrumID: ; scans: 14276

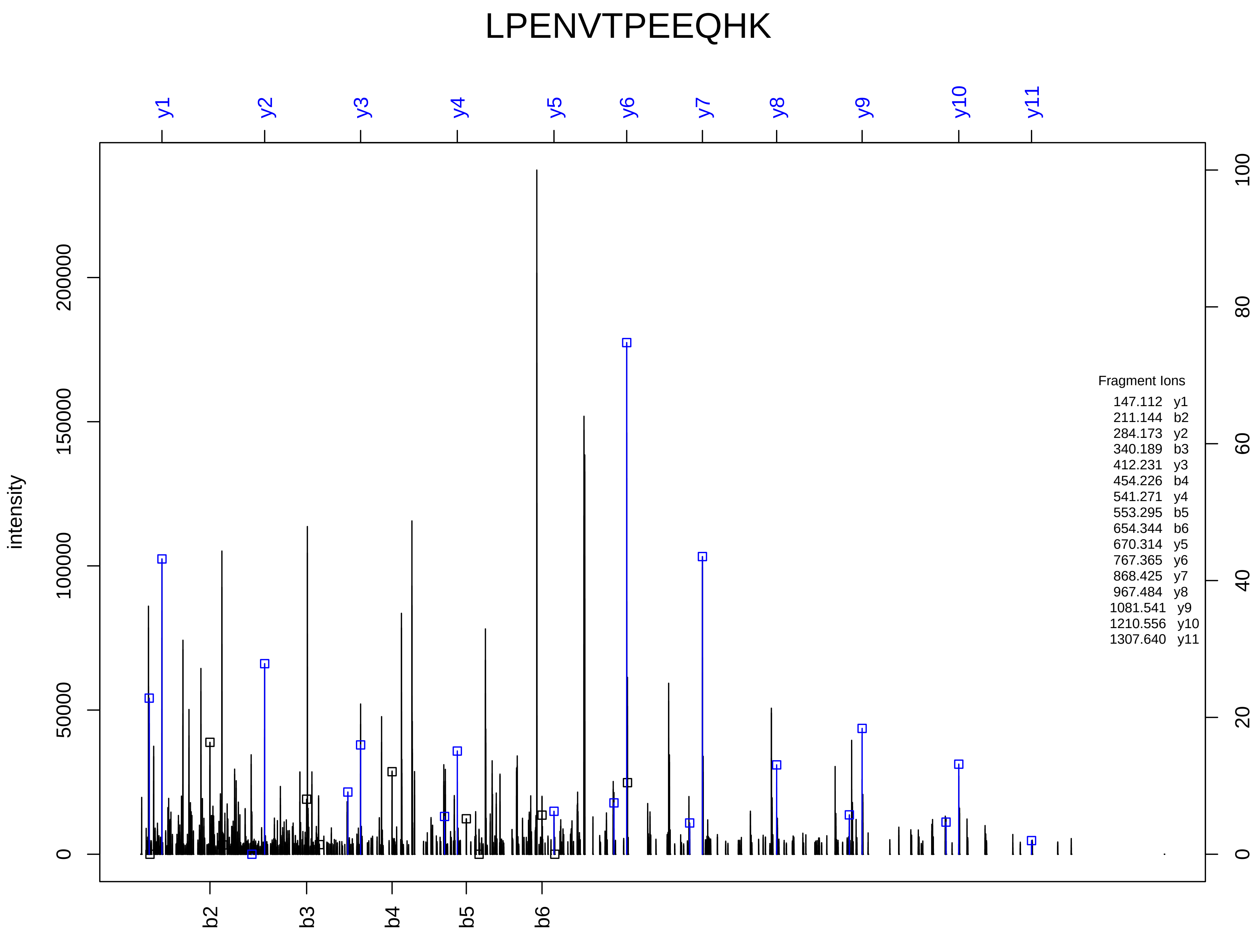

LPENVTPEEQHK / File: christine_pool_02.raw; SpectrumID: ; scans: 3393

SCCSCCPVGCAK

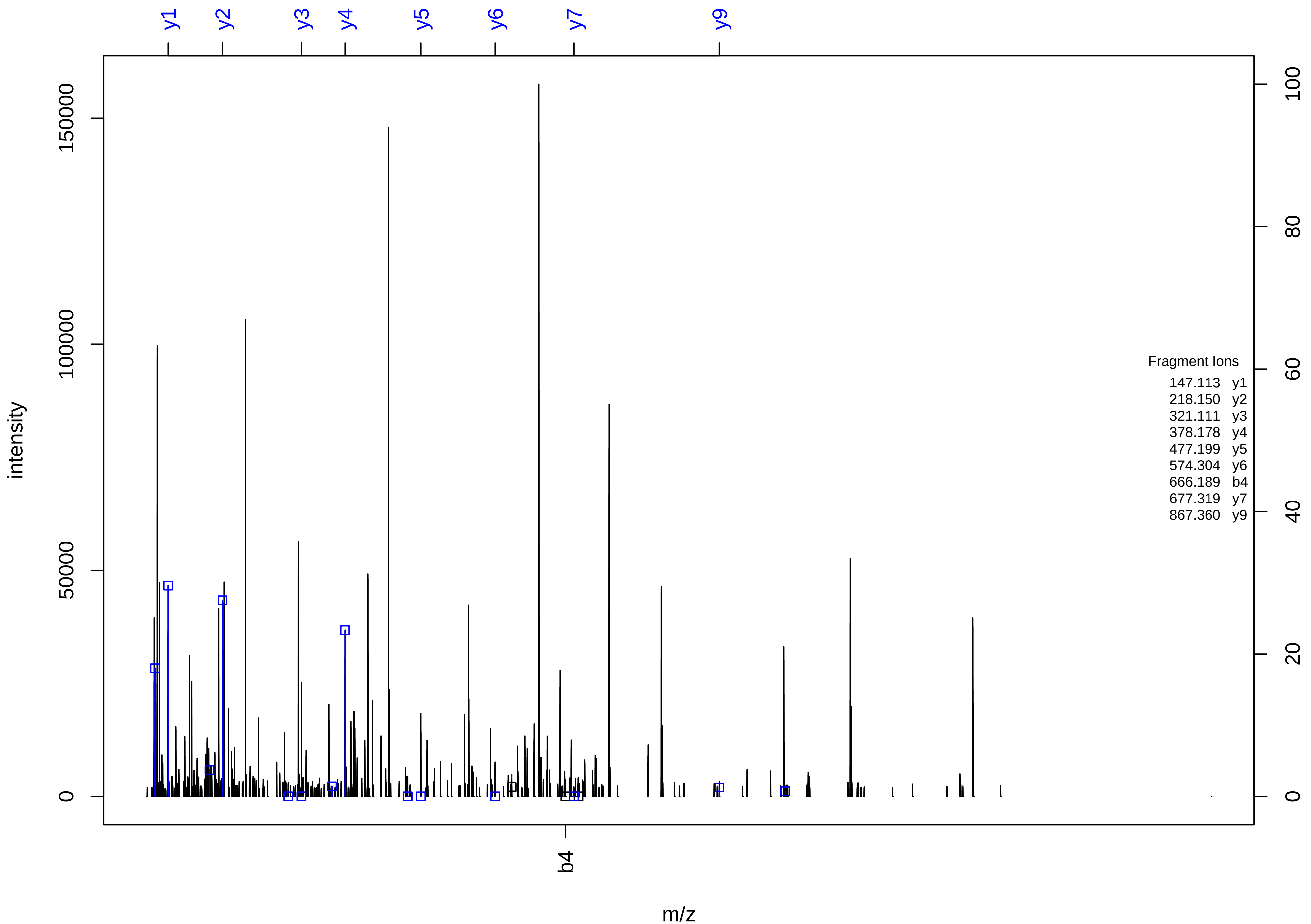

SCCSCCPVGCAK / File: christine_pool_02.raw; SpectrumID: ; scans: 2092

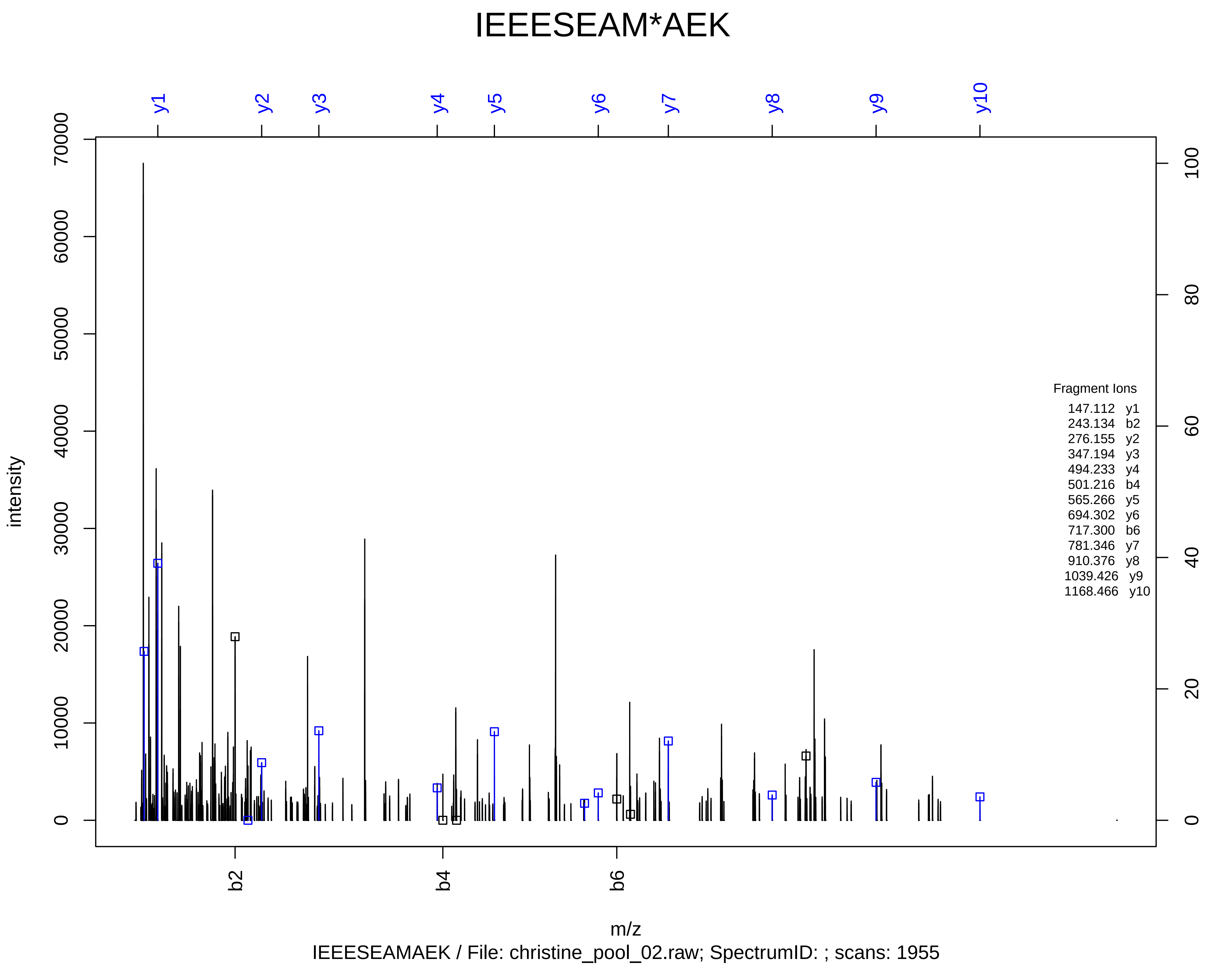
IEEESEAM*AEK
y1
y2
y3
y4
y5
y6
y7
y8
y9
y10
intensity
0
10000
20000
30000
40000
50000
60000
70000
0
20
40
60
80
100
b2
b4
b6
m/z
Fragment Ions
147.112 y1
243.134 b2
276.155 y2
347.194 y3
494.233 y4
501.216 b4
565.266 y5
694.302 y6
717.300 b6
781.346 y7
910.376 y8
1039.426 y9
1168.466 y10
IEEESEAMAEK / File: christine_pool_02.raw; SpectrumID: ; scans: 1955

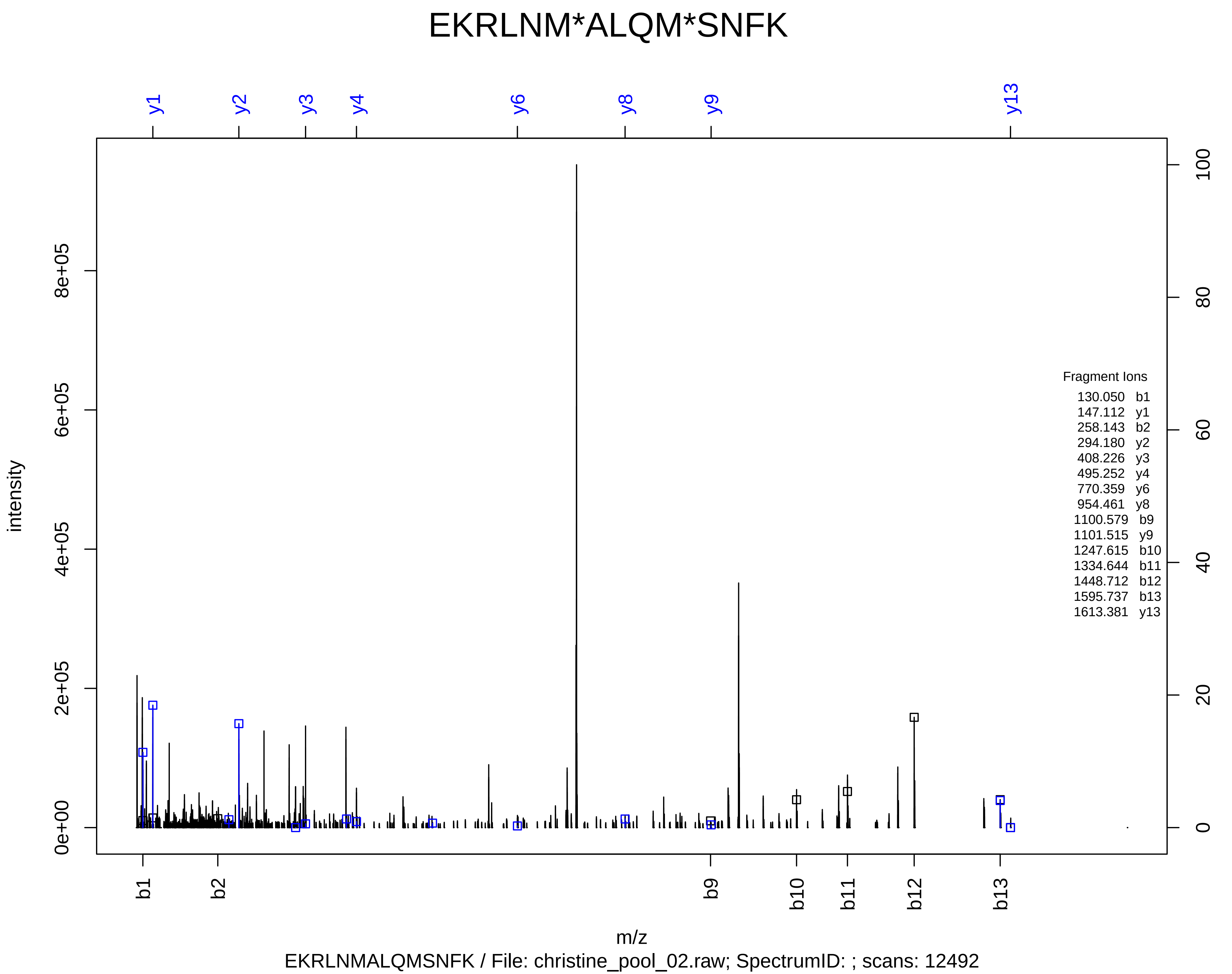

EKRLNM*ALQM*SNFK
y1
y2
y3
y4
y6
y8
y9
y13
intensity
0e+00
2e+05
4e+05
6e+05
8e+05
0
20
40
60
80
100
Fragment Ions
130.050 b1
147.112 y1
258.143 b2
294.180 y2
408.226 y3
495.252 y4
770.359 y6
954.461 y8
1100.579 b9
1101.515 y9
1247.615 b10
1334.644 b11
1448.712 b12
1595.737 b13
1613.381 y13
b1
b2
b9
b10
b11
b12
b13
m/z
EKRLNMALQMSNFK / File: christine_pool_02.raw; SpectrumID: ; scans: 12492

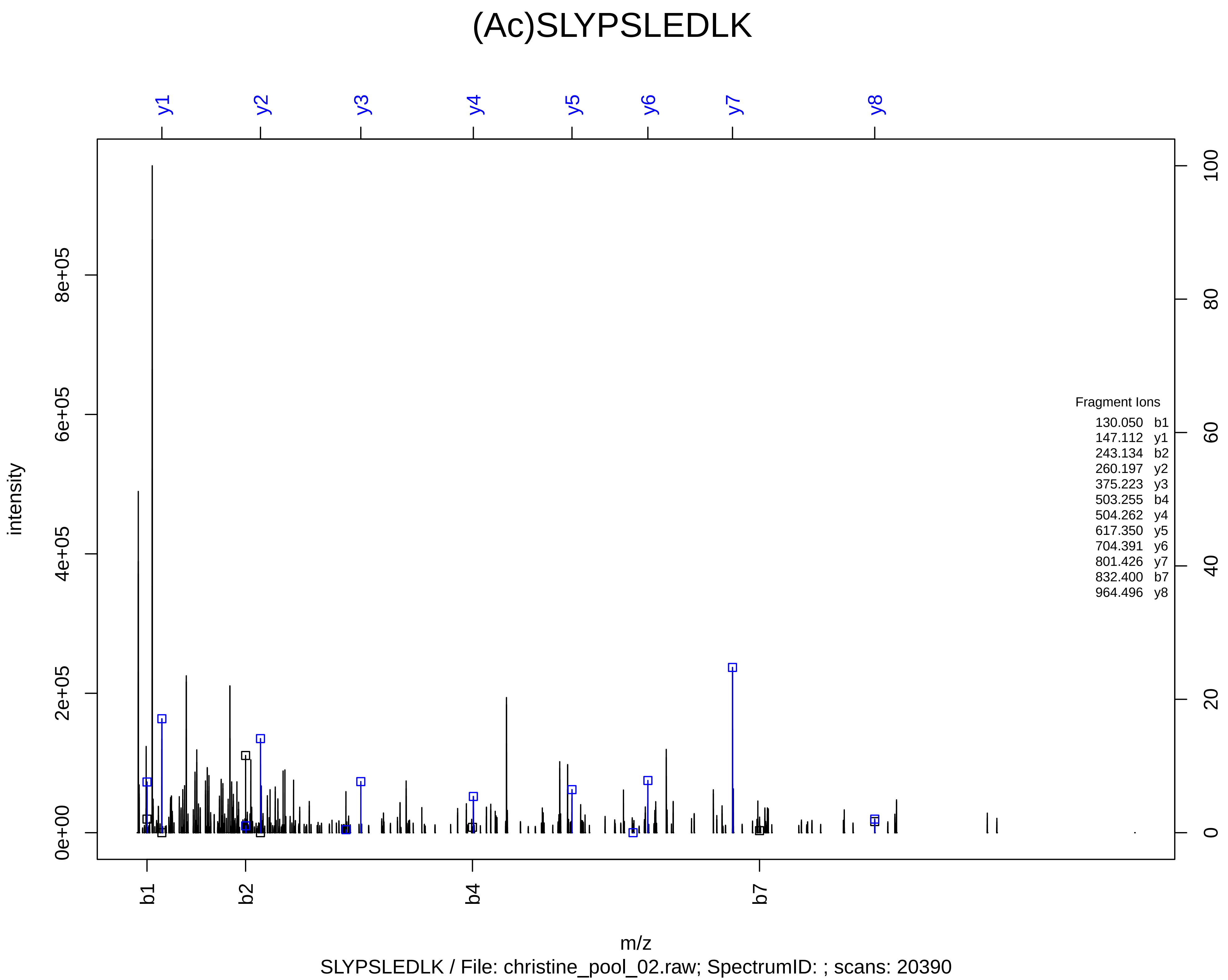
(Ac)SLYPSLEDLK
y1
y2
y3
y4
y5
y6
y7
y8
intensity
0e+00
2e+05
4e+05
6e+05
8e+05
0
20
40
60
80
100
Fragment Ions
130.050 b1
147.112 y1
243.134 b2
260.197 y2
375.223 y3
503.255 b4
504.262 y4
617.350 y5
704.391 y6
801.426 y7
832.400 b7
964.496 y8
b1
b2
b4
b7
m/z
SLYPSLEDLK / File: christine_pool_02.raw; SpectrumID: ; scans: 20390

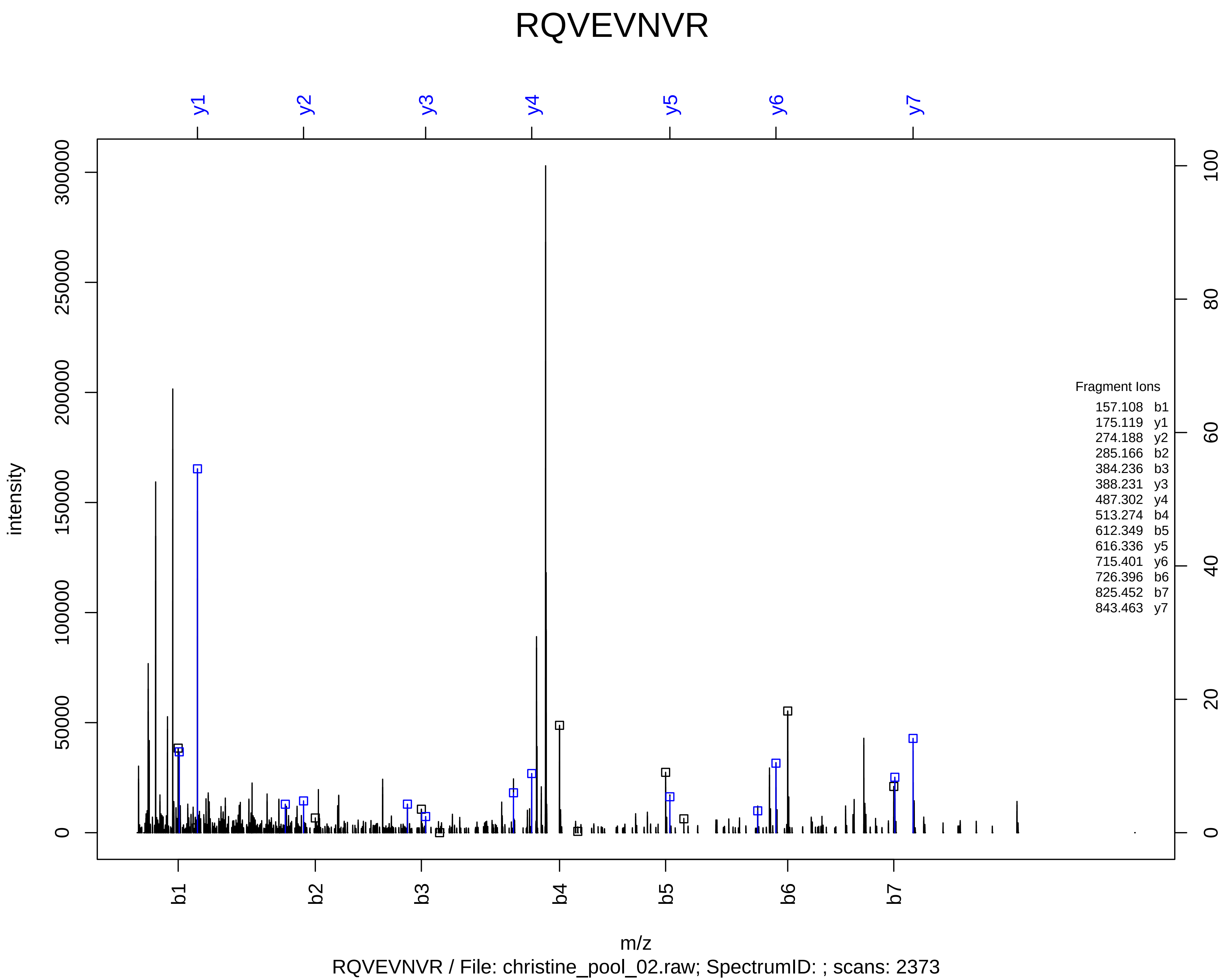

RQVEVNVR
y1
y2
y3
y4
y5
y6
y7
intensity
0
50000
100000
150000
200000
250000
300000
0
20
40
60
80
100
b1
b2
b3
b4
b5
b6
b7
m/z
Fragment Ions
157.108 b1
175.119 y1
274.188 y2
285.166 b2
384.236 b3
388.231 y3
487.302 y4
513.274 b4
612.349 b5
616.336 y5
715.401 y6
726.396 b6
825.452 b7
843.463 y7
RQVEVNVR / File: christine_pool_02.raw; SpectrumID: ; scans: 2373

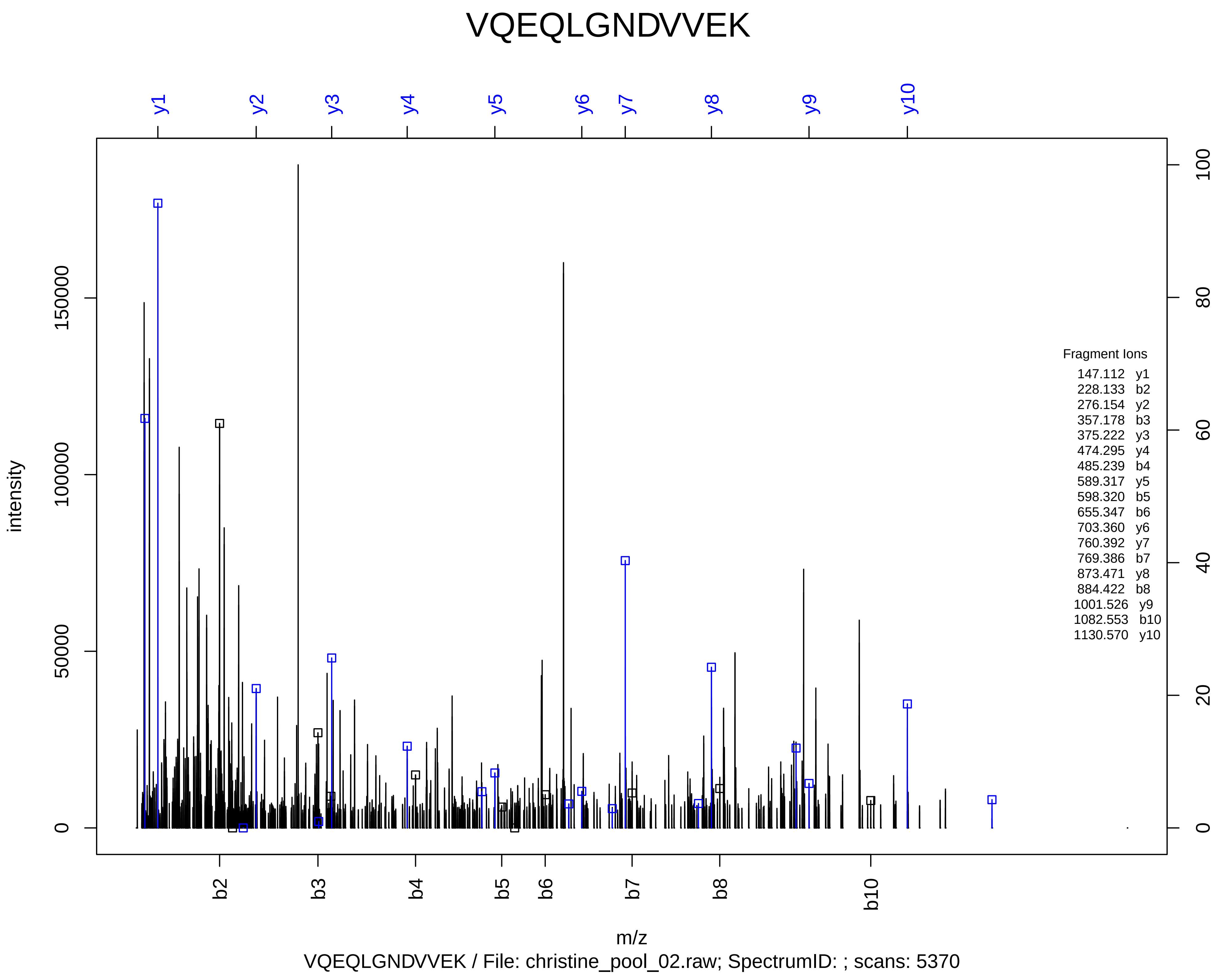

VQEQLGNDVVEK
y1
y2
y3
y4
y5
y6
y7
y8
y9
y10
b2
b3
b4
b5
b6
b7
b8
b10
intensity
m/z
Fragment Ions
147.112 y1
228.133 b2
276.154 y2
357.178 b3
375.222 y3
474.295 y4
485.239 b4
589.317 y5
598.320 b5
655.347 b6
703.360 y6
760.392 y7
769.386 b7
873.471 y8
884.422 b8
1001.526 y9
1082.553 b10
1130.570 y10
VQEQLGNDVVEK / File: christine_pool_02.raw; SpectrumID: ; scans: 5370

EFEPLLR

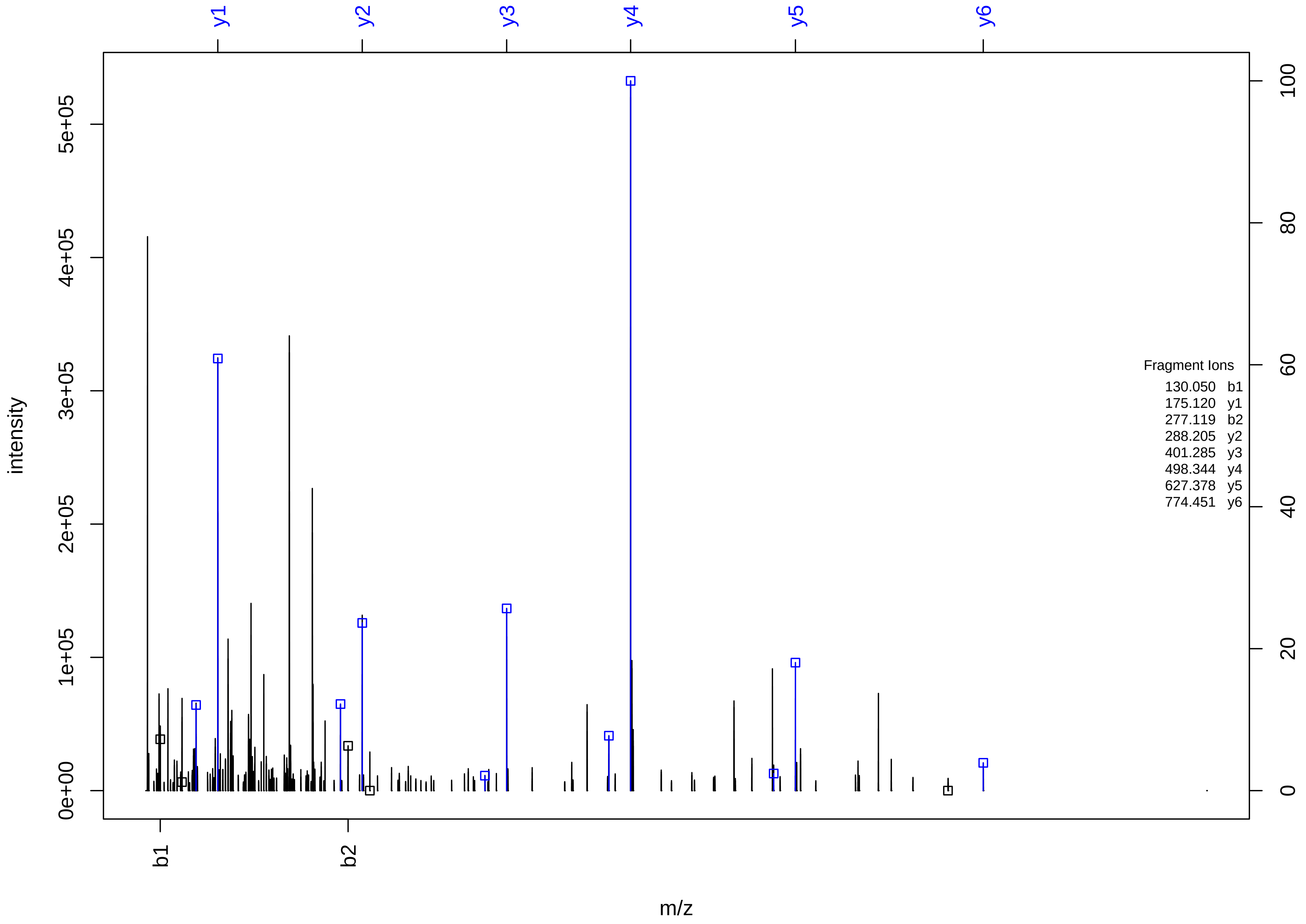

EFEPLLR / File: christine_pool_02.raw; SpectrumID: ; scans: 11914

GQNPNATFGEVSK

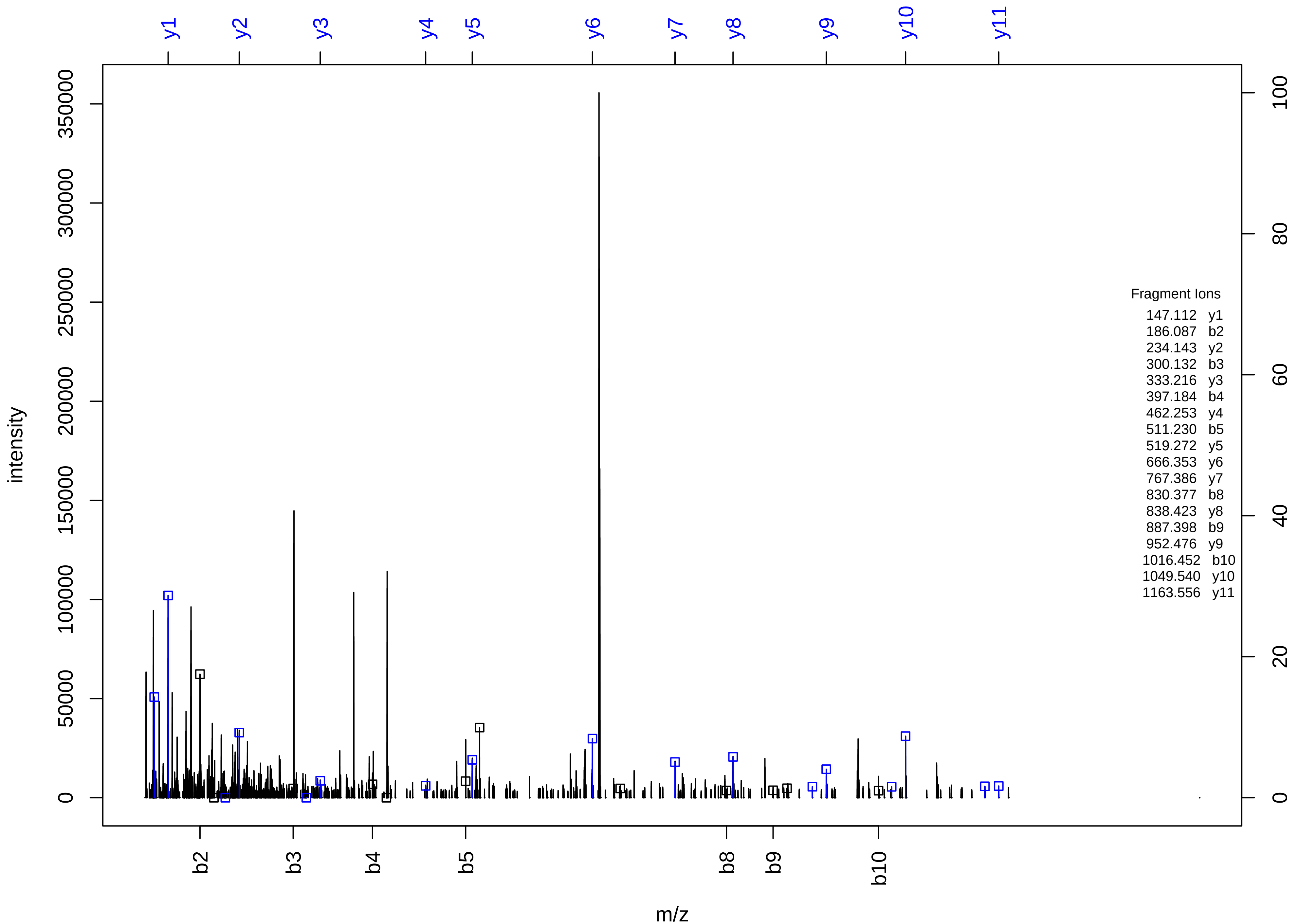

GQNPNATFGEVSK / File: christine_pool_02.raw; SpectrumID: ; scans: 4954

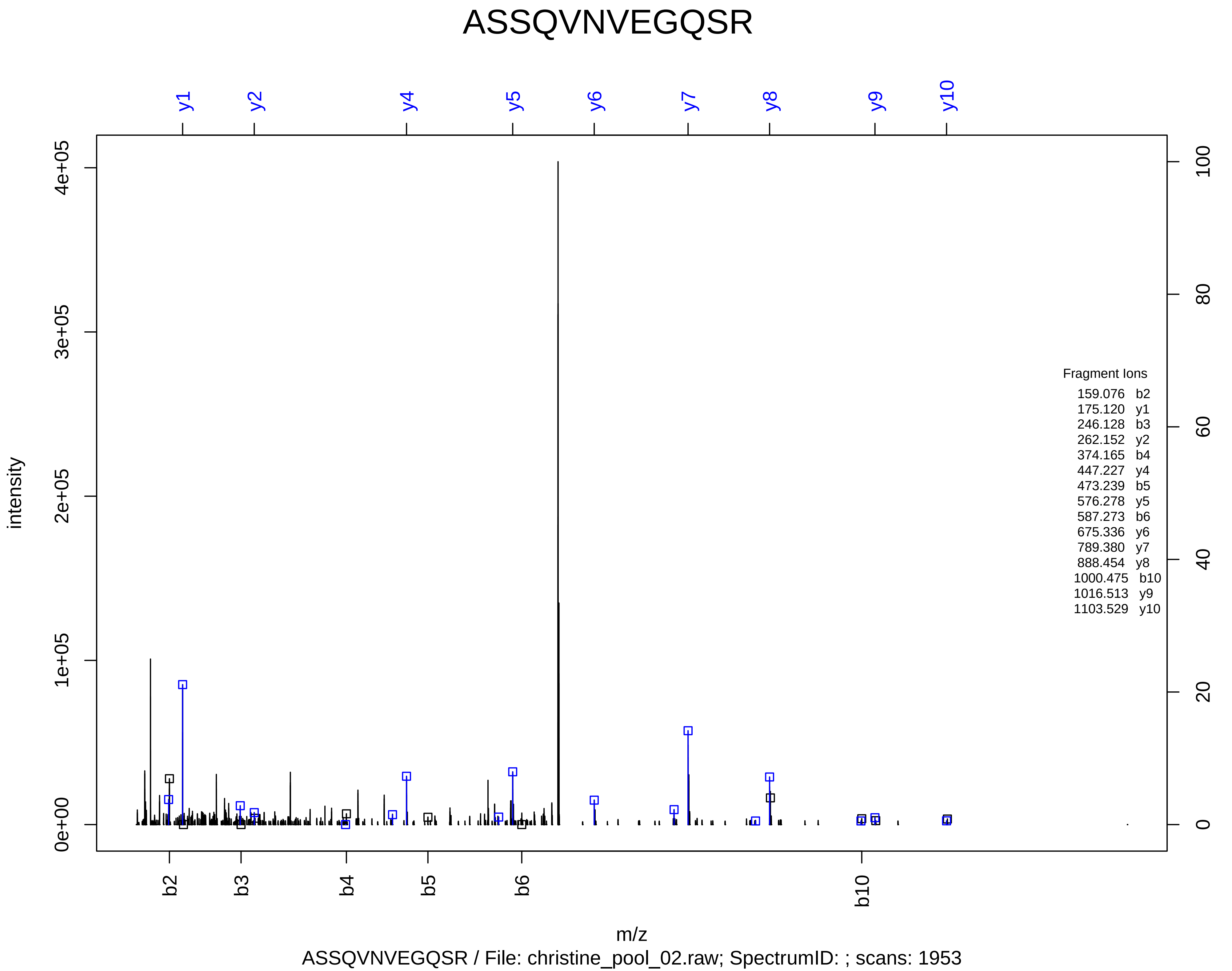

ASSQVNVEGQSR
y1
y2
y4
y5
y6
y7
y8
y9
y10
intensity
0e+00
1e+05
2e+05
3e+05
4e+05
0
20
40
60
80
100
b2
b3
b4
b5
b6
b10
m/z
Fragment Ions
159.076 b2
175.120 y1
246.128 b3
262.152 y2
374.165 b4
447.227 y4
473.239 b5
576.278 y5
587.273 b6
675.336 y6
789.380 y7
888.454 y8
1000.475 b10
1016.513 y9
1103.529 y10
ASSQVNVEGQSR / File: christine_pool_02.raw; SpectrumID: ; scans: 1953

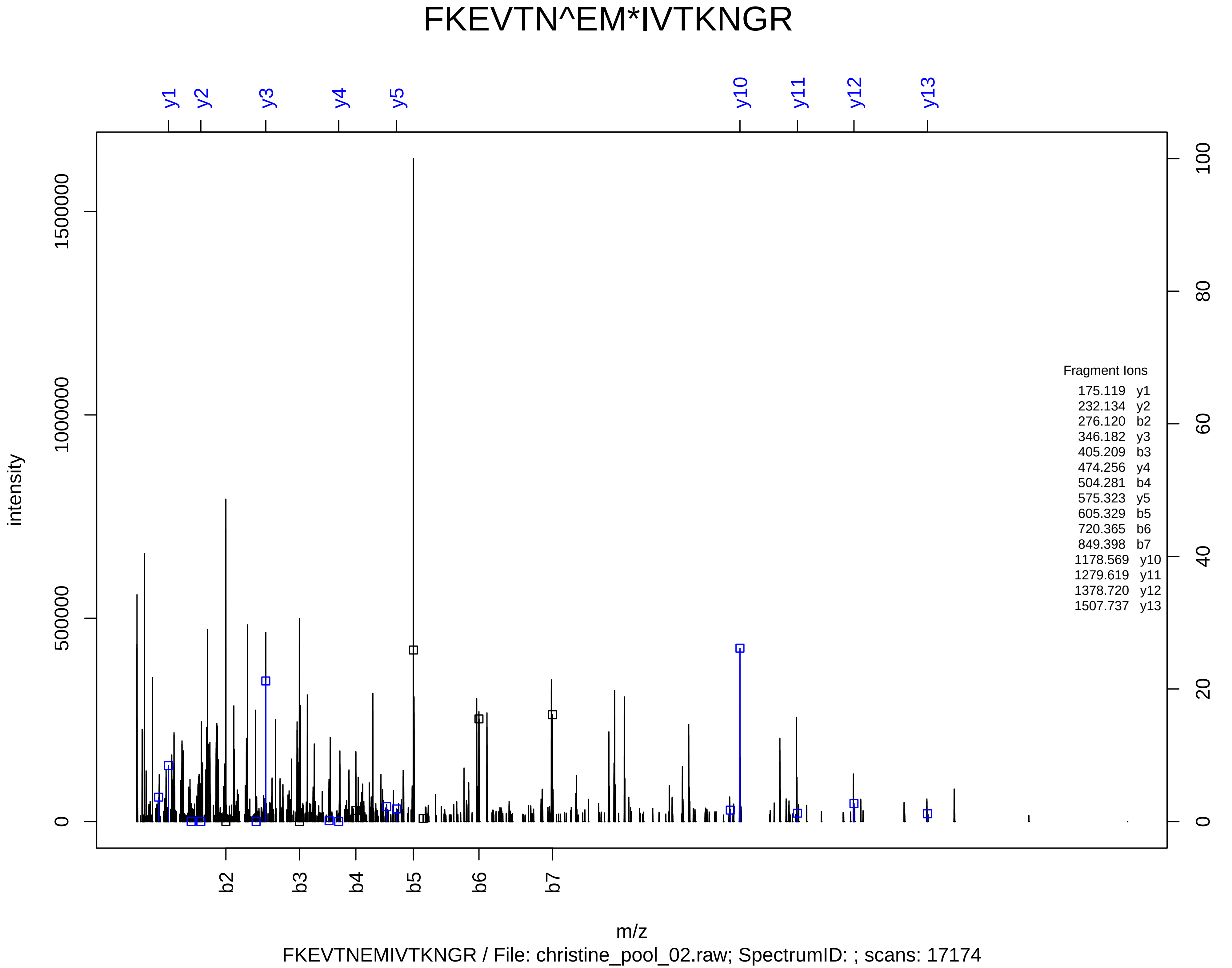
FKEVTN^EM*IVTKNGR
y1
y2
y3
y4
y5
y10
y11
y12
y13
intensity
0
500000
1000000
1500000
0
20
40
60
80
100
b2
b3
b4
b5
b6
b7
m/z
Fragment Ions
175.119 y1
232.134 y2
276.120 b2
346.182 y3
405.209 b3
474.256 y4
504.281 b4
575.323 y5
605.329 b5
720.365 b6
849.398 b7
1178.569 y10
1279.619 y11
1378.720 y12
1507.737 y13
FKEVTNEMIVTKNGR / File: christine_pool_02.raw; SpectrumID: ; scans: 17174

ALDDFVLGSAR

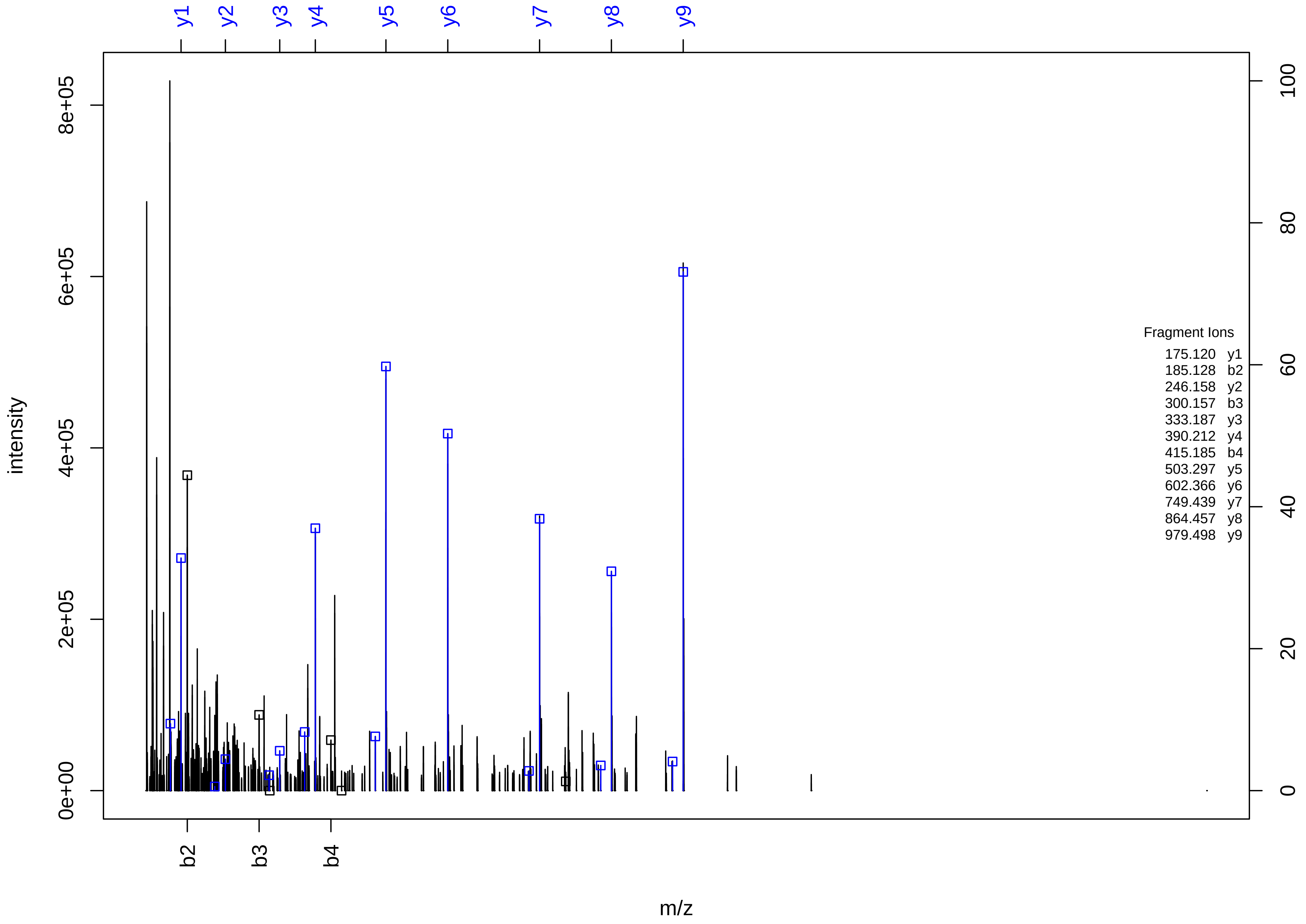

ALDDFVLGSAR / File: christine_pool_02.raw; SpectrumID: ; scans: 15087

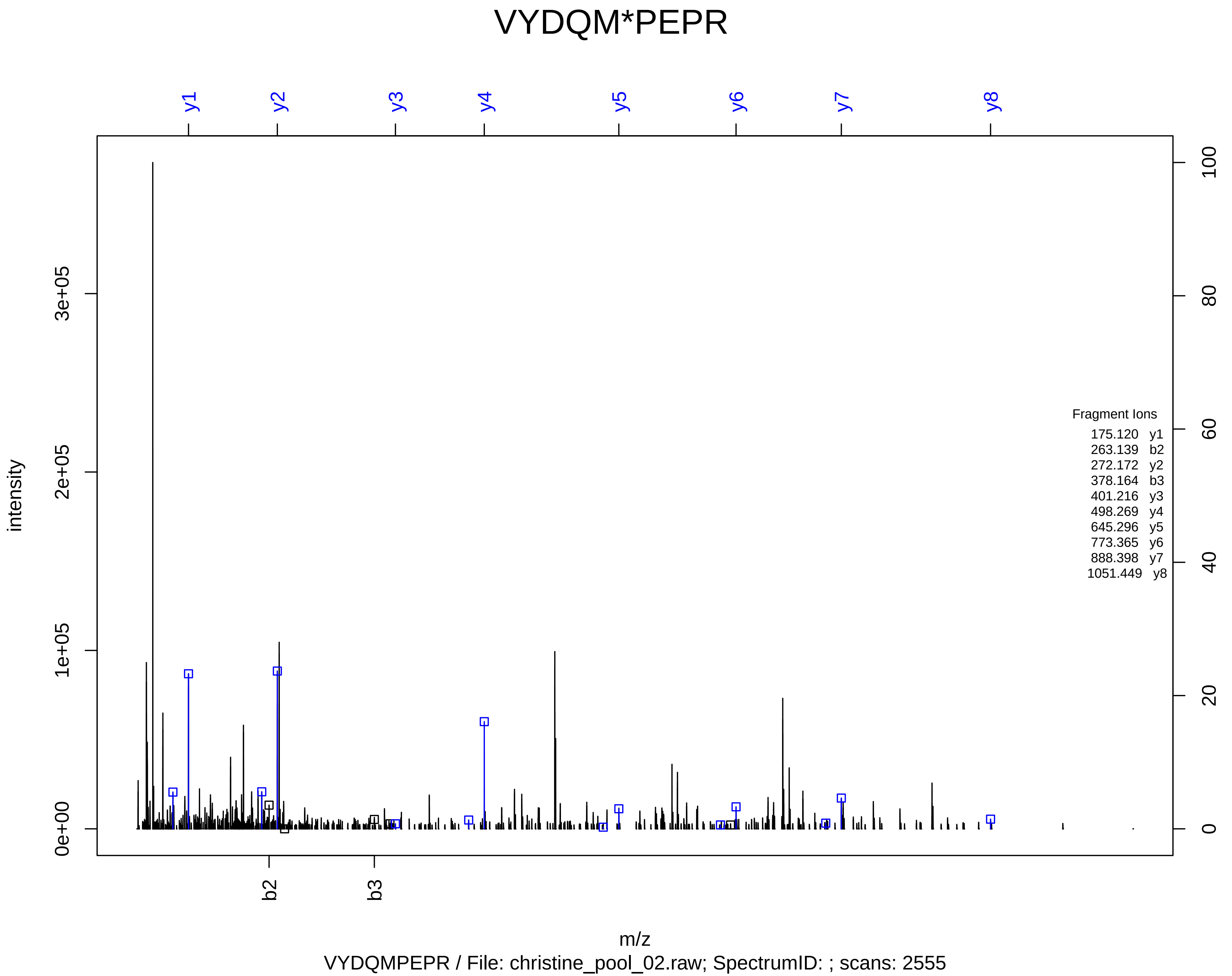
VYDQM*PEPR
y1
y2
y3
y4
y5
y6
y7
y8
b2
b3
intensity
0e+00
1e+05
2e+05
3e+05
0
20
40
60
80
100
m/z
Fragment Ions
175.120 y1
263.139 b2
272.172 y2
378.164 b3
401.216 y3
498.269 y4
645.296 y5
773.365 y6
888.398 y7
1051.449 y8
VYDQMPEPR / File: christine_pool_02.raw; SpectrumID: ; scans: 2555

NEEEGNSEEIK

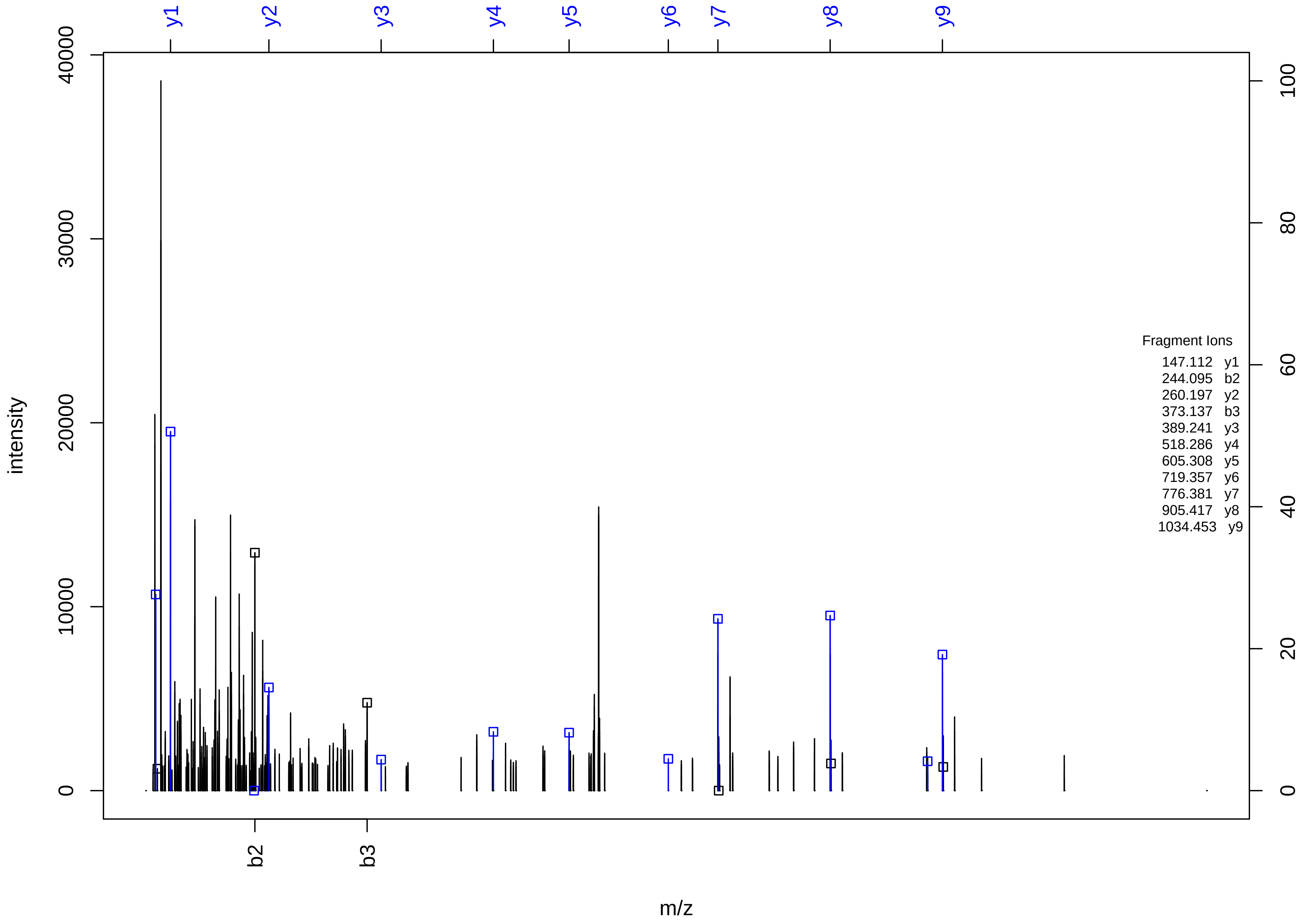

NEEEGNSEEIK / File: christine_pool_02.raw; SpectrumID: ; scans: 1716

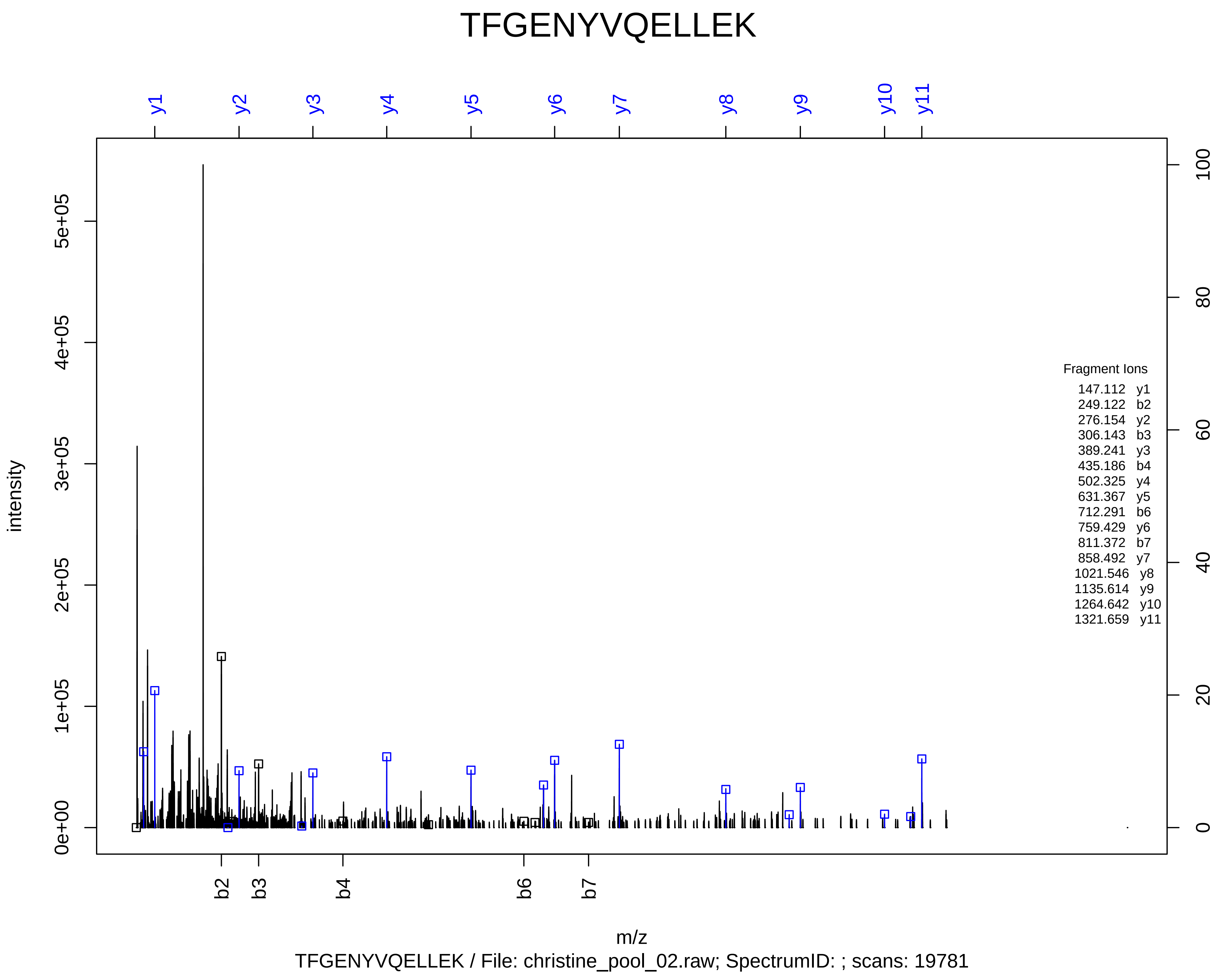

TFGENYVQELLEK
y1
y2
y3
y4
y5
y6
y7
y8
y9
y10
y11
b2
b3
b4
b6
b7
intensity
0e+00
1e+05
2e+05
3e+05
4e+05
5e+05
0
20
40
60
80
100
m/z
Fragment Ions
147.112 y1
249.122 b2
276.154 y2
306.143 b3
389.241 y3
435.186 b4
502.325 y4
631.367 y5
712.291 b6
759.429 y6
811.372 b7
858.492 y7
1021.546 y8
1135.614 y9
1264.642 y10
1321.659 y11
TFGENYVQELLEK / File: christine_pool_02.raw; SpectrumID: ; scans: 19781

VPGLYVIDSIVR

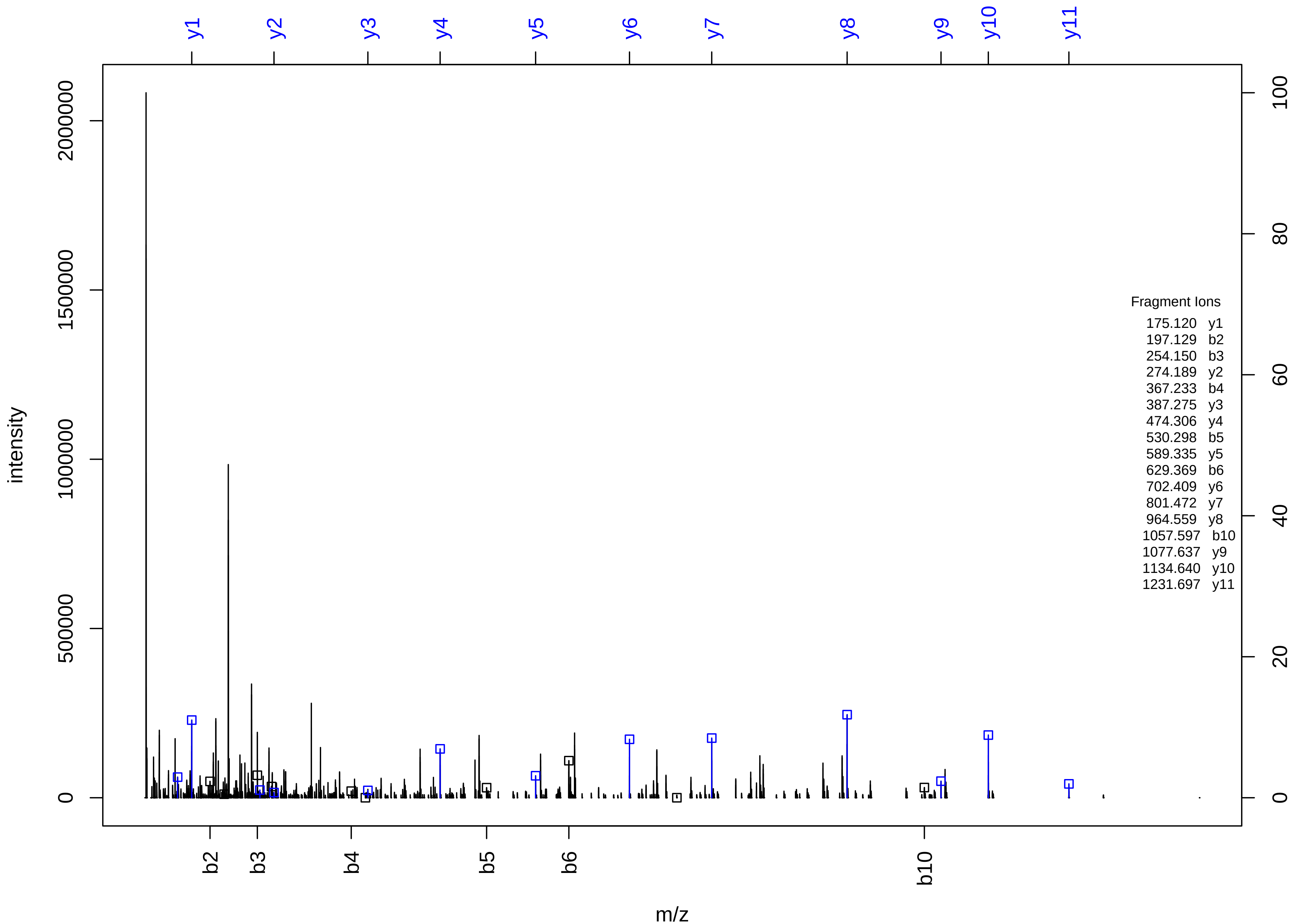

VPGLYVIDSIVR / File: christine_pool_02.raw; SpectrumID: ; scans: 21573

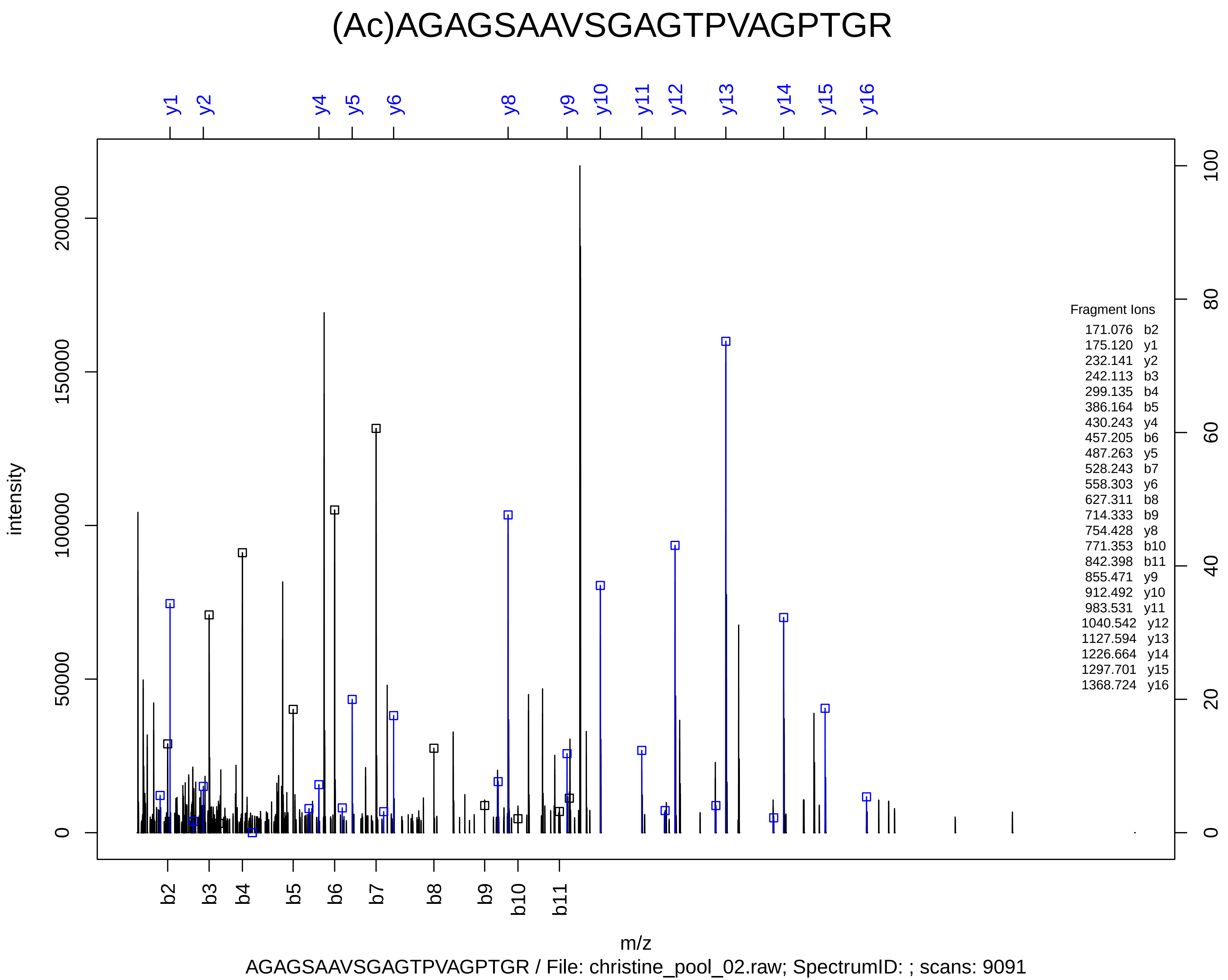
(Ac)AGAGSAAVSGAGTPVAGPTGR
y1 y2 y4 y5 y6 y8 y9 y10 y11 y12 y13 y14 y15 y16
b2 b3 b4 b5 b6 b7 b8 b9 b10 b11
intensity
0
50000
100000
150000
200000
0
20
40
60
80
100
m/z
Fragment Ions
171.076 b2
175.120 y1
232.141 y2
242.113 b3
299.135 b4
386.164 b5
430.243 y4
457.205 b6
487.263 y5
528.243 b7
558.303 y6
627.311 b8
714.333 b9
754.428 y8
771.353 b10
842.398 b11
855.471 y9
912.492 y10
983.531 y11
1040.542 y12
1127.594 y13
1226.664 y14
1297.701 y15
1368.724 y16
AGAGSAAVSGAGTPVAGPTGR / File: christine_pool_02.raw; SpectrumID: ; scans: 9091

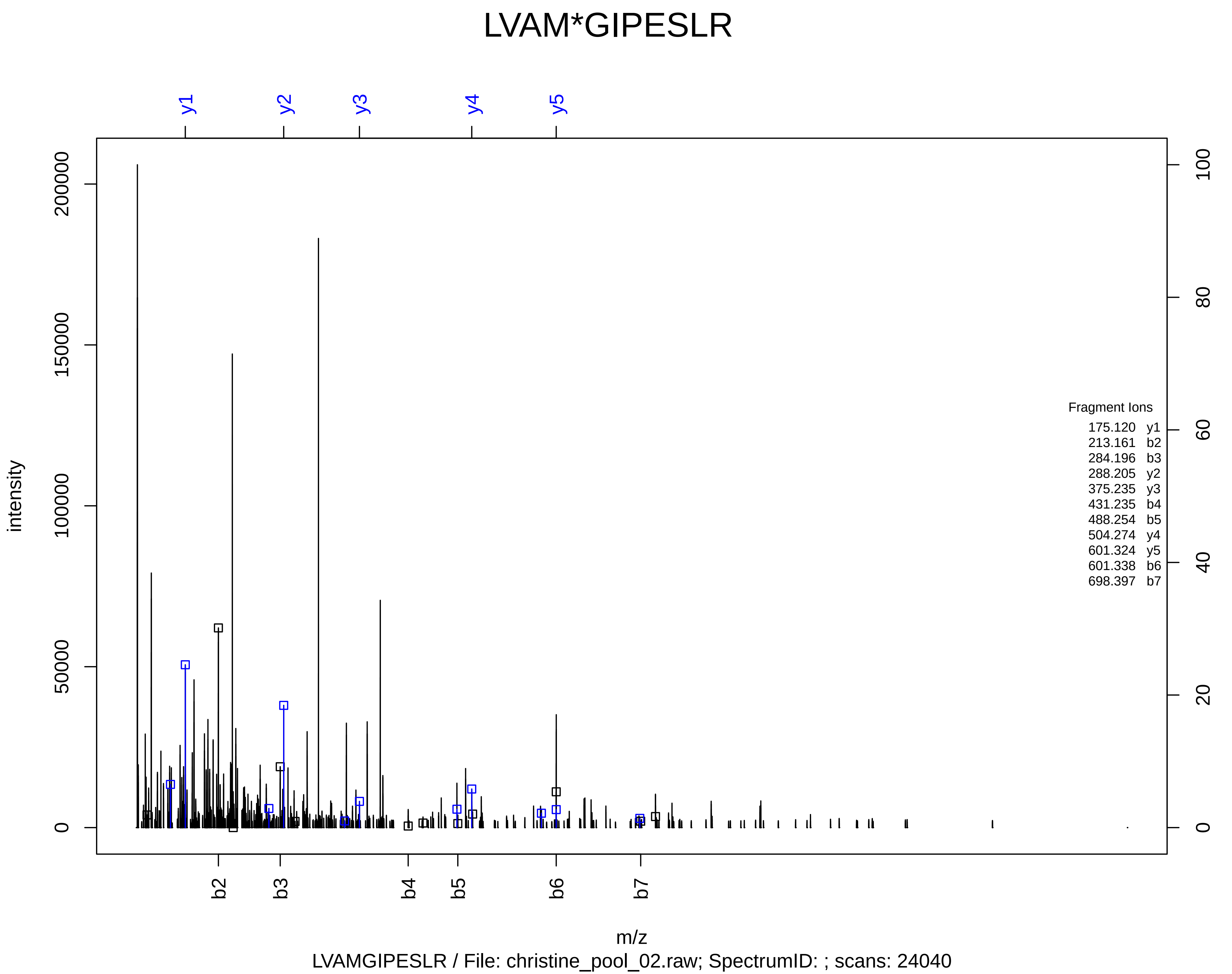
LVAM*GIPESLR
y1
y2
y3
y4
y5
intensity
0
50000
100000
150000
200000
0
20
40
60
80
100
Fragment Ions
175.120 y1
213.161 b2
284.196 b3
288.205 y2
375.235 y3
431.235 b4
488.254 b5
504.274 y4
601.324 y5
601.338 b6
698.397 b7
b2
b3
b4
b5
b6
b7
m/z
LVAMGIPESLR / File: christine_pool_02.raw; SpectrumID: ; scans: 24040

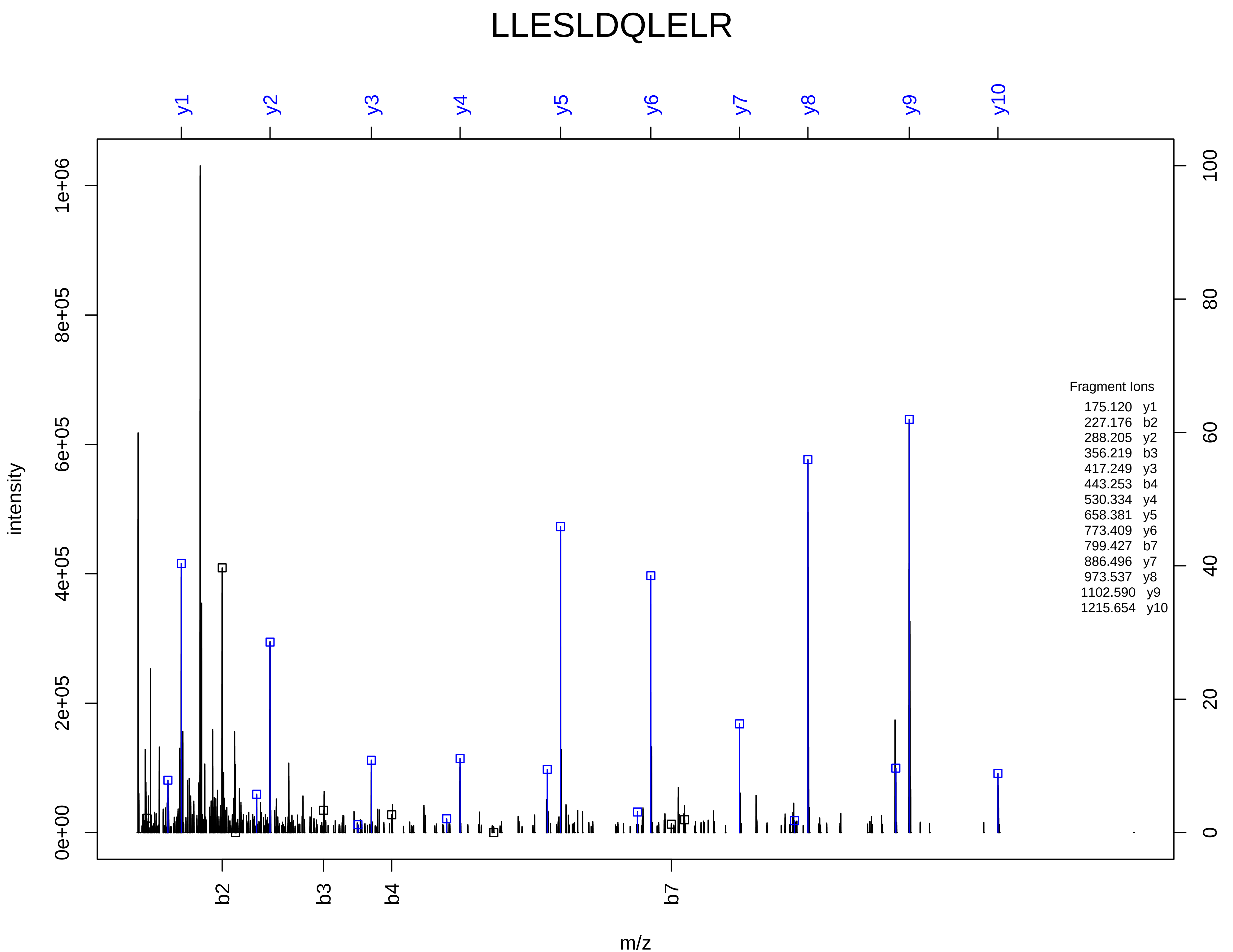

LLESLDQLELR
y1
y2
y3
y4
y5
y6
y7
y8
y9
y10
intensity
0e+00
2e+05
4e+05
6e+05
8e+05
1e+06
0
20
40
60
80
100
Fragment Ions
175.120 y1
227.176 b2
288.205 y2
356.219 b3
417.249 y3
443.253 b4
530.334 y4
658.381 y5
773.409 y6
799.427 b7
886.496 y7
973.537 y8
1102.590 y9
1215.654 y10
b2
b3
b4
b7
m/z
LLESLDQLELR / File: christine_pool_02.raw; SpectrumID: ; scans: 17866

SPWNFLGDELYEQSDEEQDSVK

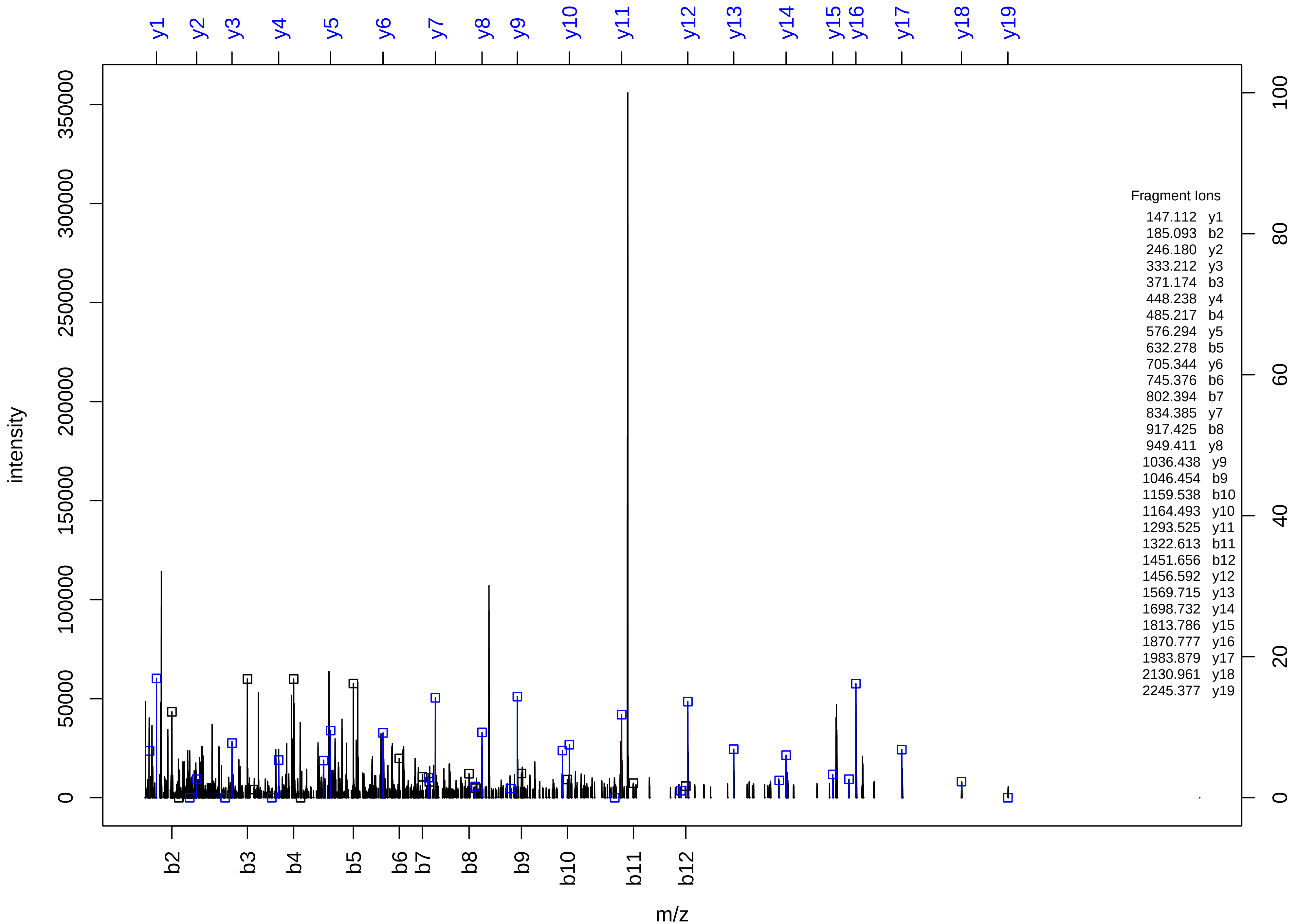

SPWNFLGDELYEQSDEEQDSVK / File: christine_pool_02.raw; SpectrumID: ; scans: 20811

(Ac)ACPLDQAIGLLVAIFHK

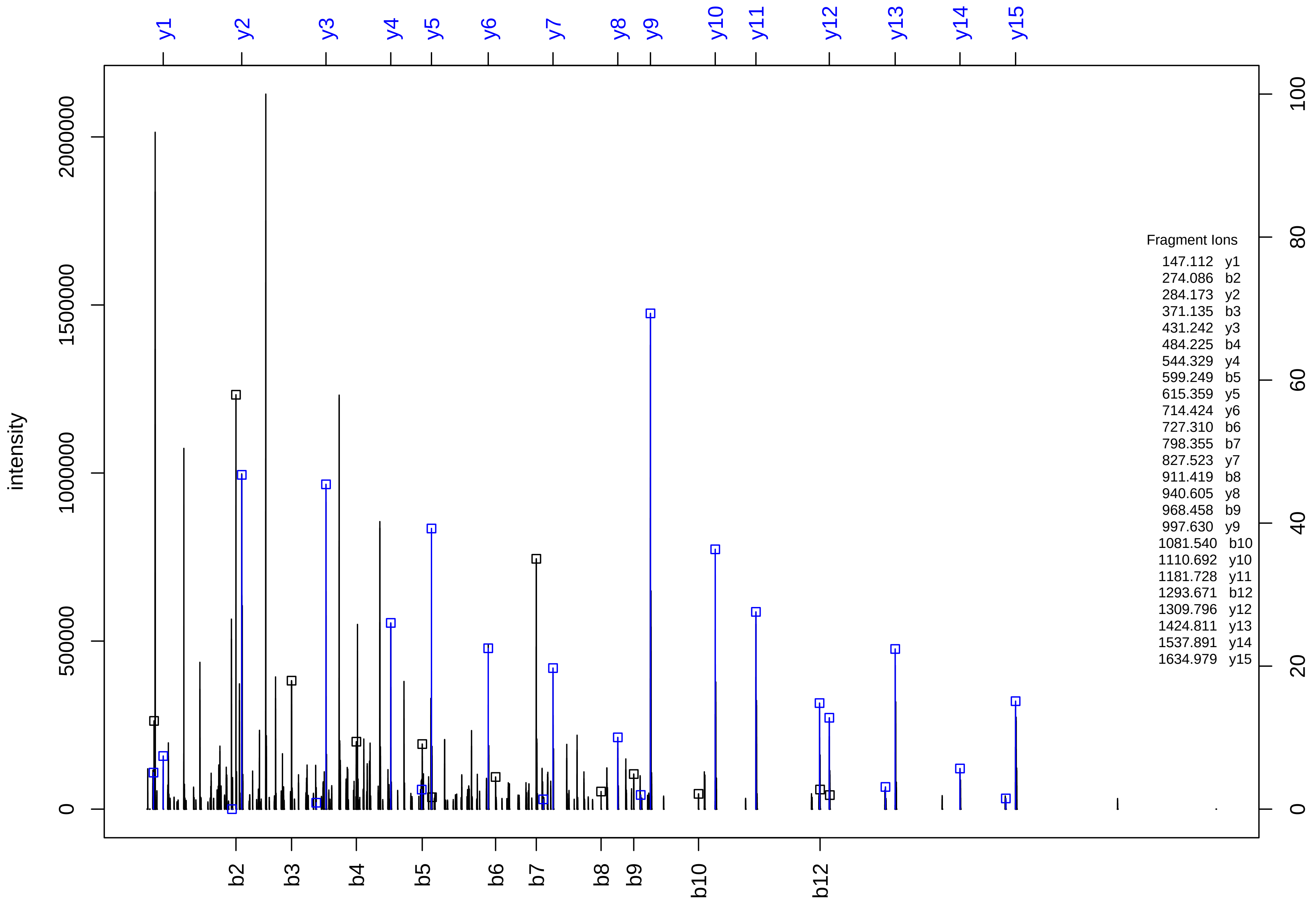

ACPLDQAIGLLVAIFHK / File: christine_pool_02.raw; SpectrumID: ; scans: 24474

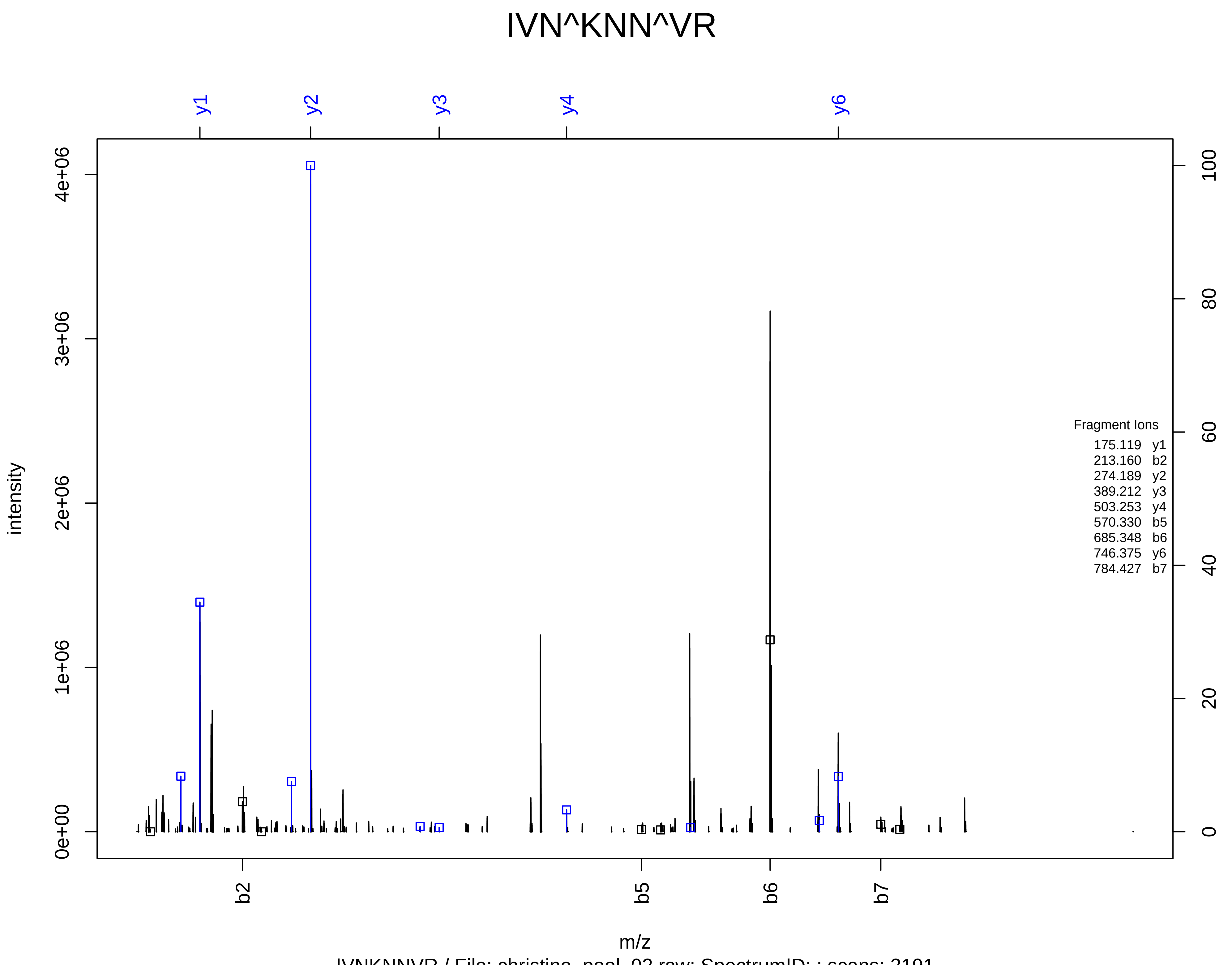
IVN^KNN^VR
y1
y2
y3
y4
y6
intensity
0e+00
1e+06
2e+06
3e+06
4e+06
0
20
40
60
80
100
Fragment Ions
175.119 y1
213.160 b2
274.189 y2
389.212 y3
503.253 y4
570.330 b5
685.348 b6
746.375 y6
784.427 b7
b2
b5
b6
b7
m/z
IVNKNNVR / File: christine_pool_02.raw; SpectrumID: ; scans: 2191

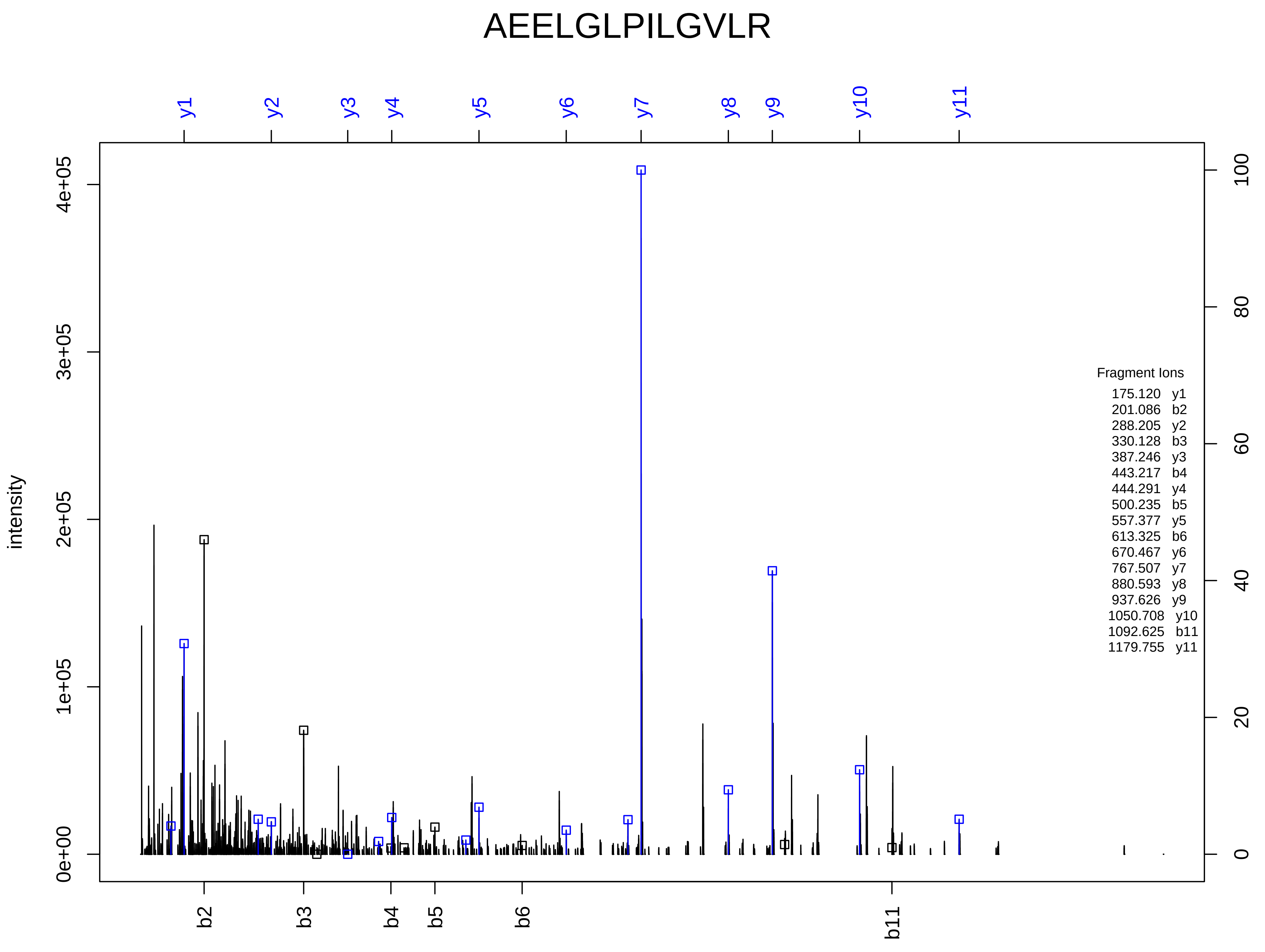

AEELGLPILGVLR / File: christine_pool_02.raw; SpectrumID: ; scans: 24113

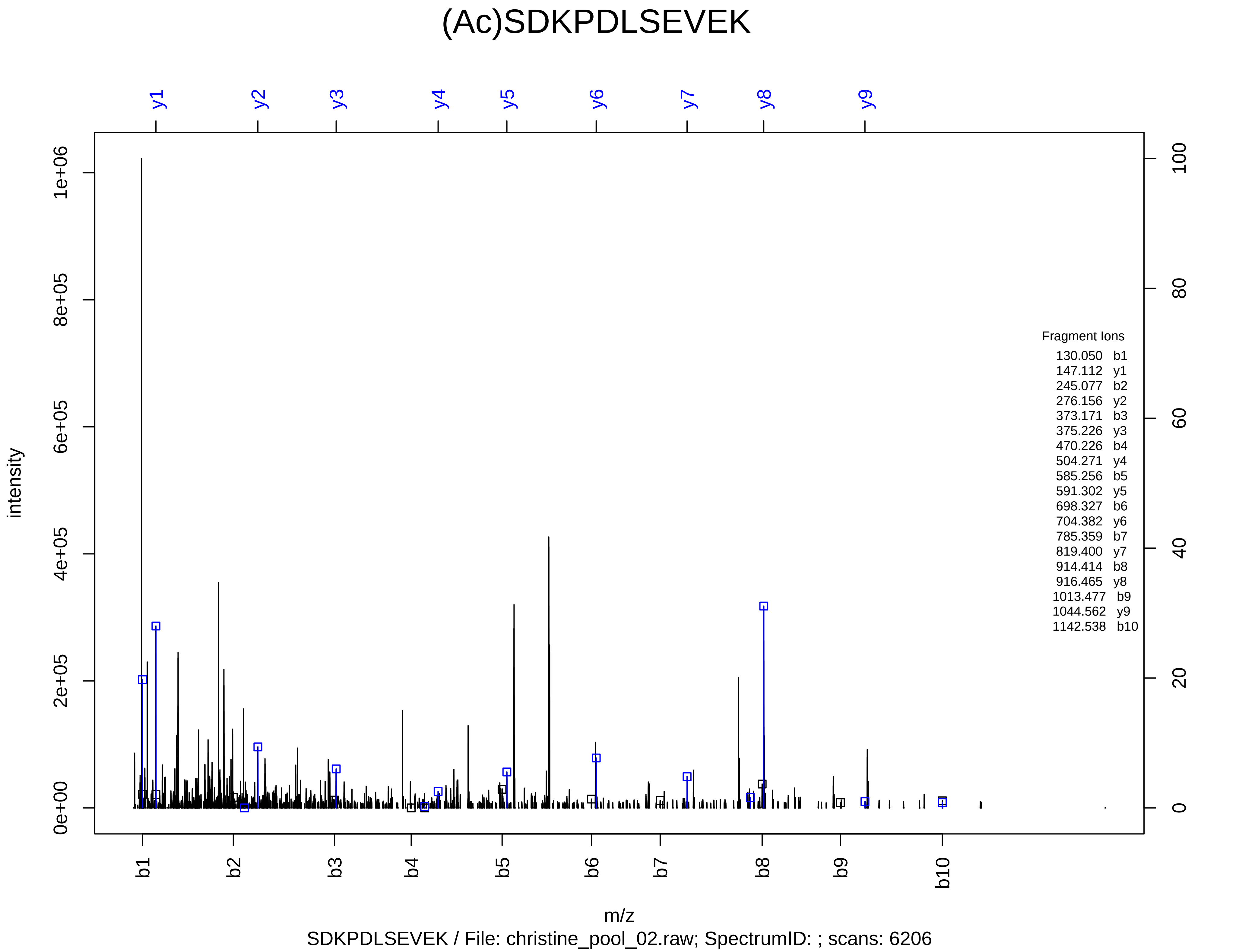
(Ac)SDKPDLSEVEK
y1 y2 y3 y4 y5 y6 y7 y8 y9
intensity
0e+00 2e+05 4e+05 6e+05 8e+05 1e+06
0 20 40 60 80 100
Fragment Ions
130.050 b1
147.112 y1
245.077 b2
276.156 y2
373.171 b3
375.226 y3
470.226 b4
504.271 y4
585.256 b5
591.302 y5
698.327 b6
704.382 y6
785.359 b7
819.400 y7
914.414 b8
916.465 y8
1013.477 b9
1044.562 y9
1142.538 b10
b1 b2 b3 b4 b5 b6 b7 b8 b9 b10
m/z
SDKPDLSEVEK / File: christine_pool_02.raw; SpectrumID: ; scans: 6206

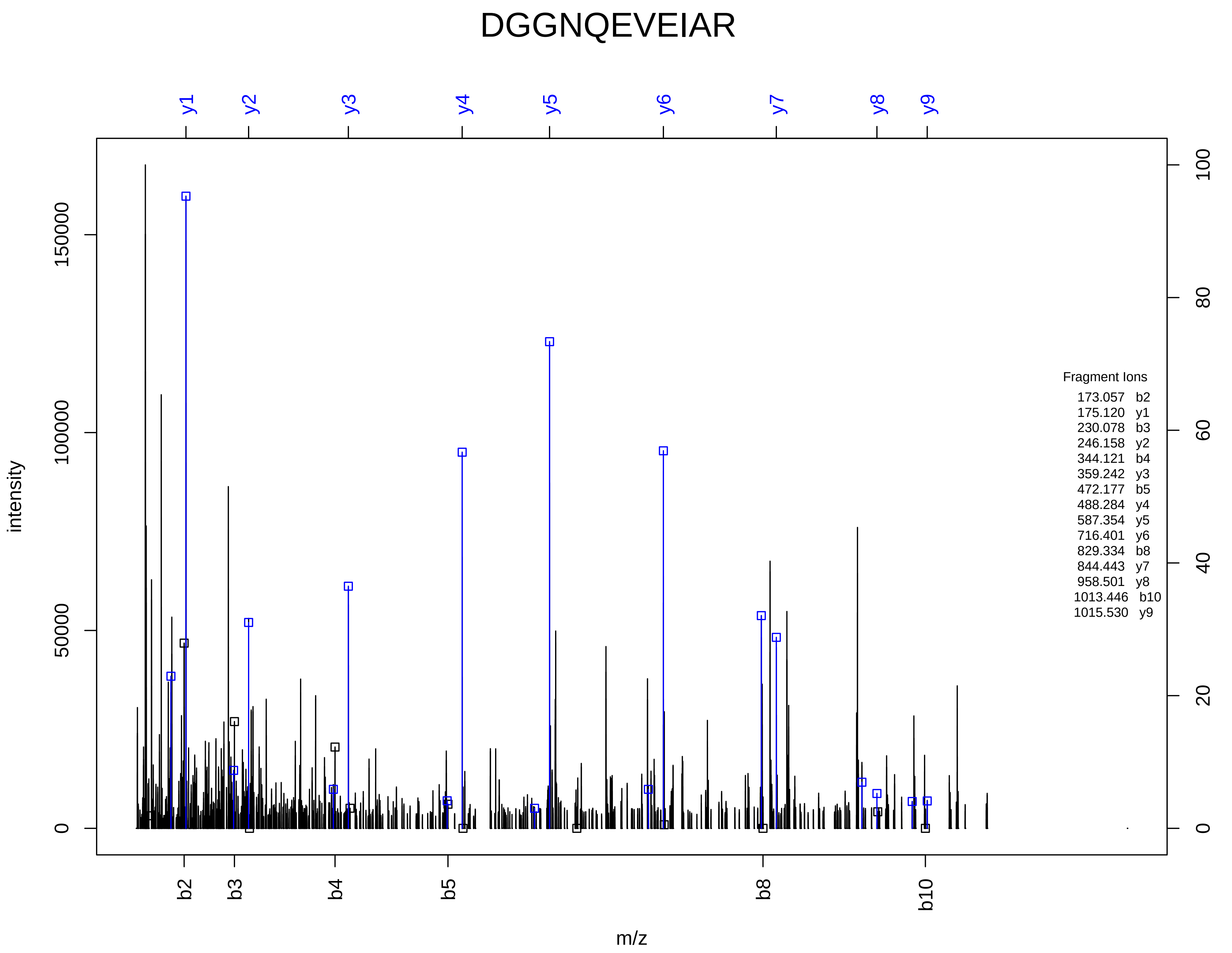

DGGNQEVEIAR
y1
y2
y3
y4
y5
y6
y7
y8
y9
intensity
0
50000
100000
150000
0
20
40
60
80
100
b2
b3
b4
b5
b8
b10
m/z
Fragment Ions
173.057 b2
175.120 y1
230.078 b3
246.158 y2
344.121 b4
359.242 y3
472.177 b5
488.284 y4
587.354 y5
716.401 y6
829.334 b8
844.443 y7
958.501 y8
1013.446 b10
1015.530 y9
DGGNQEVEIAR / File: christine_pool_02.raw; SpectrumID: ; scans: 4286

ENAEQGEVDMESHR

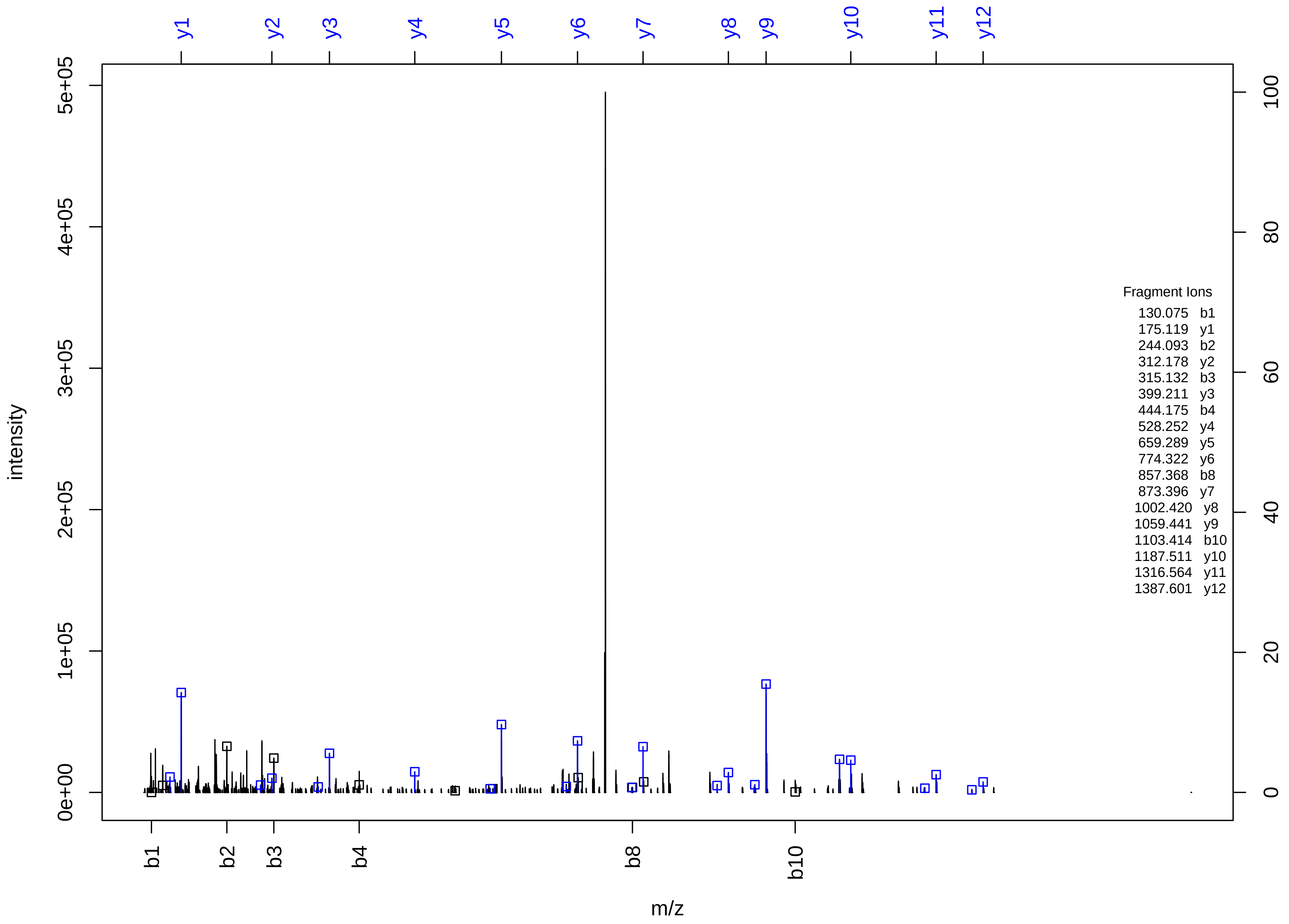

ENAEQGEVDMESHR / File: christine_pool_02.raw; SpectrumID: ; scans: 2899

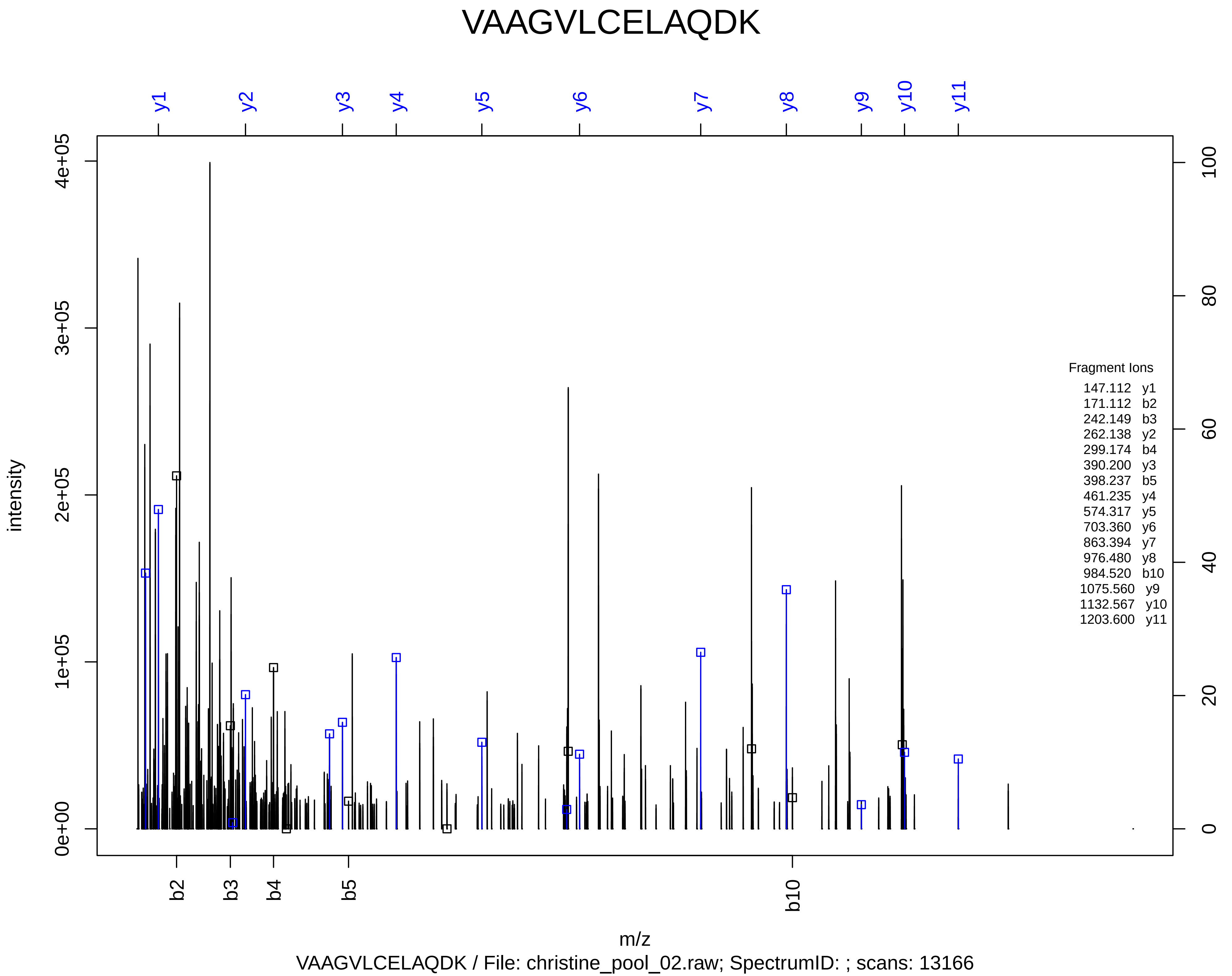

VAAGVLCELAQDK
y1
y2
y3
y4
y5
y6
y7
y8
y9
y10
y11
intensity
0e+00
1e+05
2e+05
3e+05
4e+05
0
20
40
60
80
100
b2
b3
b4
b5
b10
m/z
Fragment Ions
147.112 y1
171.112 b2
242.149 b3
262.138 y2
299.174 b4
390.200 y3
398.237 b5
461.235 y4
574.317 y5
703.360 y6
863.394 y7
976.480 y8
984.520 b10
1075.560 y9
1132.567 y10
1203.600 y11
VAAGVLCELAQDK / File: christine_pool_02.raw; SpectrumID: ; scans: 13166

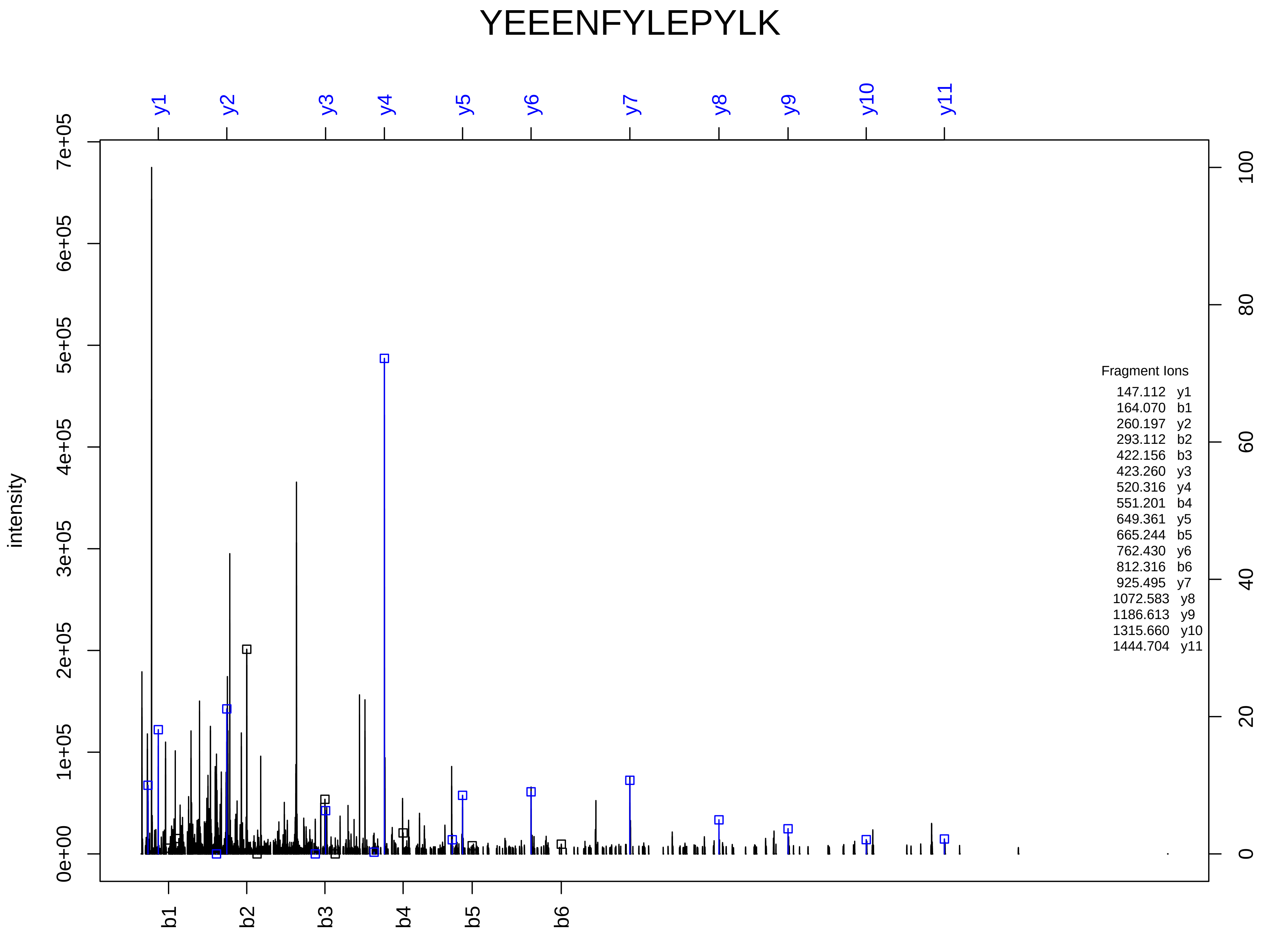

YEEENFYLEPYLK / File: christine_pool_02.raw; SpectrumID: ; scans: 17595

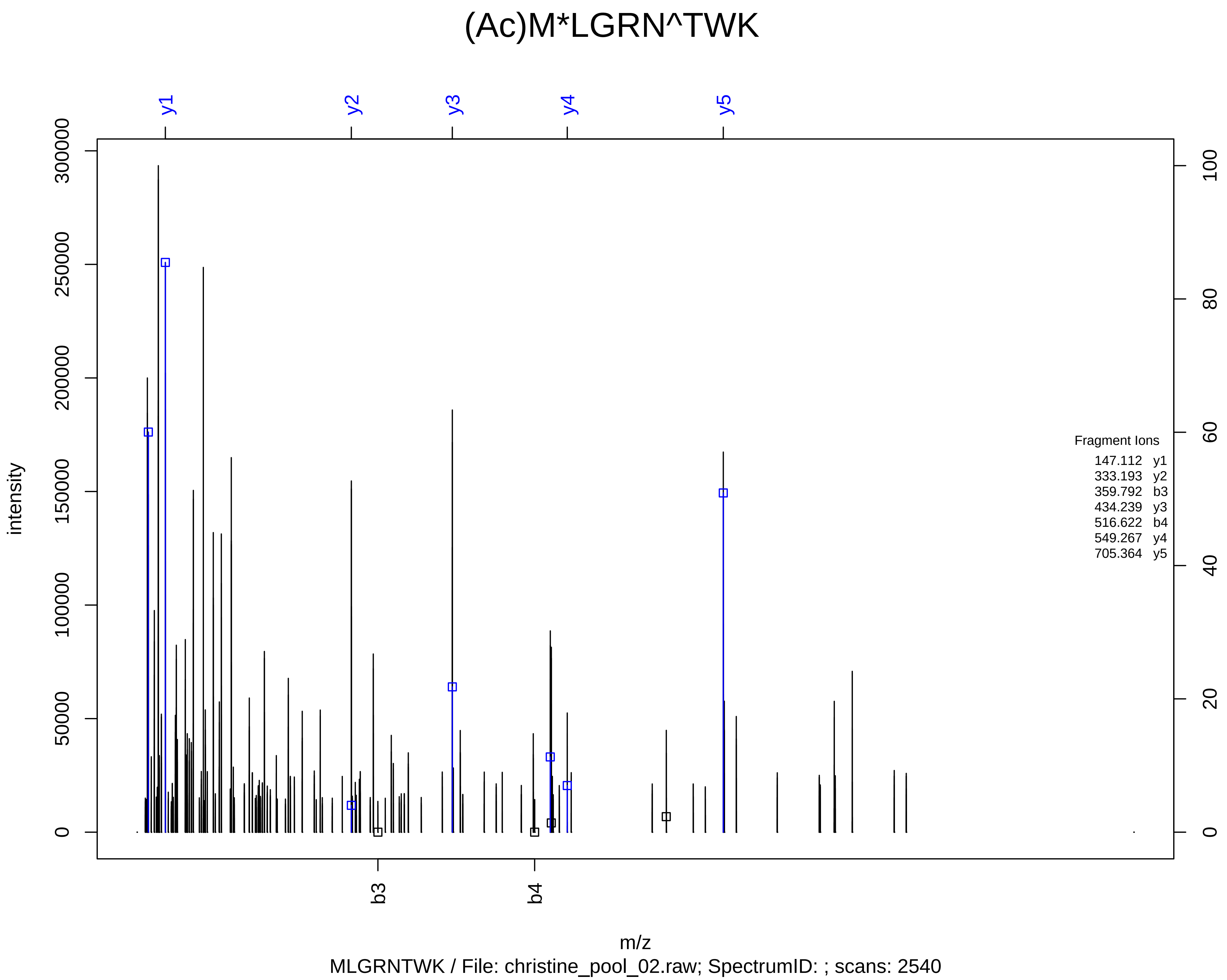

(Ac)M*LGRN^TWK
y1
y2
y3
y4
y5
b3
b4
intensity
m/z
0
50000
100000
150000
200000
250000
300000
0
20
40
60
80
100
Fragment Ions
147.112 y1
333.193 y2
359.792 b3
434.239 y3
516.622 b4
549.267 y4
705.364 y5
MLGRNTWK / File: christine_pool_02.raw; SpectrumID: ; scans: 2540

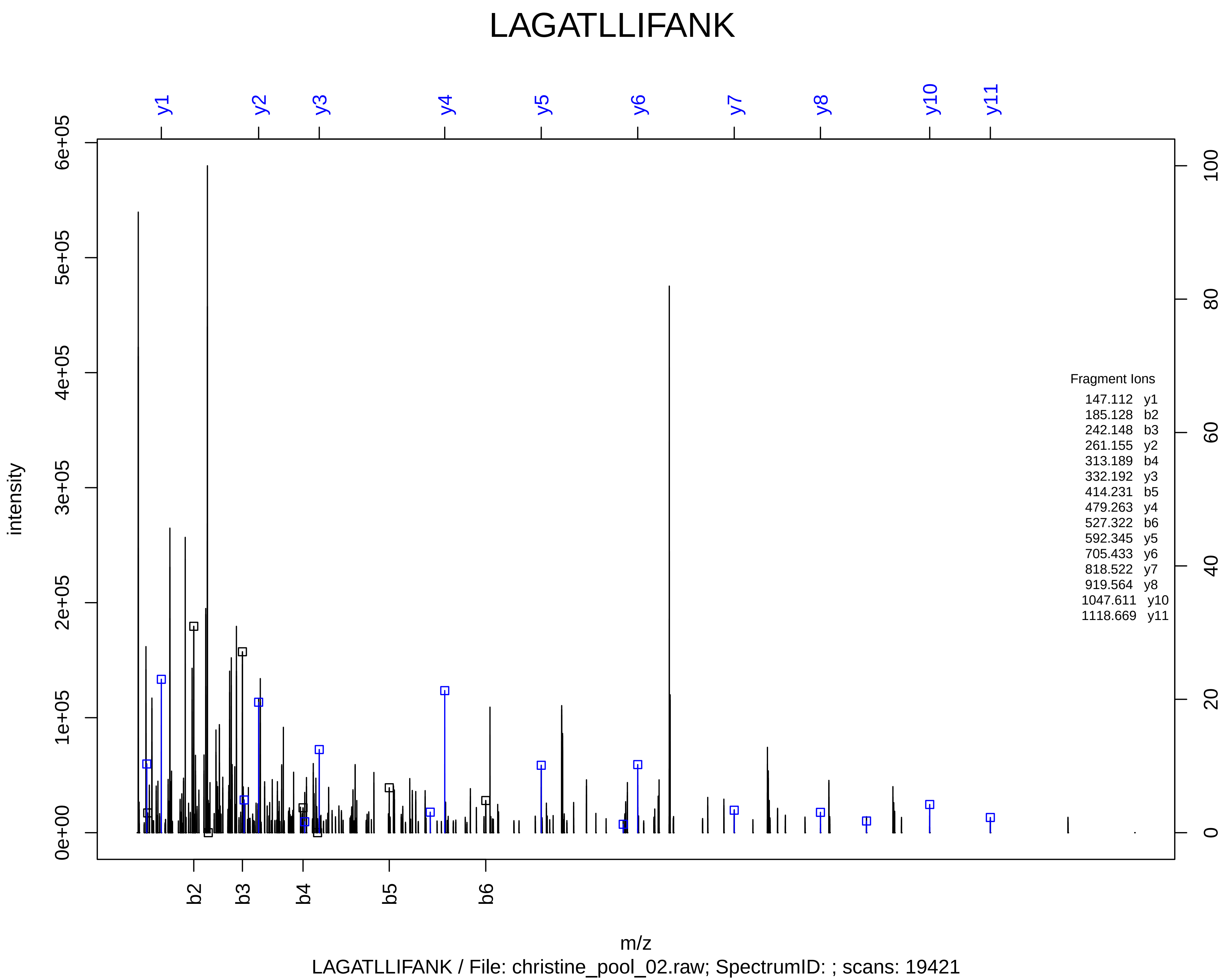

LAGATLLIFANK
intensity
m/z
y1
y2
y3
y4
y5
y6
y7
y8
y10
y11
b2
b3
b4
b5
b6
0e+00
1e+05
2e+05
3e+05
4e+05
5e+05
6e+05
0
20
40
60
80
100
Fragment Ions
147.112 y1
185.128 b2
242.148 b3
261.155 y2
313.189 b4
332.192 y3
414.231 b5
479.263 y4
527.322 b6
592.345 y5
705.433 y6
818.522 y7
919.564 y8
1047.611 y10
1118.669 y11
LAGATLLIFANK / File: christine_pool_02.raw; SpectrumID: ; scans: 19421

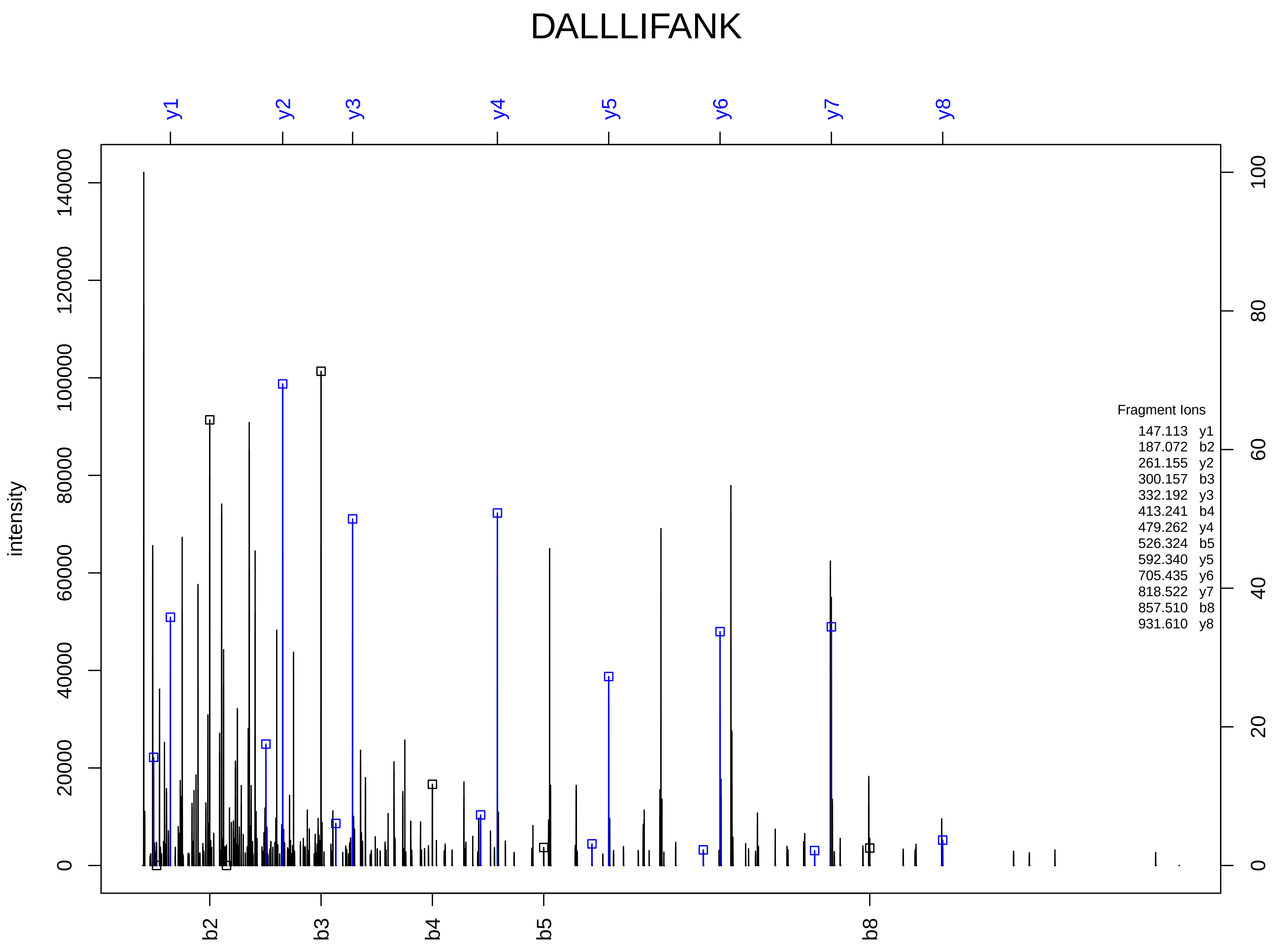

DALLLIFANK / File: christine_pool_02.raw; SpectrumID: ; scans: 21158

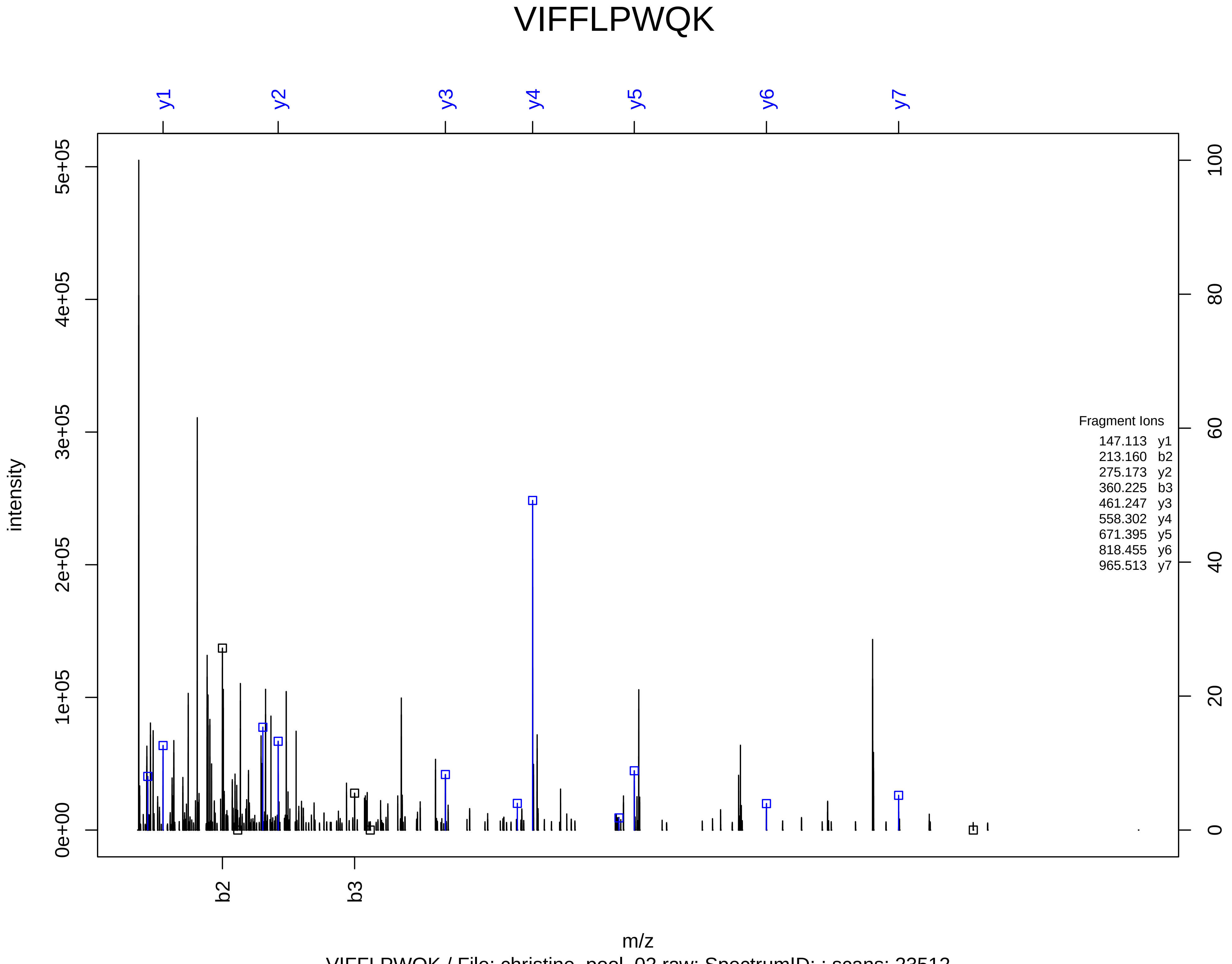

VIFFLPWQK
y1
y2
y3
y4
y5
y6
y7
b2
b3
intensity
0e+00
1e+05
2e+05
3e+05
4e+05
5e+05
0
20
40
60
80
100
m/z
Fragment Ions
147.113 y1
213.160 b2
275.173 y2
360.225 b3
461.247 y3
558.302 y4
671.395 y5
818.455 y6
965.513 y7
VIFFLPWQK / File: christine_pool_02.raw; SpectrumID: ; scans: 23512

LLFEVFDENR

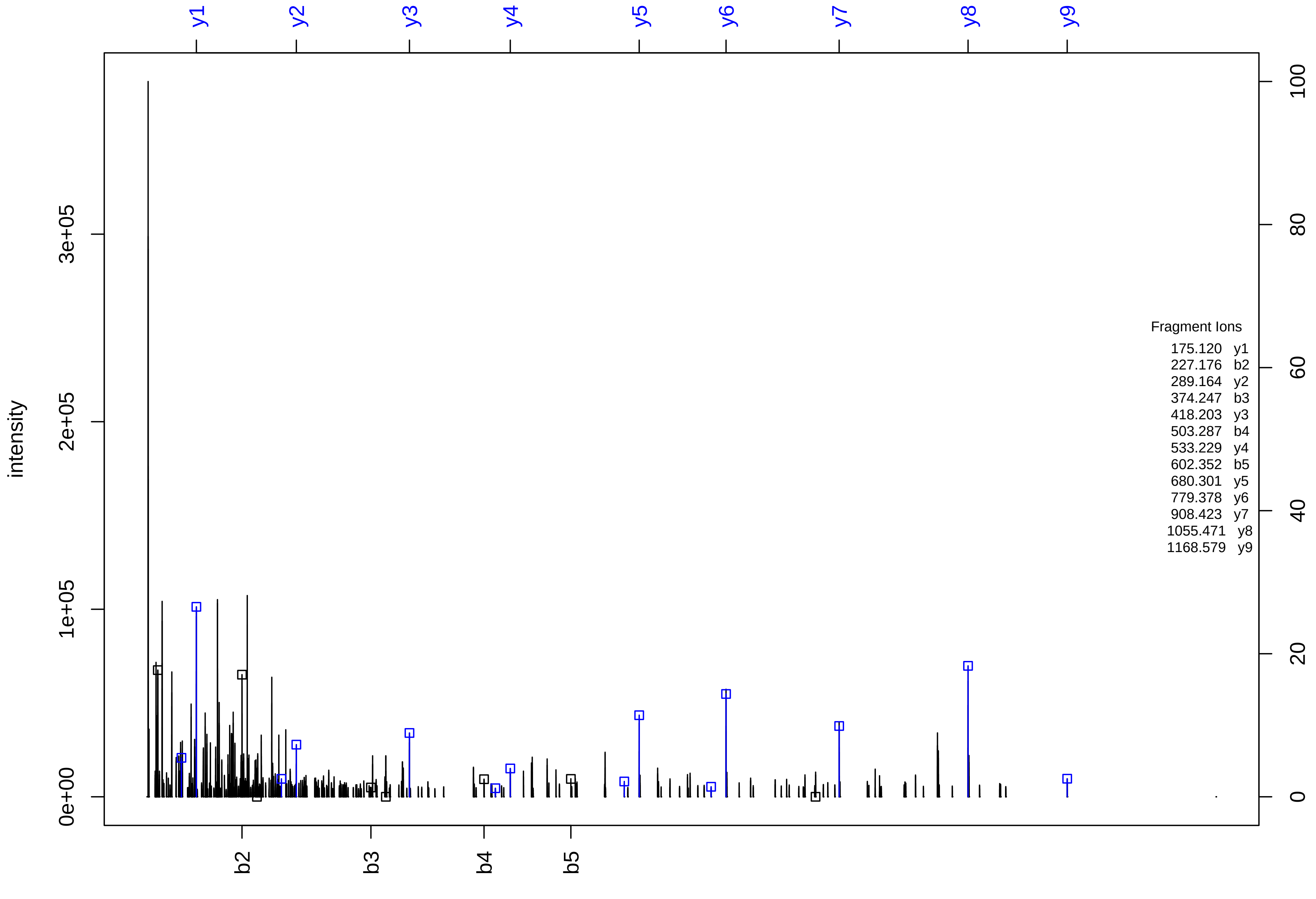

LLFEVFDENR / File: christine_pool_02.raw; SpectrumID: ; scans: 19568

DPLYIGLR

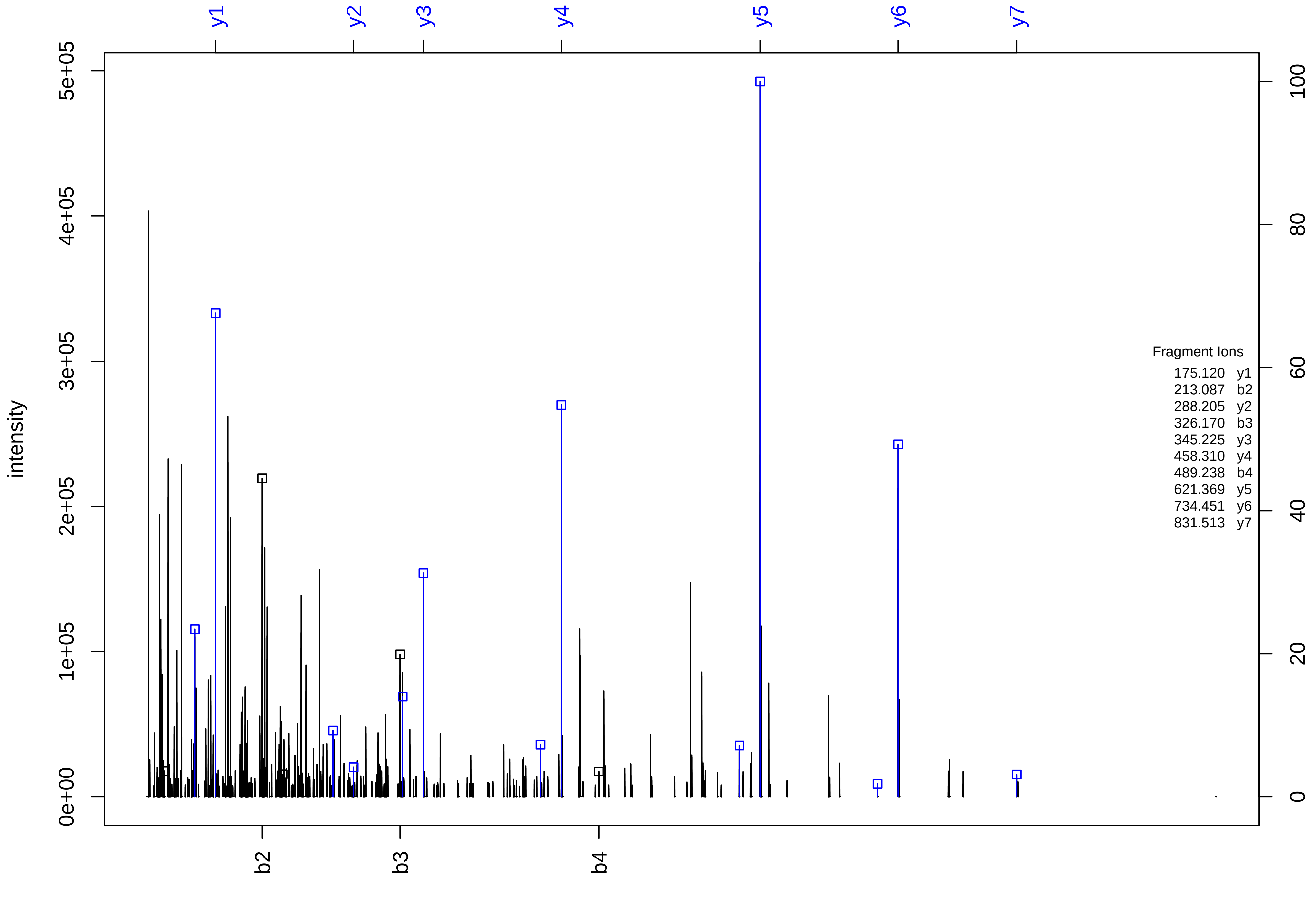

DPLYIGLR / File: christine_pool_02.raw; SpectrumID: ; scans: 14247

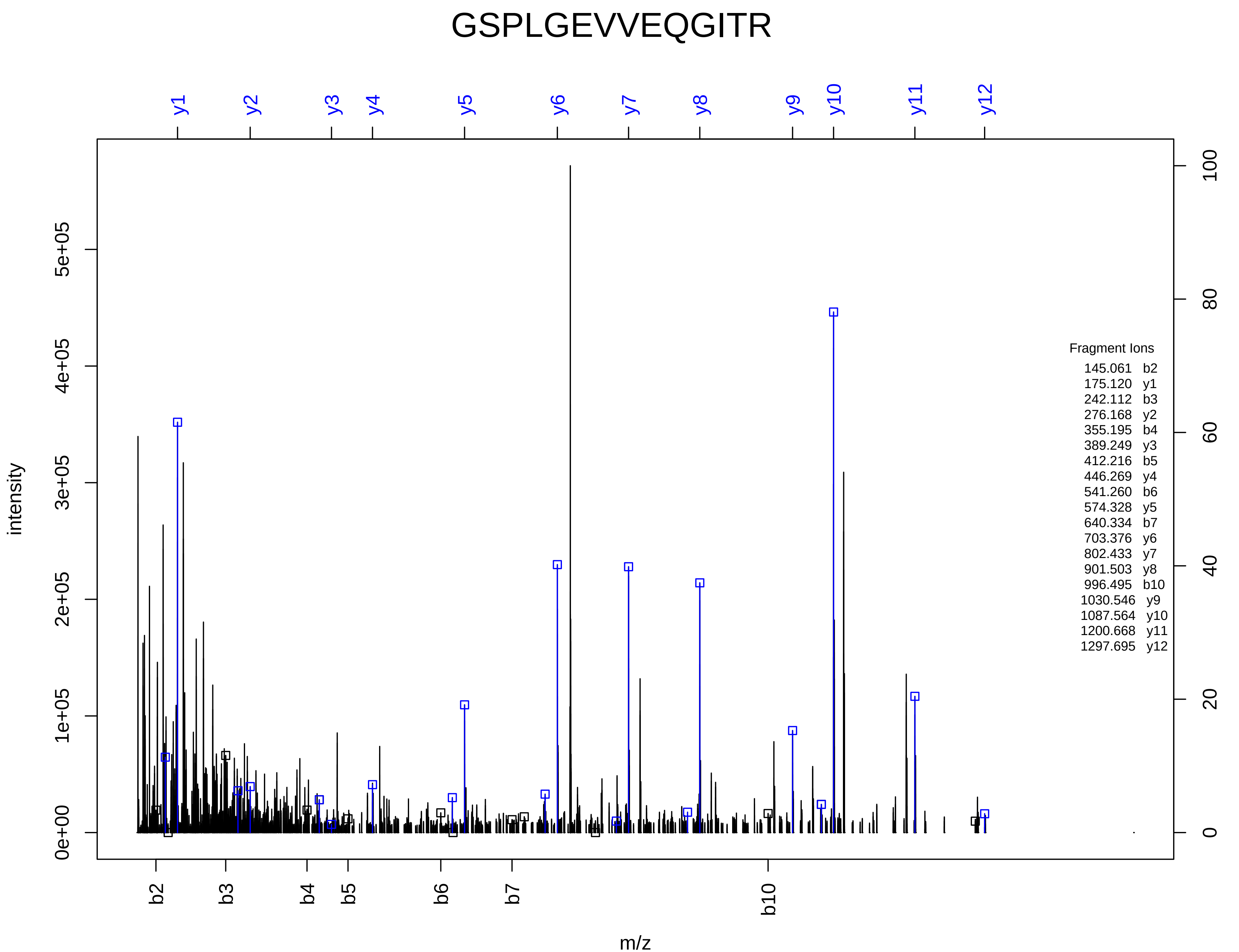
GSPLGEVVEQGITR
y1
y2
y3
y4
y5
y6
y7
y8
y9
y10
y11
y12
intensity
0e+00
1e+05
2e+05
3e+05
4e+05
5e+05
0
20
40
60
80
100
b2
b3
b4
b5
b6
b7
b10
m/z
Fragment Ions
145.061 b2
175.120 y1
242.112 b3
276.168 y2
355.195 b4
389.249 y3
412.216 b5
446.269 y4
541.260 b6
574.328 y5
640.334 b7
703.376 y6
802.433 y7
901.503 y8
996.495 b10
1030.546 y9
1087.564 y10
1200.668 y11
1297.695 y12
GSPLGEVVEQGITR / File: christine_pool_02.raw; SpectrumID: ; scans: 13562

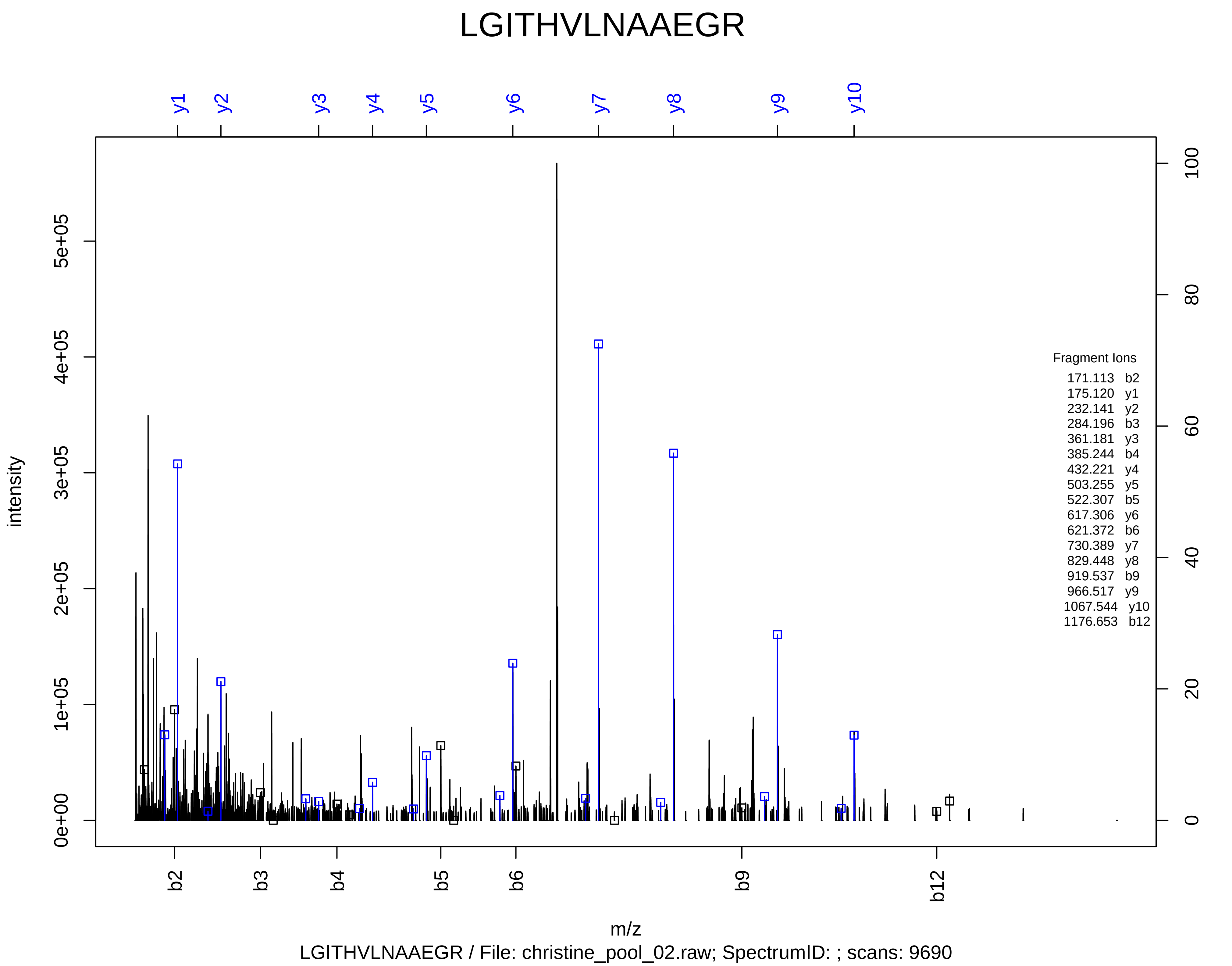

LGITHVLNAAEGR
y1
y2
y3
y4
y5
y6
y7
y8
y9
y10
intensity
0e+00
1e+05
2e+05
3e+05
4e+05
5e+05
0
20
40
60
80
100
Fragment Ions
171.113 b2
175.120 y1
232.141 y2
284.196 b3
361.181 y3
385.244 b4
432.221 y4
503.255 y5
522.307 b5
617.306 y6
621.372 b6
730.389 y7
829.448 y8
919.537 b9
966.517 y9
1067.544 y10
1176.653 b12
b2
b3
b4
b5
b6
b9
b12
m/z
LGITHVLNAAEGR / File: christine_pool_02.raw; SpectrumID: ; scans: 9690

SVIEDVINDVR

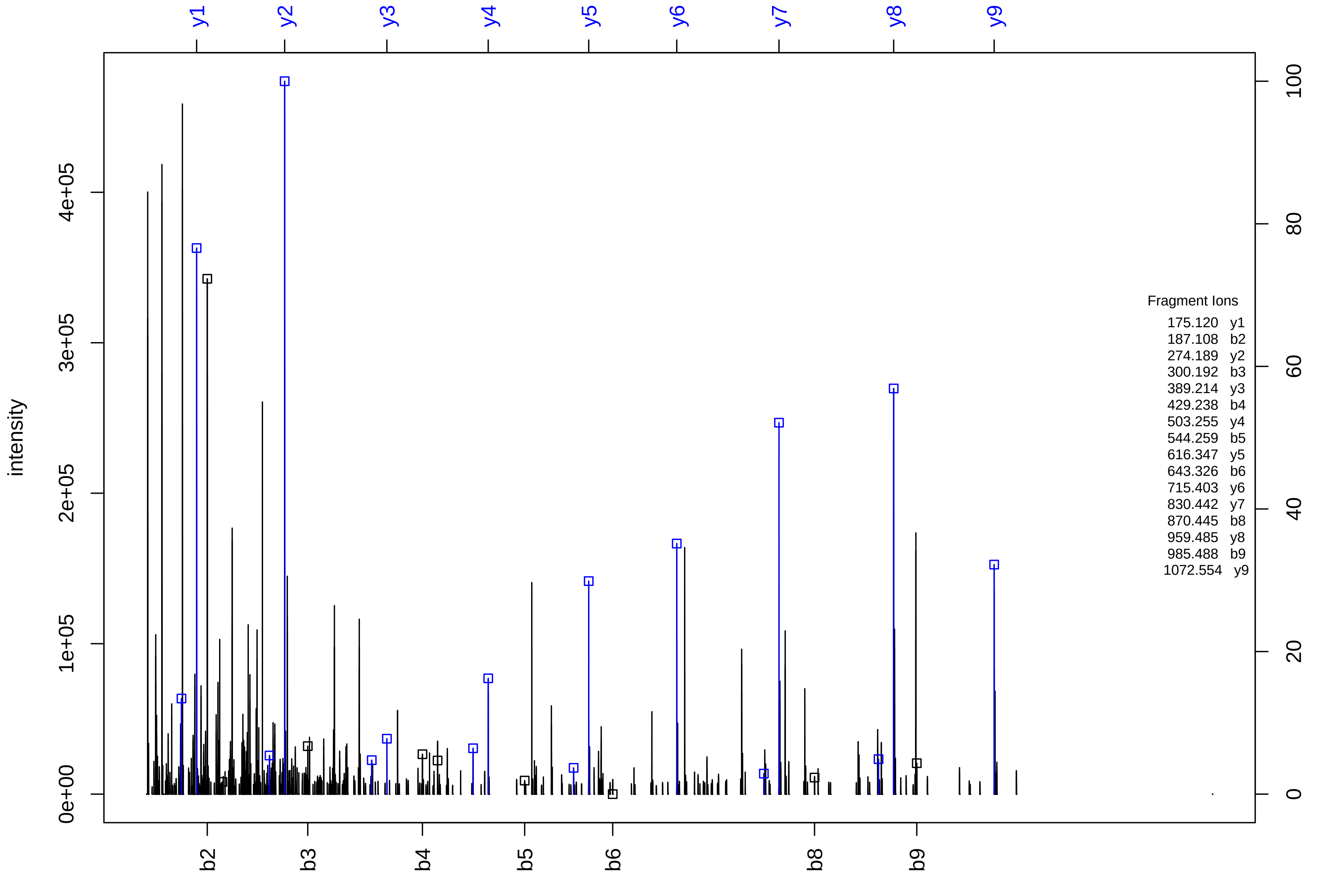

SVIEDVINDVR / File: christine_pool_02.raw; SpectrumID: ; scans: 17725

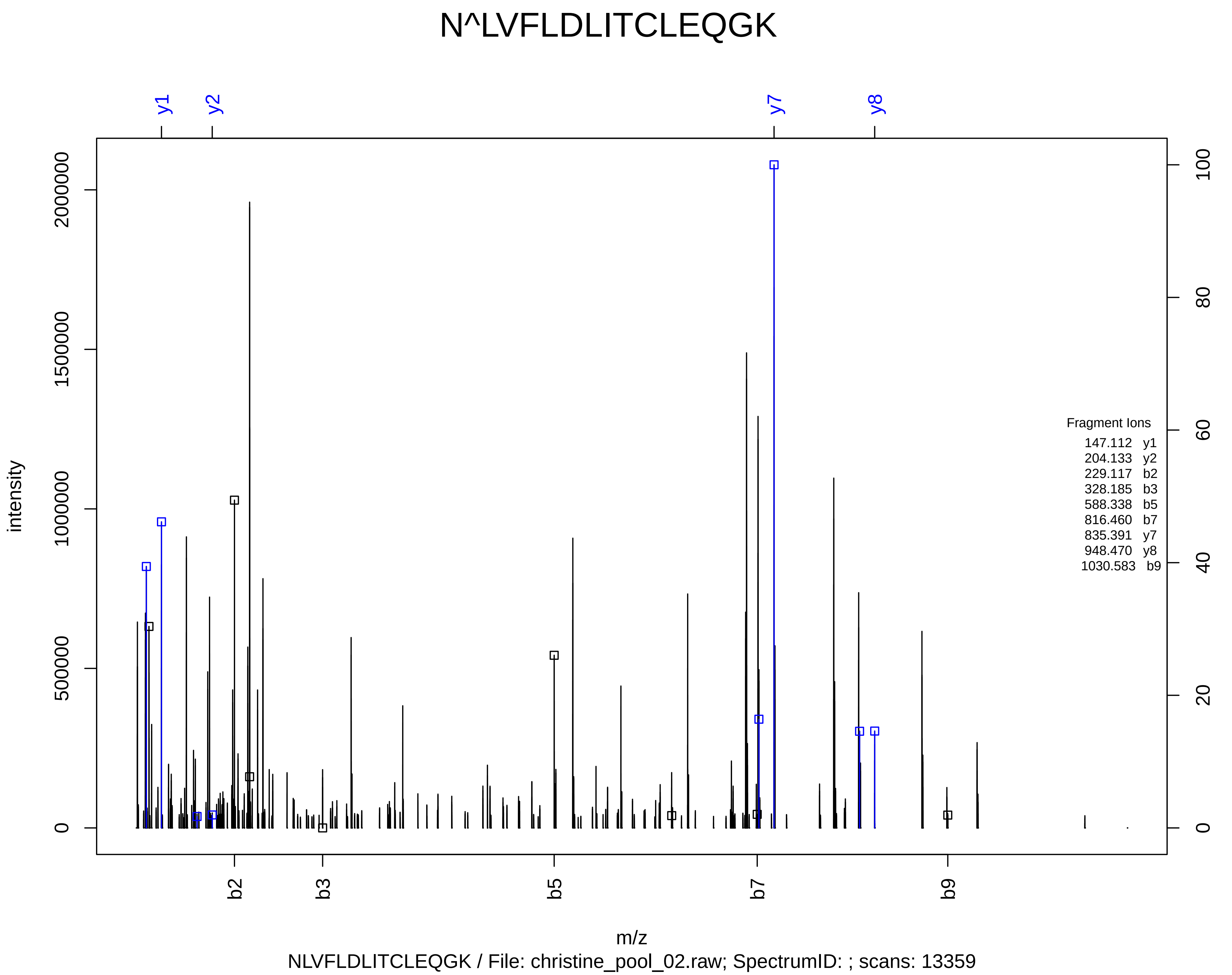

N^LVFLDLITCLEQGK
y1
y2
y7
y8
intensity
0
500000
1000000
1500000
2000000
0
20
40
60
80
100
b2
b3
b5
b7
b9
Fragment Ions
147.112 y1
204.133 y2
229.117 b2
328.185 b3
588.338 b5
816.460 b7
835.391 y7
948.470 y8
1030.583 b9
m/z
NLVFLDLITCLEQGK / File: christine_pool_02.raw; SpectrumID: ; scans: 13359

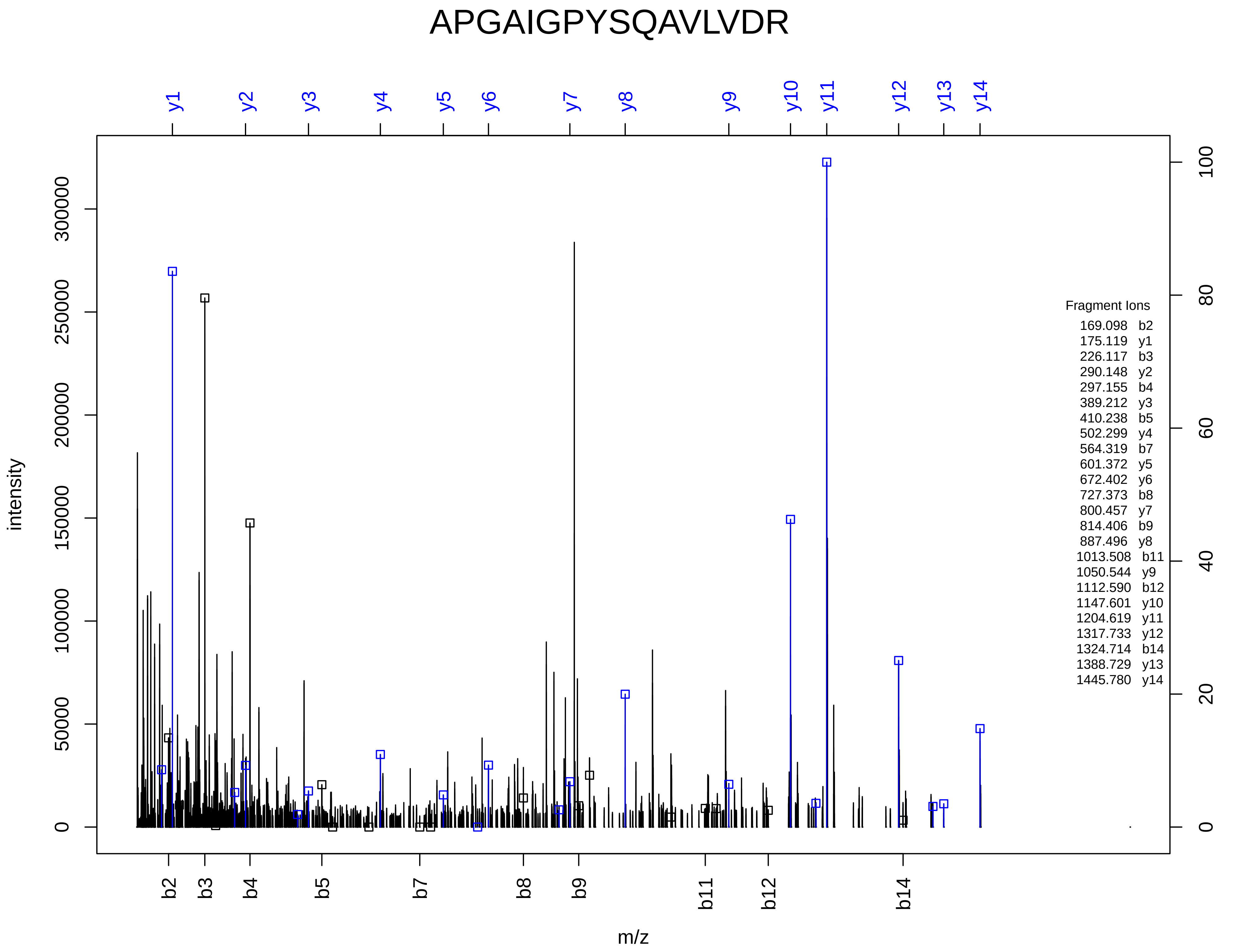

APGAIGPYSQAVLVDR / File: christine_pool_02.raw; SpectrumID: ; scans: 13131

VAGQDGSVVQFK

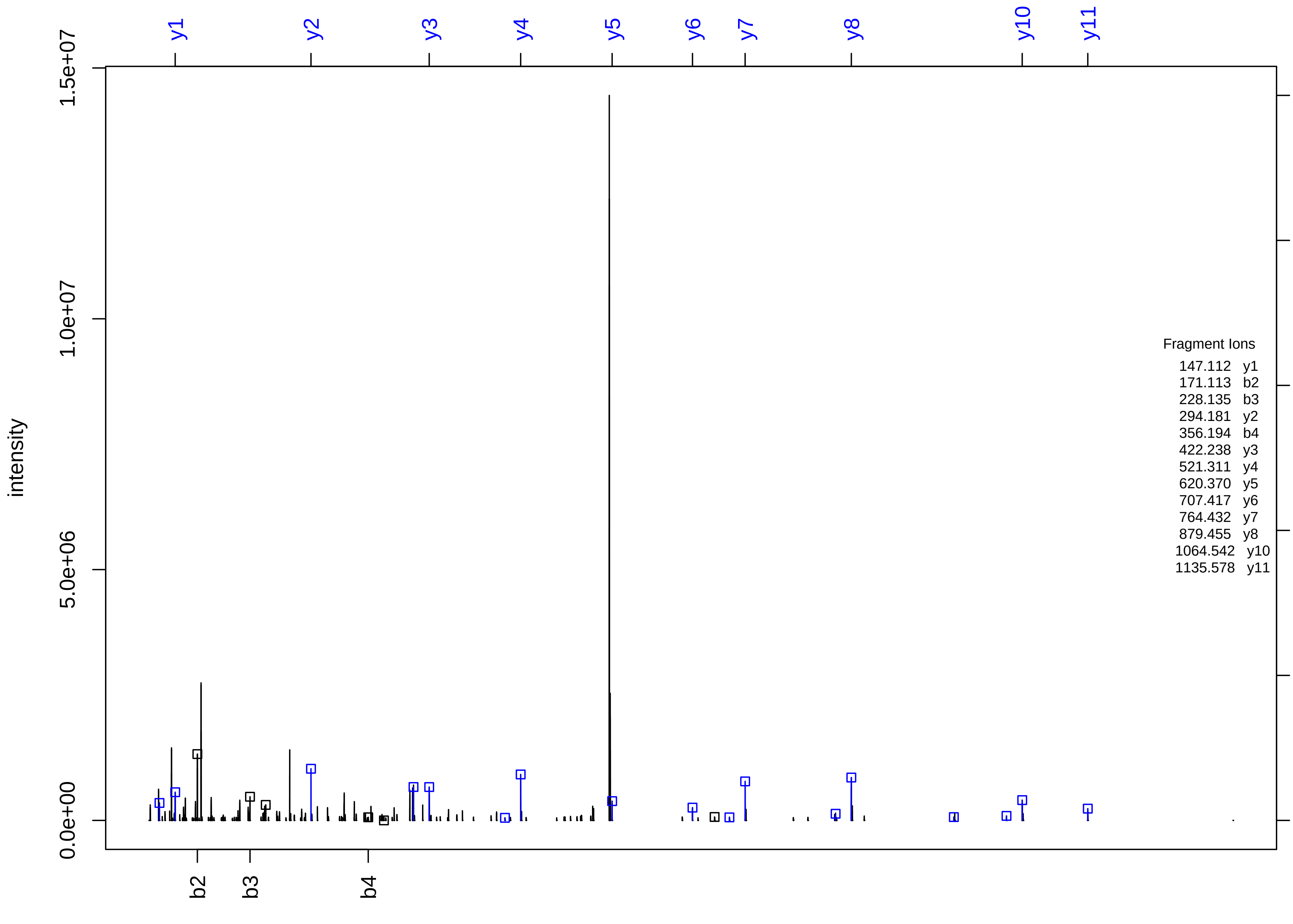

VAGQDGSVVQFK / File: christine_pool_02.raw; SpectrumID: ; scans: 6968

VVDLLVIK

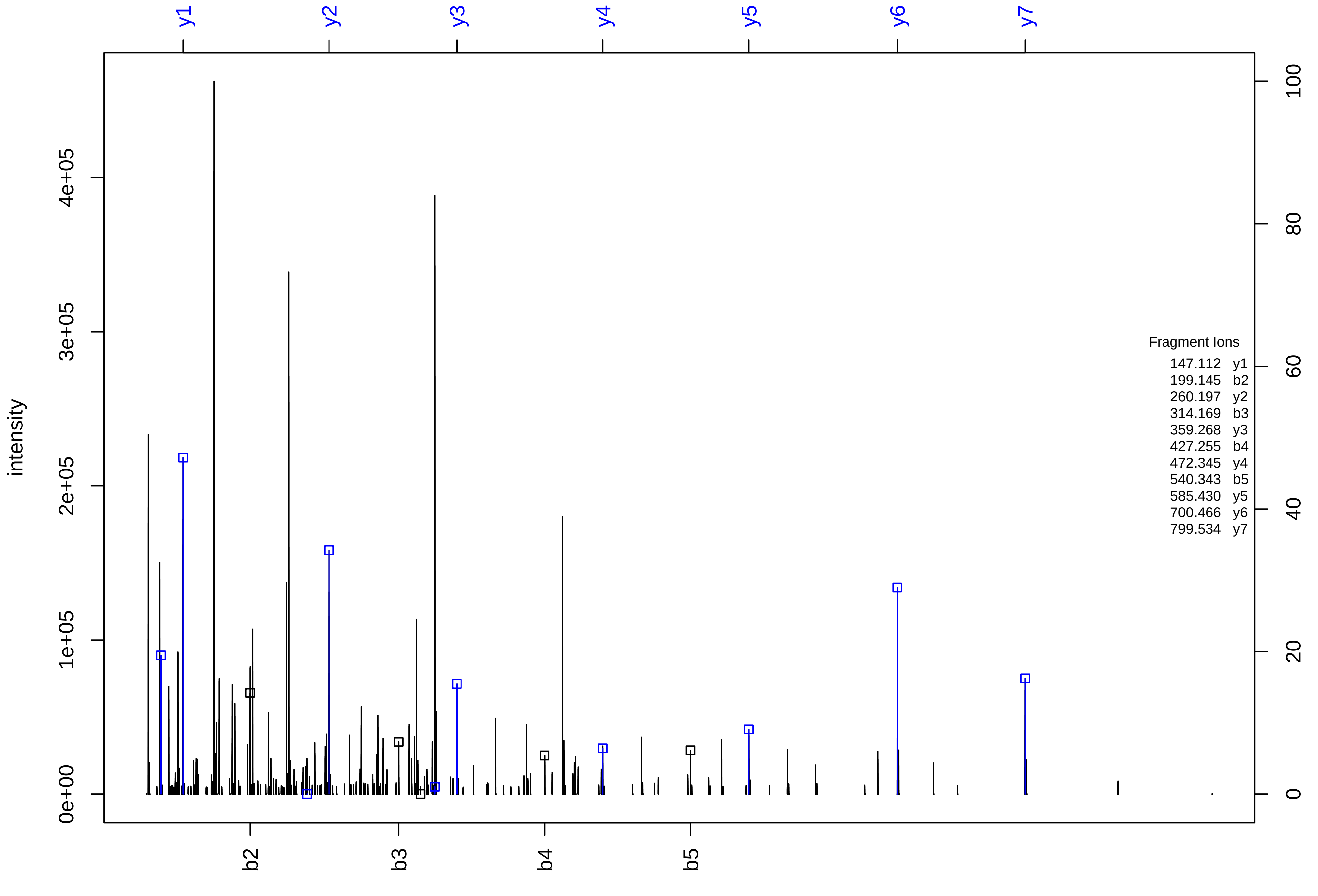

VVDLLVIK / File: christine_pool_02.raw; SpectrumID: ; scans: 16568

FIISFSR

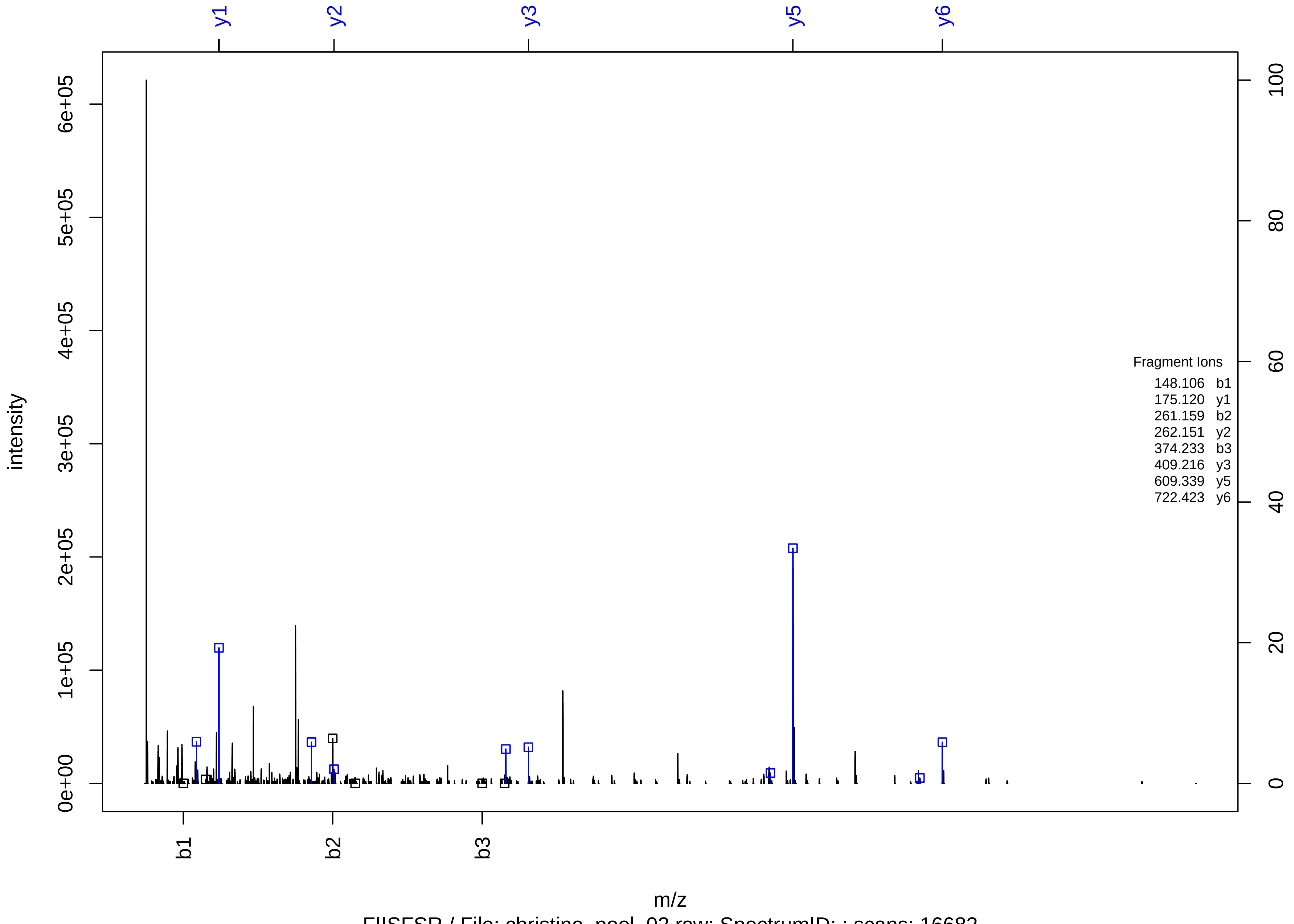

FIISFSR / File: christine_pool_02.raw; SpectrumID: ; scans: 16682

M*YGVLPWNAFPGK

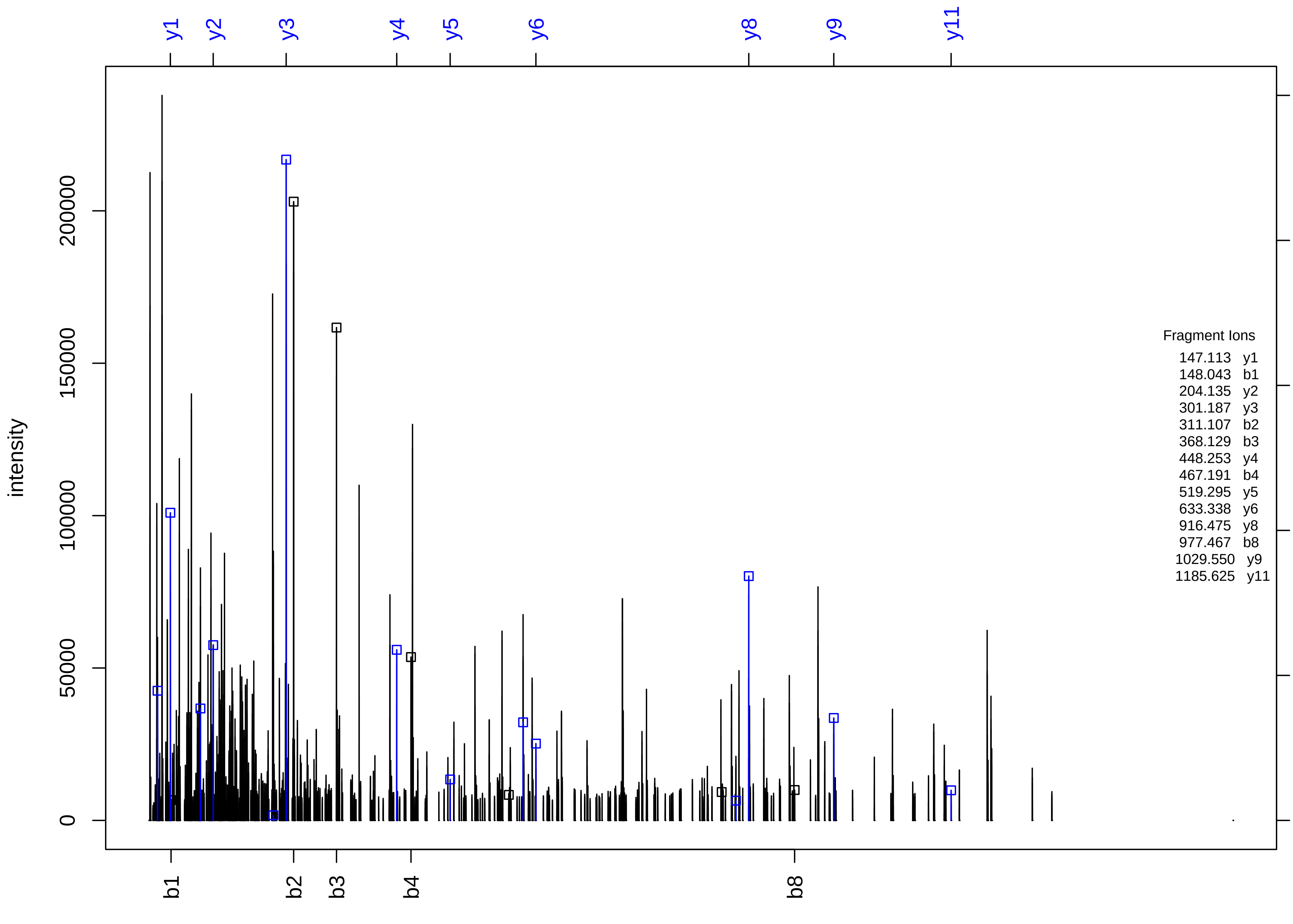

MYGVLPWNAFPGK / File: christine_pool_02.raw; SpectrumID: ; scans: 19137

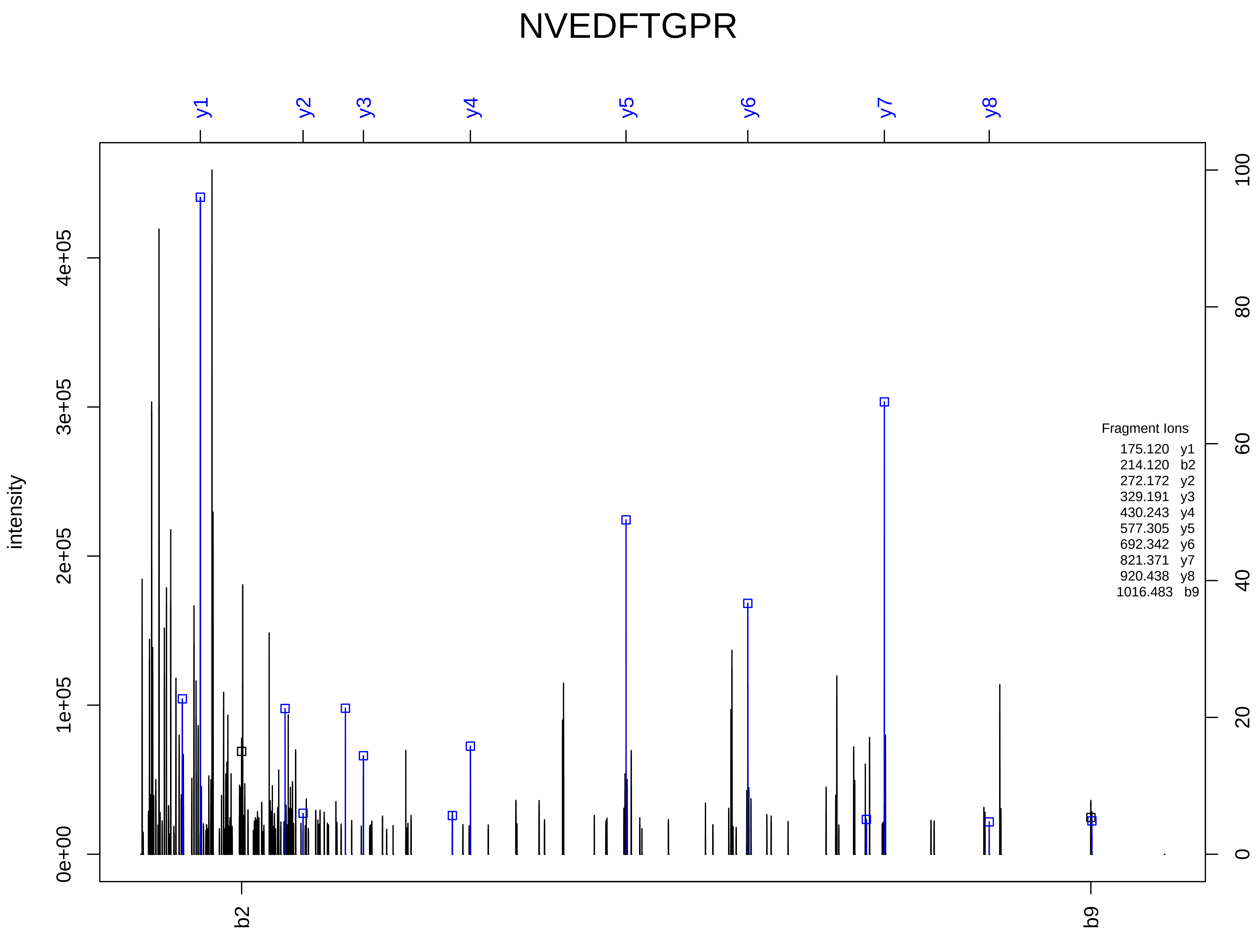

NVEDFTGPR / File: christine_pool_02.raw; SpectrumID: ; scans: 5525

(Ac)MELEAMSR

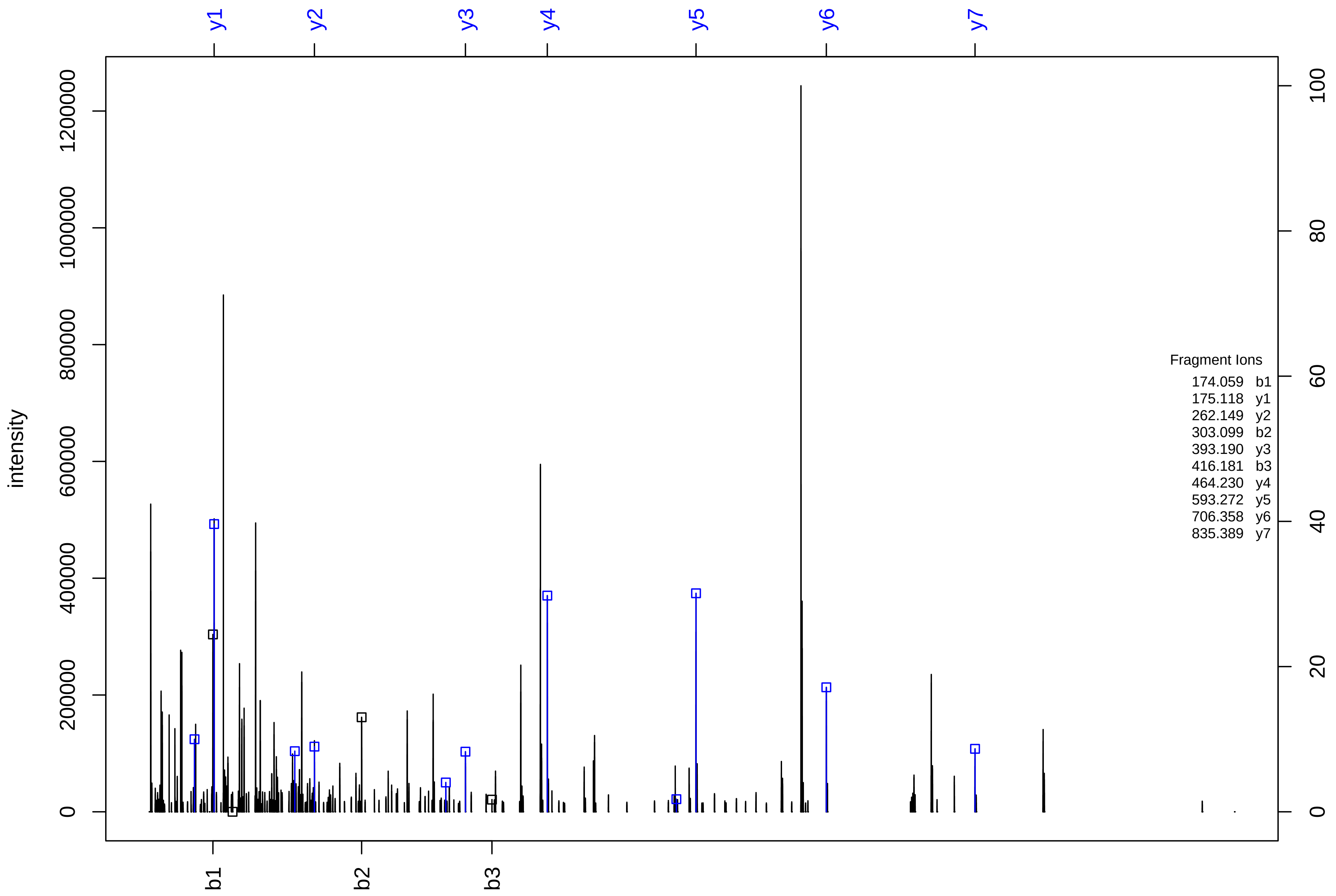

MELEAMSR / File: christine_pool_02.raw; SpectrumID: ; scans: 15558

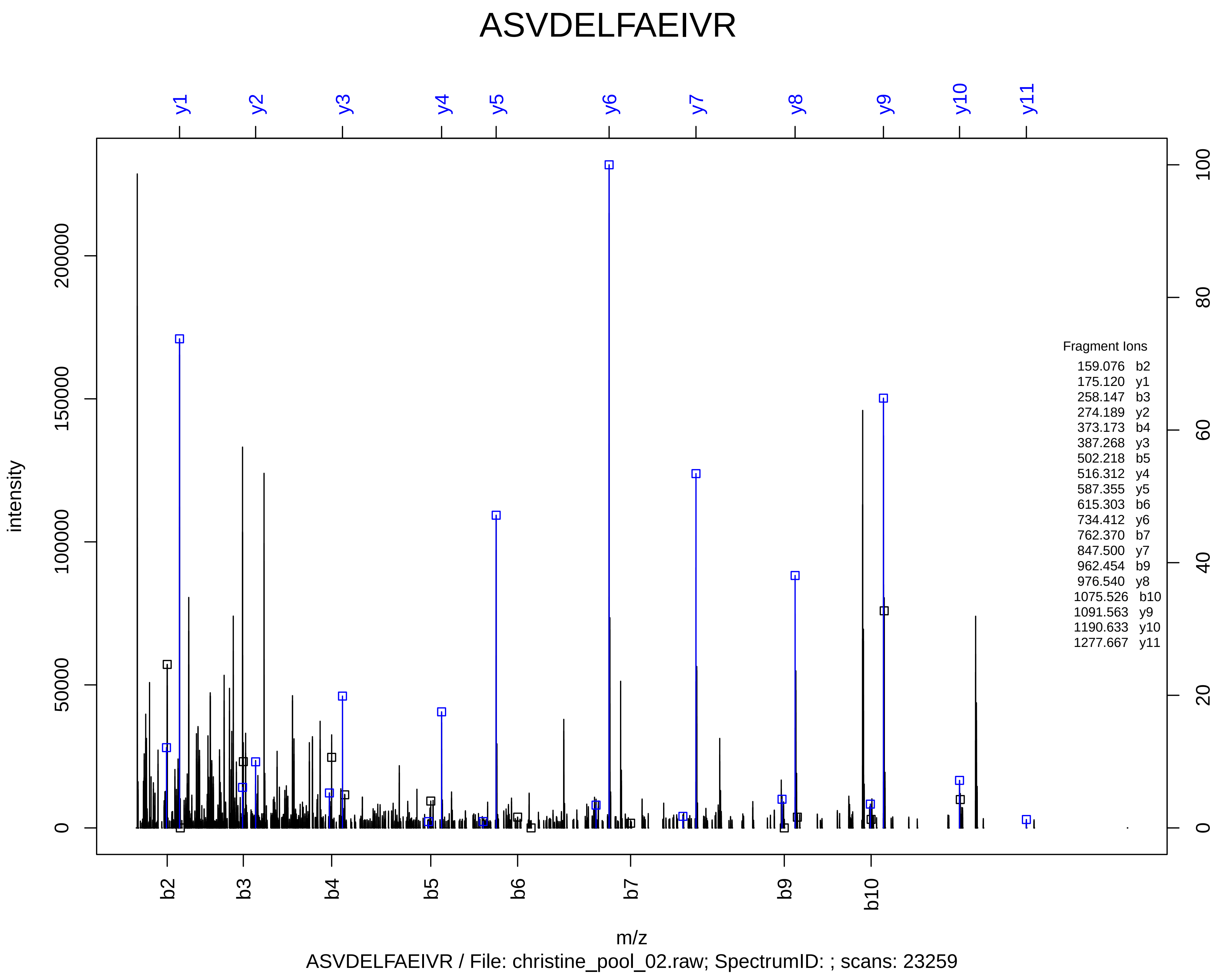
ASVDELFAEIVR
y1
y2
y3
y4
y5
y6
y7
y8
y9
y10
y11
intensity
0
50000
100000
150000
200000
0
20
40
60
80
100
b2
b3
b4
b5
b6
b7
b9
b10
m/z
Fragment Ions
159.076 b2
175.120 y1
258.147 b3
274.189 y2
373.173 b4
387.268 y3
502.218 b5
516.312 y4
587.355 y5
615.303 b6
734.412 y6
762.370 b7
847.500 y7
962.454 b9
976.540 y8
1075.526 b10
1091.563 y9
1190.633 y10
1277.667 y11
ASVDELFAEIVR / File: christine_pool_02.raw; SpectrumID: ; scans: 23259

GVYPDLLATSGDYLR

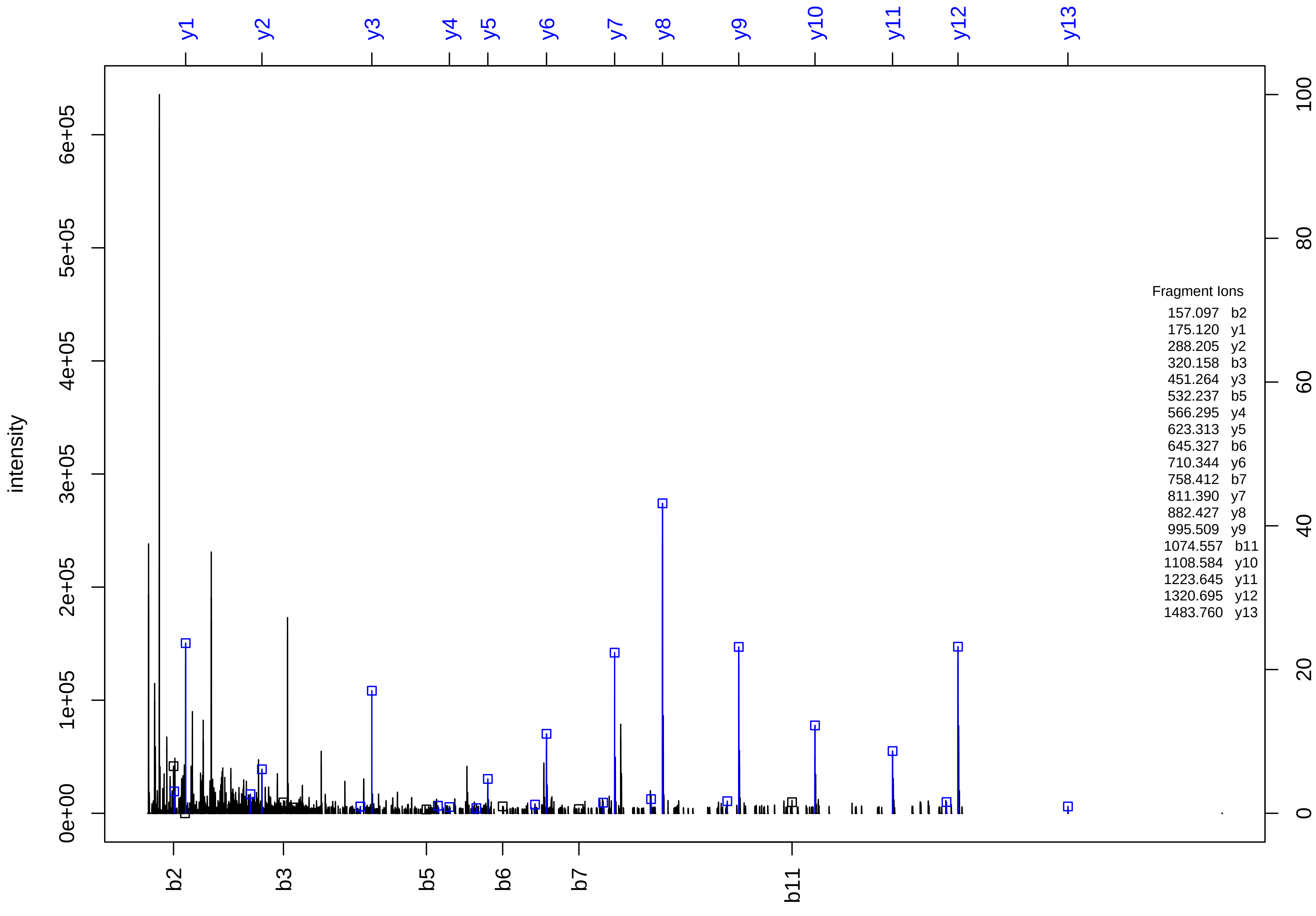

GVYPDLLATSGDYLR / File: christine_pool_02.raw; SpectrumID: ; scans: 18891

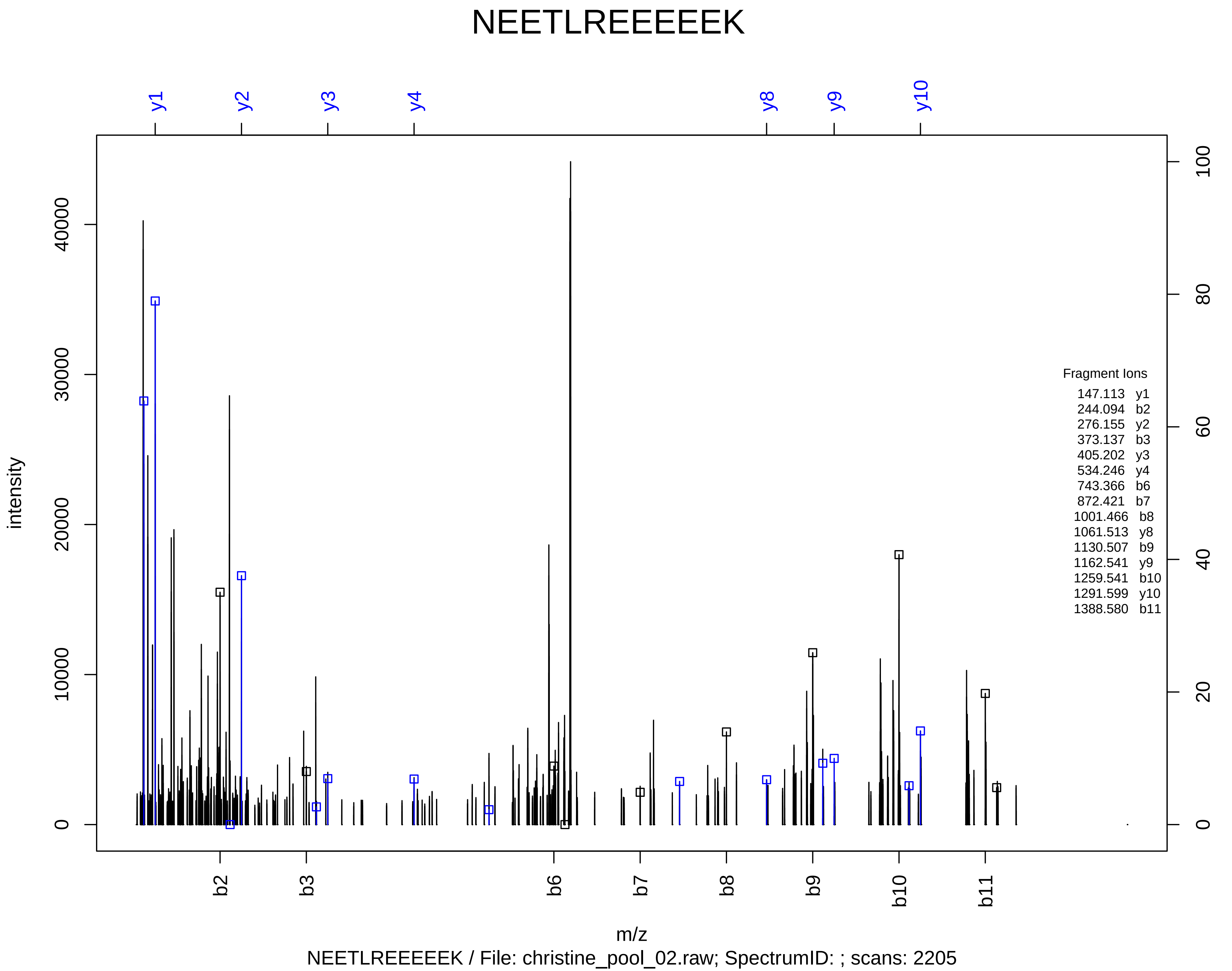
NEETLREEEEEK
y1
y2
y3
y4
y8
y9
y10
intensity
0
10000
20000
30000
40000
0
20
40
60
80
100
Fragment Ions
147.113 y1
244.094 b2
276.155 y2
373.137 b3
405.202 y3
534.246 y4
743.366 b6
872.421 b7
1001.466 b8
1061.513 y8
1130.507 b9
1162.541 y9
1259.541 b10
1291.599 y10
1388.580 b11
b2
b3
b6
b7
b8
b9
b10
b11
m/z
NEETLREEEEEK / File: christine_pool_02.raw; SpectrumID: ; scans: 2205

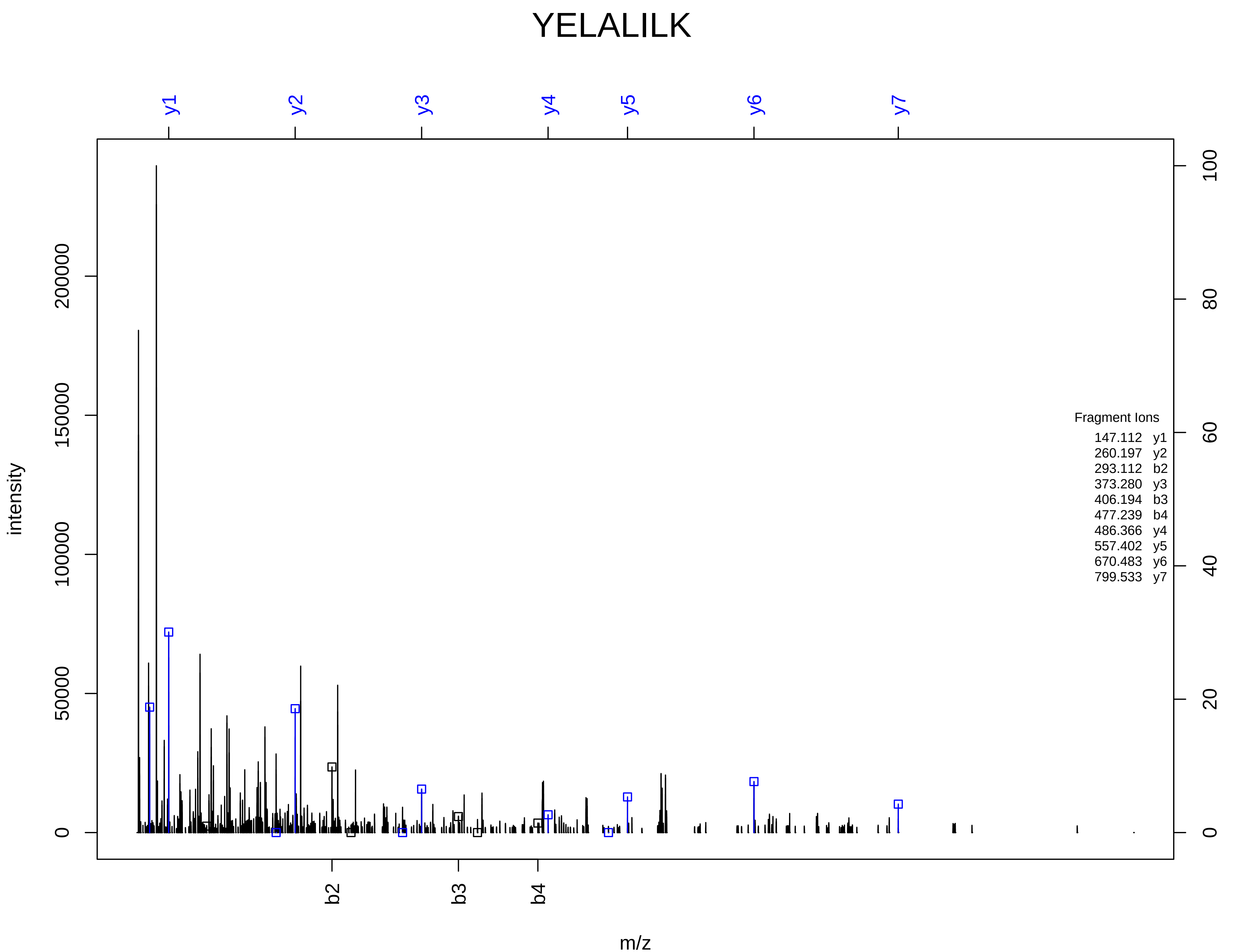

YELALILK / File: christine_pool_02.raw; SpectrumID: ; scans: 17660

VGDSQNPLLSDGDLSTMIGK

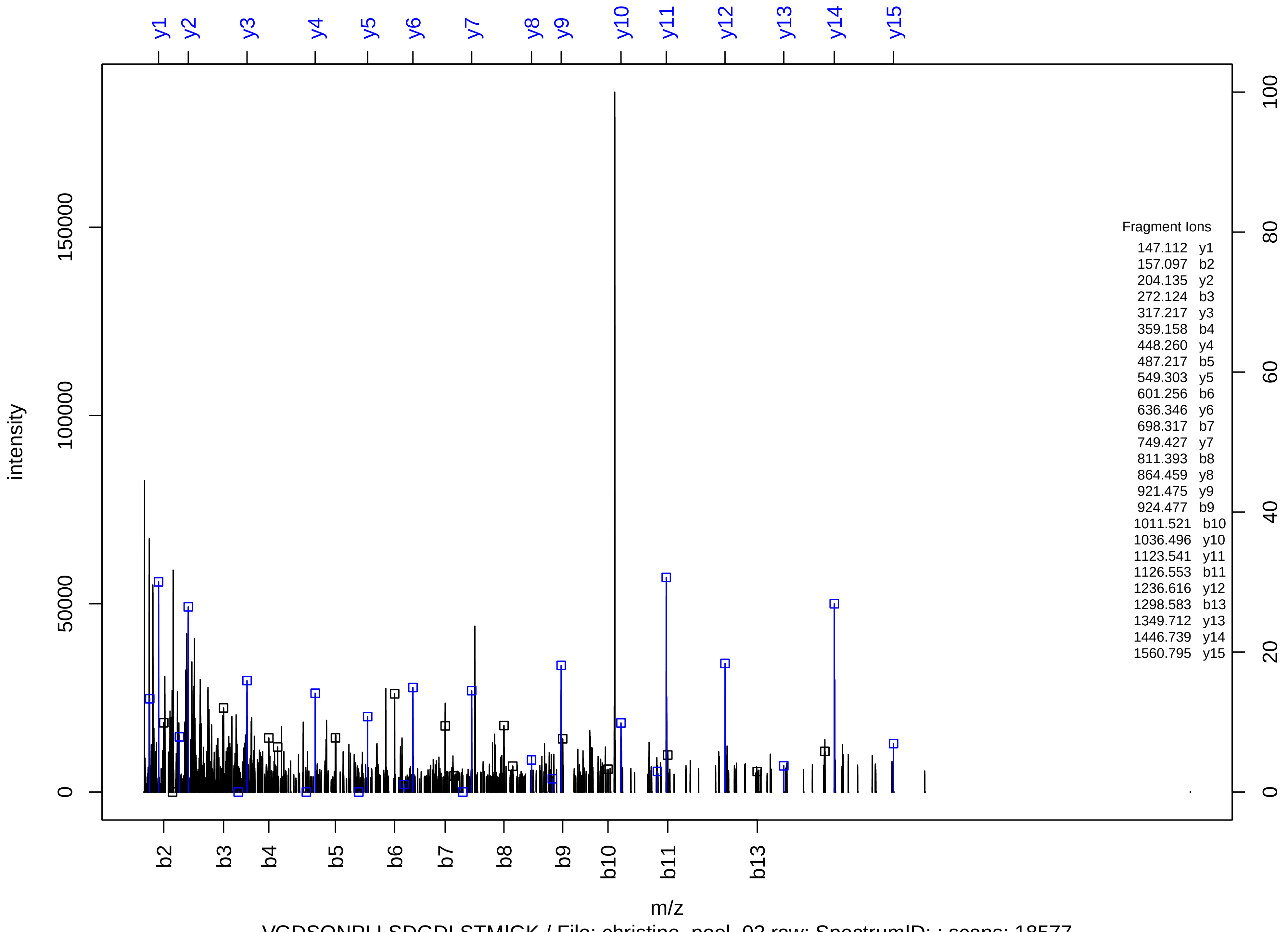

VGDSQNPLLSDGDLSTMIGK / File: christine_pool_02.raw; SpectrumID: ; scans: 18577

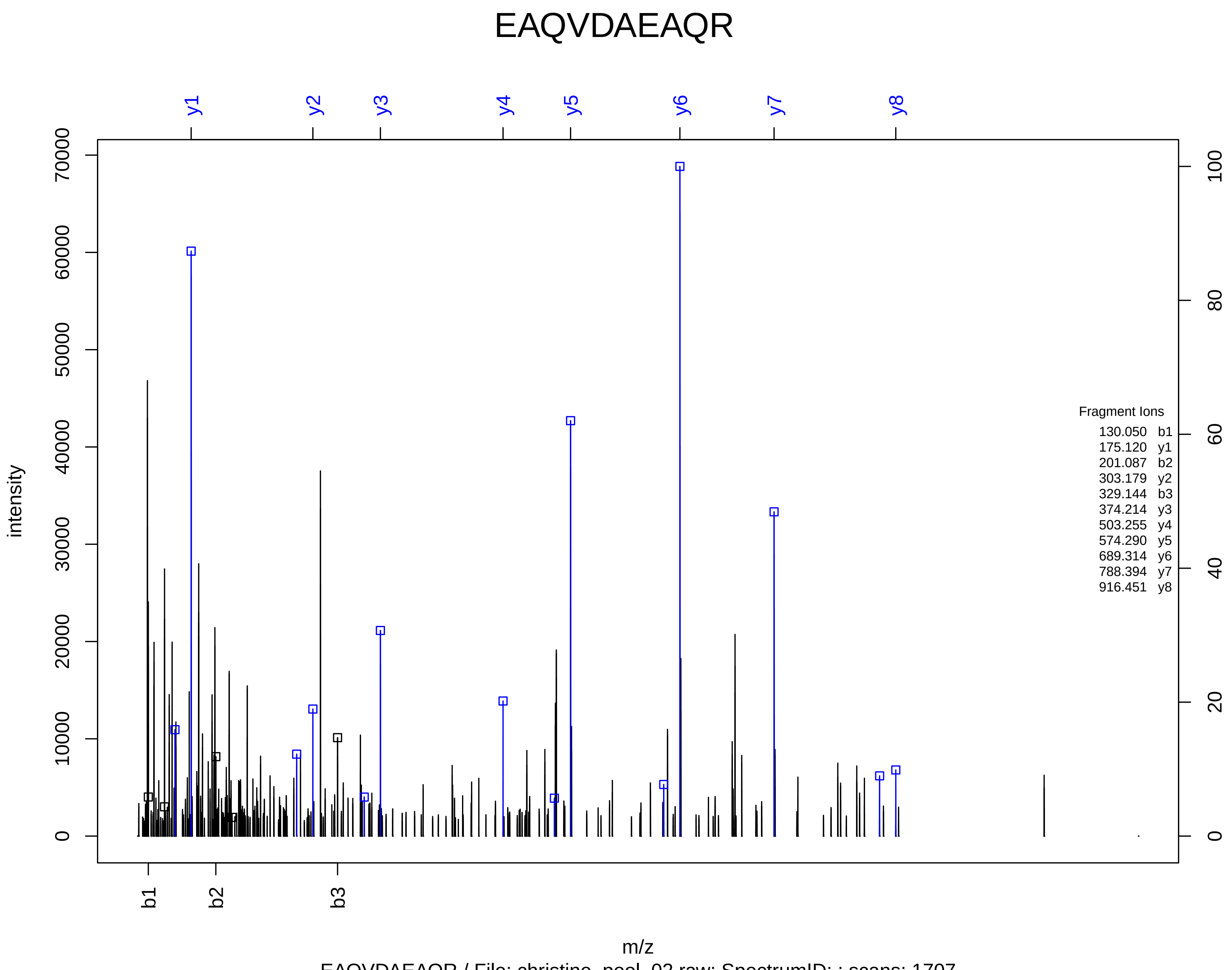
EAQVDAEAQR
y1
y2
y3
y4
y5
y6
y7
y8
b1
b2
b3
intensity
m/z
Fragment Ions
130.050 b1
175.120 y1
201.087 b2
303.179 y2
329.144 b3
374.214 y3
503.255 y4
574.290 y5
689.314 y6
788.394 y7
916.451 y8
EAQVDAEAQR / File: christine_pool_02.raw; SpectrumID: ; scans: 1707

ILADEALK

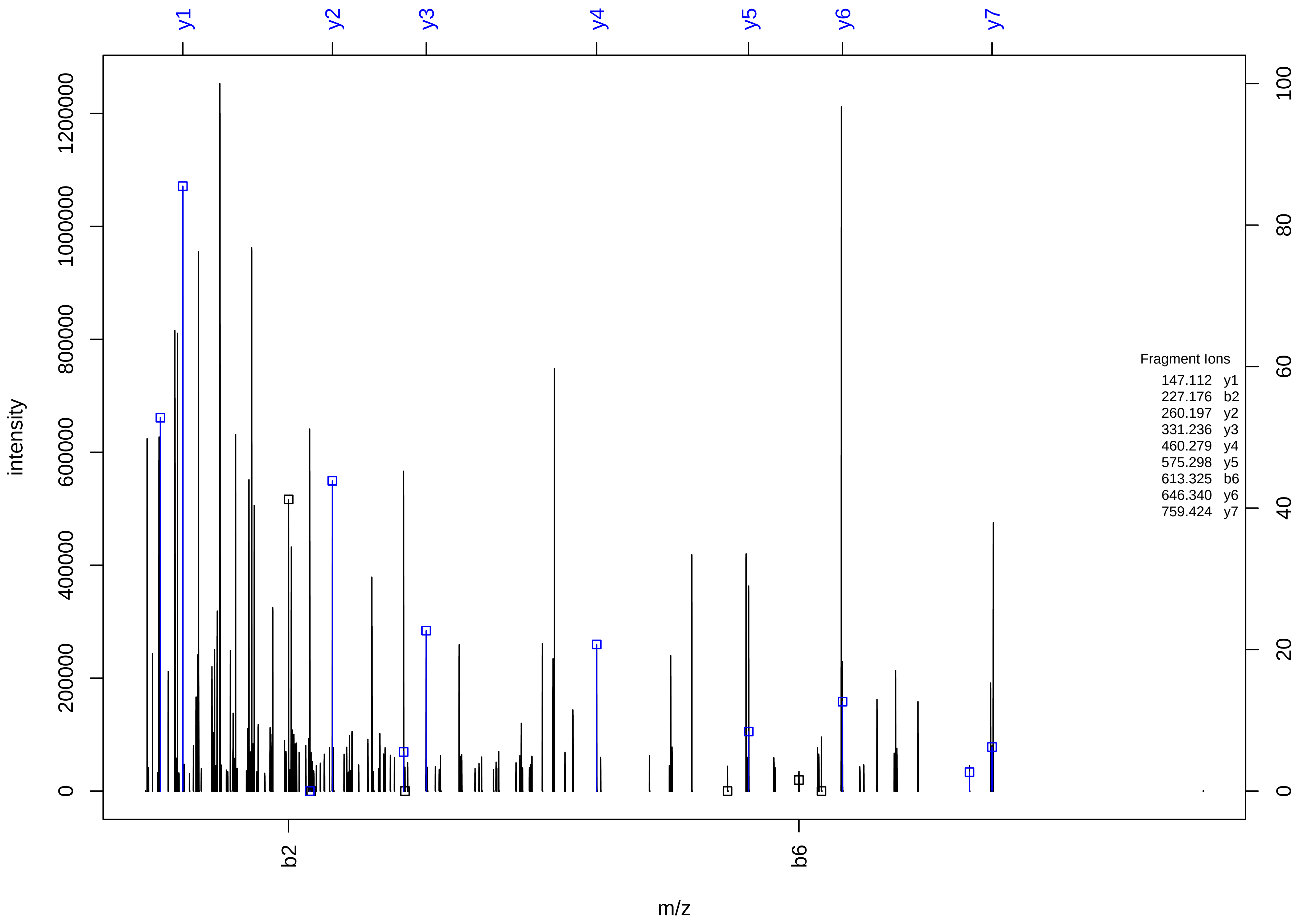

ILADEALK / File: christine_pool_02.raw; SpectrumID: ; scans: 10333

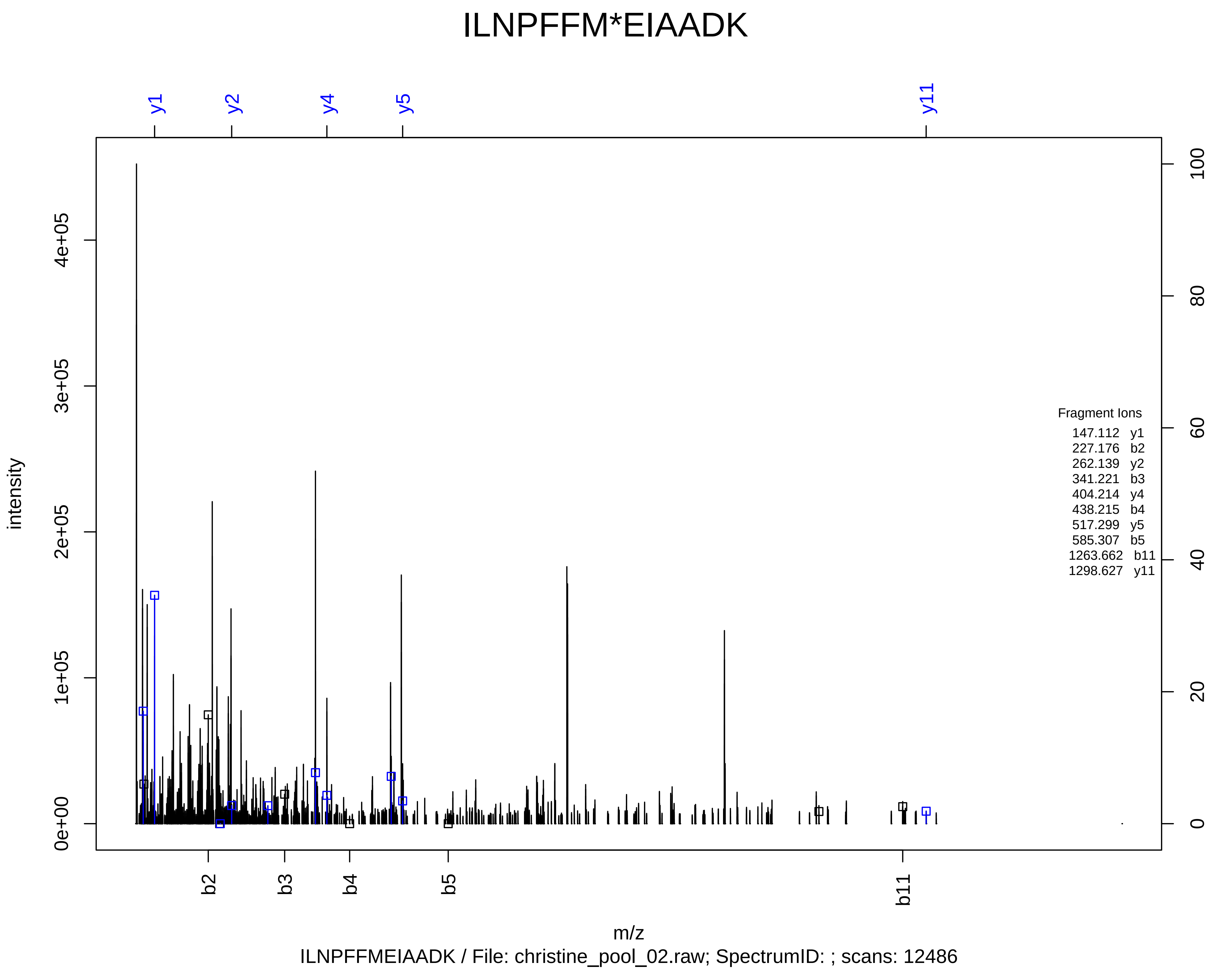

ILNPFFM*EIAADK
y1
y2
y4
y5
y11
intensity
0e+00
1e+05
2e+05
3e+05
4e+05
0
20
40
60
80
100
b2
b3
b4
b5
b11
m/z
Fragment Ions
147.112 y1
227.176 b2
262.139 y2
341.221 b3
404.214 y4
438.215 b4
517.299 y5
585.307 b5
1263.662 b11
1298.627 y11
ILNPFFMEIAADK / File: christine_pool_02.raw; SpectrumID: ; scans: 12486

AIIESDQEQGRK

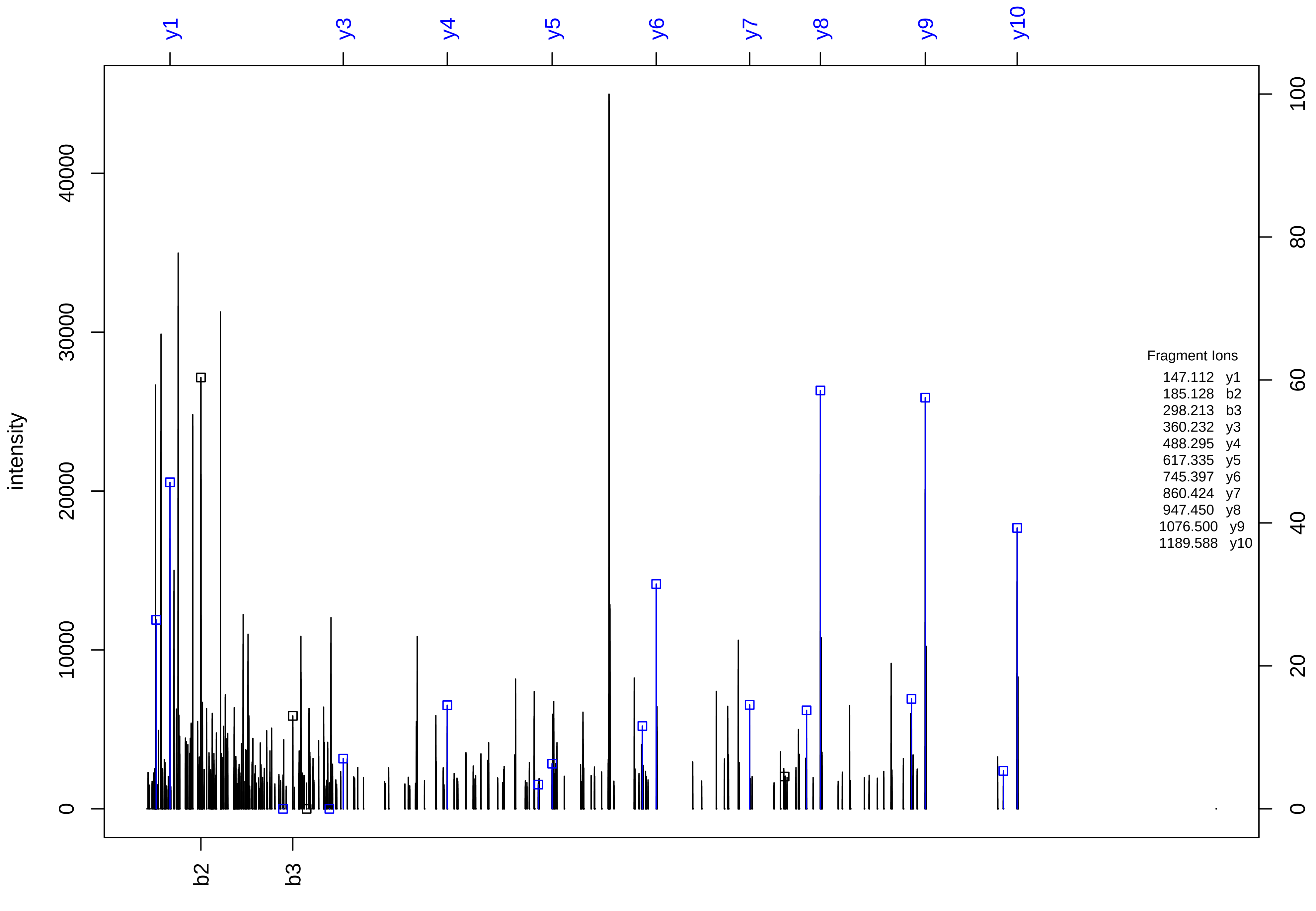

AIIESDQEQGRK / File: christine_pool_02.raw; SpectrumID: ; scans: 2144

NNYPYM*HQYM*TK

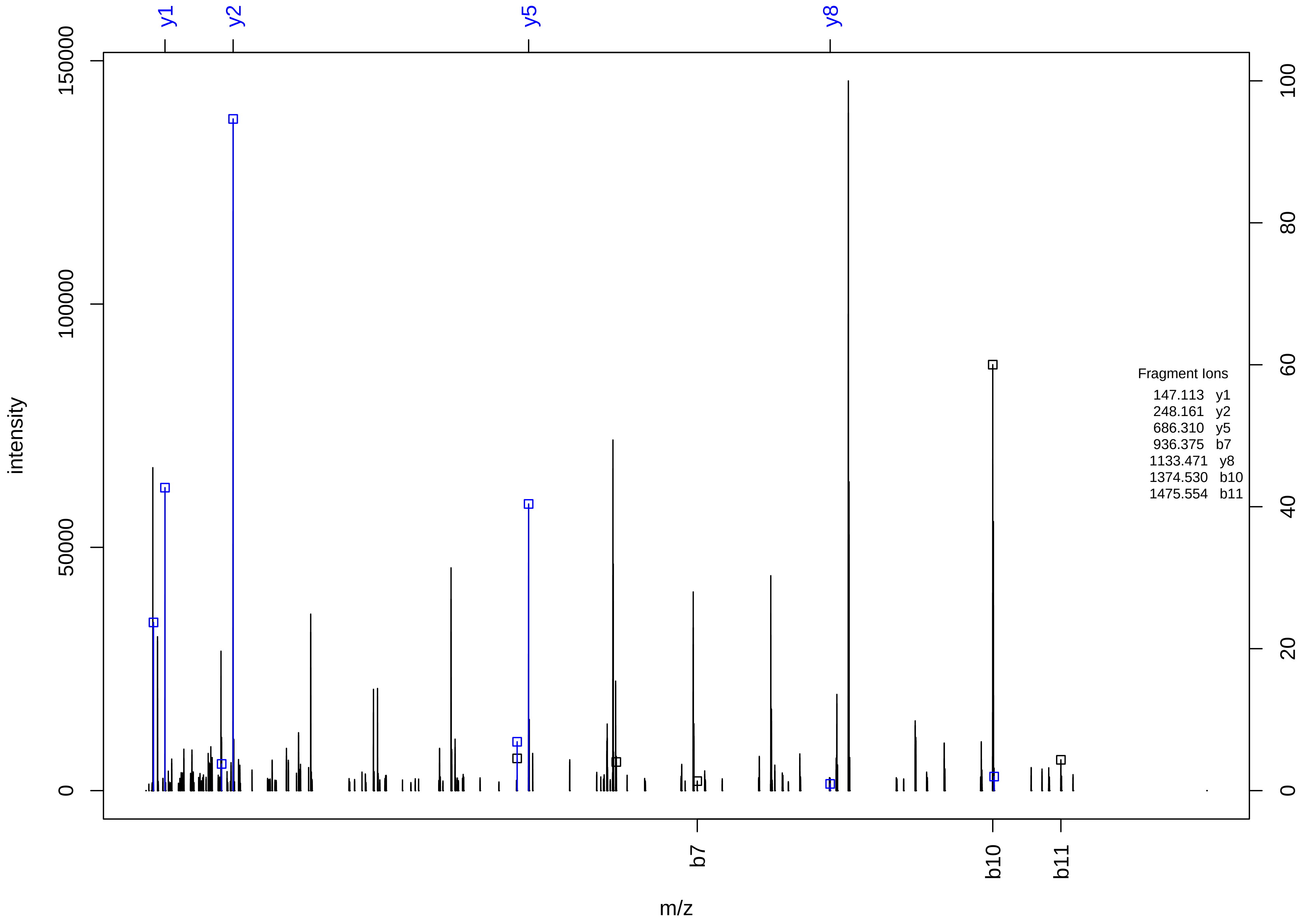

NNYPYMHQYMTK / File: christine_pool_02.raw; SpectrumID: ; scans: 1778

ILLTNIR

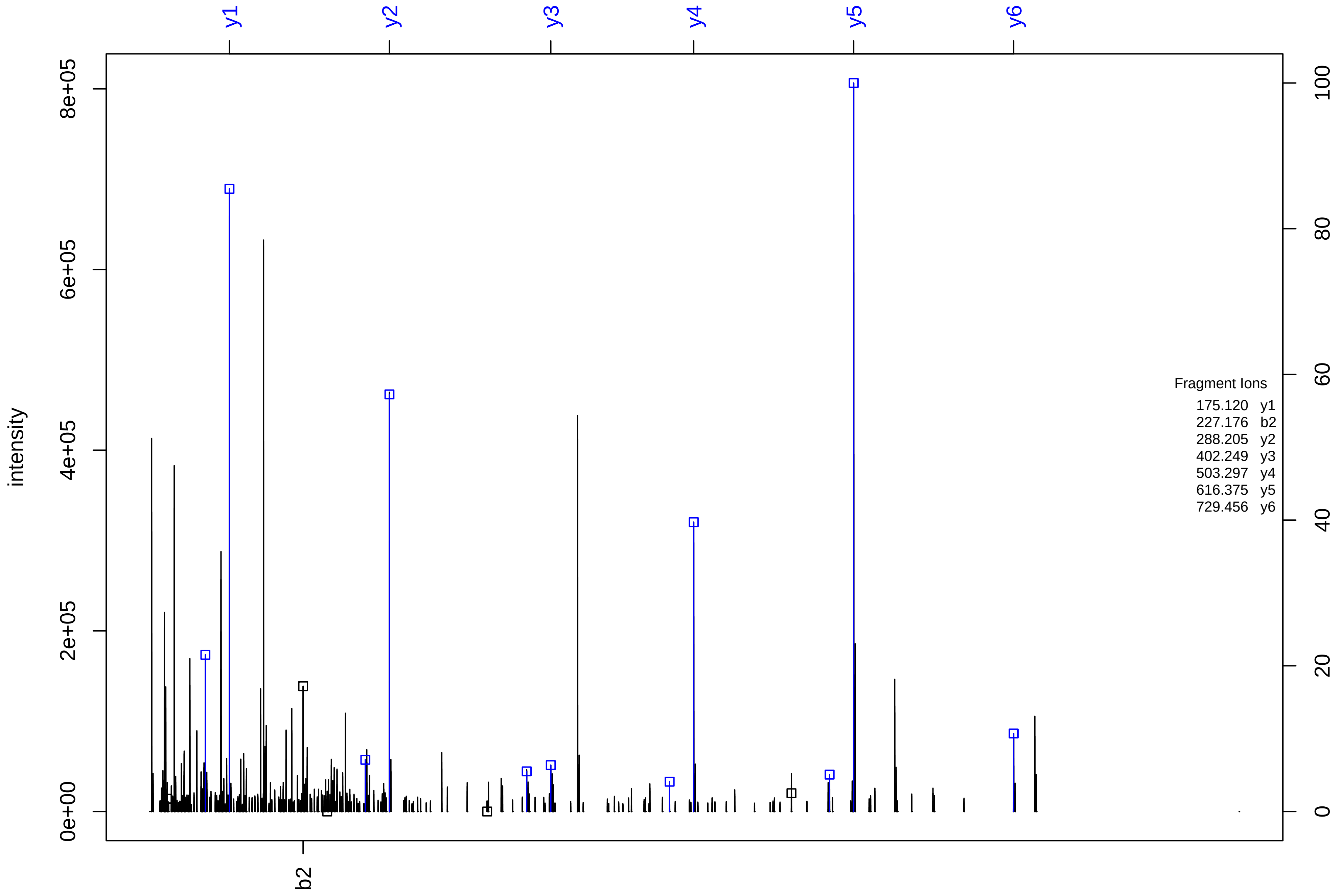

ILLTNIR / File: christine_pool_02.raw; SpectrumID: ; scans: 8216

YCALLLPLLK

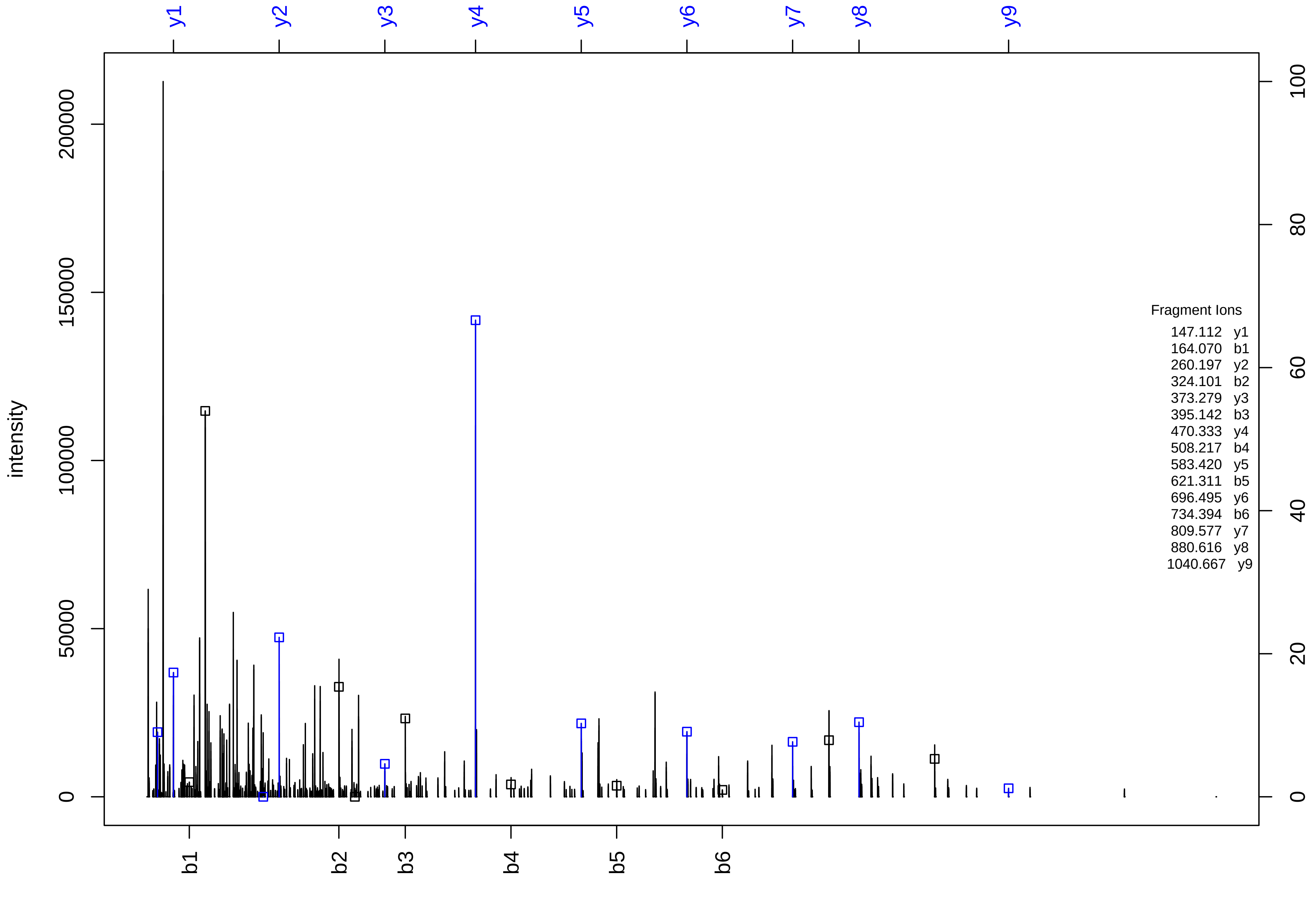

YCALLLPLLK / File: christine_pool_02.raw; SpectrumID: ; scans: 23235

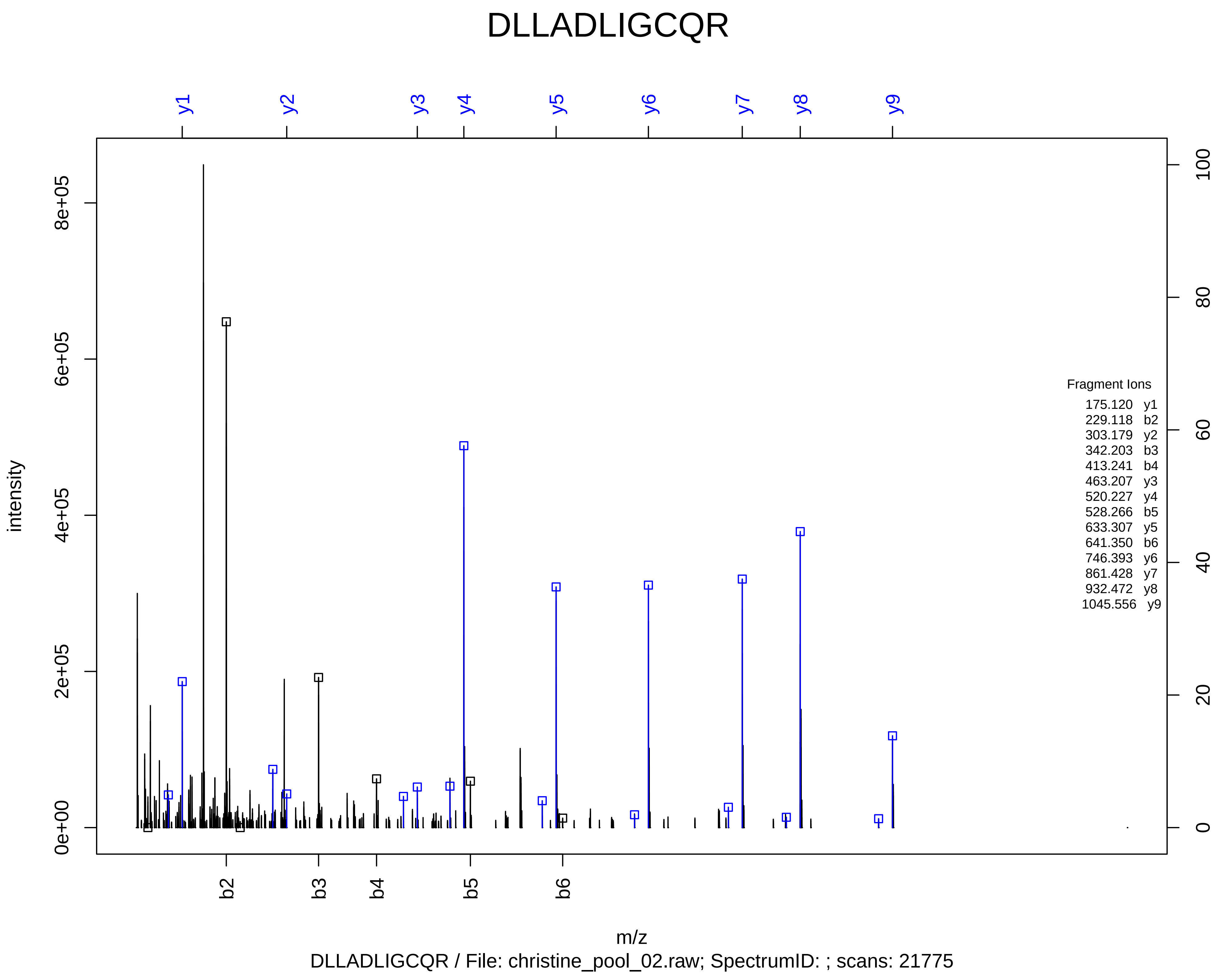
DLLADLIGCQR
y1
y2
y3
y4
y5
y6
y7
y8
y9
b2
b3
b4
b5
b6
intensity
0e+00
2e+05
4e+05
6e+05
8e+05
0
20
40
60
80
100
m/z
Fragment Ions
175.120 y1
229.118 b2
303.179 y2
342.203 b3
413.241 b4
463.207 y3
520.227 y4
528.266 b5
633.307 y5
641.350 b6
746.393 y6
861.428 y7
932.472 y8
1045.556 y9
DLLADLIGCQR / File: christine_pool_02.raw; SpectrumID: ; scans: 21775

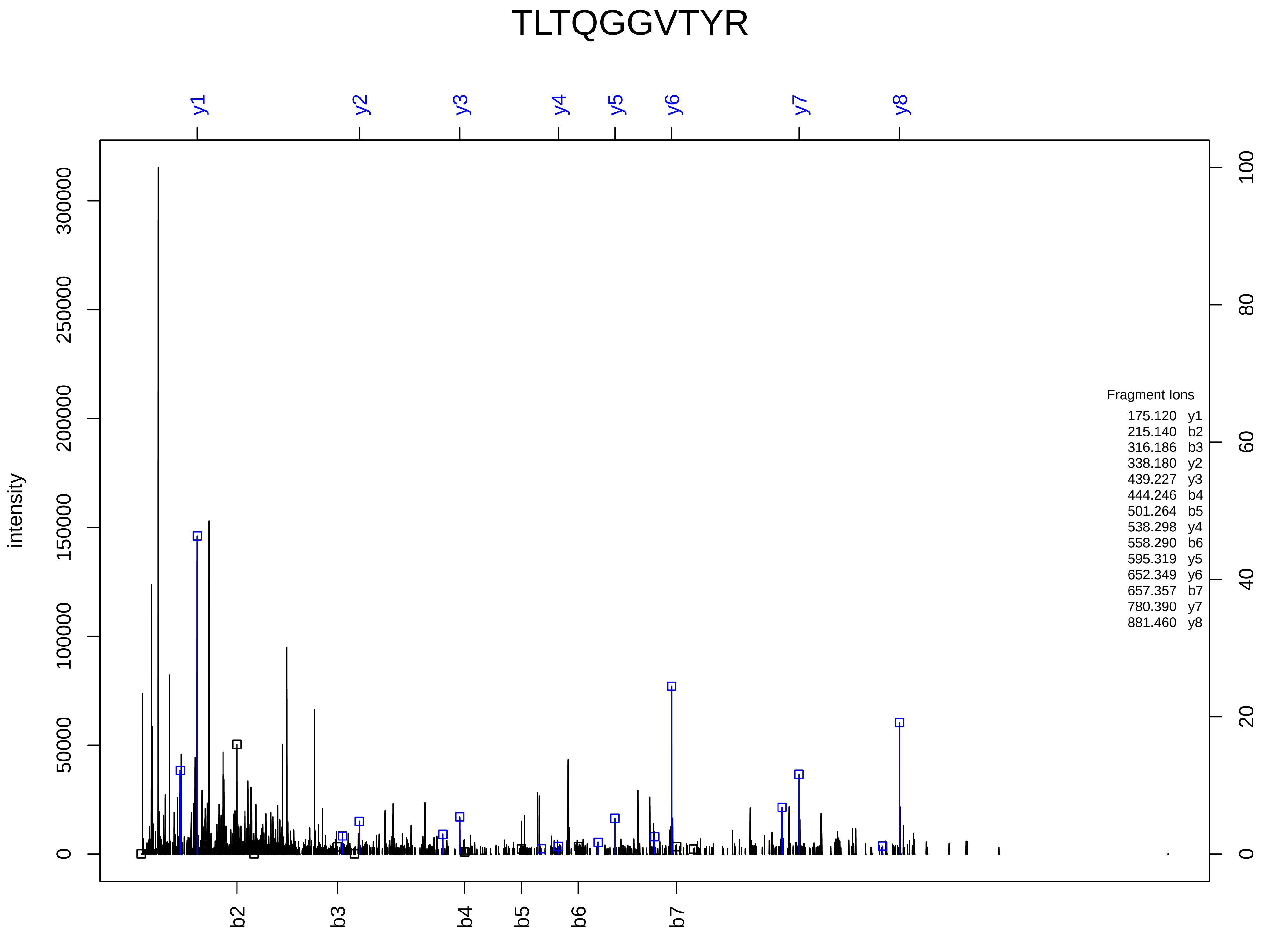

TLTQGGVTYR / File: christine_pool_02.raw; SpectrumID: ; scans: 4395

(Ac)SAPSATPIFAPGEN^CSPAWGAAPAAYDAADTHLR

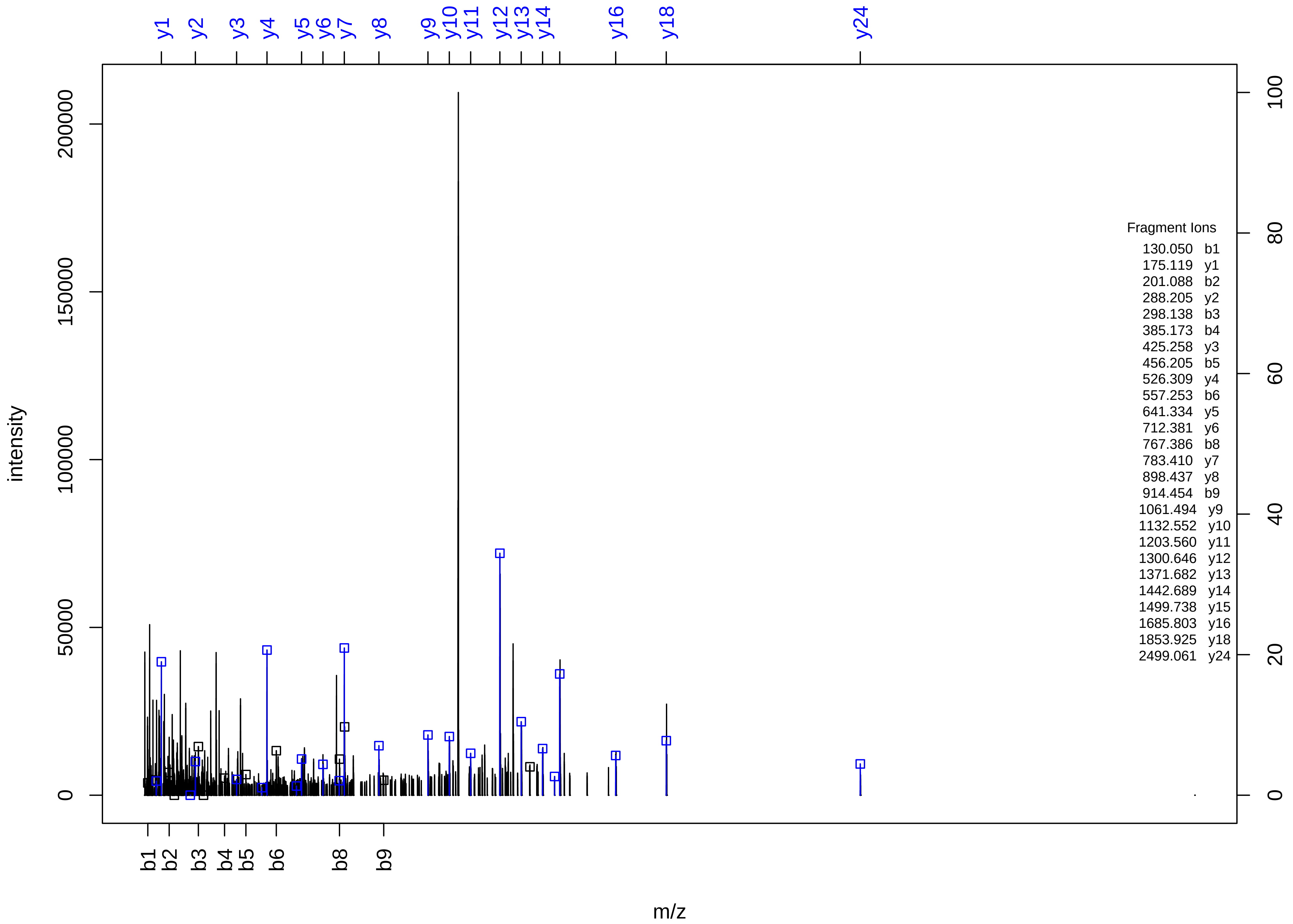

SAPSATPIFAPGENCSPAWGAAPAAYDAADTHLR / File: christine_pool_02.raw; SpectrumID: ; scans: 19748

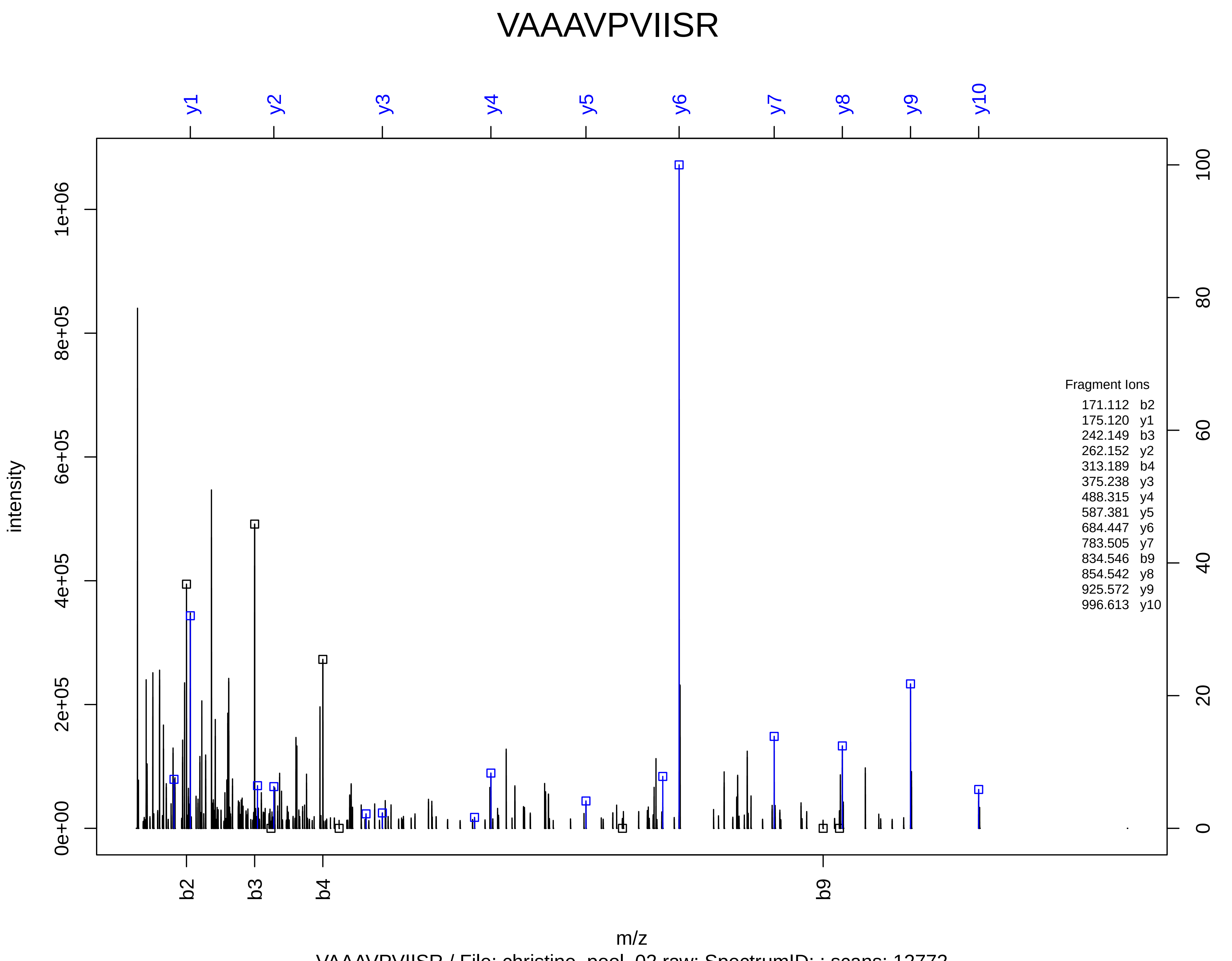
VAAAVPVIISR
y1
y2
y3
y4
y5
y6
y7
y8
y9
y10
intensity
0e+00
2e+05
4e+05
6e+05
8e+05
1e+06
0
20
40
60
80
100
b2
b3
b4
b9
m/z
Fragment Ions
171.112 b2
175.120 y1
242.149 b3
262.152 y2
313.189 b4
375.238 y3
488.315 y4
587.381 y5
684.447 y6
783.505 y7
834.546 b9
854.542 y8
925.572 y9
996.613 y10
VAAAVPVIISR / File: christine_pool_02.raw; SpectrumID: ; scans: 12772

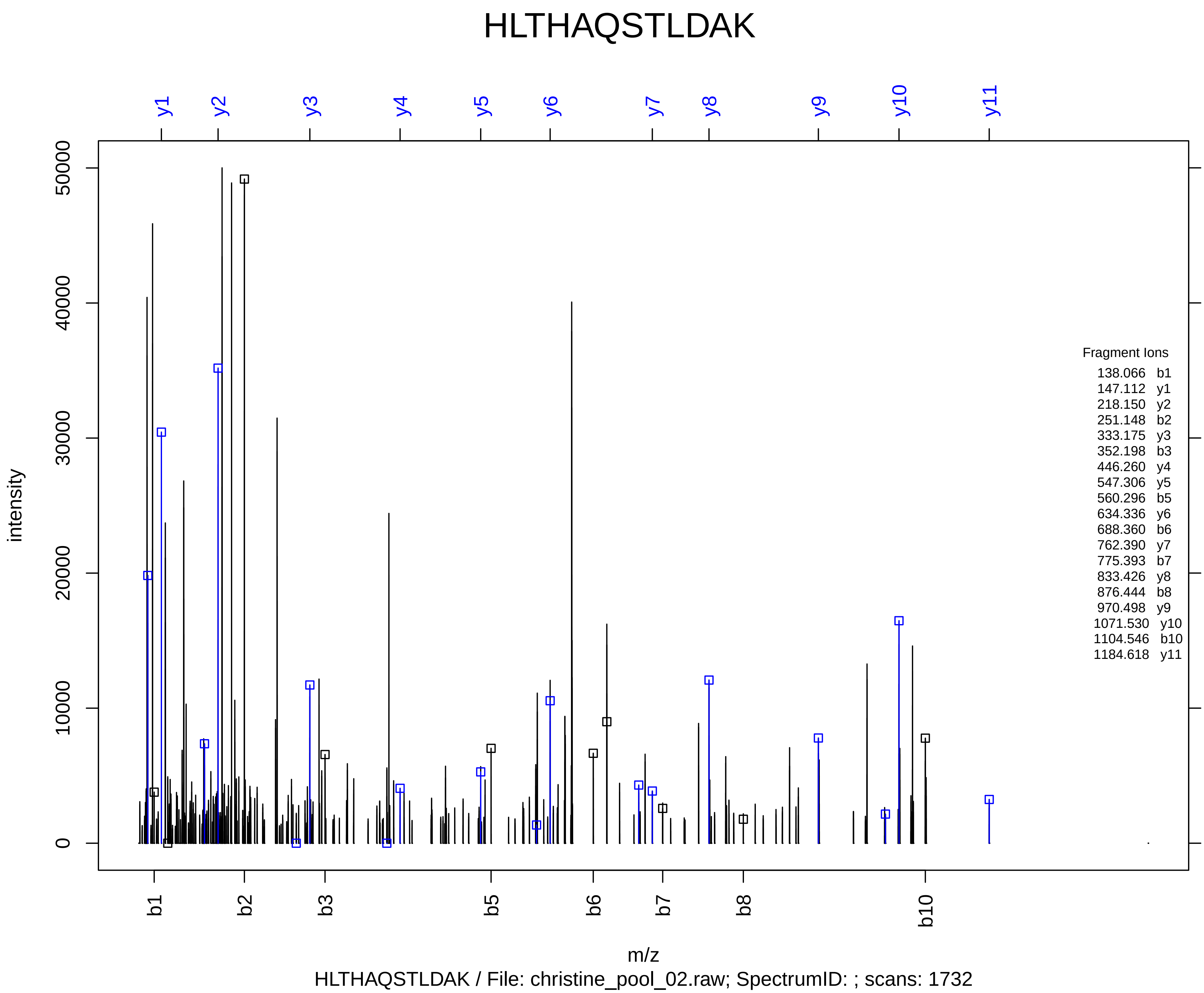
HLTHAQSTLDAK
intensity
m/z
y1 y2 y3 y4 y5 y6 y7 y8 y9 y10 y11
b1 b2 b3 b5 b6 b7 b8 b10
0 10000 20000 30000 40000 50000
0 20 40 60 80 100
Fragment Ions
138.066 b1
147.112 y1
218.150 y2
251.148 b2
333.175 y3
352.198 b3
446.260 y4
547.306 y5
560.296 b5
634.336 y6
688.360 b6
762.390 y7
775.393 b7
833.426 y8
876.444 b8
970.498 y9
1071.530 y10
1104.546 b10
1184.618 y11
HLTHAQSTLDAK / File: christine_pool_02.raw; SpectrumID: ; scans: 1732

LIDIQEK

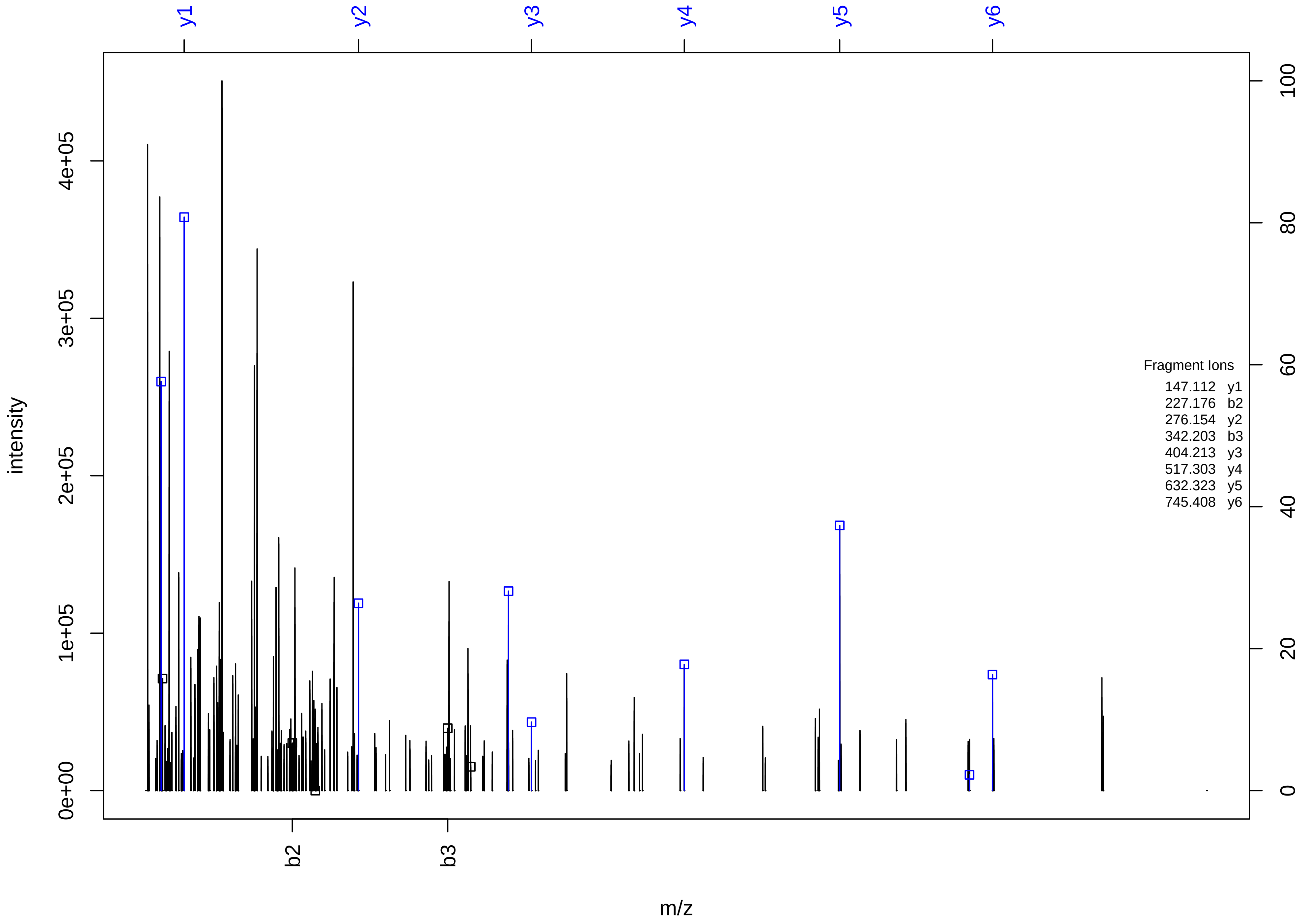

LIDIQEK / File: christine_pool_02.raw; SpectrumID: ; scans: 5499

ILDVLEEIPK

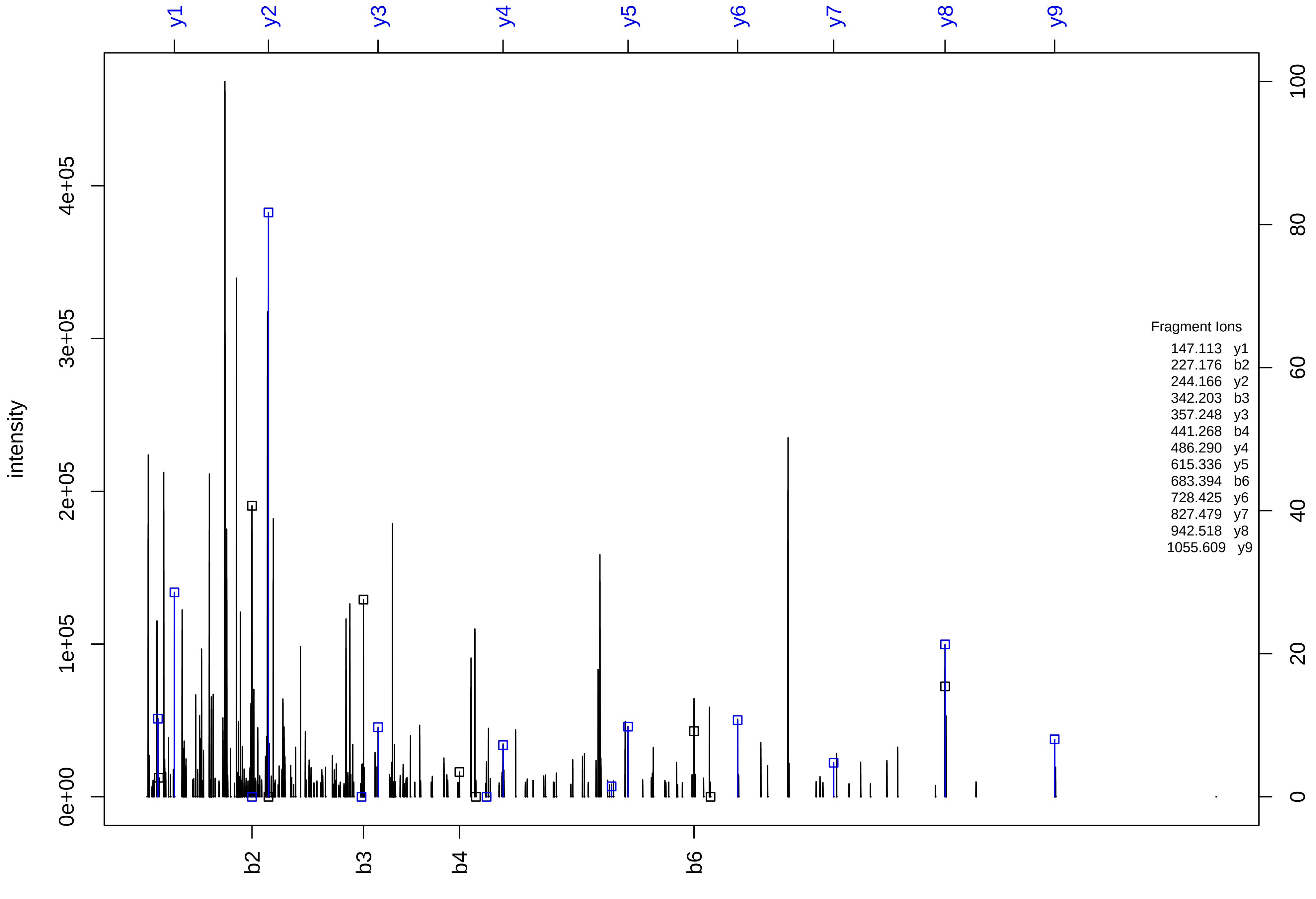

ILDVLEEIPK / File: christine_pool_02.raw; SpectrumID: ; scans: 19510

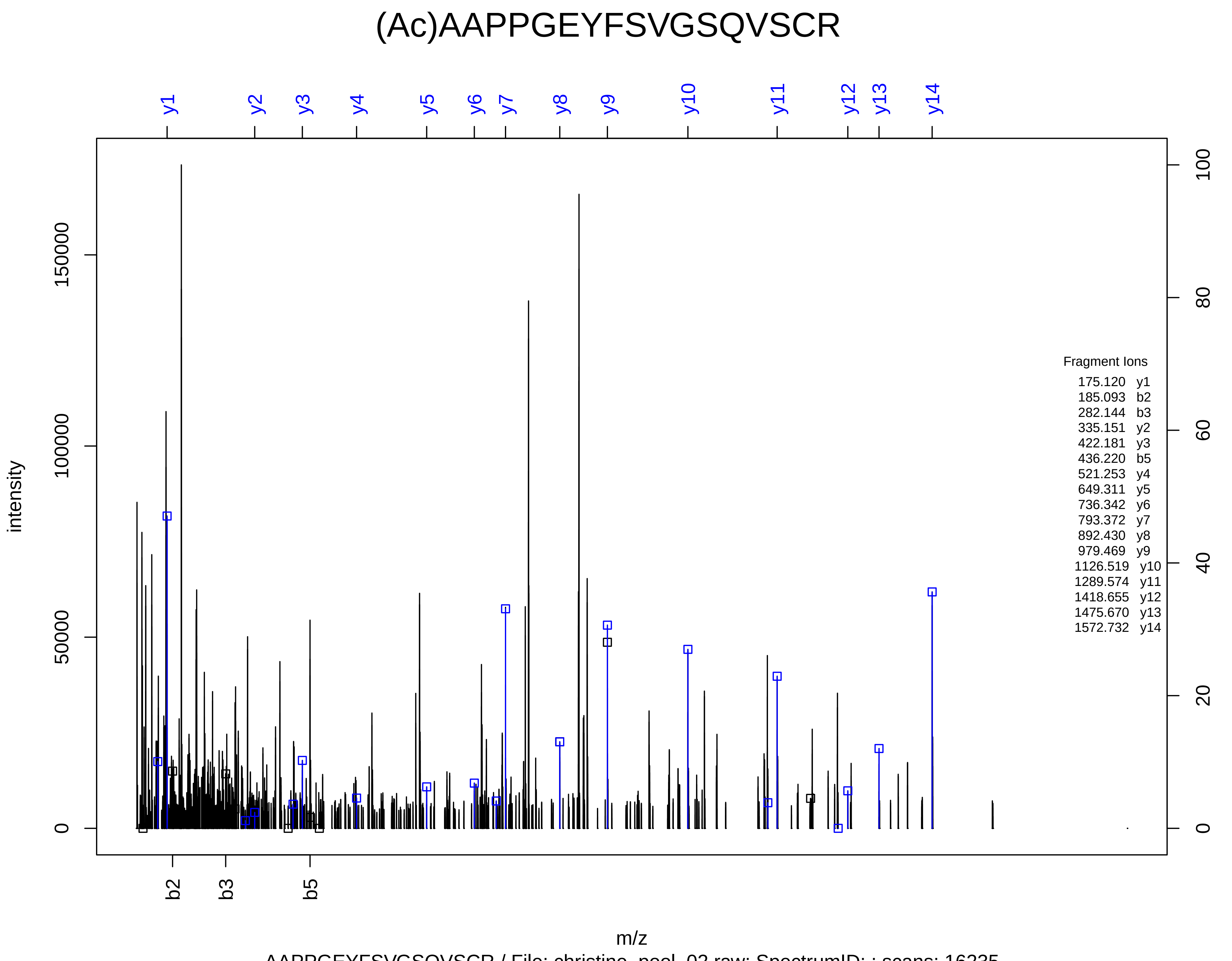
(Ac)AAPPGEYFSVGSQVSCR
y1
y2
y3
y4
y5
y6
y7
y8
y9
y10
y11
y12
y13
y14
b2
b3
b5
intensity
0
50000
100000
150000
0
20
40
60
80
100
m/z
Fragment Ions
175.120 y1
185.093 b2
282.144 b3
335.151 y2
422.181 y3
436.220 b5
521.253 y4
649.311 y5
736.342 y6
793.372 y7
892.430 y8
979.469 y9
1126.519 y10
1289.574 y11
1418.655 y12
1475.670 y13
1572.732 y14
AAPPGEYFSVGSQVSCR / File: christine_pool_02.raw; SpectrumID: ; scans: 16235

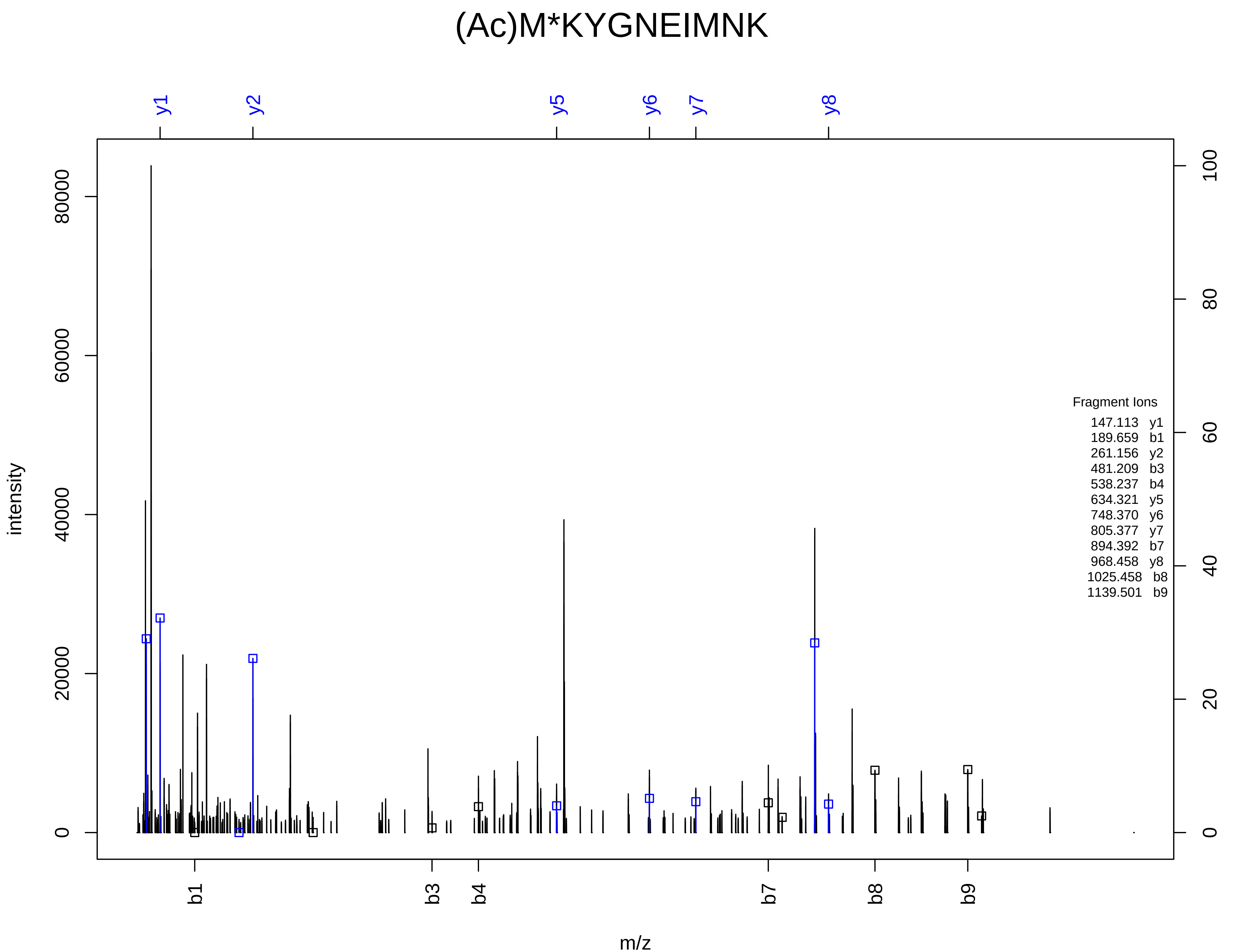

(Ac)M*KYGNEIMNK
y1
y2
y5
y6
y7
y8
intensity
0
20000
40000
60000
80000
0
20
40
60
80
100
b1
b3
b4
b7
b8
b9
m/z
Fragment Ions
147.113 y1
189.659 b1
261.156 y2
481.209 b3
538.237 b4
634.321 y5
748.370 y6
805.377 y7
894.392 b7
968.458 y8
1025.458 b8
1139.501 b9
MKYGNEIMNK / File: christine_pool_02.raw; SpectrumID: ; scans: 1796

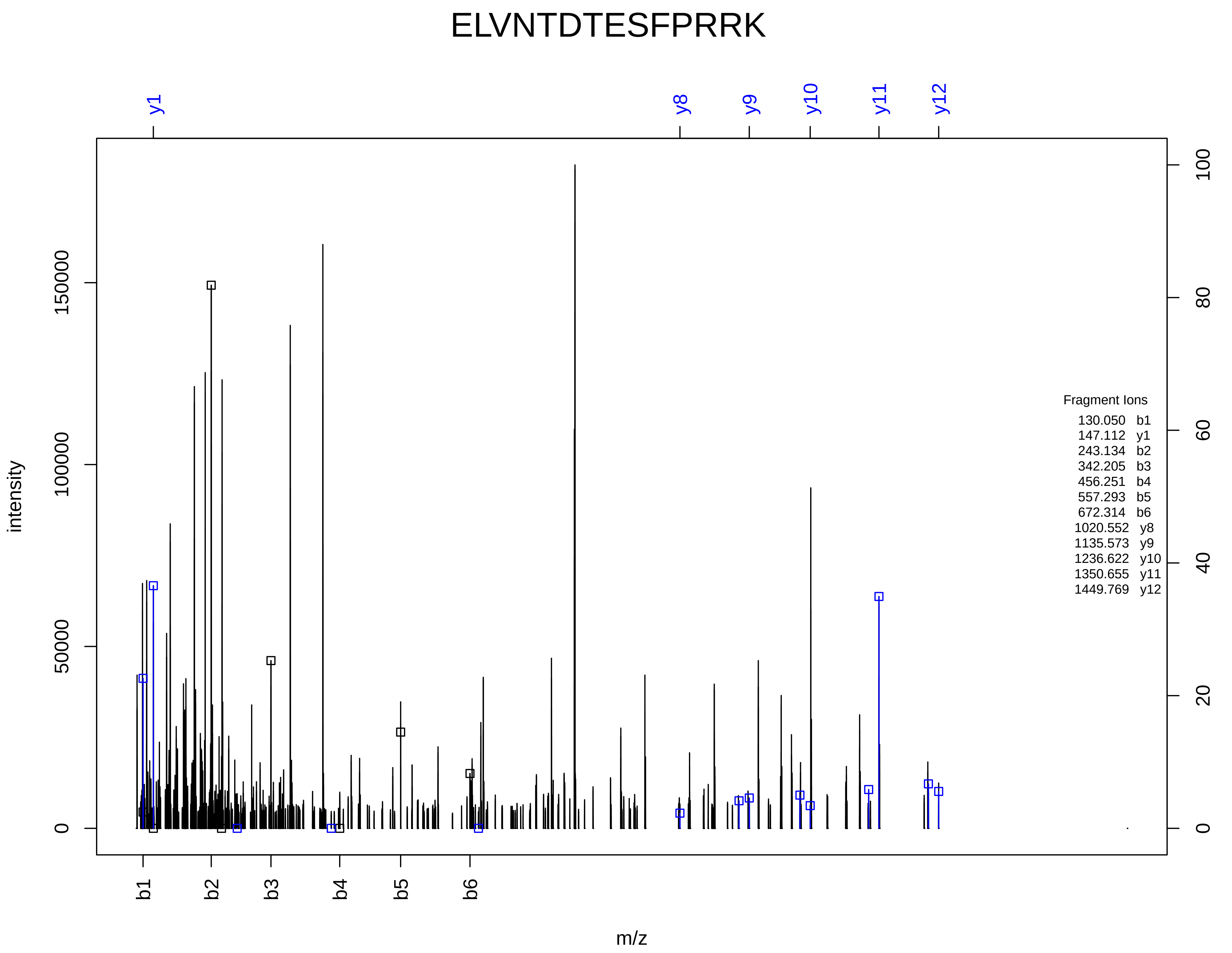

ELVNTDTESFPRRK / File: christine_pool_02.raw; SpectrumID: ; scans: 9636

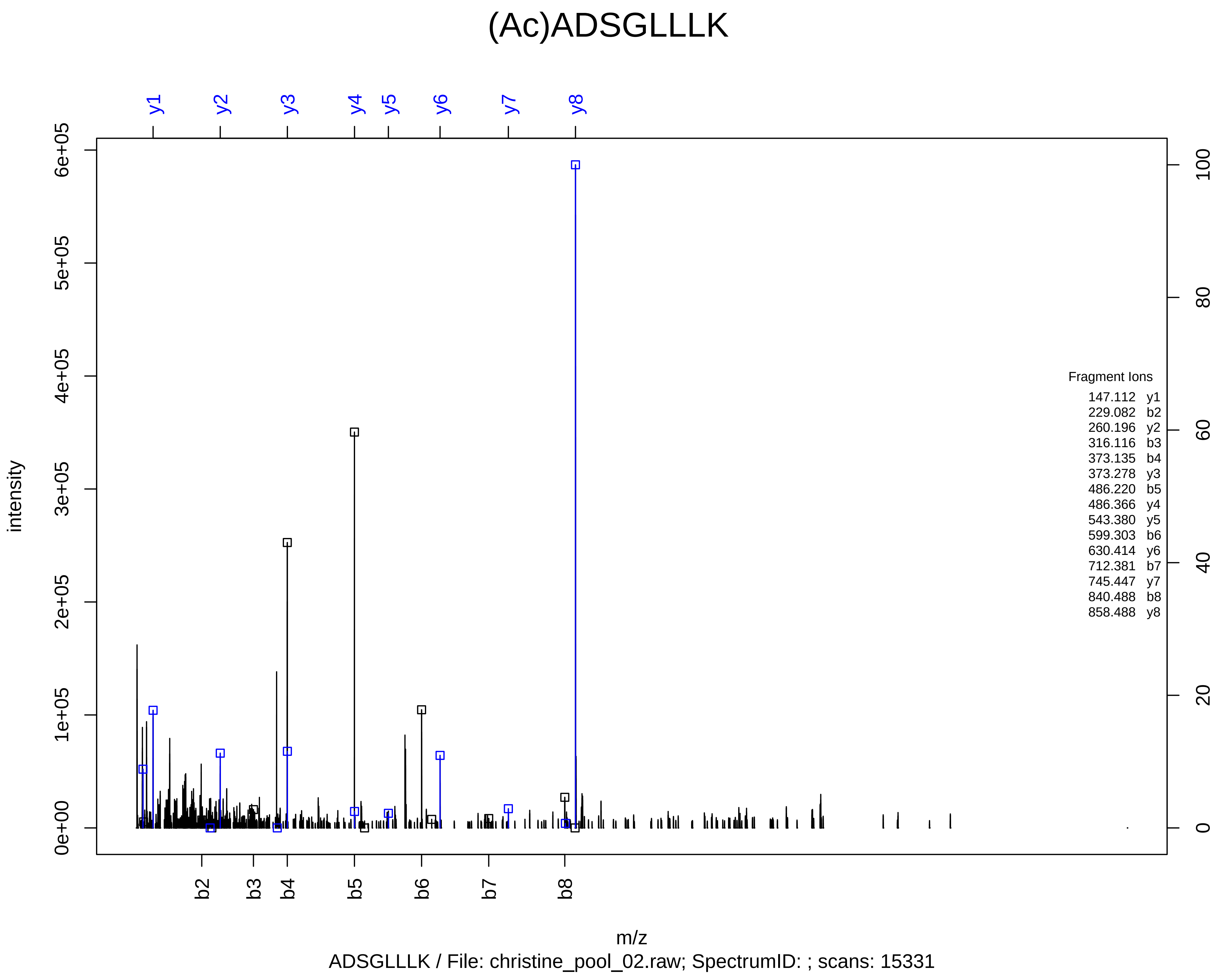
(Ac)ADSGLLLK
y1 y2 y3 y4 y5 y6 y7 y8
b2 b3 b4 b5 b6 b7 b8
intensity
0e+00 1e+05 2e+05 3e+05 4e+05 5e+05 6e+05
0 20 40 60 80 100
m/z
Fragment Ions
147.112 y1
229.082 b2
260.196 y2
316.116 b3
373.135 b4
373.278 y3
486.220 b5
486.366 y4
543.380 y5
599.303 b6
630.414 y6
712.381 b7
745.447 y7
840.488 b8
858.488 y8
ADSGLLLK / File: christine_pool_02.raw; SpectrumID: ; scans: 15331

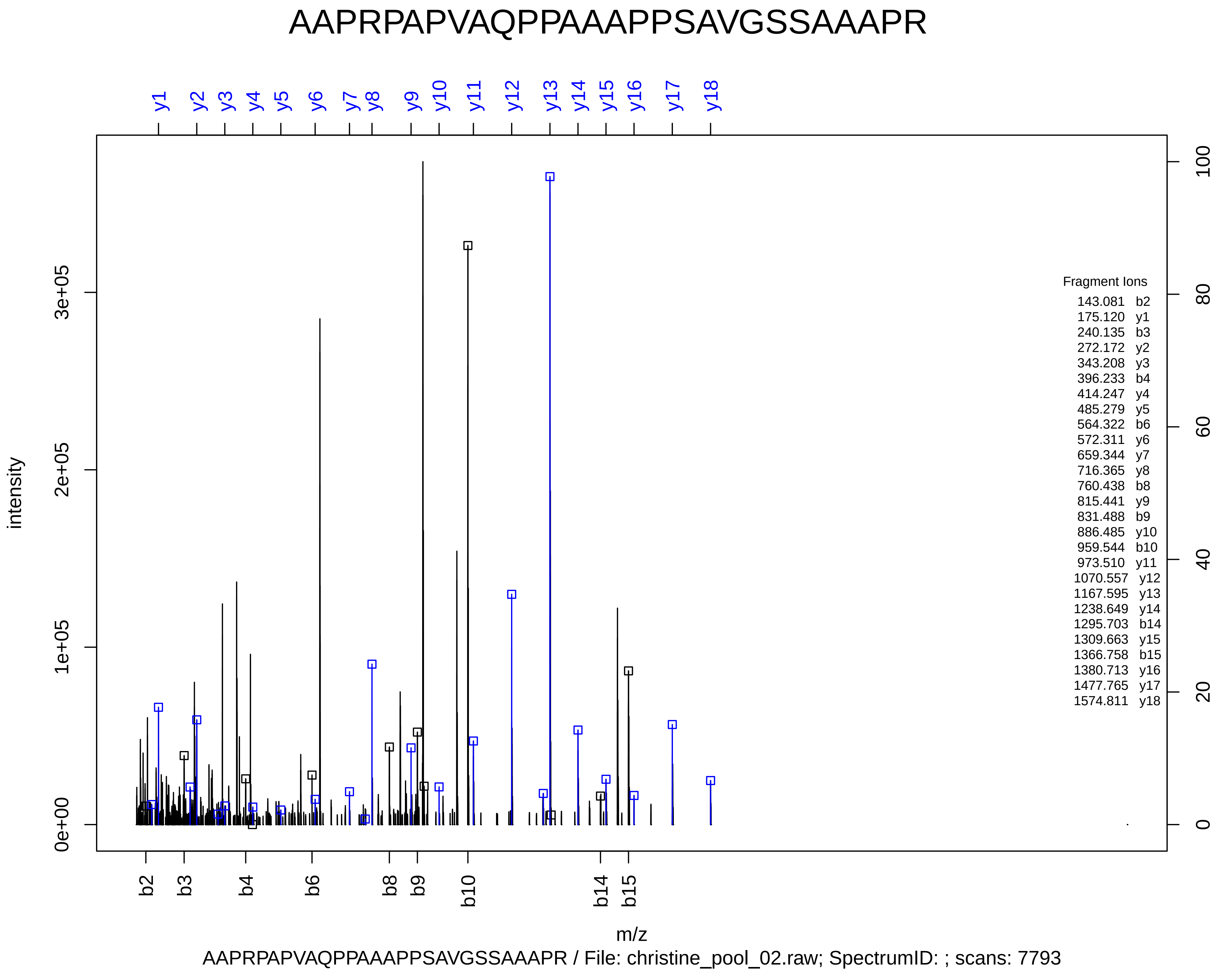
AAPRPAPVAQPPAAAPPSAVGSSAAAPR
Fragment Ions
143.081 b2
175.120 y1
240.135 b3
272.172 y2
343.208 y3
396.233 b4
414.247 y4
485.279 y5
564.322 b6
572.311 y6
659.344 y7
716.365 y8
760.438 b8
815.441 y9
831.488 b9
886.485 y10
959.544 b10
973.510 y11
1070.557 y12
1167.595 y13
1238.649 y14
1295.703 b14
1309.663 y15
1366.758 b15
1380.713 y16
1477.765 y17
1574.811 y18
intensity
m/z
AAPRPAPVAQPPAAAPPSAVGSSAAAPR / File: christine_pool_02.raw; SpectrumID: ; scans: 7793

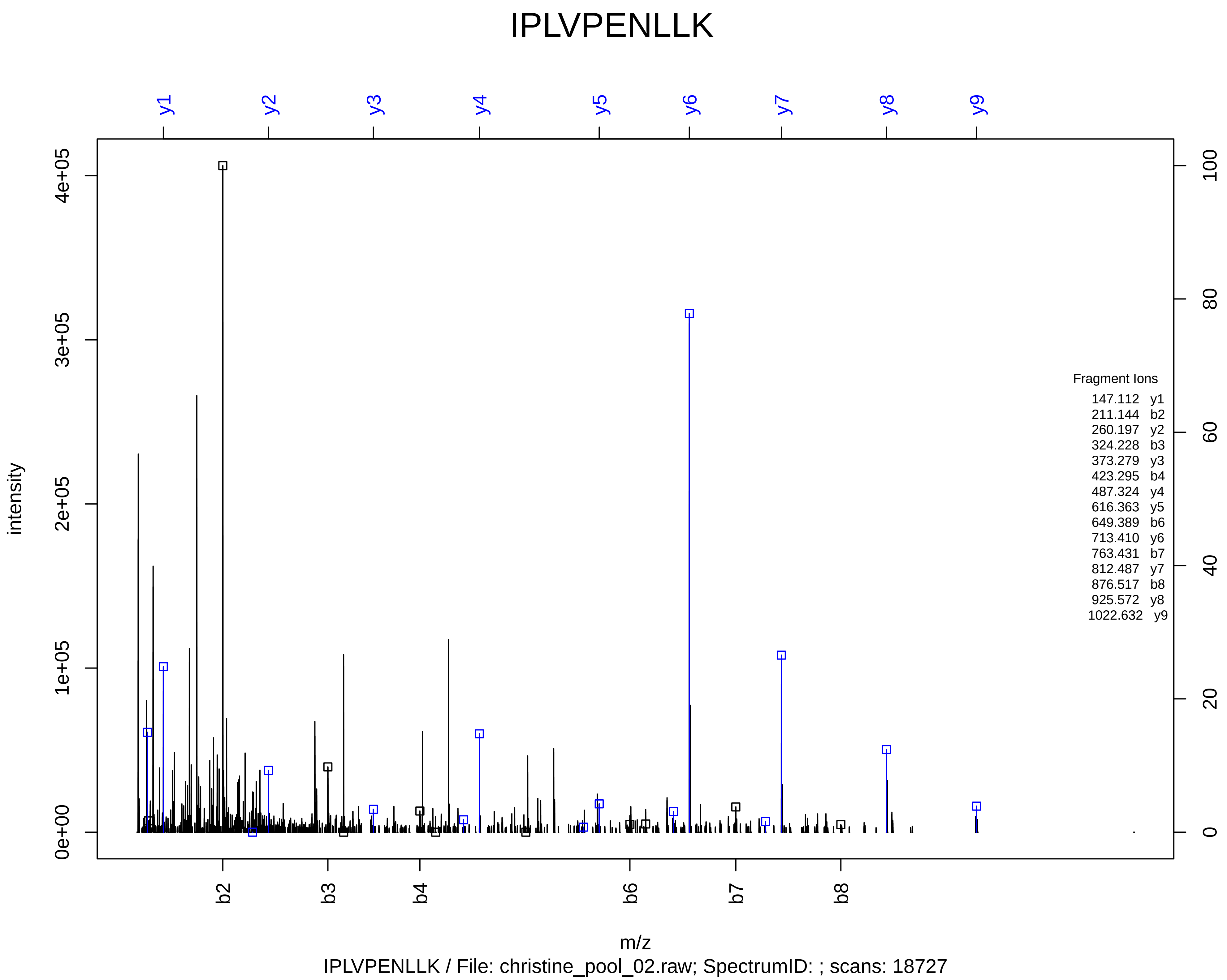
IPLVPENLLK
y1
y2
y3
y4
y5
y6
y7
y8
y9
intensity
0e+00
1e+05
2e+05
3e+05
4e+05
0
20
40
60
80
100
b2
b3
b4
b6
b7
b8
m/z
Fragment Ions
147.112 y1
211.144 b2
260.197 y2
324.228 b3
373.279 y3
423.295 b4
487.324 y4
616.363 y5
649.389 b6
713.410 y6
763.431 b7
812.487 y7
876.517 b8
925.572 y8
1022.632 y9
IPLVPENLLK / File: christine_pool_02.raw; SpectrumID: ; scans: 18727

TVLYPYCAPYALELDYR

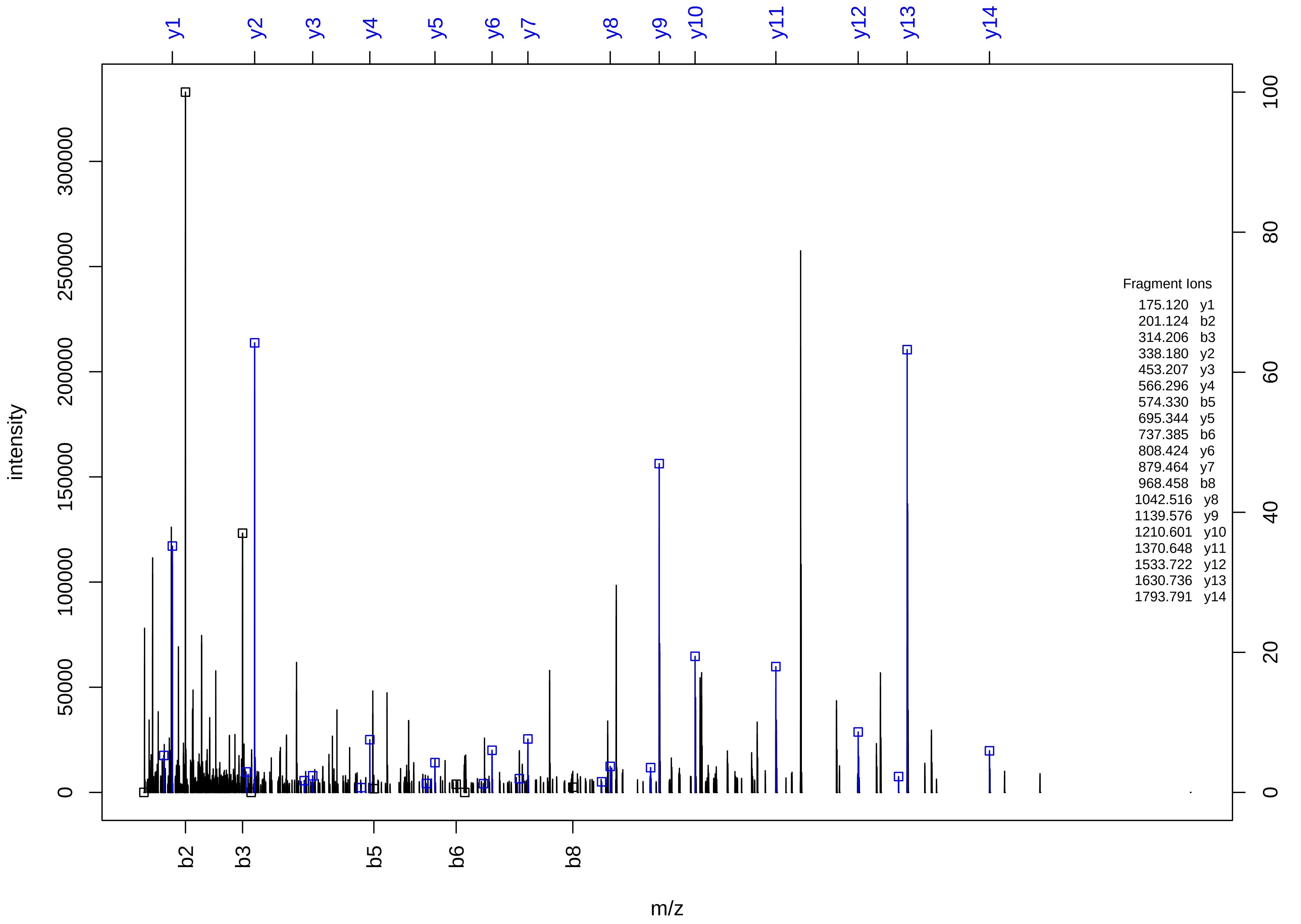

TVLYPYCAPYALELDYR / File: christine_pool_02.raw; SpectrumID: ; scans: 20792

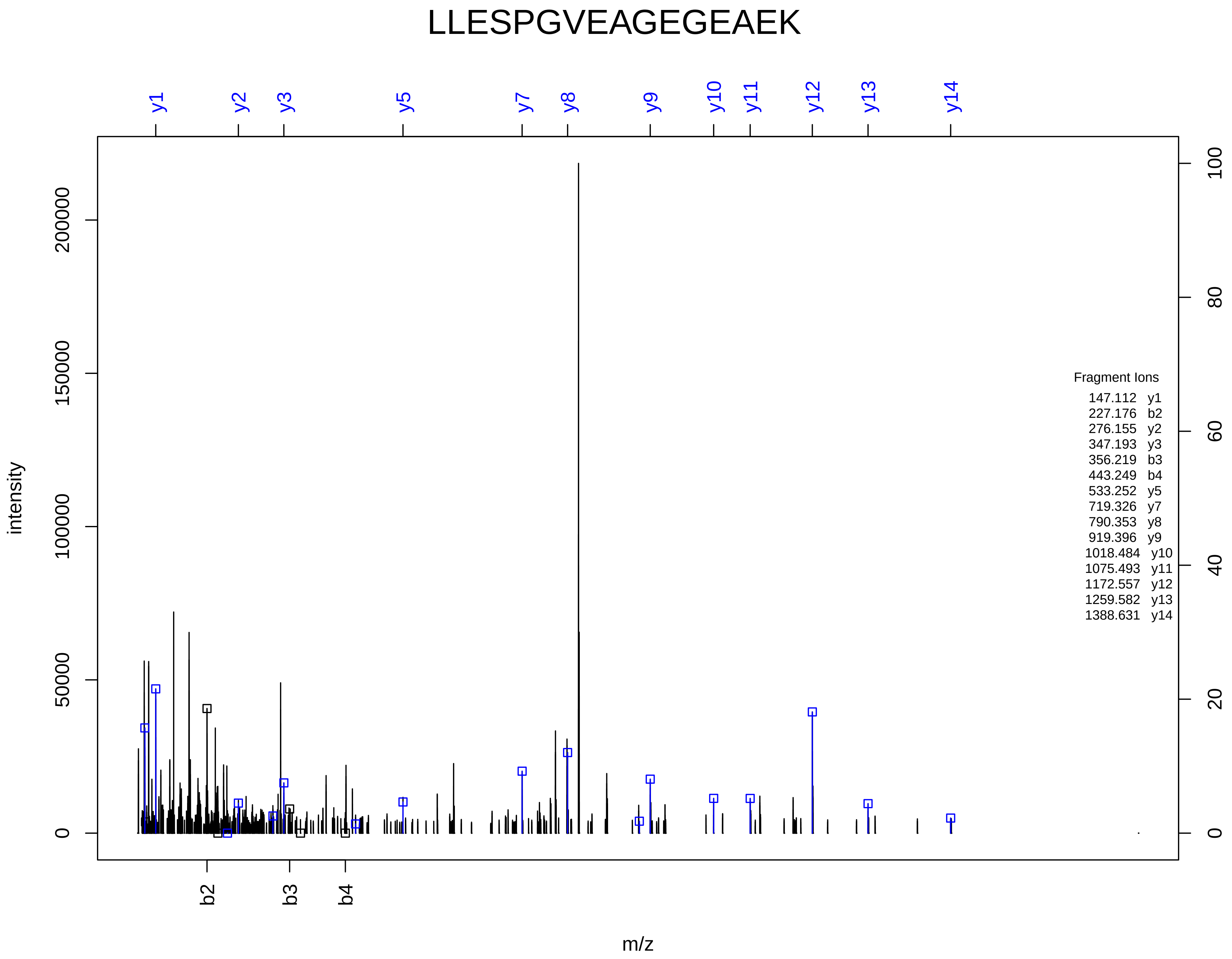

LLESPGVEAGEGEAEK
y1
y2
y3
y5
y7
y8
y9
y10
y11
y12
y13
y14
intensity
0
50000
100000
150000
200000
0
20
40
60
80
100
Fragment Ions
147.112 y1
227.176 b2
276.155 y2
347.193 y3
356.219 b3
443.249 b4
533.252 y5
719.326 y7
790.353 y8
919.396 y9
1018.484 y10
1075.493 y11
1172.557 y12
1259.582 y13
1388.631 y14
b2
b3
b4
m/z
LLESPGVEAGEGEAEK / File: christine_pool_02.raw; SpectrumID: ; scans: 7098

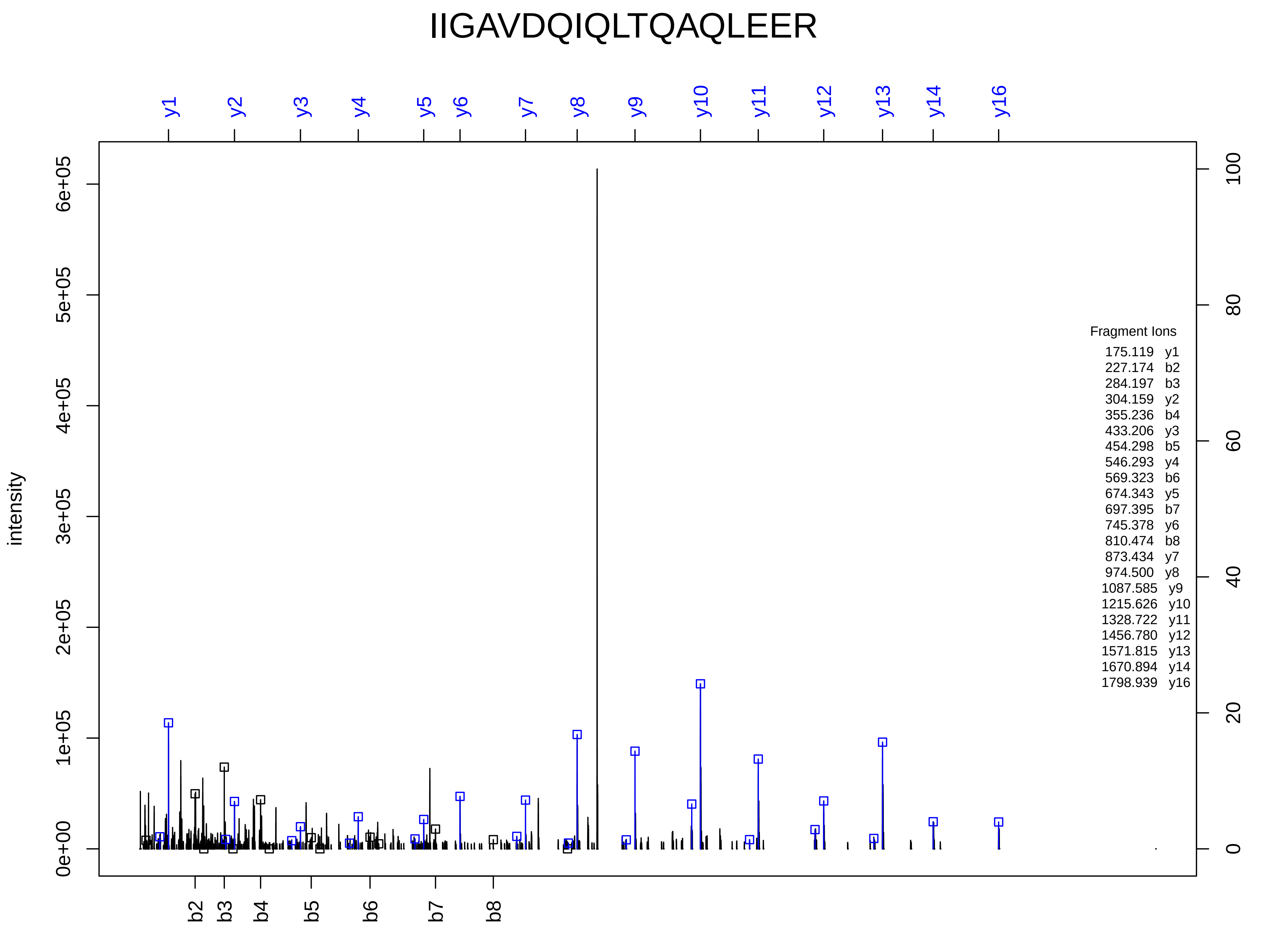

IIGAVDQIQLTQAQLEER / File: christine_pool_02.raw; SpectrumID: ; scans: 16323

RNQHMPLESR

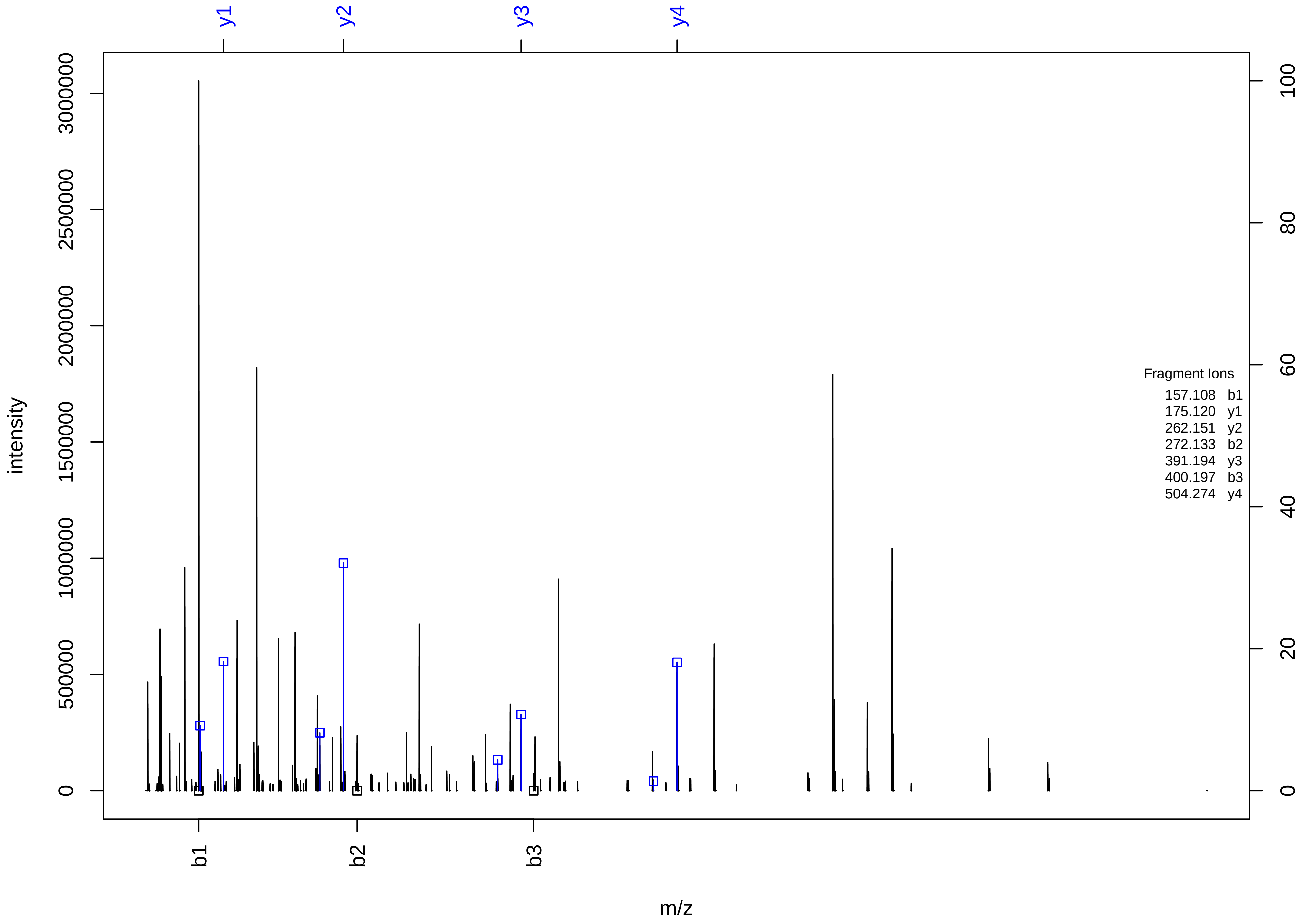

RNQHMPLESR / File: christine_pool_02.raw; SpectrumID: ; scans: 10278

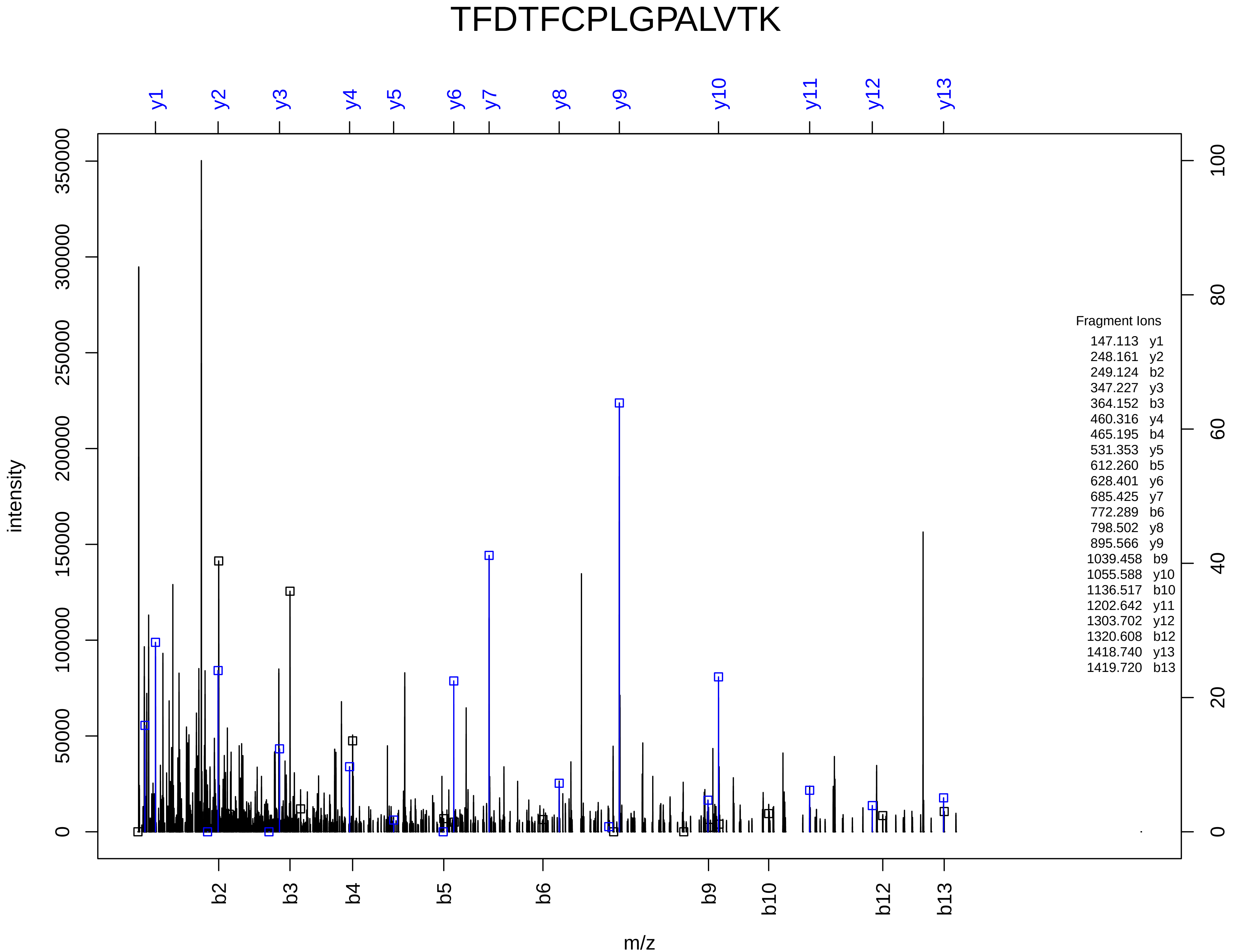

TFDTFCPLGPALVTK
y1 y2 y3 y4 y5 y6 y7 y8 y9 y10 y11 y12 y13
intensity
0
50000
100000
150000
200000
250000
300000
350000
0
20
40
60
80
100
Fragment Ions
147.113 y1
248.161 y2
249.124 b2
347.227 y3
364.152 b3
460.316 y4
465.195 b4
531.353 y5
612.260 b5
628.401 y6
685.425 y7
772.289 b6
798.502 y8
895.566 y9
1039.458 b9
1055.588 y10
1136.517 b10
1202.642 y11
1303.702 y12
1320.608 b12
1418.740 y13
1419.720 b13
b2 b3 b4 b5 b6 b9 b10 b12 b13
m/z
TFDTFCPLGPALVTK / File: christine_pool_02.raw; SpectrumID: ; scans: 19419

LILISTN^GSFIR

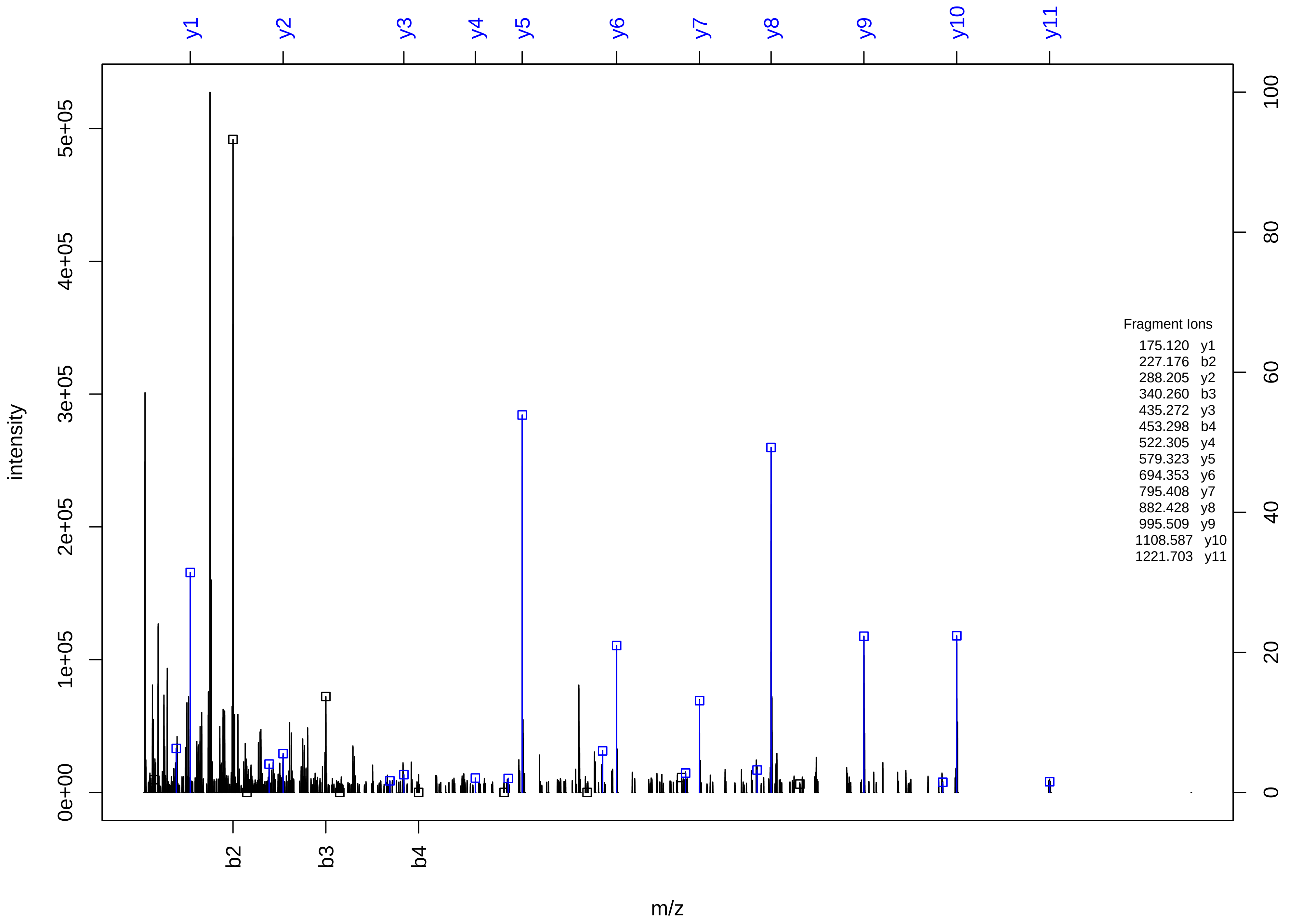

LILISTNGSFIR / File: christine_pool_02.raw; SpectrumID: ; scans: 19316

ECPGIEPVCVDLGDWEATER

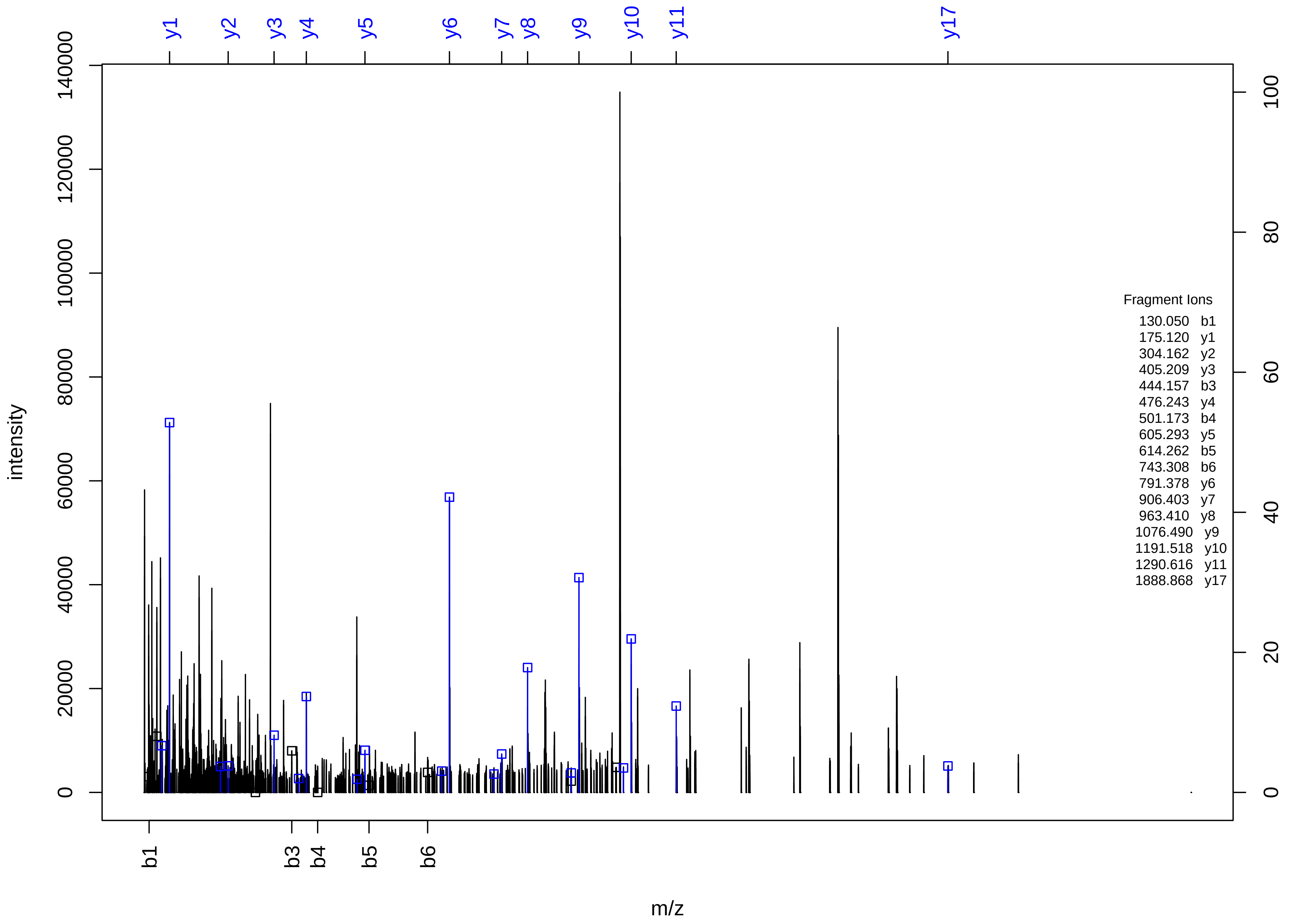

ECPGIEPVCVDLGDWEATER / File: christine_pool_02.raw; SpectrumID: ; scans: 19386

GPADSLSTAAGAAELSAEGAGK

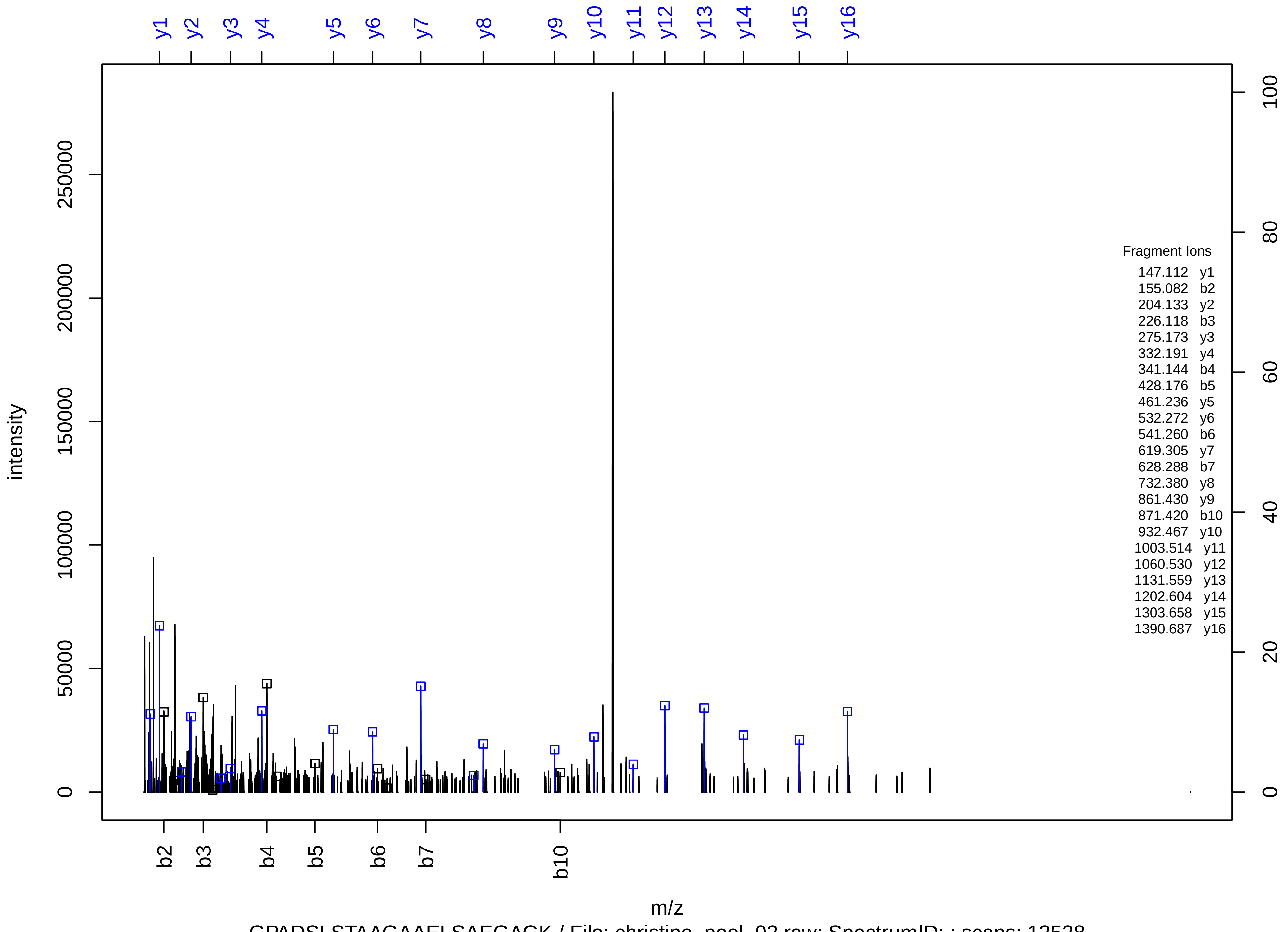

GPADSLSTAAGAAELSAEGAGK / File: christine_pool_02.raw; SpectrumID: ; scans: 12528

LLCELFIR

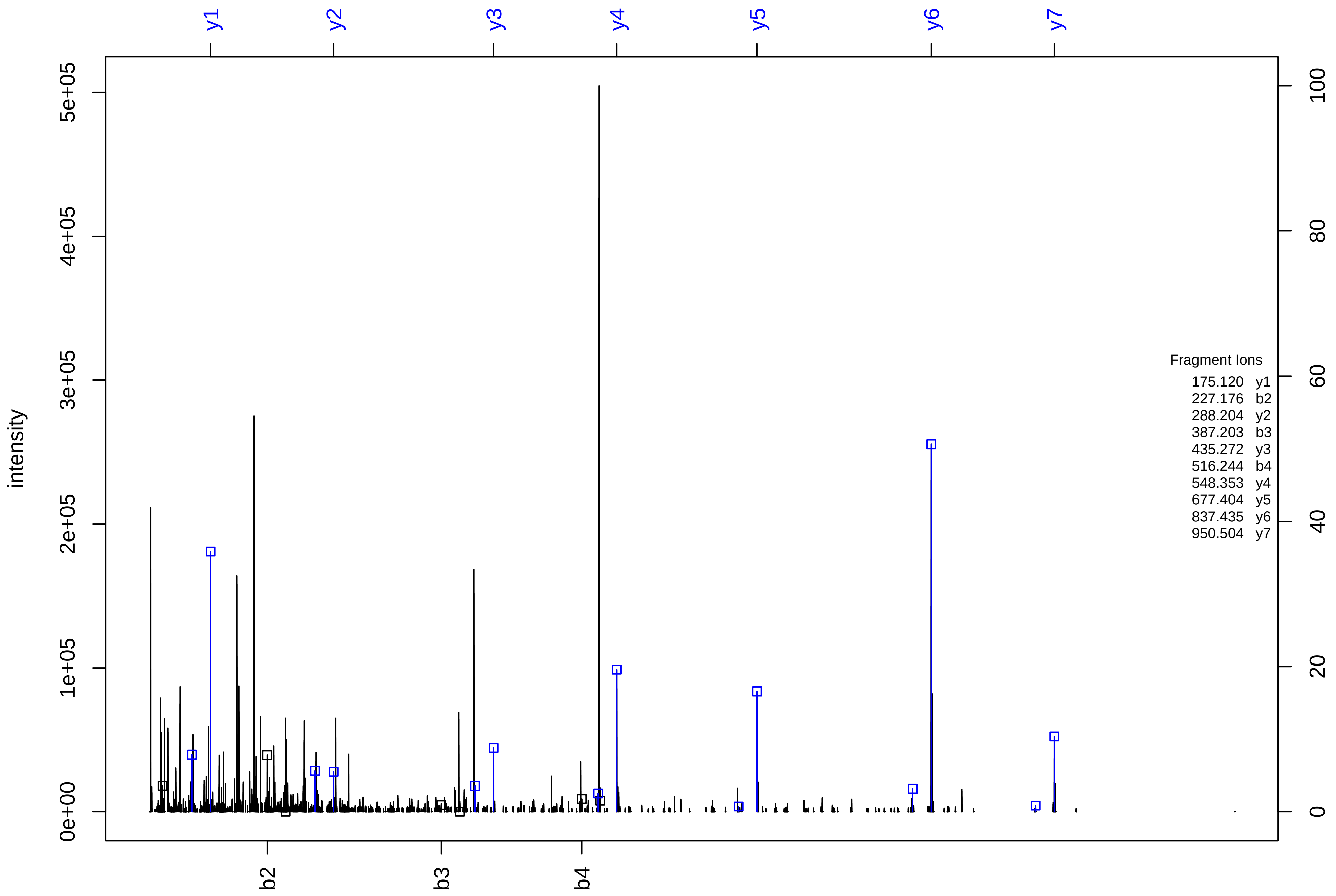

LLCELFIR / File: christine_pool_02.raw; SpectrumID: ; scans: 17870

VDDDFTAQDYR

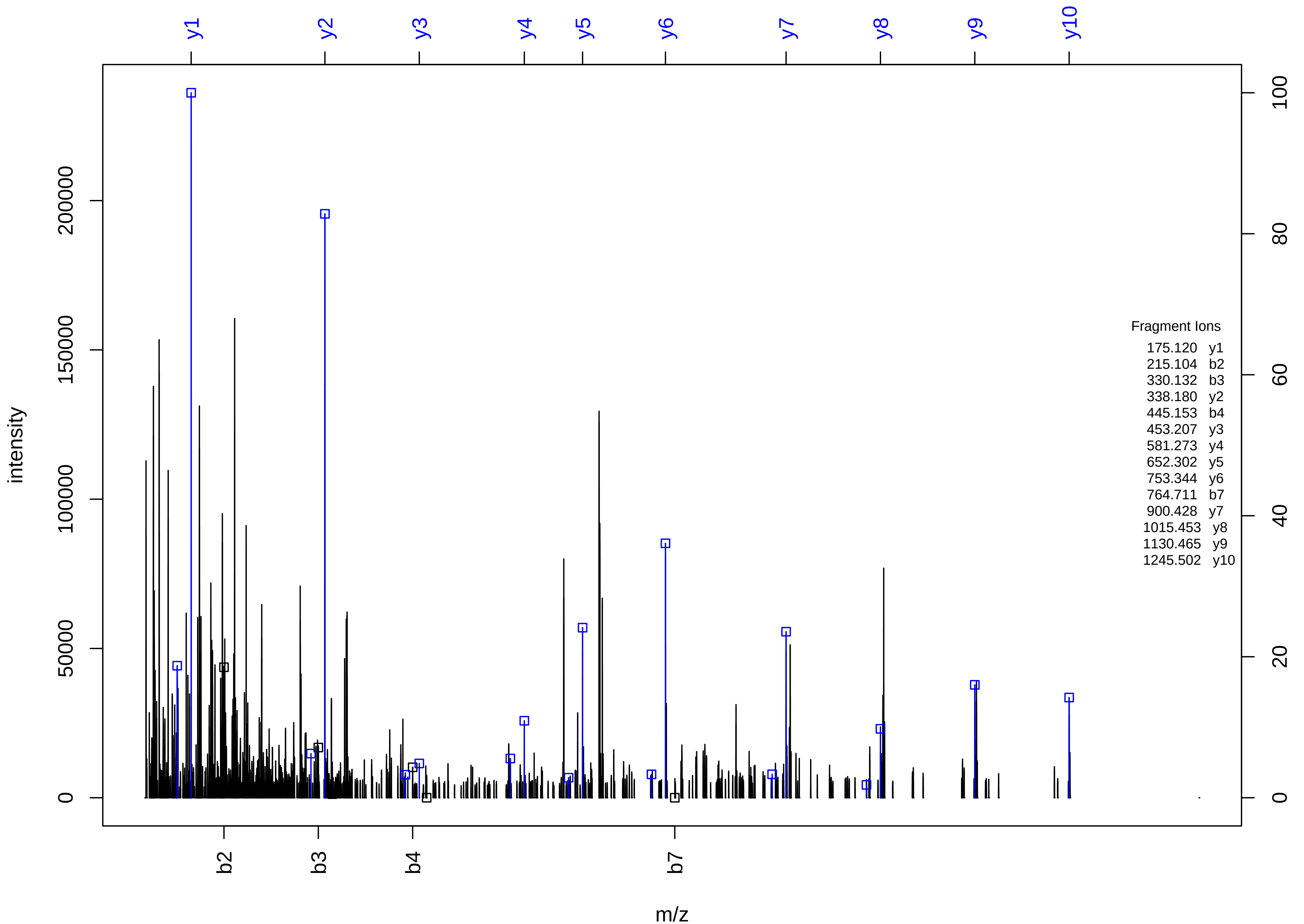

VDDDFTAQDYR / File: christine_pool_02.raw; SpectrumID: ; scans: 6794

TVPAHVETVVLFFPDVWHCLPTR

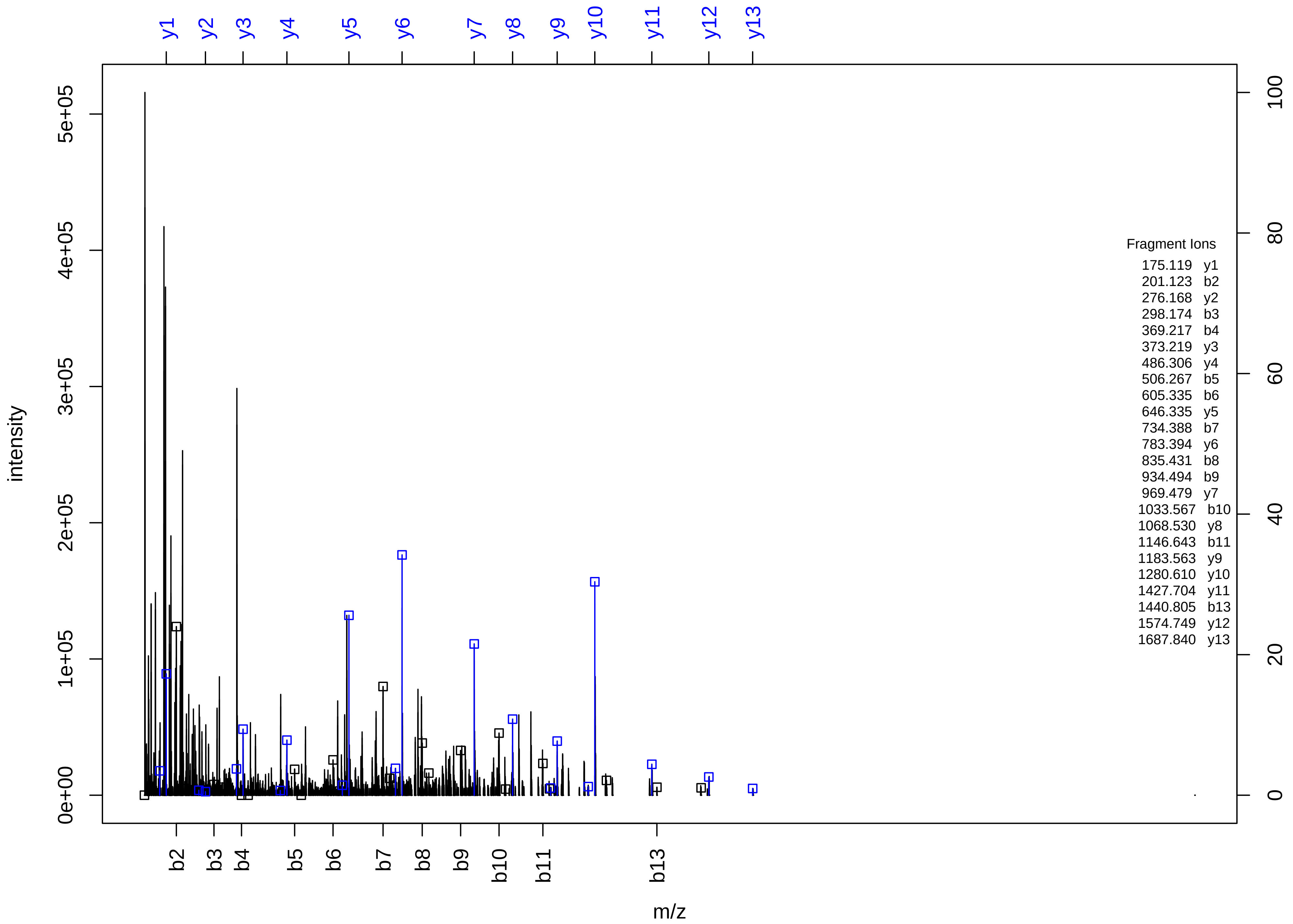

TVPAHVETVVLFFPDVWHCLPTR / File: christine_pool_02.raw; SpectrumID: ; scans: 23795

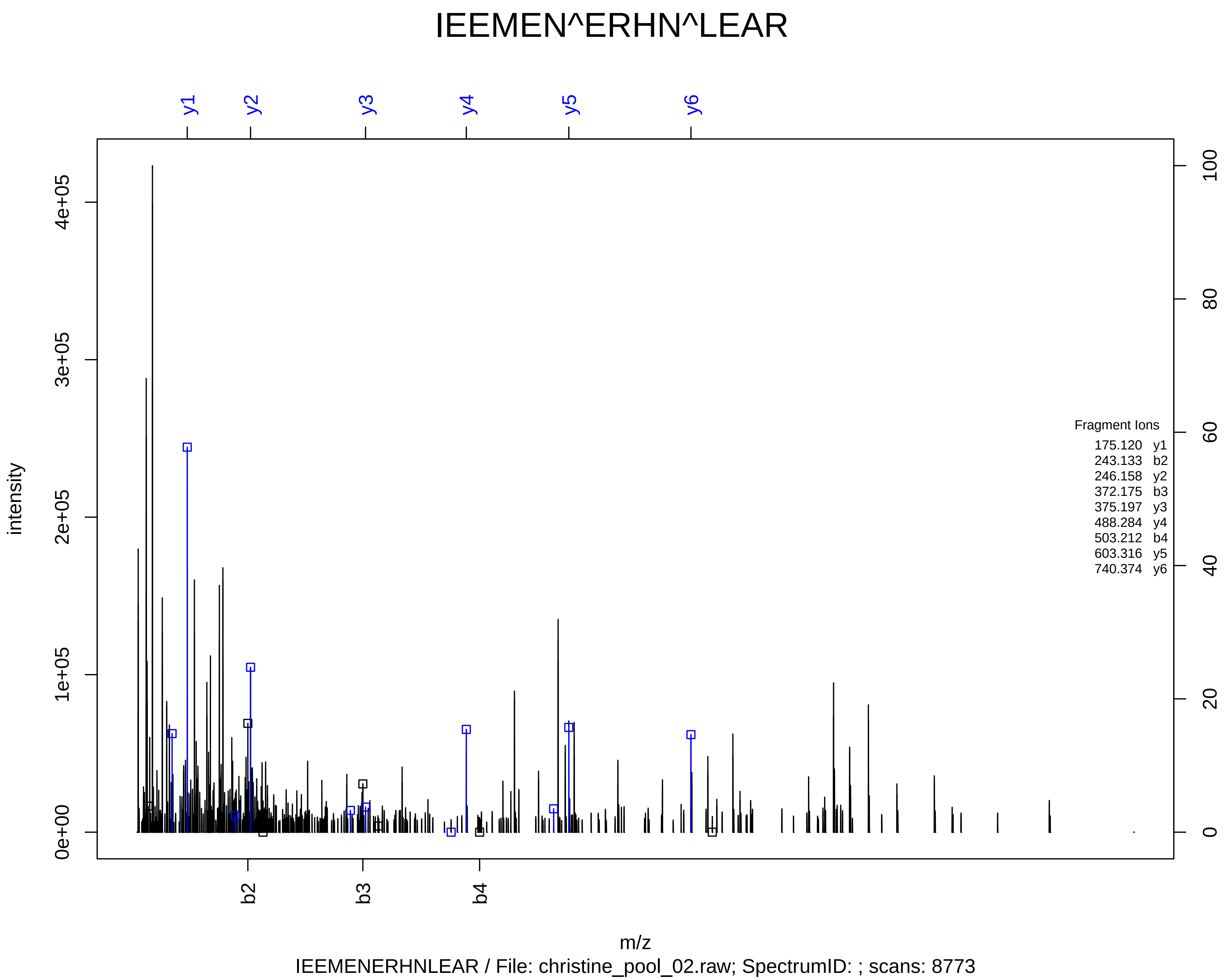
IEEMEN^ERHN^LEAR
y1
y2
y3
y4
y5
y6
b2
b3
b4
intensity
0e+00
1e+05
2e+05
3e+05
4e+05
0
20
40
60
80
100
m/z
Fragment Ions
175.120 y1
243.133 b2
246.158 y2
372.175 b3
375.197 y3
488.284 y4
503.212 b4
603.316 y5
740.374 y6
IEEMENERHNLEAR / File: christine_pool_02.raw; SpectrumID: ; scans: 8773

LM*ALDSTIAHLSLRN^N^NIDDR

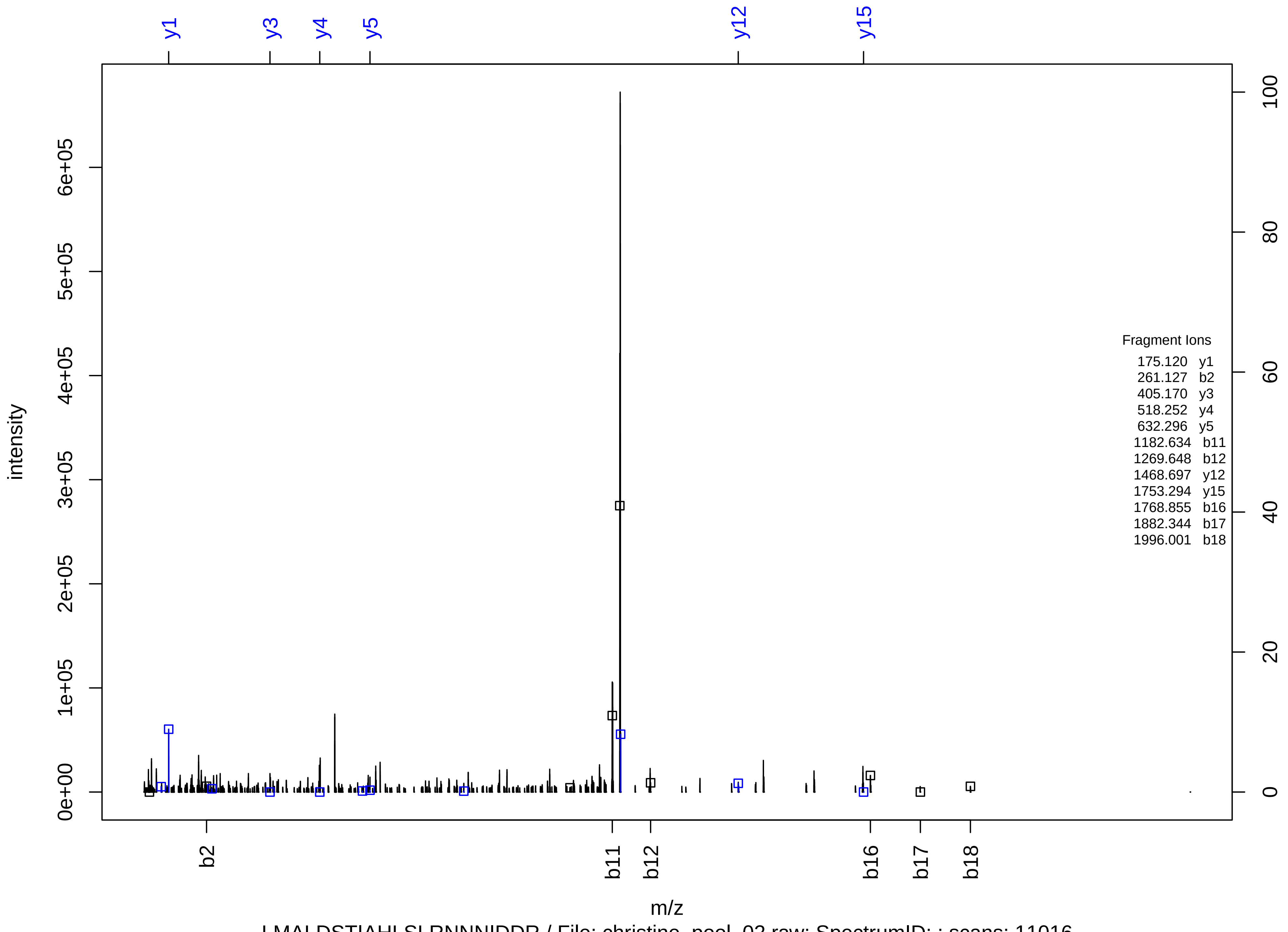

LMALDSTIAHLSLRNNNIDDR / File: christine_pool_02.raw; SpectrumID: ; scans: 11016

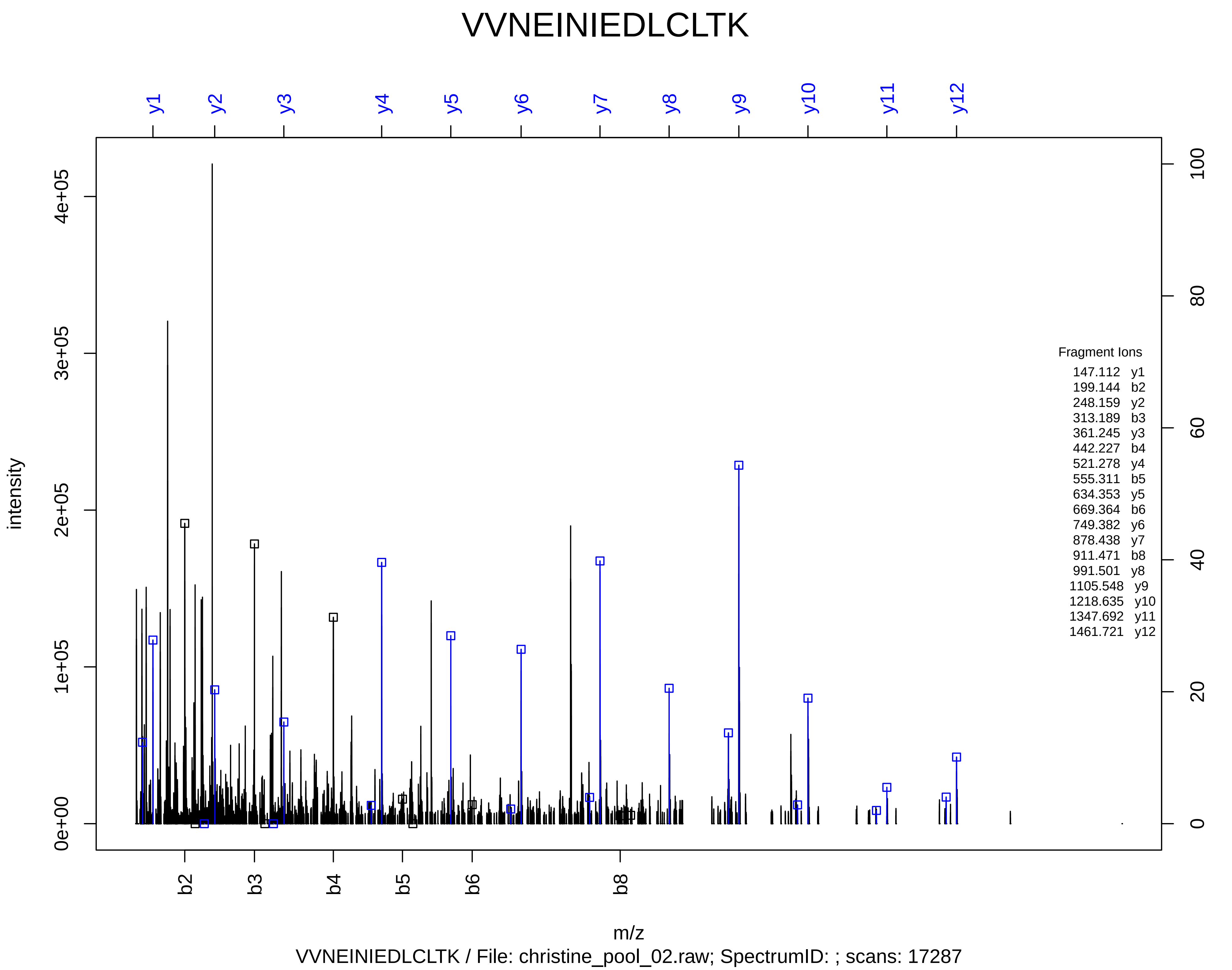
VVNEINIEDLCLTK
y1 y2 y3 y4 y5 y6 y7 y8 y9 y10 y11 y12
b2 b3 b4 b5 b6 b8
intensity
m/z
0e+00 1e+05 2e+05 3e+05 4e+05
0 20 40 60 80 100
Fragment Ions
147.112 y1
199.144 b2
248.159 y2
313.189 b3
361.245 y3
442.227 b4
521.278 y4
555.311 b5
634.353 y5
669.364 b6
749.382 y6
878.438 y7
911.471 b8
991.501 y8
1105.548 y9
1218.635 y10
1347.692 y11
1461.721 y12
VVNEINIEDLCLTK / File: christine_pool_02.raw; SpectrumID: ; scans: 17287

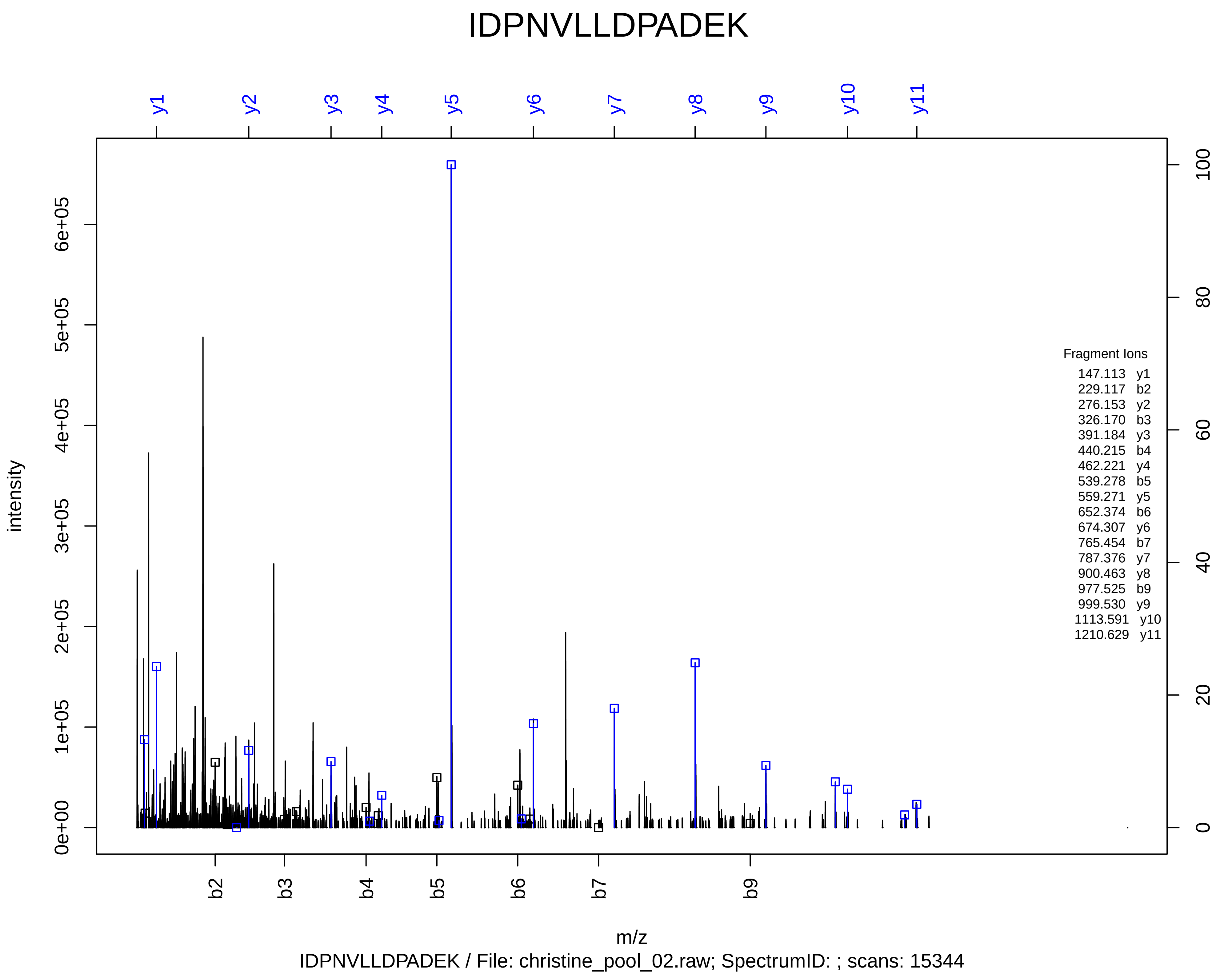
IDPNVLLDPADEK
y1 y2 y3 y4 y5 y6 y7 y8 y9 y10 y11
b2 b3 b4 b5 b6 b7 b9
intensity
m/z
0e+00 1e+05 2e+05 3e+05 4e+05 5e+05 6e+05
0 20 40 60 80 100
Fragment Ions
147.113 y1
229.117 b2
276.153 y2
326.170 b3
391.184 y3
440.215 b4
462.221 y4
539.278 b5
559.271 y5
652.374 b6
674.307 y6
765.454 b7
787.376 y7
900.463 y8
977.525 b9
999.530 y9
1113.591 y10
1210.629 y11
IDPNVLLDPADEK / File: christine_pool_02.raw; SpectrumID: ; scans: 15344

EVYEEAGVK

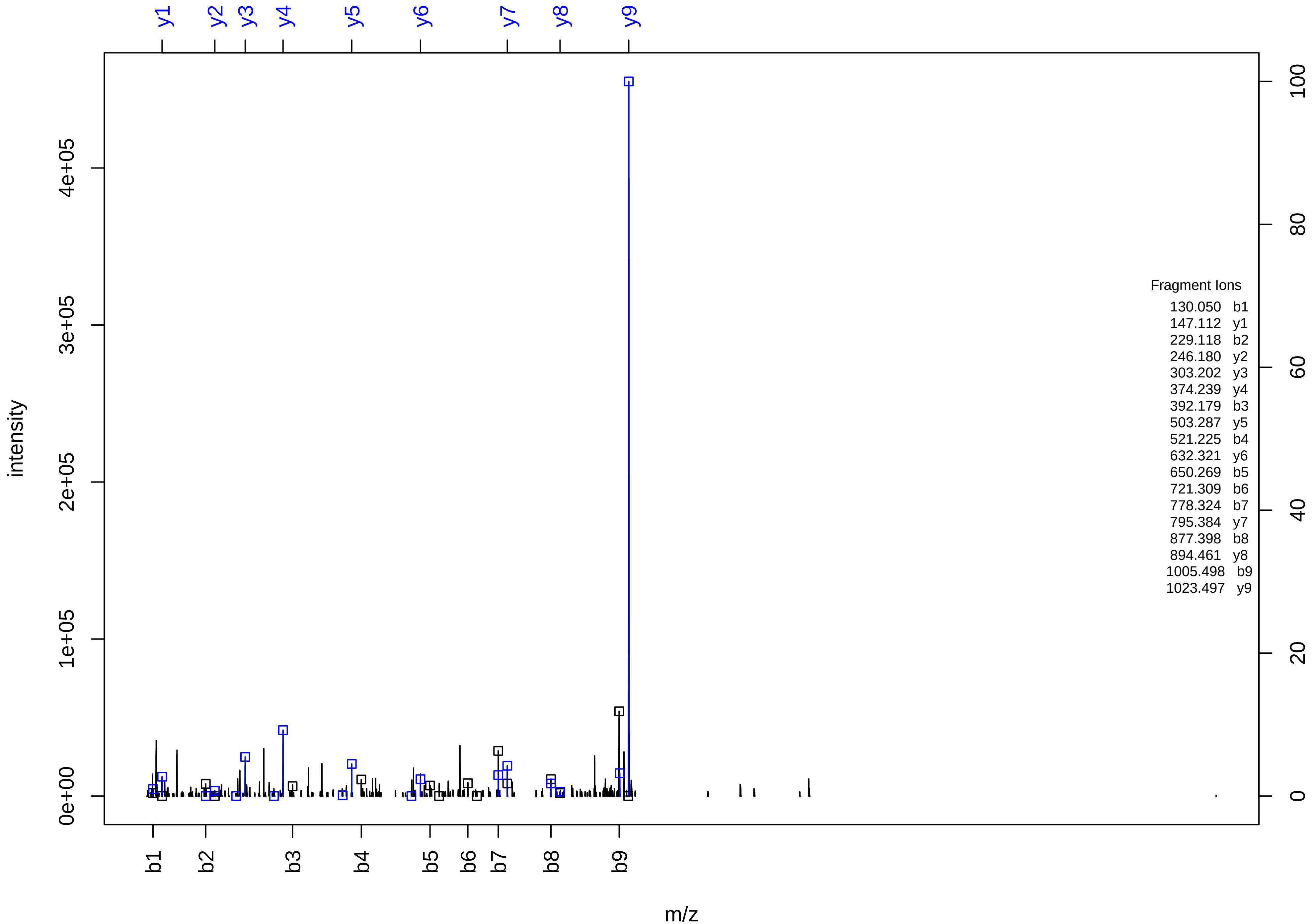

EVYEEAGVK / File: christine_pool_02.raw; SpectrumID: ; scans: 3520

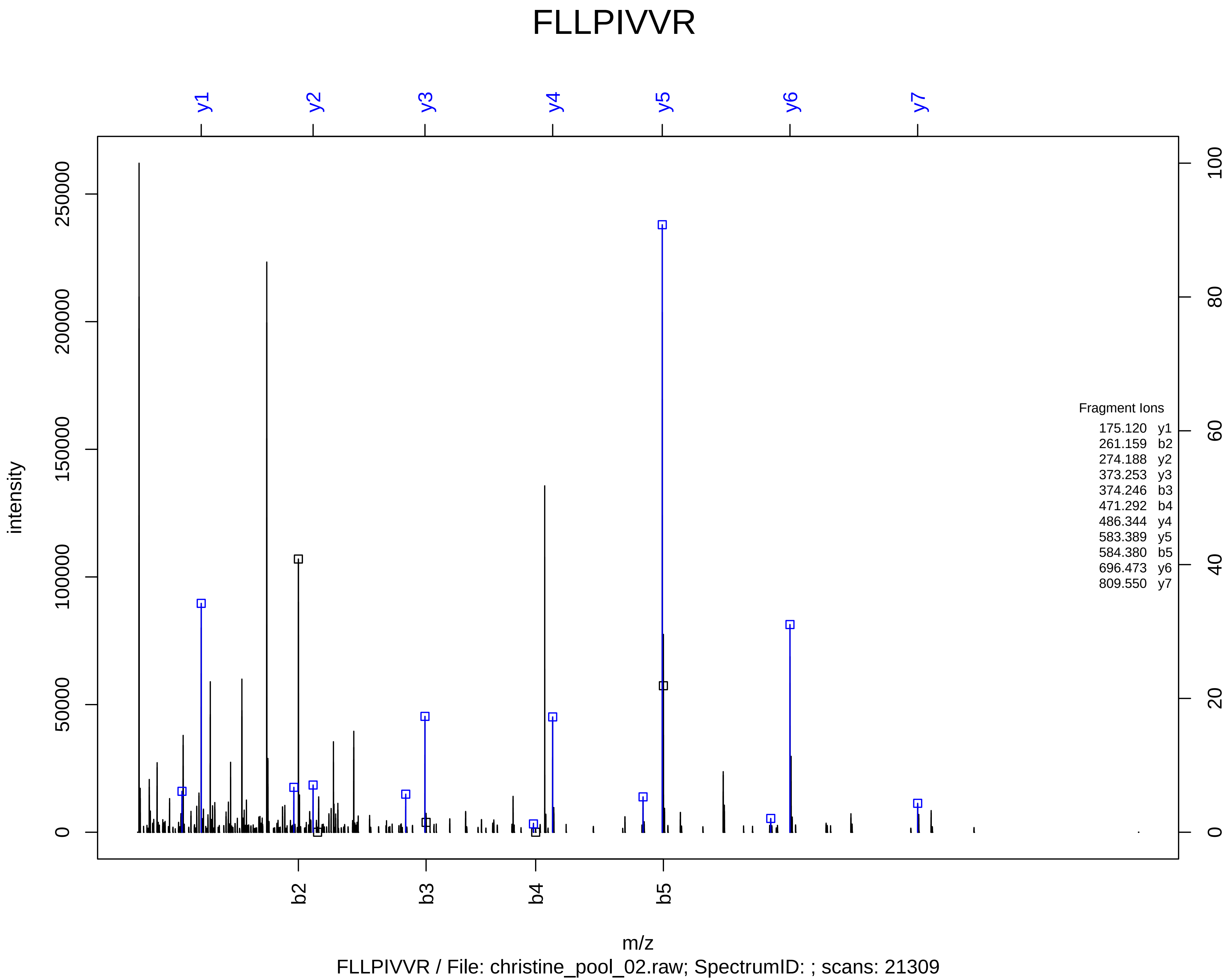

FLLPIVVR
y1
y2
y3
y4
y5
y6
y7
intensity
0
50000
100000
150000
200000
250000
0
20
40
60
80
100
b2
b3
b4
b5
m/z
Fragment Ions
175.120 y1
261.159 b2
274.188 y2
373.253 y3
374.246 b3
471.292 b4
486.344 y4
583.389 y5
584.380 b5
696.473 y6
809.550 y7
FLLPIVVR / File: christine_pool_02.raw; SpectrumID: ; scans: 21309

ENPGFDFSGAEISGNYTK

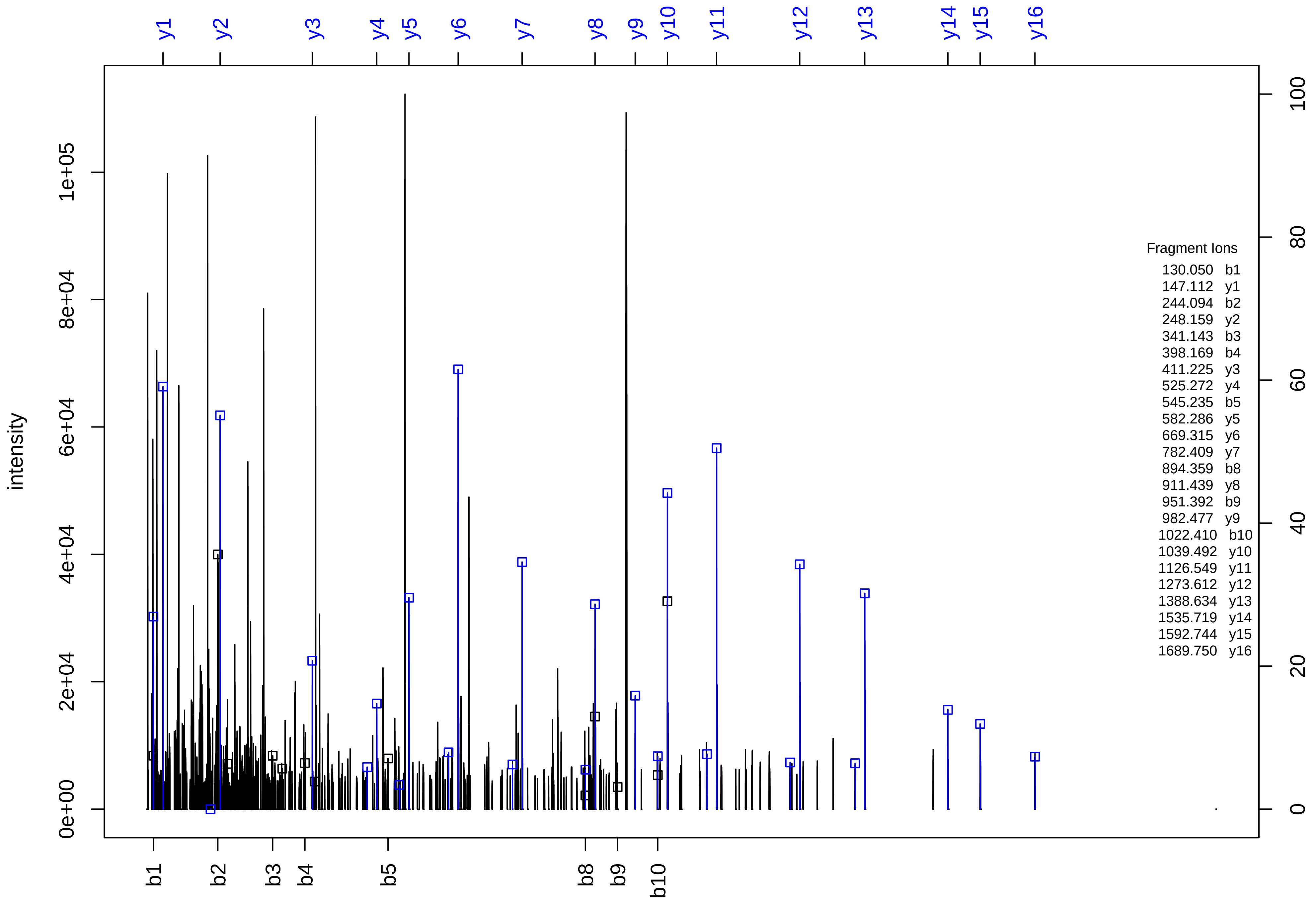

ENPGFDFSGAEISGNYTK / File: christine_pool_02.raw; SpectrumID: ; scans: 14259

VVTSEDQVQEGTK

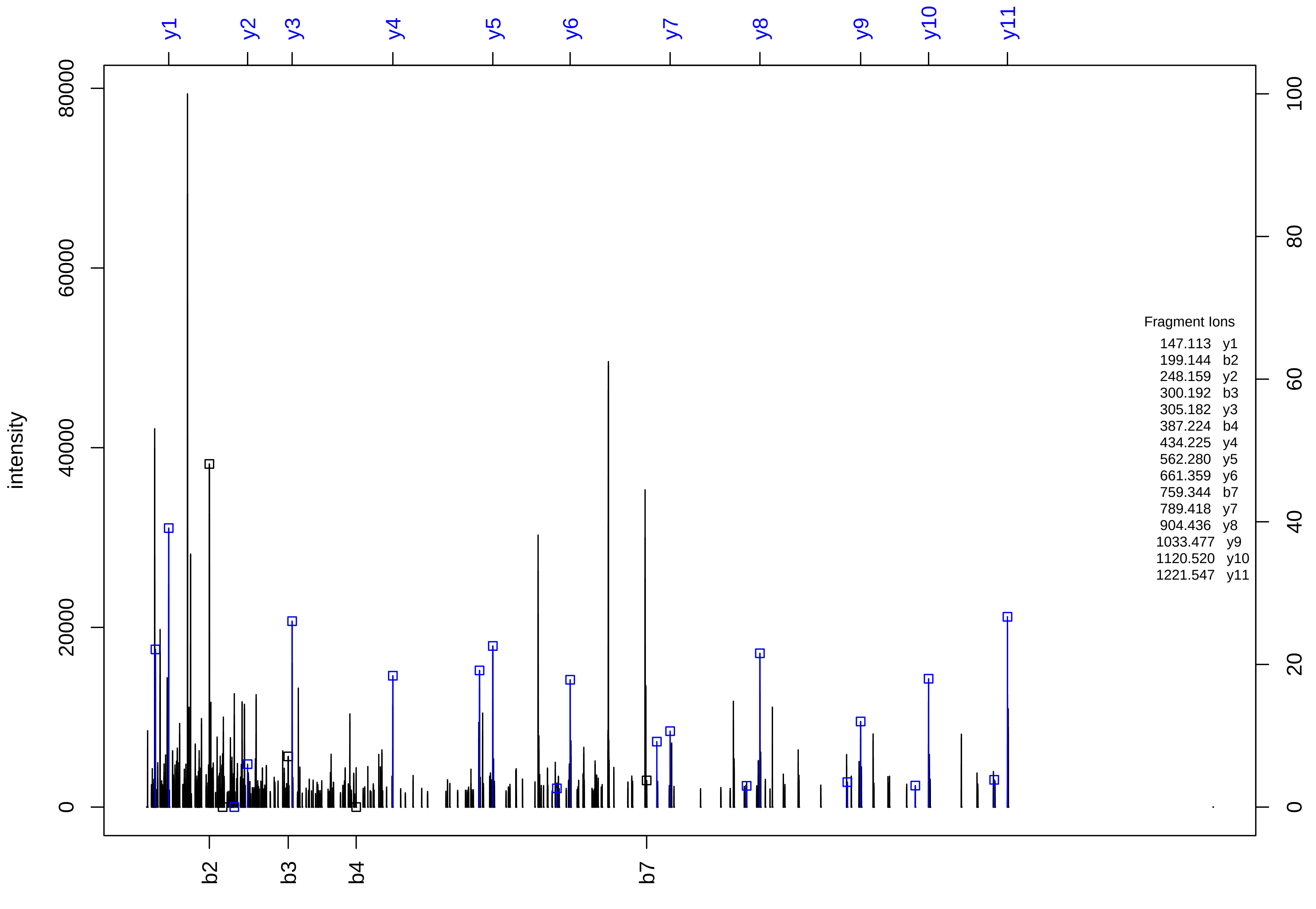

VVTSEDQVQEGTK / File: christine_pool_02.raw; SpectrumID: ; scans: 2729

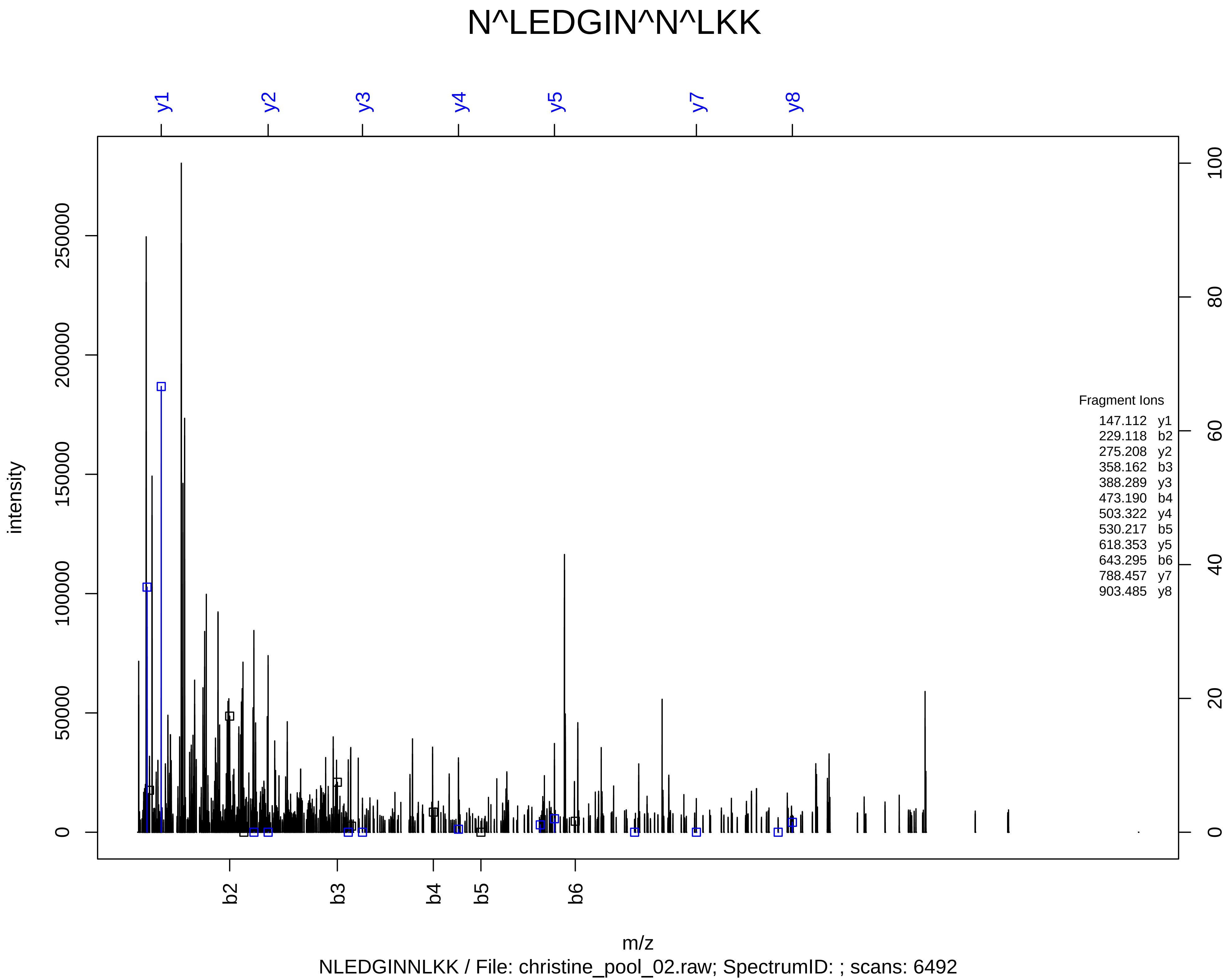
N^LEDGIN^N^LKK
y1
y2
y3
y4
y5
y7
y8
intensity
0
50000
100000
150000
200000
250000
0
20
40
60
80
100
b2
b3
b4
b5
b6
m/z
Fragment Ions
147.112 y1
229.118 b2
275.208 y2
358.162 b3
388.289 y3
473.190 b4
503.322 y4
530.217 b5
618.353 y5
643.295 b6
788.457 y7
903.485 y8
NLEDGINNLKK / File: christine_pool_02.raw; SpectrumID: ; scans: 6492

SSPSGPSNPSNPSVEEK

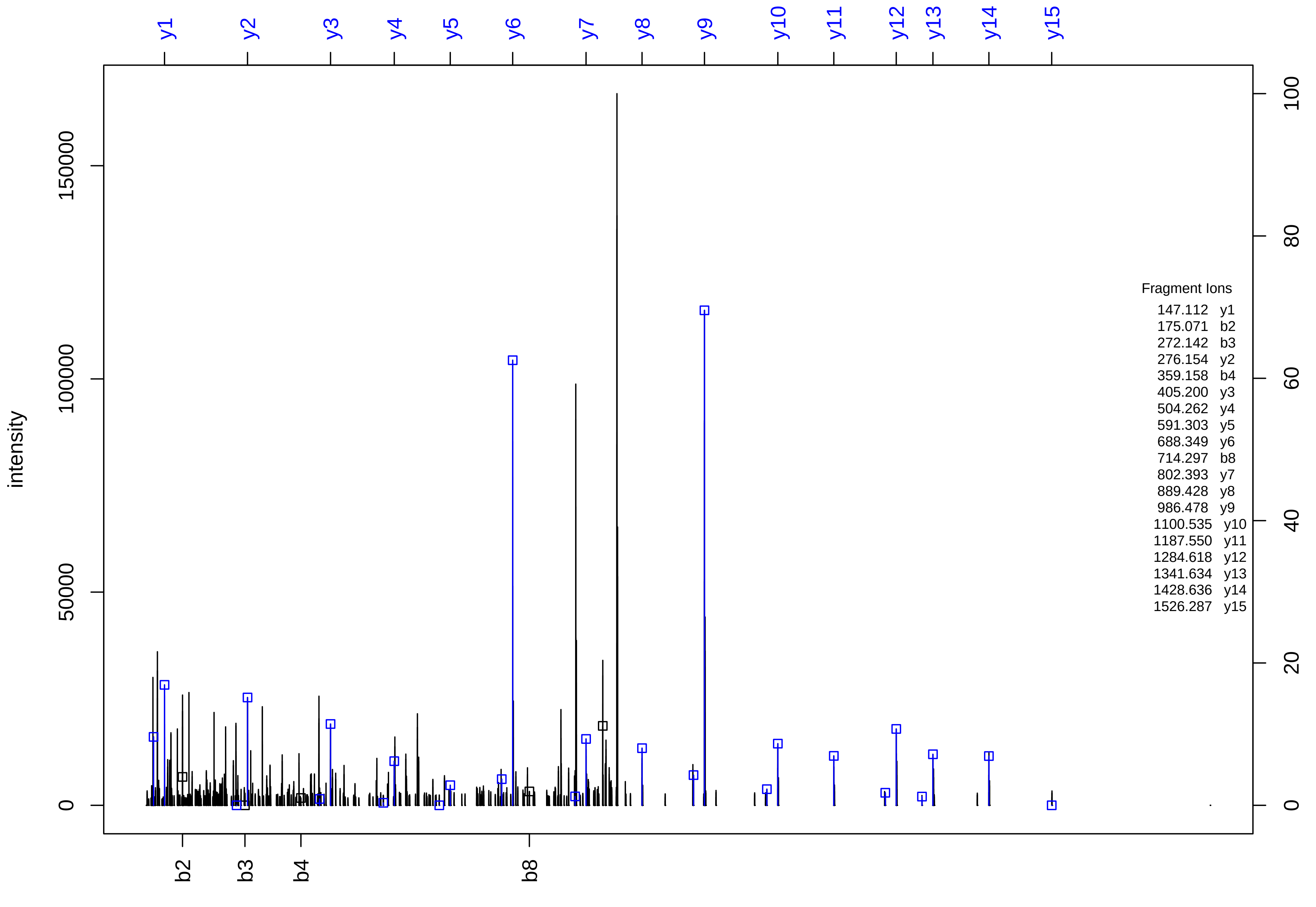

SSPSGPSNPSNPSVEEK / File: christine_pool_02.raw; SpectrumID: ; scans: 2862

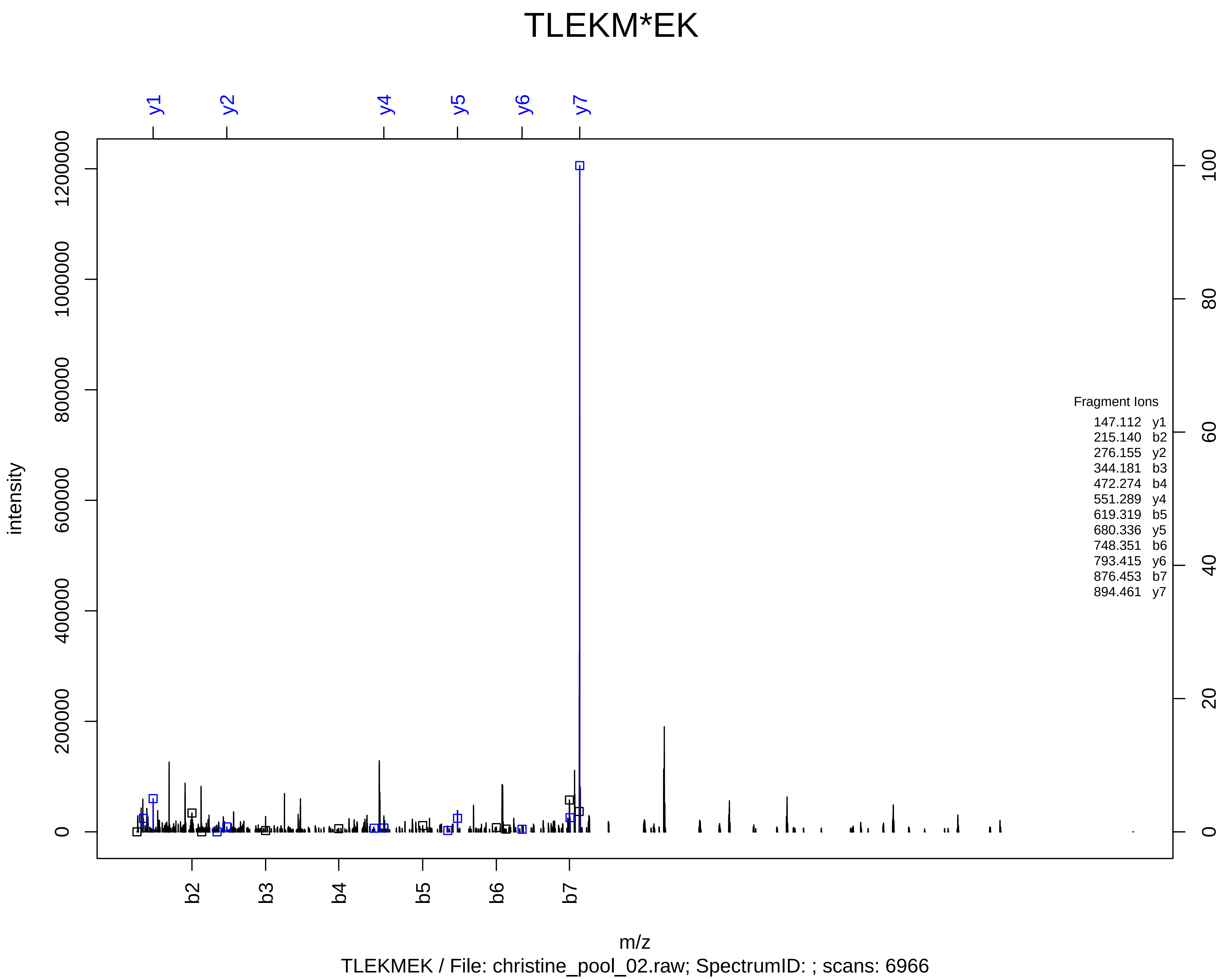
TLEKM*EK
y1
y2
y4
y5
y6
y7
intensity
1200000
1000000
800000
600000
400000
200000
0
100
80
60
40
20
0
Fragment Ions
147.112 y1
215.140 b2
276.155 y2
344.181 b3
472.274 b4
551.289 y4
619.319 b5
680.336 y5
748.351 b6
793.415 y6
876.453 b7
894.461 y7
b2
b3
b4
b5
b6
b7
m/z
TLEKMEK / File: christine_pool_02.raw; SpectrumID: ; scans: 6966

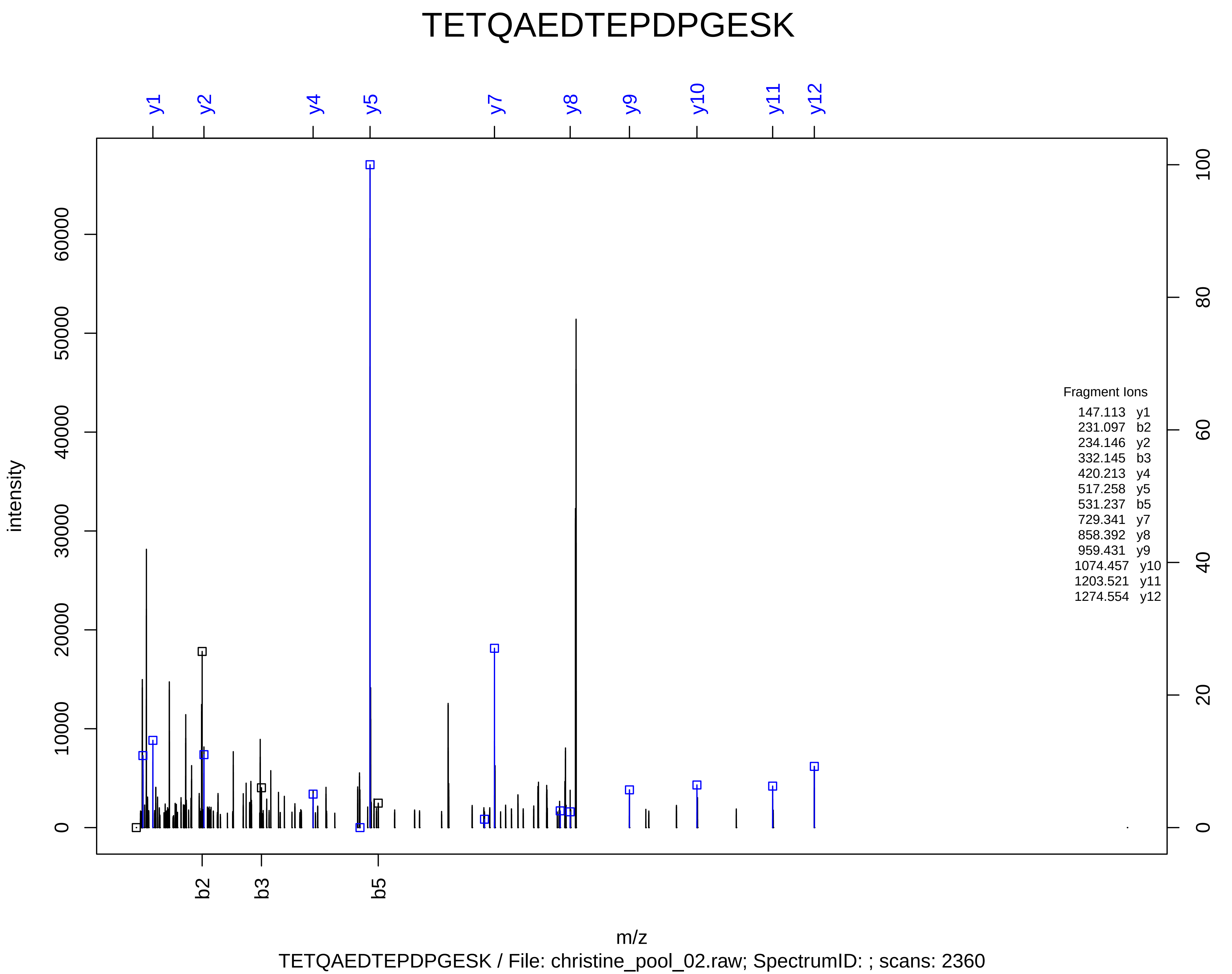
TETQAEDTEPDPGESK
y1
y2
y4
y5
y7
y8
y9
y10
y11
y12
intensity
0
10000
20000
30000
40000
50000
60000
0
20
40
60
80
100
b2
b3
b5
Fragment Ions
147.113 y1
231.097 b2
234.146 y2
332.145 b3
420.213 y4
517.258 y5
531.237 b5
729.341 y7
858.392 y8
959.431 y9
1074.457 y10
1203.521 y11
1274.554 y12
m/z
TETQAEDTEPDPGESK / File: christine_pool_02.raw; SpectrumID: ; scans: 2360

HETLTSLNLEK

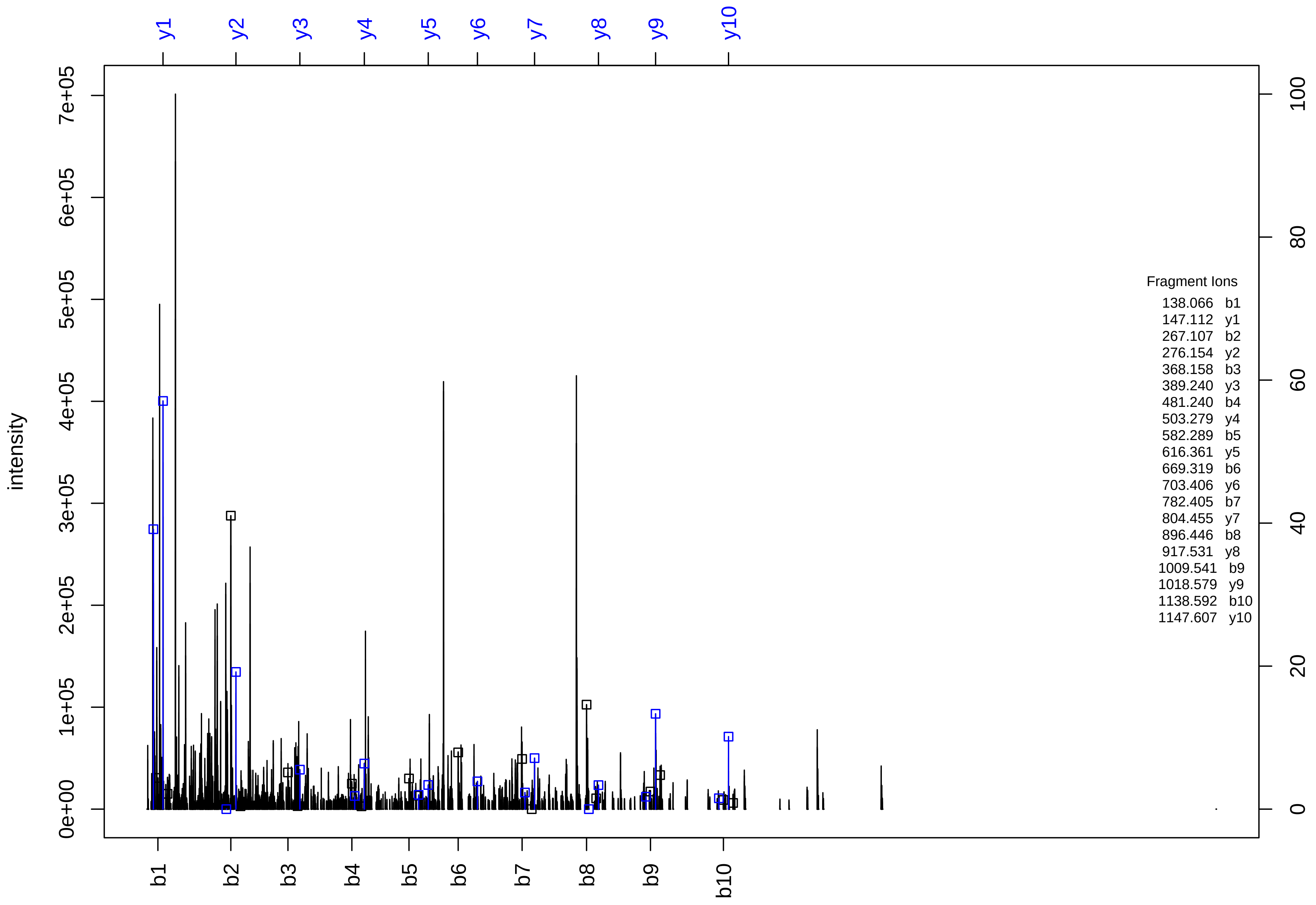

HETLTSLNLEK / File: christine_pool_02.raw; SpectrumID: ; scans: 7316

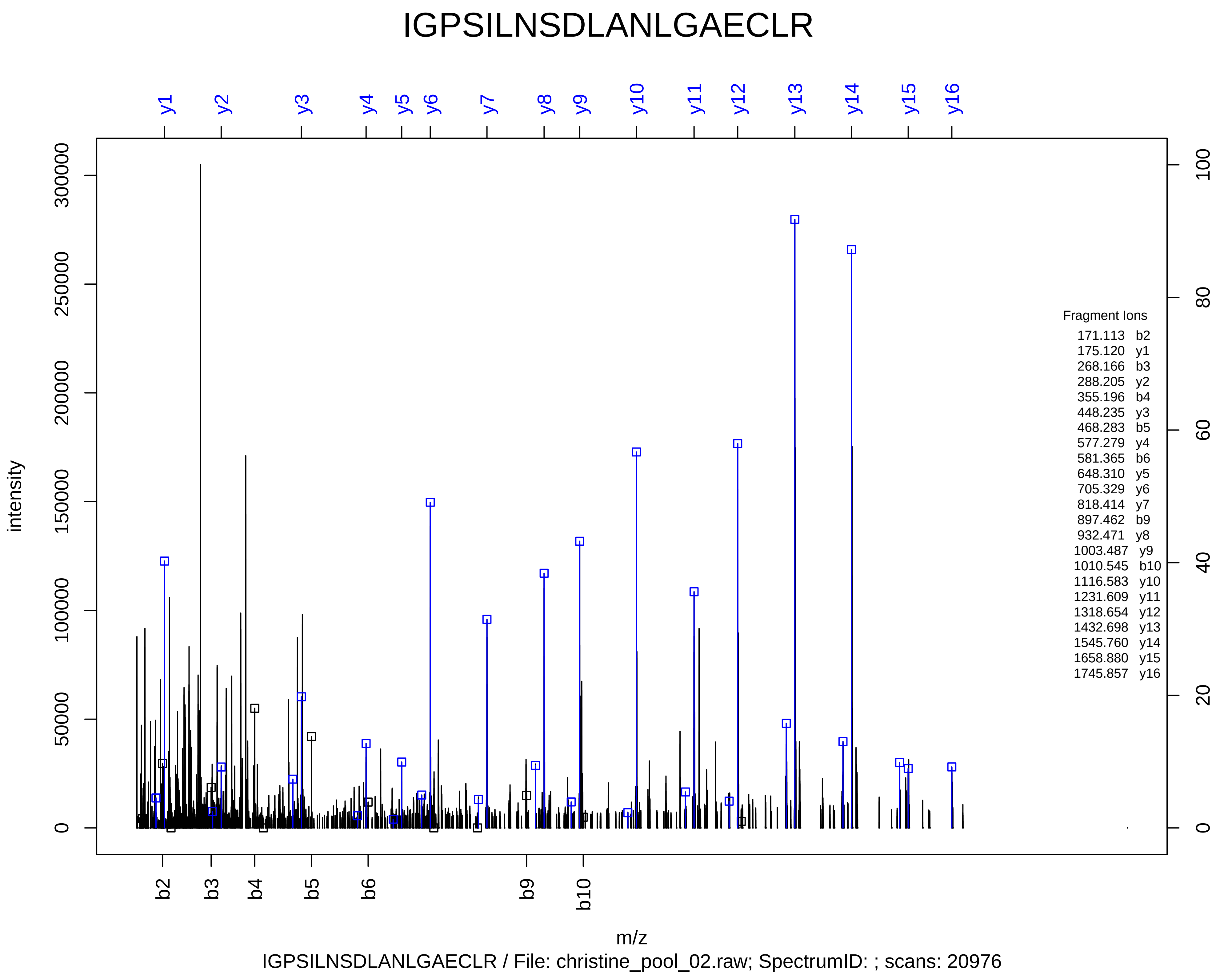
IGPSILNSDLANLGAECLR
y1 y2 y3 y4 y5 y6 y7 y8 y9 y10 y11 y12 y13 y14 y15 y16
intensity
0
50000
100000
150000
200000
250000
300000
0
20
40
60
80
100
b2 b3 b4 b5 b6 b9 b10
m/z
Fragment Ions
171.113 b2
175.120 y1
268.166 b3
288.205 y2
355.196 b4
448.235 y3
468.283 b5
577.279 y4
581.365 b6
648.310 y5
705.329 y6
818.414 y7
897.462 b9
932.471 y8
1003.487 y9
1010.545 b10
1116.583 y10
1231.609 y11
1318.654 y12
1432.698 y13
1545.760 y14
1658.880 y15
1745.857 y16
IGPSILNSDLANLGAECLR / File: christine_pool_02.raw; SpectrumID: ; scans: 20976

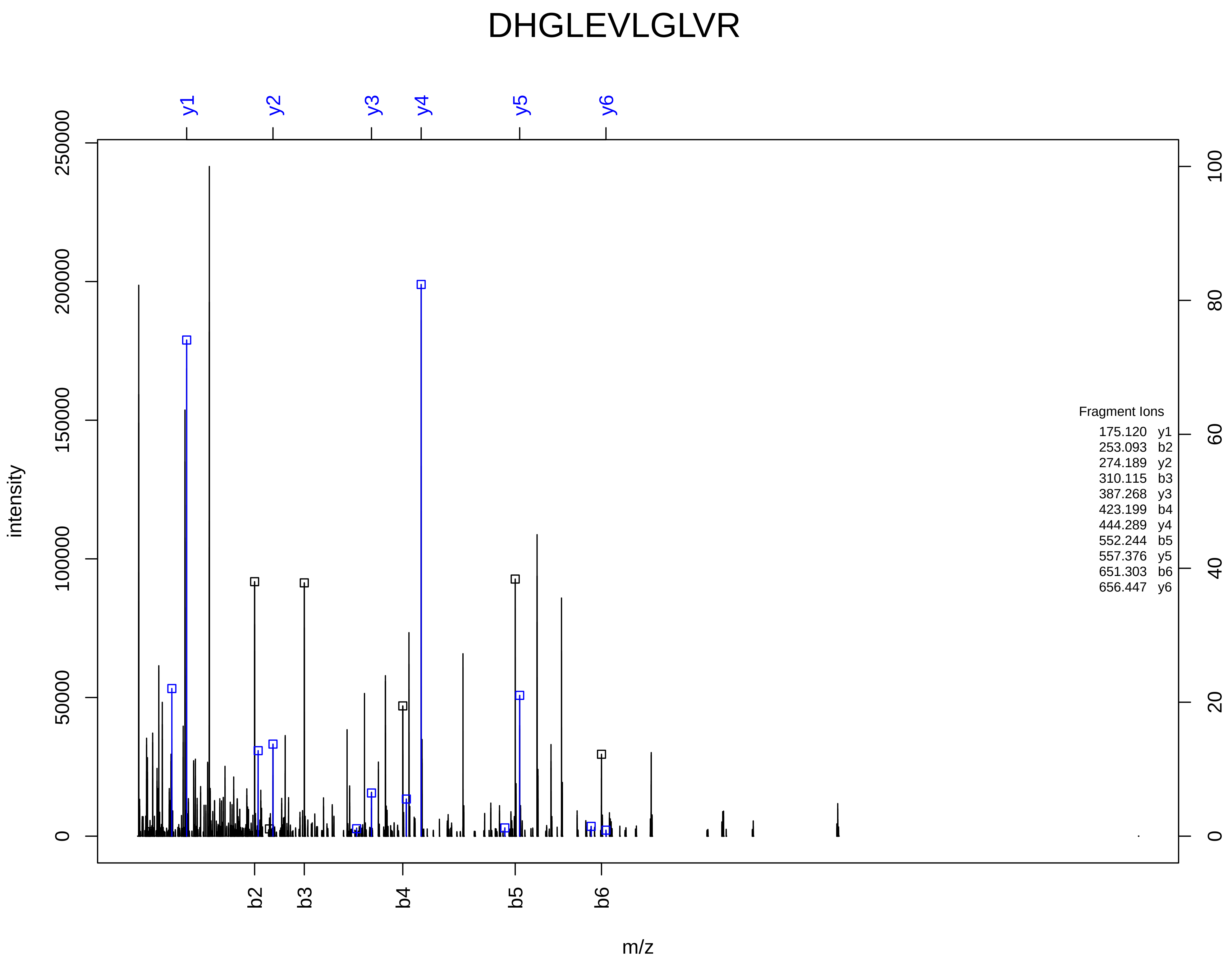

DHGLEVLGLVR
y1
y2
y3
y4
y5
y6
intensity
0
50000
100000
150000
200000
250000
0
20
40
60
80
100
b2
b3
b4
b5
b6
m/z
Fragment Ions
175.120 y1
253.093 b2
274.189 y2
310.115 b3
387.268 y3
423.199 b4
444.289 y4
552.244 b5
557.376 y5
651.303 b6
656.447 y6
DHGLEVLGLVR / File: christine_pool_02.raw; SpectrumID: ; scans: 15516

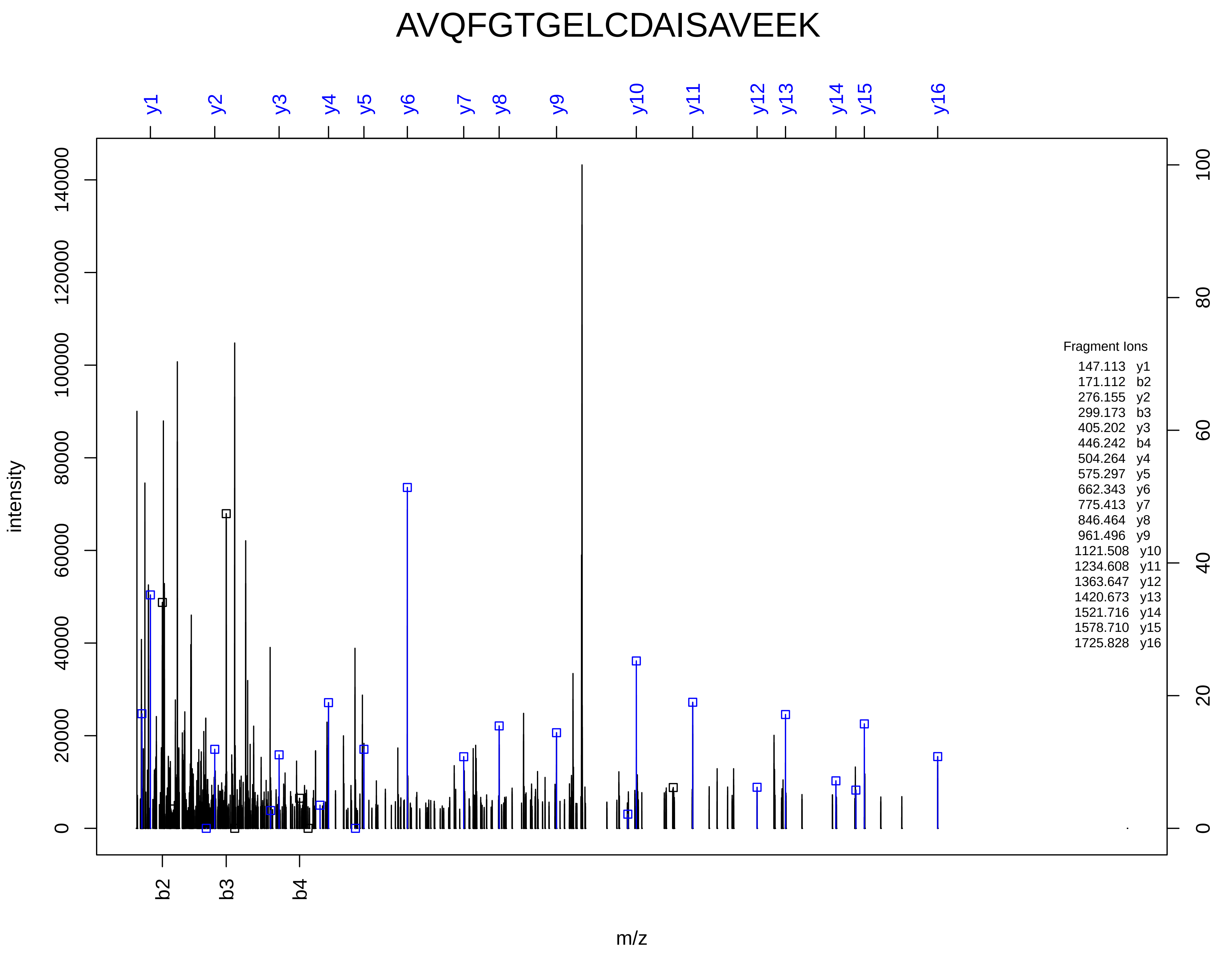
AVQFGTGELCDAISAVEEK
y1 y2 y3 y4 y5 y6 y7 y8 y9 y10 y11 y12 y13 y14 y15 y16
b2 b3 b4
intensity
m/z
Fragment Ions
147.113 y1
171.112 b2
276.155 y2
299.173 b3
405.202 y3
446.242 b4
504.264 y4
575.297 y5
662.343 y6
775.413 y7
846.464 y8
961.496 y9
1121.508 y10
1234.608 y11
1363.647 y12
1420.673 y13
1521.716 y14
1578.710 y15
1725.828 y16
AVQFGTGELCDAISAVEEK / File: christine_pool_02.raw; SpectrumID: ; scans: 19134

TGEDEDEEDNDALLK

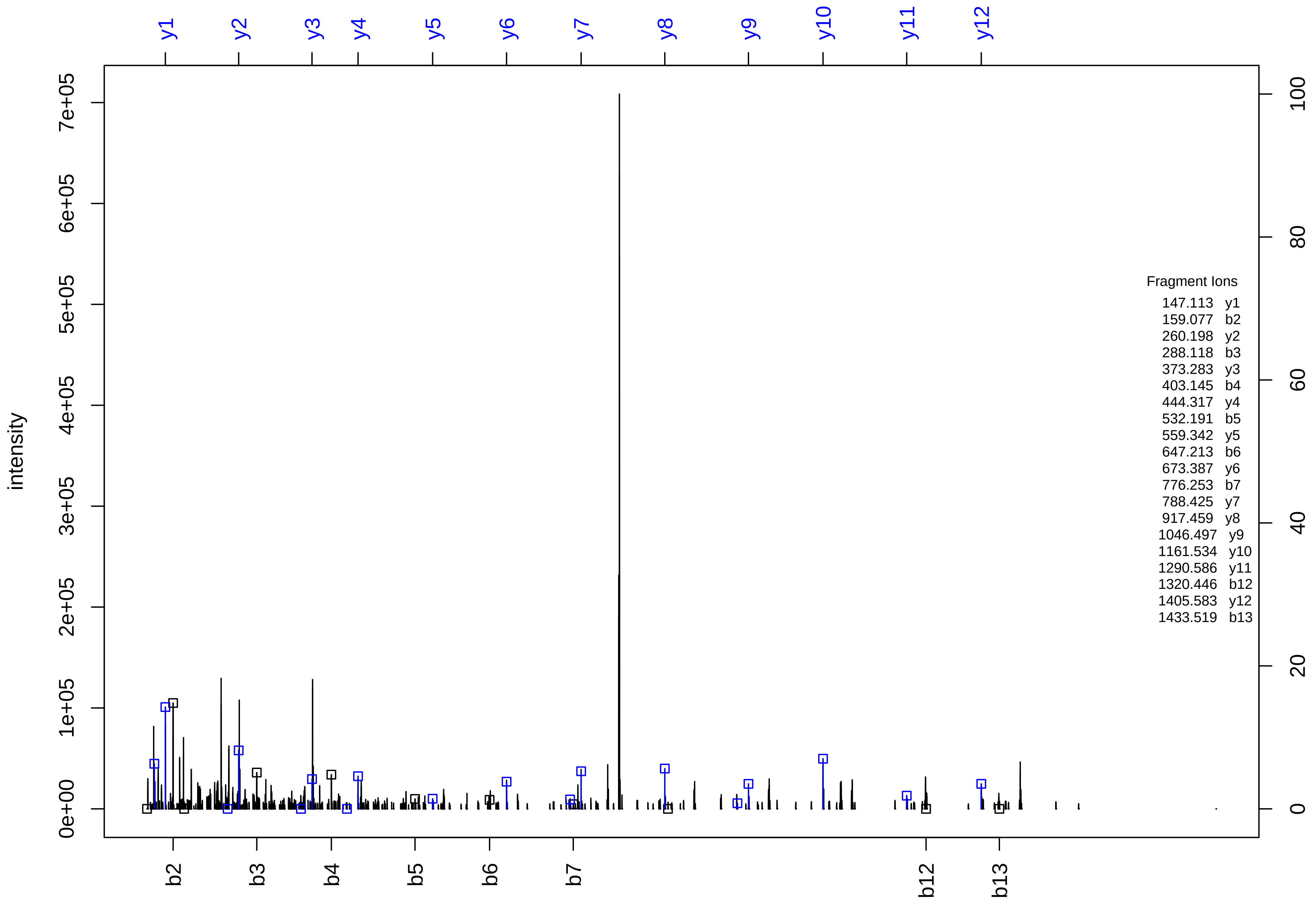

TGEDEDEEDNDALLK / File: christine_pool_02.raw; SpectrumID: ; scans: 7001

QLLLQLNQQR

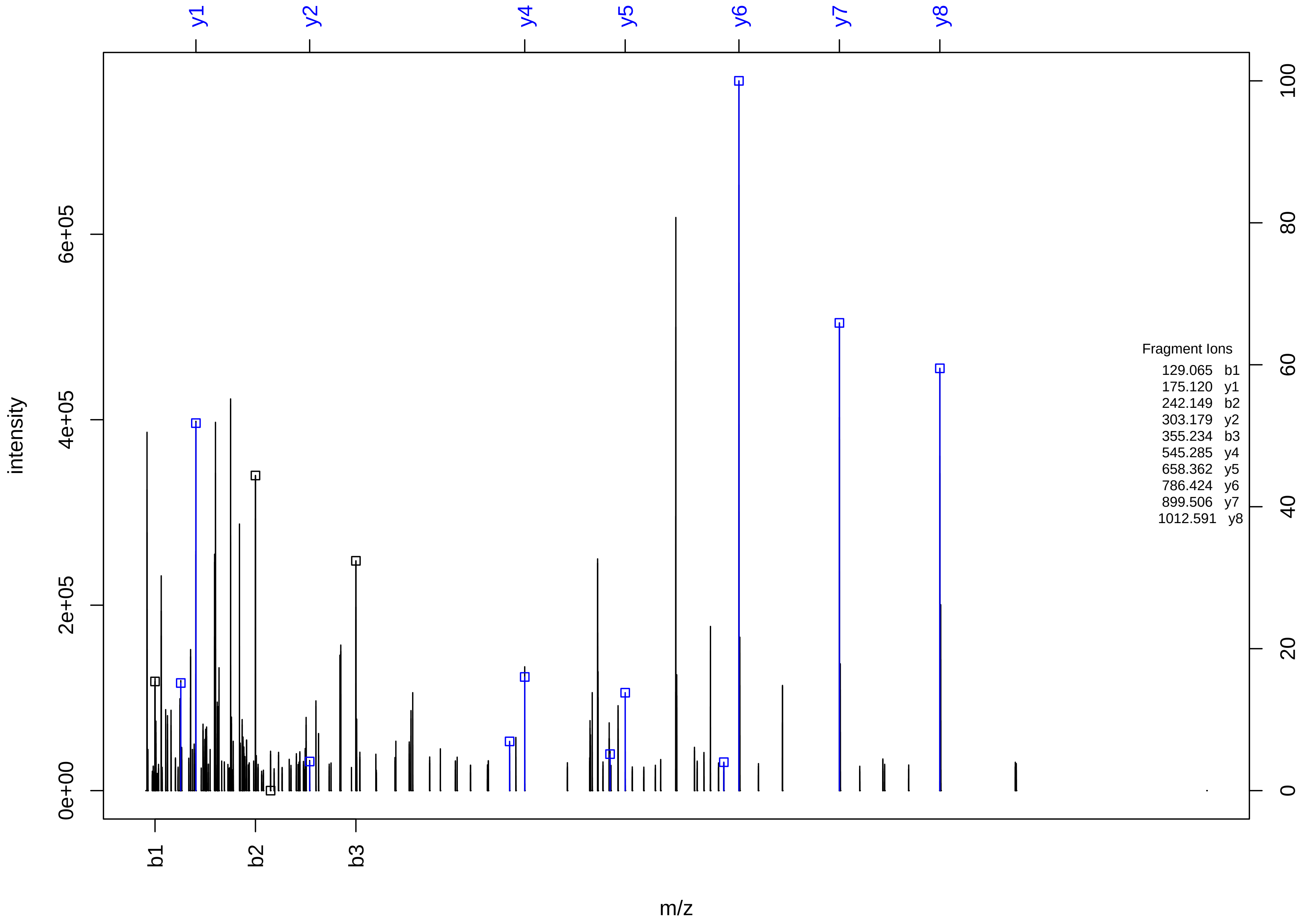

QLLLQLNQQR / File: christine_pool_02.raw; SpectrumID: ; scans: 14371

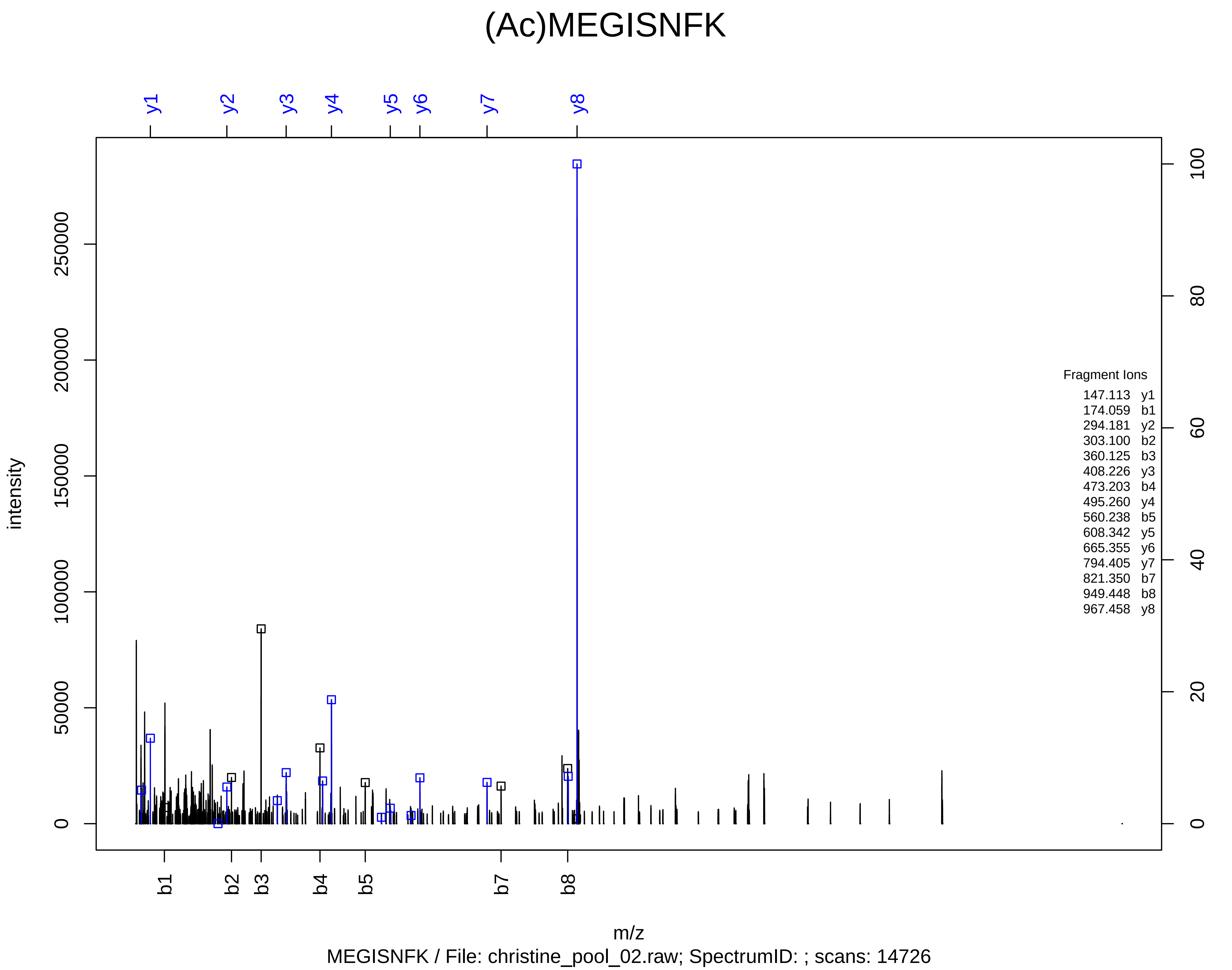

(Ac)MEGISNFK
y1
y2
y3
y4
y5
y6
y7
y8
intensity
0
50000
100000
150000
200000
250000
0
20
40
60
80
100
Fragment Ions
147.113 y1
174.059 b1
294.181 y2
303.100 b2
360.125 b3
408.226 y3
473.203 b4
495.260 y4
560.238 b5
608.342 y5
665.355 y6
794.405 y7
821.350 b7
949.448 b8
967.458 y8
b1
b2
b3
b4
b5
b7
b8
m/z
MEGISNFK / File: christine_pool_02.raw; SpectrumID: ; scans: 14726

SLLNEIESIYDER

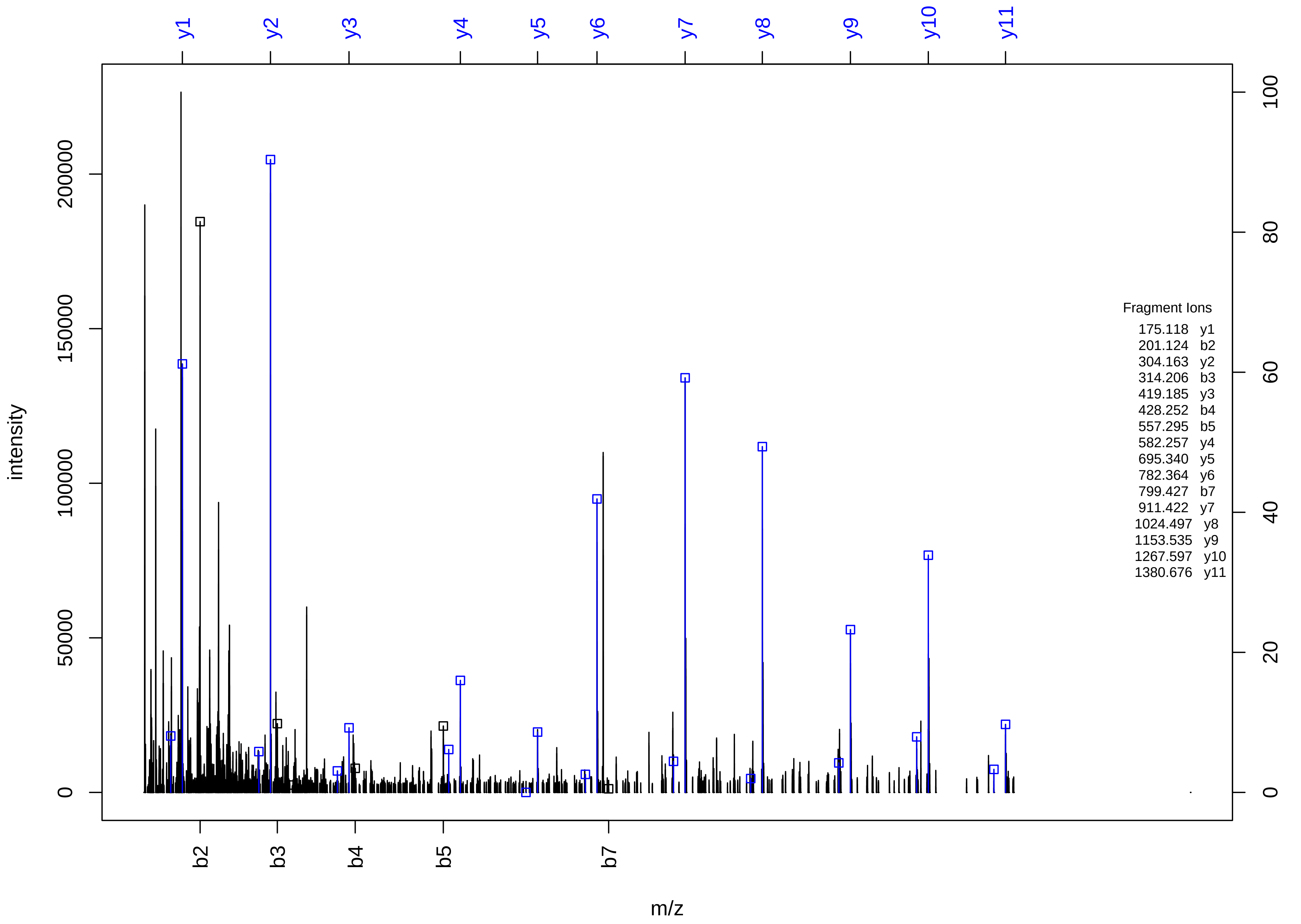

SLLNEIESIYDER / File: christine_pool_02.raw; SpectrumID: ; scans: 22608

ALCDVGTAISCSR

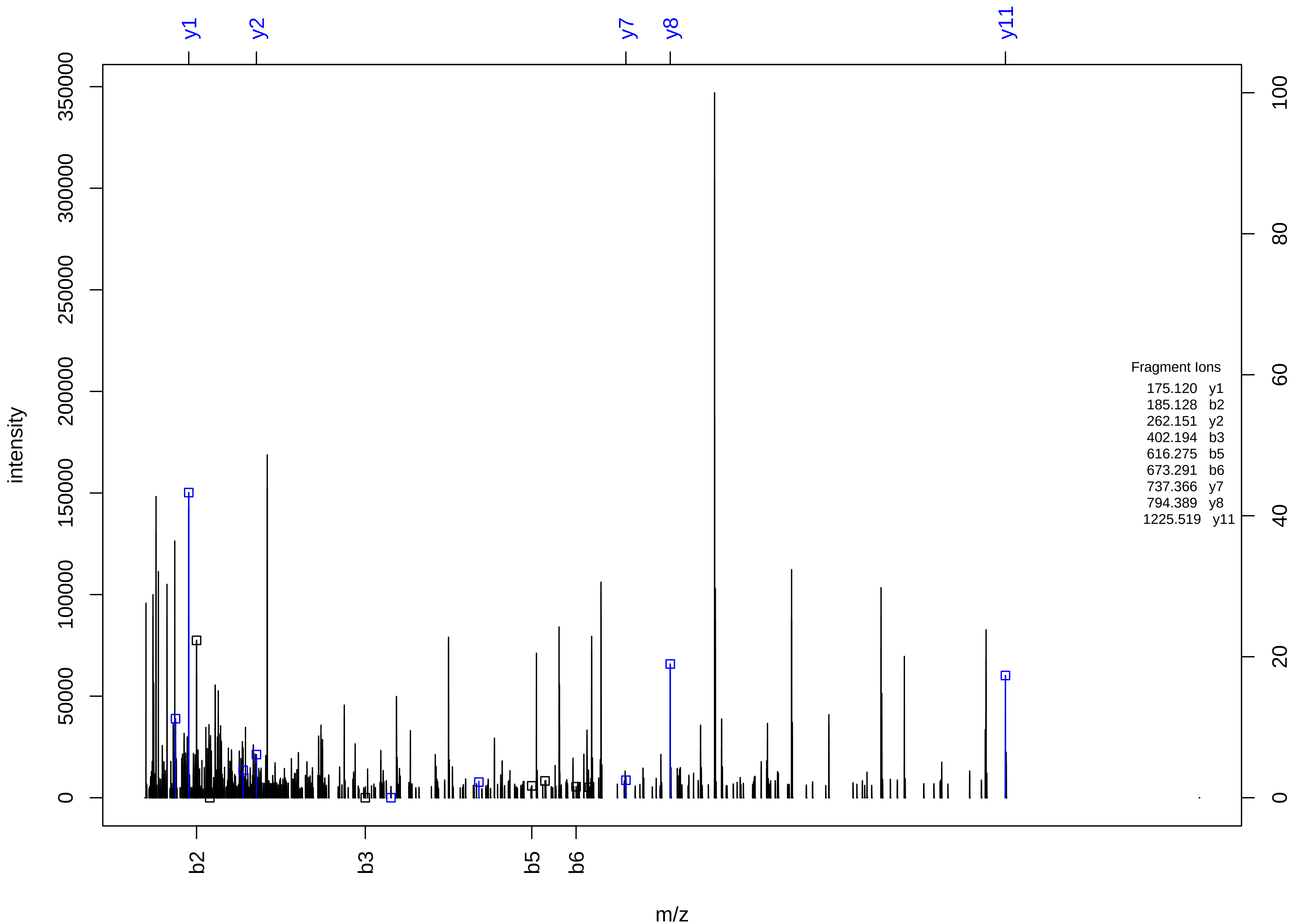

ALCDVGTAISCSR / File: christine_pool_02.raw; SpectrumID: ; scans: 8218

LLALNPDAVELFK

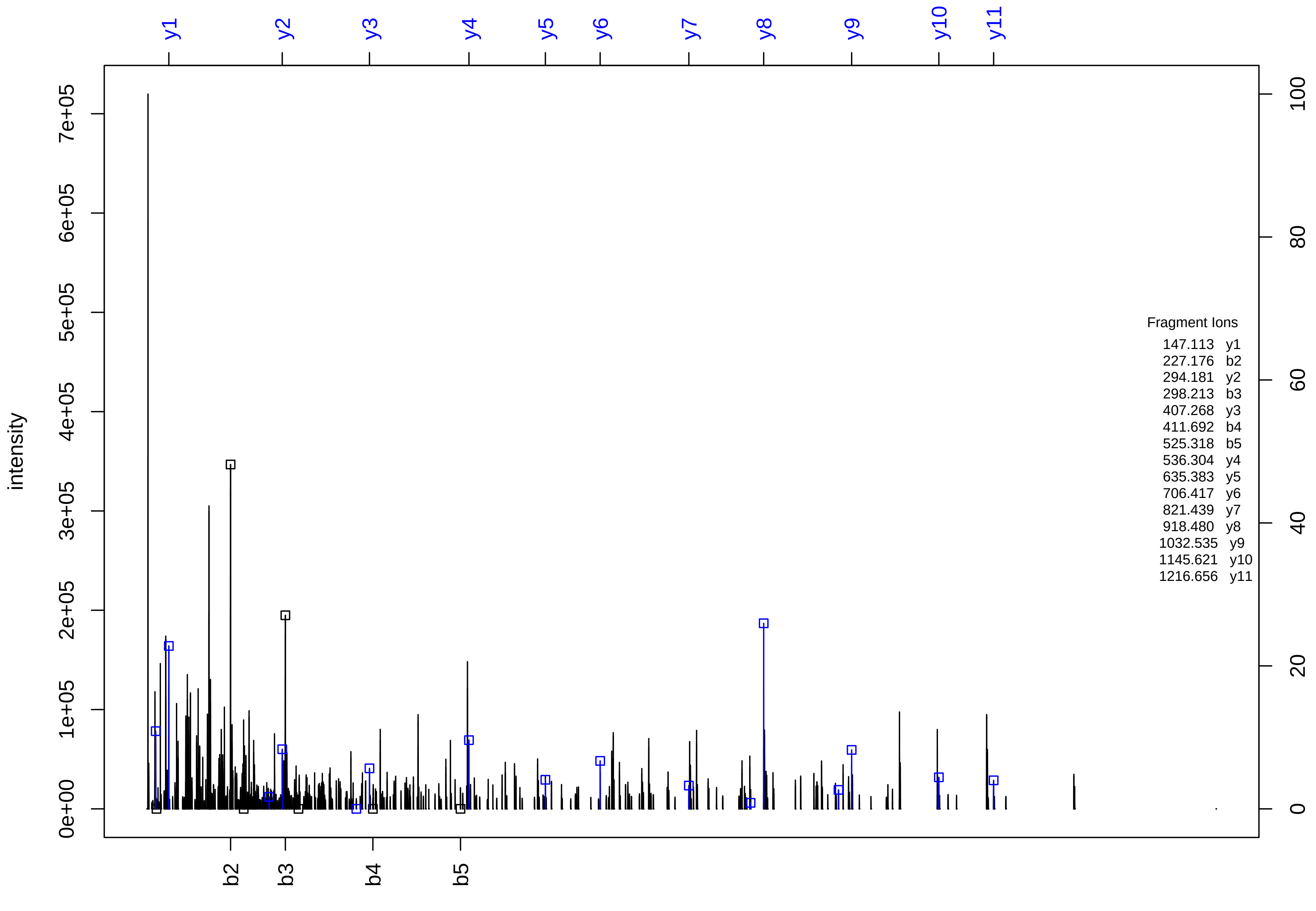

LLALNPDAVELFK / File: christine_pool_02.raw; SpectrumID: ; scans: 22564

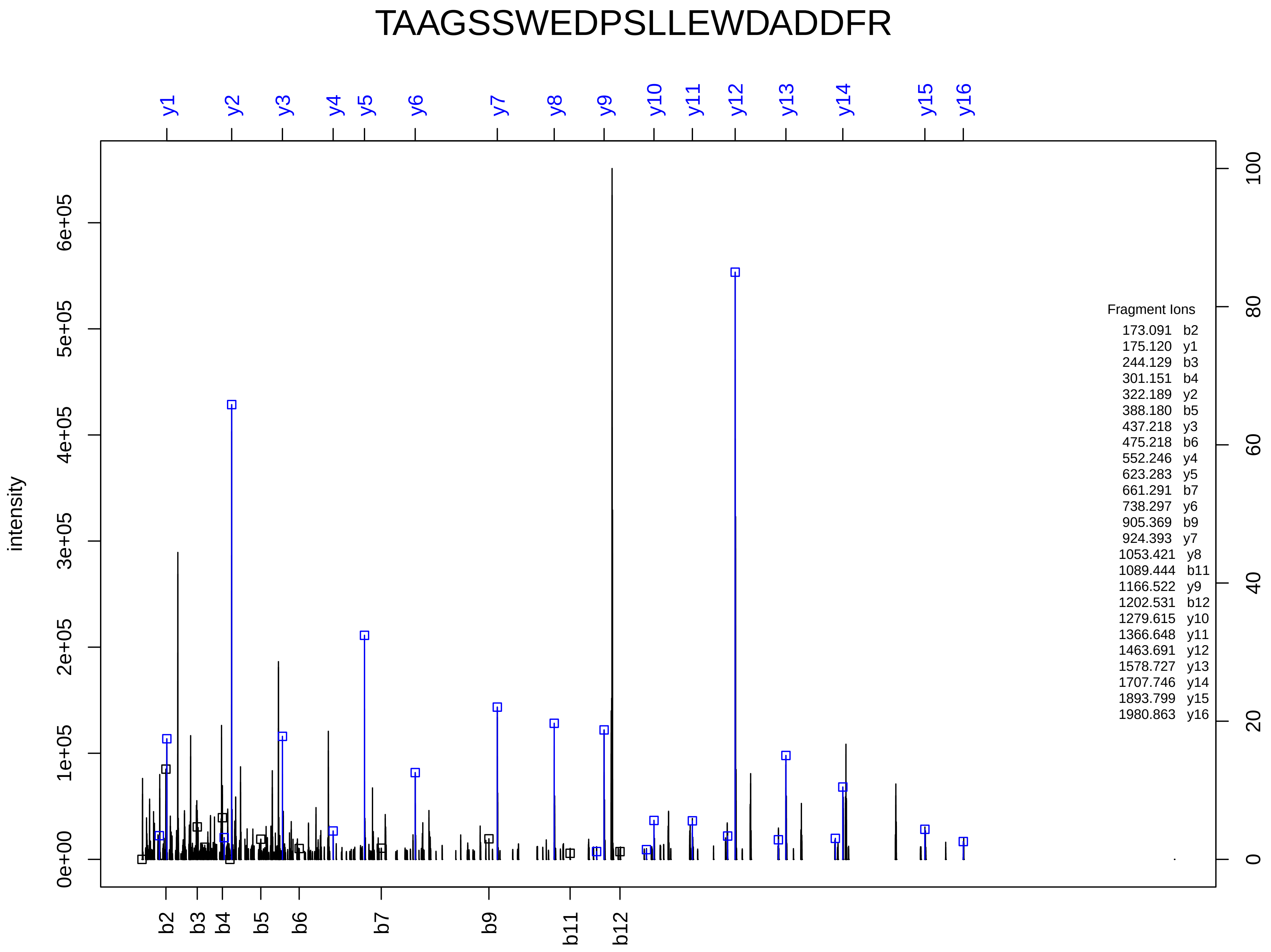

TAAGSSWEDPSLLEWDADDFR / File: christine_pool_02.raw; SpectrumID: ; scans: 22109

VIATDINESK

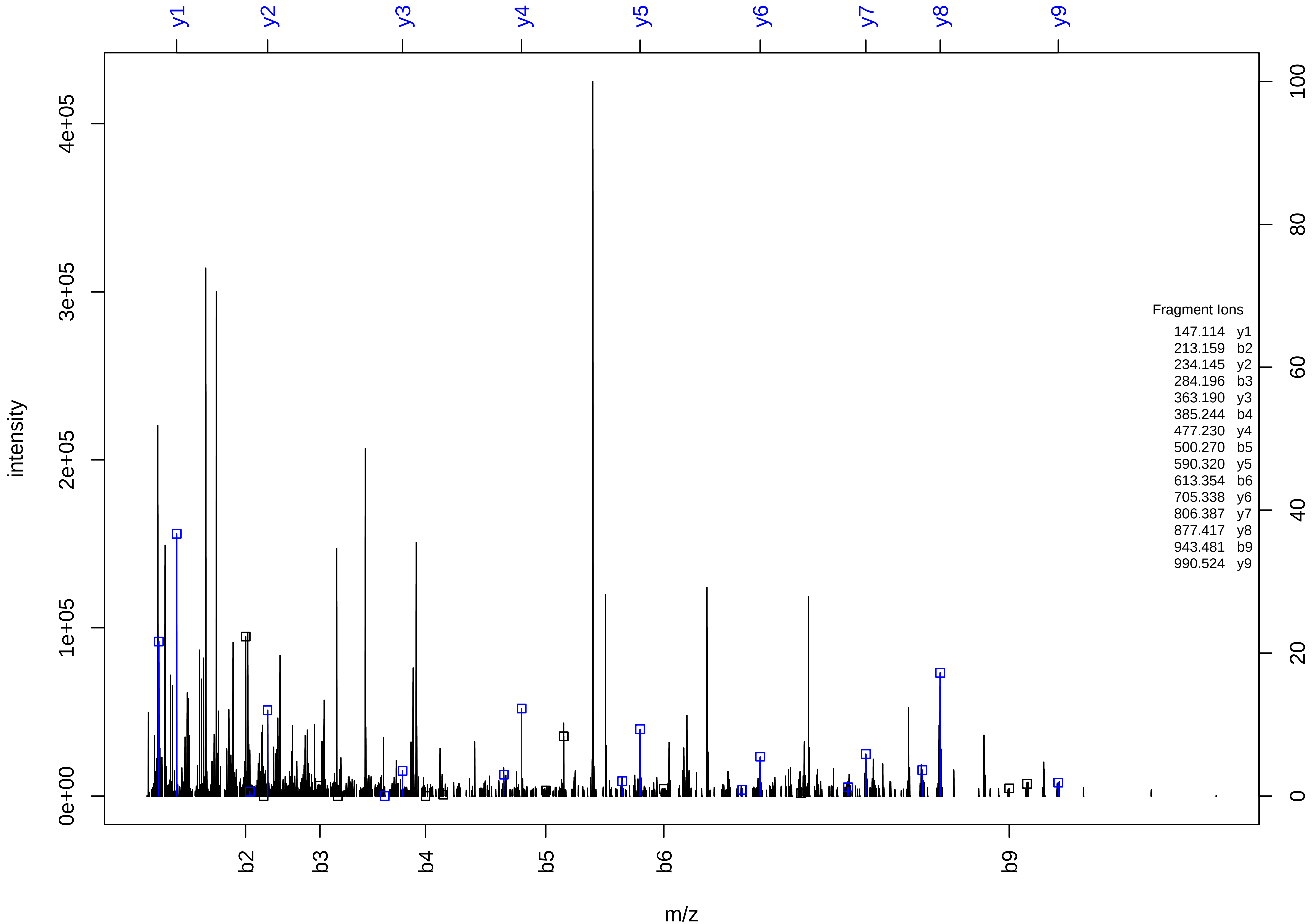

VIATDINESK / File: christine_pool_02.raw; SpectrumID: ; scans: 4261

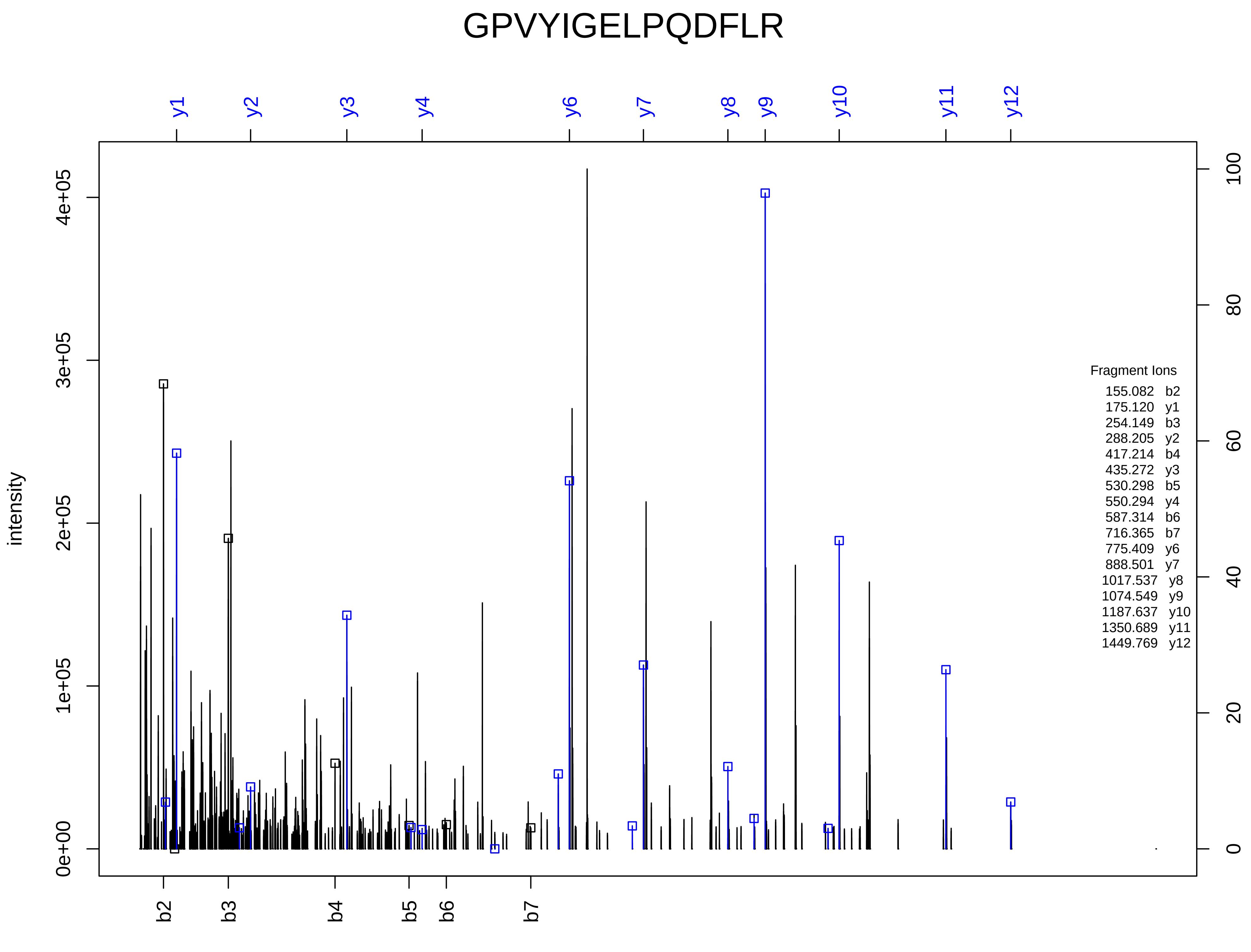

GPVYIGELPQDFLR / File: christine_pool_02.raw; SpectrumID: ; scans: 21029

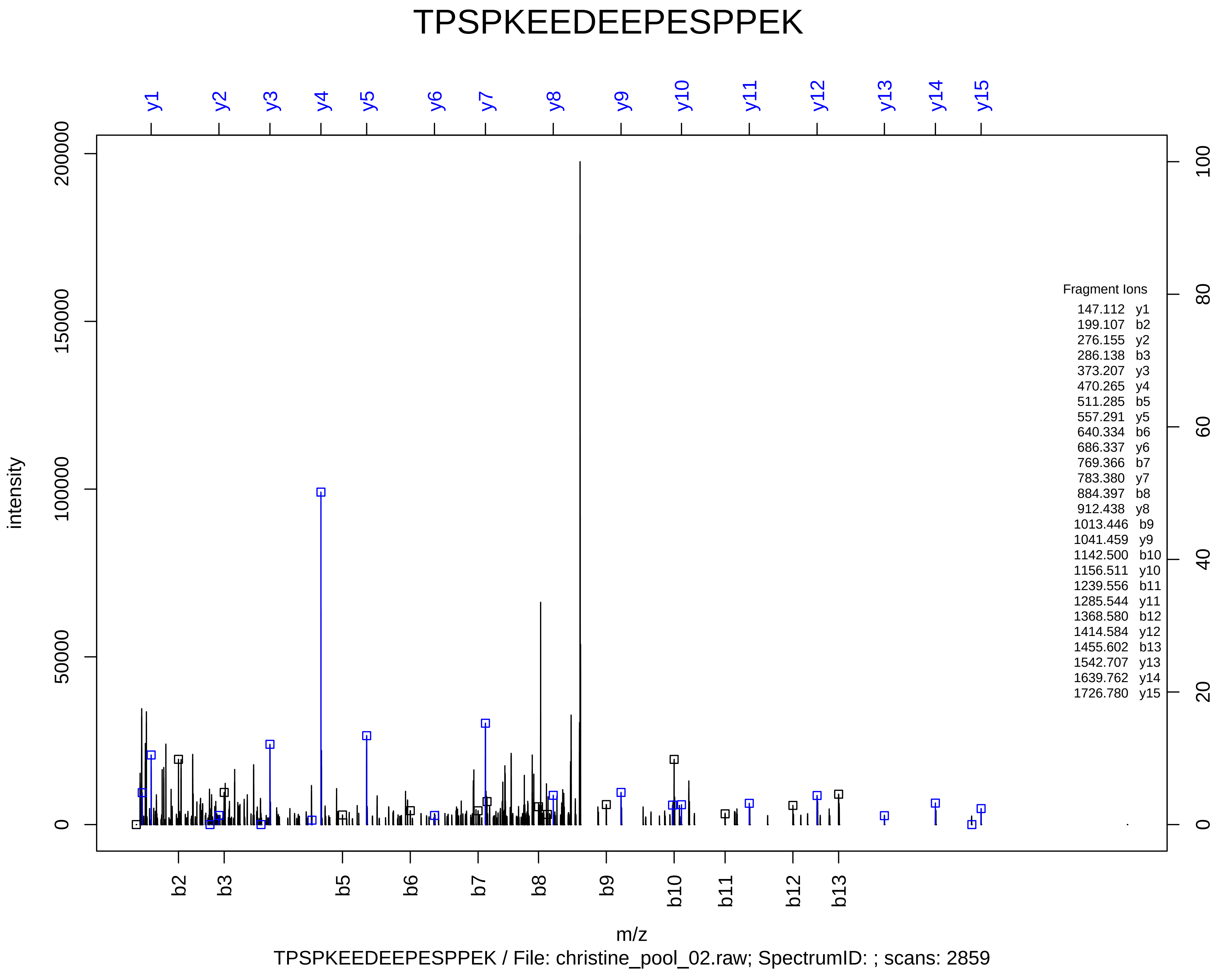
TPSPKEEDEEPESPPEK
y1
y2
y3
y4
y5
y6
y7
y8
y9
y10
y11
y12
y13
y14
y15
intensity
0
50000
100000
150000
200000
0
20
40
60
80
100
Fragment Ions
147.112 y1
199.107 b2
276.155 y2
286.138 b3
373.207 y3
470.265 y4
511.285 b5
557.291 y5
640.334 b6
686.337 y6
769.366 b7
783.380 y7
884.397 b8
912.438 y8
1013.446 b9
1041.459 y9
1142.500 b10
1156.511 y10
1239.556 b11
1285.544 y11
1368.580 b12
1414.584 y12
1455.602 b13
1542.707 y13
1639.762 y14
1726.780 y15
b2
b3
b5
b6
b7
b8
b9
b10
b11
b12
b13
m/z
TPSPKEEDEEPESPPEK / File: christine_pool_02.raw; SpectrumID: ; scans: 2859

ELEEEAEEEQR

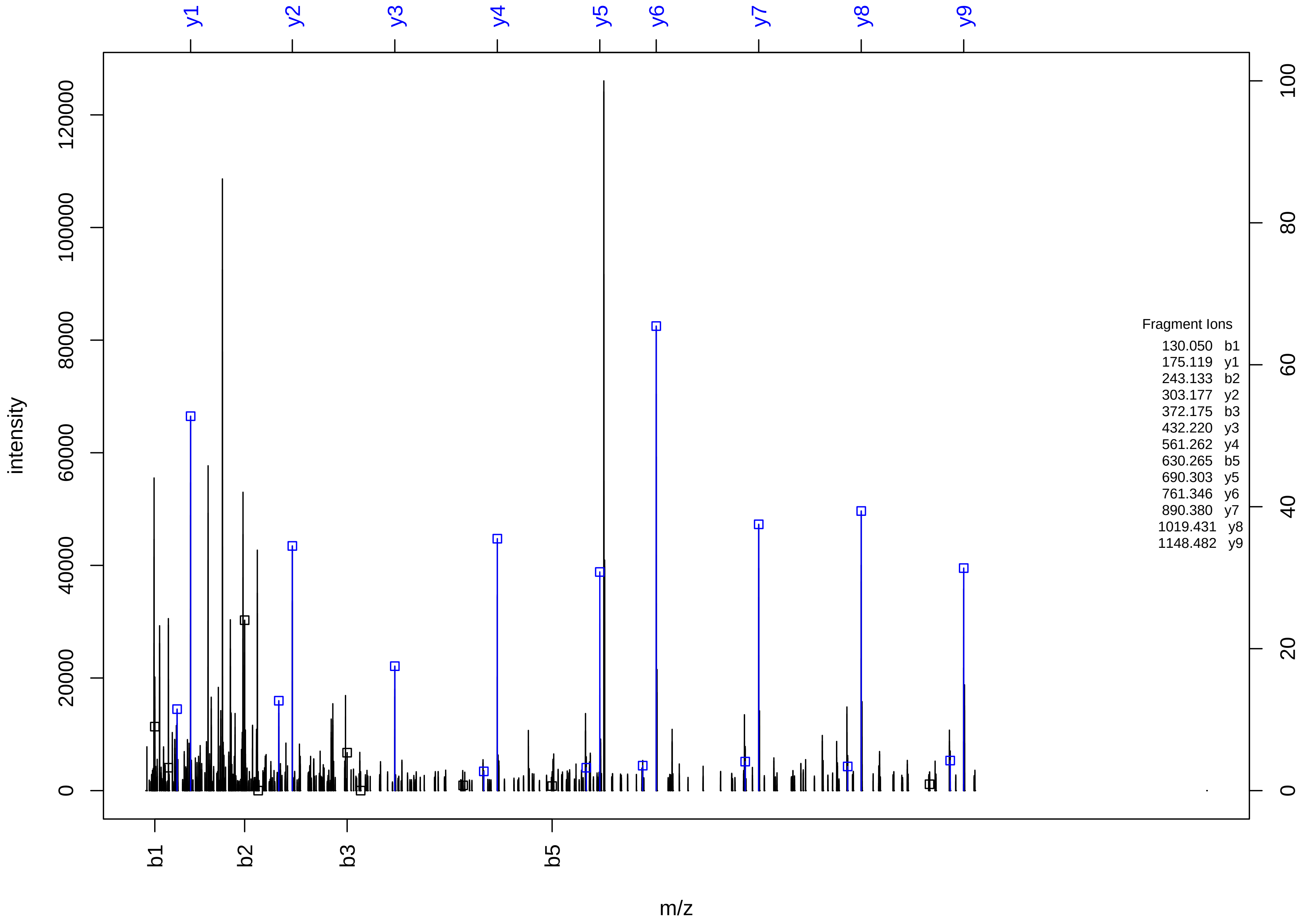

ELEEEAEEEQR / File: christine_pool_02.raw; SpectrumID: ; scans: 3156

EKIKEEMN^EDHSTPK

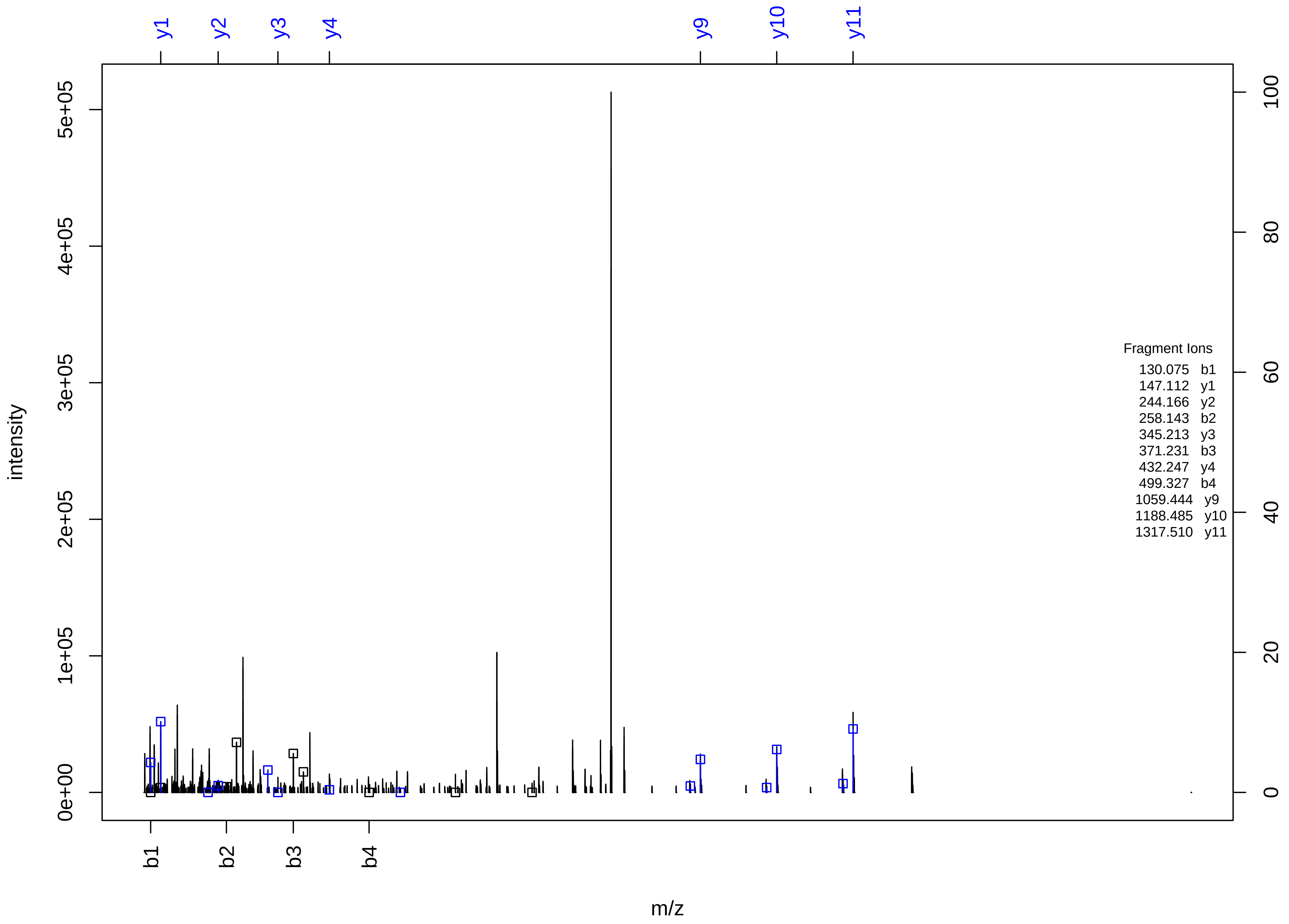

EKIKEEMNEDHSTPK / File: christine_pool_02.raw; SpectrumID: ; scans: 7917

DFINFISDK

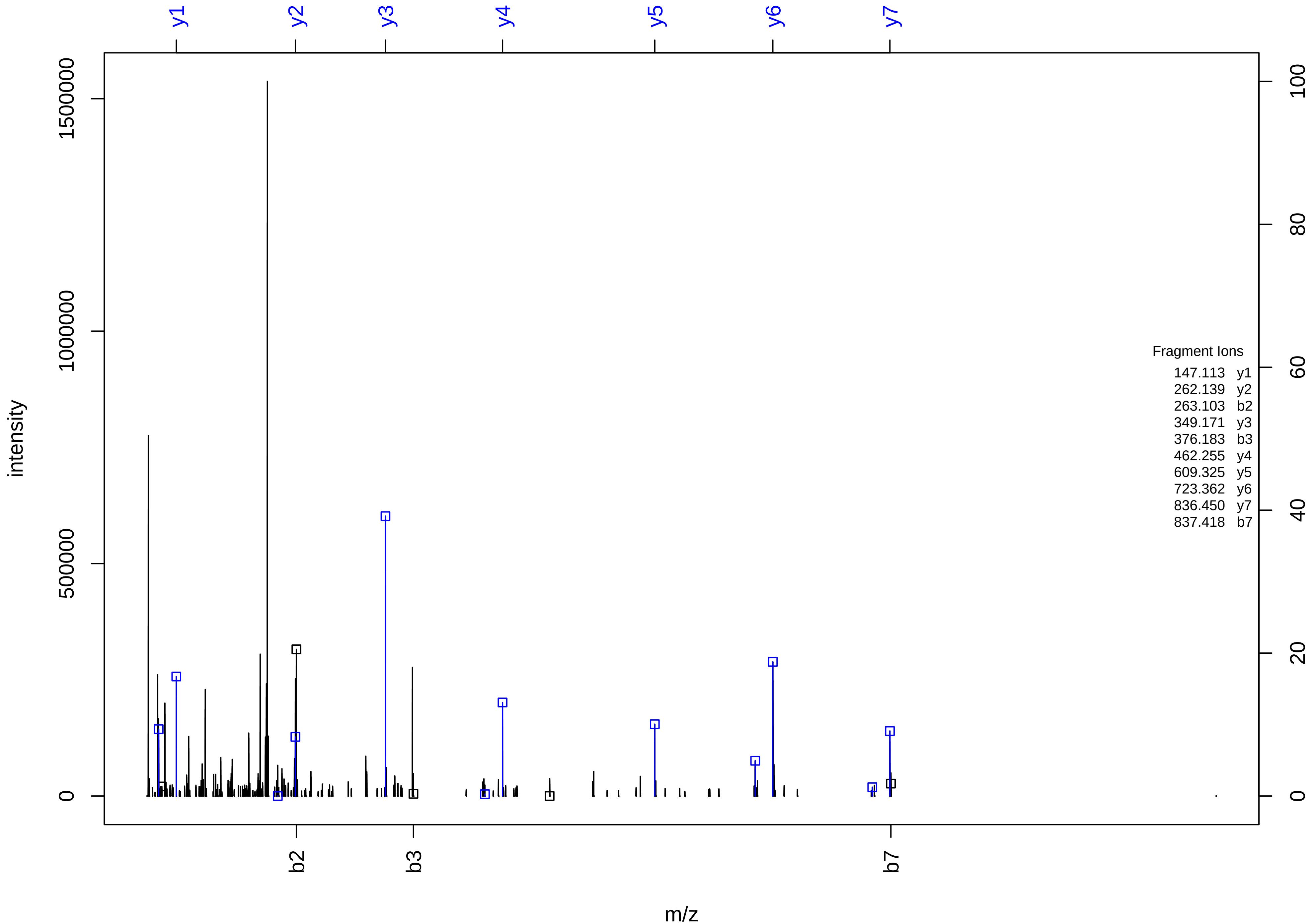

DFINFISDK / File: christine_pool_02.raw; SpectrumID: ; scans: 18197

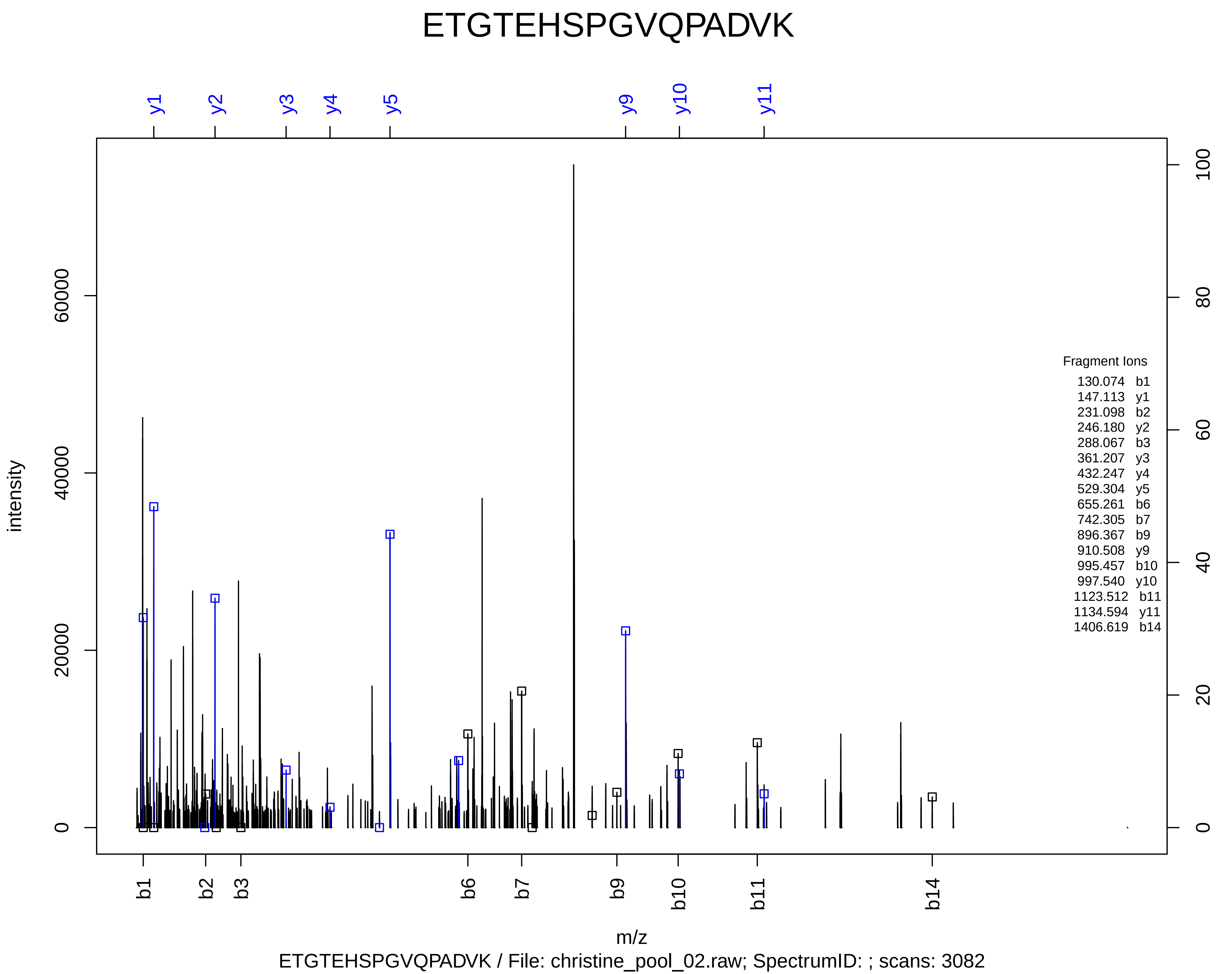
ETGTEHSPGVQPADVK
y1
y2
y3
y4
y5
y9
y10
y11
intensity
0
20000
40000
60000
0
20
40
60
80
100
Fragment Ions
130.074 b1
147.113 y1
231.098 b2
246.180 y2
288.067 b3
361.207 y3
432.247 y4
529.304 y5
655.261 b6
742.305 b7
896.367 b9
910.508 y9
995.457 b10
997.540 y10
1123.512 b11
1134.594 y11
1406.619 b14
b1
b2
b3
b6
b7
b9
b10
b11
b14
m/z
ETGTEHSPGVQPADVK / File: christine_pool_02.raw; SpectrumID: ; scans: 3082

VLEALQVLR

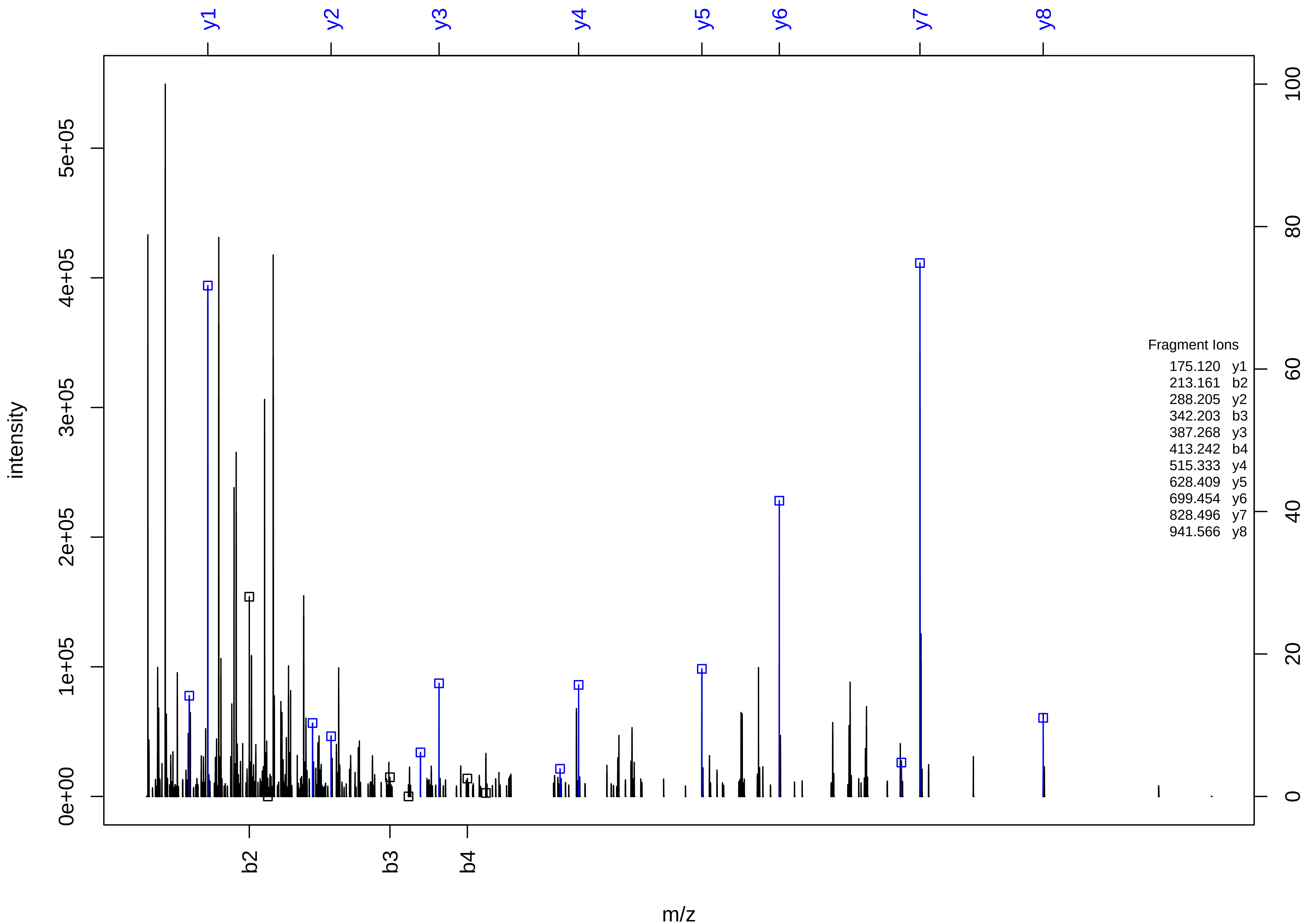

VLEALQVLR / File: christine_pool_02.raw; SpectrumID: ; scans: 16085

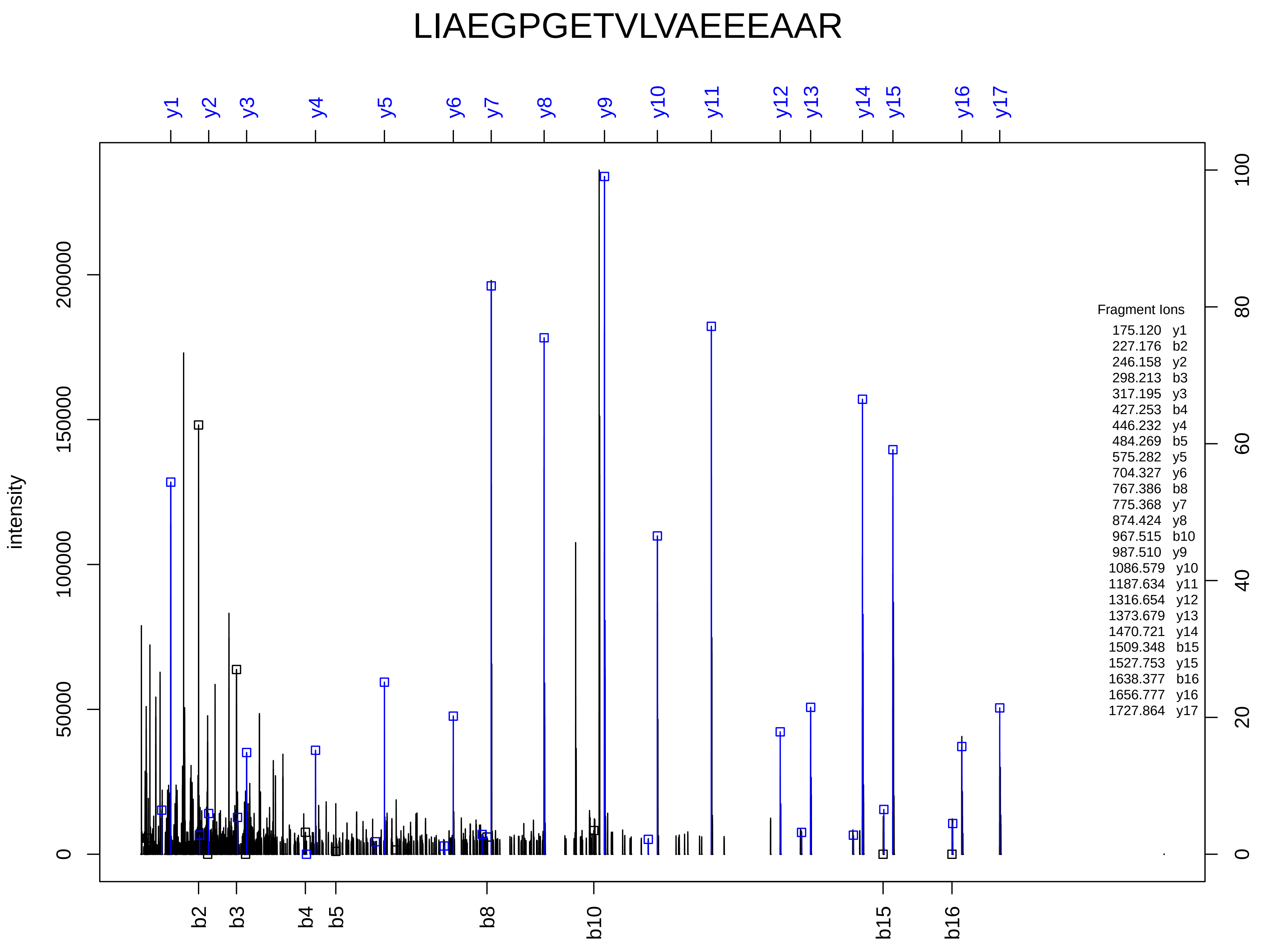

LIAEGPGETVLVAEEEAAR / File: christine_pool_02.raw; SpectrumID: ; scans: 16485

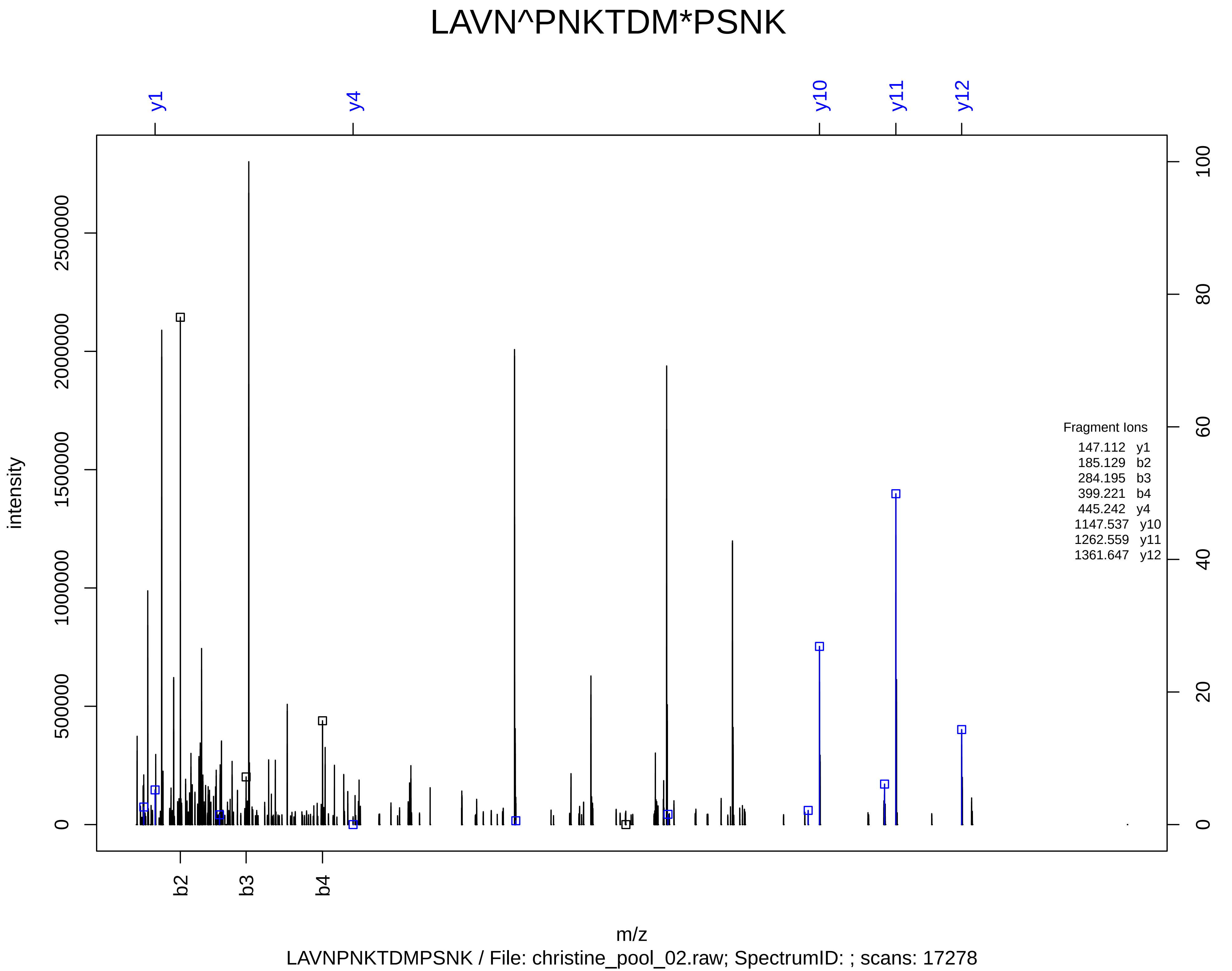
LAVN^PNKTDM*PSNK
y1
y4
y10
y11
y12
intensity
2500000
2000000
1500000
1000000
500000
0
100
80
60
40
20
0
Fragment Ions
147.112 y1
185.129 b2
284.195 b3
399.221 b4
445.242 y4
1147.537 y10
1262.559 y11
1361.647 y12
b2
b3
b4
m/z
LAVNPNKTDMPSNK / File: christine_pool_02.raw; SpectrumID: ; scans: 17278

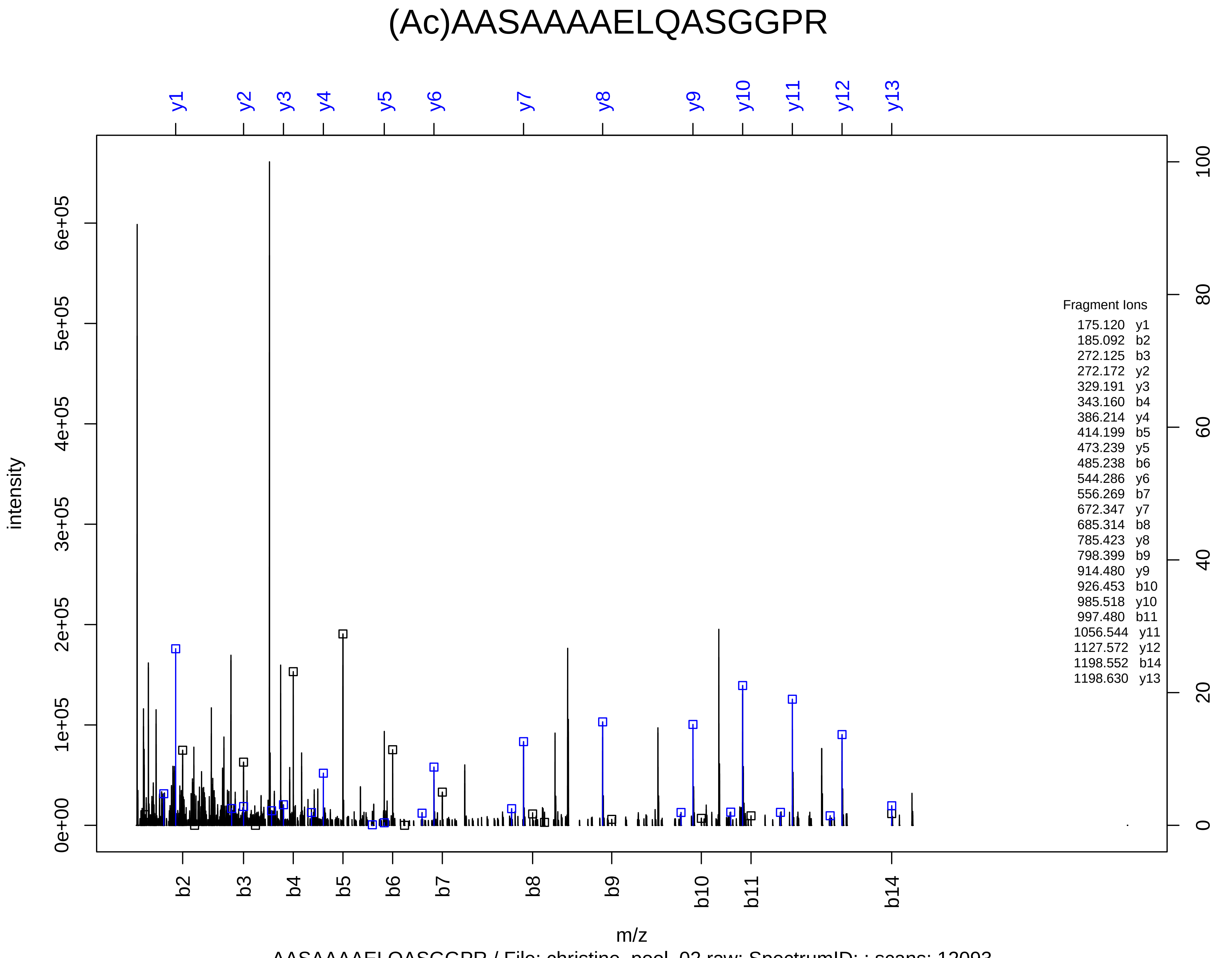

(Ac)AASAAAAELQASGGPR
y1
y2
y3
y4
y5
y6
y7
y8
y9
y10
y11
y12
y13
intensity
0e+00
1e+05
2e+05
3e+05
4e+05
5e+05
6e+05
0
20
40
60
80
100
Fragment Ions
175.120 y1
185.092 b2
272.125 b3
272.172 y2
329.191 y3
343.160 b4
386.214 y4
414.199 b5
473.239 y5
485.238 b6
544.286 y6
556.269 b7
672.347 y7
685.314 b8
785.423 y8
798.399 b9
914.480 y9
926.453 b10
985.518 y10
997.480 b11
1056.544 y11
1127.572 y12
1198.552 b14
1198.630 y13
b2
b3
b4
b5
b6
b7
b8
b9
b10
b11
b14
m/z
AASAAAAELQASGGPR / File: christine_pool_02.raw; SpectrumID: ; scans: 12093

(Ac)AGLELLSDQGYR

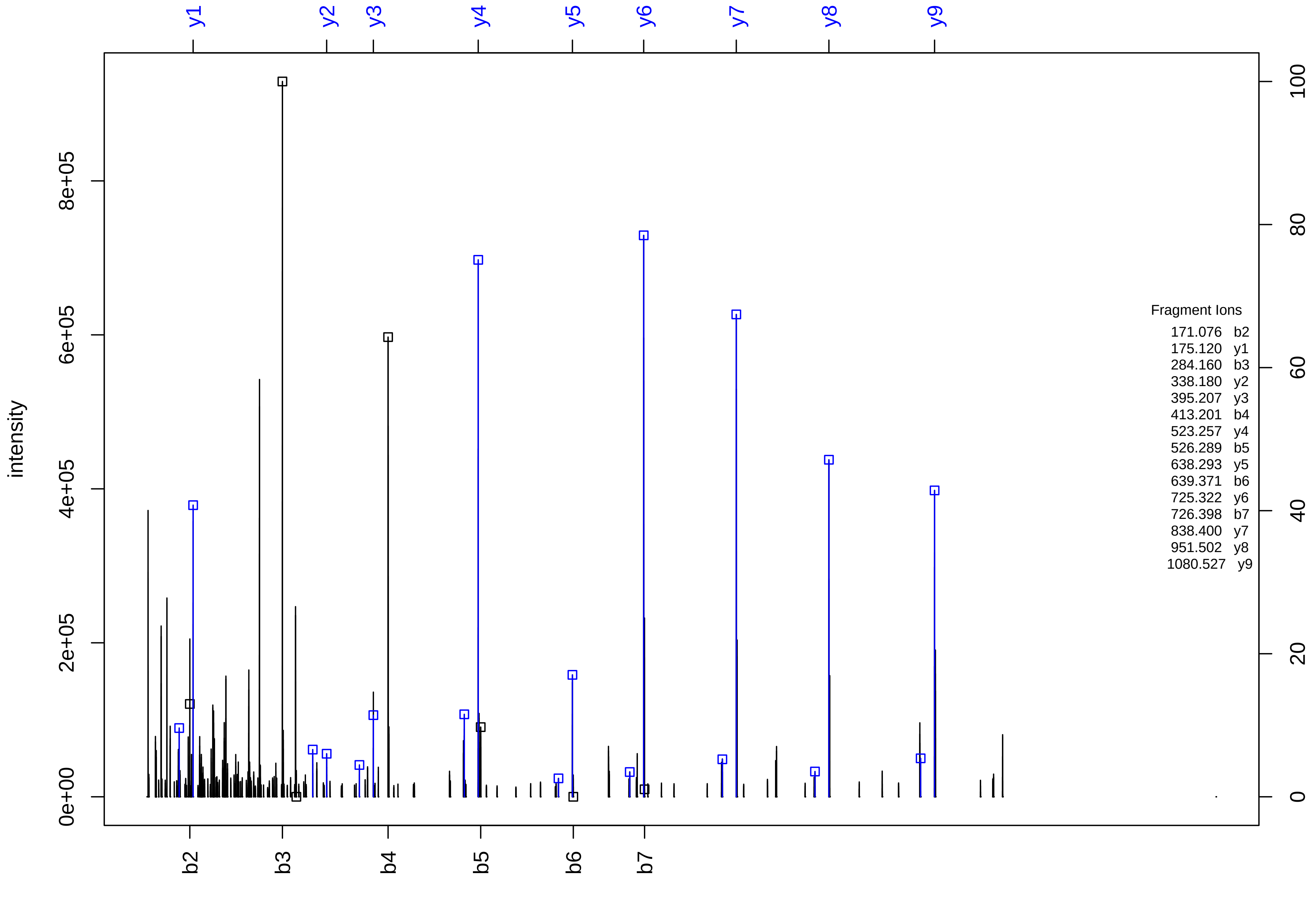

AGLELLSDQGYR / File: christine_pool_02.raw; SpectrumID: ; scans: 20545

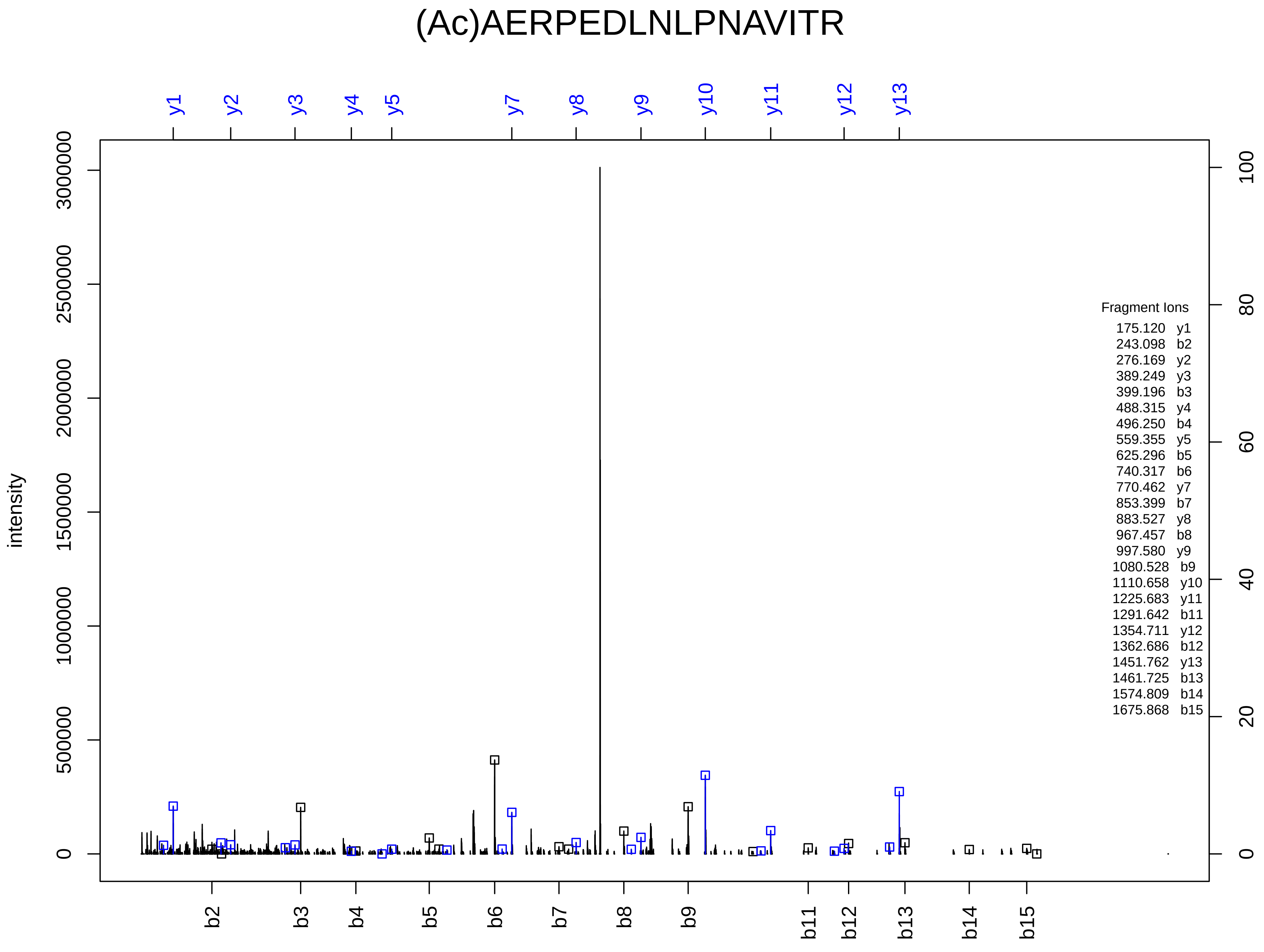

AERPEDLNLPNAVITR / File: christine_pool_02.raw; SpectrumID: ; scans: 15322

LSSSFSSR

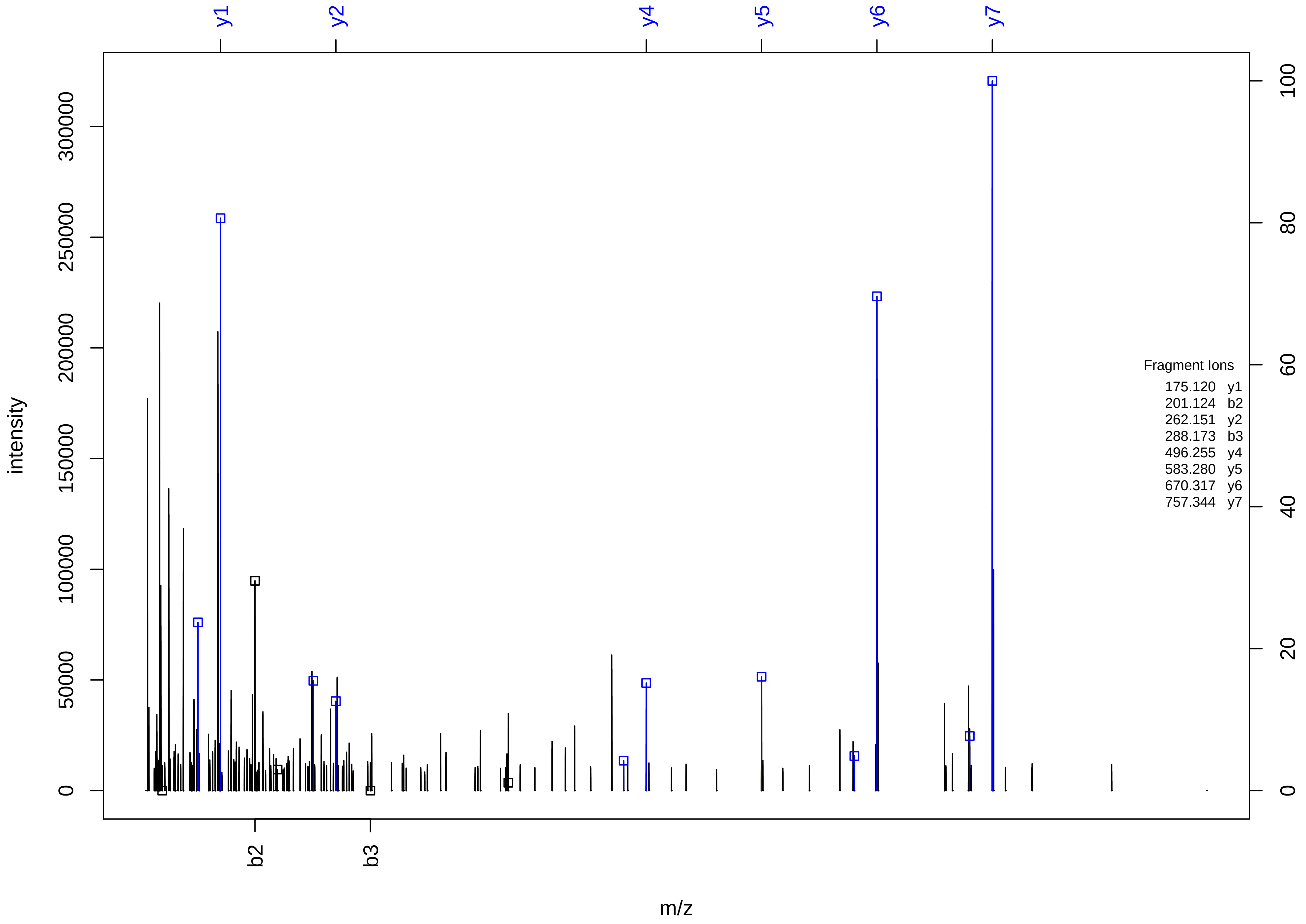

LSSSFSSR / File: christine_pool_02.raw; SpectrumID: ; scans: 2985

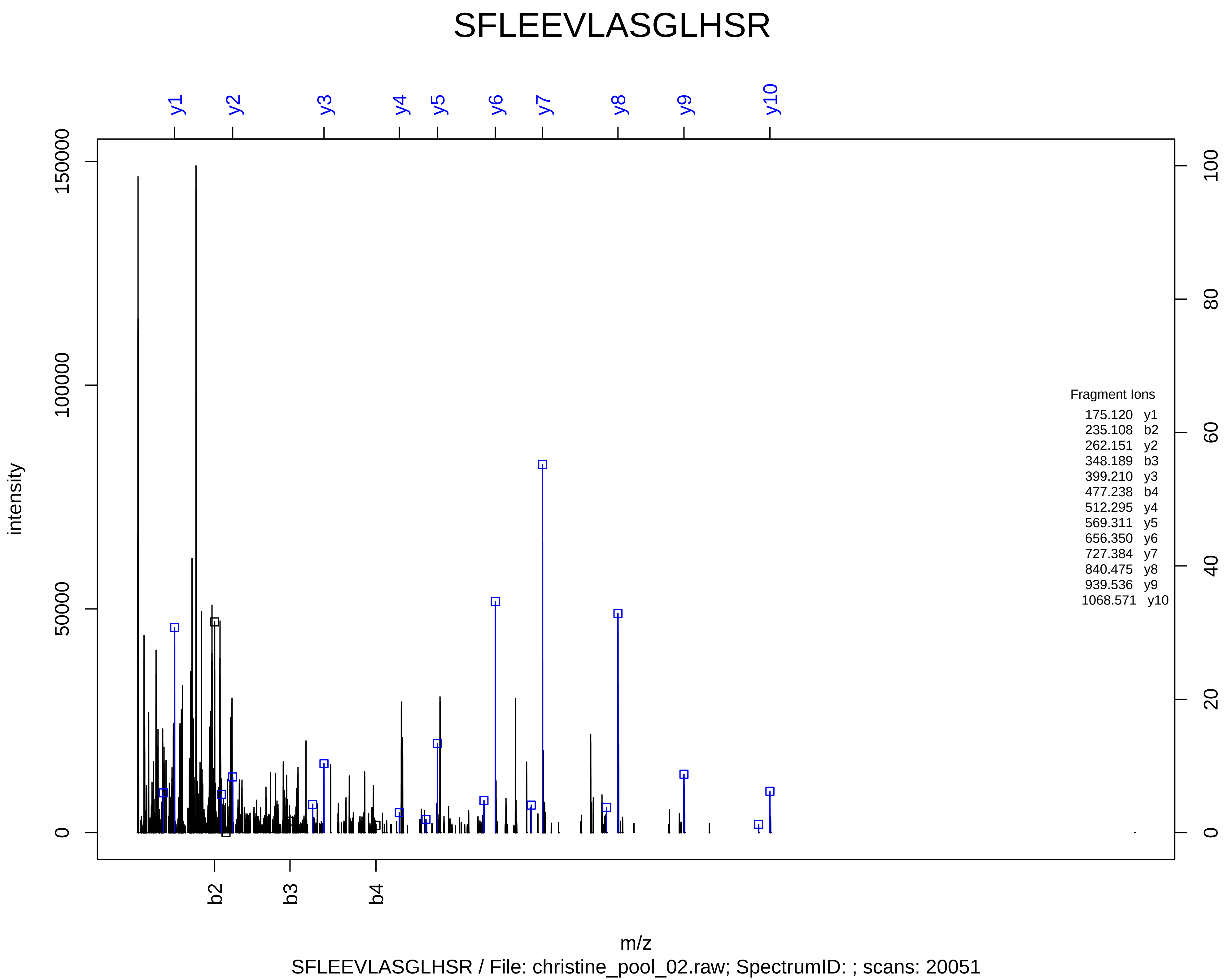

SFLEEVLASGLHSR
y1
y2
y3
y4
y5
y6
y7
y8
y9
y10
b2
b3
b4
intensity
m/z
0
50000
100000
150000
0
20
40
60
80
100
Fragment Ions
175.120 y1
235.108 b2
262.151 y2
348.189 b3
399.210 y3
477.238 b4
512.295 y4
569.311 y5
656.350 y6
727.384 y7
840.475 y8
939.536 y9
1068.571 y10
SFLEEVLASGLHSR / File: christine_pool_02.raw; SpectrumID: ; scans: 20051

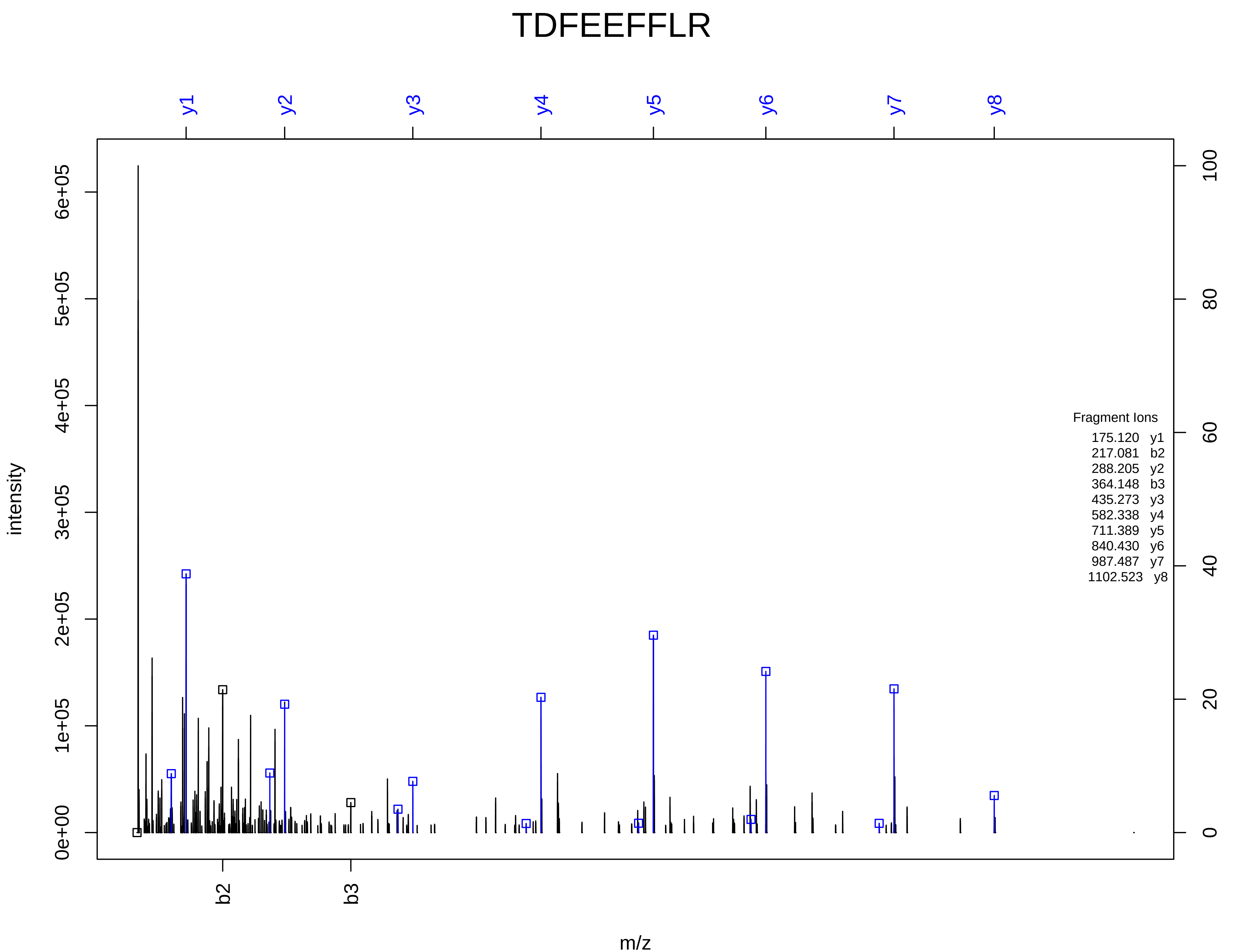

TDFEEEFFLR / File: christine_pool_02.raw; SpectrumID: ; scans: 20400

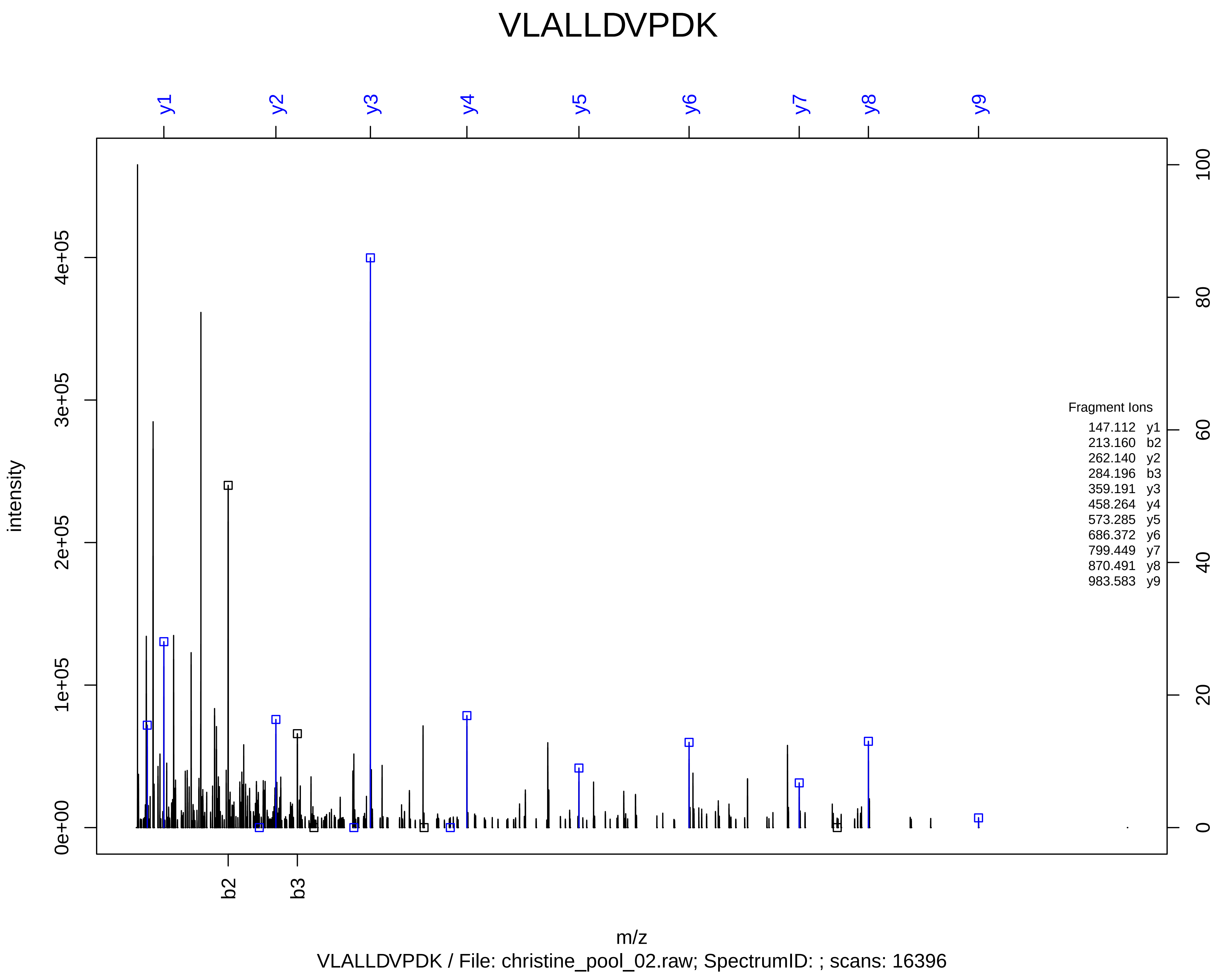

VLALLDVPDK
y1
y2
y3
y4
y5
y6
y7
y8
y9
b2
b3
intensity
0e+00
1e+05
2e+05
3e+05
4e+05
0
20
40
60
80
100
m/z
Fragment Ions
147.112 y1
213.160 b2
262.140 y2
284.196 b3
359.191 y3
458.264 y4
573.285 y5
686.372 y6
799.449 y7
870.491 y8
983.583 y9
VLALLDVPDK / File: christine_pool_02.raw; SpectrumID: ; scans: 16396

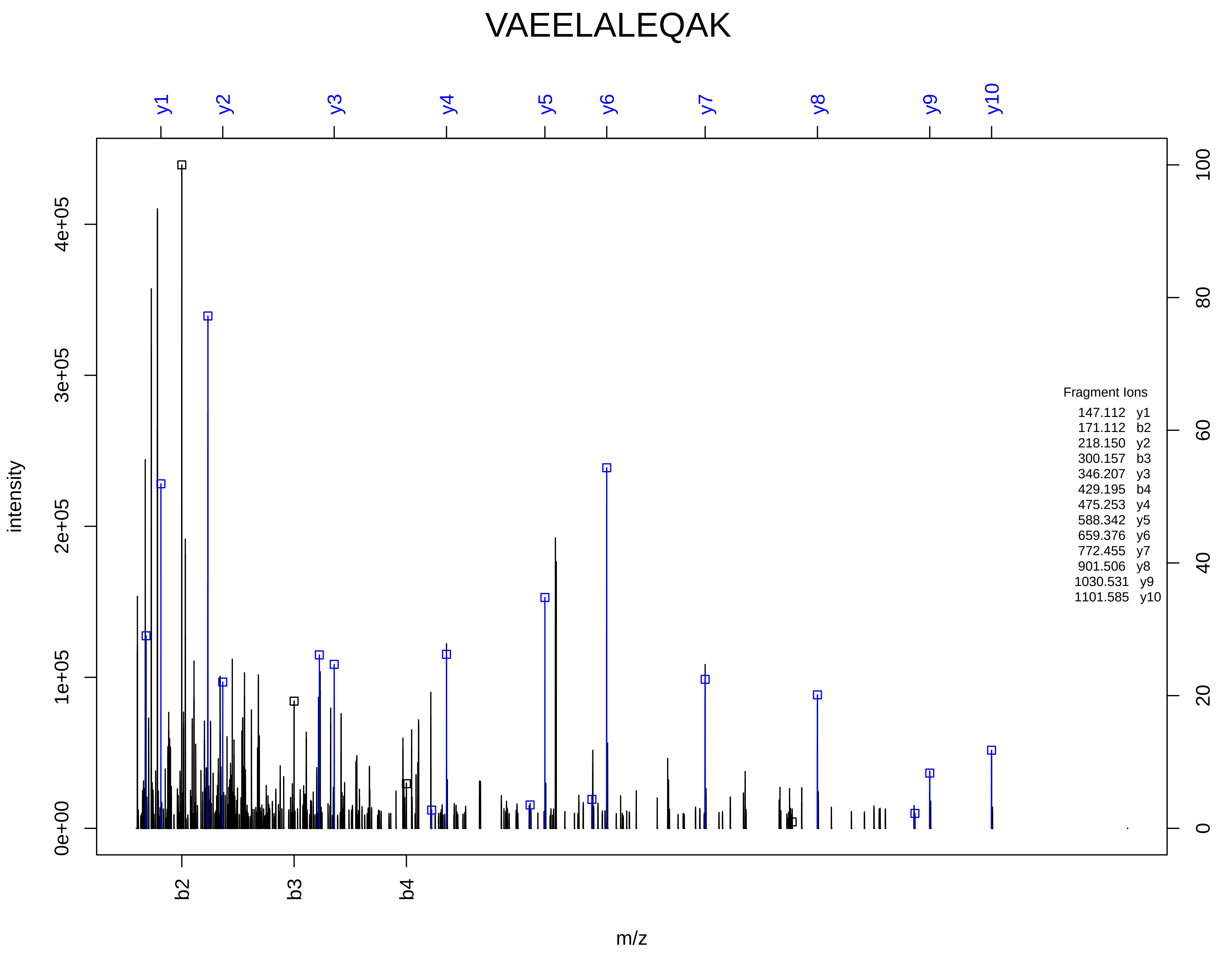

VAEELALEQAK / File: christine_pool_02.raw; SpectrumID: ; scans: 7753

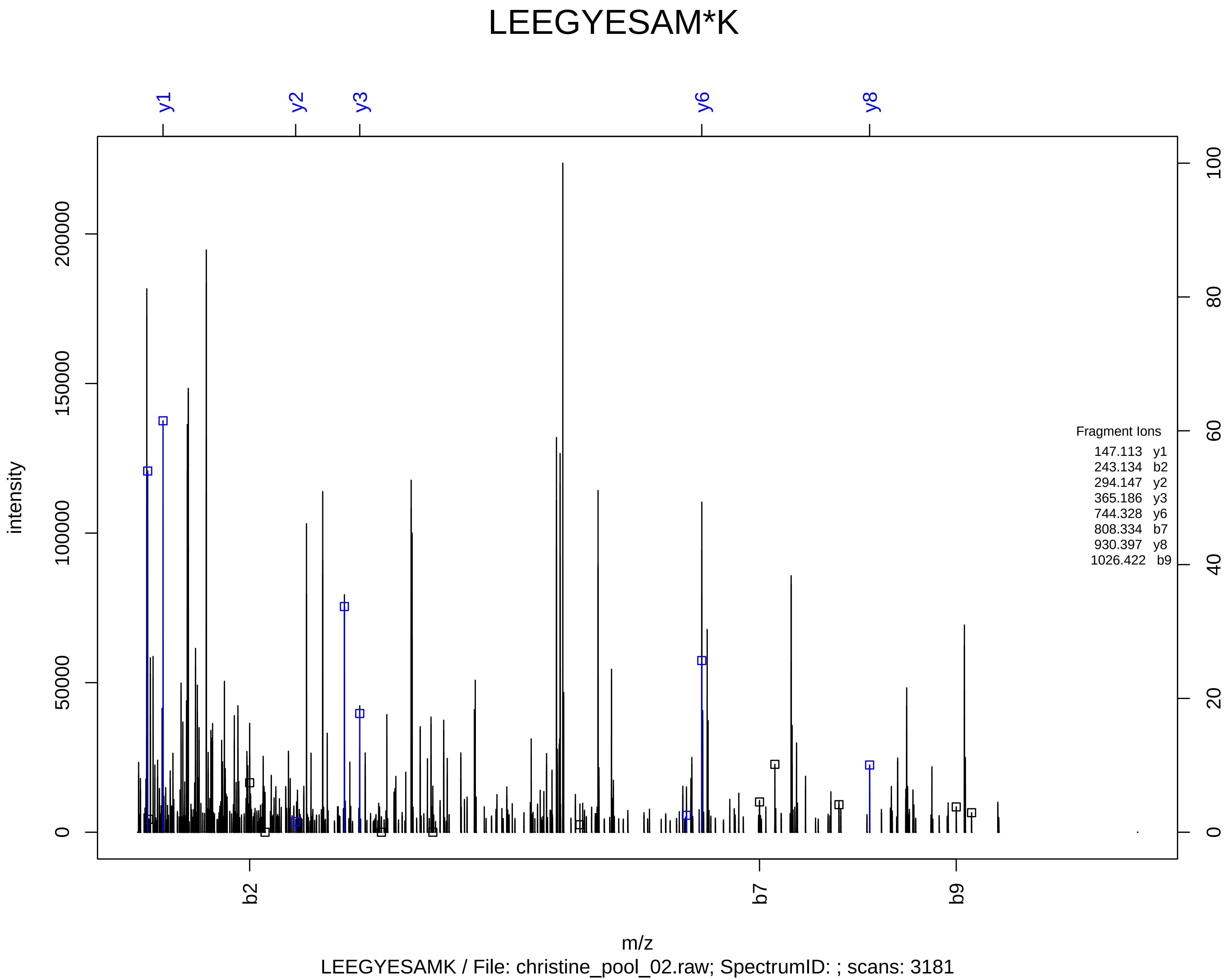
LEEGYESAM*K
y1
y2
y3
y6
y8
b2
b7
b9
intensity
0
50000
100000
150000
200000
0
20
40
60
80
100
m/z
Fragment Ions
147.113 y1
243.134 b2
294.147 y2
365.186 y3
744.328 y6
808.334 b7
930.397 y8
1026.422 b9
LEEGYESAMK / File: christine_pool_02.raw; SpectrumID: ; scans: 3181

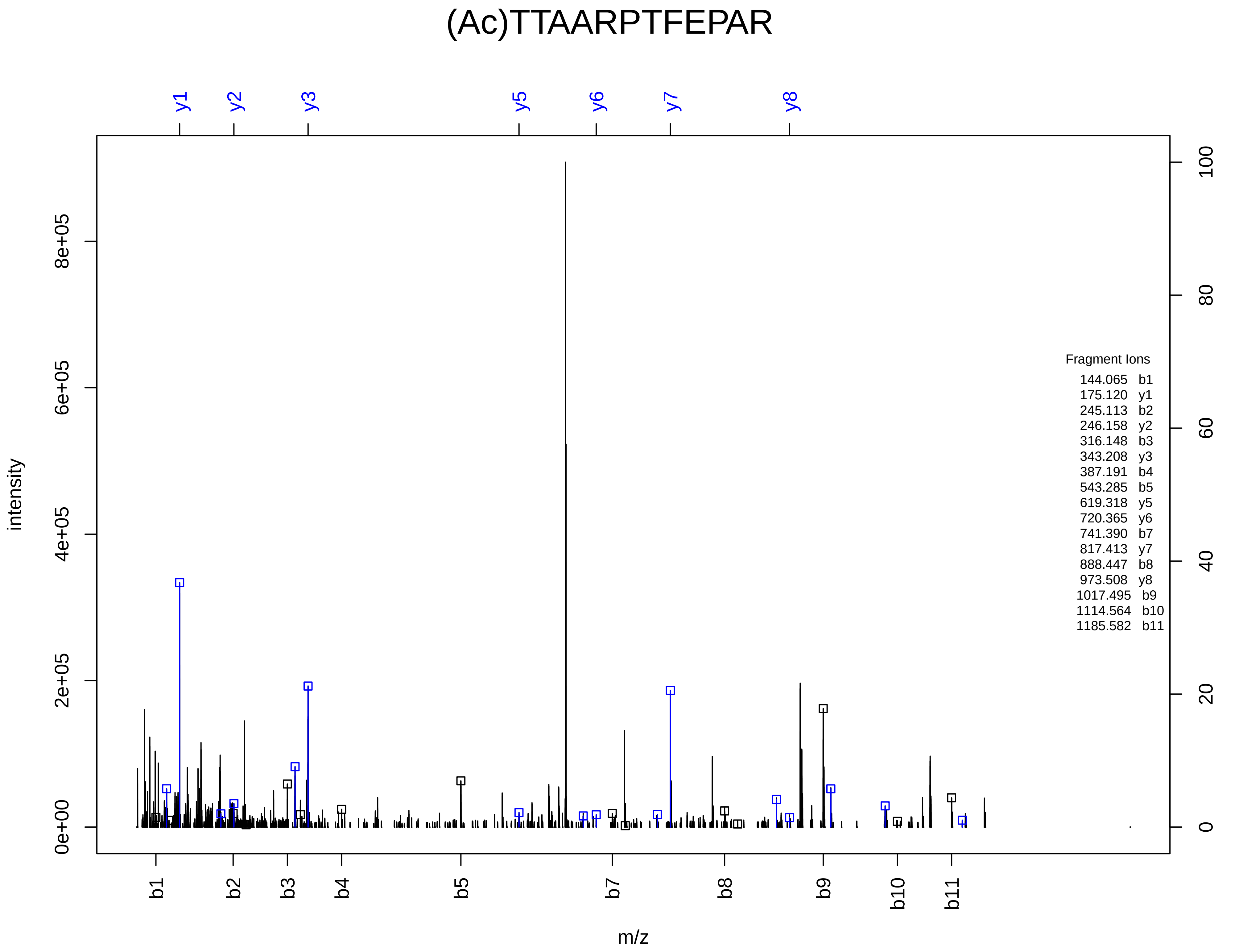

TTAARPTFEPAR / File: christine_pool_02.raw; SpectrumID: ; scans: 6740

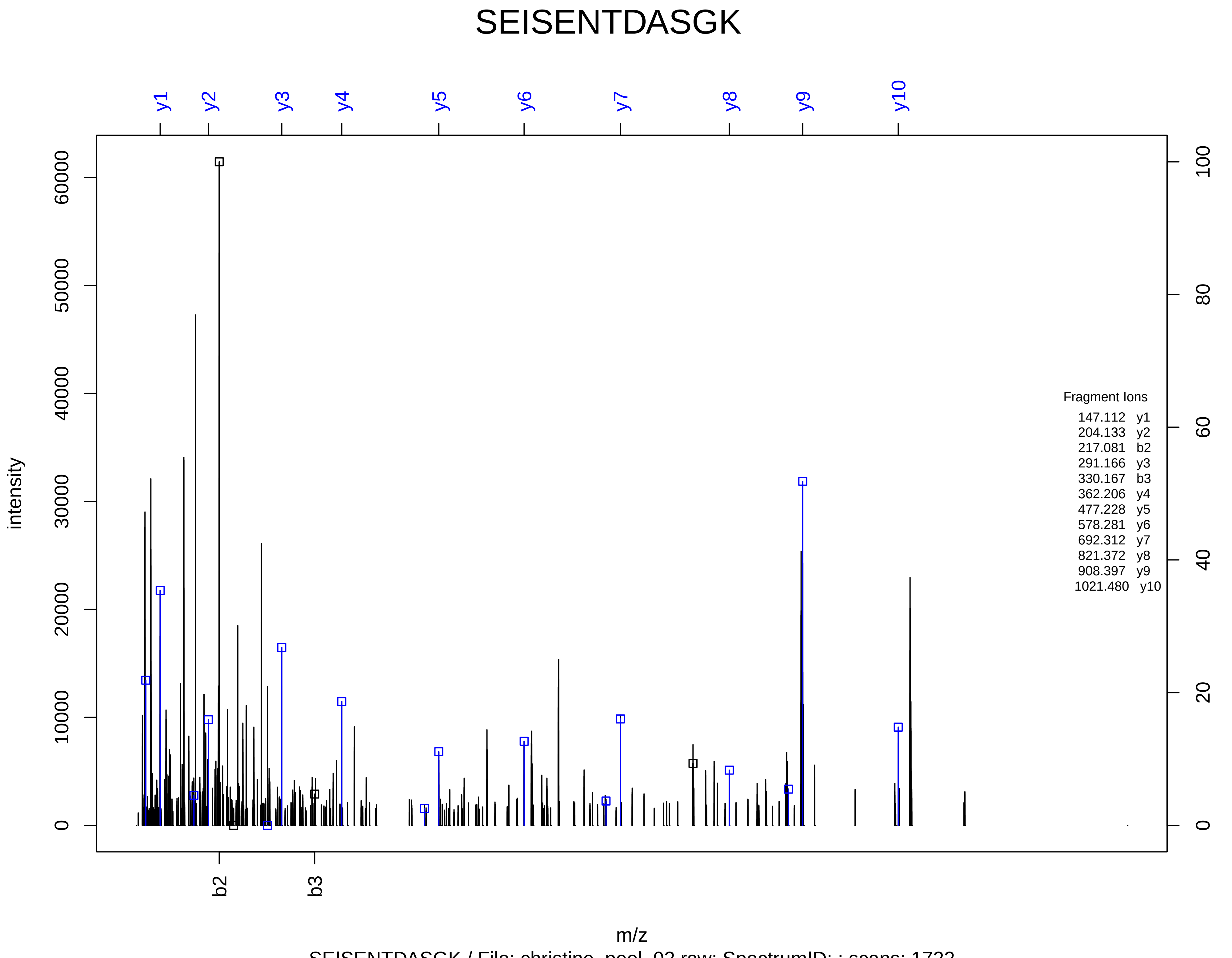
SEISENTDASGK
y1
y2
y3
y4
y5
y6
y7
y8
y9
y10
intensity
60000
50000
40000
30000
20000
10000
0
100
80
60
40
20
0
Fragment Ions
147.112 y1
204.133 y2
217.081 b2
291.166 y3
330.167 b3
362.206 y4
477.228 y5
578.281 y6
692.312 y7
821.372 y8
908.397 y9
1021.480 y10
b2
b3
m/z
SEISENTDASGK / File: christine_pool_02.raw; SpectrumID: ; scans: 1722

AVFDNLIQLEHLNIVK

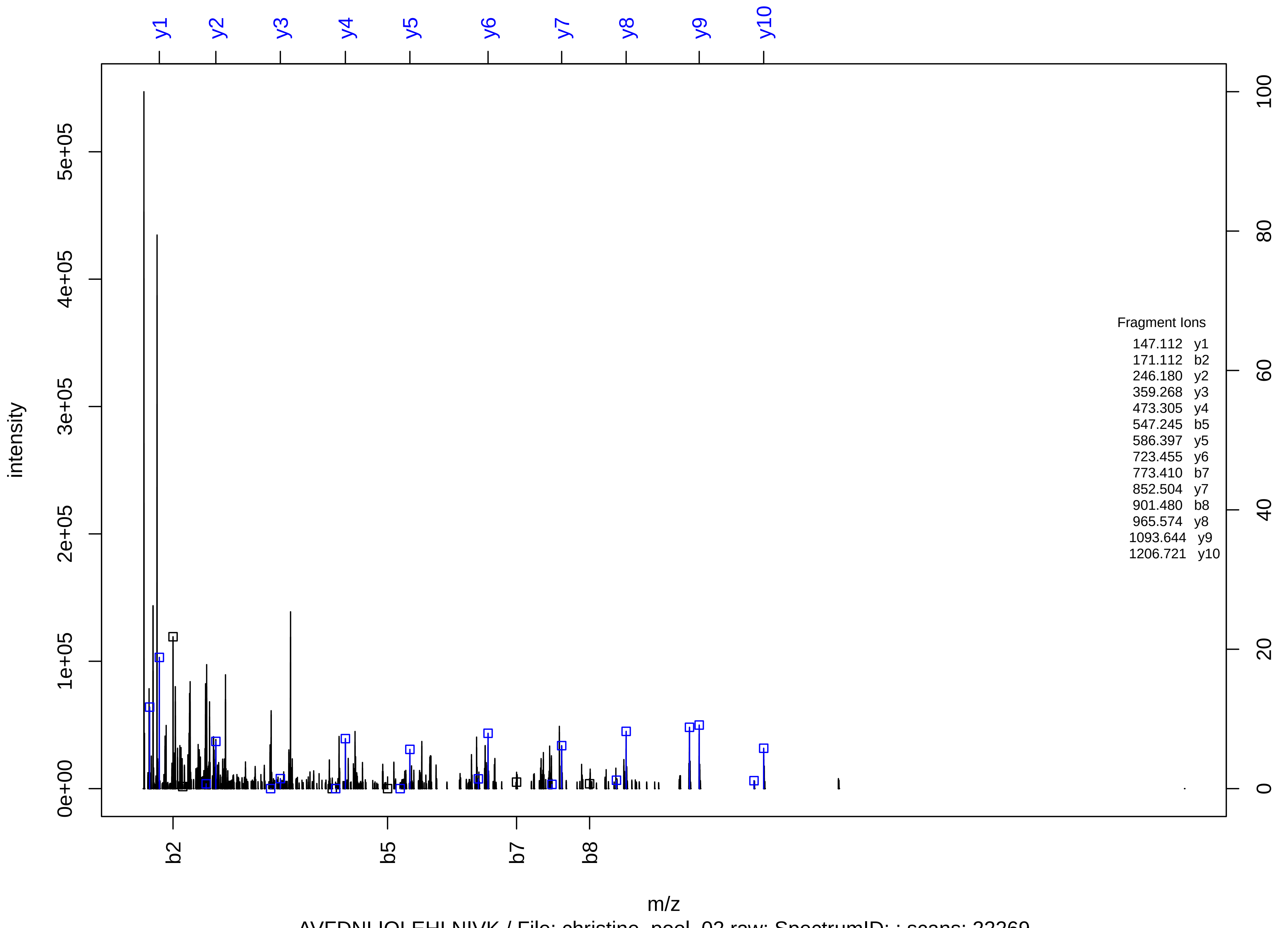

AVFDNLIQLEHLNIVK / File: christine_pool_02.raw; SpectrumID: ; scans: 22269

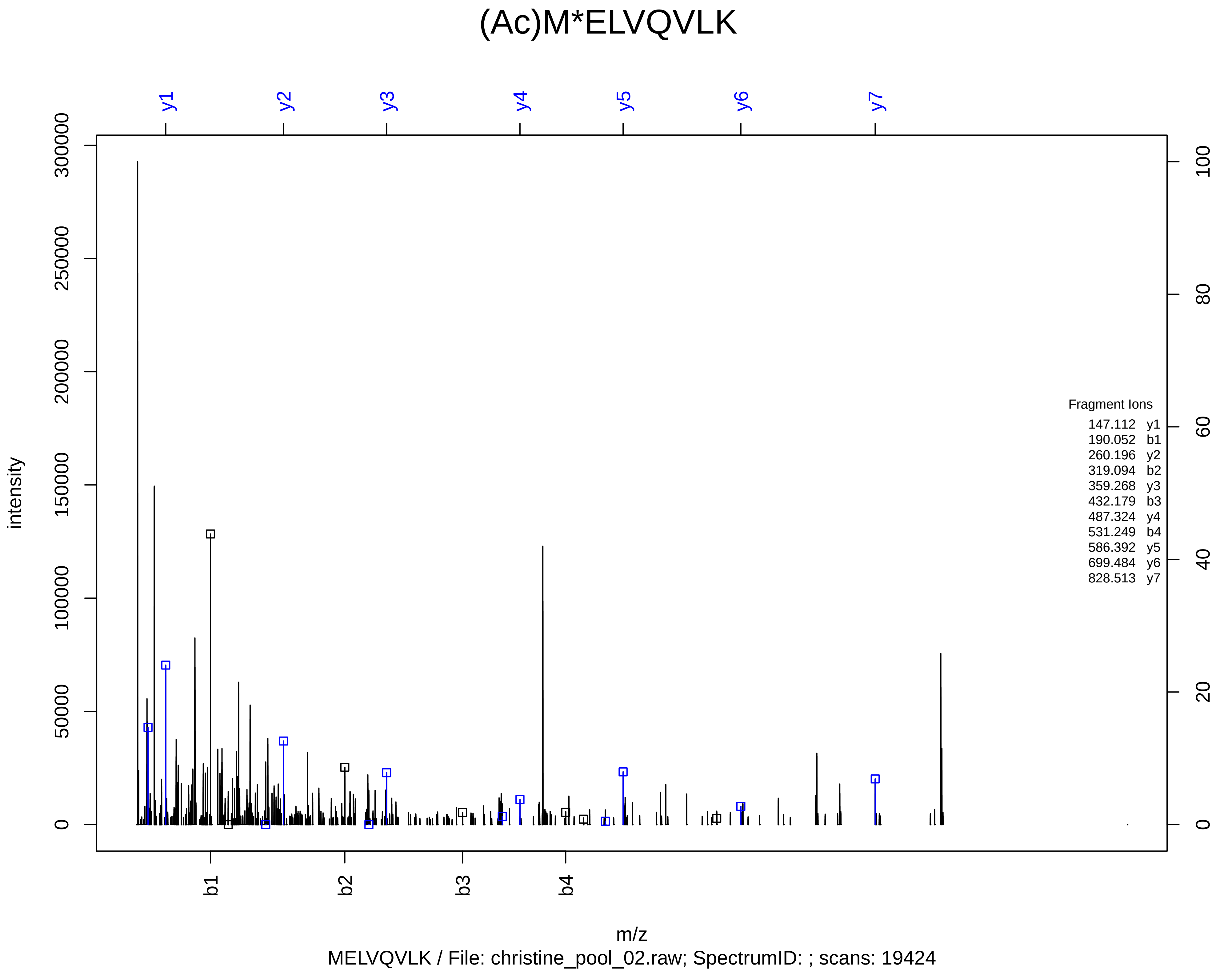
(Ac)M*ELVQVLK
y1
y2
y3
y4
y5
y6
y7
b1
b2
b3
b4
intensity
m/z
0
50000
100000
150000
200000
250000
300000
20
40
60
80
100
Fragment Ions
147.112 y1
190.052 b1
260.196 y2
319.094 b2
359.268 y3
432.179 b3
487.324 y4
531.249 b4
586.392 y5
699.484 y6
828.513 y7
MELVQVLK / File: christine_pool_02.raw; SpectrumID: ; scans: 19424

LINQVLELQHTLEDLSAR

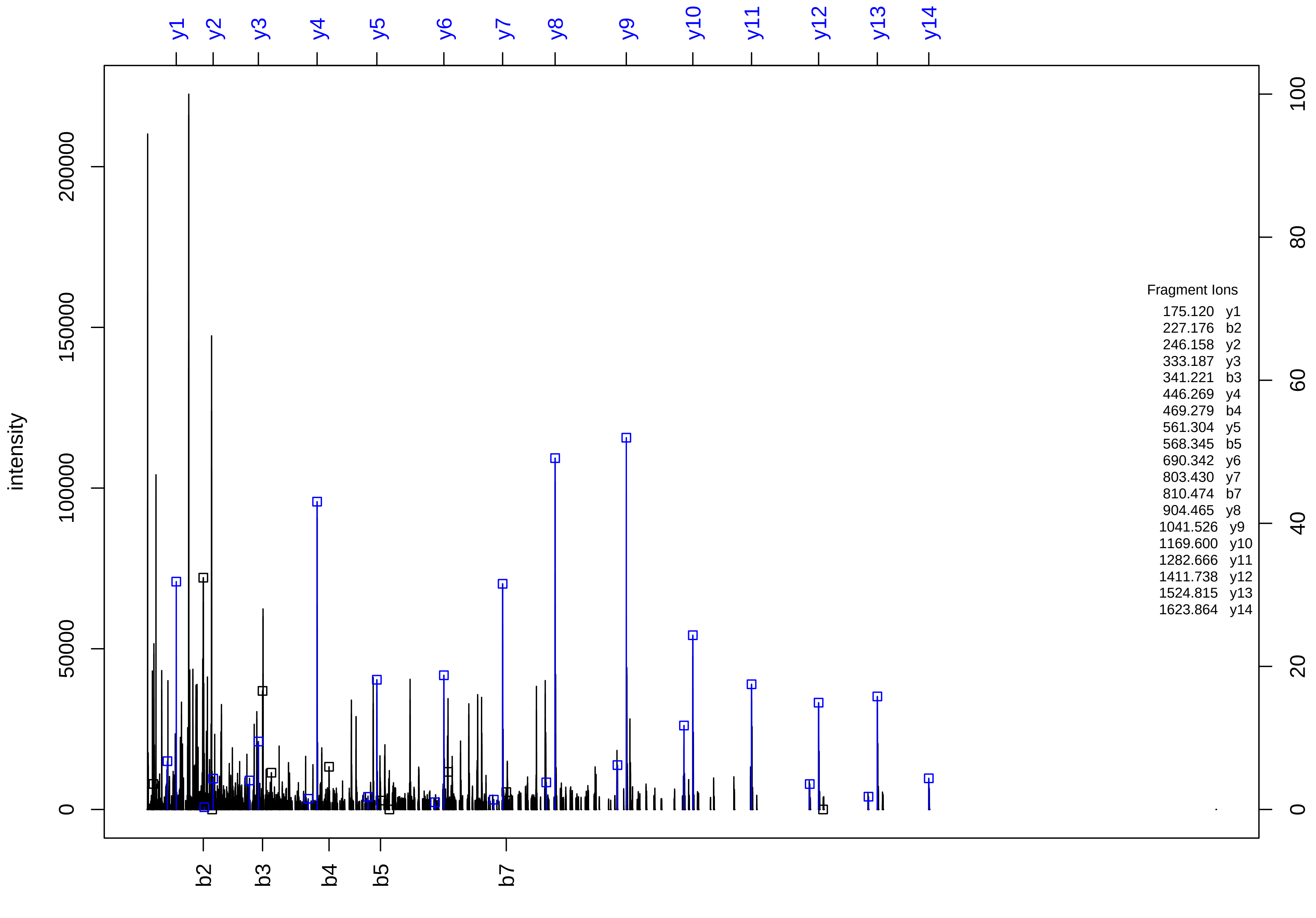

LINQVLELQHTLEDLSAR / File: christine_pool_02.raw; SpectrumID: ; scans: 22934

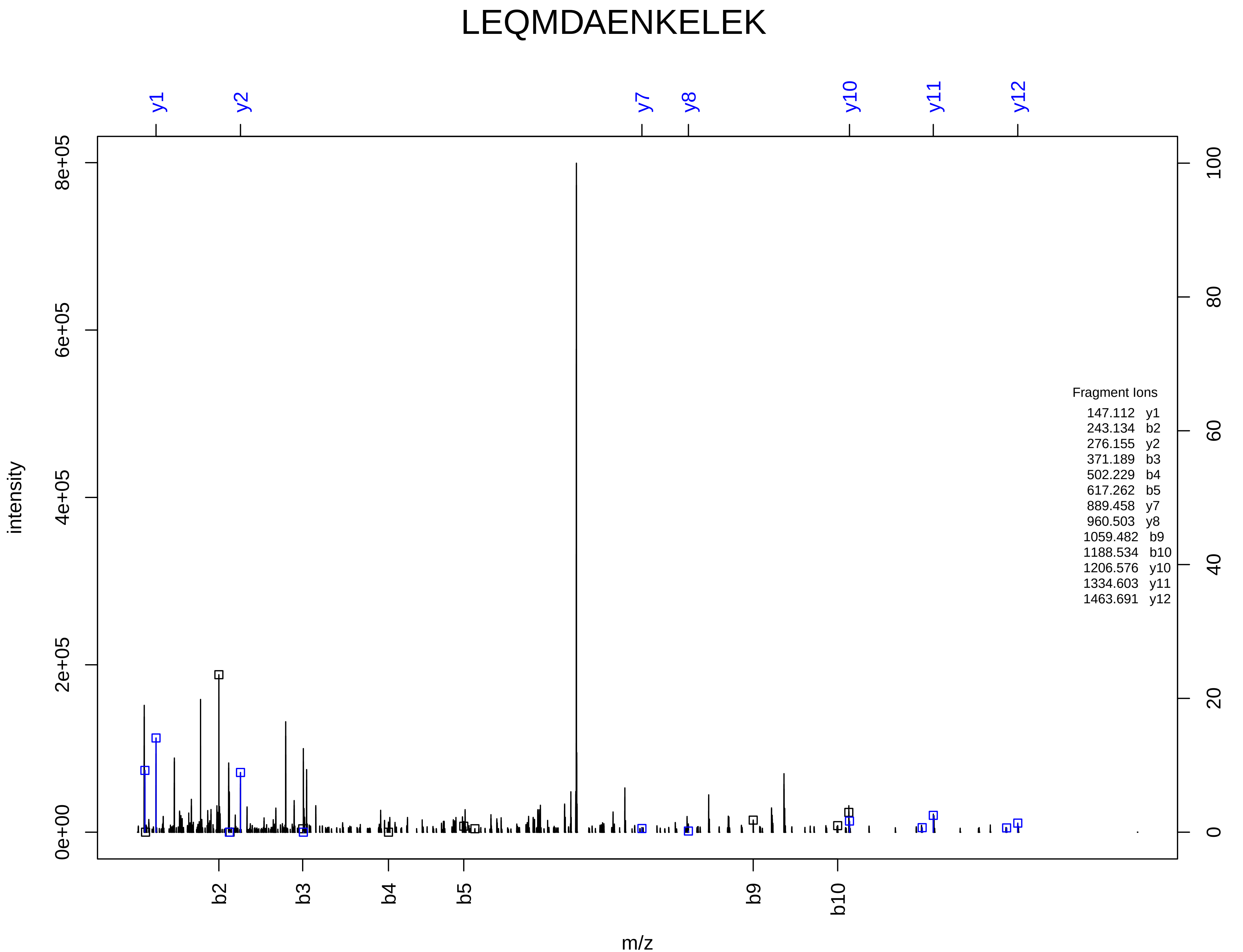

LEQMDAENKELEK / File: christine_pool_02.raw; SpectrumID: ; scans: 4266

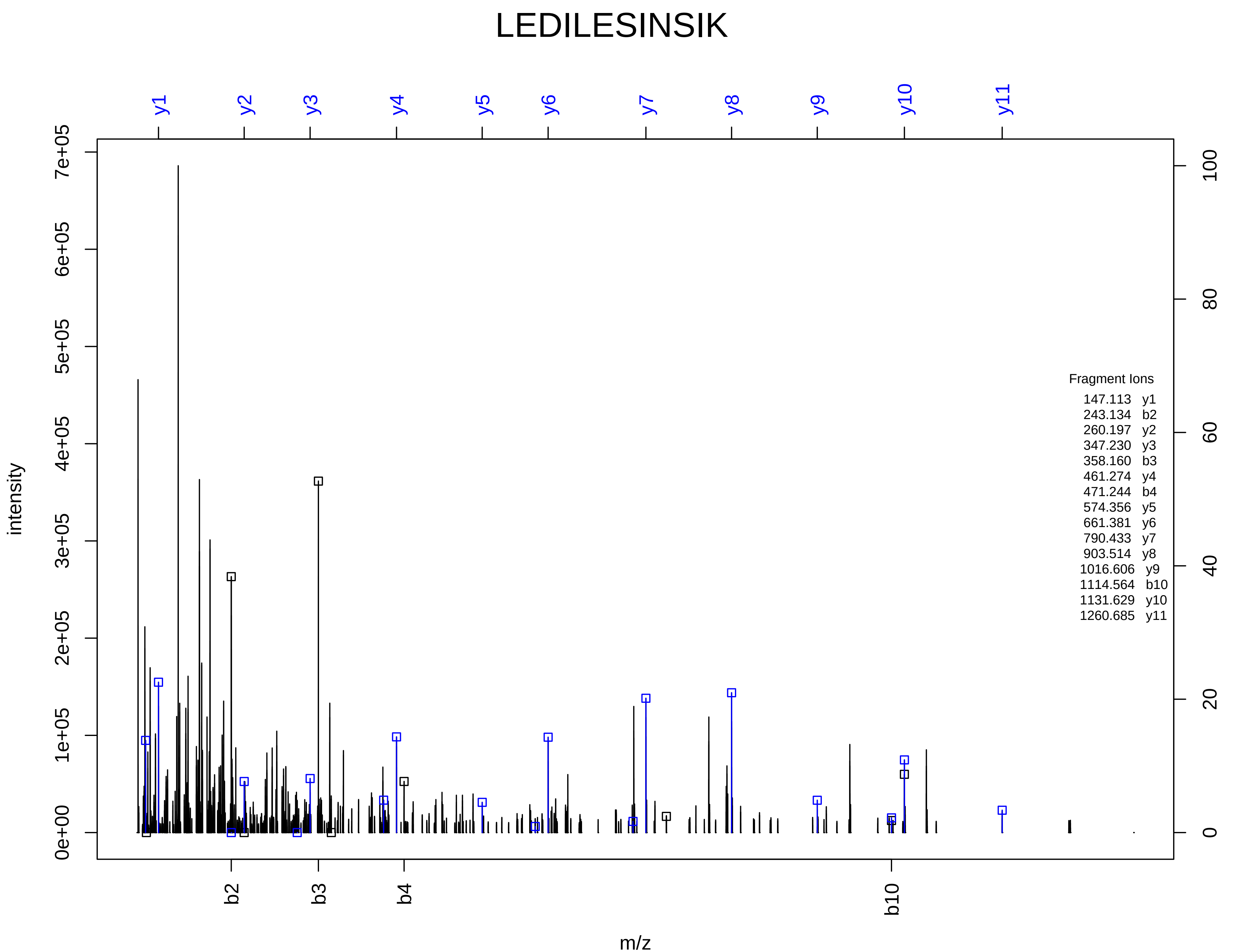

LEDILESINSIK
y1
y2
y3
y4
y5
y6
y7
y8
y9
y10
y11
intensity
0e+00
1e+05
2e+05
3e+05
4e+05
5e+05
6e+05
7e+05
0
20
40
60
80
100
b2
b3
b4
b10
m/z
Fragment Ions
147.113 y1
243.134 b2
260.197 y2
347.230 y3
358.160 b3
461.274 y4
471.244 b4
574.356 y5
661.381 y6
790.433 y7
903.514 y8
1016.606 y9
1114.564 b10
1131.629 y10
1260.685 y11
LEDILESINSIK / File: christine_pool_02.raw; SpectrumID: ; scans: 19119

AGLAMPGPPLGPVLGQR

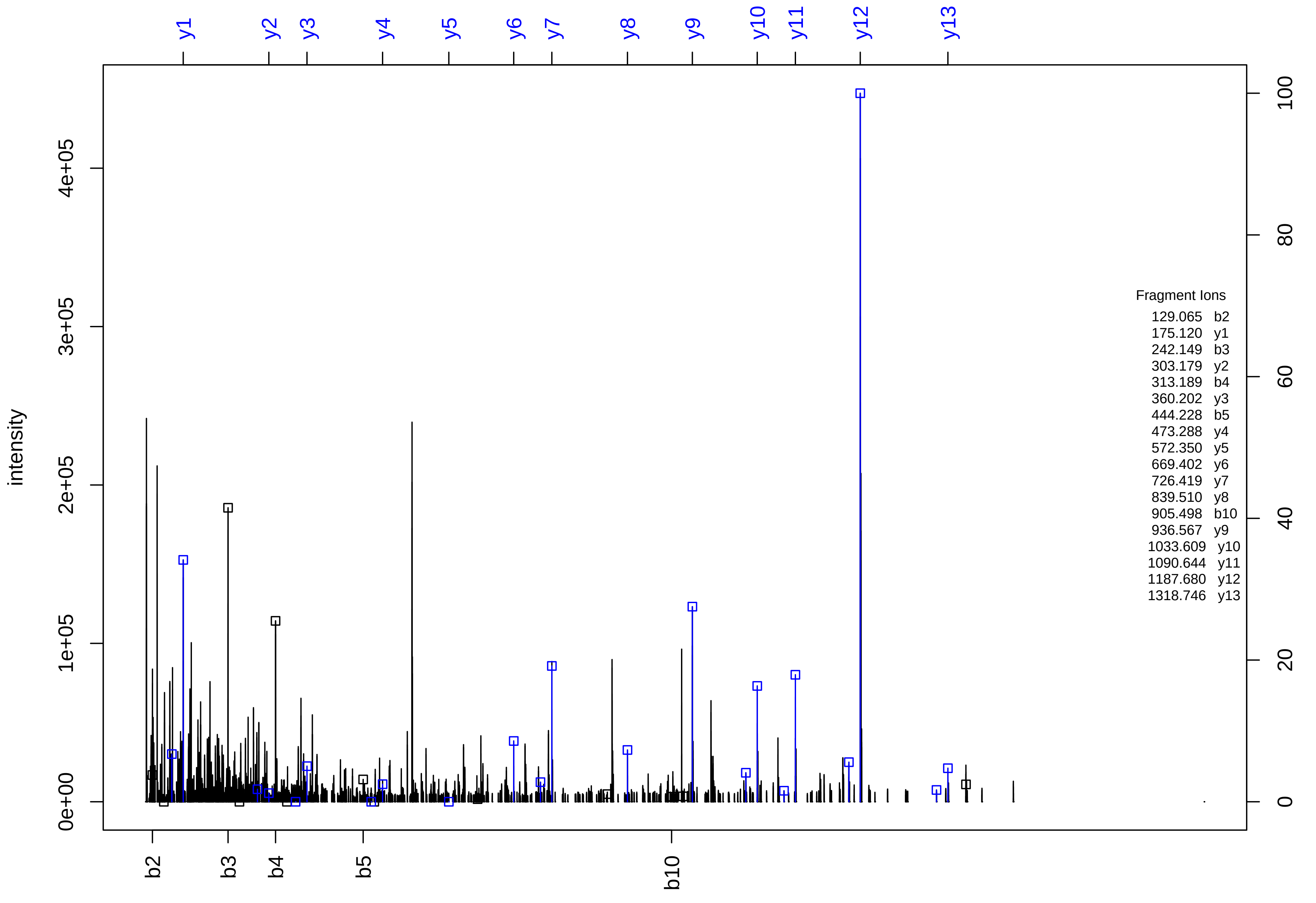

AGLAMPGPPLGPVLGQR / File: christine_pool_02.raw; SpectrumID: ; scans: 19524

APTADTQTQNVNQAK

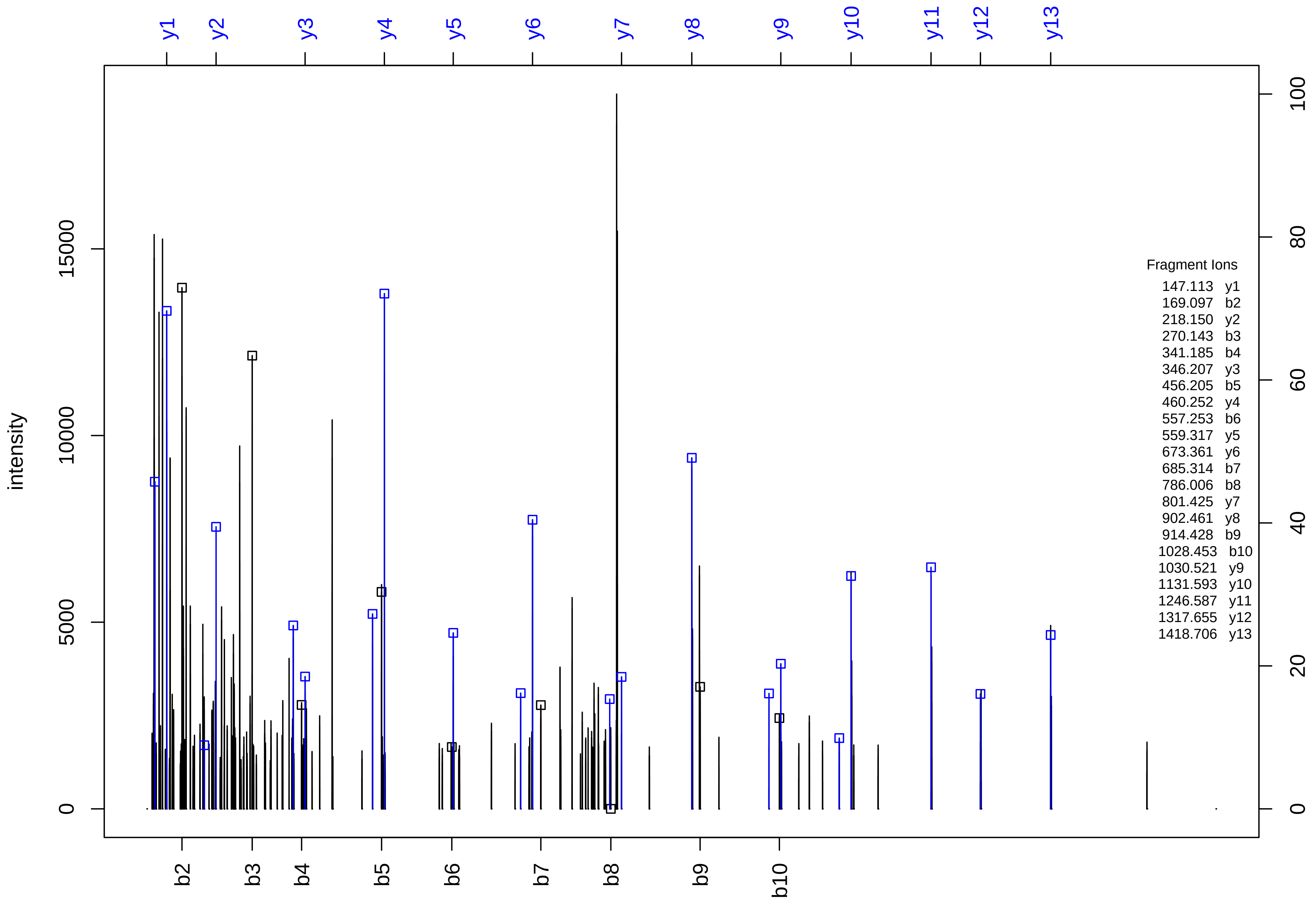

APTADTQTQNVNQAK / File: christine_pool_02.raw; SpectrumID: ; scans: 1992

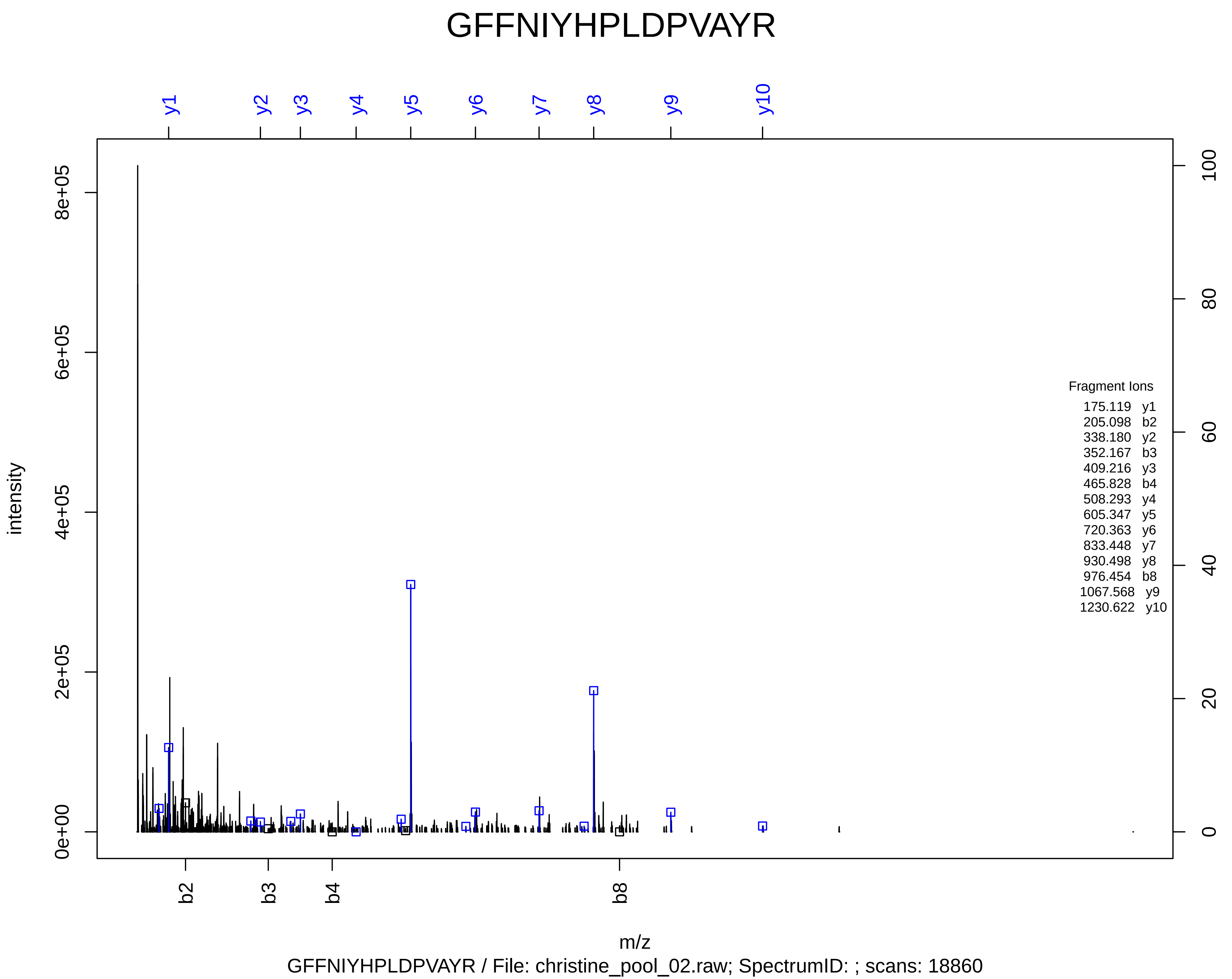
GFFNIYHPLDPVAYR
y1
y2
y3
y4
y5
y6
y7
y8
y9
y10
intensity
0e+00
2e+05
4e+05
6e+05
8e+05
0
20
40
60
80
100
b2
b3
b4
b8
m/z
Fragment Ions
175.119 y1
205.098 b2
338.180 y2
352.167 b3
409.216 y3
465.828 b4
508.293 y4
605.347 y5
720.363 y6
833.448 y7
930.498 y8
976.454 b8
1067.568 y9
1230.622 y10
GFFNIYHPLDPVAYR / File: christine_pool_02.raw; SpectrumID: ; scans: 18860

SM*GKTSNK

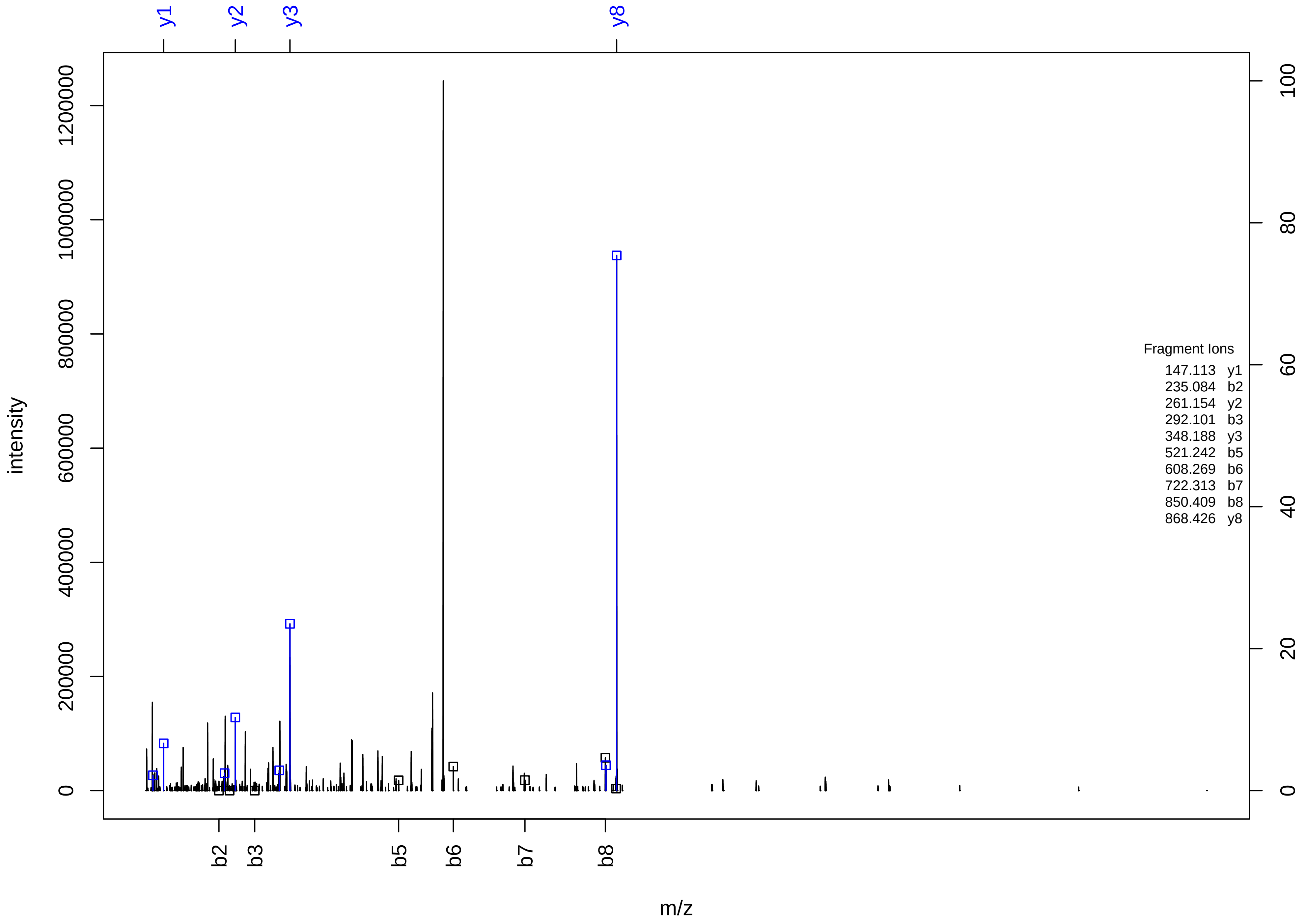

SMGKTSNK / File: christine_pool_02.raw; SpectrumID: ; scans: 6892

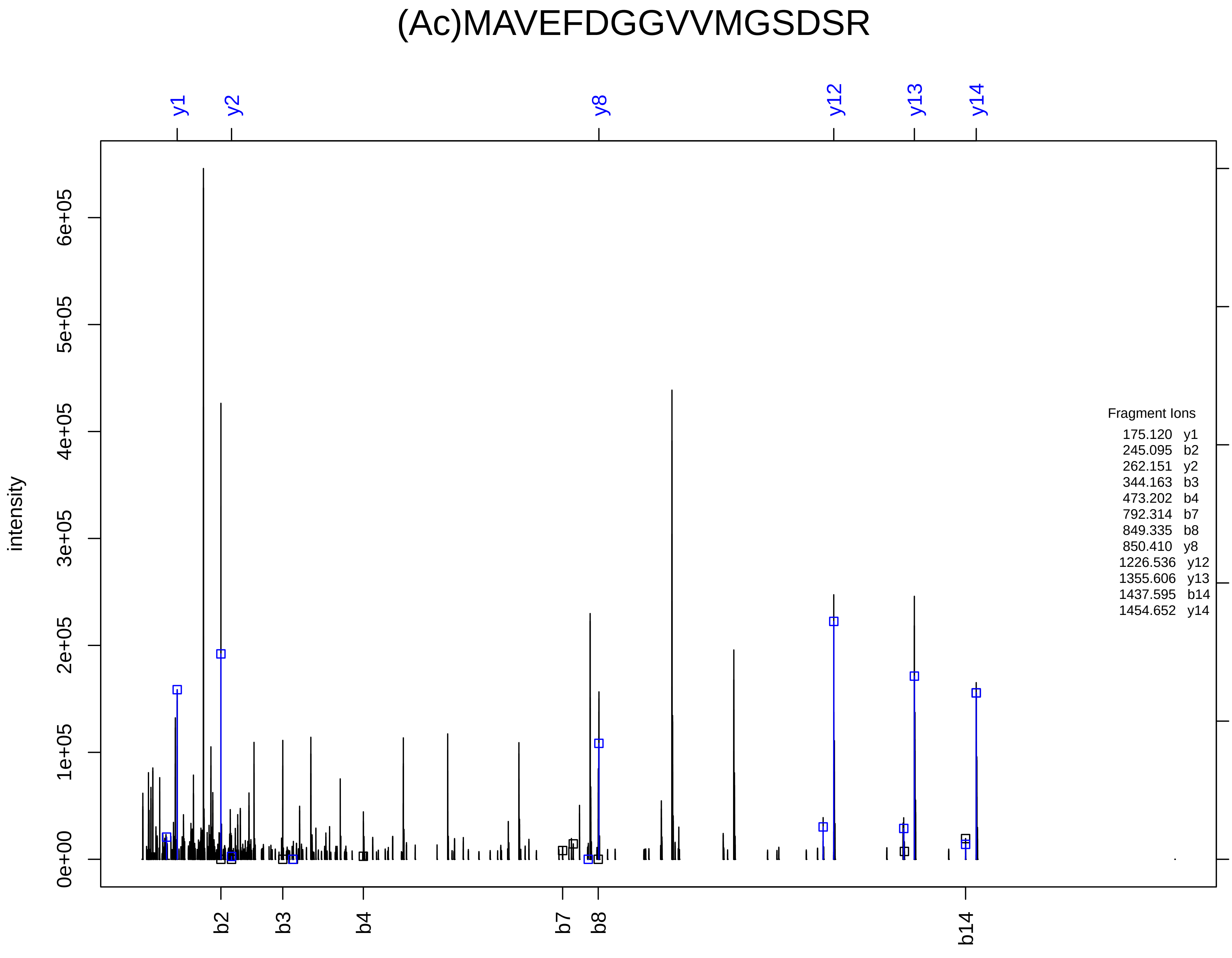
(Ac)MAVEFDGGVVMGSDSR
y1
y2
y8
y12
y13
y14
intensity
0e+00
1e+05
2e+05
3e+05
4e+05
5e+05
6e+05
0
20
40
60
80
100
Fragment Ions
175.120 y1
245.095 b2
262.151 y2
344.163 b3
473.202 b4
792.314 b7
849.335 b8
850.410 y8
1226.536 y12
1355.606 y13
1437.595 b14
1454.652 y14
b2
b3
b4
b7
b8
b14
m/z
MAVEFDGGVVMGSDSR / File: christine_pool_02.raw; SpectrumID: ; scans: 11373

DSSQGPCEPLPGPLTQPR

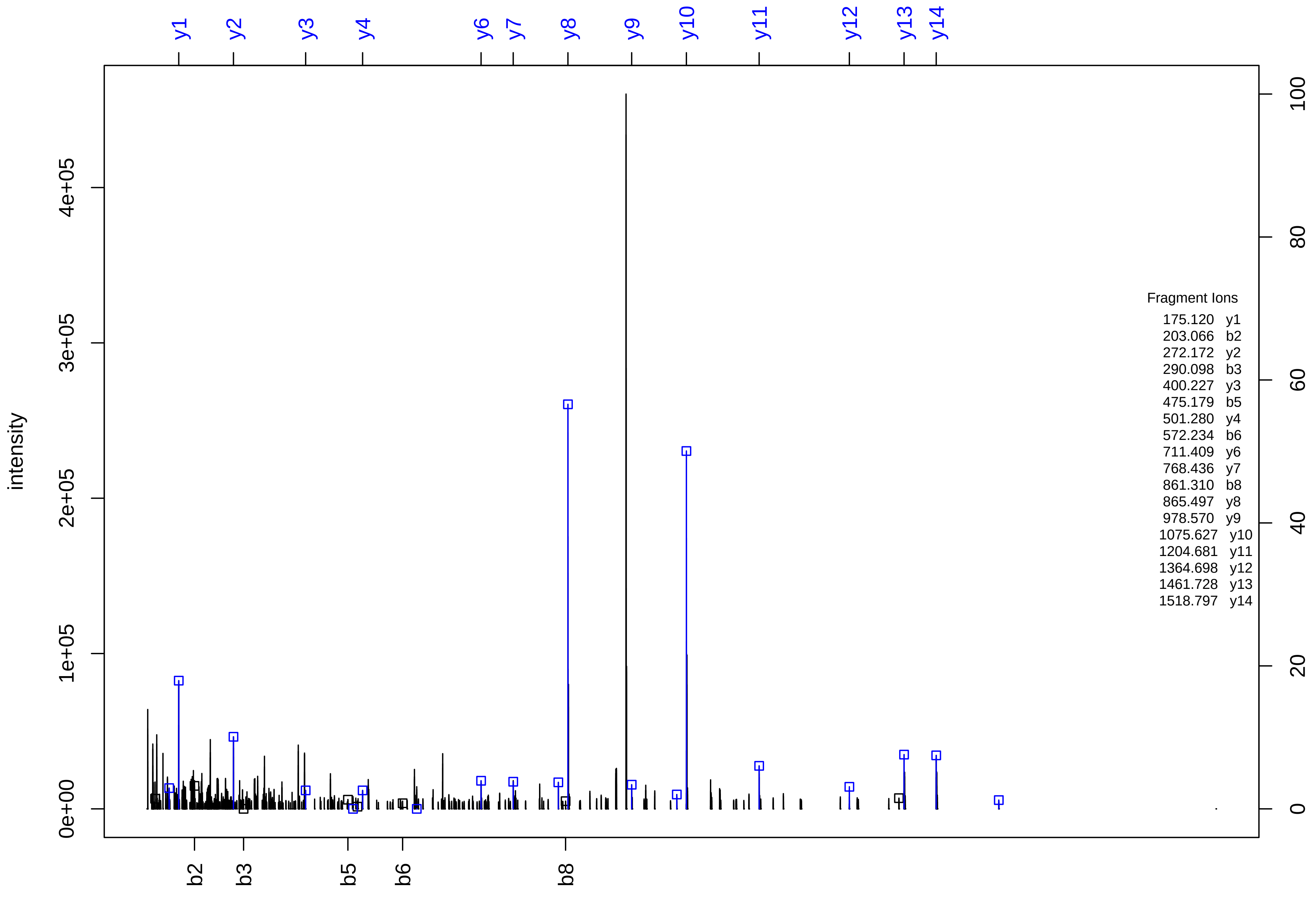

DSSQGPCEPLPGPLTQPR / File: christine_pool_02.raw; SpectrumID: ; scans: 12489

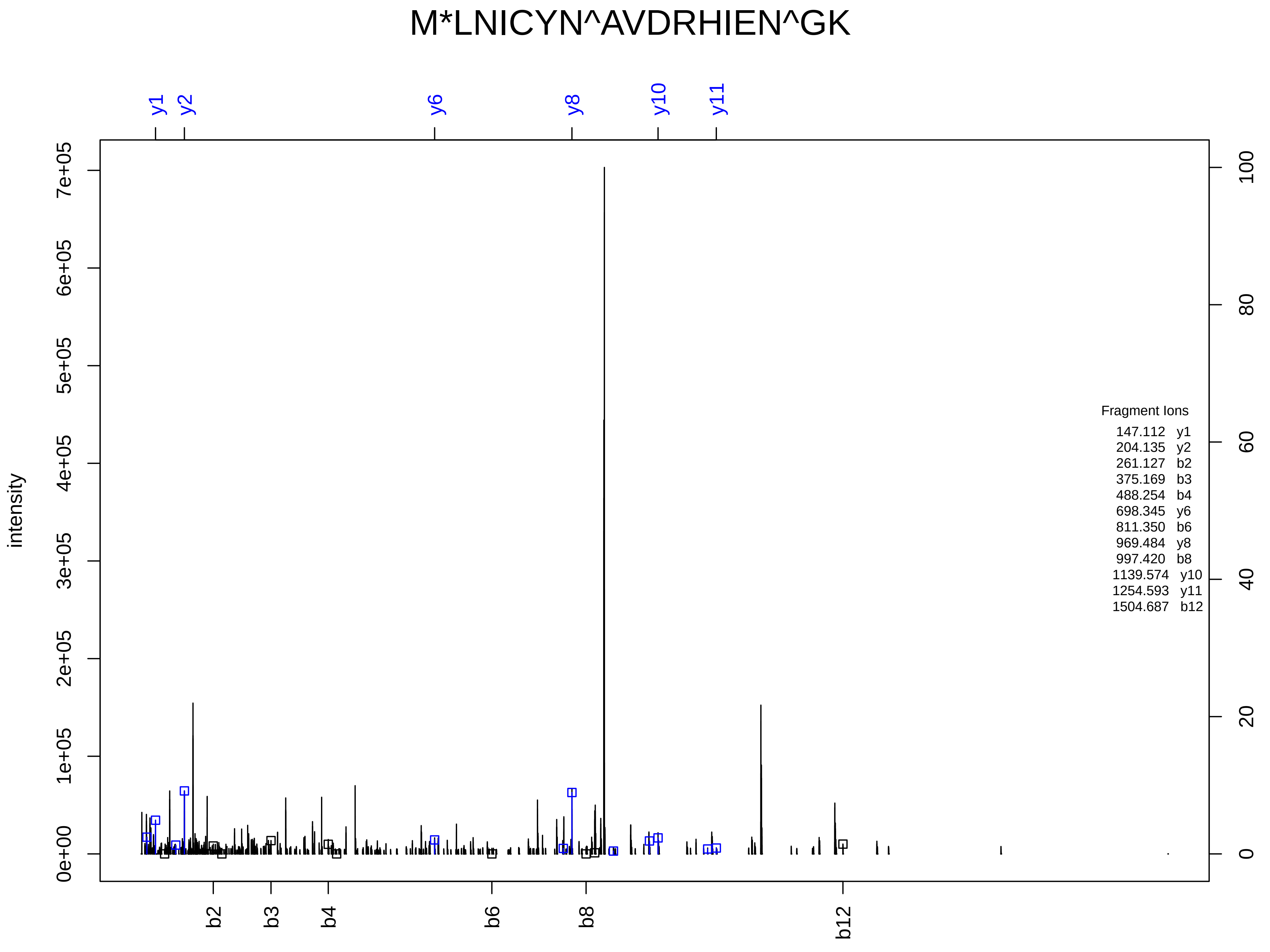

MLNICYNAVDRHIENGK / File: christine_pool_02.raw; SpectrumID: ; scans: 10528

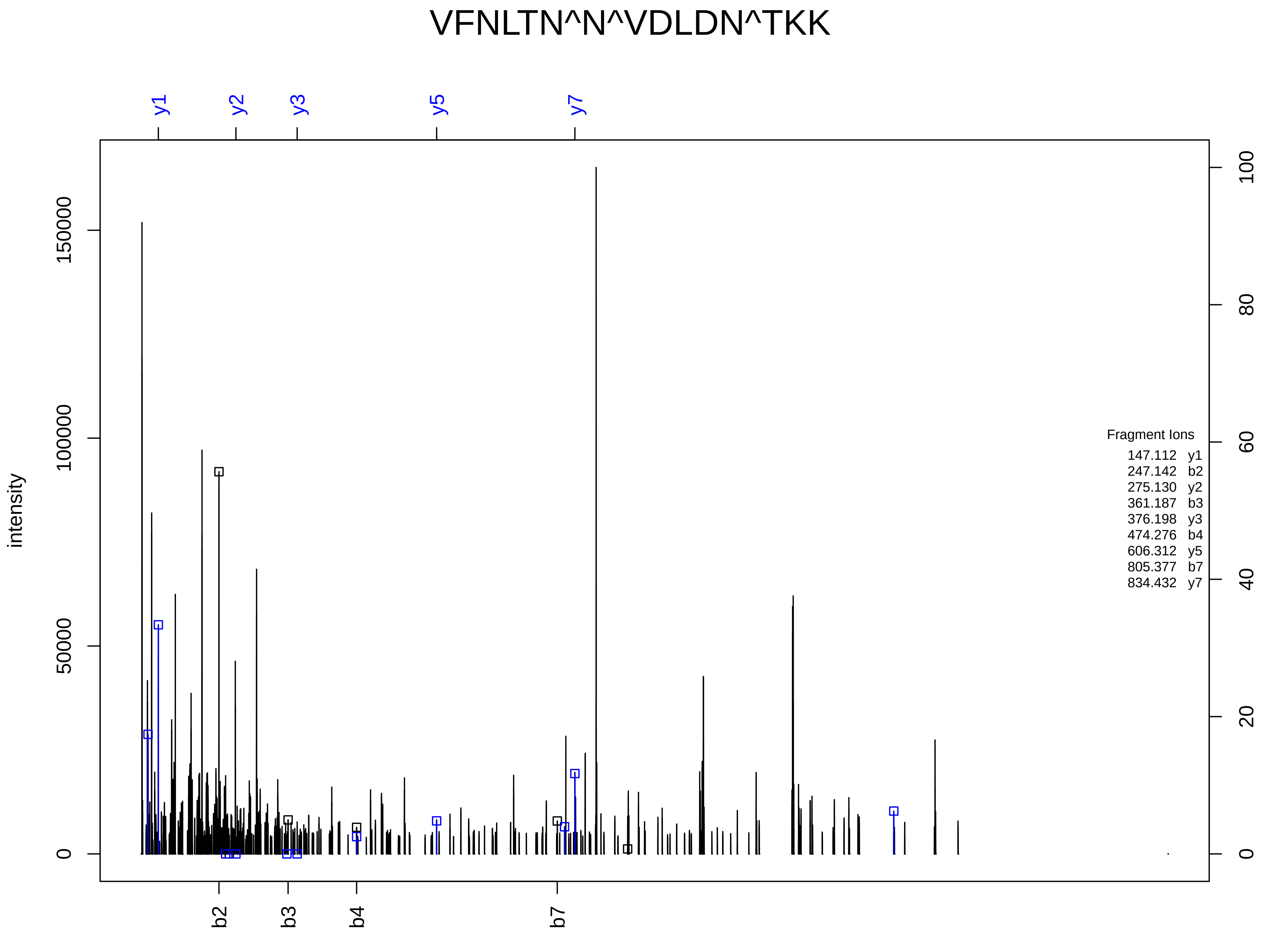

VFNLTNNVDLDNTKK / File: christine_pool_02.raw; SpectrumID: ; scans: 12300

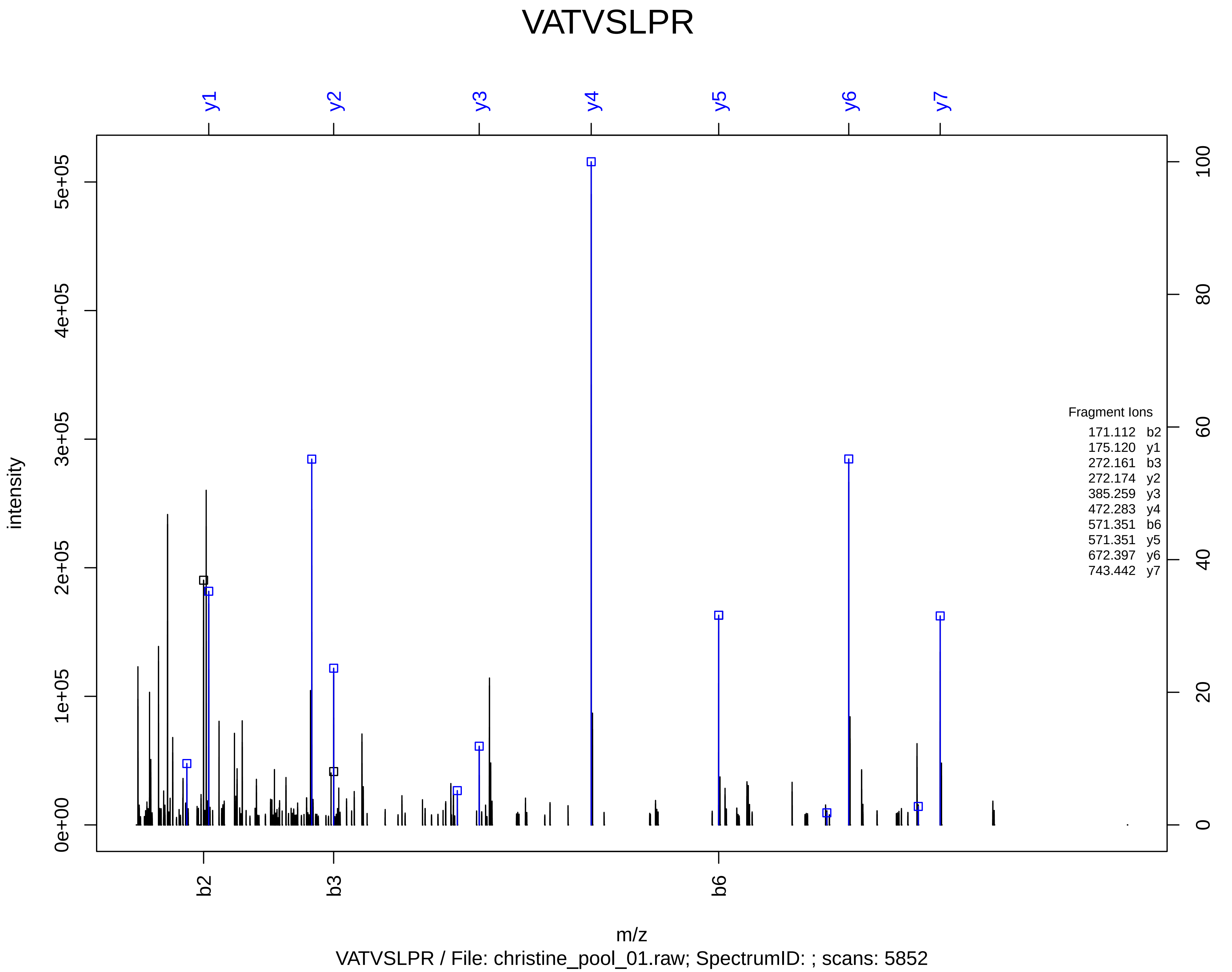

VATVSLPR
y1
y2
y3
y4
y5
y6
y7
intensity
0e+00
1e+05
2e+05
3e+05
4e+05
5e+05
0
20
40
60
80
100
Fragment Ions
171.112 b2
175.120 y1
272.161 b3
272.174 y2
385.259 y3
472.283 y4
571.351 b6
571.351 y5
672.397 y6
743.442 y7
b2
b3
b6
m/z
VATVSLPR / File: christine_pool_01.raw; SpectrumID: ; scans: 5852

MFLNLLSPR

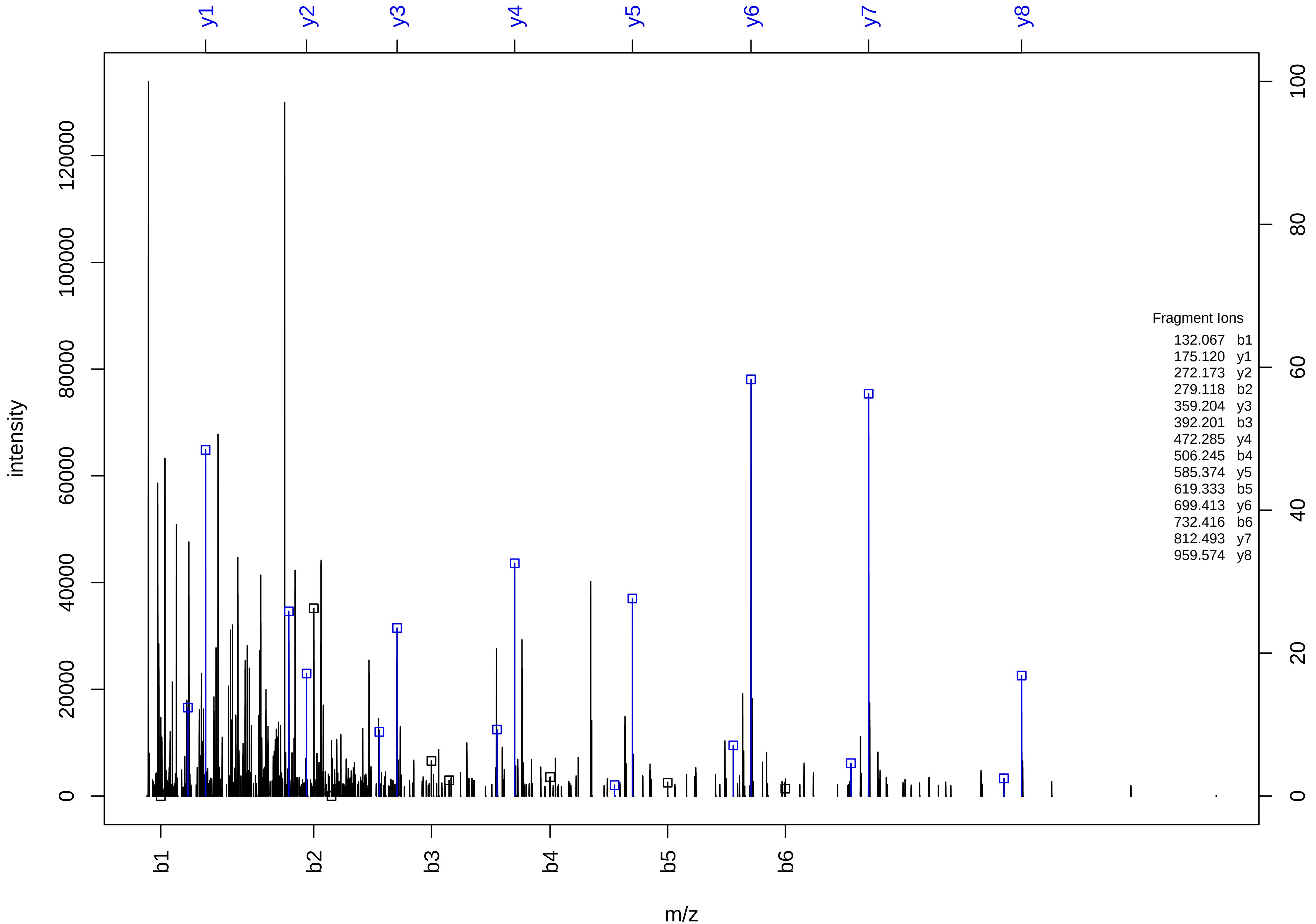

MFLNLLSPR / File: christine_pool_01.raw; SpectrumID: ; scans: 18669

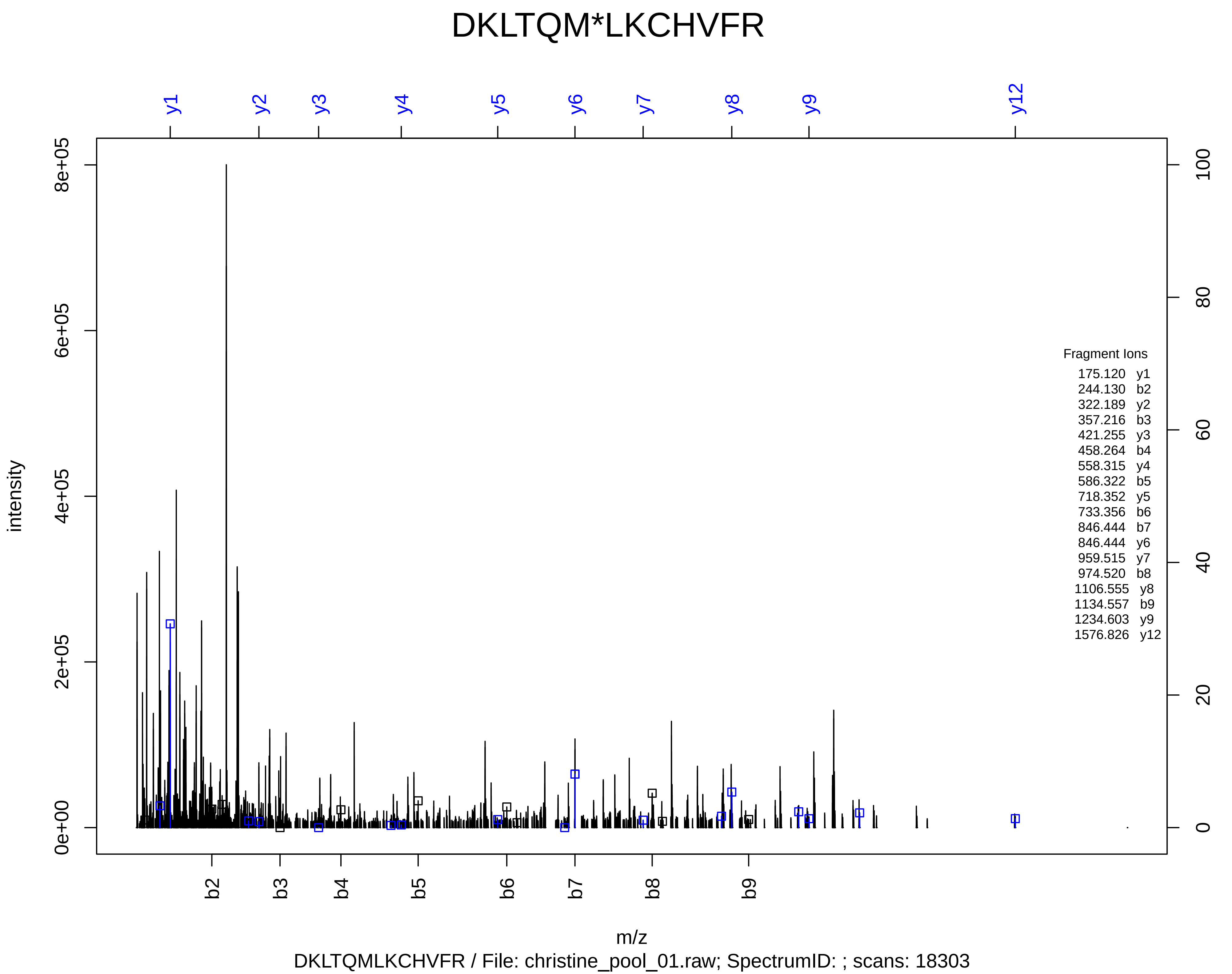
DKLTQM*LKCHVFR
intensity
m/z
y1
y2
y3
y4
y5
y6
y7
y8
y9
y12
b2
b3
b4
b5
b6
b7
b8
b9
0e+00
2e+05
4e+05
6e+05
8e+05
0
20
40
60
80
100
Fragment Ions
175.120 y1
244.130 b2
322.189 y2
357.216 b3
421.255 y3
458.264 b4
558.315 y4
586.322 b5
718.352 y5
733.356 b6
846.444 b7
846.444 y6
959.515 y7
974.520 b8
1106.555 y8
1134.557 b9
1234.603 y9
1576.826 y12
DKLTQMLKCHVFR / File: christine_pool_01.raw; SpectrumID: ; scans: 18303

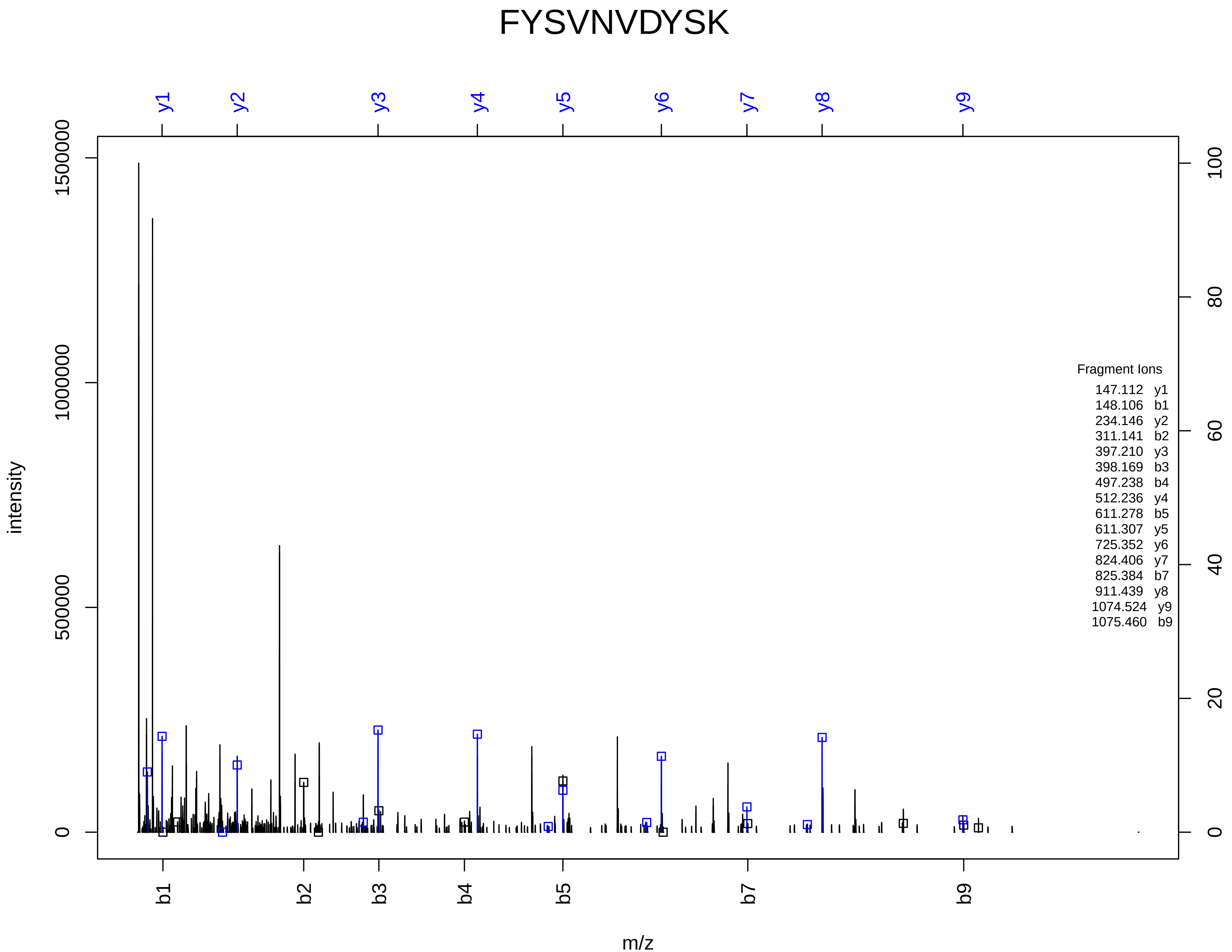
FYSVNVDYSK
y1
y2
y3
y4
y5
y6
y7
y8
y9
intensity
0
500000
1000000
1500000
0
20
40
60
80
100
b1
b2
b3
b4
b5
b7
b9
m/z
Fragment Ions
147.112 y1
148.106 b1
234.146 y2
311.141 b2
397.210 y3
398.169 b3
497.238 b4
512.236 y4
611.278 b5
611.307 y5
725.352 y6
824.406 y7
825.384 b7
911.439 y8
1074.524 y9
1075.460 b9
FYSVNVDYSK / File: christine_pool_01.raw; SpectrumID: ; scans: 7990

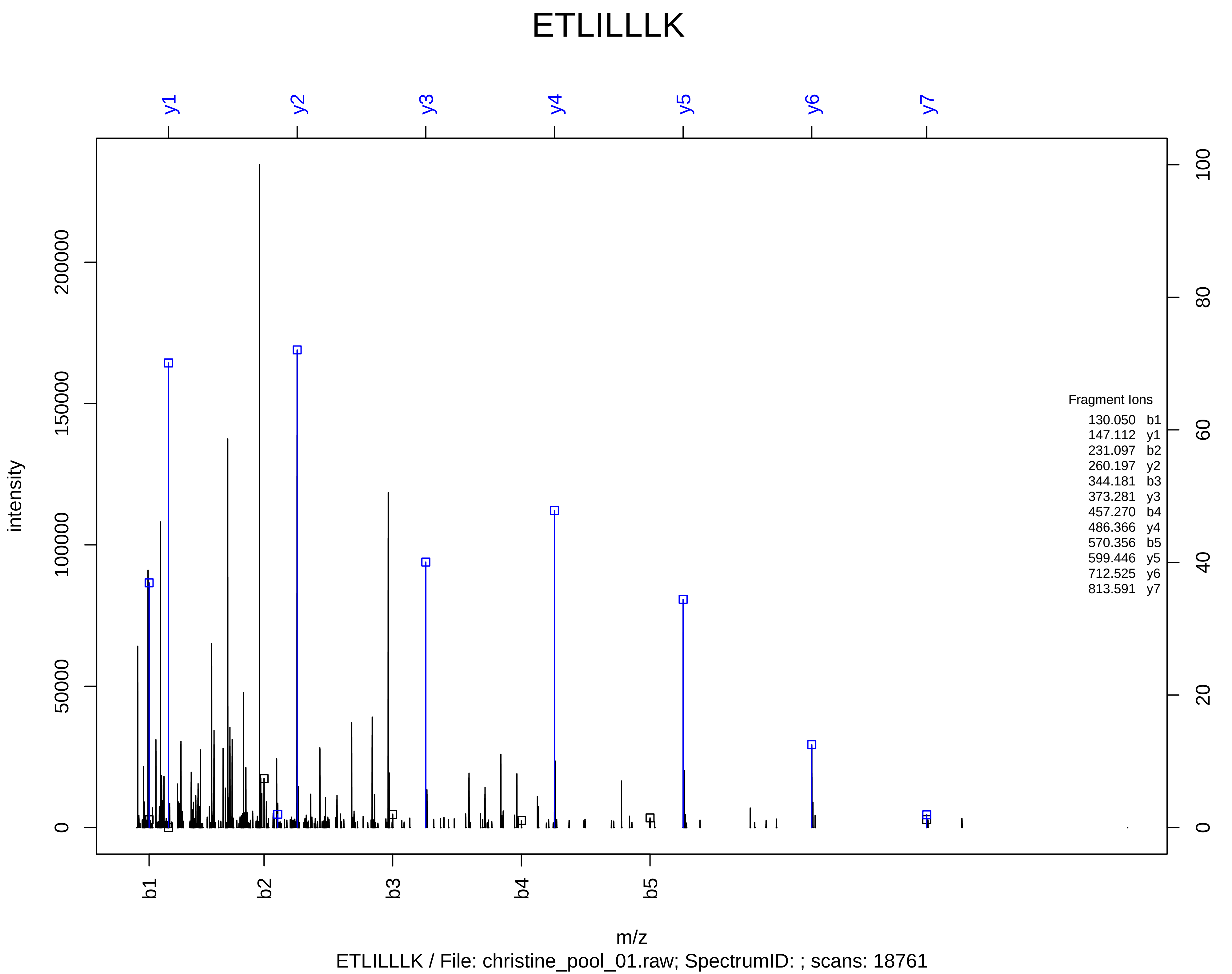

ETLILLLK
y1
y2
y3
y4
y5
y6
y7
intensity
0
50000
100000
150000
200000
0
20
40
60
80
100
Fragment Ions
130.050 b1
147.112 y1
231.097 b2
260.197 y2
344.181 b3
373.281 y3
457.270 b4
486.366 y4
570.356 b5
599.446 y5
712.525 y6
813.591 y7
b1
b2
b3
b4
b5
m/z
ETLILLLK / File: christine_pool_01.raw; SpectrumID: ; scans: 18761

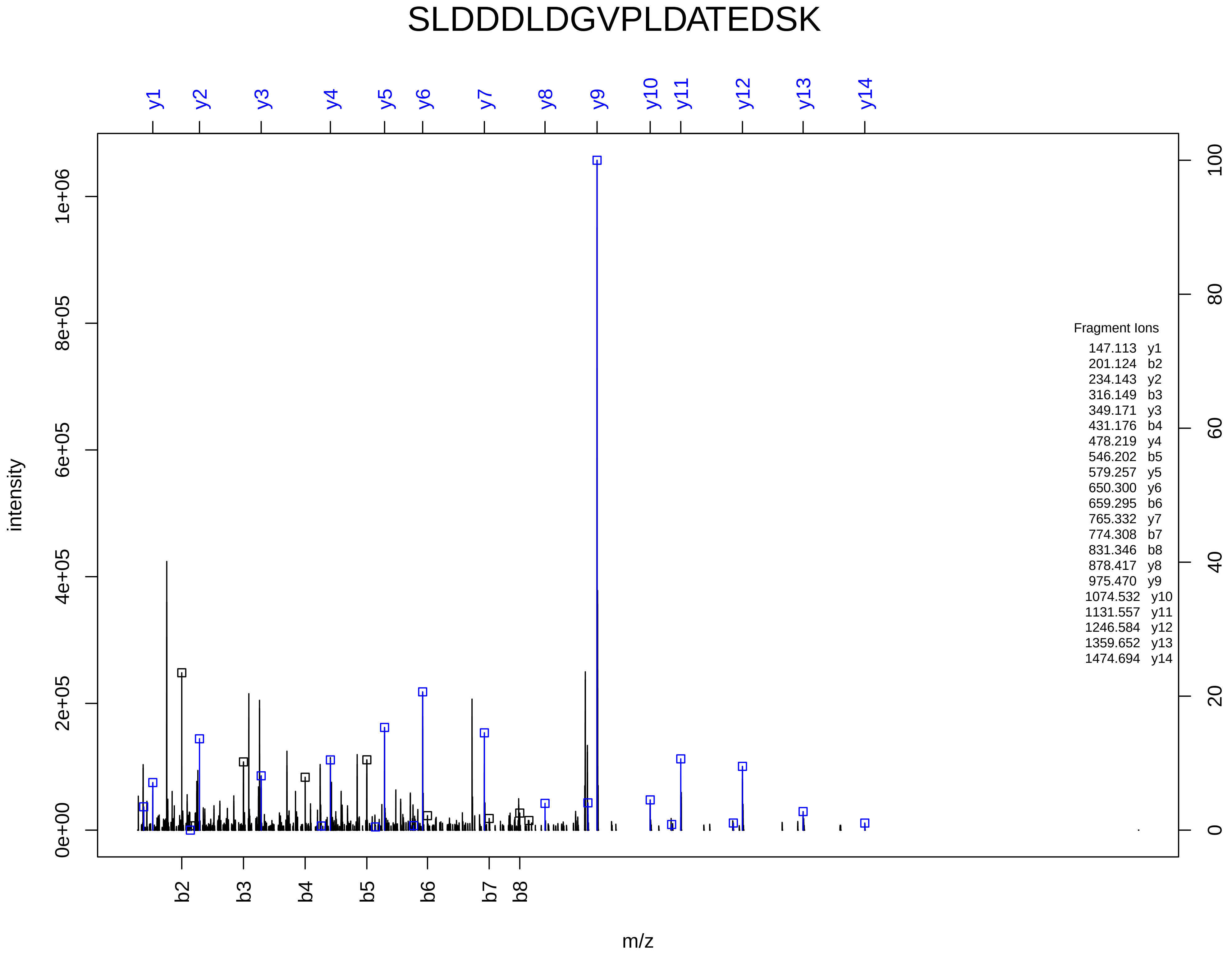

SLDDDLDGVPLDATEDSK / File: christine_pool_01.raw; SpectrumID: ; scans: 13485

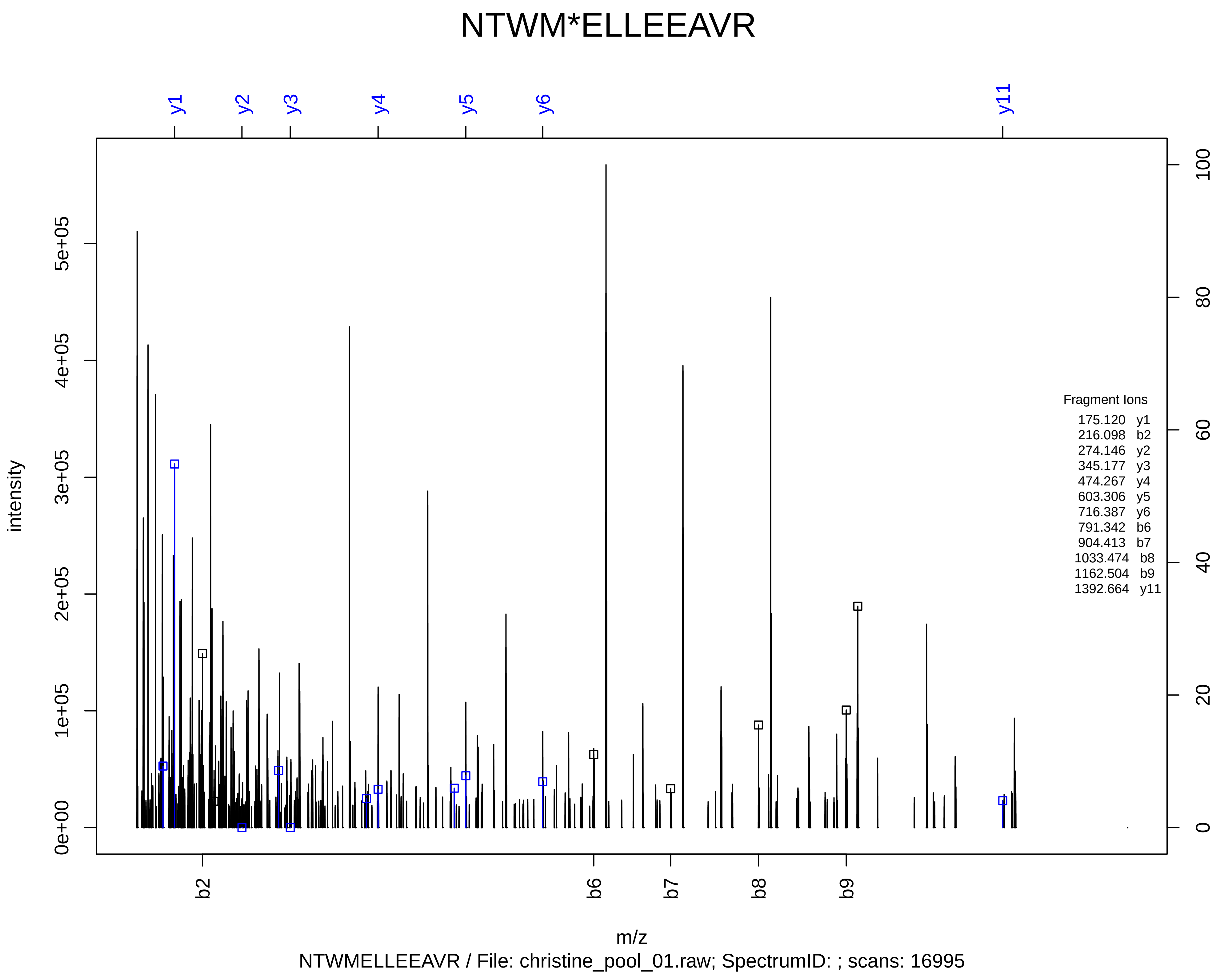
NTWM*ELLEEAVR
y1
y2
y3
y4
y5
y6
y11
intensity
0e+00
1e+05
2e+05
3e+05
4e+05
5e+05
0
20
40
60
80
100
Fragment Ions
175.120 y1
216.098 b2
274.146 y2
345.177 y3
474.267 y4
603.306 y5
716.387 y6
791.342 b6
904.413 b7
1033.474 b8
1162.504 b9
1392.664 y11
b2
b6
b7
b8
b9
m/z
NTWMELLEEAVR / File: christine_pool_01.raw; SpectrumID: ; scans: 16995

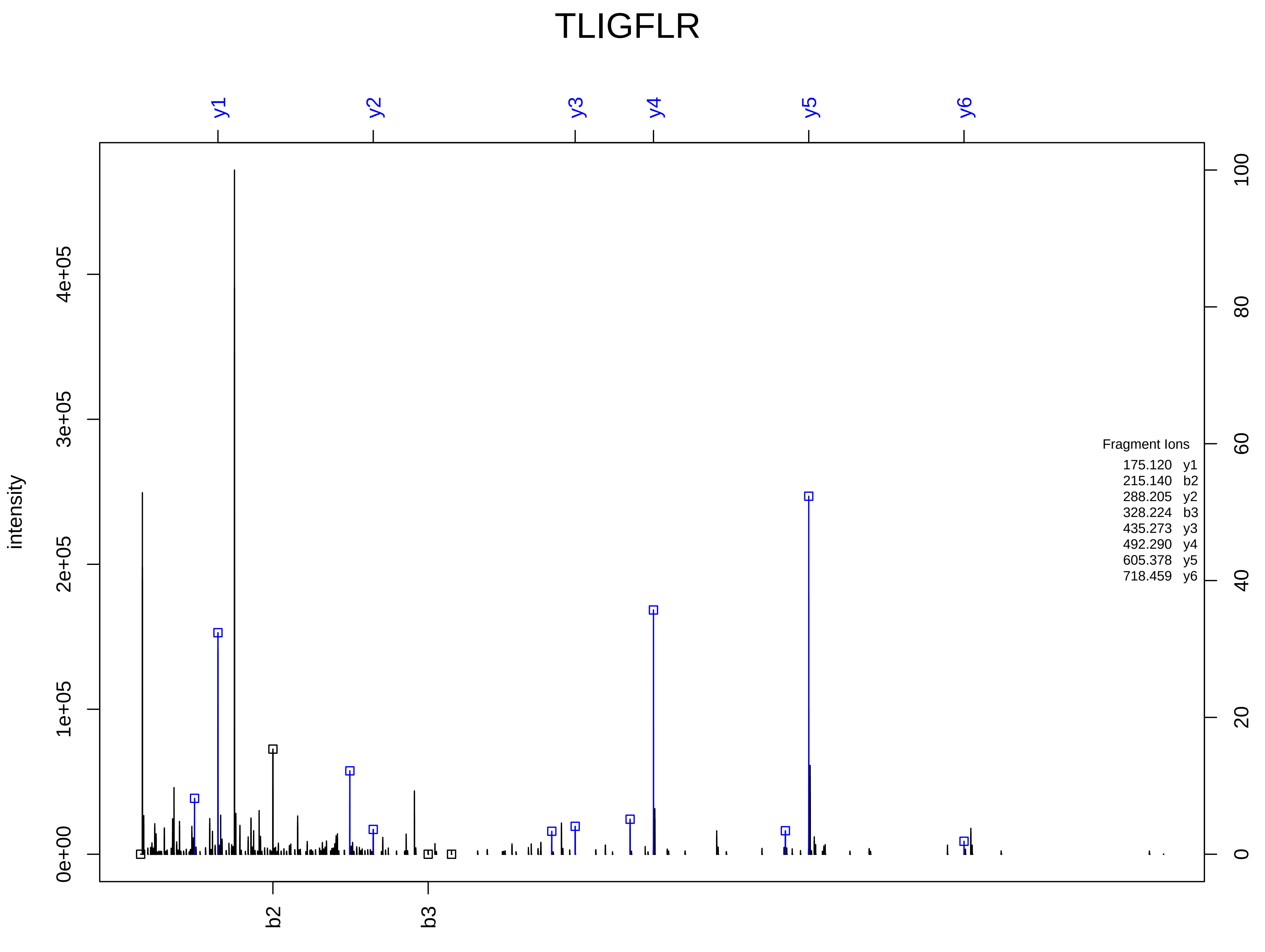

TLIGFLR / File: christine_pool_01.raw; SpectrumID: ; scans: 14455

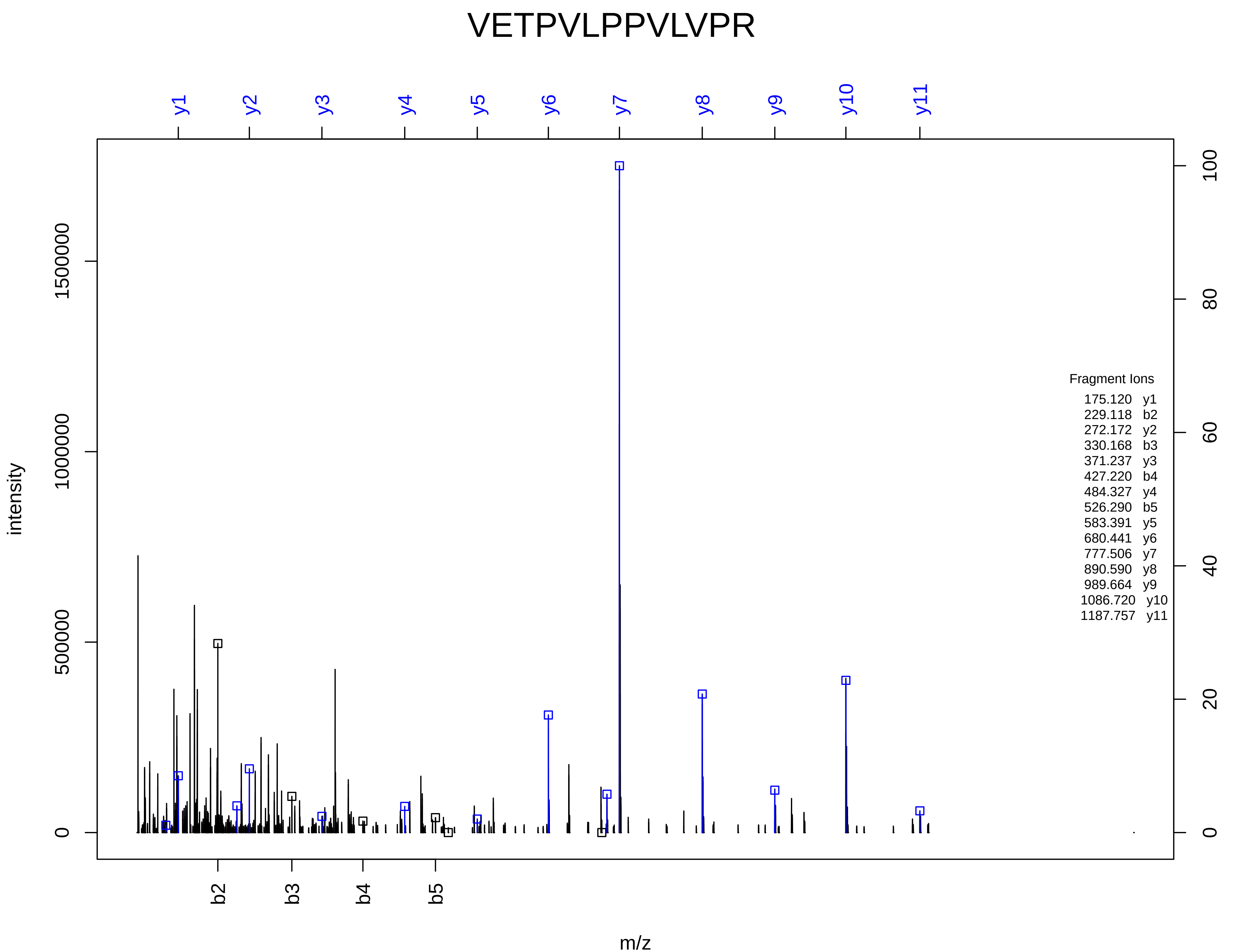

VETPVLPPVLVPR
y1
y2
y3
y4
y5
y6
y7
y8
y9
y10
y11
intensity
0
500000
1000000
1500000
0
20
40
60
80
100
b2
b3
b4
b5
m/z
Fragment Ions
175.120 y1
229.118 b2
272.172 y2
330.168 b3
371.237 y3
427.220 b4
484.327 y4
526.290 b5
583.391 y5
680.441 y6
777.506 y7
890.590 y8
989.664 y9
1086.720 y10
1187.757 y11
VETPVLPPVLVPR / File: christine_pool_01.raw; SpectrumID: ; scans: 18827

LVPLLDRPFDETTYEETED

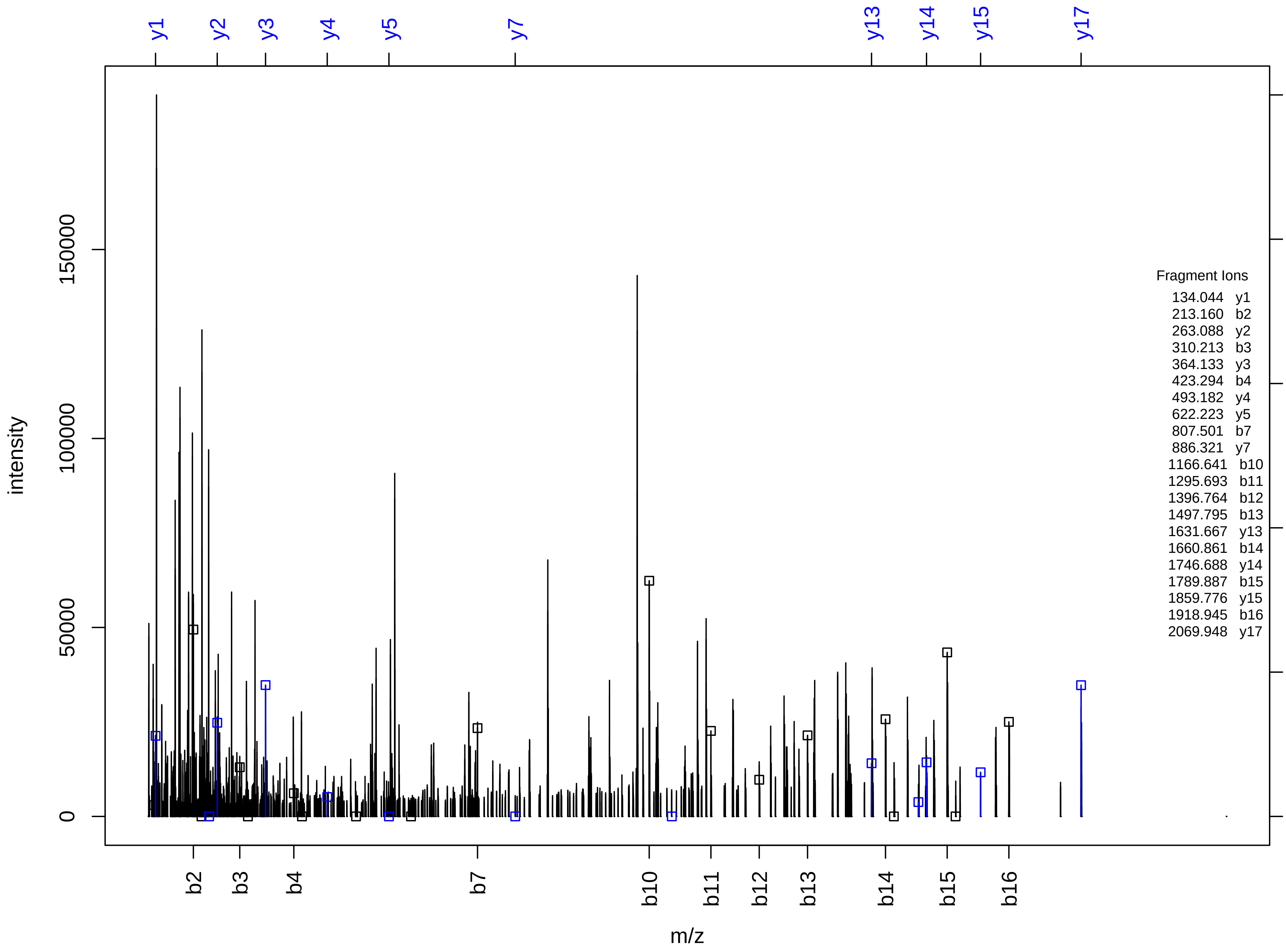

LVPLLDRPFDETTYEETED / File: christine_pool_01.raw; SpectrumID: ; scans: 18386

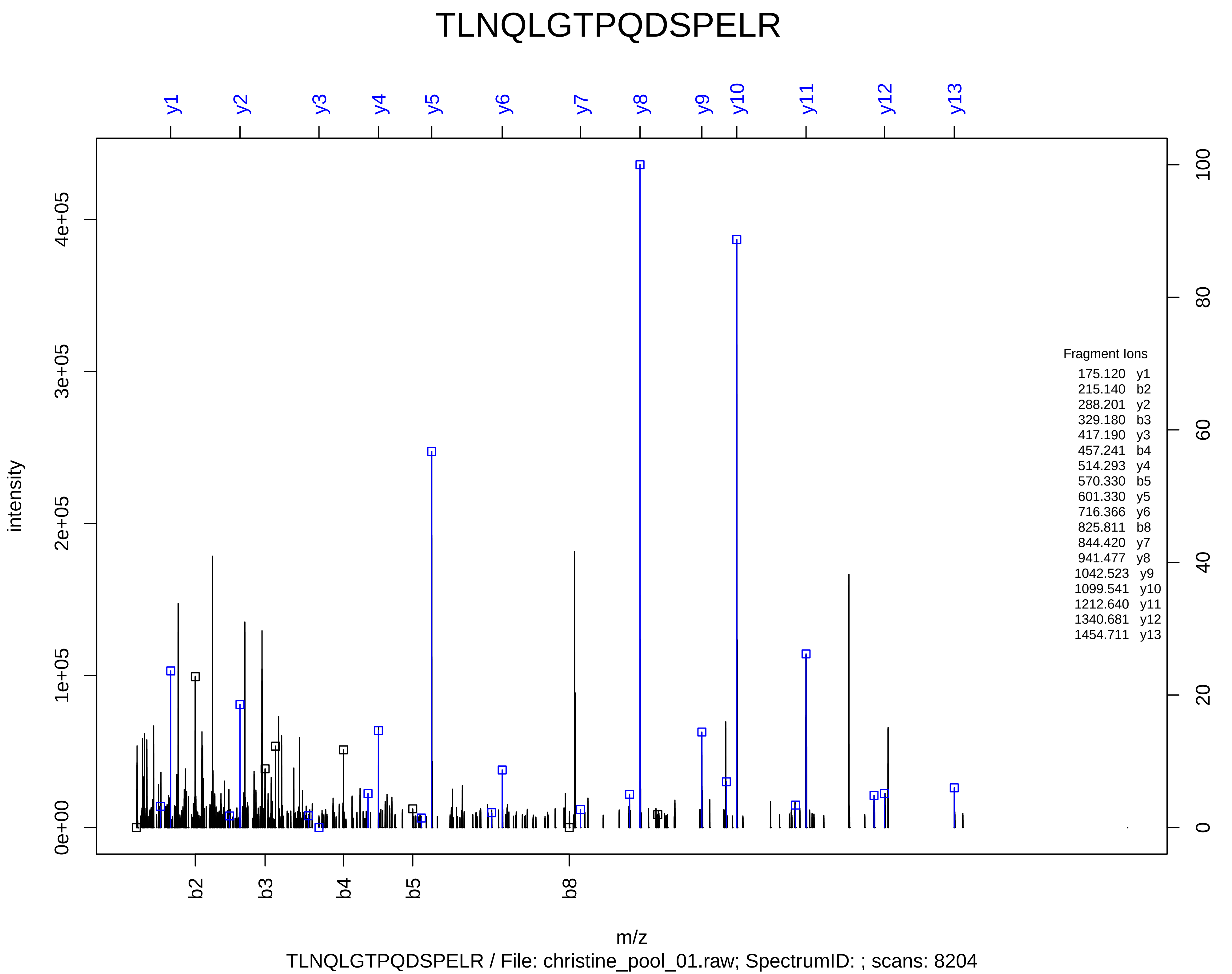
TLNQLGTPQDSPELR
y1 y2 y3 y4 y5 y6 y7 y8 y9 y10 y11 y12 y13
b2 b3 b4 b5 b8
intensity
0e+00 1e+05 2e+05 3e+05 4e+05
0 20 40 60 80 100
m/z
Fragment Ions
175.120 y1
215.140 b2
288.201 y2
329.180 b3
417.190 y3
457.241 b4
514.293 y4
570.330 b5
601.330 y5
716.366 y6
825.811 b8
844.420 y7
941.477 y8
1042.523 y9
1099.541 y10
1212.640 y11
1340.681 y12
1454.711 y13
TLNQLGTPQDSPELR / File: christine_pool_01.raw; SpectrumID: ; scans: 8204

DDDVPLMWR

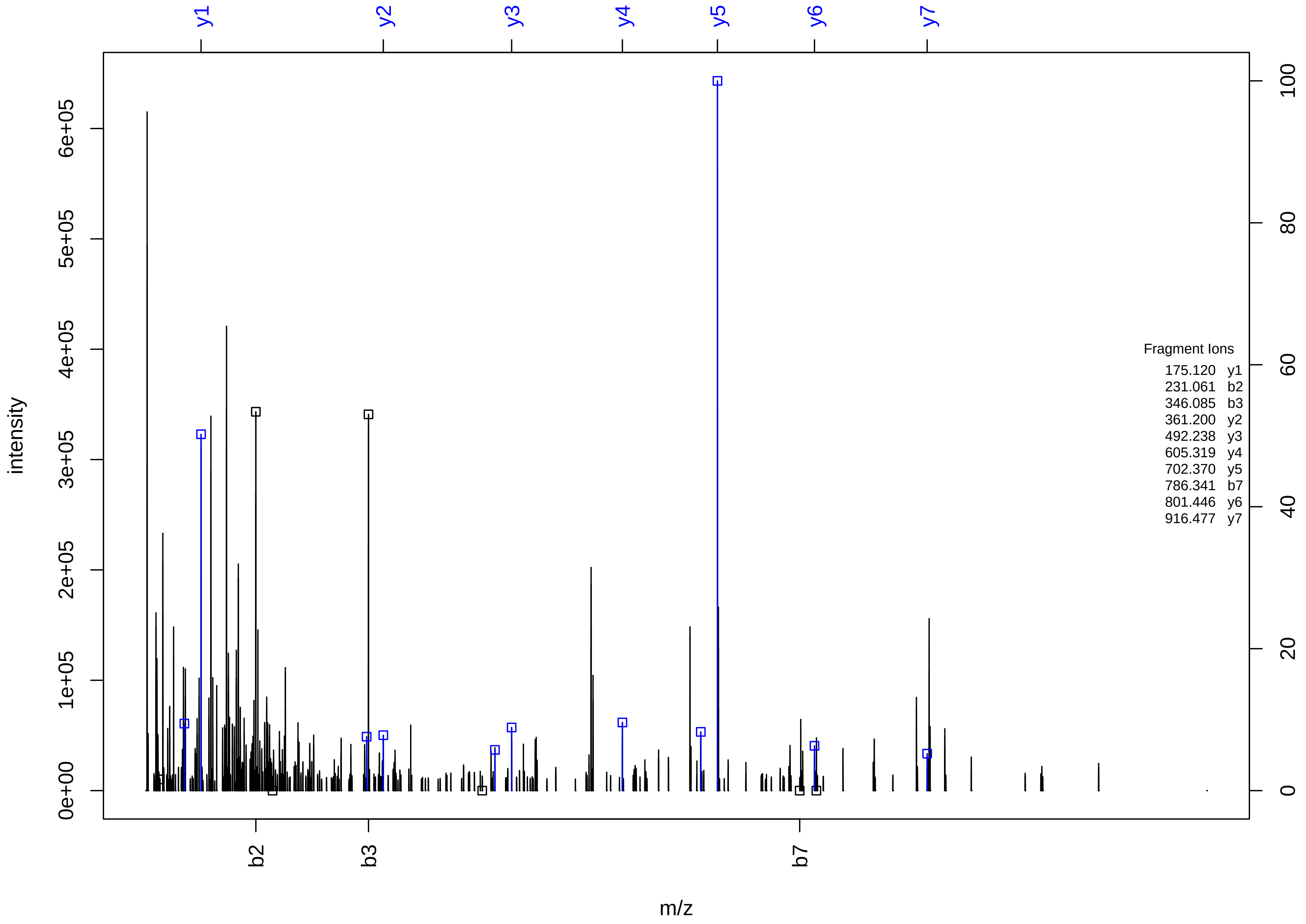

DDDVPLMWR / File: christine_pool_01.raw; SpectrumID: ; scans: 15155

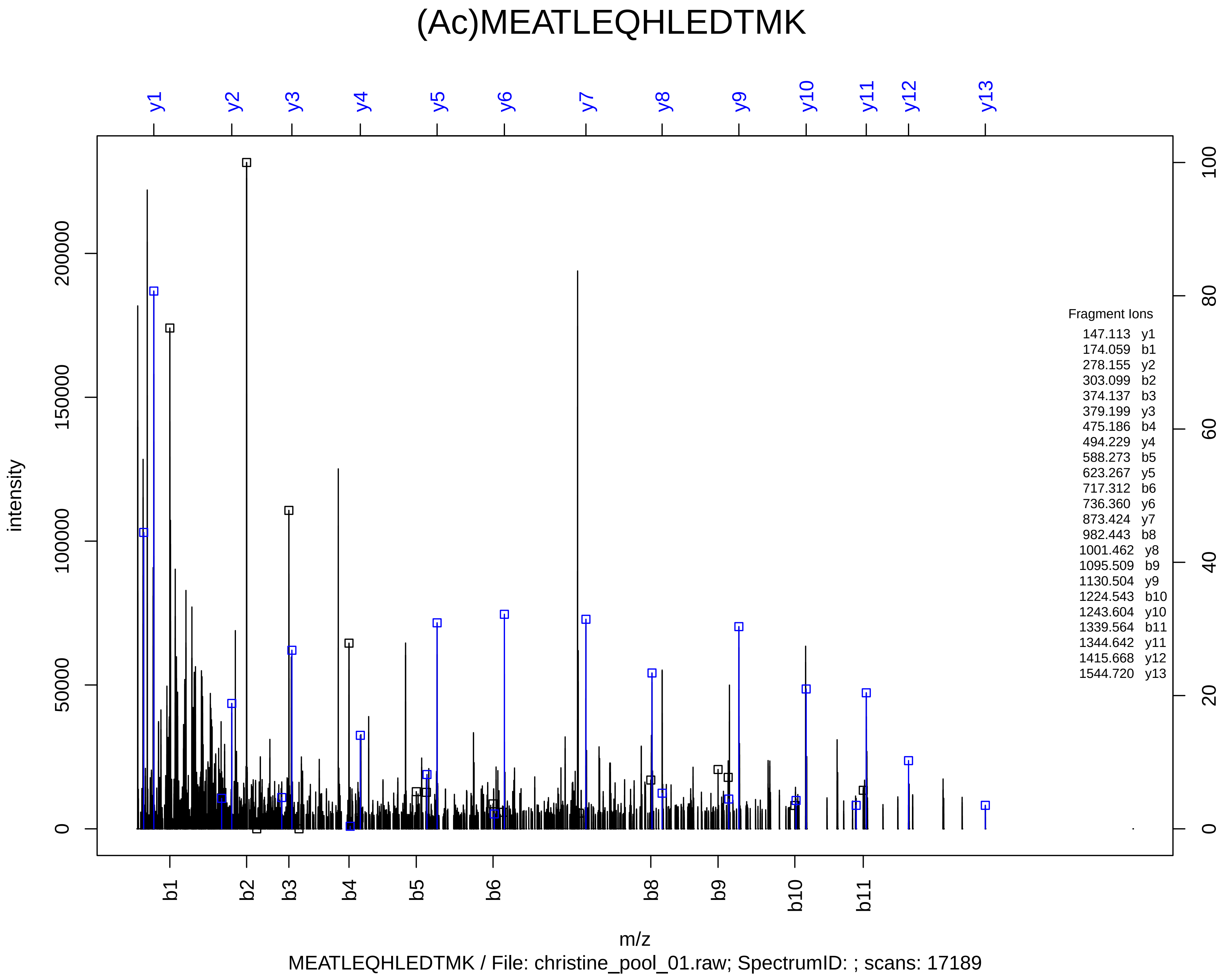
(Ac)MEATLEQHLEDTMK
intensity
m/z
Fragment Ions
147.113 y1
174.059 b1
278.155 y2
303.099 b2
374.137 b3
379.199 y3
475.186 b4
494.229 y4
588.273 b5
623.267 y5
717.312 b6
736.360 y6
873.424 y7
982.443 b8
1001.462 y8
1095.509 b9
1130.504 y9
1224.543 b10
1243.604 y10
1339.564 b11
1344.642 y11
1415.668 y12
1544.720 y13
MEATLEQHLEDTMK / File: christine_pool_01.raw; SpectrumID: ; scans: 17189

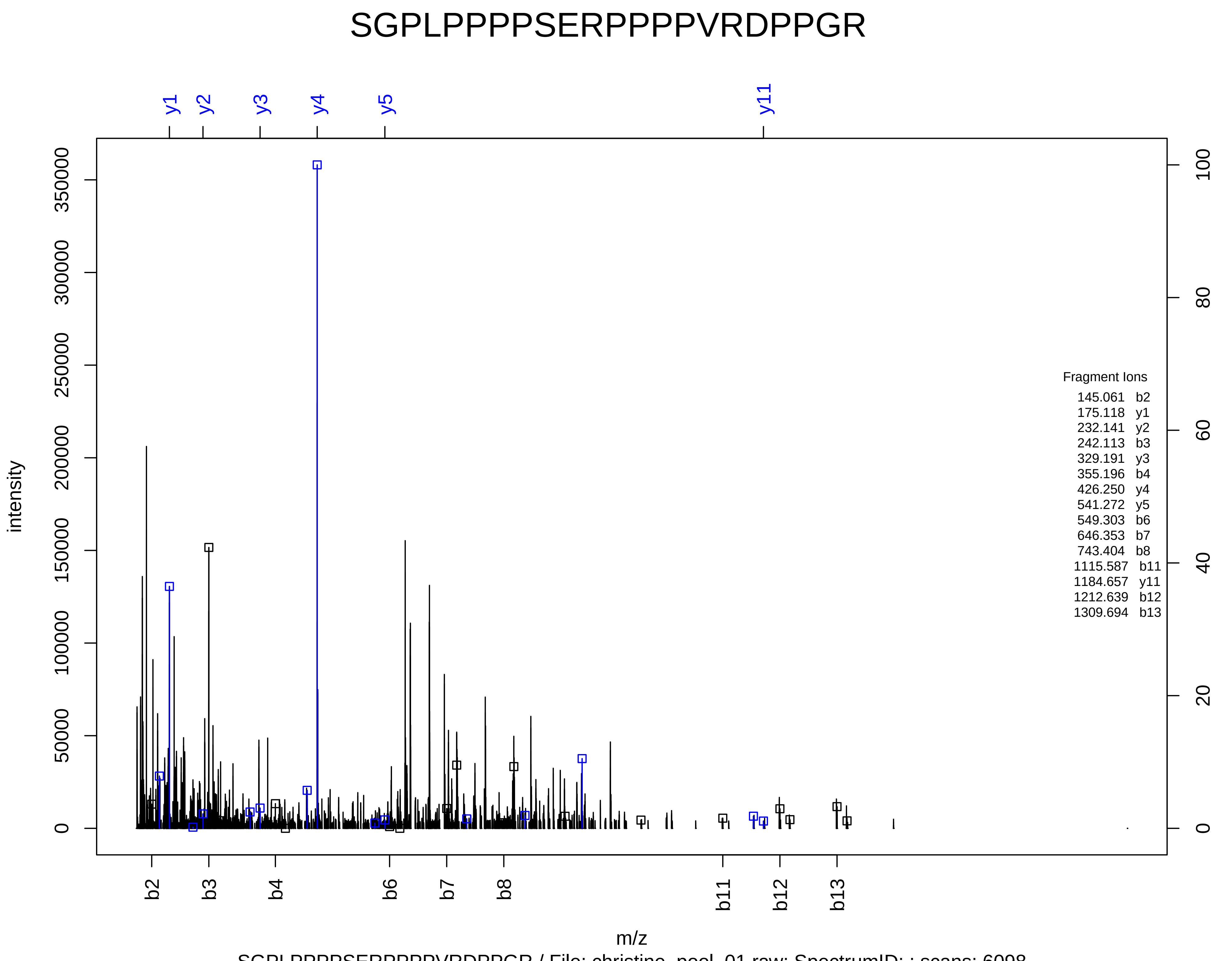
SGPLPPPPSERPPPPVRDPPGR
y1
y2
y3
y4
y5
y11
intensity
0
50000
100000
150000
200000
250000
300000
350000
0
20
40
60
80
100
Fragment Ions
145.061 b2
175.118 y1
232.141 y2
242.113 b3
329.191 y3
355.196 b4
426.250 y4
541.272 y5
549.303 b6
646.353 b7
743.404 b8
1115.587 b11
1184.657 y11
1212.639 b12
1309.694 b13
b2
b3
b4
b6
b7
b8
b11
b12
b13
m/z
SGPLPPPPSERPPPPVRDPPGR / File: christine_pool_01.raw; SpectrumID: ; scans: 6098

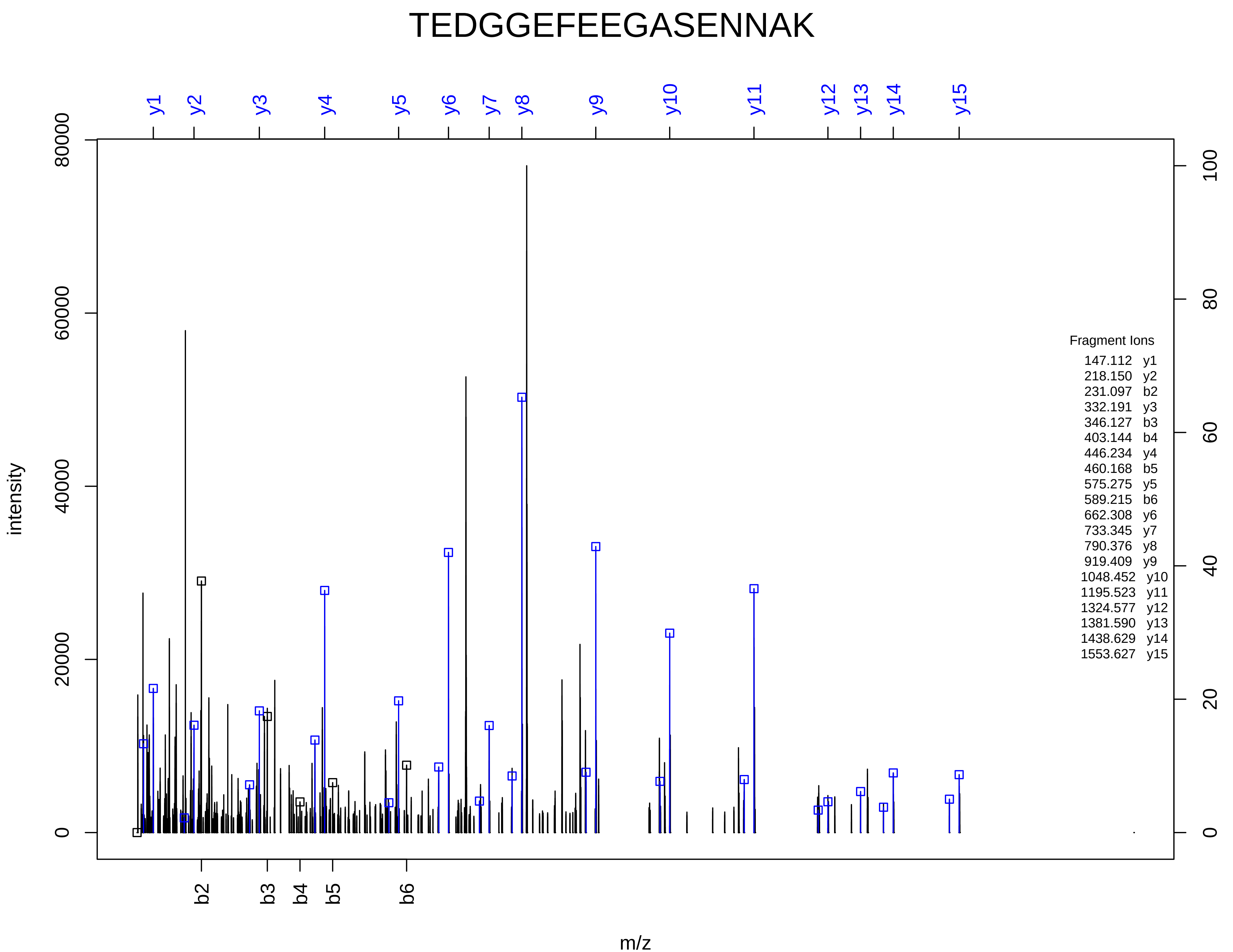
TEDGGEFEEGASENNAK
y1 y2 y3 y4 y5 y6 y7 y8 y9 y10 y11 y12 y13 y14 y15
b2 b3 b4 b5 b6
intensity
m/z
Fragment Ions
147.112 y1
218.150 y2
231.097 b2
332.191 y3
346.127 b3
403.144 b4
446.234 y4
460.168 b5
575.275 y5
589.215 b6
662.308 y6
733.345 y7
790.376 y8
919.409 y9
1048.452 y10
1195.523 y11
1324.577 y12
1381.590 y13
1438.629 y14
1553.627 y15
TEDGGEFEEGASENNAK / File: christine_pool_01.raw; SpectrumID: ; scans: 2728

DFLAMFSPK

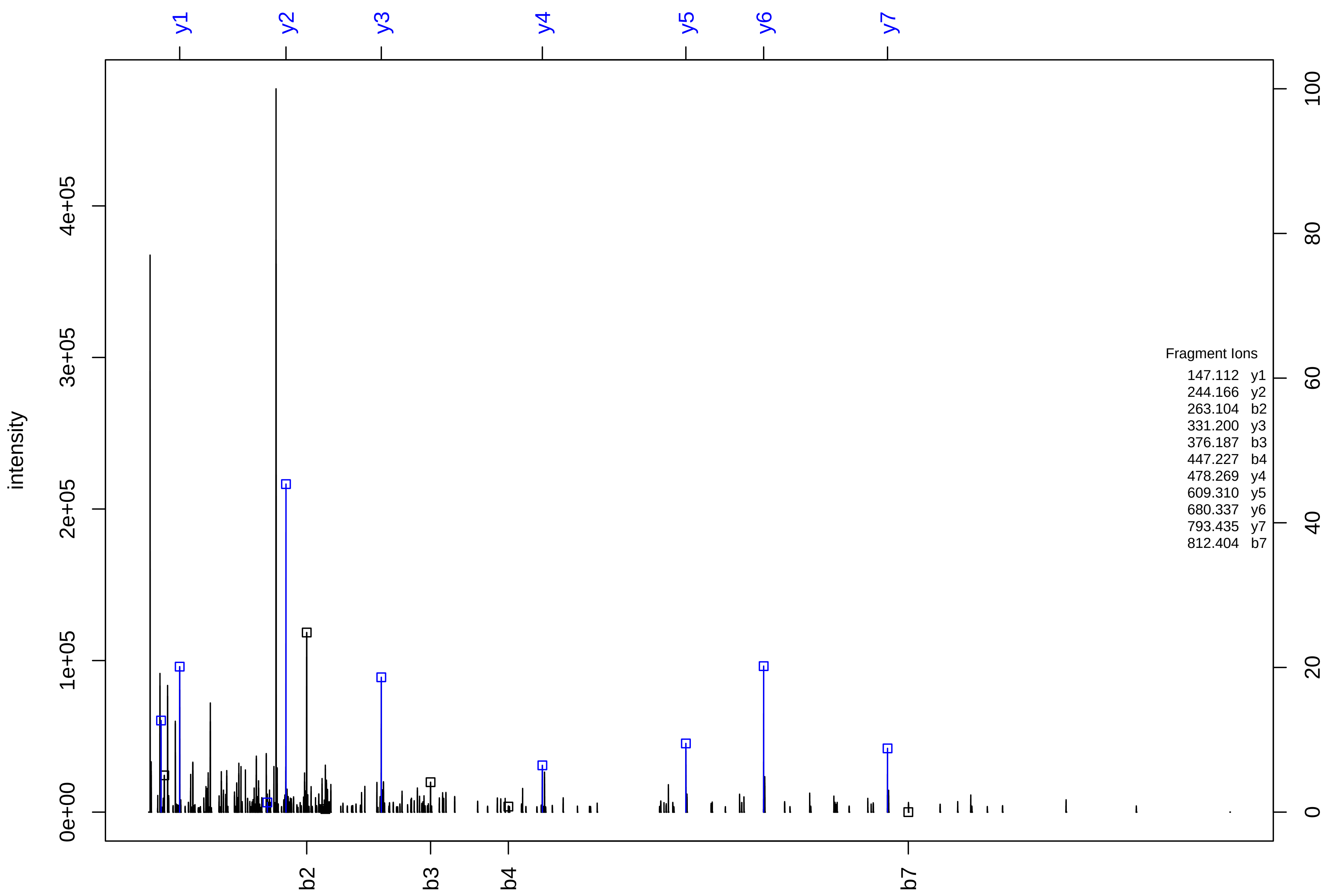

DFLAMFSPK / File: christine_pool_01.raw; SpectrumID: ; scans: 19748

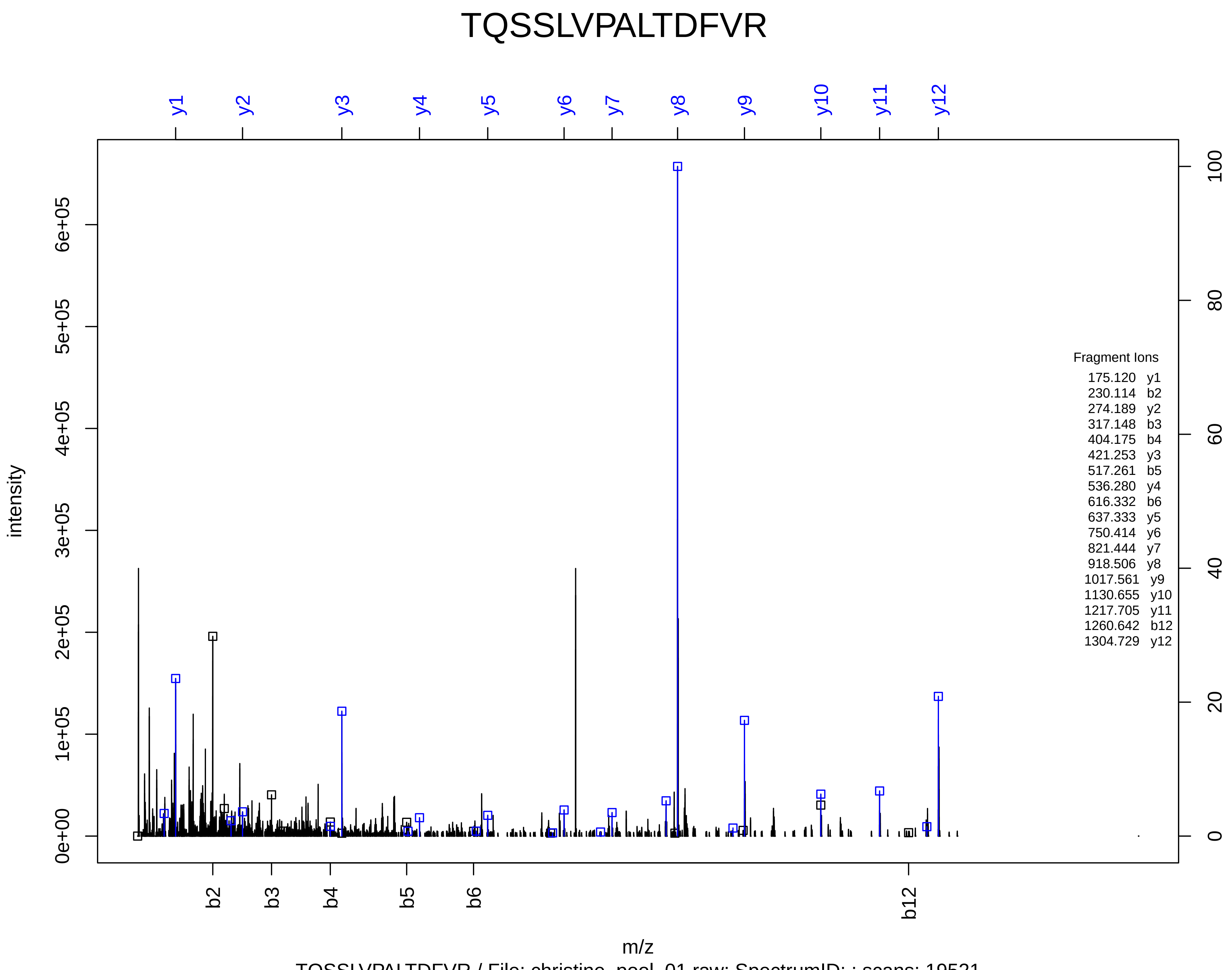
TQSSLVPALTDFVR
y1
y2
y3
y4
y5
y6
y7
y8
y9
y10
y11
y12
intensity
0e+00
1e+05
2e+05
3e+05
4e+05
5e+05
6e+05
0
20
40
60
80
100
b2
b3
b4
b5
b6
b12
m/z
Fragment Ions
175.120 y1
230.114 b2
274.189 y2
317.148 b3
404.175 b4
421.253 y3
517.261 b5
536.280 y4
616.332 b6
637.333 y5
750.414 y6
821.444 y7
918.506 y8
1017.561 y9
1130.655 y10
1217.705 y11
1260.642 b12
1304.729 y12
TQSSLVPALTDFVR / File: christine_pool_01.raw; SpectrumID: ; scans: 19521

HIGTSALMDLLLR

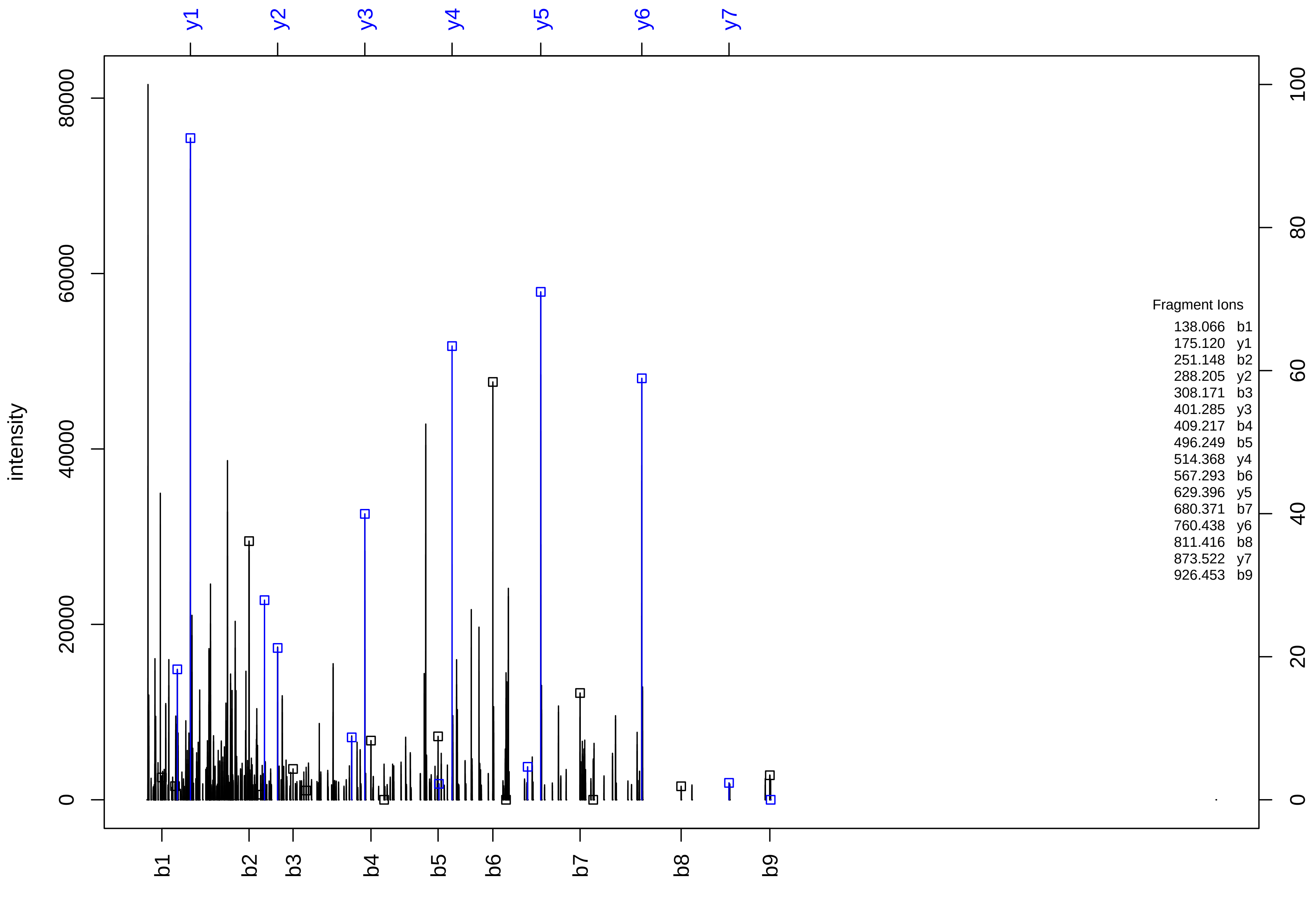

HIGTSALMDLLLR / File: christine_pool_01.raw; SpectrumID: ; scans: 20038

VEVEEDGQLK

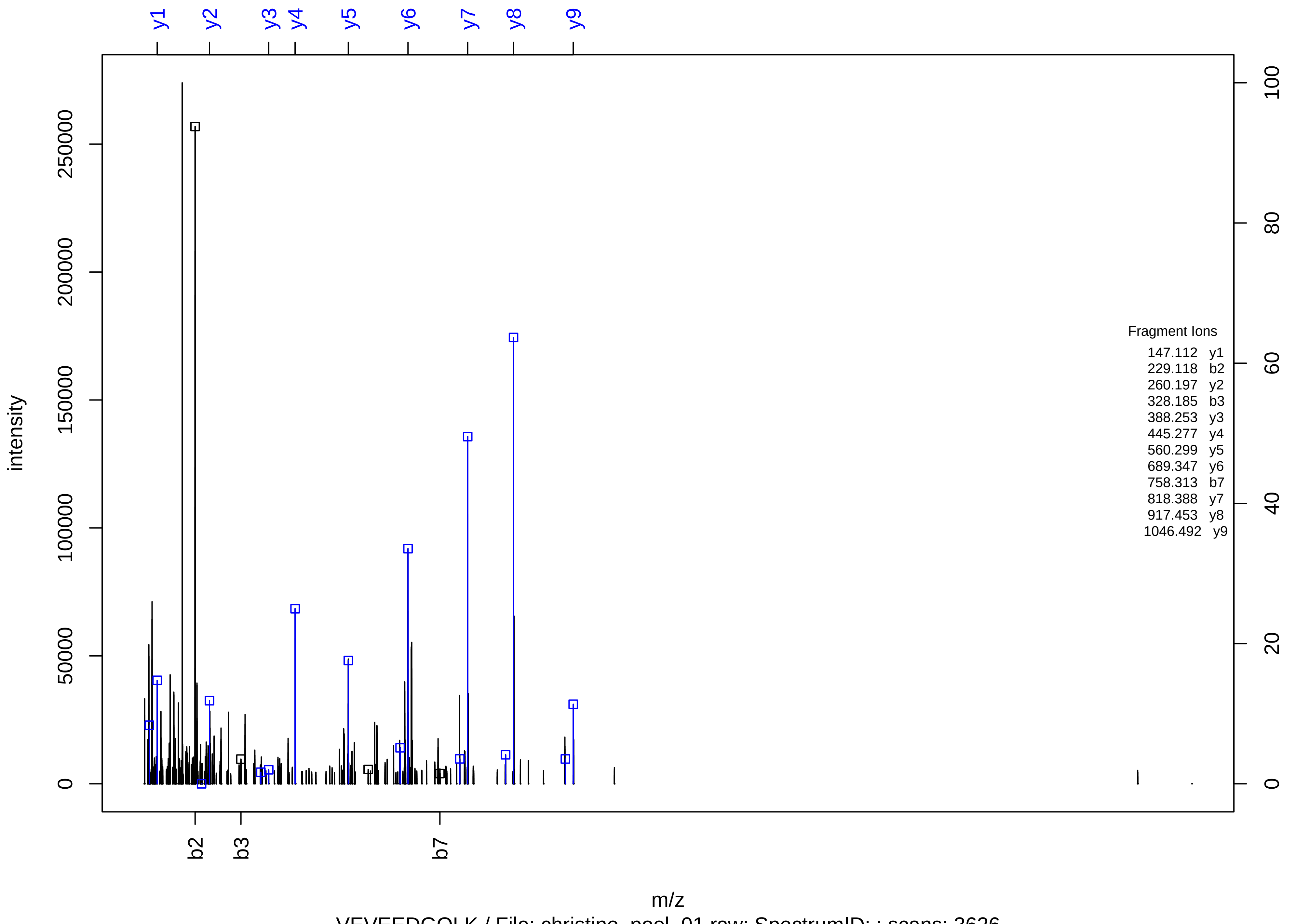

VEVEEDGQLK / File: christine_pool_01.raw; SpectrumID: ; scans: 3626

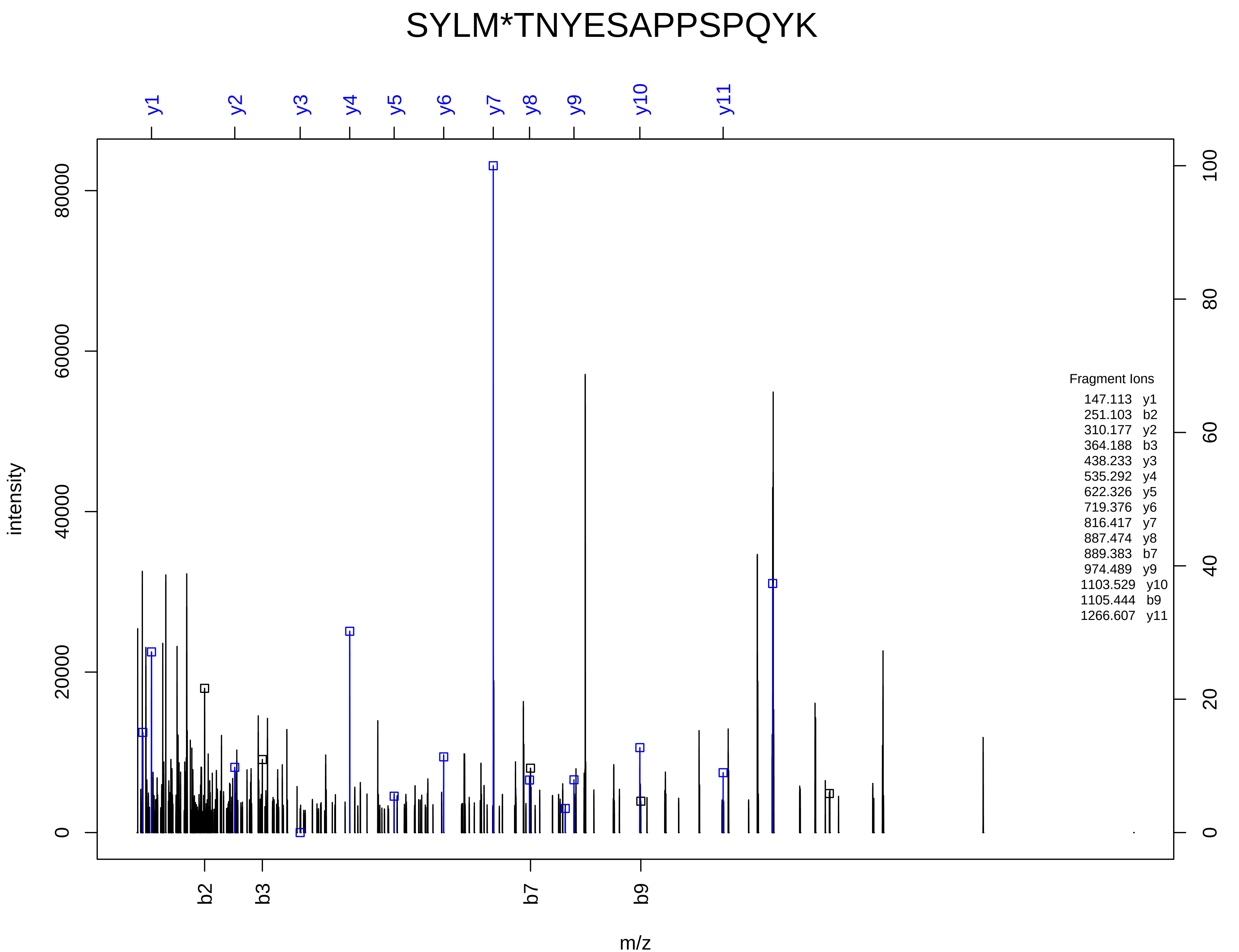

SYLM*TNYESAPPSPQYK
y1
y2
y3
y4
y5
y6
y7
y8
y9
y10
y11
intensity
0
20000
40000
60000
80000
0
20
40
60
80
100
b2
b3
b7
b9
m/z
Fragment Ions
147.113 y1
251.103 b2
310.177 y2
364.188 b3
438.233 y3
535.292 y4
622.326 y5
719.376 y6
816.417 y7
887.474 y8
889.383 b7
974.489 y9
1103.529 y10
1105.444 b9
1266.607 y11
SYLMTNYESAPPSPQYK / File: christine_pool_01.raw; SpectrumID: ; scans: 7759

GLGLGIVAGSLLVK

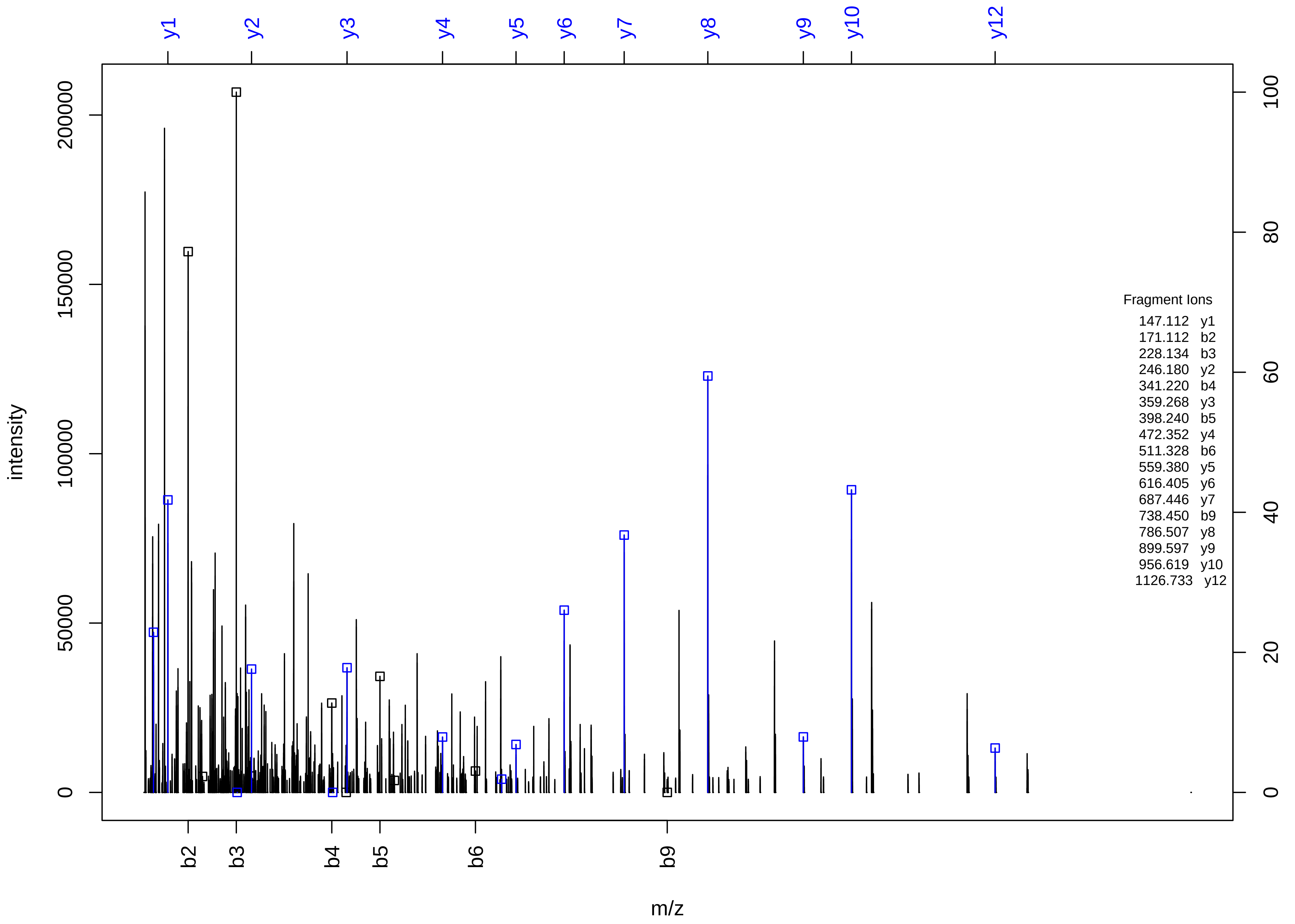

GLGLGIVAGSLLVK / File: christine_pool_01.raw; SpectrumID: ; scans: 21361

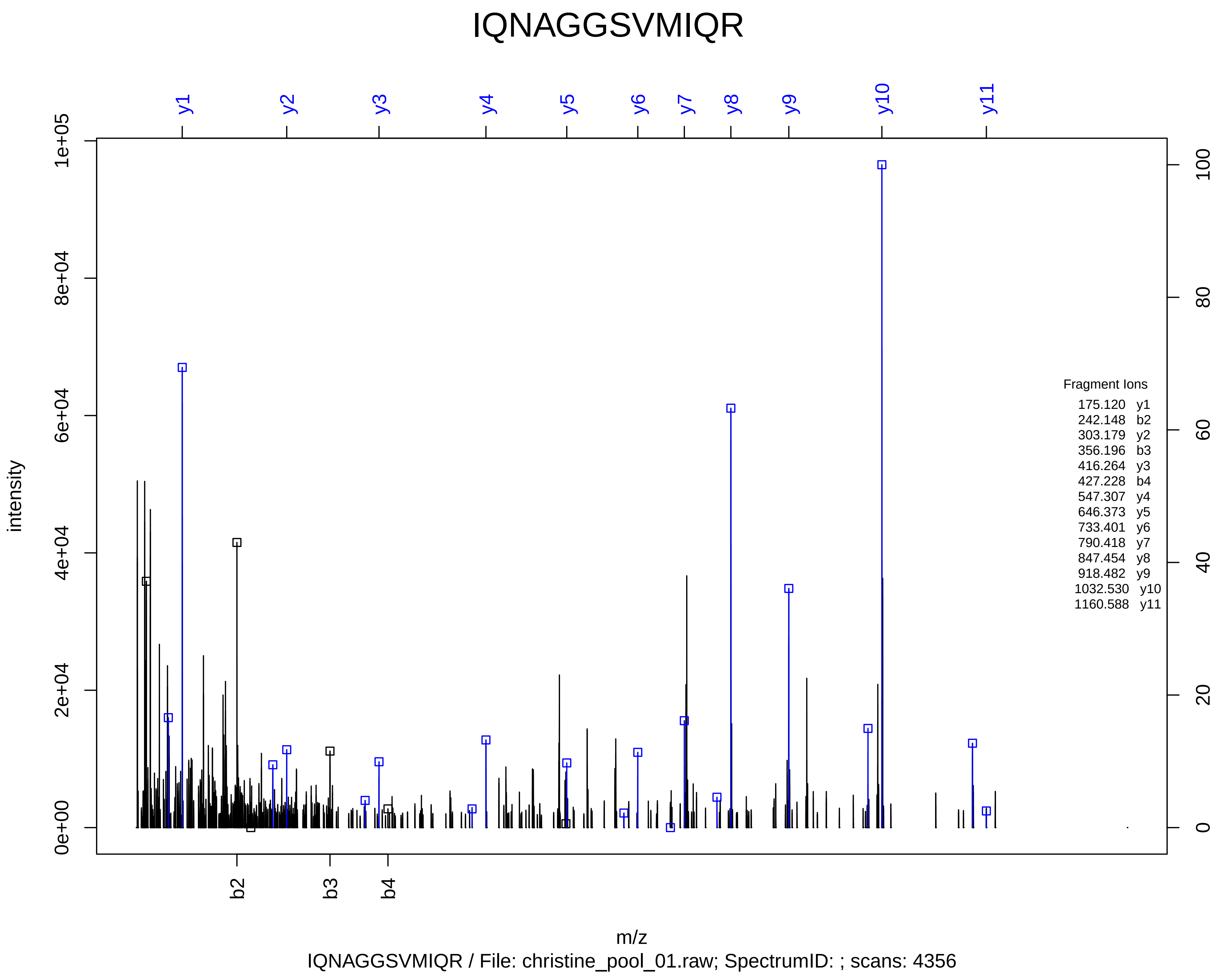

IQNAGGSVMIQR
y1
y2
y3
y4
y5
y6
y7
y8
y9
y10
y11
1e+05
8e+04
6e+04
4e+04
2e+04
0e+00
intensity
100
80
60
40
20
0
b2
b3
b4
Fragment Ions
175.120 y1
242.148 b2
303.179 y2
356.196 b3
416.264 y3
427.228 b4
547.307 y4
646.373 y5
733.401 y6
790.418 y7
847.454 y8
918.482 y9
1032.530 y10
1160.588 y11
m/z
IQNAGGSVMIQR / File: christine_pool_01.raw; SpectrumID: ; scans: 4356

YTAQVDAEEK

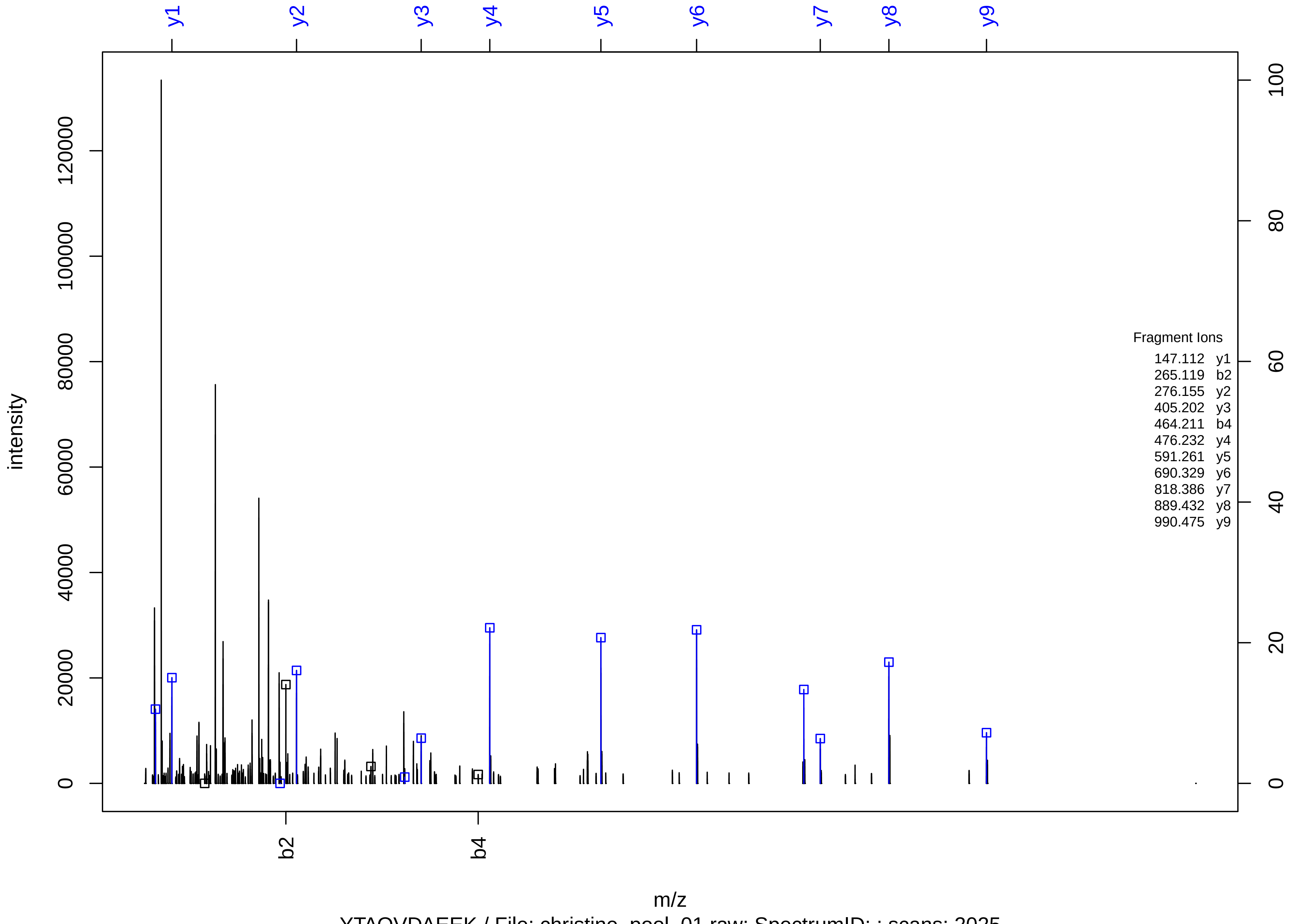

YTAQVDAEEK / File: christine_pool_01.raw; SpectrumID: ; scans: 2025

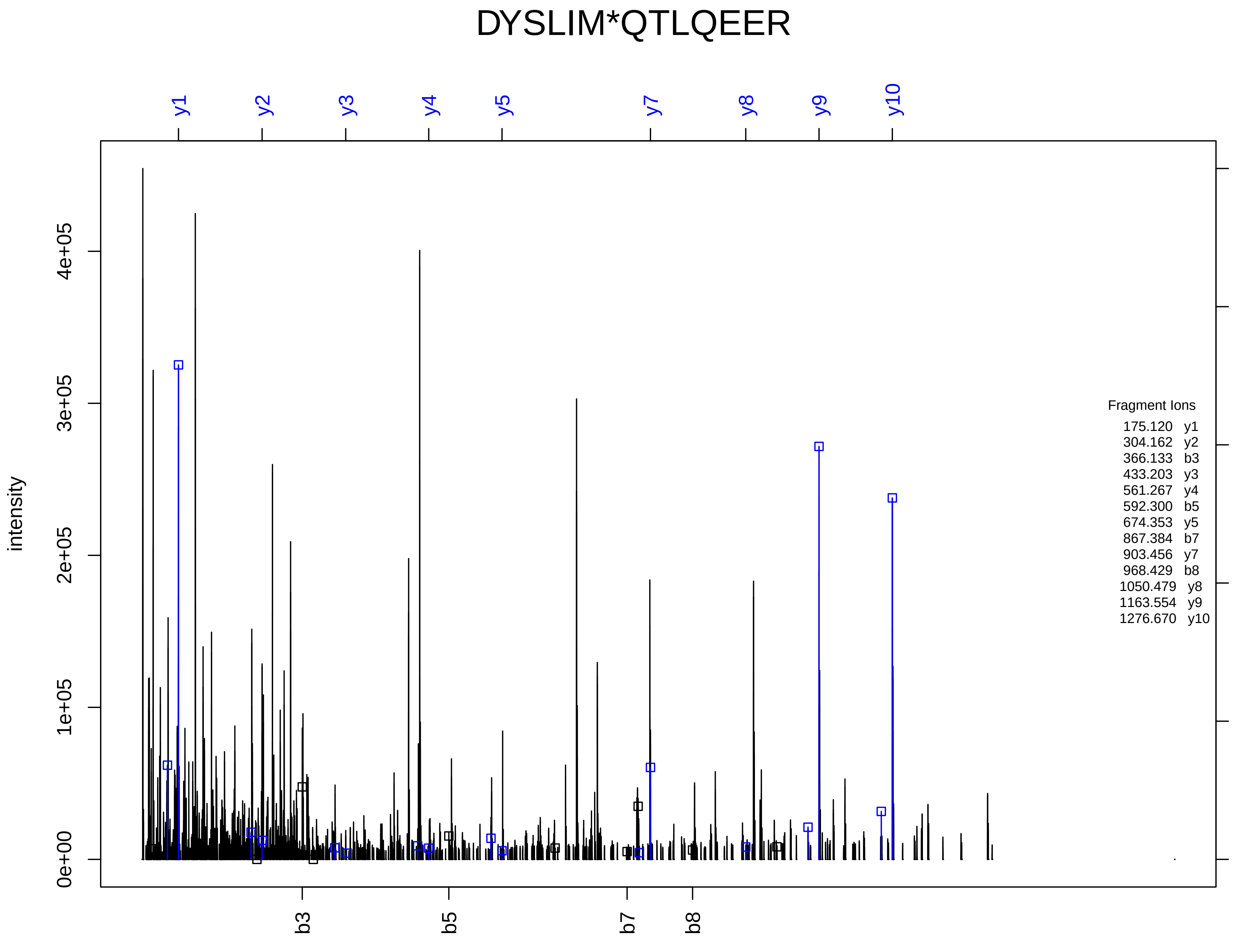

DYSLIMQTLQEER / File: christine_pool_01.raw; SpectrumID: ; scans: 17648

GNEATEGSGLLLN^STGDLM*KK

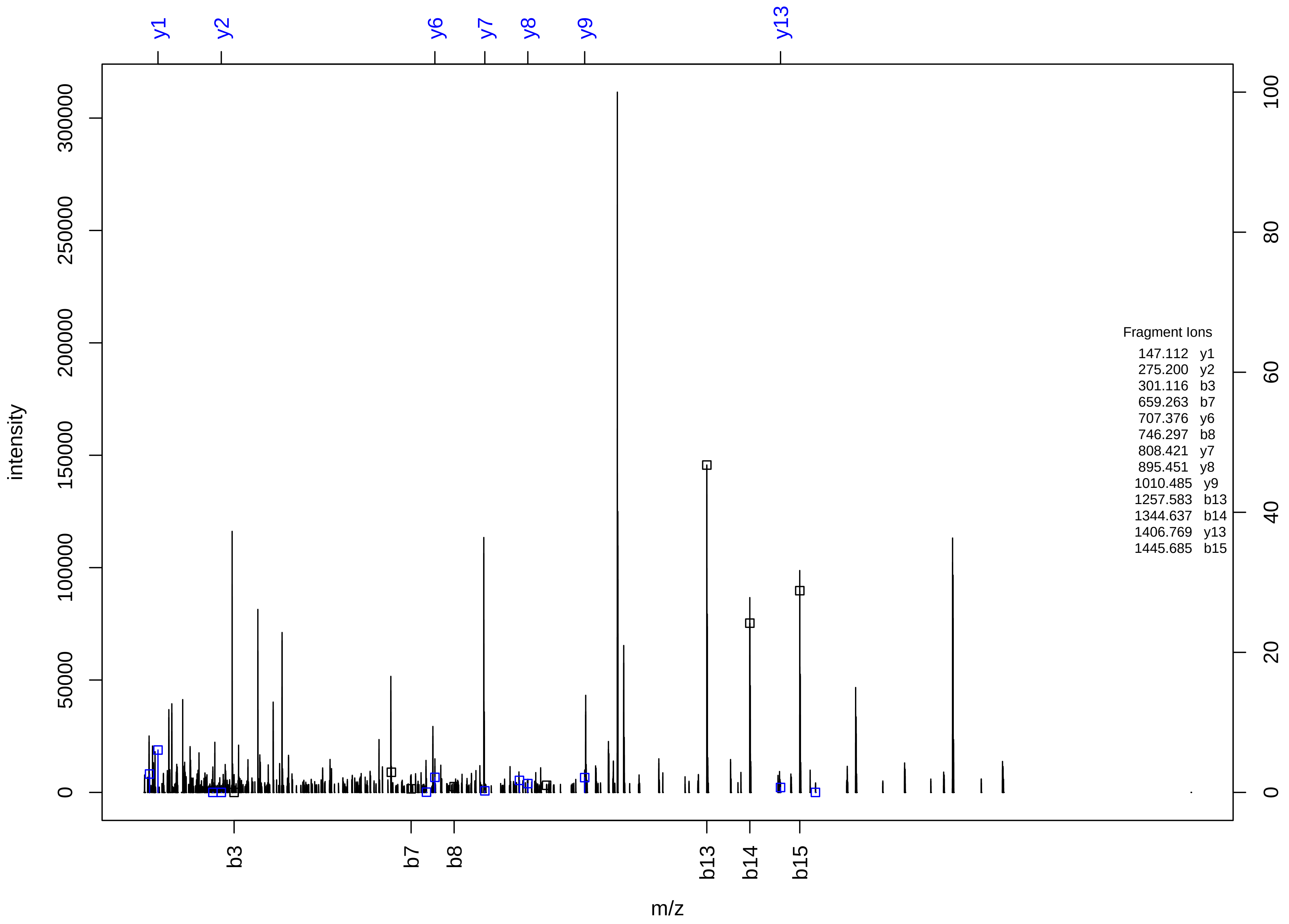

GNEATEGSGLLLNSTGDLMKK / File: christine_pool_01.raw; SpectrumID: ; scans: 7152

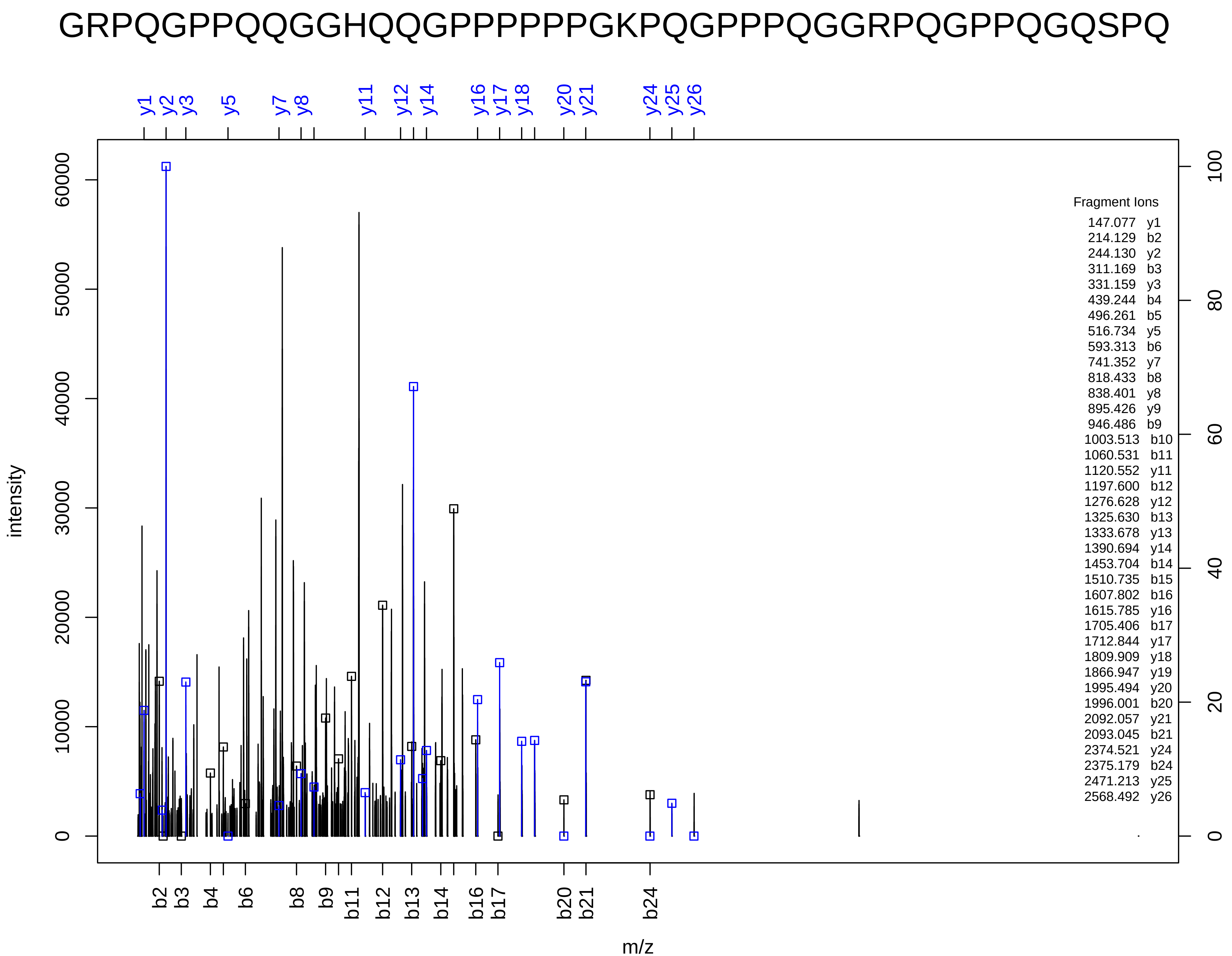

GRPQGPPQQGGHQQGPPPPPPGKPQGPPPQGGRPQGPPQGQSPQ / File: christine_pool_01.raw; SpectrumID: ; scans: 3354

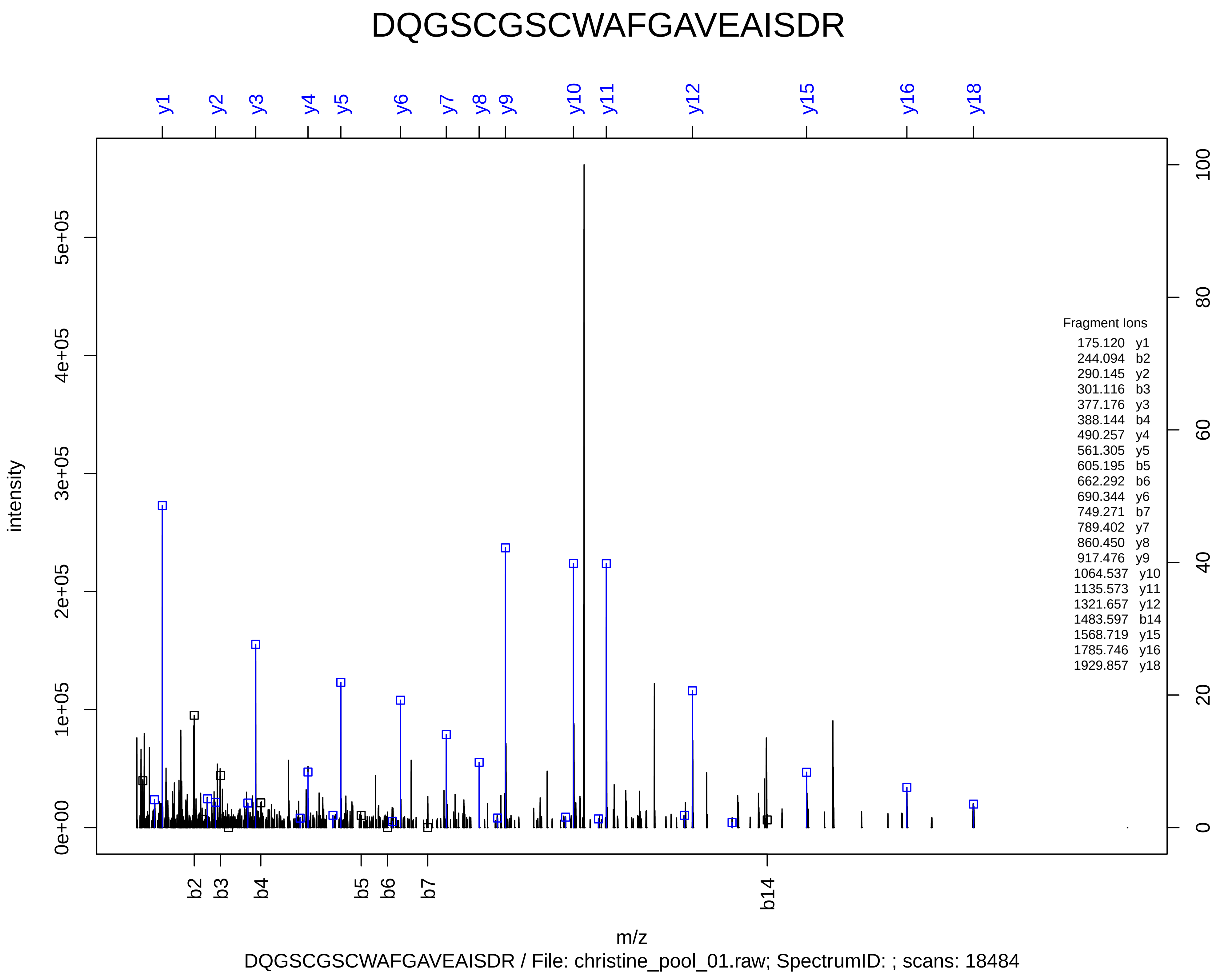

DQGSCGSCWAFGAVEAISDR
y1 y2 y3 y4 y5 y6 y7 y8 y9 y10 y11 y12 y15 y16 y18
b2 b3 b4 b5 b6 b7 b14
intensity
0e+00 1e+05 2e+05 3e+05 4e+05 5e+05
0 20 40 60 80 100
m/z
Fragment Ions
175.120 y1
244.094 b2
290.145 y2
301.116 b3
377.176 y3
388.144 b4
490.257 y4
561.305 y5
605.195 b5
662.292 b6
690.344 y6
749.271 b7
789.402 y7
860.450 y8
917.476 y9
1064.537 y10
1135.573 y11
1321.657 y12
1483.597 b14
1568.719 y15
1785.746 y16
1929.857 y18
DQGSCGSCWAFGAVEAISDR / File: christine_pool_01.raw; SpectrumID: ; scans: 18484

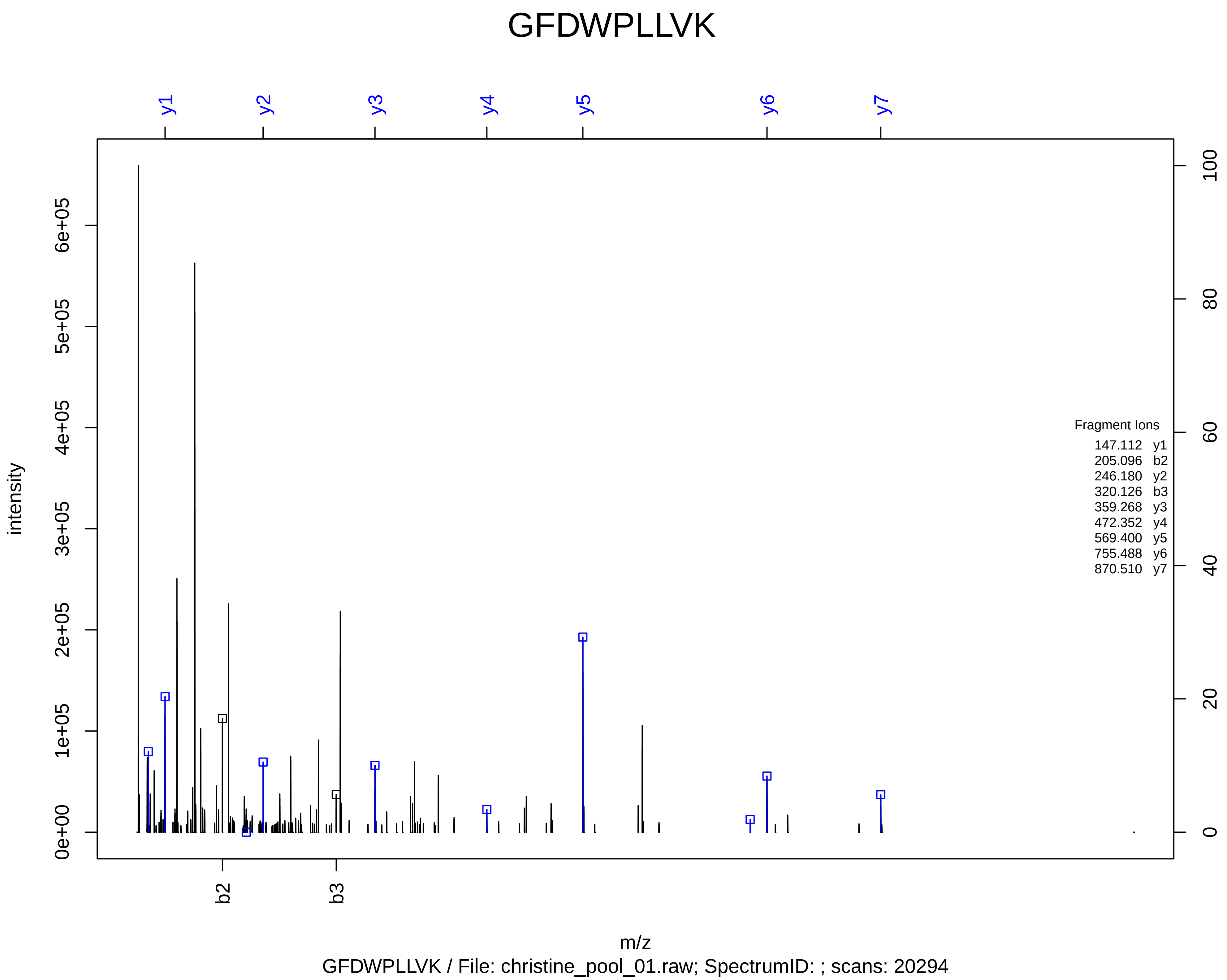
GFDWPLLVK
y1
y2
y3
y4
y5
y6
y7
b2
b3
intensity
0e+00
1e+05
2e+05
3e+05
4e+05
5e+05
6e+05
0
20
40
60
80
100
m/z
Fragment Ions
147.112 y1
205.096 b2
246.180 y2
320.126 b3
359.268 y3
472.352 y4
569.400 y5
755.488 y6
870.510 y7
GFDWPLLVK / File: christine_pool_01.raw; SpectrumID: ; scans: 20294

TQTVTISDNANAVK

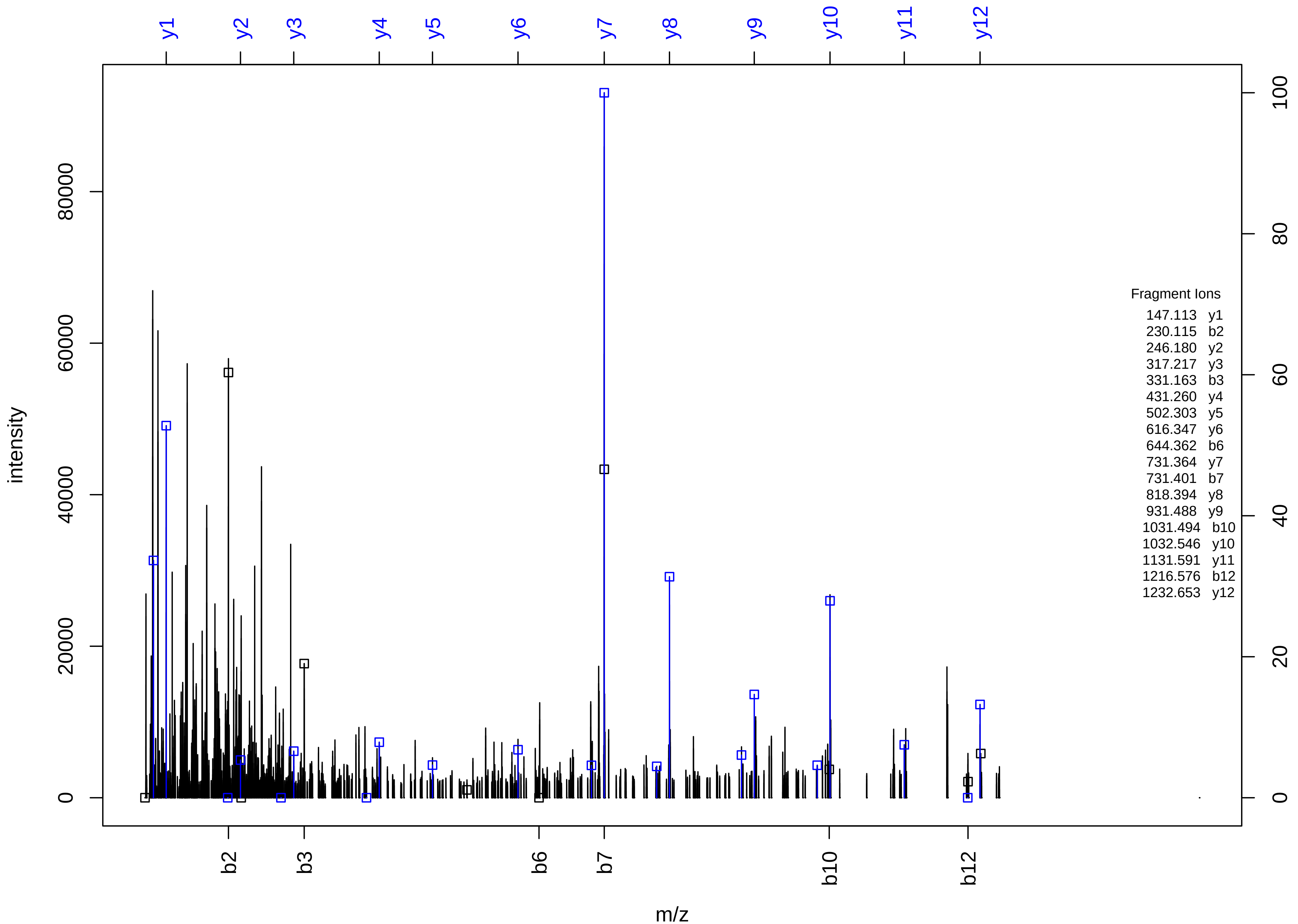

TQTVTISDNANAVK / File: christine_pool_01.raw; SpectrumID: ; scans: 4532

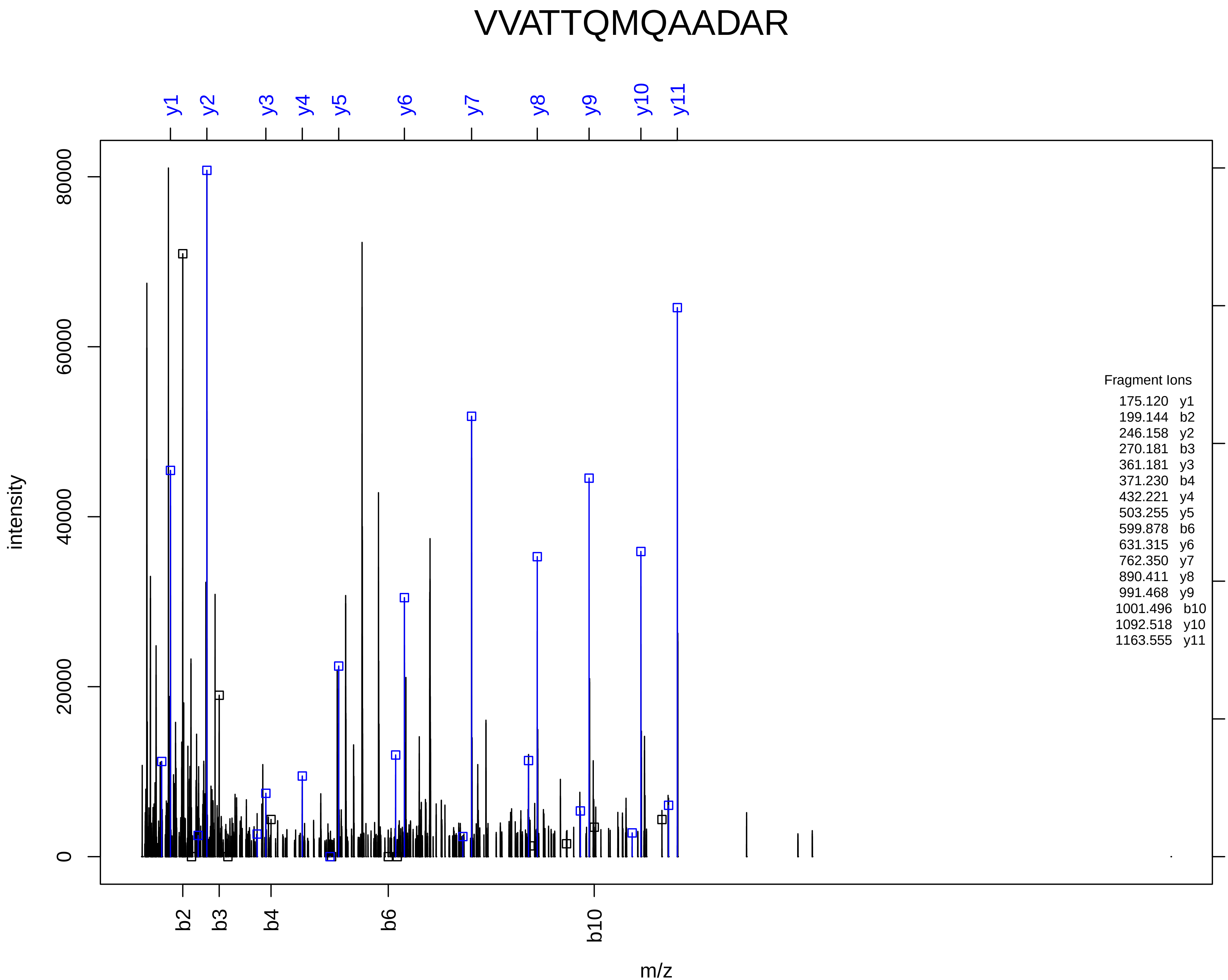

VVATTQMQAADAR
y1 y2 y3 y4 y5 y6 y7 y8 y9 y10 y11
intensity
0
20000
40000
60000
80000
0
20
40
60
80
100
b2 b3 b4 b6 b10
Fragment Ions
175.120 y1
199.144 b2
246.158 y2
270.181 b3
361.181 y3
371.230 b4
432.221 y4
503.255 y5
599.878 b6
631.315 y6
762.350 y7
890.411 y8
991.468 y9
1001.496 b10
1092.518 y10
1163.555 y11
m/z
VVATTQMQAADAR / File: christine_pool_01.raw; SpectrumID: ; scans: 3385

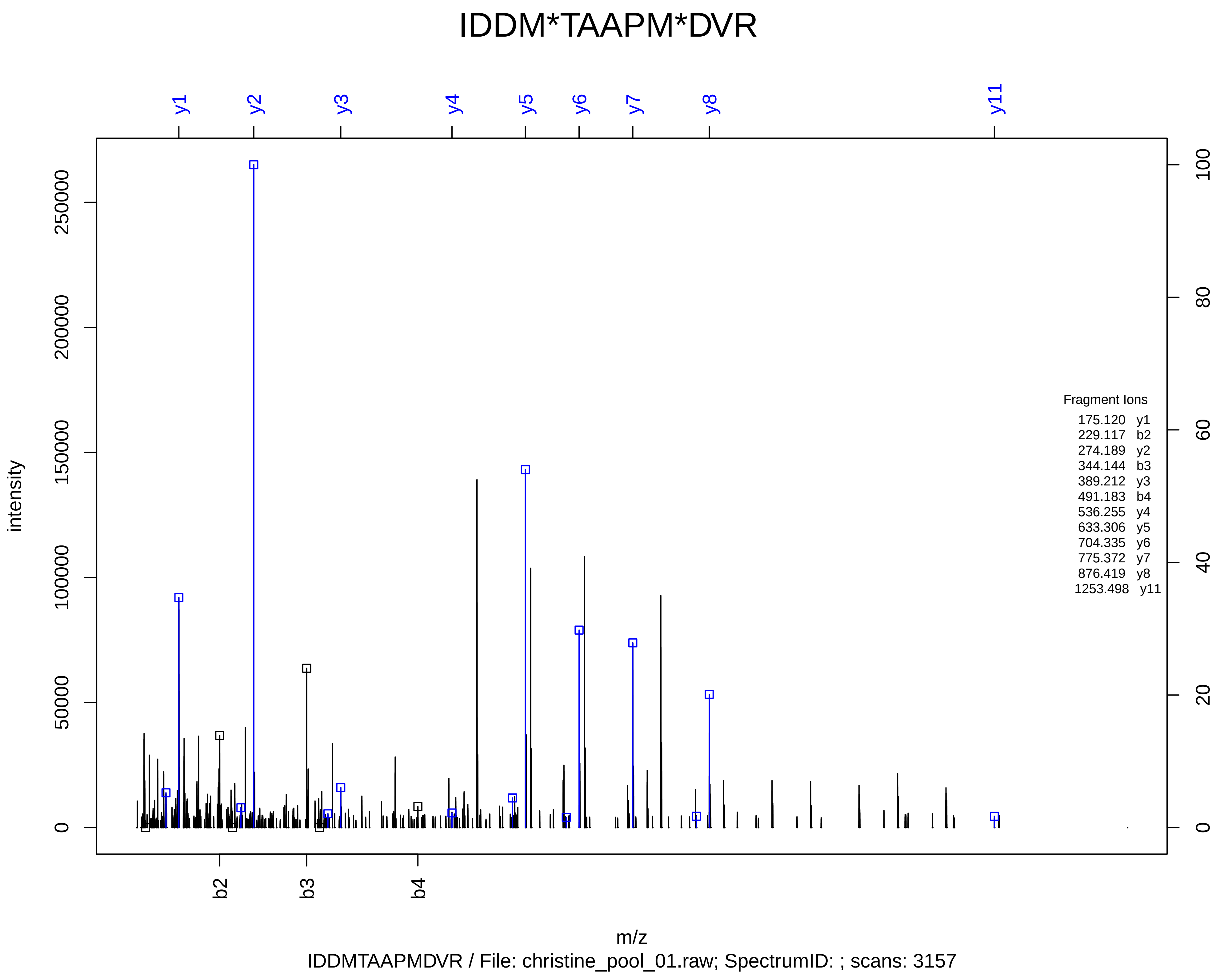
IDDM*TAAPM*DVR
y1
y2
y3
y4
y5
y6
y7
y8
y11
intensity
0
50000
100000
150000
200000
250000
0
20
40
60
80
100
b2
b3
b4
Fragment Ions
175.120 y1
229.117 b2
274.189 y2
344.144 b3
389.212 y3
491.183 b4
536.255 y4
633.306 y5
704.335 y6
775.372 y7
876.419 y8
1253.498 y11
m/z
IDDMTAAPMDVR / File: christine_pool_01.raw; SpectrumID: ; scans: 3157

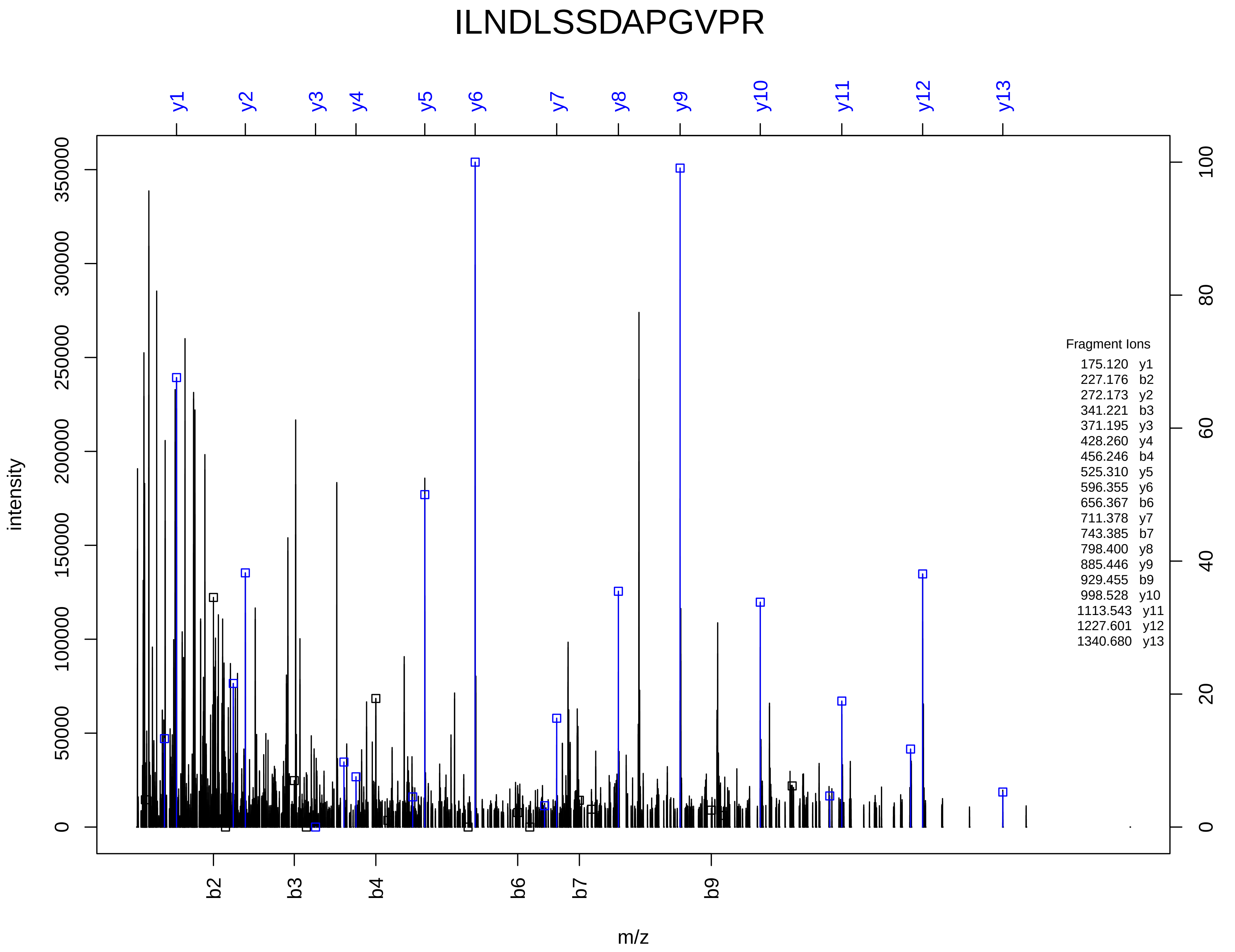

ILNDLSSDAPGVPR / File: christine_pool_01.raw; SpectrumID: ; scans: 10070

HLVSPEALDLLDK

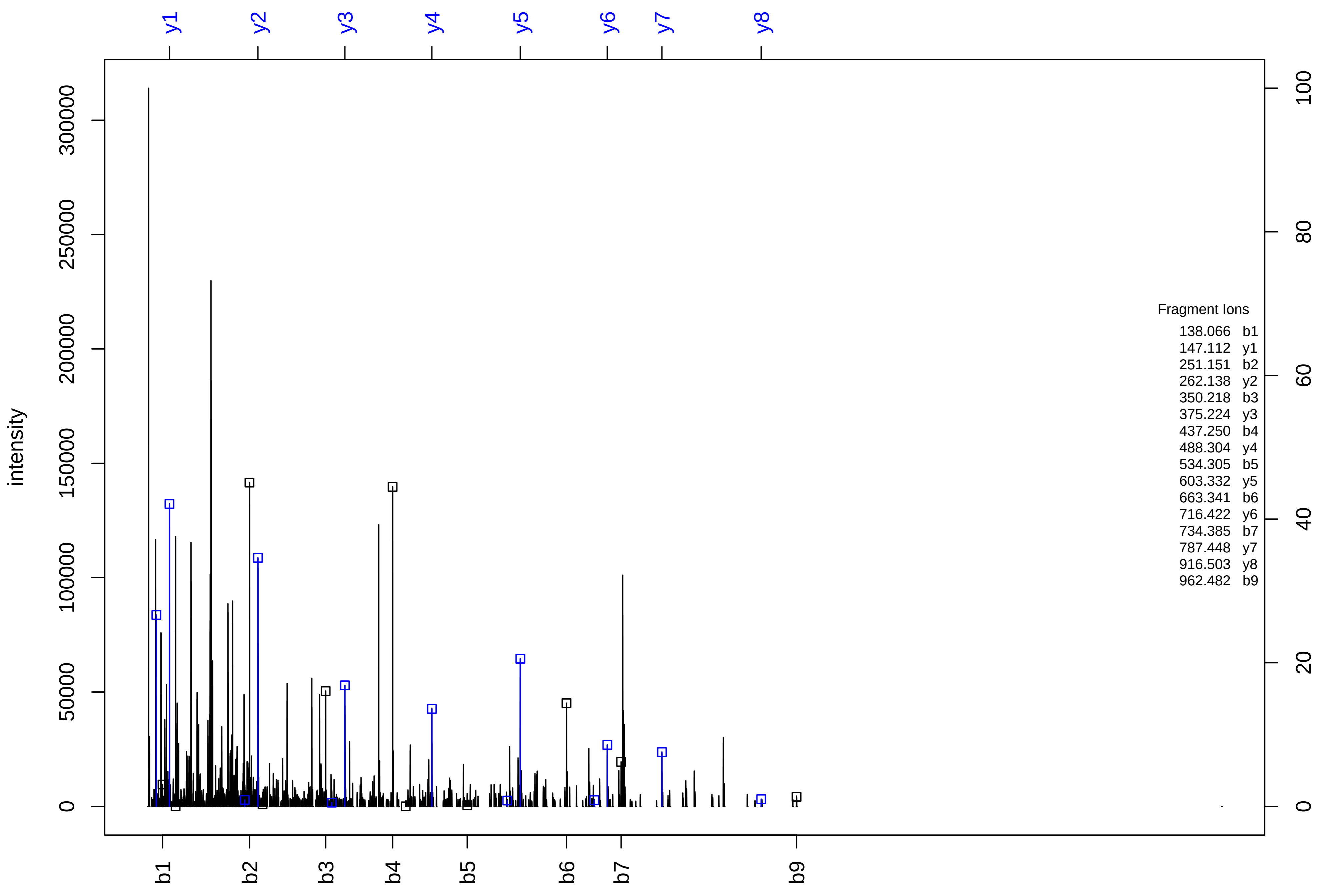

HLVSPEALDLLDK / File: christine_pool_01.raw; SpectrumID: ; scans: 14225

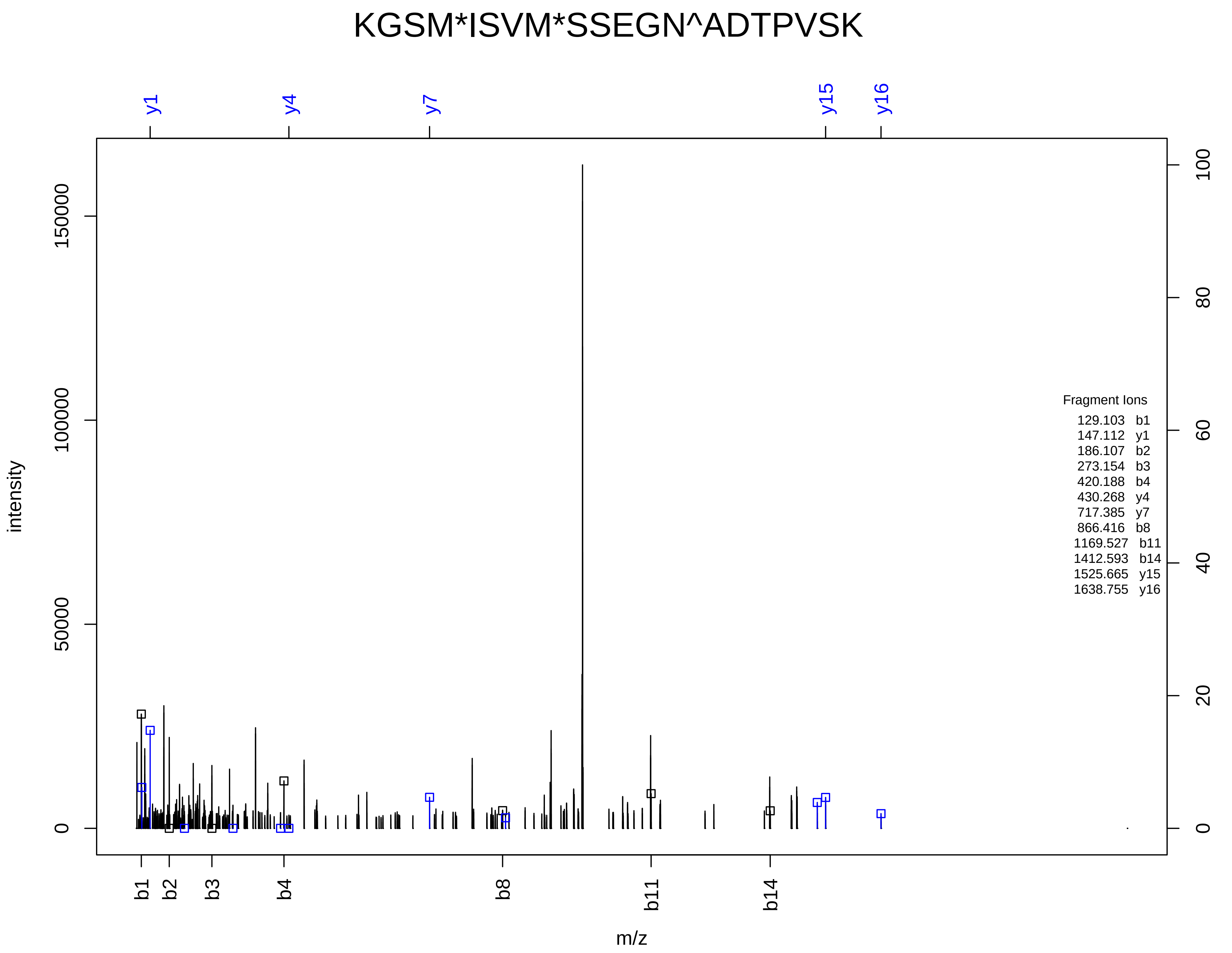
KGSM*ISVM*SSEGN^ADTPVSK
y1
y4
y7
y15
y16
intensity
150000
100000
50000
0
100
80
60
40
20
0
Fragment Ions
129.103 b1
147.112 y1
186.107 b2
273.154 b3
420.188 b4
430.268 y4
717.385 y7
866.416 b8
1169.527 b11
1412.593 b14
1525.665 y15
1638.755 y16
b1
b2
b3
b4
b8
b11
b14
m/z
KGSMISVMSSEGNADTPVSK / File: christine_pool_01.raw; SpectrumID: ; scans: 6992

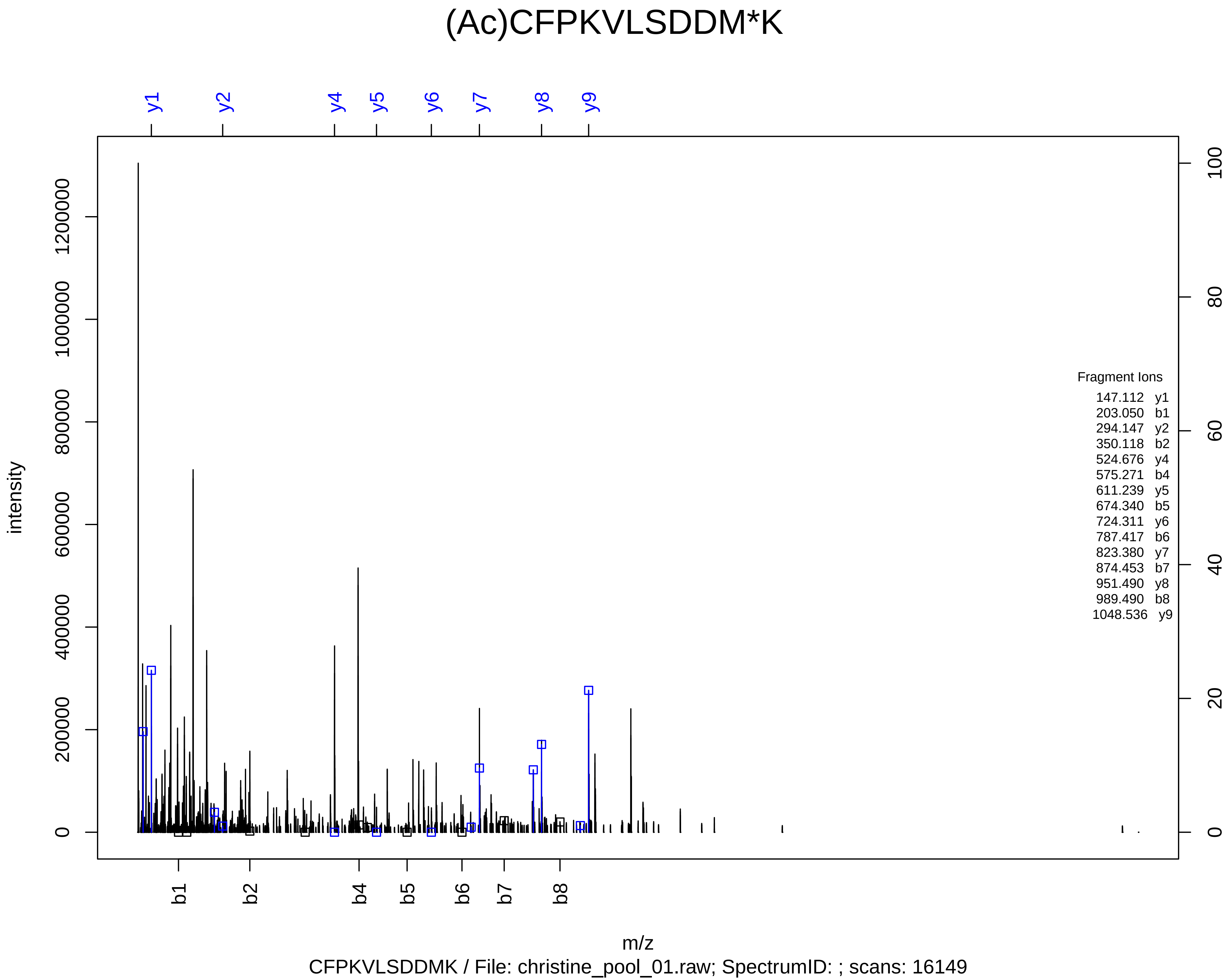
(Ac)CFPKVLSDDM*K
y1
y2
y4
y5
y6
y7
y8
y9
intensity
0
200000
400000
600000
800000
1000000
1200000
0
20
40
60
80
100
Fragment Ions
147.112 y1
203.050 b1
294.147 y2
350.118 b2
524.676 y4
575.271 b4
611.239 y5
674.340 b5
724.311 y6
787.417 b6
823.380 y7
874.453 b7
951.490 y8
989.490 b8
1048.536 y9
b1
b2
b4
b5
b6
b7
b8
m/z
CFPKVLSDDMK / File: christine_pool_01.raw; SpectrumID: ; scans: 16149

SLLVPDHVITR

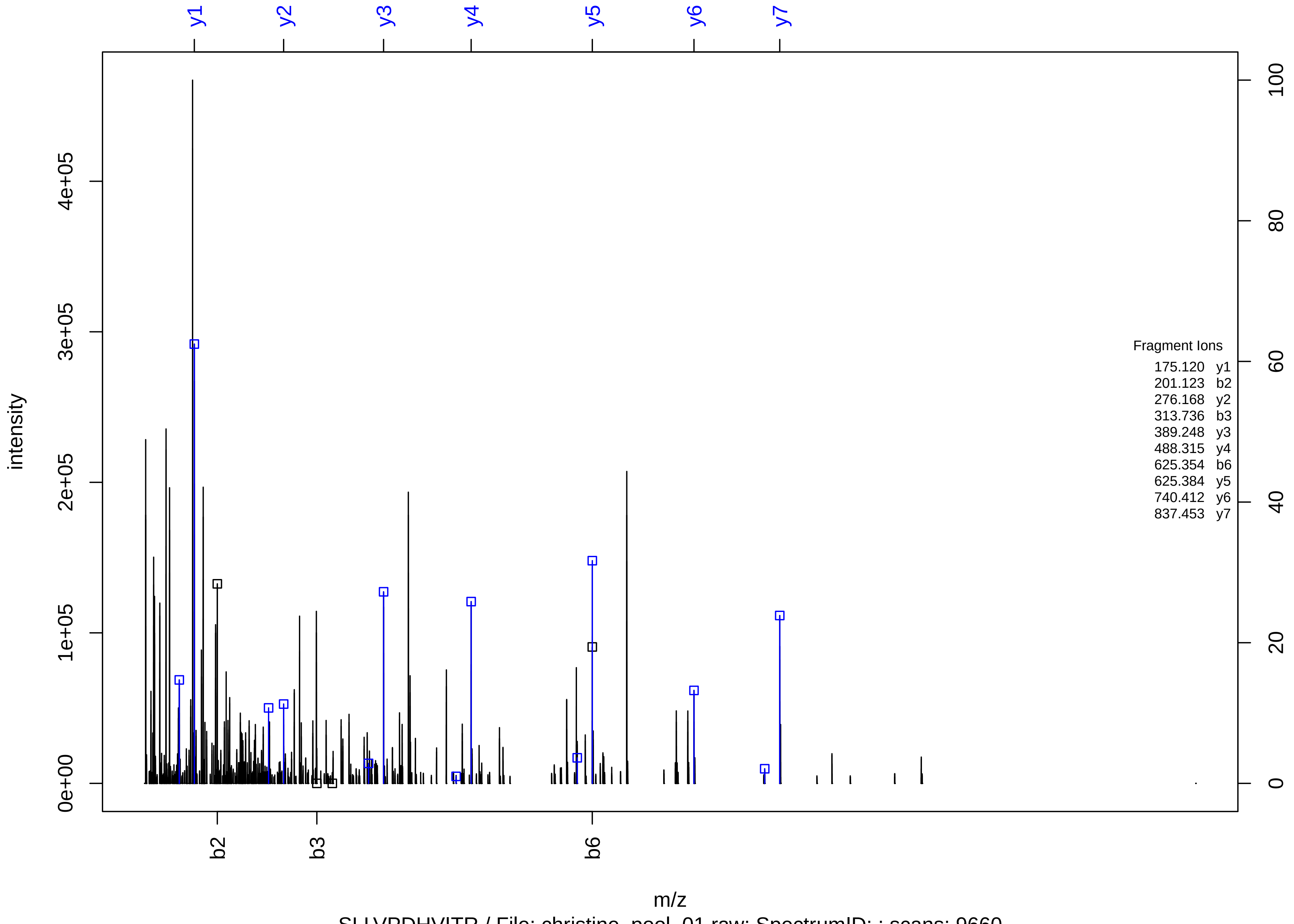

SLLVPDHVITR / File: christine_pool_01.raw; SpectrumID: ; scans: 9660

ISIEGNIAAGK

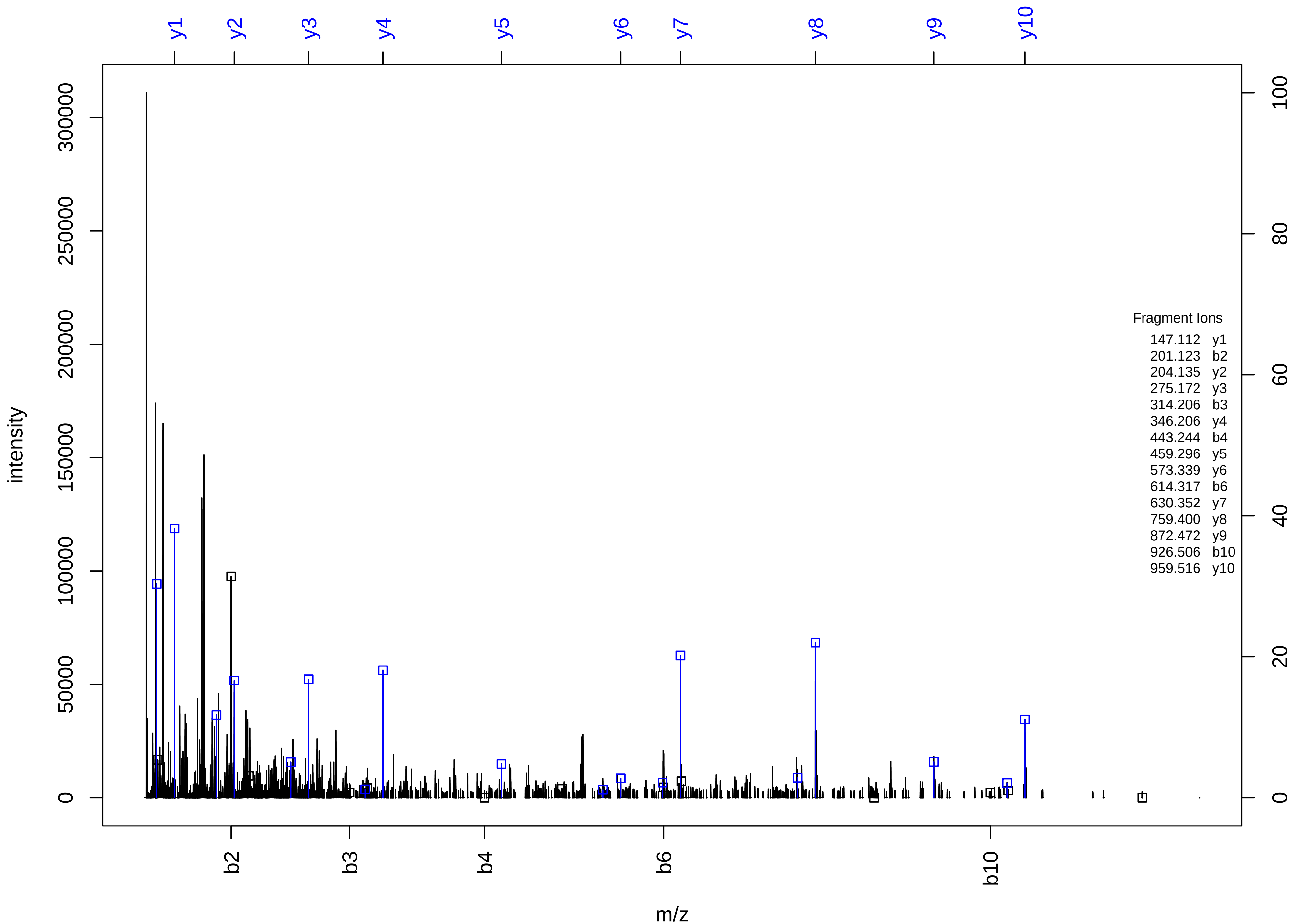

ISIEGNIAAGK / File: christine_pool_01.raw; SpectrumID: ; scans: 6704

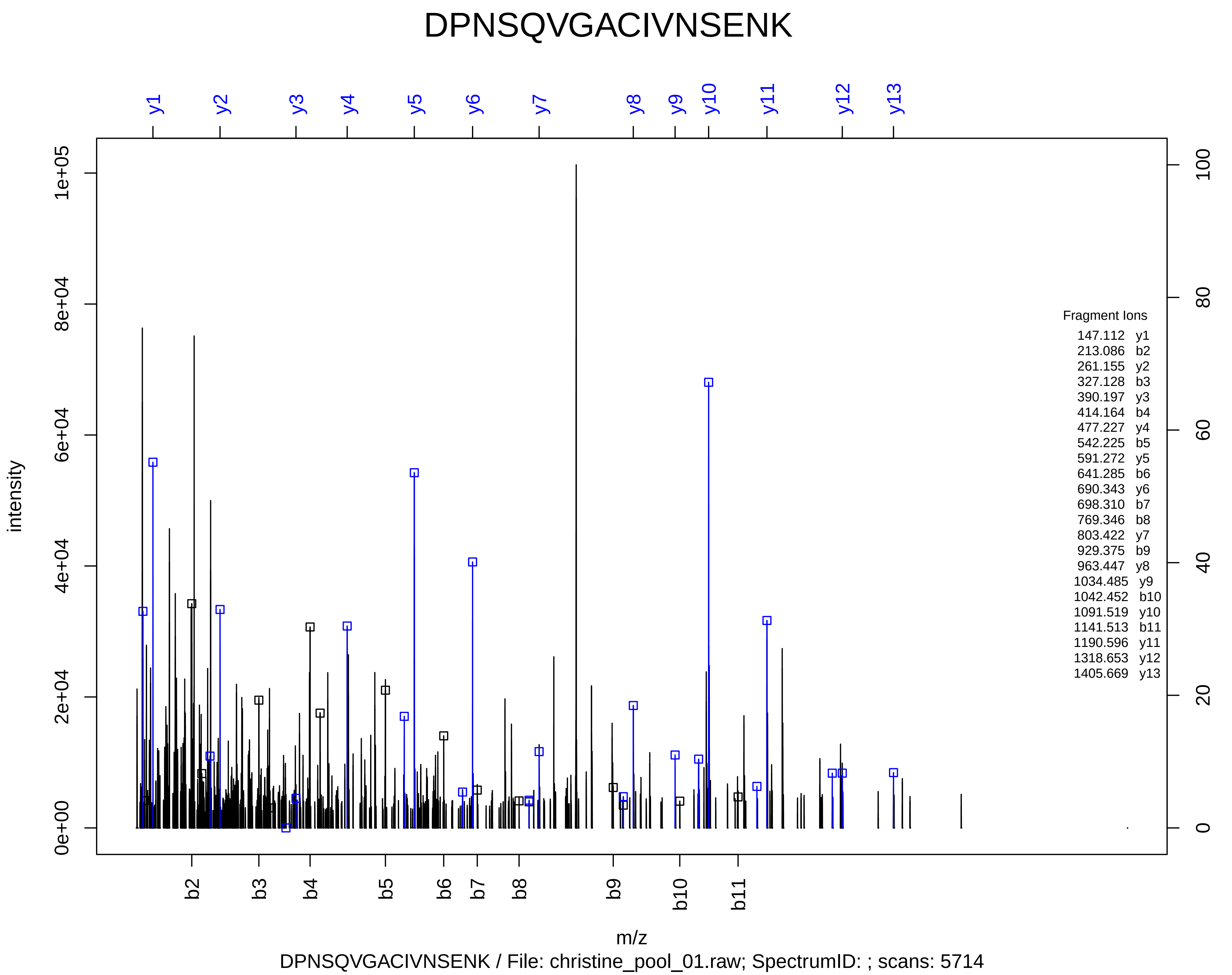

DPNSQVGACIVNSENK
y1 y2 y3 y4 y5 y6 y7 y8 y9 y10 y11 y12 y13
b2 b3 b4 b5 b6 b7 b8 b9 b10 b11
intensity
0e+00
2e+04
4e+04
6e+04
8e+04
1e+05
0
20
40
60
80
100
m/z
Fragment Ions
147.112 y1
213.086 b2
261.155 y2
327.128 b3
390.197 y3
414.164 b4
477.227 y4
542.225 b5
591.272 y5
641.285 b6
690.343 y6
698.310 b7
769.346 b8
803.422 y7
929.375 b9
963.447 y8
1034.485 y9
1042.452 b10
1091.519 y10
1141.513 b11
1190.596 y11
1318.653 y12
1405.669 y13
DPNSQVGACIVNSENK / File: christine_pool_01.raw; SpectrumID: ; scans: 5714

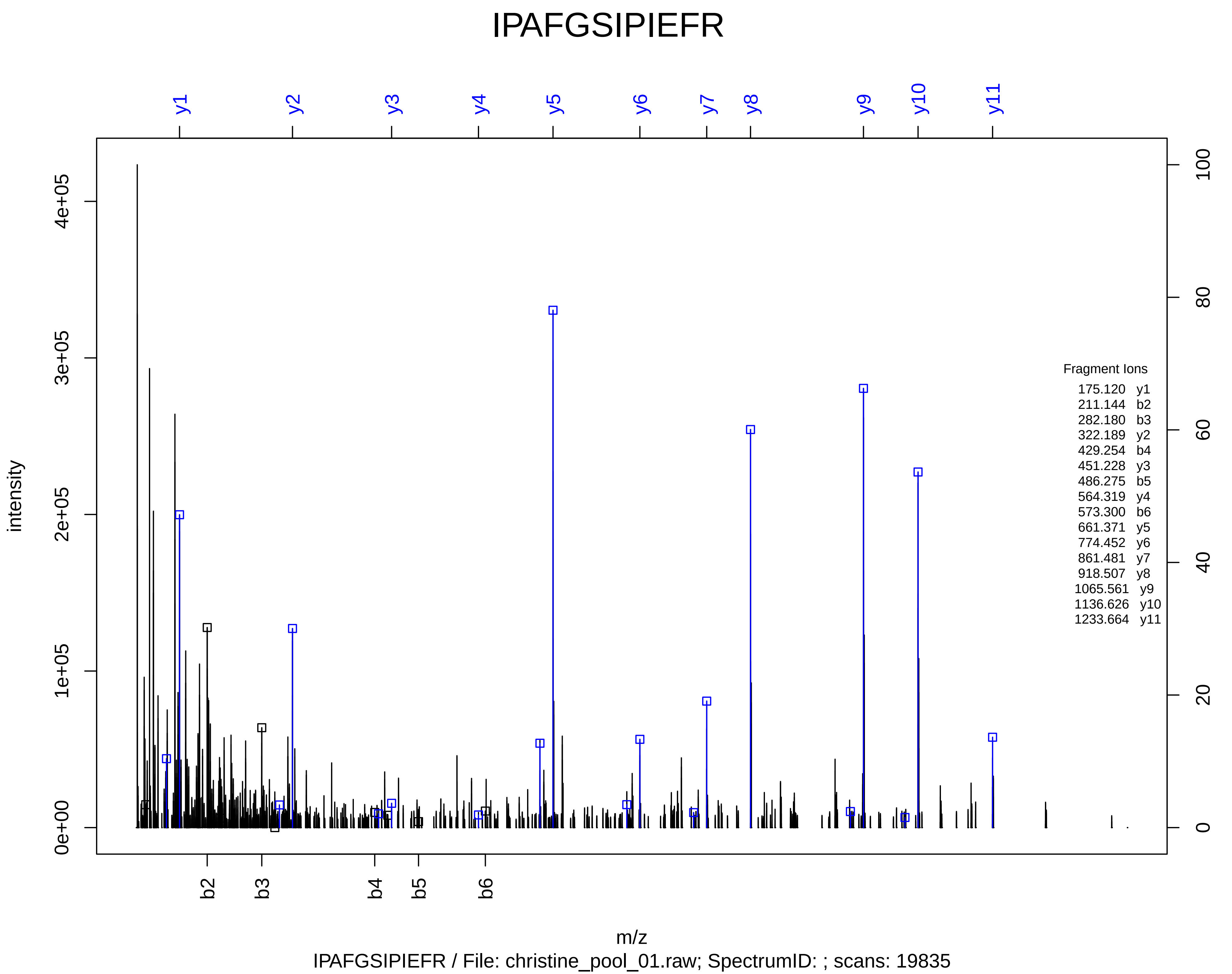

IPAFGSIPIEFR
y1
y2
y3
y4
y5
y6
y7
y8
y9
y10
y11
intensity
0e+00
1e+05
2e+05
3e+05
4e+05
0
20
40
60
80
100
b2
b3
b4
b5
b6
Fragment Ions
175.120 y1
211.144 b2
282.180 b3
322.189 y2
429.254 b4
451.228 y3
486.275 b5
564.319 y4
573.300 b6
661.371 y5
774.452 y6
861.481 y7
918.507 y8
1065.561 y9
1136.626 y10
1233.664 y11
m/z
IPAFGSIPIEFR / File: christine_pool_01.raw; SpectrumID: ; scans: 19835

LPREEEEEEEEDDDEEEK

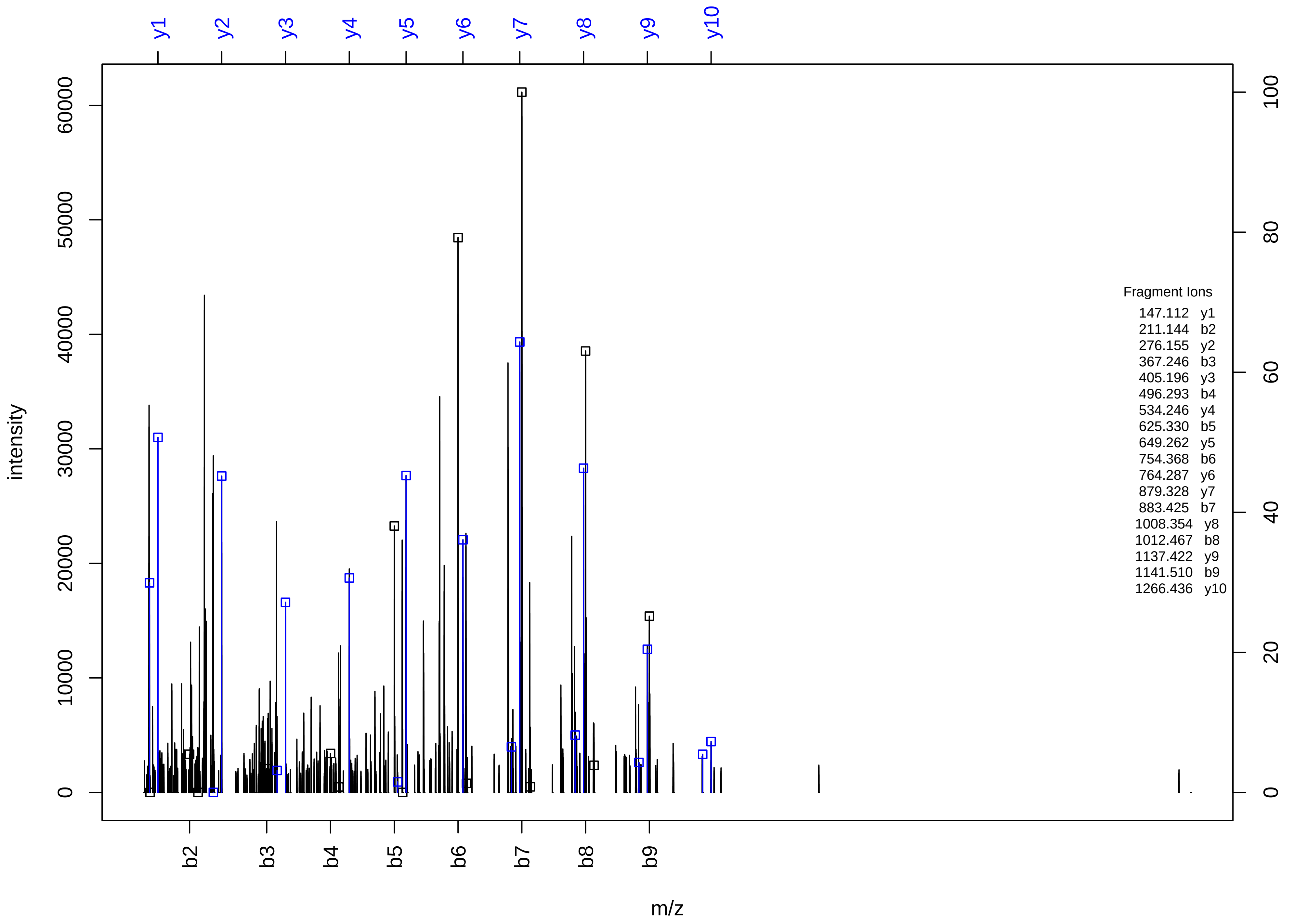

LPREEEEEEEEDDDEEEK / File: christine_pool_01.raw; SpectrumID: ; scans: 2077

IQAAASTPTNATAASDANTGDR

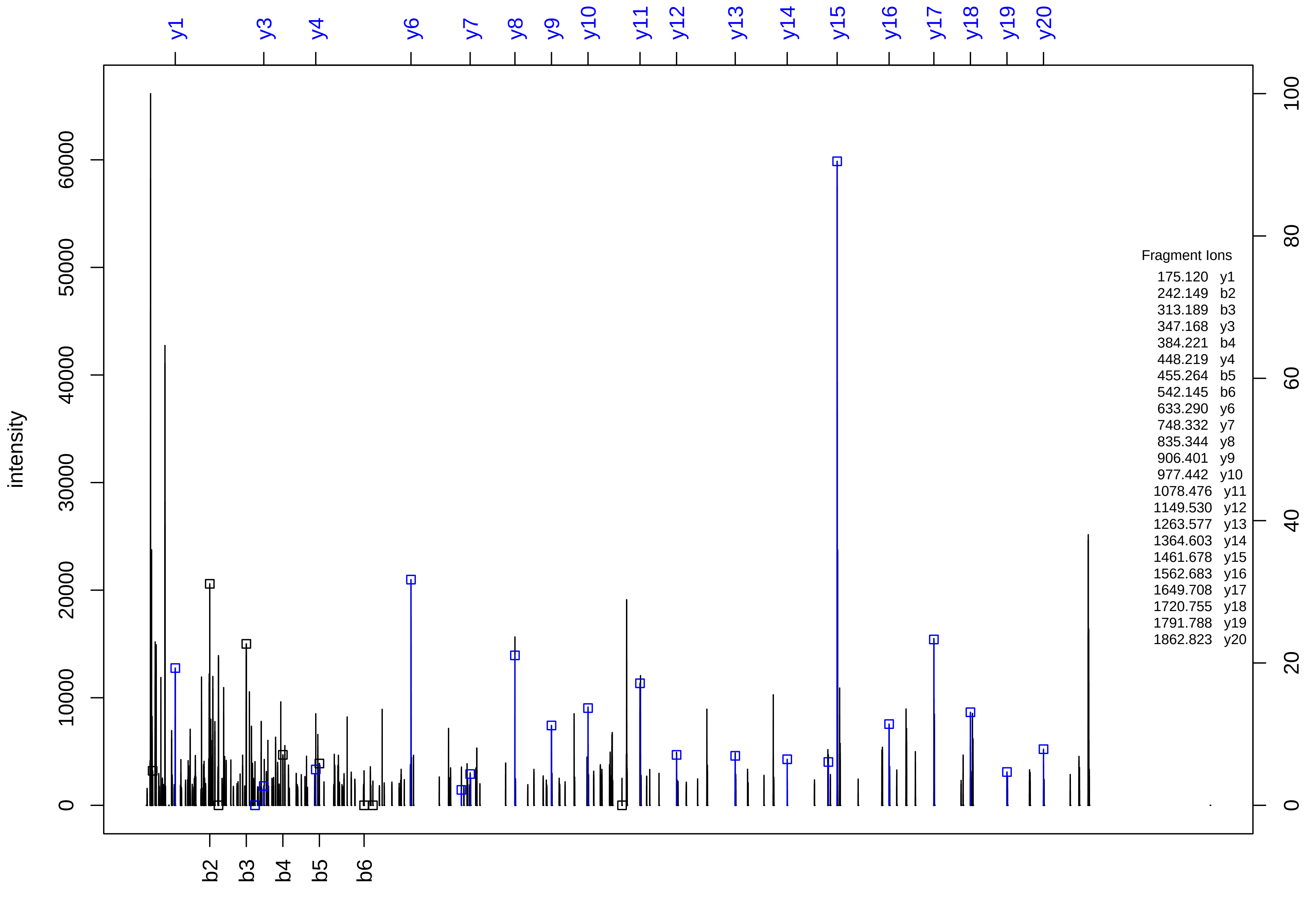

IQAAASTPTNATAASDANTGDR / File: christine_pool_01.raw; SpectrumID: ; scans: 3363

HLDSSSNERPDISSIQR

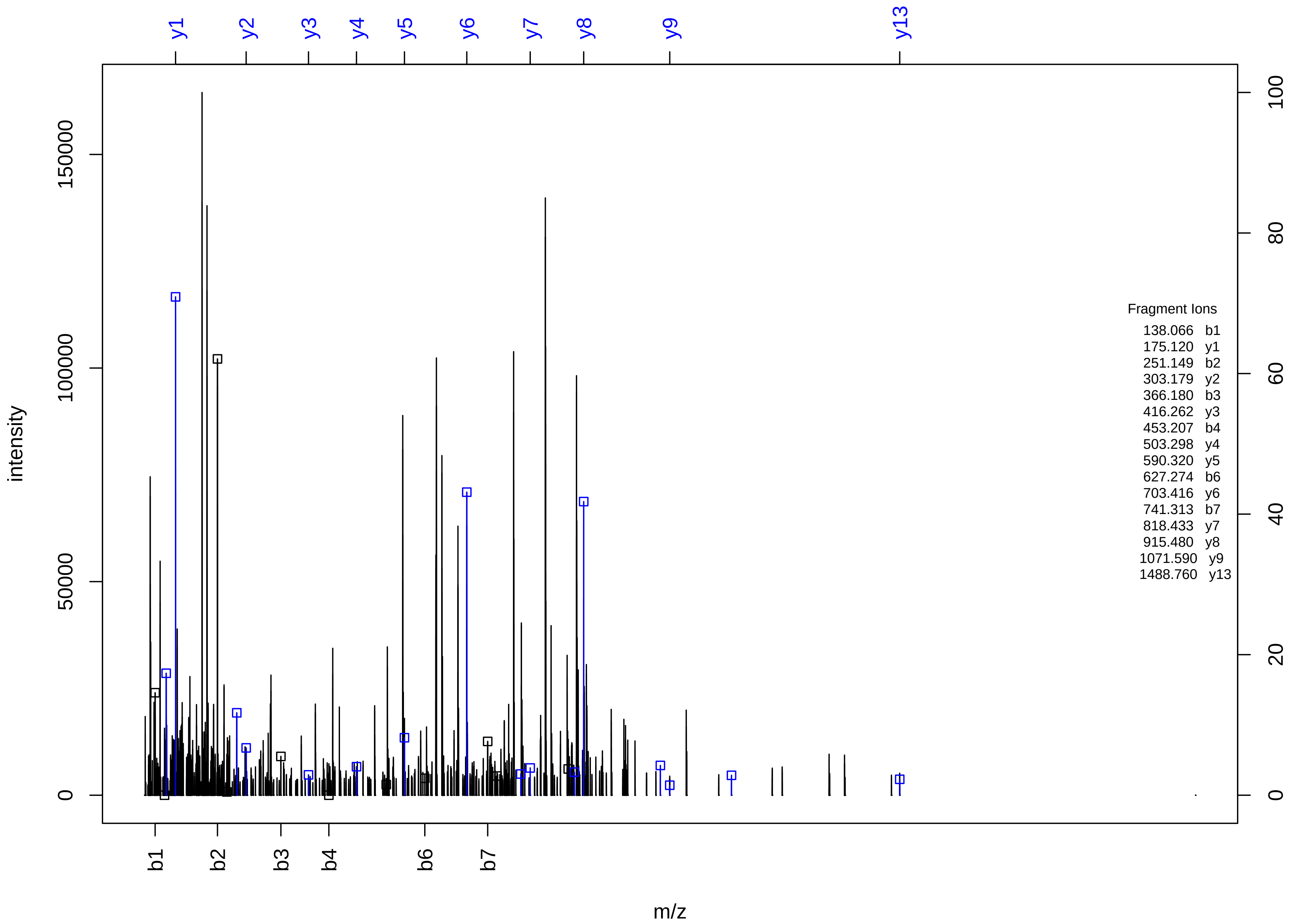

HLDSSSNERPDISSIQR / File: christine_pool_01.raw; SpectrumID: ; scans: 3978

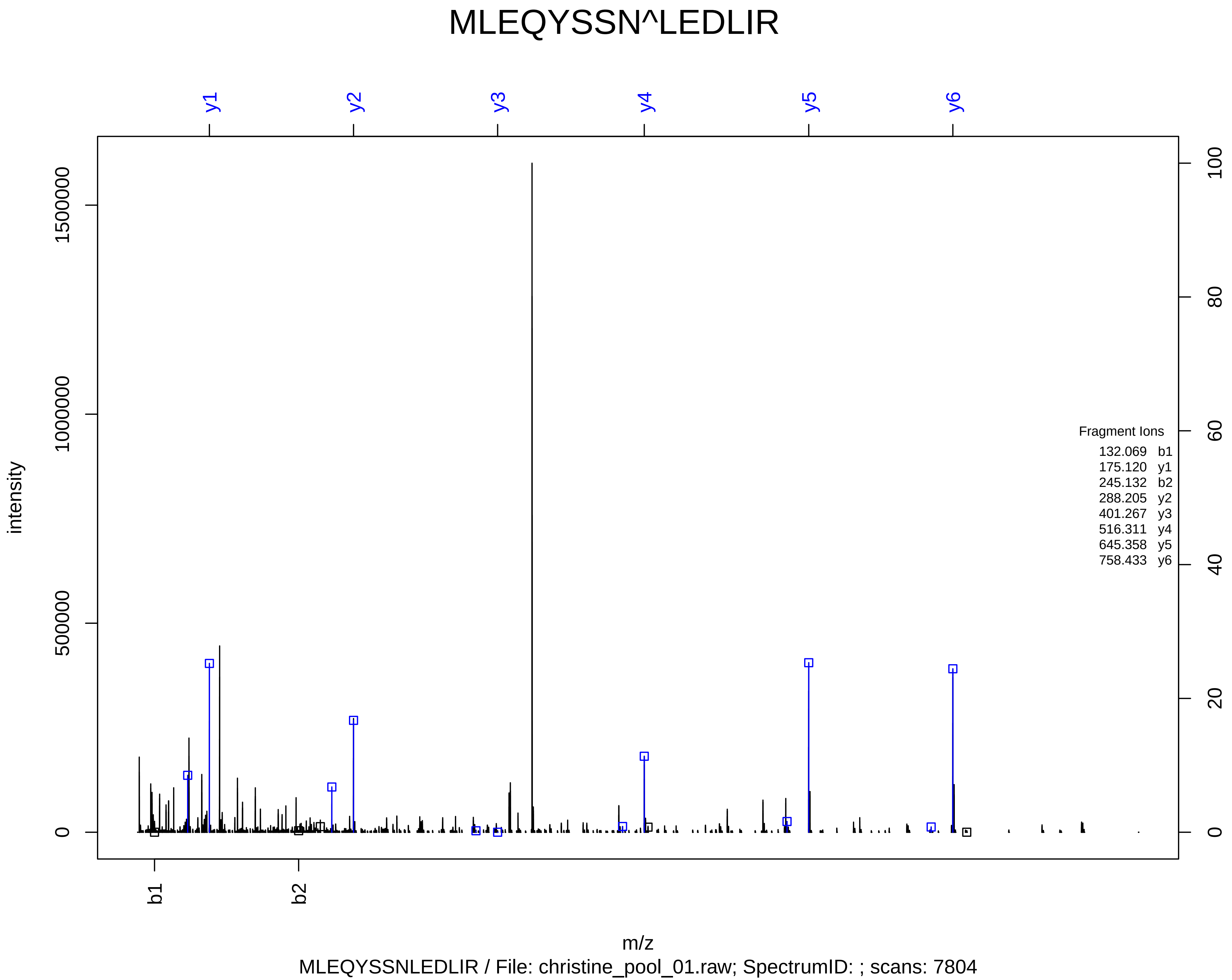
MLEQYSSN^LEDLIR
y1
y2
y3
y4
y5
y6
intensity
1500000
1000000
500000
0
100
80
60
40
20
0
Fragment Ions
132.069 b1
175.120 y1
245.132 b2
288.205 y2
401.267 y3
516.311 y4
645.358 y5
758.433 y6
b1
b2
m/z
MLEQYSSNLEDLIR / File: christine_pool_01.raw; SpectrumID: ; scans: 7804

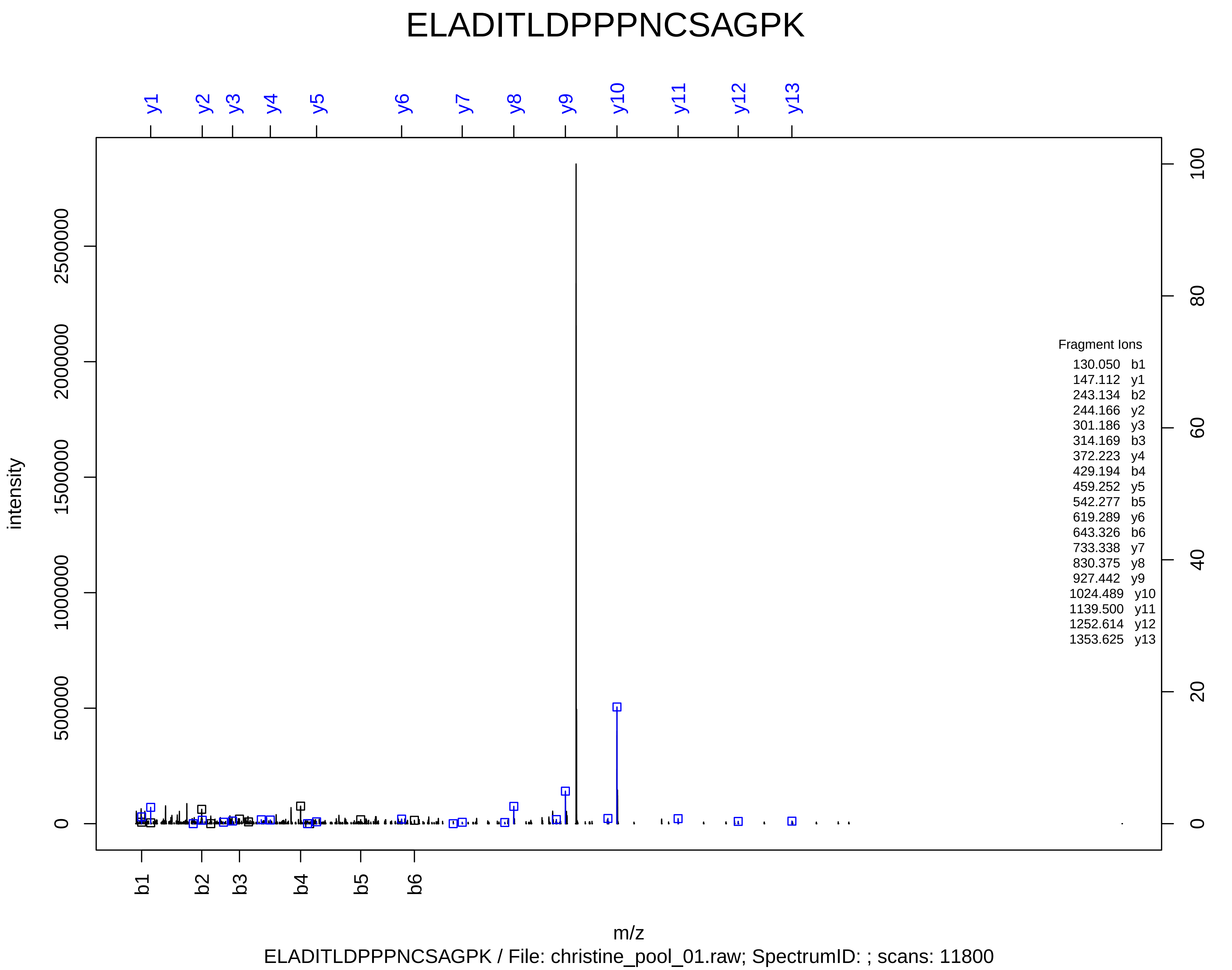
ELADITLDPPPNCSAGPK
y1 y2 y3 y4 y5 y6 y7 y8 y9 y10 y11 y12 y13
intensity
0
500000
1000000
1500000
2000000
2500000
0 20 40 60 80 100
b1 b2 b3 b4 b5 b6
m/z
Fragment Ions
130.050 b1
147.112 y1
243.134 b2
244.166 y2
301.186 y3
314.169 b3
372.223 y4
429.194 b4
459.252 y5
542.277 b5
619.289 y6
643.326 b6
733.338 y7
830.375 y8
927.442 y9
1024.489 y10
1139.500 y11
1252.614 y12
1353.625 y13
ELADITLDPPPNCSAGPK / File: christine_pool_01.raw; SpectrumID: ; scans: 11800

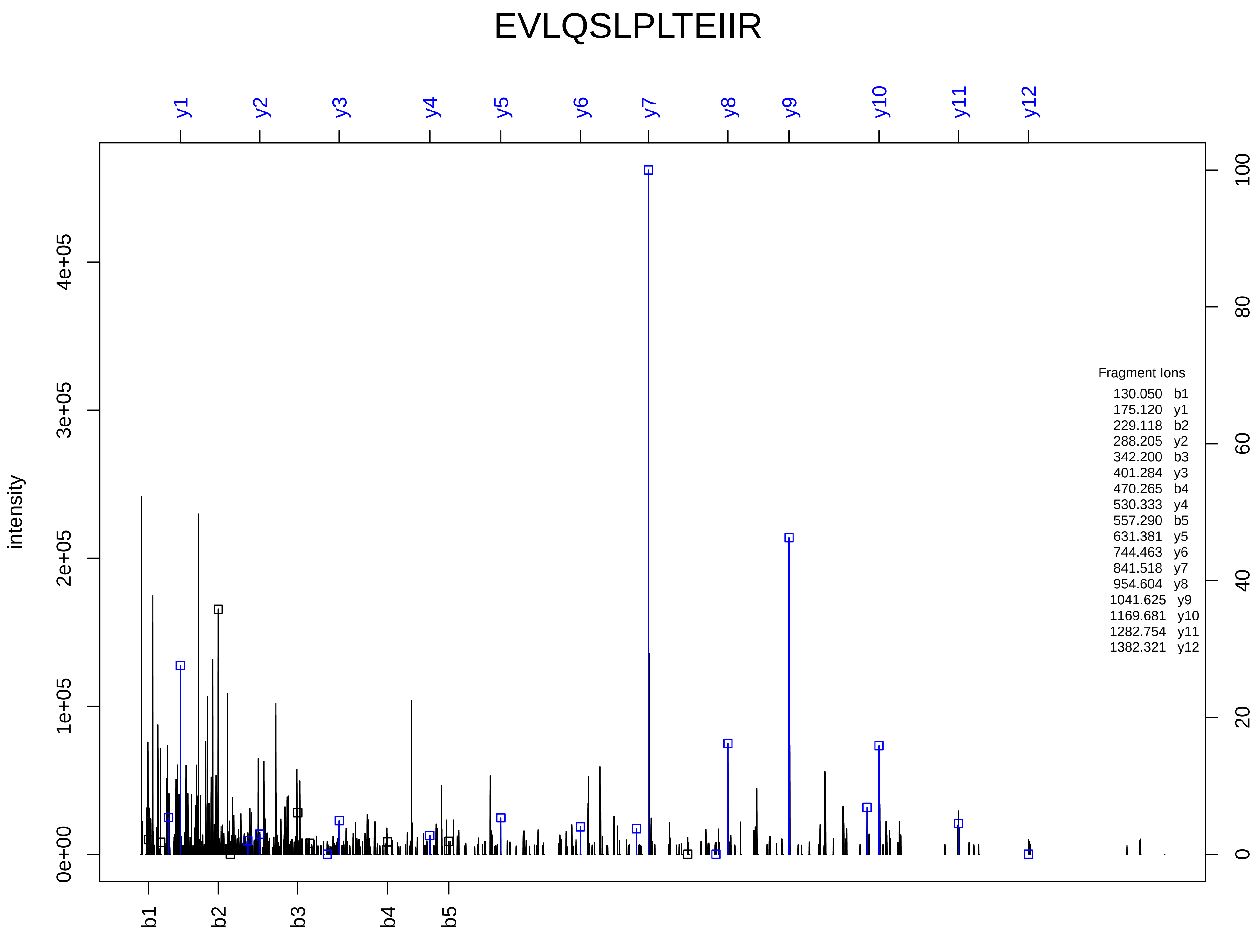

EVLQSLPLTEIIR / File: christine_pool_01.raw; SpectrumID: ; scans: 21055

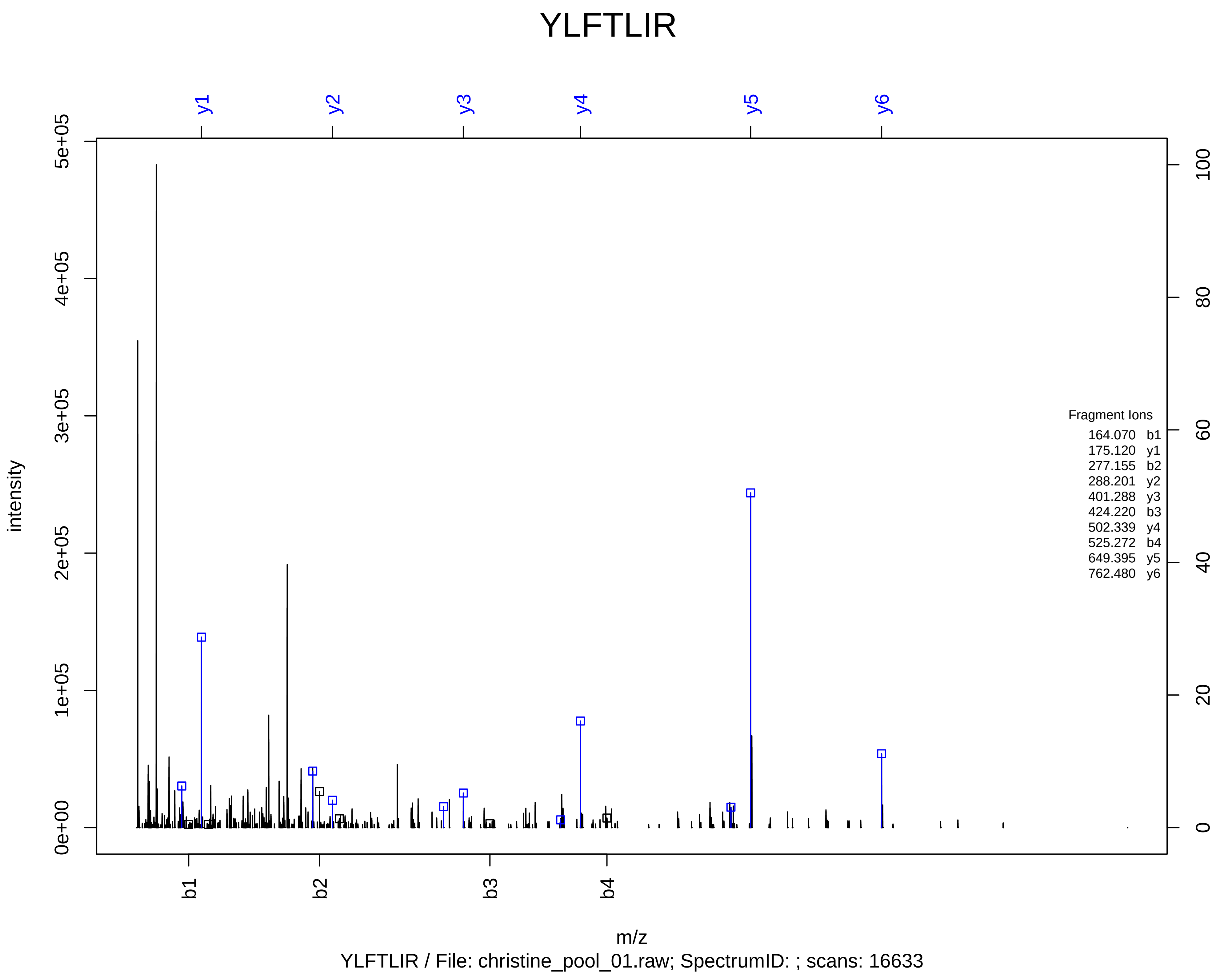

YLFTLIR
y1
y2
y3
y4
y5
y6
intensity
0e+00
1e+05
2e+05
3e+05
4e+05
5e+05
0
20
40
60
80
100
Fragment Ions
164.070 b1
175.120 y1
277.155 b2
288.201 y2
401.288 y3
424.220 b3
502.339 y4
525.272 b4
649.395 y5
762.480 y6
b1
b2
b3
b4
m/z
YLFTLIR / File: christine_pool_01.raw; SpectrumID: ; scans: 16633

PVAVGPYGQSQPSCFDR

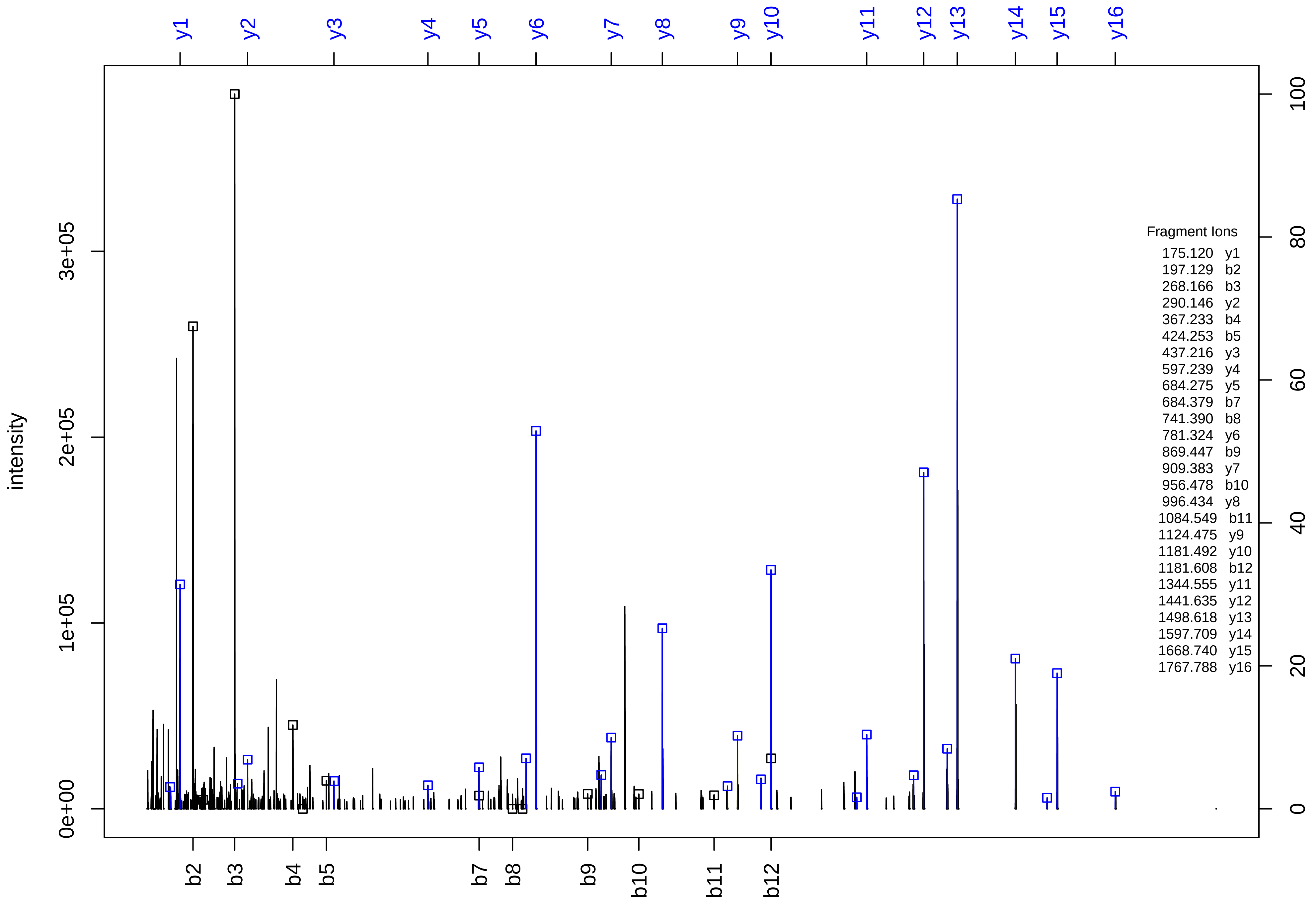

PVAVGPYGQSQPSCFDR / File: christine_pool_01.raw; SpectrumID: ; scans: 7930

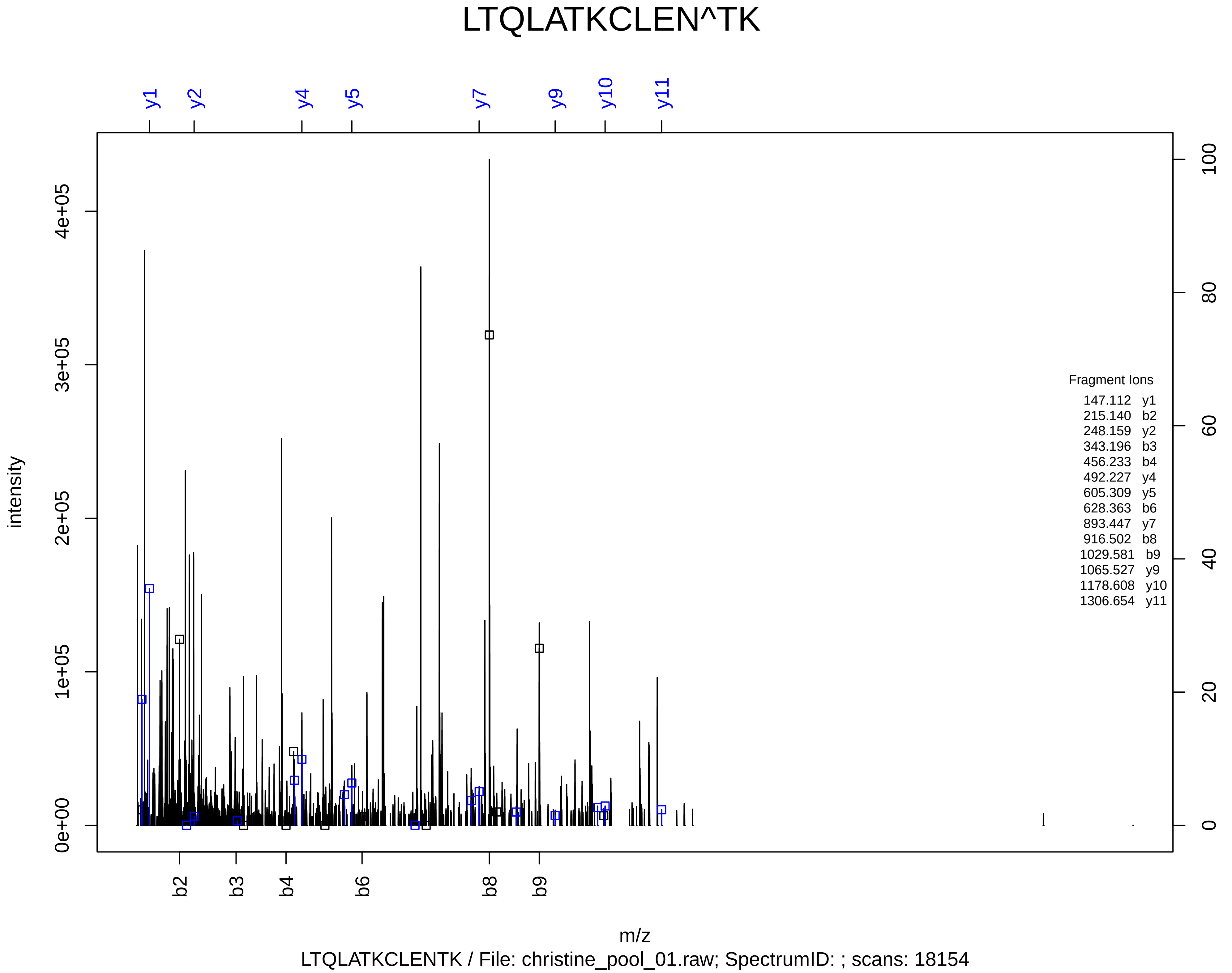
LTQLATKCLEN^TK
y1
y2
y4
y5
y7
y9
y10
y11
intensity
0e+00
1e+05
2e+05
3e+05
4e+05
0
20
40
60
80
100
b2
b3
b4
b6
b8
b9
m/z
Fragment Ions
147.112 y1
215.140 b2
248.159 y2
343.196 b3
456.233 b4
492.227 y4
605.309 y5
628.363 b6
893.447 y7
916.502 b8
1029.581 b9
1065.527 y9
1178.608 y10
1306.654 y11
LTQLATKCLENTK / File: christine_pool_01.raw; SpectrumID: ; scans: 18154

DCDLQEDACYNCGR

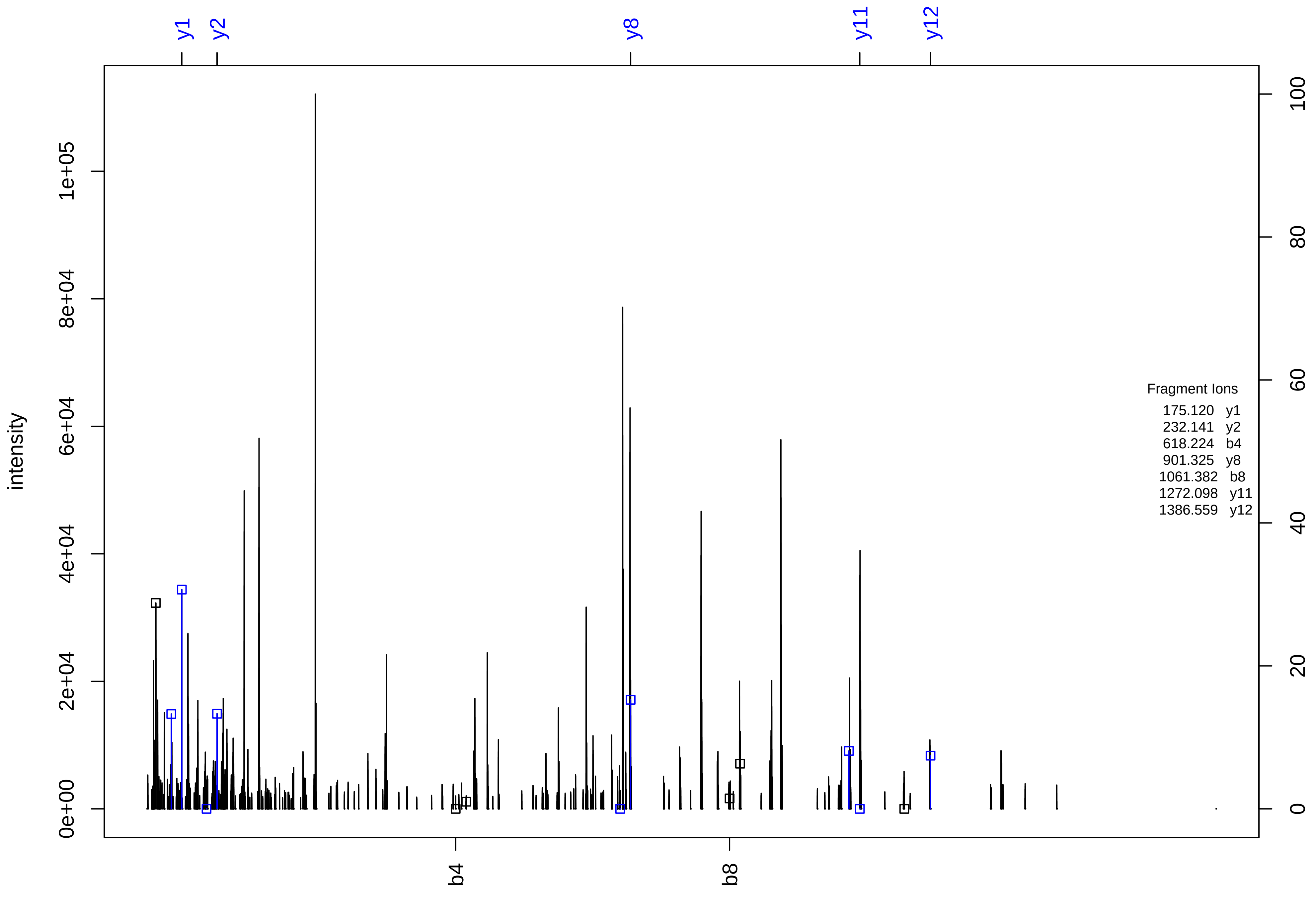

DCDLQEDACYNCGR / File: christine_pool_01.raw; SpectrumID: ; scans: 4175

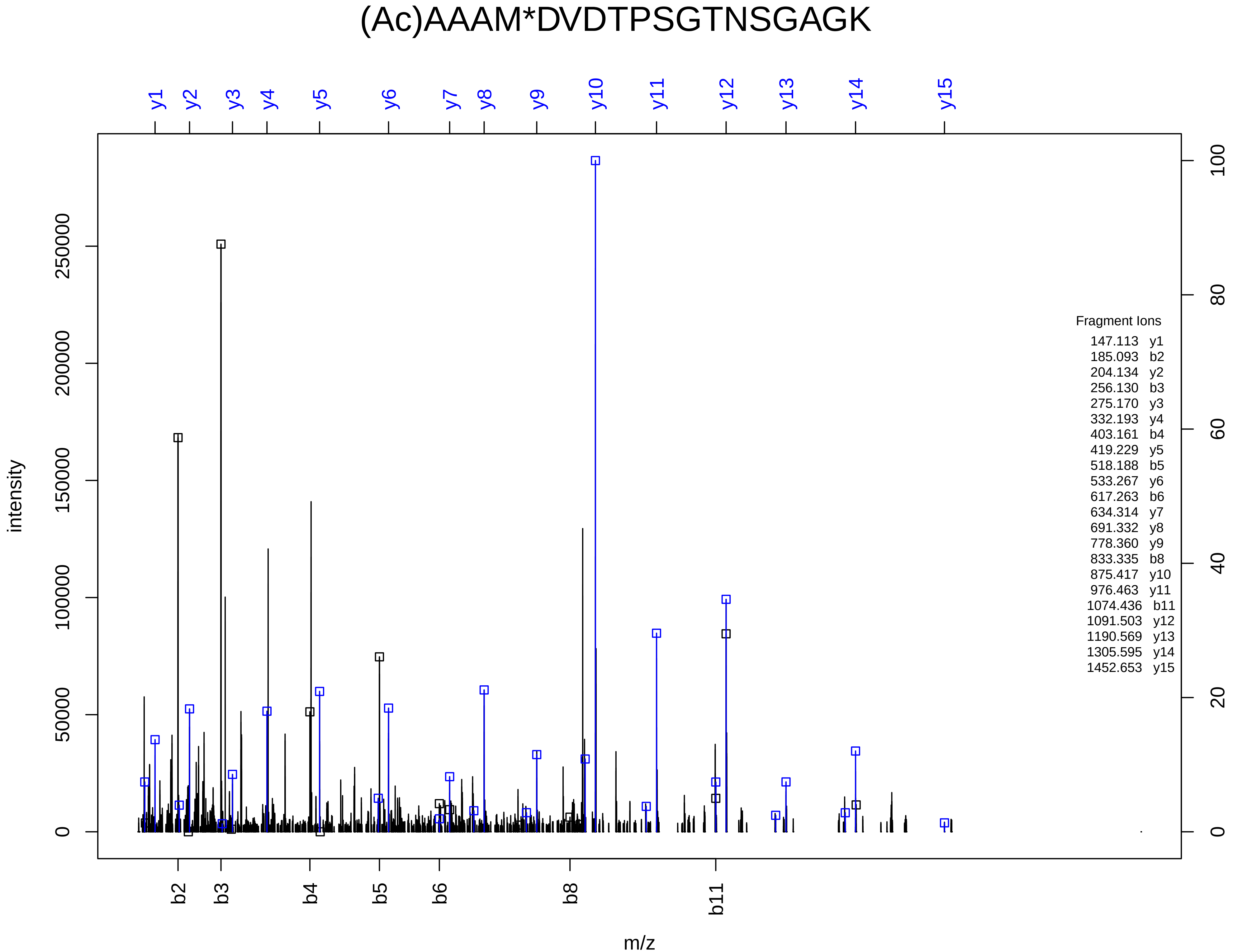

AAAMDVDTPSGTNSGAGK / File: christine_pool_01.raw; SpectrumID: ; scans: 3975

LYTLVTYVPVTTFK

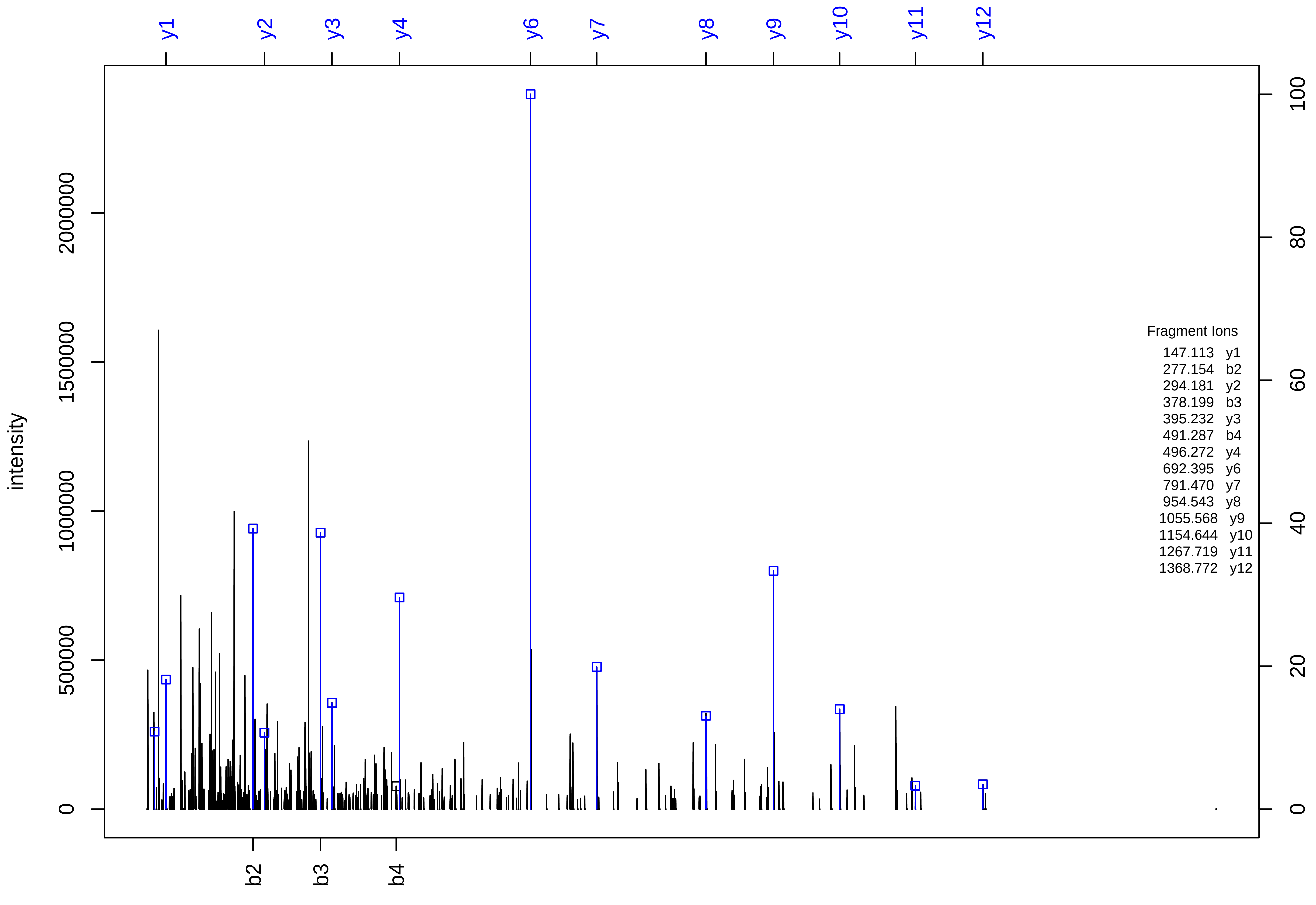

LYTLVTYVPVTTFK / File: christine_pool_01.raw; SpectrumID: ; scans: 20311

LECVEPNCR

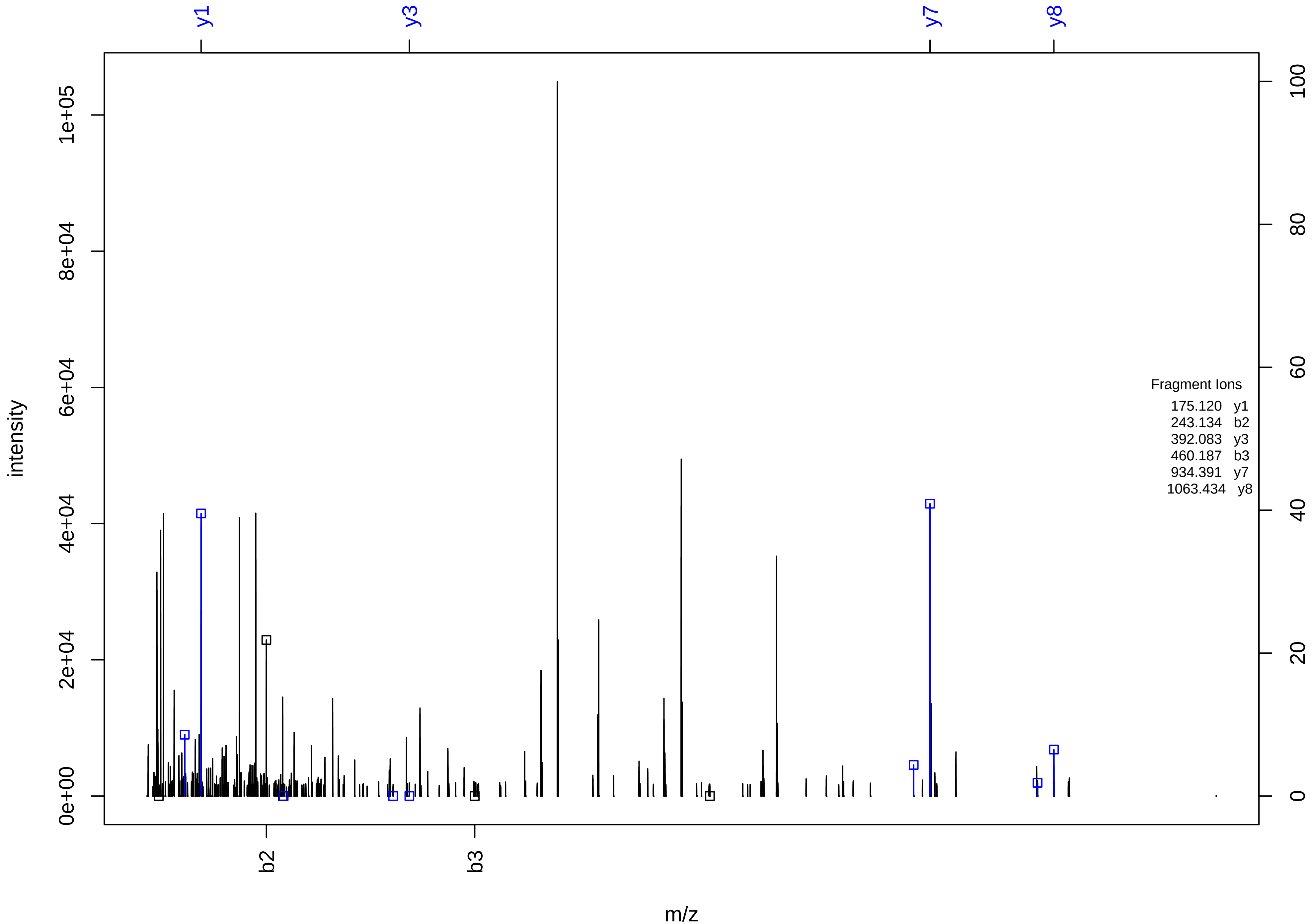

LECVEPNCR / File: christine_pool_01.raw; SpectrumID: ; scans: 2659

EQGQAPITPQQGQALAK

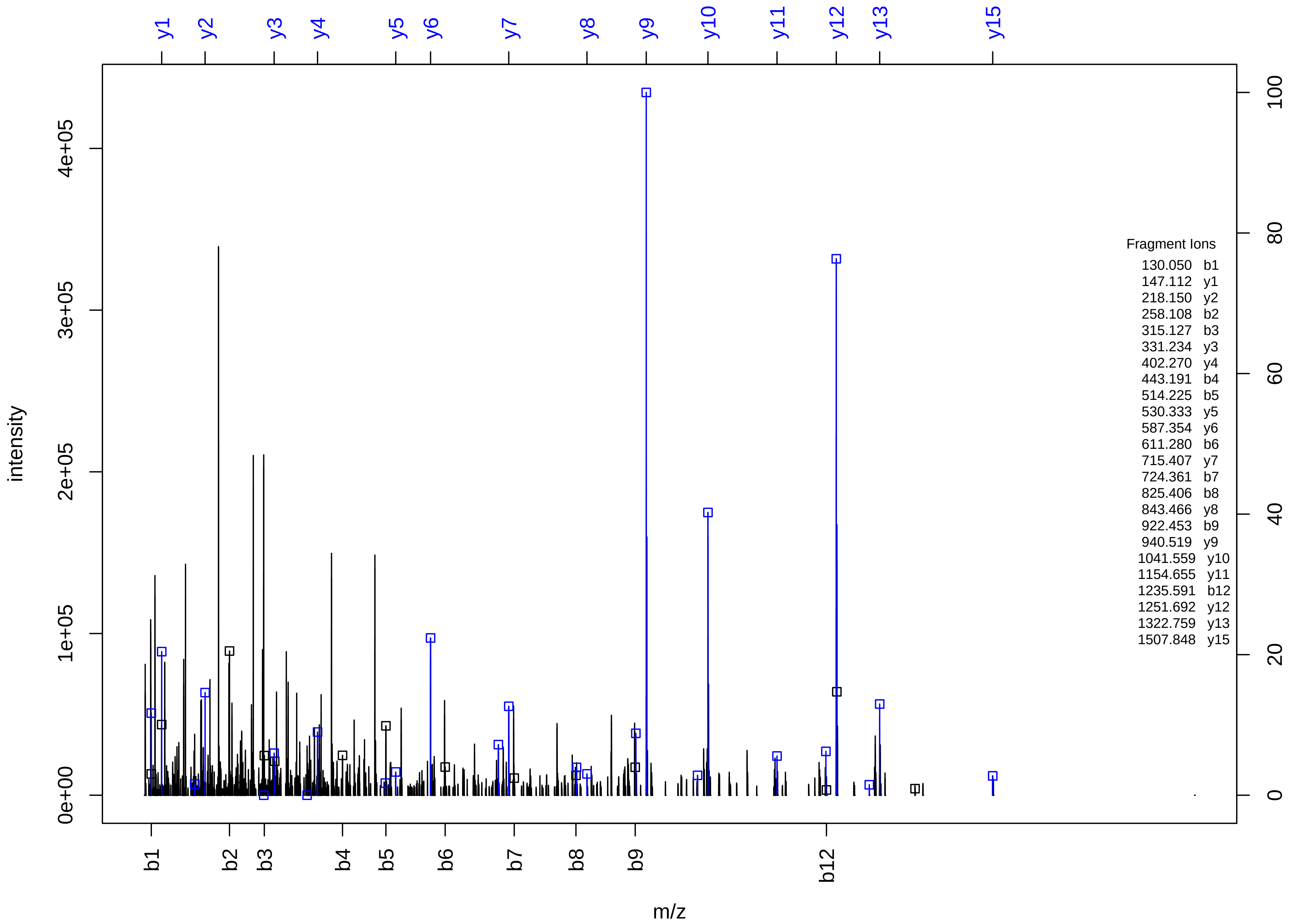

EQGQAPITPQQGQALAK / File: christine_pool_01.raw; SpectrumID: ; scans: 5297

TVEVVESIEK

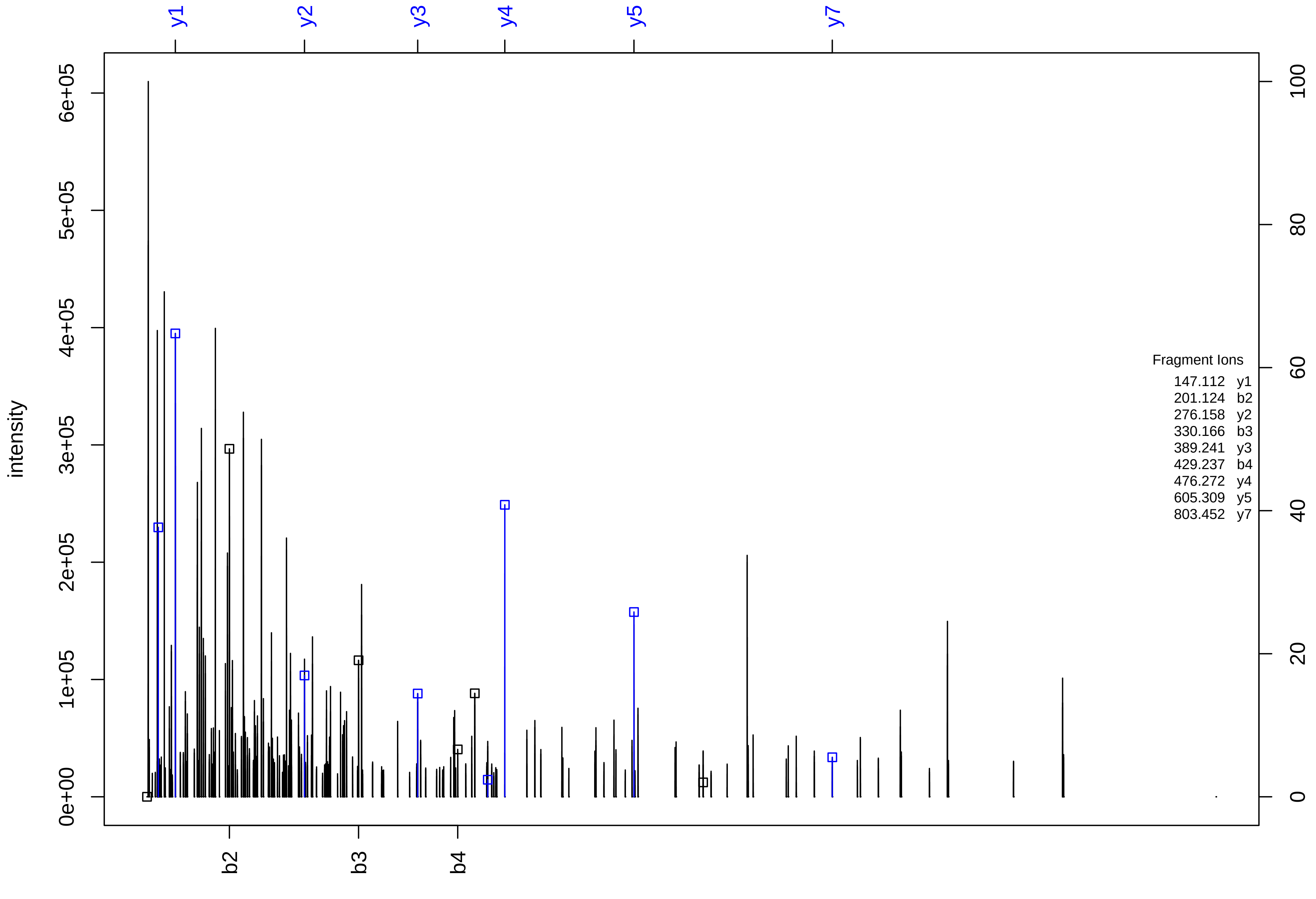

TVEVVESIEK / File: christine_pool_01.raw; SpectrumID: ; scans: 13581

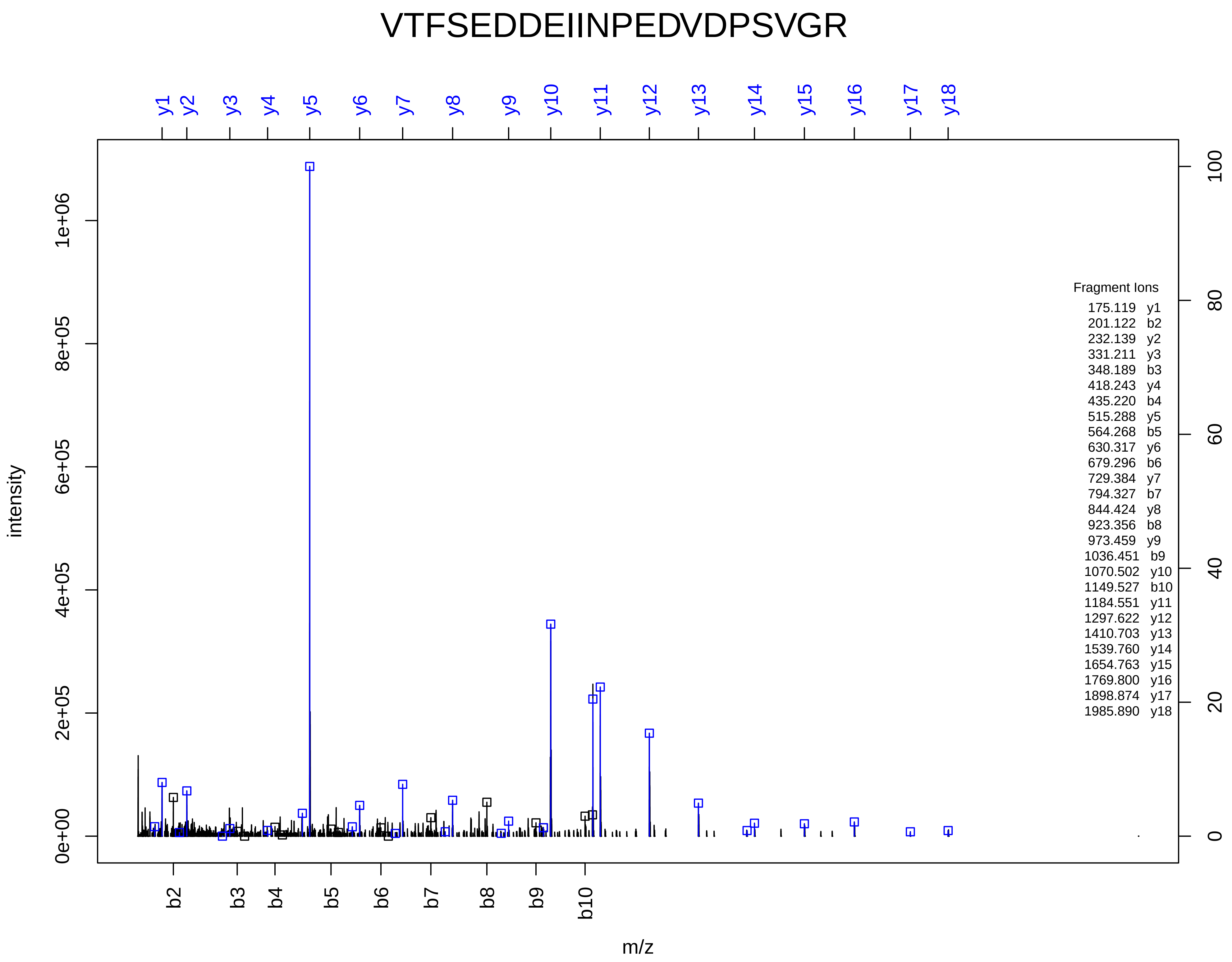
VTFSEDDEIINPEDVDPSVGR
y1 y2 y3 y4 y5 y6 y7 y8 y9 y10 y11 y12 y13 y14 y15 y16 y17 y18
intensity
0e+00 2e+05 4e+05 6e+05 8e+05 1e+06
0 20 40 60 80 100
b2 b3 b4 b5 b6 b7 b8 b9 b10
m/z
Fragment Ions
175.119 y1
201.122 b2
232.139 y2
331.211 y3
348.189 b3
418.243 y4
435.220 b4
515.288 y5
564.268 b5
630.317 y6
679.296 b6
729.384 y7
794.327 b7
844.424 y8
923.356 b8
973.459 y9
1036.451 b9
1070.502 y10
1149.527 b10
1184.551 y11
1297.622 y12
1410.703 y13
1539.760 y14
1654.763 y15
1769.800 y16
1898.874 y17
1985.890 y18
VTFSEDDEIINPEDVDPSVGR / File: christine_pool_01.raw; SpectrumID: ; scans: 15775

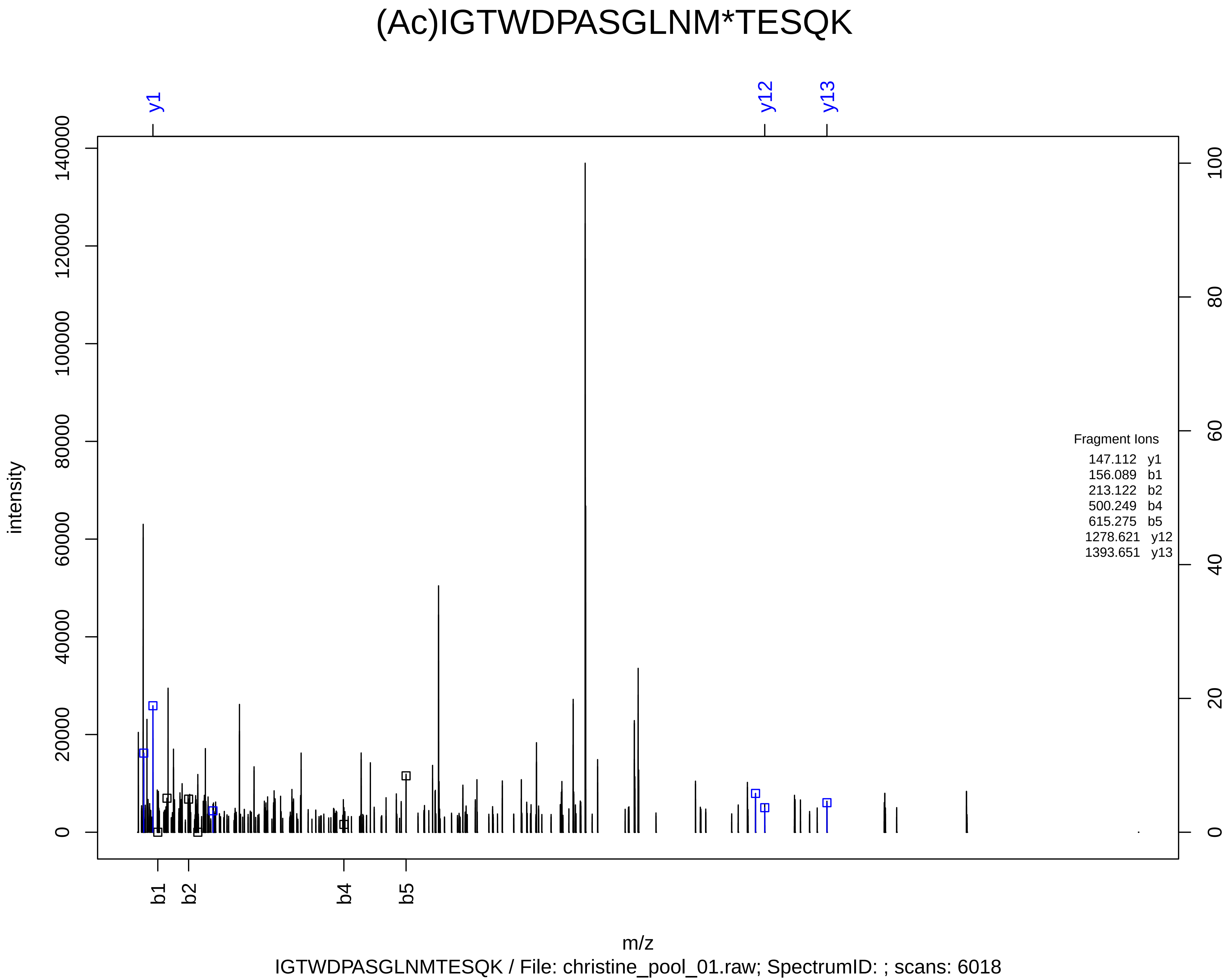

(Ac)IGTWDPASGLNM*TESQK
y1
y12
y13
intensity
0
20000
40000
60000
80000
100000
120000
140000
0
20
40
60
80
100
b1
b2
b4
b5
m/z
Fragment Ions
147.112 y1
156.089 b1
213.122 b2
500.249 b4
615.275 b5
1278.621 y12
1393.651 y13
IGTWDPASGLNMTESQK / File: christine_pool_01.raw; SpectrumID: ; scans: 6018

IGIIGGTGLDDPEILEGR

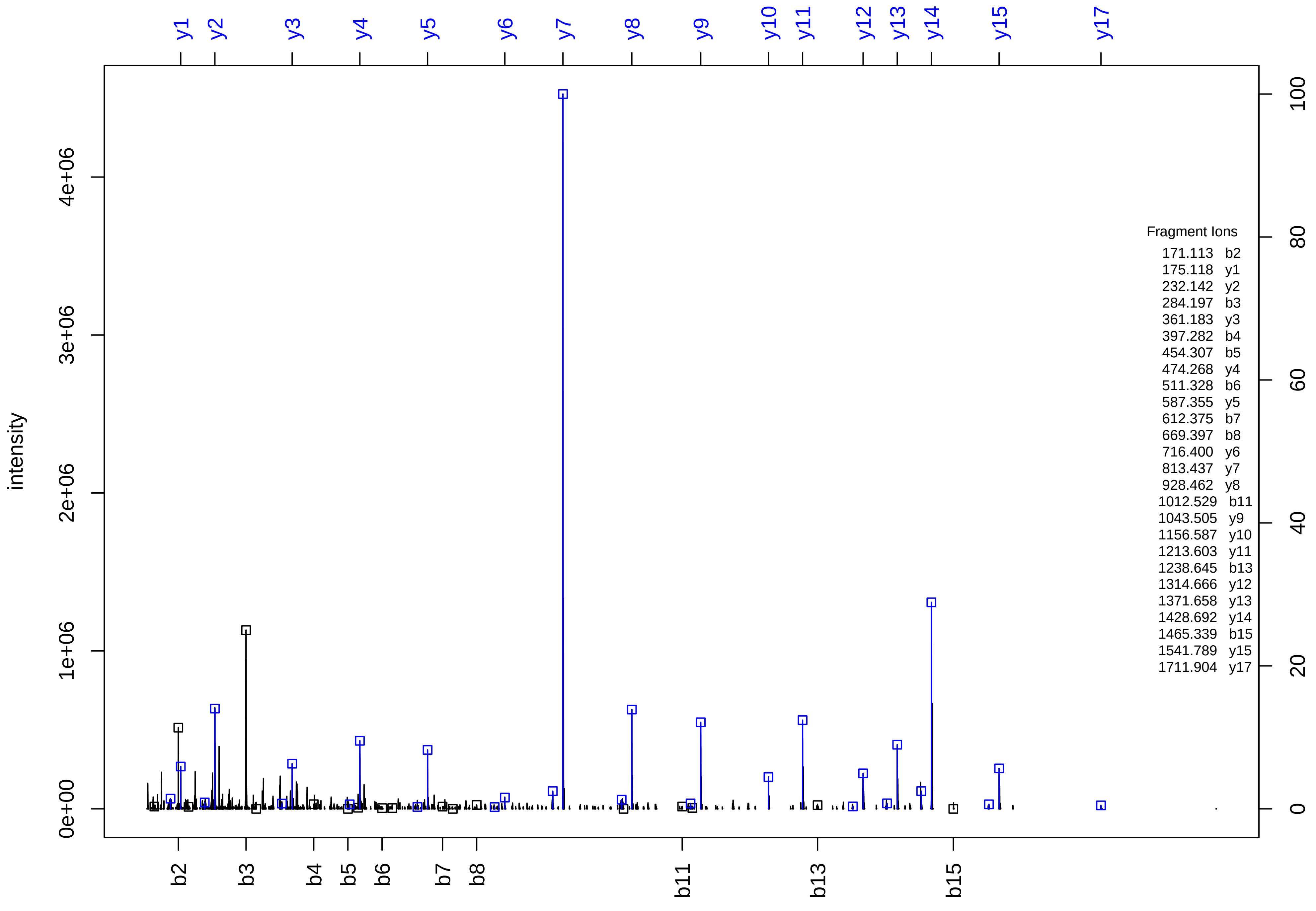

IGIIGGTGLDDPEILEGR / File: christine_pool_01.raw; SpectrumID: ; scans: 18675

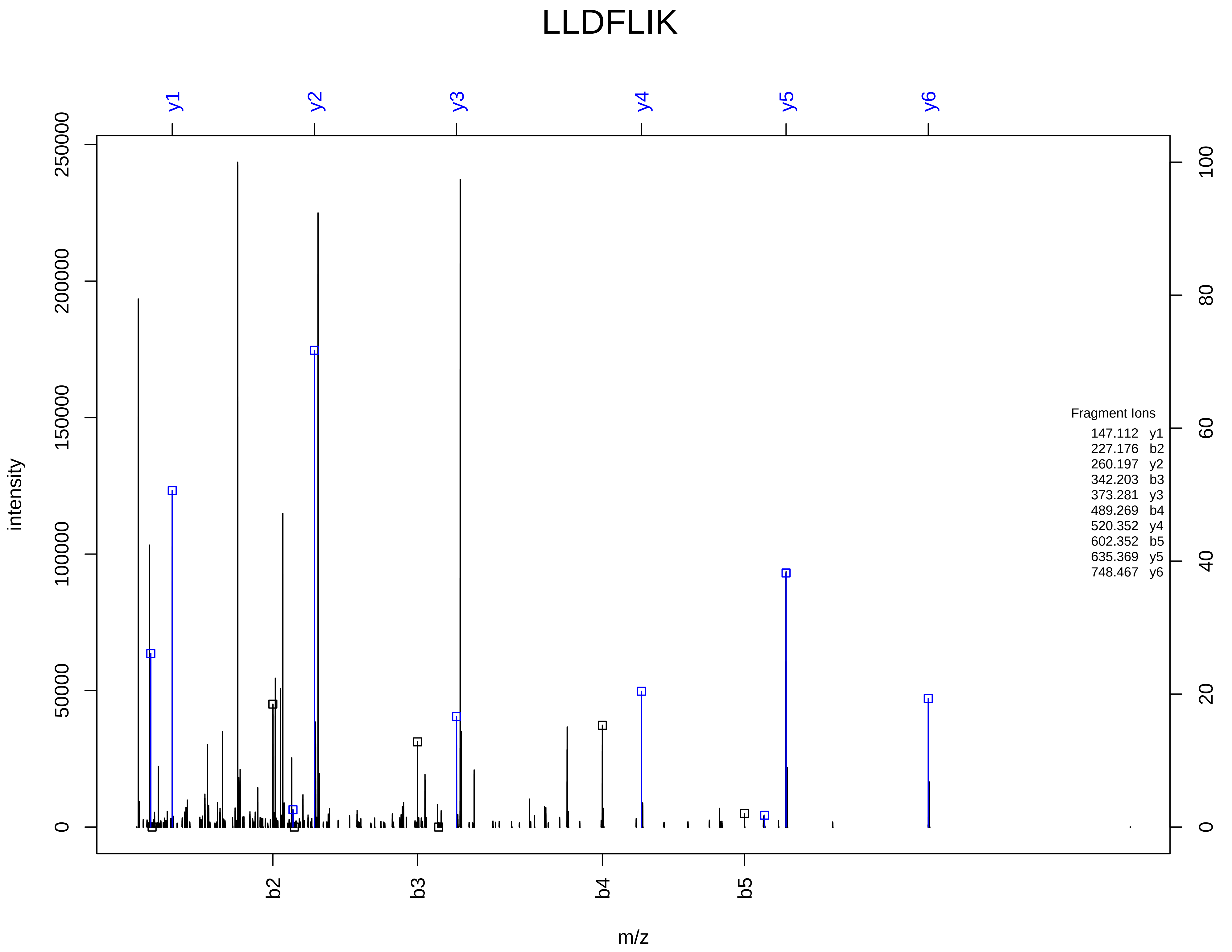
LLDFLIK
y1
y2
y3
y4
y5
y6
intensity
0
50000
100000
150000
200000
250000
0
20
40
60
80
100
Fragment Ions
147.112 y1
227.176 b2
260.197 y2
342.203 b3
373.281 y3
489.269 b4
520.352 y4
602.352 b5
635.369 y5
748.467 y6
b2
b3
b4
b5
m/z
LLDFLIK / File: christine_pool_01.raw; SpectrumID: ; scans: 18245

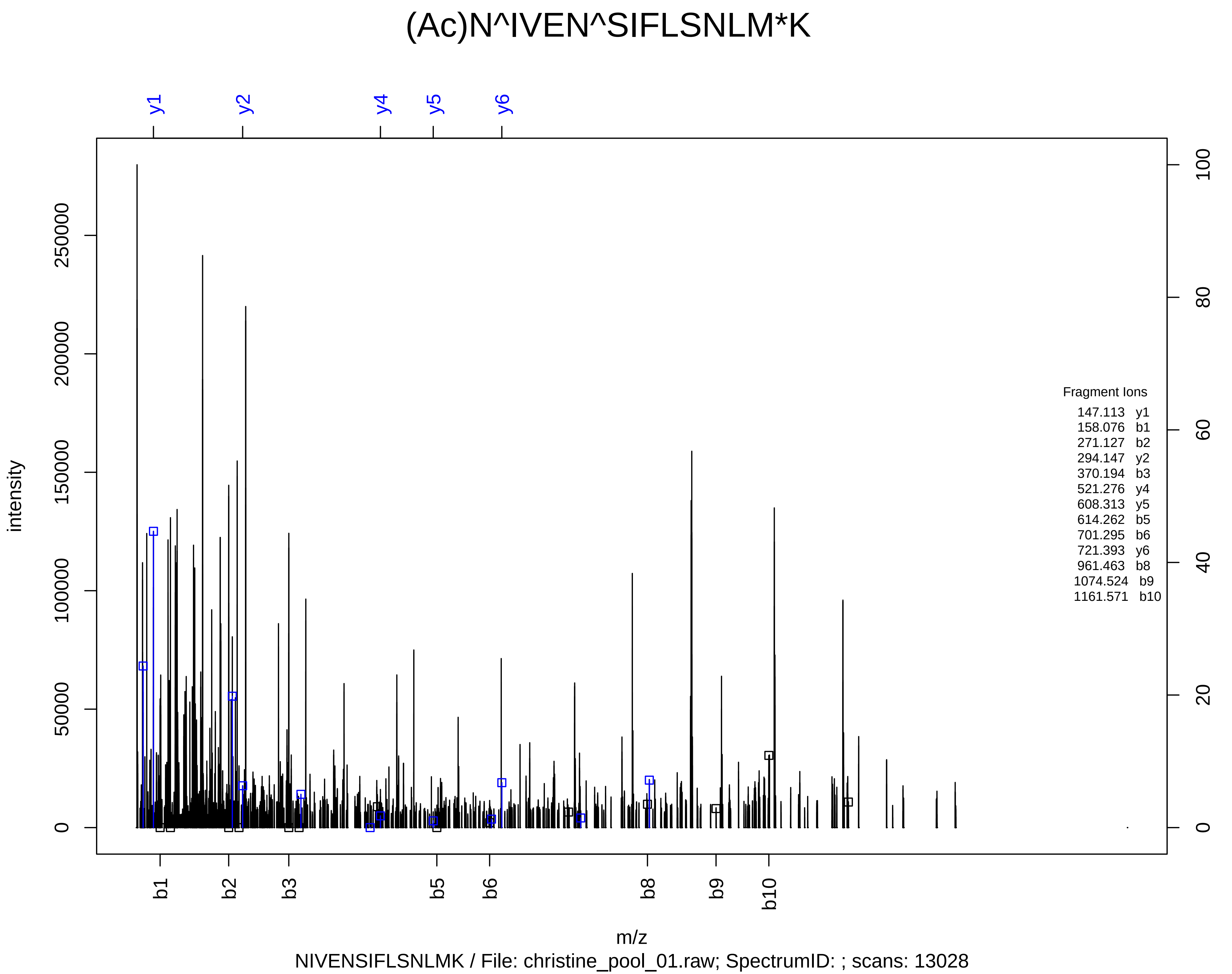
(Ac)N^IVEN^SIFLSNLM*K
y1
y2
y4
y5
y6
intensity
0
50000
100000
150000
200000
250000
0
20
40
60
80
100
b1
b2
b3
b5
b6
b8
b9
b10
m/z
Fragment Ions
147.113 y1
158.076 b1
271.127 b2
294.147 y2
370.194 b3
521.276 y4
608.313 y5
614.262 b5
701.295 b6
721.393 y6
961.463 b8
1074.524 b9
1161.571 b10
NIVENSIFLSNLMK / File: christine_pool_01.raw; SpectrumID: ; scans: 13028

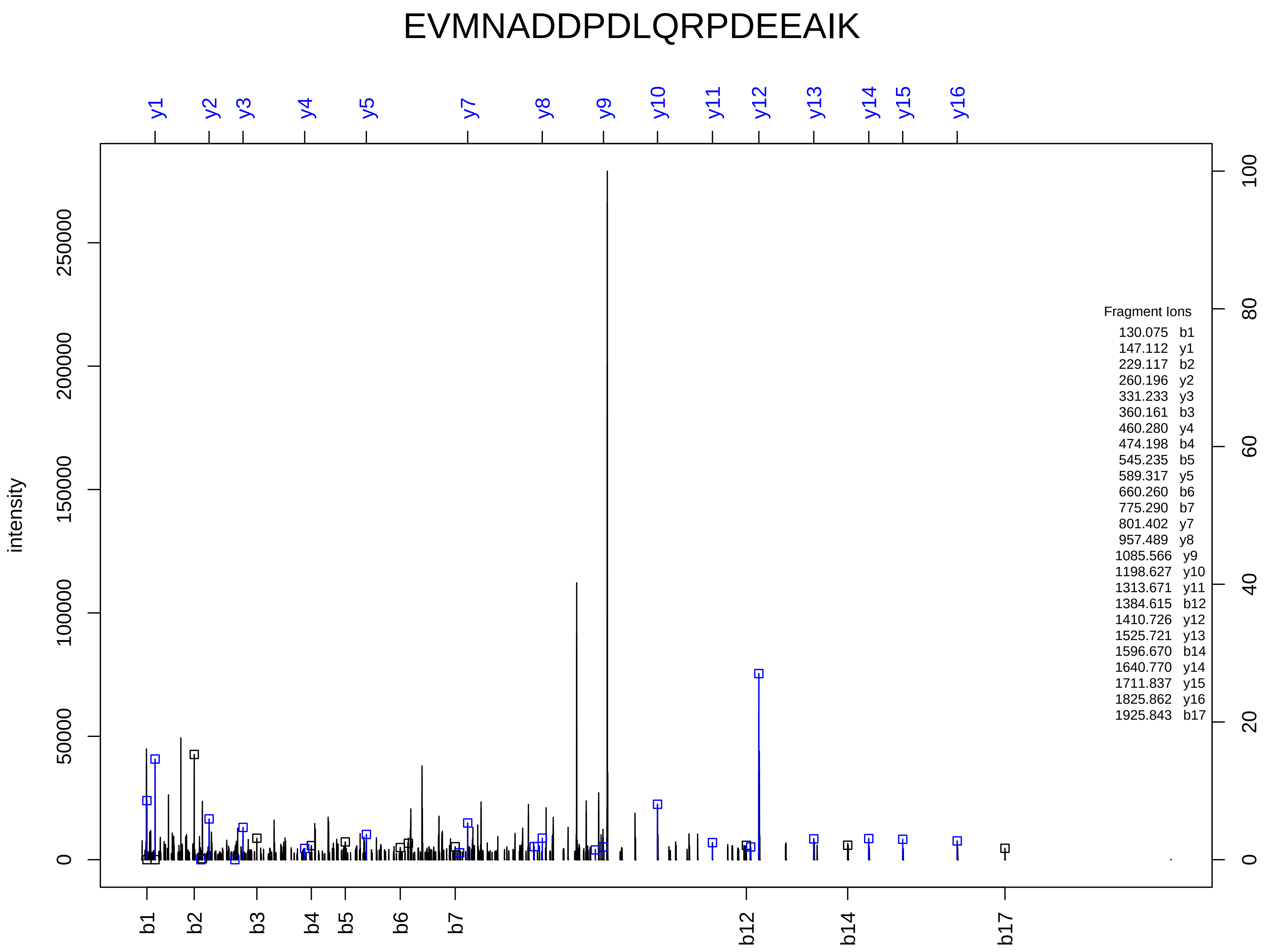

EVMNADDPDLQRPDEEAIK / File: christine_pool_01.raw; SpectrumID: ; scans: 7101

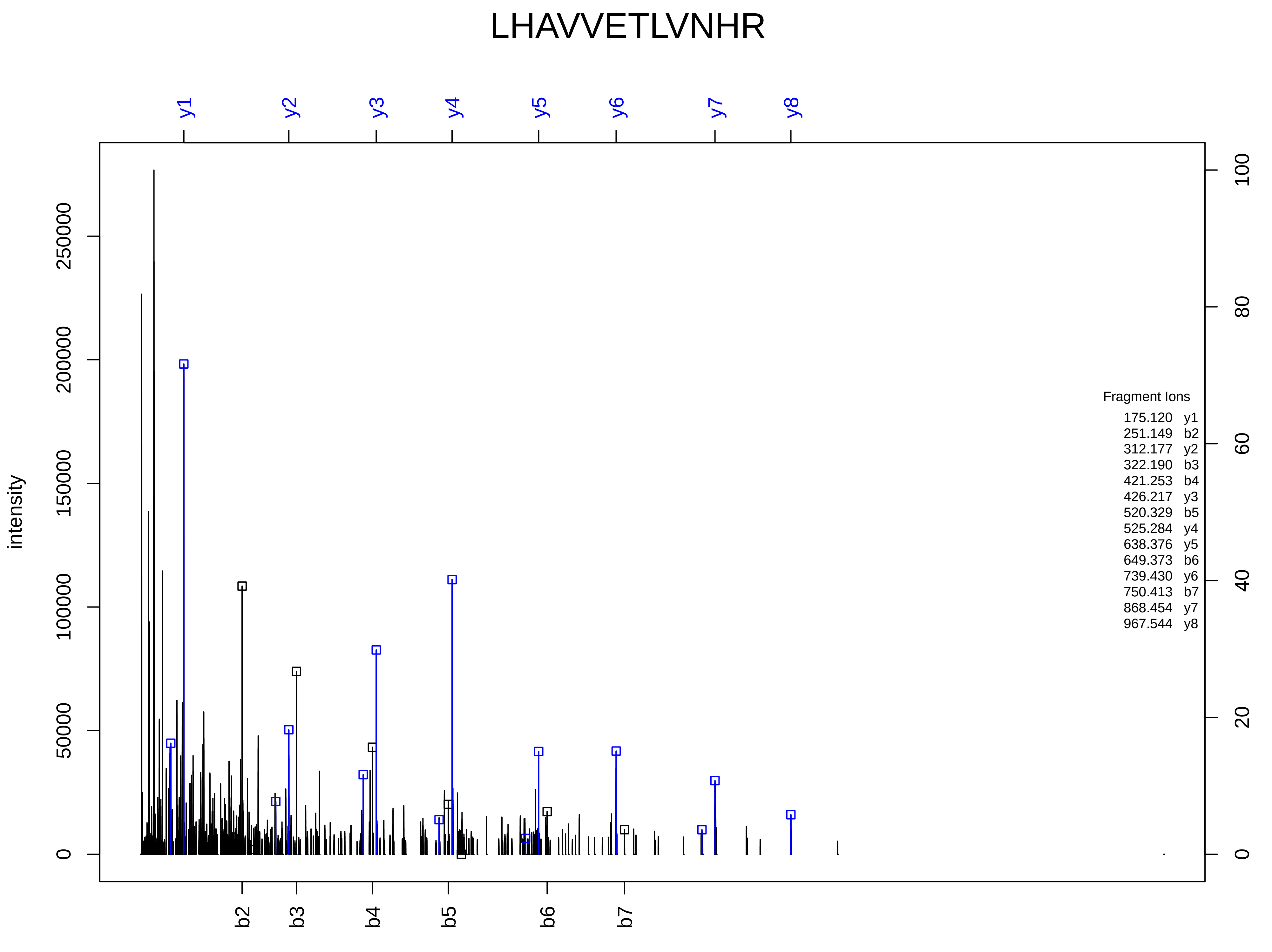

LHAVVETLVNHR / File: christine_pool_01.raw; SpectrumID: ; scans: 6028

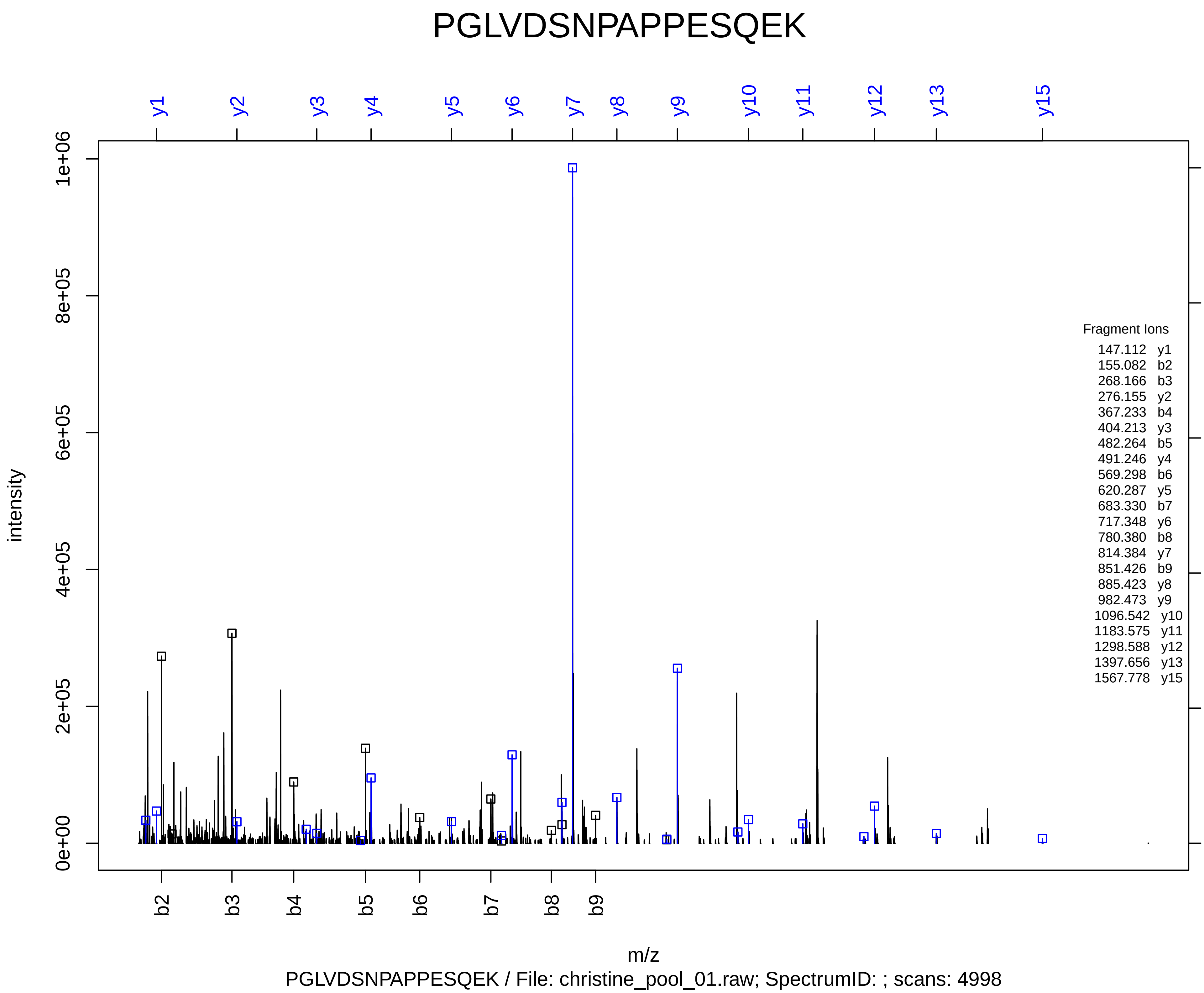

PGLVDSNPAPPESQEK
y1
y2
y3
y4
y5
y6
y7
y8
y9
y10
y11
y12
y13
y15
intensity
0e+00
2e+05
4e+05
6e+05
8e+05
1e+06
0
20
40
60
80
100
b2
b3
b4
b5
b6
b7
b8
b9
m/z
Fragment Ions
147.112 y1
155.082 b2
268.166 b3
276.155 y2
367.233 b4
404.213 y3
482.264 b5
491.246 y4
569.298 b6
620.287 y5
683.330 b7
717.348 y6
780.380 b8
814.384 y7
851.426 b9
885.423 y8
982.473 y9
1096.542 y10
1183.575 y11
1298.588 y12
1397.656 y13
1567.778 y15
PGLVDSNPAPPESQEK / File: christine_pool_01.raw; SpectrumID: ; scans: 4998

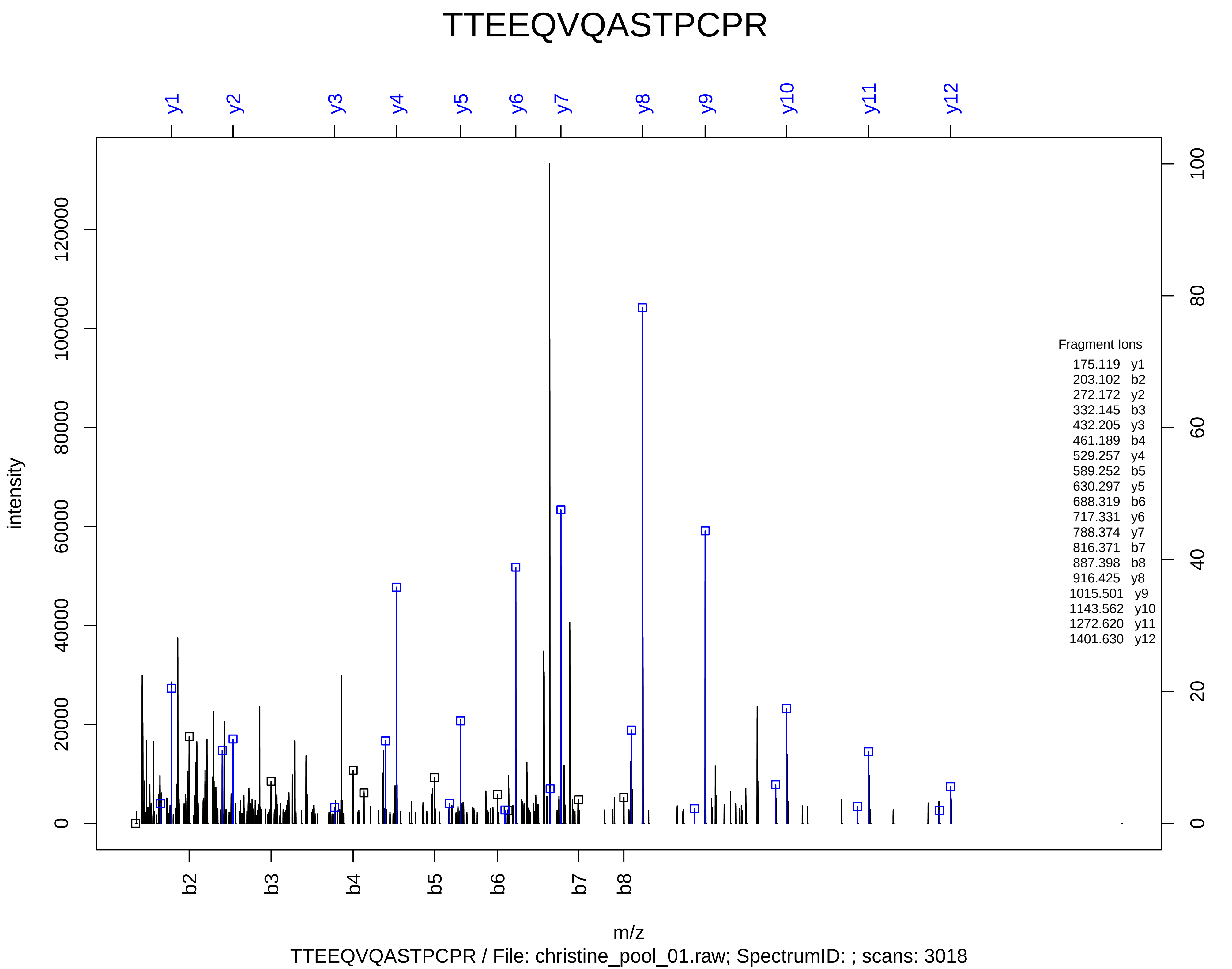
TTEEQVQASTPCPR
y1
y2
y3
y4
y5
y6
y7
y8
y9
y10
y11
y12
intensity
0
20000
40000
60000
80000
100000
120000
0
20
40
60
80
100
Fragment Ions
175.119 y1
203.102 b2
272.172 y2
332.145 b3
432.205 y3
461.189 b4
529.257 y4
589.252 b5
630.297 y5
688.319 b6
717.331 y6
788.374 y7
816.371 b7
887.398 b8
916.425 y8
1015.501 y9
1143.562 y10
1272.620 y11
1401.630 y12
b2
b3
b4
b5
b6
b7
b8
m/z
TTEEQVQASTPCPR / File: christine_pool_01.raw; SpectrumID: ; scans: 3018

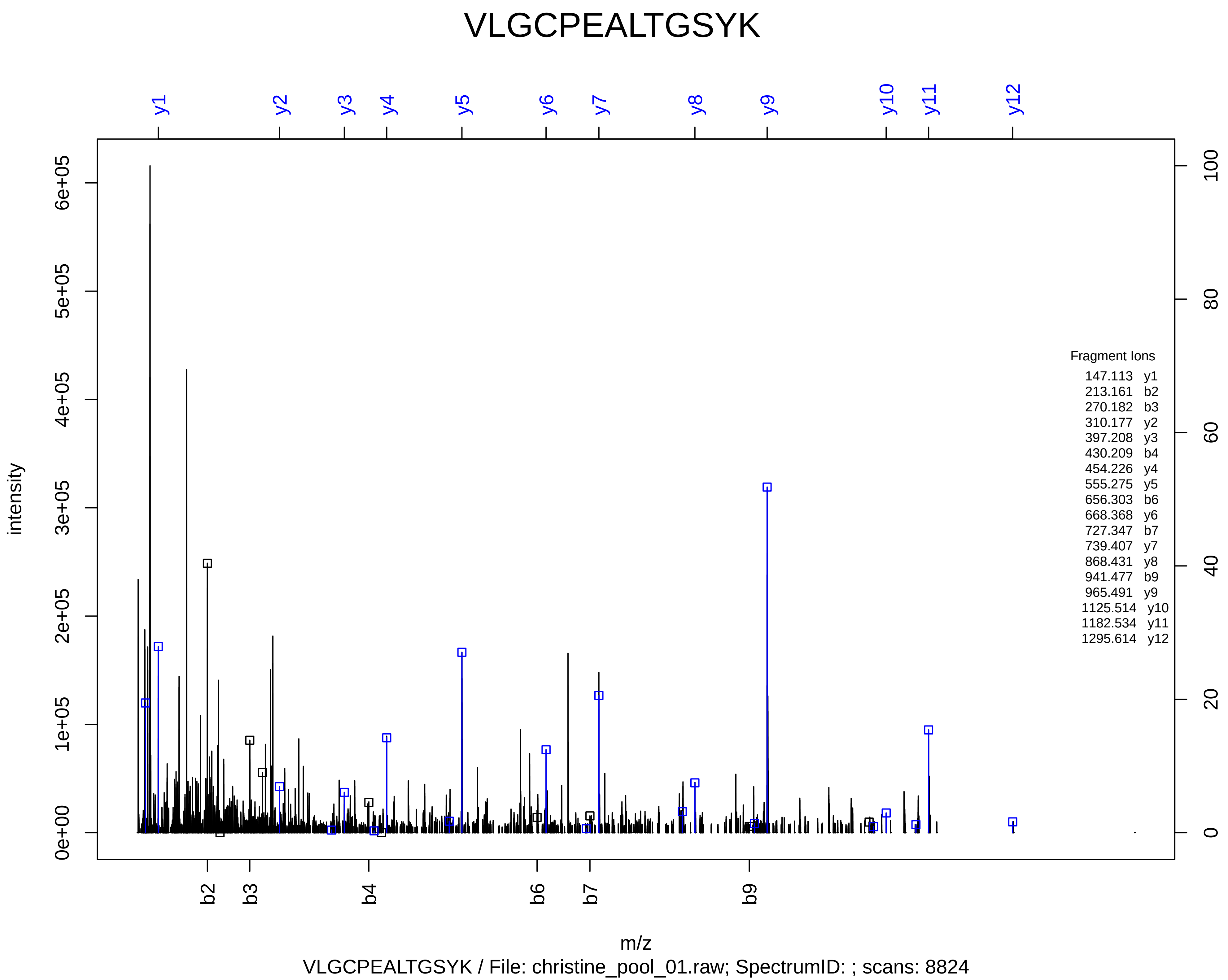

VLGCPEALTGSYK
y1
y2
y3
y4
y5
y6
y7
y8
y9
y10
y11
y12
intensity
6e+05
5e+05
4e+05
3e+05
2e+05
1e+05
0e+00
100
80
60
40
20
0
Fragment Ions
147.113 y1
213.161 b2
270.182 b3
310.177 y2
397.208 y3
430.209 b4
454.226 y4
555.275 y5
656.303 b6
668.368 y6
727.347 b7
739.407 y7
868.431 y8
941.477 b9
965.491 y9
1125.514 y10
1182.534 y11
1295.614 y12
b2
b3
b4
b6
b7
b9
m/z
VLGCPEALTGSYK / File: christine_pool_01.raw; SpectrumID: ; scans: 8824

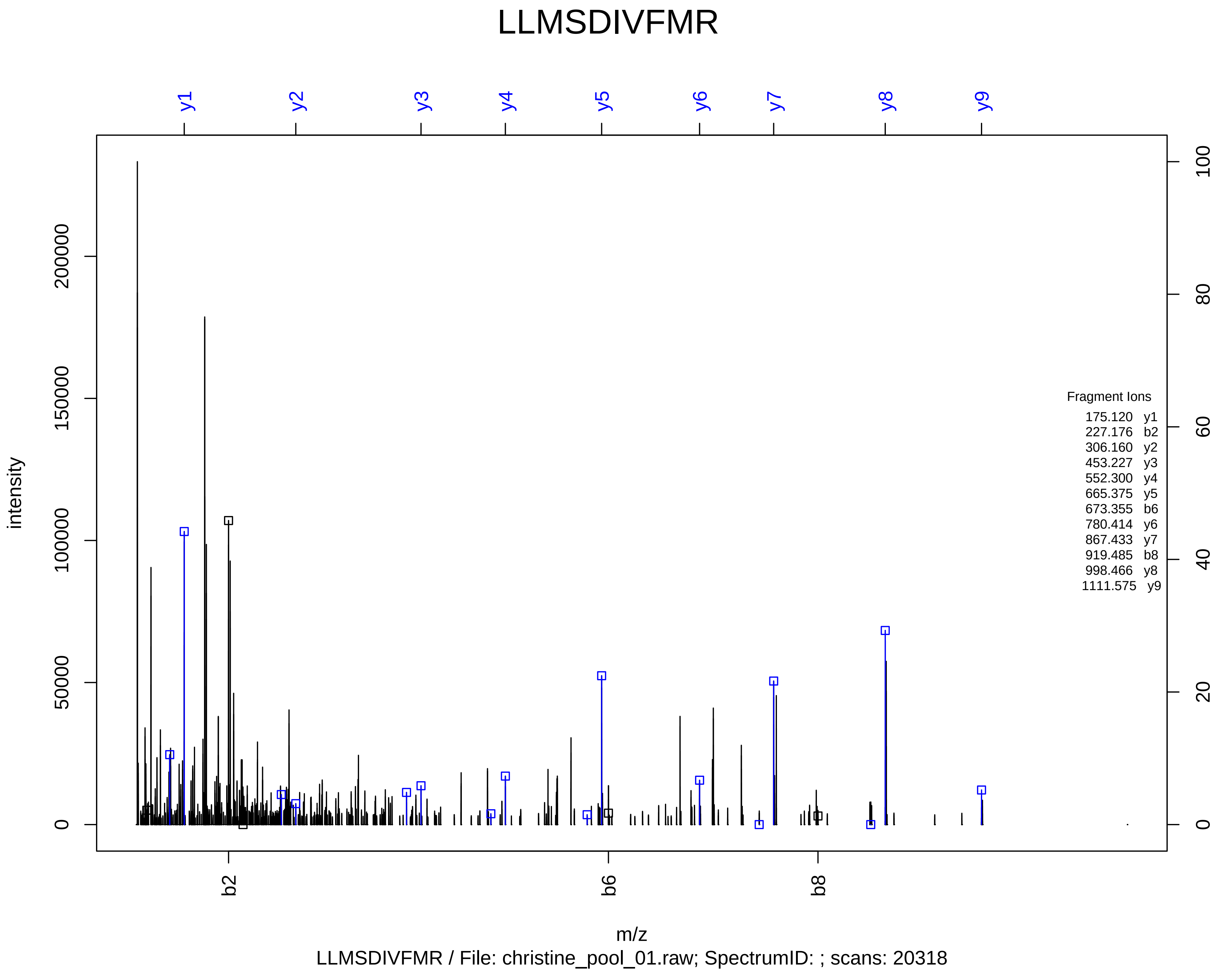
LLMSDIVFMR
y1
y2
y3
y4
y5
y6
y7
y8
y9
intensity
0
50000
100000
150000
200000
0
20
40
60
80
100
Fragment Ions
175.120 y1
227.176 b2
306.160 y2
453.227 y3
552.300 y4
665.375 y5
673.355 b6
780.414 y6
867.433 y7
919.485 b8
998.466 y8
1111.575 y9
b2
b6
b8
m/z
LLMSDIVFMR / File: christine_pool_01.raw; SpectrumID: ; scans: 20318

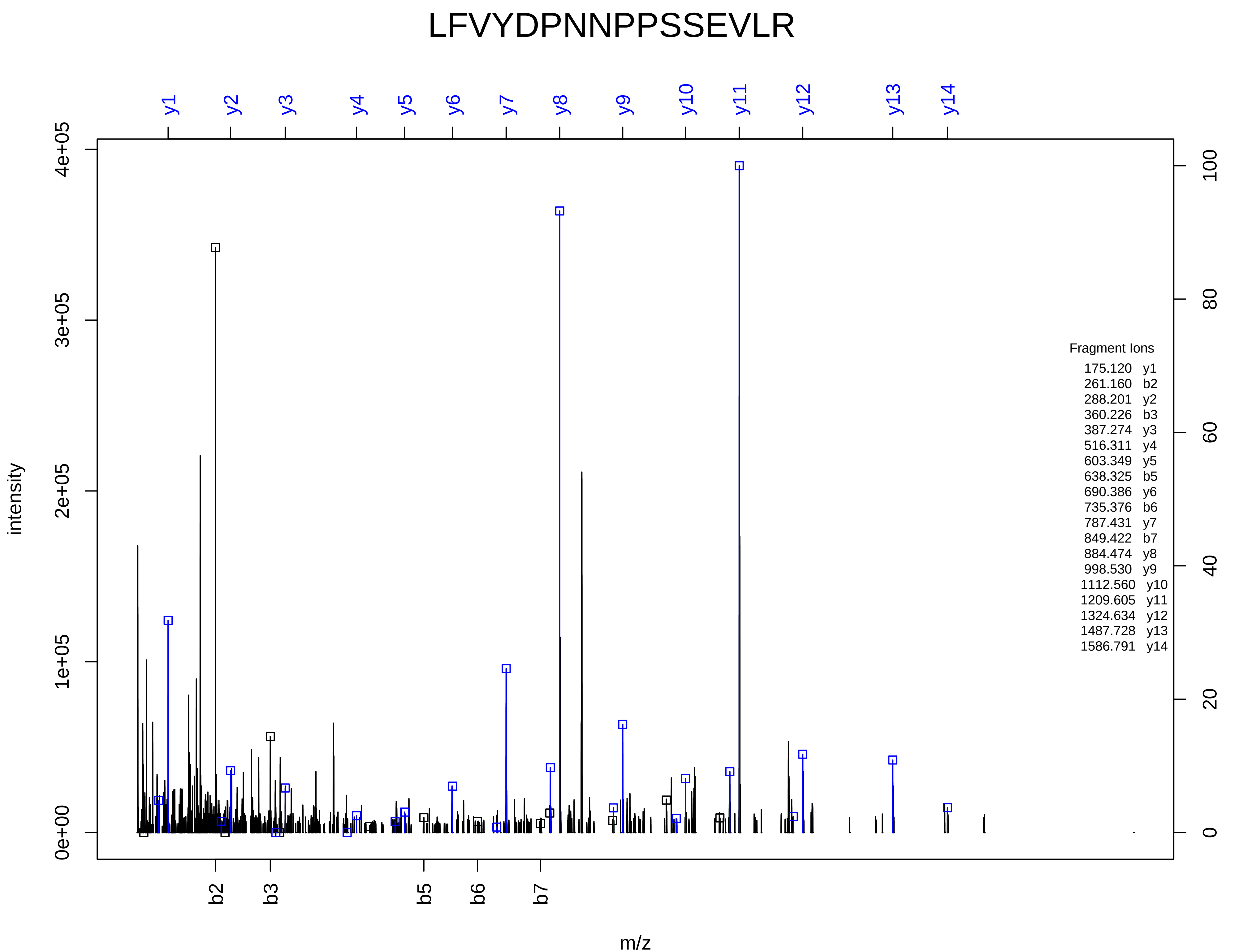
LFVYDPNNPPSSEVLR
y1 y2 y3 y4 y5 y6 y7 y8 y9 y10 y11 y12 y13 y14
b2 b3 b5 b6 b7
intensity
m/z
Fragment Ions
175.120 y1
261.160 b2
288.201 y2
360.226 b3
387.274 y3
516.311 y4
603.349 y5
638.325 b5
690.386 y6
735.376 b6
787.431 y7
849.422 b7
884.474 y8
998.530 y9
1112.560 y10
1209.605 y11
1324.634 y12
1487.728 y13
1586.791 y14
LFVYDPNNPPSSEVLR / File: christine_pool_01.raw; SpectrumID: ; scans: 14450

TLGECGFTSQTARPQAPATVGLAFR

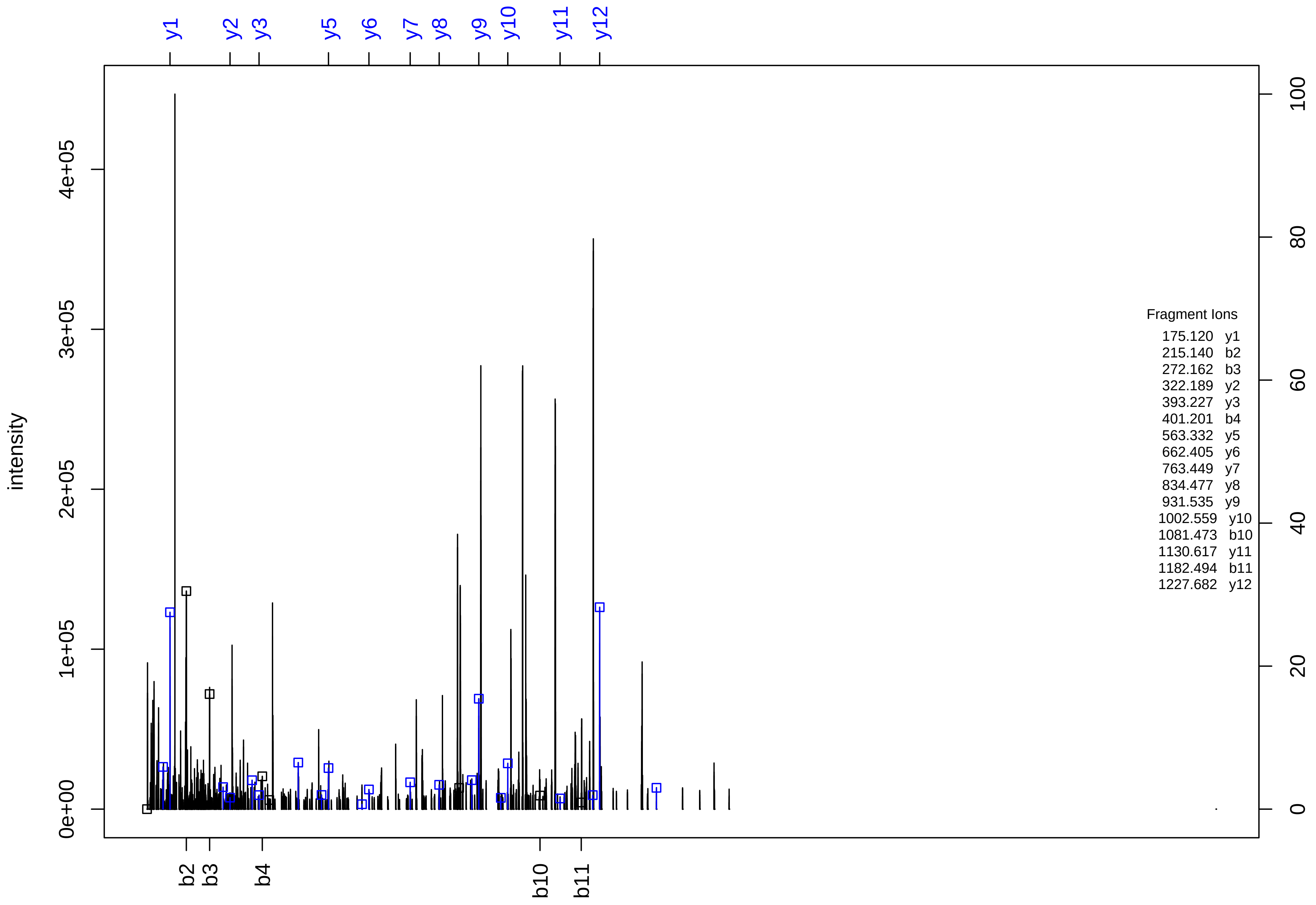

TLGECGFTSQTARPQAPATVGLAFR / File: christine_pool_01.raw; SpectrumID: ; scans: 13075

GYGYGQGAGTLNMDR

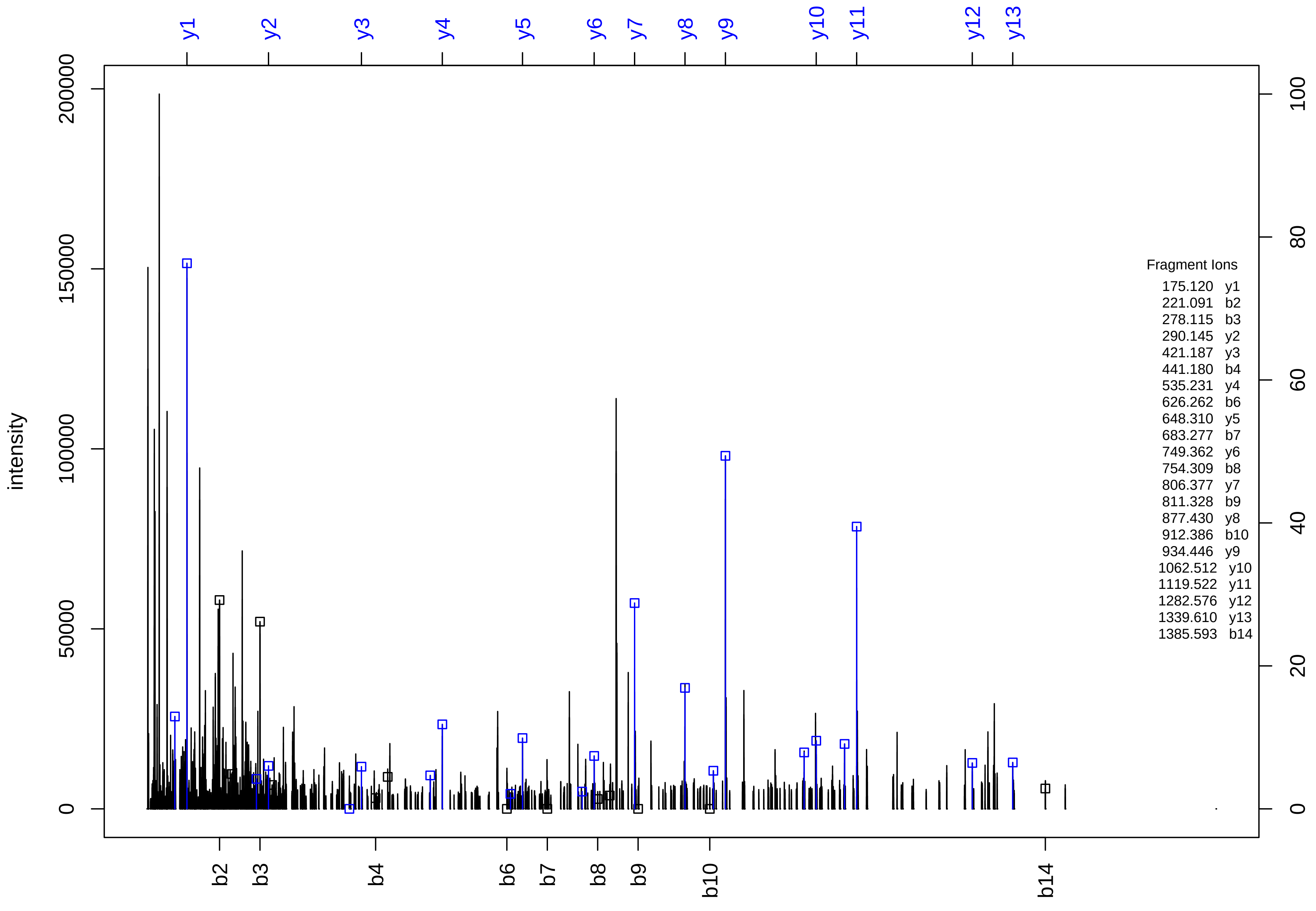

GYGYGQGAGTLNMDR / File: christine_pool_01.raw; SpectrumID: ; scans: 6677

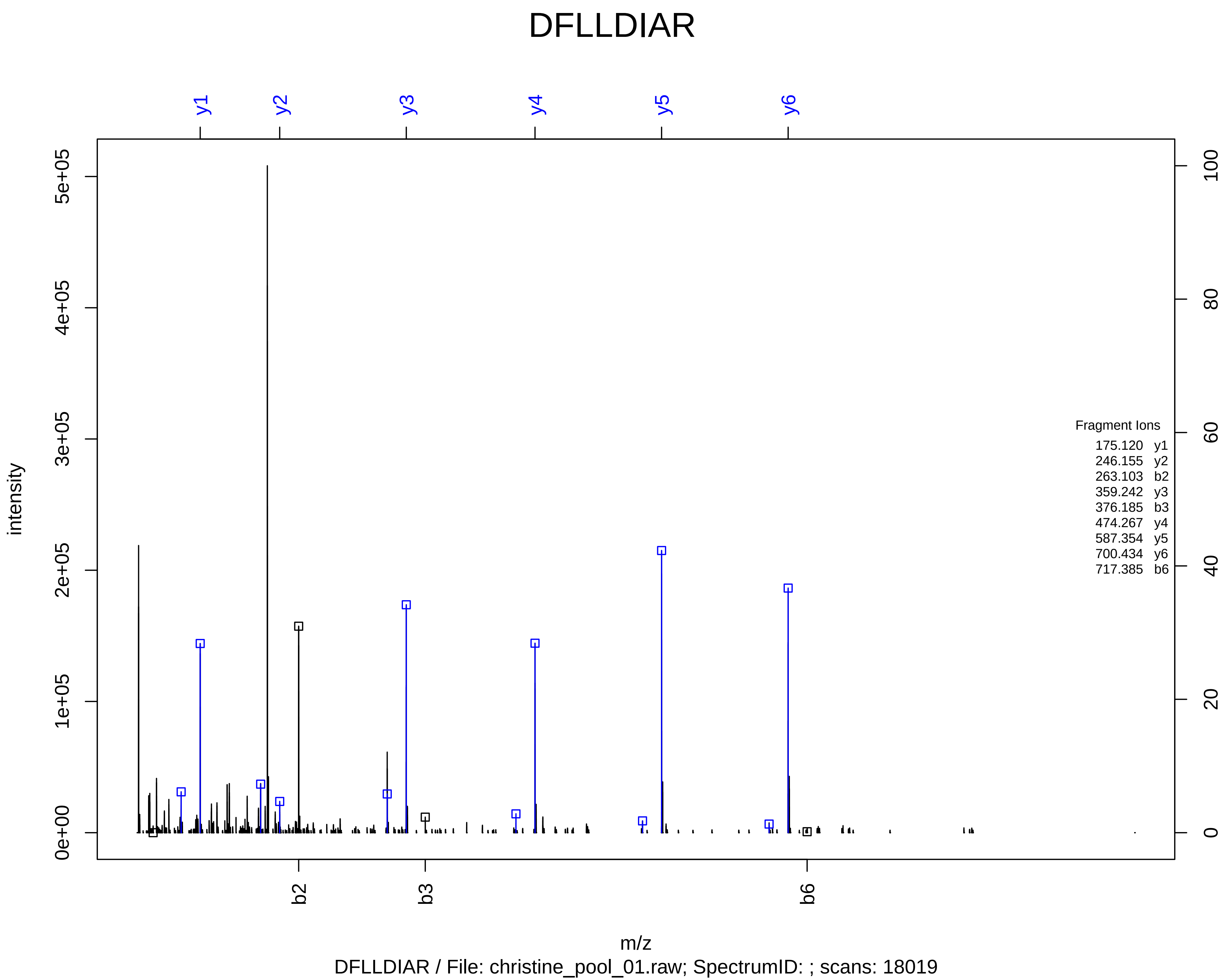
DFLLDIAR
y1
y2
y3
y4
y5
y6
intensity
0e+00
1e+05
2e+05
3e+05
4e+05
5e+05
0
20
40
60
80
100
Fragment Ions
175.120 y1
246.155 y2
263.103 b2
359.242 y3
376.185 b3
474.267 y4
587.354 y5
700.434 y6
717.385 b6
b2
b3
b6
m/z
DFLLDIAR / File: christine_pool_01.raw; SpectrumID: ; scans: 18019

SAPWNSFLPPPPPMPGPR

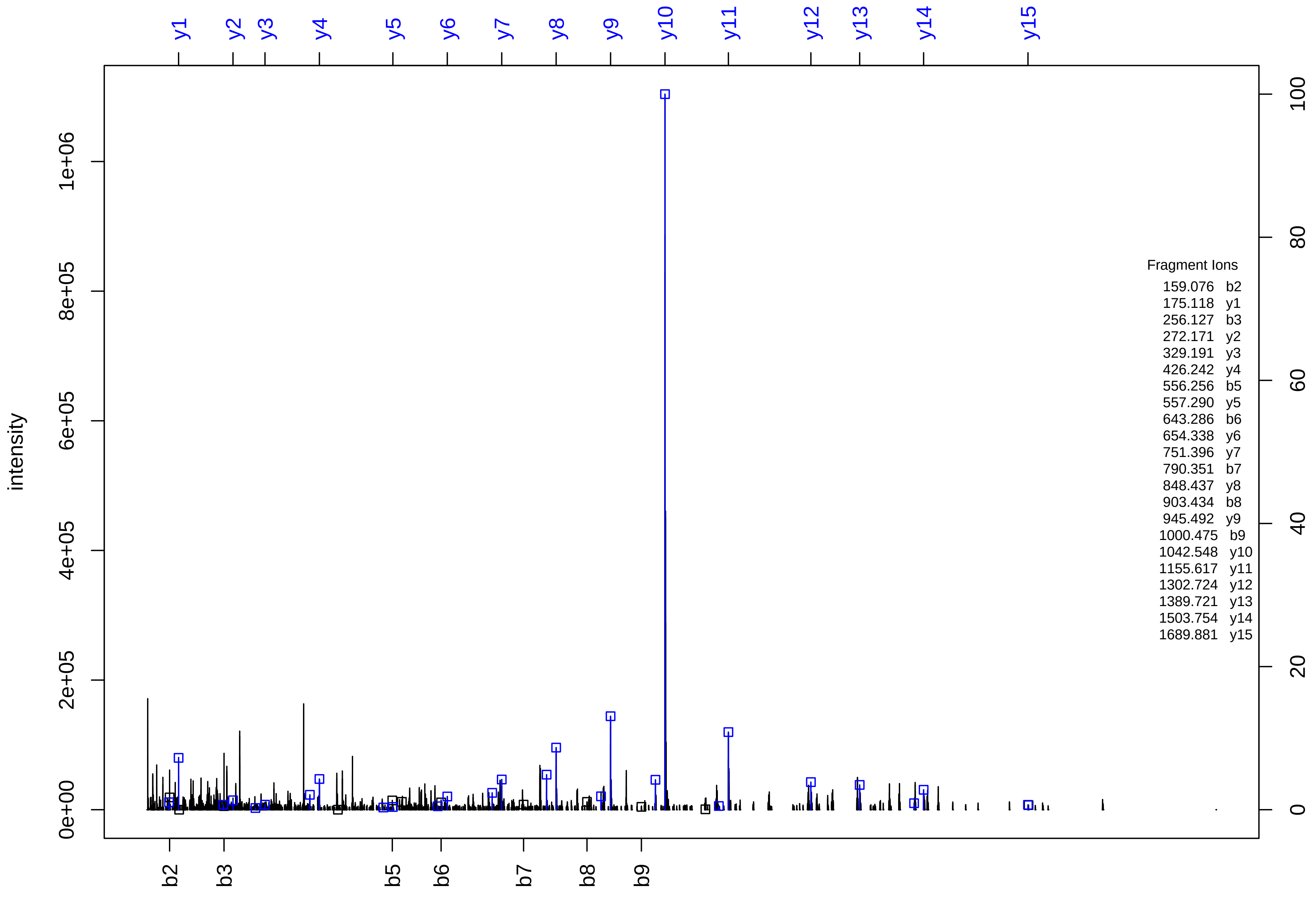

SAPWNSFLPPPPPMPGPR / File: christine_pool_01.raw; SpectrumID: ; scans: 20417

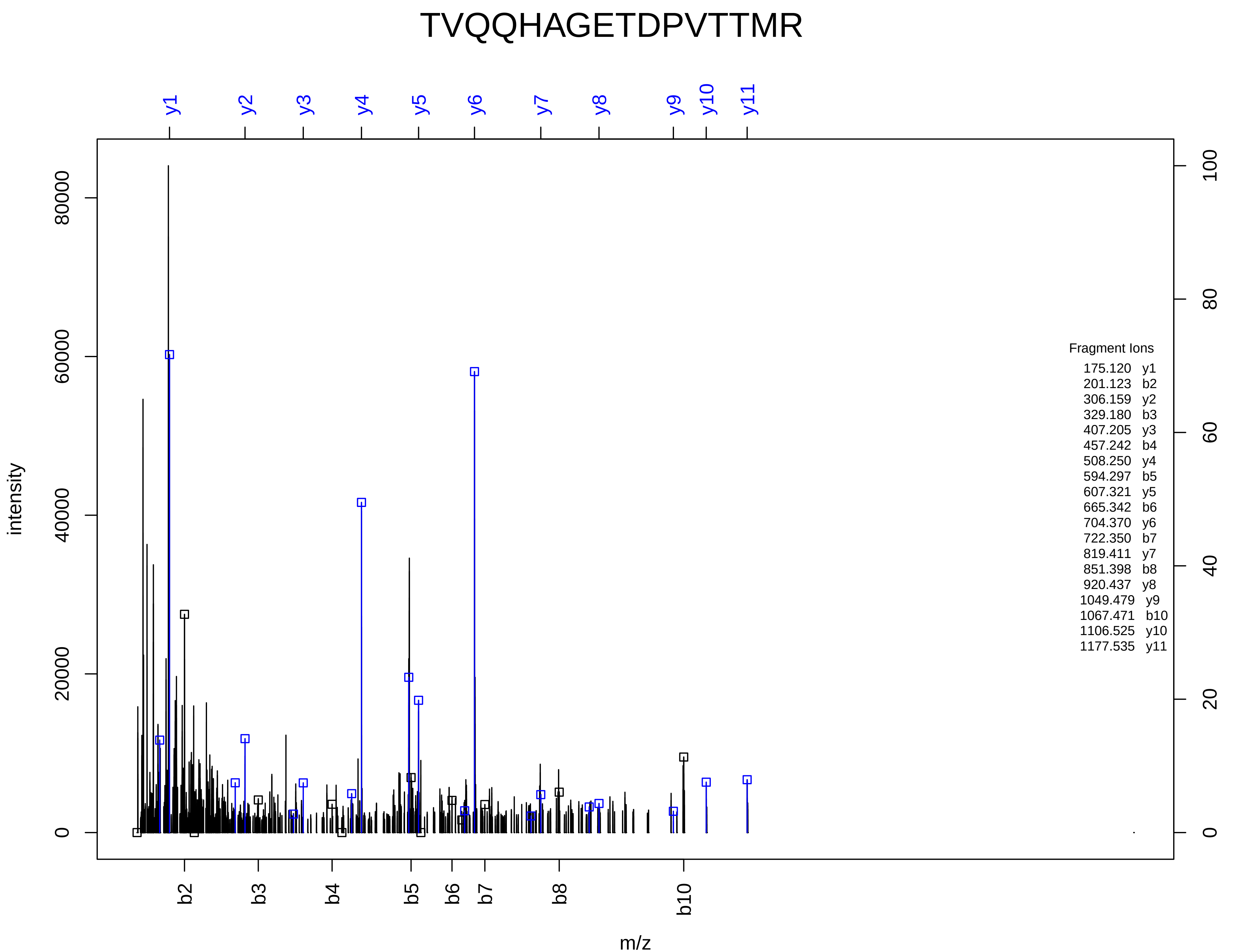

TVQQHAGETDPVTTMR / File: christine_pool_01.raw; SpectrumID: ; scans: 3467

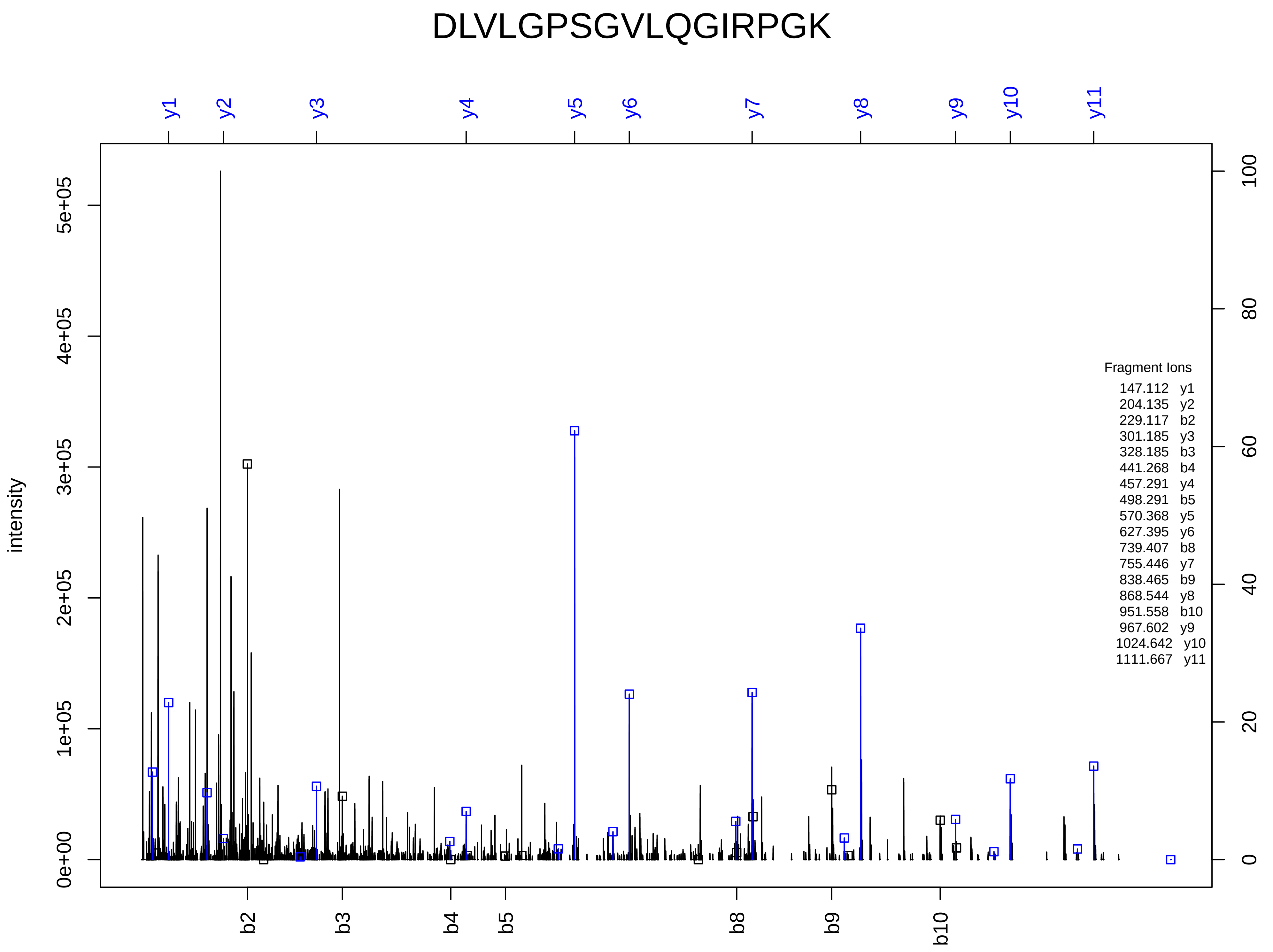

DLVLGPSGVLQGIRPGK / File: christine_pool_01.raw; SpectrumID: ; scans: 16013

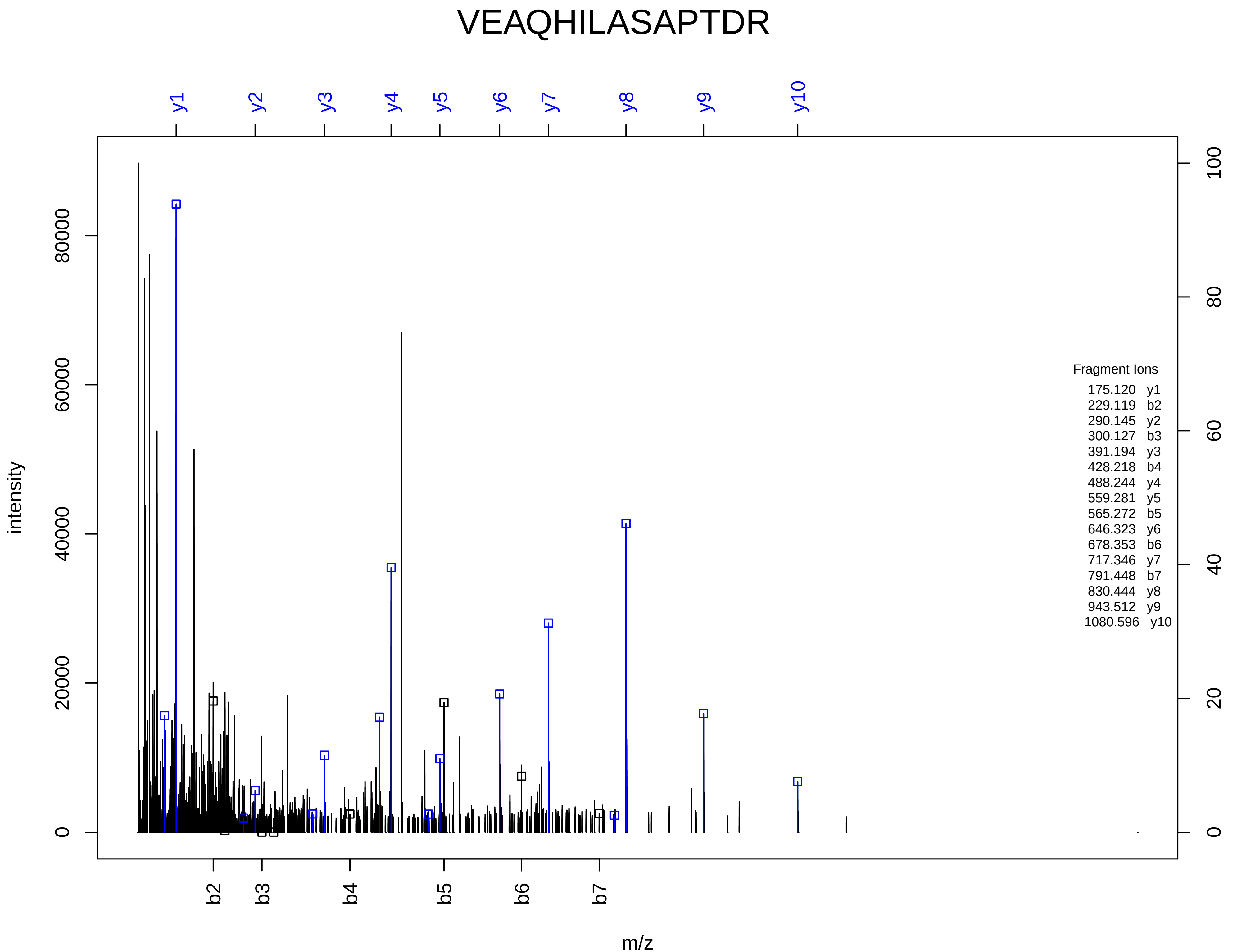

VEAQHILASAPTDR / File: christine_pool_01.raw; SpectrumID: ; scans: 5216

ELWTILLGR

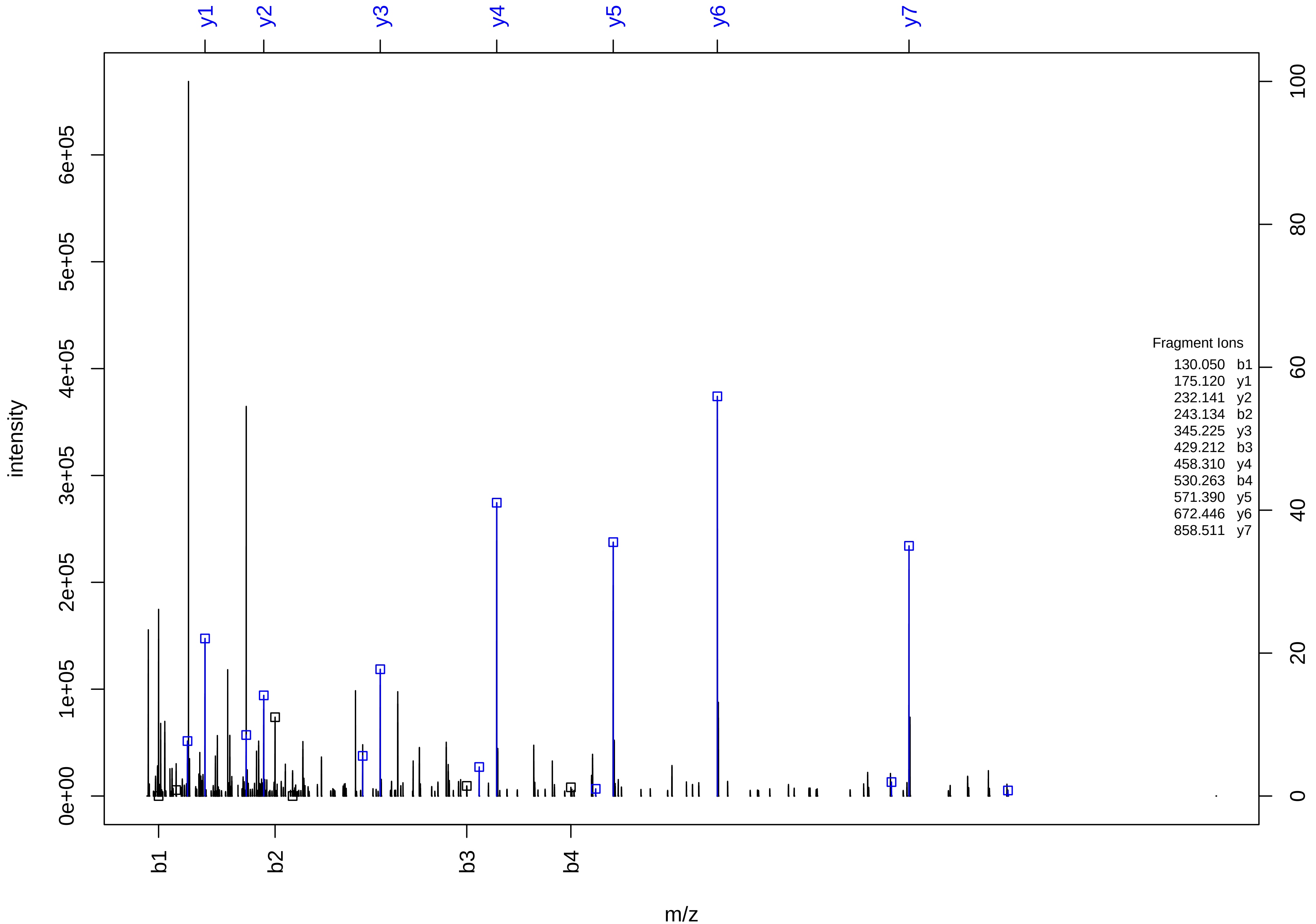

ELWTILLGR / File: christine_pool_01.raw; SpectrumID: ; scans: 22159

TLVLVSPGDM*KK

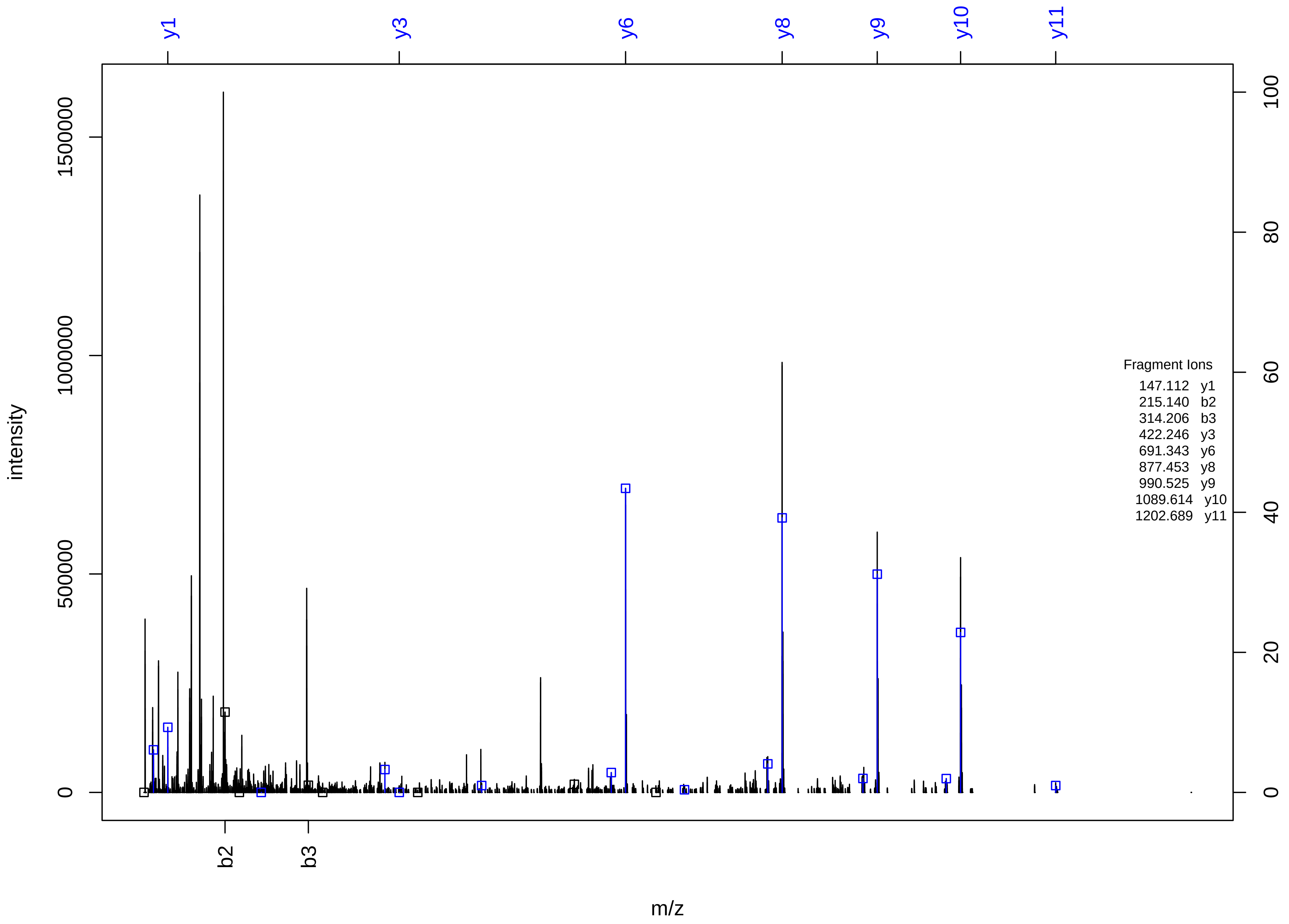

TLVLVSPGDMKK / File: christine_pool_01.raw; SpectrumID: ; scans: 13267

NLSIN^NDLNLR

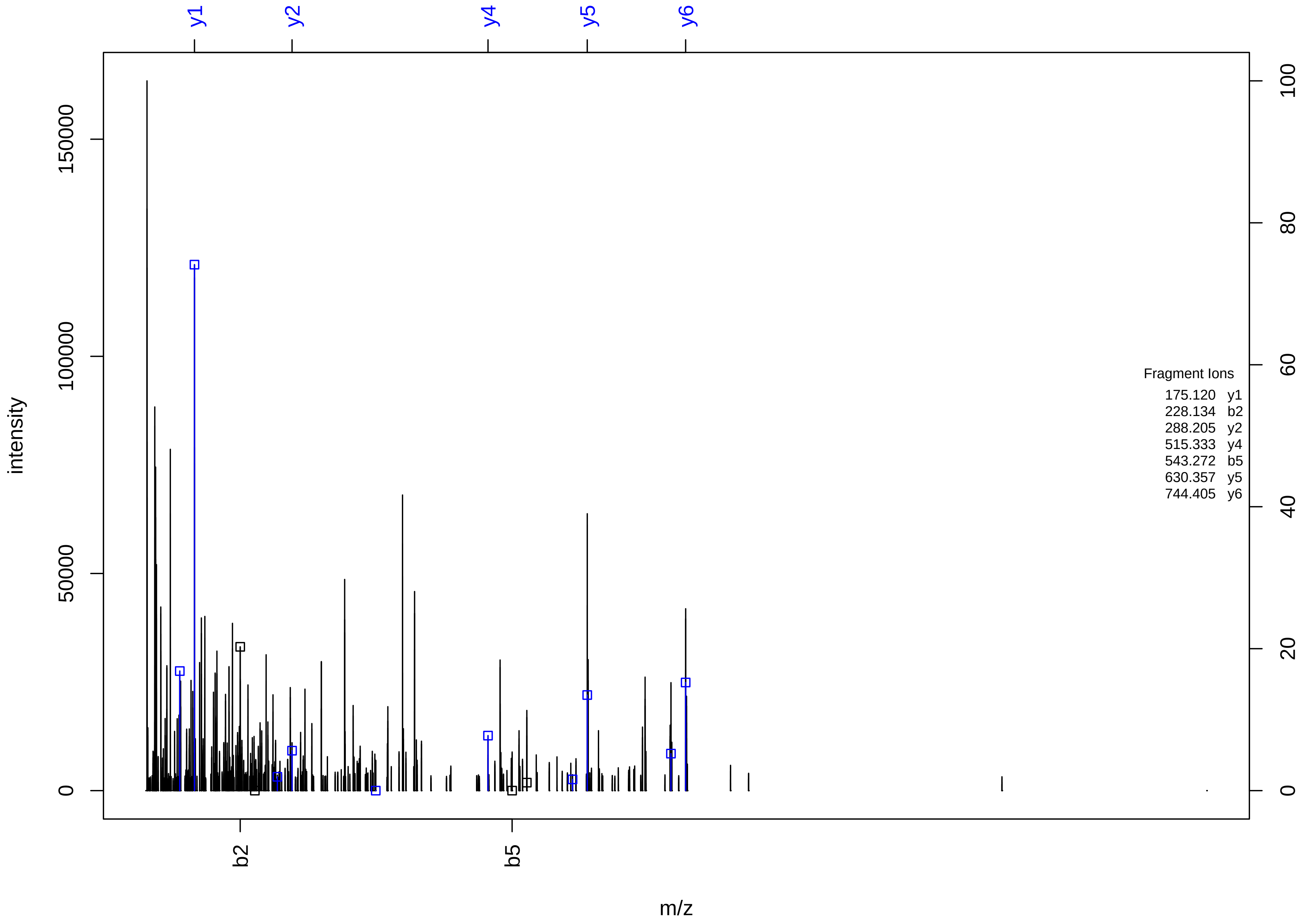

NLSINNDLNLR / File: christine_pool_01.raw; SpectrumID: ; scans: 5637

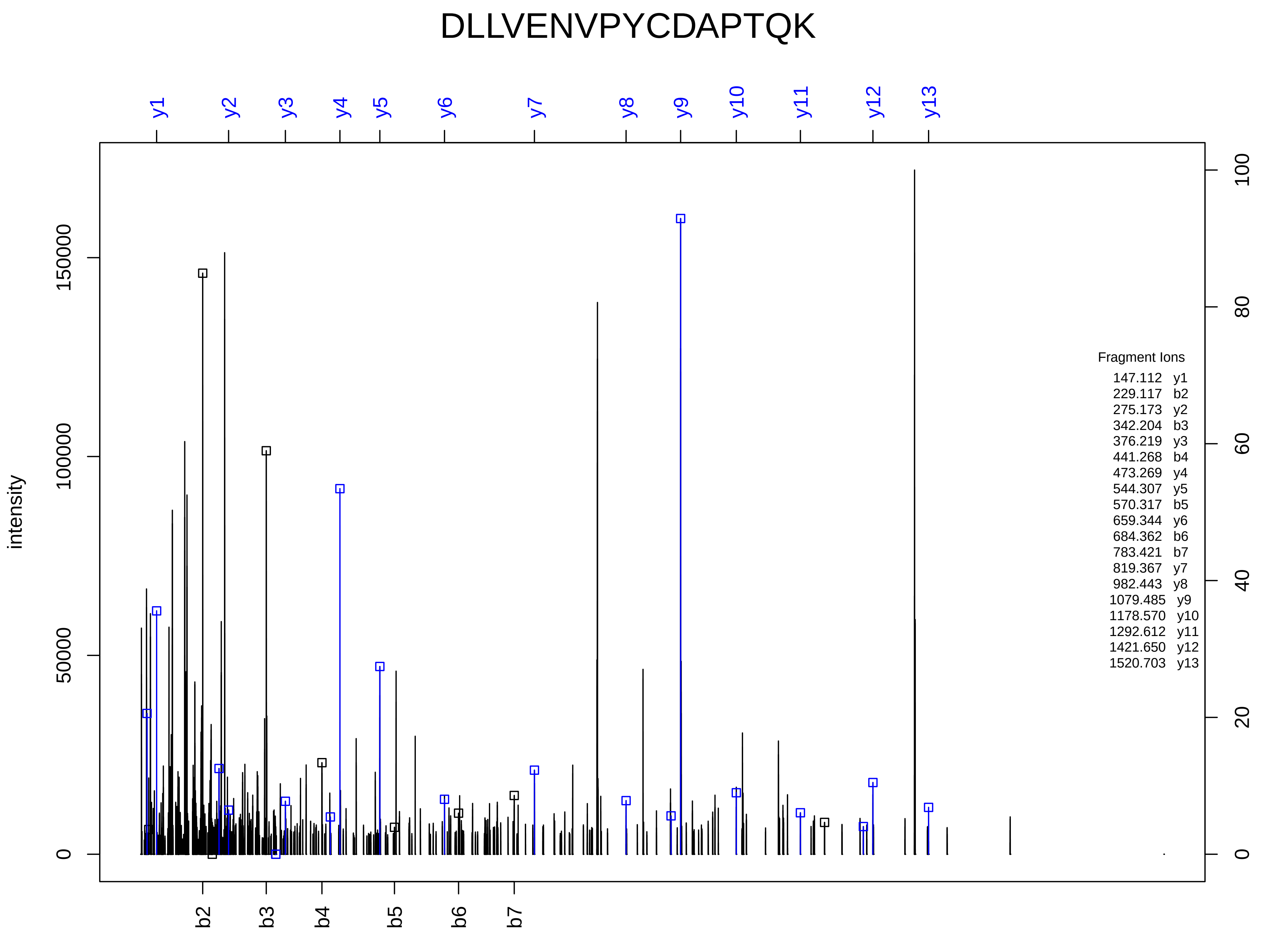

DLLVENVPYCDAPTQK / File: christine_pool_01.raw; SpectrumID: ; scans: 12910

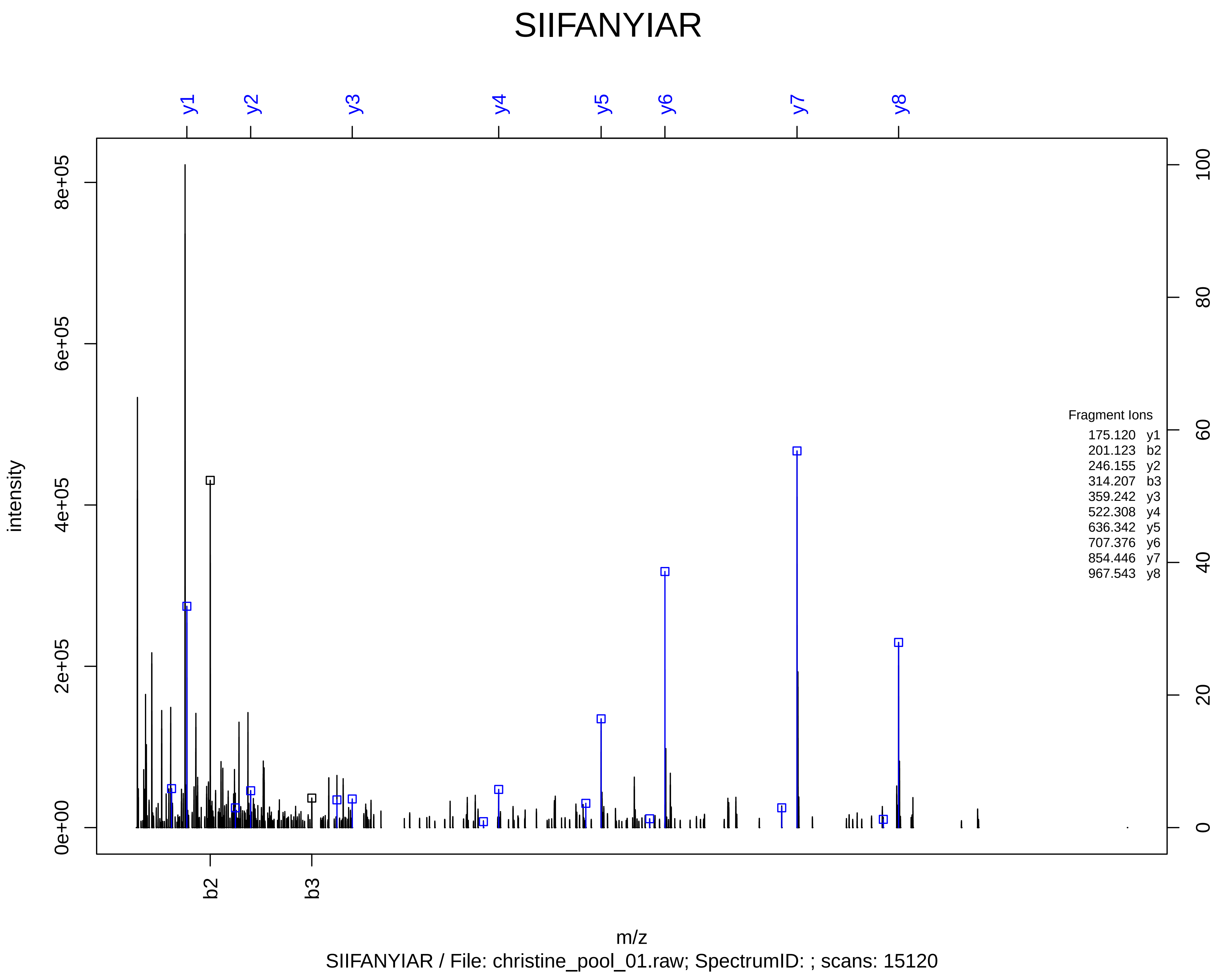
SIIFANYIAR
y1
y2
y3
y4
y5
y6
y7
y8
b2
b3
intensity
0e+00
2e+05
4e+05
6e+05
8e+05
0
20
40
60
80
100
Fragment Ions
175.120 y1
201.123 b2
246.155 y2
314.207 b3
359.242 y3
522.308 y4
636.342 y5
707.376 y6
854.446 y7
967.543 y8
m/z
SIIFANYIAR / File: christine_pool_01.raw; SpectrumID: ; scans: 15120

VVGALVDQGIFEELTR

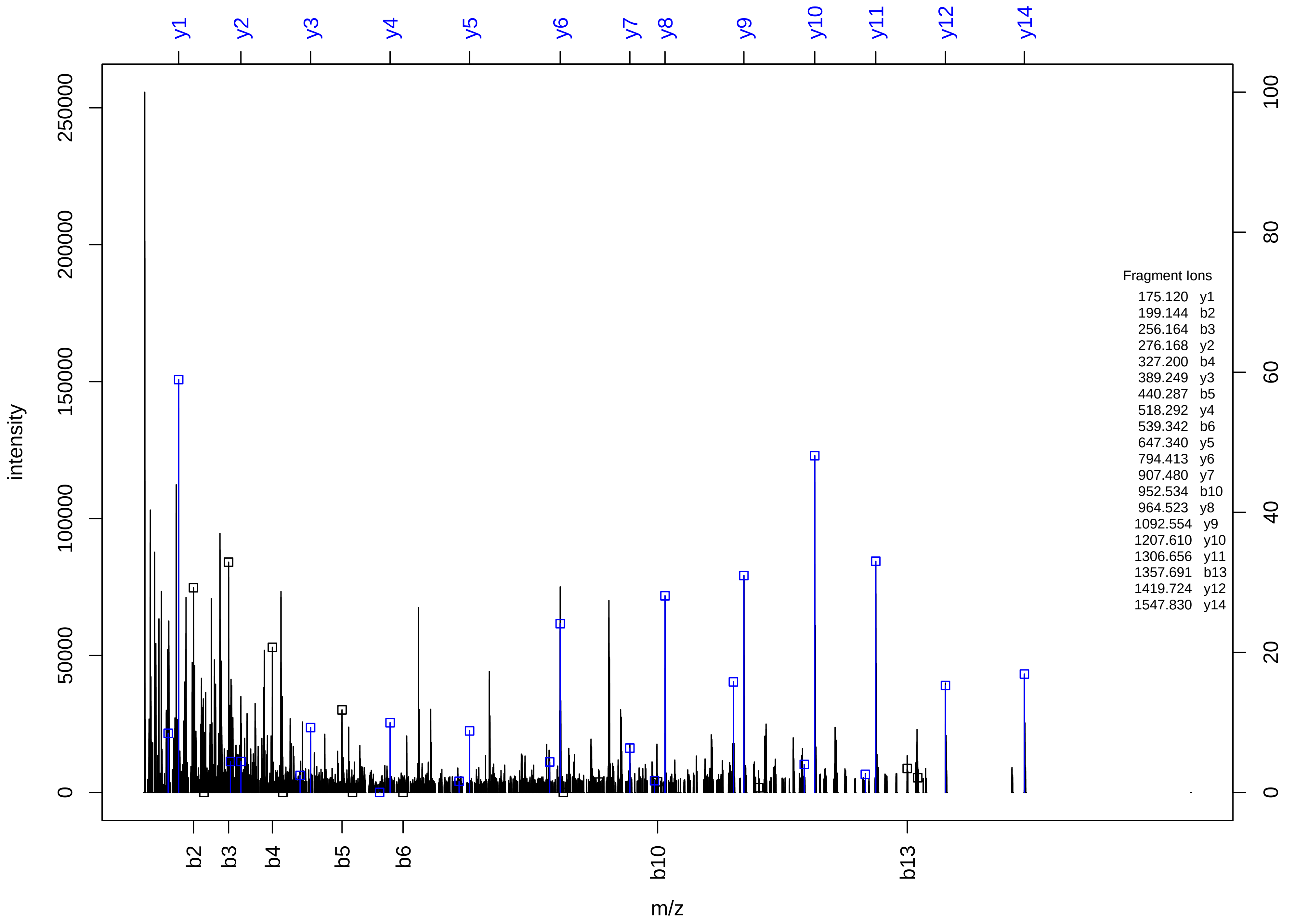

VVGALVDQGIFEELTR / File: christine_pool_01.raw; SpectrumID: ; scans: 21577

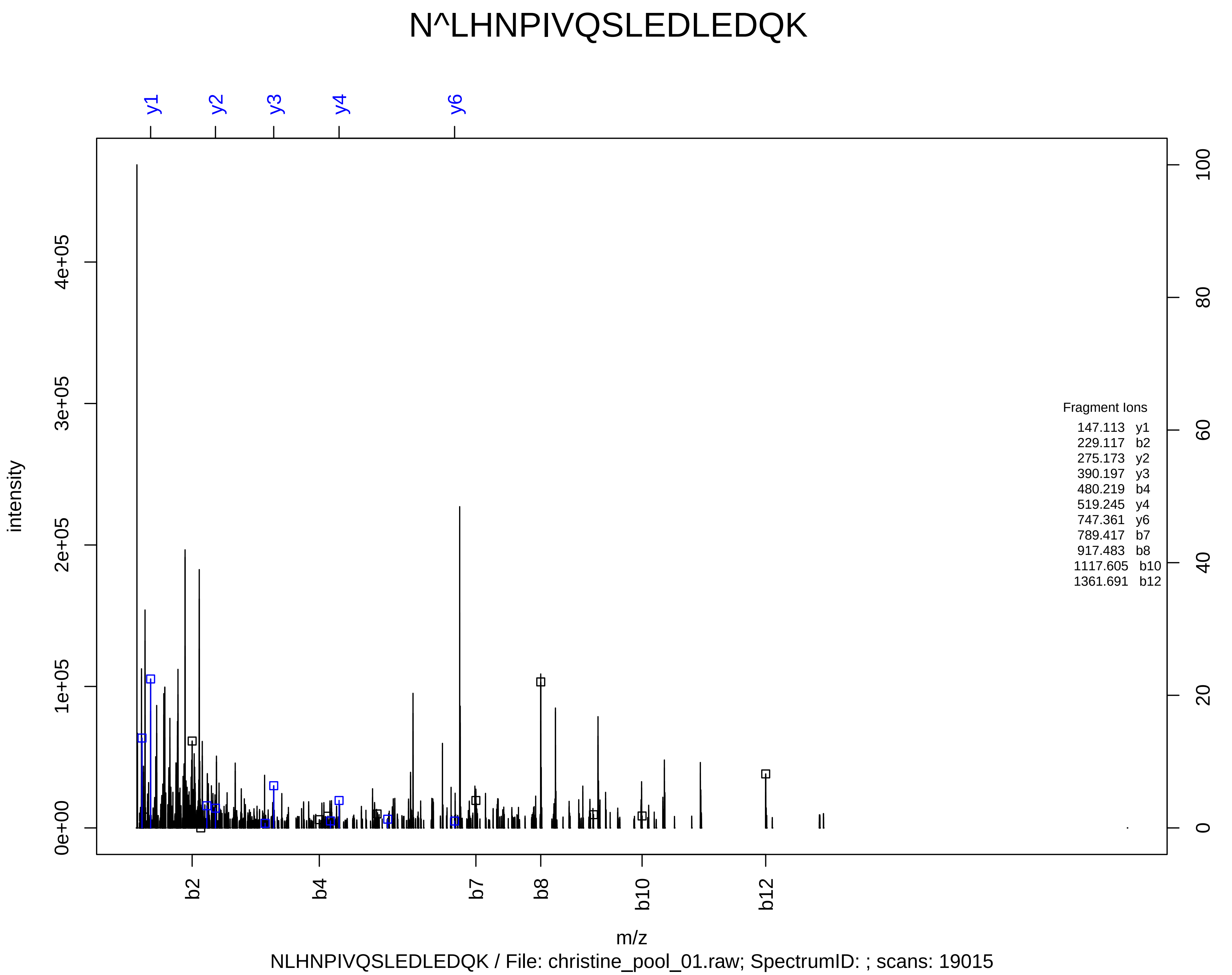
N^LHNPIVQSLEDLEDQK
y1
y2
y3
y4
y6
intensity
0e+00
1e+05
2e+05
3e+05
4e+05
0
20
40
60
80
100
Fragment Ions
147.113 y1
229.117 b2
275.173 y2
390.197 y3
480.219 b4
519.245 y4
747.361 y6
789.417 b7
917.483 b8
1117.605 b10
1361.691 b12
b2
b4
b7
b8
b10
b12
m/z
NLHNPIVQSLEDLEDQK / File: christine_pool_01.raw; SpectrumID: ; scans: 19015

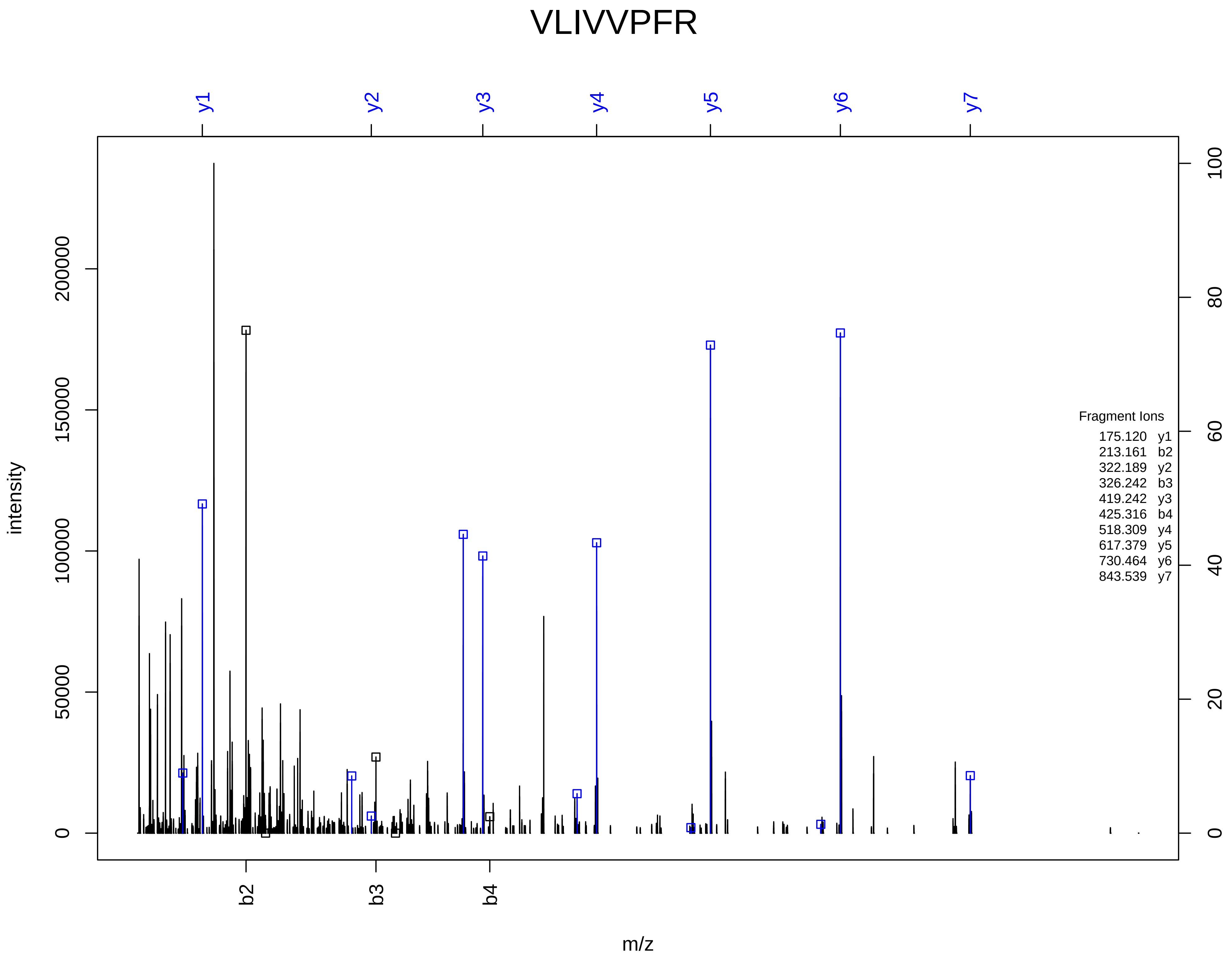

VLIVVPFR / File: christine_pool_01.raw; SpectrumID: ; scans: 17214

DGTDGETEVGEIQQNK

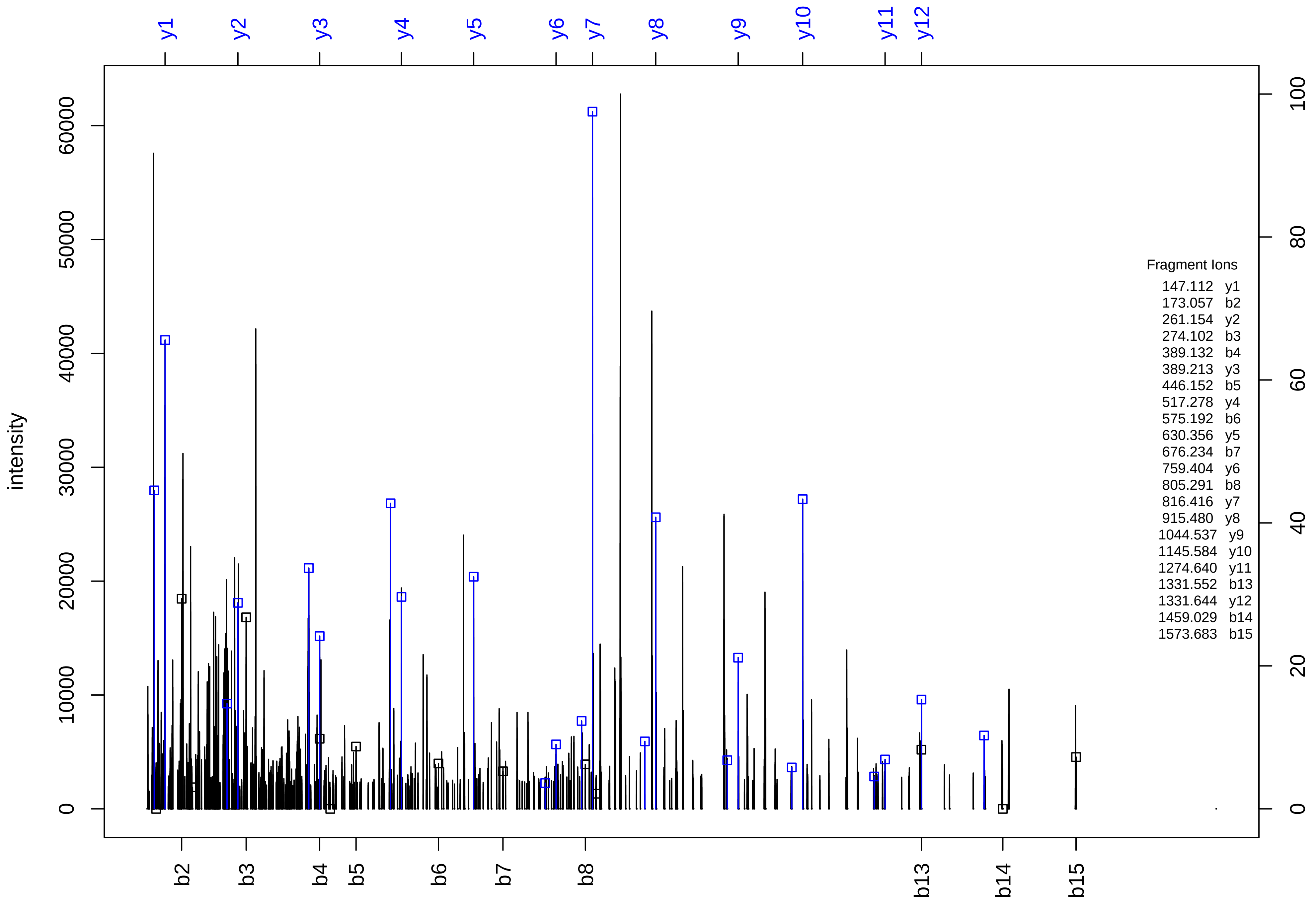

DGTDGETEVGEIQQNK / File: christine_pool_01.raw; SpectrumID: ; scans: 4204

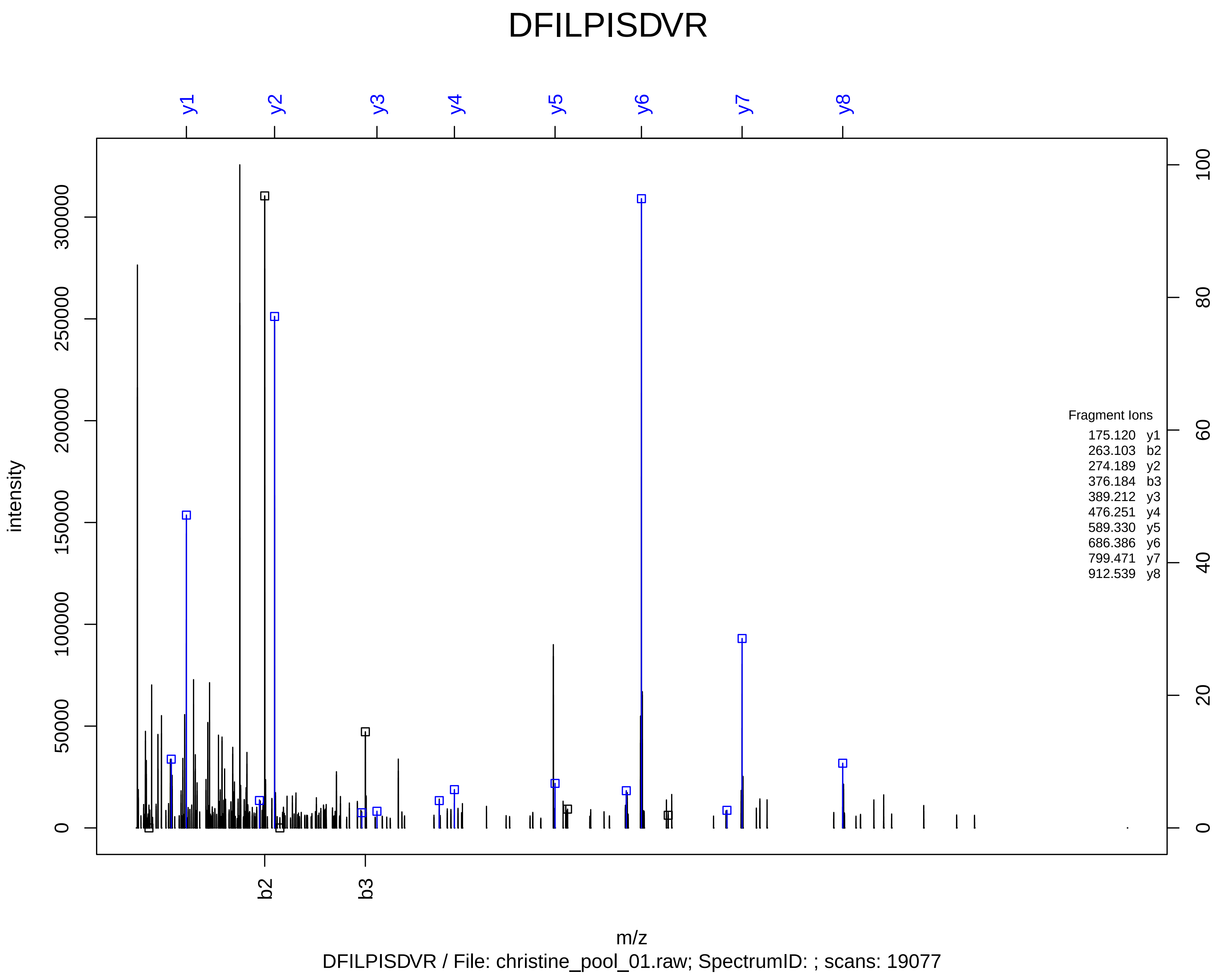

DFILPISDVR
y1
y2
y3
y4
y5
y6
y7
y8
b2
b3
intensity
m/z
Fragment Ions
175.120 y1
263.103 b2
274.189 y2
376.184 b3
389.212 y3
476.251 y4
589.330 y5
686.386 y6
799.471 y7
912.539 y8
DFILPISDVR / File: christine_pool_01.raw; SpectrumID: ; scans: 19077

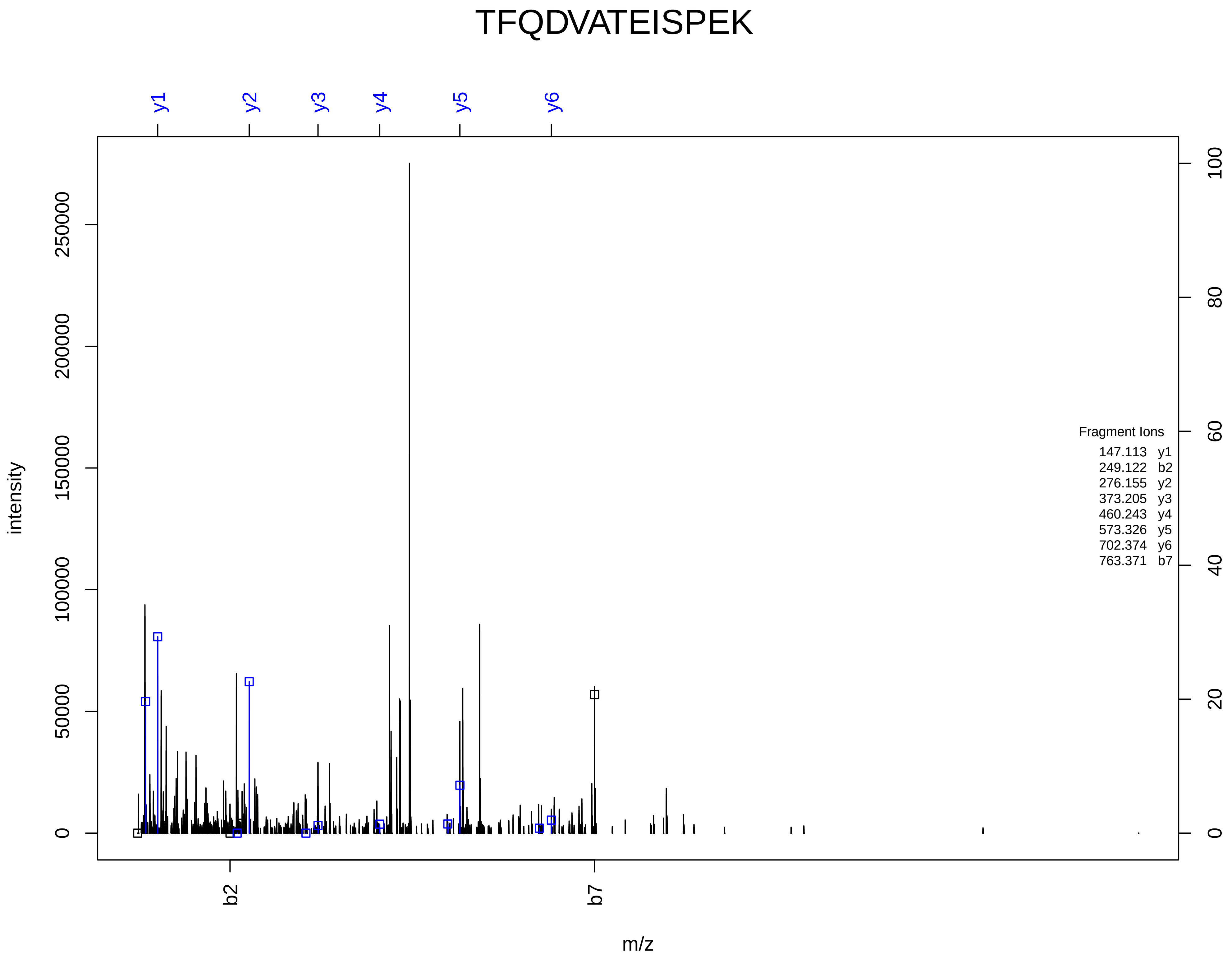

TFQDVATEISPEK / File: christine_pool_01.raw; SpectrumID: ; scans: 2254

GFGLLGSIFGK

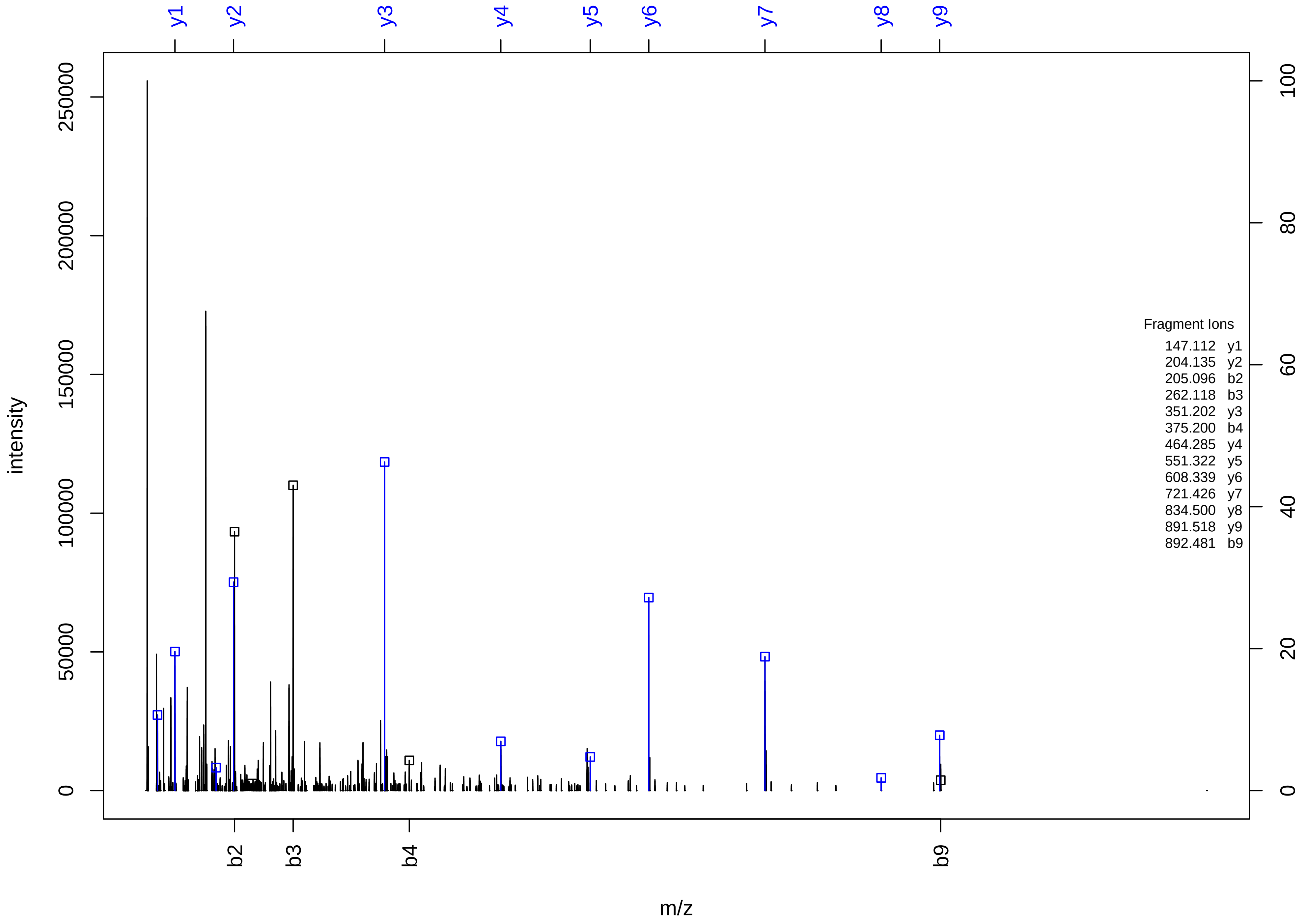

GFGLLGSIFGK / File: christine_pool_01.raw; SpectrumID: ; scans: 22549

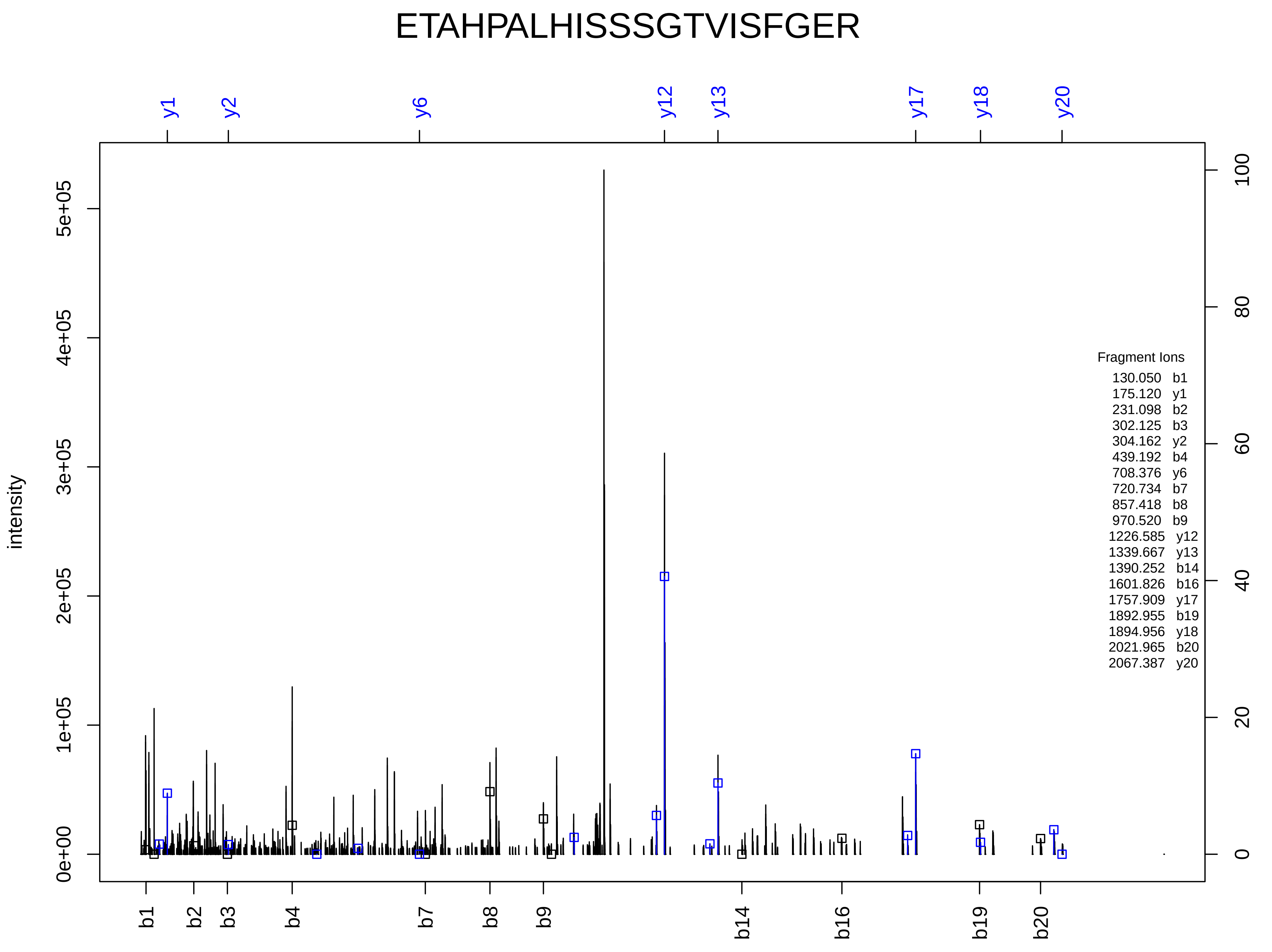

ETAHPALHISSSGTVISFGER / File: christine_pool_01.raw; SpectrumID: ; scans: 11648

ALEEGDGSVSGSSPR

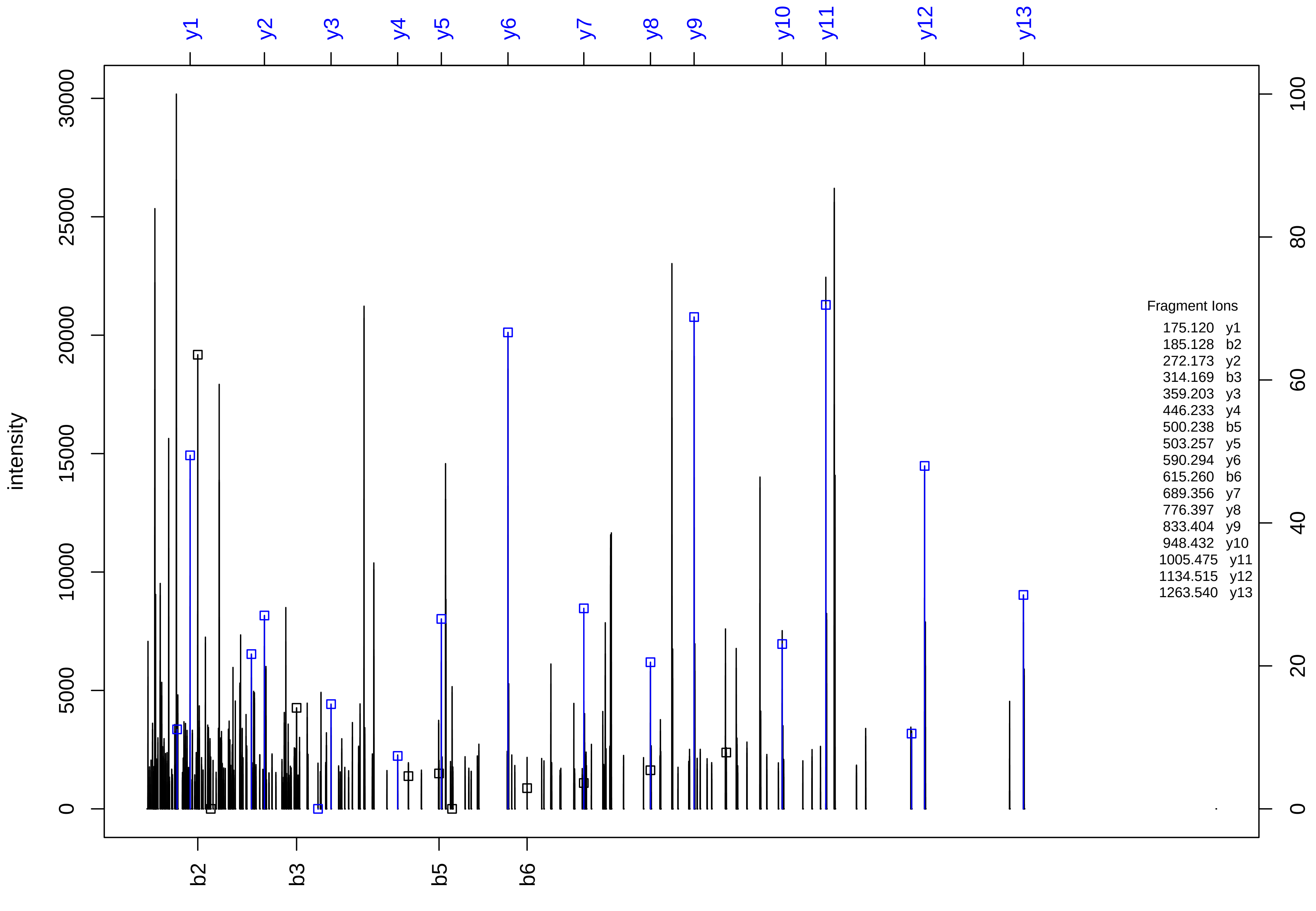

ALEEGDGSVSGSSPR / File: christine_pool_01.raw; SpectrumID: ; scans: 2482

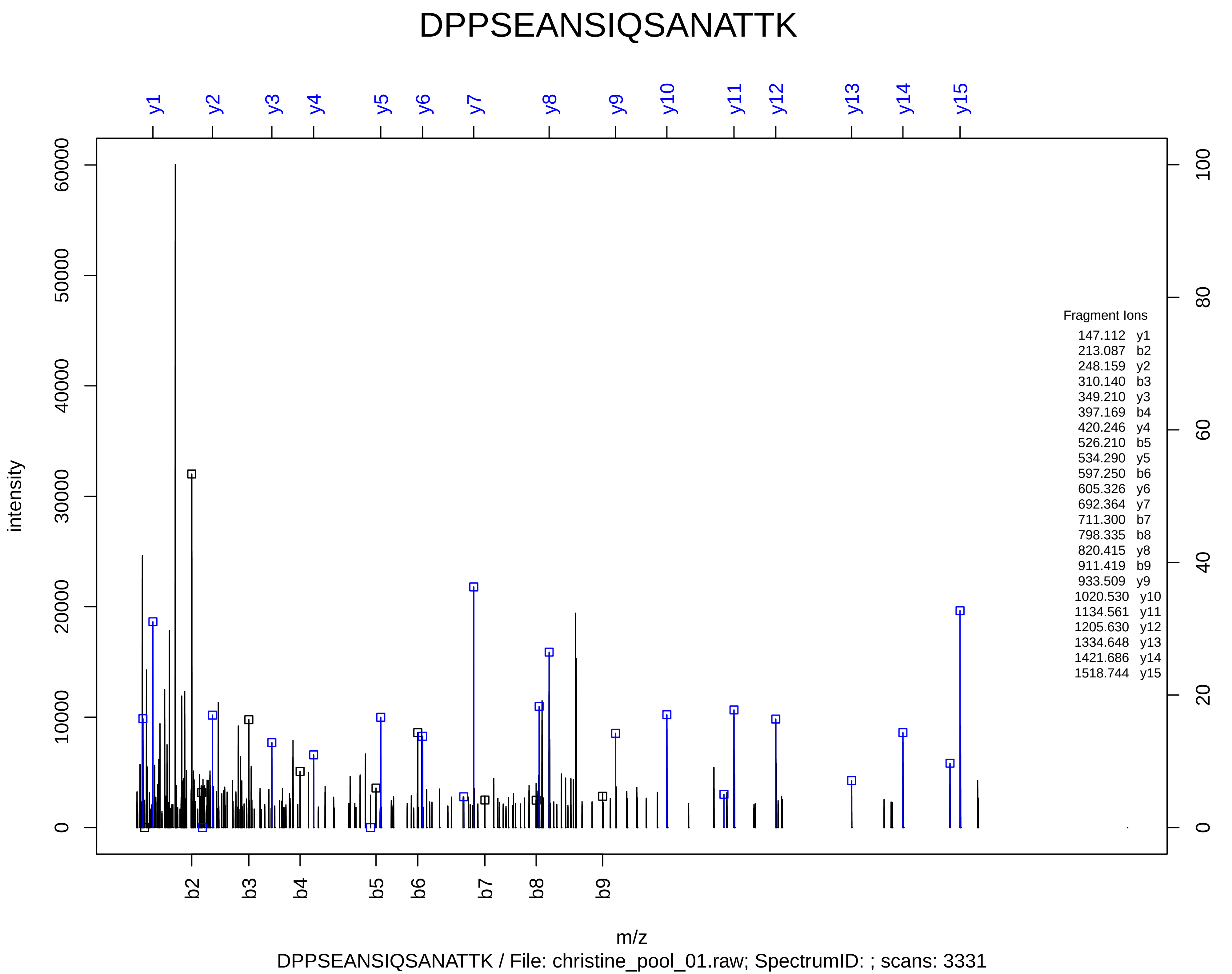

DPPSEANSIQSANATTK
y1
y2
y3
y4
y5
y6
y7
y8
y9
y10
y11
y12
y13
y14
y15
intensity
0
10000
20000
30000
40000
50000
60000
0
20
40
60
80
100
Fragment Ions
147.112 y1
213.087 b2
248.159 y2
310.140 b3
349.210 y3
397.169 b4
420.246 y4
526.210 b5
534.290 y5
597.250 b6
605.326 y6
692.364 y7
711.300 b7
798.335 b8
820.415 y8
911.419 b9
933.509 y9
1020.530 y10
1134.561 y11
1205.630 y12
1334.648 y13
1421.686 y14
1518.744 y15
b2
b3
b4
b5
b6
b7
b8
b9
m/z
DPPSEANSIQSANATTK / File: christine_pool_01.raw; SpectrumID: ; scans: 3331

(Ac)SDEFSLADALPEHSPAK

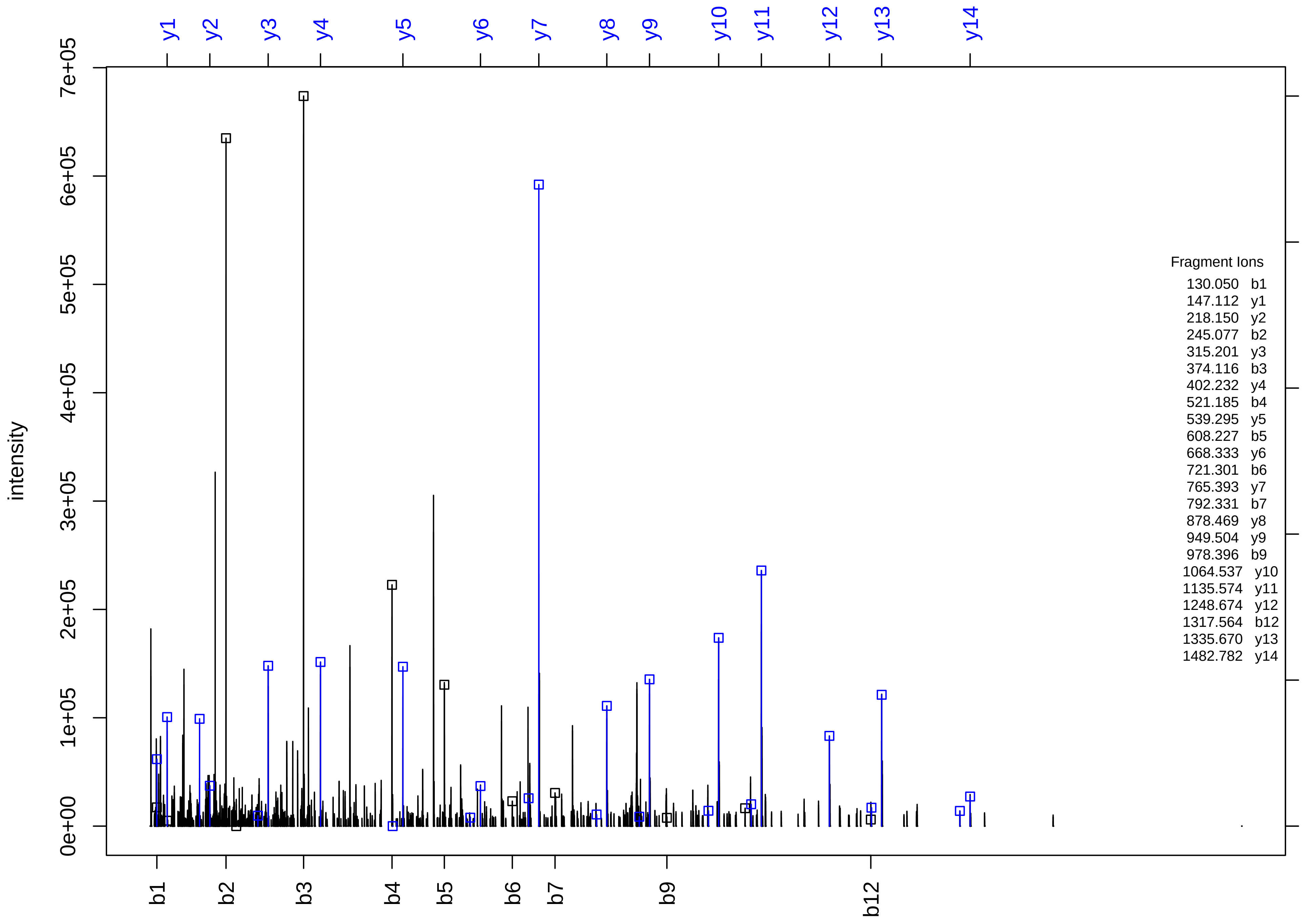

SDEFSLADALPEHSPAK / File: christine_pool_01.raw; SpectrumID: ; scans: 15487

(Ac)ERNHN^PDN^CN^VLN^FFFADKK

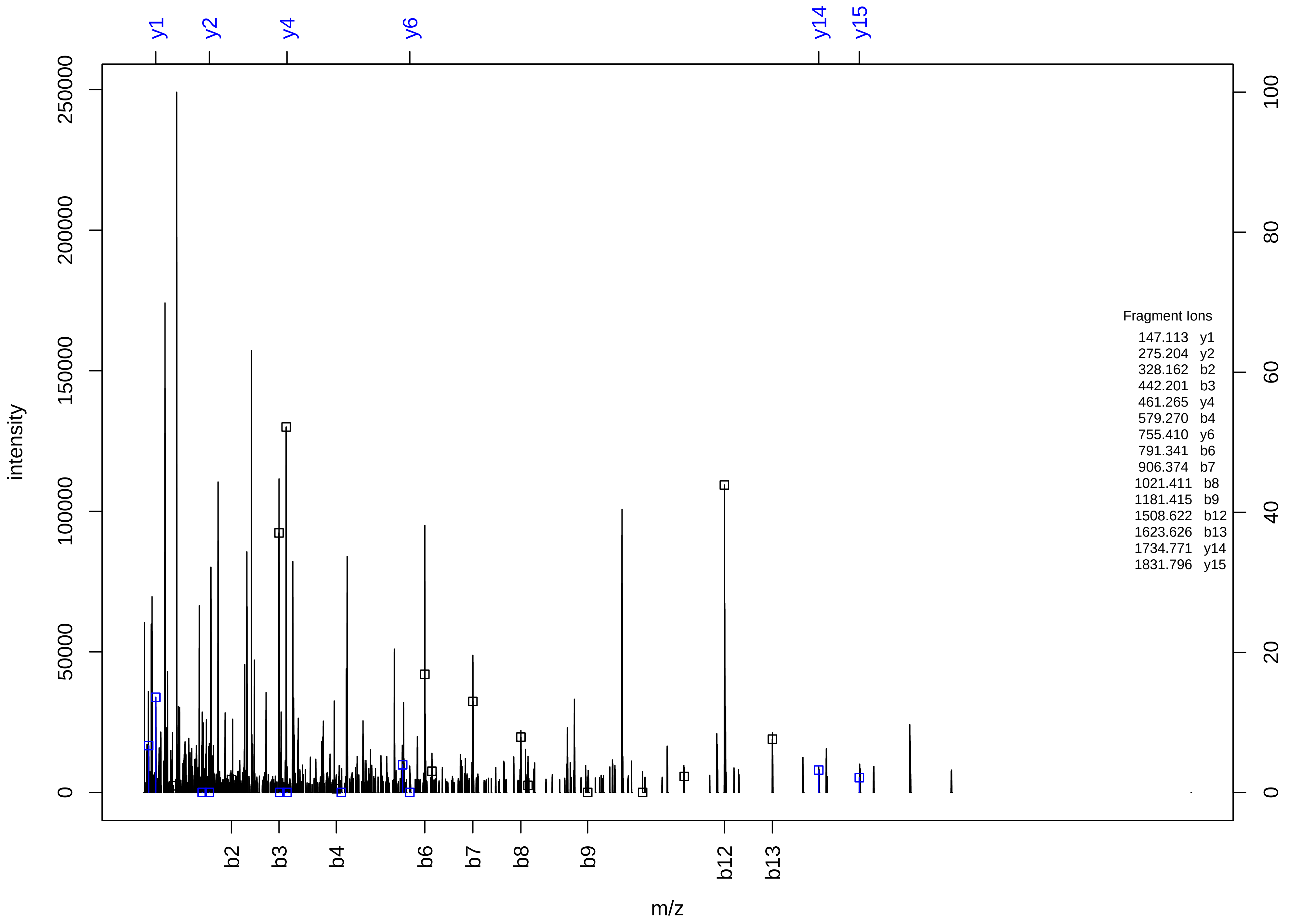

ERNHNPDNCNVLNFFFADKK / File: christine_pool_01.raw; SpectrumID: ; scans: 21502

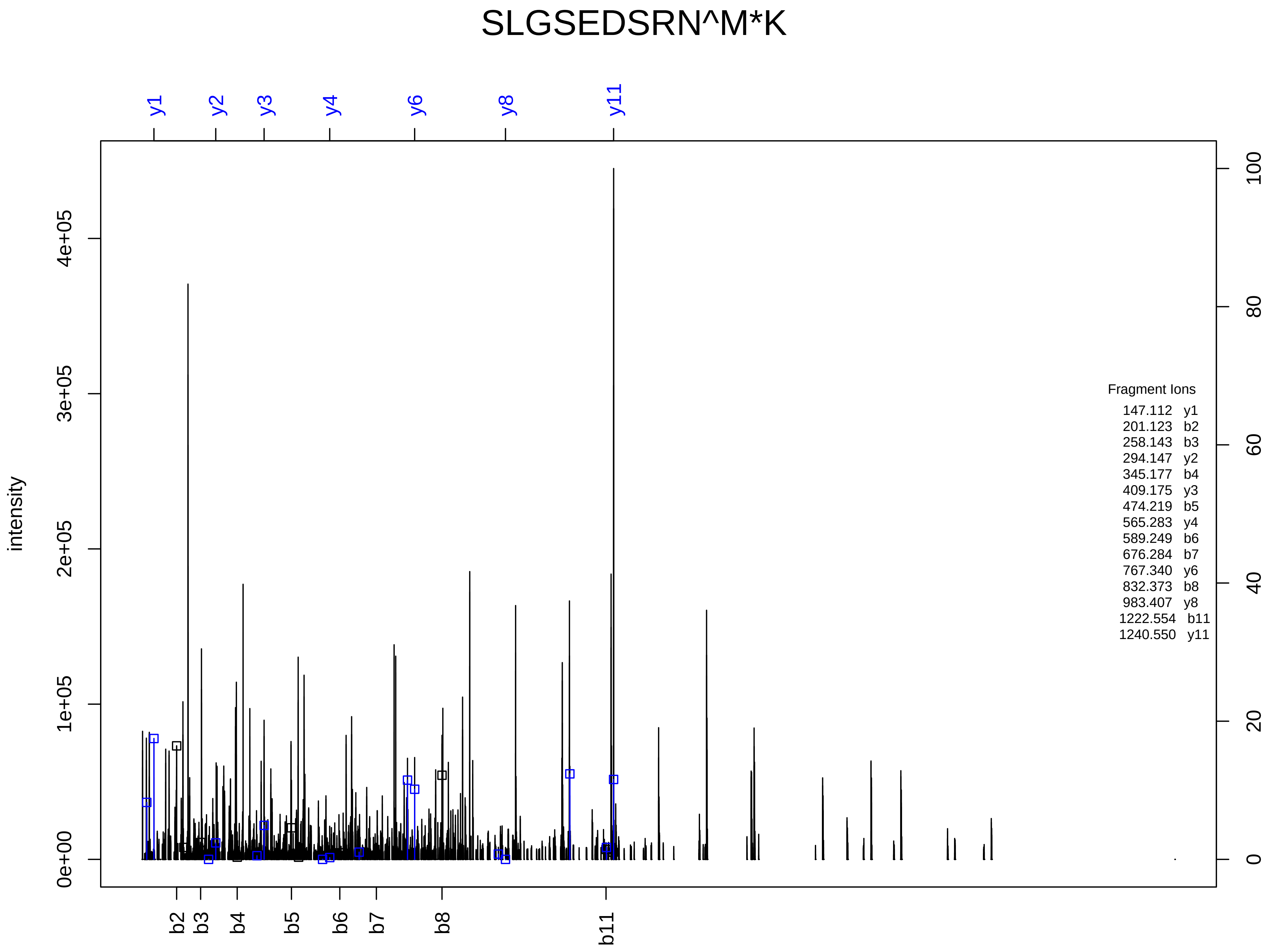

SLGSEDSRNMK / File: christine_pool_01.raw; SpectrumID: ; scans: 21934

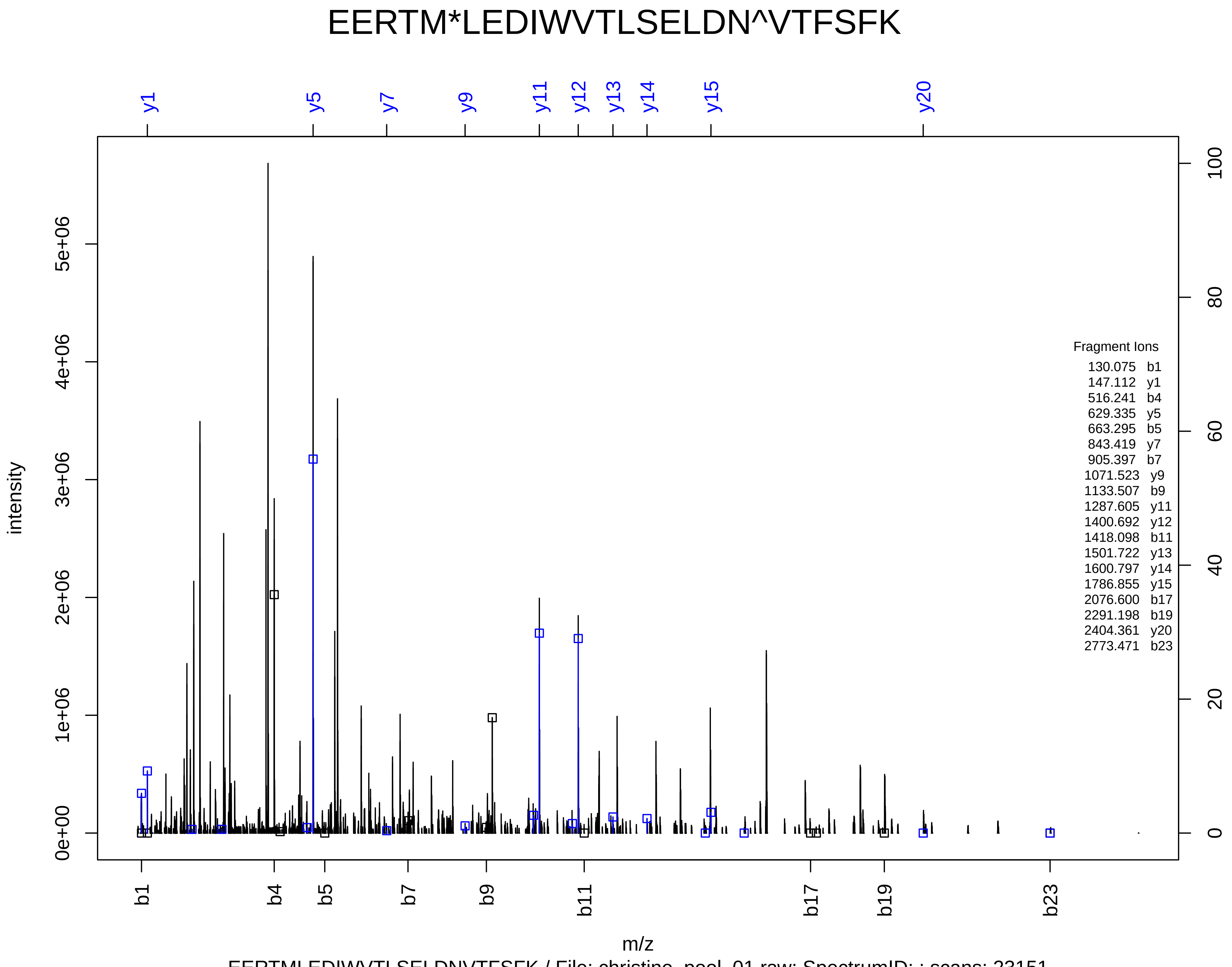

EERTM*LEDIWVTLSELDN^VTFSFK
y1
y5
y7
y9
y11
y12
y13
y14
y15
y20
intensity
0e+00
1e+06
2e+06
3e+06
4e+06
5e+06
0
20
40
60
80
100
Fragment Ions
130.075 b1
147.112 y1
516.241 b4
629.335 y5
663.295 b5
843.419 y7
905.397 b7
1071.523 y9
1133.507 b9
1287.605 y11
1400.692 y12
1418.098 b11
1501.722 y13
1600.797 y14
1786.855 y15
2076.600 b17
2291.198 b19
2404.361 y20
2773.471 b23
b1
b4
b5
b7
b9
b11
b17
b19
b23
m/z
EERTMLEDIWVTLSELDNVTFSFK / File: christine_pool_01.raw; SpectrumID: ; scans: 23151

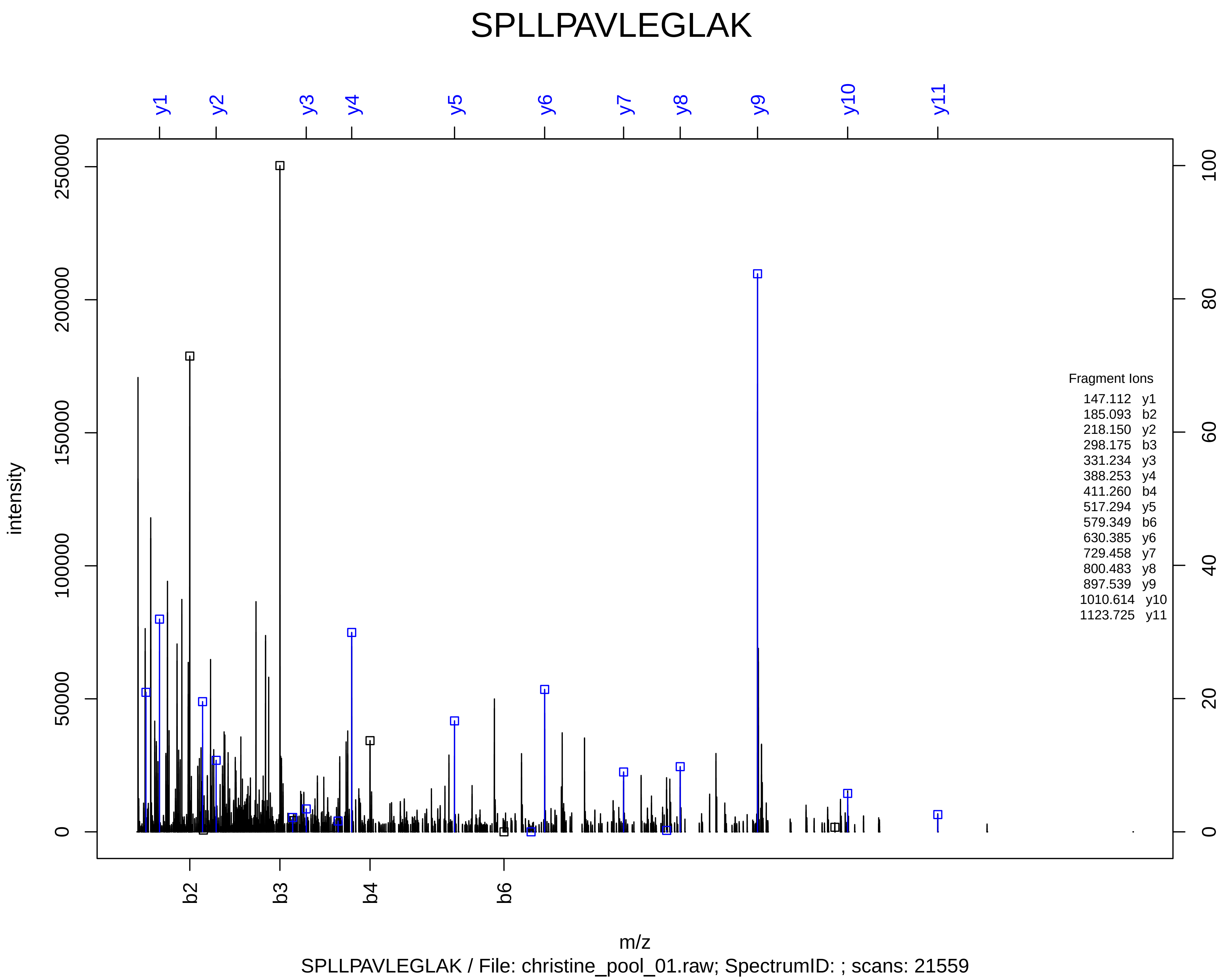

SPLLPAVLEGLAK
y1
y2
y3
y4
y5
y6
y7
y8
y9
y10
y11
intensity
0
50000
100000
150000
200000
250000
0
20
40
60
80
100
b2
b3
b4
b6
m/z
Fragment Ions
147.112 y1
185.093 b2
218.150 y2
298.175 b3
331.234 y3
388.253 y4
411.260 b4
517.294 y5
579.349 b6
630.385 y6
729.458 y7
800.483 y8
897.539 y9
1010.614 y10
1123.725 y11
SPLLPAVLEGLAK / File: christine_pool_01.raw; SpectrumID: ; scans: 21559

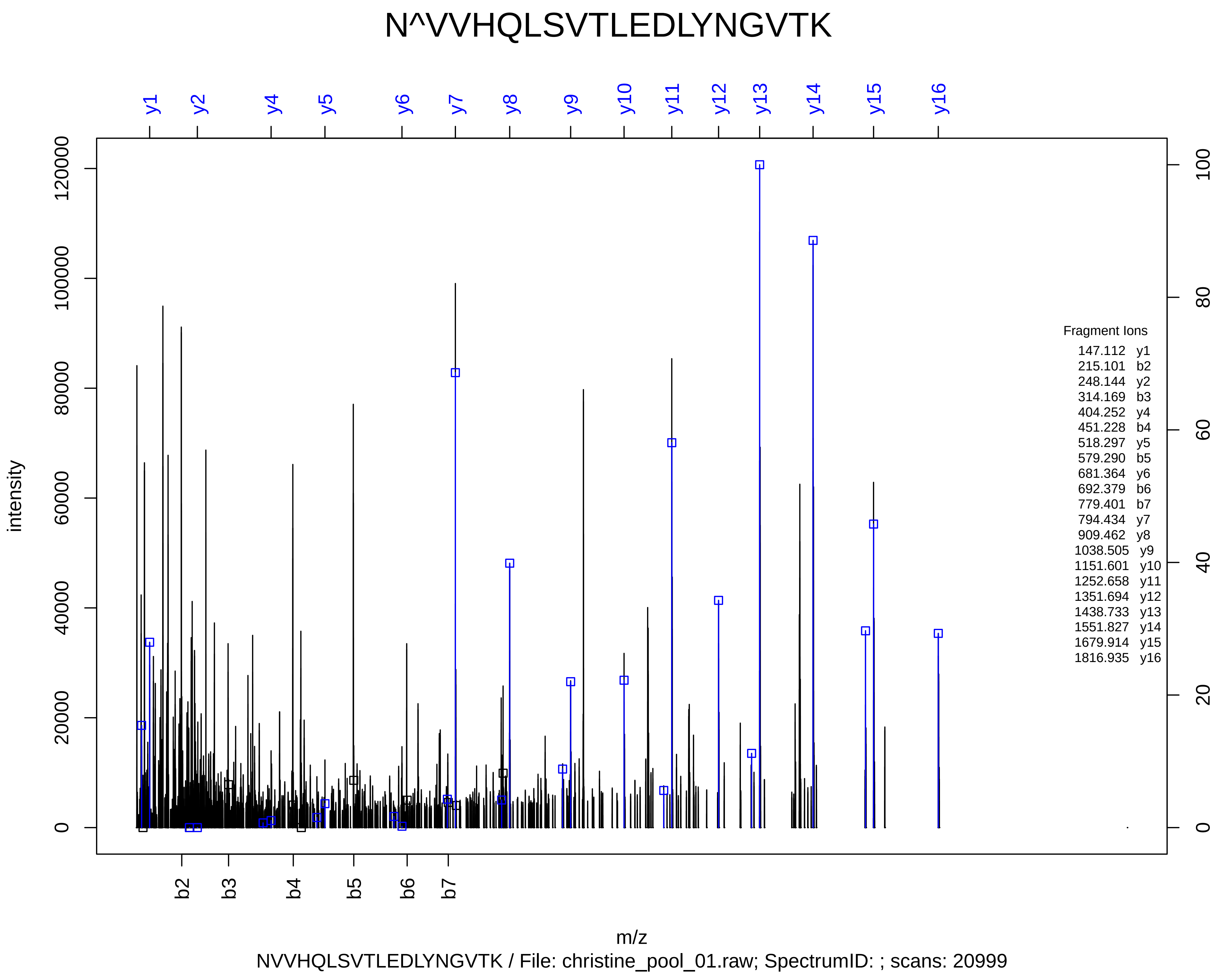
N^VVHQLSVTLEDLYNGVTK
y1 y2 y4 y5 y6 y7 y8 y9 y10 y11 y12 y13 y14 y15 y16
b2 b3 b4 b5 b6 b7
intensity
0 20000 40000 60000 80000 100000 120000
0 20 40 60 80 100
m/z
Fragment Ions
147.112 y1
215.101 b2
248.144 y2
314.169 b3
404.252 y4
451.228 b4
518.297 y5
579.290 b5
681.364 y6
692.379 b6
779.401 b7
794.434 y7
909.462 y8
1038.505 y9
1151.601 y10
1252.658 y11
1351.694 y12
1438.733 y13
1551.827 y14
1679.914 y15
1816.935 y16
NVVHQLSVTLEDLYNGVTK / File: christine_pool_01.raw; SpectrumID: ; scans: 20999

(Ac)AGLEVLFASAAPAITCR

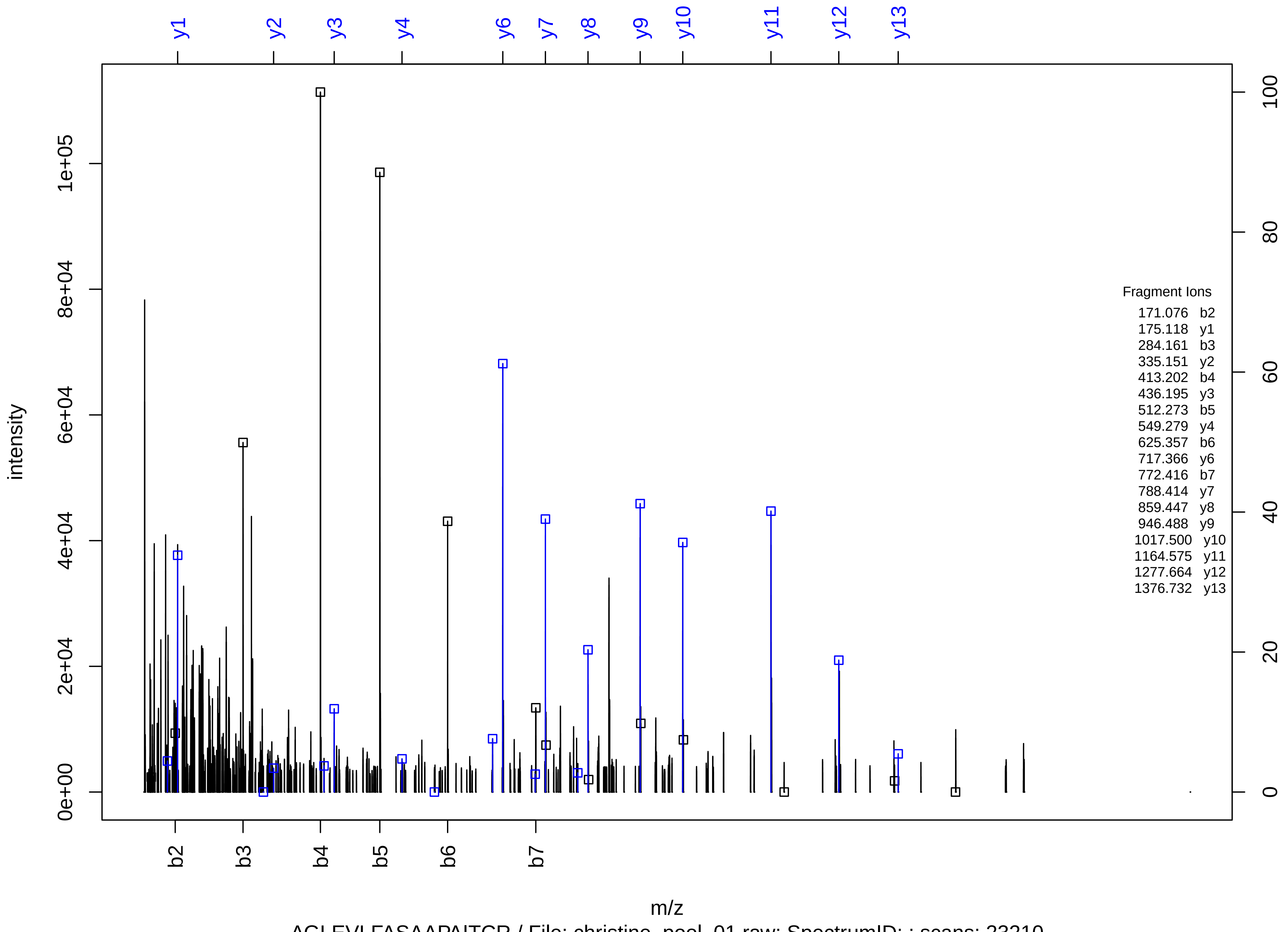

AGLEVLFASAAPAITCR / File: christine_pool_01.raw; SpectrumID: ; scans: 23210

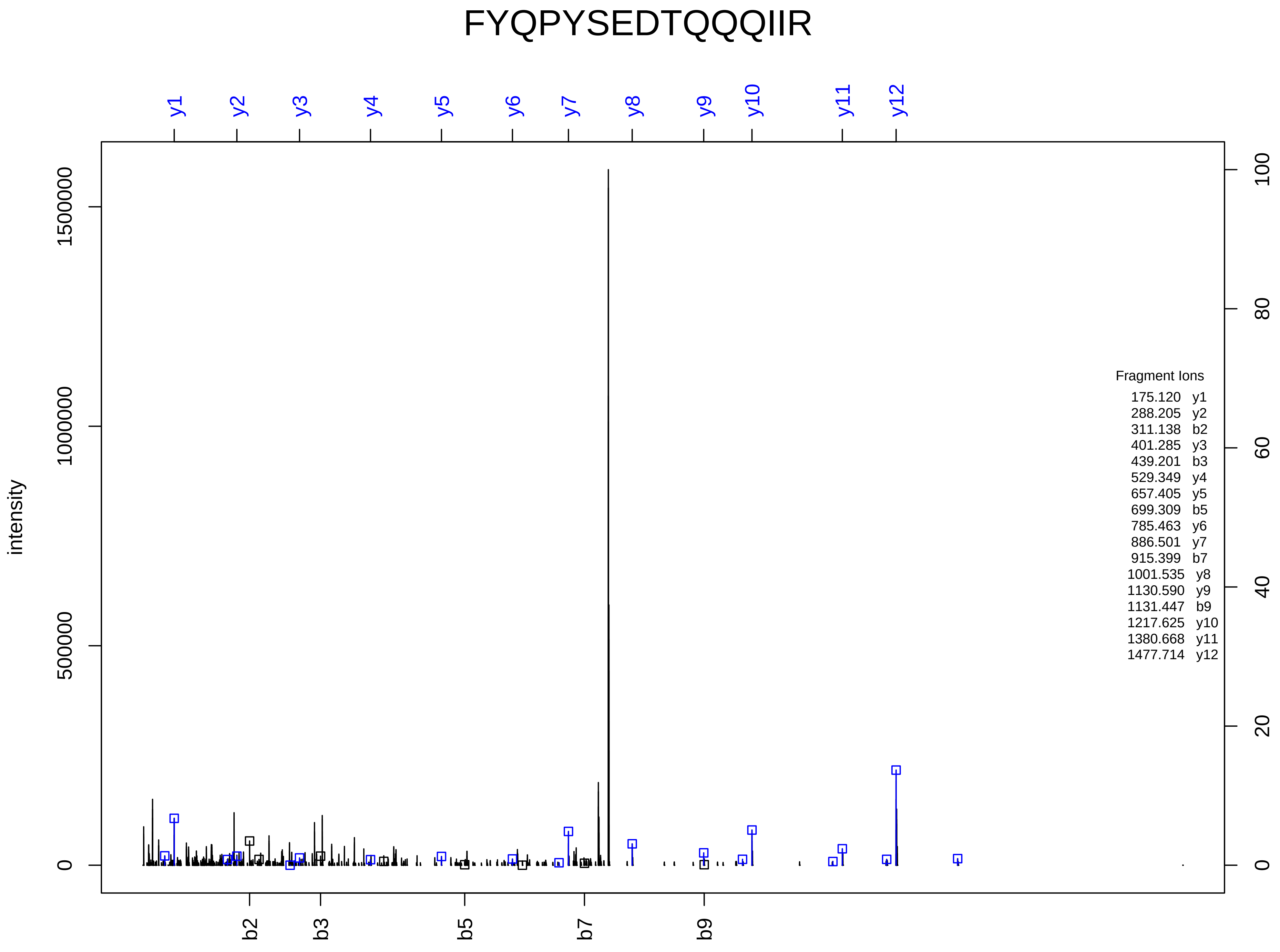

FYQPYSEDTQQQIIR / File: christine_pool_01.raw; SpectrumID: ; scans: 9960

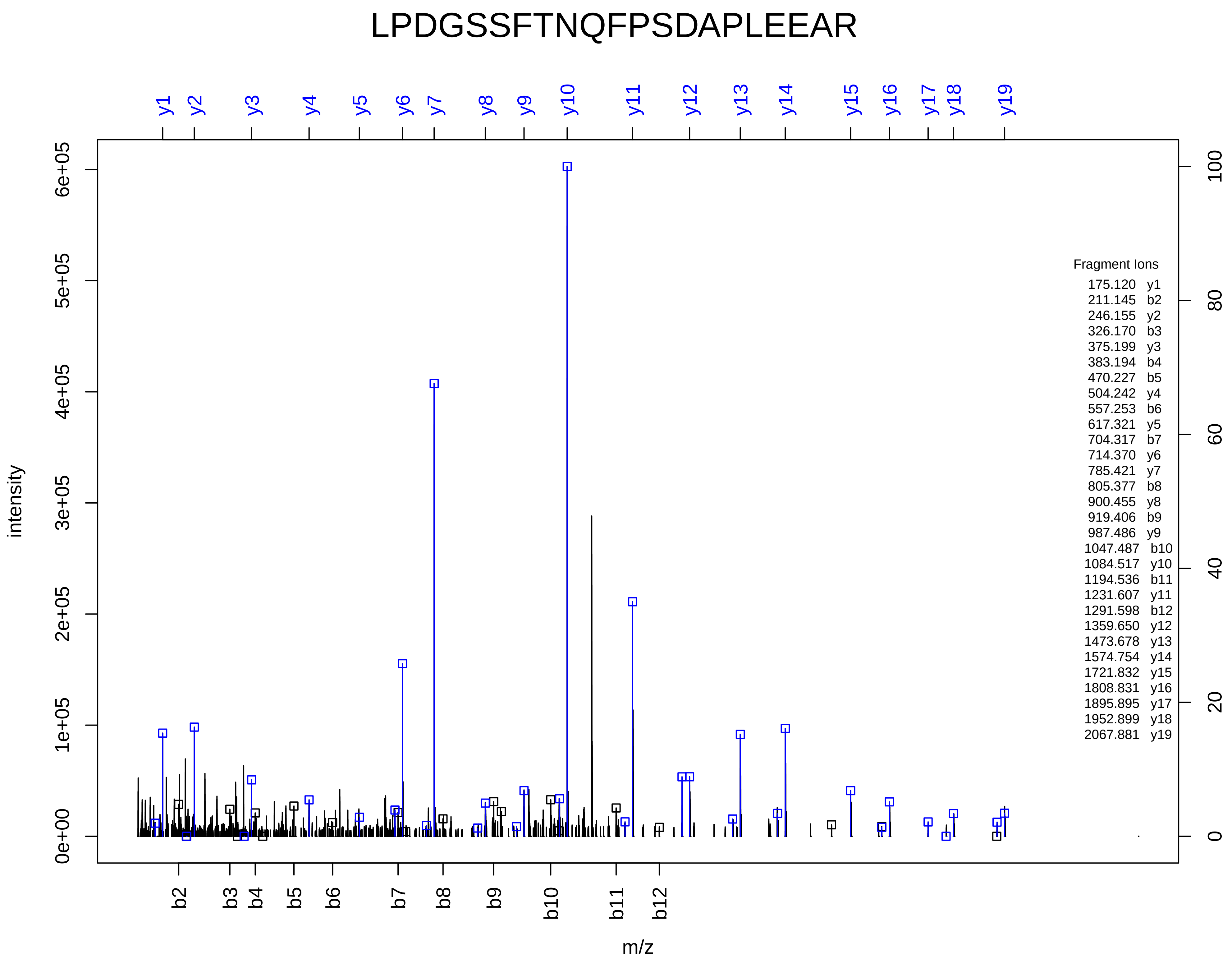

LPDGSSFTNQFPSDAPLEEAR / File: christine_pool_01.raw; SpectrumID: ; scans: 14779

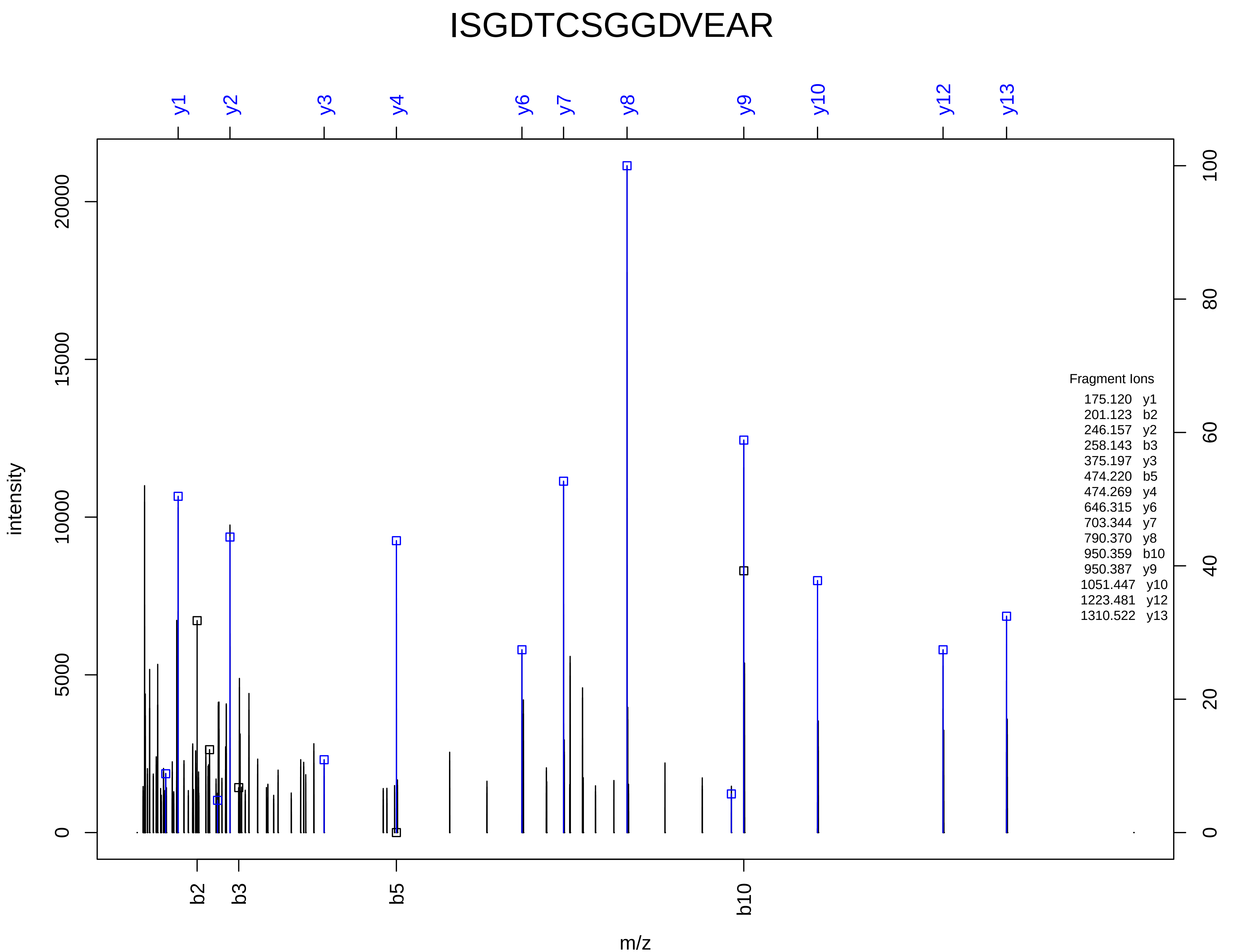

ISGDTCSGGDVEAR
y1
y2
y3
y4
y6
y7
y8
y9
y10
y12
y13
intensity
0
5000
10000
15000
20000
0
20
40
60
80
100
b2
b3
b5
b10
m/z
Fragment Ions
175.120 y1
201.123 b2
246.157 y2
258.143 b3
375.197 y3
474.220 b5
474.269 y4
646.315 y6
703.344 y7
790.370 y8
950.359 b10
950.387 y9
1051.447 y10
1223.481 y12
1310.522 y13
ISGDTCSGGDVEAR / File: christine_pool_01.raw; SpectrumID: ; scans: 1793

(Ac)AVEELQSIIK

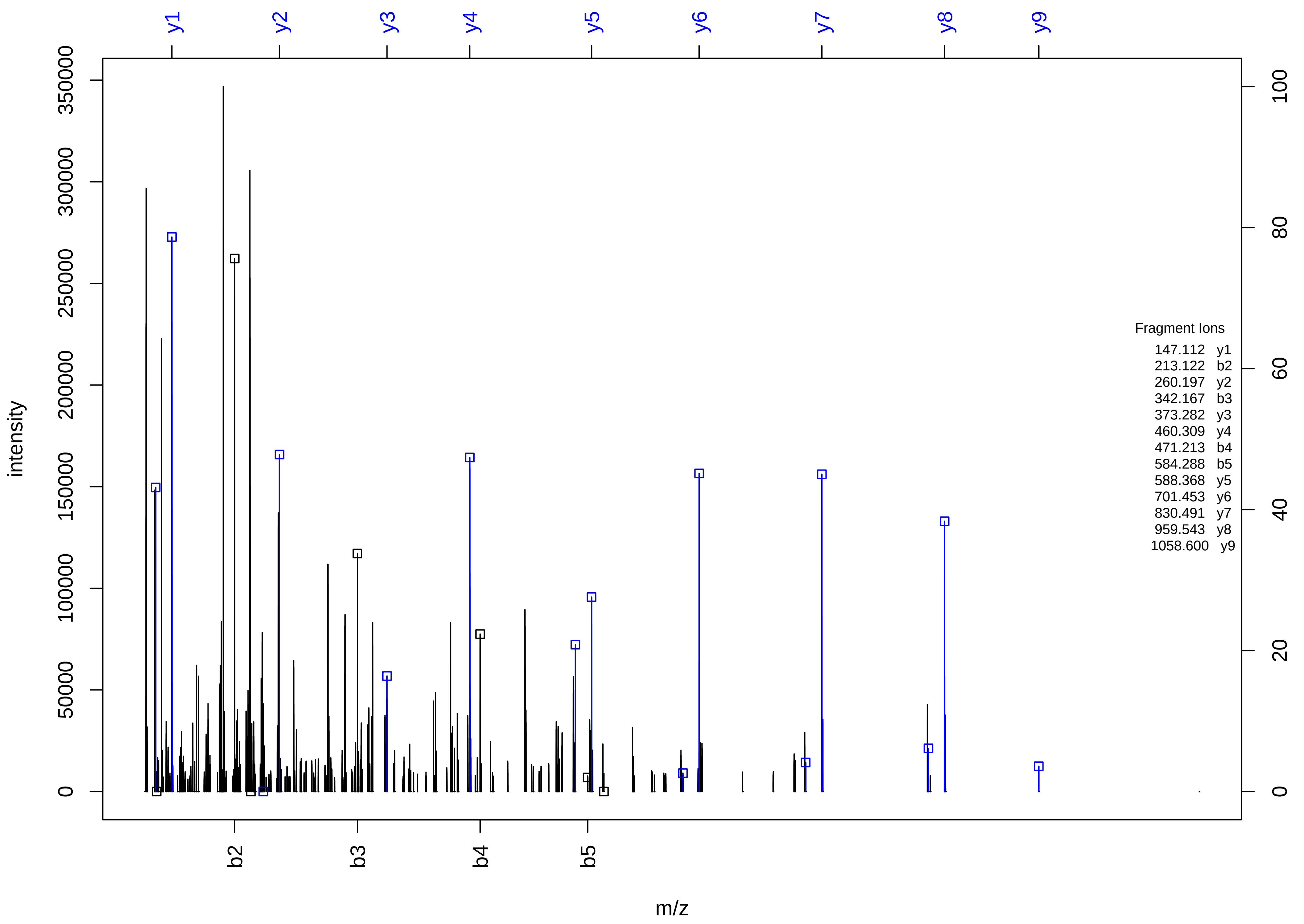

AVEELQSIIK / File: christine_pool_01.raw; SpectrumID: ; scans: 22247

DHSFFIPDIEYLSDIK

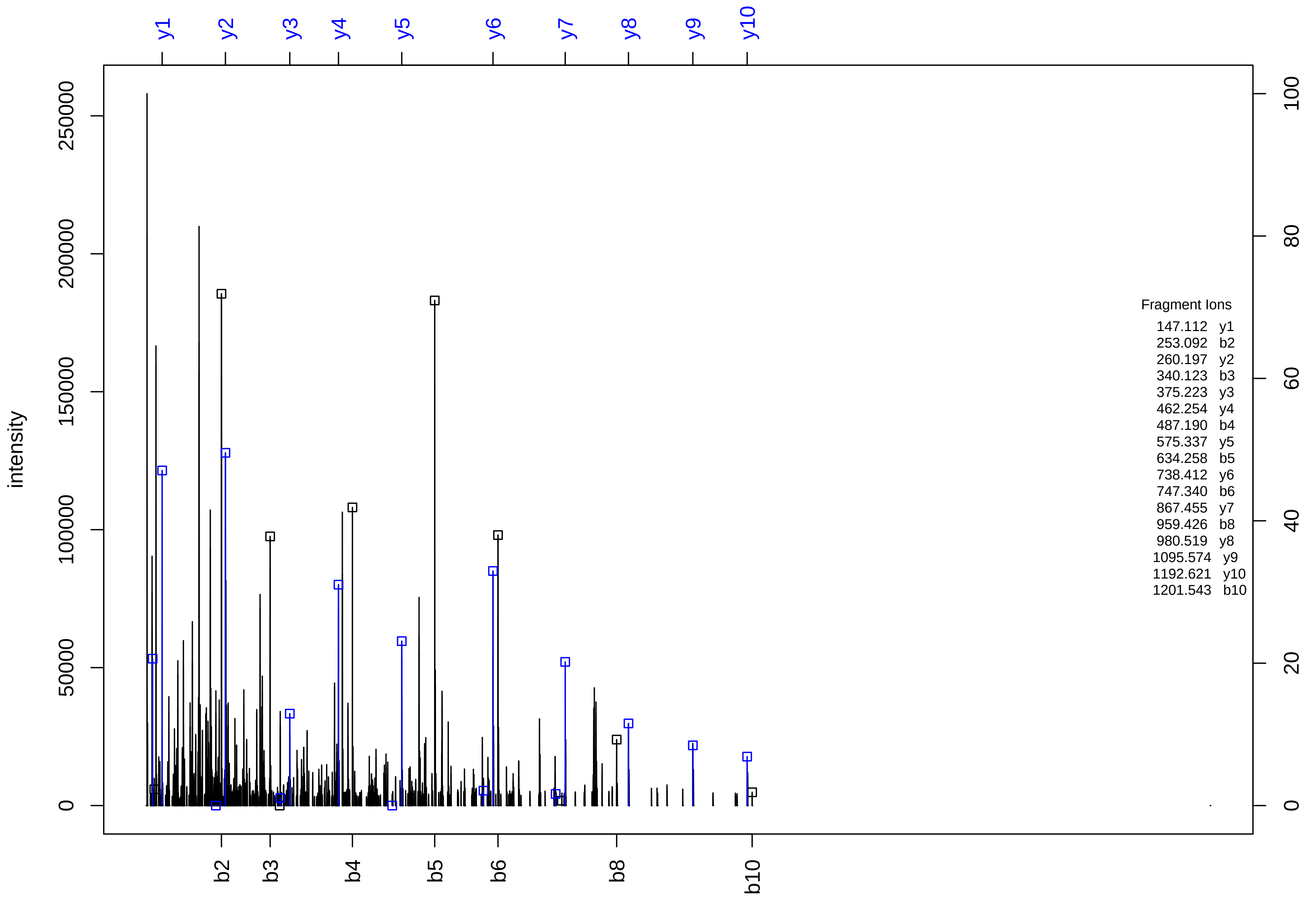

DHSFFIPDIEYLSDIK / File: christine_pool_01.raw; SpectrumID: ; scans: 22576

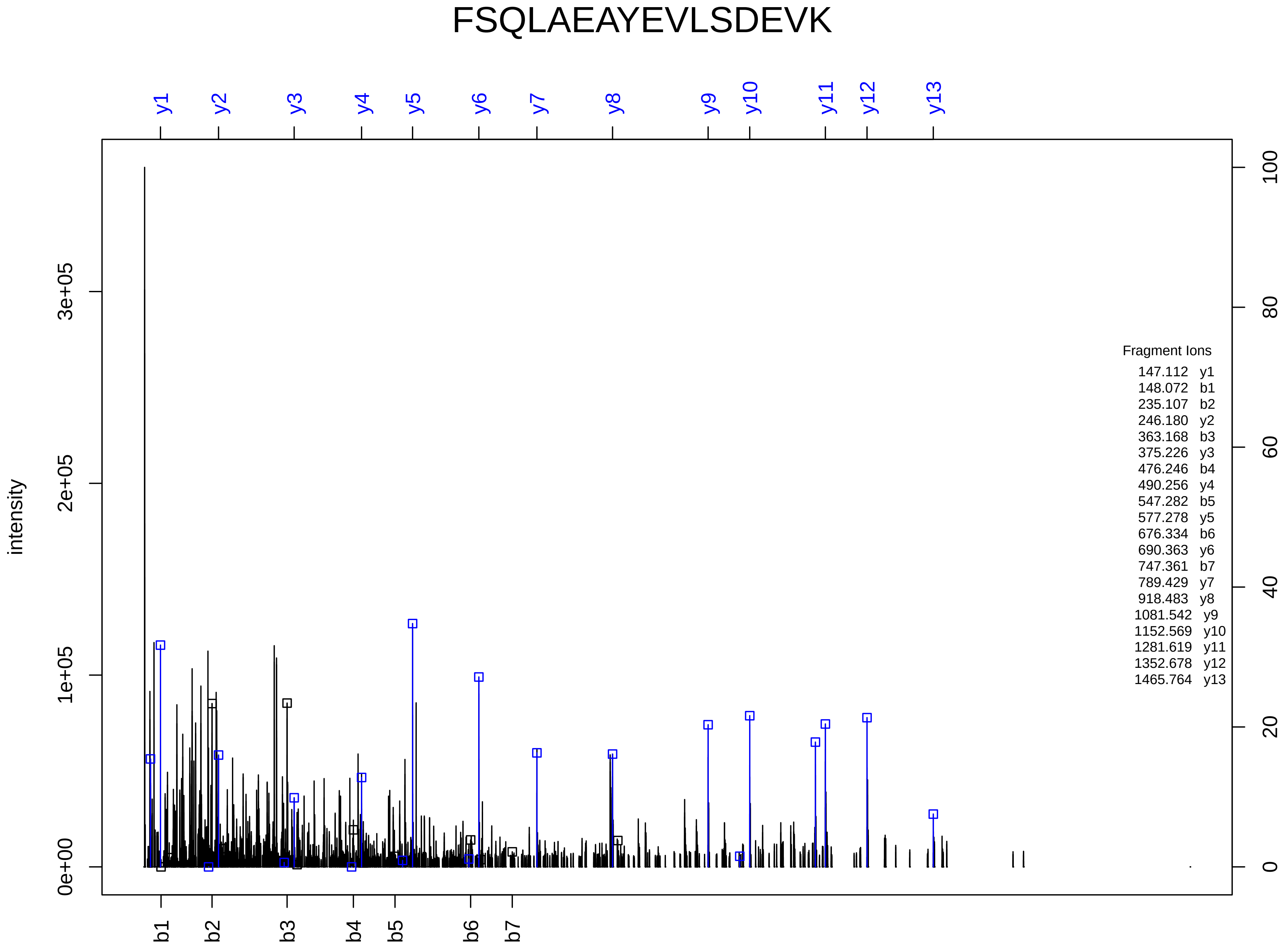

FSQLAEAYEVLSDEVK / File: christine_pool_01.raw; SpectrumID: ; scans: 20135

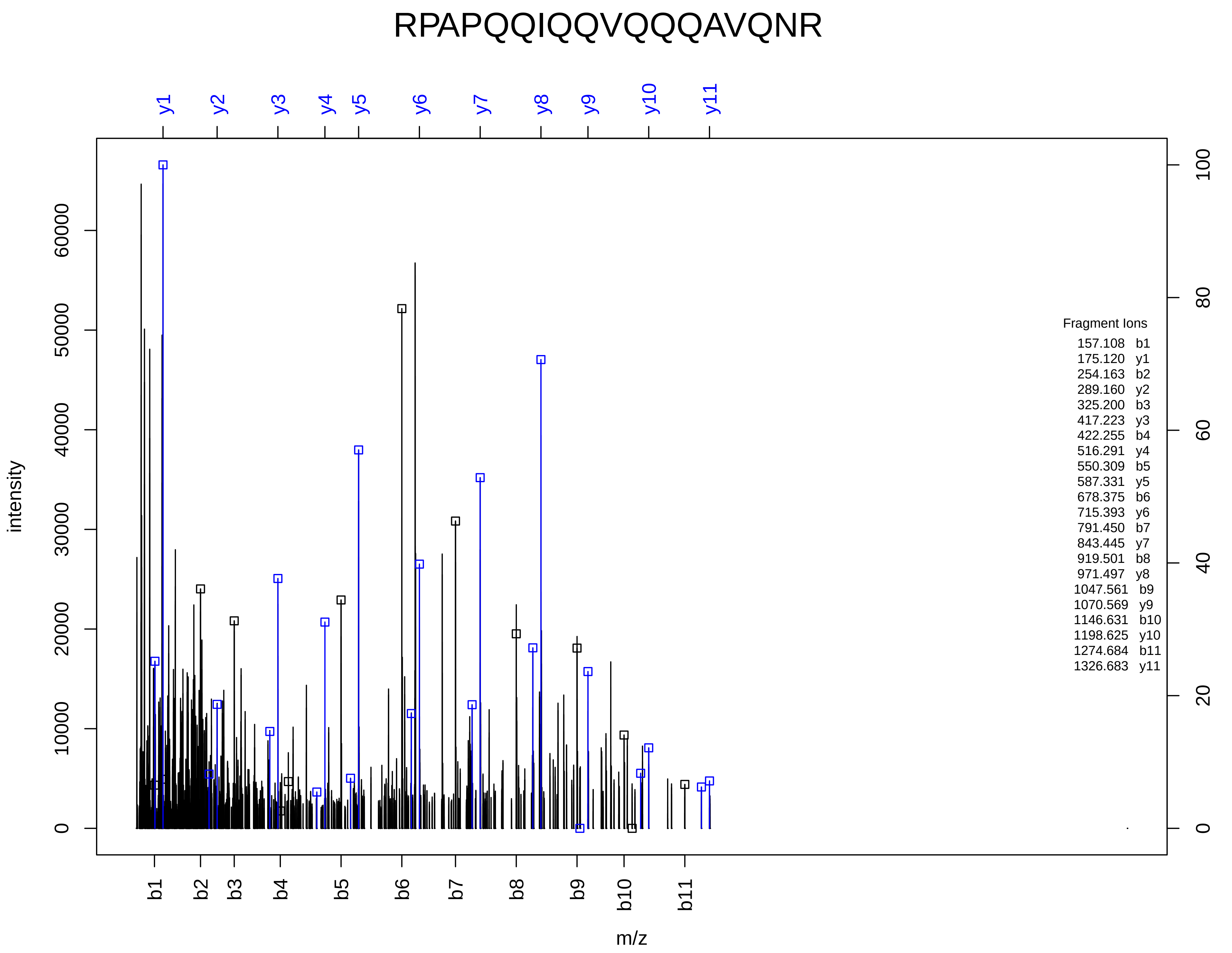

RPAPQQIQQVQQQAVQNR
y1 y2 y3 y4 y5 y6 y7 y8 y9 y10 y11
b1 b2 b3 b4 b5 b6 b7 b8 b9 b10 b11
intensity
m/z
Fragment Ions
157.108 b1
175.120 y1
254.163 b2
289.160 y2
325.200 b3
417.223 y3
422.255 b4
516.291 y4
550.309 b5
587.331 y5
678.375 b6
715.393 y6
791.450 b7
843.445 y7
919.501 b8
971.497 y8
1047.561 b9
1070.569 y9
1146.631 b10
1198.625 y10
1274.684 b11
1326.683 y11
RPAPQQIQQVQQQAVQNR / File: christine_pool_01.raw; SpectrumID: ; scans: 4512

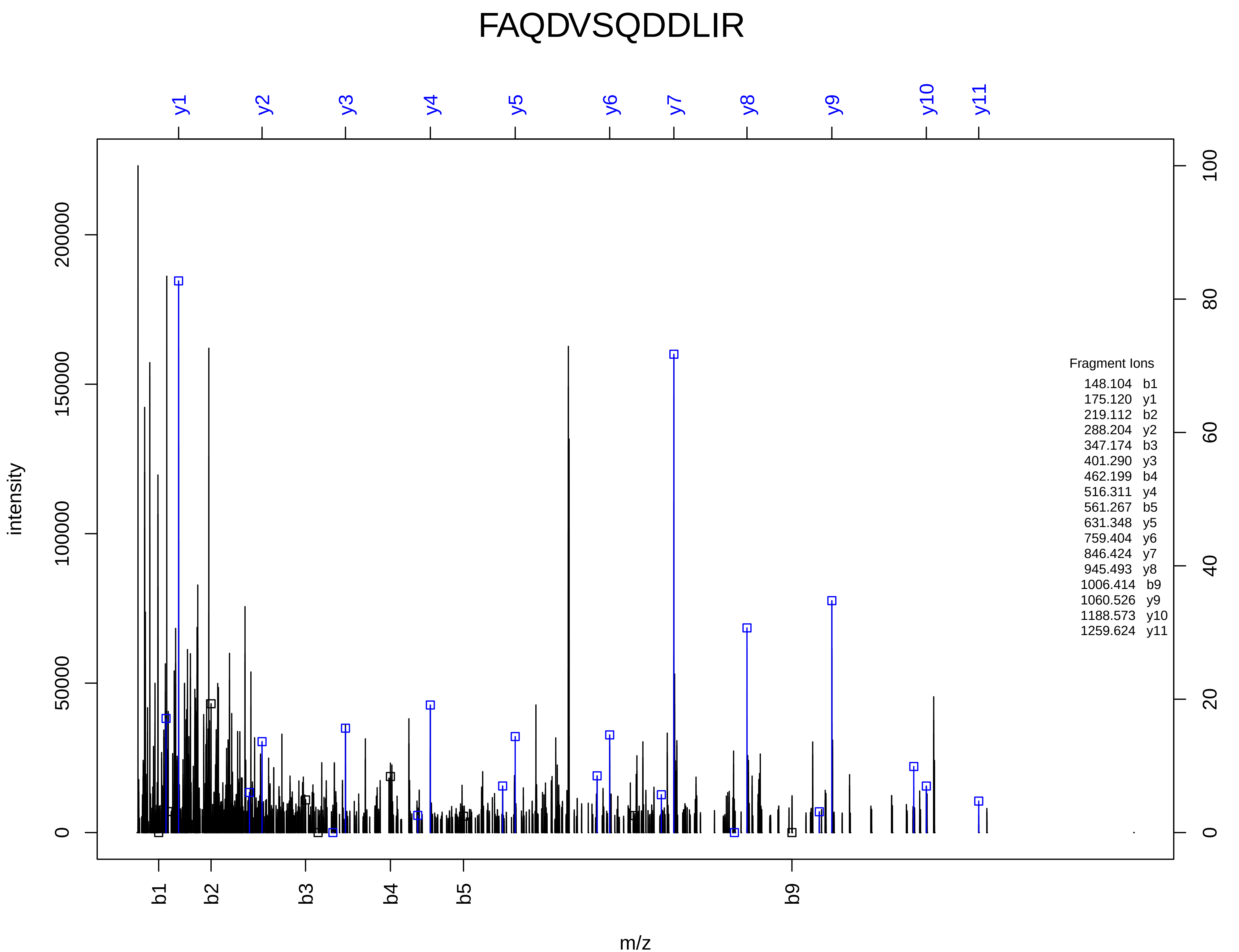

FAQDVSQDDLIR / File: christine_pool_01.raw; SpectrumID: ; scans: 8770

AFLLESLLK

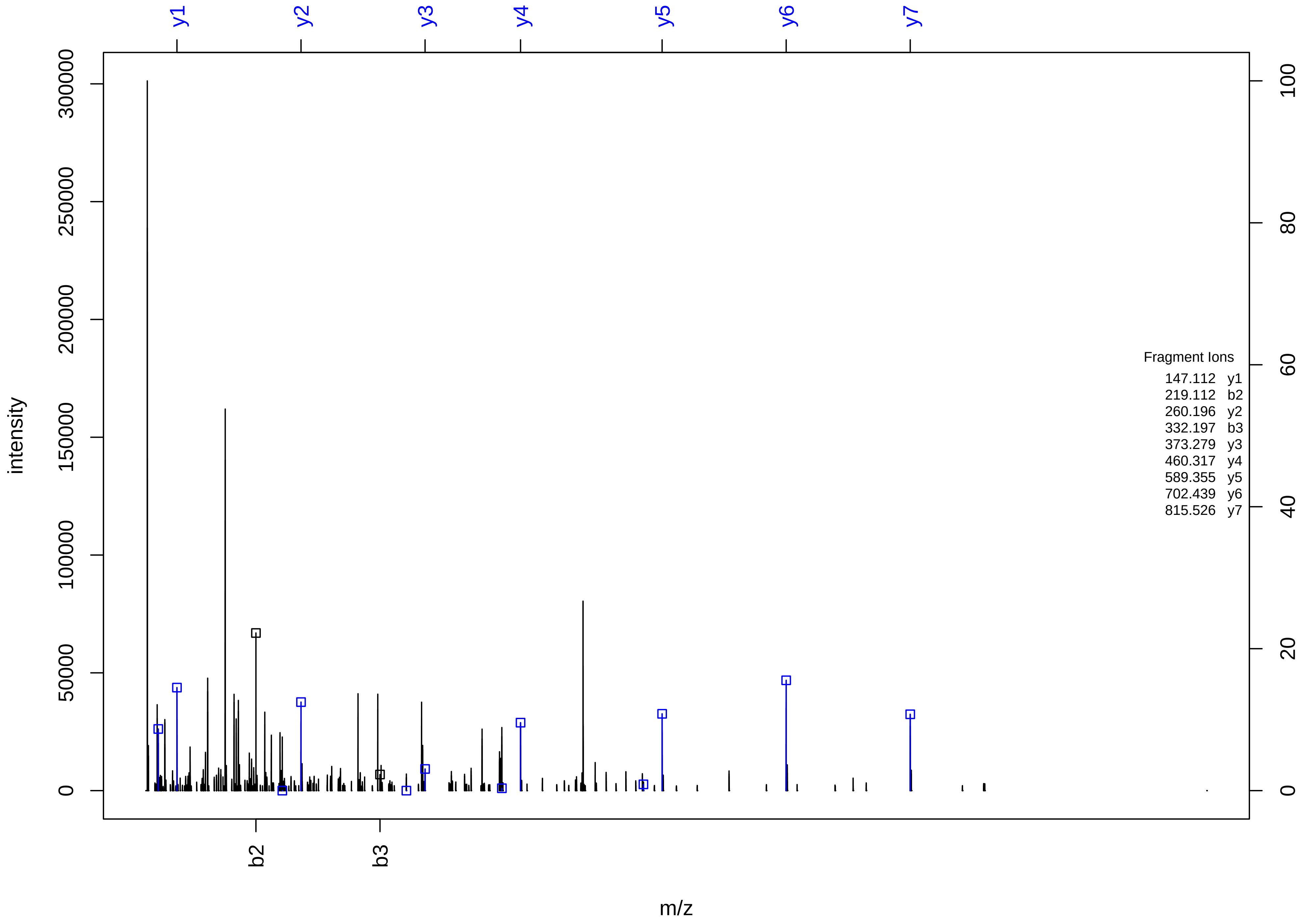

AFLLESLLK / File: christine_pool_01.raw; SpectrumID: ; scans: 21730

(Ac)MLLLPSAADGR

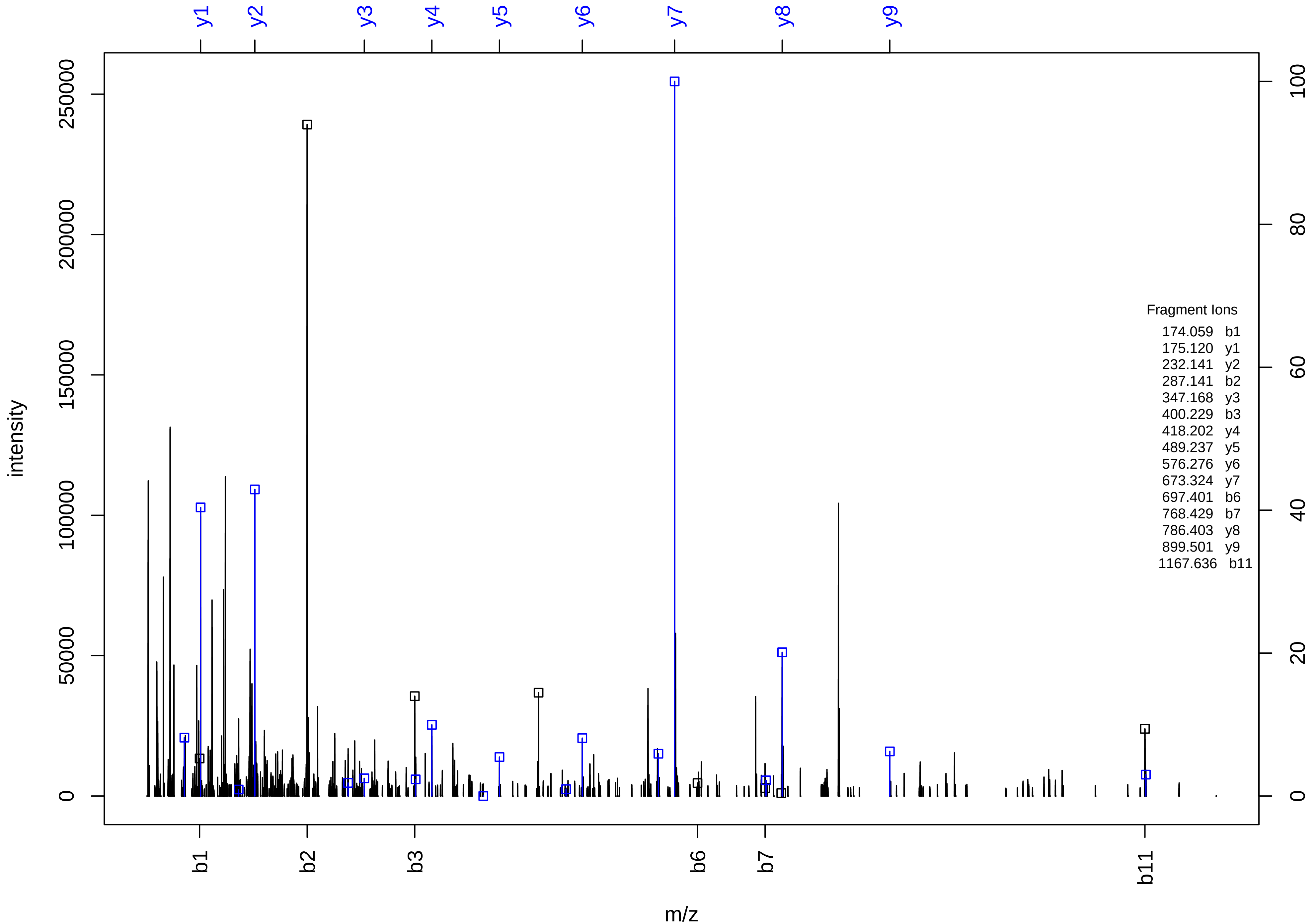

MLLLPSAADGR / File: christine_pool_01.raw; SpectrumID: ; scans: 22615

VTPPAVTGSPEFER

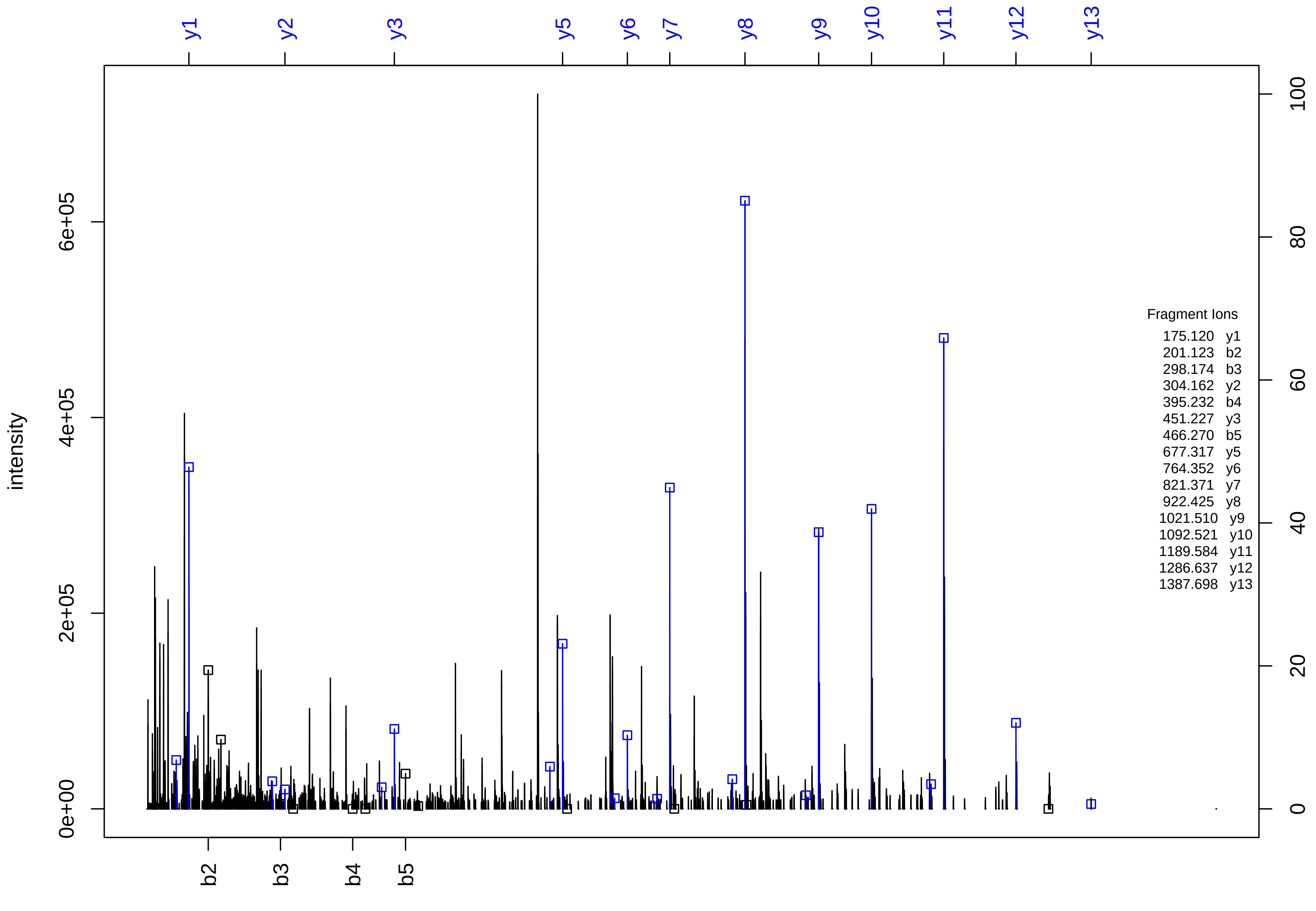

VTPPAVTGSPEFER / File: christine_pool_01.raw; SpectrumID: ; scans: 8165

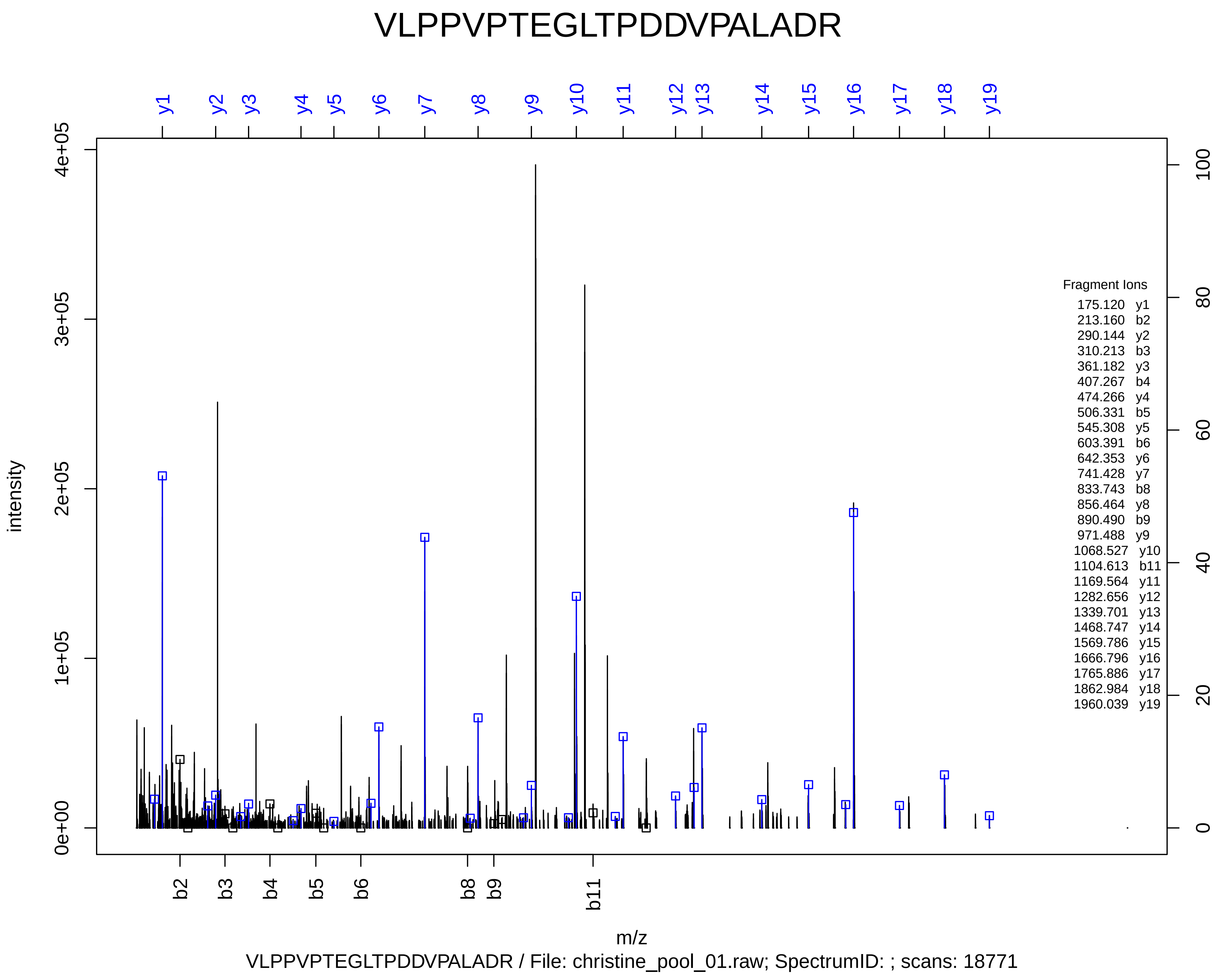
VLPPVPTEGLTPDDVPALADR
y1 y2 y3 y4 y5 y6 y7 y8 y9 y10 y11 y12 y13 y14 y15 y16 y17 y18 y19
b2 b3 b4 b5 b6 b8 b9 b11
intensity
0e+00
1e+05
2e+05
3e+05
4e+05
0
20
40
60
80
100
m/z
Fragment Ions
175.120 y1
213.160 b2
290.144 y2
310.213 b3
361.182 y3
407.267 b4
474.266 y4
506.331 b5
545.308 y5
603.391 b6
642.353 y6
741.428 y7
833.743 b8
856.464 y8
890.490 b9
971.488 y9
1068.527 y10
1104.613 b11
1169.564 y11
1282.656 y12
1339.701 y13
1468.747 y14
1569.786 y15
1666.796 y16
1765.886 y17
1862.984 y18
1960.039 y19
VLPPVPTEGLTPDDVPALADR / File: christine_pool_01.raw; SpectrumID: ; scans: 18771

ISAEGGEQVER

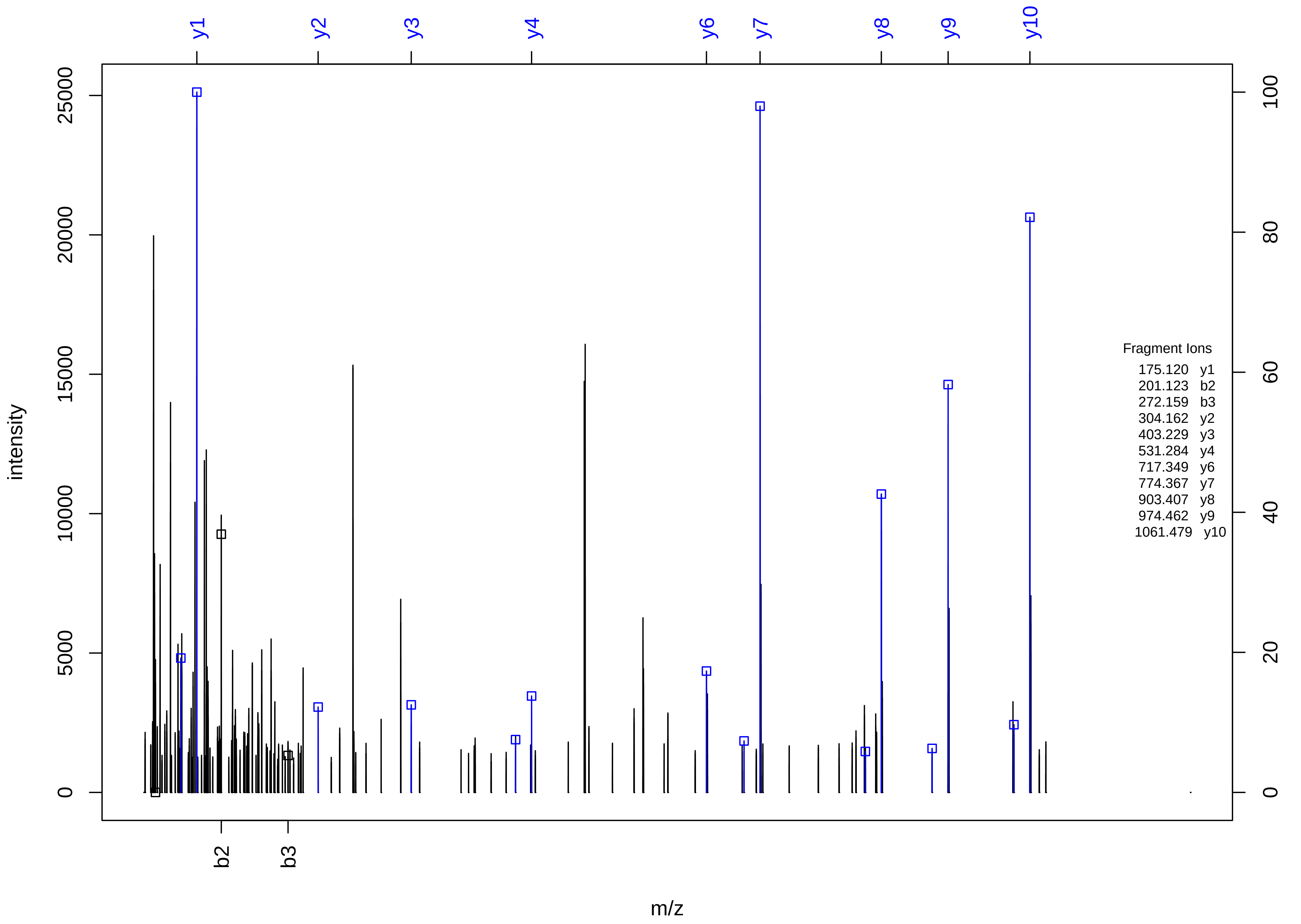

ISAEGGEQVER / File: christine_pool_01.raw; SpectrumID: ; scans: 2007

DSGAASEQATAAPNPCSSSSR

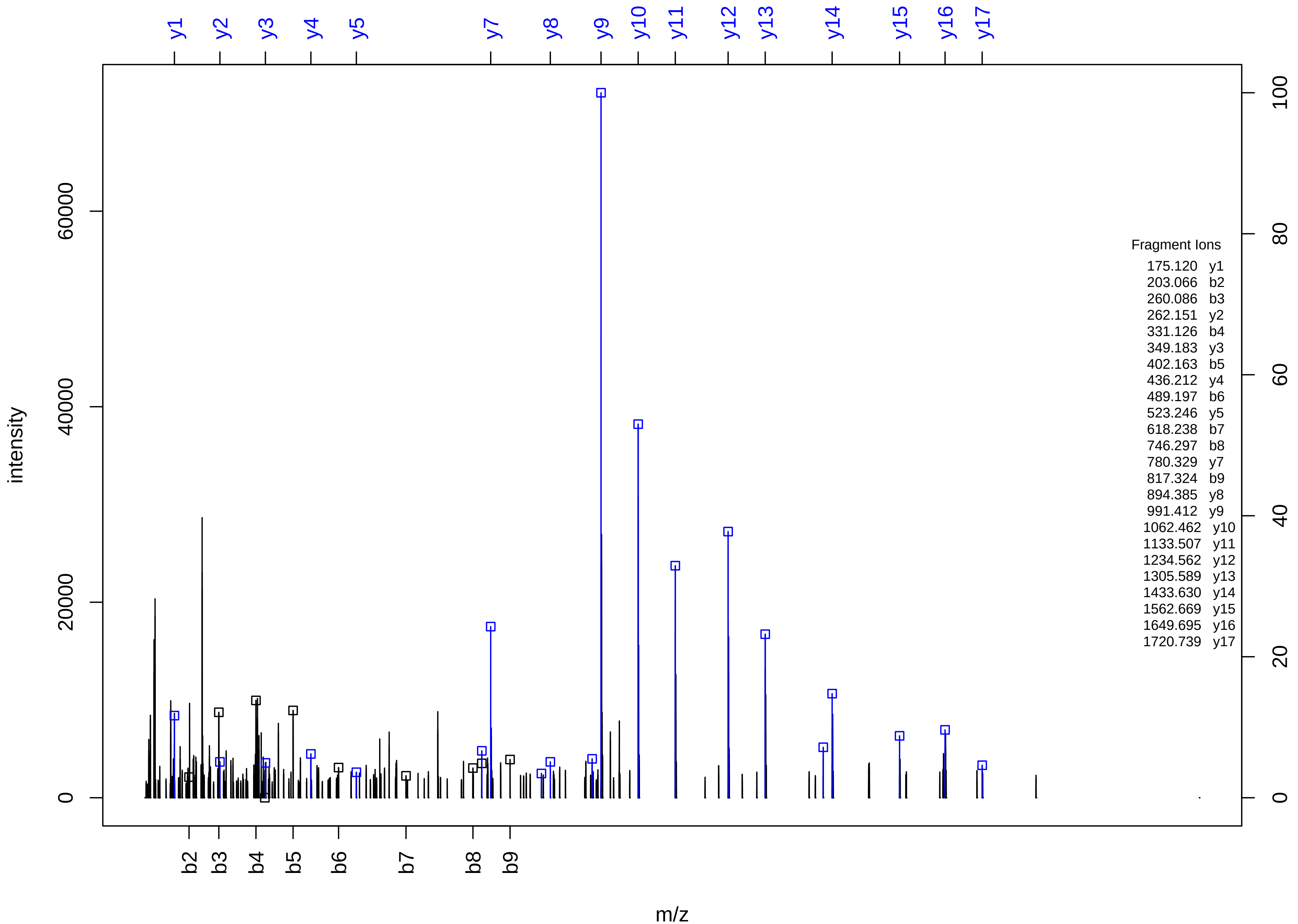

DSGAASEQATAAPNPCSSSSR / File: christine_pool_01.raw; SpectrumID: ; scans: 2620

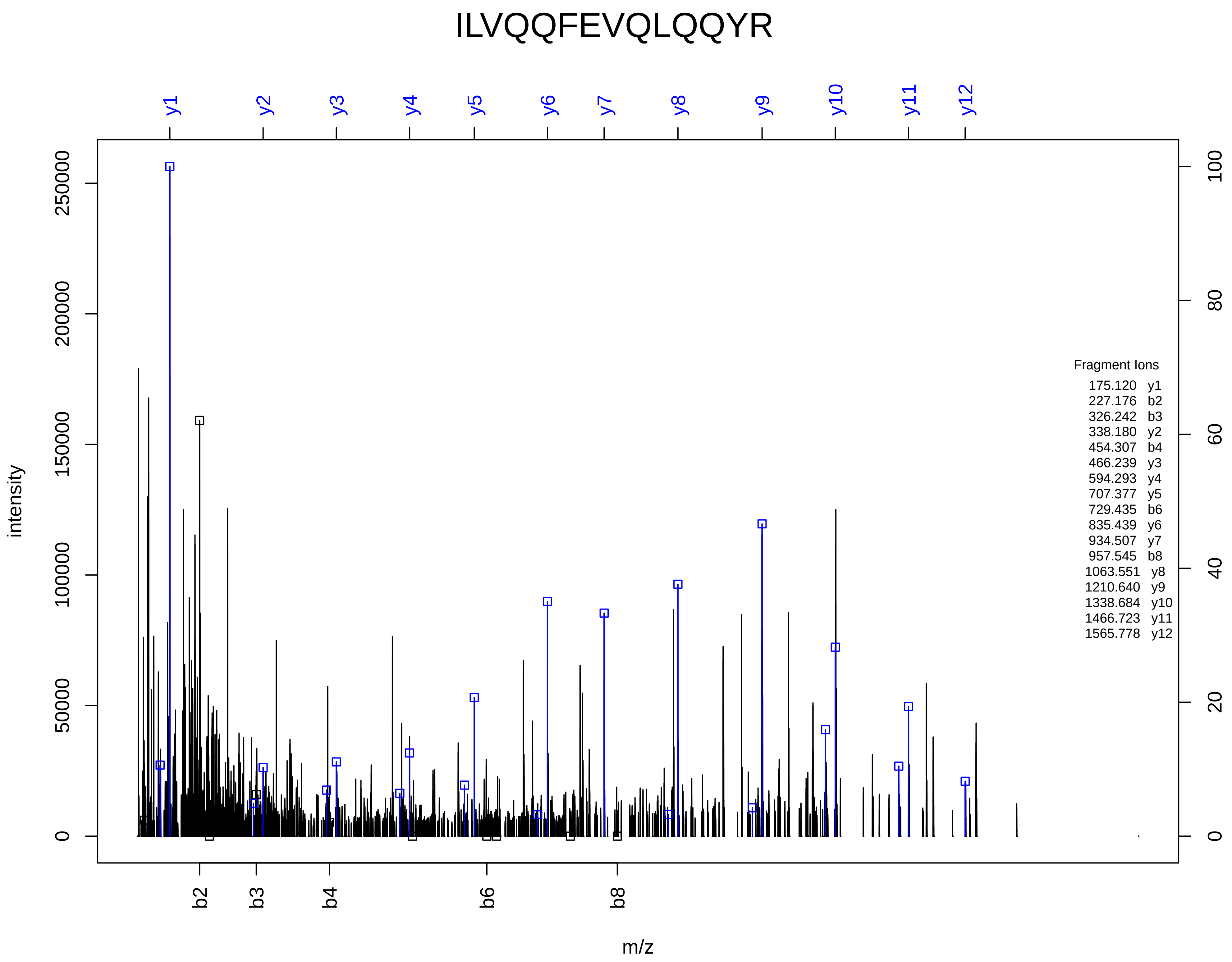

ILVQQFEVQLQQYR / File: christine_pool_01.raw; SpectrumID: ; scans: 17194

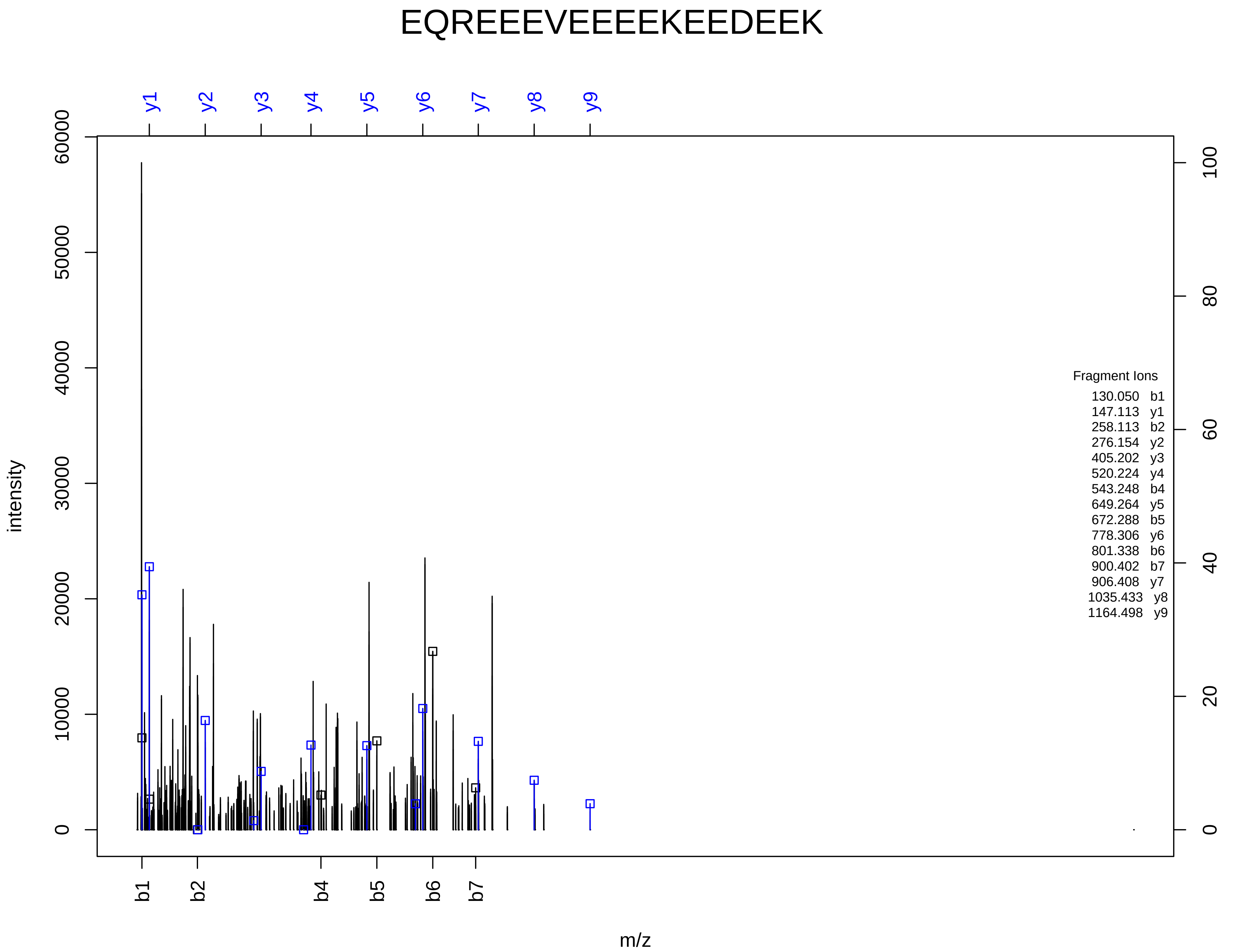

EQREEEVEEEEKEEDEEK / File: christine_pool_01.raw; SpectrumID: ; scans: 2114

VYNALVAFIR

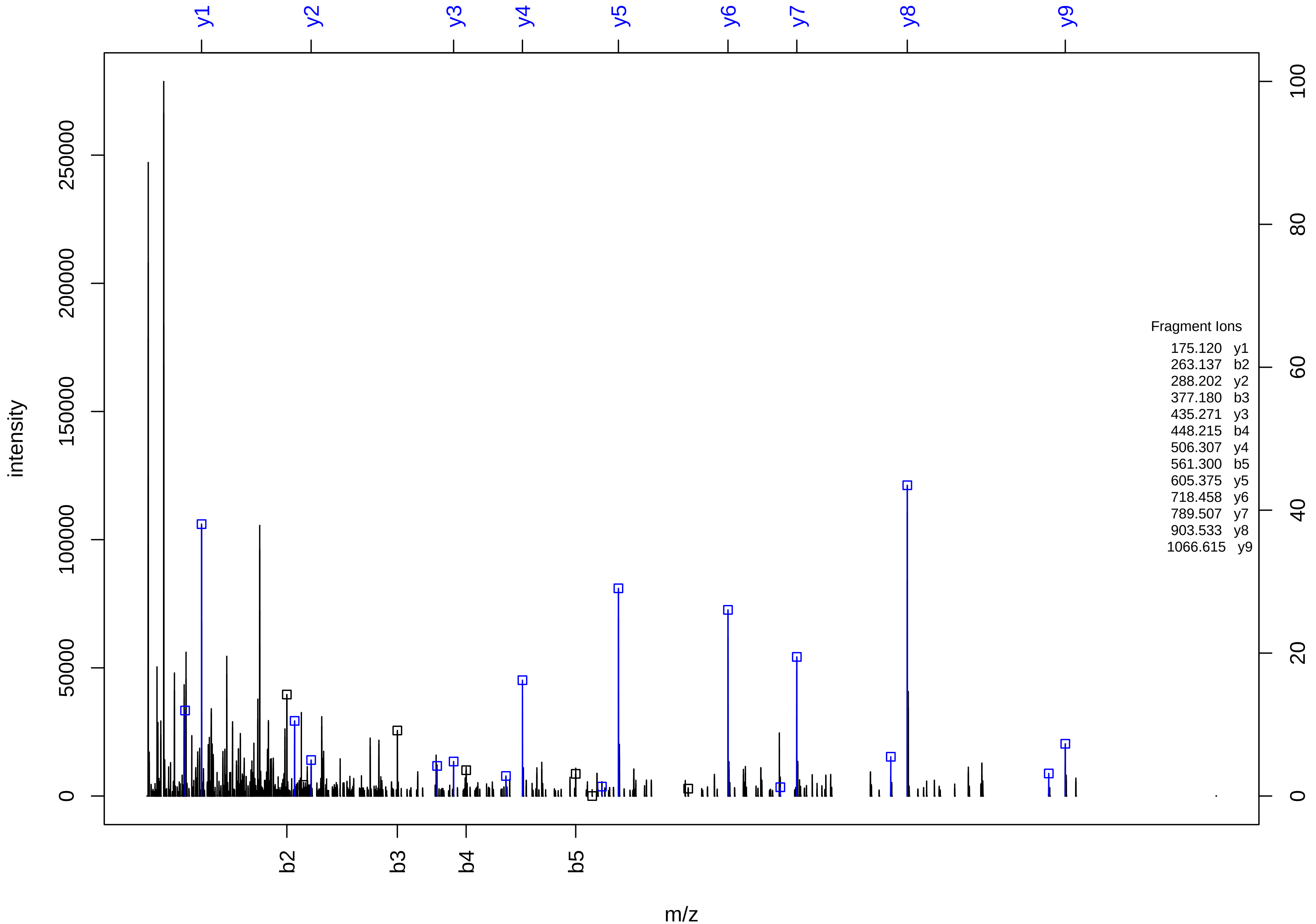

VYNALVAFIR / File: christine_pool_01.raw; SpectrumID: ; scans: 18349

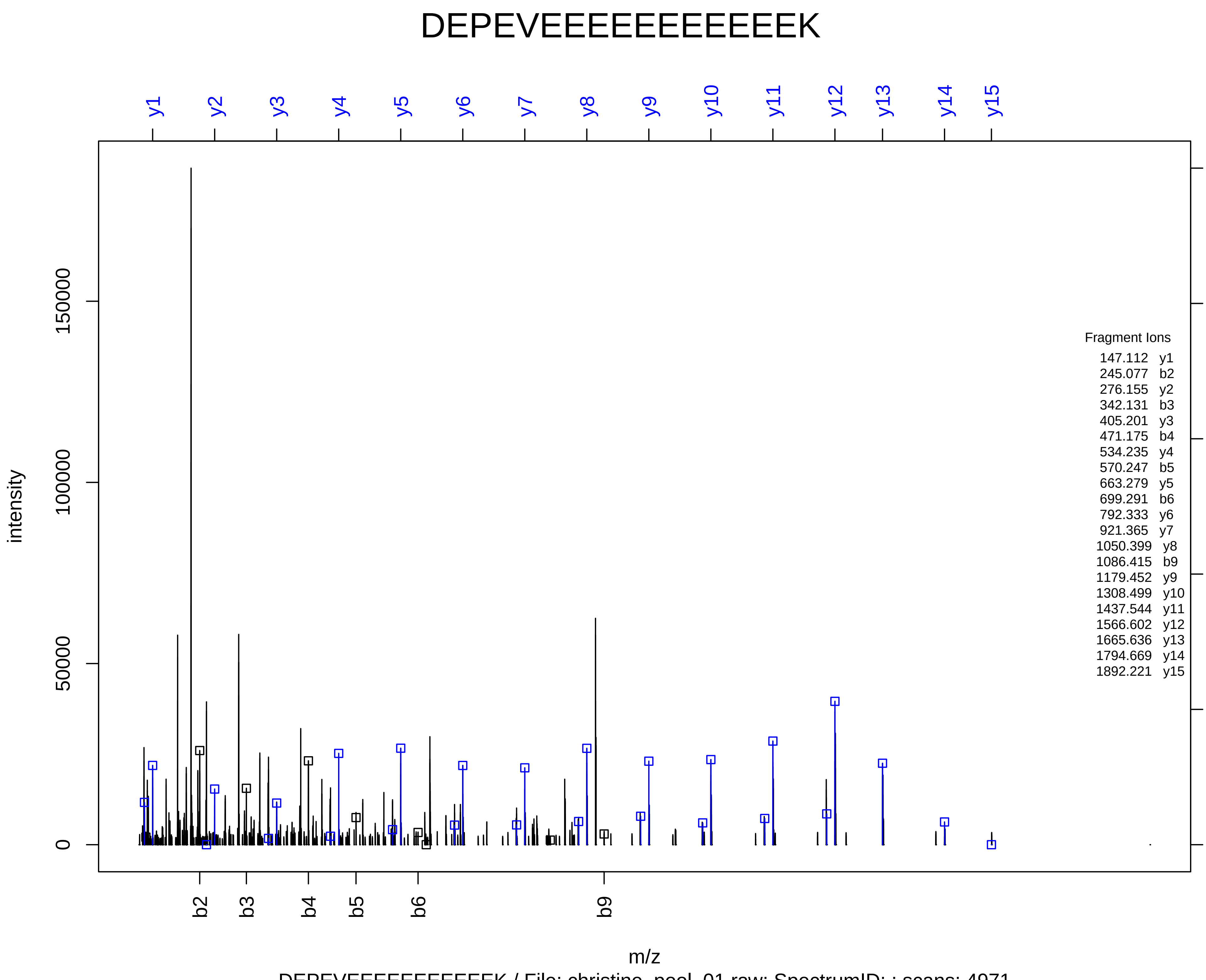
DEPEVEEEEEEEEEEEK
y1 y2 y3 y4 y5 y6 y7 y8 y9 y10 y11 y12 y13 y14 y15
b2 b3 b4 b5 b6 b9
intensity
0
50000
100000
150000
0
20
40
60
80
100
m/z
Fragment Ions
147.112 y1
245.077 b2
276.155 y2
342.131 b3
405.201 y3
471.175 b4
534.235 y4
570.247 b5
663.279 y5
699.291 b6
792.333 y6
921.365 y7
1050.399 y8
1086.415 b9
1179.452 y9
1308.499 y10
1437.544 y11
1566.602 y12
1665.636 y13
1794.669 y14
1892.221 y15
DEPEVEEEEEEEEEEEK / File: christine_pool_01.raw; SpectrumID: ; scans: 4971

FDIELGR

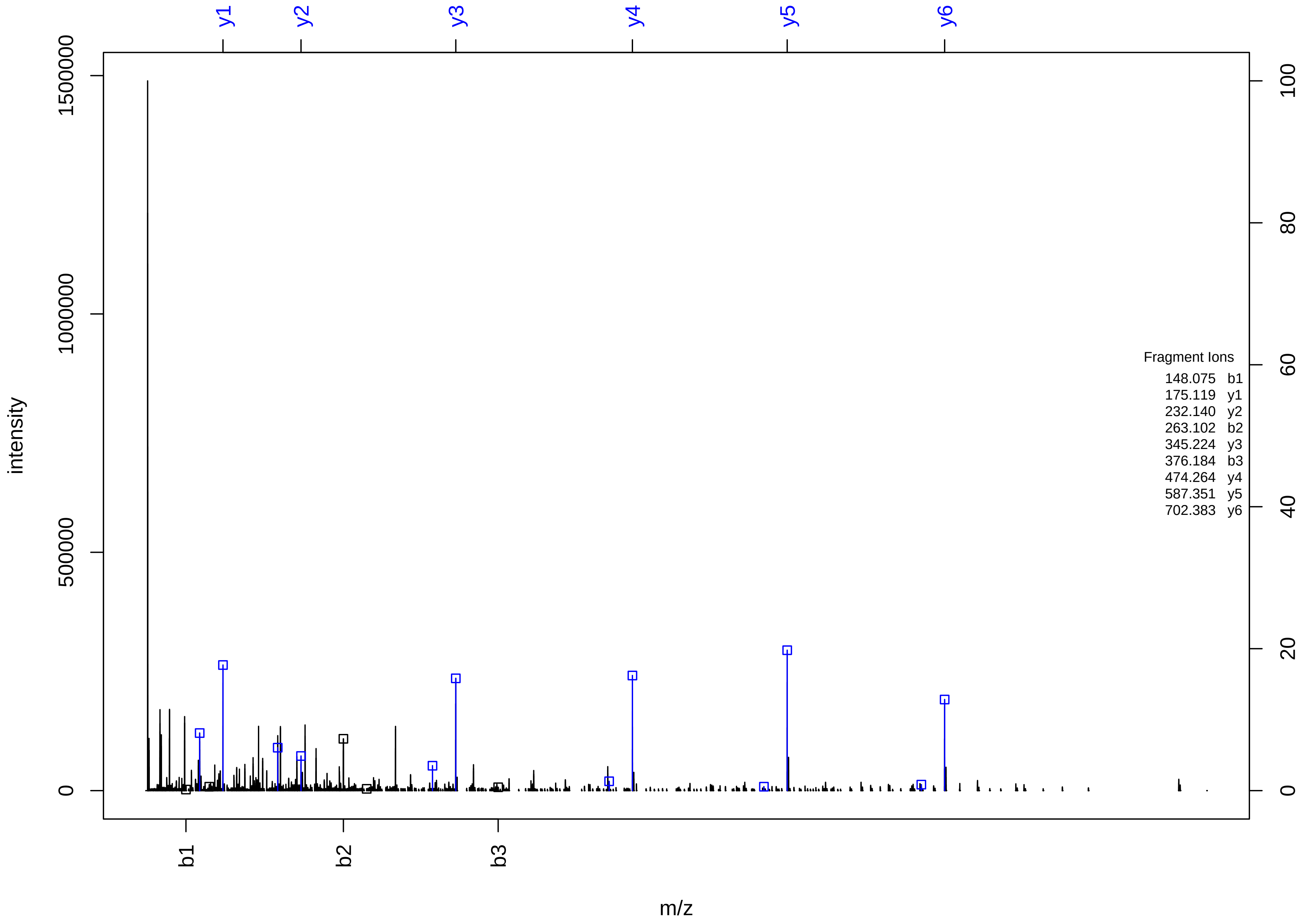

FDIELGR / File: christine_pool_01.raw; SpectrumID: ; scans: 10453

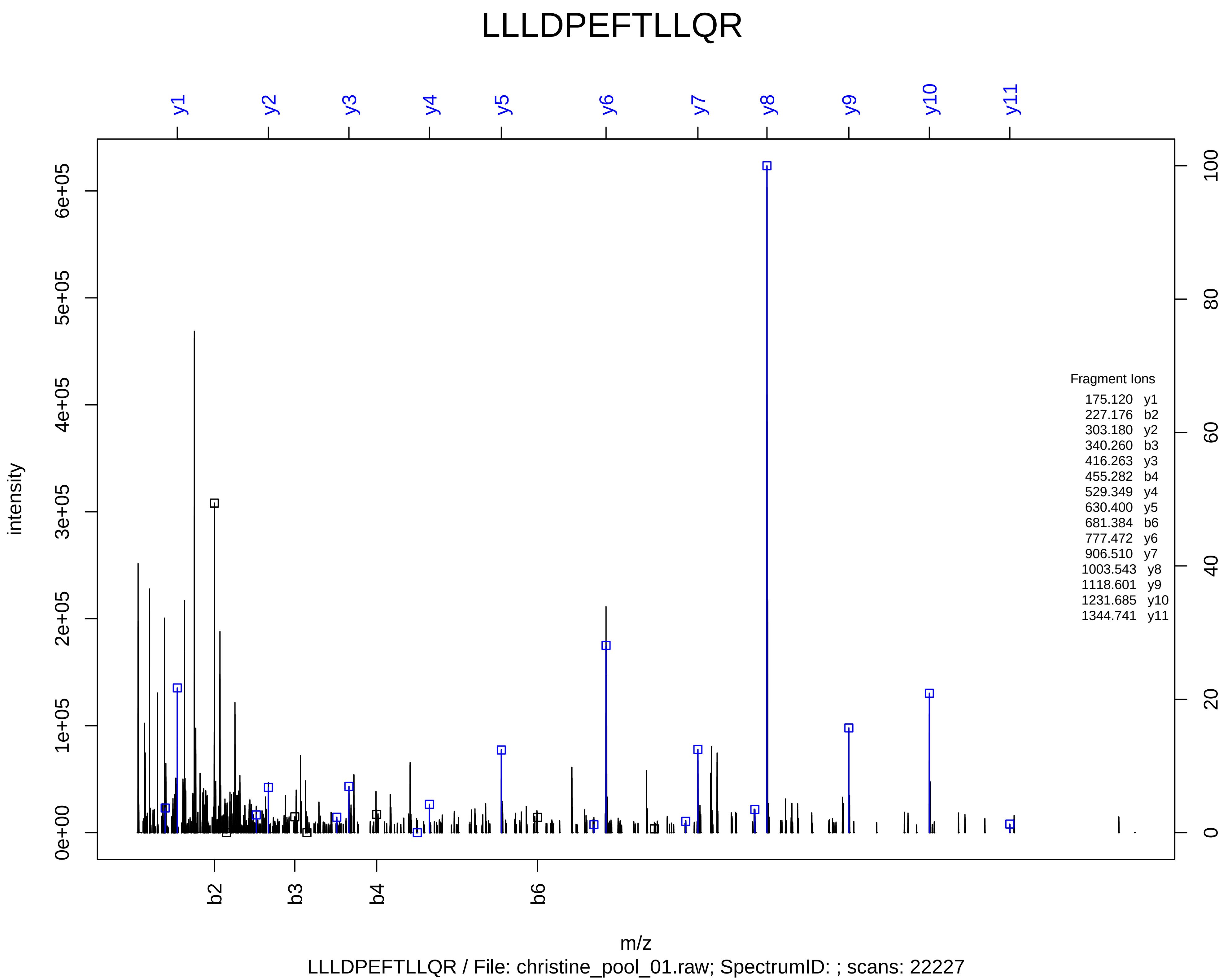
LLLDPEFTLLQR
y1 y2 y3 y4 y5 y6 y7 y8 y9 y10 y11
intensity
0e+00 1e+05 2e+05 3e+05 4e+05 5e+05 6e+05
0 20 40 60 80 100
b2 b3 b4 b6
m/z
Fragment Ions
175.120 y1
227.176 b2
303.180 y2
340.260 b3
416.263 y3
455.282 b4
529.349 y4
630.400 y5
681.384 b6
777.472 y6
906.510 y7
1003.543 y8
1118.601 y9
1231.685 y10
1344.741 y11
LLLDPEFTLLQR / File: christine_pool_01.raw; SpectrumID: ; scans: 22227

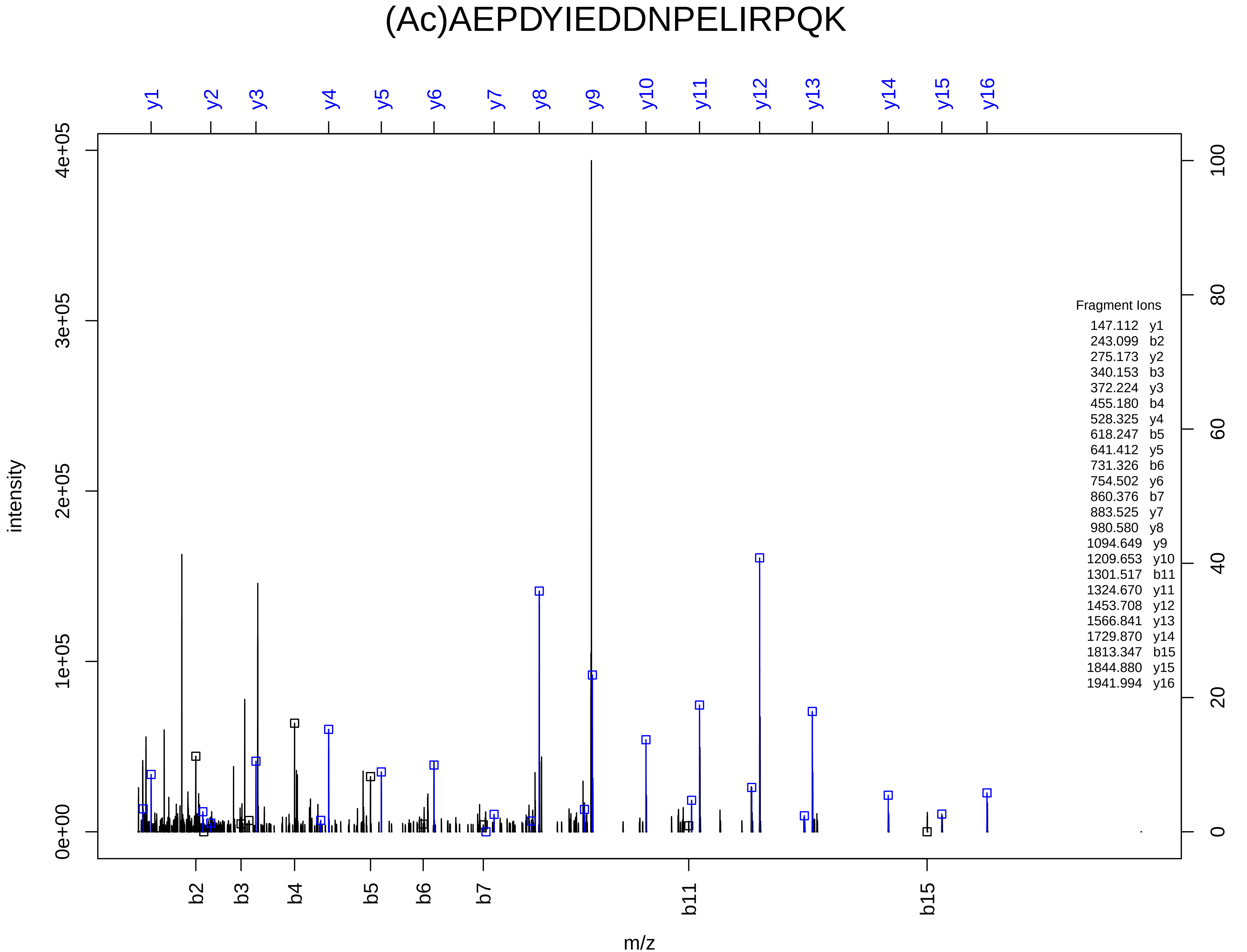

AEPDYIEDDNPELIRPQK / File: christine_pool_01.raw; SpectrumID: ; scans: 12586

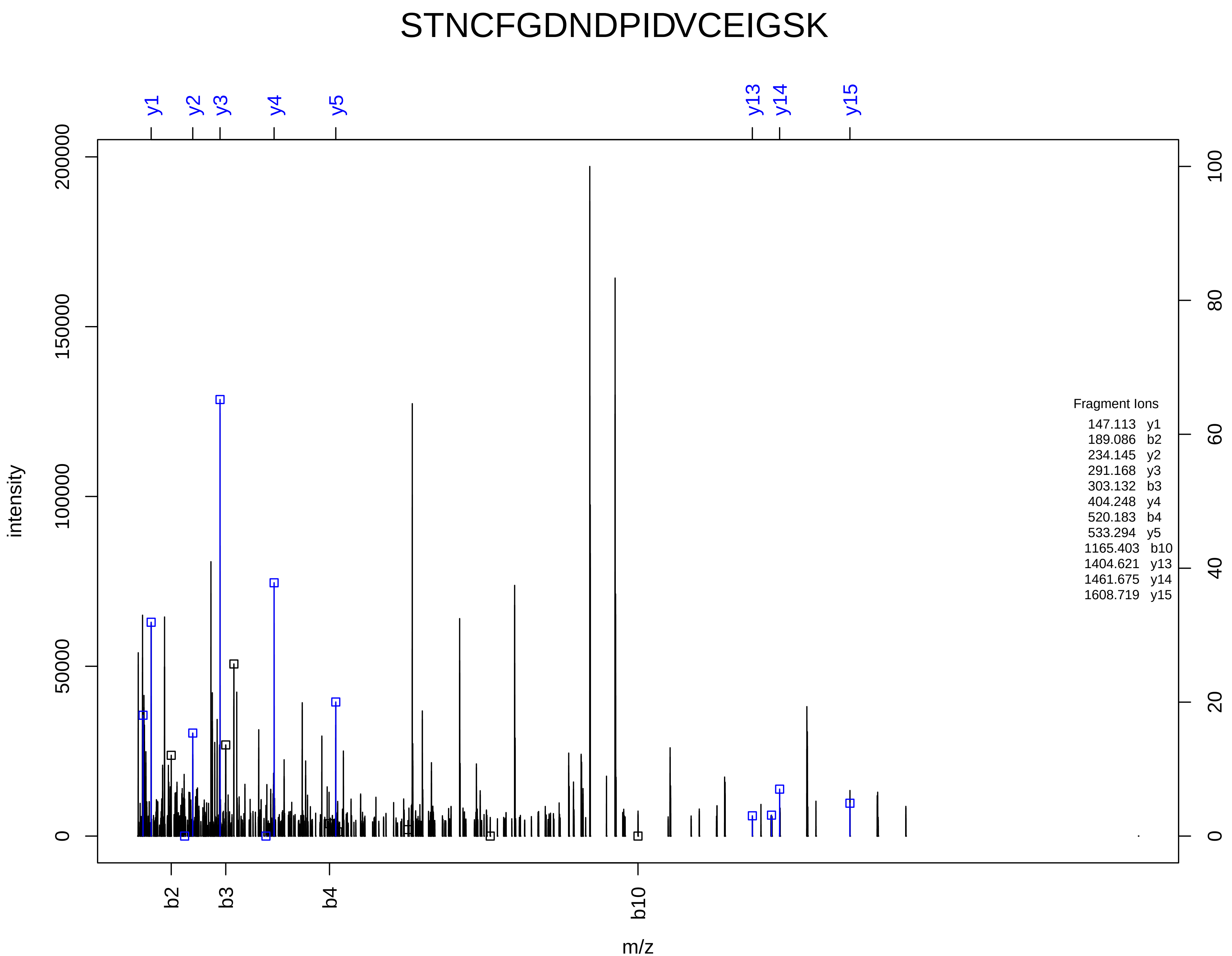
STNCFGDNDPIDVCEIGSK
intensity
m/z
y1
y2
y3
y4
y5
y13
y14
y15
b2
b3
b4
b10
Fragment Ions
147.113 y1
189.086 b2
234.145 y2
291.168 y3
303.132 b3
404.248 y4
520.183 b4
533.294 y5
1165.403 b10
1404.621 y13
1461.675 y14
1608.719 y15
STNCFGDNDPIDVCEIGSK / File: christine_pool_01.raw; SpectrumID: ; scans: 11985

SQQQDDIEELETK

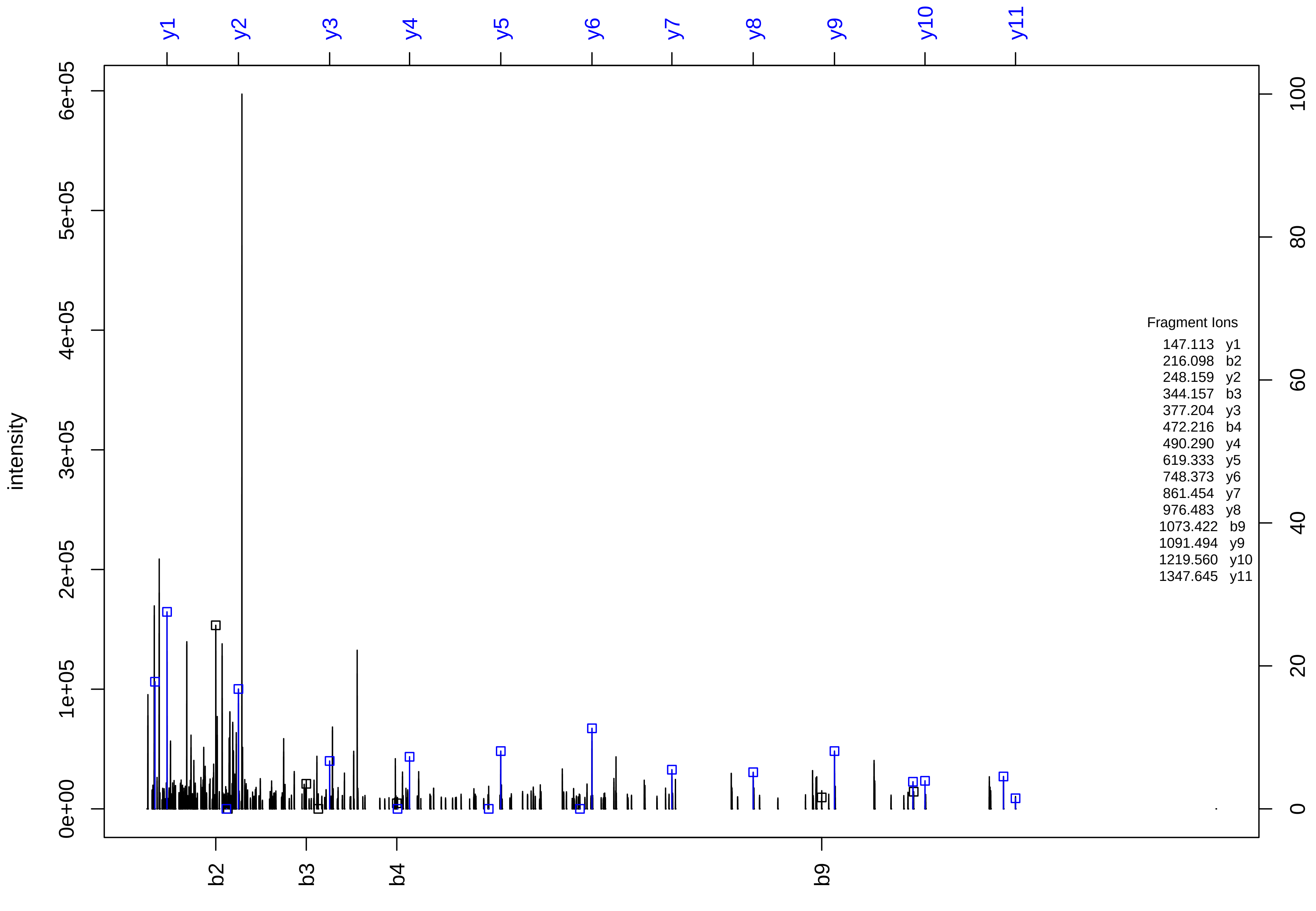

SQQQDDIEELETK / File: christine_pool_01.raw; SpectrumID: ; scans: 6009

EFQTVPFYIFSESYGGK

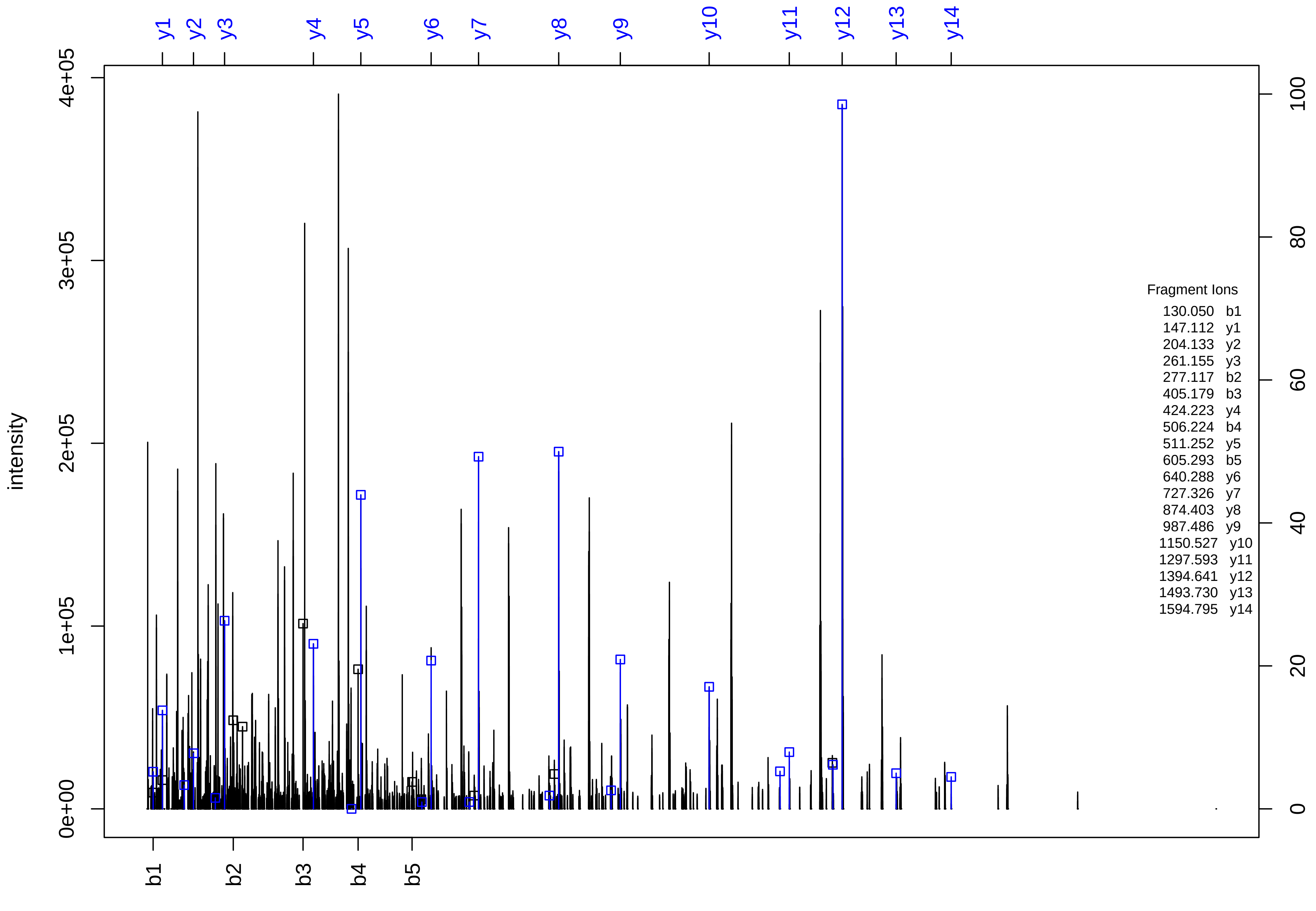

EFQTVPFYIFSESYGGK / File: christine_pool_01.raw; SpectrumID: ; scans: 22285

AAAEDVNVTFEDQQK

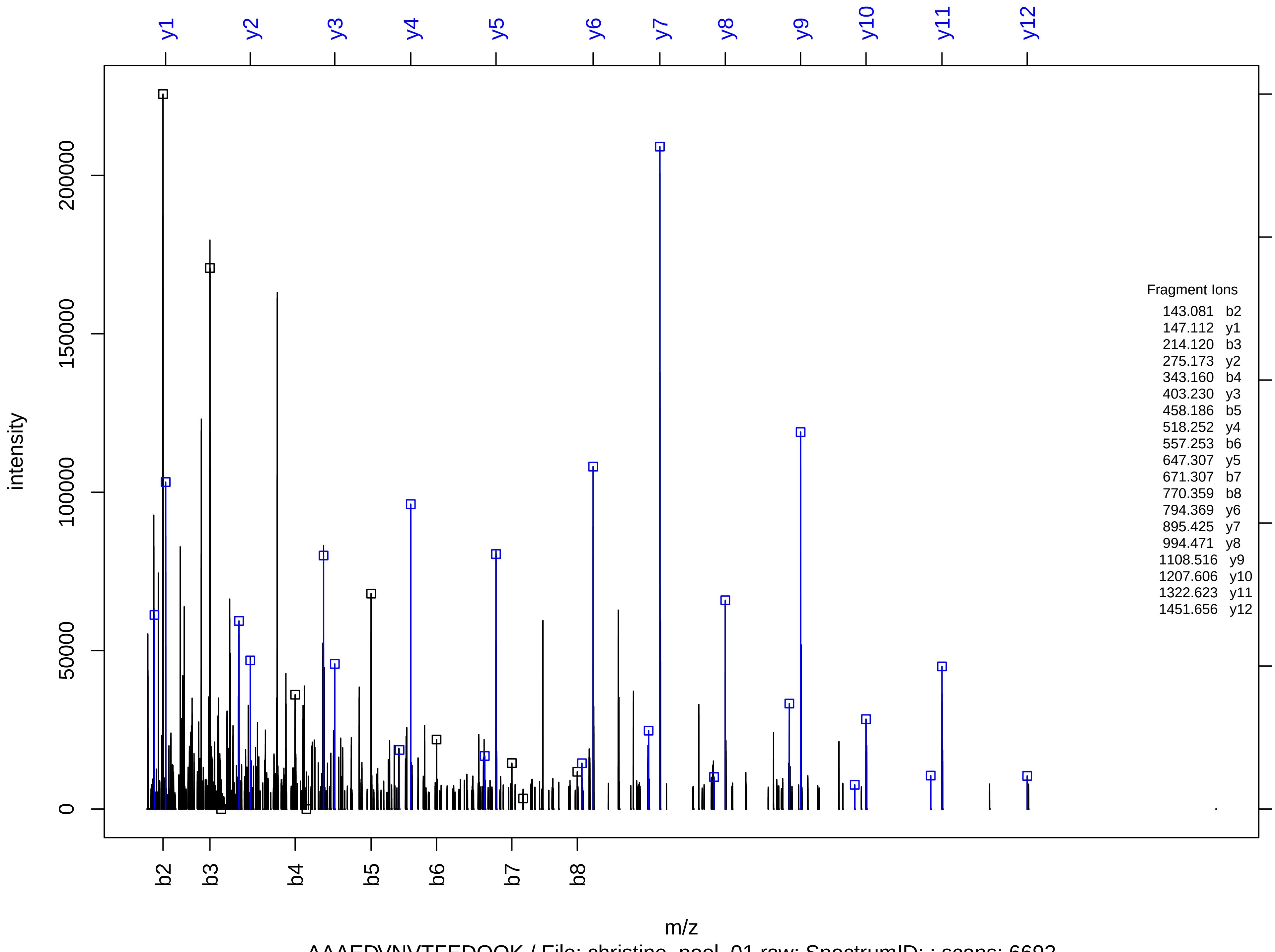

AAAEDVNVTFEDQQK / File: christine_pool_01.raw; SpectrumID: ; scans: 6692

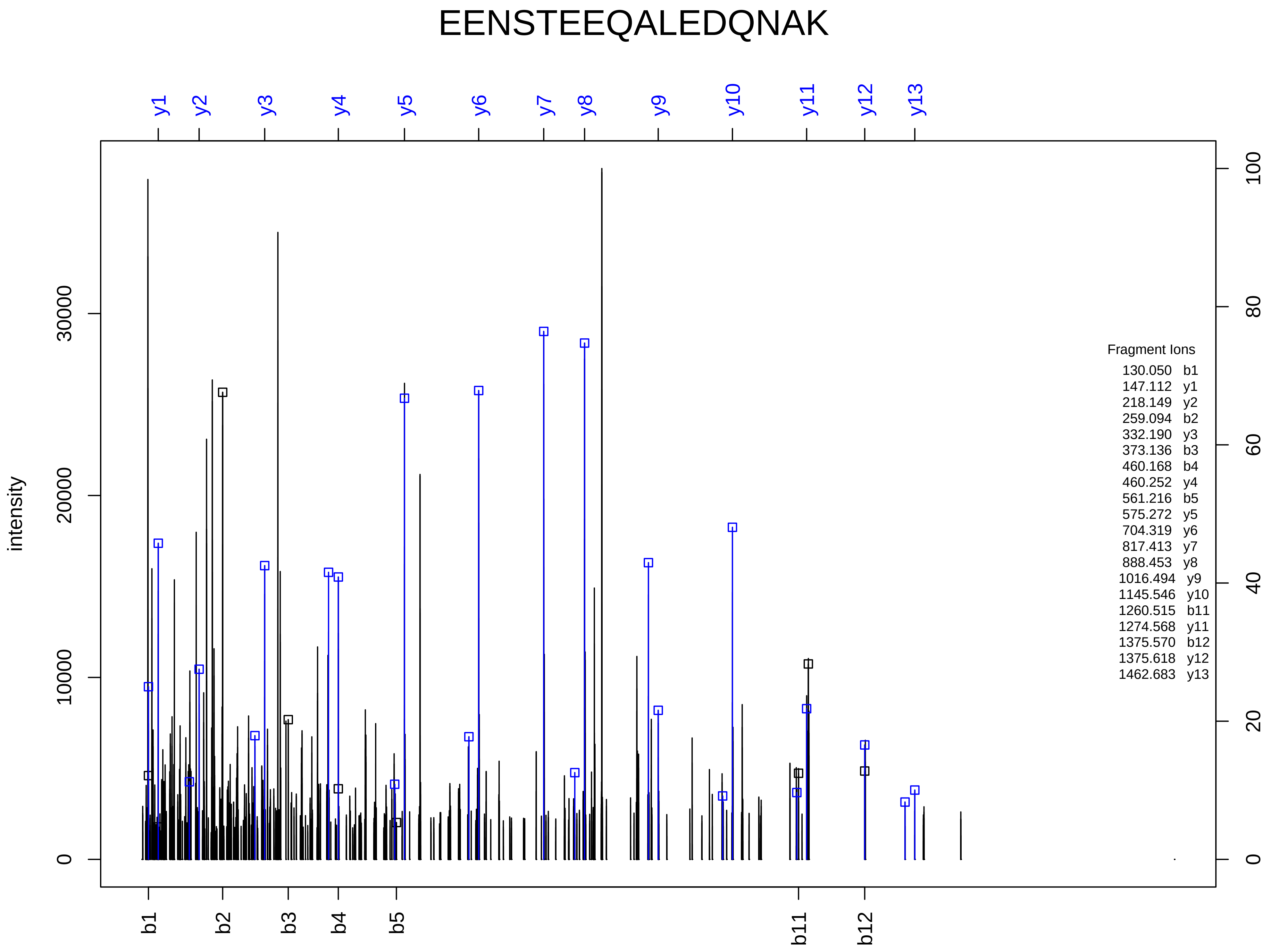

EENSTEEQALEDQNAK / File: christine_pool_01.raw; SpectrumID: ; scans: 3091

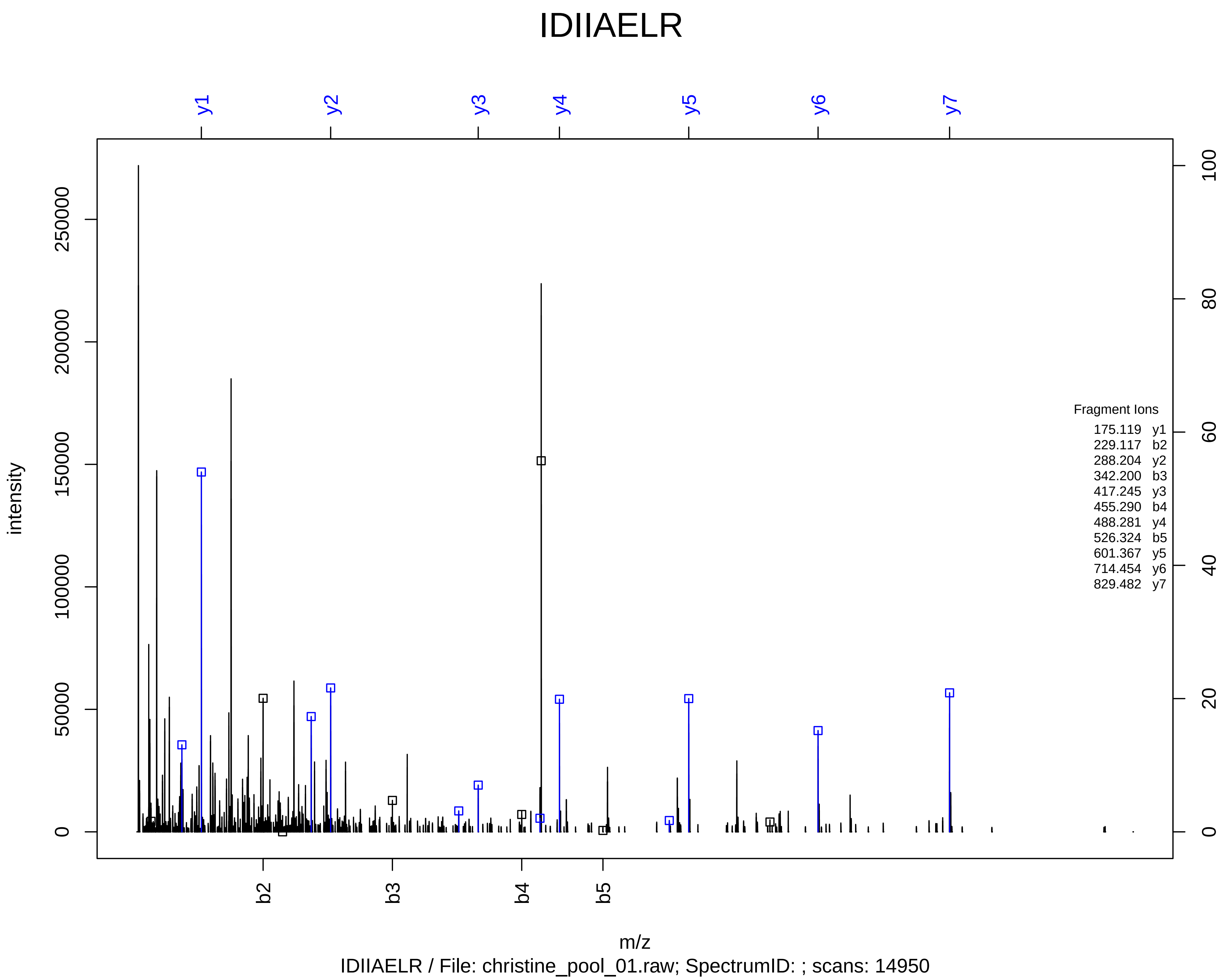
IDIIAELR
y1
y2
y3
y4
y5
y6
y7
b2
b3
b4
b5
intensity
m/z
Fragment Ions
175.119 y1
229.117 b2
288.204 y2
342.200 b3
417.245 y3
455.290 b4
488.281 y4
526.324 b5
601.367 y5
714.454 y6
829.482 y7
IDIIAELR / File: christine_pool_01.raw; SpectrumID: ; scans: 14950

ICFQGDEGACPTR

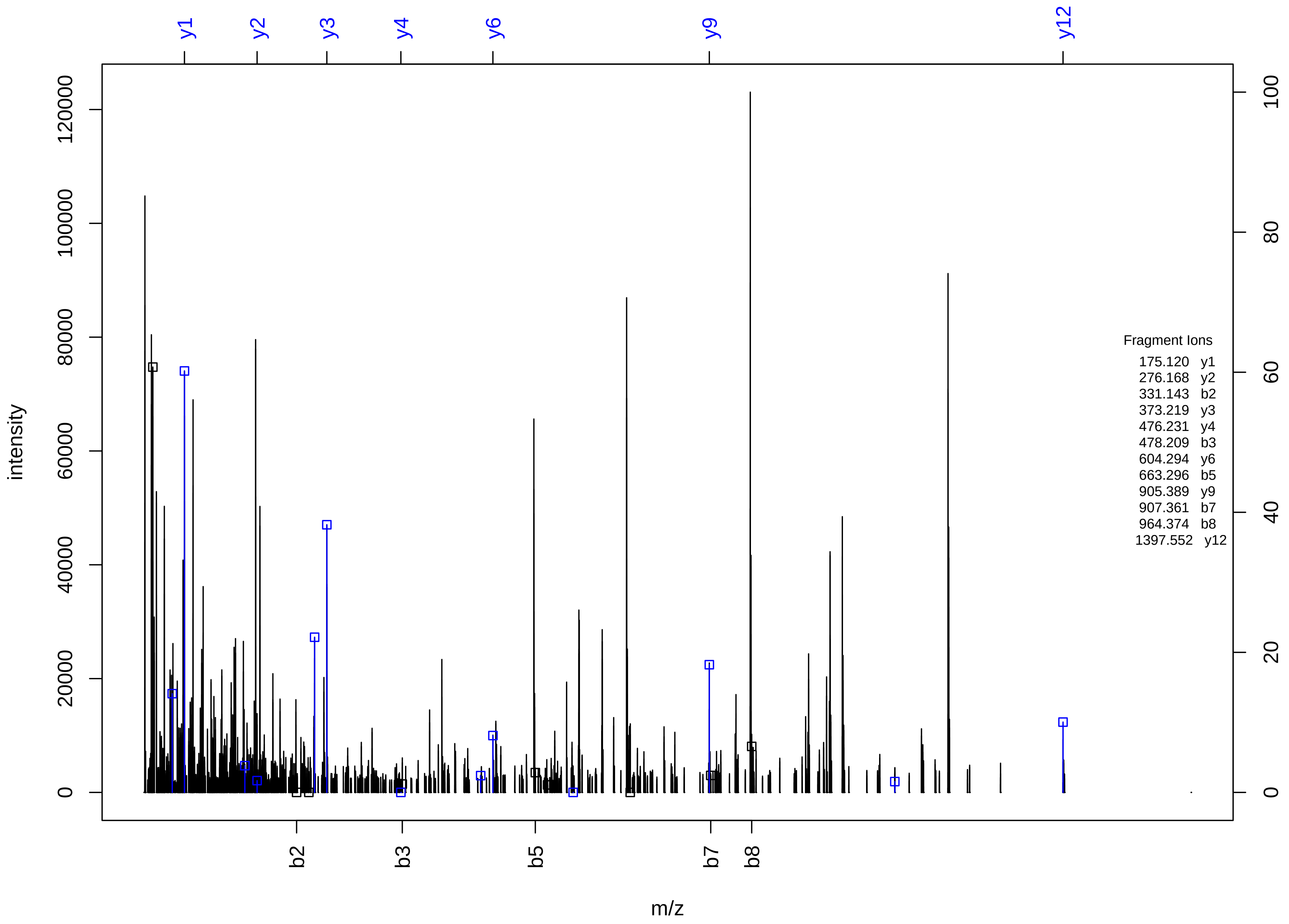

ICFQGDEGACPTR / File: christine_pool_01.raw; SpectrumID: ; scans: 4966

LALSVGTDK

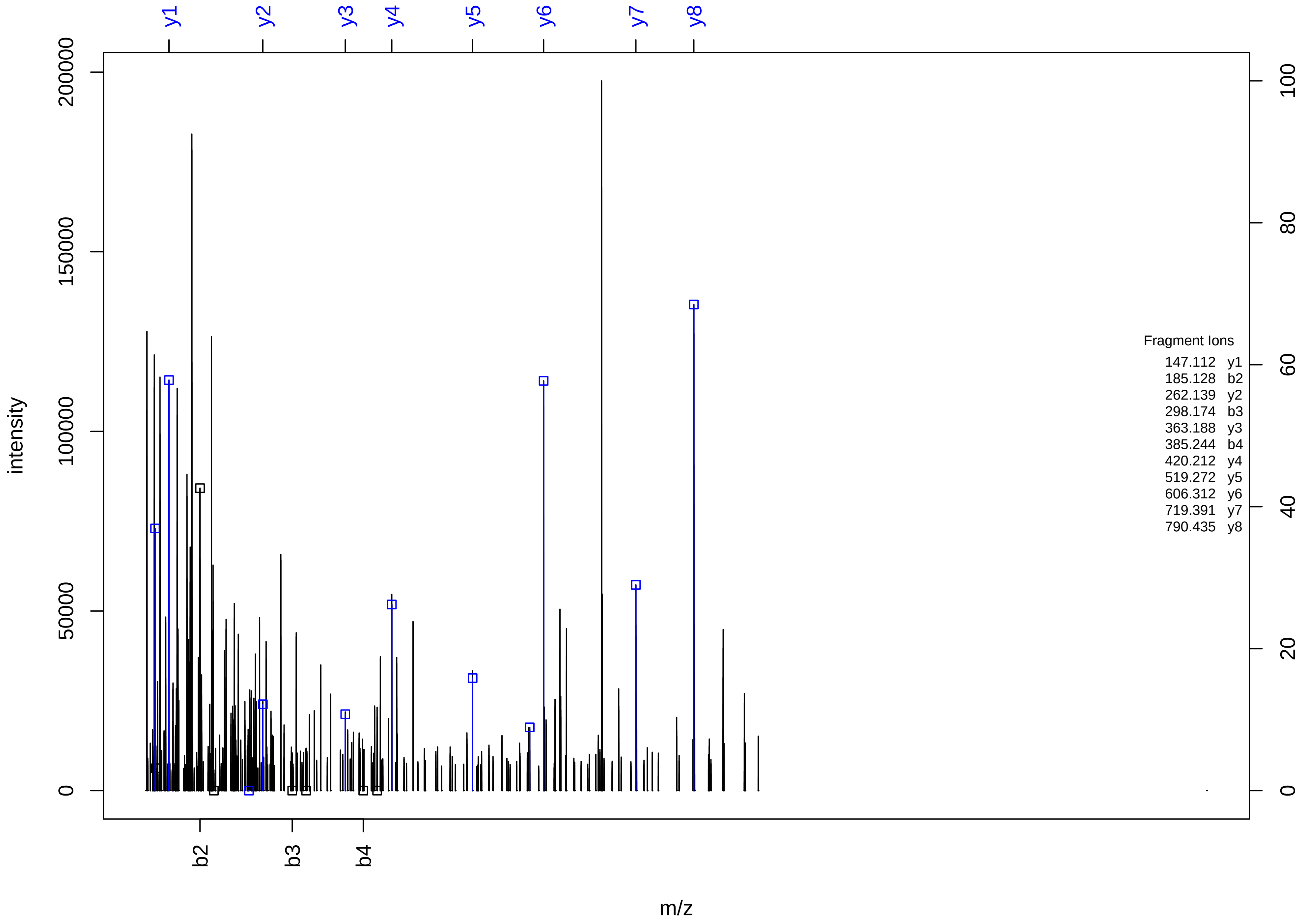

LALSVGTDK / File: christine_pool_01.raw; SpectrumID: ; scans: 6199

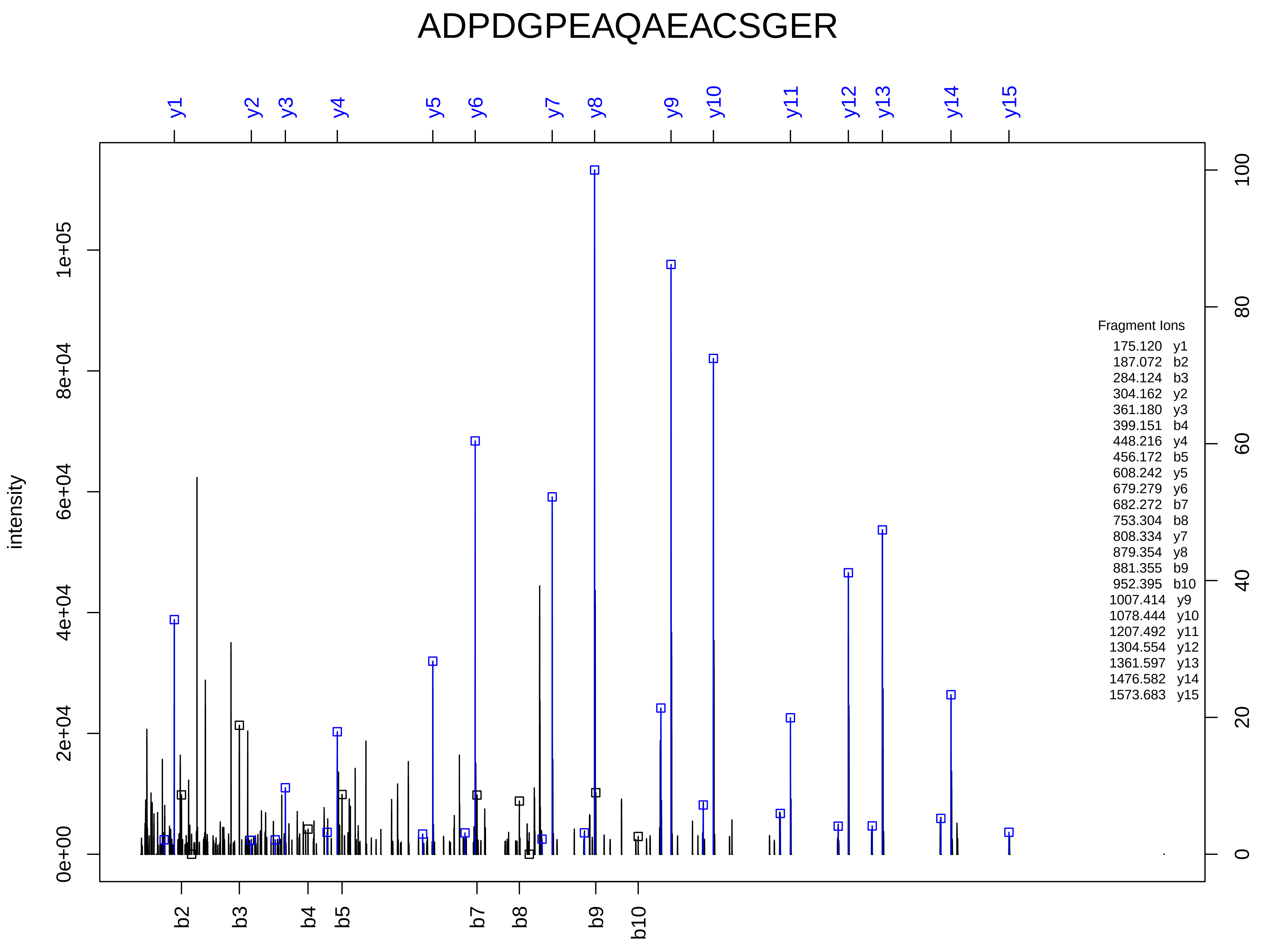

ADPDGPEAQAEACSGER / File: christine_pool_01.raw; SpectrumID: ; scans: 2633

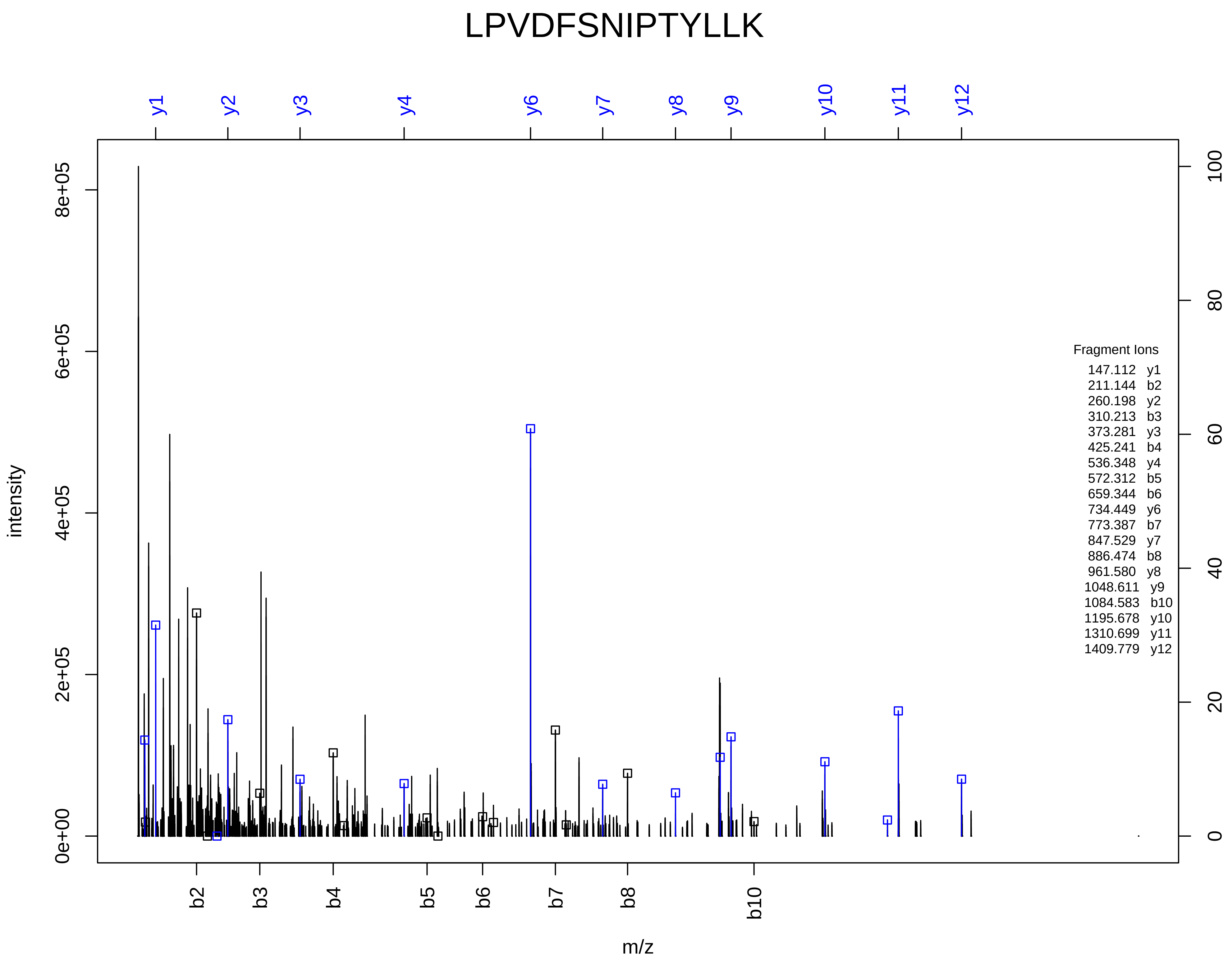

LPVDFSNIPTYLLK / File: christine_pool_01.raw; SpectrumID: ; scans: 22526

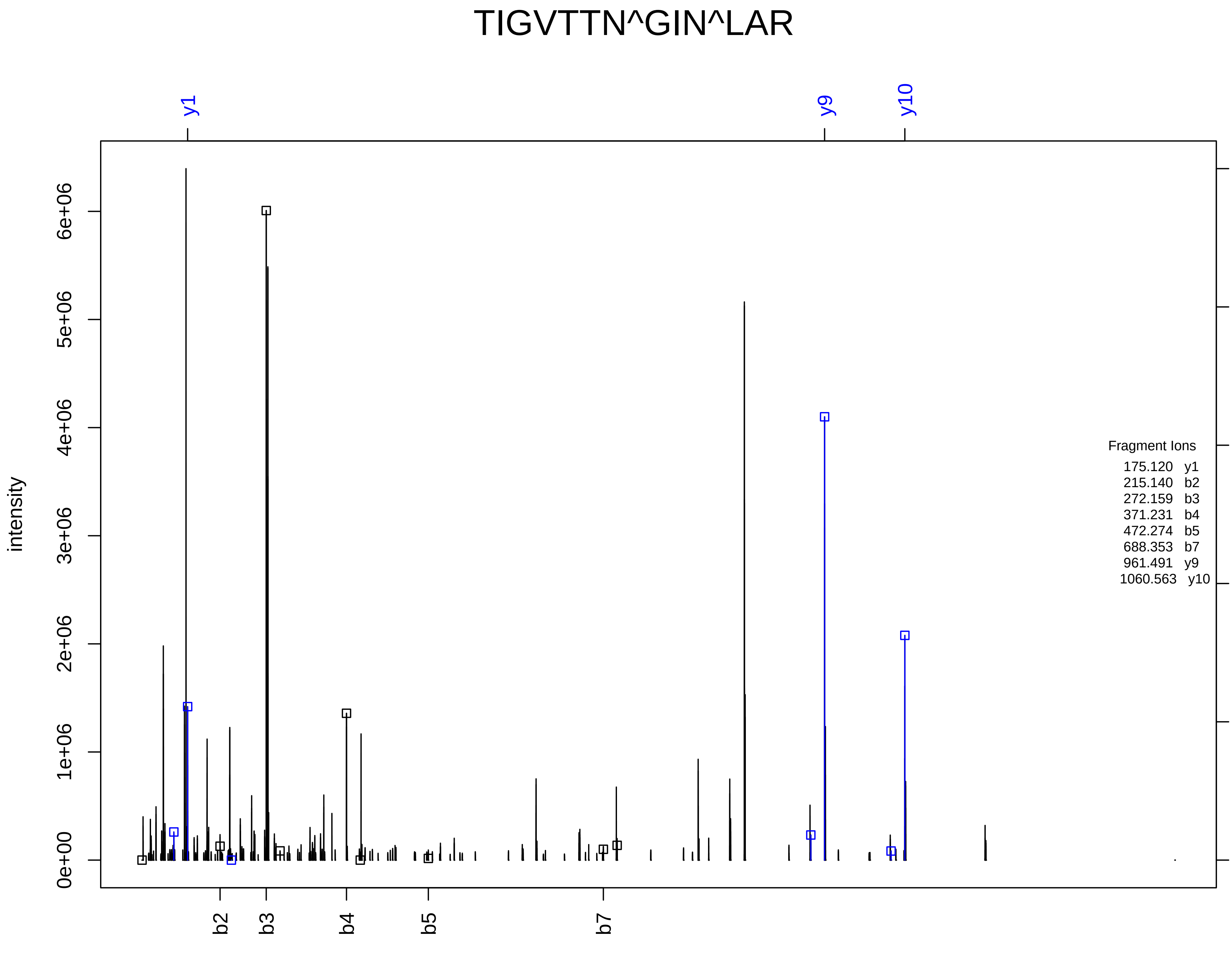

TIGVTTN^GIN^LAR
y1
y9
y10
intensity
0e+00
1e+06
2e+06
3e+06
4e+06
5e+06
6e+06
0
20
40
60
80
100
Fragment Ions
175.120 y1
215.140 b2
272.159 b3
371.231 b4
472.274 b5
688.353 b7
961.491 y9
1060.563 y10
b2
b3
b4
b5
b7
m/z
TIGVTTNGINLAR / File: christine_pool_01.raw; SpectrumID: ; scans: 8233

EQSICAAEEQPAEDGQGETNK

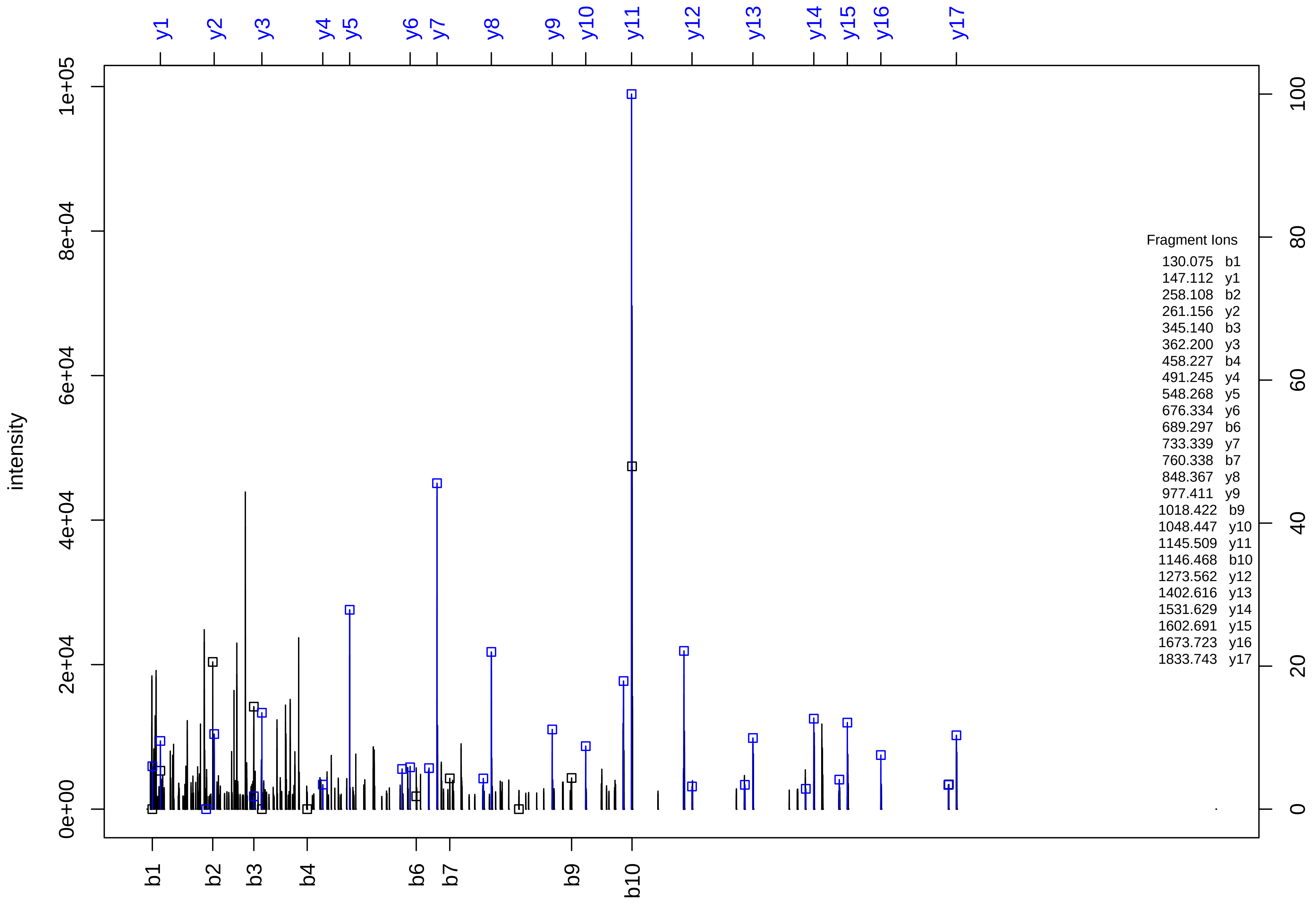

EQSICAAEEQPAEDGQGETNK / File: christine_pool_01.raw; SpectrumID: ; scans: 3376

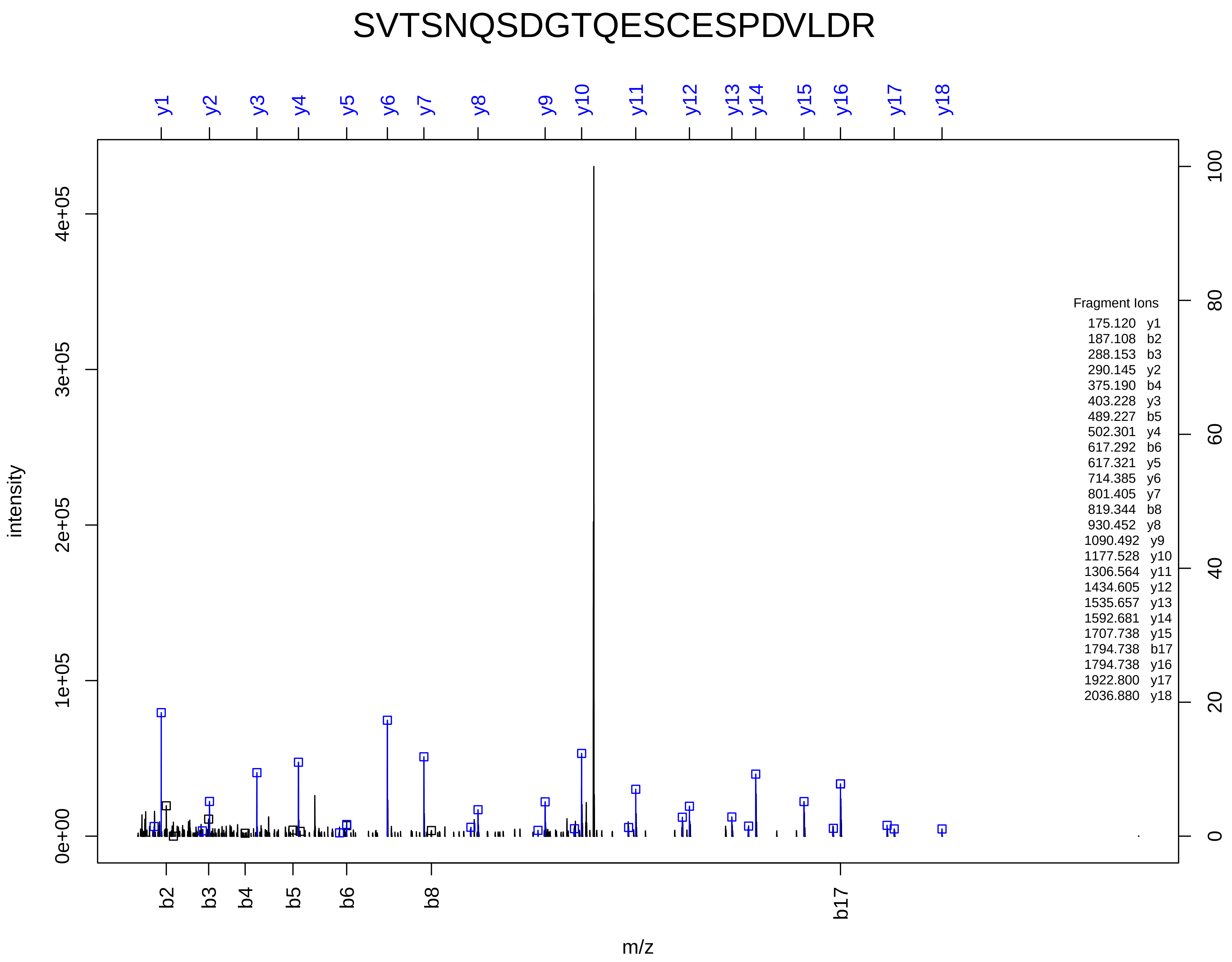

SVTSNQSDGTQESCESPDVLDR / File: christine_pool_01.raw; SpectrumID: ; scans: 5580

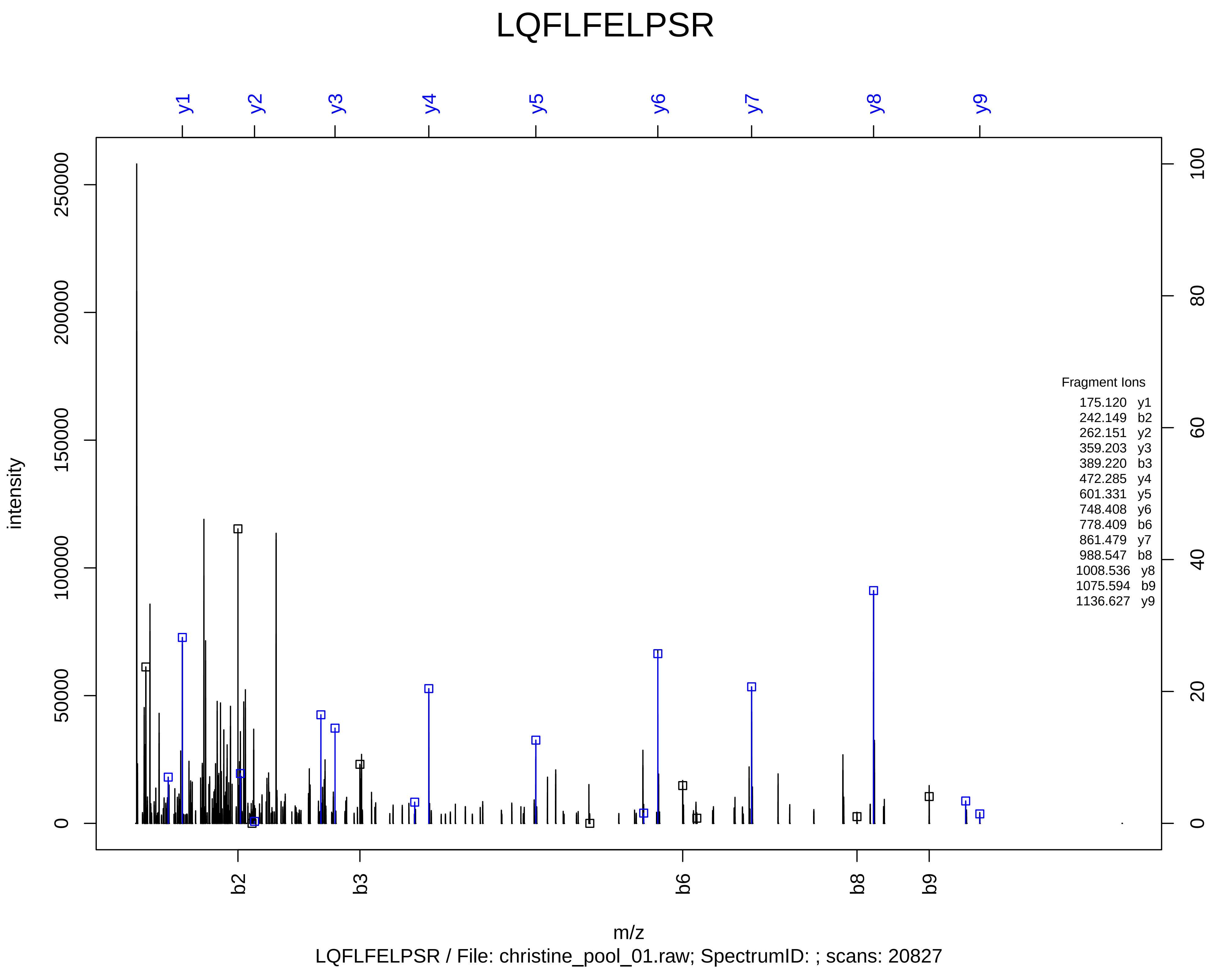
LQFLFELPSR
y1
y2
y3
y4
y5
y6
y7
y8
y9
intensity
250000
200000
150000
100000
50000
0
100
80
60
40
20
0
Fragment Ions
175.120 y1
242.149 b2
262.151 y2
359.203 y3
389.220 b3
472.285 y4
601.331 y5
748.408 y6
778.409 b6
861.479 y7
988.547 b8
1008.536 y8
1075.594 b9
1136.627 y9
b2
b3
b6
b8
b9
m/z
LQFLFELPSR / File: christine_pool_01.raw; SpectrumID: ; scans: 20827

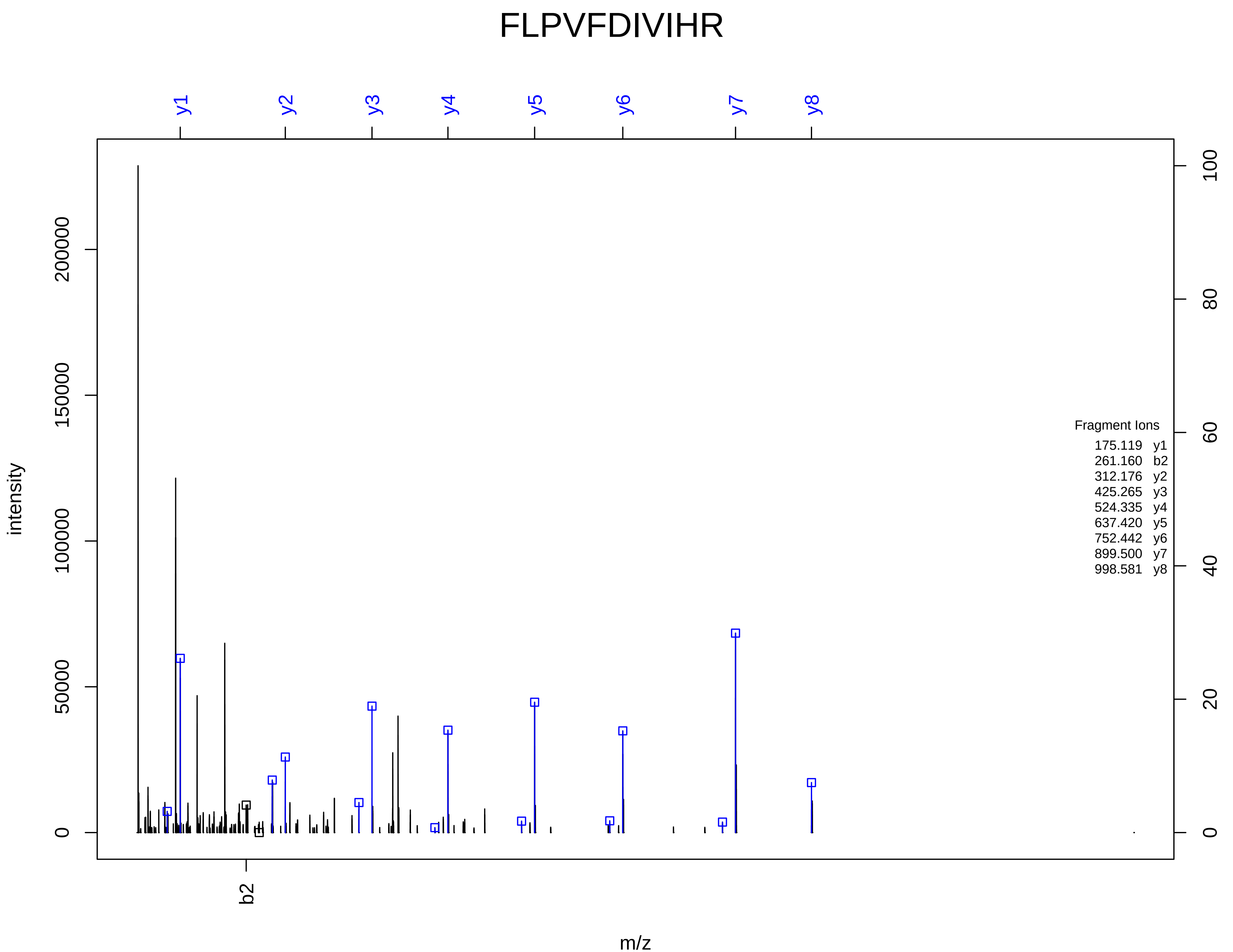
FLPVFDIVIHR
y1
y2
y3
y4
y5
y6
y7
y8
b2
intensity
0
50000
100000
150000
200000
0
20
40
60
80
100
m/z
Fragment Ions
175.119 y1
261.160 b2
312.176 y2
425.265 y3
524.335 y4
637.420 y5
752.442 y6
899.500 y7
998.581 y8
FLPVFDIVIHR / File: christine_pool_01.raw; SpectrumID: ; scans: 21351

NPPPPINFQEWDGLVR

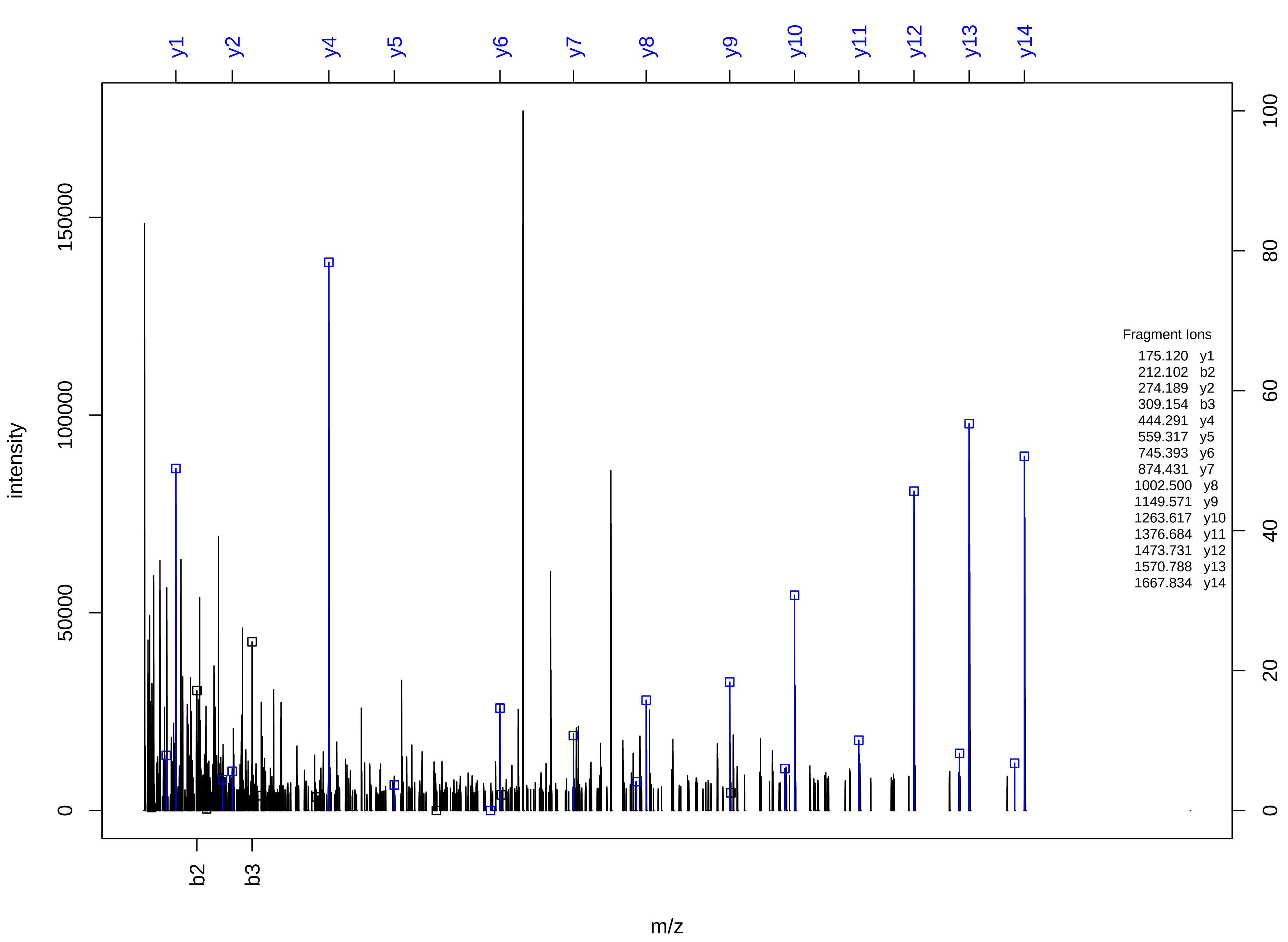

NPPPPINFQEWDGLVR / File: christine_pool_01.raw; SpectrumID: ; scans: 19645

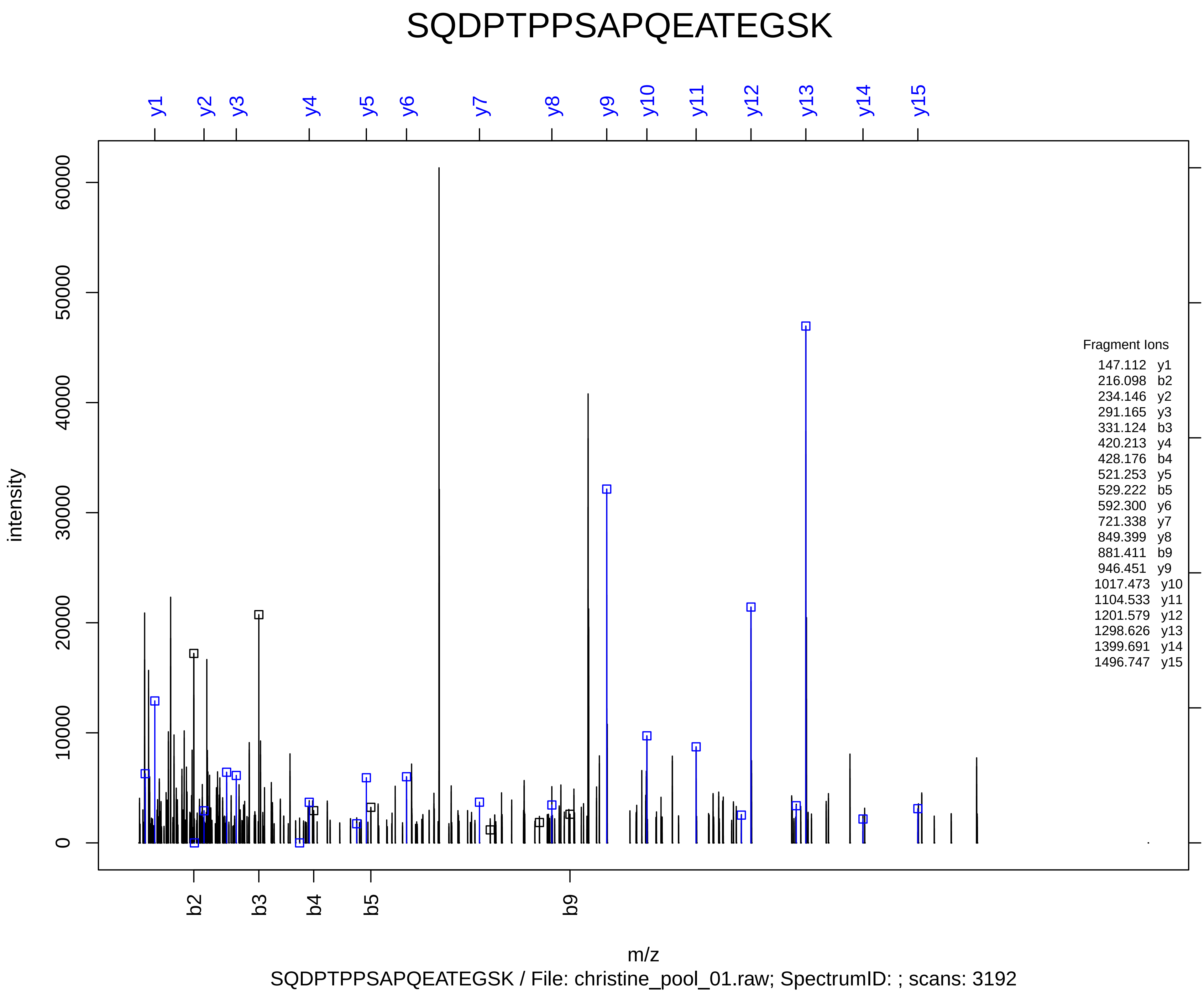

SQDPTPPSAPQEATEGSK
y1 y2 y3 y4 y5 y6 y7 y8 y9 y10 y11 y12 y13 y14 y15
b2 b3 b4 b5 b9
intensity
m/z
0
10000
20000
30000
40000
50000
60000
0
20
40
60
80
100
Fragment Ions
147.112 y1
216.098 b2
234.146 y2
291.165 y3
331.124 b3
420.213 y4
428.176 b4
521.253 y5
529.222 b5
592.300 y6
721.338 y7
849.399 y8
881.411 b9
946.451 y9
1017.473 y10
1104.533 y11
1201.579 y12
1298.626 y13
1399.691 y14
1496.747 y15
SQDPTPPSAPQEATEGSK / File: christine_pool_01.raw; SpectrumID: ; scans: 3192

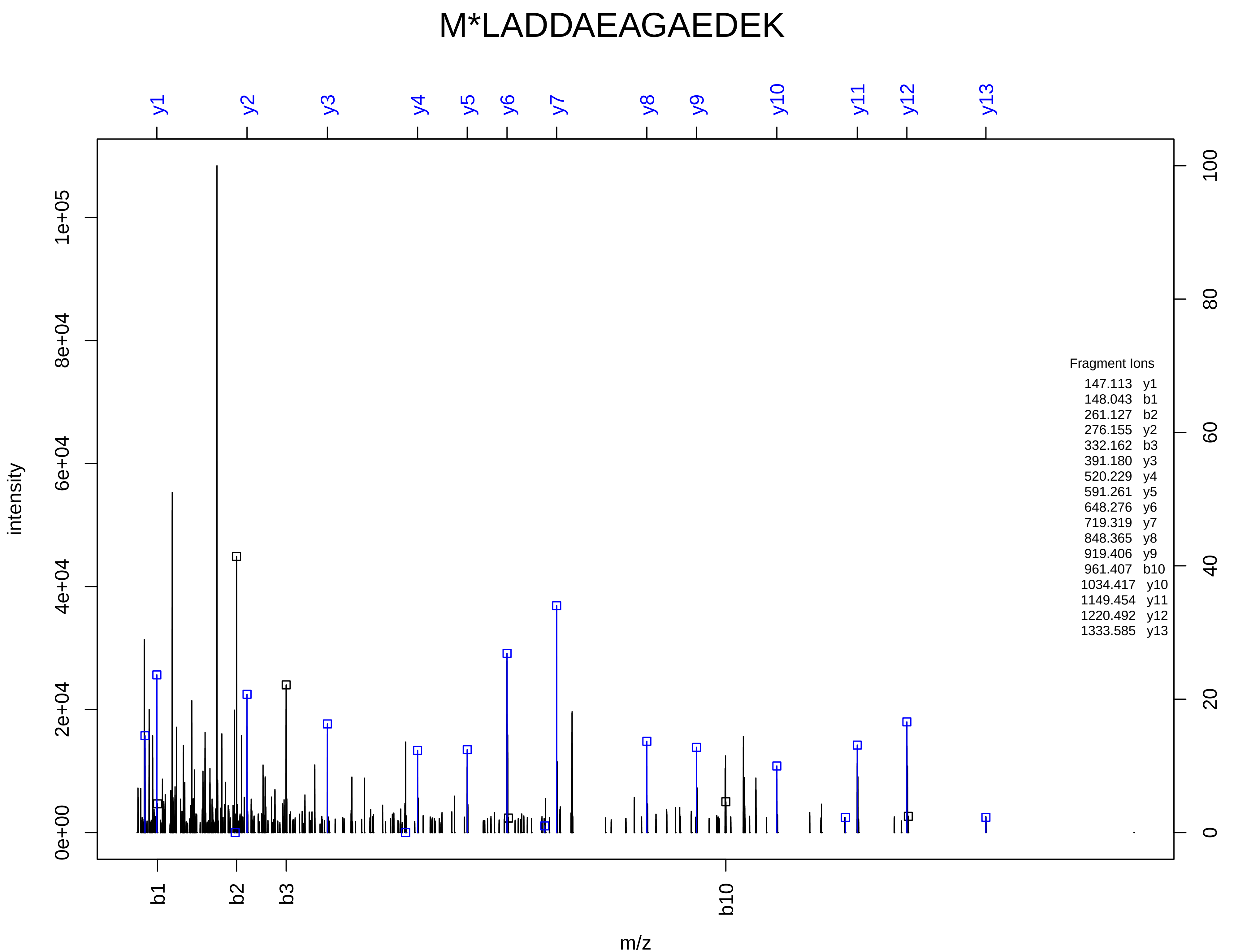

MLADDAEAGAEDEK / File: christine_pool_01.raw; SpectrumID: ; scans: 2805

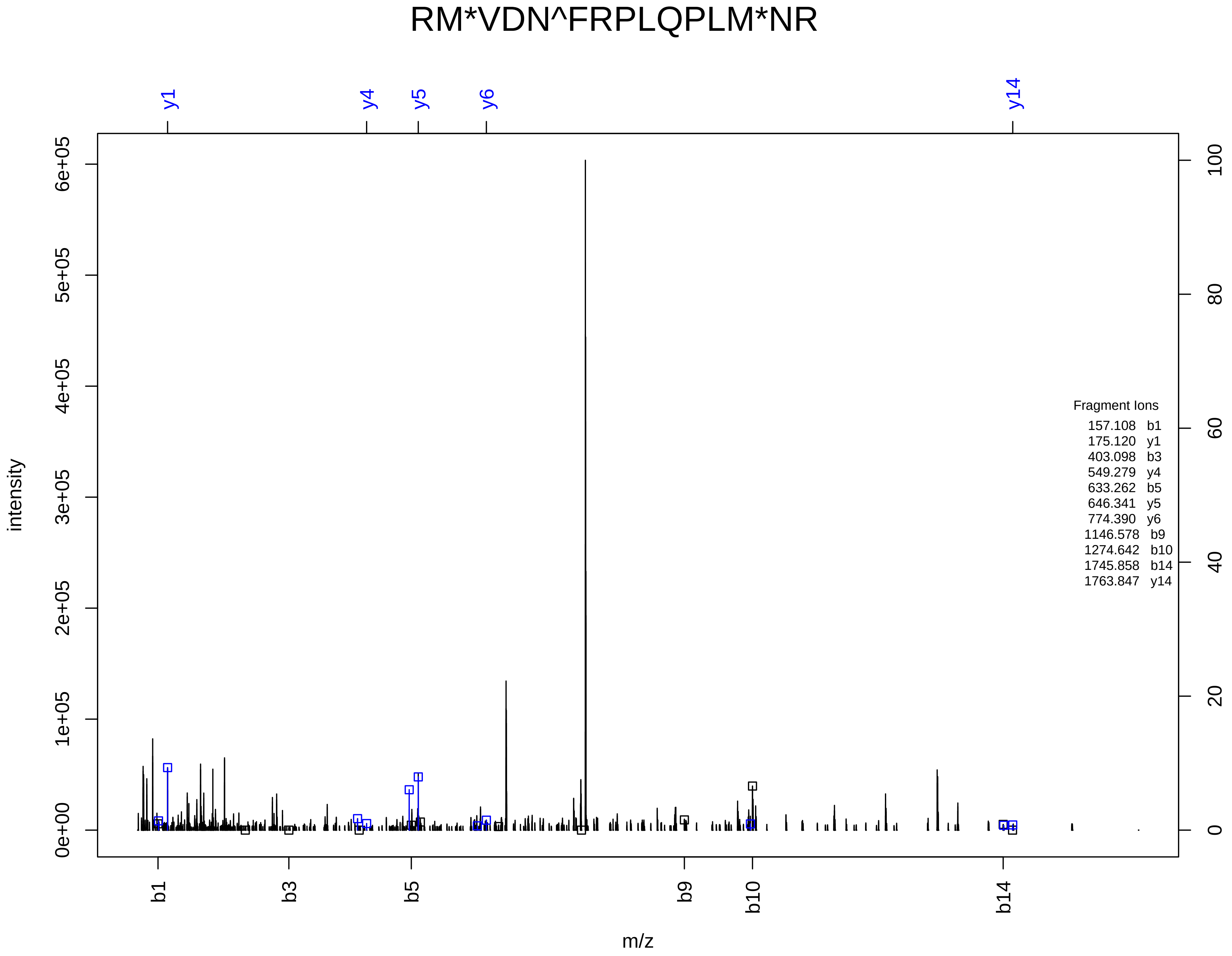
RM*VDN^FRPLQPLM*NR
y1
y4
y5
y6
y14
intensity
0e+00
1e+05
2e+05
3e+05
4e+05
5e+05
6e+05
0
20
40
60
80
100
b1
b3
b5
b9
b10
b14
m/z
Fragment Ions
157.108 b1
175.120 y1
403.098 b3
549.279 y4
633.262 b5
646.341 y5
774.390 y6
1146.578 b9
1274.642 b10
1745.858 b14
1763.847 y14
RMVDNFRPLQPLMNR / File: christine_pool_01.raw; SpectrumID: ; scans: 5893

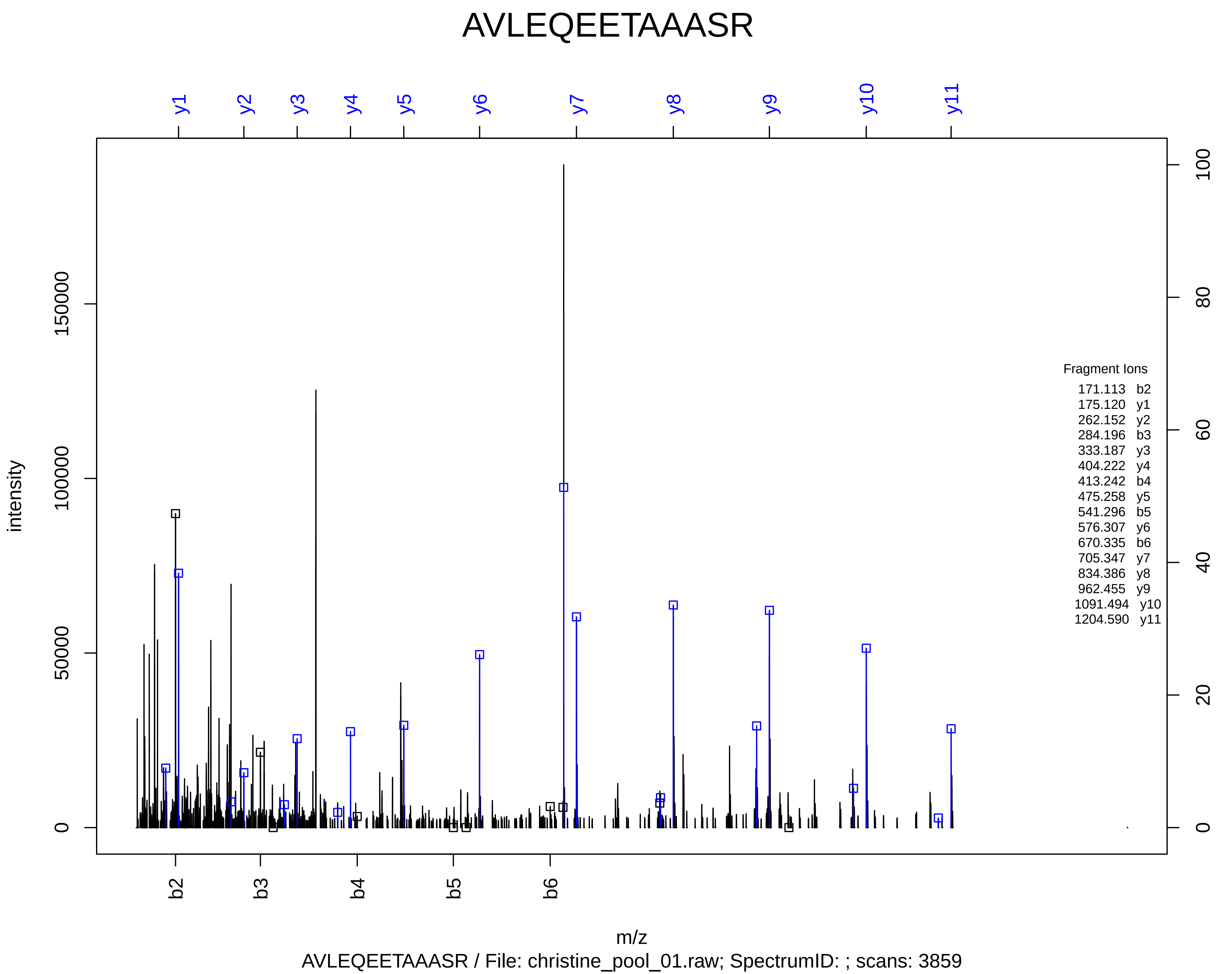
AVLEQEETAAASR
y1
y2
y3
y4
y5
y6
y7
y8
y9
y10
y11
intensity
0
50000
100000
150000
0
20
40
60
80
100
b2
b3
b4
b5
b6
m/z
Fragment Ions
171.113 b2
175.120 y1
262.152 y2
284.196 b3
333.187 y3
404.222 y4
413.242 b4
475.258 y5
541.296 b5
576.307 y6
670.335 b6
705.347 y7
834.386 y8
962.455 y9
1091.494 y10
1204.590 y11
AVLEQEETAAASR / File: christine_pool_01.raw; SpectrumID: ; scans: 3859

LENLDSDVVQLR

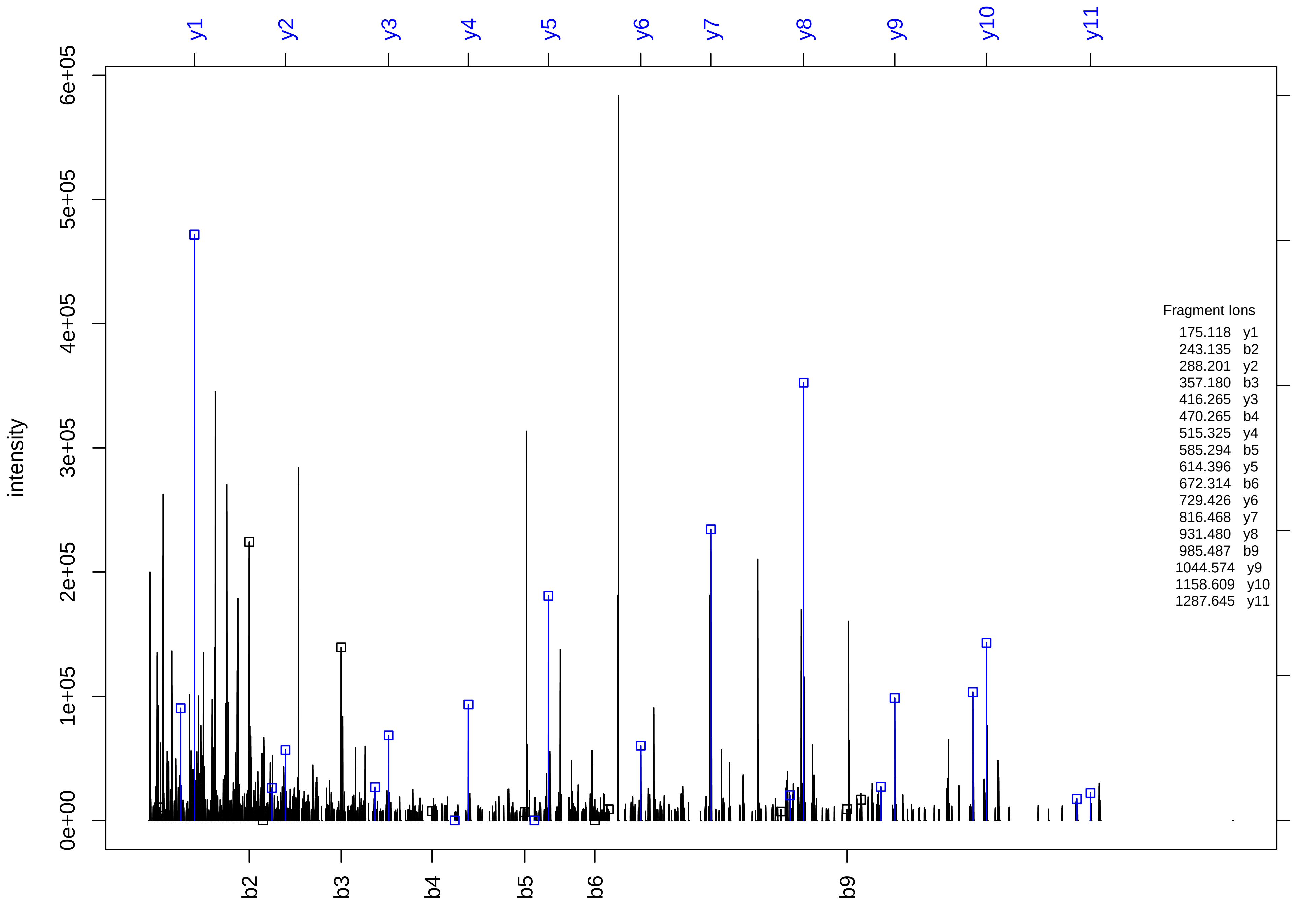

LENLDSDVVQLR / File: christine_pool_01.raw; SpectrumID: ; scans: 11674

DILVLPLDLTDTGSHEAATK

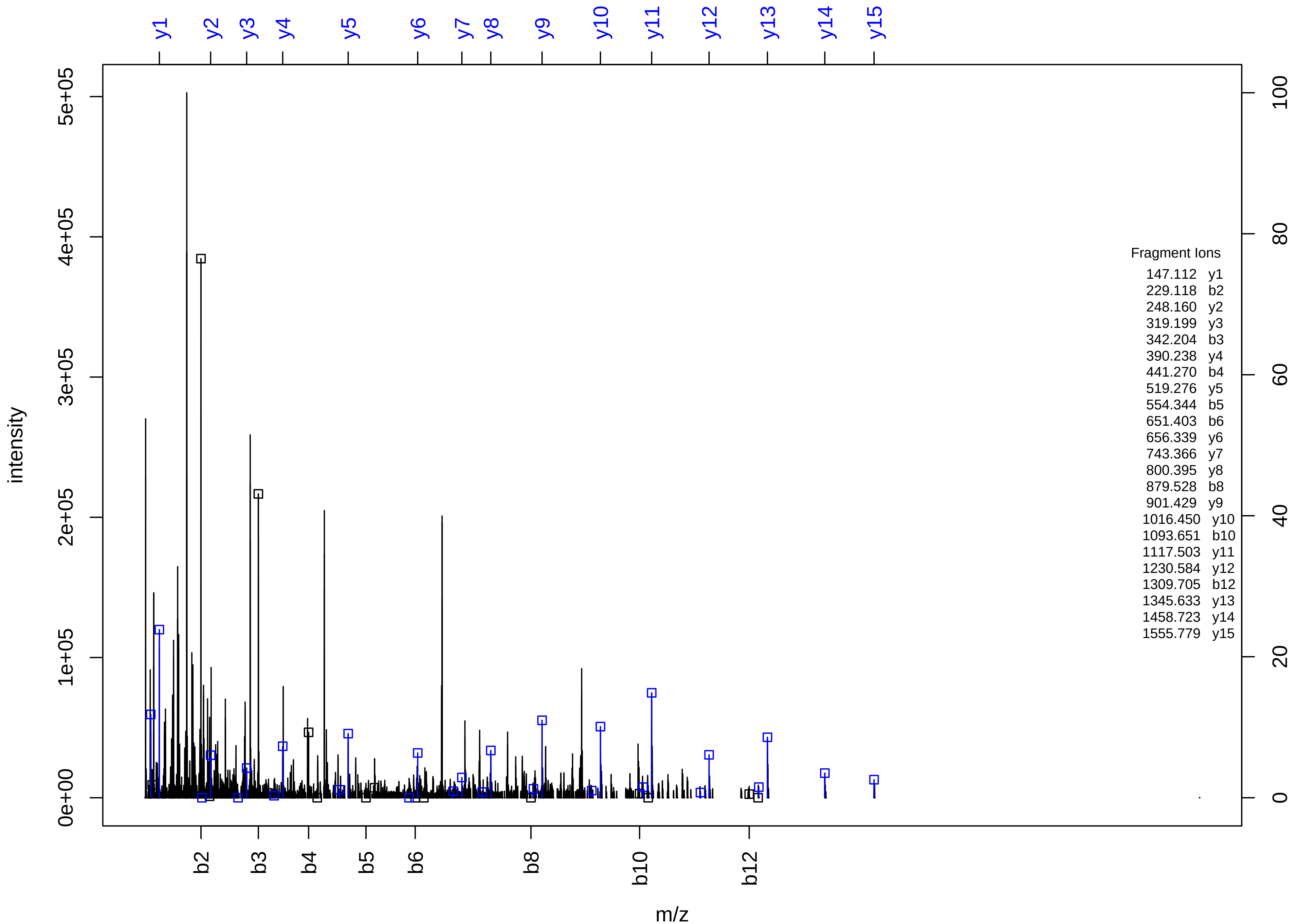

DILVLPLDLTDTGSHEAATK / File: christine_pool_01.raw; SpectrumID: ; scans: 20488

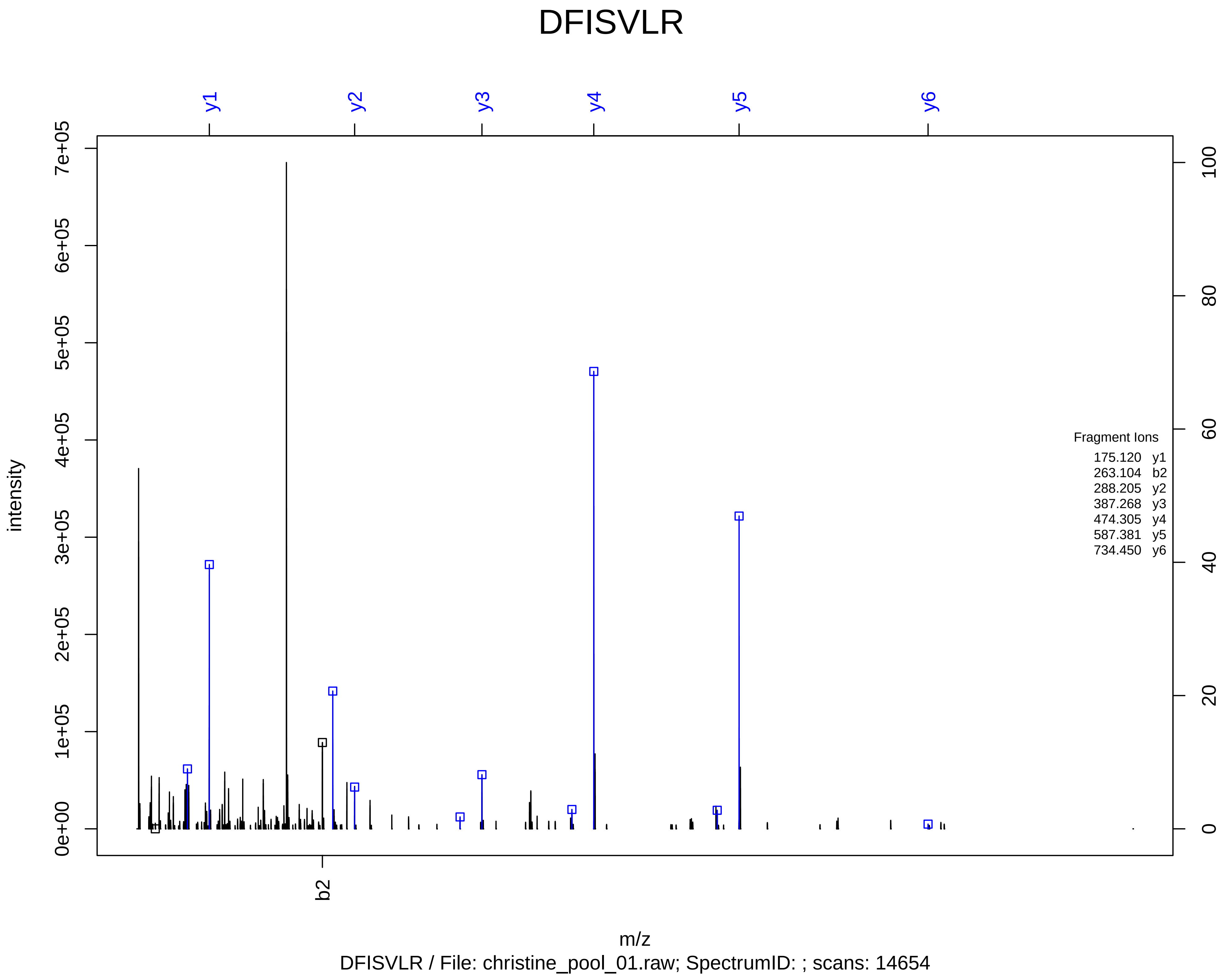

DFISVLR
y1
y2
y3
y4
y5
y6
b2
intensity
0e+00
1e+05
2e+05
3e+05
4e+05
5e+05
6e+05
7e+05
0
20
40
60
80
100
Fragment Ions
175.120 y1
263.104 b2
288.205 y2
387.268 y3
474.305 y4
587.381 y5
734.450 y6
m/z
DFISVLR / File: christine_pool_01.raw; SpectrumID: ; scans: 14654

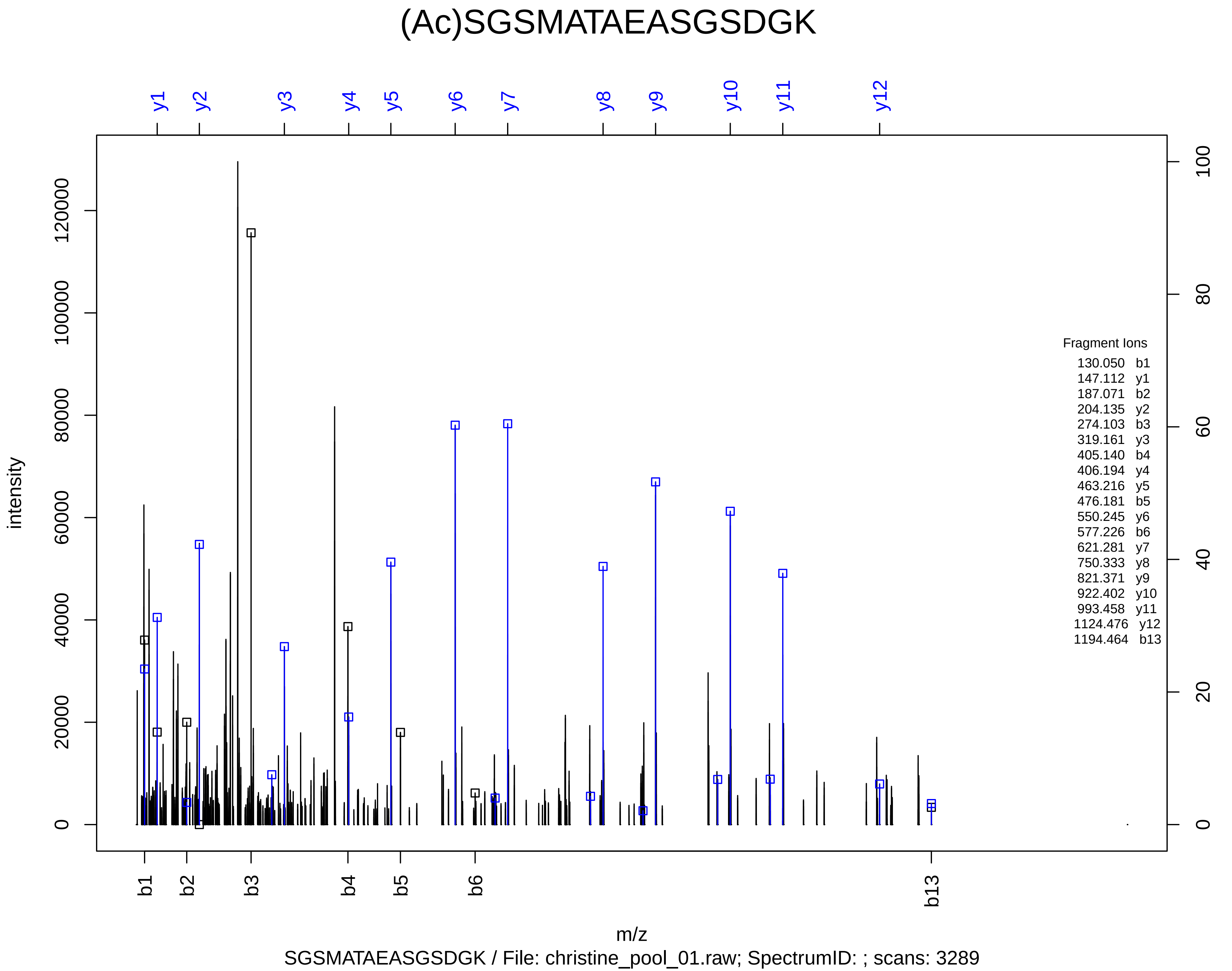
(Ac)SGSMATAEASGSDGK
y1 y2 y3 y4 y5 y6 y7 y8 y9 y10 y11 y12
b1 b2 b3 b4 b5 b6 b13
intensity
0 20000 40000 60000 80000 100000 120000
0 20 40 60 80 100
m/z
Fragment Ions
130.050 b1
147.112 y1
187.071 b2
204.135 y2
274.103 b3
319.161 y3
405.140 b4
406.194 y4
463.216 y5
476.181 b5
550.245 y6
577.226 b6
621.281 y7
750.333 y8
821.371 y9
922.402 y10
993.458 y11
1124.476 y12
1194.464 b13
SGSMATAEASGSDGK / File: christine_pool_01.raw; SpectrumID: ; scans: 3289

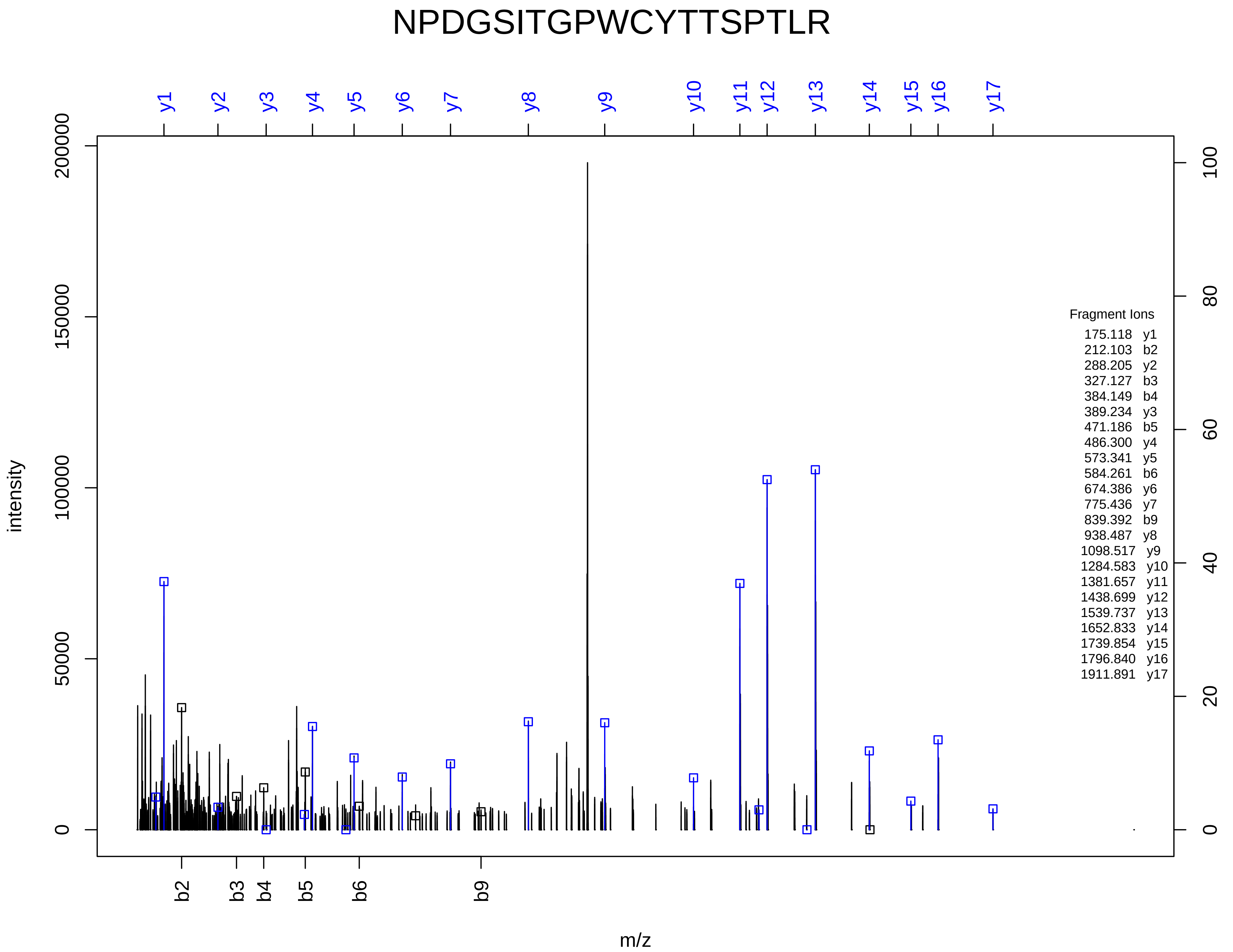

NPDGSITGPWCYTTSPTLR / File: christine_pool_03.raw; SpectrumID: ; scans: 15806

GSYSCEVTHEGSTVTK

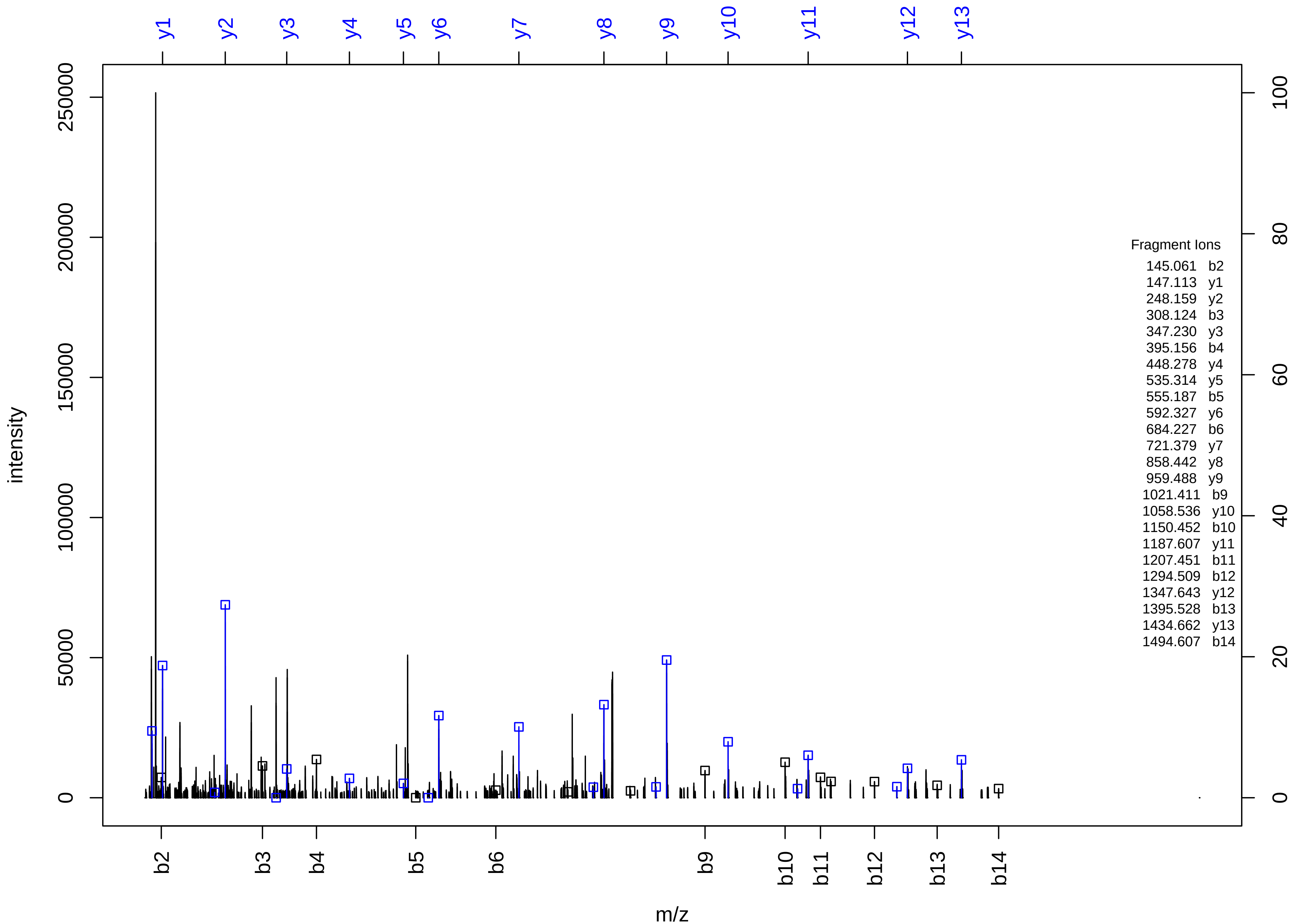

GSYSCEVTHEGSTVTK / File: christine_pool_03.raw; SpectrumID: ; scans: 3316

FLLPVPQLK

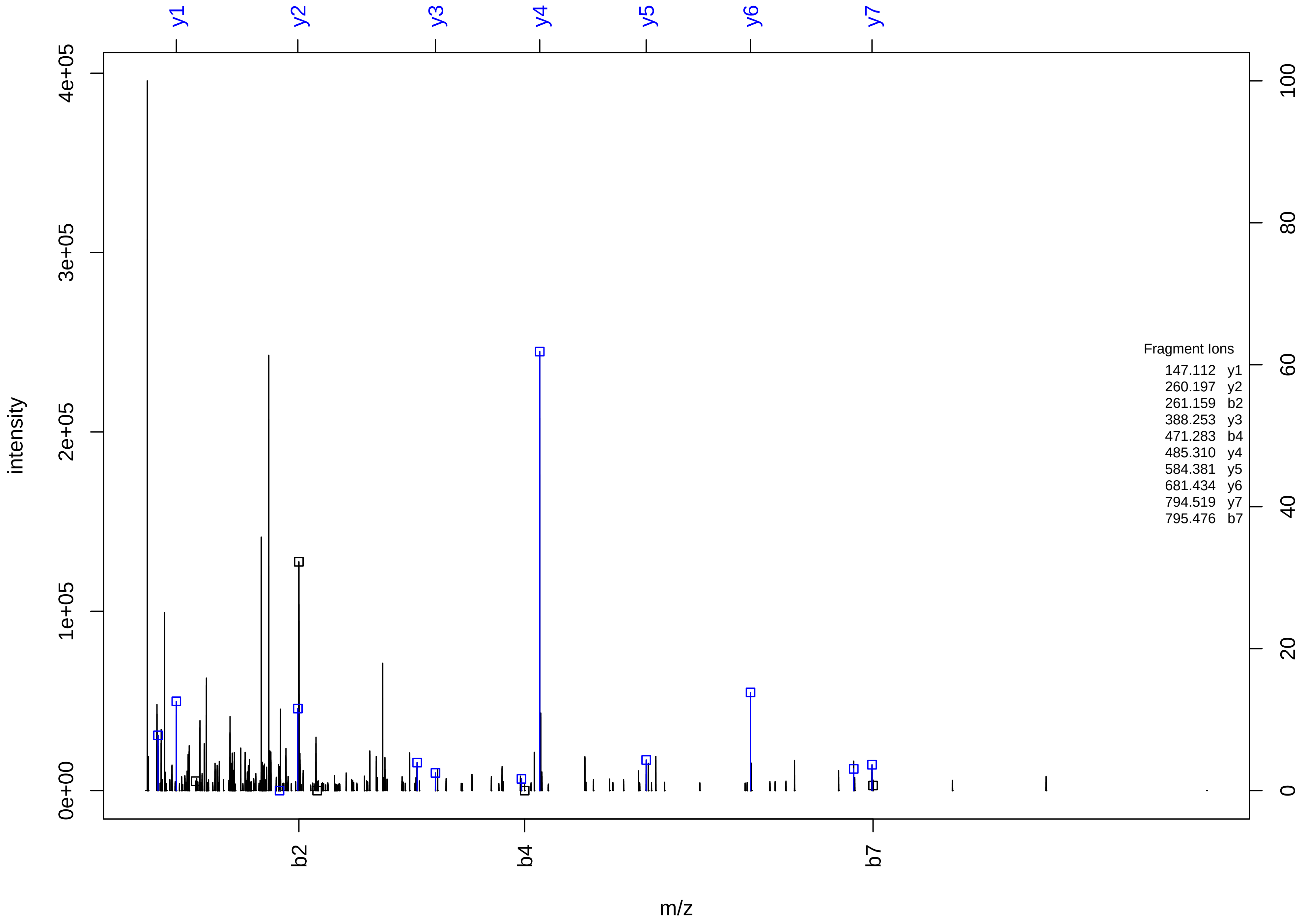

FLLPVPQLK / File: christine_pool_03.raw; SpectrumID: ; scans: 20414

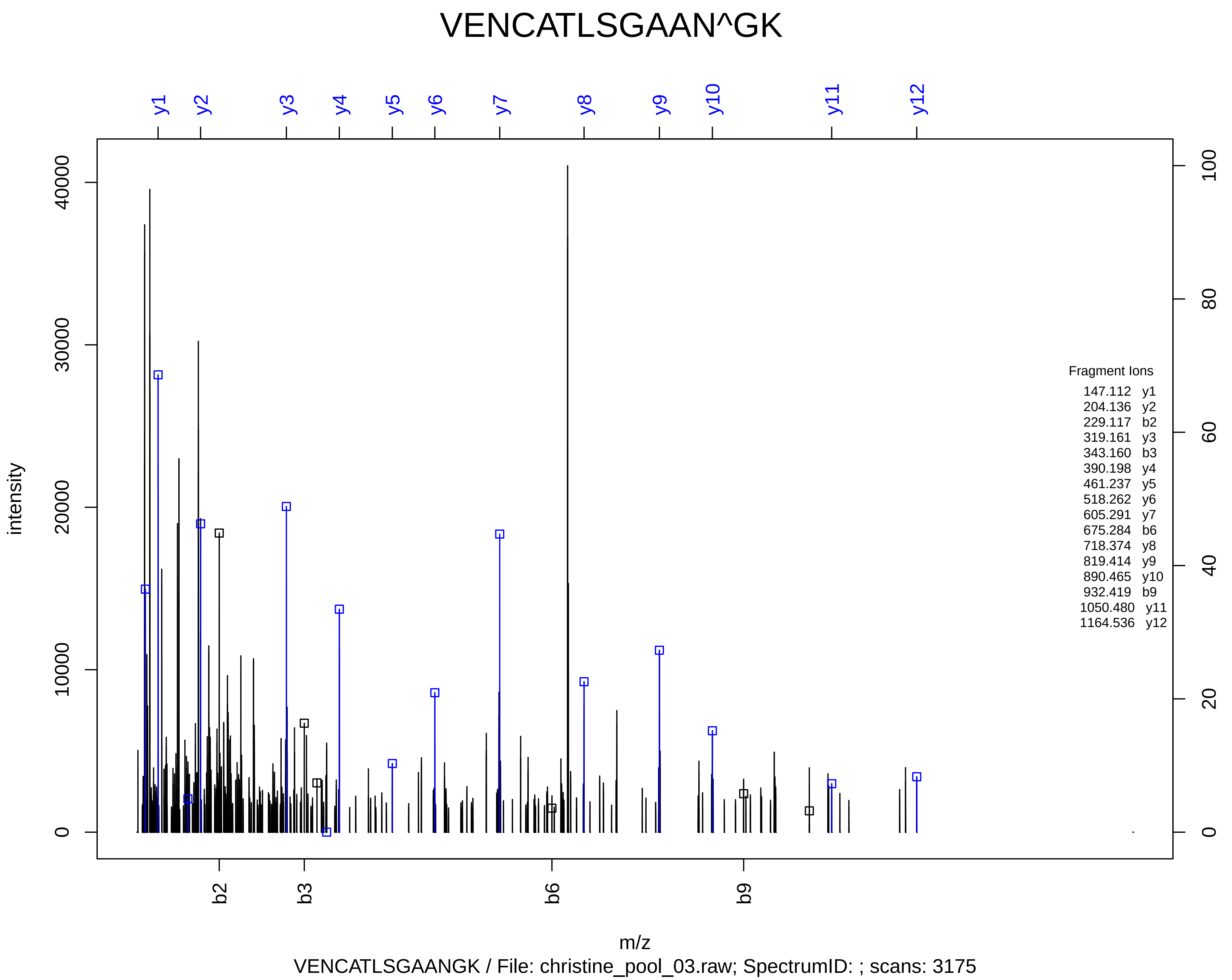
VENCATLSGAAN^GK
y1
y2
y3
y4
y5
y6
y7
y8
y9
y10
y11
y12
b2
b3
b6
b9
intensity
m/z
Fragment Ions
147.112 y1
204.136 y2
229.117 b2
319.161 y3
343.160 b3
390.198 y4
461.237 y5
518.262 y6
605.291 y7
675.284 b6
718.374 y8
819.414 y9
890.465 y10
932.419 b9
1050.480 y11
1164.536 y12
VENCATLSGAANGK / File: christine_pool_03.raw; SpectrumID: ; scans: 3175

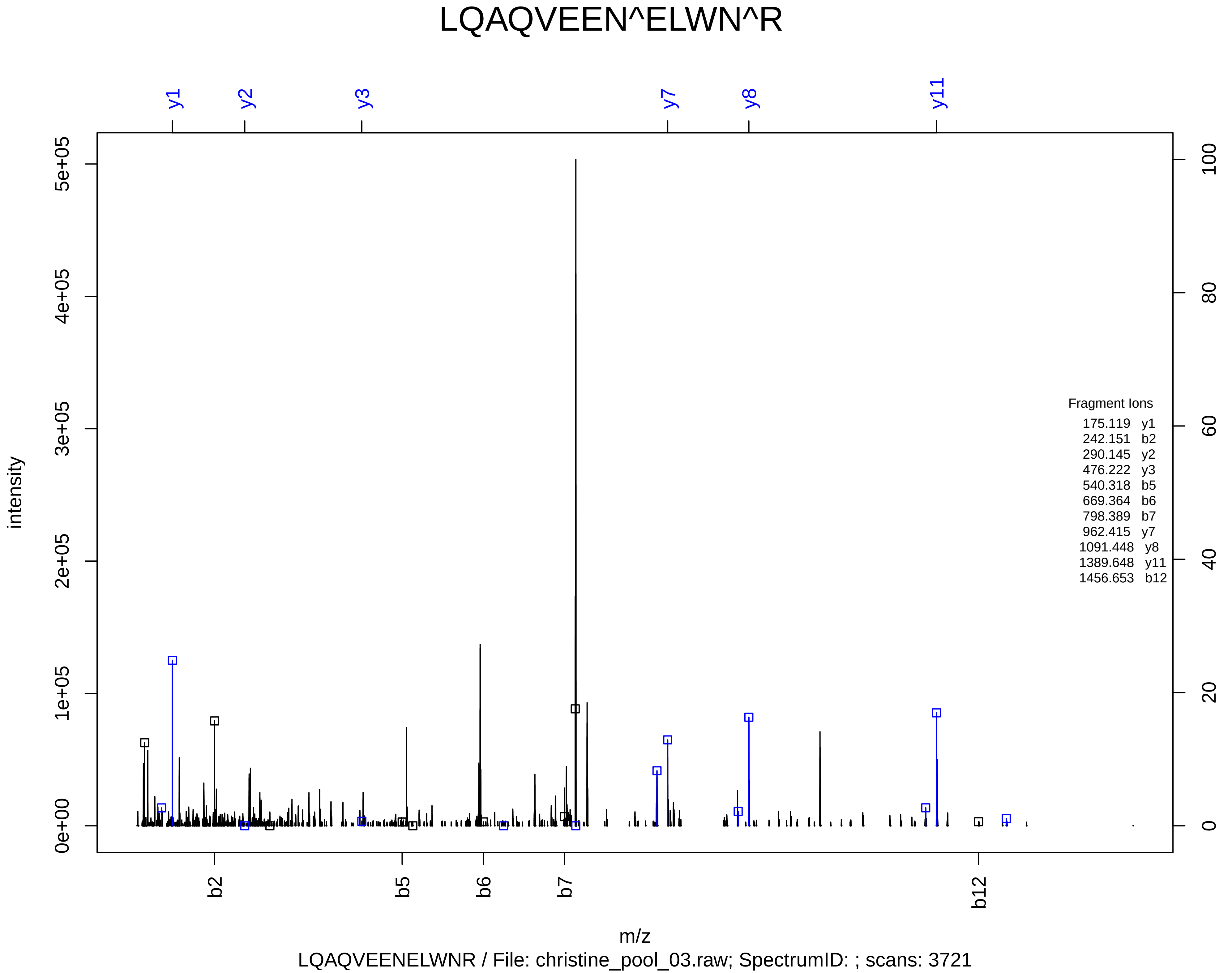
LQAQVEEN^ELWN^R
y1
y2
y3
y7
y8
y11
intensity
0e+00
1e+05
2e+05
3e+05
4e+05
5e+05
0
20
40
60
80
100
b2
b5
b6
b7
b12
m/z
Fragment Ions
175.119 y1
242.151 b2
290.145 y2
476.222 y3
540.318 b5
669.364 b6
798.389 b7
962.415 y7
1091.448 y8
1389.648 y11
1456.653 b12
LQAQVEENELWNR / File: christine_pool_03.raw; SpectrumID: ; scans: 3721

ILGLAIESQDAGIK

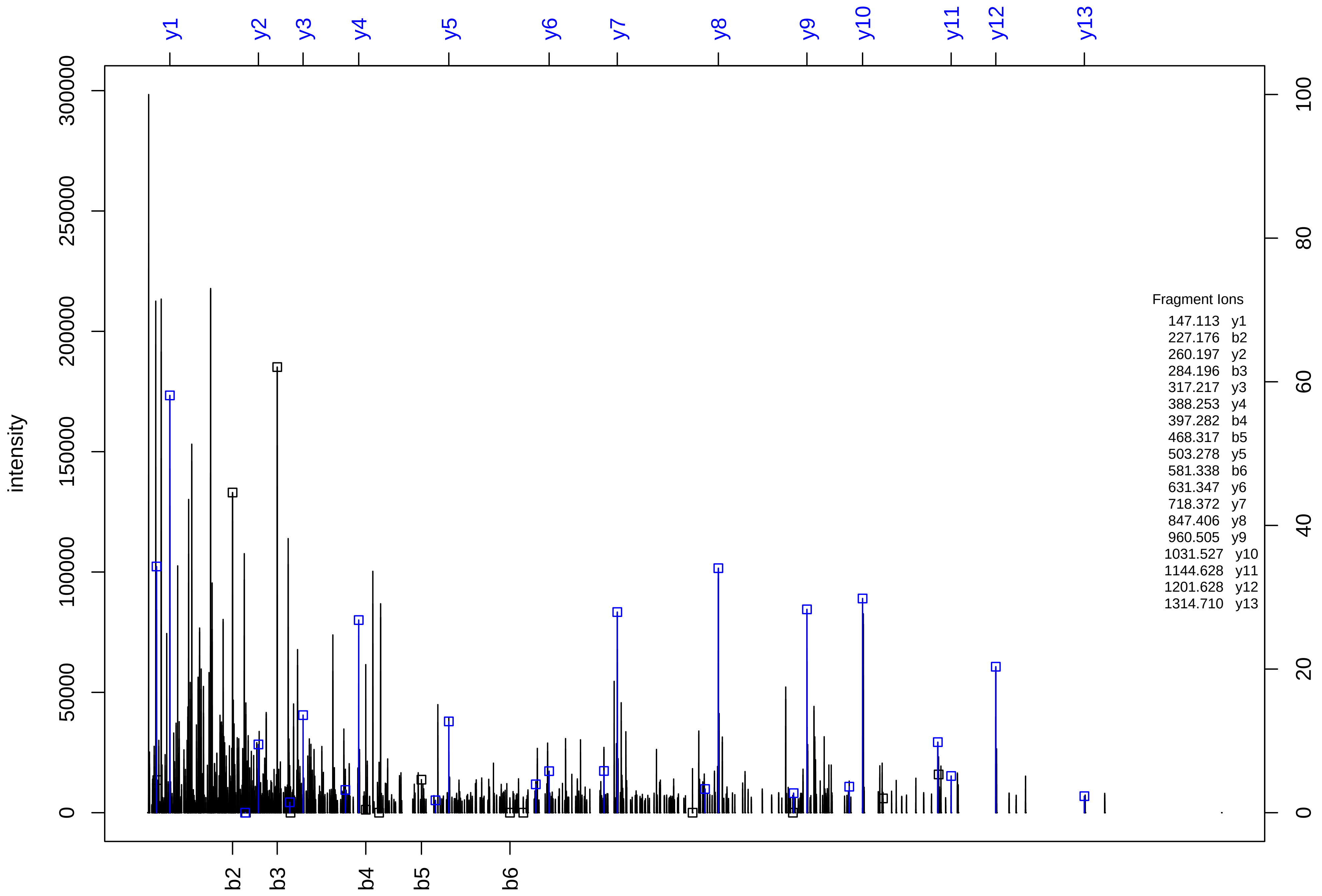

ILGLAIESQDAGIK / File: christine_pool_03.raw; SpectrumID: ; scans: 15561

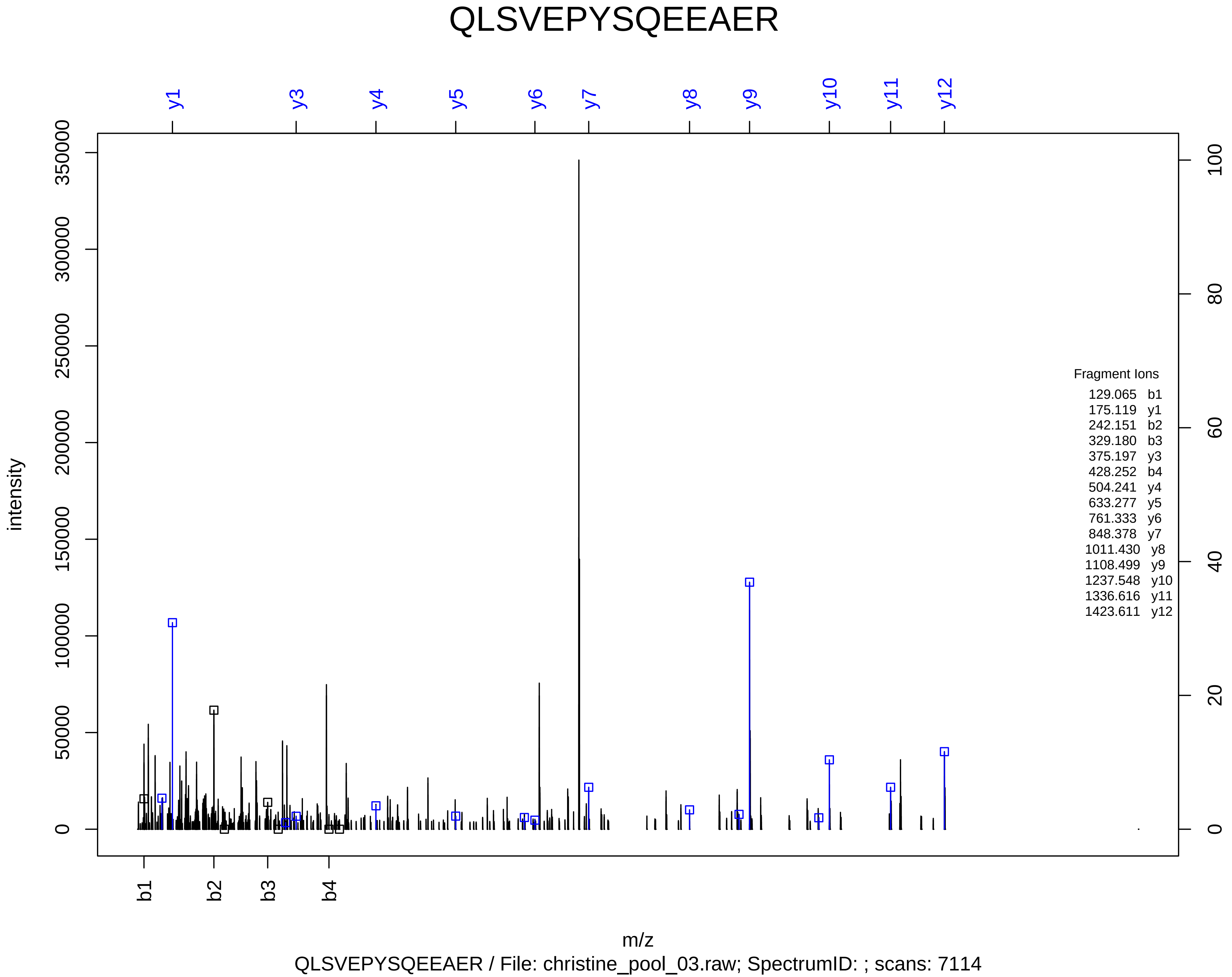

QLSVEPYSQEEAER
intensity
m/z
y1
y3
y4
y5
y6
y7
y8
y9
y10
y11
y12
b1
b2
b3
b4
Fragment Ions
129.065 b1
175.119 y1
242.151 b2
329.180 b3
375.197 y3
428.252 b4
504.241 y4
633.277 y5
761.333 y6
848.378 y7
1011.430 y8
1108.499 y9
1237.548 y10
1336.616 y11
1423.611 y12
QLSVEPYSQEEAER / File: christine_pool_03.raw; SpectrumID: ; scans: 7114

ELEPFLLR

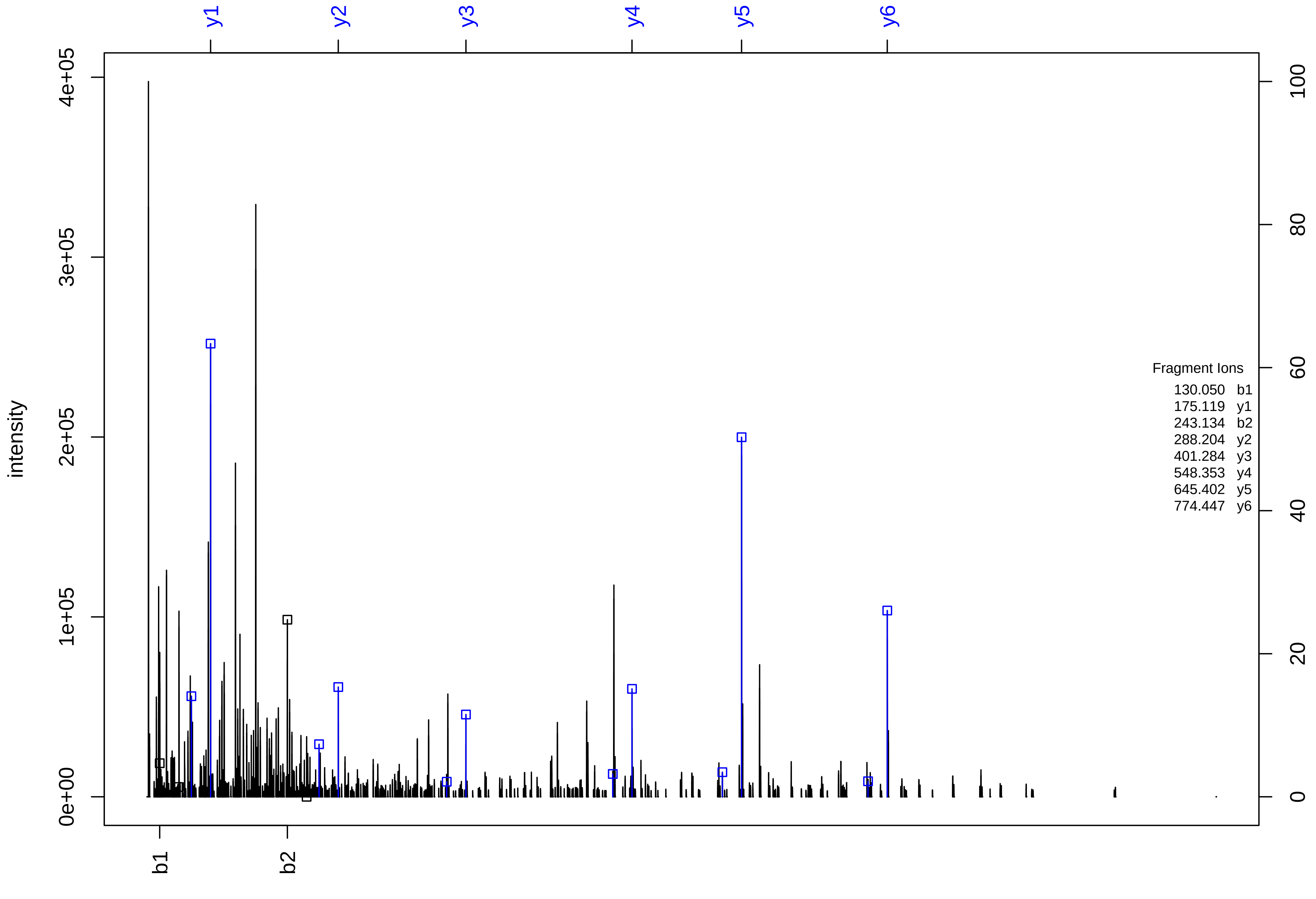

ELEPFLLR / File: christine_pool_03.raw; SpectrumID: ; scans: 16746

VAAPEIVSGVSGESEQK

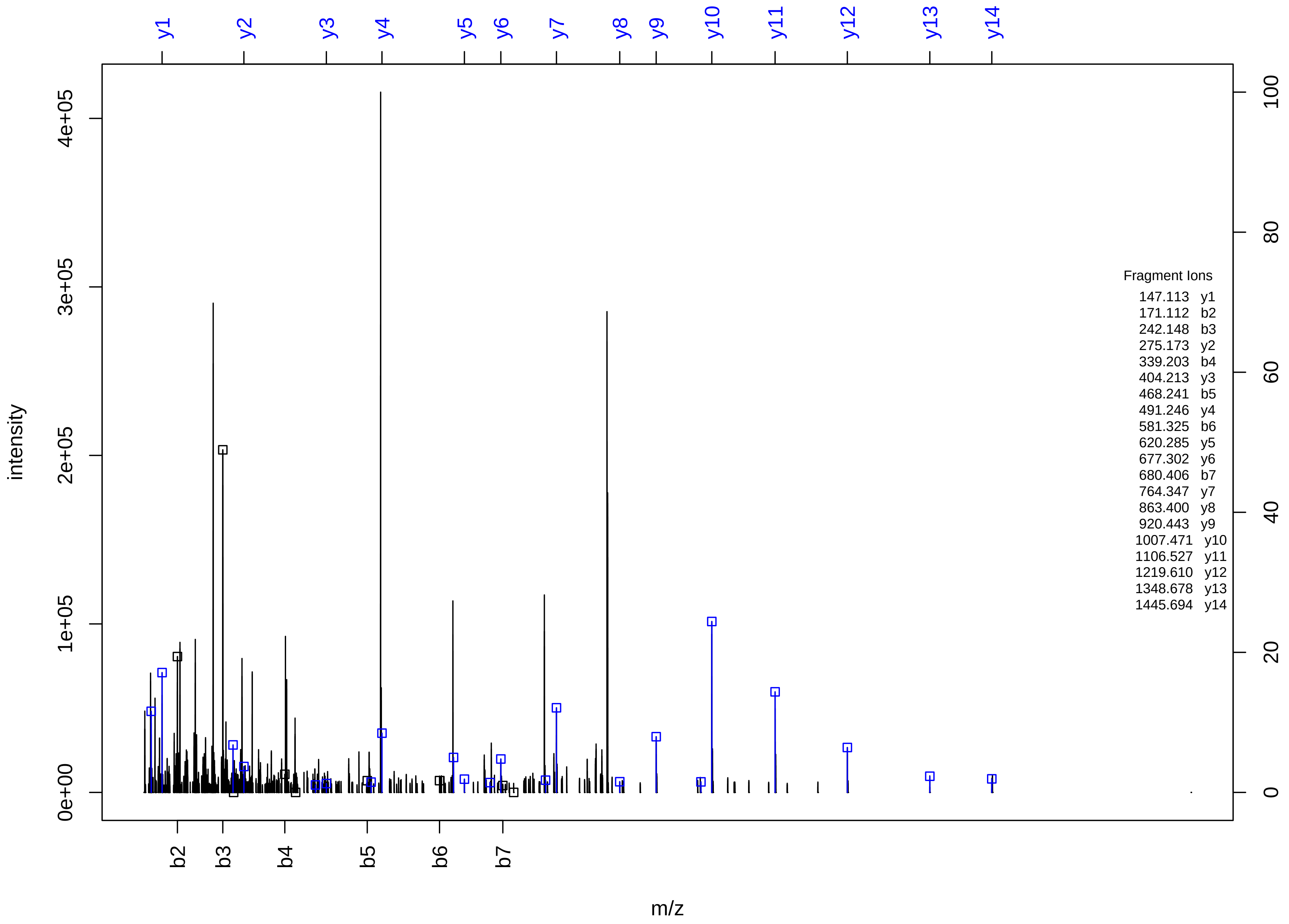

VAAPEIVSGVSGESEQK / File: christine_pool_03.raw; SpectrumID: ; scans: 9158

ILESSQTLLSVLK

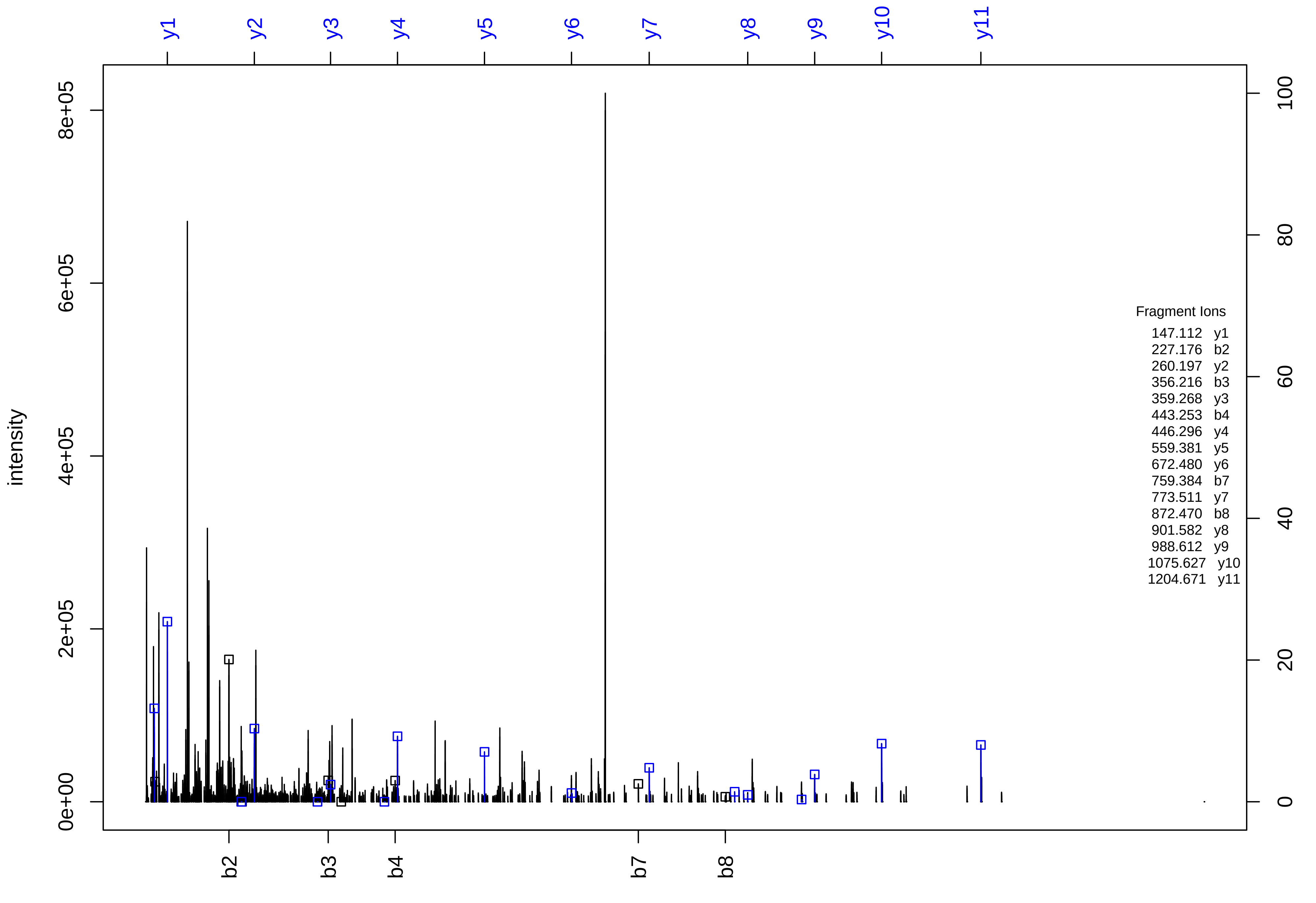

ILESSQTLLSVLK / File: christine_pool_03.raw; SpectrumID: ; scans: 19282

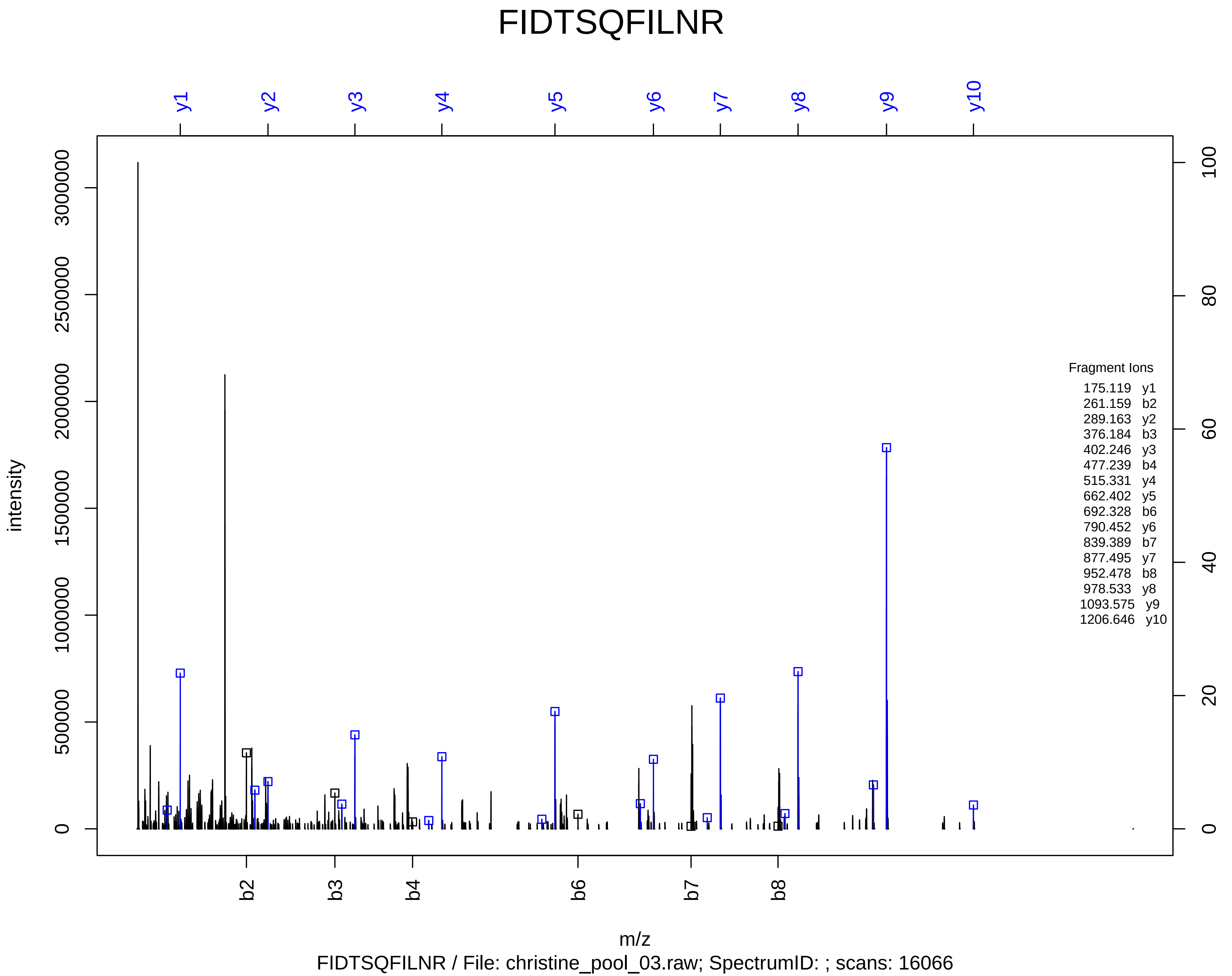
FIDTSQFILNR
y1
y2
y3
y4
y5
y6
y7
y8
y9
y10
intensity
0
500000
1000000
1500000
2000000
2500000
3000000
0
20
40
60
80
100
Fragment Ions
175.119 y1
261.159 b2
289.163 y2
376.184 b3
402.246 y3
477.239 b4
515.331 y4
662.402 y5
692.328 b6
790.452 y6
839.389 b7
877.495 y7
952.478 b8
978.533 y8
1093.575 y9
1206.646 y10
b2
b3
b4
b6
b7
b8
m/z
FIDTSQFILNR / File: christine_pool_03.raw; SpectrumID: ; scans: 16066

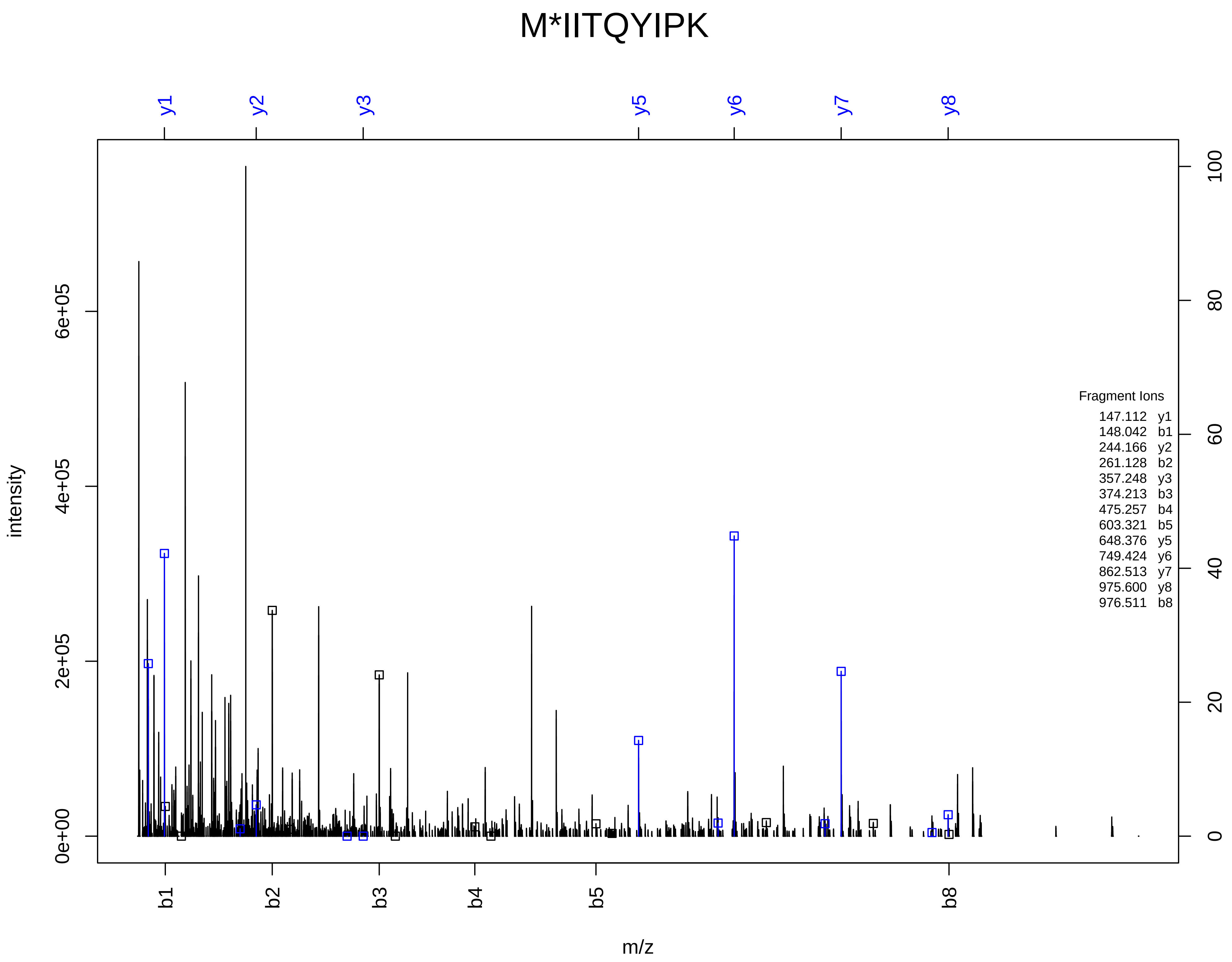

MIITQYIPK / File: christine_pool_03.raw; SpectrumID: ; scans: 14101

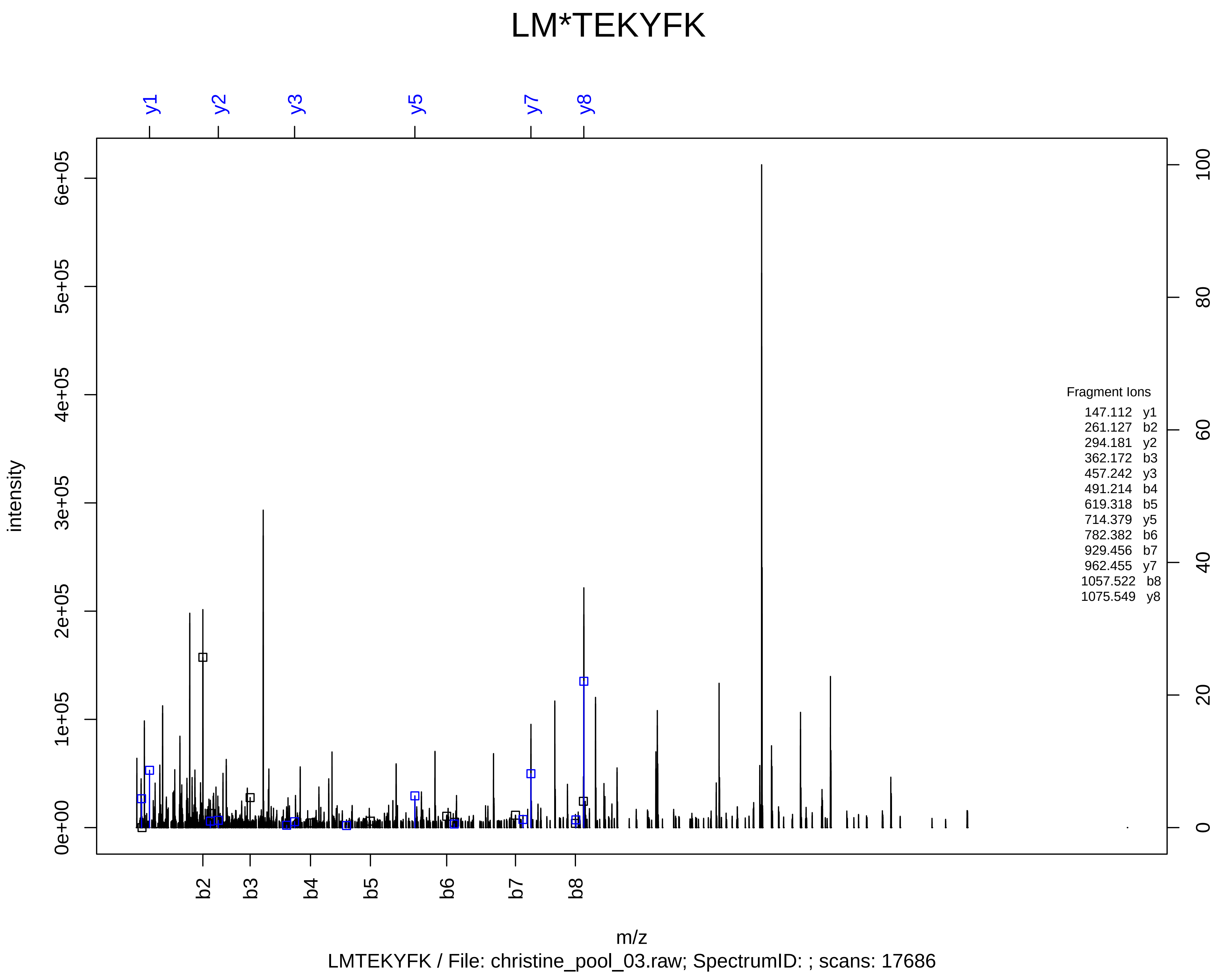
LM*TEKYFK
y1
y2
y3
y5
y7
y8
intensity
0e+00
1e+05
2e+05
3e+05
4e+05
5e+05
6e+05
0
20
40
60
80
100
b2
b3
b4
b5
b6
b7
b8
Fragment Ions
147.112 y1
261.127 b2
294.181 y2
362.172 b3
457.242 y3
491.214 b4
619.318 b5
714.379 y5
782.382 b6
929.456 b7
962.455 y7
1057.522 b8
1075.549 y8
m/z
LMTEKYFK / File: christine_pool_03.raw; SpectrumID: ; scans: 17686

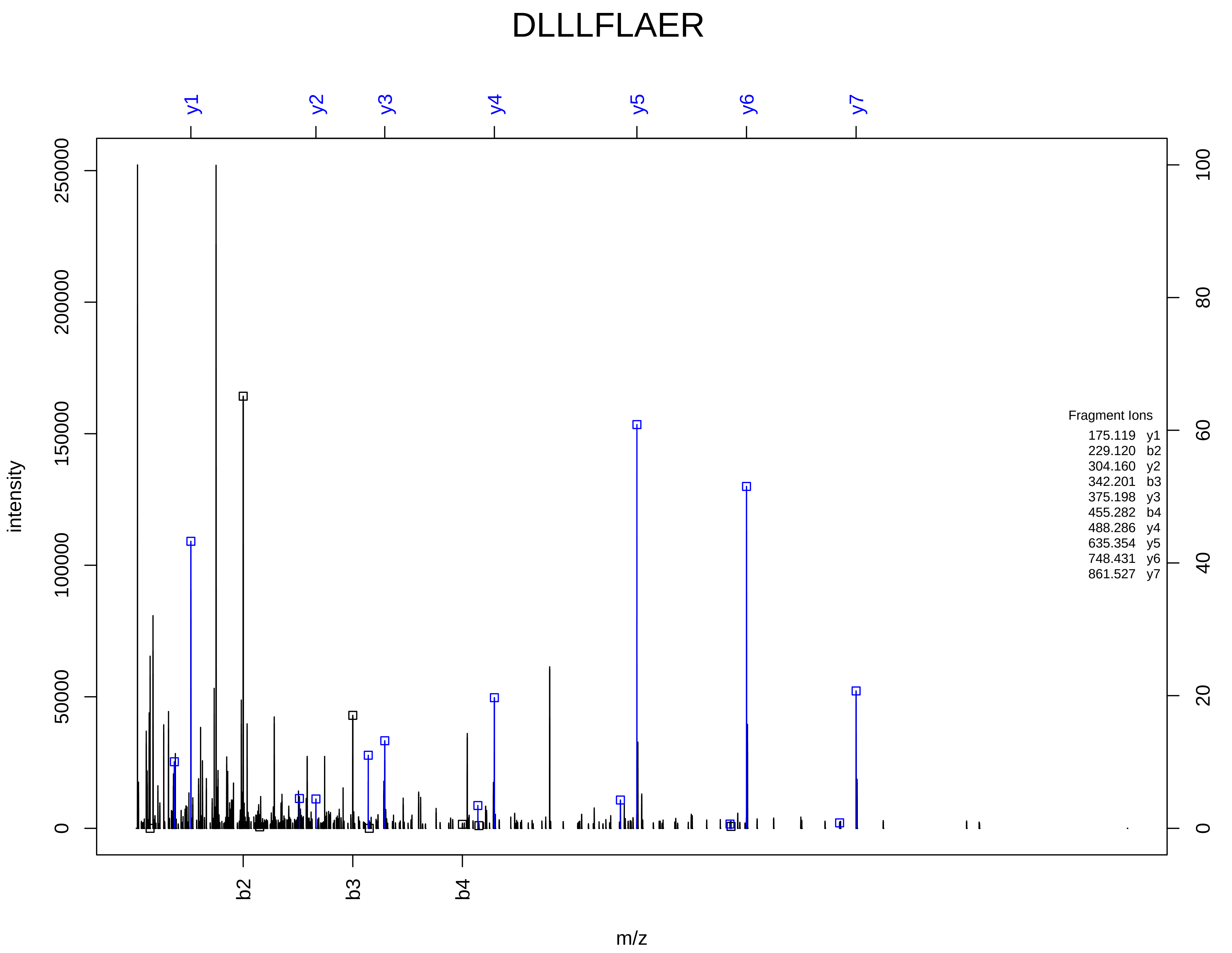
DLLLFLAER
y1
y2
y3
y4
y5
y6
y7
b2
b3
b4
intensity
m/z
0
50000
100000
150000
200000
250000
0
20
40
60
80
100
Fragment Ions
175.119 y1
229.120 b2
304.160 y2
342.201 b3
375.198 y3
455.282 b4
488.286 y4
635.354 y5
748.431 y6
861.527 y7
DLLLFLAER / File: christine_pool_03.raw; SpectrumID: ; scans: 23322

VACAEEWQESR

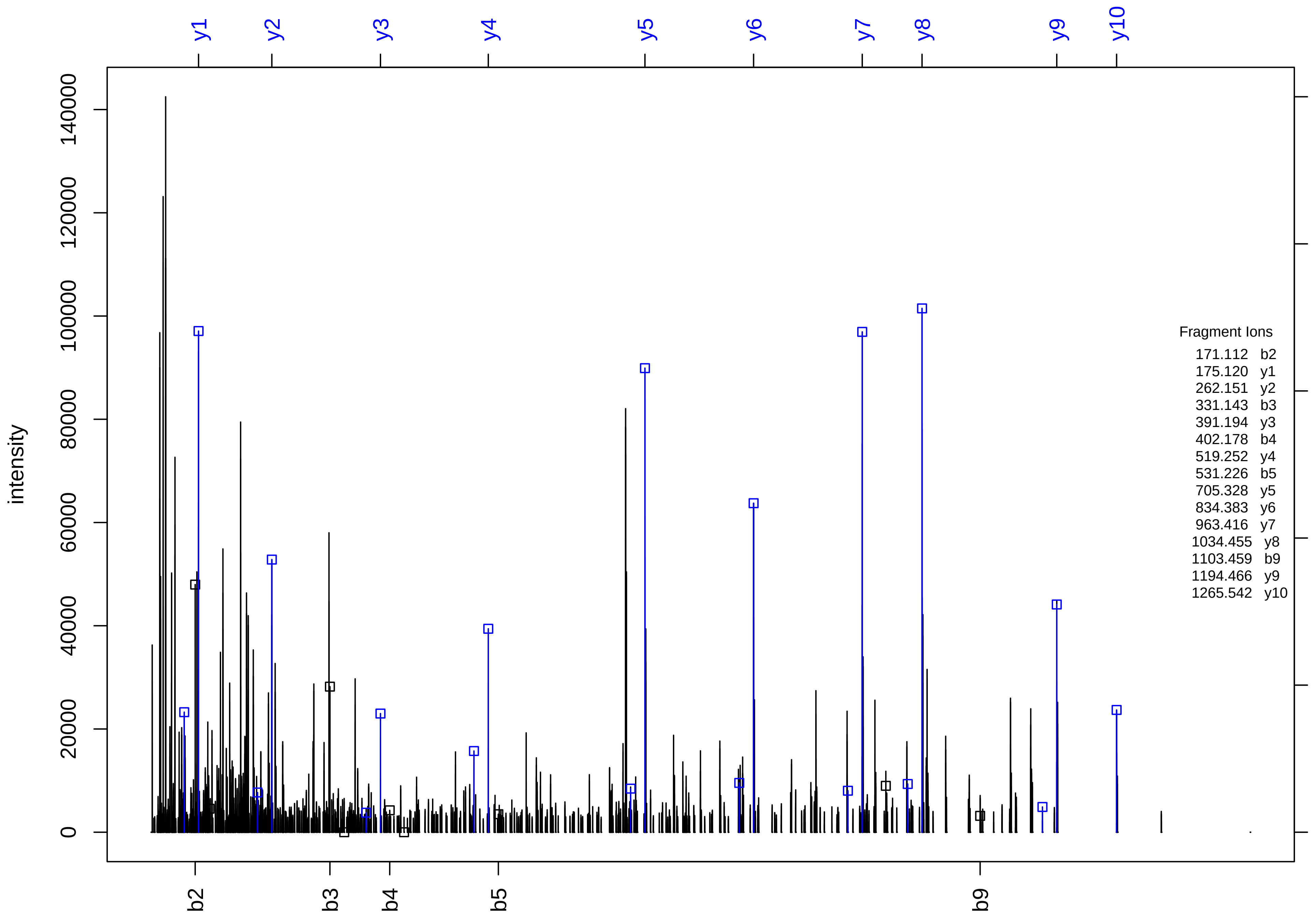

VACAEEWQESR / File: christine_pool_03.raw; SpectrumID: ; scans: 4396

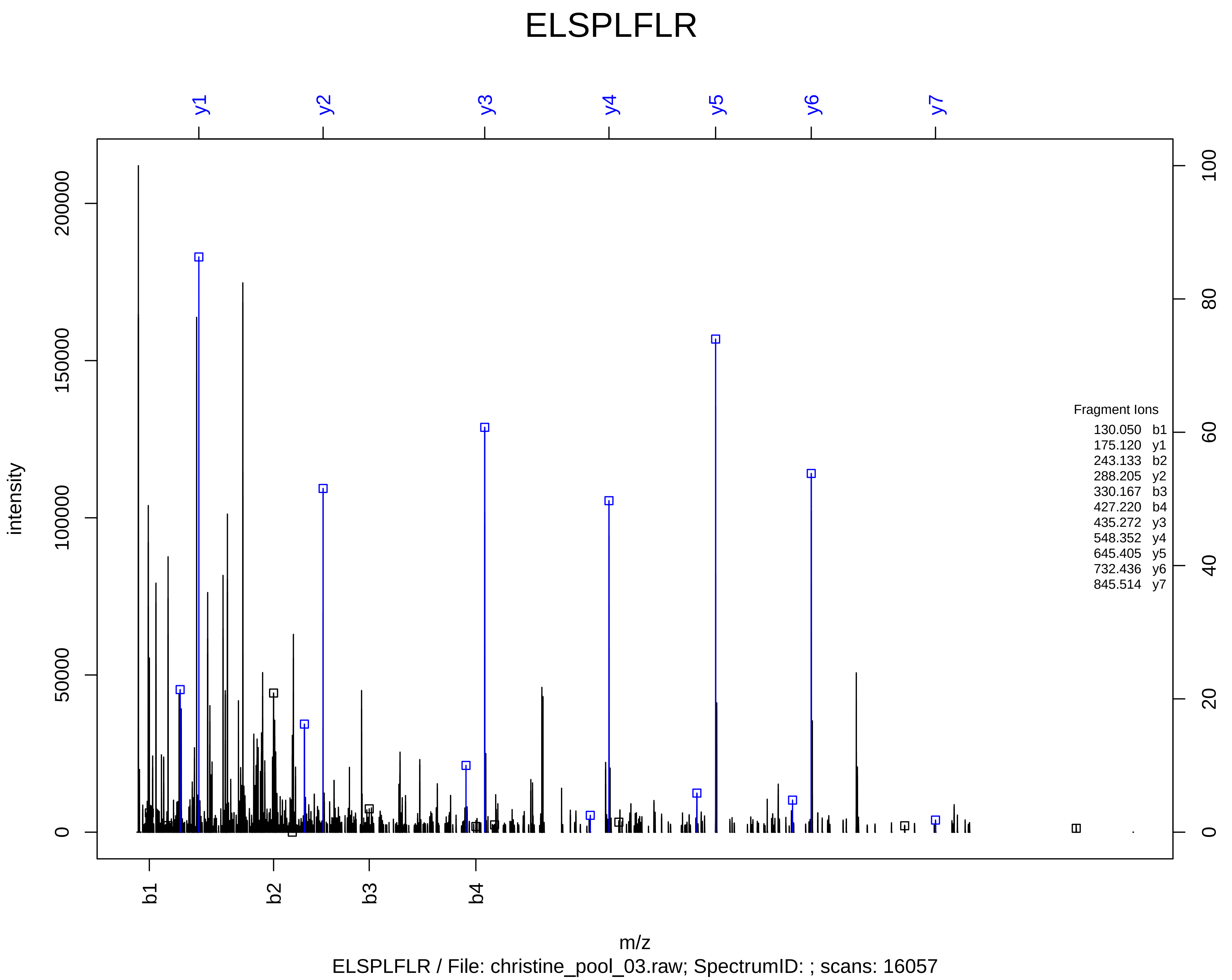

ELSPLFLR
y1
y2
y3
y4
y5
y6
y7
intensity
0
50000
100000
150000
200000
0
20
40
60
80
100
Fragment Ions
130.050 b1
175.120 y1
243.133 b2
288.205 y2
330.167 b3
427.220 b4
435.272 y3
548.352 y4
645.405 y5
732.436 y6
845.514 y7
b1
b2
b3
b4
m/z
ELSPLFLR / File: christine_pool_03.raw; SpectrumID: ; scans: 16057

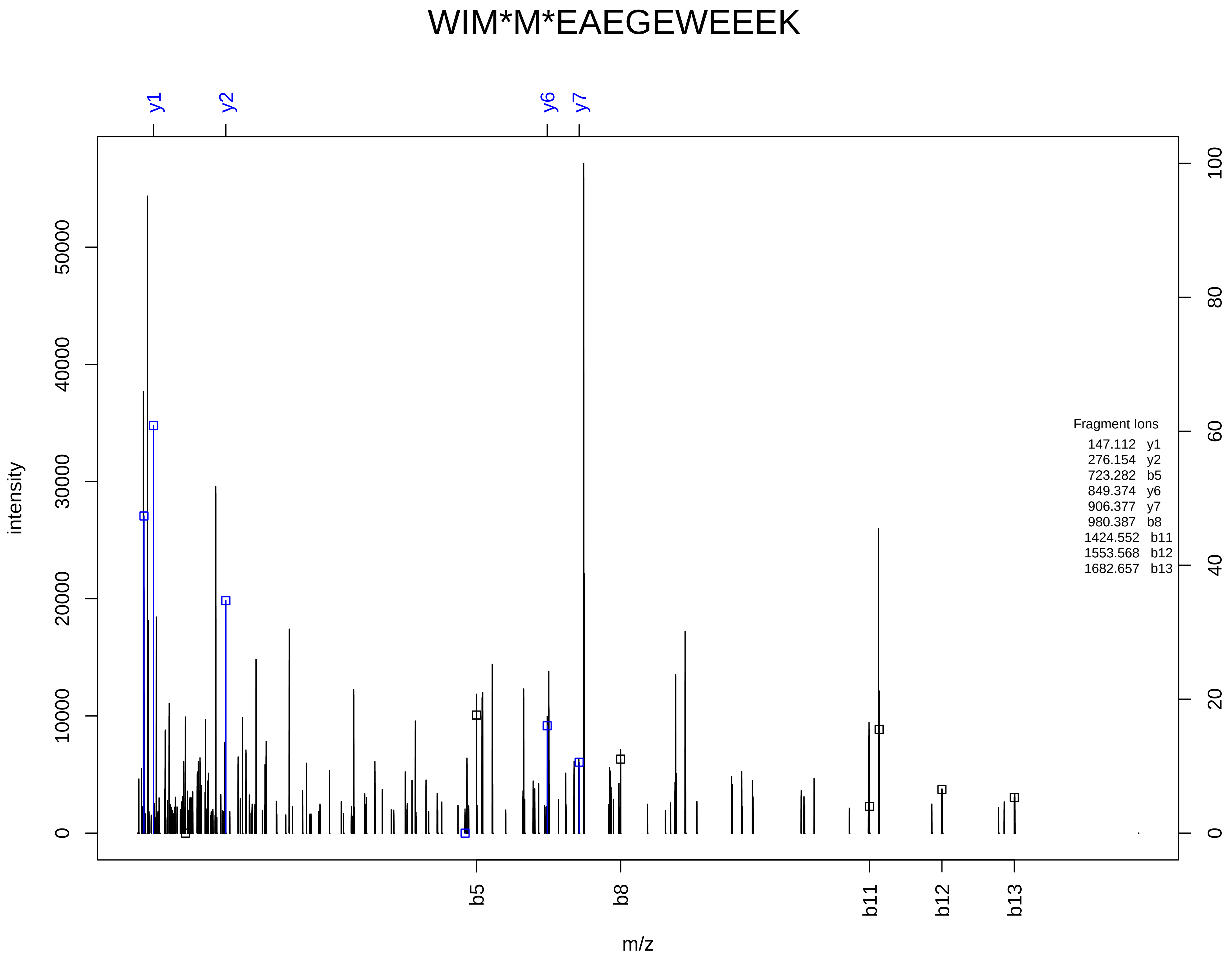

WIMMEAEGEWEEEK / File: christine_pool_03.raw; SpectrumID: ; scans: 3095

ENPDIVNQSQQAQAR

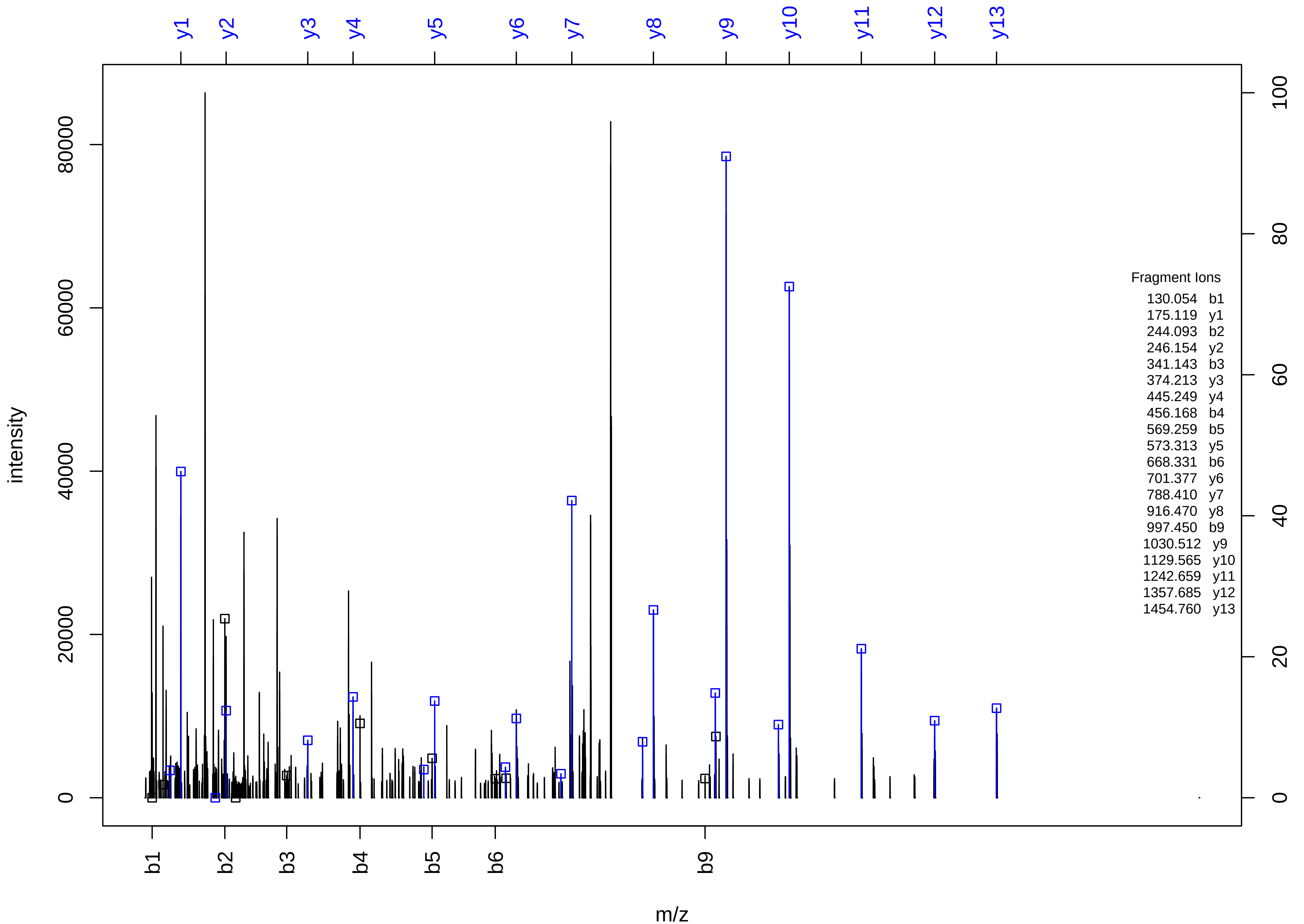

ENPDIVNQSQQAQAR / File: christine_pool_03.raw; SpectrumID: ; scans: 3883

LTPEELER

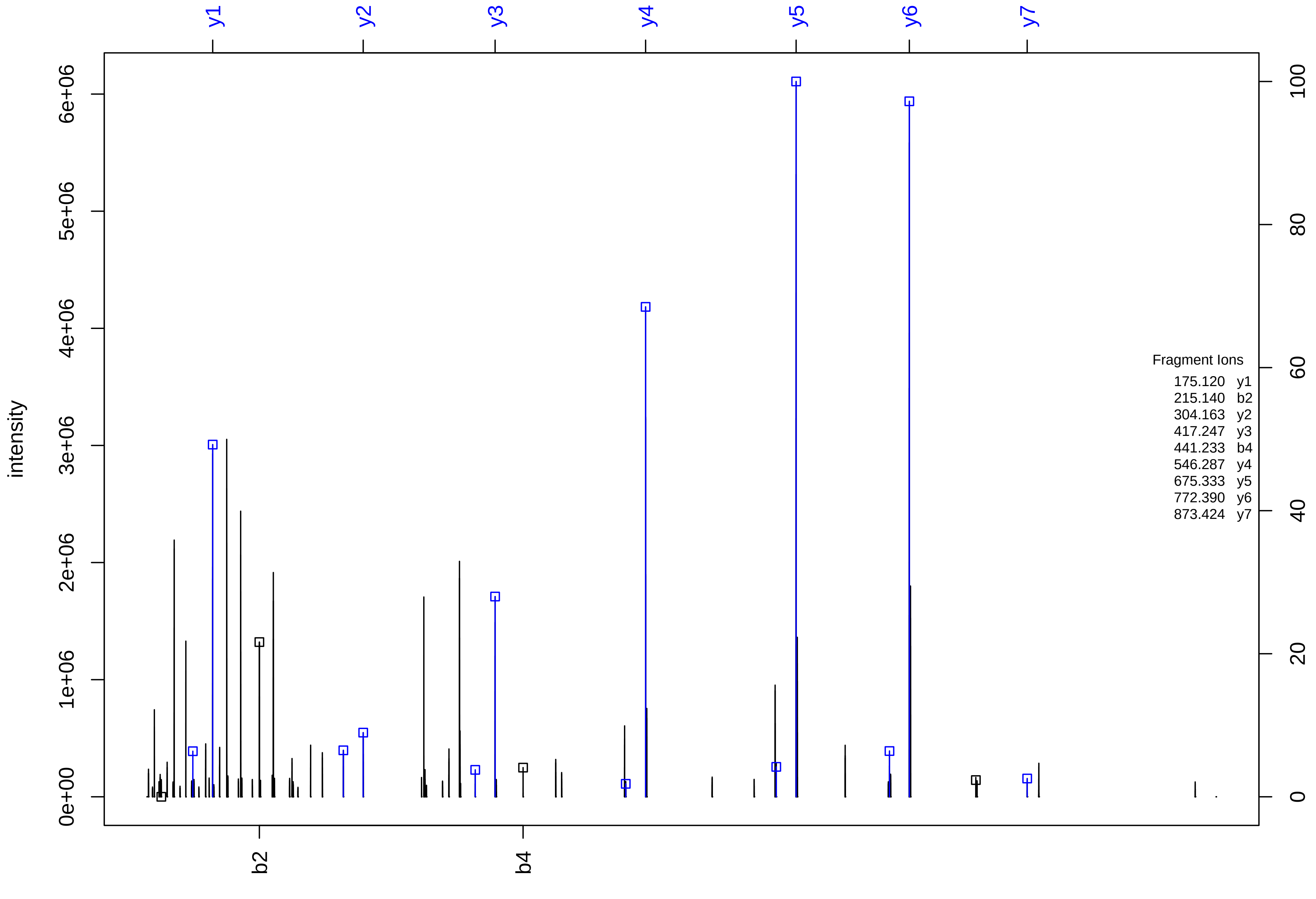

LTPEELER / File: christine_pool_03.raw; SpectrumID: ; scans: 5201

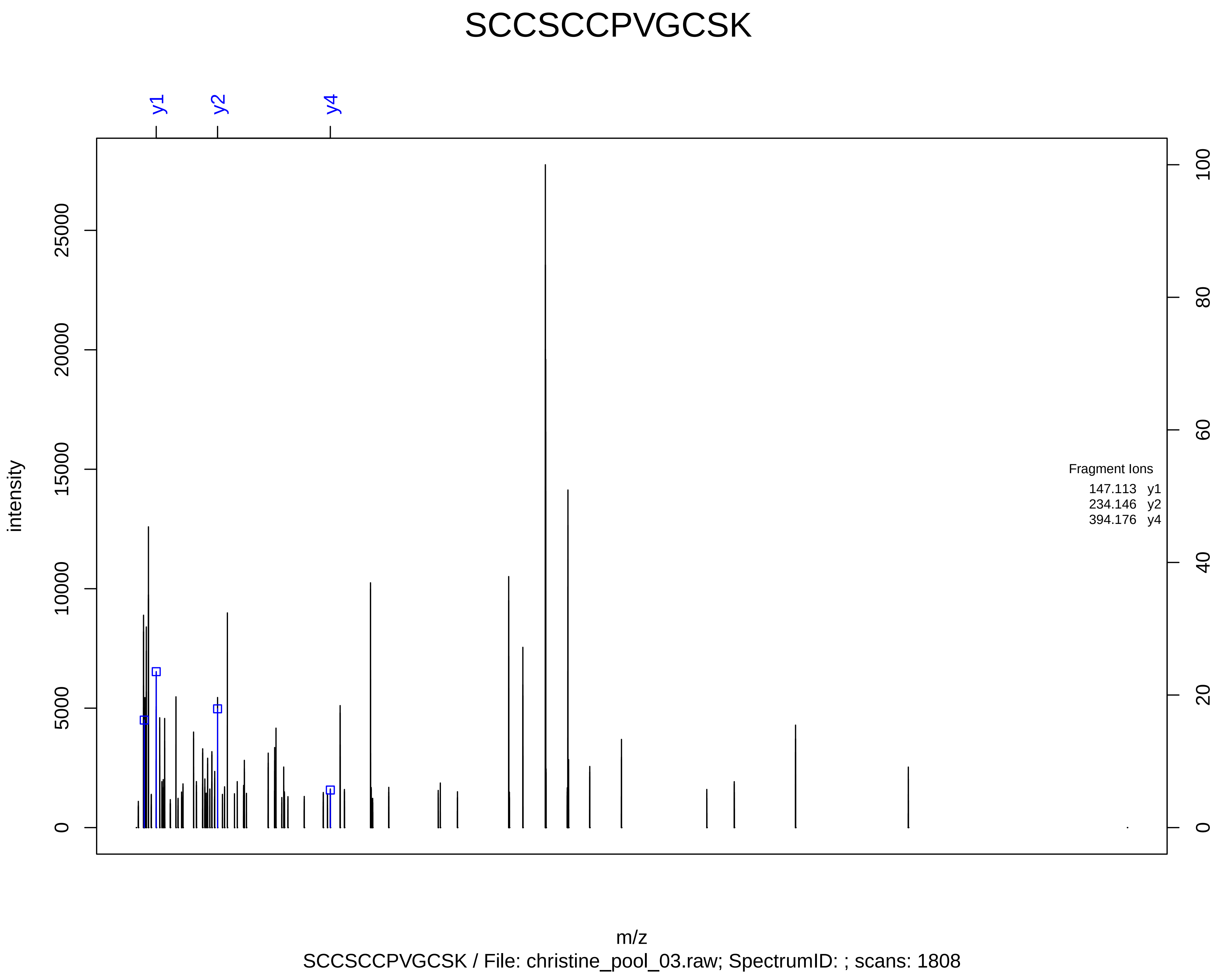
SCCSCCPVGCSK
y1
y2
y4
intensity
0
5000
10000
15000
20000
25000
0
20
40
60
80
100
Fragment Ions
147.113 y1
234.146 y2
394.176 y4
m/z
SCCSCCPVGCSK / File: christine_pool_03.raw; SpectrumID: ; scans: 1808

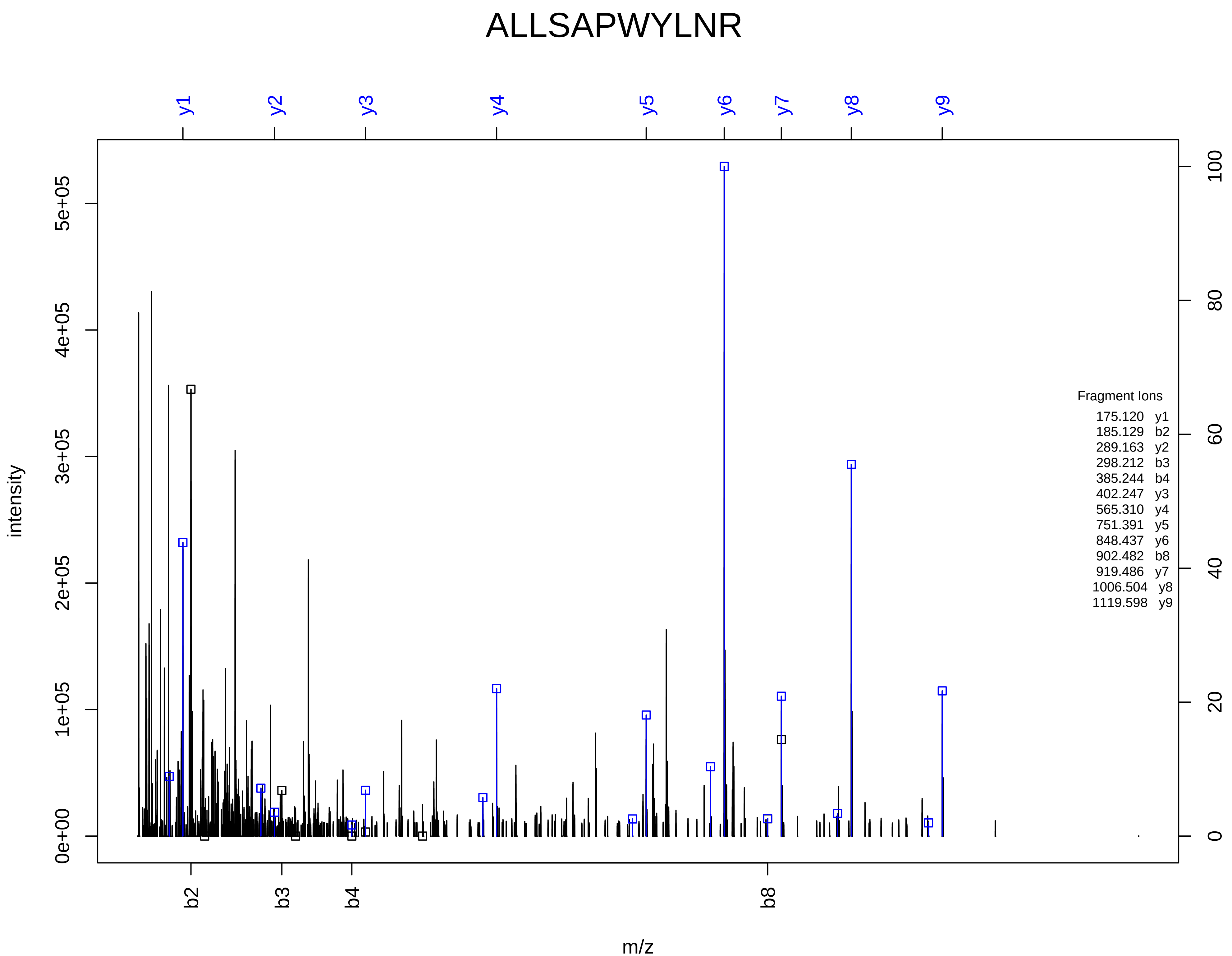

ALLSAPWYLNR / File: christine_pool_03.raw; SpectrumID: ; scans: 17746

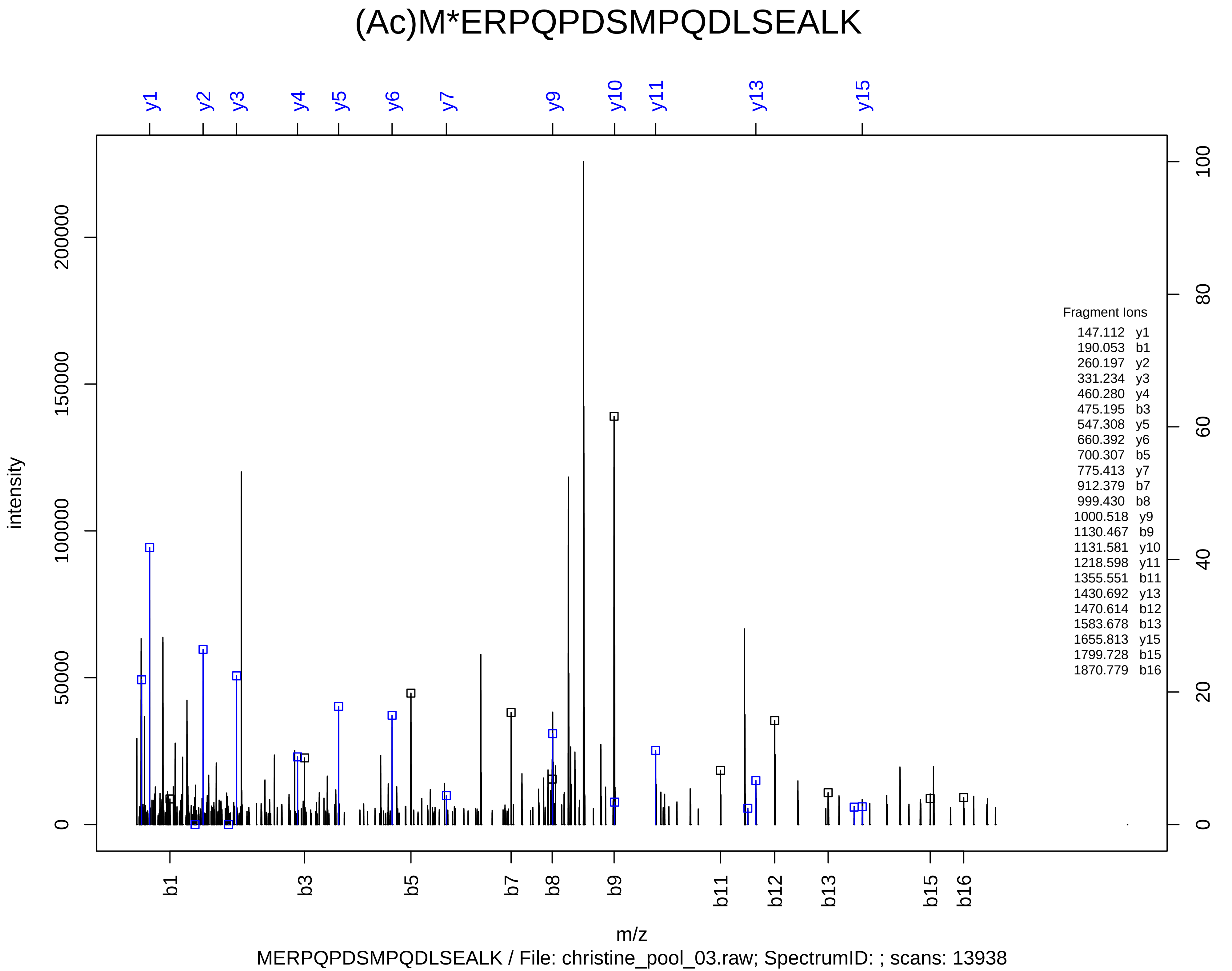
(Ac)M*ERPQPDSMPQDLSEALK
y1
y2
y3
y4
y5
y6
y7
y9
y10
y11
y13
y15
intensity
0
50000
100000
150000
200000
0
20
40
60
80
100
Fragment Ions
147.112 y1
190.053 b1
260.197 y2
331.234 y3
460.280 y4
475.195 b3
547.308 y5
660.392 y6
700.307 b5
775.413 y7
912.379 b7
999.430 b8
1000.518 y9
1130.467 b9
1131.581 y10
1218.598 y11
1355.551 b11
1430.692 y13
1470.614 b12
1583.678 b13
1655.813 y15
1799.728 b15
1870.779 b16
b1
b3
b5
b7
b8
b9
b11
b12
b13
b15
b16
m/z
MERPQPDSMPQDLSEALK / File: christine_pool_03.raw; SpectrumID: ; scans: 13938

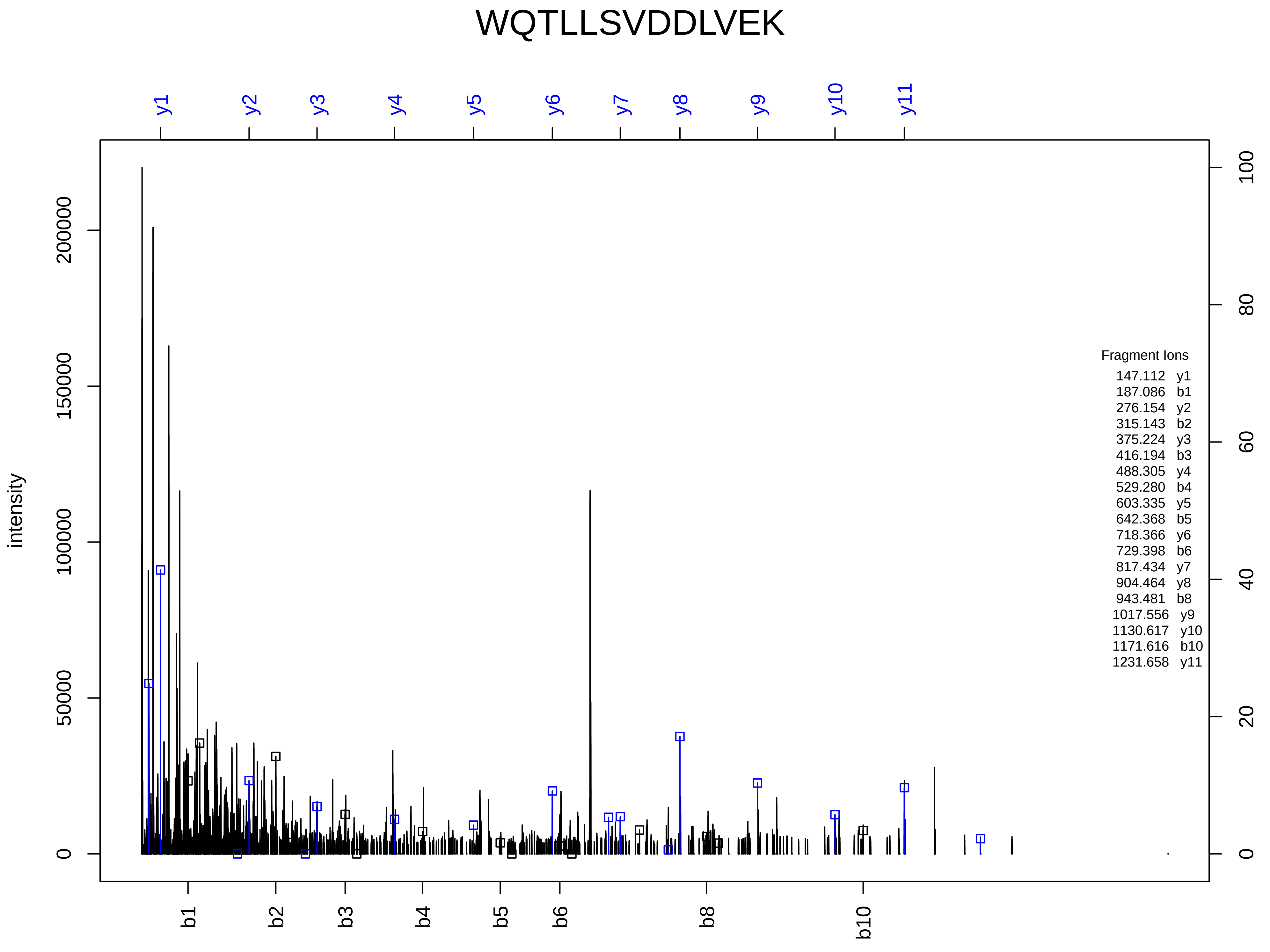

WQTLLSVDDLVEK / File: christine_pool_03.raw; SpectrumID: ; scans: 20210

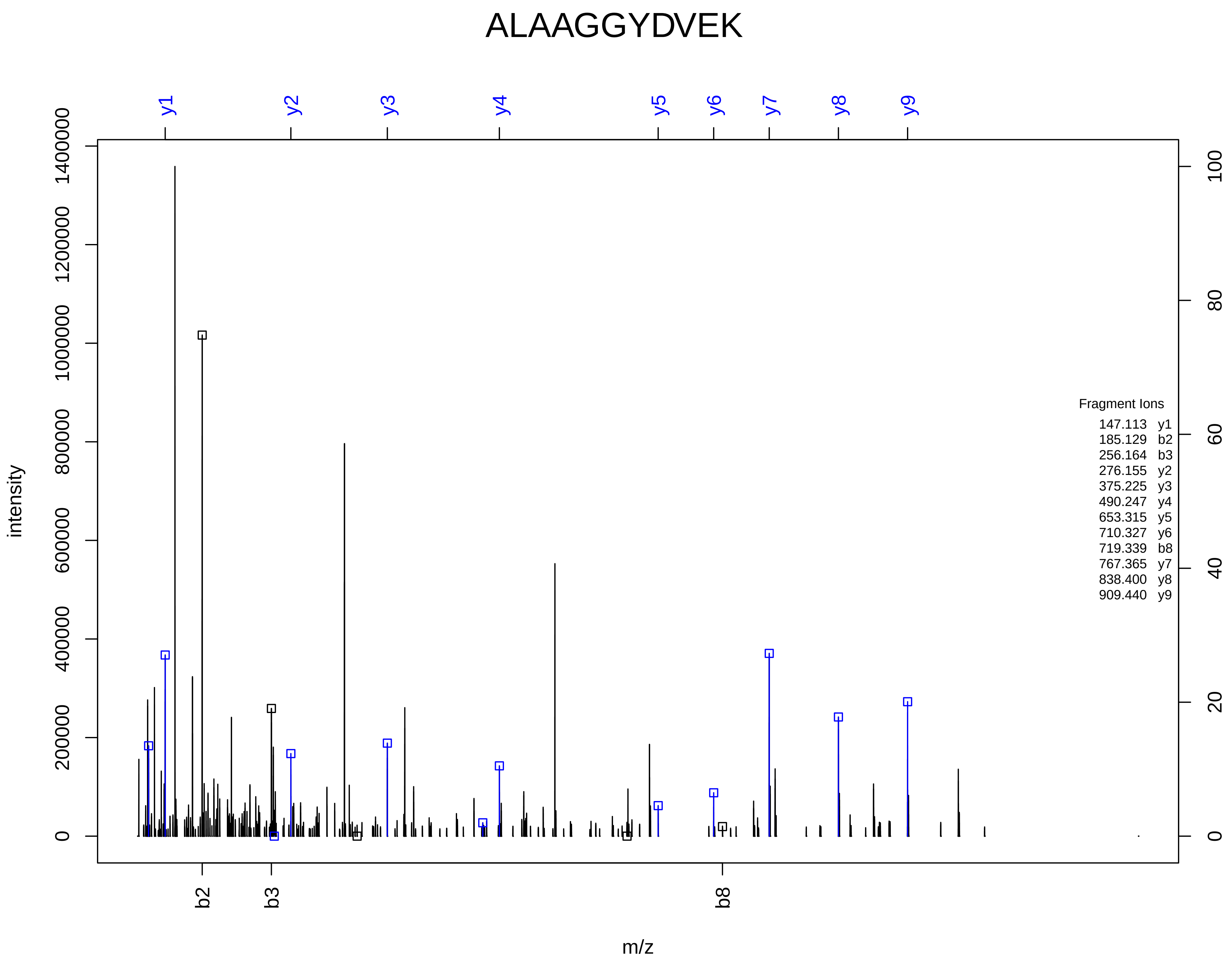

ALAAGGYDVEK
y1
y2
y3
y4
y5
y6
y7
y8
y9
intensity
0
200000
400000
600000
800000
1000000
1200000
1400000
0
20
40
60
80
100
b2
b3
b8
m/z
Fragment Ions
147.113 y1
185.129 b2
256.164 b3
276.155 y2
375.225 y3
490.247 y4
653.315 y5
710.327 y6
719.339 b8
767.365 y7
838.400 y8
909.440 y9
ALAAGGYDVEK / File: christine_pool_03.raw; SpectrumID: ; scans: 5046

ILFDGIPLEK

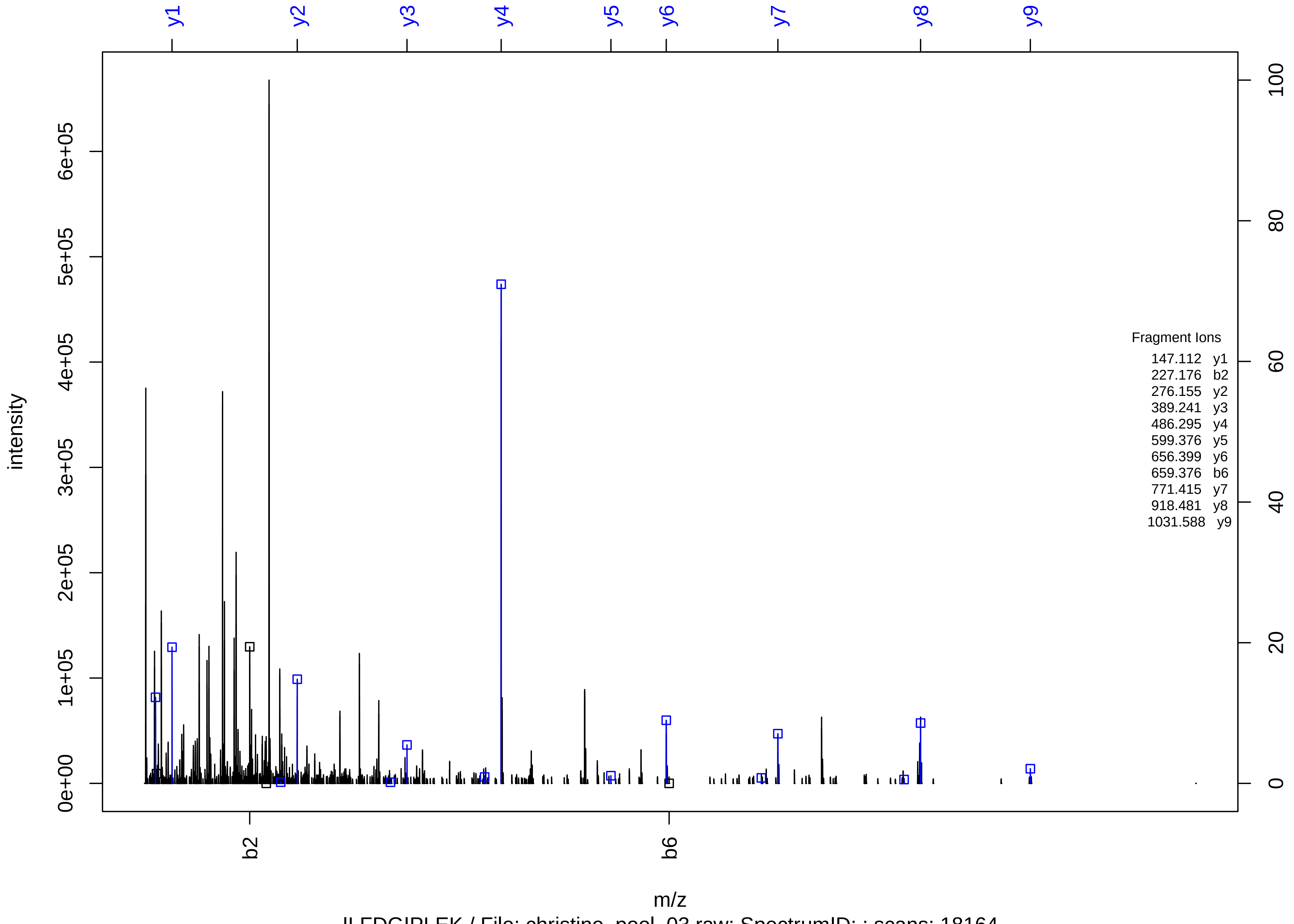

ILFDGIPLEK / File: christine_pool_03.raw; SpectrumID: ; scans: 18164

IPLPGAEMLEEEPLYVNAK

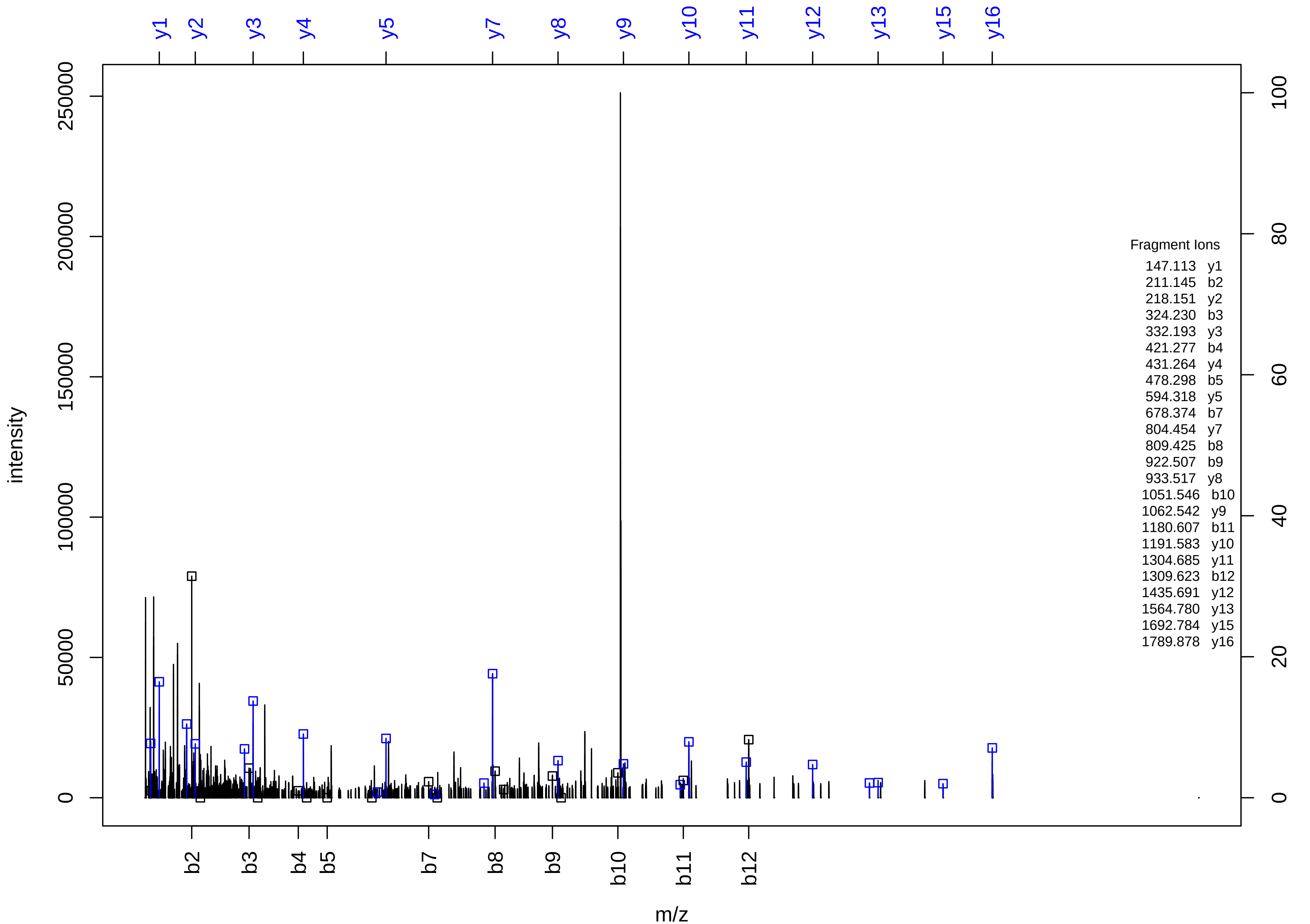

IPLPGAEMLEEEPLYVNAK / File: christine_pool_03.raw; SpectrumID: ; scans: 21856

AVLGPLVGAVDQGTSSTR

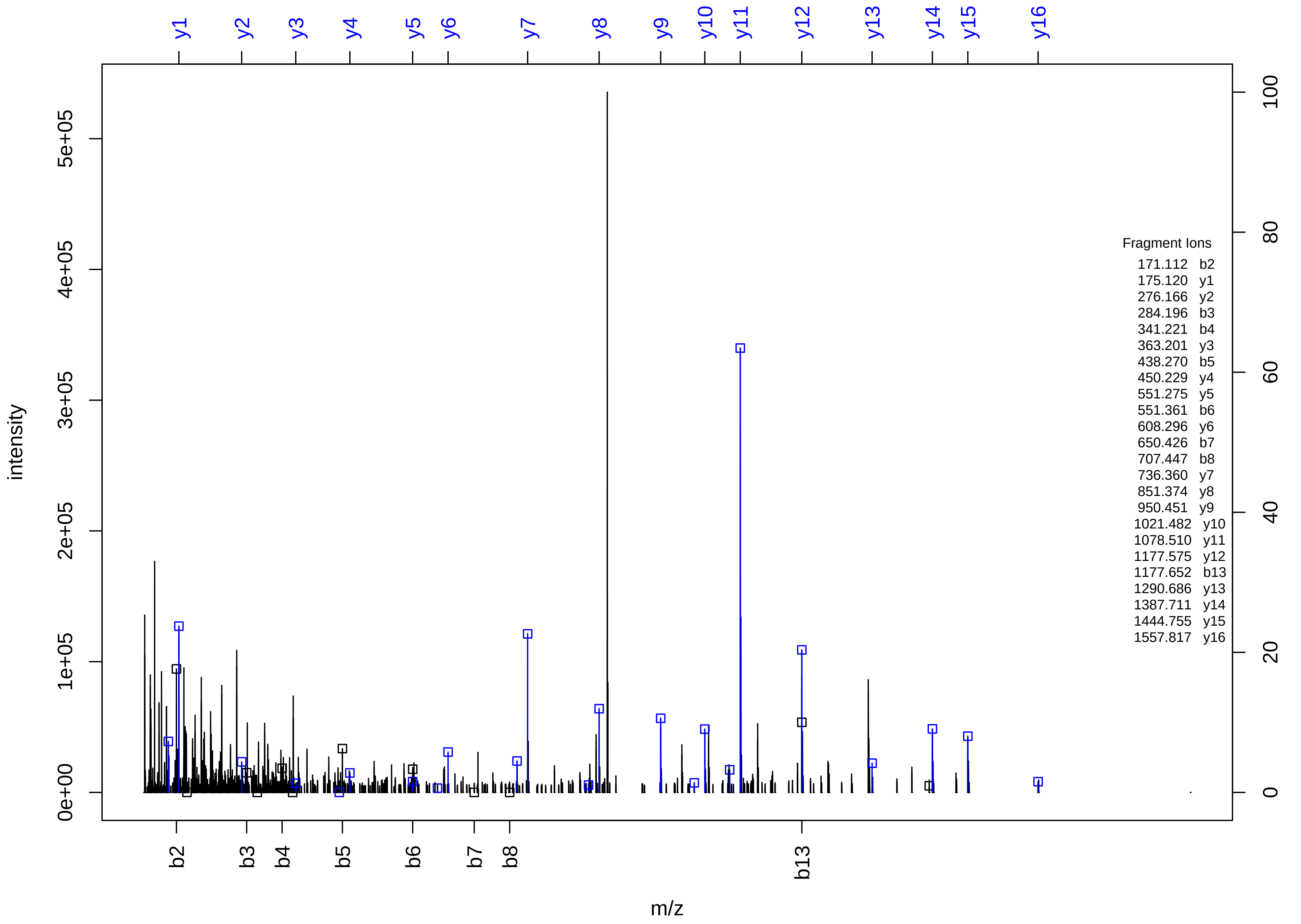

AVLGPLVGAVDQGTSSTR / File: christine_pool_03.raw; SpectrumID: ; scans: 14768

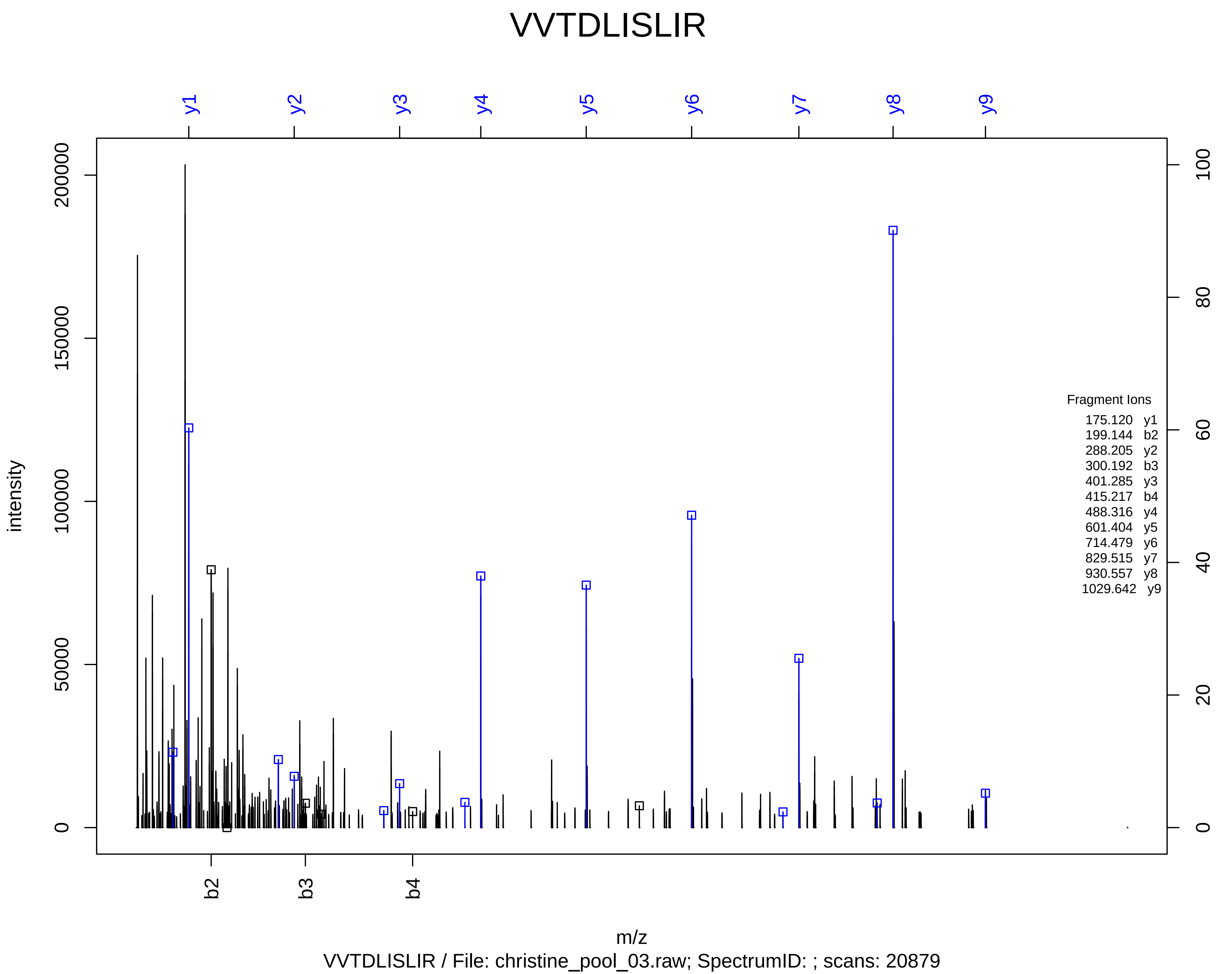

VVTDLISLIR
y1
y2
y3
y4
y5
y6
y7
y8
y9
b2
b3
b4
intensity
0
50000
100000
150000
200000
0
20
40
60
80
100
Fragment Ions
175.120 y1
199.144 b2
288.205 y2
300.192 b3
401.285 y3
415.217 b4
488.316 y4
601.404 y5
714.479 y6
829.515 y7
930.557 y8
1029.642 y9
m/z
VVTDLISLIR / File: christine_pool_03.raw; SpectrumID: ; scans: 20879

AQAAAPASVPAQAPK

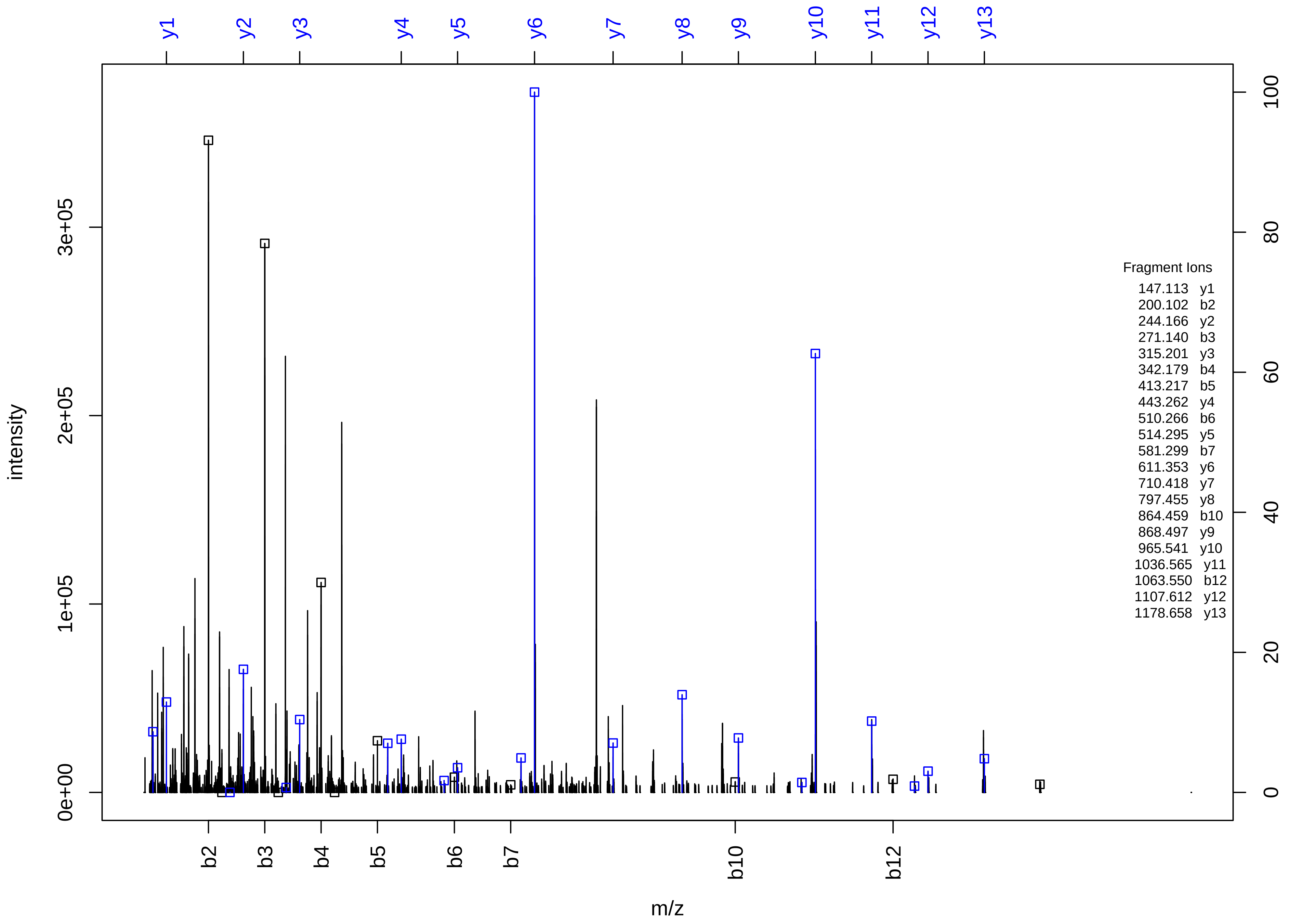

AQAAAPASVPAQAPK / File: christine_pool_03.raw; SpectrumID: ; scans: 4216

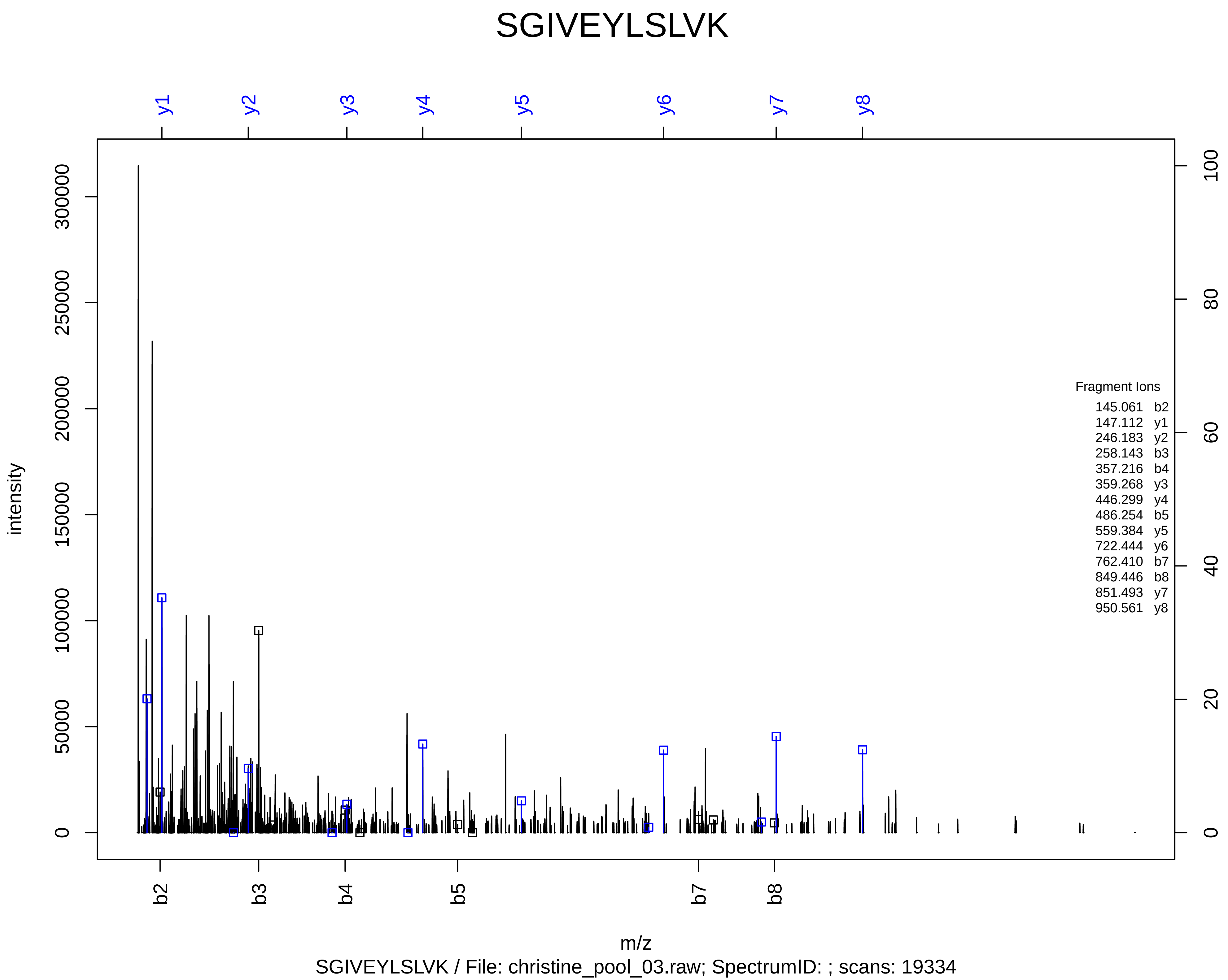
SGIVEYLSLVK
y1
y2
y3
y4
y5
y6
y7
y8
b2
b3
b4
b5
b7
b8
intensity
m/z
Fragment Ions
145.061 b2
147.112 y1
246.183 y2
258.143 b3
357.216 b4
359.268 y3
446.299 y4
486.254 b5
559.384 y5
722.444 y6
762.410 b7
849.446 b8
851.493 y7
950.561 y8
SGIVEYLSLVK / File: christine_pool_03.raw; SpectrumID: ; scans: 19334

SLETSLVPLSDPK

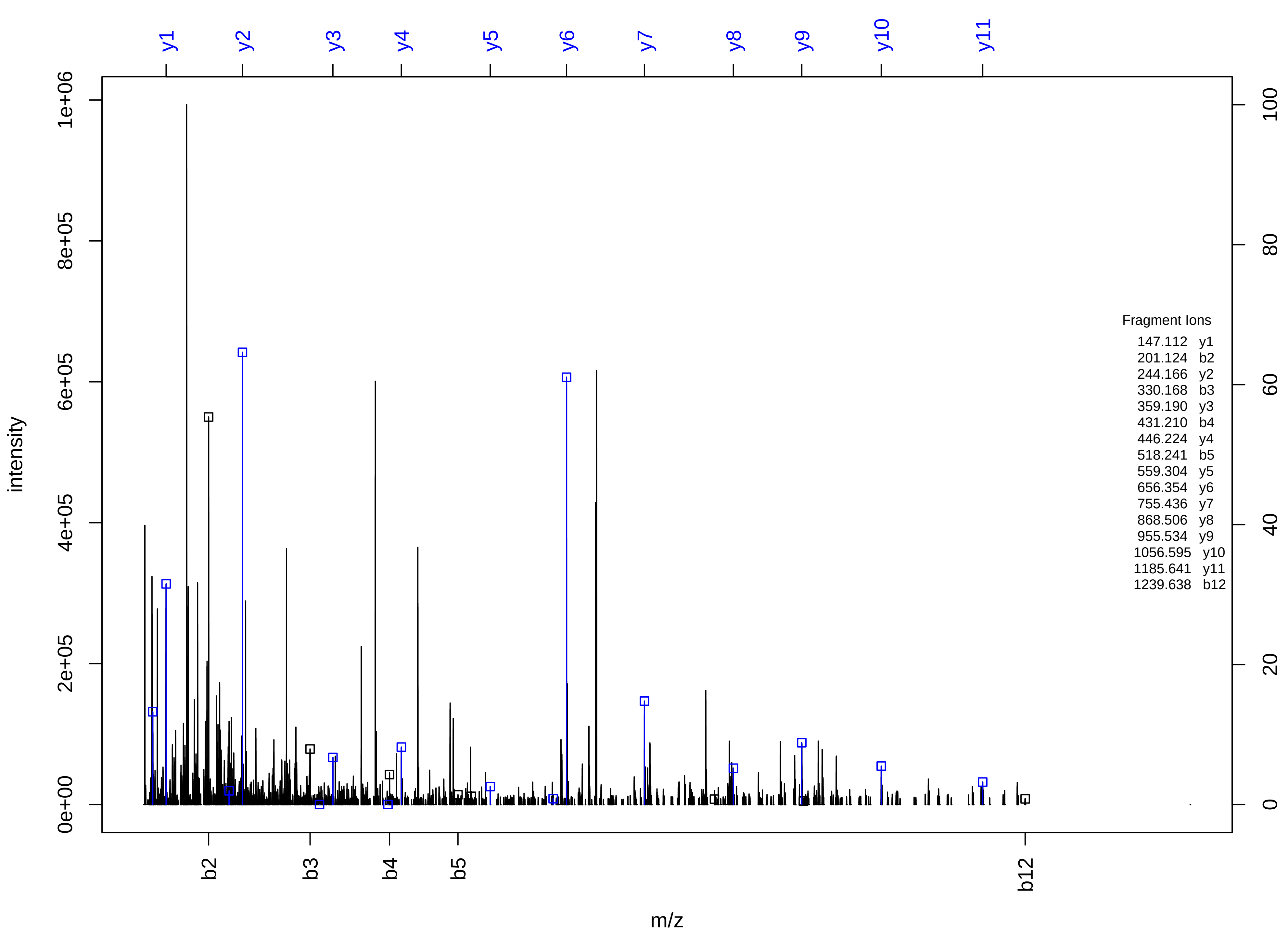

SLETSLVPLSDPK / File: christine_pool_03.raw; SpectrumID: ; scans: 14464

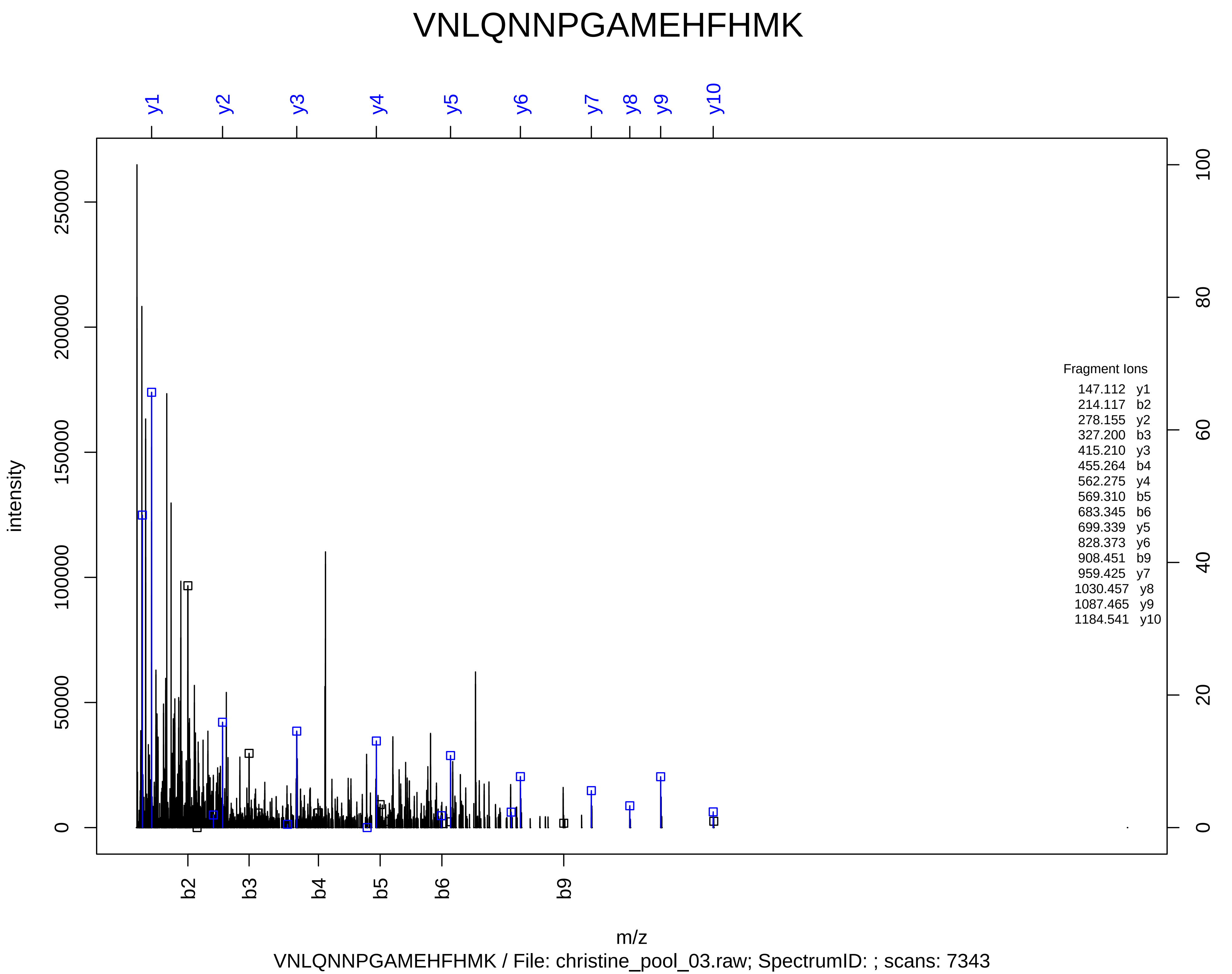
VNLQNNPGAMEHFHMK
y1
y2
y3
y4
y5
y6
y7
y8
y9
y10
intensity
0
50000
100000
150000
200000
250000
0
20
40
60
80
100
b2
b3
b4
b5
b6
b9
m/z
Fragment Ions
147.112 y1
214.117 b2
278.155 y2
327.200 b3
415.210 y3
455.264 b4
562.275 y4
569.310 b5
683.345 b6
699.339 y5
828.373 y6
908.451 b9
959.425 y7
1030.457 y8
1087.465 y9
1184.541 y10
VNLQNNPGAMEHFHMK / File: christine_pool_03.raw; SpectrumID: ; scans: 7343

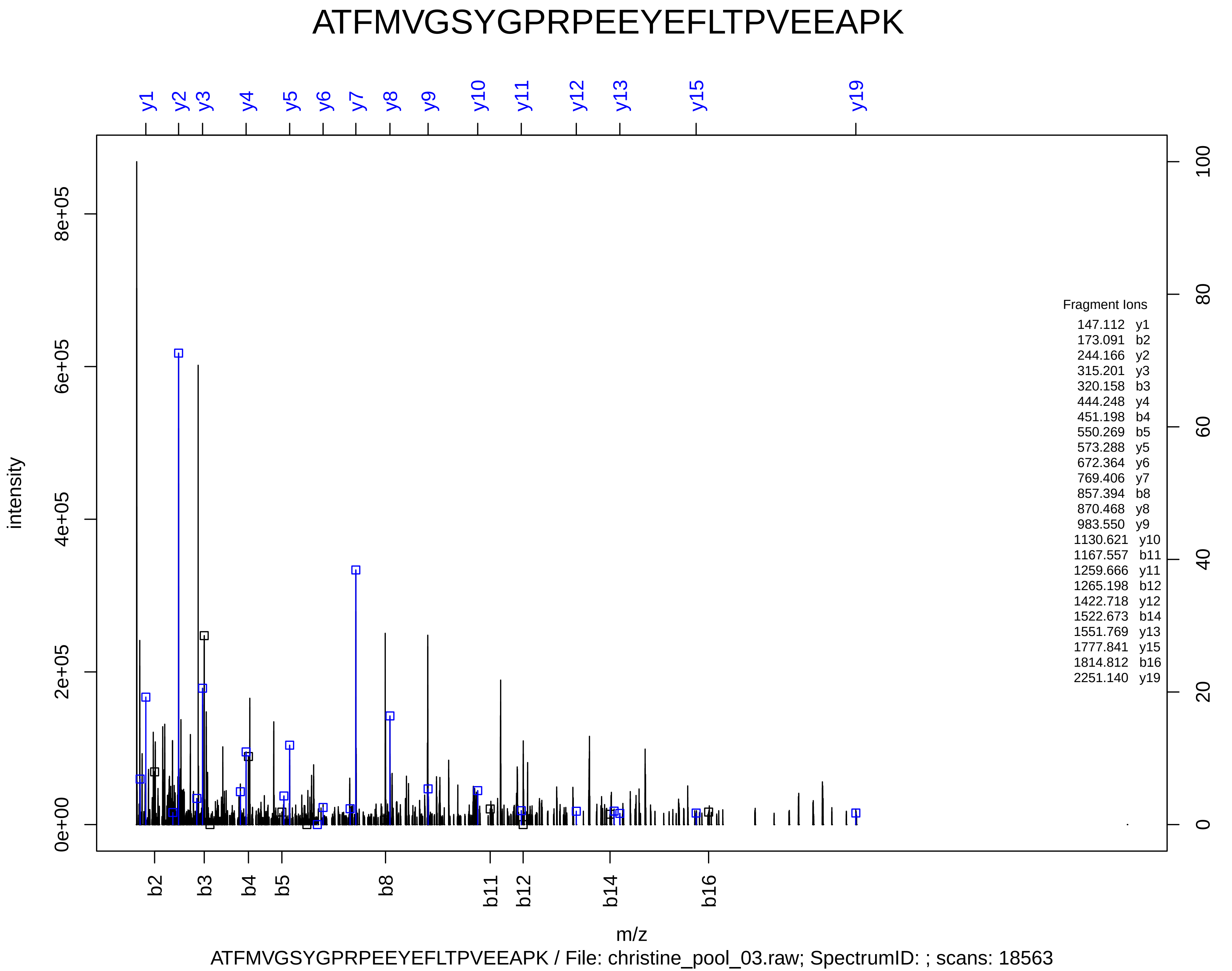
ATFMVGSYGPRPEEYEFLTPVEEAPK
intensity
m/z
Fragment Ions
147.112 y1
173.091 b2
244.166 y2
315.201 y3
320.158 b3
444.248 y4
451.198 b4
550.269 b5
573.288 y5
672.364 y6
769.406 y7
857.394 b8
870.468 y8
983.550 y9
1130.621 y10
1167.557 b11
1259.666 y11
1265.198 b12
1422.718 y12
1522.673 b14
1551.769 y13
1777.841 y15
1814.812 b16
2251.140 y19
ATFMVGSYGPRPEEYEFLTPVEEAPK / File: christine_pool_03.raw; SpectrumID: ; scans: 18563

QIWLGLFDDR

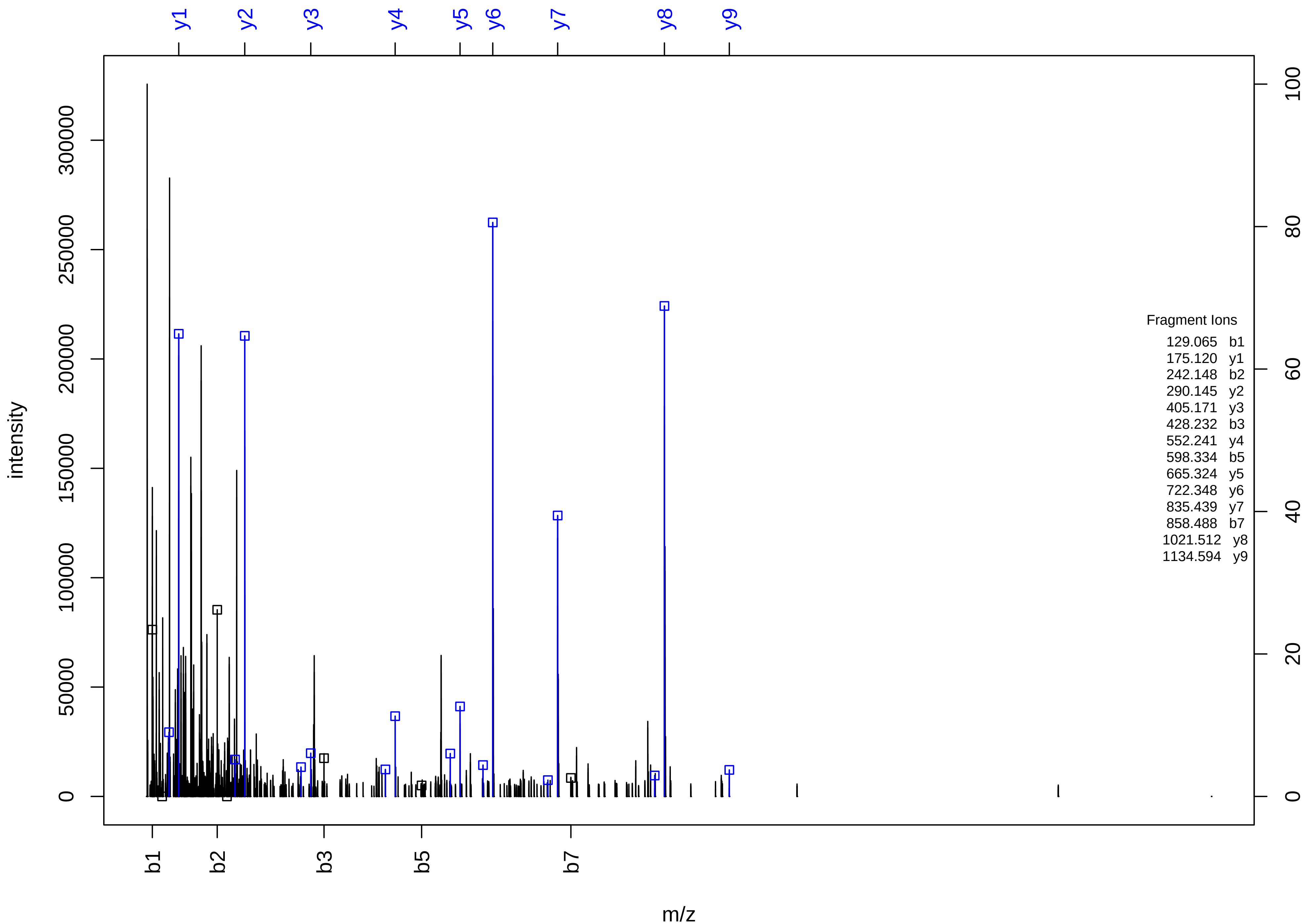

QIWLGLFDDR / File: christine_pool_03.raw; SpectrumID: ; scans: 21451

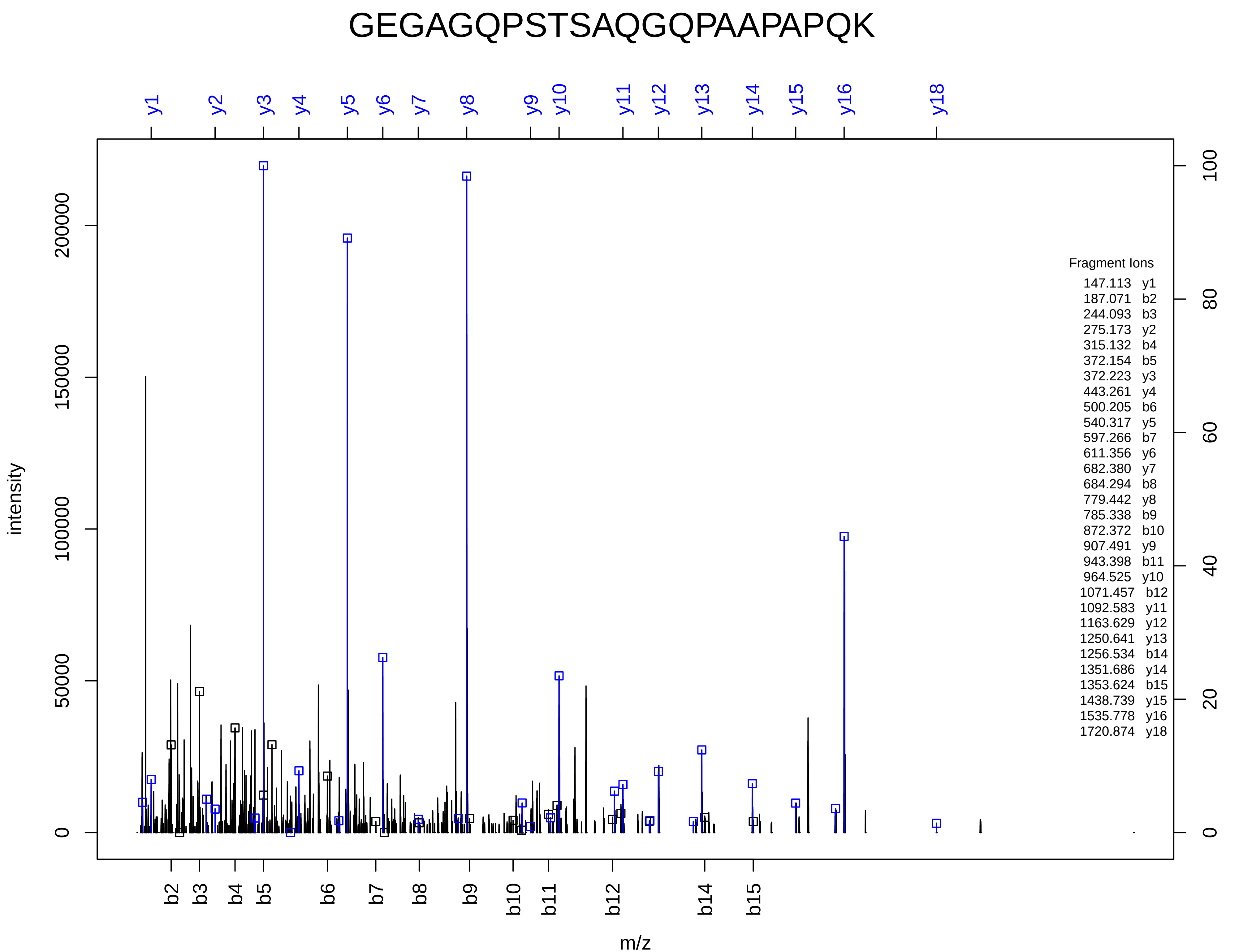

GEGAGQPSTSAQGQPAAPAPQK / File: christine_pool_03.raw; SpectrumID: ; scans: 2810

LAAQLDALR

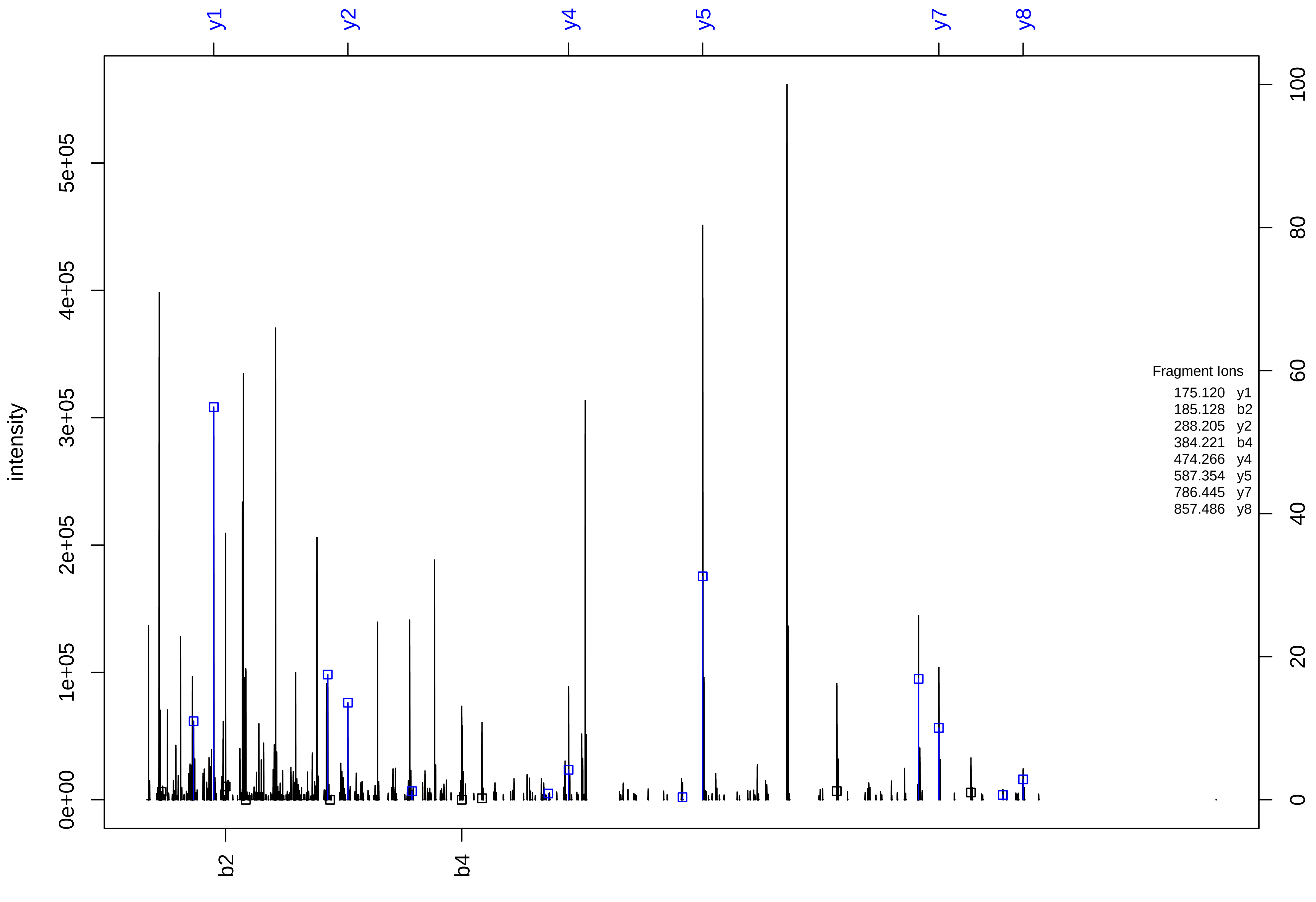

LAAQLDALR / File: christine_pool_03.raw; SpectrumID: ; scans: 17487

EQEAEPEEQEEDSSSDPR

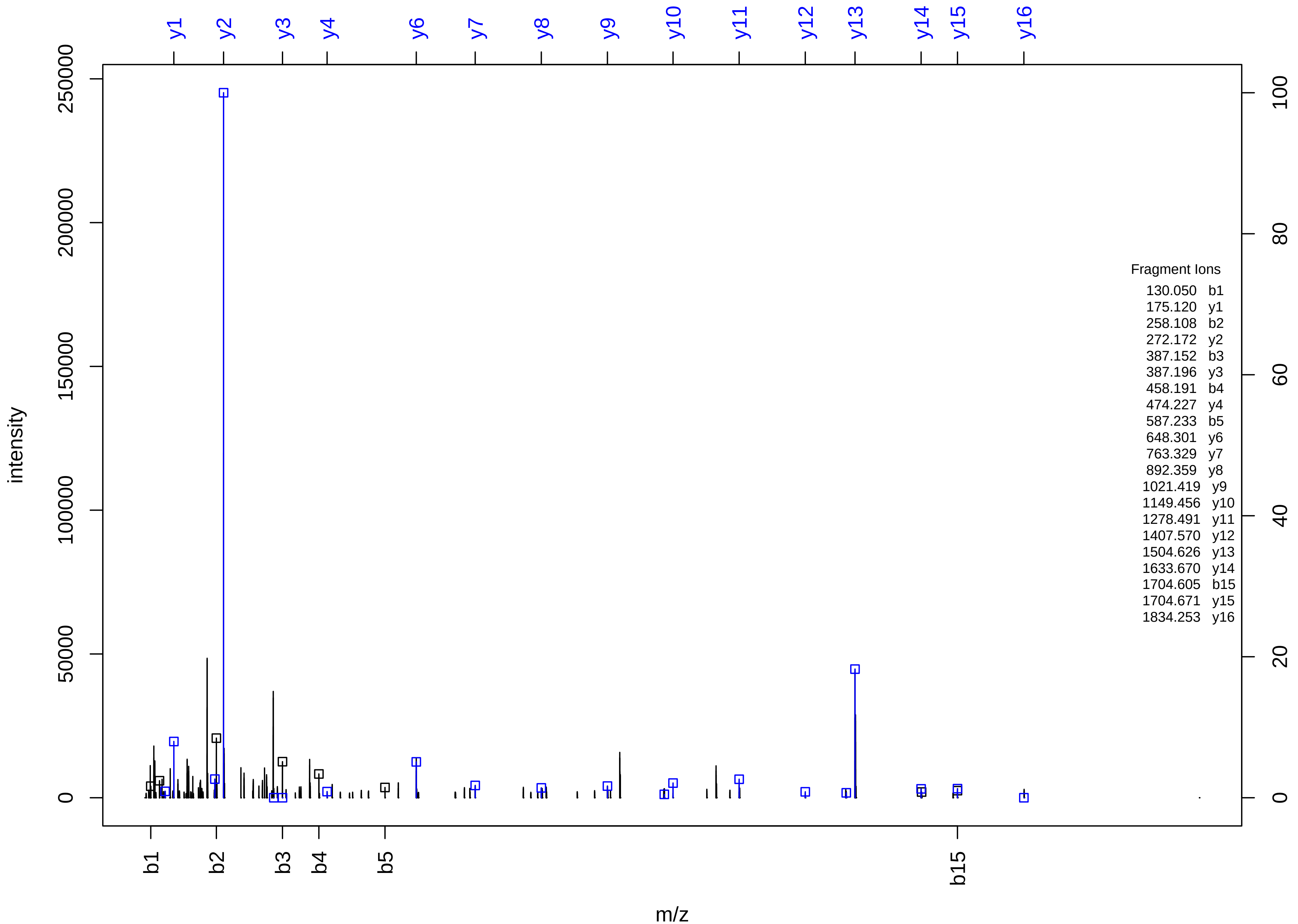

EQEAEPEEQEEDSSSDPR / File: christine_pool_03.raw; SpectrumID: ; scans: 2361

ALLANALTSALR

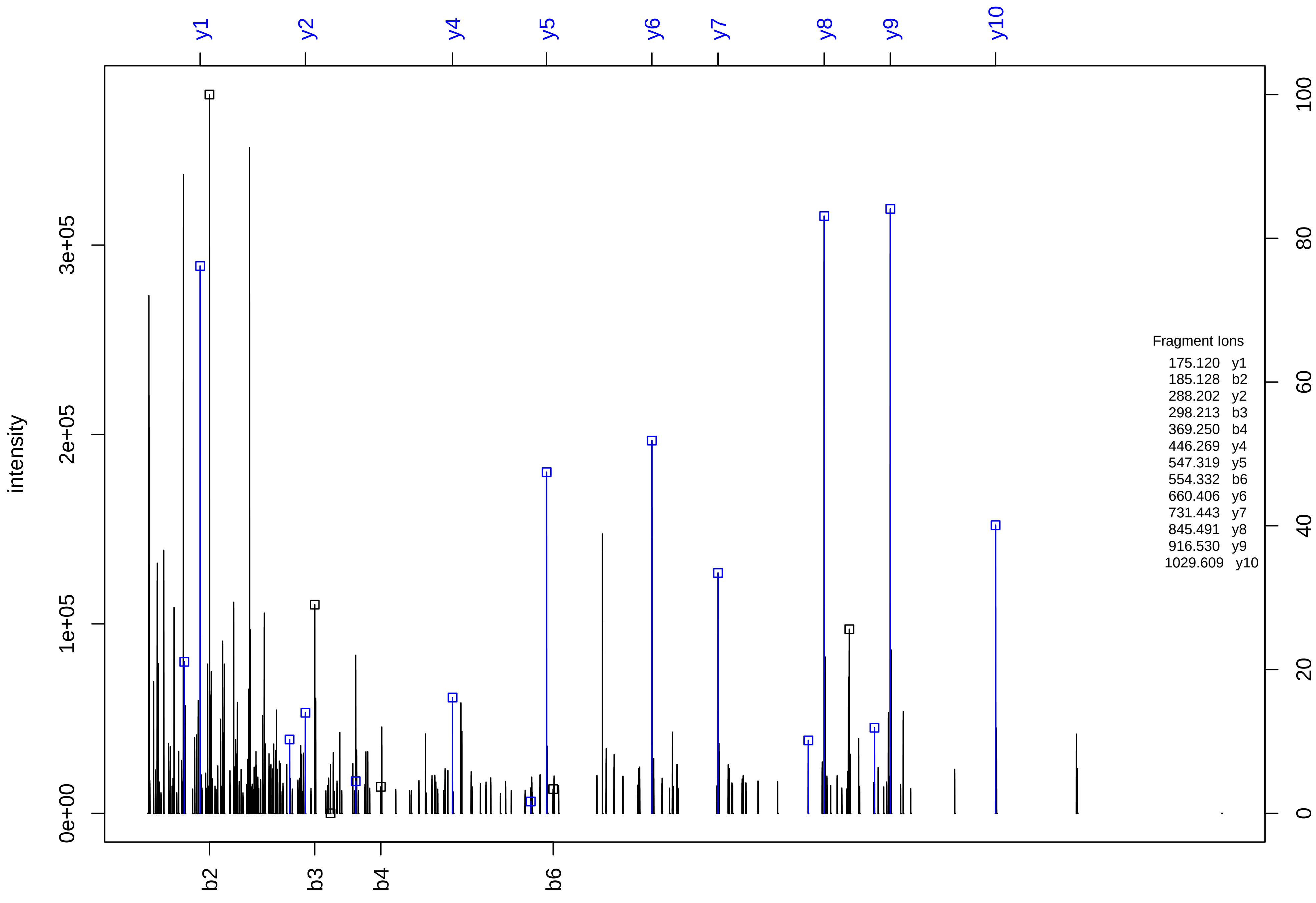

ALLANALTSALR / File: christine_pool_03.raw; SpectrumID: ; scans: 17725

DINLASFIEQVAVSMT

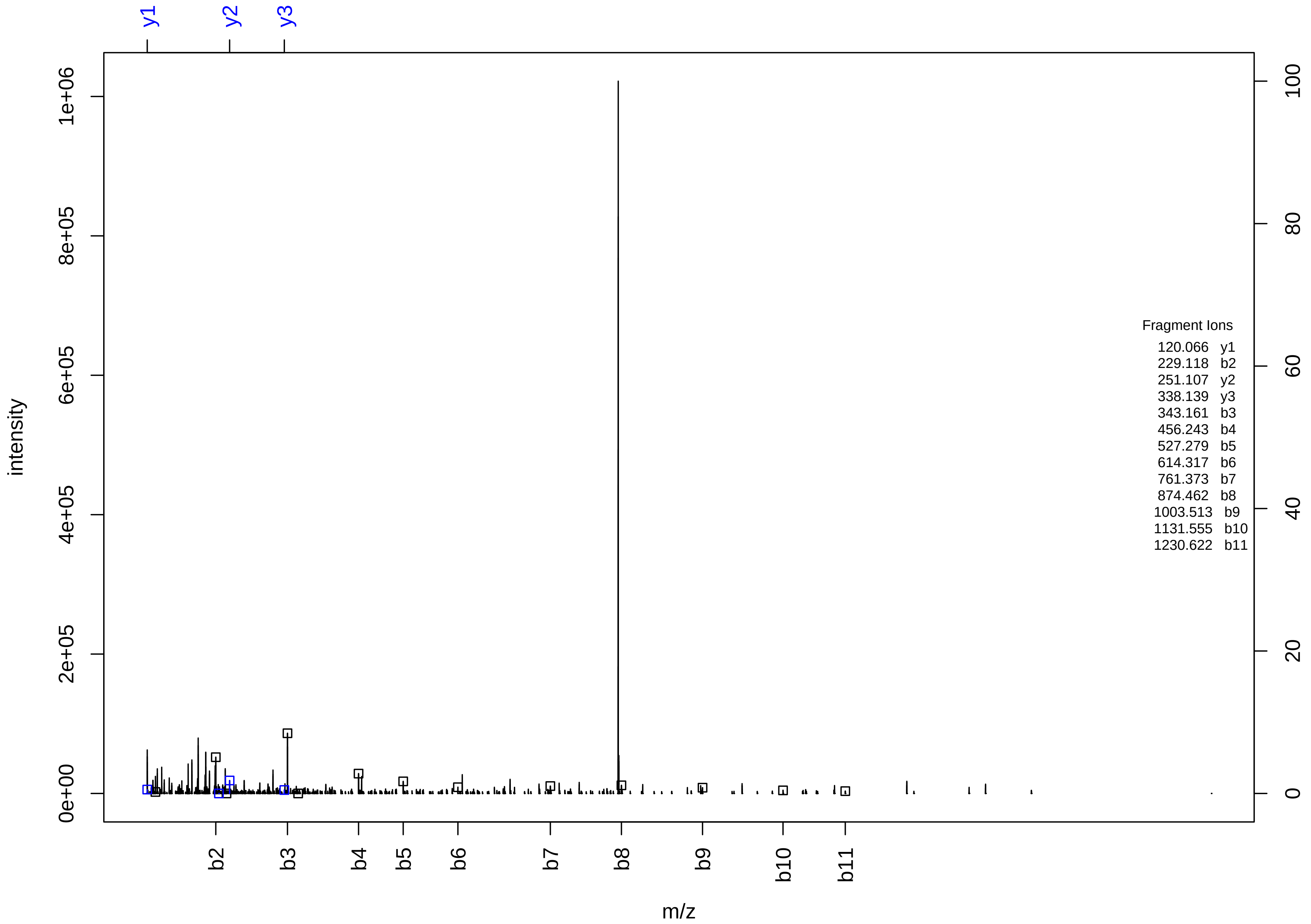

DINLASFIEQVAVSMT / File: christine_pool_03.raw; SpectrumID: ; scans: 24238

LMELLEEISER

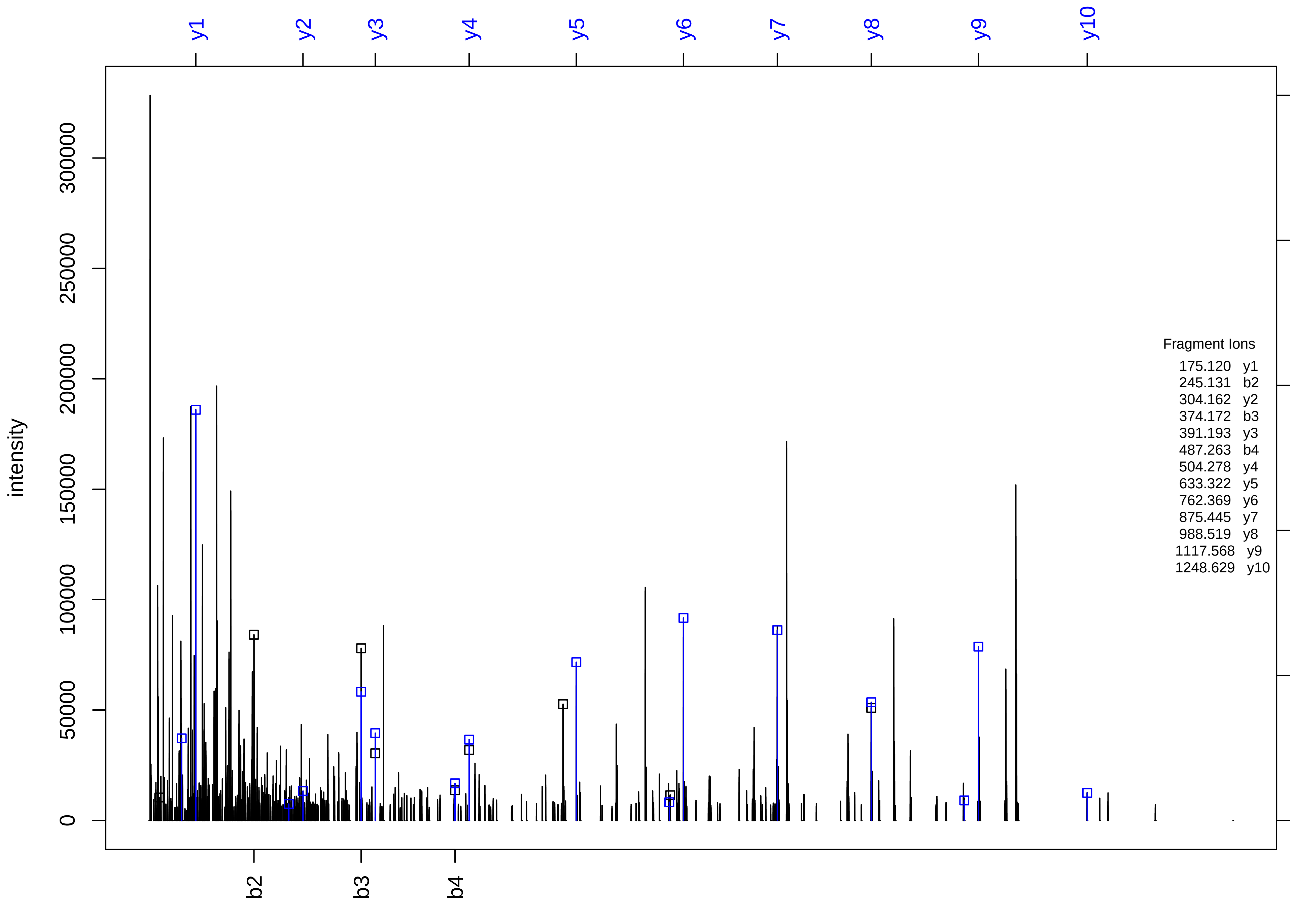

LMELLEEISER / File: christine_pool_03.raw; SpectrumID: ; scans: 20033

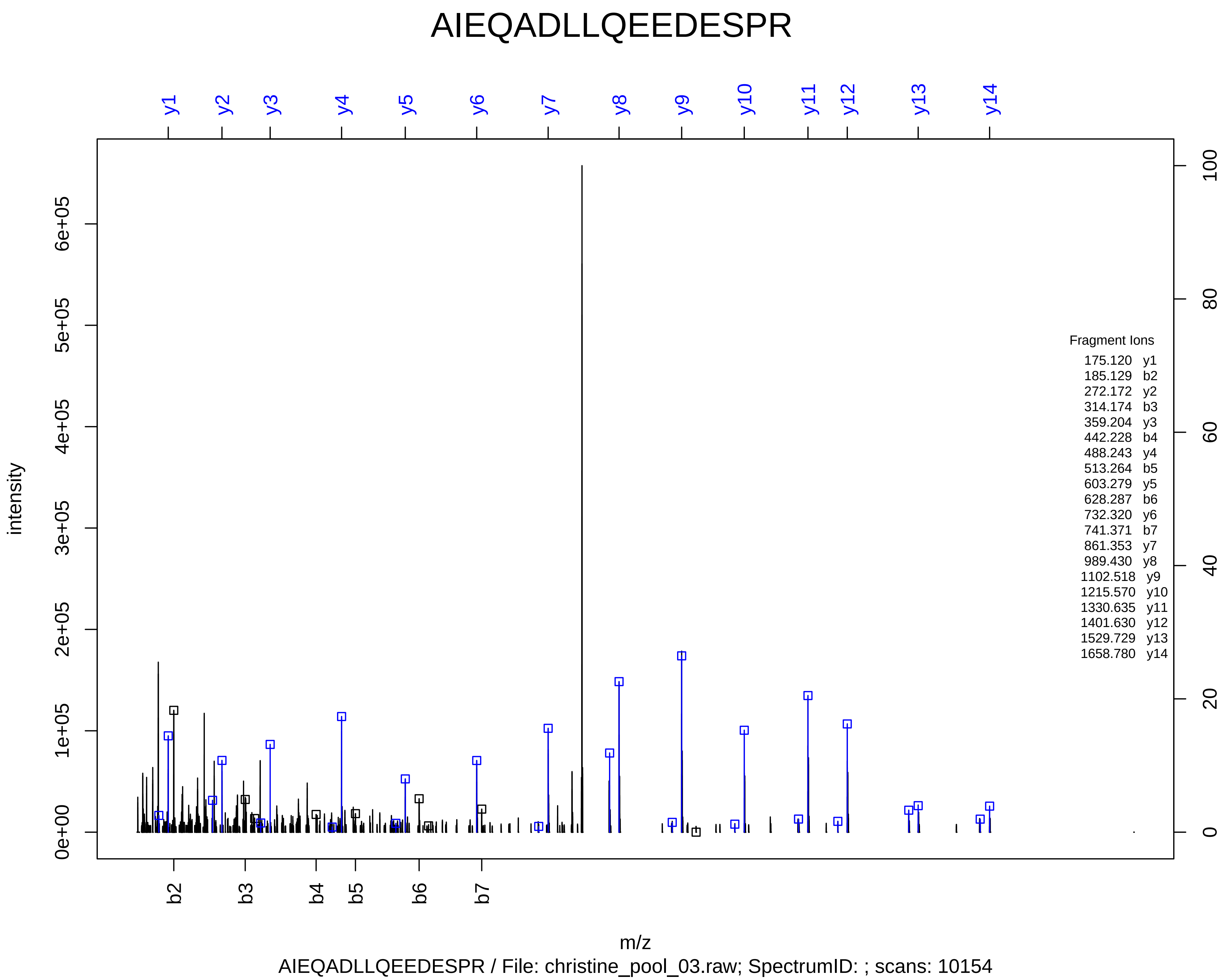

AIEQADLLQEEDESPR
y1
y2
y3
y4
y5
y6
y7
y8
y9
y10
y11
y12
y13
y14
intensity
0e+00
1e+05
2e+05
3e+05
4e+05
5e+05
6e+05
0
20
40
60
80
100
Fragment Ions
175.120 y1
185.129 b2
272.172 y2
314.174 b3
359.204 y3
442.228 b4
488.243 y4
513.264 b5
603.279 y5
628.287 b6
732.320 y6
741.371 b7
861.353 y7
989.430 y8
1102.518 y9
1215.570 y10
1330.635 y11
1401.630 y12
1529.729 y13
1658.780 y14
b2
b3
b4
b5
b6
b7
m/z
AIEQADLLQEEDESPR / File: christine_pool_03.raw; SpectrumID: ; scans: 10154

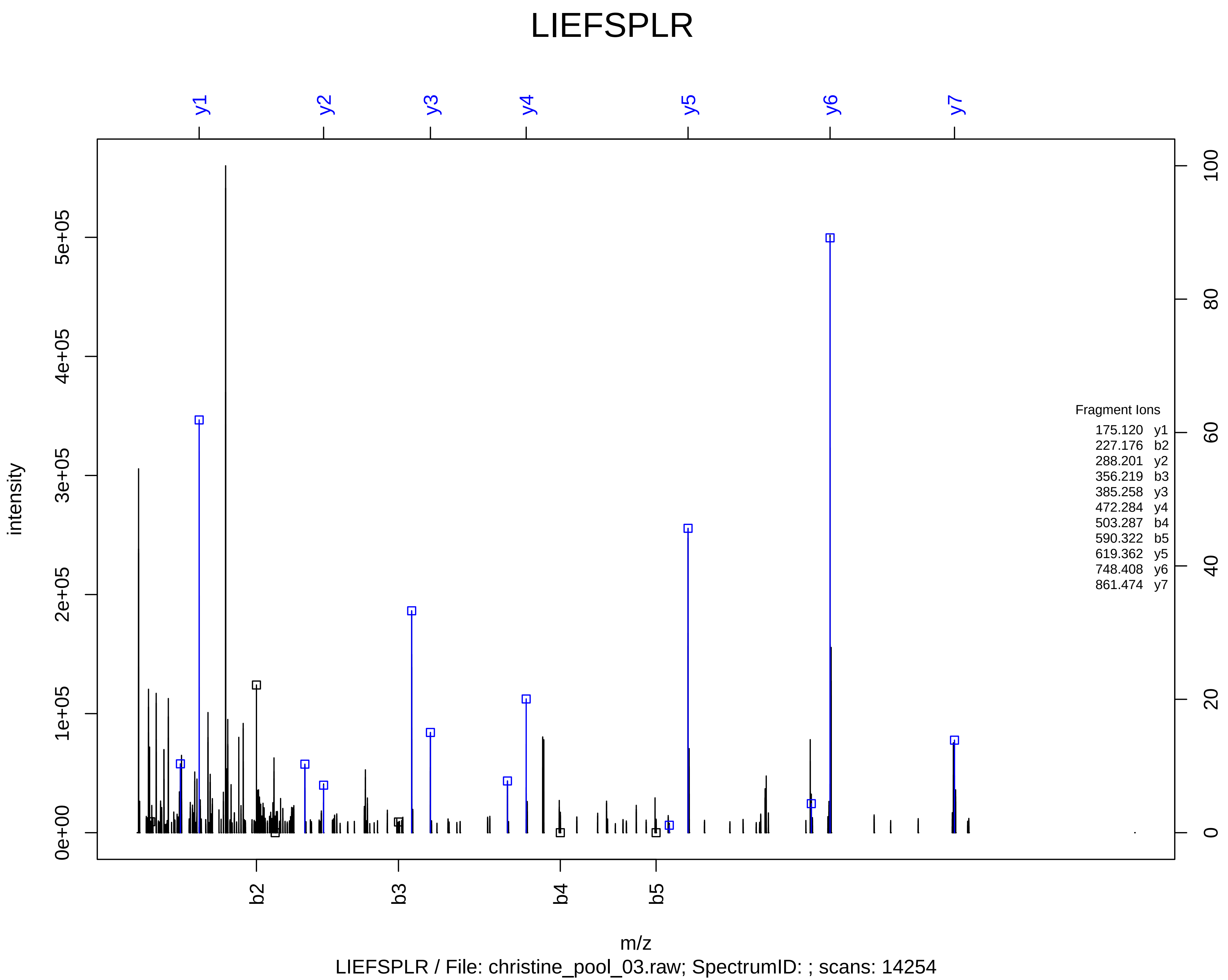

LIEFSPLR
y1
y2
y3
y4
y5
y6
y7
intensity
0e+00
1e+05
2e+05
3e+05
4e+05
5e+05
0
20
40
60
80
100
b2
b3
b4
b5
m/z
Fragment Ions
175.120 y1
227.176 b2
288.201 y2
356.219 b3
385.258 y3
472.284 y4
503.287 b4
590.322 b5
619.362 y5
748.408 y6
861.474 y7
LIEFSPLR / File: christine_pool_03.raw; SpectrumID: ; scans: 14254

SGDSEVYQLGDVSQK

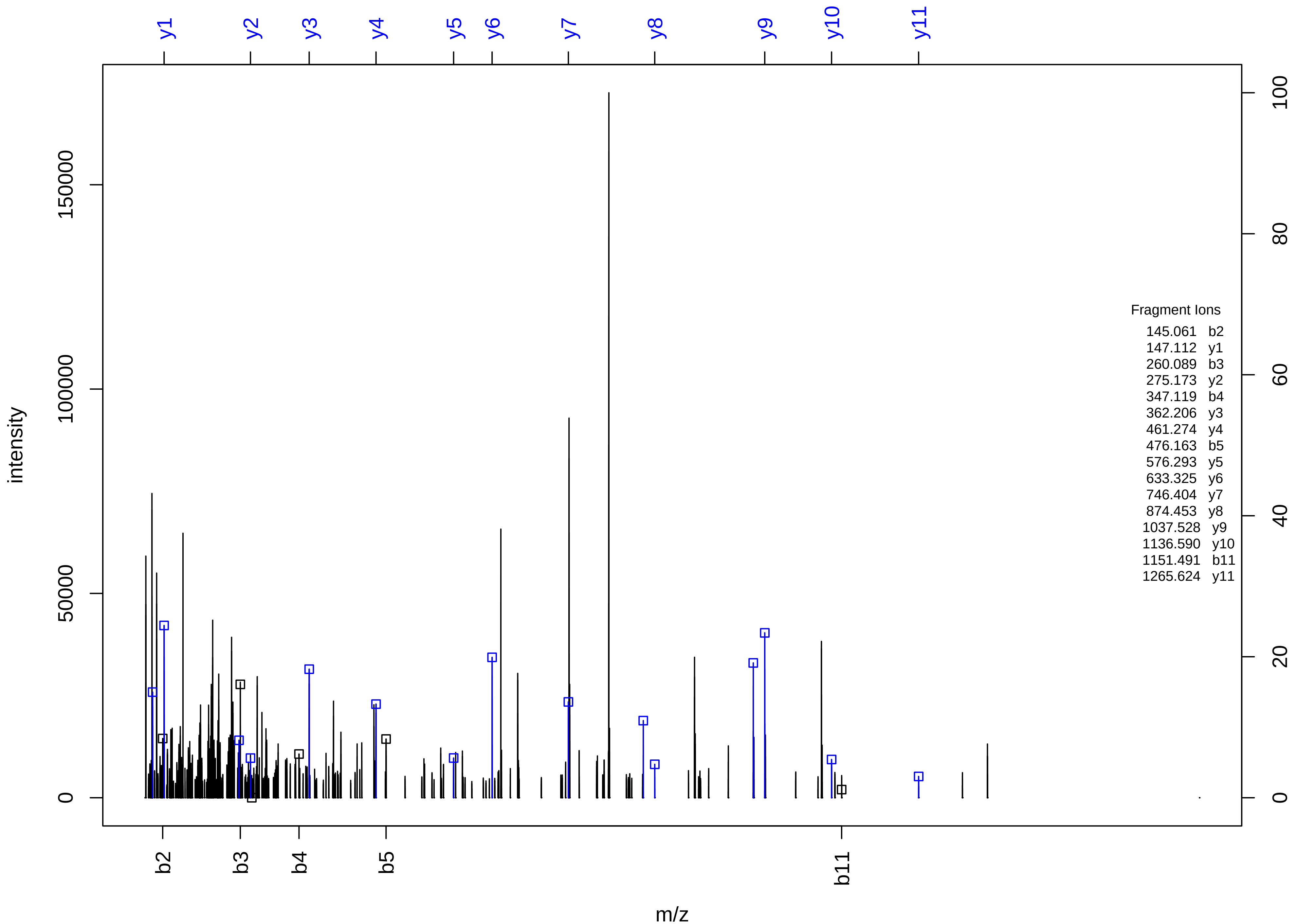

SGDSEVYQLGDVSQK / File: christine_pool_03.raw; SpectrumID: ; scans: 8229

VIQIVLMR

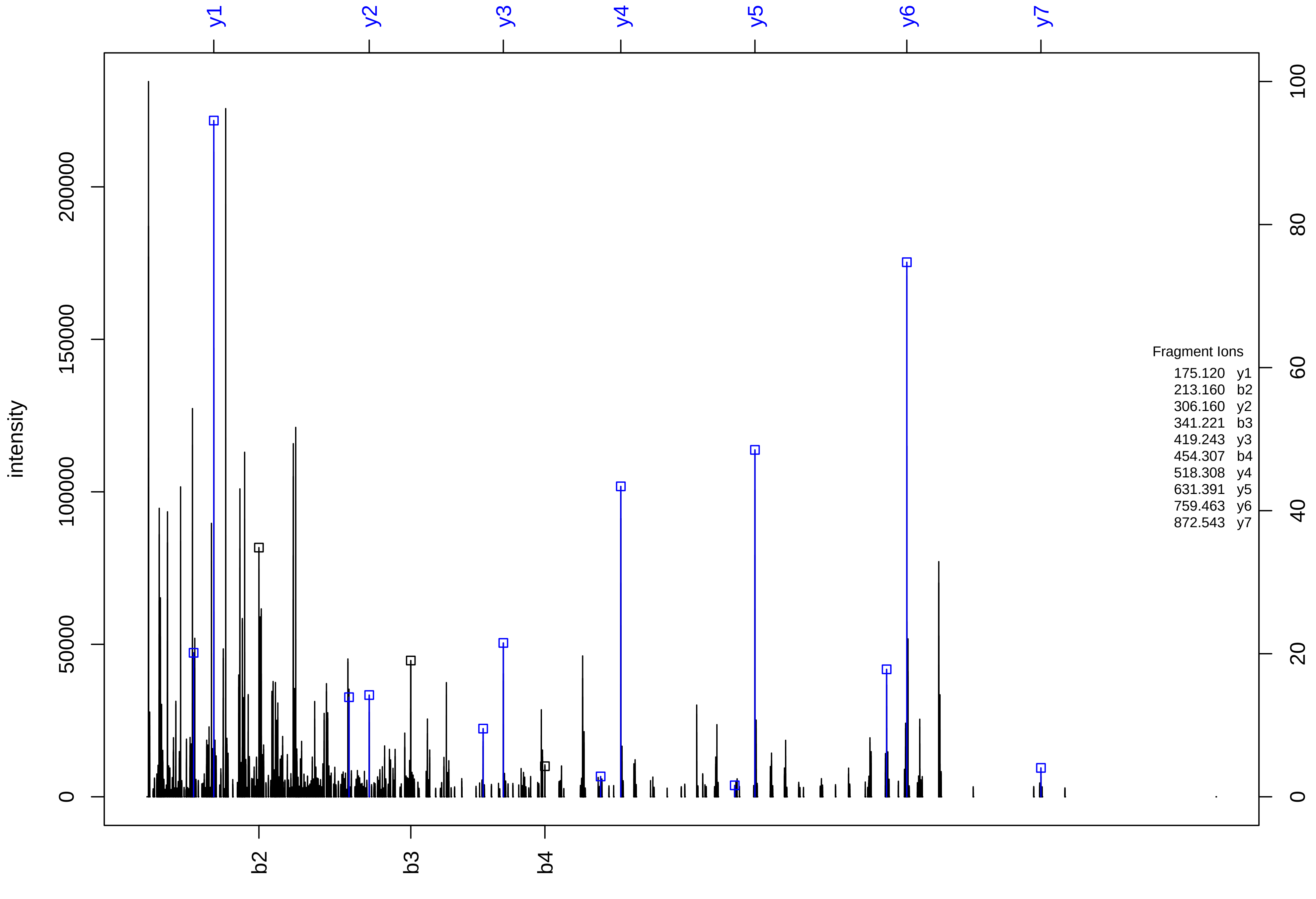

VIQIVLMR / File: christine_pool_03.raw; SpectrumID: ; scans: 15246

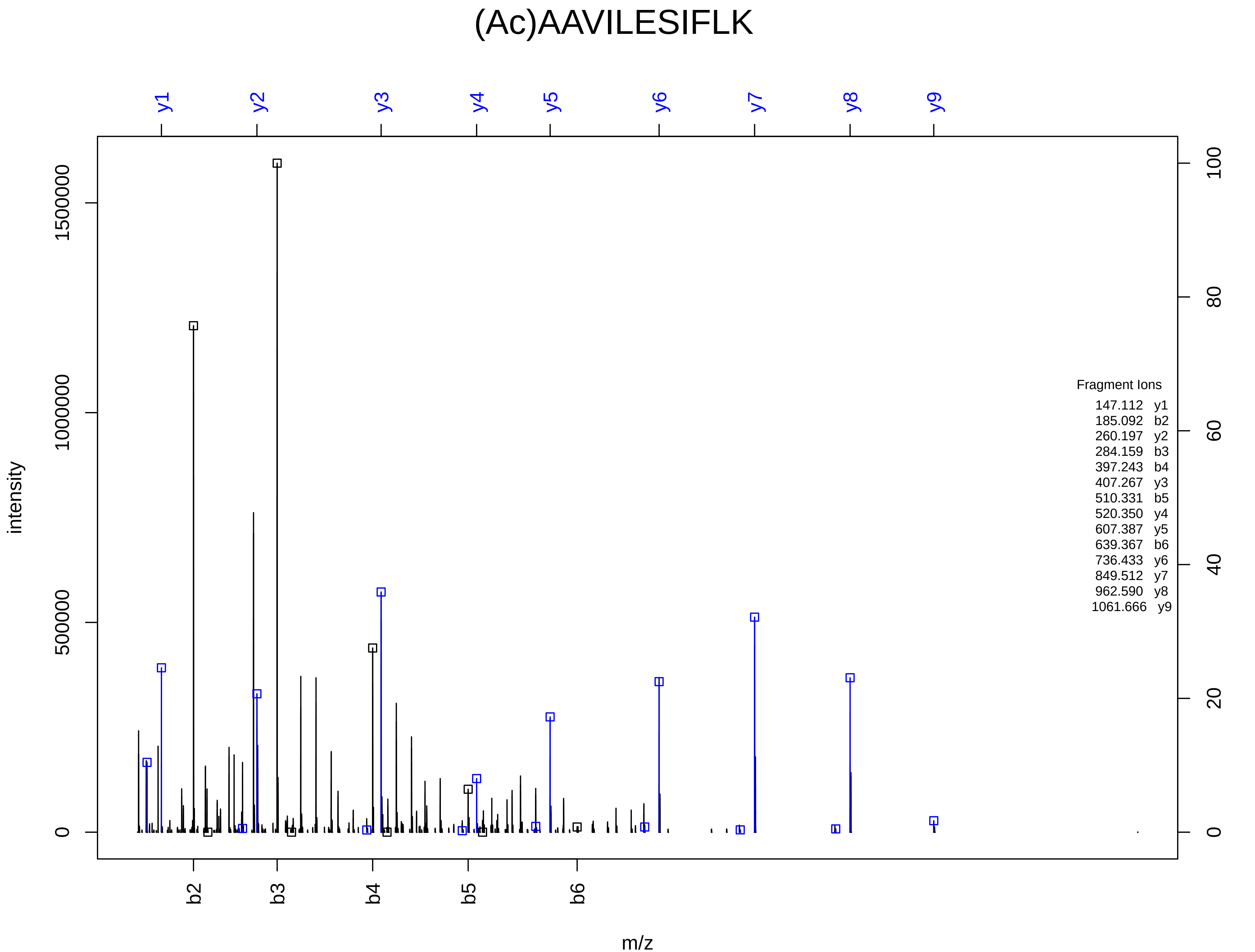

AAVILESIFLK / File: christine_pool_03.raw; SpectrumID: ; scans: 24214

GFLIASFR

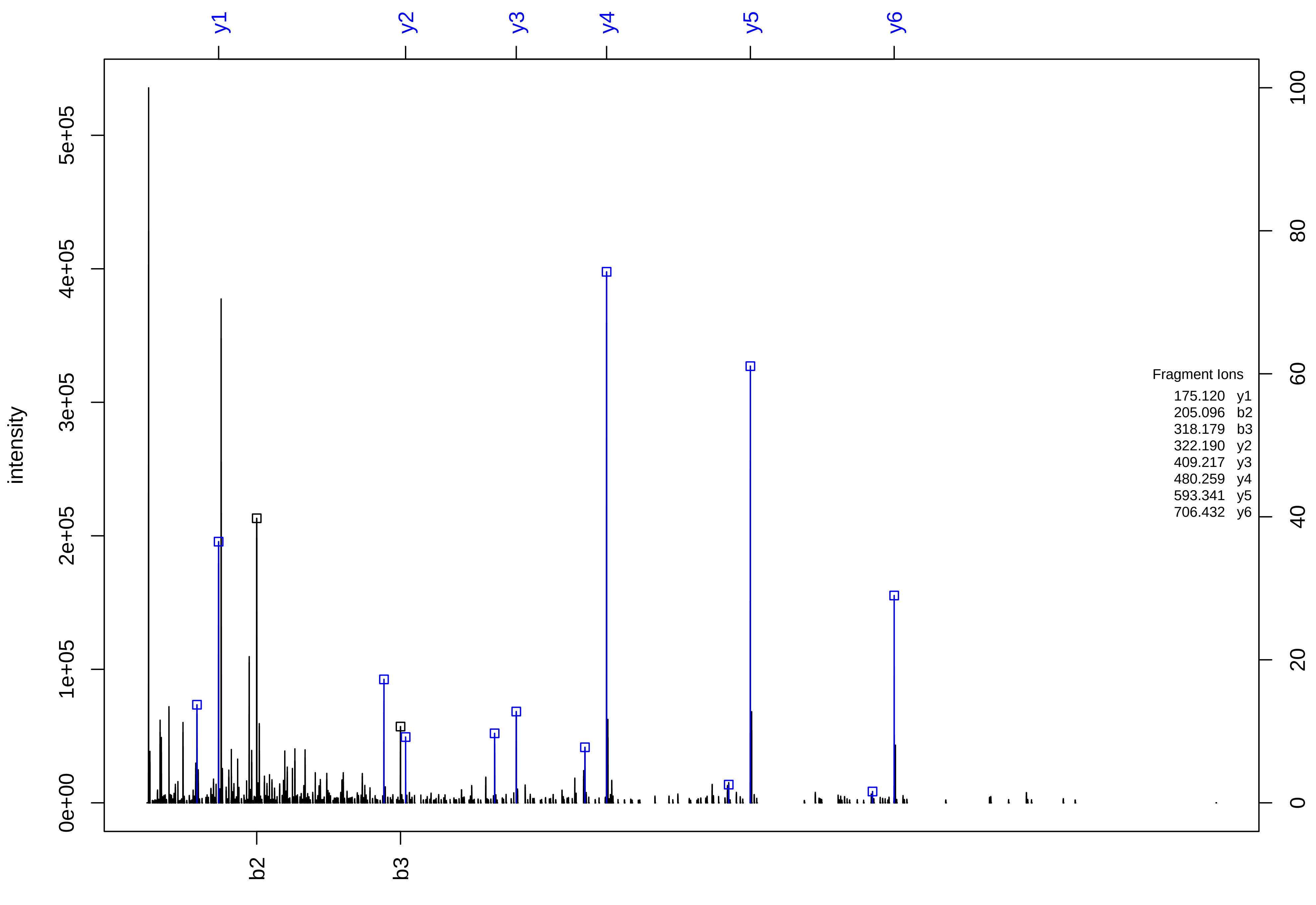

GFLIASFR / File: christine_pool_03.raw; SpectrumID: ; scans: 16023

GFAFVYFER

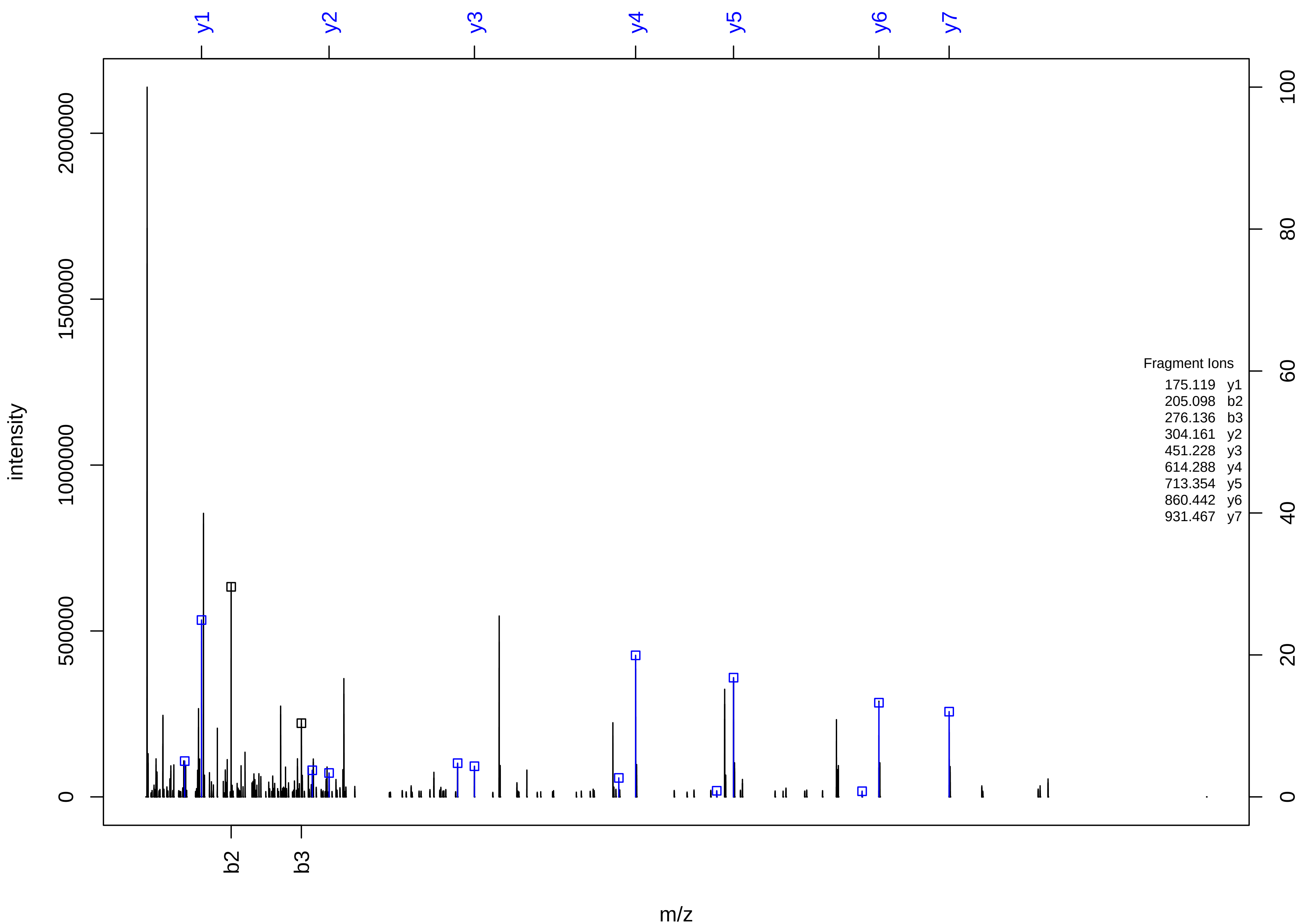

GFAFVYFER / File: christine_pool_03.raw; SpectrumID: ; scans: 17973

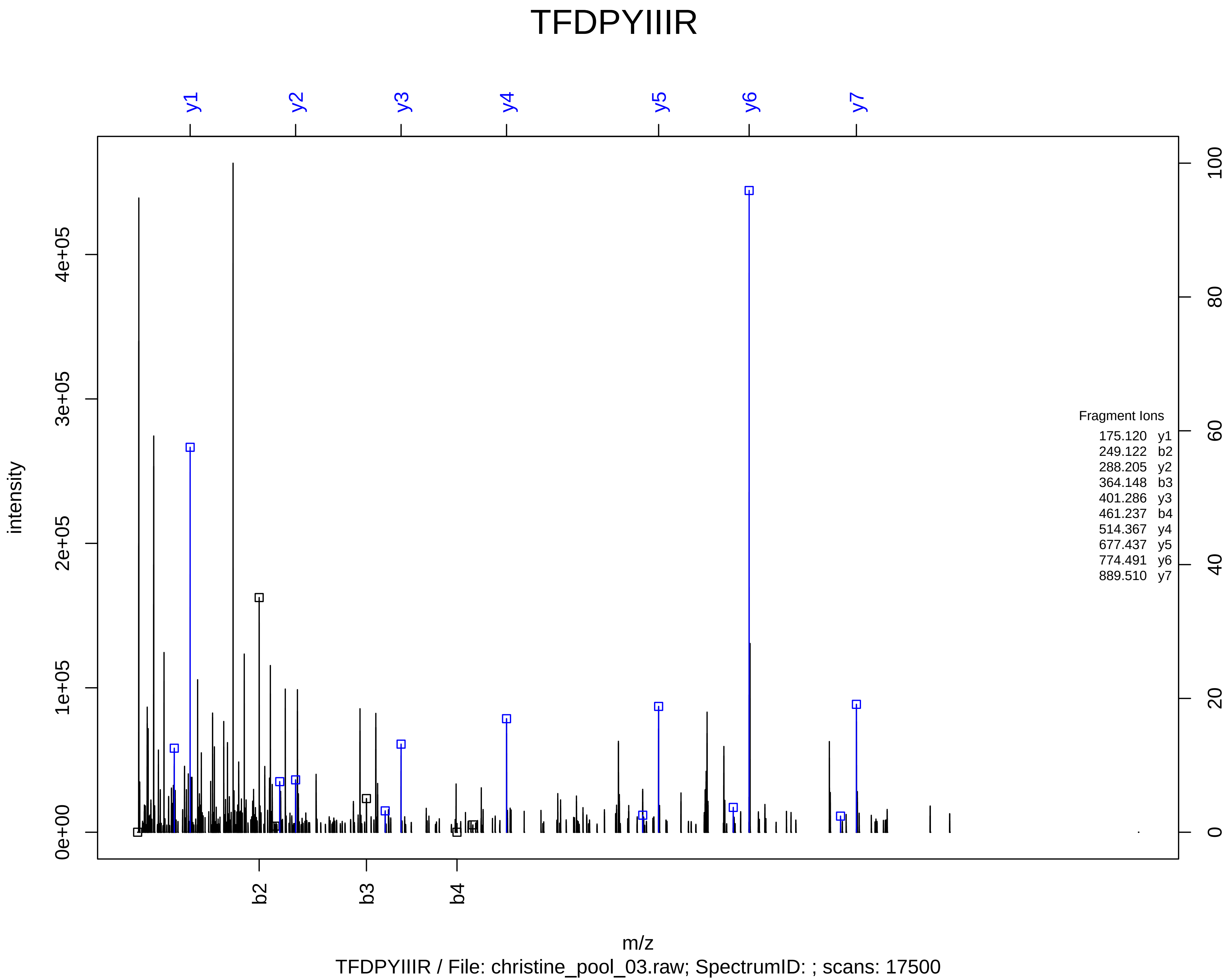

TFDPYIIIR
y1
y2
y3
y4
y5
y6
y7
intensity
0e+00
1e+05
2e+05
3e+05
4e+05
0
20
40
60
80
100
b2
b3
b4
m/z
Fragment Ions
175.120 y1
249.122 b2
288.205 y2
364.148 b3
401.286 y3
461.237 b4
514.367 y4
677.437 y5
774.491 y6
889.510 y7
TFDPYIIIR / File: christine_pool_03.raw; SpectrumID: ; scans: 17500

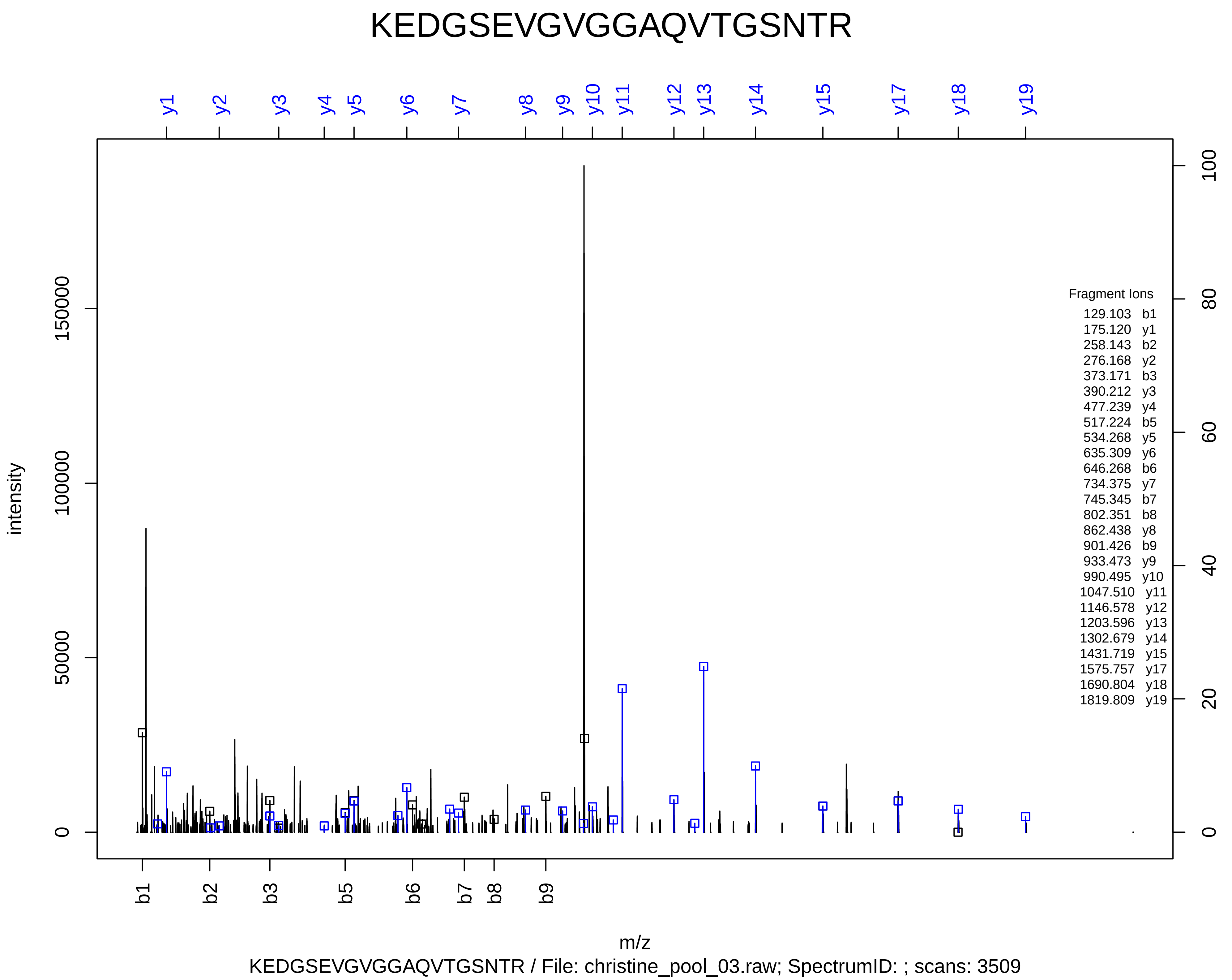
KEDGSEVGVGGAQVTGSNTR
y1 y2 y3 y4 y5 y6 y7 y8 y9 y10 y11 y12 y13 y14 y15 y17 y18 y19
b1 b2 b3 b5 b6 b7 b8 b9
intensity
m/z
Fragment Ions
129.103 b1
175.120 y1
258.143 b2
276.168 y2
373.171 b3
390.212 y3
477.239 y4
517.224 b5
534.268 y5
635.309 y6
646.268 b6
734.375 y7
745.345 b7
802.351 b8
862.438 y8
901.426 b9
933.473 y9
990.495 y10
1047.510 y11
1146.578 y12
1203.596 y13
1302.679 y14
1431.719 y15
1575.757 y17
1690.804 y18
1819.809 y19
KEDGSEVGVGGAQVTGSNTR / File: christine_pool_03.raw; SpectrumID: ; scans: 3509

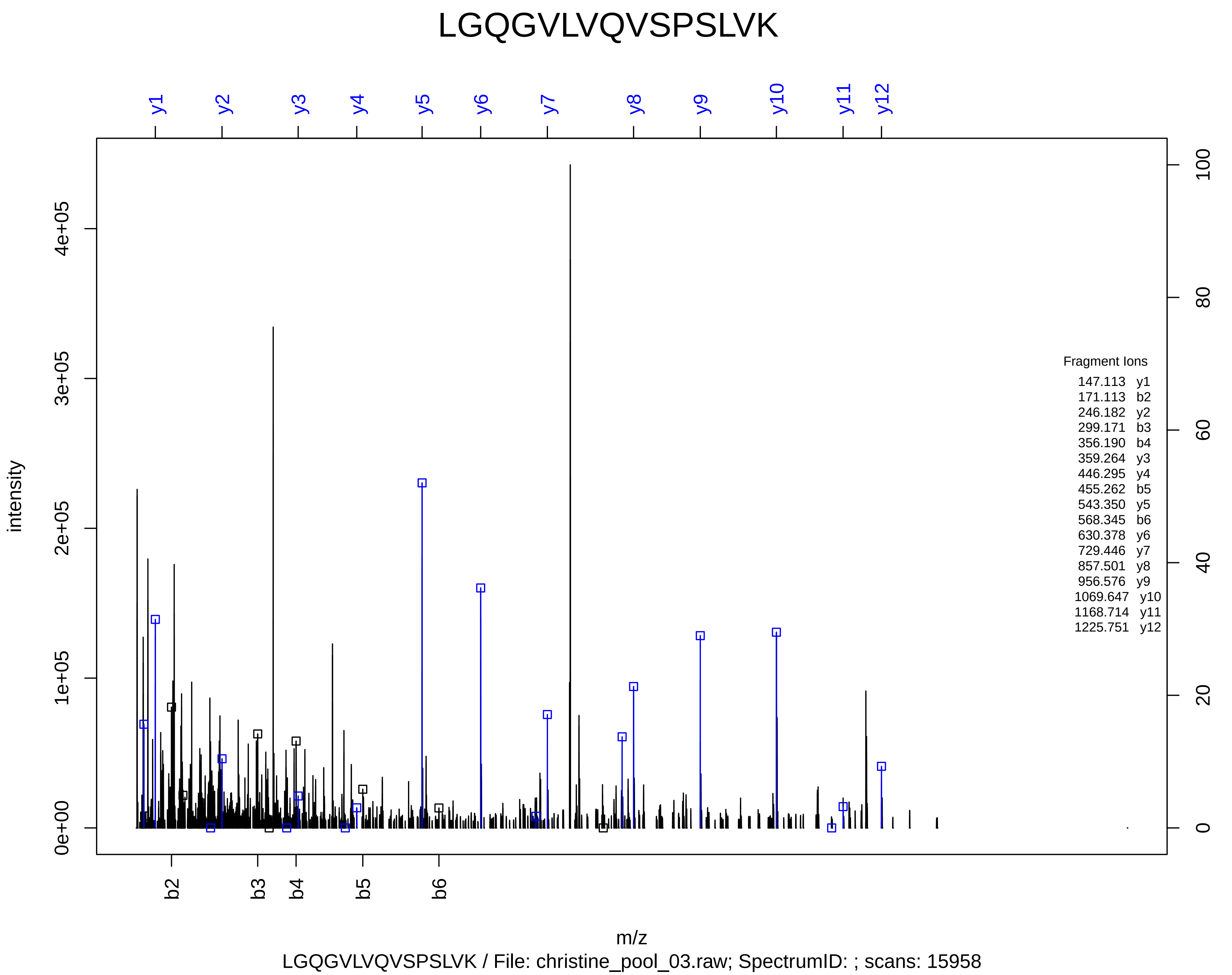
LGQGVLVQVSPSLVK
y1
y2
y3
y4
y5
y6
y7
y8
y9
y10
y11
y12
b2
b3
b4
b5
b6
intensity
0e+00
1e+05
2e+05
3e+05
4e+05
0
20
40
60
80
100
m/z
Fragment Ions
147.113 y1
171.113 b2
246.182 y2
299.171 b3
356.190 b4
359.264 y3
446.295 y4
455.262 b5
543.350 y5
568.345 b6
630.378 y6
729.446 y7
857.501 y8
956.576 y9
1069.647 y10
1168.714 y11
1225.751 y12
LGQGVLVQVSPSLVK / File: christine_pool_03.raw; SpectrumID: ; scans: 15958

AQQEDALAQQAFEEAR

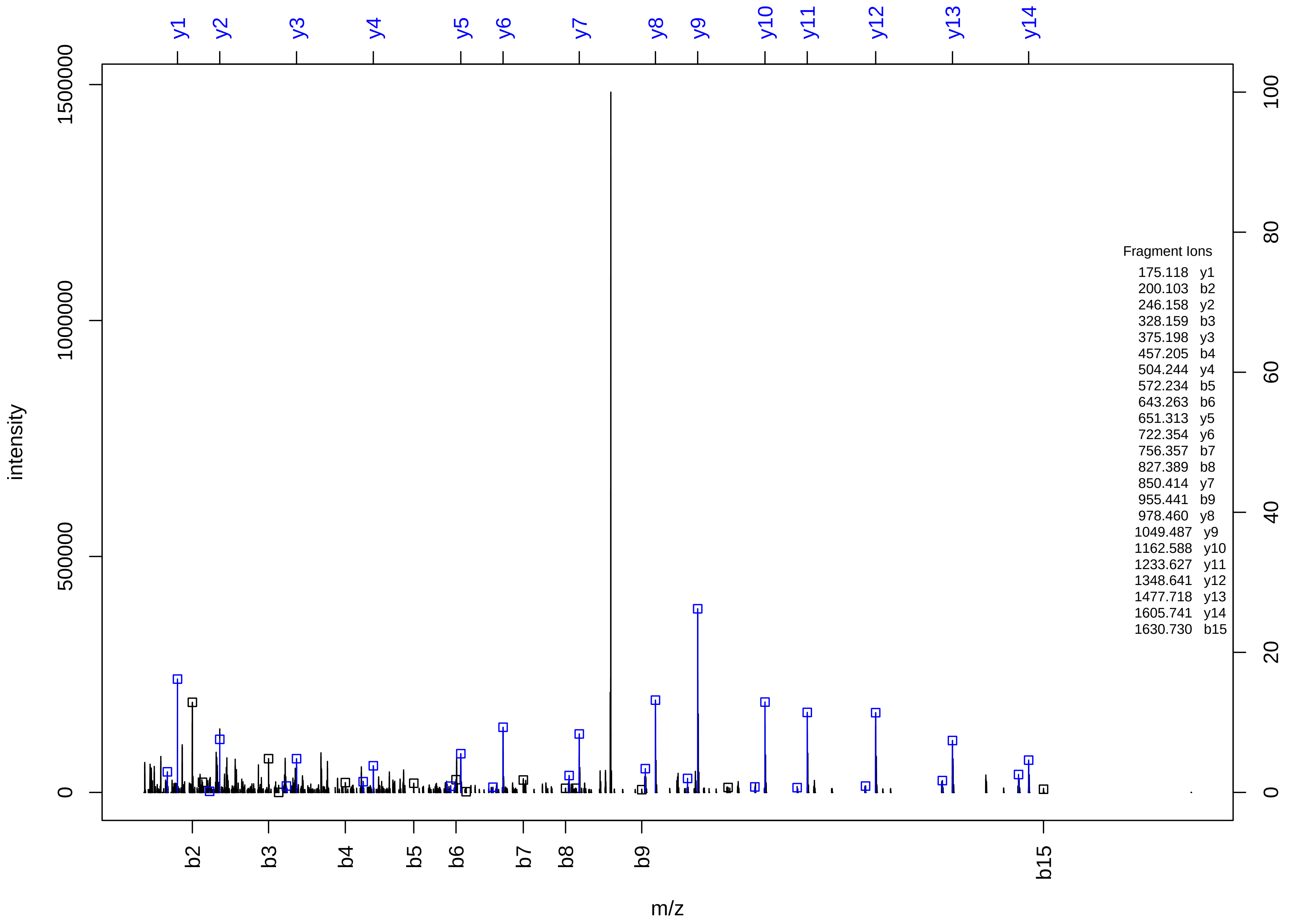

AQQEDALAQQAFEEAR / File: christine_pool_03.raw; SpectrumID: ; scans: 10712

VPLILNLIR

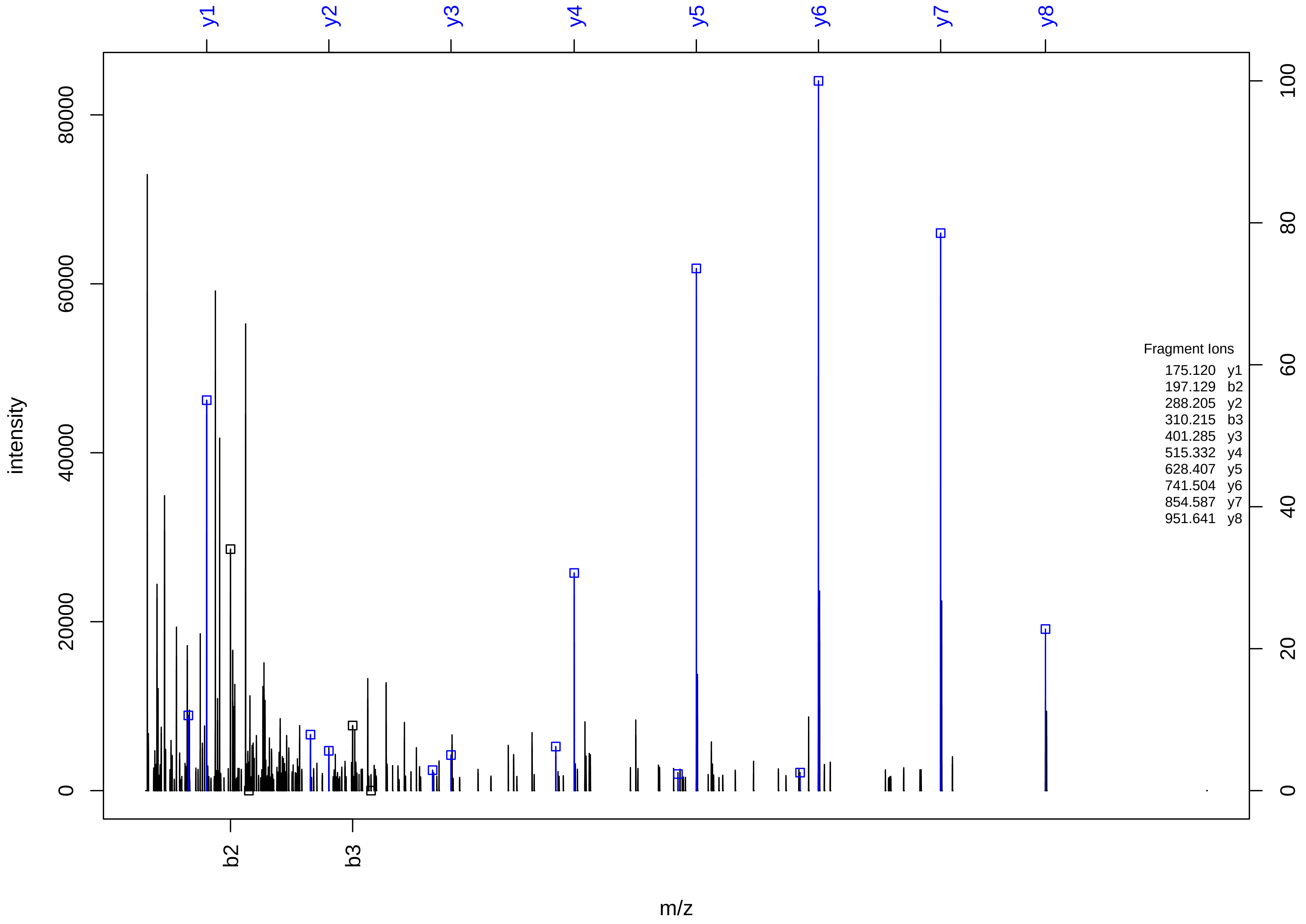

VPLILNLIR / File: christine_pool_03.raw; SpectrumID: ; scans: 22626

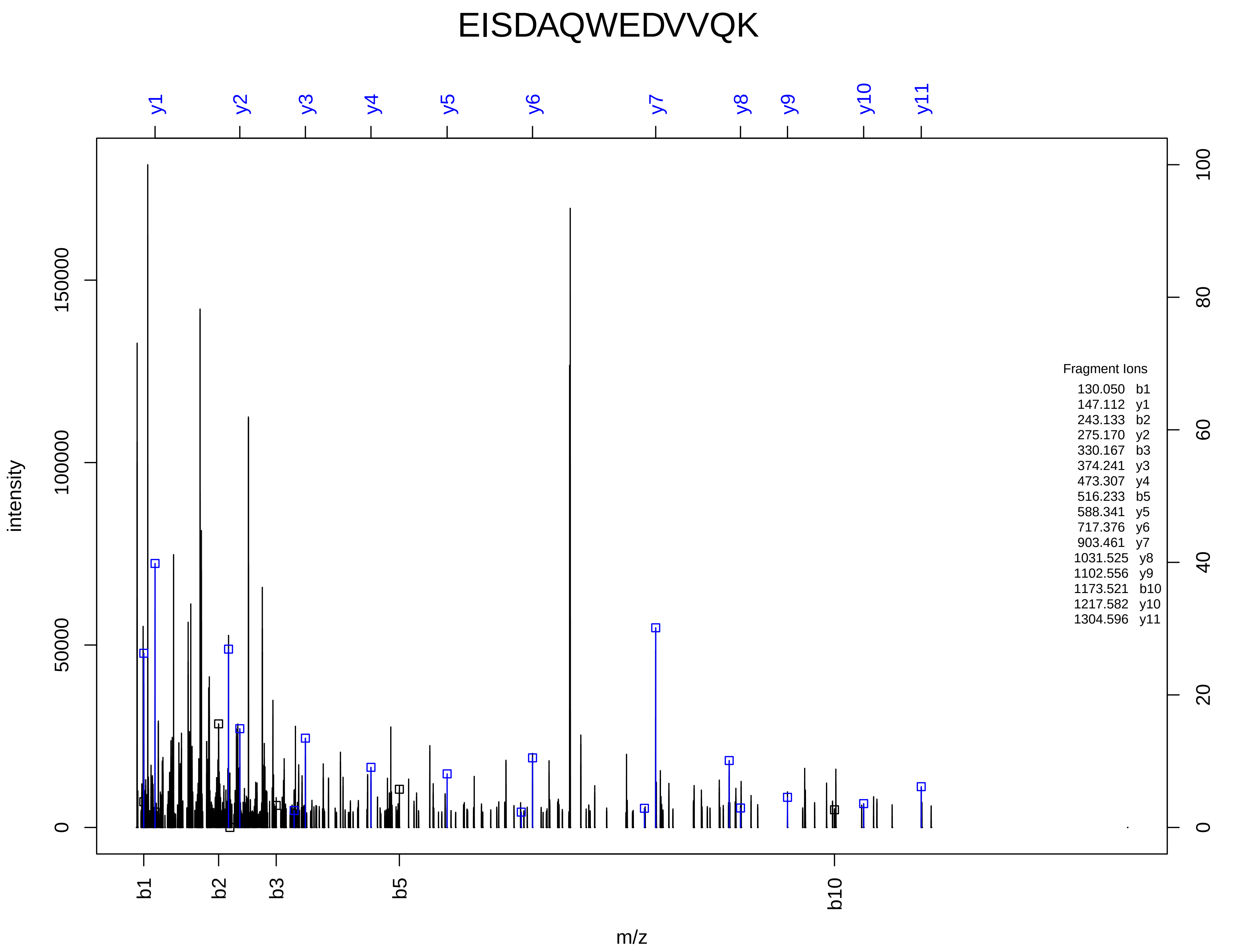

EISDAQWEDVVQK / File: christine_pool_03.raw; SpectrumID: ; scans: 11485

EILLTTQLDSLER

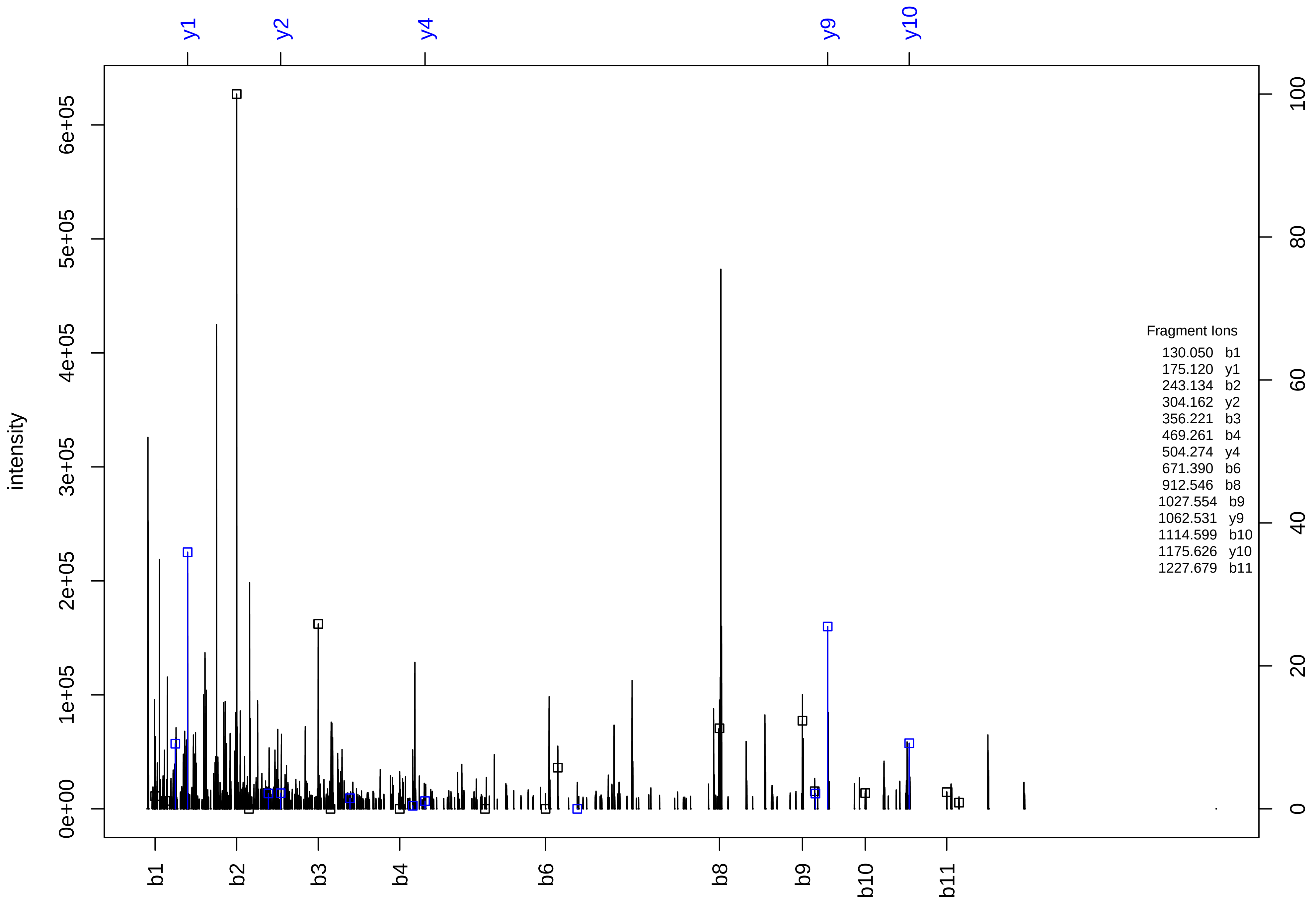

EILLTTQLDSLER / File: christine_pool_03.raw; SpectrumID: ; scans: 17896

SAVEAQNEVTENPK

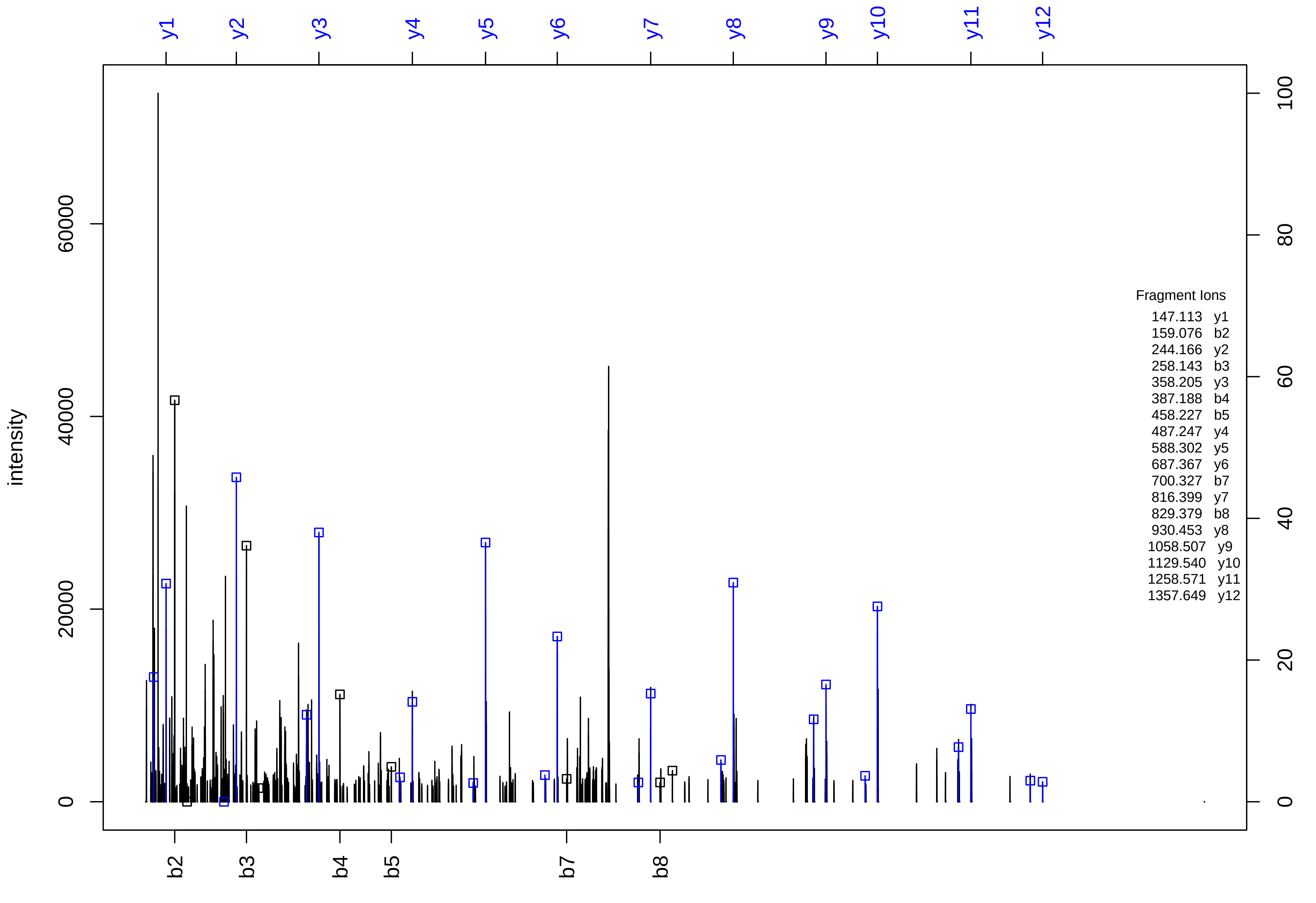

SAVEAQNEVTENPK / File: christine_pool_03.raw; SpectrumID: ; scans: 3183

LPLPAFR

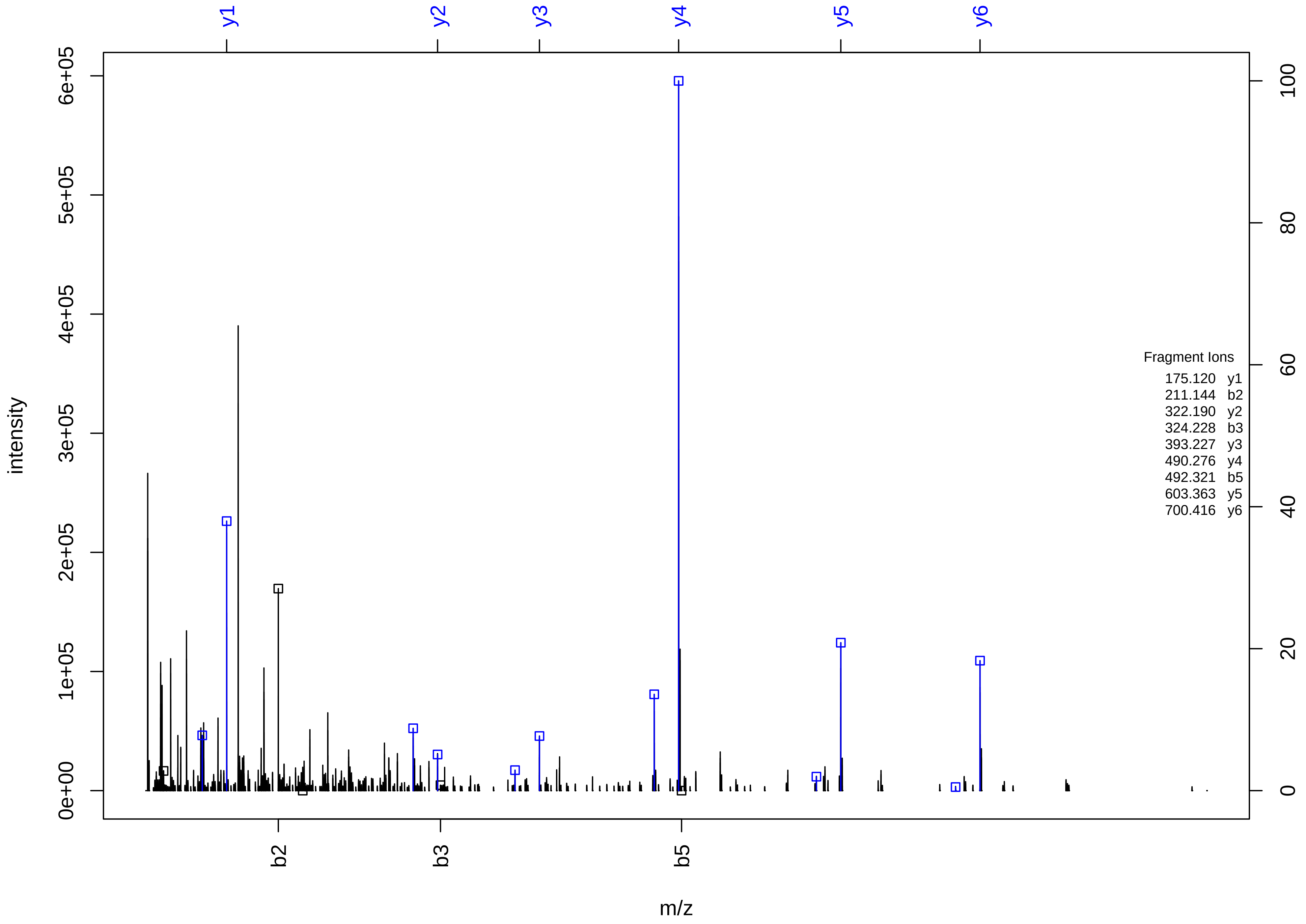

LPLPAFR / File: christine_pool_03.raw; SpectrumID: ; scans: 13336

AIEFLNNPPEEAPR

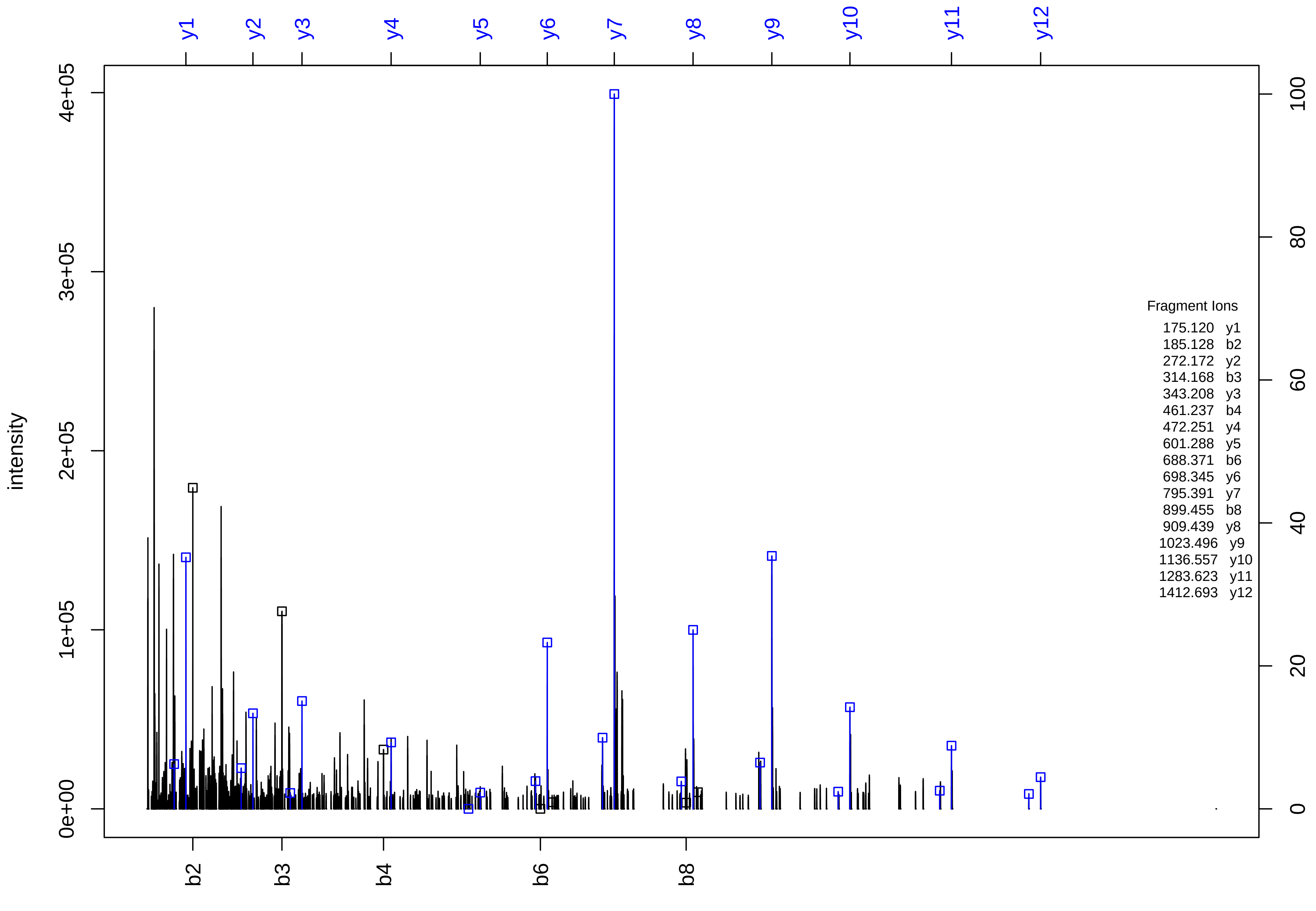

AIEFLNNPPEEAPR / File: christine_pool_03.raw; SpectrumID: ; scans: 12294

LVQTAAQQVAEDK

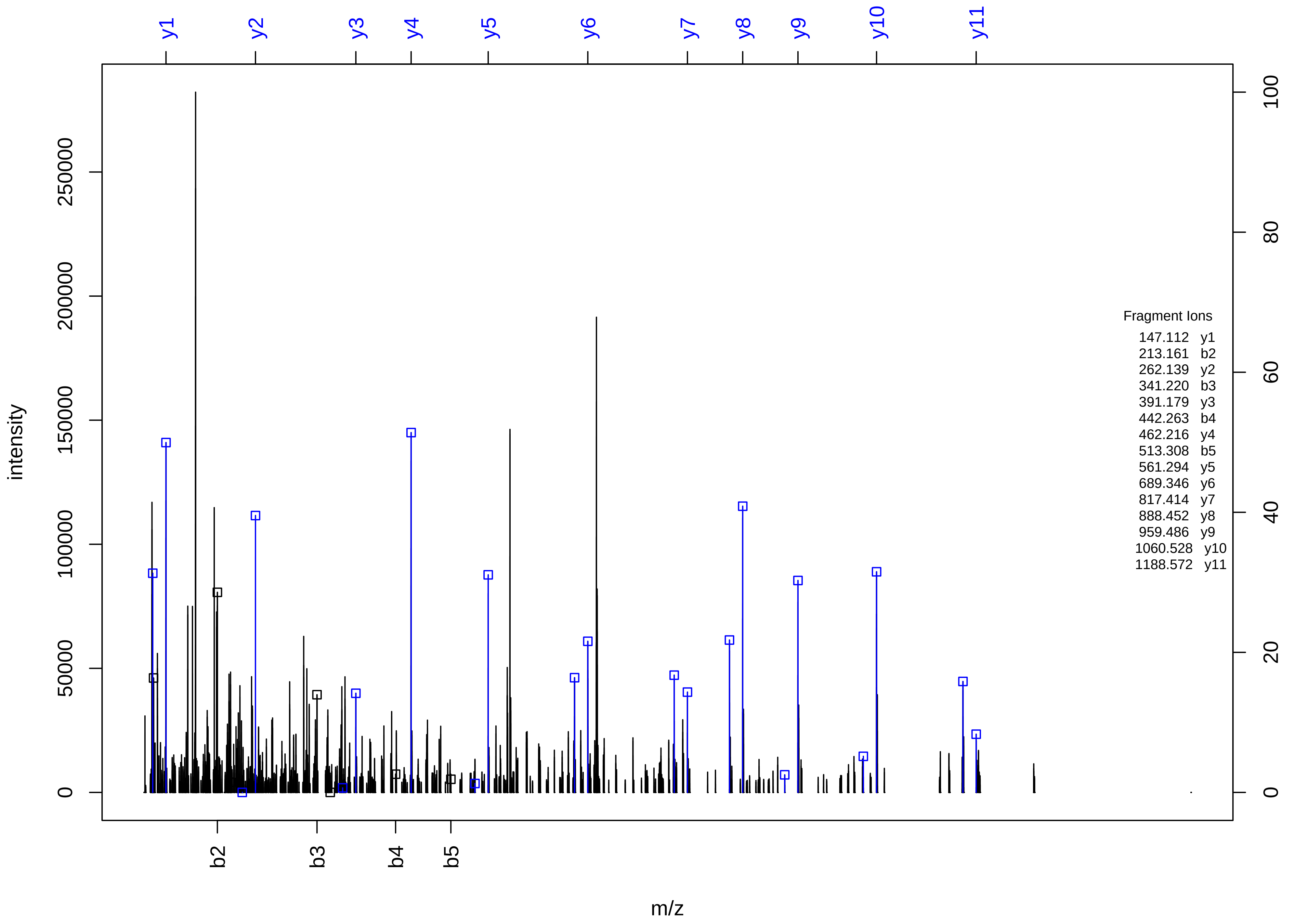

LVQTAAQQVAEDK / File: christine_pool_03.raw; SpectrumID: ; scans: 4736

VLFILFR

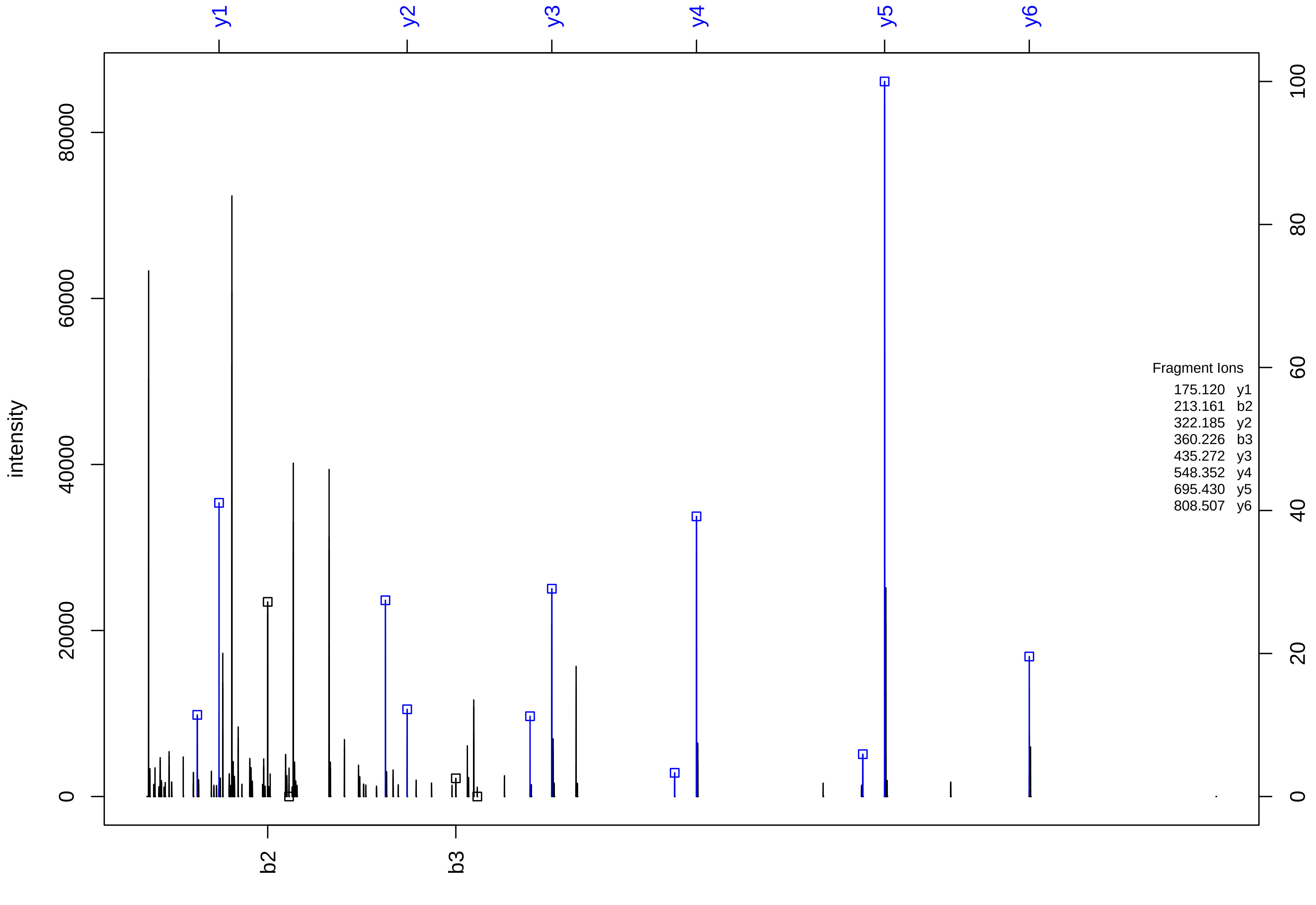

VLFILFR / File: christine_pool_03.raw; SpectrumID: ; scans: 22852

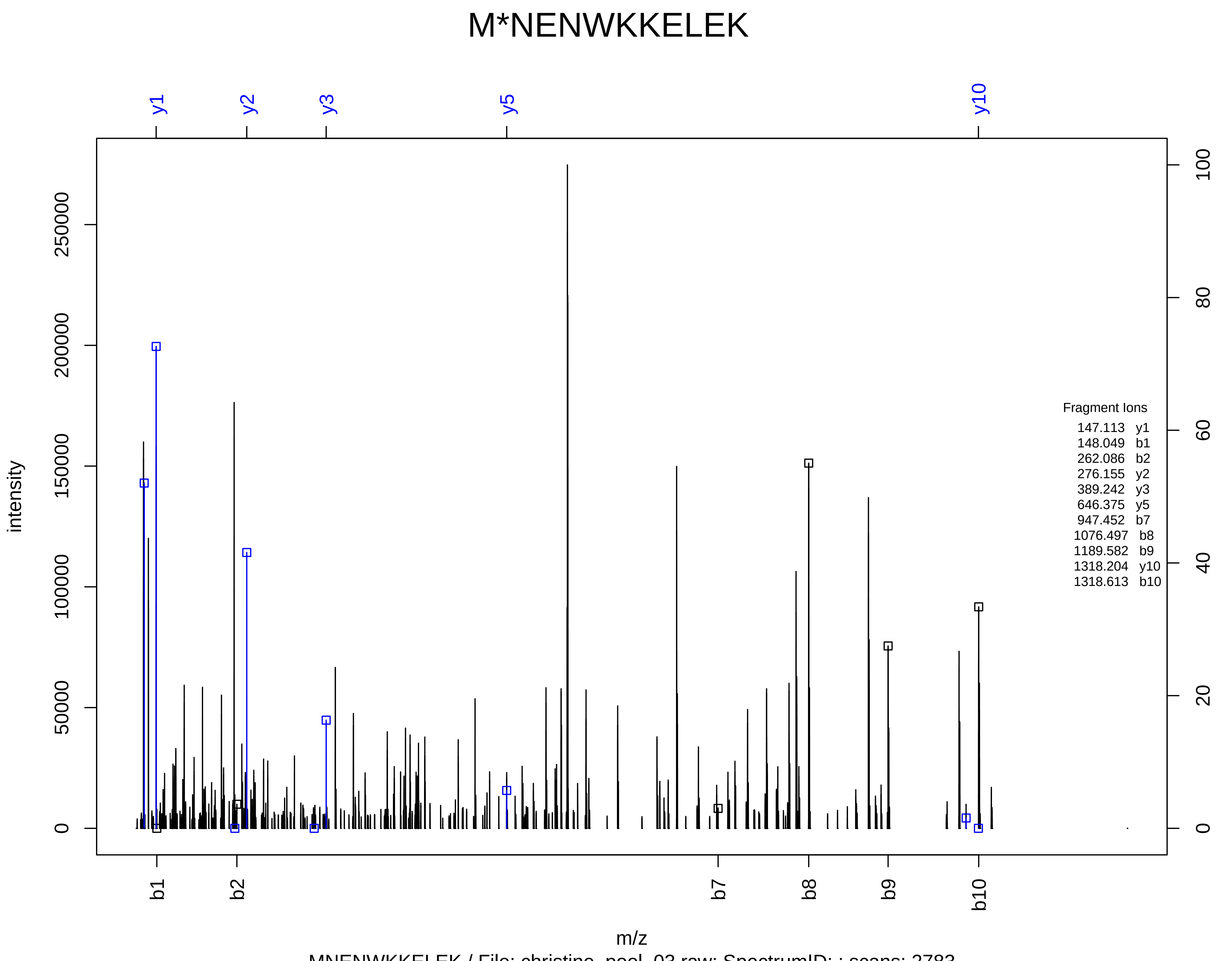

M*NENWKKELEK
y1
y2
y3
y5
y10
intensity
0
50000
100000
150000
200000
250000
0
20
40
60
80
100
Fragment Ions
147.113 y1
148.049 b1
262.086 b2
276.155 y2
389.242 y3
646.375 y5
947.452 b7
1076.497 b8
1189.582 b9
1318.204 y10
1318.613 b10
b1
b2
b7
b8
b9
b10
m/z
MNENWKKELEK / File: christine_pool_03.raw; SpectrumID: ; scans: 2783

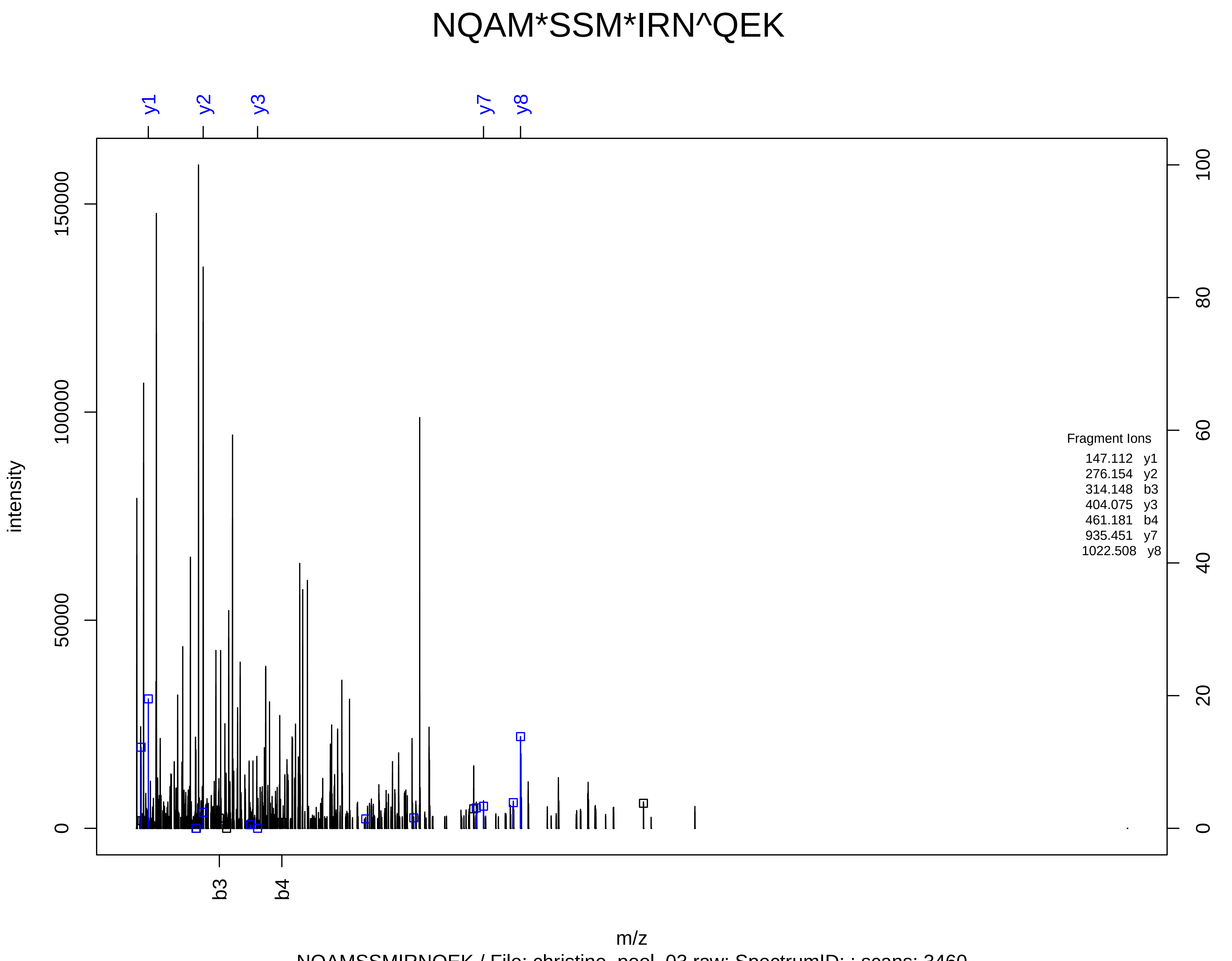
NQAM*SSM*IRN^QEK
y1
y2
y3
y7
y8
intensity
0
50000
100000
150000
0
20
40
60
80
100
b3
b4
m/z
Fragment Ions
147.112 y1
276.154 y2
314.148 b3
404.075 y3
461.181 b4
935.451 y7
1022.508 y8
NQAMSSMIRNQEK / File: christine_pool_03.raw; SpectrumID: ; scans: 3460

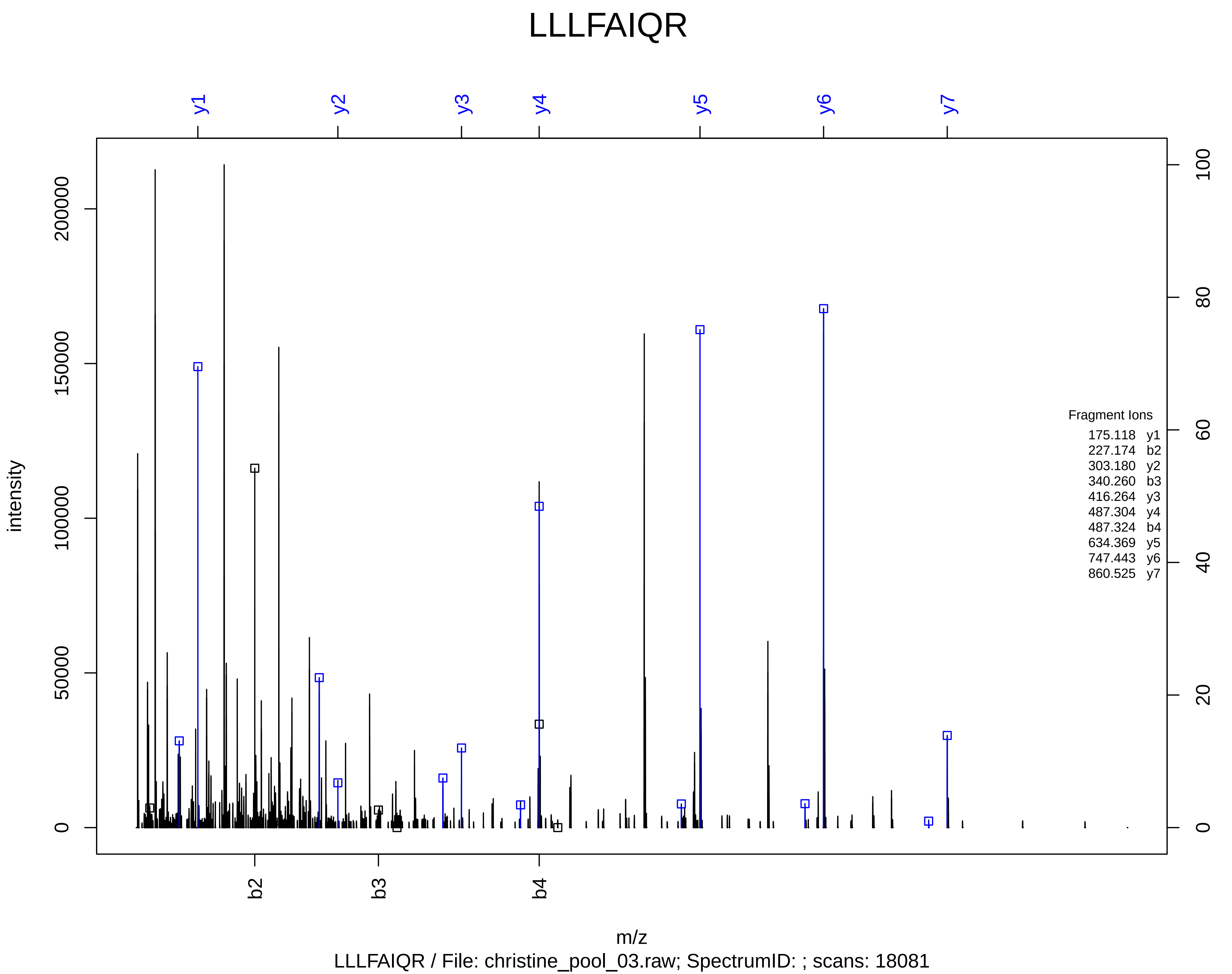
LLLFAIQR
y1
y2
y3
y4
y5
y6
y7
b2
b3
b4
intensity
m/z
Fragment Ions
175.118 y1
227.174 b2
303.180 y2
340.260 b3
416.264 y3
487.304 y4
487.324 b4
634.369 y5
747.443 y6
860.525 y7
LLLFAIQR / File: christine_pool_03.raw; SpectrumID: ; scans: 18081

LILPELGPVFK

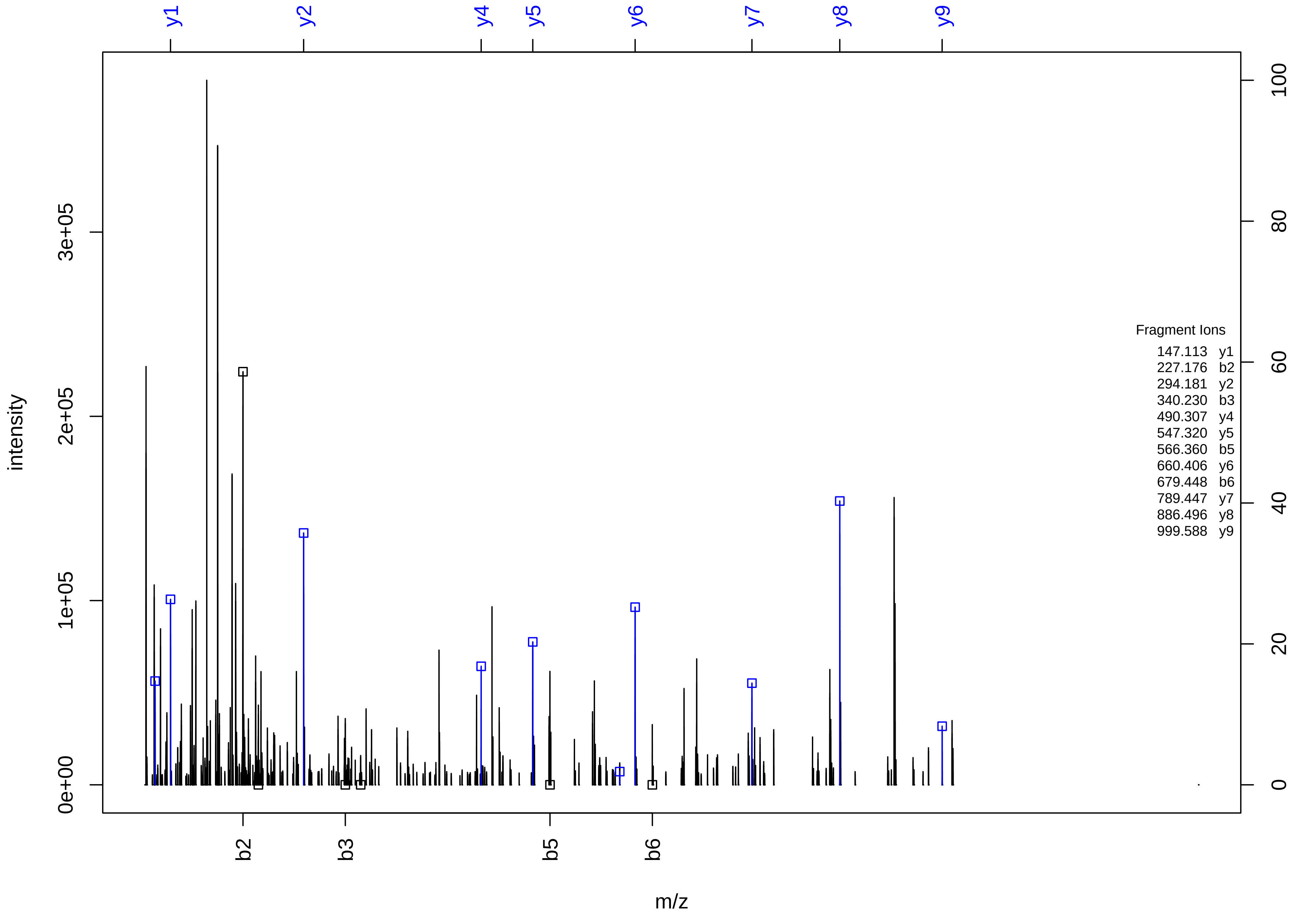

LILPELGPVFK / File: christine_pool_03.raw; SpectrumID: ; scans: 22452

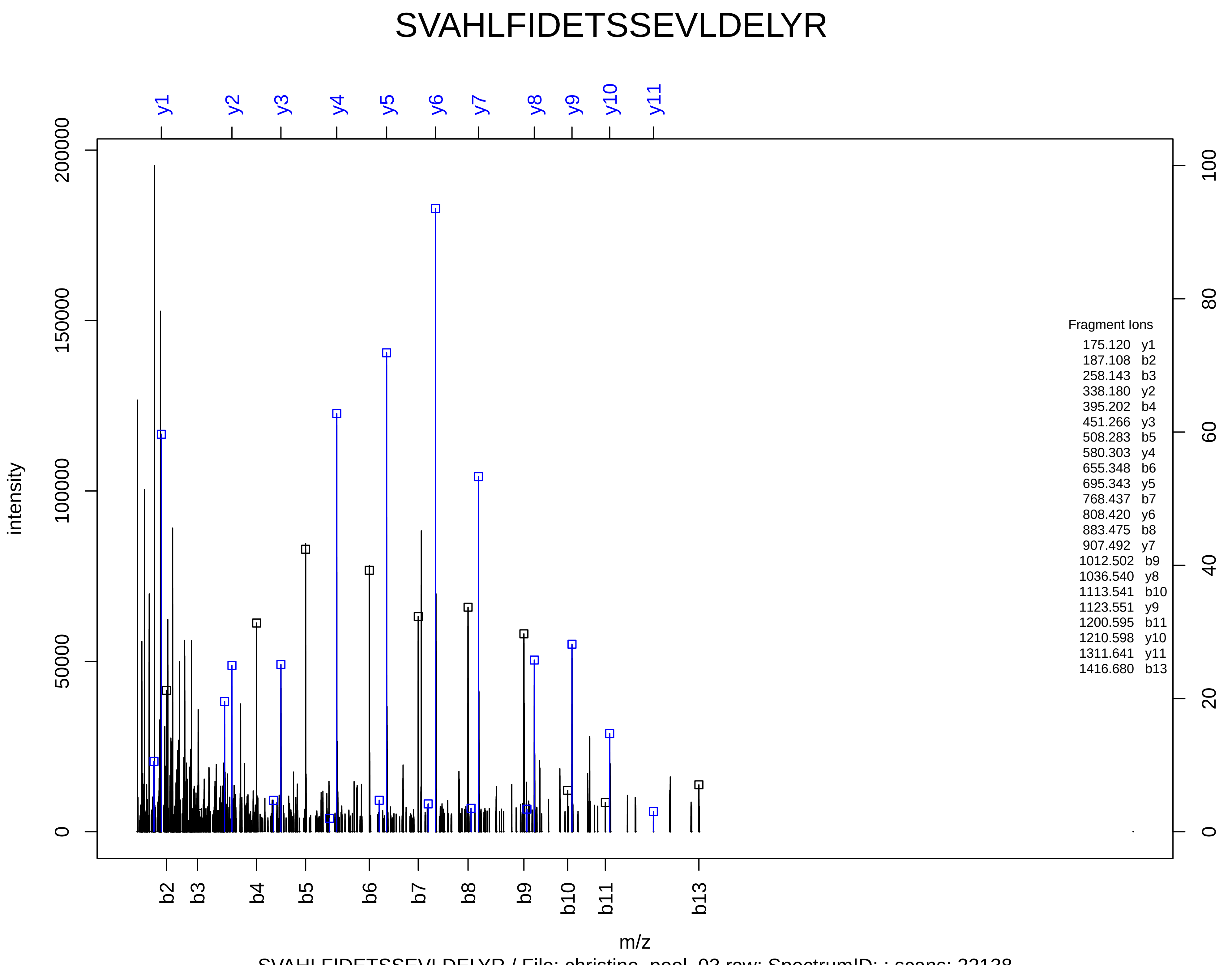
SVAHLFIDETSSEVLDELYR
y1
y2
y3
y4
y5
y6
y7
y8
y9
y10
y11
intensity
0
50000
100000
150000
200000
0
20
40
60
80
100
b2
b3
b4
b5
b6
b7
b8
b9
b10
b11
b13
m/z
Fragment Ions
175.120 y1
187.108 b2
258.143 b3
338.180 y2
395.202 b4
451.266 y3
508.283 b5
580.303 y4
655.348 b6
695.343 y5
768.437 b7
808.420 y6
883.475 b8
907.492 y7
1012.502 b9
1036.540 y8
1113.541 b10
1123.551 y9
1200.595 b11
1210.598 y10
1311.641 y11
1416.680 b13
SVAHLFIDETSSEVLDELYR / File: christine_pool_03.raw; SpectrumID: ; scans: 22138

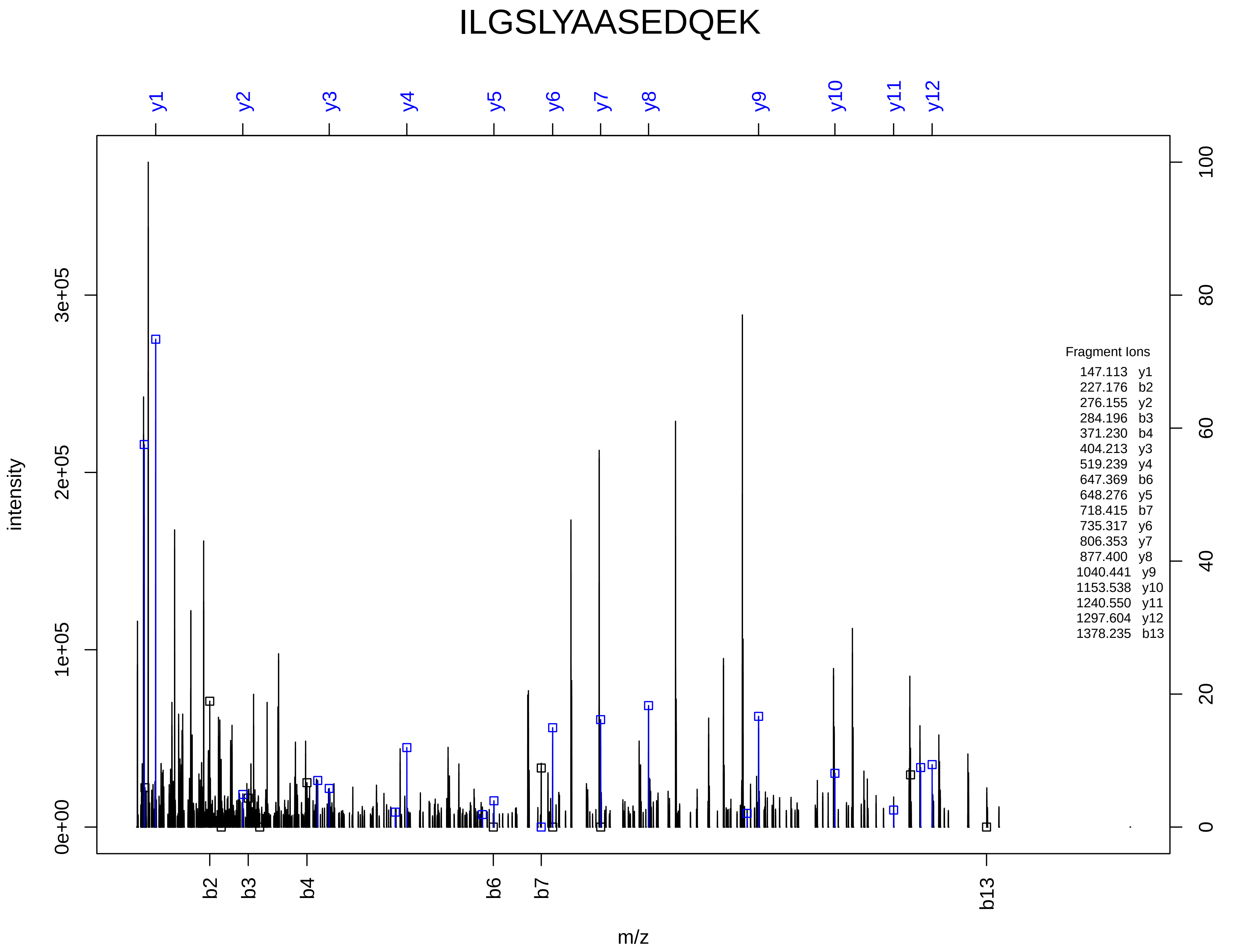

ILGSLYAASEDQEK
y1
y2
y3
y4
y5
y6
y7
y8
y9
y10
y11
y12
b2
b3
b4
b6
b7
b13
intensity
0e+00
1e+05
2e+05
3e+05
0
20
40
60
80
100
m/z
Fragment Ions
147.113 y1
227.176 b2
276.155 y2
284.196 b3
371.230 b4
404.213 y3
519.239 y4
647.369 b6
648.276 y5
718.415 b7
735.317 y6
806.353 y7
877.400 y8
1040.441 y9
1153.538 y10
1240.550 y11
1297.604 y12
1378.235 b13
ILGSLYAASEDQEK / File: christine_pool_03.raw; SpectrumID: ; scans: 9678

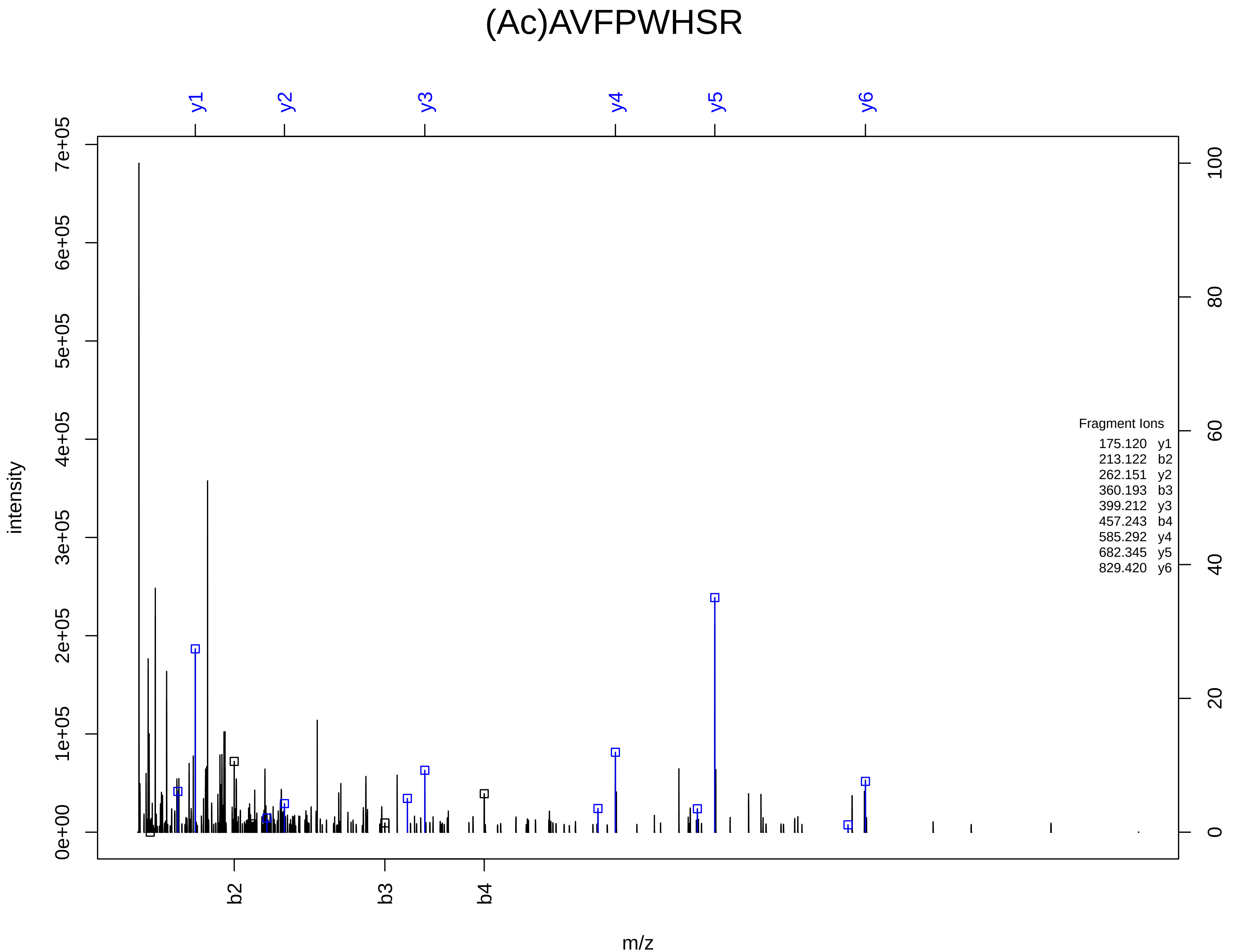
(Ac)AVFPWHSR
y1
y2
y3
y4
y5
y6
intensity
0e+00
1e+05
2e+05
3e+05
4e+05
5e+05
6e+05
7e+05
0
20
40
60
80
100
b2
b3
b4
Fragment Ions
175.120 y1
213.122 b2
262.151 y2
360.193 b3
399.212 y3
457.243 b4
585.292 y4
682.345 y5
829.420 y6
m/z
AVFPWHSR / File: christine_pool_03.raw; SpectrumID: ; scans: 14512

LGDPLEAFPVFK

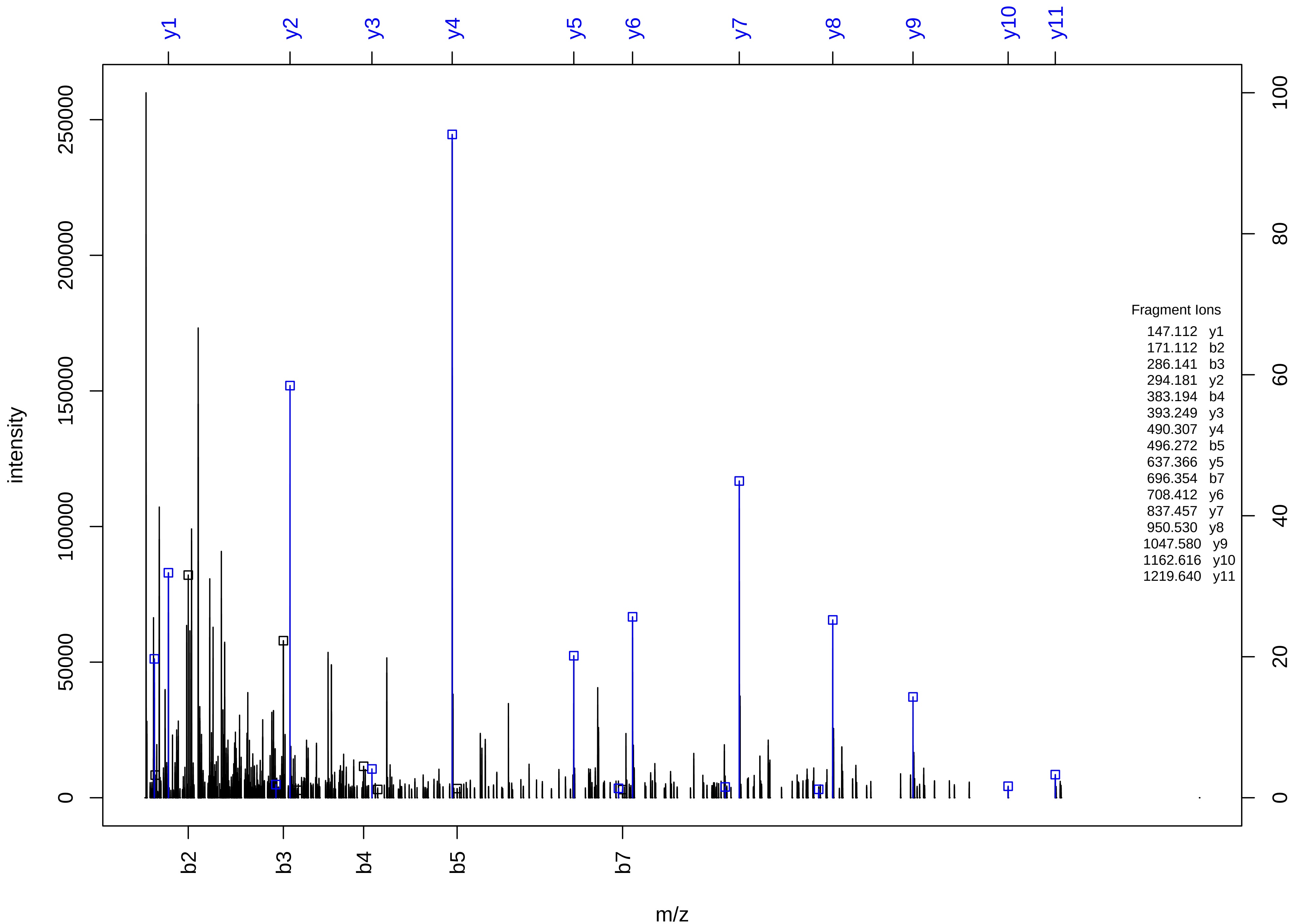

LGDPLEAFPVFK / File: christine_pool_03.raw; SpectrumID: ; scans: 22350

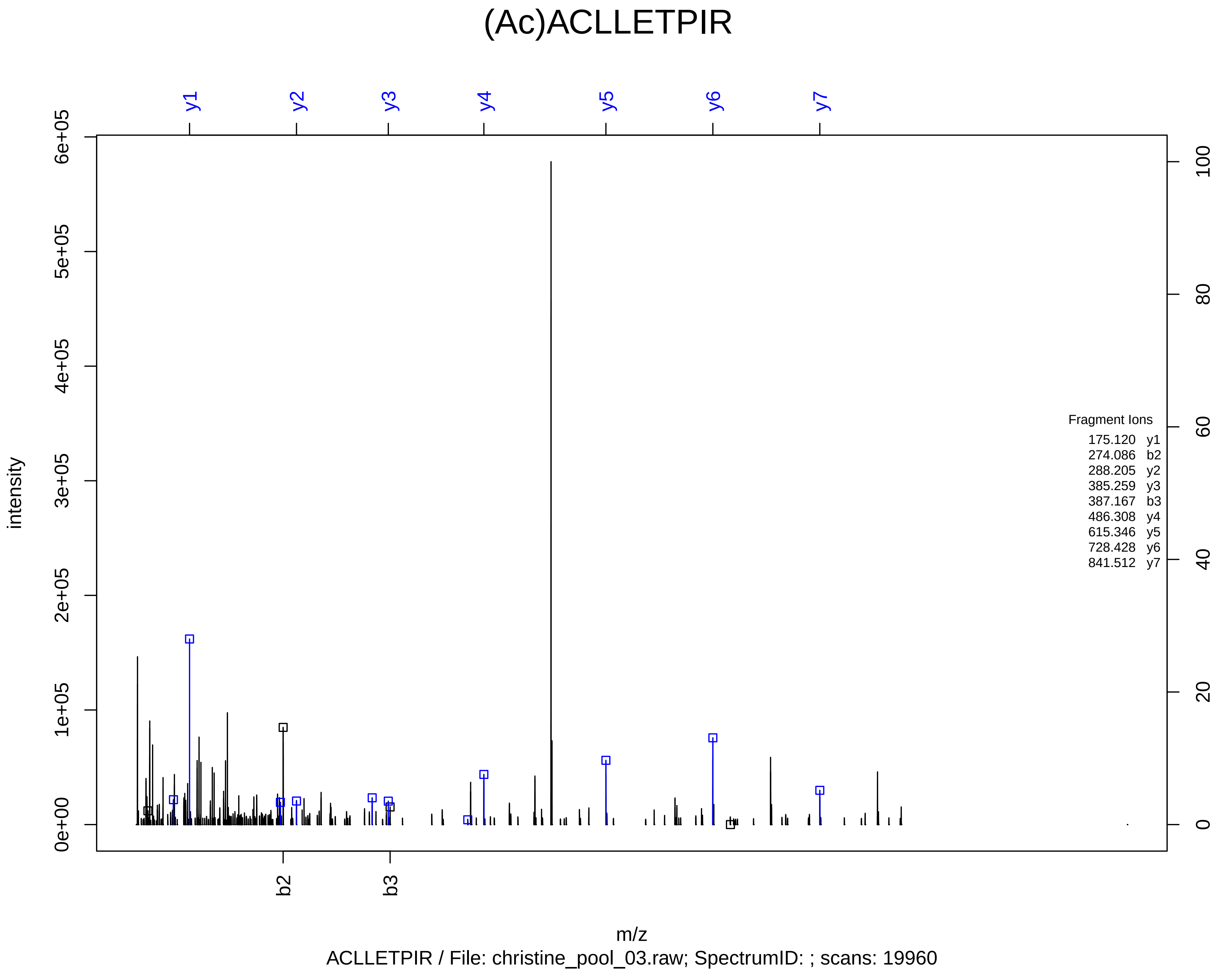
(Ac)ACLLETPIR
y1
y2
y3
y4
y5
y6
y7
b2
b3
intensity
m/z
0e+00
1e+05
2e+05
3e+05
4e+05
5e+05
6e+05
0
20
40
60
80
100
Fragment Ions
175.120 y1
274.086 b2
288.205 y2
385.259 y3
387.167 b3
486.308 y4
615.346 y5
728.428 y6
841.512 y7
ACLLETPIR / File: christine_pool_03.raw; SpectrumID: ; scans: 19960

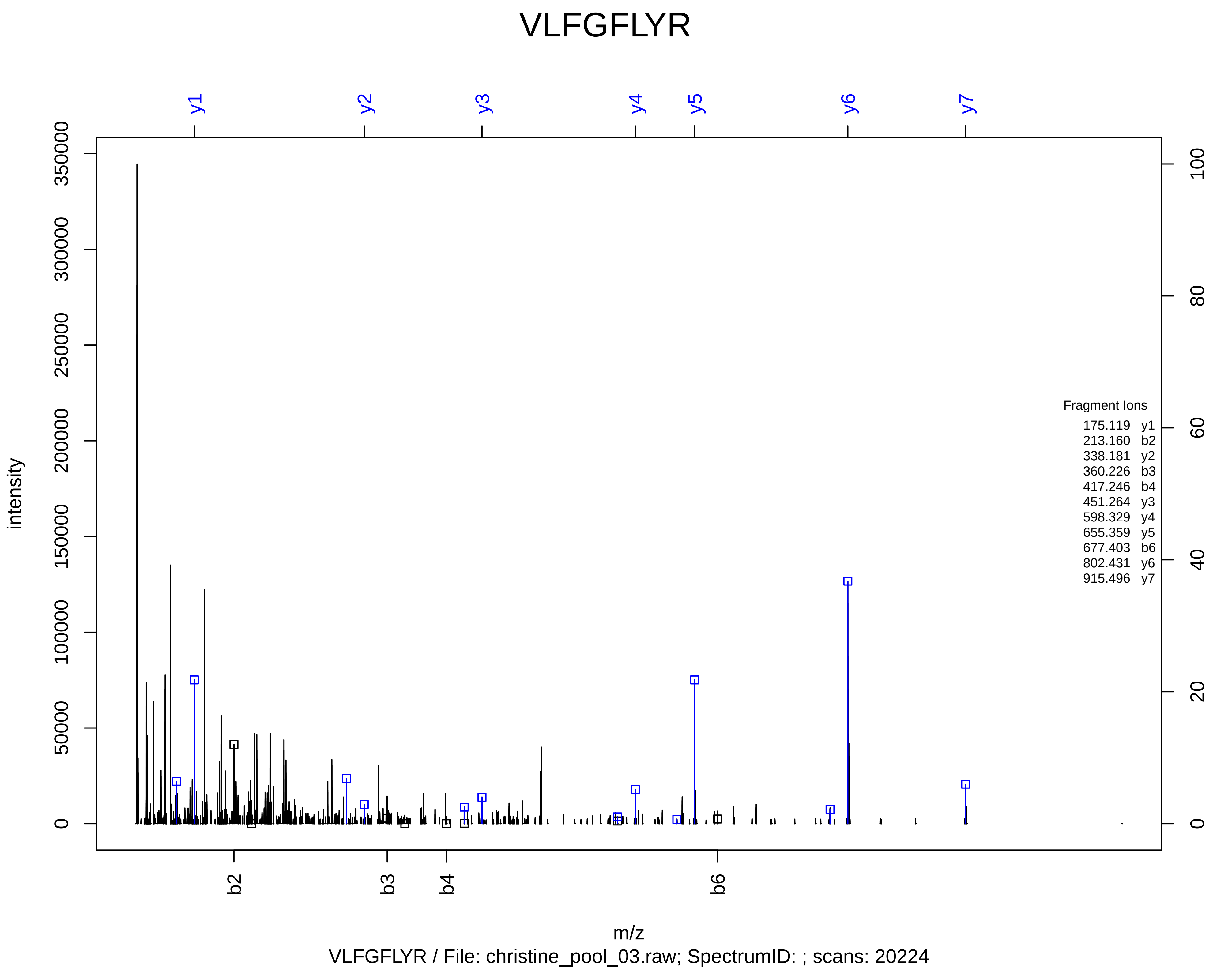
VLFGFLYR
y1
y2
y3
y4
y5
y6
y7
b2
b3
b4
b6
intensity
m/z
Fragment Ions
175.119 y1
213.160 b2
338.181 y2
360.226 b3
417.246 b4
451.264 y3
598.329 y4
655.359 y5
677.403 b6
802.431 y6
915.496 y7
0
50000
100000
150000
200000
250000
300000
350000
0
20
40
60
80
100
VLFGFLYR / File: christine_pool_03.raw; SpectrumID: ; scans: 20224

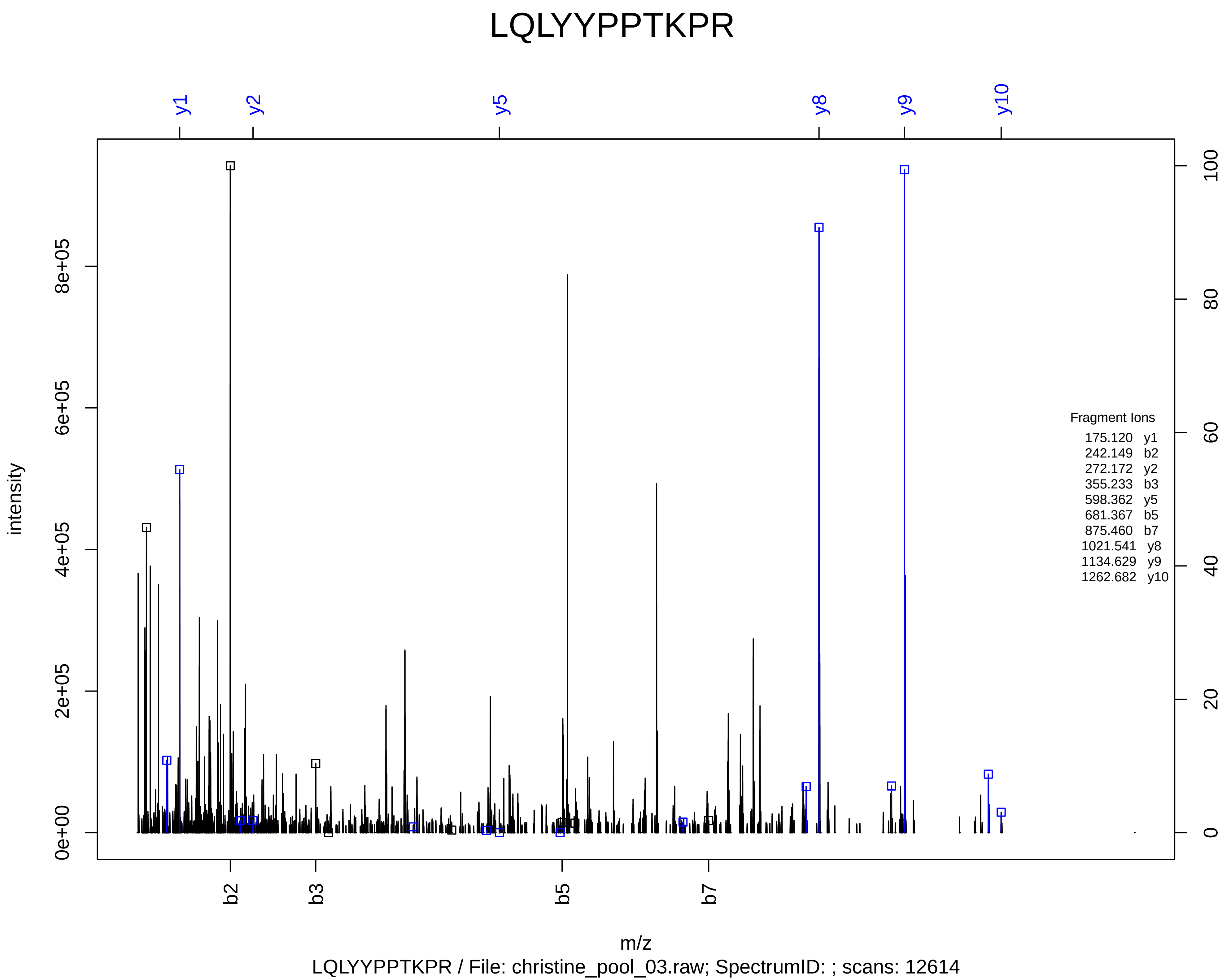

LQLYYPPTKPR
y1
y2
y5
y8
y9
y10
b2
b3
b5
b7
intensity
0e+00
2e+05
4e+05
6e+05
8e+05
0
20
40
60
80
100
Fragment Ions
175.120 y1
242.149 b2
272.172 y2
355.233 b3
598.362 y5
681.367 b5
875.460 b7
1021.541 y8
1134.629 y9
1262.682 y10
m/z
LQLYYPPTKPR / File: christine_pool_03.raw; SpectrumID: ; scans: 12614

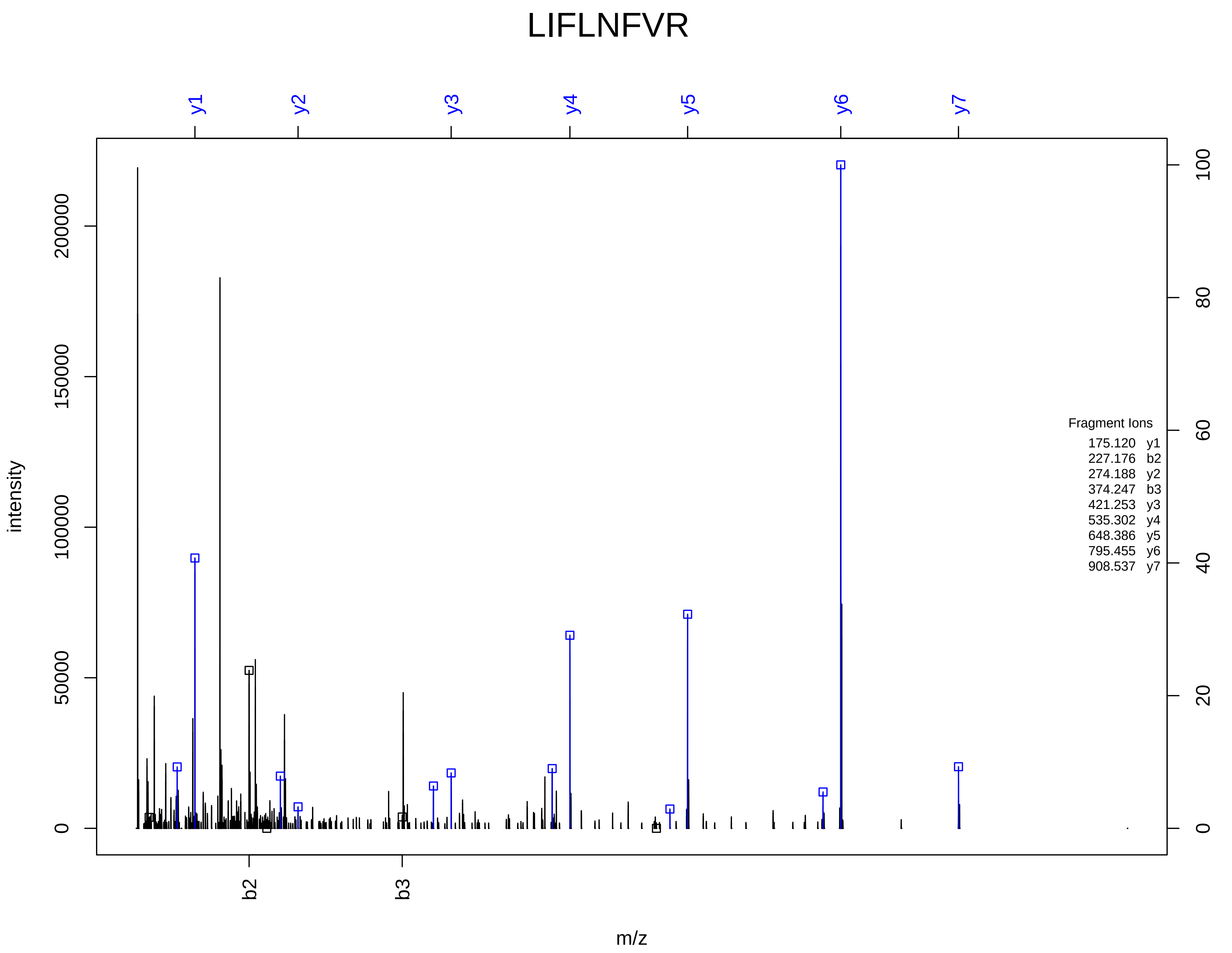

LIFLNFVR / File: christine_pool_03.raw; SpectrumID: ; scans: 21346

TVEINNTDAEGR

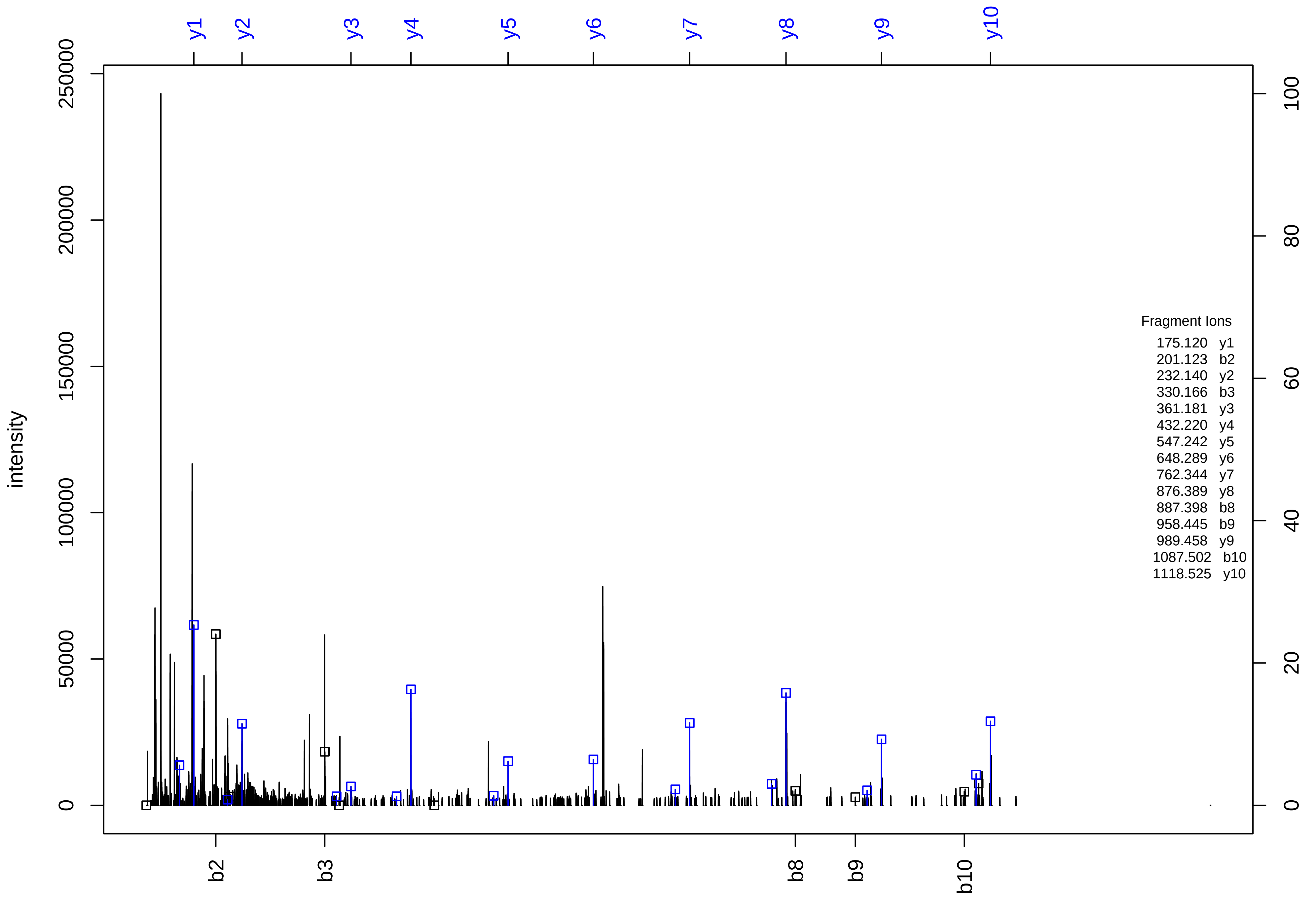

TVEINNTDAEGR / File: christine_pool_03.raw; SpectrumID: ; scans: 3318

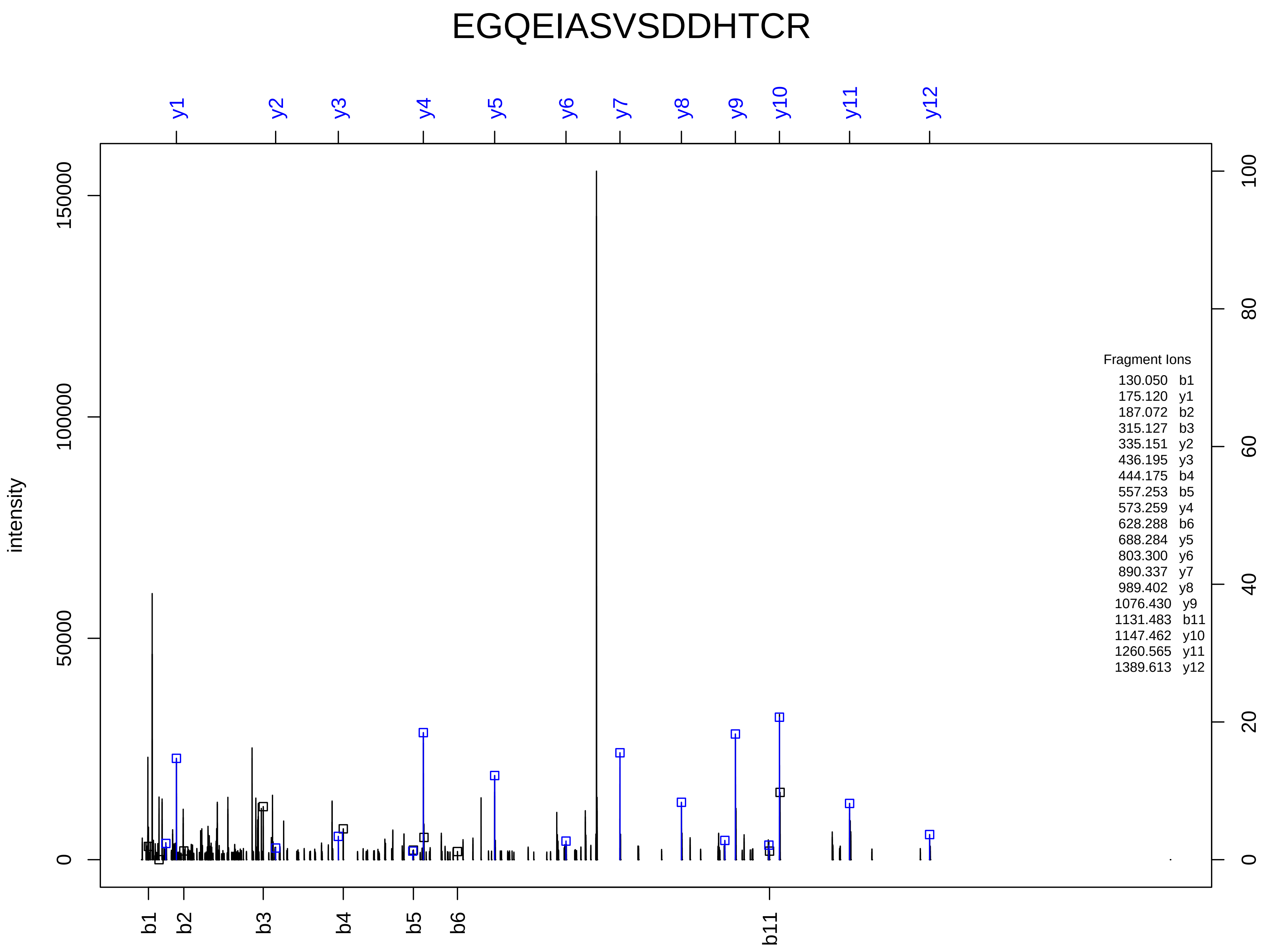

EGQEIASVSDDHTCR / File: christine_pool_03.raw; SpectrumID: ; scans: 3388

SIYDDISSPGLGSTPLTSR

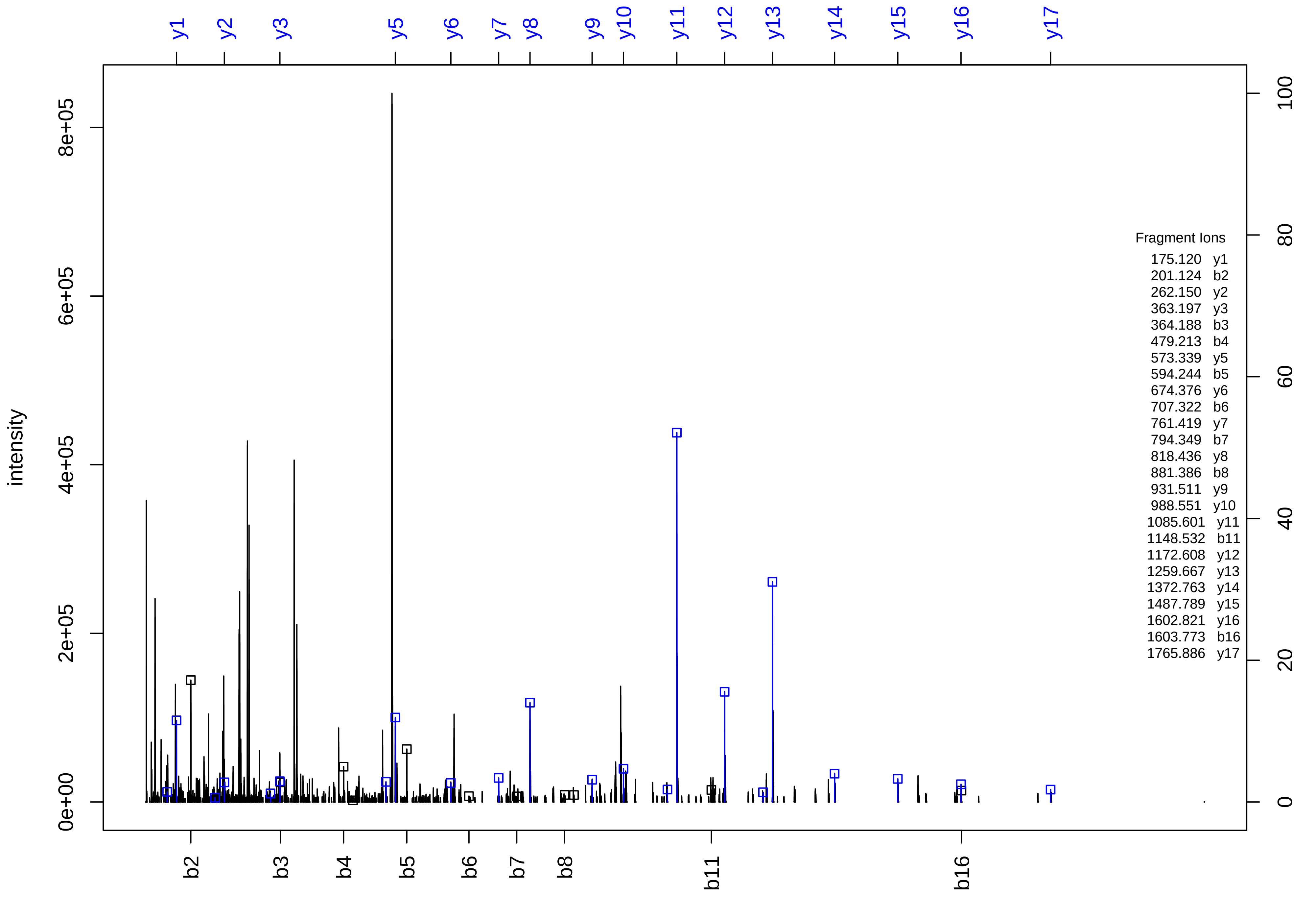

SIYDDISSPGLGSTPLTSR / File: christine_pool_03.raw; SpectrumID: ; scans: 16147

IAKNEAATN^ELM*R

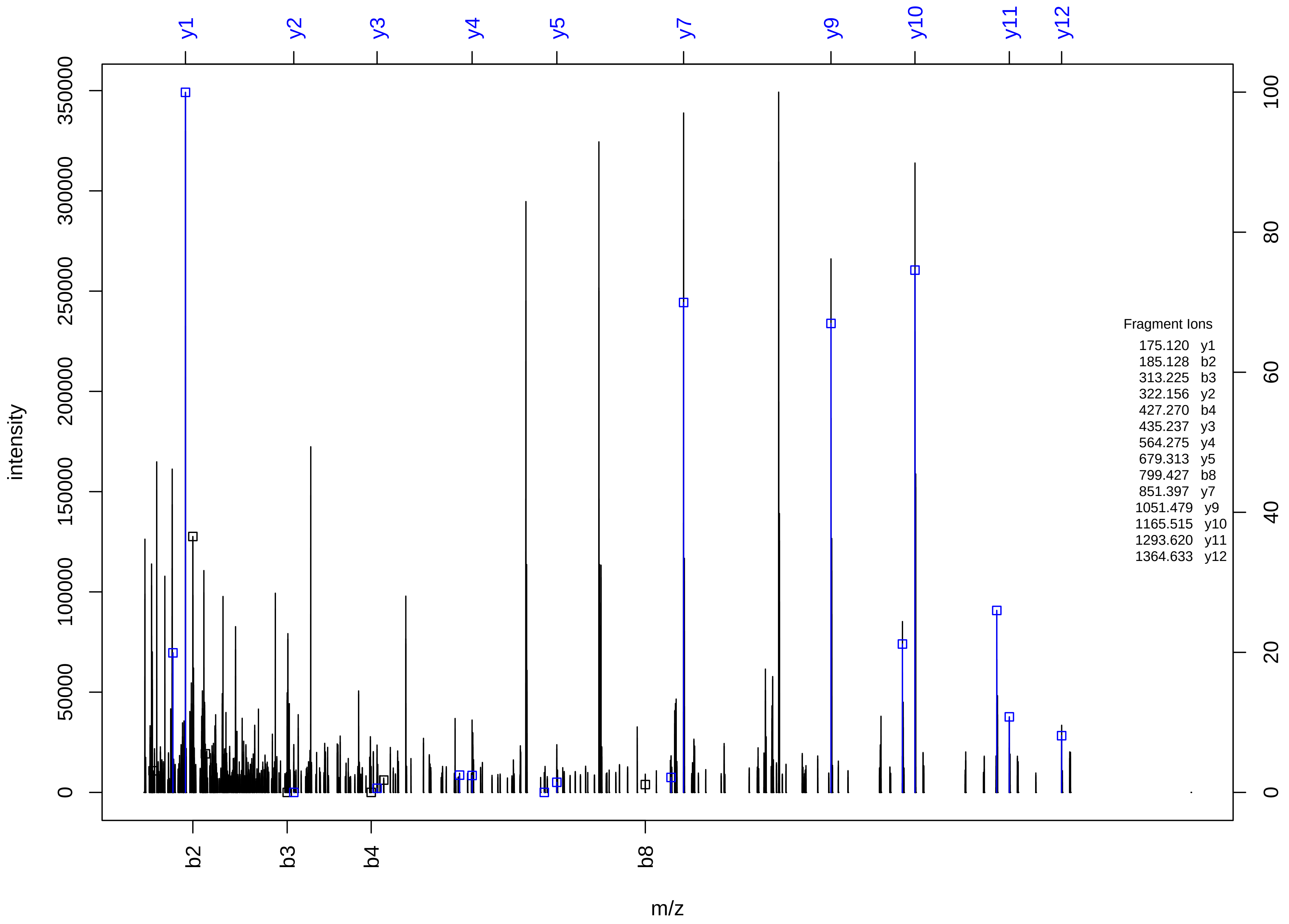

IAKNEAATNELMR / File: christine_pool_03.raw; SpectrumID: ; scans: 9008

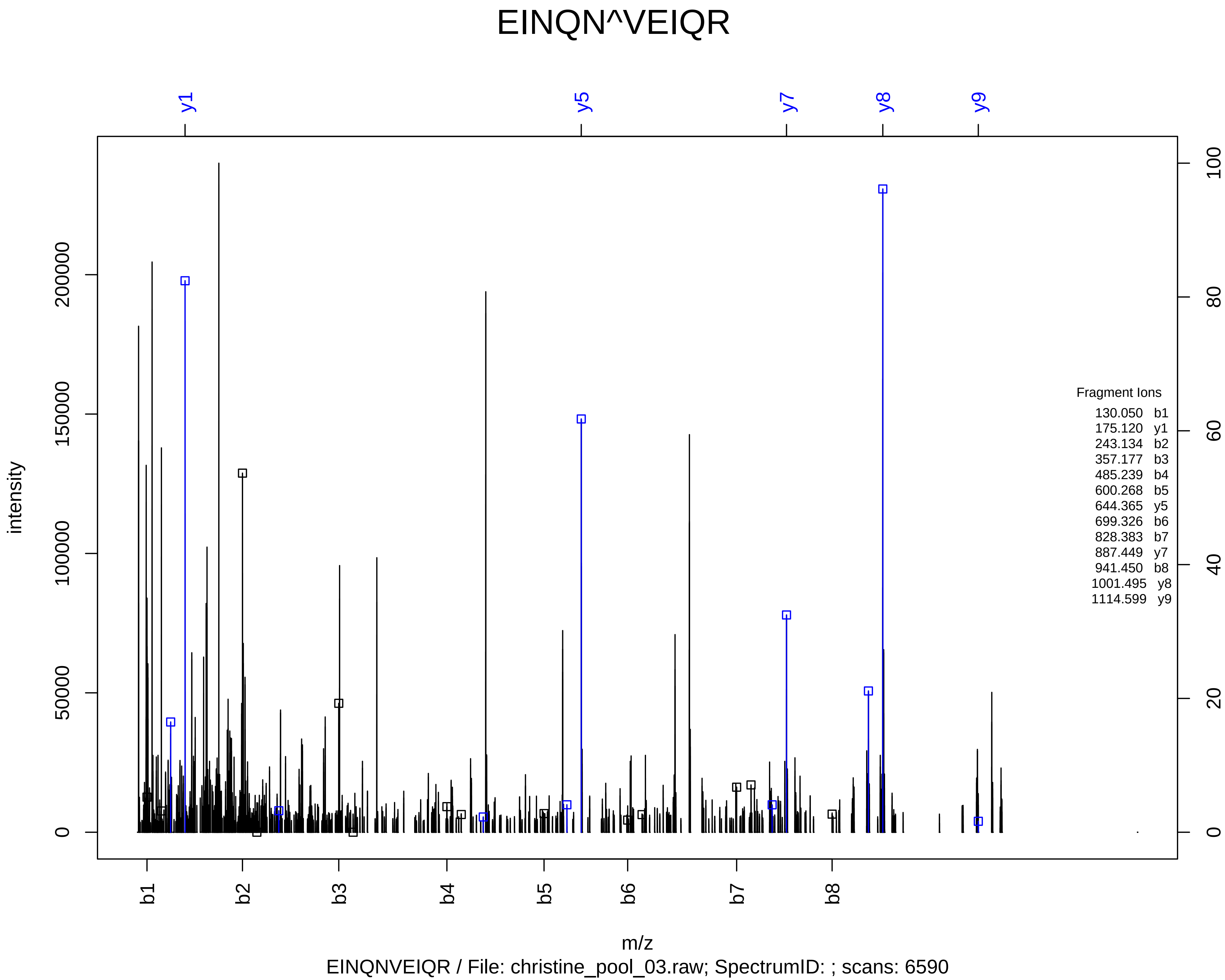
EINQN^VEIQR
y1
y5
y7
y8
y9
intensity
0
50000
100000
150000
200000
0
20
40
60
80
100
Fragment Ions
130.050 b1
175.120 y1
243.134 b2
357.177 b3
485.239 b4
600.268 b5
644.365 y5
699.326 b6
828.383 b7
887.449 y7
941.450 b8
1001.495 y8
1114.599 y9
b1
b2
b3
b4
b5
b6
b7
b8
m/z
EINQNVEIQR / File: christine_pool_03.raw; SpectrumID: ; scans: 6590

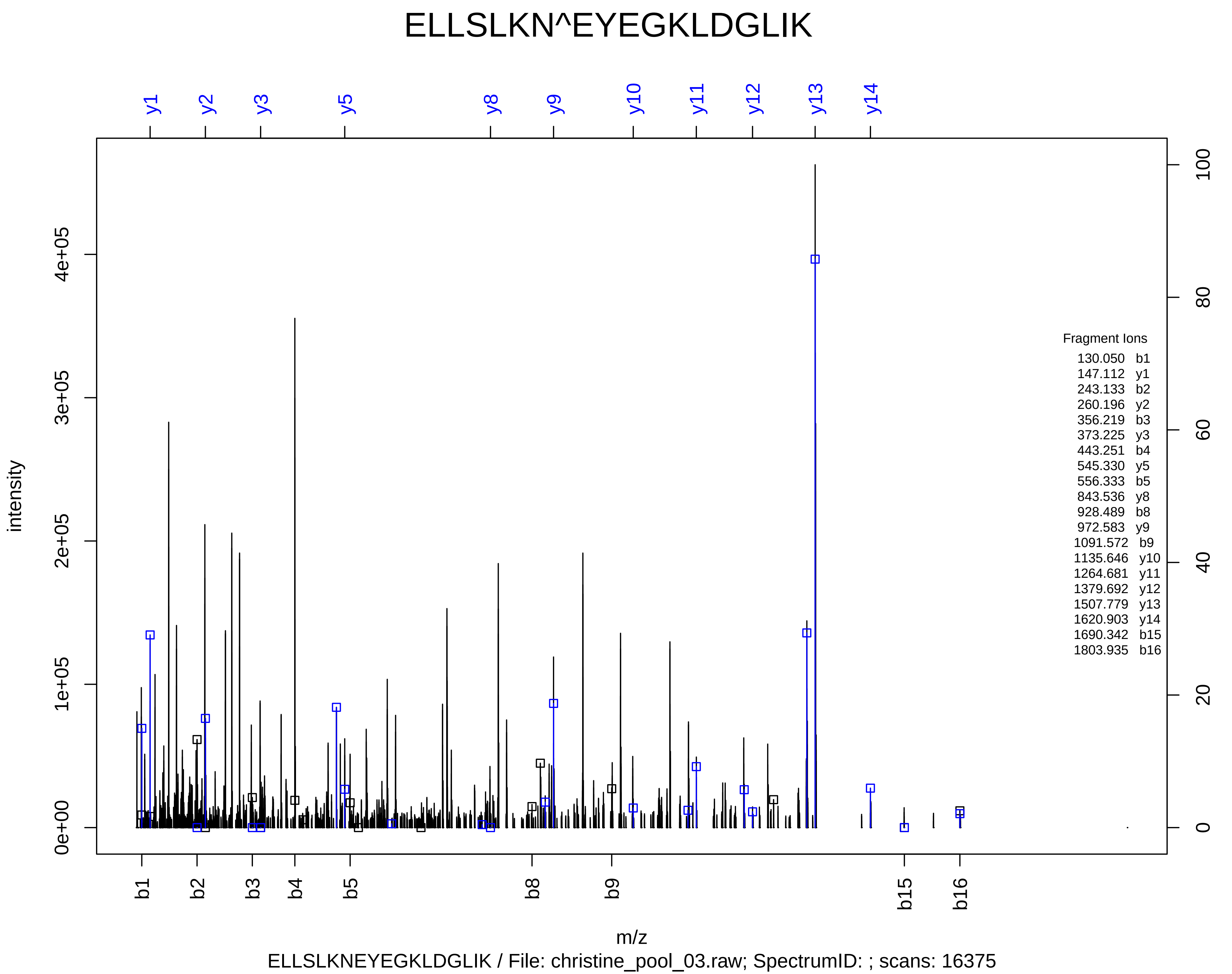

ELLSLKN^EYEGKLDGLIK
y1 y2 y3 y5 y8 y9 y10 y11 y12 y13 y14
b1 b2 b3 b4 b5 b8 b9 b15 b16
intensity
m/z
Fragment Ions
130.050 b1
147.112 y1
243.133 b2
260.196 y2
356.219 b3
373.225 y3
443.251 b4
545.330 y5
556.333 b5
843.536 y8
928.489 b8
972.583 y9
1091.572 b9
1135.646 y10
1264.681 y11
1379.692 y12
1507.779 y13
1620.903 y14
1690.342 b15
1803.935 b16
ELLSLKNEYEGKLDGLIK / File: christine_pool_03.raw; SpectrumID: ; scans: 16375

SASSGAEGDVSSEREP

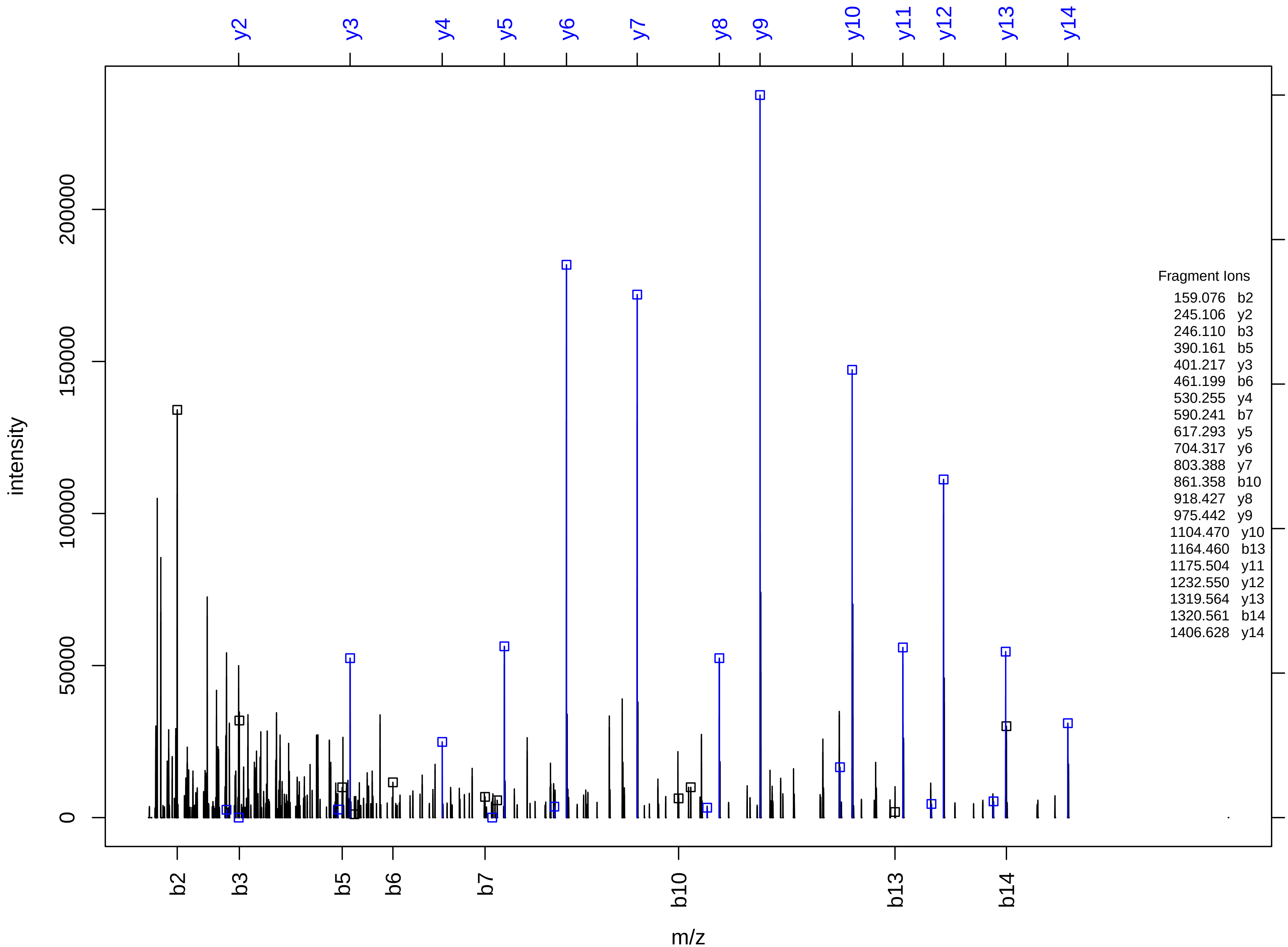

SASSGAEGDVSSEREP / File: christine_pool_03.raw; SpectrumID: ; scans: 2746

ALDLENIM*R

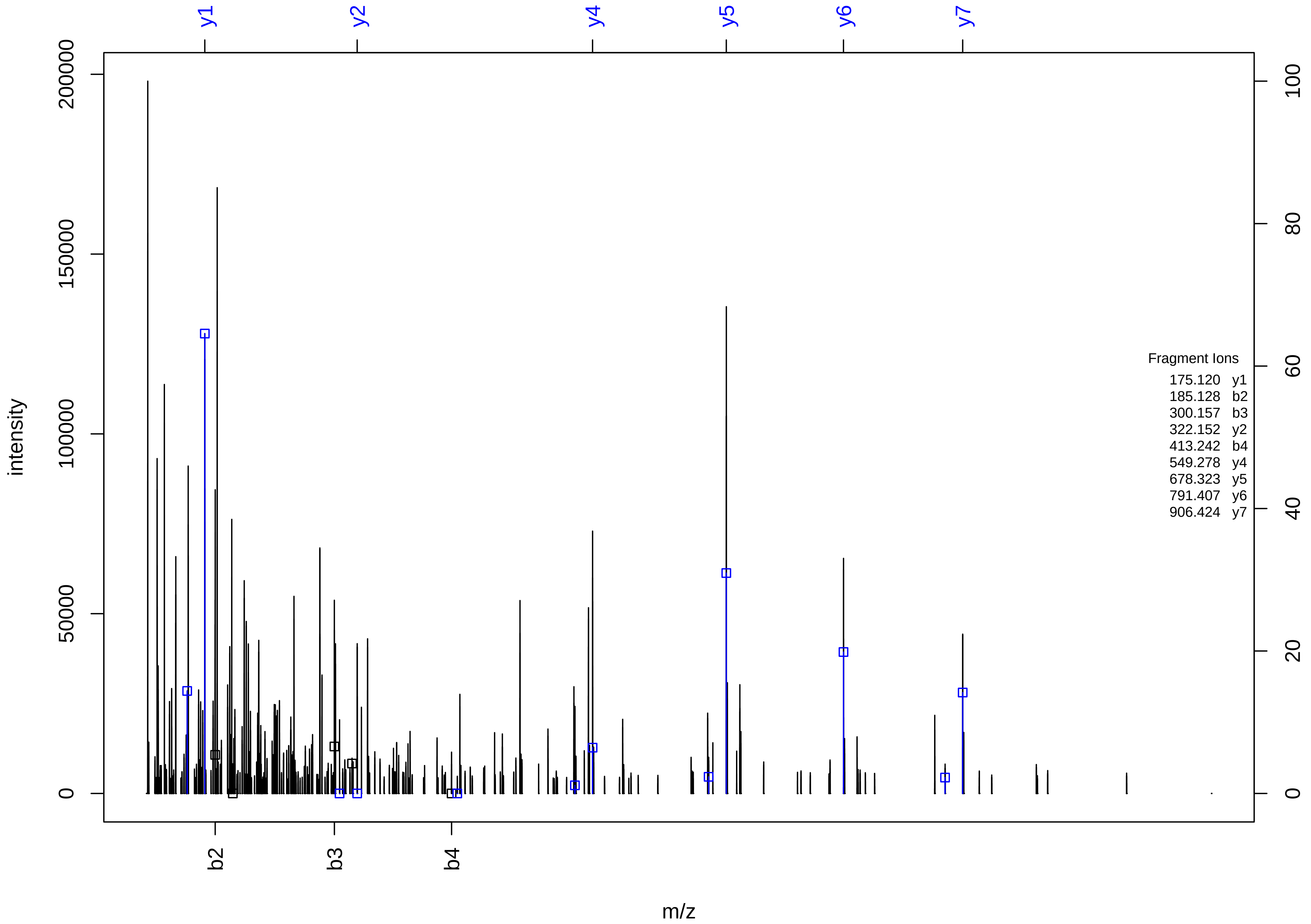

ALDLENIMR / File: christine_pool_03.raw; SpectrumID: ; scans: 19790

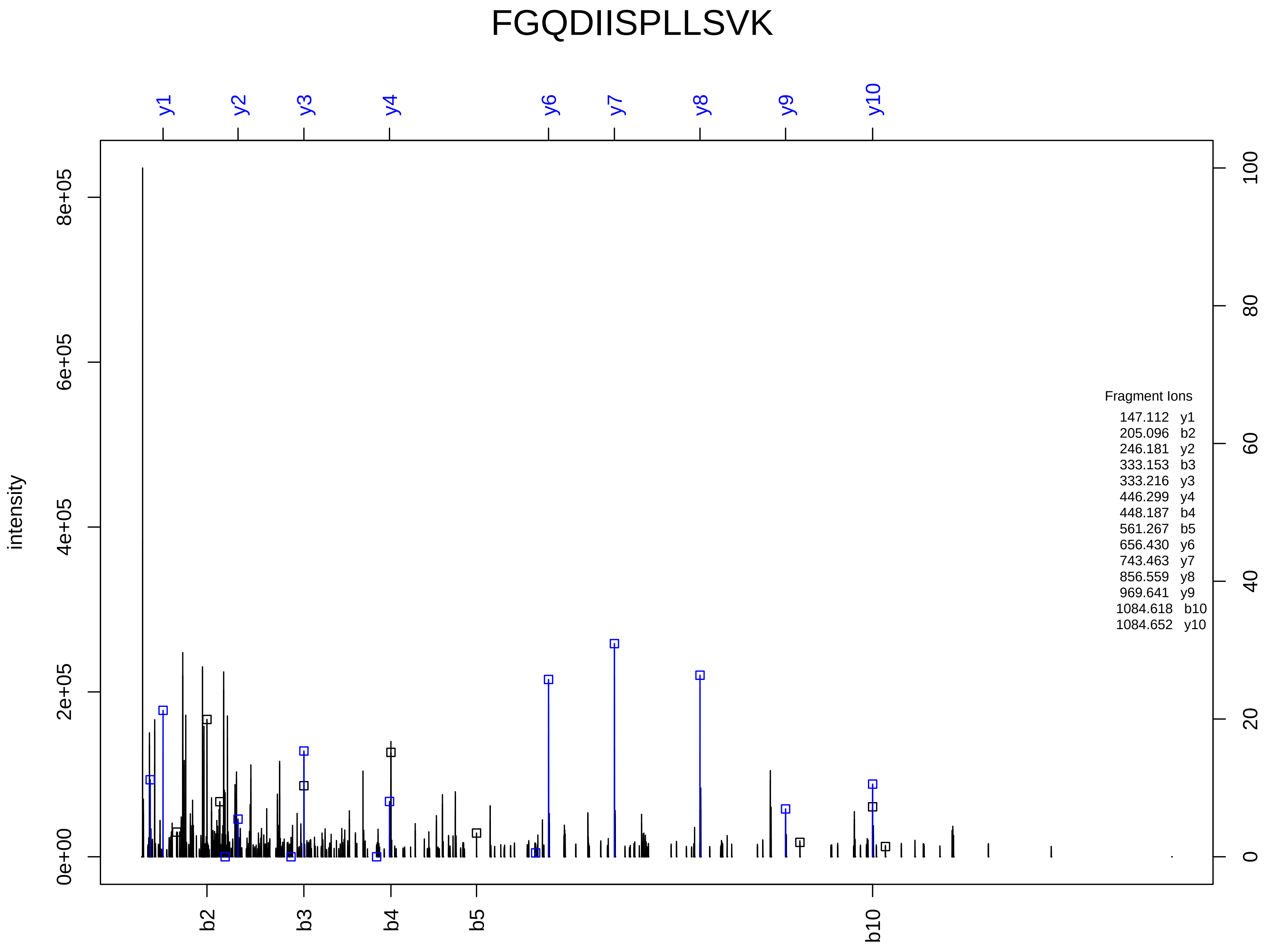

FGQDIISPLLSVK / File: christine_pool_03.raw; SpectrumID: ; scans: 20582

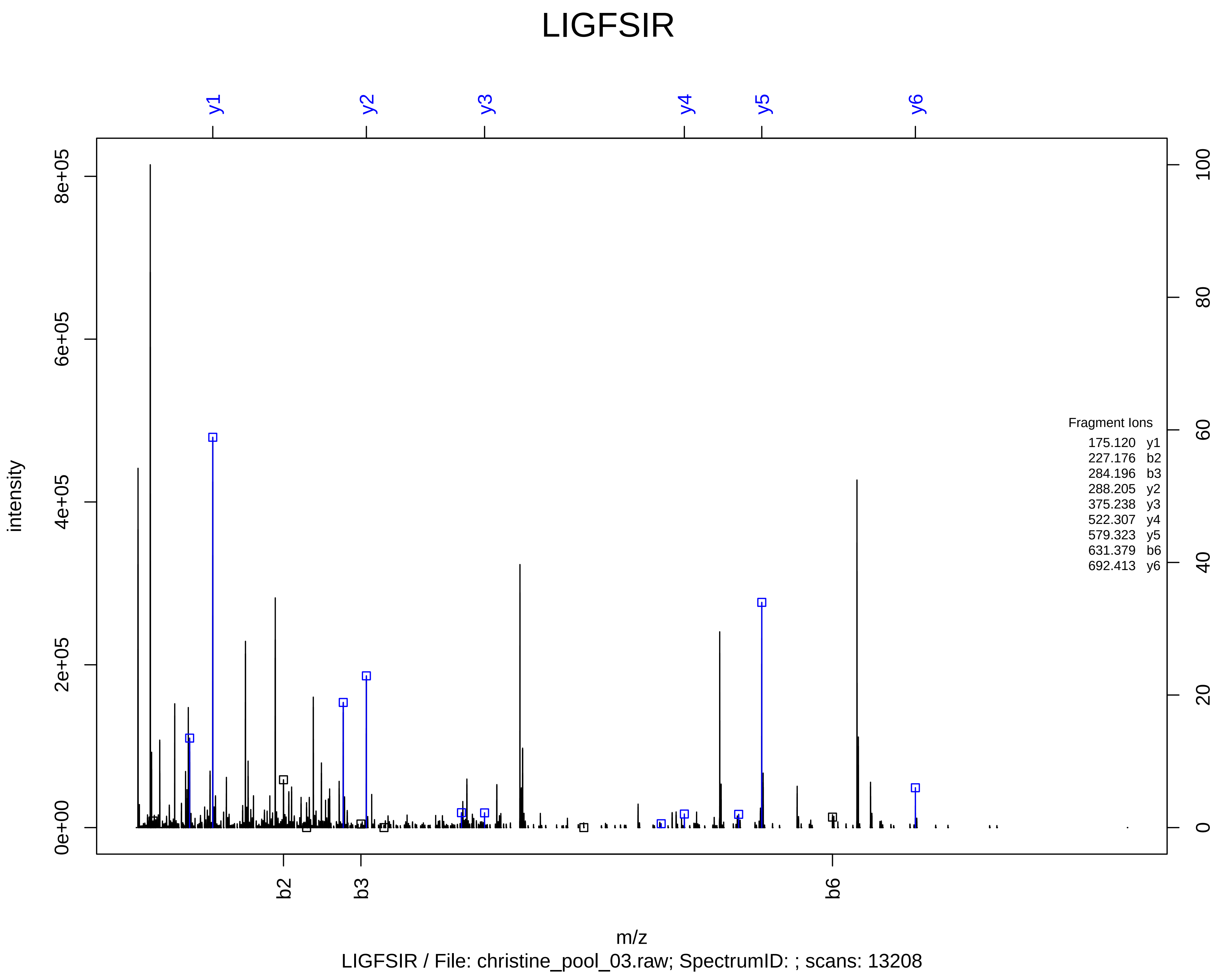

LIGFSIR
y1
y2
y3
y4
y5
y6
intensity
0e+00
2e+05
4e+05
6e+05
8e+05
0
20
40
60
80
100
Fragment Ions
175.120 y1
227.176 b2
284.196 b3
288.205 y2
375.238 y3
522.307 y4
579.323 y5
631.379 b6
692.413 y6
b2
b3
b6
m/z
LIGFSIR / File: christine_pool_03.raw; SpectrumID: ; scans: 13208

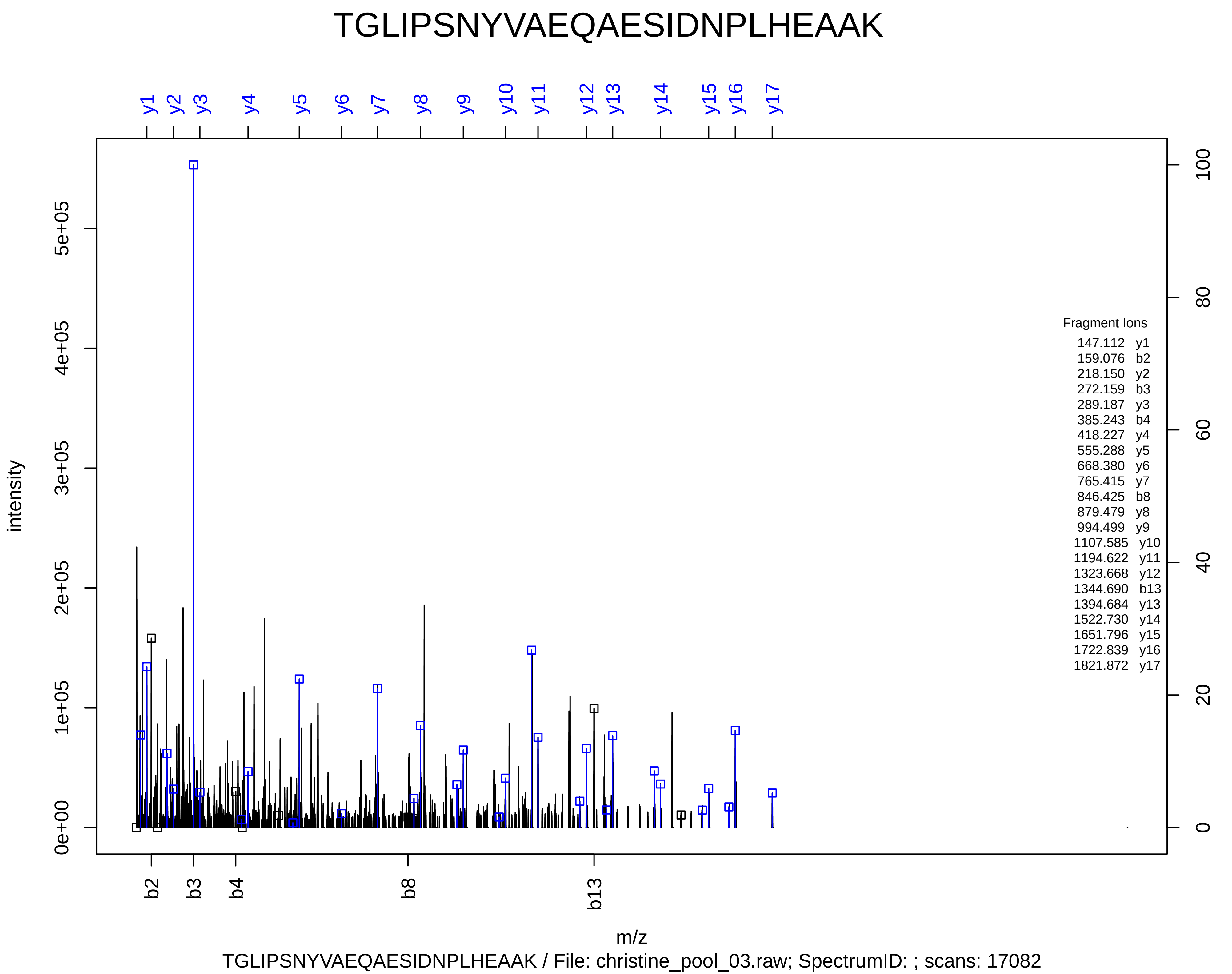
TGLIPSNYVAEQAESIDNPLHEAAK
intensity
m/z
Fragment Ions
147.112 y1
159.076 b2
218.150 y2
272.159 b3
289.187 y3
385.243 b4
418.227 y4
555.288 y5
668.380 y6
765.415 y7
846.425 b8
879.479 y8
994.499 y9
1107.585 y10
1194.622 y11
1323.668 y12
1344.690 b13
1394.684 y13
1522.730 y14
1651.796 y15
1722.839 y16
1821.872 y17
TGLIPSNYVAEQAESIDNPLHEAAK / File: christine_pool_03.raw; SpectrumID: ; scans: 17082

ATEAGSLEAR

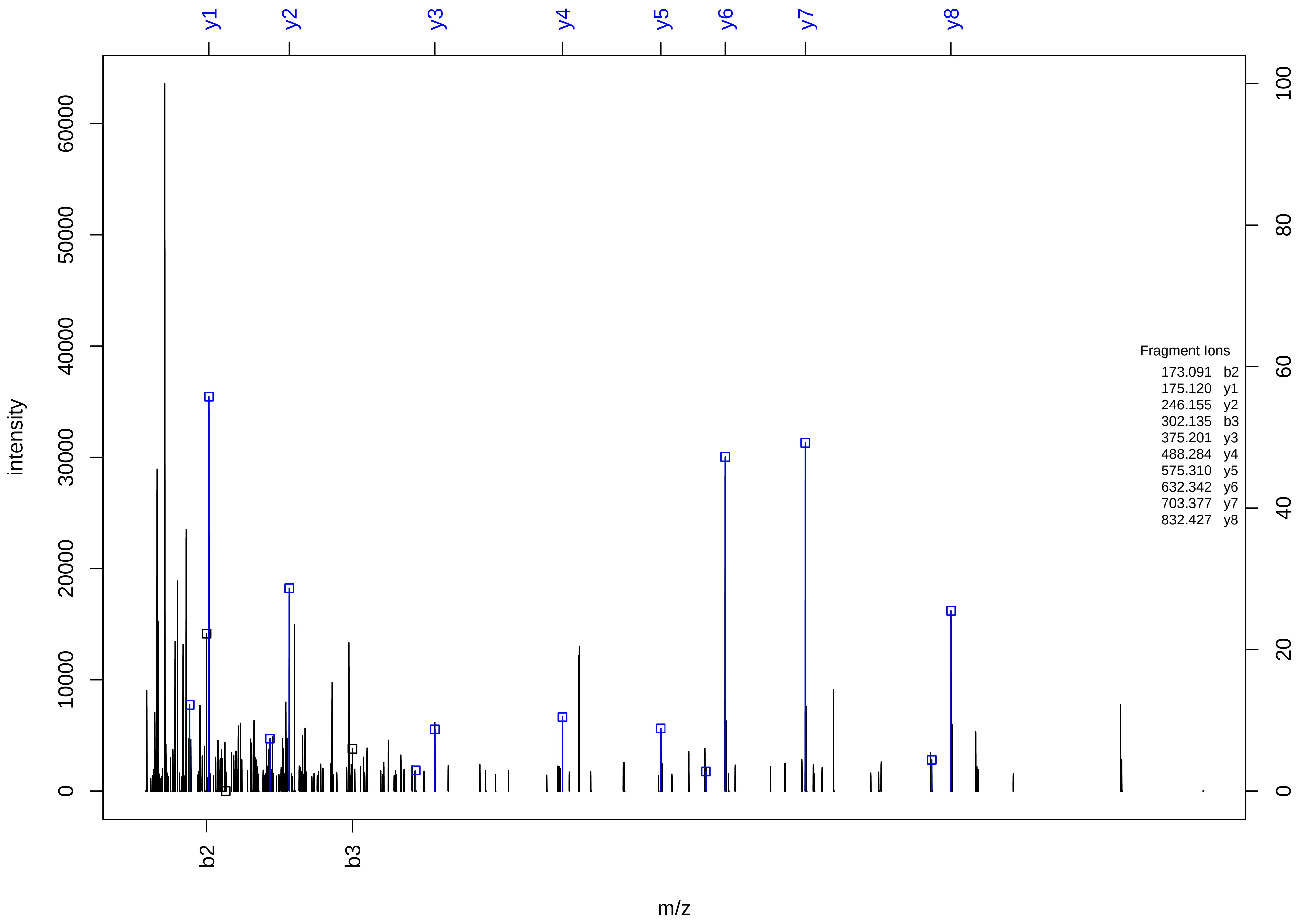

ATEAGSLEAR / File: christine_pool_03.raw; SpectrumID: ; scans: 2400

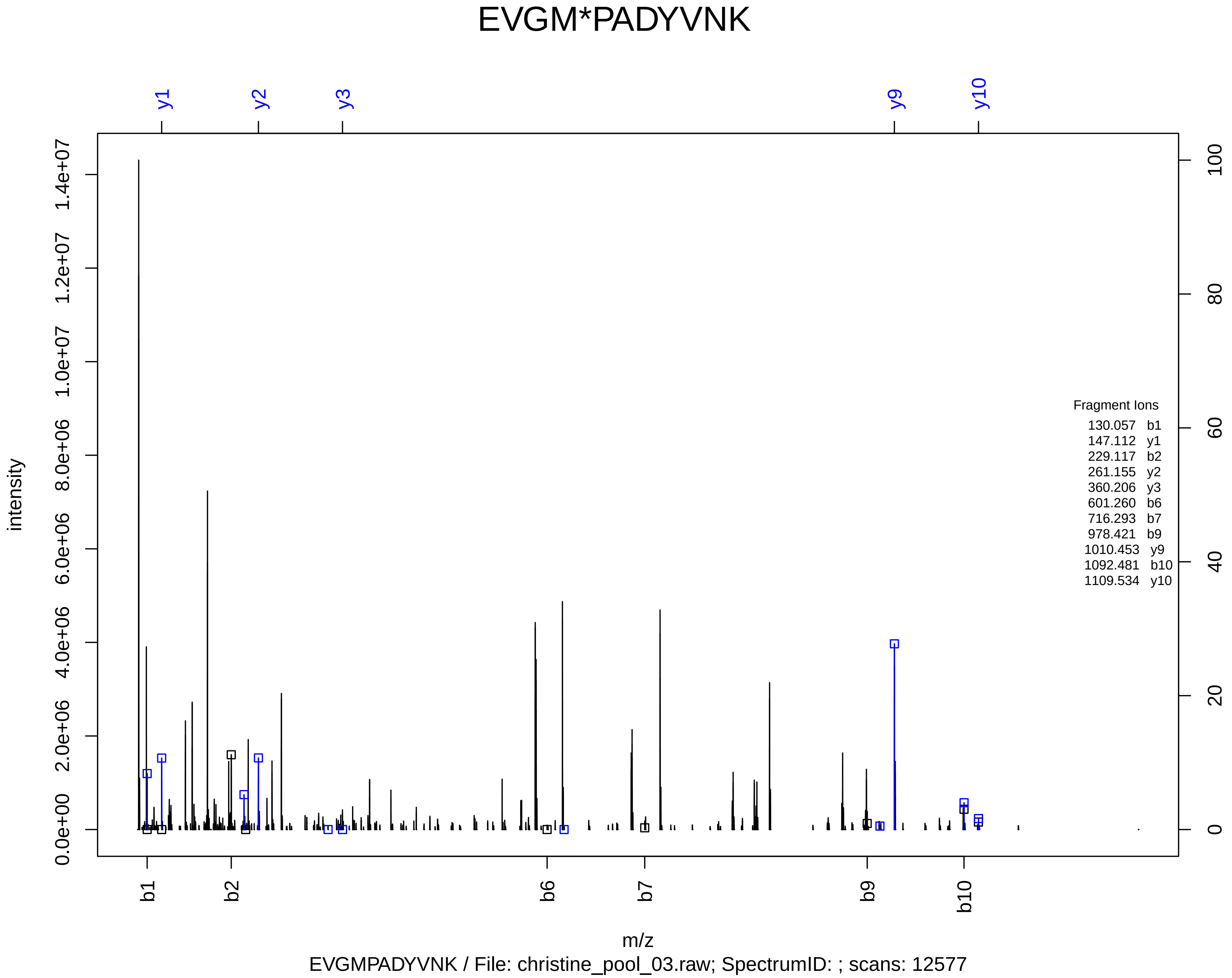
EVGM*PADYVNK
y1
y2
y3
y9
y10
intensity
0.0e+00
2.0e+06
4.0e+06
6.0e+06
8.0e+06
1.0e+07
1.2e+07
1.4e+07
0
20
40
60
80
100
b1
b2
b6
b7
b9
b10
m/z
Fragment Ions
130.057 b1
147.112 y1
229.117 b2
261.155 y2
360.206 y3
601.260 b6
716.293 b7
978.421 b9
1010.453 y9
1092.481 b10
1109.534 y10
EVGMPADYVNK / File: christine_pool_03.raw; SpectrumID: ; scans: 12577

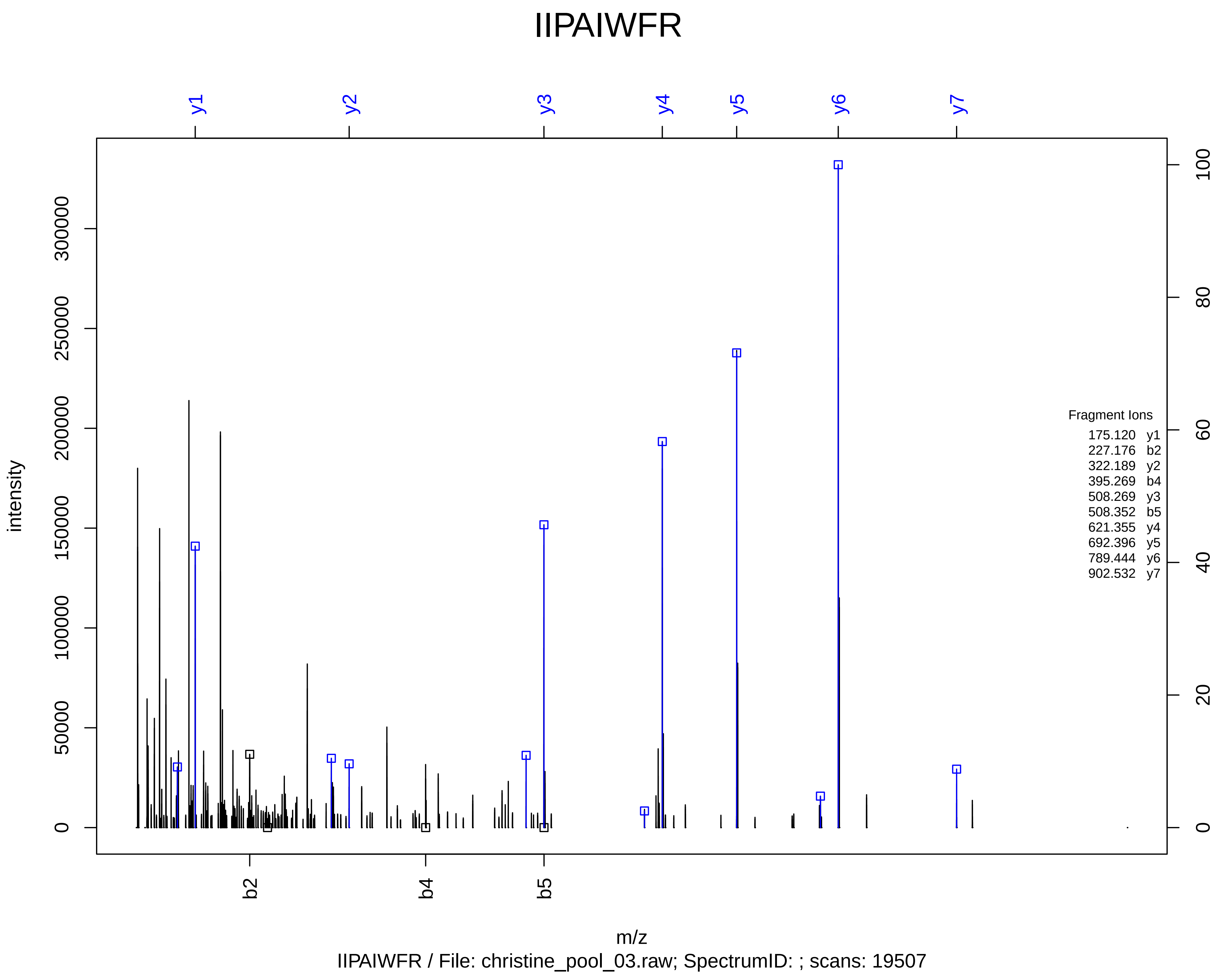
IIPAIWFR
y1
y2
y3
y4
y5
y6
y7
intensity
0
50000
100000
150000
200000
250000
300000
0
20
40
60
80
100
b2
b4
b5
m/z
Fragment Ions
175.120 y1
227.176 b2
322.189 y2
395.269 b4
508.269 y3
508.352 b5
621.355 y4
692.396 y5
789.444 y6
902.532 y7
IIPAIWFR / File: christine_pool_03.raw; SpectrumID: ; scans: 19507

LEILQLLK

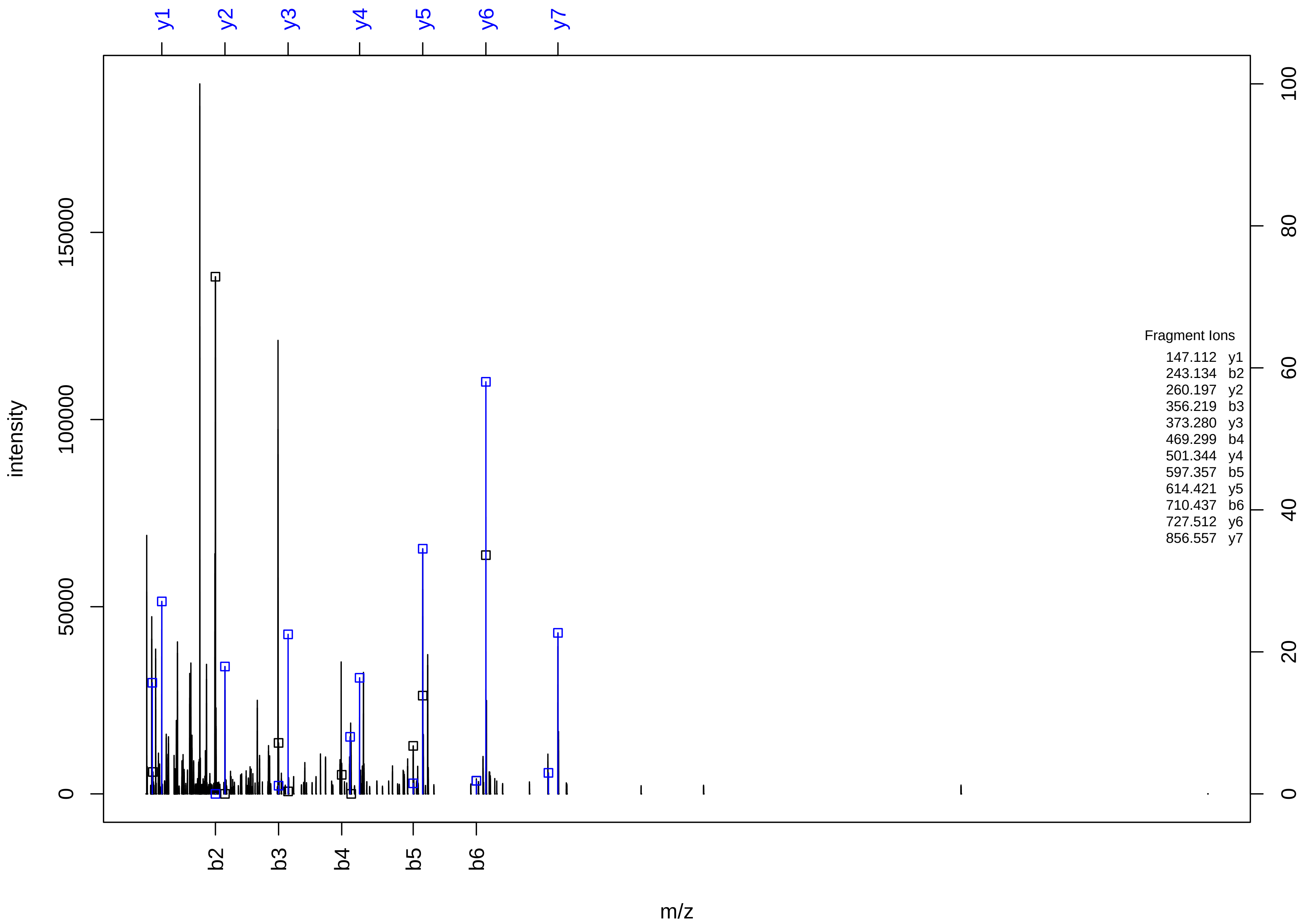

LEILQLLK / File: christine_pool_03.raw; SpectrumID: ; scans: 20802

EEVMAATQAEGPK

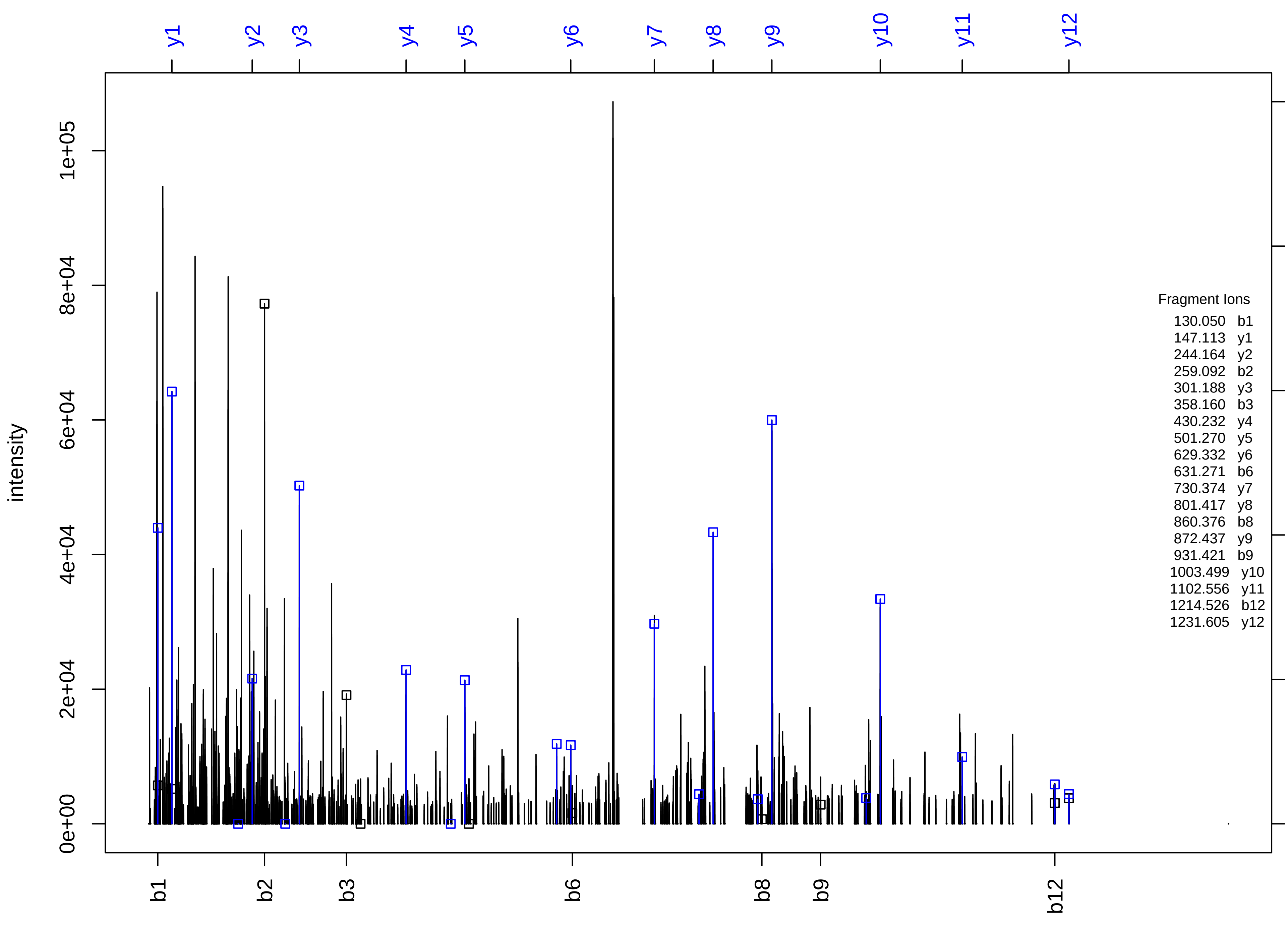

EEVMAATQAEGPK / File: christine_pool_03.raw; SpectrumID: ; scans: 4374

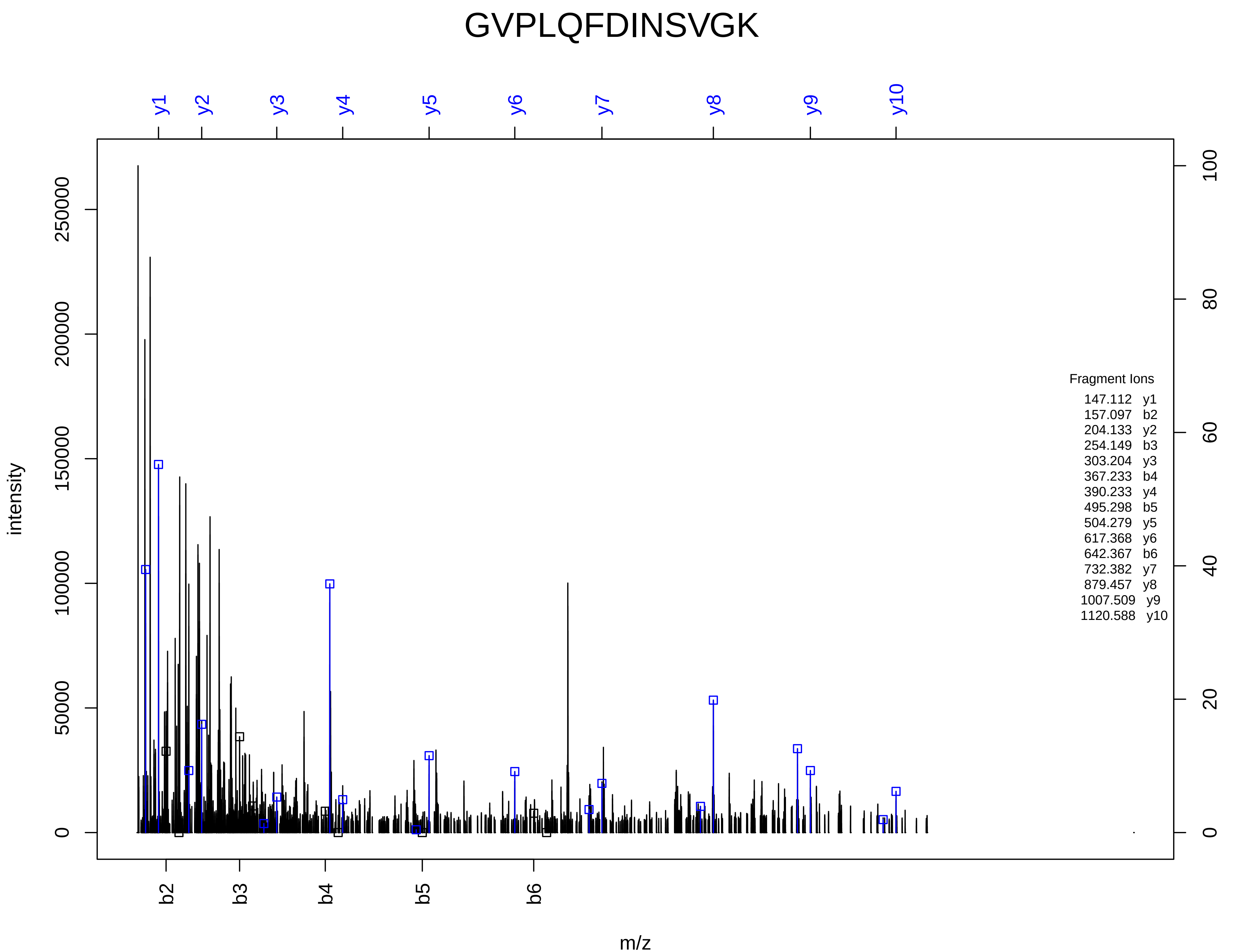
GVPLQFDINSVGK
y1
y2
y3
y4
y5
y6
y7
y8
y9
y10
intensity
0
50000
100000
150000
200000
250000
0
20
40
60
80
100
b2
b3
b4
b5
b6
m/z
Fragment Ions
147.112 y1
157.097 b2
204.133 y2
254.149 b3
303.204 y3
367.233 b4
390.233 y4
495.298 b5
504.279 y5
617.368 y6
642.367 b6
732.382 y7
879.457 y8
1007.509 y9
1120.588 y10
GVPLQFDINSVGK / File: christine_pool_03.raw; SpectrumID: ; scans: 15881

TILFIDEIHR

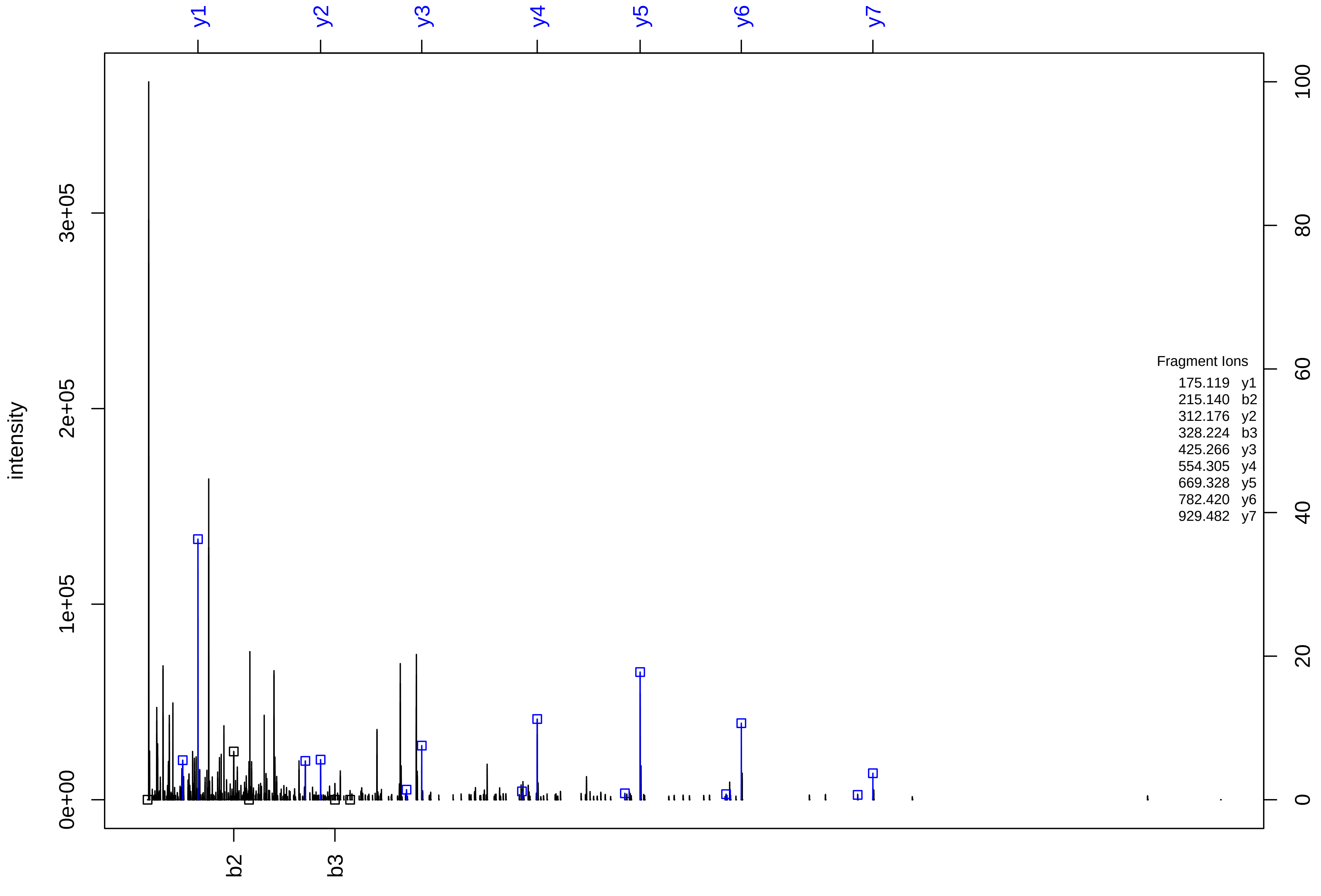

TILFIDEIHR / File: christine_pool_03.raw; SpectrumID: ; scans: 14792

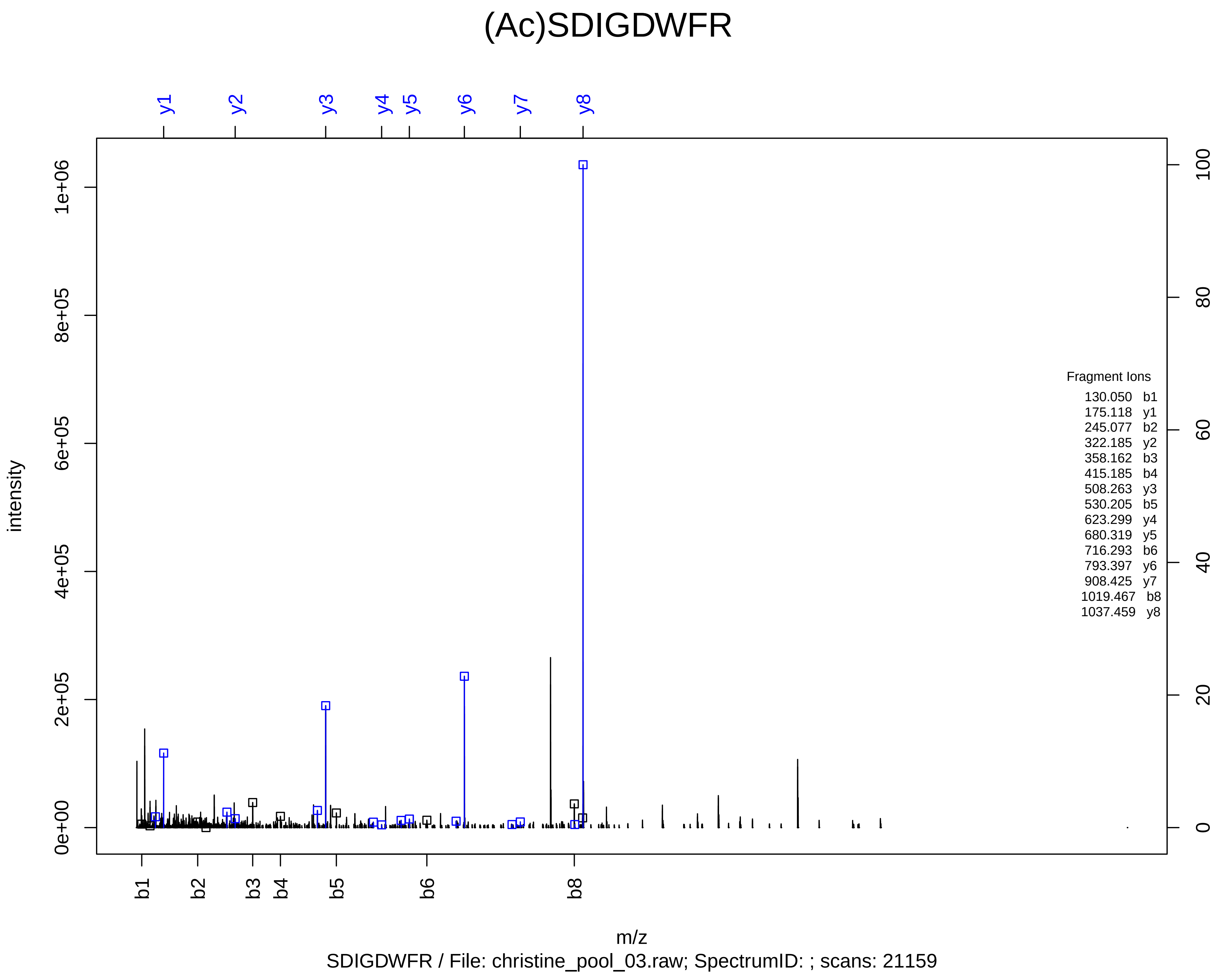
(Ac)SDIGDWFR
y1
y2
y3
y4
y5
y6
y7
y8
intensity
0e+00
2e+05
4e+05
6e+05
8e+05
1e+06
0
20
40
60
80
100
b1
b2
b3
b4
b5
b6
b8
Fragment Ions
130.050 b1
175.118 y1
245.077 b2
322.185 y2
358.162 b3
415.185 b4
508.263 y3
530.205 b5
623.299 y4
680.319 y5
716.293 b6
793.397 y6
908.425 y7
1019.467 b8
1037.459 y8
m/z
SDIGDWFR / File: christine_pool_03.raw; SpectrumID: ; scans: 21159

NVLDSEDEIEELSK

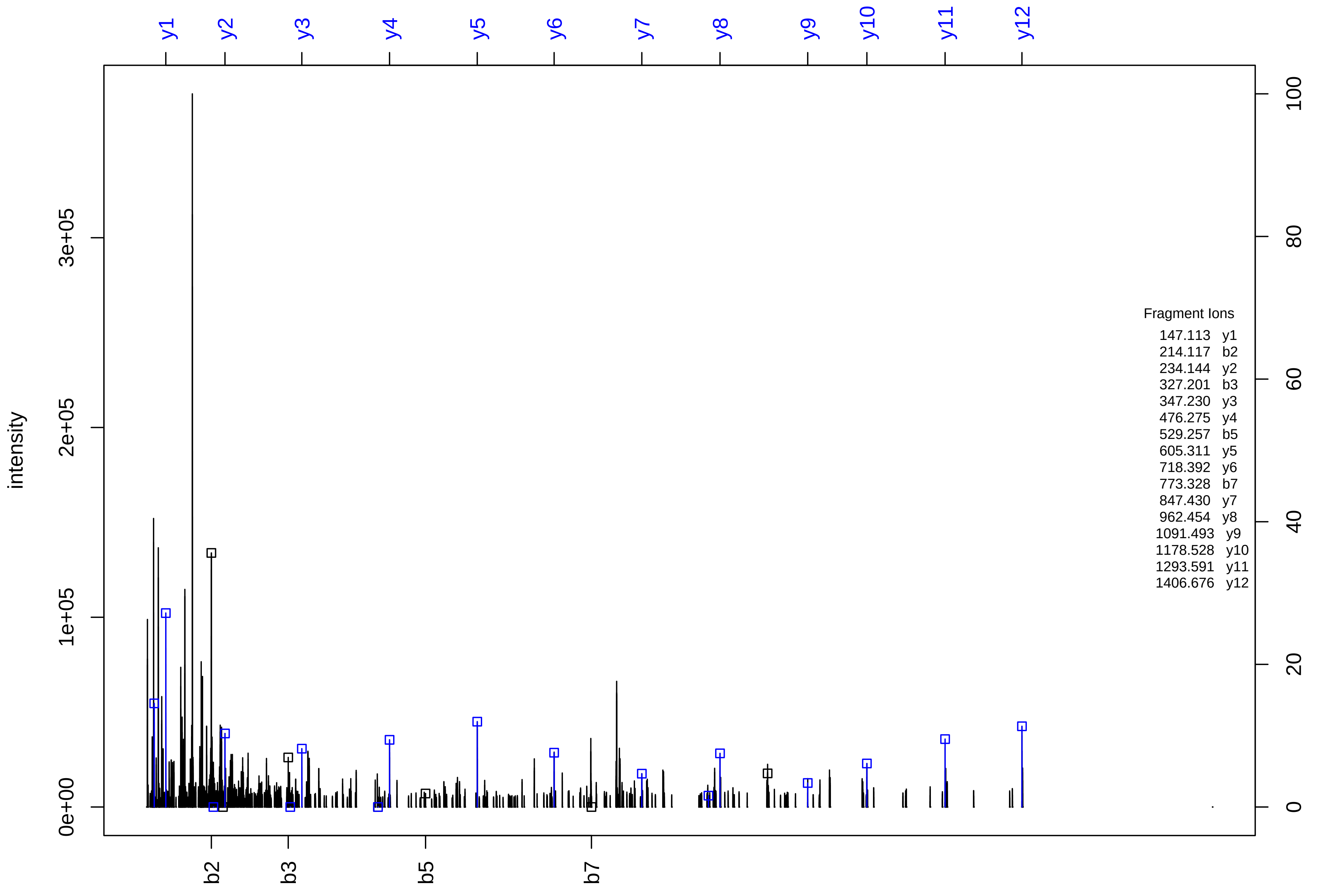

NVLDSEDEIEELSK / File: christine_pool_03.raw; SpectrumID: ; scans: 13720

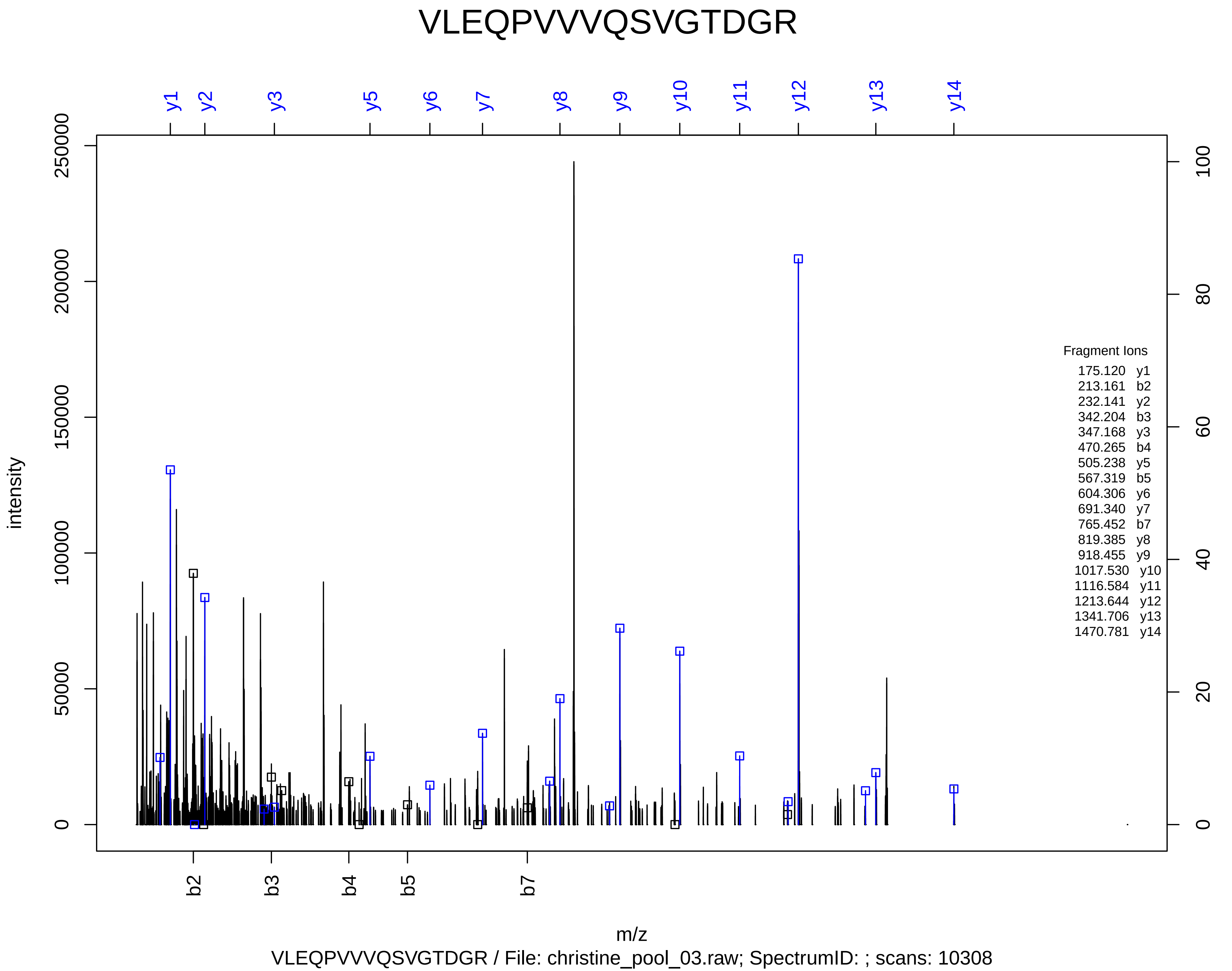

VLEQPVVVQSVGTDGR
y1
y2
y3
y5
y6
y7
y8
y9
y10
y11
y12
y13
y14
b2
b3
b4
b5
b7
intensity
m/z
Fragment Ions
175.120 y1
213.161 b2
232.141 y2
342.204 b3
347.168 y3
470.265 b4
505.238 y5
567.319 b5
604.306 y6
691.340 y7
765.452 b7
819.385 y8
918.455 y9
1017.530 y10
1116.584 y11
1213.644 y12
1341.706 y13
1470.781 y14
VLEQPVVVQSVGTDGR / File: christine_pool_03.raw; SpectrumID: ; scans: 10308

VFNVLYPM*PADQRR

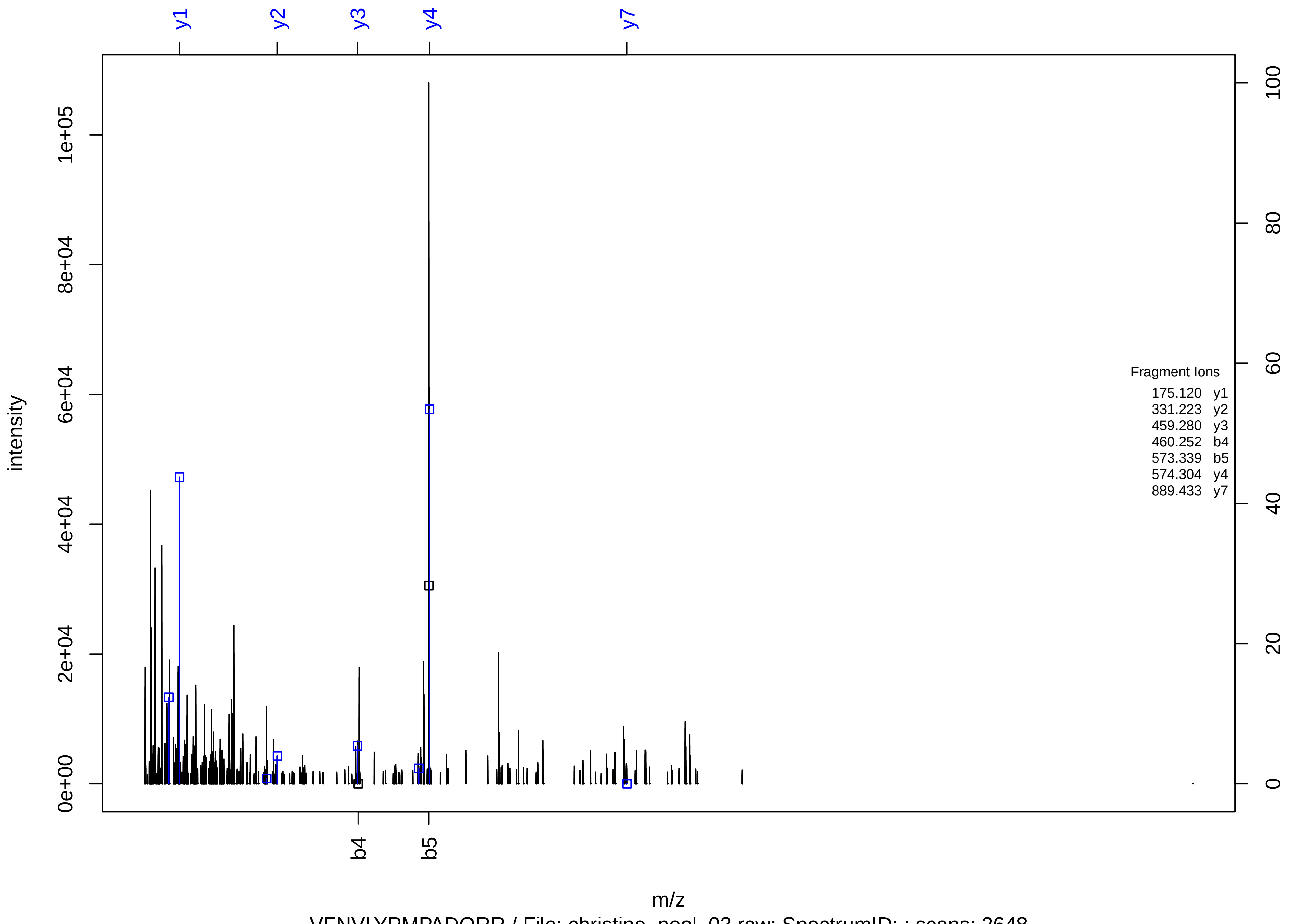

VFNVLYPMPADQRR / File: christine_pool_03.raw; SpectrumID: ; scans: 2648

LLSMGQLDLLR

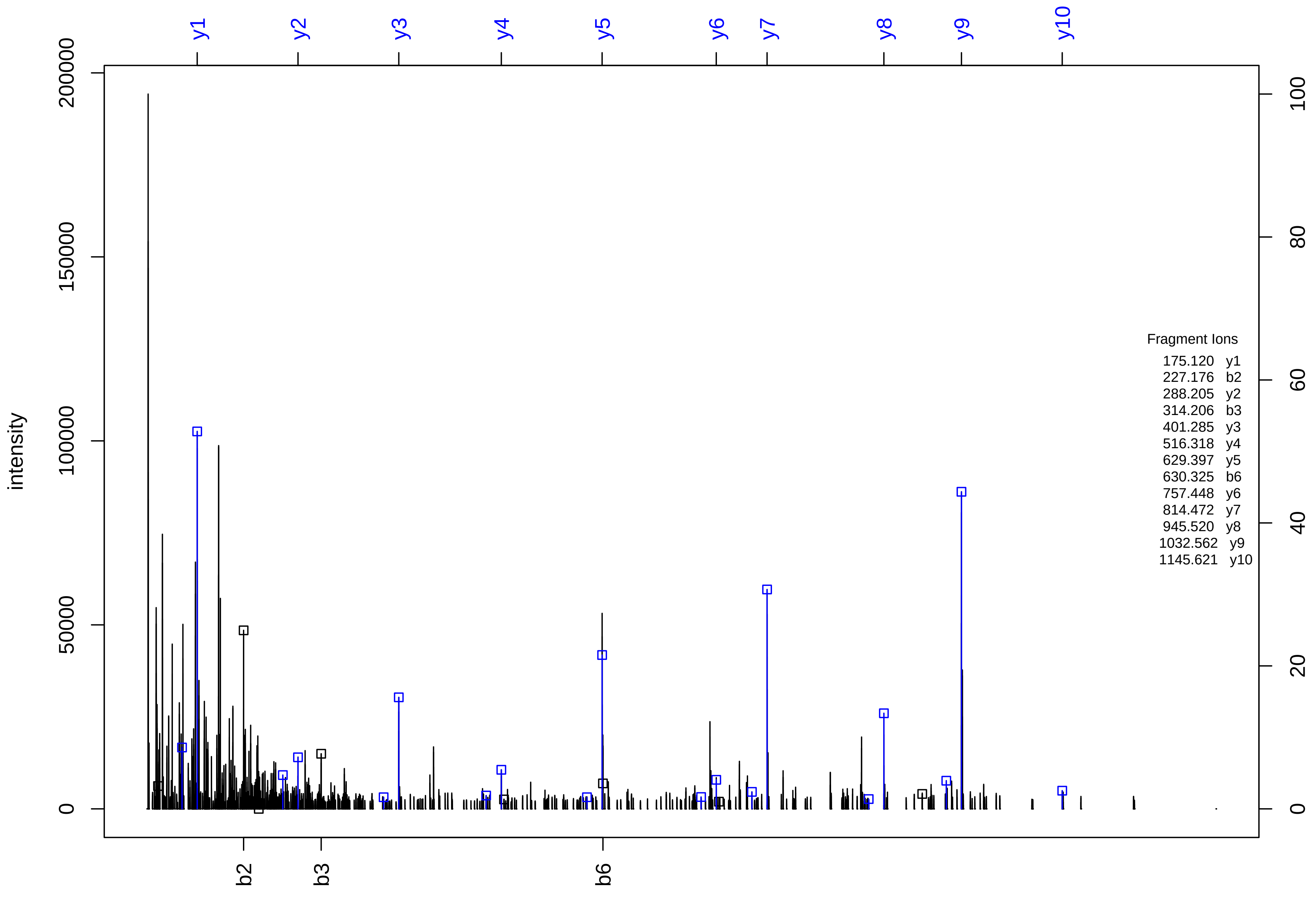

LLSMGQLDLLR / File: christine_pool_03.raw; SpectrumID: ; scans: 20039

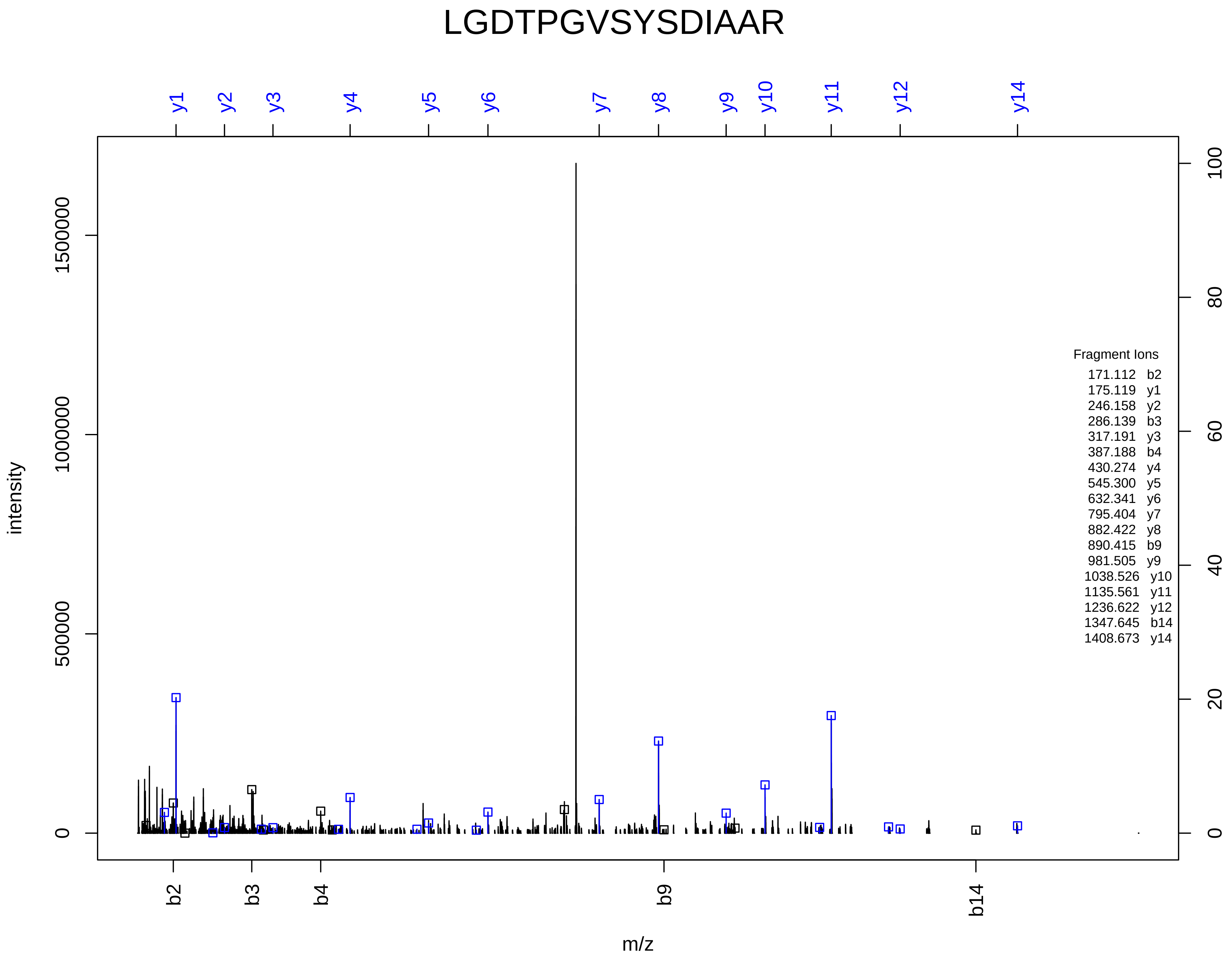

LGDTPGVSYSDIAAR / File: christine_pool_03.raw; SpectrumID: ; scans: 9967

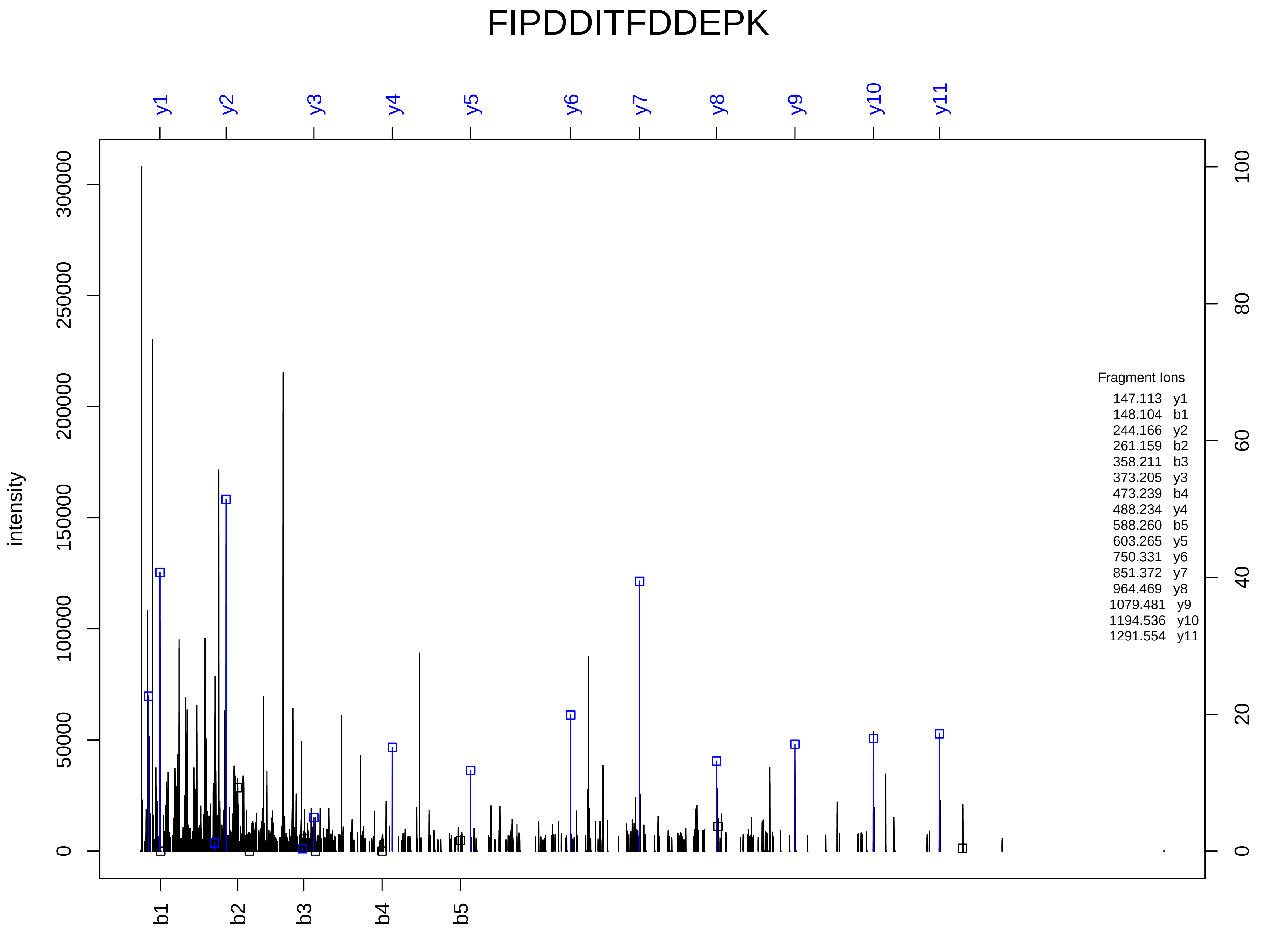

FIPDDITFDDEPK / File: christine_pool_03.raw; SpectrumID: ; scans: 15329

LIVDAFLQQLPNCVNR

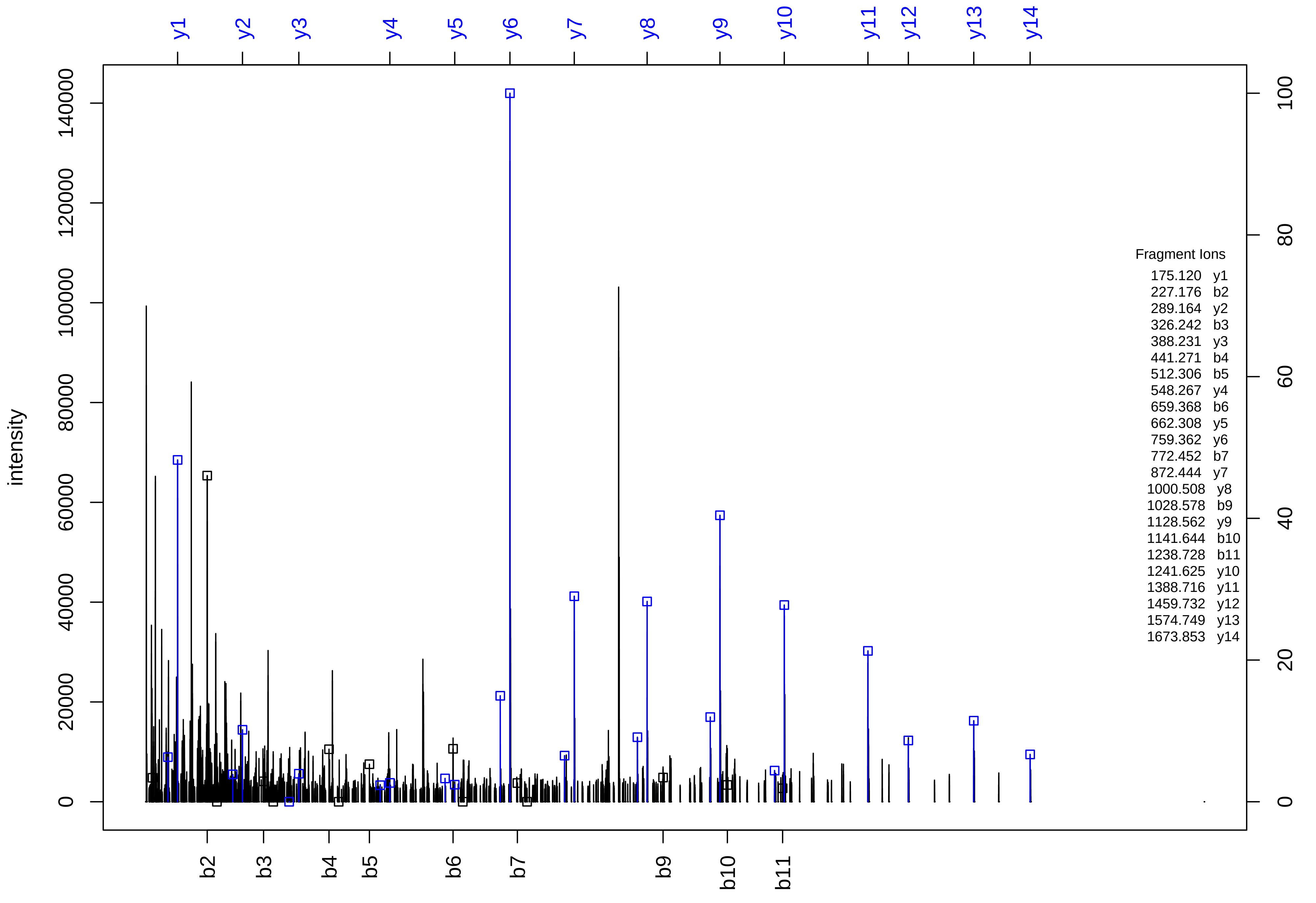

LIVDAFLQQLPNCVNR / File: christine_pool_03.raw; SpectrumID: ; scans: 22442

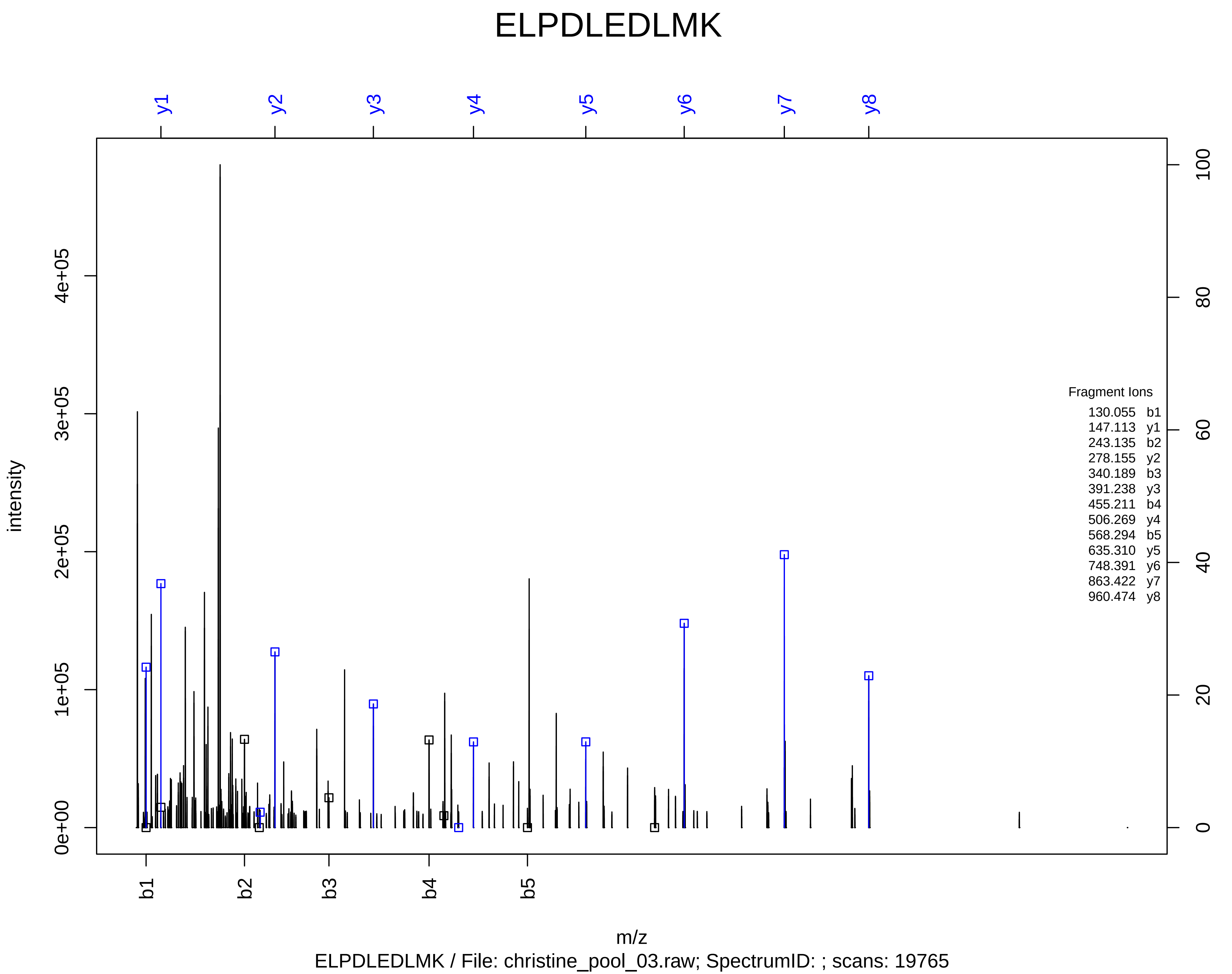

ELPDLEDLMK
intensity
m/z
y1
y2
y3
y4
y5
y6
y7
y8
b1
b2
b3
b4
b5
0e+00
1e+05
2e+05
3e+05
4e+05
0
20
40
60
80
100
Fragment Ions
130.055 b1
147.113 y1
243.135 b2
278.155 y2
340.189 b3
391.238 y3
455.211 b4
506.269 y4
568.294 b5
635.310 y5
748.391 y6
863.422 y7
960.474 y8
ELPDLEDLMK / File: christine_pool_03.raw; SpectrumID: ; scans: 19765

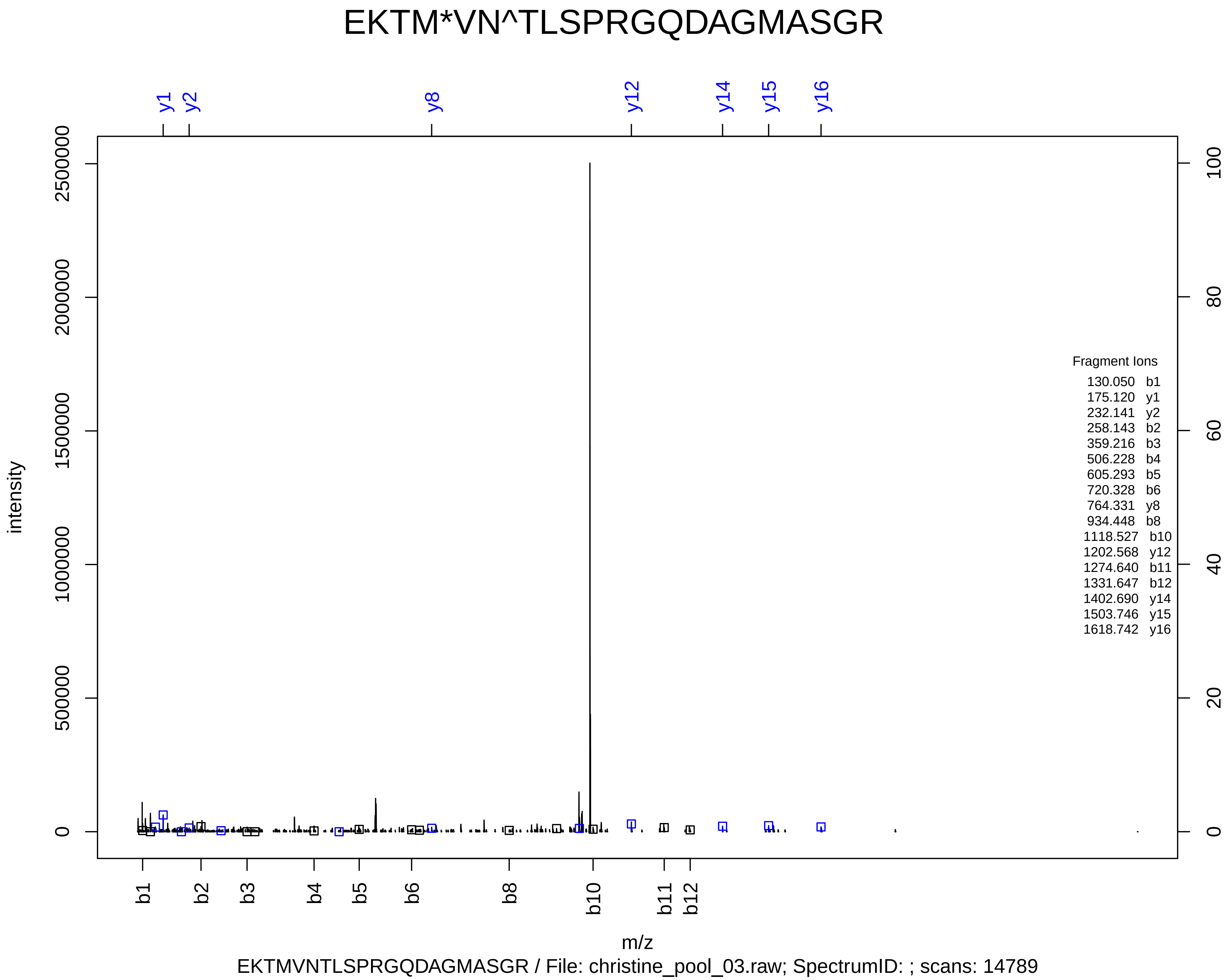
EKTM*VN^TLSPRGQDAGMASGR
y1
y2
y8
y12
y14
y15
y16
intensity
0
500000
1000000
1500000
2000000
2500000
0
20
40
60
80
100
Fragment Ions
130.050 b1
175.120 y1
232.141 y2
258.143 b2
359.216 b3
506.228 b4
605.293 b5
720.328 b6
764.331 y8
934.448 b8
1118.527 b10
1202.568 y12
1274.640 b11
1331.647 b12
1402.690 y14
1503.746 y15
1618.742 y16
b1
b2
b3
b4
b5
b6
b8
b10
b11
b12
m/z
EKTMVNTLSPRGQDAGMASGR / File: christine_pool_03.raw; SpectrumID: ; scans: 14789

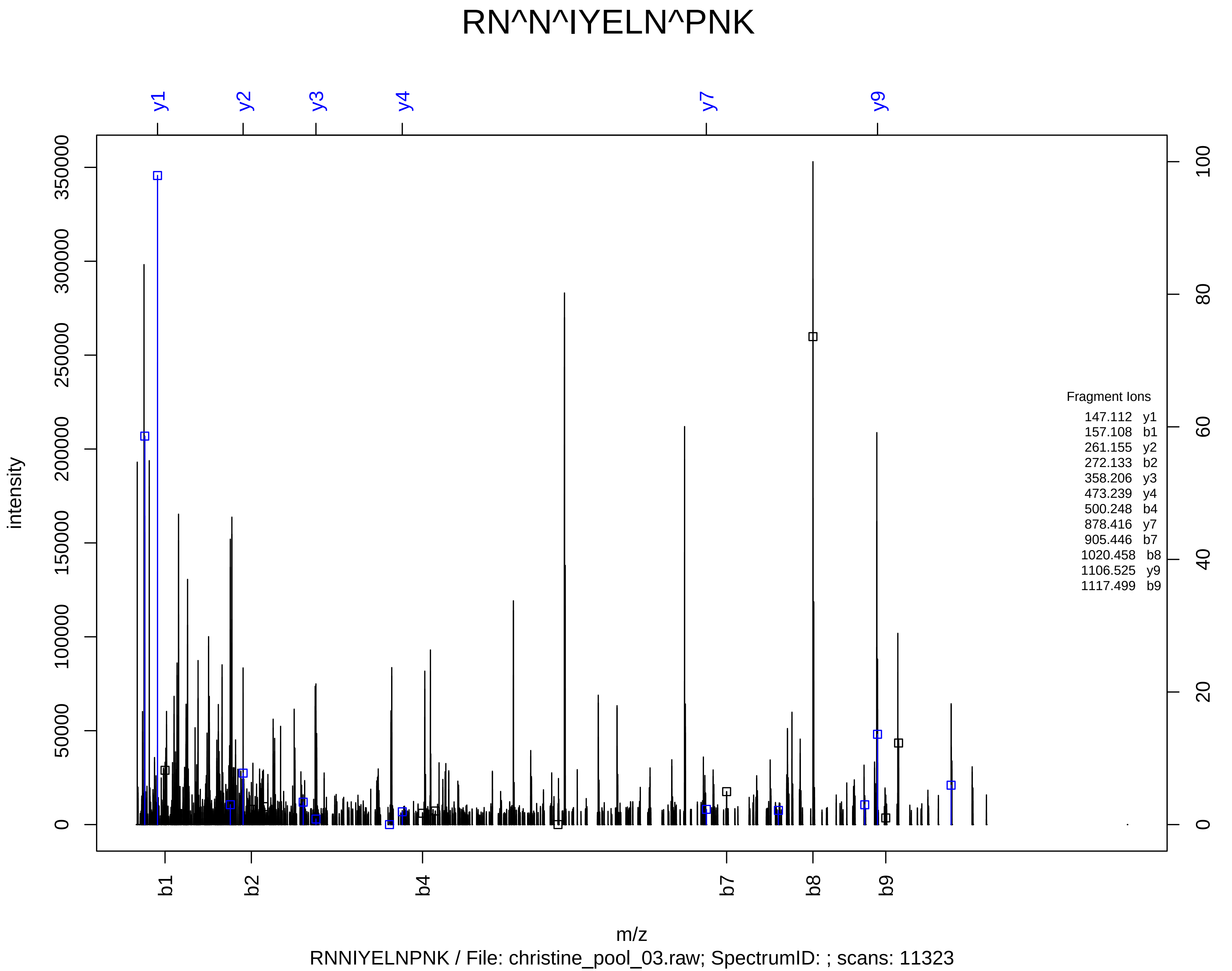
RN^N^IYELN^PNK
y1
y2
y3
y4
y7
y9
intensity
350000
300000
250000
200000
150000
100000
50000
0
100
80
60
40
20
0
Fragment Ions
147.112 y1
157.108 b1
261.155 y2
272.133 b2
358.206 y3
473.239 y4
500.248 b4
878.416 y7
905.446 b7
1020.458 b8
1106.525 y9
1117.499 b9
b1
b2
b4
b7
b8
b9
m/z
RNNIYELNPNK / File: christine_pool_03.raw; SpectrumID: ; scans: 11323

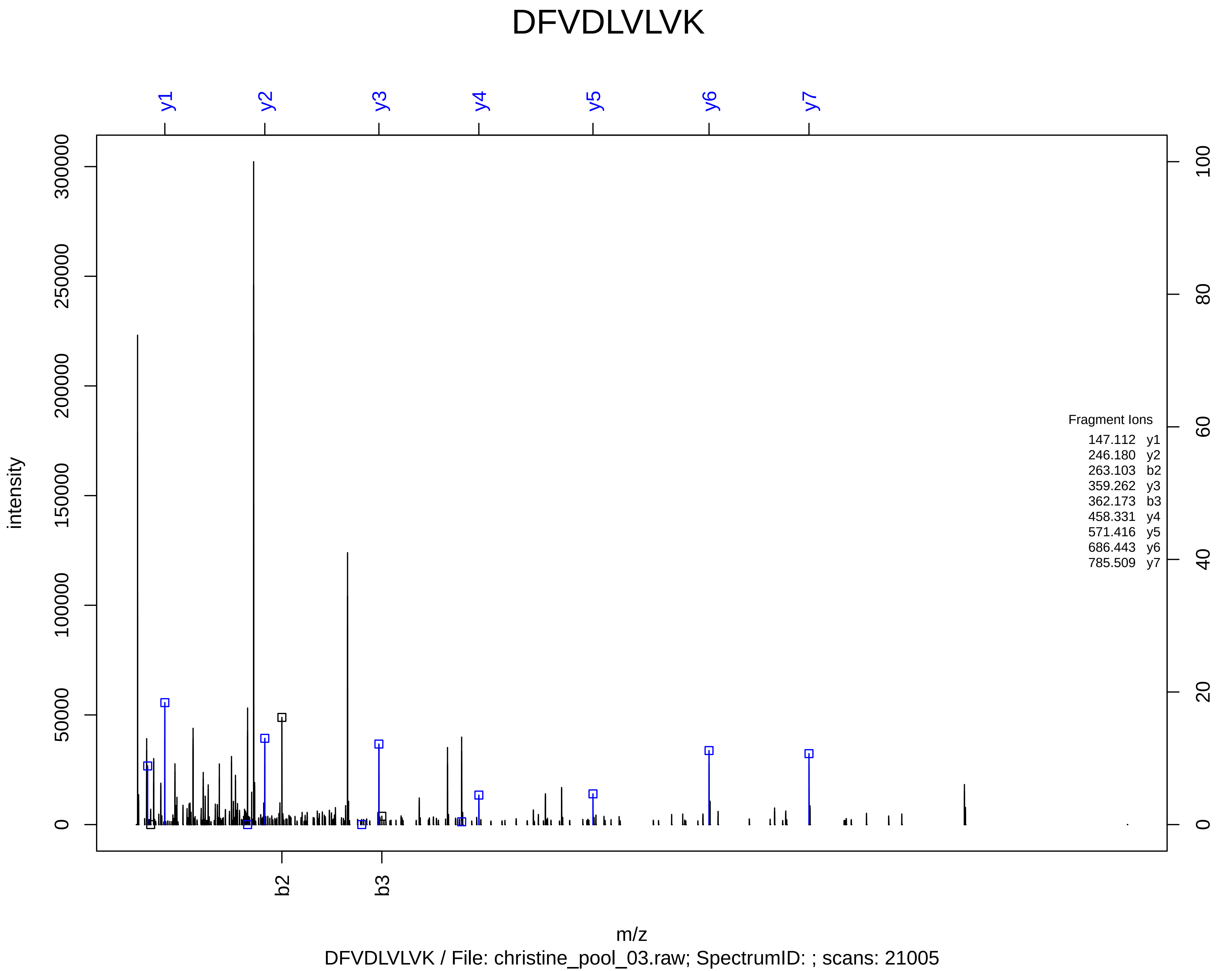
DFVDLVLVK
y1
y2
y3
y4
y5
y6
y7
intensity
300000
250000
200000
150000
100000
50000
0
100
80
60
40
20
0
Fragment Ions
147.112 y1
246.180 y2
263.103 b2
359.262 y3
362.173 b3
458.331 y4
571.416 y5
686.443 y6
785.509 y7
b2
b3
m/z
DFVDLVLVK / File: christine_pool_03.raw; SpectrumID: ; scans: 21005

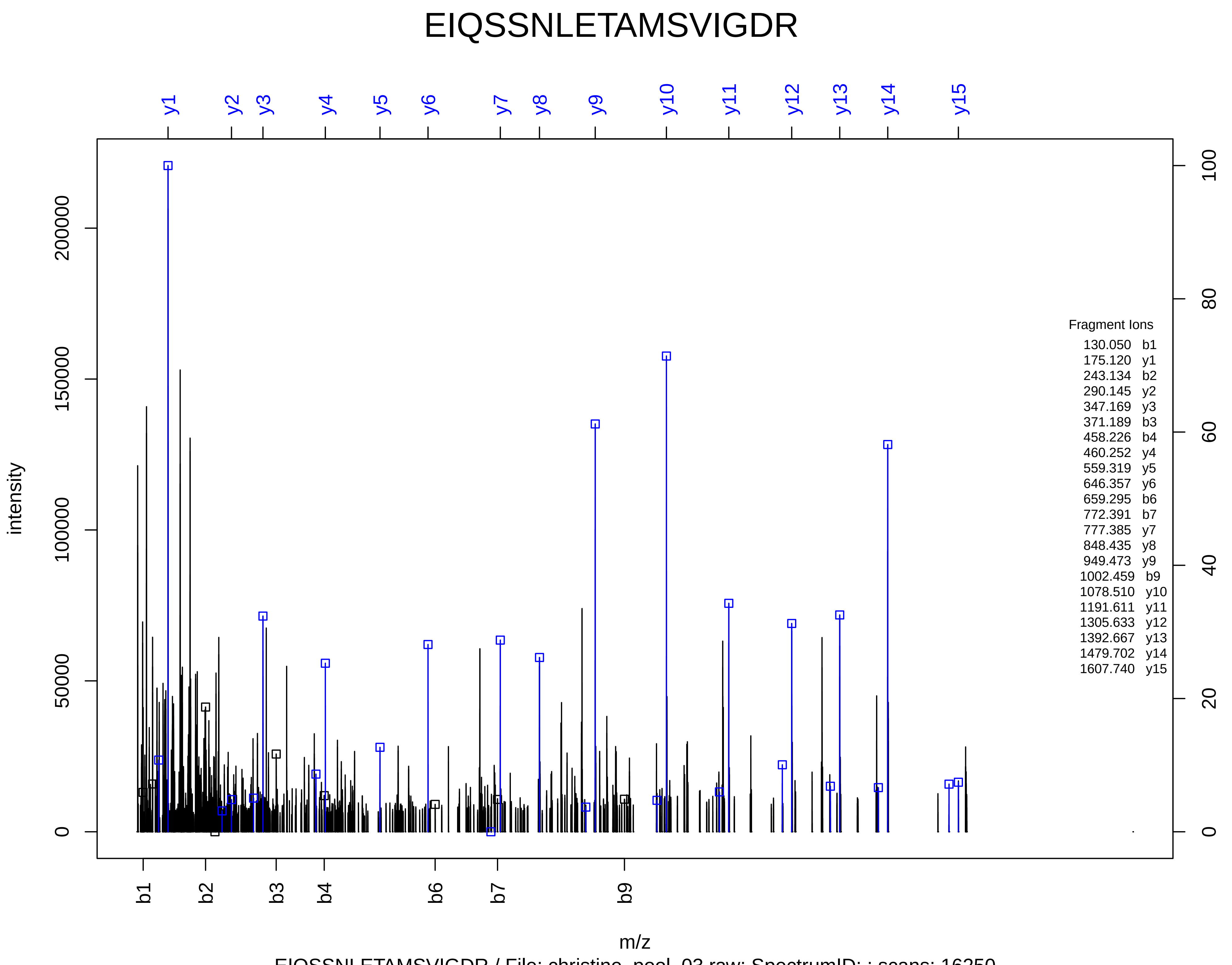

EIQSSNLETAMSVIGDR / File: christine_pool_03.raw; SpectrumID: ; scans: 16250

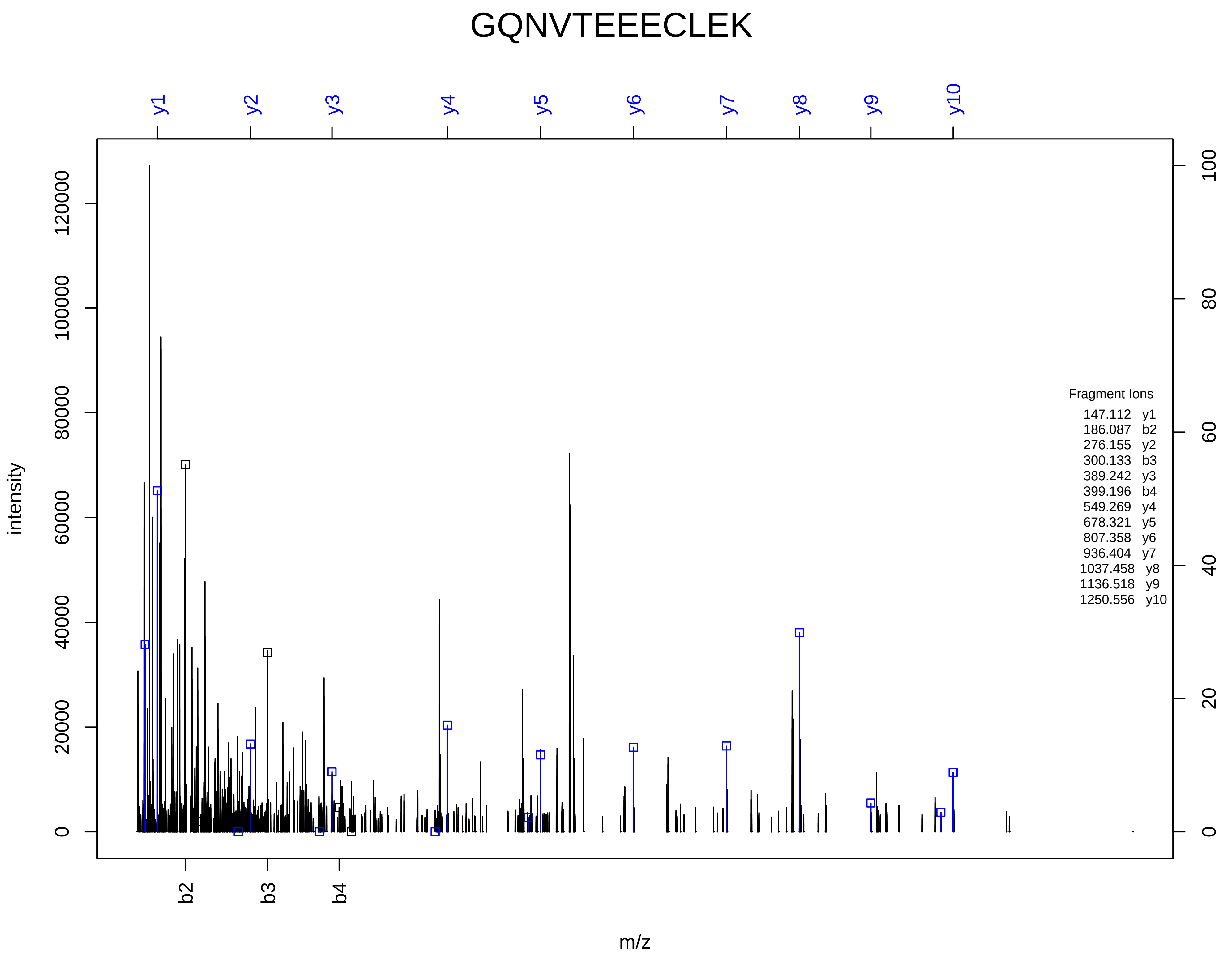

GQNVTEEECLEK / File: christine_pool_03.raw; SpectrumID: ; scans: 3797

VPFPLTLR

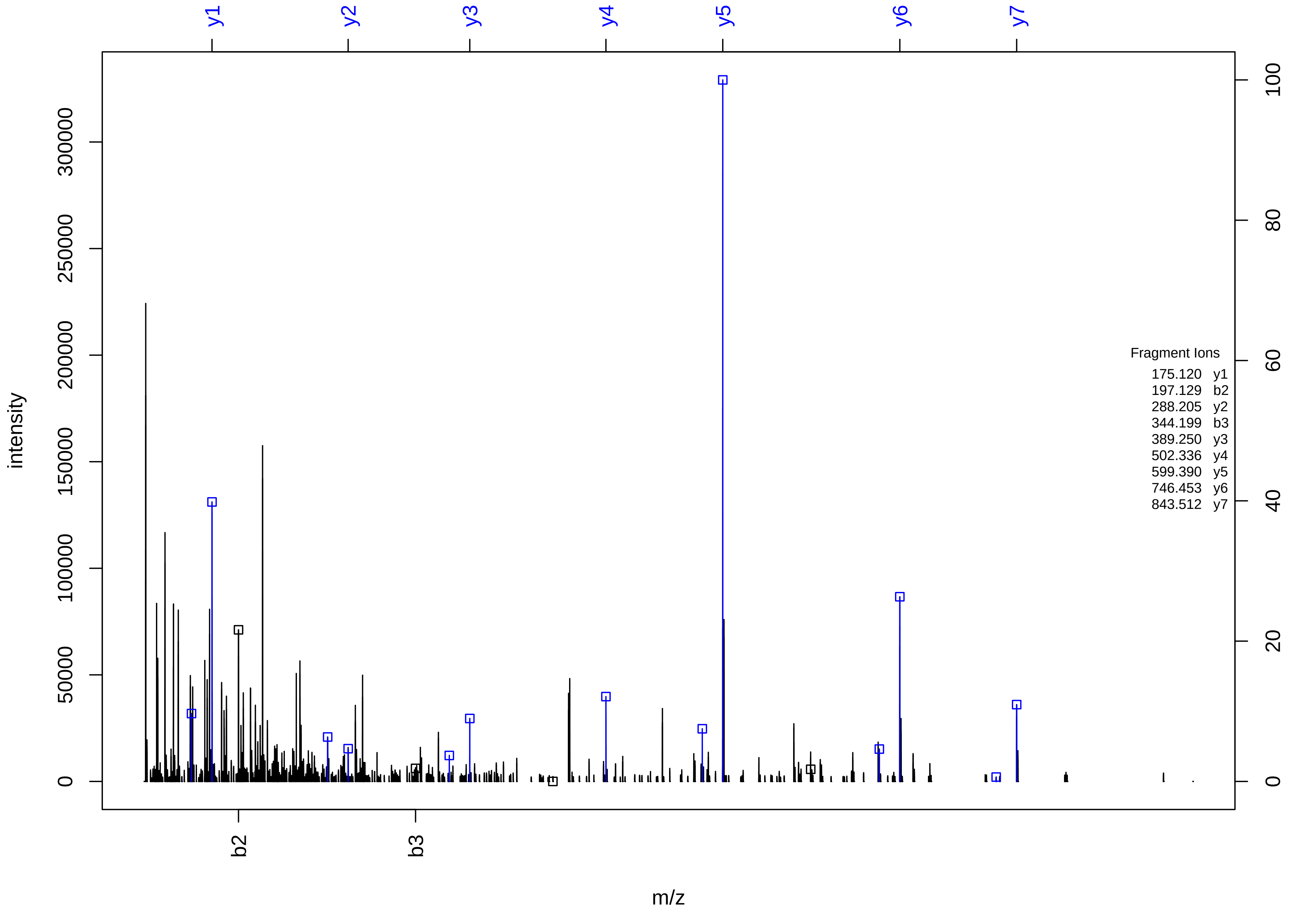

VPFPLTLR / File: christine_pool_03.raw; SpectrumID: ; scans: 16494

VQTDAFVSNELDDPDDLQCK

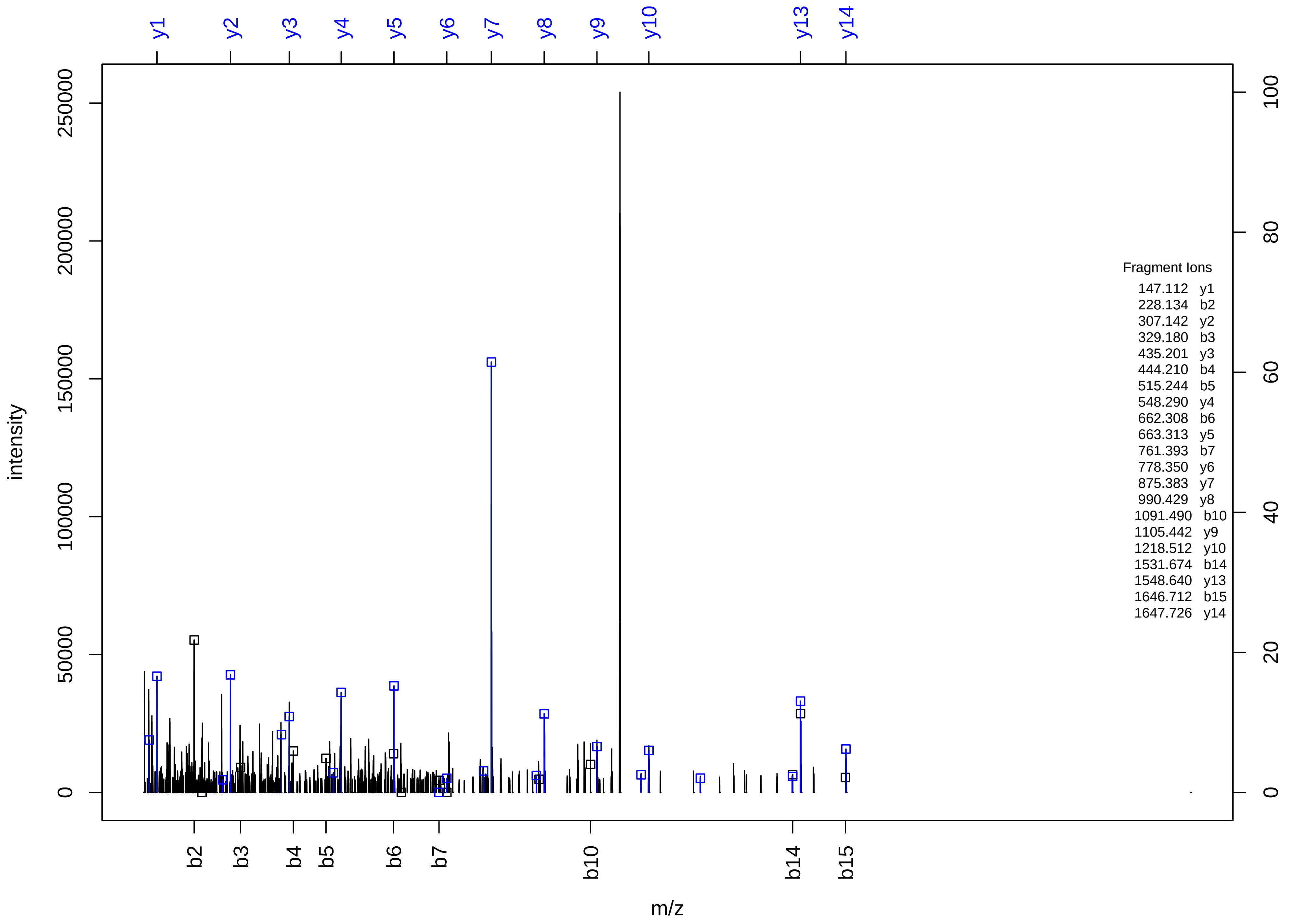

VQTDAFVSNELDDPDDLQCK / File: christine_pool_03.raw; SpectrumID: ; scans: 13578

SFLSGLYEFPLNK

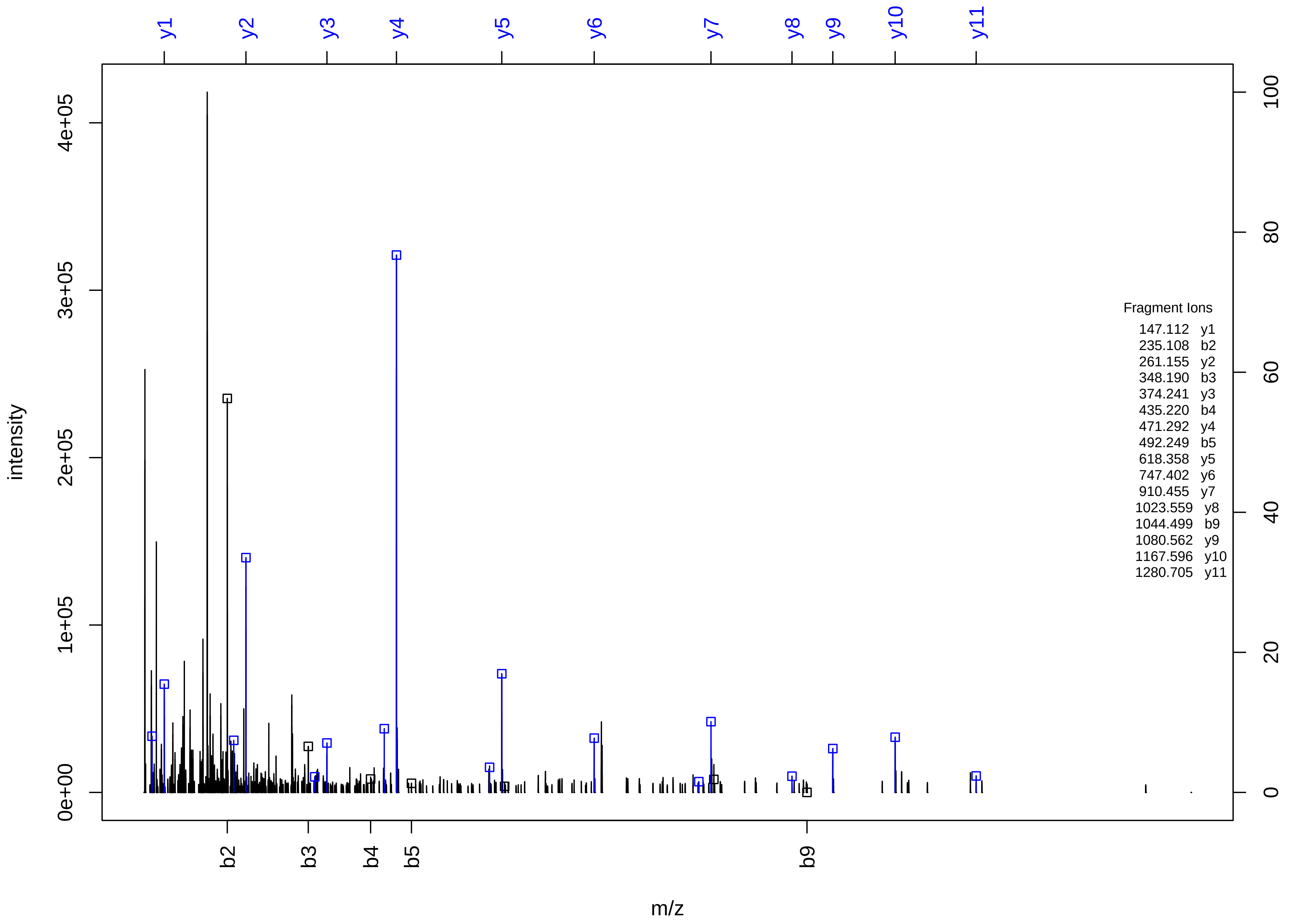

SFLSGLYEFPLNK / File: christine_pool_03.raw; SpectrumID: ; scans: 22137

ELNESNSQM*EADM*IKLR

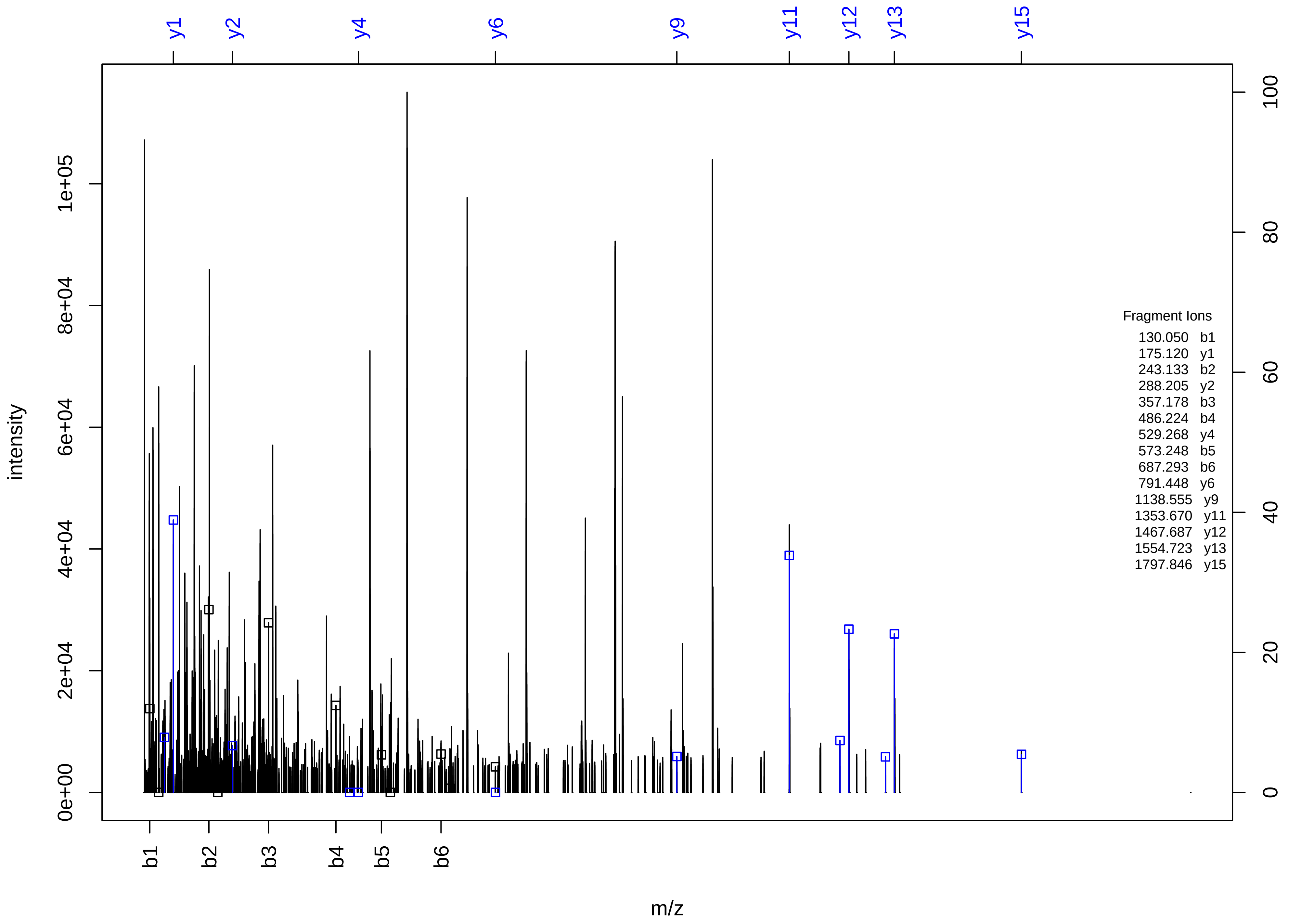

ELNESNSQMEADMIKLR / File: christine_pool_03.raw; SpectrumID: ; scans: 18547

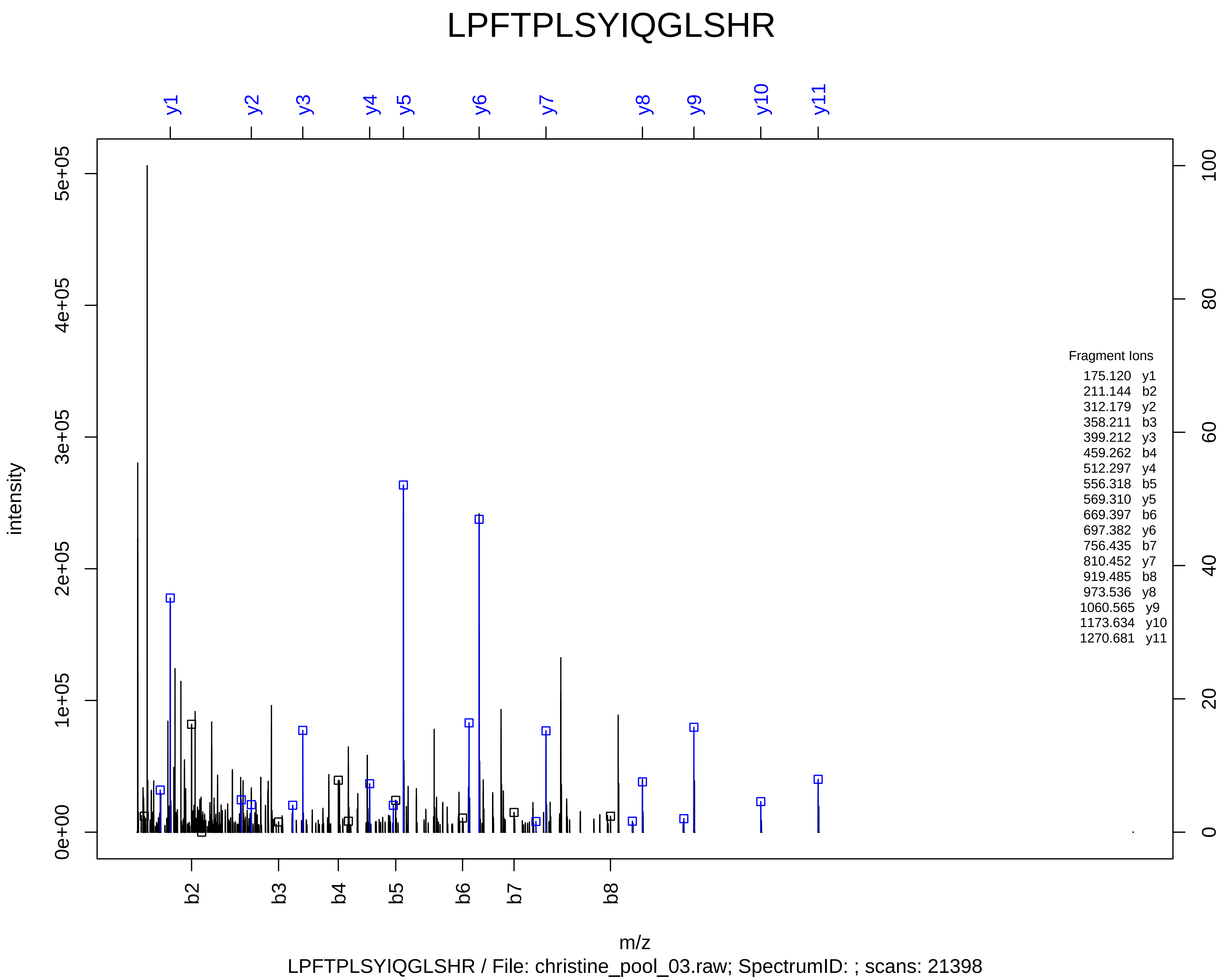

LPFTPLSYIQGLSHR
y1
y2
y3
y4
y5
y6
y7
y8
y9
y10
y11
intensity
0e+00
1e+05
2e+05
3e+05
4e+05
5e+05
0
20
40
60
80
100
b2
b3
b4
b5
b6
b7
b8
m/z
Fragment Ions
175.120 y1
211.144 b2
312.179 y2
358.211 b3
399.212 y3
459.262 b4
512.297 y4
556.318 b5
569.310 y5
669.397 b6
697.382 y6
756.435 b7
810.452 y7
919.485 b8
973.536 y8
1060.565 y9
1173.634 y10
1270.681 y11
LPFTPLSYIQGLSHR / File: christine_pool_03.raw; SpectrumID: ; scans: 21398

SVFFGPDFITVTK

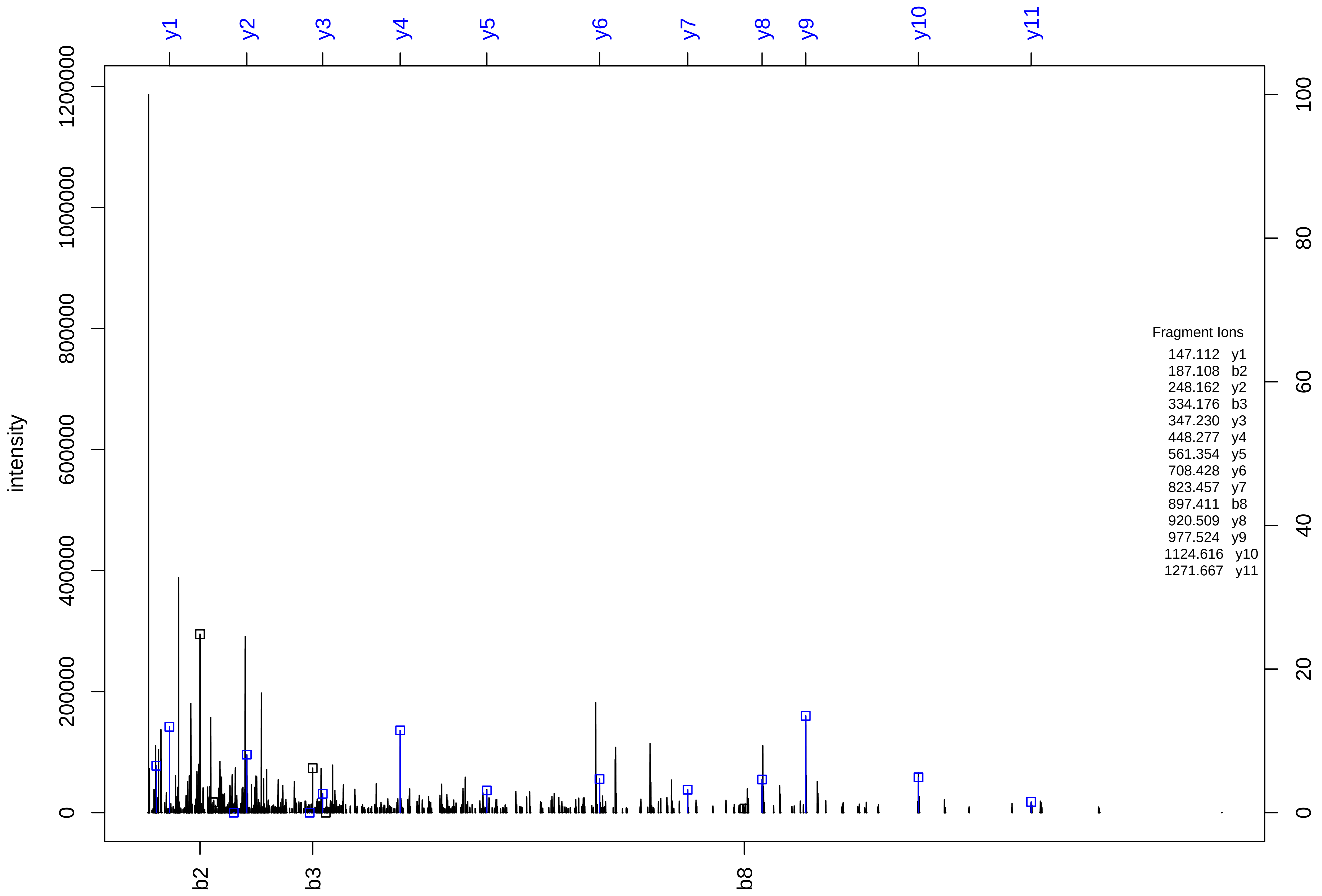

SVFFGPDFITVTK / File: christine_pool_03.raw; SpectrumID: ; scans: 21043

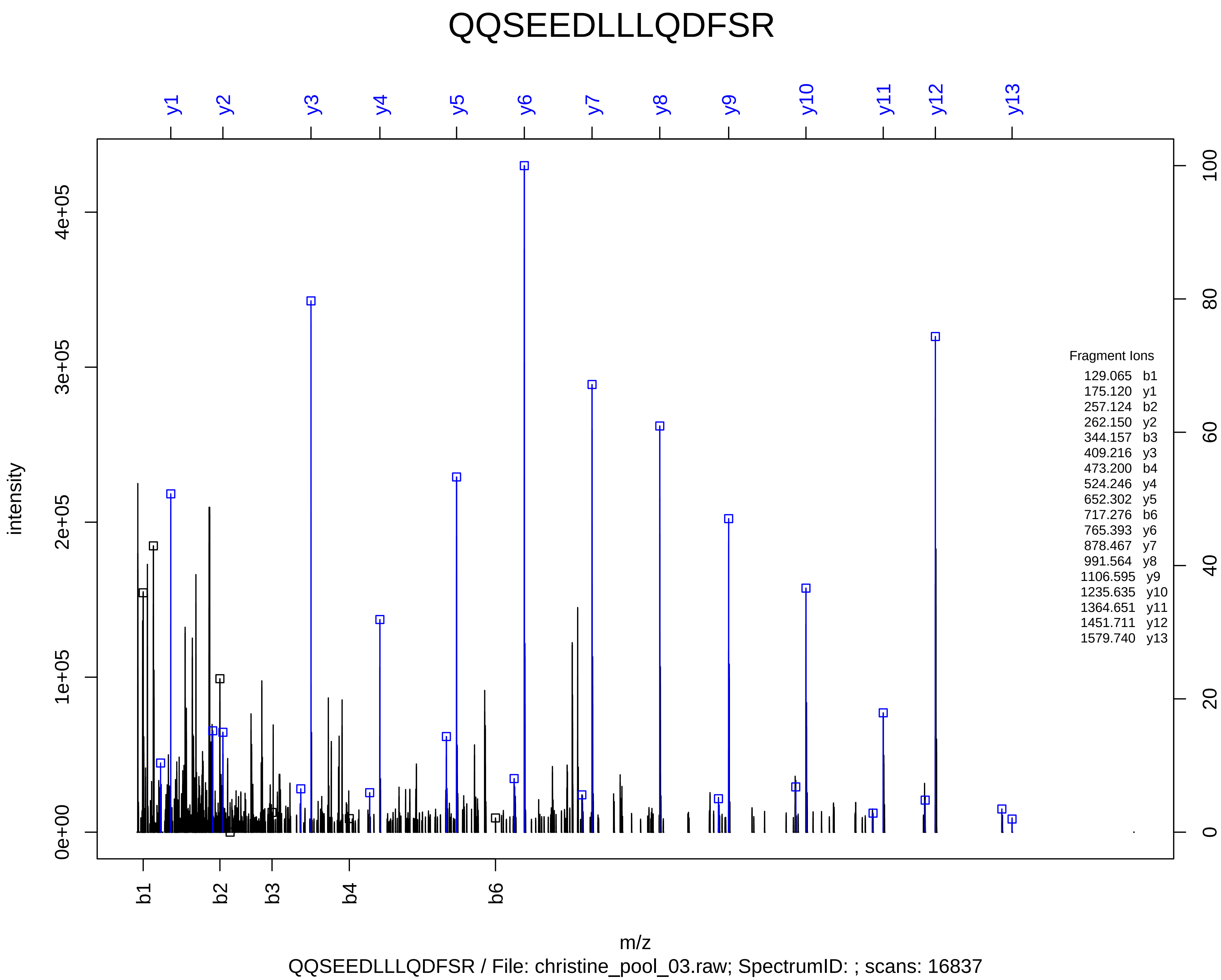
QQSEEDLLLQDFSR
y1
y2
y3
y4
y5
y6
y7
y8
y9
y10
y11
y12
y13
intensity
0e+00
1e+05
2e+05
3e+05
4e+05
0
20
40
60
80
100
b1
b2
b3
b4
b6
m/z
Fragment Ions
129.065 b1
175.120 y1
257.124 b2
262.150 y2
344.157 b3
409.216 y3
473.200 b4
524.246 y4
652.302 y5
717.276 b6
765.393 y6
878.467 y7
991.564 y8
1106.595 y9
1235.635 y10
1364.651 y11
1451.711 y12
1579.740 y13
QQSEEDLLLQDFSR / File: christine_pool_03.raw; SpectrumID: ; scans: 16837

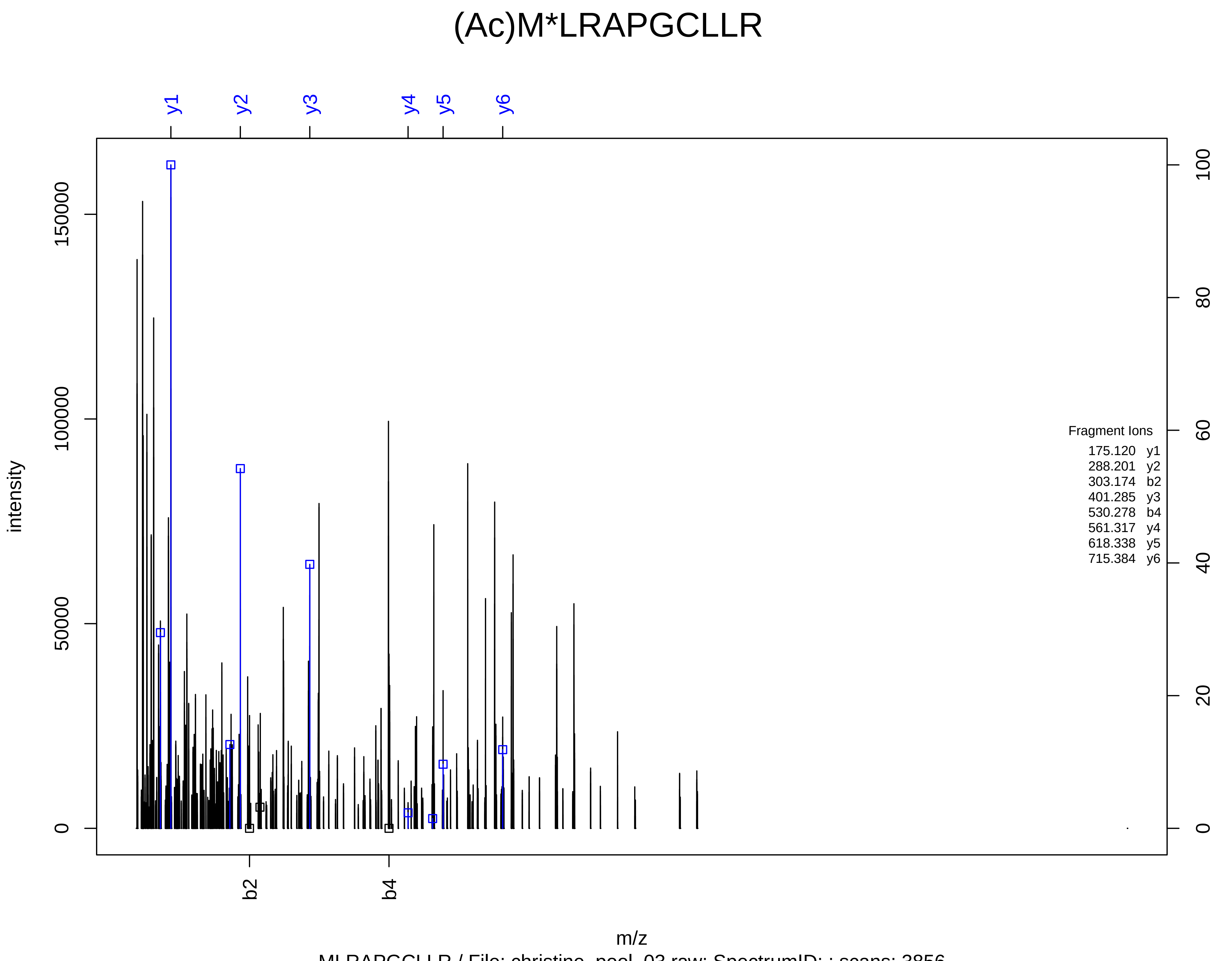
(Ac)M*LRAPGCLLR
y1
y2
y3
y4
y5
y6
b2
b4
intensity
0
50000
100000
150000
0
20
40
60
80
100
Fragment Ions
175.120 y1
288.201 y2
303.174 b2
401.285 y3
530.278 b4
561.317 y4
618.338 y5
715.384 y6
m/z
MLRAPGCLLR / File: christine_pool_03.raw; SpectrumID: ; scans: 3856

(Ac)ATQAHSLSYAGCNFLR

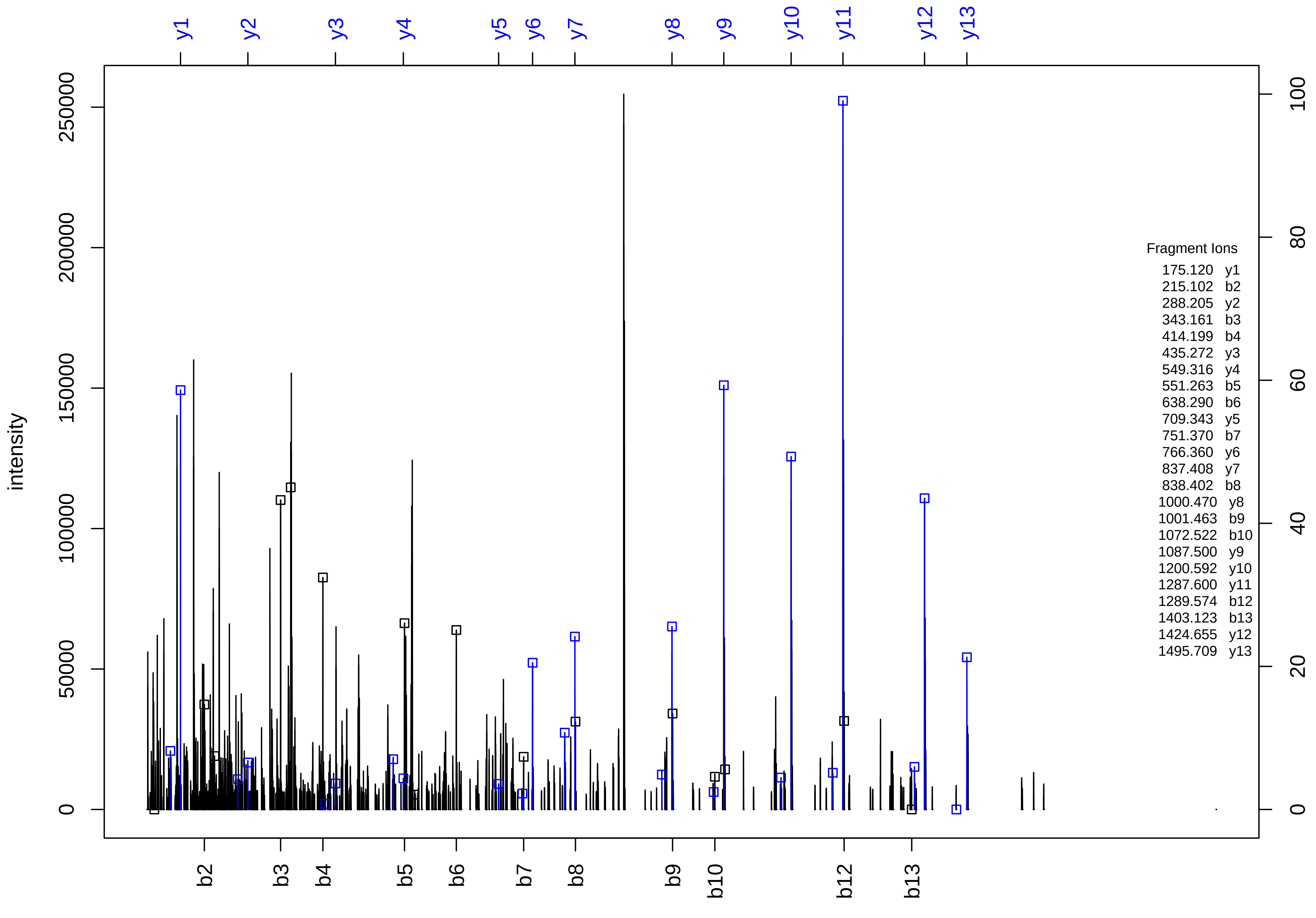

ATQAHSLSYAGCNFLR / File: christine_pool_03.raw; SpectrumID: ; scans: 13252

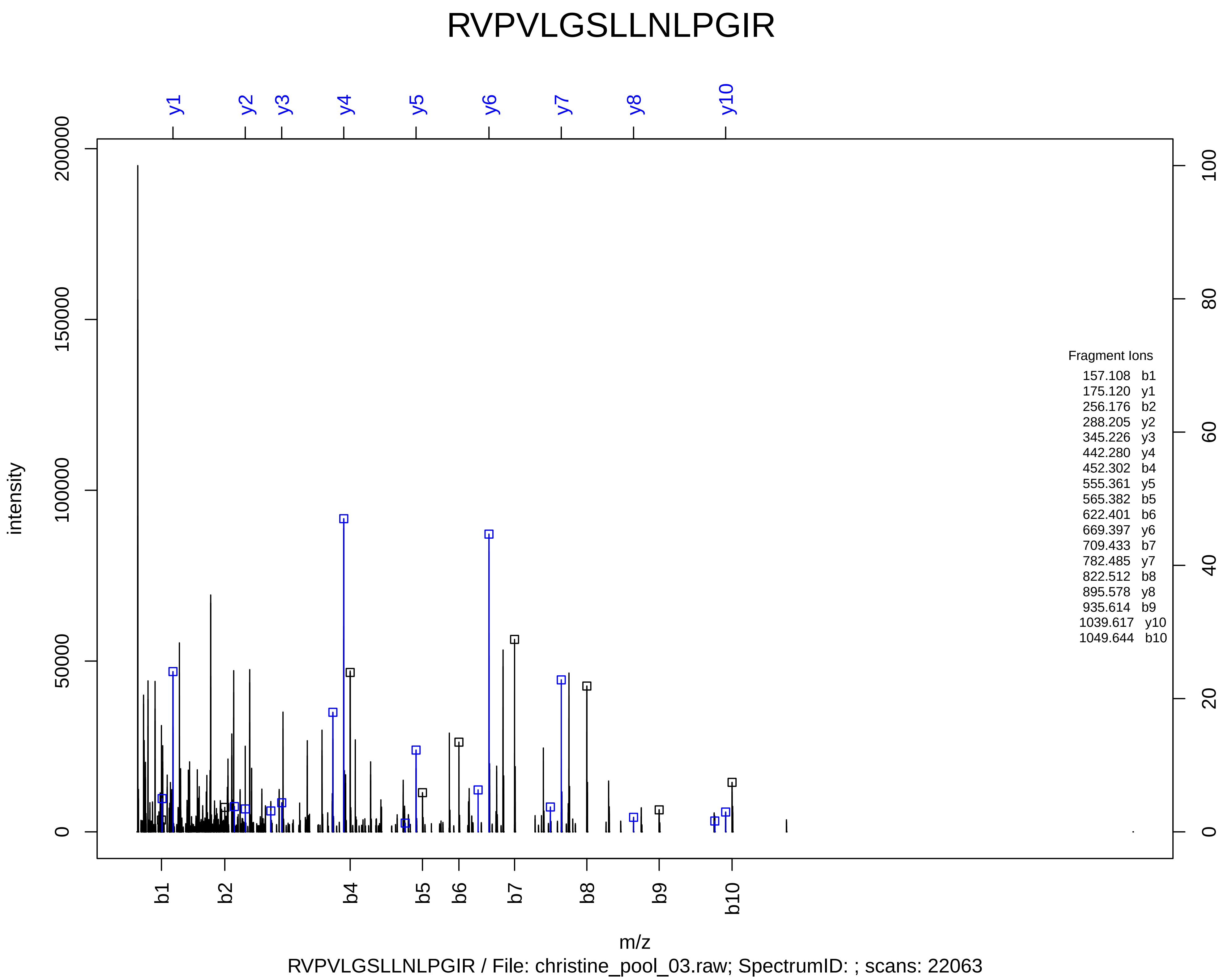

RVPVLGSLLNLPGIR
y1
y2
y3
y4
y5
y6
y7
y8
y10
intensity
0
50000
100000
150000
200000
0
20
40
60
80
100
Fragment Ions
157.108 b1
175.120 y1
256.176 b2
288.205 y2
345.226 y3
442.280 y4
452.302 b4
555.361 y5
565.382 b5
622.401 b6
669.397 y6
709.433 b7
782.485 y7
822.512 b8
895.578 y8
935.614 b9
1039.617 y10
1049.644 b10
b1
b2
b4
b5
b6
b7
b8
b9
b10
m/z
RVPVLGSLLNLPGIR / File: christine_pool_03.raw; SpectrumID: ; scans: 22063

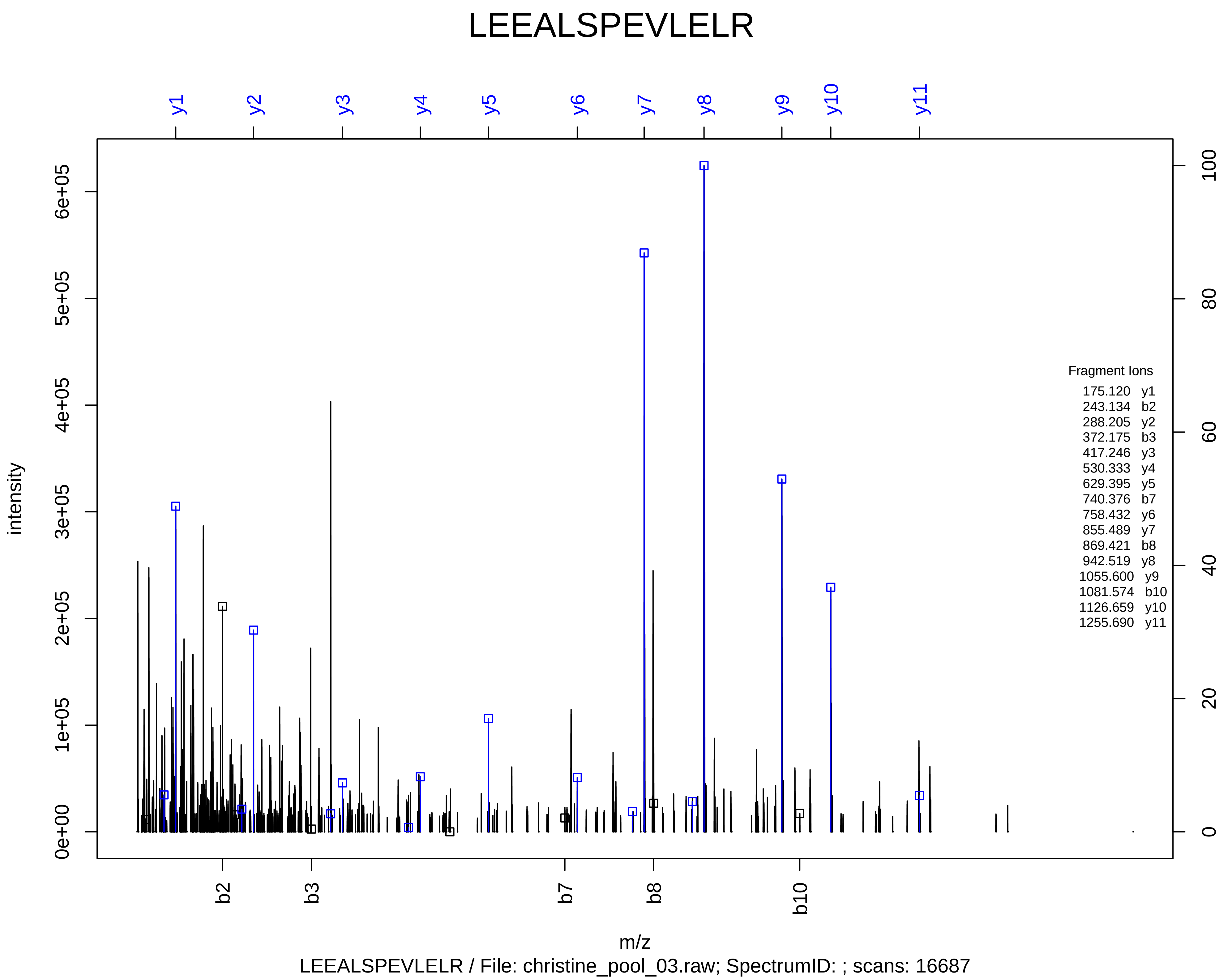
LEEALSPEVLELR
intensity
m/z
y1 y2 y3 y4 y5 y6 y7 y8 y9 y10 y11
b2 b3 b7 b8 b10
0e+00 1e+05 2e+05 3e+05 4e+05 5e+05 6e+05
0 20 40 60 80 100
Fragment Ions
175.120 y1
243.134 b2
288.205 y2
372.175 b3
417.246 y3
530.333 y4
629.395 y5
740.376 b7
758.432 y6
855.489 y7
869.421 b8
942.519 y8
1055.600 y9
1081.574 b10
1126.659 y10
1255.690 y11
LEEALSPEVLELR / File: christine_pool_03.raw; SpectrumID: ; scans: 16687

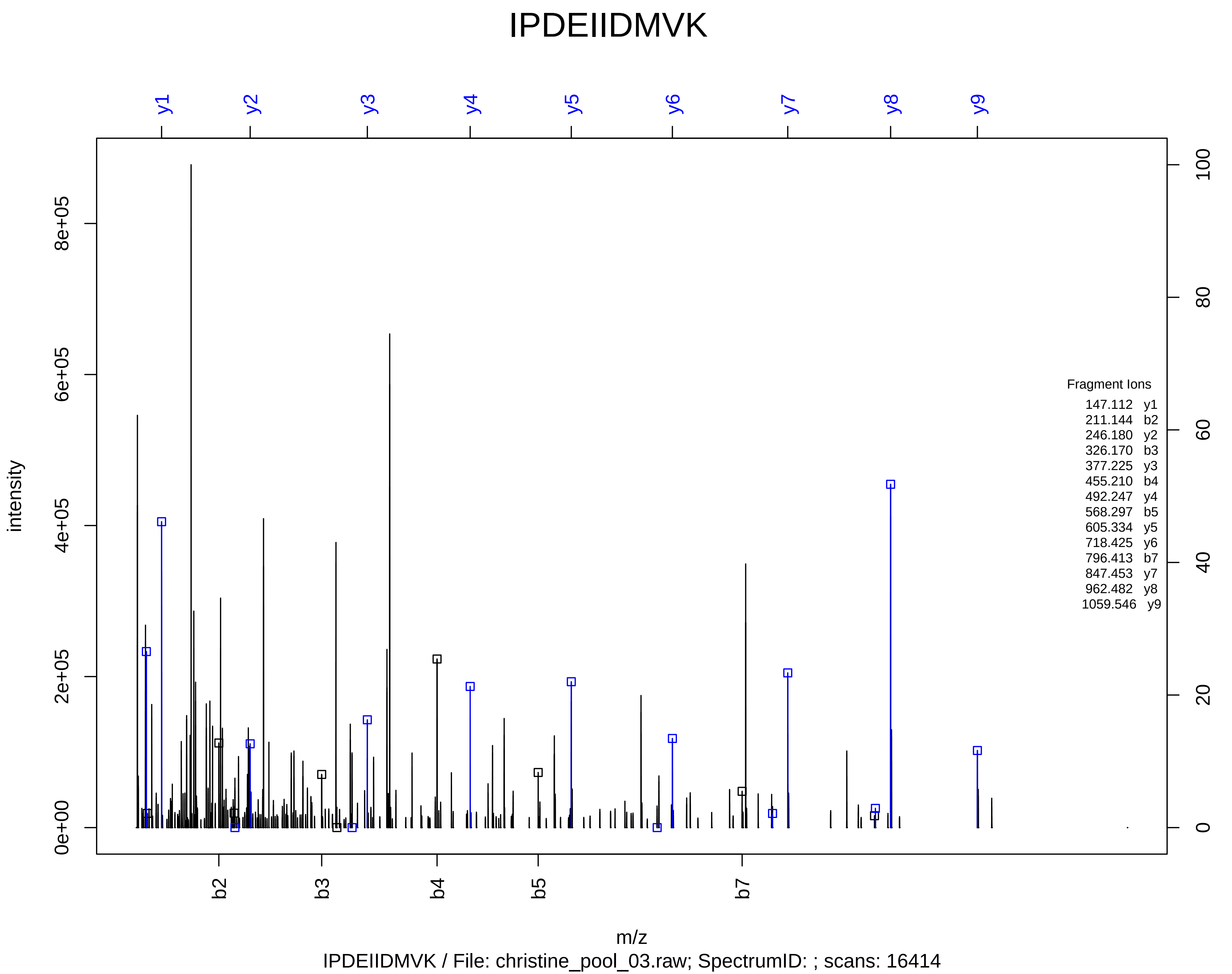
IPDEIIDMVK
y1
y2
y3
y4
y5
y6
y7
y8
y9
intensity
0e+00
2e+05
4e+05
6e+05
8e+05
0
20
40
60
80
100
b2
b3
b4
b5
b7
m/z
Fragment Ions
147.112 y1
211.144 b2
246.180 y2
326.170 b3
377.225 y3
455.210 b4
492.247 y4
568.297 b5
605.334 y5
718.425 y6
796.413 b7
847.453 y7
962.482 y8
1059.546 y9
IPDEIIDMVK / File: christine_pool_03.raw; SpectrumID: ; scans: 16414

AIDNAADLLIFGK

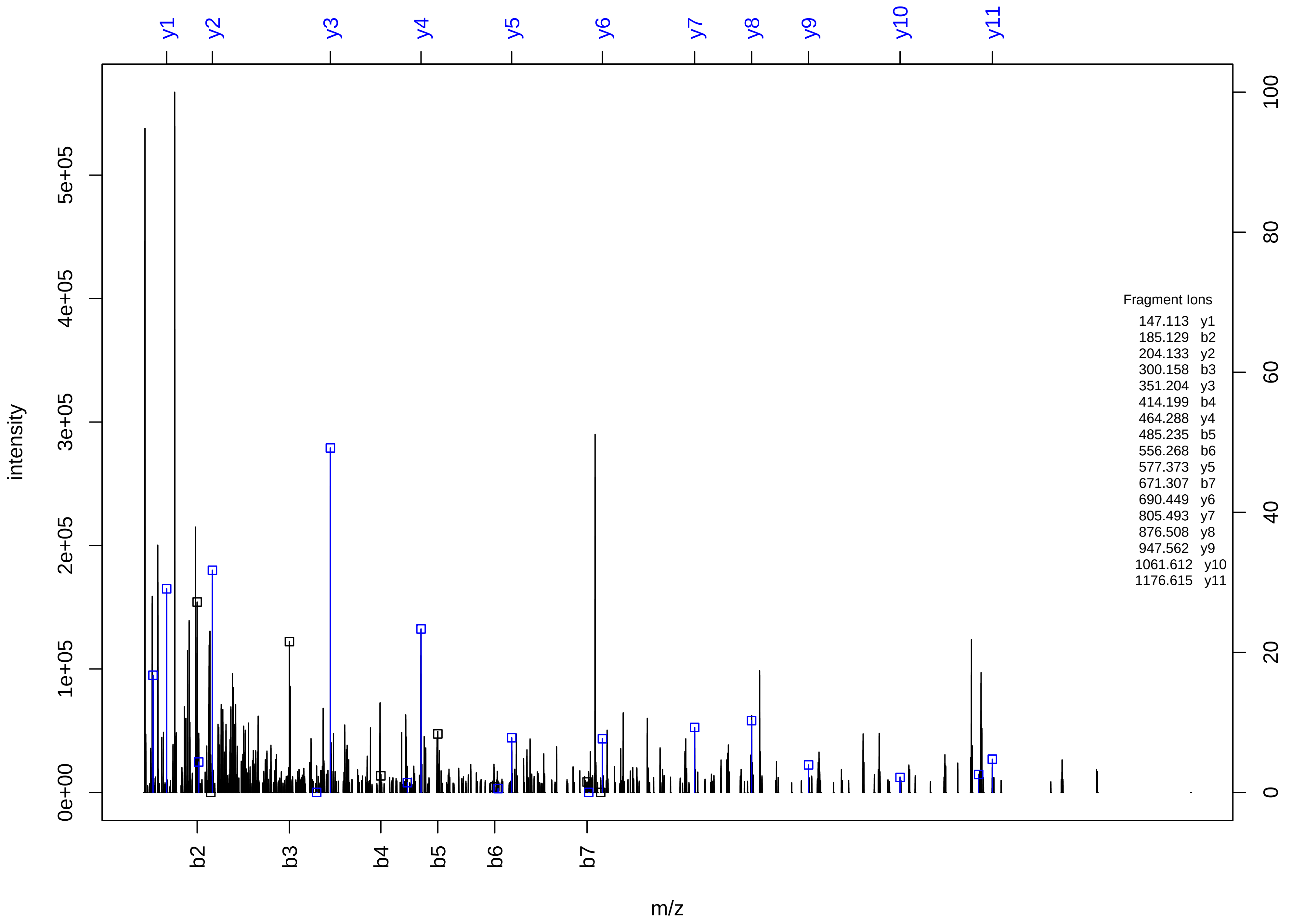

AIDNAADLLIFGK / File: christine_pool_03.raw; SpectrumID: ; scans: 20277

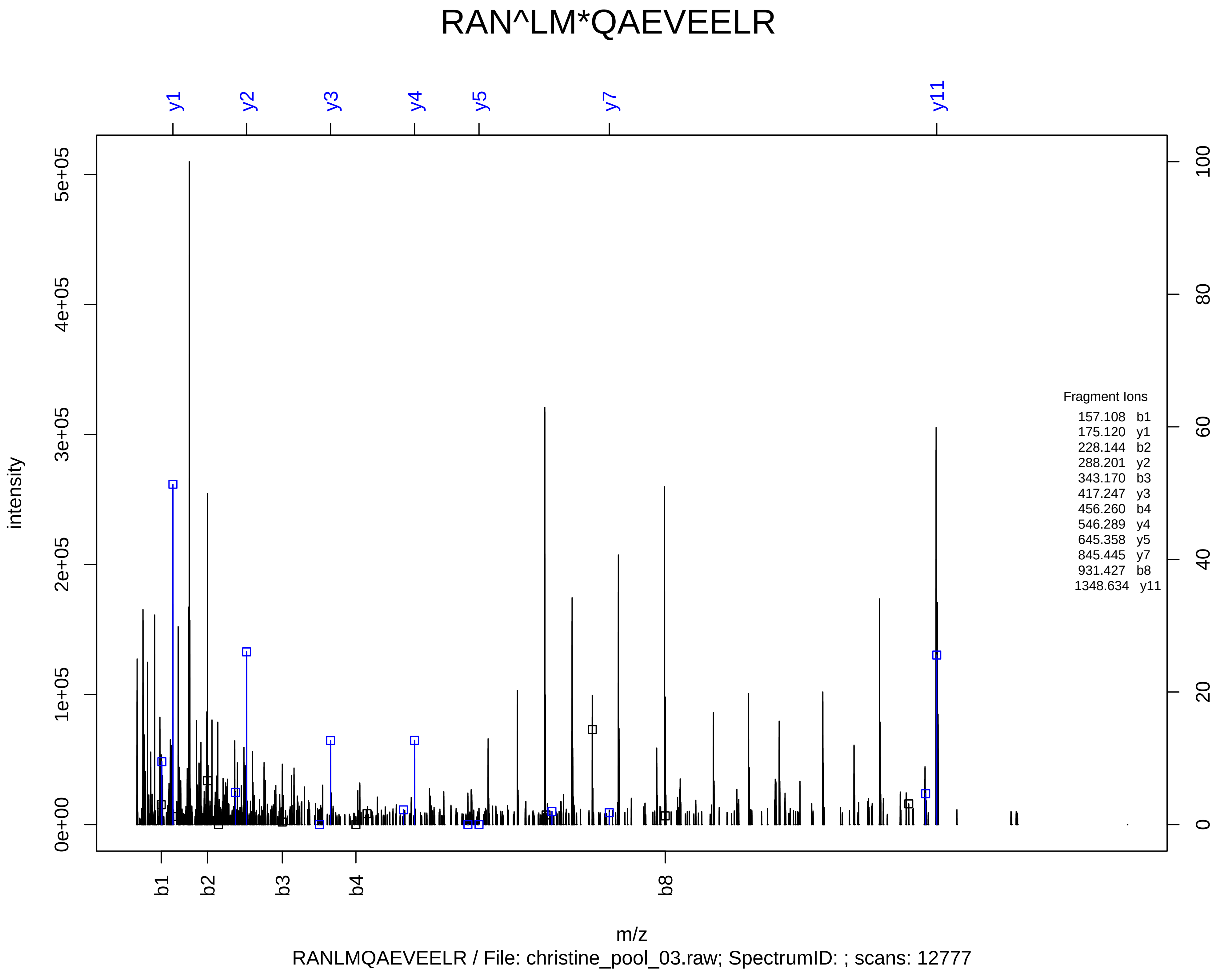
RAN^LM*QAEVEELR
y1
y2
y3
y4
y5
y7
y11
intensity
0e+00
1e+05
2e+05
3e+05
4e+05
5e+05
0
20
40
60
80
100
Fragment Ions
157.108 b1
175.120 y1
228.144 b2
288.201 y2
343.170 b3
417.247 y3
456.260 b4
546.289 y4
645.358 y5
845.445 y7
931.427 b8
1348.634 y11
b1
b2
b3
b4
b8
m/z
RANLMQAEVEELR / File: christine_pool_03.raw; SpectrumID: ; scans: 12777

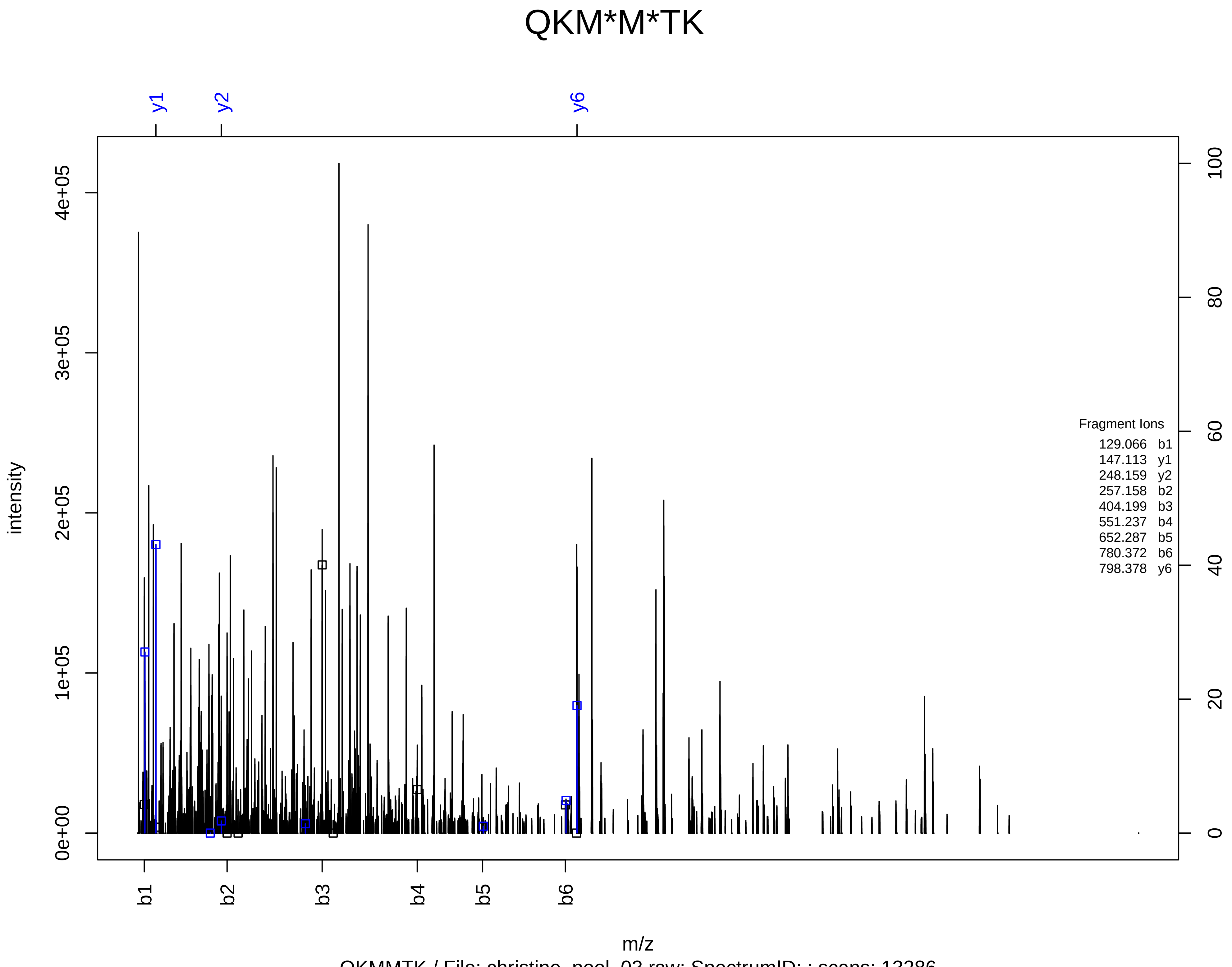

QKM*M*TK
y1
y2
y6
intensity
0e+00
1e+05
2e+05
3e+05
4e+05
0
20
40
60
80
100
Fragment Ions
129.066 b1
147.113 y1
248.159 y2
257.158 b2
404.199 b3
551.237 b4
652.287 b5
780.372 b6
798.378 y6
b1
b2
b3
b4
b5
b6
m/z
QKMMTK / File: christine_pool_03.raw; SpectrumID: ; scans: 13286

(Ac)PITEKSN^PLTQDLDK

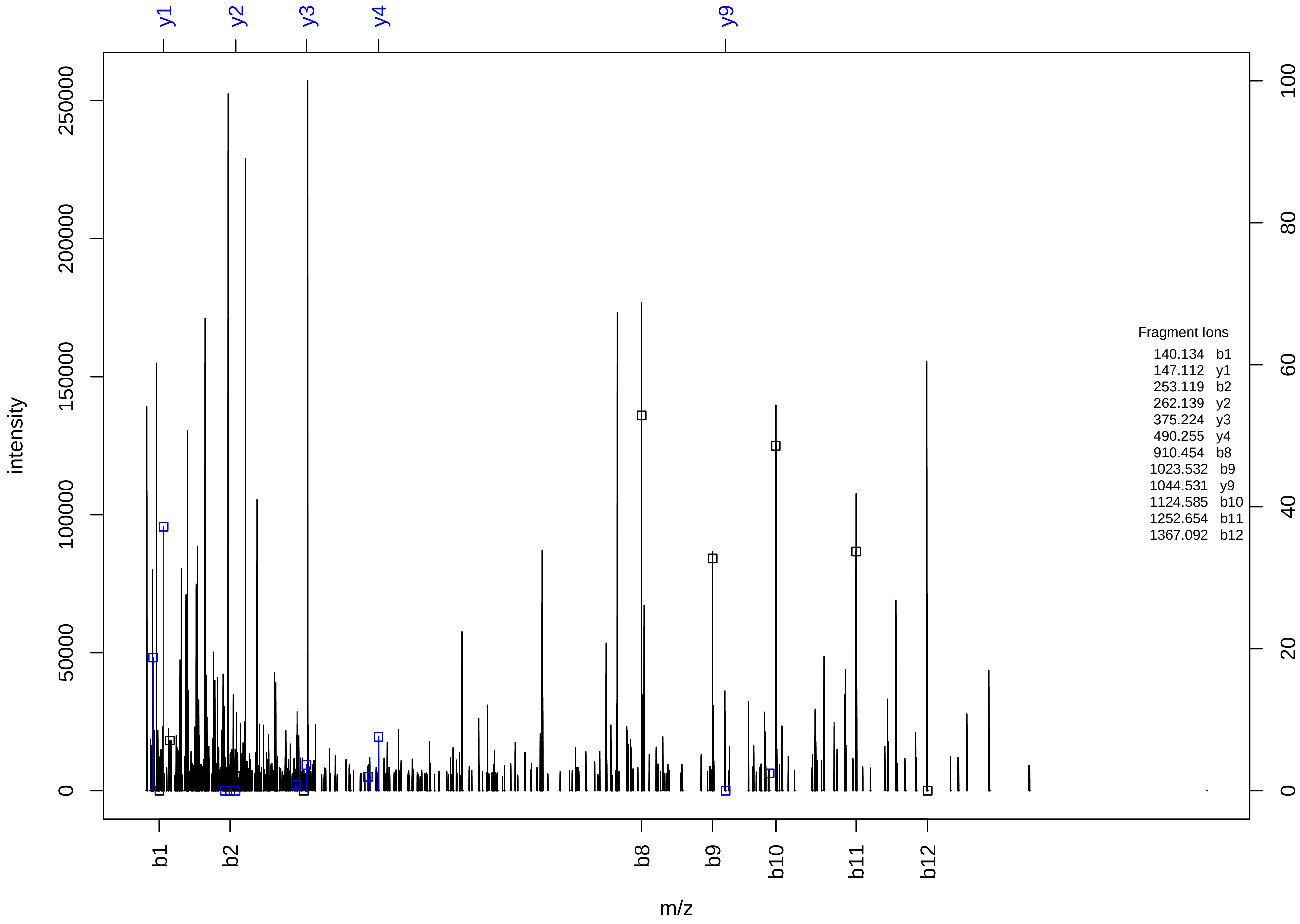

PITEKSNPLTQDLDK / File: christine_pool_03.raw; SpectrumID: ; scans: 13597

(Ac)VTLRN^PGSSGRK

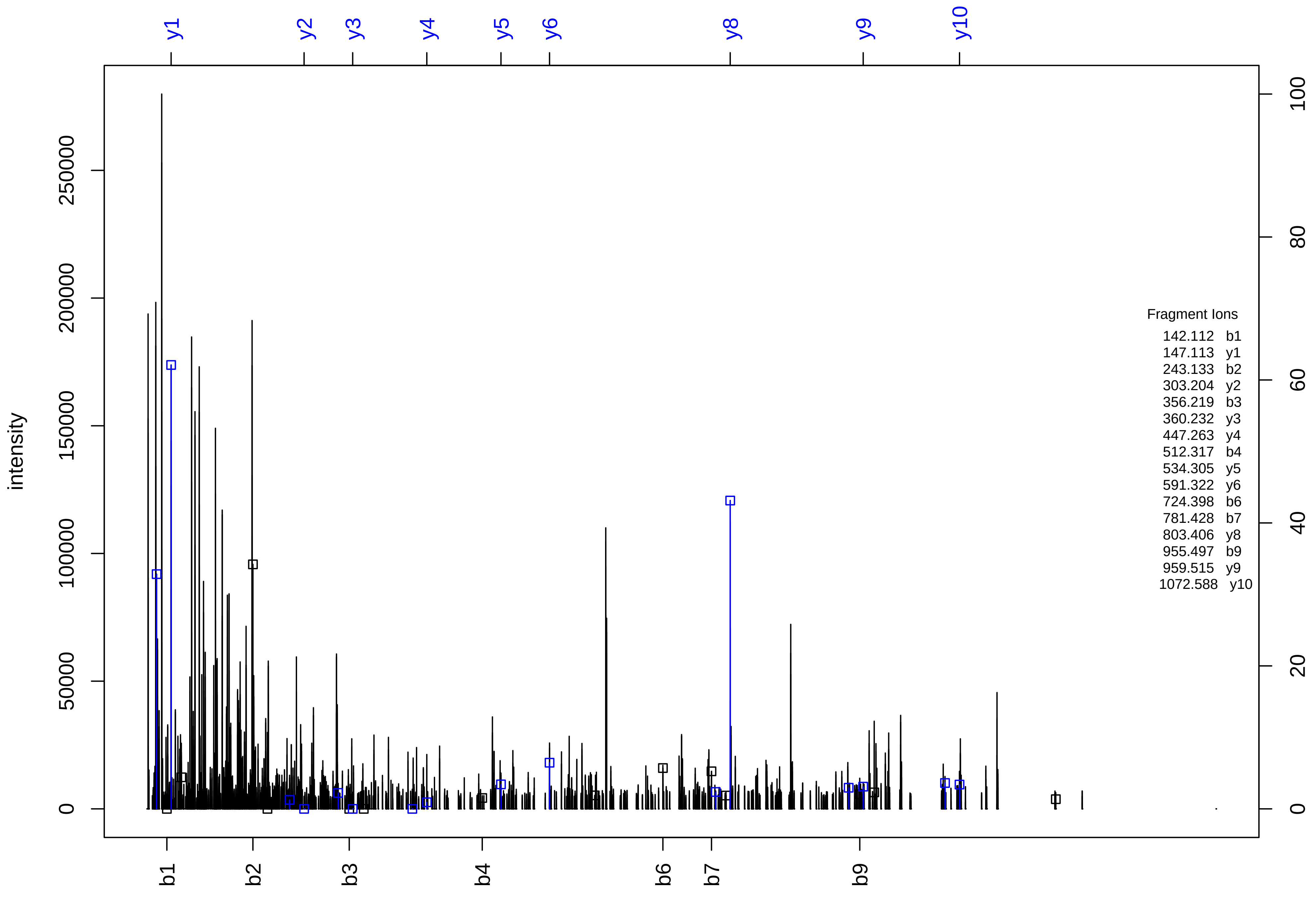

VTLRNPGSSGRK / File: christine_pool_03.raw; SpectrumID: ; scans: 10080

(Ac)VLN^EVVIDR

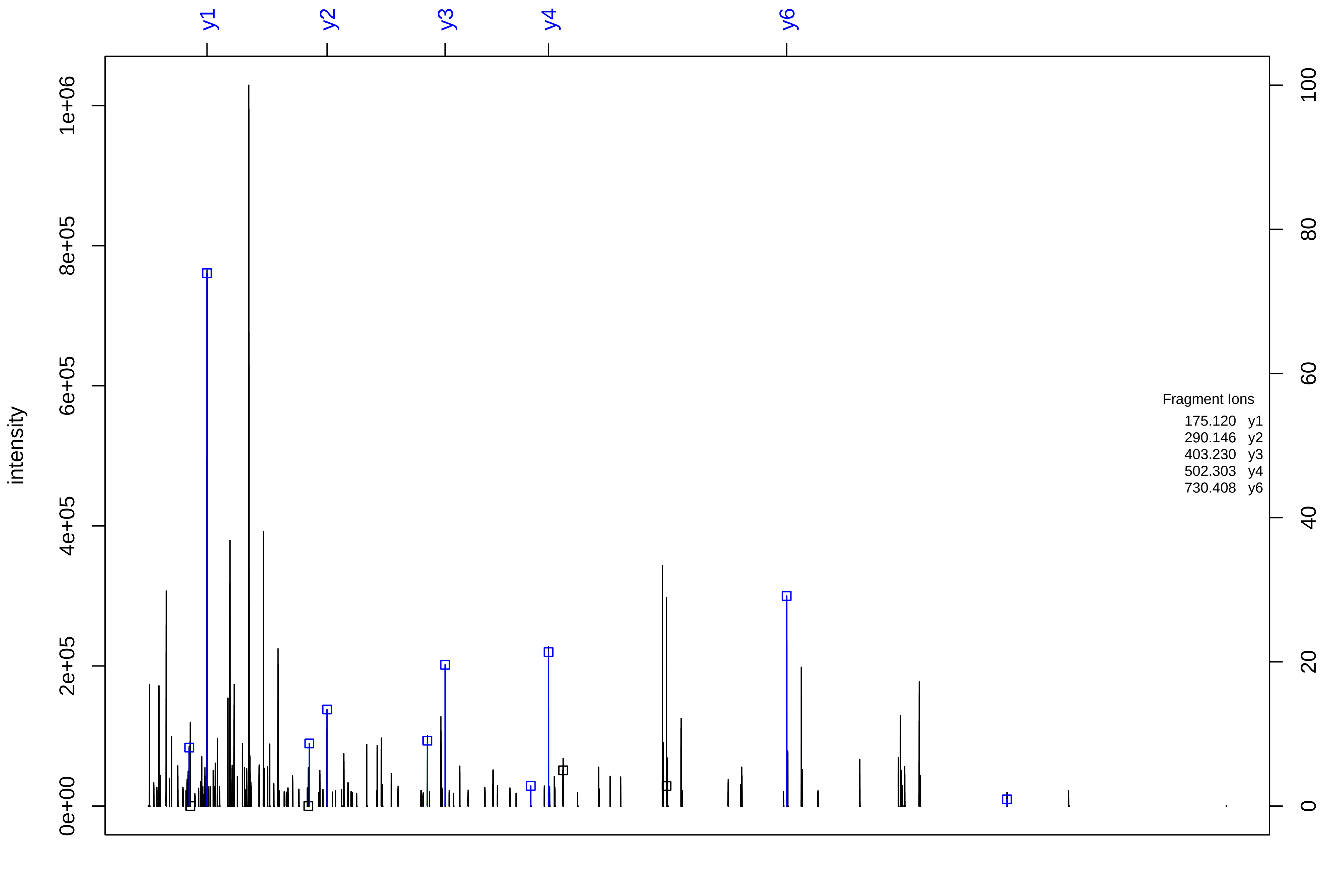

VLNEVVIDR / File: christine_pool_03.raw; SpectrumID: ; scans: 18314

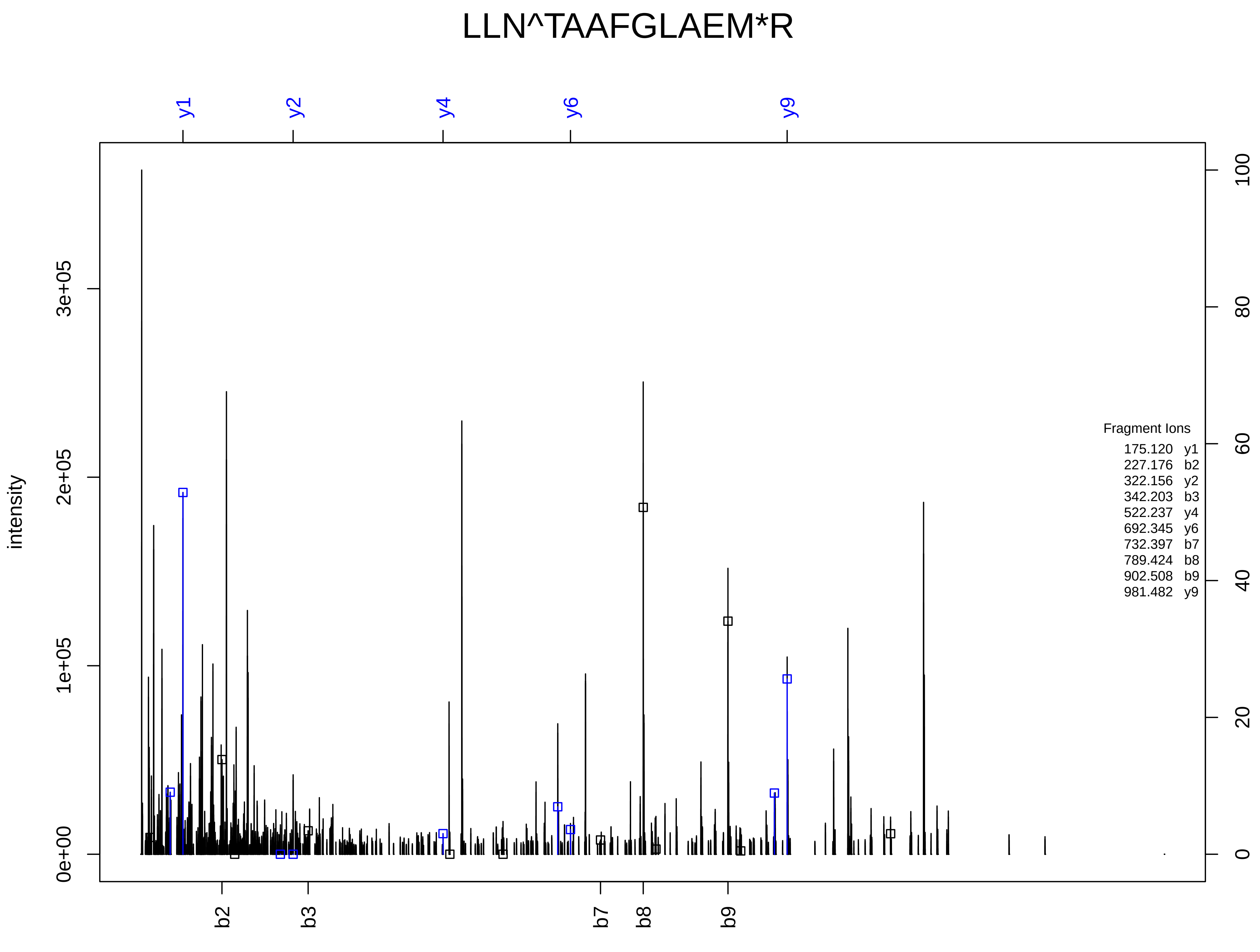
LLN^TAAFGLAEM*R
y1
y2
y4
y6
y9
intensity
0e+00
1e+05
2e+05
3e+05
0
20
40
60
80
100
b2
b3
b7
b8
b9
Fragment Ions
175.120 y1
227.176 b2
322.156 y2
342.203 b3
522.237 y4
692.345 y6
732.397 b7
789.424 b8
902.508 b9
981.482 y9
m/z
LLNTAAFGLAEMR / File: christine_pool_03.raw; SpectrumID: ; scans: 12190

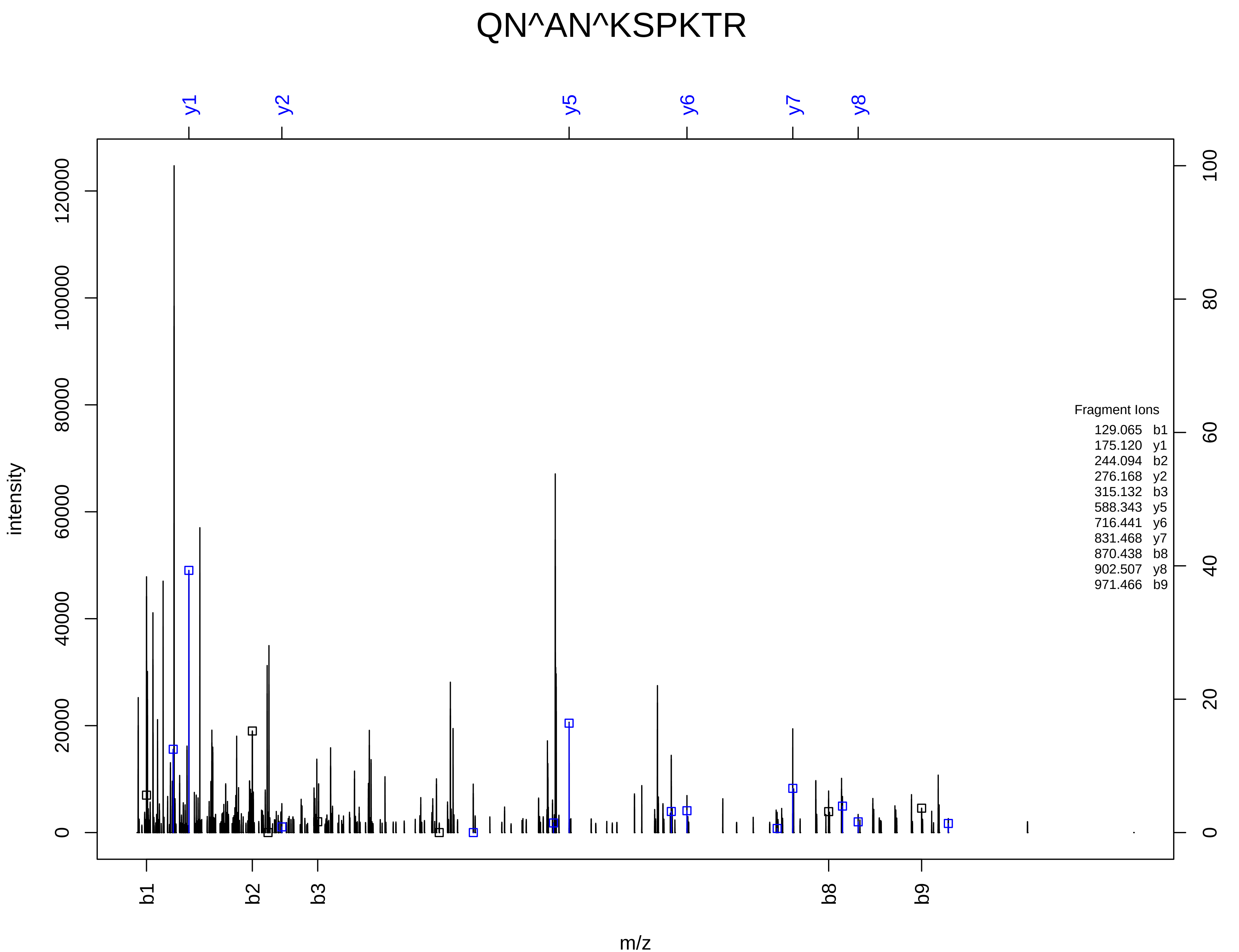

QNANKSPKTR / File: christine_pool_03.raw; SpectrumID: ; scans: 2708
