# Supplementary material for: Proximity Labeling Proteomics Reveals Kv1.3 Potassium Channel Immune Interactors in Microglia
Source: Mol Cell Proteomics. 2024 Jun 25;23(8):100809. doi: 10.1016/j.mcpro.2024.100809 (PMC11780389; doi:10.1016/j.mcpro.2024.100809)
Supplement: OHW_BV2_lysate [file mmc9.pdf]

ALIGFQQAPFSELPPPIHVLQESGLPDADPSQPPGAPR

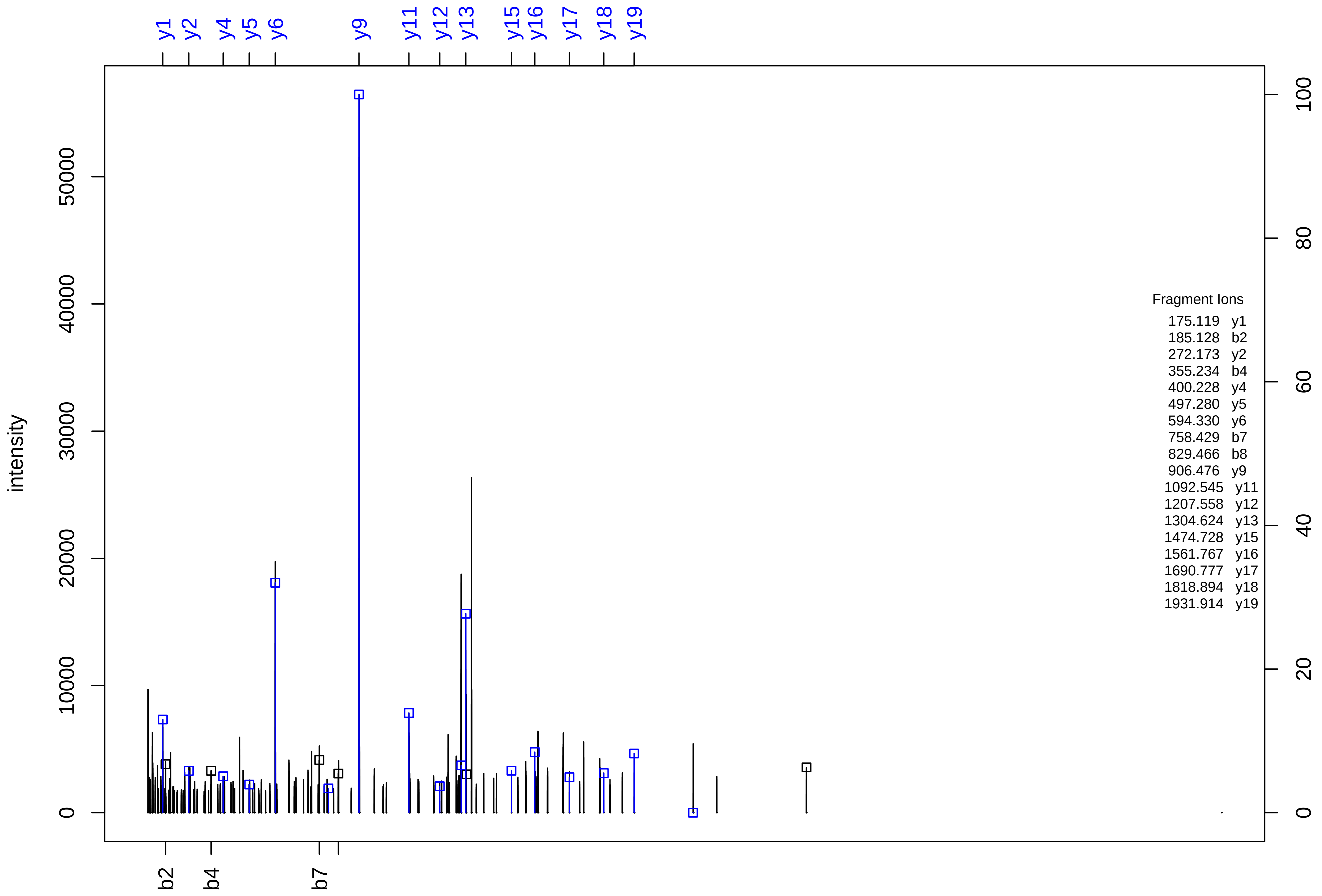

ALIGFQQAPFSELPPPIHVLQESGLPDADPSQPPGAPR / File: christine_10pools_06.raw; SpectrumID: ; scans: 37282

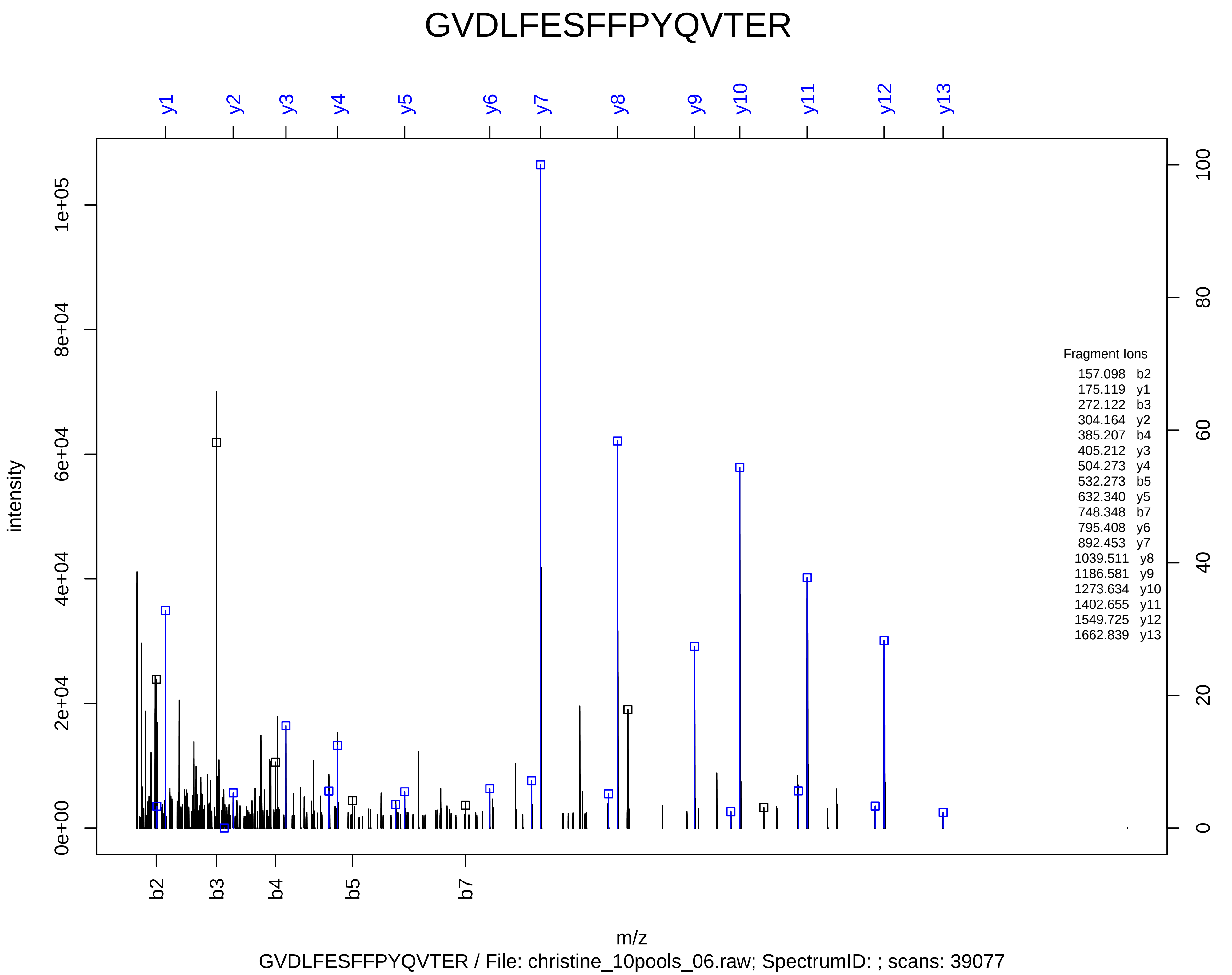
GVDLFESFFFPYQVTER
y1
y2
y3
y4
y5
y6
y7
y8
y9
y10
y11
y12
y13
intensity
0e+00
2e+04
4e+04
6e+04
8e+04
1e+05
0
20
40
60
80
100
b2
b3
b4
b5
b7
m/z
Fragment Ions
157.098 b2
175.119 y1
272.122 b3
304.164 y2
385.207 b4
405.212 y3
504.273 y4
532.273 b5
632.340 y5
748.348 b7
795.408 y6
892.453 y7
1039.511 y8
1186.581 y9
1273.634 y10
1402.655 y11
1549.725 y12
1662.839 y13
GVDLFESFFFPYQVTER / File: christine_10pools_06.raw; SpectrumID: ; scans: 39077

LQSGELSPEAVLFTYLGK

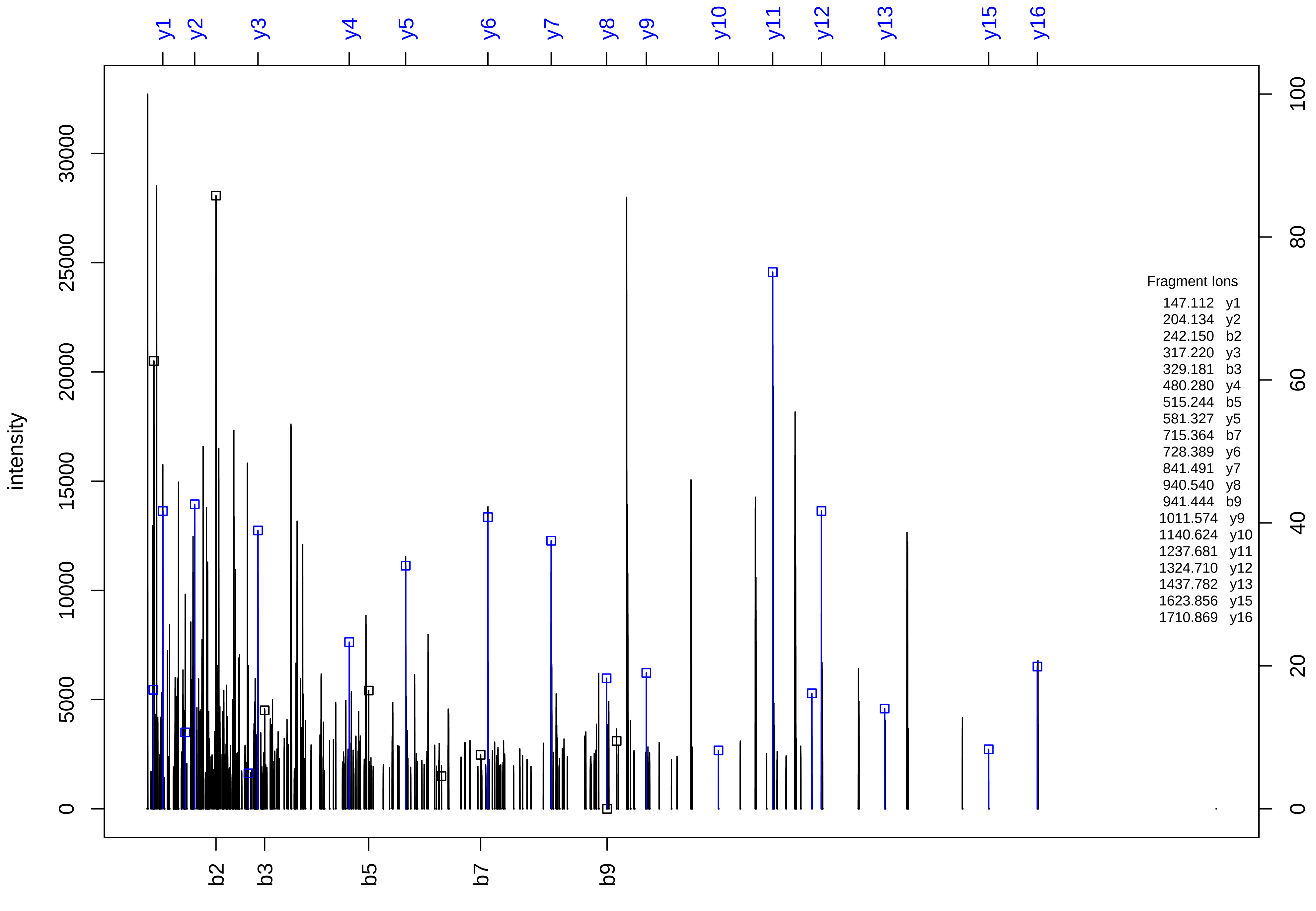

LQSGELSPEAVLFTYLGK / File: christine_10pools_06.raw; SpectrumID: ; scans: 39253

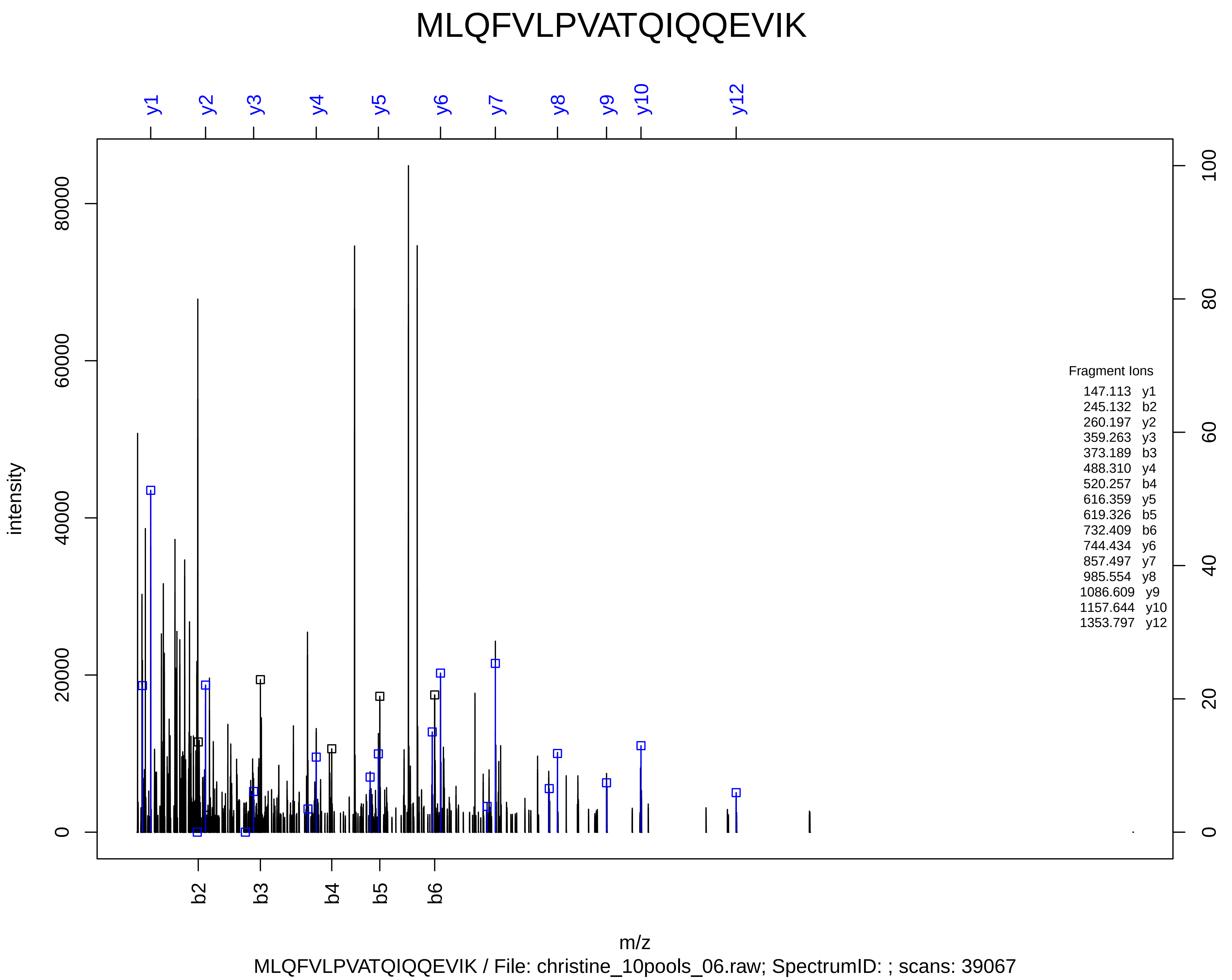

MLQFVLPVATQIQQEVIK
y1
y2
y3
y4
y5
y6
y7
y8
y9
y10
y12
intensity
0
20000
40000
60000
80000
0
20
40
60
80
100
b2
b3
b4
b5
b6
m/z
Fragment Ions
147.113 y1
245.132 b2
260.197 y2
359.263 y3
373.189 b3
488.310 y4
520.257 b4
616.359 y5
619.326 b5
732.409 b6
744.434 y6
857.497 y7
985.554 y8
1086.609 y9
1157.644 y10
1353.797 y12
MLQFVLPVATQIQQEVIK / File: christine_10pools_06.raw; SpectrumID: ; scans: 39067

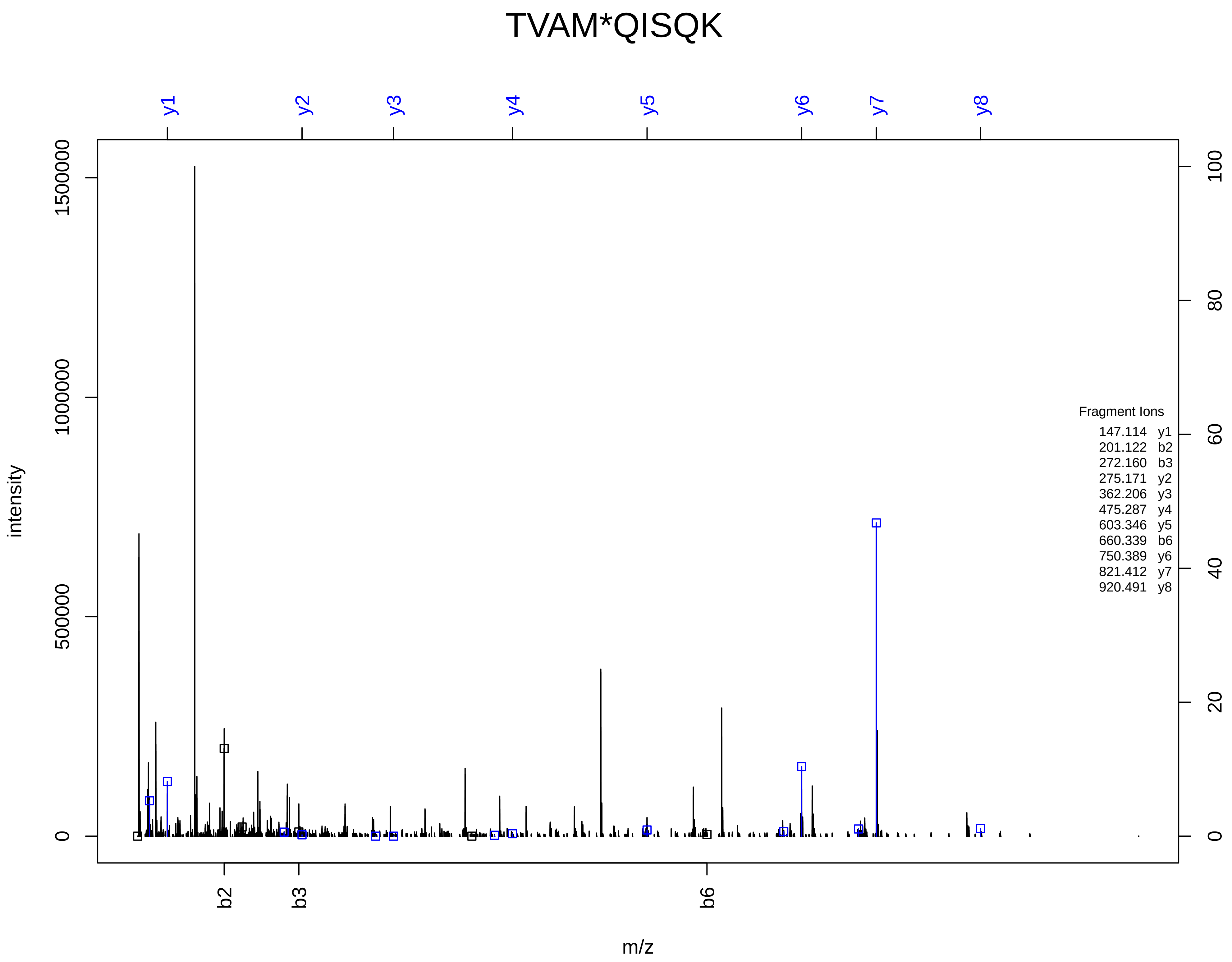

TVAMQISQK / File: christine_10pools_06.raw; SpectrumID: ; scans: 13238

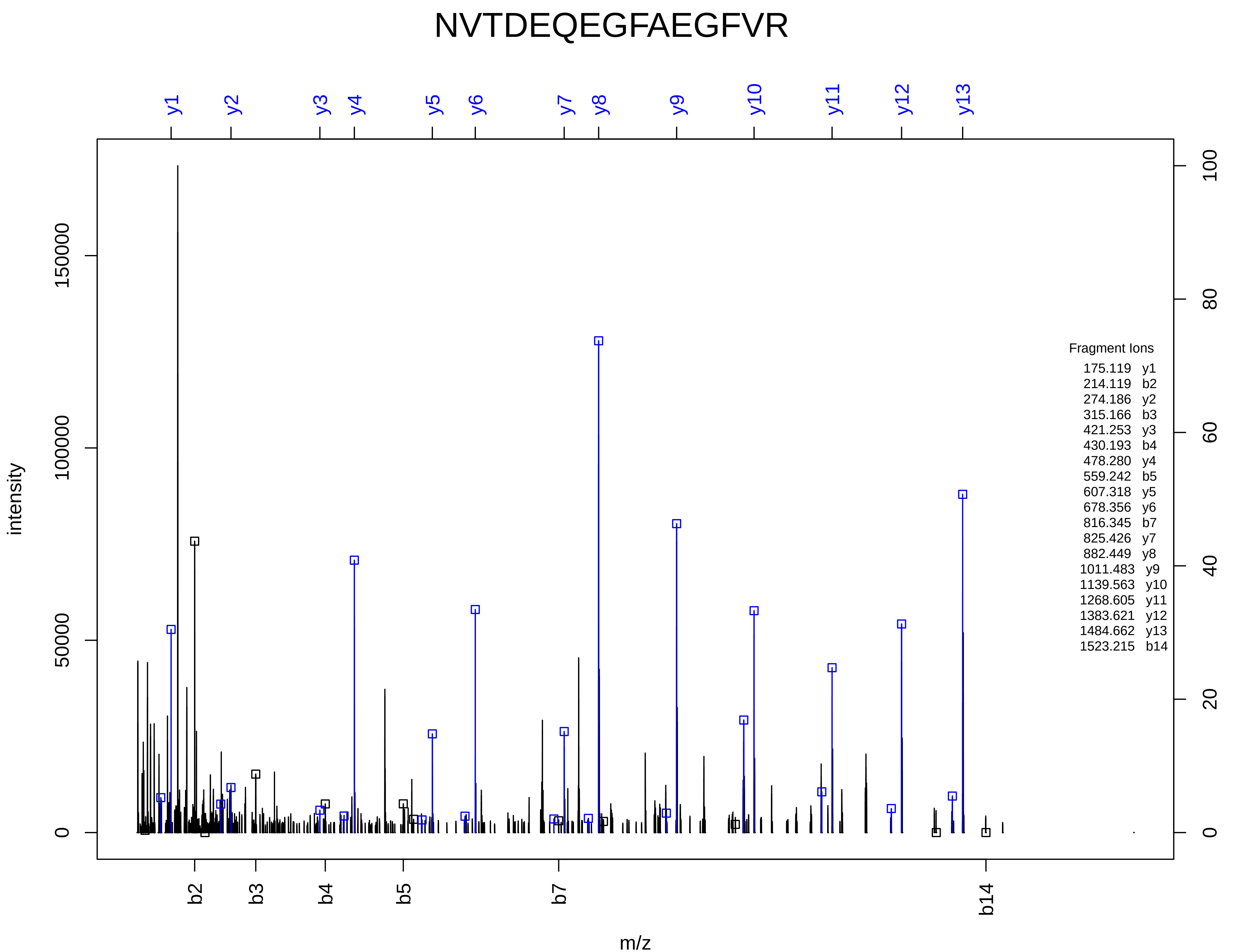

NVTDEQEGFAEGFVR / File: christine_10pools_06.raw; SpectrumID: ; scans: 16445

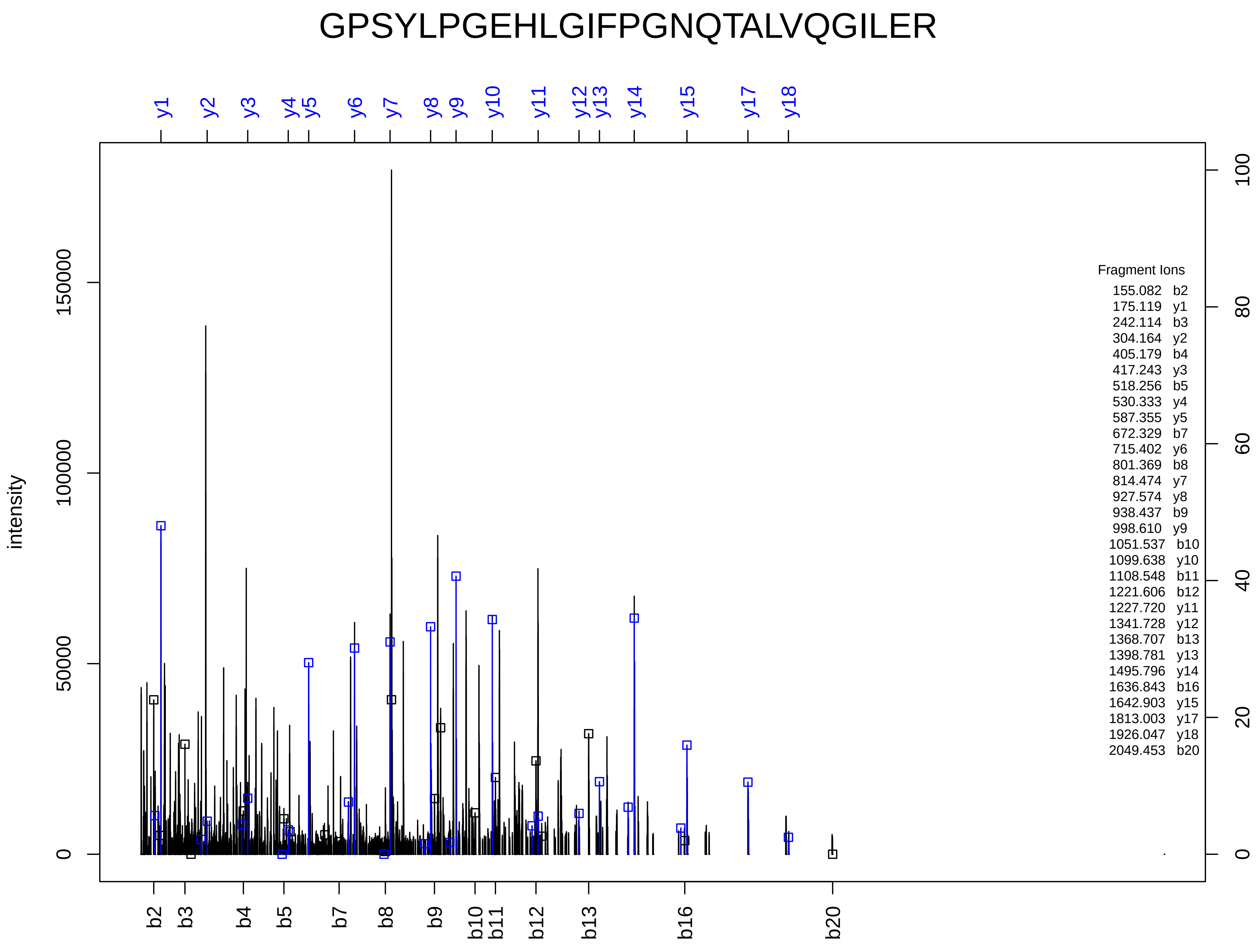

GPSYLPGEHLGIFPGNQTALVQGILER / File: christine_10pools_06.raw; SpectrumID: ; scans: 32667

ILGGSVLHLVLALR

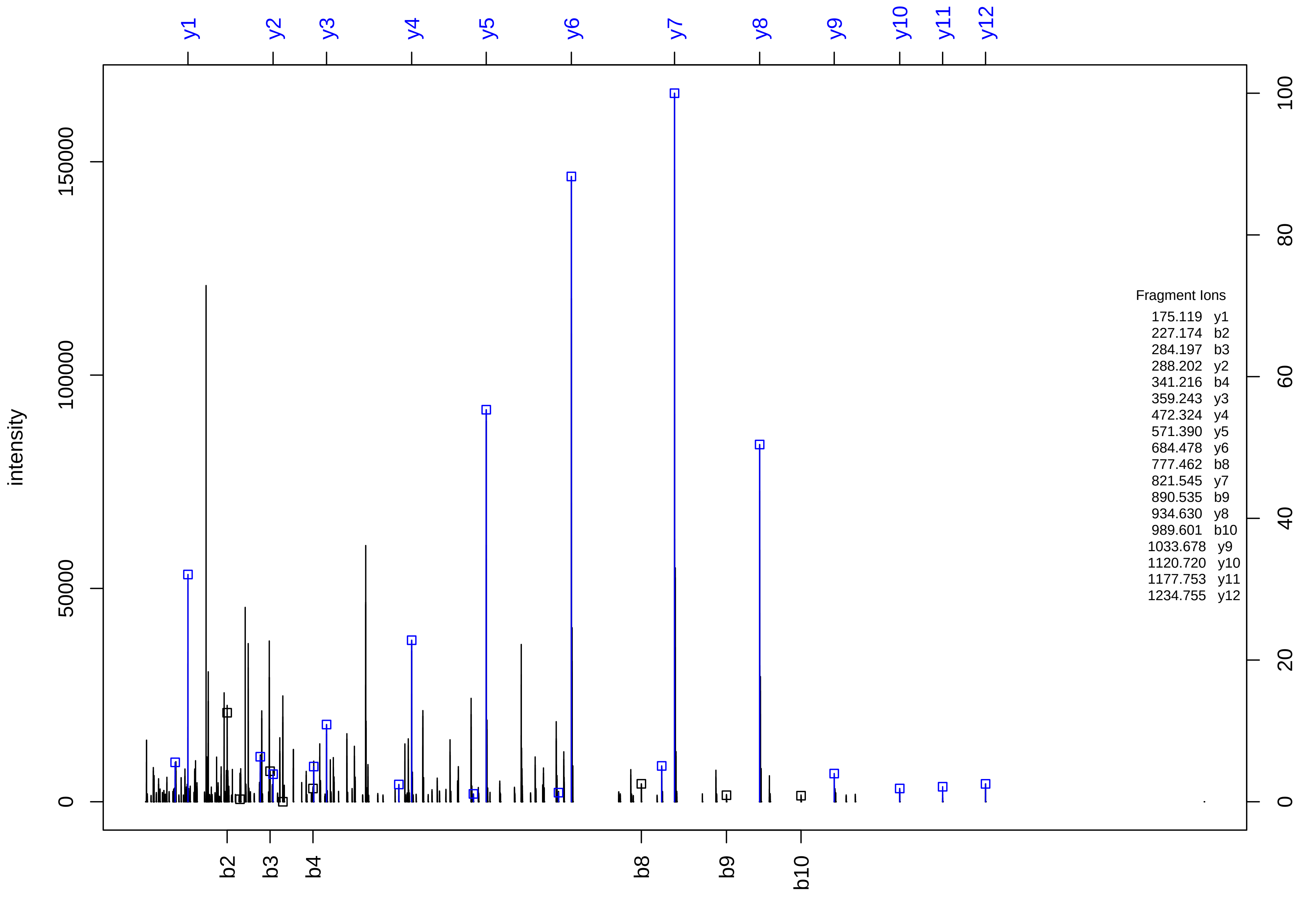

ILGGSVLHLVLALR / File: christine_10pools_06.raw; SpectrumID: ; scans: 37663

YGLSSMQGWR

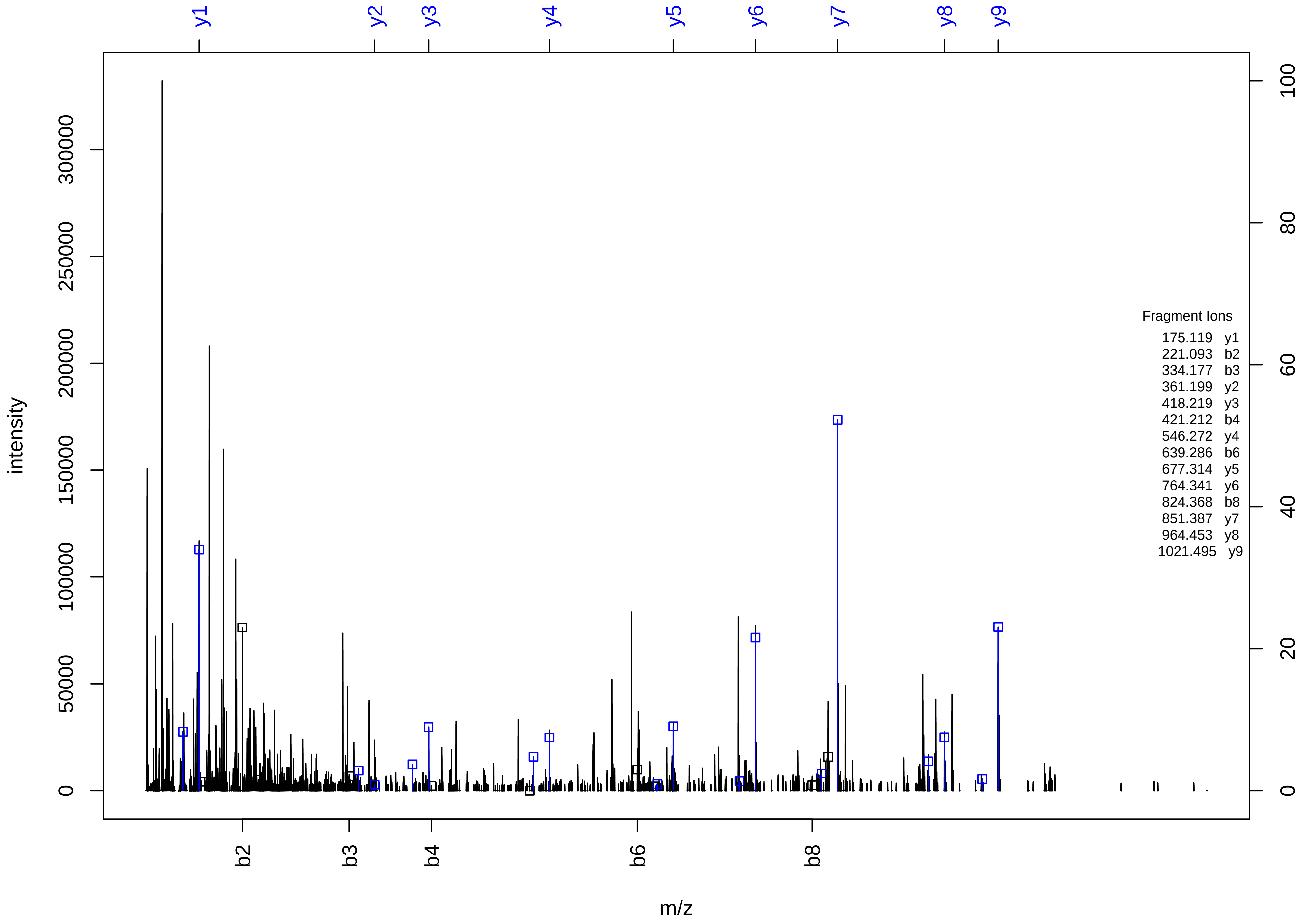

YGLSSMQGWR / File: christine_10pools_06.raw; SpectrumID: ; scans: 13336

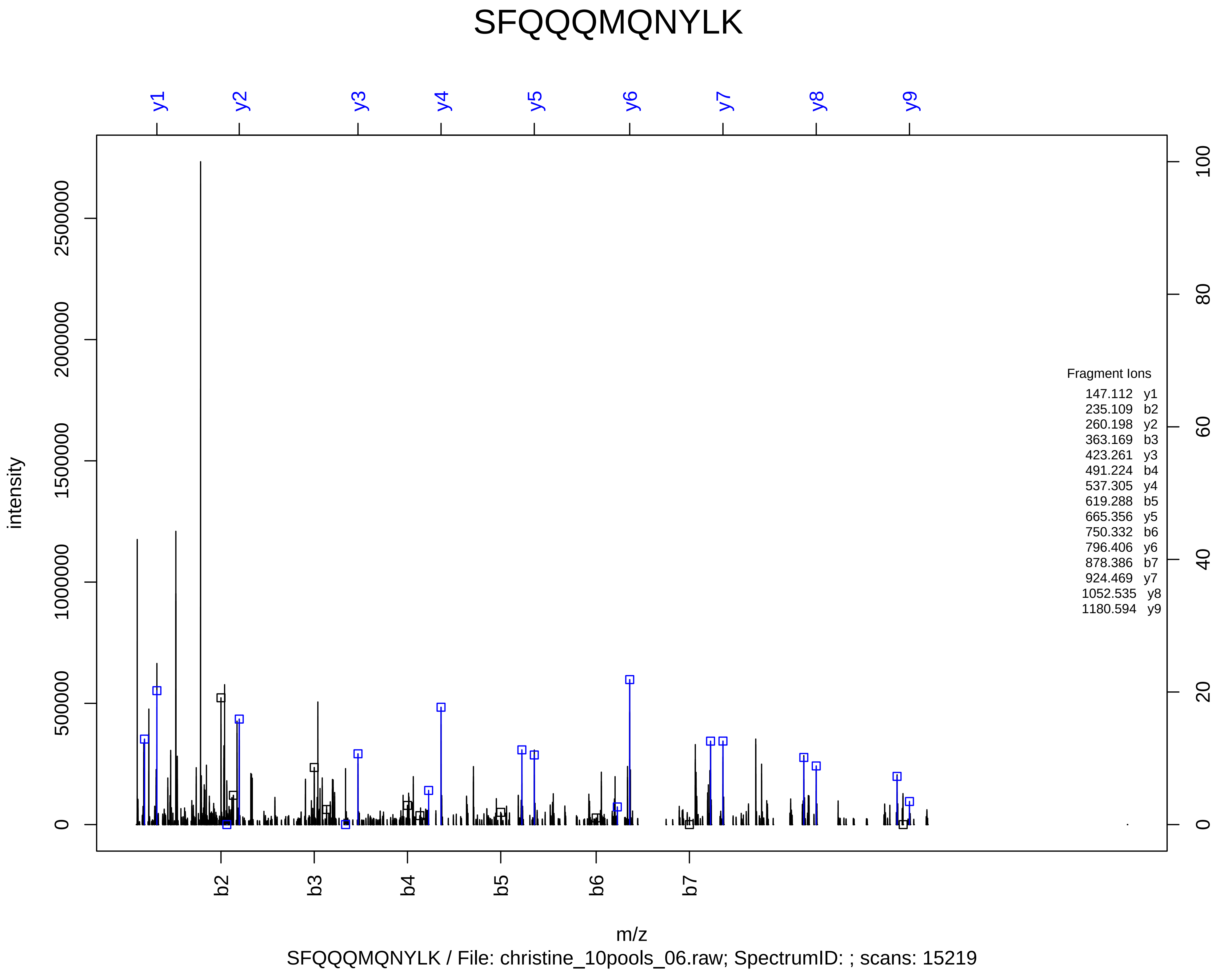
SFQQQMQNYLK
y1
y2
y3
y4
y5
y6
y7
y8
y9
intensity
0
500000
1000000
1500000
2000000
2500000
0
20
40
60
80
100
Fragment Ions
147.112 y1
235.109 b2
260.198 y2
363.169 b3
423.261 y3
491.224 b4
537.305 y4
619.288 b5
665.356 y5
750.332 b6
796.406 y6
878.386 b7
924.469 y7
1052.535 y8
1180.594 y9
b2
b3
b4
b5
b6
b7
m/z
SFQQQMQNYLK / File: christine_10pools_06.raw; SpectrumID: ; scans: 15219

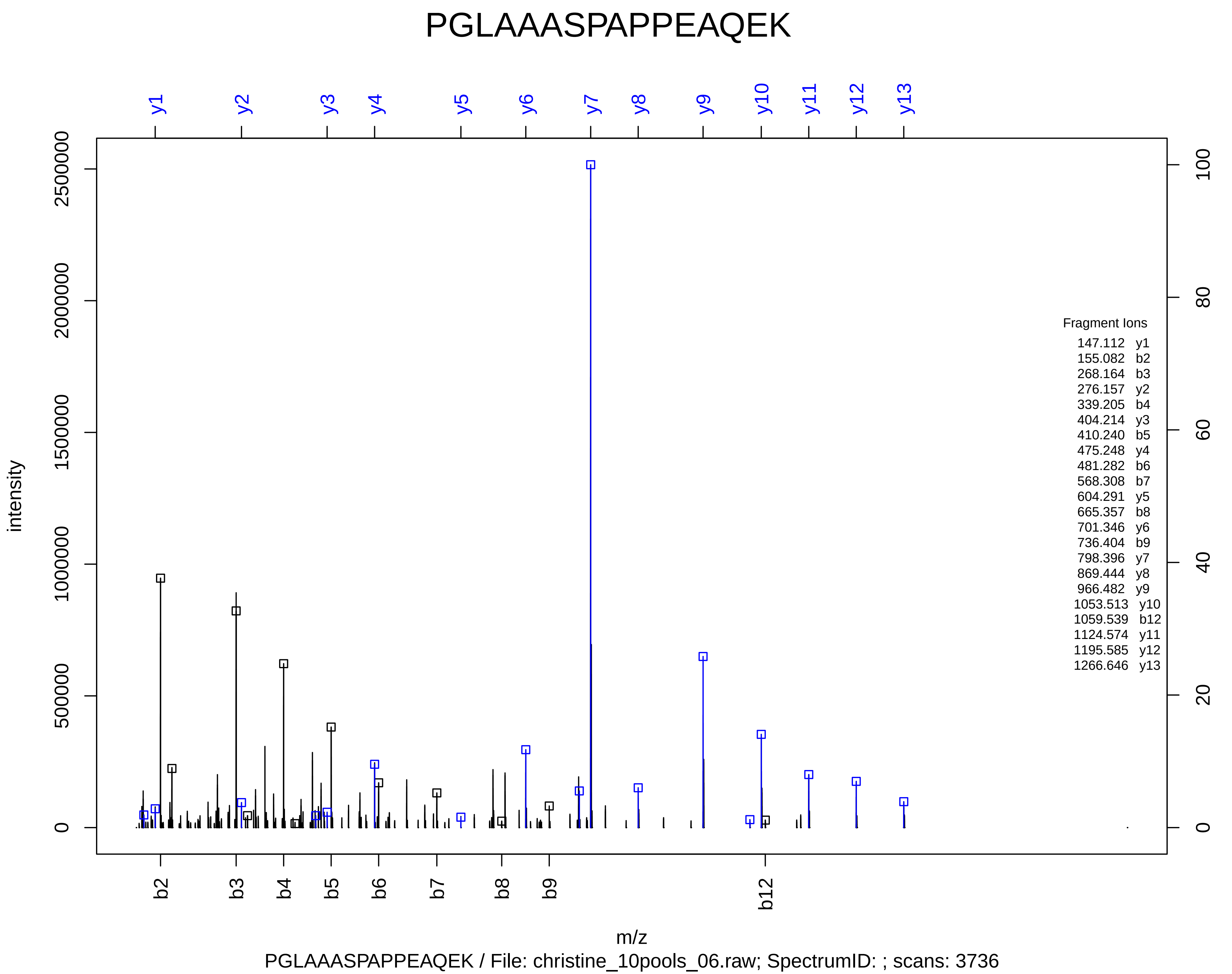

PGLAAASPAPPEAQEK
y1
y2
y3
y4
y5
y6
y7
y8
y9
y10
y11
y12
y13
intensity
0
500000
1000000
1500000
2000000
2500000
0
20
40
60
80
100
Fragment Ions
147.112 y1
155.082 b2
268.164 b3
276.157 y2
339.205 b4
404.214 y3
410.240 b5
475.248 y4
481.282 b6
568.308 b7
604.291 y5
665.357 b8
701.346 y6
736.404 b9
798.396 y7
869.444 y8
966.482 y9
1053.513 y10
1059.539 b12
1124.574 y11
1195.585 y12
1266.646 y13
b2
b3
b4
b5
b6
b7
b8
b9
b12
m/z
PGLAAASPAPPEAQEK / File: christine_10pools_06.raw; SpectrumID: ; scans: 3736

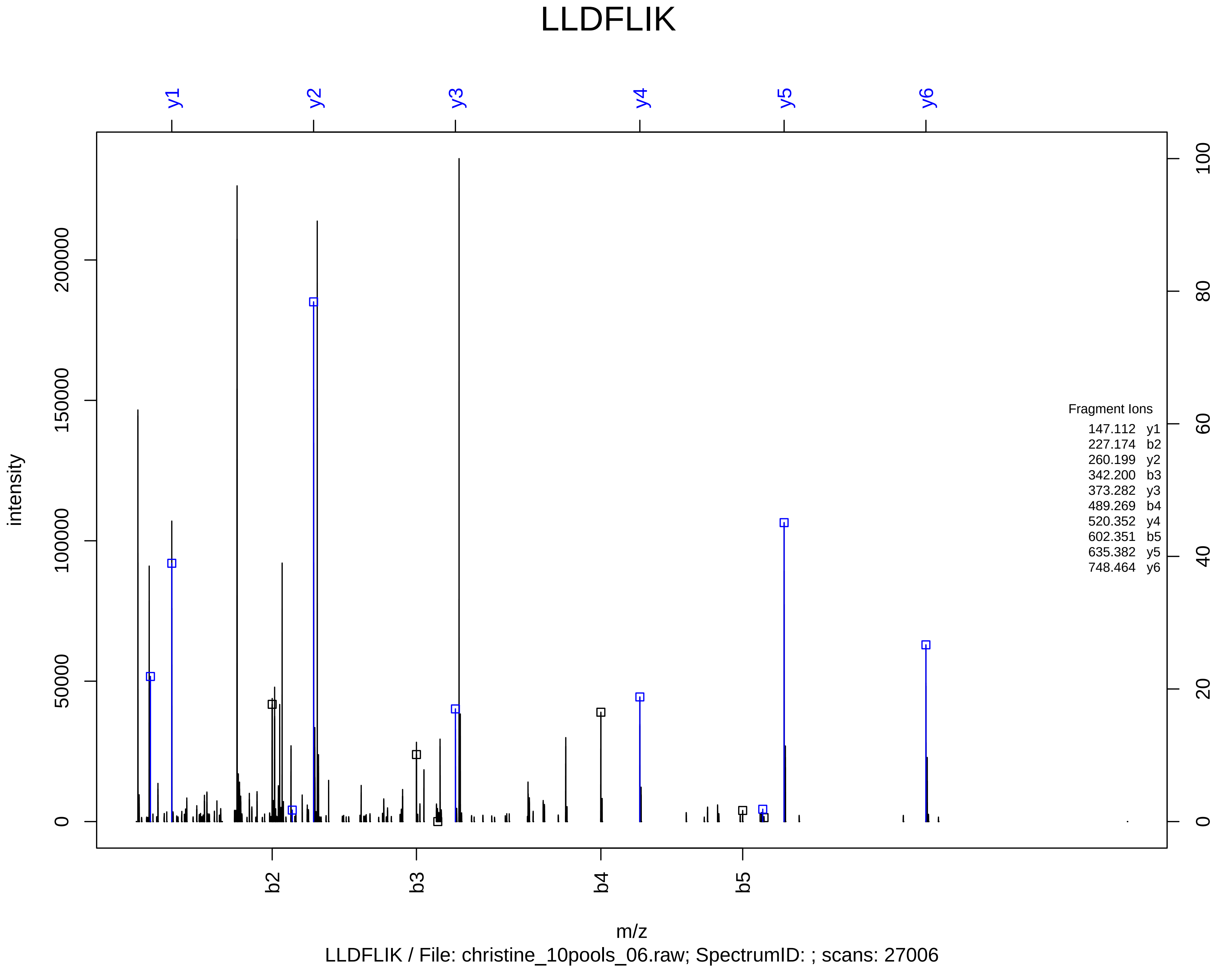

LLDFLIK
y1
y2
y3
y4
y5
y6
intensity
0
50000
100000
150000
200000
0
20
40
60
80
100
Fragment Ions
147.112 y1
227.174 b2
260.199 y2
342.200 b3
373.282 y3
489.269 b4
520.352 y4
602.351 b5
635.382 y5
748.464 y6
b2
b3
b4
b5
m/z
LLDFLIK / File: christine_10pools_06.raw; SpectrumID: ; scans: 27006

APIASIHSVDLDLL

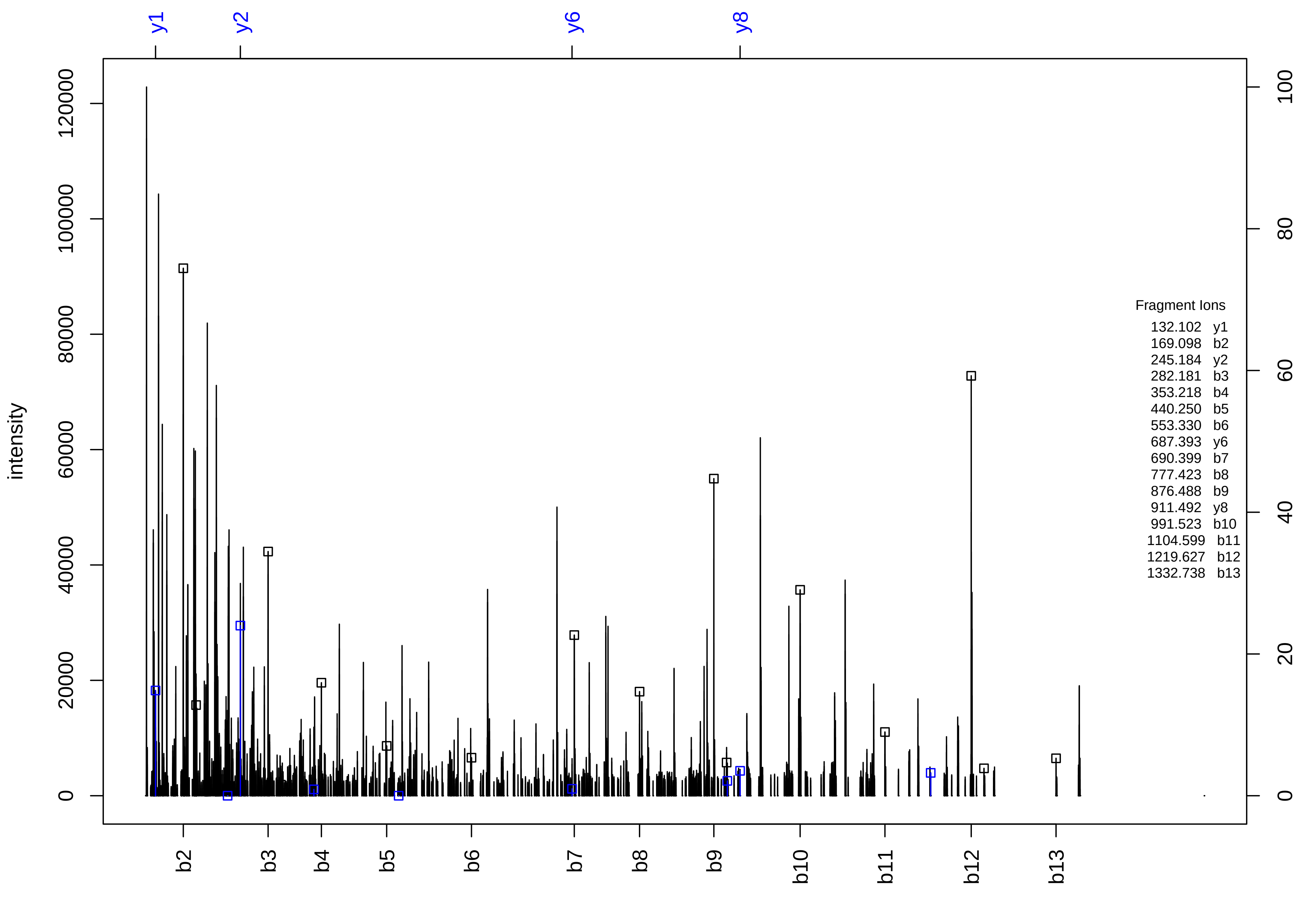

APIASIHSVDLDLL / File: christine_10pools_06.raw; SpectrumID: ; scans: 30094

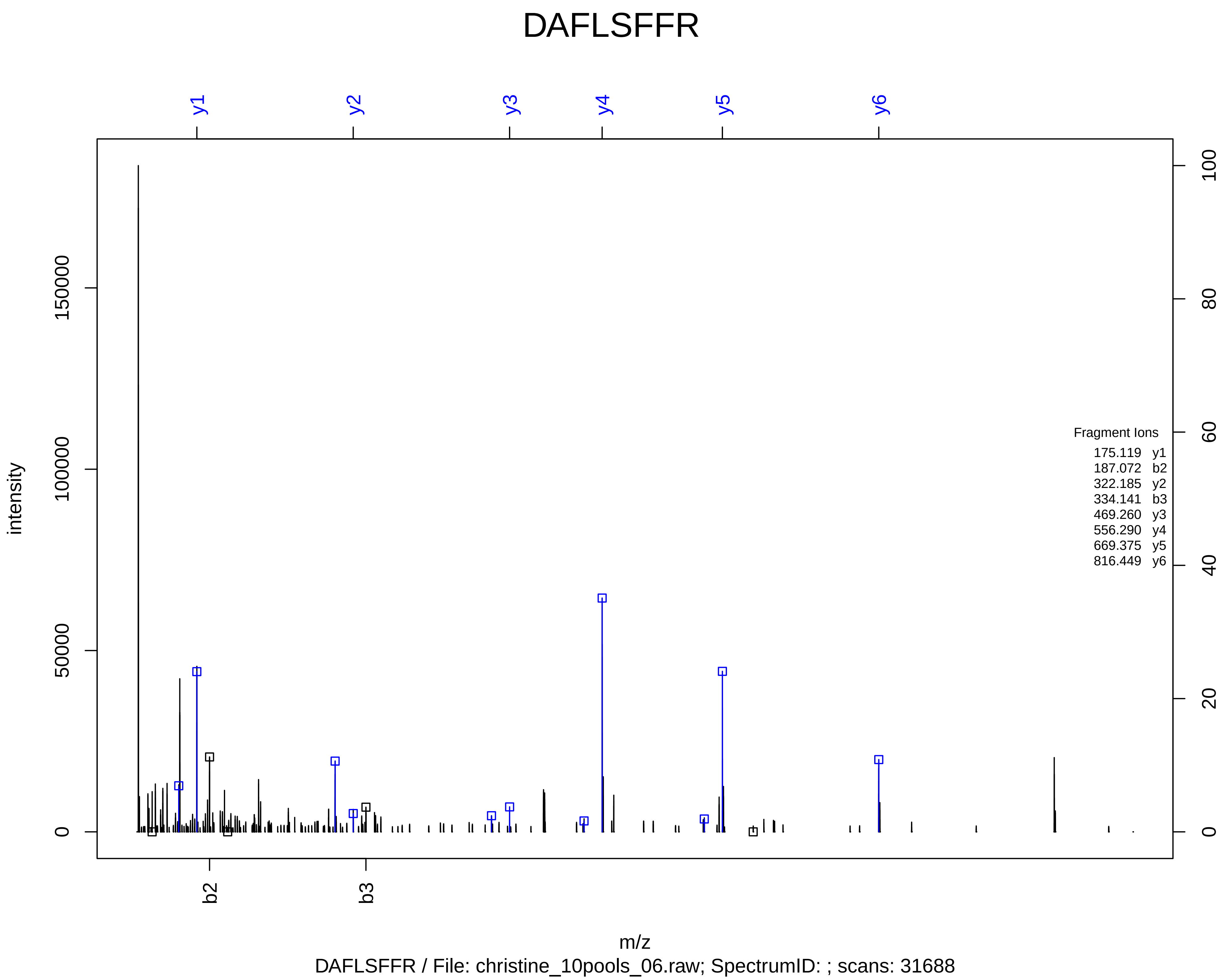

DAFLSFFR
y1
y2
y3
y4
y5
y6
b2
b3
intensity
0
50000
100000
150000
0
20
40
60
80
100
m/z
Fragment Ions
175.119 y1
187.072 b2
322.185 y2
334.141 b3
469.260 y3
556.290 y4
669.375 y5
816.449 y6
DAFLSFFR / File: christine_10pools_06.raw; SpectrumID: ; scans: 31688

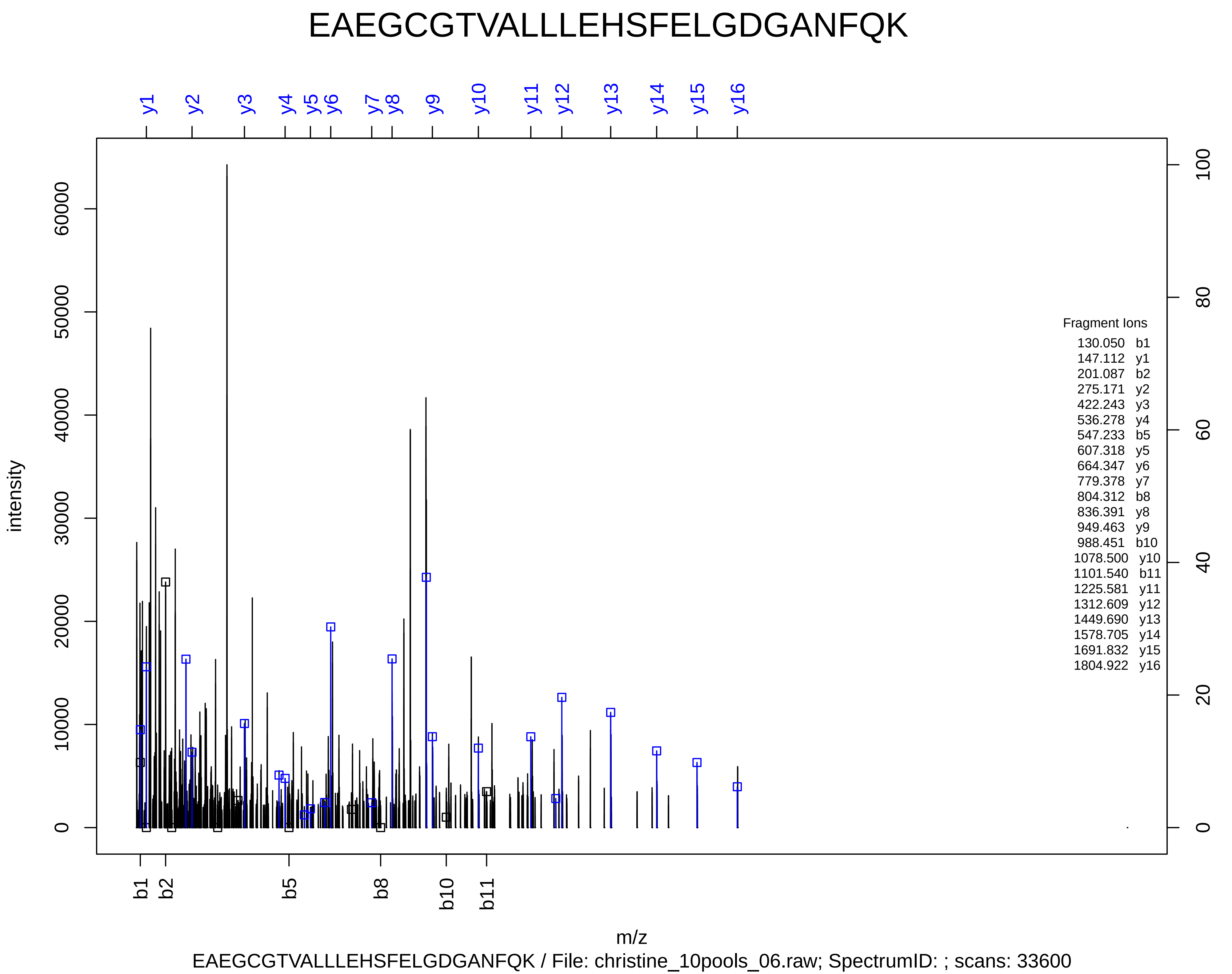
EAEGCGTVALLLEHSFELGDGANFQK
y1 y2 y3 y4 y5 y6 y7 y8 y9 y10 y11 y12 y13 y14 y15 y16
b1 b2 b5 b8 b10 b11
intensity
m/z
Fragment Ions
130.050 b1
147.112 y1
201.087 b2
275.171 y2
422.243 y3
536.278 y4
547.233 b5
607.318 y5
664.347 y6
779.378 y7
804.312 b8
836.391 y8
949.463 y9
988.451 b10
1078.500 y10
1101.540 b11
1225.581 y11
1312.609 y12
1449.690 y13
1578.705 y14
1691.832 y15
1804.922 y16
EAEGCGTVALLLEHSFELGDGANFQK / File: christine_10pools_06.raw; SpectrumID: ; scans: 33600

GTVYPVVYVDDSAILDCQFSEFYHTPPPGFEK

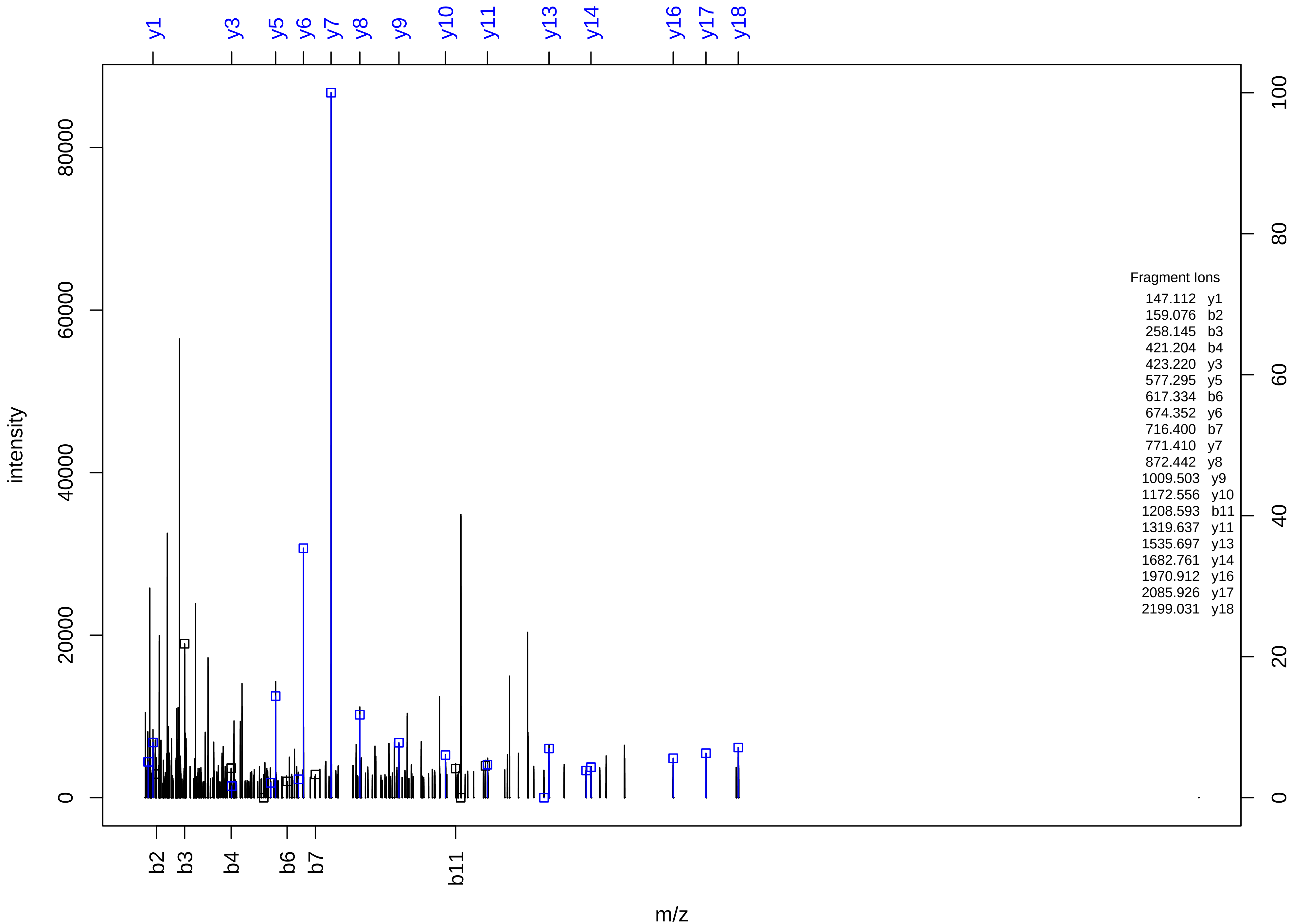

GTVYPVVYVDDSAILDCQFSEFYHTPPPGFEK / File: christine_10pools_06.raw; SpectrumID: ; scans: 36487

ALFEQFAEVFPLK

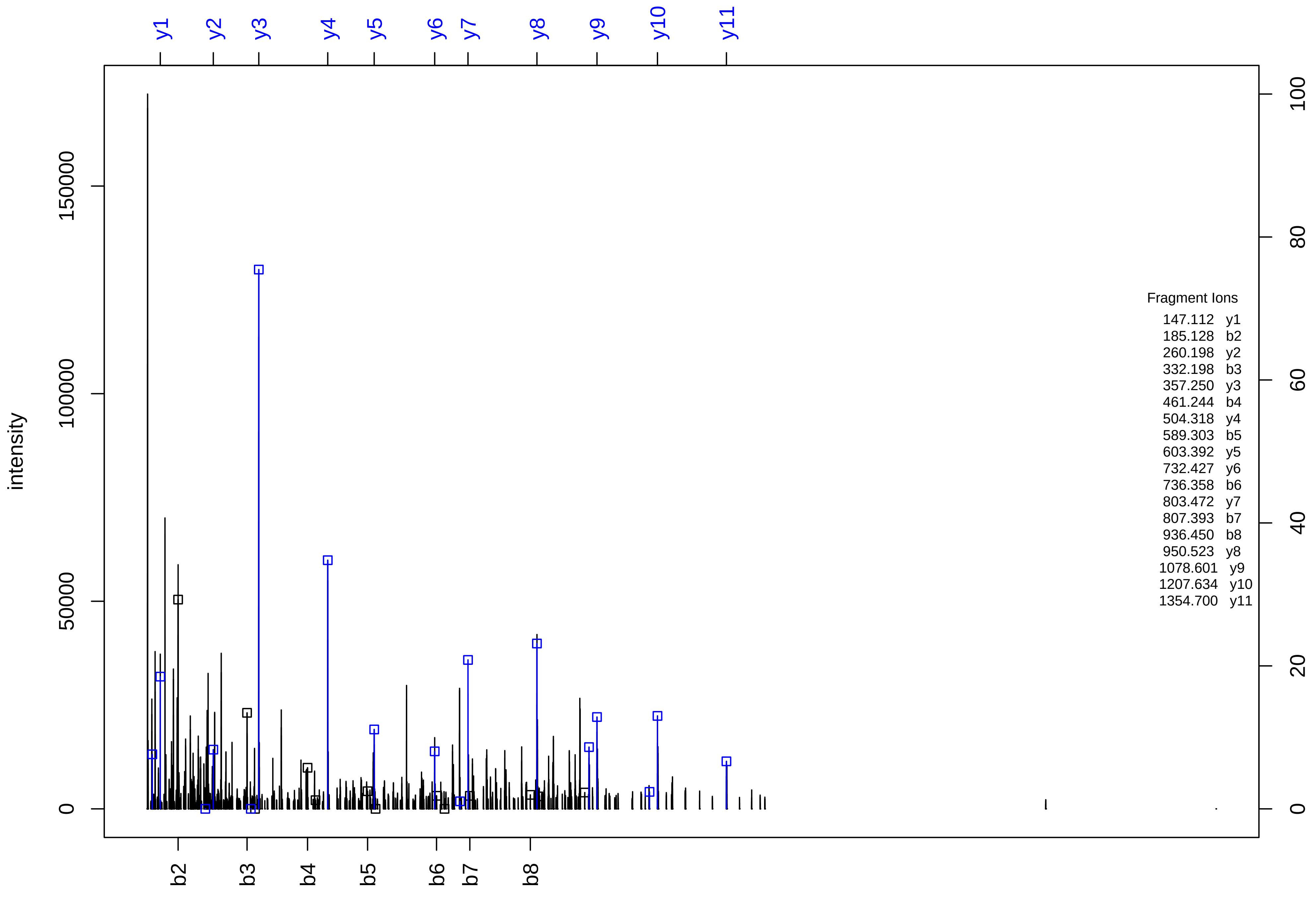

ALFEQFAEVFPLK / File: christine_10pools_06.raw; SpectrumID: ; scans: 38537

VIQEQLQVNDNTK

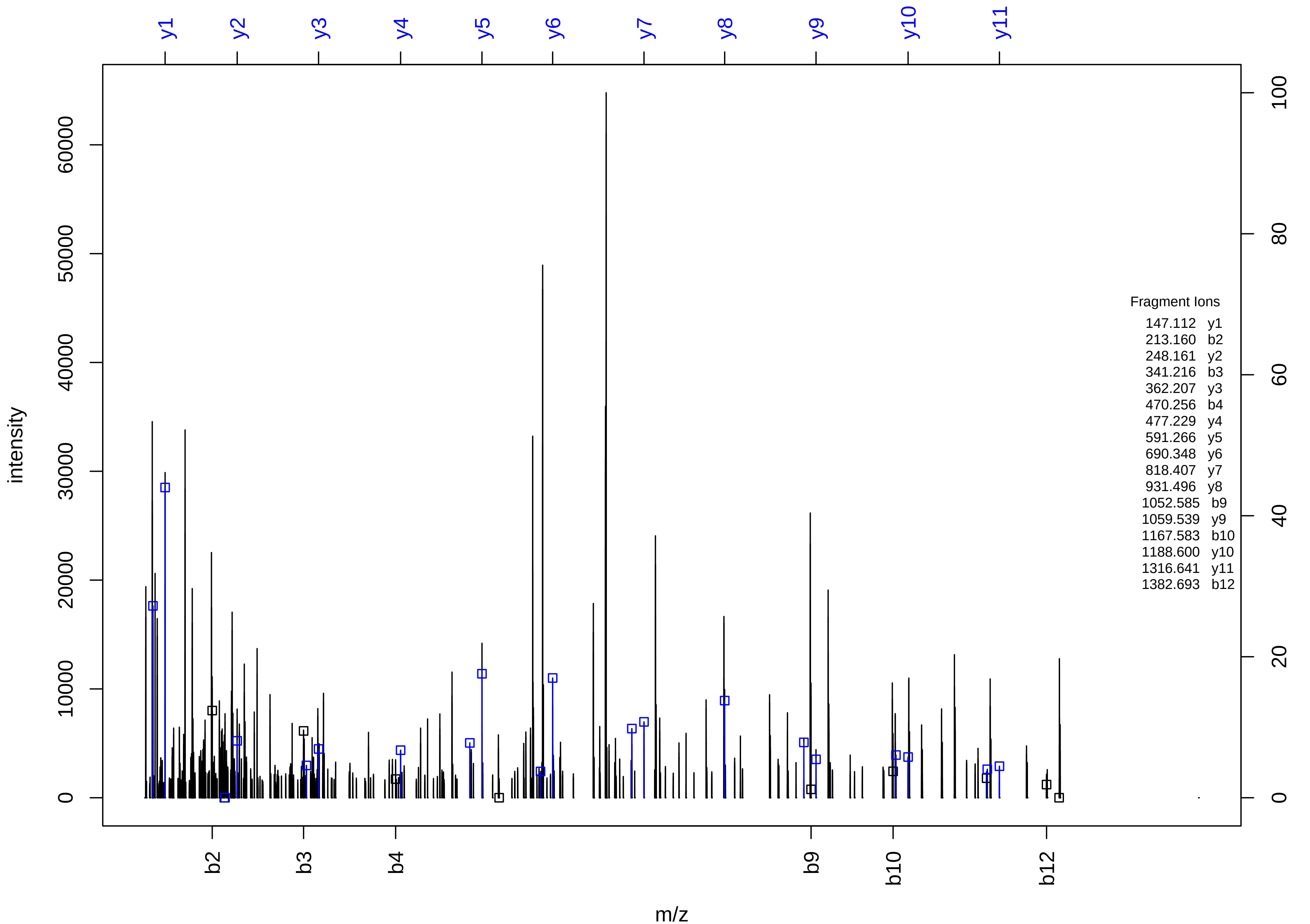

VIQEQLQVNDNTK / File: christine_10pools_06.raw; SpectrumID: ; scans: 3644

ILSLLQNLGISK

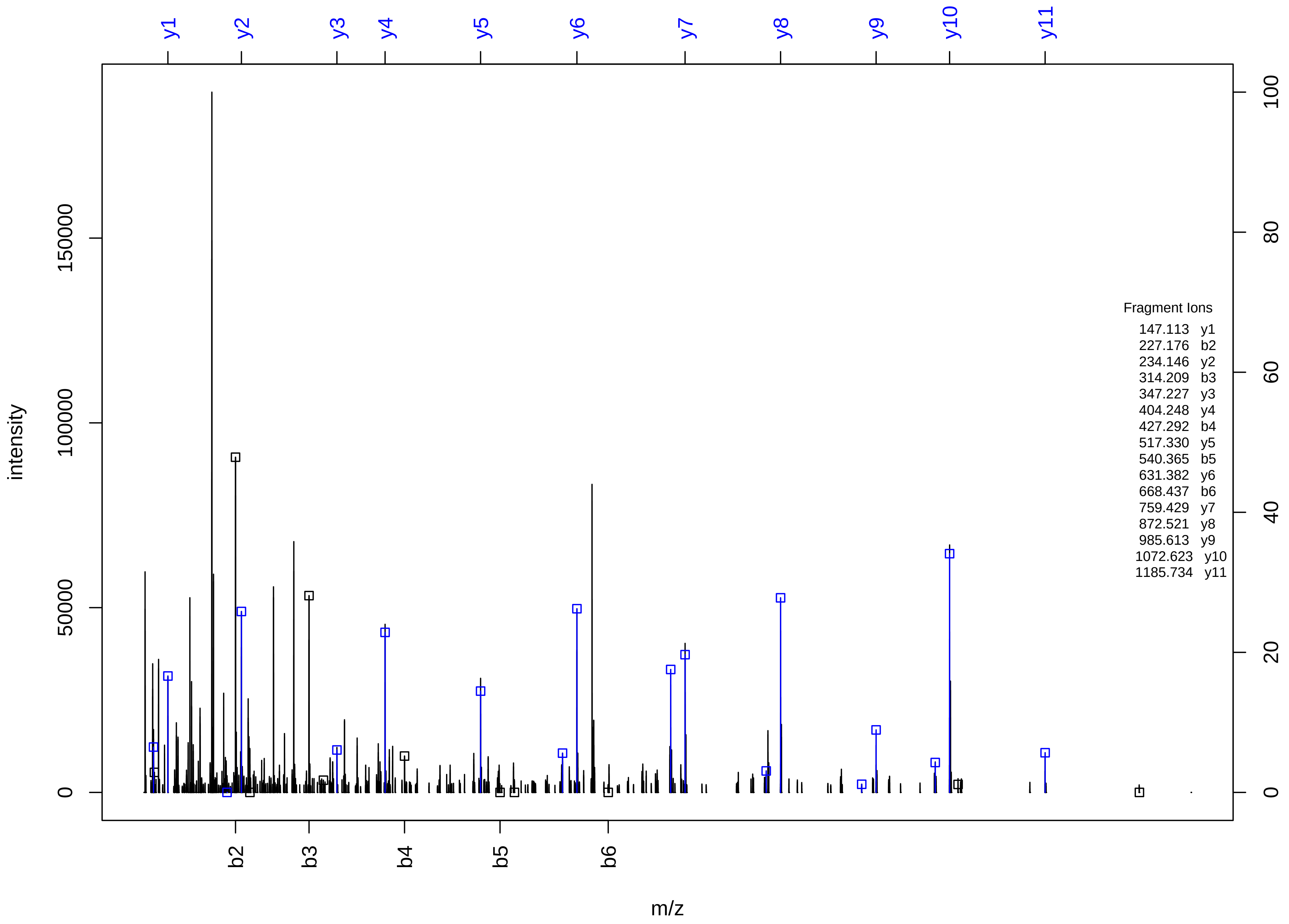

ILSLLQNLGISK / File: christine_10pools_06.raw; SpectrumID: ; scans: 30615

ILLPLPILNEHQEK

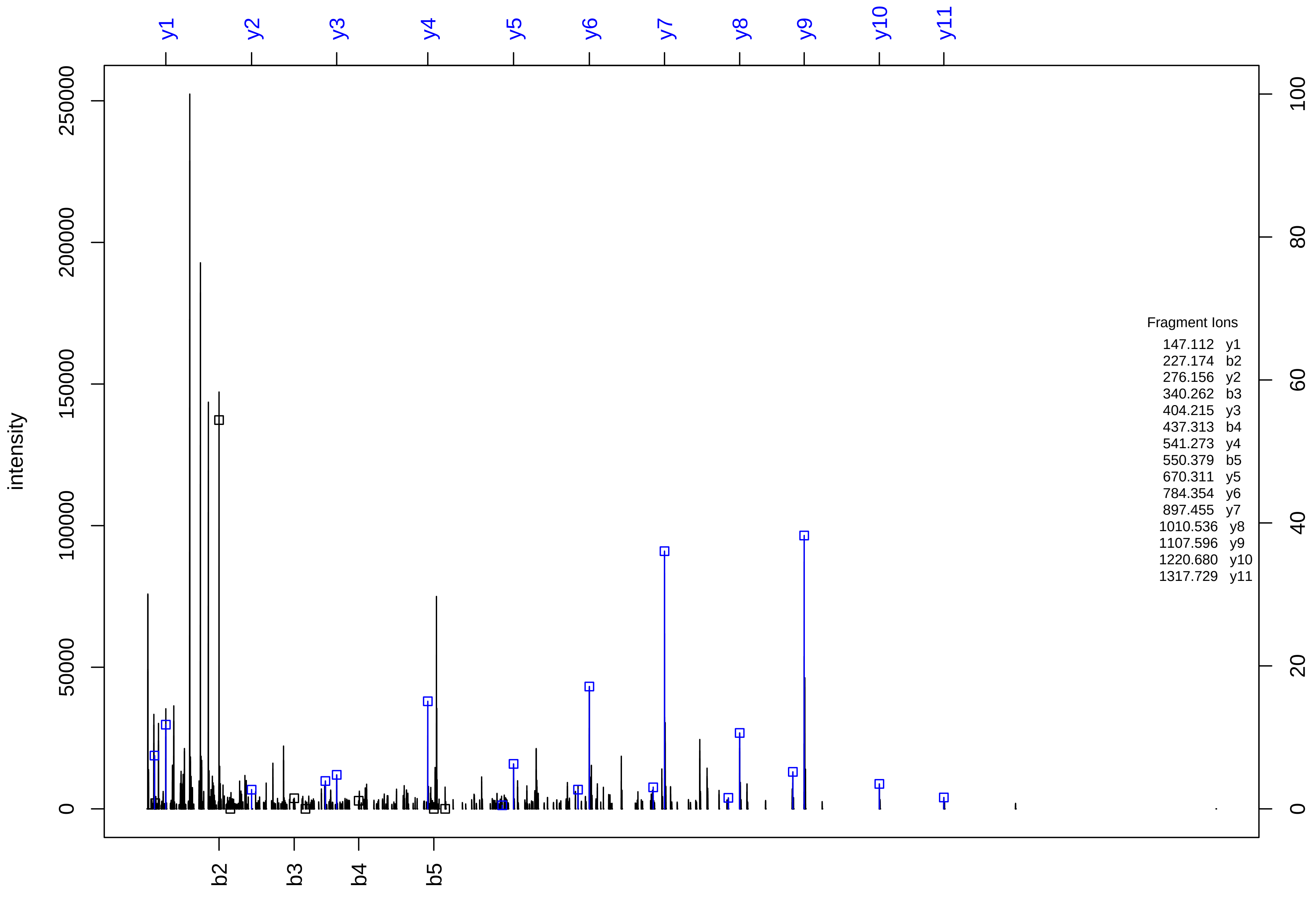

ILLPLPILNEHQEK / File: christine_10pools_06.raw; SpectrumID: ; scans: 27381

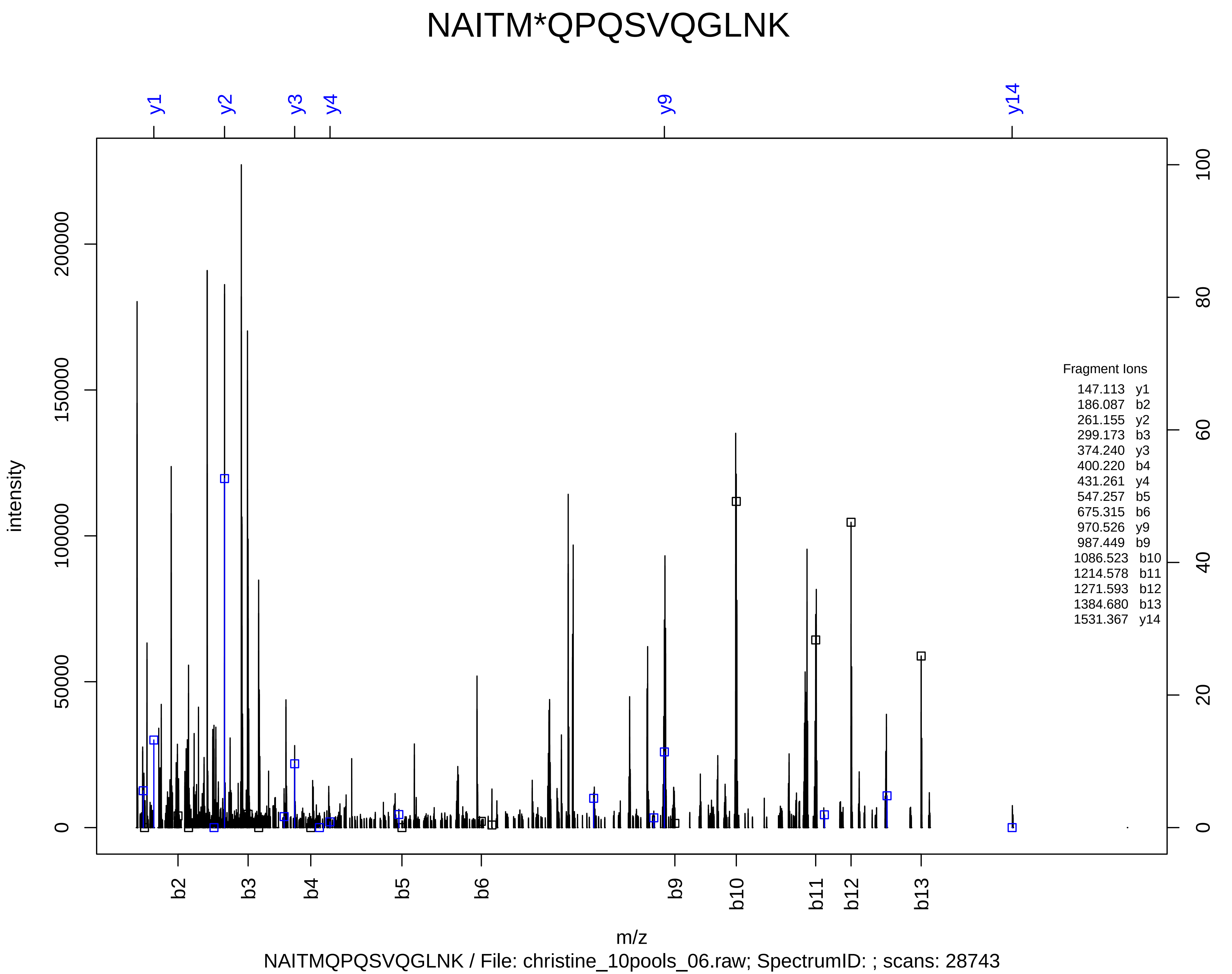
NAITM*QPQSVQGLNK
y1
y2
y3
y4
y9
y14
intensity
0
50000
100000
150000
200000
0
20
40
60
80
100
Fragment Ions
147.113 y1
186.087 b2
261.155 y2
299.173 b3
374.240 y3
400.220 b4
431.261 y4
547.257 b5
675.315 b6
970.526 y9
987.449 b9
1086.523 b10
1214.578 b11
1271.593 b12
1384.680 b13
1531.367 y14
b2
b3
b4
b5
b6
b9
b10
b11
b12
b13
m/z
NAITMQPQSVQGLNK / File: christine_10pools_06.raw; SpectrumID: ; scans: 28743

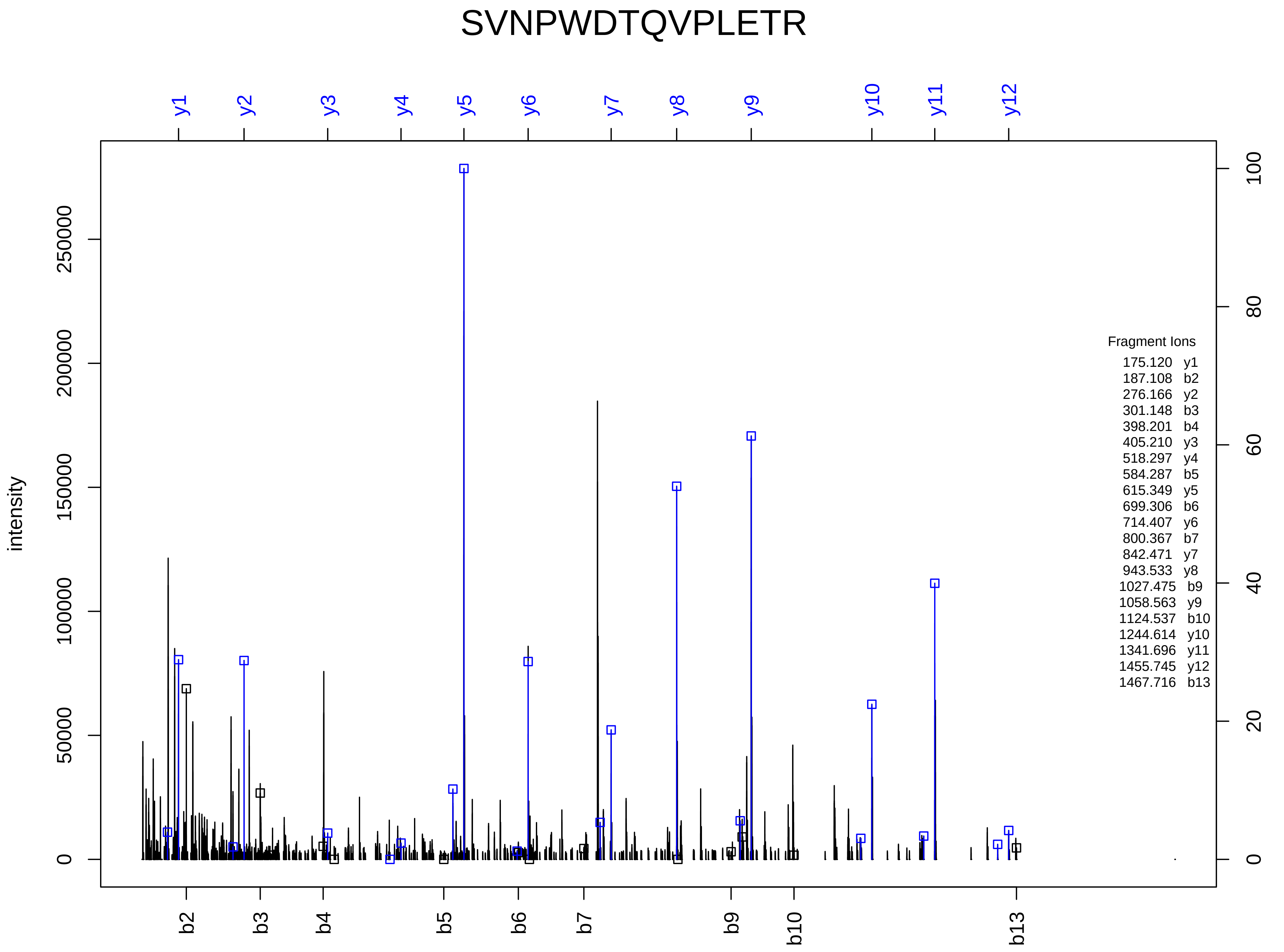

SVNPWDTQVPLETR / File: christine_10pools_06.raw; SpectrumID: ; scans: 18718

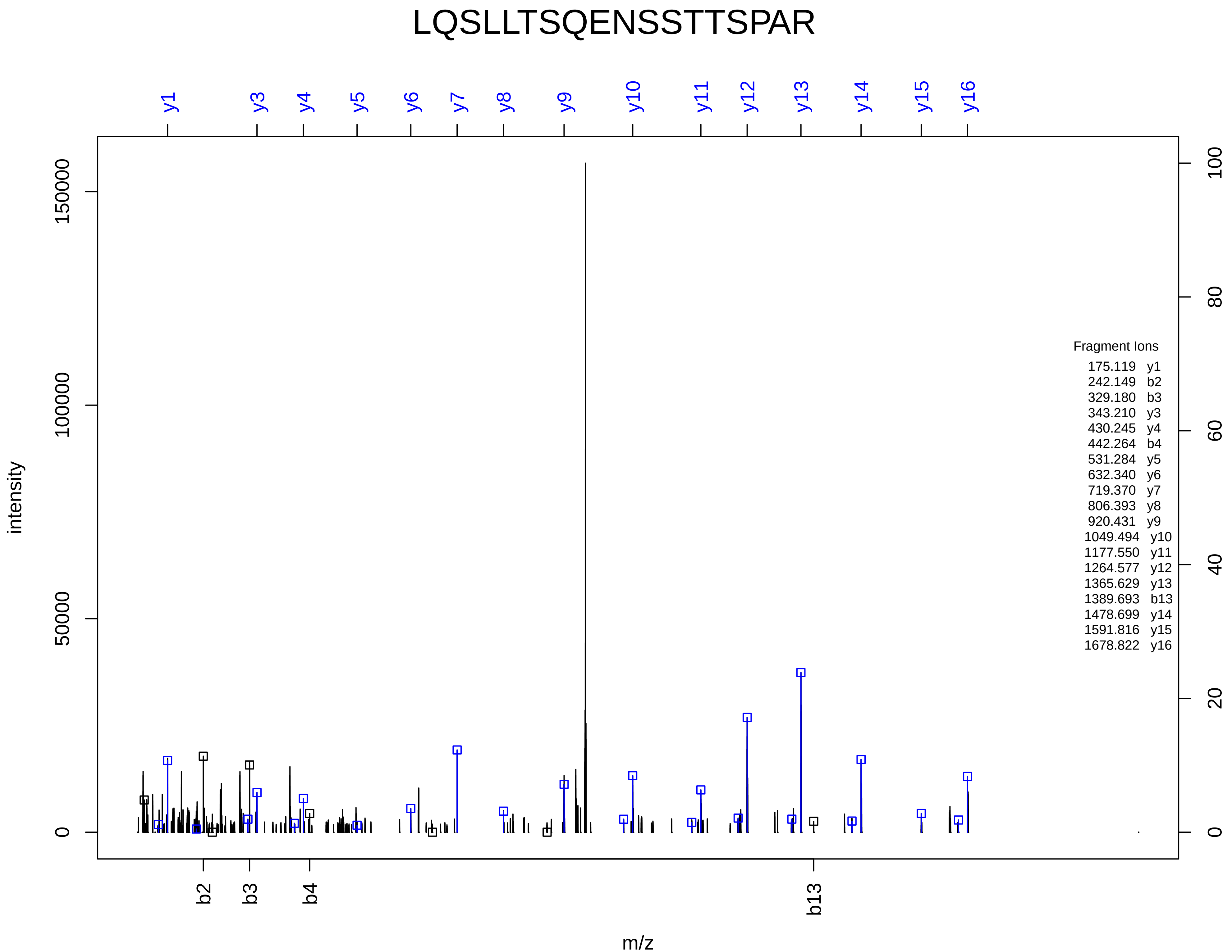

LQSLLTSQENSSTTSPAR / File: christine_10pools_06.raw; SpectrumID: ; scans: 7681

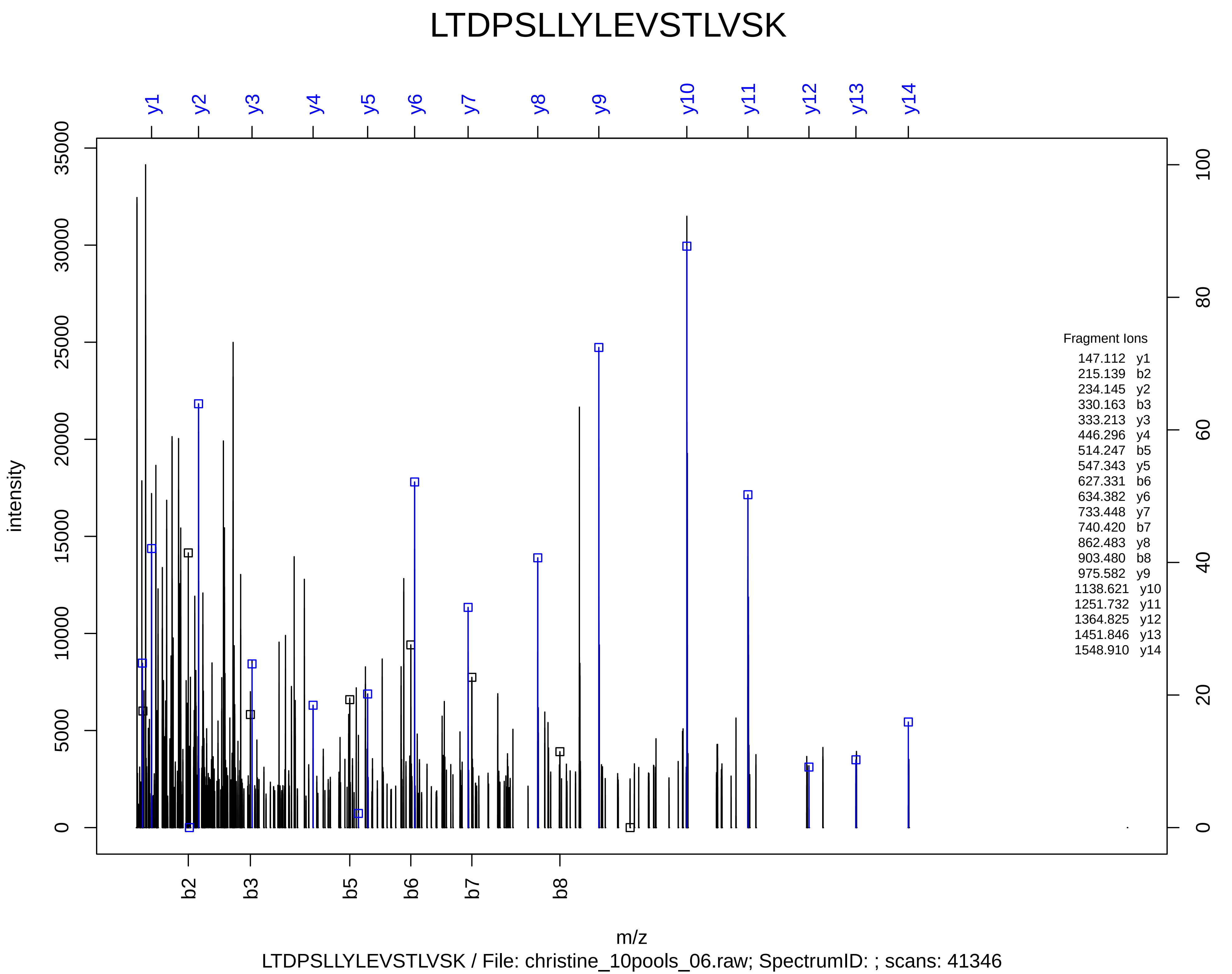
LTDPSLLYLEVSTLVSK
y1 y2 y3 y4 y5 y6 y7 y8 y9 y10 y11 y12 y13 y14
b2 b3 b5 b6 b7 b8
intensity
m/z
Fragment Ions
147.112 y1
215.139 b2
234.145 y2
330.163 b3
333.213 y3
446.296 y4
514.247 b5
547.343 y5
627.331 b6
634.382 y6
733.448 y7
740.420 b7
862.483 y8
903.480 b8
975.582 y9
1138.621 y10
1251.732 y11
1364.825 y12
1451.846 y13
1548.910 y14
LTDPSLLYLEVSTLVSK / File: christine_10pools_06.raw; SpectrumID: ; scans: 41346

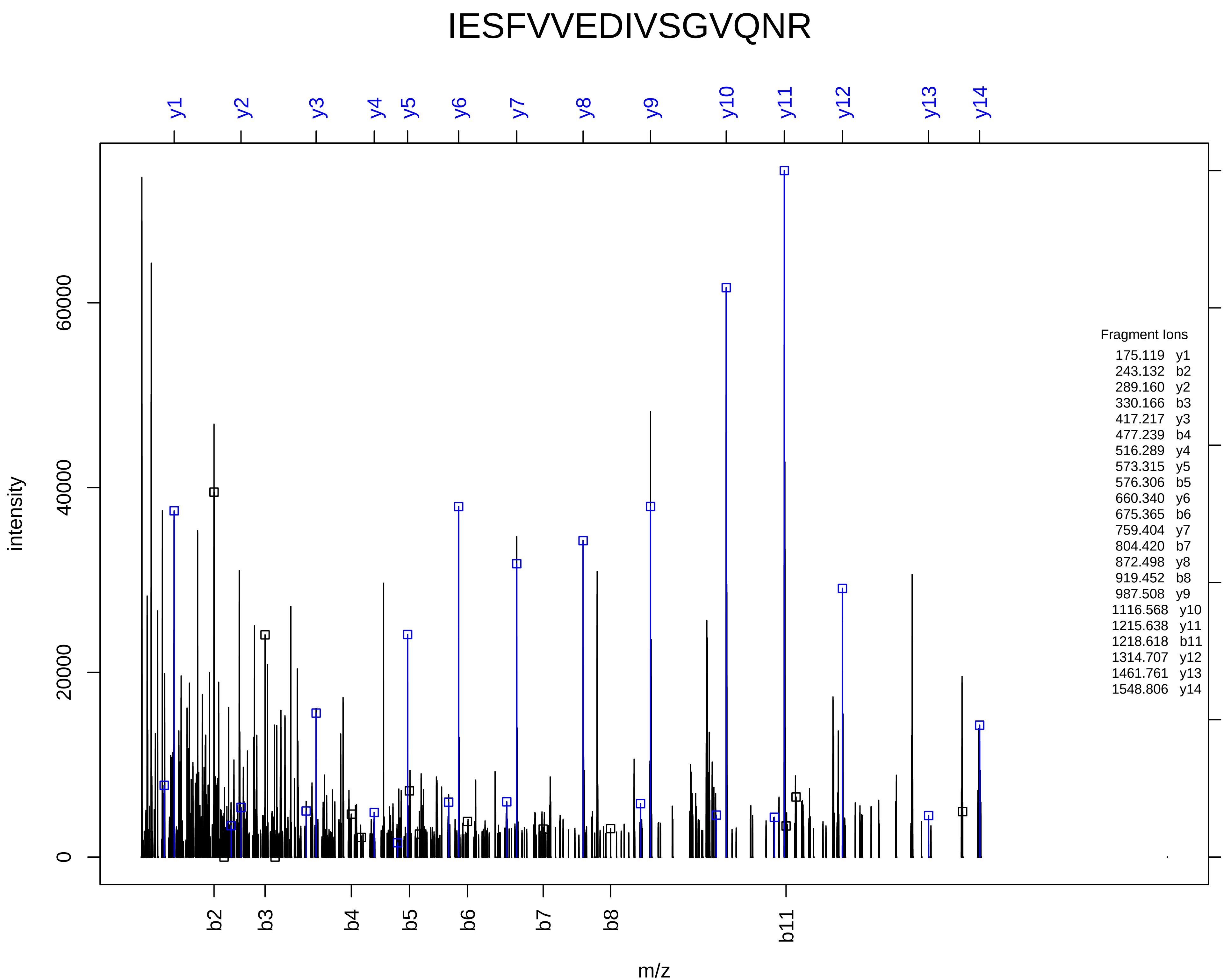

IESFVVEDIVSGVQNR / File: christine_10pools_06.raw; SpectrumID: ; scans: 28240

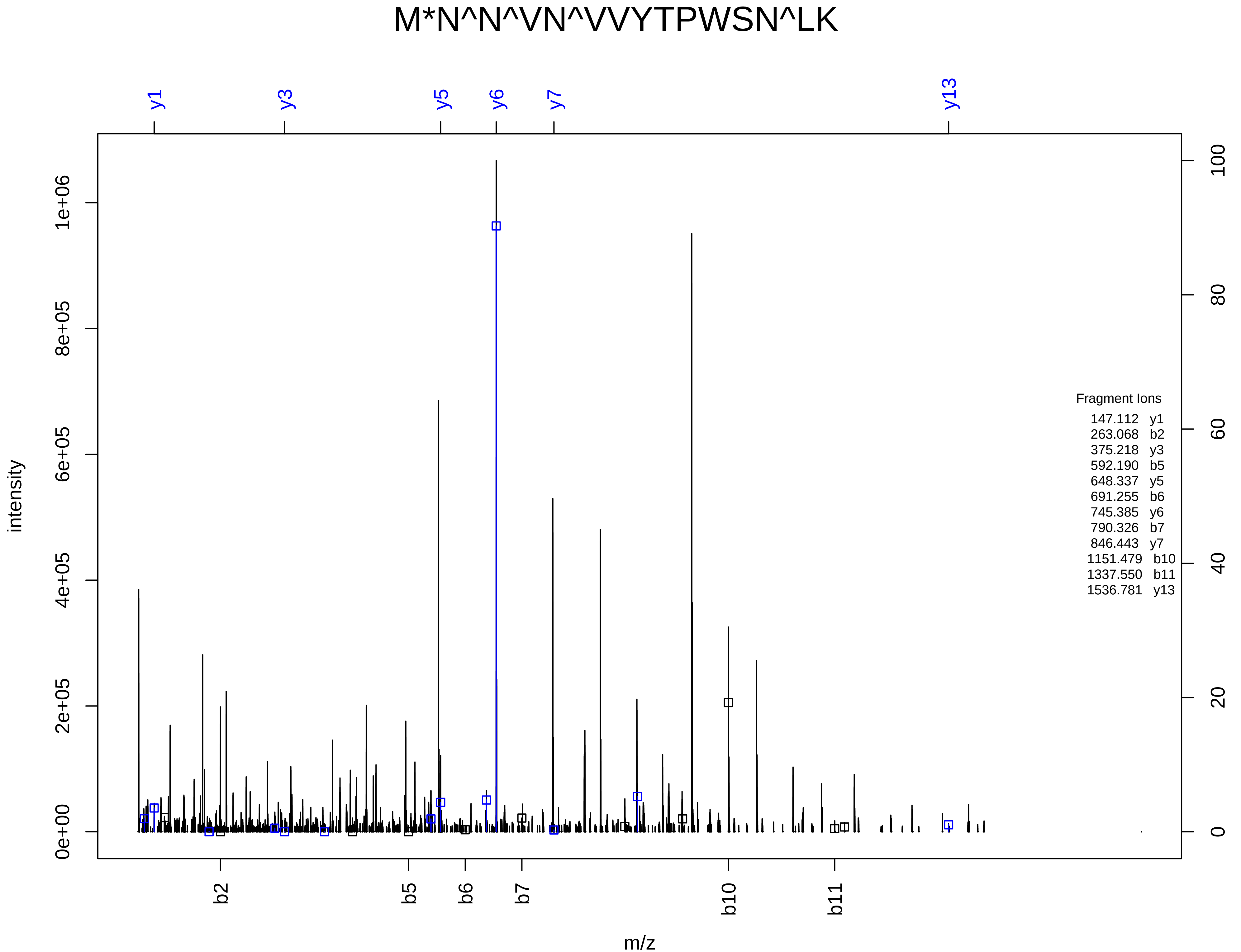

MNNVNVVYTPWSNLK / File: christine_10pools_06.raw; SpectrumID: ; scans: 15367

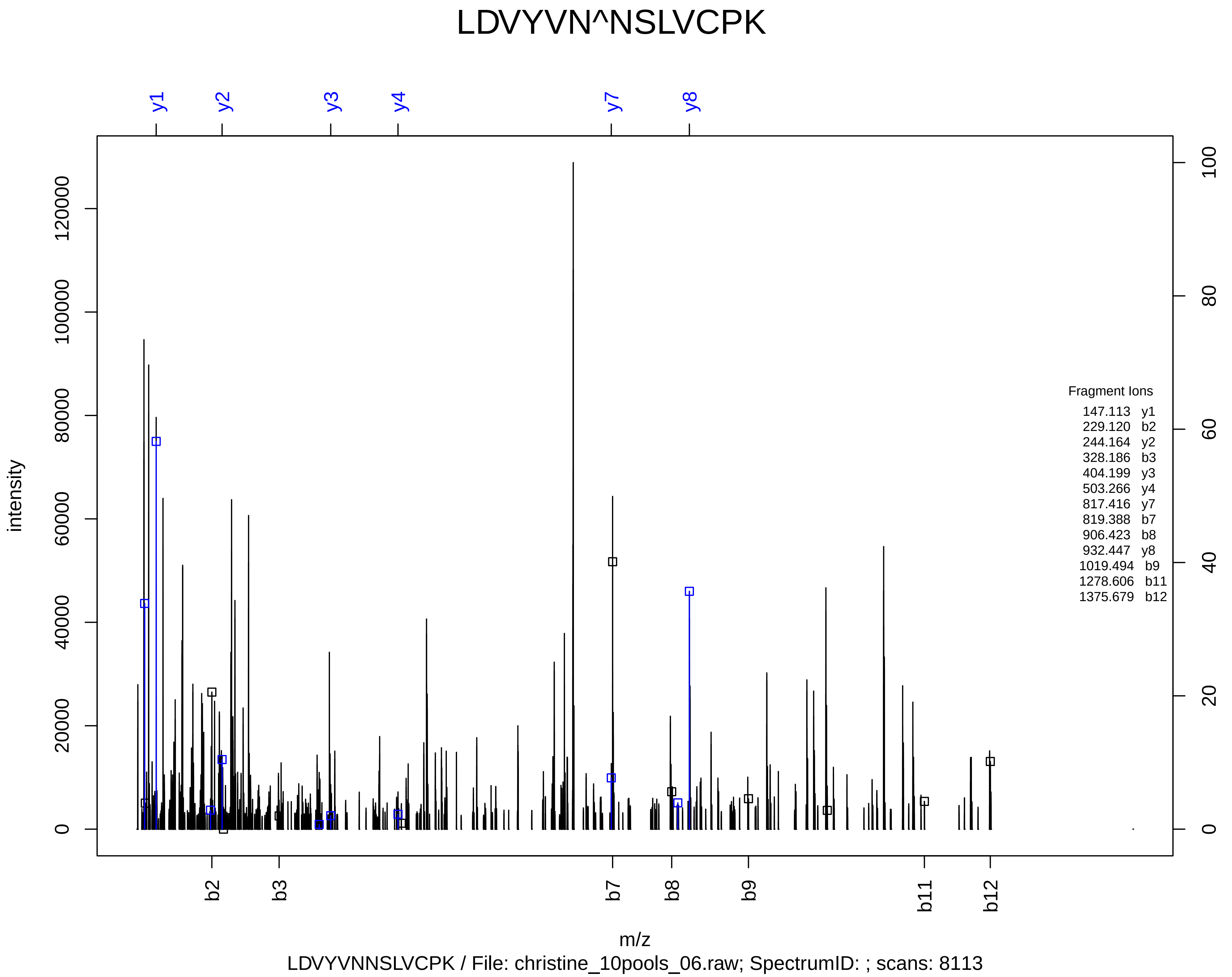

LDVYVN^NSLVCPK
y1
y2
y3
y4
y7
y8
intensity
0
20000
40000
60000
80000
100000
120000
0
20
40
60
80
100
Fragment Ions
147.113 y1
229.120 b2
244.164 y2
328.186 b3
404.199 y3
503.266 y4
817.416 y7
819.388 b7
906.423 b8
932.447 y8
1019.494 b9
1278.606 b11
1375.679 b12
b2
b3
b7
b8
b9
b11
b12
m/z
LDVYVNNSLVCPK / File: christine_10pools_06.raw; SpectrumID: ; scans: 8113

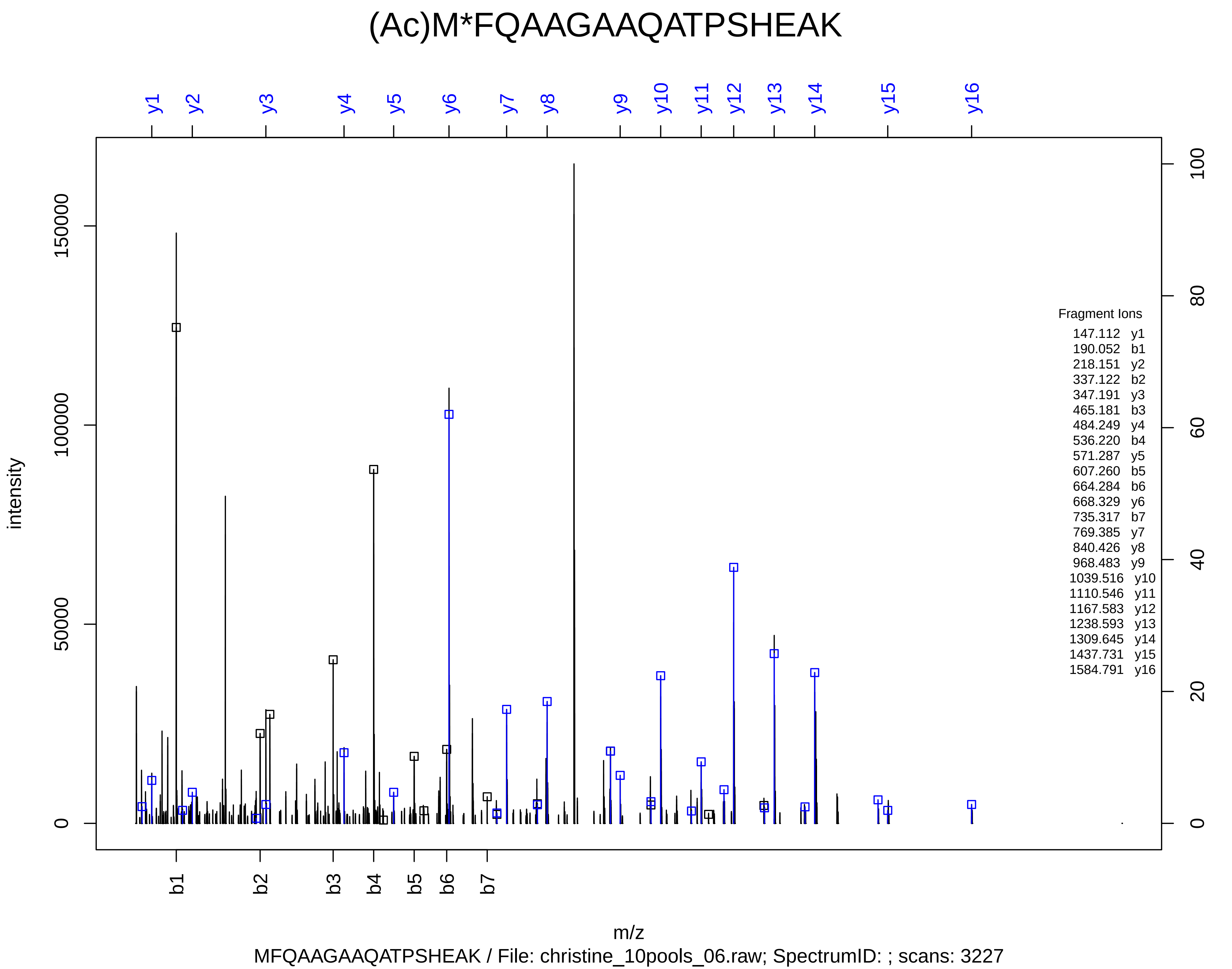

(Ac)M*FQAAGAAQATPSHEAK
y1 y2 y3 y4 y5 y6 y7 y8 y9 y10 y11 y12 y13 y14 y15 y16
b1 b2 b3 b4 b5 b6 b7
intensity
0
50000
100000
150000
0
20
40
60
80
100
m/z
Fragment Ions
147.112 y1
190.052 b1
218.151 y2
337.122 b2
347.191 y3
465.181 b3
484.249 y4
536.220 b4
571.287 y5
607.260 b5
664.284 b6
668.329 y6
735.317 b7
769.385 y7
840.426 y8
968.483 y9
1039.516 y10
1110.546 y11
1167.583 y12
1238.593 y13
1309.645 y14
1437.731 y15
1584.791 y16
MFQAAGAAQATPSHEAK / File: christine_10pools_06.raw; SpectrumID: ; scans: 3227

LNLLQSHPQEVMYFQPGEPPGPVEDDHIPFLR

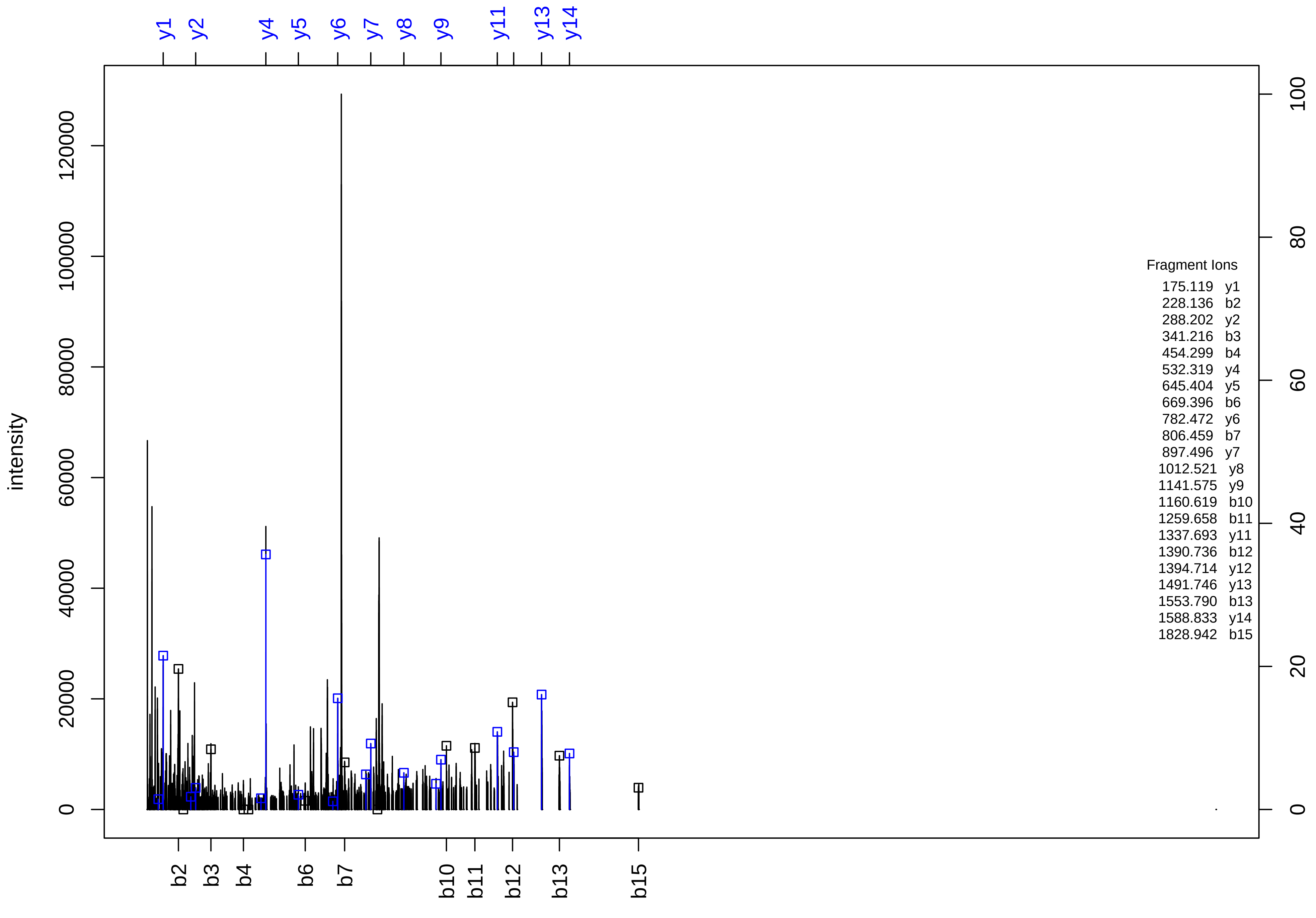

LNLLQSHPQEVMYFQPGEPPGPVEDDHIPFLR / File: christine_10pools_06.raw; SpectrumID: ; scans: 30598

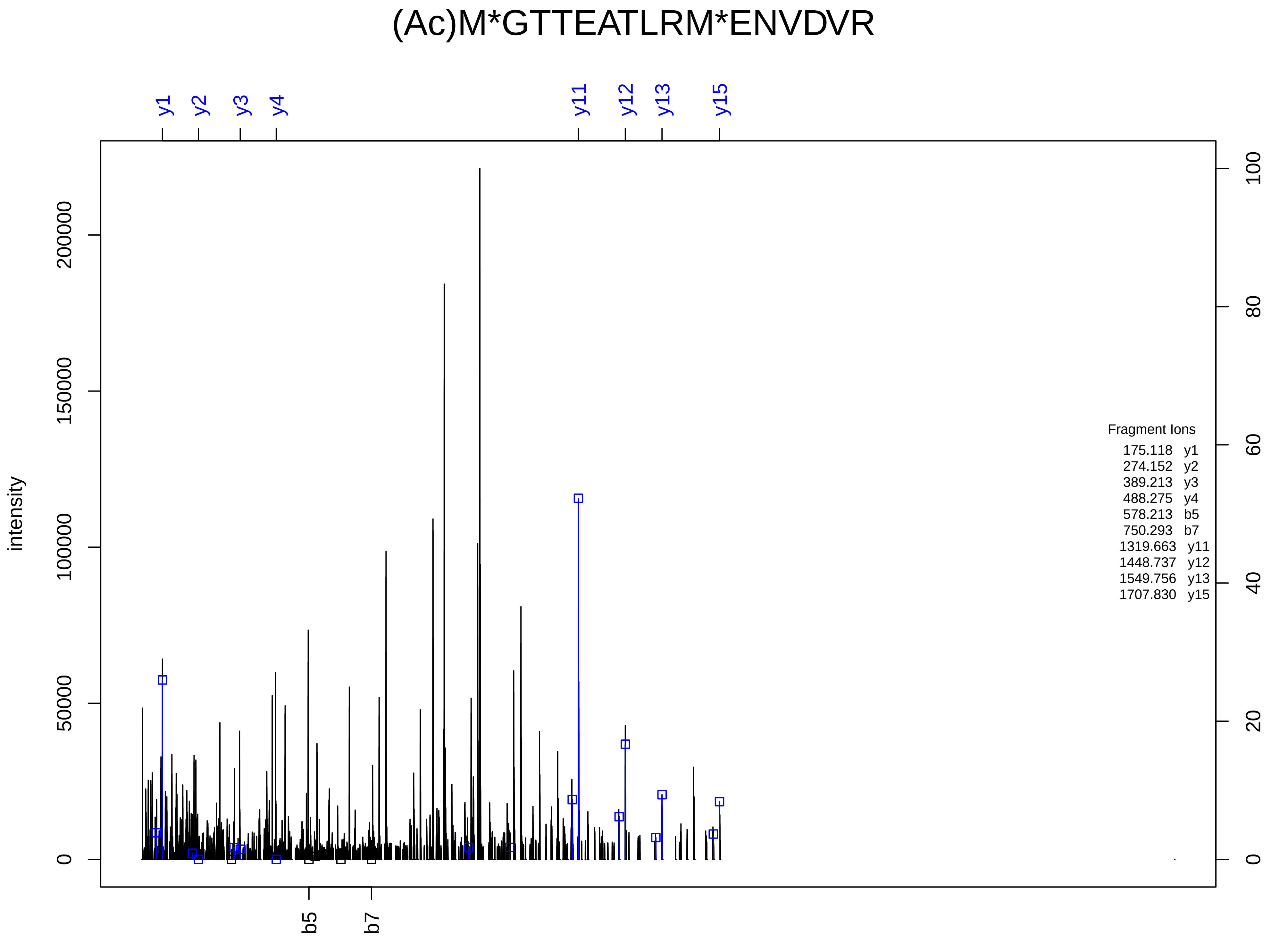

MGTTEATLRMENVDVR / File: christine_10pools_06.raw; SpectrumID: ; scans: 30841

LLGSEN^DTGSPDFYTPR

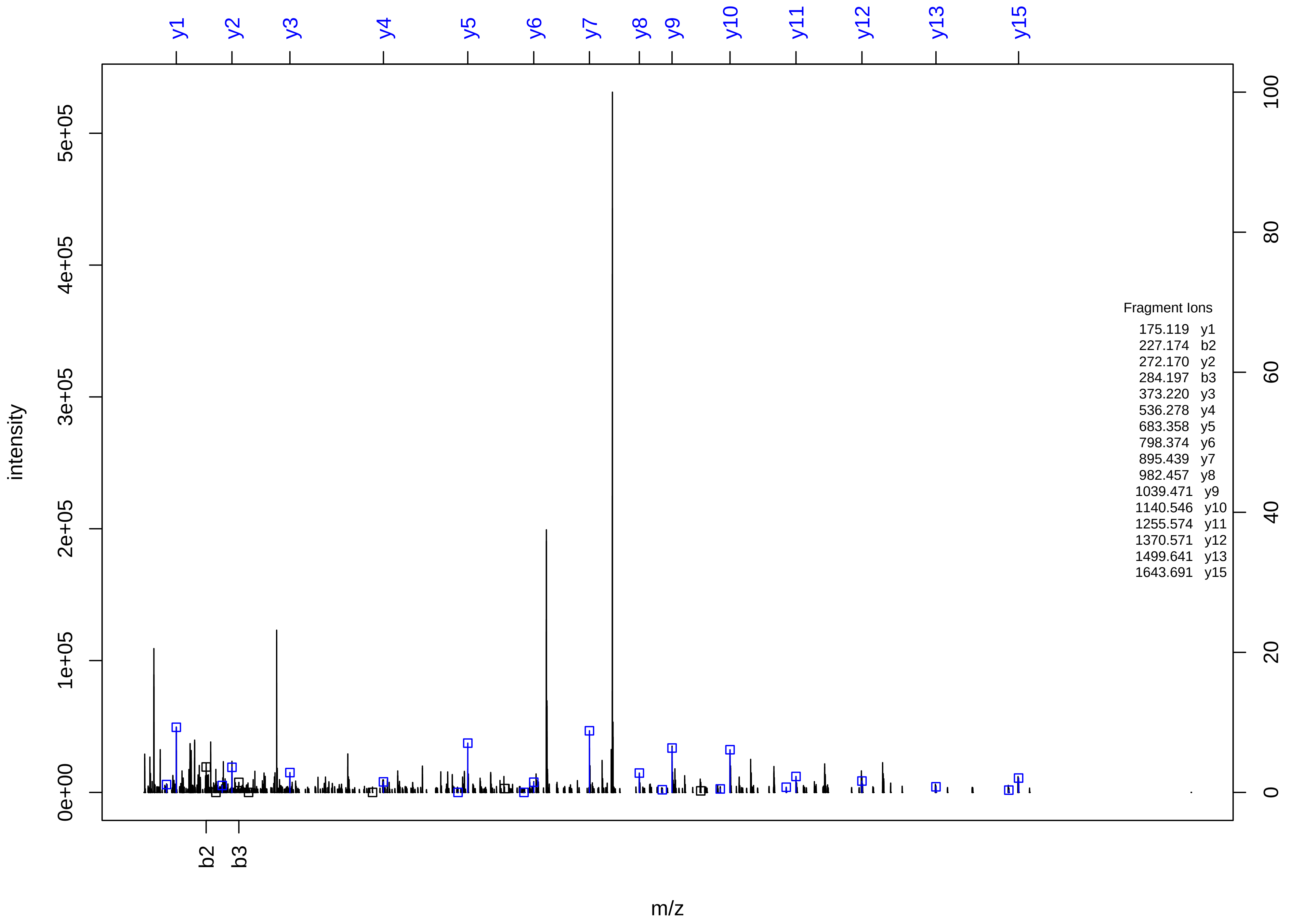

LLGSENDTGSPDFYTPR / File: christine_10pools_06.raw; SpectrumID: ; scans: 15289

TSLEGYPAGGSLPYSIQTAEK

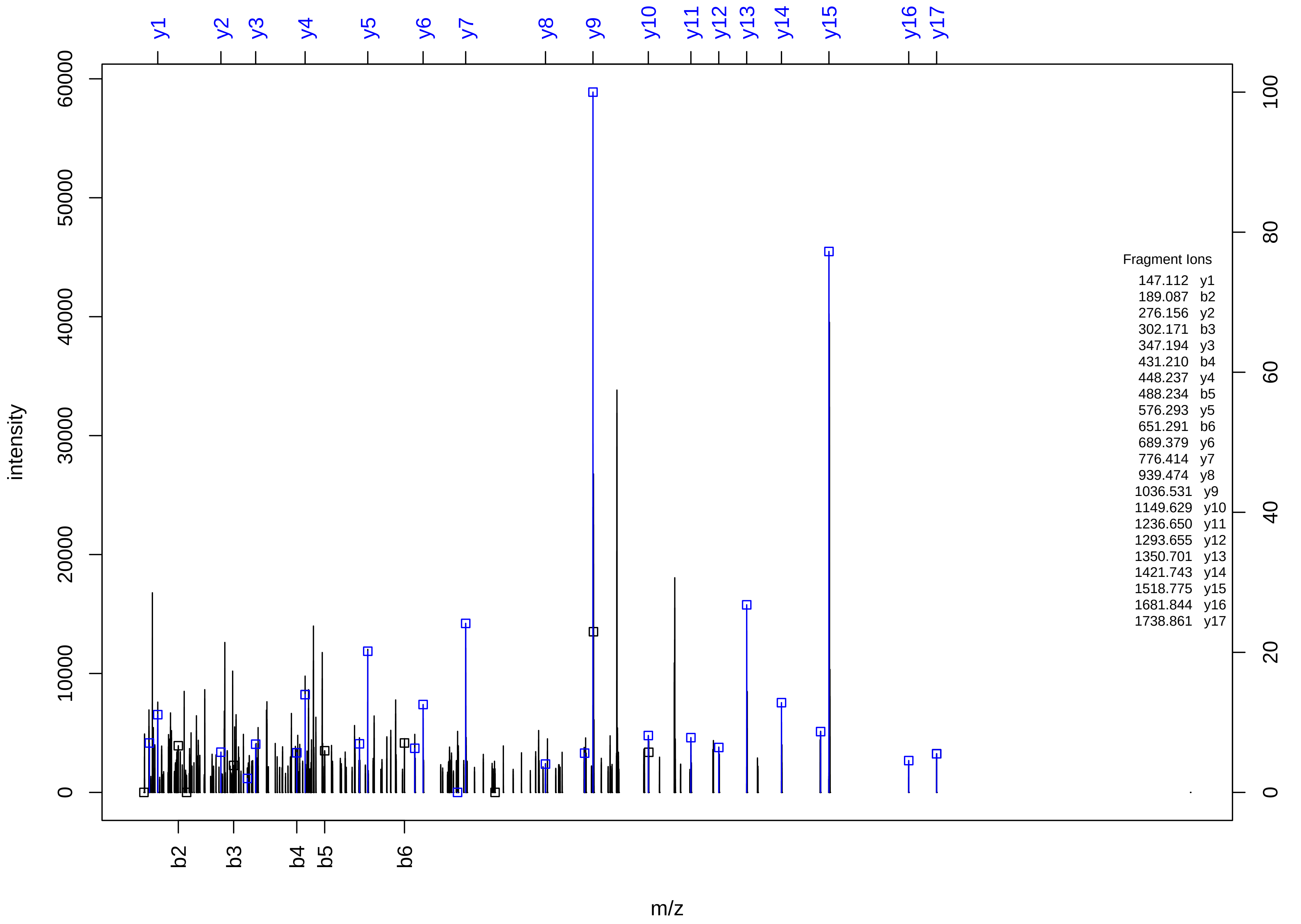

TSLEGYPAGGSLPYSIQTAEK / File: christine_10pools_06.raw; SpectrumID: ; scans: 19024

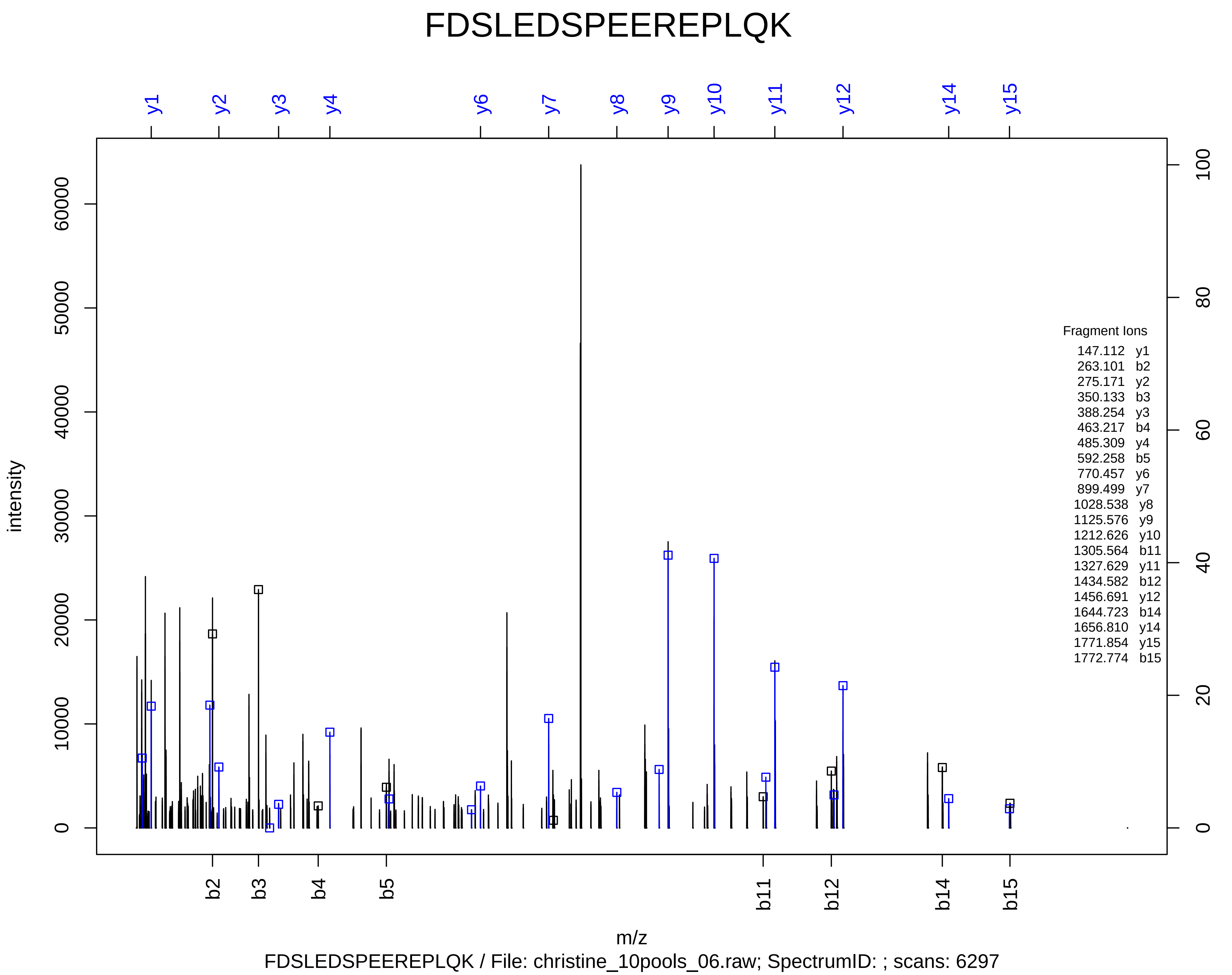
FDSLEDSPEEREPLQK
intensity
m/z
y1
y2
y3
y4
y6
y7
y8
y9
y10
y11
y12
y14
y15
b2
b3
b4
b5
b11
b12
b14
b15
Fragment Ions
147.112 y1
263.101 b2
275.171 y2
350.133 b3
388.254 y3
463.217 b4
485.309 y4
592.258 b5
770.457 y6
899.499 y7
1028.538 y8
1125.576 y9
1212.626 y10
1305.564 b11
1327.629 y11
1434.582 b12
1456.691 y12
1644.723 b14
1656.810 y14
1771.854 y15
1772.774 b15
FDSLEDSPEEREPLQK / File: christine_10pools_06.raw; SpectrumID: ; scans: 6297

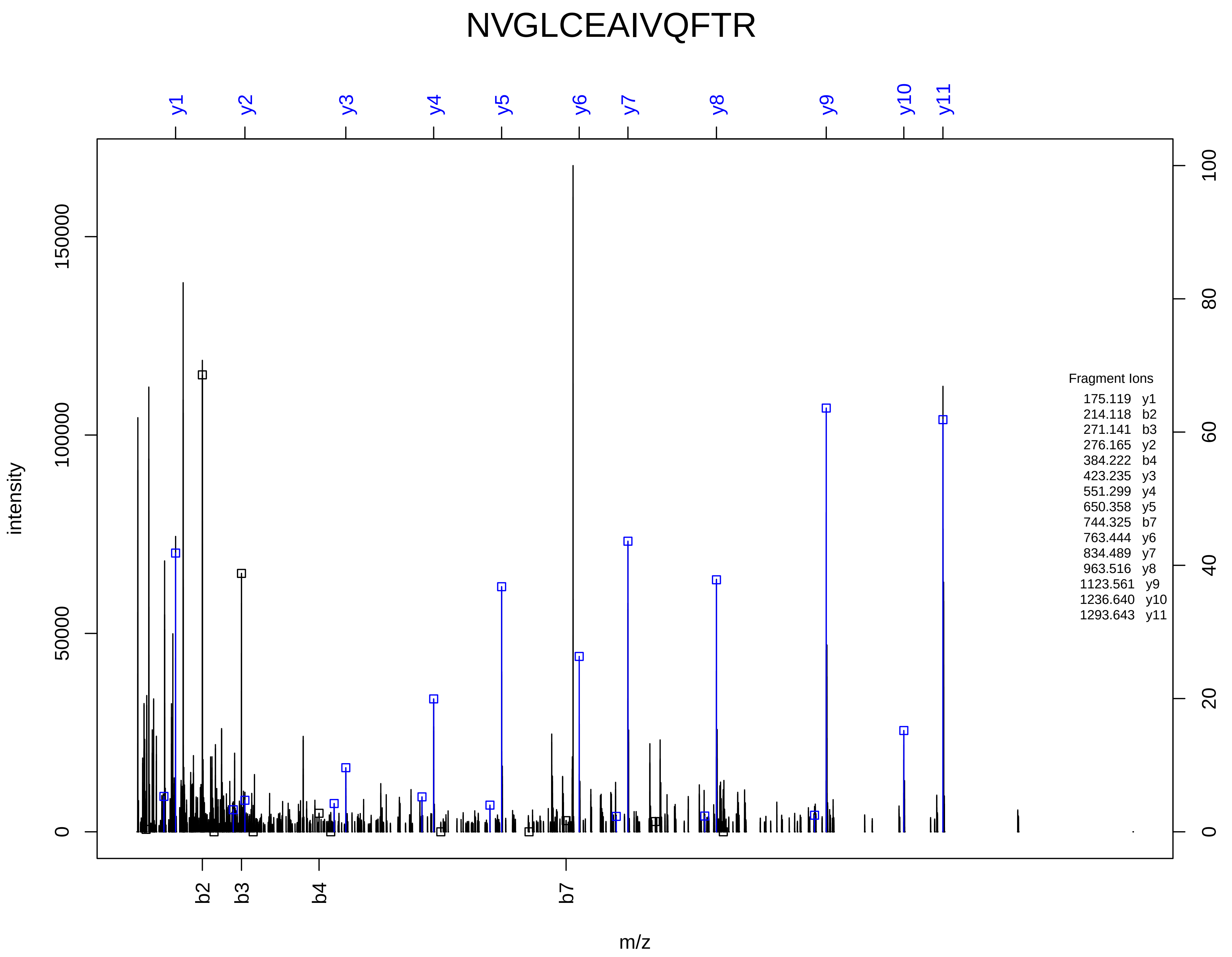

NVGLCEAIVQFTR / File: christine_10pools_06.raw; SpectrumID: ; scans: 26201

DMIEIPLPPWQER

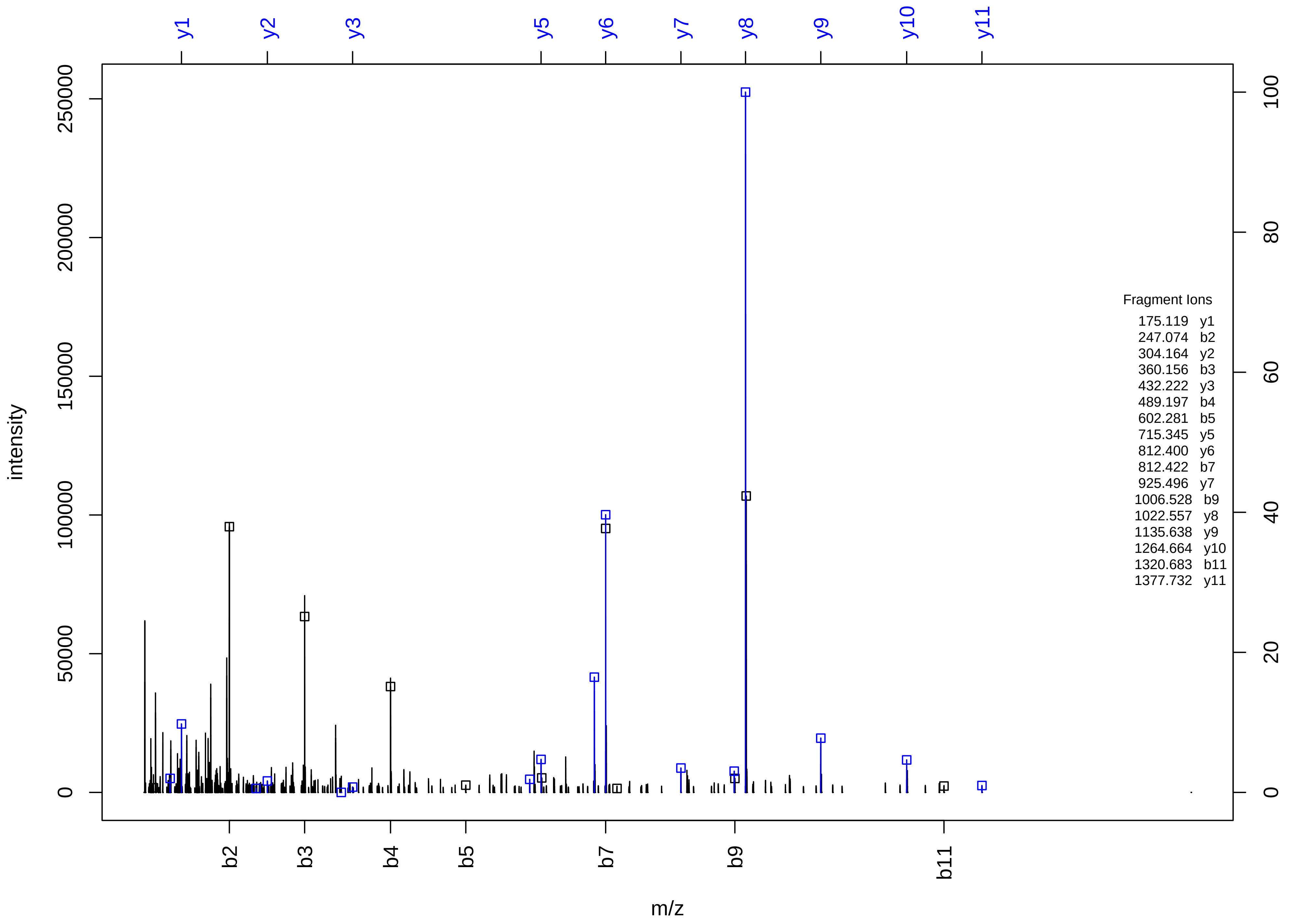

DMIEIPLPPWQER / File: christine_10pools_06.raw; SpectrumID: ; scans: 35050

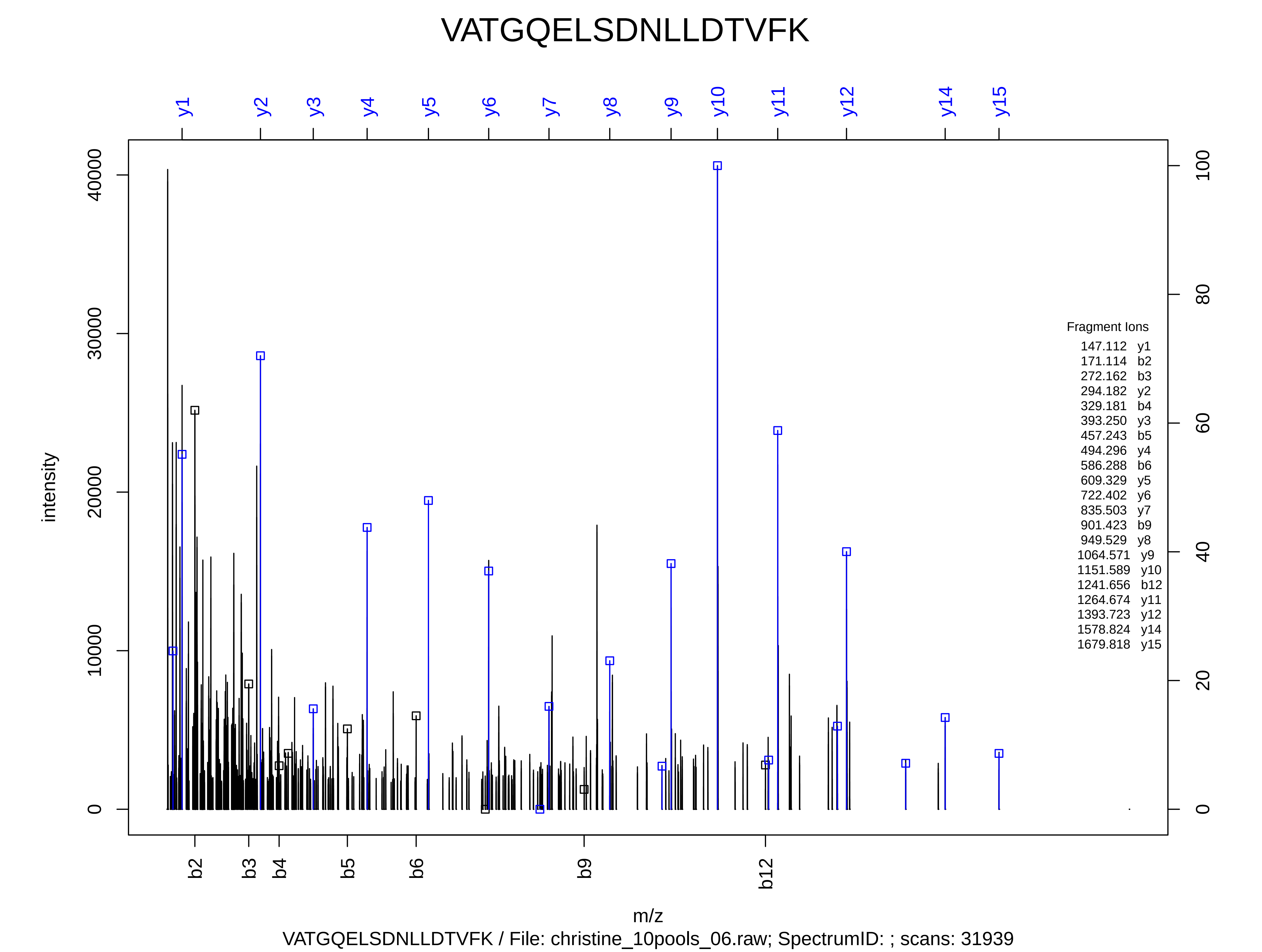

VATGQELSDNLLDTVFK
intensity
m/z
Fragment Ions
147.112 y1
171.114 b2
272.162 b3
294.182 y2
329.181 b4
393.250 y3
457.243 b5
494.296 y4
586.288 b6
609.329 y5
722.402 y6
835.503 y7
901.423 b9
949.529 y8
1064.571 y9
1151.589 y10
1241.656 b12
1264.674 y11
1393.723 y12
1578.824 y14
1679.818 y15
VATGQELSDNLLDTVFK / File: christine_10pools_06.raw; SpectrumID: ; scans: 31939

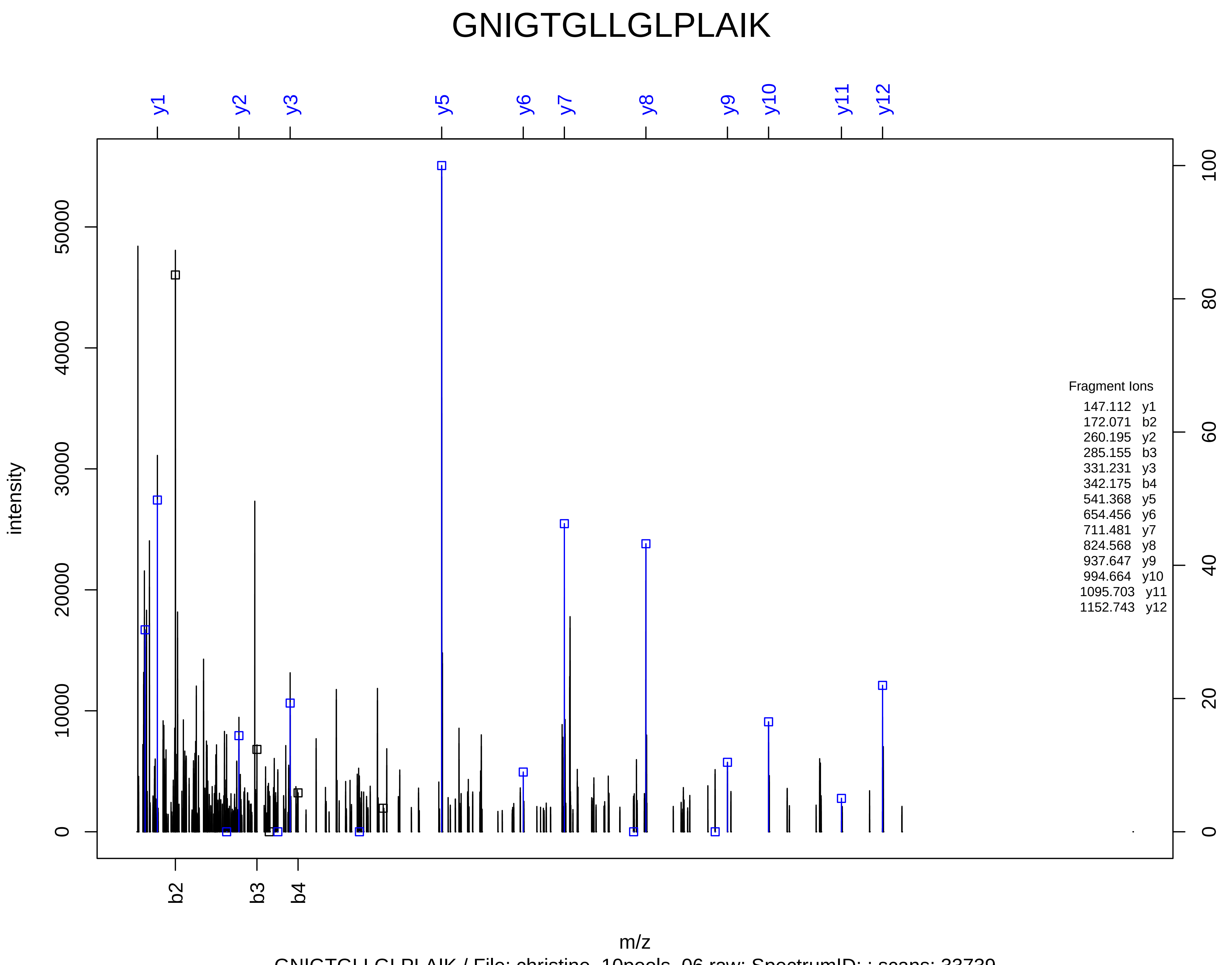
GNIGTGLLGLPLAIK
y1
y2
y3
y5
y6
y7
y8
y9
y10
y11
y12
b2
b3
b4
intensity
m/z
Fragment Ions
147.112 y1
172.071 b2
260.195 y2
285.155 b3
331.231 y3
342.175 b4
541.368 y5
654.456 y6
711.481 y7
824.568 y8
937.647 y9
994.664 y10
1095.703 y11
1152.743 y12
GNIGTGLLGLPLAIK / File: christine_10pools_06.raw; SpectrumID: ; scans: 33739

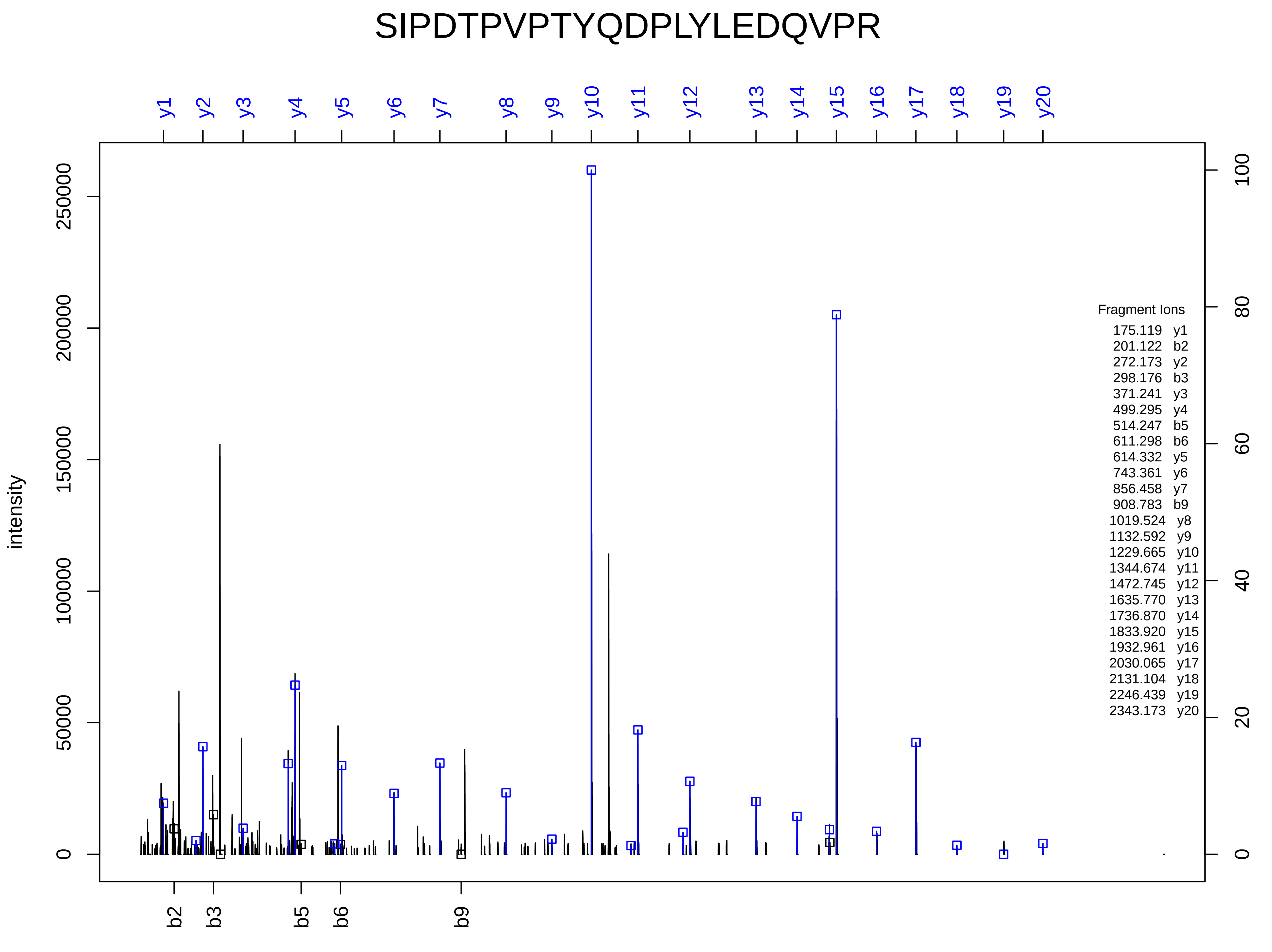

SIPDTPVPTYQDPLYLEDQVPR / File: christine_10pools_06.raw; SpectrumID: ; scans: 27587

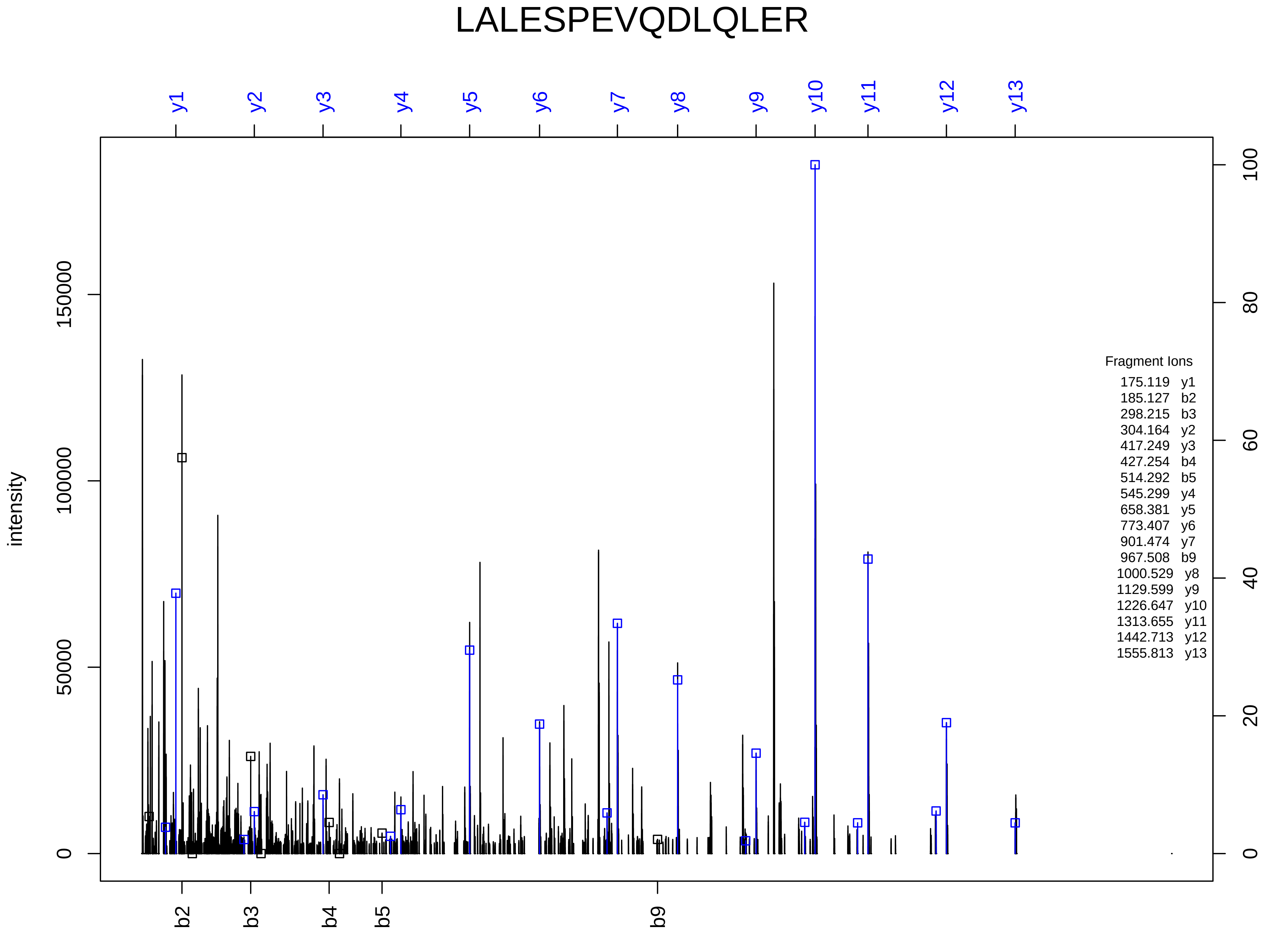

LALESPEVQDLQLER / File: christine_10pools_06.raw; SpectrumID: ; scans: 21254

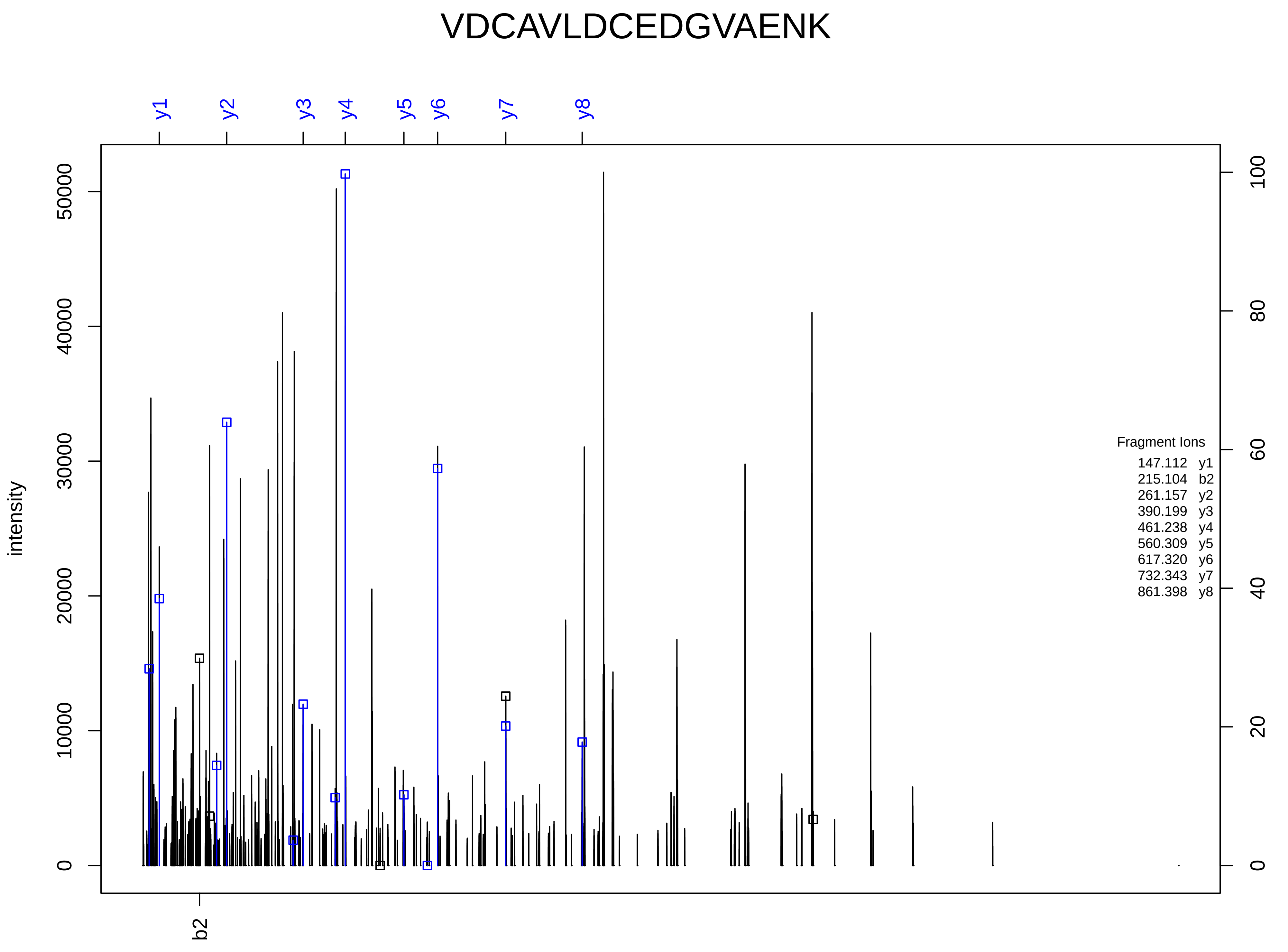

VDCAVLDCEDGVAENK / File: christine_10pools_06.raw; SpectrumID: ; scans: 8379

NLFTQTLGLGSQK

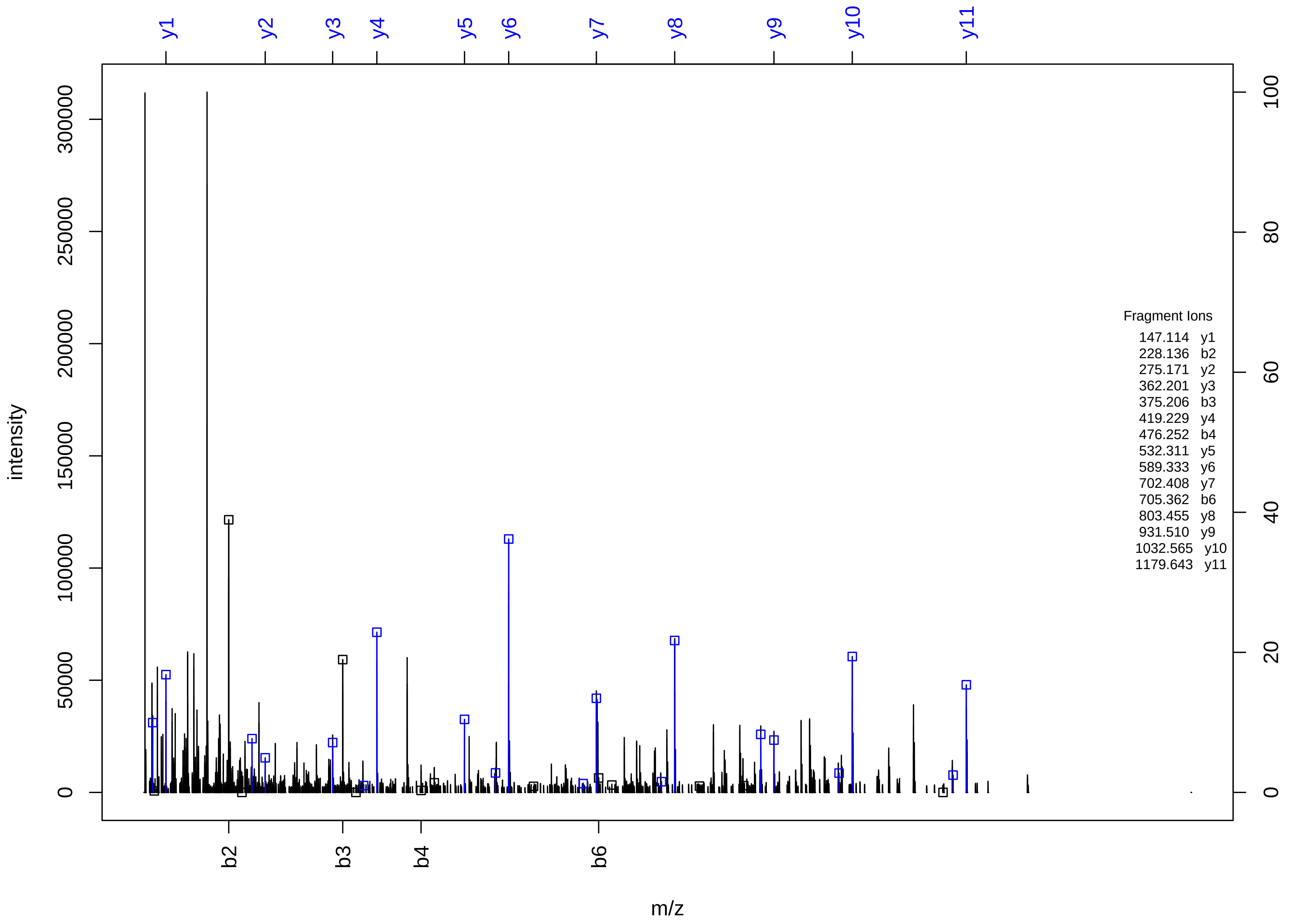

NLFTQTLGLGSQK / File: christine_10pools_06.raw; SpectrumID: ; scans: 23933

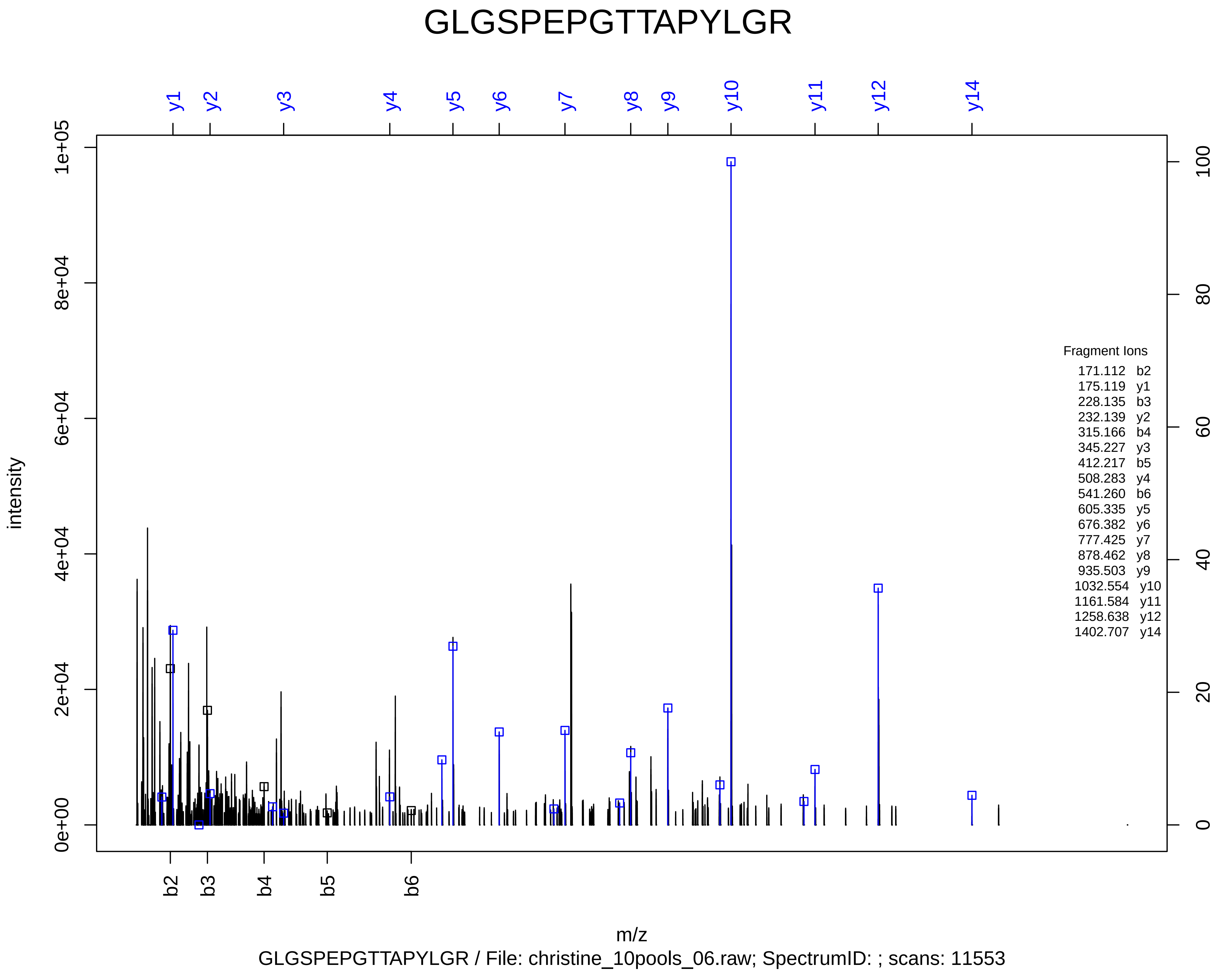
GLGSPEPGTTAPYLGR
y1
y2
y3
y4
y5
y6
y7
y8
y9
y10
y11
y12
y14
intensity
0e+00
2e+04
4e+04
6e+04
8e+04
1e+05
0
20
40
60
80
100
b2
b3
b4
b5
b6
m/z
Fragment Ions
171.112 b2
175.119 y1
228.135 b3
232.139 y2
315.166 b4
345.227 y3
412.217 b5
508.283 y4
541.260 b6
605.335 y5
676.382 y6
777.425 y7
878.462 y8
935.503 y9
1032.554 y10
1161.584 y11
1258.638 y12
1402.707 y14
GLGSPEPGTTAPYLGR / File: christine_10pools_06.raw; SpectrumID: ; scans: 11553

AATPSPSGAIGGLLEQFAR

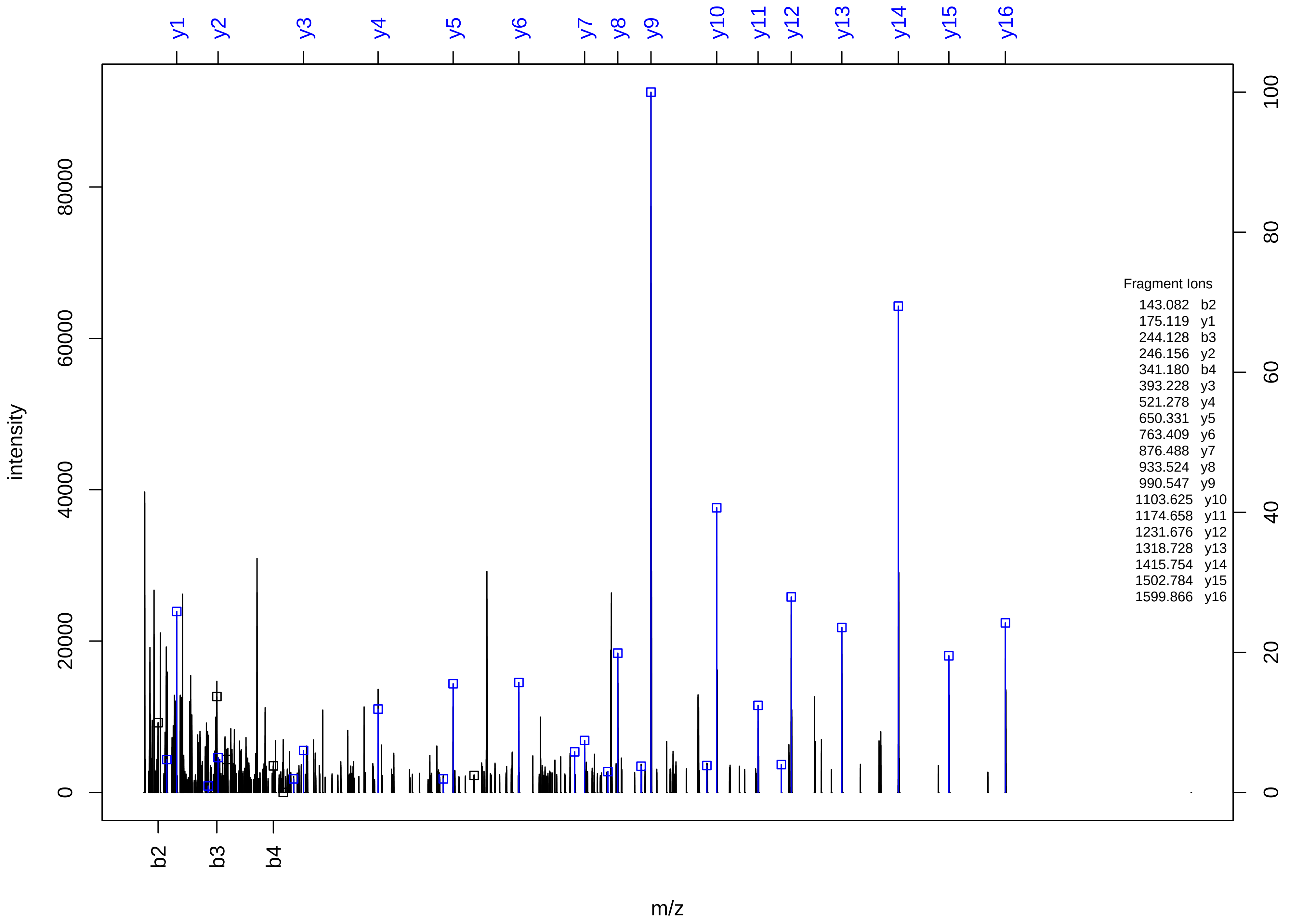

AATPSPSGAIGGLLEQFAR / File: christine_10pools_06.raw; SpectrumID: ; scans: 34358

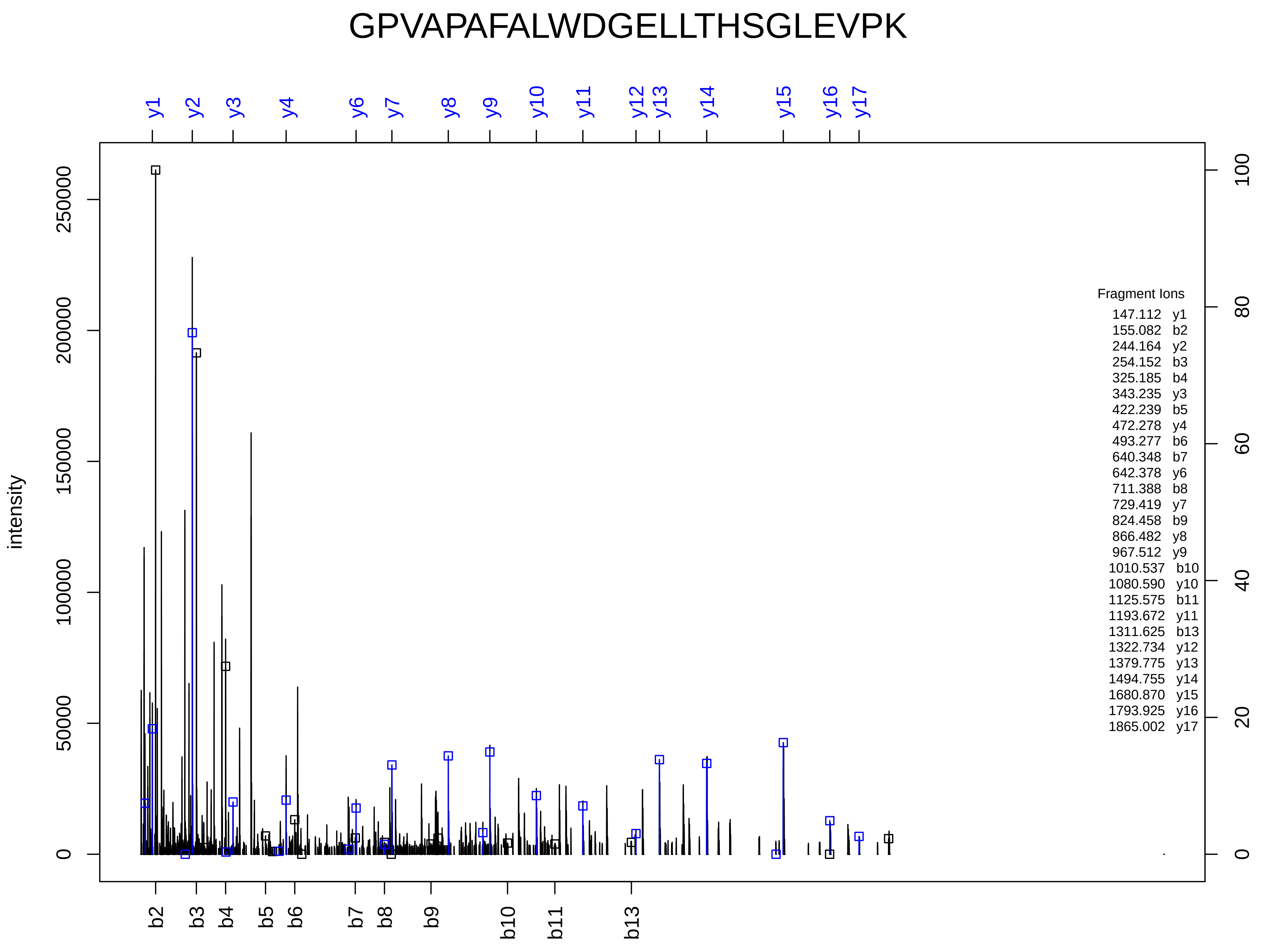

GPVAPAFALWDGELLTHSGLEVPK / File: christine_10pools_06.raw; SpectrumID: ; scans: 36525

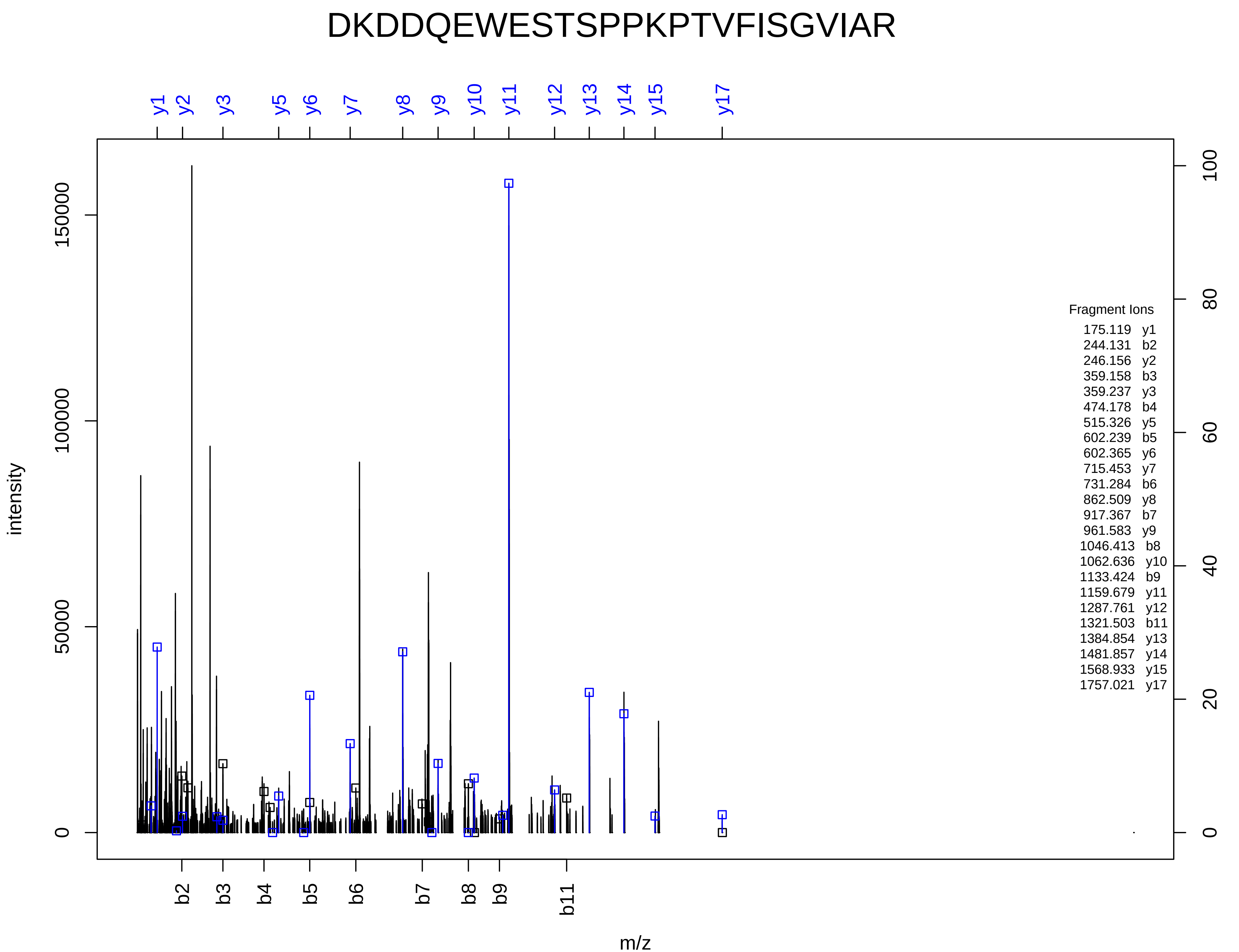

DKDDQEWESTSPPKPTVFISGVIAR / File: christine_10pools_06.raw; SpectrumID: ; scans: 22761

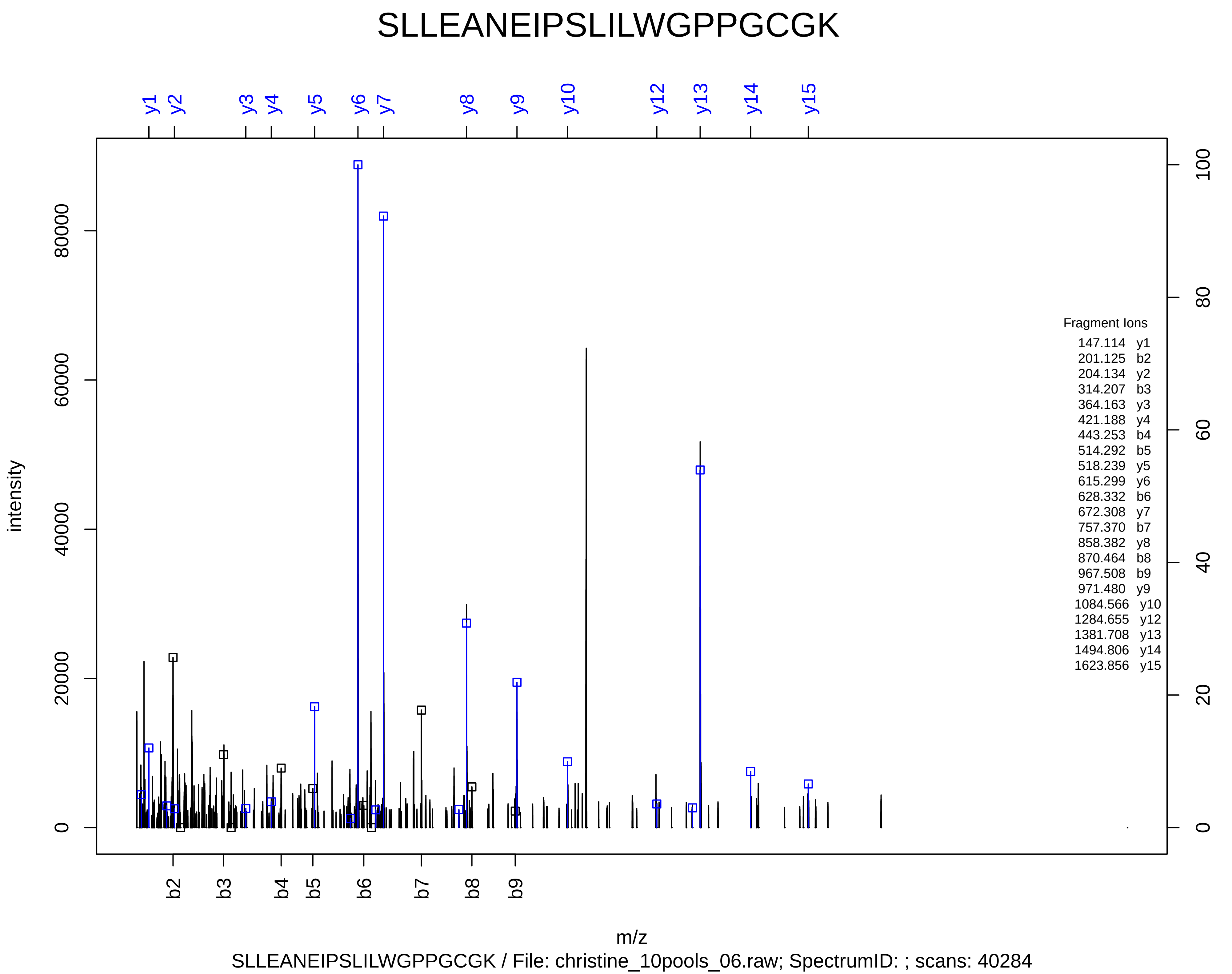

SLLEANEIPSLILWGPPGCGK
y1
y2
y3
y4
y5
y6
y7
y8
y9
y10
y12
y13
y14
y15
intensity
0
20000
40000
60000
80000
100
80
60
40
20
0
Fragment Ions
147.114 y1
201.125 b2
204.134 y2
314.207 b3
364.163 y3
421.188 y4
443.253 b4
514.292 b5
518.239 y5
615.299 y6
628.332 b6
672.308 y7
757.370 b7
858.382 y8
870.464 b8
967.508 b9
971.480 y9
1084.566 y10
1284.655 y12
1381.708 y13
1494.806 y14
1623.856 y15
b2
b3
b4
b5
b6
b7
b8
b9
m/z
SLLEANEIPSLILWGPPGCGK / File: christine_10pools_06.raw; SpectrumID: ; scans: 40284

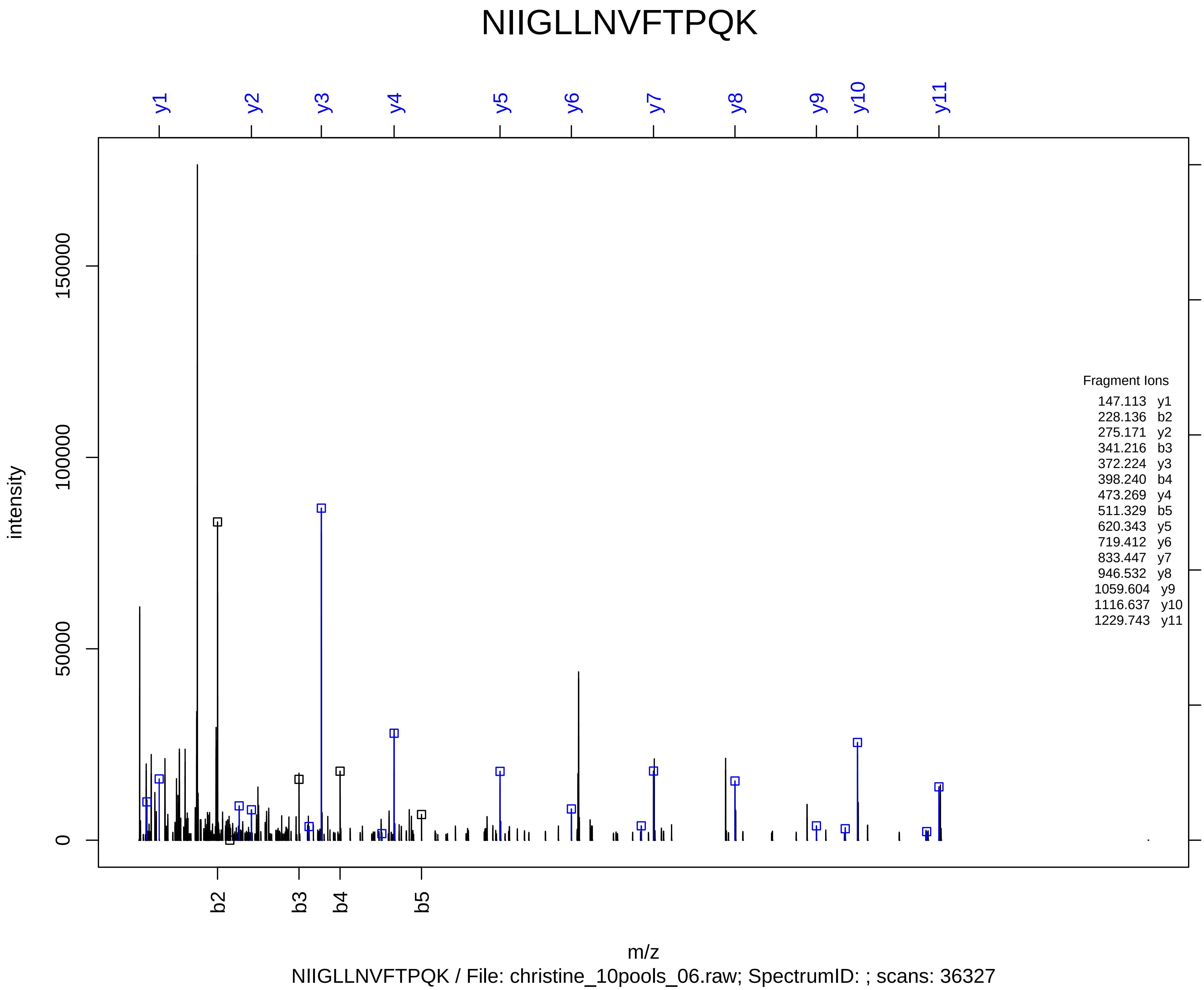
NIIGLLNVFTPQK
y1
y2
y3
y4
y5
y6
y7
y8
y9
y10
y11
intensity
0
50000
100000
150000
0
20
40
60
80
100
b2
b3
b4
b5
m/z
Fragment Ions
147.113 y1
228.136 b2
275.171 y2
341.216 b3
372.224 y3
398.240 b4
473.269 y4
511.329 b5
620.343 y5
719.412 y6
833.447 y7
946.532 y8
1059.604 y9
1116.637 y10
1229.743 y11
NIIGLLNVFTPQK / File: christine_10pools_06.raw; SpectrumID: ; scans: 36327

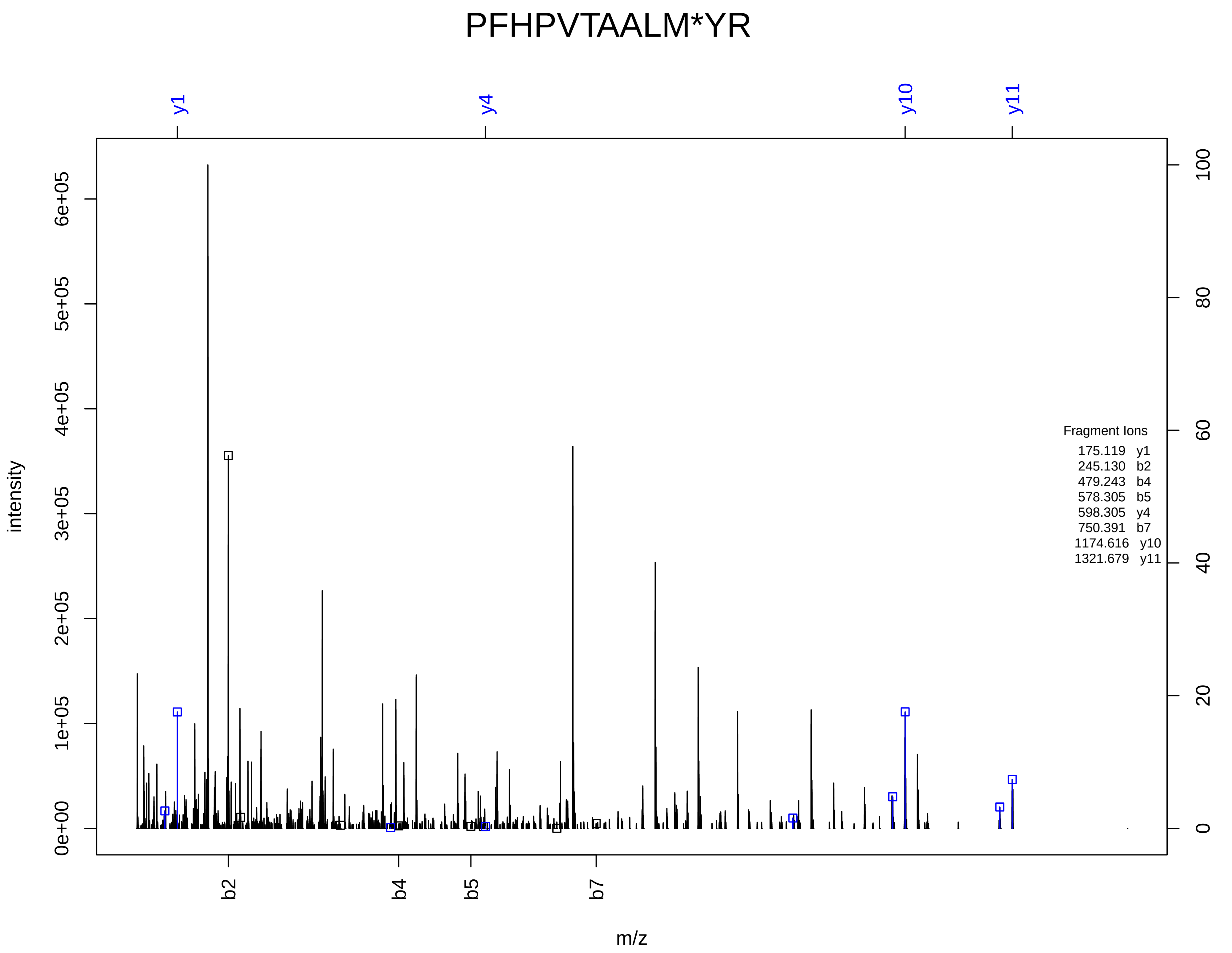

PFHPVTAALM*YR
y1
y4
y10
y11
intensity
0e+00
1e+05
2e+05
3e+05
4e+05
5e+05
6e+05
0
20
40
60
80
100
b2
b4
b5
b7
m/z
Fragment Ions
175.119 y1
245.130 b2
479.243 b4
578.305 b5
598.305 y4
750.391 b7
1174.616 y10
1321.679 y11
PFHPVTAALMYR / File: christine_10pools_06.raw; SpectrumID: ; scans: 12046

(Ac)AAAVAGPLAAGGEEAAASVSLPGSPGLPGSR

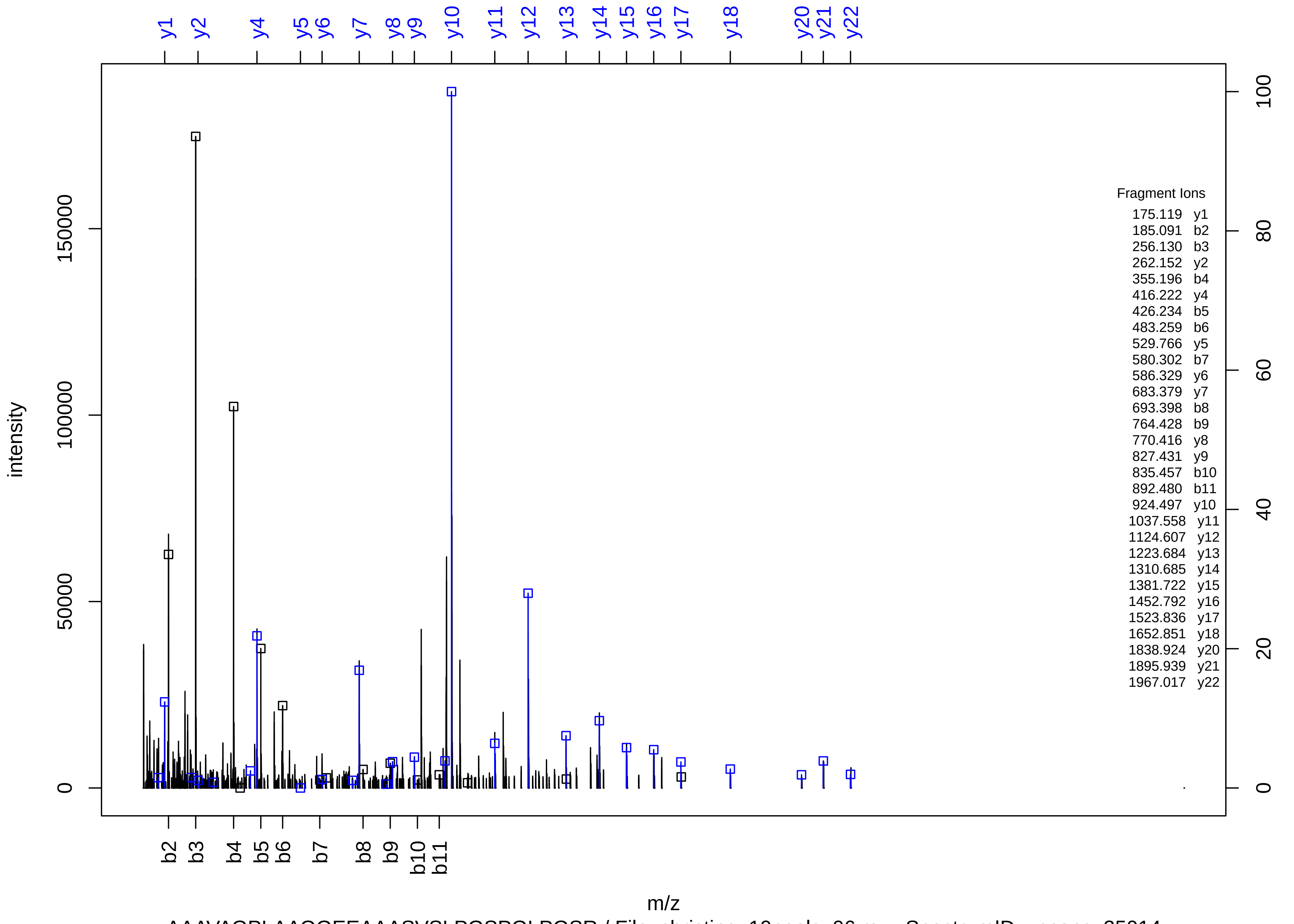

AAAVAGPLAAGGEEAAASVSLPGSPGLPGSR / File: christine_10pools_06.raw; SpectrumID: ; scans: 35014

(Ac)MDDTLFQLK

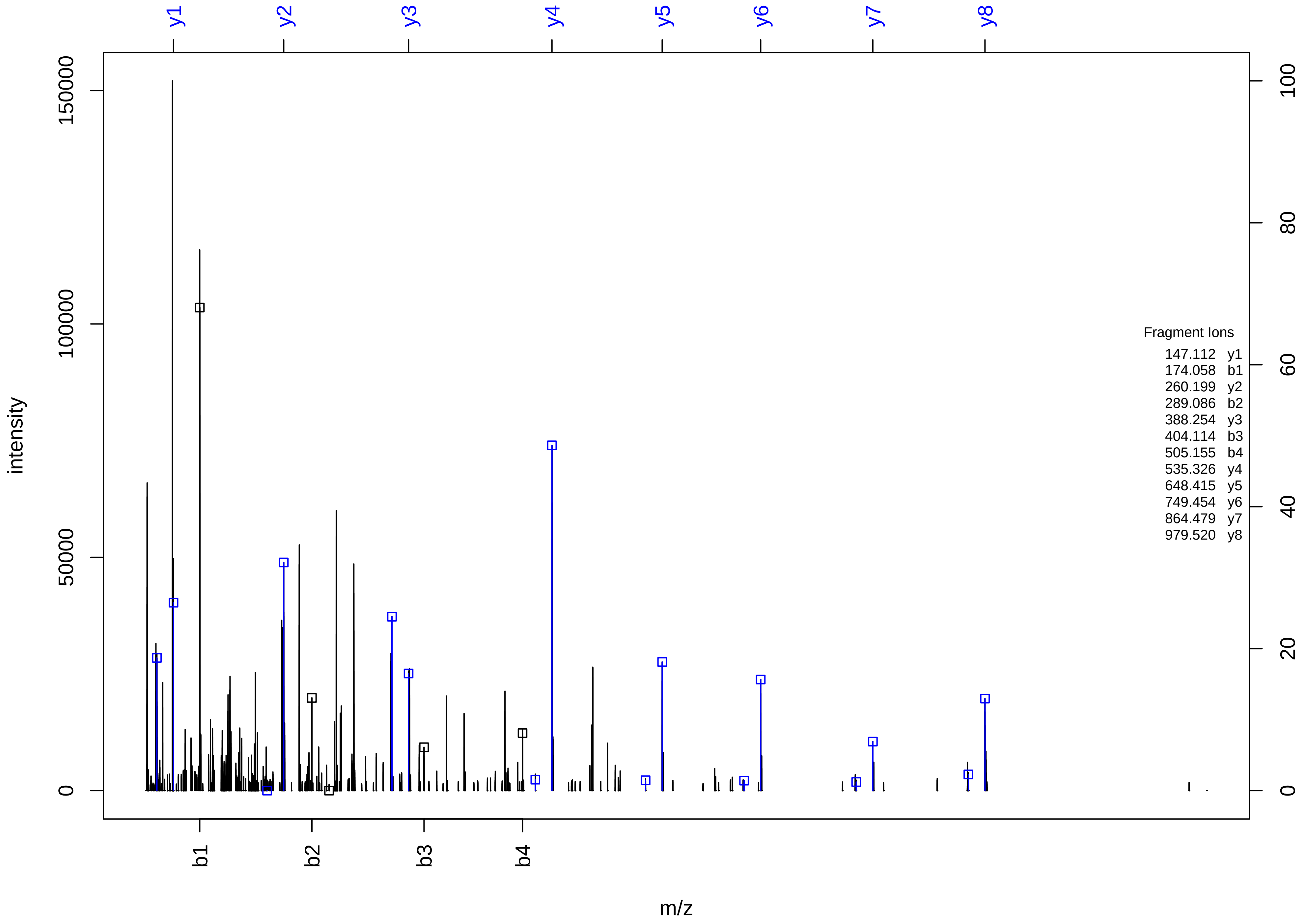

MDDTLFQLK / File: christine_10pools_06.raw; SpectrumID: ; scans: 33005

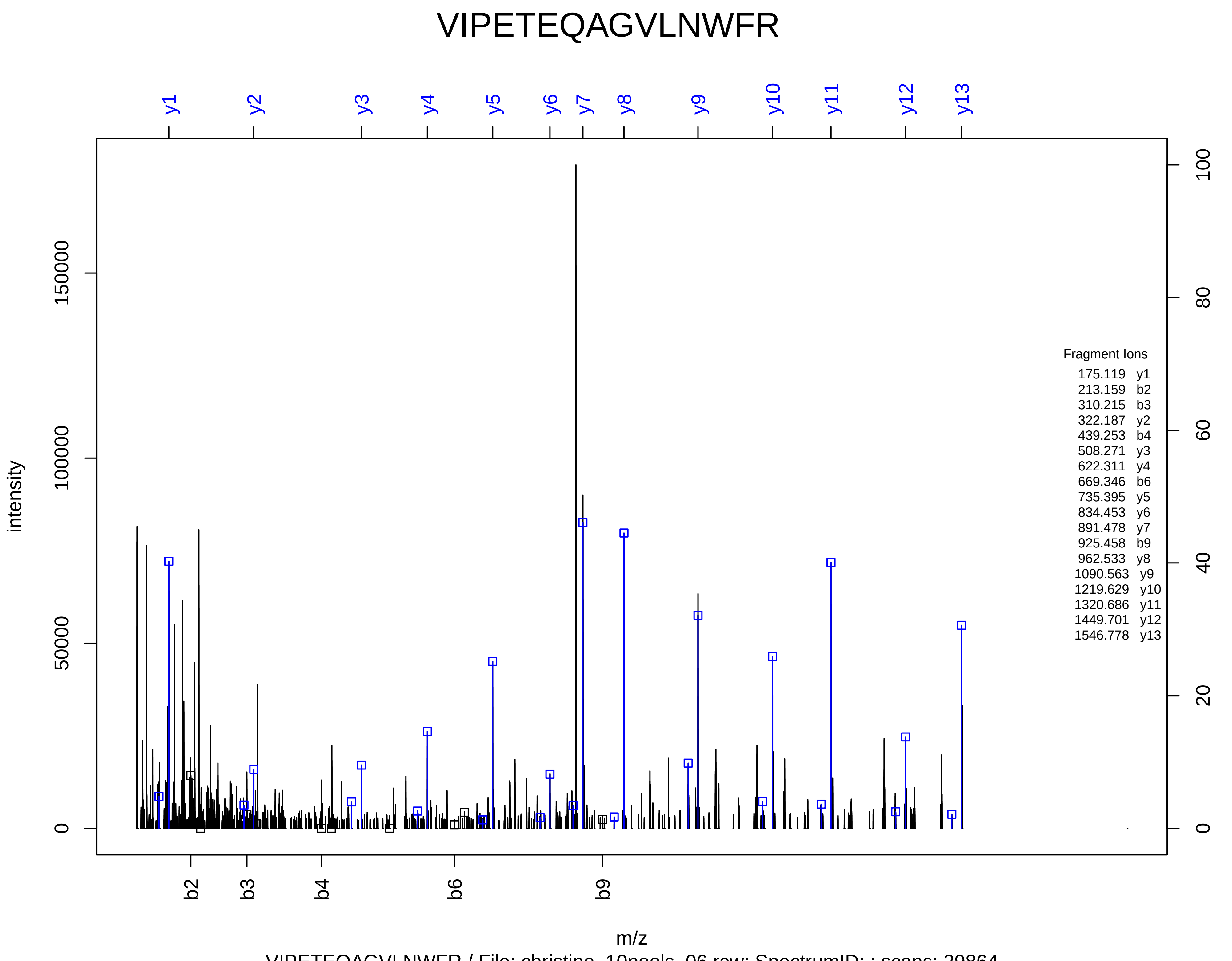
VIPETEQAGVLNWFR
y1 y2 y3 y4 y5 y6 y7 y8 y9 y10 y11 y12 y13
intensity
0
50000
100000
150000
0 20 40 60 80 100
b2 b3 b4 b6 b9
m/z
Fragment Ions
175.119 y1
213.159 b2
310.215 b3
322.187 y2
439.253 b4
508.271 y3
622.311 y4
669.346 b6
735.395 y5
834.453 y6
891.478 y7
925.458 b9
962.533 y8
1090.563 y9
1219.629 y10
1320.686 y11
1449.701 y12
1546.778 y13
VIPETEQAGVLNWFR / File: christine_10pools_06.raw; SpectrumID: ; scans: 29864

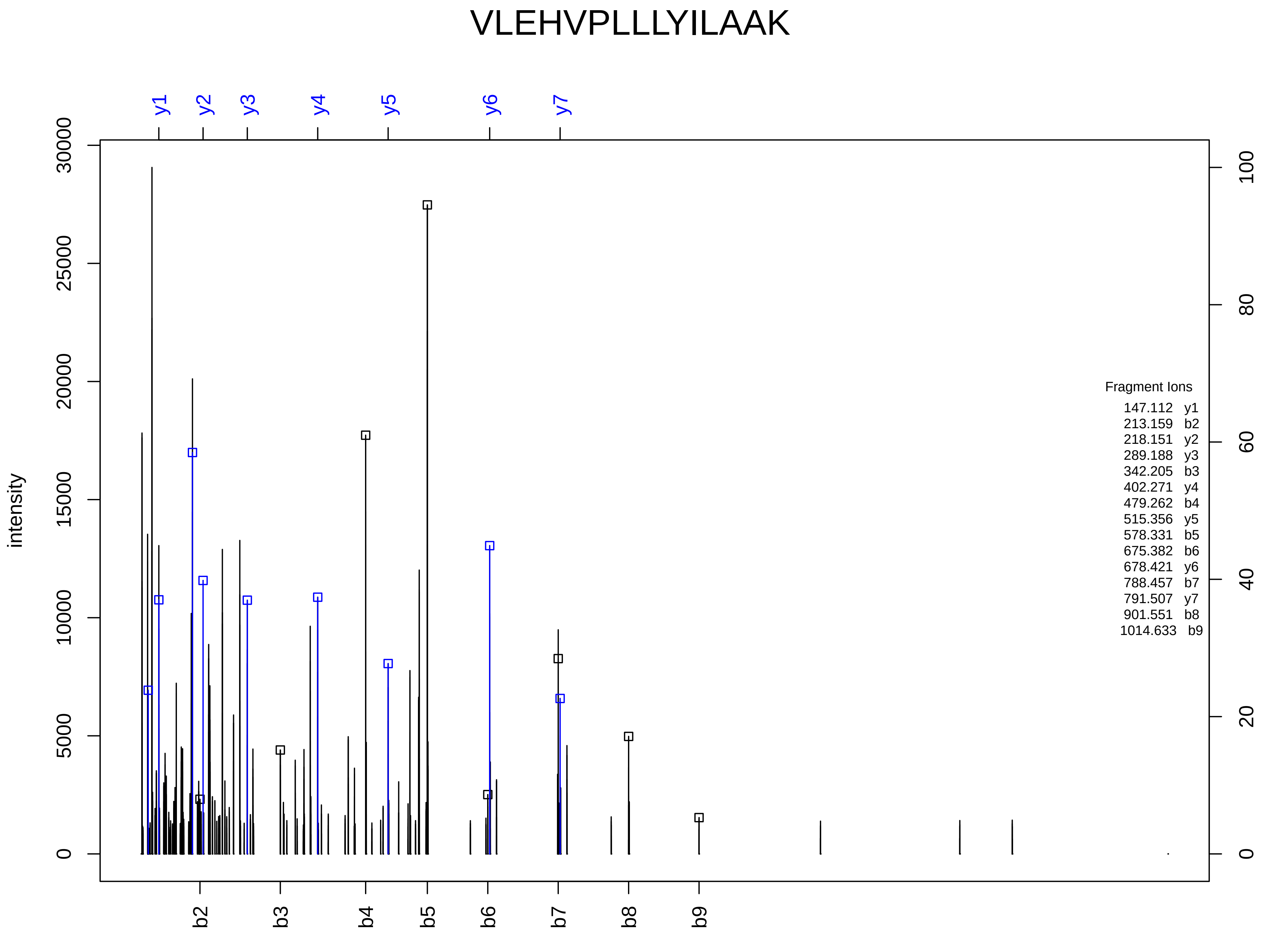

VLEHVPLLLYILAAK / File: christine_10pools_06.raw; SpectrumID: ; scans: 40194

AQGVMDDVVNNVIDHIRPGPWR

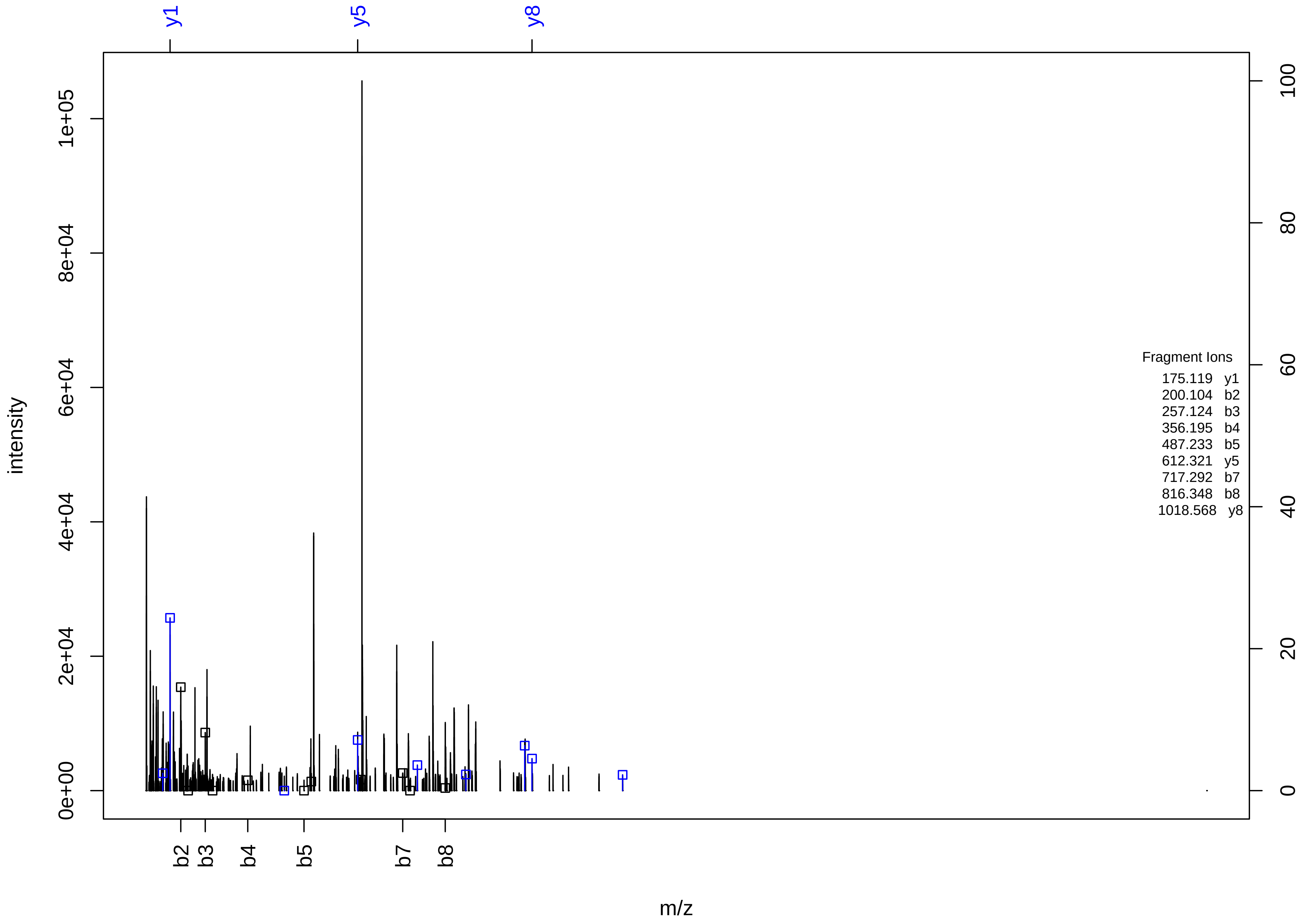

AQGVMDDVVNNVIDHIRPGPWR / File: christine_10pools_06.raw; SpectrumID: ; scans: 32003

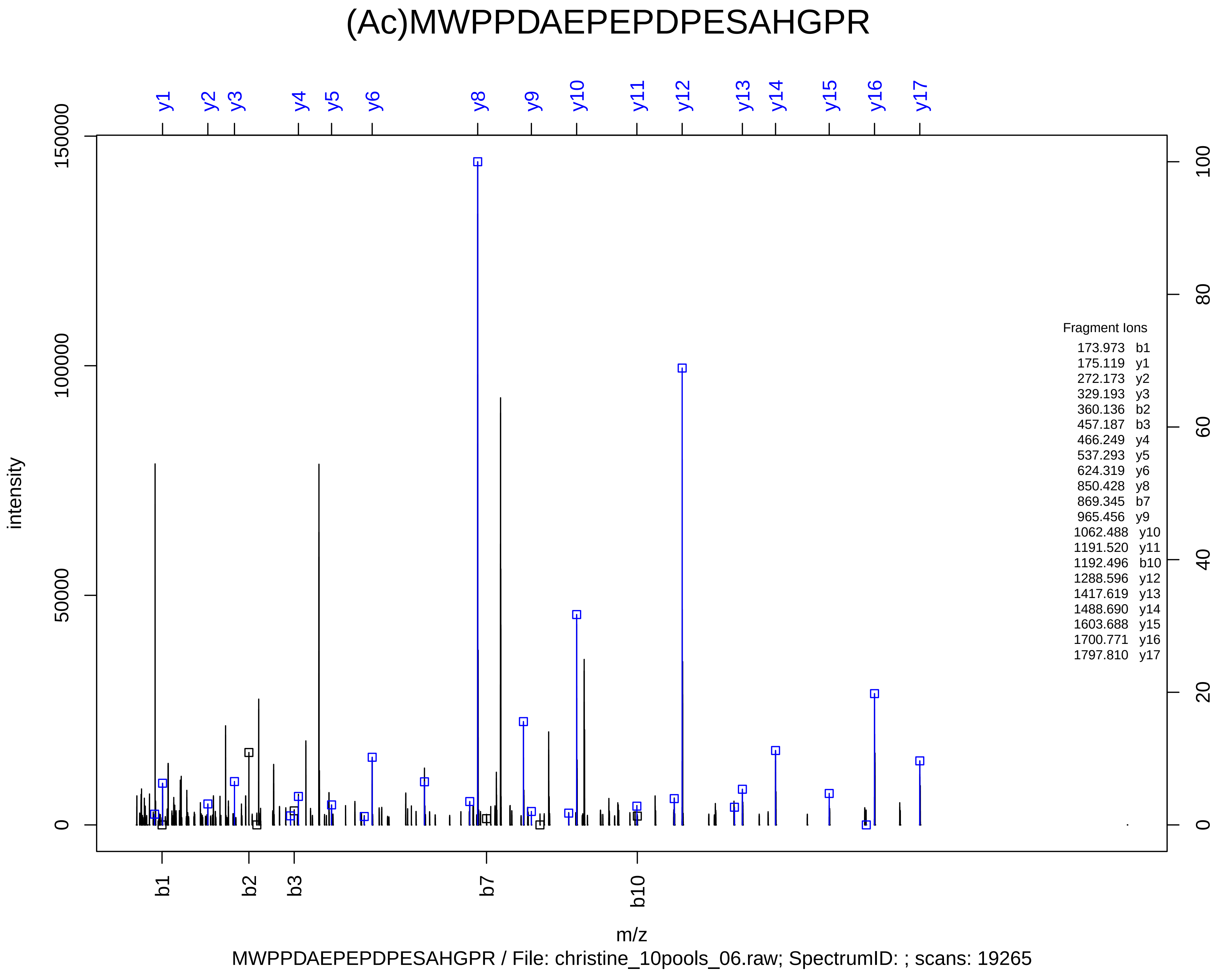
(Ac)MWPPDAEPEPDPESAHGPR
y1 y2 y3 y4 y5 y6 y8 y9 y10 y11 y12 y13 y14 y15 y16 y17
b1 b2 b3 b7 b10
intensity
0
50000
100000
150000
0
20
40
60
80
100
m/z
Fragment Ions
173.973 b1
175.119 y1
272.173 y2
329.193 y3
360.136 b2
457.187 b3
466.249 y4
537.293 y5
624.319 y6
850.428 y8
869.345 b7
965.456 y9
1062.488 y10
1191.520 y11
1192.496 b10
1288.596 y12
1417.619 y13
1488.690 y14
1603.688 y15
1700.771 y16
1797.810 y17
MWPPDAEPEPDPESAHGPR / File: christine_10pools_06.raw; SpectrumID: ; scans: 19265

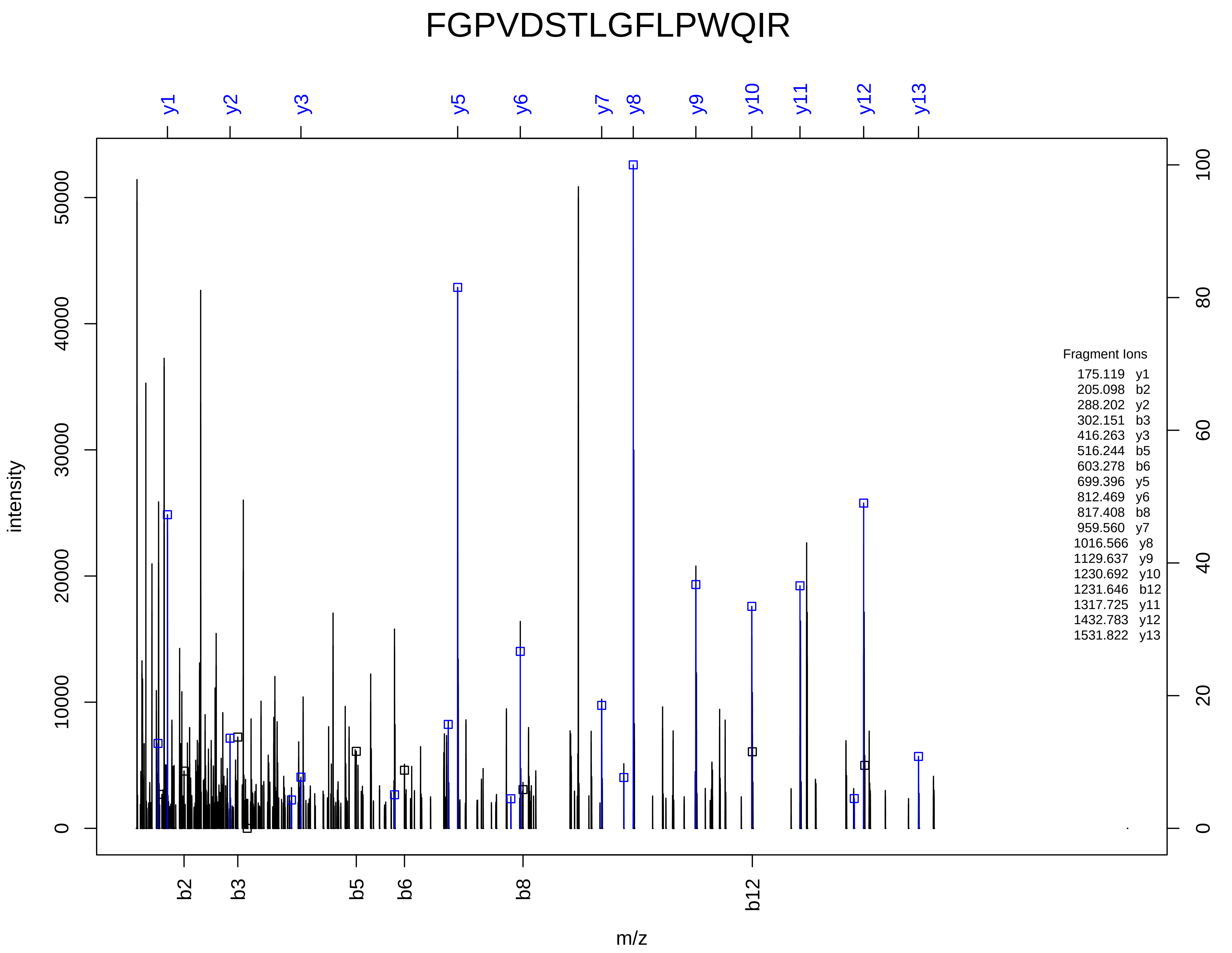

FGPVDSTLGFLPWQIR / File: christine_10pools_06.raw; SpectrumID: ; scans: 39959

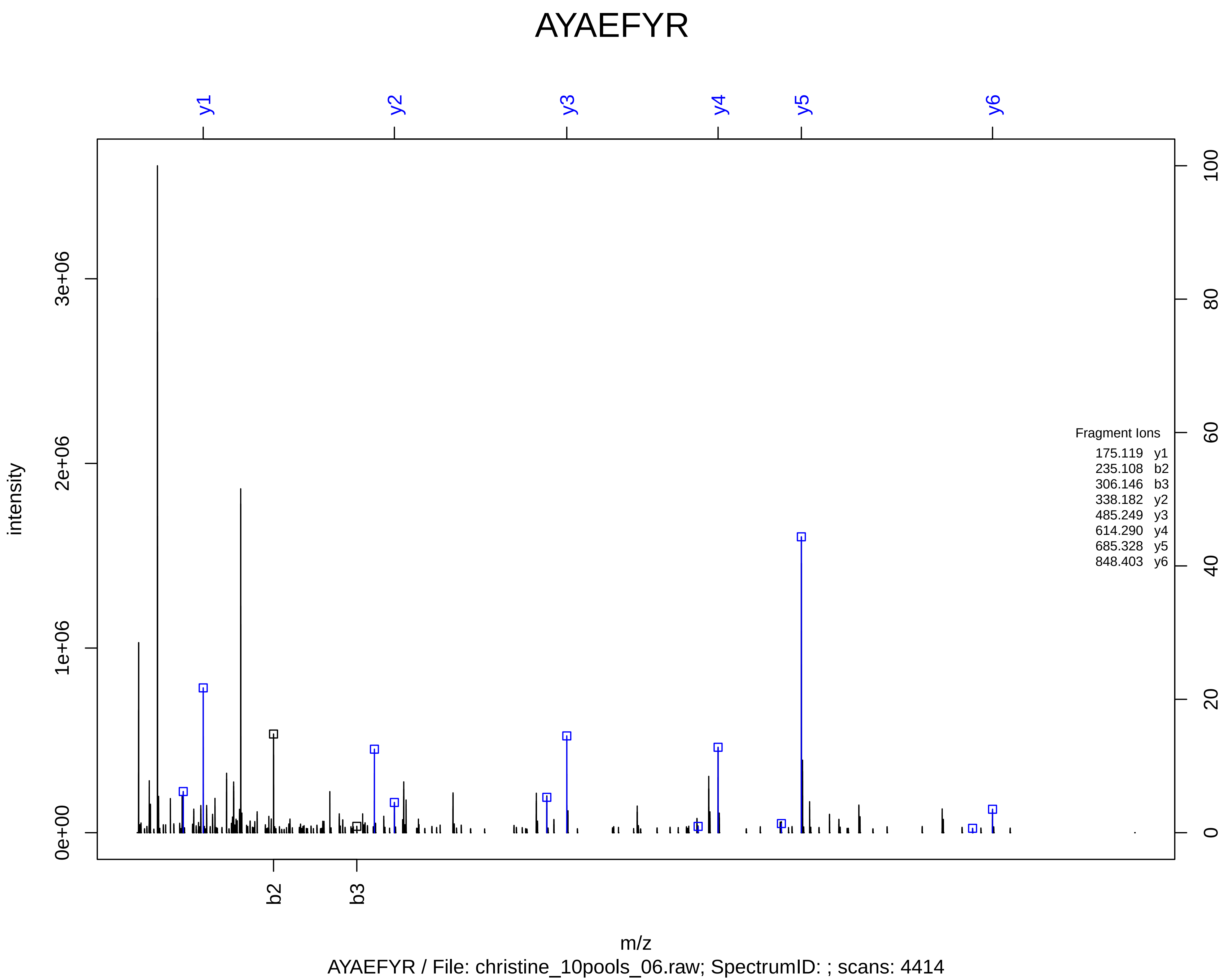

AYAEFYR
y1
y2
y3
y4
y5
y6
b2
b3
intensity
0e+00
1e+06
2e+06
3e+06
0
20
40
60
80
100
Fragment Ions
175.119 y1
235.108 b2
306.146 b3
338.182 y2
485.249 y3
614.290 y4
685.328 y5
848.403 y6
m/z
AYAEFYR / File: christine_10pools_06.raw; SpectrumID: ; scans: 4414

LASGAQSIPADIPAHAEGPSSEEEGFAVEK

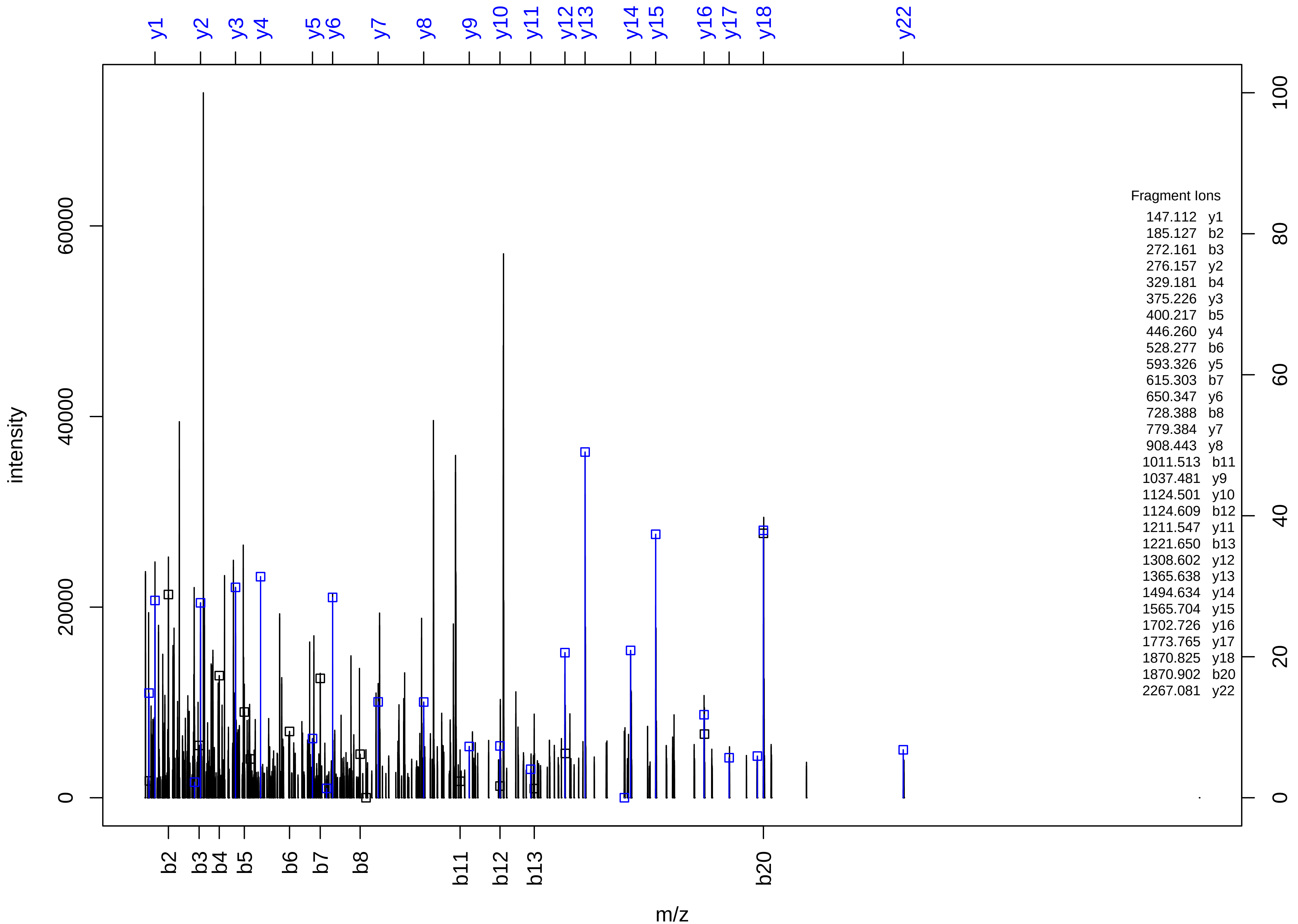

LASGAQSIPADIPAHAEGPSSEEEGFAVEK / File: christine_10pools_06.raw; SpectrumID: ; scans: 15006

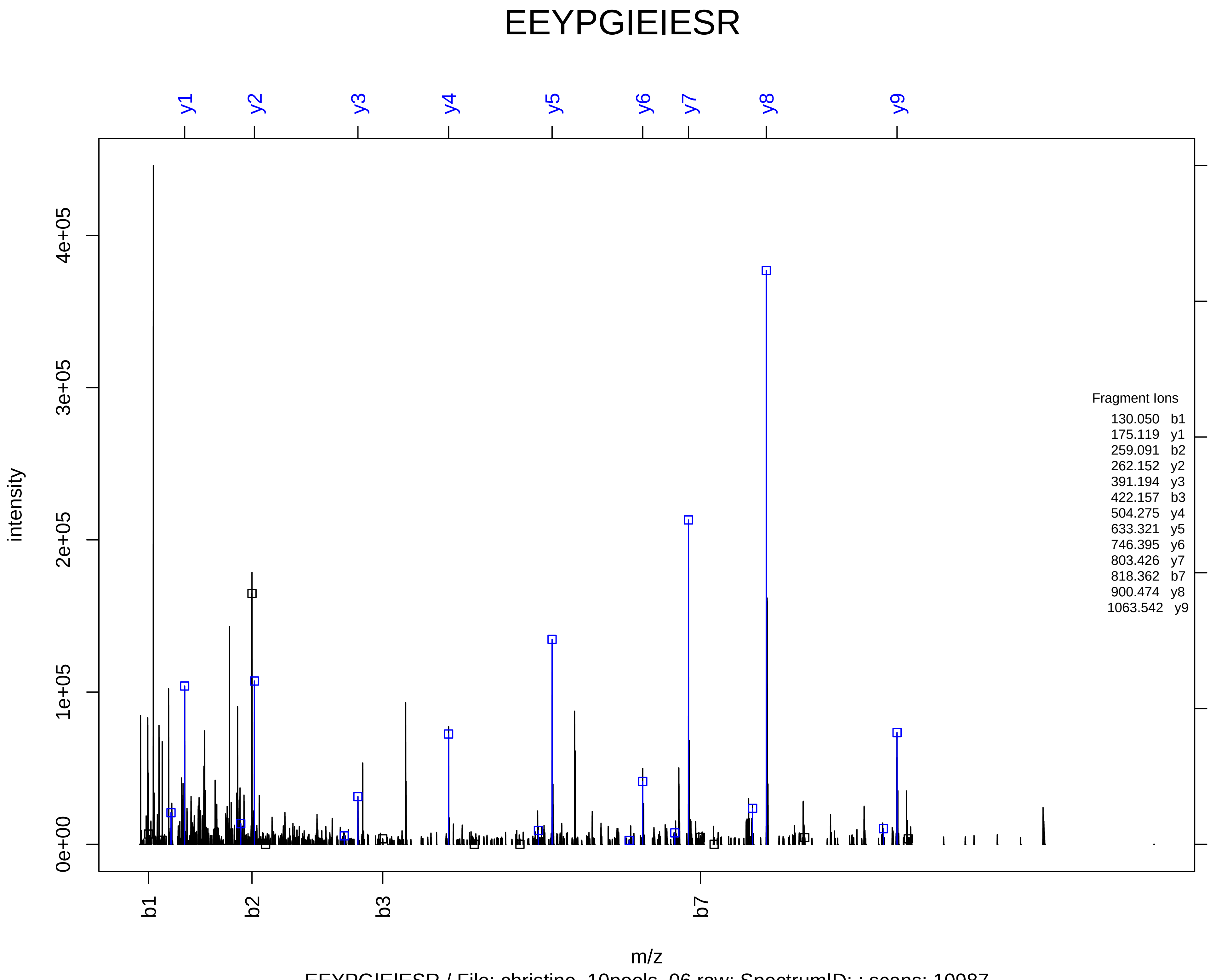

EEYPGIEIESR
y1
y2
y3
y4
y5
y6
y7
y8
y9
intensity
0e+00
1e+05
2e+05
3e+05
4e+05
0
20
40
60
80
100
b1
b2
b3
b7
m/z
Fragment Ions
130.050 b1
175.119 y1
259.091 b2
262.152 y2
391.194 y3
422.157 b3
504.275 y4
633.321 y5
746.395 y6
803.426 y7
818.362 b7
900.474 y8
1063.542 y9
EEYPGIEIESR / File: christine_10pools_06.raw; SpectrumID: ; scans: 10987

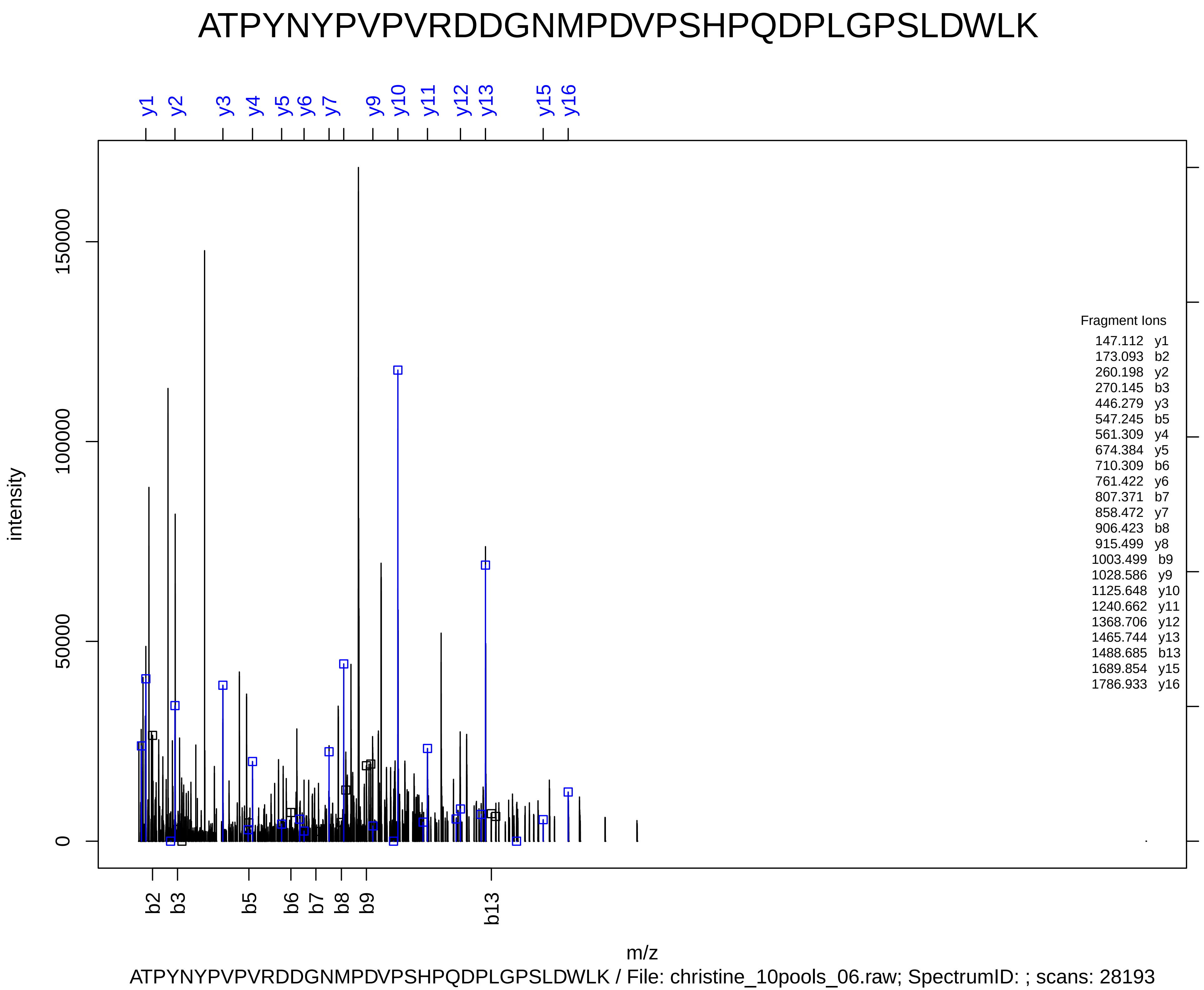
ATPYNYPVPVRDDGNMPDVPSHPQDPLGPSLDWLK
y1 y2 y3 y4 y5 y6 y7 y9 y10 y11 y12 y13 y15 y16
b2 b3 b5 b6 b7 b8 b9 b13
intensity
0
50000
100000
150000
0
20
40
60
80
100
m/z
Fragment Ions
147.112 y1
173.093 b2
260.198 y2
270.145 b3
446.279 y3
547.245 b5
561.309 y4
674.384 y5
710.309 b6
761.422 y6
807.371 b7
858.472 y7
906.423 b8
915.499 y8
1003.499 b9
1028.586 y9
1125.648 y10
1240.662 y11
1368.706 y12
1465.744 y13
1488.685 b13
1689.854 y15
1786.933 y16
ATPYNYPVPVRDDGNMPDVPSHPQDPLGPSLDWLK / File: christine_10pools_06.raw; SpectrumID: ; scans: 28193

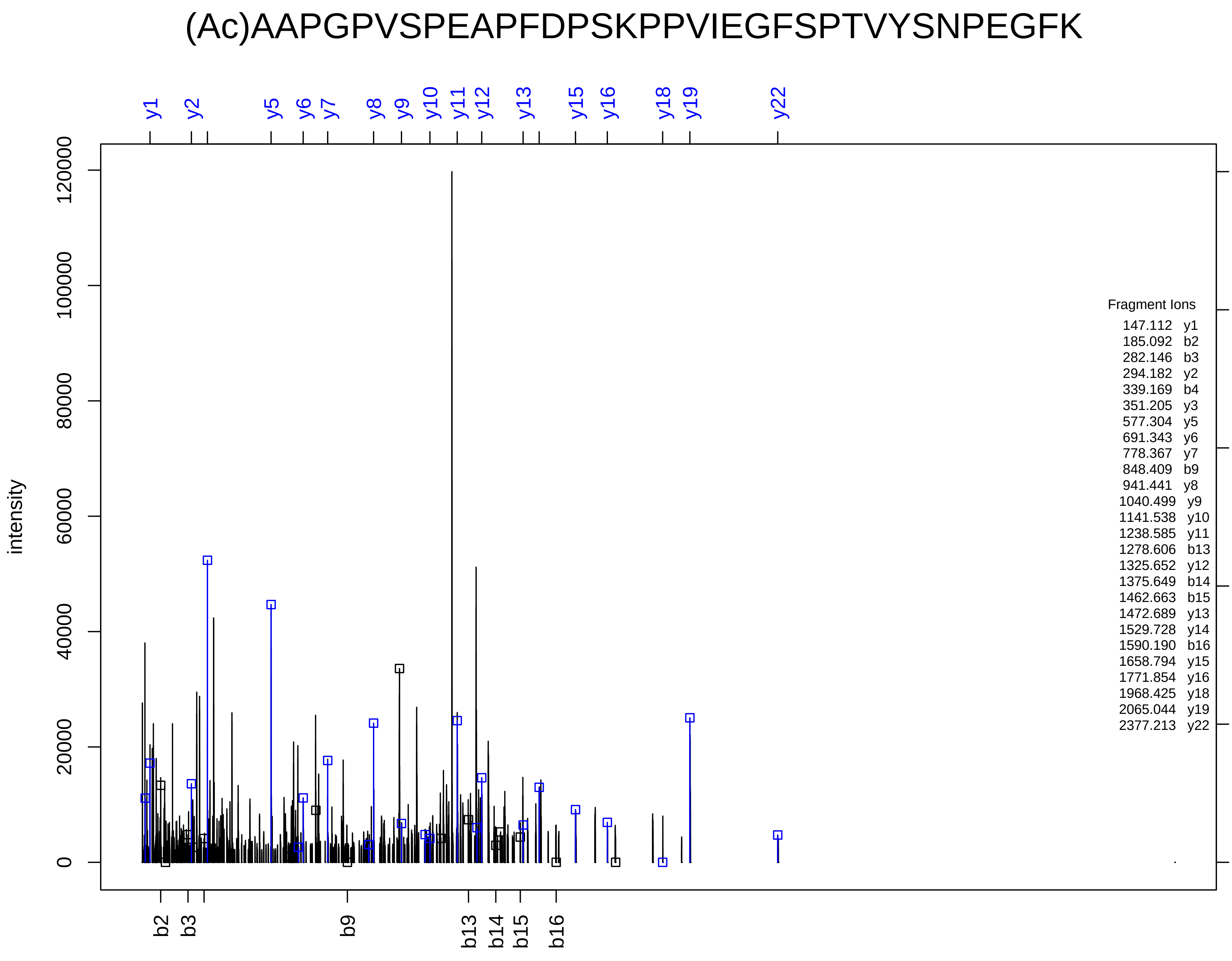

AAPGPVSPEAPFDPSKPPVIEGFSPTVYSNPEGFK / File: christine_10pools_06.raw; SpectrumID: ; scans: 34504

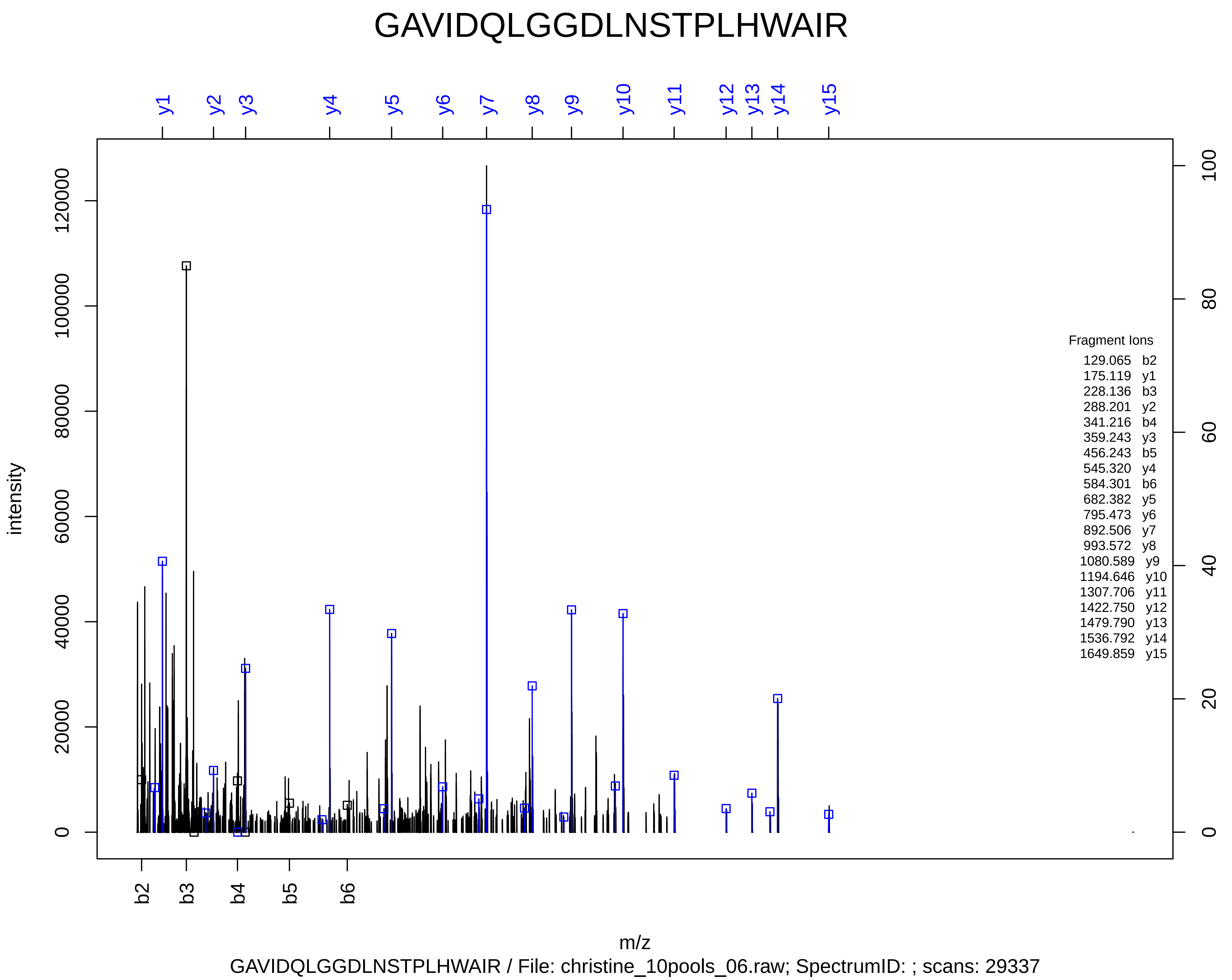

GAVIDQLGGDLNSTPLHWAIR
y1 y2 y3 y4 y5 y6 y7 y8 y9 y10 y11 y12 y13 y14 y15
intensity
0 20000 40000 60000 80000 100000 120000
0 20 40 60 80 100
b2 b3 b4 b5 b6
m/z
Fragment Ions
129.065 b2
175.119 y1
228.136 b3
288.201 y2
341.216 b4
359.243 y3
456.243 b5
545.320 y4
584.301 b6
682.382 y5
795.473 y6
892.506 y7
993.572 y8
1080.589 y9
1194.646 y10
1307.706 y11
1422.750 y12
1479.790 y13
1536.792 y14
1649.859 y15
GAVIDQLGGDLNSTPLHWAIR / File: christine_10pools_06.raw; SpectrumID: ; scans: 29337

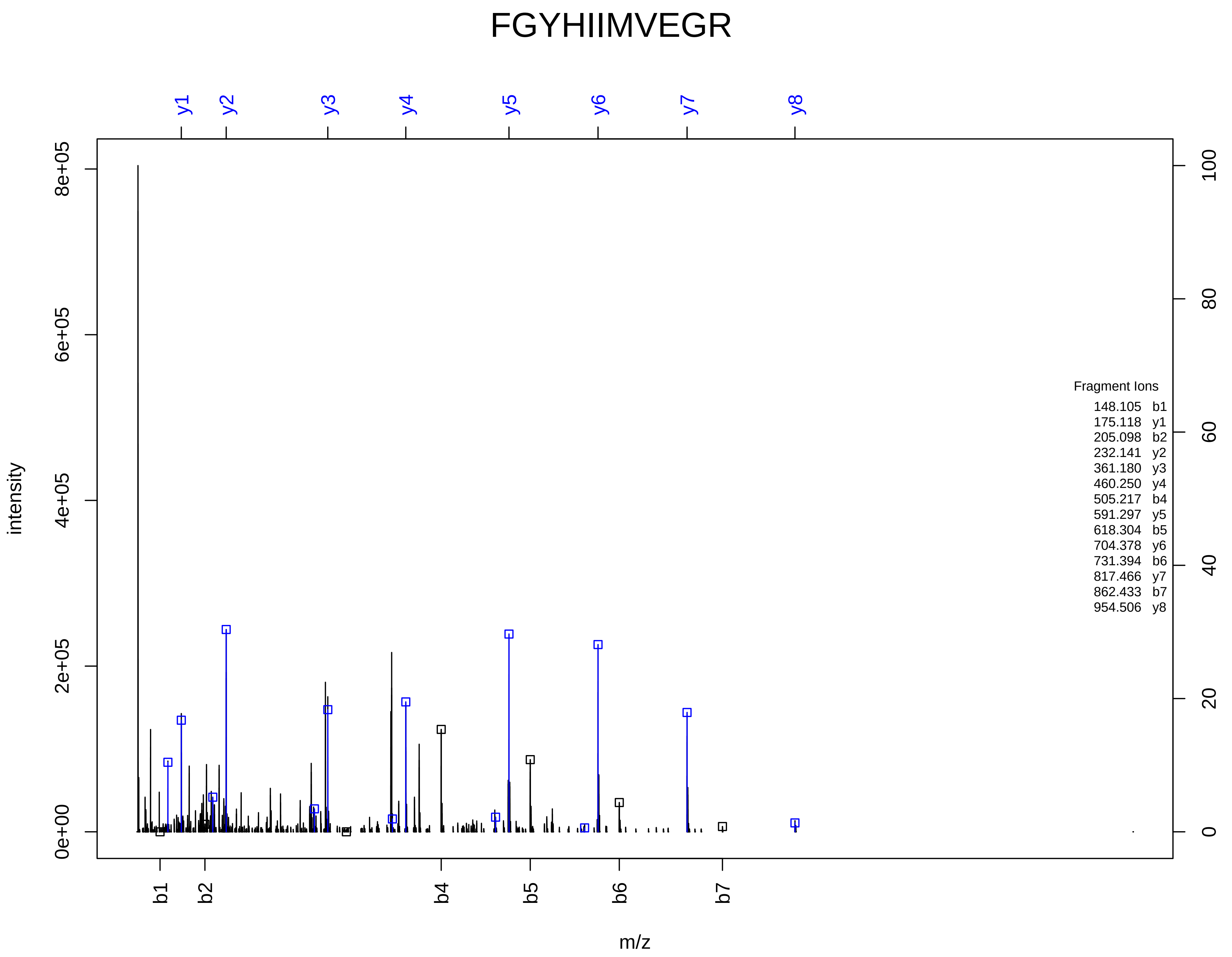

FGYHIIMVEGR
y1
y2
y3
y4
y5
y6
y7
y8
intensity
0e+00
2e+05
4e+05
6e+05
8e+05
0
20
40
60
80
100
b1
b2
b4
b5
b6
b7
m/z
Fragment Ions
148.105 b1
175.118 y1
205.098 b2
232.141 y2
361.180 y3
460.250 y4
505.217 b4
591.297 y5
618.304 b5
704.378 y6
731.394 b6
817.466 y7
862.433 b7
954.506 y8
FGYHIIMVEGR / File: christine_10pools_06.raw; SpectrumID: ; scans: 13982

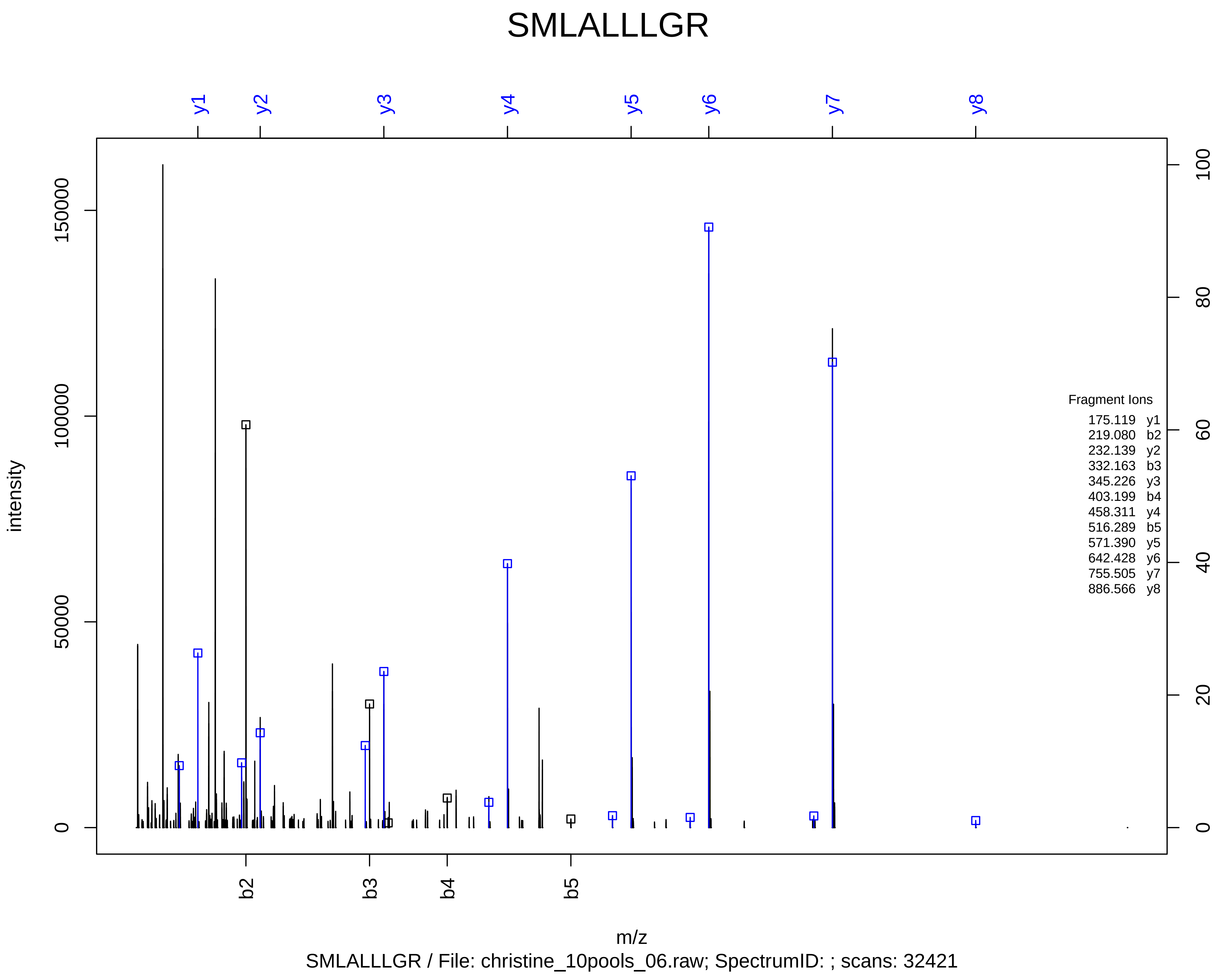
SMLALLLGR
y1
y2
y3
y4
y5
y6
y7
y8
intensity
0
50000
100000
150000
0
20
40
60
80
100
Fragment Ions
175.119 y1
219.080 b2
232.139 y2
332.163 b3
345.226 y3
403.199 b4
458.311 y4
516.289 b5
571.390 y5
642.428 y6
755.505 y7
886.566 y8
b2
b3
b4
b5
m/z
SMLALLLGR / File: christine_10pools_06.raw; SpectrumID: ; scans: 32421

HDGTVGLLTYPVLQAADILCYK

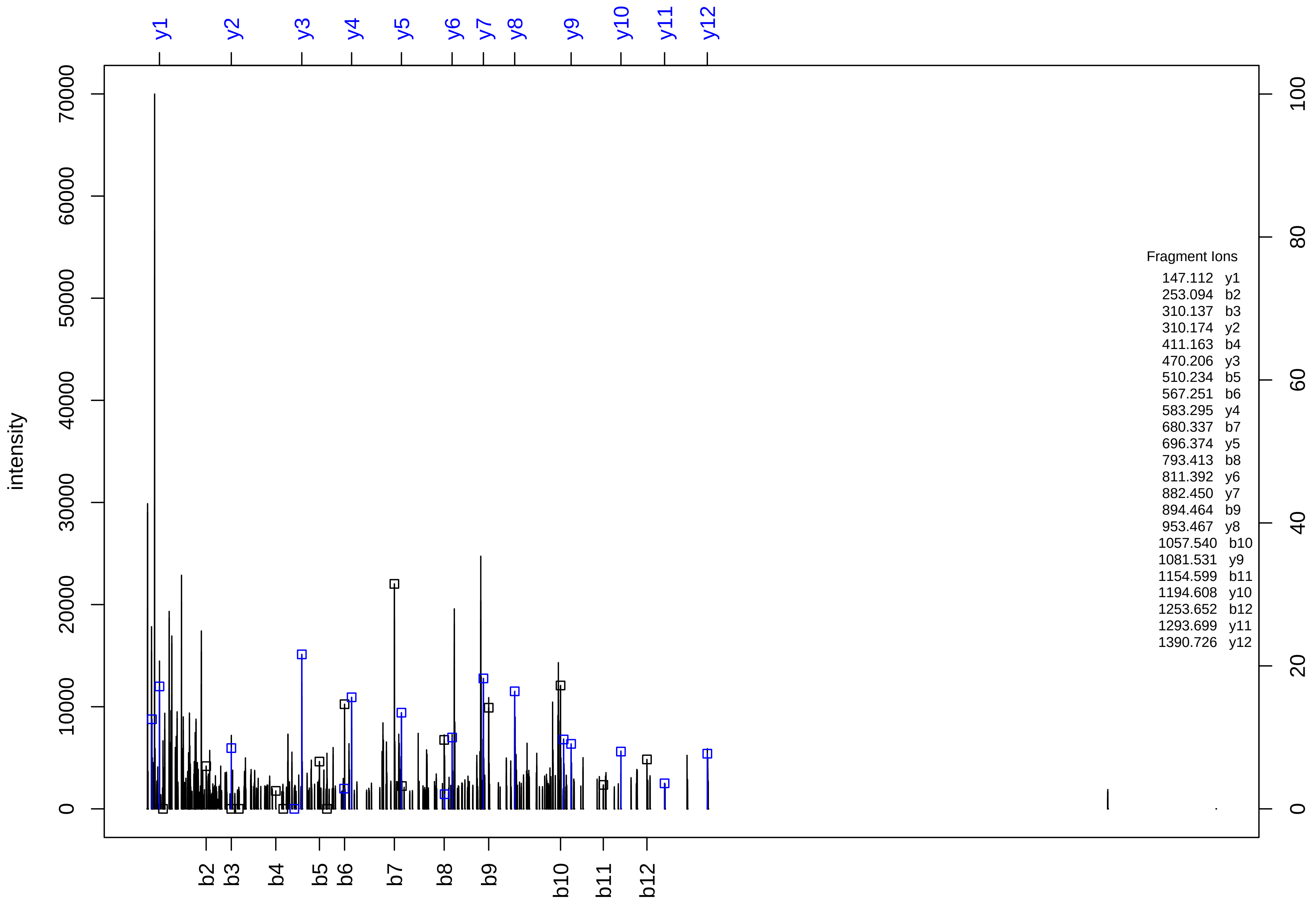

HDGTVGLLTYPVLQAADILCYK / File: christine_10pools_06.raw; SpectrumID: ; scans: 40011

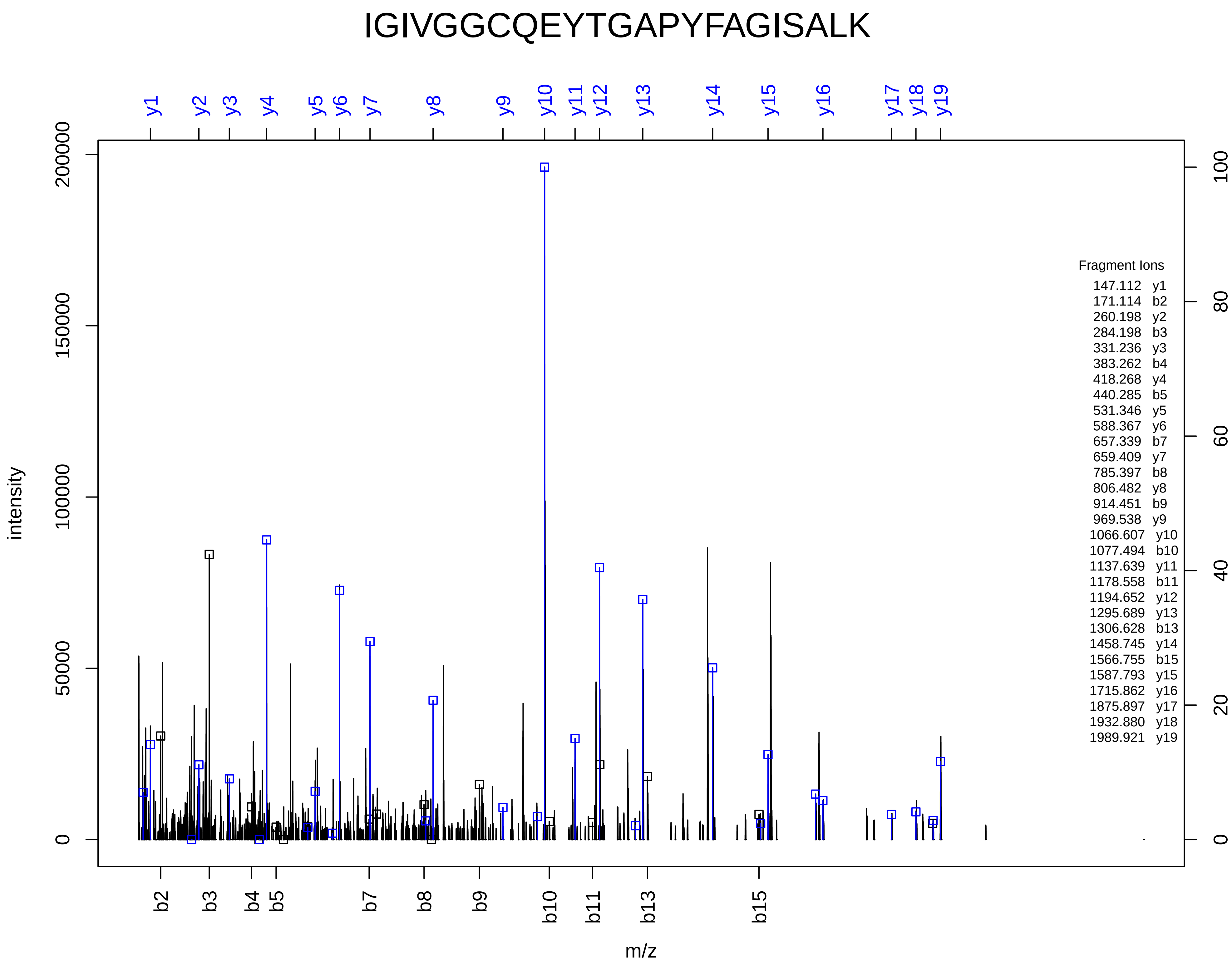

IGIVGGCQEYTGAPYFAGISALK / File: christine_10pools_06.raw; SpectrumID: ; scans: 30188

GAGVNSEHQGAPAPGDSNQGK

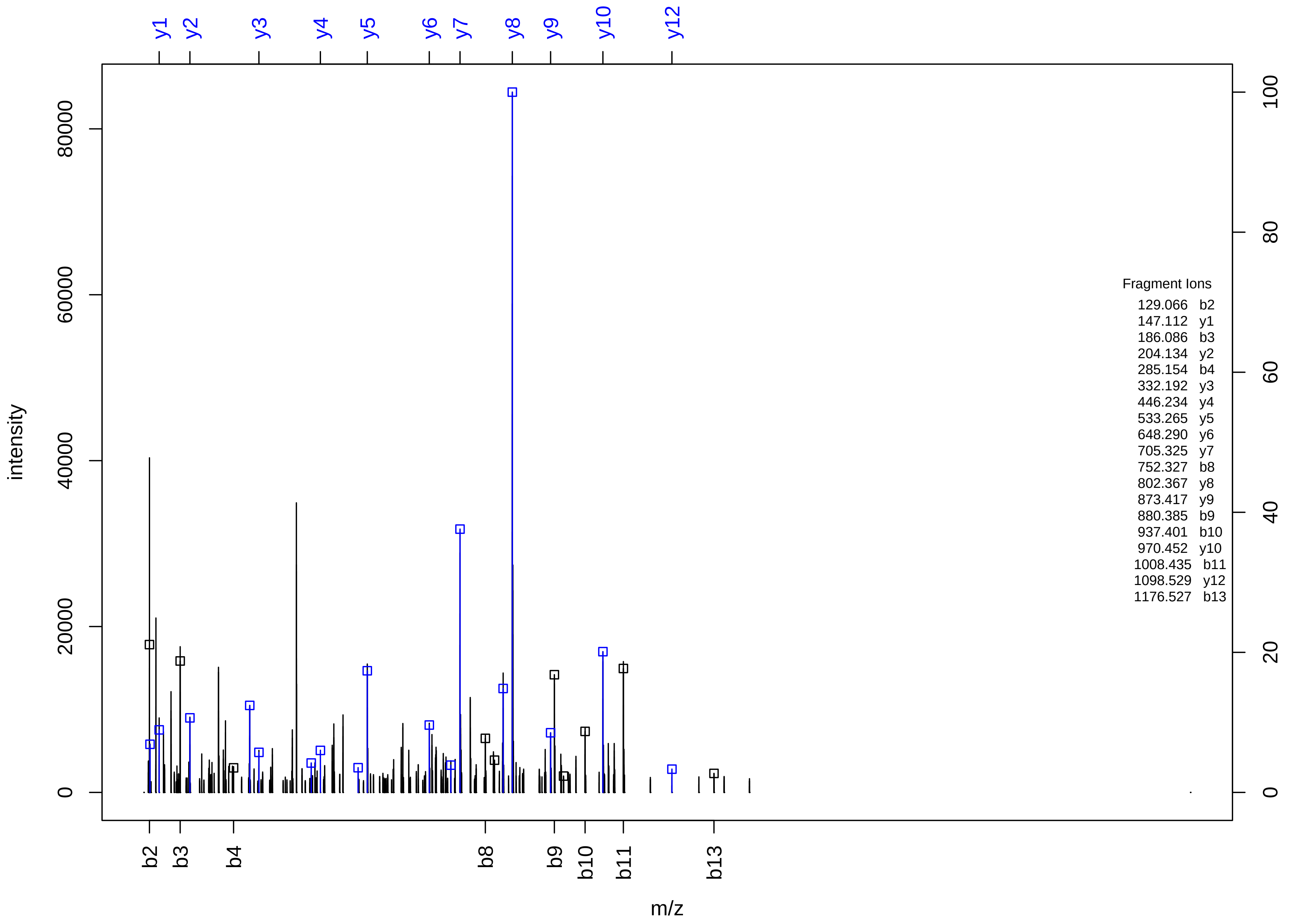

GAGVNSEHQGAPAPGDSNQGK / File: christine_10pools_06.raw; SpectrumID: ; scans: 930

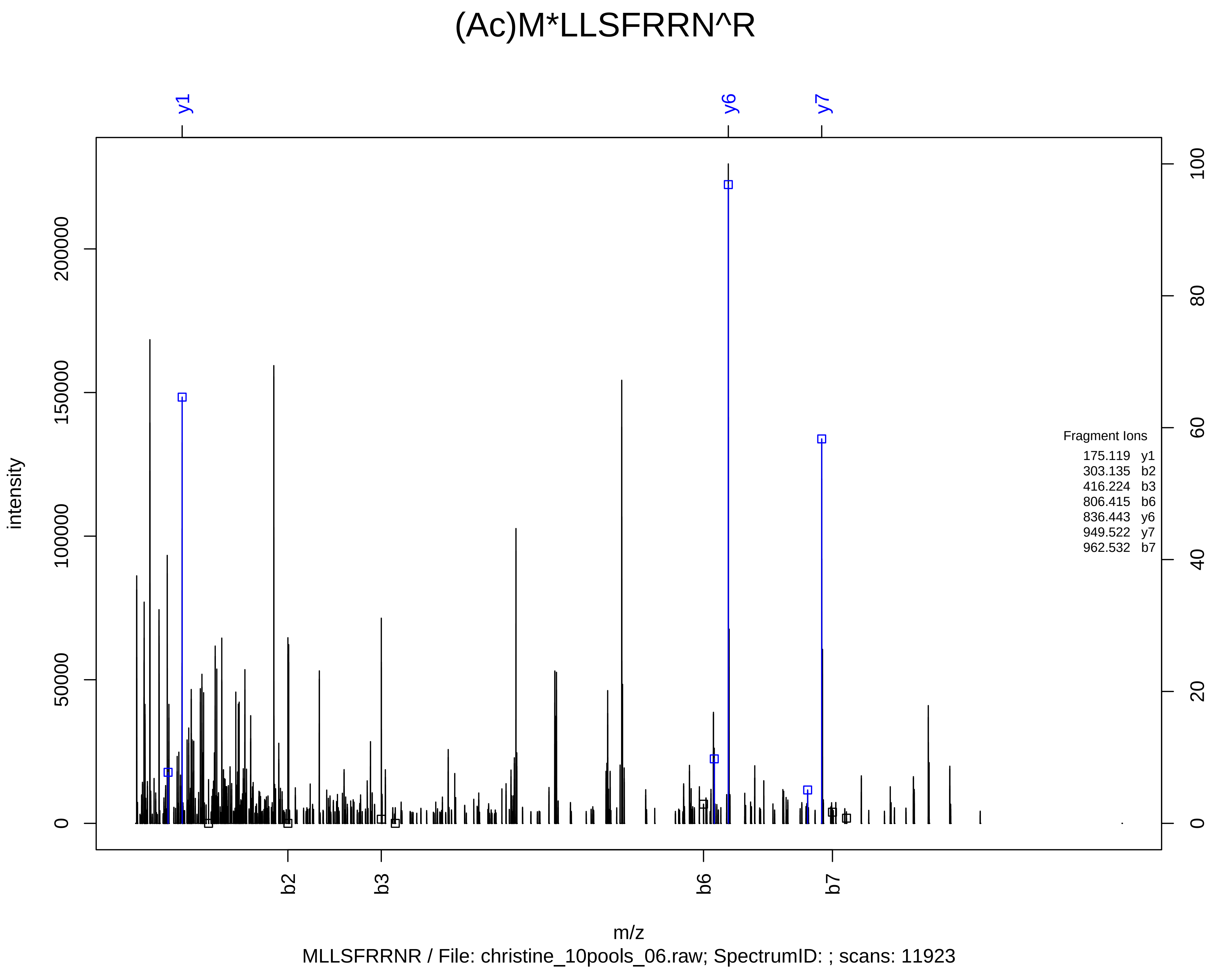

(Ac)M*LLSFRRN^R
y1
y6
y7
intensity
0
50000
100000
150000
200000
0
20
40
60
80
100
Fragment Ions
175.119 y1
303.135 b2
416.224 b3
806.415 b6
836.443 y6
949.522 y7
962.532 b7
b2
b3
b6
b7
m/z
MLLSFRRNR / File: christine_10pools_06.raw; SpectrumID: ; scans: 11923

MELLHPLGSDSPITGFLQK

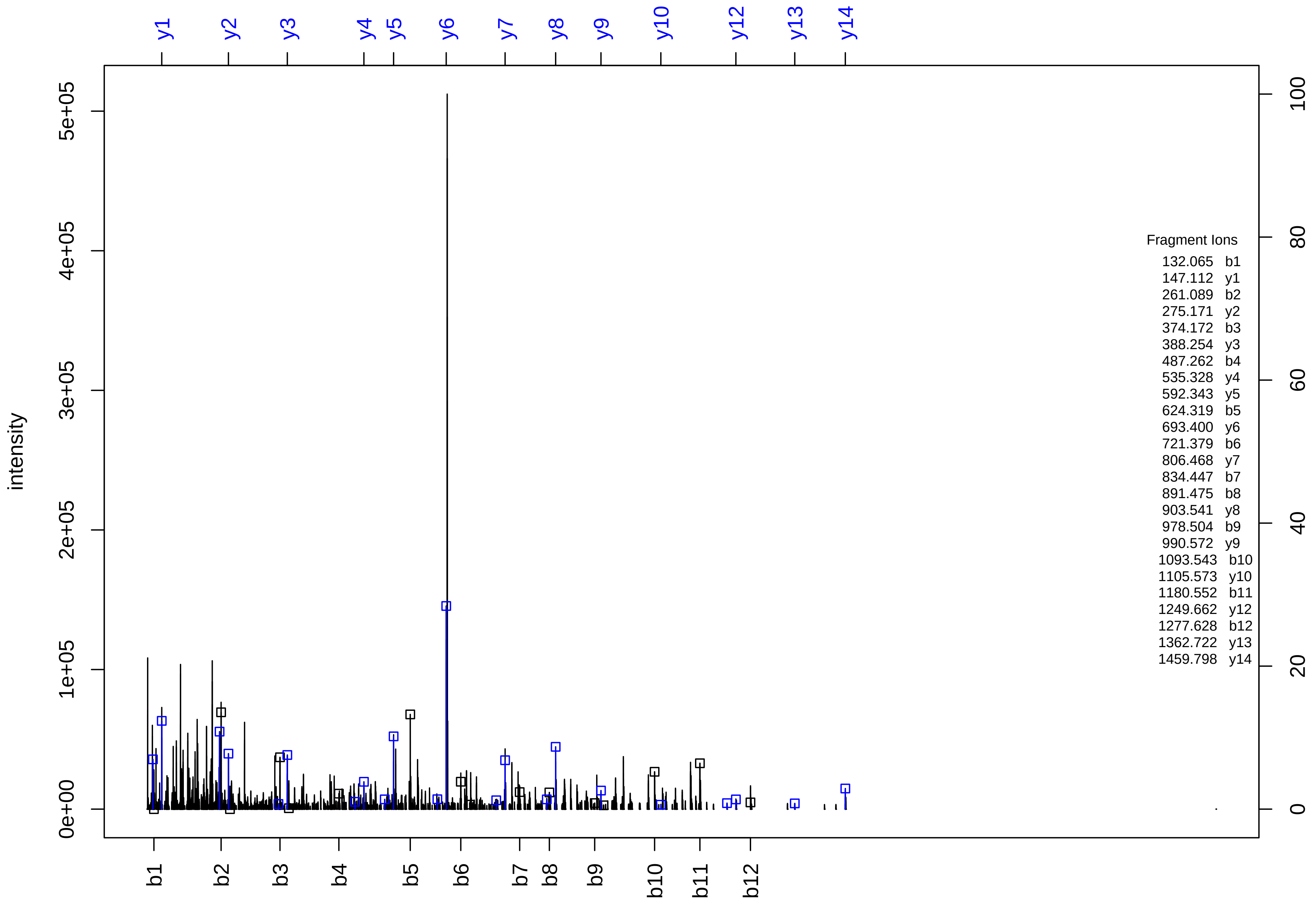

MELLHPLGSDSPITGFLQK / File: christine_10pools_06.raw; SpectrumID: ; scans: 29205

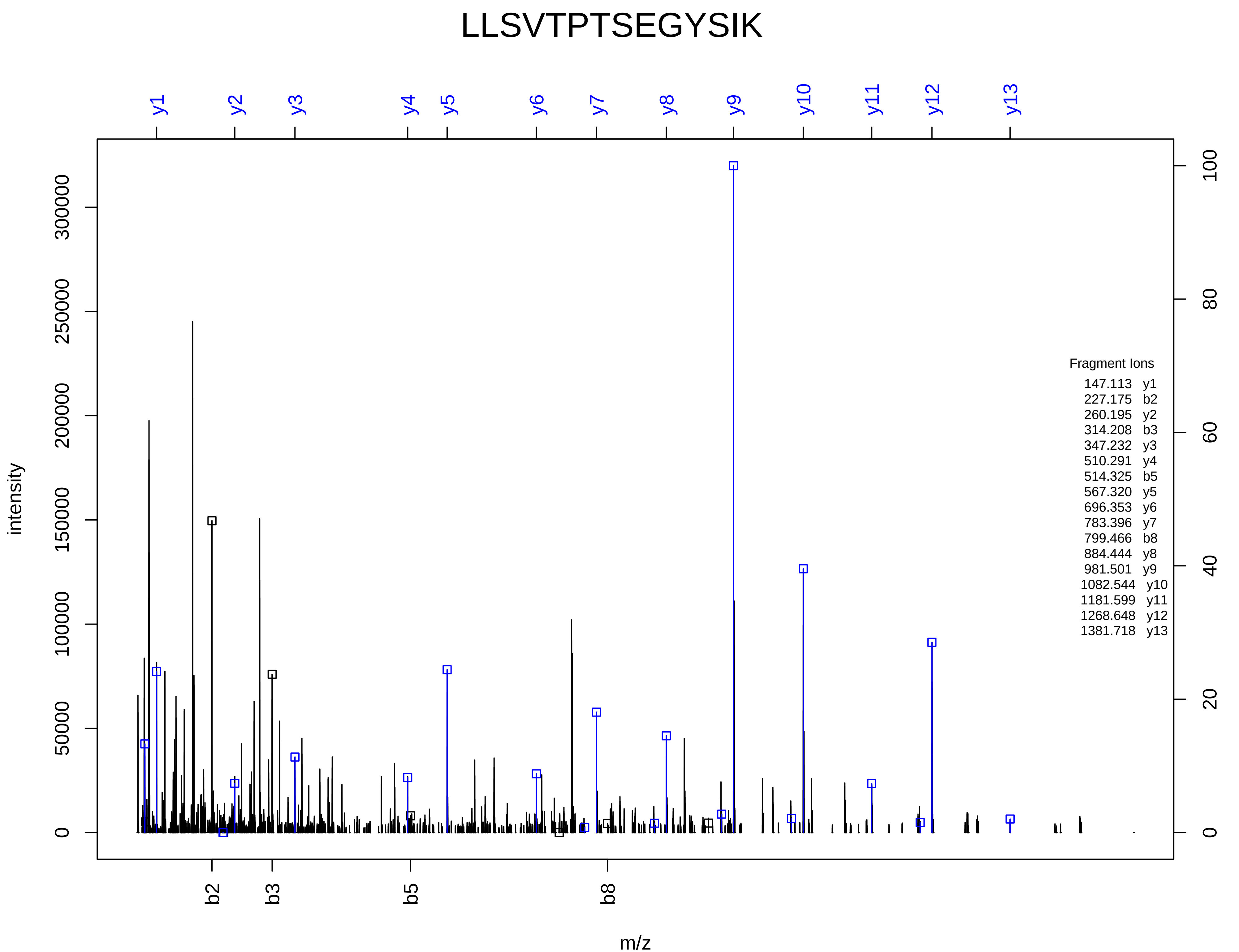

LLSVTPTSEGYSIK / File: christine_10pools_06.raw; SpectrumID: ; scans: 14954

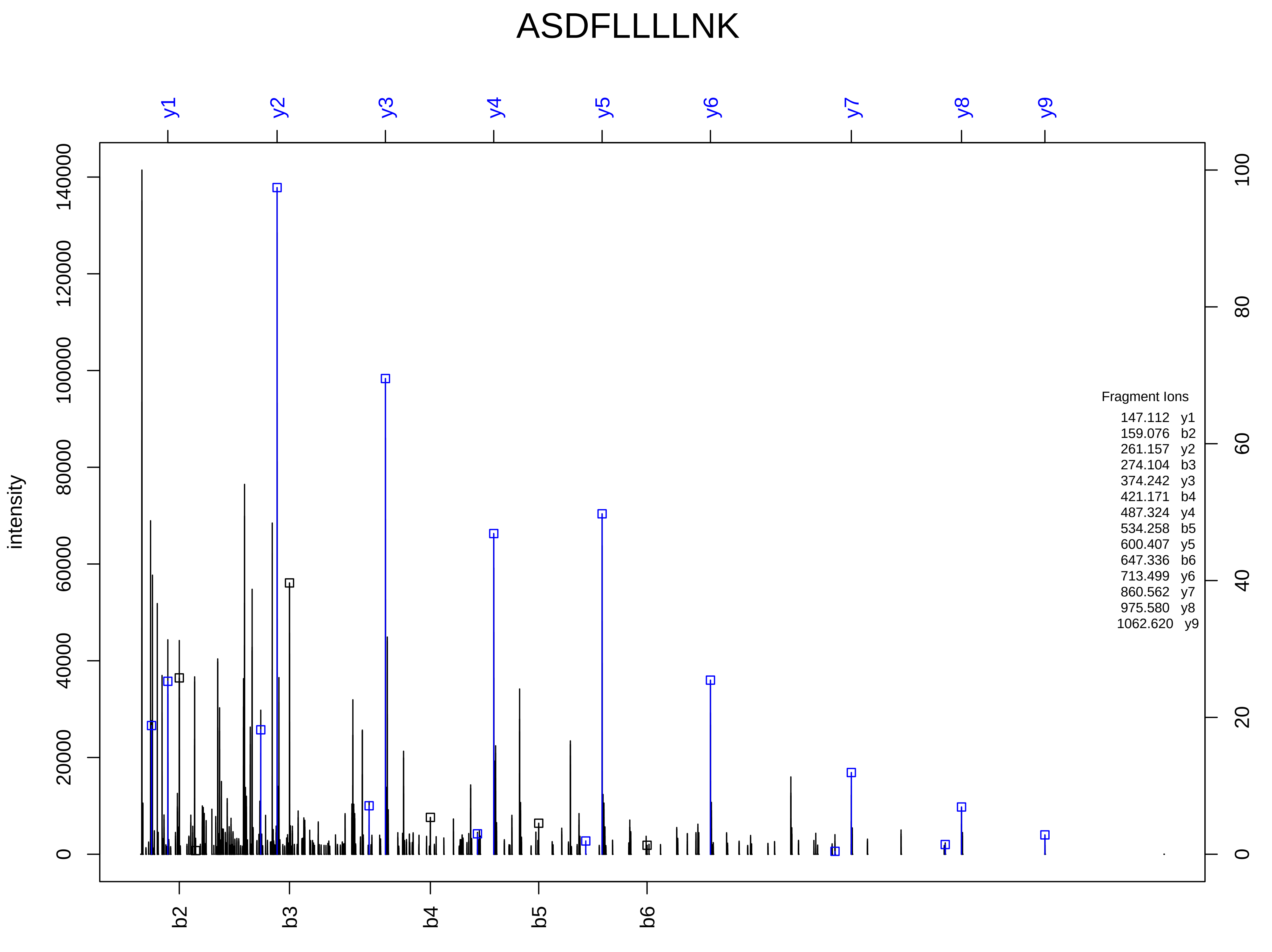

ASDFLLLLNK / File: christine_10pools_06.raw; SpectrumID: ; scans: 27365

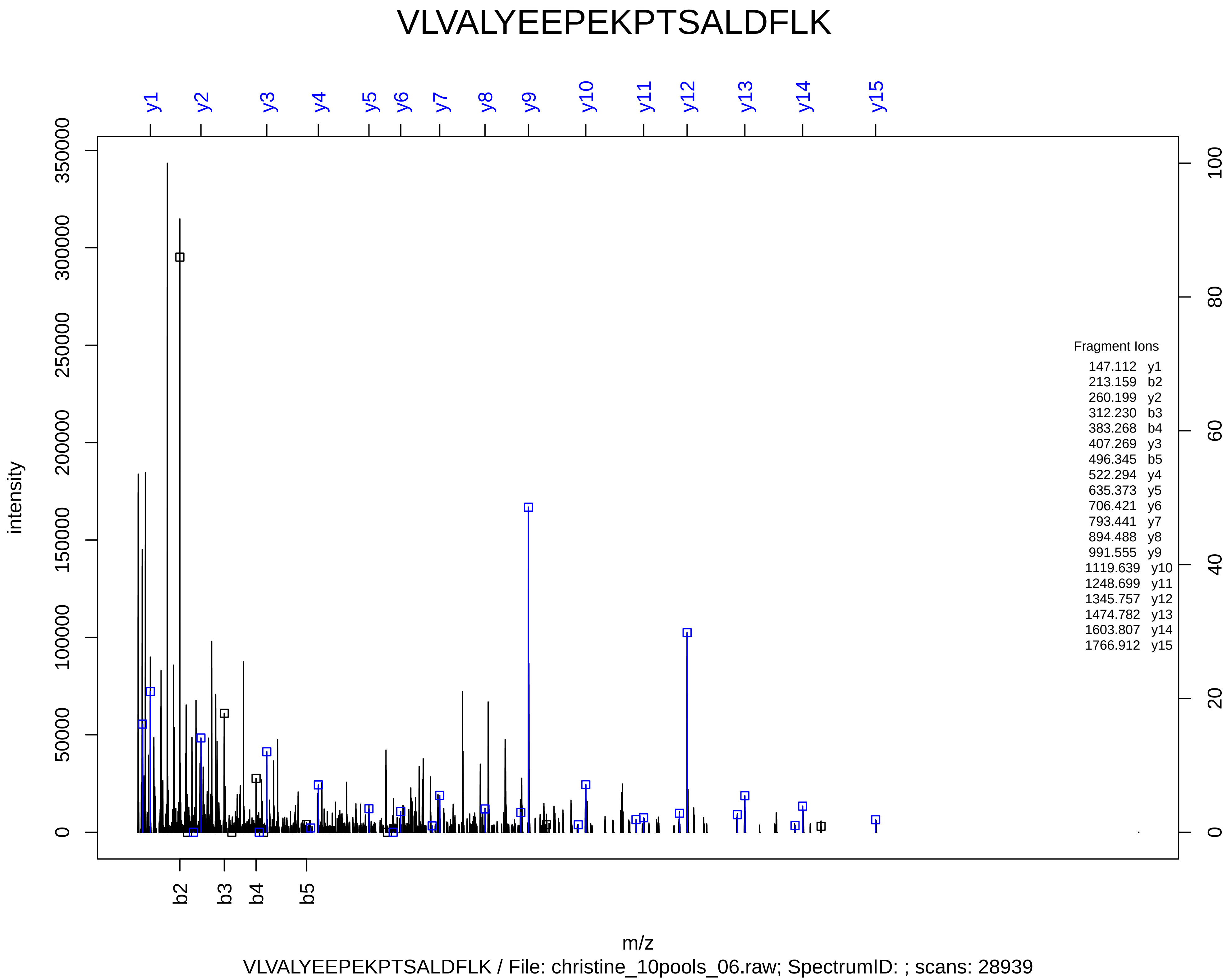

VLVALYEEPEKPTSALDFLK
y1
y2
y3
y4
y5
y6
y7
y8
y9
y10
y11
y12
y13
y14
y15
intensity
0
50000
100000
150000
200000
250000
300000
350000
0
20
40
60
80
100
b2
b3
b4
b5
Fragment Ions
147.112 y1
213.159 b2
260.199 y2
312.230 b3
383.268 b4
407.269 y3
496.345 b5
522.294 y4
635.373 y5
706.421 y6
793.441 y7
894.488 y8
991.555 y9
1119.639 y10
1248.699 y11
1345.757 y12
1474.782 y13
1603.807 y14
1766.912 y15
m/z
VLVALYEEPEKPTSALDFLK / File: christine_10pools_06.raw; SpectrumID: ; scans: 28939

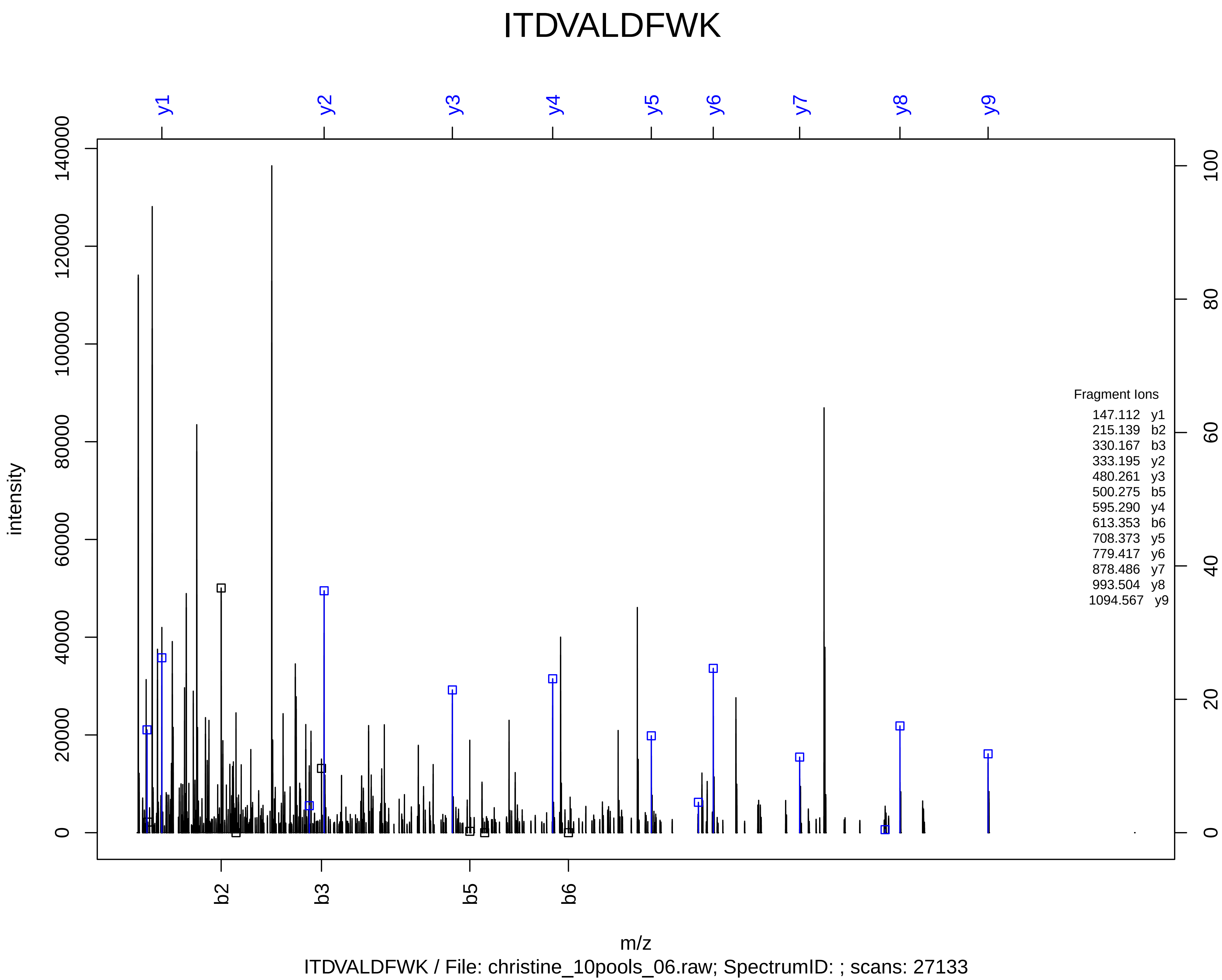
ITDVALDFWK
y1
y2
y3
y4
y5
y6
y7
y8
y9
b2
b3
b5
b6
intensity
m/z
Fragment Ions
147.112 y1
215.139 b2
330.167 b3
333.195 y2
480.261 y3
500.275 b5
595.290 y4
613.353 b6
708.373 y5
779.417 y6
878.486 y7
993.504 y8
1094.567 y9
ITDVALDFWK / File: christine_10pools_06.raw; SpectrumID: ; scans: 27133

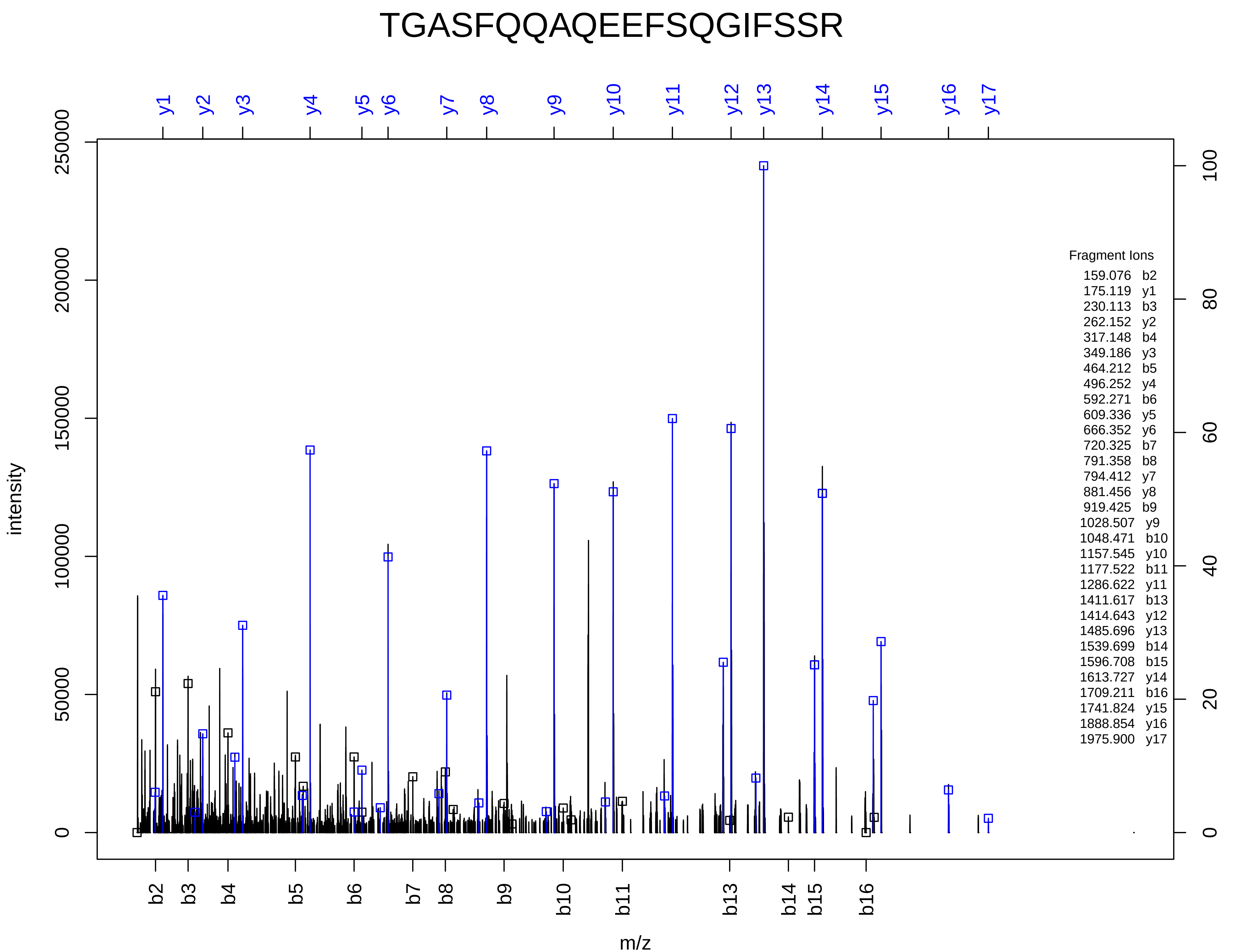
TGASFQQAQEEFSQGIFSSR
y1 y2 y3 y4 y5 y6 y7 y8 y9 y10 y11 y12 y13 y14 y15 y16 y17
b2 b3 b4 b5 b6 b7 b8 b9 b10 b11 b13 b14 b15 b16
intensity
m/z
Fragment Ions
159.076 b2
175.119 y1
230.113 b3
262.152 y2
317.148 b4
349.186 y3
464.212 b5
496.252 y4
592.271 b6
609.336 y5
666.352 y6
720.325 b7
791.358 b8
794.412 y7
881.456 y8
919.425 b9
1028.507 y9
1048.471 b10
1157.545 y10
1177.522 b11
1286.622 y11
1411.617 b13
1414.643 y12
1485.696 y13
1539.699 b14
1596.708 b15
1613.727 y14
1709.211 b16
1741.824 y15
1888.854 y16
1975.900 y17
TGASFQQAQEEFSQGIFSSR / File: christine_10pools_06.raw; SpectrumID: ; scans: 24534

AIASASASGPCIHQQPPGQPPPPQLSSR

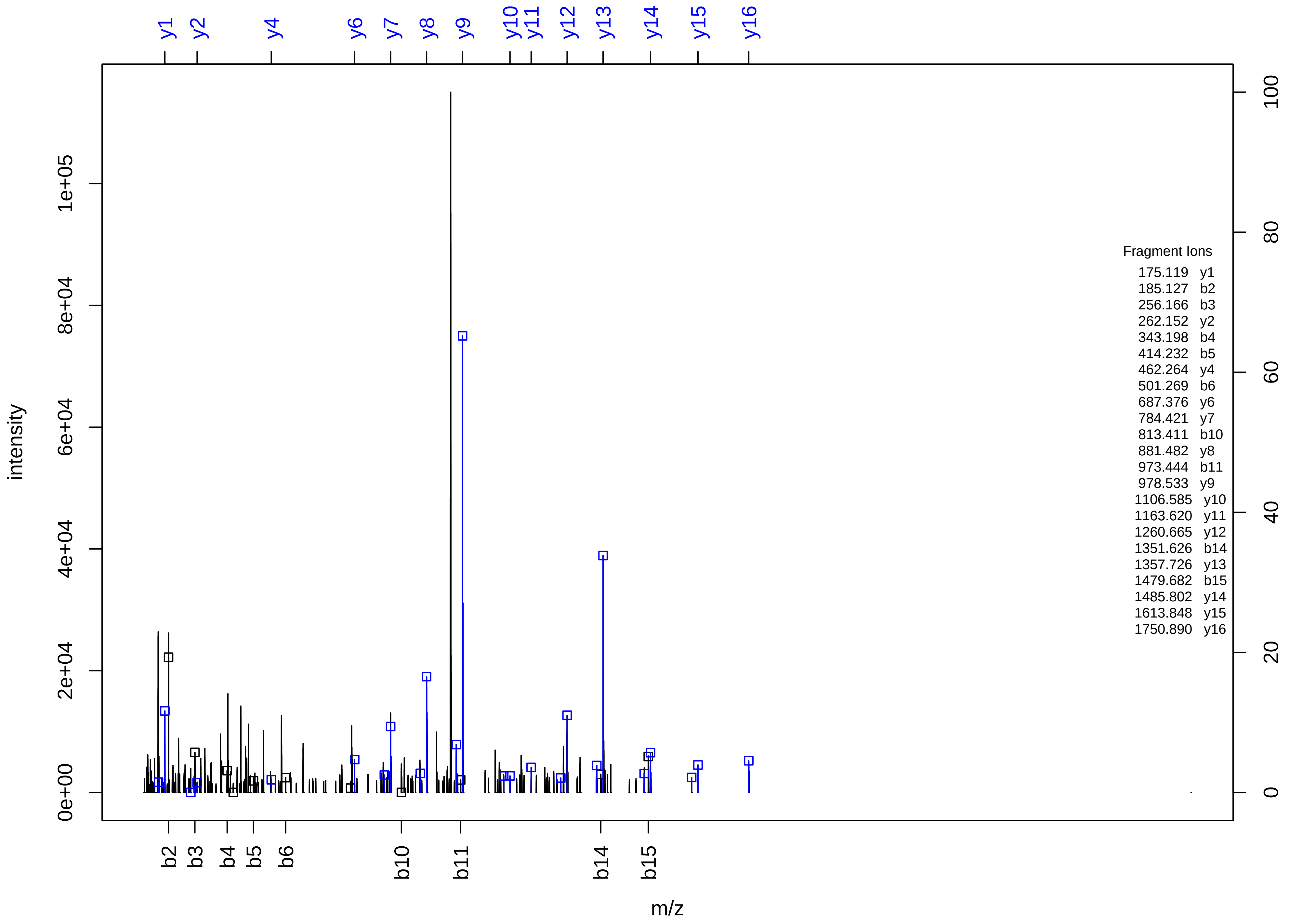

AIASASASGPCIHQQPPGQPPPPQLSSR / File: christine_10pools_06.raw; SpectrumID: ; scans: 6573

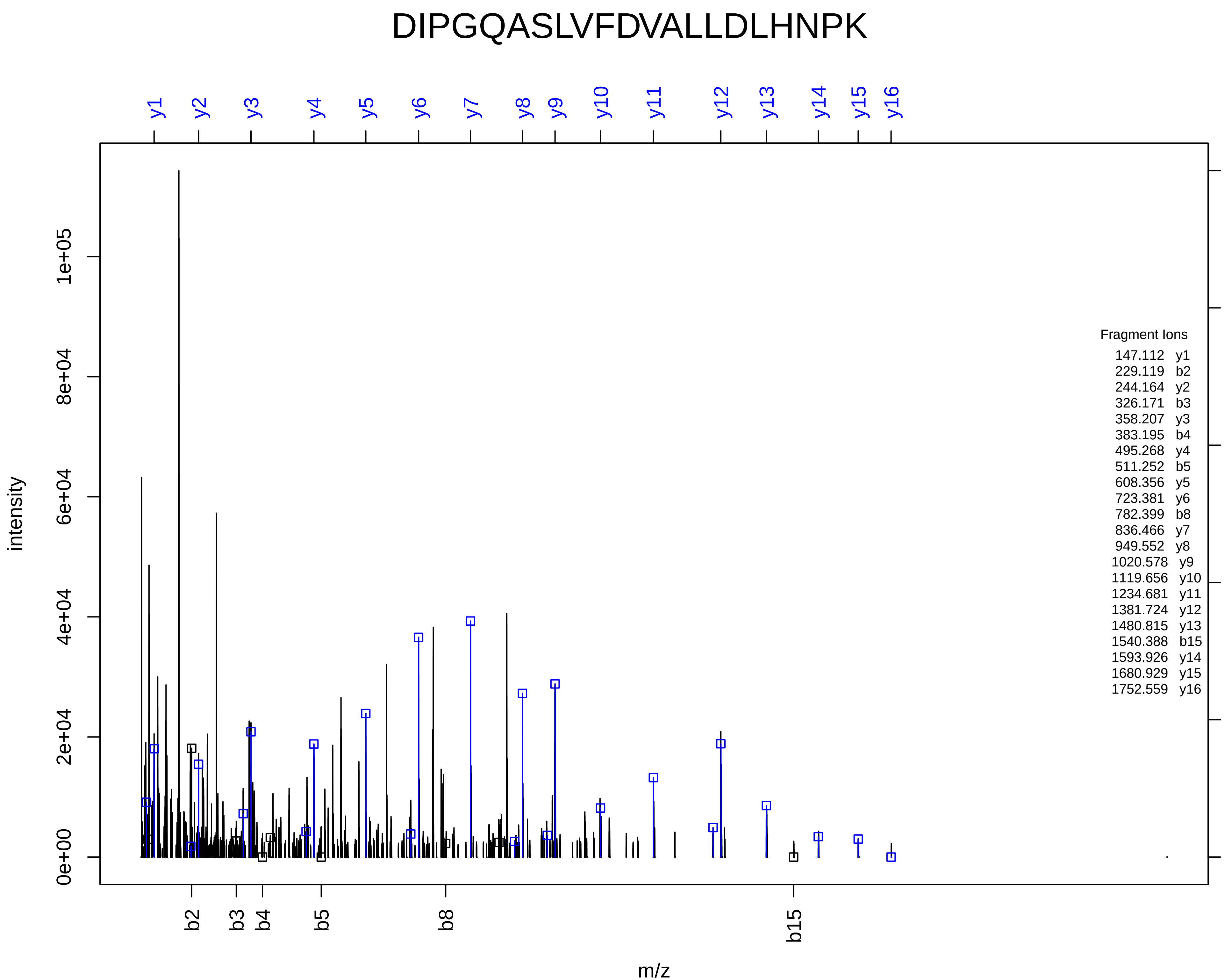

DIPGQASLVFDVALLDLHNPK / File: christine_10pools_06.raw; SpectrumID: ; scans: 38788

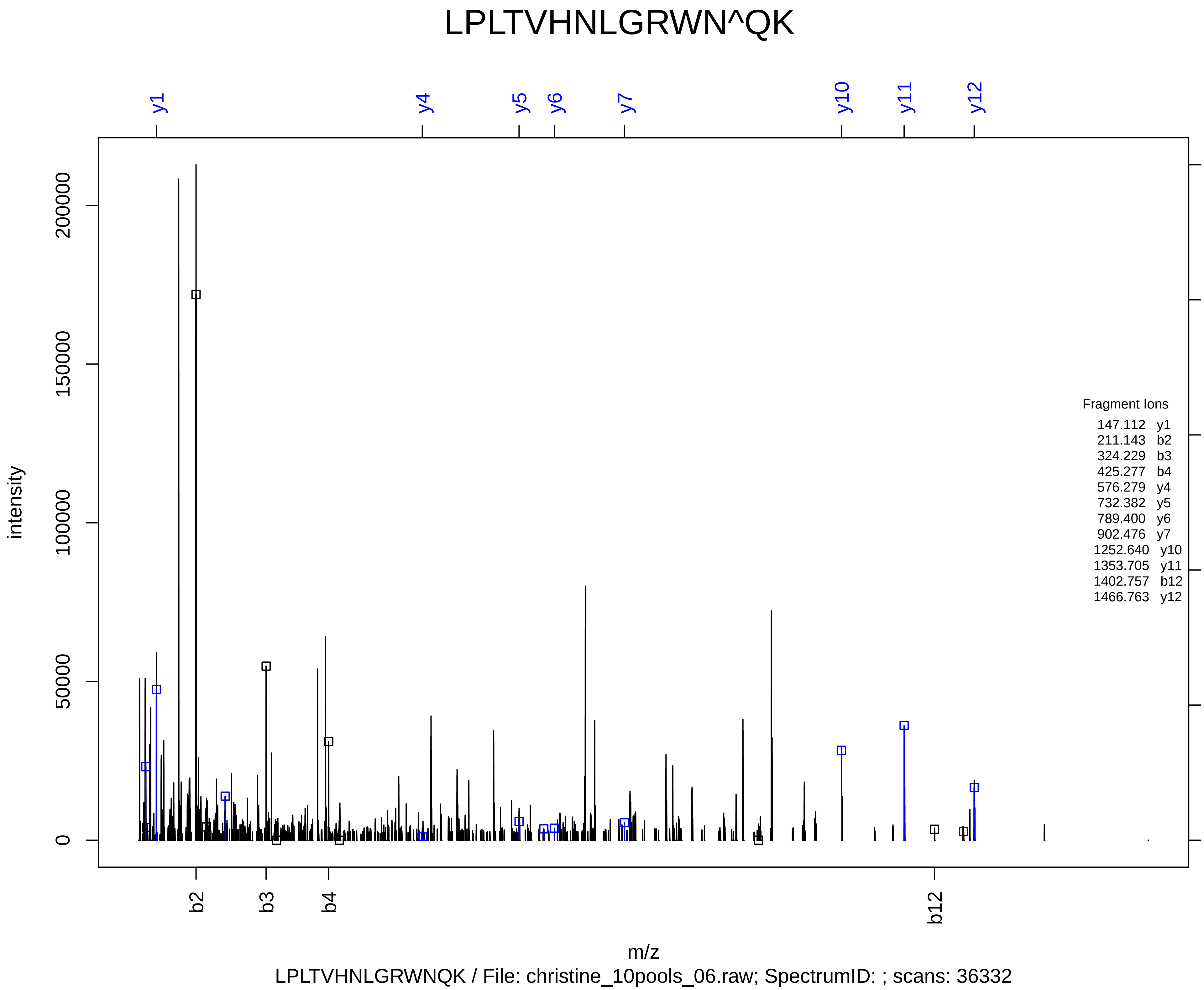

LPLTVHNLGRWN^QK
y1
y4
y5
y6
y7
y10
y11
y12
intensity
0
50000
100000
150000
200000
0
20
40
60
80
100
b2
b3
b4
b12
m/z
Fragment Ions
147.112 y1
211.143 b2
324.229 b3
425.277 b4
576.279 y4
732.382 y5
789.400 y6
902.476 y7
1252.640 y10
1353.705 y11
1402.757 b12
1466.763 y12
LPLTVHNLGRWNQK / File: christine_10pools_06.raw; SpectrumID: ; scans: 36332

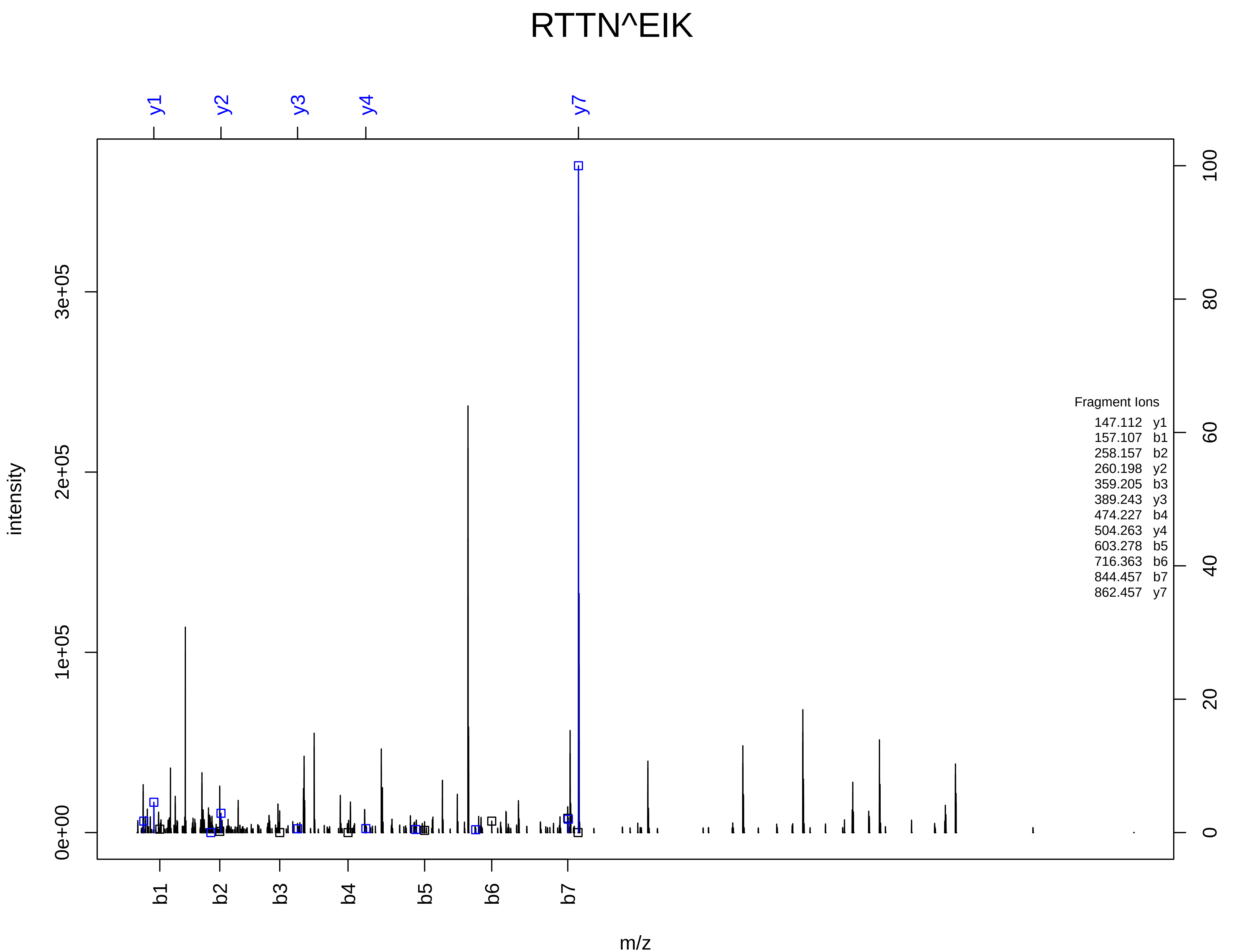

RTTN^EIK
y1
y2
y3
y4
y7
intensity
0e+00
1e+05
2e+05
3e+05
0
20
40
60
80
100
b1
b2
b3
b4
b5
b6
b7
m/z
Fragment Ions
147.112 y1
157.107 b1
258.157 b2
260.198 y2
359.205 b3
389.243 y3
474.227 b4
504.263 y4
603.278 b5
716.363 b6
844.457 b7
862.457 y7
RTTNEIK / File: christine_10pools_06.raw; SpectrumID: ; scans: 6320

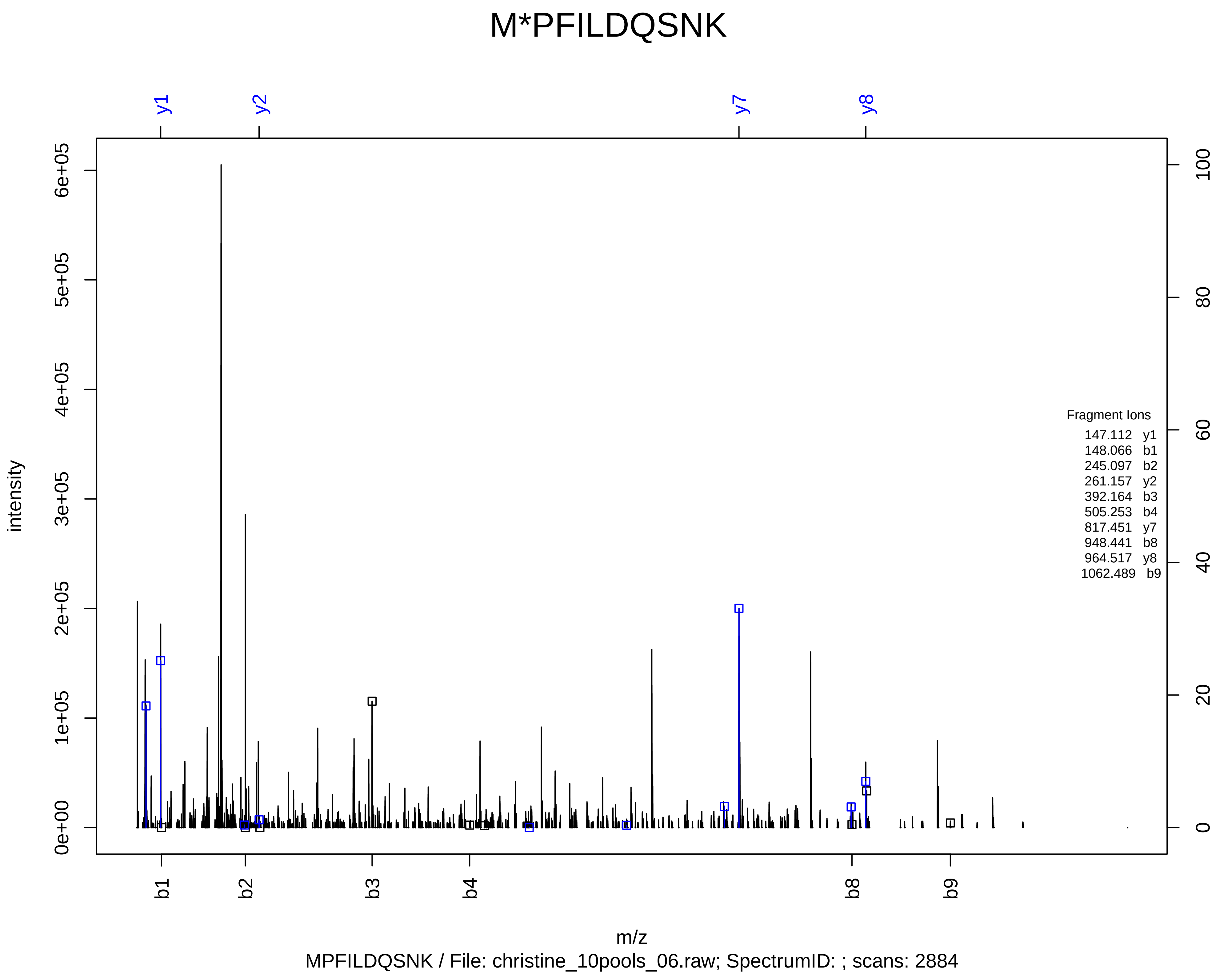
M*PFILDQSNK
y1
y2
y7
y8
intensity
6e+05
5e+05
4e+05
3e+05
2e+05
1e+05
0e+00
100
80
60
40
20
0
Fragment Ions
147.112 y1
148.066 b1
245.097 b2
261.157 y2
392.164 b3
505.253 b4
817.451 y7
948.441 b8
964.517 y8
1062.489 b9
b1
b2
b3
b4
b8
b9
m/z
MPFILDQSNK / File: christine_10pools_06.raw; SpectrumID: ; scans: 2884

LGEHNIN^VLEGNEQFIDAANIIKHPK

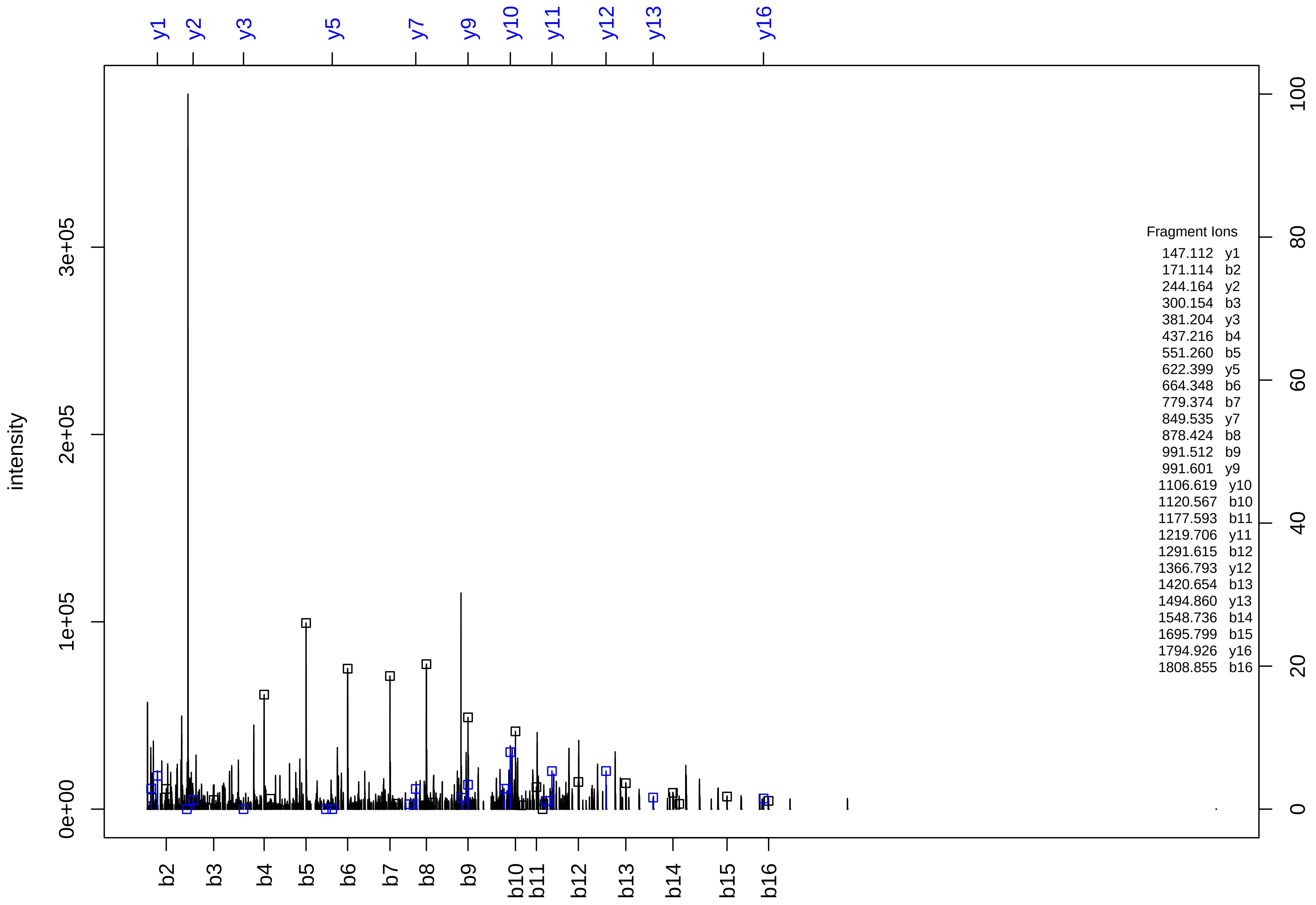

LGEHNINVLEGNEQFIDAANIIKHPK / File: christine_10pools_06.raw; SpectrumID: ; scans: 26305

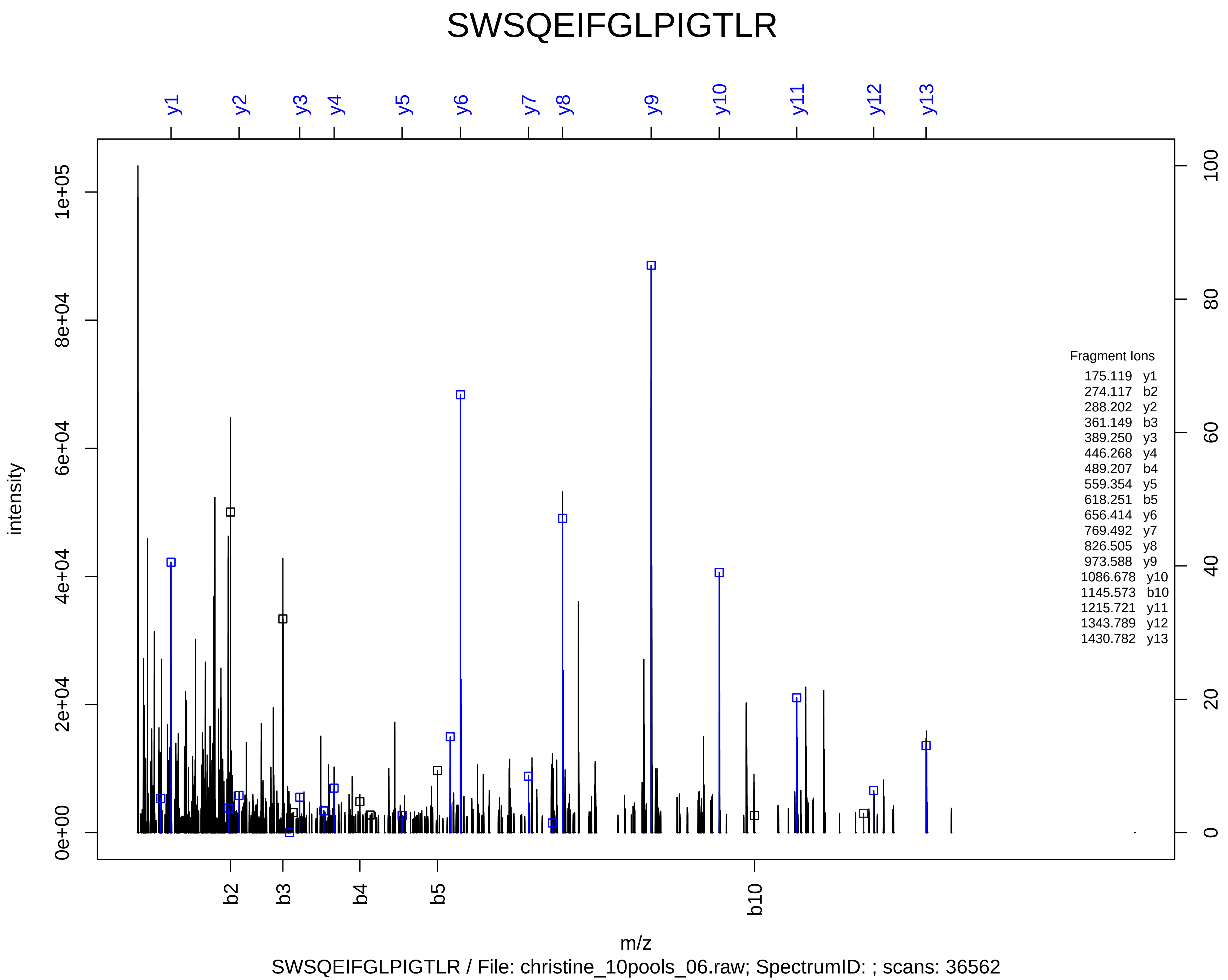
SWSQEIFGLPIGTLR
y1
y2
y3
y4
y5
y6
y7
y8
y9
y10
y11
y12
y13
intensity
0e+00
2e+04
4e+04
6e+04
8e+04
1e+05
0
20
40
60
80
100
b2
b3
b4
b5
b10
m/z
Fragment Ions
175.119 y1
274.117 b2
288.202 y2
361.149 b3
389.250 y3
446.268 y4
489.207 b4
559.354 y5
618.251 b5
656.414 y6
769.492 y7
826.505 y8
973.588 y9
1086.678 y10
1145.573 b10
1215.721 y11
1343.789 y12
1430.782 y13
SWSQEIFGLPIGTLR / File: christine_10pools_06.raw; SpectrumID: ; scans: 36562

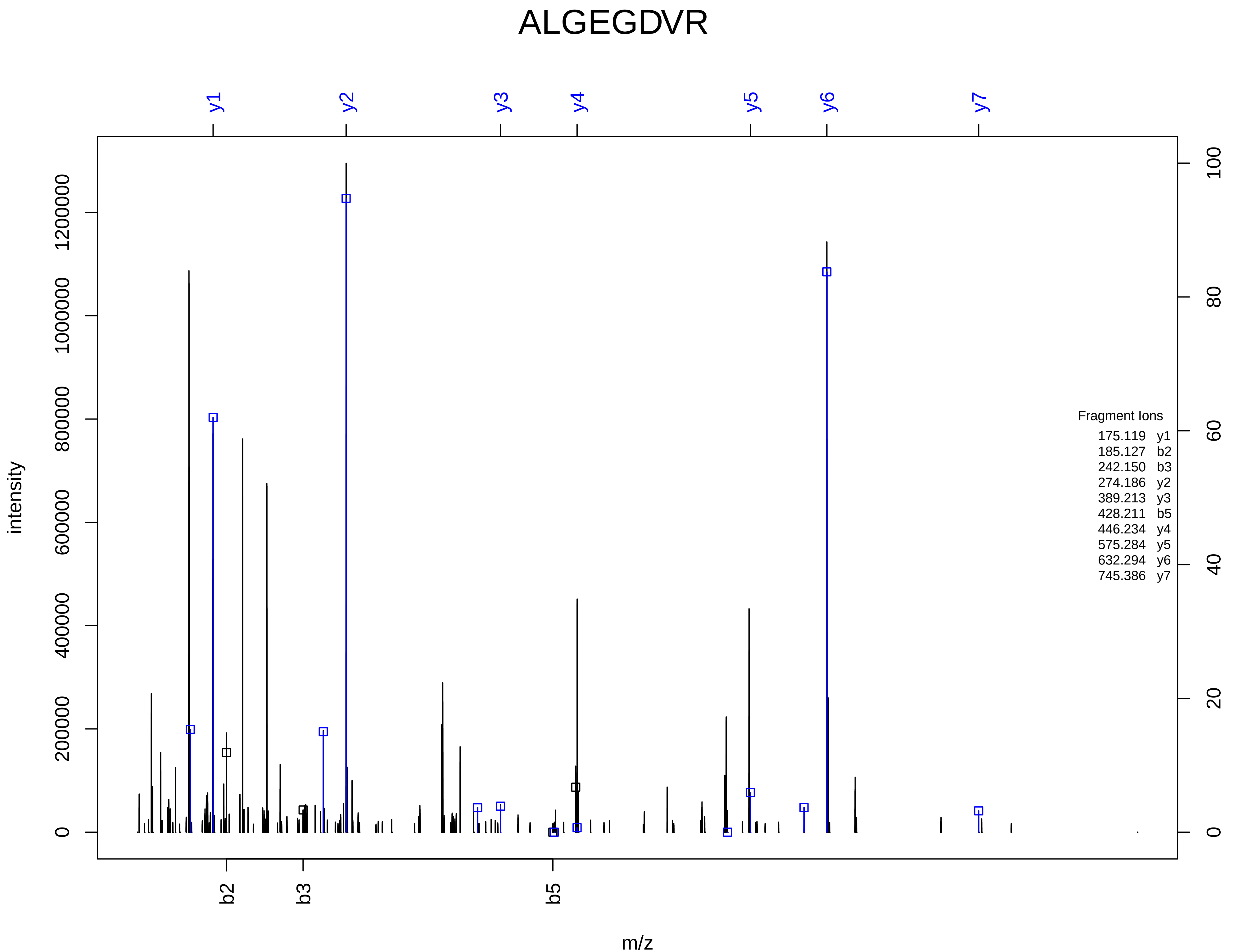
ALGEGDVR
y1
y2
y3
y4
y5
y6
y7
b2
b3
b5
intensity
m/z
0
200000
400000
600000
800000
1000000
1200000
0
20
40
60
80
100
Fragment Ions
175.119 y1
185.127 b2
242.150 b3
274.186 y2
389.213 y3
428.211 b5
446.234 y4
575.284 y5
632.294 y6
745.386 y7
ALGEGDVR / File: christine_10pools_06.raw; SpectrumID: ; scans: 1325

ITLAEALLHPFFAGLTPEER

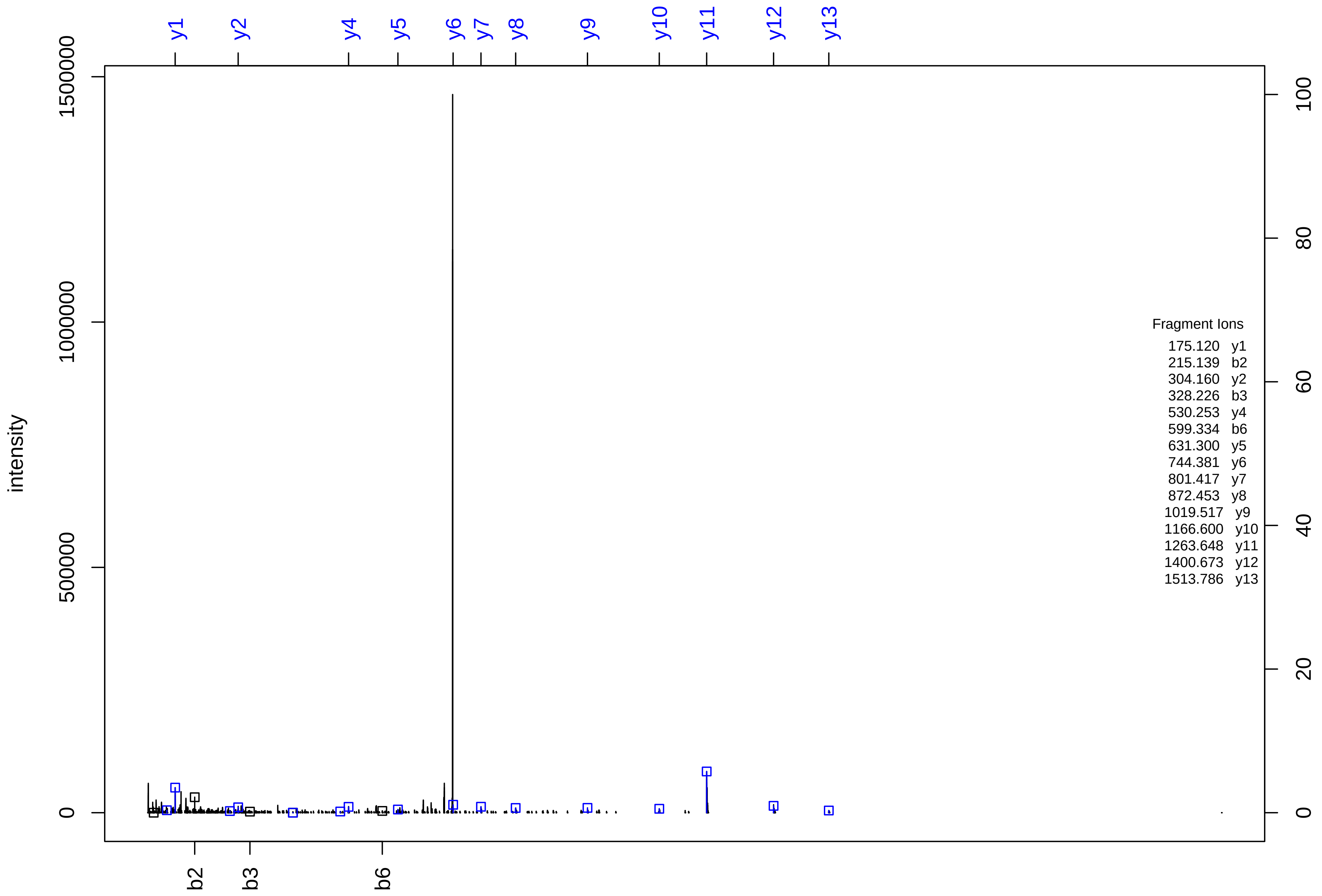

ITLAEALLHPFFAGLTPEER / File: christine_10pools_09.raw; SpectrumID: ; scans: 41542

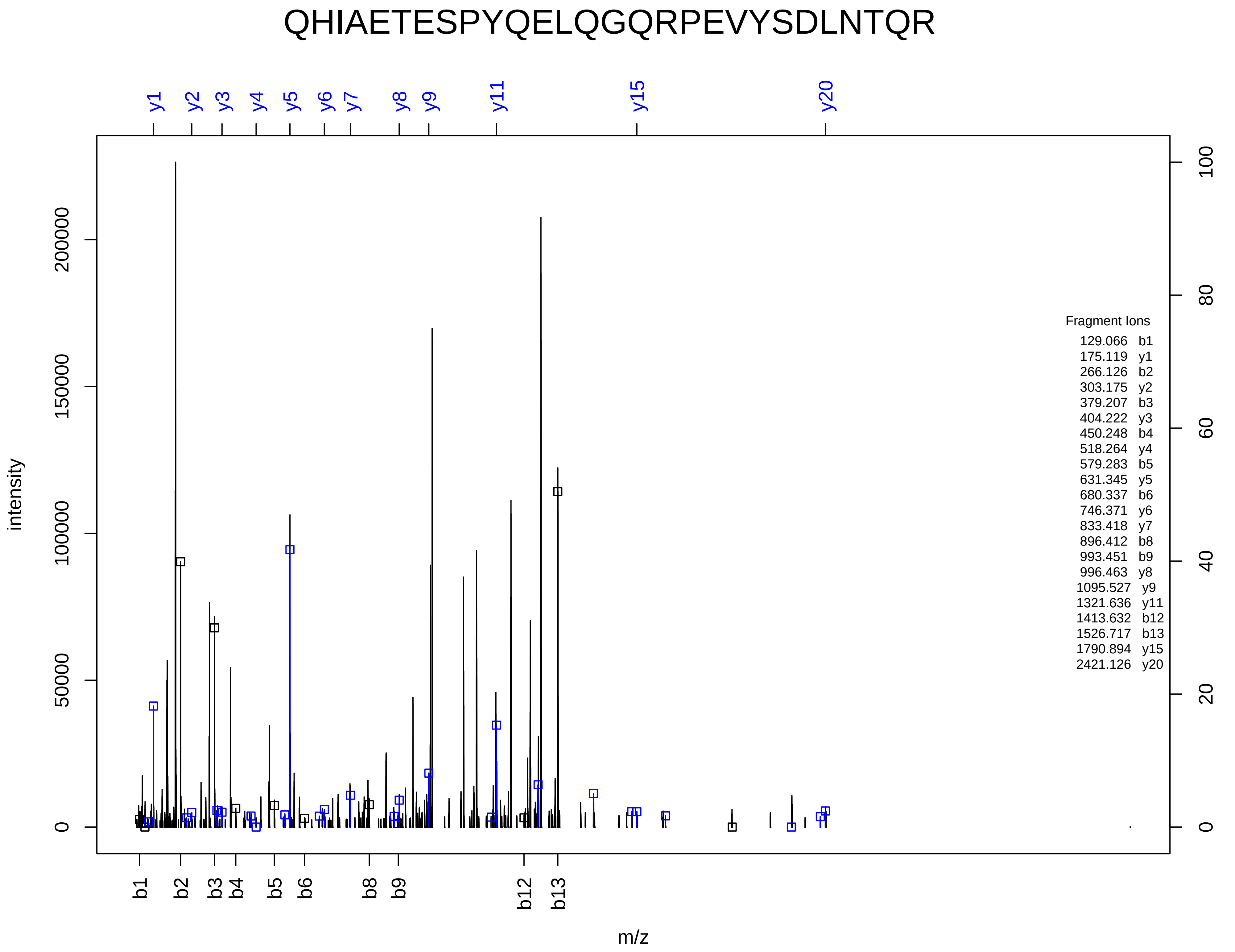
QHIAETESPYQELQGQRPEVYSDLNTQR
y1
y2
y3
y4
y5
y6
y7
y8
y9
y11
y15
y20
intensity
0
50000
100000
150000
200000
0
20
40
60
80
100
Fragment Ions
129.066 b1
175.119 y1
266.126 b2
303.175 y2
379.207 b3
404.222 y3
450.248 b4
518.264 y4
579.283 b5
631.345 y5
680.337 b6
746.371 y6
833.418 y7
896.412 b8
993.451 b9
996.463 y8
1095.527 y9
1321.636 y11
1413.632 b12
1526.717 b13
1790.894 y15
2421.126 y20
b1
b2
b3
b4
b5
b6
b8
b9
b12
b13
m/z
QHIAETESPYQELQGQRPEVYSDLNTQR / File: christine_10pools_09.raw; SpectrumID: ; scans: 11990

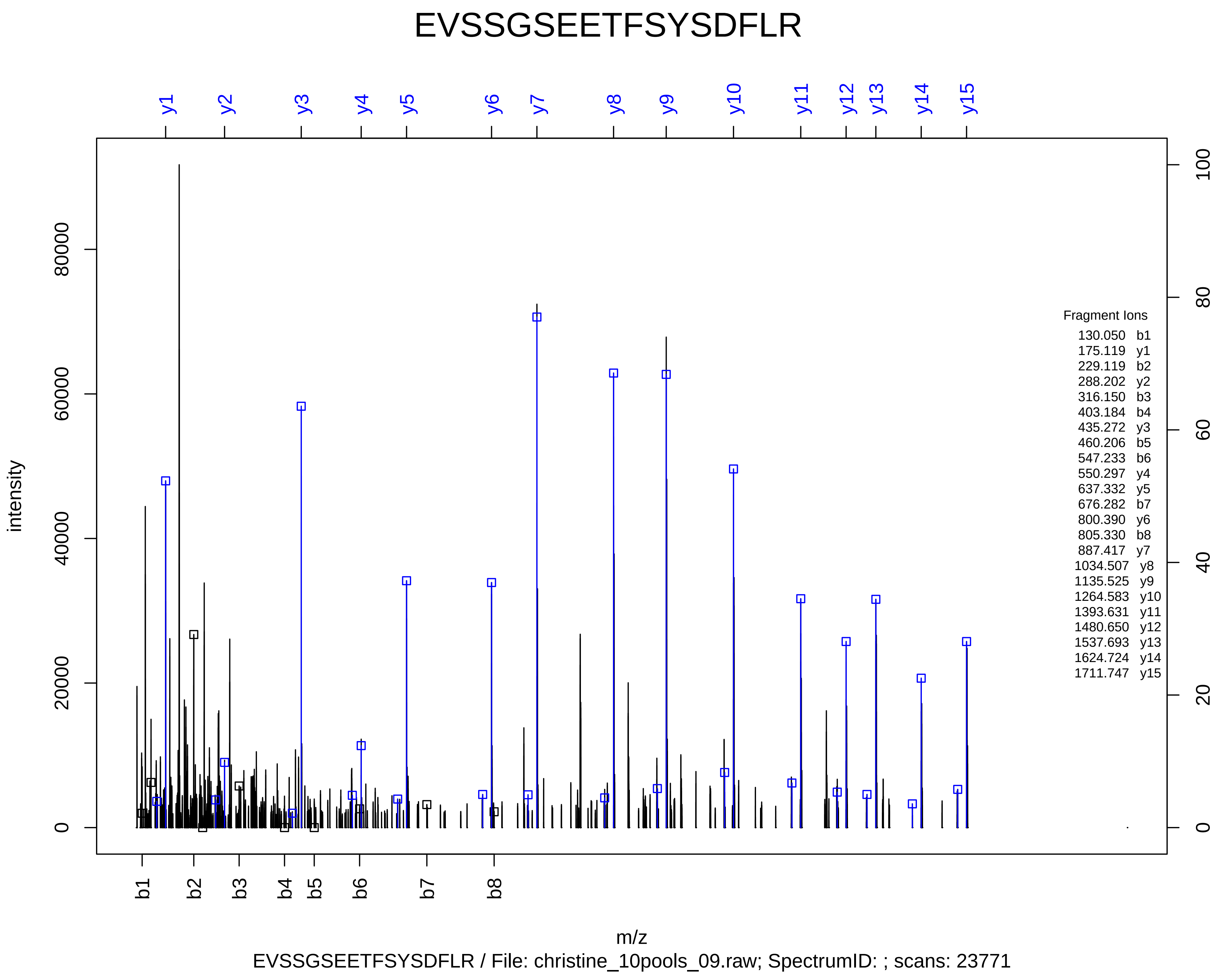
EVSSGSEETFSYSDFLR
y1 y2 y3 y4 y5 y6 y7 y8 y9 y10 y11 y12 y13 y14 y15
b1 b2 b3 b4 b5 b6 b7 b8
intensity
0 20000 40000 60000 80000
0 20 40 60 80 100
m/z
Fragment Ions
130.050 b1
175.119 y1
229.119 b2
288.202 y2
316.150 b3
403.184 b4
435.272 y3
460.206 b5
547.233 b6
550.297 y4
637.332 y5
676.282 b7
800.390 y6
805.330 b8
887.417 y7
1034.507 y8
1135.525 y9
1264.583 y10
1393.631 y11
1480.650 y12
1537.693 y13
1624.724 y14
1711.747 y15
EVSSGSEETFSYSDFLR / File: christine_10pools_09.raw; SpectrumID: ; scans: 23771

NWPFLEDCACTPER

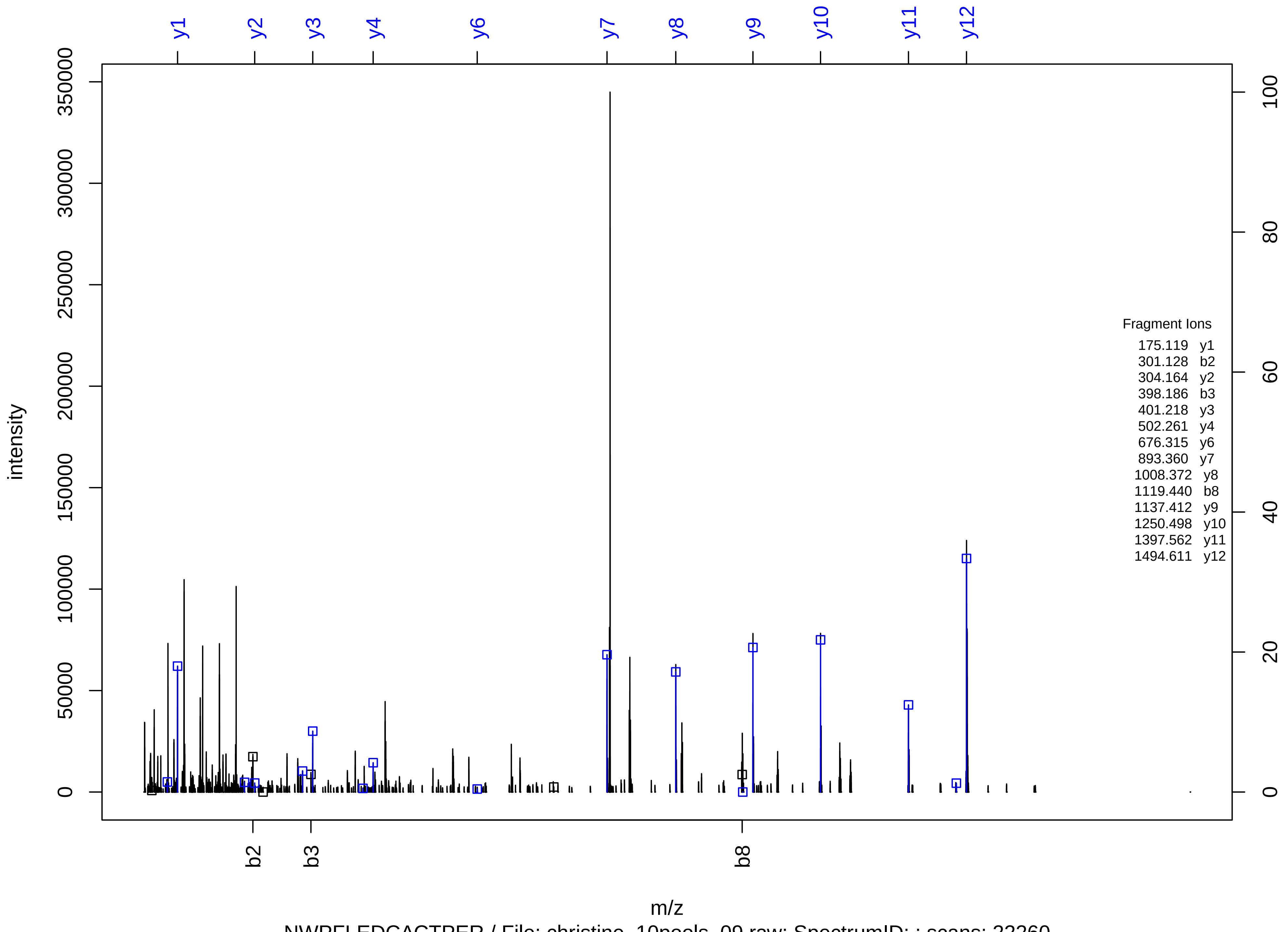

NWPFLEDCACTPER / File: christine_10pools_09.raw; SpectrumID: ; scans: 22260

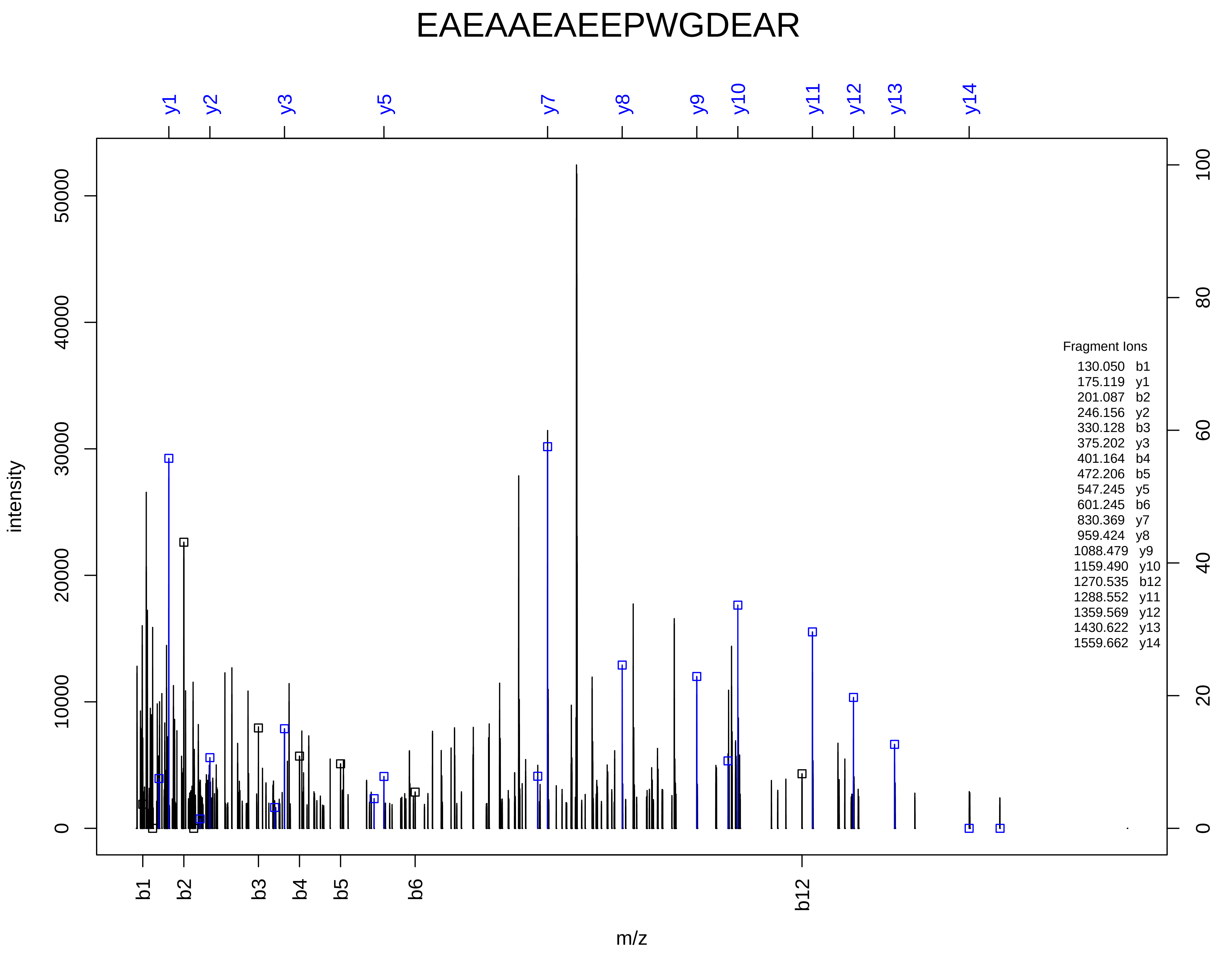

EAEAAEAEEPWGDEAR / File: christine_10pools_09.raw; SpectrumID: ; scans: 7869

ILDETQEAVEYQR

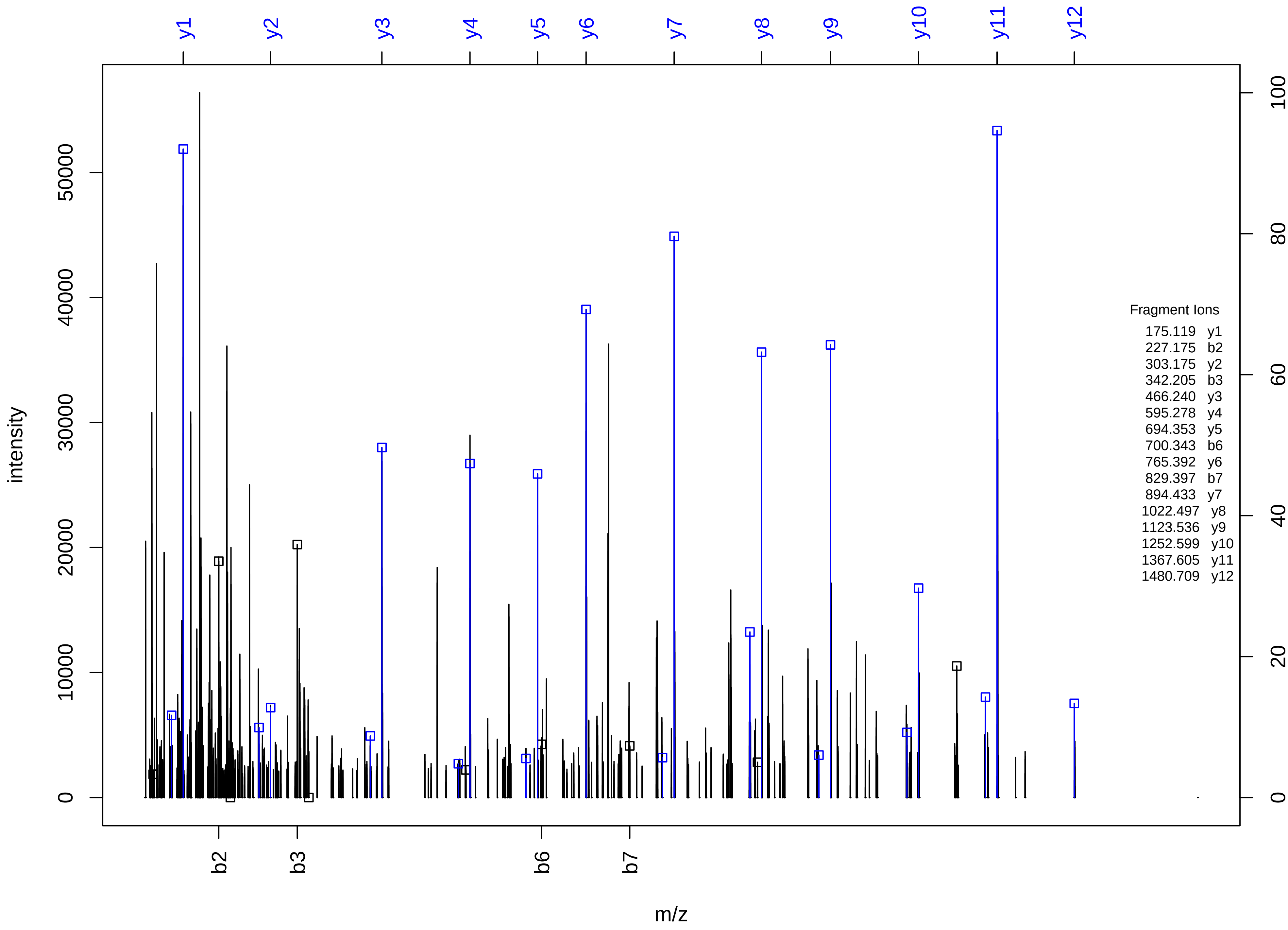

ILDETQEAVEYQR / File: christine_10pools_09.raw; SpectrumID: ; scans: 7987

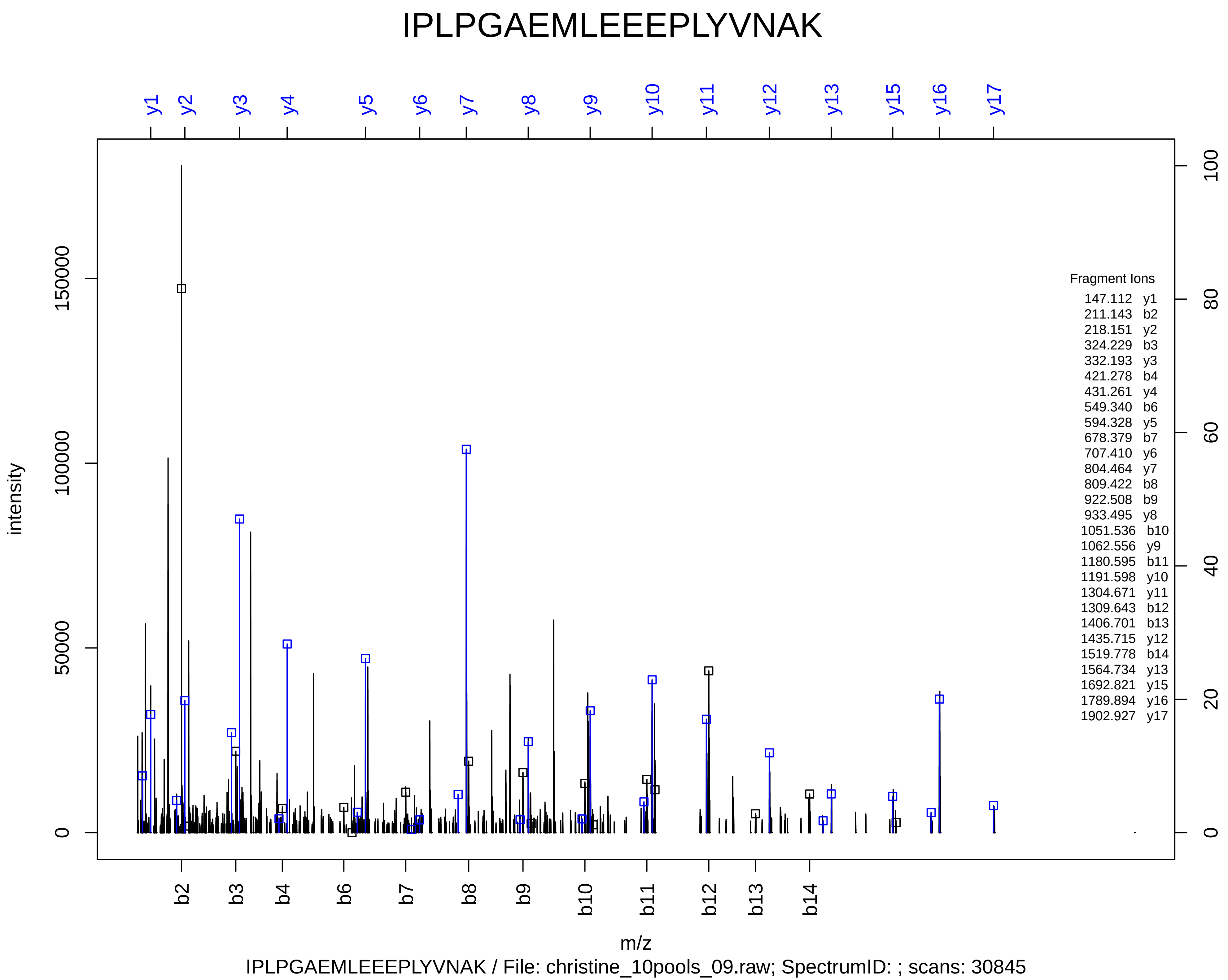

IPLPGAEMLEEEPLYVNAK
intensity
m/z
Fragment Ions
147.112 y1
211.143 b2
218.151 y2
324.229 b3
332.193 y3
421.278 b4
431.261 y4
549.340 b6
594.328 y5
678.379 b7
707.410 y6
804.464 y7
809.422 b8
922.508 b9
933.495 y8
1051.536 b10
1062.556 y9
1180.595 b11
1191.598 y10
1304.671 y11
1309.643 b12
1406.701 b13
1435.715 y12
1519.778 b14
1564.734 y13
1692.821 y15
1789.894 y16
1902.927 y17
IPLPGAEMLEEEPLYVNAK / File: christine_10pools_09.raw; SpectrumID: ; scans: 30845

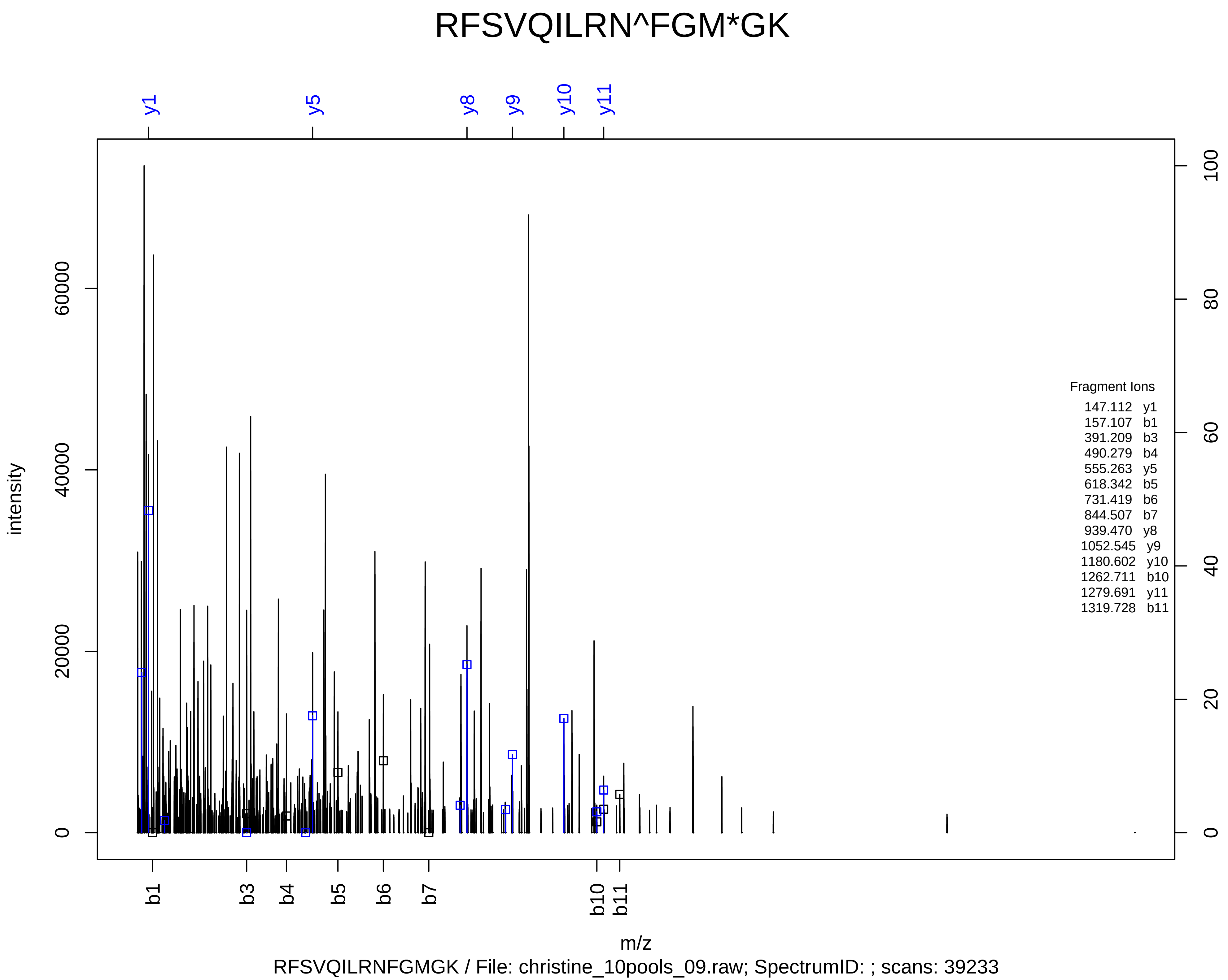

RFSVQILRN^FGM*GK
y1
y5
y8
y9
y10
y11
intensity
0
20000
40000
60000
0
20
40
60
80
100
Fragment Ions
147.112 y1
157.107 b1
391.209 b3
490.279 b4
555.263 y5
618.342 b5
731.419 b6
844.507 b7
939.470 y8
1052.545 y9
1180.602 y10
1262.711 b10
1279.691 y11
1319.728 b11
b1
b3
b4
b5
b6
b7
b10
b11
m/z
RFSVQILRNFGMGK / File: christine_10pools_09.raw; SpectrumID: ; scans: 39233

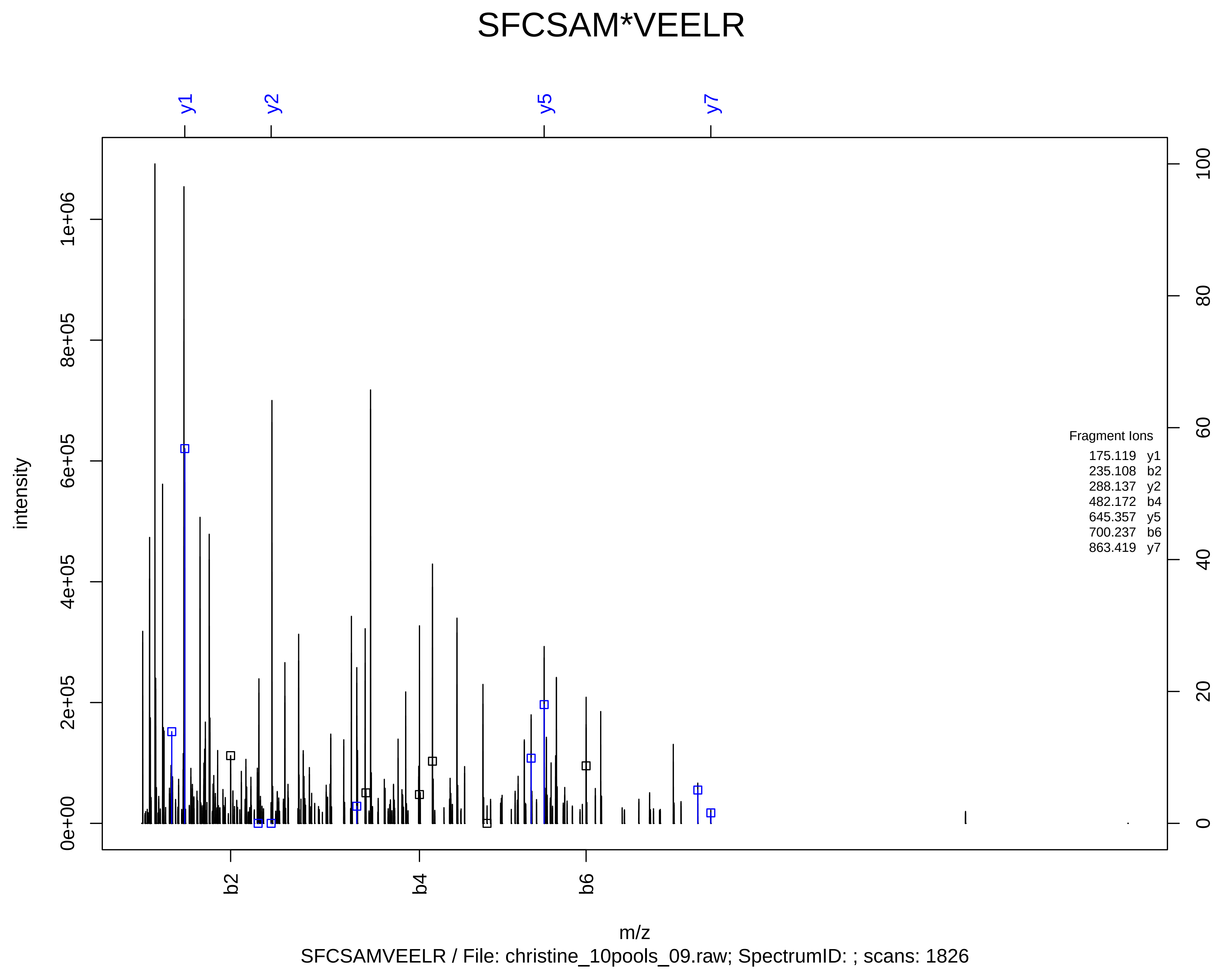
SFCSAM*VEELR
y1
y2
y5
y7
intensity
0e+00
2e+05
4e+05
6e+05
8e+05
1e+06
0
20
40
60
80
100
Fragment Ions
175.119 y1
235.108 b2
288.137 y2
482.172 b4
645.357 y5
700.237 b6
863.419 y7
b2
b4
b6
m/z
SFCSAMVEELR / File: christine_10pools_09.raw; SpectrumID: ; scans: 1826

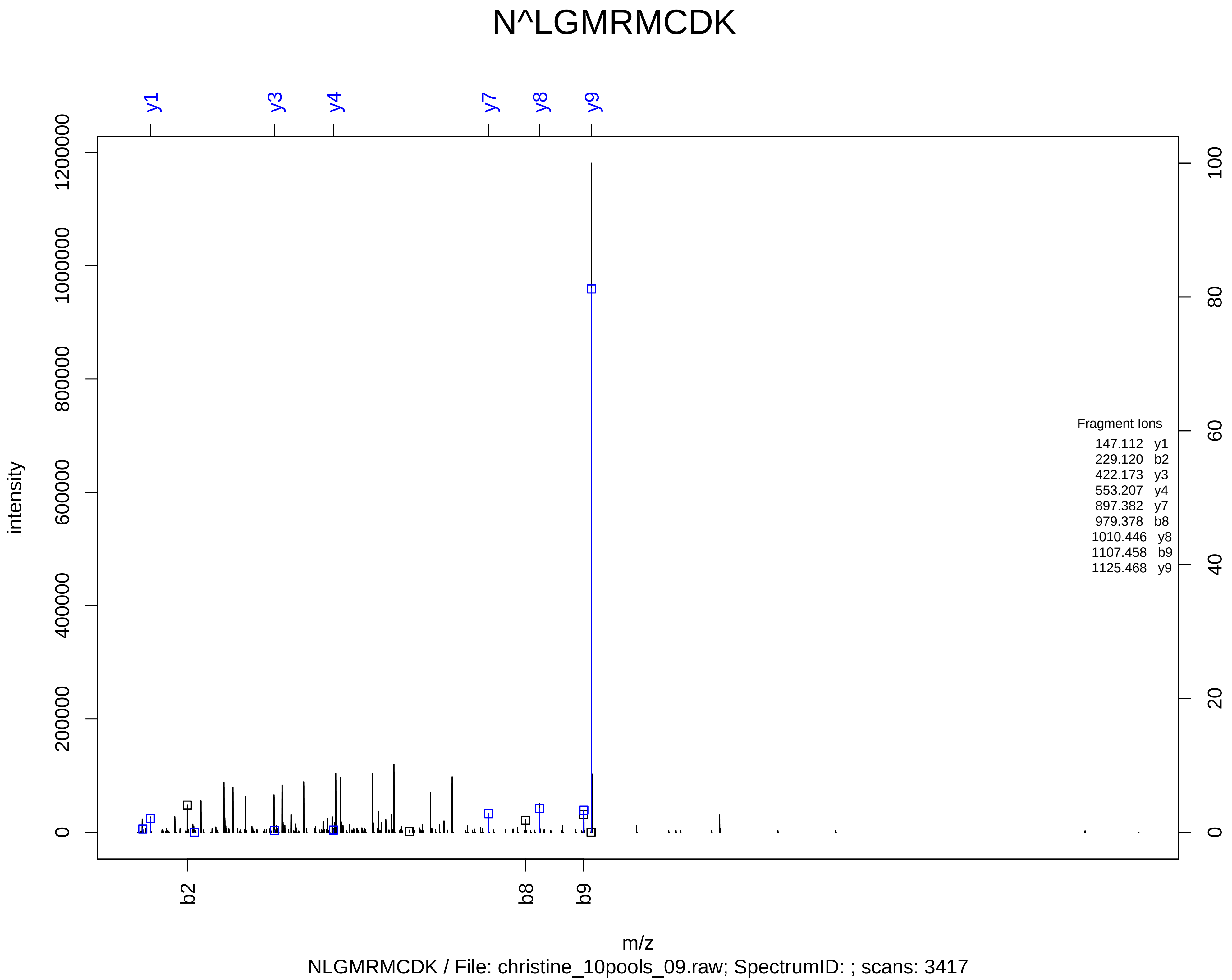

N^LGMRMCDK
y1
y3
y4
y7
y8
y9
intensity
0
200000
400000
600000
800000
1000000
1200000
0
20
40
60
80
100
b2
b8
b9
Fragment Ions
147.112 y1
229.120 b2
422.173 y3
553.207 y4
897.382 y7
979.378 b8
1010.446 y8
1107.458 b9
1125.468 y9
m/z
NLGMRMCDK / File: christine_10pools_09.raw; SpectrumID: ; scans: 3417

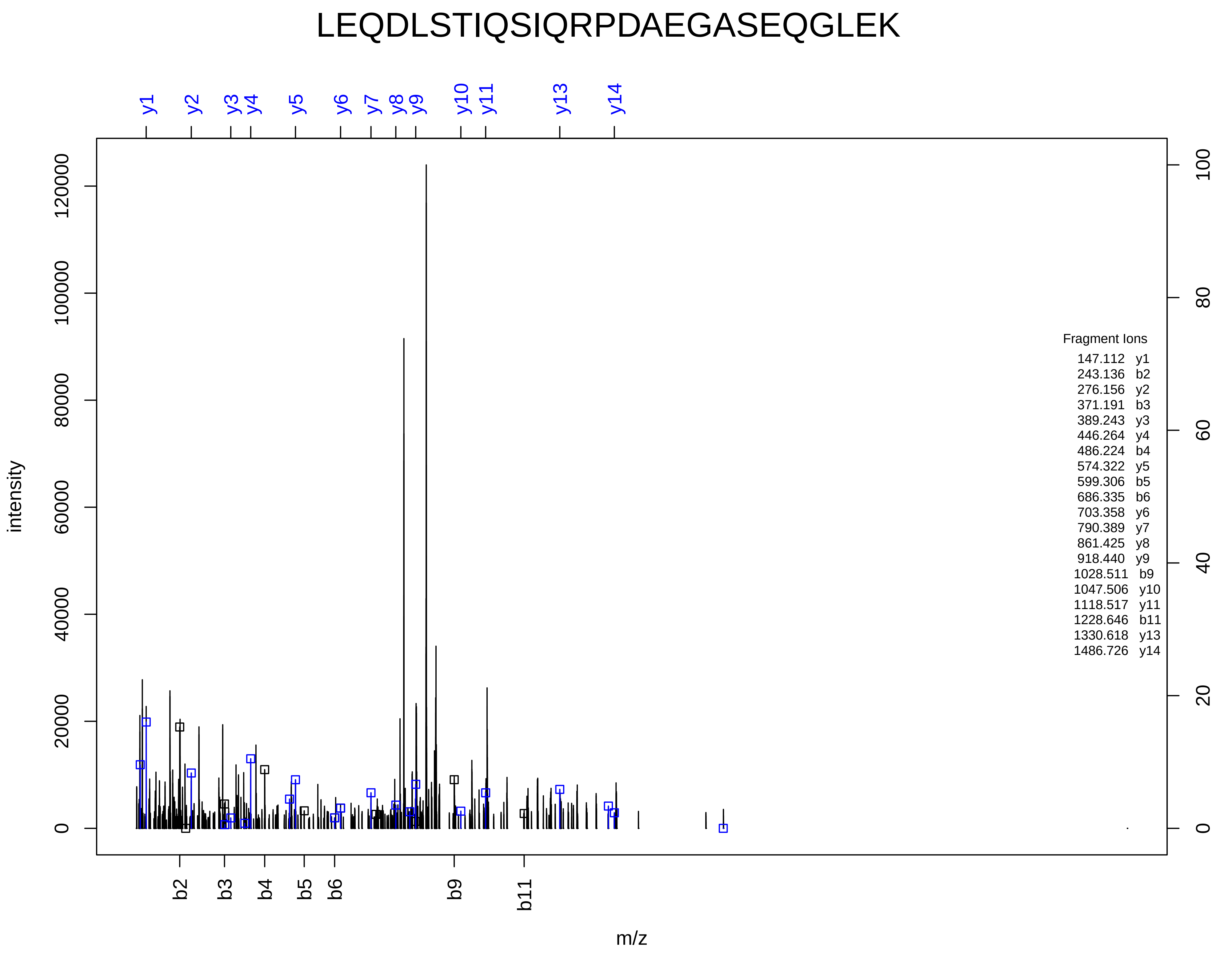
LEQDLSTIQSIQRPDAEGASEQGLEK
y1 y2 y3 y4 y5 y6 y7 y8 y9 y10 y11 y13 y14
b2 b3 b4 b5 b6 b9 b11
intensity
m/z
Fragment Ions
147.112 y1
243.136 b2
276.156 y2
371.191 b3
389.243 y3
446.264 y4
486.224 b4
574.322 y5
599.306 b5
686.335 b6
703.358 y6
790.389 y7
861.425 y8
918.440 y9
1028.511 b9
1047.506 y10
1118.517 y11
1228.646 b11
1330.618 y13
1486.726 y14
LEQDLSTIQSIQRPDAEGASEQGLEK / File: christine_10pools_09.raw; SpectrumID: ; scans: 13230

DEGSSDLAIVSDPSQQLPSPNVNNFLNPAPQENPLK

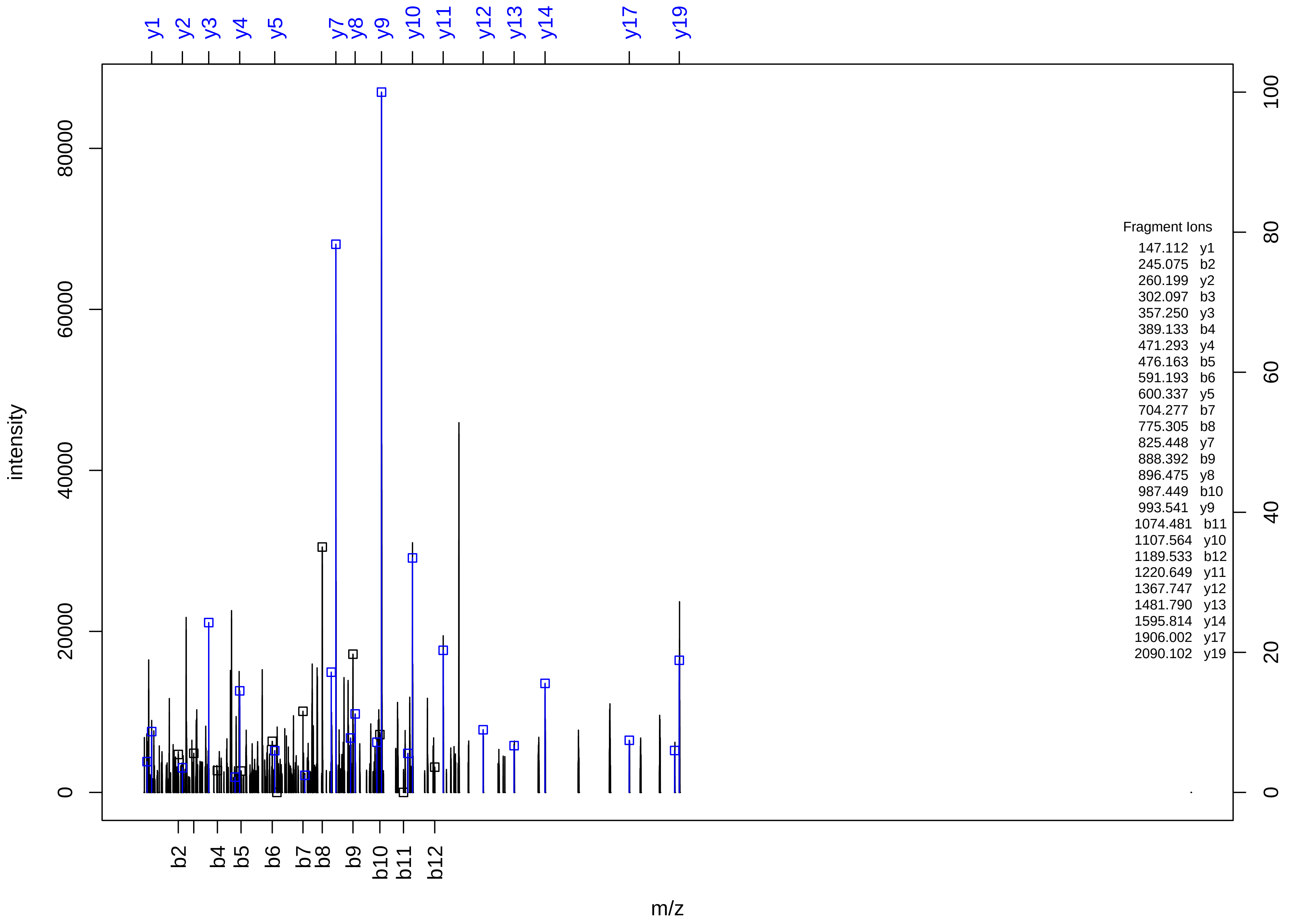

DEGSSDLAIVSDPSQQLPSPNVNNFLNPAPQENPLK / File: christine_10pools_09.raw; SpectrumID: ; scans: 33771

NQNTVSPGNSGDRPQQVSQVSVEAFSGLR

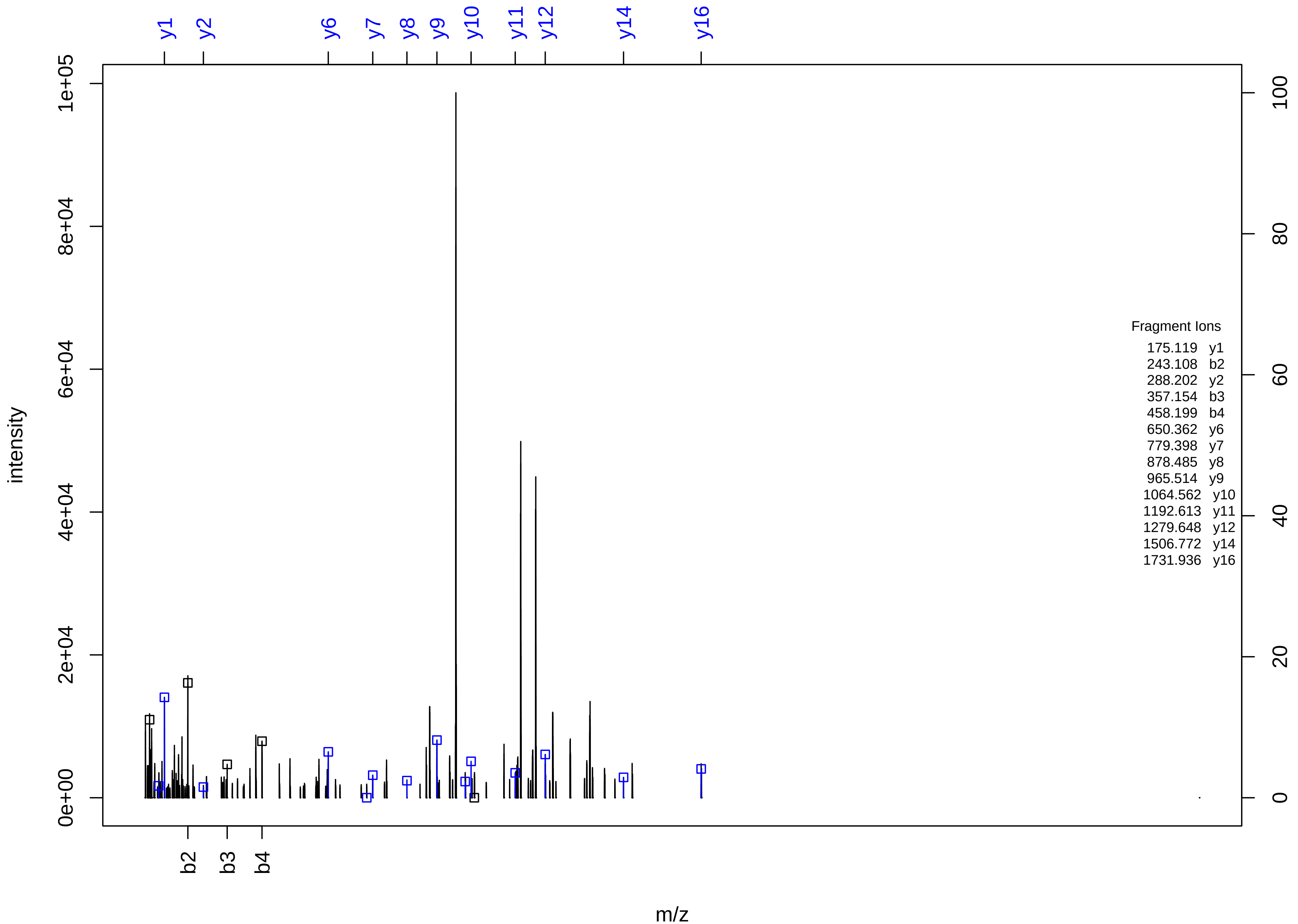

NQNTVSPGNSGDRPQQVSQVSVEAFSGLR / File: christine_10pools_09.raw; SpectrumID: ; scans: 15879

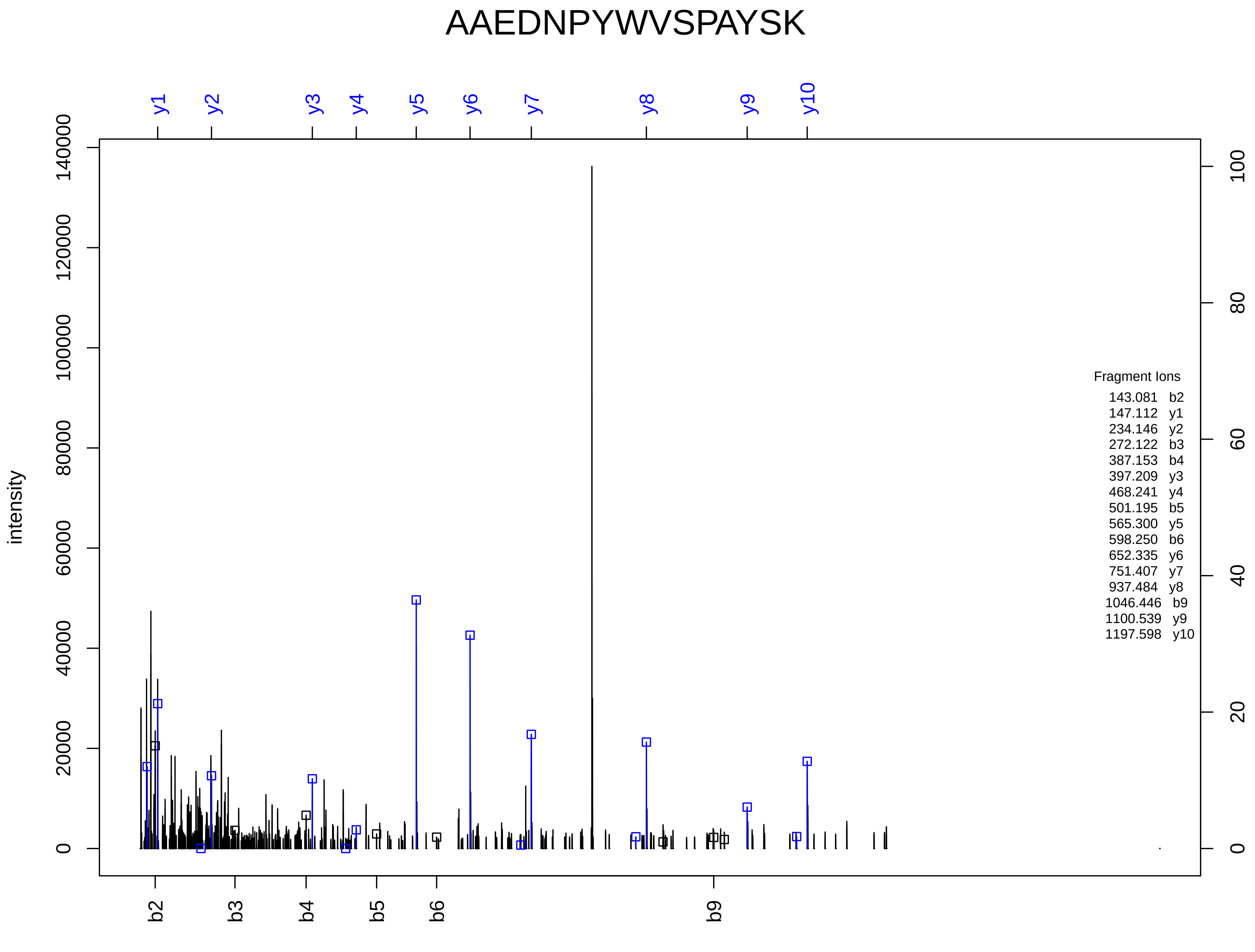

AAEDNPYWVSPAYSK / File: christine_10pools_09.raw; SpectrumID: ; scans: 13101

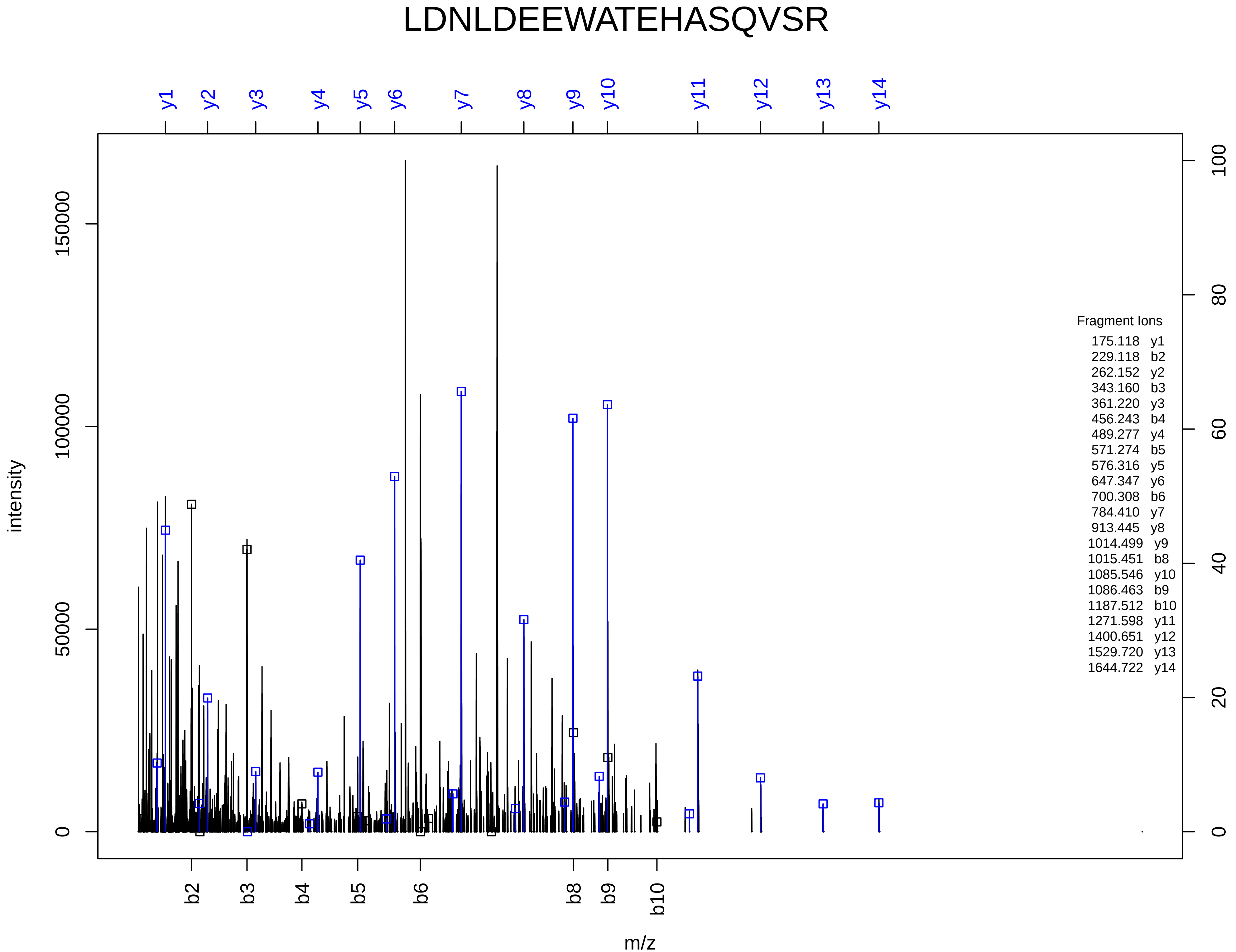

LDNLDEEWATEHASQVSR / File: christine_10pools_09.raw; SpectrumID: ; scans: 10974

DWSDLVTPQGIMGIFAKPDPVK

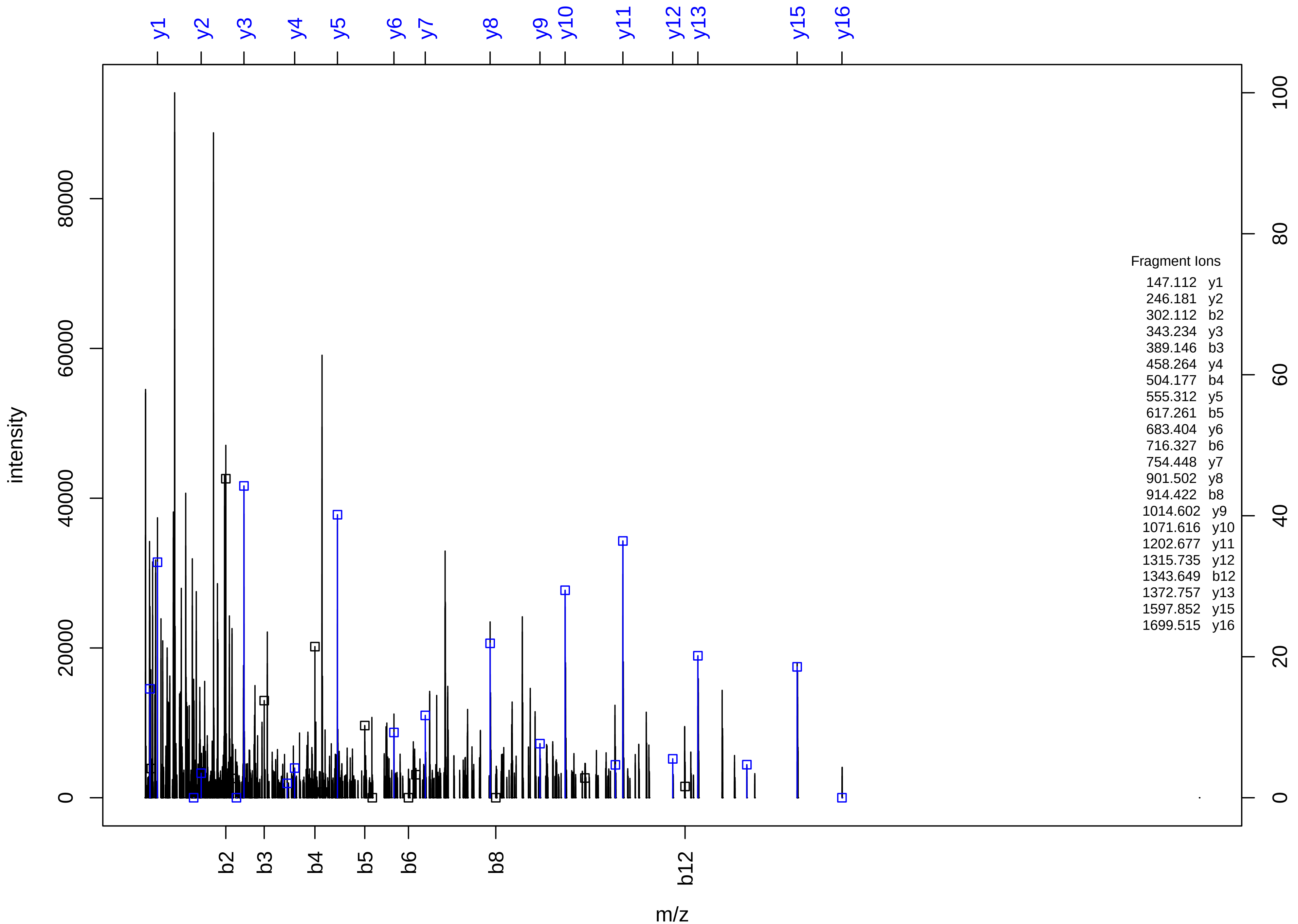

DWSDLVTPQGIMGIFAKPDPVK / File: christine_10pools_09.raw; SpectrumID: ; scans: 41218

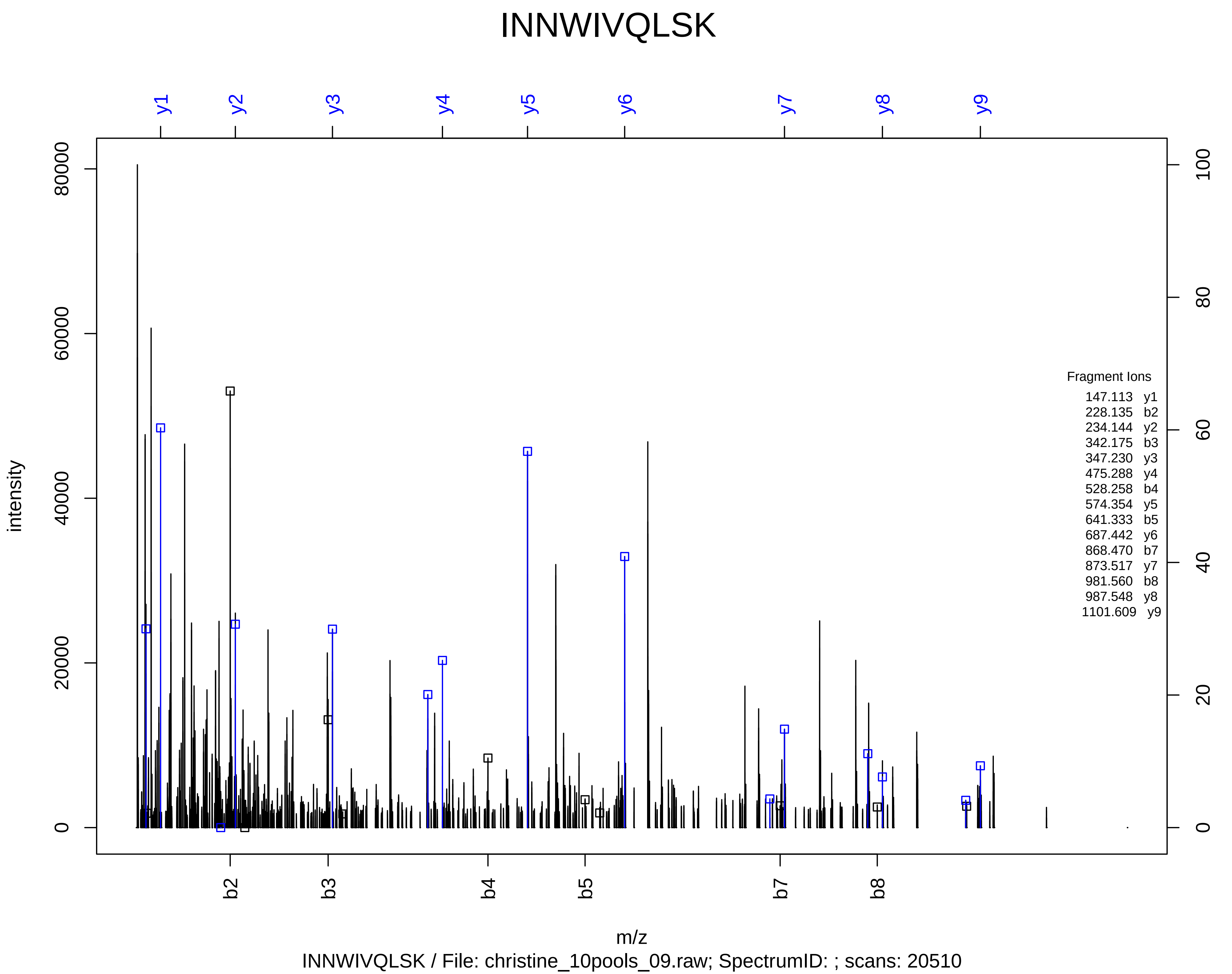
INNWIVQLSK
y1
y2
y3
y4
y5
y6
y7
y8
y9
intensity
80000
60000
40000
20000
0
100
80
60
40
20
0
b2
b3
b4
b5
b7
b8
m/z
Fragment Ions
147.113 y1
228.135 b2
234.144 y2
342.175 b3
347.230 y3
475.288 y4
528.258 b4
574.354 y5
641.333 b5
687.442 y6
868.470 b7
873.517 y7
981.560 b8
987.548 y8
1101.609 y9
INNWIVQLSK / File: christine_10pools_09.raw; SpectrumID: ; scans: 20510

SLQTGFAVGVQQLHPTLIQR

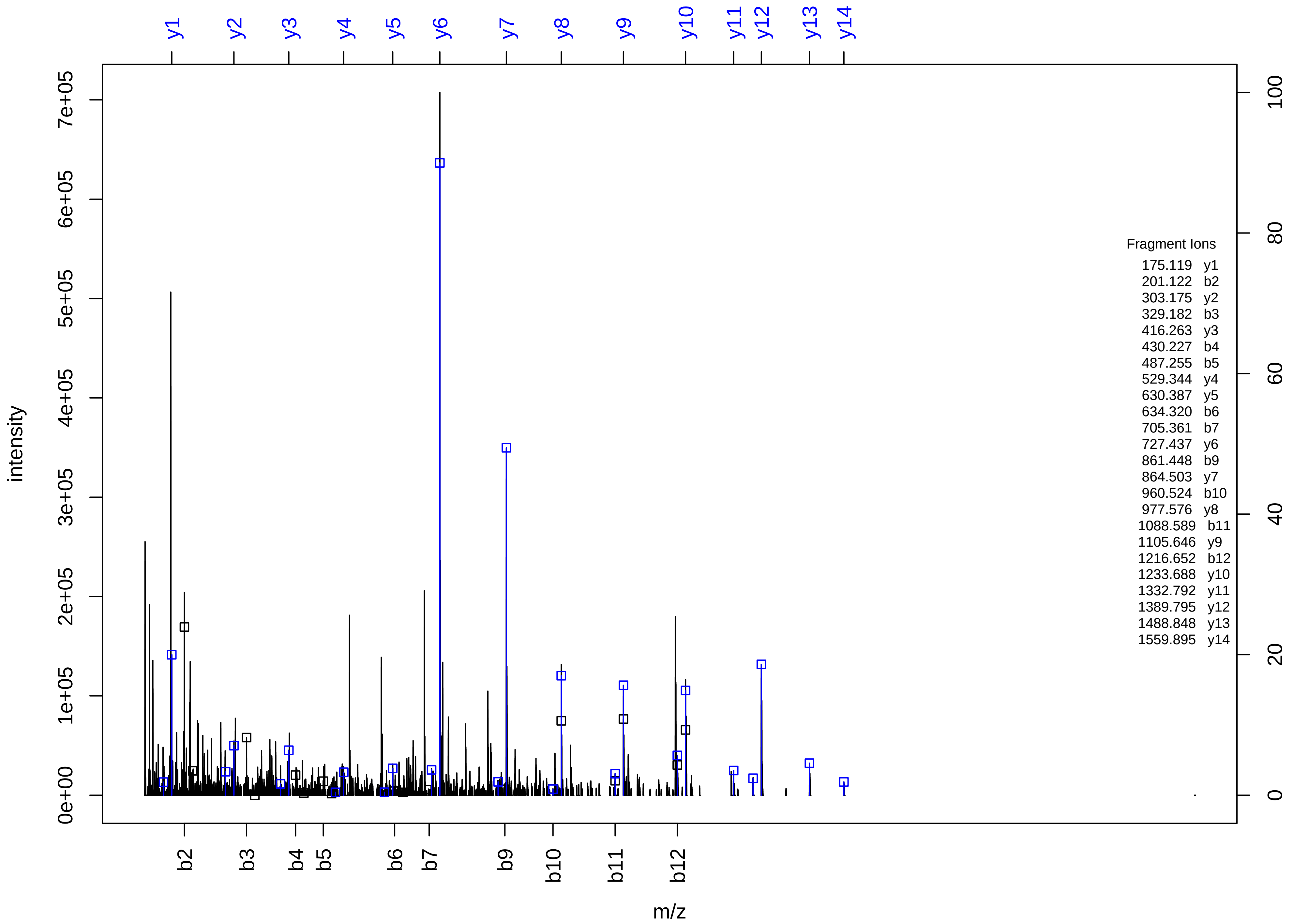

SLQTGFAVGVQQLHPTLIQR / File: christine_10pools_09.raw; SpectrumID: ; scans: 21430

LYSIILSAPIPEPK

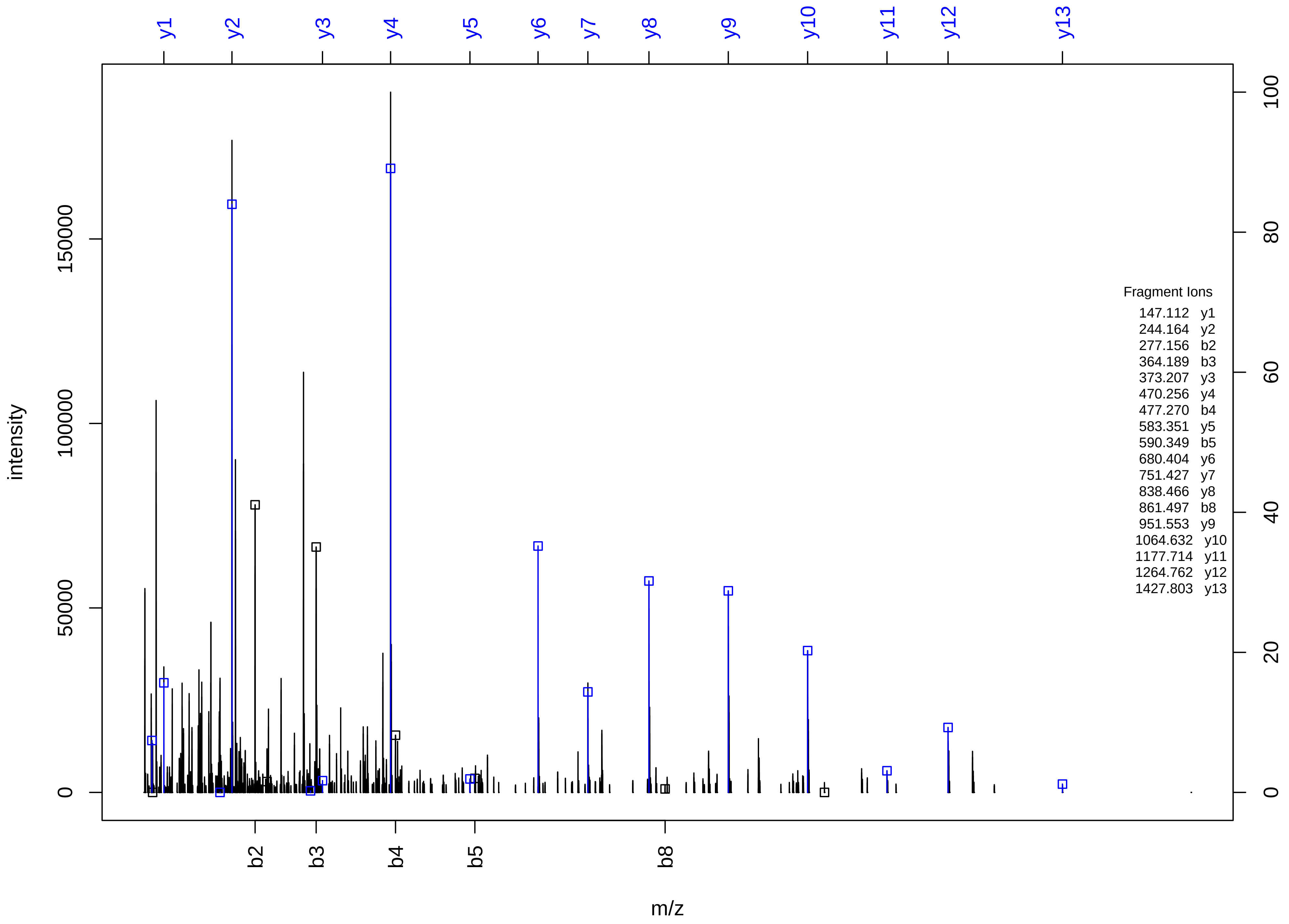

LYSIILSAPIPEPK / File: christine_10pools_09.raw; SpectrumID: ; scans: 27993

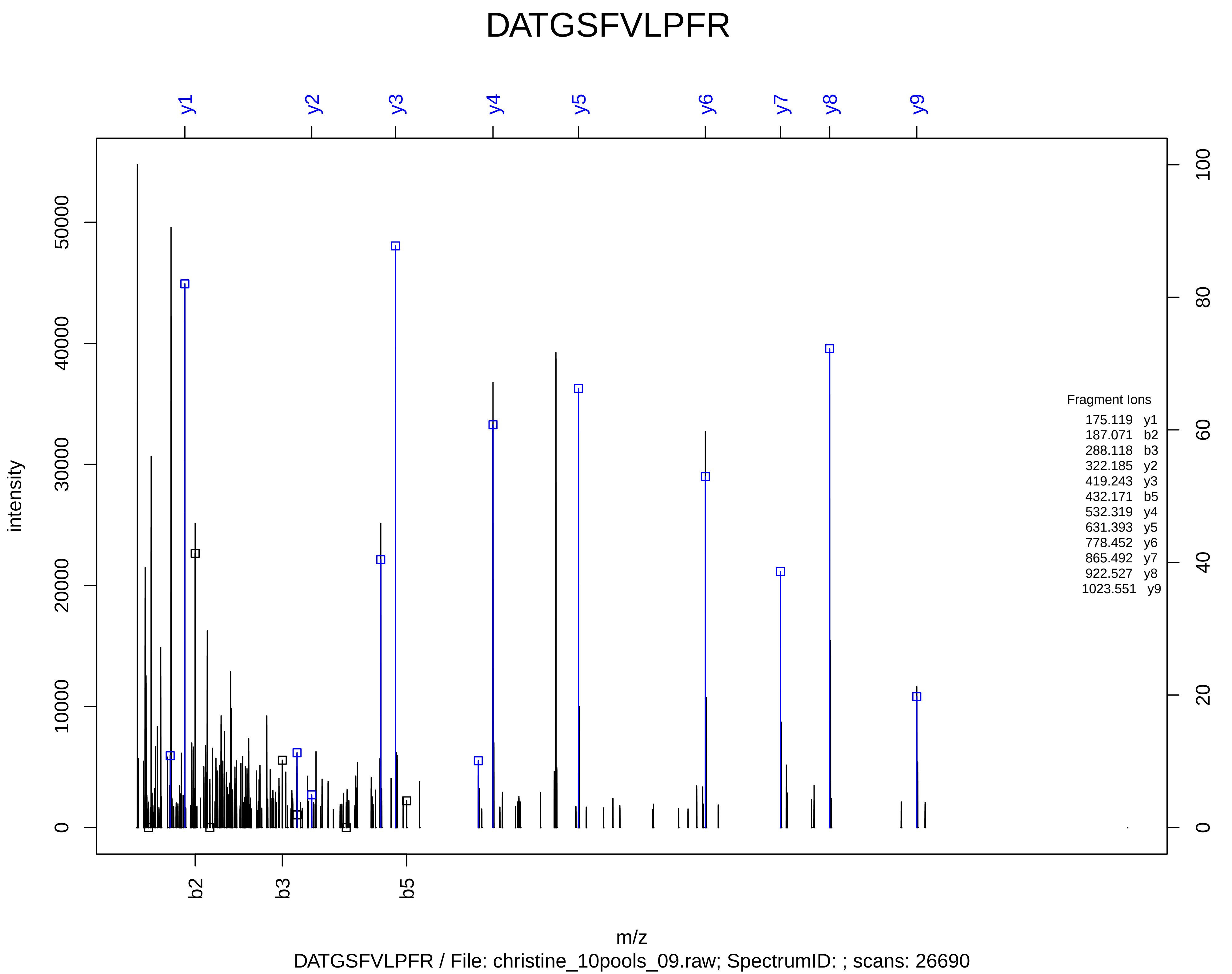
DATGSFVLPFR
y1 y2 y3 y4 y5 y6 y7 y8 y9
b2 b3 b5
intensity
m/z
0
10000
20000
30000
40000
50000
0
20
40
60
80
100
Fragment Ions
175.119 y1
187.071 b2
288.118 b3
322.185 y2
419.243 y3
432.171 b5
532.319 y4
631.393 y5
778.452 y6
865.492 y7
922.527 y8
1023.551 y9
DATGSFVLPFR / File: christine_10pools_09.raw; SpectrumID: ; scans: 26690

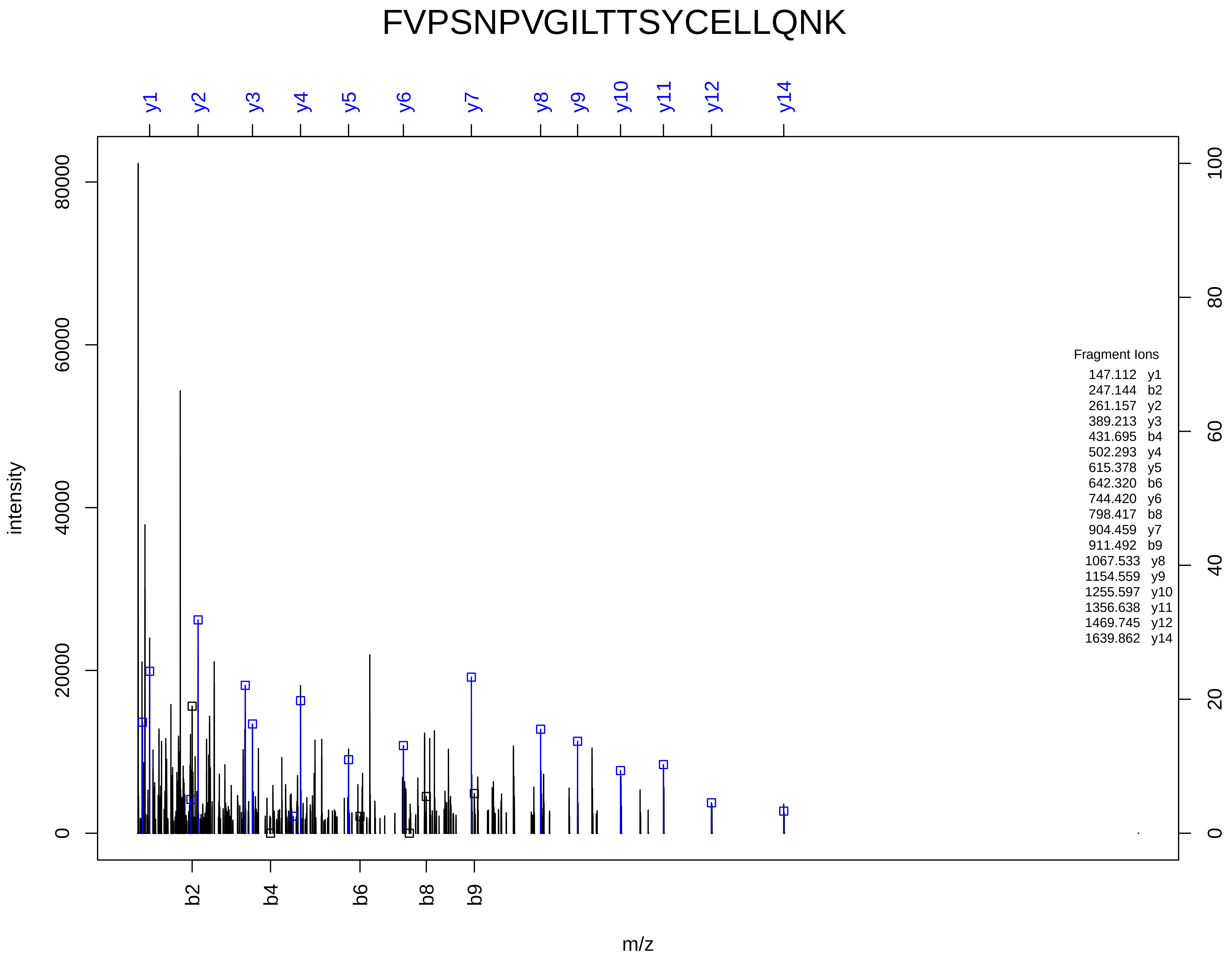

FVPSNPVGILTTSYCELLQNK / File: christine_10pools_09.raw; SpectrumID: ; scans: 40049

LAADPSVVNLGQGFPDISPPSYVK

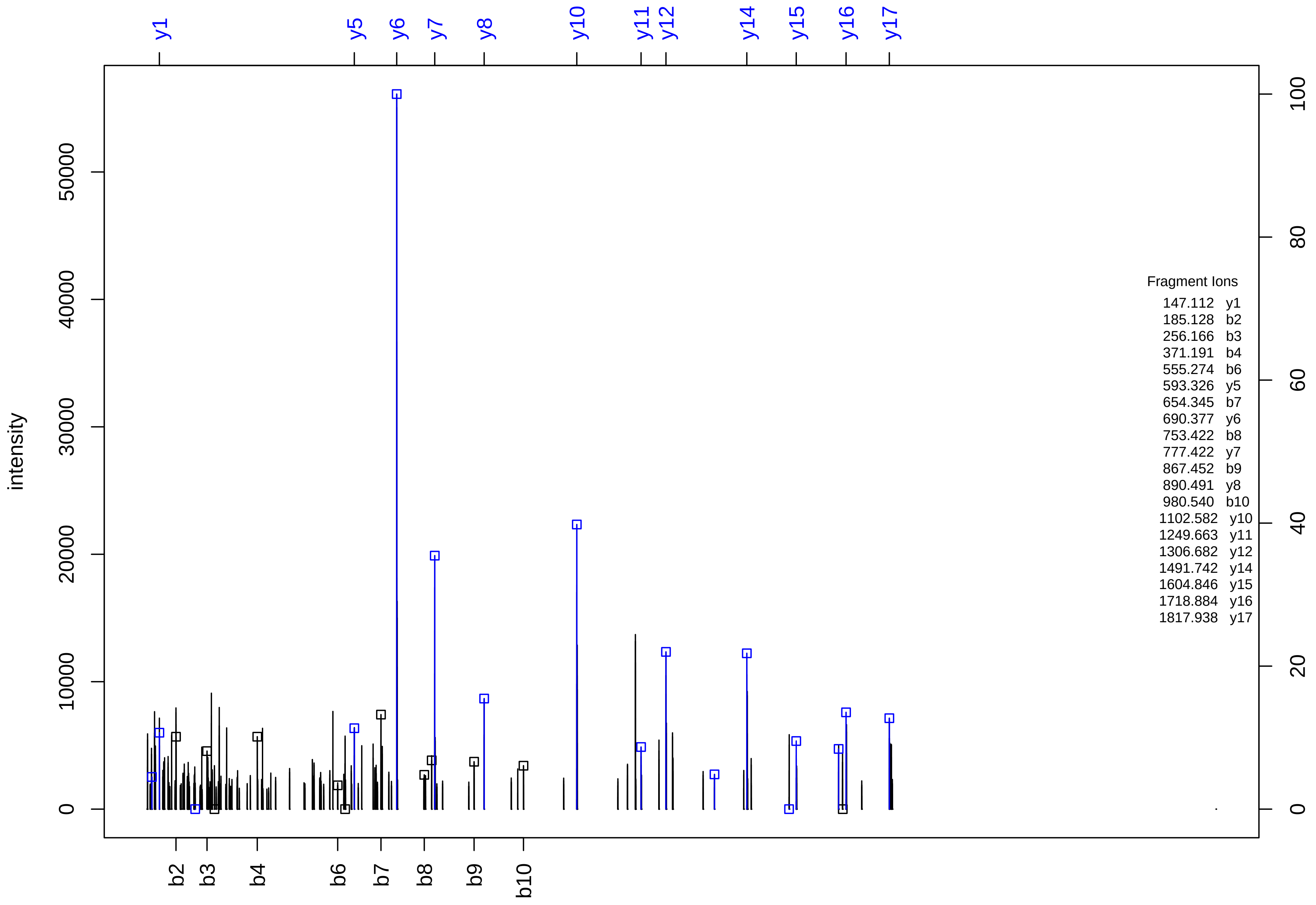

LAADPSVVNLGQGFPDISPPSYVK / File: christine_10pools_09.raw; SpectrumID: ; scans: 30456

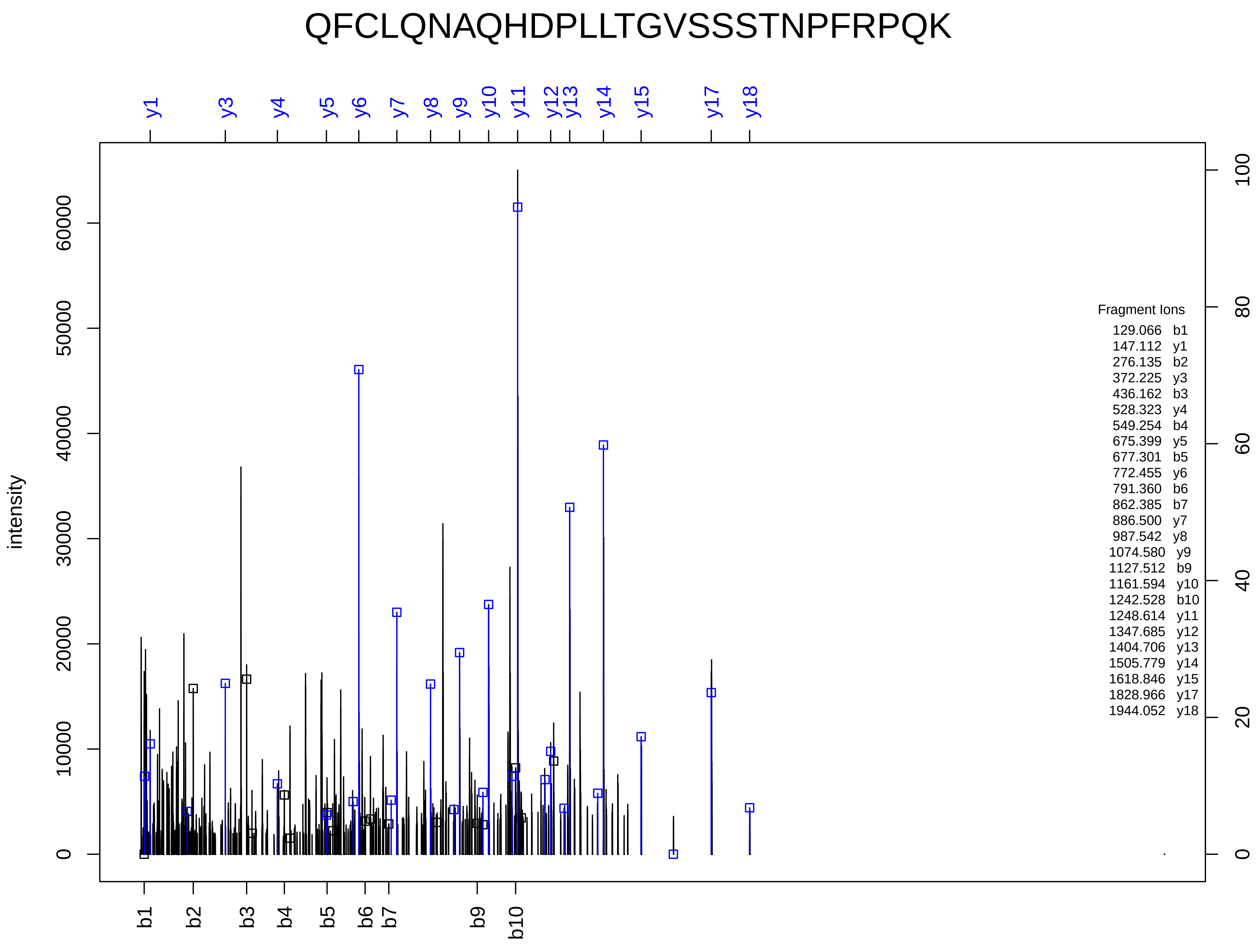

QFCLQNAQHDPLLTGVSSSTNPFRPQK / File: christine_10pools_09.raw; SpectrumID: ; scans: 19615

TPGLAPTPQAYLVPENFR

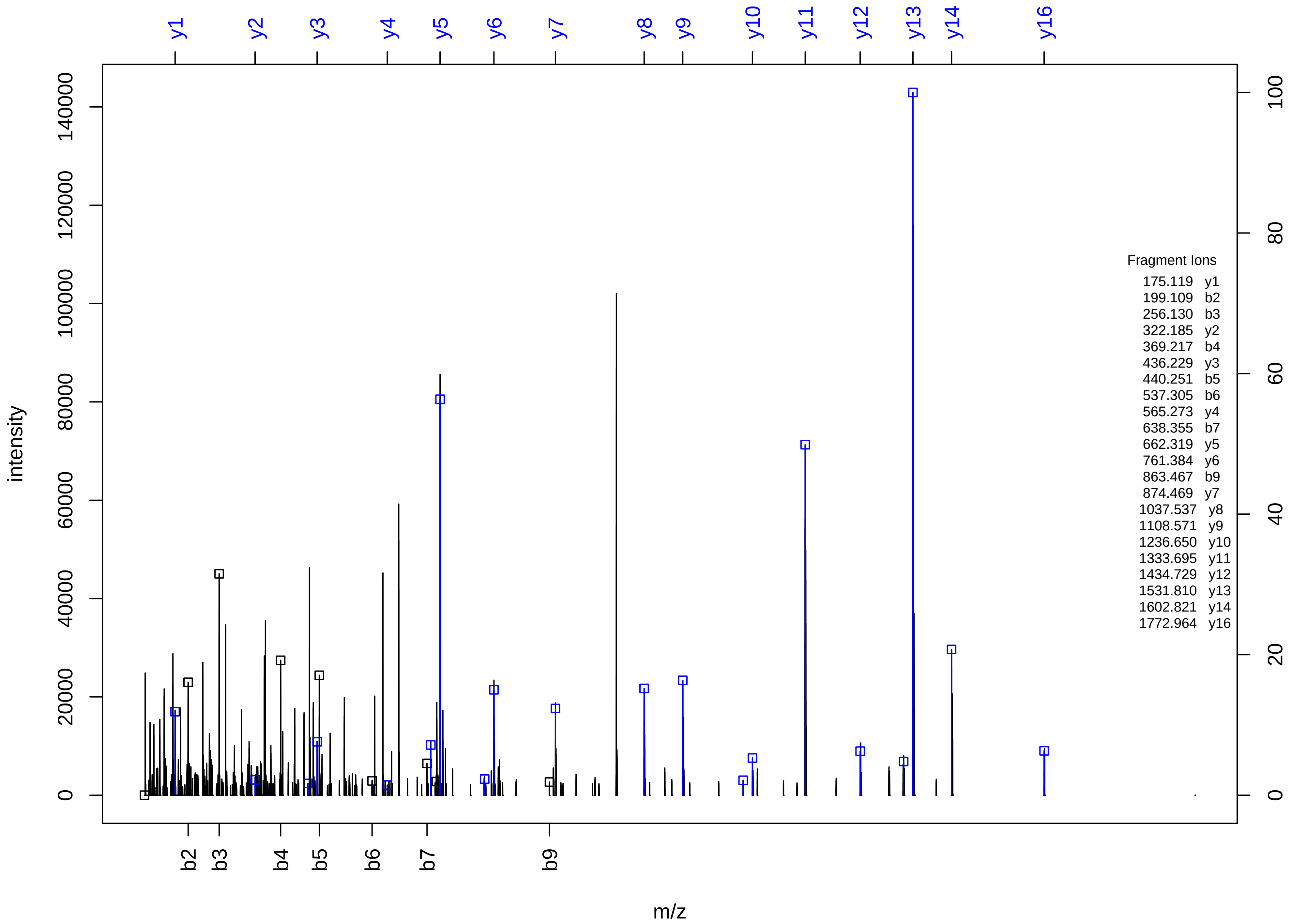

TPGLAPTPQAYLVPENFR / File: christine_10pools_09.raw; SpectrumID: ; scans: 25035

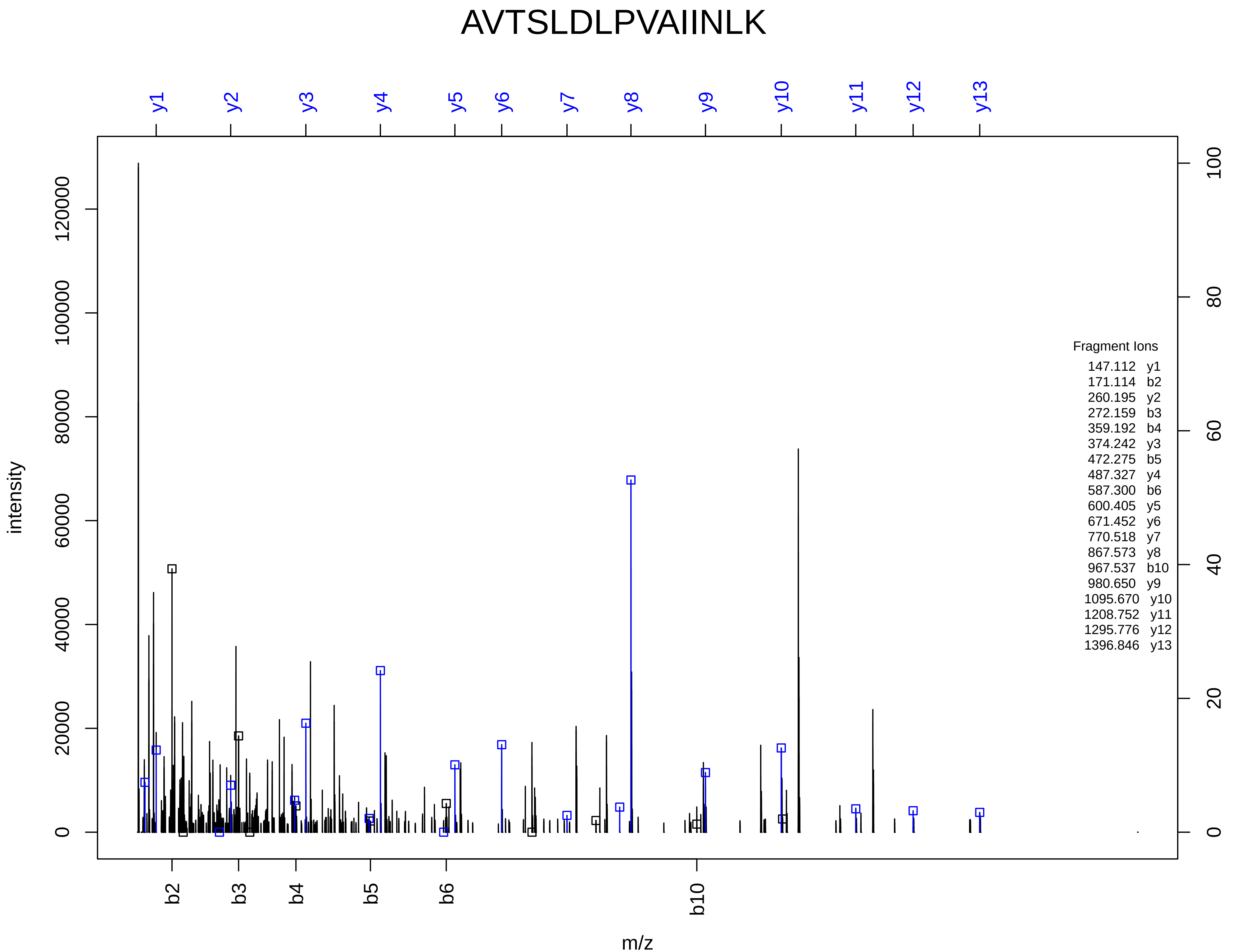

AVTSLDLPVAIINLK / File: christine_10pools_09.raw; SpectrumID: ; scans: 34617

LN^FSTSPEIFR

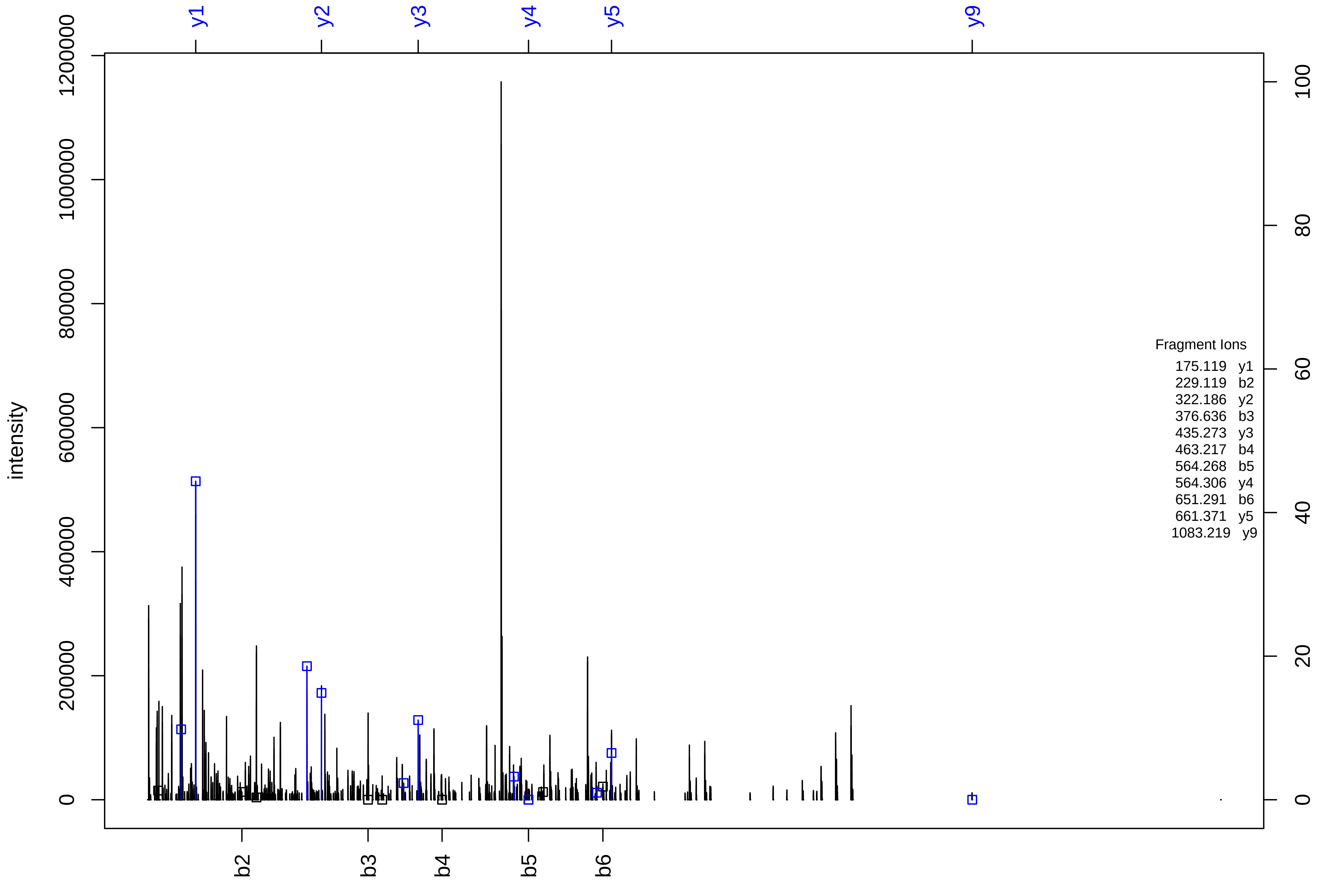

LNFSTSPEIFR / File: christine_10pools_09.raw; SpectrumID: ; scans: 7438

GPAGNDDEESSDSTPLLPGAR

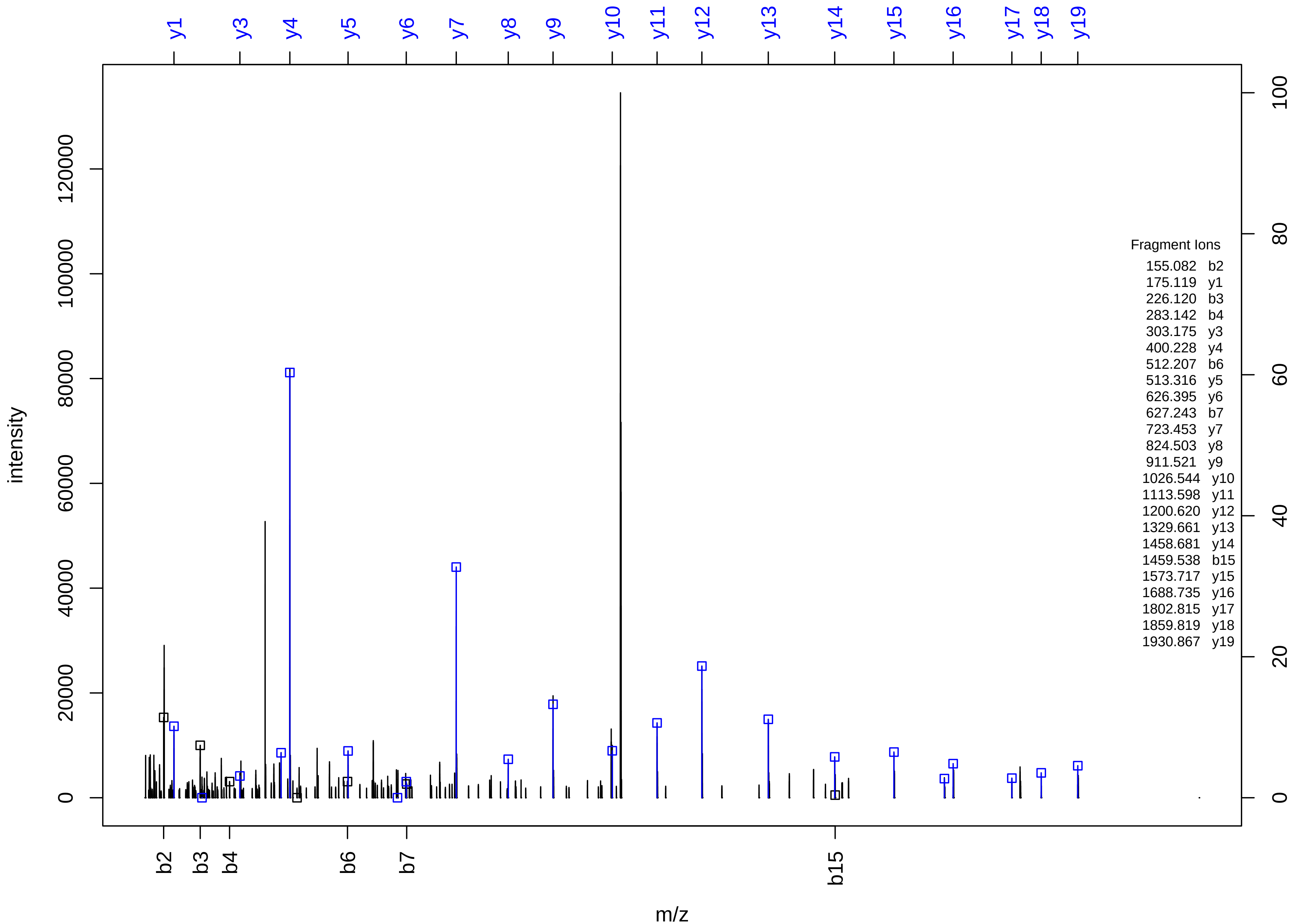

GPAGNDDEESSDSTPLLPGAR / File: christine_10pools_09.raw; SpectrumID: ; scans: 10637

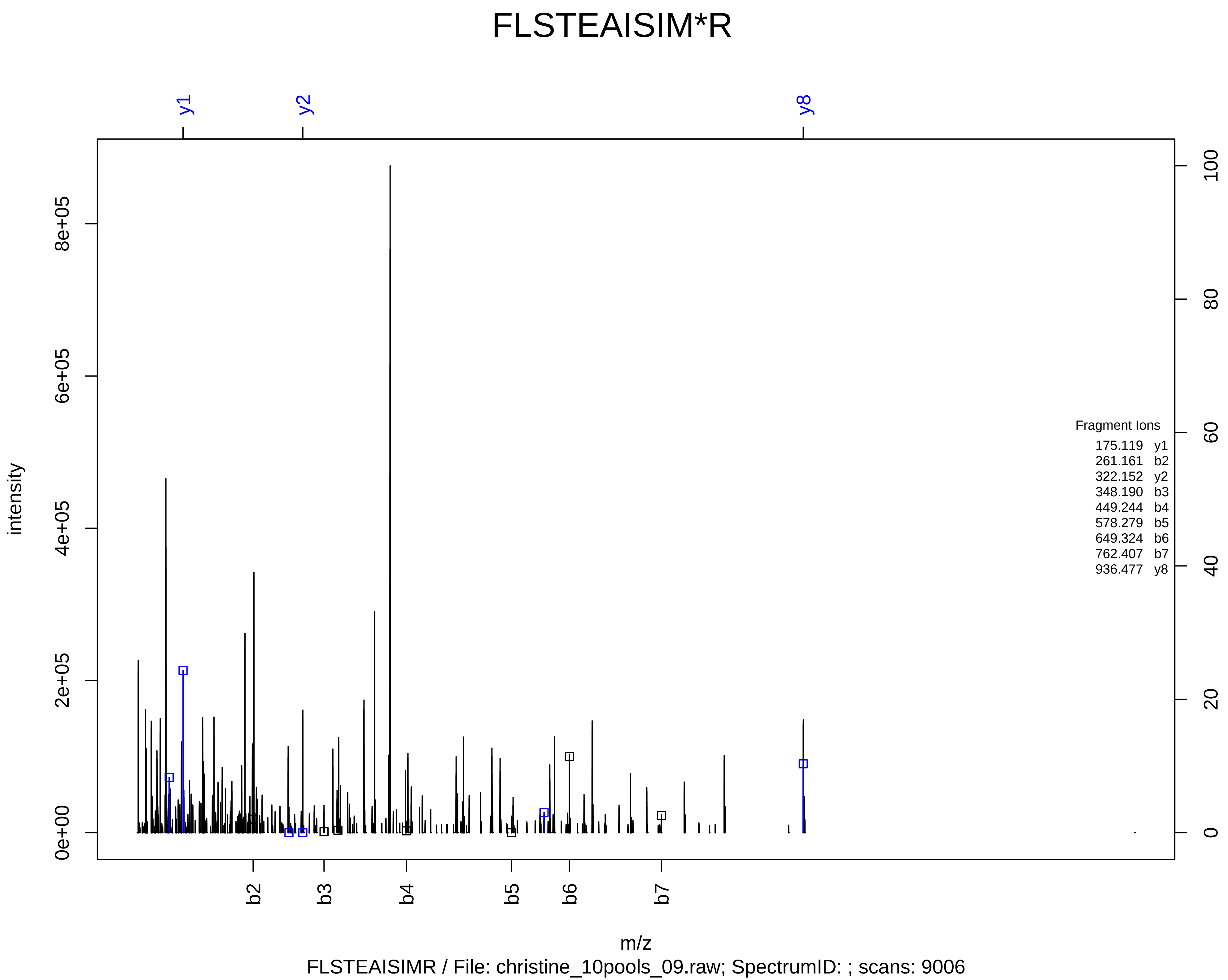
FLSTEAISIM*R
y1
y2
y8
intensity
0e+00
2e+05
4e+05
6e+05
8e+05
0
20
40
60
80
100
Fragment Ions
175.119 y1
261.161 b2
322.152 y2
348.190 b3
449.244 b4
578.279 b5
649.324 b6
762.407 b7
936.477 y8
b2
b3
b4
b5
b6
b7
m/z
FLSTEAISIMR / File: christine_10pools_09.raw; SpectrumID: ; scans: 9006

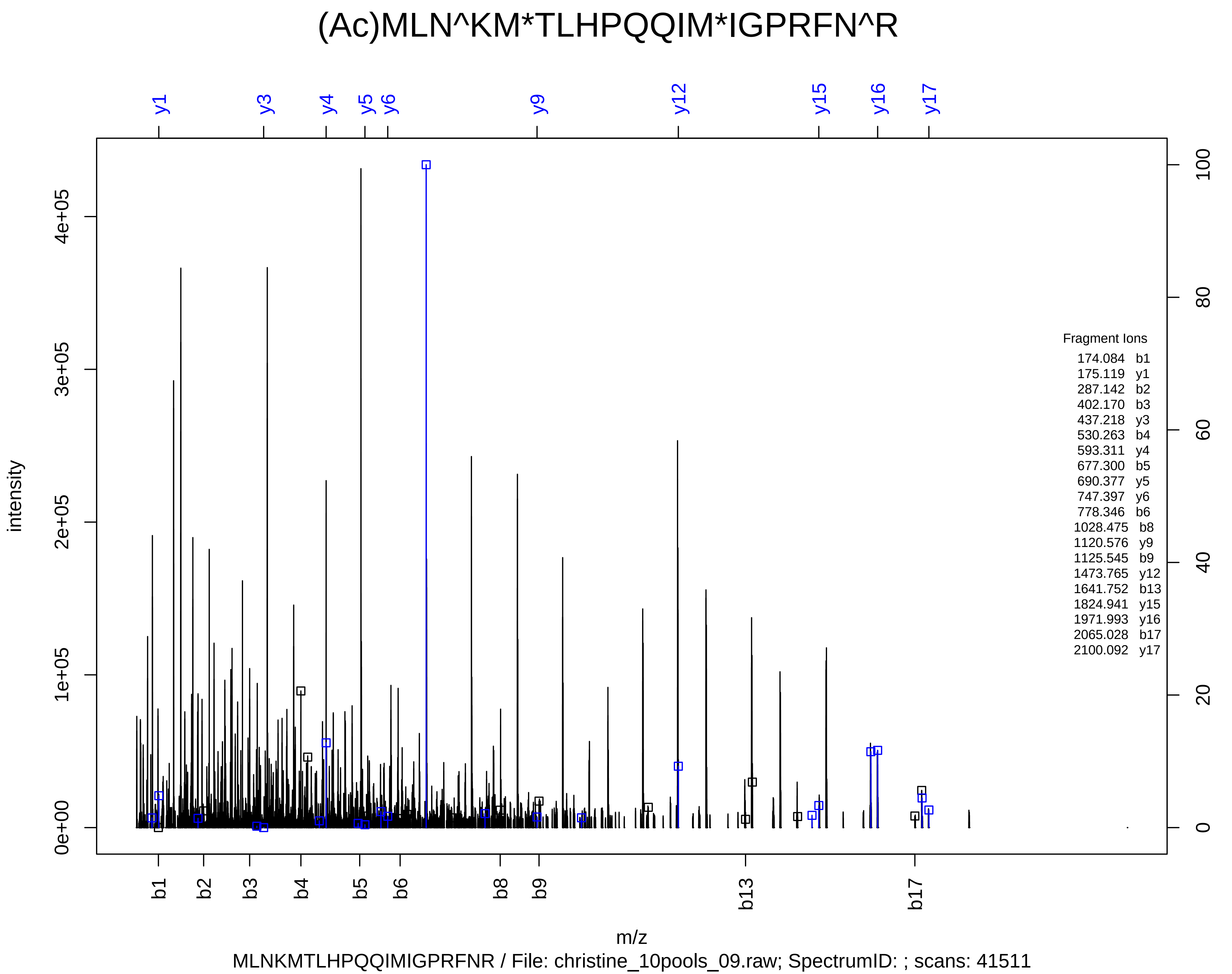

(Ac)MLN^KM*TLHPQQIM*IGPRFN^R
y1
y3
y4
y5
y6
y9
y12
y15
y16
y17
b1
b2
b3
b4
b5
b6
b8
b9
b13
b17
intensity
0e+00
1e+05
2e+05
3e+05
4e+05
0
20
40
60
80
100
m/z
Fragment Ions
174.084 b1
175.119 y1
287.142 b2
402.170 b3
437.218 y3
530.263 b4
593.311 y4
677.300 b5
690.377 y5
747.397 y6
778.346 b6
1028.475 b8
1120.576 y9
1125.545 b9
1473.765 y12
1641.752 b13
1824.941 y15
1971.993 y16
2065.028 b17
2100.092 y17
MLNKMTLHPQQIMIGPRFNR / File: christine_10pools_09.raw; SpectrumID: ; scans: 41511

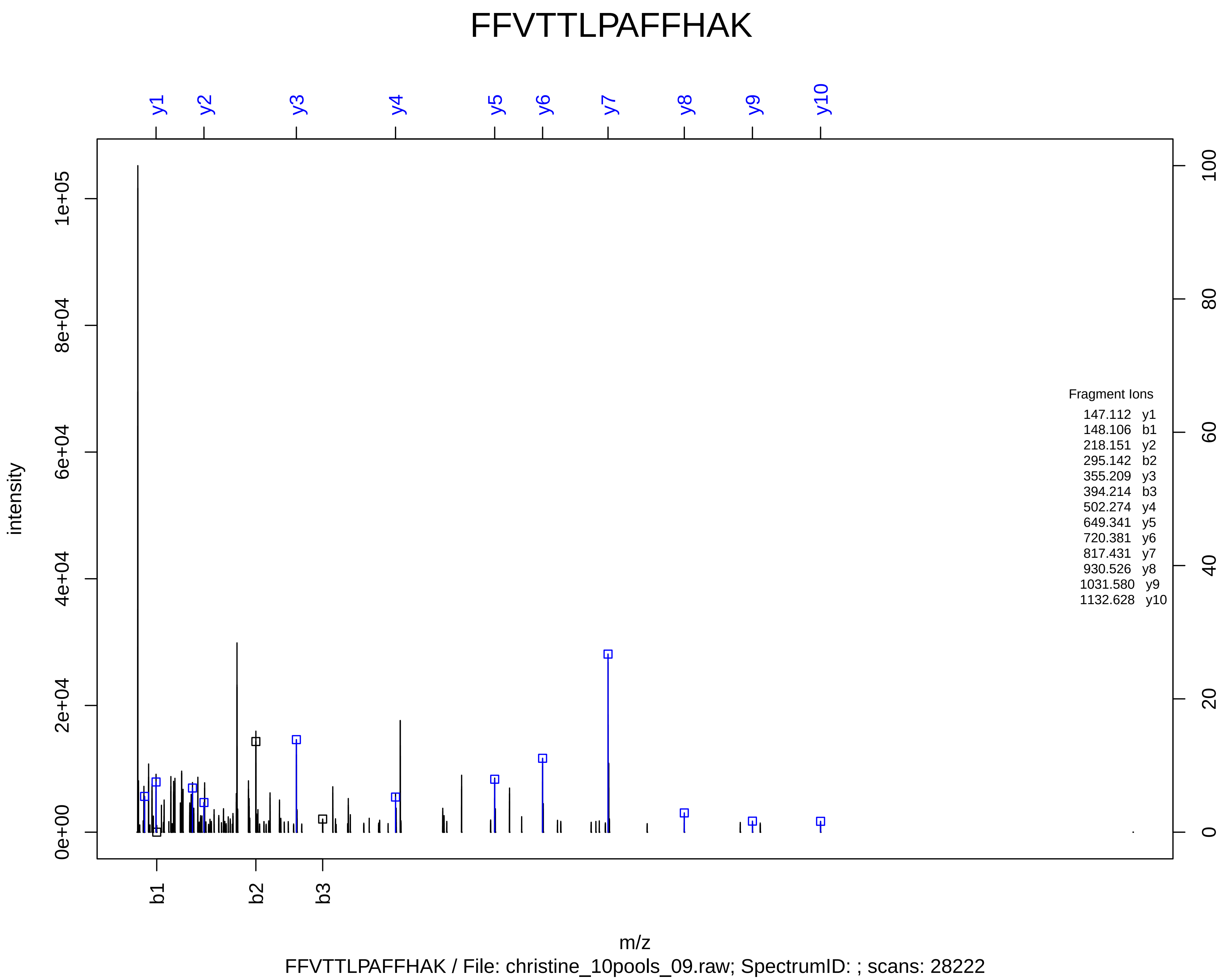

FFVTTLPAFFHAK
y1
y2
y3
y4
y5
y6
y7
y8
y9
y10
intensity
0e+00
2e+04
4e+04
6e+04
8e+04
1e+05
0
20
40
60
80
100
Fragment Ions
147.112 y1
148.106 b1
218.151 y2
295.142 b2
355.209 y3
394.214 b3
502.274 y4
649.341 y5
720.381 y6
817.431 y7
930.526 y8
1031.580 y9
1132.628 y10
b1
b2
b3
m/z
FFVTTLPAFFHAK / File: christine_10pools_09.raw; SpectrumID: ; scans: 28222

ATNAFHLPEDPSAPIIM*VGPGTGVAPFVGFLQHR

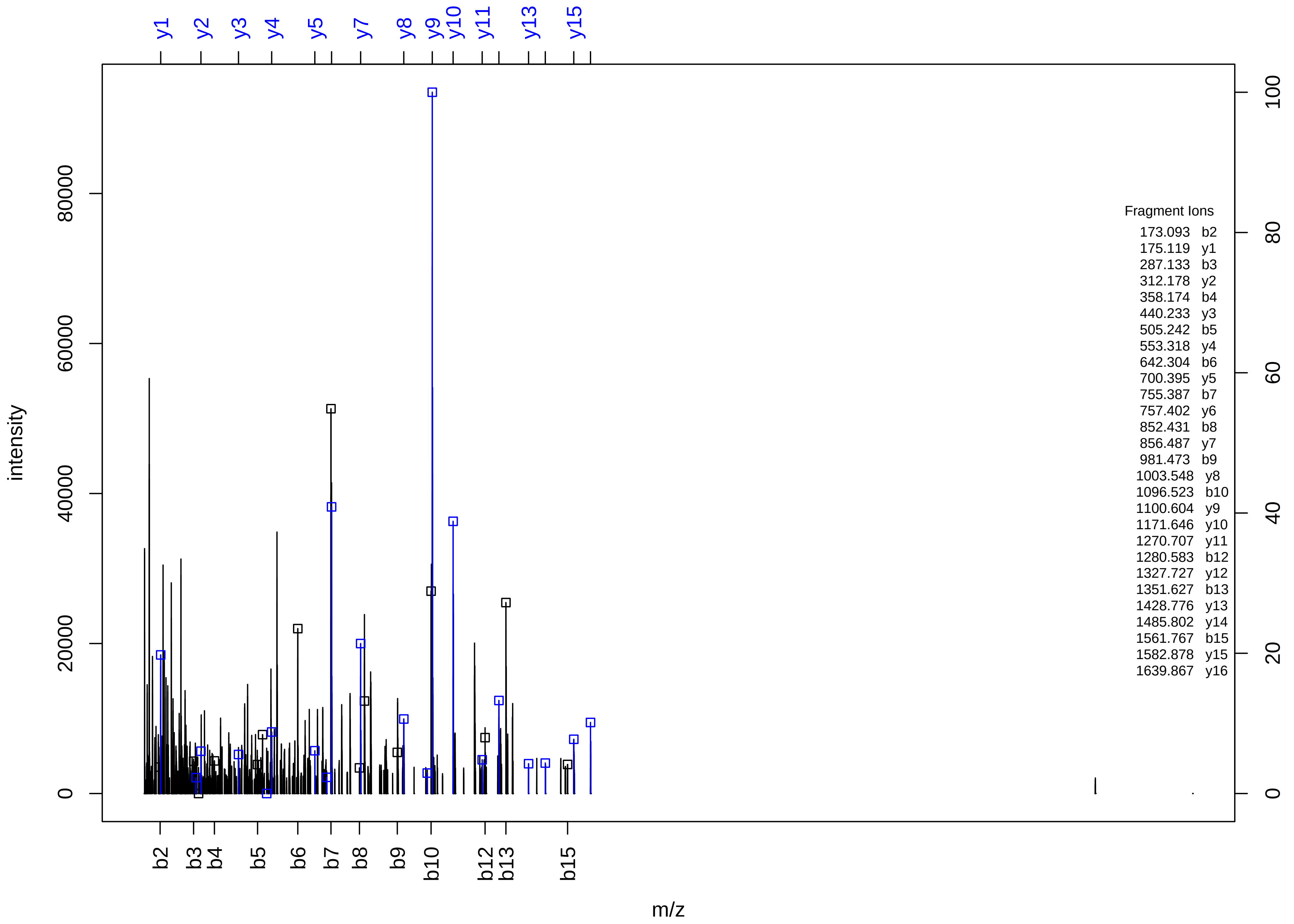

ATNAFHLPEDPSAPIIMVGPGTGVAPFVGFLQHR / File: christine_10pools_09.raw; SpectrumID: ; scans: 34767

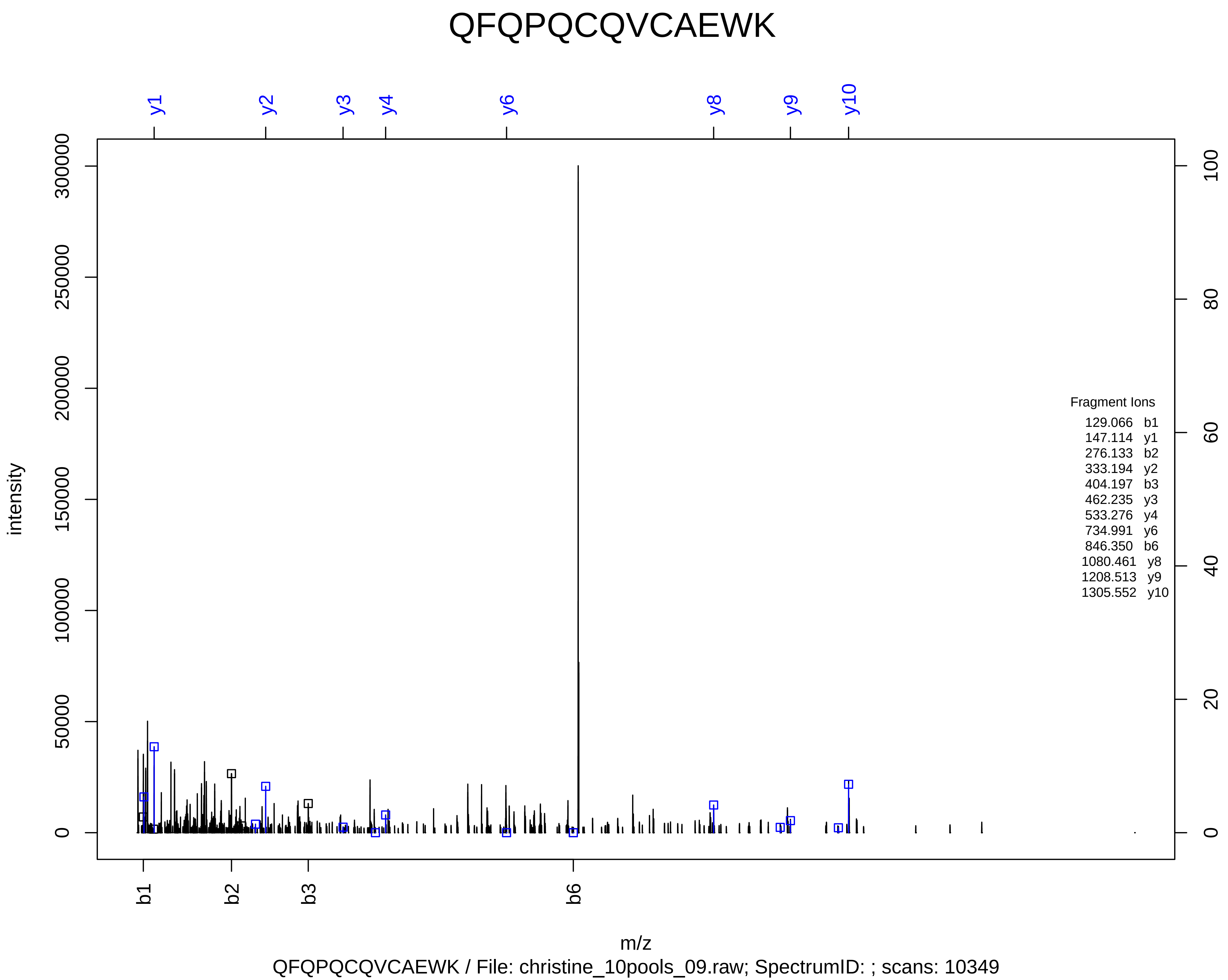

QFQPQCQVCAEWK
y1
y2
y3
y4
y6
y8
y9
y10
intensity
0
50000
100000
150000
200000
250000
300000
0
20
40
60
80
100
Fragment Ions
129.066 b1
147.114 y1
276.133 b2
333.194 y2
404.197 b3
462.235 y3
533.276 y4
734.991 y6
846.350 b6
1080.461 y8
1208.513 y9
1305.552 y10
b1
b2
b3
b6
m/z
QFQPQCQVCAEWK / File: christine_10pools_09.raw; SpectrumID: ; scans: 10349

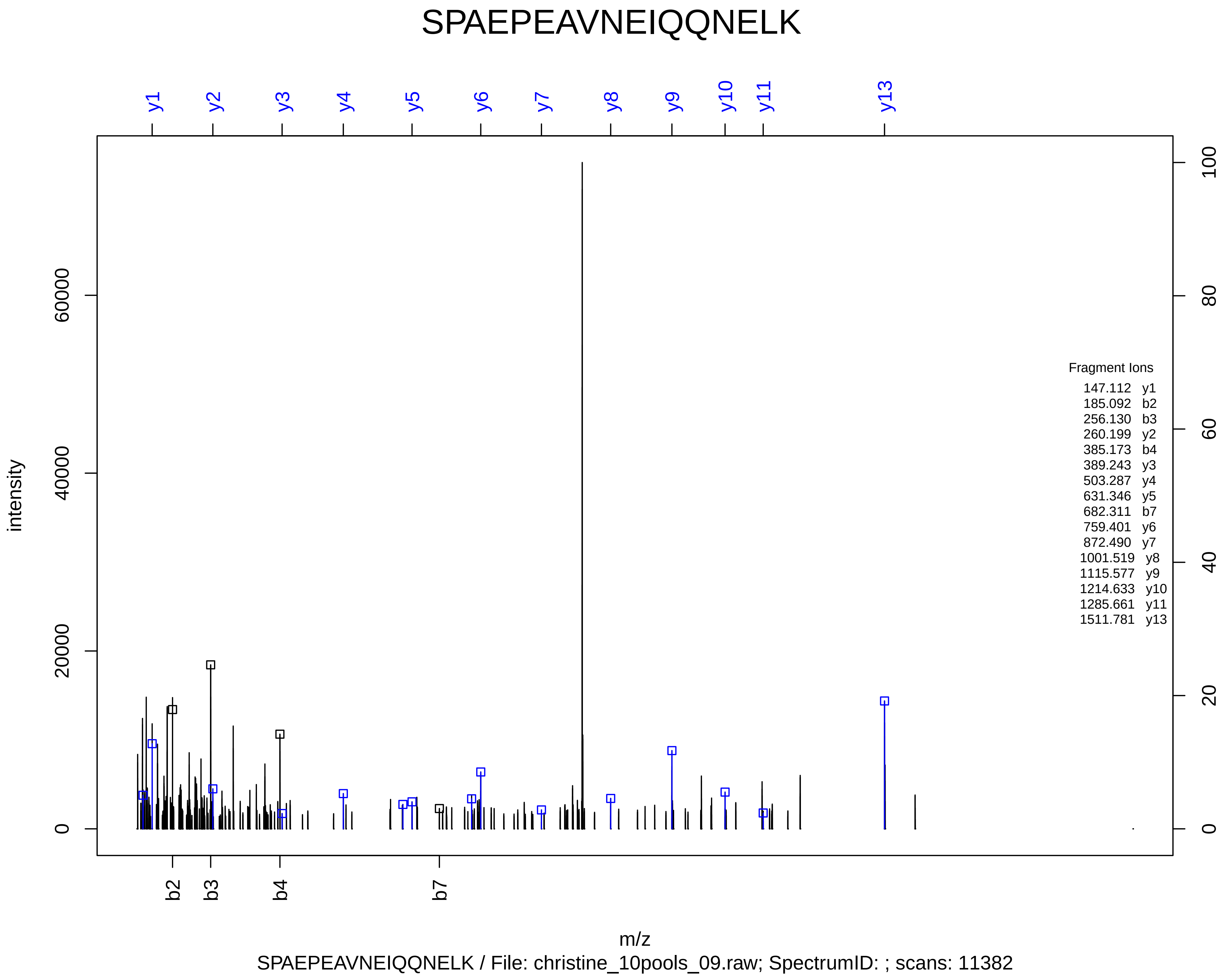

SPAEPEAVNEIQQNELK
y1
y2
y3
y4
y5
y6
y7
y8
y9
y10
y11
y13
intensity
0
20000
40000
60000
0
20
40
60
80
100
Fragment Ions
147.112 y1
185.092 b2
256.130 b3
260.199 y2
385.173 b4
389.243 y3
503.287 y4
631.346 y5
682.311 b7
759.401 y6
872.490 y7
1001.519 y8
1115.577 y9
1214.633 y10
1285.661 y11
1511.781 y13
b2
b3
b4
b7
m/z
SPAEPEAVNEIQQNELK / File: christine_10pools_09.raw; SpectrumID: ; scans: 11382

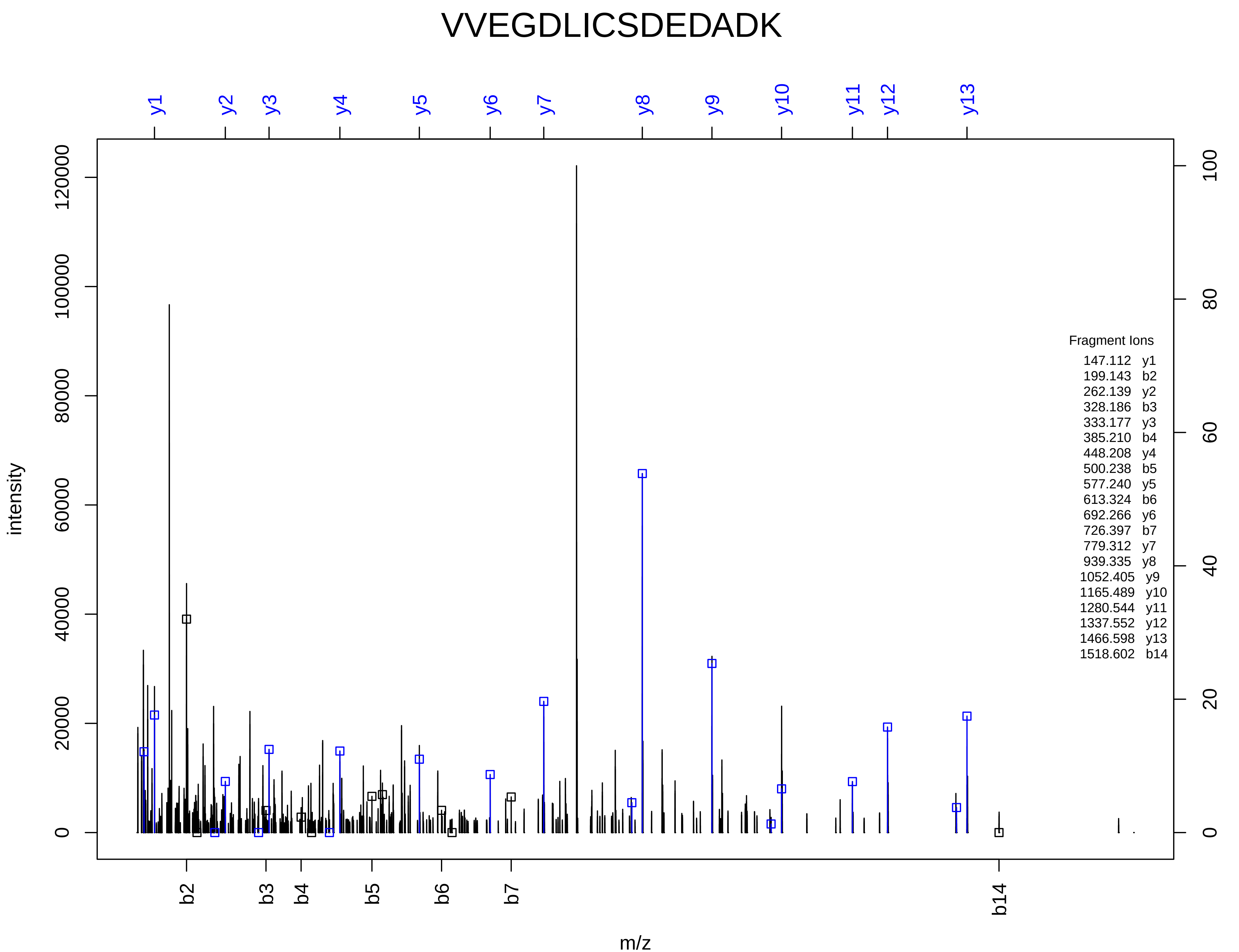

VVEGDLICSDEDADK / File: christine_10pools_09.raw; SpectrumID: ; scans: 8270

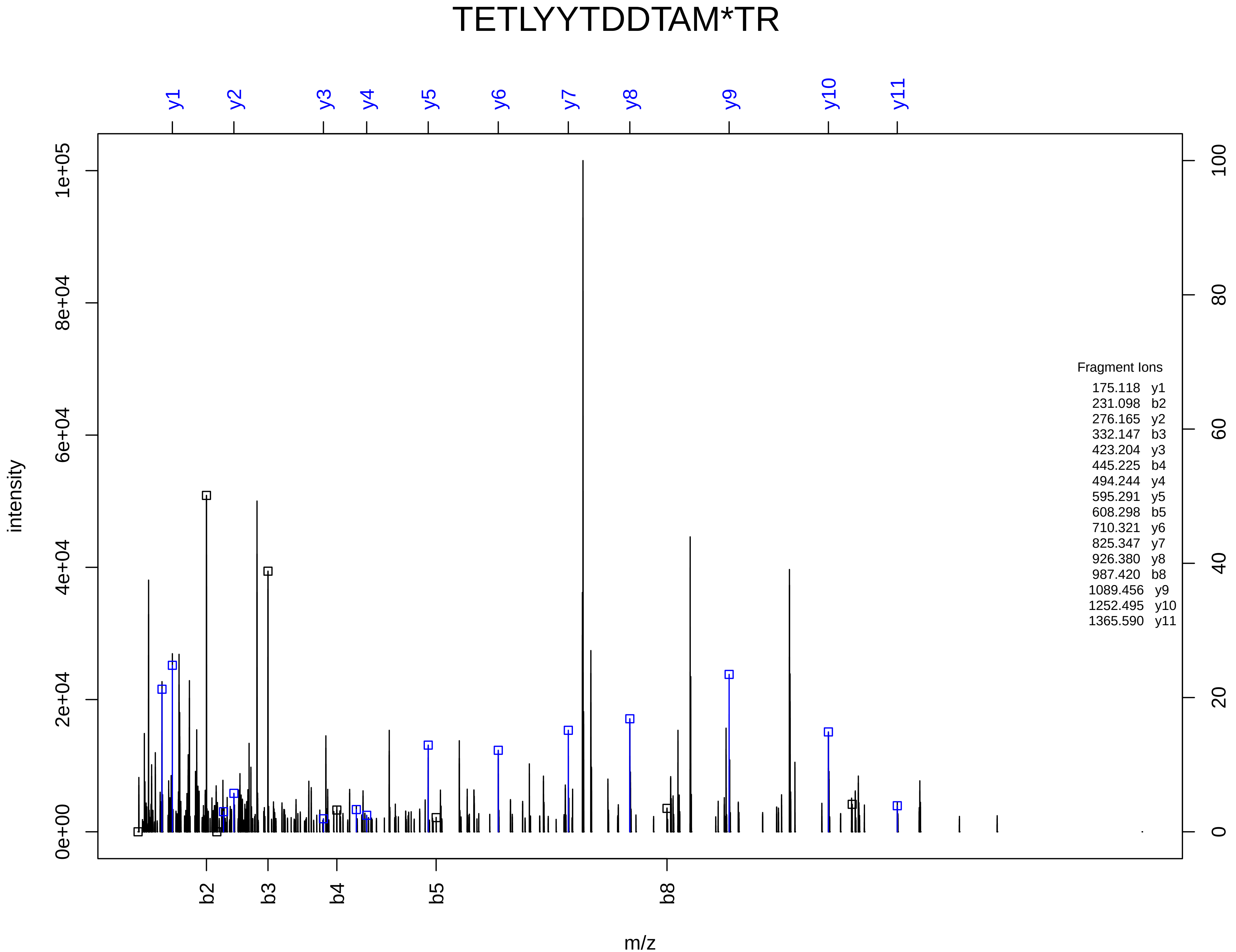

TETLYYTDDTAMTR / File: christine_10pools_09.raw; SpectrumID: ; scans: 6036

APTGVDSTGVIAGEGPGDDK

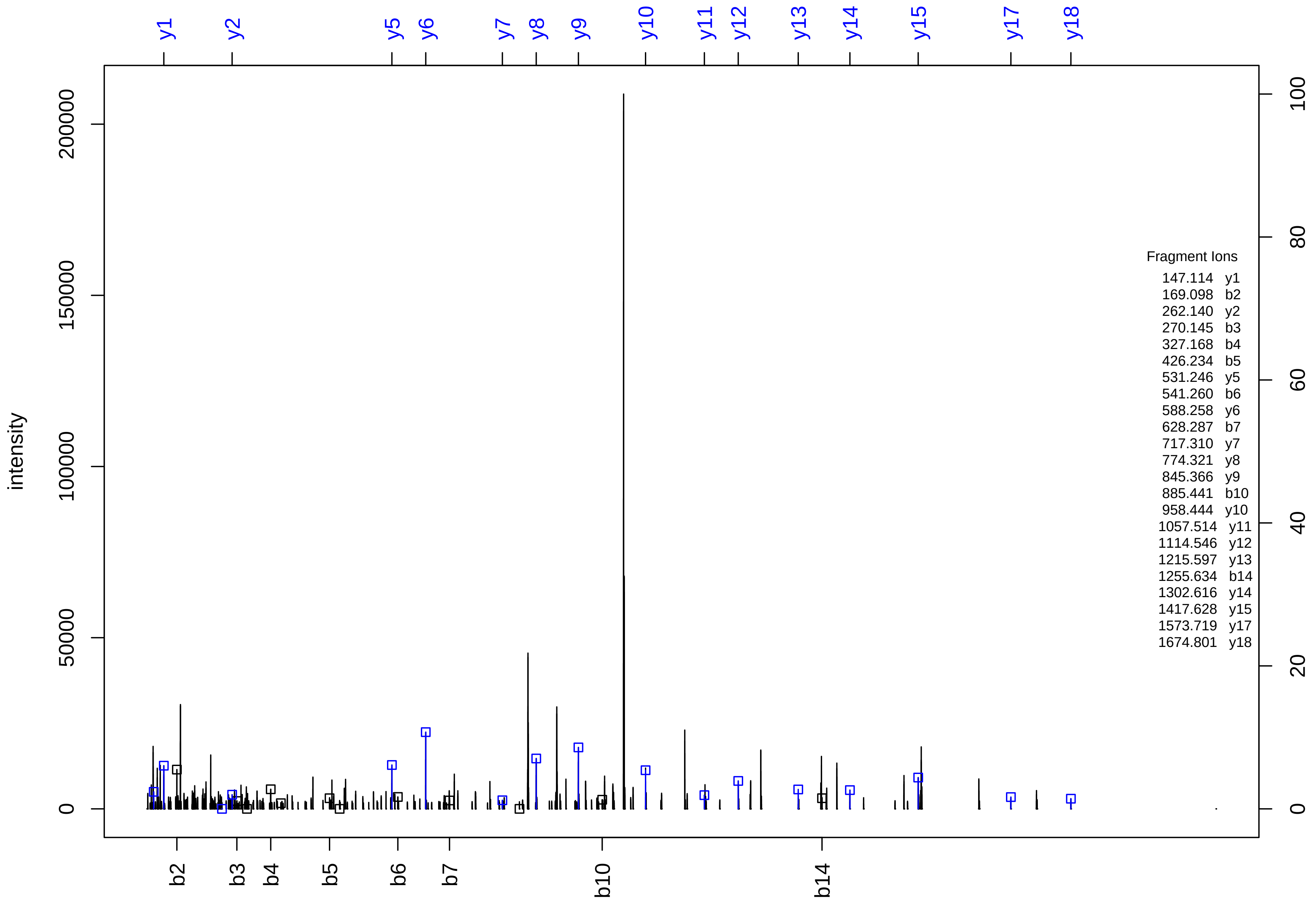

APTGVDSTGVIAGEGPGDDK / File: christine_10pools_09.raw; SpectrumID: ; scans: 6959

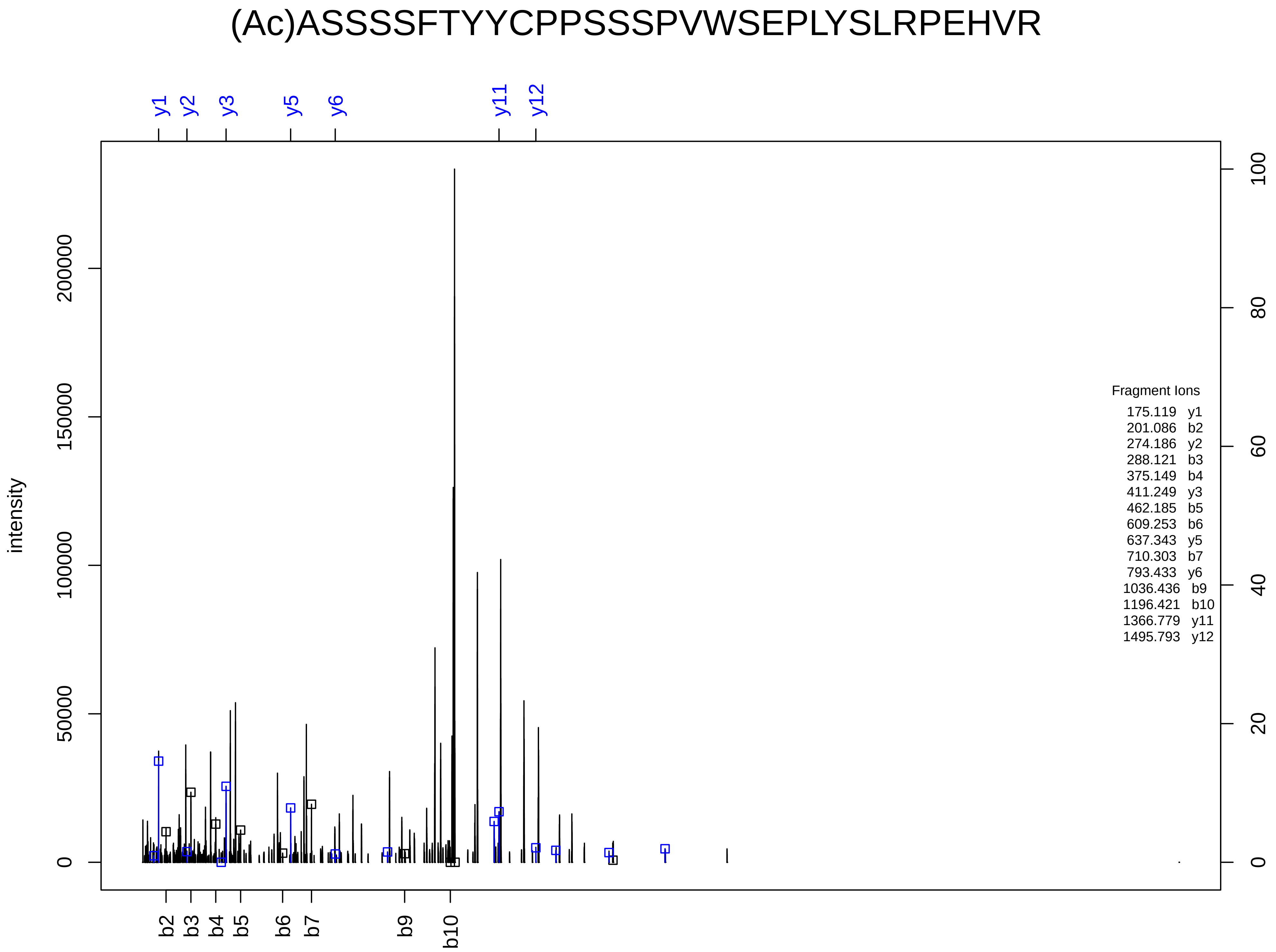

ASSSSFTYYCPPSSSPVWSEPLYSLRPEHVR / File: christine_10pools_09.raw; SpectrumID: ; scans: 28618

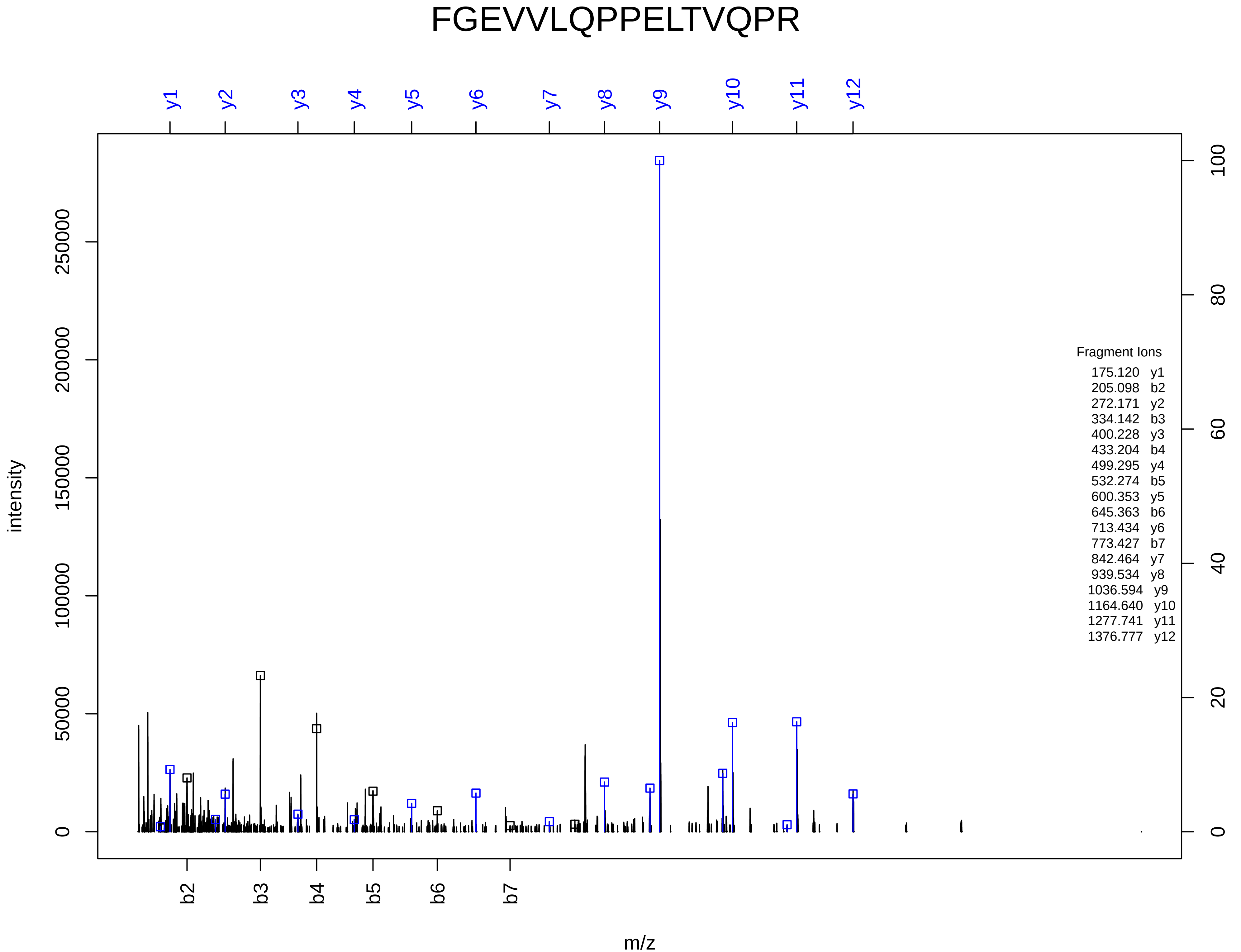

FGEVVLQPPELTVQPR / File: christine_10pools_09.raw; SpectrumID: ; scans: 25618

APSGTSLEVPIPEGLN^GQK

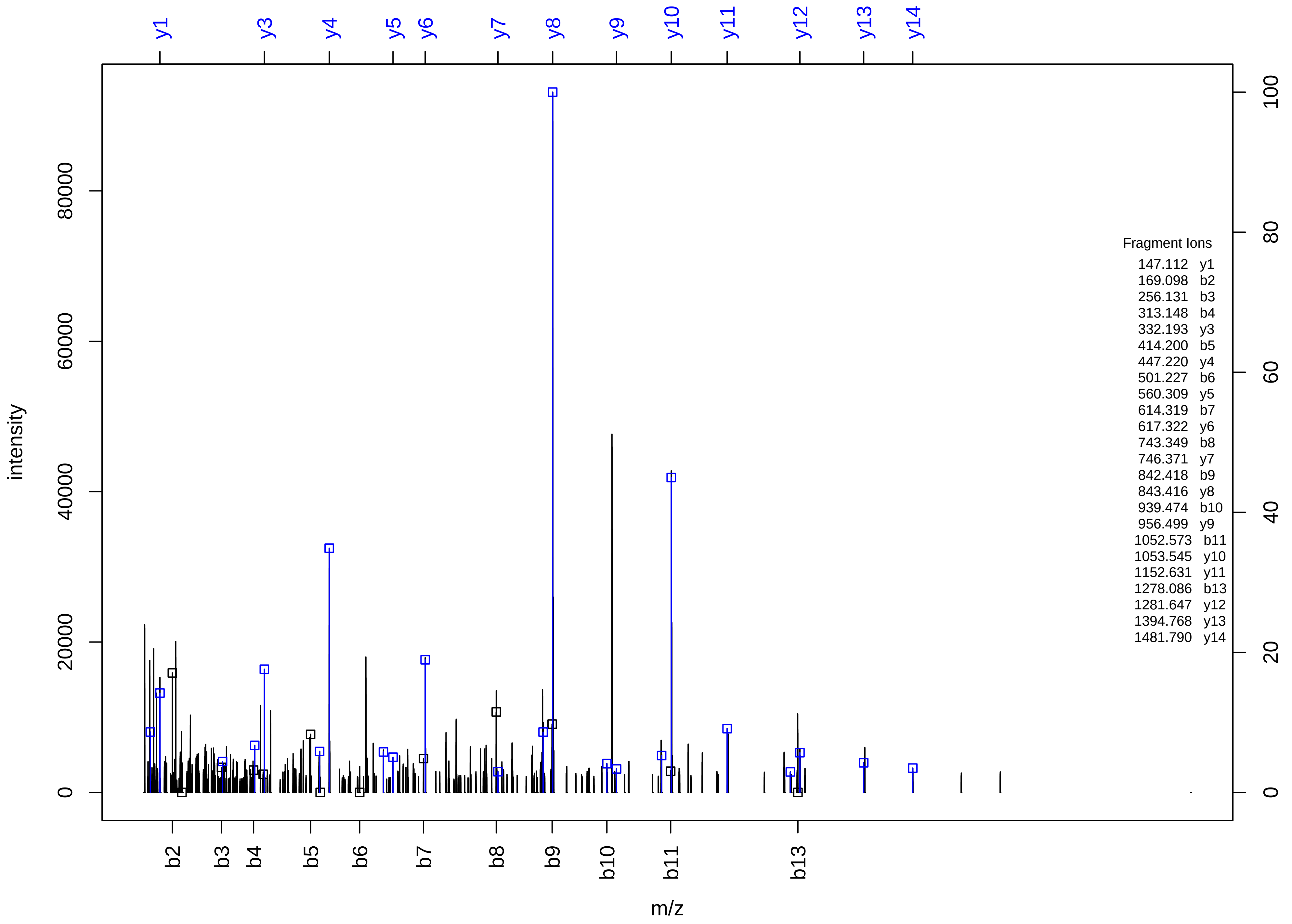

APSGTSLEVPIPEGLNGQK / File: christine_10pools_09.raw; SpectrumID: ; scans: 21734

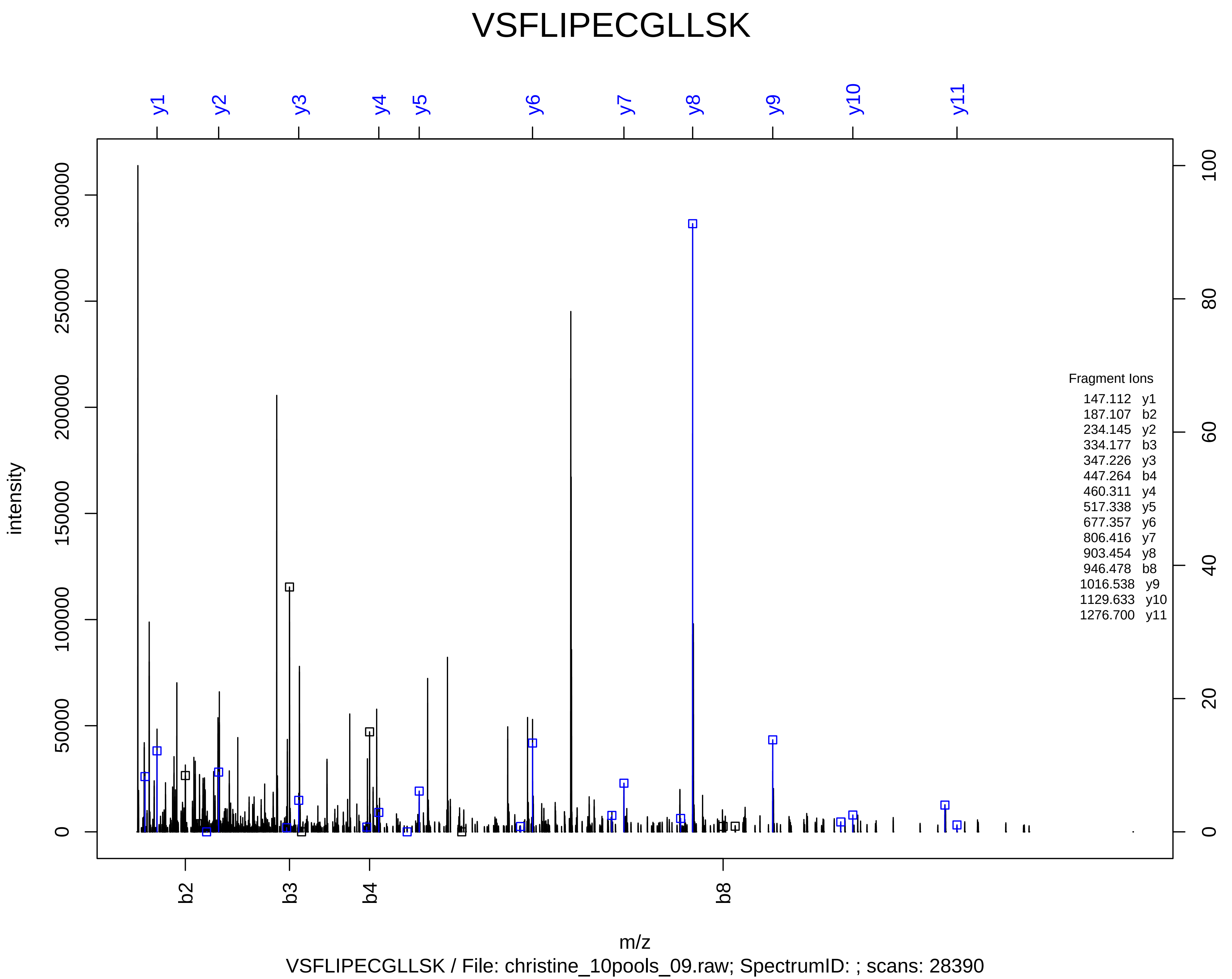

VSFLIPECGLLSK
y1
y2
y3
y4
y5
y6
y7
y8
y9
y10
y11
intensity
0
50000
100000
150000
200000
250000
300000
0
20
40
60
80
100
b2
b3
b4
b8
m/z
Fragment Ions
147.112 y1
187.107 b2
234.145 y2
334.177 b3
347.226 y3
447.264 b4
460.311 y4
517.338 y5
677.357 y6
806.416 y7
903.454 y8
946.478 b8
1016.538 y9
1129.633 y10
1276.700 y11
VSFLIPECGLLSK / File: christine_10pools_09.raw; SpectrumID: ; scans: 28390

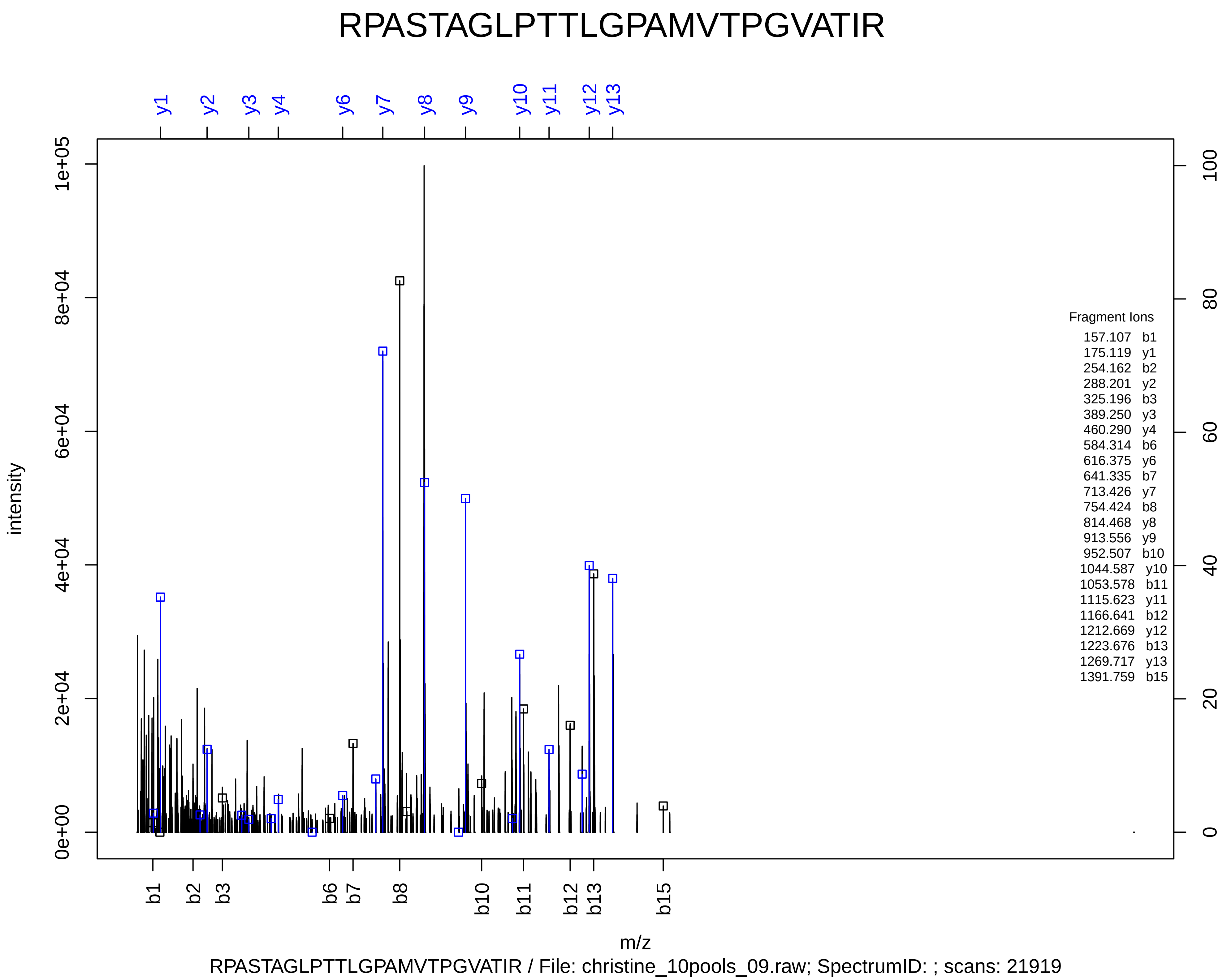
RPASTAGLPTTLGPAMVTPGVATIR
intensity
m/z
y1 y2 y3 y4 y6 y7 y8 y9 y10 y11 y12 y13
b1 b2 b3 b6 b7 b8 b10 b11 b12 b13 b15
0e+00 2e+04 4e+04 6e+04 8e+04 1e+05
0 20 40 60 80 100
Fragment Ions
157.107 b1
175.119 y1
254.162 b2
288.201 y2
325.196 b3
389.250 y3
460.290 y4
584.314 b6
616.375 y6
641.335 b7
713.426 y7
754.424 b8
814.468 y8
913.556 y9
952.507 b10
1044.587 y10
1053.578 b11
1115.623 y11
1166.641 b12
1212.669 y12
1223.676 b13
1269.717 y13
1391.759 b15
RPASTAGLPTTLGPAMVTPGVATIR / File: christine_10pools_09.raw; SpectrumID: ; scans: 21919

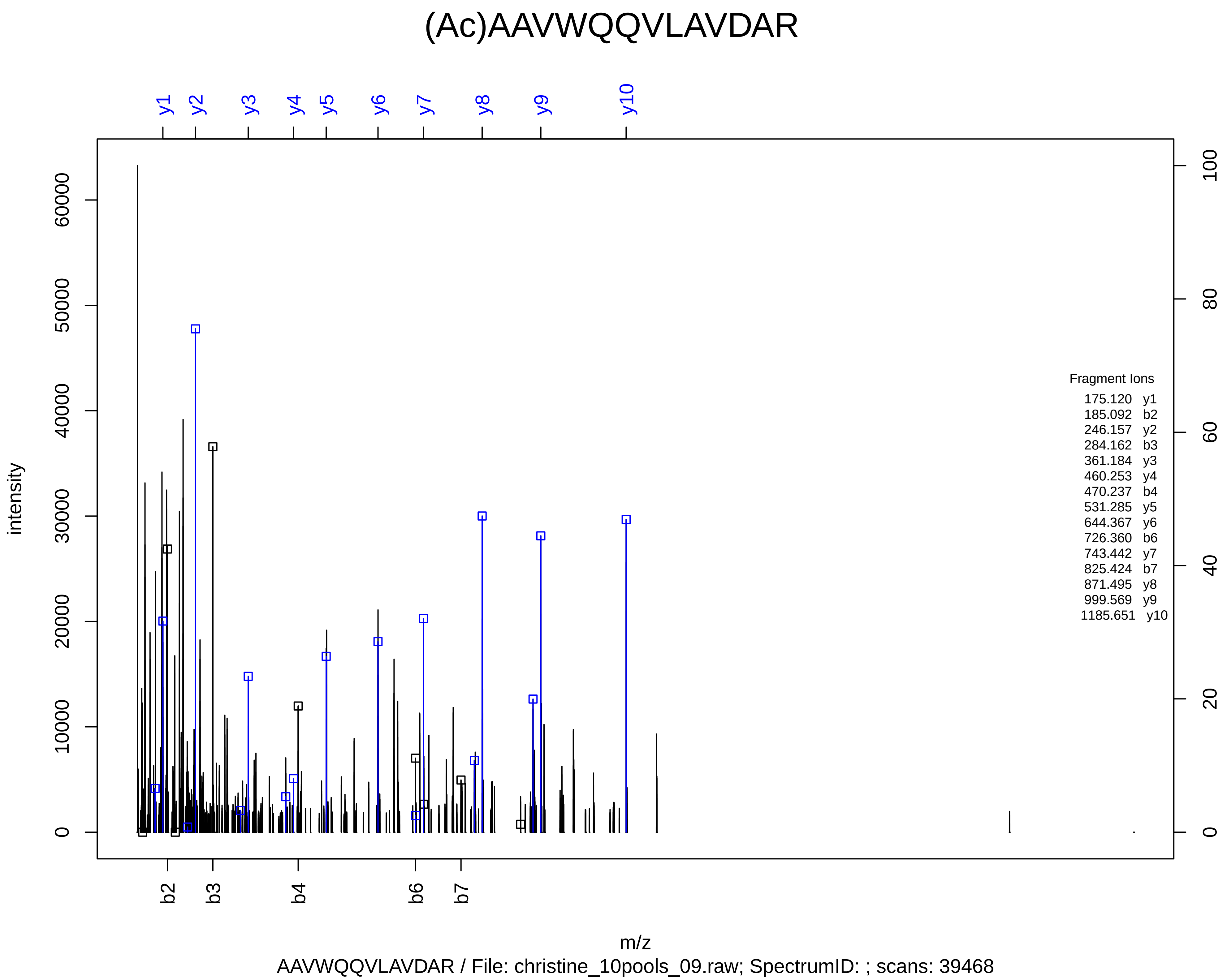
(Ac)AAVWQQVLAVDAR
y1
y2
y3
y4
y5
y6
y7
y8
y9
y10
intensity
0
10000
20000
30000
40000
50000
60000
0
20
40
60
80
100
b2
b3
b4
b6
b7
Fragment Ions
175.120 y1
185.092 b2
246.157 y2
284.162 b3
361.184 y3
460.253 y4
470.237 b4
531.285 y5
644.367 y6
726.360 b6
743.442 y7
825.424 b7
871.495 y8
999.569 y9
1185.651 y10
m/z
AAVWQQVLAVDAR / File: christine_10pools_09.raw; SpectrumID: ; scans: 39468

IYAPQGLLLTDPIER

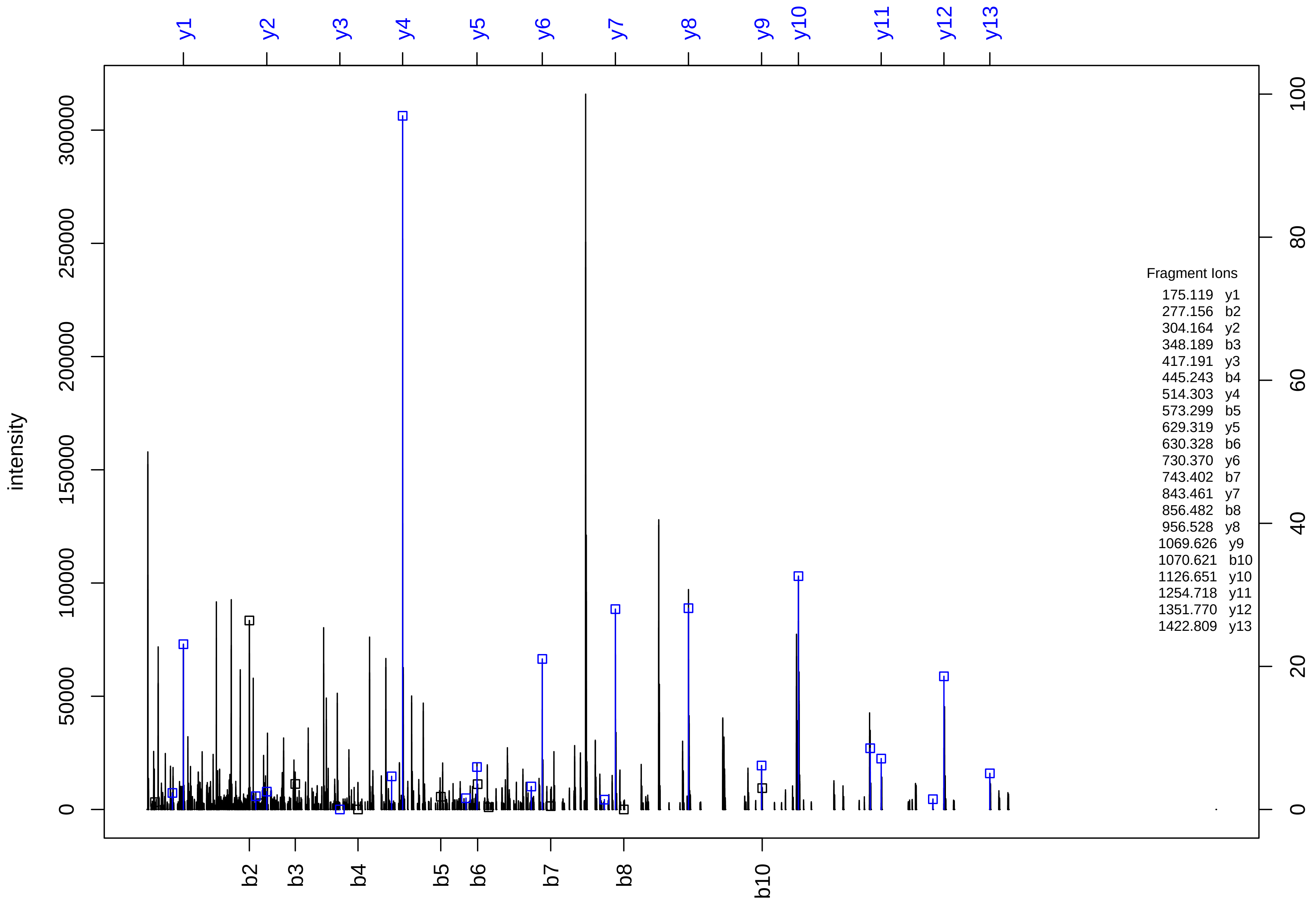

IYAPQGLLLTDPIER / File: christine_10pools_09.raw; SpectrumID: ; scans: 27197

GVTSNAVHPGNM*MYSAIHR

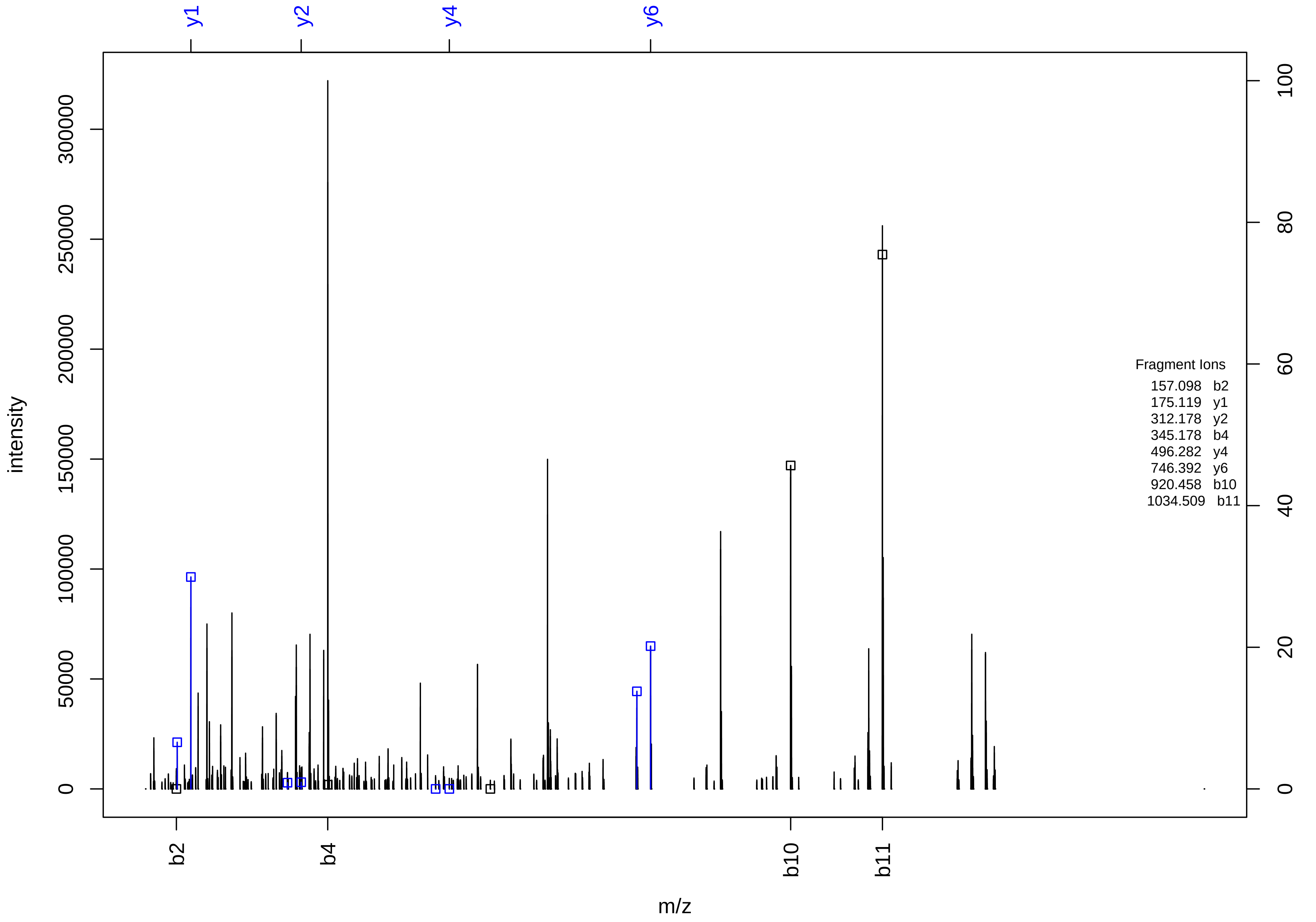

GVTSNAVHPGNMMYSAIHR / File: christine_10pools_09.raw; SpectrumID: ; scans: 980

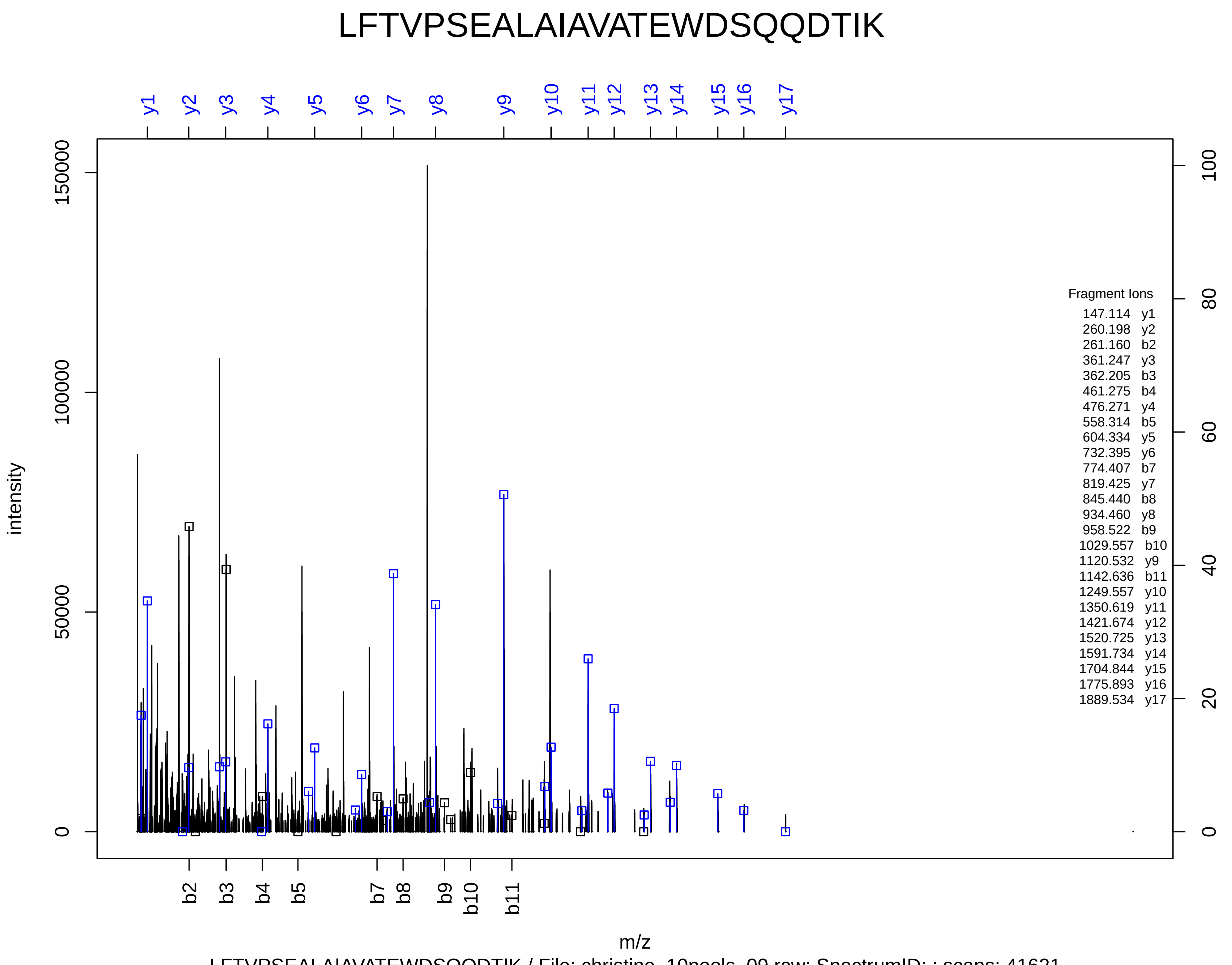

LFTVPSEALAIAVATEWDSQQDTIK
y1 y2 y3 y4 y5 y6 y7 y8 y9 y10 y11 y12 y13 y14 y15 y16 y17
intensity
0
50000
100000
150000
0
20
40
60
80
100
Fragment Ions
147.114 y1
260.198 y2
261.160 b2
361.247 y3
362.205 b3
461.275 b4
476.271 y4
558.314 b5
604.334 y5
732.395 y6
774.407 b7
819.425 y7
845.440 b8
934.460 y8
958.522 b9
1029.557 b10
1120.532 y9
1142.636 b11
1249.557 y10
1350.619 y11
1421.674 y12
1520.725 y13
1591.734 y14
1704.844 y15
1775.893 y16
1889.534 y17
b2 b3 b4 b5 b7 b8 b9 b10 b11
m/z
LFTVPSEALAIAVATEWDSQQDTIK / File: christine_10pools_09.raw; SpectrumID: ; scans: 41621

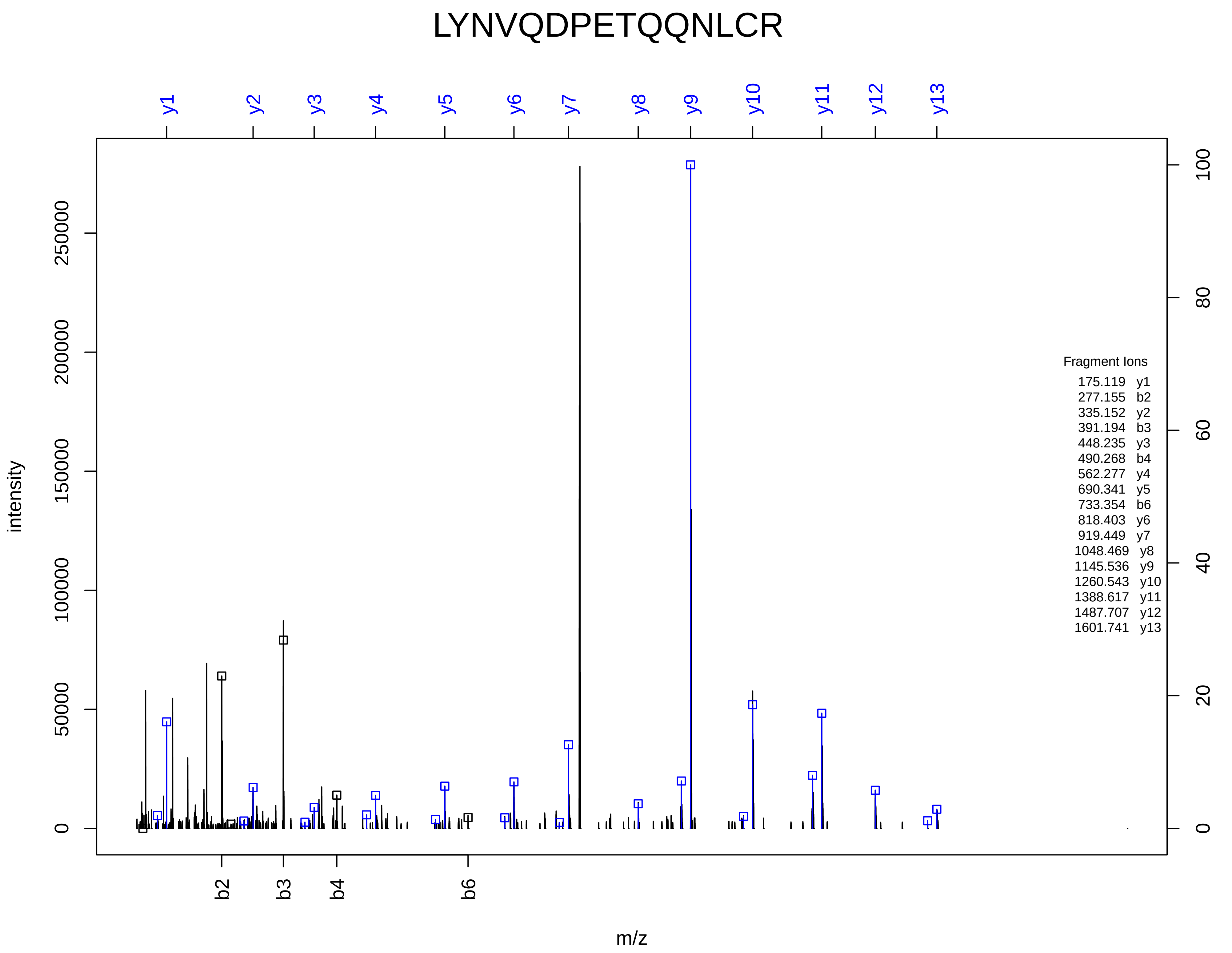

LYNVQDPETQQNLCR
y1
y2
y3
y4
y5
y6
y7
y8
y9
y10
y11
y12
y13
b2
b3
b4
b6
intensity
0
50000
100000
150000
200000
250000
0
20
40
60
80
100
m/z
Fragment Ions
175.119 y1
277.155 b2
335.152 y2
391.194 b3
448.235 y3
490.268 b4
562.277 y4
690.341 y5
733.354 b6
818.403 y6
919.449 y7
1048.469 y8
1145.536 y9
1260.543 y10
1388.617 y11
1487.707 y12
1601.741 y13
LYNVQDPETQQNLCR / File: christine_10pools_09.raw; SpectrumID: ; scans: 7444

AIQTVSCLLQGPCDAGNR

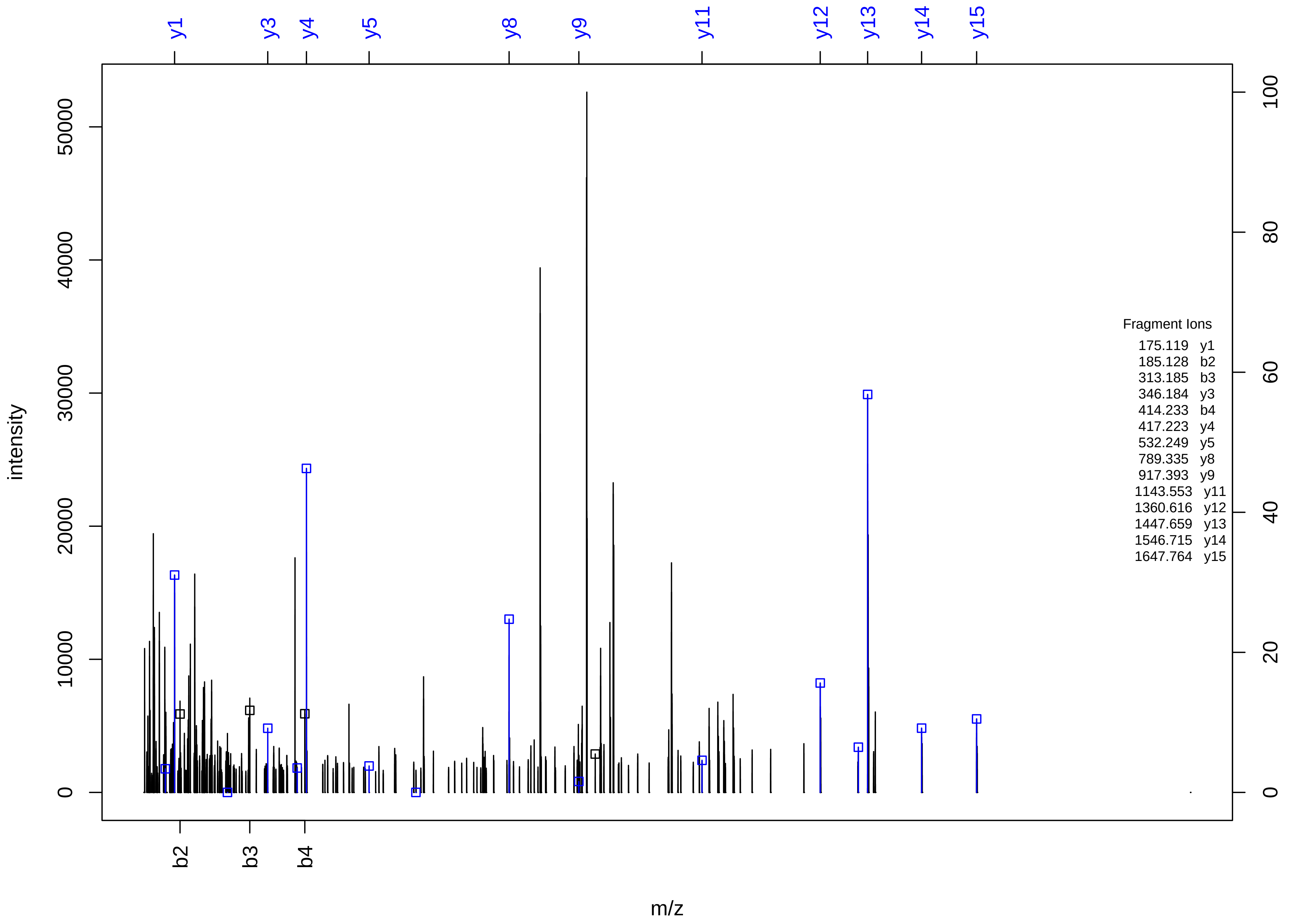

AIQTVSCLLQGPCDAGNR / File: christine_10pools_09.raw; SpectrumID: ; scans: 17835

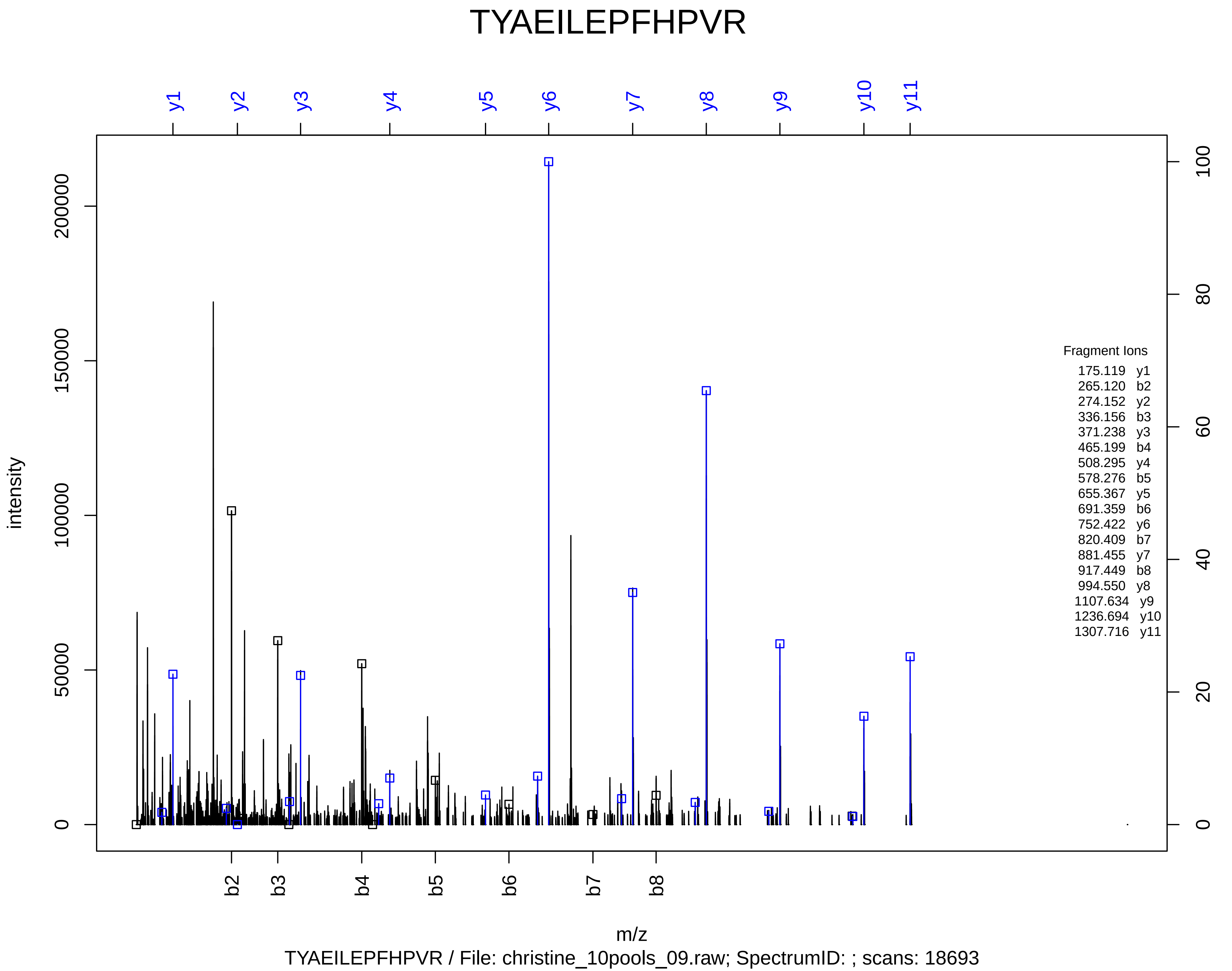

TYAEILEPFHPVR
y1
y2
y3
y4
y5
y6
y7
y8
y9
y10
y11
b2
b3
b4
b5
b6
b7
b8
intensity
0
50000
100000
150000
200000
0
20
40
60
80
100
m/z
Fragment Ions
175.119 y1
265.120 b2
274.152 y2
336.156 b3
371.238 y3
465.199 b4
508.295 y4
578.276 b5
655.367 y5
691.359 b6
752.422 y6
820.409 b7
881.455 y7
917.449 b8
994.550 y8
1107.634 y9
1236.694 y10
1307.716 y11
TYAEILEPFHPVR / File: christine_10pools_09.raw; SpectrumID: ; scans: 18693

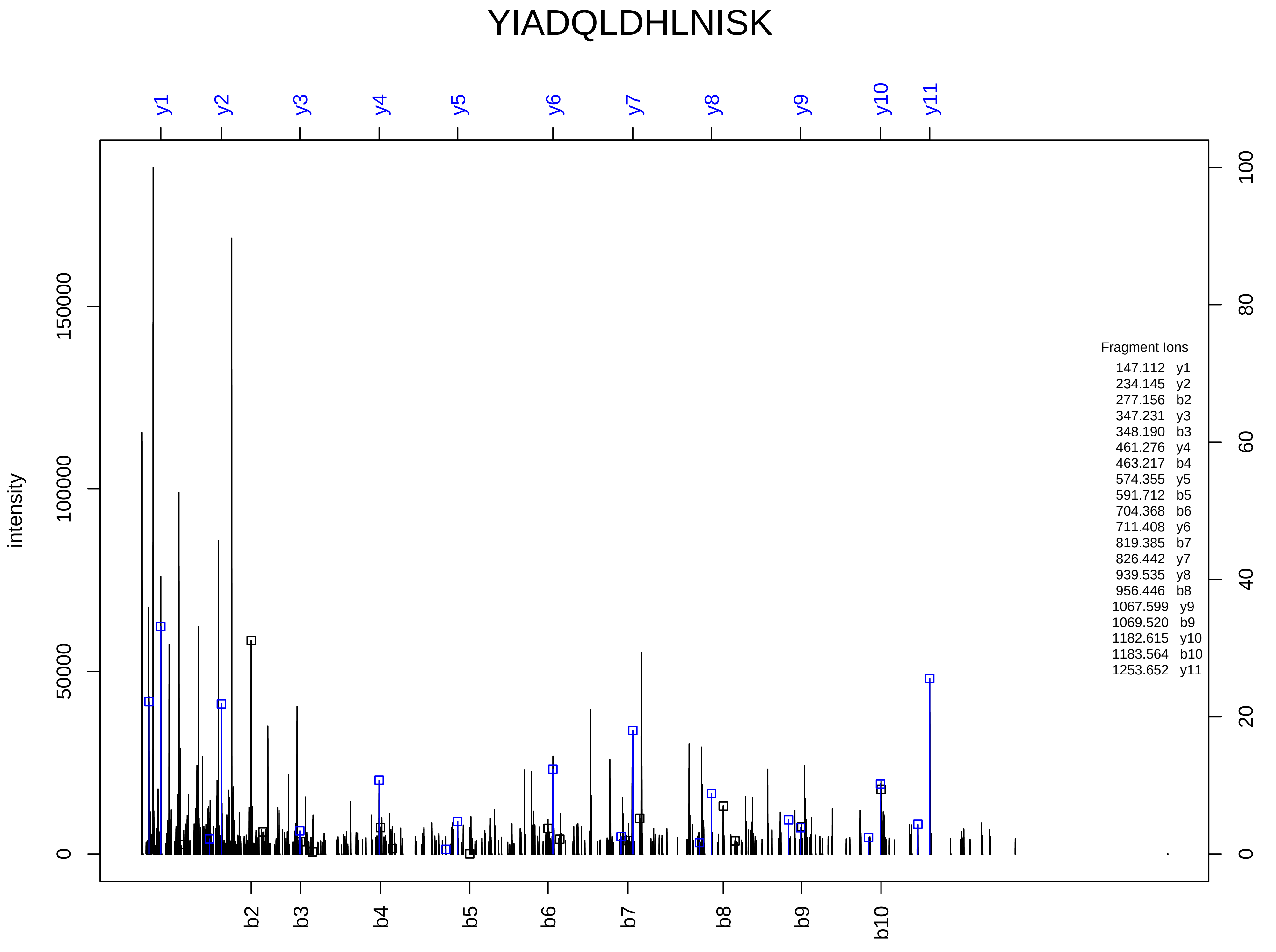
YIADQLDHLNISK
y1 y2 y3 y4 y5 y6 y7 y8 y9 y10 y11
b2 b3 b4 b5 b6 b7 b8 b9 b10
intensity
0
50000
100000
150000
0
20
40
60
80
100
m/z
Fragment Ions
147.112 y1
234.145 y2
277.156 b2
347.231 y3
348.190 b3
461.276 y4
463.217 b4
574.355 y5
591.712 b5
704.368 b6
711.408 y6
819.385 b7
826.442 y7
939.535 y8
956.446 b8
1067.599 y9
1069.520 b9
1182.615 y10
1183.564 b10
1253.652 y11
YIADQLDHLNISK / File: christine_10pools_09.raw; SpectrumID: ; scans: 13749

AVAPLRPGELLFR

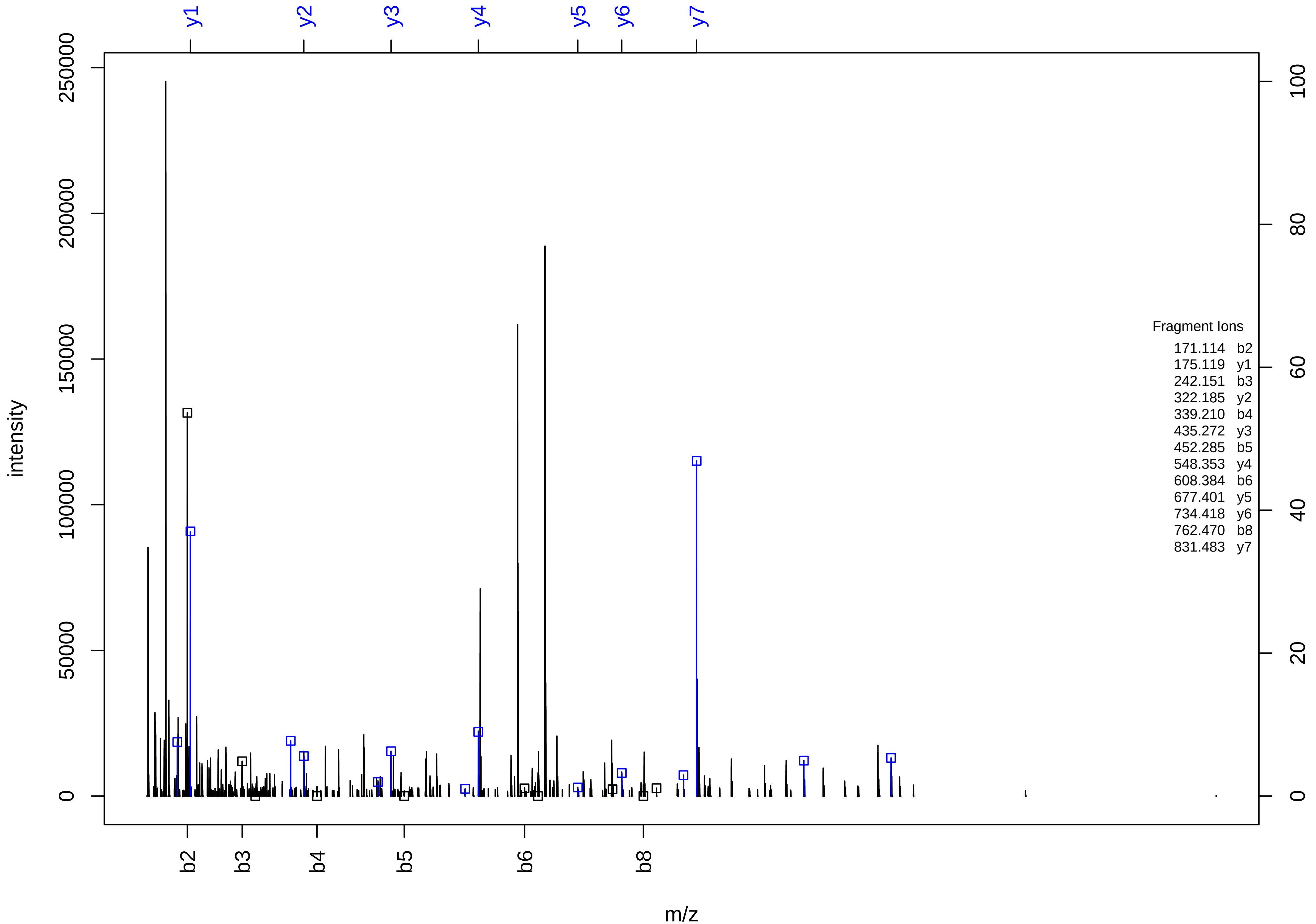

AVAPLRPGELLFR / File: christine_10pools_09.raw; SpectrumID: ; scans: 19553

DGLSYQIFPDPSDFDR

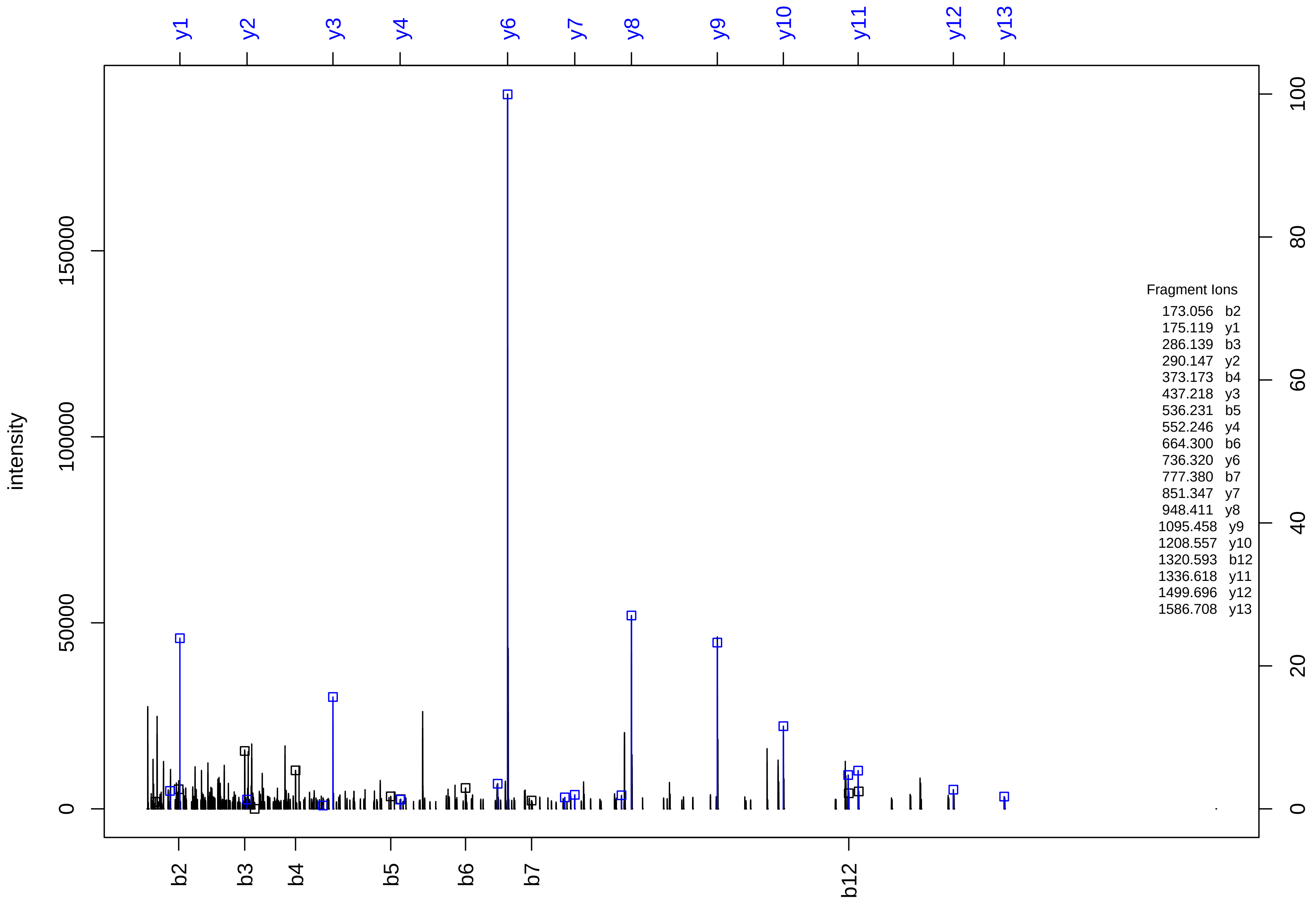

DGLSYQIFPDPSDFDR / File: christine_10pools_09.raw; SpectrumID: ; scans: 31273

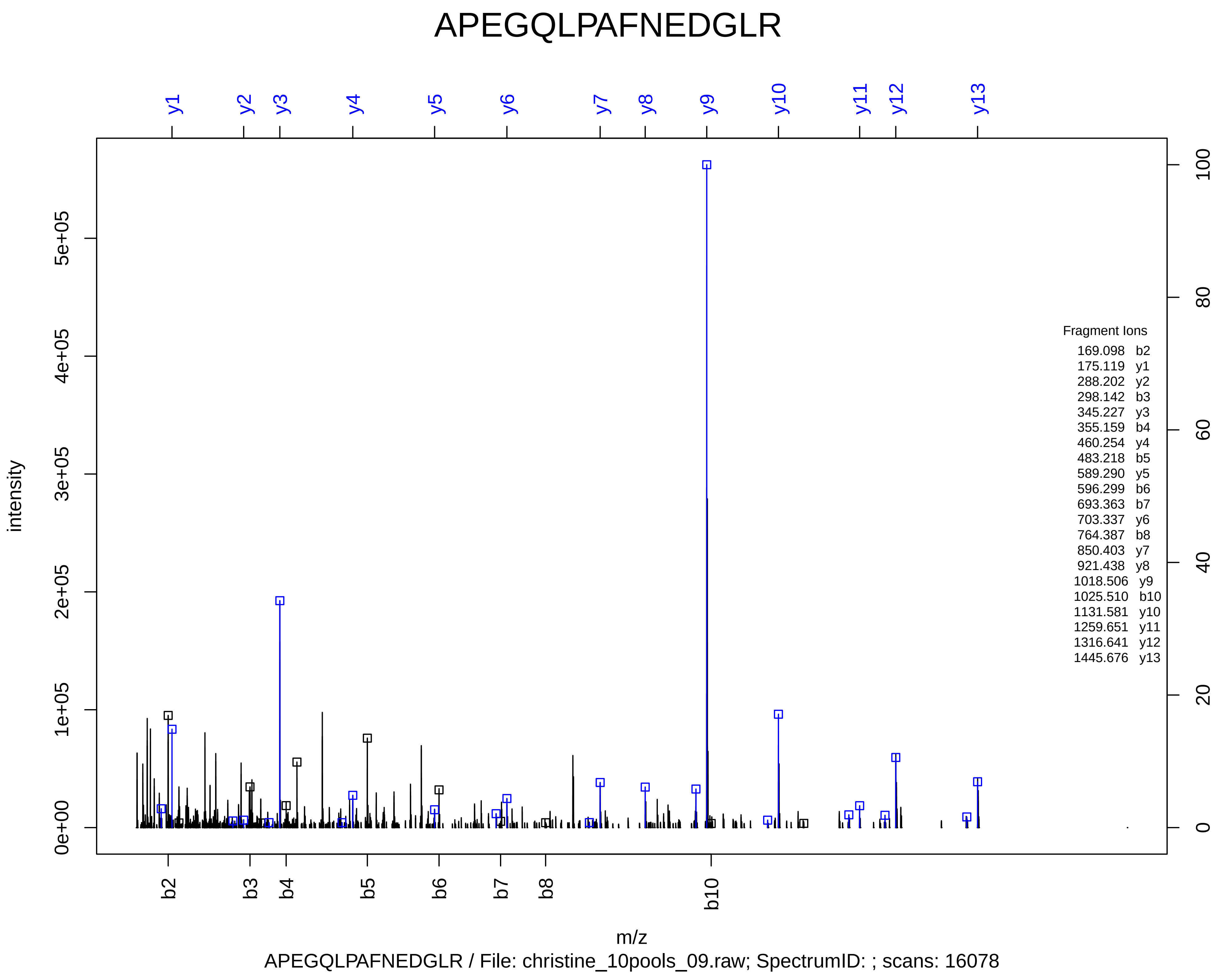
APEGQLPAFNEDGLR
y1
y2
y3
y4
y5
y6
y7
y8
y9
y10
y11
y12
y13
intensity
0e+00
1e+05
2e+05
3e+05
4e+05
5e+05
0
20
40
60
80
100
Fragment Ions
169.098 b2
175.119 y1
288.202 y2
298.142 b3
345.227 y3
355.159 b4
460.254 y4
483.218 b5
589.290 y5
596.299 b6
693.363 b7
703.337 y6
764.387 b8
850.403 y7
921.438 y8
1018.506 y9
1025.510 b10
1131.581 y10
1259.651 y11
1316.641 y12
1445.676 y13
b2
b3
b4
b5
b6
b7
b8
b10
m/z
APEGQLPAFNEDGLR / File: christine_10pools_09.raw; SpectrumID: ; scans: 16078

(Ac)ASGRPEELWEAVVGAAER

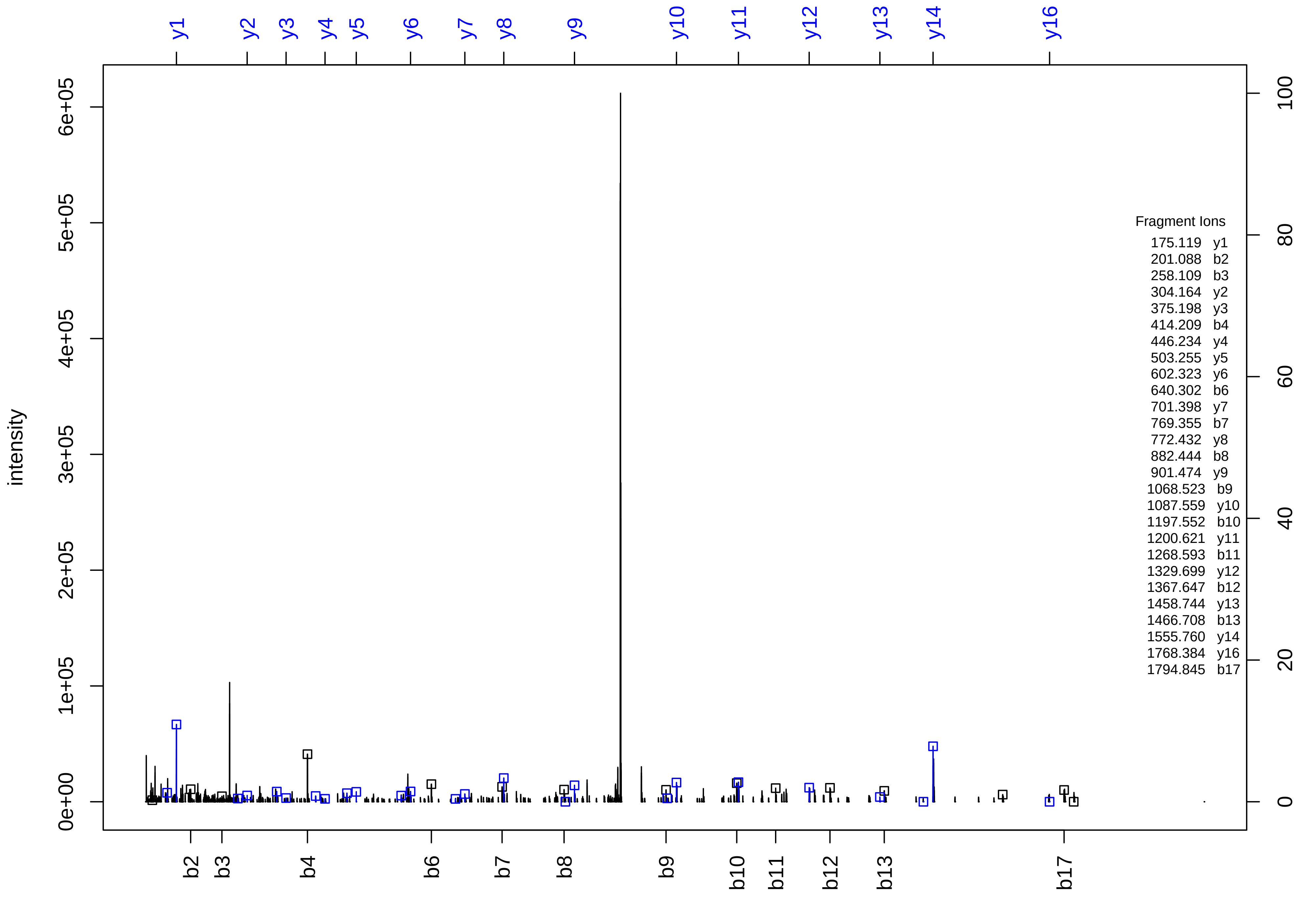

ASGRPEELWEAVVGAAER / File: christine_10pools_09.raw; SpectrumID: ; scans: 33287

QELIQQWEN^TIEQM*QRR

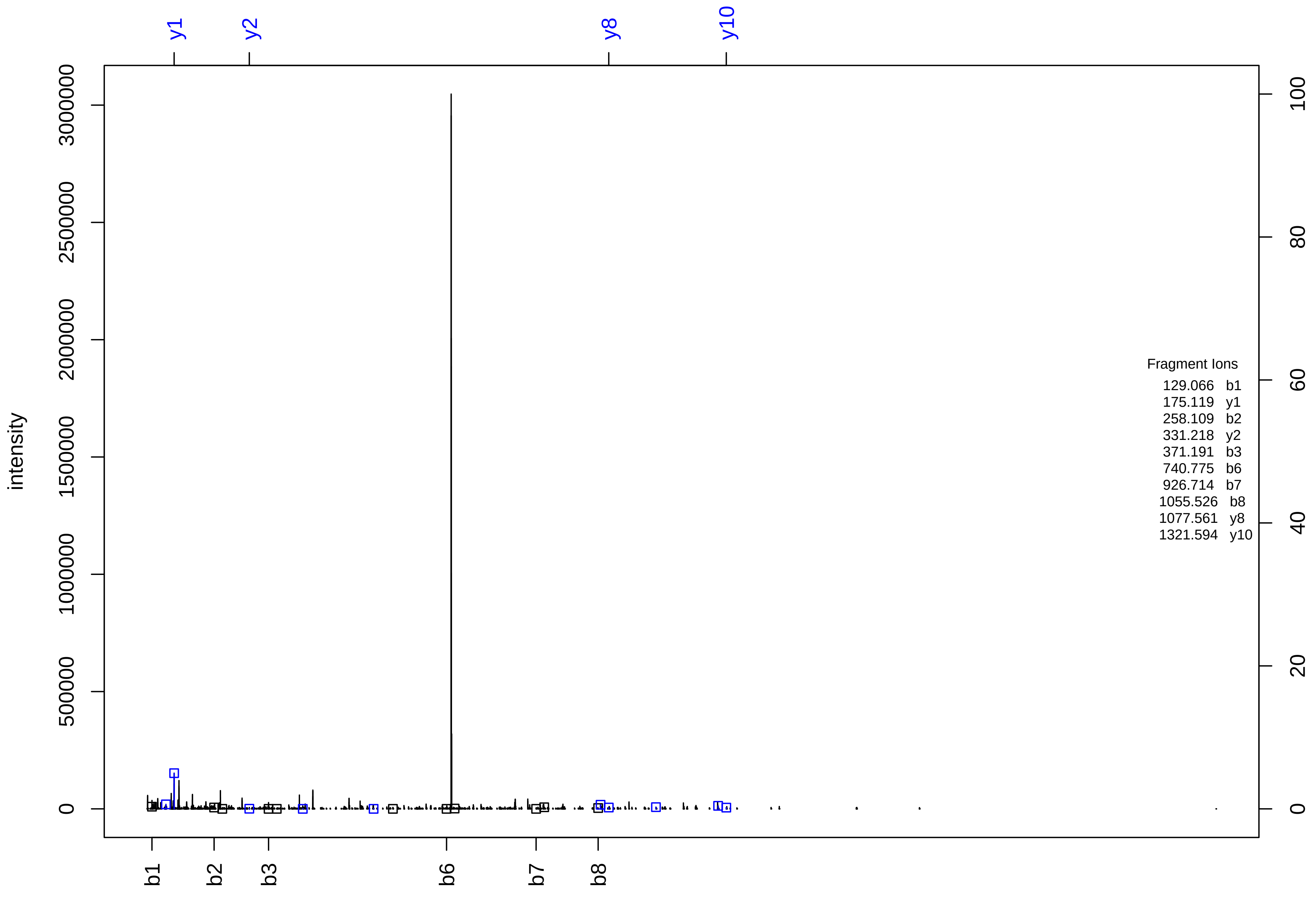

QELIQQWENTIEQMQRR / File: christine_10pools_09.raw; SpectrumID: ; scans: 16127

AAAFGPDQSLLSPVHVLDLEPR

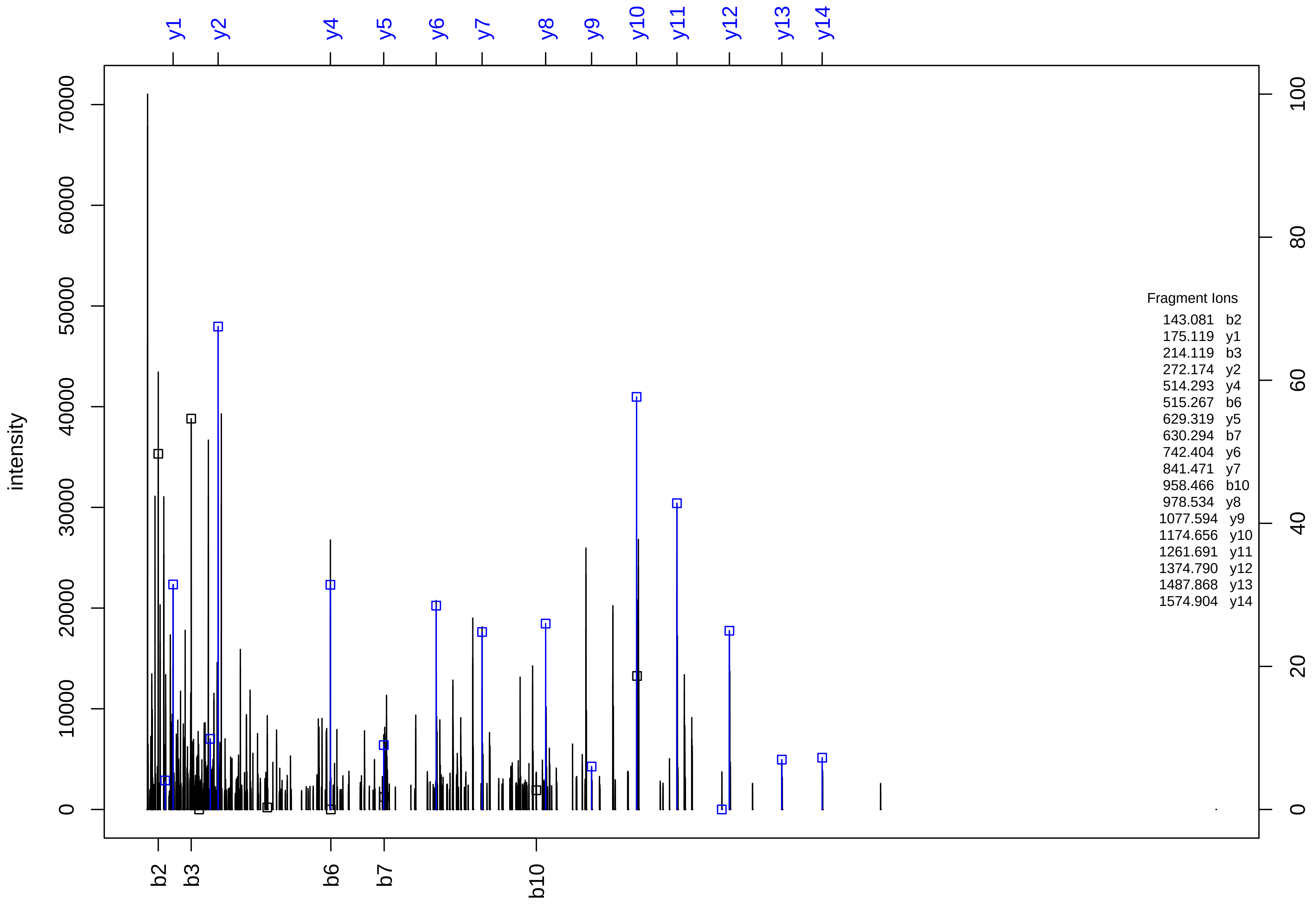

AAAFGPDQSLLSPVHVLDLEPR / File: christine_10pools_09.raw; SpectrumID: ; scans: 30463

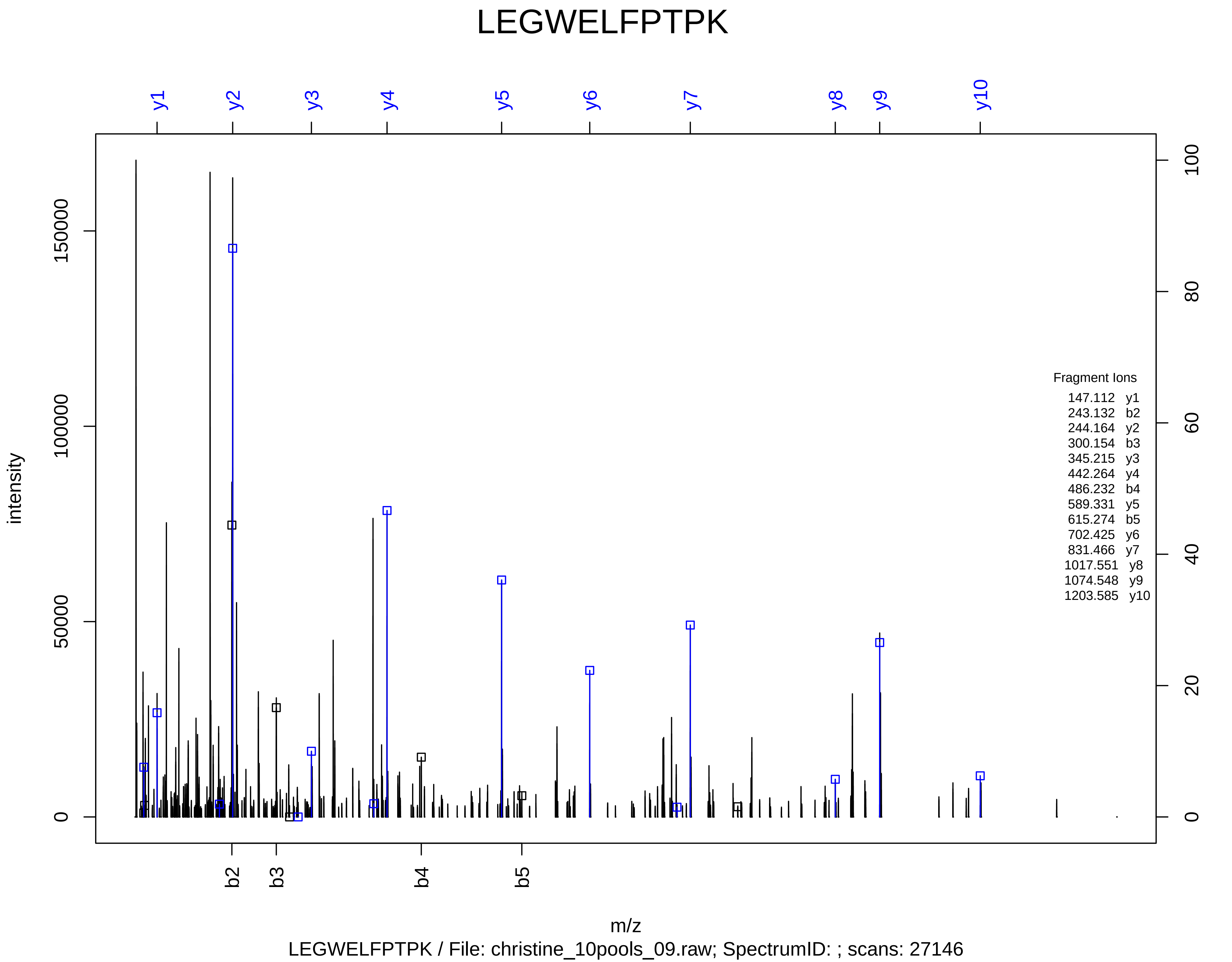

LEGWELFPTPK
y1
y2
y3
y4
y5
y6
y7
y8
y9
y10
b2
b3
b4
b5
intensity
0
50000
100000
150000
0
20
40
60
80
100
m/z
Fragment Ions
147.112 y1
243.132 b2
244.164 y2
300.154 b3
345.215 y3
442.264 y4
486.232 b4
589.331 y5
615.274 b5
702.425 y6
831.466 y7
1017.551 y8
1074.548 y9
1203.585 y10
LEGWELFPTPK / File: christine_10pools_09.raw; SpectrumID: ; scans: 27146

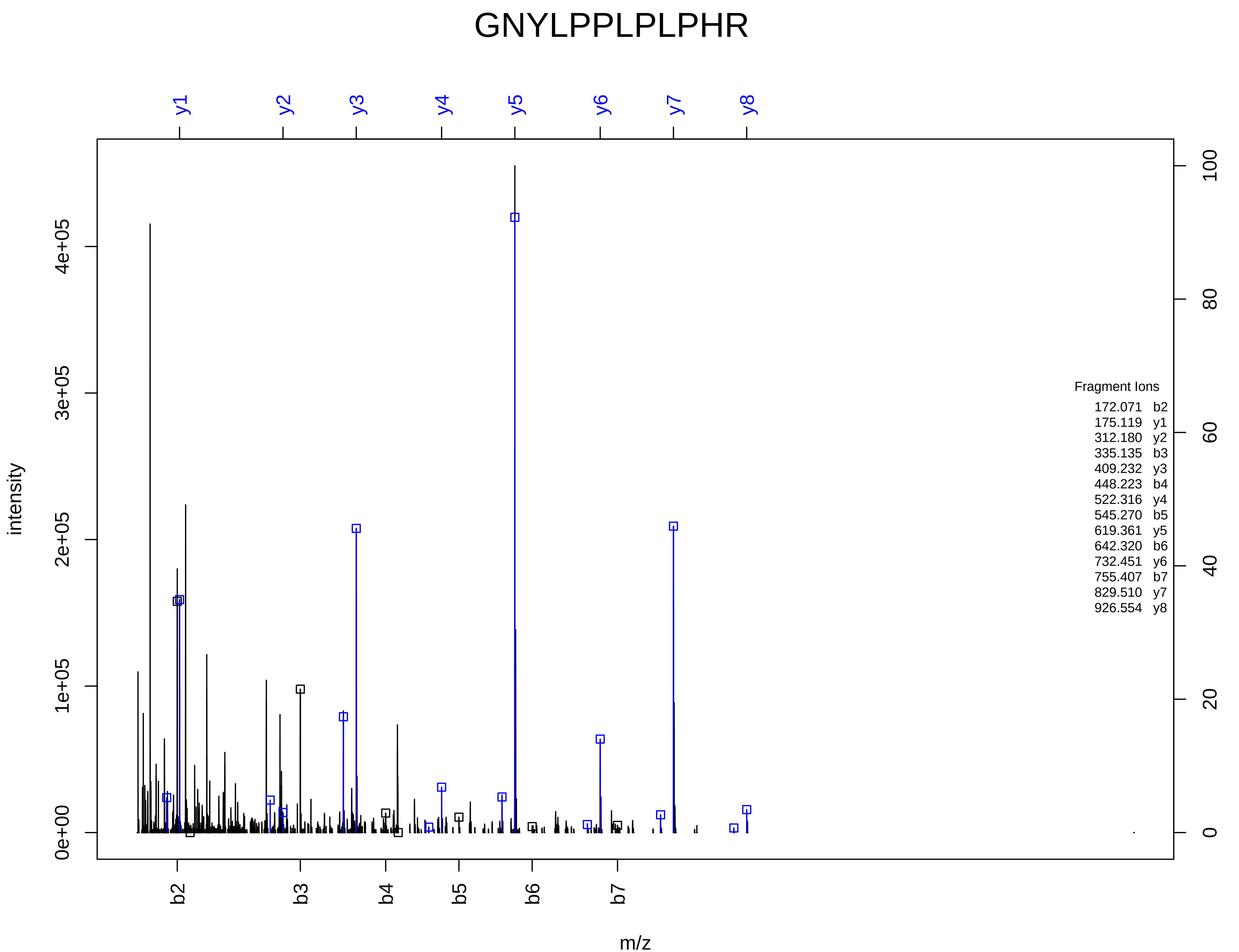
GNYLPPLPLPHR
y1
y2
y3
y4
y5
y6
y7
y8
intensity
0e+00
1e+05
2e+05
3e+05
4e+05
0
20
40
60
80
100
Fragment Ions
172.071 b2
175.119 y1
312.180 y2
335.135 b3
409.232 y3
448.223 b4
522.316 y4
545.270 b5
619.361 y5
642.320 b6
732.451 y6
755.407 b7
829.510 y7
926.554 y8
b2
b3
b4
b5
b6
b7
m/z
GNYLPPLPLPHR / File: christine_10pools_09.raw; SpectrumID: ; scans: 18411

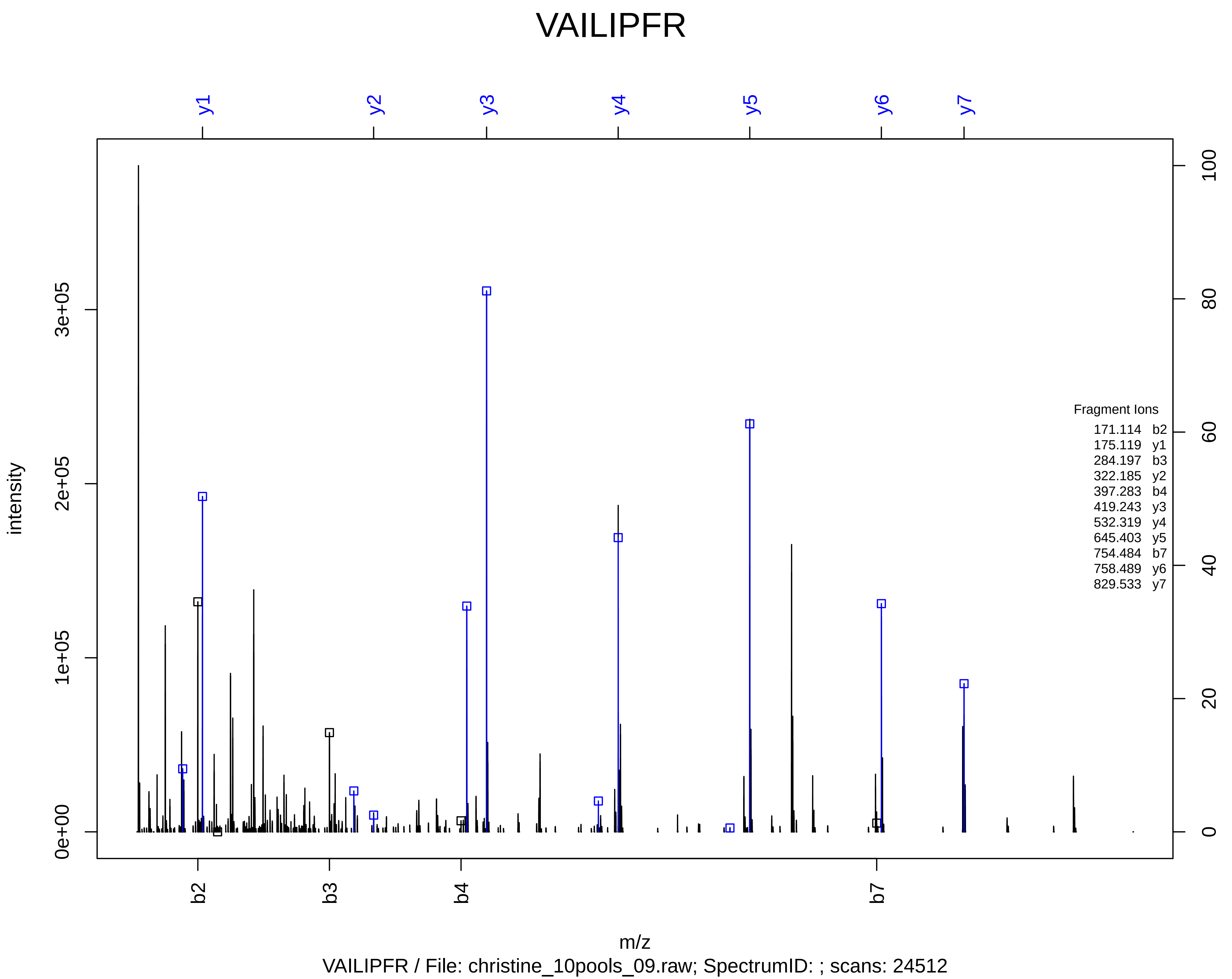
VAILIPFR
y1
y2
y3
y4
y5
y6
y7
intensity
0e+00
1e+05
2e+05
3e+05
0
20
40
60
80
100
Fragment Ions
171.114 b2
175.119 y1
284.197 b3
322.185 y2
397.283 b4
419.243 y3
532.319 y4
645.403 y5
754.484 b7
758.489 y6
829.533 y7
b2
b3
b4
b7
m/z
VAILIPFR / File: christine_10pools_09.raw; SpectrumID: ; scans: 24512

VLHAAFIPFGDITDIQIPLDYETEK

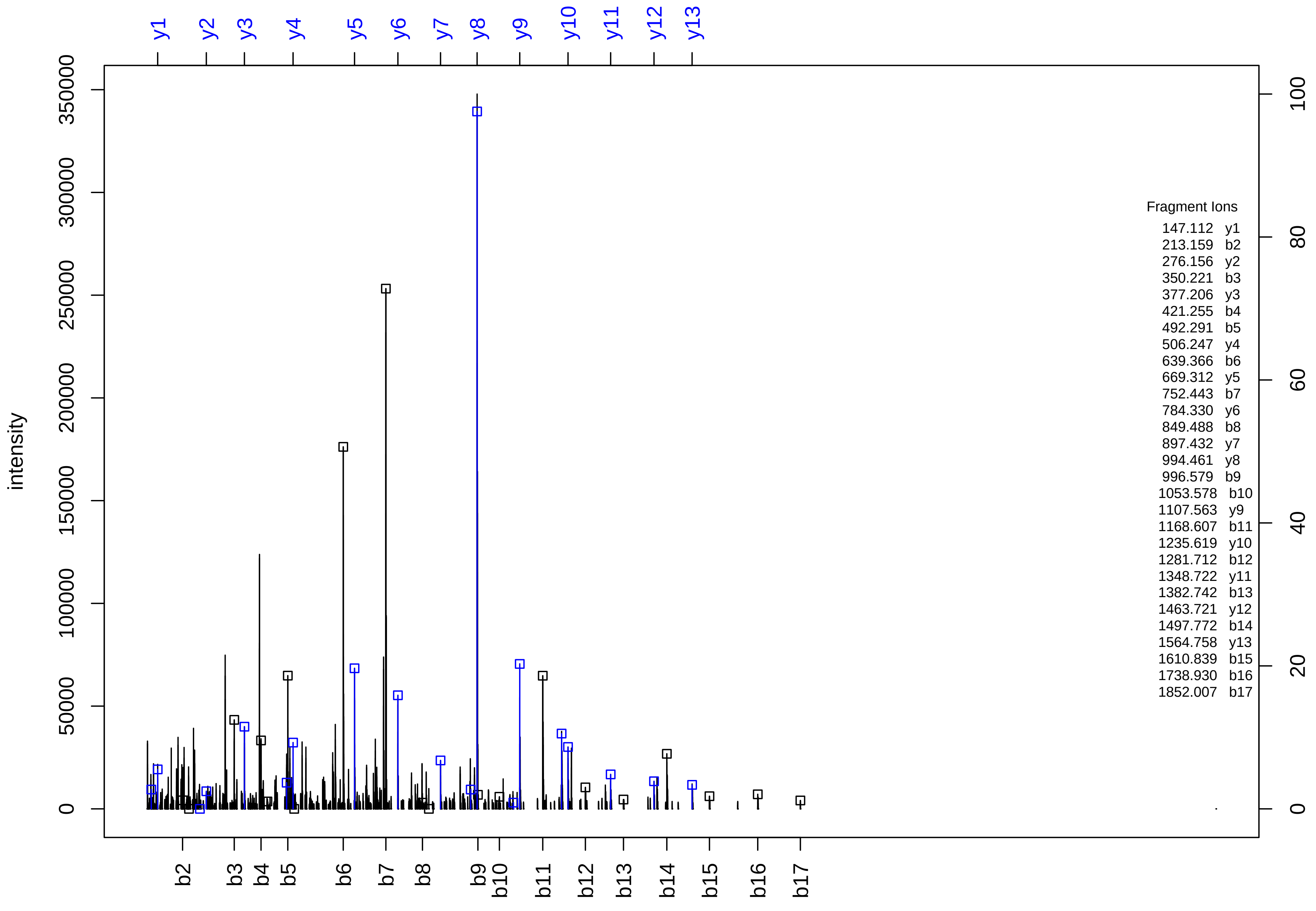

VLHAAFIPFGDITDIQIPLDYETEK / File: christine_10pools_09.raw; SpectrumID: ; scans: 39541

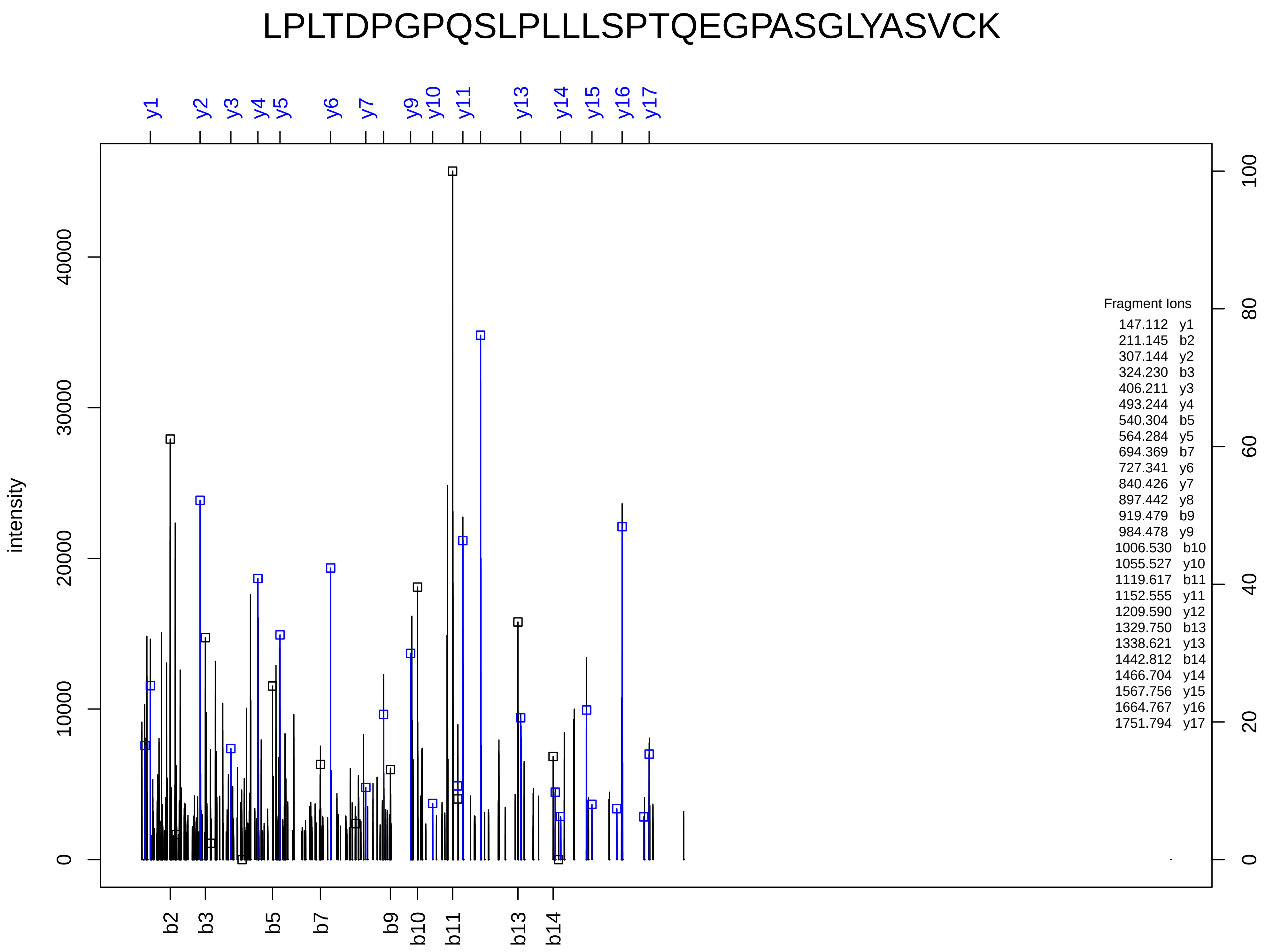

LPLTDPGPQSLPLLLSPTQEGPASGLYASVCK / File: christine_10pools_09.raw; SpectrumID: ; scans: 38219

IPNAPYVLEDFVDNVK

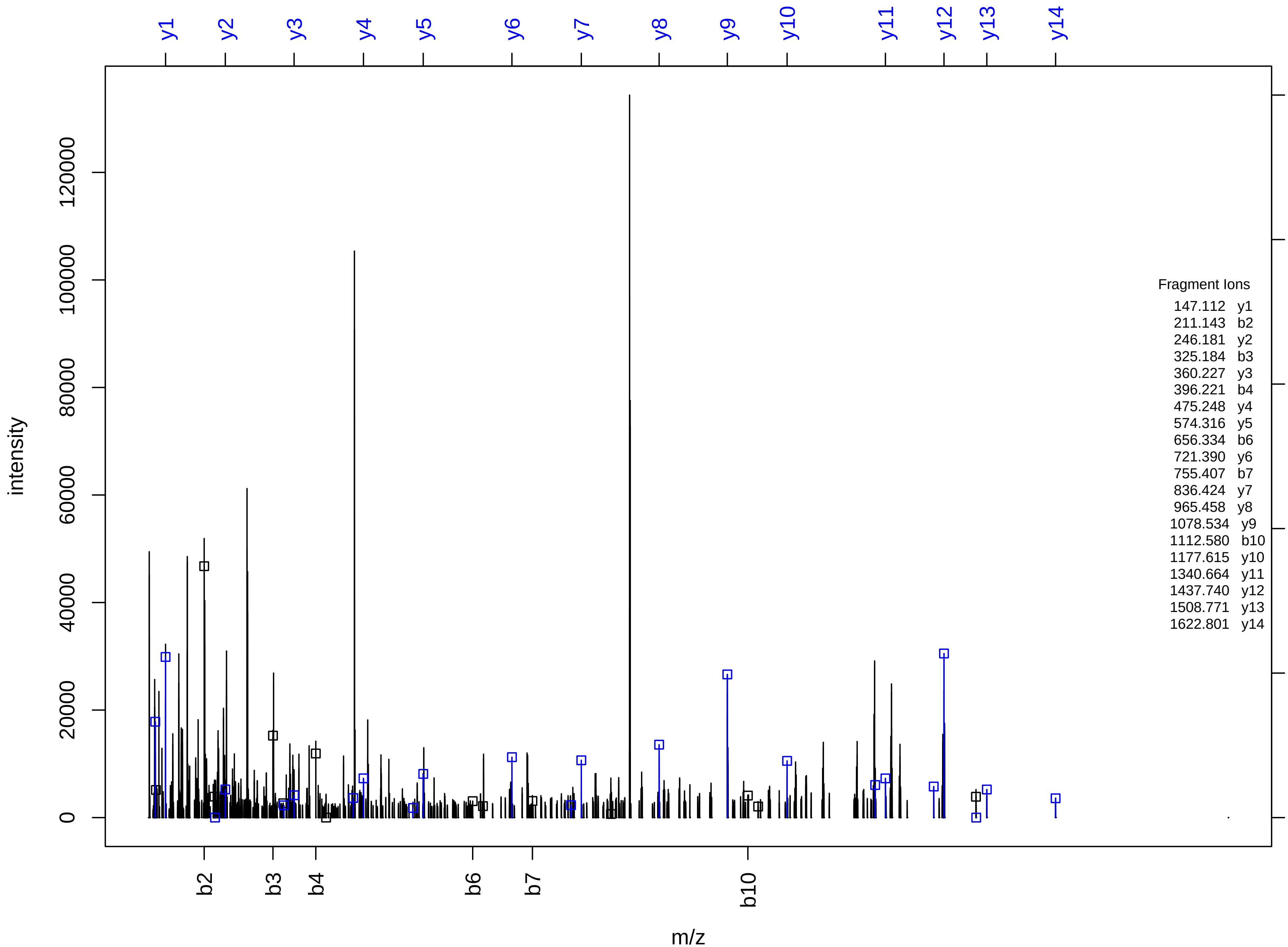

IPNAPYVLEDFVDNVK / File: christine_10pools_09.raw; SpectrumID: ; scans: 33121

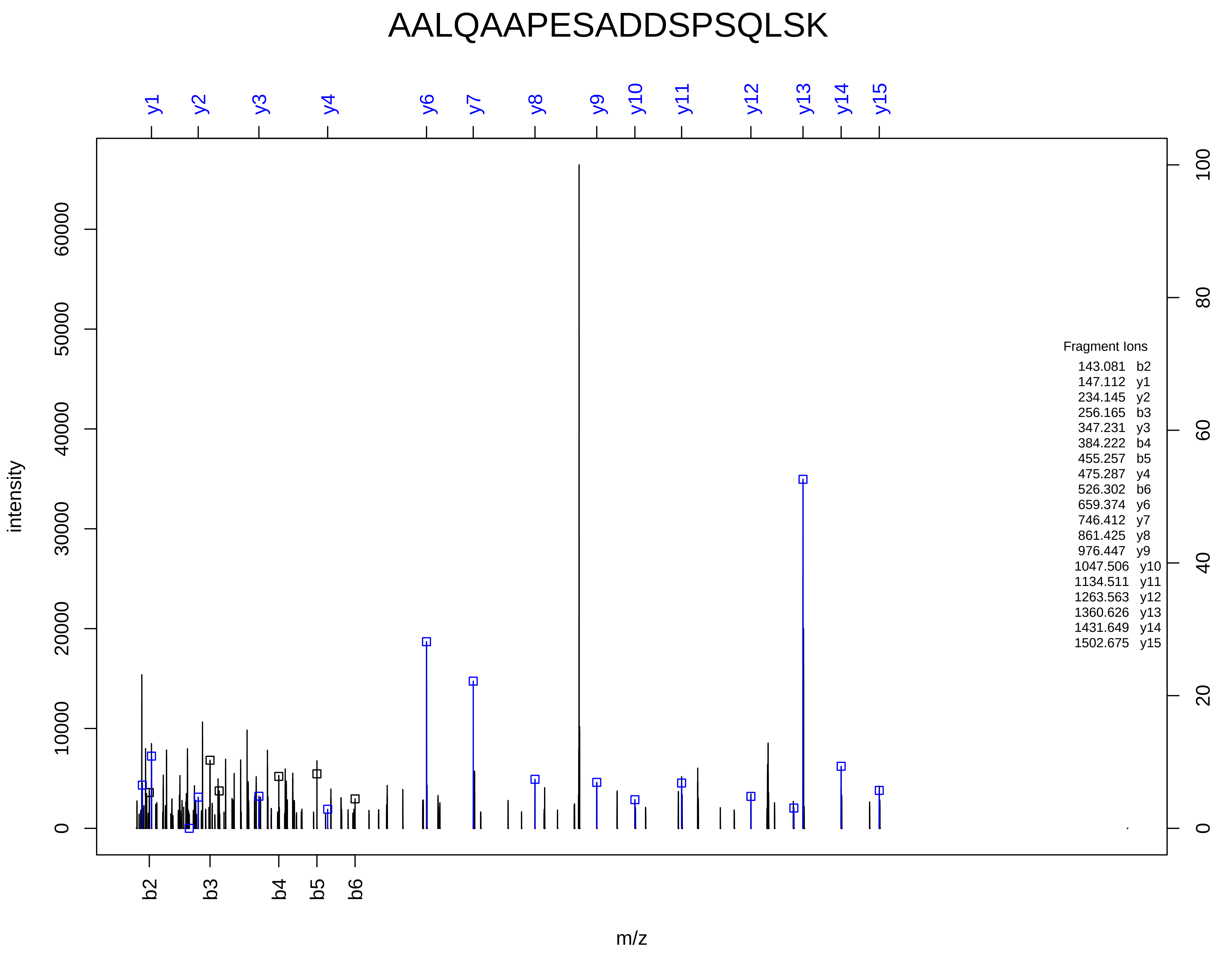

AALQAAPESADDSPSQLSK / File: christine_10pools_09.raw; SpectrumID: ; scans: 5246

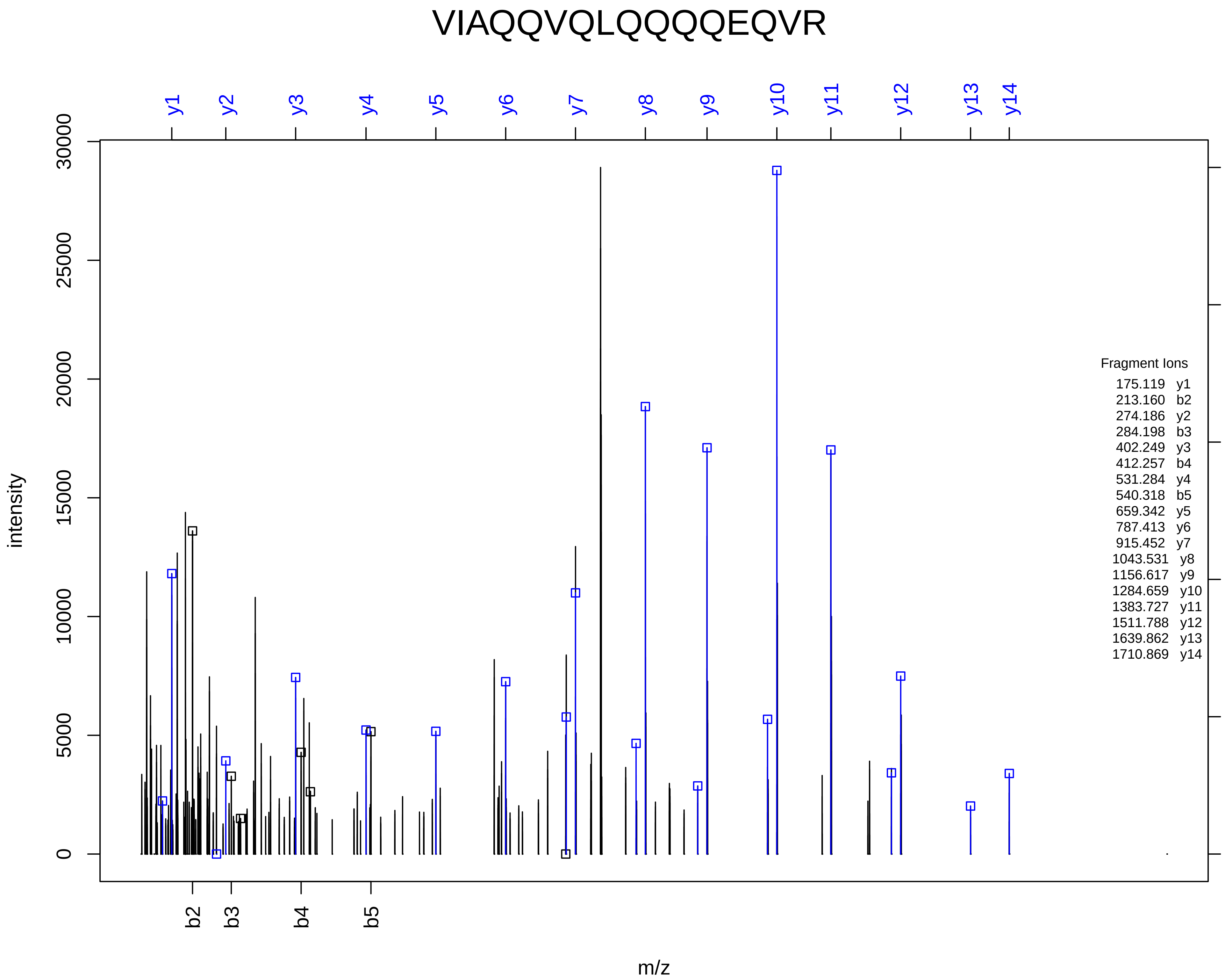

VIAQQVQLQQQEQVR / File: christine_10pools_09.raw; SpectrumID: ; scans: 5537

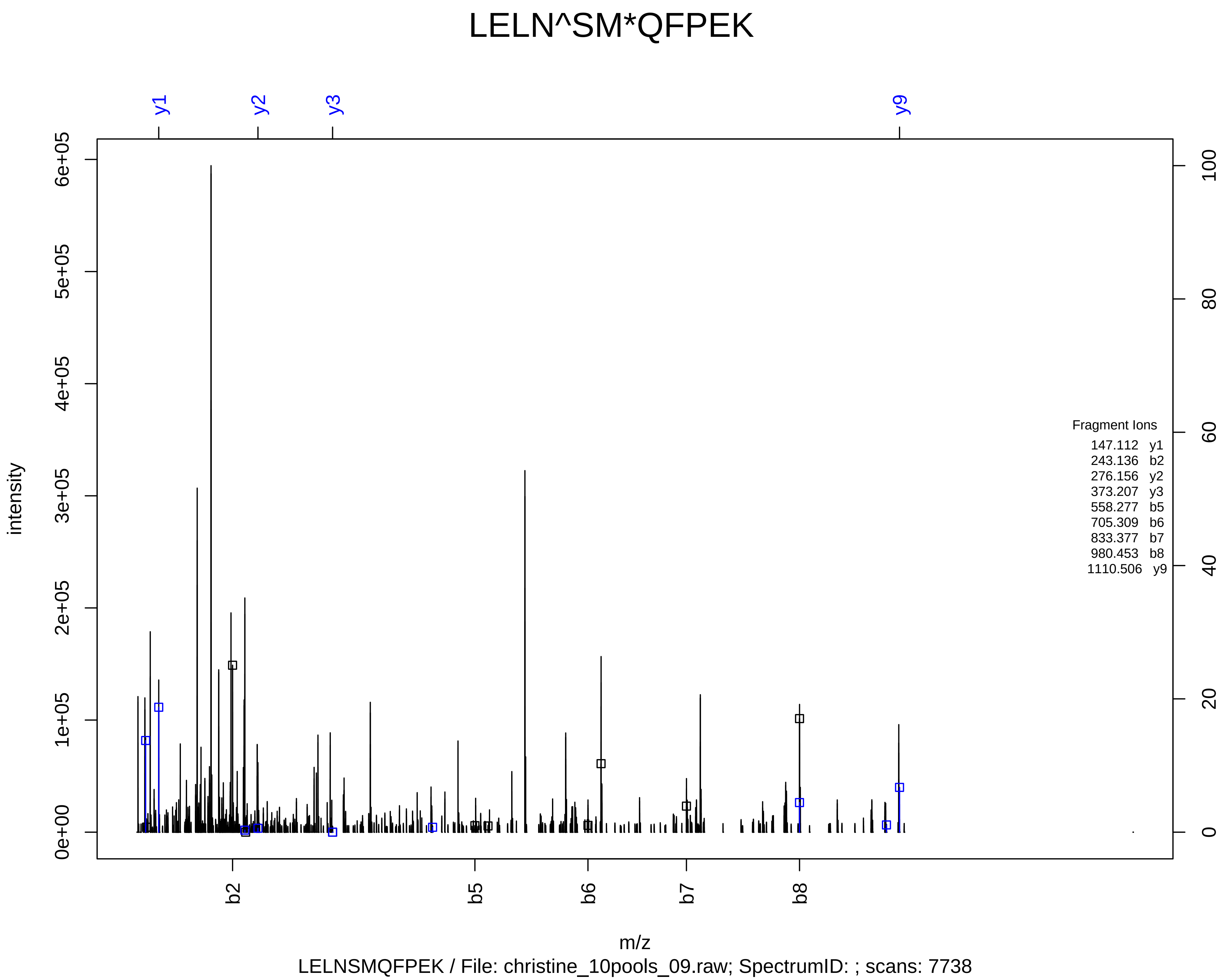
LELN^SM*QFPEK
y1
y2
y3
y9
intensity
0e+00
1e+05
2e+05
3e+05
4e+05
5e+05
6e+05
0
20
40
60
80
100
b2
b5
b6
b7
b8
m/z
Fragment Ions
147.112 y1
243.136 b2
276.156 y2
373.207 y3
558.277 b5
705.309 b6
833.377 b7
980.453 b8
1110.506 y9
LELNSMQFPEK / File: christine_10pools_09.raw; SpectrumID: ; scans: 7738

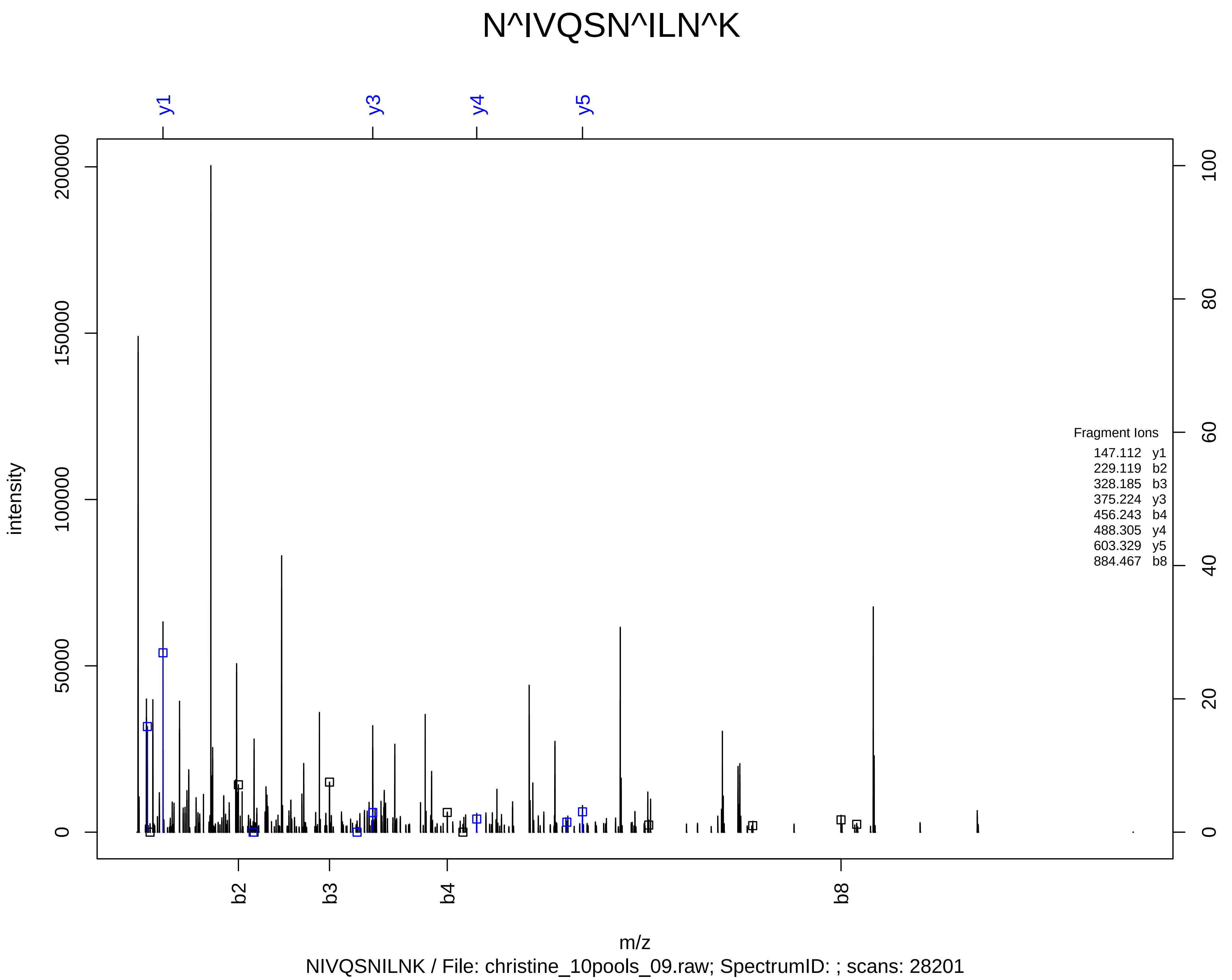

N^IVQSN^ILN^K
y1
y3
y4
y5
intensity
200000
150000
100000
50000
0
100
80
60
40
20
0
Fragment Ions
147.112 y1
229.119 b2
328.185 b3
375.224 y3
456.243 b4
488.305 y4
603.329 y5
884.467 b8
b2
b3
b4
b8
m/z
NIVQSNILNK / File: christine_10pools_09.raw; SpectrumID: ; scans: 28201

RQKN^VYN^N^IPSN^GQHR

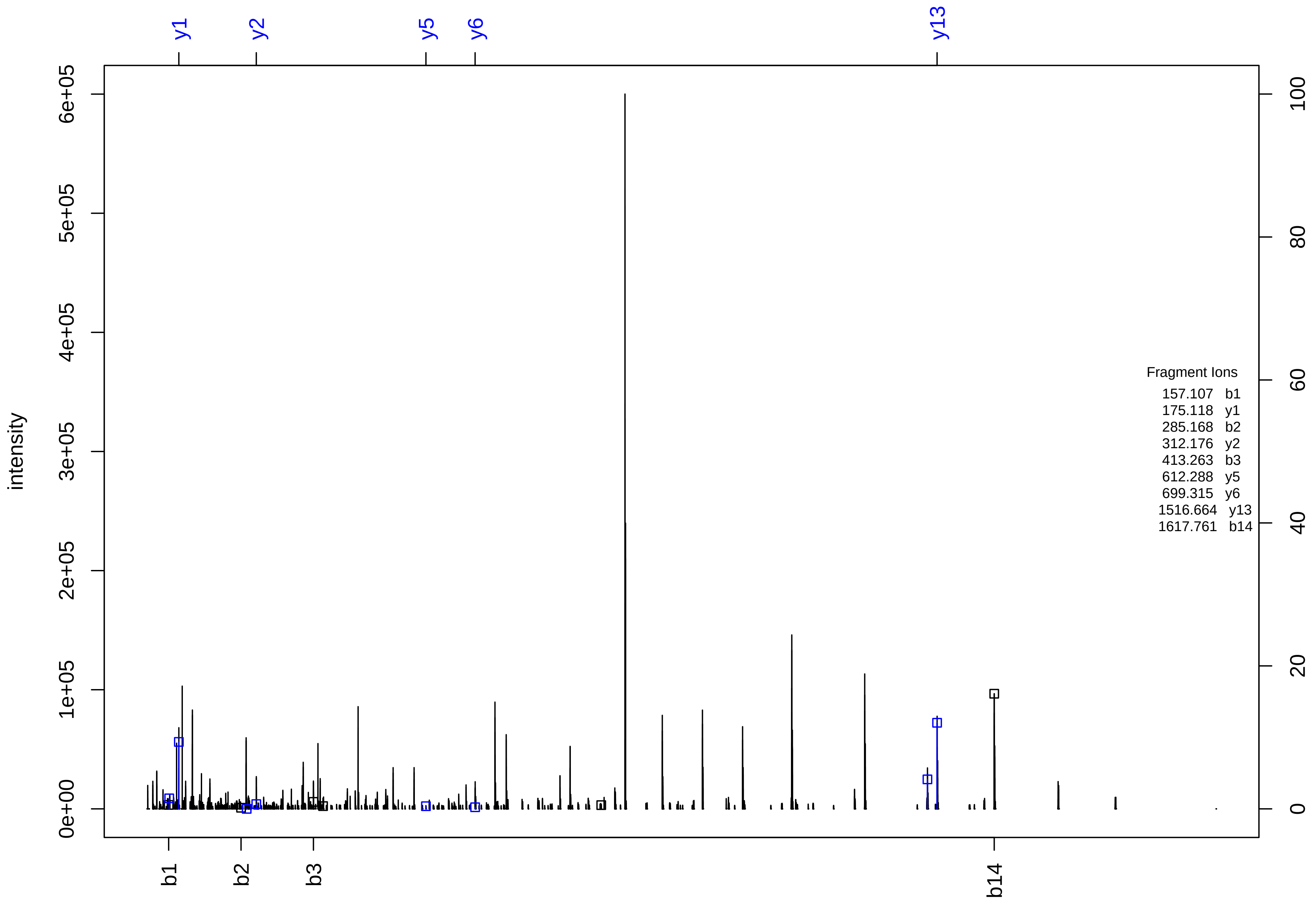

RQKNVYNNIPSNGQHR / File: christine_10pools_09.raw; SpectrumID: ; scans: 19798

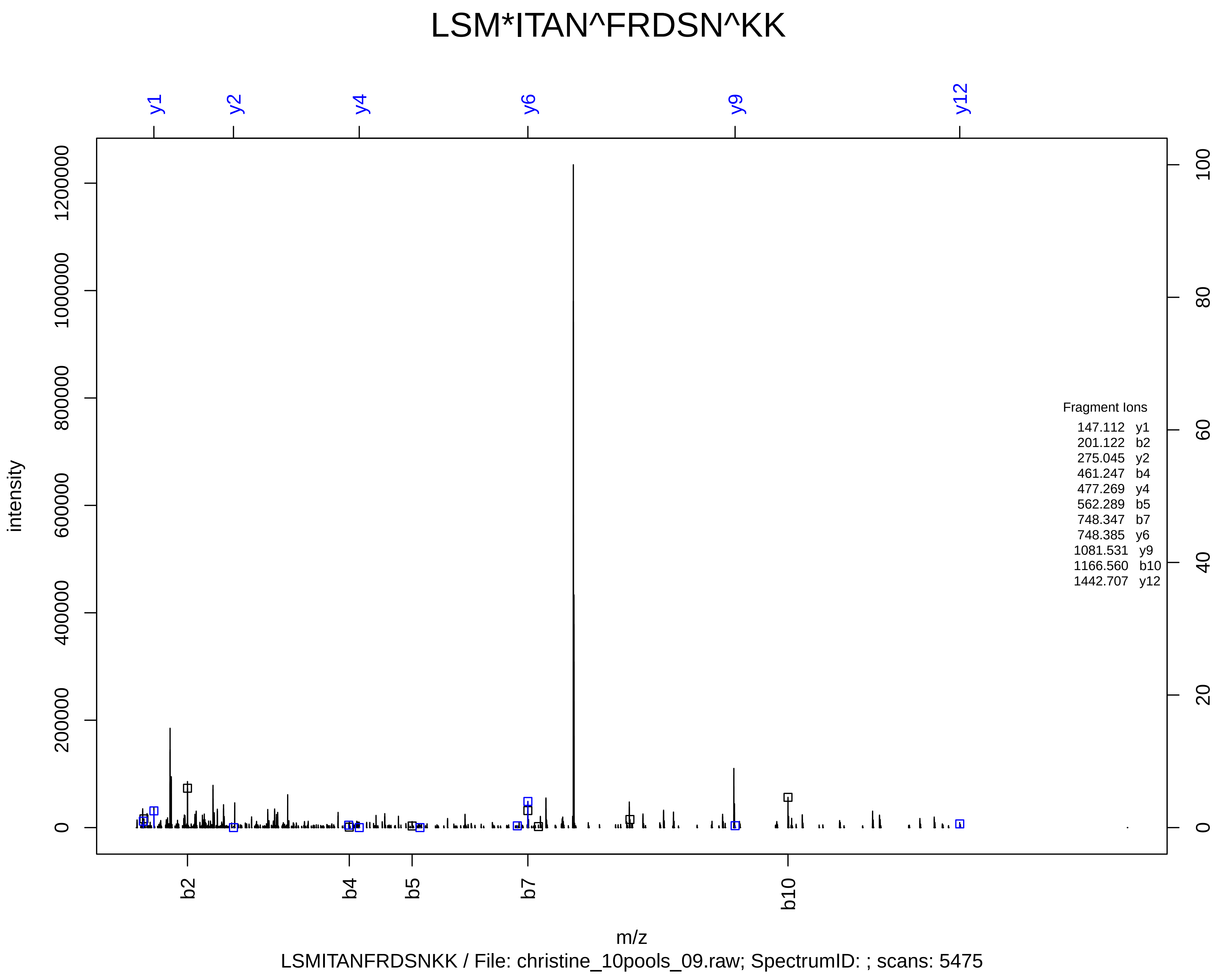
LSM*ITAN^FRDSN^KK
y1
y2
y4
y6
y9
y12
intensity
0
200000
400000
600000
800000
1000000
1200000
0
20
40
60
80
100
b2
b4
b5
b7
b10
m/z
Fragment Ions
147.112 y1
201.122 b2
275.045 y2
461.247 b4
477.269 y4
562.289 b5
748.347 b7
748.385 y6
1081.531 y9
1166.560 b10
1442.707 y12
LSMITANFRDSNKK / File: christine_10pools_09.raw; SpectrumID: ; scans: 5475

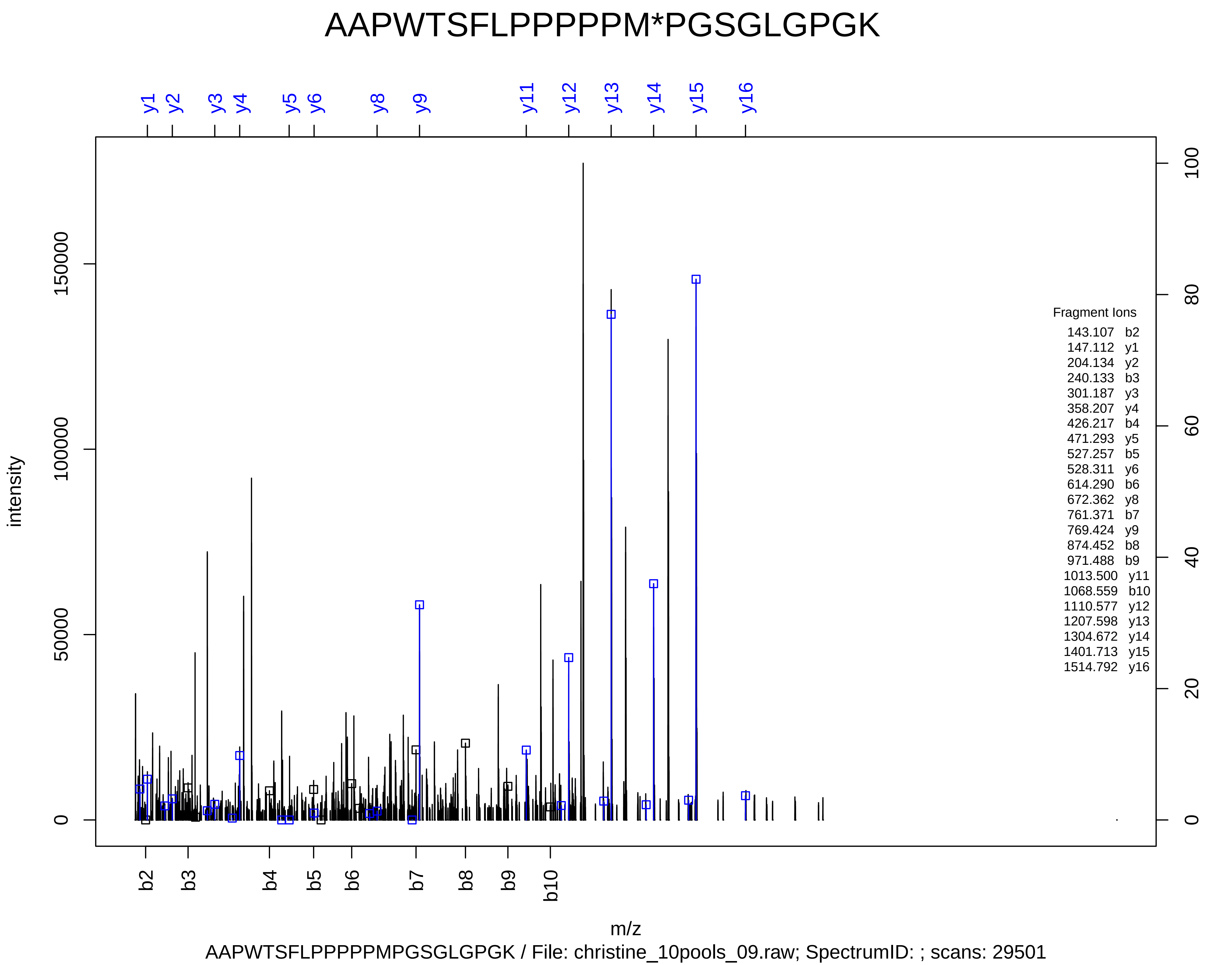

AAPWTSFLPPPPPM*PGSGLGPGK
y1
y2
y3
y4
y5
y6
y8
y9
y11
y12
y13
y14
y15
y16
intensity
0
50000
100000
150000
0
20
40
60
80
100
Fragment Ions
143.107 b2
147.112 y1
204.134 y2
240.133 b3
301.187 y3
358.207 y4
426.217 b4
471.293 y5
527.257 b5
528.311 y6
614.290 b6
672.362 y8
761.371 b7
769.424 y9
874.452 b8
971.488 b9
1013.500 y11
1068.559 b10
1110.577 y12
1207.598 y13
1304.672 y14
1401.713 y15
1514.792 y16
b2
b3
b4
b5
b6
b7
b8
b9
b10
m/z
AAPWTSFLPPPPPMPGSGLGPGK / File: christine_10pools_09.raw; SpectrumID: ; scans: 29501

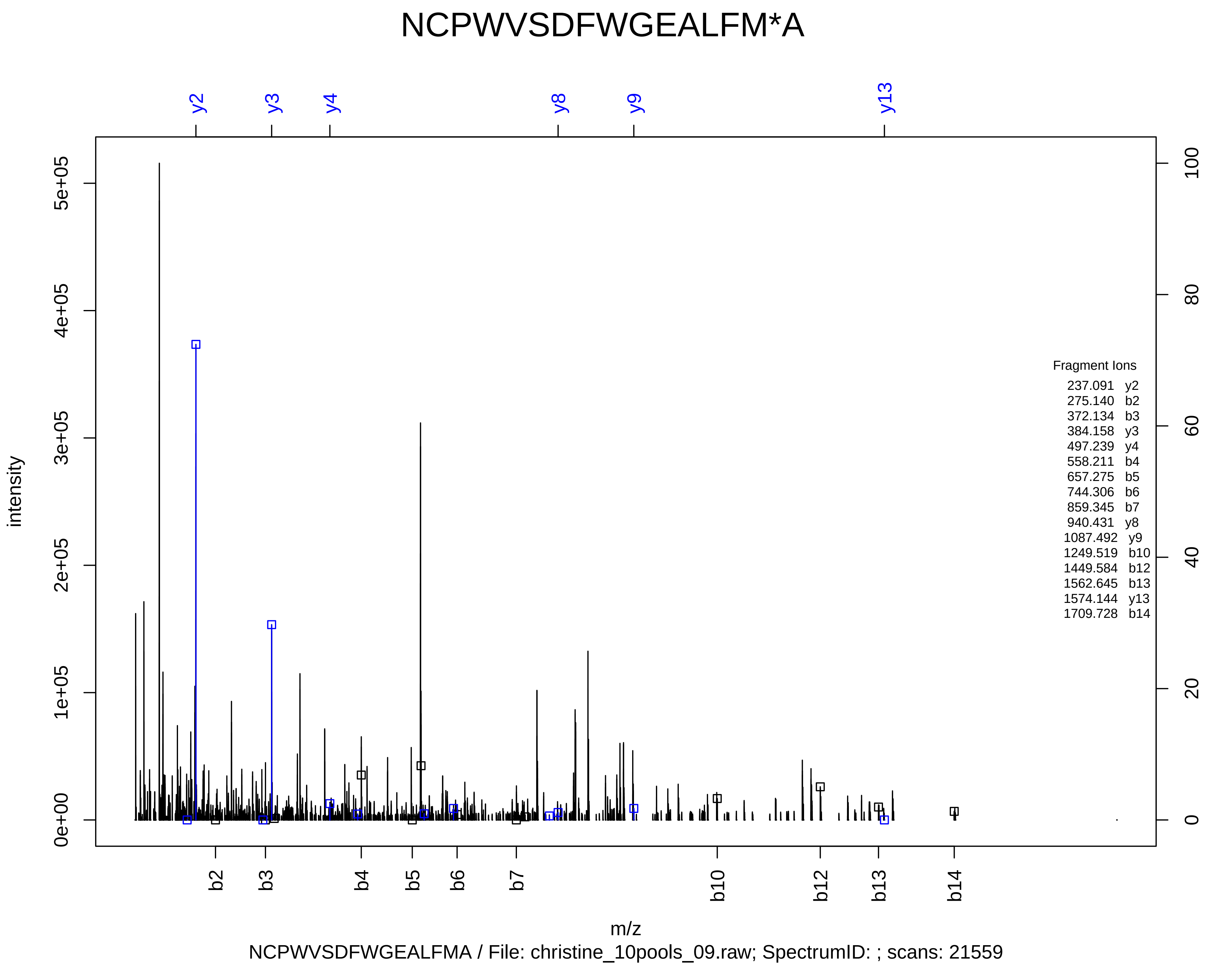

NCPWVSDFWGEALFM*A
y2
y3
y4
y8
y9
y13
intensity
0e+00
1e+05
2e+05
3e+05
4e+05
5e+05
0
20
40
60
80
100
Fragment Ions
237.091 y2
275.140 b2
372.134 b3
384.158 y3
497.239 y4
558.211 b4
657.275 b5
744.306 b6
859.345 b7
940.431 y8
1087.492 y9
1249.519 b10
1449.584 b12
1562.645 b13
1574.144 y13
1709.728 b14
b2
b3
b4
b5
b6
b7
b10
b12
b13
b14
m/z
NCPWVSDFWGEALFMA / File: christine_10pools_09.raw; SpectrumID: ; scans: 21559

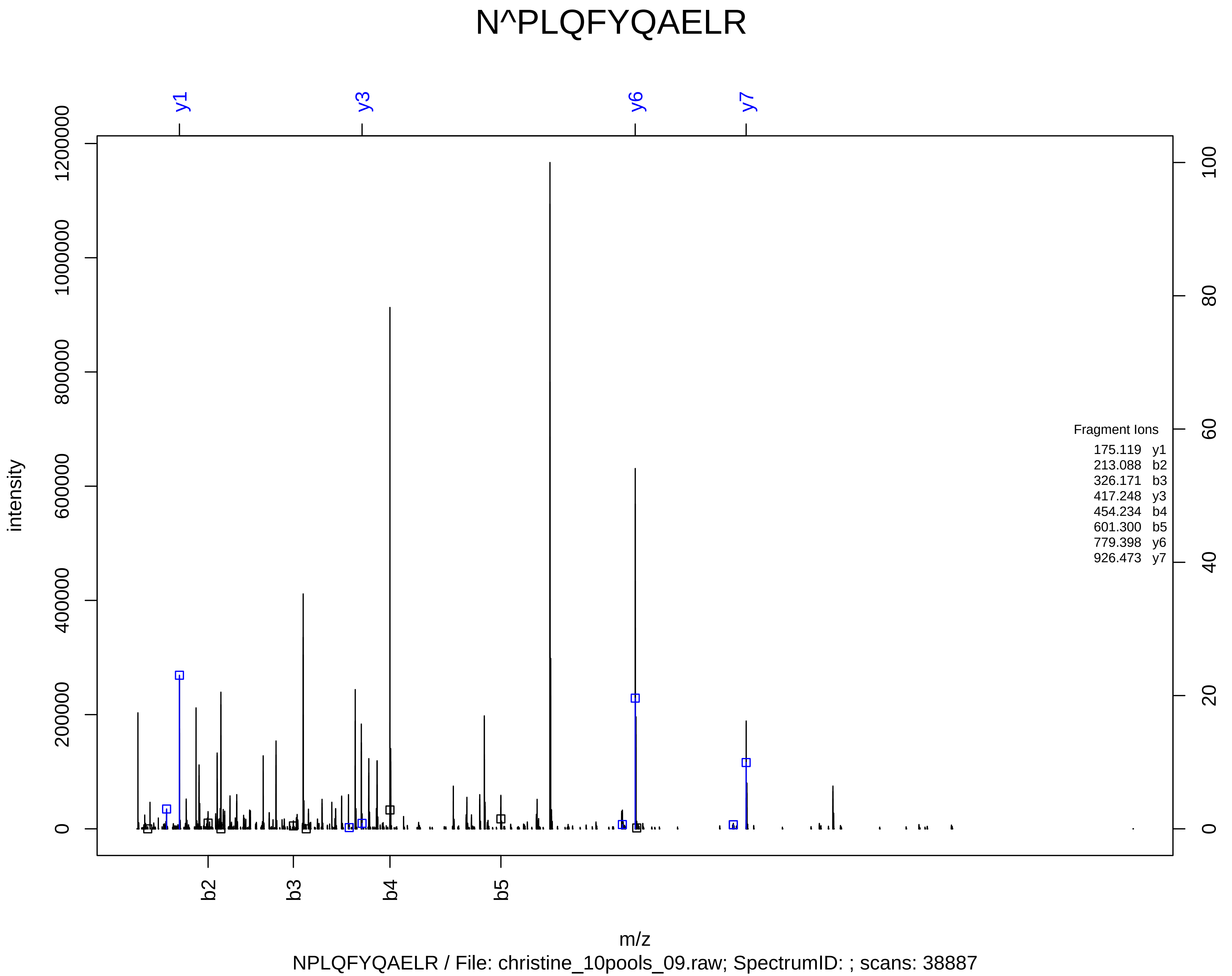

N^PLQFYQAELR
y1
y3
y6
y7
intensity
0
200000
400000
600000
800000
1000000
1200000
0
20
40
60
80
100
b2
b3
b4
b5
m/z
Fragment Ions
175.119 y1
213.088 b2
326.171 b3
417.248 y3
454.234 b4
601.300 b5
779.398 y6
926.473 y7
NPLQFYQAELR / File: christine_10pools_09.raw; SpectrumID: ; scans: 38887

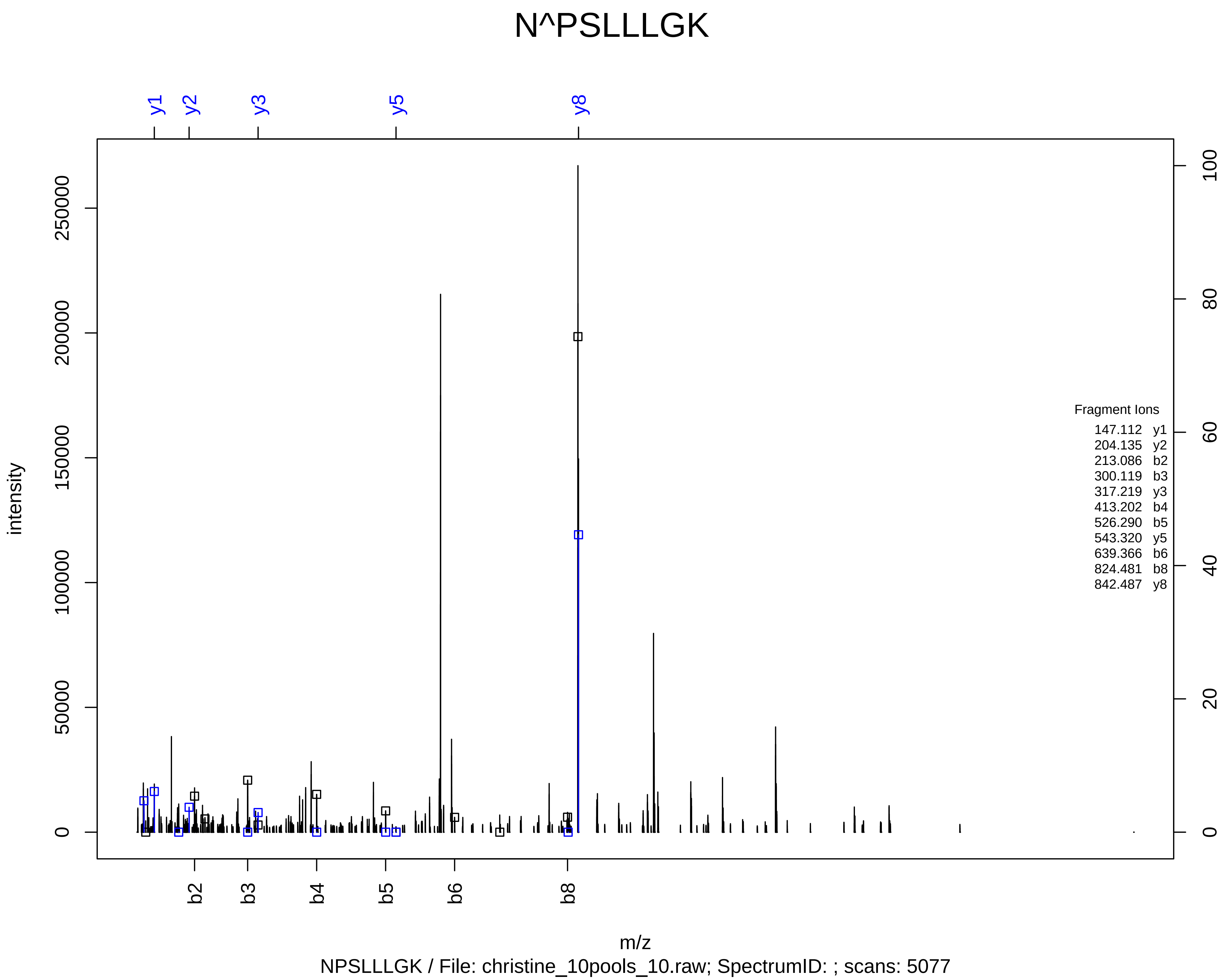

N^PSLLLGK
y1
y2
y3
y5
y8
intensity
0
50000
100000
150000
200000
250000
0
20
40
60
80
100
b2
b3
b4
b5
b6
b8
Fragment Ions
147.112 y1
204.135 y2
213.086 b2
300.119 b3
317.219 y3
413.202 b4
526.290 b5
543.320 y5
639.366 b6
824.481 b8
842.487 y8
m/z
NPSLLLGK / File: christine_10pools_10.raw; SpectrumID: ; scans: 5077

LALLQDAPIHSPM*VPR

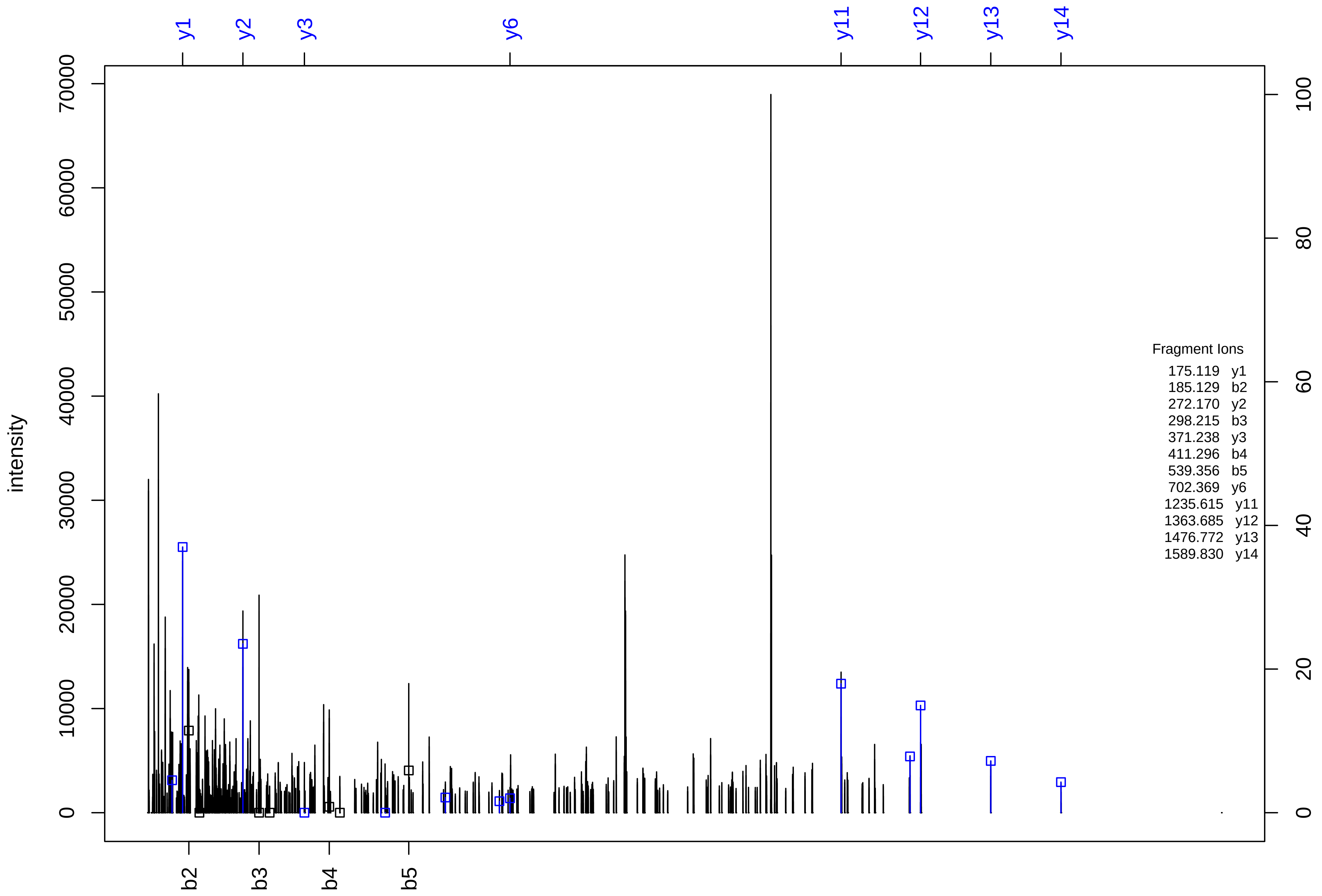

LALLQDAPIHSPMVPR / File: christine_10pools_10.raw; SpectrumID: ; scans: 21252

NITFDELVDAYQEQAK

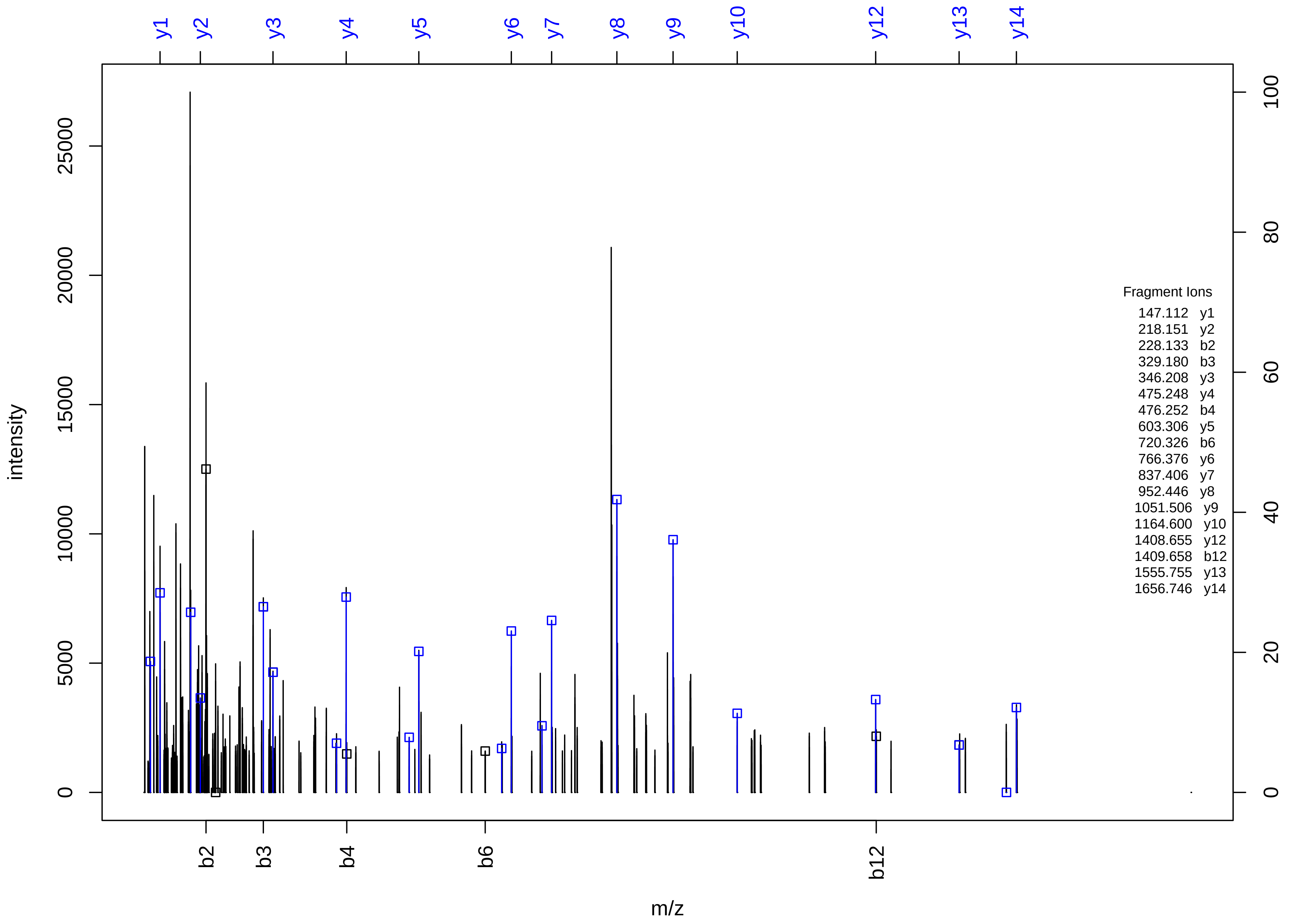

NITFDELVDAYQEQAK / File: christine_10pools_10.raw; SpectrumID: ; scans: 27460

IDLENTLEQEQEALVNR

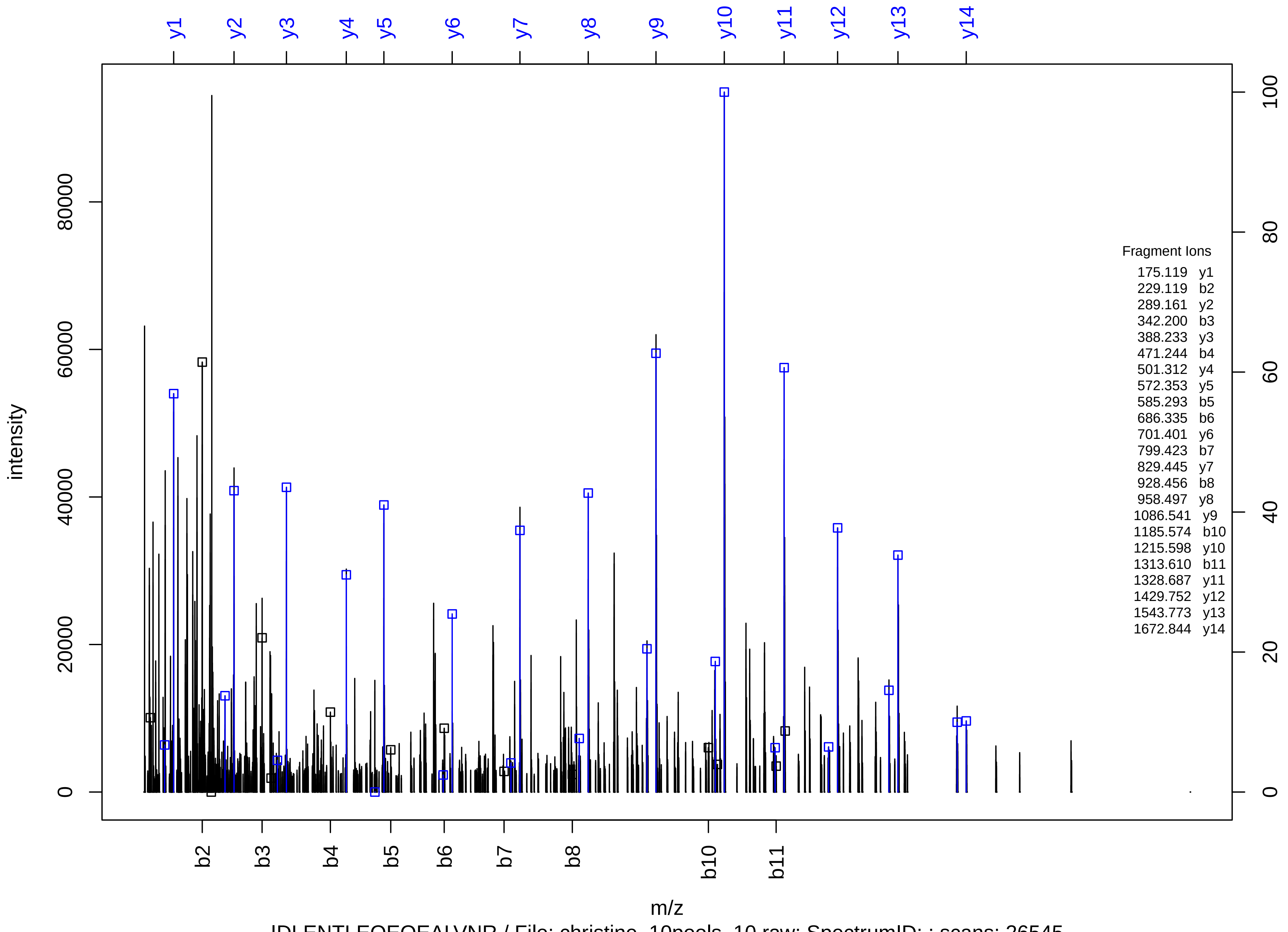

IDLENTLEQEQEALVNR / File: christine_10pools_10.raw; SpectrumID: ; scans: 26545

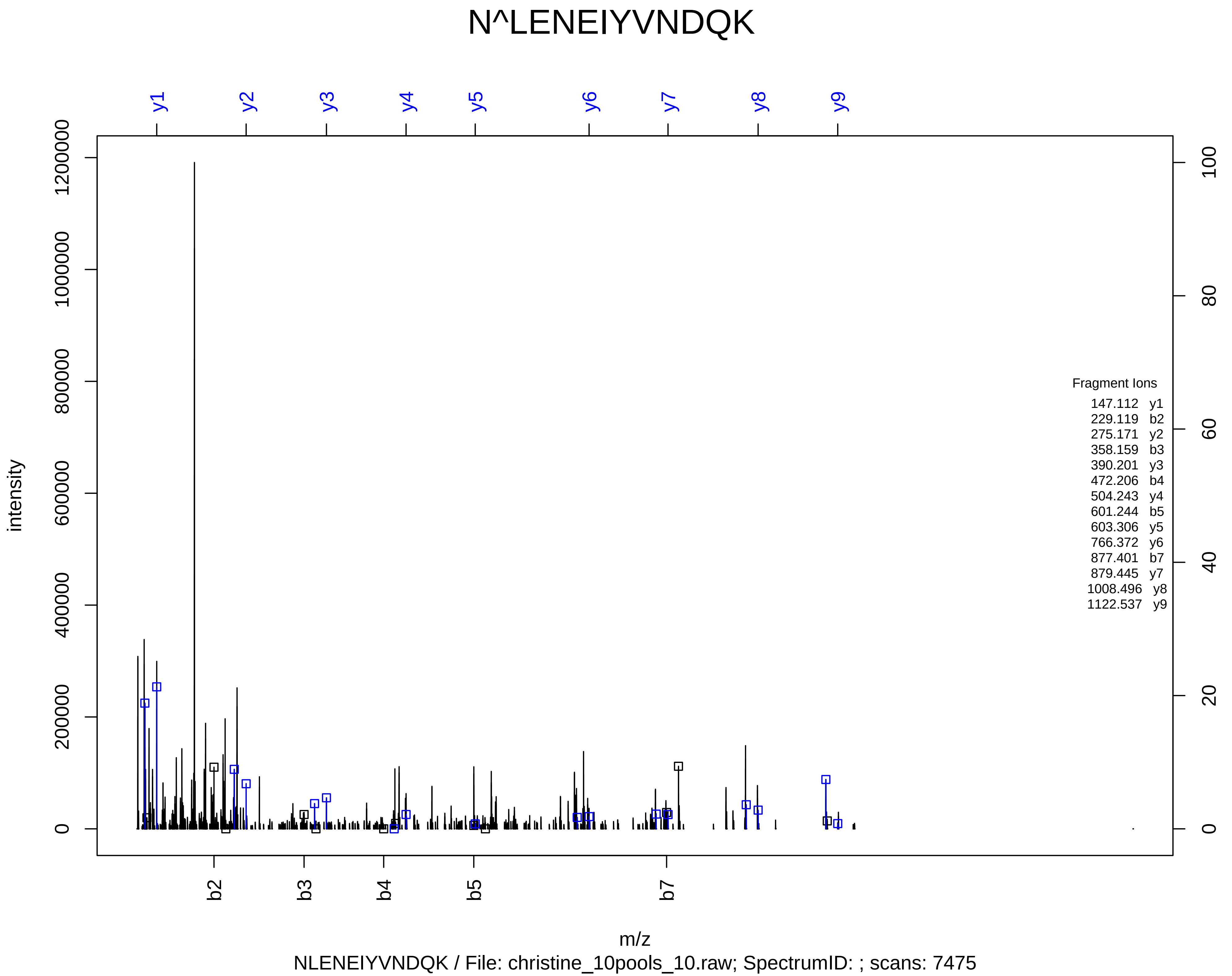

N^LENEIYVNDQK
y1
y2
y3
y4
y5
y6
y7
y8
y9
intensity
0
200000
400000
600000
800000
1000000
1200000
0
20
40
60
80
100
b2
b3
b4
b5
b7
m/z
Fragment Ions
147.112 y1
229.119 b2
275.171 y2
358.159 b3
390.201 y3
472.206 b4
504.243 y4
601.244 b5
603.306 y5
766.372 y6
877.401 b7
879.445 y7
1008.496 y8
1122.537 y9
NLENEIYVNDQK / File: christine_10pools_10.raw; SpectrumID: ; scans: 7475

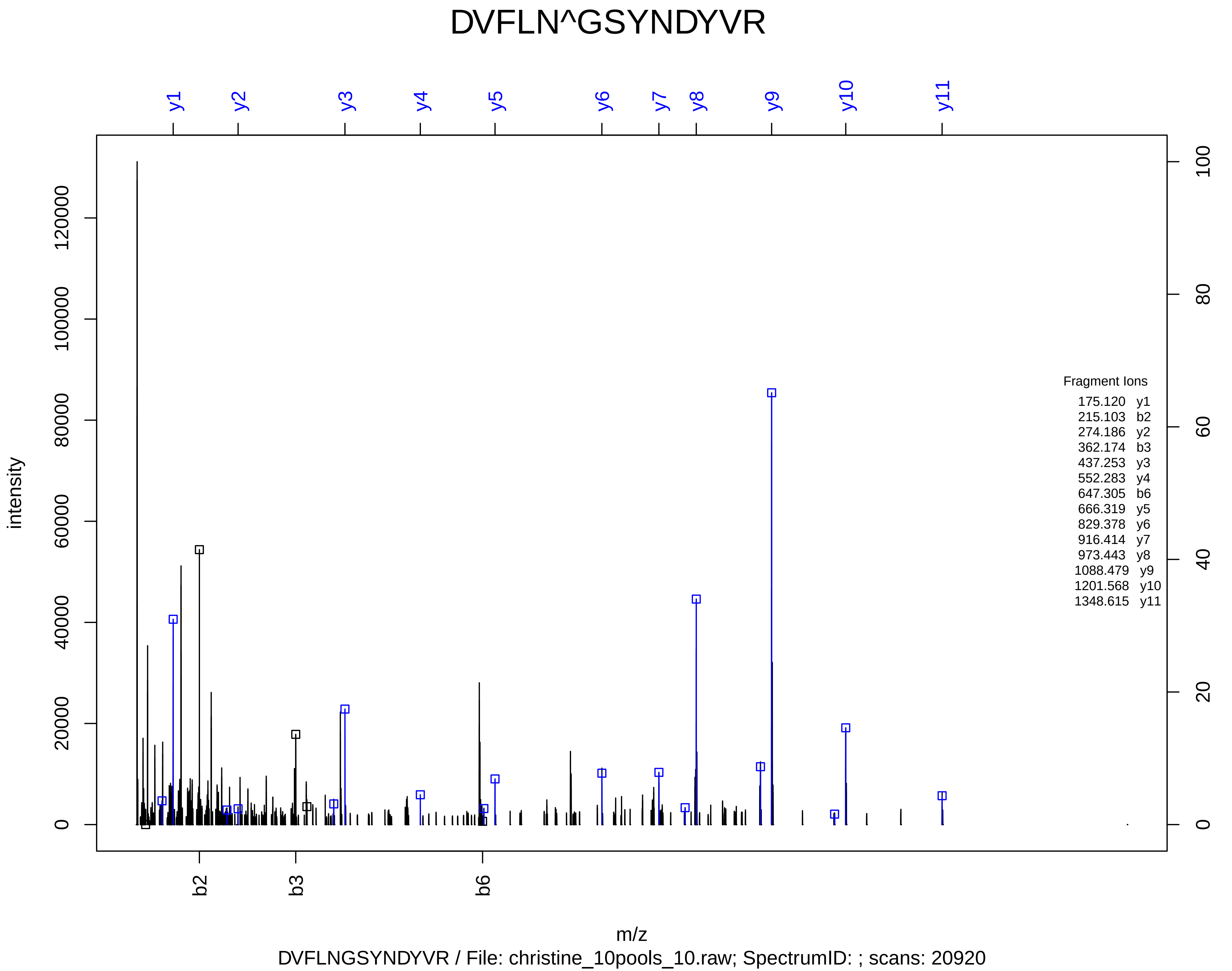
DVFLN^GSYNDYVR
y1
y2
y3
y4
y5
y6
y7
y8
y9
y10
y11
intensity
0
20000
40000
60000
80000
100000
120000
0
20
40
60
80
100
b2
b3
b6
m/z
Fragment Ions
175.120 y1
215.103 b2
274.186 y2
362.174 b3
437.253 y3
552.283 y4
647.305 b6
666.319 y5
829.378 y6
916.414 y7
973.443 y8
1088.479 y9
1201.568 y10
1348.615 y11
DVFLNGSYNDYVR / File: christine_10pools_10.raw; SpectrumID: ; scans: 20920

M*GNIIPLNVPR

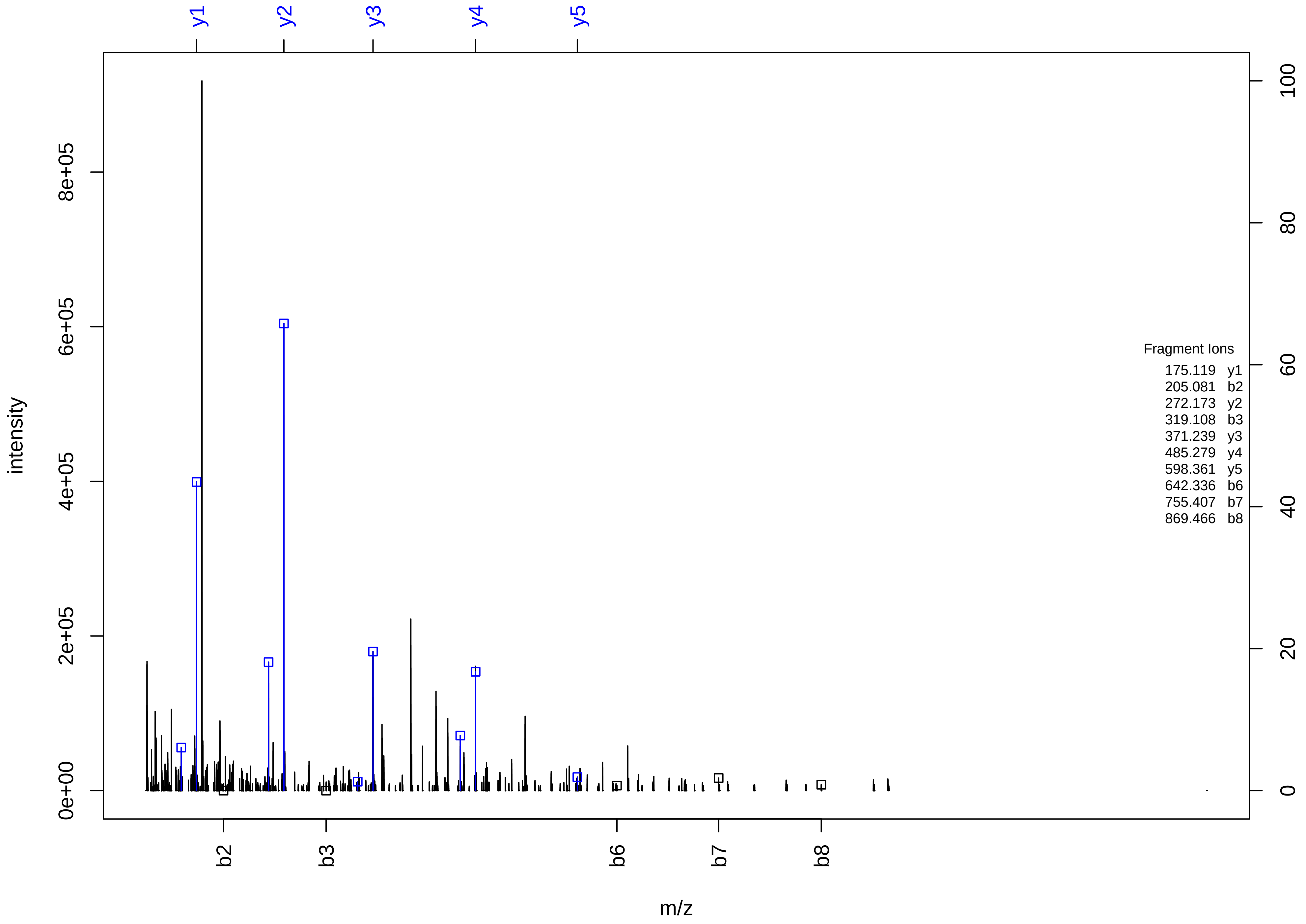

MGNIIPLNVPR / File: christine_10pools_10.raw; SpectrumID: ; scans: 5916

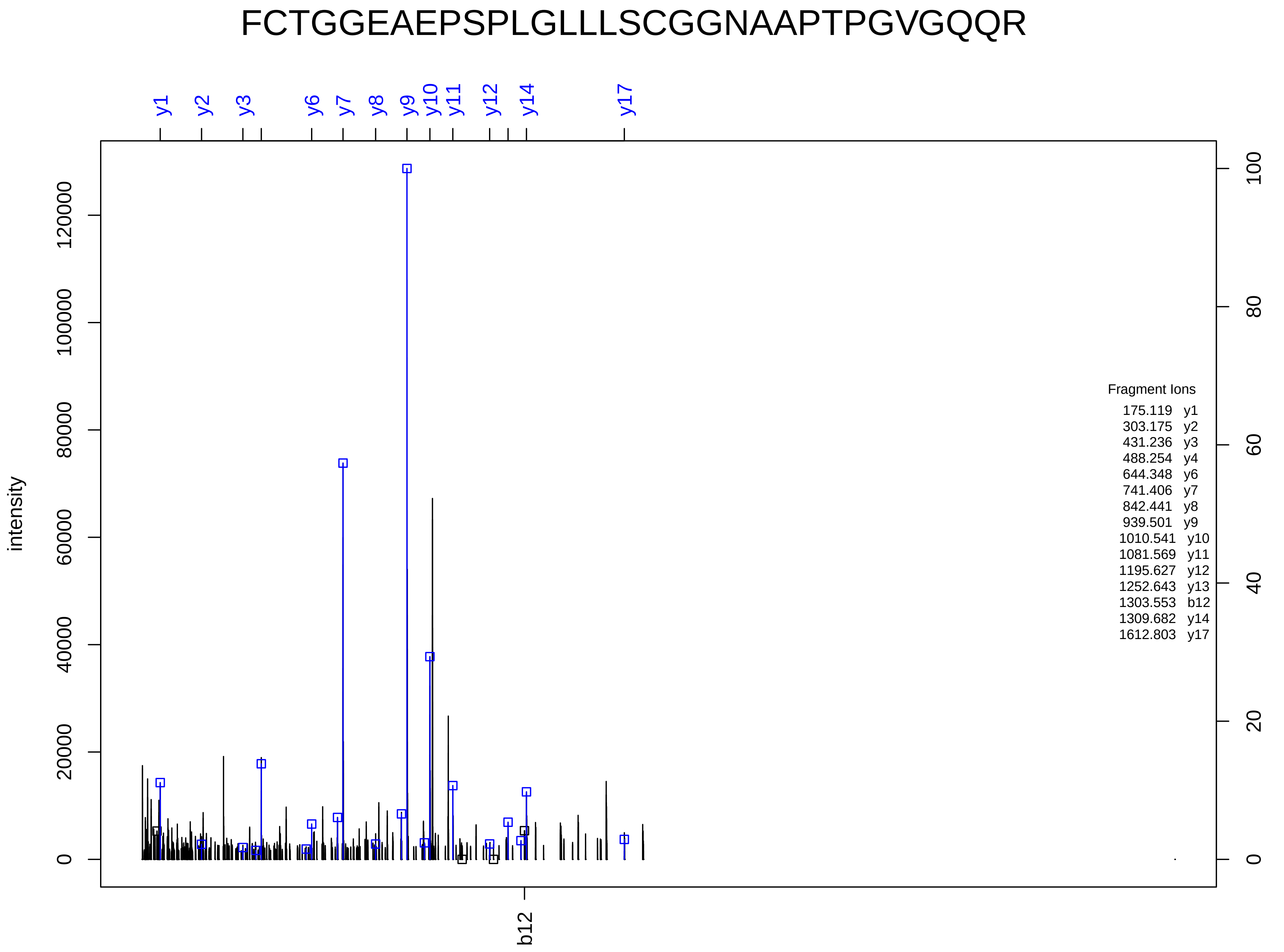

FCTGGEAEPSPLGLLLSCGGNAAPTPGVGQQR / File: christine_10pools_10.raw; SpectrumID: ; scans: 31880

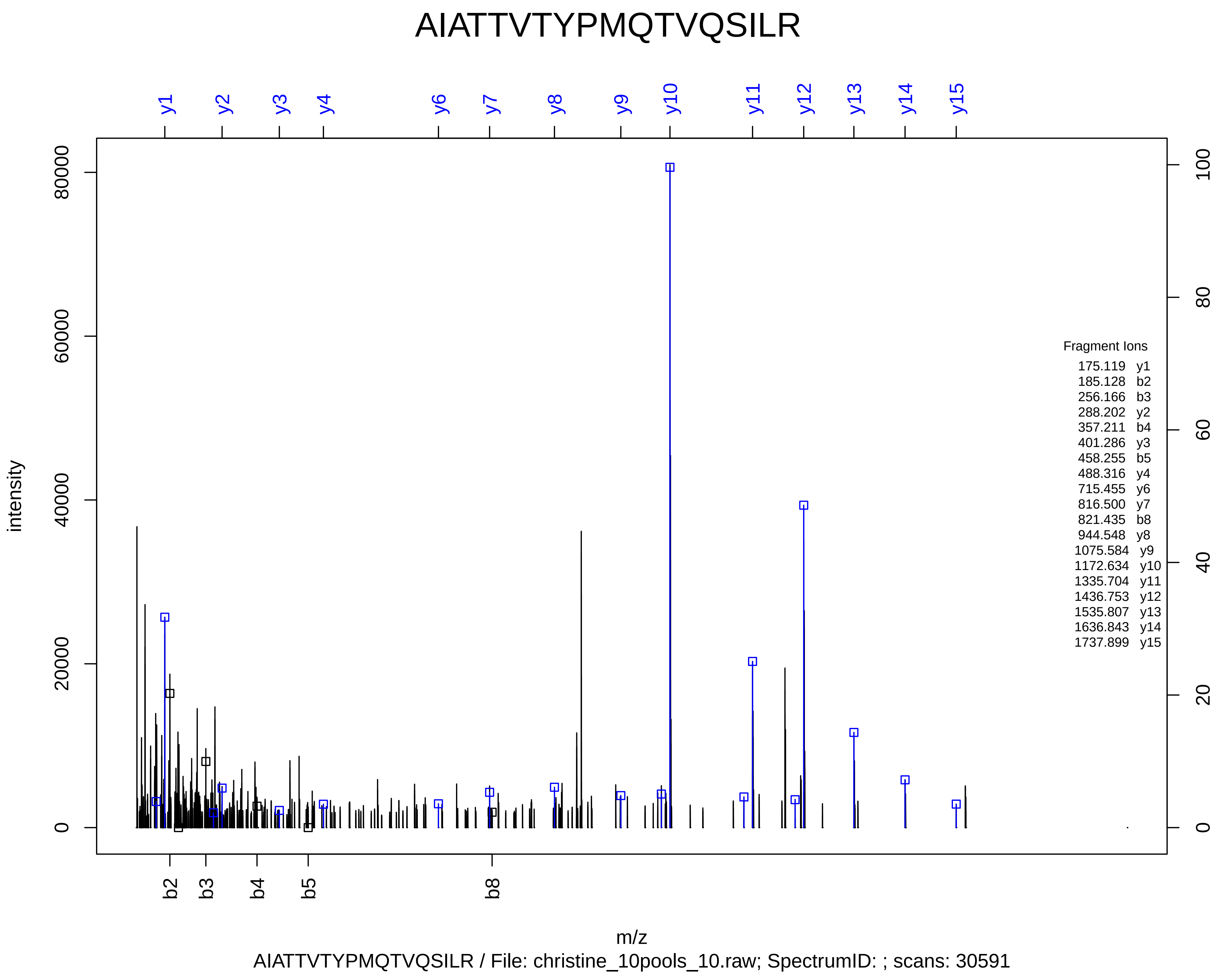
AIATTVTYPMQTVQSILR
y1 y2 y3 y4 y6 y7 y8 y9 y10 y11 y12 y13 y14 y15
b2 b3 b4 b5 b8
intensity
m/z
Fragment Ions
175.119 y1
185.128 b2
256.166 b3
288.202 y2
357.211 b4
401.286 y3
458.255 b5
488.316 y4
715.455 y6
816.500 y7
821.435 b8
944.548 y8
1075.584 y9
1172.634 y10
1335.704 y11
1436.753 y12
1535.807 y13
1636.843 y14
1737.899 y15
AIATTVTYPMQTVQSILR / File: christine_10pools_10.raw; SpectrumID: ; scans: 30591

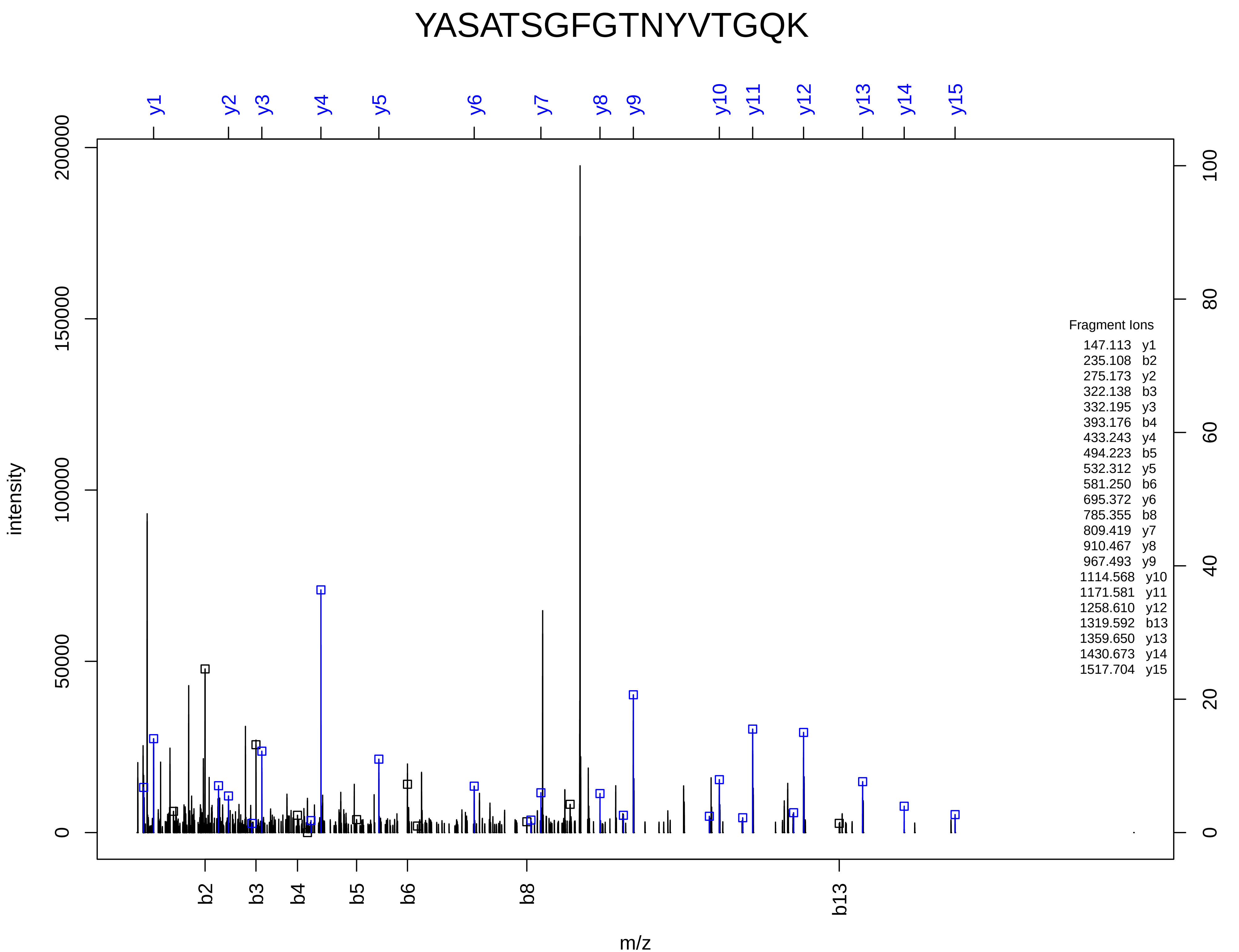

YASATSGFGTNYVTGQK / File: christine_10pools_10.raw; SpectrumID: ; scans: 8441

CLVGEFVSDALLVPDK

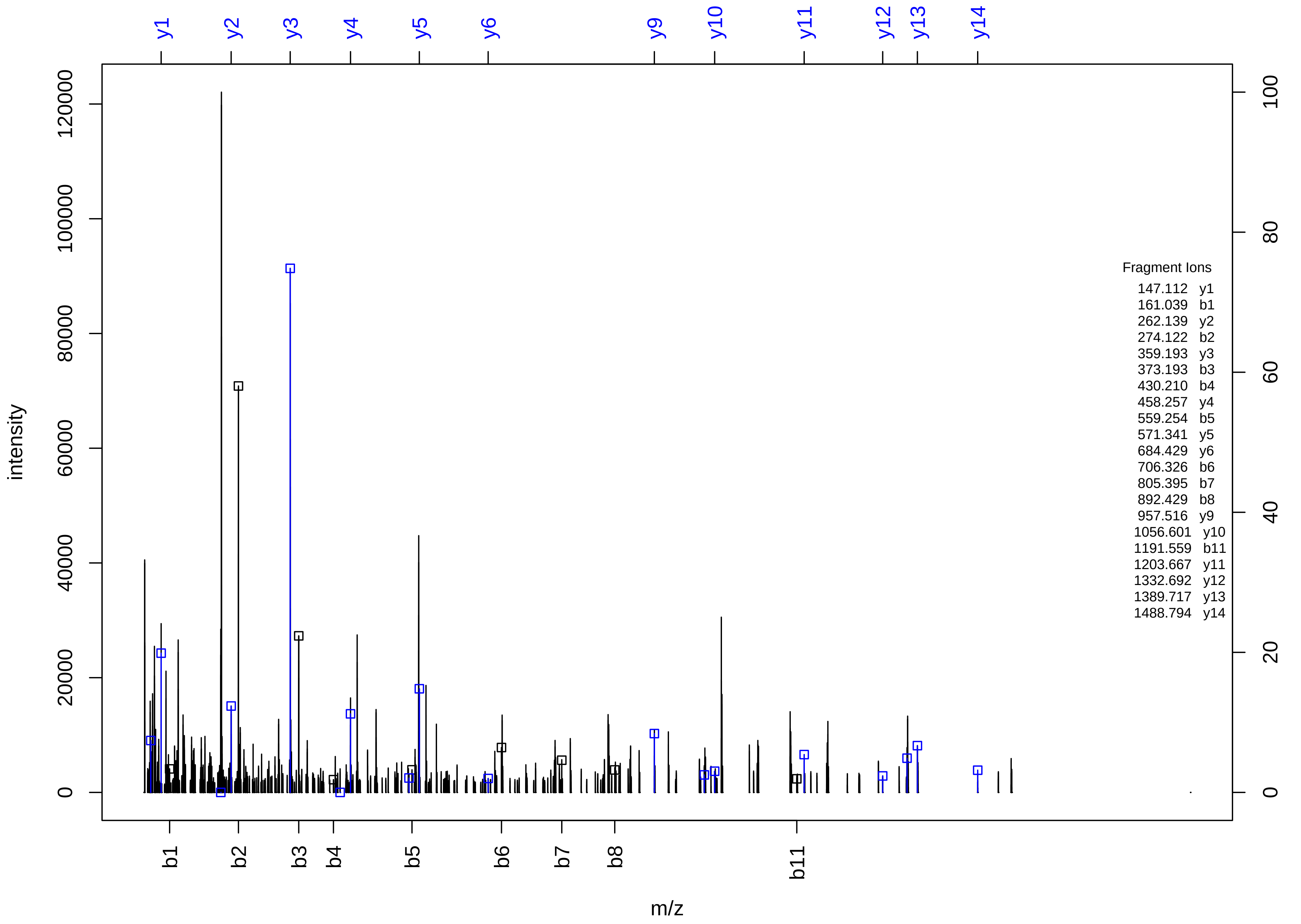

CLVGEFVSDALLVPDK / File: christine_10pools_10.raw; SpectrumID: ; scans: 33263

FCSSTHNAECECIEGFHCLGPQCTR

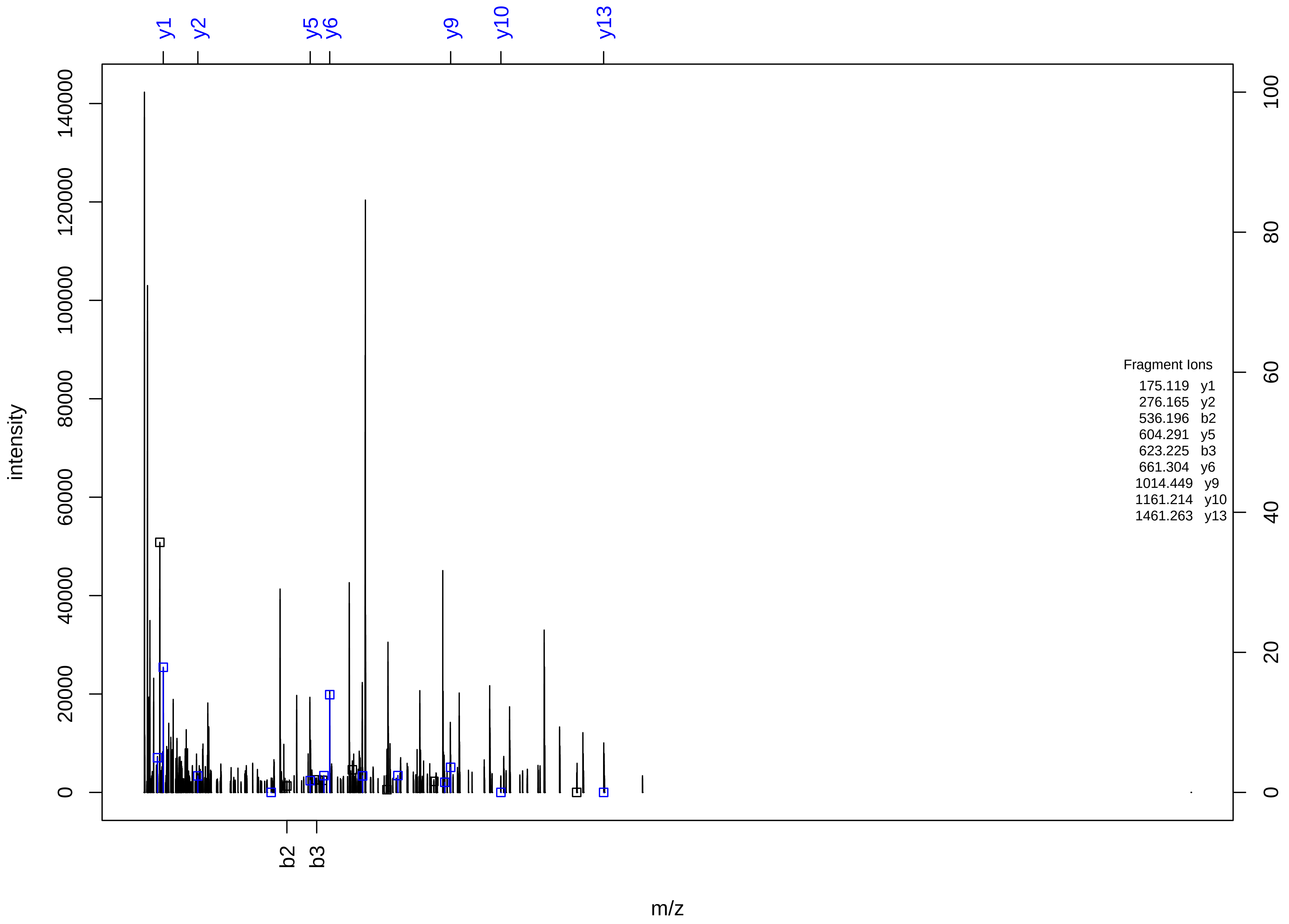

FCSSTHNAECECIEGFHCLGPQCTR / File: christine_10pools_10.raw; SpectrumID: ; scans: 7801

TEETCAPSVSYFK

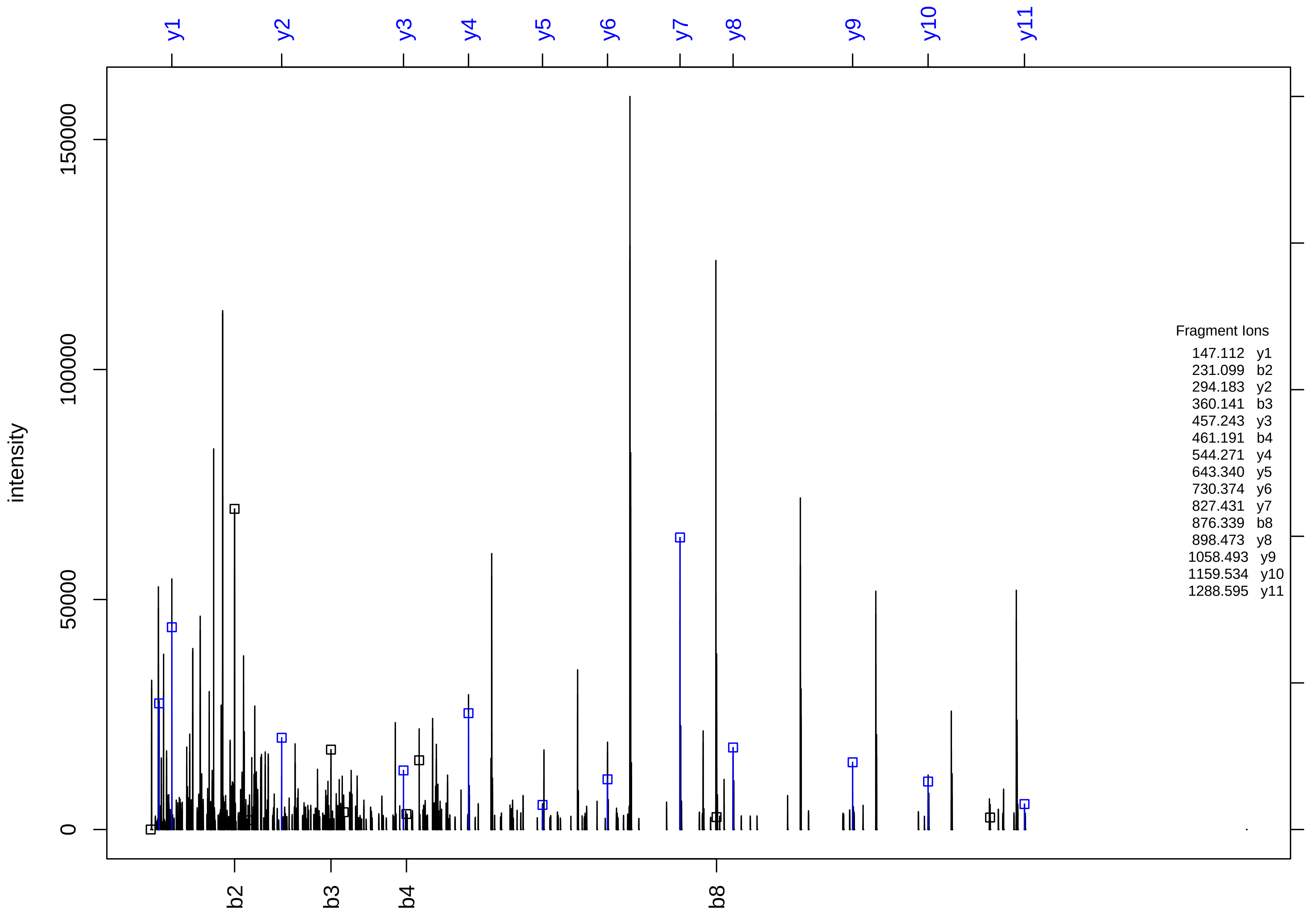

TEETCAPSVSYFK / File: christine_10pools_10.raw; SpectrumID: ; scans: 9290

RQDPQEMEDYPGHNTAAPVQETLHGCQPVTQEDGK

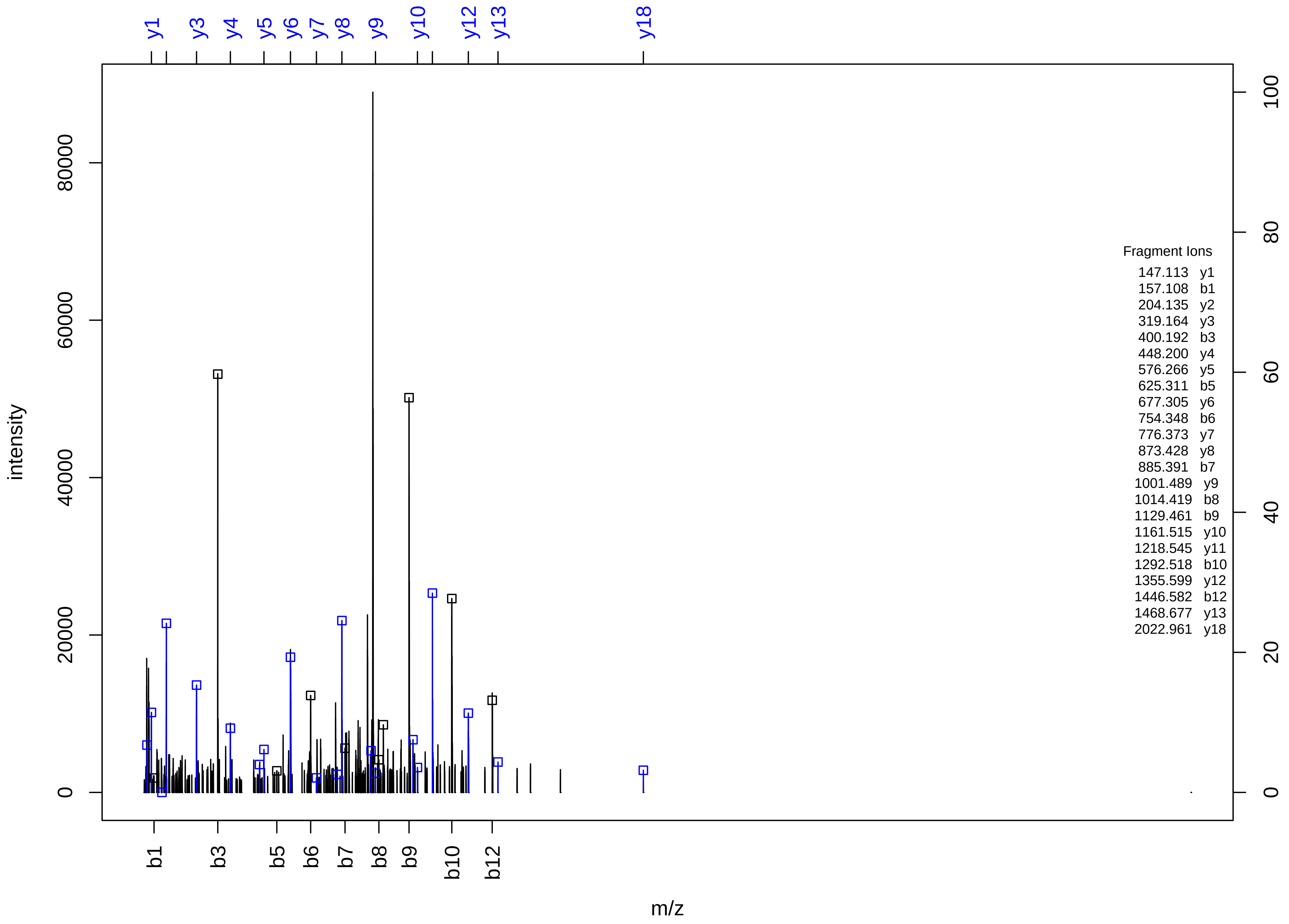

RQDPQEMEDYPGHNTAAPVQETLHGCQPVTQEDGK / File: christine_10pools_10.raw; SpectrumID: ; scans: 6548

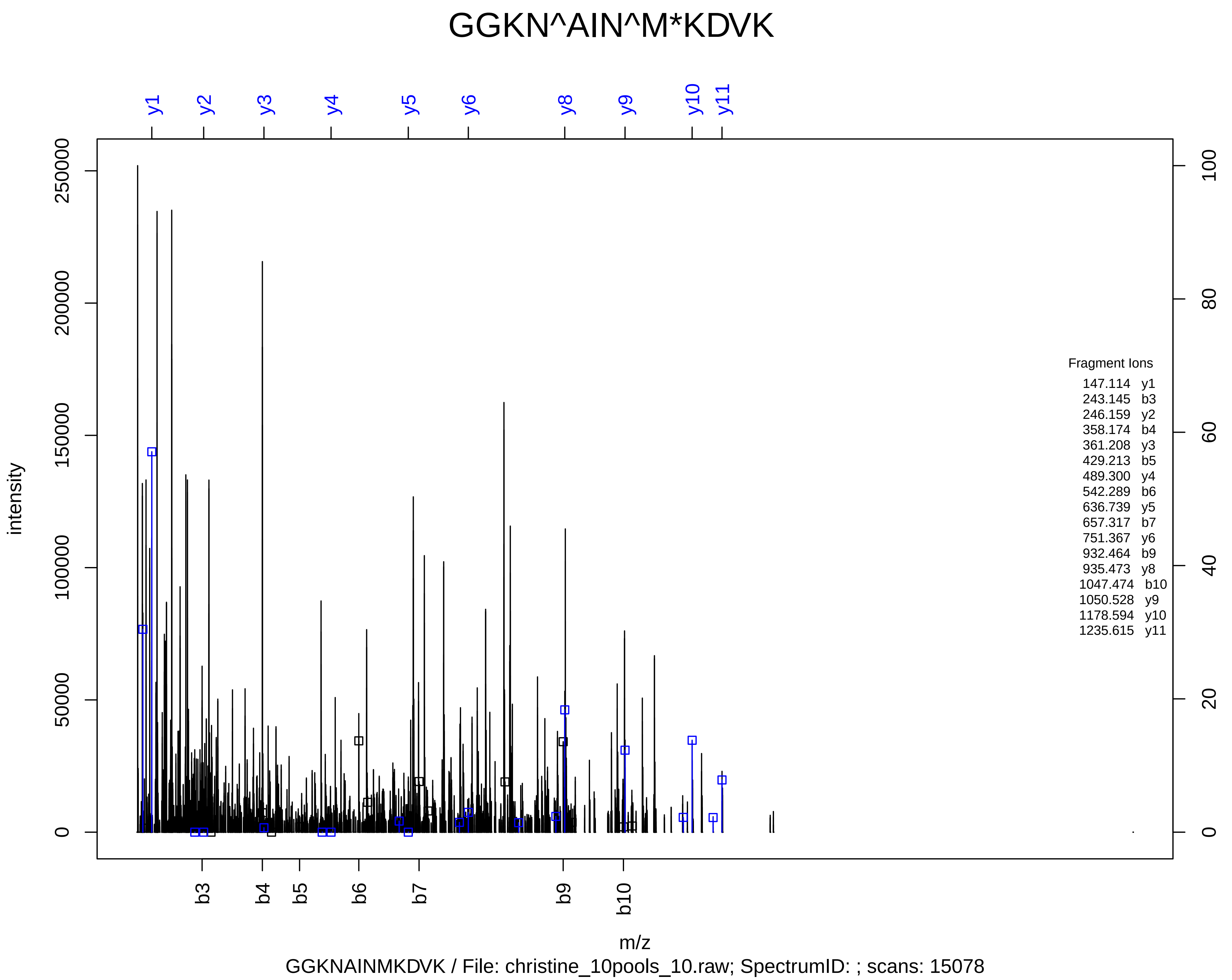
GGKN^AIN^M*KDVK
y1
y2
y3
y4
y5
y6
y8
y9
y10
y11
intensity
0
50000
100000
150000
200000
250000
0
20
40
60
80
100
b3
b4
b5
b6
b7
b9
b10
m/z
Fragment Ions
147.114 y1
243.145 b3
246.159 y2
358.174 b4
361.208 y3
429.213 b5
489.300 y4
542.289 b6
636.739 y5
657.317 b7
751.367 y6
932.464 b9
935.473 y8
1047.474 b10
1050.528 y9
1178.594 y10
1235.615 y11
GGKNAINMKDVK / File: christine_10pools_10.raw; SpectrumID: ; scans: 15078

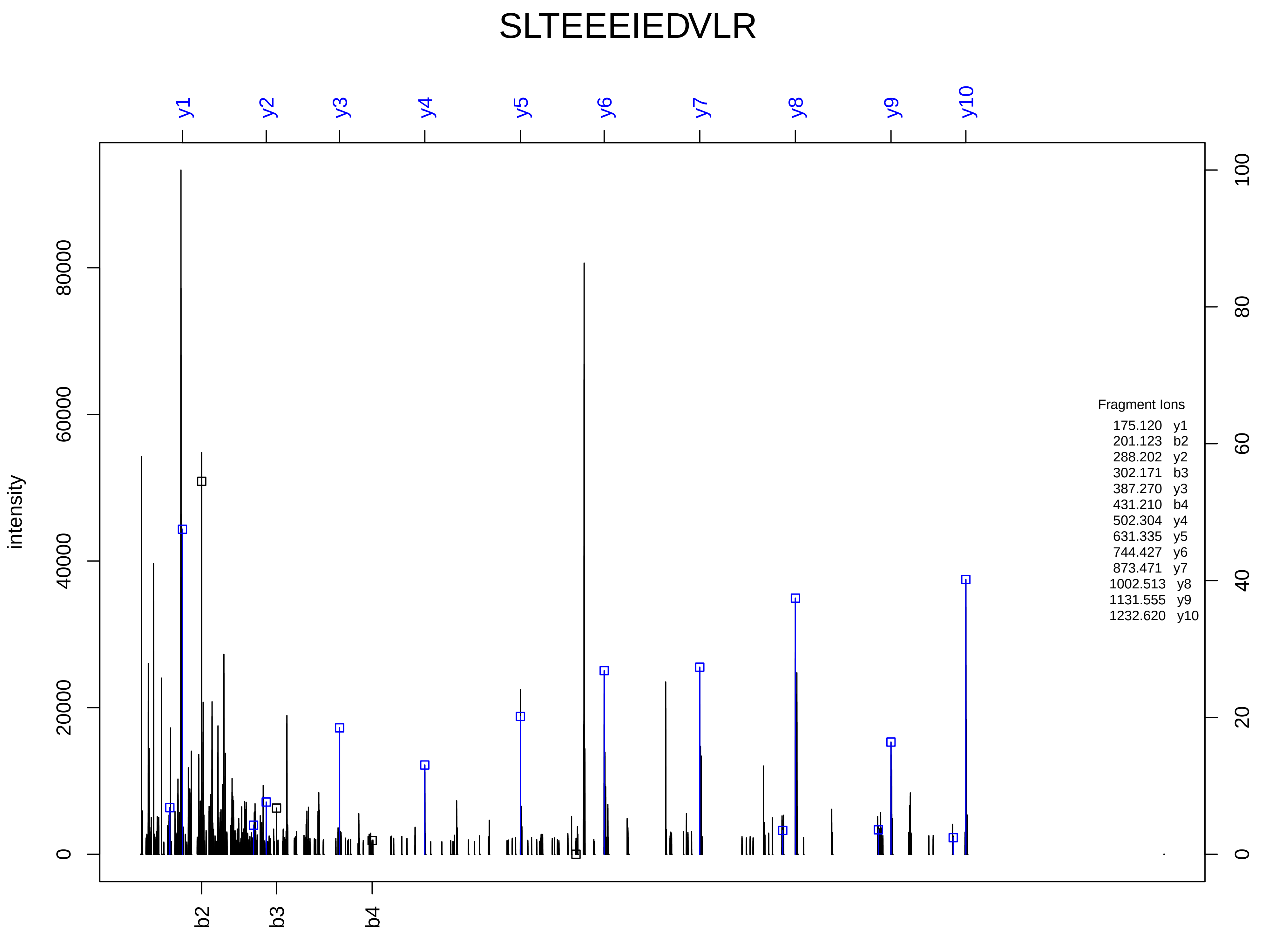
SLTEEEIEDVLR
y1
y2
y3
y4
y5
y6
y7
y8
y9
y10
b2
b3
b4
intensity
m/z
Fragment Ions
175.120 y1
201.123 b2
288.202 y2
302.171 b3
387.270 y3
431.210 b4
502.304 y4
631.335 y5
744.427 y6
873.471 y7
1002.513 y8
1131.555 y9
1232.620 y10
SLTEEEIEDVLR / File: christine_10pools_10.raw; SpectrumID: ; scans: 23247

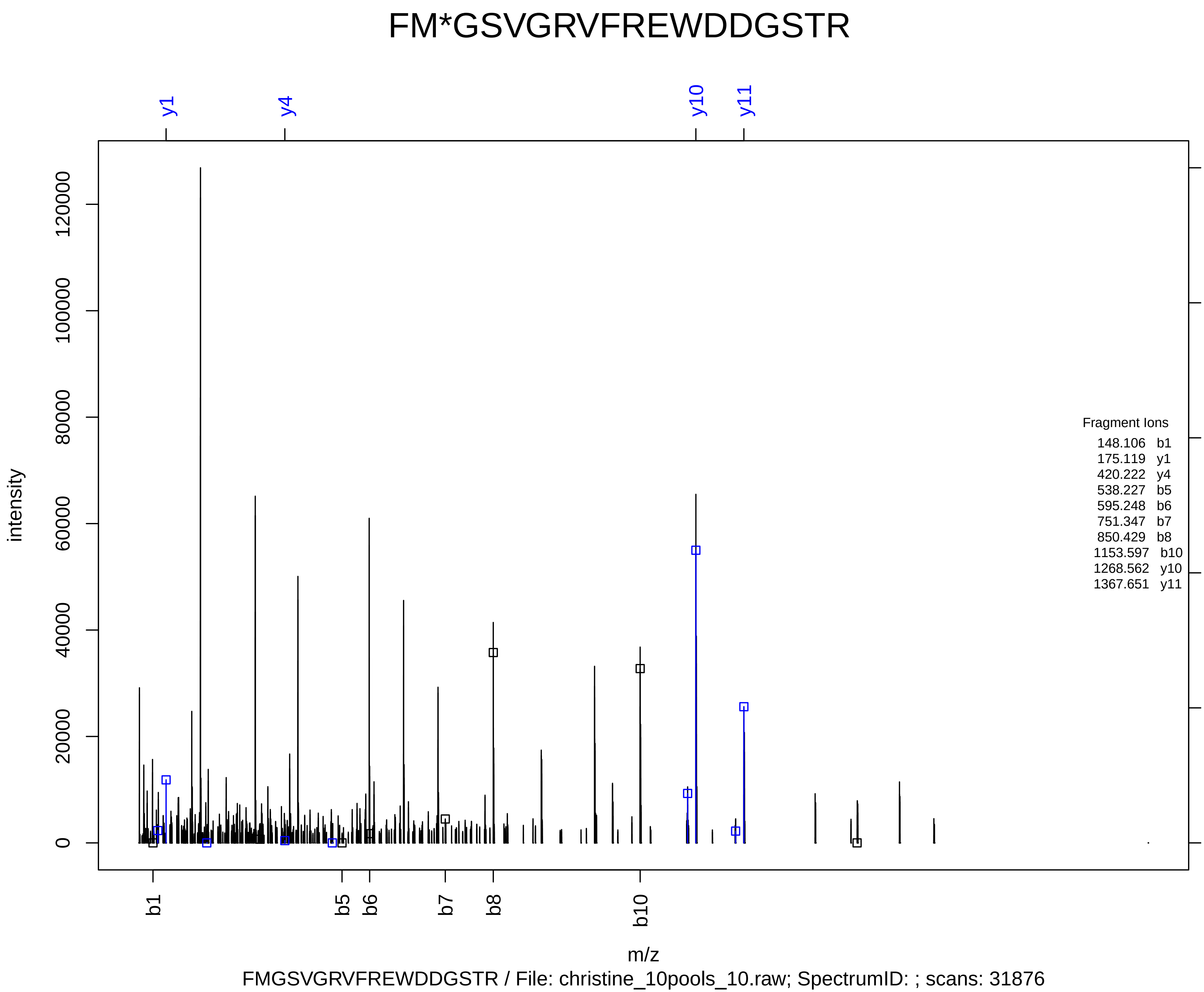

FM*GSVGRVFREWDDGSTR
y1
y4
y10
y11
intensity
0
20000
40000
60000
80000
100000
120000
0
20
40
60
80
100
Fragment Ions
148.106 b1
175.119 y1
420.222 y4
538.227 b5
595.248 b6
751.347 b7
850.429 b8
1153.597 b10
1268.562 y10
1367.651 y11
b1
b5
b6
b7
b8
b10
m/z
FMGSVGRVFREWDDGSTR / File: christine_10pools_10.raw; SpectrumID: ; scans: 31876

AAAAVASAASSCRPLGSGTAPNPTAAAPASSPAPGPGPVGK

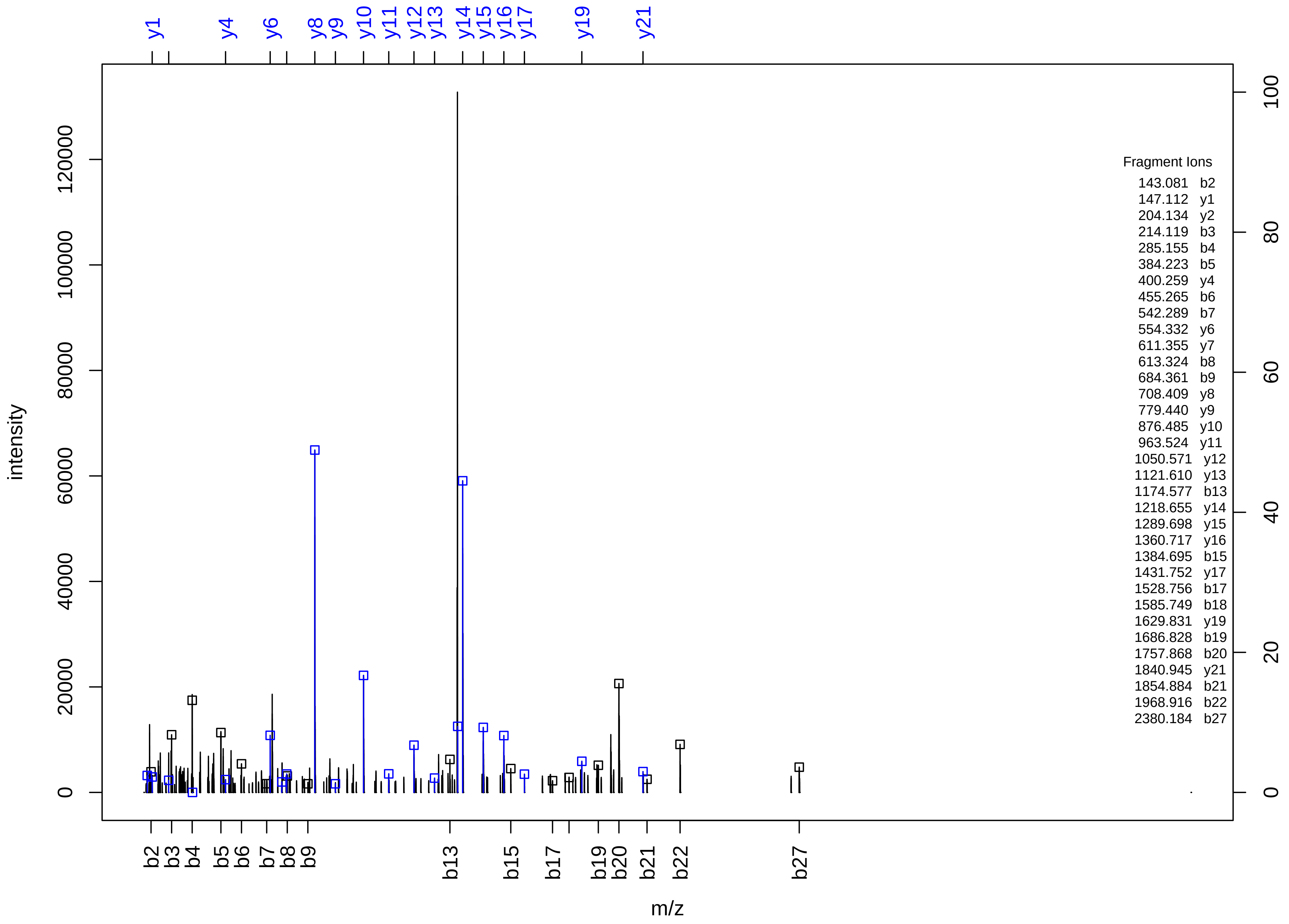

AAAAVASAASSCRPLGSGTAPNPTAAAPASSPAPGPGPVGK / File: christine_10pools_10.raw; SpectrumID: ; scans: 9908

VDPTVENEETFM*NR

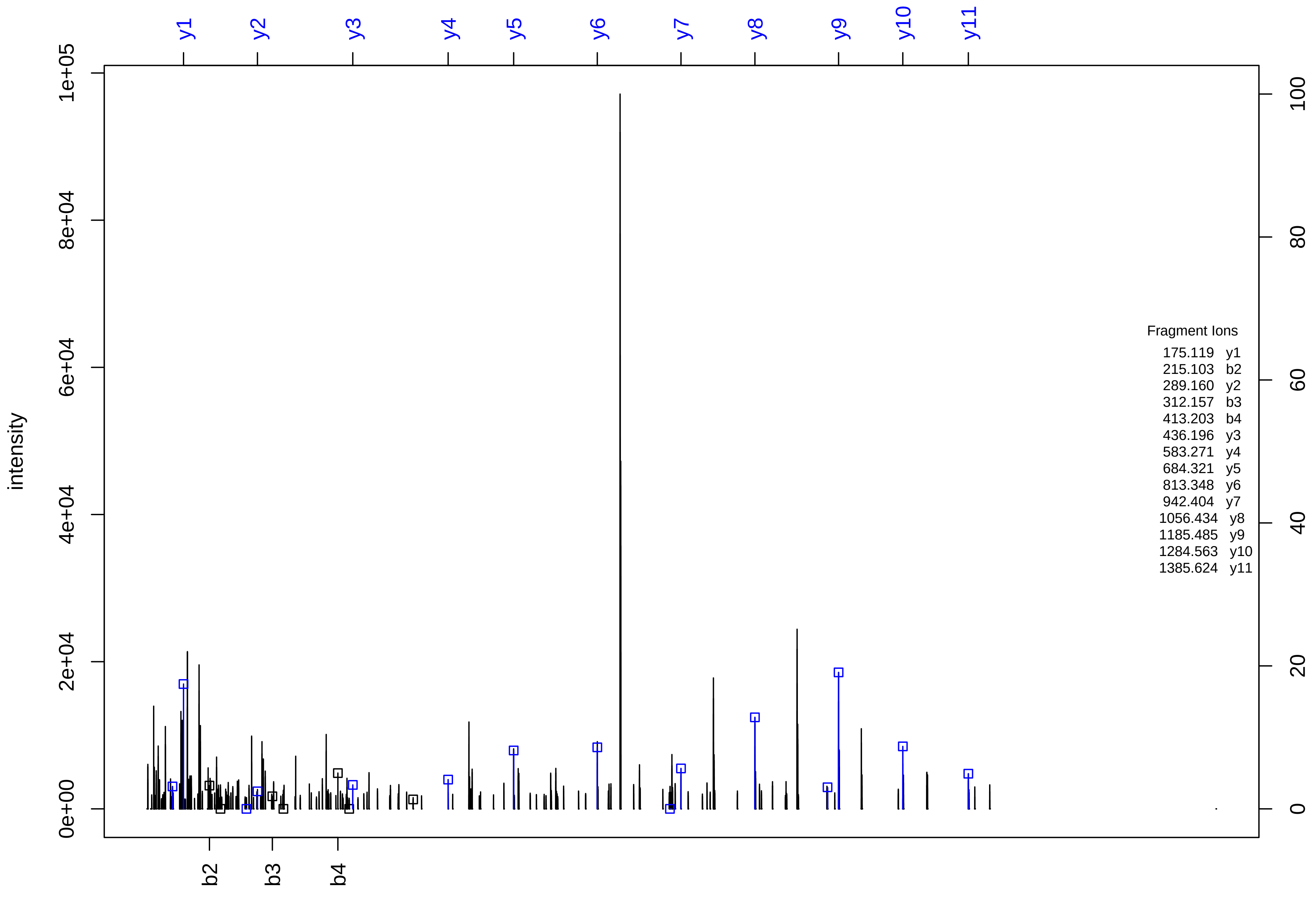

VDPTVENEETFMNR / File: christine_10pools_10.raw; SpectrumID: ; scans: 5567

KWENEEEEEEPAPTSAEGEGNAEGPDAEAGSASTPR

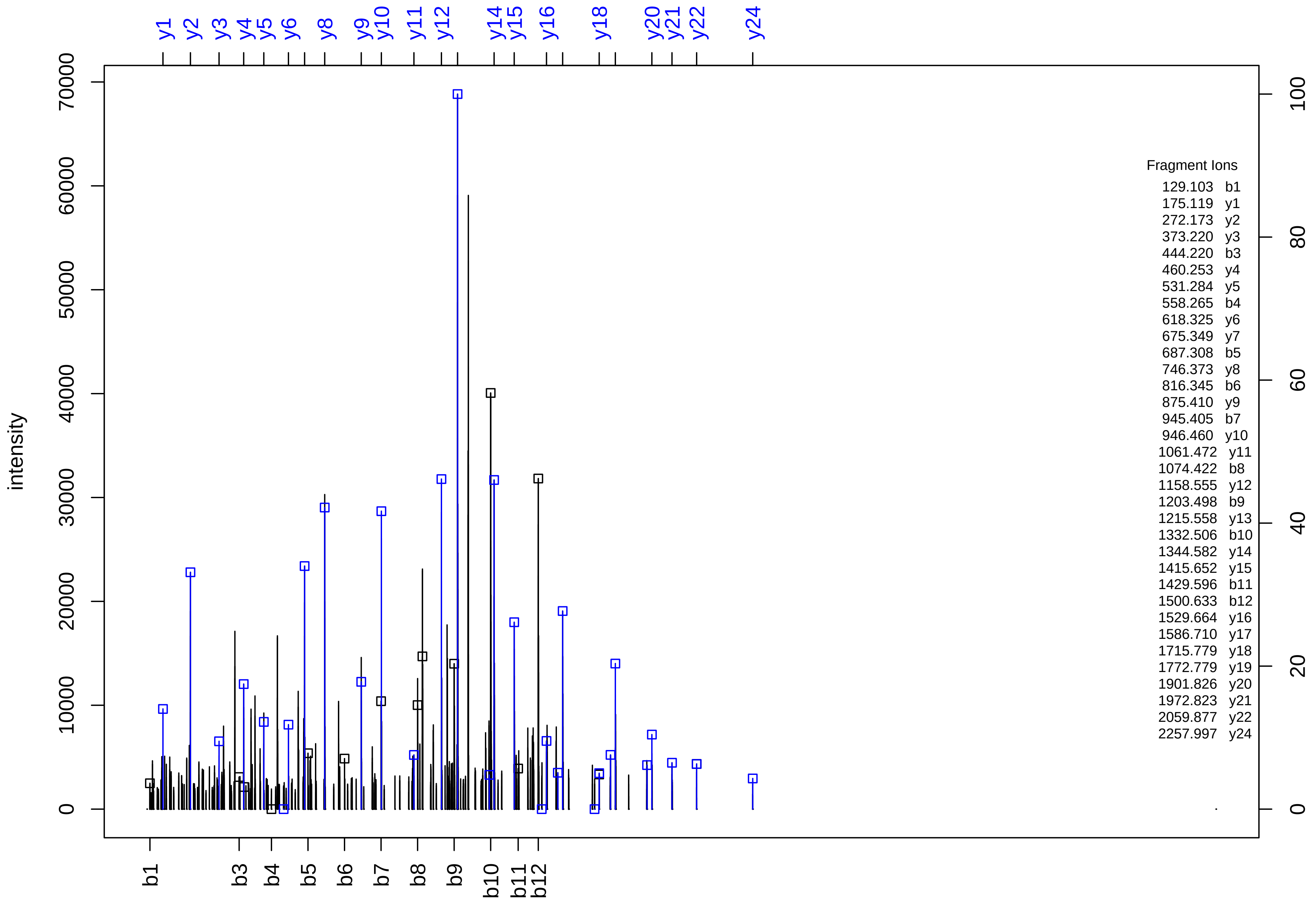

KWENEEEEEEPAPTSAEGEGNAEGPDAEAGSASTPR / File: christine_10pools_10.raw; SpectrumID: ; scans: 5209

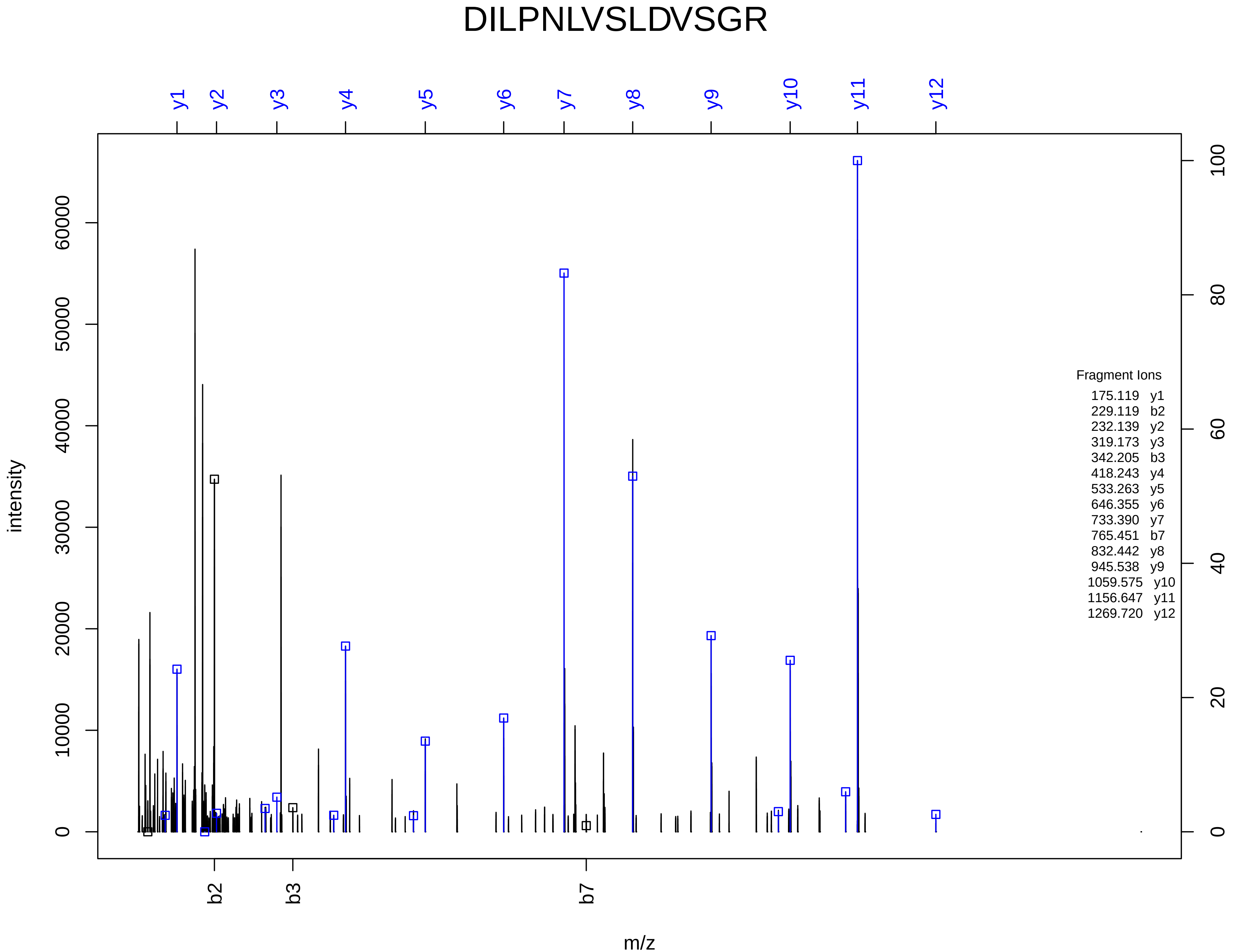

DILPNLVSLDVSGR / File: christine_10pools_10.raw; SpectrumID: ; scans: 31494

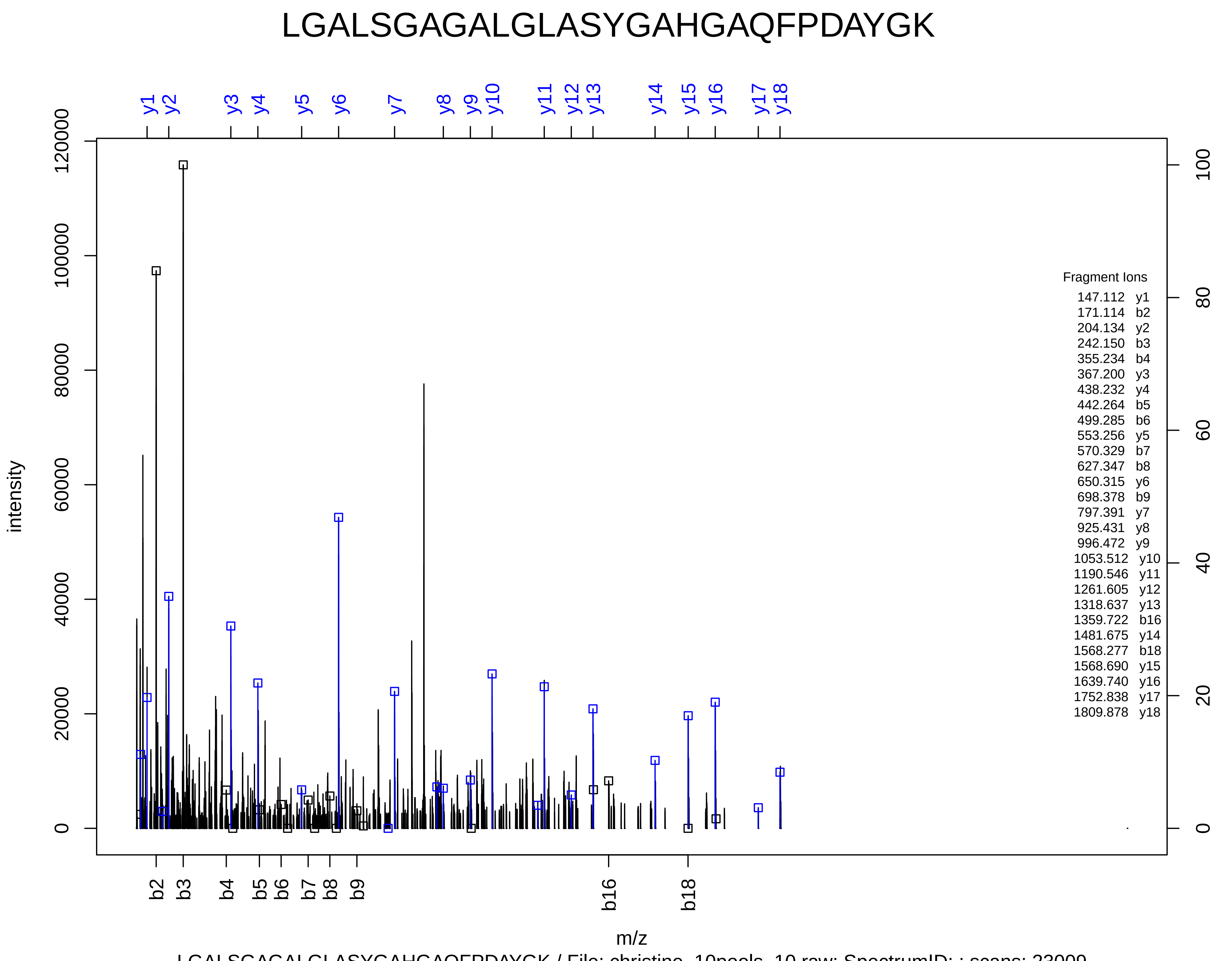

LGALSGAGALGLASYGAHGAQFPDAYGK
y1 y2 y3 y4 y5 y6 y7 y8 y9 y10 y11 y12 y13 y14 y15 y16 y17 y18
intensity
0
20000
40000
60000
80000
100000
120000
0
20
40
60
80
100
b2 b3 b4 b5 b6 b7 b8 b9 b16 b18
m/z
Fragment Ions
147.112 y1
171.114 b2
204.134 y2
242.150 b3
355.234 b4
367.200 y3
438.232 y4
442.264 b5
499.285 b6
553.256 y5
570.329 b7
627.347 b8
650.315 y6
698.378 b9
797.391 y7
925.431 y8
996.472 y9
1053.512 y10
1190.546 y11
1261.605 y12
1318.637 y13
1359.722 b16
1481.675 y14
1568.277 b18
1568.690 y15
1639.740 y16
1752.838 y17
1809.878 y18
LGALSGAGALGLASYGAHGAQFPDAYGK / File: christine_10pools_10.raw; SpectrumID: ; scans: 23009

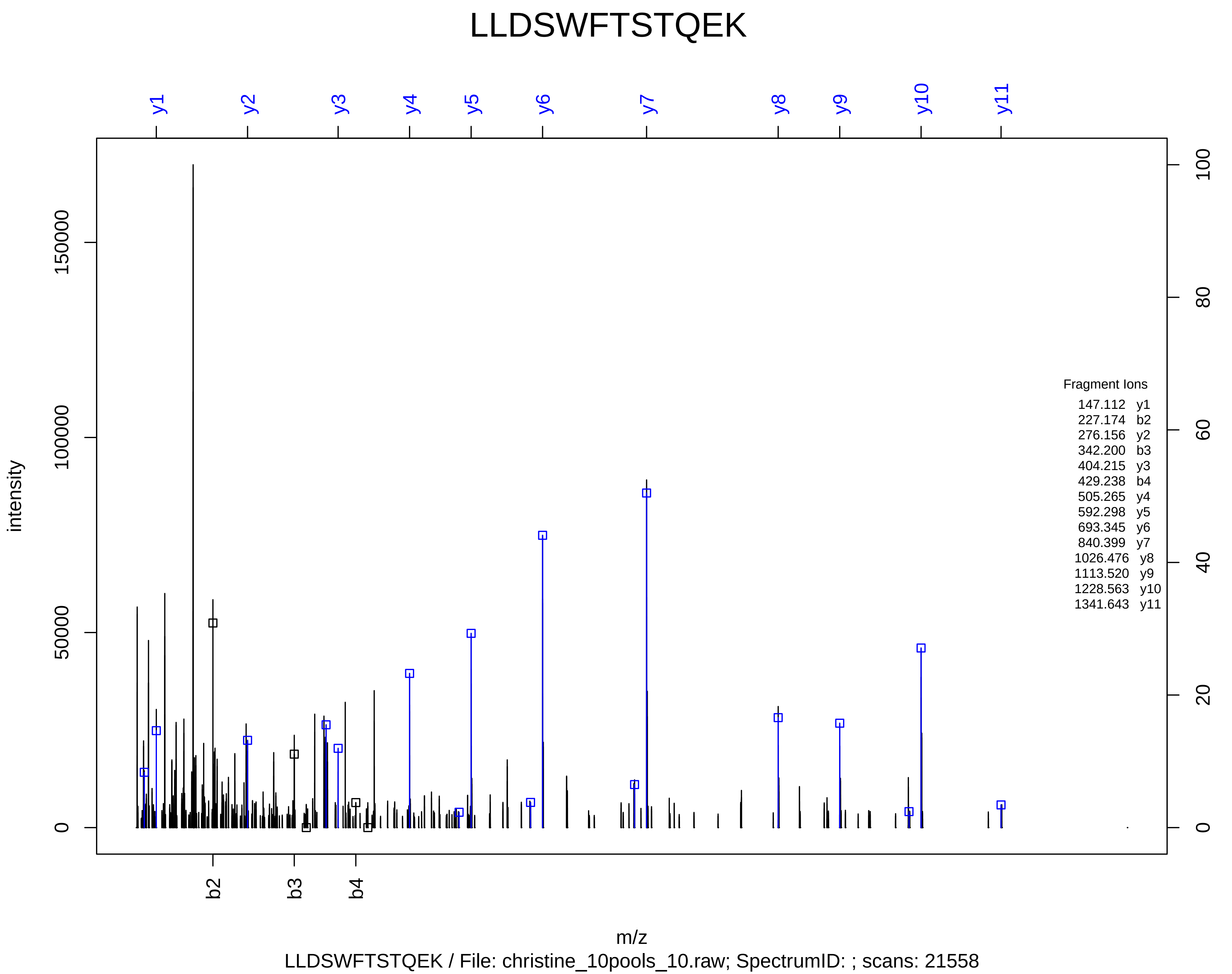
LLDSWFTSTQEK
y1
y2
y3
y4
y5
y6
y7
y8
y9
y10
y11
b2
b3
b4
intensity
0
50000
100000
150000
0
20
40
60
80
100
Fragment Ions
147.112 y1
227.174 b2
276.156 y2
342.200 b3
404.215 y3
429.238 b4
505.265 y4
592.298 y5
693.345 y6
840.399 y7
1026.476 y8
1113.520 y9
1228.563 y10
1341.643 y11
m/z
LLDSWFTSTQEK / File: christine_10pools_10.raw; SpectrumID: ; scans: 21558

FGAPLFLSHPHFYNADPVLSEAVLGLNPNPK

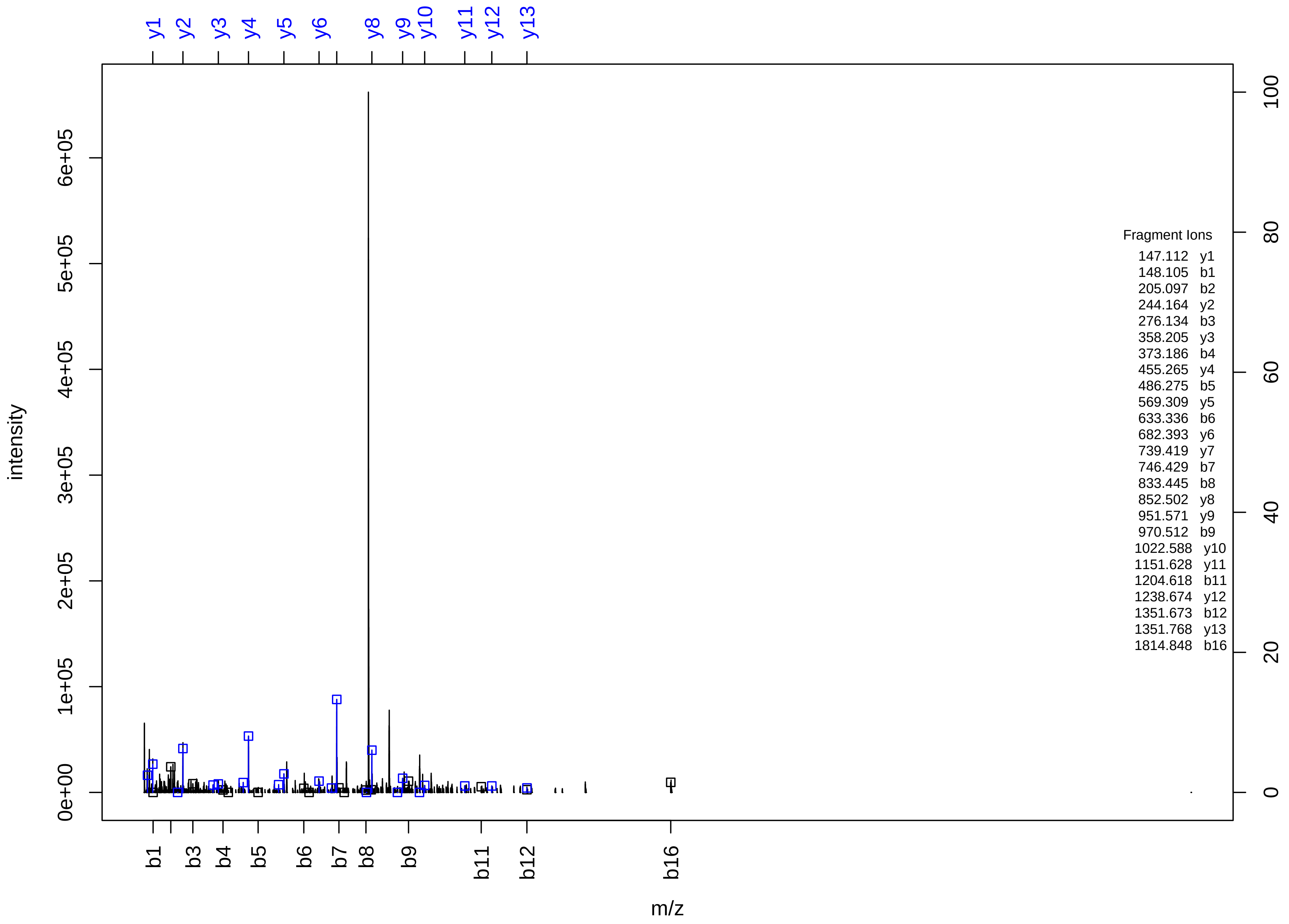

FGAPLFLSHPHFYNADPVLSEAVLGLNPNPK / File: christine_10pools_10.raw; SpectrumID: ; scans: 33903

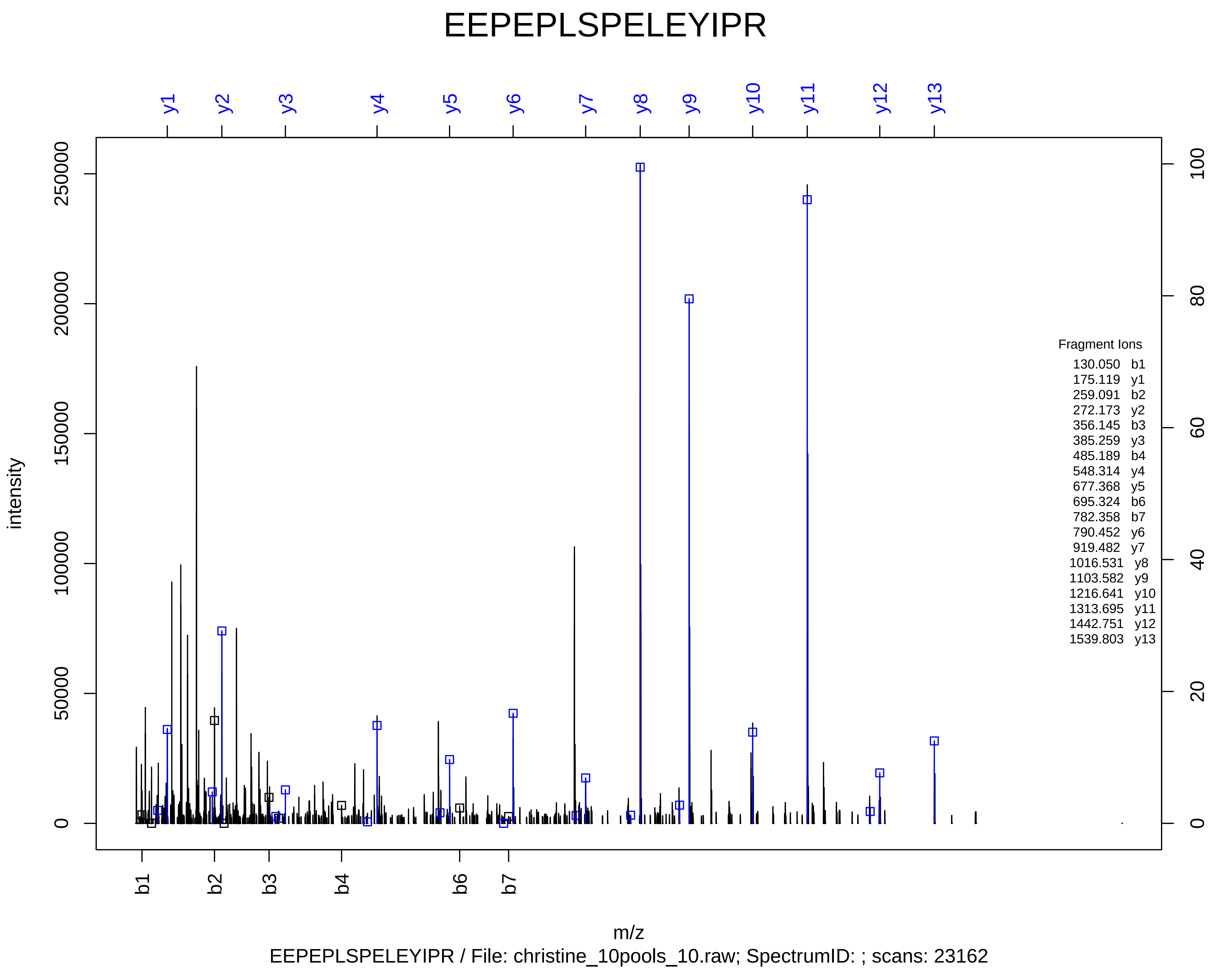
EEPEPLSPELEYIPR
y1 y2 y3 y4 y5 y6 y7 y8 y9 y10 y11 y12 y13
b1 b2 b3 b4 b6 b7
intensity
0 50000 100000 150000 200000 250000
0 20 40 60 80 100
m/z
Fragment Ions
130.050 b1
175.119 y1
259.091 b2
272.173 y2
356.145 b3
385.259 y3
485.189 b4
548.314 y4
677.368 y5
695.324 b6
782.358 b7
790.452 y6
919.482 y7
1016.531 y8
1103.582 y9
1216.641 y10
1313.695 y11
1442.751 y12
1539.803 y13
EEPEPLSPELEYIPR / File: christine_10pools_10.raw; SpectrumID: ; scans: 23162

LDTSGLAELPLFPCR

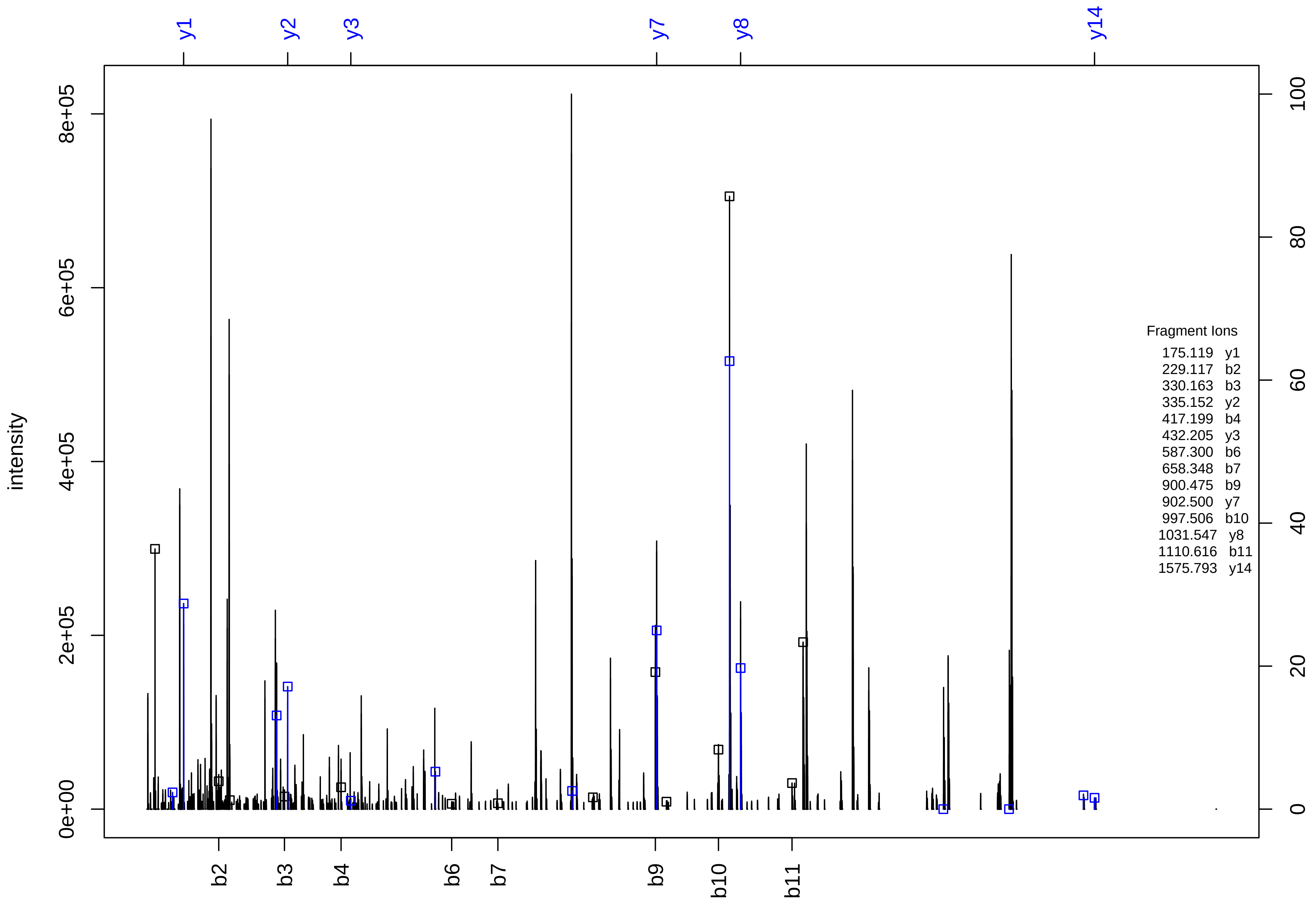

LDTSGLAELPLFPCR / File: christine_10pools_10.raw; SpectrumID: ; scans: 23551

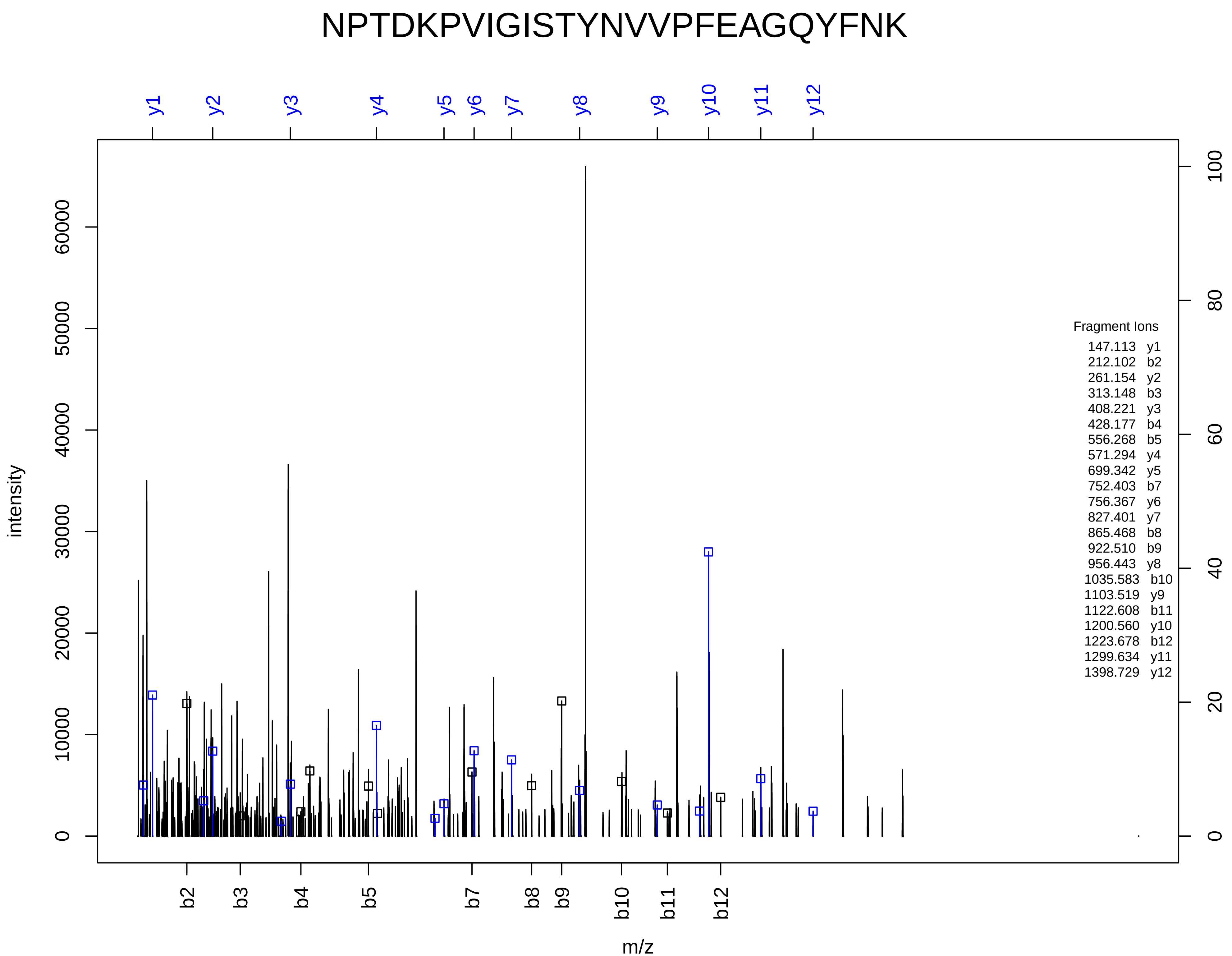

NPTDKPVIGISTYNVVPFEAGQYFNK / File: christine_10pools_10.raw; SpectrumID: ; scans: 27614

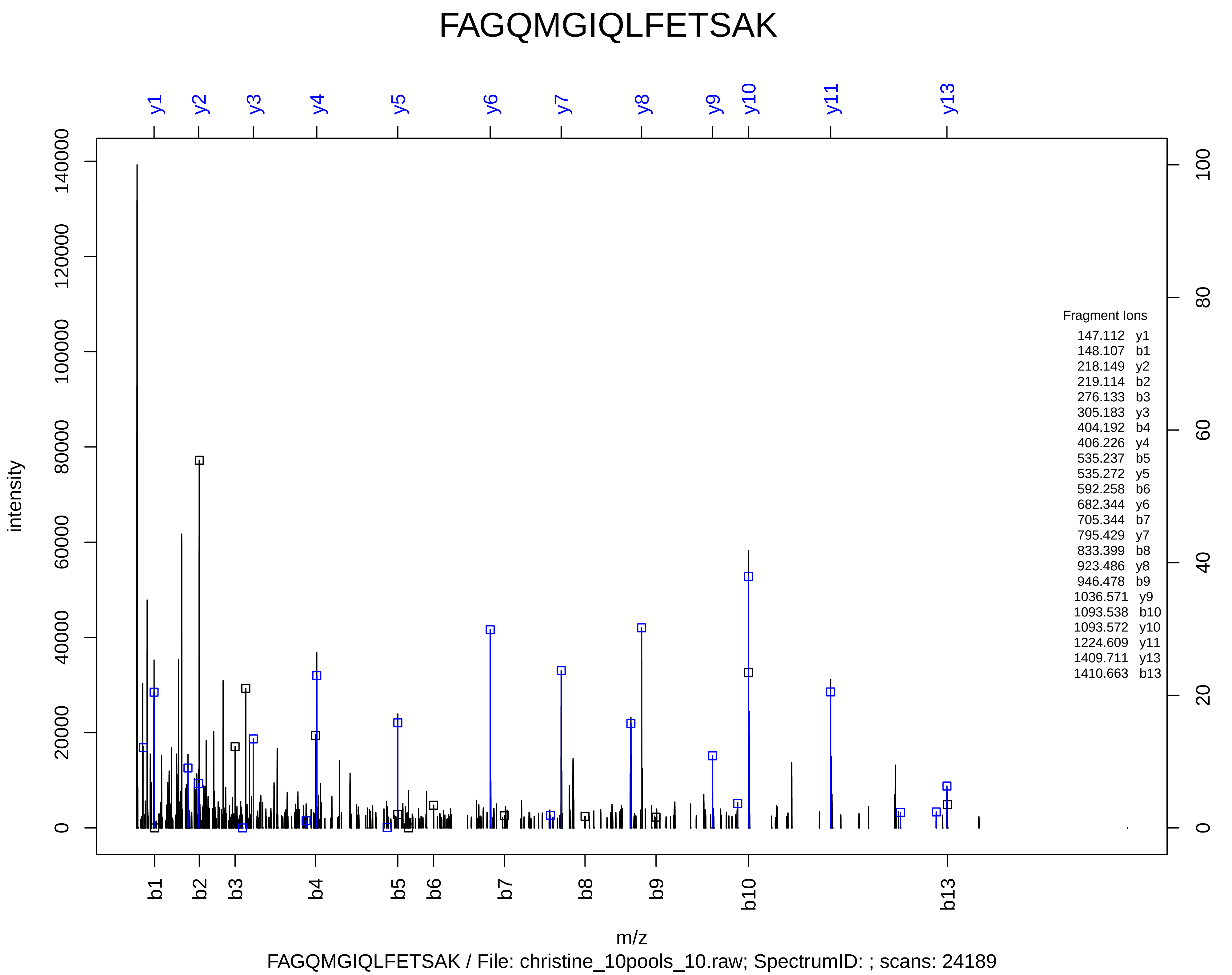
FAGQMGIQLFETSAK
y1 y2 y3 y4 y5 y6 y7 y8 y9 y10 y11 y13
b1 b2 b3 b4 b5 b6 b7 b8 b9 b10 b13
intensity
m/z
Fragment Ions
147.112 y1
148.107 b1
218.149 y2
219.114 b2
276.133 b3
305.183 y3
404.192 b4
406.226 y4
535.237 b5
535.272 y5
592.258 b6
682.344 y6
705.344 b7
795.429 y7
833.399 b8
923.486 y8
946.478 b9
1036.571 y9
1093.538 b10
1093.572 y10
1224.609 y11
1409.711 y13
1410.663 b13
FAGQMGIQLFETSAK / File: christine_10pools_10.raw; SpectrumID: ; scans: 24189

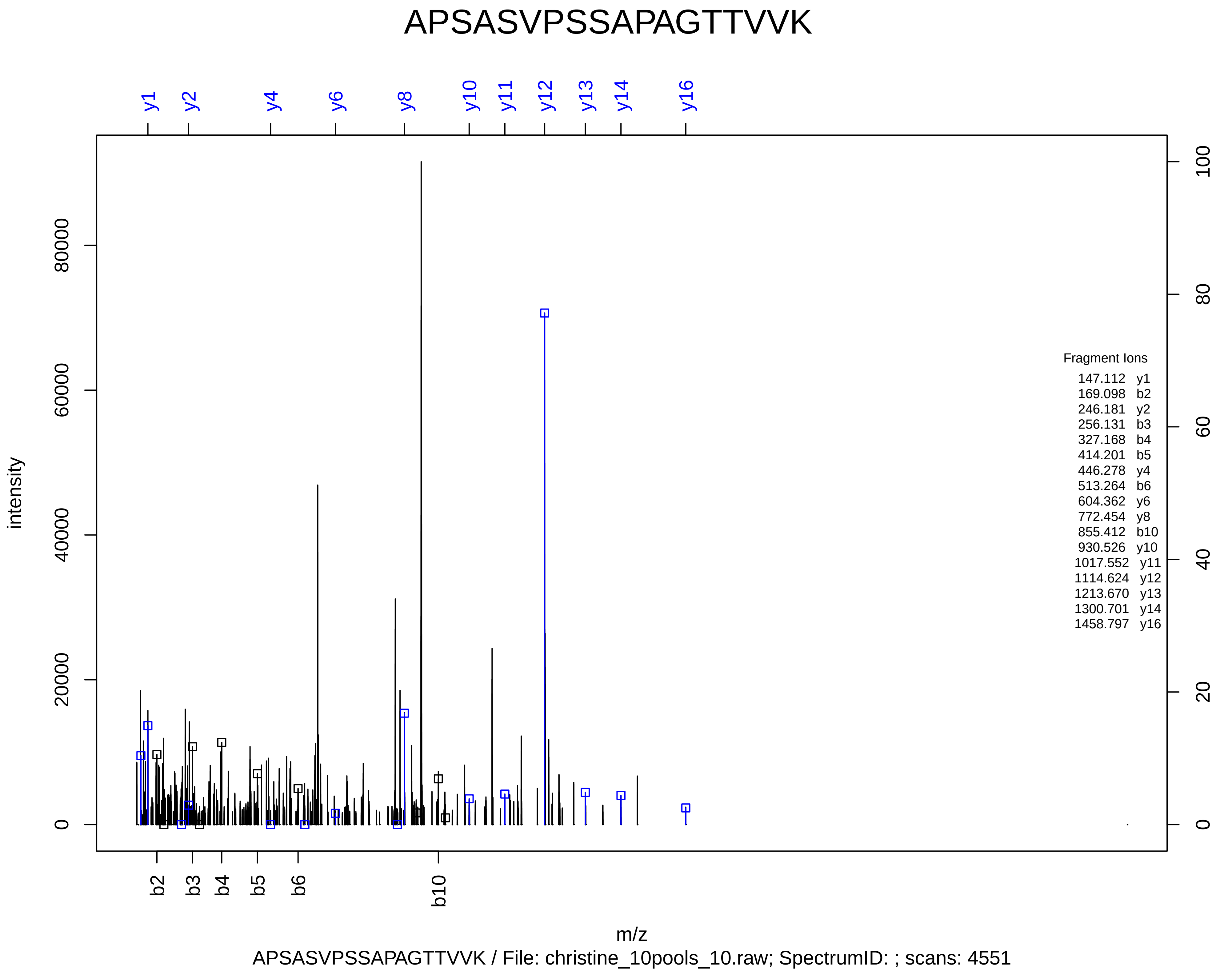

APSASVPSSAPAGTTVVK
y1 y2 y4 y6 y8 y10 y11 y12 y13 y14 y16
intensity
0 20000 40000 60000 80000
0 20 40 60 80 100
b2 b3 b4 b5 b6 b10
Fragment Ions
147.112 y1
169.098 b2
246.181 y2
256.131 b3
327.168 b4
414.201 b5
446.278 y4
513.264 b6
604.362 y6
772.454 y8
855.412 b10
930.526 y10
1017.552 y11
1114.624 y12
1213.670 y13
1300.701 y14
1458.797 y16
m/z
APSASVPSSAPAGTTVVK / File: christine_10pools_10.raw; SpectrumID: ; scans: 4551

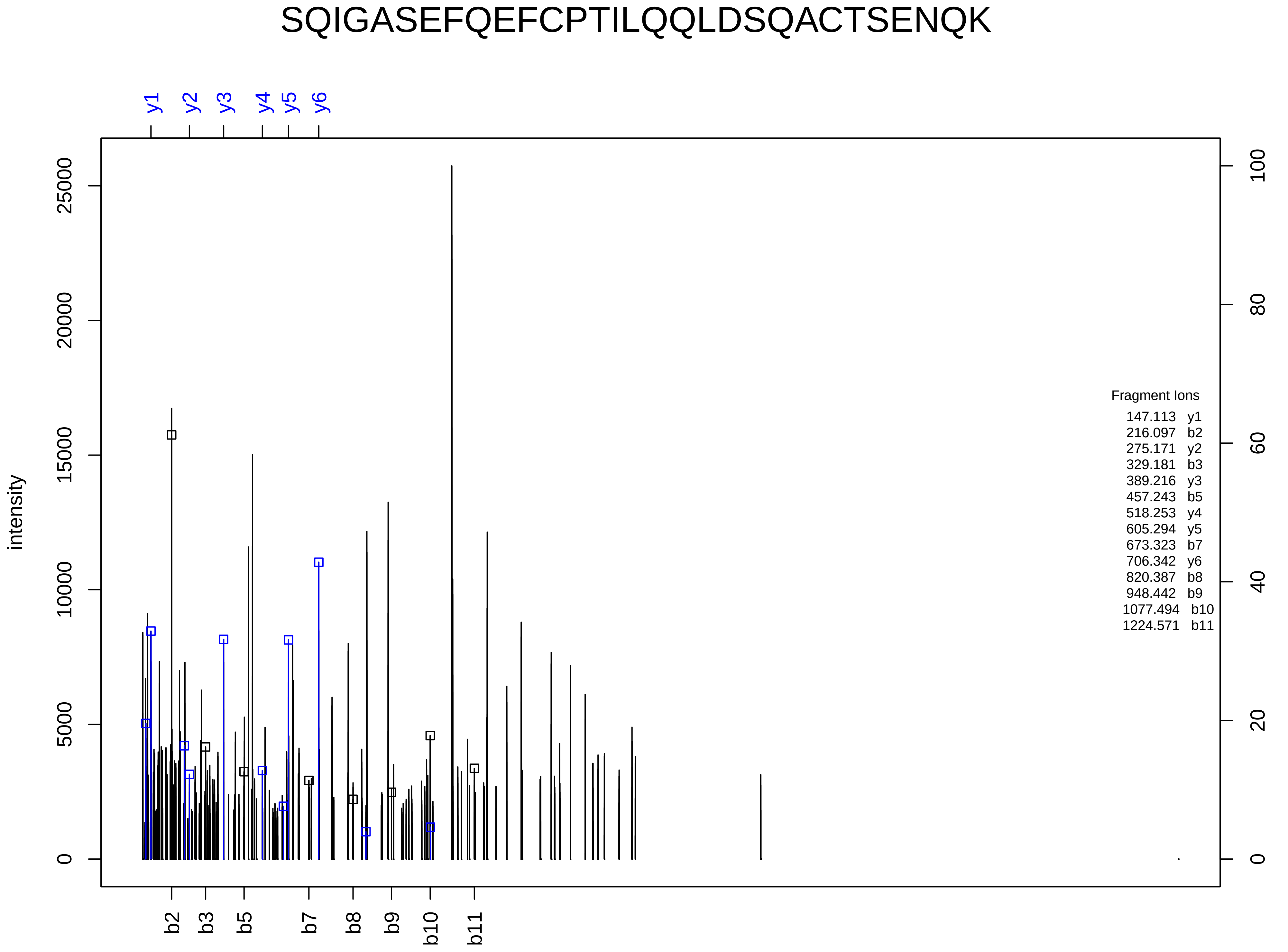

SQIGASEFQEFCPTILQQLDSQACTSENQK / File: christine_10pools_10.raw; SpectrumID: ; scans: 31438

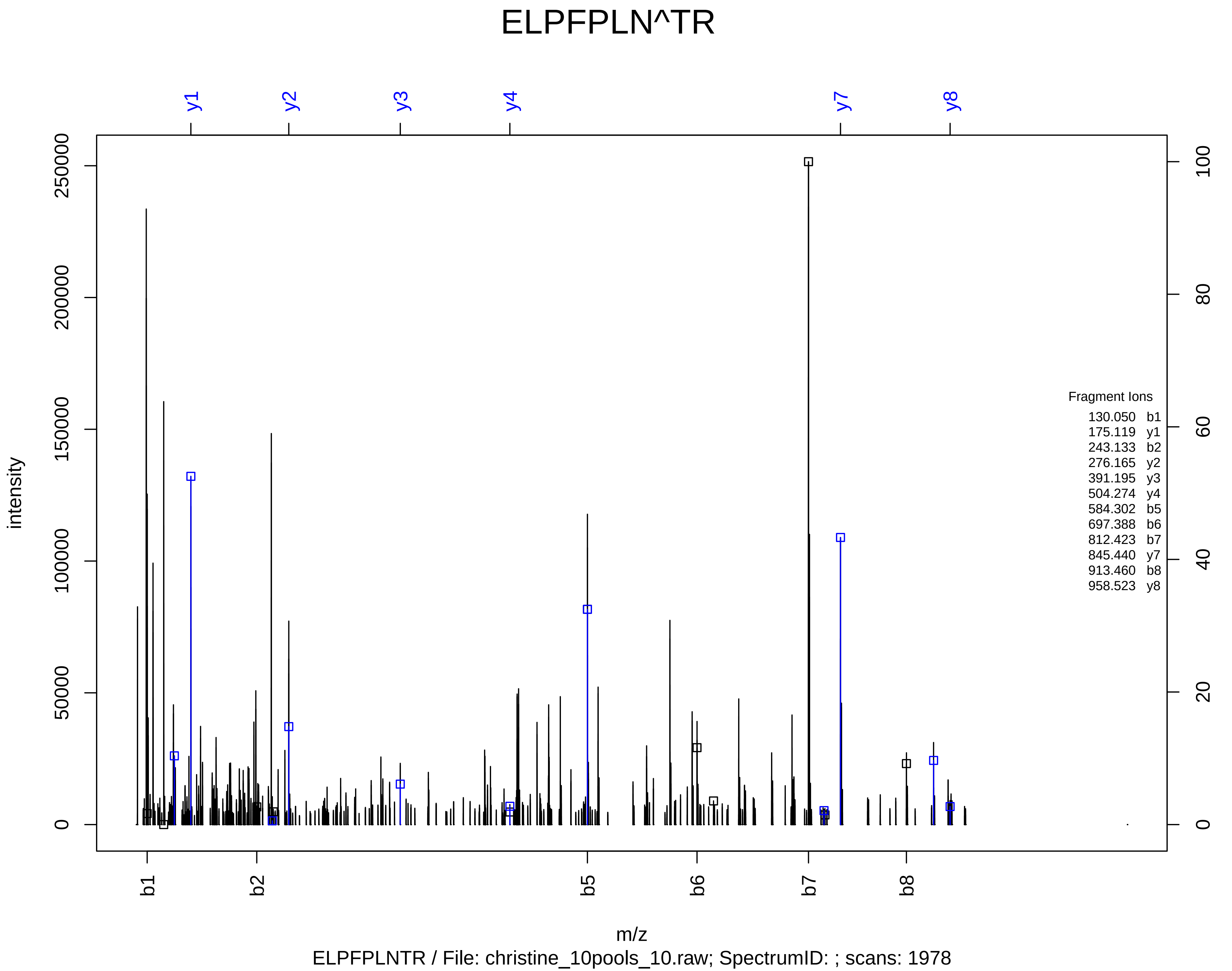

ELPFPLN^TR
y1
y2
y3
y4
y7
y8
intensity
0
50000
100000
150000
200000
250000
0
20
40
60
80
100
b1
b2
b5
b6
b7
b8
m/z
Fragment Ions
130.050 b1
175.119 y1
243.133 b2
276.165 y2
391.195 y3
504.274 y4
584.302 b5
697.388 b6
812.423 b7
845.440 y7
913.460 b8
958.523 y8
ELPFPLNTR / File: christine_10pools_10.raw; SpectrumID: ; scans: 1978

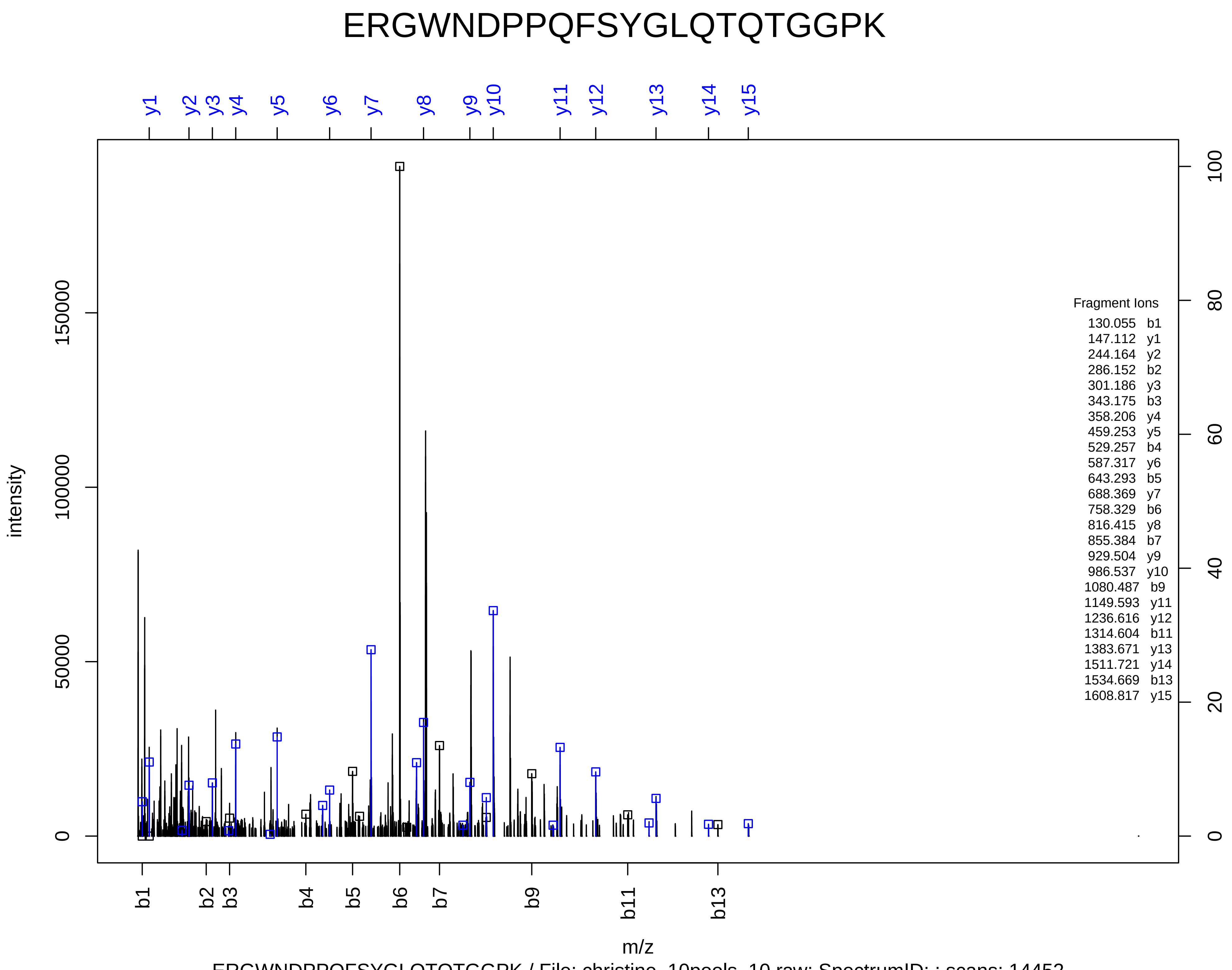
ERGWNDPPQFSYGLQTQTGGPK
y1 y2 y3 y4 y5 y6 y7 y8 y9 y10 y11 y12 y13 y14 y15
intensity
0
50000
100000
150000
0
20
40
60
80
100
Fragment Ions
130.055 b1
147.112 y1
244.164 y2
286.152 b2
301.186 y3
343.175 b3
358.206 y4
459.253 y5
529.257 b4
587.317 y6
643.293 b5
688.369 y7
758.329 b6
816.415 y8
855.384 b7
929.504 y9
986.537 y10
1080.487 b9
1149.593 y11
1236.616 y12
1314.604 b11
1383.671 y13
1511.721 y14
1534.669 b13
1608.817 y15
b1 b2 b3 b4 b5 b6 b7 b9 b11 b13
m/z
ERGWNDPPQFSYGLQTQTGGPK / File: christine_10pools_10.raw; SpectrumID: ; scans: 14452

LQEAQLELADIK

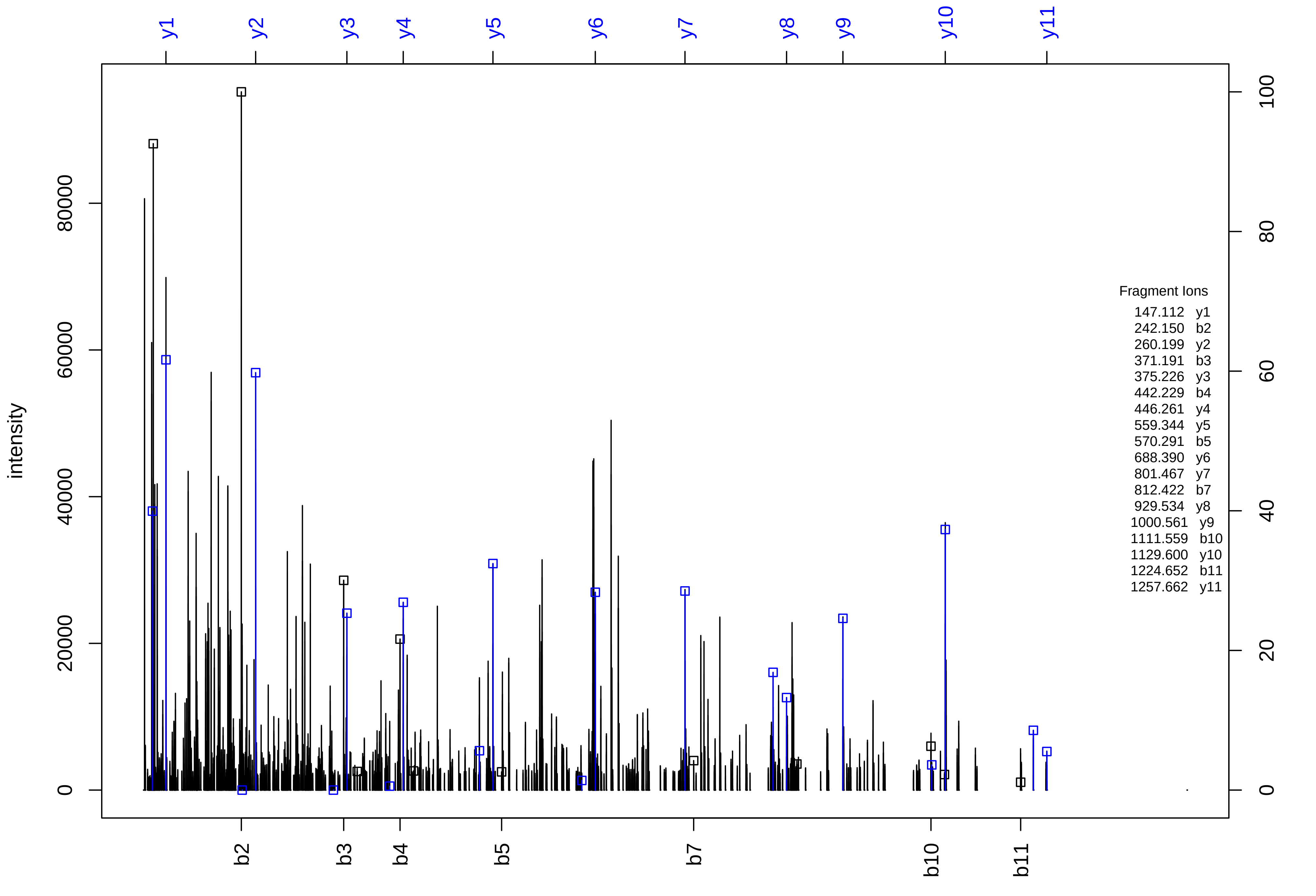

LQEAQLELADIK / File: christine_10pools_10.raw; SpectrumID: ; scans: 16419

LPVWVVEDHQEVLPFIYR

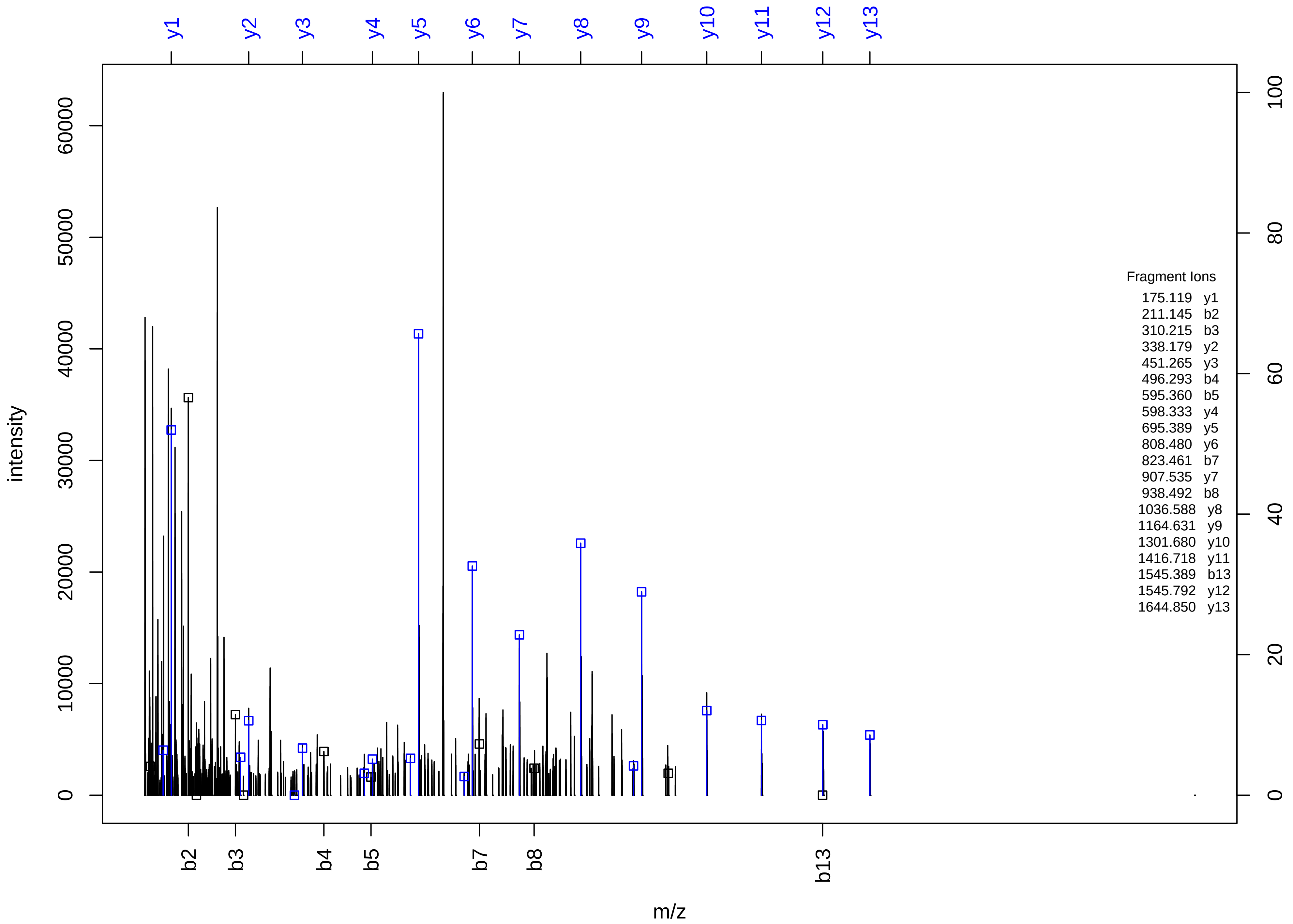

LPVWVVEDHQEVLPFIYR / File: christine_10pools_10.raw; SpectrumID: ; scans: 31579

ALIAPDHVVPAPEECYVYSPLGSAYK

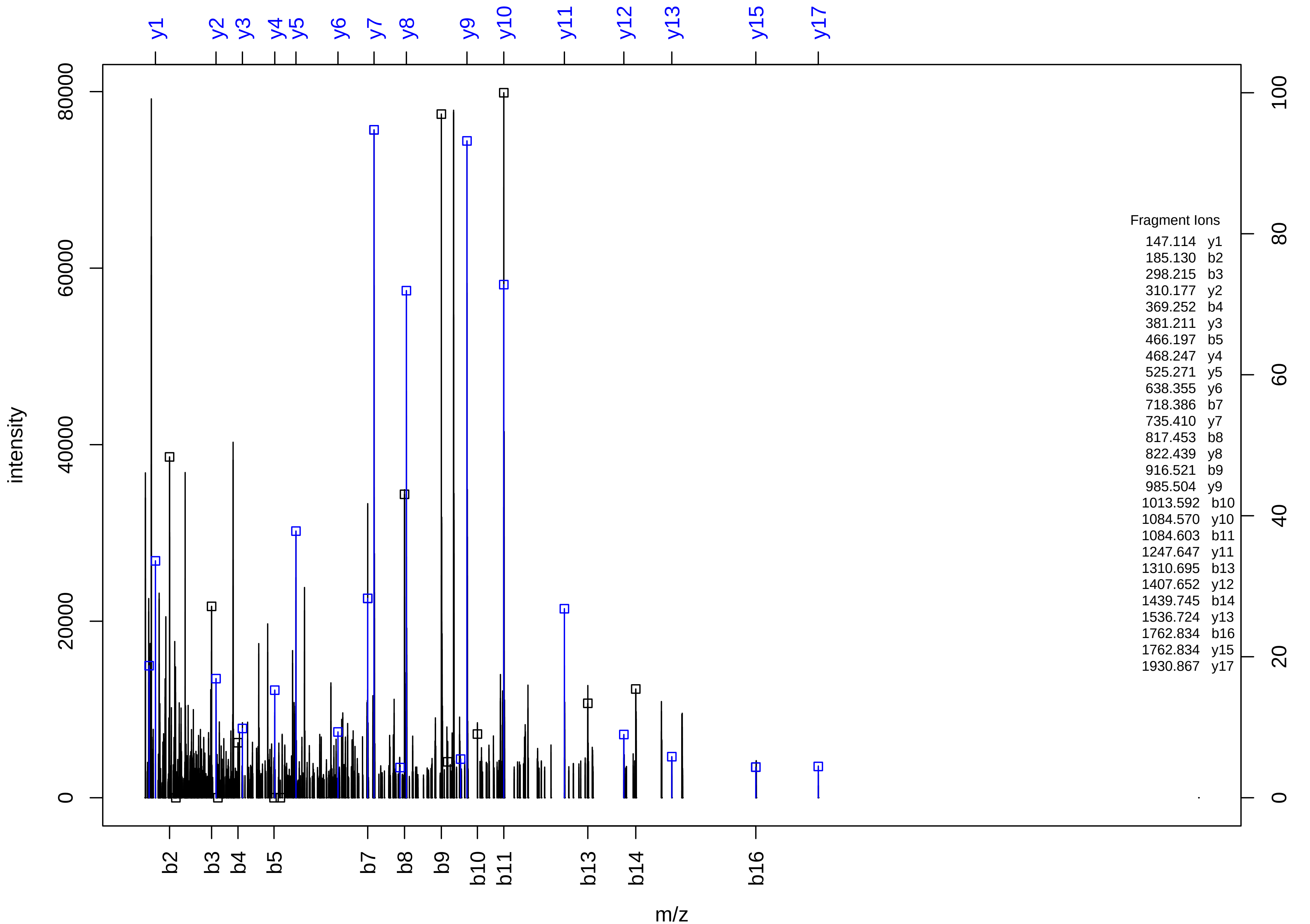

ALIAPDHVVPAPEECYVYSPLGSAYK / File: christine_10pools_10.raw; SpectrumID: ; scans: 24336

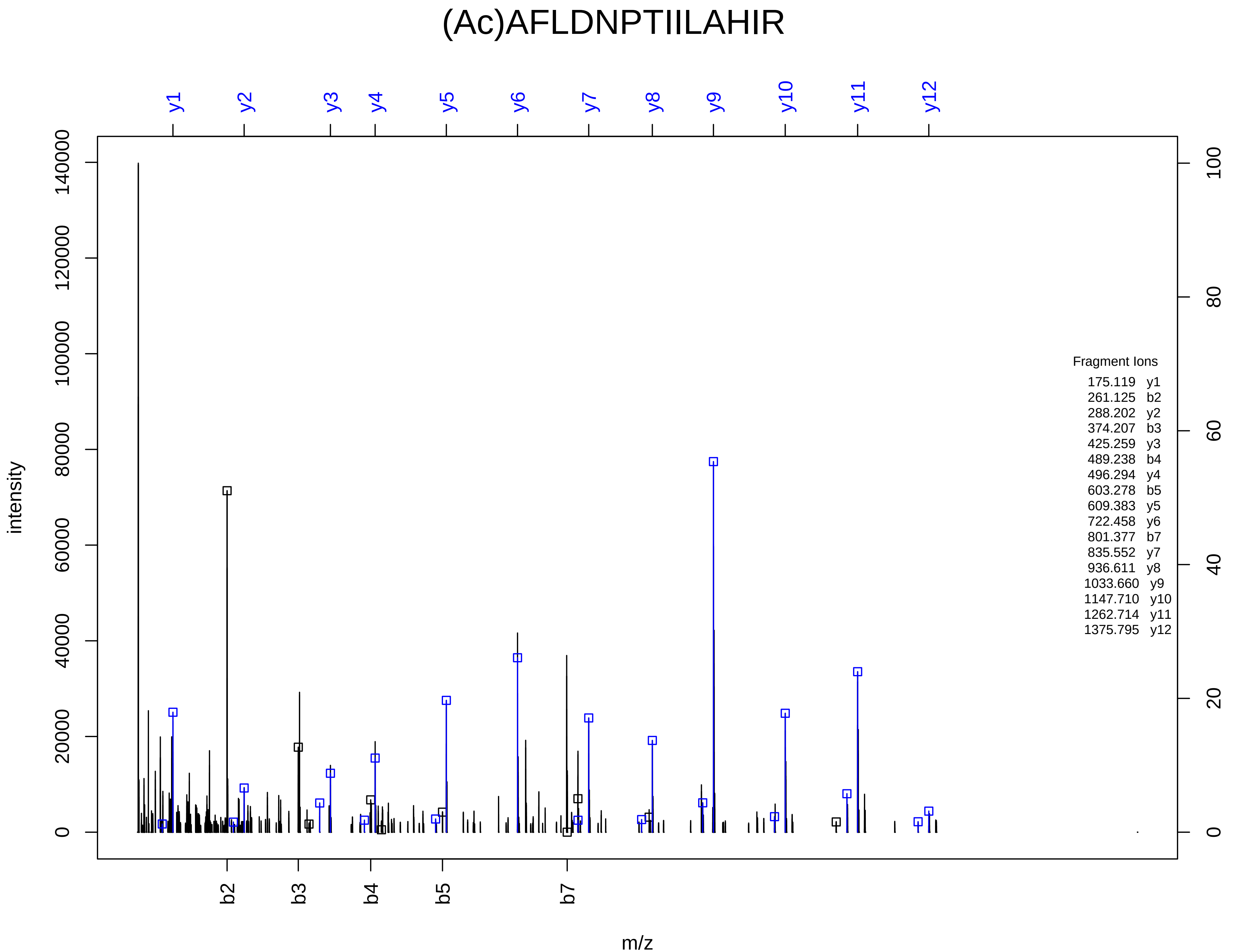

AFLDNPTIILAHIR / File: christine_10pools_10.raw; SpectrumID: ; scans: 39479

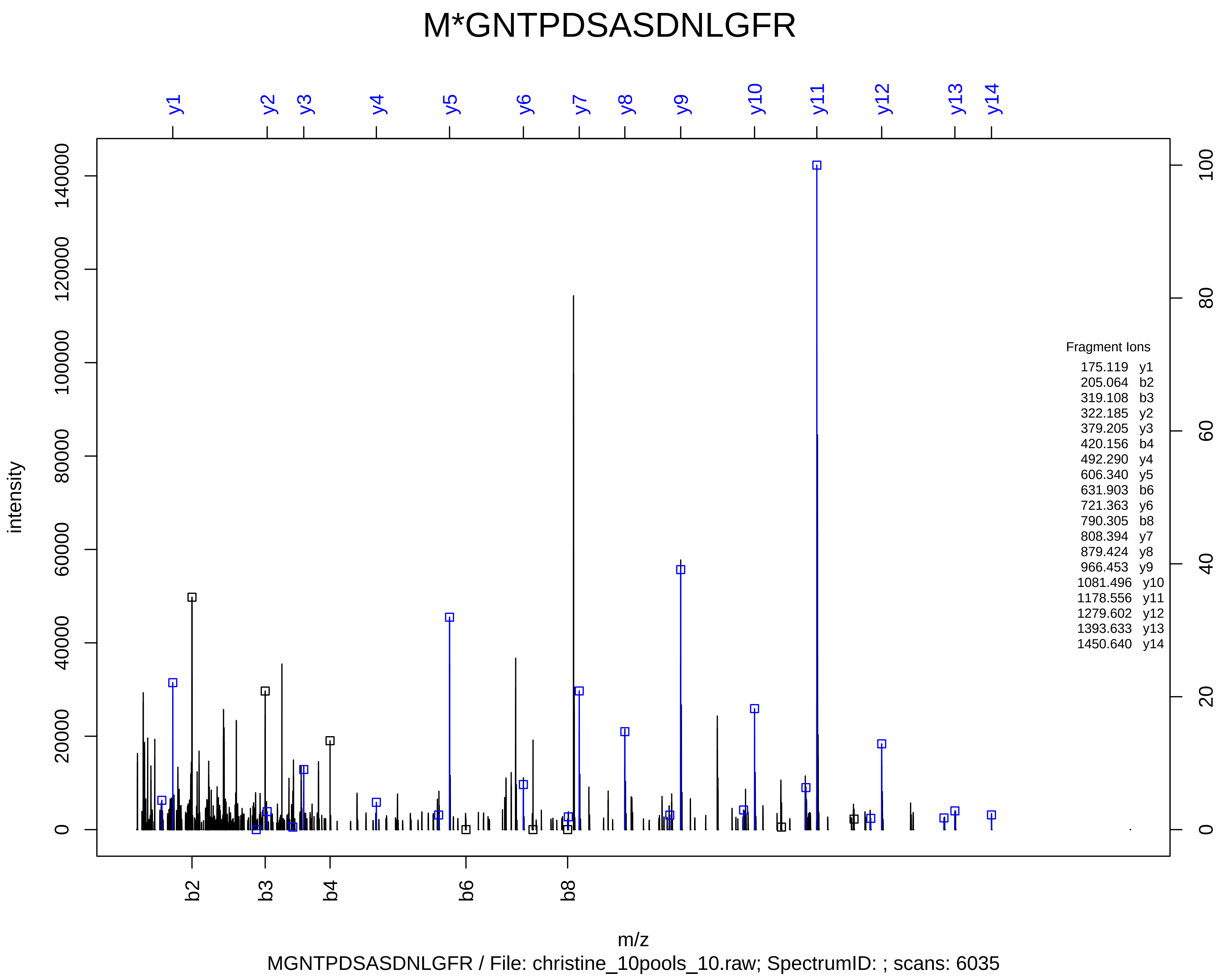
M*GNTPDSASDNLGFR
y1 y2 y3 y4 y5 y6 y7 y8 y9 y10 y11 y12 y13 y14
b2 b3 b4 b6 b8
intensity
m/z
Fragment Ions
175.119 y1
205.064 b2
319.108 b3
322.185 y2
379.205 y3
420.156 b4
492.290 y4
606.340 y5
631.903 b6
721.363 y6
790.305 b8
808.394 y7
879.424 y8
966.453 y9
1081.496 y10
1178.556 y11
1279.602 y12
1393.633 y13
1450.640 y14
MGNTPDSASDNLGFR / File: christine_10pools_10.raw; SpectrumID: ; scans: 6035

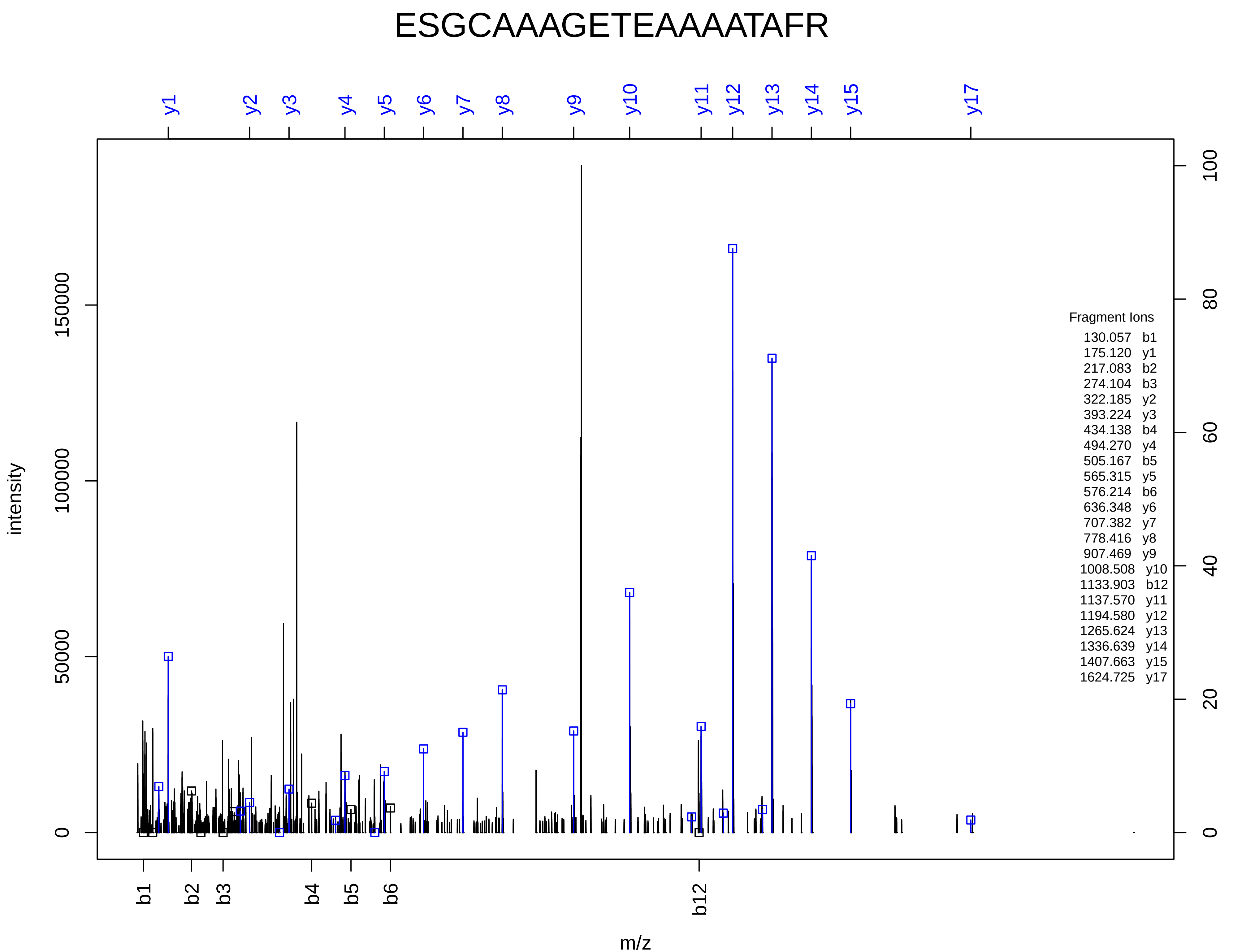
ESGCAAAGETEAAAATAFR
y1 y2 y3 y4 y5 y6 y7 y8 y9 y10 y11 y12 y13 y14 y15 y17
b1 b2 b3 b4 b5 b6 b12
intensity
m/z
Fragment Ions
130.057 b1
175.120 y1
217.083 b2
274.104 b3
322.185 y2
393.224 y3
434.138 b4
494.270 y4
505.167 b5
565.315 y5
576.214 b6
636.348 y6
707.382 y7
778.416 y8
907.469 y9
1008.508 y10
1133.903 b12
1137.570 y11
1194.580 y12
1265.624 y13
1336.639 y14
1407.663 y15
1624.725 y17
ESGCAAAGETEAAAATAFR / File: christine_10pools_10.raw; SpectrumID: ; scans: 10725

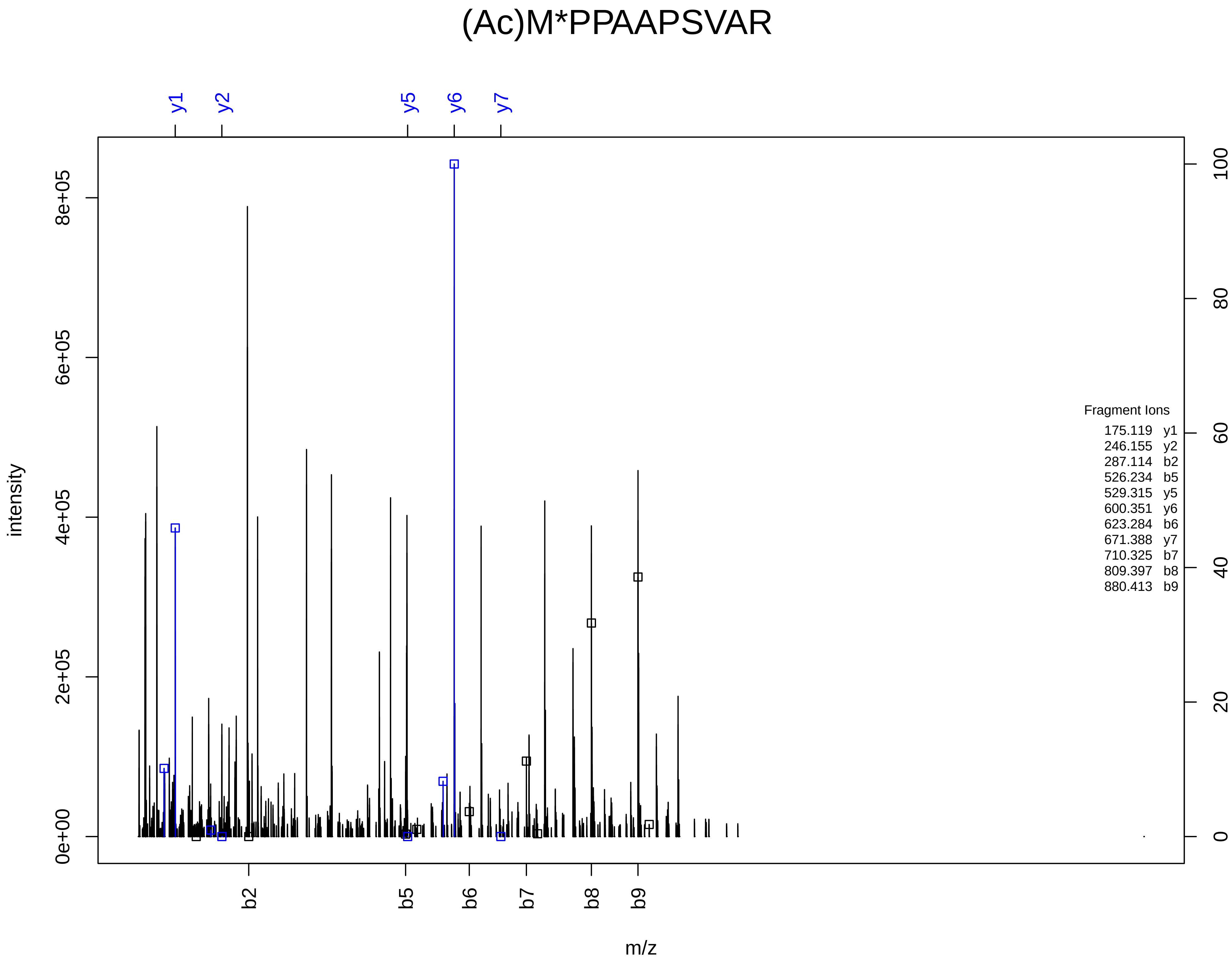

MPPAAPSVAR / File: christine_10pools_10.raw; SpectrumID: ; scans: 8842

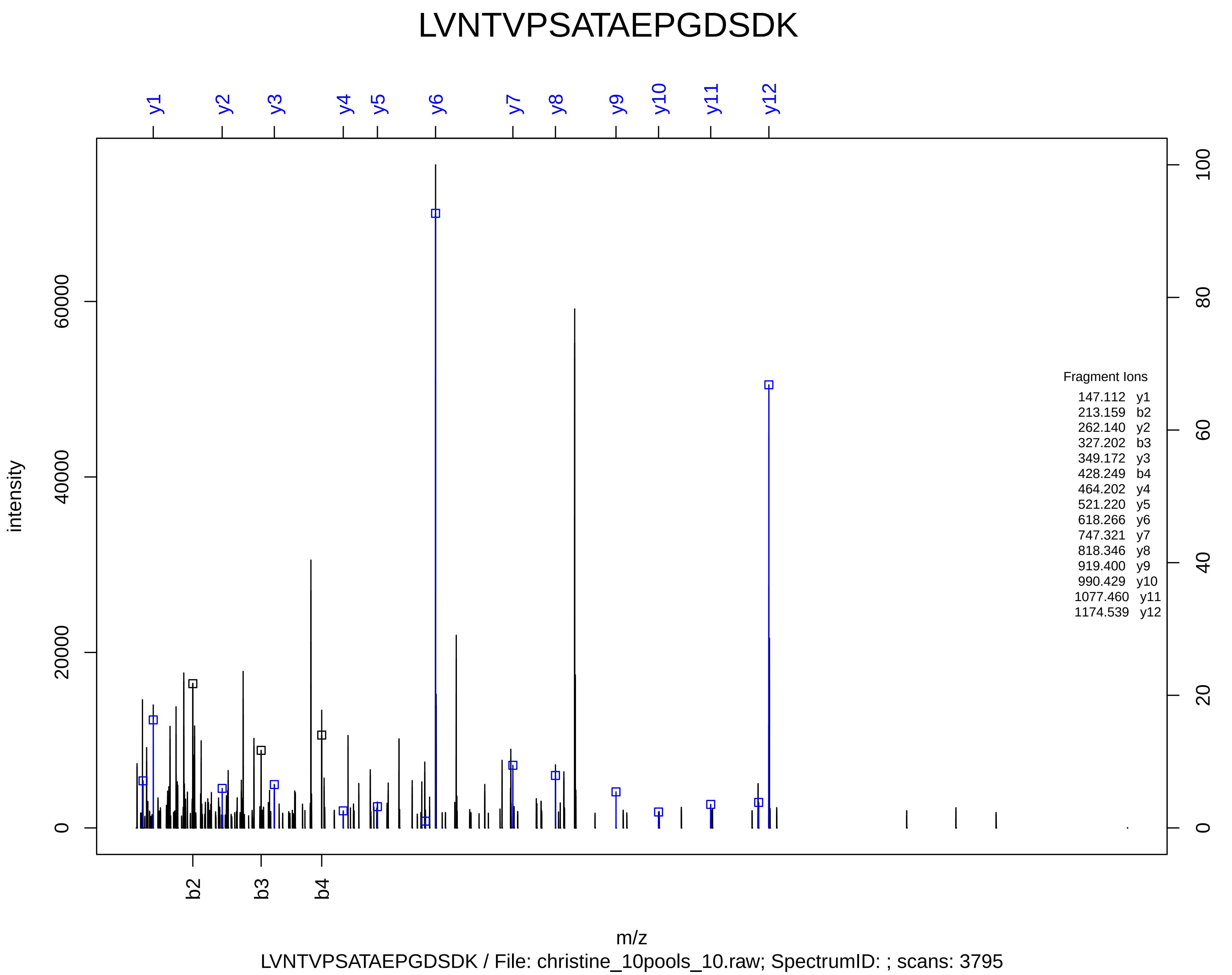

LVNTVPSATAEPGDSDK
y1
y2
y3
y4
y5
y6
y7
y8
y9
y10
y11
y12
b2
b3
b4
intensity
0
20000
40000
60000
0
20
40
60
80
100
m/z
Fragment Ions
147.112 y1
213.159 b2
262.140 y2
327.202 b3
349.172 y3
428.249 b4
464.202 y4
521.220 y5
618.266 y6
747.321 y7
818.346 y8
919.400 y9
990.429 y10
1077.460 y11
1174.539 y12
LVNTVPSATAEPGDSDK / File: christine_10pools_10.raw; SpectrumID: ; scans: 3795

VLFRPSDATNSSNLDALSSNTSLK

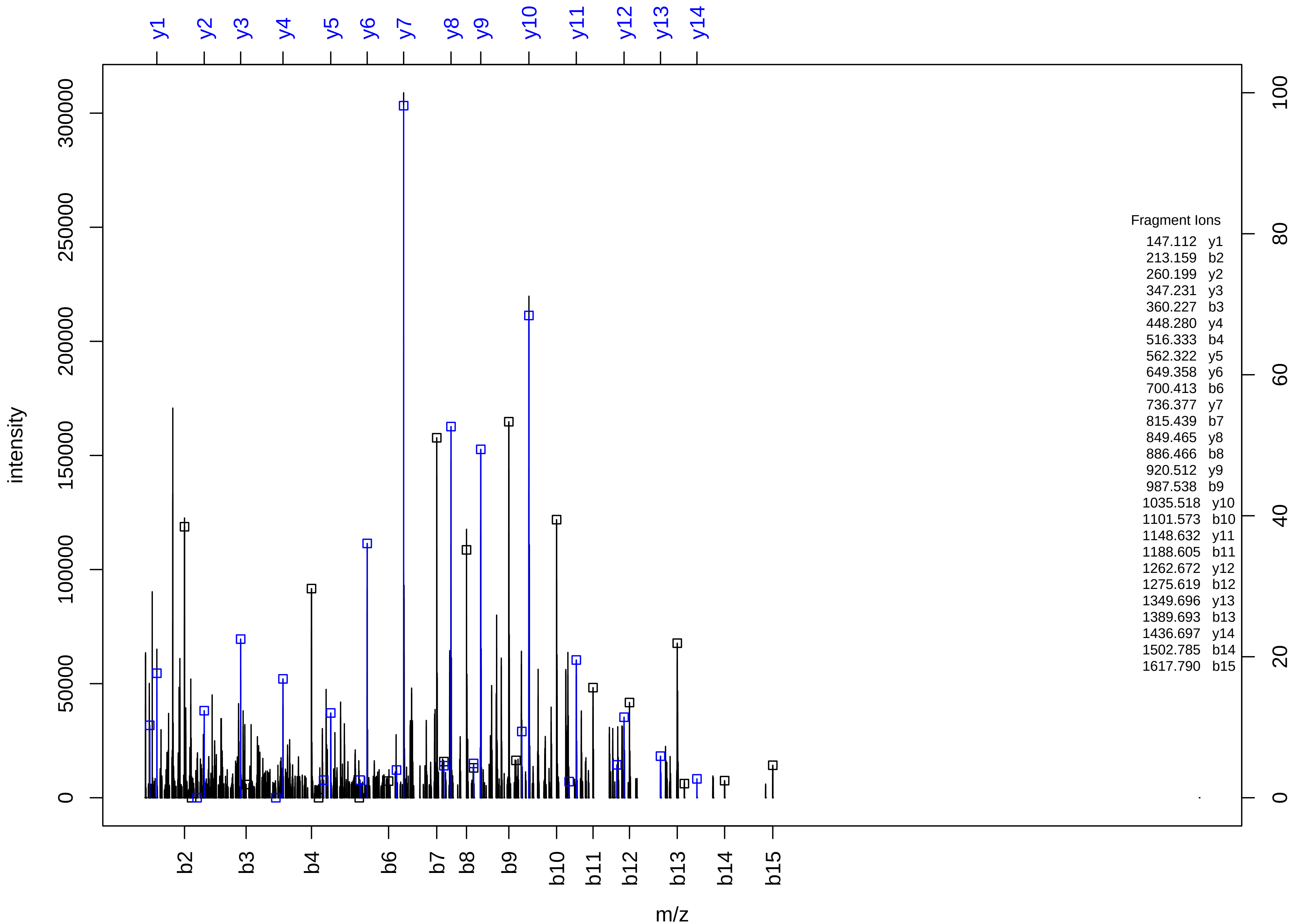

VLFRPSDATNSSNLDALSSNTSLK / File: christine_10pools_10.raw; SpectrumID: ; scans: 16855

DPSSQVGACIVNTENK

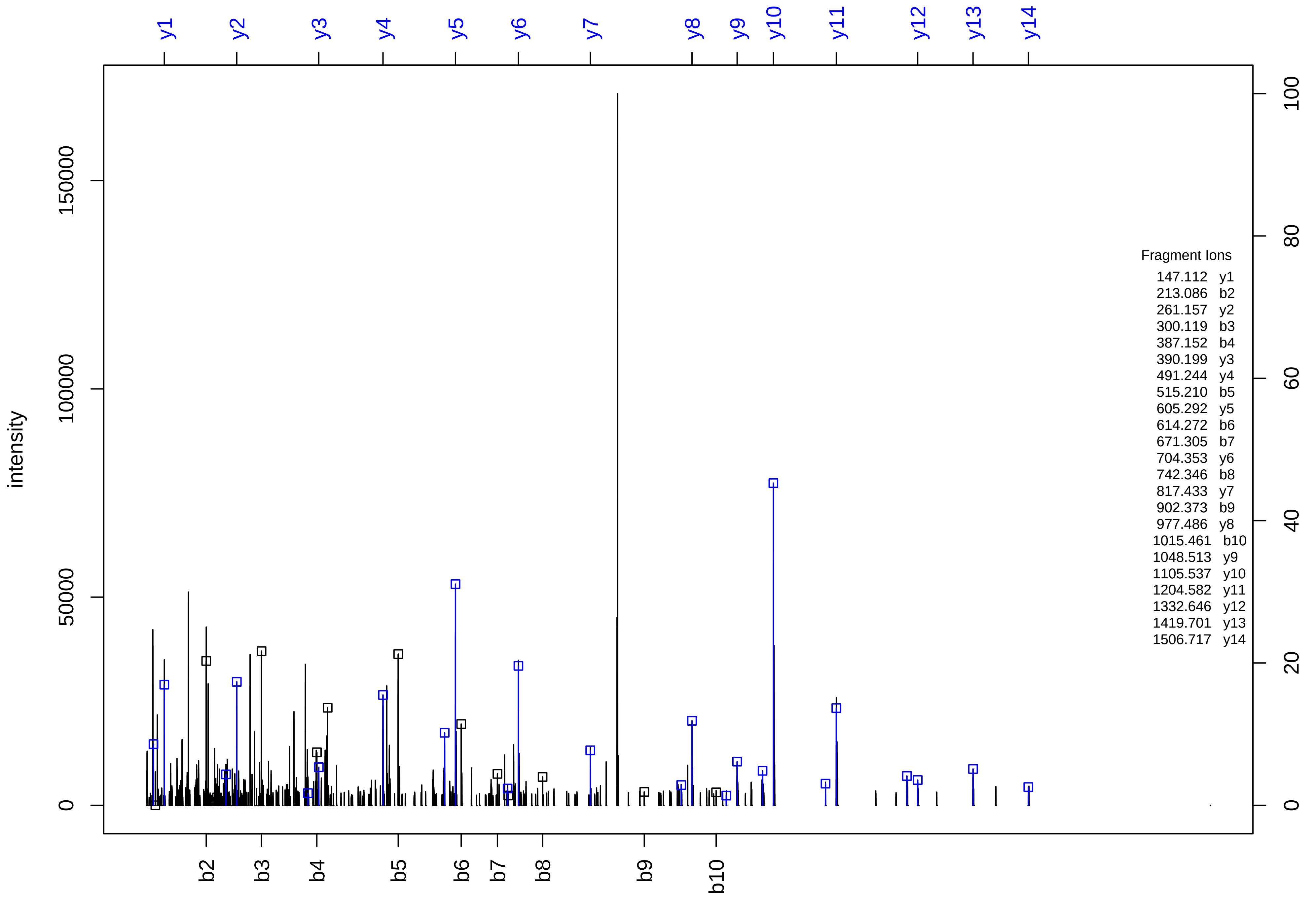

DPSSQVGACIVNTENK / File: christine_10pools_10.raw; SpectrumID: ; scans: 8139

M*VLSLILQLSTLWPACR

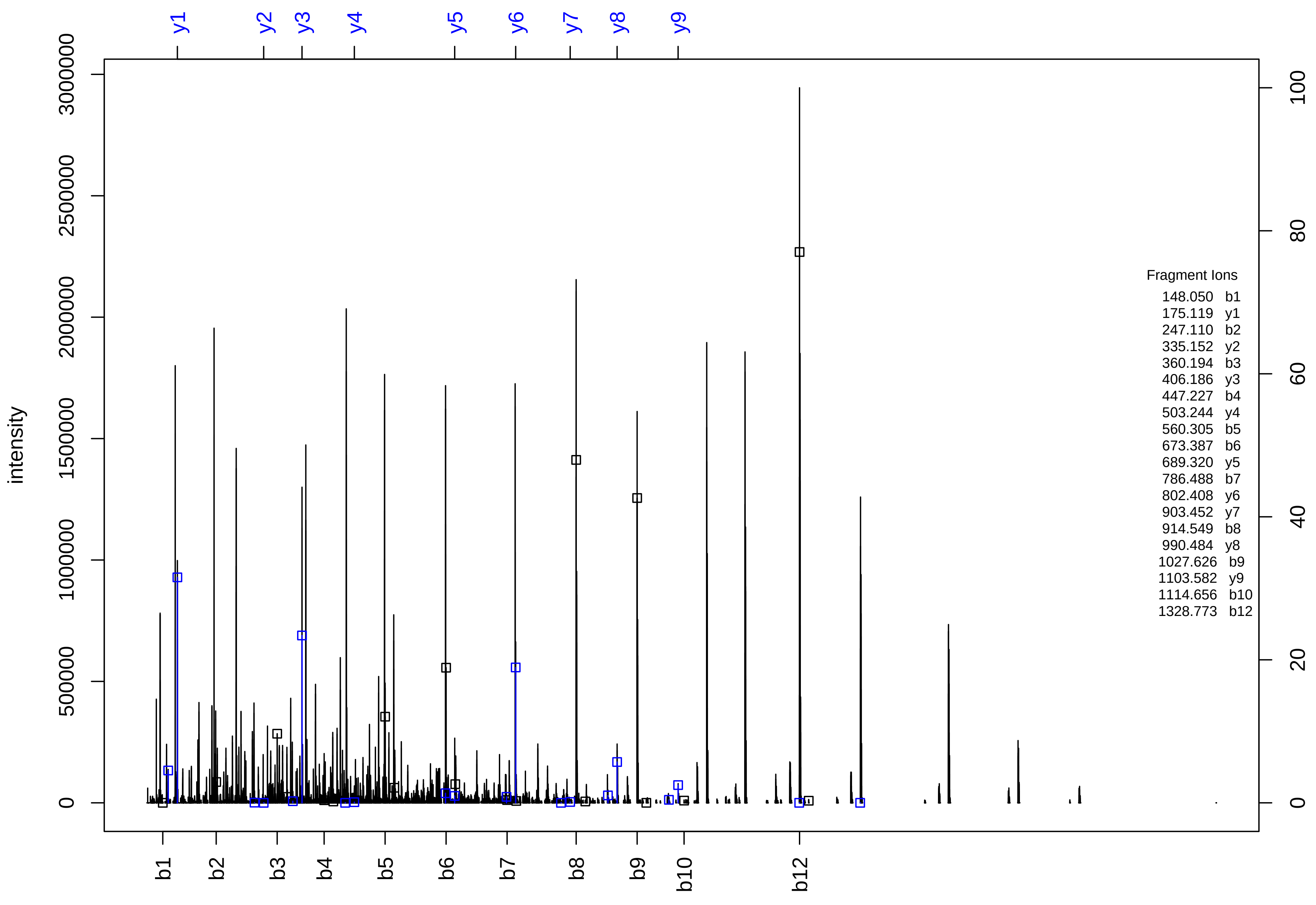

MVLSLILQLSTLWPACR / File: christine_10pools_10.raw; SpectrumID: ; scans: 41584

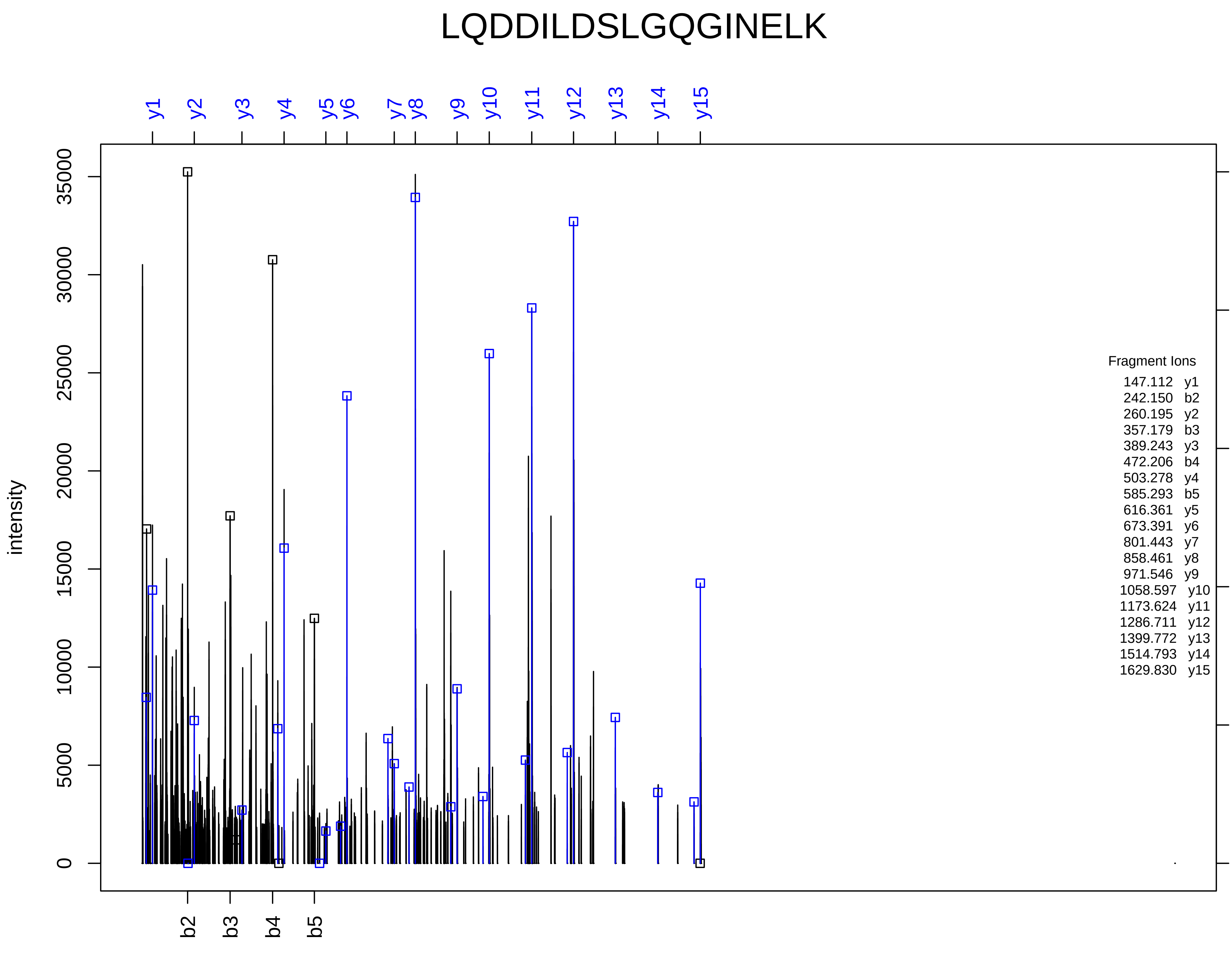

LQDDILDSLGQGINELK / File: christine_10pools_10.raw; SpectrumID: ; scans: 32425

LLYEANLPENFR

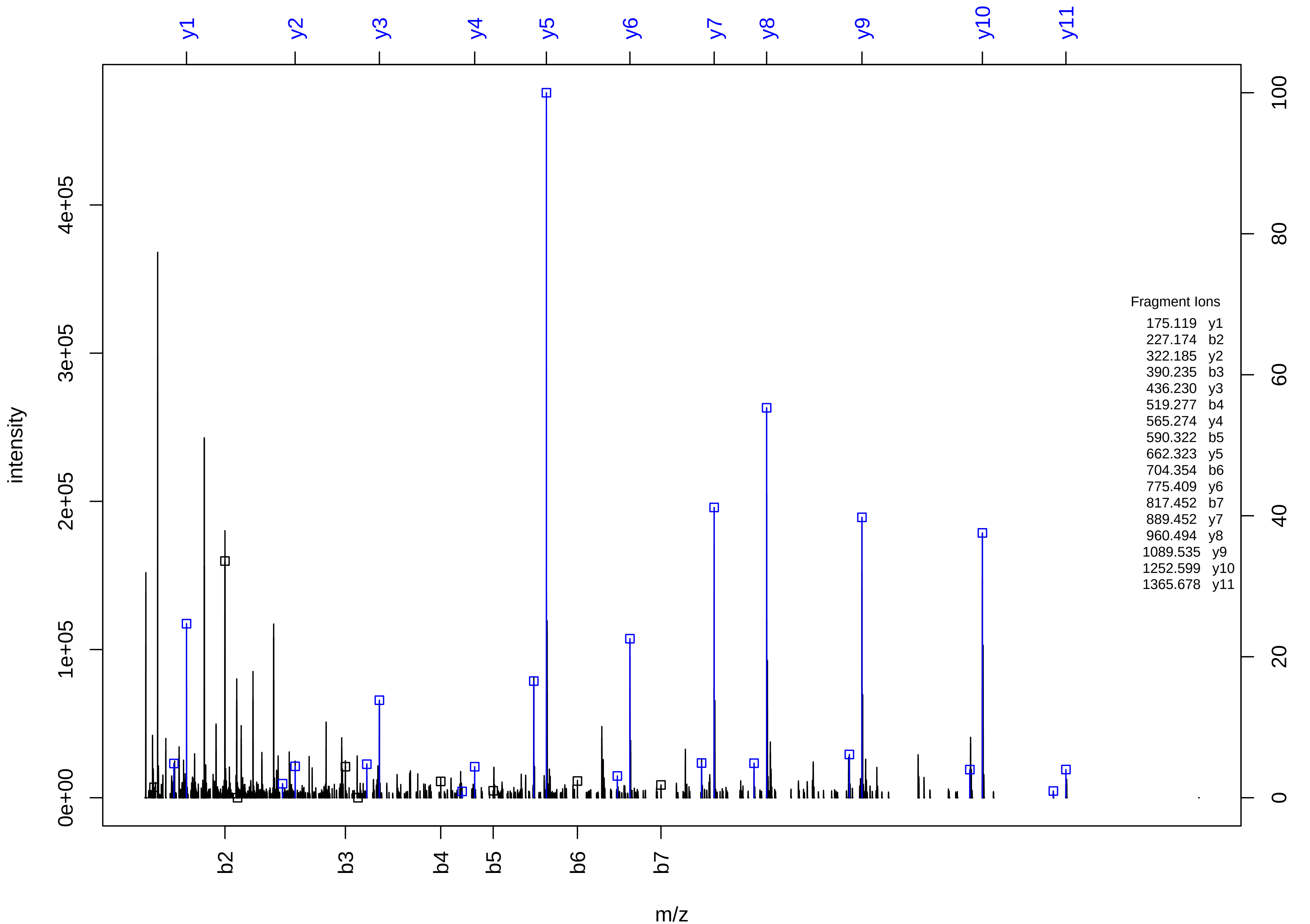

LLYEANLPENFR / File: christine_10pools_10.raw; SpectrumID: ; scans: 19561

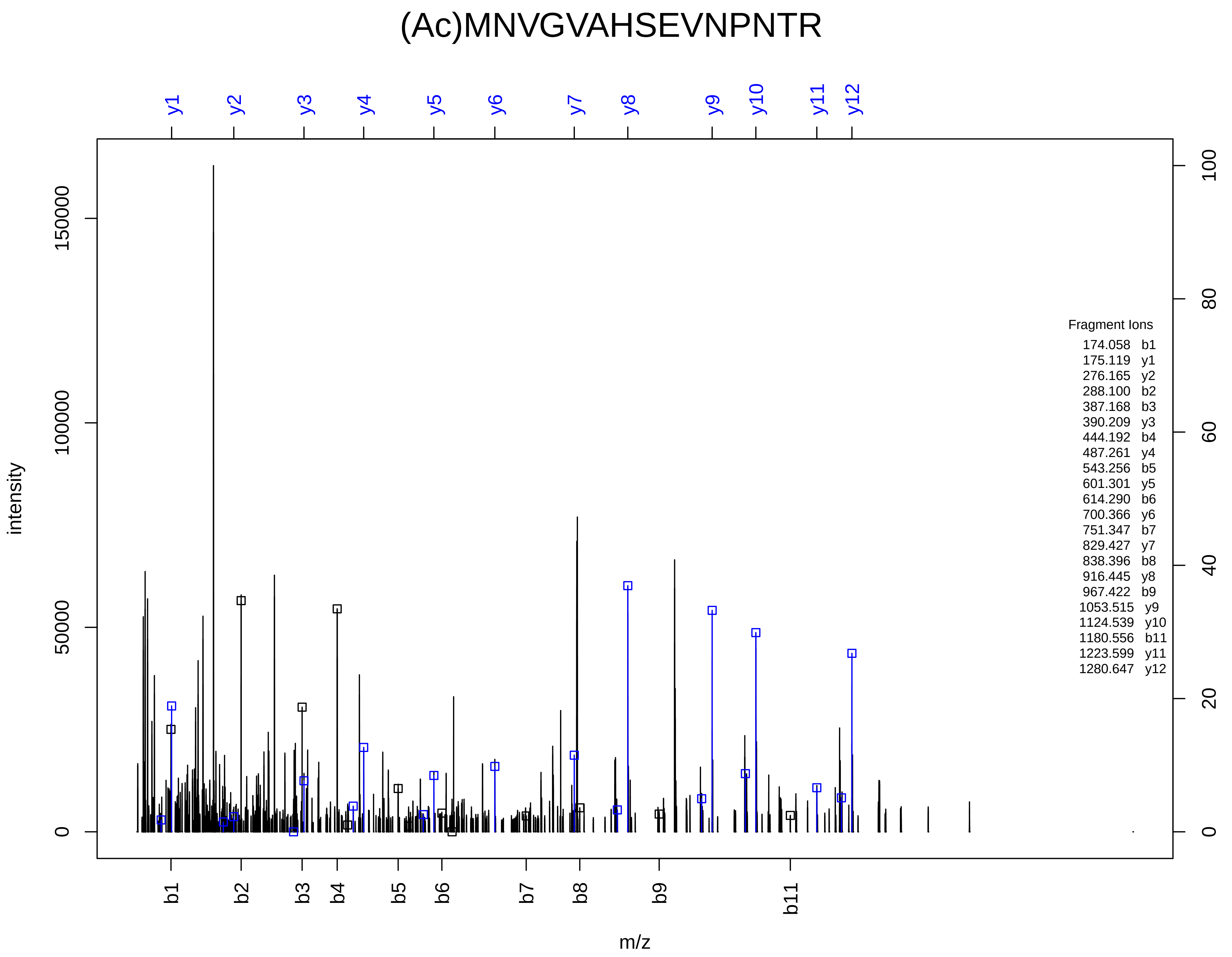

(Ac)MNVGVAHSEVNPNTR
y1
y2
y3
y4
y5
y6
y7
y8
y9
y10
y11
y12
intensity
150000
100000
50000
0
100
80
60
40
20
0
Fragment Ions
174.058 b1
175.119 y1
276.165 y2
288.100 b2
387.168 b3
390.209 y3
444.192 b4
487.261 y4
543.256 b5
601.301 y5
614.290 b6
700.366 y6
751.347 b7
829.427 y7
838.396 b8
916.445 y8
967.422 b9
1053.515 y9
1124.539 y10
1180.556 b11
1223.599 y11
1280.647 y12
b1
b2
b3
b4
b5
b6
b7
b8
b9
b11
m/z
MNVGVAHSEVNPNTR / File: christine_10pools_10.raw; SpectrumID: ; scans: 7290

IDHLSFGELVPGIINPLDGTEK

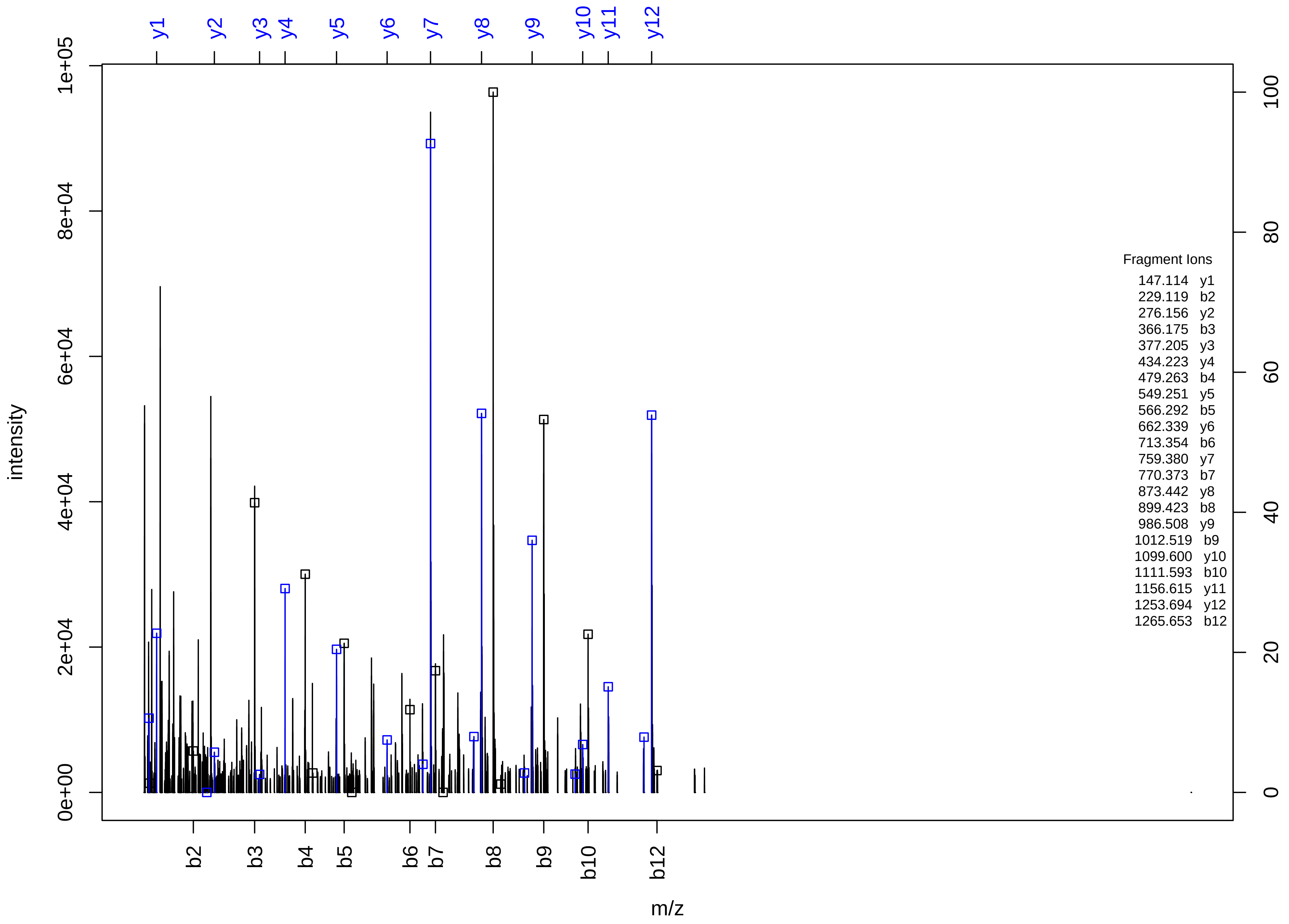

IDHLSFGELVPGIINPLDGTEK / File: christine_10pools_10.raw; SpectrumID: ; scans: 34382

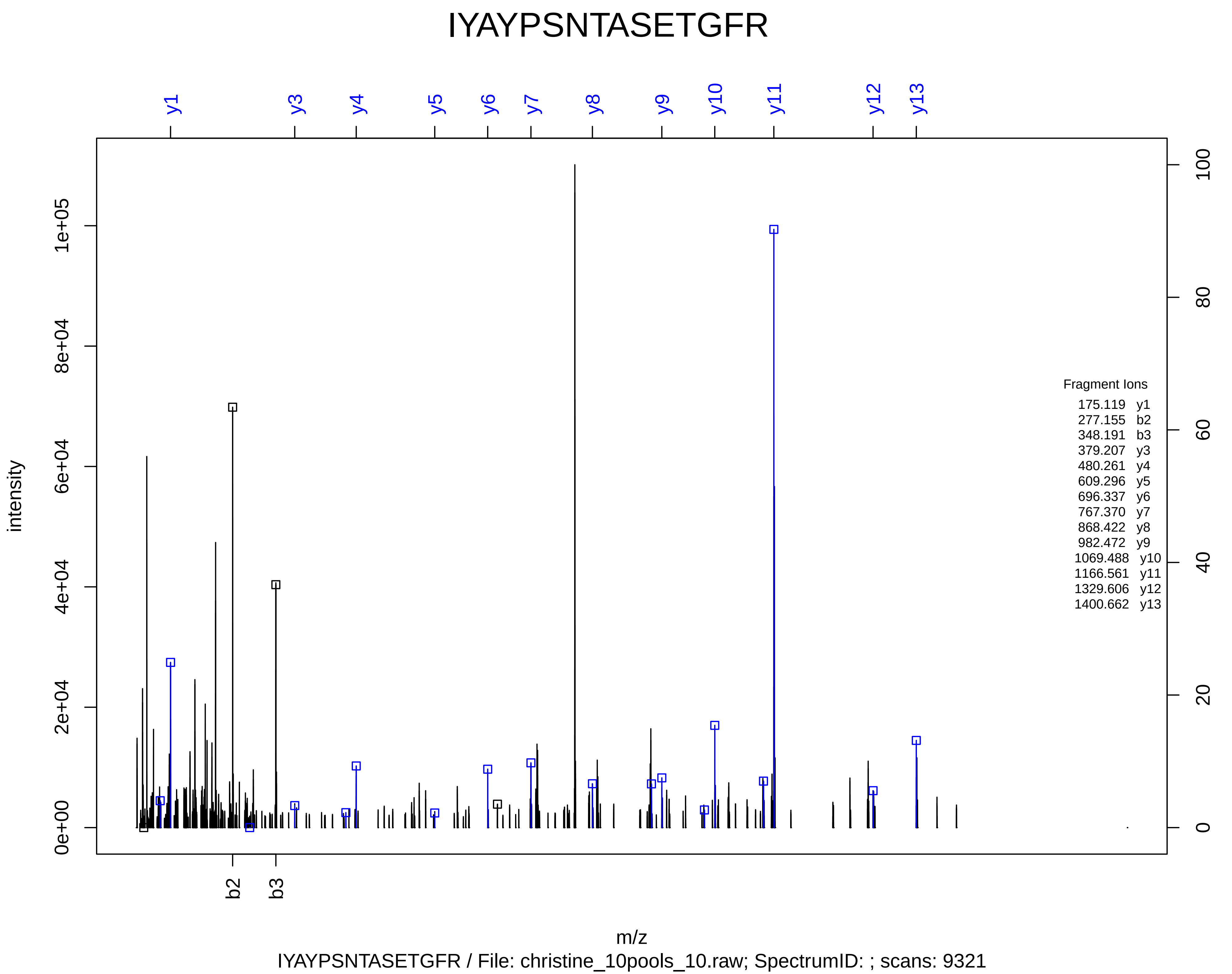
IYAYPSNTASETGFR
y1
y3
y4
y5
y6
y7
y8
y9
y10
y11
y12
y13
b2
b3
intensity
0e+00
2e+04
4e+04
6e+04
8e+04
1e+05
0
20
40
60
80
100
m/z
Fragment Ions
175.119 y1
277.155 b2
348.191 b3
379.207 y3
480.261 y4
609.296 y5
696.337 y6
767.370 y7
868.422 y8
982.472 y9
1069.488 y10
1166.561 y11
1329.606 y12
1400.662 y13
IYAYPSNTASETGFR / File: christine_10pools_10.raw; SpectrumID: ; scans: 9321

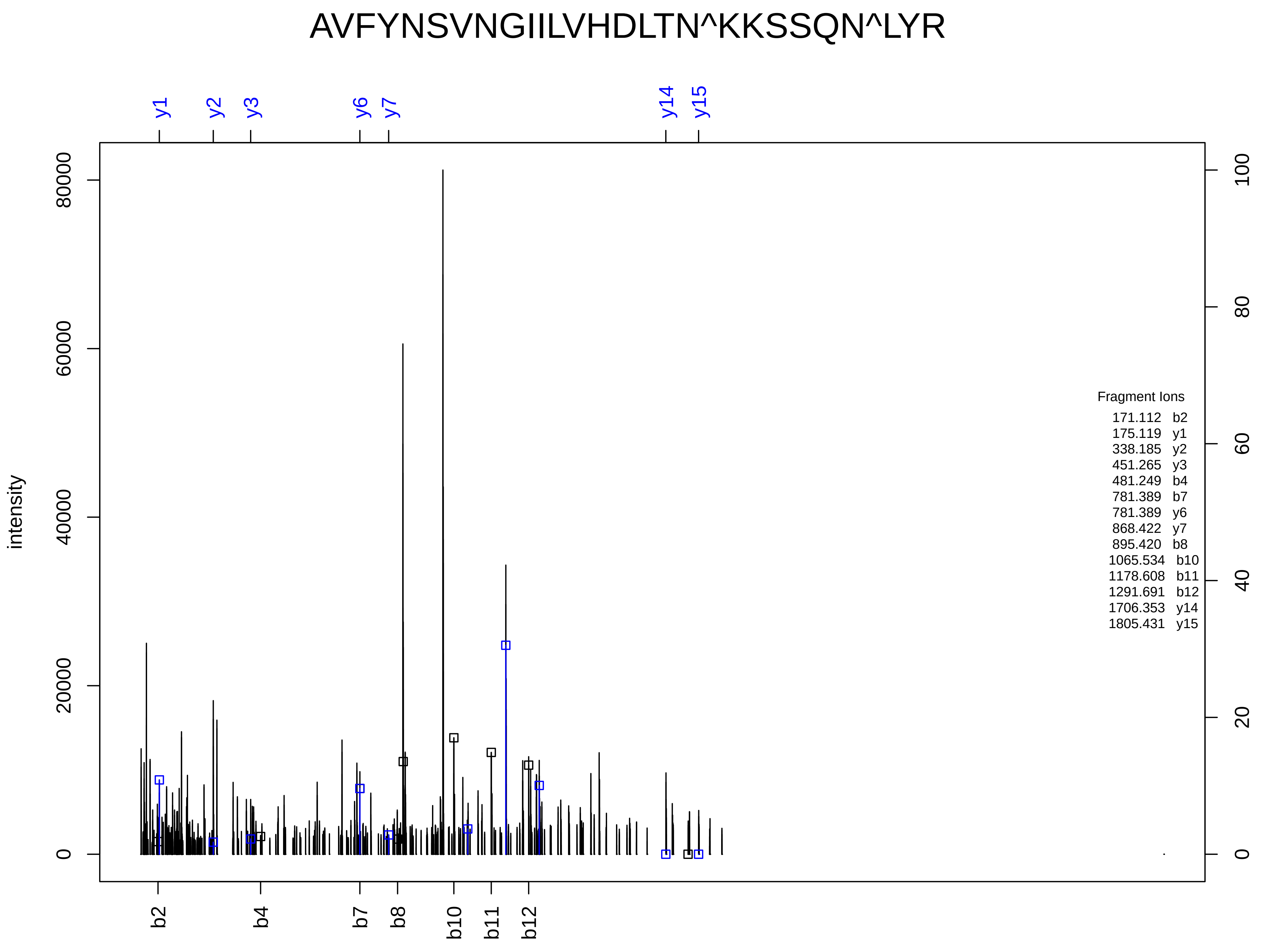

AVFYNSVNGIILVHDLTNKKSSQNLYR / File: christine_10pools_10.raw; SpectrumID: ; scans: 37005

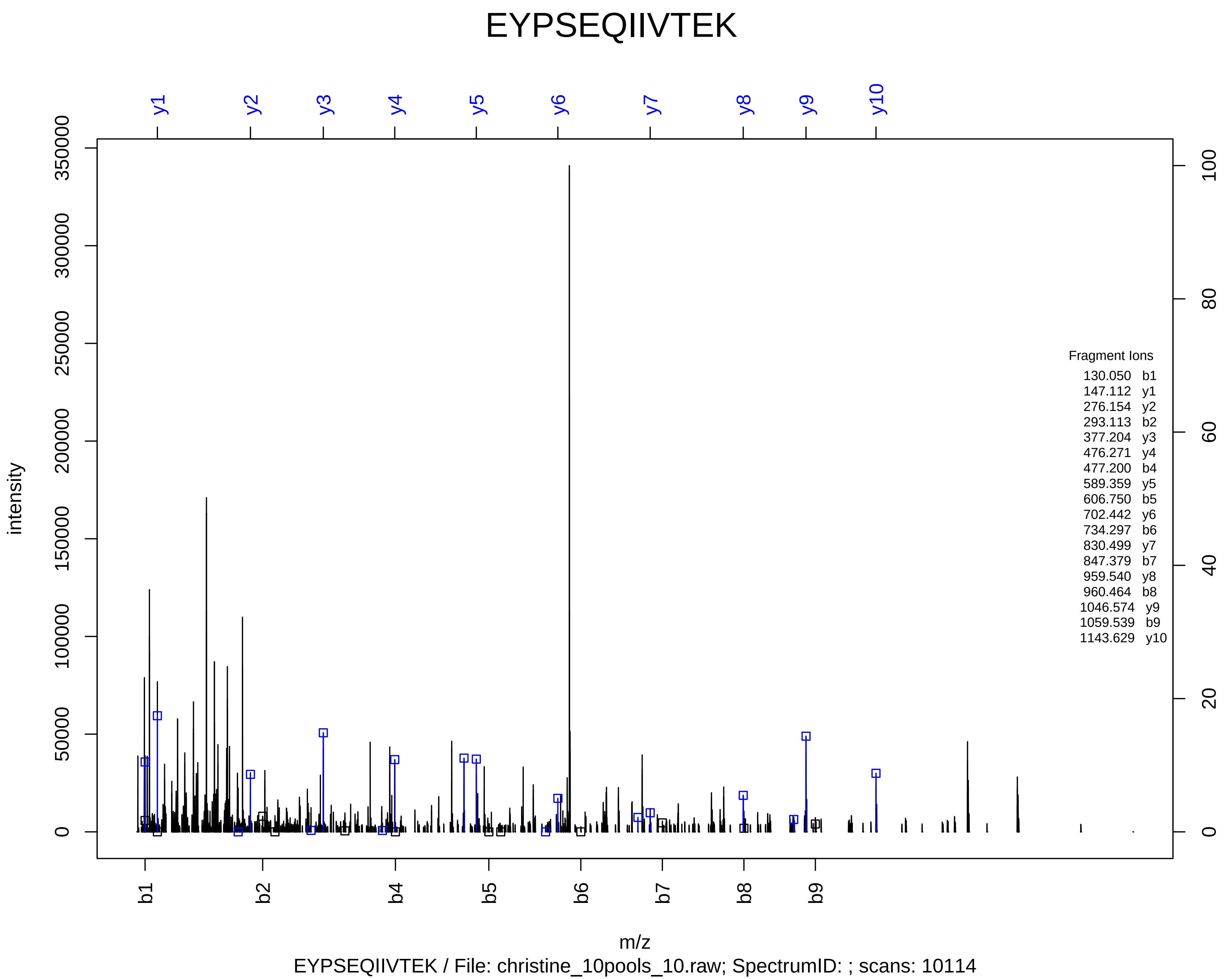
EYPSEQIIVTEK
y1
y2
y3
y4
y5
y6
y7
y8
y9
y10
intensity
0
50000
100000
150000
200000
250000
300000
350000
0
20
40
60
80
100
Fragment Ions
130.050 b1
147.112 y1
276.154 y2
293.113 b2
377.204 y3
476.271 y4
477.200 b4
589.359 y5
606.750 b5
702.442 y6
734.297 b6
830.499 y7
847.379 b7
959.540 y8
960.464 b8
1046.574 y9
1059.539 b9
1143.629 y10
b1
b2
b4
b5
b6
b7
b8
b9
m/z
EYPSEQIIVTEK / File: christine_10pools_10.raw; SpectrumID: ; scans: 10114

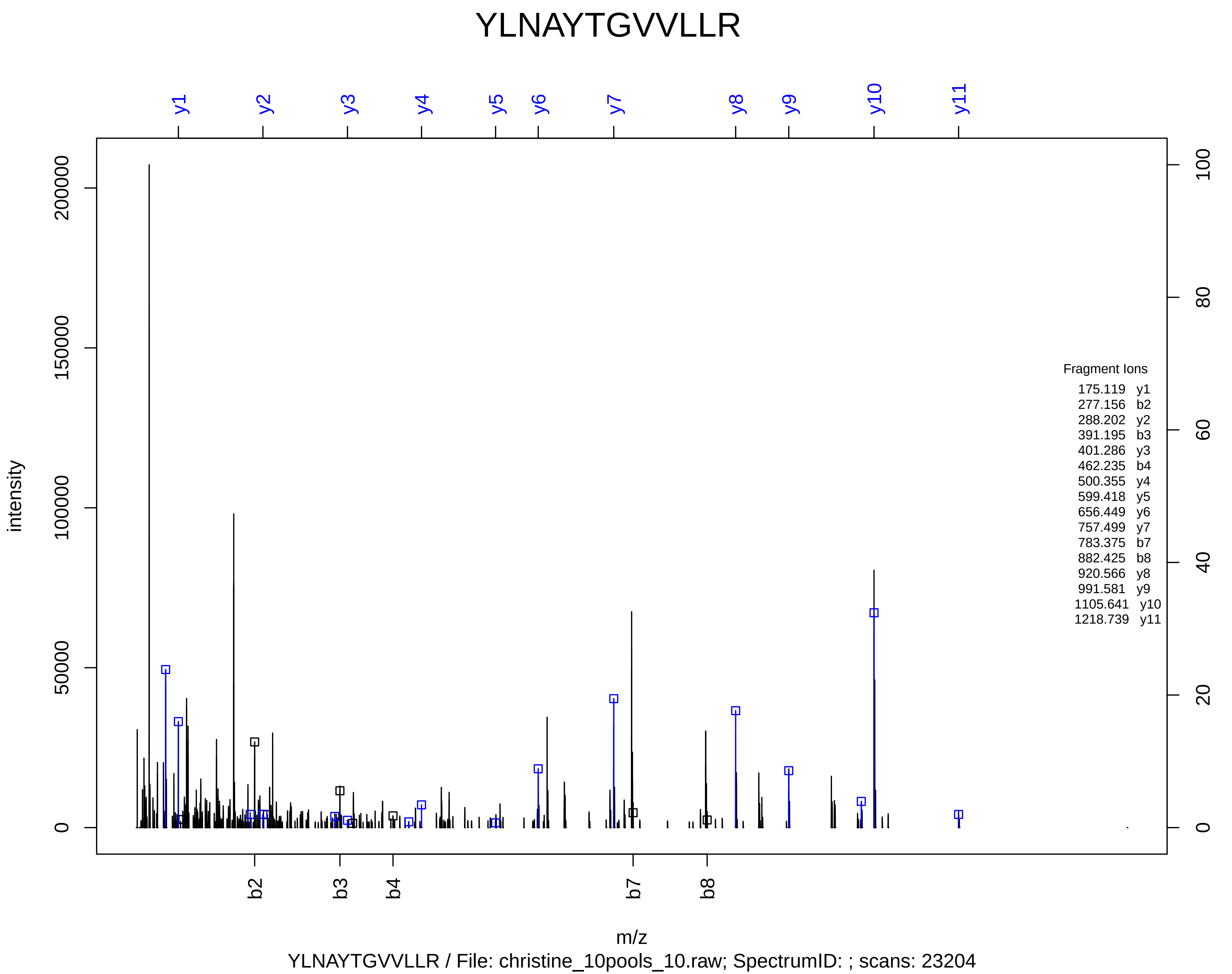
YLNAYTGVVLLR
y1
y2
y3
y4
y5
y6
y7
y8
y9
y10
y11
intensity
0
50000
100000
150000
200000
0
20
40
60
80
100
Fragment Ions
175.119 y1
277.156 b2
288.202 y2
391.195 b3
401.286 y3
462.235 b4
500.355 y4
599.418 y5
656.449 y6
757.499 y7
783.375 b7
882.425 b8
920.566 y8
991.581 y9
1105.641 y10
1218.739 y11
b2
b3
b4
b7
b8
m/z
YLNAYTGVVLLR / File: christine_10pools_10.raw; SpectrumID: ; scans: 23204

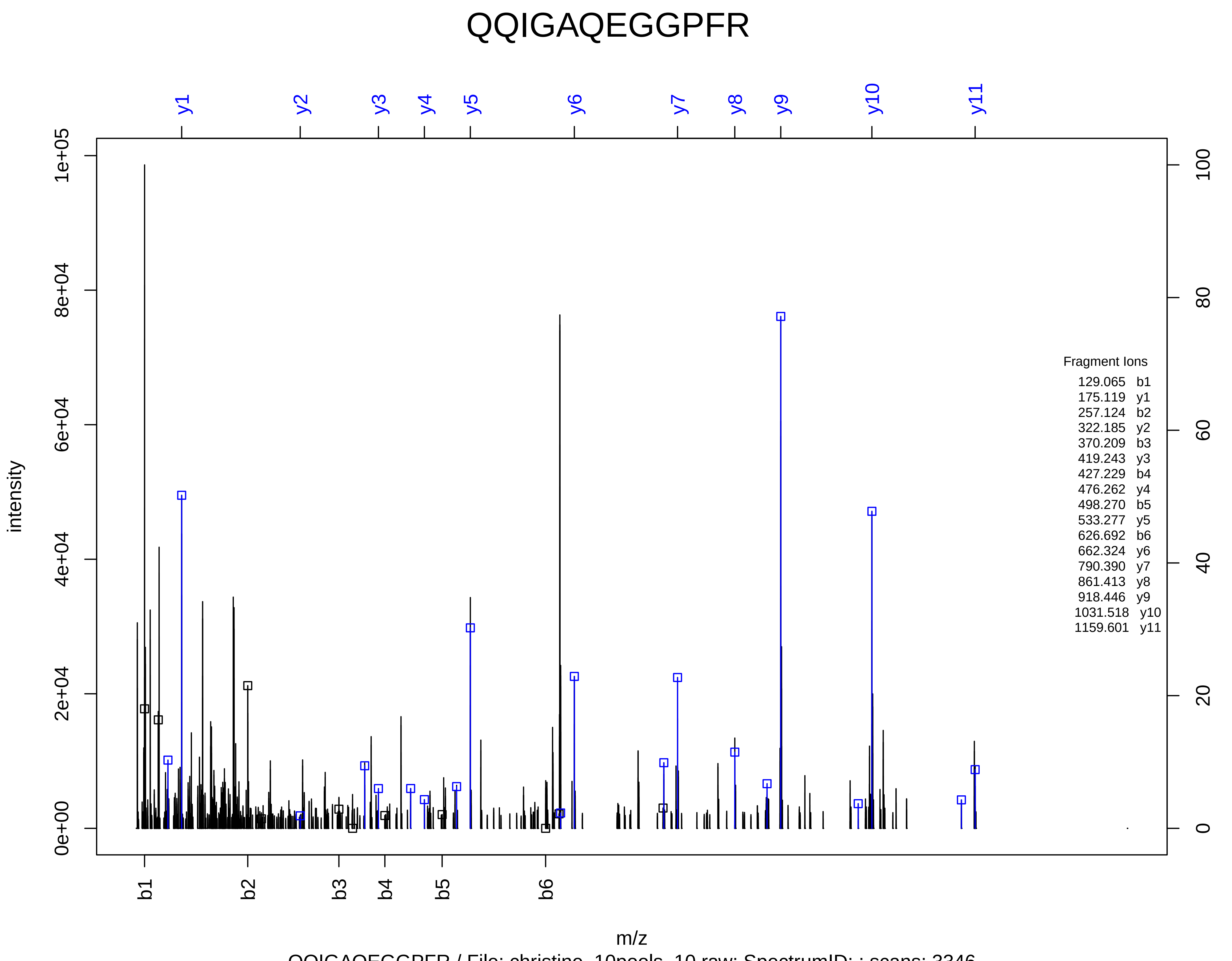
QQIGAQEGGPFR
intensity
m/z
y1 y2 y3 y4 y5 y6 y7 y8 y9 y10 y11
b1 b2 b3 b4 b5 b6
0e+00 2e+04 4e+04 6e+04 8e+04 1e+05
0 20 40 60 80 100
Fragment Ions
129.065 b1
175.119 y1
257.124 b2
322.185 y2
370.209 b3
419.243 y3
427.229 b4
476.262 y4
498.270 b5
533.277 y5
626.692 b6
662.324 y6
790.390 y7
861.413 y8
918.446 y9
1031.518 y10
1159.601 y11
QQIGAQEGGPFR / File: christine_10pools_10.raw; SpectrumID: ; scans: 3346

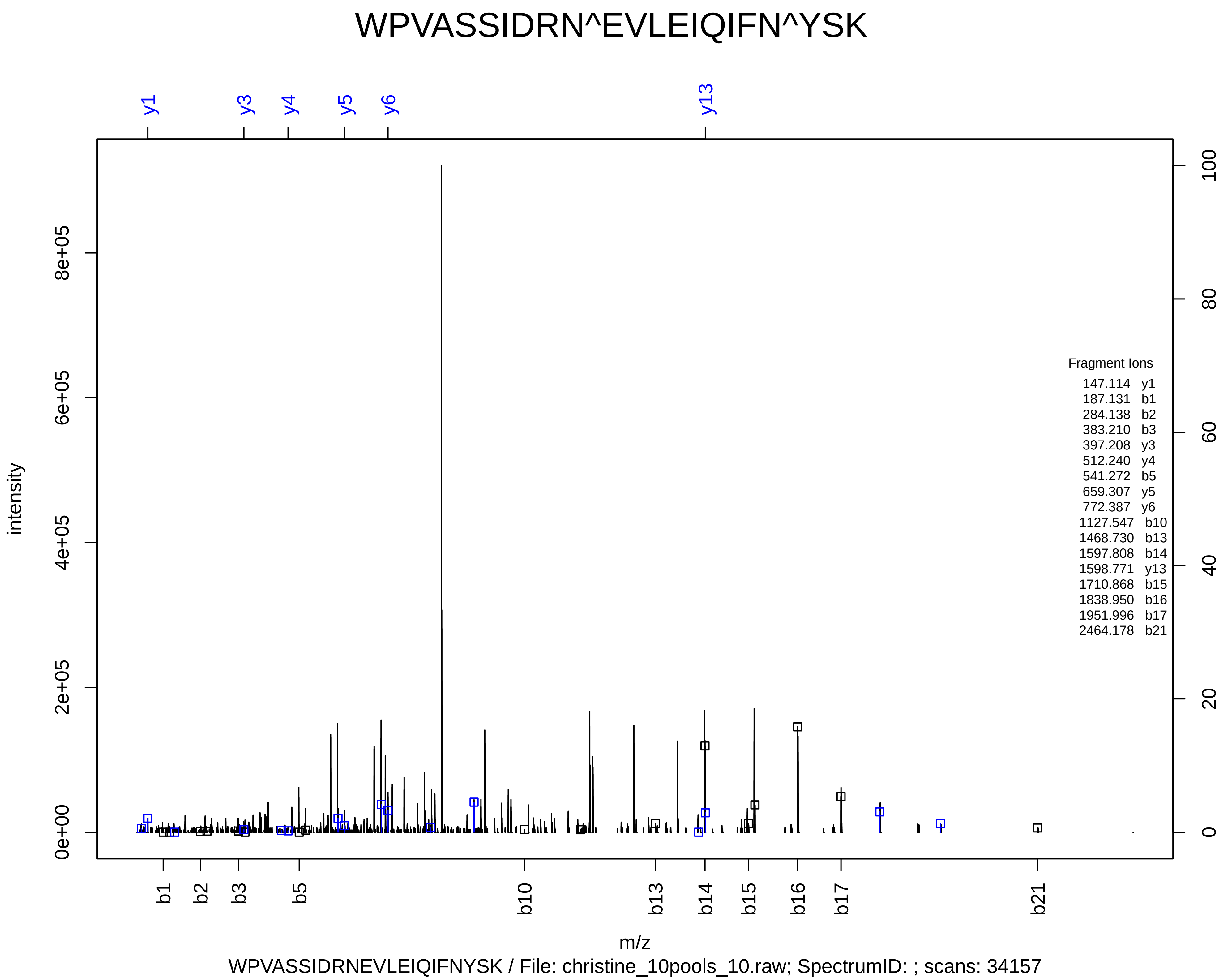
WPVASSIDRN^EVLEIQIFN^YSK
y1
y3
y4
y5
y6
y13
intensity
0e+00
2e+05
4e+05
6e+05
8e+05
0
20
40
60
80
100
Fragment Ions
147.114 y1
187.131 b1
284.138 b2
383.210 b3
397.208 y3
512.240 y4
541.272 b5
659.307 y5
772.387 y6
1127.547 b10
1468.730 b13
1597.808 b14
1598.771 y13
1710.868 b15
1838.950 b16
1951.996 b17
2464.178 b21
b1
b2
b3
b5
b10
b13
b14
b15
b16
b17
b21
m/z
WPVASSIDRNEVLEIQIFNYSK / File: christine_10pools_10.raw; SpectrumID: ; scans: 34157

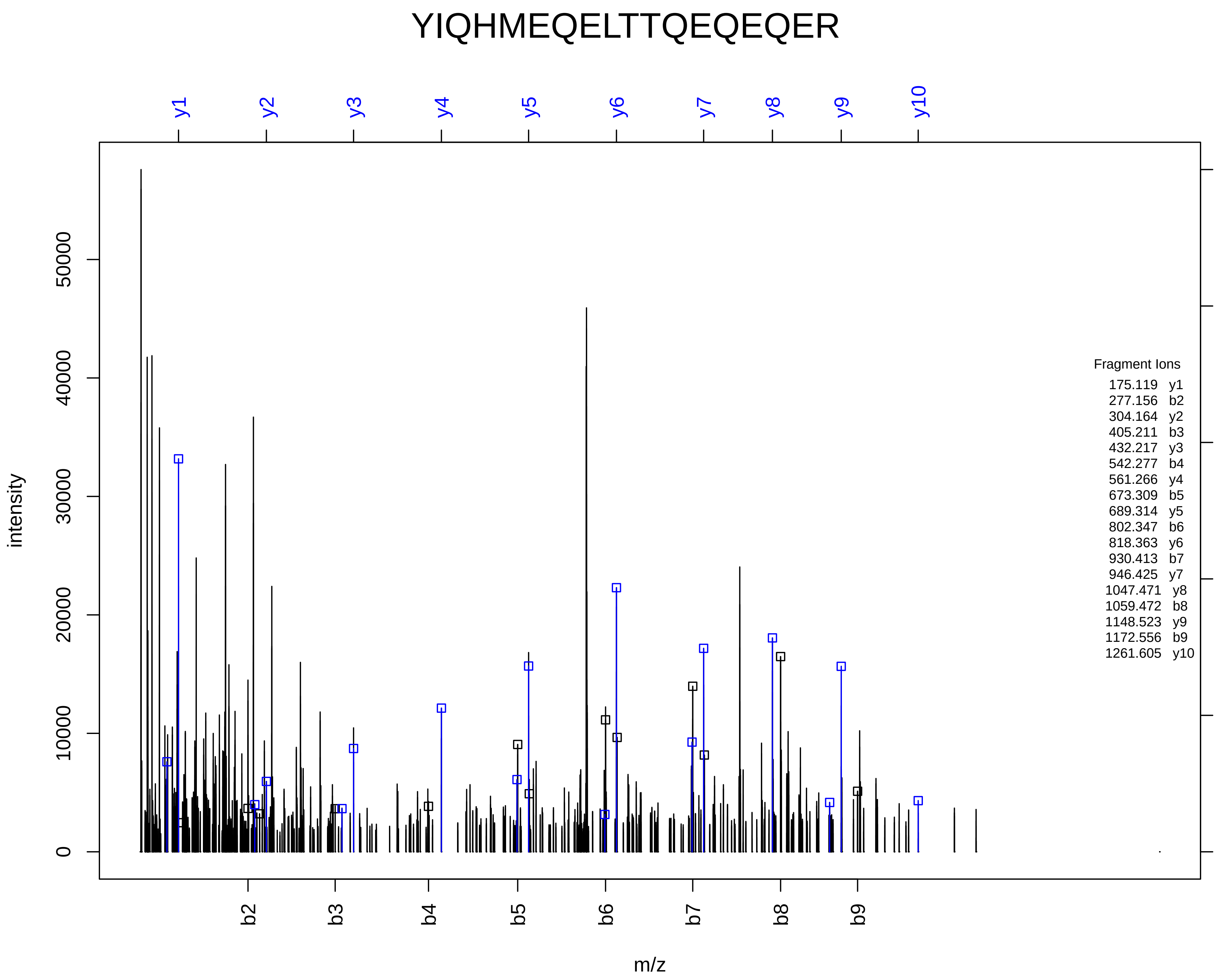
YIQHMEQELTTQEQEQER
y1
y2
y3
y4
y5
y6
y7
y8
y9
y10
b2
b3
b4
b5
b6
b7
b8
b9
intensity
m/z
Fragment Ions
175.119 y1
277.156 b2
304.164 y2
405.211 b3
432.217 y3
542.277 b4
561.266 y4
673.309 b5
689.314 y5
802.347 b6
818.363 y6
930.413 b7
946.425 y7
1047.471 y8
1059.472 b8
1148.523 y9
1172.556 b9
1261.605 y10
YIQHMEQELTTQEQEQER / File: christine_10pools_10.raw; SpectrumID: ; scans: 5781

TM*LDM*N^KMTQSIIGSM*QYSK

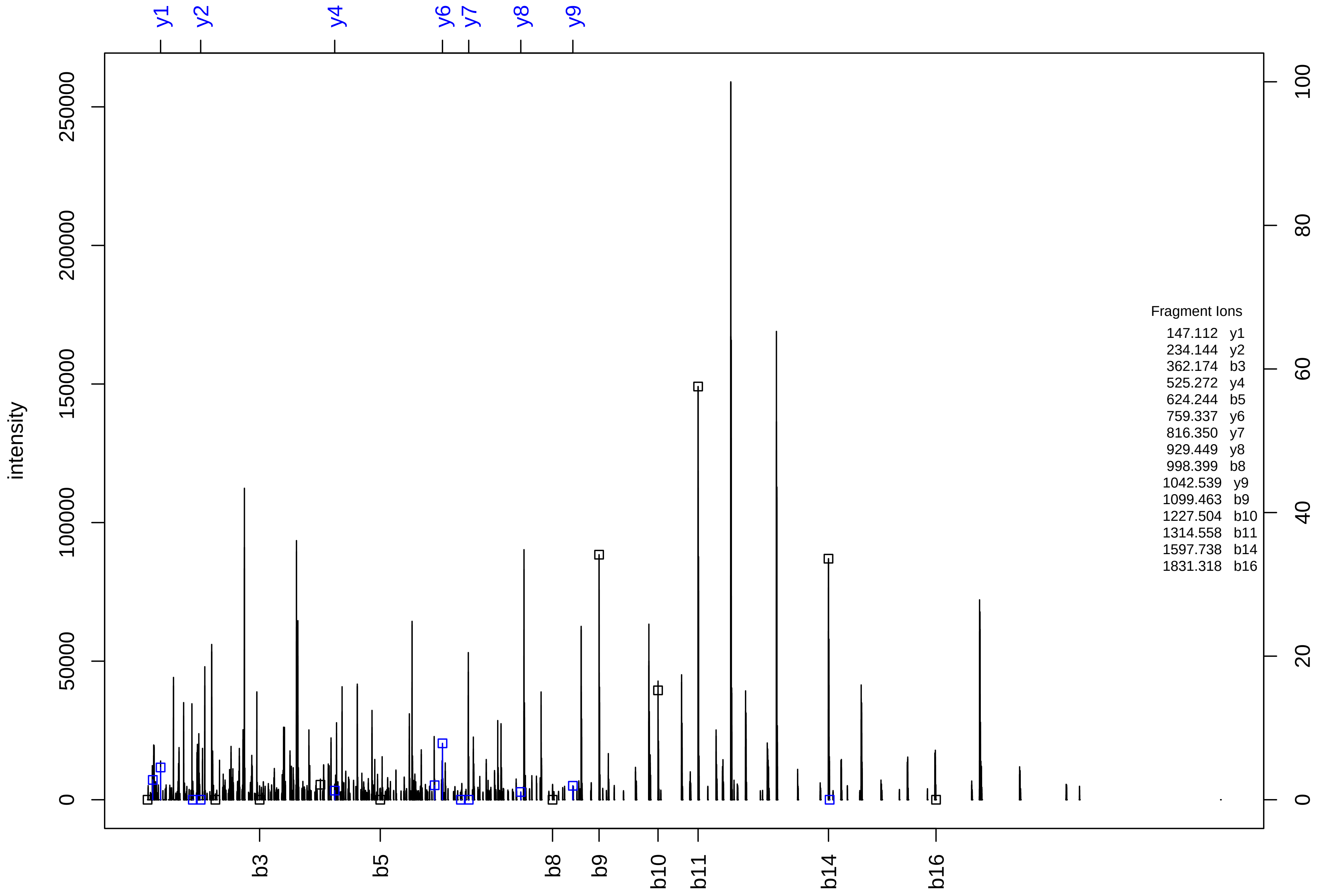

TMLDMNKMTQSIIGSMQYSK / File: christine_10pools_10.raw; SpectrumID: ; scans: 21729

NTAEALEPSPTLILQDVAK

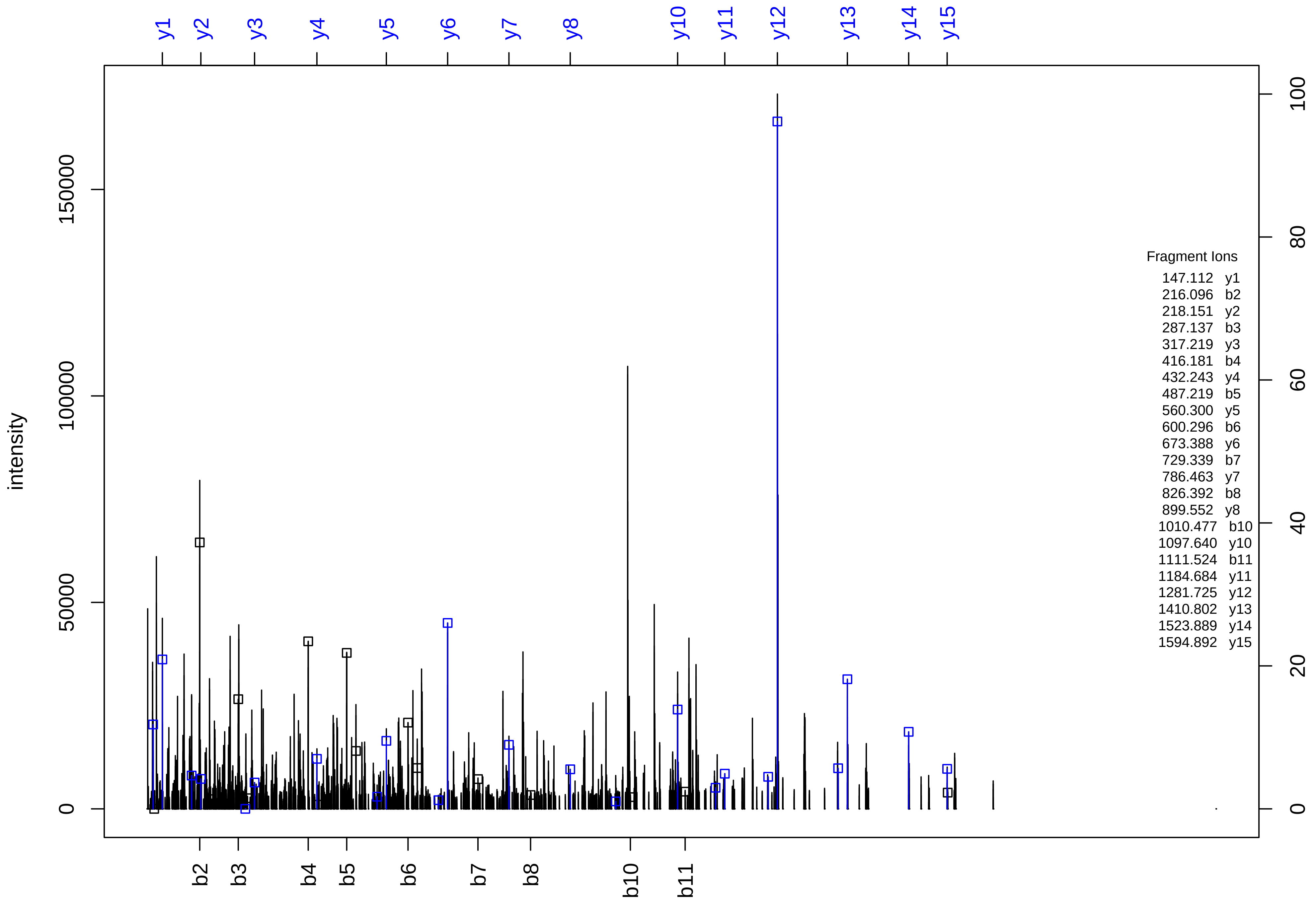

NTAEALEPSPTLILQDVAK / File: christine_10pools_10.raw; SpectrumID: ; scans: 28453

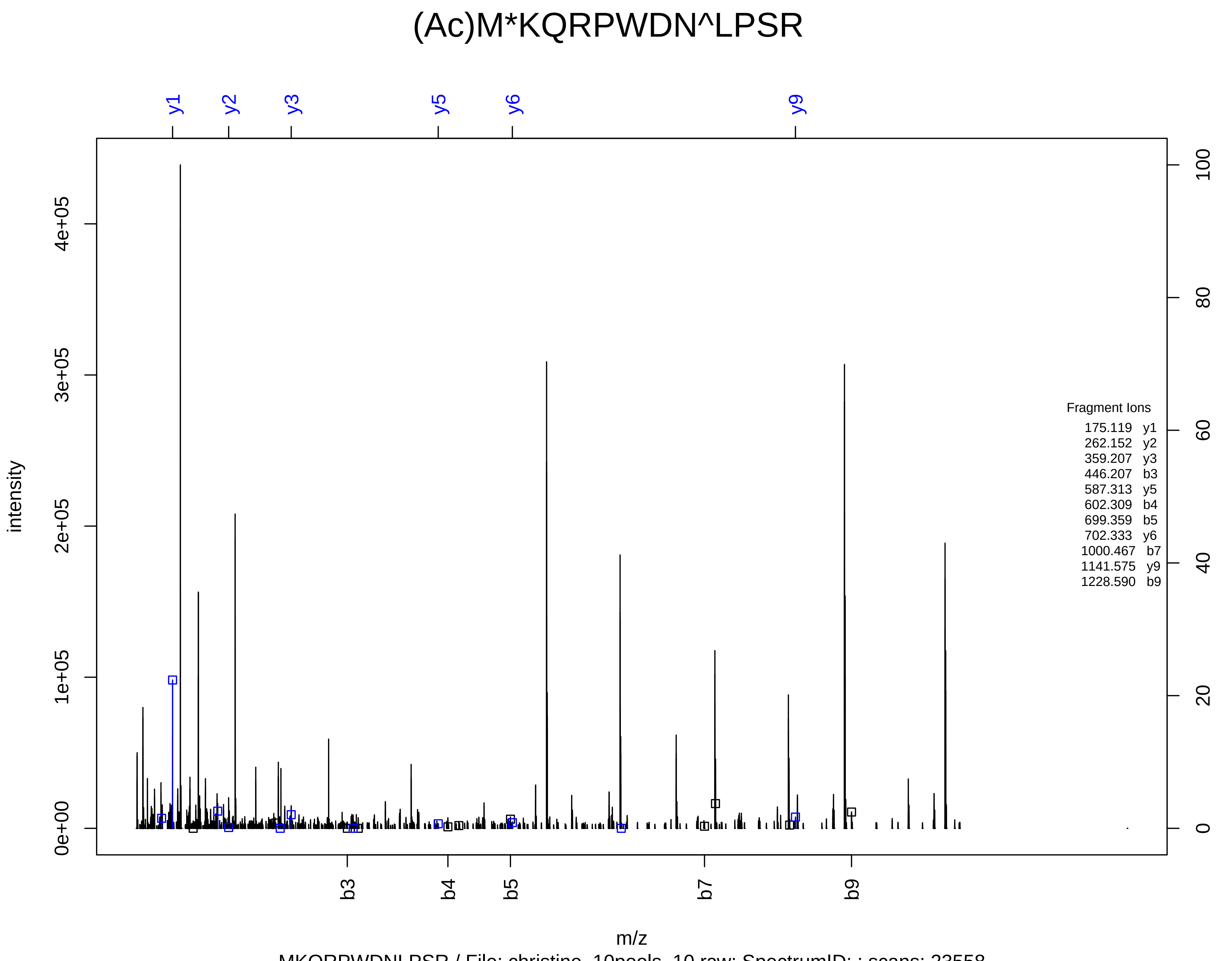
(Ac)M*KQRPWDN^LPSR
y1
y2
y3
y5
y6
y9
intensity
0e+00
1e+05
2e+05
3e+05
4e+05
0
20
40
60
80
100
Fragment Ions
175.119 y1
262.152 y2
359.207 y3
446.207 b3
587.313 y5
602.309 b4
699.359 b5
702.333 y6
1000.467 b7
1141.575 y9
1228.590 b9
b3
b4
b5
b7
b9
m/z
MKQRPWDNLPSR / File: christine_10pools_10.raw; SpectrumID: ; scans: 23558

MLPVDEFLPVM*FDR

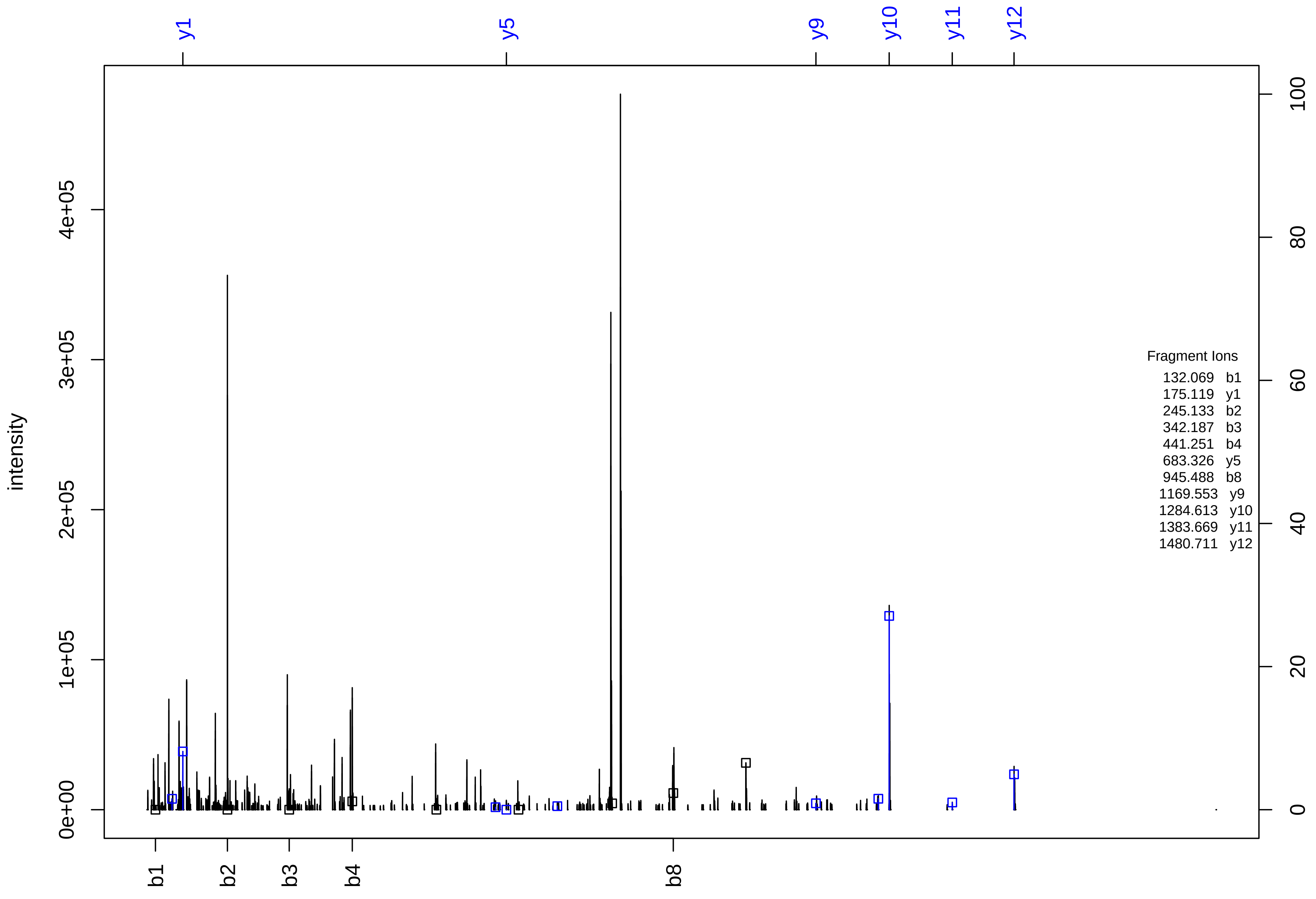

MLPVDEFLPVMFDR / File: christine_10pools_08.raw; SpectrumID: ; scans: 12078

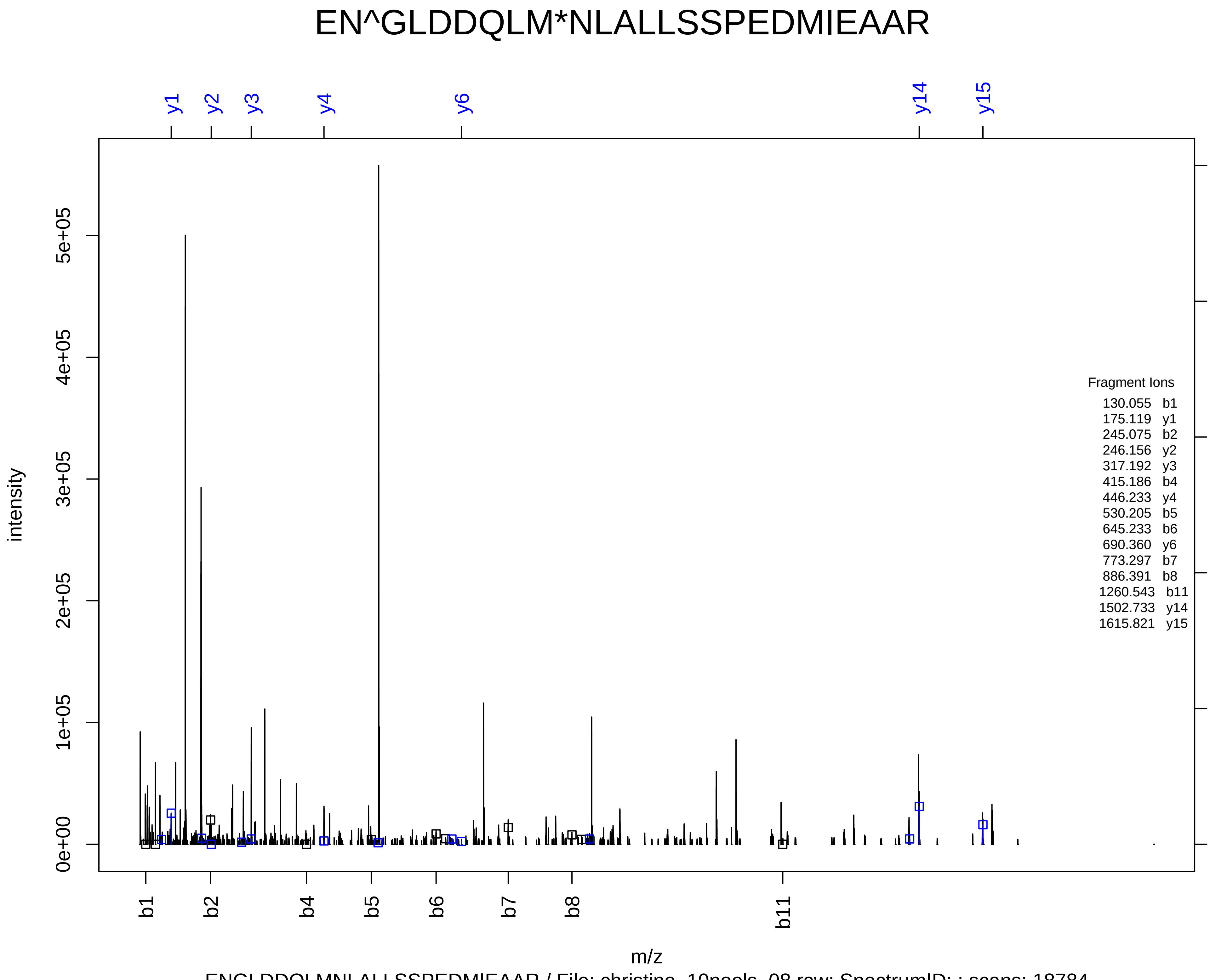

EN^GLDDQLM*NLALLSSPEDMIEAAR
y1
y2
y3
y4
y6
y14
y15
intensity
0e+00
1e+05
2e+05
3e+05
4e+05
5e+05
0
20
40
60
80
100
Fragment Ions
130.055 b1
175.119 y1
245.075 b2
246.156 y2
317.192 y3
415.186 b4
446.233 y4
530.205 b5
645.233 b6
690.360 y6
773.297 b7
886.391 b8
1260.543 b11
1502.733 y14
1615.821 y15
b1
b2
b4
b5
b6
b7
b8
b11
m/z
ENGLDDQLMNLALLSSPEDMIEAAR / File: christine_10pools_08.raw; SpectrumID: ; scans: 18784

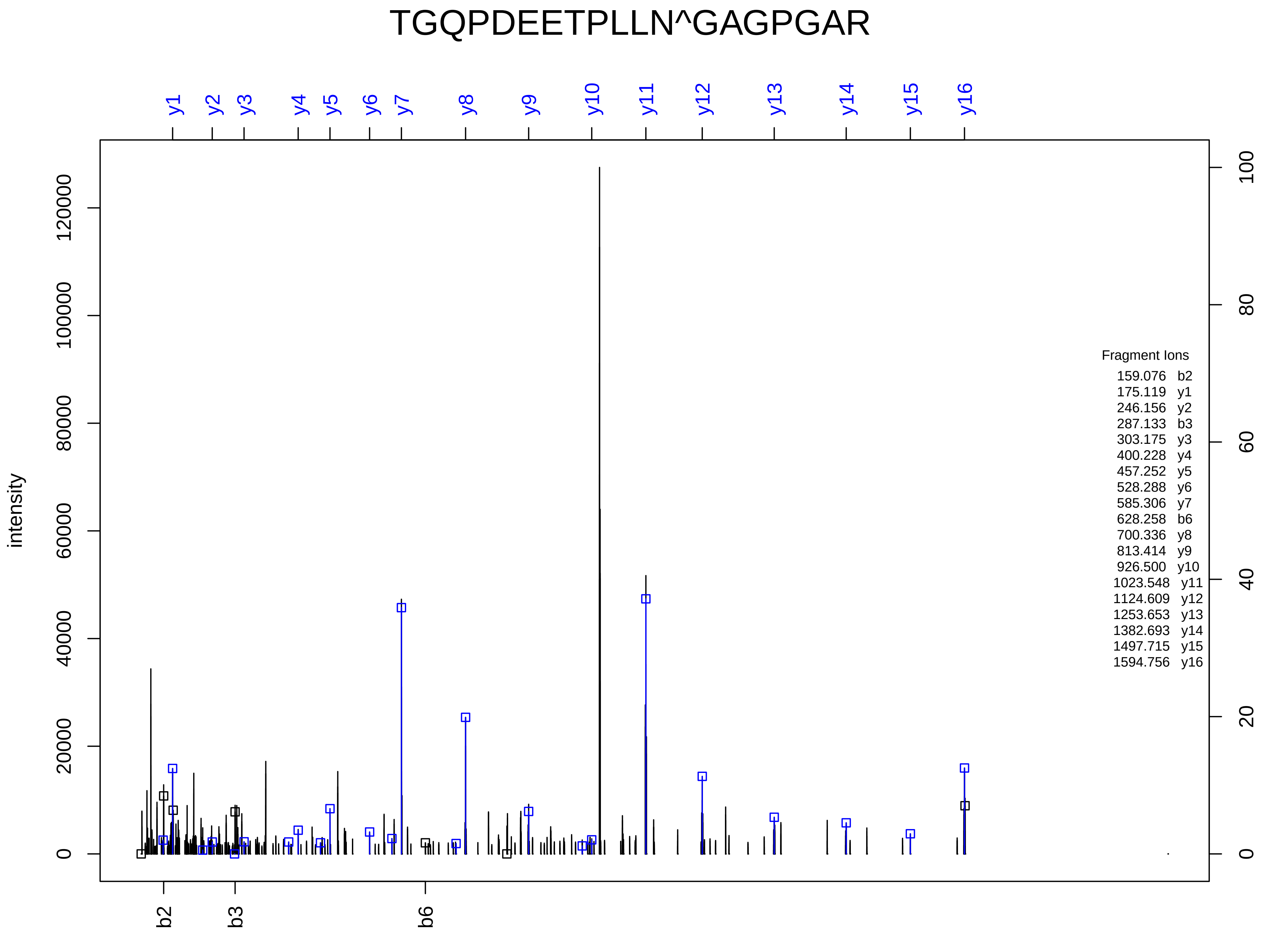

TGQPDEETPLLNGAGPGAR / File: christine_10pools_08.raw; SpectrumID: ; scans: 10192

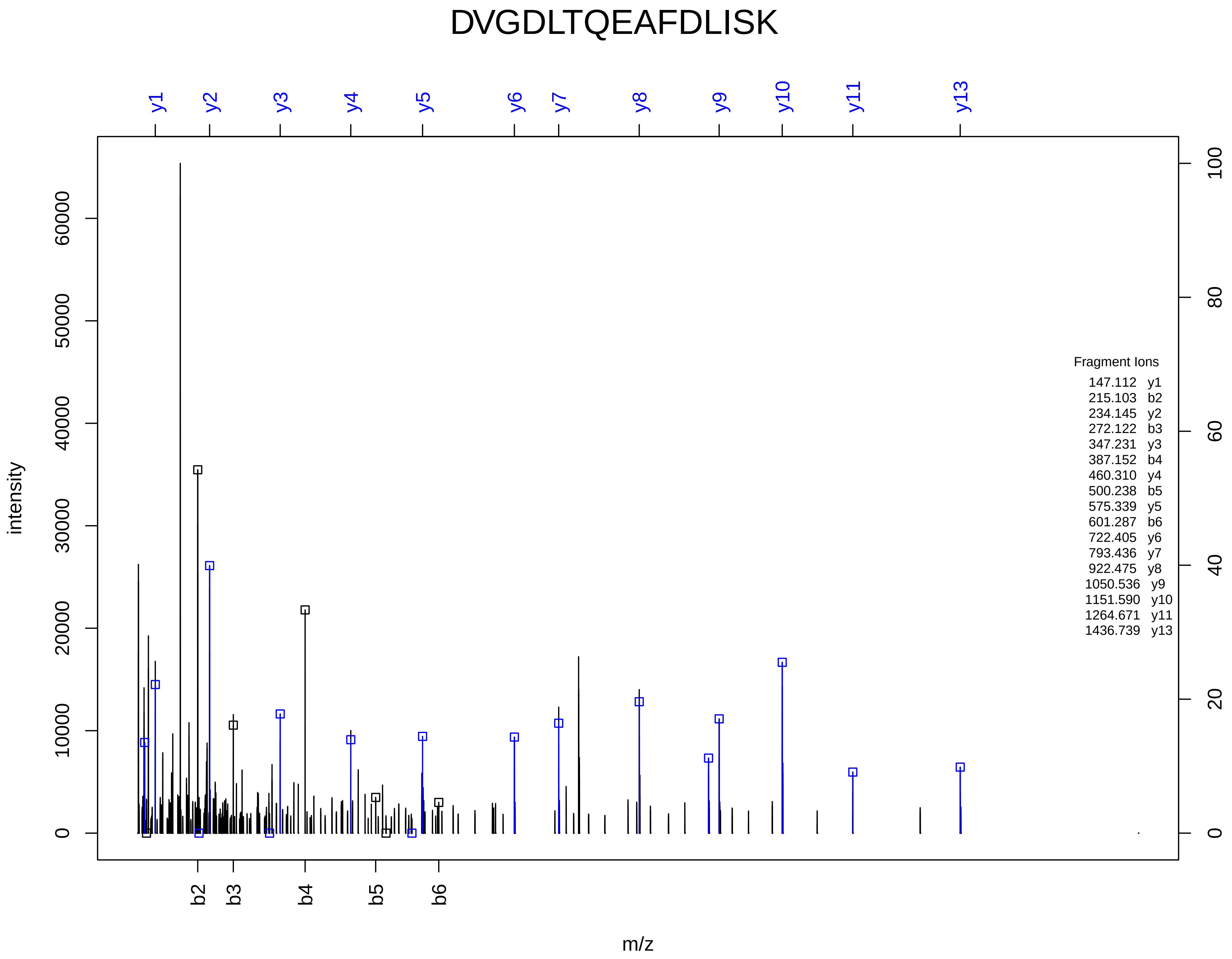

DVGDLTQEAFDLISK / File: christine_10pools_08.raw; SpectrumID: ; scans: 37844

TCLSCSSSCTTDQVEIR

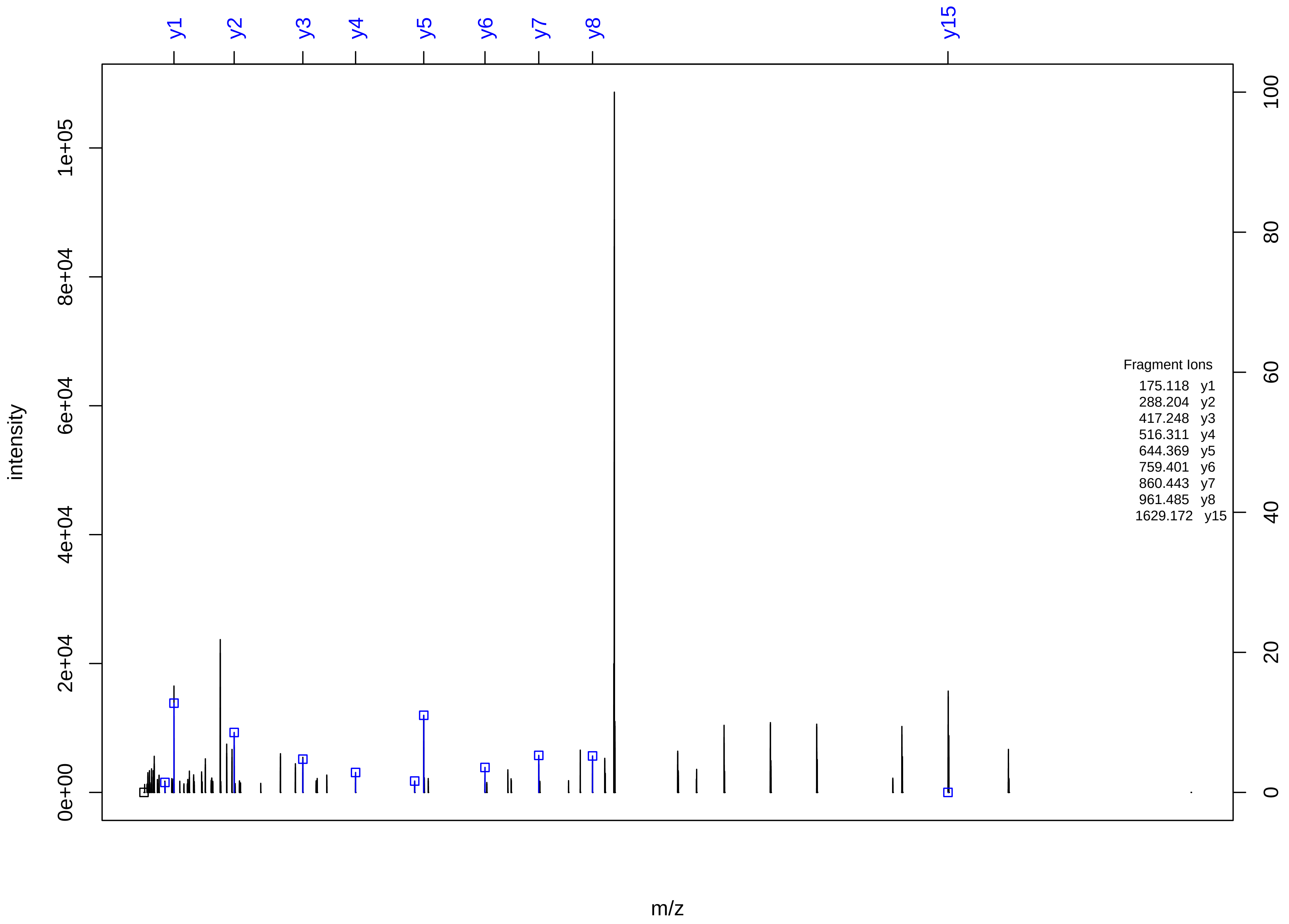

TCLSCSSSCTTDQVEIR / File: christine_10pools_08.raw; SpectrumID: ; scans: 5296

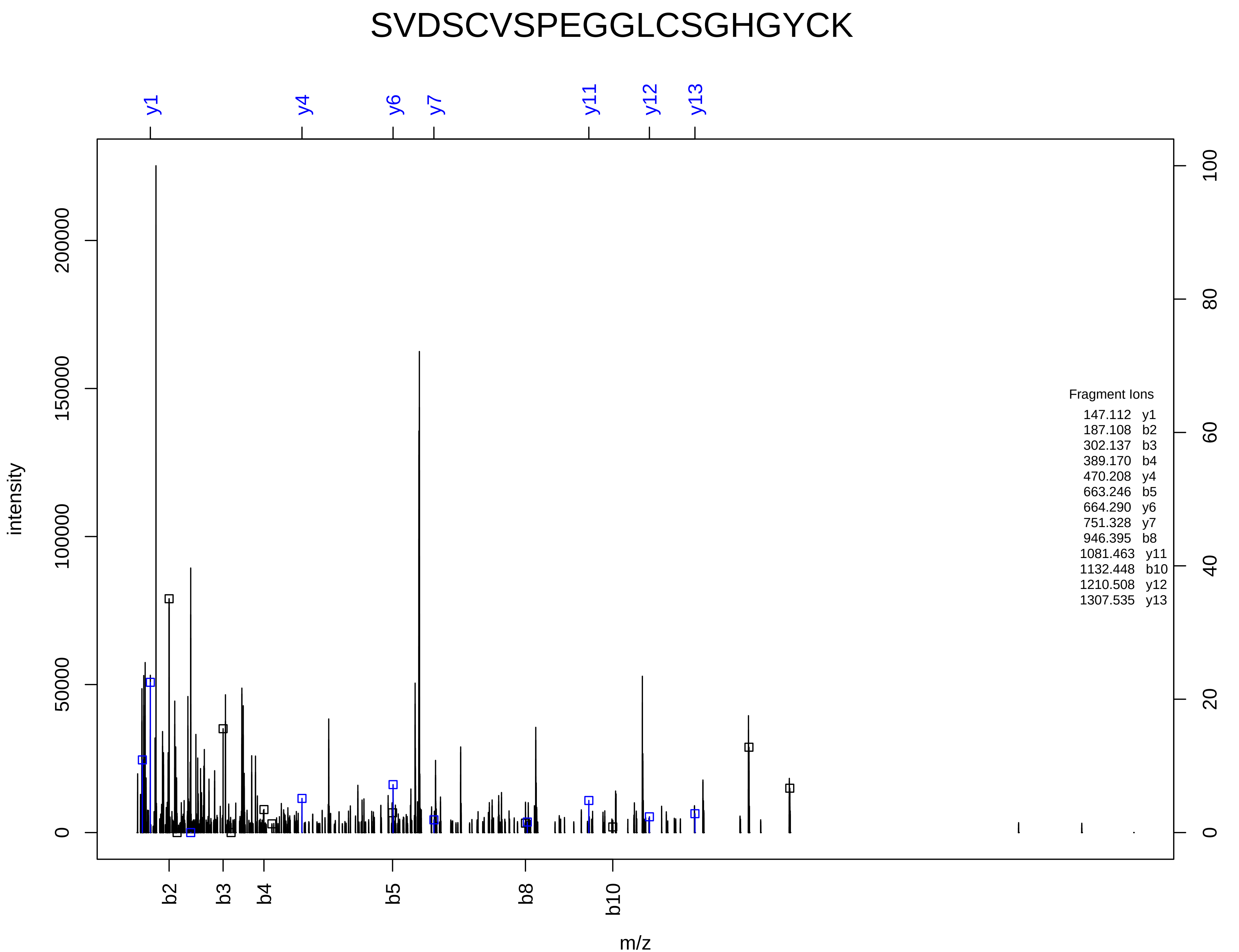

SVDSCVSPEGGLCSGHGYCK
y1
y4
y6
y7
y11
y12
y13
intensity
0
50000
100000
150000
200000
0
20
40
60
80
100
b2
b3
b4
b5
b8
b10
m/z
Fragment Ions
147.112 y1
187.108 b2
302.137 b3
389.170 b4
470.208 y4
663.246 b5
664.290 y6
751.328 y7
946.395 b8
1081.463 y11
1132.448 b10
1210.508 y12
1307.535 y13
SVDSCVSPEGGLCSGHGYCK / File: christine_10pools_08.raw; SpectrumID: ; scans: 2967

EELM*KMRLPTR

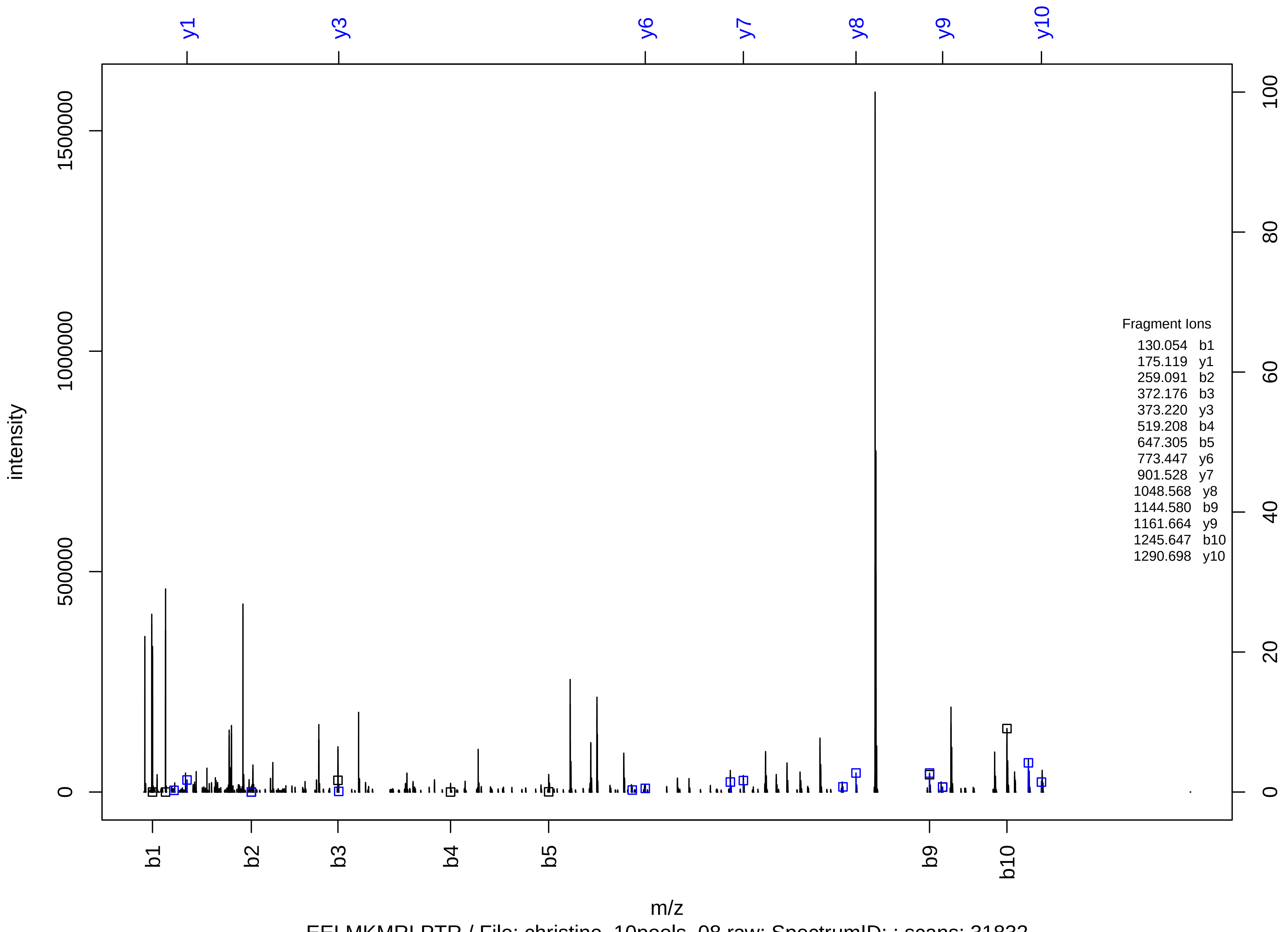

EELMKMRLPTR / File: christine_10pools_08.raw; SpectrumID: ; scans: 31832

(Ac)MAQN^FSRR

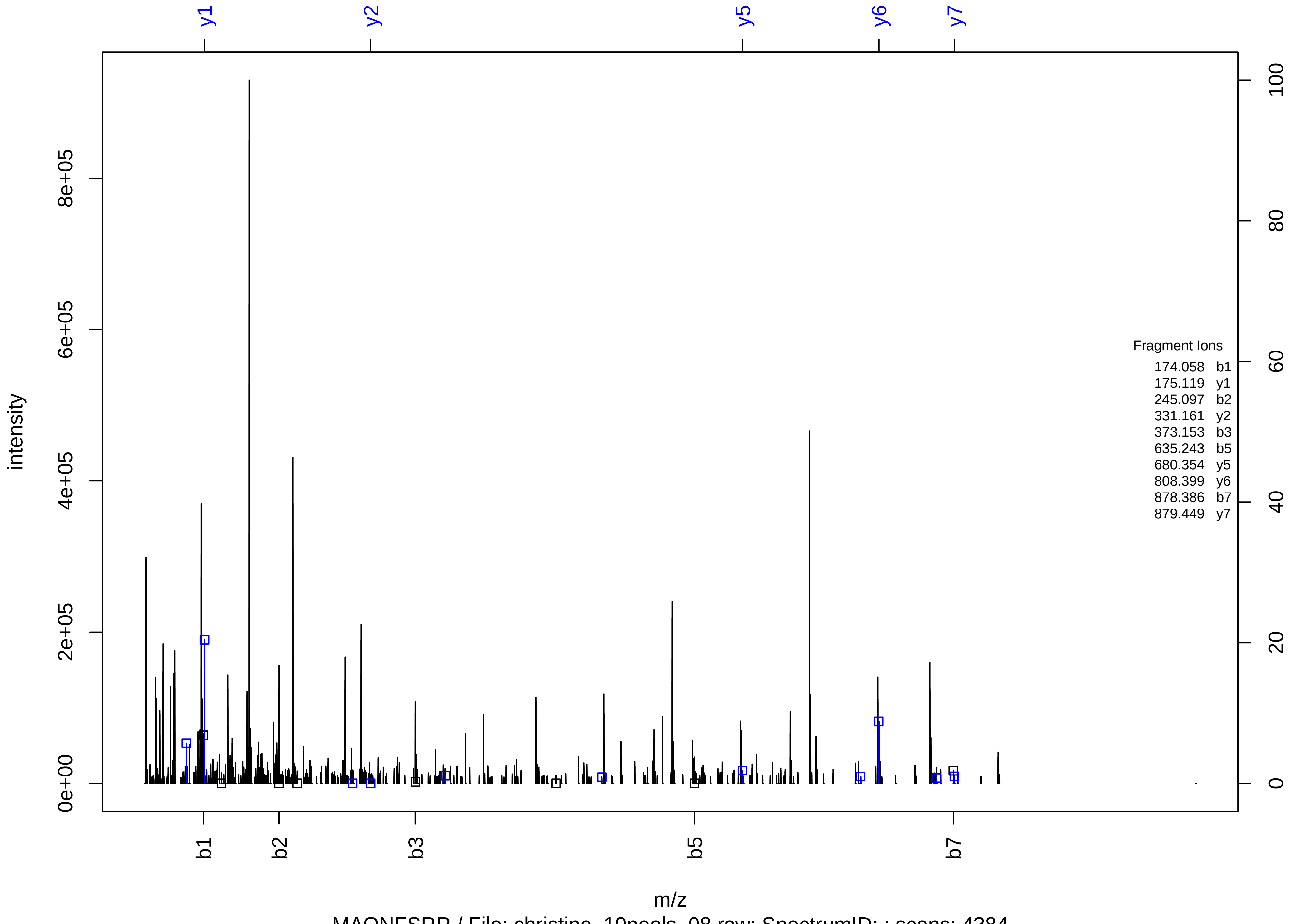

MAQNFSRR / File: christine_10pools_08.raw; SpectrumID: ; scans: 4384

ADLGN^IM*MN^LLSQR

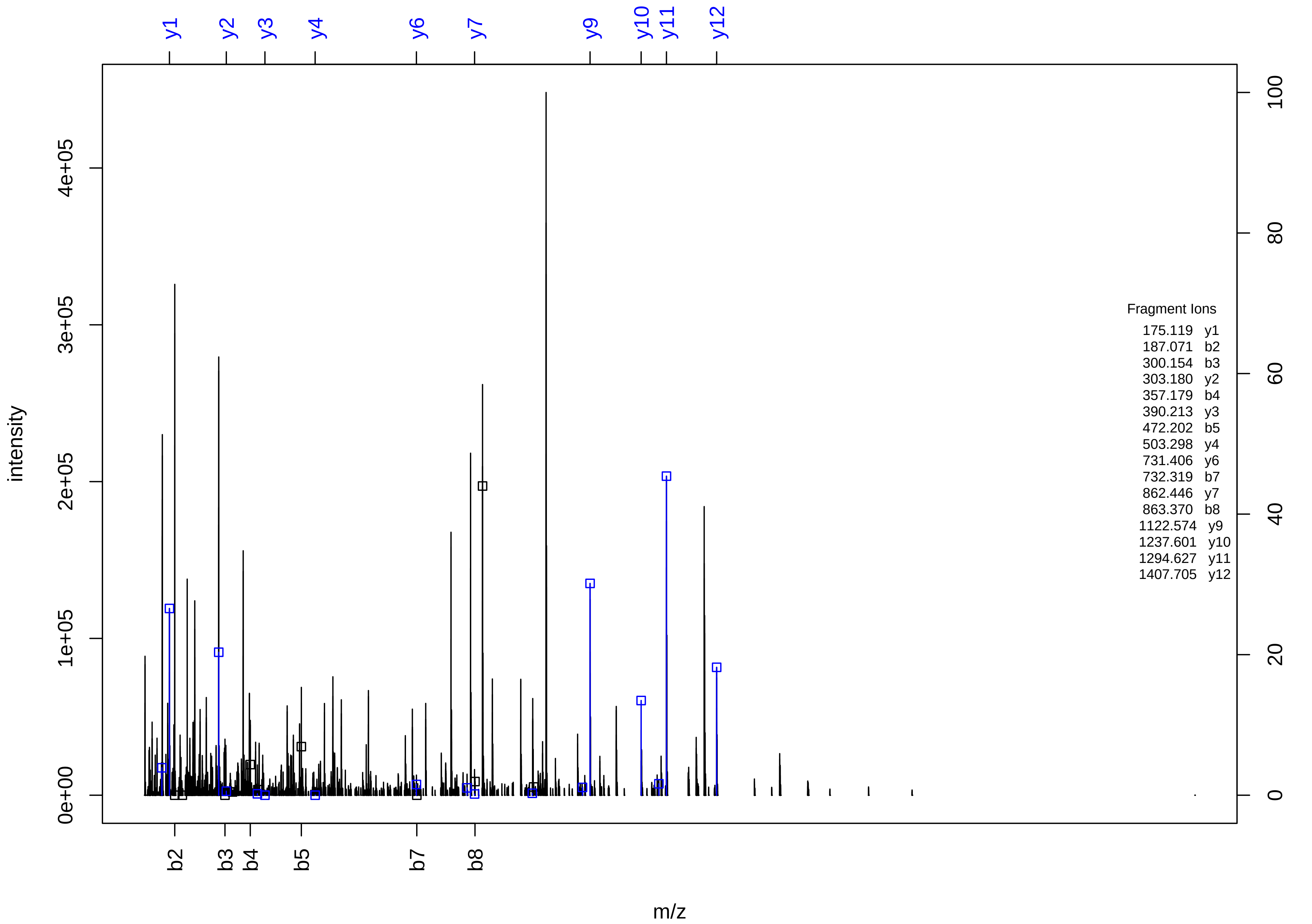

ADLGNIMMNLLSQR / File: christine_10pools_08.raw; SpectrumID: ; scans: 28281

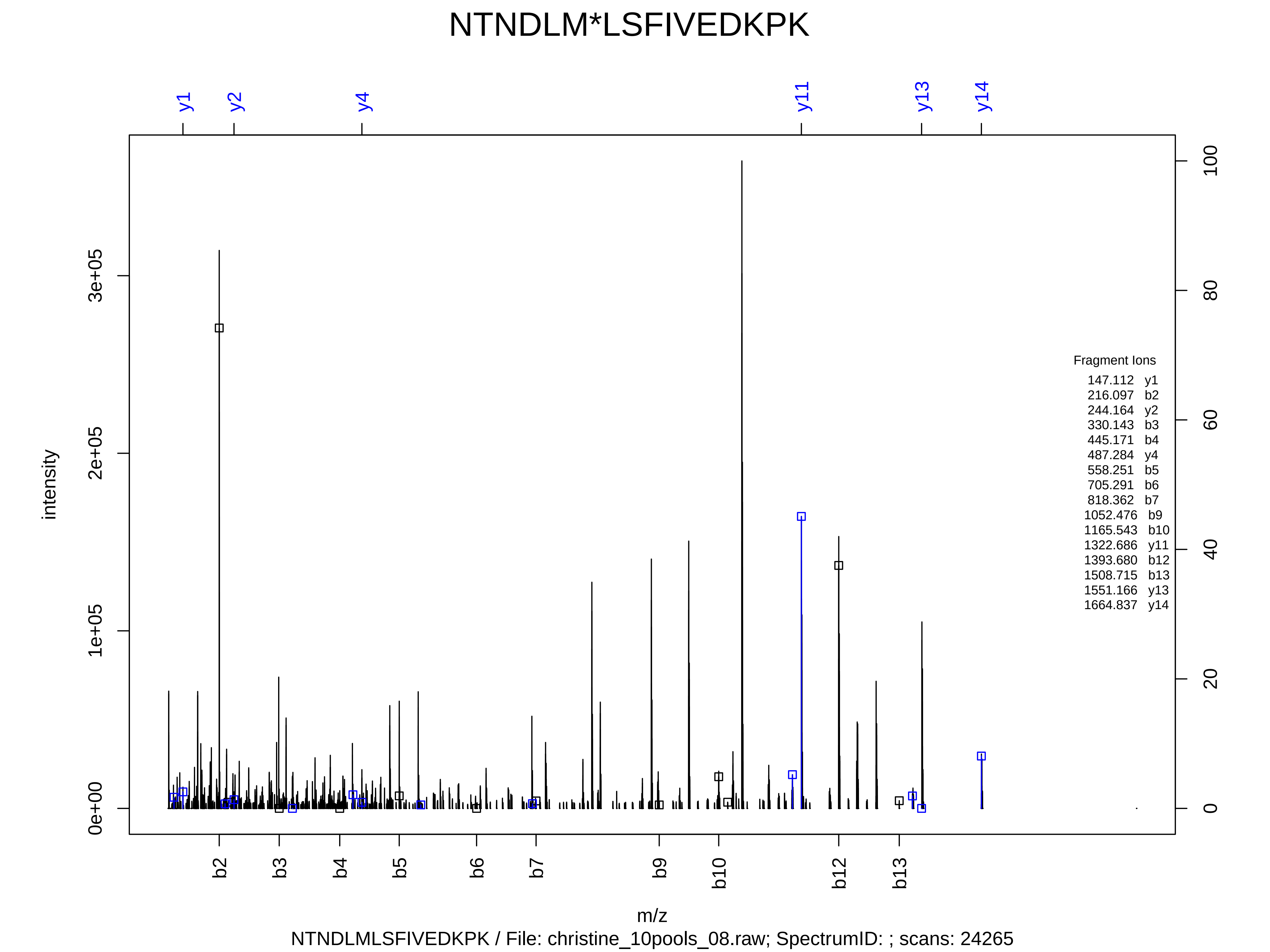
NTNDLM*LSFIVEDKPK
y1
y2
y4
y11
y13
y14
intensity
0e+00
1e+05
2e+05
3e+05
0
20
40
60
80
100
b2
b3
b4
b5
b6
b7
b9
b10
b12
b13
m/z
Fragment Ions
147.112 y1
216.097 b2
244.164 y2
330.143 b3
445.171 b4
487.284 y4
558.251 b5
705.291 b6
818.362 b7
1052.476 b9
1165.543 b10
1322.686 y11
1393.680 b12
1508.715 b13
1551.166 y13
1664.837 y14
NTNDLMLSFIVEDKPK / File: christine_10pools_08.raw; SpectrumID: ; scans: 24265

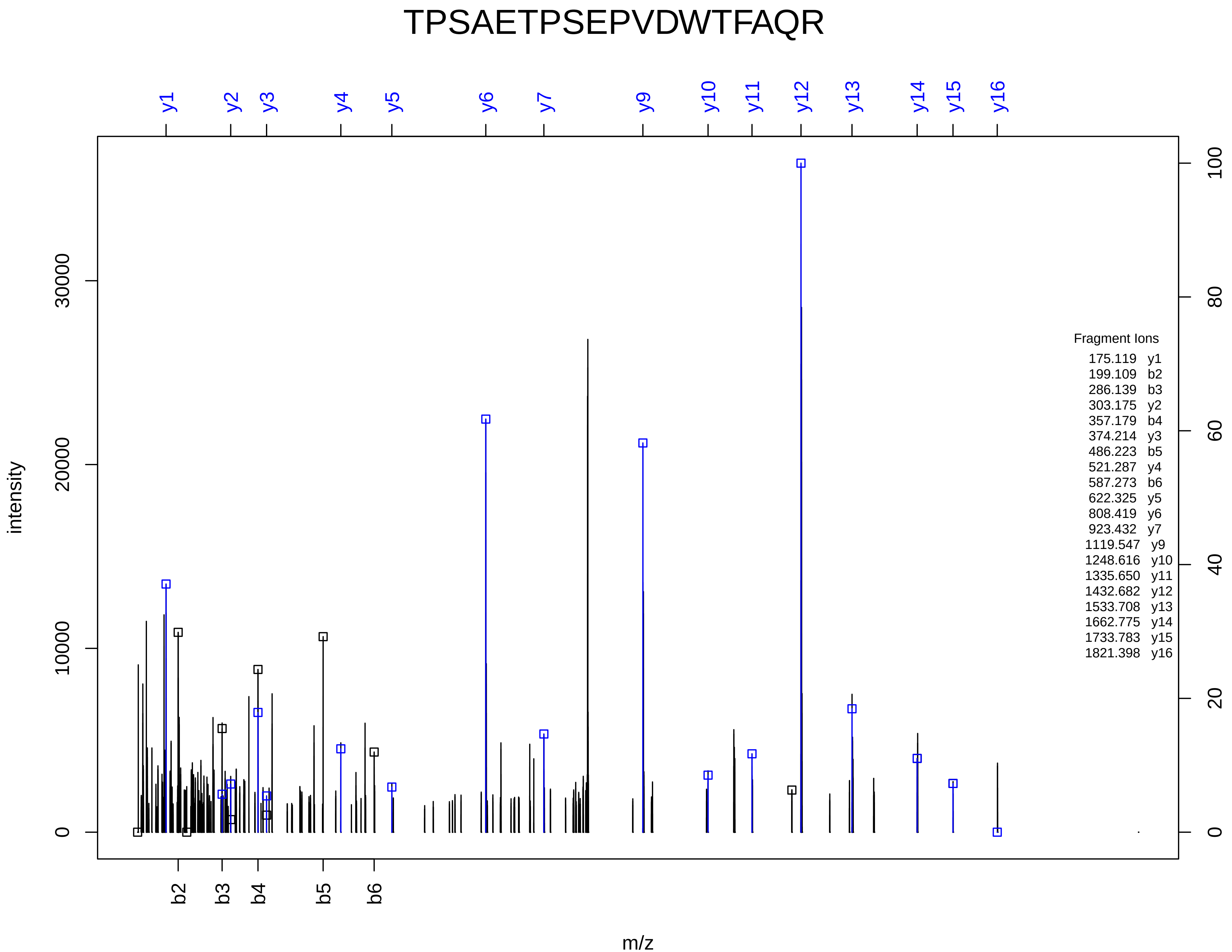

TPSAETPSEPVDWTFAQR / File: christine_10pools_08.raw; SpectrumID: ; scans: 18633

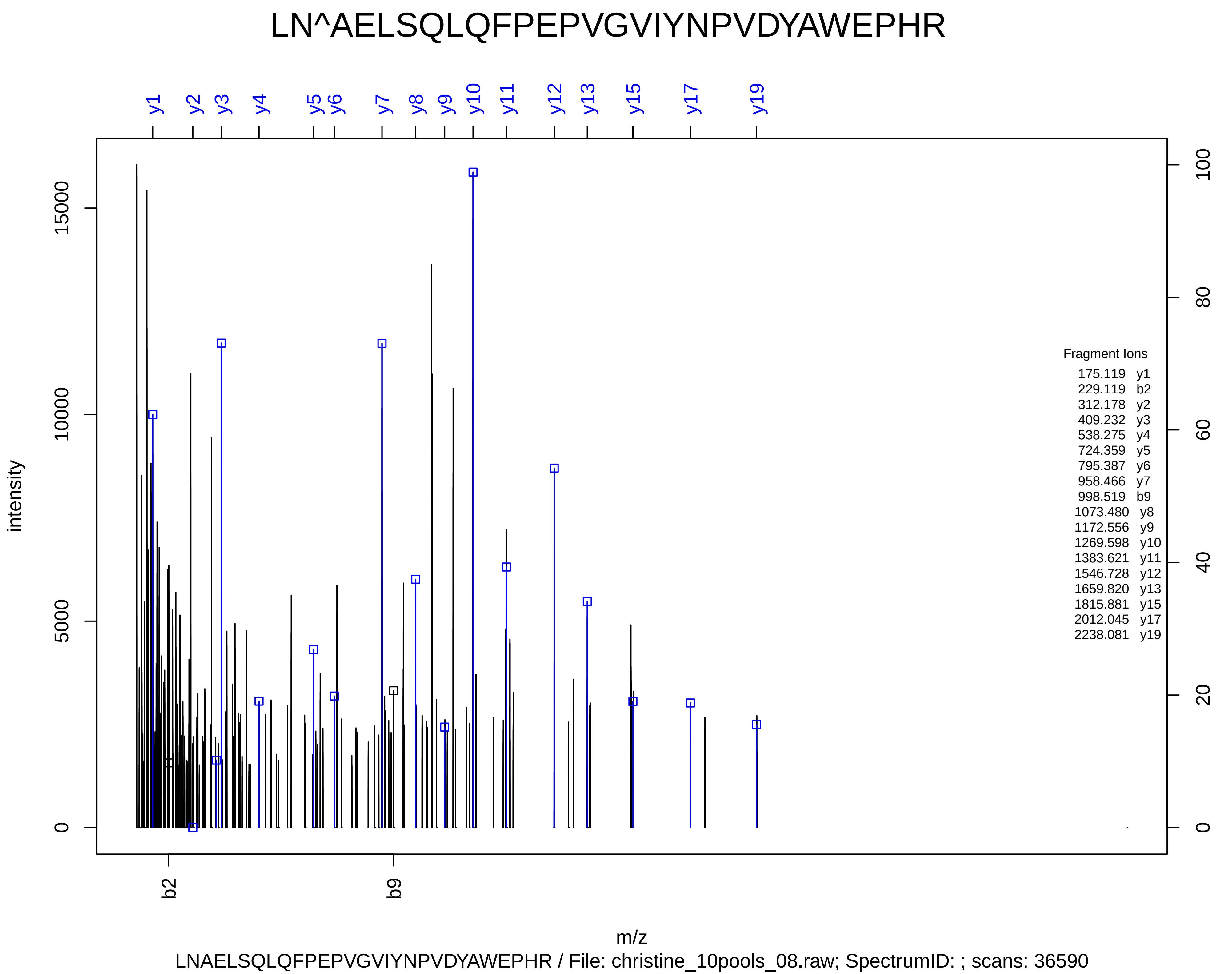

LN^AELSQLQFPEPVGVIYNPVDYAWEPHR
y1
y2
y3
y4
y5
y6
y7
y8
y9
y10
y11
y12
y13
y15
y17
y19
intensity
0
5000
10000
15000
0
20
40
60
80
100
b2
b9
m/z
Fragment Ions
175.119 y1
229.119 b2
312.178 y2
409.232 y3
538.275 y4
724.359 y5
795.387 y6
958.466 y7
998.519 b9
1073.480 y8
1172.556 y9
1269.598 y10
1383.621 y11
1546.728 y12
1659.820 y13
1815.881 y15
2012.045 y17
2238.081 y19
LNAELSQLQFPEPVGVIYNPVDYAWEPHR / File: christine_10pools_08.raw; SpectrumID: ; scans: 36590

SSQPQQVTQRPEEGK

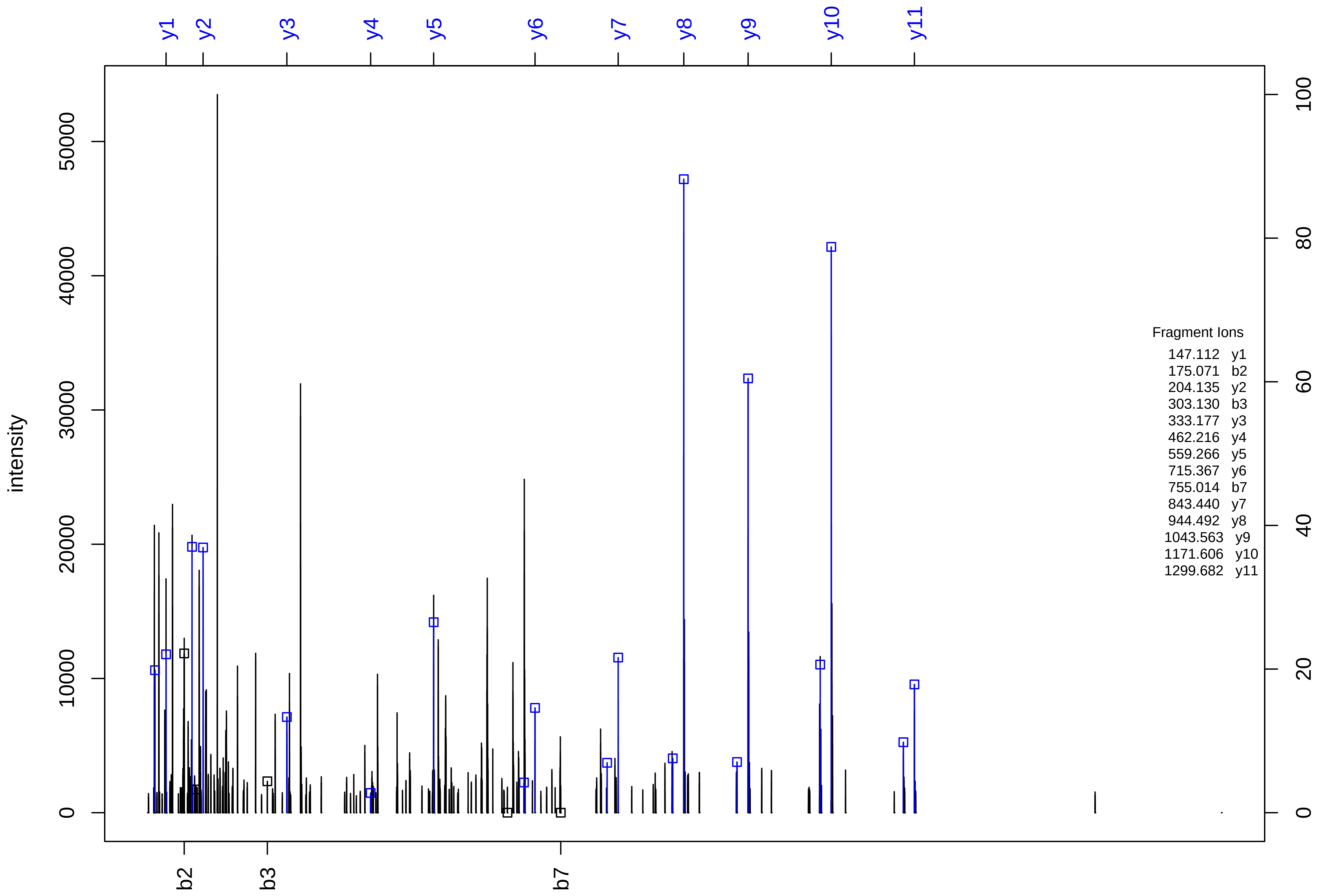

SSQPQQVTQRPEEGK / File: christine_10pools_08.raw; SpectrumID: ; scans: 884

(Ac)AGYEYVSPEQLSGFDK

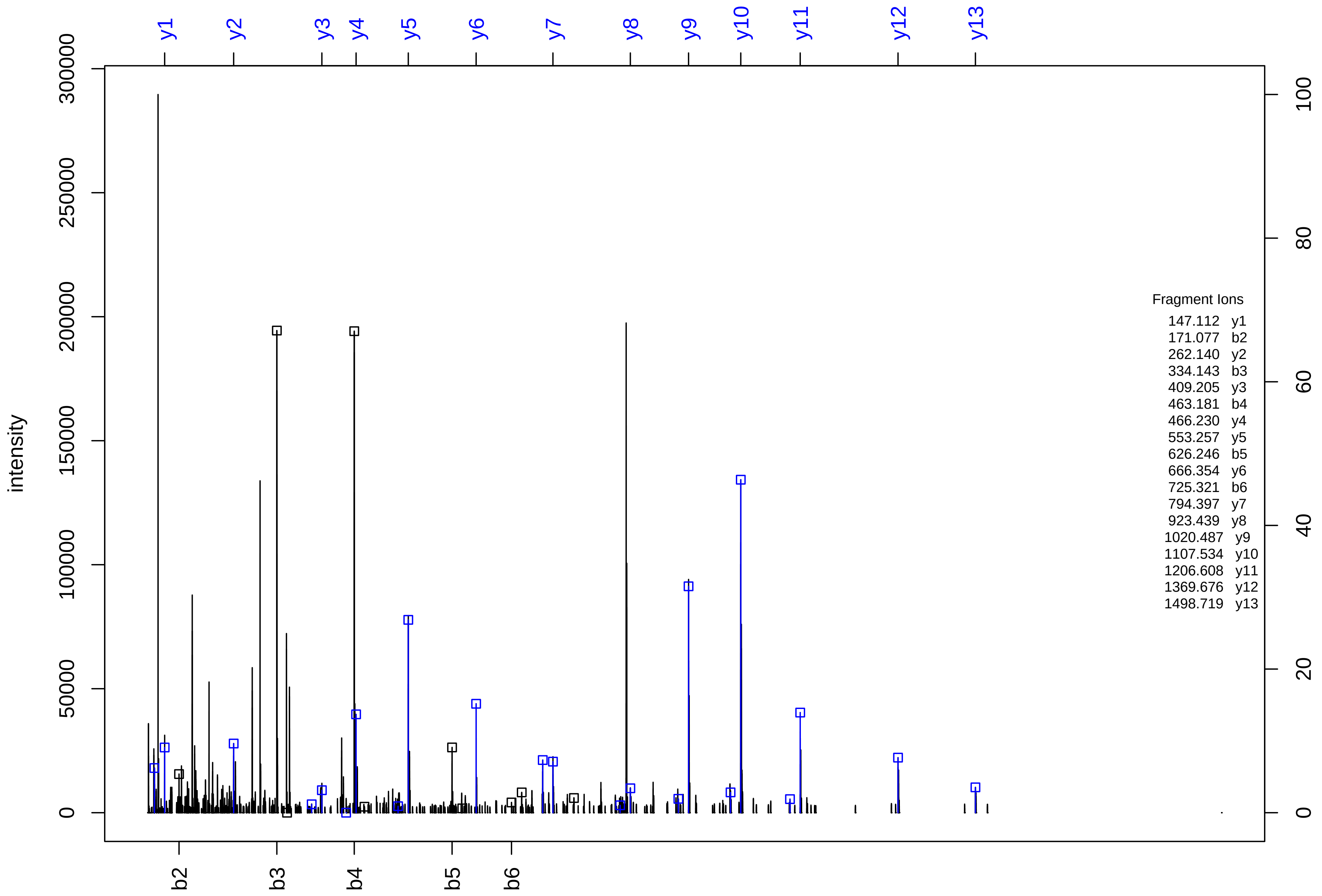

AGYEYVSPEQLSGFDK / File: christine_10pools_08.raw; SpectrumID: ; scans: 27260

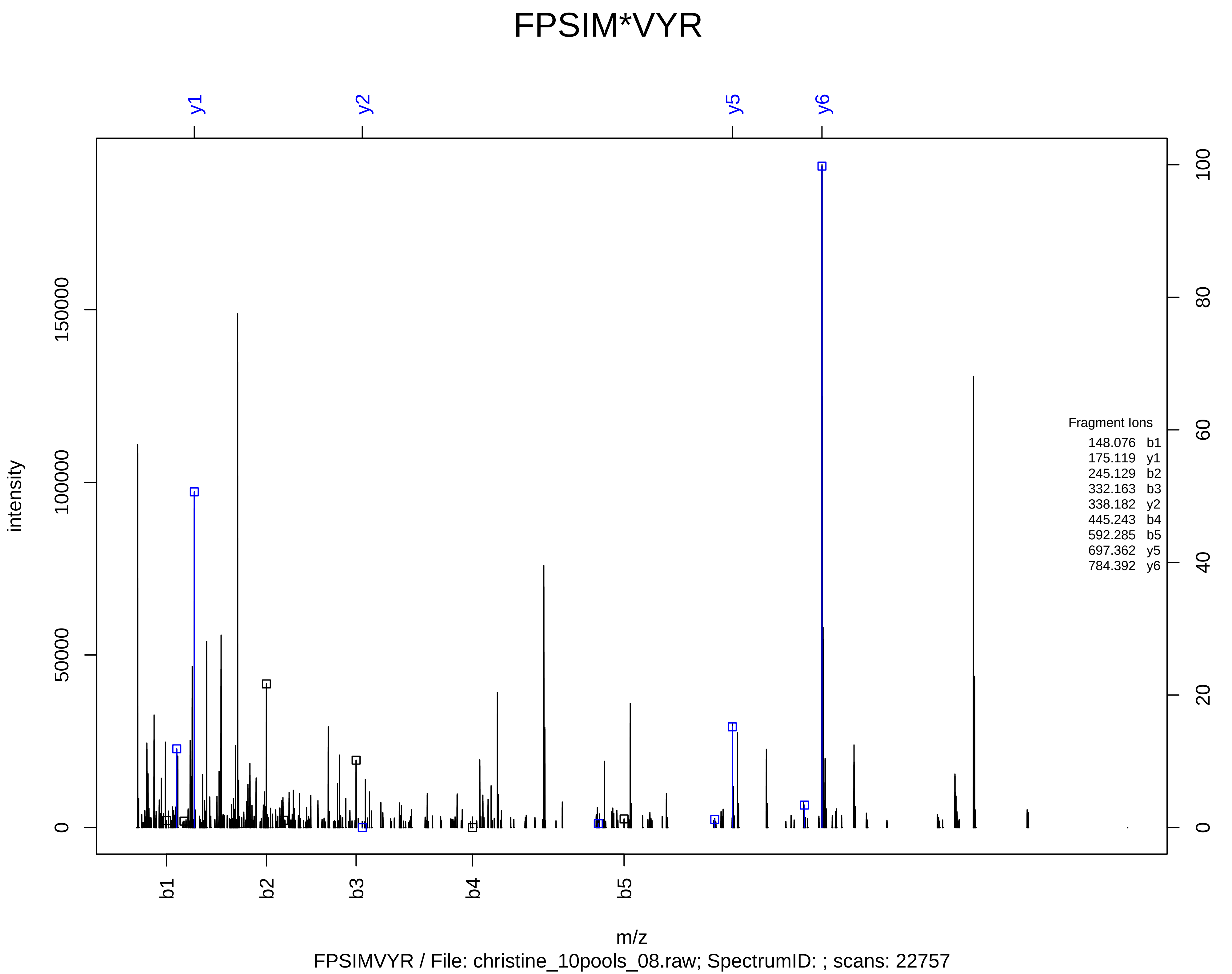
FPSIM*VYR
y1
y2
y5
y6
intensity
0
50000
100000
150000
0
20
40
60
80
100
b1
b2
b3
b4
b5
m/z
Fragment Ions
148.076 b1
175.119 y1
245.129 b2
332.163 b3
338.182 y2
445.243 b4
592.285 b5
697.362 y5
784.392 y6
FPSIMVYR / File: christine_10pools_08.raw; SpectrumID: ; scans: 22757

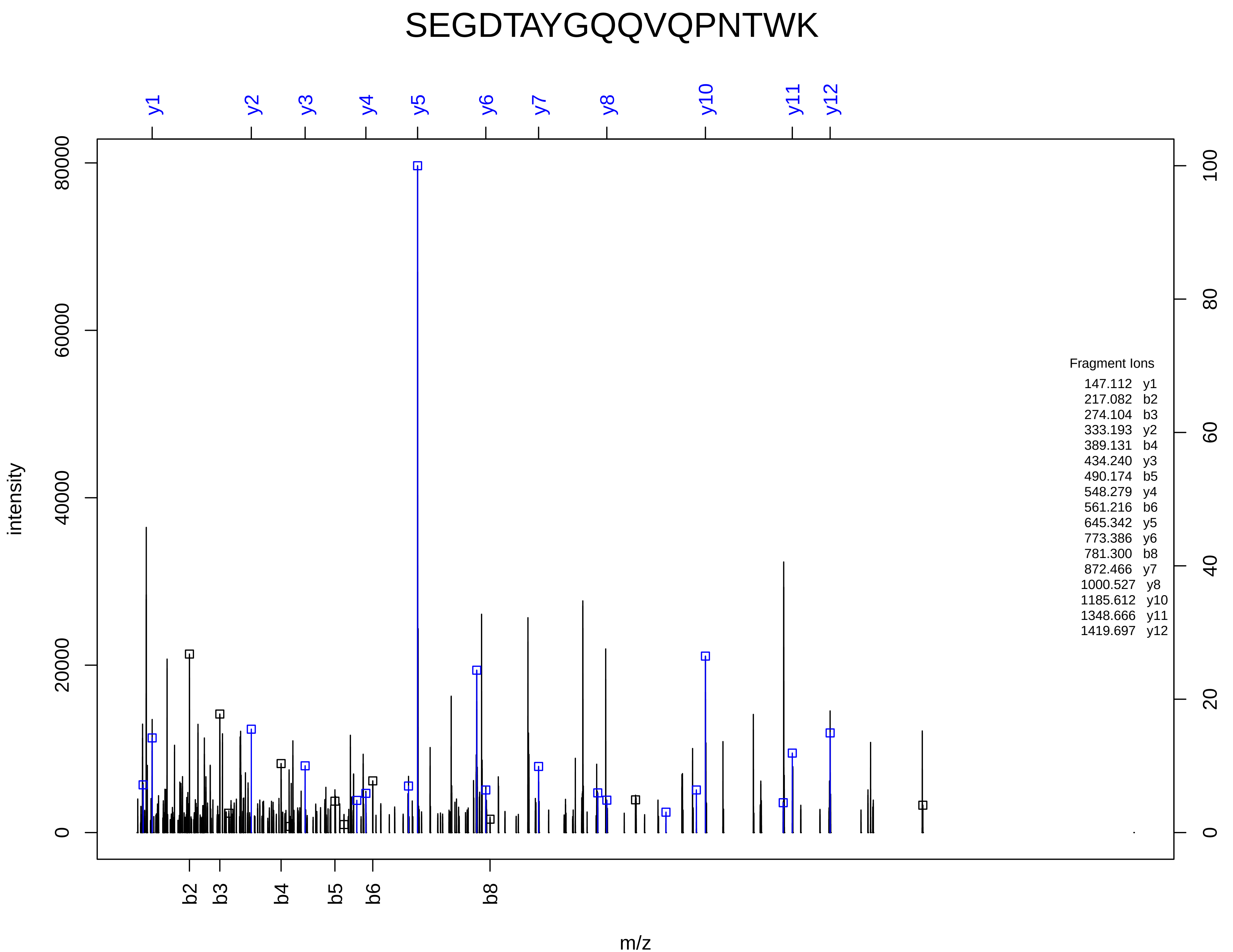

SEGDTAYGQQVQPNTWK / File: christine_10pools_08.raw; SpectrumID: ; scans: 6546

NDAVDFSPTLPVTCGK

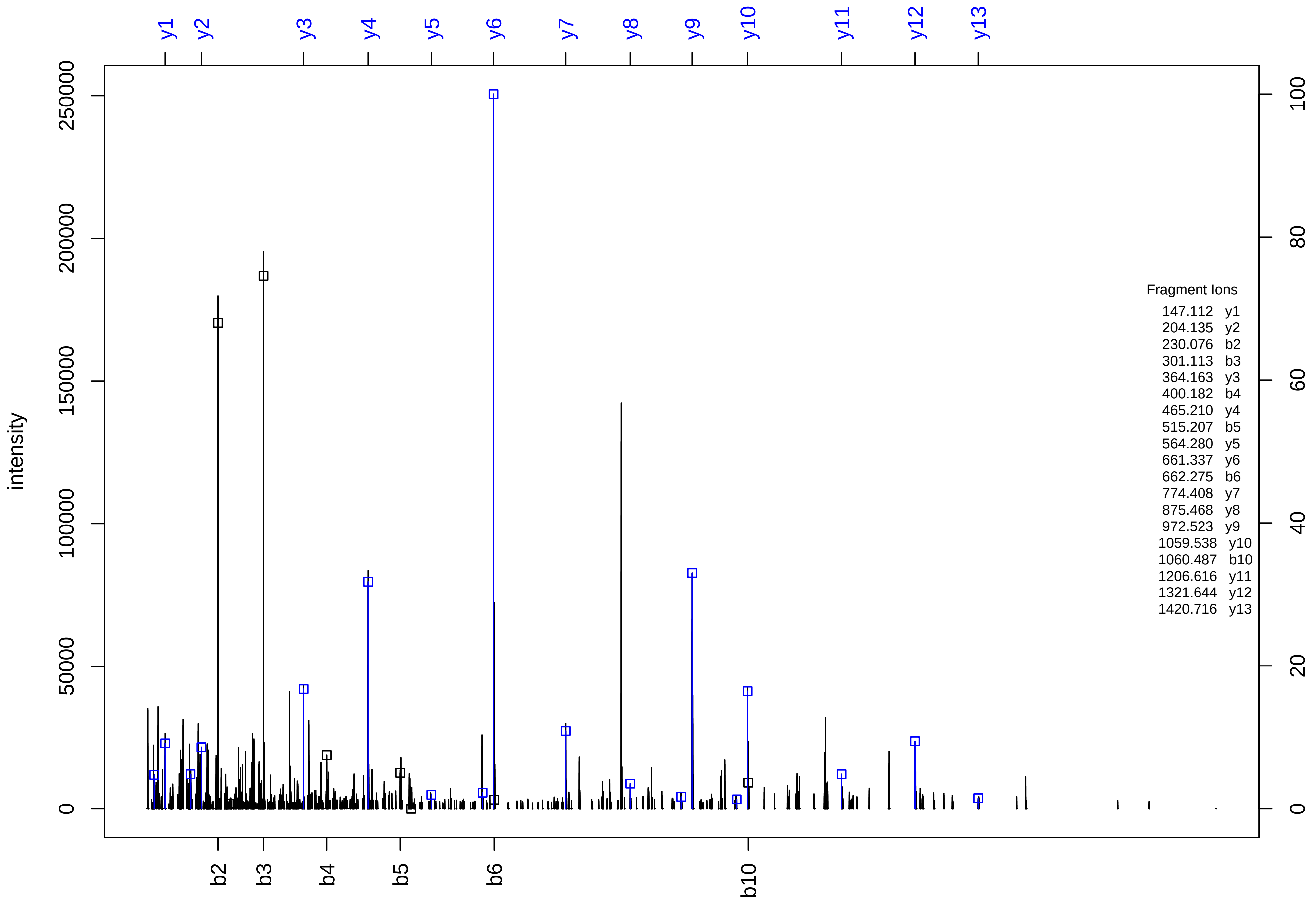

NDAVDFSPTLPVTCGK / File: christine_10pools_08.raw; SpectrumID: ; scans: 18819

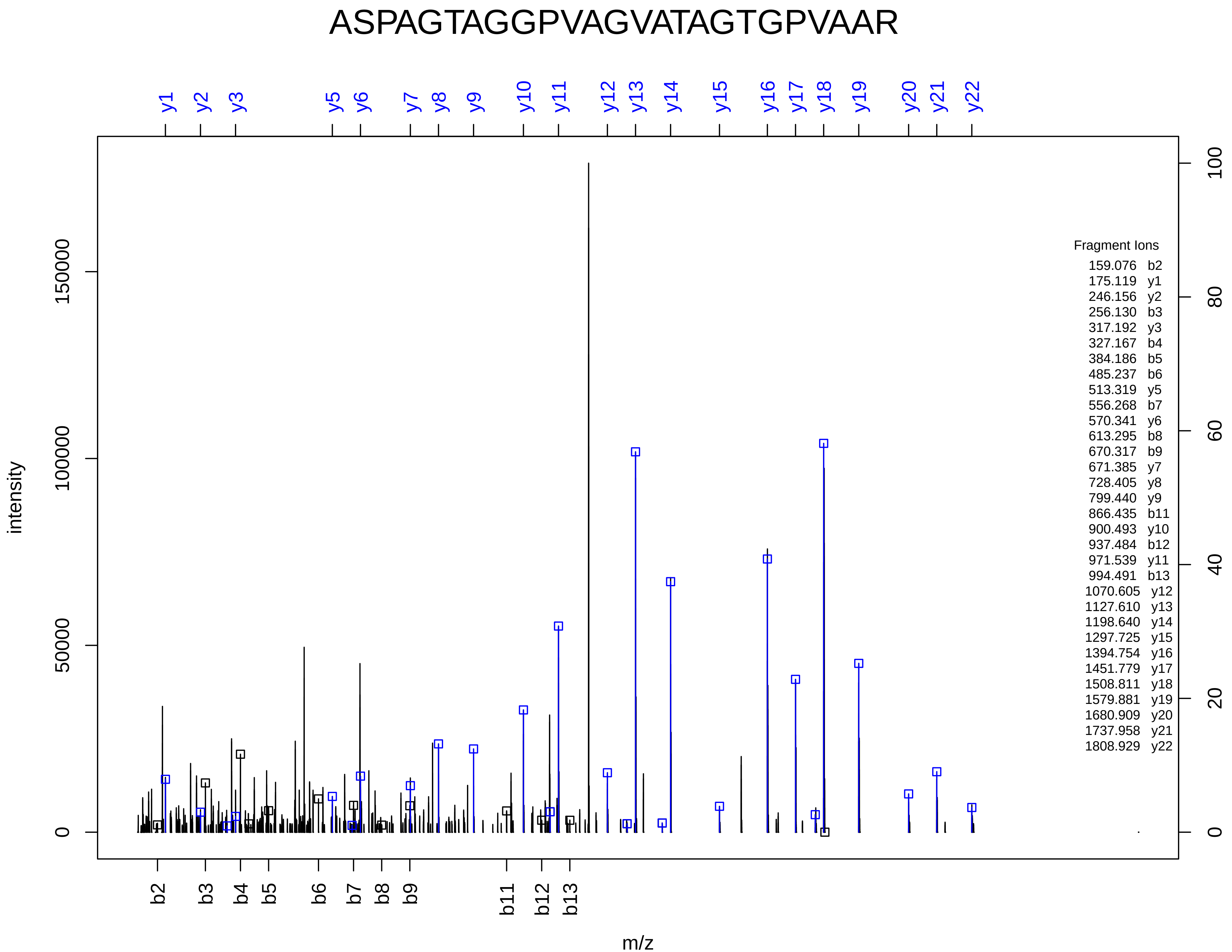

ASPAGTAGGPVAGVATAGTGPVAAR / File: christine_10pools_08.raw; SpectrumID: ; scans: 9295

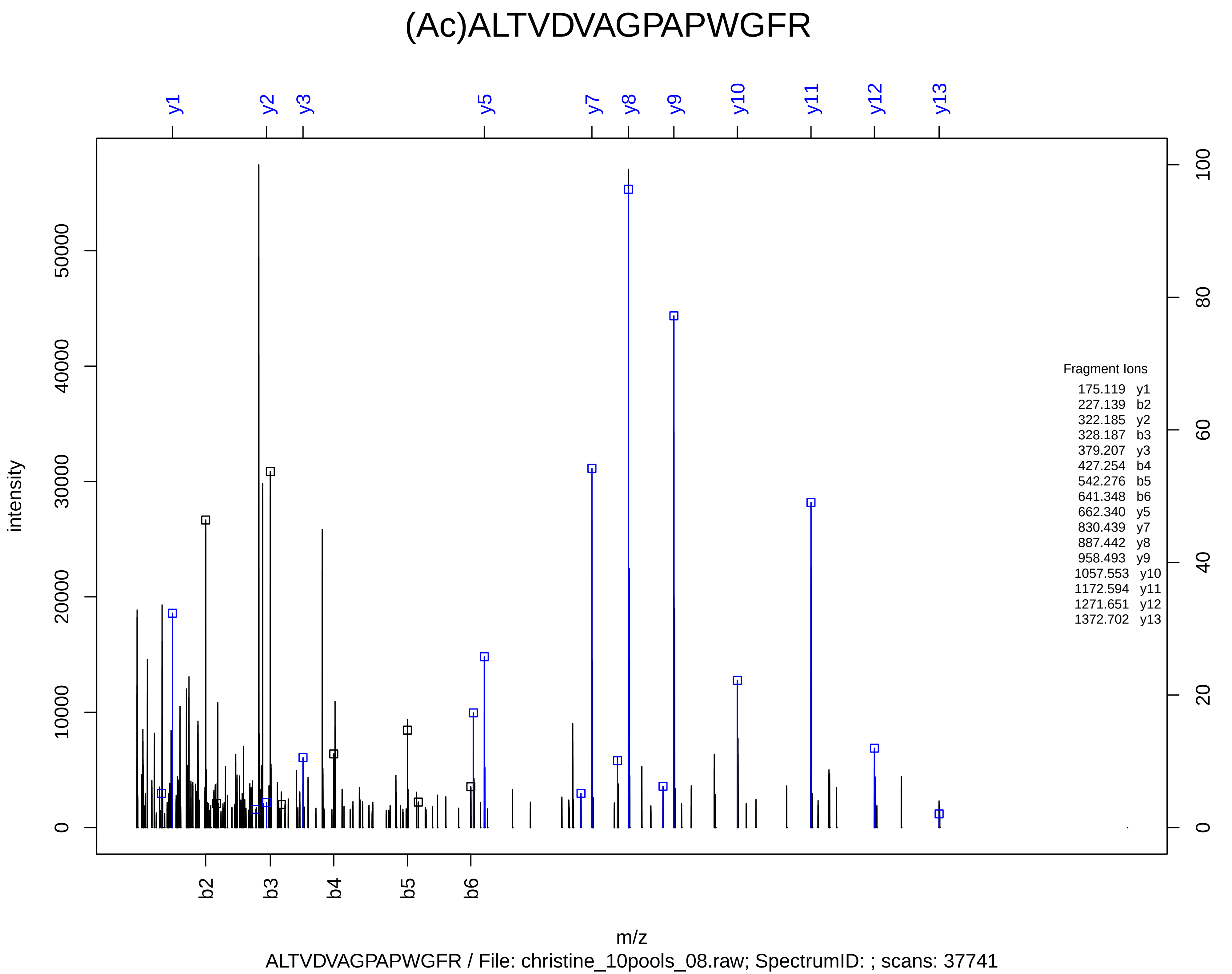

(Ac)ALTVDVAGPAPWGFR
y1
y2
y3
y5
y7
y8
y9
y10
y11
y12
y13
intensity
0
10000
20000
30000
40000
50000
0
20
40
60
80
100
b2
b3
b4
b5
b6
m/z
Fragment Ions
175.119 y1
227.139 b2
322.185 y2
328.187 b3
379.207 y3
427.254 b4
542.276 b5
641.348 b6
662.340 y5
830.439 y7
887.442 y8
958.493 y9
1057.553 y10
1172.594 y11
1271.651 y12
1372.702 y13
ALTVDVAGPAPWGFR / File: christine_10pools_08.raw; SpectrumID: ; scans: 37741

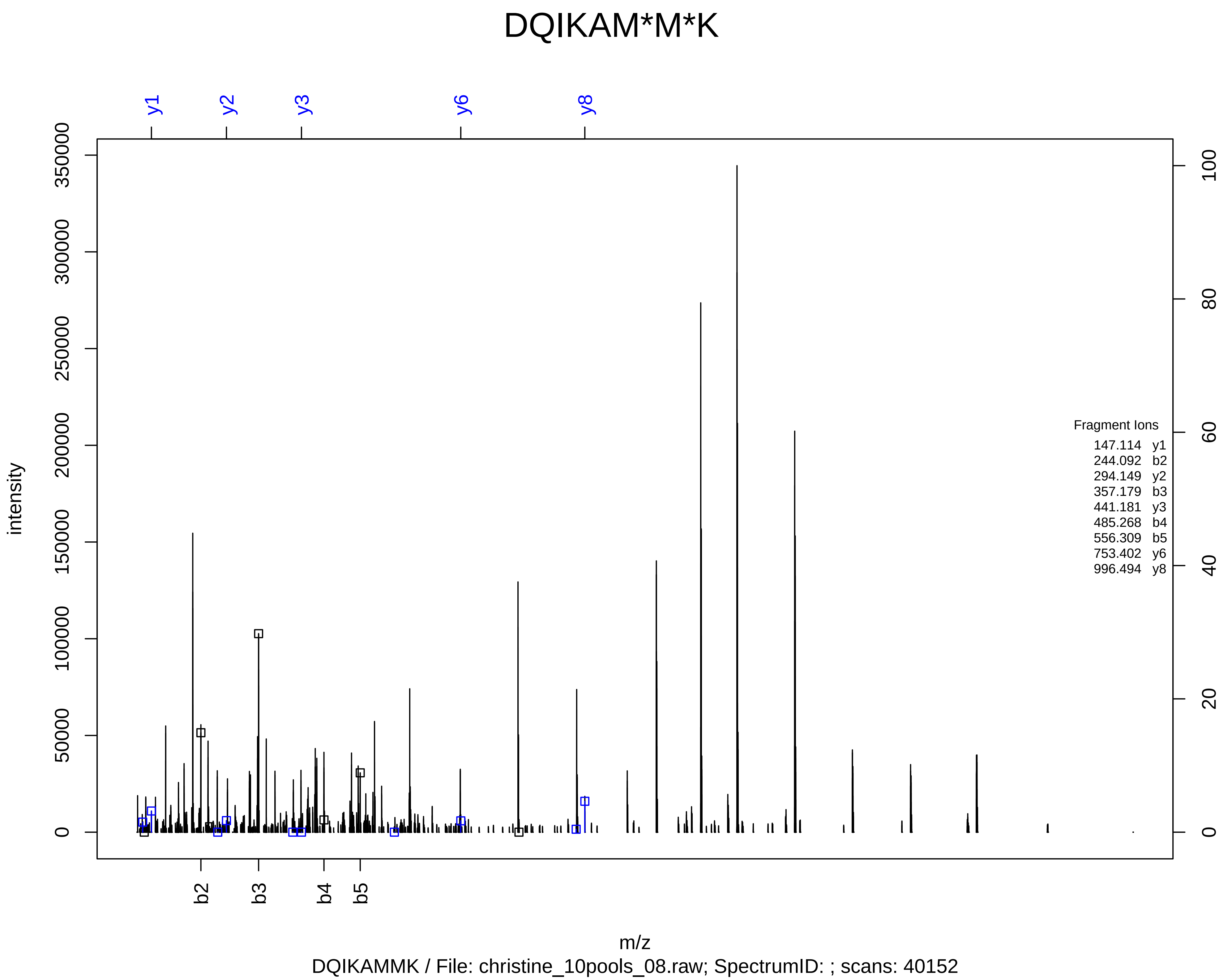
DQIKAM*M*K
y1
y2
y3
y6
y8
b2
b3
b4
b5
intensity
m/z
Fragment Ions
147.114 y1
244.092 b2
294.149 y2
357.179 b3
441.181 y3
485.268 b4
556.309 b5
753.402 y6
996.494 y8
DQIKAMMK / File: christine_10pools_08.raw; SpectrumID: ; scans: 40152

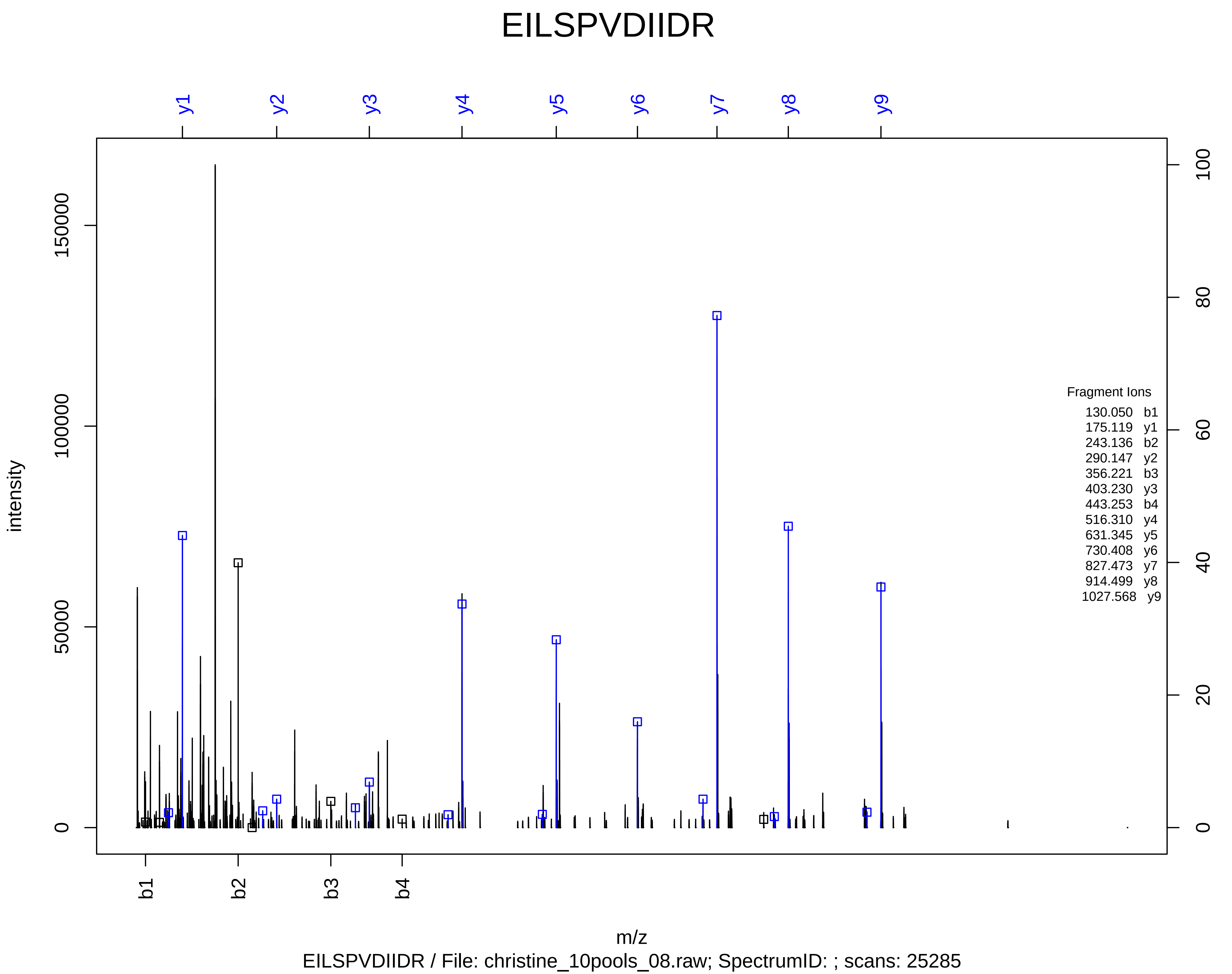
EILSPVDIIDR
y1
y2
y3
y4
y5
y6
y7
y8
y9
intensity
0
50000
100000
150000
0
20
40
60
80
100
b1
b2
b3
b4
m/z
Fragment Ions
130.050 b1
175.119 y1
243.136 b2
290.147 y2
356.221 b3
403.230 y3
443.253 b4
516.310 y4
631.345 y5
730.408 y6
827.473 y7
914.499 y8
1027.568 y9
EILSPVDIIDR / File: christine_10pools_08.raw; SpectrumID: ; scans: 25285

(Ac)AAAAAAGSGTPREEEAPGGEAAASQAQAPTSAPGGVR

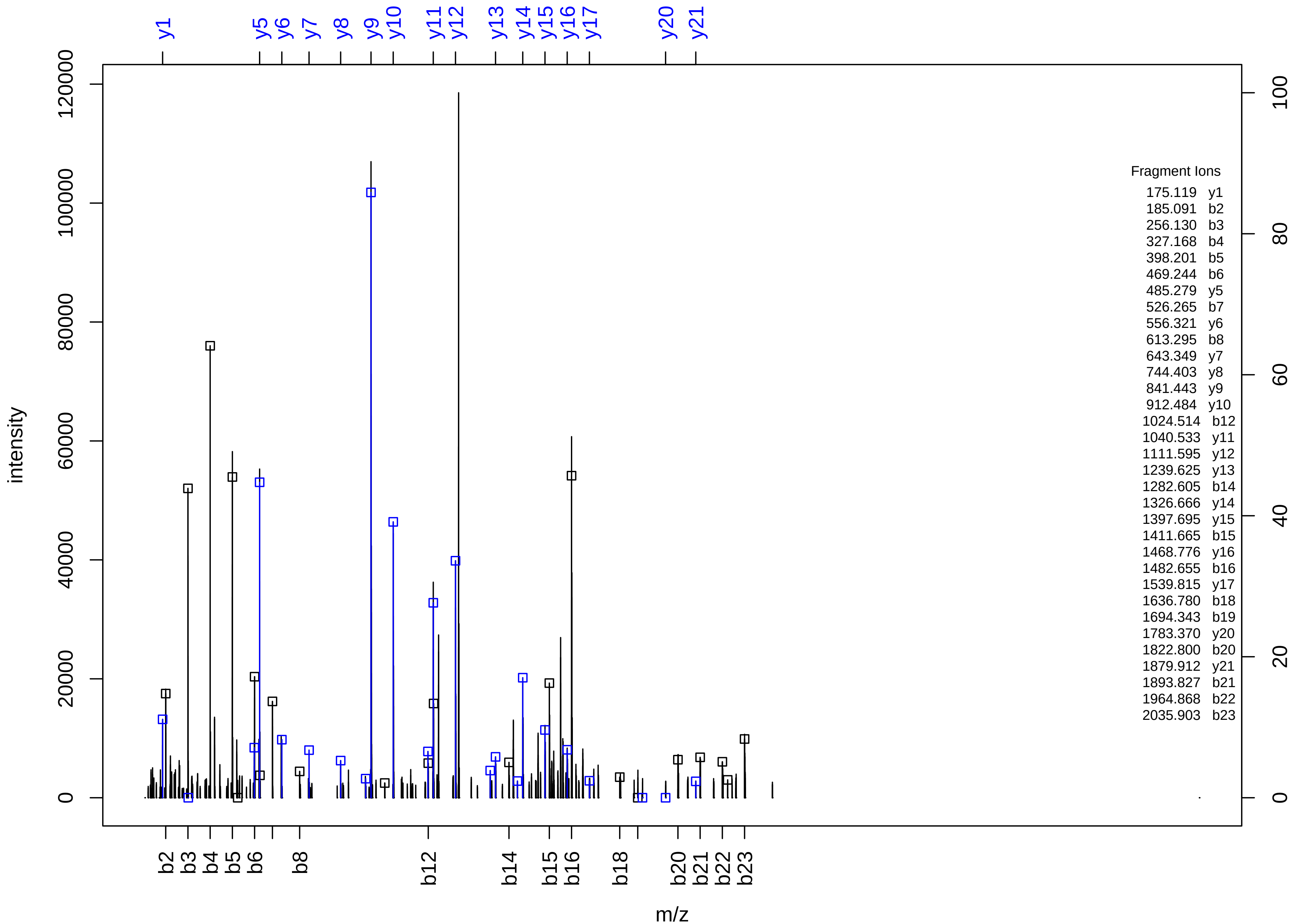

AAAAAAGSGTPREEEAPGGEAAASQAQAPTSAPGGVR / File: christine_10pools_08.raw; SpectrumID: ; scans: 7759

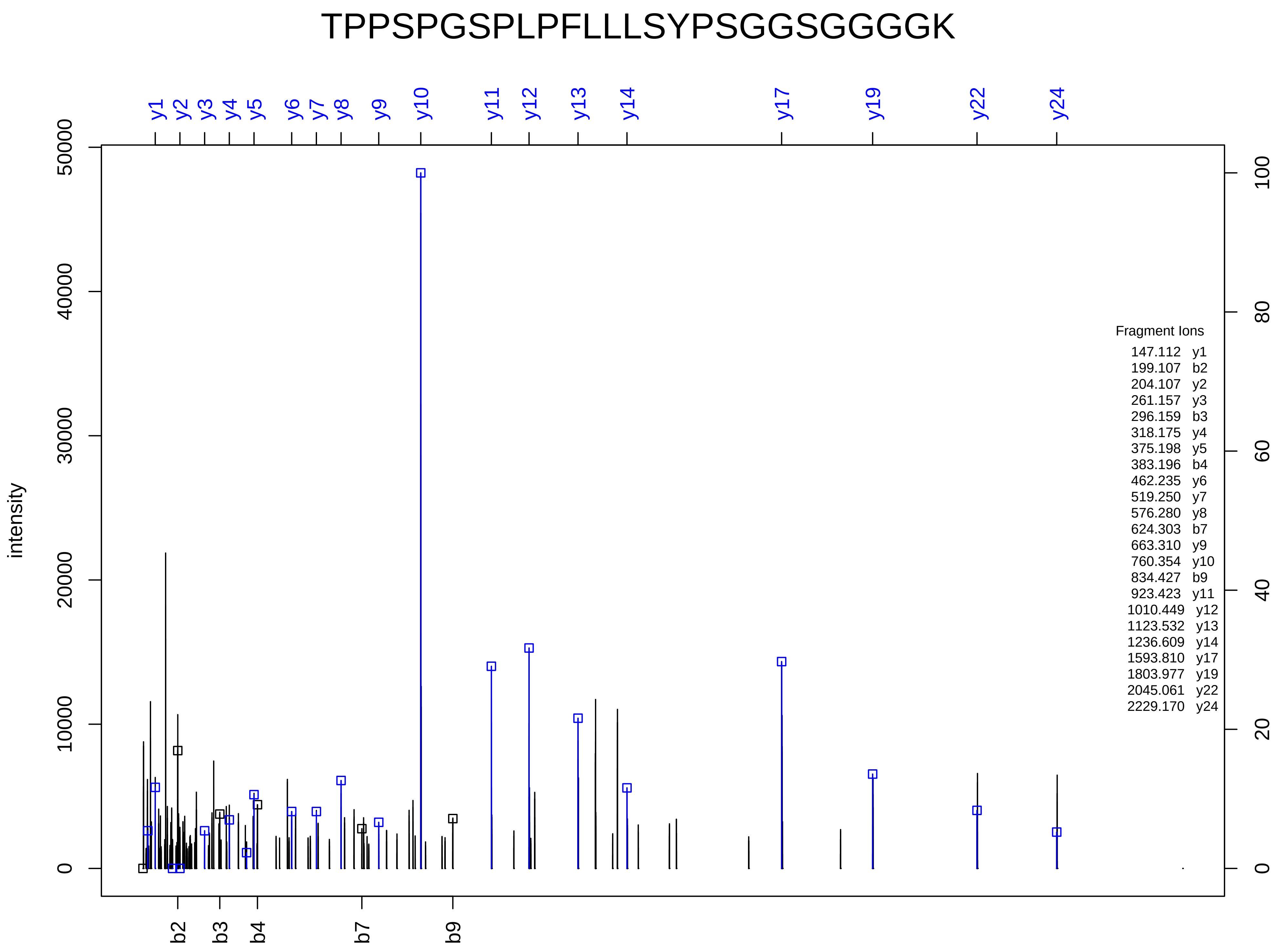

TPPSPGSPLPFLLLSYPSGGSGGGGK / File: christine_10pools_08.raw; SpectrumID: ; scans: 35523

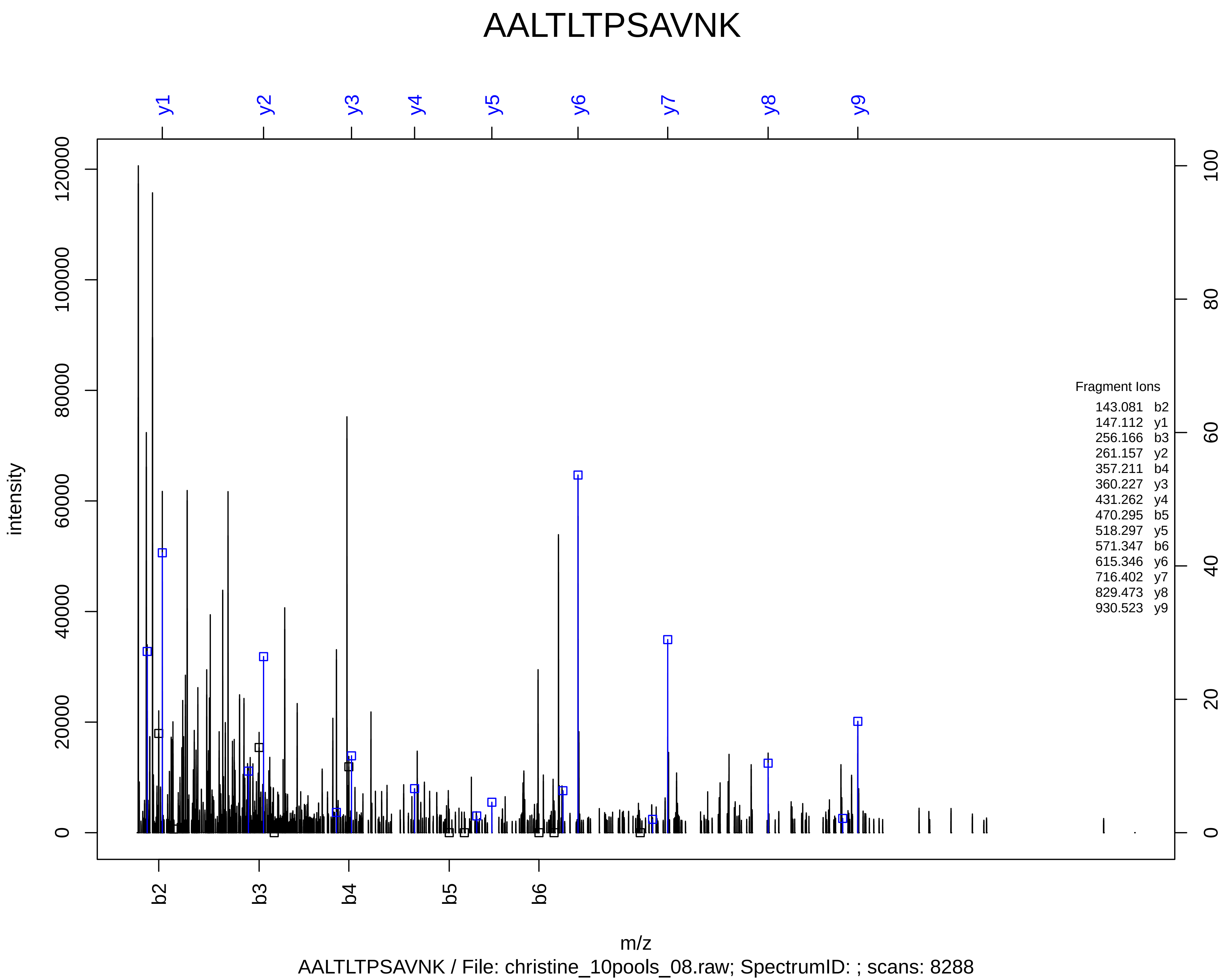

AALTLTPSAVNK
y1
y2
y3
y4
y5
y6
y7
y8
y9
intensity
120000
100000
80000
60000
40000
20000
0
100
80
60
40
20
0
Fragment Ions
143.081 b2
147.112 y1
256.166 b3
261.157 y2
357.211 b4
360.227 y3
431.262 y4
470.295 b5
518.297 y5
571.347 b6
615.346 y6
716.402 y7
829.473 y8
930.523 y9
b2
b3
b4
b5
b6
m/z
AALTLTPSAVNK / File: christine_10pools_08.raw; SpectrumID: ; scans: 8288

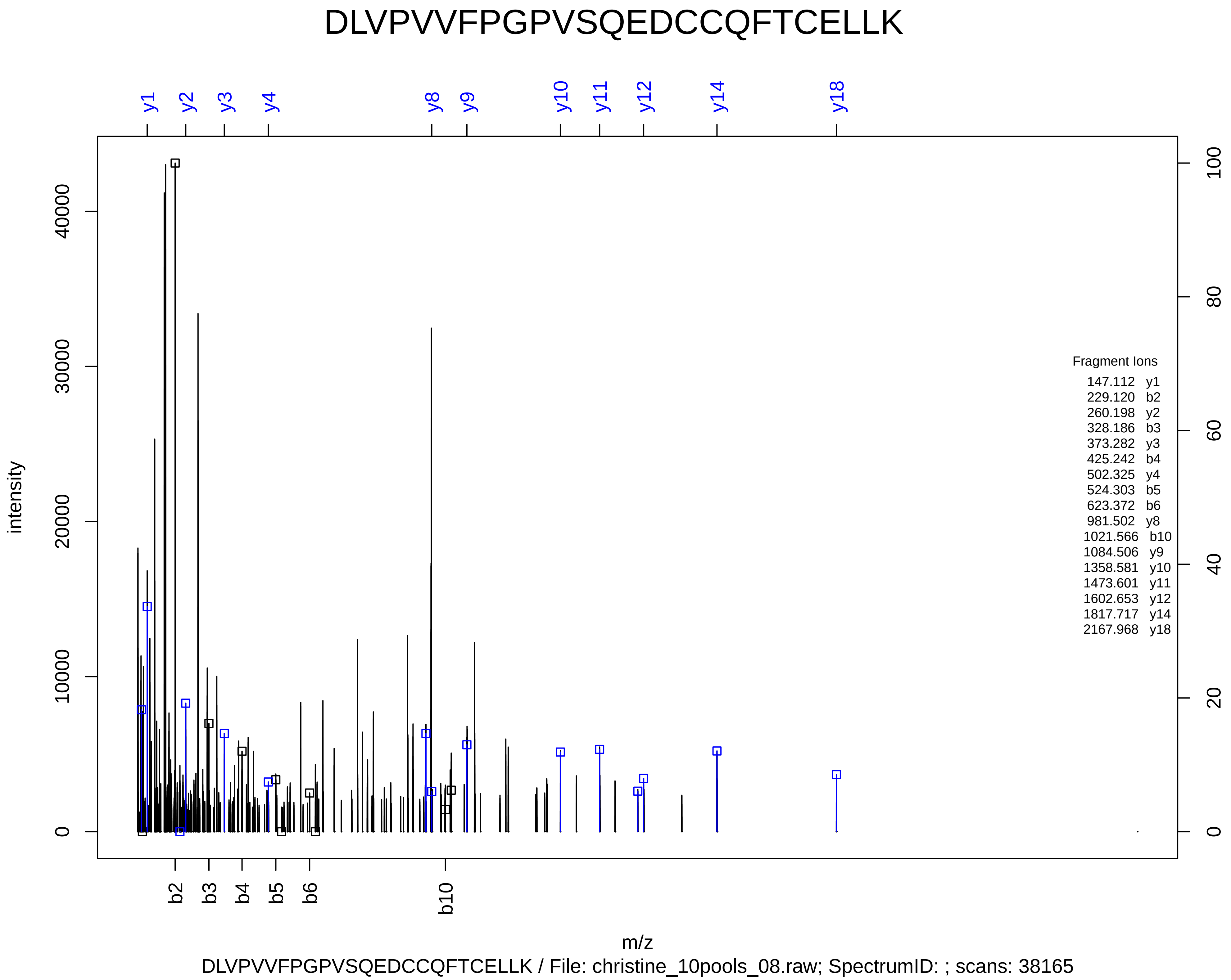
DLVPVVFPGPVSQEDCCQFTCELLK
y1
y2
y3
y4
y8
y9
y10
y11
y12
y14
y18
intensity
0
10000
20000
30000
40000
0
20
40
60
80
100
b2
b3
b4
b5
b6
b10
m/z
Fragment Ions
147.112 y1
229.120 b2
260.198 y2
328.186 b3
373.282 y3
425.242 b4
502.325 y4
524.303 b5
623.372 b6
981.502 y8
1021.566 b10
1084.506 y9
1358.581 y10
1473.601 y11
1602.653 y12
1817.717 y14
2167.968 y18
DLVPVVFPGPVSQEDCCQFTCELLK / File: christine_10pools_08.raw; SpectrumID: ; scans: 38165

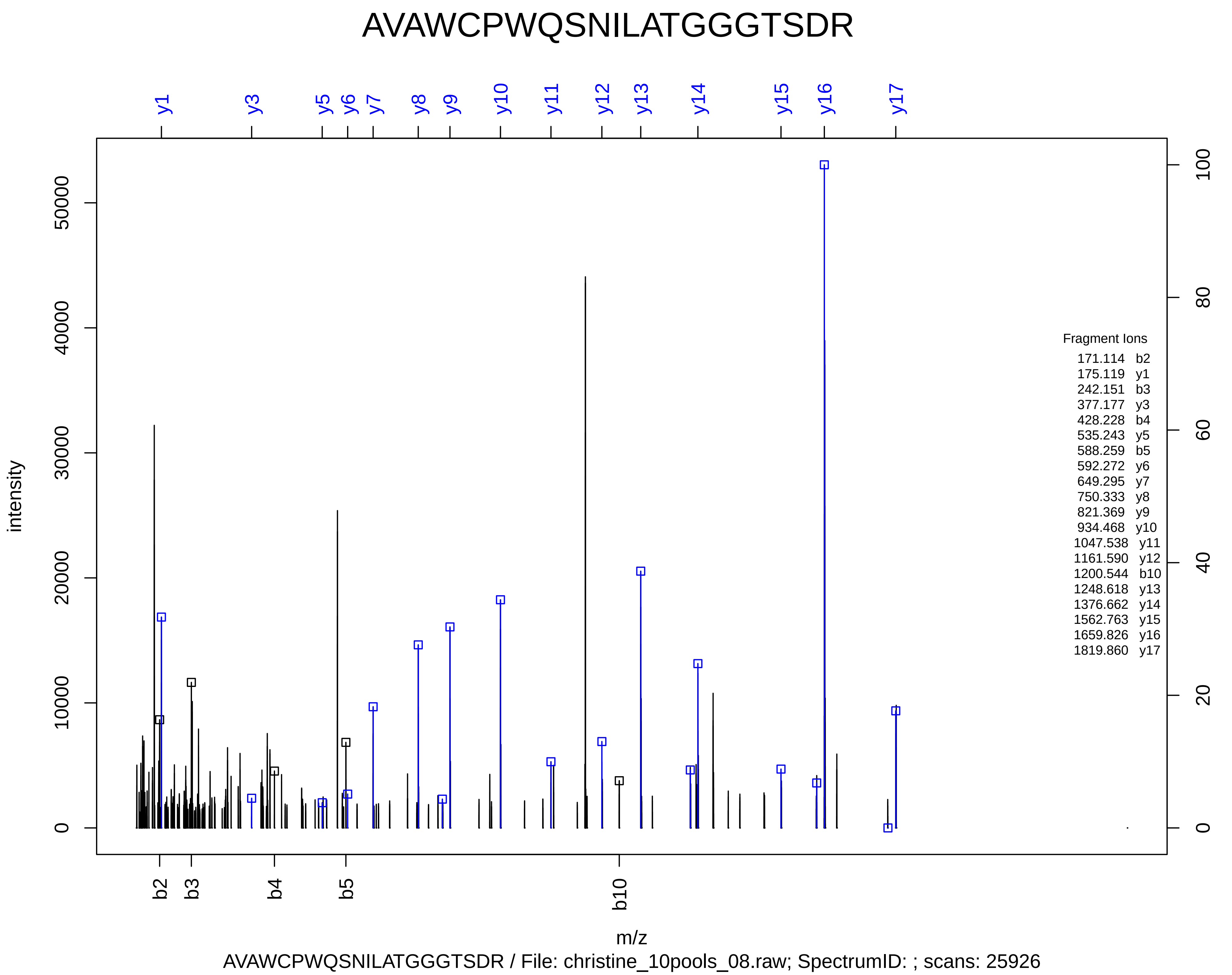
AVAWCPWQSNILATGGGTSDR
y1
y3
y5
y6
y7
y8
y9
y10
y11
y12
y13
y14
y15
y16
y17
b2
b3
b4
b5
b10
intensity
0
10000
20000
30000
40000
50000
0
20
40
60
80
100
m/z
Fragment Ions
171.114 b2
175.119 y1
242.151 b3
377.177 y3
428.228 b4
535.243 y5
588.259 b5
592.272 y6
649.295 y7
750.333 y8
821.369 y9
934.468 y10
1047.538 y11
1161.590 y12
1200.544 b10
1248.618 y13
1376.662 y14
1562.763 y15
1659.826 y16
1819.860 y17
AVAWCPWQSNILATGGGTSDR / File: christine_10pools_08.raw; SpectrumID: ; scans: 25926

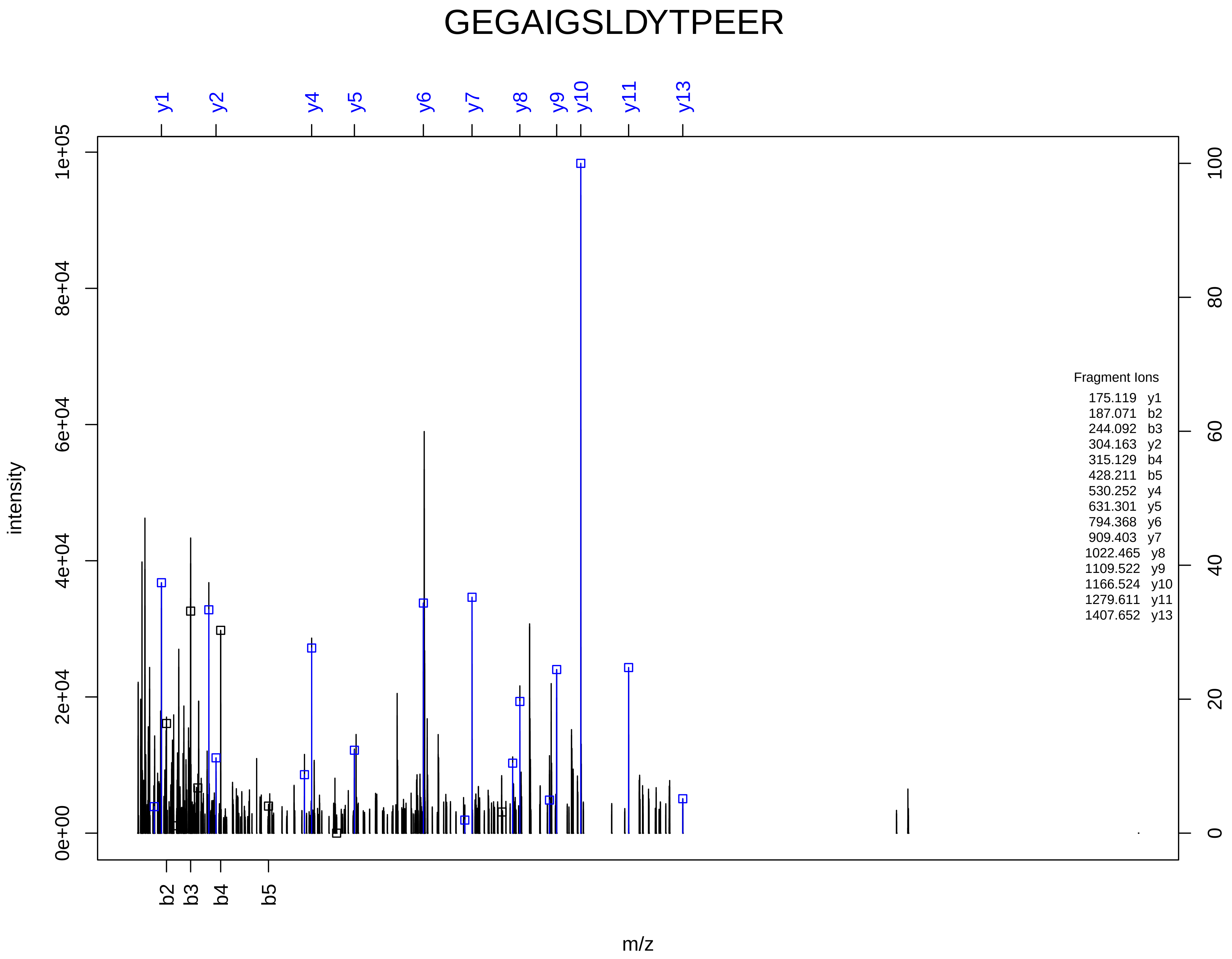

GEGAIGSLDYTPEER / File: christine_10pools_08.raw; SpectrumID: ; scans: 10950

LEILQLLK

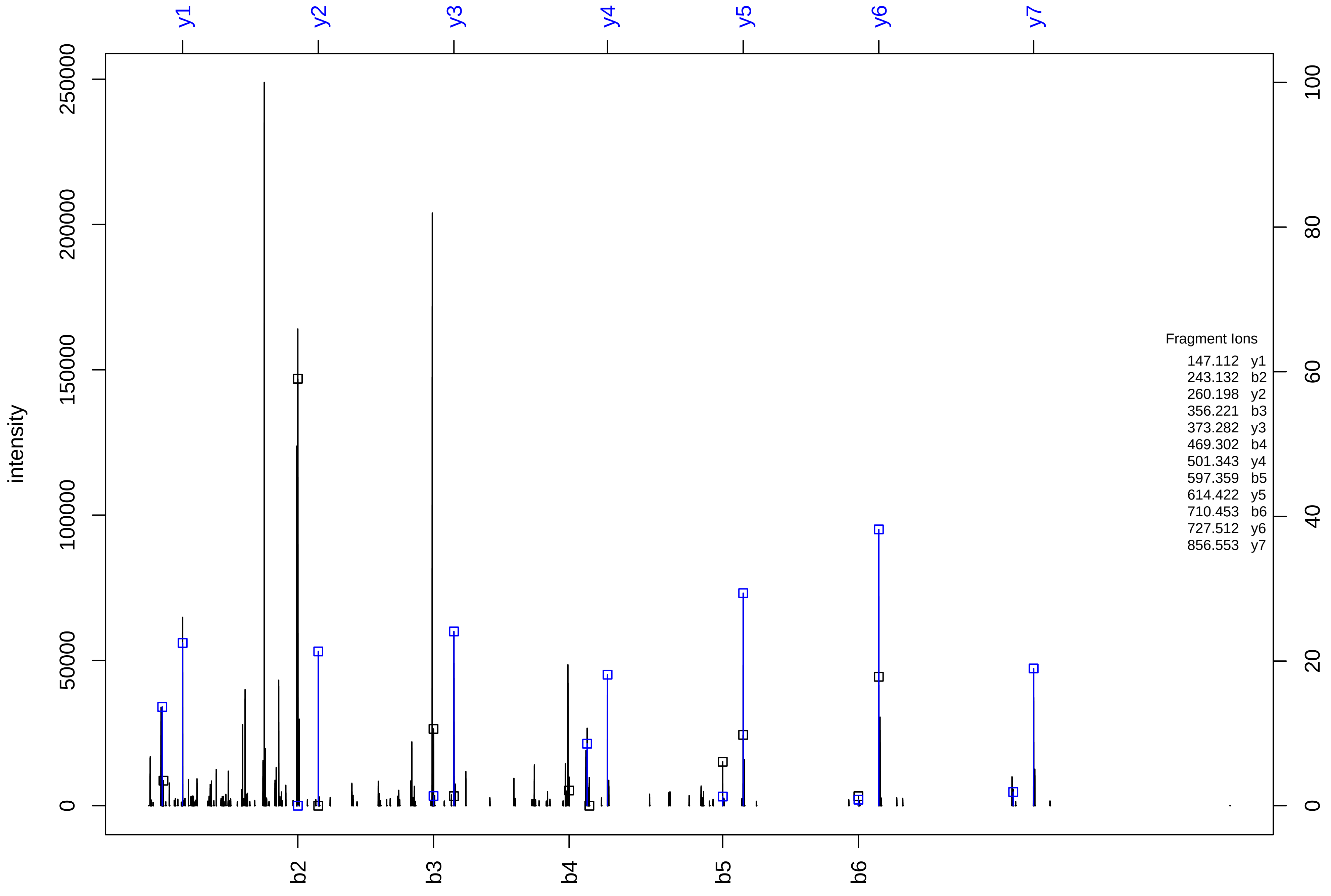

LEILQLLK / File: christine_10pools_08.raw; SpectrumID: ; scans: 28796

(Ac)SAPGPYQAAAGPSVVPTAPPTYEETVGVNSYYPTPPAPMPGPATGLITGPDGK

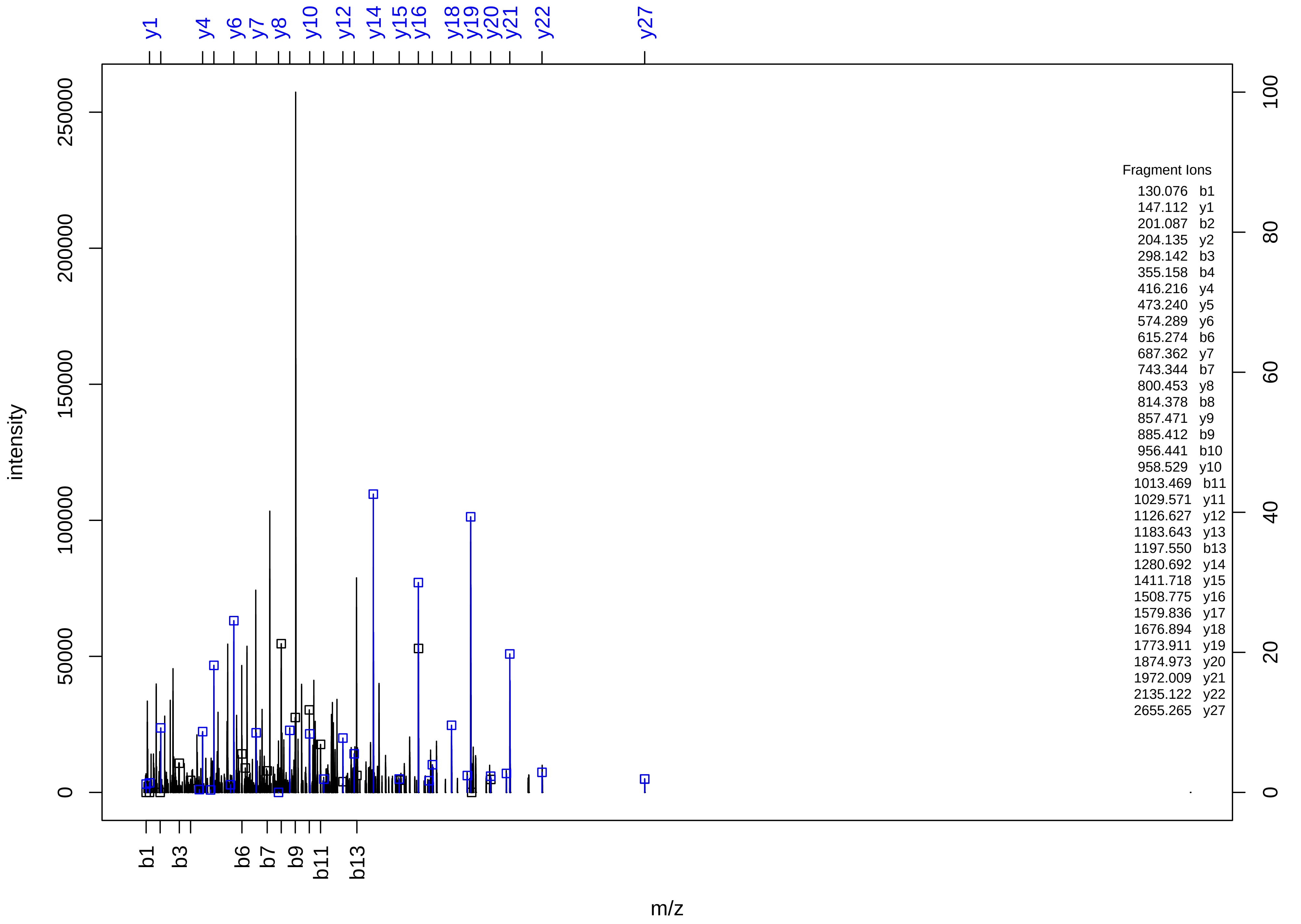

SAPGPYQAAAGPSVVPTAPPTYEETVGVNSYYPTPPAPMPGPATGLITGPDGK / File: christine_10pools_08.raw; SpectrumID: ; scans: 37[illegible]

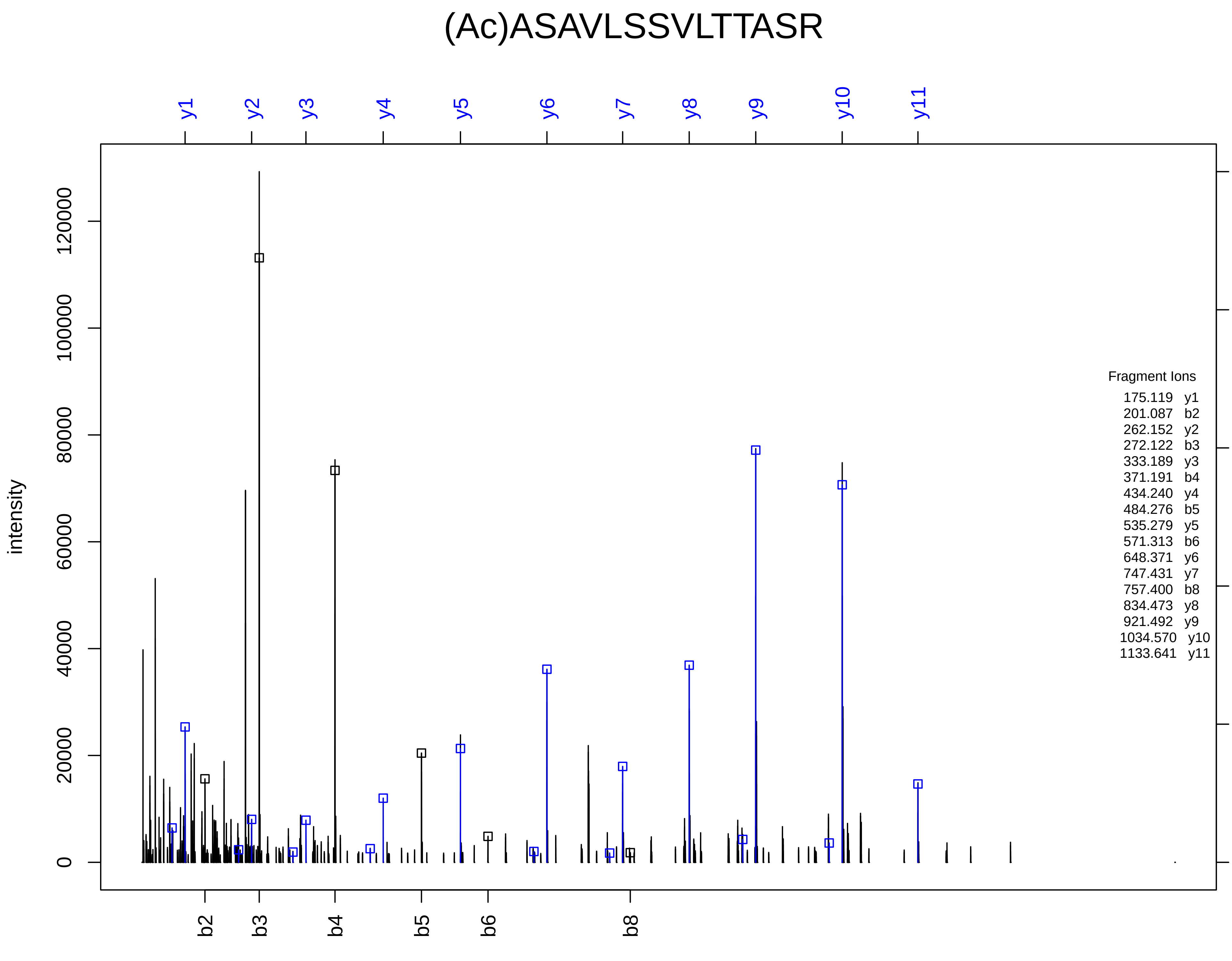

ASAVLSSVLTTASR / File: christine_10pools_08.raw; SpectrumID: ; scans: 36601

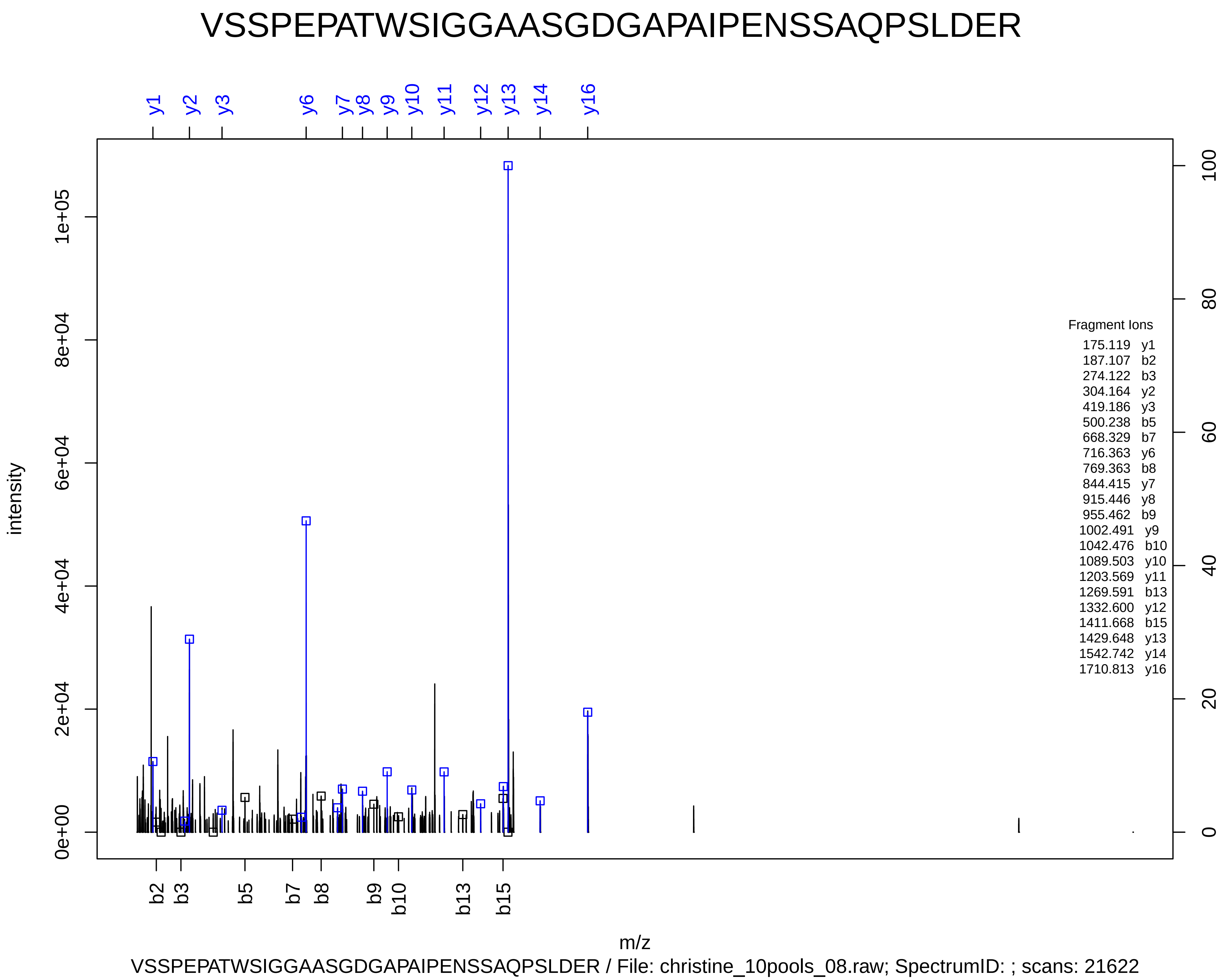
VSSPEPATWSIGGAASGDGAPAIPENSSAQPSLDER
y1 y2 y3 y6 y7 y8 y9 y10 y11 y12 y13 y14 y16
b2 b3 b5 b7 b8 b9 b10 b13 b15
intensity
0e+00 2e+04 4e+04 6e+04 8e+04 1e+05
0 20 40 60 80 100
m/z
Fragment Ions
175.119 y1
187.107 b2
274.122 b3
304.164 y2
419.186 y3
500.238 b5
668.329 b7
716.363 y6
769.363 b8
844.415 y7
915.446 y8
955.462 b9
1002.491 y9
1042.476 b10
1089.503 y10
1203.569 y11
1269.591 b13
1332.600 y12
1411.668 b15
1429.648 y13
1542.742 y14
1710.813 y16
VSSPEPATWSIGGAASGDGAPAIPENSSAQPSLDER / File: christine_10pools_08.raw; SpectrumID: ; scans: 21622

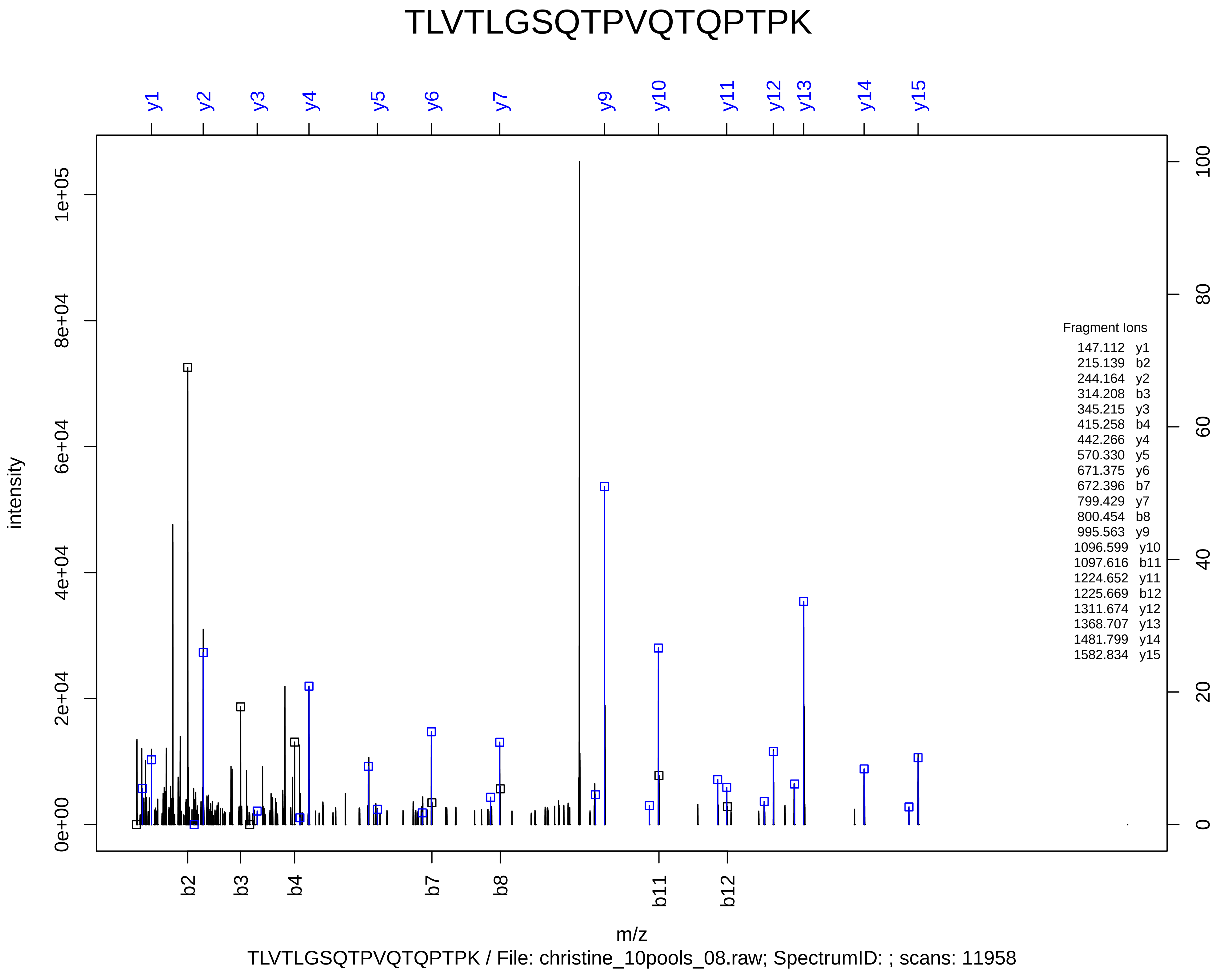

TLVTLGSQTPVQTQPTPK
y1
y2
y3
y4
y5
y6
y7
y9
y10
y11
y12
y13
y14
y15
intensity
0e+00
2e+04
4e+04
6e+04
8e+04
1e+05
0
20
40
60
80
100
Fragment Ions
147.112 y1
215.139 b2
244.164 y2
314.208 b3
345.215 y3
415.258 b4
442.266 y4
570.330 y5
671.375 y6
672.396 b7
799.429 y7
800.454 b8
995.563 y9
1096.599 y10
1097.616 b11
1224.652 y11
1225.669 b12
1311.674 y12
1368.707 y13
1481.799 y14
1582.834 y15
b2
b3
b4
b7
b8
b11
b12
m/z
TLVTLGSQTPVQTQPTPK / File: christine_10pools_08.raw; SpectrumID: ; scans: 11958

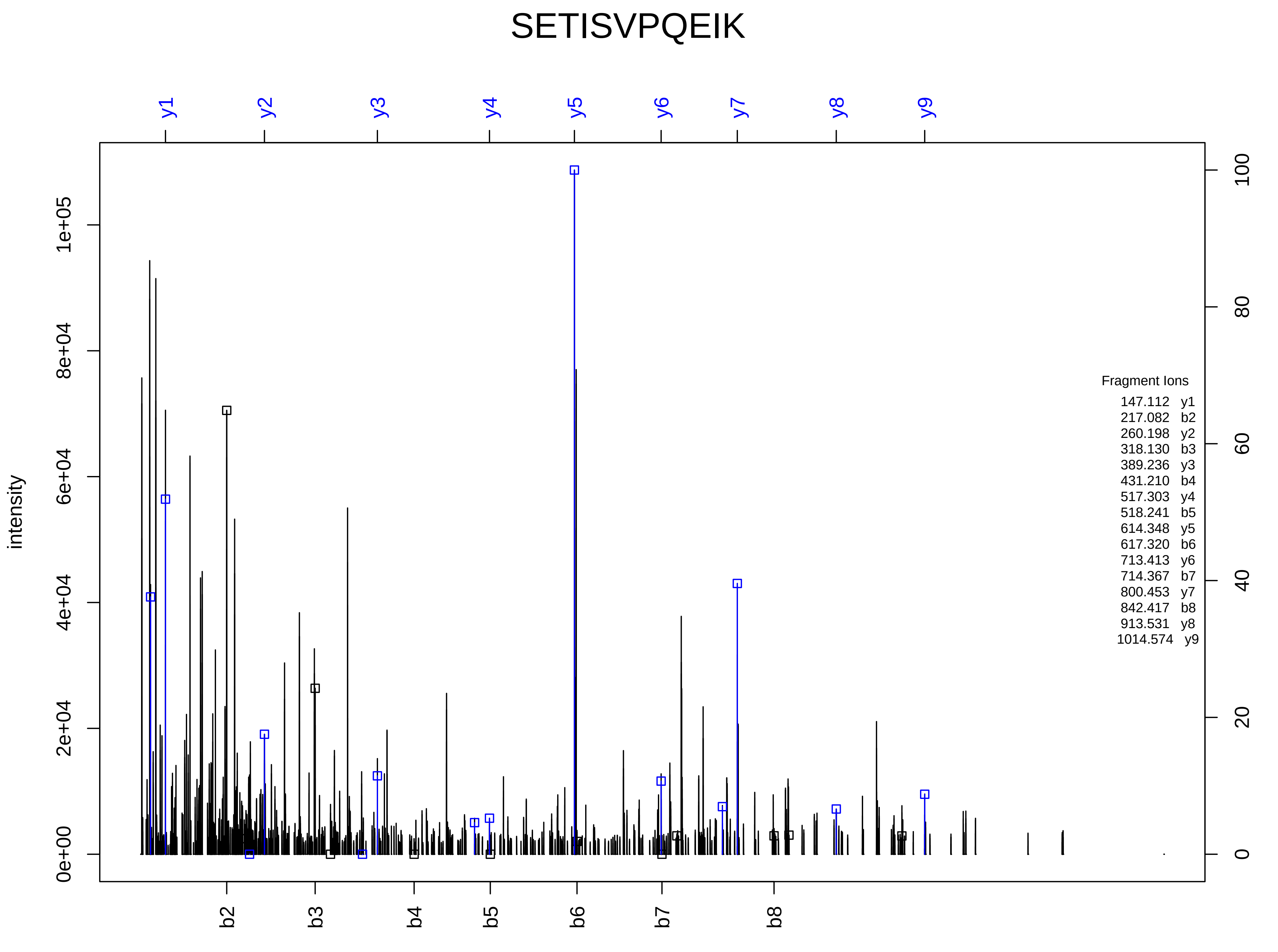

SETISVPQEIK / File: christine_10pools_08.raw; SpectrumID: ; scans: 8811

YFYNREENLR

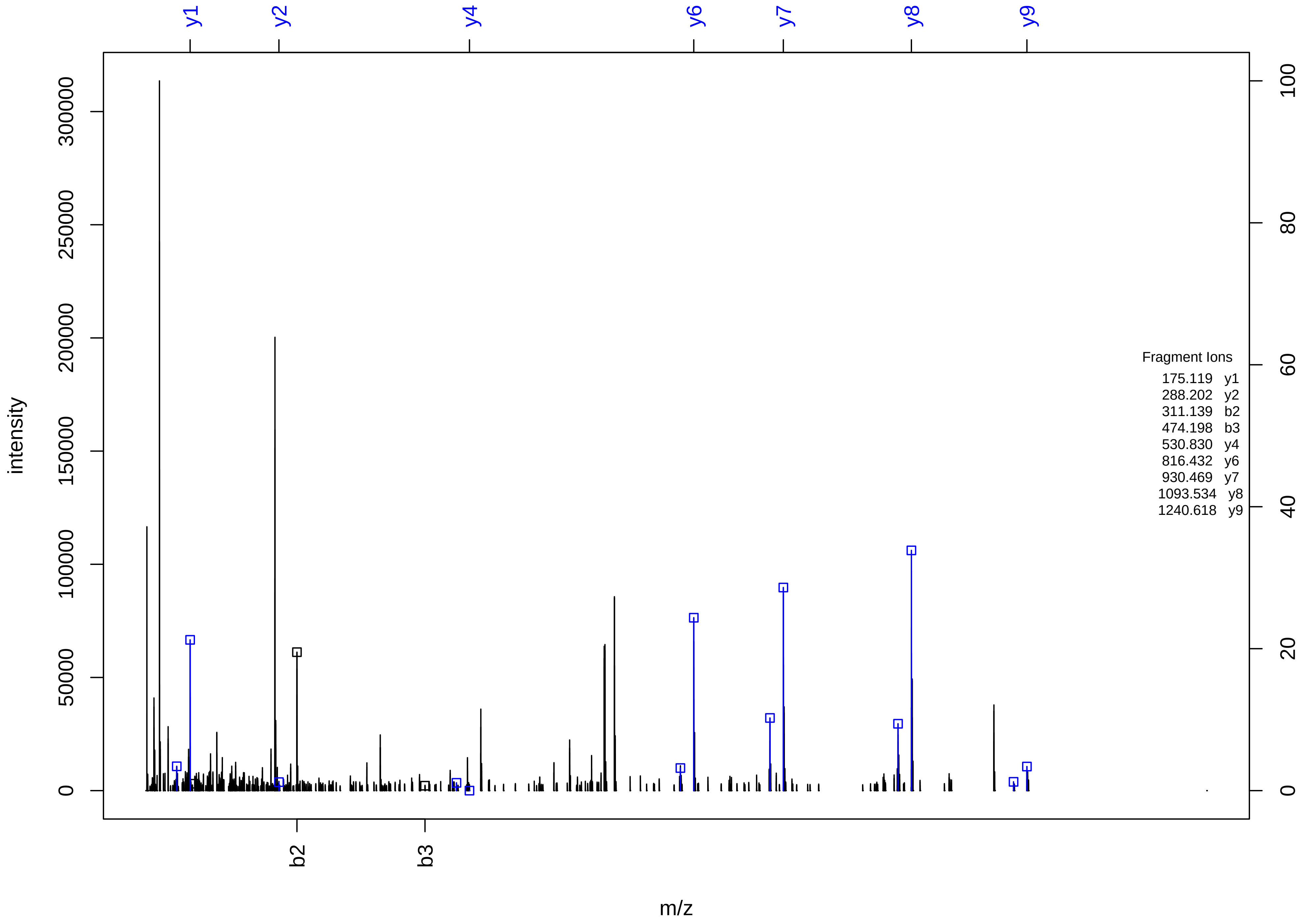

YFYNREENLR / File: christine_10pools_08.raw; SpectrumID: ; scans: 6913

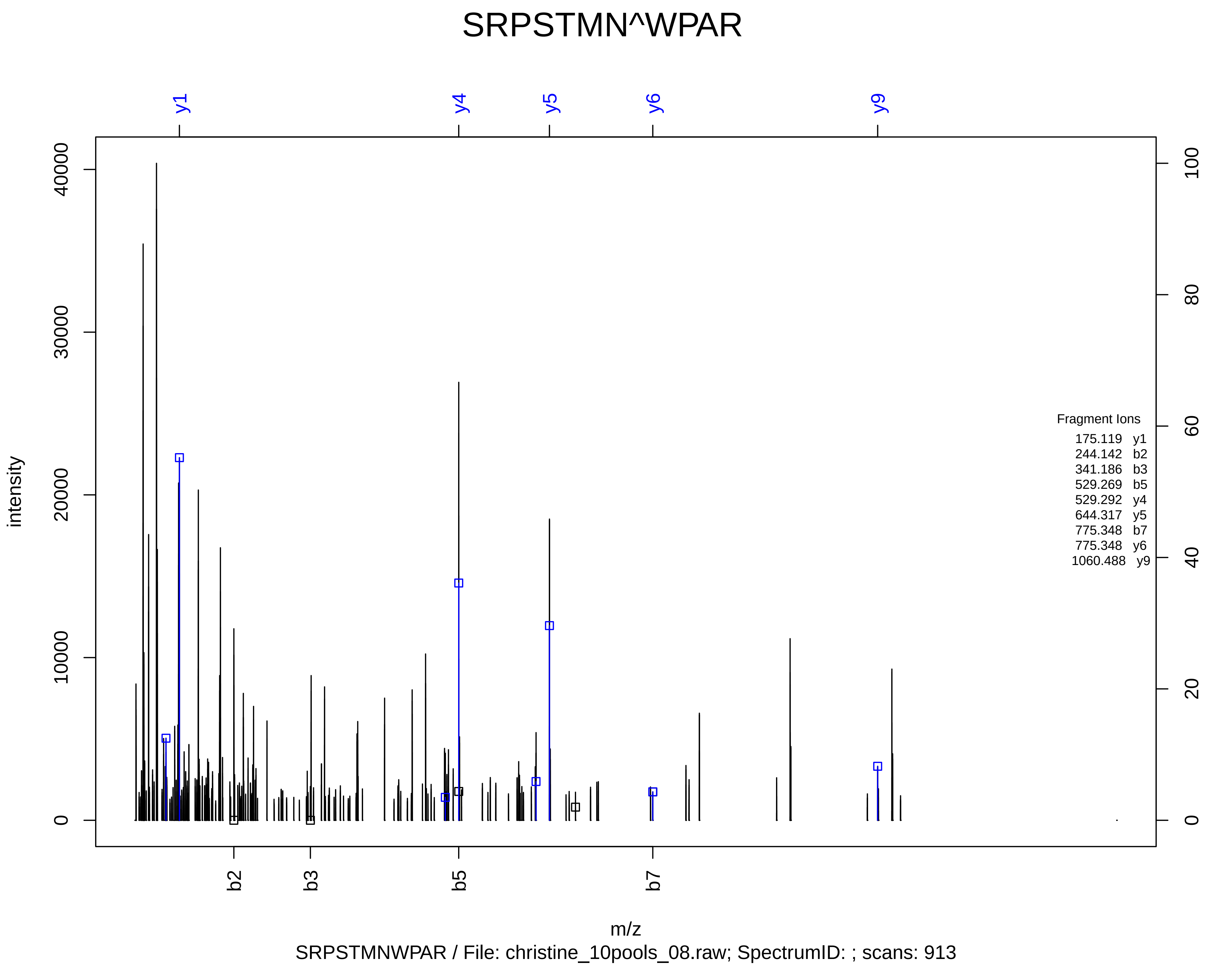
SRPSTMN^WPAR
y1
y4
y5
y6
y9
intensity
0
10000
20000
30000
40000
0
20
40
60
80
100
b2
b3
b5
b7
m/z
Fragment Ions
175.119 y1
244.142 b2
341.186 b3
529.269 b5
529.292 y4
644.317 y5
775.348 b7
775.348 y6
1060.488 y9
SRPSTMNWPAR / File: christine_10pools_08.raw; SpectrumID: ; scans: 913

AIVPILLDANVSTYDK

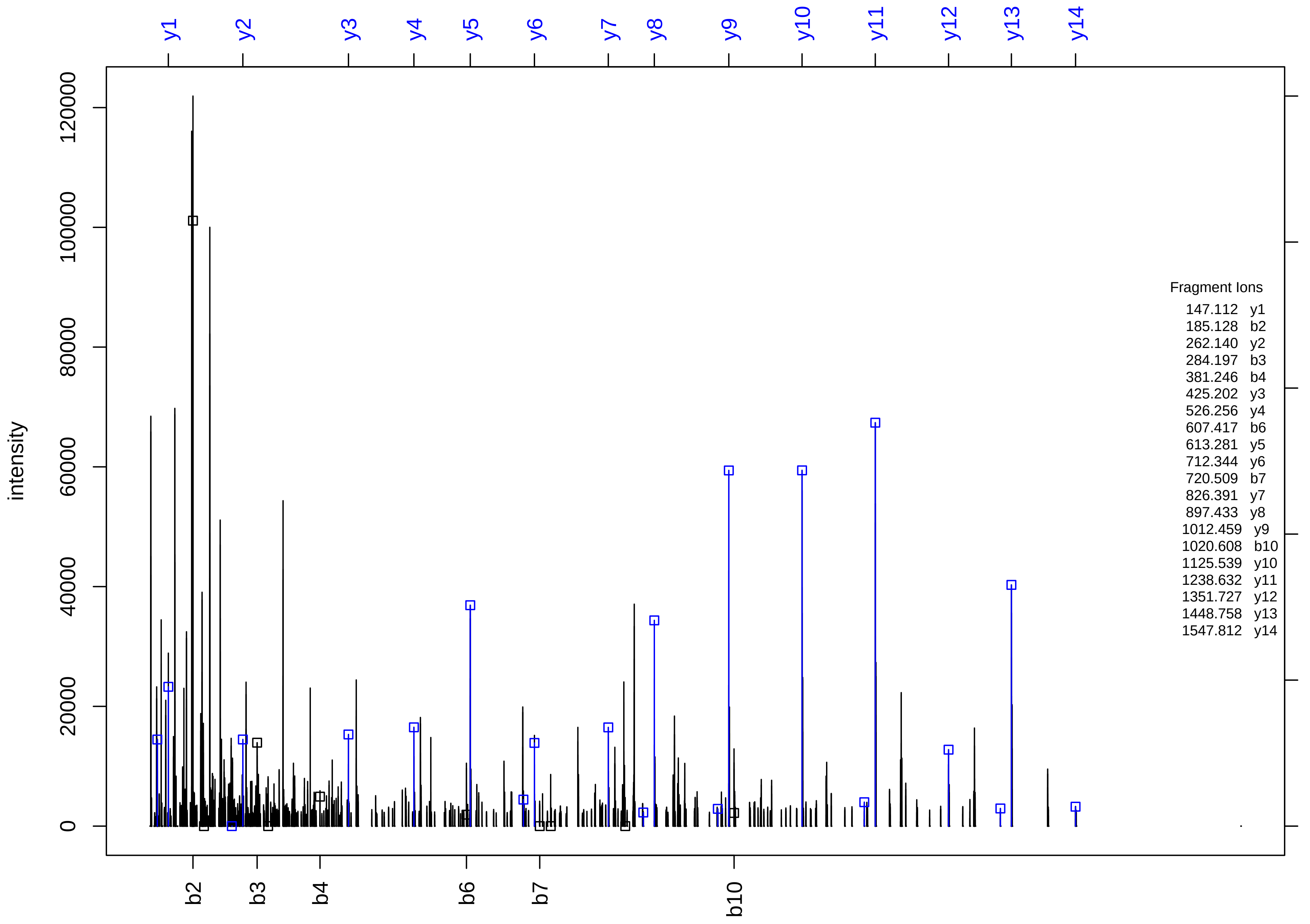

AIVPILLDANVSTYDK / File: christine_10pools_07.raw; SpectrumID: ; scans: 28914

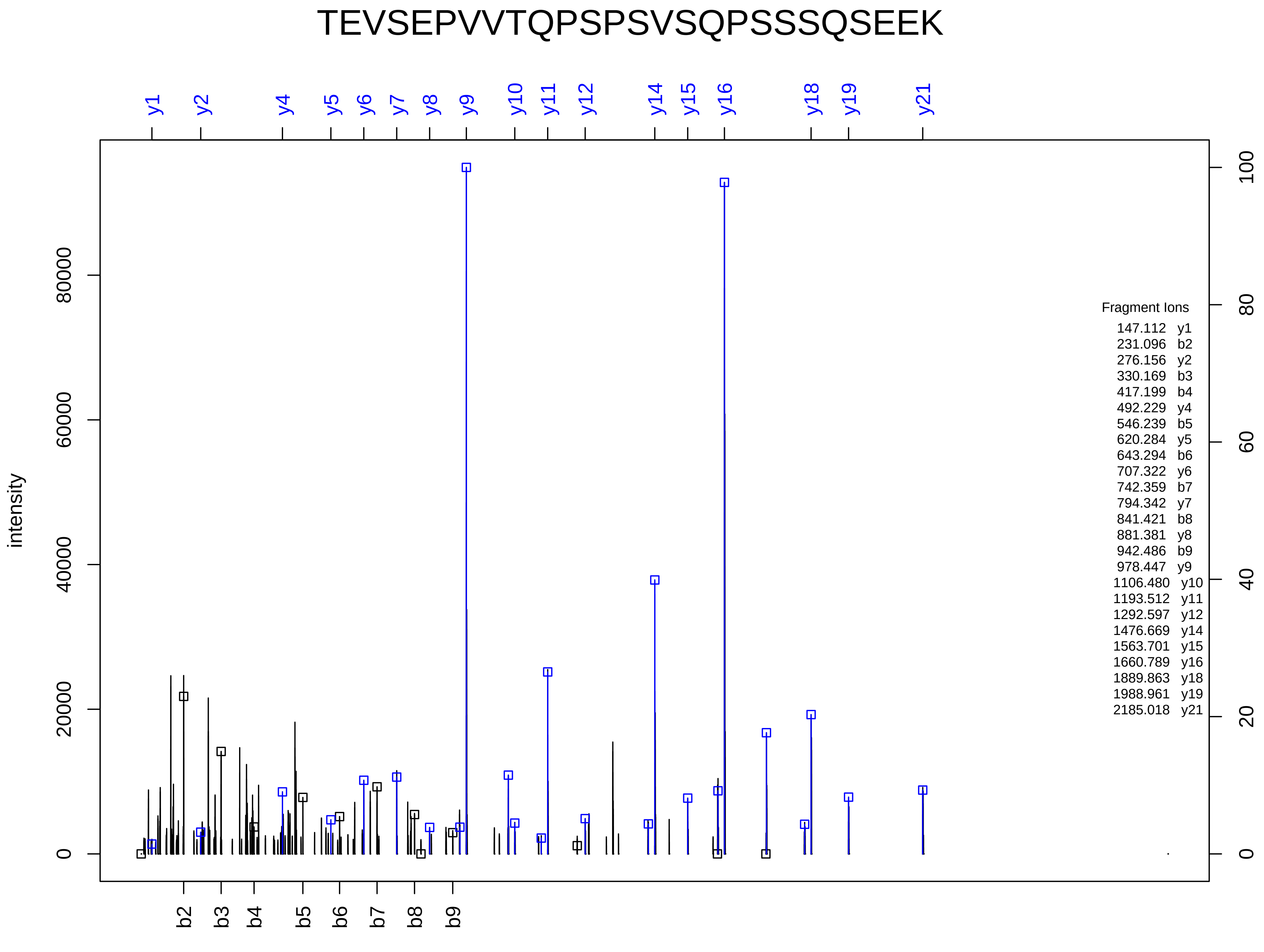

TEVSEPVVTQPSPSVSQPSSSQSEEK / File: christine_10pools_07.raw; SpectrumID: ; scans: 7072

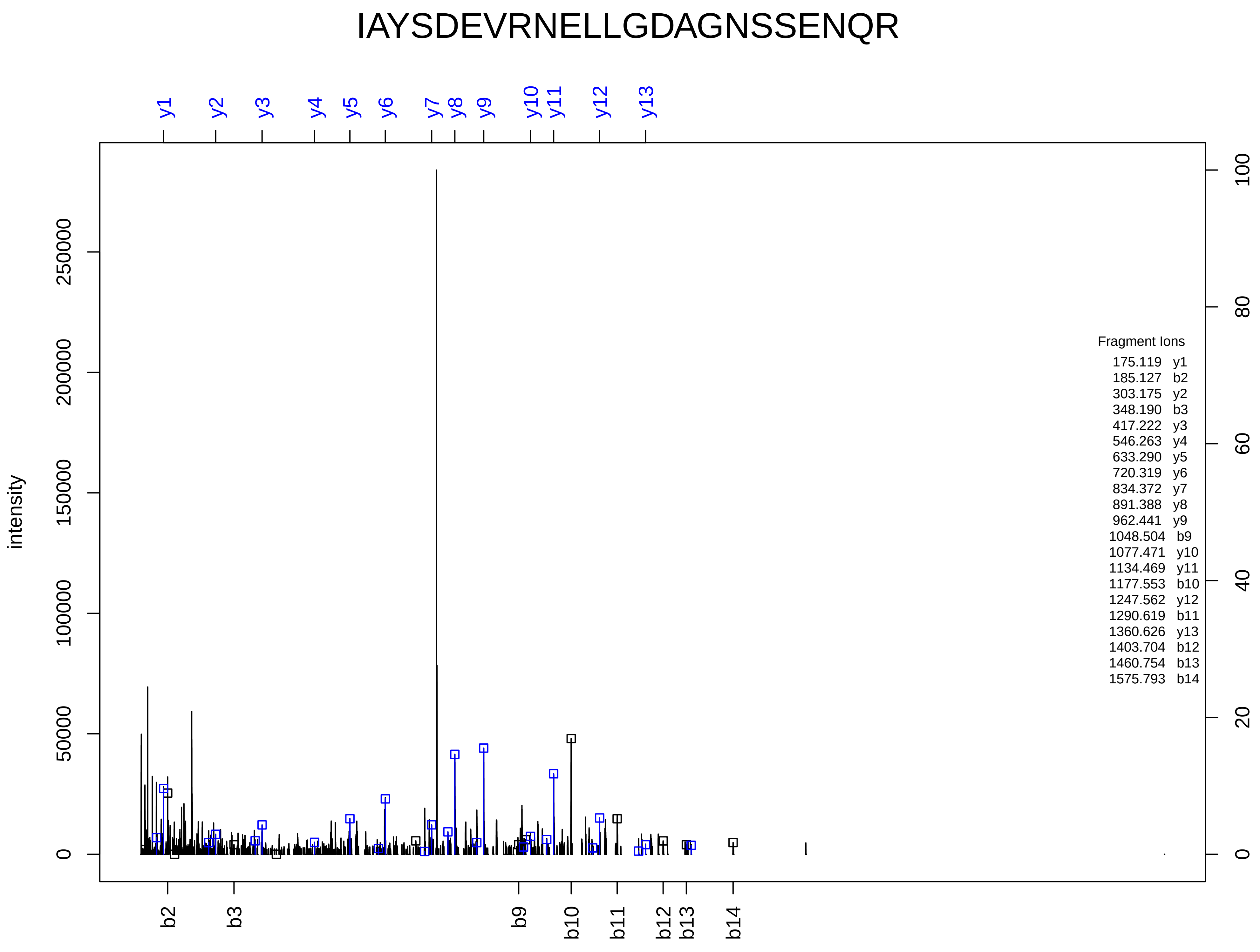

IAYSDEVRNELLGDAGNSSENQR / File: christine_10pools_07.raw; SpectrumID: ; scans: 15109

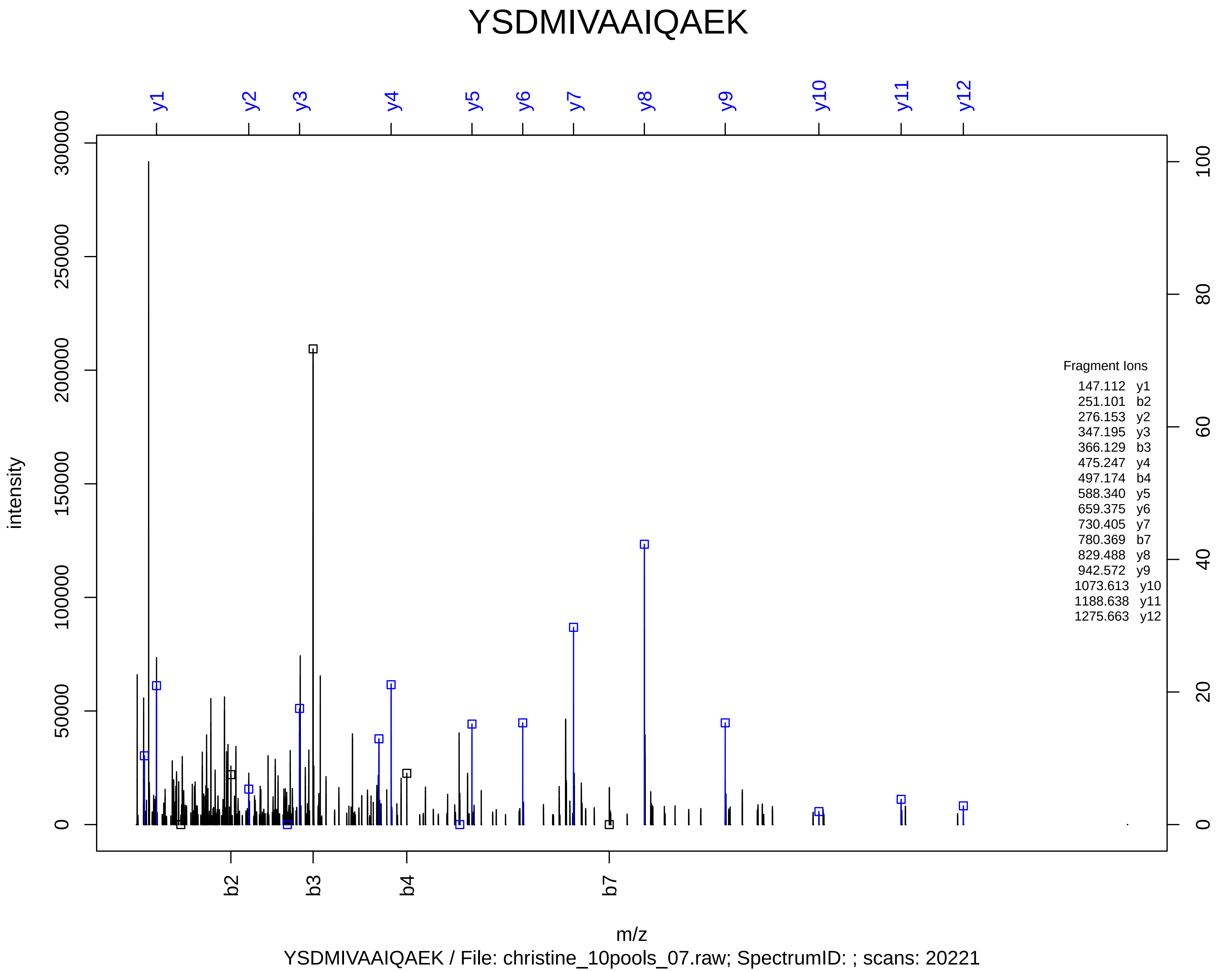
YSDMIVAAIQAEK
y1
y2
y3
y4
y5
y6
y7
y8
y9
y10
y11
y12
b2
b3
b4
b7
intensity
m/z
Fragment Ions
147.112 y1
251.101 b2
276.153 y2
347.195 y3
366.129 b3
475.247 y4
497.174 b4
588.340 y5
659.375 y6
730.405 y7
780.369 b7
829.488 y8
942.572 y9
1073.613 y10
1188.638 y11
1275.663 y12
YSDMIVAAIQAEK / File: christine_10pools_07.raw; SpectrumID: ; scans: 20221

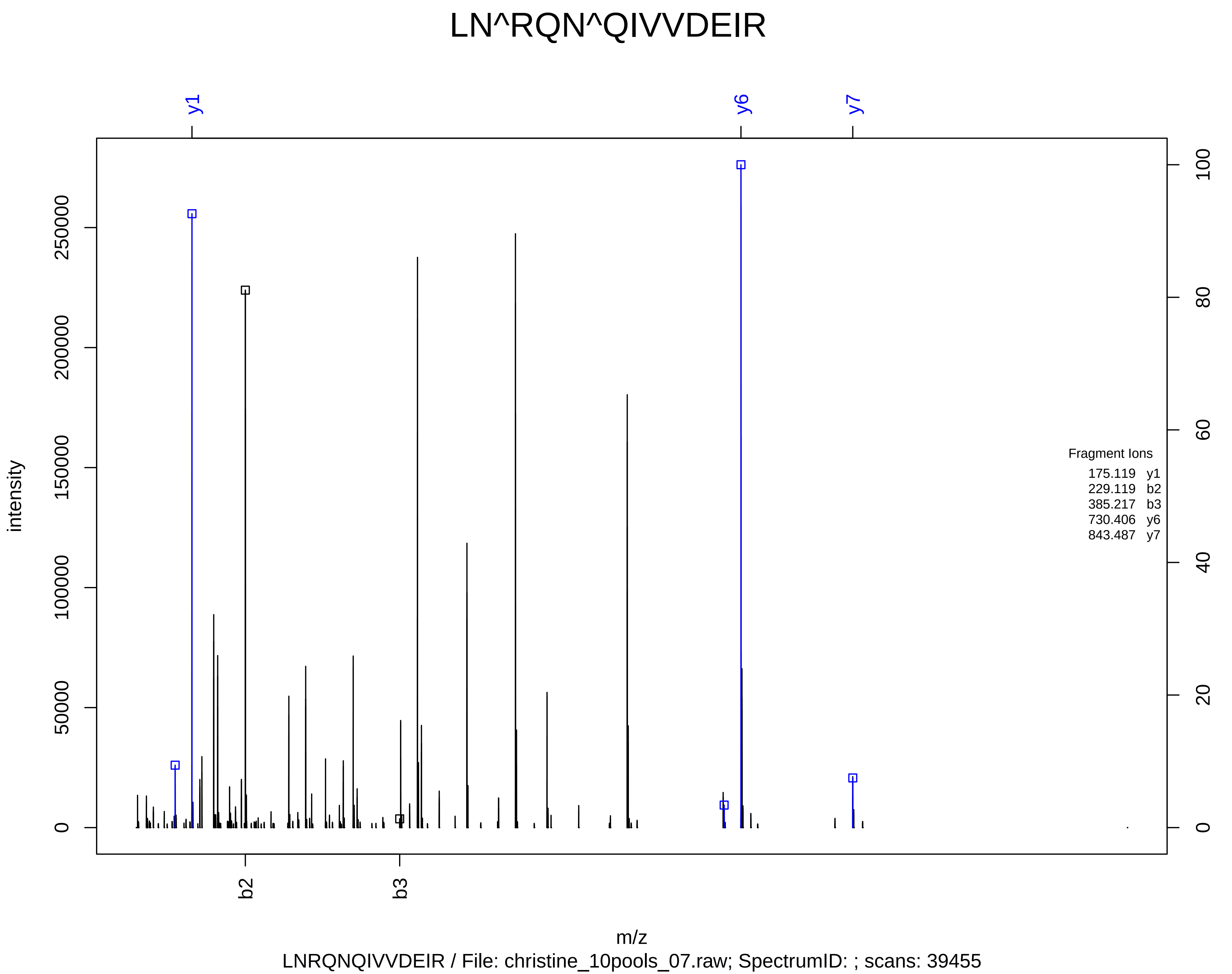

LN^RQN^QIVVDEIR
y1
y6
y7
intensity
0
50000
100000
150000
200000
250000
0
20
40
60
80
100
Fragment Ions
175.119 y1
229.119 b2
385.217 b3
730.406 y6
843.487 y7
b2
b3
m/z
LNRQNQIVVDEIR / File: christine_10pools_07.raw; SpectrumID: ; scans: 39455

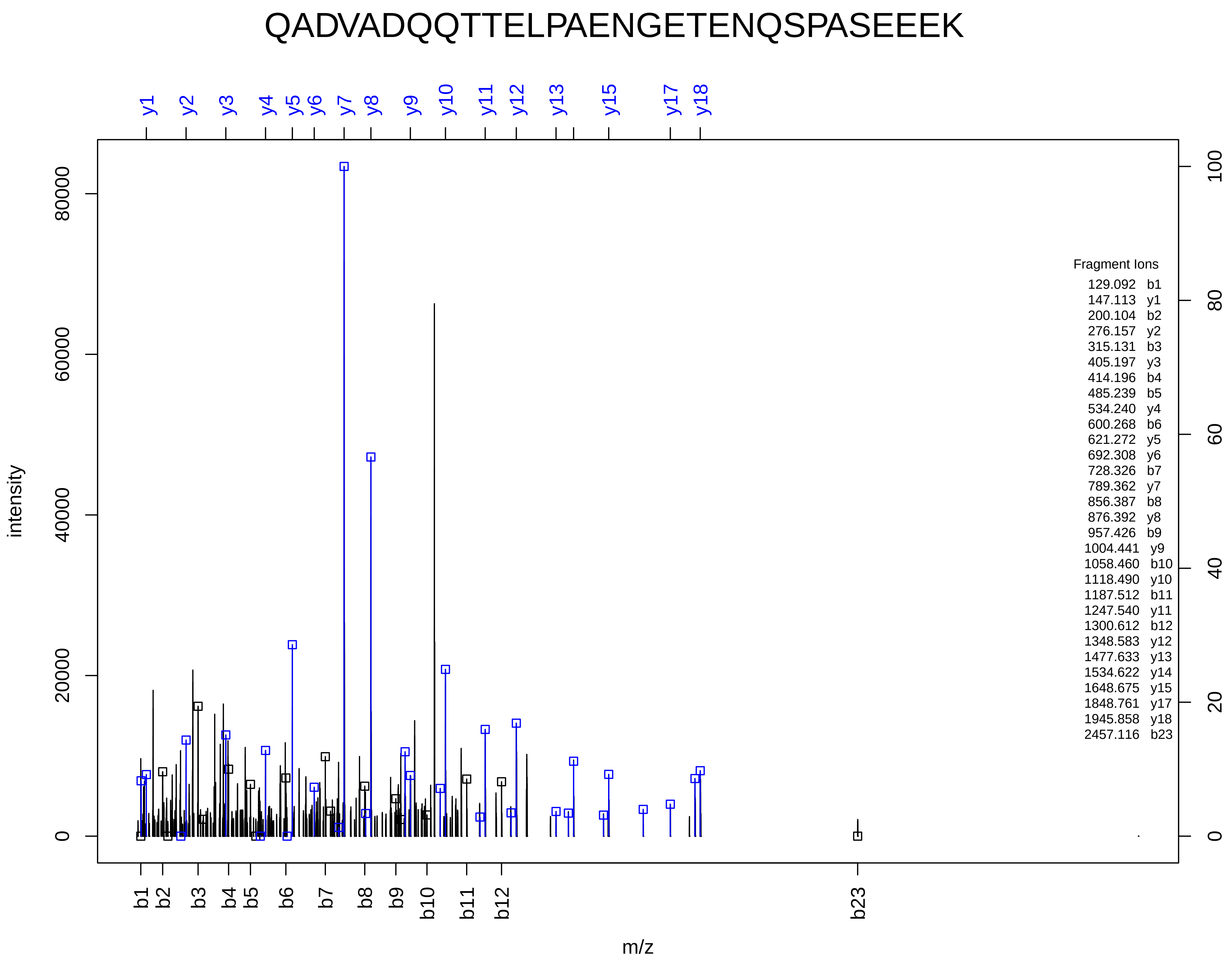

QADVADQQTTELPAENGETENQSPASEEEK / File: christine_10pools_07.raw; SpectrumID: ; scans: 6352

IGTSGGIGLEPGSVVITQQAVNECFKPEFEQIVLGK

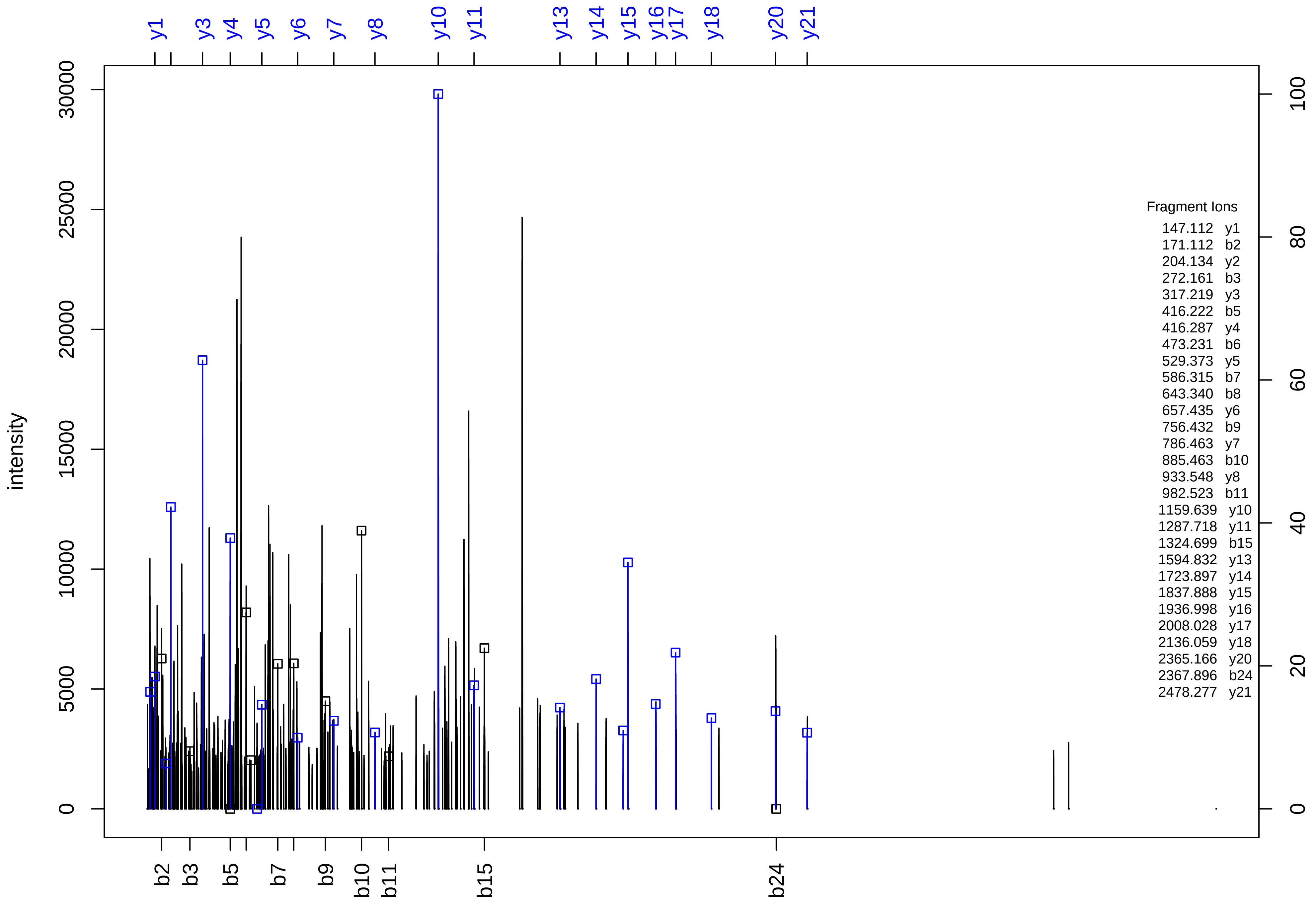

IGTSGGIGLEPGSVVITQQAVNECFKPEFEQIVLGK / File: christine_10pools_07.raw; SpectrumID: ; scans: 35185

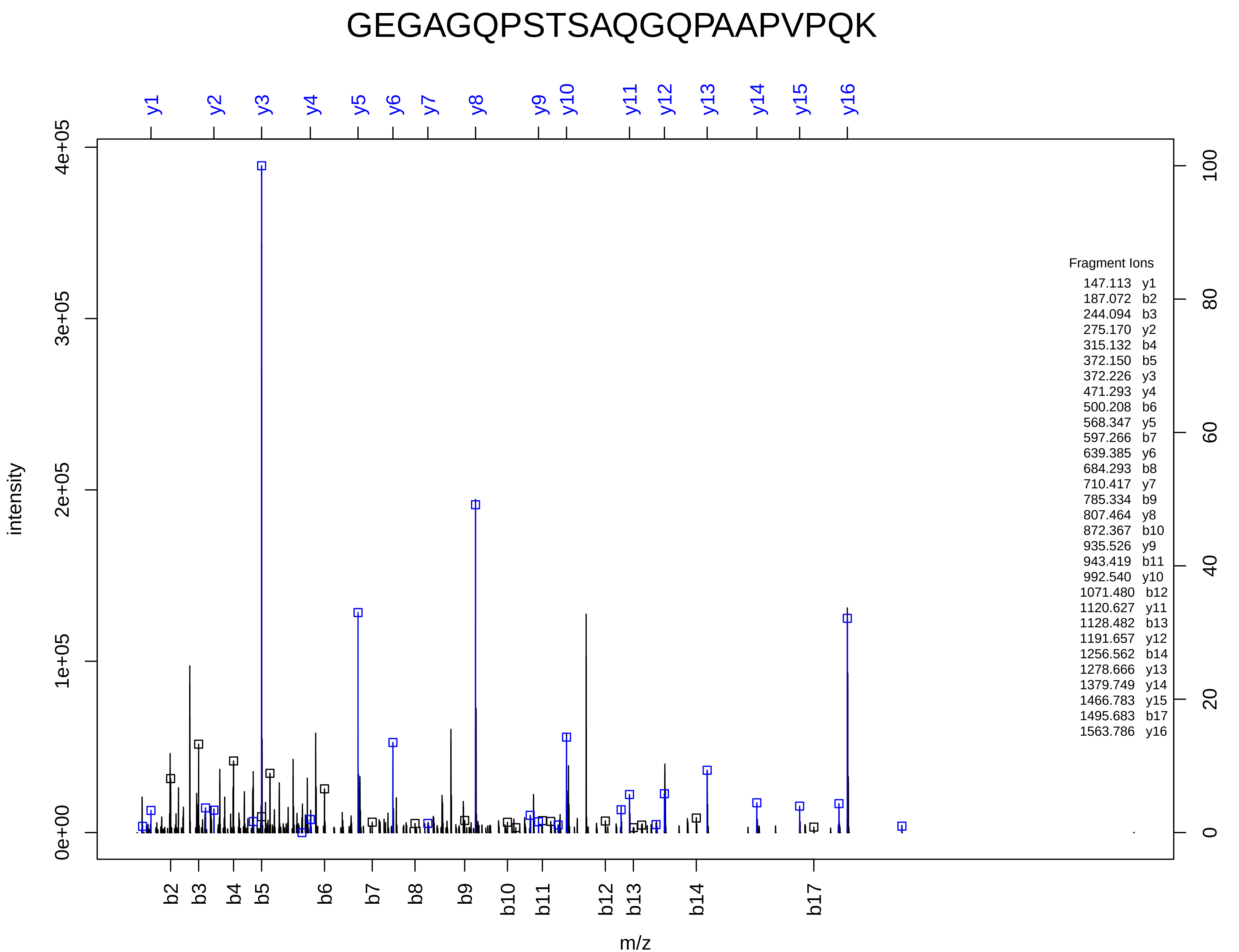

GEGAGQPSTSAQGQPAAPVPQK / File: christine_10pools_07.raw; SpectrumID: ; scans: 2067

DLFLEHAHSLADNVQPISTETTATNSDNPGQ

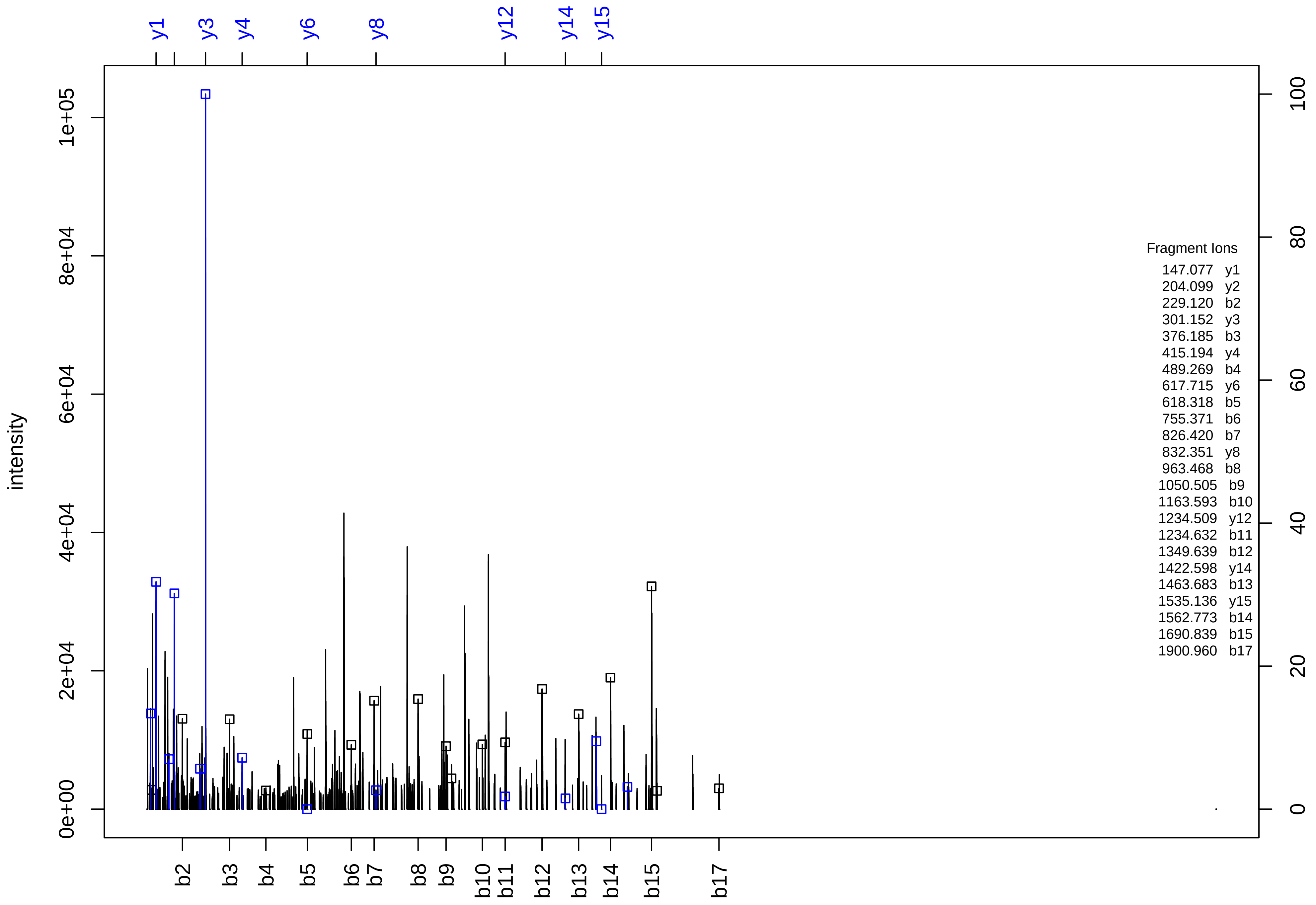

DLFLEHAHSLADNVQPISTETTATNSDNPGQ / File: christine_10pools_07.raw; SpectrumID: ; scans: 22689

N^KKIYDGGAR

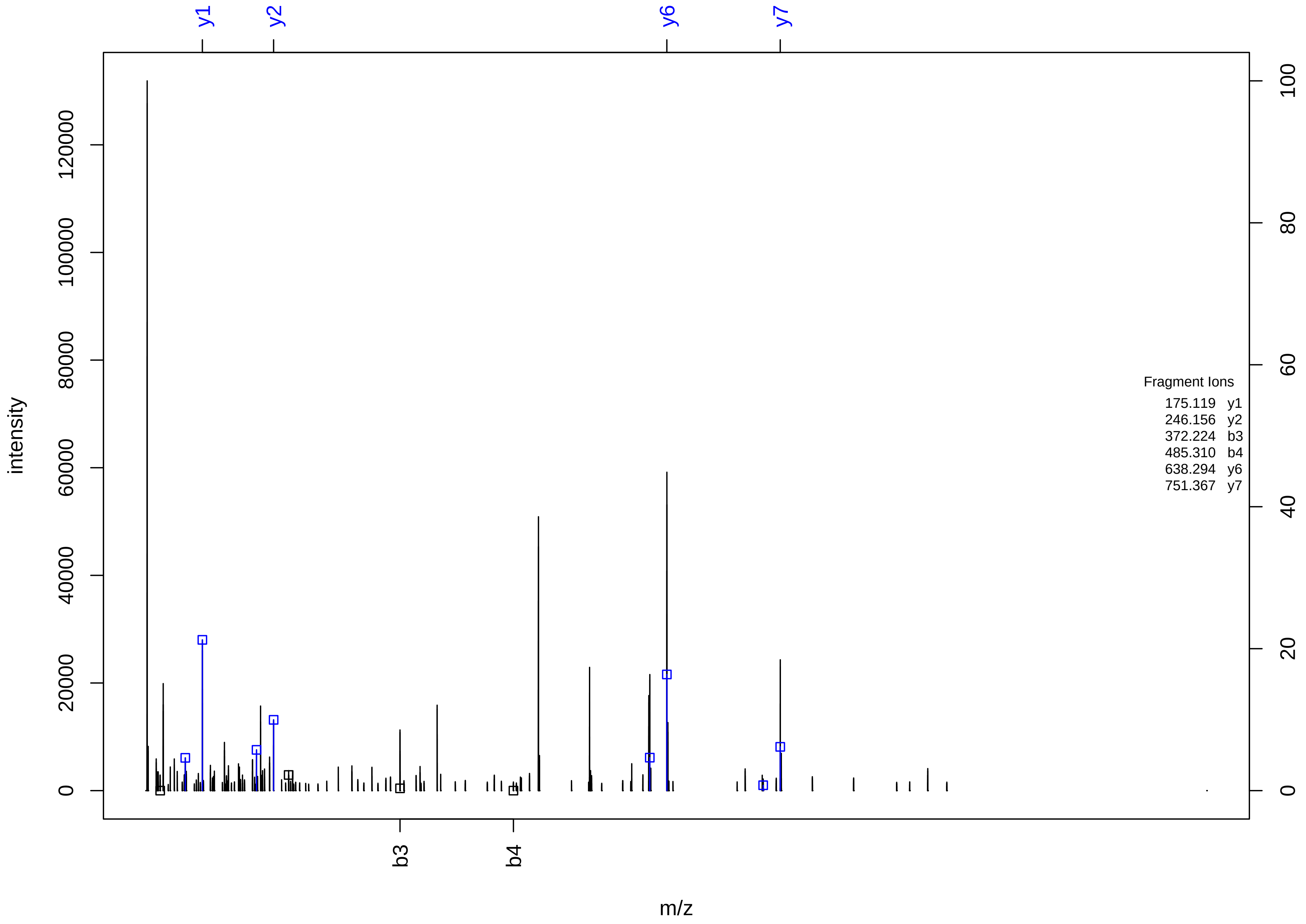

NKKIYDGGAR / File: christine_10pools_07.raw; SpectrumID: ; scans: 28315

SVLVTSVLNLEPLDEDLYR

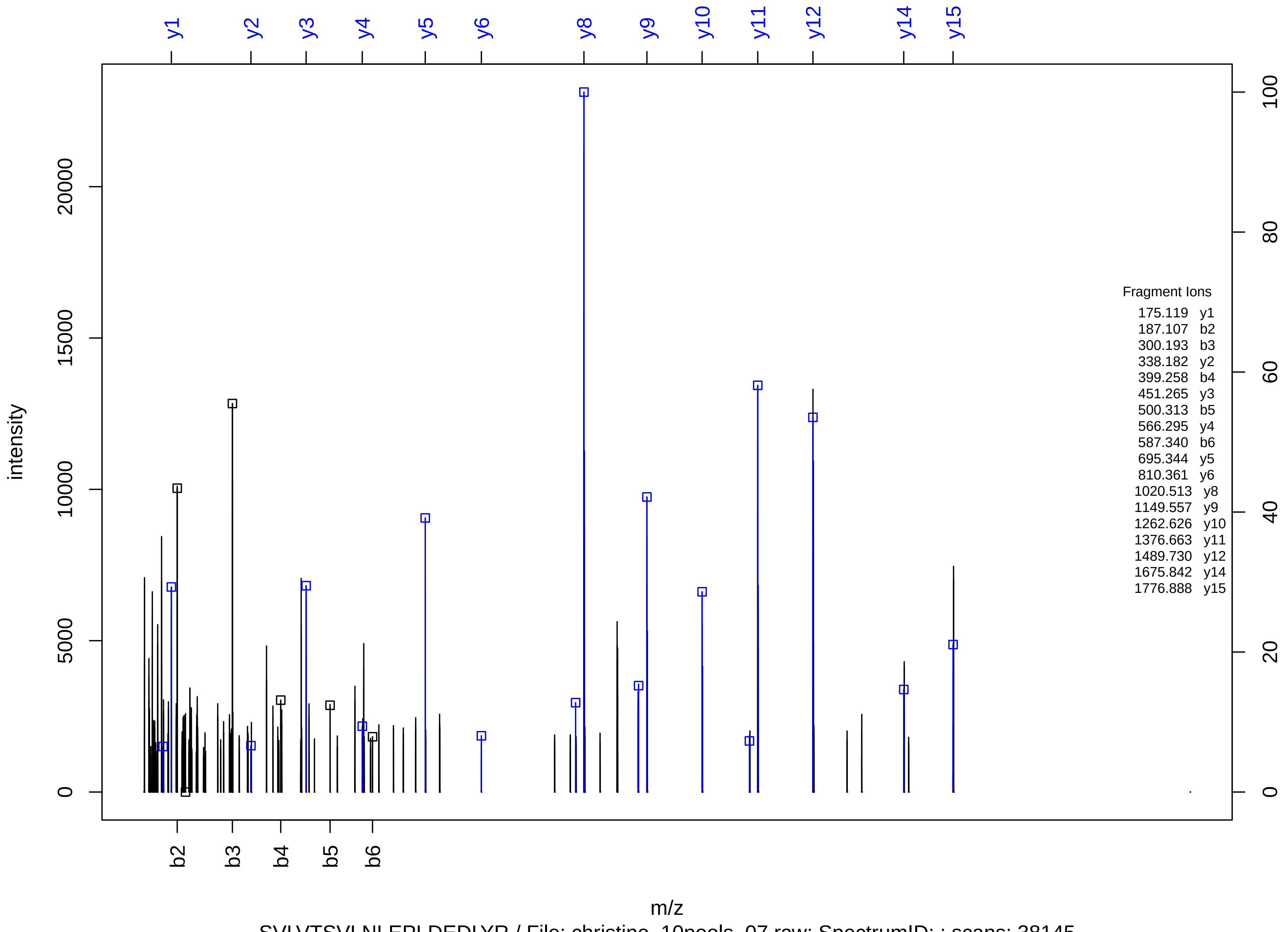

SVLVTSVLNLEPLDEDLYR / File: christine_10pools_07.raw; SpectrumID: ; scans: 38145

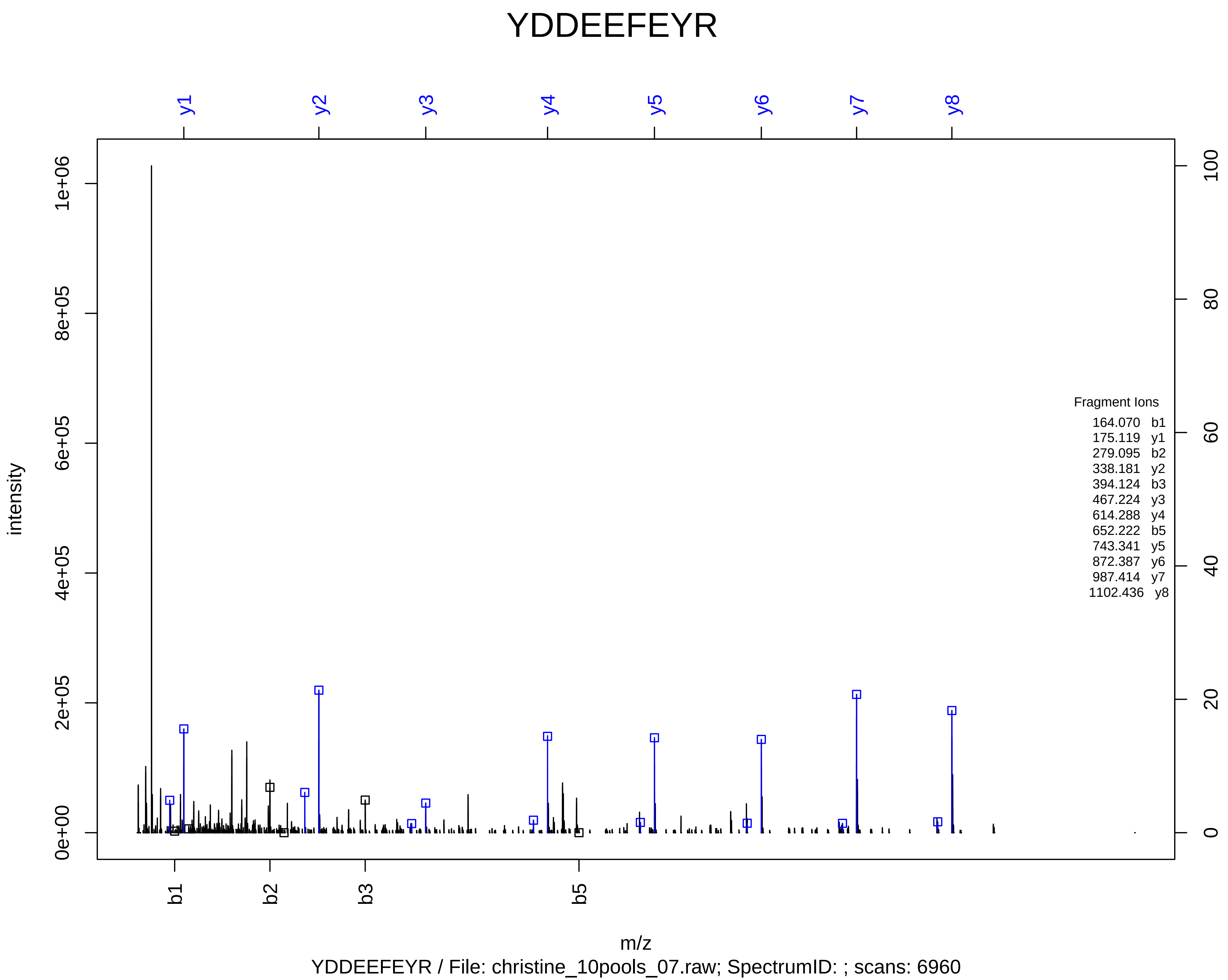

YDDEEFEYR
y1
y2
y3
y4
y5
y6
y7
y8
intensity
0e+00
2e+05
4e+05
6e+05
8e+05
1e+06
0
20
40
60
80
100
b1
b2
b3
b5
m/z
Fragment Ions
164.070 b1
175.119 y1
279.095 b2
338.181 y2
394.124 b3
467.224 y3
614.288 y4
652.222 b5
743.341 y5
872.387 y6
987.414 y7
1102.436 y8
YDDEEFEYR / File: christine_10pools_07.raw; SpectrumID: ; scans: 6960

TPISLLQEYGTR

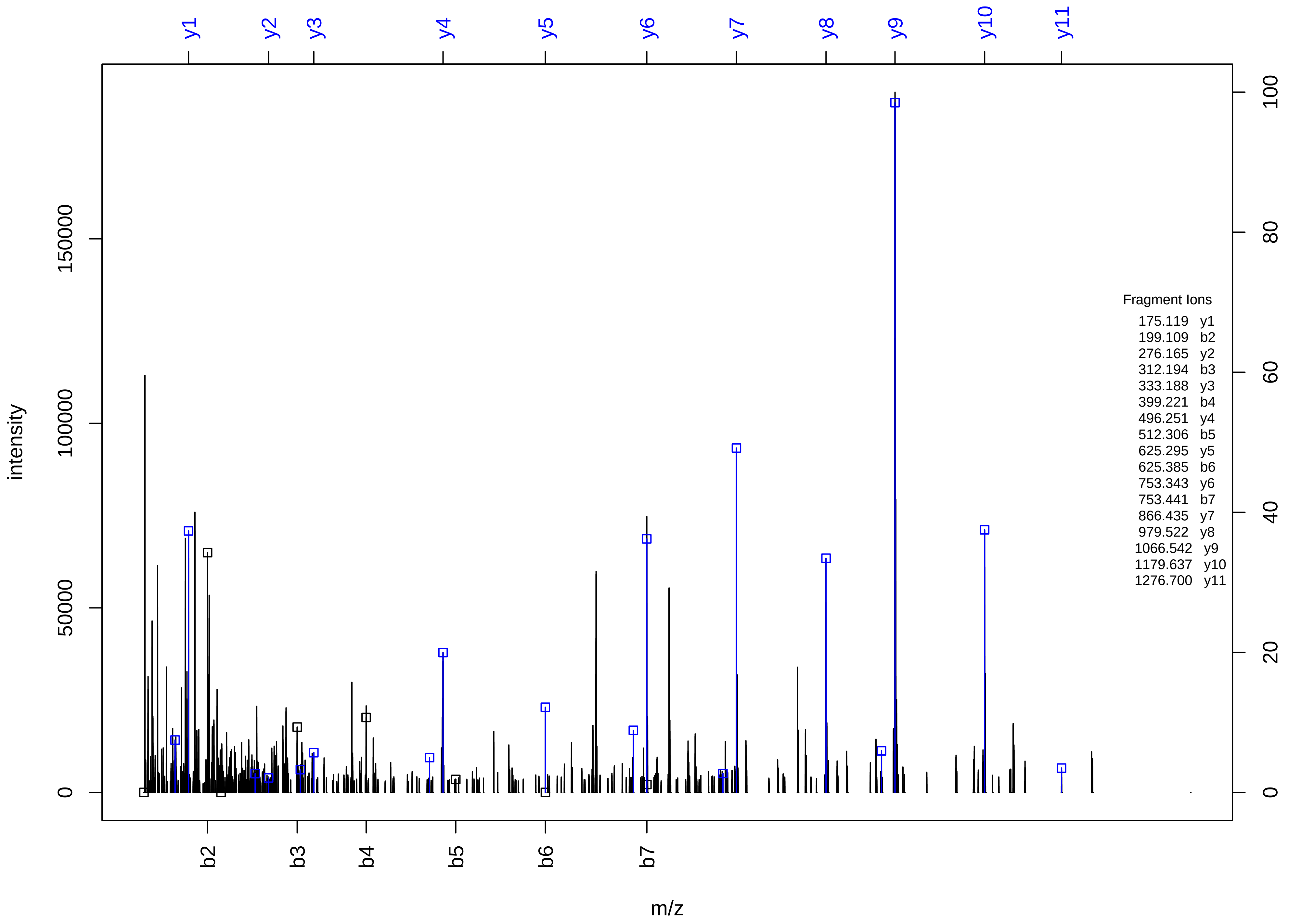

TPISLLQEYGTR / File: christine_10pools_07.raw; SpectrumID: ; scans: 19281

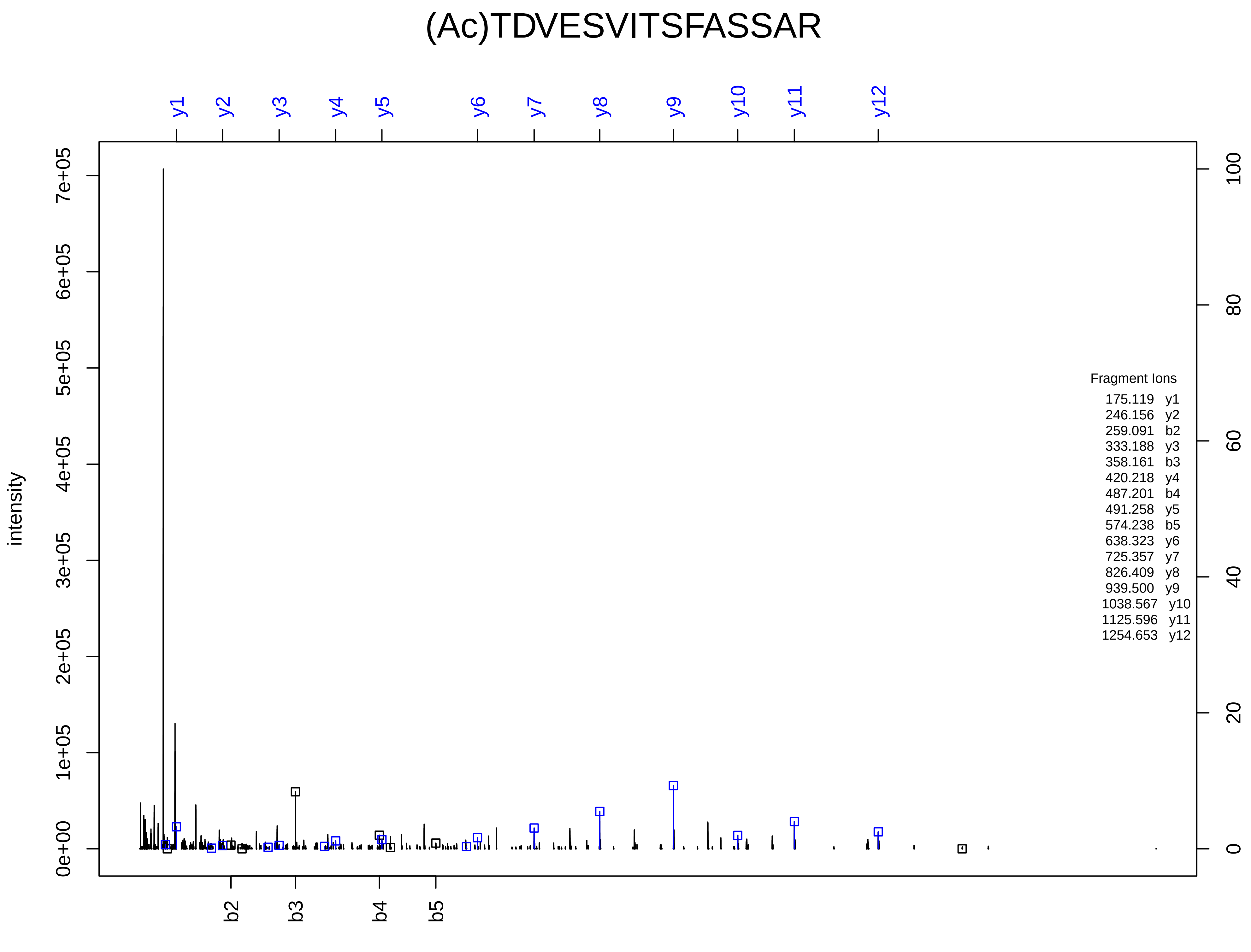

TDVESVITSFASSAR / File: christine_10pools_07.raw; SpectrumID: ; scans: 39614

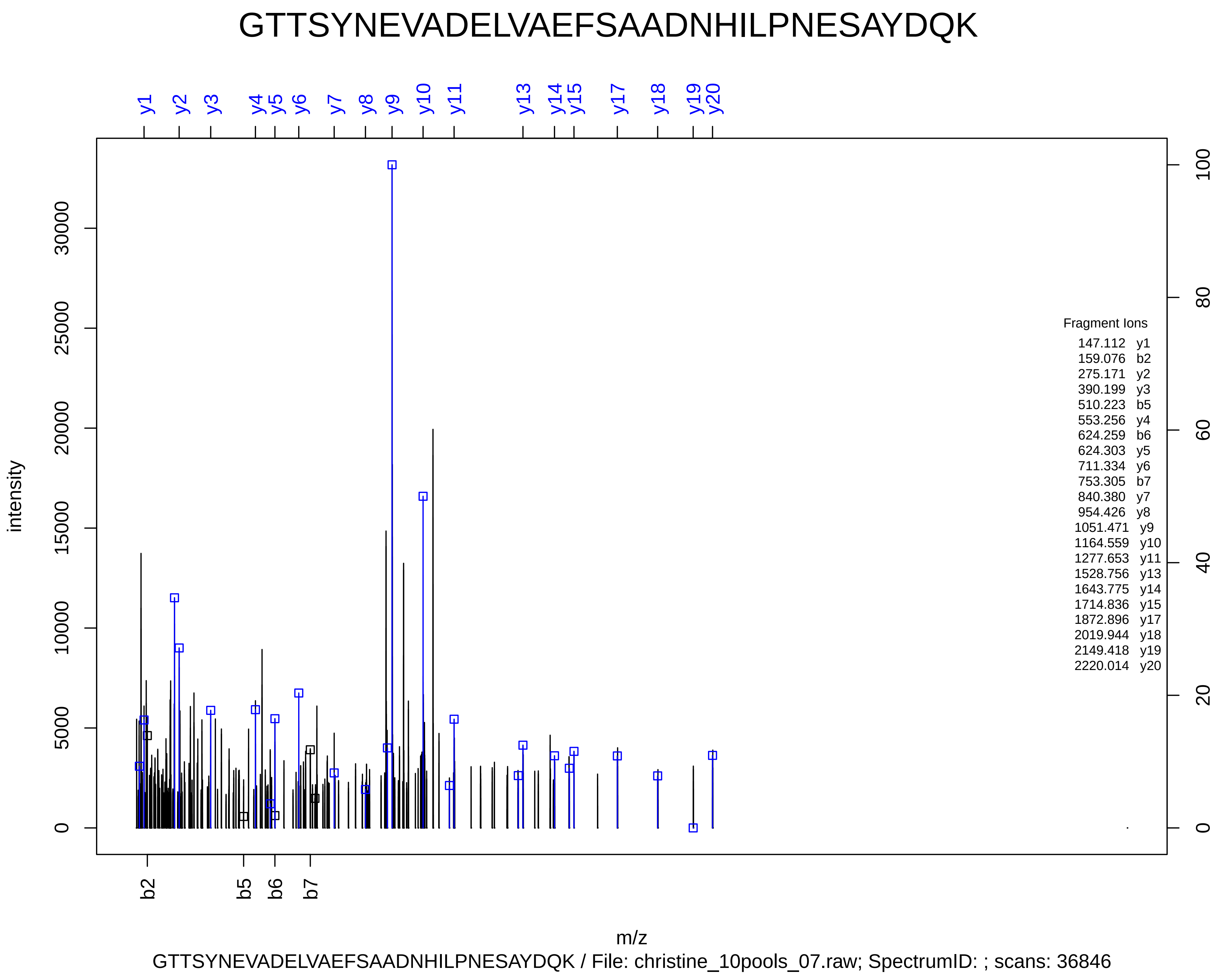
GTTSYNEVADELVAEFSAADNHILPNESAYDQK
y1 y2 y3 y4 y5 y6 y7 y8 y9 y10 y11 y13 y14 y15 y17 y18 y19 y20
b2 b5 b6 b7
intensity
m/z
Fragment Ions
147.112 y1
159.076 b2
275.171 y2
390.199 y3
510.223 b5
553.256 y4
624.259 b6
624.303 y5
711.334 y6
753.305 b7
840.380 y7
954.426 y8
1051.471 y9
1164.559 y10
1277.653 y11
1528.756 y13
1643.775 y14
1714.836 y15
1872.896 y17
2019.944 y18
2149.418 y19
2220.014 y20
GTTSYNEVADELVAEFSAADNHILPNESAYDQK / File: christine_10pools_07.raw; SpectrumID: ; scans: 36846

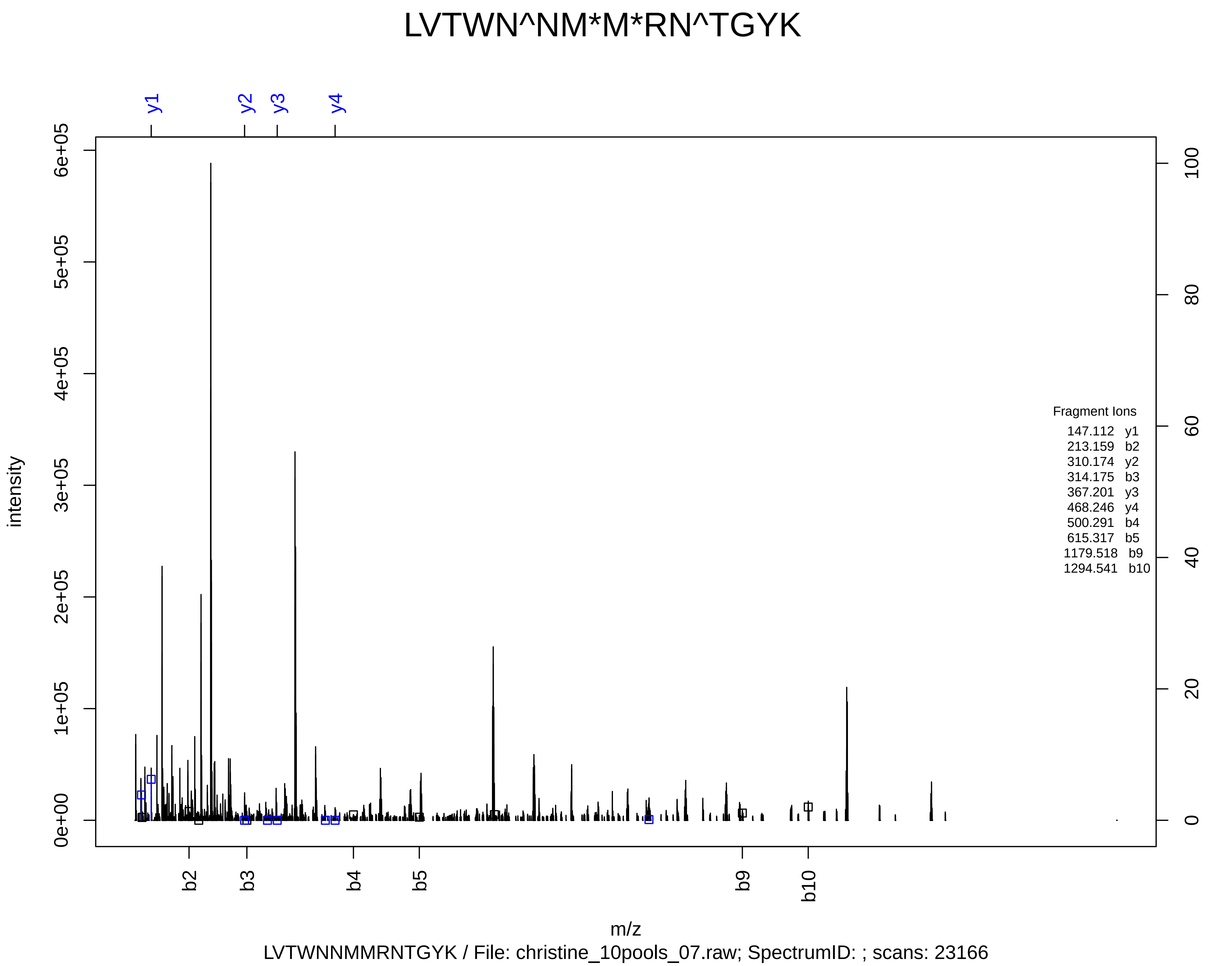

LVTWN^NM*M*RN^TGYK
y1
y2
y3
y4
intensity
0e+00
1e+05
2e+05
3e+05
4e+05
5e+05
6e+05
0
20
40
60
80
100
Fragment Ions
147.112 y1
213.159 b2
310.174 y2
314.175 b3
367.201 y3
468.246 y4
500.291 b4
615.317 b5
1179.518 b9
1294.541 b10
b2
b3
b4
b5
b9
b10
m/z
LVTWNNMMRNTGYK / File: christine_10pools_07.raw; SpectrumID: ; scans: 23166

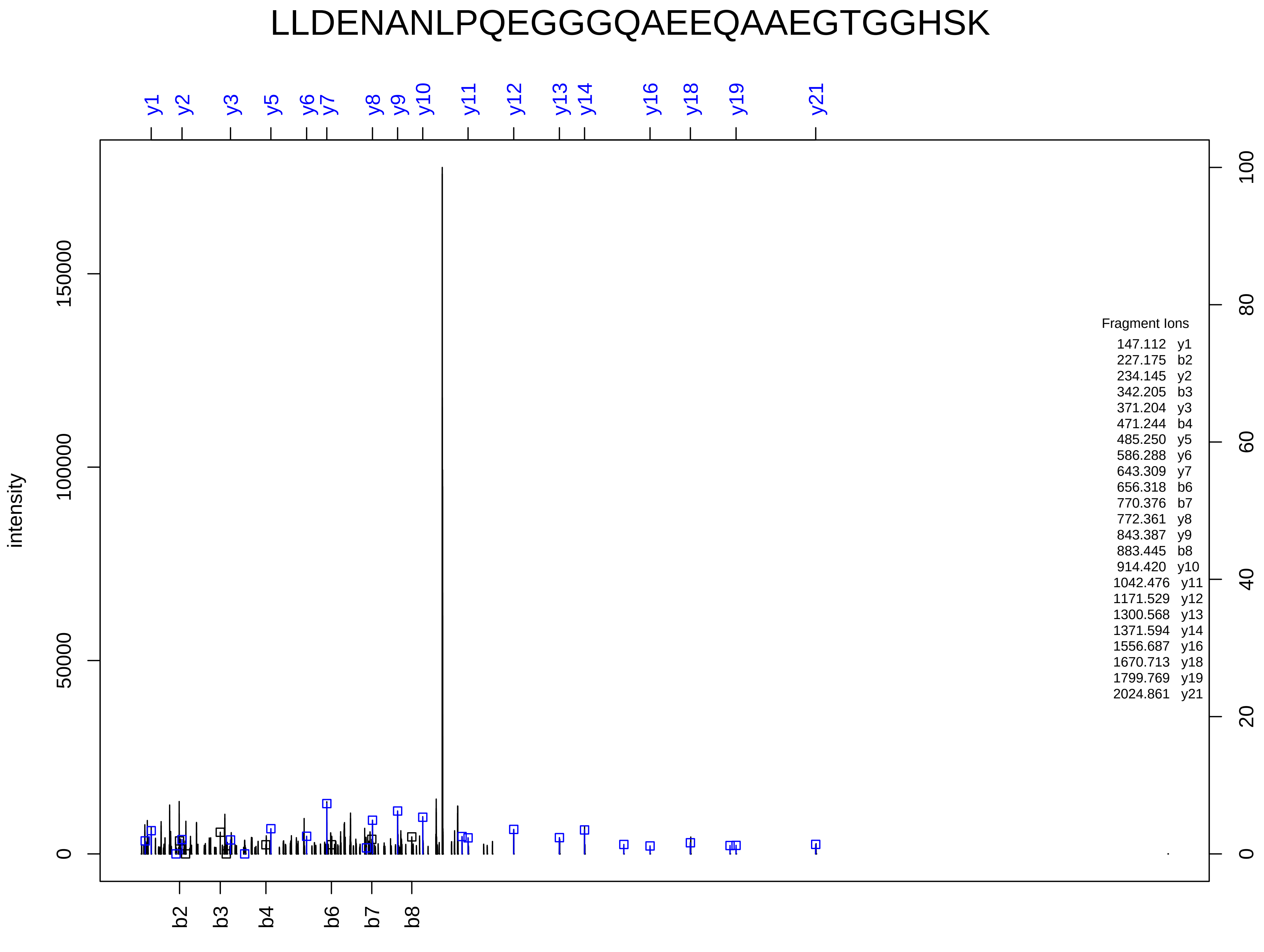

LLDENANLPQEGGGQAEEQAAEGTGGHSK / File: christine_10pools_07.raw; SpectrumID: ; scans: 6696

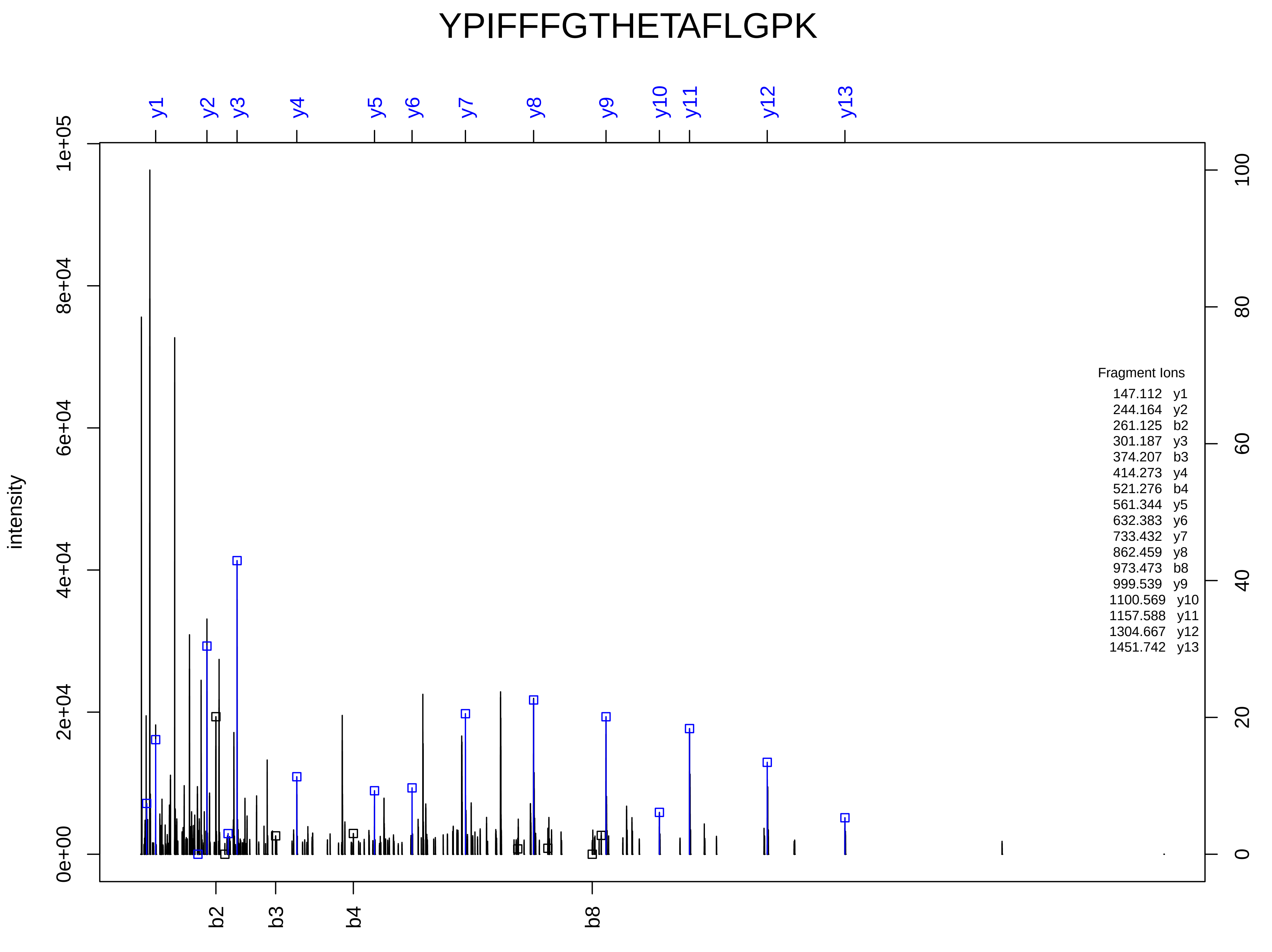

YPIFFFGTHETAFLGPK / File: christine_10pools_07.raw; SpectrumID: ; scans: 29329

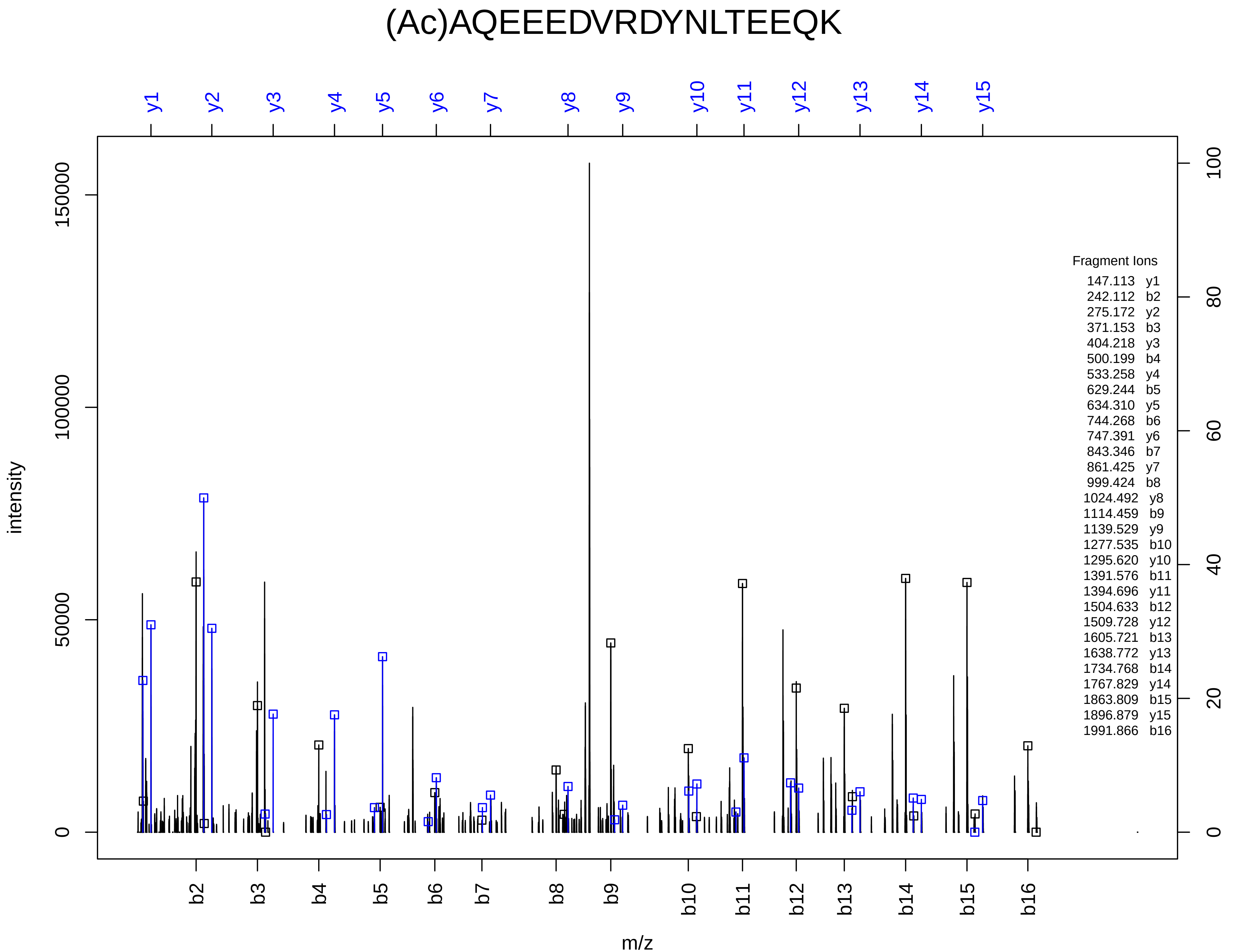

AQEEEDVRDYNLTEEQK / File: christine_10pools_07.raw; SpectrumID: ; scans: 10002

NGQLIYTPFTEDTPSVGQR

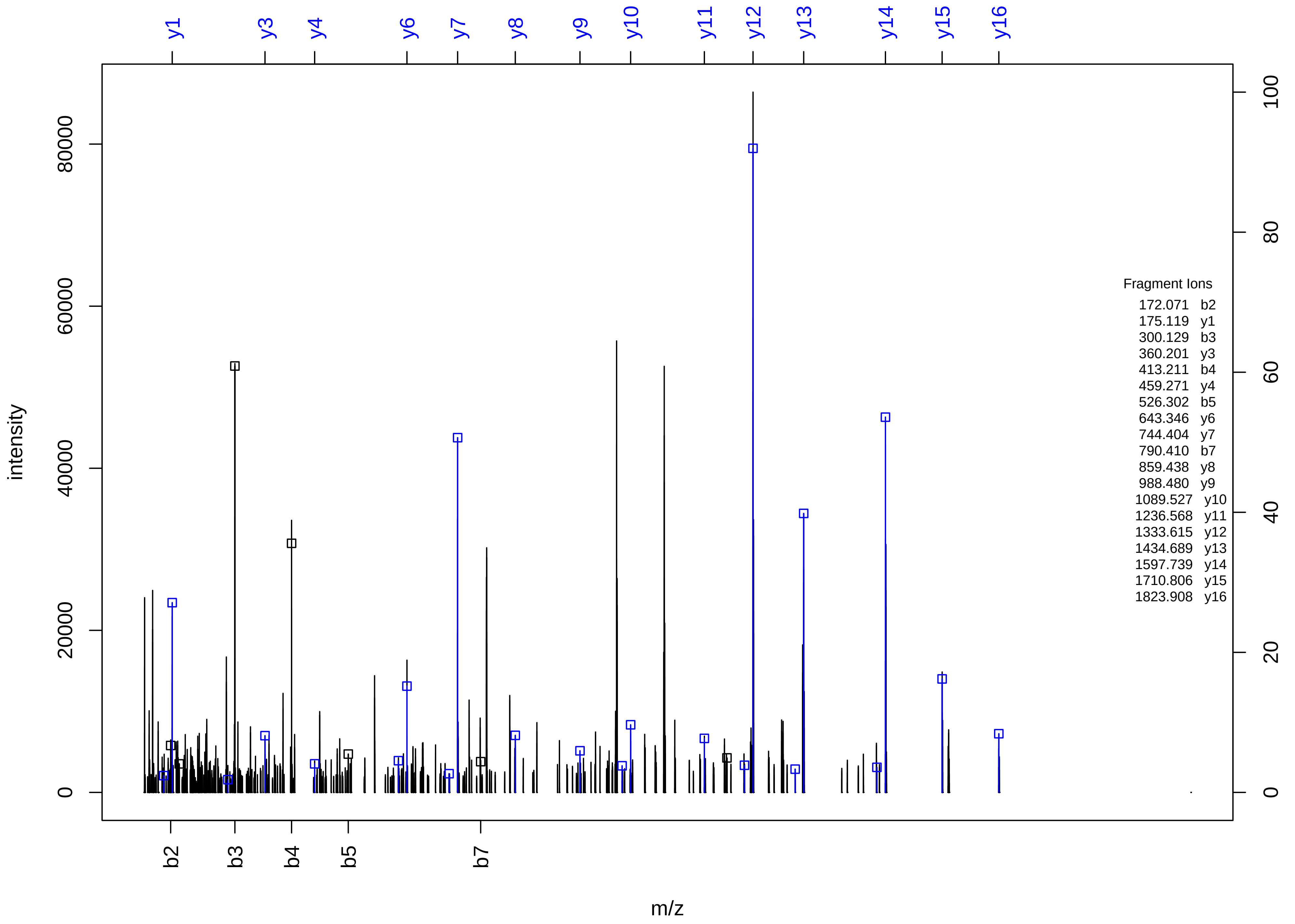

NGQLIYTPFTEDTPSVGQR / File: christine_10pools_07.raw; SpectrumID: ; scans: 22243

GPGTSAEFSEFPLVNVNDNR

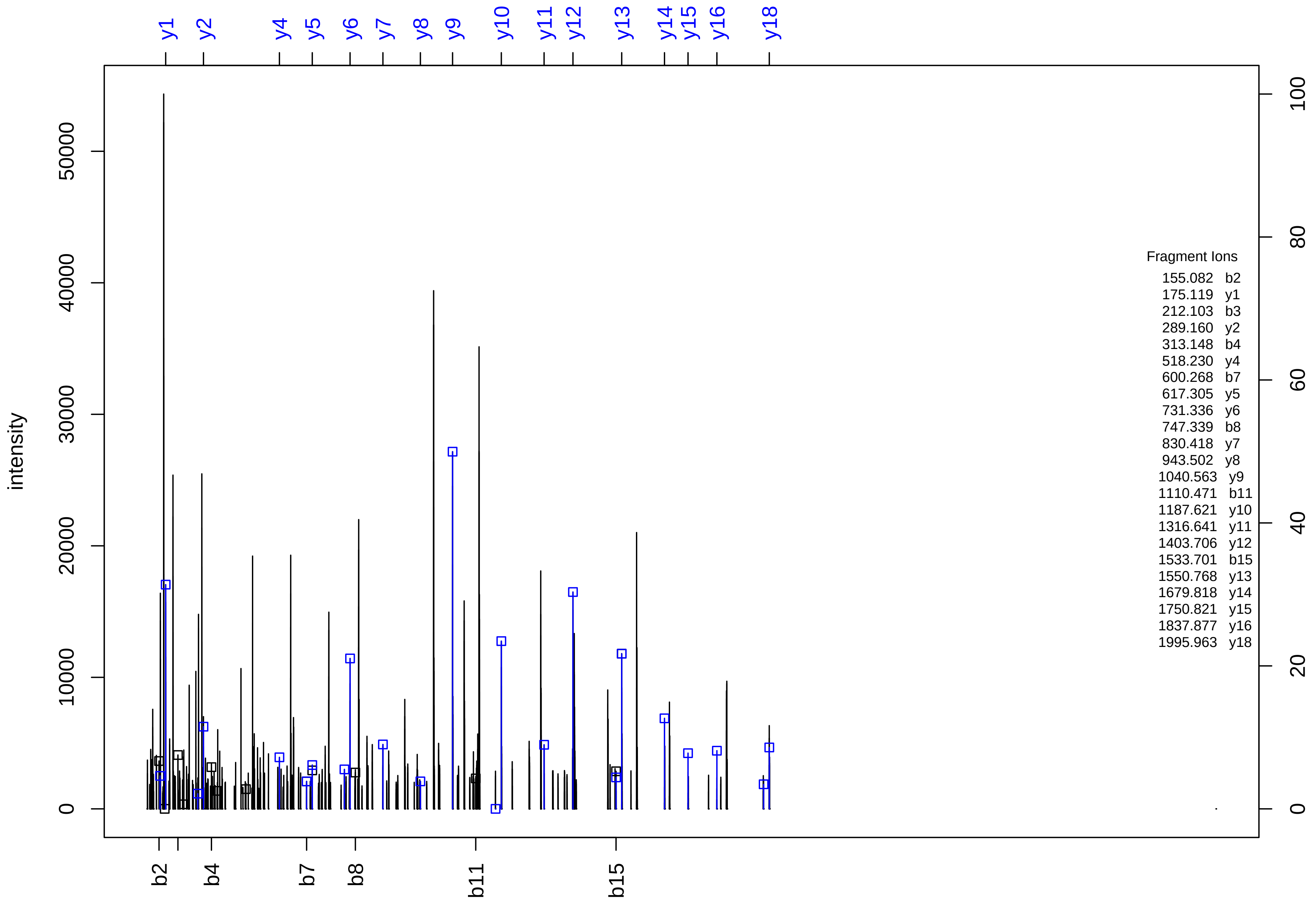

GPGTSAEFSEFPLVNVNDNR / File: christine_10pools_07.raw; SpectrumID: ; scans: 24536

IWRN^YLN^M*N^KYK

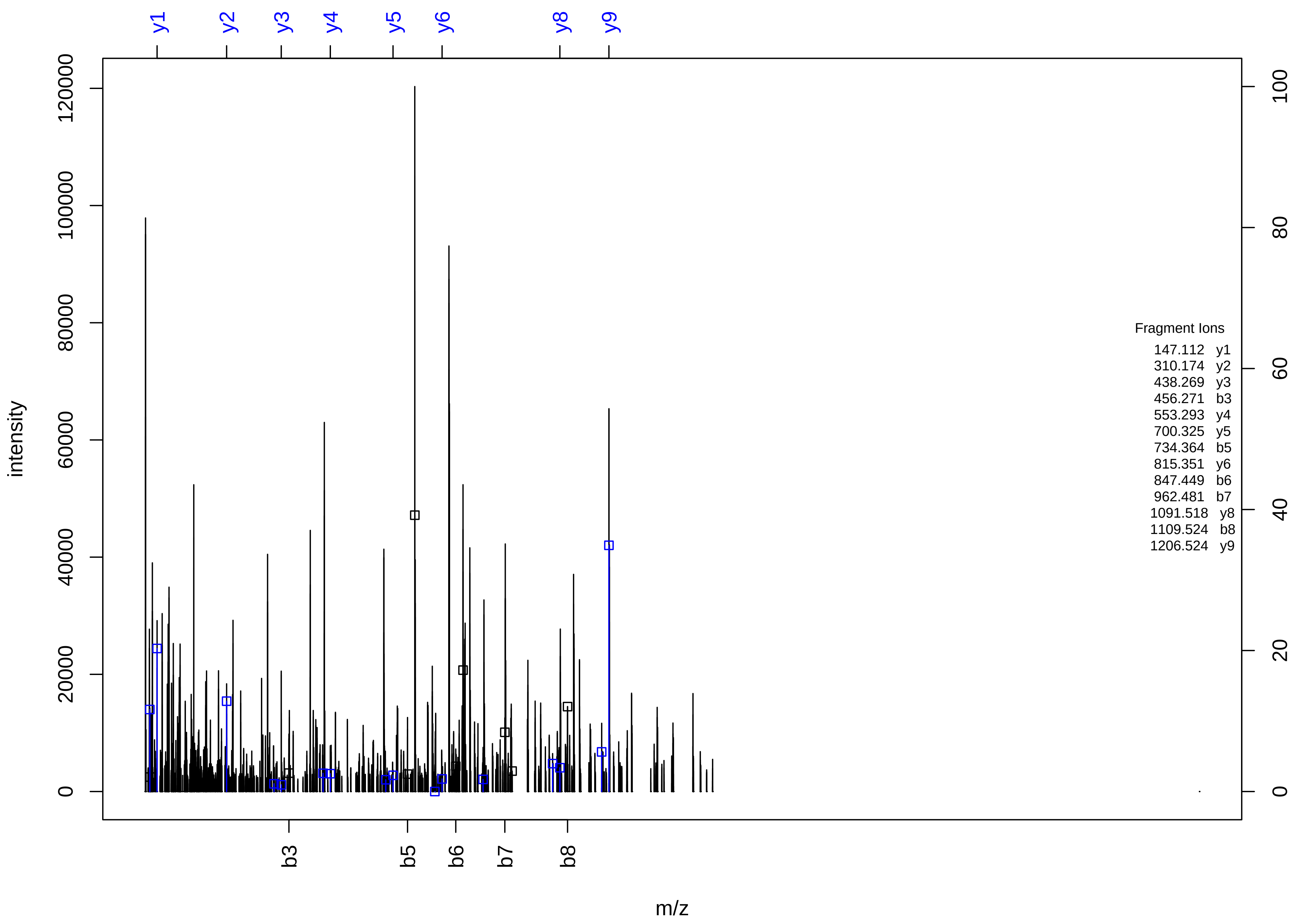

IWRNYLNMNKYK / File: christine_10pools_07.raw; SpectrumID: ; scans: 15844

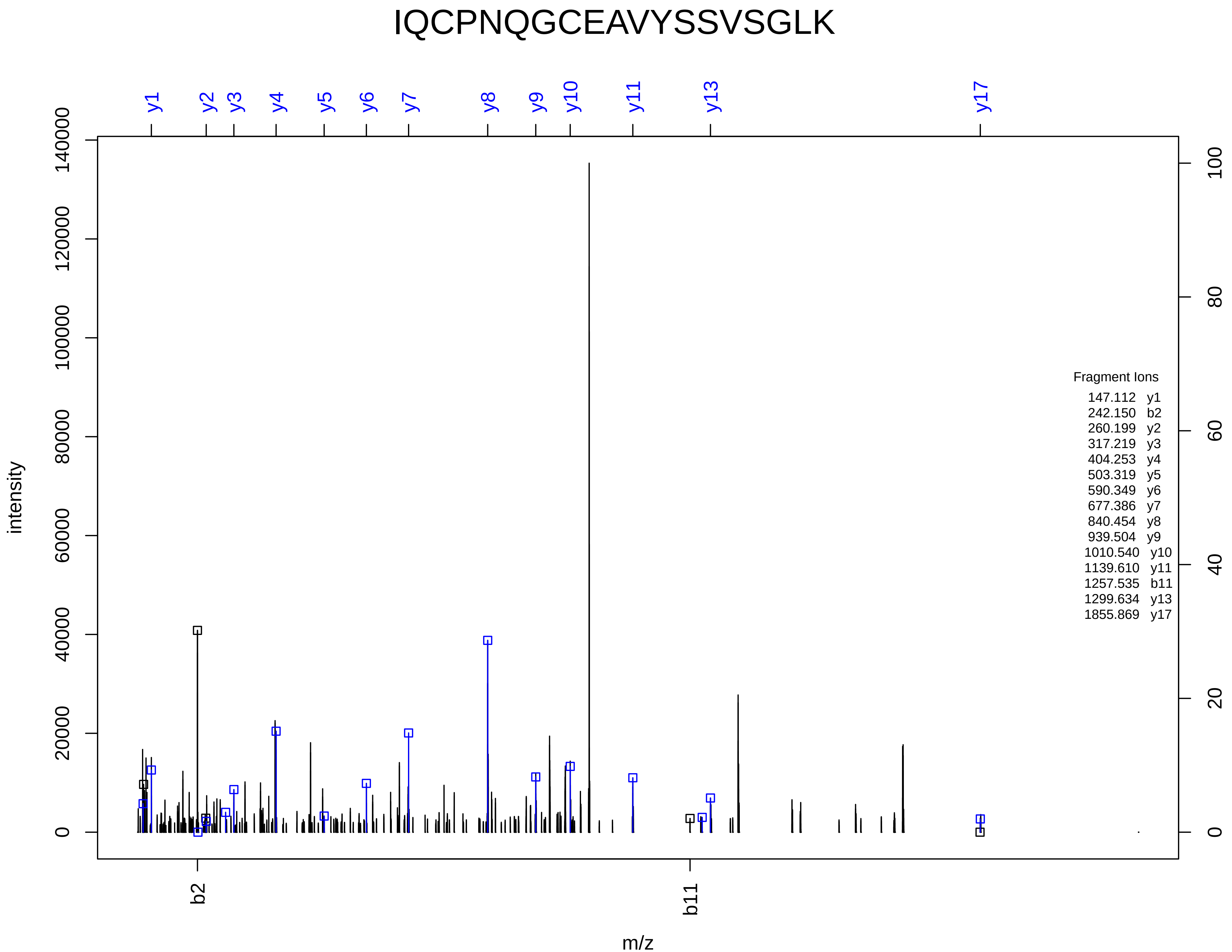

IQCPNQGCEAVYSSVSGLK / File: christine_10pools_07.raw; SpectrumID: ; scans: 12309

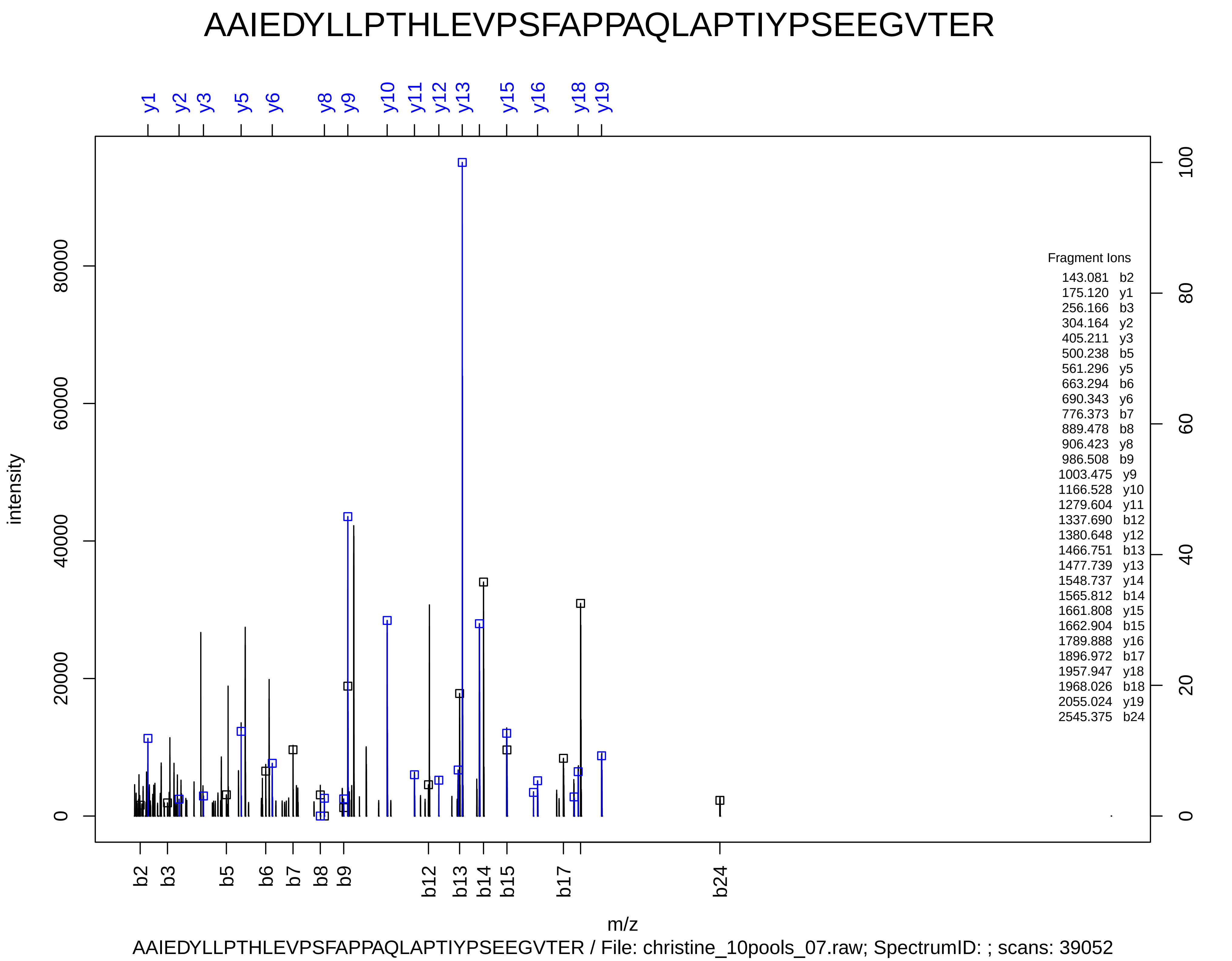
AAIEDYLLPTHLEVPSFAPPAQLAPTIYPSEEGVTER
y1 y2 y3 y5 y6 y8 y9 y10 y11 y12 y13 y15 y16 y18 y19
intensity
0 20000 40000 60000 80000
0 20 40 60 80 100
b2 b3 b5 b6 b7 b8 b9 b12 b13 b14 b15 b17 b24
m/z
Fragment Ions
143.081 b2
175.120 y1
256.166 b3
304.164 y2
405.211 y3
500.238 b5
561.296 y5
663.294 b6
690.343 y6
776.373 b7
889.478 b8
906.423 y8
986.508 b9
1003.475 y9
1166.528 y10
1279.604 y11
1337.690 b12
1380.648 y12
1466.751 b13
1477.739 y13
1548.737 y14
1565.812 b14
1661.808 y15
1662.904 b15
1789.888 y16
1896.972 b17
1957.947 y18
1968.026 b18
2055.024 y19
2545.375 b24
AAIEDYLLPTHLEVPSFAPPAQLAPTIYPSEEGVTER / File: christine_10pools_07.raw; SpectrumID: ; scans: 39052

QMCDGVLN^N^PTVM*ERLQK

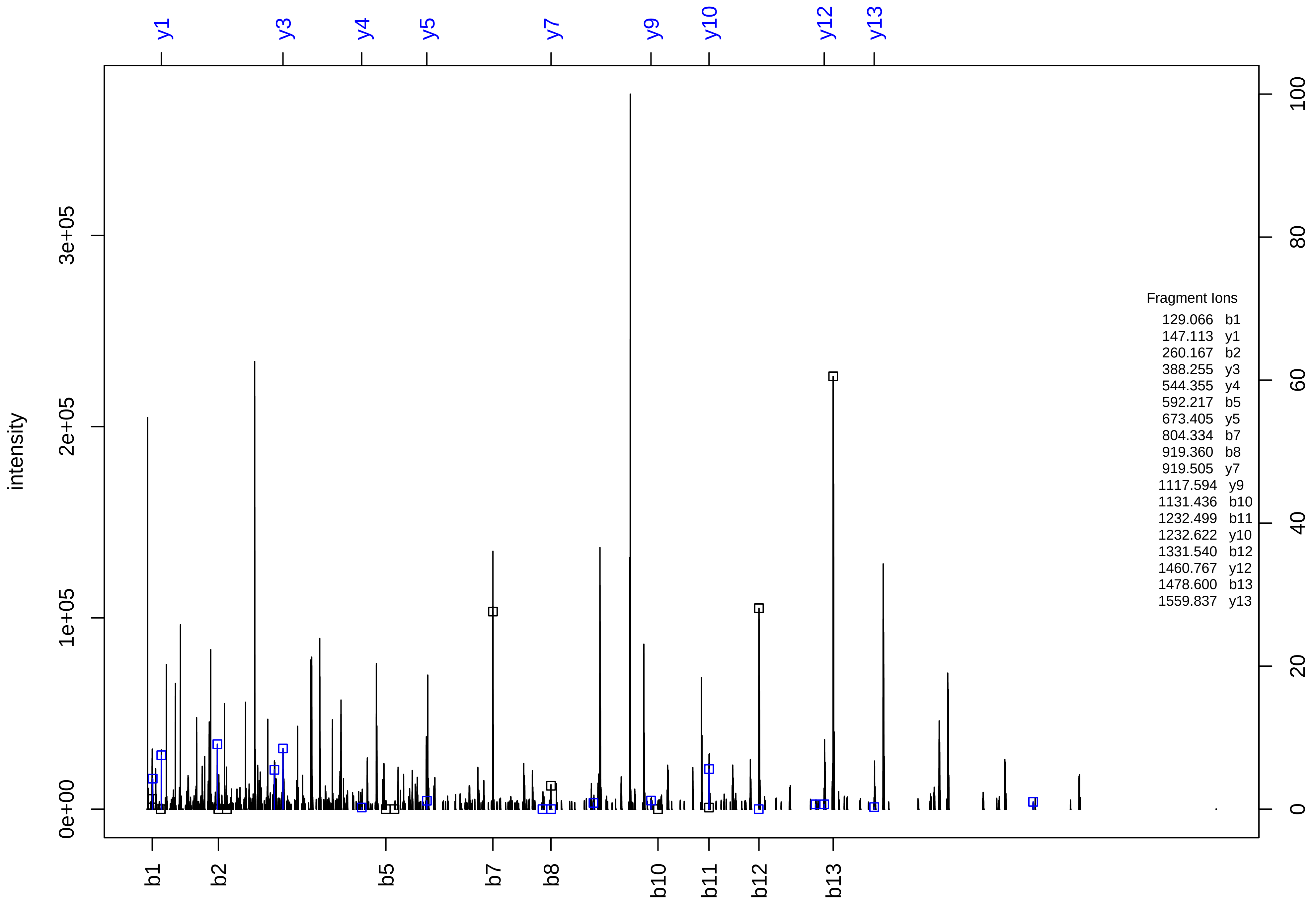

QMCDGVLNNPTVMERLQK / File: christine_10pools_07.raw; SpectrumID: ; scans: 24158

LIDFGLAR

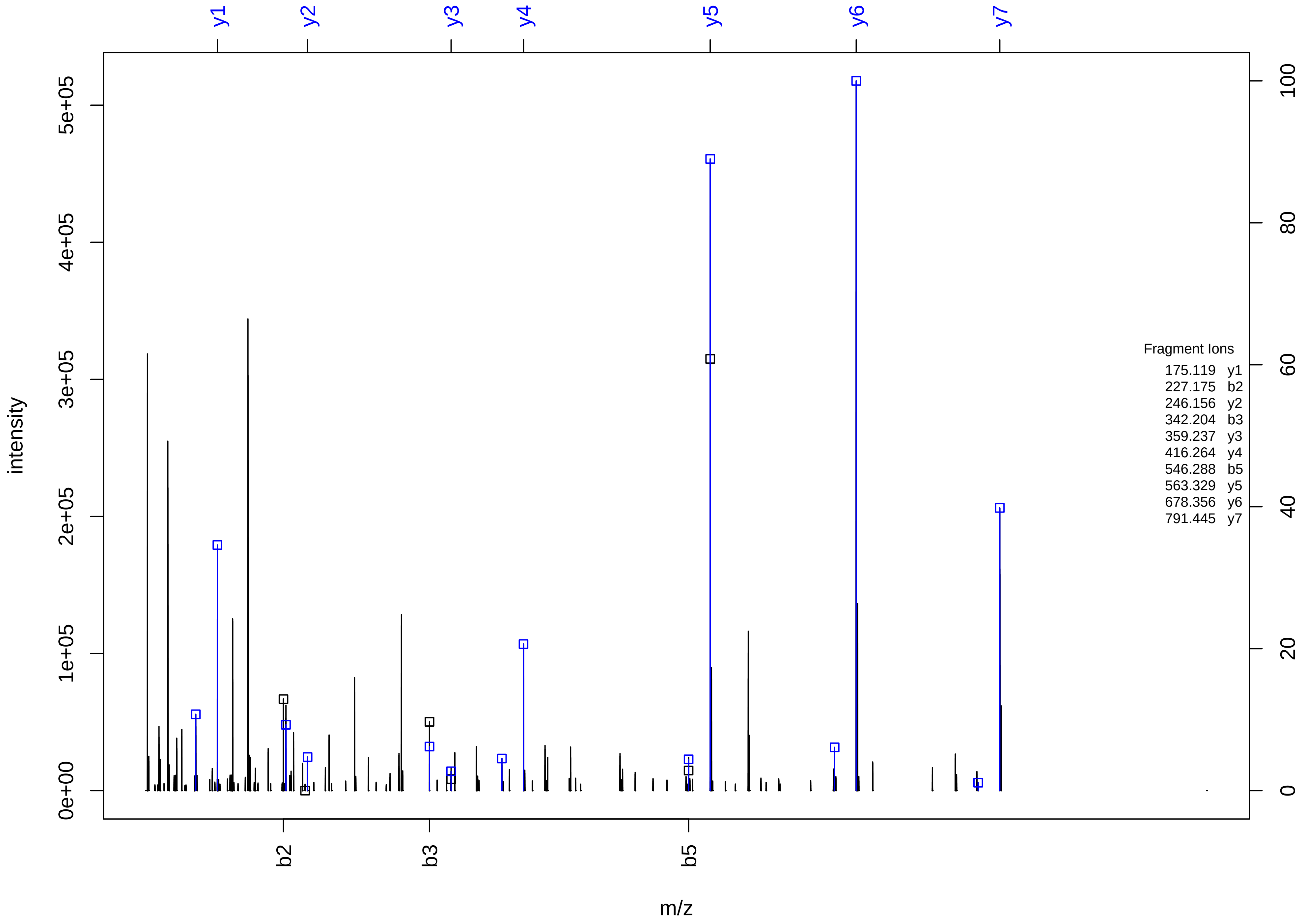

LIDFGLAR / File: christine_10pools_07.raw; SpectrumID: ; scans: 18858

YNEQLAQEQALTEHANATK

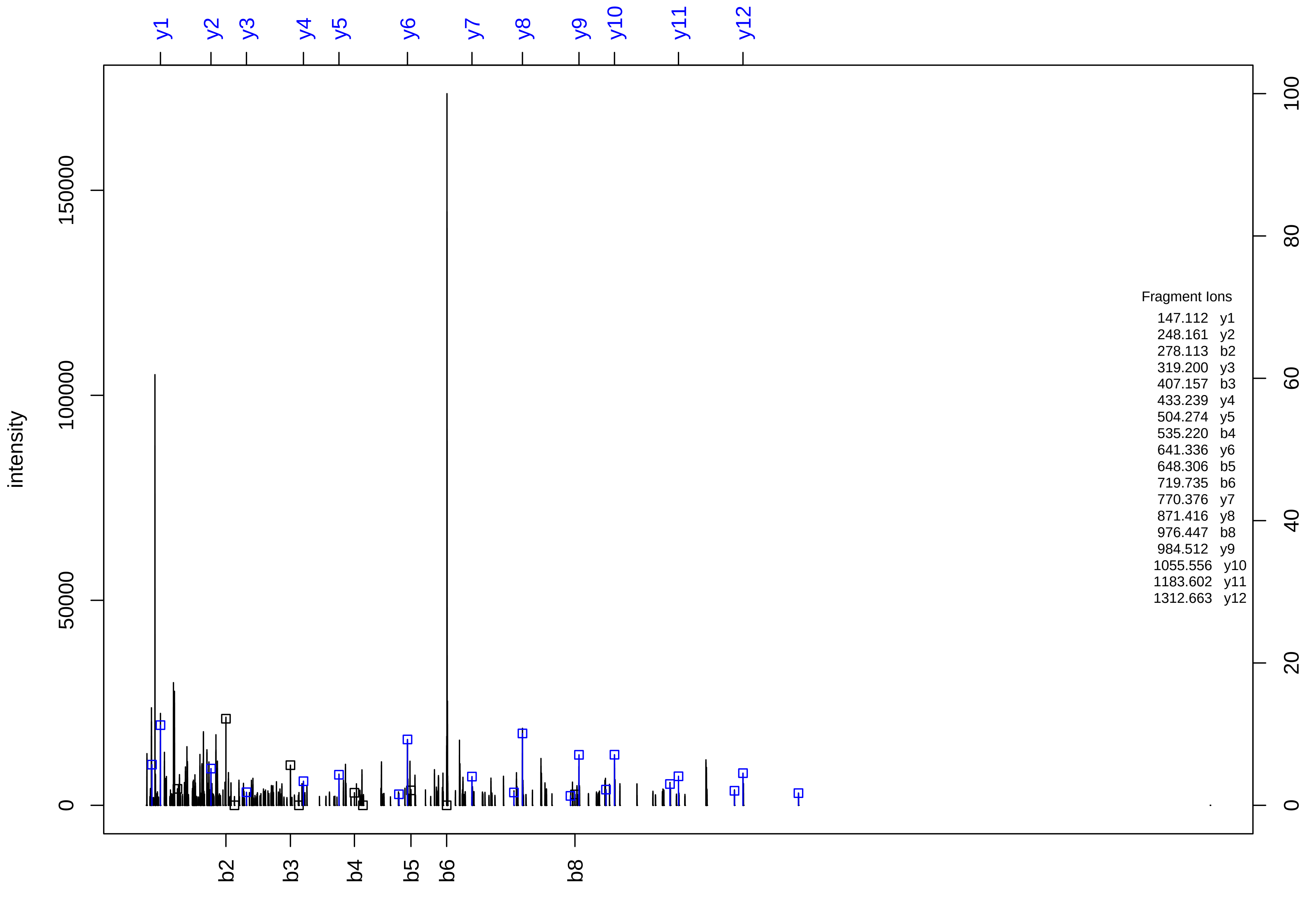

YNEQLAQEQALTEHANATK / File: christine_10pools_07.raw; SpectrumID: ; scans: 4492

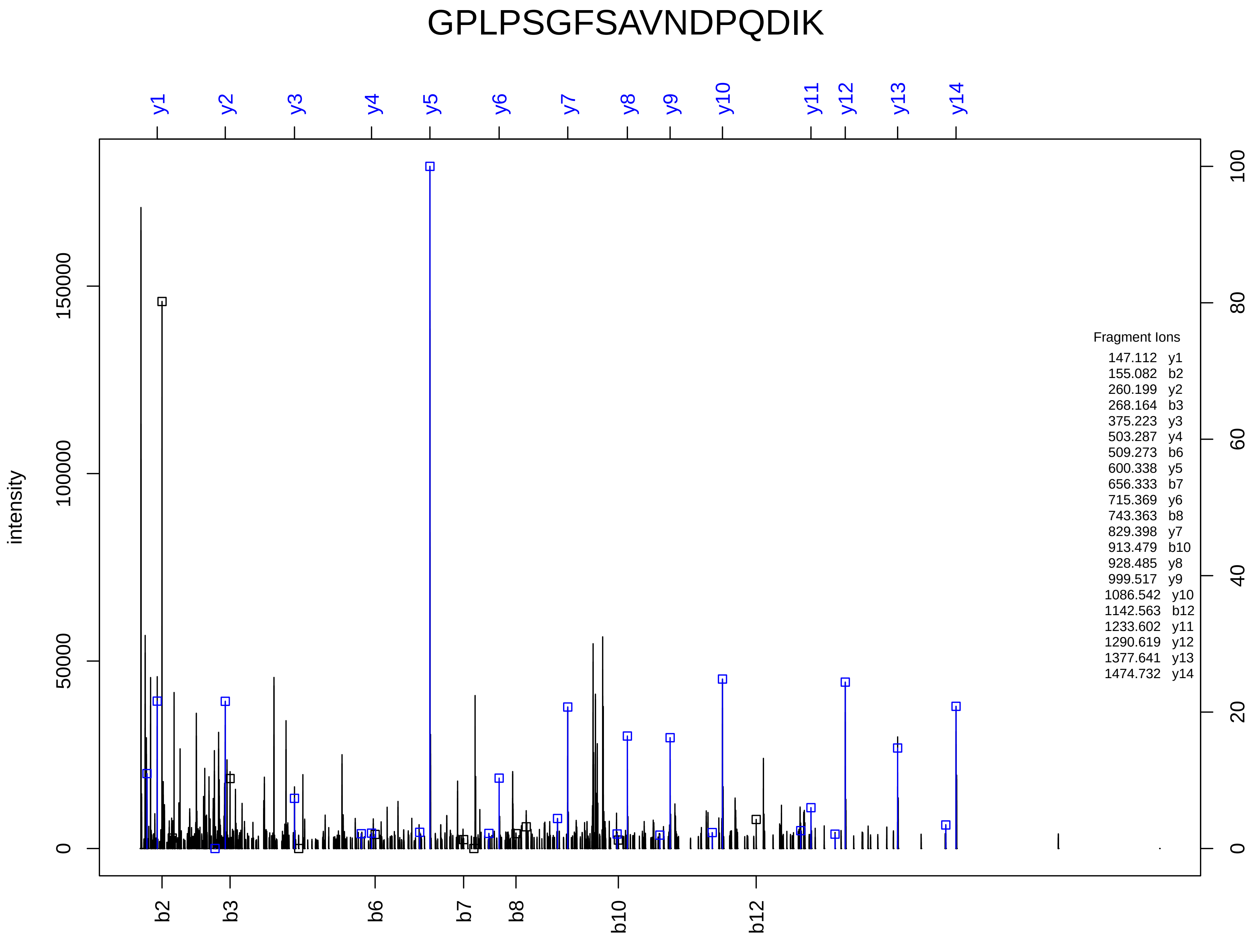

GPLPSGFSAVNDPQDIK / File: christine_10pools_07.raw; SpectrumID: ; scans: 16656

LGATIPLSVDEGPELQGPVIDRR

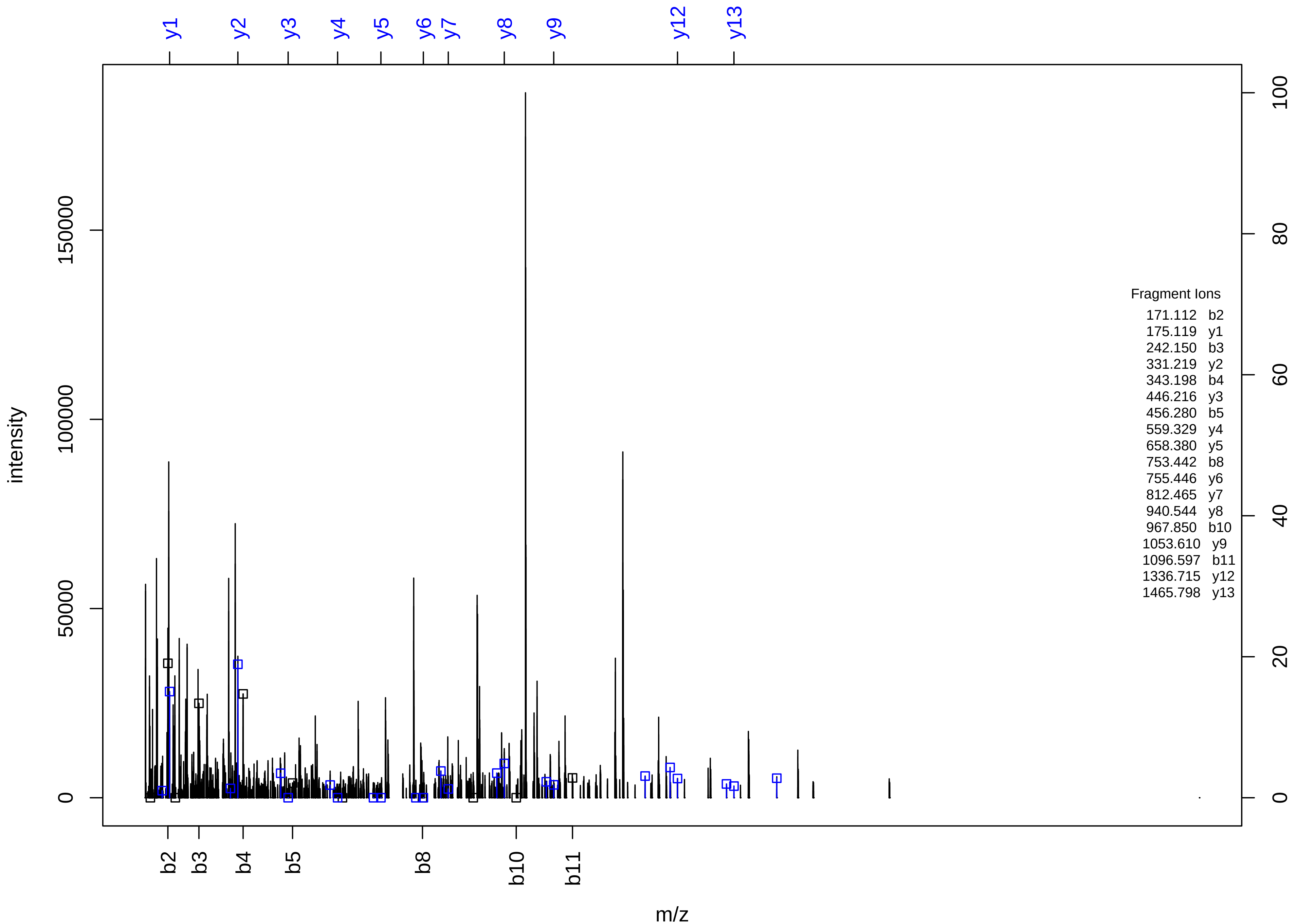

LGATIPLSVDEGPELQGPVIDRR / File: christine_10pools_07.raw; SpectrumID: ; scans: 22667

FGIDDQDYQNSVTR

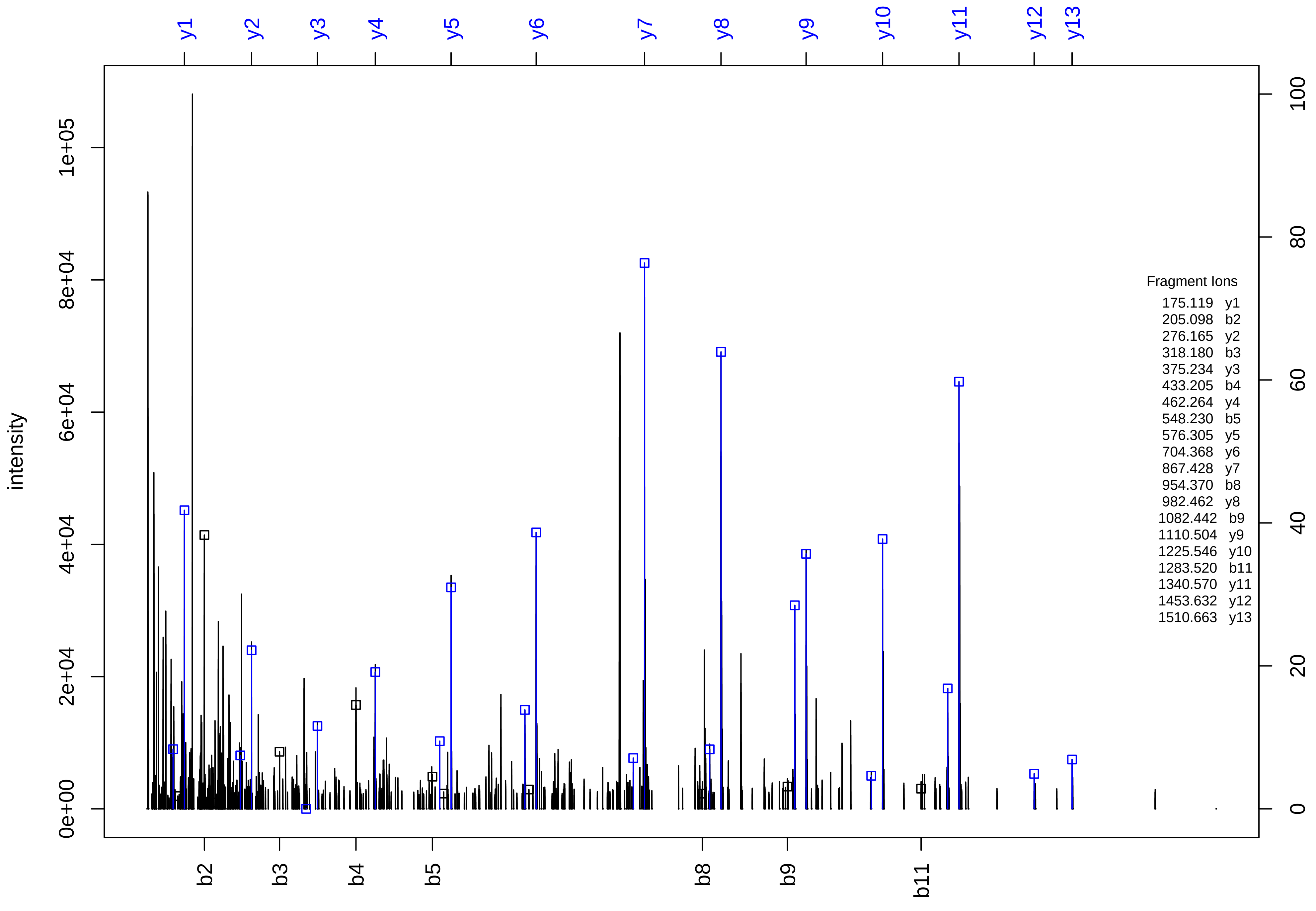

FGIDDQDYQNSVTR / File: christine_10pools_07.raw; SpectrumID: ; scans: 10817

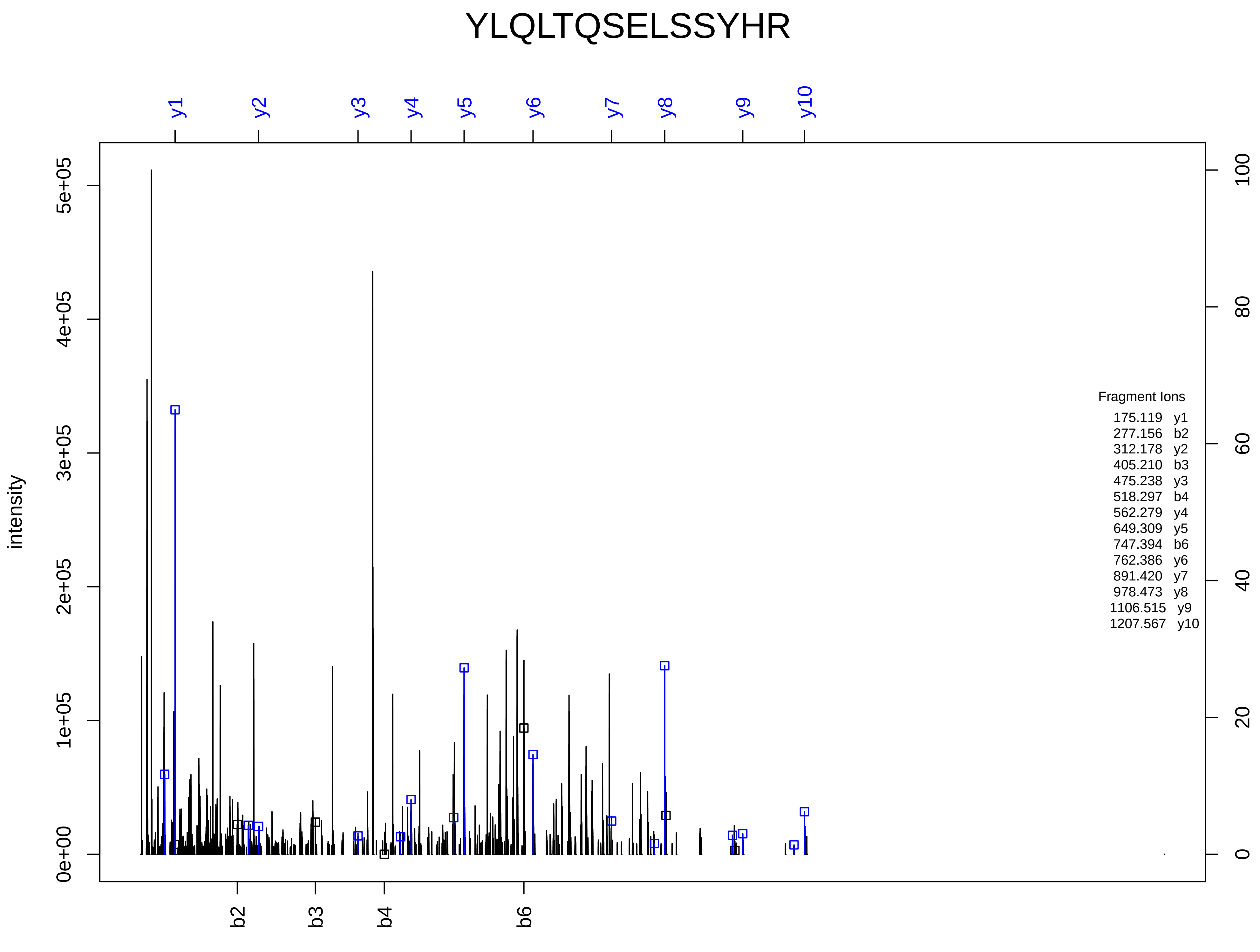

YLQLTQSELSSYHR / File: christine_10pools_07.raw; SpectrumID: ; scans: 9832

QVTPGPATRPGYSDYTQGDSWGEGEGDEDEGCDQVAR

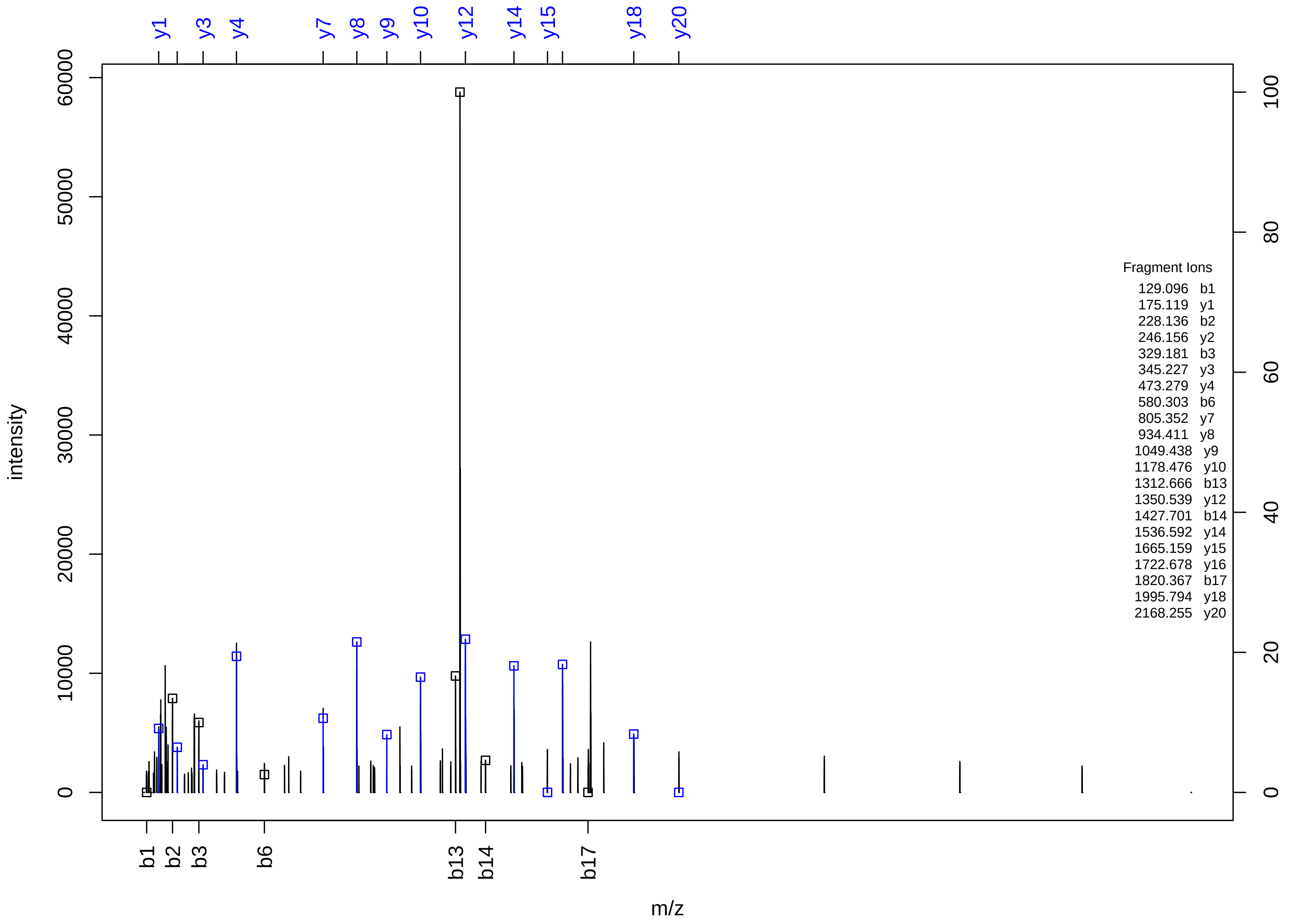

QVTPGPATRPGYSDYTQGDSWGEGEGDEDEGCDQVAR / File: christine_10pools_07.raw; SpectrumID: ; scans: 10577

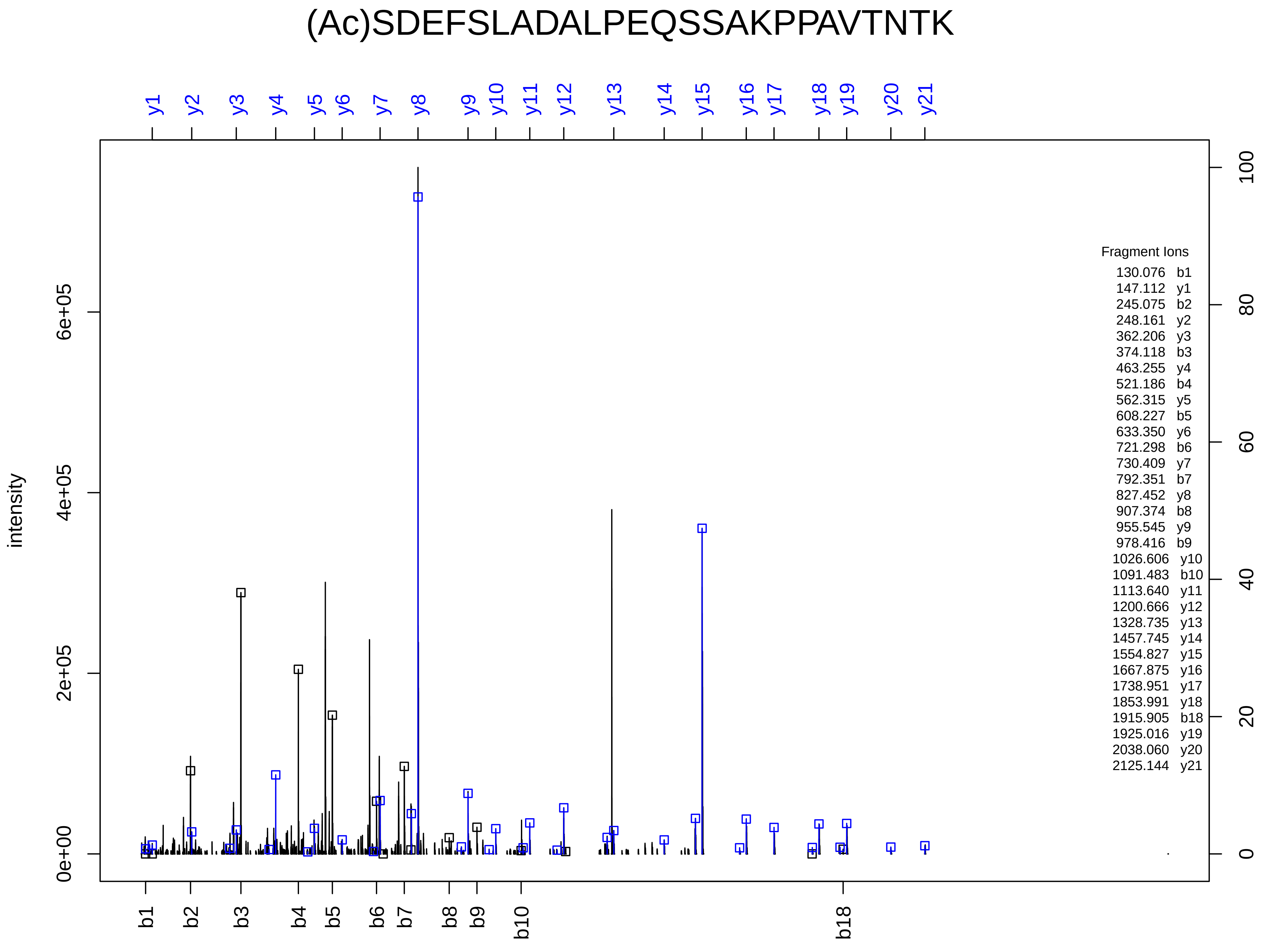

SDEFSLADALPEQSSAKPPAVTNTK / File: christine_10pools_07.raw; SpectrumID: ; scans: 23250

ANLFPFVGEQQSEFSTFDK

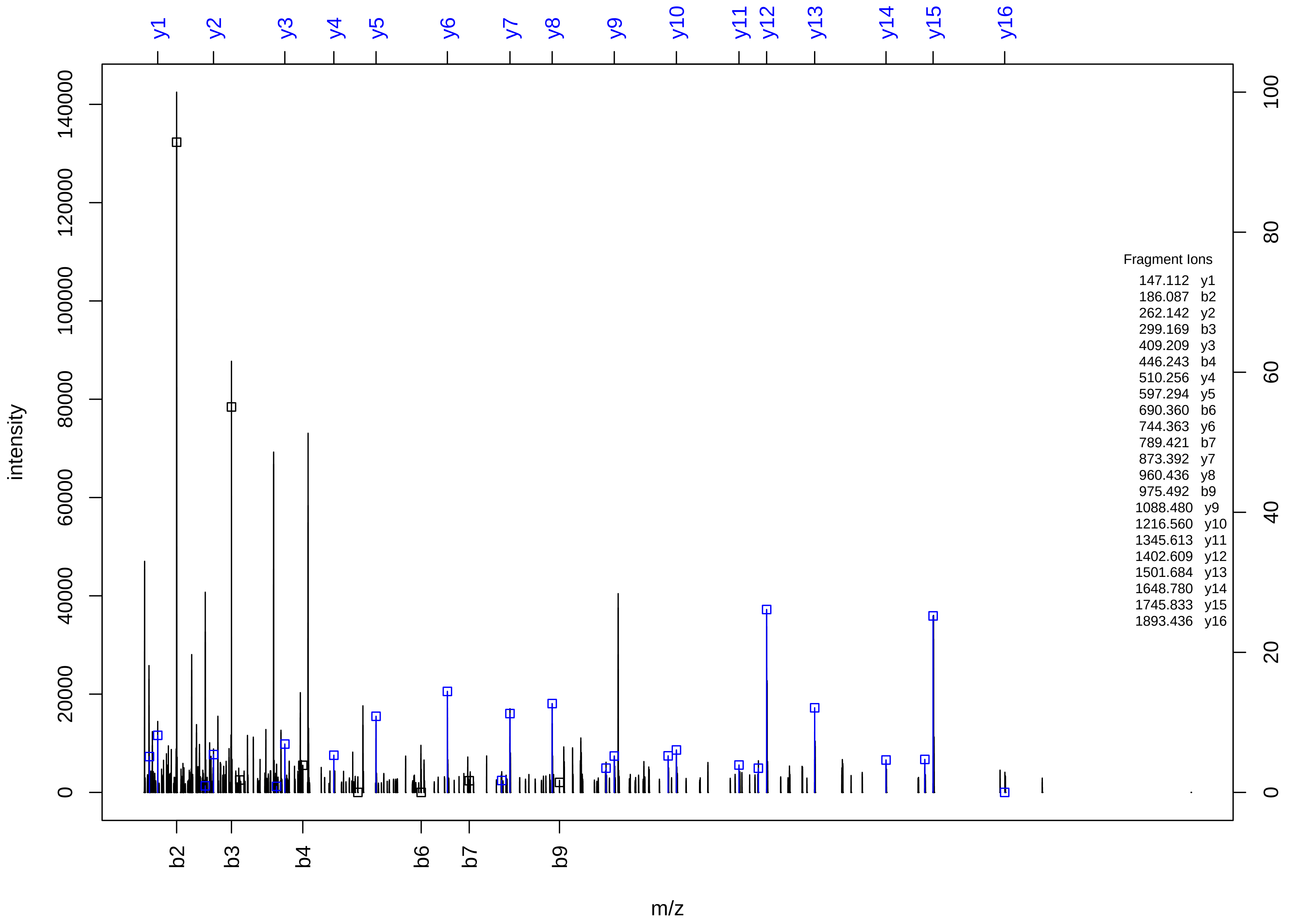

ANLFPFVGEQQSEFSTFDK / File: christine_10pools_07.raw; SpectrumID: ; scans: 30915

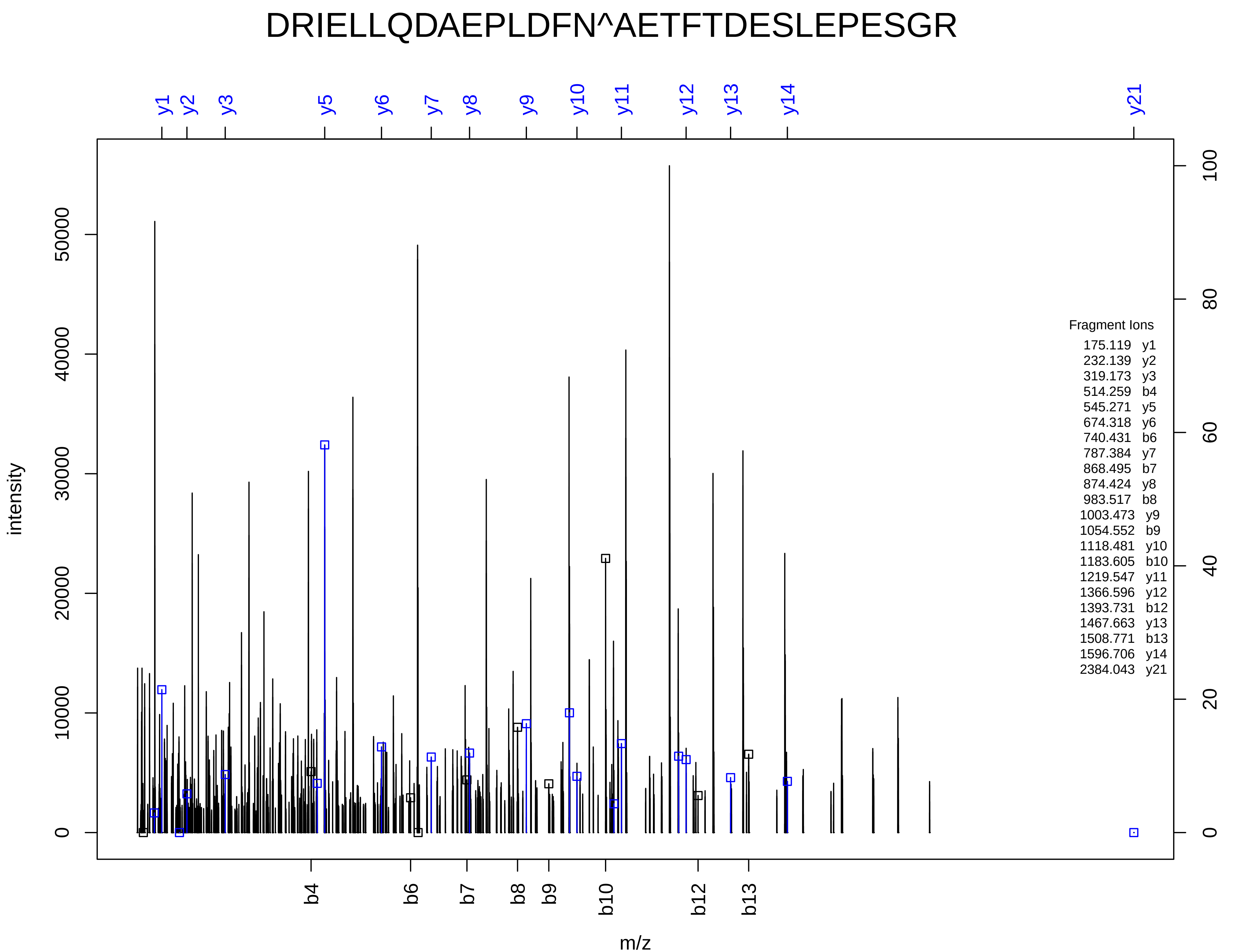

DRIELLQDAEPLDFNAETFTDESLEPESGR / File: christine_10pools_07.raw; SpectrumID: ; scans: 33082

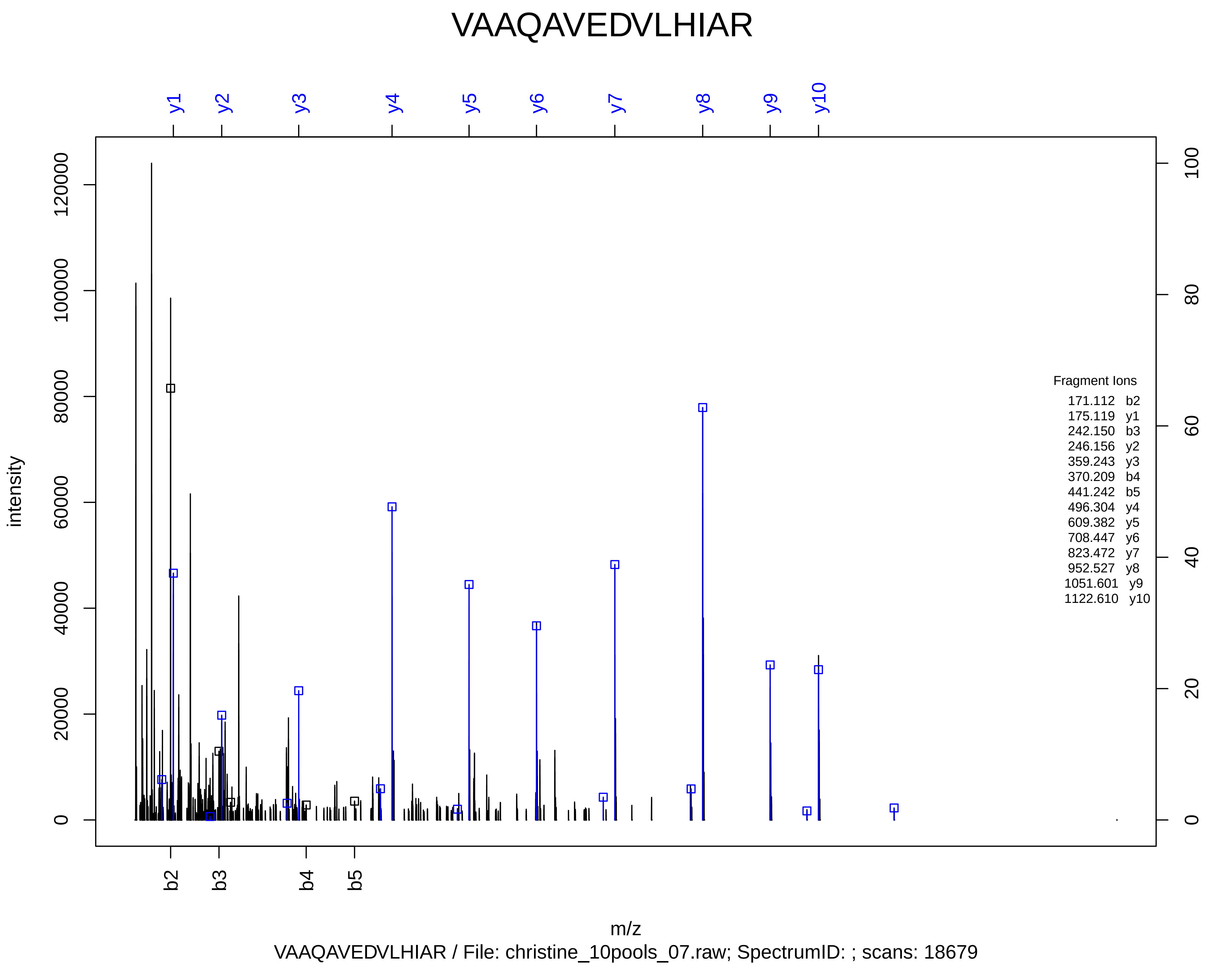
VAAQAVEDVLHIAR
y1
y2
y3
y4
y5
y6
y7
y8
y9
y10
intensity
120000
100000
80000
60000
40000
20000
0
100
80
60
40
20
0
b2
b3
b4
b5
m/z
Fragment Ions
171.112 b2
175.119 y1
242.150 b3
246.156 y2
359.243 y3
370.209 b4
441.242 b5
496.304 y4
609.382 y5
708.447 y6
823.472 y7
952.527 y8
1051.601 y9
1122.610 y10
VAAQAVEDVLHIAR / File: christine_10pools_07.raw; SpectrumID: ; scans: 18679

DQVLDFMLGAVNNIR

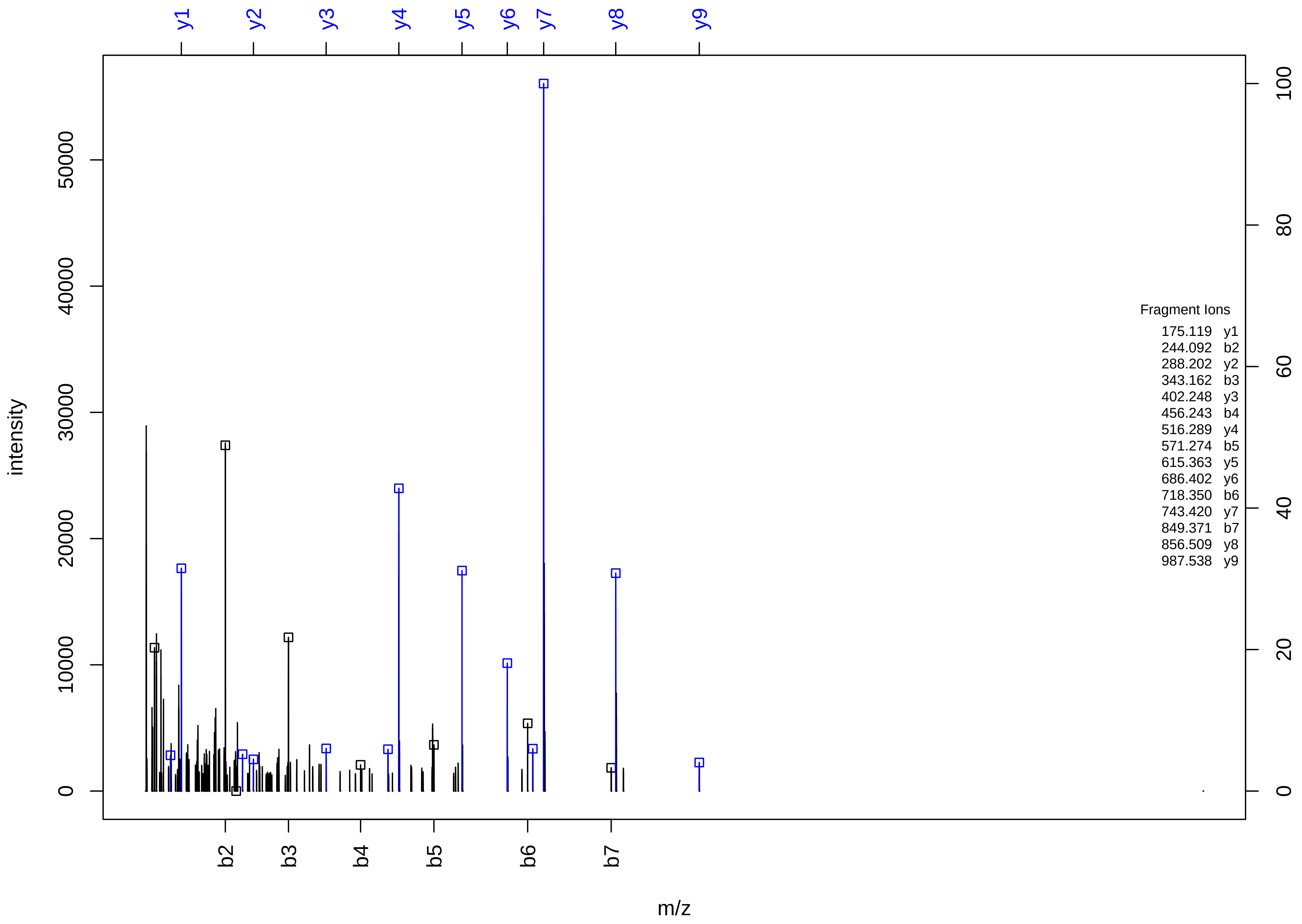

DQVLDFMLGAVNNIR / File: christine_10pools_07.raw; SpectrumID: ; scans: 40301

AIAFADQLHAVDTDGVEPLESVLEDR

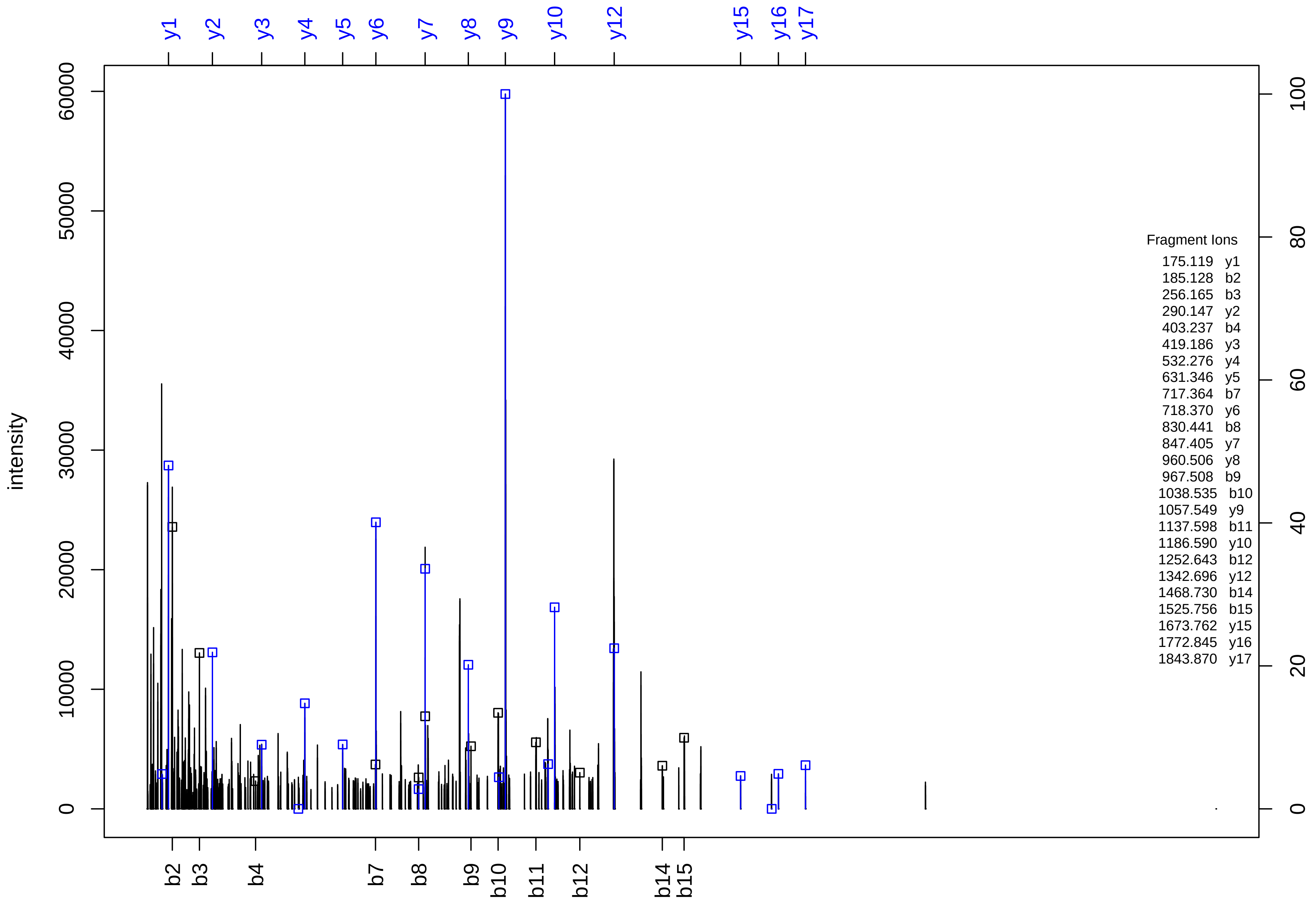

AIAFADQLHAVDTDGVEPLESVLEDR / File: christine_10pools_07.raw; SpectrumID: ; scans: 32550

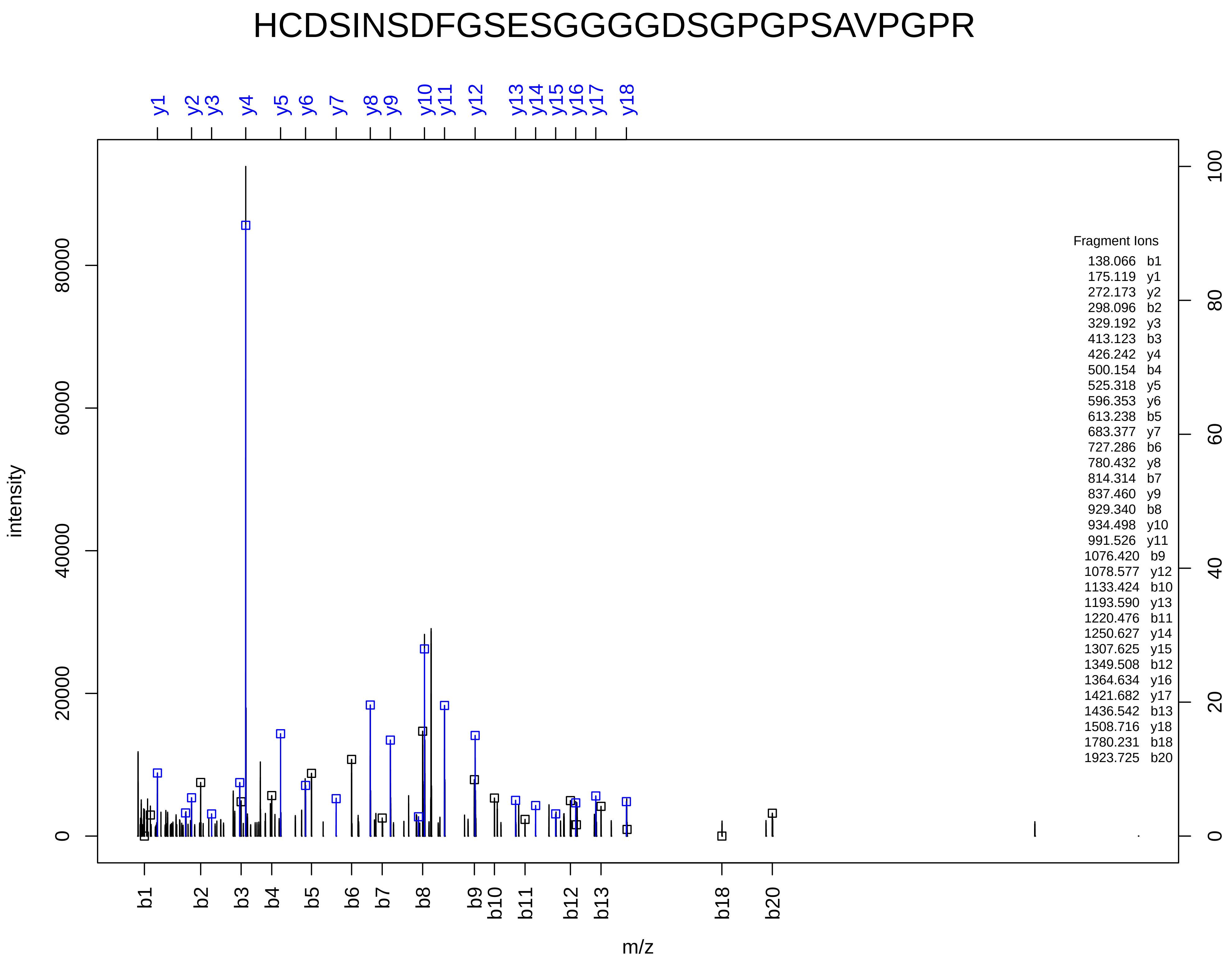

HCDSINSDFGSESGGGGDSGPGPSAVPGPR / File: christine_10pools_07.raw; SpectrumID: ; scans: 7968

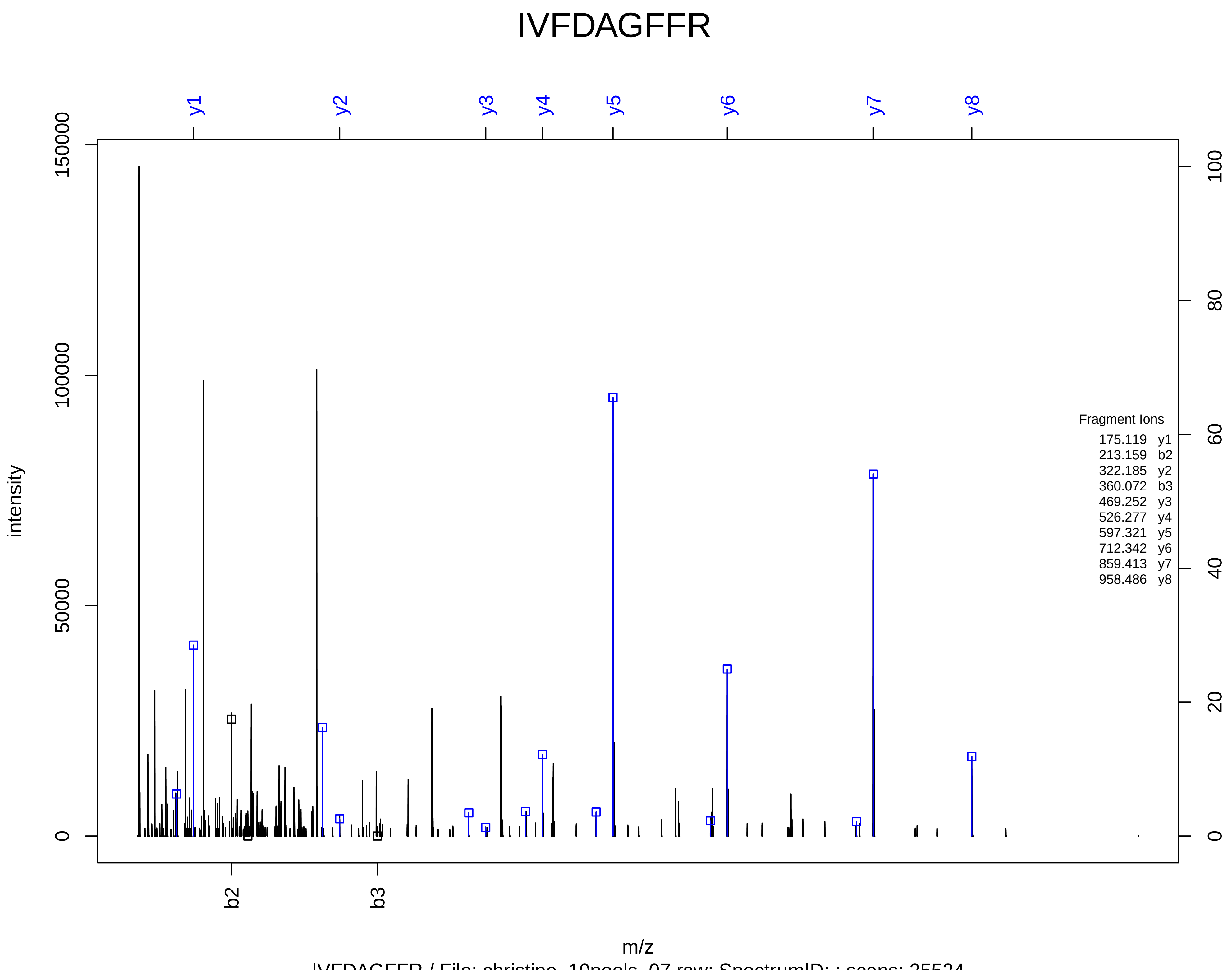
IVFDAGFFR
y1
y2
y3
y4
y5
y6
y7
y8
b2
b3
intensity
m/z
0
50000
100000
150000
0
20
40
60
80
100
Fragment Ions
175.119 y1
213.159 b2
322.185 y2
360.072 b3
469.252 y3
526.277 y4
597.321 y5
712.342 y6
859.413 y7
958.486 y8
IVFDAGFFR / File: christine_10pools_07.raw; SpectrumID: ; scans: 25524

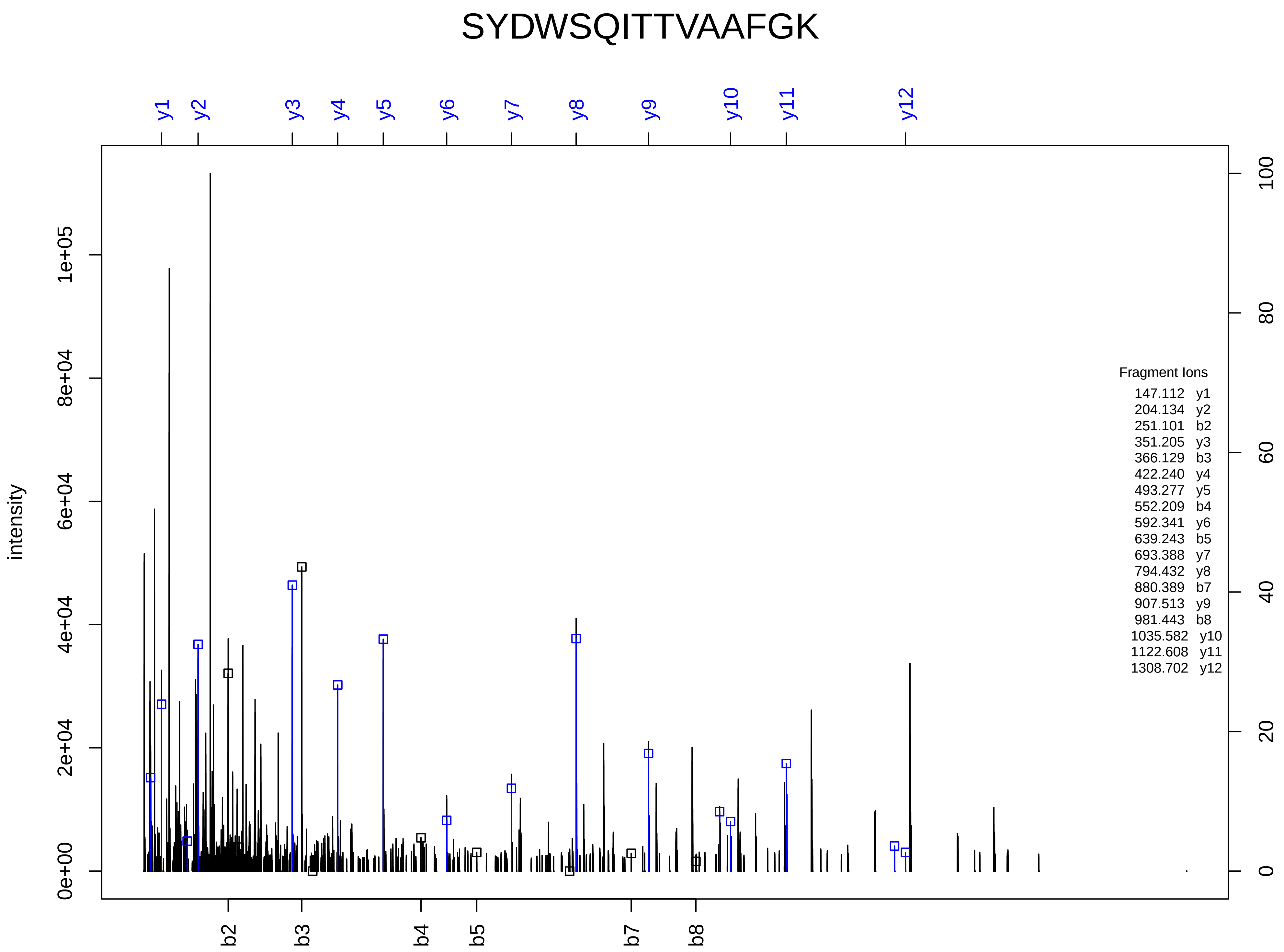

SYDWSQITTVAAFGK / File: christine_10pools_07.raw; SpectrumID: ; scans: 27176

YQLPIYCLSPPVNLLLEHTEEESLEPPEPTPSVR

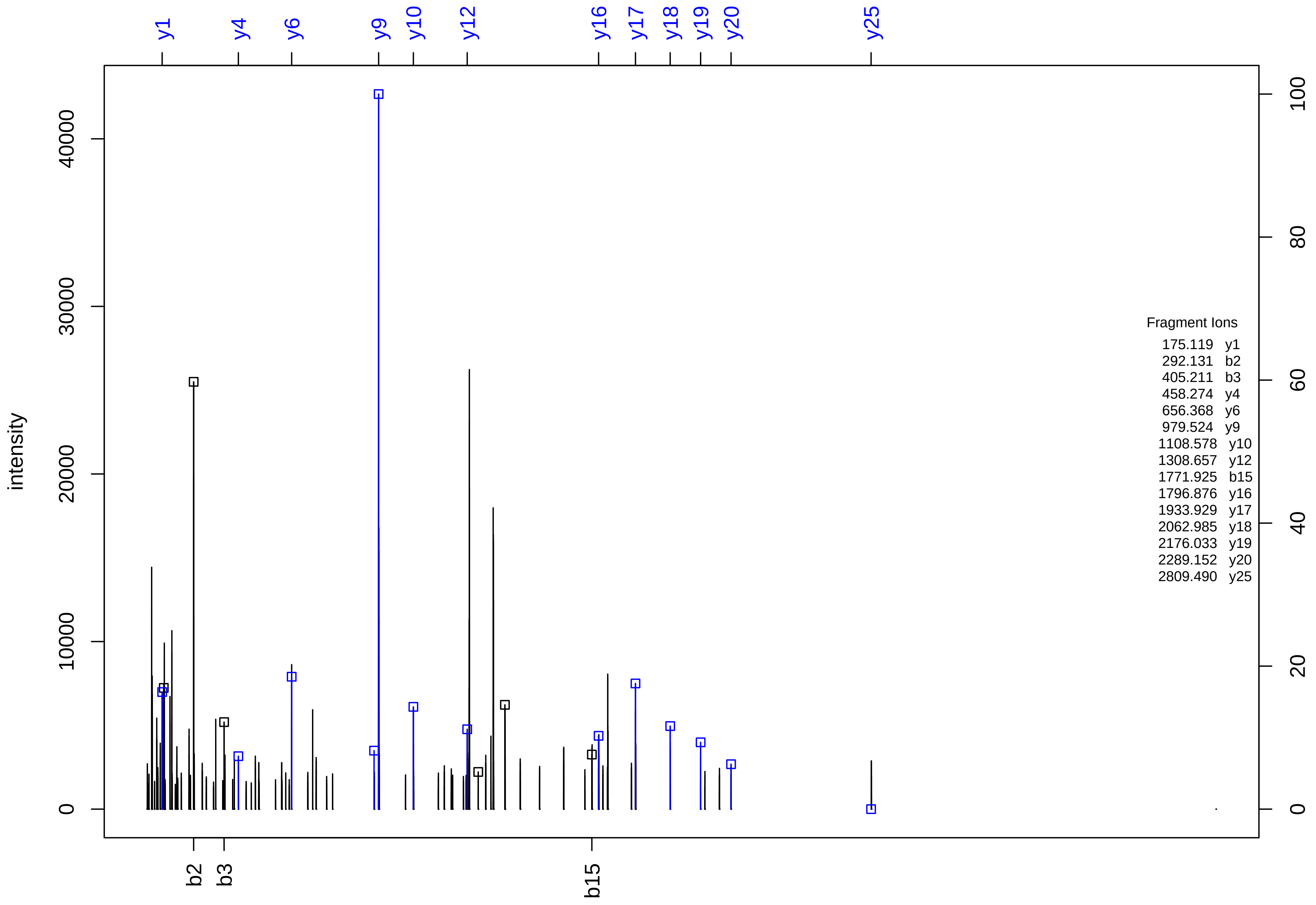

YQLPIYCLSPPVNLLLEHTEEESLEPPEPTPSVR / File: christine_10pools_07.raw; SpectrumID: ; scans: 39078

RLDQEPWEPGGQEEEEAEDGEPAPAYLGQATELITQALR

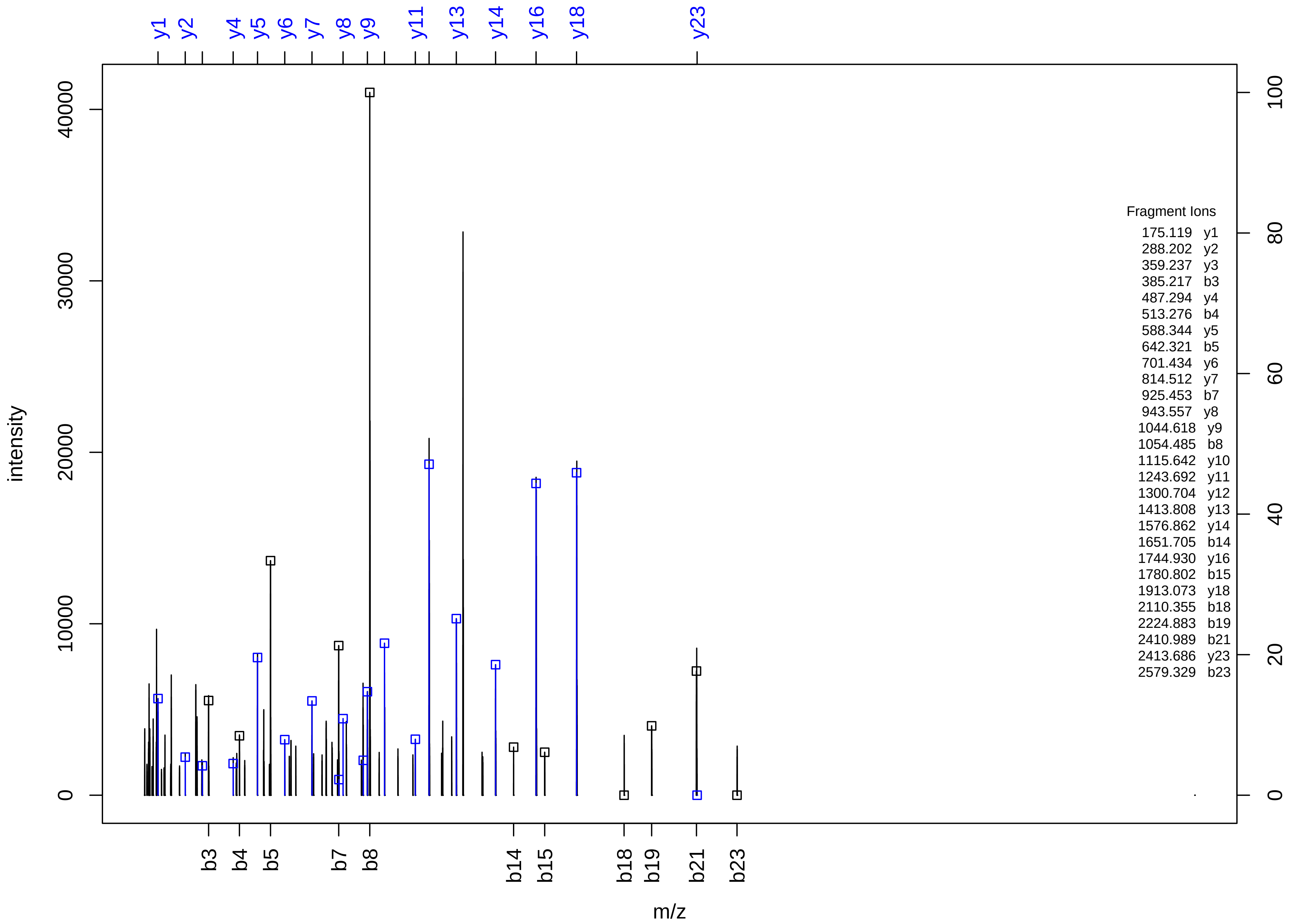

RLDQEPWEPGGQEEEEAEDGEPAPAYLGQATELITQALR / File: christine_10pools_07.raw; SpectrumID: ; scans: 40118

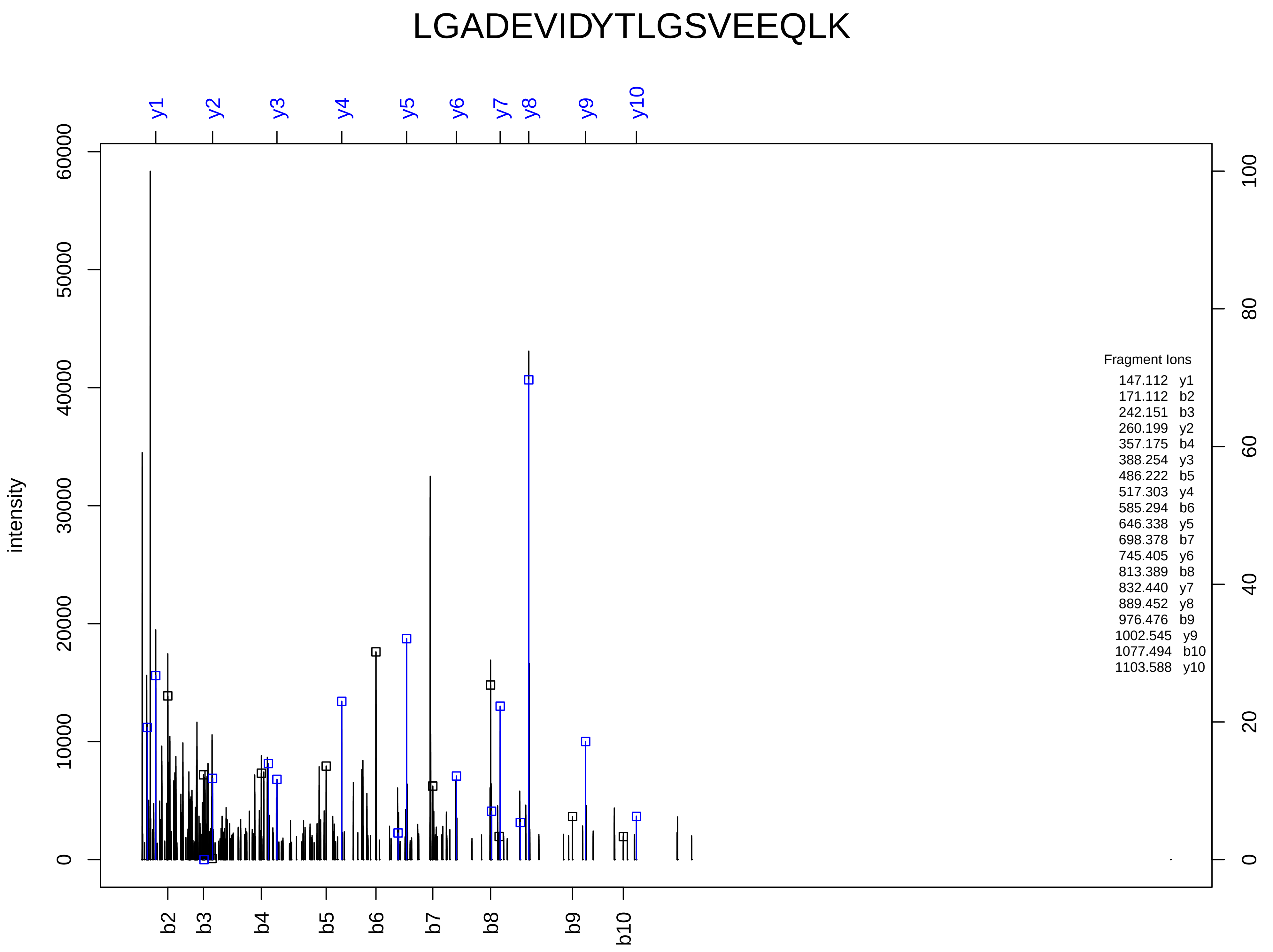

LGADEVIDYTLGSVEEQLK / File: christine_10pools_07.raw; SpectrumID: ; scans: 39293

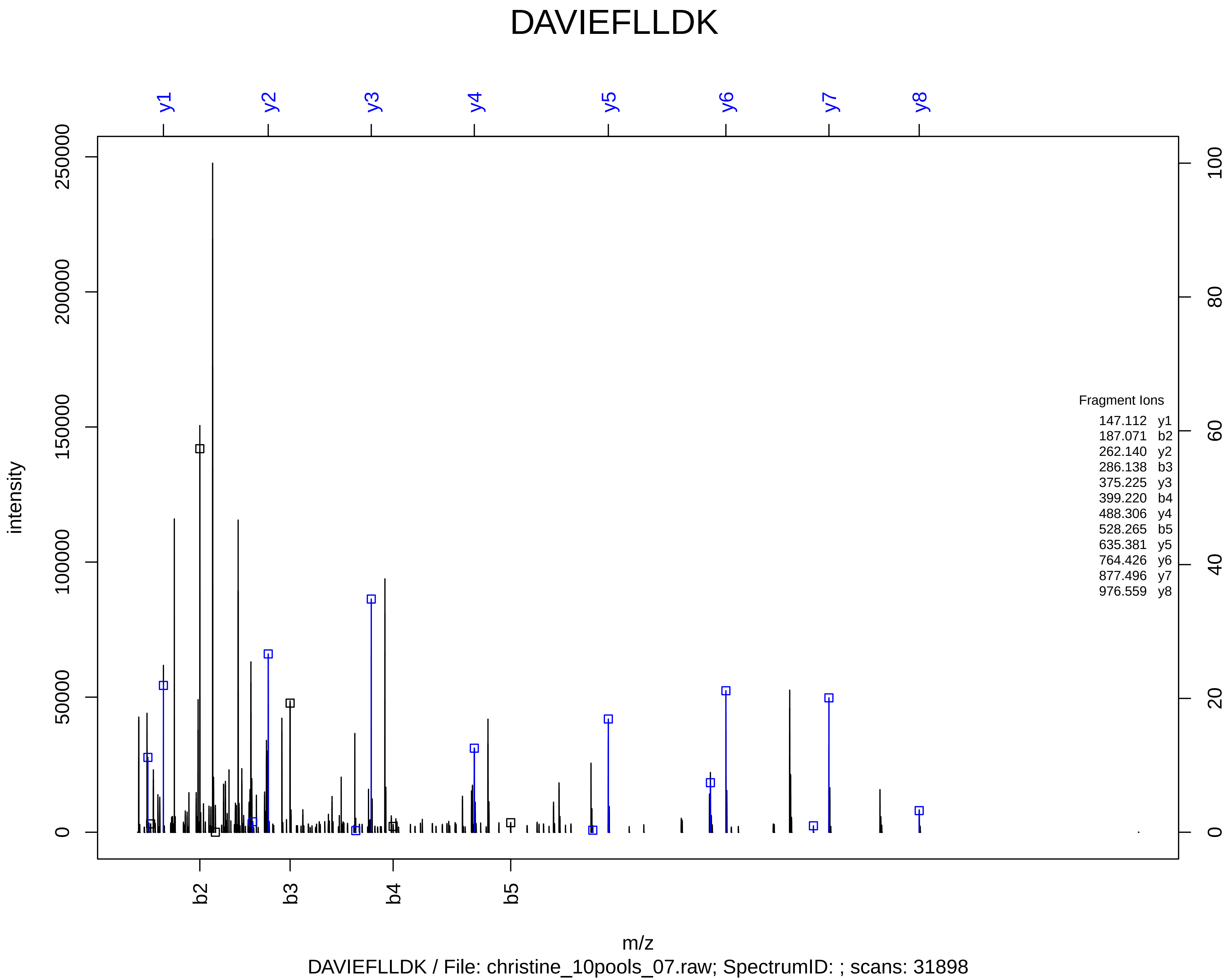
DAVIEFLLDK
y1
y2
y3
y4
y5
y6
y7
y8
250000
200000
150000
100000
50000
0
intensity
100
80
60
40
20
0
Fragment Ions
147.112 y1
187.071 b2
262.140 y2
286.138 b3
375.225 y3
399.220 b4
488.306 y4
528.265 b5
635.381 y5
764.426 y6
877.496 y7
976.559 y8
b2
b3
b4
b5
m/z
DAVIEFLLDK / File: christine_10pools_07.raw; SpectrumID: ; scans: 31898

ELTVDNSFEFEK

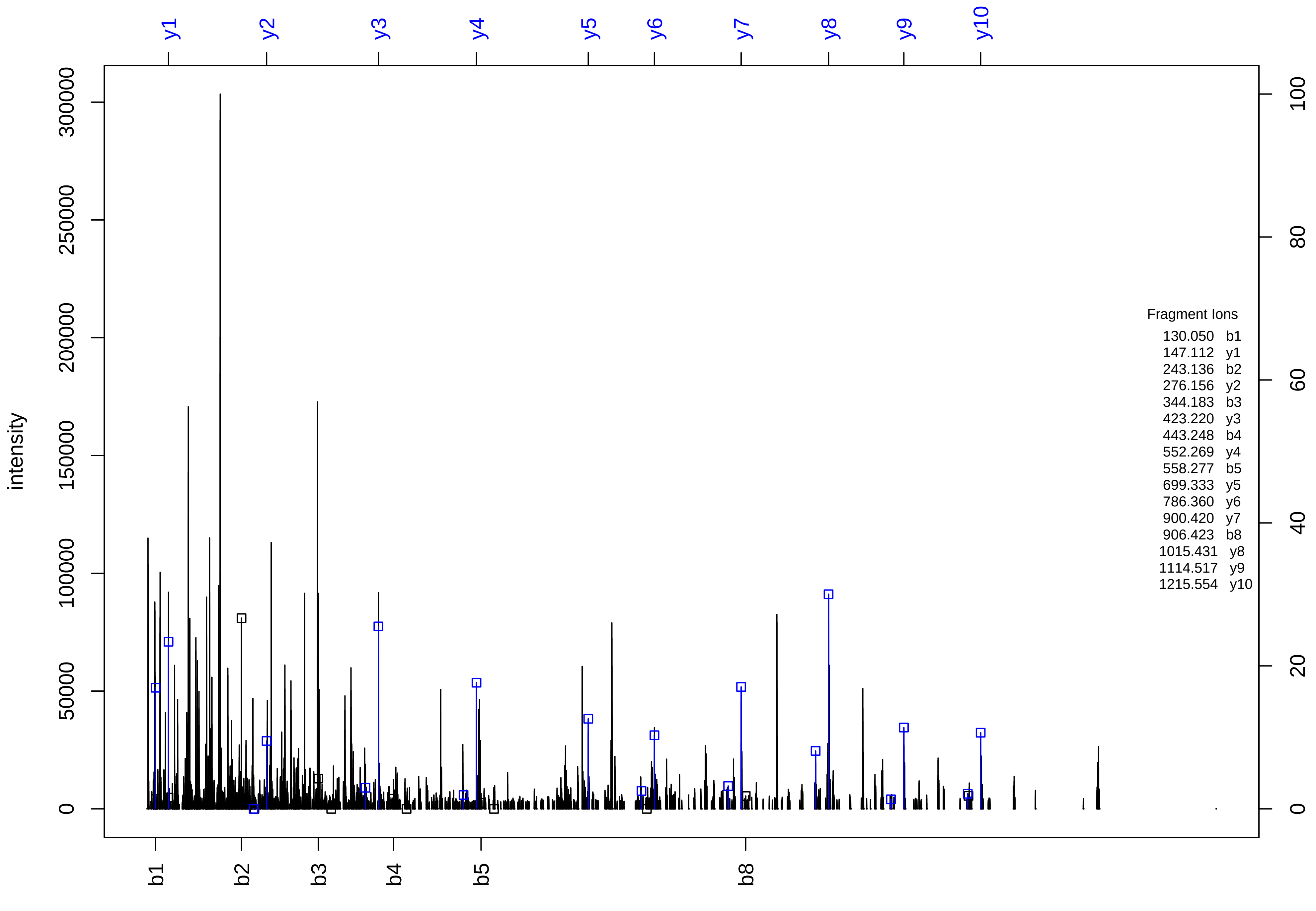

ELTVDNSFEFEK / File: christine_10pools_07.raw; SpectrumID: ; scans: 16810

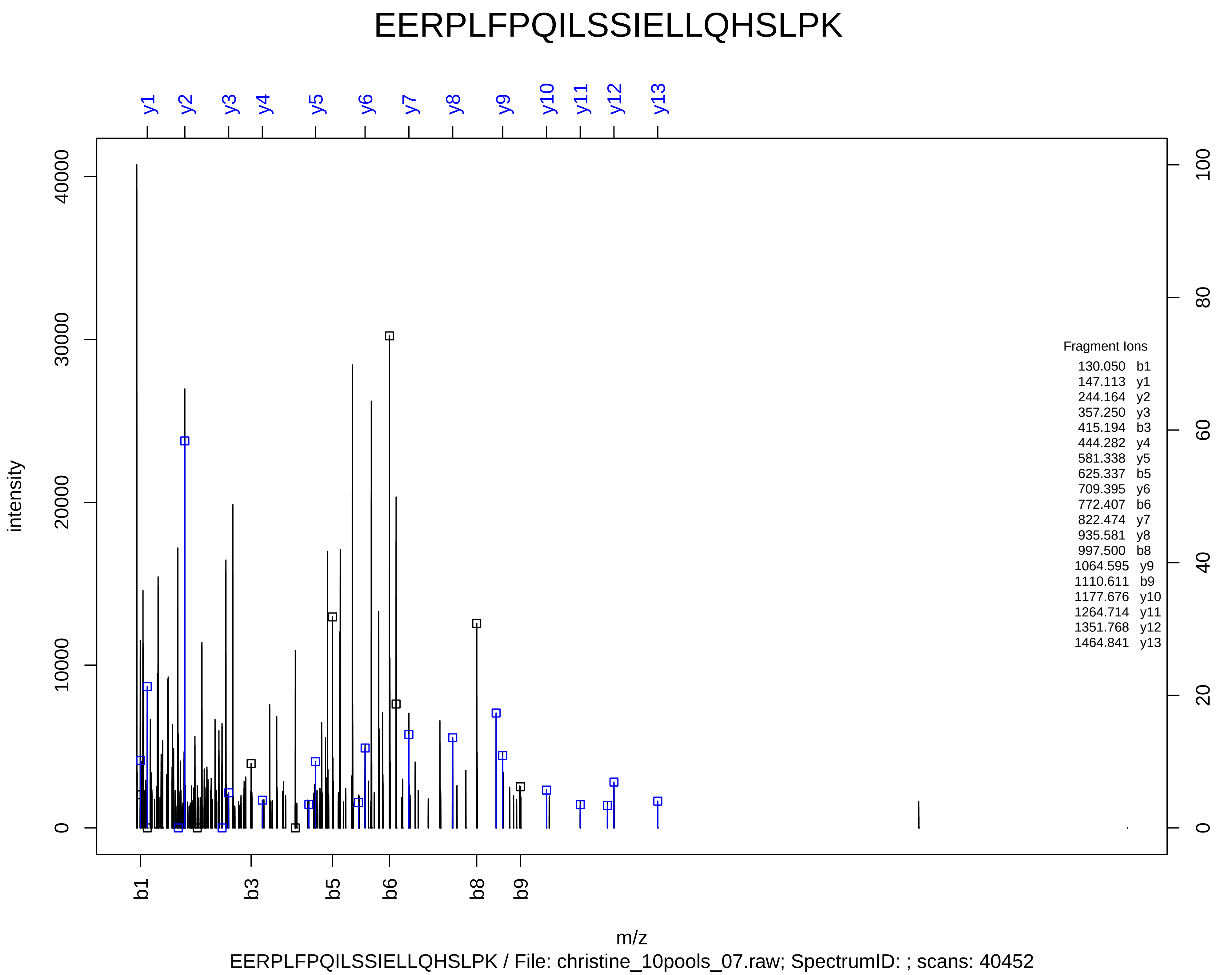
EERPLFPQILSSIELLQHSLPK
y1 y2 y3 y4 y5 y6 y7 y8 y9 y10 y11 y12 y13
intensity
0
10000
20000
30000
40000
0
20
40
60
80
100
b1 b3 b5 b6 b8 b9
m/z
Fragment Ions
130.050 b1
147.113 y1
244.164 y2
357.250 y3
415.194 b3
444.282 y4
581.338 y5
625.337 b5
709.395 y6
772.407 b6
822.474 y7
935.581 y8
997.500 b8
1064.595 y9
1110.611 b9
1177.676 y10
1264.714 y11
1351.768 y12
1464.841 y13
EERPLFPQILSSIELLQHSLPK / File: christine_10pools_07.raw; SpectrumID: ; scans: 40452

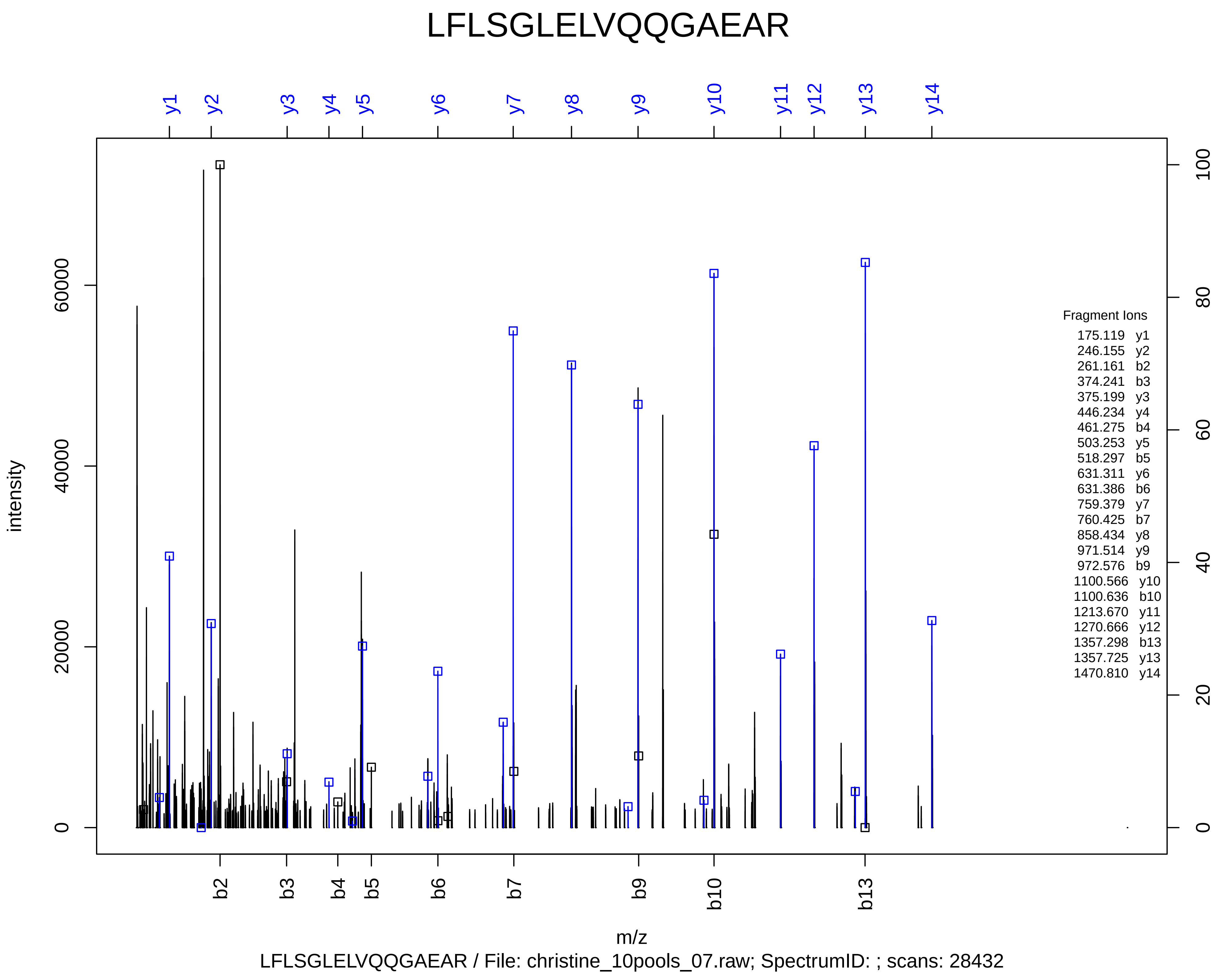

LFLSGLELVQQGAEAR
y1
y2
y3
y4
y5
y6
y7
y8
y9
y10
y11
y12
y13
y14
intensity
0
20000
40000
60000
0
20
40
60
80
100
Fragment Ions
175.119 y1
246.155 y2
261.161 b2
374.241 b3
375.199 y3
446.234 y4
461.275 b4
503.253 y5
518.297 b5
631.311 y6
631.386 b6
759.379 y7
760.425 b7
858.434 y8
971.514 y9
972.576 b9
1100.566 y10
1100.636 b10
1213.670 y11
1270.666 y12
1357.298 b13
1357.725 y13
1470.810 y14
b2
b3
b4
b5
b6
b7
b9
b10
b13
m/z
LFLSGLELVQQGAEAR / File: christine_10pools_07.raw; SpectrumID: ; scans: 28432

WAFSCGTWLPSR

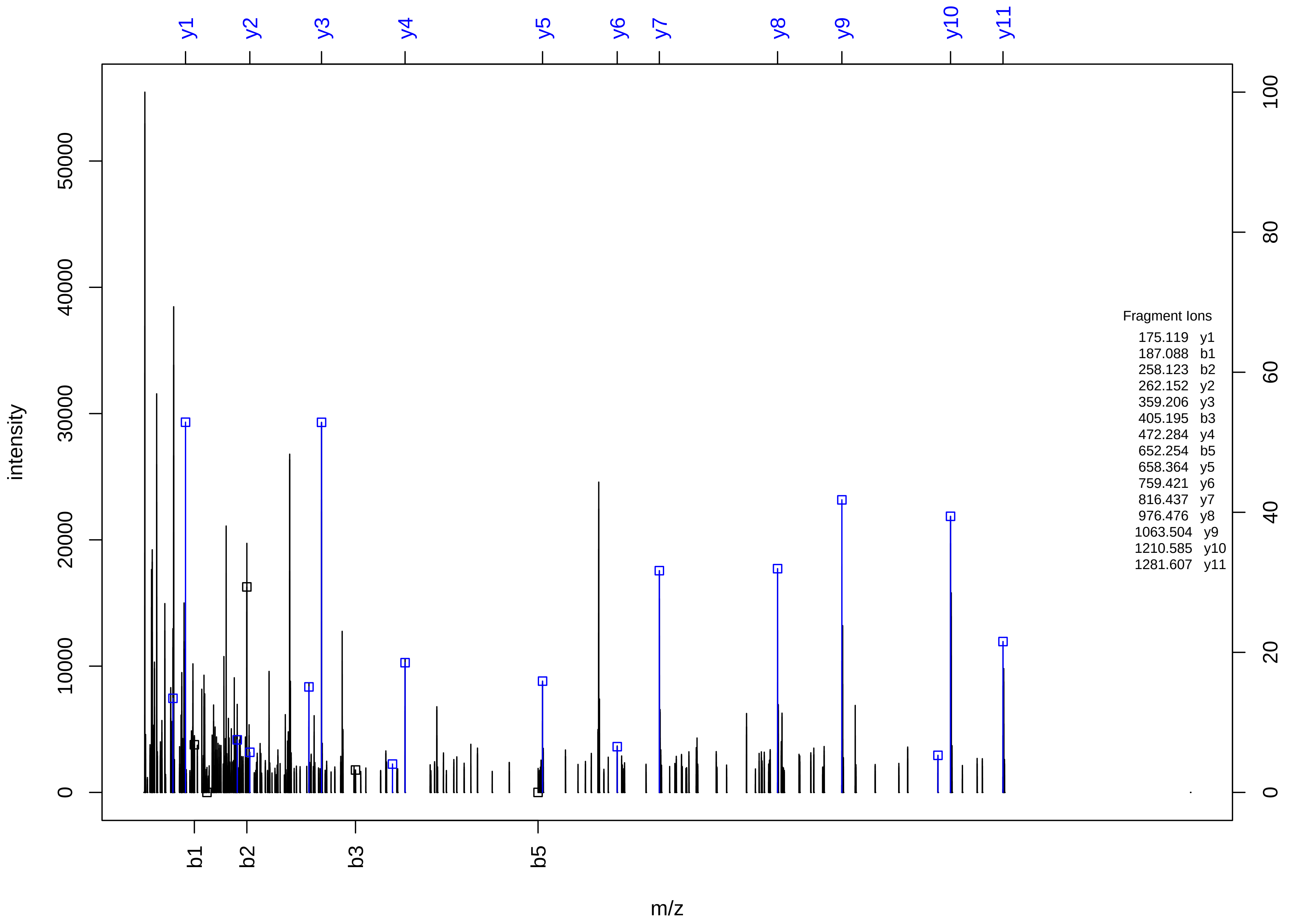

WAFSCGTWLPSR / File: christine_10pools_07.raw; SpectrumID: ; scans: 24528

QADEEFQILANSWR

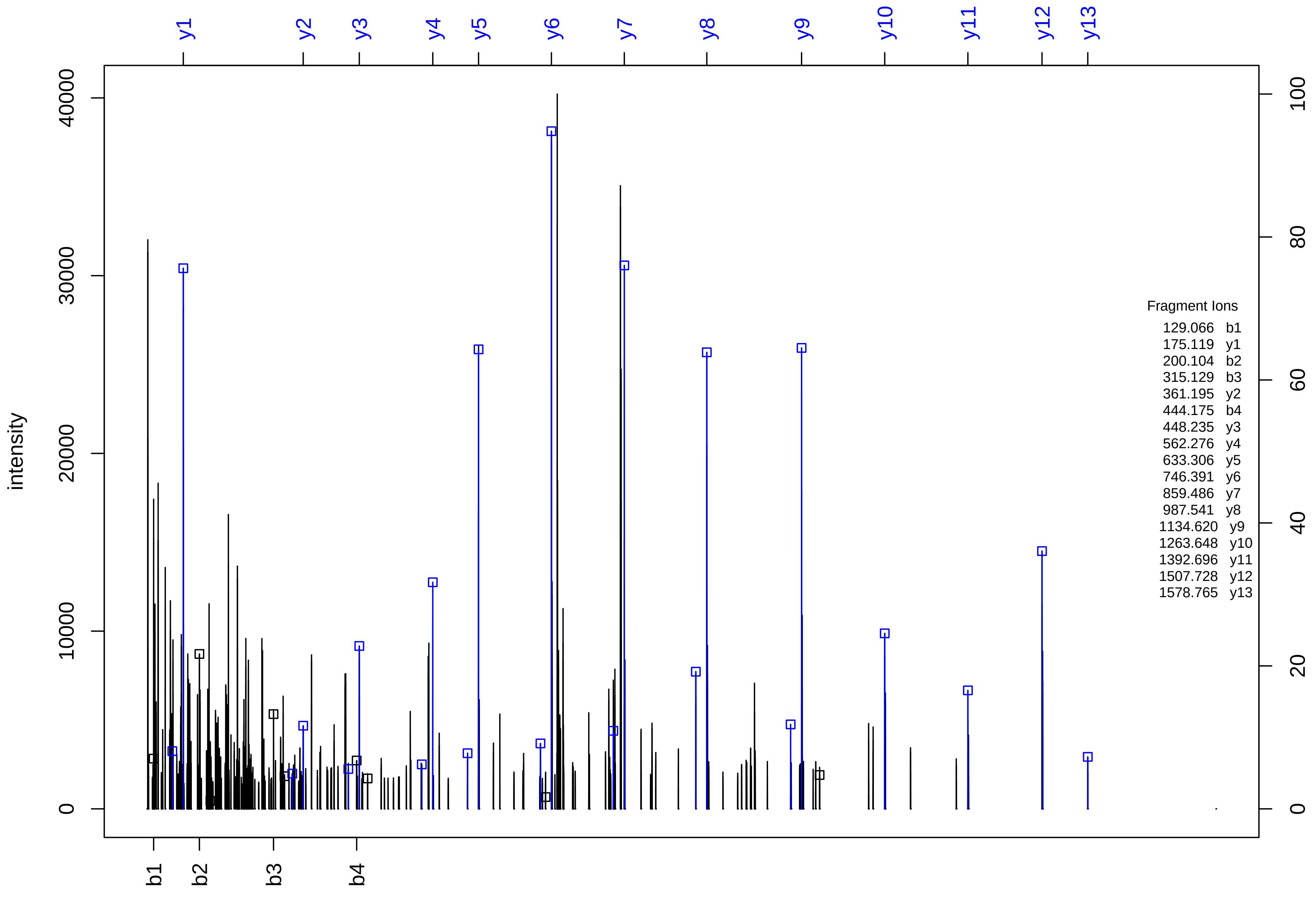

QADEEFQILANSWR / File: christine_10pools_07.raw; SpectrumID: ; scans: 26617

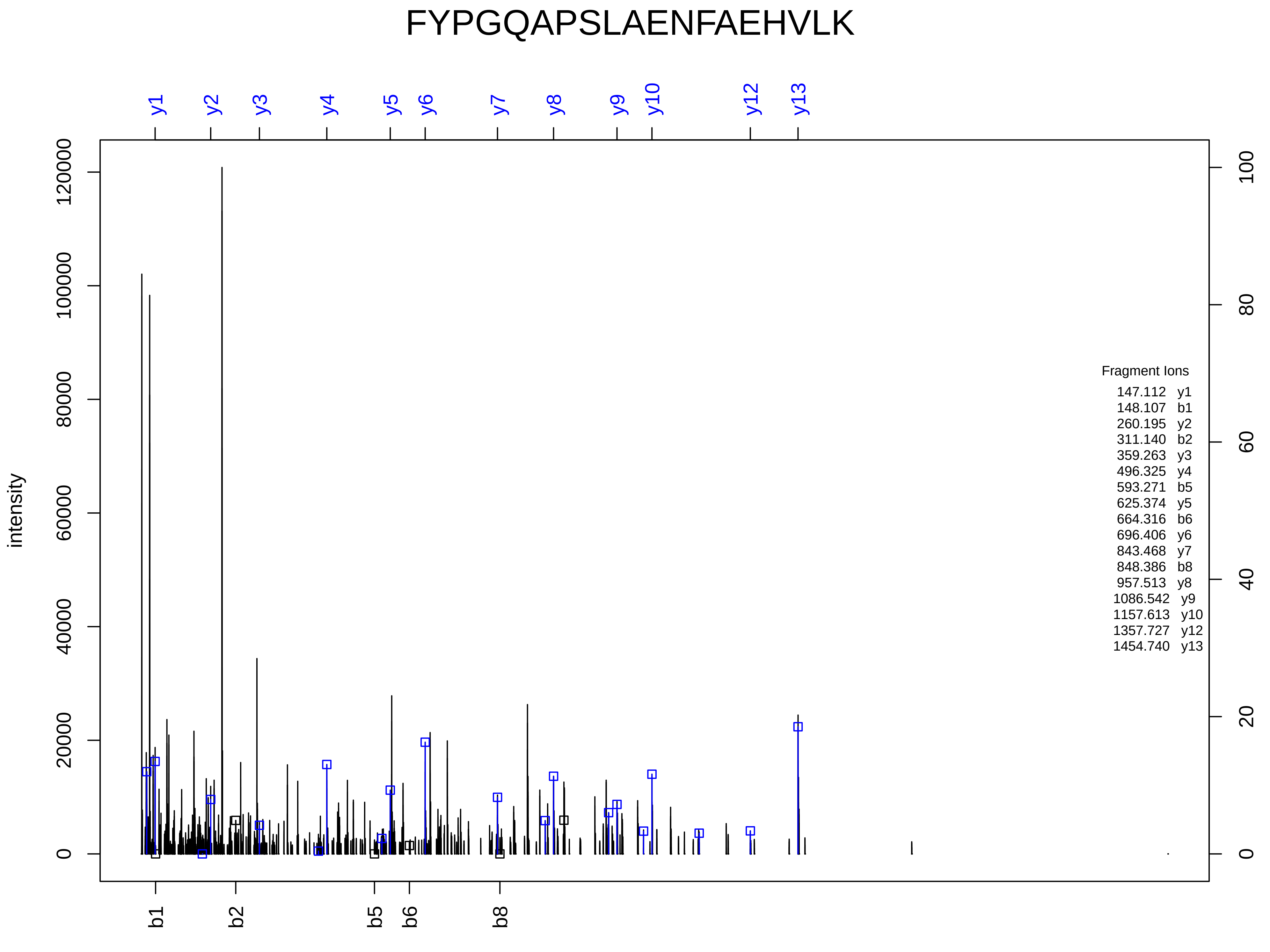

FYPGQAPSLAENFAEHVLK / File: christine_10pools_07.raw; SpectrumID: ; scans: 28037

(Ac)M*KEN^VAEFLR

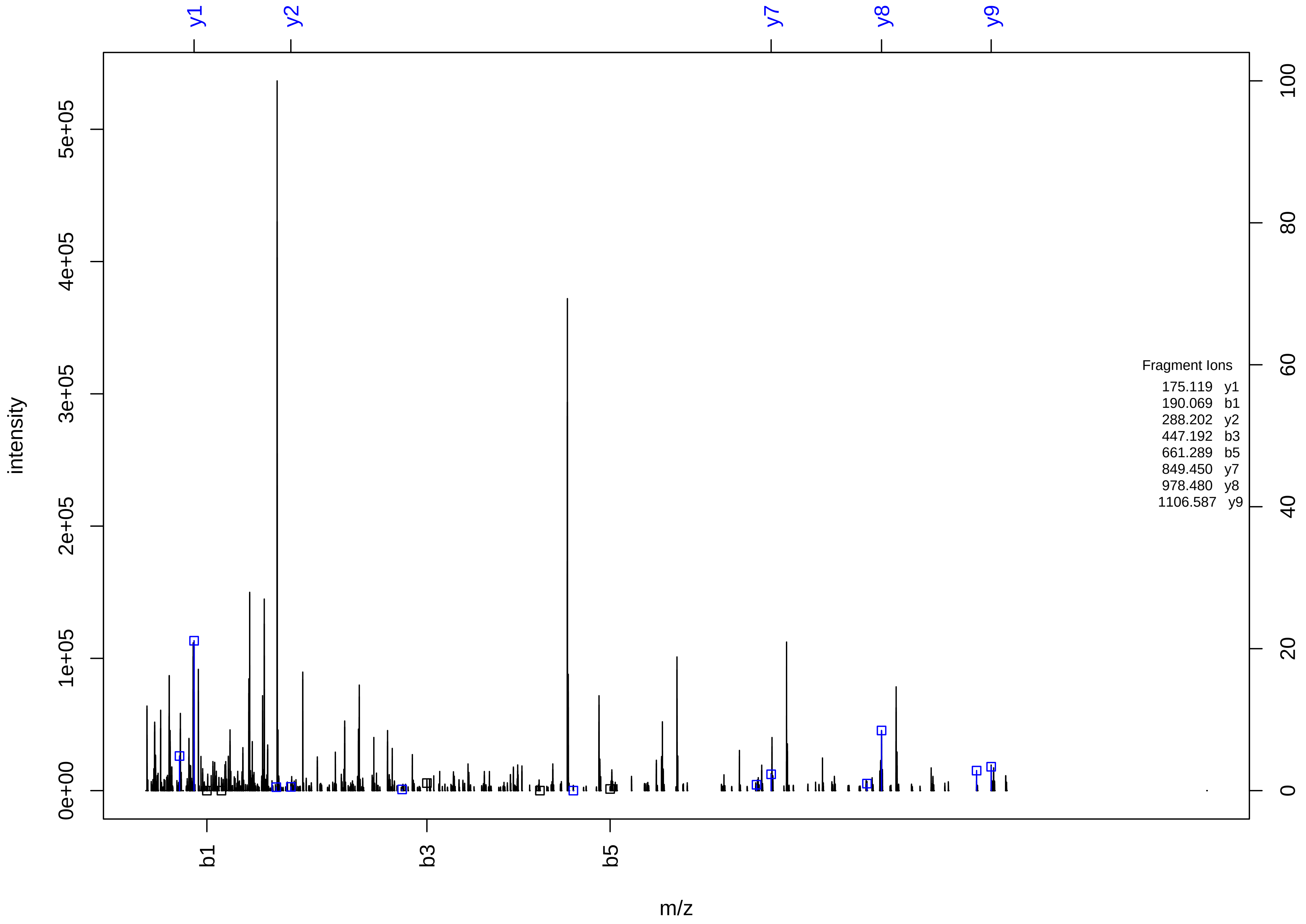

MKENVAEFLR / File: christine_10pools_07.raw; SpectrumID: ; scans: 8557

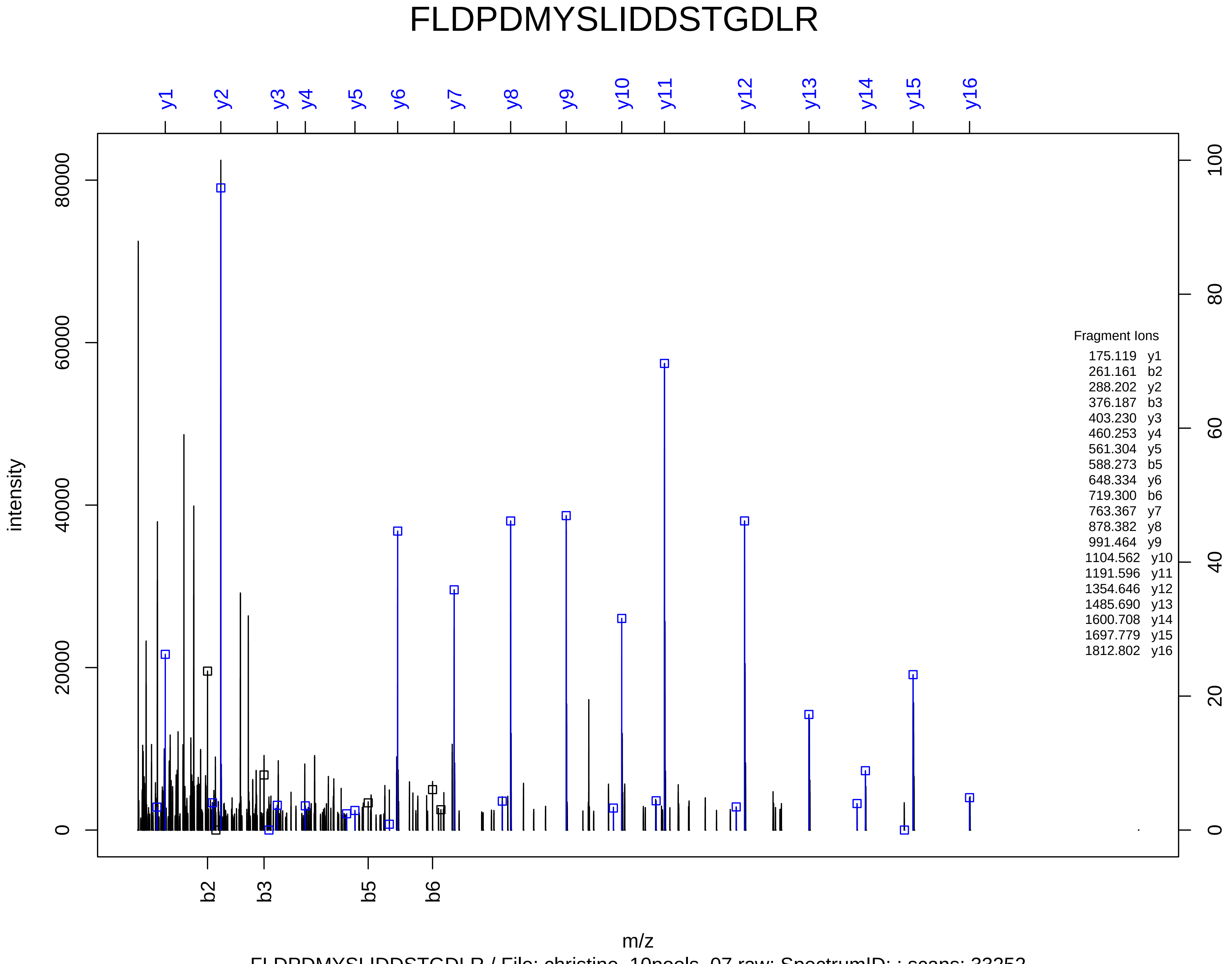
FLDPDMYSLIDDSTGDLR
intensity
m/z
y1
y2
y3
y4
y5
y6
y7
y8
y9
y10
y11
y12
y13
y14
y15
y16
b2
b3
b5
b6
Fragment Ions
175.119 y1
261.161 b2
288.202 y2
376.187 b3
403.230 y3
460.253 y4
561.304 y5
588.273 b5
648.334 y6
719.300 b6
763.367 y7
878.382 y8
991.464 y9
1104.562 y10
1191.596 y11
1354.646 y12
1485.690 y13
1600.708 y14
1697.779 y15
1812.802 y16
FLDPDMYSLIDDSTGDLR / File: christine_10pools_07.raw; SpectrumID: ; scans: 33252

DAGVSPEQISYVNAHATSTPLGDAAENR

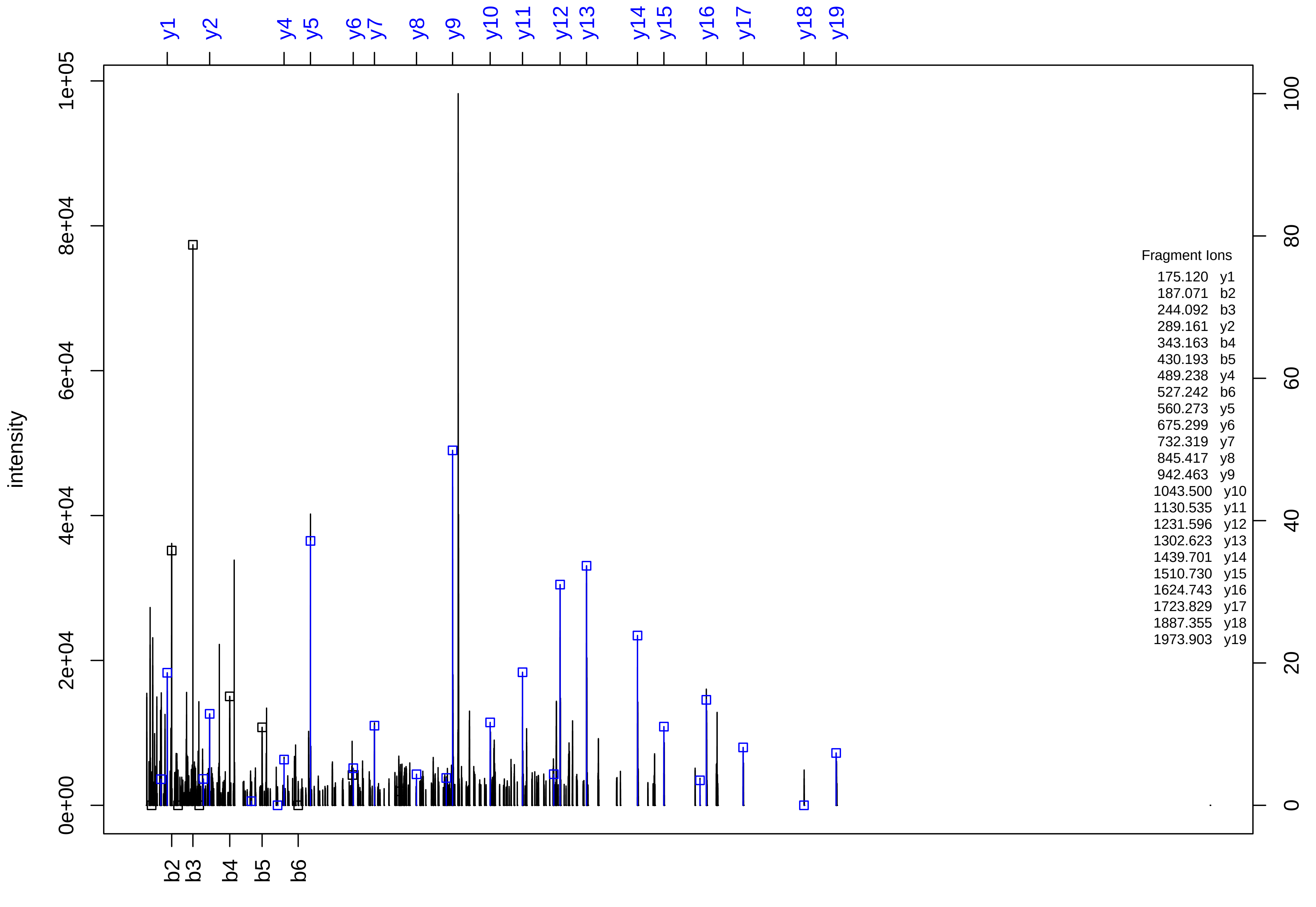

DAGVSPEQISYVNAHATSTPLGDAAENR / File: christine_10pools_07.raw; SpectrumID: ; scans: 15568

TLLDQHGQYPVWMNQR

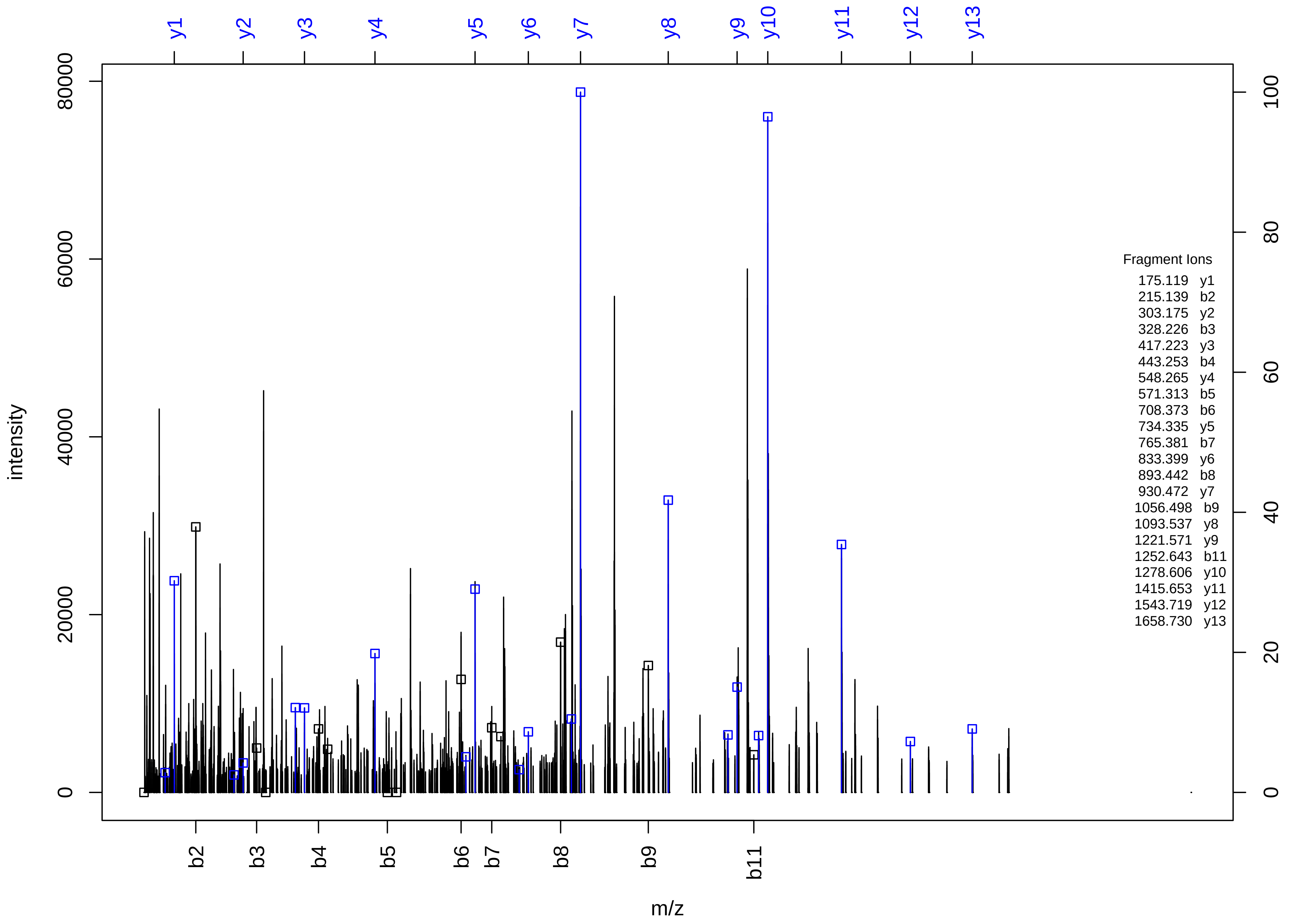

TLLDQHGQYPVWMNQR / File: christine_10pools_07.raw; SpectrumID: ; scans: 16540

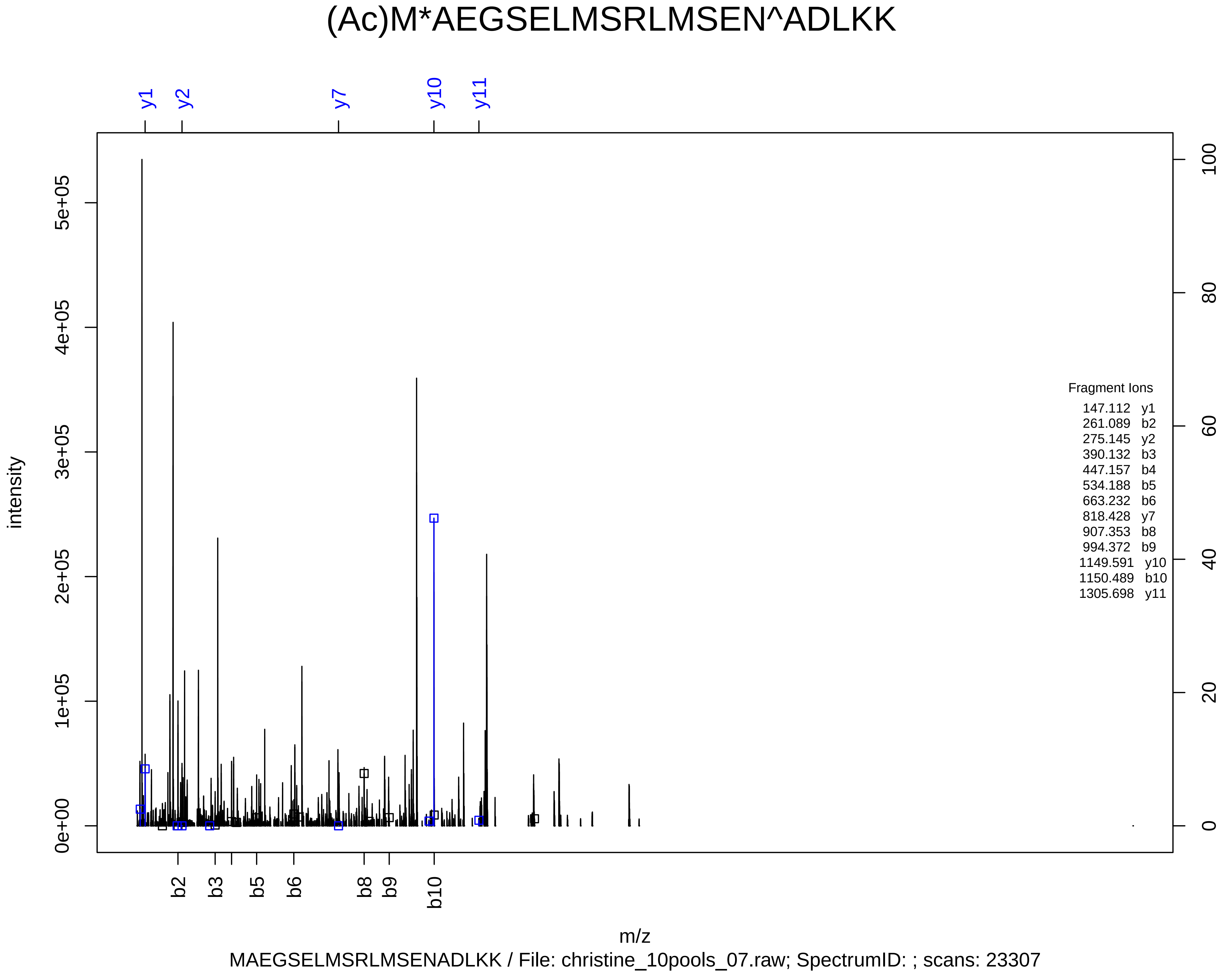
(Ac)M*AEGSELMSRLMSEN^ADLKK
y1
y2
y7
y10
y11
intensity
5e+05
4e+05
3e+05
2e+05
1e+05
0e+00
100
80
60
40
20
0
b2
b3
b5
b6
b8
b9
b10
m/z
Fragment Ions
147.112 y1
261.089 b2
275.145 y2
390.132 b3
447.157 b4
534.188 b5
663.232 b6
818.428 y7
907.353 b8
994.372 b9
1149.591 y10
1150.489 b10
1305.698 y11
MAEGSELMSRLMSENADLKK / File: christine_10pools_07.raw; SpectrumID: ; scans: 23307

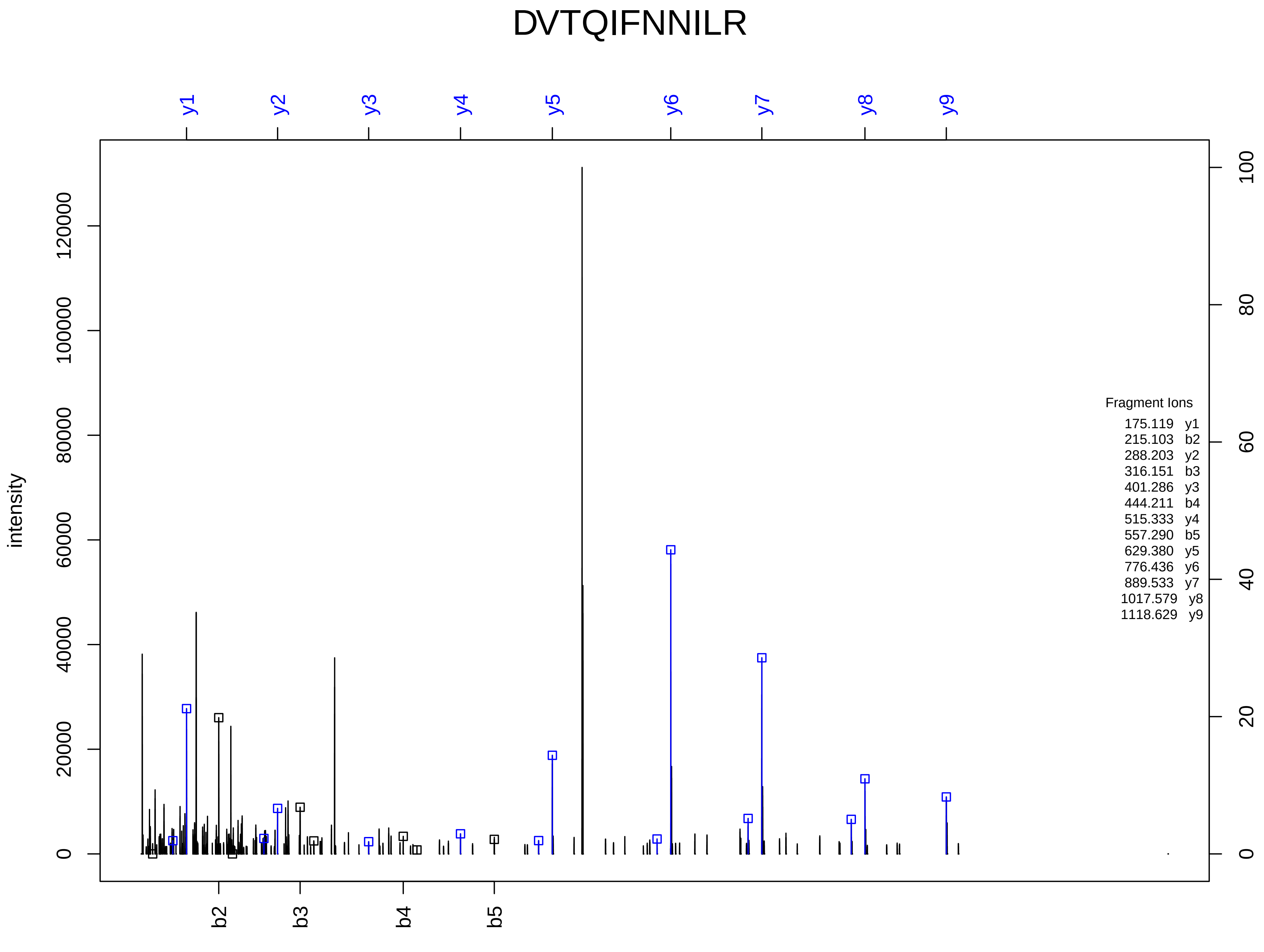

DVTQIFNNILR / File: christine_10pools_07.raw; SpectrumID: ; scans: 34336

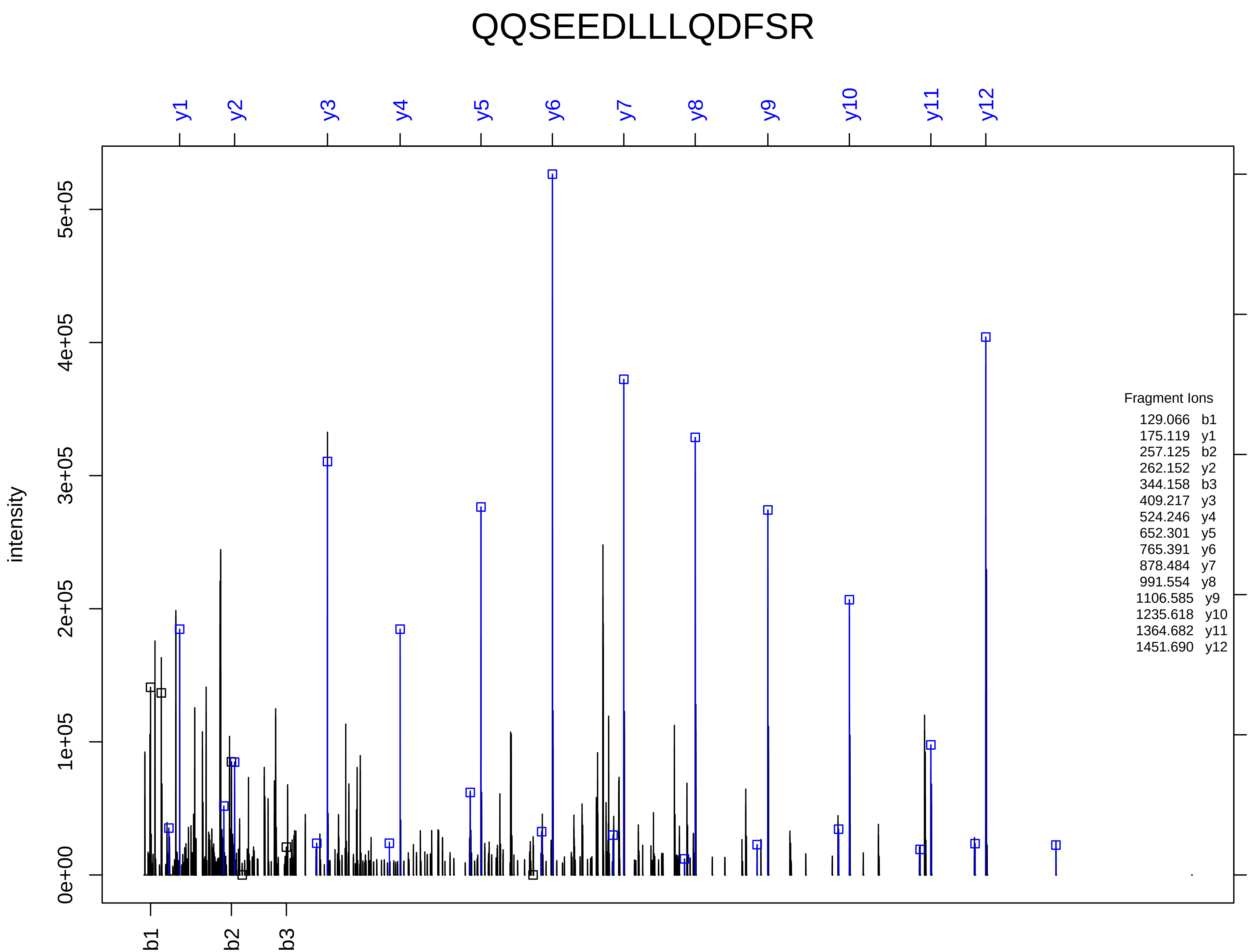

QQSEEDLLLQDFSR / File: christine_10pools_07.raw; SpectrumID: ; scans: 23747

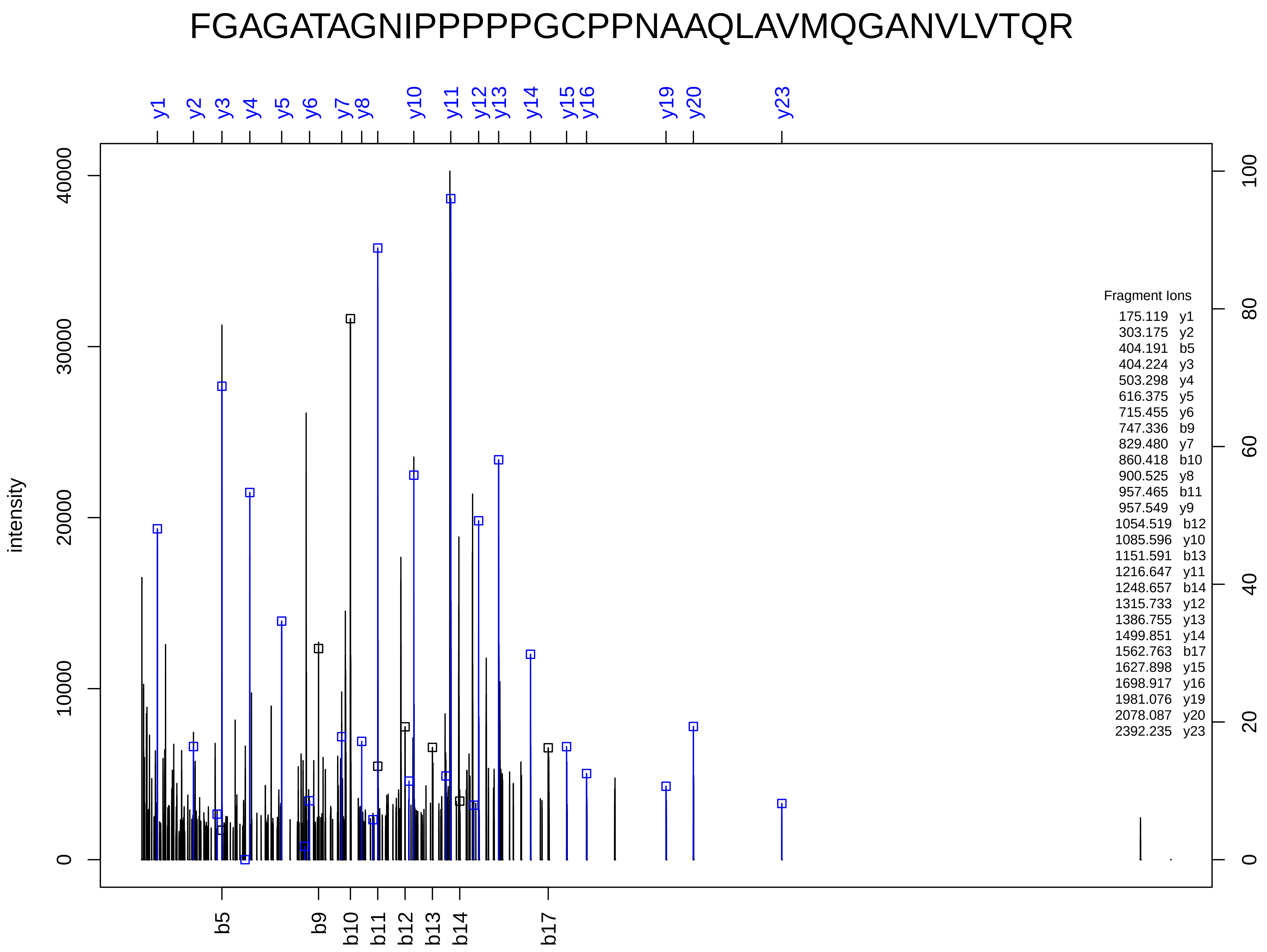

FGAGATAGNIPPPPPGCPPNAAQLAVMQGANVLVTQR / File: christine_10pools_07.raw; SpectrumID: ; scans: 29493

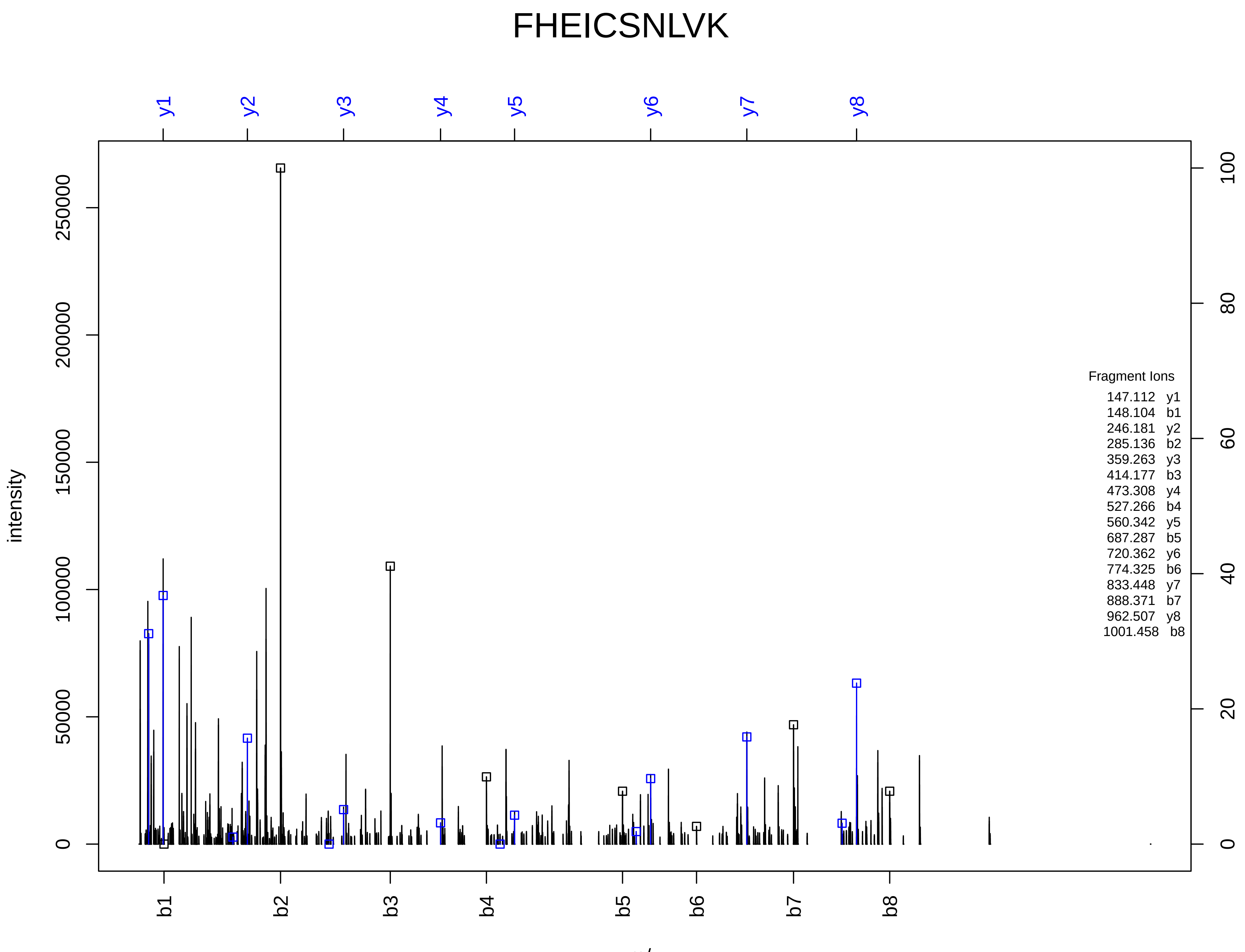

FHEICSNLVK / File: christine_10pools_07.raw; SpectrumID: ; scans: 3472

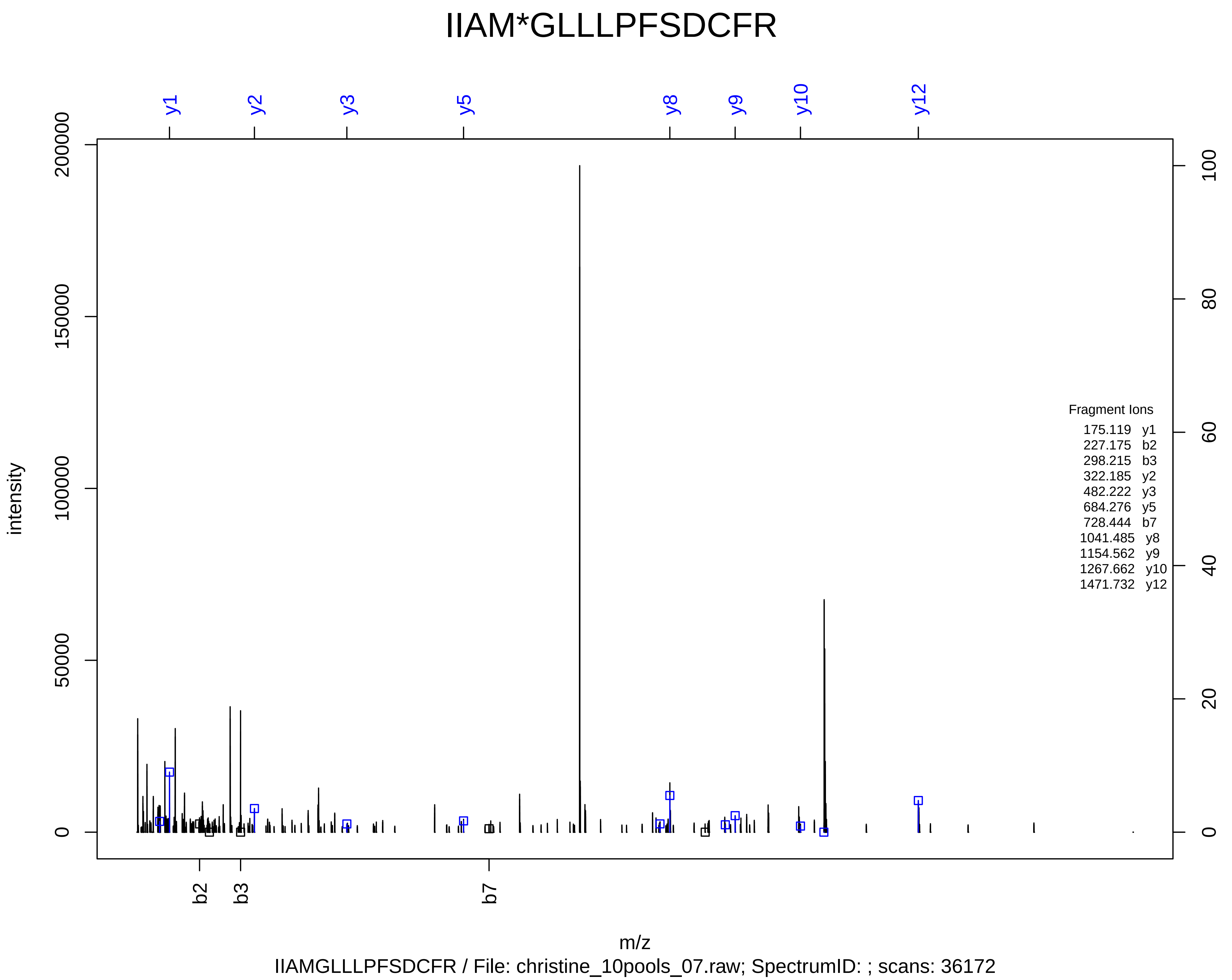
IIAM*GLLLPFSDCFR
y1
y2
y3
y5
y8
y9
y10
y12
intensity
200000
150000
100000
50000
0
100
80
60
40
20
0
Fragment Ions
175.119 y1
227.175 b2
298.215 b3
322.185 y2
482.222 y3
684.276 y5
728.444 b7
1041.485 y8
1154.562 y9
1267.662 y10
1471.732 y12
b2
b3
b7
m/z
IIAMGLLLPFSDCFR / File: christine_10pools_07.raw; SpectrumID: ; scans: 36172

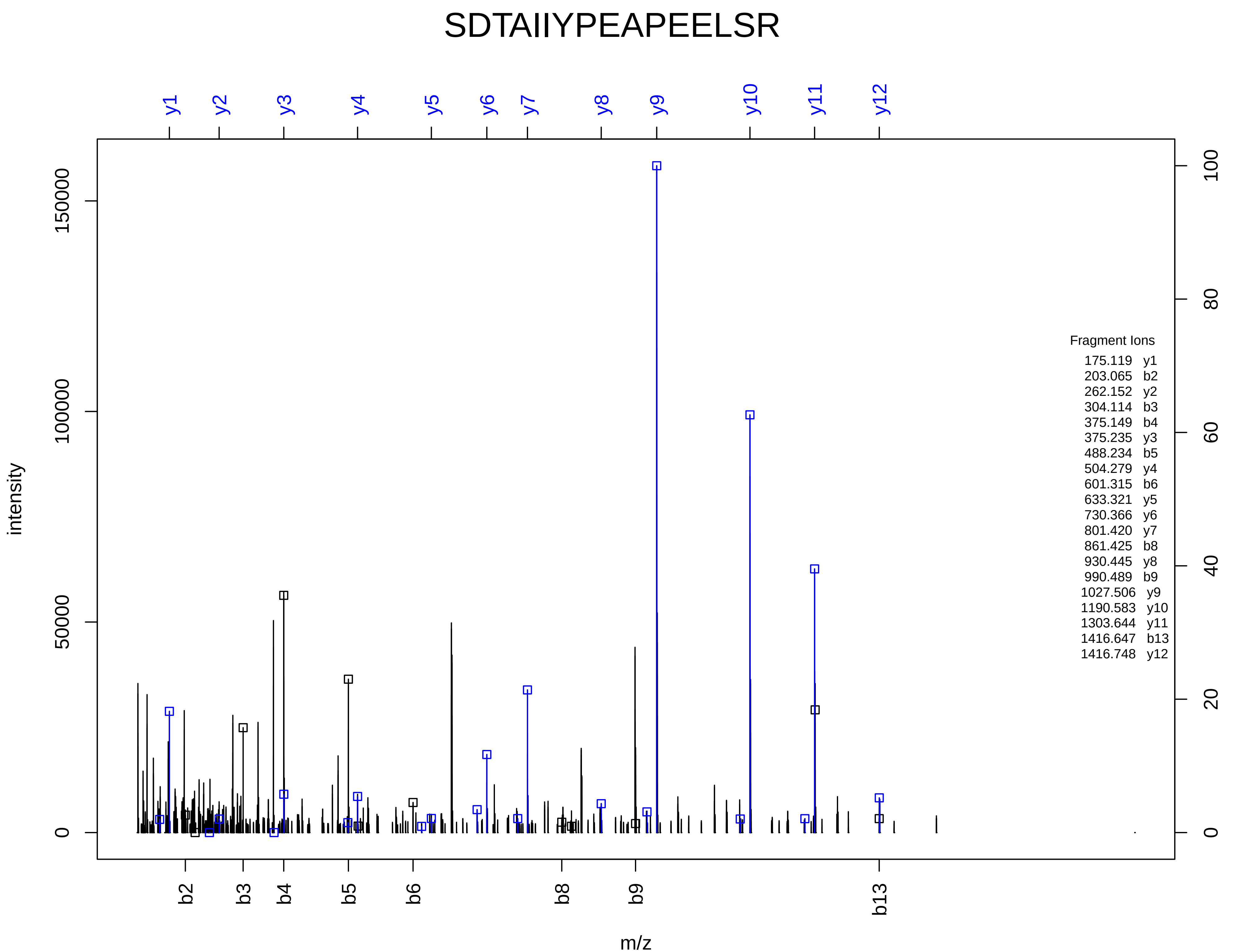

SDTAIIYPEAPEELSR / File: christine_10pools_07.raw; SpectrumID: ; scans: 17794

QGTFCVDCETYHTAASTLGSQGQAGK

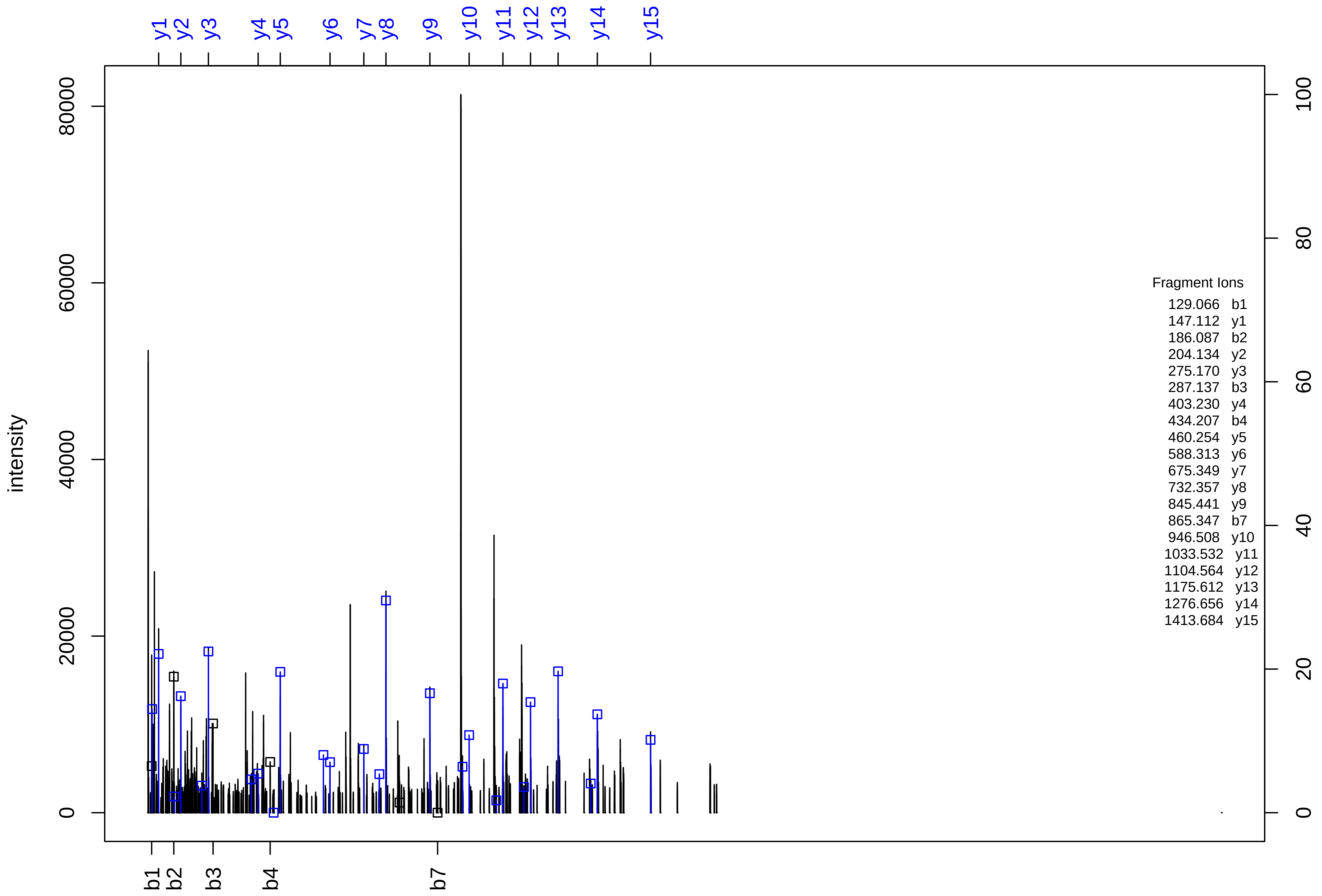

QGTFCVDCETYHTAASTLGSQGQAGK / File: christine_10pools_07.raw; SpectrumID: ; scans: 11094

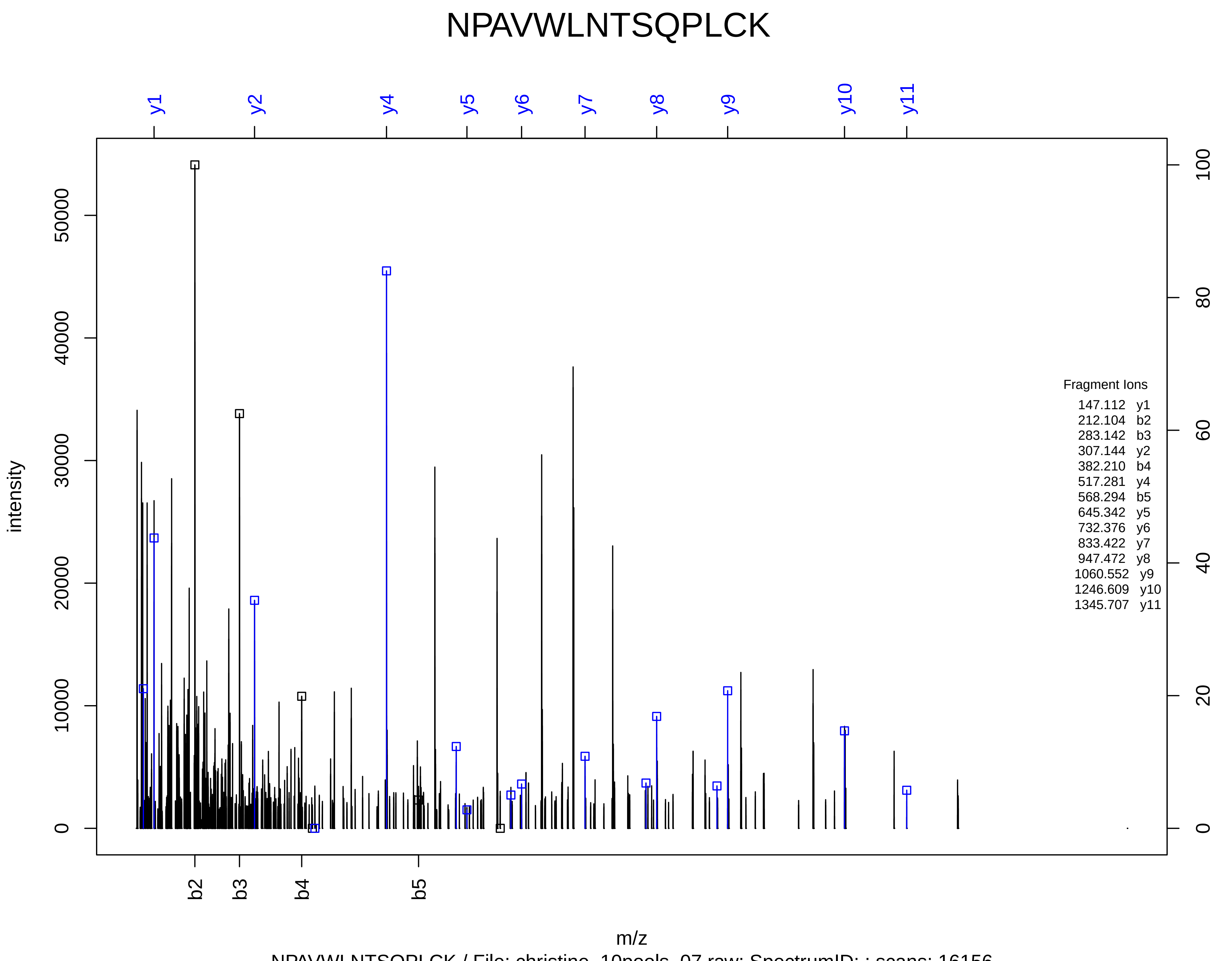

NPAVWLNTSQPLCK
y1
y2
y4
y5
y6
y7
y8
y9
y10
y11
intensity
0
10000
20000
30000
40000
50000
0
20
40
60
80
100
b2
b3
b4
b5
m/z
Fragment Ions
147.112 y1
212.104 b2
283.142 b3
307.144 y2
382.210 b4
517.281 y4
568.294 b5
645.342 y5
732.376 y6
833.422 y7
947.472 y8
1060.552 y9
1246.609 y10
1345.707 y11
NPAVWLNTSQPLCK / File: christine_10pools_07.raw; SpectrumID: ; scans: 16156

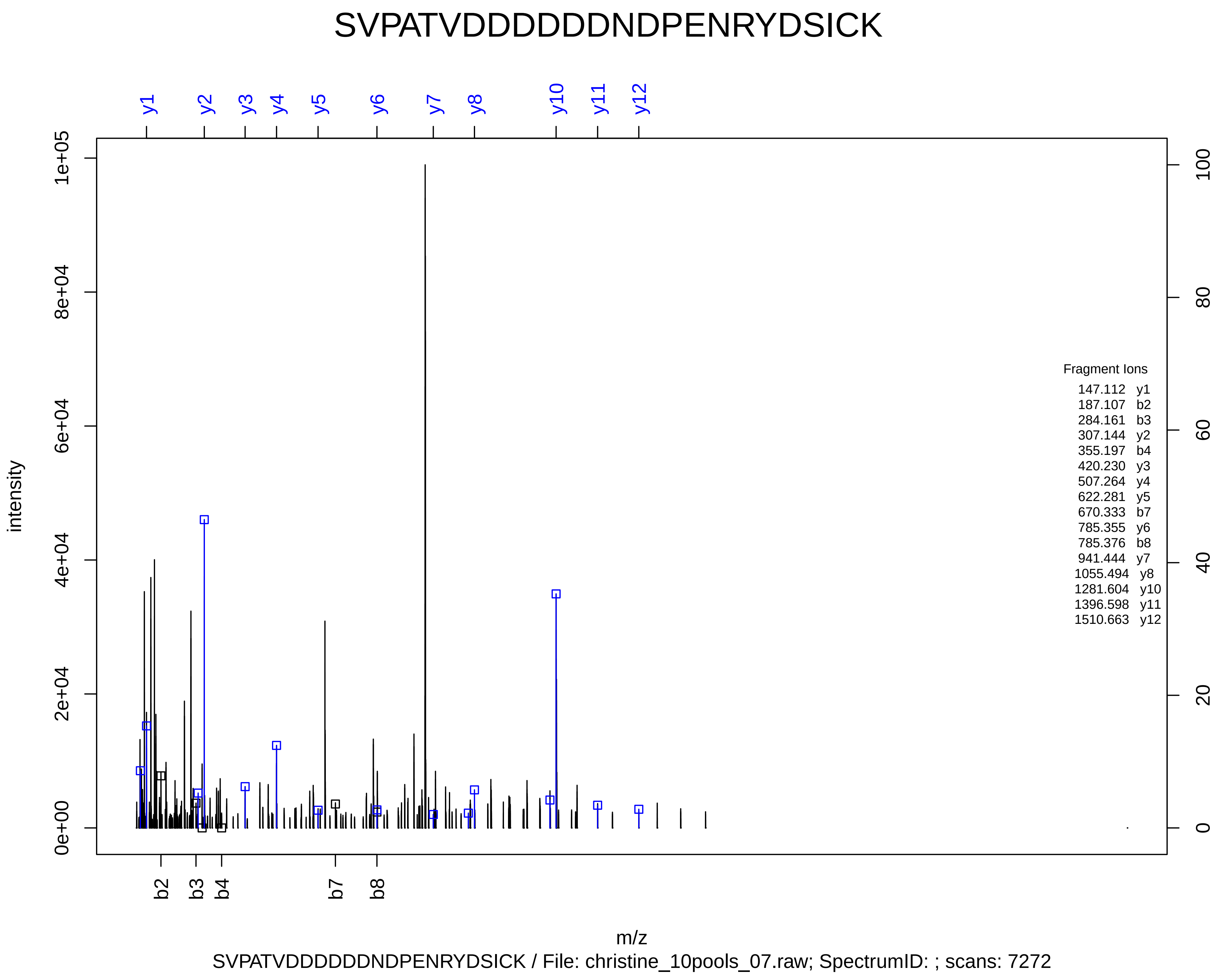

SVPATVDDDDDDNDPENRYDSICK
y1
y2
y3
y4
y5
y6
y7
y8
y10
y11
y12
intensity
0e+00
2e+04
4e+04
6e+04
8e+04
1e+05
0
20
40
60
80
100
b2
b3
b4
b7
b8
m/z
Fragment Ions
147.112 y1
187.107 b2
284.161 b3
307.144 y2
355.197 b4
420.230 y3
507.264 y4
622.281 y5
670.333 b7
785.355 y6
785.376 b8
941.444 y7
1055.494 y8
1281.604 y10
1396.598 y11
1510.663 y12
SVPATVDDDDDDNDPENRYDSICK / File: christine_10pools_07.raw; SpectrumID: ; scans: 7272

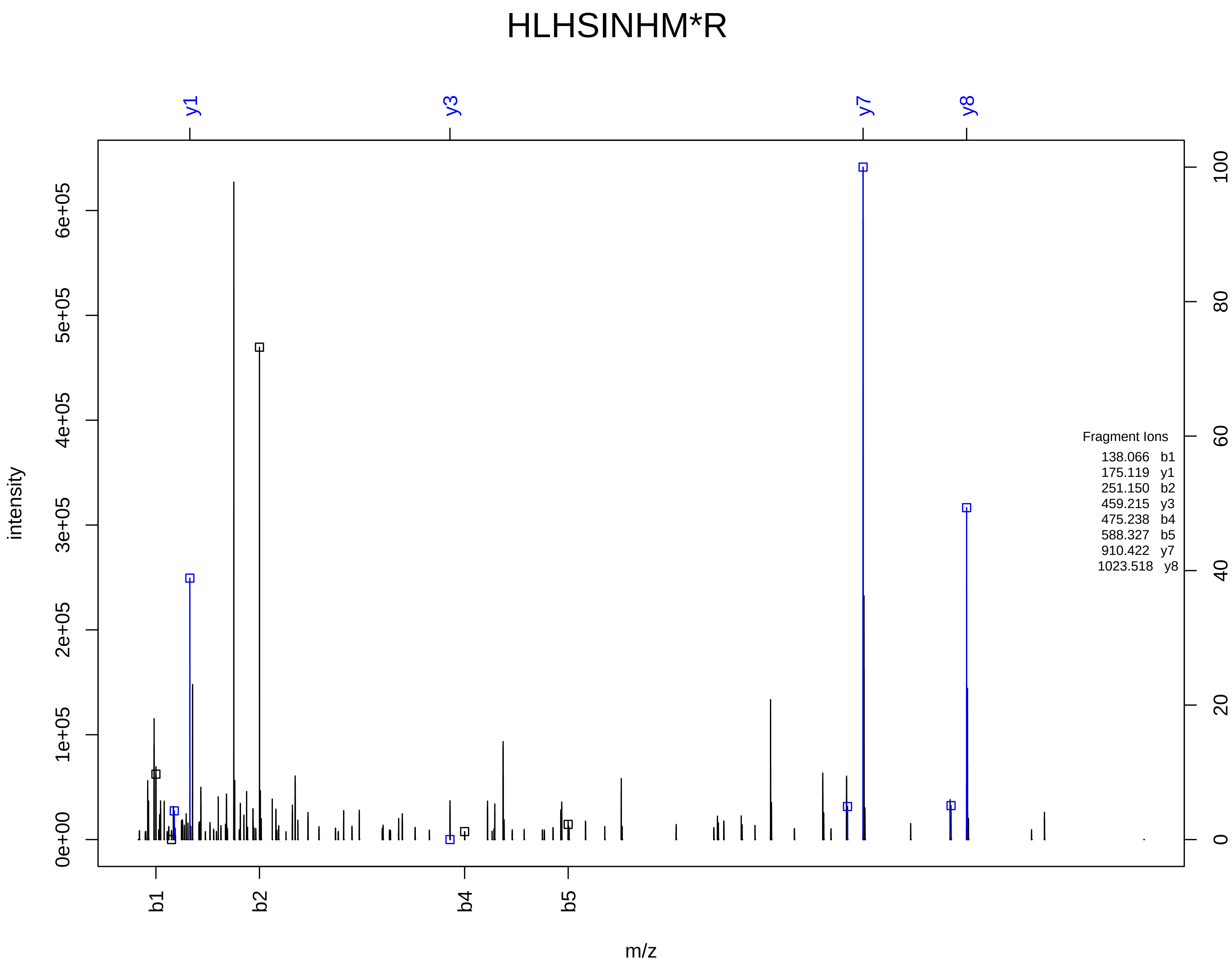
HLHSINHM*R
y1
y3
y7
y8
intensity
0e+00
1e+05
2e+05
3e+05
4e+05
5e+05
6e+05
0
20
40
60
80
100
Fragment Ions
138.066 b1
175.119 y1
251.150 b2
459.215 y3
475.238 b4
588.327 b5
910.422 y7
1023.518 y8
b1
b2
b4
b5
m/z
HLHSINHMR / File: christine_10pools_07.raw; SpectrumID: ; scans: 1499

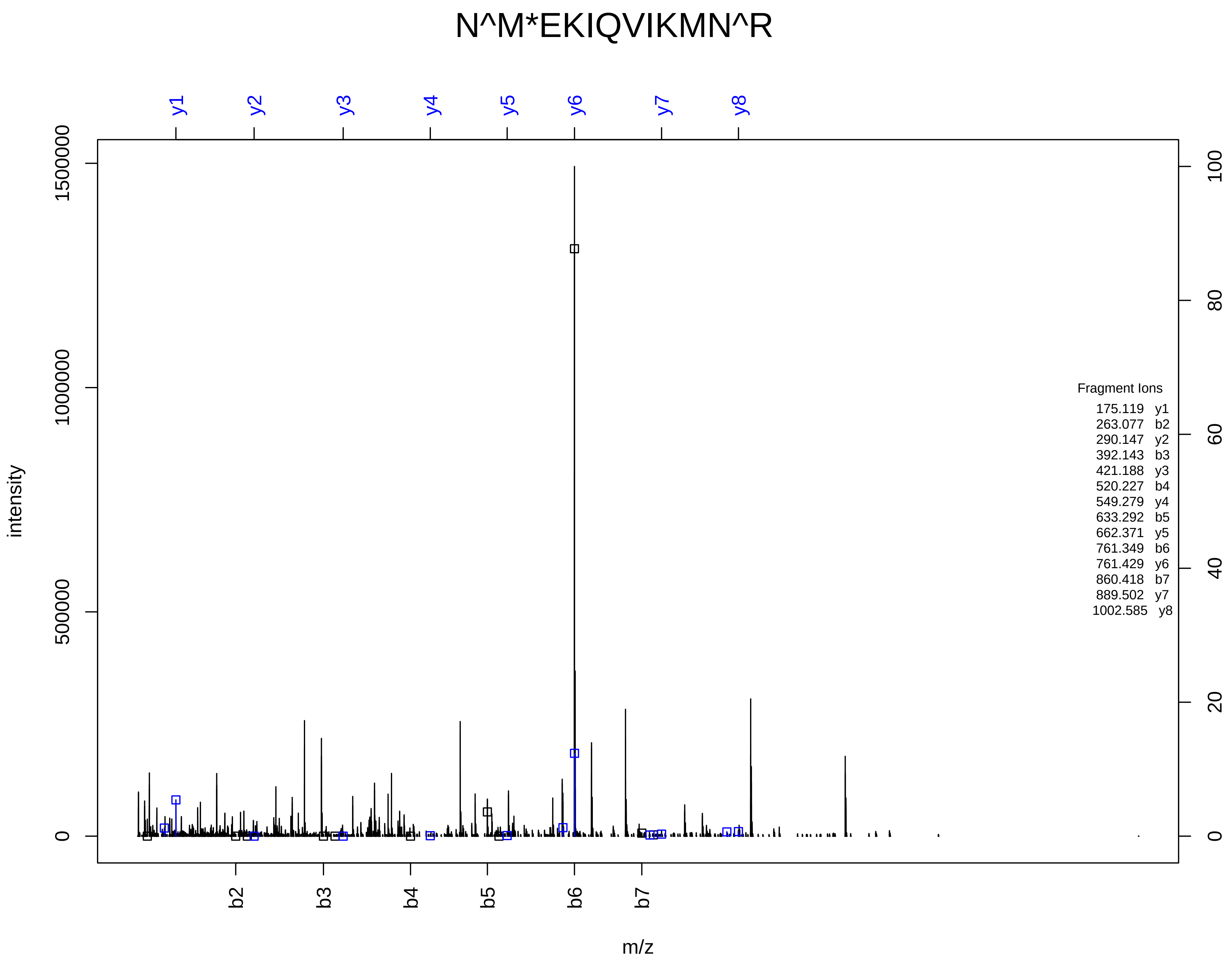
N^M*EKIQVIKMN^R
y1
y2
y3
y4
y5
y6
y7
y8
b2
b3
b4
b5
b6
b7
intensity
0
500000
1000000
1500000
0
20
40
60
80
100
m/z
Fragment Ions
175.119 y1
263.077 b2
290.147 y2
392.143 b3
421.188 y3
520.227 b4
549.279 y4
633.292 b5
662.371 y5
761.349 b6
761.429 y6
860.418 b7
889.502 y7
1002.585 y8
NMEKIQVIKMNR / File: christine_10pools_07.raw; SpectrumID: ; scans: 13715

(Ac)MVQPSLGAM*ALKR

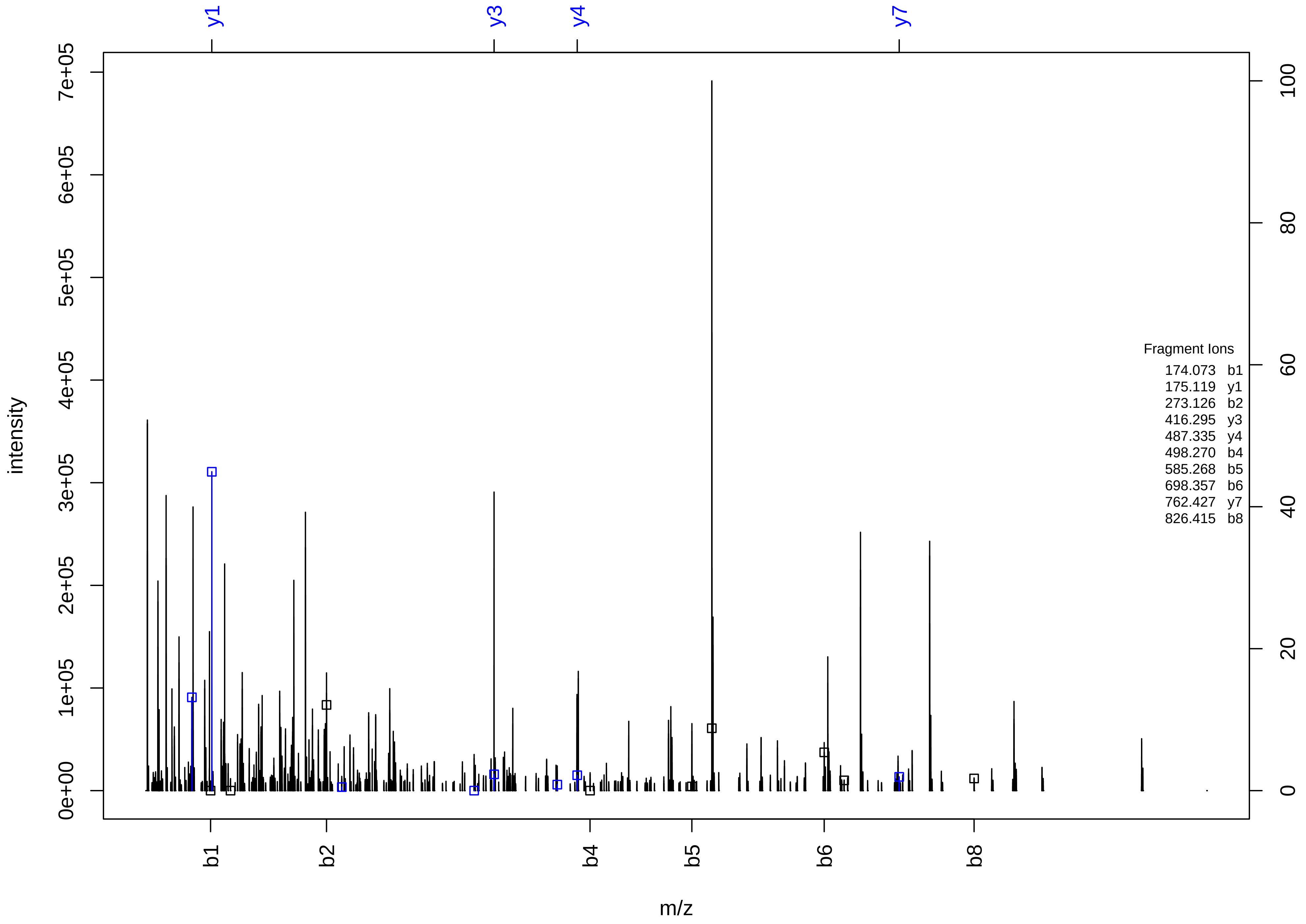

MVQPSLGAMALKR / File: christine_10pools_07.raw; SpectrumID: ; scans: 10932

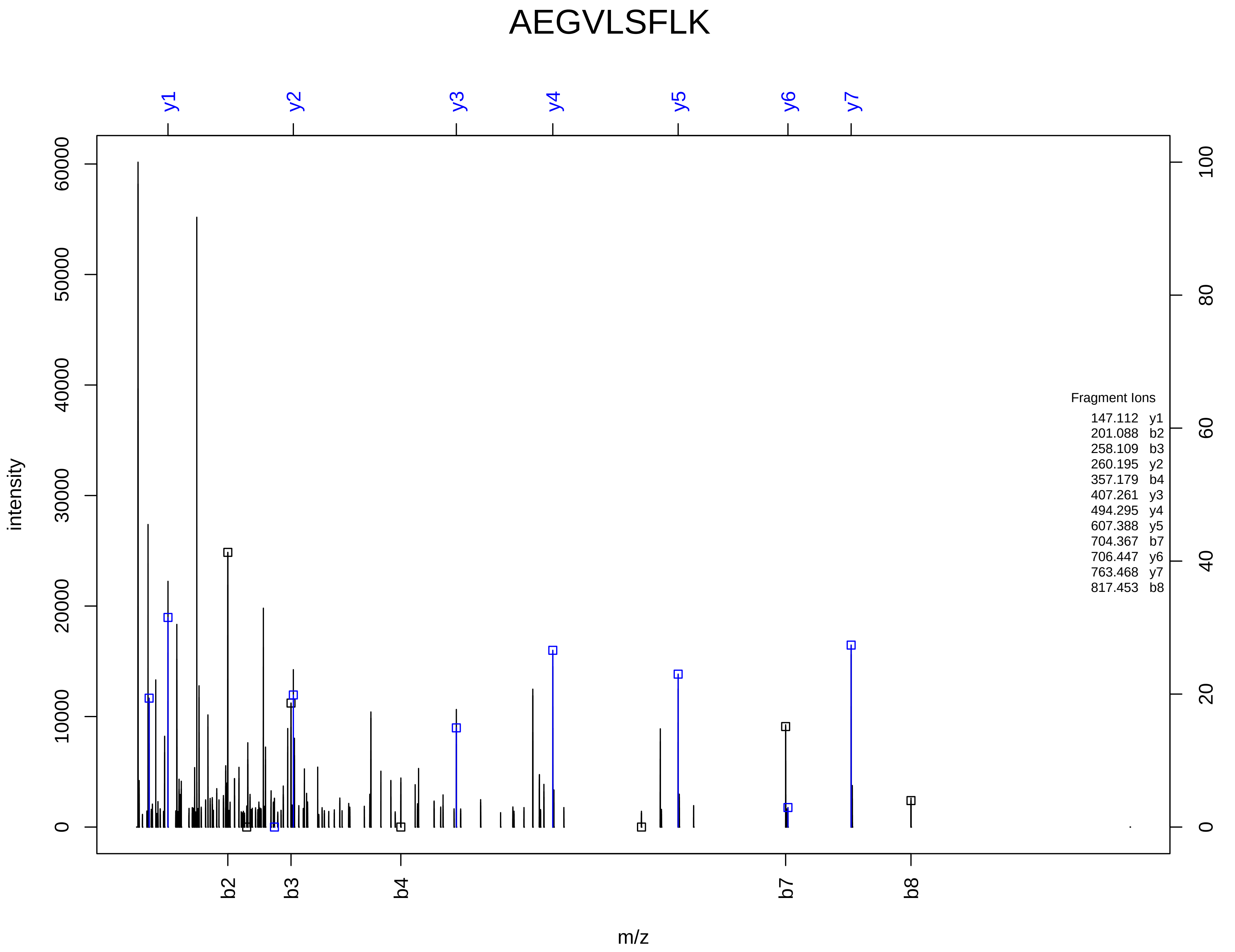
AEGVLSFLK
y1
y2
y3
y4
y5
y6
y7
intensity
0
10000
20000
30000
40000
50000
60000
0
20
40
60
80
100
Fragment Ions
147.112 y1
201.088 b2
258.109 b3
260.195 y2
357.179 b4
407.261 y3
494.295 y4
607.388 y5
704.367 b7
706.447 y6
763.468 y7
817.453 b8
b2
b3
b4
b7
b8
m/z
AEGVLSFLK / File: christine_10pools_07.raw; SpectrumID: ; scans: 22374

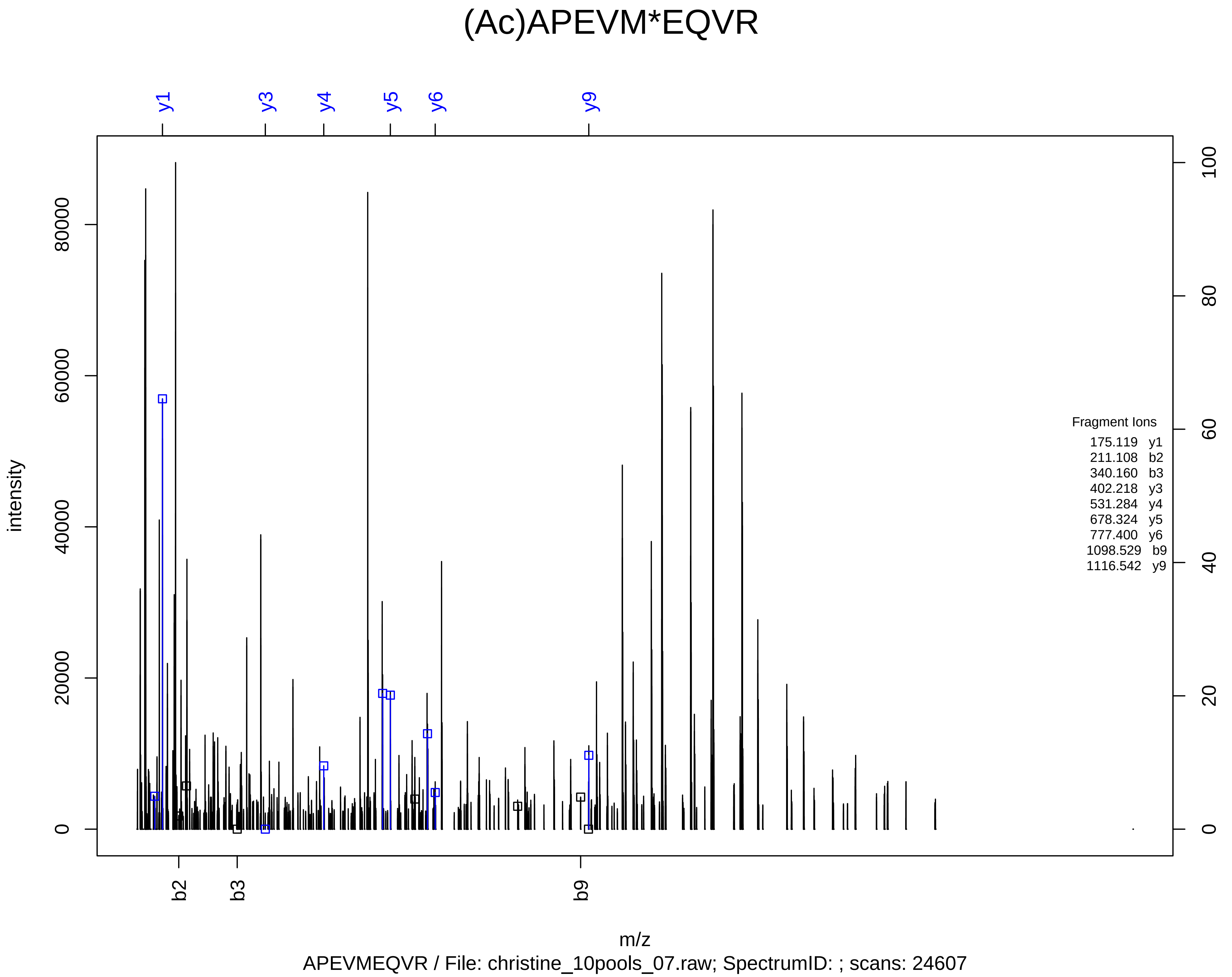

(Ac)APEVM*EQVR
y1
y3
y4
y5
y6
y9
intensity
0
20000
40000
60000
80000
0
20
40
60
80
100
b2
b3
b9
m/z
Fragment Ions
175.119 y1
211.108 b2
340.160 b3
402.218 y3
531.284 y4
678.324 y5
777.400 y6
1098.529 b9
1116.542 y9
APEVMEQVR / File: christine_10pools_07.raw; SpectrumID: ; scans: 24607

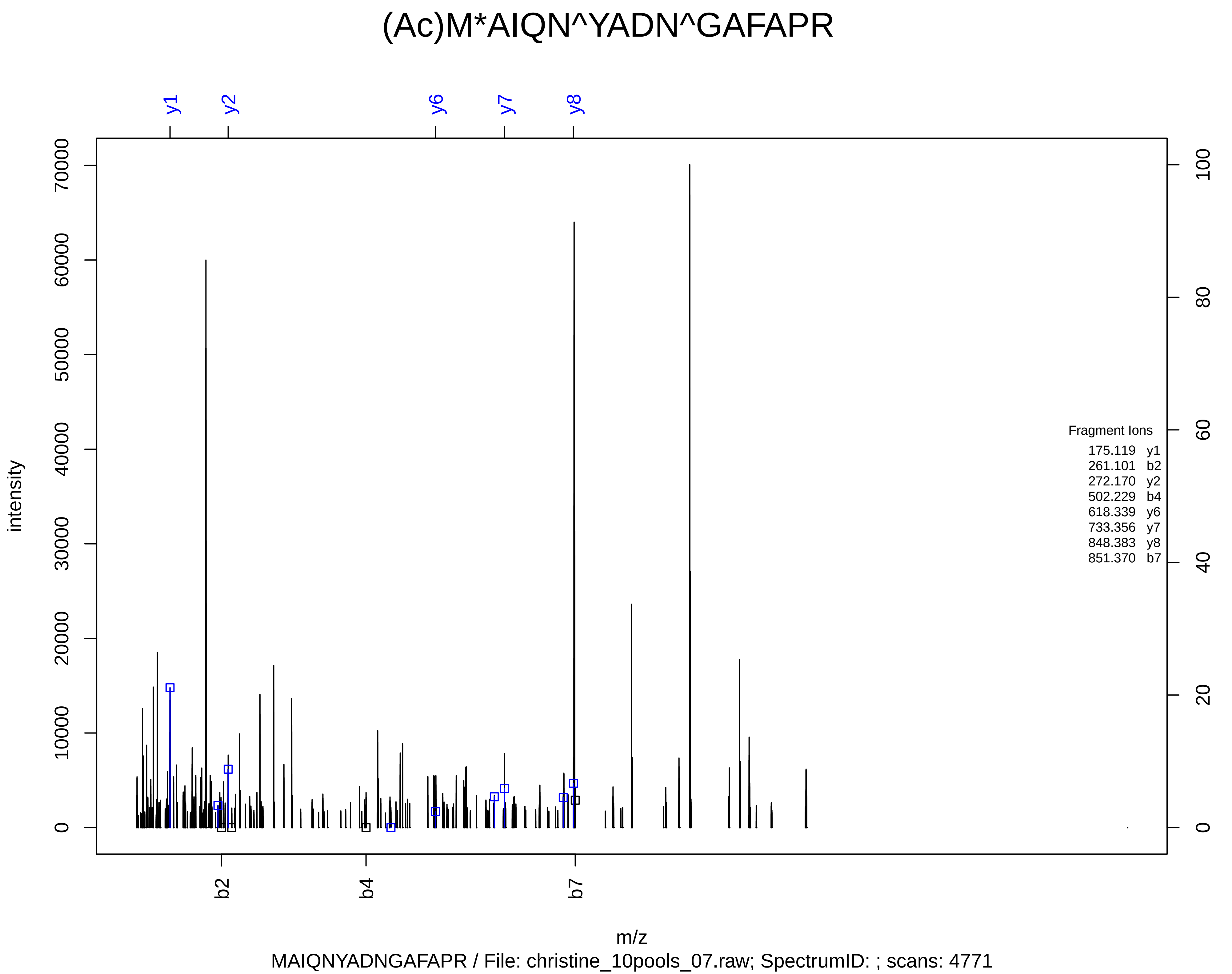

(Ac)M*AIQN^YADN^GAFAPR
y1
y2
y6
y7
y8
intensity
70000
60000
50000
40000
30000
20000
10000
0
100
80
60
40
20
0
Fragment Ions
175.119 y1
261.101 b2
272.170 y2
502.229 b4
618.339 y6
733.356 y7
848.383 y8
851.370 b7
b2
b4
b7
m/z
MAIQNYADNGAFAPR / File: christine_10pools_07.raw; SpectrumID: ; scans: 4771

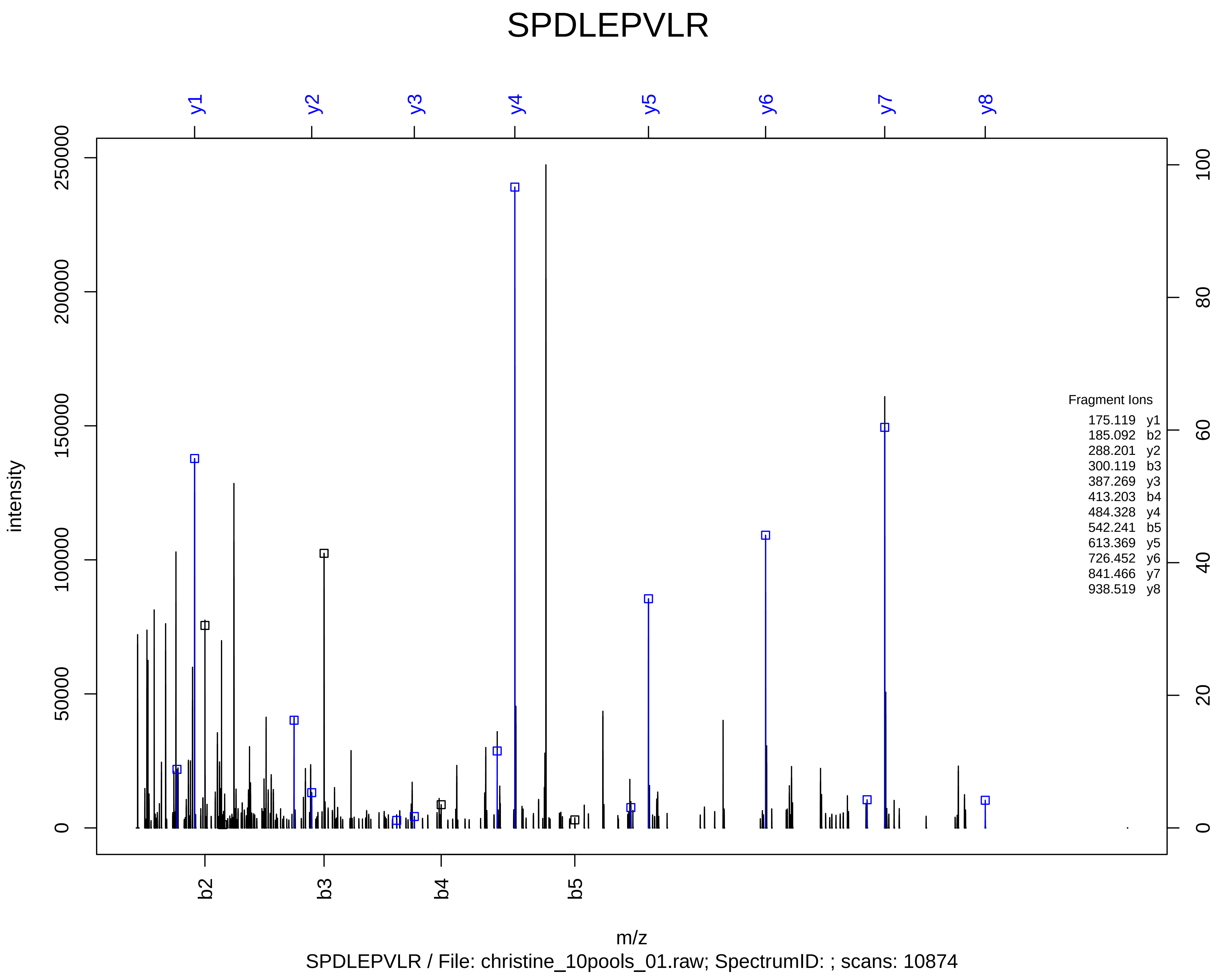

SPDLEPVLR
y1
y2
y3
y4
y5
y6
y7
y8
intensity
0
50000
100000
150000
200000
250000
0
20
40
60
80
100
b2
b3
b4
b5
m/z
Fragment Ions
175.119 y1
185.092 b2
288.201 y2
300.119 b3
387.269 y3
413.203 b4
484.328 y4
542.241 b5
613.369 y5
726.452 y6
841.466 y7
938.519 y8
SPDLEPVLR / File: christine_10pools_01.raw; SpectrumID: ; scans: 10874

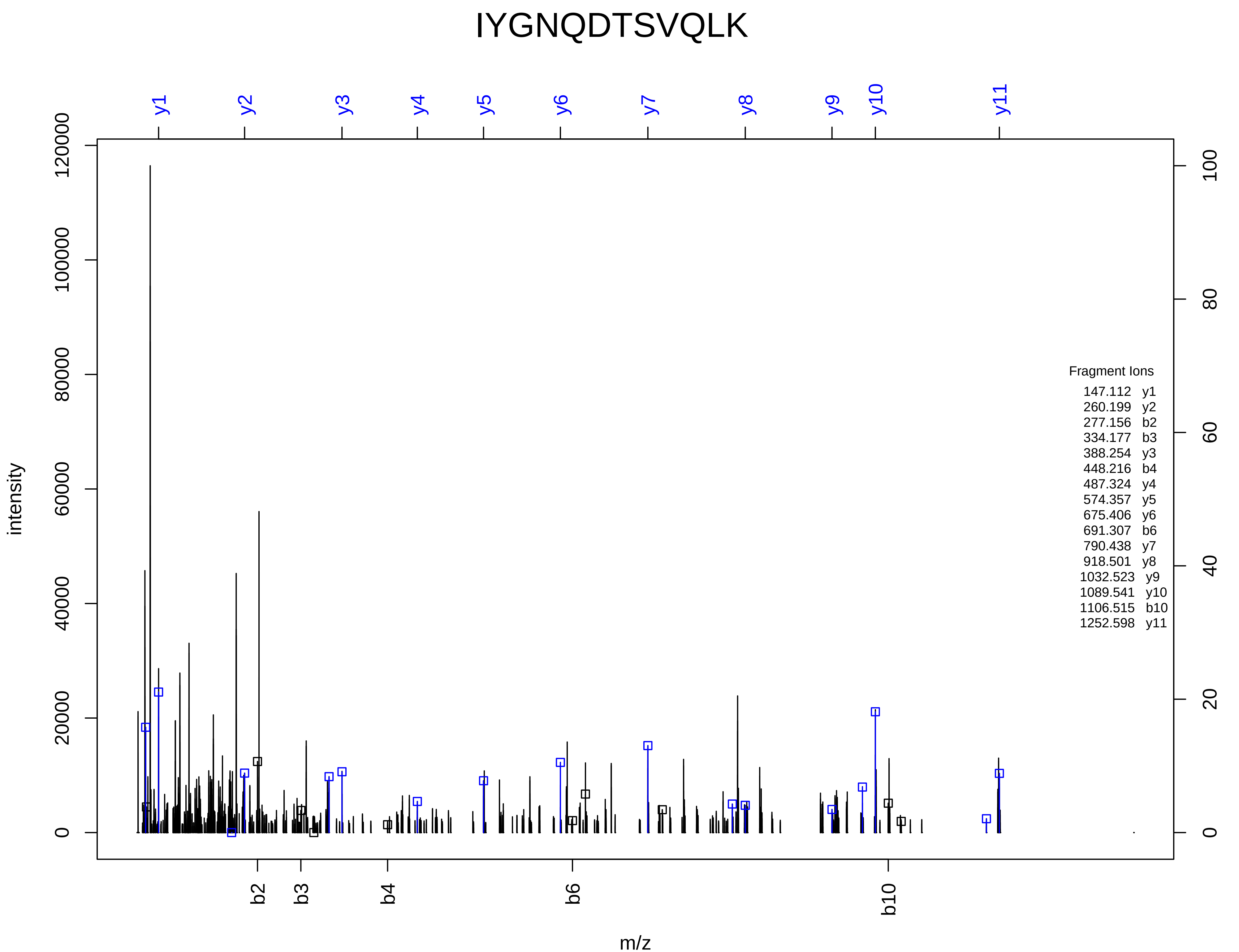

IYGNQDTSVQLK
intensity
m/z
y1 y2 y3 y4 y5 y6 y7 y8 y9 y10 y11
b2 b3 b4 b6 b10
Fragment Ions
147.112 y1
260.199 y2
277.156 b2
334.177 b3
388.254 y3
448.216 b4
487.324 y4
574.357 y5
675.406 y6
691.307 b6
790.438 y7
918.501 y8
1032.523 y9
1089.541 y10
1106.515 b10
1252.598 y11
IYGNQDTSVQLK / File: christine_10pools_01.raw; SpectrumID: ; scans: 4033

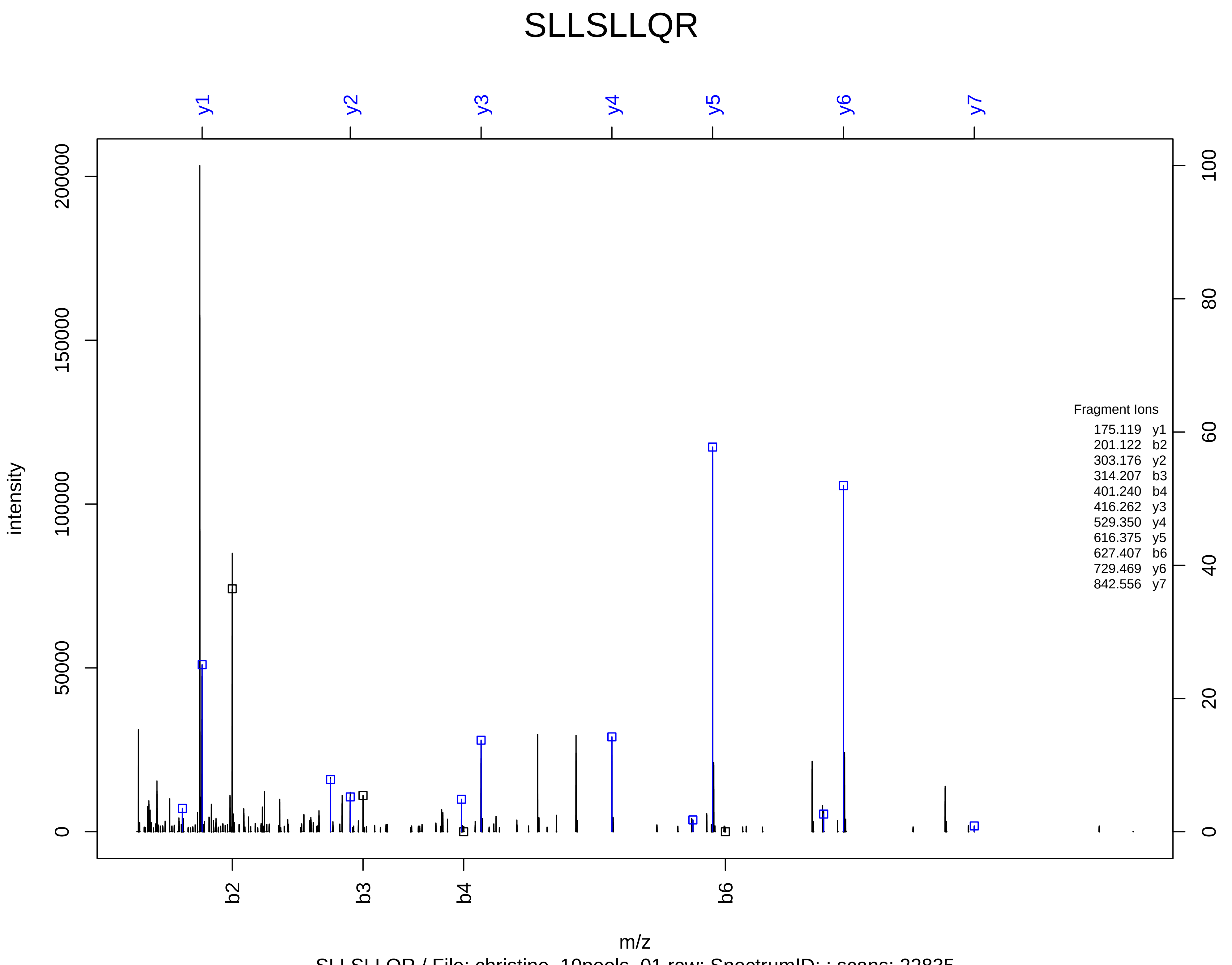
SLLSLLQR
y1
y2
y3
y4
y5
y6
y7
b2
b3
b4
b6
intensity
0
50000
100000
150000
200000
0
20
40
60
80
100
m/z
Fragment Ions
175.119 y1
201.122 b2
303.176 y2
314.207 b3
401.240 b4
416.262 y3
529.350 y4
616.375 y5
627.407 b6
729.469 y6
842.556 y7
SLLSLLQR / File: christine_10pools_01.raw; SpectrumID: ; scans: 22835

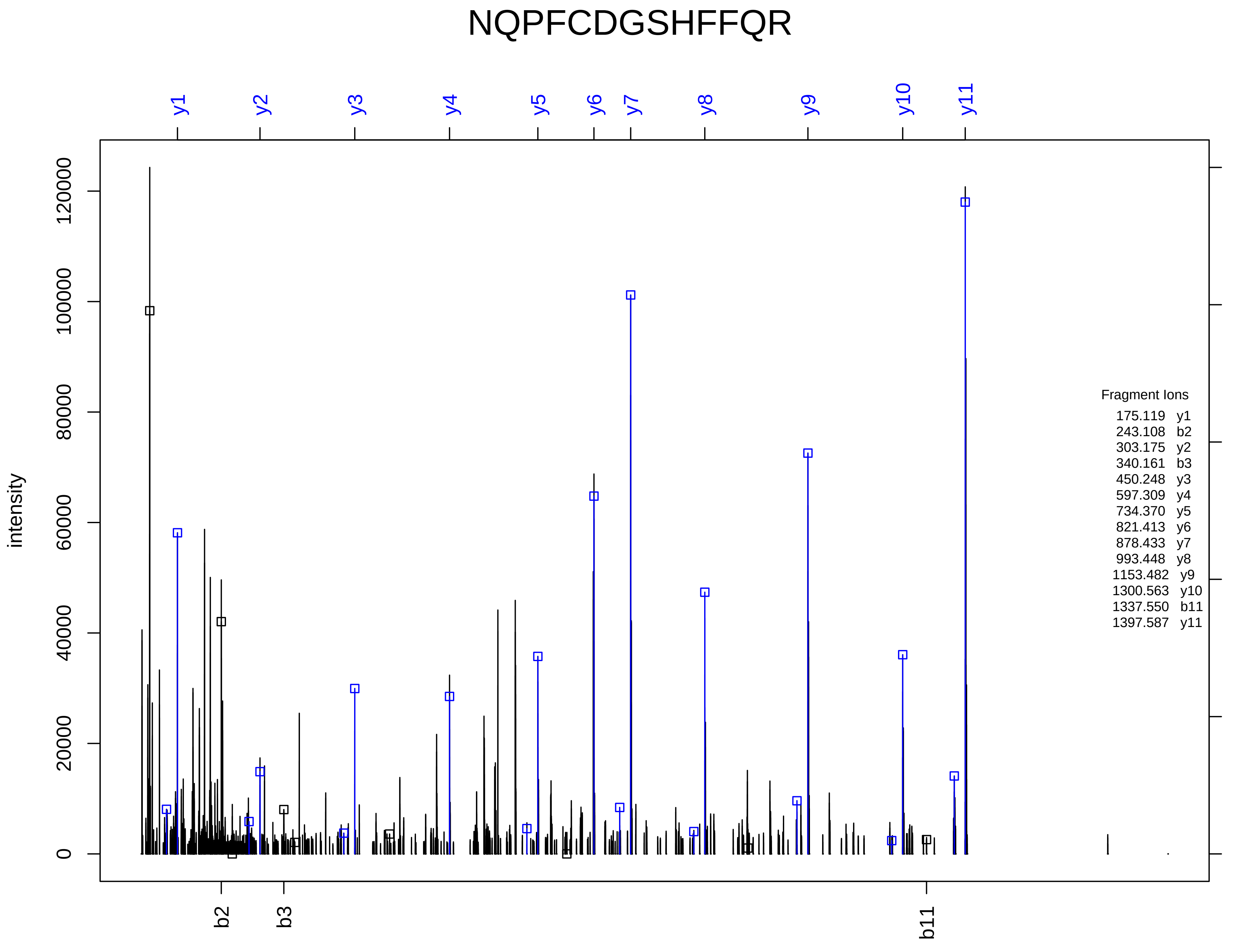
NQPFCDGSHFFQR
y1
y2
y3
y4
y5
y6
y7
y8
y9
y10
y11
b2
b3
b11
intensity
0
20000
40000
60000
80000
100000
120000
0
20
40
60
80
100
m/z
Fragment Ions
175.119 y1
243.108 b2
303.175 y2
340.161 b3
450.248 y3
597.309 y4
734.370 y5
821.413 y6
878.433 y7
993.448 y8
1153.482 y9
1300.563 y10
1337.550 b11
1397.587 y11
NQPFCDGSHFFQR / File: christine_10pools_01.raw; SpectrumID: ; scans: 9528

LLPAGDGEDVPCPACPPR

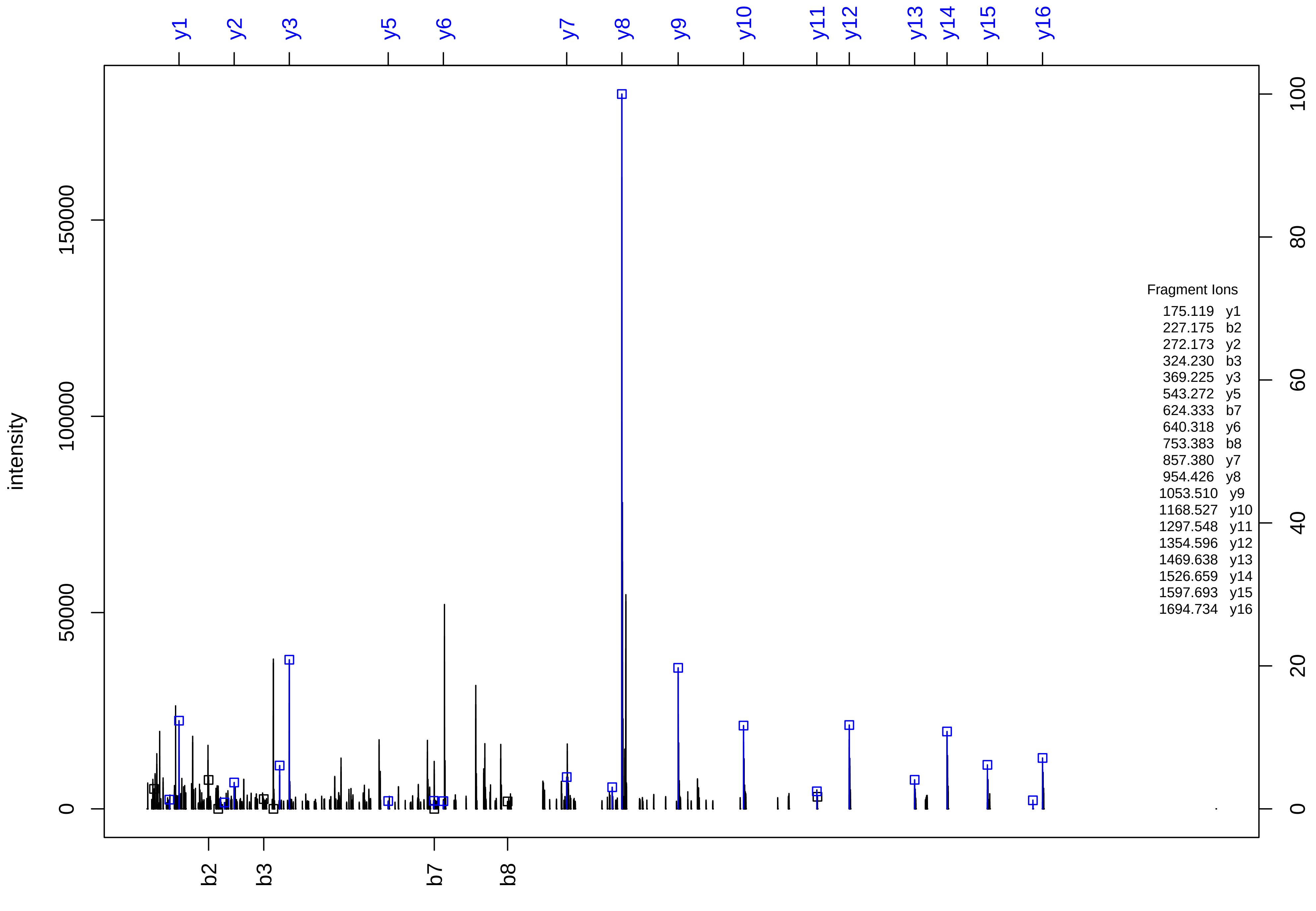

LLPAGDGEDVPCPACPPR / File: christine_10pools_01.raw; SpectrumID: ; scans: 11592

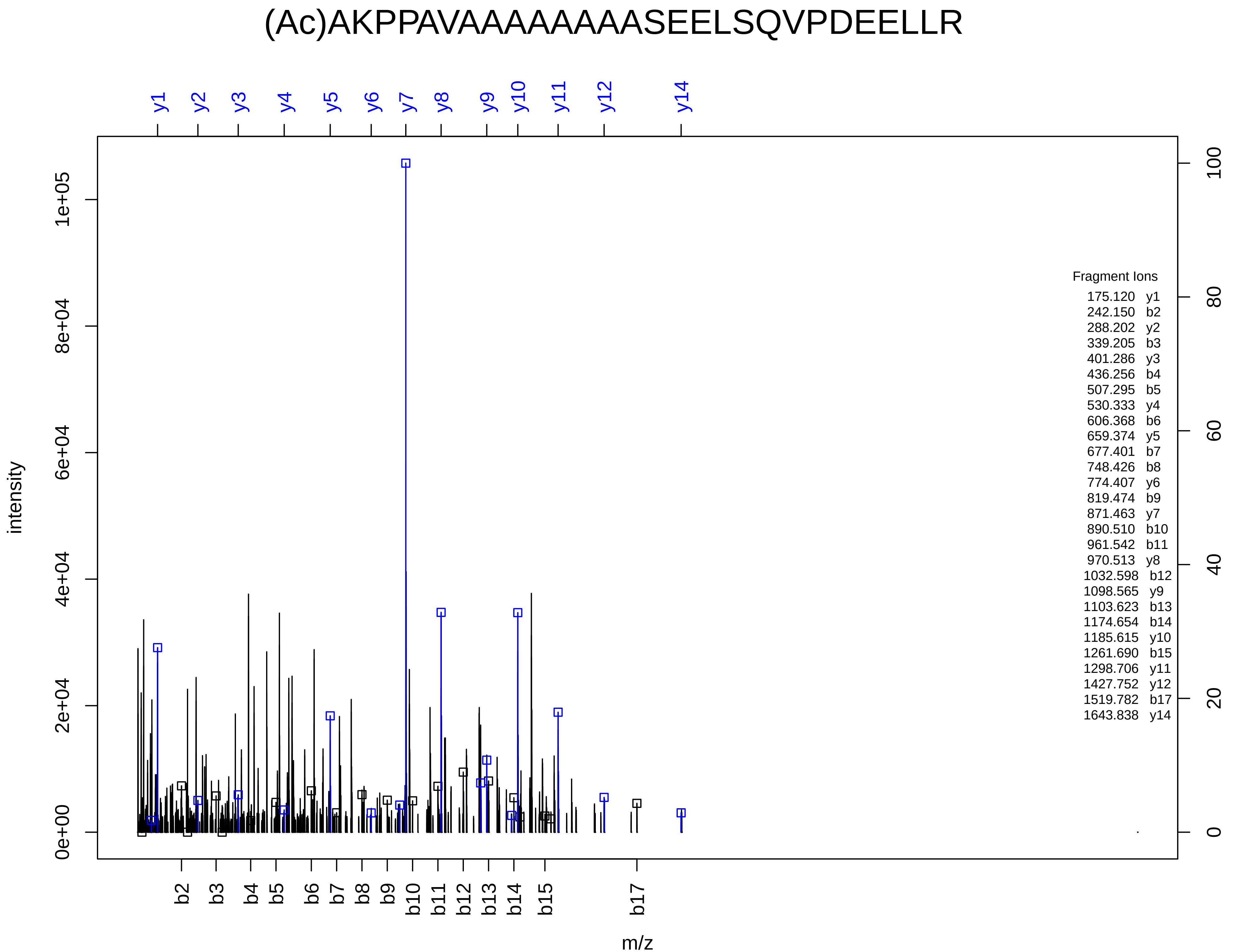

AKPPAVAAAAAAAASEELSQVPDEELLR / File: christine_10pools_01.raw; SpectrumID: ; scans: 37170

(Ac)SIETLLEAAR

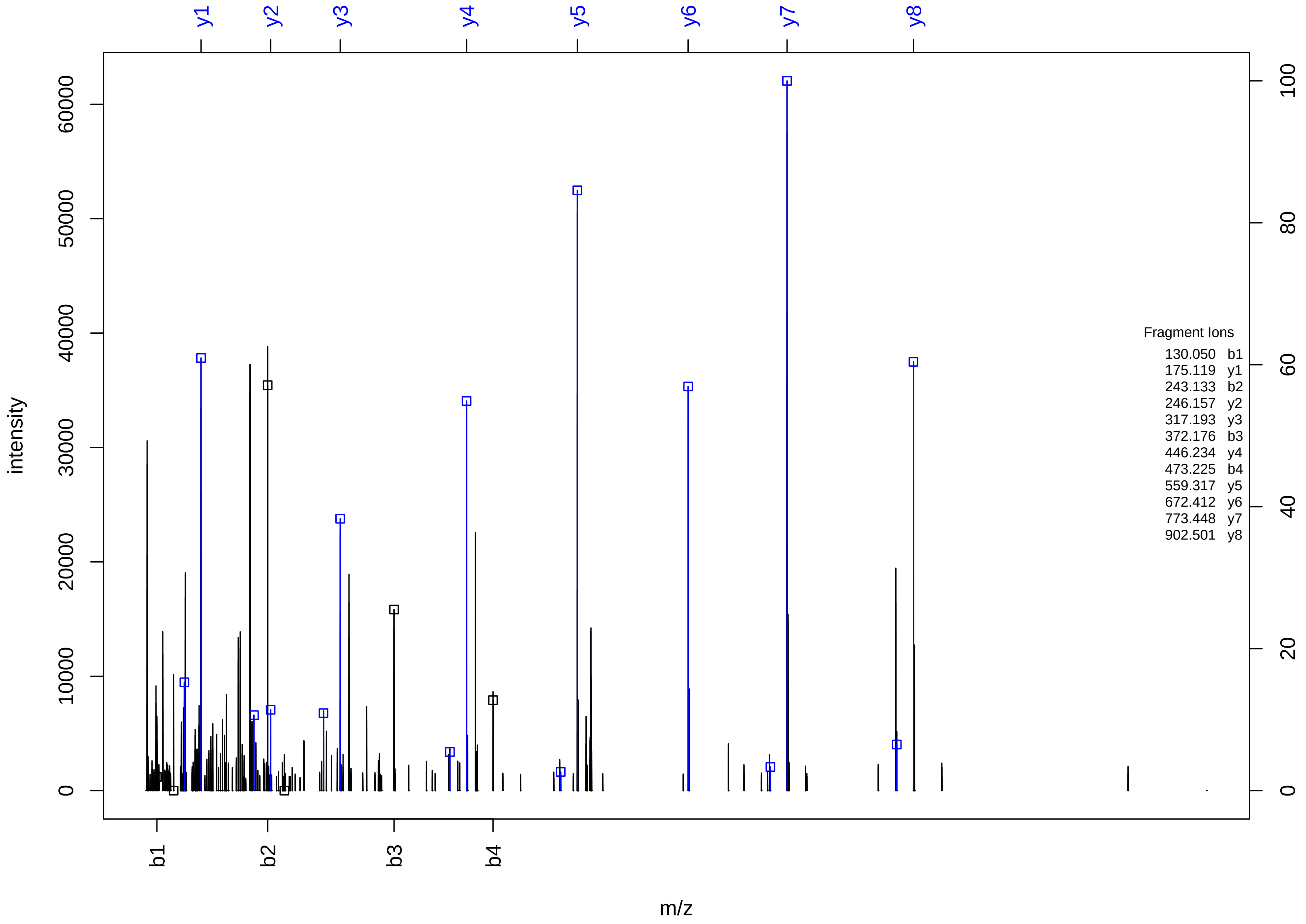

SIETLLEAAR / File: christine_10pools_01.raw; SpectrumID: ; scans: 35515

LVDTVPTDELEALNCGTELLQQR

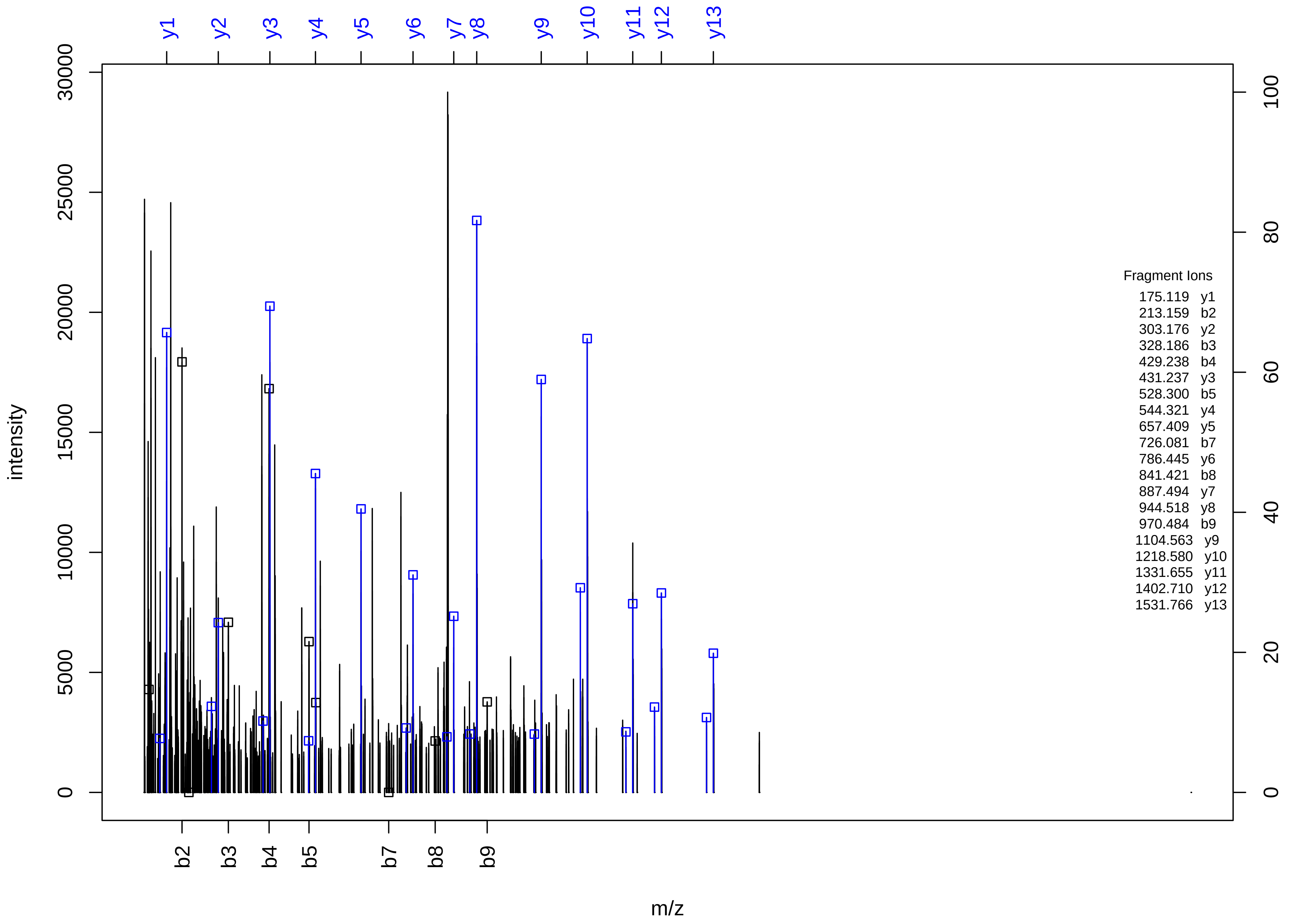

LVDTVPTDELEALNCGTELLQQR / File: christine_10pools_01.raw; SpectrumID: ; scans: 31835

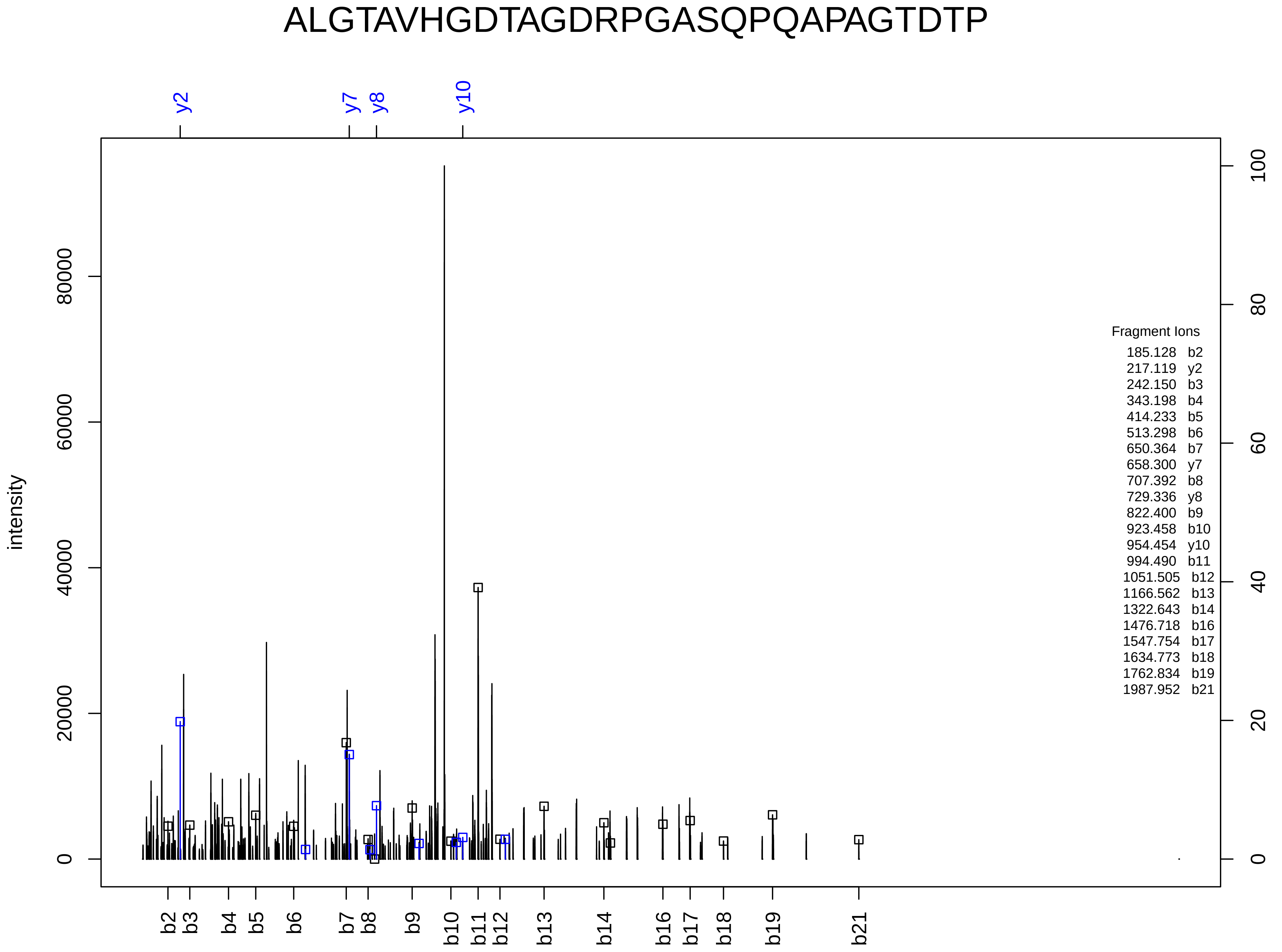

ALGTAVHGDTAGDRPGASQPQAPAGTDTP / File: christine_10pools_01.raw; SpectrumID: ; scans: 3469

ANCPHIVQFGSTDDPFLPWK

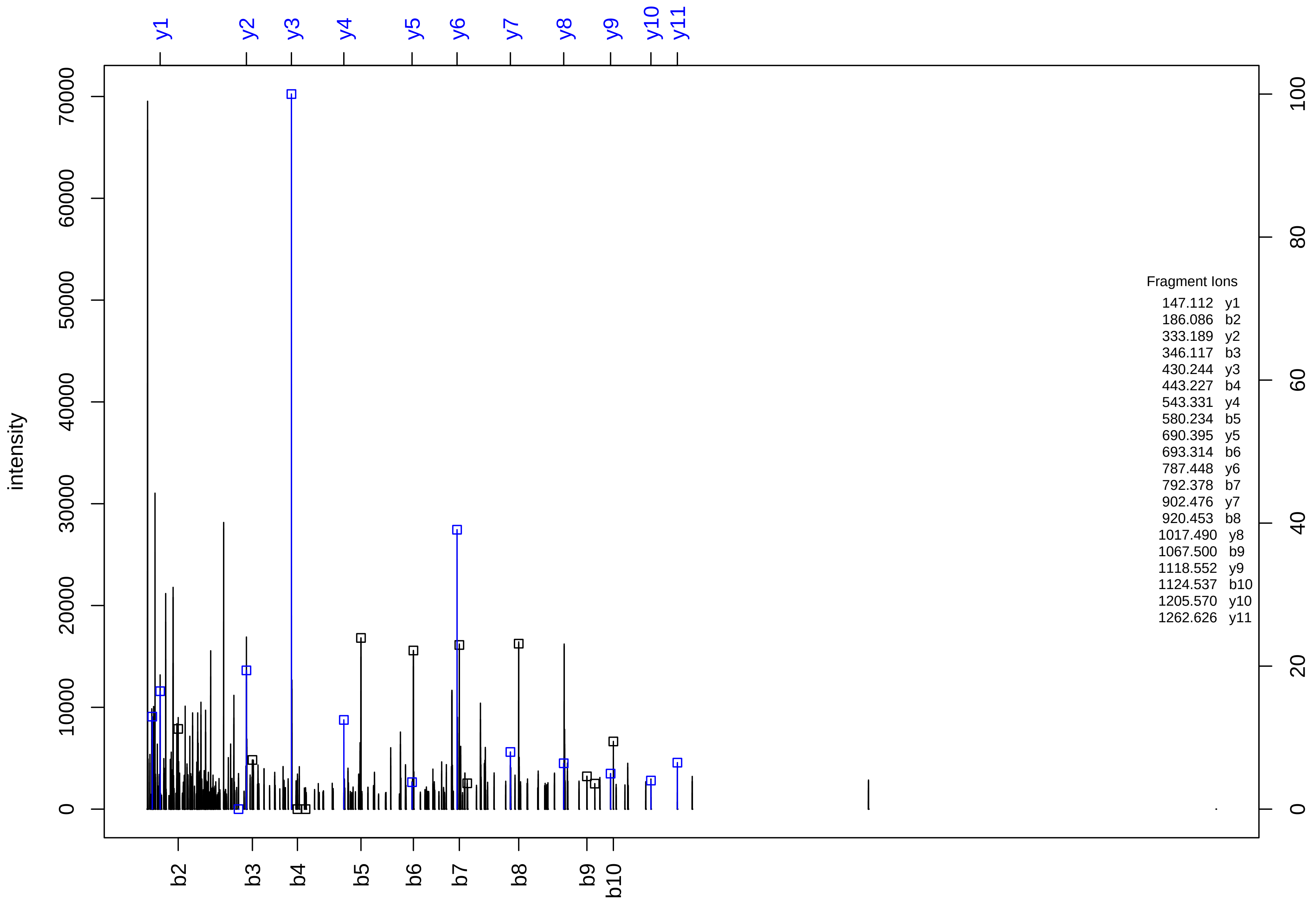

ANCPHIVQFGSTDDPFLPWK / File: christine_10pools_01.raw; SpectrumID: ; scans: 26569

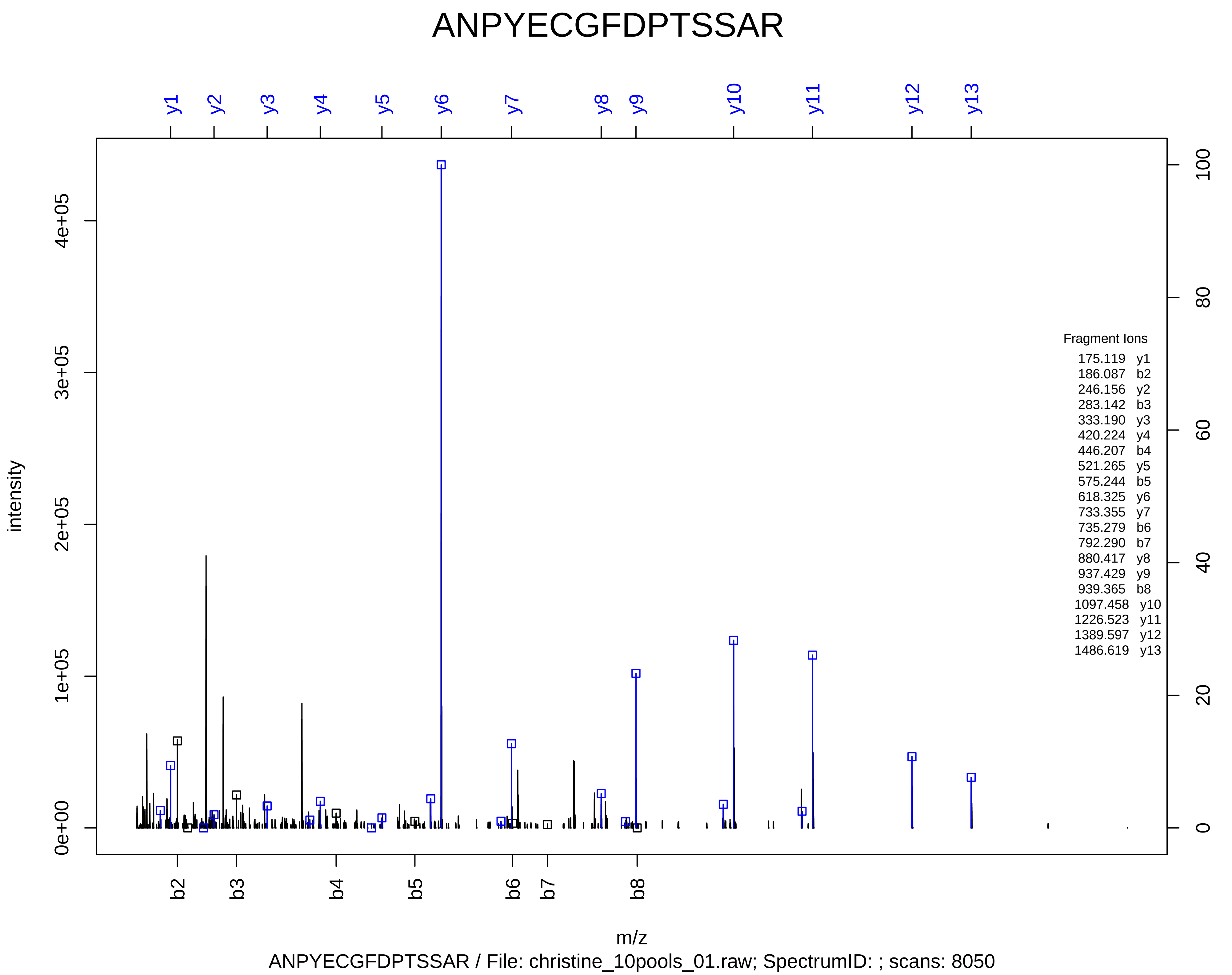

ANPYECGFDPTSSAR
y1
y2
y3
y4
y5
y6
y7
y8
y9
y10
y11
y12
y13
b2
b3
b4
b5
b6
b7
b8
intensity
0e+00
1e+05
2e+05
3e+05
4e+05
0
20
40
60
80
100
m/z
Fragment Ions
175.119 y1
186.087 b2
246.156 y2
283.142 b3
333.190 y3
420.224 y4
446.207 b4
521.265 y5
575.244 b5
618.325 y6
733.355 y7
735.279 b6
792.290 b7
880.417 y8
937.429 y9
939.365 b8
1097.458 y10
1226.523 y11
1389.597 y12
1486.619 y13
ANPYECGFDPTSSAR / File: christine_10pools_01.raw; SpectrumID: ; scans: 8050

ADAVYTGLNTR

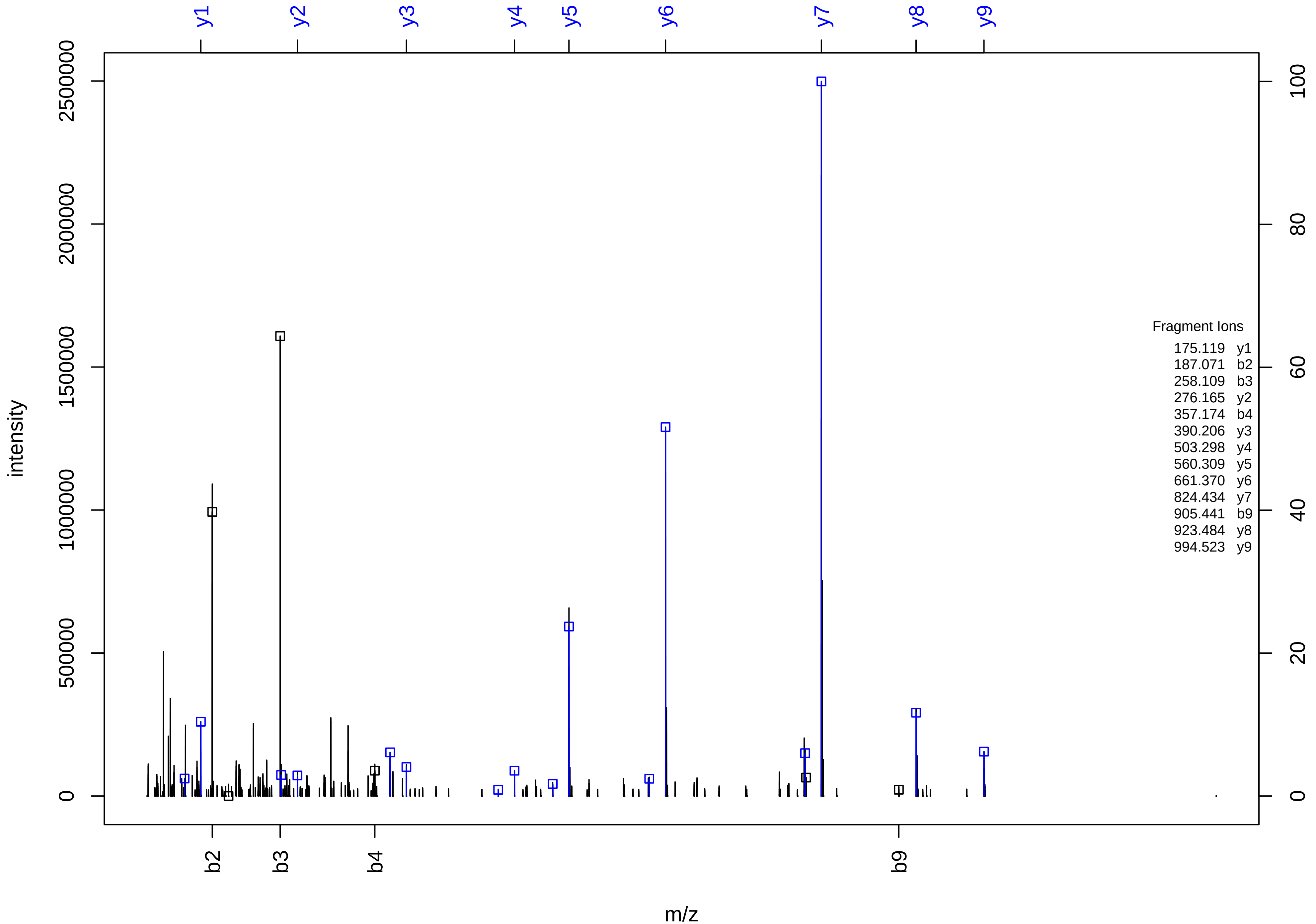

ADAVYTGLNTR / File: christine_10pools_01.raw; SpectrumID: ; scans: 4084

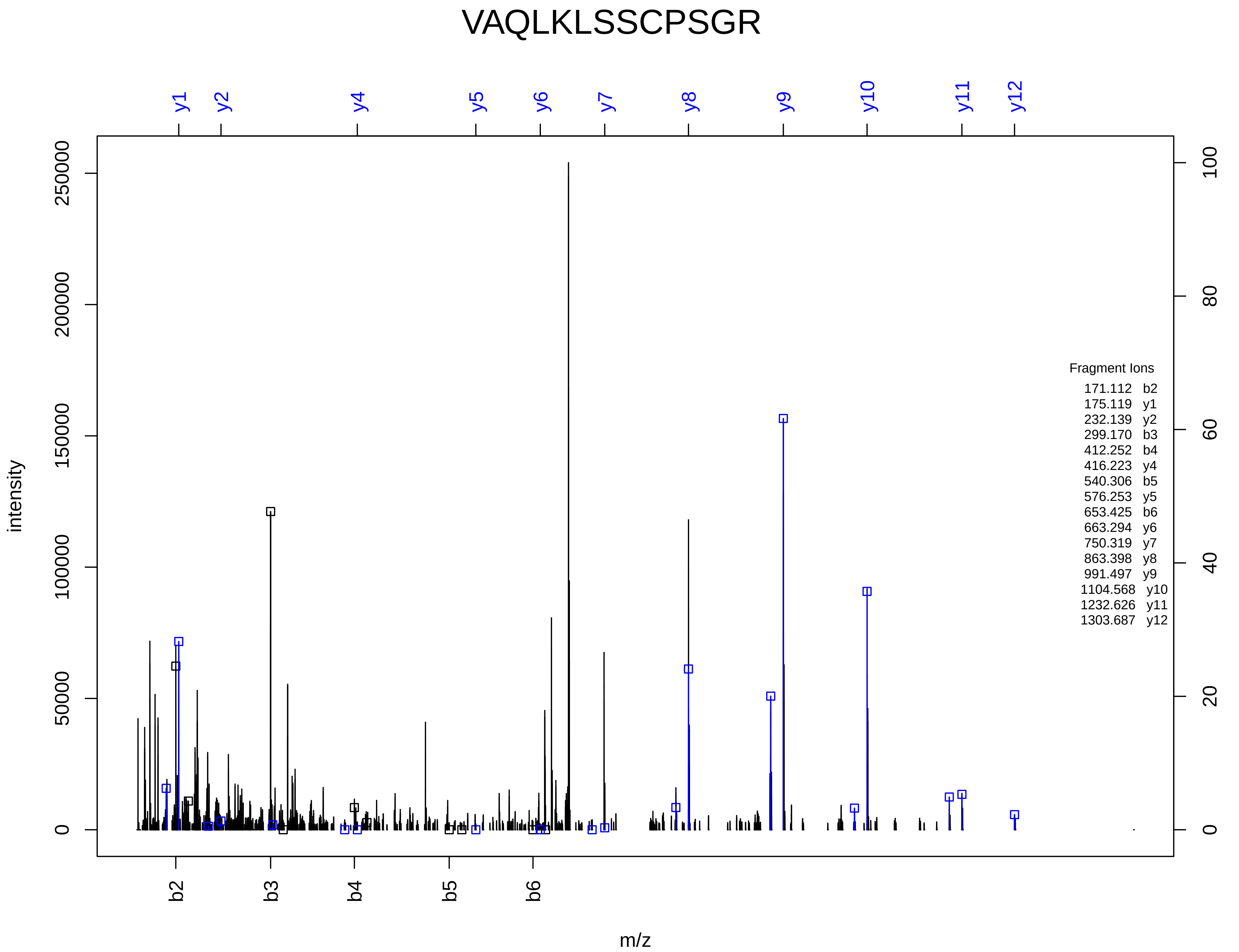

VAQLKLSSCPSGR / File: christine_10pools_01.raw; SpectrumID: ; scans: 11906

QAGLSYIR

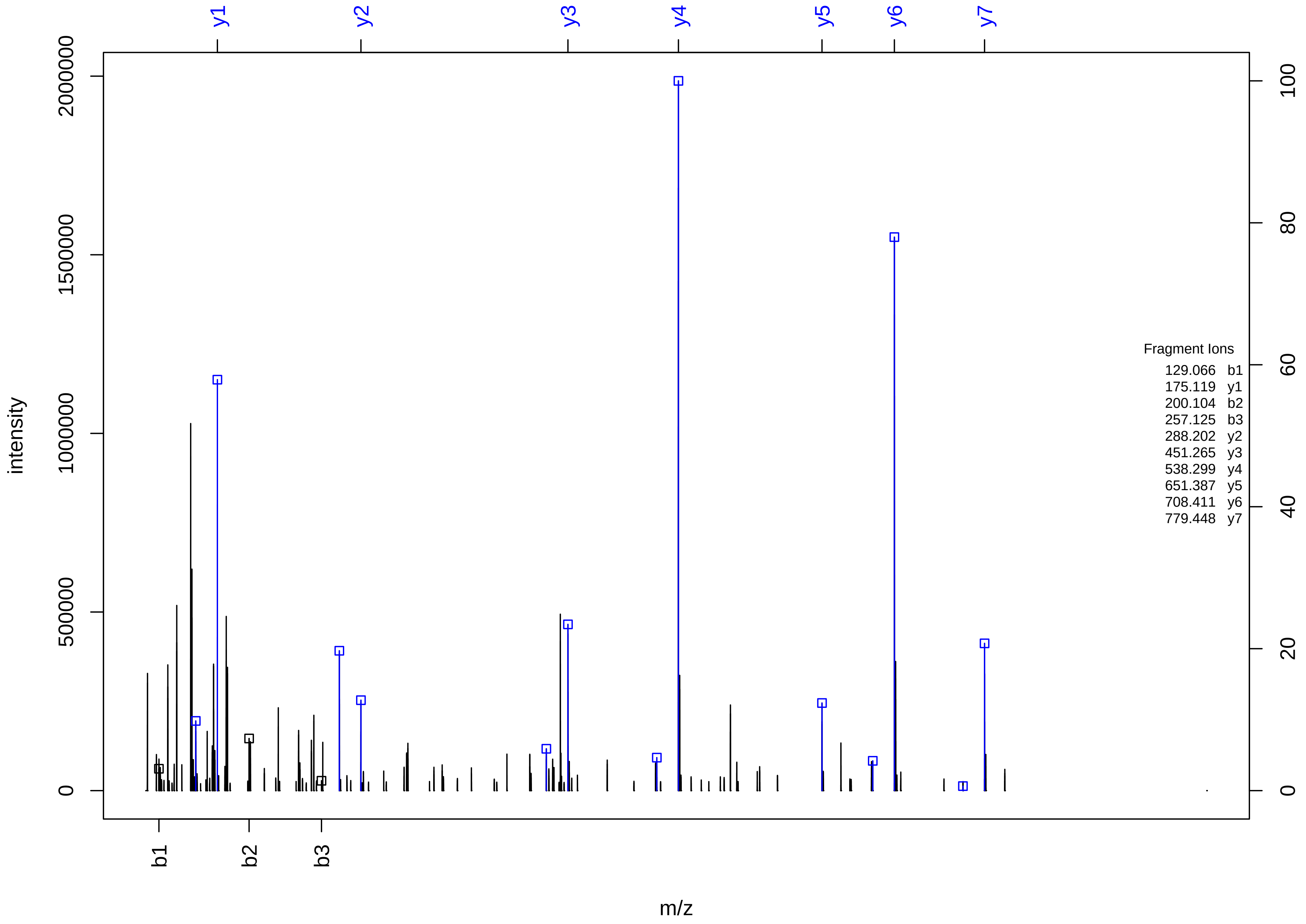

QAGLSYIR / File: christine_10pools_01.raw; SpectrumID: ; scans: 3812

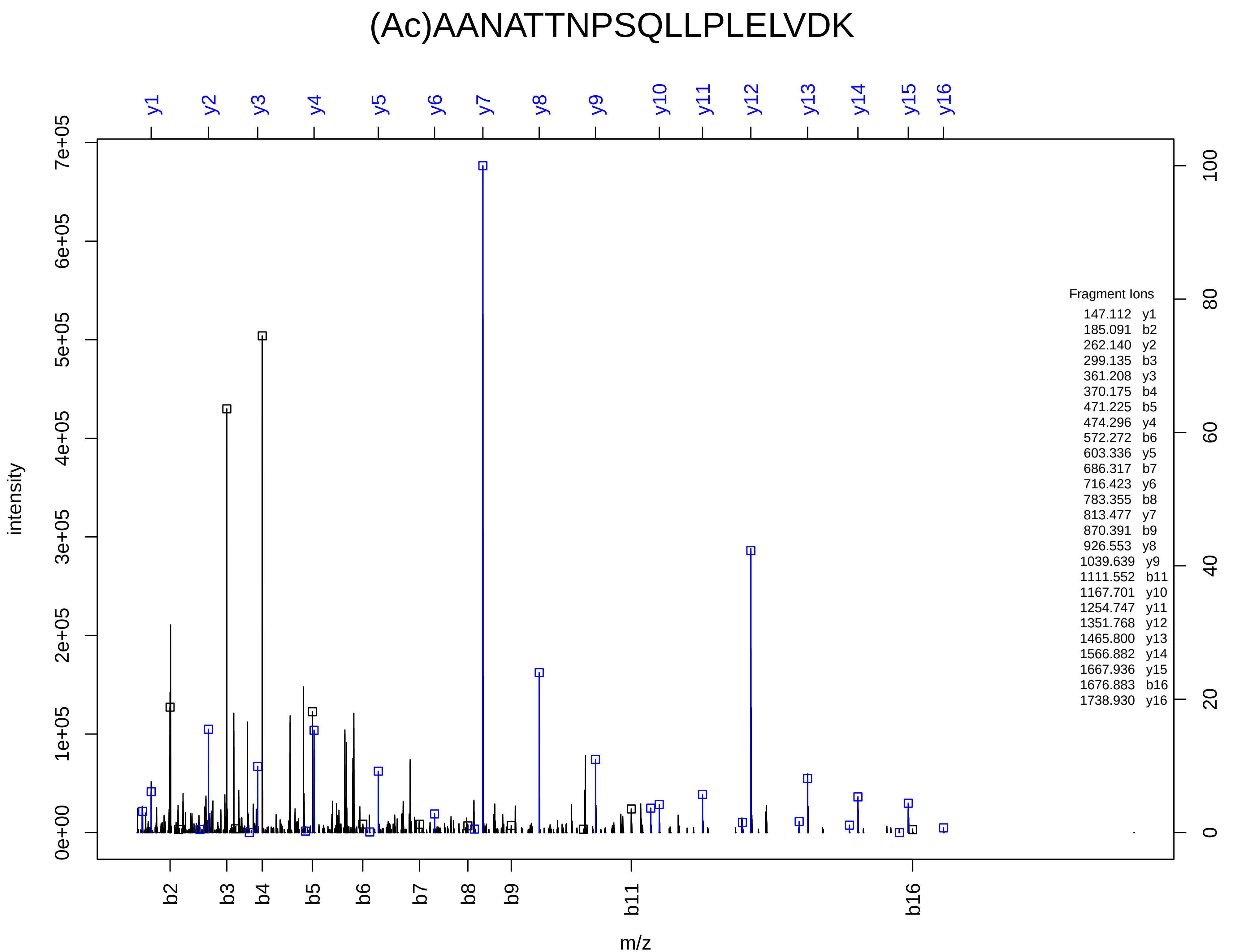

AANATTNPSQLLPLELVDK / File: christine_10pools_01.raw; SpectrumID: ; scans: 37003

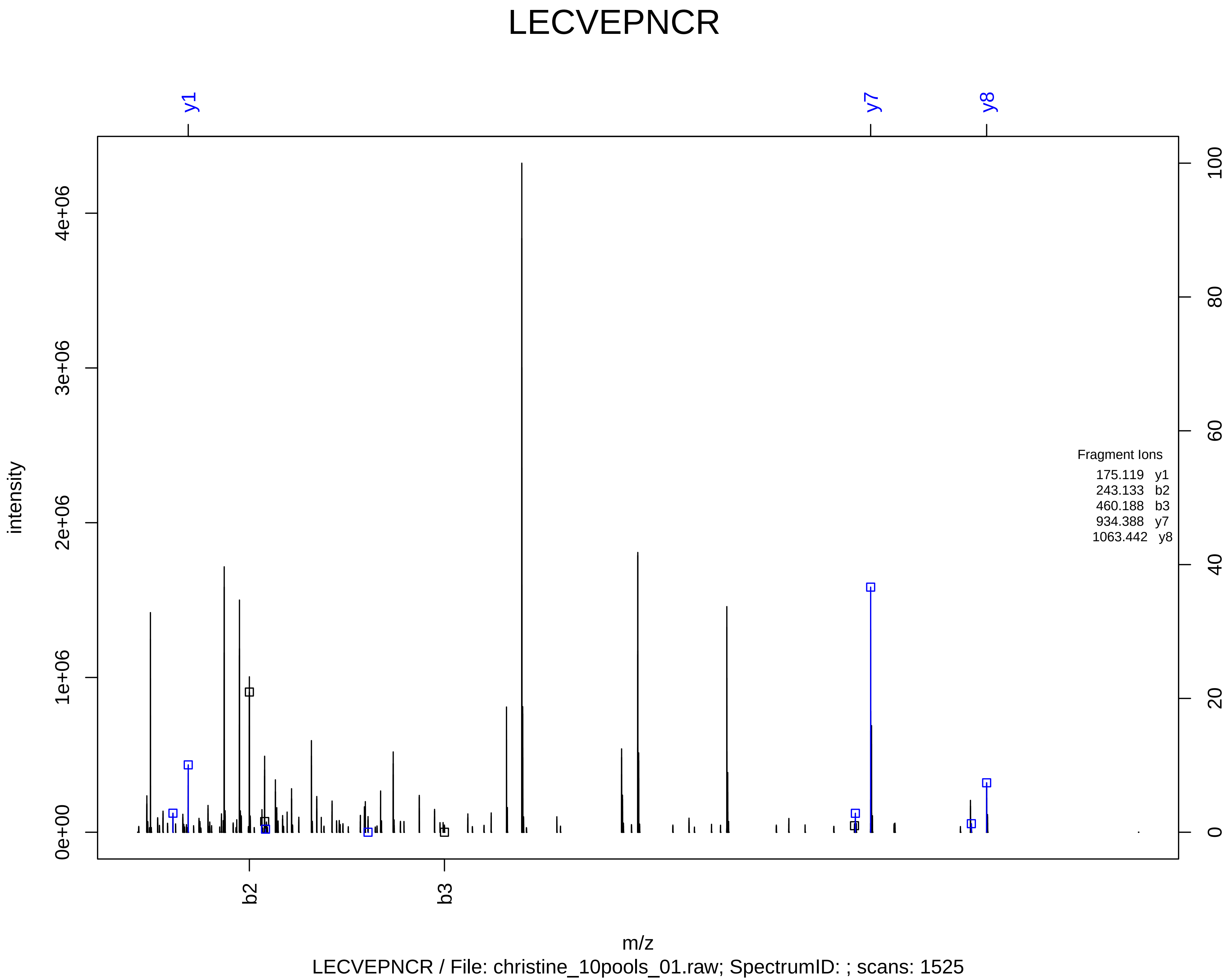
LECVEPNCR
y1
y7
y8
intensity
0e+00
1e+06
2e+06
3e+06
4e+06
0
20
40
60
80
100
Fragment Ions
175.119 y1
243.133 b2
460.188 b3
934.388 y7
1063.442 y8
b2
b3
m/z
LECVEPNCR / File: christine_10pools_01.raw; SpectrumID: ; scans: 1525

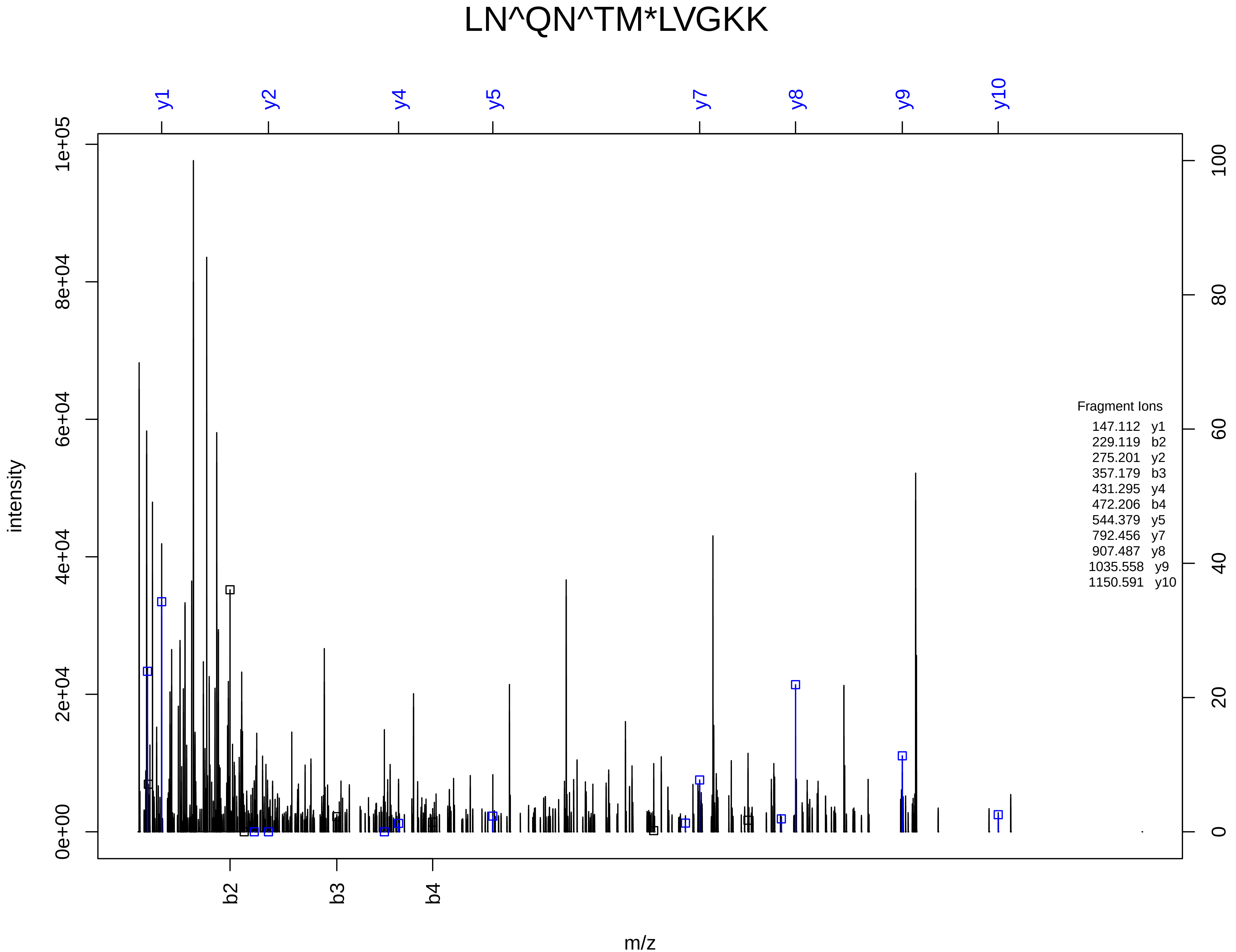
LN^QN^TM*LVGKK
y1
y2
y4
y5
y7
y8
y9
y10
intensity
1e+05
8e+04
6e+04
4e+04
2e+04
0e+00
100
80
60
40
20
0
Fragment Ions
147.112 y1
229.119 b2
275.201 y2
357.179 b3
431.295 y4
472.206 b4
544.379 y5
792.456 y7
907.487 y8
1035.558 y9
1150.591 y10
b2
b3
b4
m/z
LNQNTMLVGKK / File: christine_10pools_01.raw; SpectrumID: ; scans: 15926

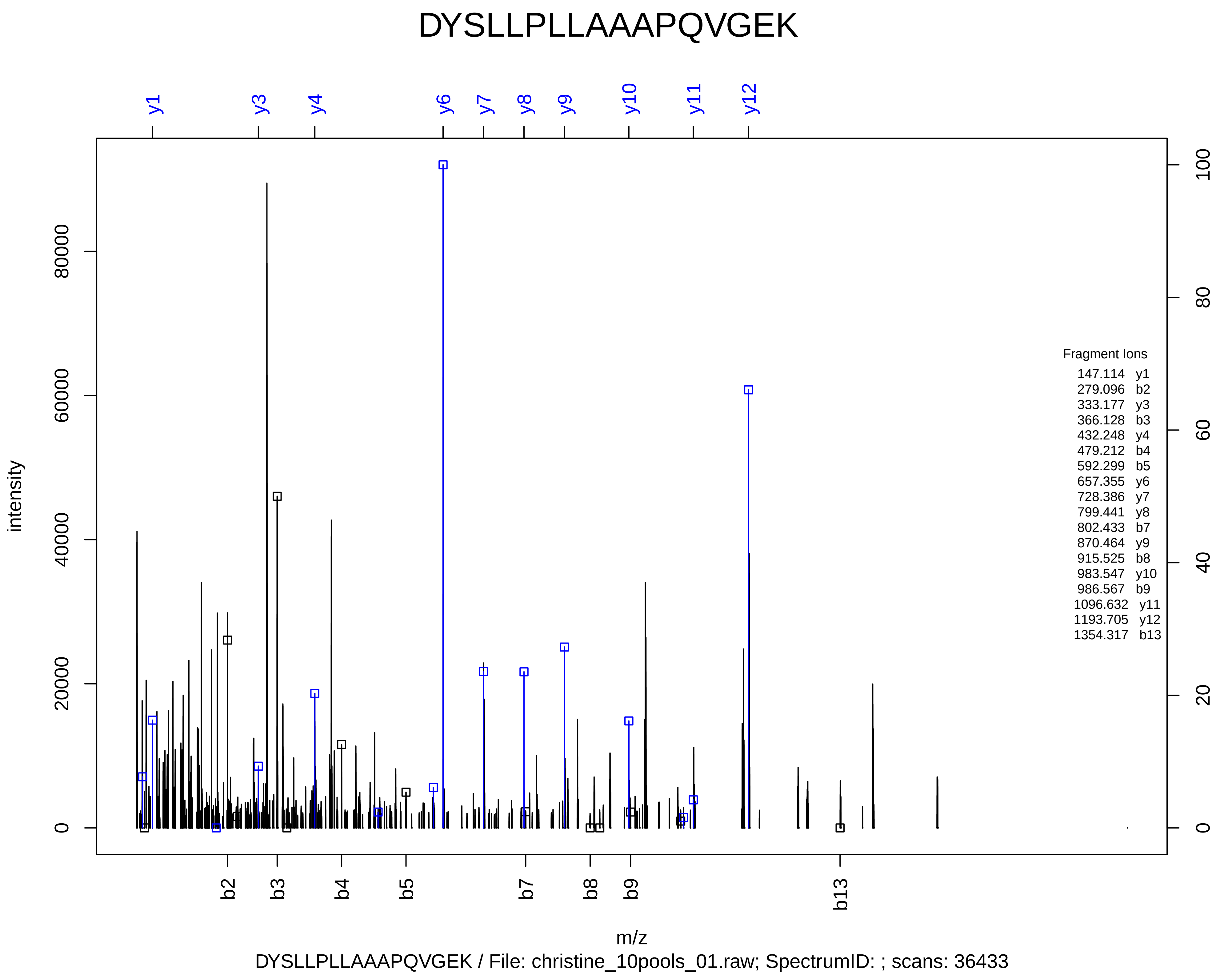

DYSLLPLLAAAPQVGEK
y1
y3
y4
y6
y7
y8
y9
y10
y11
y12
intensity
0
20000
40000
60000
80000
0
20
40
60
80
100
b2
b3
b4
b5
b7
b8
b9
b13
m/z
Fragment Ions
147.114 y1
279.096 b2
333.177 y3
366.128 b3
432.248 y4
479.212 b4
592.299 b5
657.355 y6
728.386 y7
799.441 y8
802.433 b7
870.464 y9
915.525 b8
983.547 y10
986.567 b9
1096.632 y11
1193.705 y12
1354.317 b13
DYSLLPLLAAAPQVGEK / File: christine_10pools_01.raw; SpectrumID: ; scans: 36433

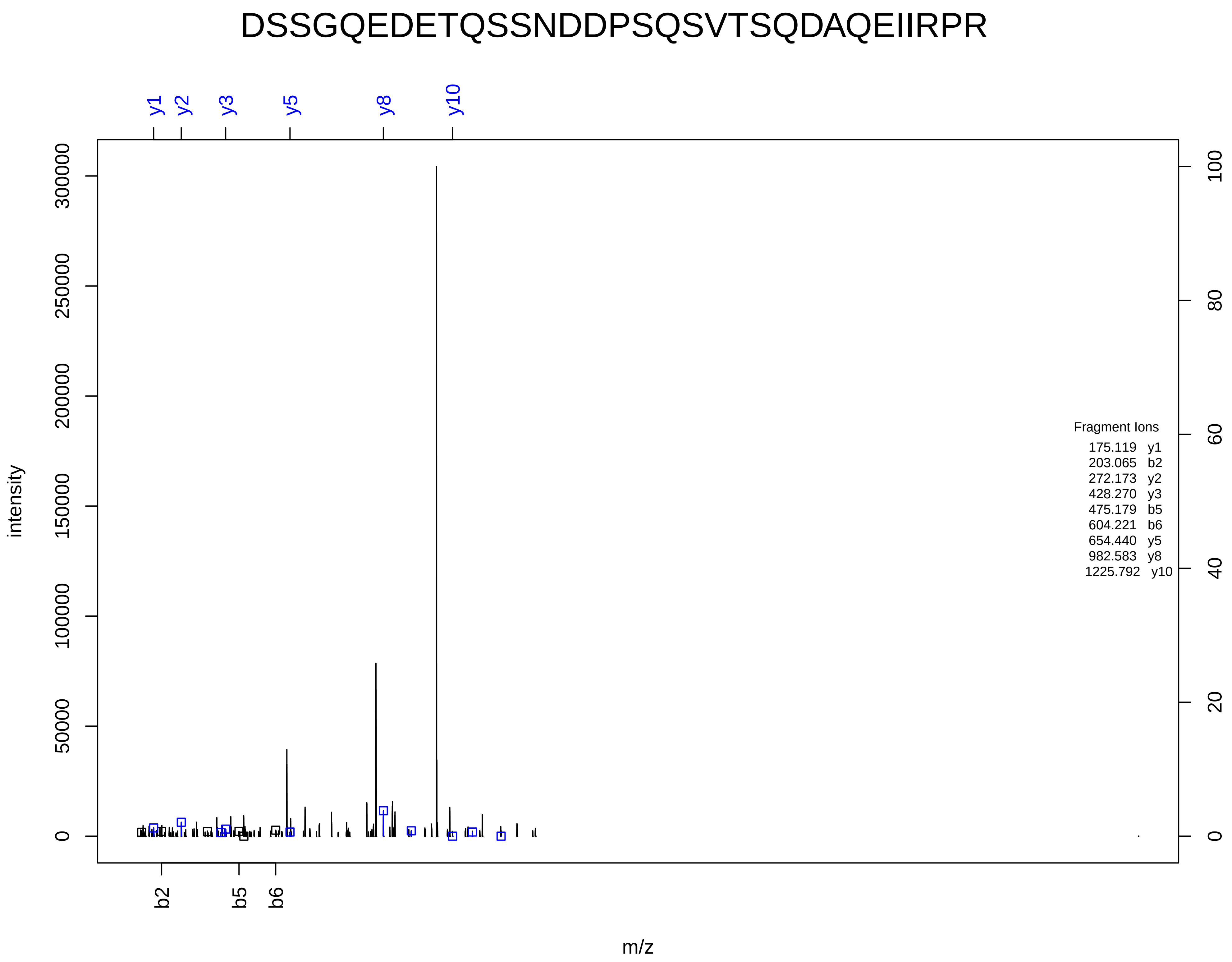
DSSGQEDETQSSNDDPSQSVTSQDAQEIIRPR
y1
y2
y3
y5
y8
y10
intensity
0
50000
100000
150000
200000
250000
300000
0
20
40
60
80
100
Fragment Ions
175.119 y1
203.065 b2
272.173 y2
428.270 y3
475.179 b5
604.221 b6
654.440 y5
982.583 y8
1225.792 y10
b2
b5
b6
m/z
DSSGQEDETQSSNDDPSQSVTSQDAQEIIRPR / File: christine_10pools_01.raw; SpectrumID: ; scans: 10982

LNLVADLVEEAQESVSK

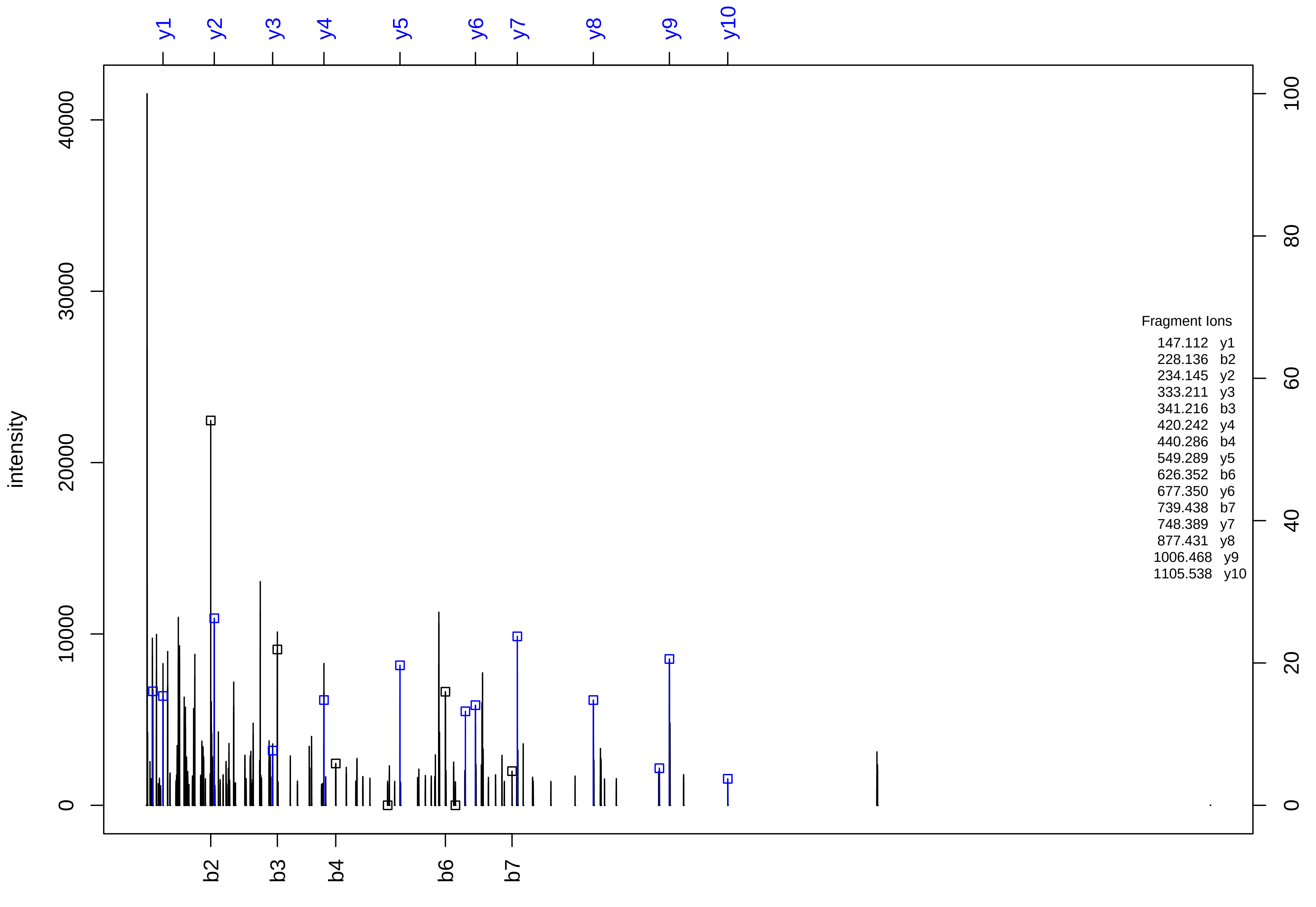

LNLVADLVEEAQESVSK / File: christine_10pools_01.raw; SpectrumID: ; scans: 38599

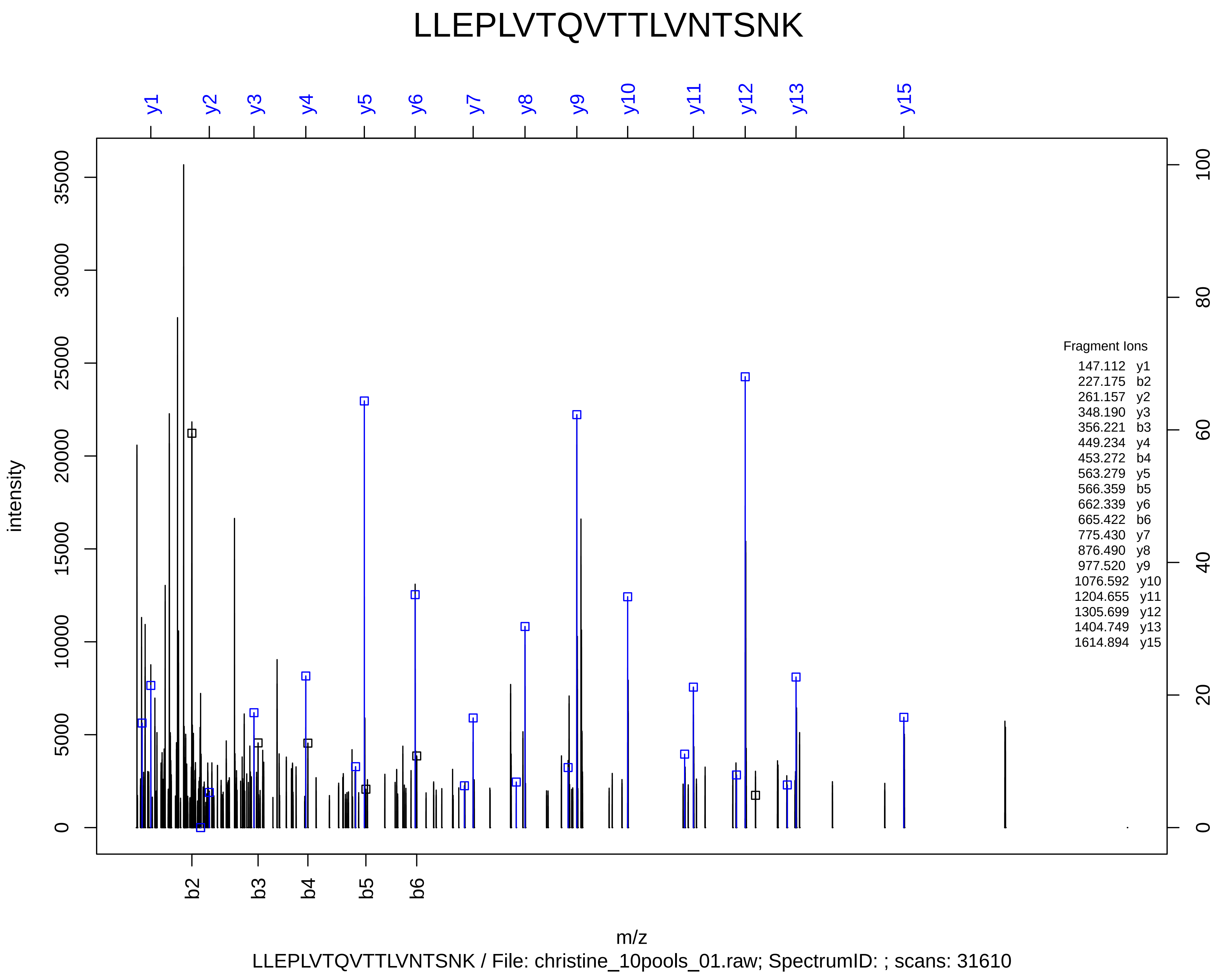
LLEPLVTQVTTLVNTSNK
y1 y2 y3 y4 y5 y6 y7 y8 y9 y10 y11 y12 y13 y15
intensity
0 5000 10000 15000 20000 25000 30000 35000
0 20 40 60 80 100
b2 b3 b4 b5 b6
m/z
Fragment Ions
147.112 y1
227.175 b2
261.157 y2
348.190 y3
356.221 b3
449.234 y4
453.272 b4
563.279 y5
566.359 b5
662.339 y6
665.422 b6
775.430 y7
876.490 y8
977.520 y9
1076.592 y10
1204.655 y11
1305.699 y12
1404.749 y13
1614.894 y15
LLEPLVTQVTTLVNTSNK / File: christine_10pools_01.raw; SpectrumID: ; scans: 31610

TPNVVEATNKPDLYNK

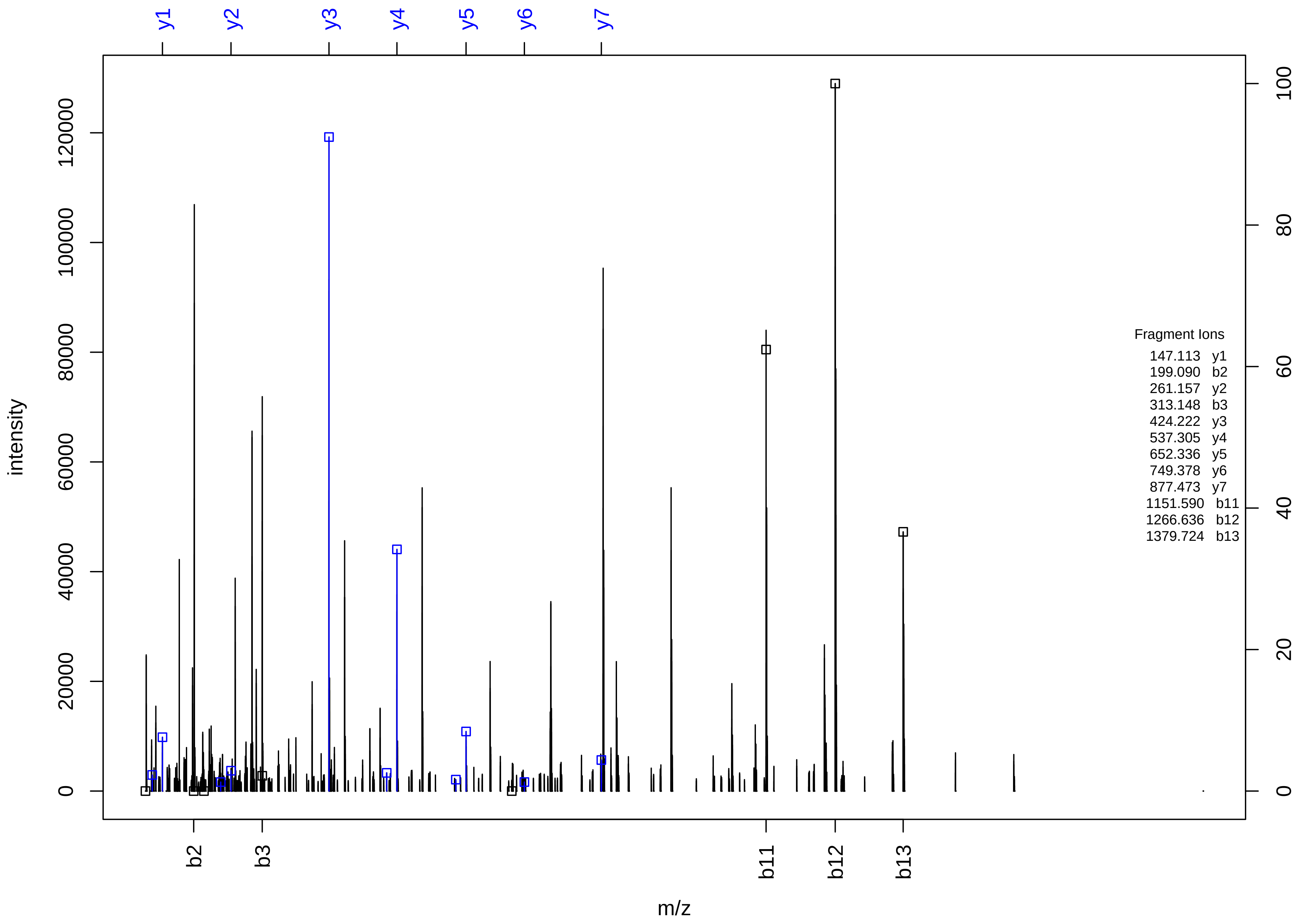

TPNVVEATNKPDLYNK / File: christine_10pools_01.raw; SpectrumID: ; scans: 36303

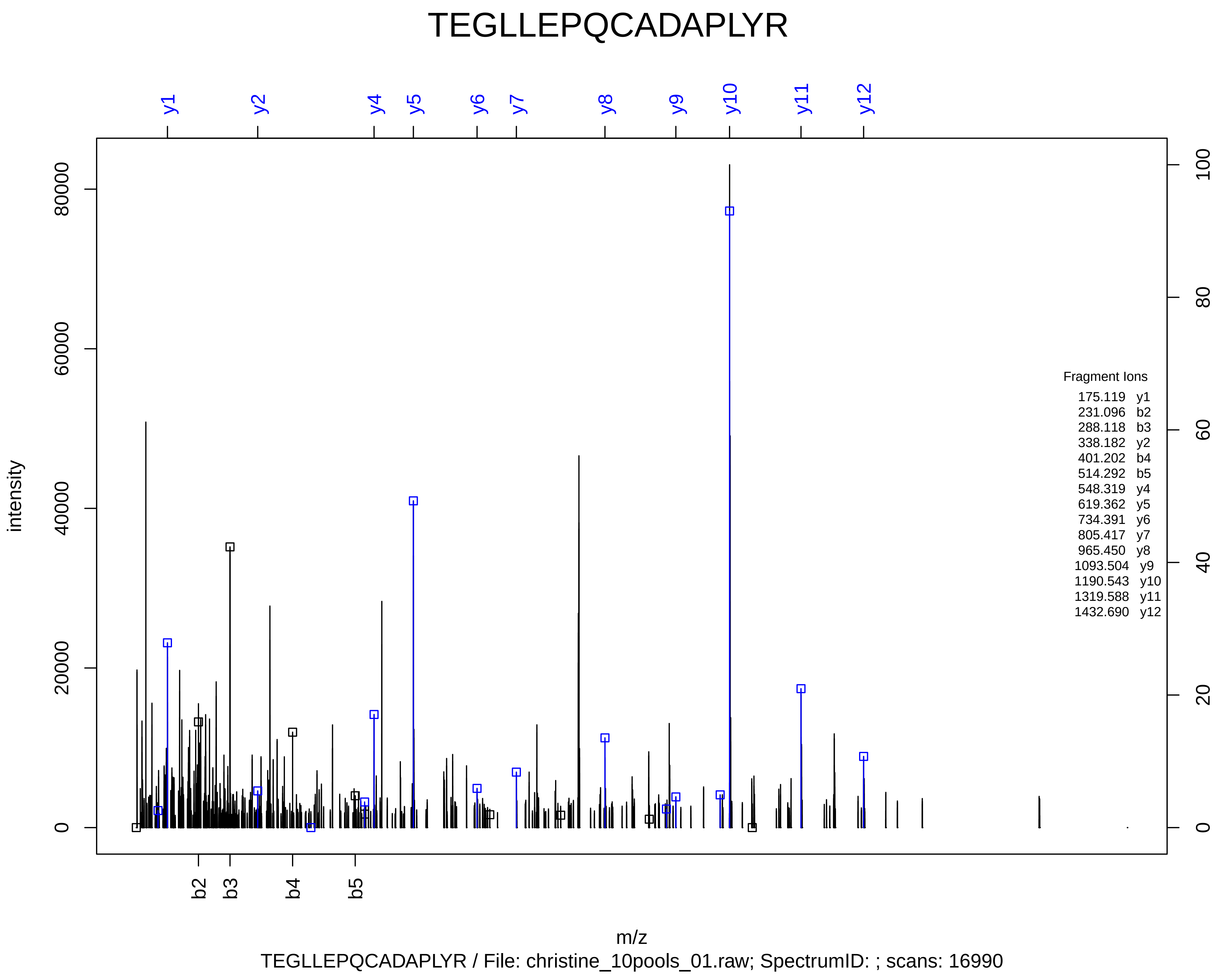

TEGLLEPQCADAPLYR
y1
y2
y4
y5
y6
y7
y8
y9
y10
y11
y12
intensity
0
20000
40000
60000
80000
0
20
40
60
80
100
b2
b3
b4
b5
Fragment Ions
175.119 y1
231.096 b2
288.118 b3
338.182 y2
401.202 b4
514.292 b5
548.319 y4
619.362 y5
734.391 y6
805.417 y7
965.450 y8
1093.504 y9
1190.543 y10
1319.588 y11
1432.690 y12
m/z
TEGLLEPQCADAPLYR / File: christine_10pools_01.raw; SpectrumID: ; scans: 16990

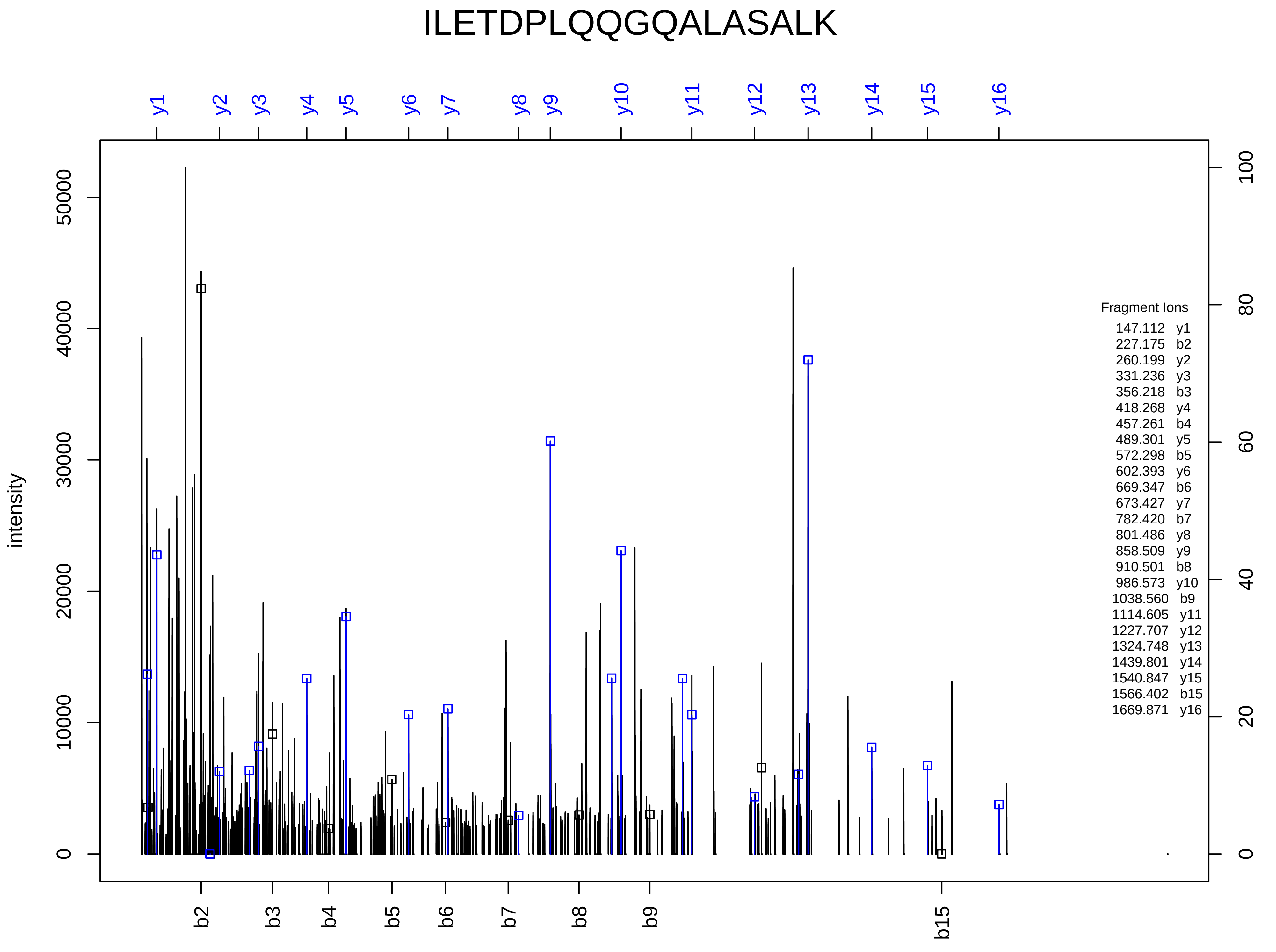

ILETDPLQQGQALASALK / File: christine_10pools_01.raw; SpectrumID: ; scans: 26207

TQETLQVECTQLK

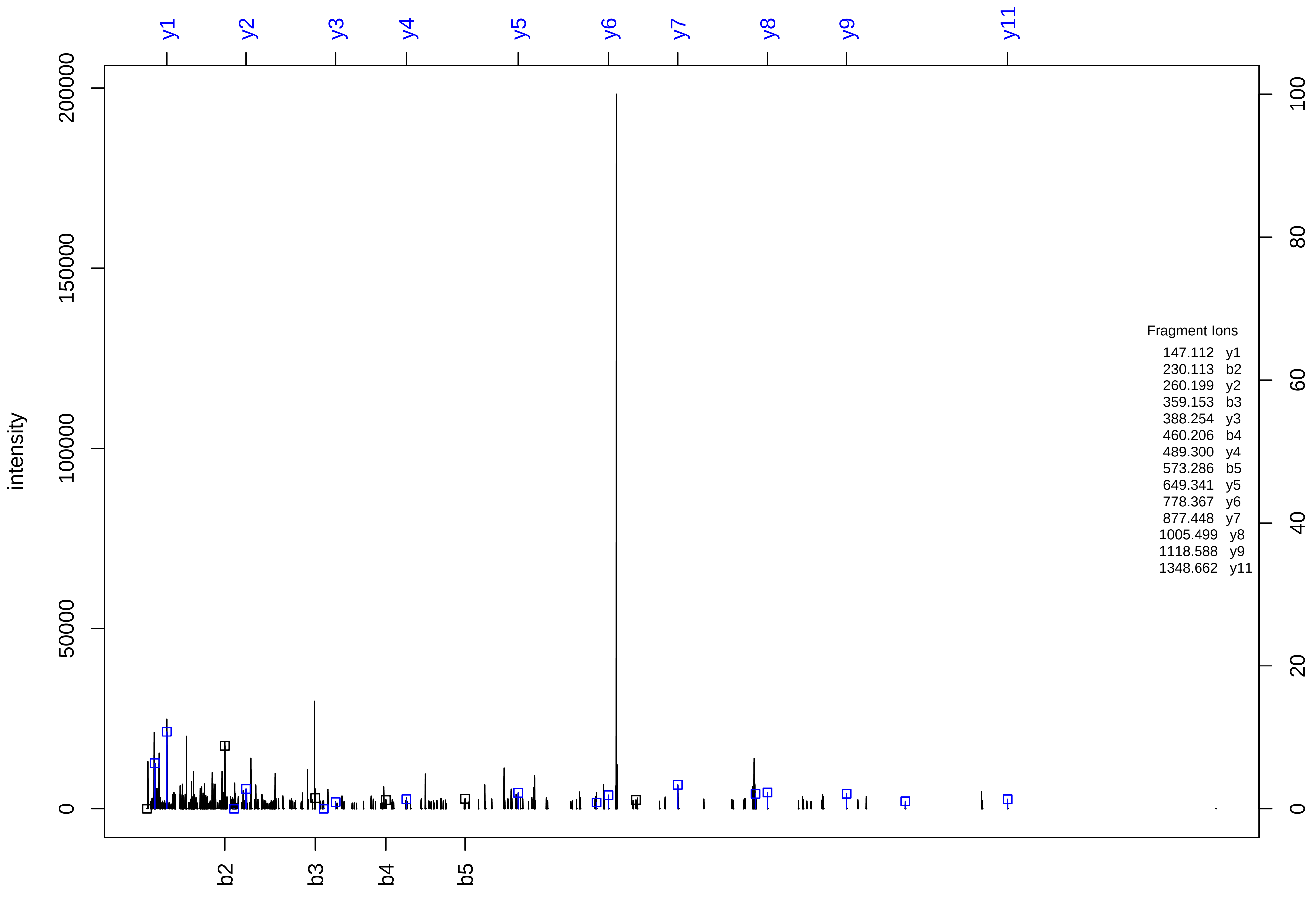

TQETLQVECTQLK / File: christine_10pools_01.raw; SpectrumID: ; scans: 6946

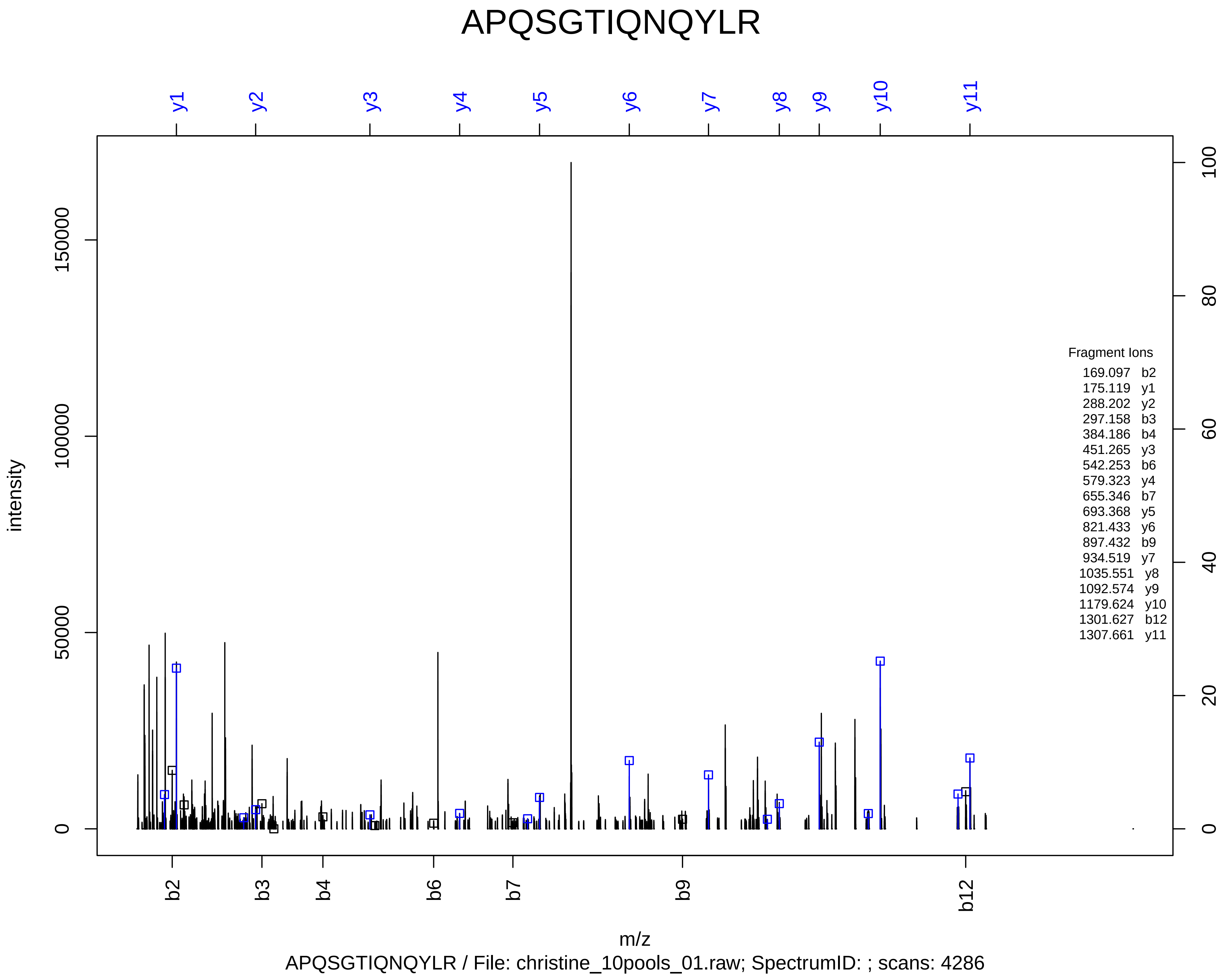
APQSGTIQNQYLR
y1
y2
y3
y4
y5
y6
y7
y8
y9
y10
y11
intensity
150000
100000
50000
0
100
80
60
40
20
0
Fragment Ions
169.097 b2
175.119 y1
288.202 y2
297.158 b3
384.186 b4
451.265 y3
542.253 b6
579.323 y4
655.346 b7
693.368 y5
821.433 y6
897.432 b9
934.519 y7
1035.551 y8
1092.574 y9
1179.624 y10
1301.627 b12
1307.661 y11
b2
b3
b4
b6
b7
b9
b12
m/z
APQSGTIQNQYLR / File: christine_10pools_01.raw; SpectrumID: ; scans: 4286

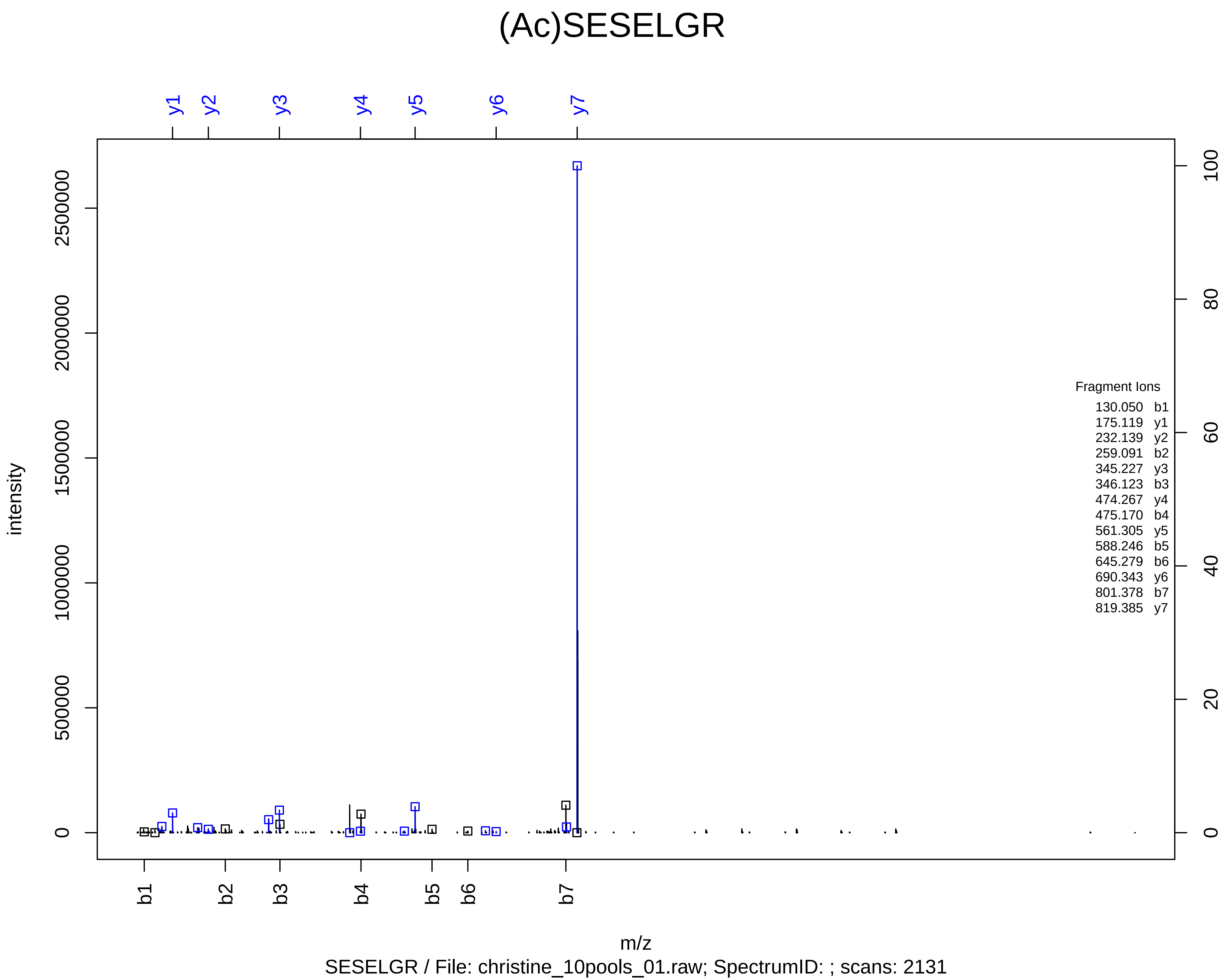

(Ac)SESELGR
y1
y2
y3
y4
y5
y6
y7
b1
b2
b3
b4
b5
b6
b7
intensity
0
500000
1000000
1500000
2000000
2500000
0
20
40
60
80
100
m/z
Fragment Ions
130.050 b1
175.119 y1
232.139 y2
259.091 b2
345.227 y3
346.123 b3
474.267 y4
475.170 b4
561.305 y5
588.246 b5
645.279 b6
690.343 y6
801.378 b7
819.385 y7
SESELGR / File: christine_10pools_01.raw; SpectrumID: ; scans: 2131

LPIVPSLQR

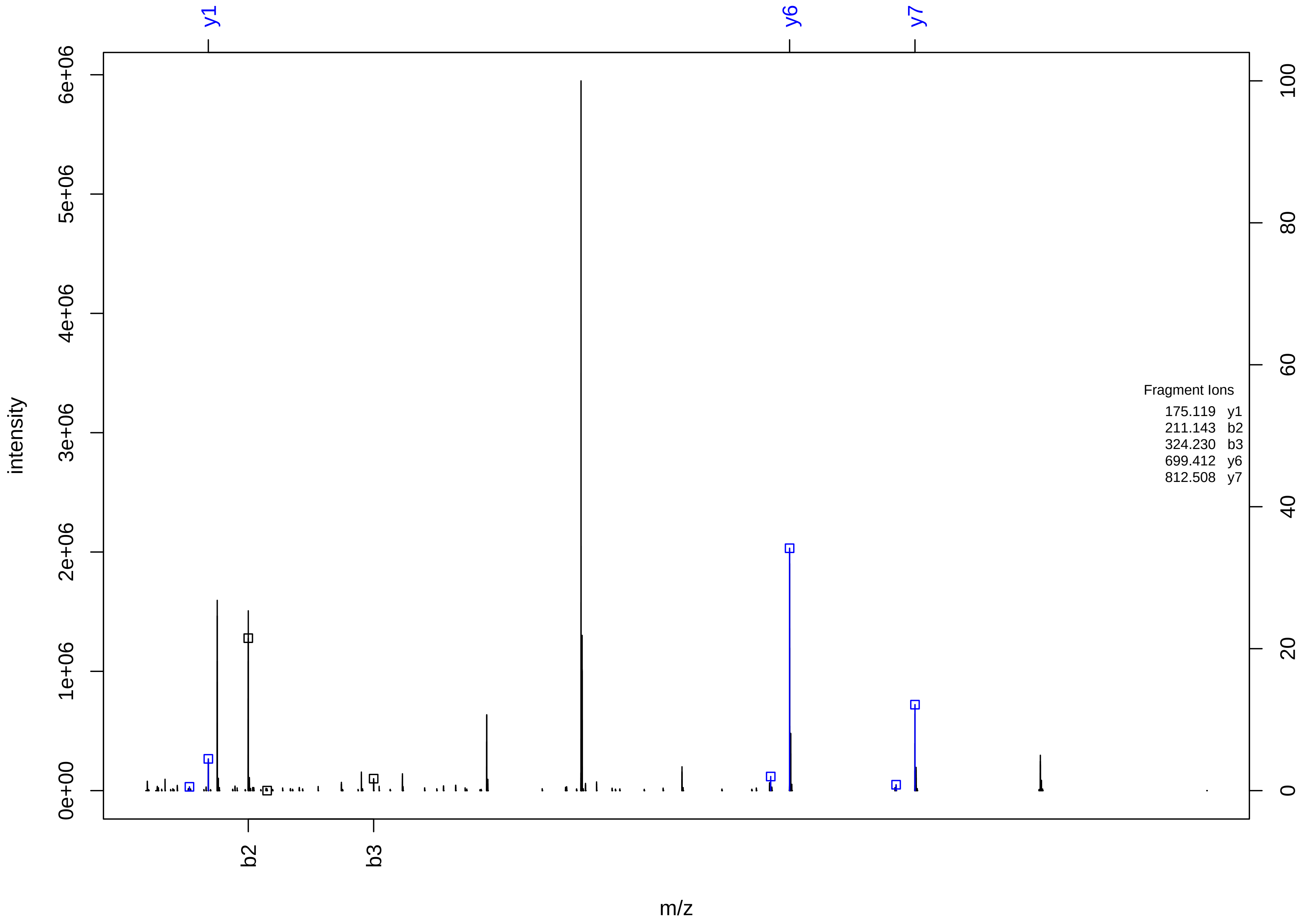

LPIVPSLQR / File: christine_10pools_01.raw; SpectrumID: ; scans: 16102

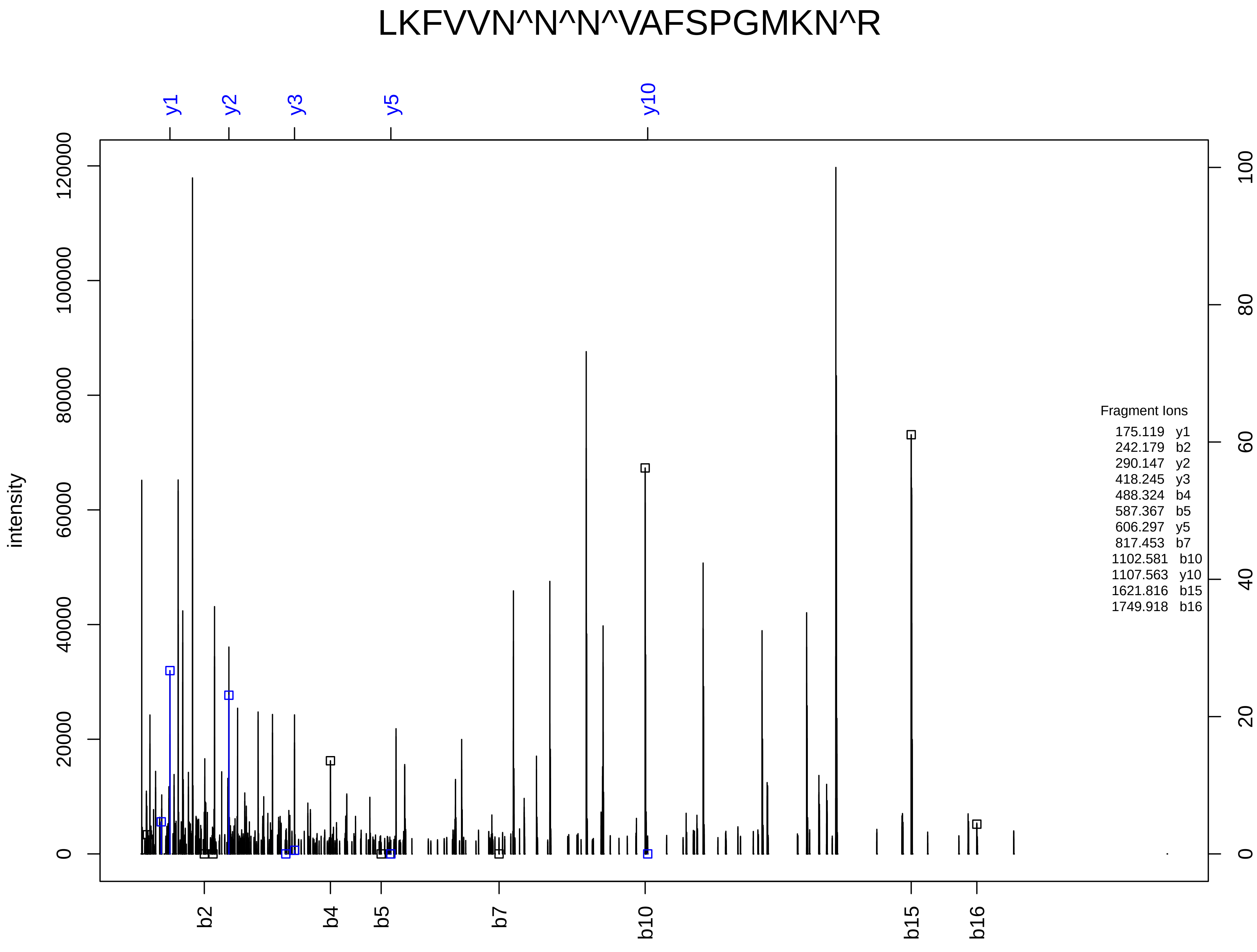

LKFVVNNNVAFSPGMKNR / File: christine_10pools_01.raw; SpectrumID: ; scans: 31417

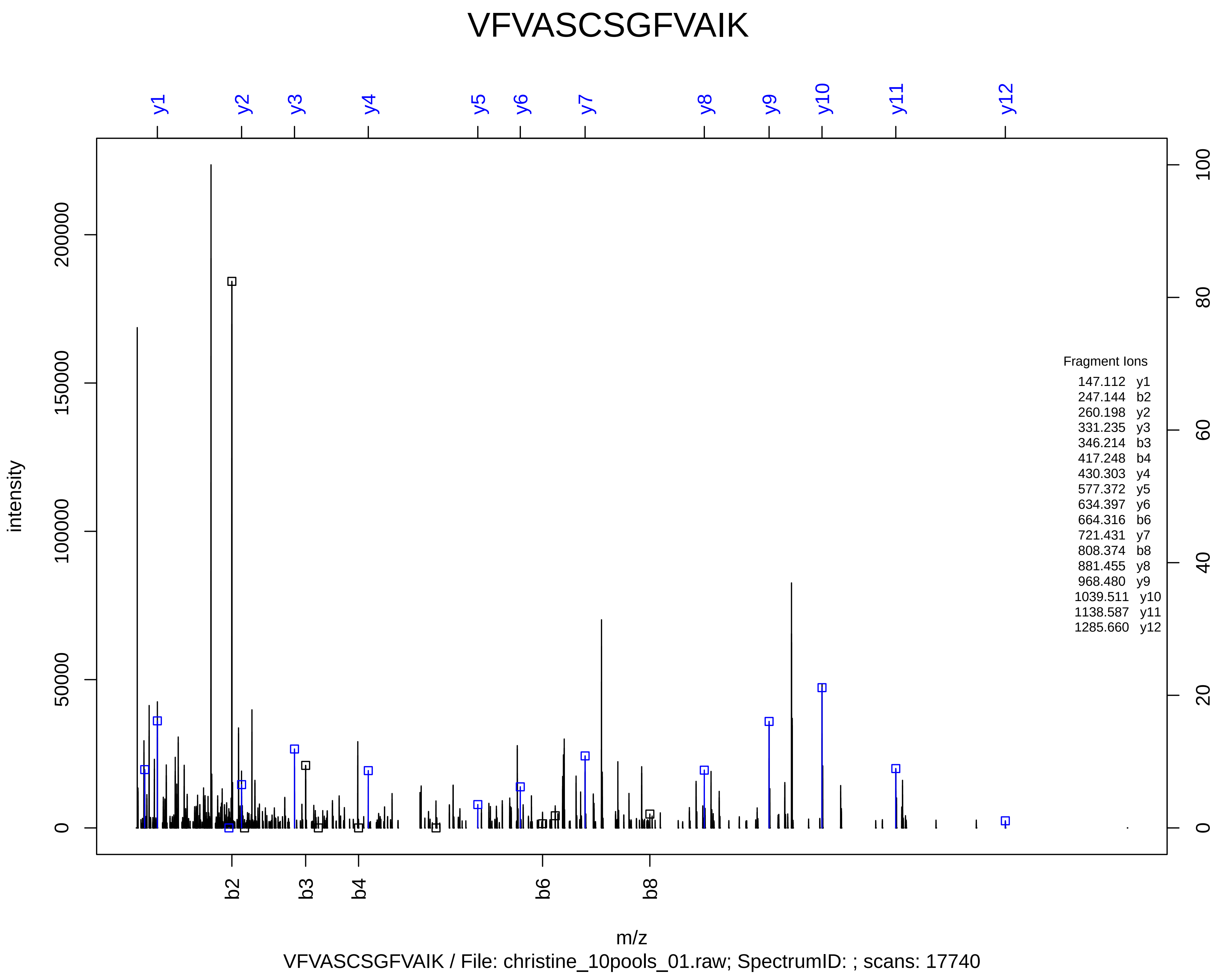
VFVASCSGFVAIK
y1
y2
y3
y4
y5
y6
y7
y8
y9
y10
y11
y12
intensity
0
50000
100000
150000
200000
0
20
40
60
80
100
b2
b3
b4
b6
b8
m/z
Fragment Ions
147.112 y1
247.144 b2
260.198 y2
331.235 y3
346.214 b3
417.248 b4
430.303 y4
577.372 y5
634.397 y6
664.316 b6
721.431 y7
808.374 b8
881.455 y8
968.480 y9
1039.511 y10
1138.587 y11
1285.660 y12
VFVASCSGFVAIK / File: christine_10pools_01.raw; SpectrumID: ; scans: 17740

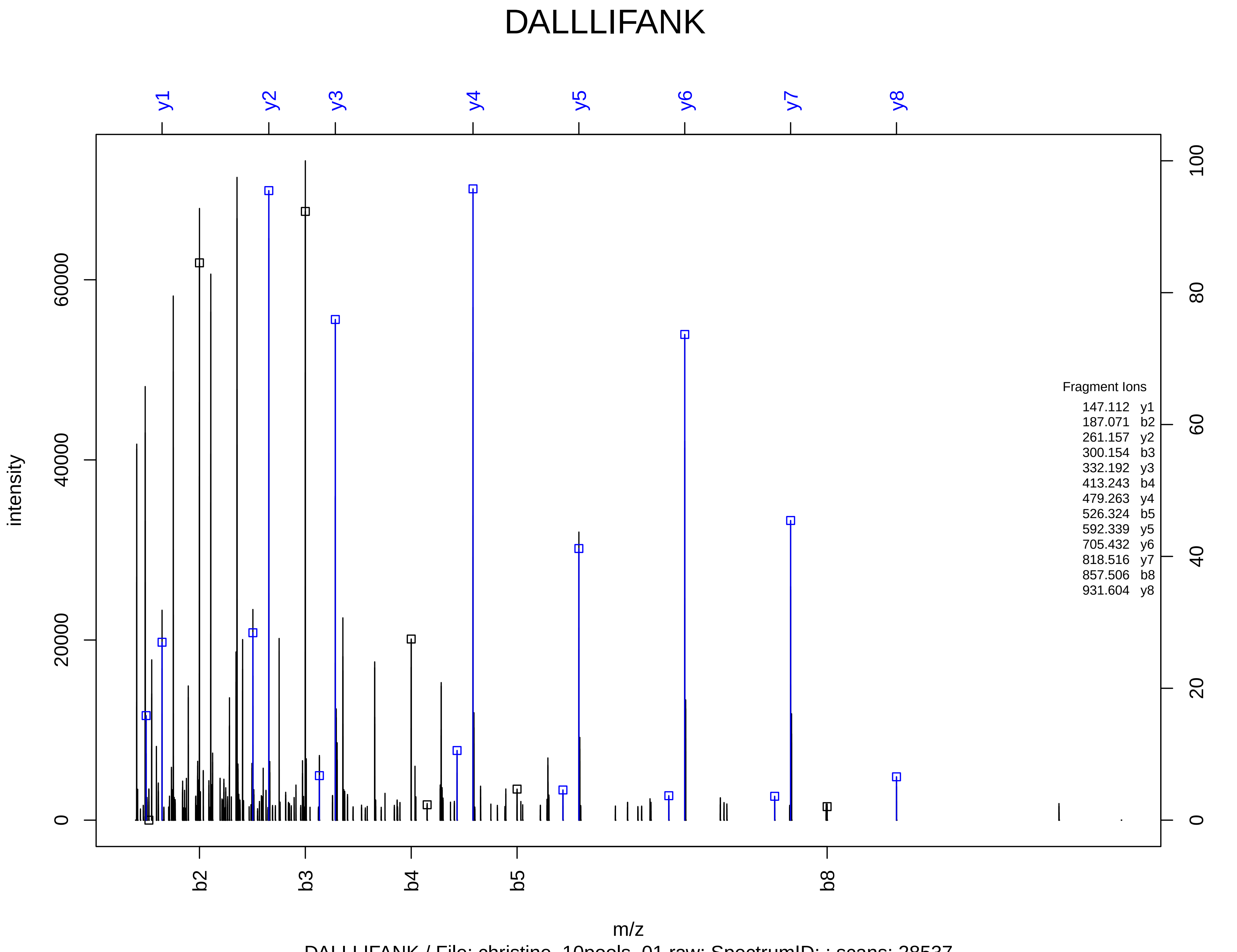
DALLLIFANK
y1
y2
y3
y4
y5
y6
y7
y8
intensity
0
20000
40000
60000
0
20
40
60
80
100
b2
b3
b4
b5
b8
m/z
Fragment Ions
147.112 y1
187.071 b2
261.157 y2
300.154 b3
332.192 y3
413.243 b4
479.263 y4
526.324 b5
592.339 y5
705.432 y6
818.516 y7
857.506 b8
931.604 y8
DALLLIFANK / File: christine_10pools_01.raw; SpectrumID: ; scans: 28537

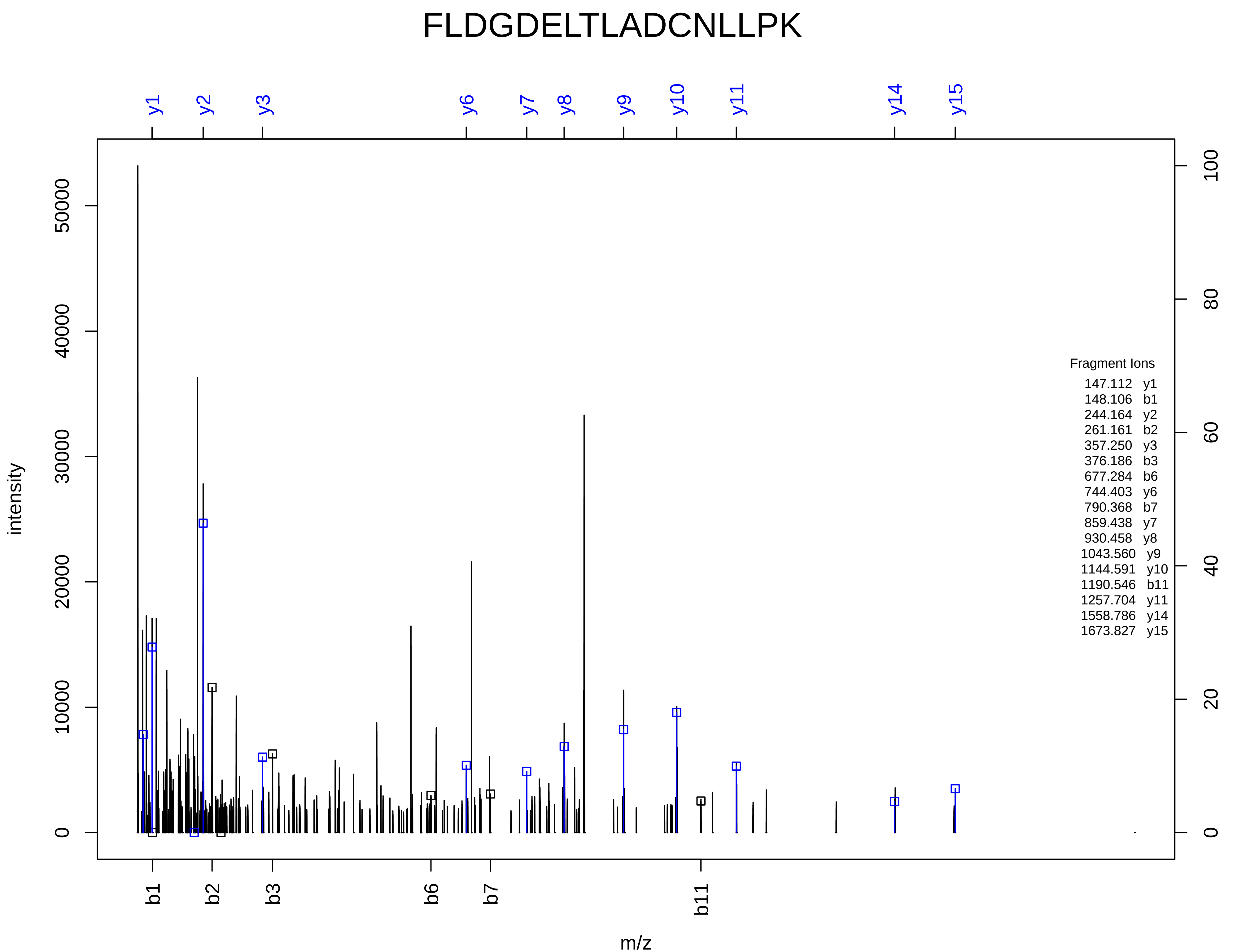

FLDGDELTLADCNLLPK / File: christine_10pools_01.raw; SpectrumID: ; scans: 30894

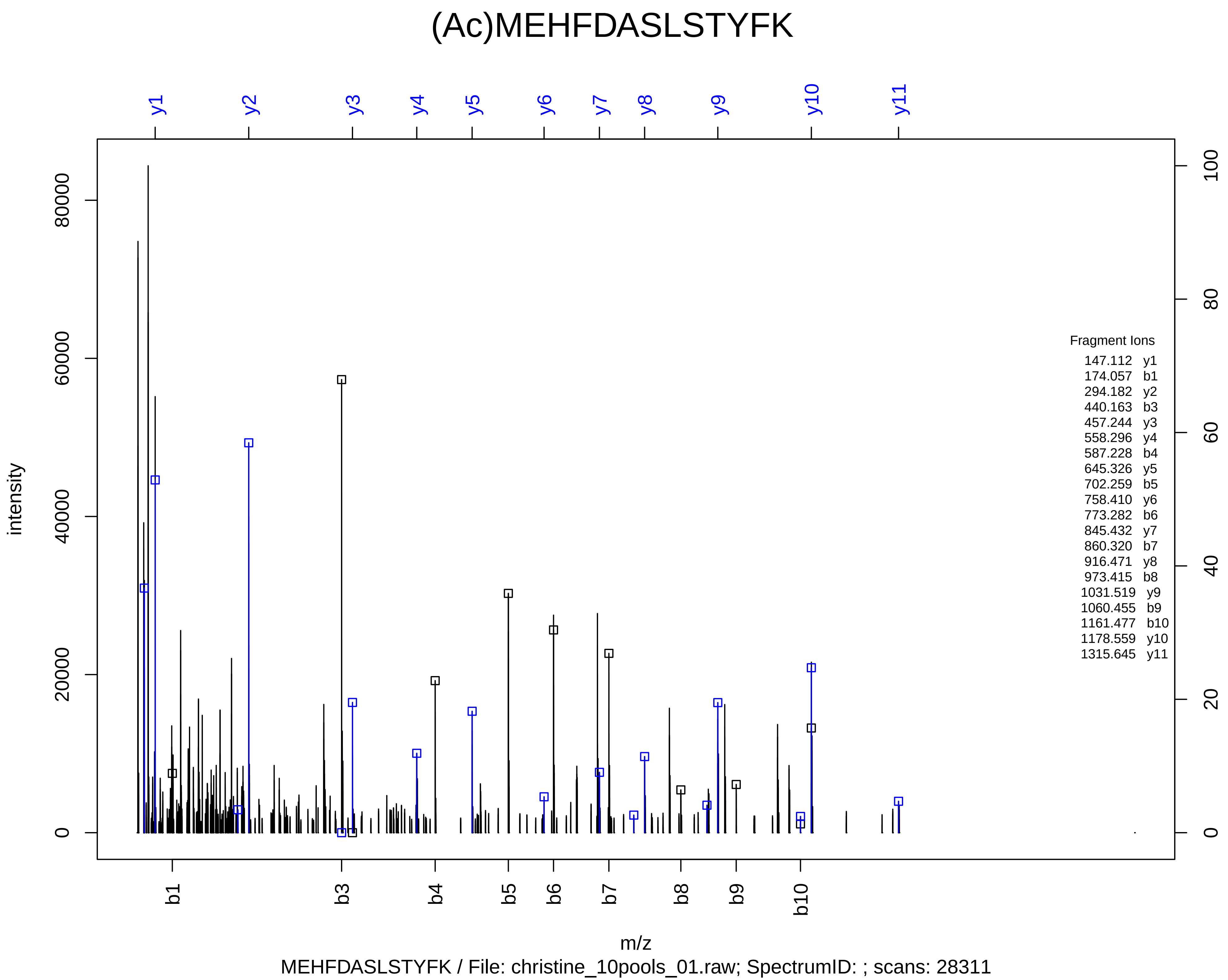

(Ac)MEHFDASLSTYFK
y1
y2
y3
y4
y5
y6
y7
y8
y9
y10
y11
intensity
0
20000
40000
60000
80000
0
20
40
60
80
100
b1
b3
b4
b5
b6
b7
b8
b9
b10
m/z
Fragment Ions
147.112 y1
174.057 b1
294.182 y2
440.163 b3
457.244 y3
558.296 y4
587.228 b4
645.326 y5
702.259 b5
758.410 y6
773.282 b6
845.432 y7
860.320 b7
916.471 y8
973.415 b8
1031.519 y9
1060.455 b9
1161.477 b10
1178.559 y10
1315.645 y11
MEHFDASLSTYFK / File: christine_10pools_01.raw; SpectrumID: ; scans: 28311

TIFDTPDEDPNYNPLPEERPGGFAWGEGQR

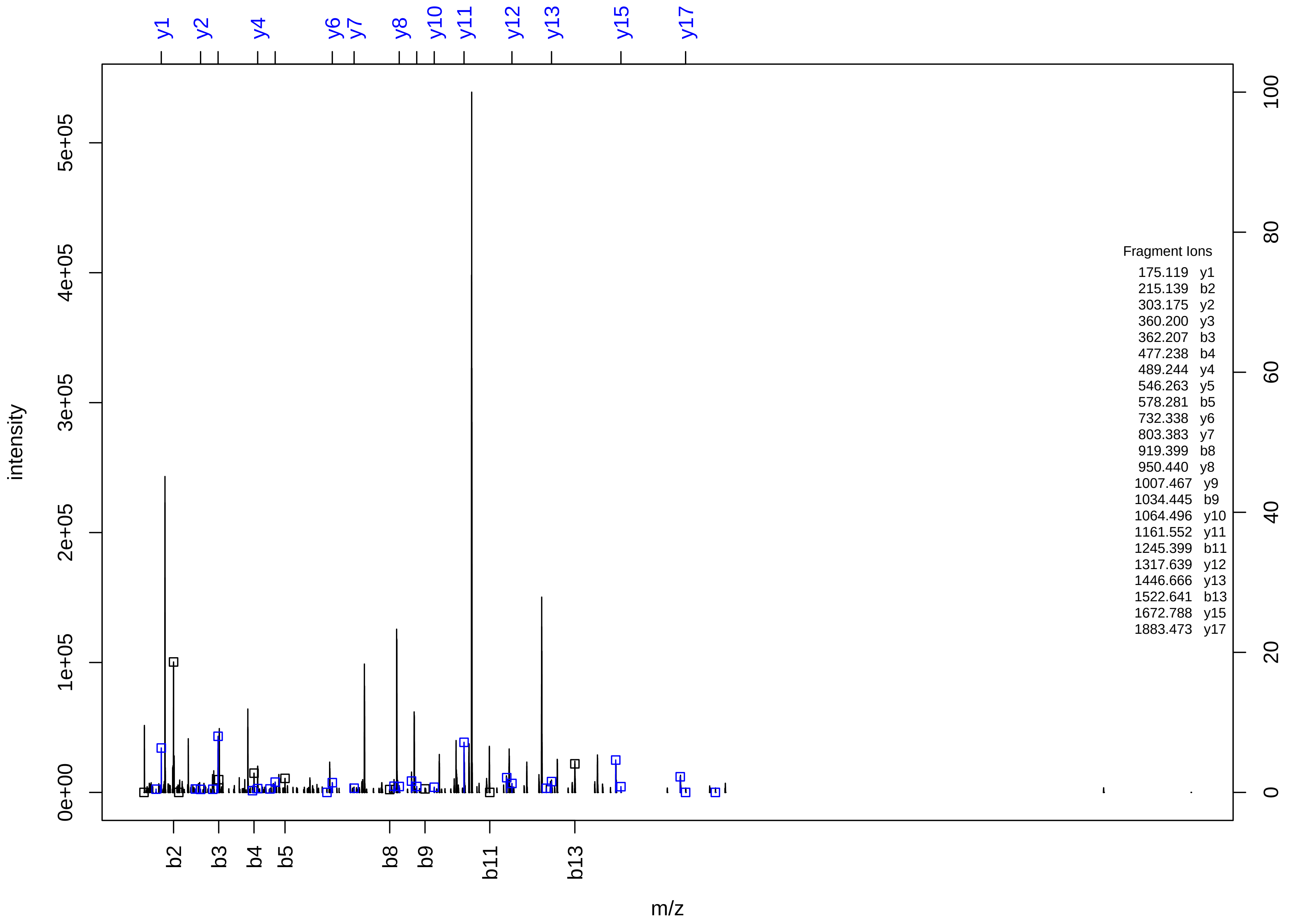

TIFDTPDEDPNYNPLPEERPGGFAWGEGQR / File: christine_10pools_01.raw; SpectrumID: ; scans: 24826

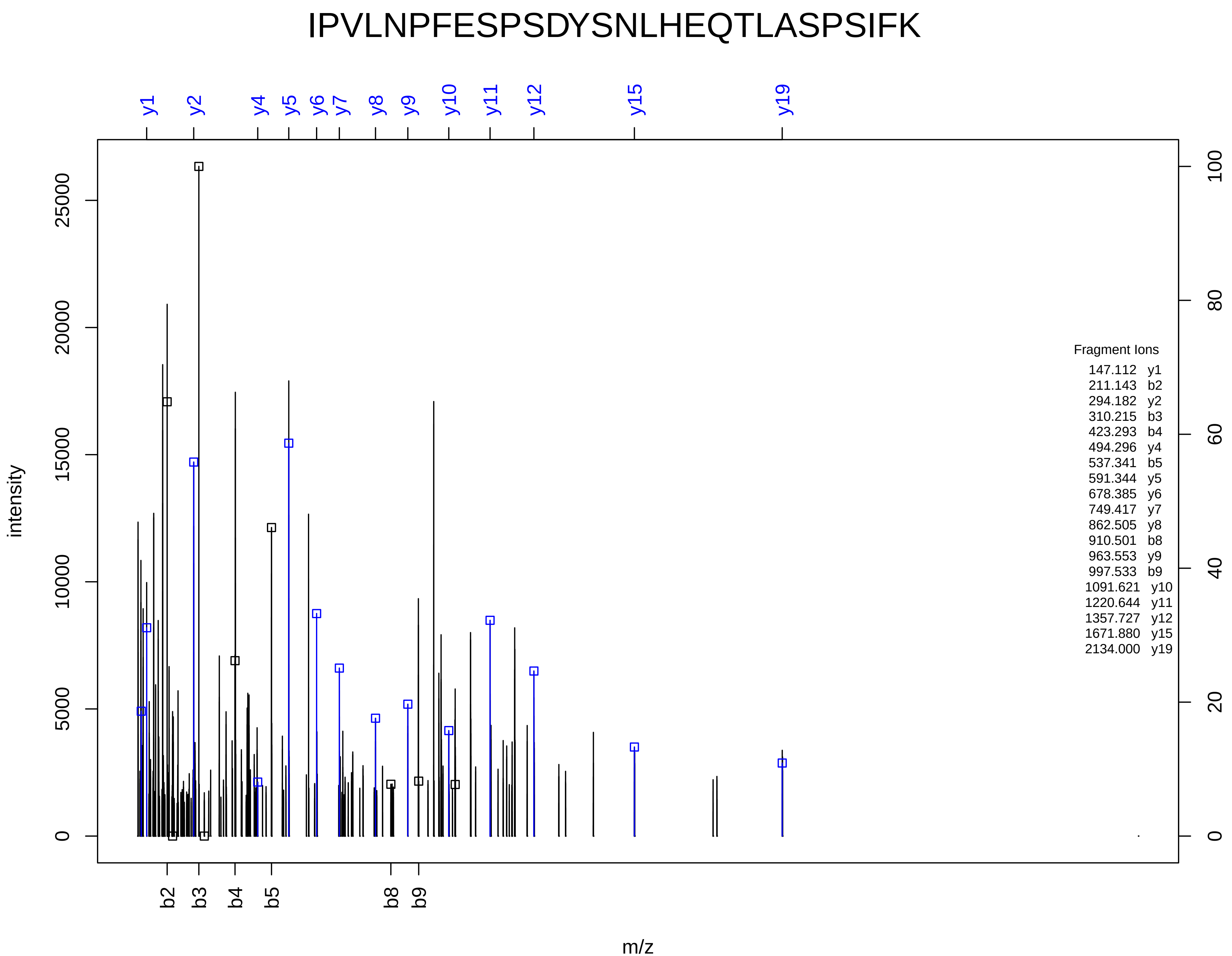

IPVLNPFESPSDYSNLHEQTLASPSIFK / File: christine_10pools_01.raw; SpectrumID: ; scans: 30076

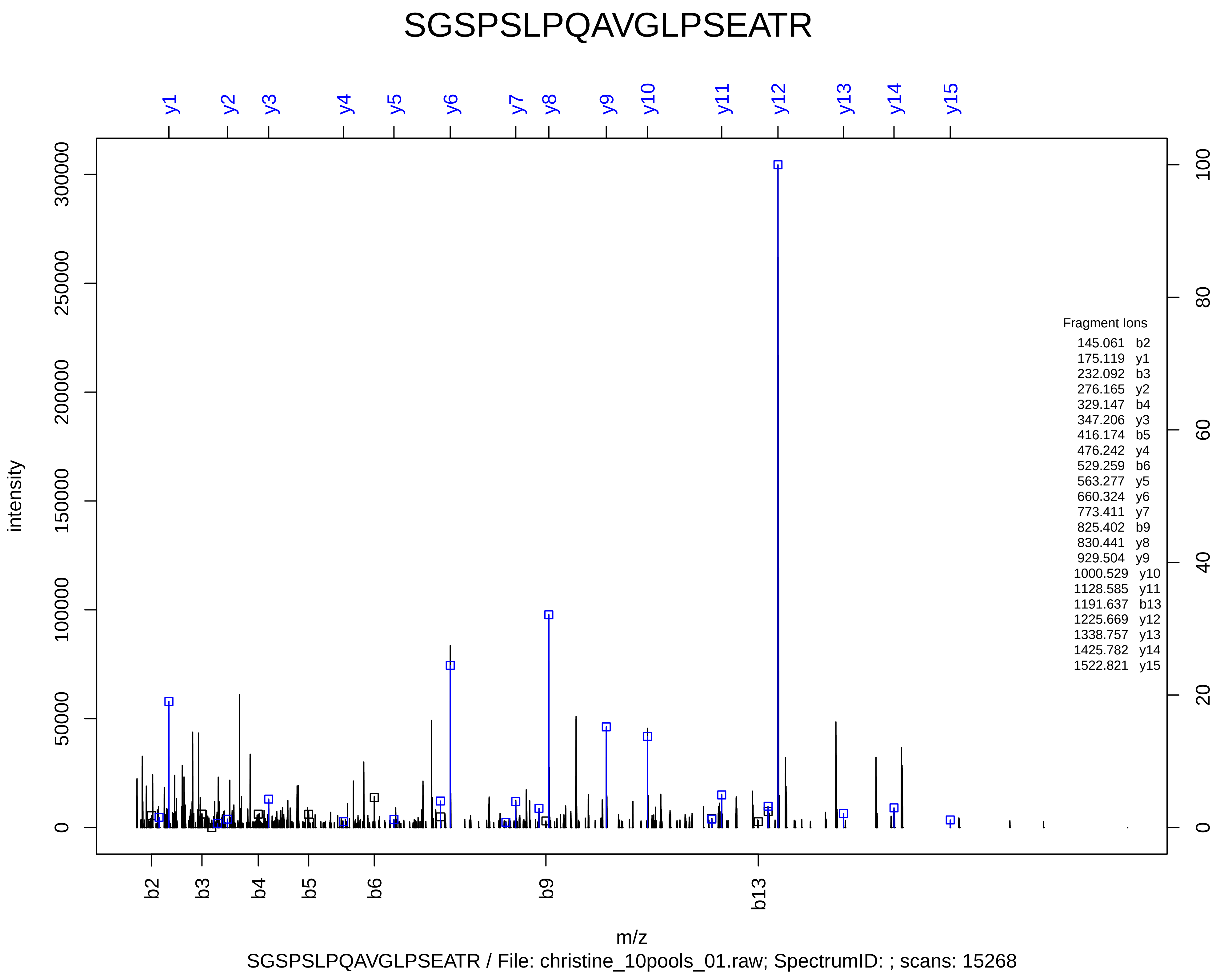
SGSPSLPQAVGLPSEATR
y1 y2 y3 y4 y5 y6 y7 y8 y9 y10 y11 y12 y13 y14 y15
intensity
0 50000 100000 150000 200000 250000 300000
0 20 40 60 80 100
b2 b3 b4 b5 b6 b9 b13
m/z
Fragment Ions
145.061 b2
175.119 y1
232.092 b3
276.165 y2
329.147 b4
347.206 y3
416.174 b5
476.242 y4
529.259 b6
563.277 y5
660.324 y6
773.411 y7
825.402 b9
830.441 y8
929.504 y9
1000.529 y10
1128.585 y11
1191.637 b13
1225.669 y12
1338.757 y13
1425.782 y14
1522.821 y15
SGSPSLPQAVGLPSEATR / File: christine_10pools_01.raw; SpectrumID: ; scans: 15268

SALLPTIPETEDEISPDK

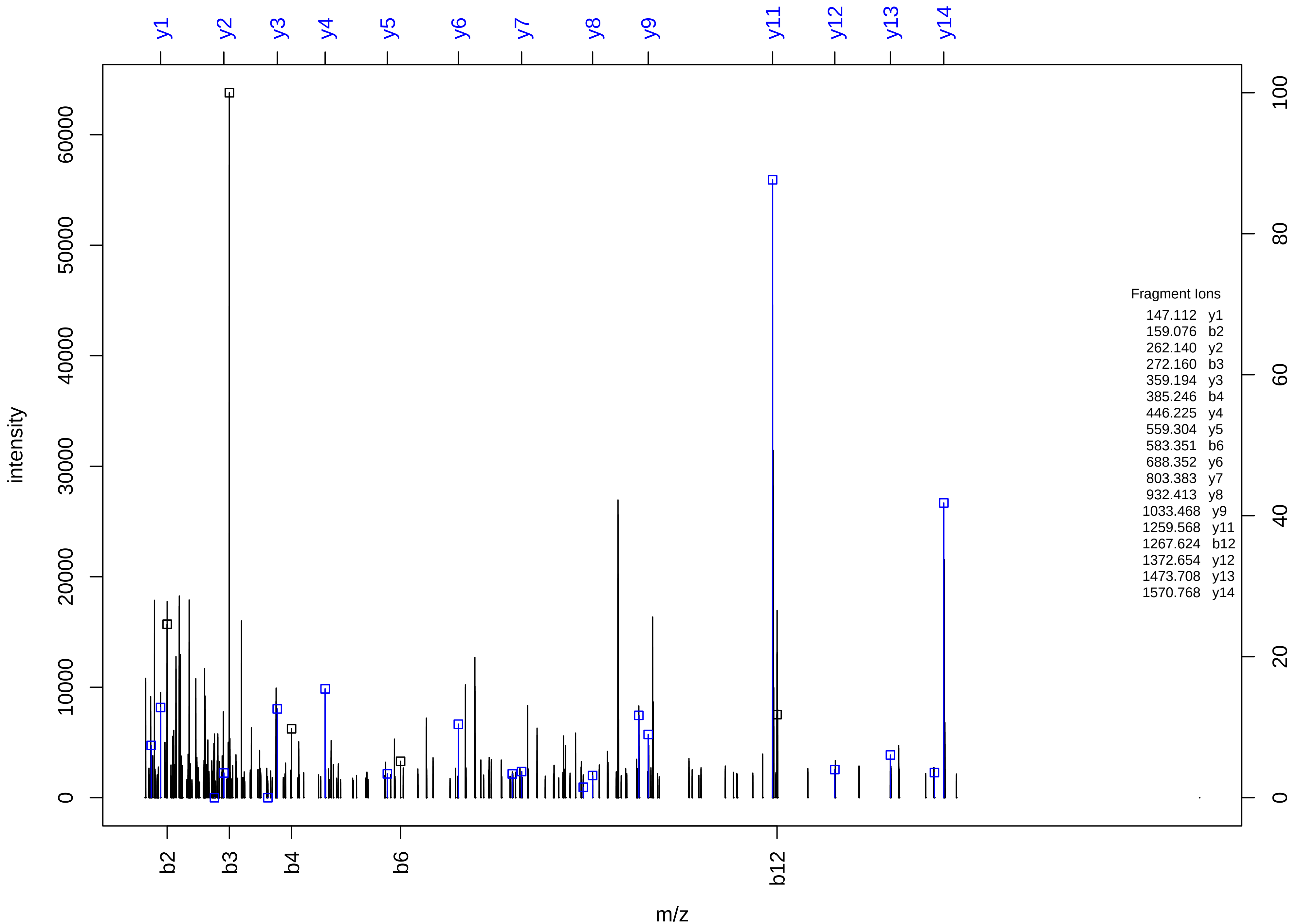

SALLPTIPETEDEISPDK / File: christine_10pools_01.raw; SpectrumID: ; scans: 22913

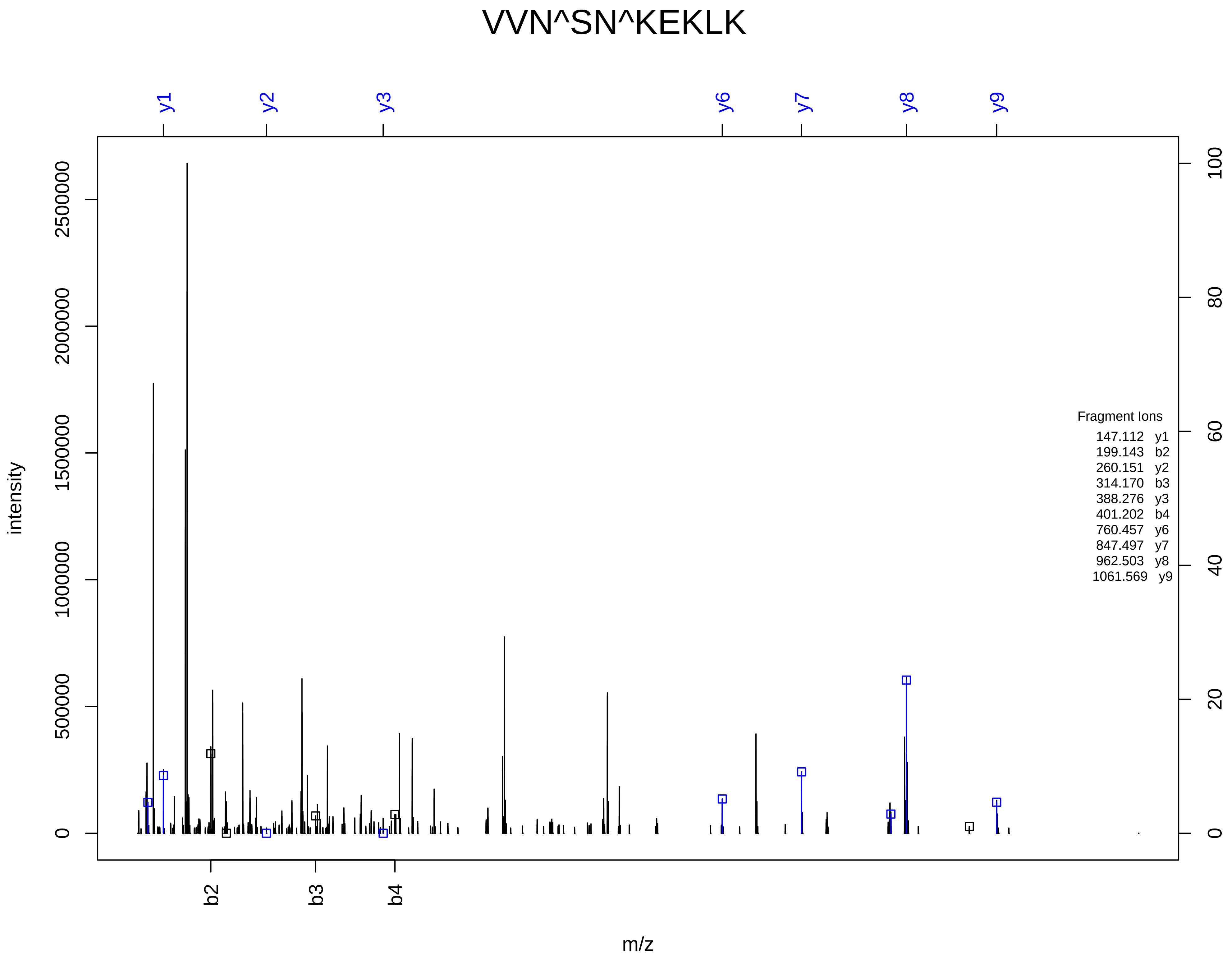

VVNSNKEKLK / File: christine_10pools_01.raw; SpectrumID: ; scans: 7123

ATVLQNQLDESQQER

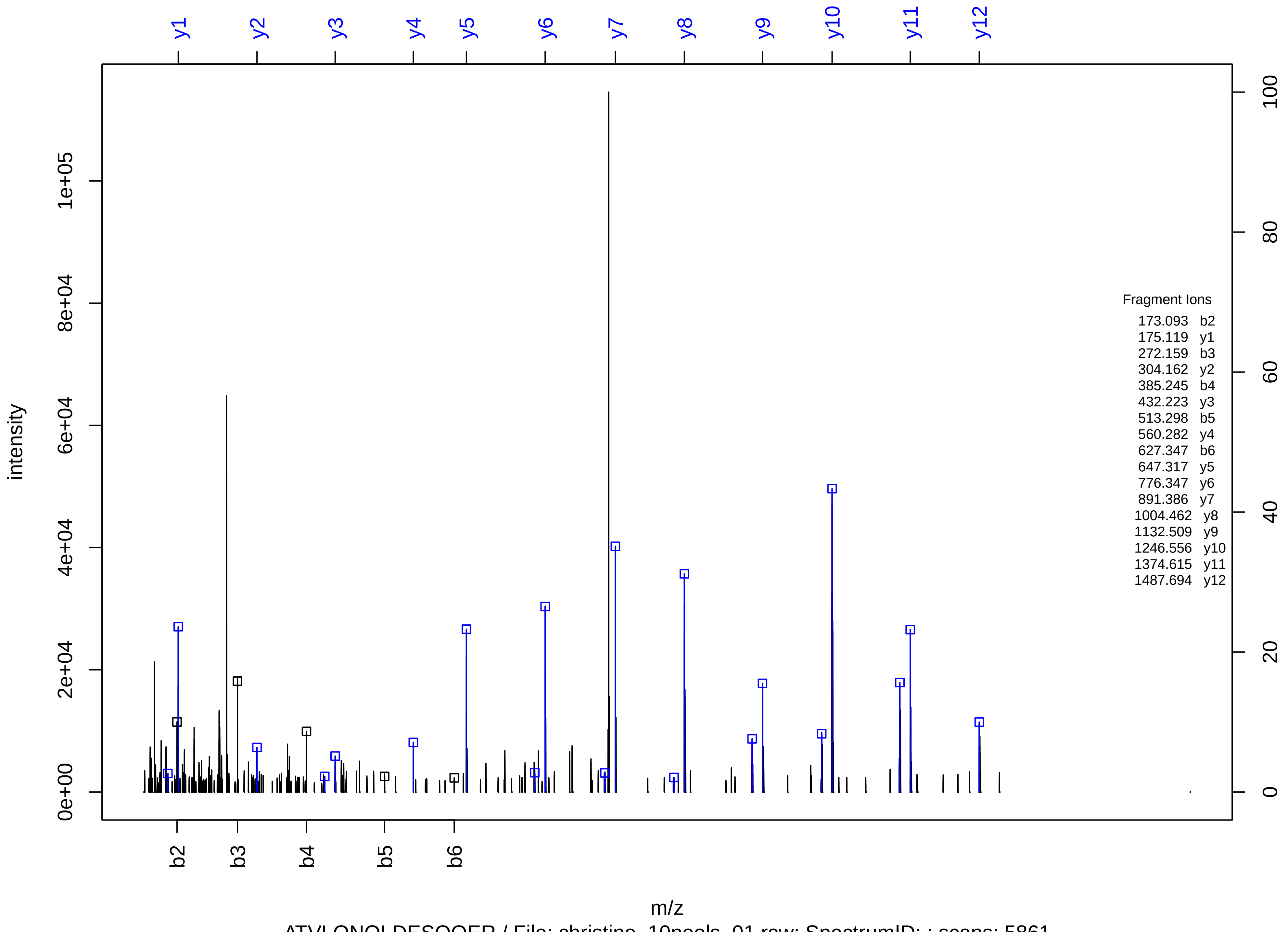

ATVLQNQLDESQQER / File: christine_10pools_01.raw; SpectrumID: ; scans: 5861

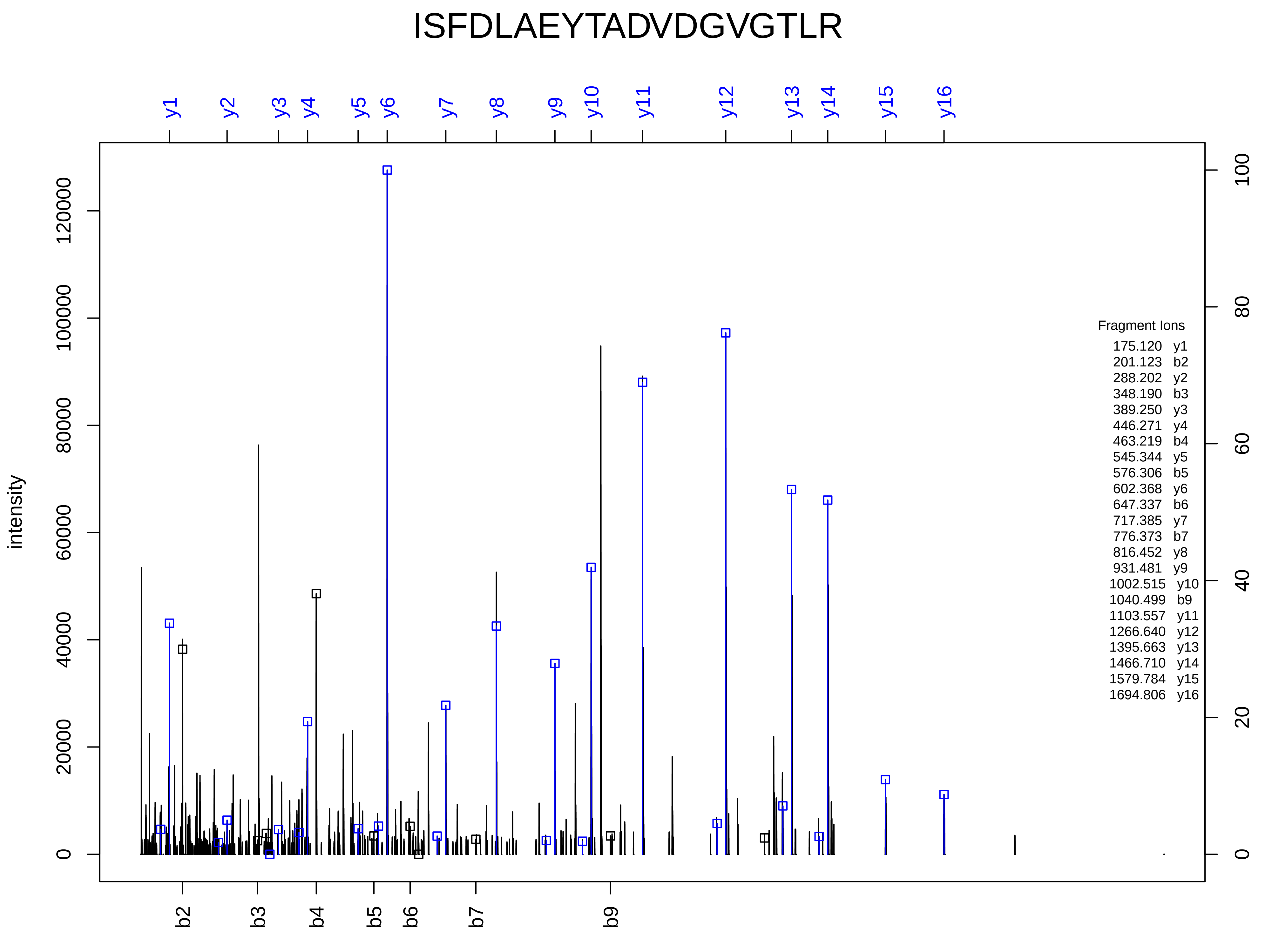

ISFDLAEYTADVDGVGTLR / File: christine_10pools_01.raw; SpectrumID: ; scans: 31657

EEAQAGEQDLNHEAAADPAPSPPNDFQNNVQVK

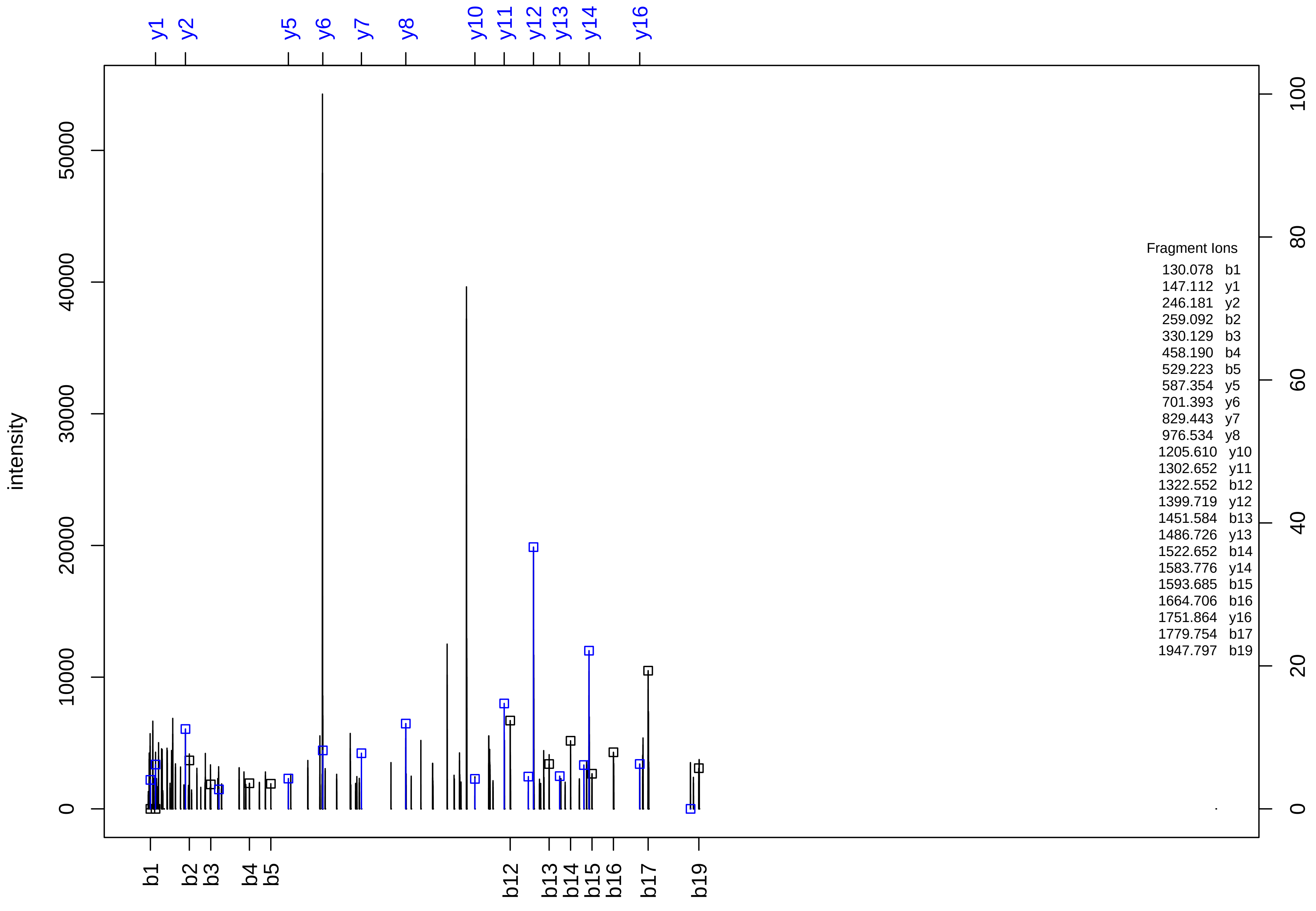

EEAQAGEQDLNHEAAADPAPSPPNDFQNNVQVK / File: christine_10pools_01.raw; SpectrumID: ; scans: 10467

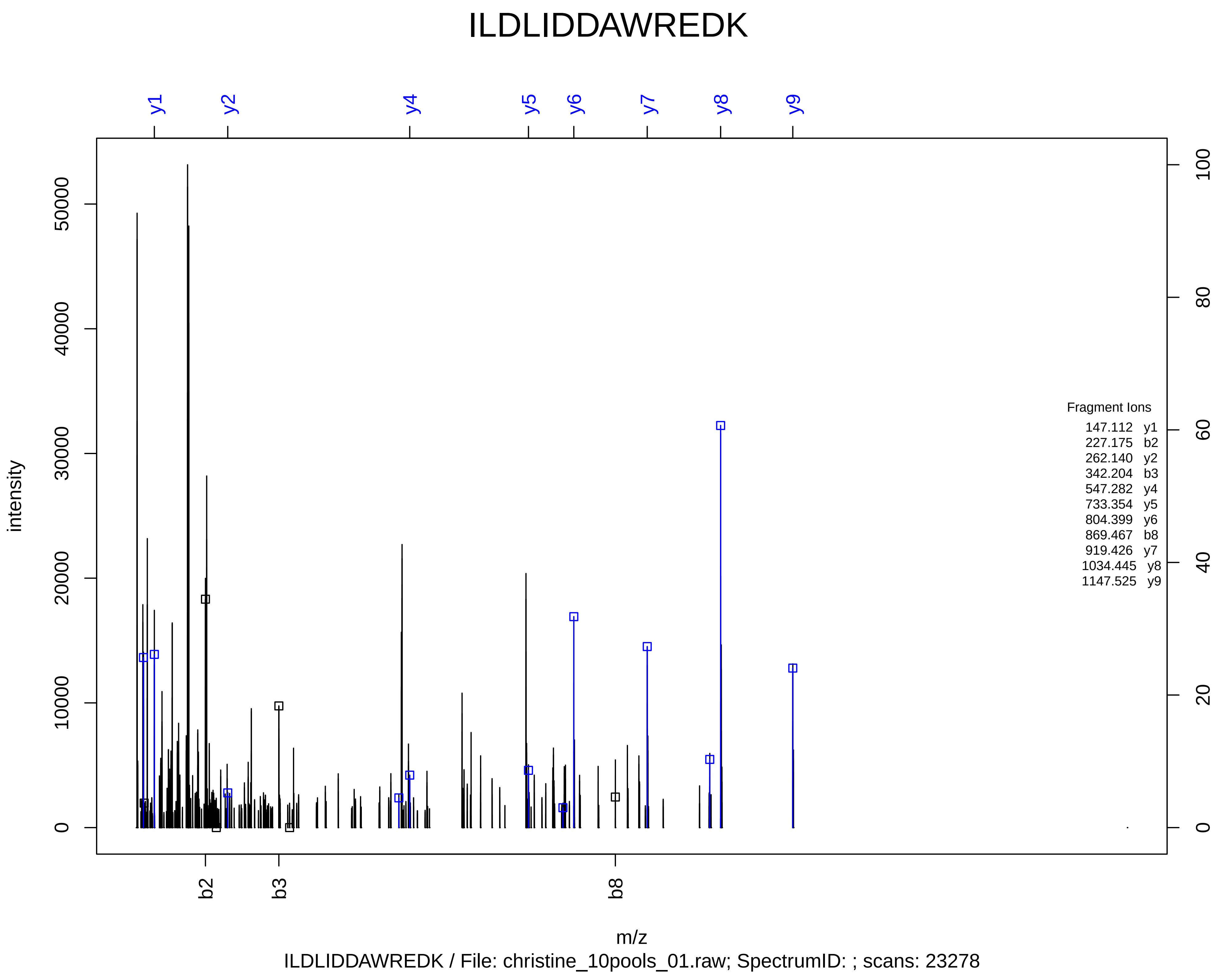
ILDLIDDAWREDK
y1
y2
y4
y5
y6
y7
y8
y9
b2
b3
b8
intensity
m/z
Fragment Ions
147.112 y1
227.175 b2
262.140 y2
342.204 b3
547.282 y4
733.354 y5
804.399 y6
869.467 b8
919.426 y7
1034.445 y8
1147.525 y9
ILDLIDDAWREDK / File: christine_10pools_01.raw; SpectrumID: ; scans: 23278

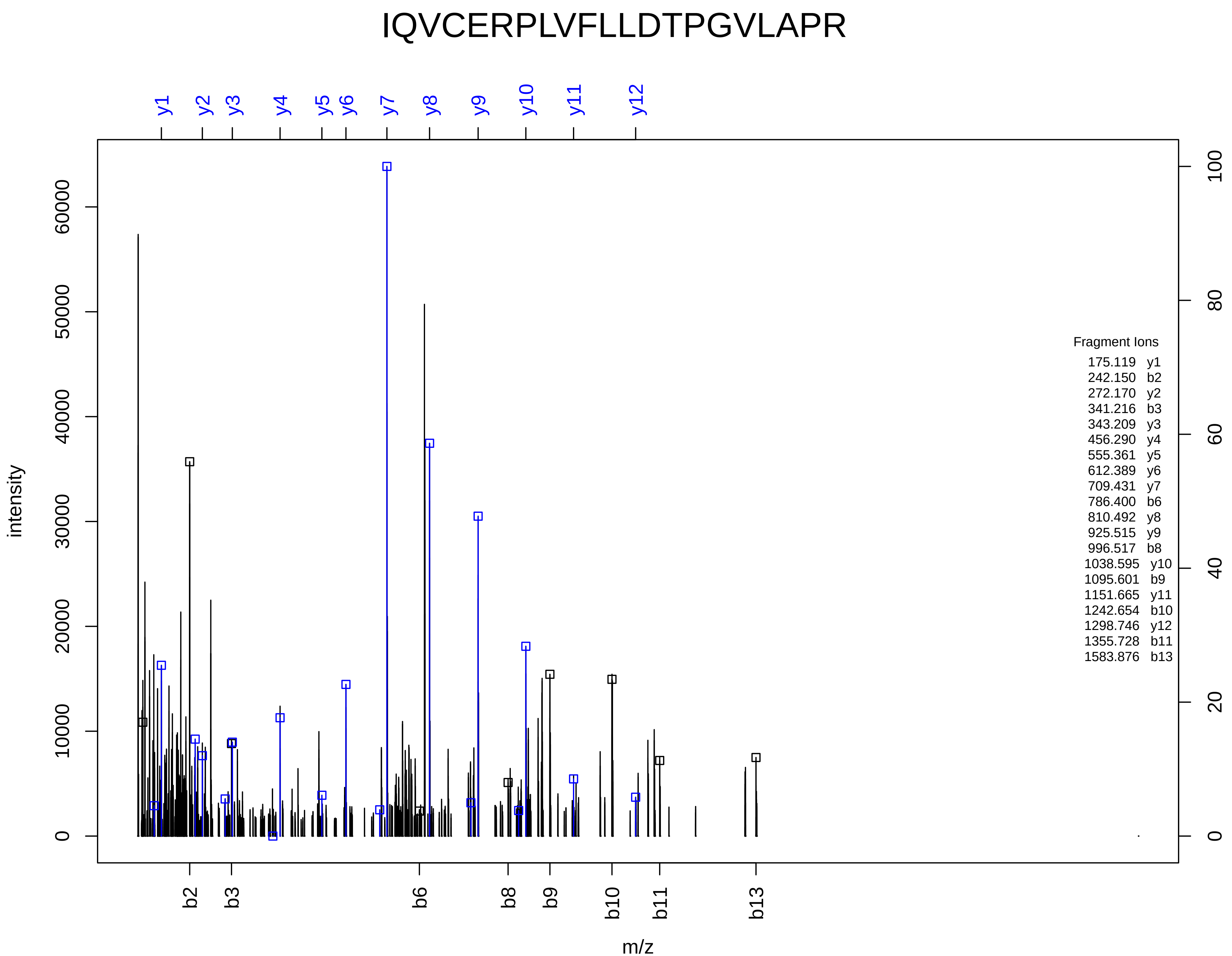

IQVCERPLVFLLDTPGVLAPR / File: christine_10pools_01.raw; SpectrumID: ; scans: 34357

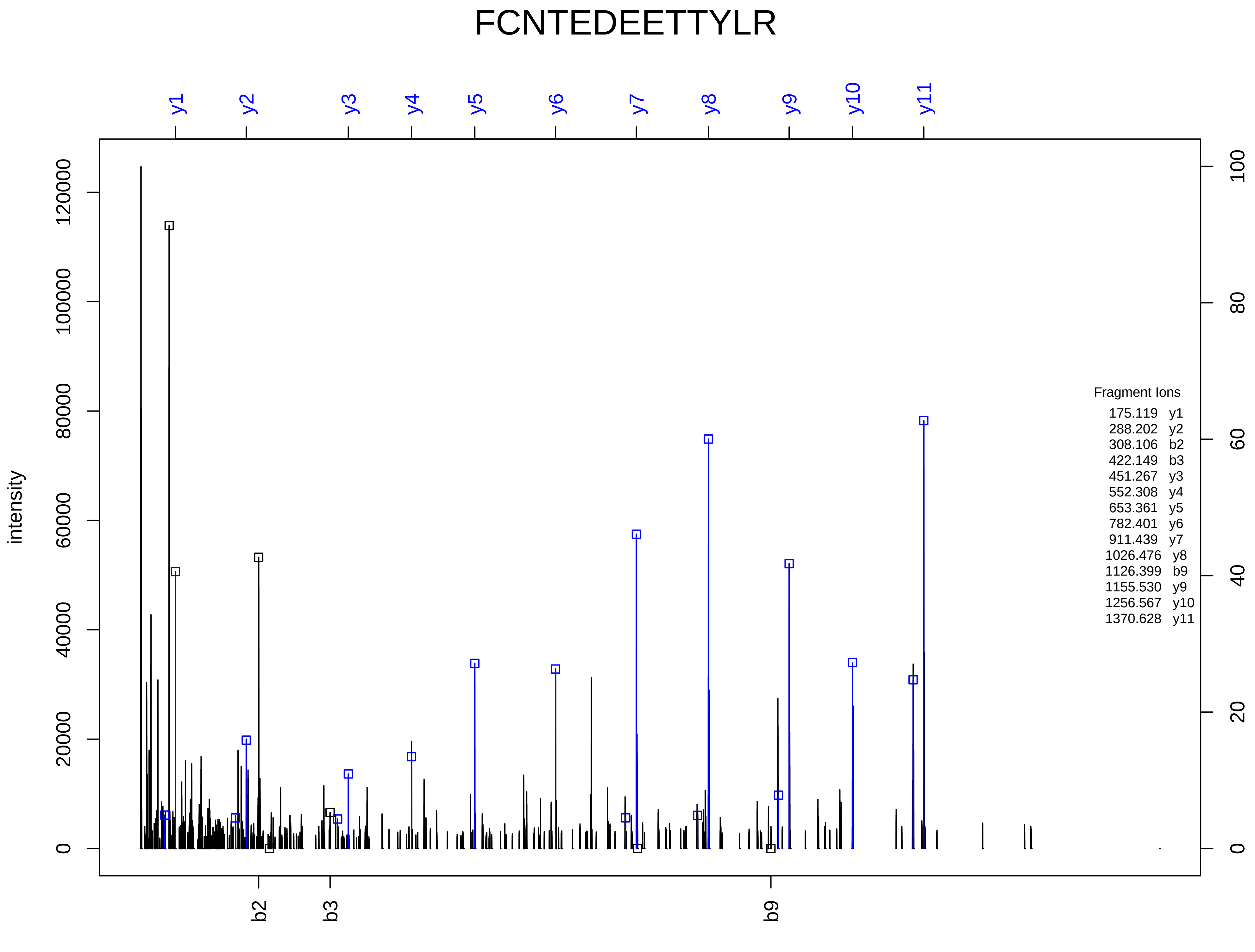

FCNTEDEETTYLR / File: christine_10pools_01.raw; SpectrumID: ; scans: 7506

AEDASGEAAAMLNNM*R

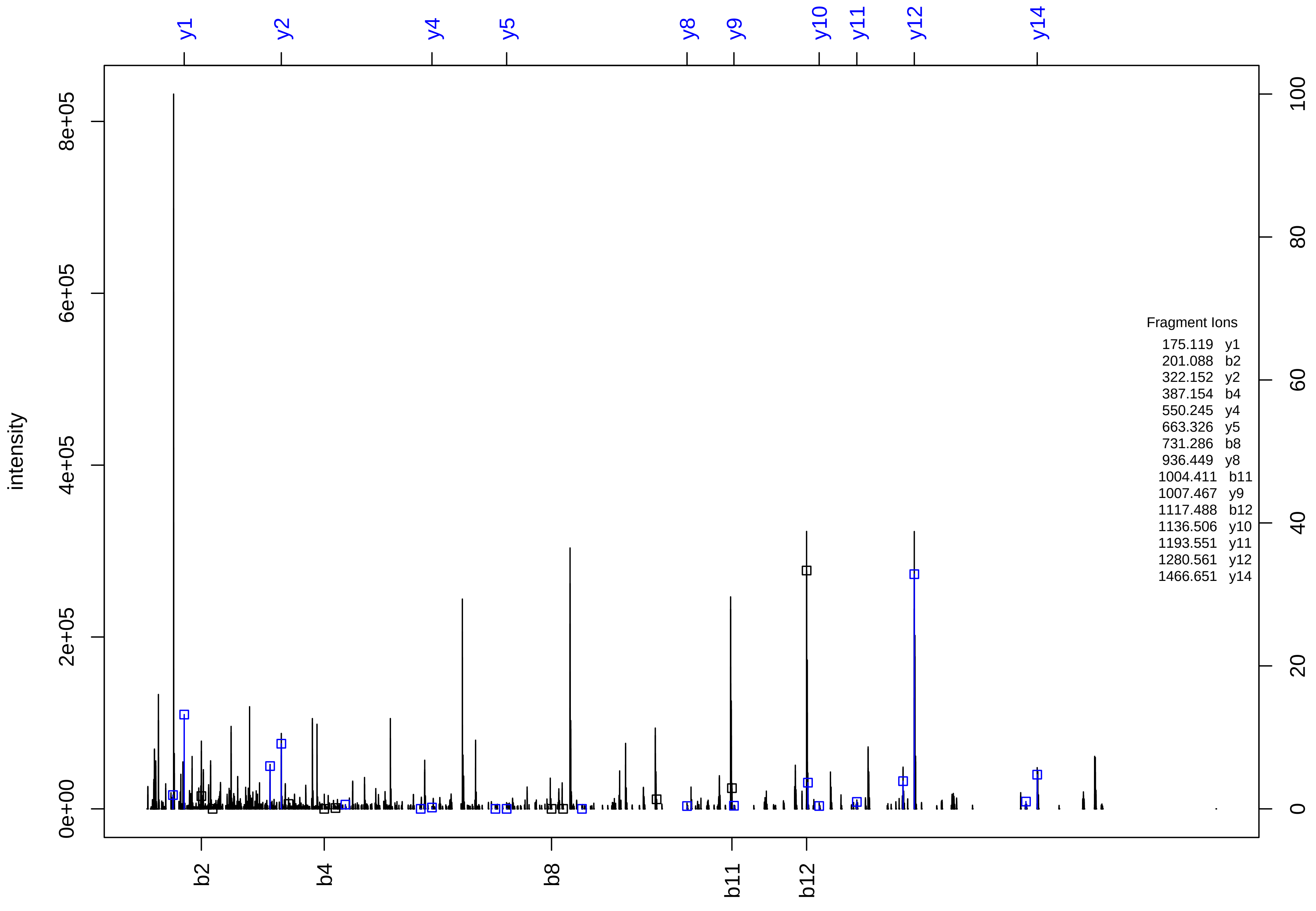

AEDASGEAAAMLNNMR / File: christine_10pools_01.raw; SpectrumID: ; scans: 13425

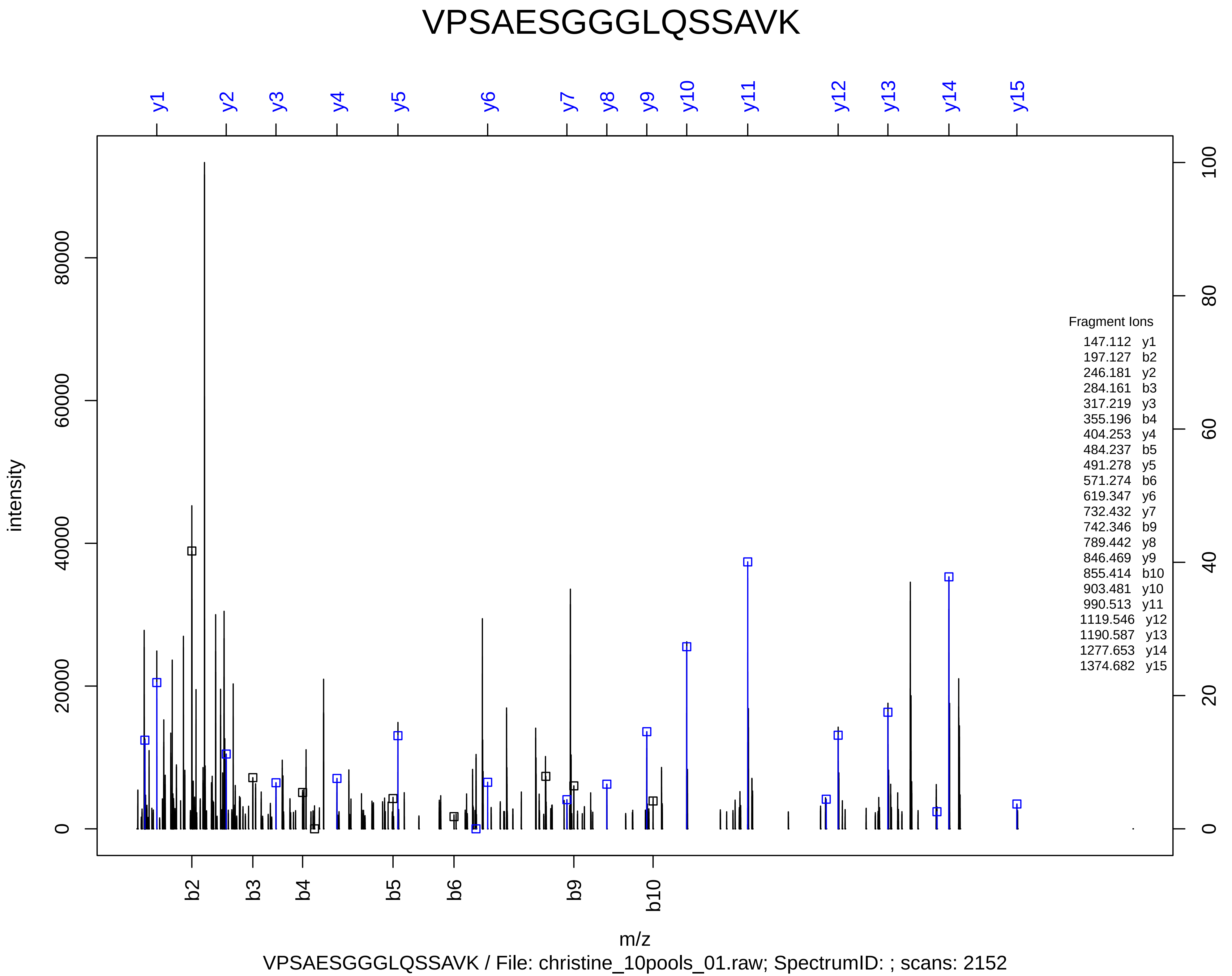
VPSAESGGGLQSSAVK
y1
y2
y3
y4
y5
y6
y7
y8
y9
y10
y11
y12
y13
y14
y15
b2
b3
b4
b5
b6
b9
b10
intensity
m/z
0
20000
40000
60000
80000
0
20
40
60
80
100
Fragment Ions
147.112 y1
197.127 b2
246.181 y2
284.161 b3
317.219 y3
355.196 b4
404.253 y4
484.237 b5
491.278 y5
571.274 b6
619.347 y6
732.432 y7
742.346 b9
789.442 y8
846.469 y9
855.414 b10
903.481 y10
990.513 y11
1119.546 y12
1190.587 y13
1277.653 y14
1374.682 y15
VPSAESGGGLQSSAVK / File: christine_10pools_01.raw; SpectrumID: ; scans: 2152

AVYDEQGTVDEDSAGLNQDRDWDAYWR

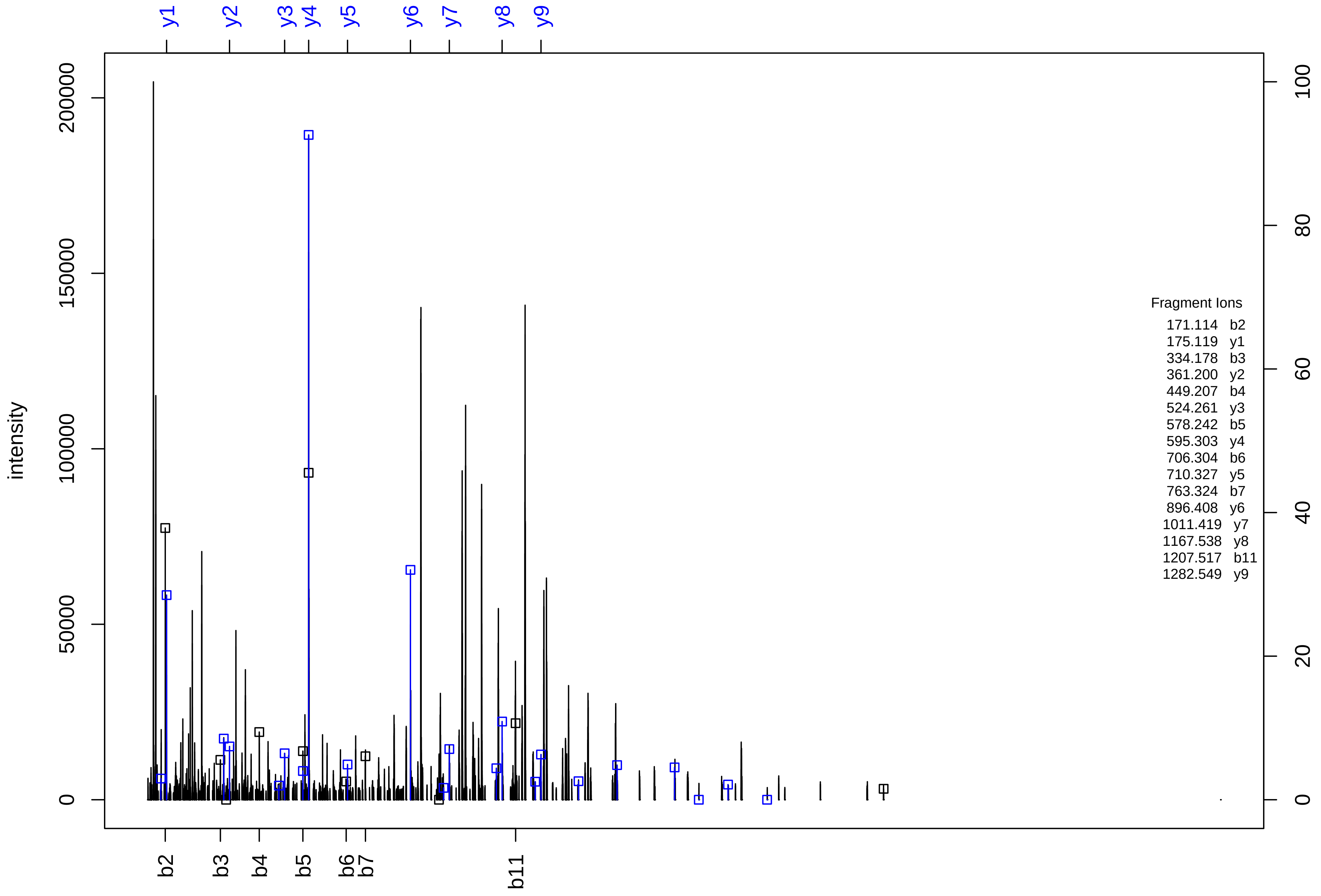

AVYDEQGTVDEDSAGLNQDRDWDAYWR / File: christine_10pools_01.raw; SpectrumID: ; scans: 20660

LTLPDGLTSAASPEEGLSAELLEAQTEEPADTASLDCR

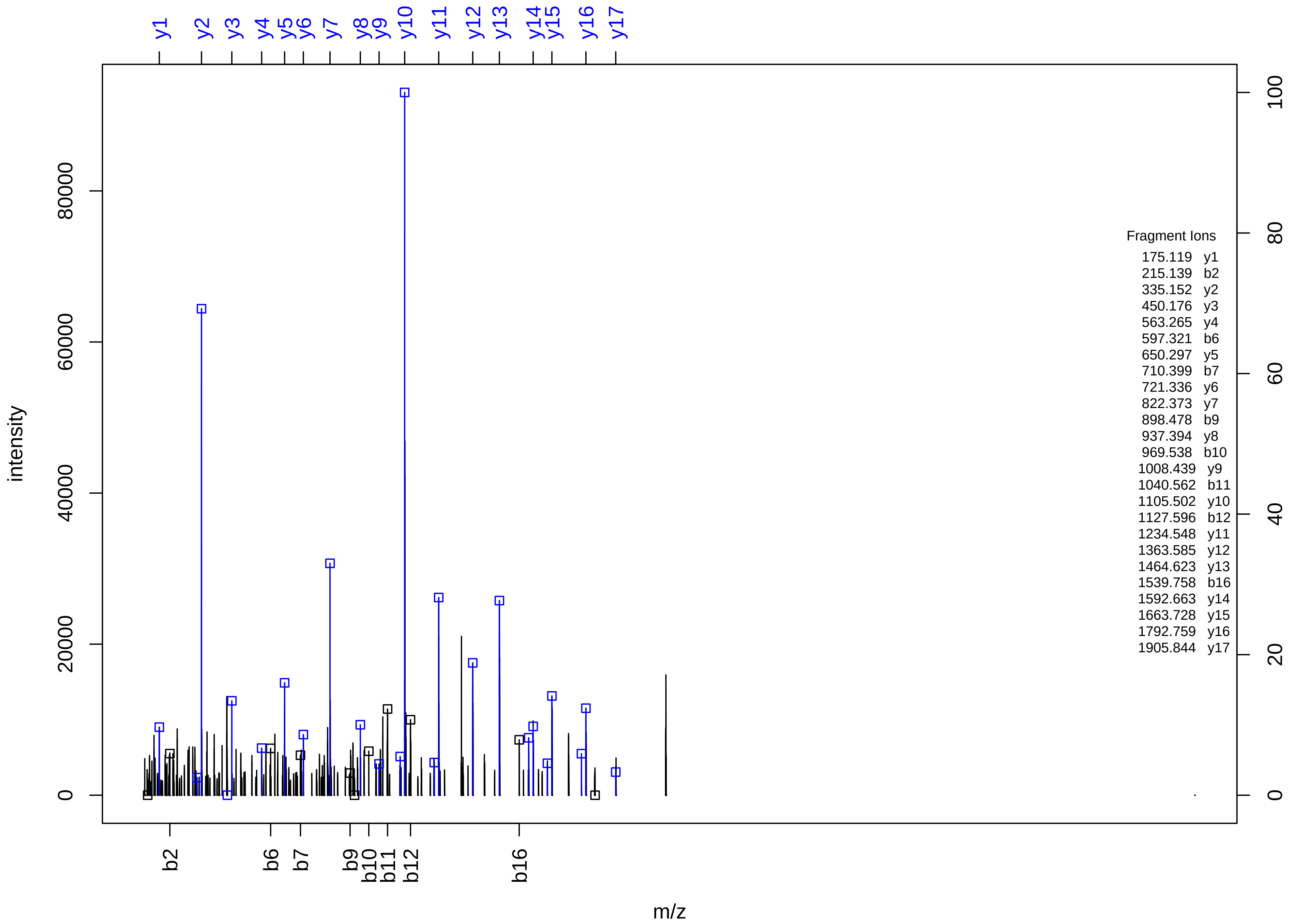

LTLPDGLTSAASPEEGLSAELLEAQTEEPADTASLDCR / File: christine_10pools_01.raw; SpectrumID: ; scans: 34605

GPDLAAAPYSTQNSSVLPLYENTFQELQVVR

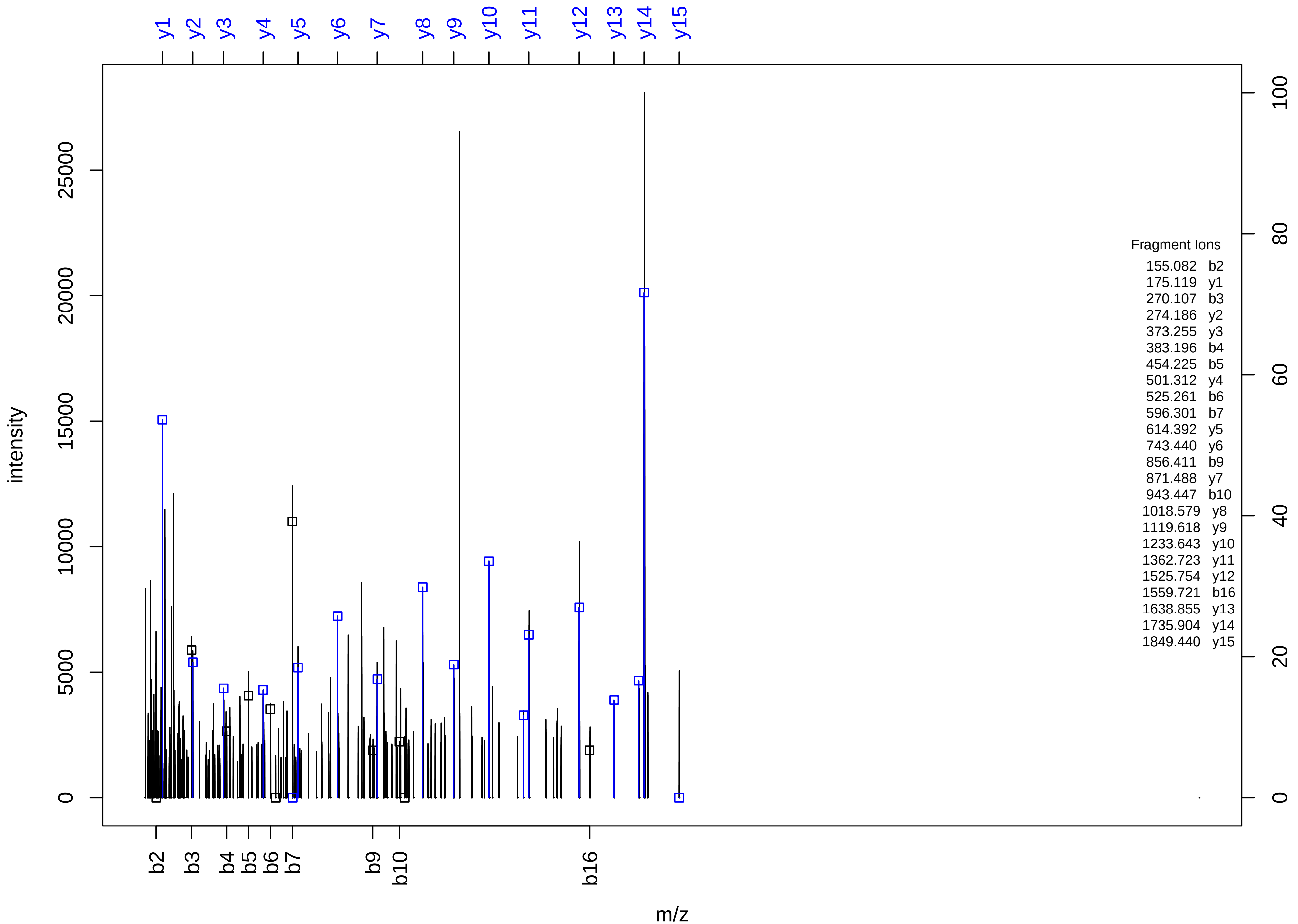

GPDLAAAPYSTQNSSVLPLYENTFQELQVVR / File: christine_10pools_01.raw; SpectrumID: ; scans: 36487

ELFDFVTDPSITADNMDAYLEK

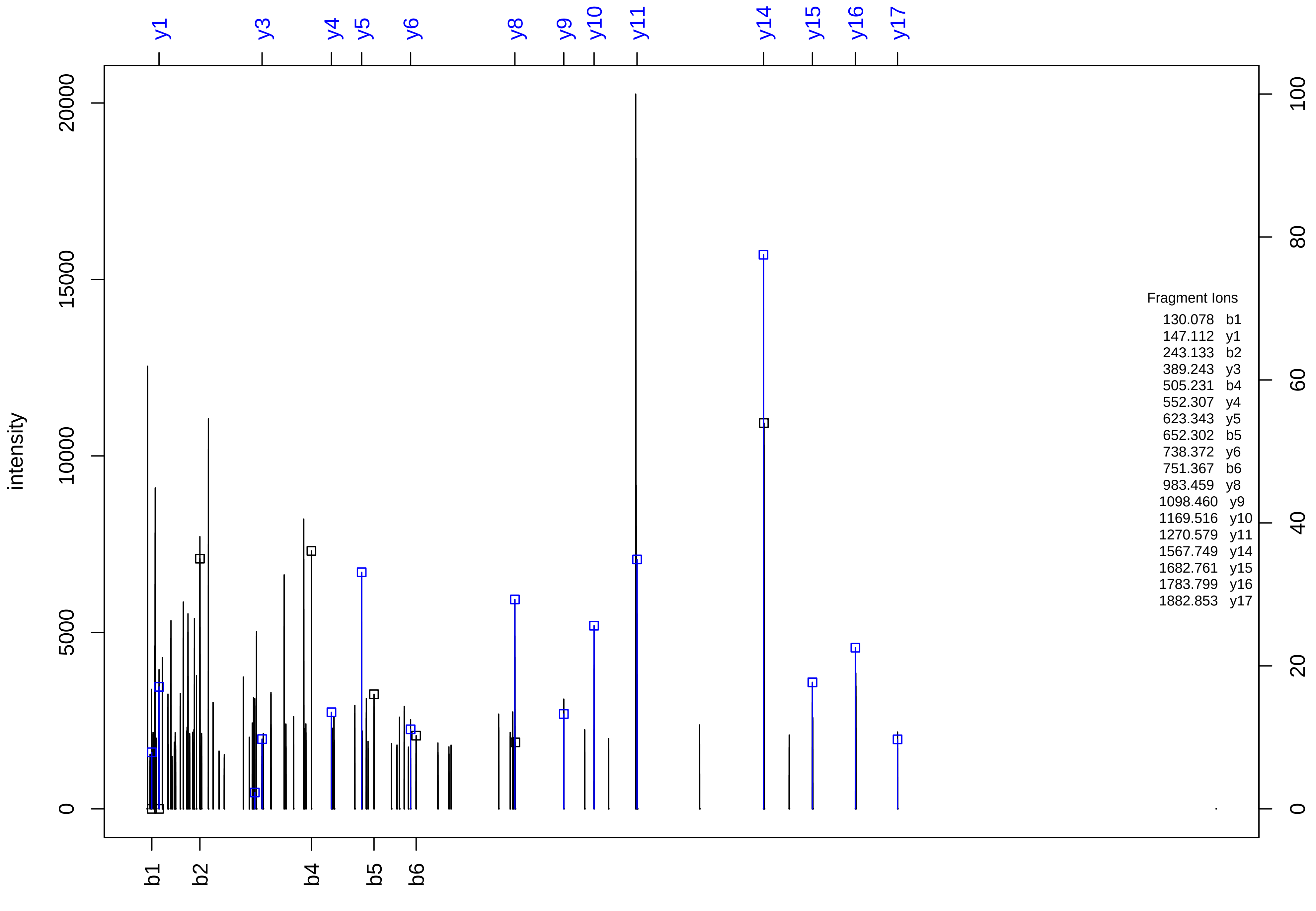

ELFDFVTDPSITADNMDAYLEK / File: christine_10pools_01.raw; SpectrumID: ; scans: 37531

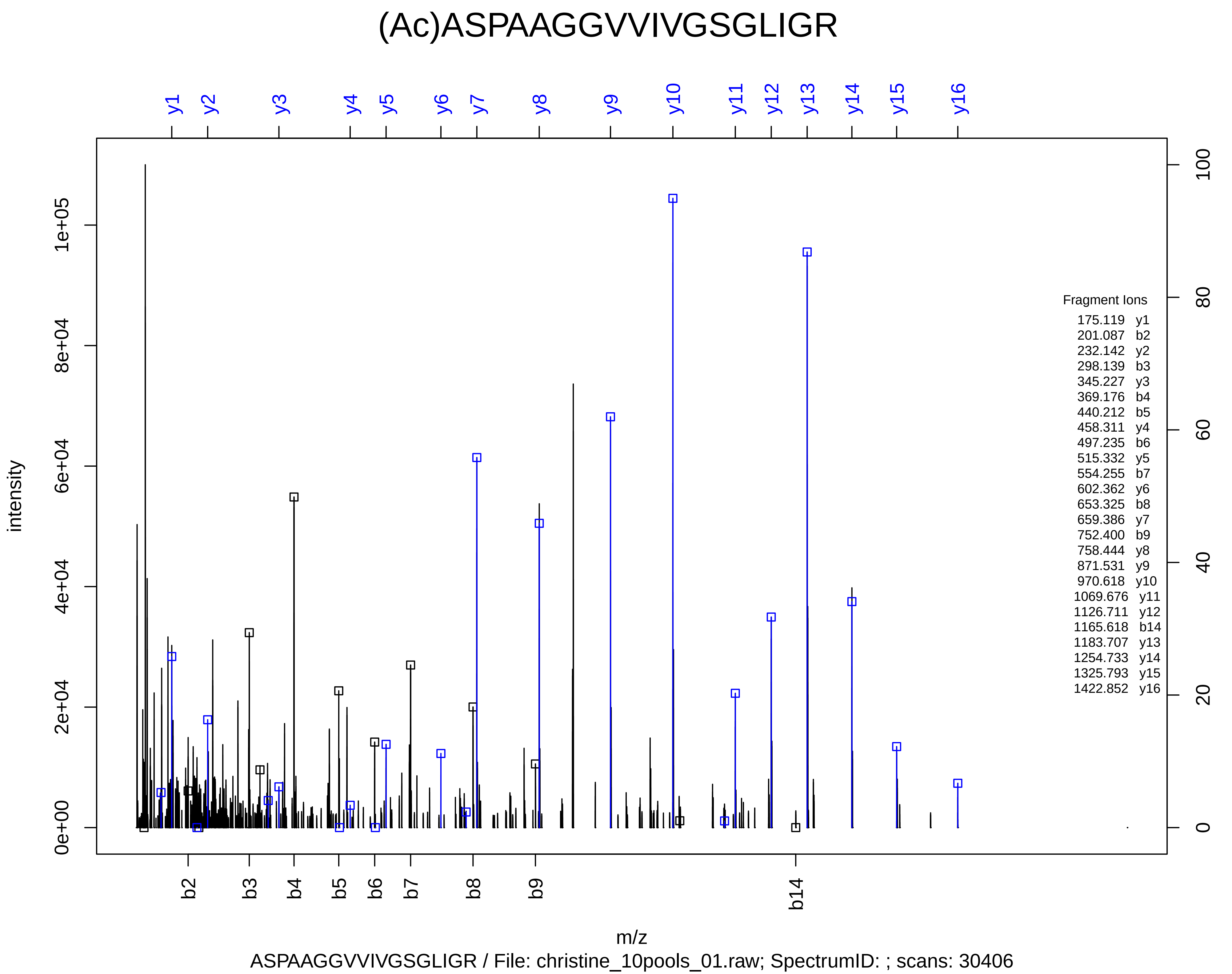

(Ac)ASPAAGGVVIVGSGLIGR
intensity
m/z
Fragment Ions
175.119 y1
201.087 b2
232.142 y2
298.139 b3
345.227 y3
369.176 b4
440.212 b5
458.311 y4
497.235 b6
515.332 y5
554.255 b7
602.362 y6
653.325 b8
659.386 y7
752.400 b9
758.444 y8
871.531 y9
970.618 y10
1069.676 y11
1126.711 y12
1165.618 b14
1183.707 y13
1254.733 y14
1325.793 y15
1422.852 y16
ASPAAGGVVIVGSGLIGR / File: christine_10pools_01.raw; SpectrumID: ; scans: 30406

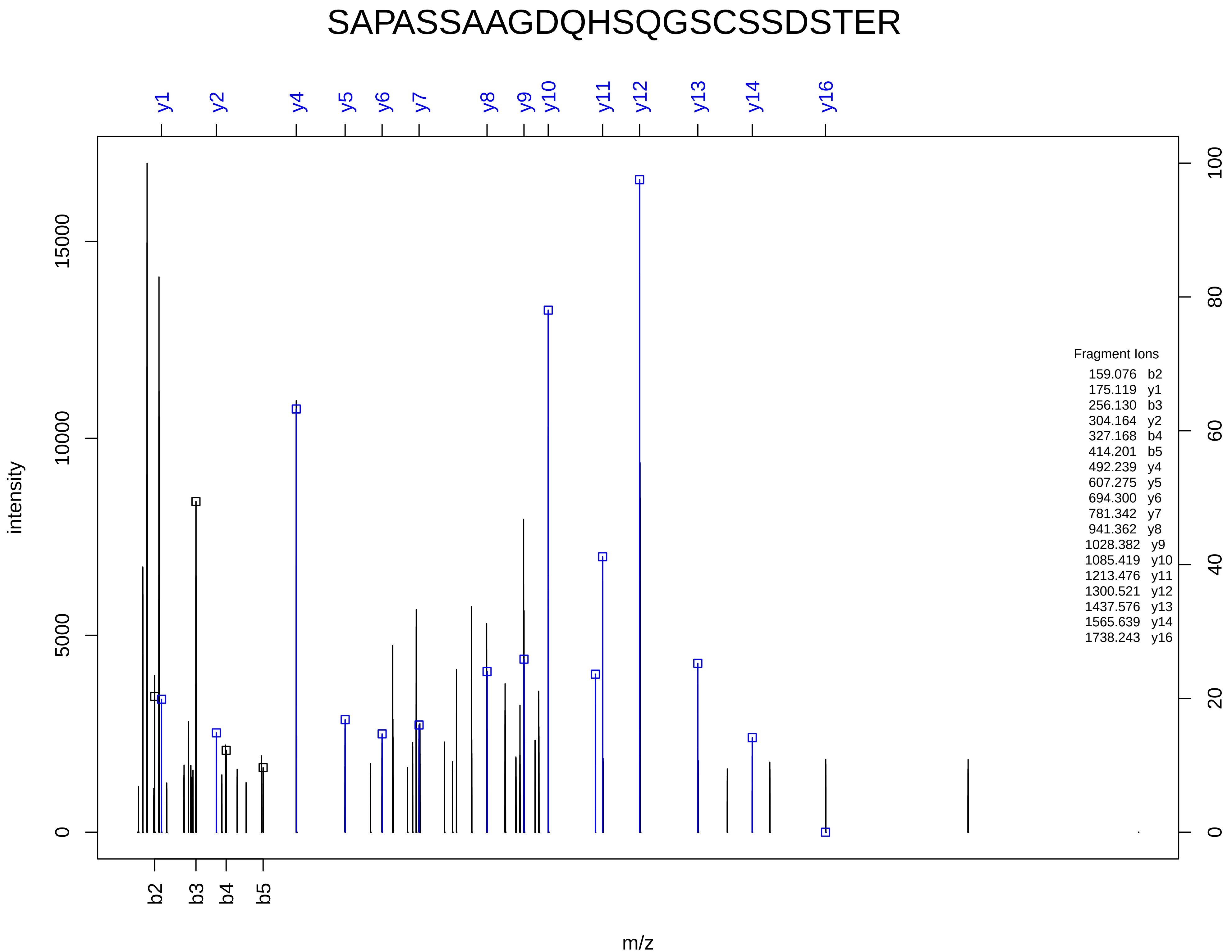

SAPASSAAGDQHSQGSCSSDSTER / File: christine_10pools_01.raw; SpectrumID: ; scans: 651

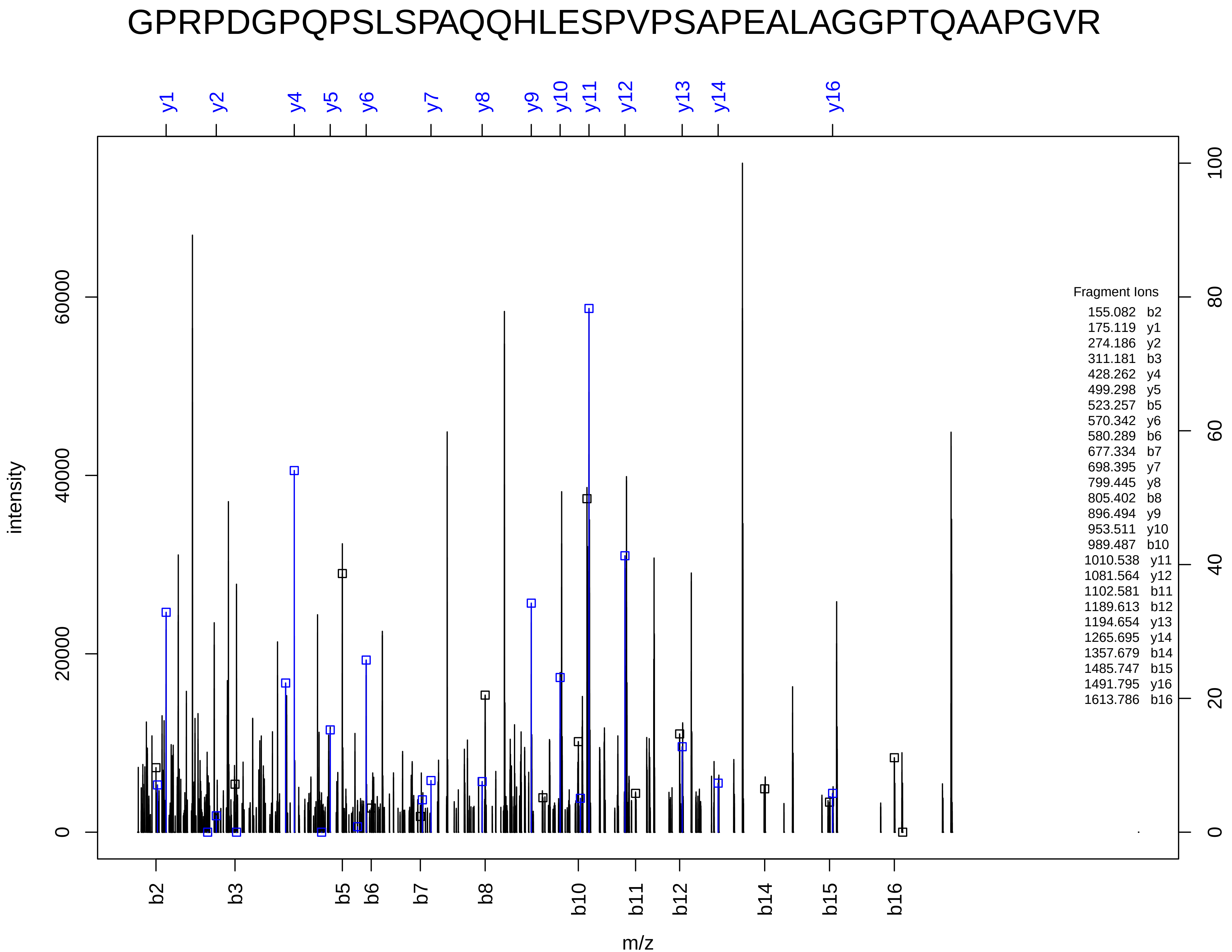

GPRPDGPQPSLSPAQQHLESPVPSAPEALAGGPTQAAPGVR / File: christine_10pools_01.raw; SpectrumID: ; scans: 15939

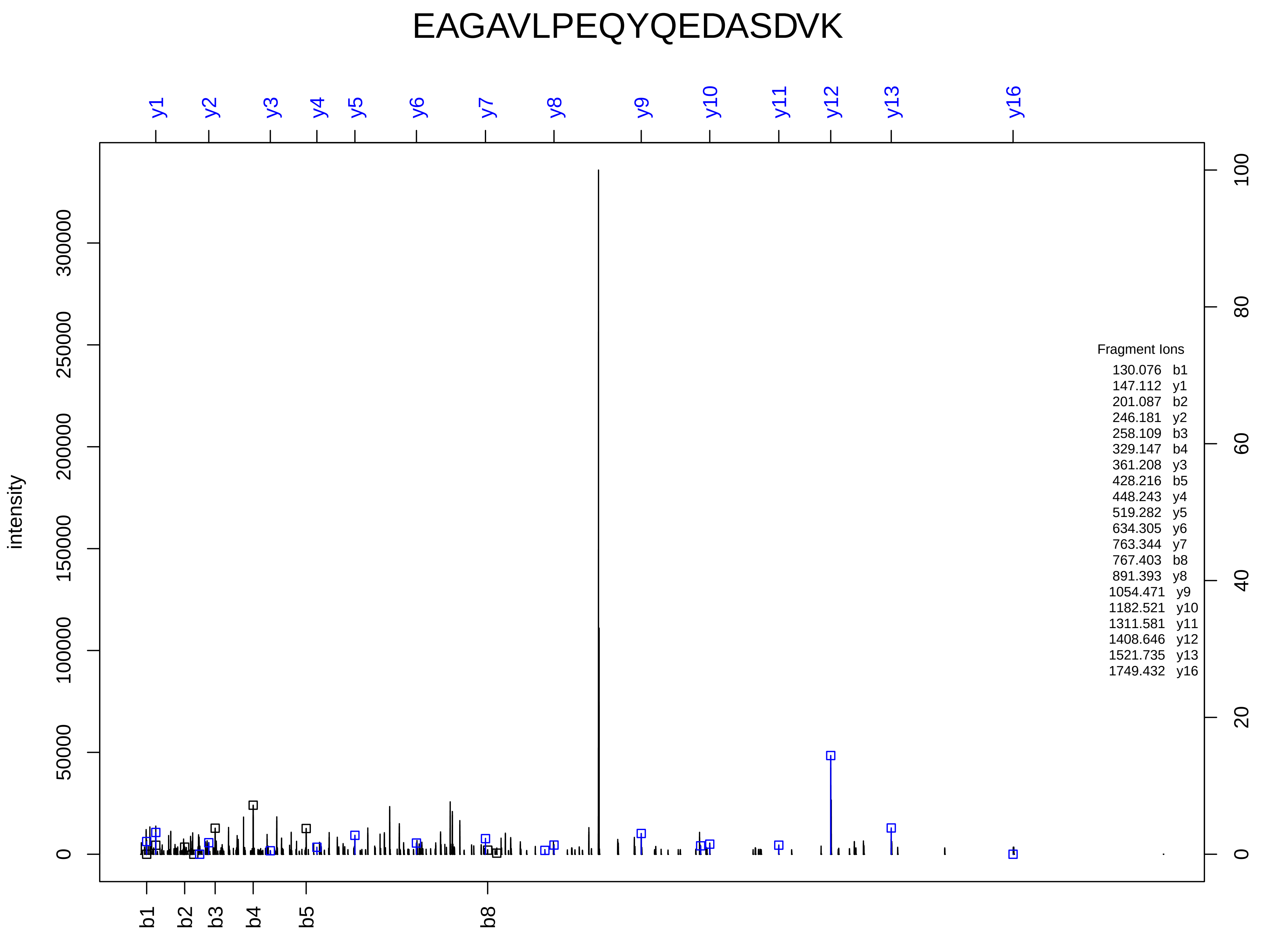

EAGAVLPEQYQEDASDVK / File: christine_10pools_01.raw; SpectrumID: ; scans: 11198

ADSEYPTWLFQVNLGPPK

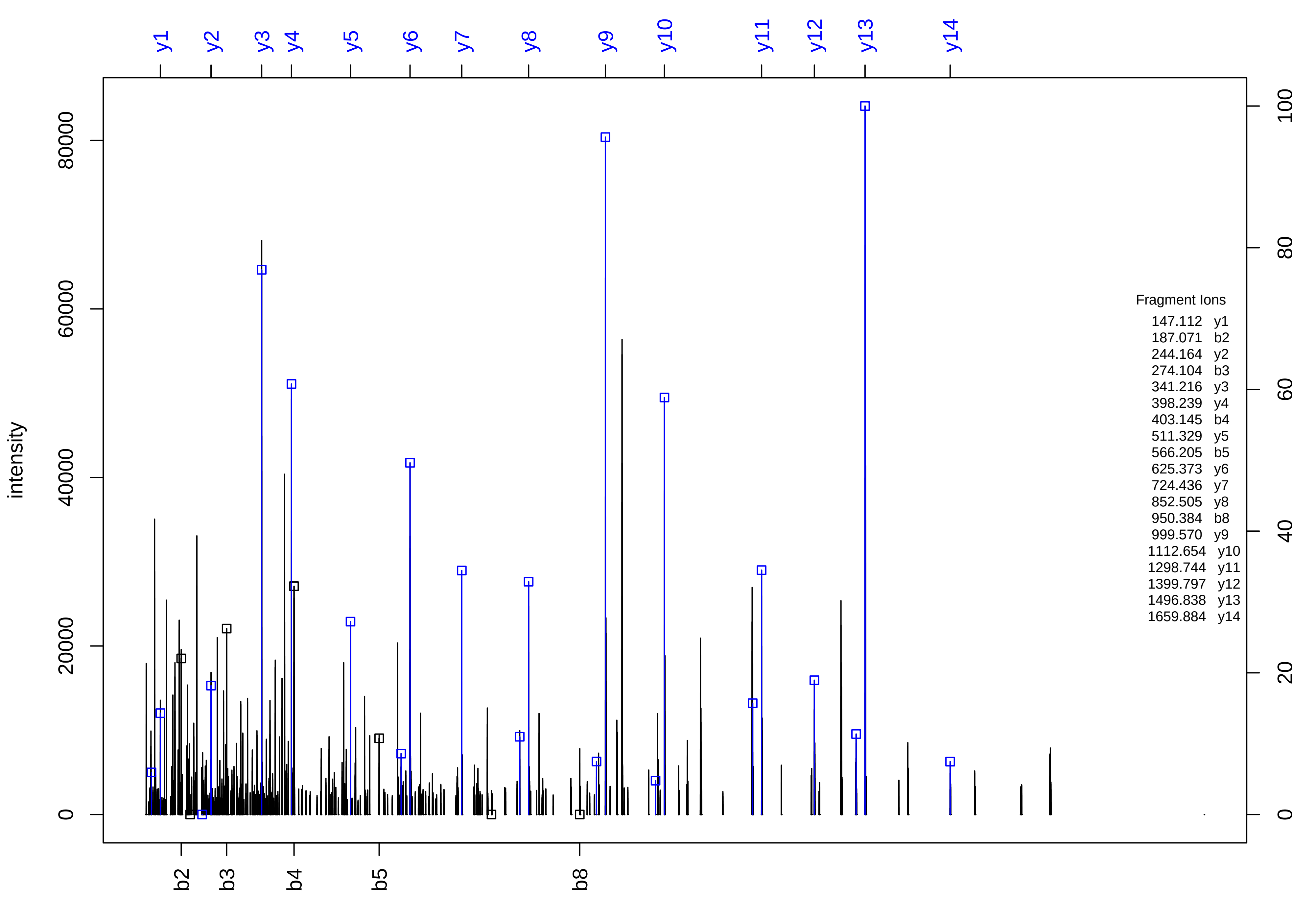

ADSEYPTWLFQVNLGPPK / File: christine_10pools_01.raw; SpectrumID: ; scans: 33553

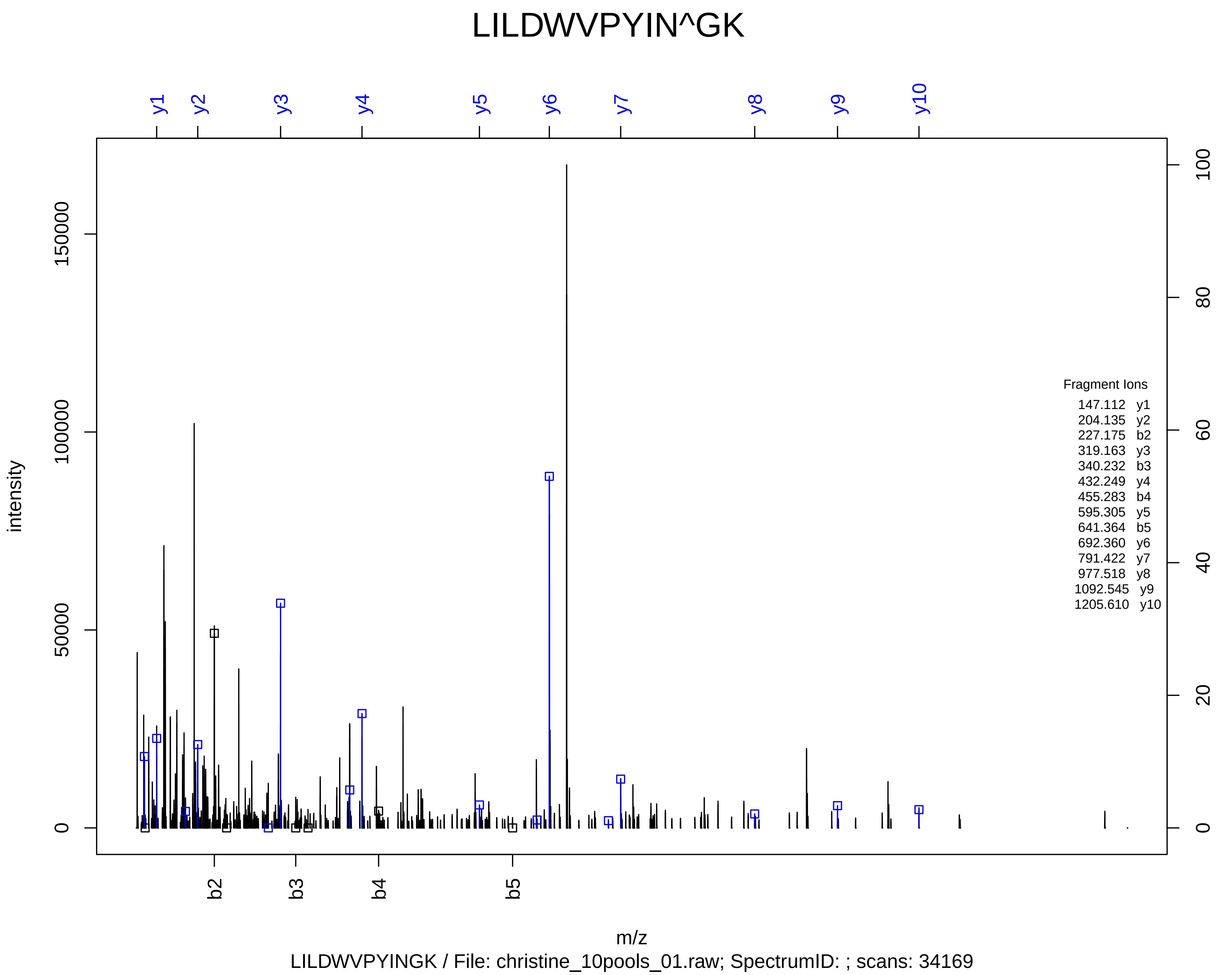

LILDWVPYIN^GK
y1
y2
y3
y4
y5
y6
y7
y8
y9
y10
b2
b3
b4
b5
intensity
m/z
Fragment Ions
147.112 y1
204.135 y2
227.175 b2
319.163 y3
340.232 b3
432.249 y4
455.283 b4
595.305 y5
641.364 b5
692.360 y6
791.422 y7
977.518 y8
1092.545 y9
1205.610 y10
LILDWVPYINGK / File: christine_10pools_01.raw; SpectrumID: ; scans: 34169

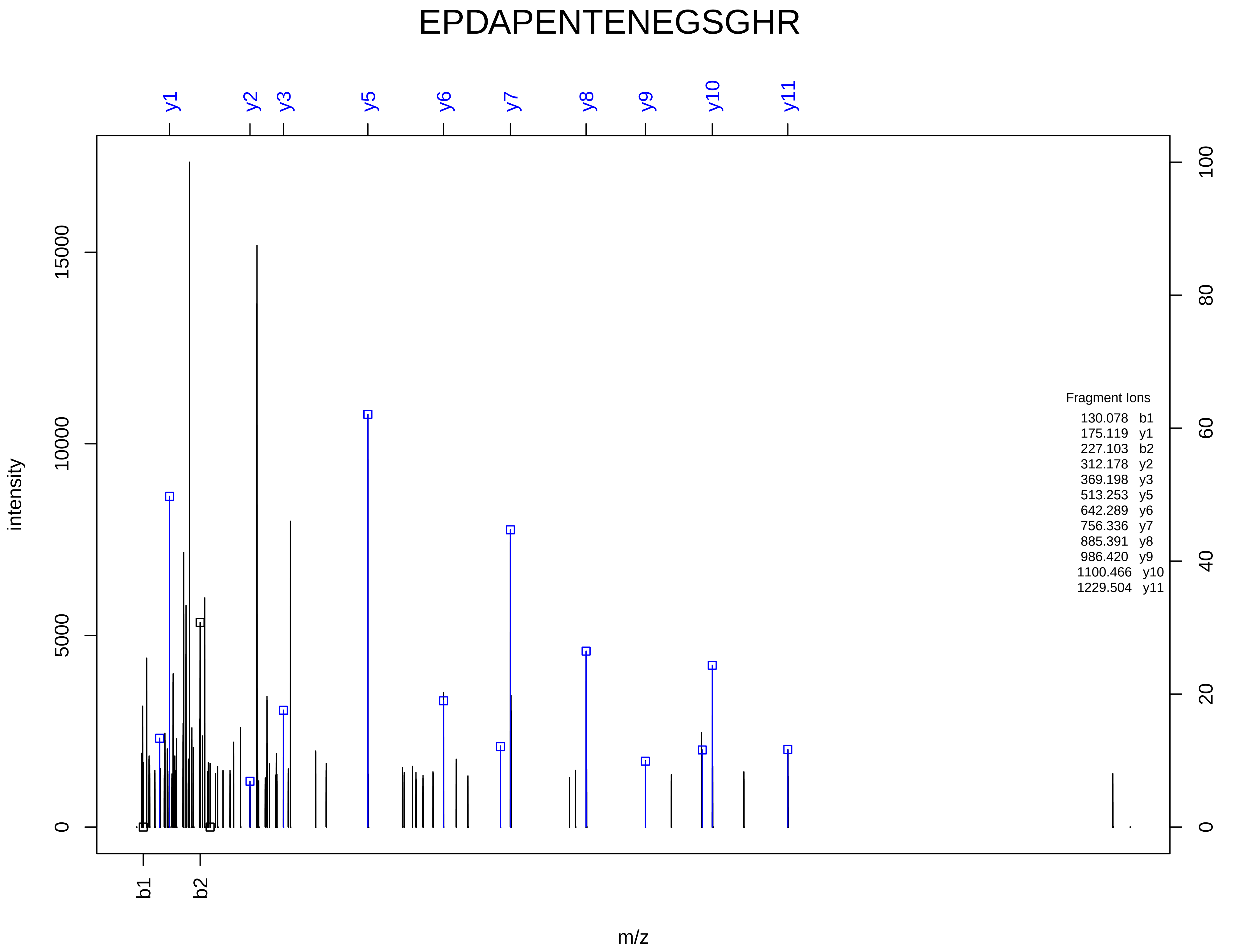

EPDAPENTENEGSGHR / File: christine_10pools_01.raw; SpectrumID: ; scans: 783

HLDTLTEHYDIPK

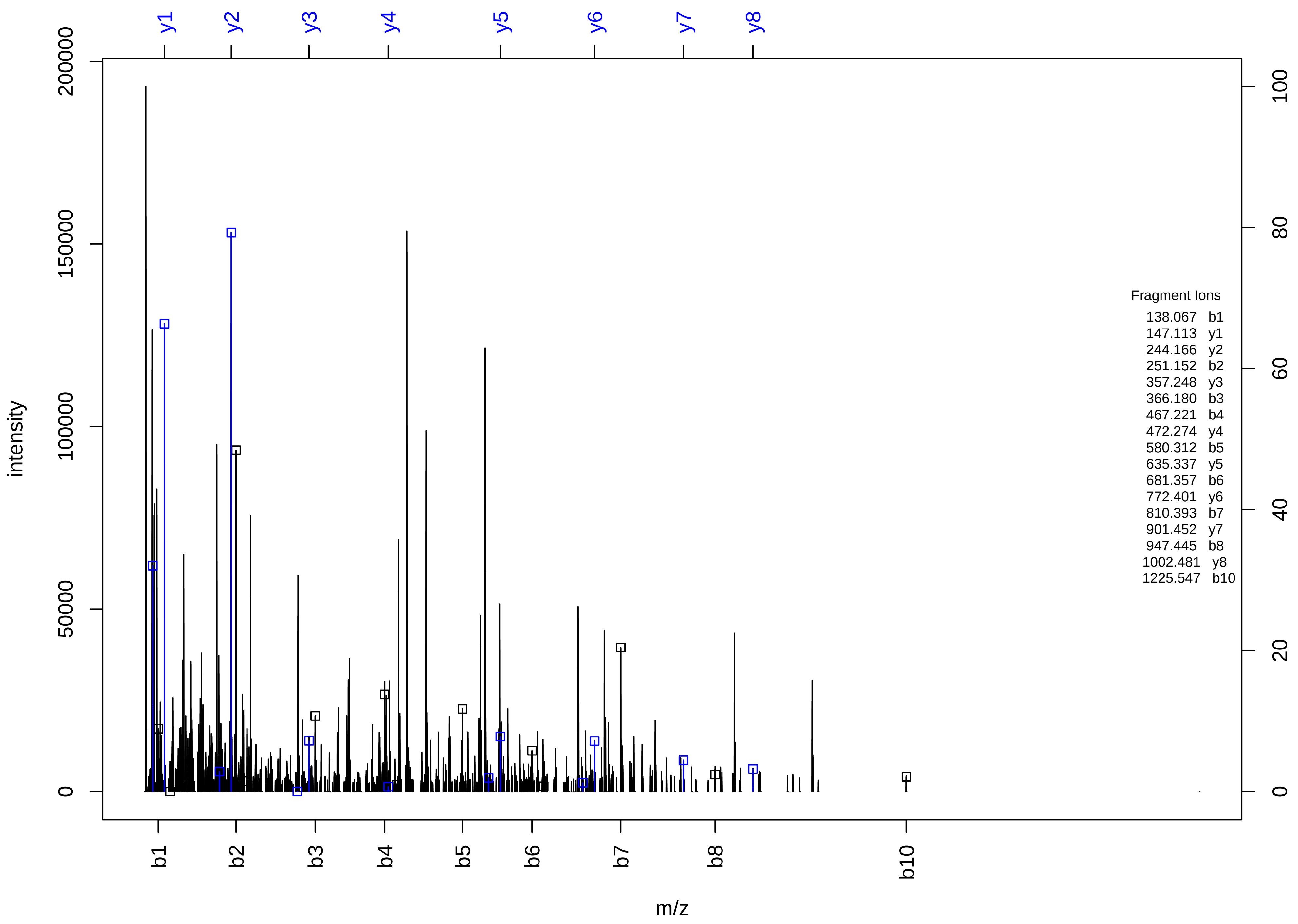

HLDTLTEHYDIPK / File: christine_10pools_01.raw; SpectrumID: ; scans: 6811

GHGFITPADGGPDIFLHISDVEGEYVPVEGDEVTYK

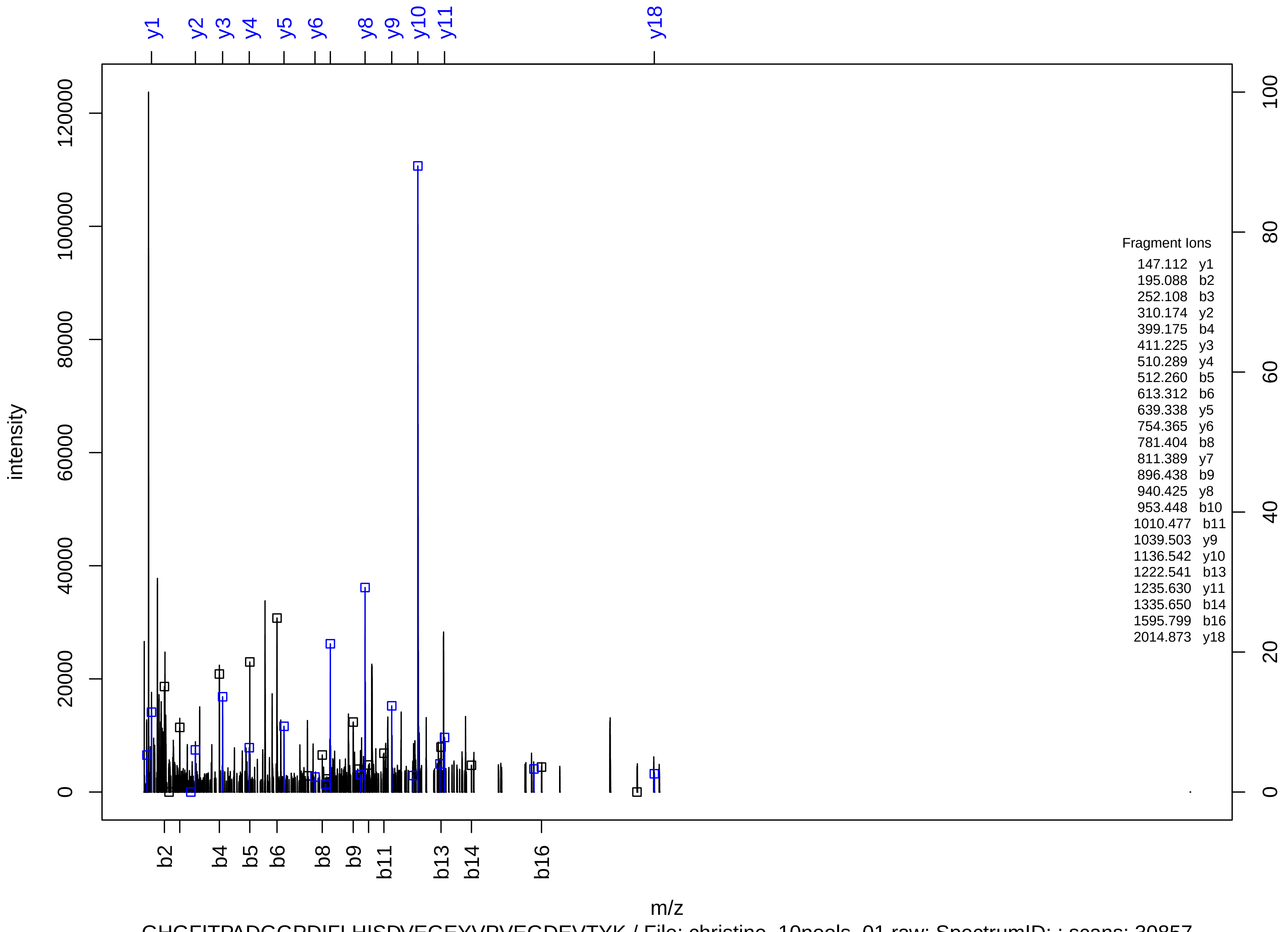

GHGFITPADGGPDIFLHISDVEGEYVPVEGDEVTYK / File: christine_10pools_01.raw; SpectrumID: ; scans: 30857

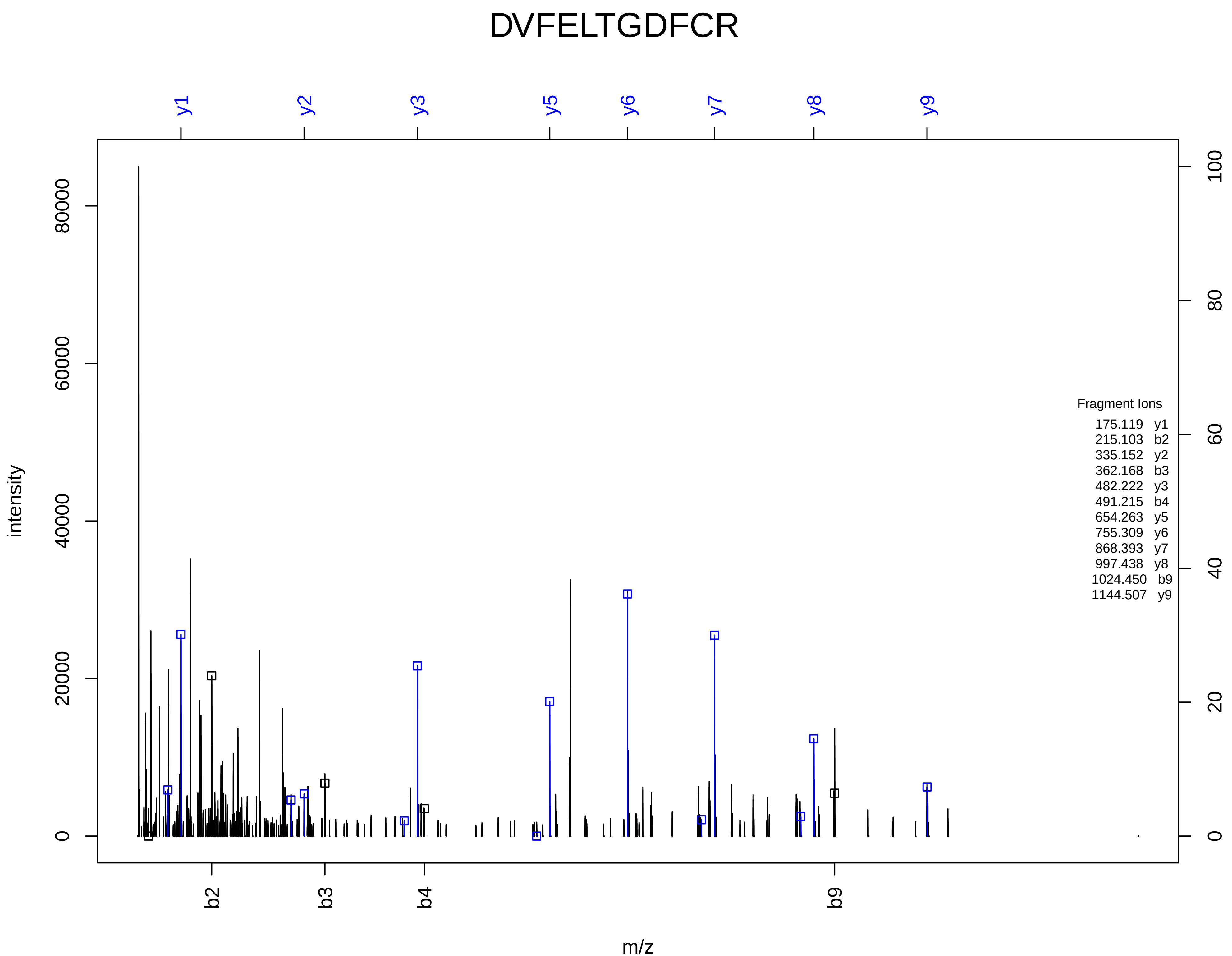

DVFELTGDFCR / File: christine_10pools_01.raw; SpectrumID: ; scans: 24302

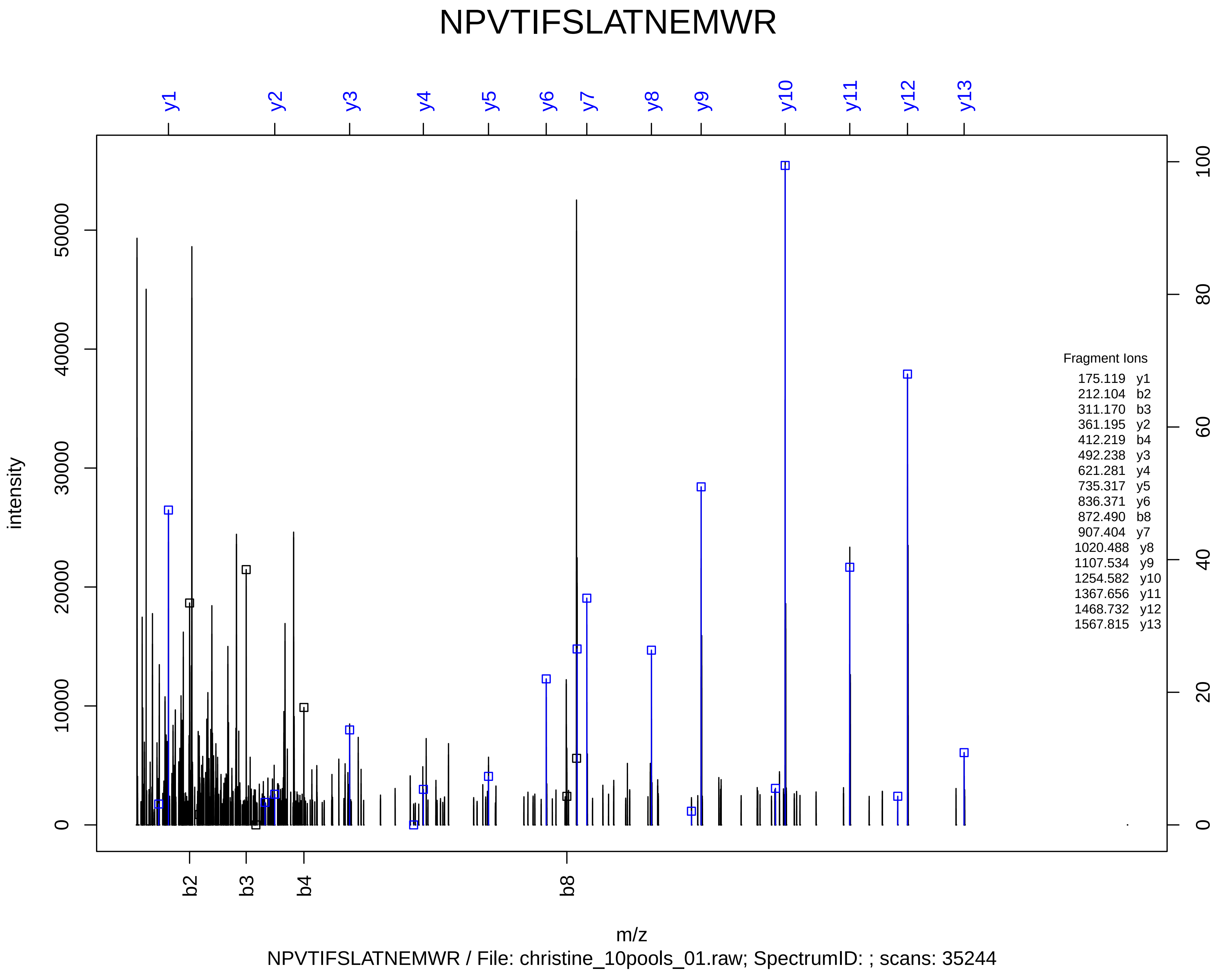
NPVTIFSLATNEMWR
intensity
m/z
y1 y2 y3 y4 y5 y6 y7 y8 y9 y10 y11 y12 y13
b2 b3 b4 b8
Fragment Ions
175.119 y1
212.104 b2
311.170 b3
361.195 y2
412.219 b4
492.238 y3
621.281 y4
735.317 y5
836.371 y6
872.490 b8
907.404 y7
1020.488 y8
1107.534 y9
1254.582 y10
1367.656 y11
1468.732 y12
1567.815 y13
NPVTIFSLATNEMWR / File: christine_10pools_01.raw; SpectrumID: ; scans: 35244

GIHHGPTVAQPIHLDSAQLSPK

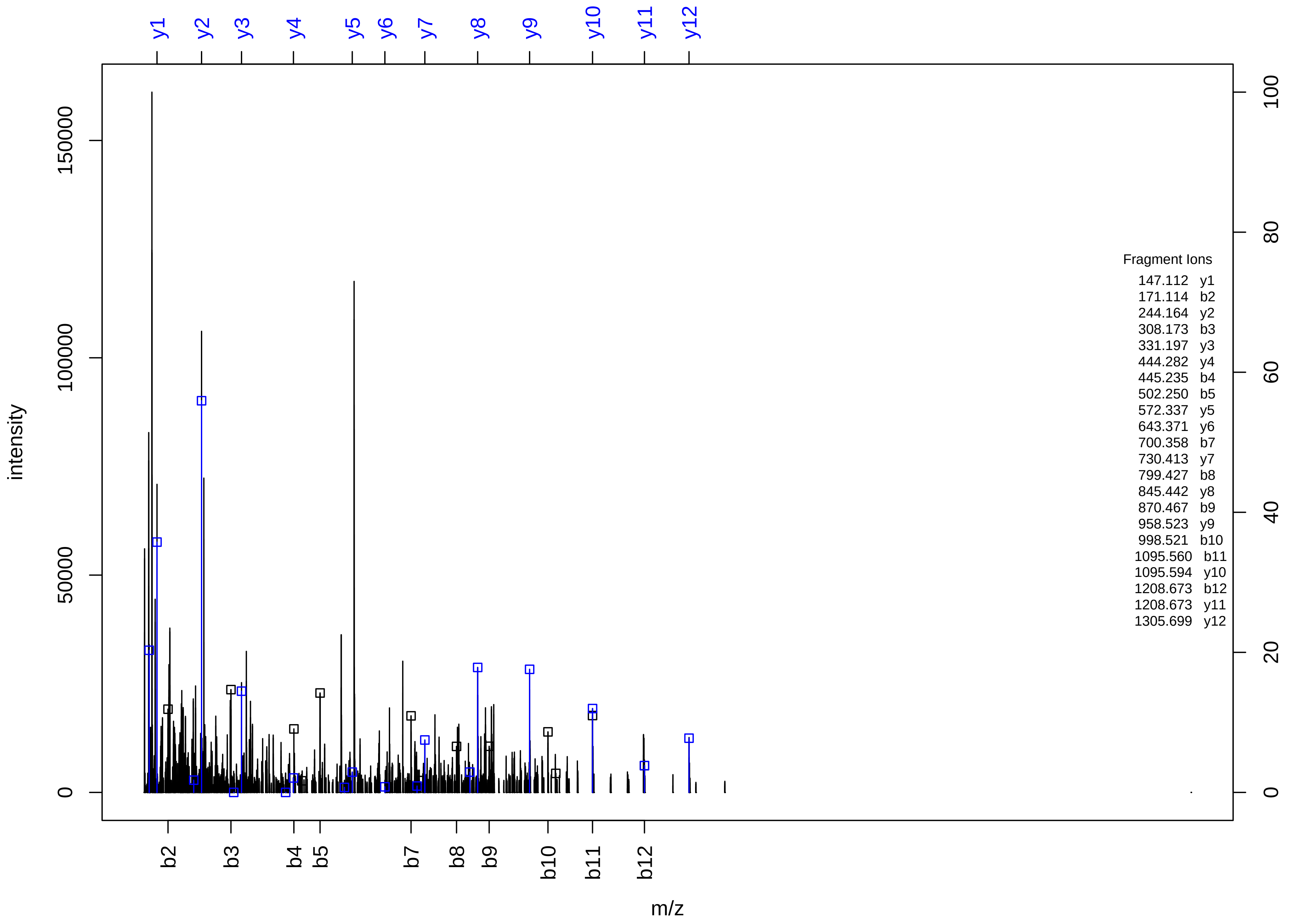

GIHHGPTVAQPIHLDSAQLSPK / File: christine_10pools_01.raw; SpectrumID: ; scans: 6557

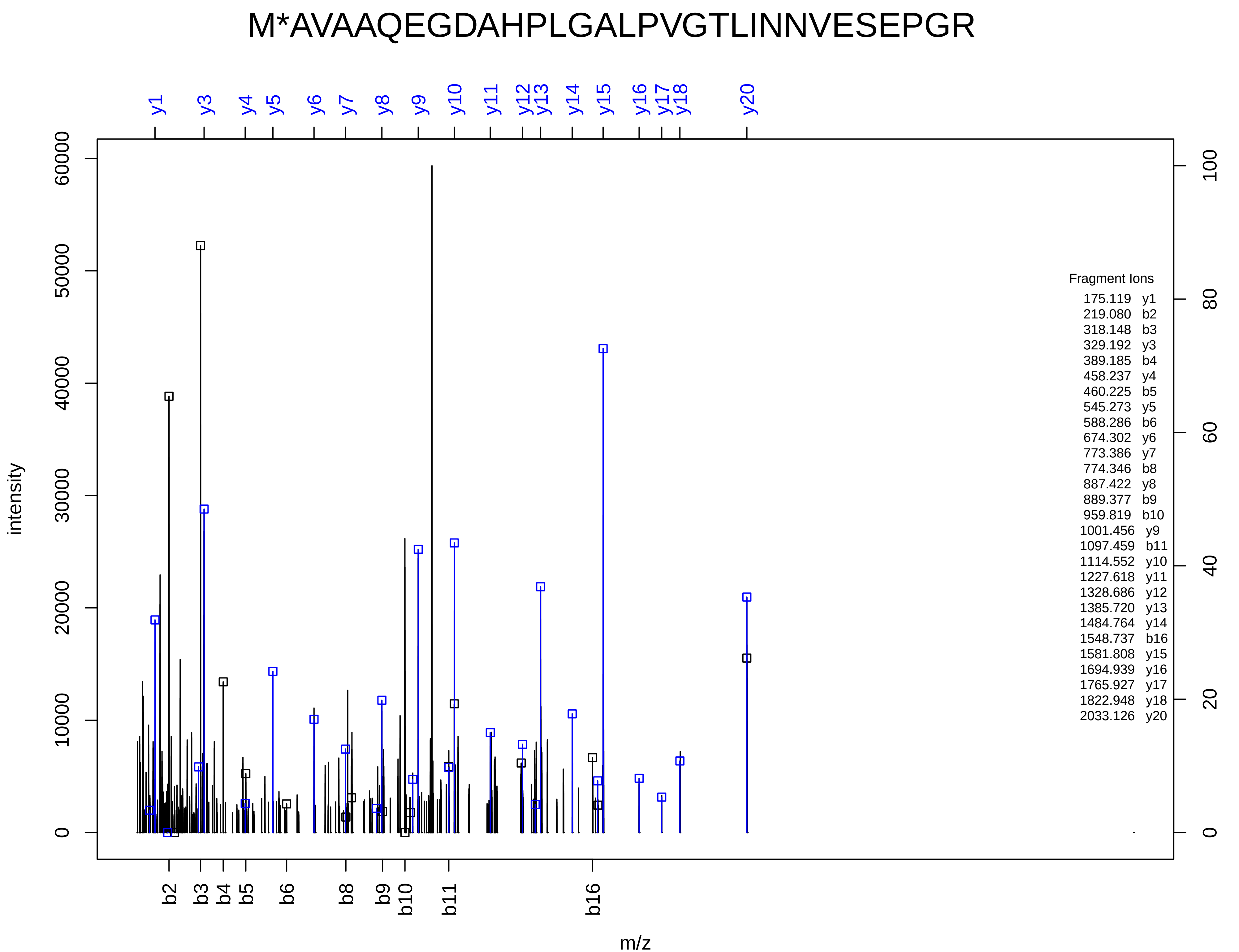

MAVAAQEGDAHPLGALPVGTLINNVESEPGR / File: christine_10pools_01.raw; SpectrumID: ; scans: 25168

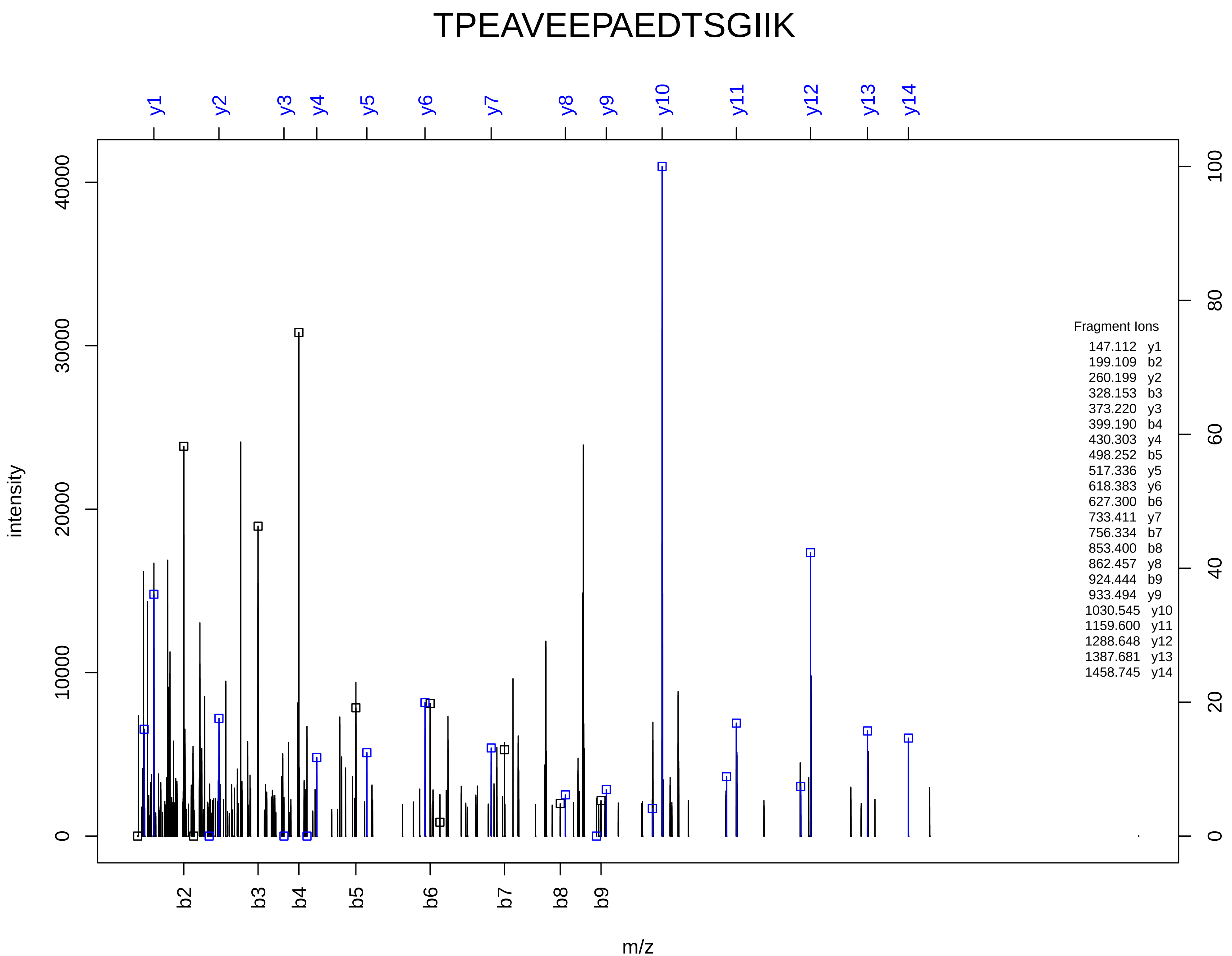

TPEAVEEPAEDTSGIIK / File: christine_10pools_01.raw; SpectrumID: ; scans: 11028

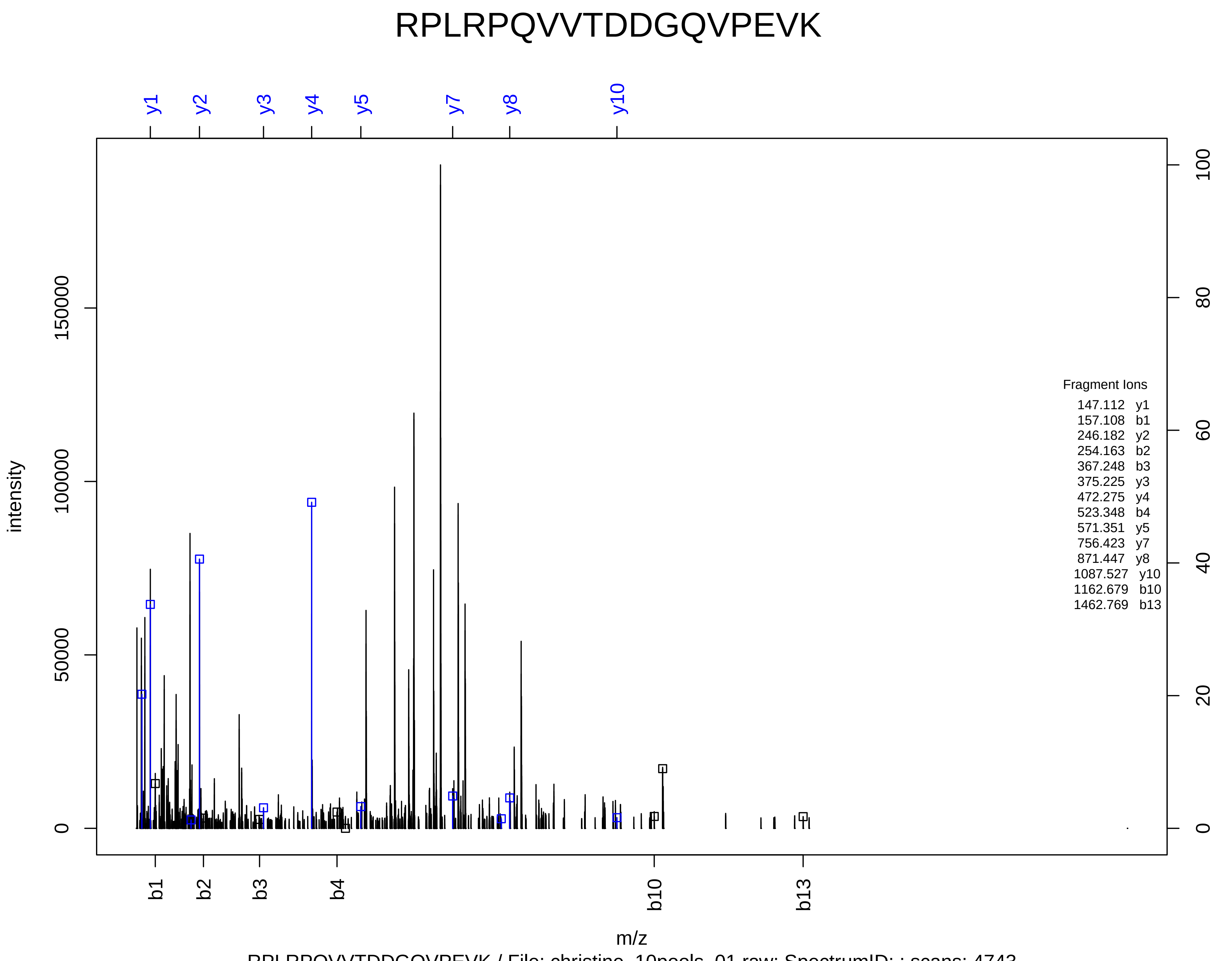

RPLRPQVVTDDGQVPEVK
y1
y2
y3
y4
y5
y7
y8
y10
intensity
0
50000
100000
150000
0
20
40
60
80
100
Fragment Ions
147.112 y1
157.108 b1
246.182 y2
254.163 b2
367.248 b3
375.225 y3
472.275 y4
523.348 b4
571.351 y5
756.423 y7
871.447 y8
1087.527 y10
1162.679 b10
1462.769 b13
b1
b2
b3
b4
b10
b13
m/z
RPLRPQVVTDDGQVPEVK / File: christine_10pools_01.raw; SpectrumID: ; scans: 4743

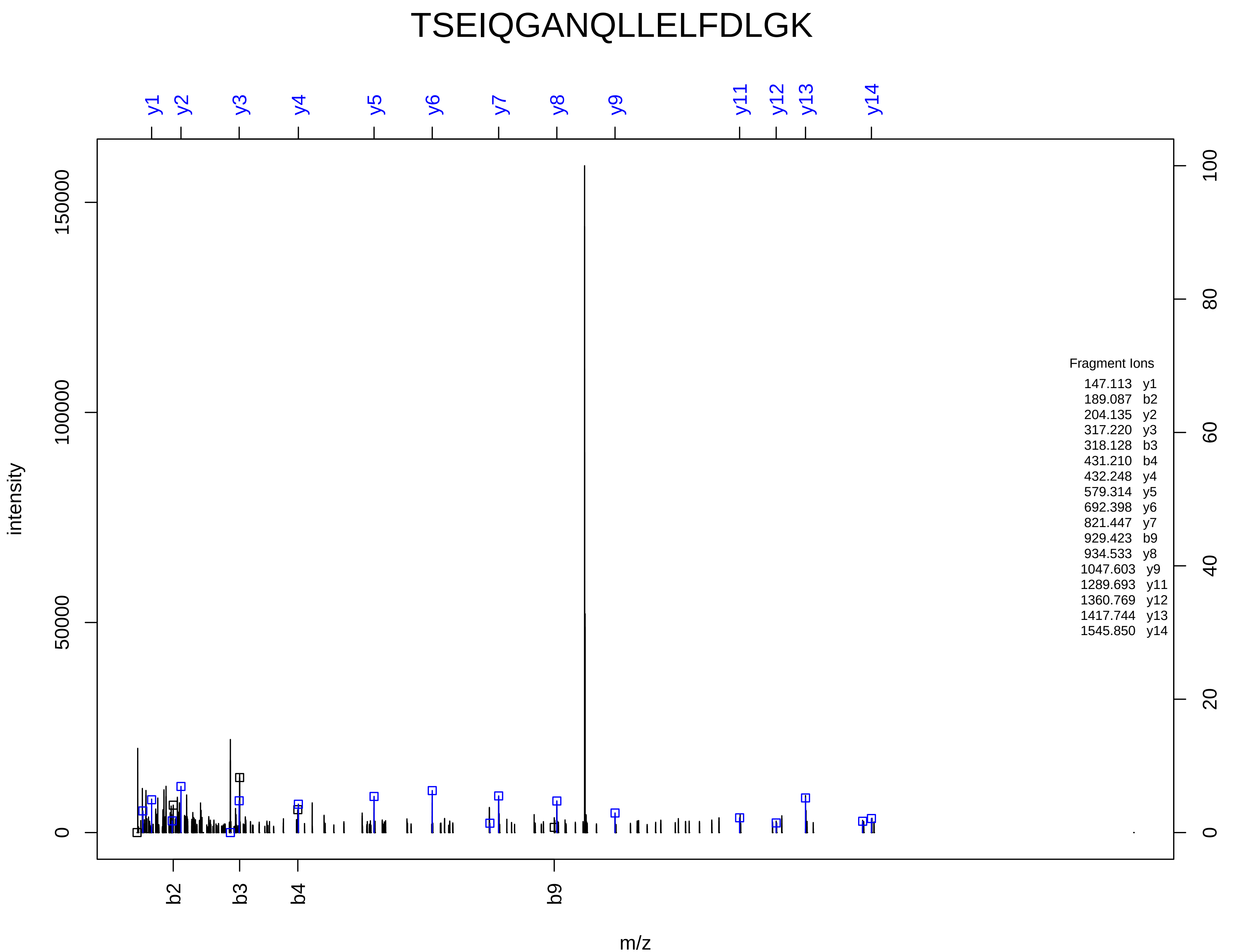
TSEIQGANQLLELFDLGK
y1
y2
y3
y4
y5
y6
y7
y8
y9
y11
y12
y13
y14
b2
b3
b4
b9
intensity
m/z
Fragment Ions
147.113 y1
189.087 b2
204.135 y2
317.220 y3
318.128 b3
431.210 b4
432.248 y4
579.314 y5
692.398 y6
821.447 y7
929.423 b9
934.533 y8
1047.603 y9
1289.693 y11
1360.769 y12
1417.744 y13
1545.850 y14
TSEIQGANQLLELFDLGK / File: christine_10pools_01.raw; SpectrumID: ; scans: 39679

TTCSSGGALGPGQPSEGLLDR

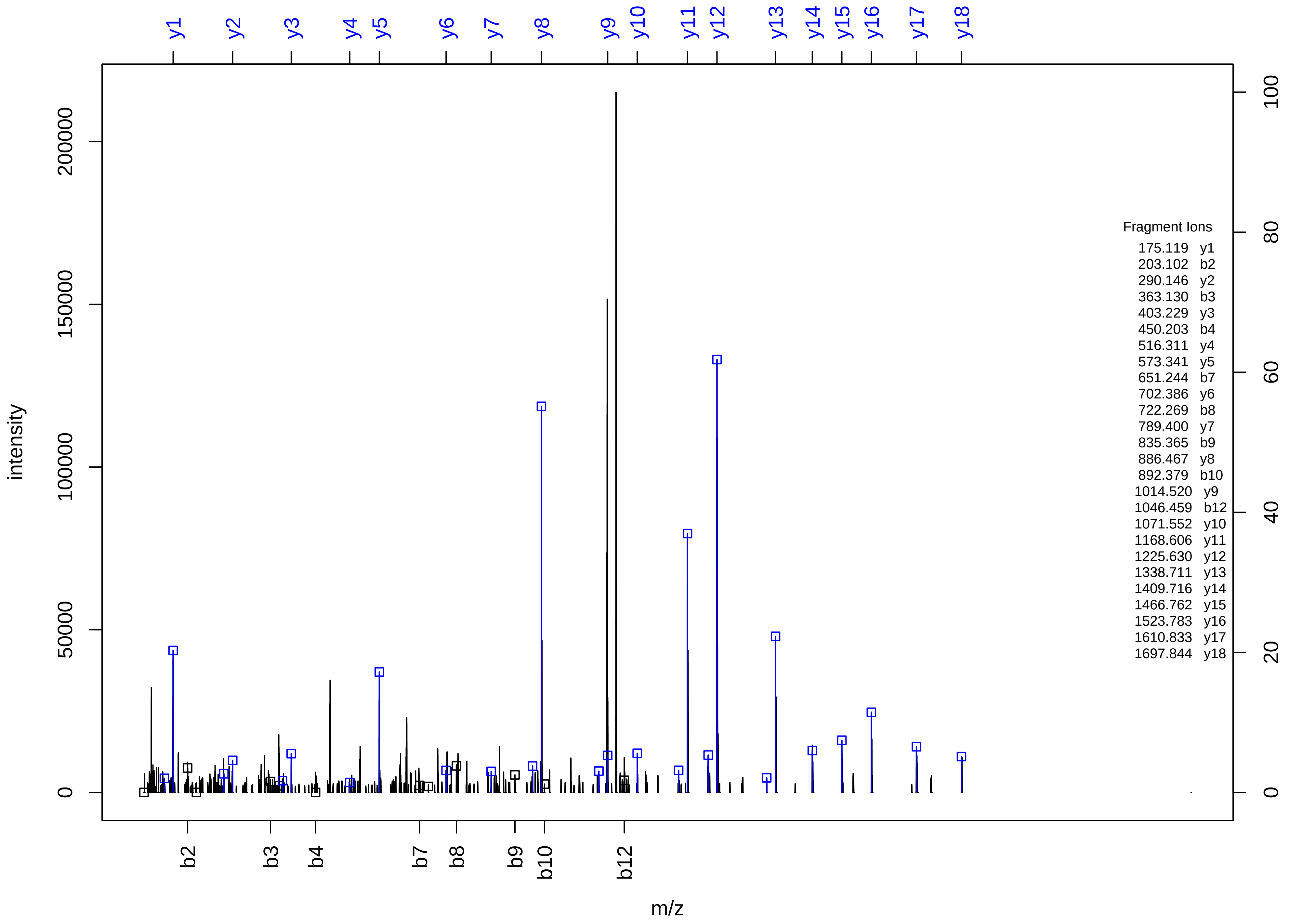

TTCSSGGALGPGQPSEGLLDR / File: christine_10pools_01.raw; SpectrumID: ; scans: 11461

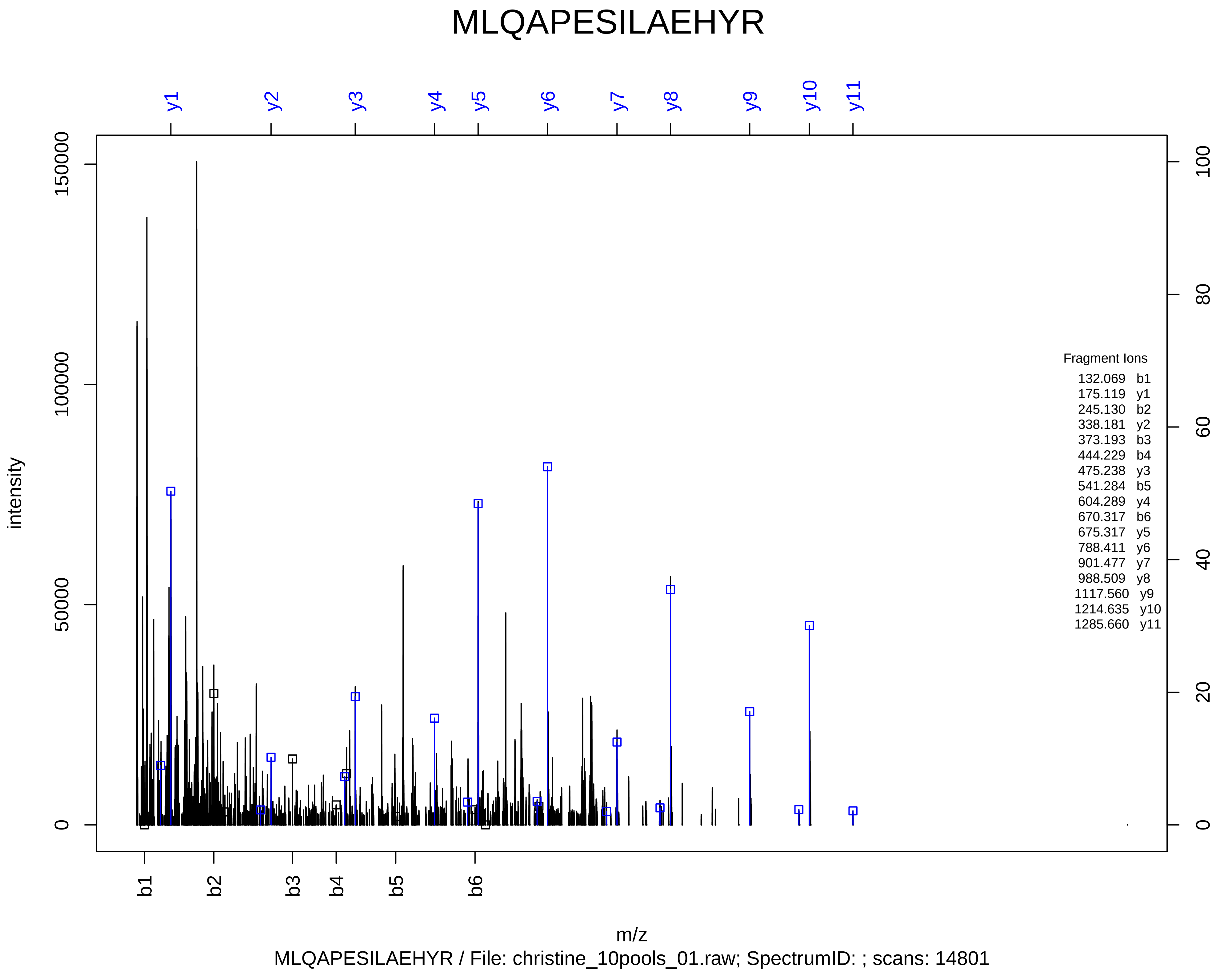

MLQAPESILAEHYR
y1
y2
y3
y4
y5
y6
y7
y8
y9
y10
y11
intensity
0
50000
100000
150000
0
20
40
60
80
100
Fragment Ions
132.069 b1
175.119 y1
245.130 b2
338.181 y2
373.193 b3
444.229 b4
475.238 y3
541.284 b5
604.289 y4
670.317 b6
675.317 y5
788.411 y6
901.477 y7
988.509 y8
1117.560 y9
1214.635 y10
1285.660 y11
b1
b2
b3
b4
b5
b6
m/z
MLQAPESILAEHYR / File: christine_10pools_01.raw; SpectrumID: ; scans: 14801

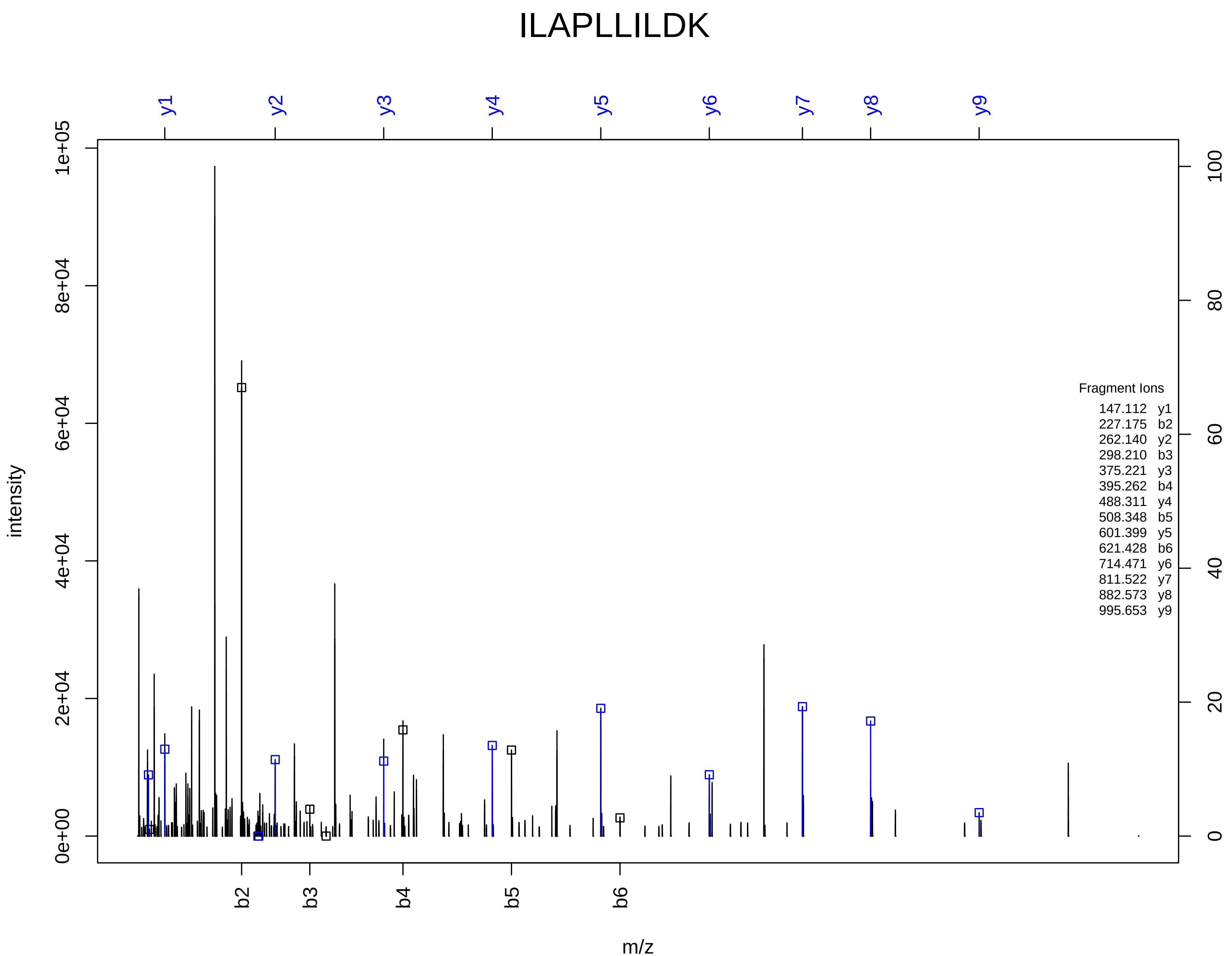
ILAPLLILDK
y1
y2
y3
y4
y5
y6
y7
y8
y9
b2
b3
b4
b5
b6
intensity
m/z
Fragment Ions
147.112 y1
227.175 b2
262.140 y2
298.210 b3
375.221 y3
395.262 b4
488.311 y4
508.348 b5
601.399 y5
621.428 b6
714.471 y6
811.522 y7
882.573 y8
995.653 y9
ILAPLLILDK / File: christine_10pools_01.raw; SpectrumID: ; scans: 31274

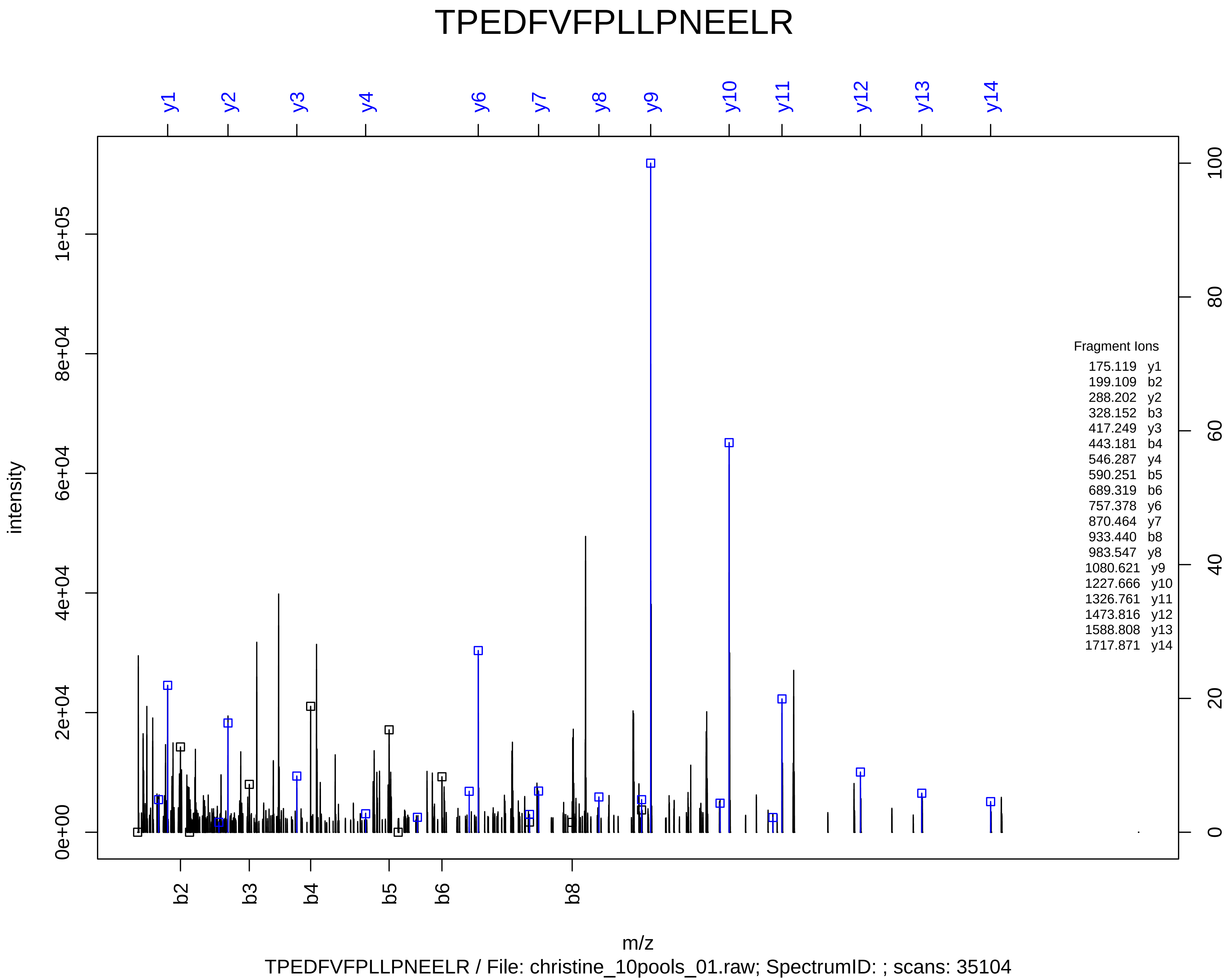

TPEDFVFPLLPNEELR
y1
y2
y3
y4
y6
y7
y8
y9
y10
y11
y12
y13
y14
b2
b3
b4
b5
b6
b8
intensity
0e+00
2e+04
4e+04
6e+04
8e+04
1e+05
0
20
40
60
80
100
Fragment Ions
175.119 y1
199.109 b2
288.202 y2
328.152 b3
417.249 y3
443.181 b4
546.287 y4
590.251 b5
689.319 b6
757.378 y6
870.464 y7
933.440 b8
983.547 y8
1080.621 y9
1227.666 y10
1326.761 y11
1473.816 y12
1588.808 y13
1717.871 y14
m/z
TPEDFVFPLLPNEELR / File: christine_10pools_01.raw; SpectrumID: ; scans: 35104

RSM*SLQGLM*M*KR

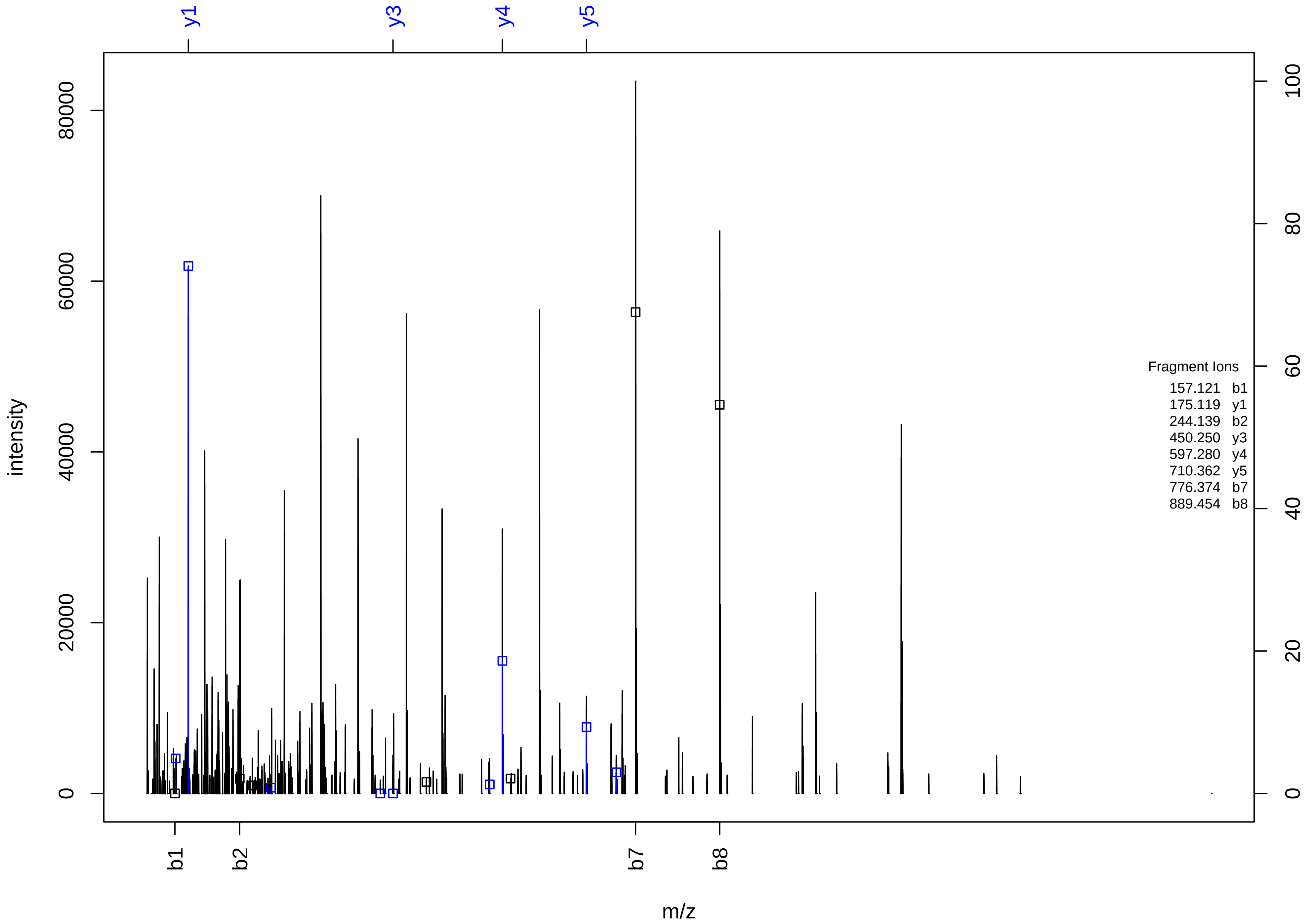

RSMSLQGLMMKR / File: christine_10pools_01.raw; SpectrumID: ; scans: 39428

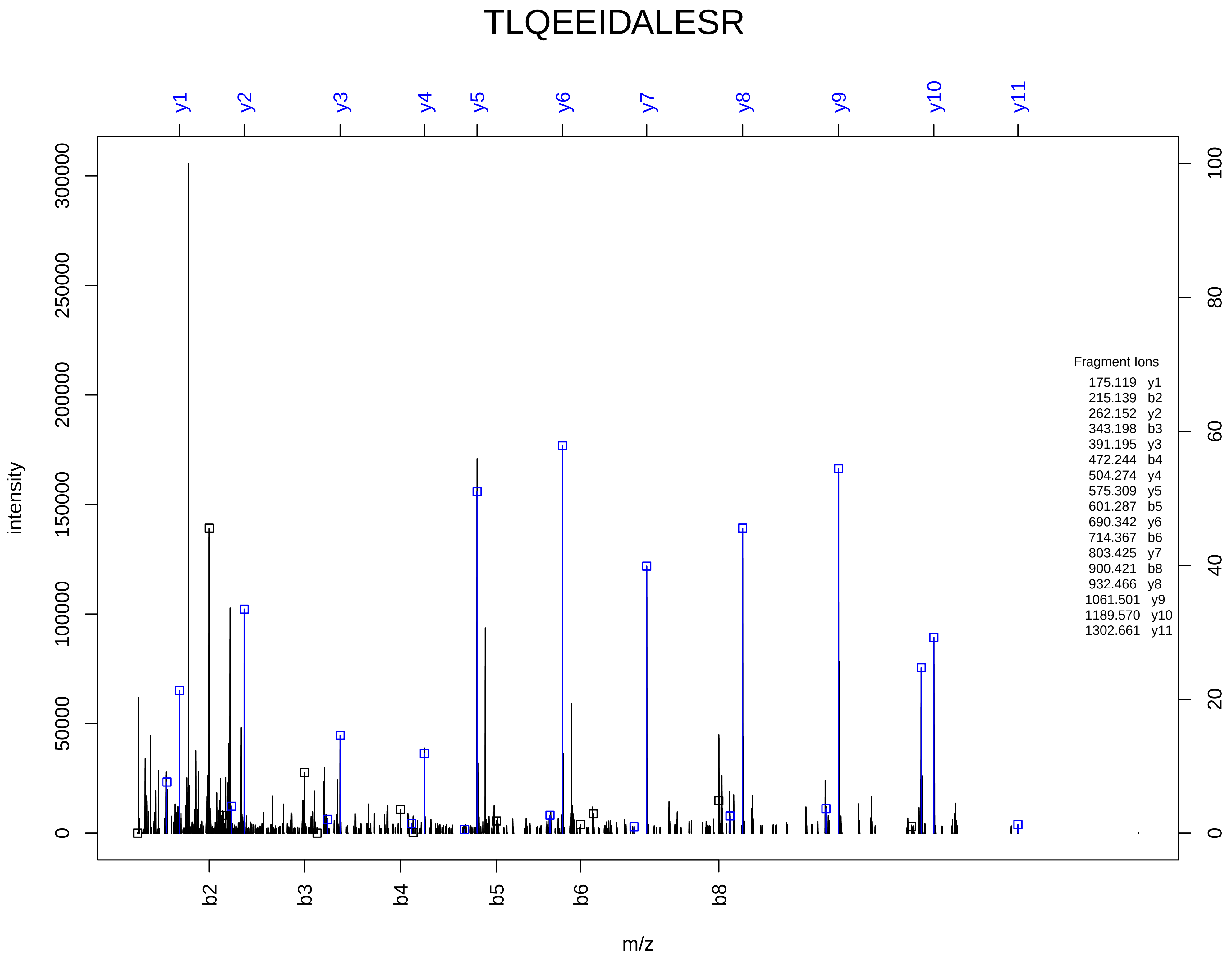

TLQEEIDALESR / File: christine_10pools_01.raw; SpectrumID: ; scans: 20361

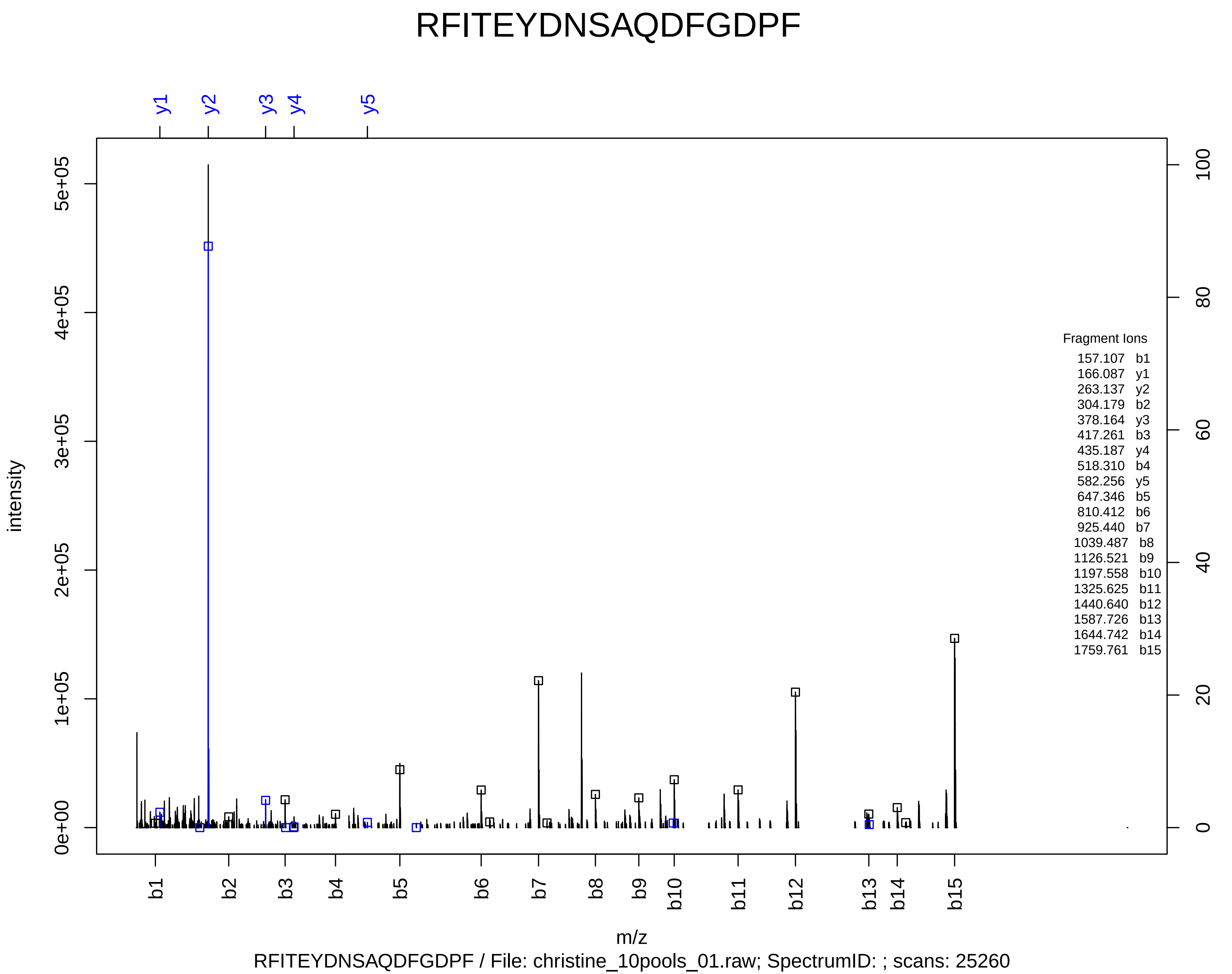

RFITEYDNSAQDFGDPF
y1
y2
y3
y4
y5
intensity
0e+00
1e+05
2e+05
3e+05
4e+05
5e+05
0
20
40
60
80
100
Fragment Ions
157.107 b1
166.087 y1
263.137 y2
304.179 b2
378.164 y3
417.261 b3
435.187 y4
518.310 b4
582.256 y5
647.346 b5
810.412 b6
925.440 b7
1039.487 b8
1126.521 b9
1197.558 b10
1325.625 b11
1440.640 b12
1587.726 b13
1644.742 b14
1759.761 b15
b1
b2
b3
b4
b5
b6
b7
b8
b9
b10
b11
b12
b13
b14
b15
m/z
RFITEYDNSAQDFGDPF / File: christine_10pools_01.raw; SpectrumID: ; scans: 25260

M*ISNGIGTVTAGK

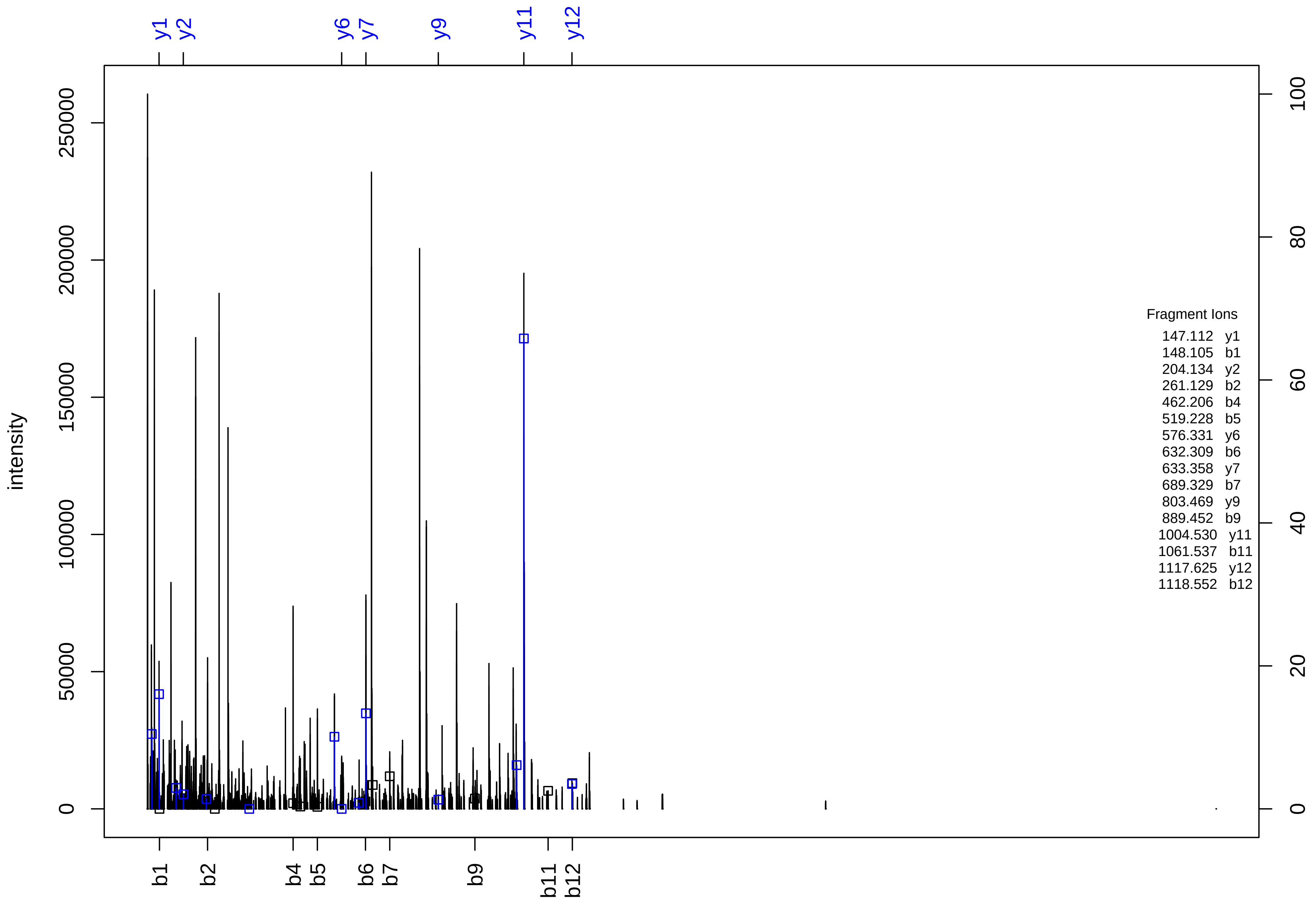

MISNGIGTVTAGK / File: christine_10pools_05.raw; SpectrumID: ; scans: 13583

APPN^YAM*KK

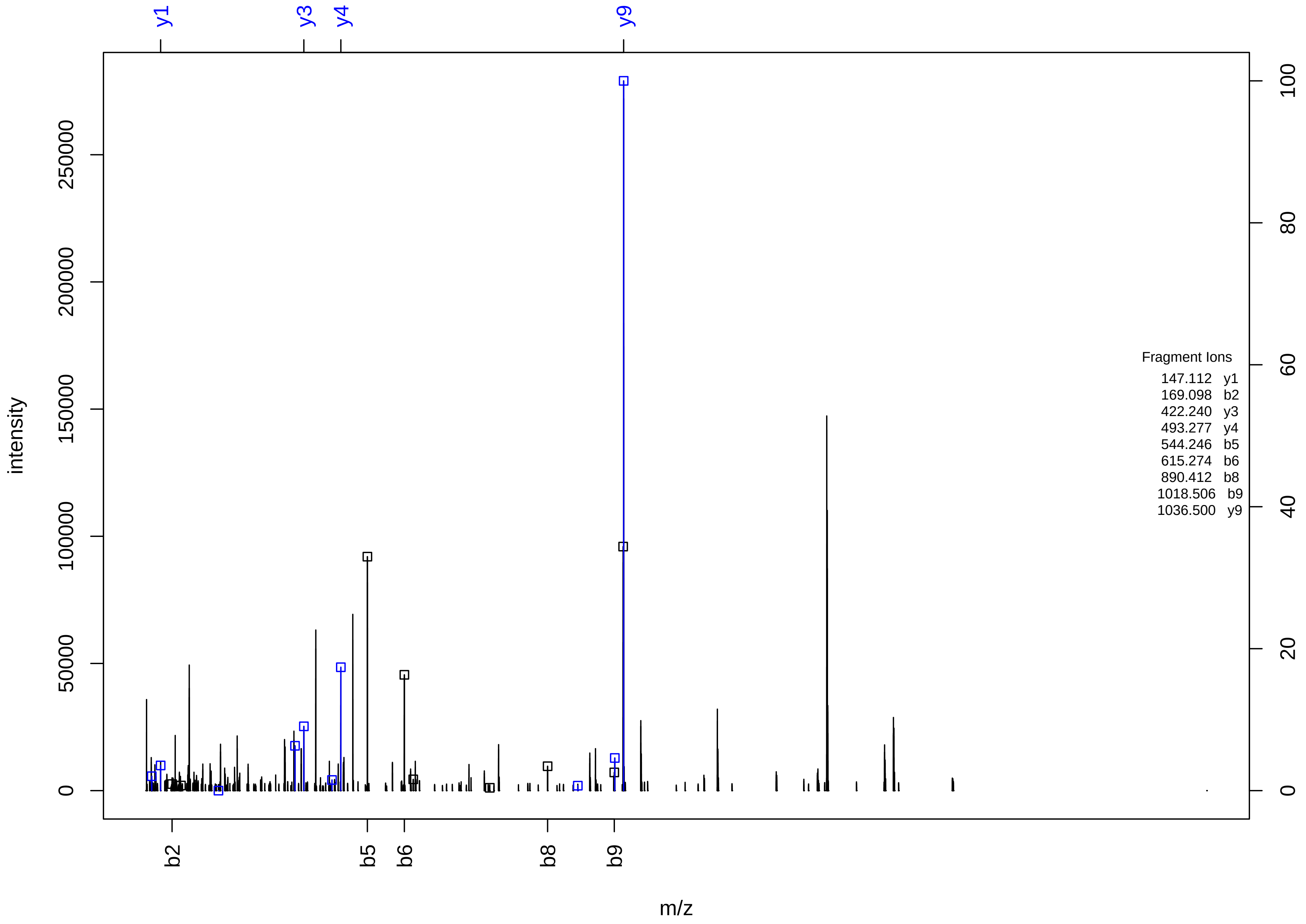

APPNYAMKK / File: christine_10pools_05.raw; SpectrumID: ; scans: 20381

LQLGPETLLQDNPK

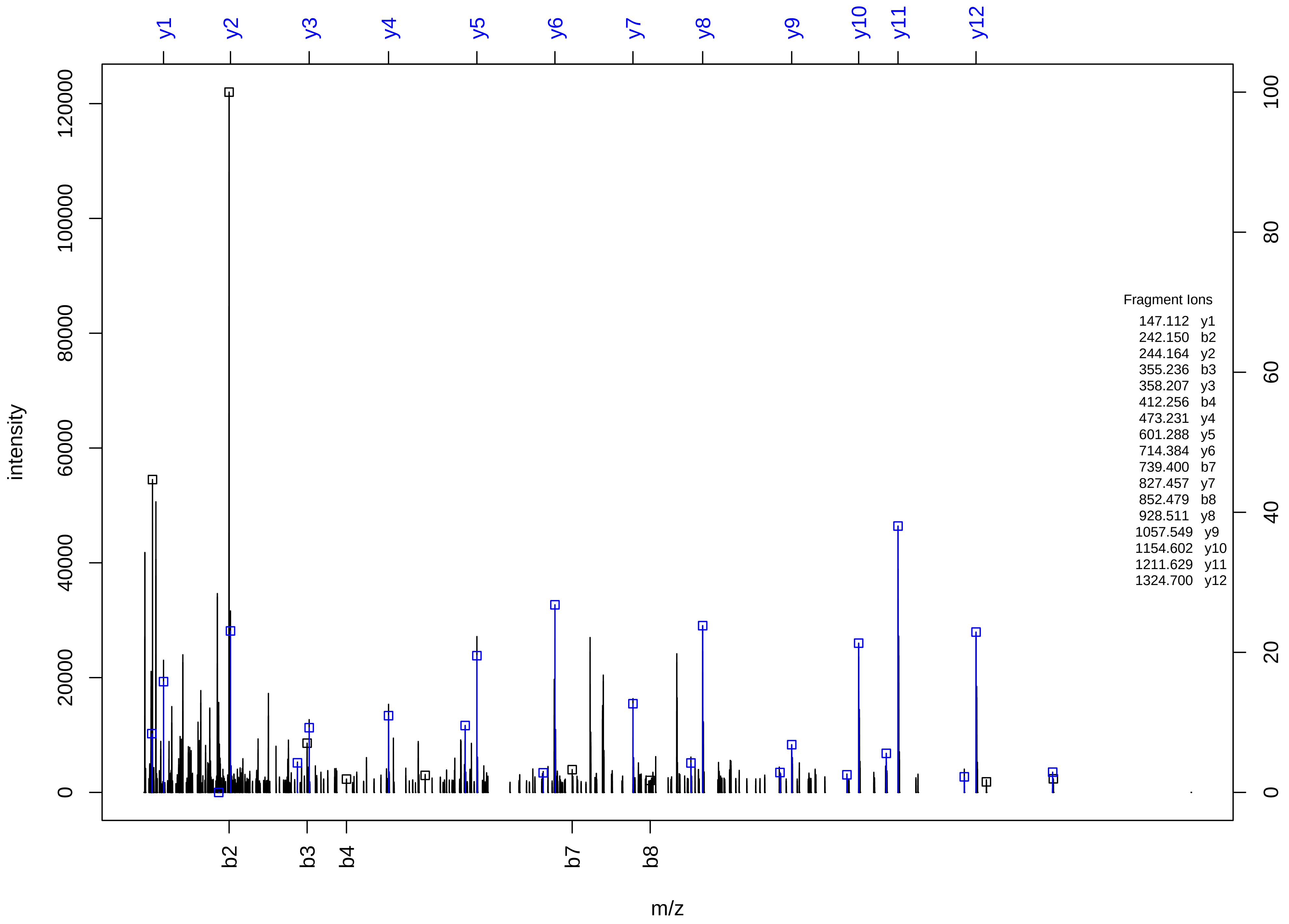

LQLGPETLLQDNPK / File: christine_10pools_05.raw; SpectrumID: ; scans: 20754

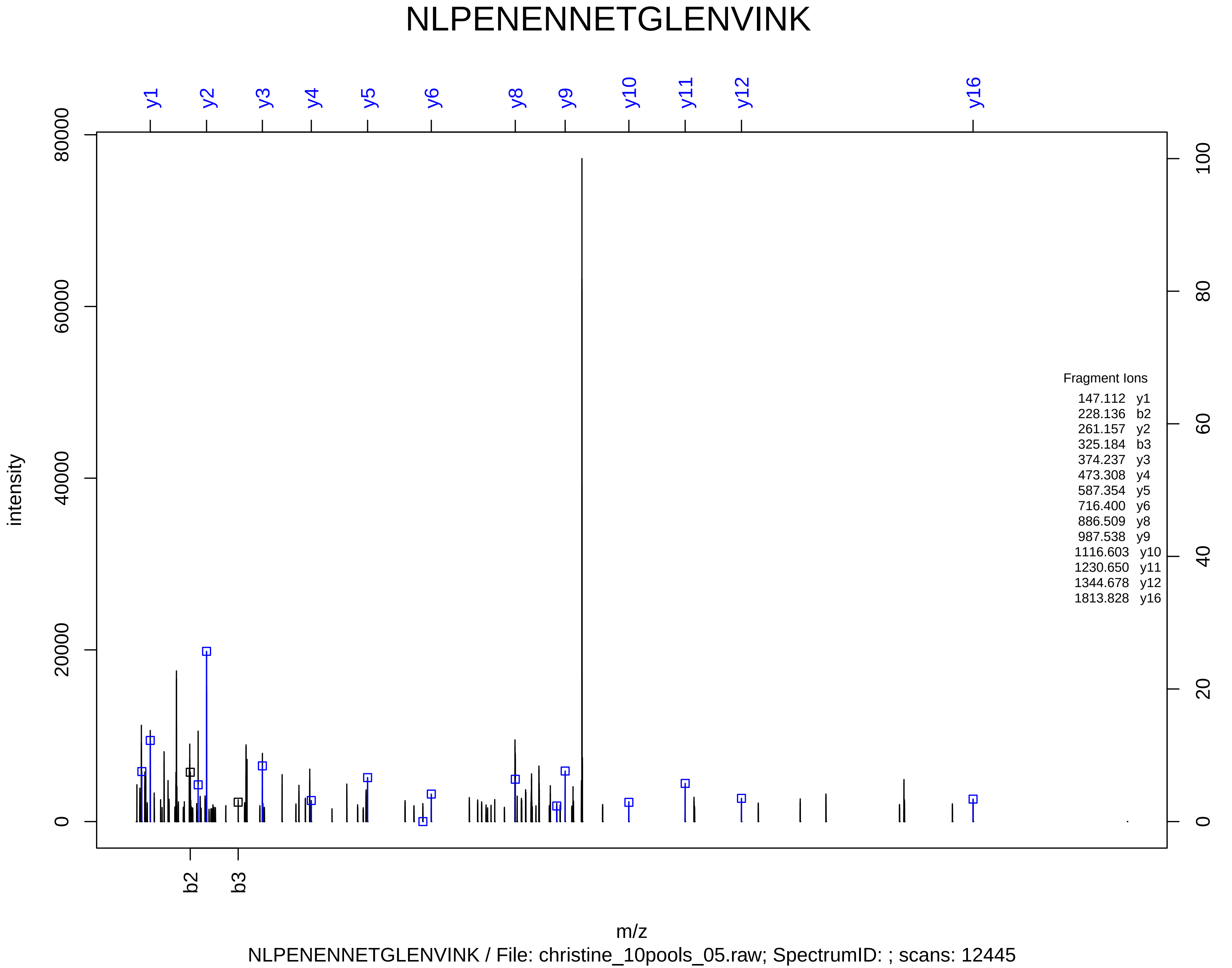
NLPENENNETGLENVINK
y1
y2
y3
y4
y5
y6
y8
y9
y10
y11
y12
y16
intensity
0
20000
40000
60000
80000
0
20
40
60
80
100
Fragment Ions
147.112 y1
228.136 b2
261.157 y2
325.184 b3
374.237 y3
473.308 y4
587.354 y5
716.400 y6
886.509 y8
987.538 y9
1116.603 y10
1230.650 y11
1344.678 y12
1813.828 y16
b2
b3
m/z
NLPENENNETGLENVINK / File: christine_10pools_05.raw; SpectrumID: ; scans: 12445

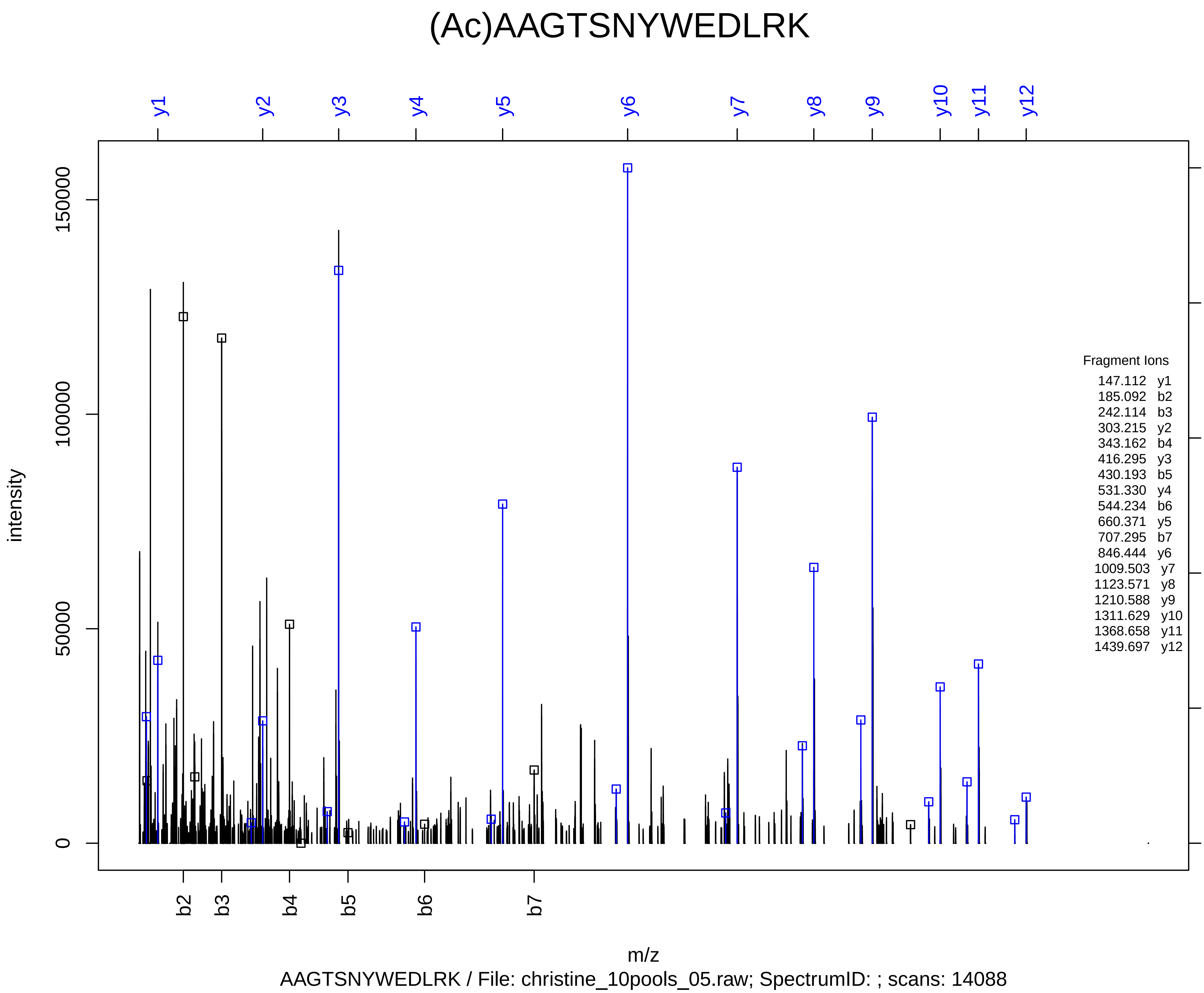

(Ac)AAGTSNYWEDLRK
intensity
m/z
Fragment Ions
147.112 y1
185.092 b2
242.114 b3
303.215 y2
343.162 b4
416.295 y3
430.193 b5
531.330 y4
544.234 b6
660.371 y5
707.295 b7
846.444 y6
1009.503 y7
1123.571 y8
1210.588 y9
1311.629 y10
1368.658 y11
1439.697 y12
y1 y2 y3 y4 y5 y6 y7 y8 y9 y10 y11 y12
b2 b3 b4 b5 b6 b7
0 50000 100000 150000
0 20 40 60 80 100
AAGTSNYWEDLRK / File: christine_10pools_05.raw; SpectrumID: ; scans: 14088

APQSCPPERPGPPQR

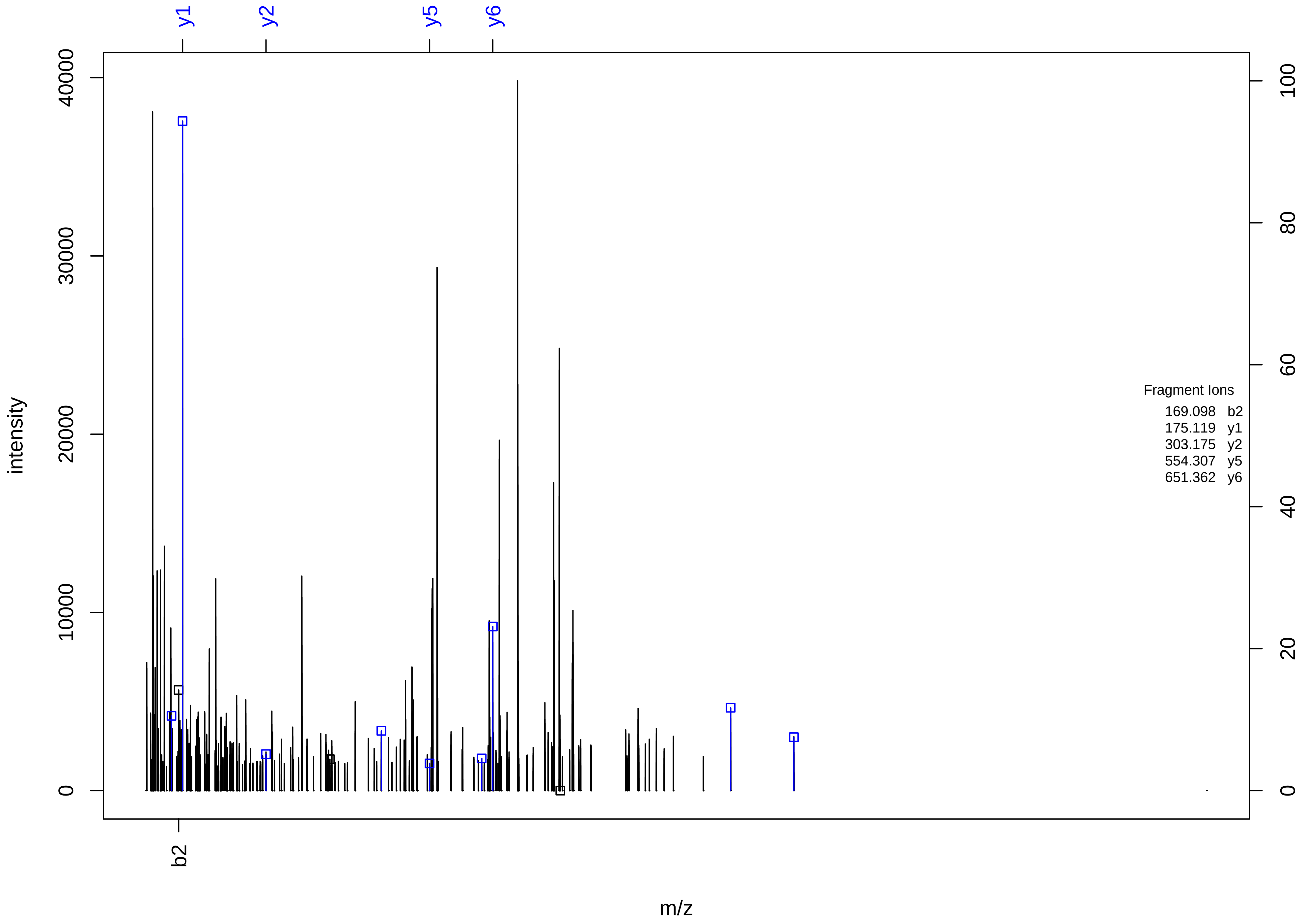

APQSCPPERPGPPQR / File: christine_10pools_05.raw; SpectrumID: ; scans: 1135

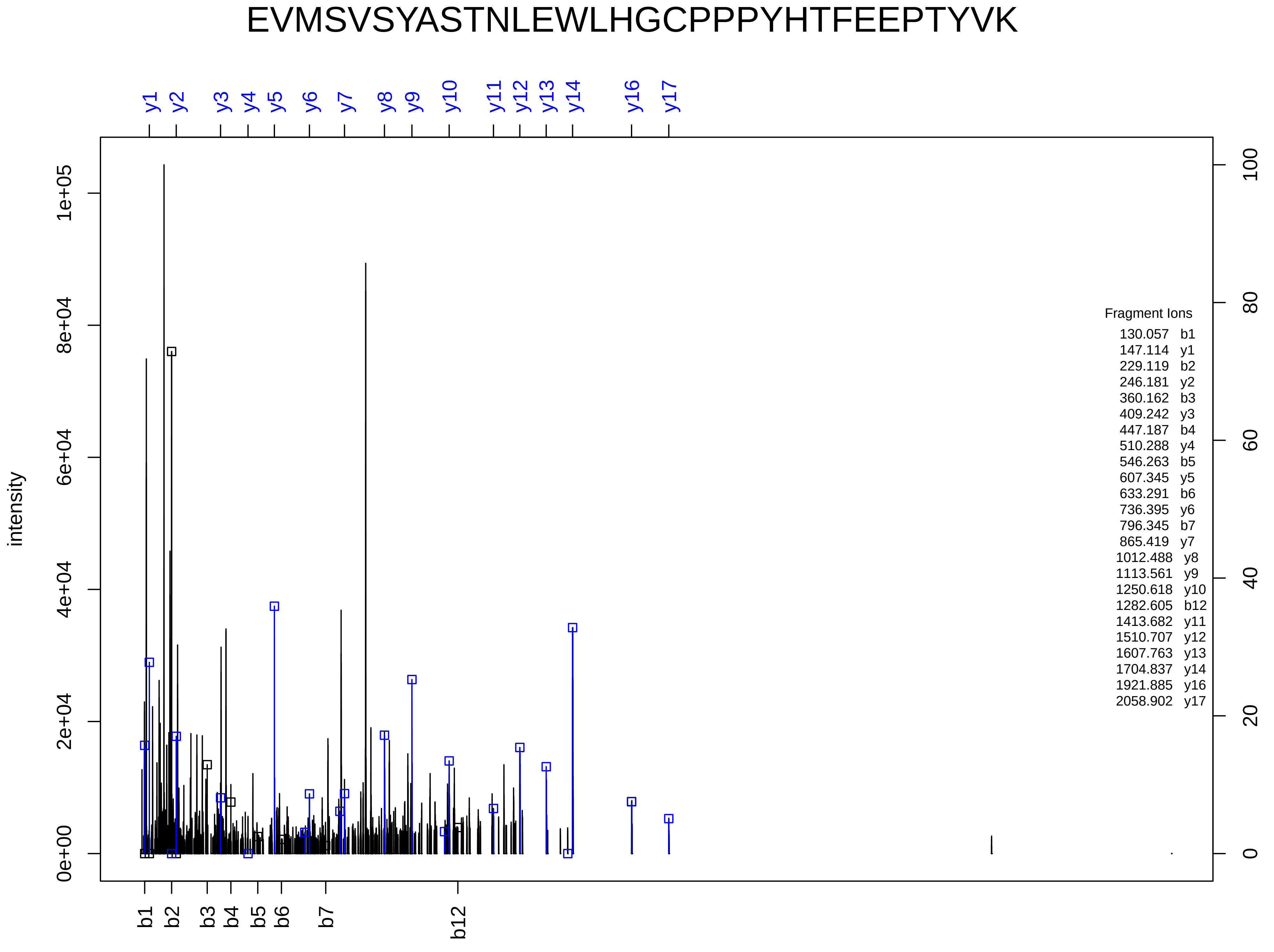

EVMSVSYASTNLEWLHGCPPPYHTFEEPTYVK / File: christine_10pools_05.raw; SpectrumID: ; scans: 24246

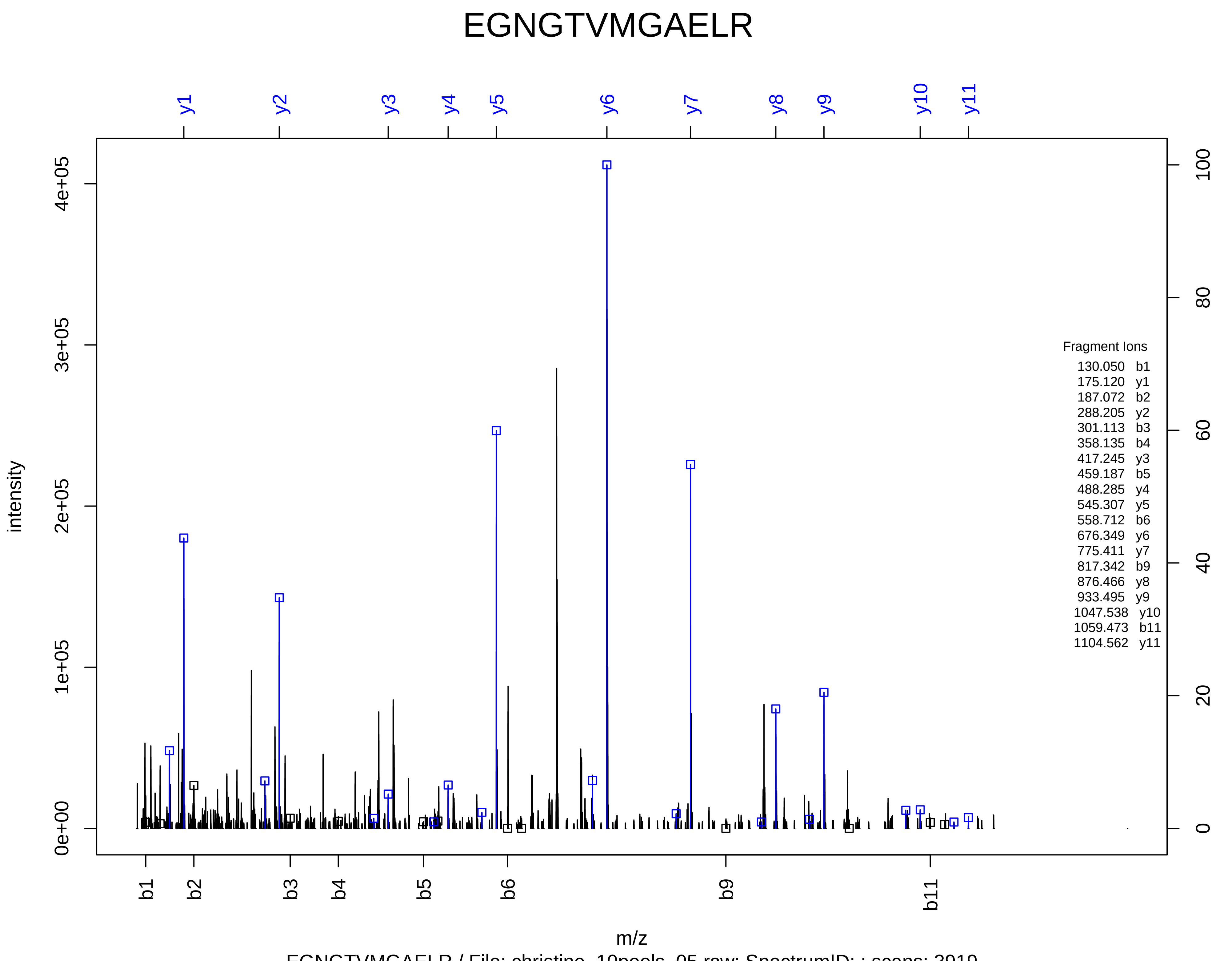
EGNGTVMGAELR
y1
y2
y3
y4
y5
y6
y7
y8
y9
y10
y11
intensity
4e+05
3e+05
2e+05
1e+05
0e+00
100
80
60
40
20
0
Fragment Ions
130.050 b1
175.120 y1
187.072 b2
288.205 y2
301.113 b3
358.135 b4
417.245 y3
459.187 b5
488.285 y4
545.307 y5
558.712 b6
676.349 y6
775.411 y7
817.342 b9
876.466 y8
933.495 y9
1047.538 y10
1059.473 b11
1104.562 y11
b1
b2
b3
b4
b5
b6
b9
b11
m/z
EGNGTVMGAELR / File: christine_10pools_05.raw; SpectrumID: ; scans: 3919

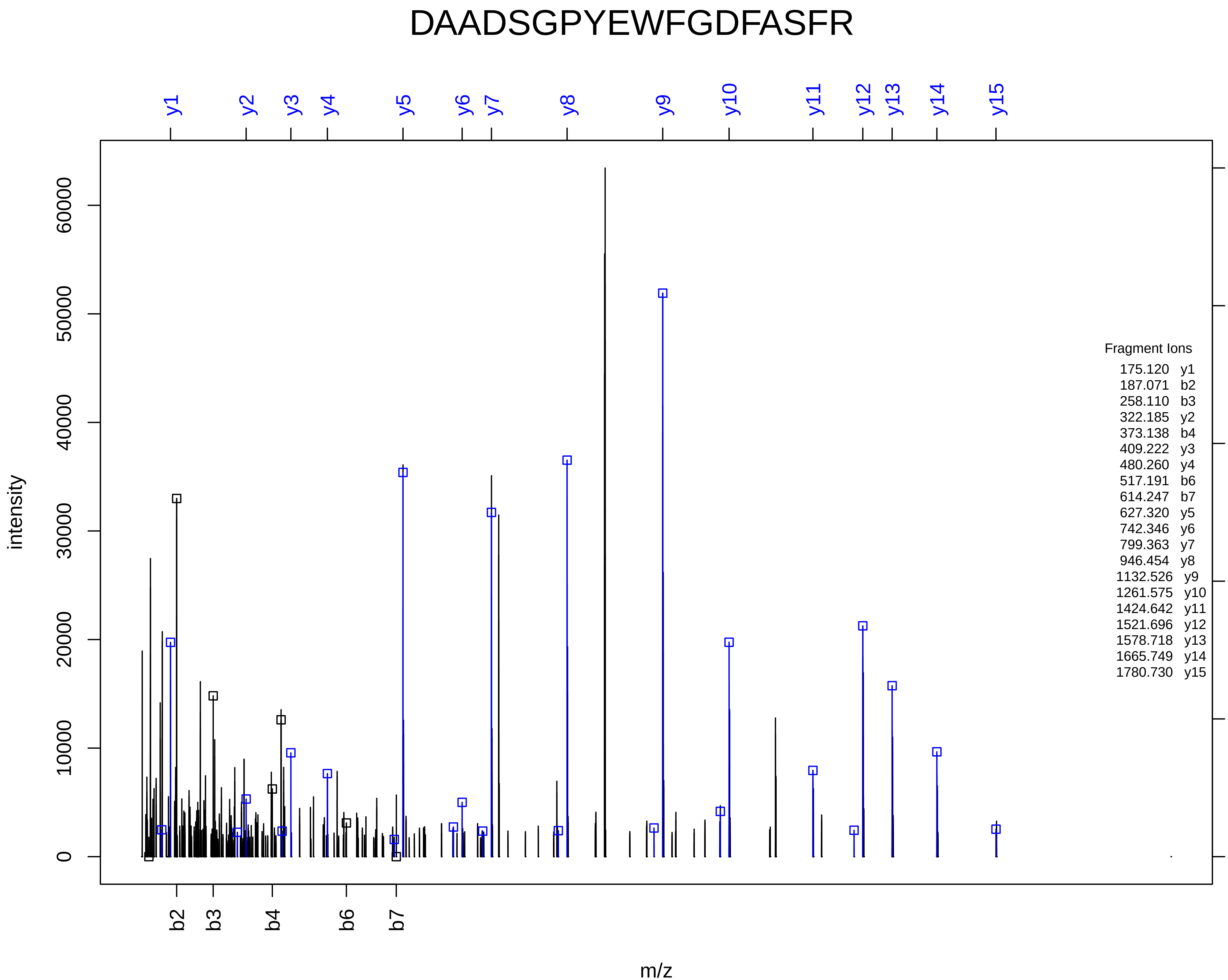
DAADSGPYEWFGDFASFR
y1 y2 y3 y4 y5 y6 y7 y8 y9 y10 y11 y12 y13 y14 y15
b2 b3 b4 b6 b7
intensity
m/z
Fragment Ions
175.120 y1
187.071 b2
258.110 b3
322.185 y2
373.138 b4
409.222 y3
480.260 y4
517.191 b6
614.247 b7
627.320 y5
742.346 y6
799.363 y7
946.454 y8
1132.526 y9
1261.575 y10
1424.642 y11
1521.696 y12
1578.718 y13
1665.749 y14
1780.730 y15
DAADSGPYEWFGDFASFR / File: christine_10pools_05.raw; SpectrumID: ; scans: 38866

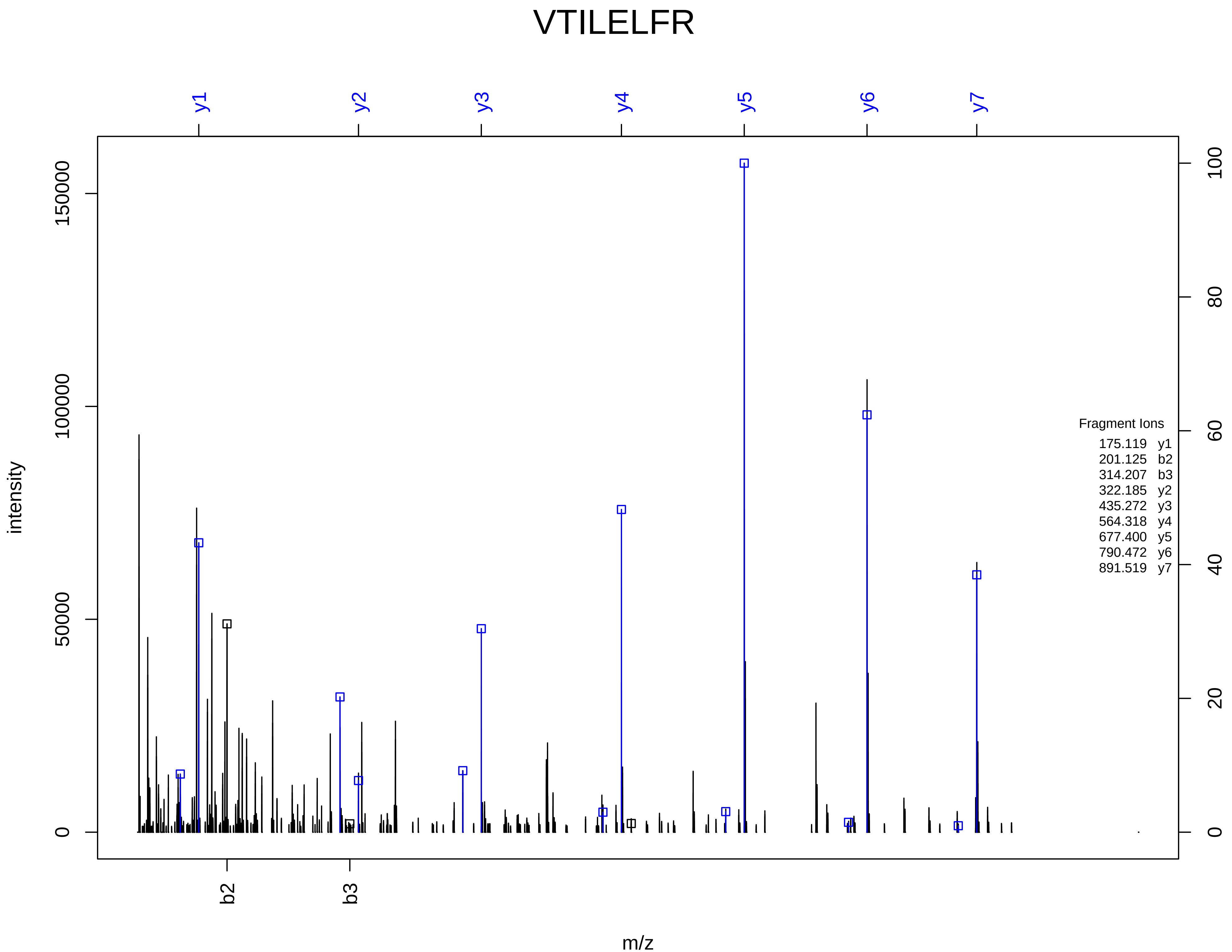

VTILELFR / File: christine_10pools_05.raw; SpectrumID: ; scans: 29953

SQGGLAEVAAAPADREEATQPAEK

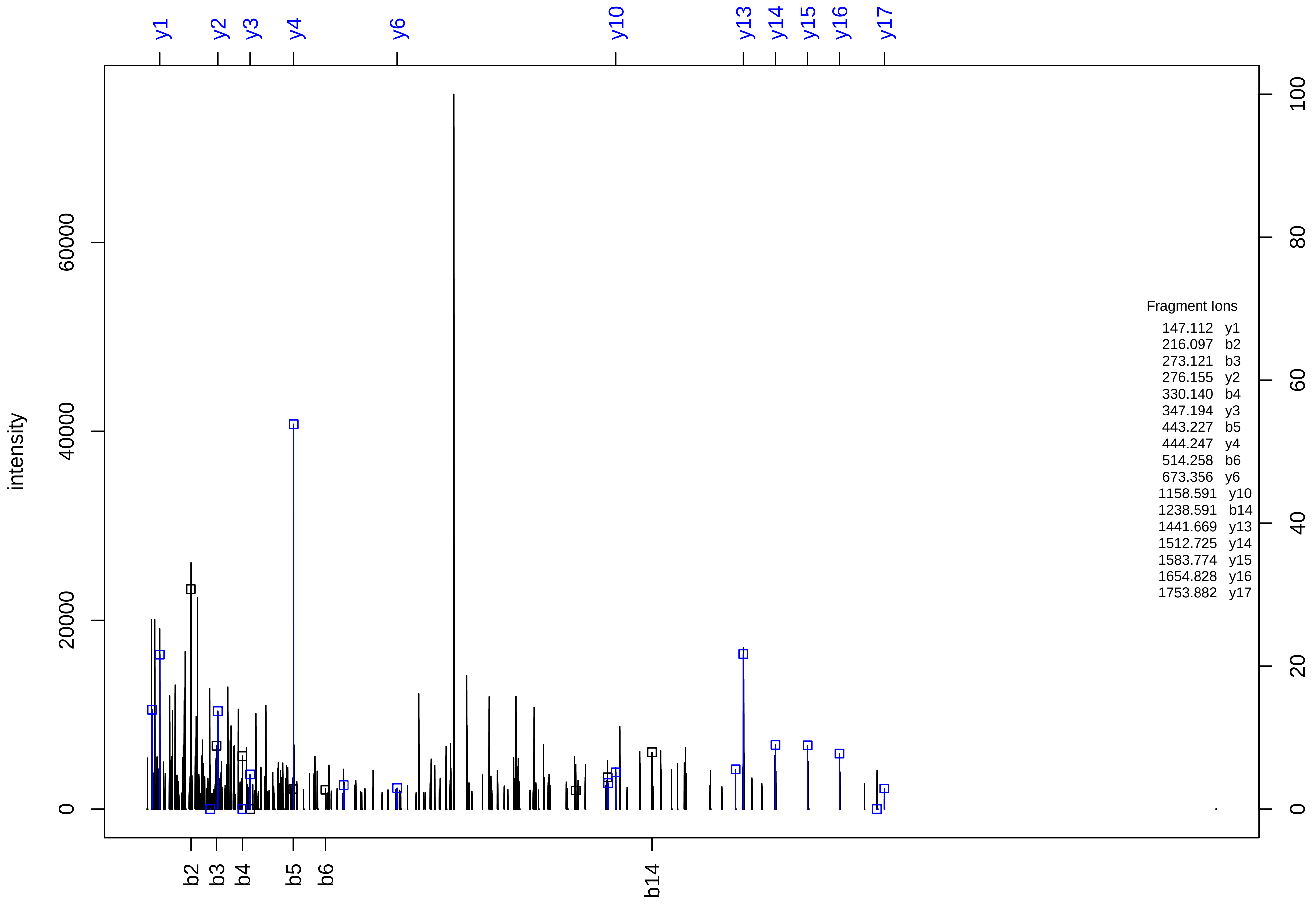

SQGGLAEVAAAPADREEATQPAEK / File: christine_10pools_05.raw; SpectrumID: ; scans: 4530

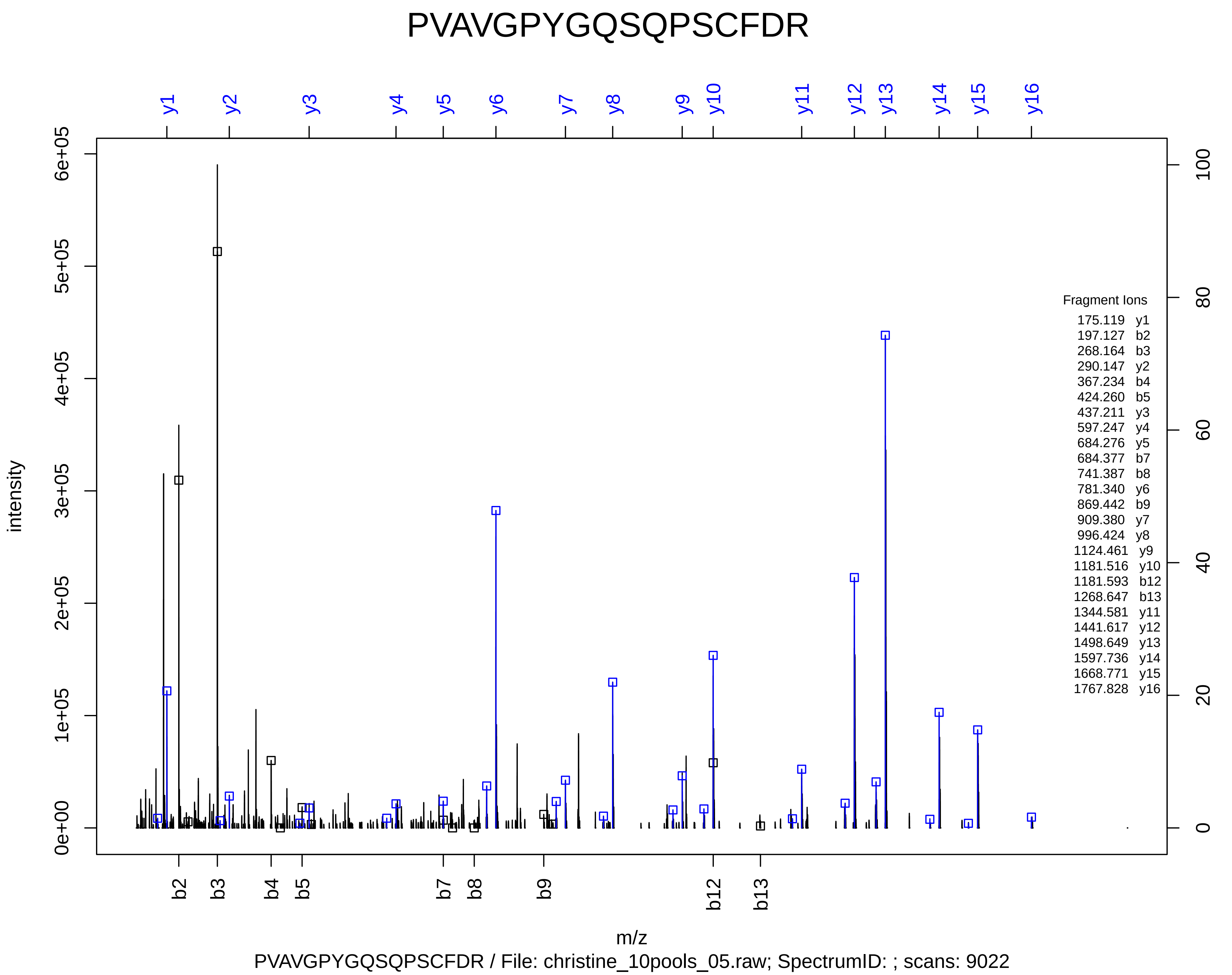

PVAVGPYGQSQPSCFDR
intensity
m/z
y1 y2 y3 y4 y5 y6 y7 y8 y9 y10 y11 y12 y13 y14 y15 y16
b2 b3 b4 b5 b7 b8 b9 b12 b13
0e+00 1e+05 2e+05 3e+05 4e+05 5e+05 6e+05
0 20 40 60 80 100
Fragment Ions
175.119 y1
197.127 b2
268.164 b3
290.147 y2
367.234 b4
424.260 b5
437.211 y3
597.247 y4
684.276 y5
684.377 b7
741.387 b8
781.340 y6
869.442 b9
909.380 y7
996.424 y8
1124.461 y9
1181.516 y10
1181.593 b12
1268.647 b13
1344.581 y11
1441.617 y12
1498.649 y13
1597.736 y14
1668.771 y15
1767.828 y16
PVAVGPYGQSQPSCFDR / File: christine_10pools_05.raw; SpectrumID: ; scans: 9022

LFLVQTVQQASK

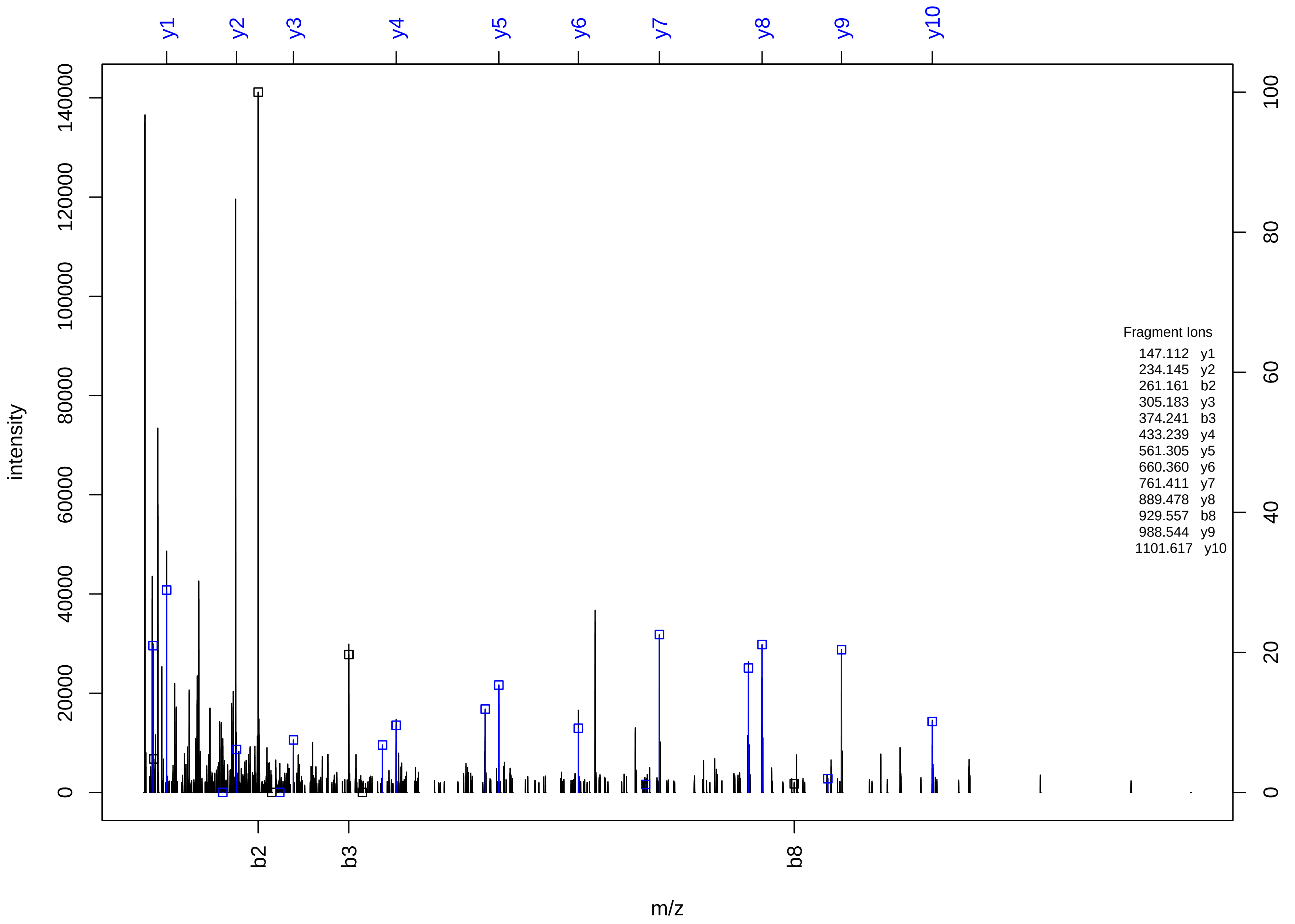

LFLVQTVQQASK / File: christine_10pools_05.raw; SpectrumID: ; scans: 16893

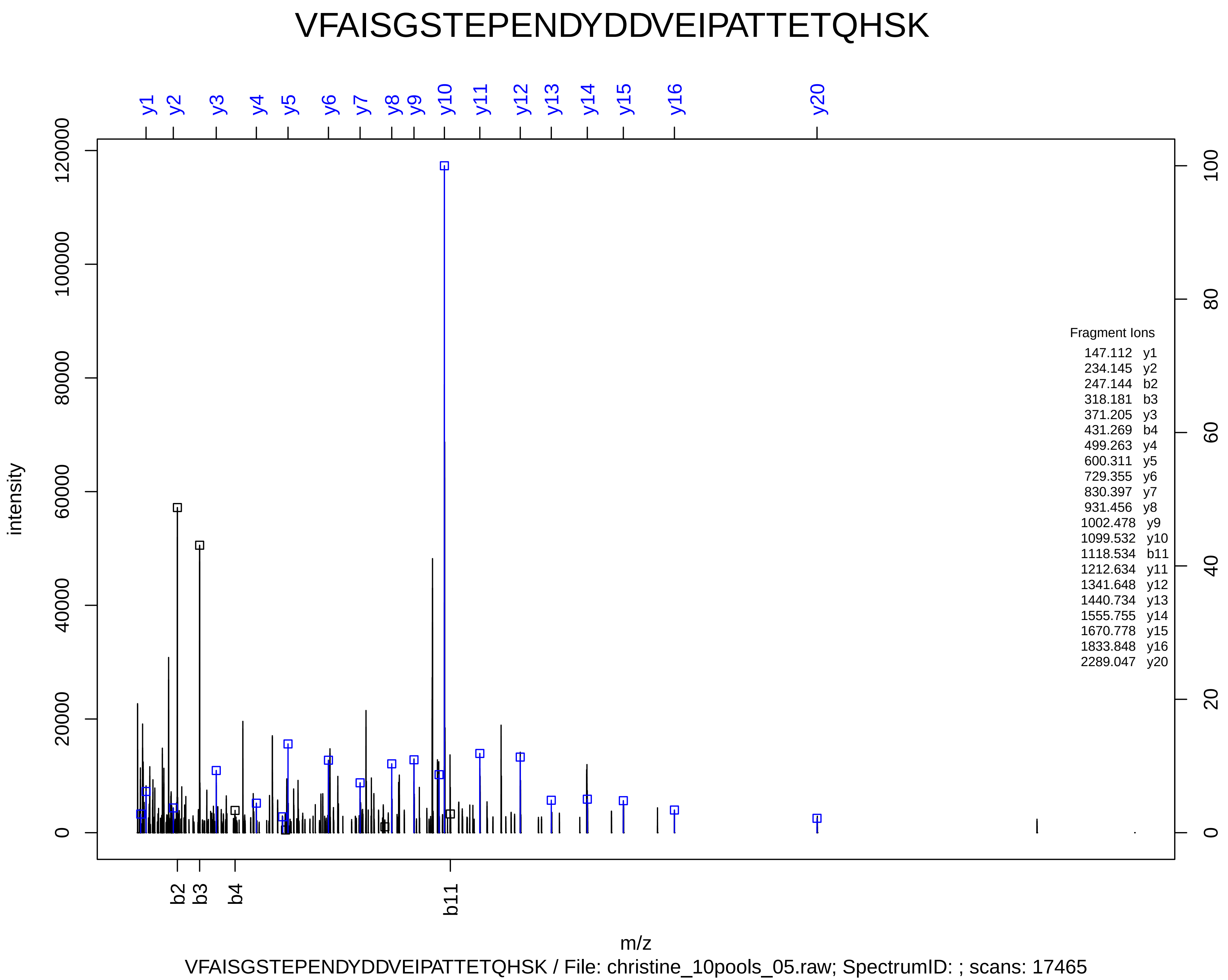

VFAISGSTEPENDYDDVEIPATTETQHSK
y1 y2 y3 y4 y5 y6 y7 y8 y9 y10 y11 y12 y13 y14 y15 y16 y20
intensity
0 20000 40000 60000 80000 100000 120000
0 20 40 60 80 100
b2 b3 b4 b11
m/z
Fragment Ions
147.112 y1
234.145 y2
247.144 b2
318.181 b3
371.205 y3
431.269 b4
499.263 y4
600.311 y5
729.355 y6
830.397 y7
931.456 y8
1002.478 y9
1099.532 y10
1118.534 b11
1212.634 y11
1341.648 y12
1440.734 y13
1555.755 y14
1670.778 y15
1833.848 y16
2289.047 y20
VFAISGSTEPENDYDDVEIPATTETQHSK / File: christine_10pools_05.raw; SpectrumID: ; scans: 17465

LPPPVDCPAPLYELM*K

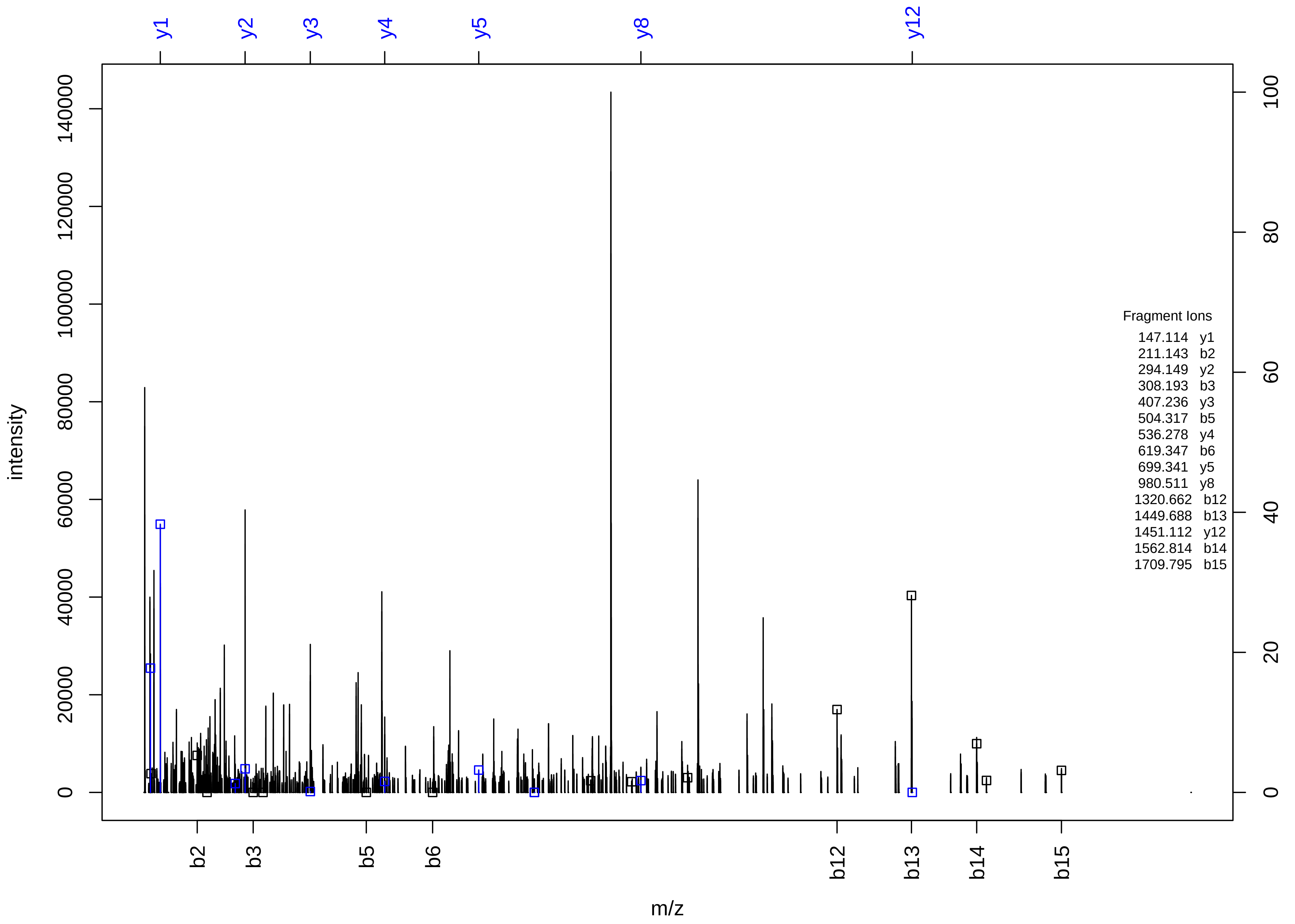

LPPPVDCPAPLYELMK / File: christine_10pools_05.raw; SpectrumID: ; scans: 29319

NDNAEEHLLEAENK

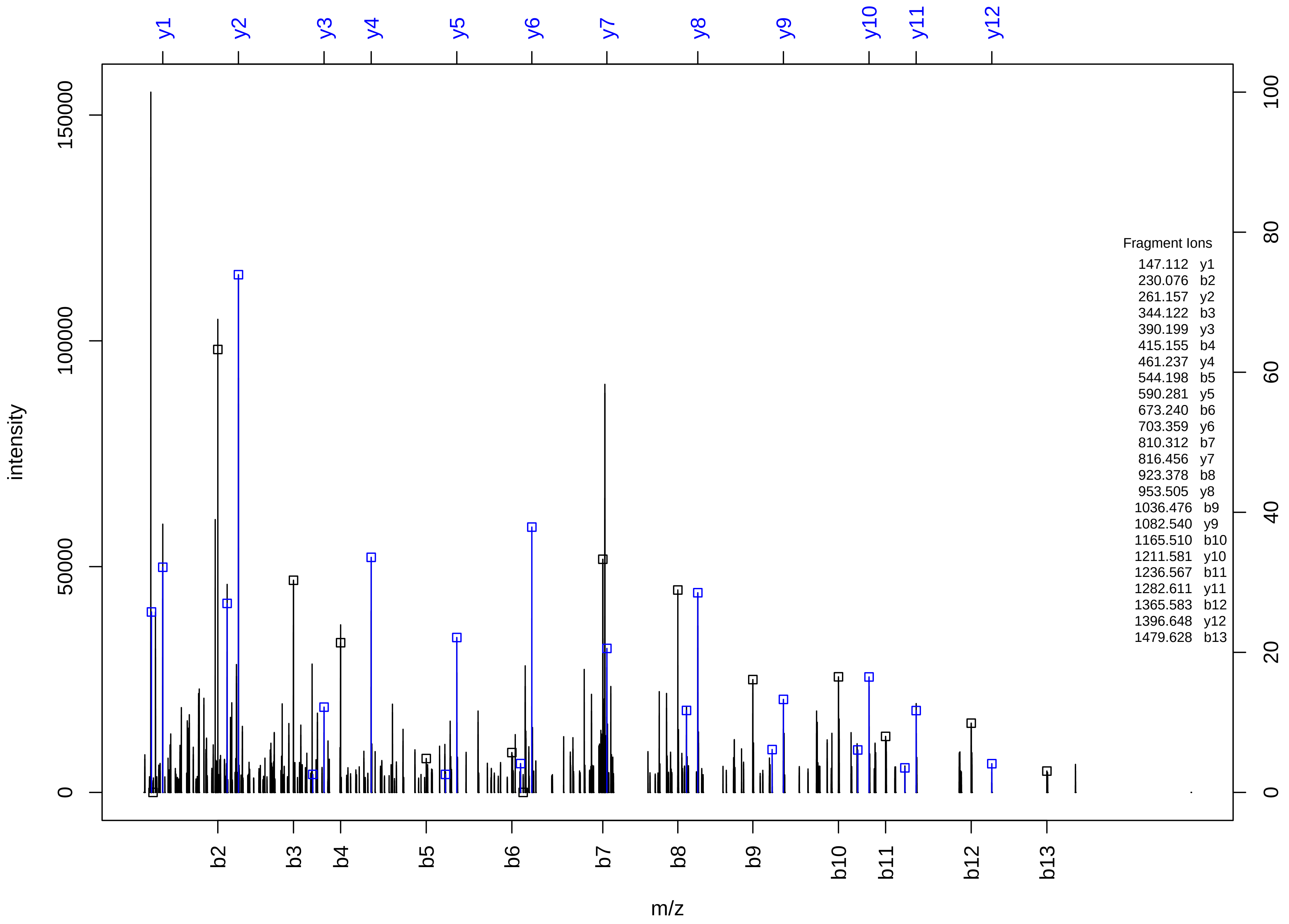

NDNAEEHLLEAENK / File: christine_10pools_05.raw; SpectrumID: ; scans: 3145

MDNFLRGDN^SRK

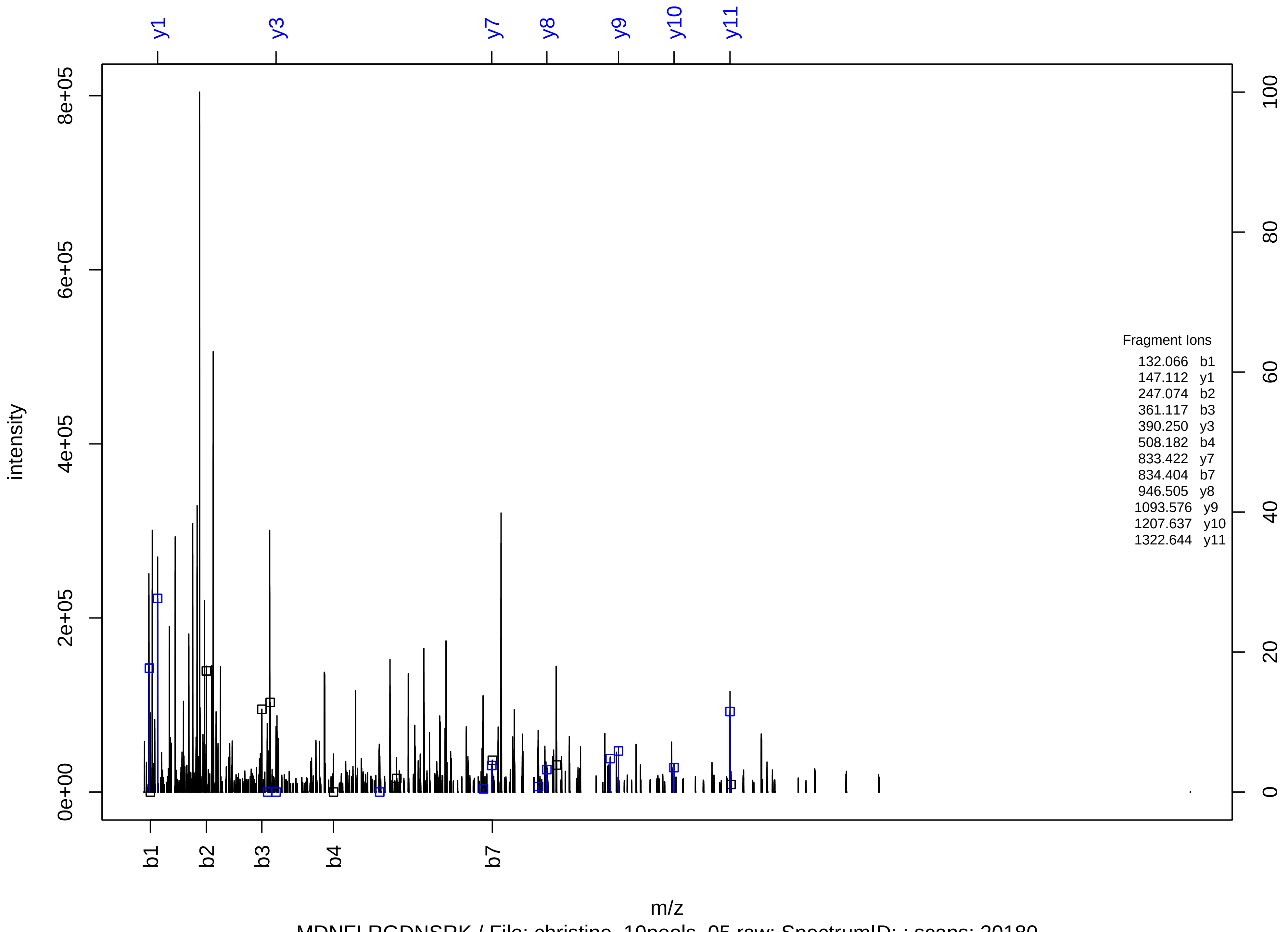

MDNFLRGDNSRK / File: christine_10pools_05.raw; SpectrumID: ; scans: 20180

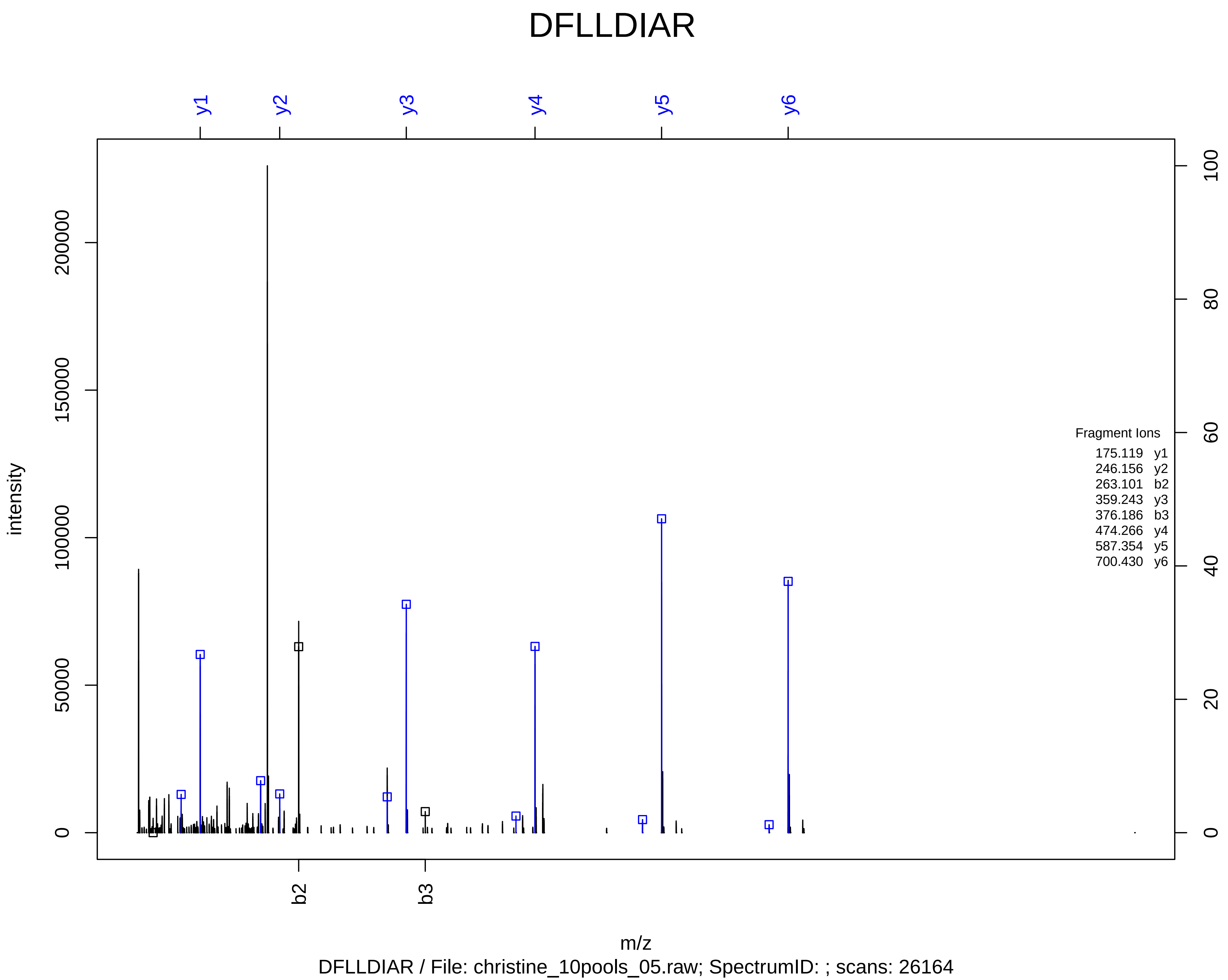
DFLLDIAR
y1
y2
y3
y4
y5
y6
b2
b3
intensity
m/z
0
50000
100000
150000
200000
0
20
40
60
80
100
Fragment Ions
175.119 y1
246.156 y2
263.101 b2
359.243 y3
376.186 b3
474.266 y4
587.354 y5
700.430 y6
DFLLDIAR / File: christine_10pools_05.raw; SpectrumID: ; scans: 26164

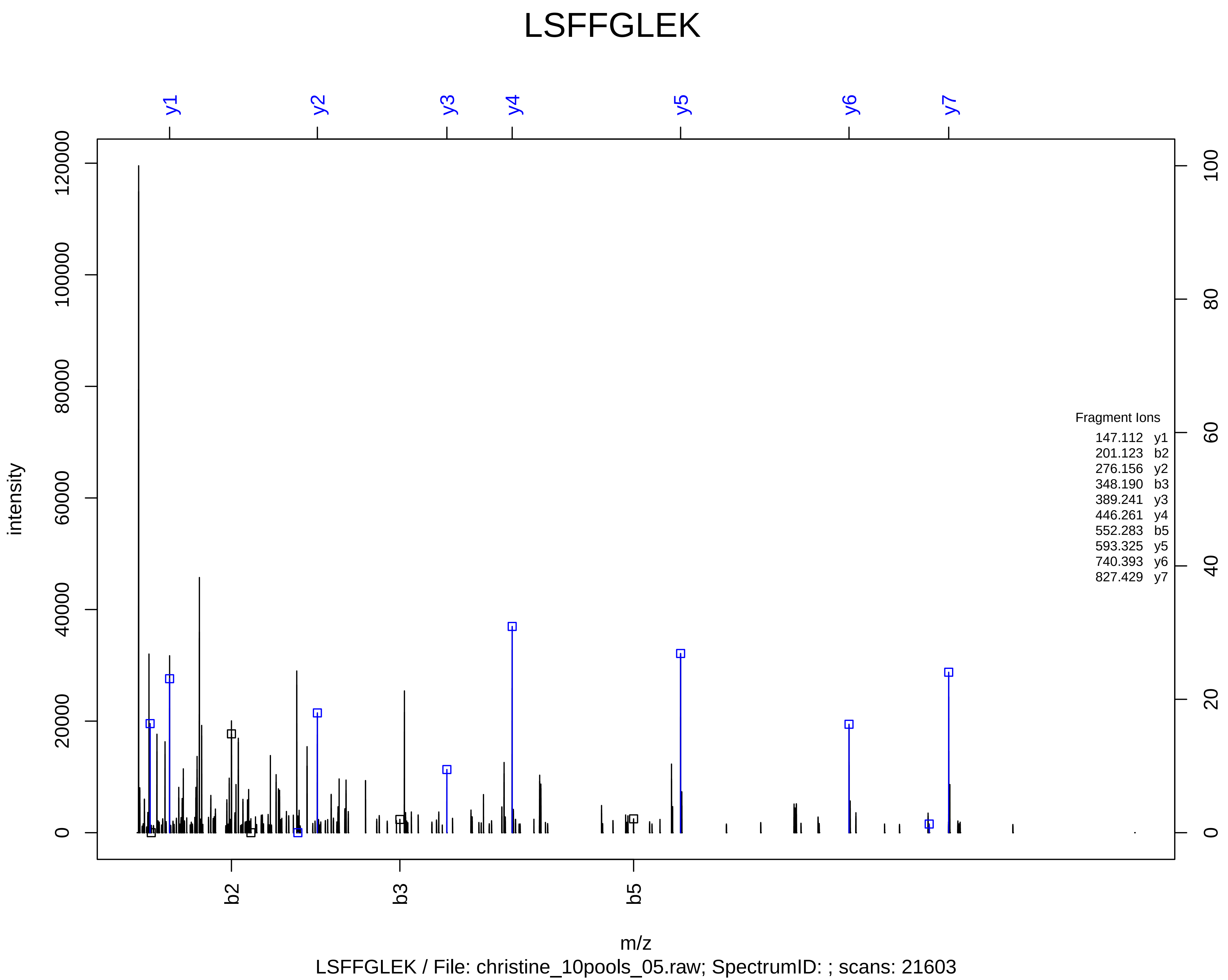

LSFFGLEK
y1
y2
y3
y4
y5
y6
y7
b2
b3
b5
intensity
m/z
0
20000
40000
60000
80000
100000
120000
0
20
40
60
80
100
Fragment Ions
147.112 y1
201.123 b2
276.156 y2
348.190 b3
389.241 y3
446.261 y4
552.283 b5
593.325 y5
740.393 y6
827.429 y7
LSFFGLEK / File: christine_10pools_05.raw; SpectrumID: ; scans: 21603

VVNALQTLCALIR

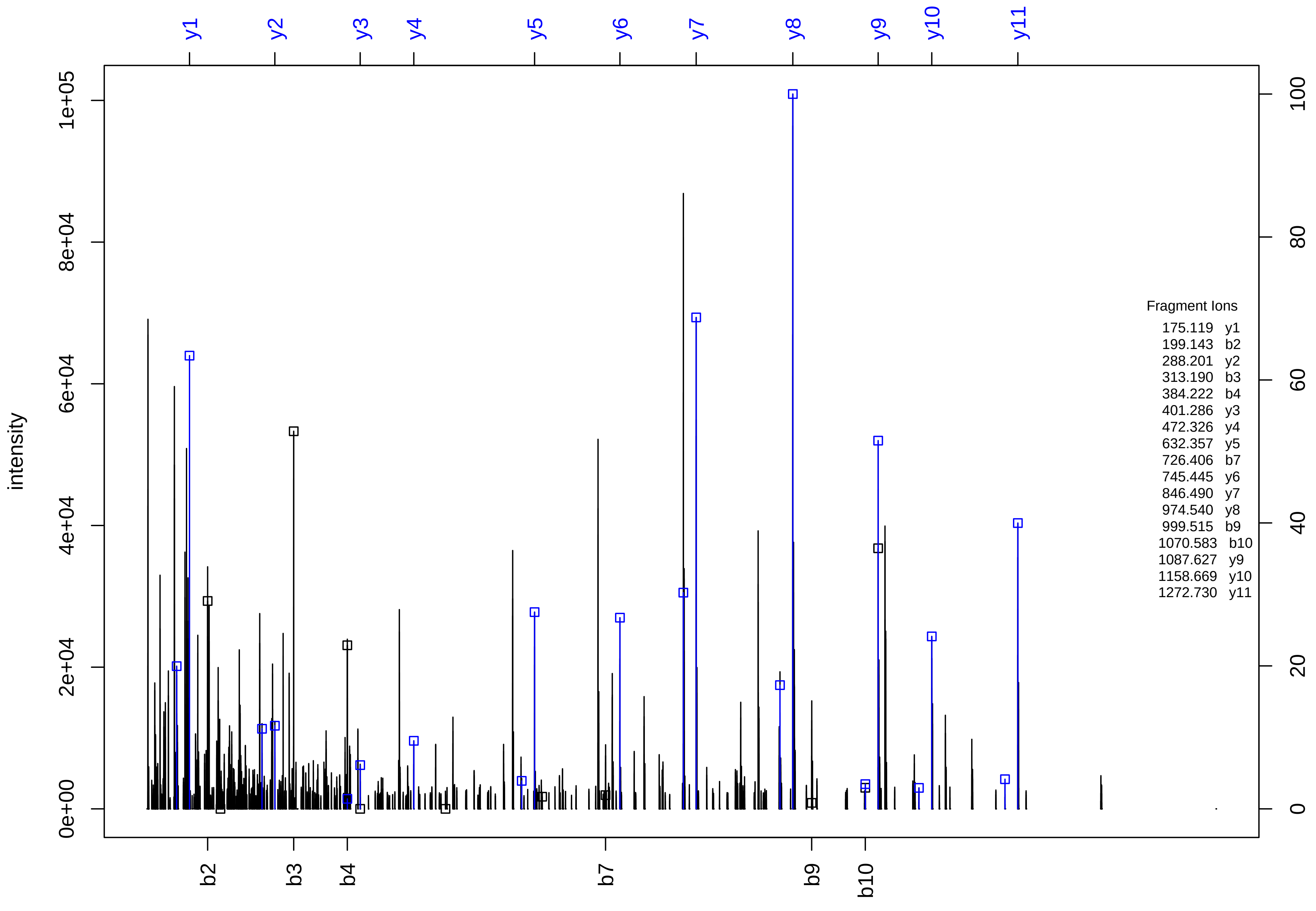

VVNALQTLCALIR / File: christine_10pools_05.raw; SpectrumID: ; scans: 34912

LINQVLELQHTLEDLSAR

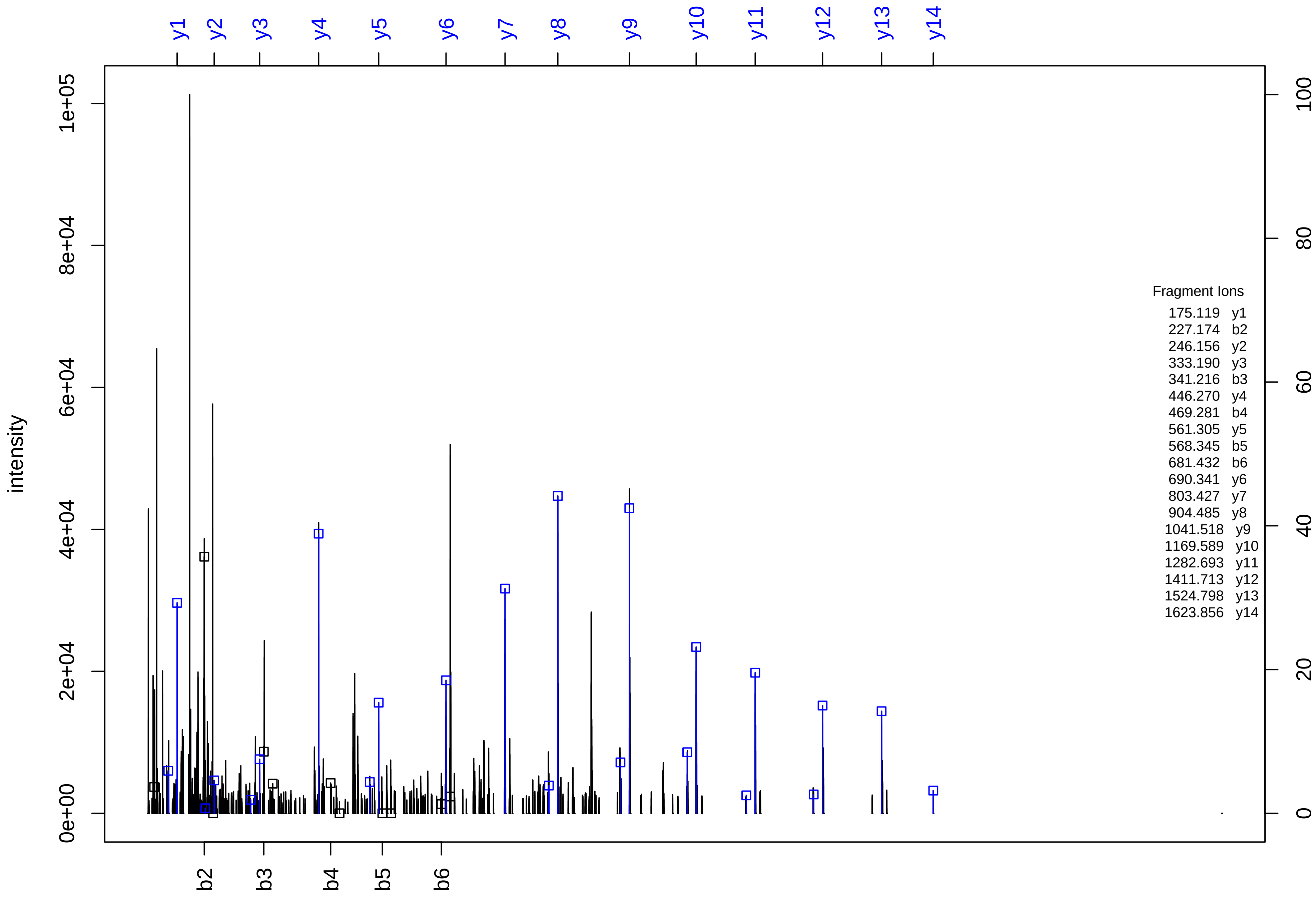

LINQVLELQHTLEDLSAR / File: christine_10pools_05.raw; SpectrumID: ; scans: 31935

EQAASVGTQVEEAAER

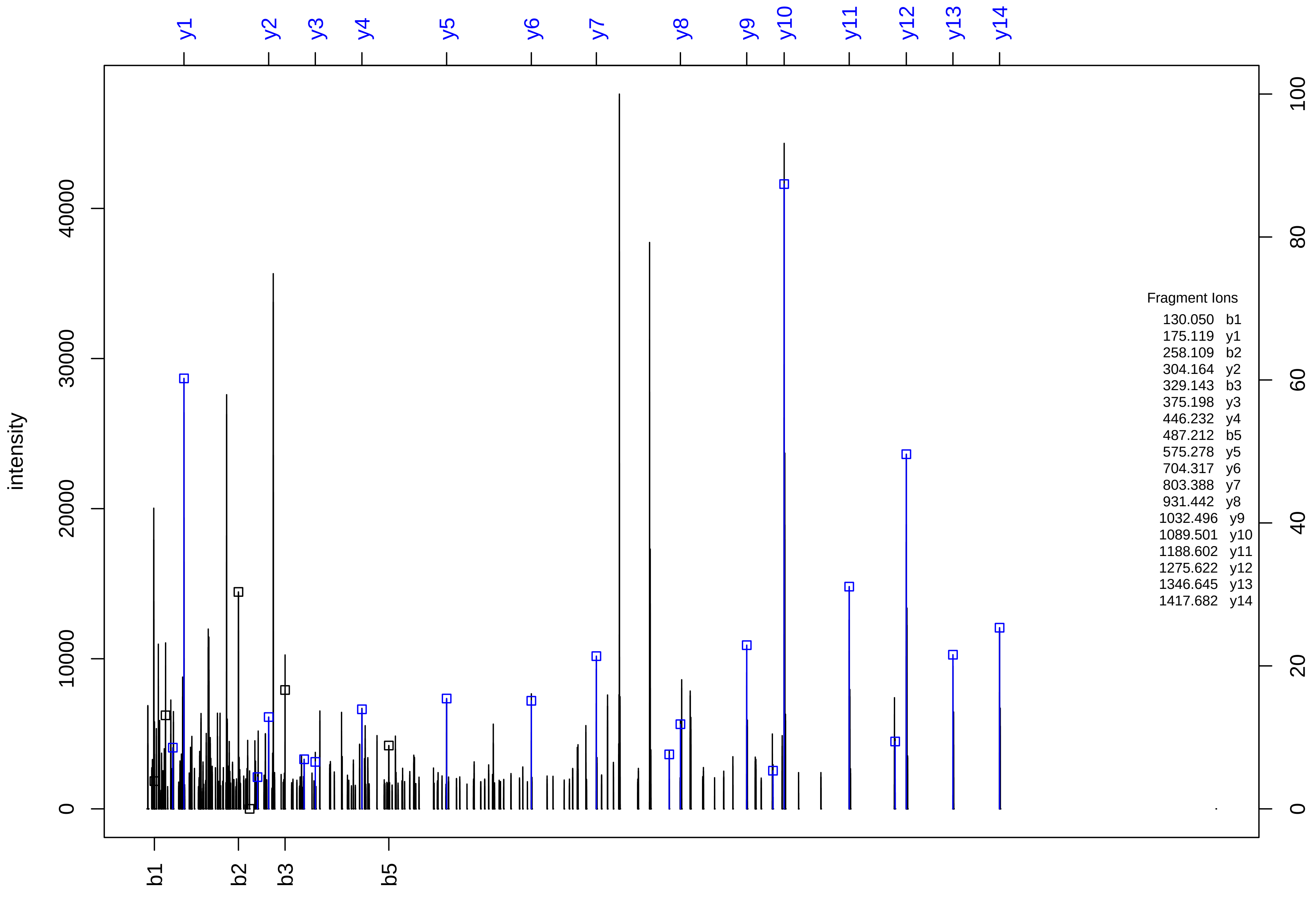

EQAASVGTQVEEAAER / File: christine_10pools_05.raw; SpectrumID: ; scans: 6178

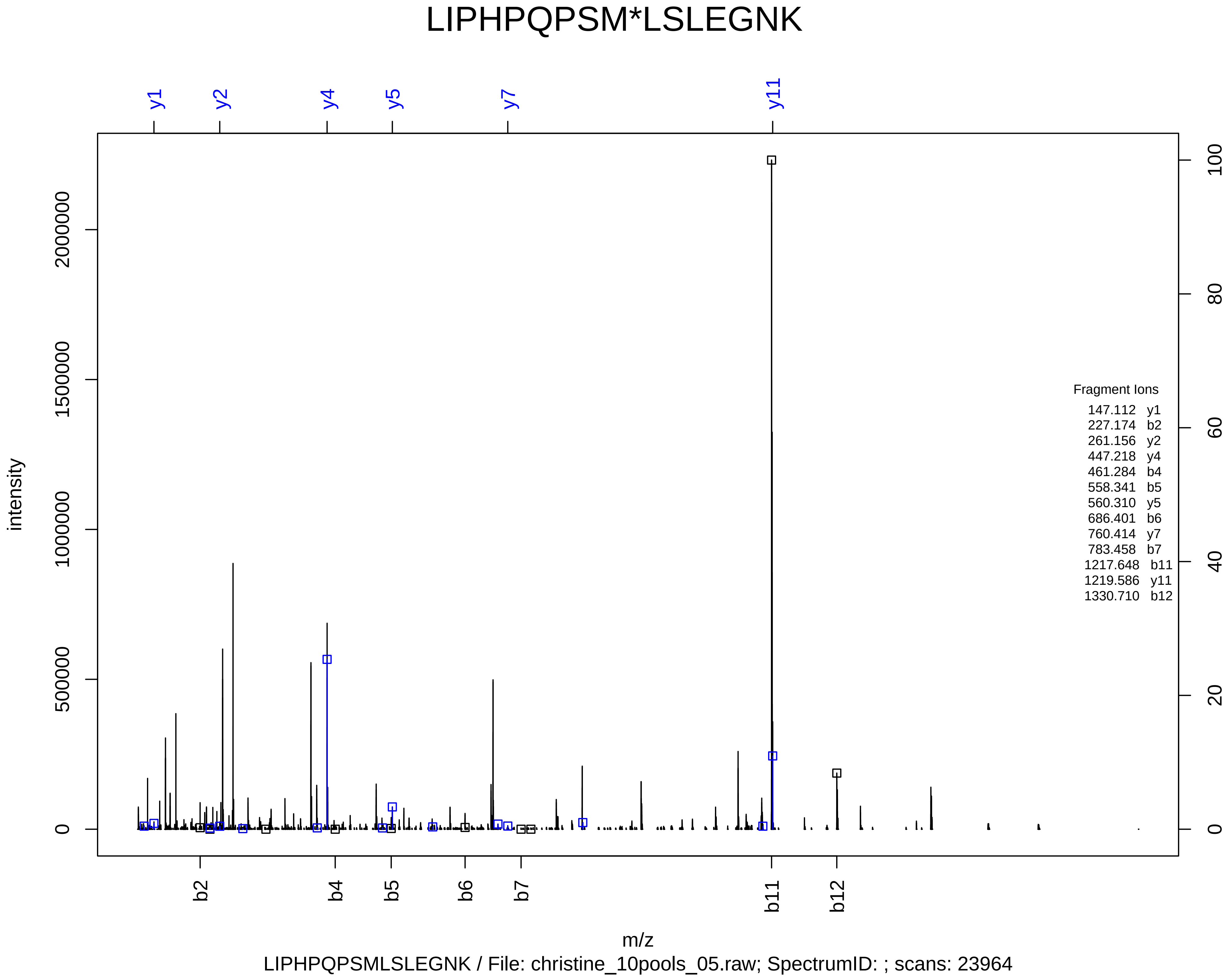

LIPHPQPSM*LSLEGNK
y1
y2
y4
y5
y7
y11
intensity
0
500000
1000000
1500000
2000000
0
20
40
60
80
100
Fragment Ions
147.112 y1
227.174 b2
261.156 y2
447.218 y4
461.284 b4
558.341 b5
560.310 y5
686.401 b6
760.414 y7
783.458 b7
1217.648 b11
1219.586 y11
1330.710 b12
b2
b4
b5
b6
b7
b11
b12
m/z
LIPHPQPSMLSLEGNK / File: christine_10pools_05.raw; SpectrumID: ; scans: 23964

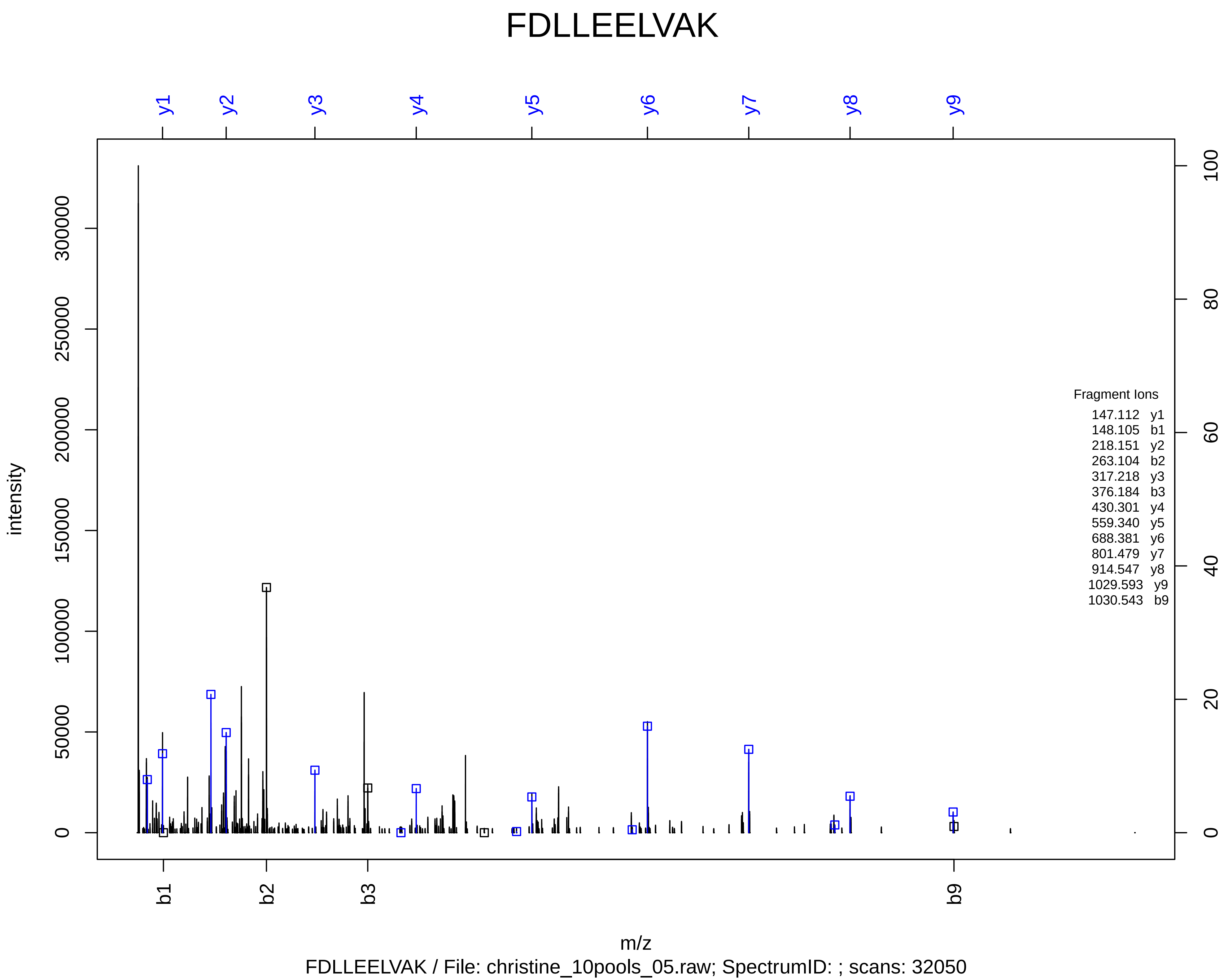

FDLLEELVAK
y1
y2
y3
y4
y5
y6
y7
y8
y9
intensity
0
50000
100000
150000
200000
250000
300000
0
20
40
60
80
100
Fragment Ions
147.112 y1
148.105 b1
218.151 y2
263.104 b2
317.218 y3
376.184 b3
430.301 y4
559.340 y5
688.381 y6
801.479 y7
914.547 y8
1029.593 y9
1030.543 b9
b1
b2
b3
b9
m/z
FDLLEELVAK / File: christine_10pools_05.raw; SpectrumID: ; scans: 32050

AQESVGIFEVTHQFVK

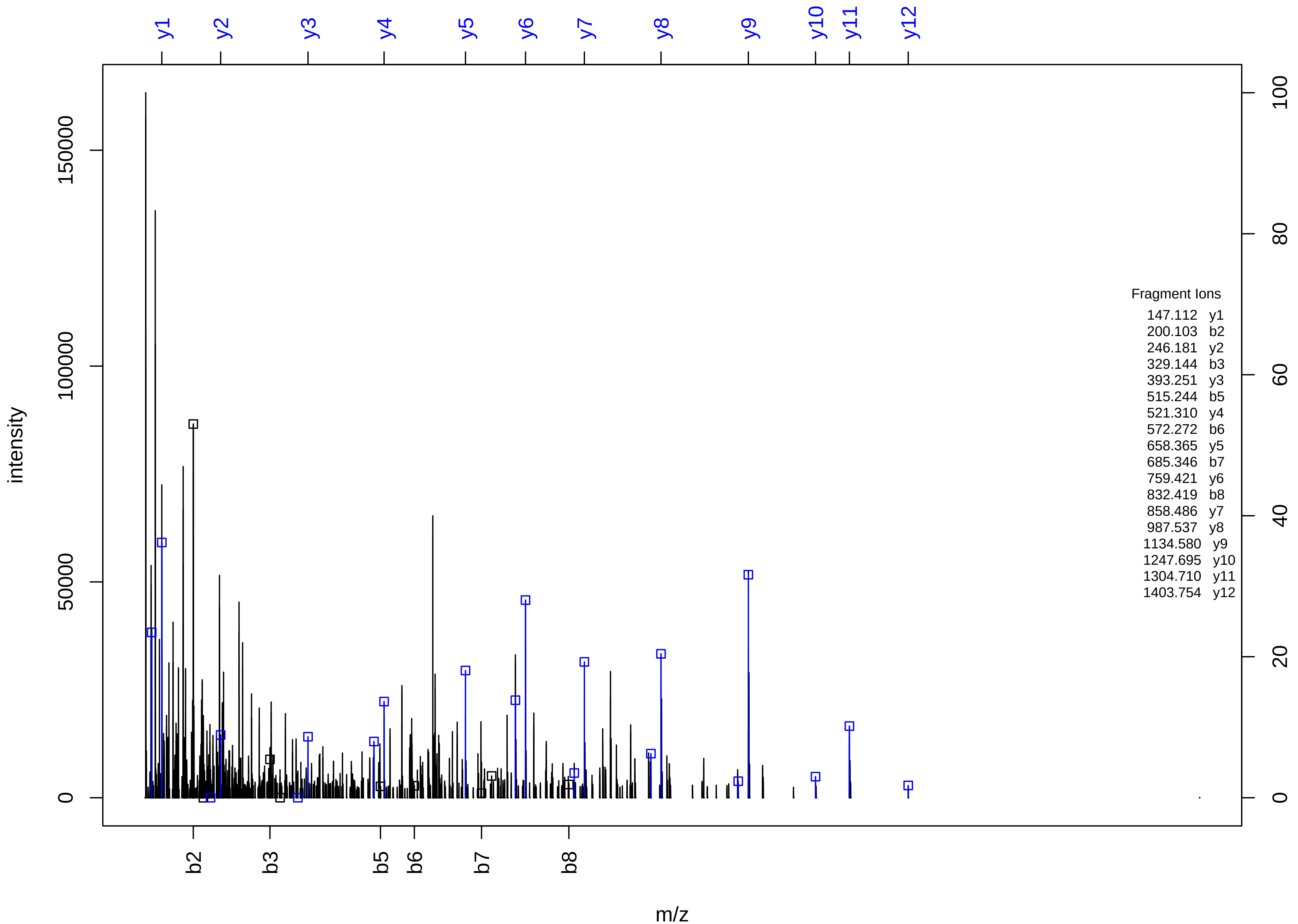

AQESVGIFEVTHQFVK / File: christine_10pools_05.raw; SpectrumID: ; scans: 20486

VLPAQGEPLIQLAHNLGLR

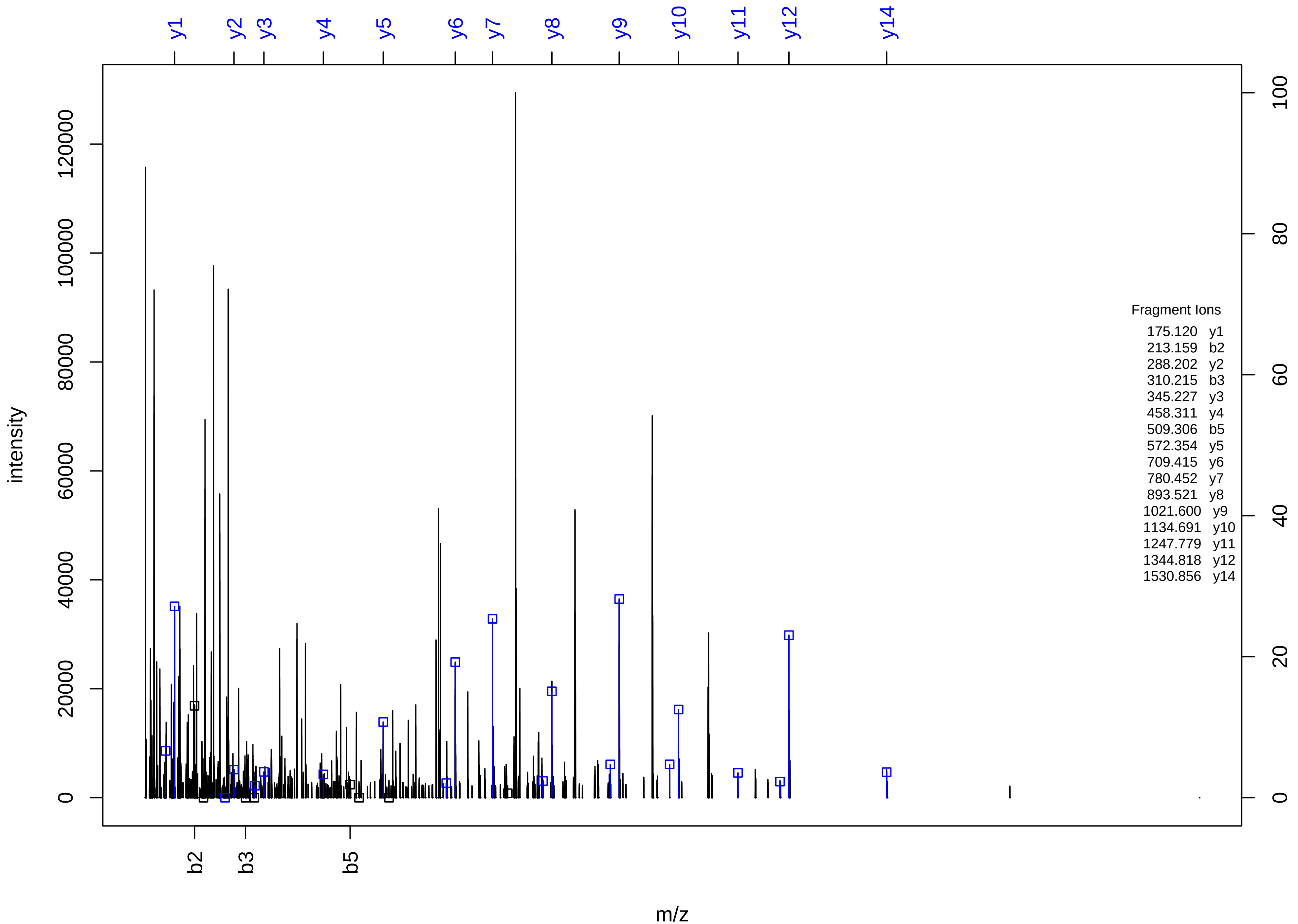

VLPAQGEPLIQLAHNLGLR / File: christine_10pools_05.raw; SpectrumID: ; scans: 33187

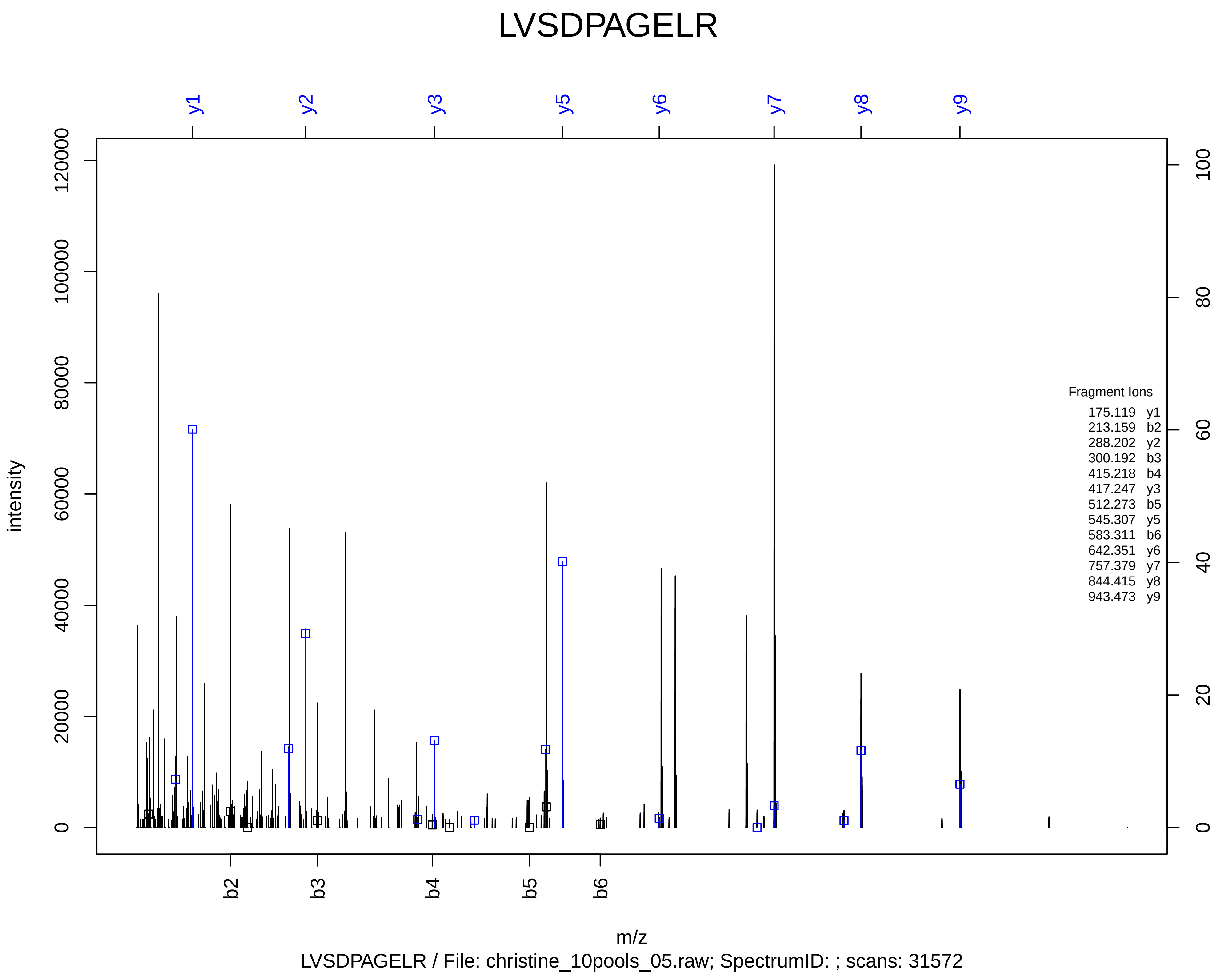

LVSDPAGELR
y1
y2
y3
y5
y6
y7
y8
y9
b2
b3
b4
b5
b6
intensity
m/z
Fragment Ions
175.119 y1
213.159 b2
288.202 y2
300.192 b3
415.218 b4
417.247 y3
512.273 b5
545.307 y5
583.311 b6
642.351 y6
757.379 y7
844.415 y8
943.473 y9
LVSDPAGELR / File: christine_10pools_05.raw; SpectrumID: ; scans: 31572

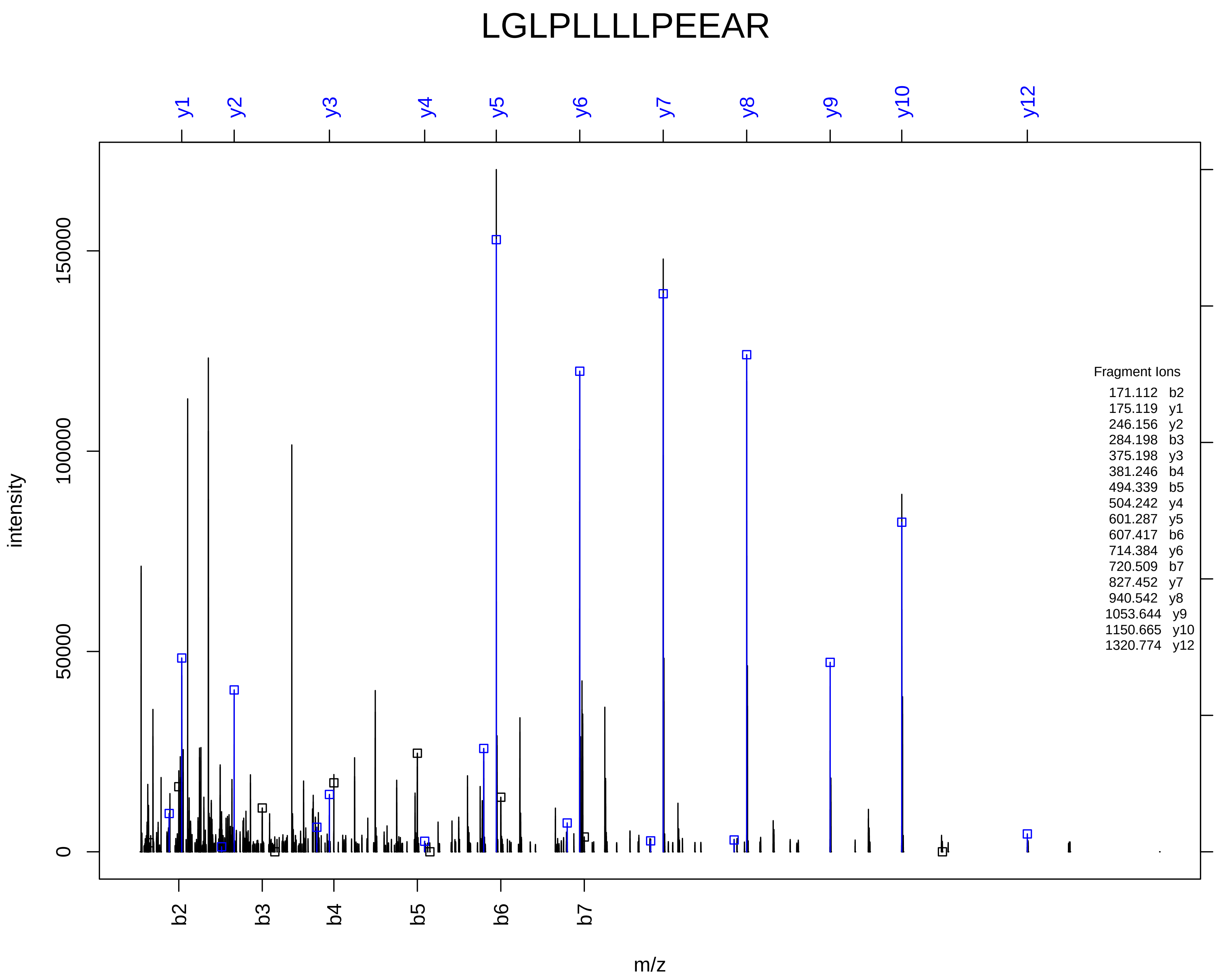

LGLPLLLLPEEAR / File: christine_10pools_05.raw; SpectrumID: ; scans: 36950

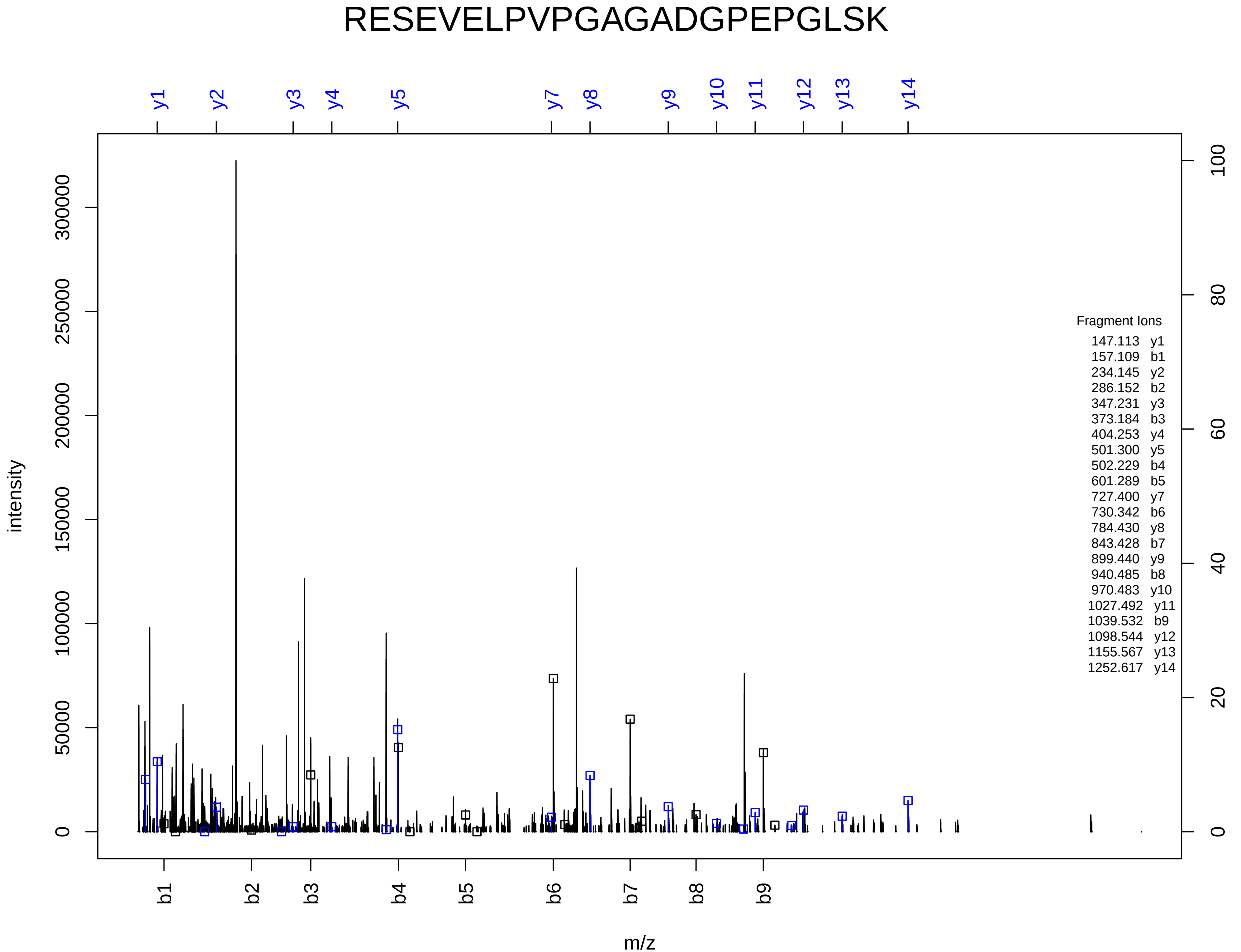

RESEVELPVPGAGADGPEPGLSK / File: christine_10pools_05.raw; SpectrumID: ; scans: 14673

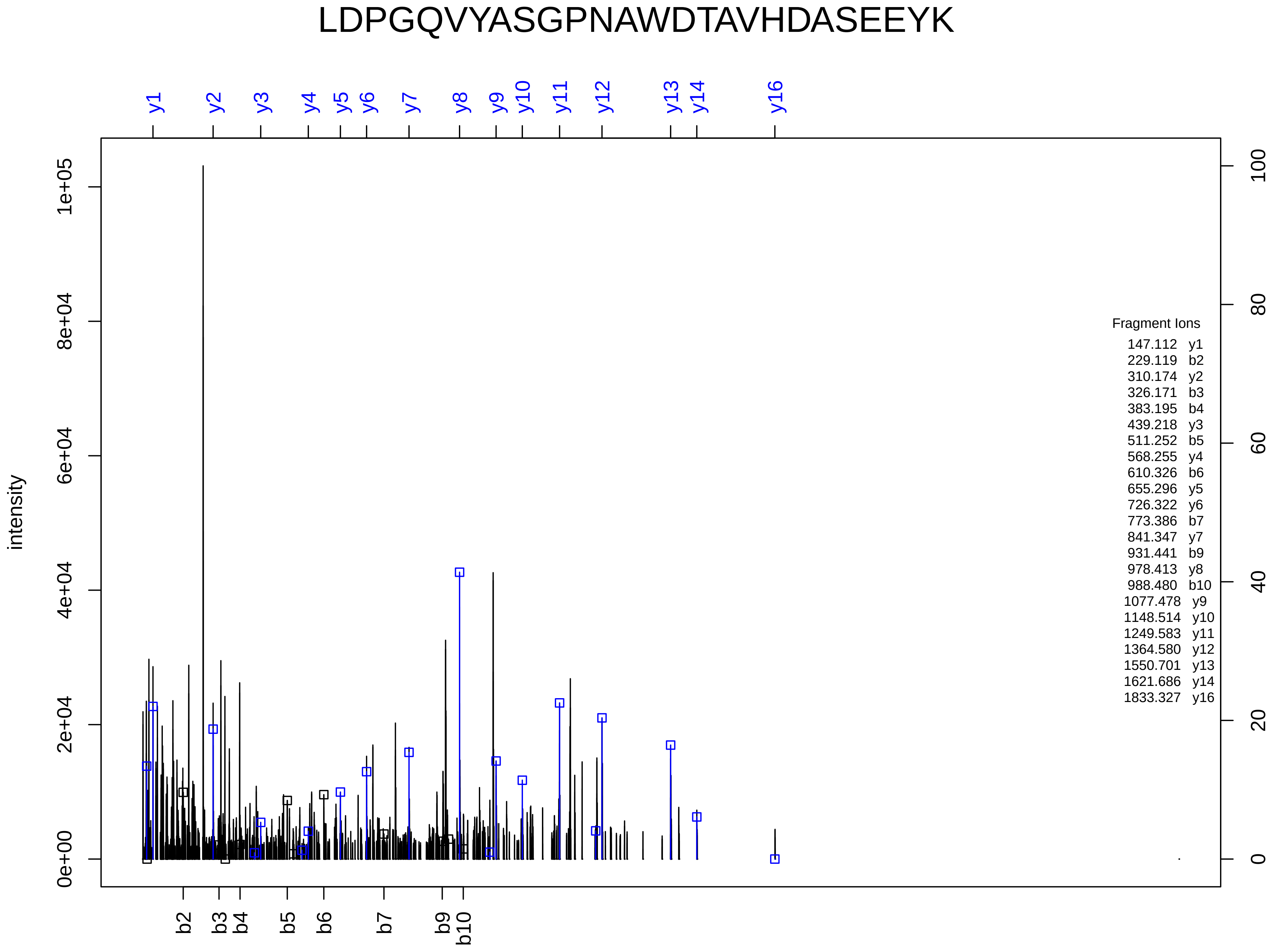

LDPGQVYASGPNAWDTAVHDASEEYK / File: christine_10pools_05.raw; SpectrumID: ; scans: 22163

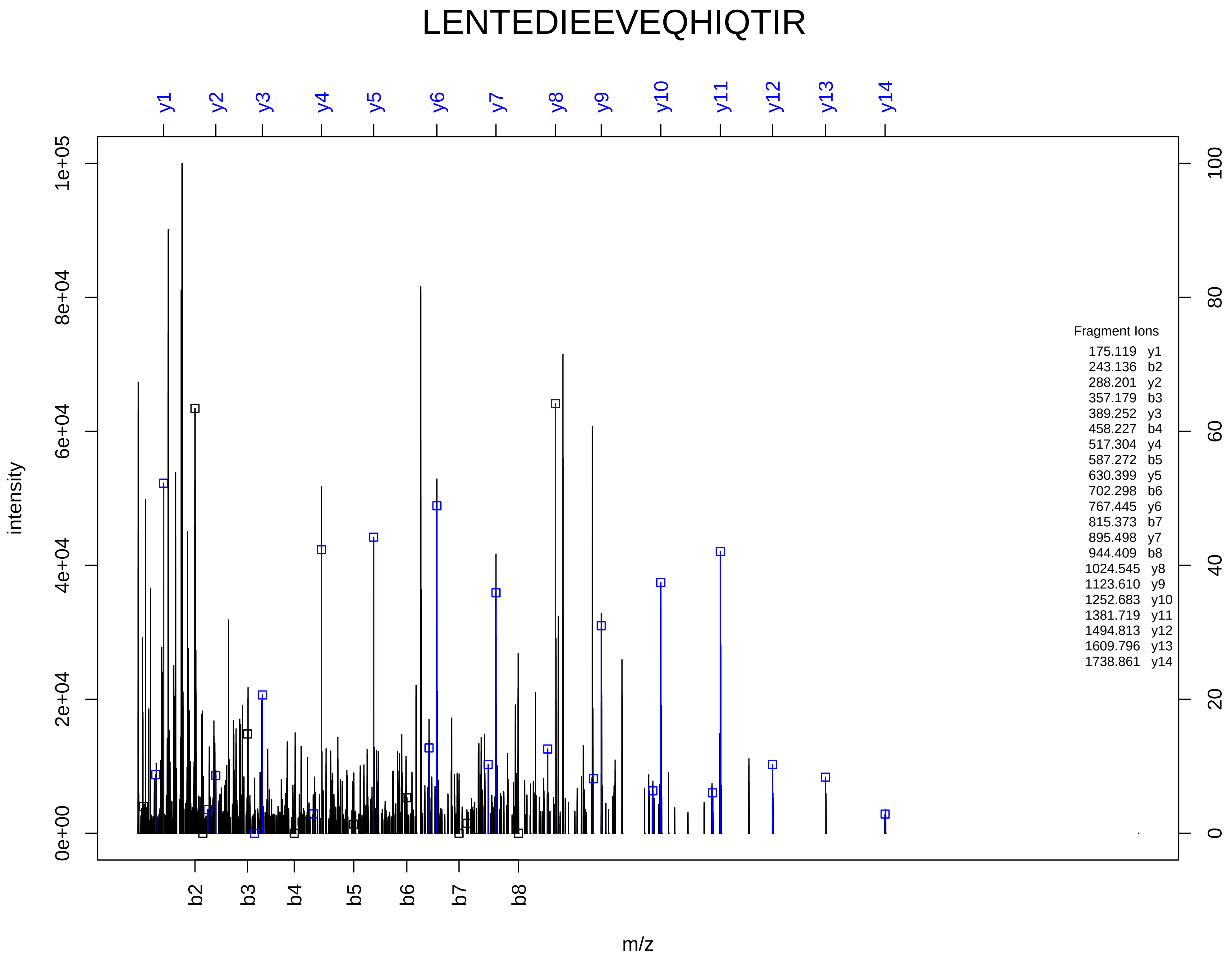

LENTEDIEEVEQHIQTIR / File: christine_10pools_05.raw; SpectrumID: ; scans: 18765

LIPLVLGLQEQLR

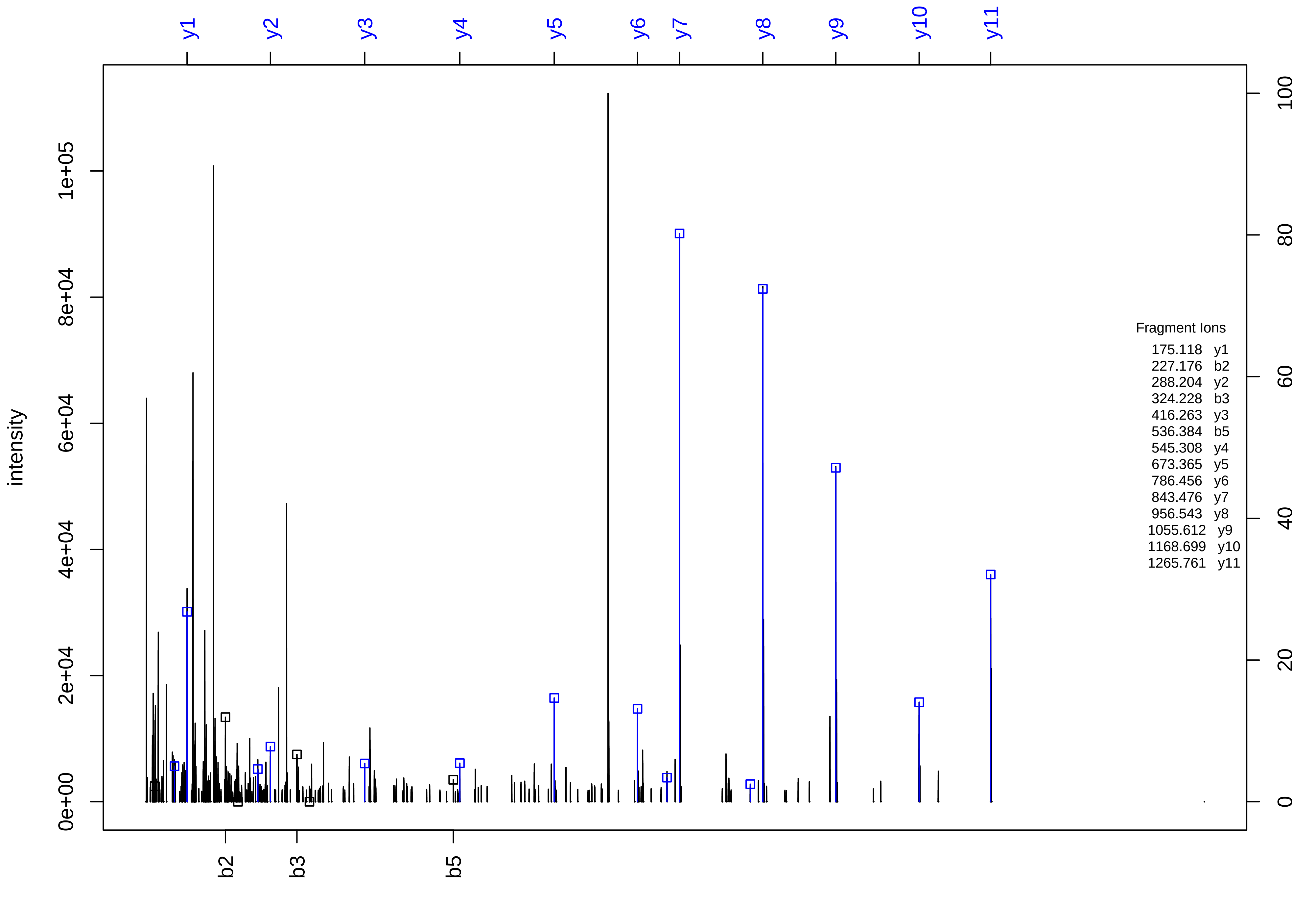

LIPLVLGLQEQLR / File: christine_10pools_05.raw; SpectrumID: ; scans: 32840

ANVDPALGEFPFAK

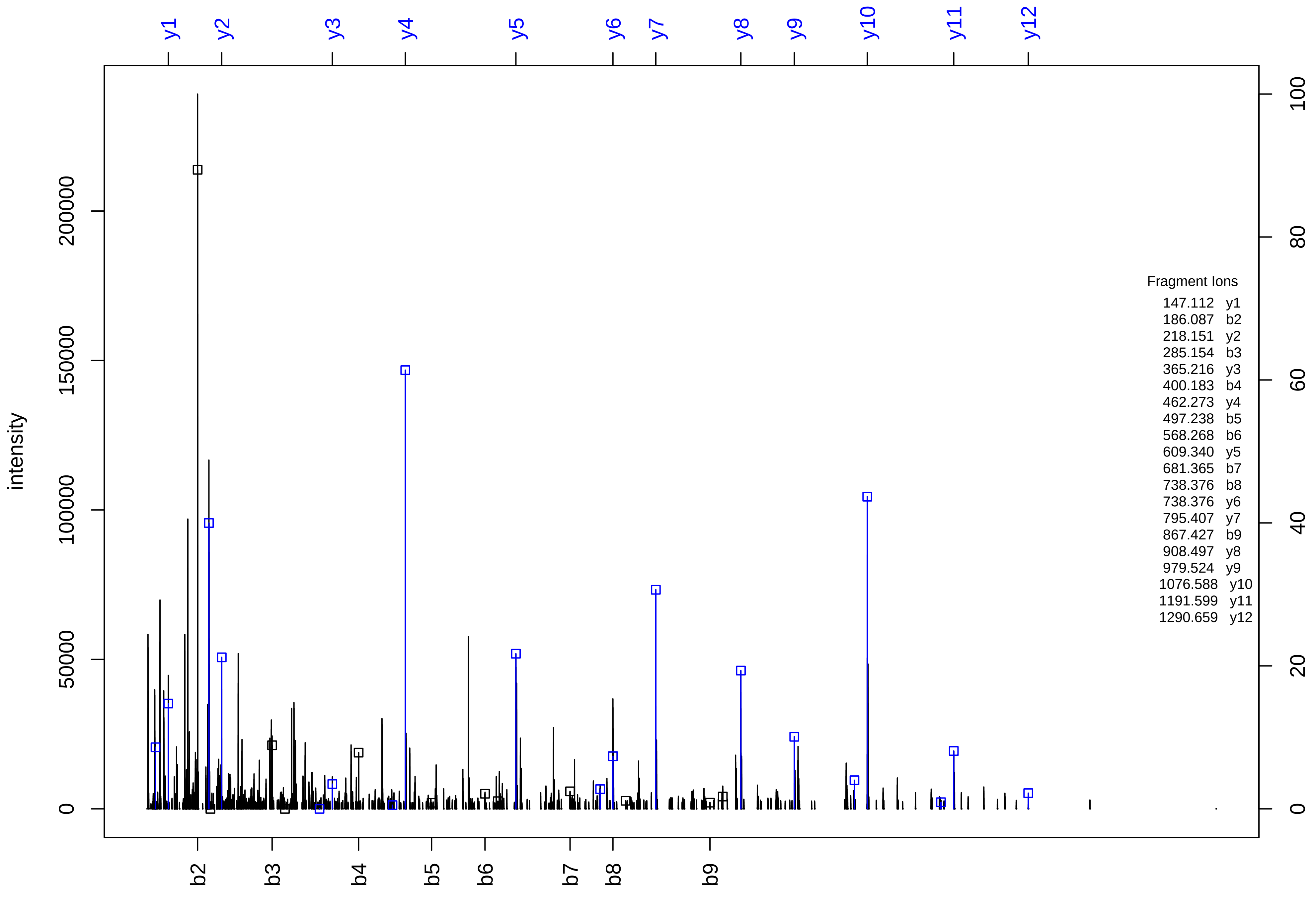

ANVDPALGEFPFAK / File: christine_10pools_05.raw; SpectrumID: ; scans: 23254

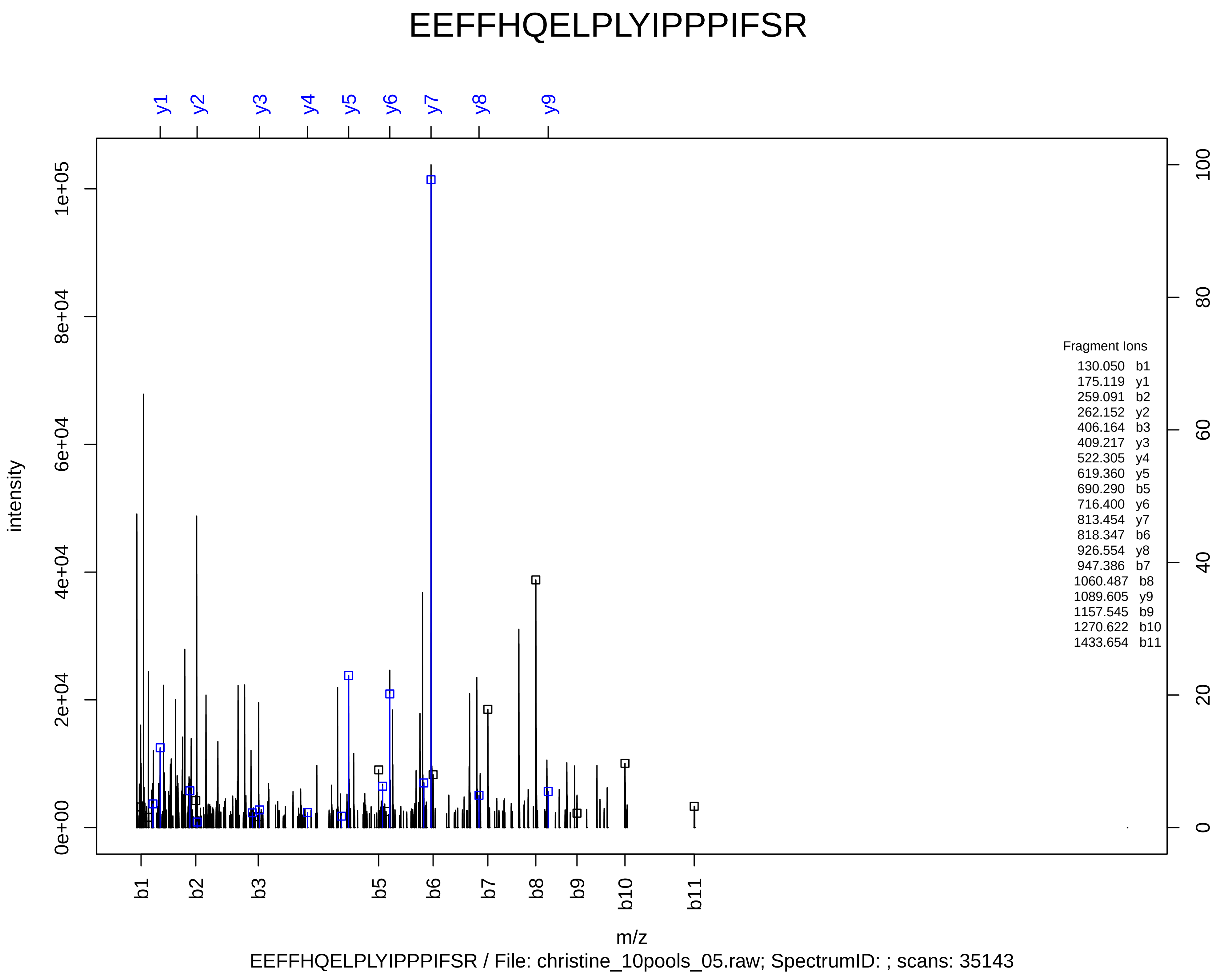

EEFFHQELPLYIPPPIFSR
y1 y2 y3 y4 y5 y6 y7 y8 y9
intensity
0e+00 2e+04 4e+04 6e+04 8e+04 1e+05
0 20 40 60 80 100
Fragment Ions
130.050 b1
175.119 y1
259.091 b2
262.152 y2
406.164 b3
409.217 y3
522.305 y4
619.360 y5
690.290 b5
716.400 y6
813.454 y7
818.347 b6
926.554 y8
947.386 b7
1060.487 b8
1089.605 y9
1157.545 b9
1270.622 b10
1433.654 b11
b1 b2 b3 b5 b6 b7 b8 b9 b10 b11
m/z
EEFFHQELPLYIPPPIFSR / File: christine_10pools_05.raw; SpectrumID: ; scans: 35143

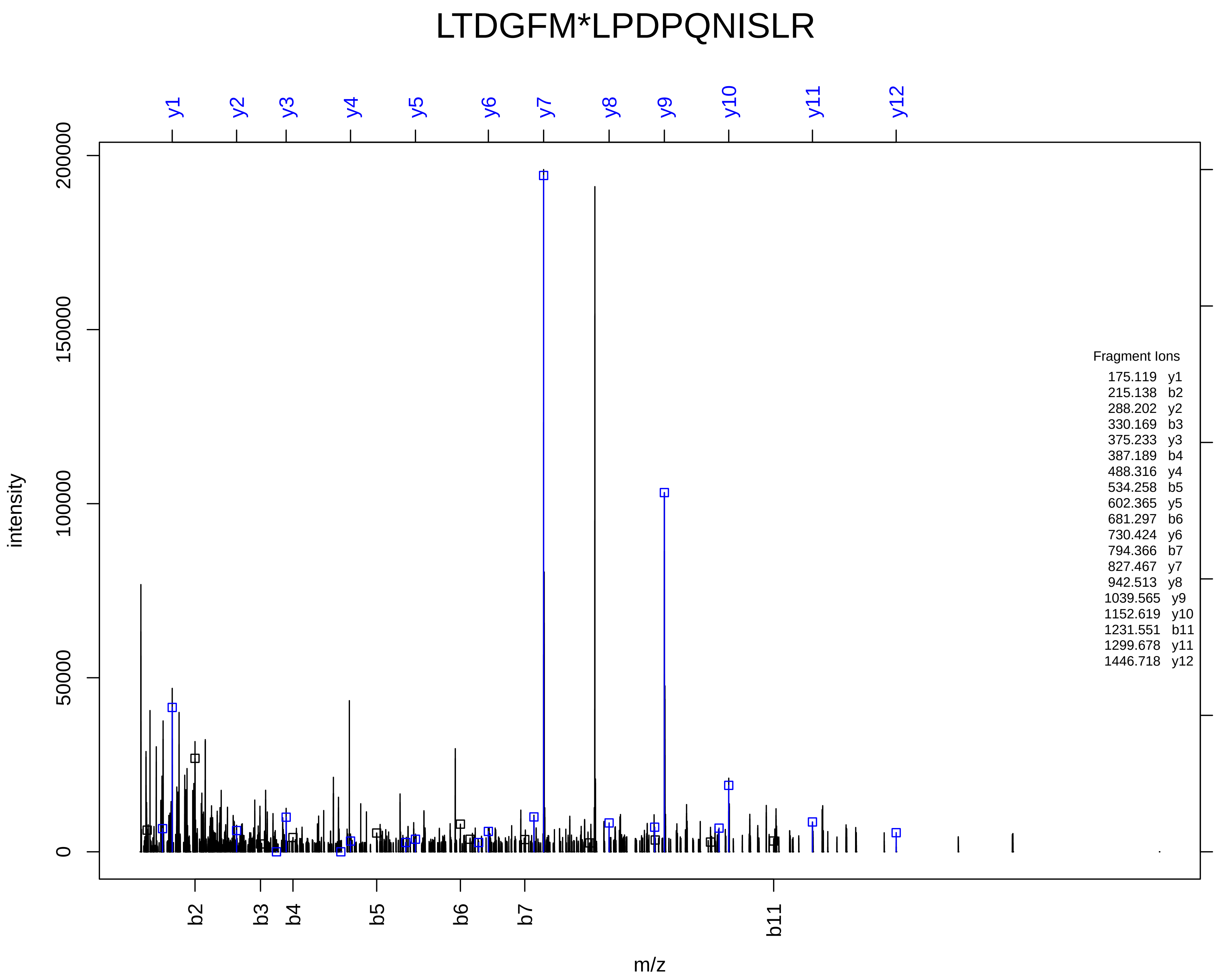
LTDGFM*LPDPQNISLR
y1
y2
y3
y4
y5
y6
y7
y8
y9
y10
y11
y12
b2
b3
b4
b5
b6
b7
b11
intensity
m/z
0
50000
100000
150000
200000
0
20
40
60
80
100
Fragment Ions
175.119 y1
215.138 b2
288.202 y2
330.169 b3
375.233 y3
387.189 b4
488.316 y4
534.258 b5
602.365 y5
681.297 b6
730.424 y6
794.366 b7
827.467 y7
942.513 y8
1039.565 y9
1152.619 y10
1231.551 b11
1299.678 y11
1446.718 y12
LTDGFMLPDPQNISLR / File: christine_10pools_05.raw; SpectrumID: ; scans: 26431

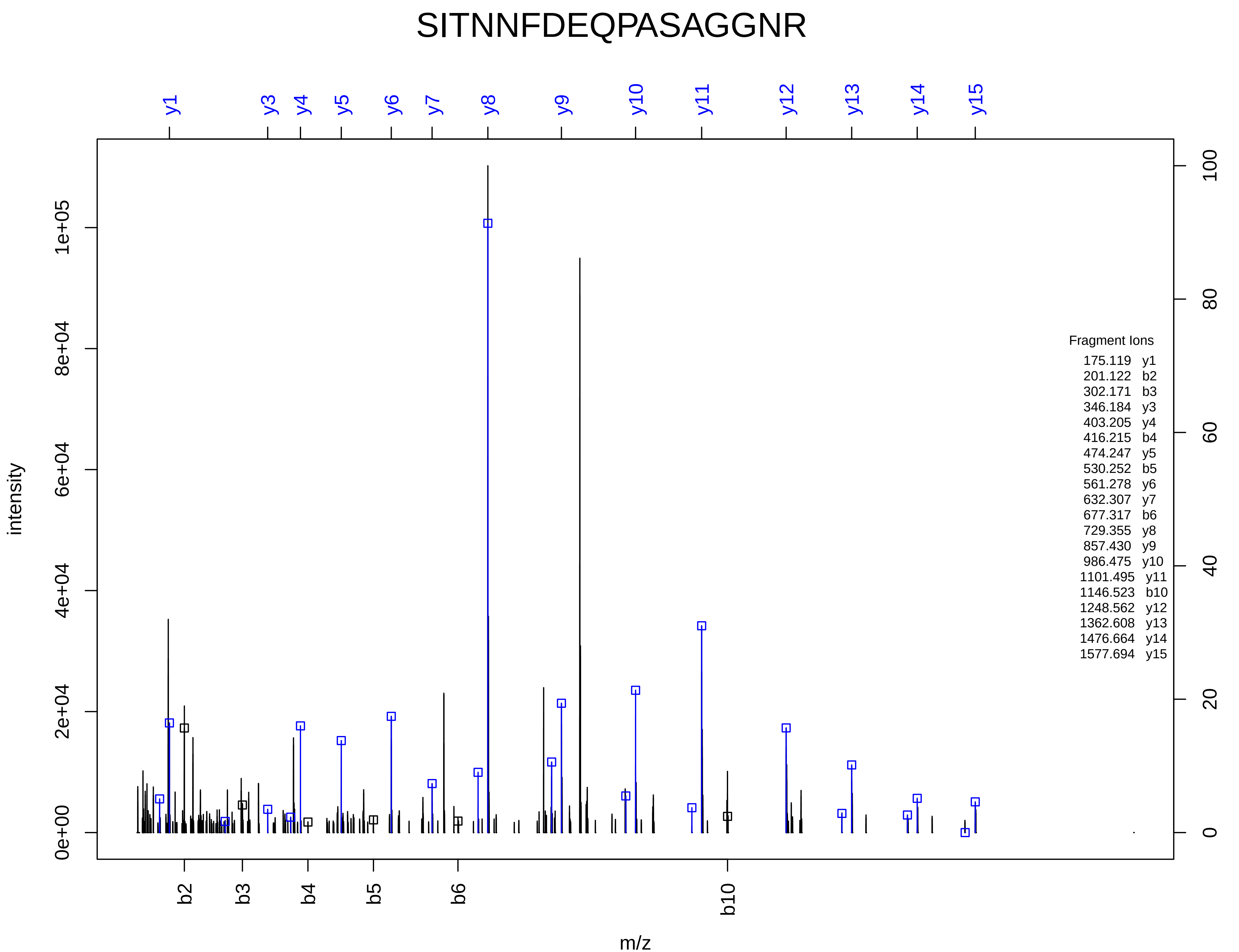

SITNNFDEQPASAGGNR / File: christine_10pools_05.raw; SpectrumID: ; scans: 3671

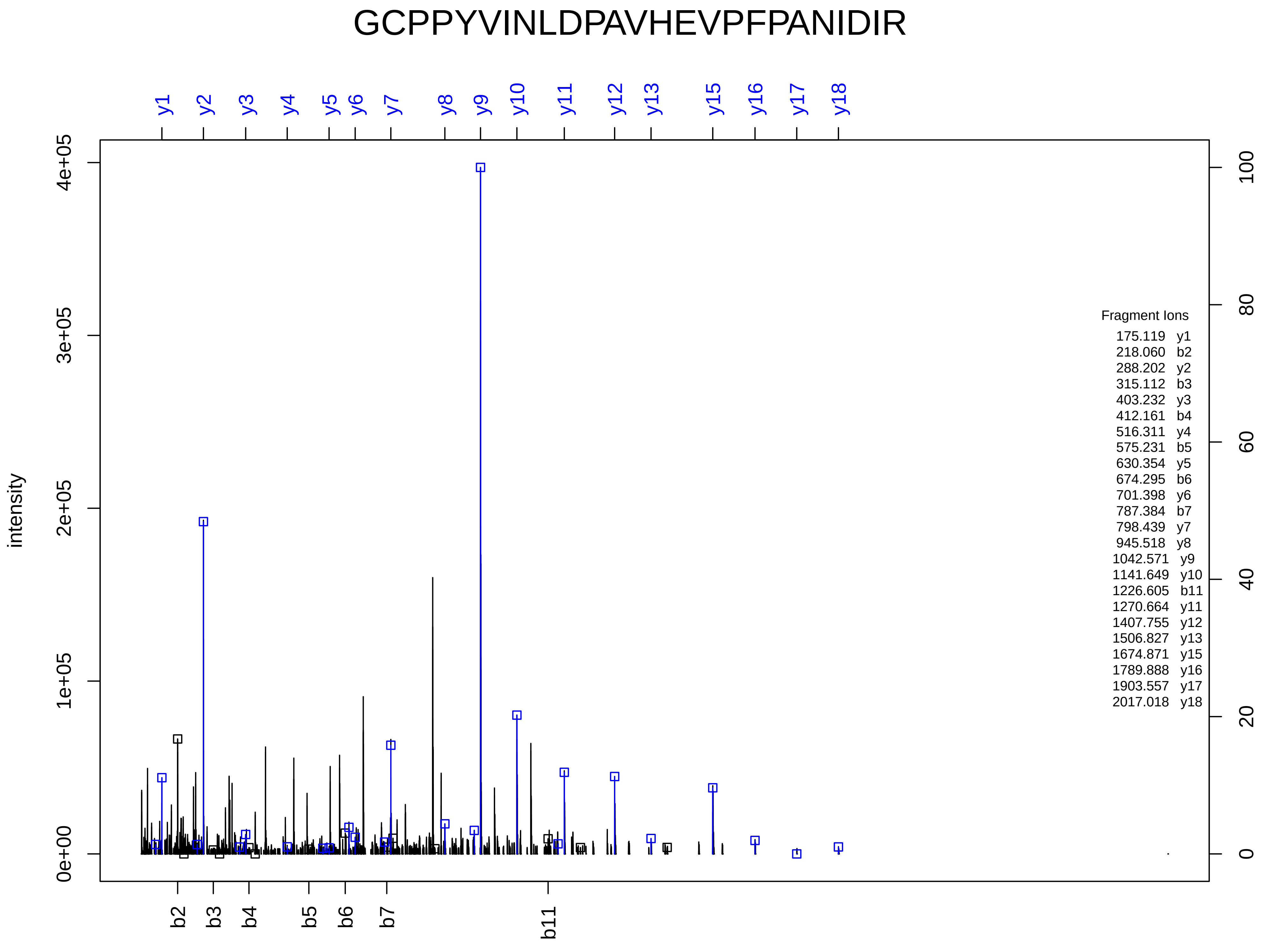

GCPPYVINLDPAVHEVPFPANIDIR / File: christine_10pools_05.raw; SpectrumID: ; scans: 31270

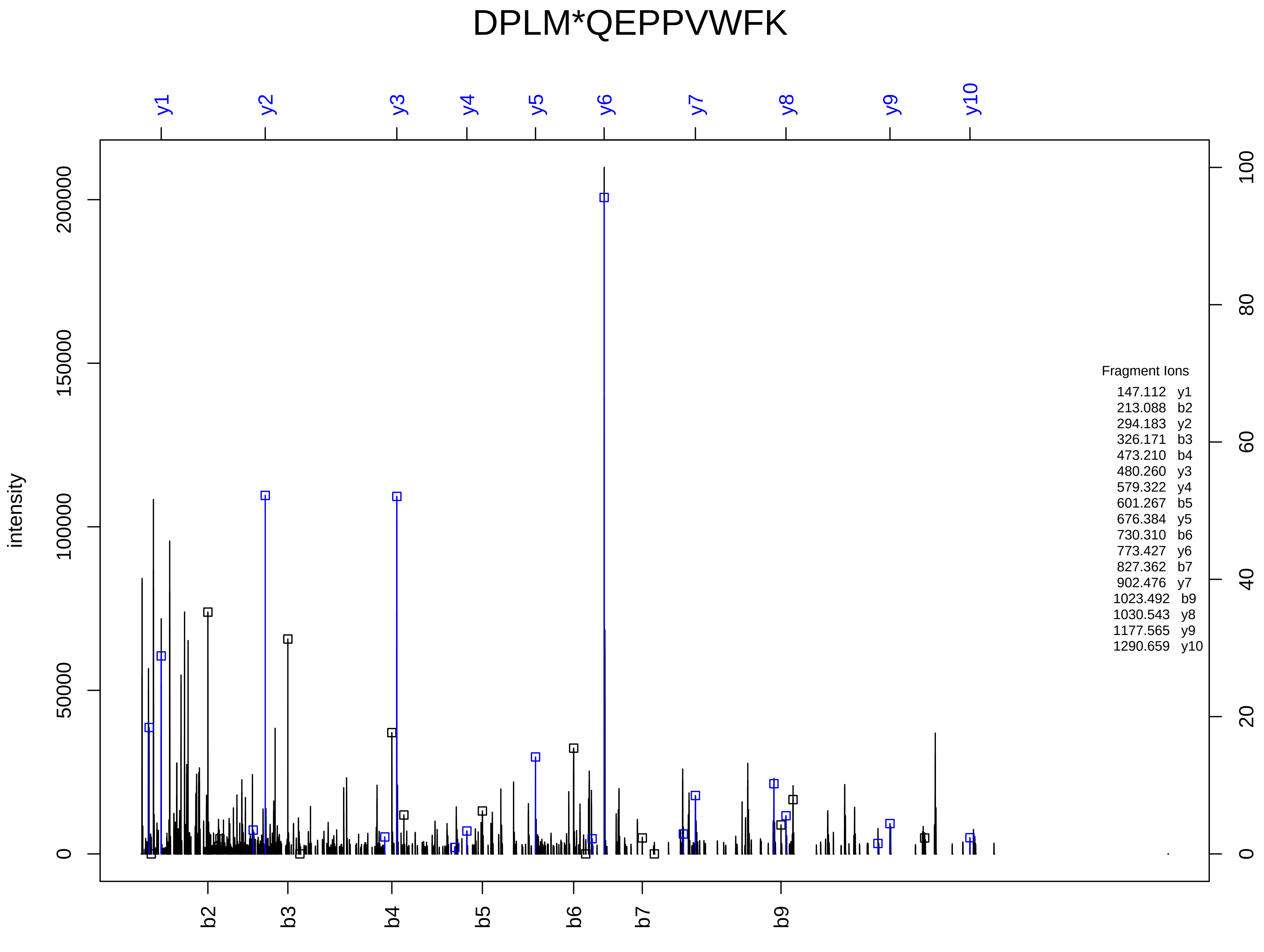

DPLMQEPPVWFK / File: christine_10pools_05.raw; SpectrumID: ; scans: 23184

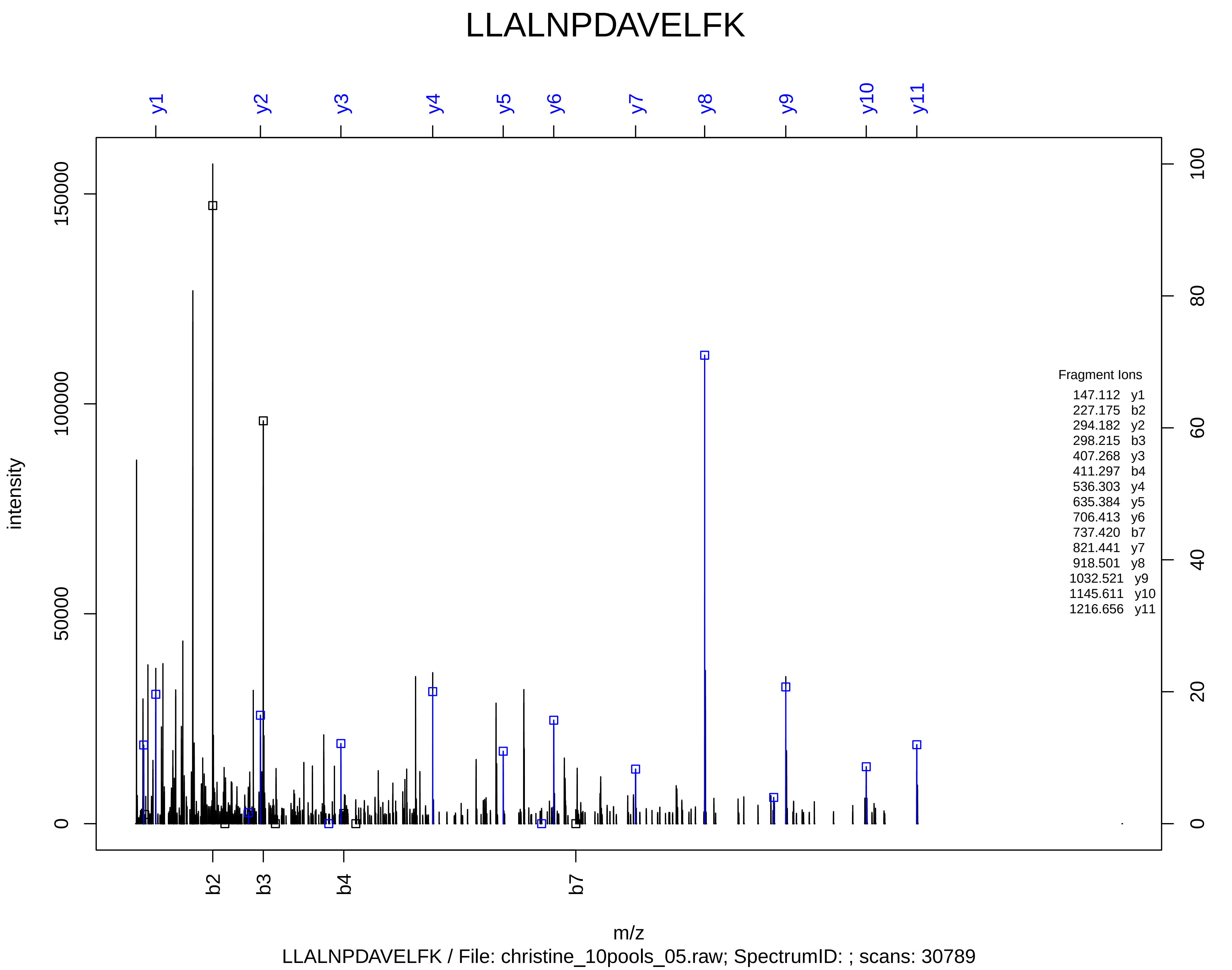
LLALNPDAVELFK
y1
y2
y3
y4
y5
y6
y7
y8
y9
y10
y11
intensity
0
50000
100000
150000
0
20
40
60
80
100
Fragment Ions
147.112 y1
227.175 b2
294.182 y2
298.215 b3
407.268 y3
411.297 b4
536.303 y4
635.384 y5
706.413 y6
737.420 b7
821.441 y7
918.501 y8
1032.521 y9
1145.611 y10
1216.656 y11
b2
b3
b4
b7
m/z
LLALNPDAVELFK / File: christine_10pools_05.raw; SpectrumID: ; scans: 30789

DILDLVHEFPEK

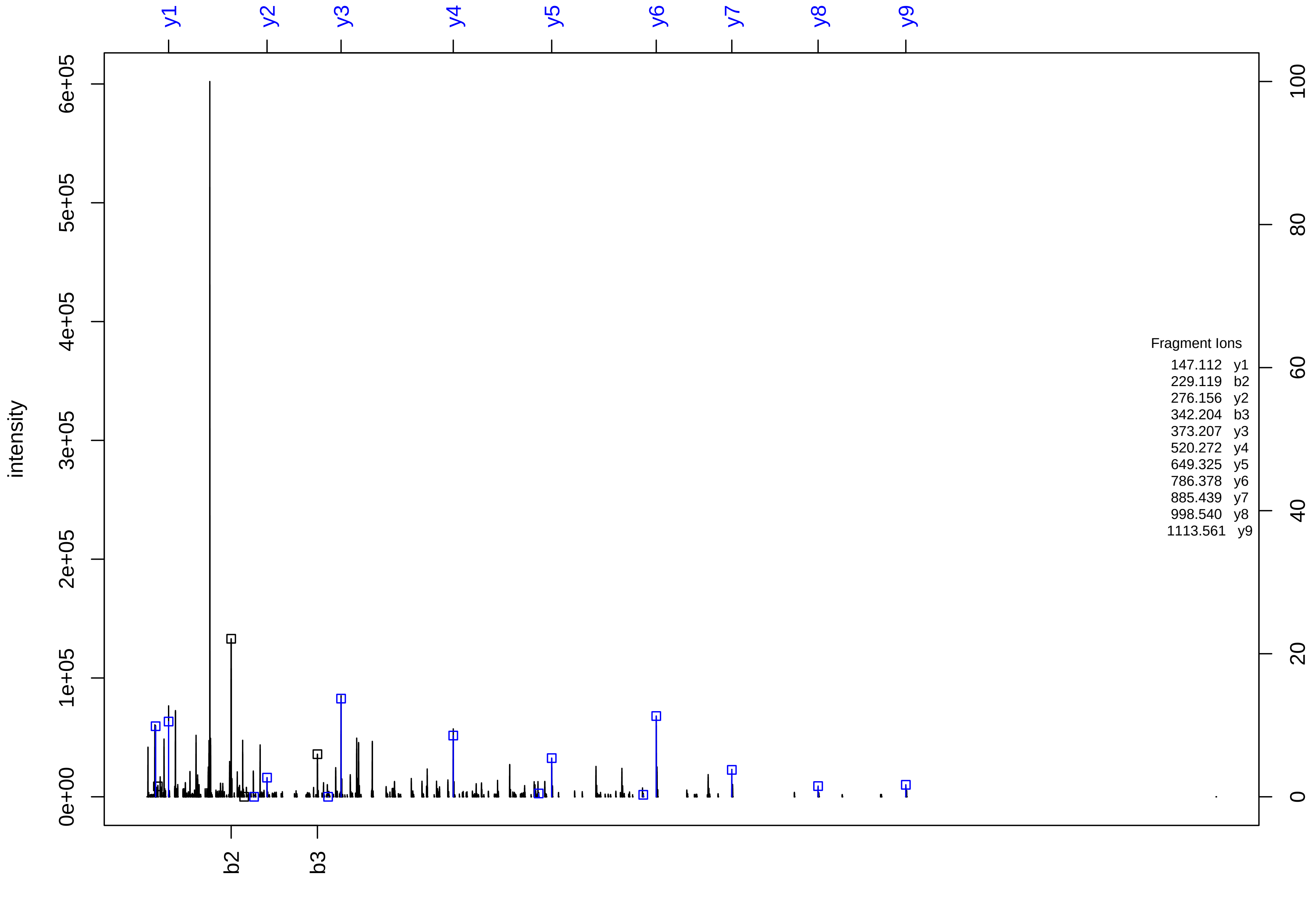

DILDLVHEFPEK / File: christine_10pools_05.raw; SpectrumID: ; scans: 26972

VDTYVQFPLR

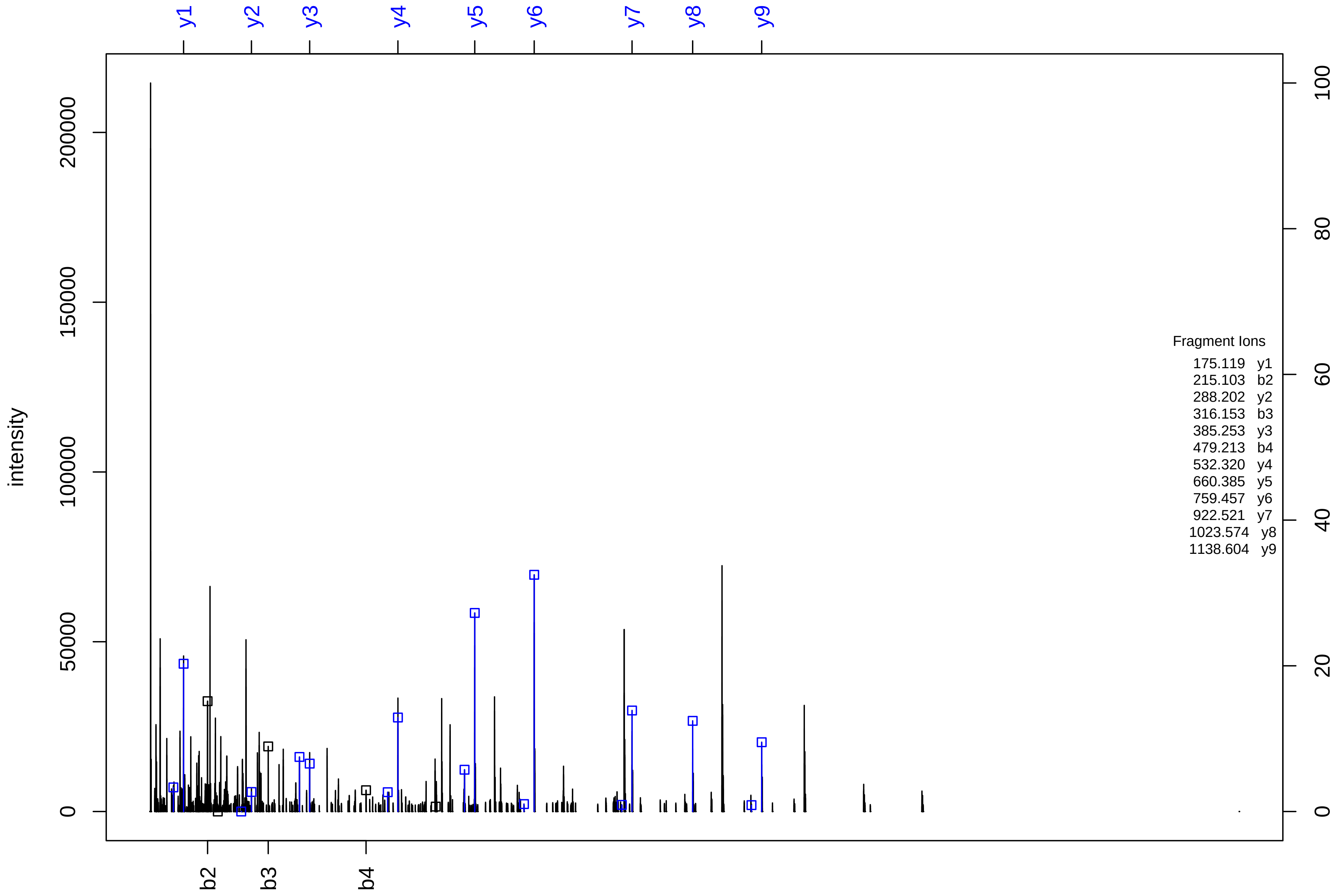

VDTYVQFPLR / File: christine_10pools_05.raw; SpectrumID: ; scans: 19165

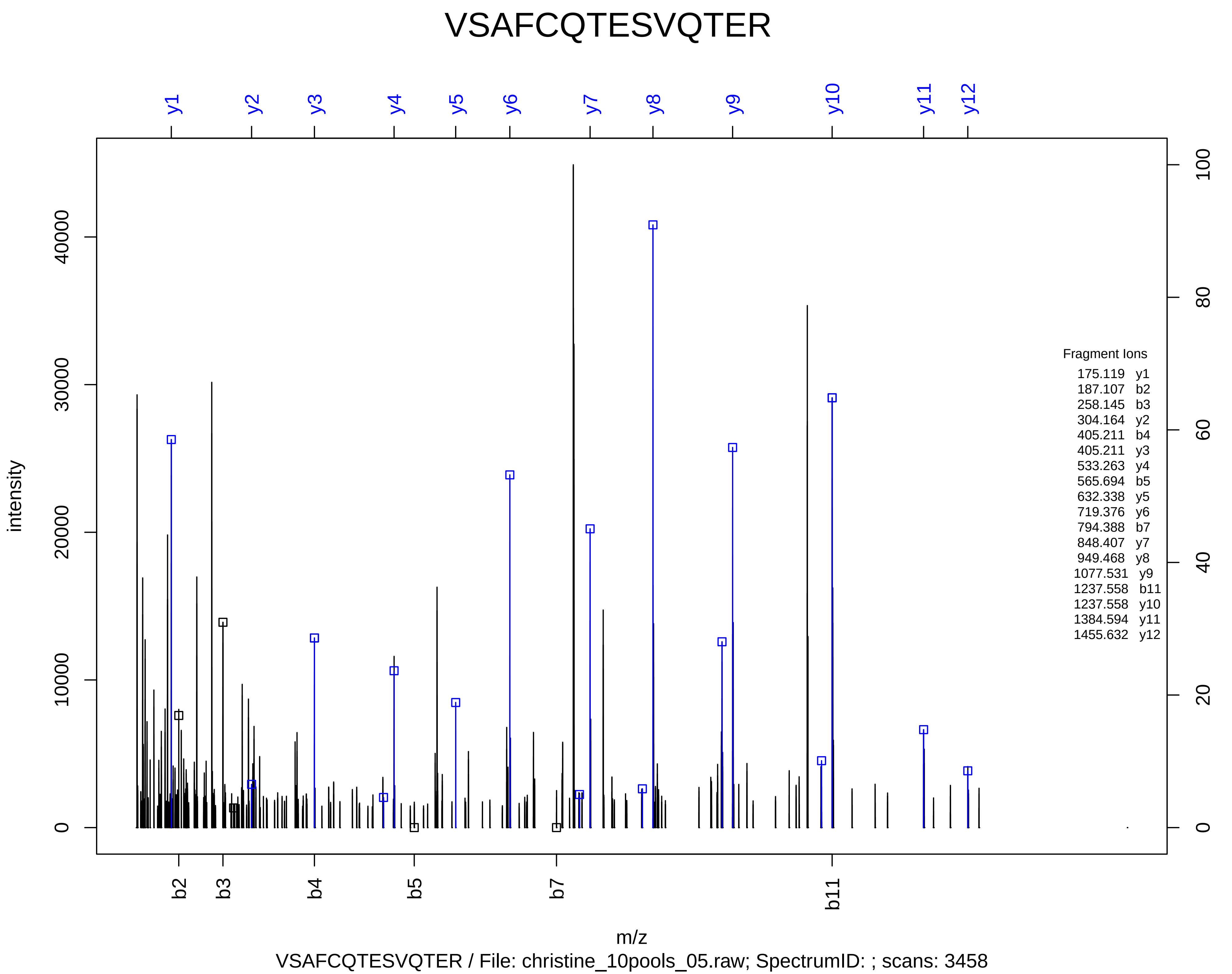
VSAFCQTESVQTER
y1
y2
y3
y4
y5
y6
y7
y8
y9
y10
y11
y12
intensity
0
10000
20000
30000
40000
0
20
40
60
80
100
Fragment Ions
175.119 y1
187.107 b2
258.145 b3
304.164 y2
405.211 b4
405.211 y3
533.263 y4
565.694 b5
632.338 y5
719.376 y6
794.388 b7
848.407 y7
949.468 y8
1077.531 y9
1237.558 b11
1237.558 y10
1384.594 y11
1455.632 y12
b2
b3
b4
b5
b7
b11
m/z
VSAFCQTESVQTER / File: christine_10pools_05.raw; SpectrumID: ; scans: 3458

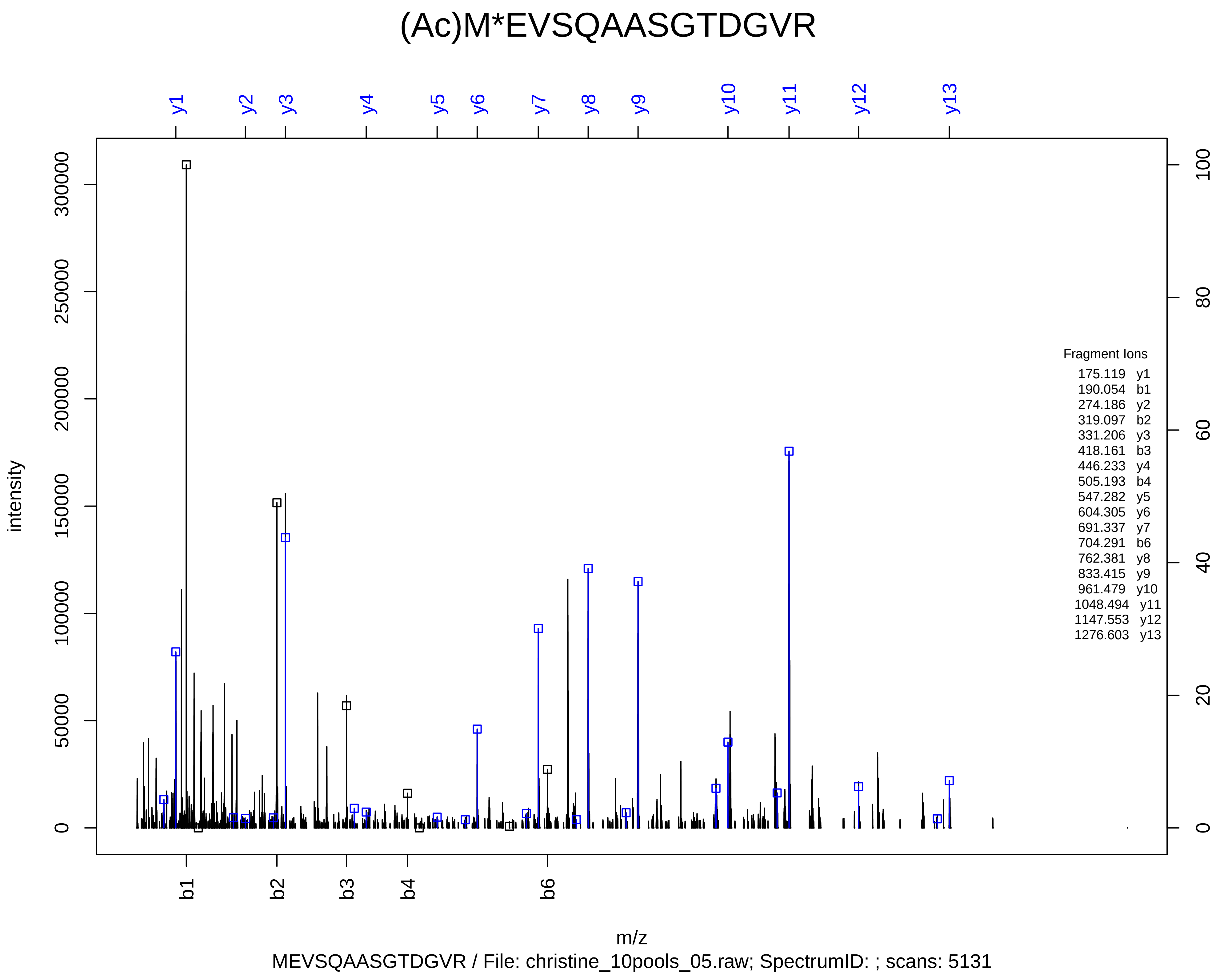

(Ac)M*EVSQAASGTDGVR
y1
y2
y3
y4
y5
y6
y7
y8
y9
y10
y11
y12
y13
intensity
0
50000
100000
150000
200000
250000
300000
0
20
40
60
80
100
Fragment Ions
175.119 y1
190.054 b1
274.186 y2
319.097 b2
331.206 y3
418.161 b3
446.233 y4
505.193 b4
547.282 y5
604.305 y6
691.337 y7
704.291 b6
762.381 y8
833.415 y9
961.479 y10
1048.494 y11
1147.553 y12
1276.603 y13
b1
b2
b3
b4
b6
m/z
MEVSQAASGTDGVR / File: christine_10pools_05.raw; SpectrumID: ; scans: 5131

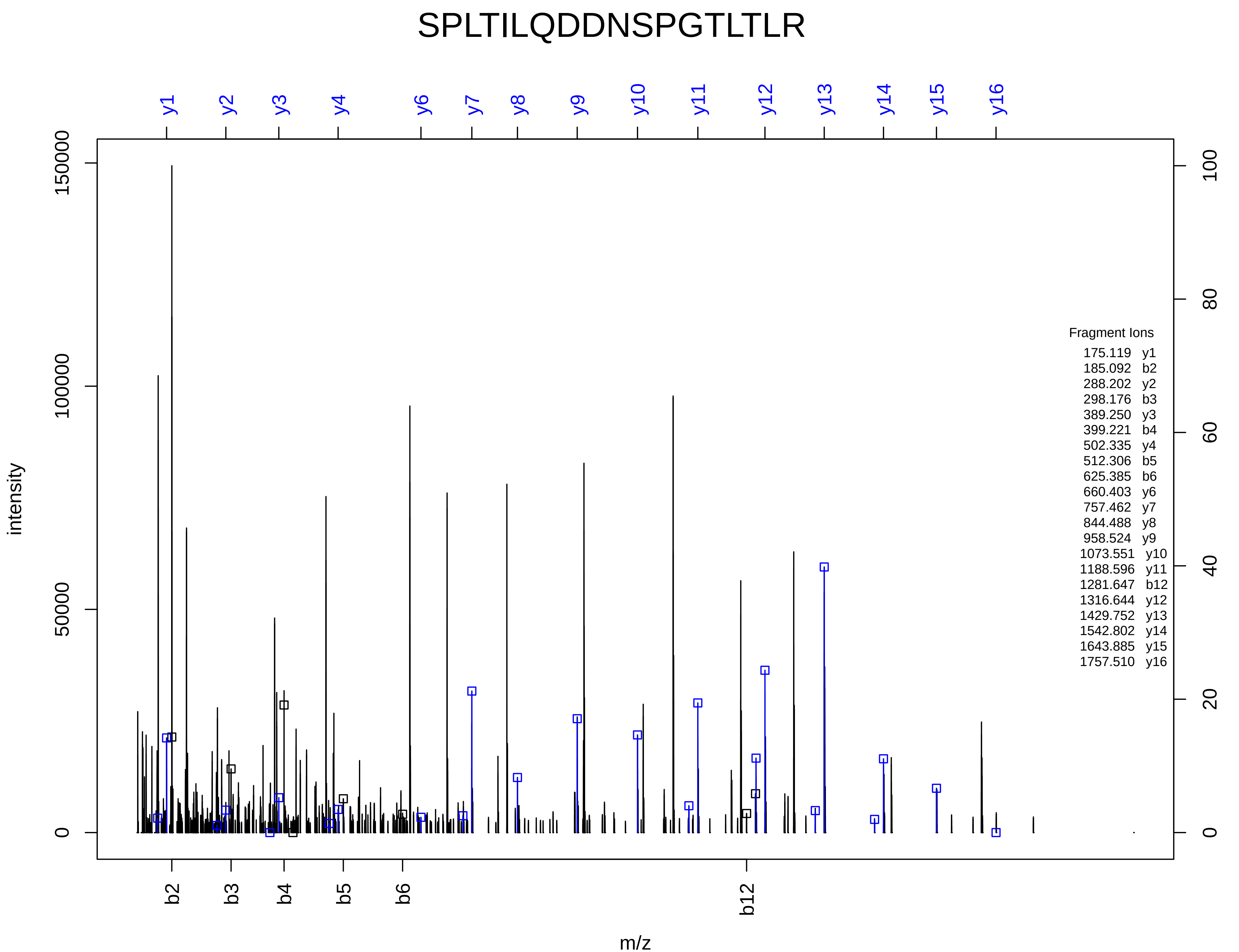

SPLTILQDDNSPGTLTLR / File: christine_10pools_05.raw; SpectrumID: ; scans: 24978

AVYAAYILGVLQEEEEEEK

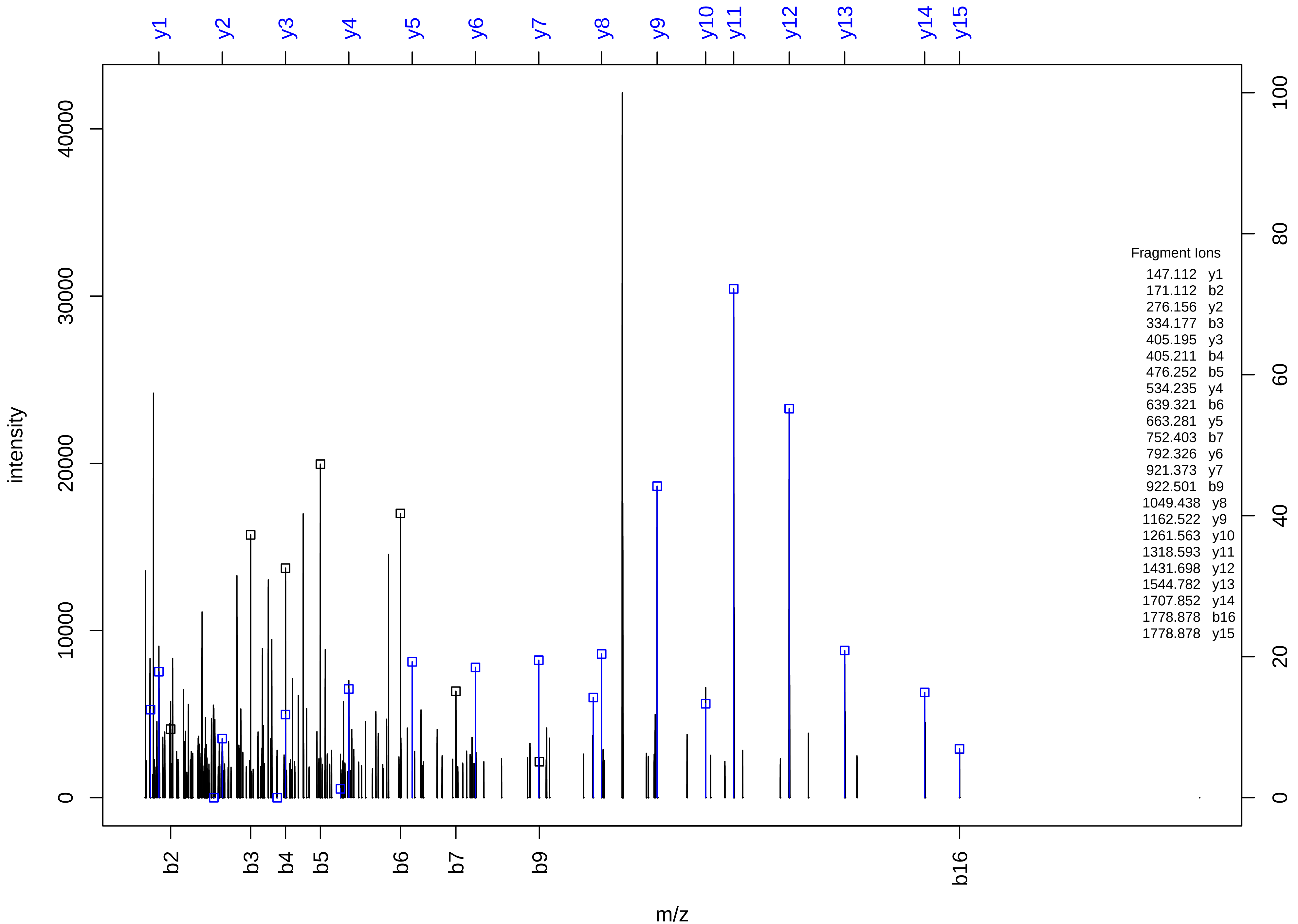

AVYAAYILGVLQEEEEEEK / File: christine_10pools_05.raw; SpectrumID: ; scans: 35845

TGLLGIFWK

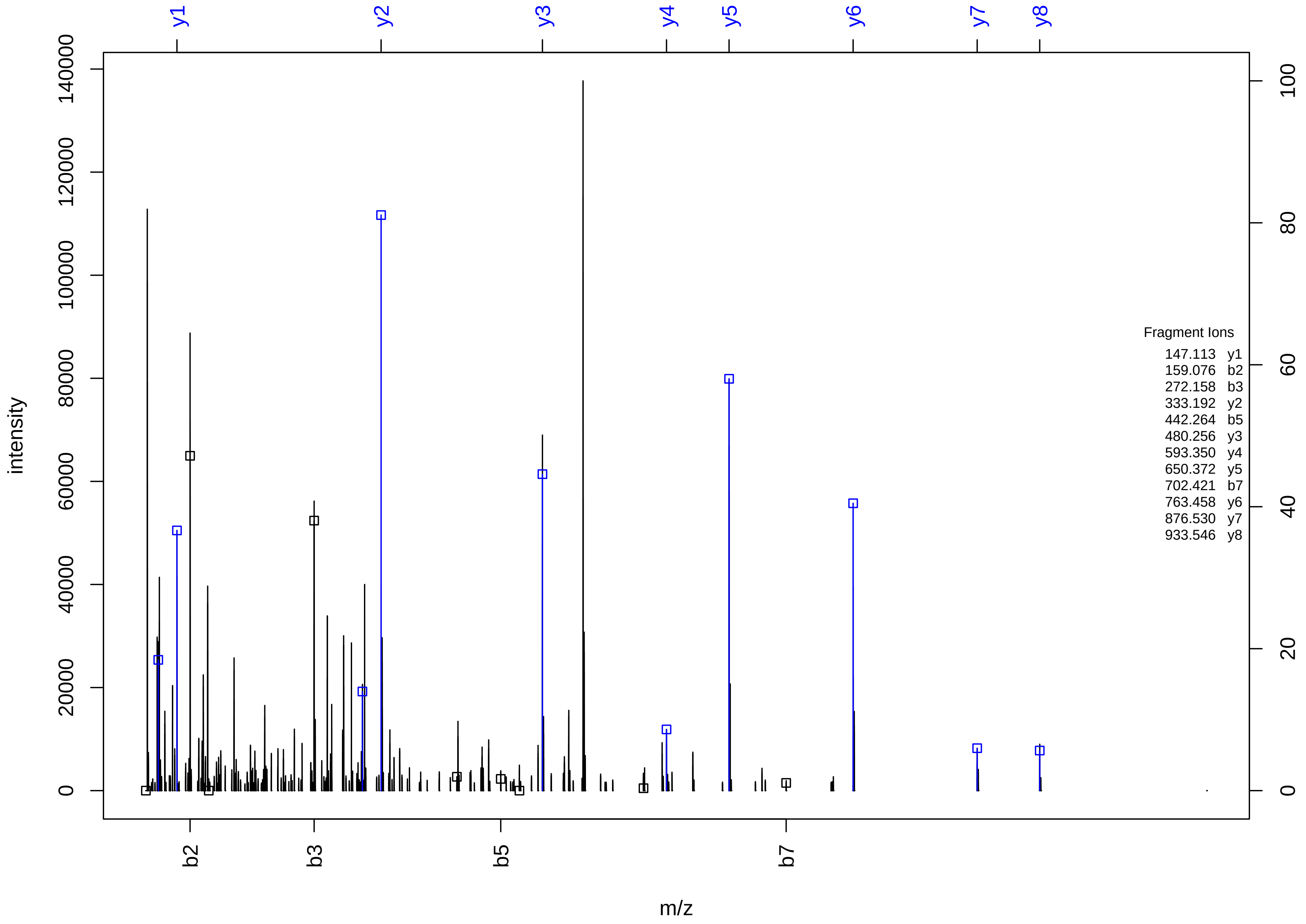

TGLLGIFWK / File: christine_10pools_05.raw; SpectrumID: ; scans: 32093

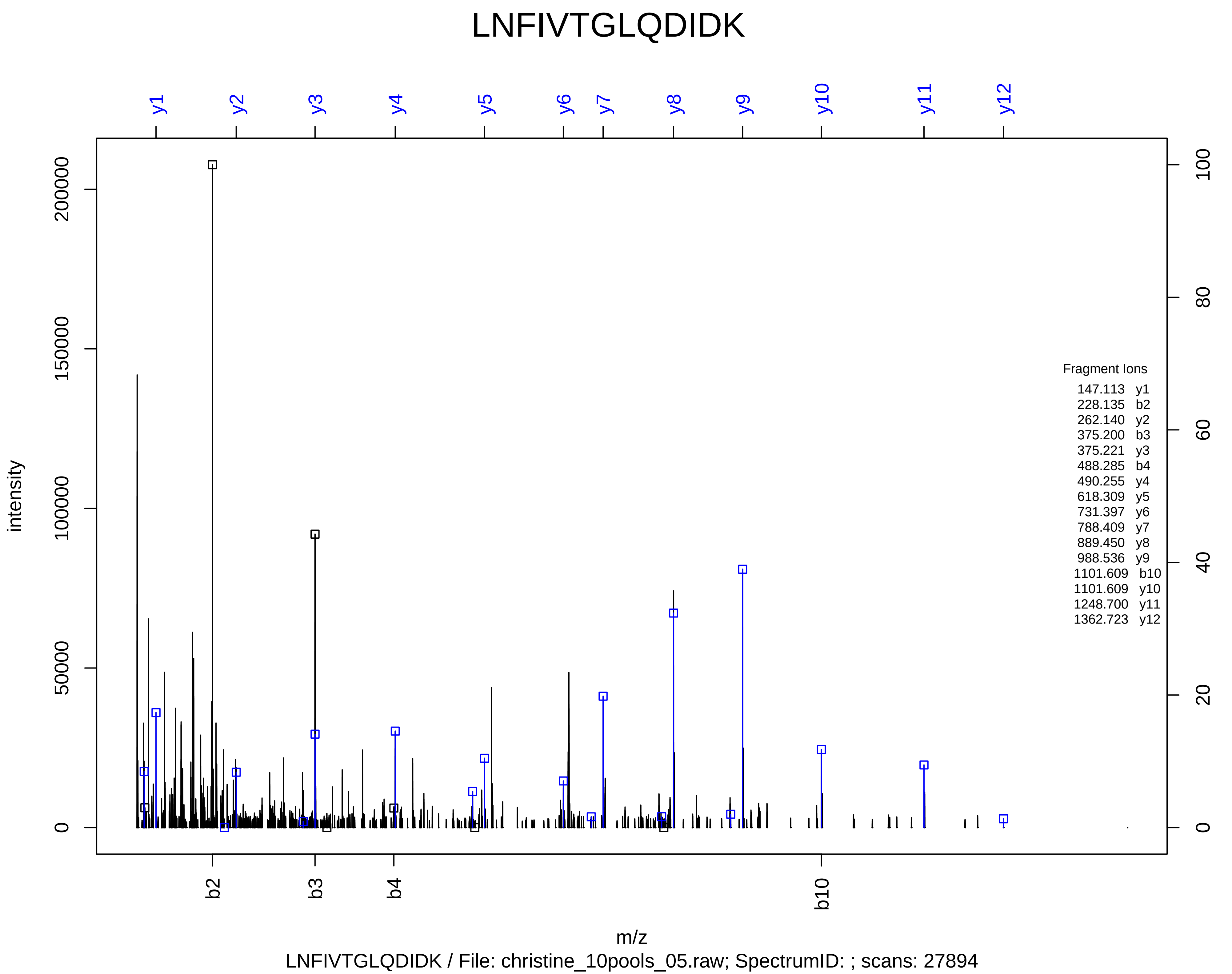

LNFIVTGLQDIDK
y1
y2
y3
y4
y5
y6
y7
y8
y9
y10
y11
y12
intensity
0
50000
100000
150000
200000
0
20
40
60
80
100
Fragment Ions
147.113 y1
228.135 b2
262.140 y2
375.200 b3
375.221 y3
488.285 b4
490.255 y4
618.309 y5
731.397 y6
788.409 y7
889.450 y8
988.536 y9
1101.609 b10
1101.609 y10
1248.700 y11
1362.723 y12
b2
b3
b4
b10
m/z
LNFIVTGLQDIDK / File: christine_10pools_05.raw; SpectrumID: ; scans: 27894

NAAPCAVSYLLFDQSDR

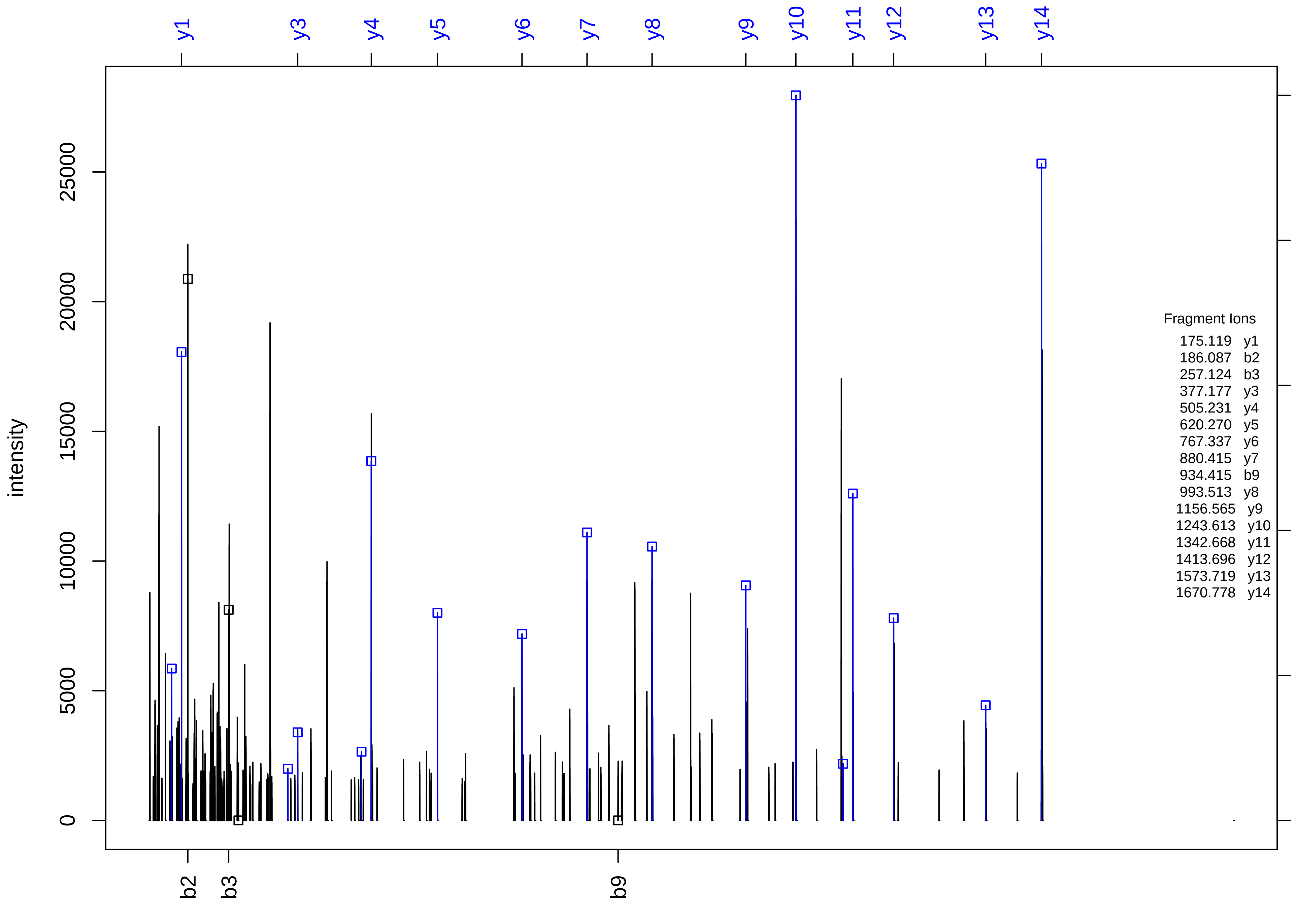

NAAPCAVSYLLFDQSDR / File: christine_10pools_05.raw; SpectrumID: ; scans: 25526

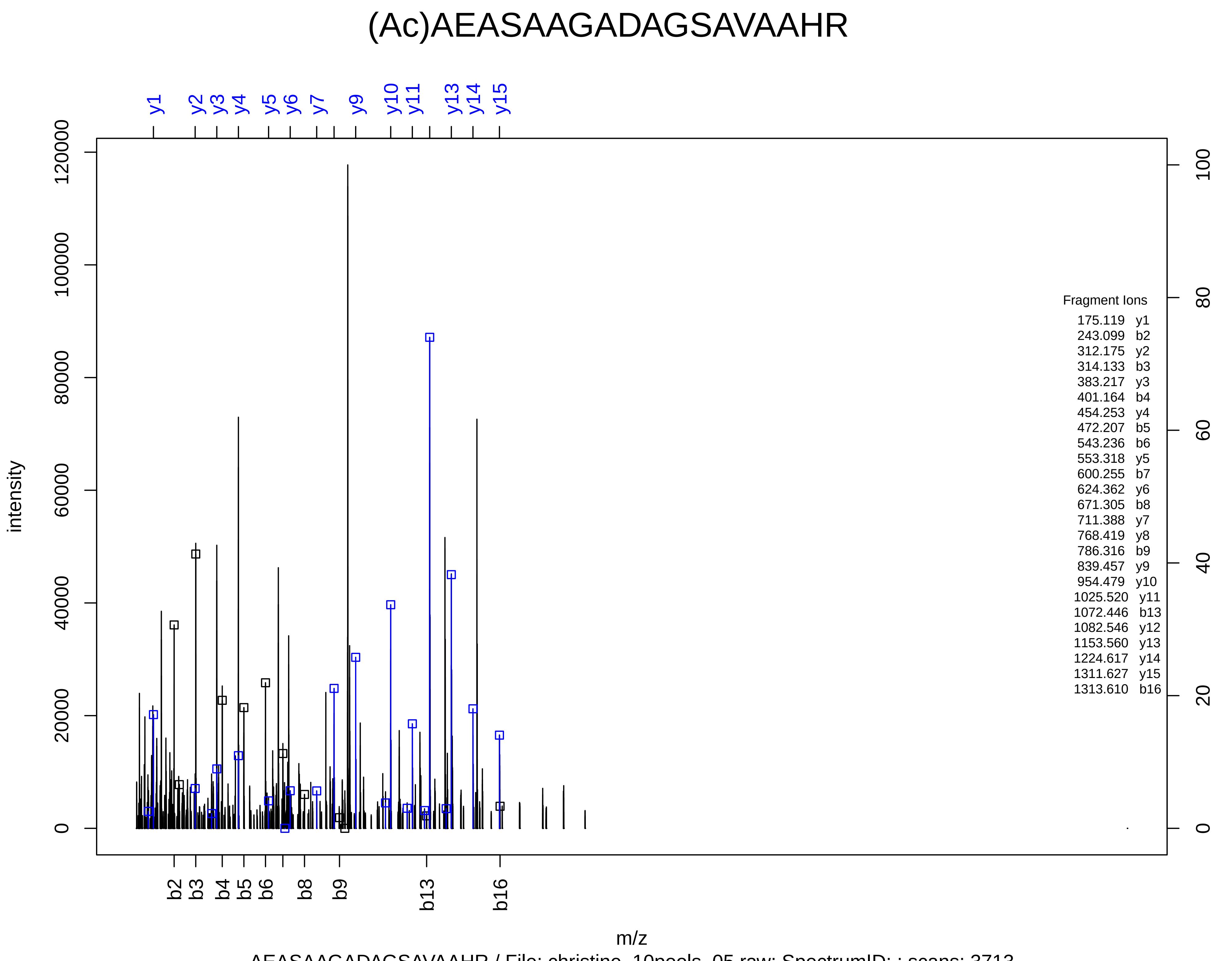

(Ac)AEASAAGADAGSAVAAHR
y1
y2
y3
y4
y5
y6
y7
y9
y10
y11
y13
y14
y15
intensity
0
20000
40000
60000
80000
100000
120000
0
20
40
60
80
100
b2
b3
b4
b5
b6
b8
b9
b13
b16
m/z
Fragment Ions
175.119 y1
243.099 b2
312.175 y2
314.133 b3
383.217 y3
401.164 b4
454.253 y4
472.207 b5
543.236 b6
553.318 y5
600.255 b7
624.362 y6
671.305 b8
711.388 y7
768.419 y8
786.316 b9
839.457 y9
954.479 y10
1025.520 y11
1072.446 b13
1082.546 y12
1153.560 y13
1224.617 y14
1311.627 y15
1313.610 b16
AEASAAGADAGSAVAAHR / File: christine_10pools_05.raw; SpectrumID: ; scans: 3713

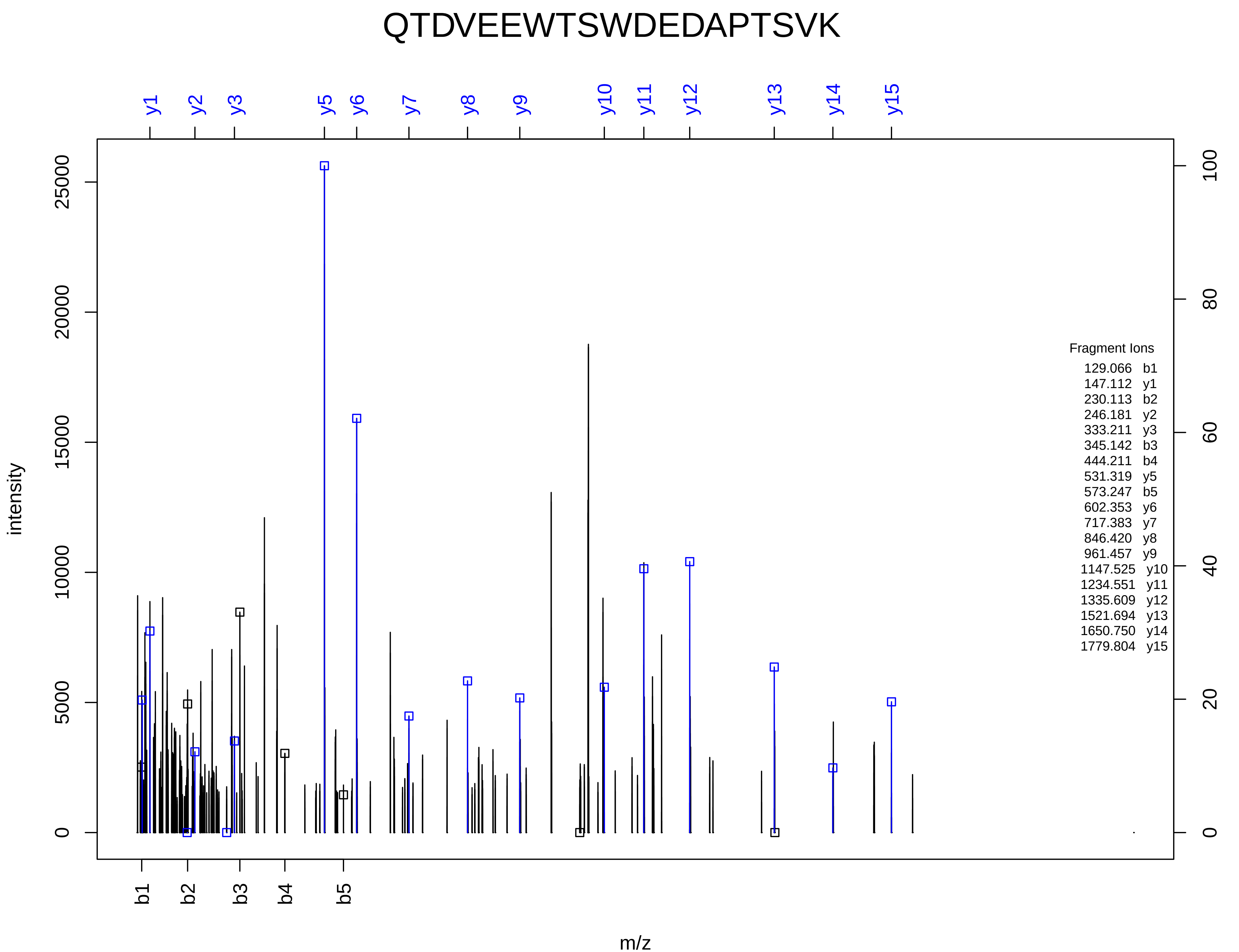

QTDVEEWTSWDEDAPTSVK / File: christine_10pools_05.raw; SpectrumID: ; scans: 21176

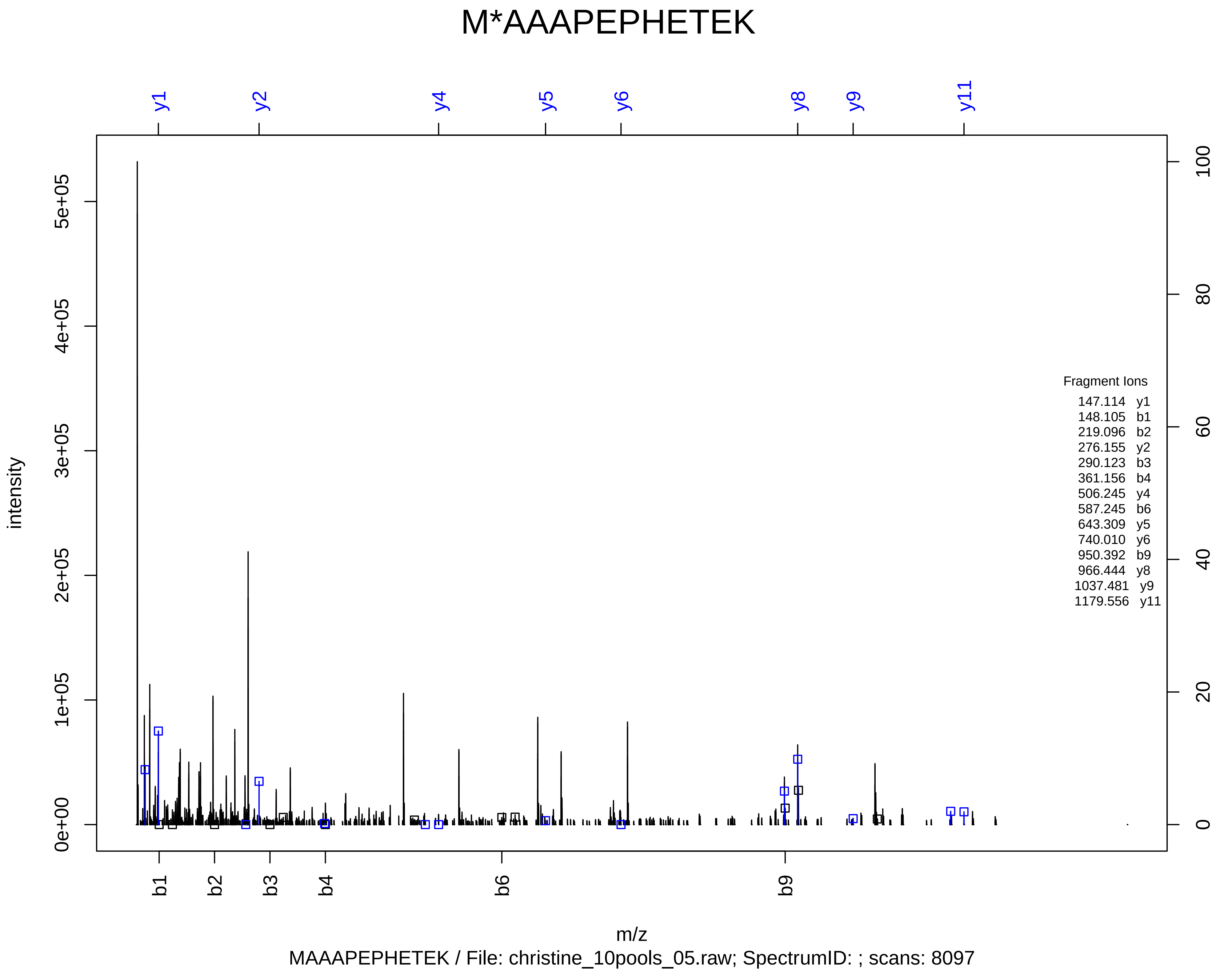
M*AAAPEPHETEK
y1
y2
y4
y5
y6
y8
y9
y11
intensity
0e+00
1e+05
2e+05
3e+05
4e+05
5e+05
0
20
40
60
80
100
Fragment Ions
147.114 y1
148.105 b1
219.096 b2
276.155 y2
290.123 b3
361.156 b4
506.245 y4
587.245 b6
643.309 y5
740.010 y6
950.392 b9
966.444 y8
1037.481 y9
1179.556 y11
b1
b2
b3
b4
b6
b9
m/z
MAAAPEPHETEK / File: christine_10pools_05.raw; SpectrumID: ; scans: 8097

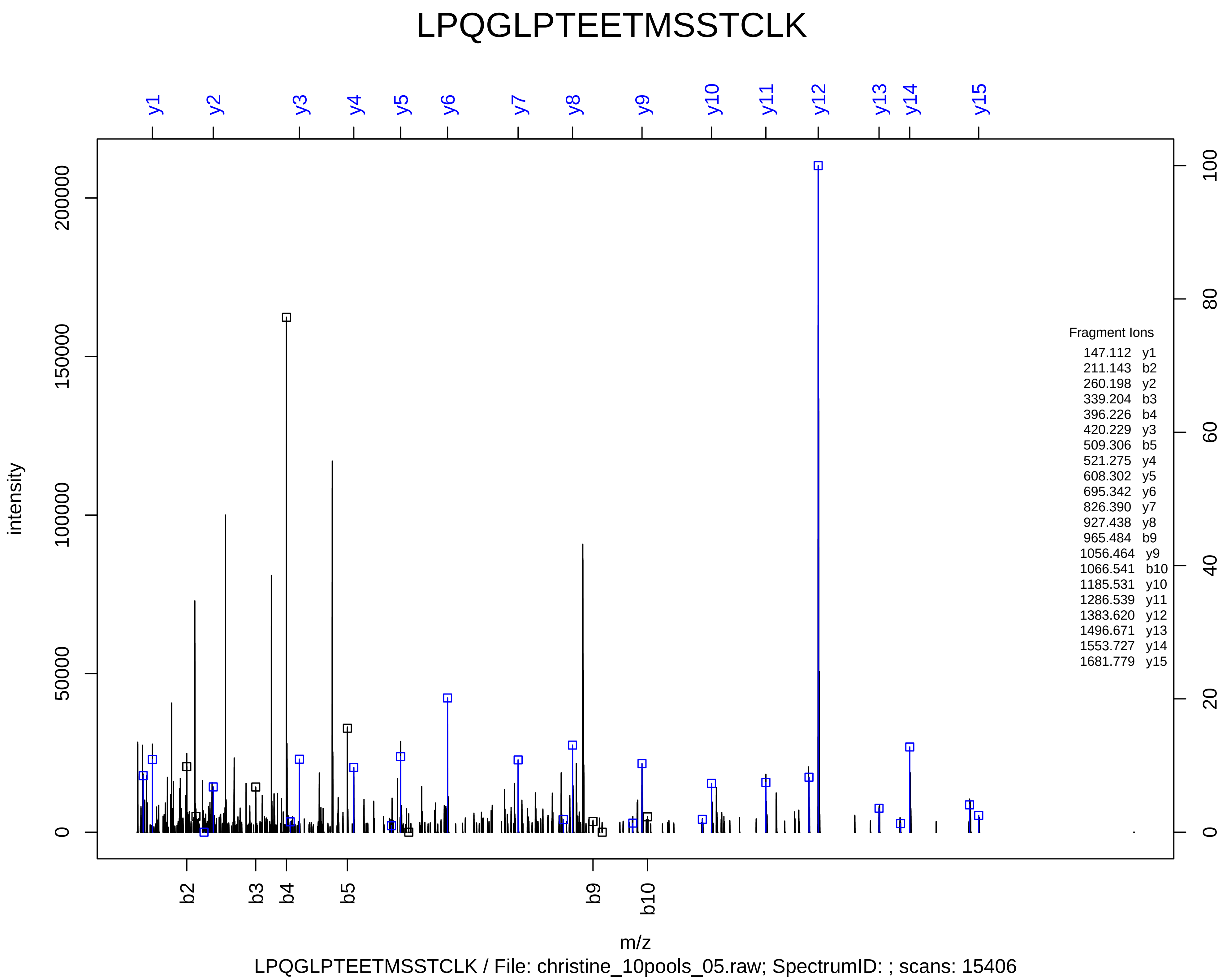
LPQGLPTEETMSSTCLK
y1
y2
y3
y4
y5
y6
y7
y8
y9
y10
y11
y12
y13
y14
y15
intensity
0
50000
100000
150000
200000
0
20
40
60
80
100
Fragment Ions
147.112 y1
211.143 b2
260.198 y2
339.204 b3
396.226 b4
420.229 y3
509.306 b5
521.275 y4
608.302 y5
695.342 y6
826.390 y7
927.438 y8
965.484 b9
1056.464 y9
1066.541 b10
1185.531 y10
1286.539 y11
1383.620 y12
1496.671 y13
1553.727 y14
1681.779 y15
b2
b3
b4
b5
b9
b10
m/z
LPQGLPTEETMSSTCLK / File: christine_10pools_05.raw; SpectrumID: ; scans: 15406

SGSPGTPAYPLSDLLFQSYQER

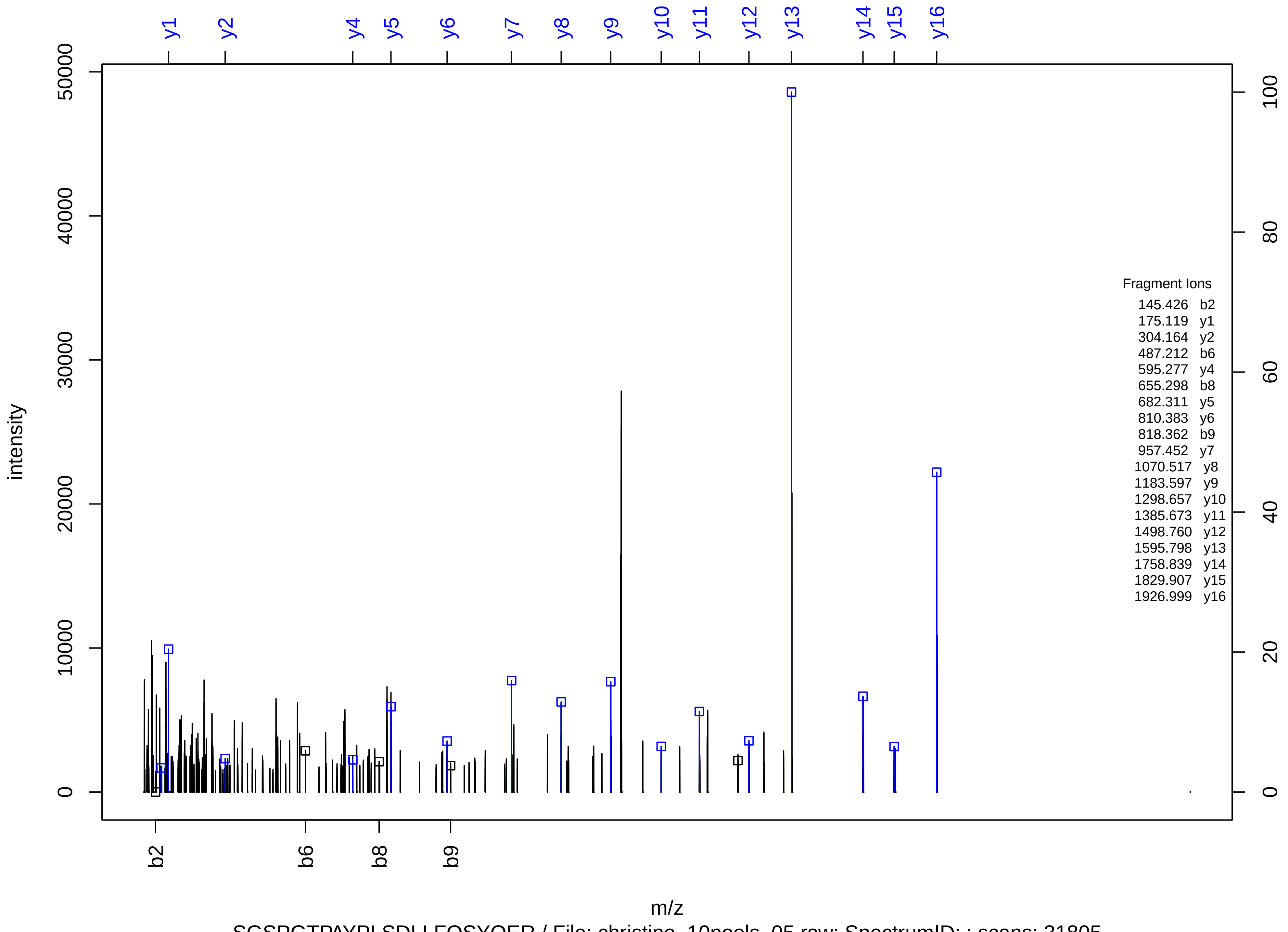

SGSPGTPAYPLSDLLFQSYQER / File: christine_10pools_05.raw; SpectrumID: ; scans: 31805

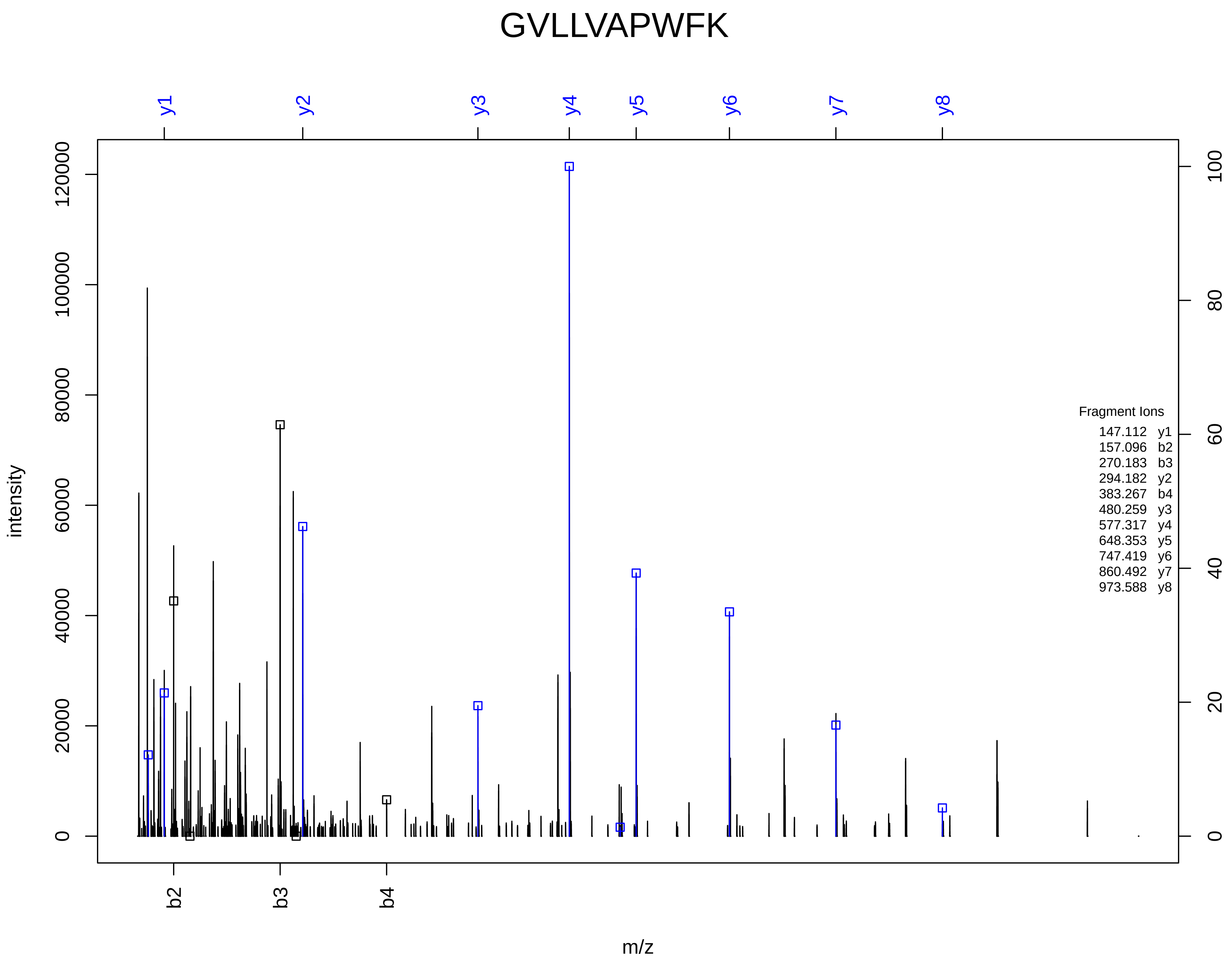

GVLLVAPWFK / File: christine_10pools_05.raw; SpectrumID: ; scans: 30984

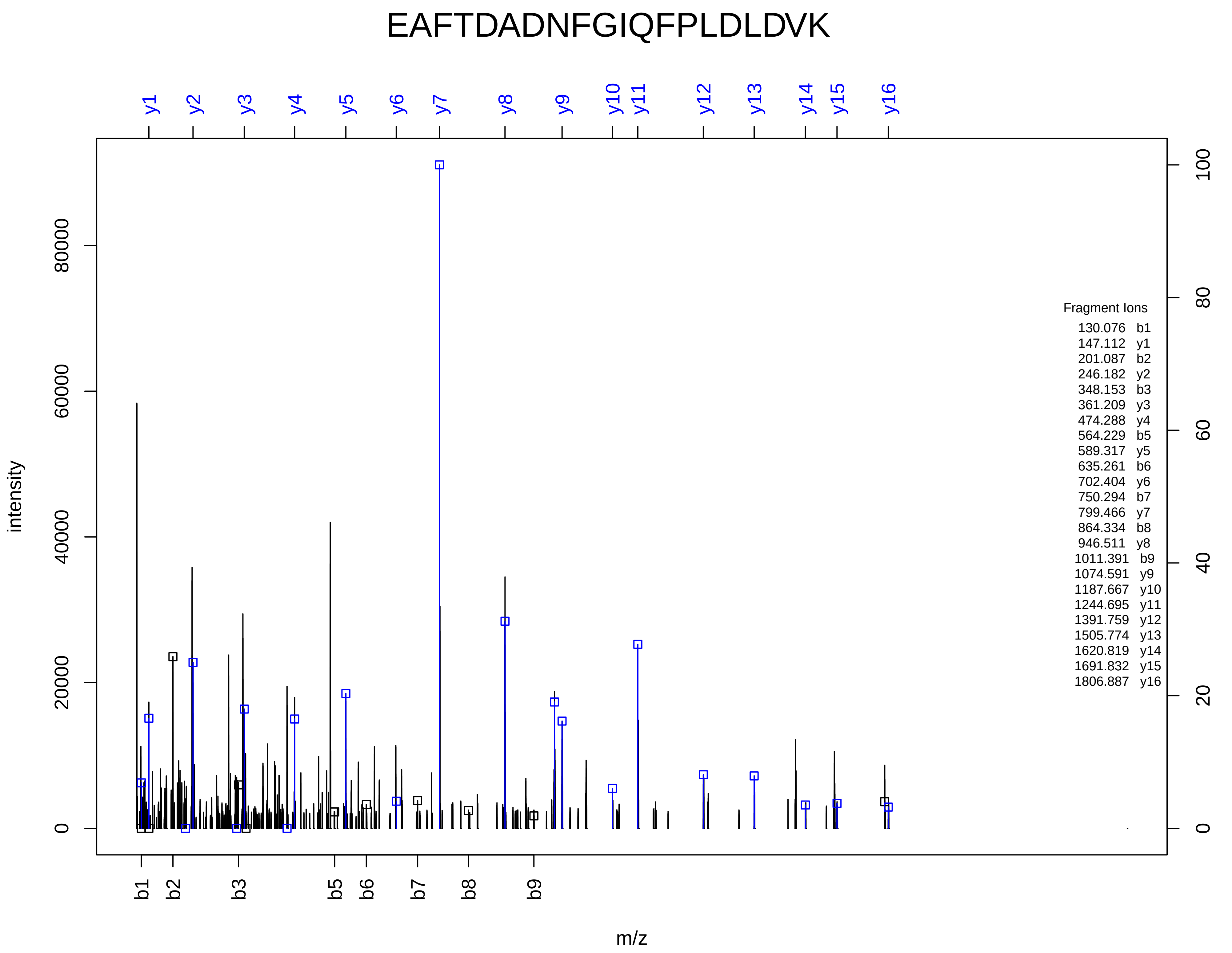

EAFTDADNFGIQFPLDLDVK / File: christine_10pools_05.raw; SpectrumID: ; scans: 38447

DFIPGVAIAANIIQEGFHK

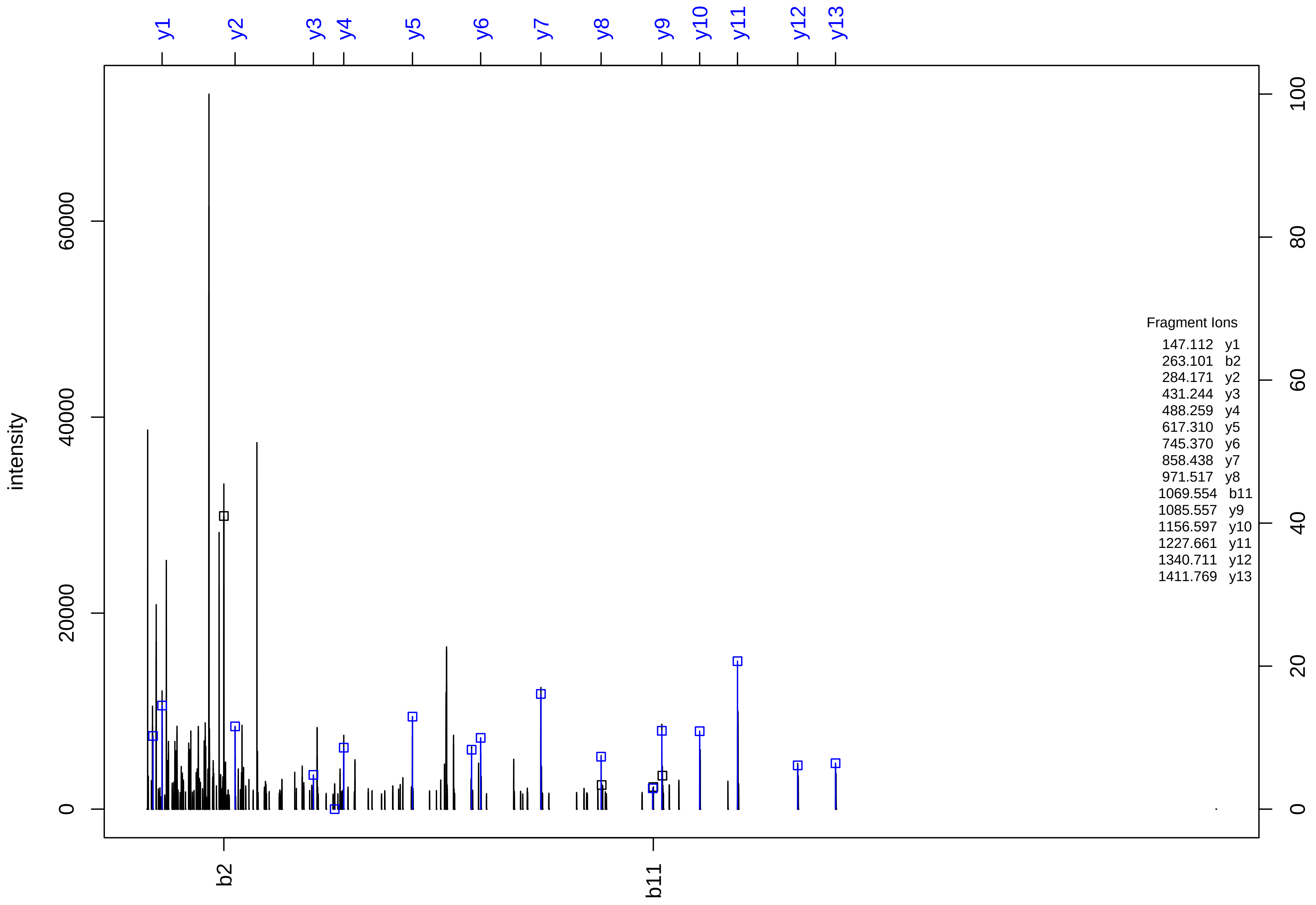

DFIPGVAIAANIIQEGFHK / File: christine_10pools_05.raw; SpectrumID: ; scans: 40066

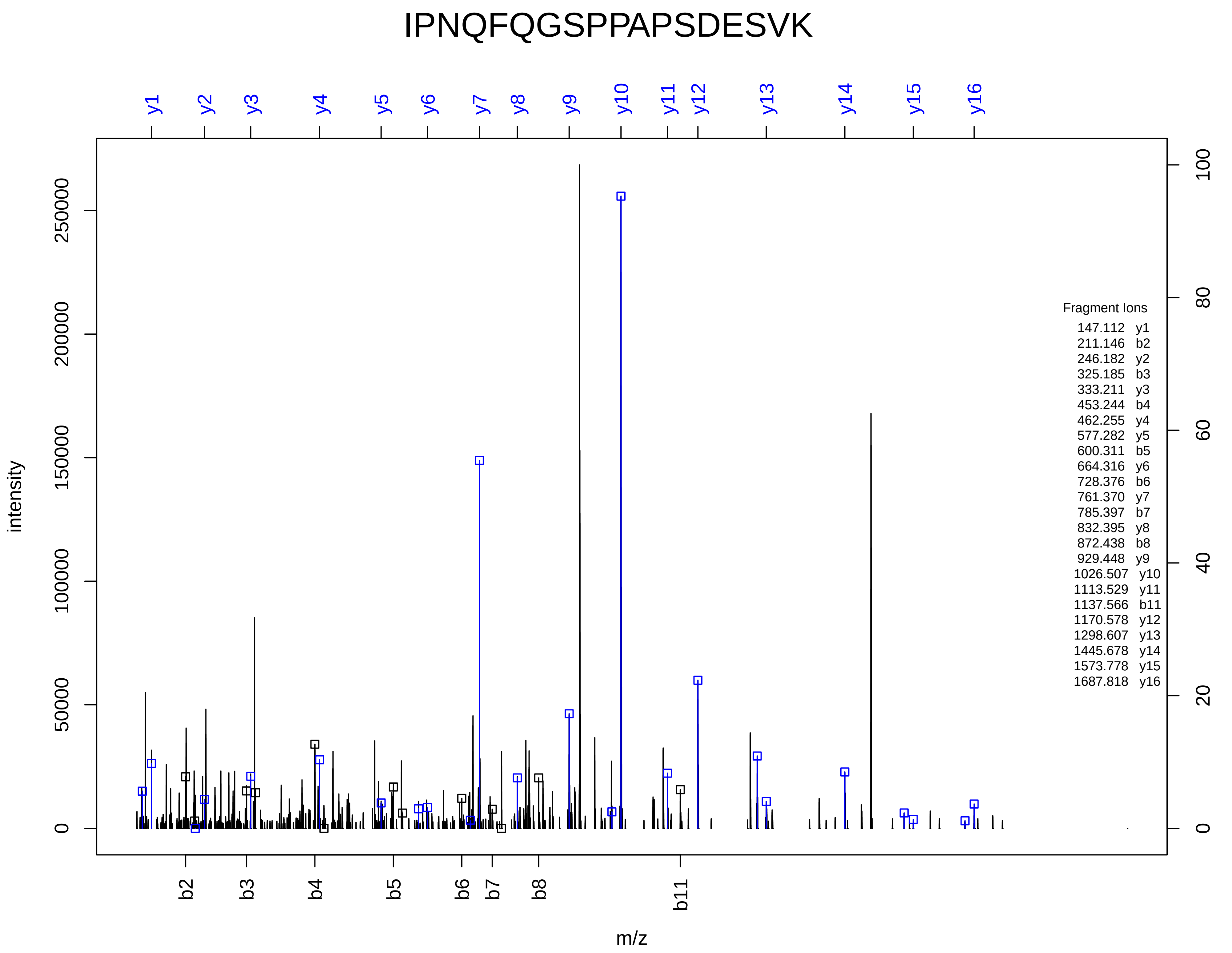
IPNQFQGSPPAPSDESVK
intensity
m/z
y1 y2 y3 y4 y5 y6 y7 y8 y9 y10 y11 y12 y13 y14 y15 y16
b2 b3 b4 b5 b6 b7 b8 b11
0 50000 100000 150000 200000 250000
0 20 40 60 80 100
Fragment Ions
147.112 y1
211.146 b2
246.182 y2
325.185 b3
333.211 y3
453.244 b4
462.255 y4
577.282 y5
600.311 b5
664.316 y6
728.376 b6
761.370 y7
785.397 b7
832.395 y8
872.438 b8
929.448 y9
1026.507 y10
1113.529 y11
1137.566 b11
1170.578 y12
1298.607 y13
1445.678 y14
1573.778 y15
1687.818 y16
IPNQFQGSPPAPSDESVK / File: christine_10pools_05.raw; SpectrumID: ; scans: 6978

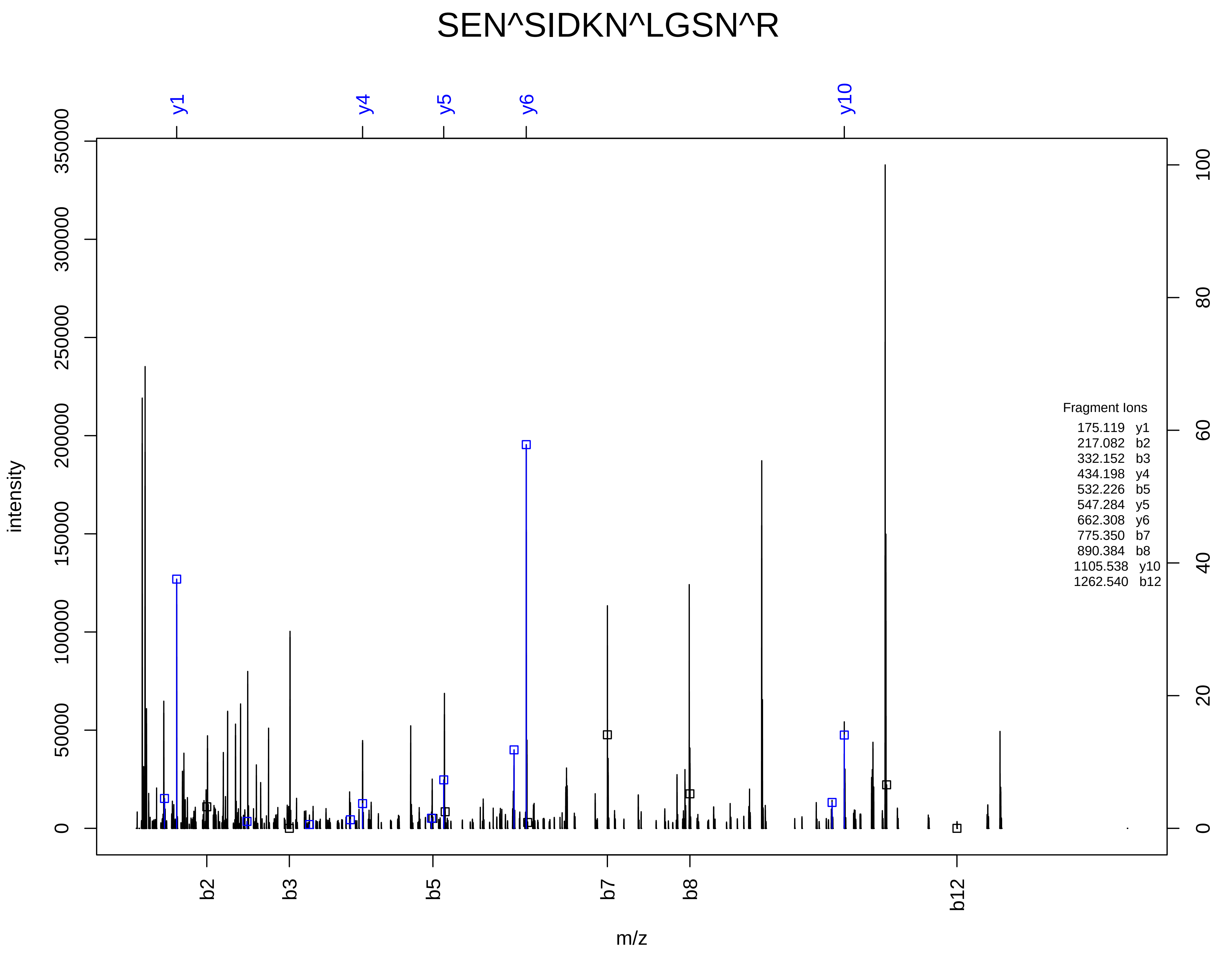
SEN^SIDKN^LGSN^R
y1
y4
y5
y6
y10
intensity
0
50000
100000
150000
200000
250000
300000
350000
0
20
40
60
80
100
b2
b3
b5
b7
b8
b12
m/z
Fragment Ions
175.119 y1
217.082 b2
332.152 b3
434.198 y4
532.226 b5
547.284 y5
662.308 y6
775.350 b7
890.384 b8
1105.538 y10
1262.540 b12
SENSIDKNLGSNR / File: christine_10pools_05.raw; SpectrumID: ; scans: 2596

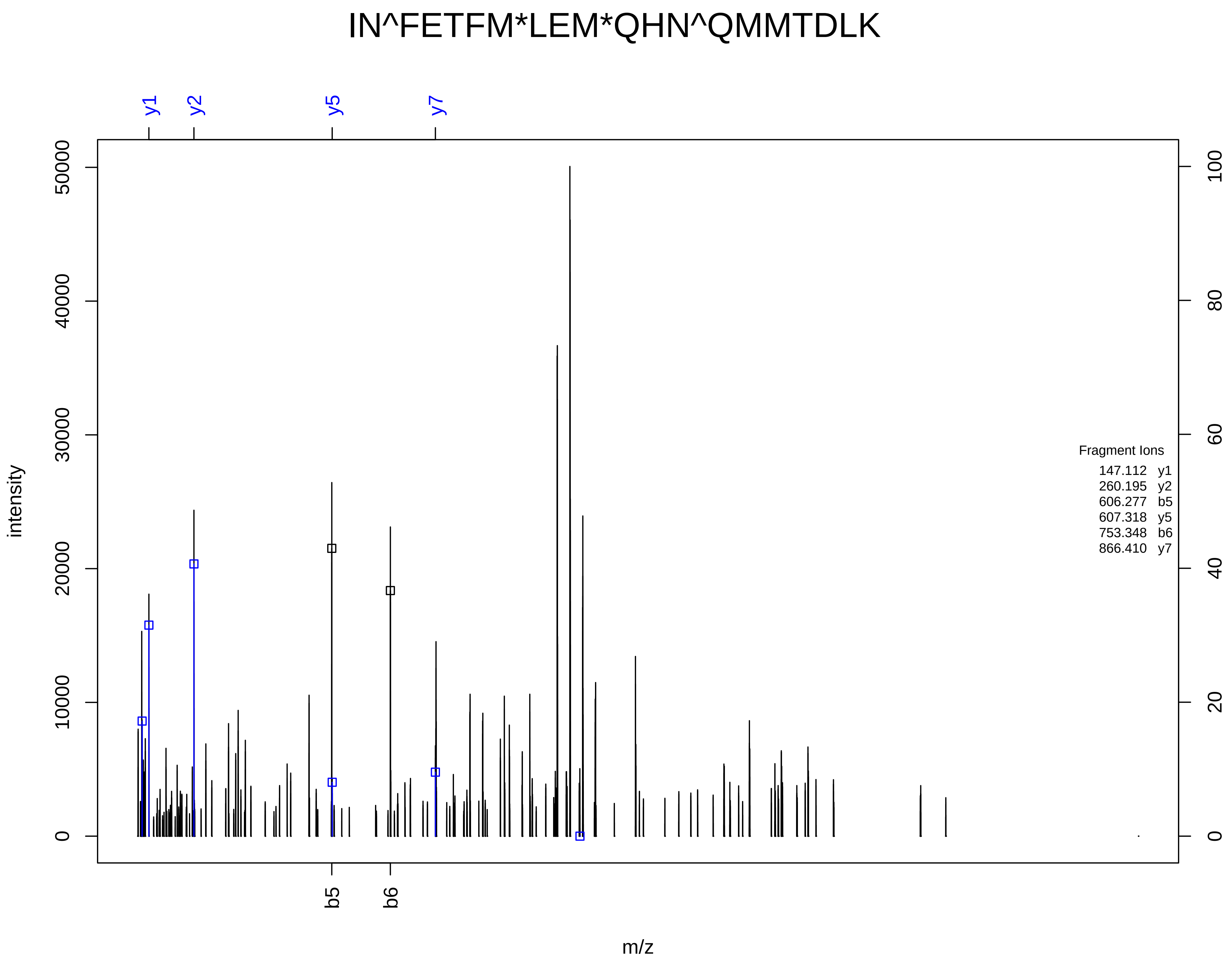
IN^FETFM*LEM*QHN^QMMTDLK
y1
y2
y5
y7
b5
b6
intensity
m/z
Fragment Ions
147.112 y1
260.195 y2
606.277 b5
607.318 y5
753.348 b6
866.410 y7
INFETFMLEMQHNQMMTDLK / File: christine_10pools_05.raw; SpectrumID: ; scans: 25165

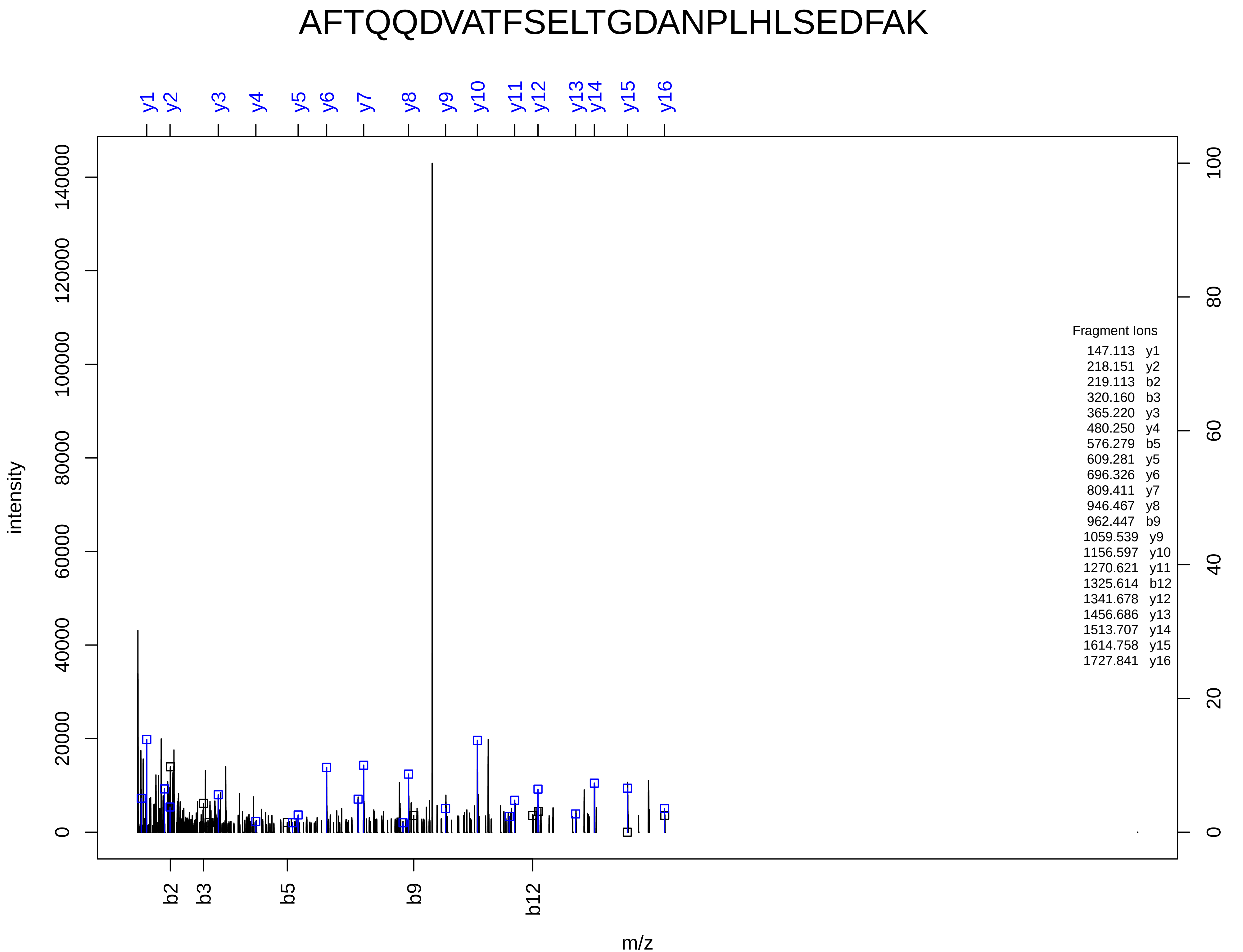
AFTQQDVATFSELTGDANPLHLSEDFAK
y1
y2
y3
y4
y5
y6
y7
y8
y9
y10
y11
y12
y13
y14
y15
y16
intensity
0
20000
40000
60000
80000
100000
120000
140000
0
20
40
60
80
100
b2
b3
b5
b9
b12
m/z
Fragment Ions
147.113 y1
218.151 y2
219.113 b2
320.160 b3
365.220 y3
480.250 y4
576.279 b5
609.281 y5
696.326 y6
809.411 y7
946.467 y8
962.447 b9
1059.539 y9
1156.597 y10
1270.621 y11
1325.614 b12
1341.678 y12
1456.686 y13
1513.707 y14
1614.758 y15
1727.841 y16
AFTQQDVATFSELTGDANPLHLSEDFAK / File: christine_10pools_05.raw; SpectrumID: ; scans: 32428

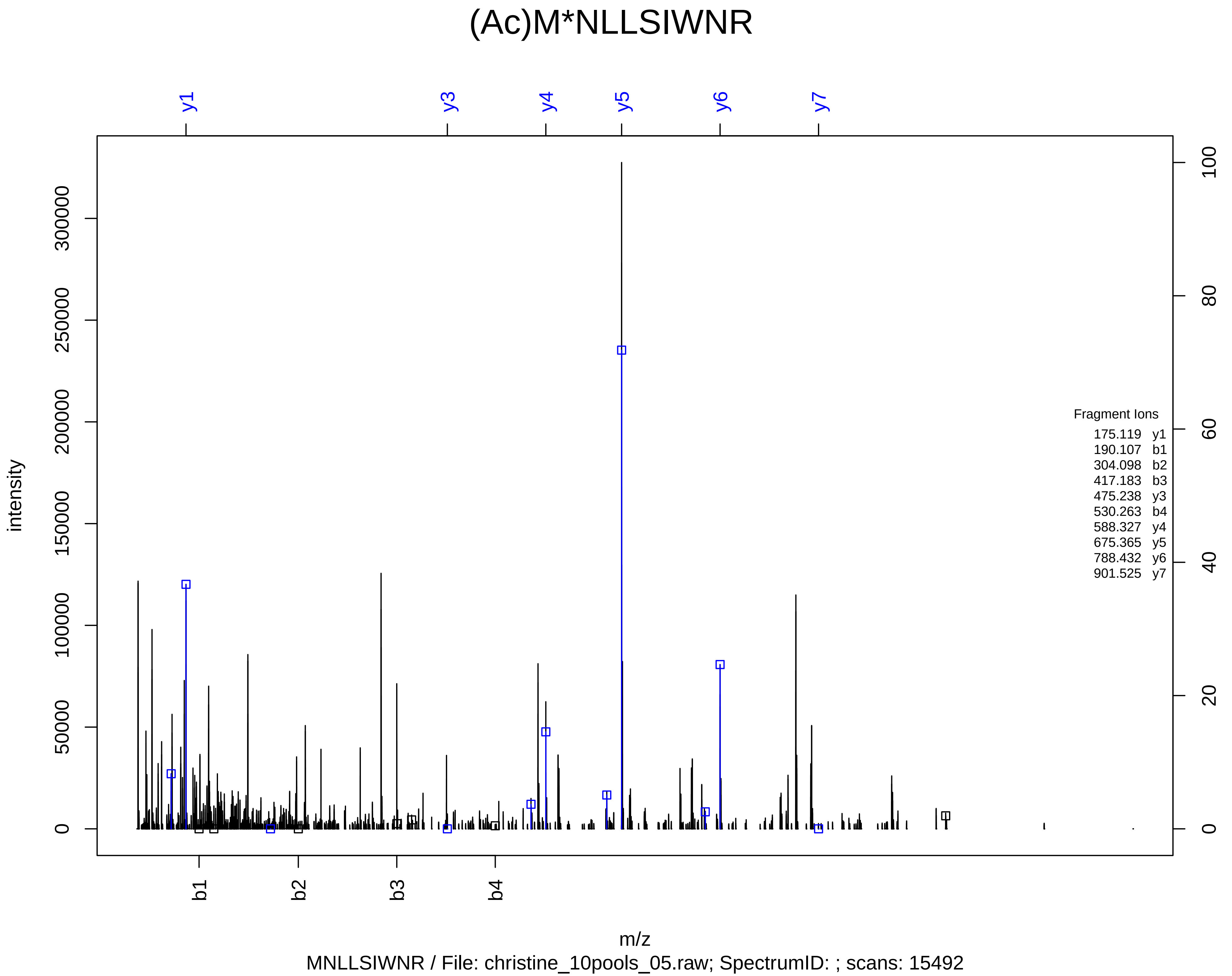

(Ac)M*NLLSIWNR
y1
y3
y4
y5
y6
y7
intensity
0
50000
100000
150000
200000
250000
300000
0
20
40
60
80
100
Fragment Ions
175.119 y1
190.107 b1
304.098 b2
417.183 b3
475.238 y3
530.263 b4
588.327 y4
675.365 y5
788.432 y6
901.525 y7
b1
b2
b3
b4
m/z
MNLLSIWNR / File: christine_10pools_05.raw; SpectrumID: ; scans: 15492

LALPELR

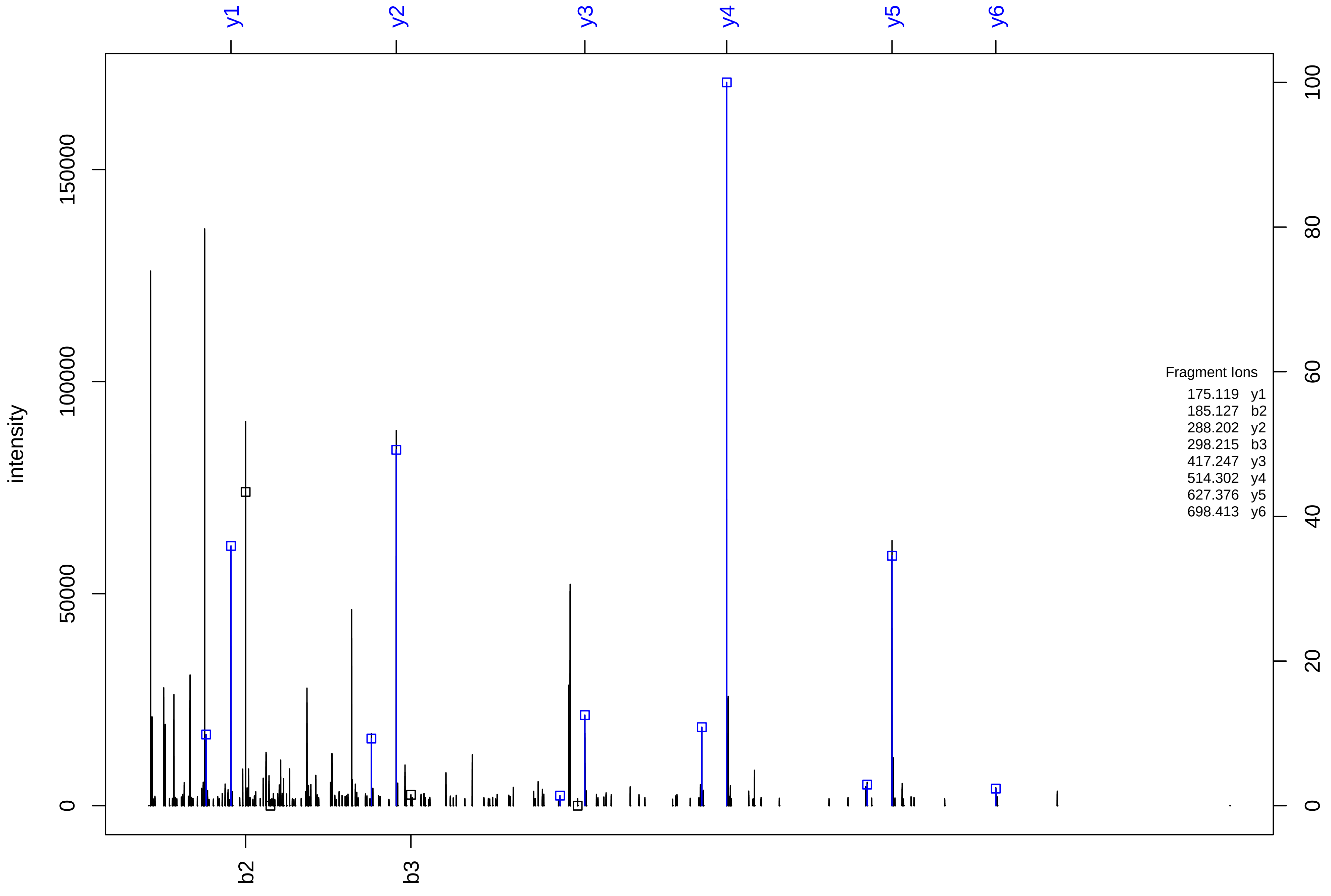

LALPELR / File: christine_10pools_05.raw; SpectrumID: ; scans: 14607

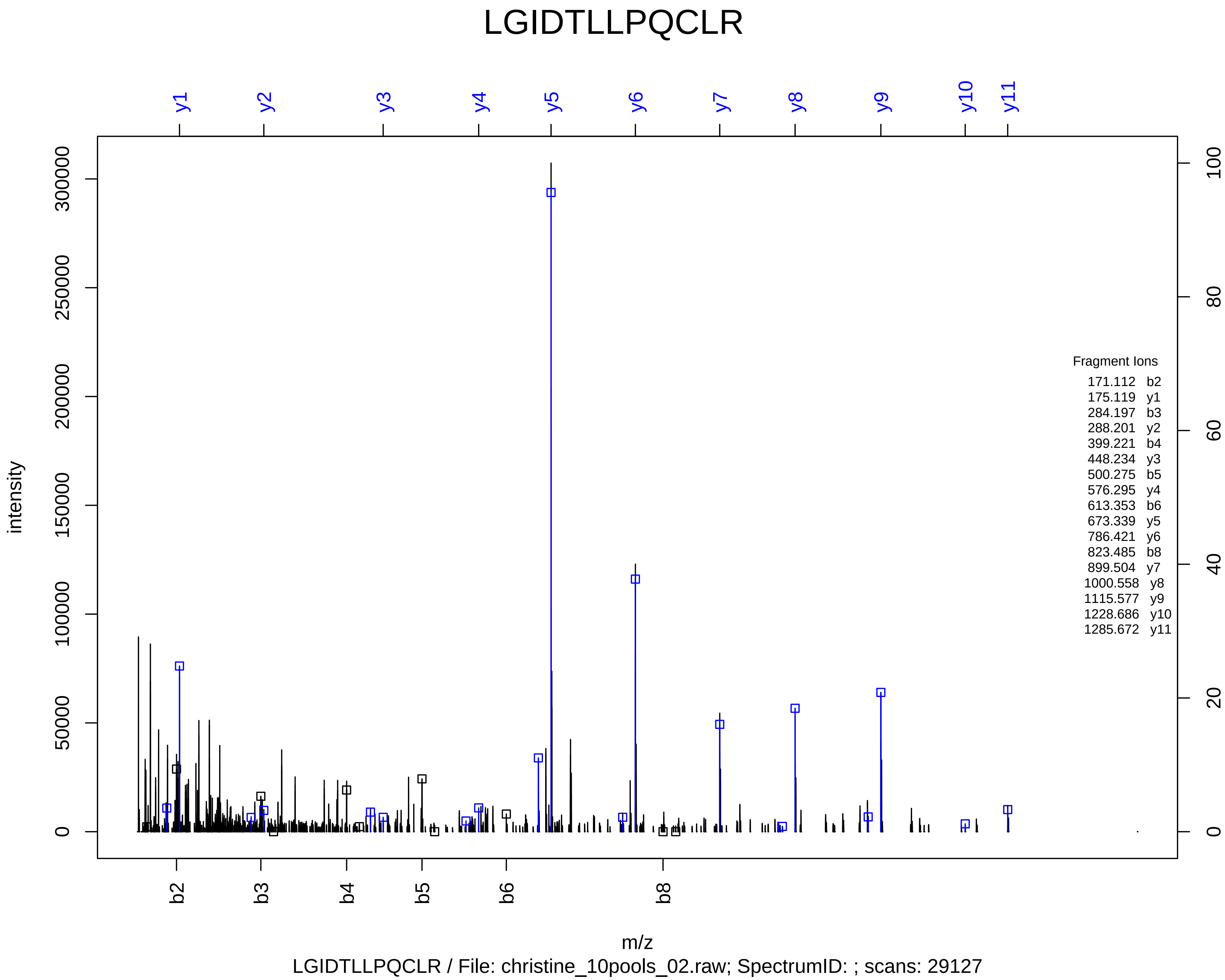
LGIDTLLPQCLR
y1
y2
y3
y4
y5
y6
y7
y8
y9
y10
y11
intensity
300000
250000
200000
150000
100000
50000
0
100
80
60
40
20
0
Fragment Ions
171.112 b2
175.119 y1
284.197 b3
288.201 y2
399.221 b4
448.234 y3
500.275 b5
576.295 y4
613.353 b6
673.339 y5
786.421 y6
823.485 b8
899.504 y7
1000.558 y8
1115.577 y9
1228.686 y10
1285.672 y11
b2
b3
b4
b5
b6
b8
m/z
LGIDTLLPQCLR / File: christine_10pools_02.raw; SpectrumID: ; scans: 29127

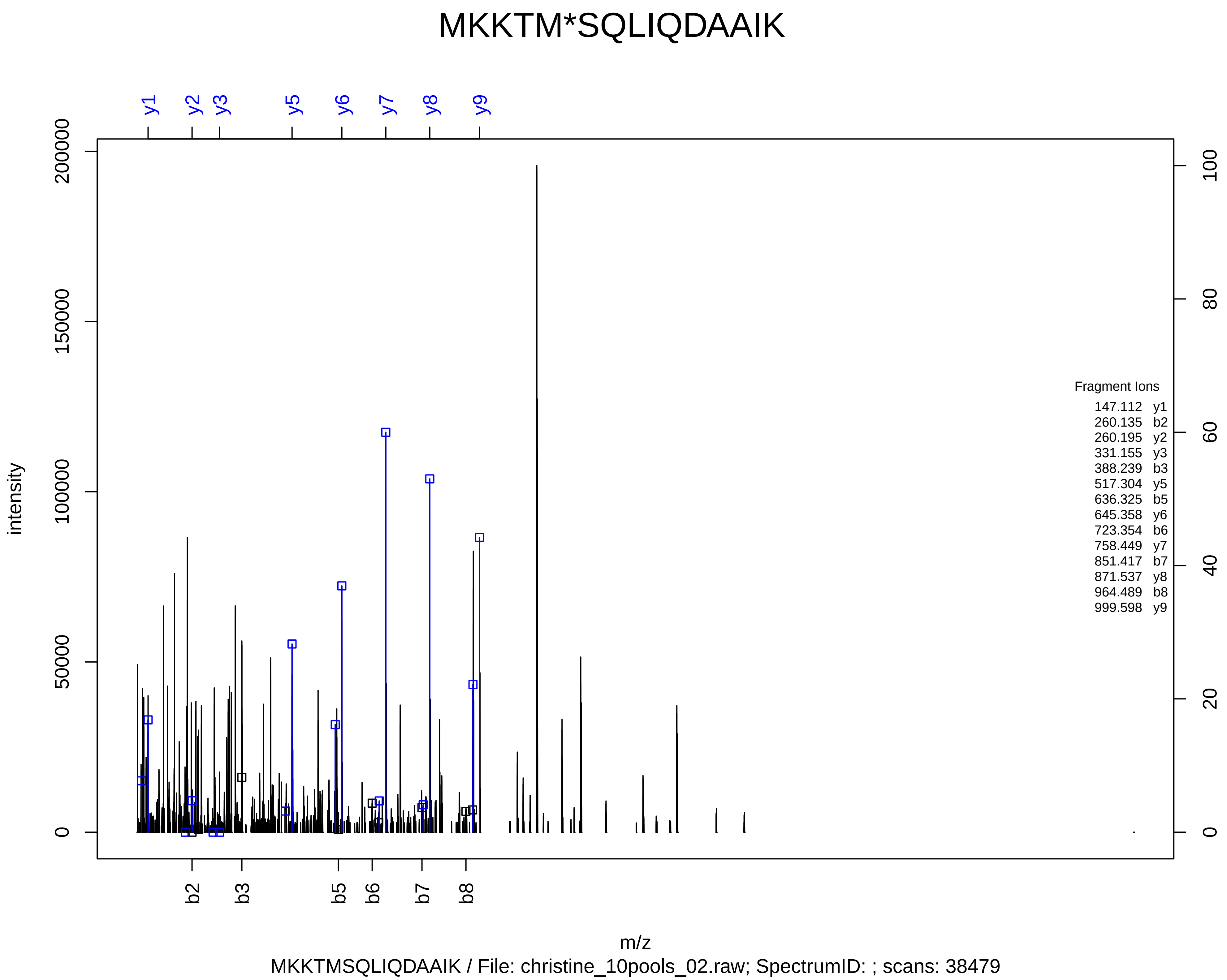
MKKTM*SQLIQDAAIK
y1
y2
y3
y5
y6
y7
y8
y9
b2
b3
b5
b6
b7
b8
intensity
m/z
0
50000
100000
150000
200000
0
20
40
60
80
100
Fragment Ions
147.112 y1
260.135 b2
260.195 y2
331.155 y3
388.239 b3
517.304 y5
636.325 b5
645.358 y6
723.354 b6
758.449 y7
851.417 b7
871.537 y8
964.489 b8
999.598 y9
MKKTMSQLIQDAAIK / File: christine_10pools_02.raw; SpectrumID: ; scans: 38479

SACGALRNLVYGKAN^DDNK

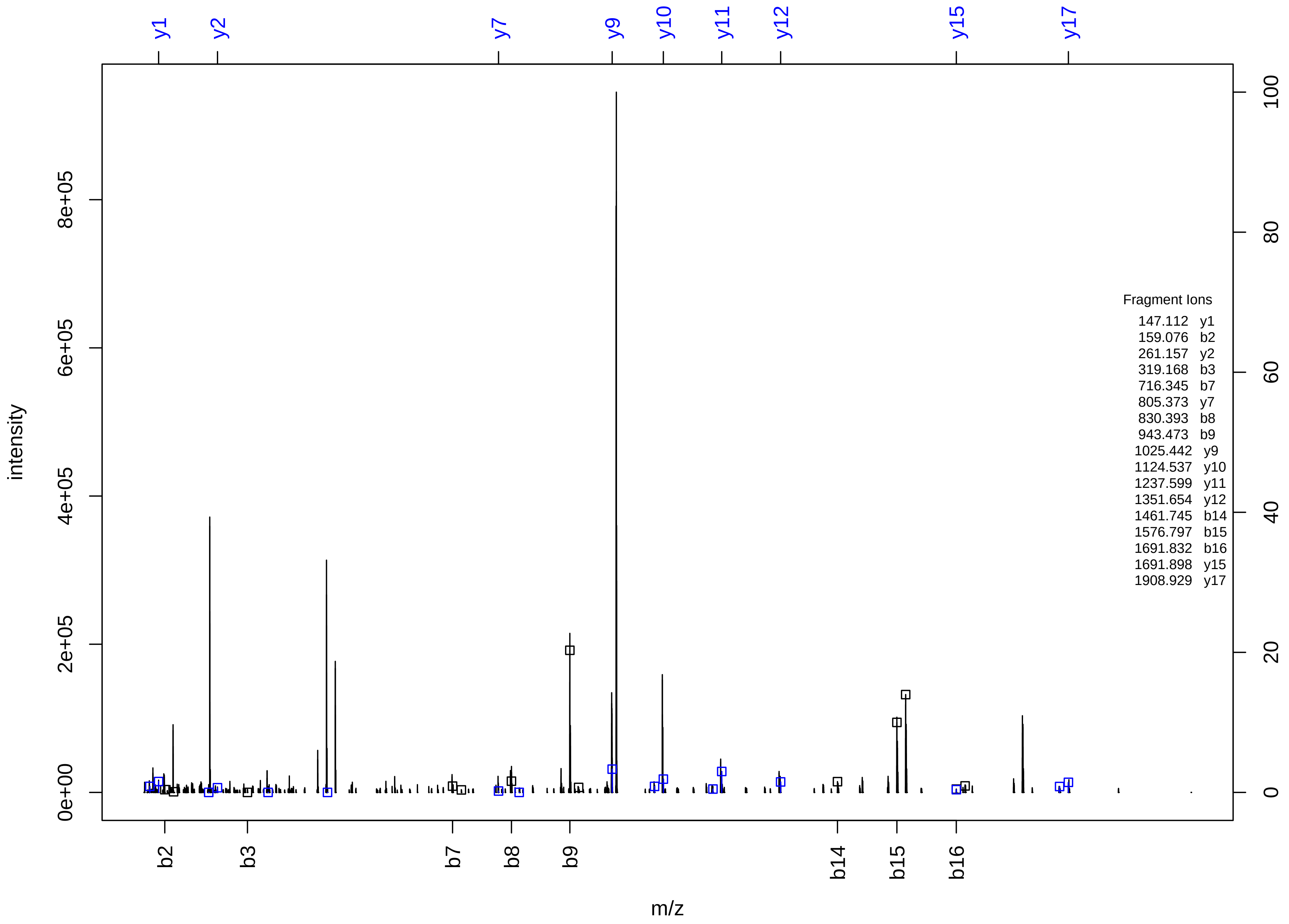

SACGALRNLVYGKANDDNK / File: christine_10pools_02.raw; SpectrumID: ; scans: 16344

AGVDAFIQNYVDPTPDPNTAYASYPSASVENYQQPPFTQNVETTEGYQPPPVY

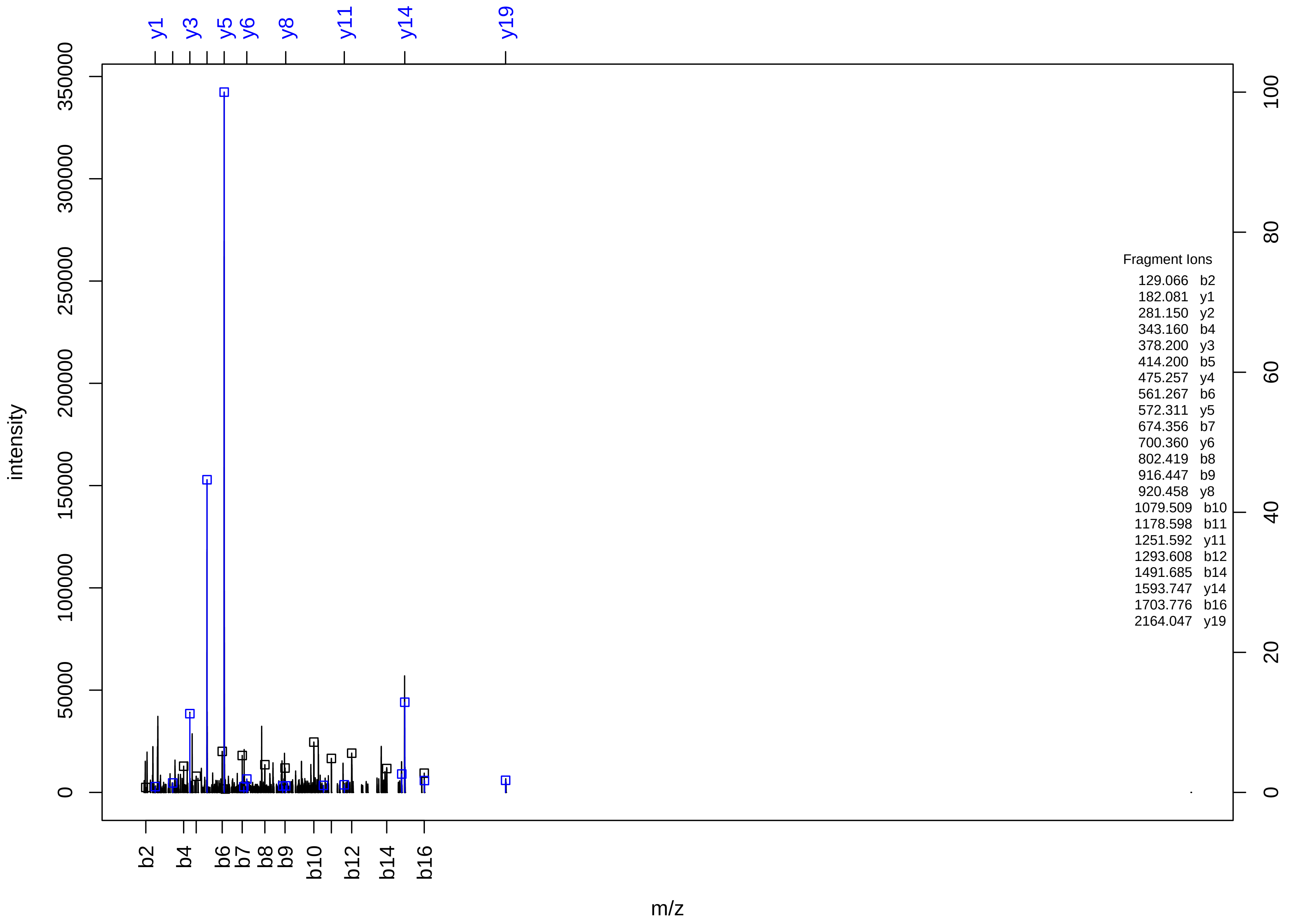

AGVDAFIQNYVDPTPDPNTAYASYPSASVENYQQPPFTQNVETTEGYQPPPVY / File: christine_10pools_02.raw; SpectrumID: ; scans: 40[illegible]

M*MAIEIM*AWGLR

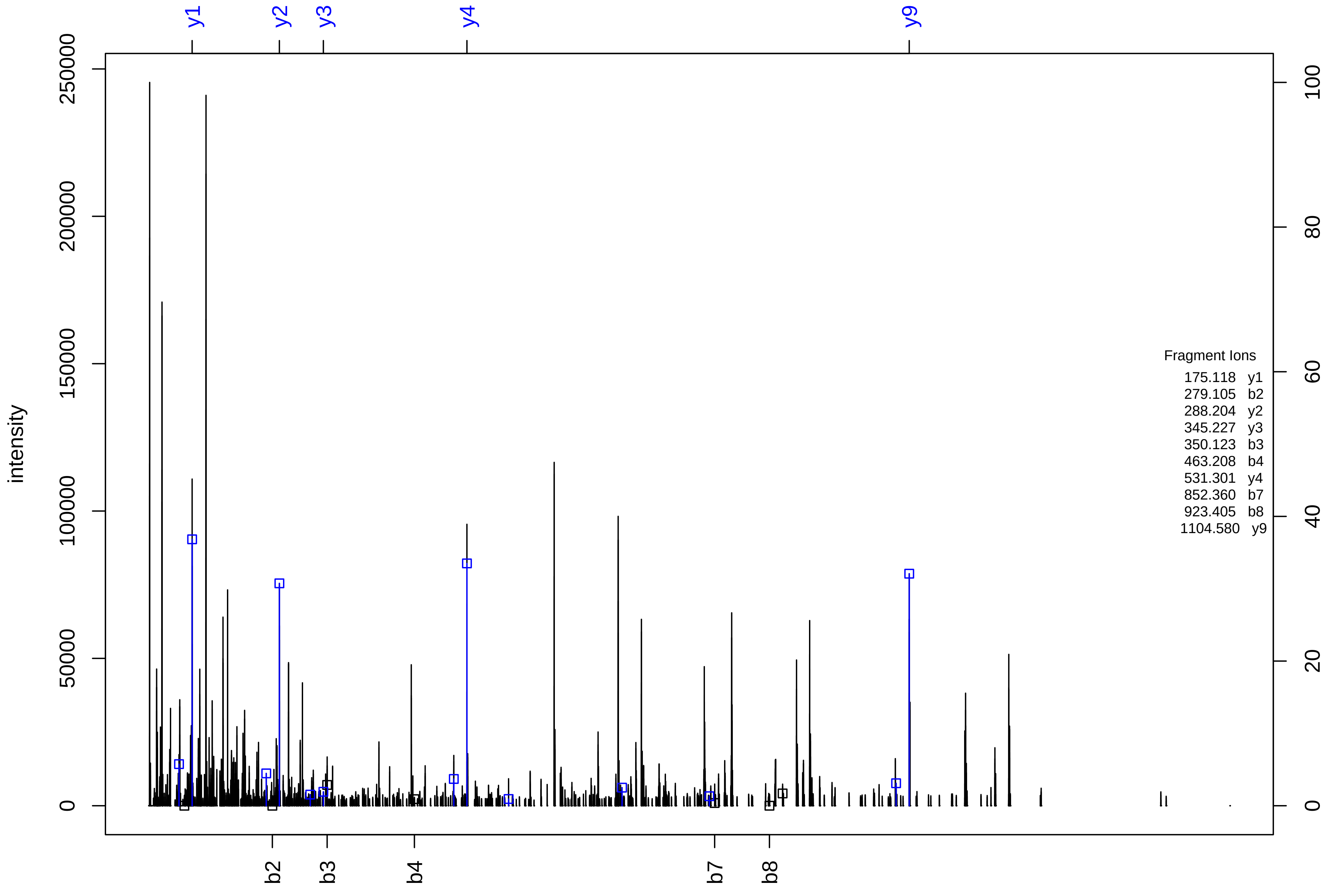

MMAIEIMAWGLR / File: christine_10pools_02.raw; SpectrumID: ; scans: 26031

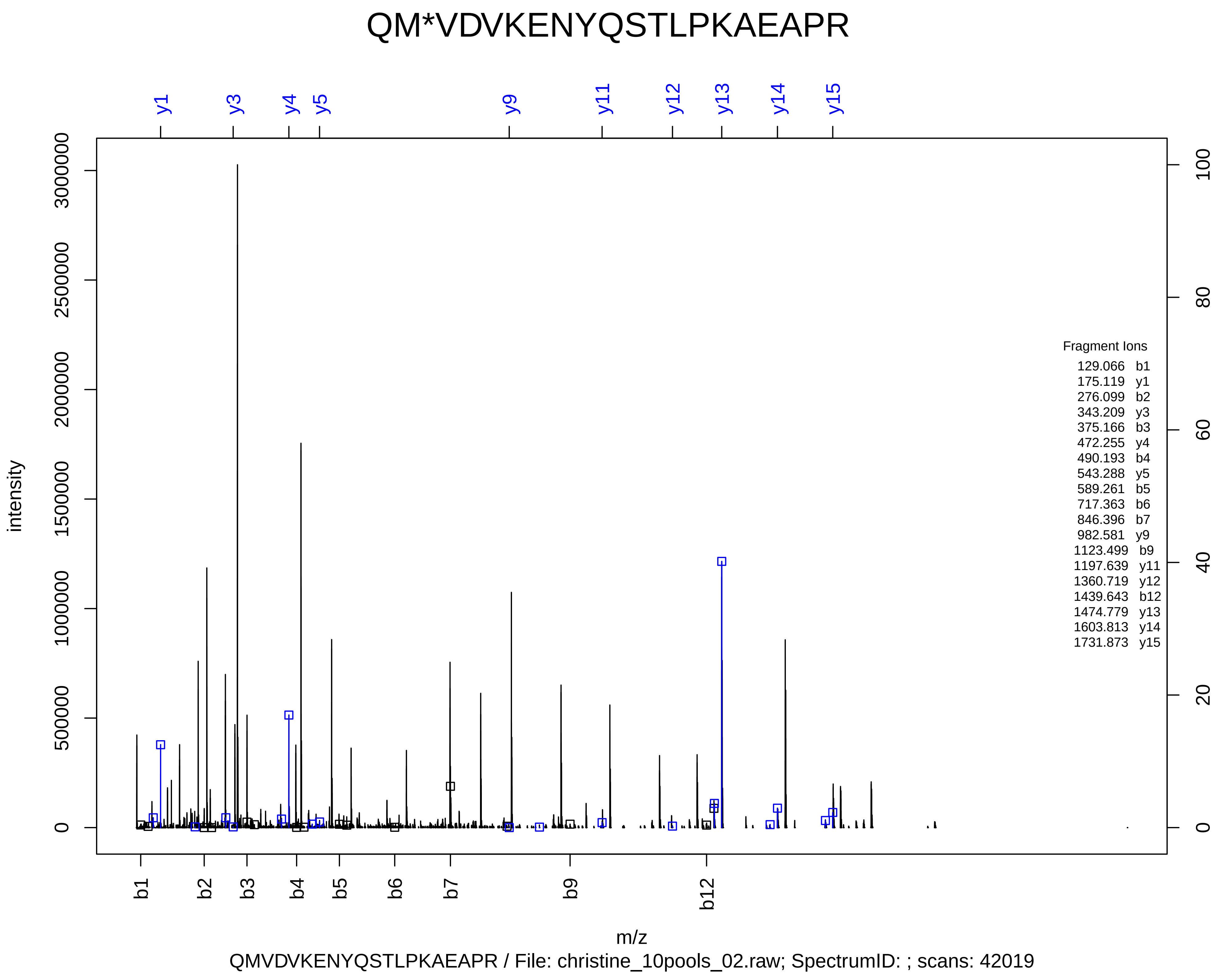

QM*VDVKENYQSTLPKAEAPR
y1
y3
y4
y5
y9
y11
y12
y13
y14
y15
intensity
0
500000
1000000
1500000
2000000
2500000
3000000
0
20
40
60
80
100
Fragment Ions
129.066 b1
175.119 y1
276.099 b2
343.209 y3
375.166 b3
472.255 y4
490.193 b4
543.288 y5
589.261 b5
717.363 b6
846.396 b7
982.581 y9
1123.499 b9
1197.639 y11
1360.719 y12
1439.643 b12
1474.779 y13
1603.813 y14
1731.873 y15
b1
b2
b3
b4
b5
b6
b7
b9
b12
m/z
QMVDVKENYQSTLPKAEAPR / File: christine_10pools_02.raw; SpectrumID: ; scans: 42019

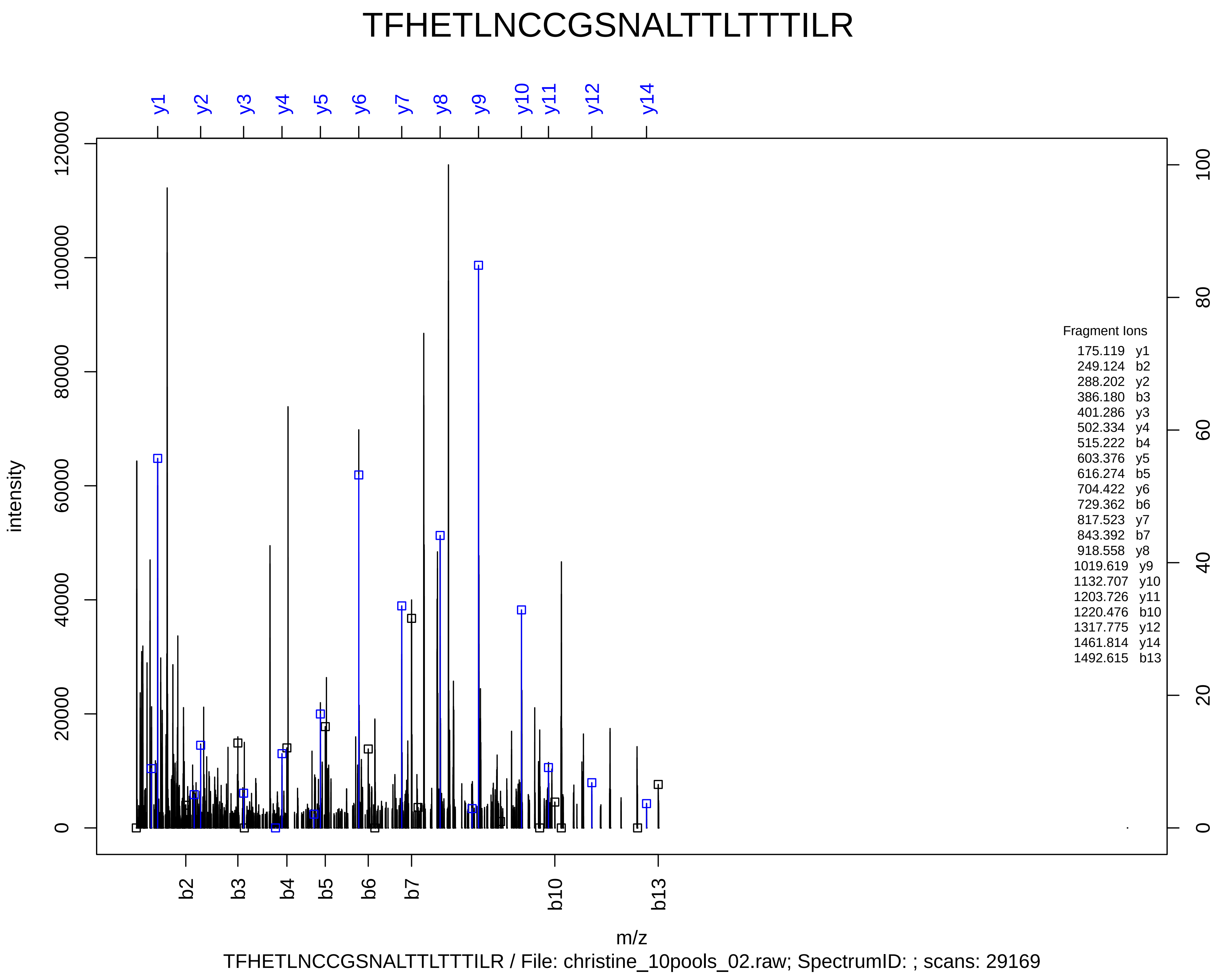
TFHETLNCCGSNALTTLTTTILR
y1
y2
y3
y4
y5
y6
y7
y8
y9
y10
y11
y12
y14
intensity
0
20000
40000
60000
80000
100000
120000
0
20
40
60
80
100
b2
b3
b4
b5
b6
b7
b10
b13
m/z
Fragment Ions
175.119 y1
249.124 b2
288.202 y2
386.180 b3
401.286 y3
502.334 y4
515.222 b4
603.376 y5
616.274 b5
704.422 y6
729.362 b6
817.523 y7
843.392 b7
918.558 y8
1019.619 y9
1132.707 y10
1203.726 y11
1220.476 b10
1317.775 y12
1461.814 y14
1492.615 b13
TFHETLNCCGSNALTTLTTTILR / File: christine_10pools_02.raw; SpectrumID: ; scans: 29169

MFQTLIQK

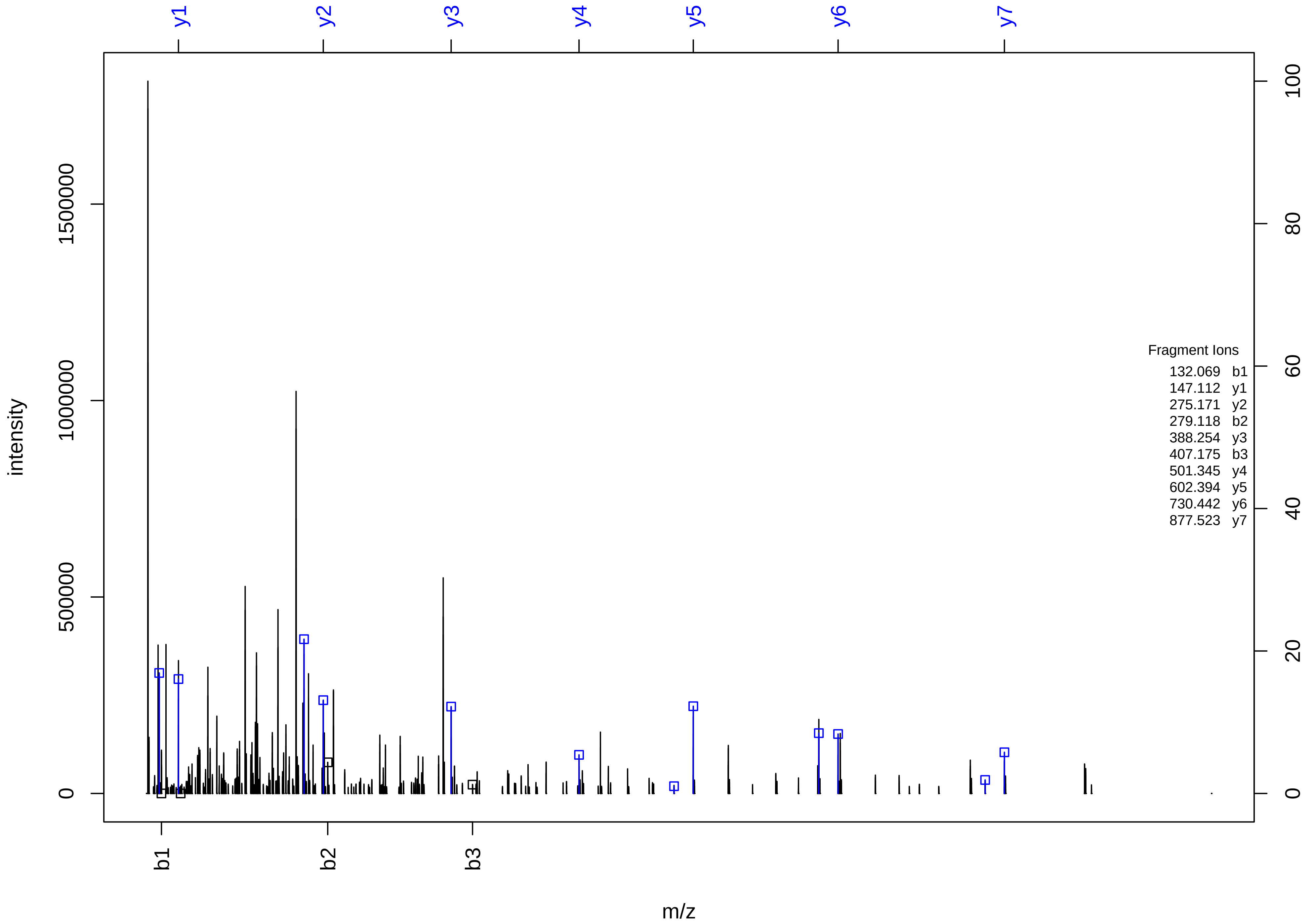

MFQTLIQK / File: christine_10pools_02.raw; SpectrumID: ; scans: 10941

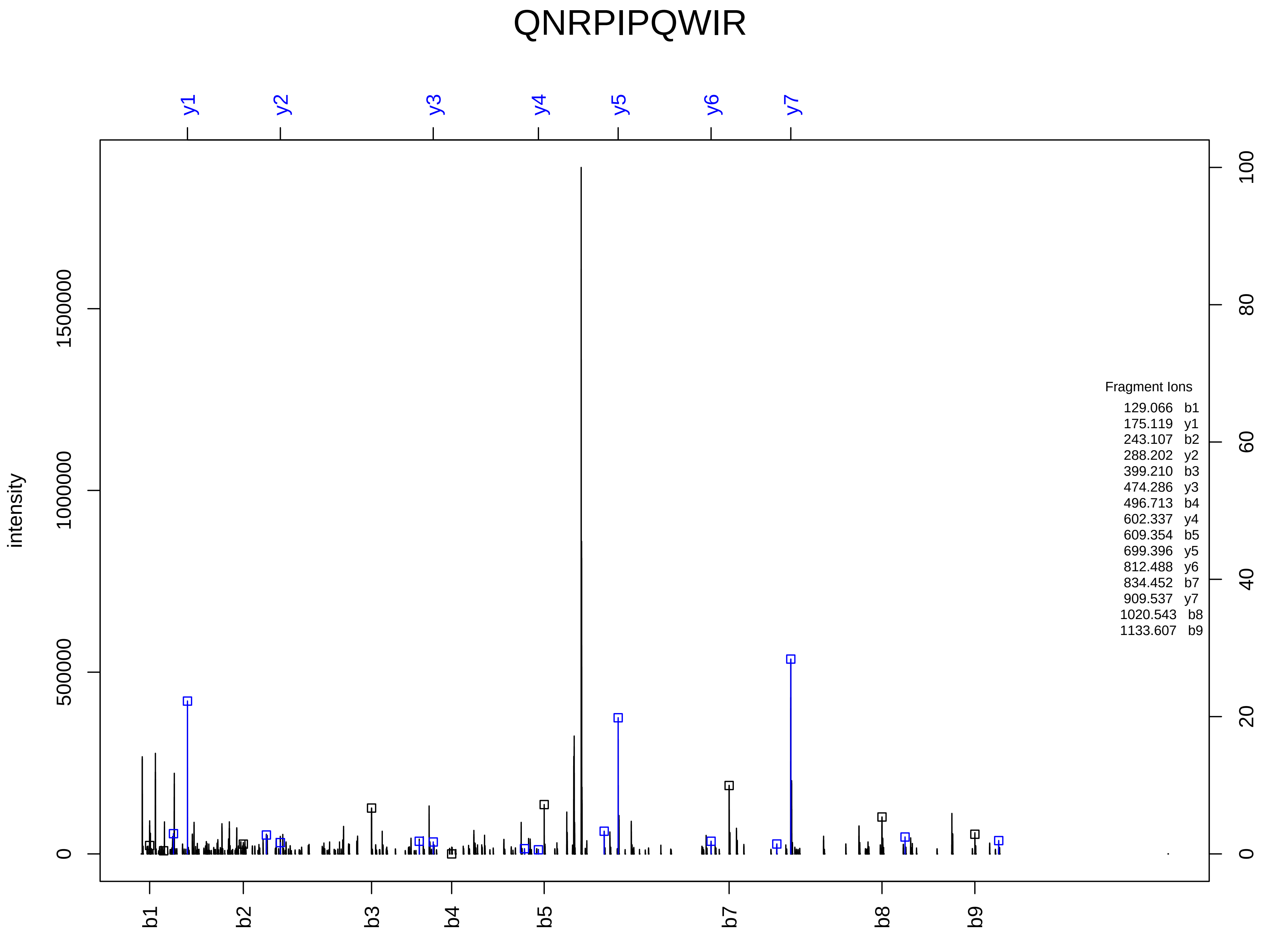

QNRPIPQWIR / File: christine_10pools_02.raw; SpectrumID: ; scans: 7002

VVMDGVISDDECQELQR

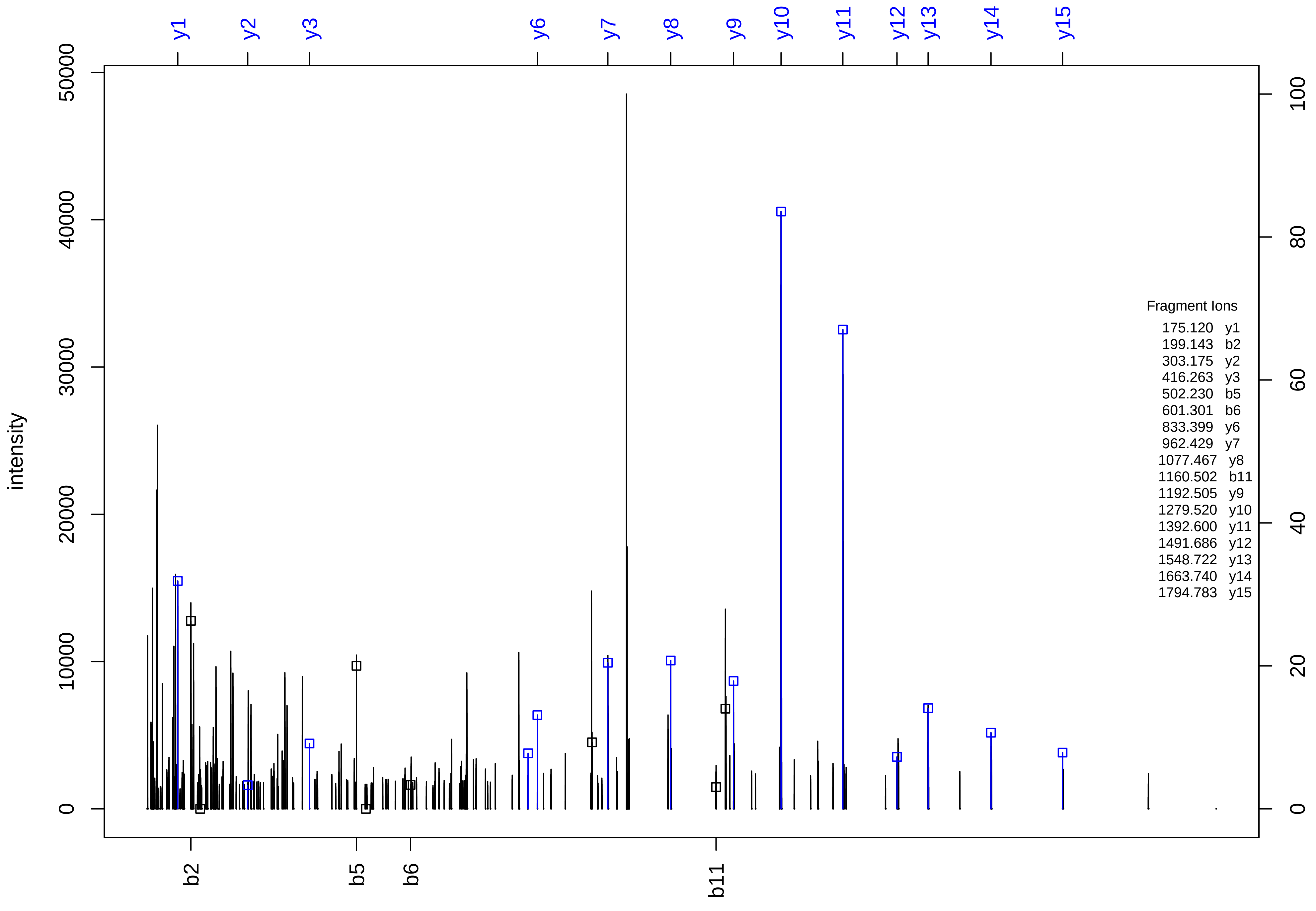

VVMDGVISDDECQELQR / File: christine_10pools_02.raw; SpectrumID: ; scans: 14995

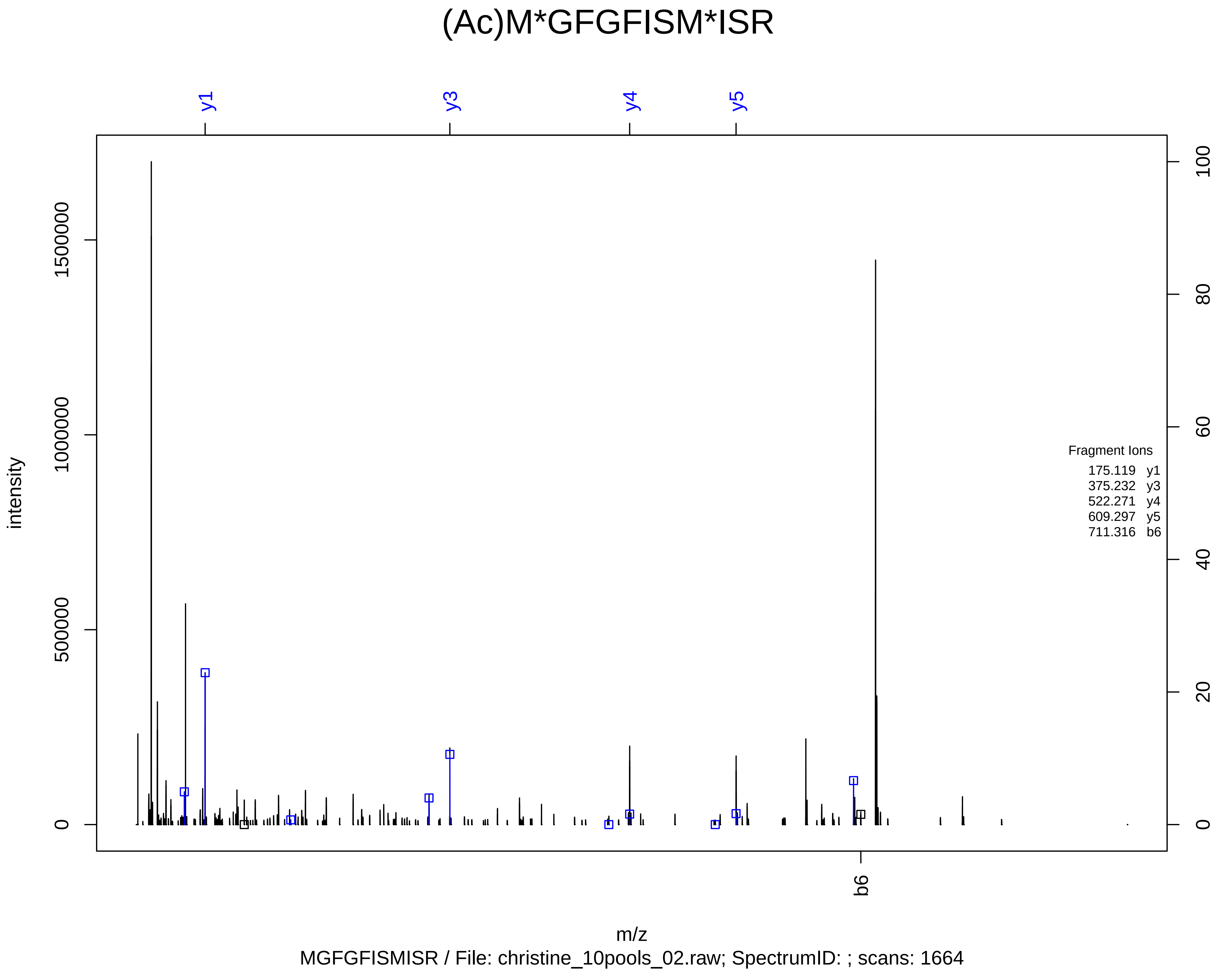
(Ac)M*GFGFISM*ISR
y1
y3
y4
y5
b6
intensity
0
500000
1000000
1500000
0
20
40
60
80
100
Fragment Ions
175.119 y1
375.232 y3
522.271 y4
609.297 y5
711.316 b6
m/z
MGFGFISMISR / File: christine_10pools_02.raw; SpectrumID: ; scans: 1664

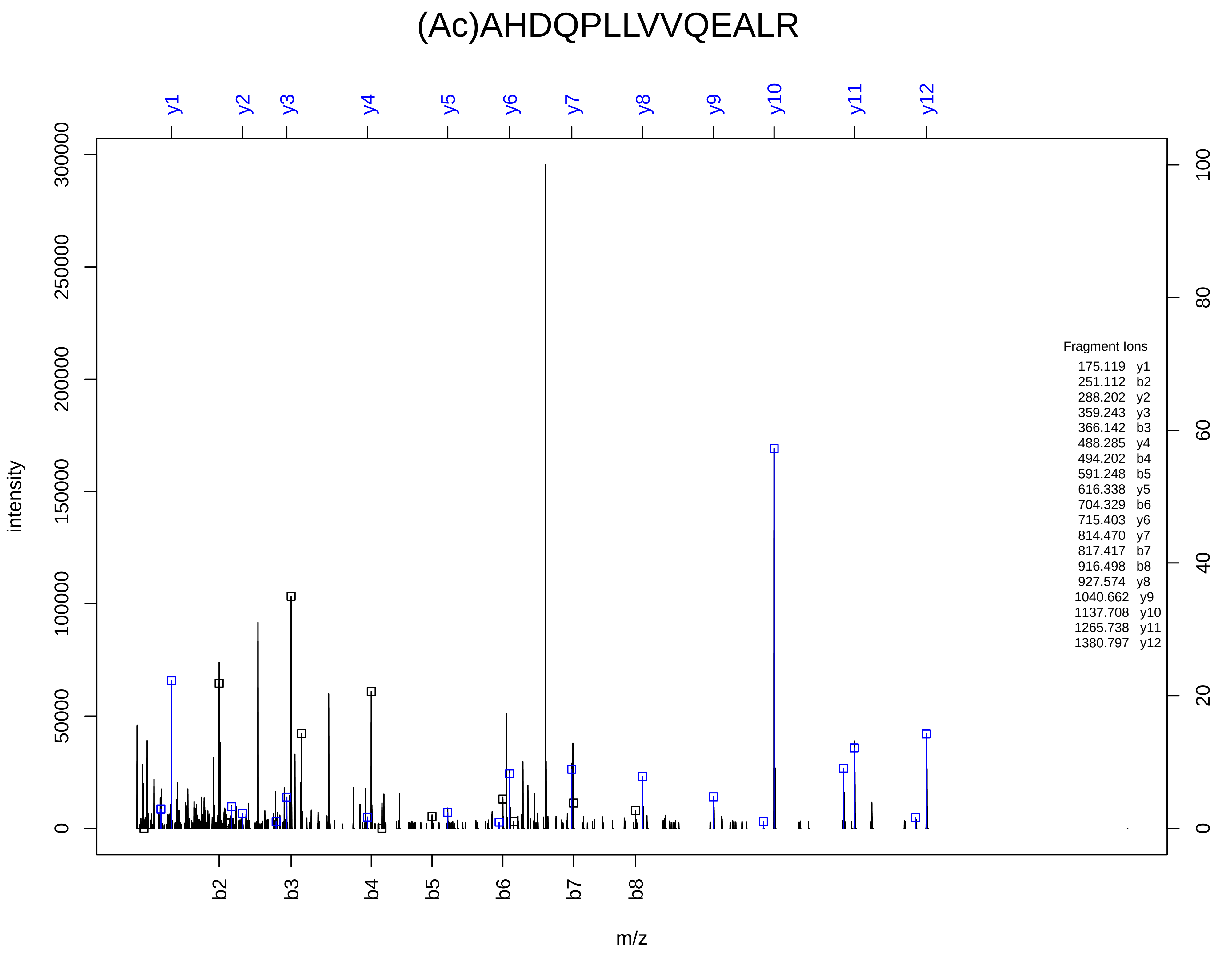

(Ac)AHDQPLLVVQEALR
y1
y2
y3
y4
y5
y6
y7
y8
y9
y10
y11
y12
intensity
0
50000
100000
150000
200000
250000
300000
0
20
40
60
80
100
b2
b3
b4
b5
b6
b7
b8
m/z
Fragment Ions
175.119 y1
251.112 b2
288.202 y2
359.243 y3
366.142 b3
488.285 y4
494.202 b4
591.248 b5
616.338 y5
704.329 b6
715.403 y6
814.470 y7
817.417 b7
916.498 b8
927.574 y8
1040.662 y9
1137.708 y10
1265.738 y11
1380.797 y12
AHDQPLLVVQEALR / File: christine_10pools_02.raw; SpectrumID: ; scans: 28232

ALSSYGGGSGNIWLDNVNCR

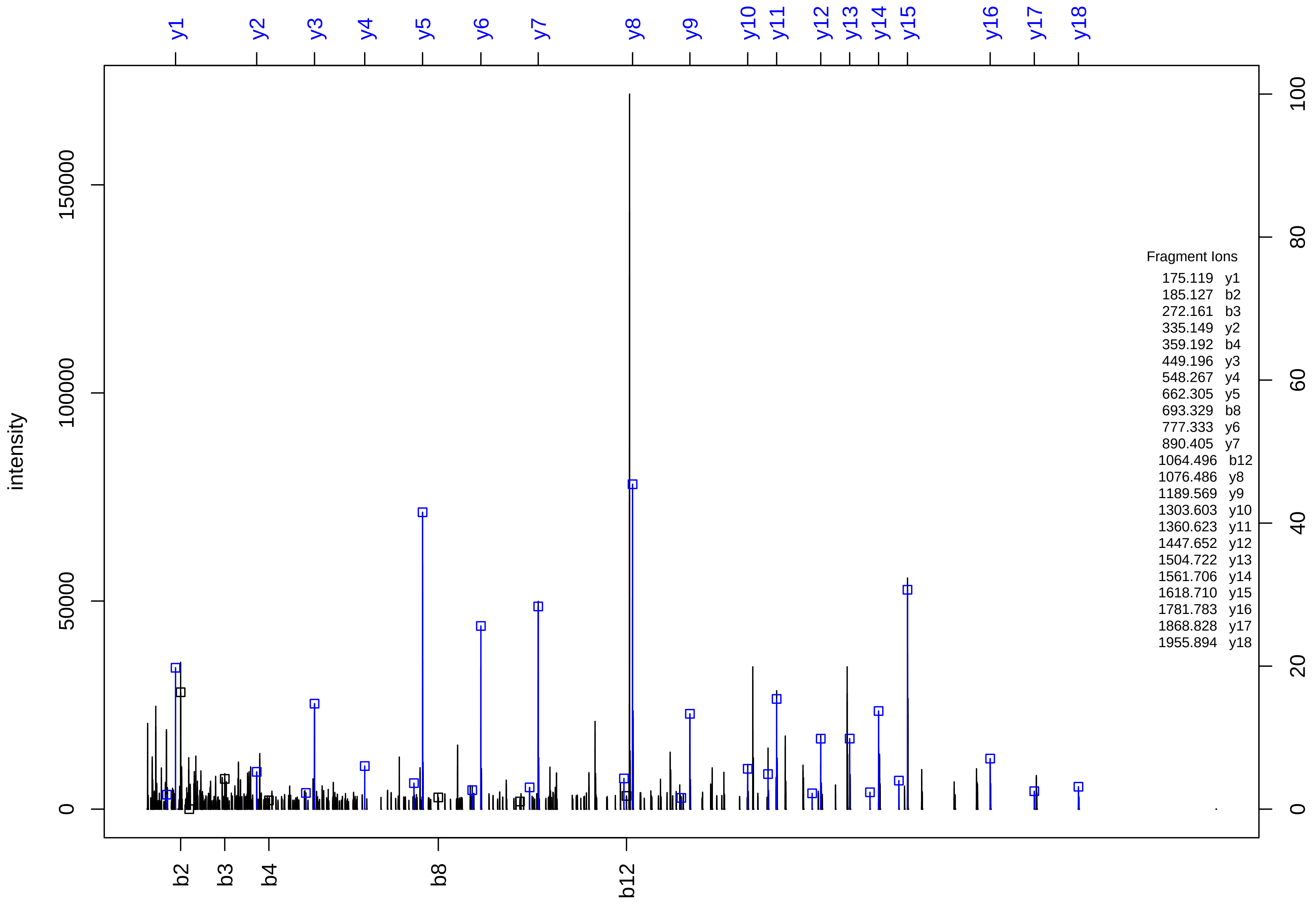

ALSSYGGGSGNIWLDNVNCR / File: christine_10pools_02.raw; SpectrumID: ; scans: 19128

EGLAESEPMICHTLPLPEGFQVVK

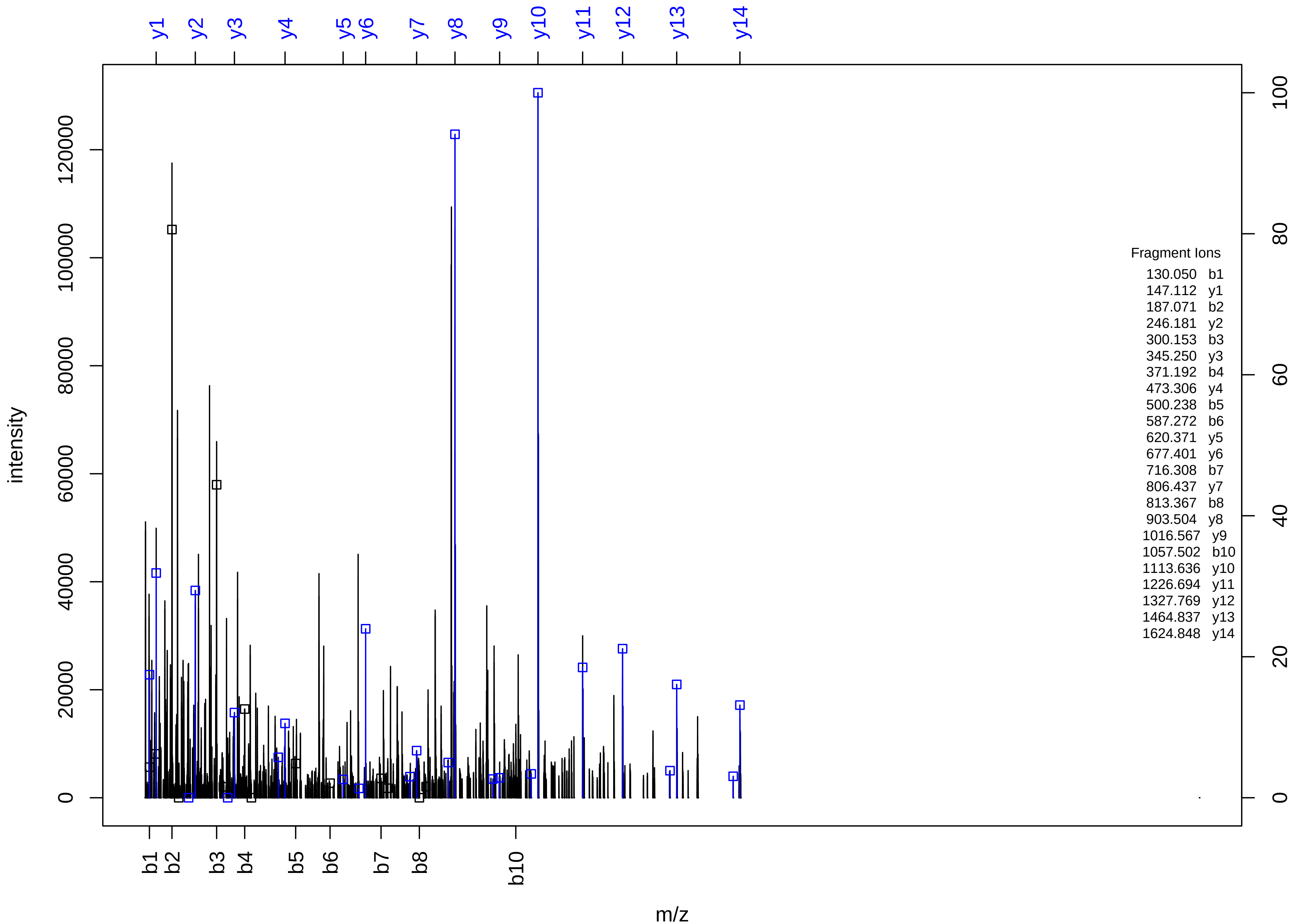

EGLAESEPMICHTLPLPEGFQVVK / File: christine_10pools_02.raw; SpectrumID: ; scans: 28167

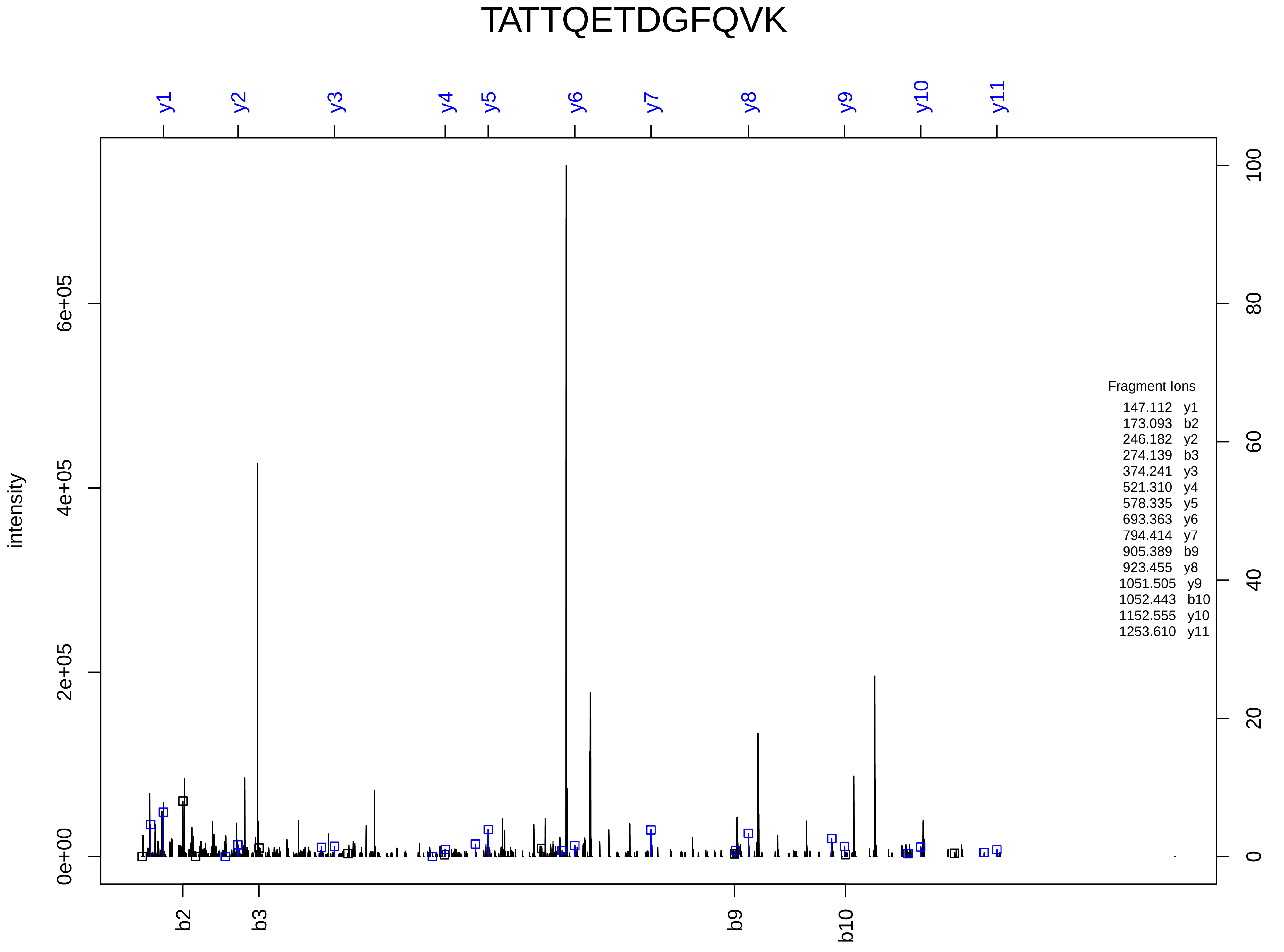

TATTQETDGFQVK / File: christine_10pools_02.raw; SpectrumID: ; scans: 2907

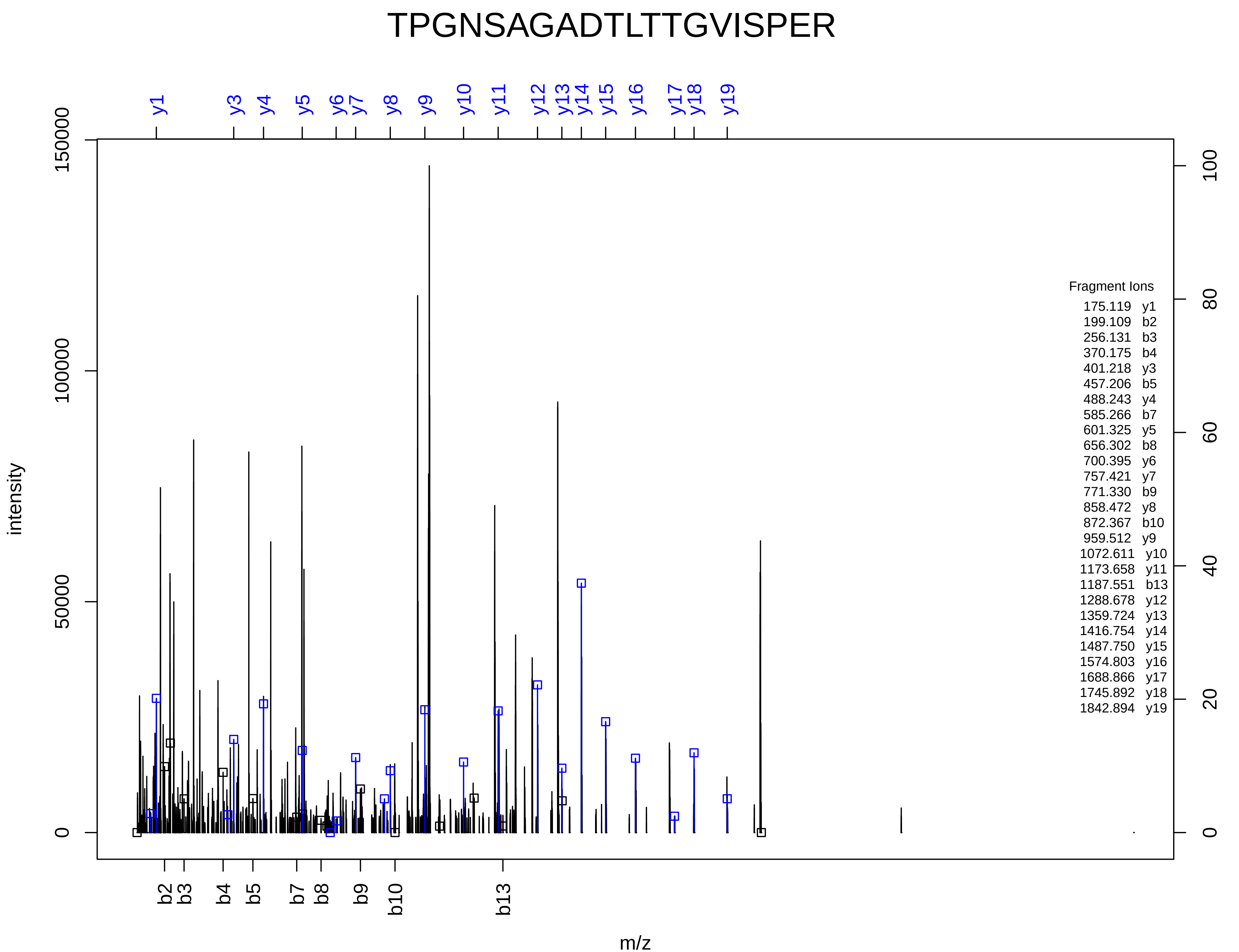

TPGNSAGADTLTTGVISPER / File: christine_10pools_02.raw; SpectrumID: ; scans: 11690

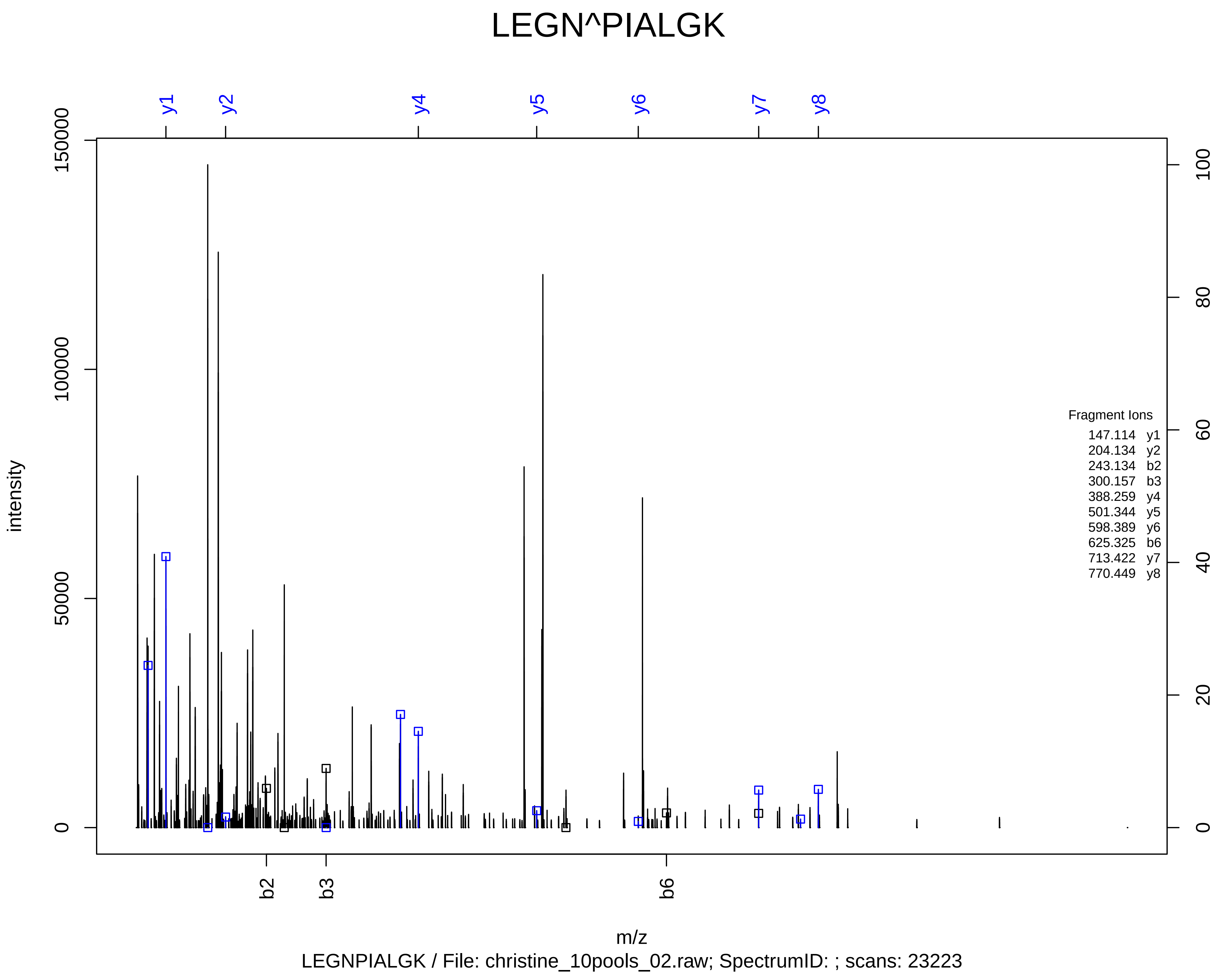
LEGN^PIALGK
y1
y2
y4
y5
y6
y7
y8
intensity
150000
100000
50000
0
100
80
60
40
20
0
Fragment Ions
147.114 y1
204.134 y2
243.134 b2
300.157 b3
388.259 y4
501.344 y5
598.389 y6
625.325 b6
713.422 y7
770.449 y8
b2
b3
b6
m/z
LEGNPIALGK / File: christine_10pools_02.raw; SpectrumID: ; scans: 23223

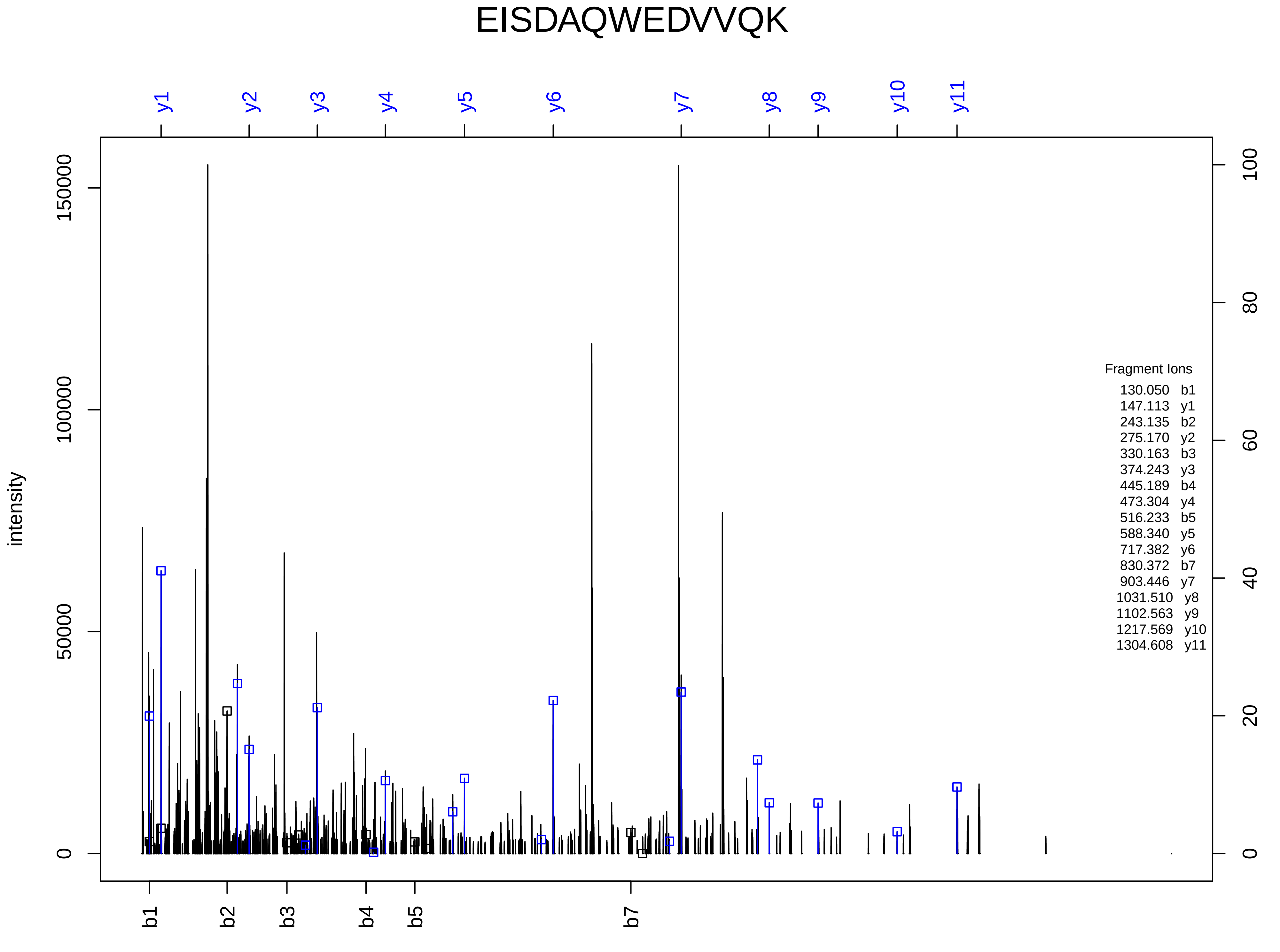

EISDAQWEDVVQK / File: christine_10pools_02.raw; SpectrumID: ; scans: 14571

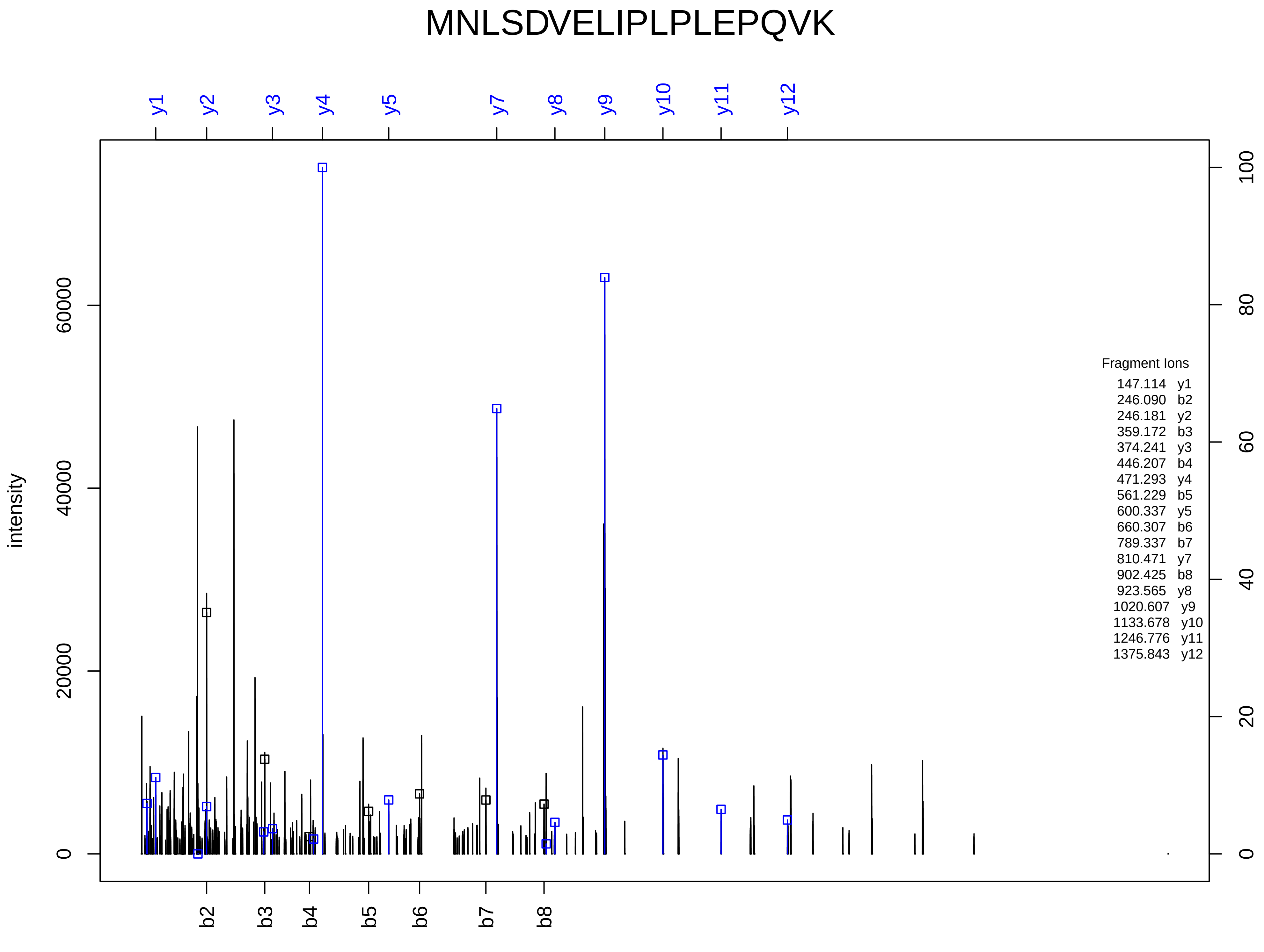

MNLSDVELIPLPLEPQVK / File: christine_10pools_02.raw; SpectrumID: ; scans: 38835

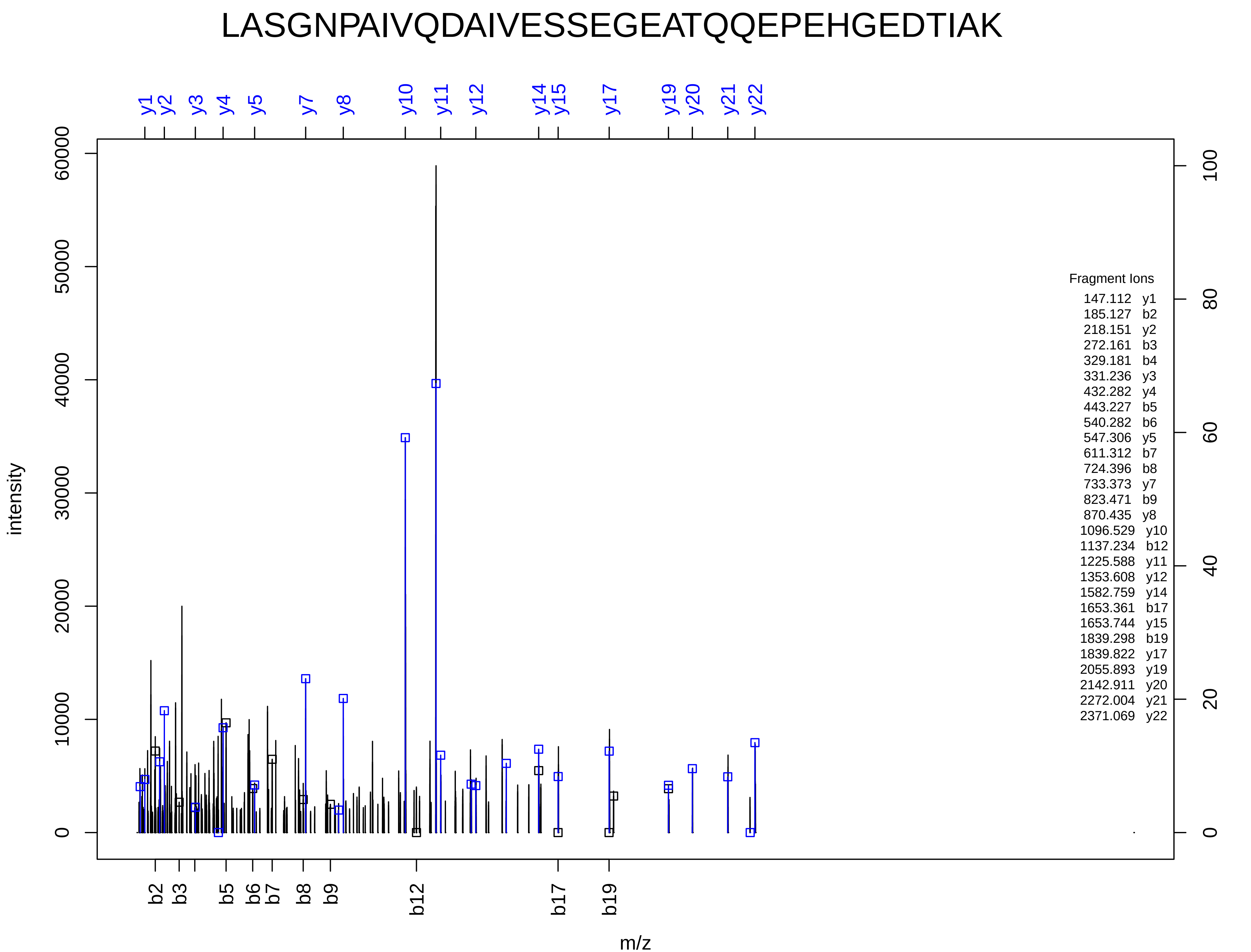

LASGNPAIVQDAIVESSEGEATQQEPEHGEDTIAK / File: christine_10pools_02.raw; SpectrumID: ; scans: 17508

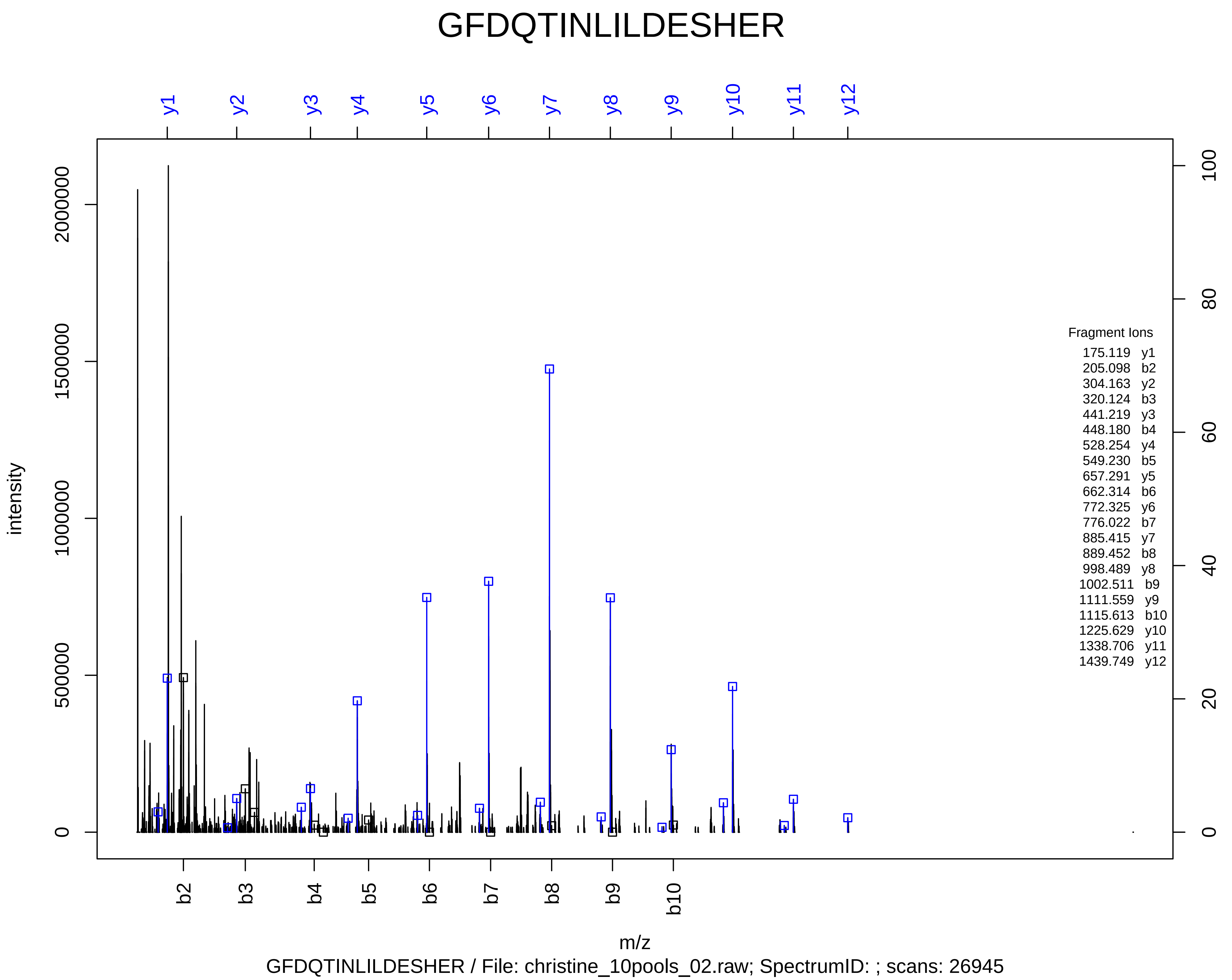
GFDQTINLILDESHER
y1 y2 y3 y4 y5 y6 y7 y8 y9 y10 y11 y12
b2 b3 b4 b5 b6 b7 b8 b9 b10
intensity
0
500000
1000000
1500000
2000000
0
20
40
60
80
100
m/z
Fragment Ions
175.119 y1
205.098 b2
304.163 y2
320.124 b3
441.219 y3
448.180 b4
528.254 y4
549.230 b5
657.291 y5
662.314 b6
772.325 y6
776.022 b7
885.415 y7
889.452 b8
998.489 y8
1002.511 b9
1111.559 y9
1115.613 b10
1225.629 y10
1338.706 y11
1439.749 y12
GFDQTINLILDESHER / File: christine_10pools_02.raw; SpectrumID: ; scans: 26945

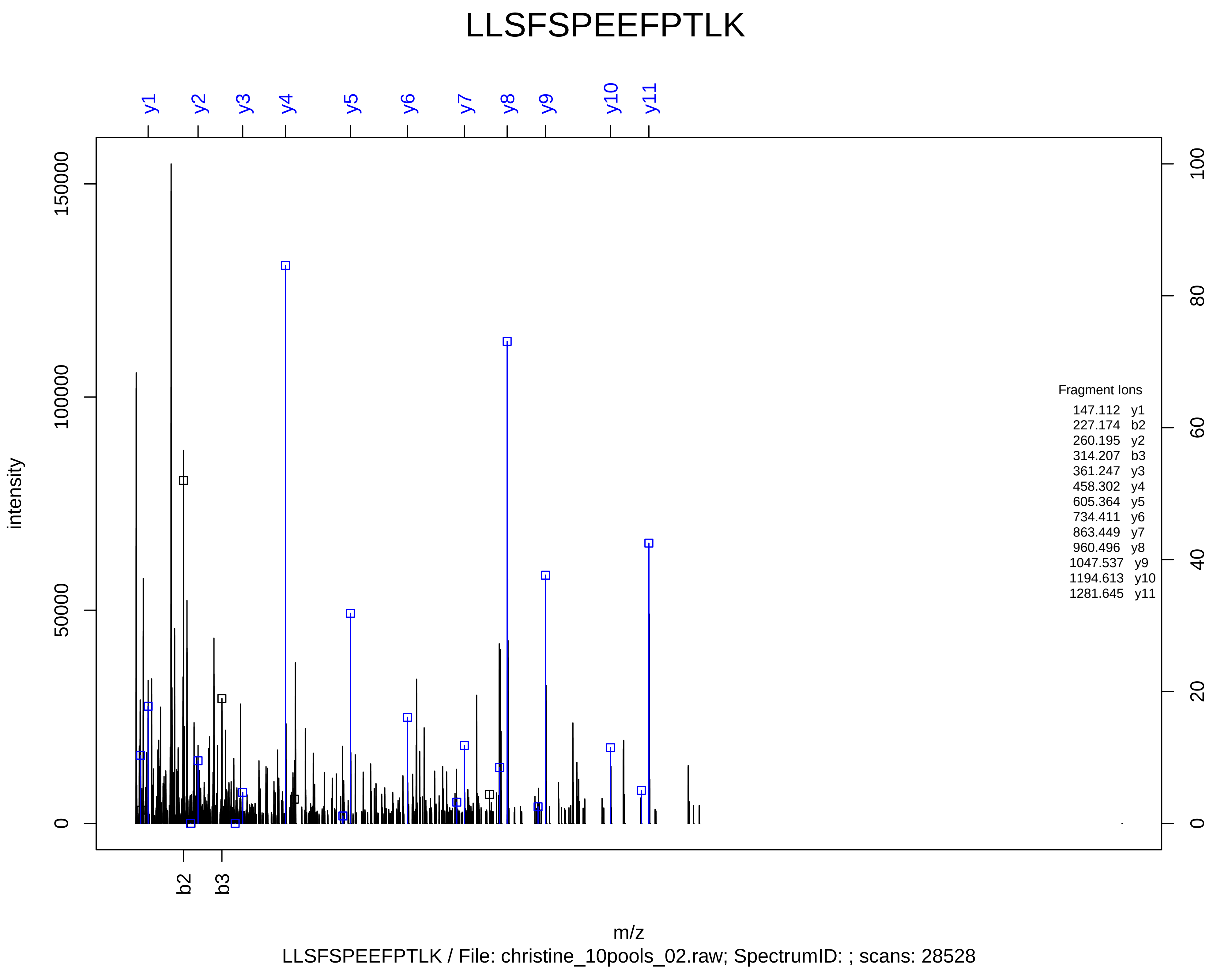

LLSFSPEEFPTLK
y1
y2
y3
y4
y5
y6
y7
y8
y9
y10
y11
intensity
0
50000
100000
150000
0
20
40
60
80
100
b2
b3
m/z
Fragment Ions
147.112 y1
227.174 b2
260.195 y2
314.207 b3
361.247 y3
458.302 y4
605.364 y5
734.411 y6
863.449 y7
960.496 y8
1047.537 y9
1194.613 y10
1281.645 y11
LLSFSPEEFPTLK / File: christine_10pools_02.raw; SpectrumID: ; scans: 28528

KDLN^ELIM*EERSVK

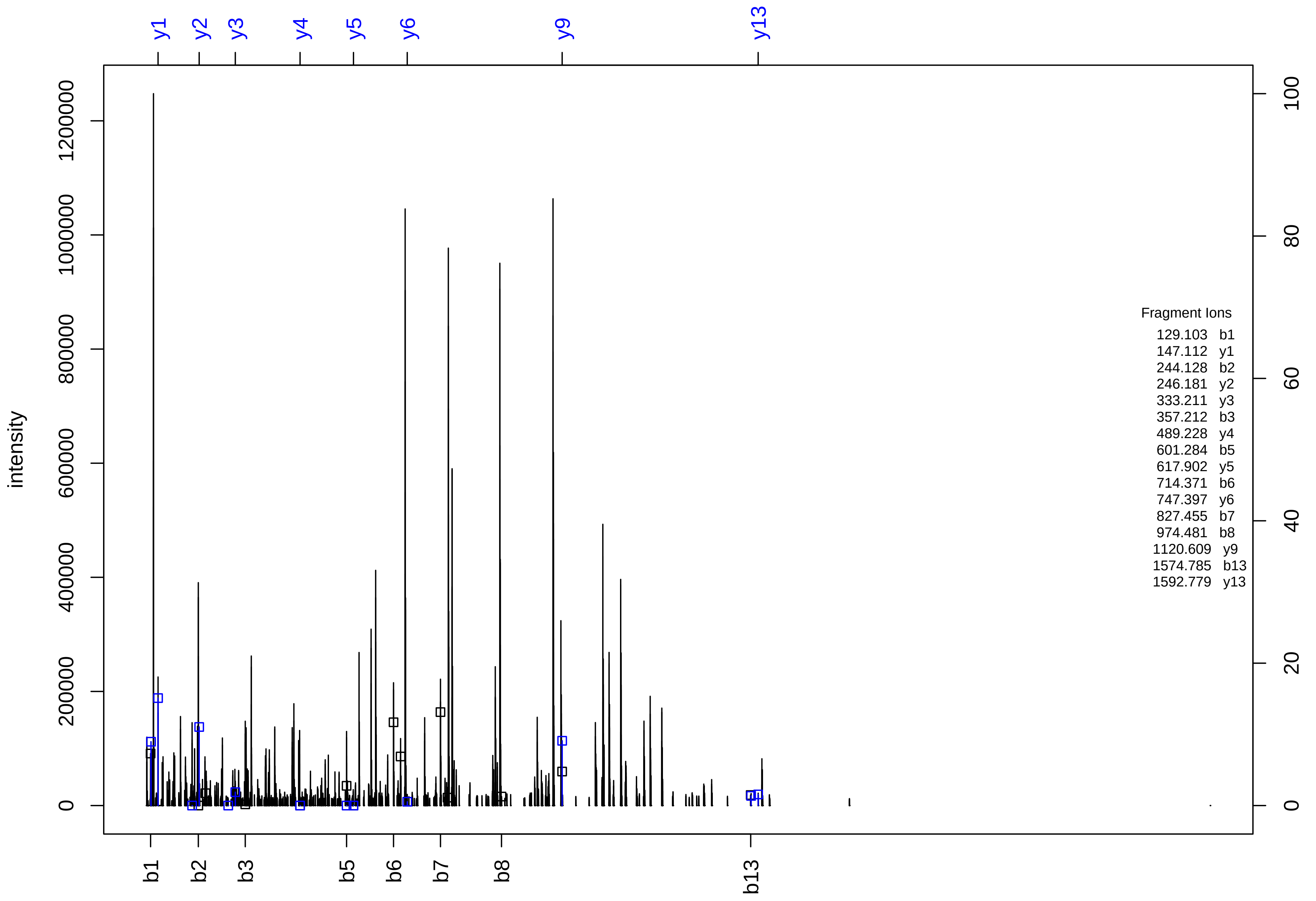

KDLNELIMEERSVK / File: christine_10pools_02.raw; SpectrumID: ; scans: 29760

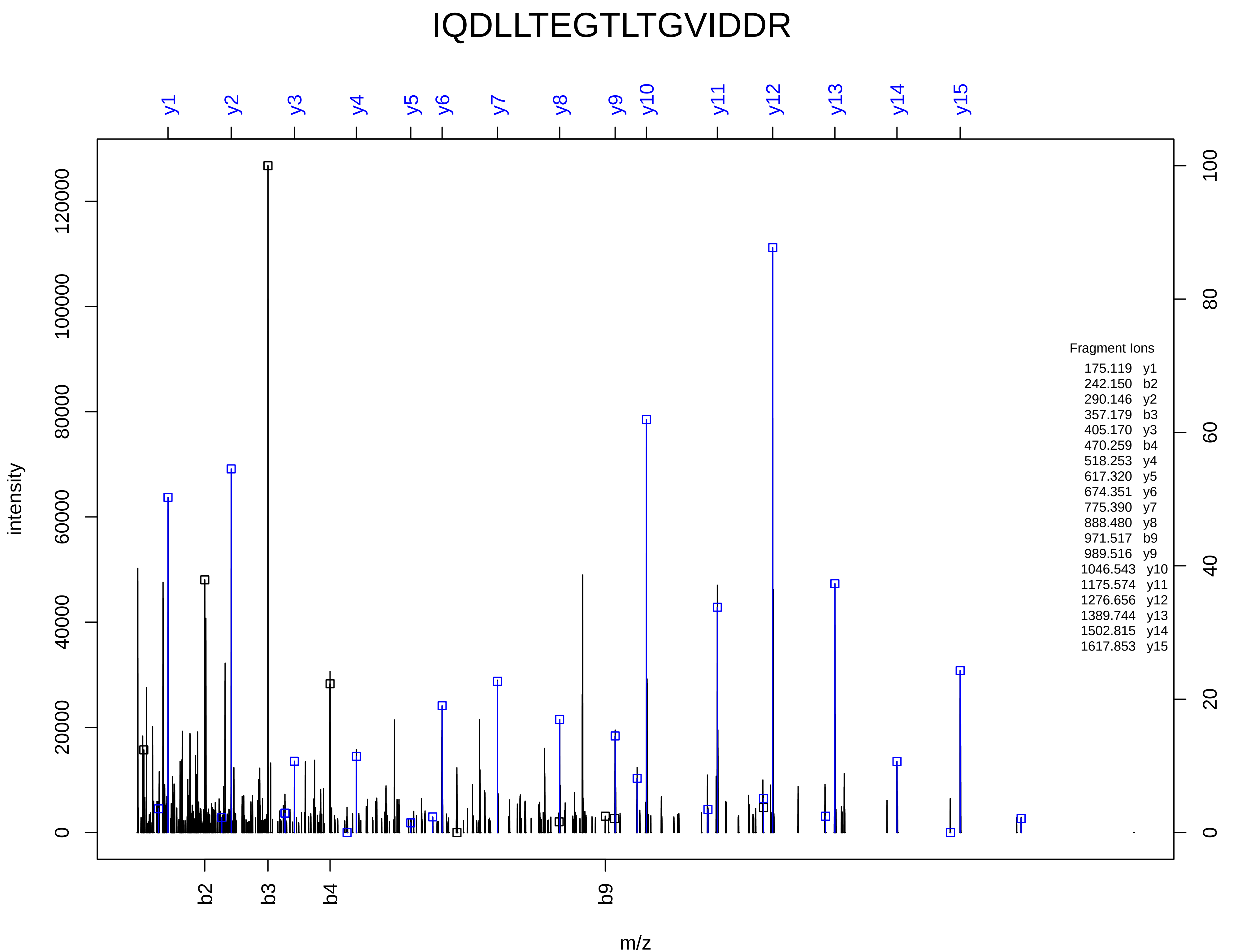

IQDLLTEGTLTGVIDDR / File: christine_10pools_02.raw; SpectrumID: ; scans: 28049

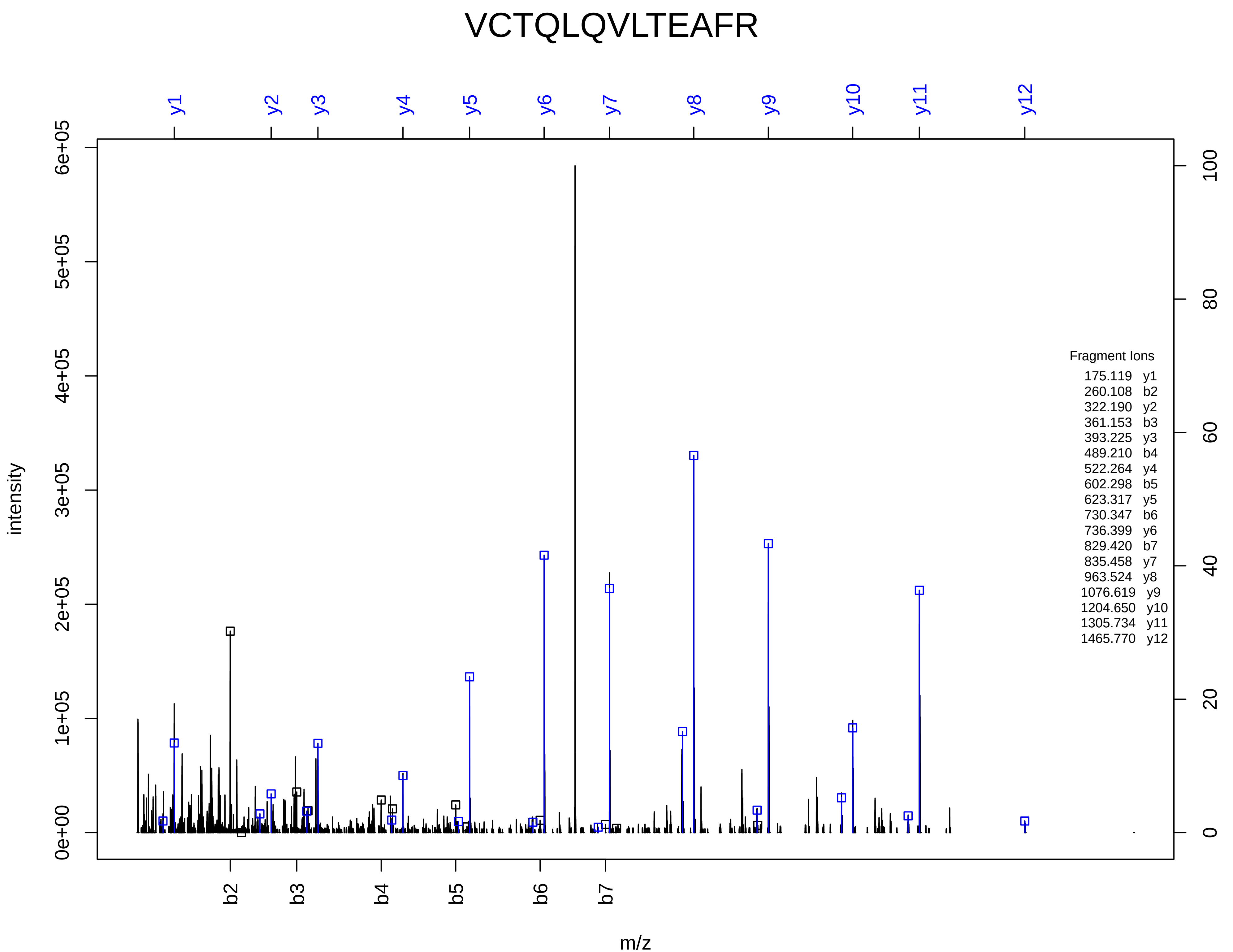

VCTQLQVLTEAFR / File: christine_10pools_02.raw; SpectrumID: ; scans: 26639

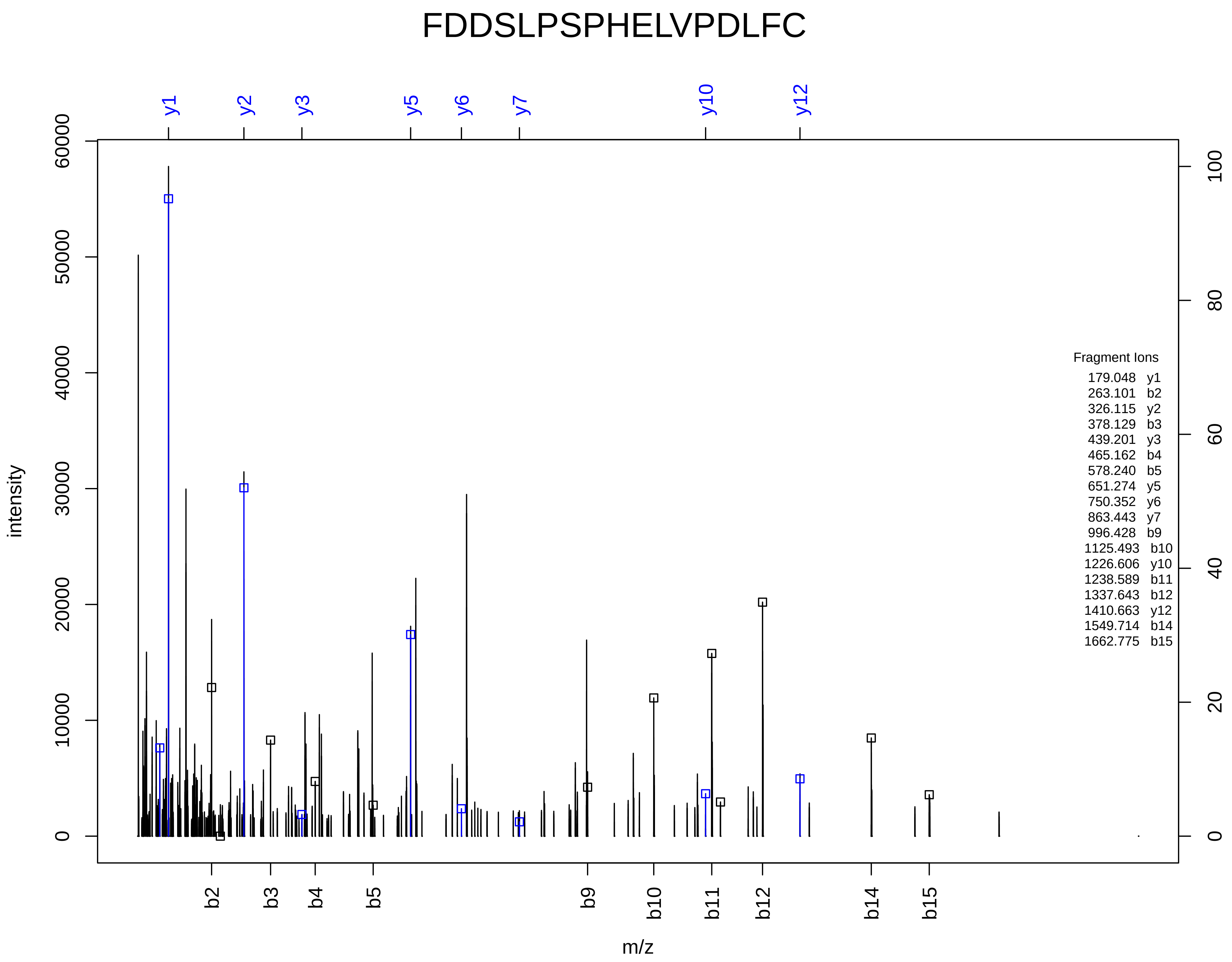
FDDSLPSPHELVPDLFC
y1
y2
y3
y5
y6
y7
y10
y12
intensity
0
10000
20000
30000
40000
50000
60000
0
20
40
60
80
100
Fragment Ions
179.048 y1
263.101 b2
326.115 y2
378.129 b3
439.201 y3
465.162 b4
578.240 b5
651.274 y5
750.352 y6
863.443 y7
996.428 b9
1125.493 b10
1226.606 y10
1238.589 b11
1337.643 b12
1410.663 y12
1549.714 b14
1662.775 b15
b2
b3
b4
b5
b9
b10
b11
b12
b14
b15
m/z
FDDSLPSPHELVPDLFC / File: christine_10pools_02.raw; SpectrumID: ; scans: 37121

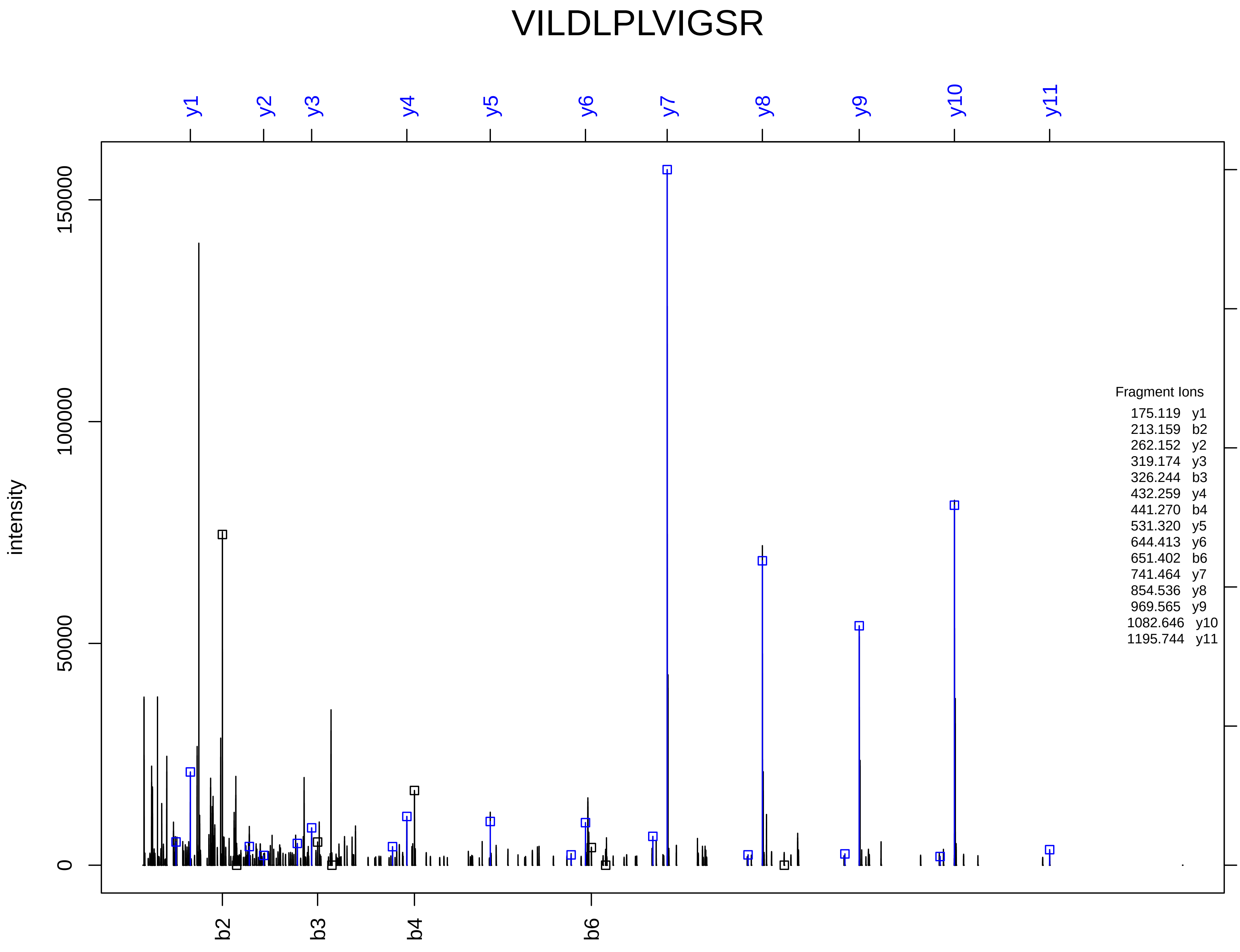

VILDLPLVIGSR / File: christine_10pools_02.raw; SpectrumID: ; scans: 33083

KVLN^DIISTMFN^R

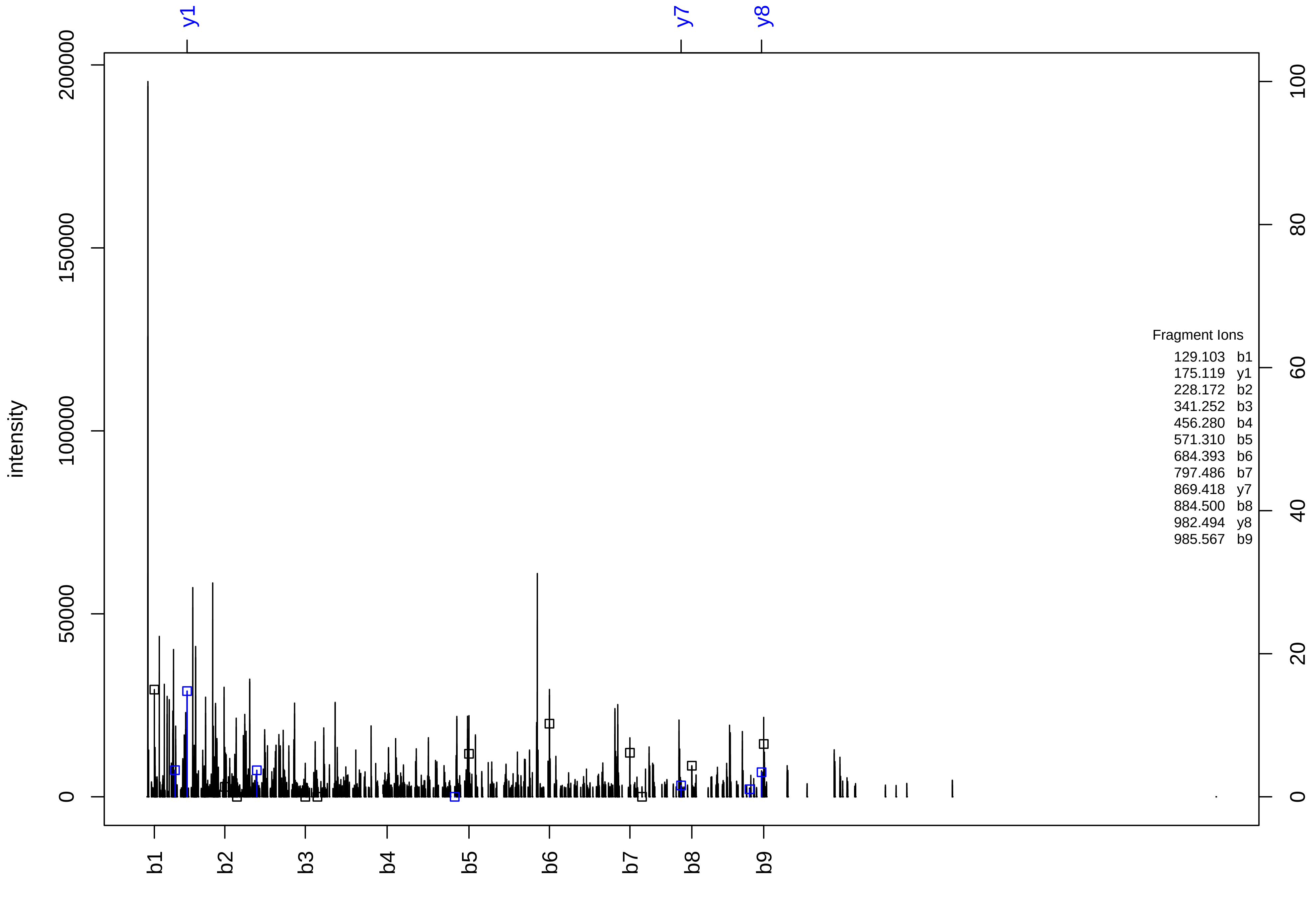

KVLNDIISTMFNR / File: christine_10pools_02.raw; SpectrumID: ; scans: 41528

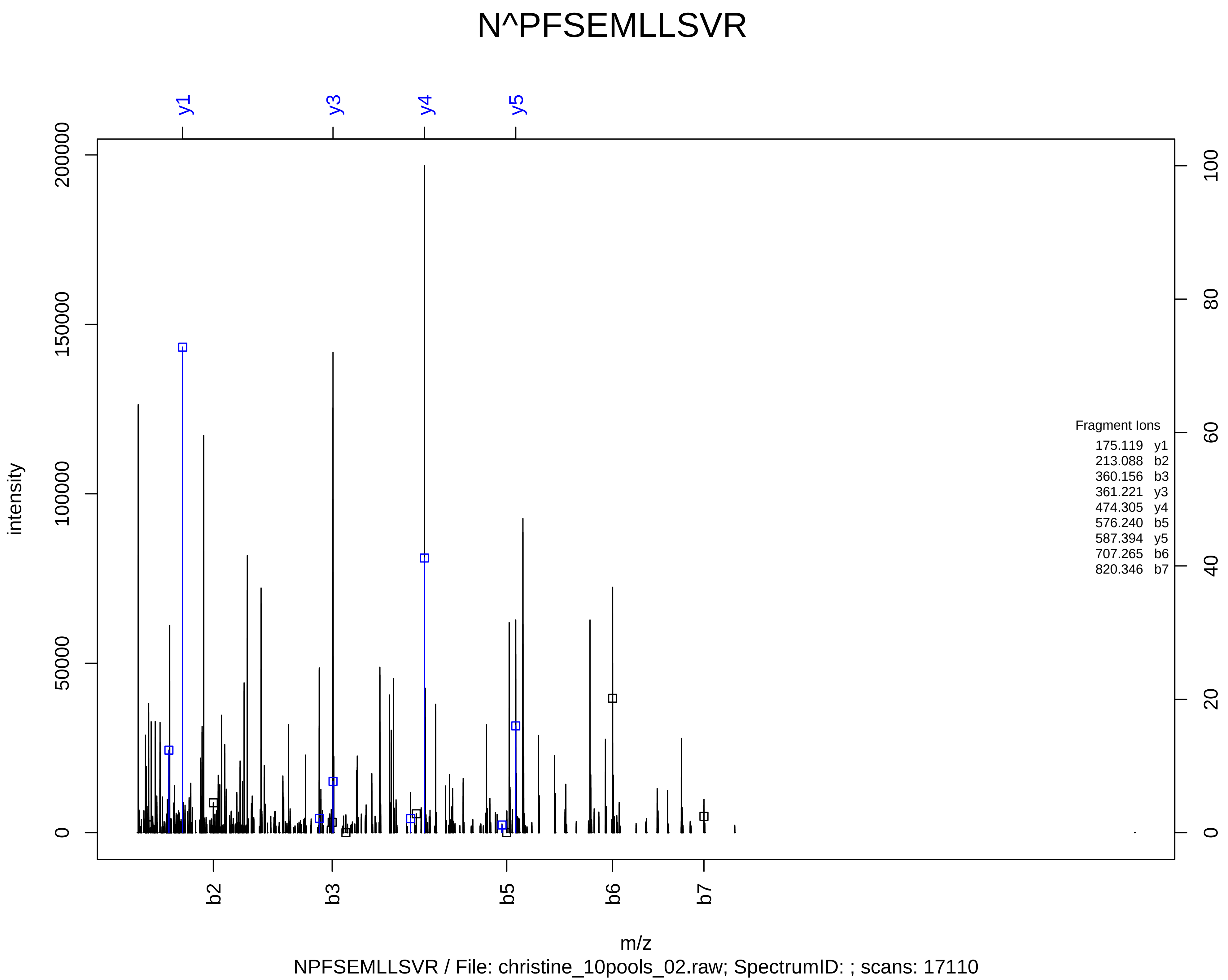

N^PFSEMLLSVR
y1
y3
y4
y5
intensity
0
50000
100000
150000
200000
0
20
40
60
80
100
b2
b3
b5
b6
b7
m/z
Fragment Ions
175.119 y1
213.088 b2
360.156 b3
361.221 y3
474.305 y4
576.240 b5
587.394 y5
707.265 b6
820.346 b7
NPFSEMLLSVR / File: christine_10pools_02.raw; SpectrumID: ; scans: 17110

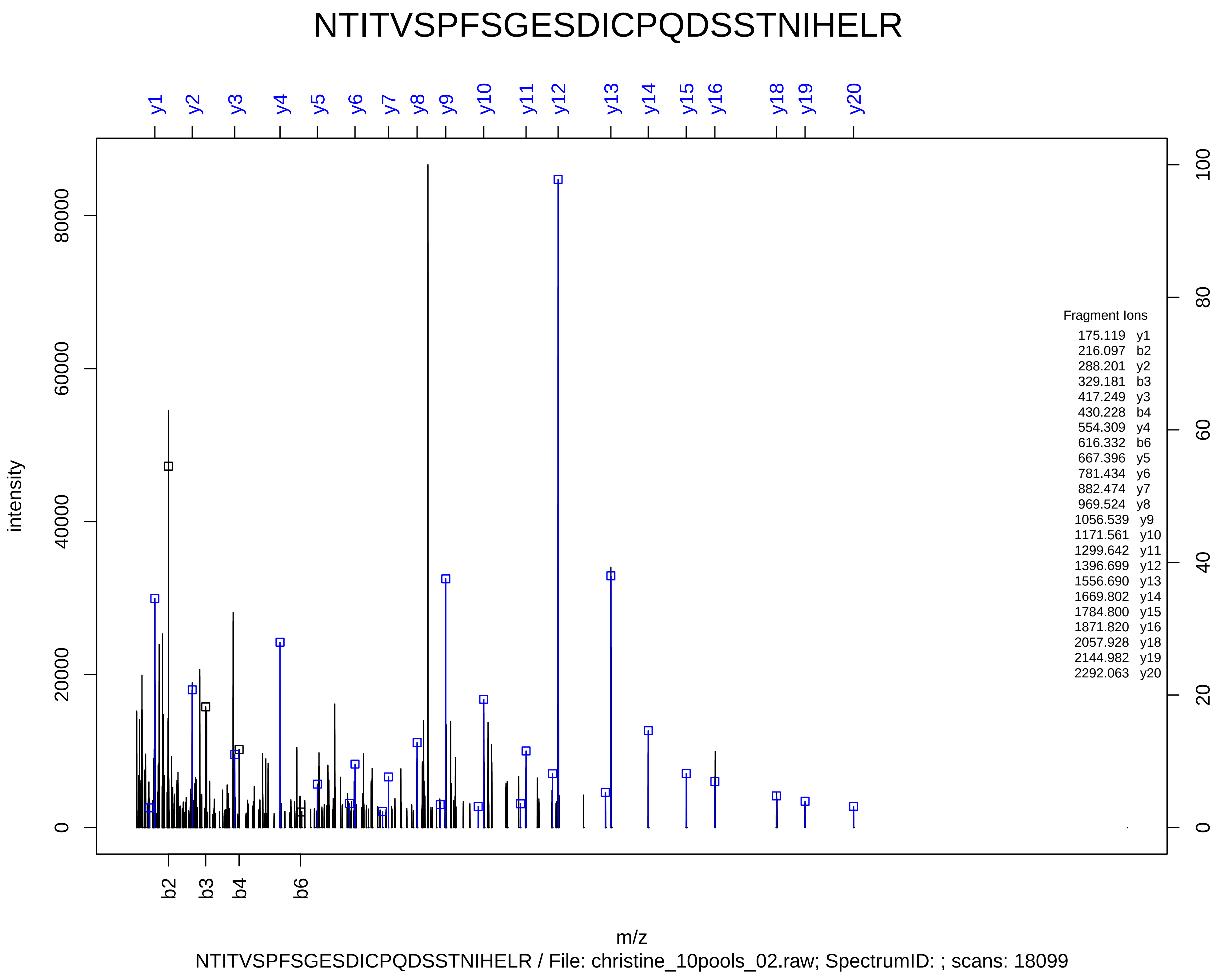
NTITVSPFSGESDICPQDSSTNIHELR
y1 y2 y3 y4 y5 y6 y7 y8 y9 y10 y11 y12 y13 y14 y15 y16 y18 y19 y20
b2 b3 b4 b6
intensity
0
20000
40000
60000
80000
0
20
40
60
80
100
m/z
Fragment Ions
175.119 y1
216.097 b2
288.201 y2
329.181 b3
417.249 y3
430.228 b4
554.309 y4
616.332 b6
667.396 y5
781.434 y6
882.474 y7
969.524 y8
1056.539 y9
1171.561 y10
1299.642 y11
1396.699 y12
1556.690 y13
1669.802 y14
1784.800 y15
1871.820 y16
2057.928 y18
2144.982 y19
2292.063 y20
NTITVSPFSGESDICPQDSSTNIHELR / File: christine_10pools_02.raw; SpectrumID: ; scans: 18099

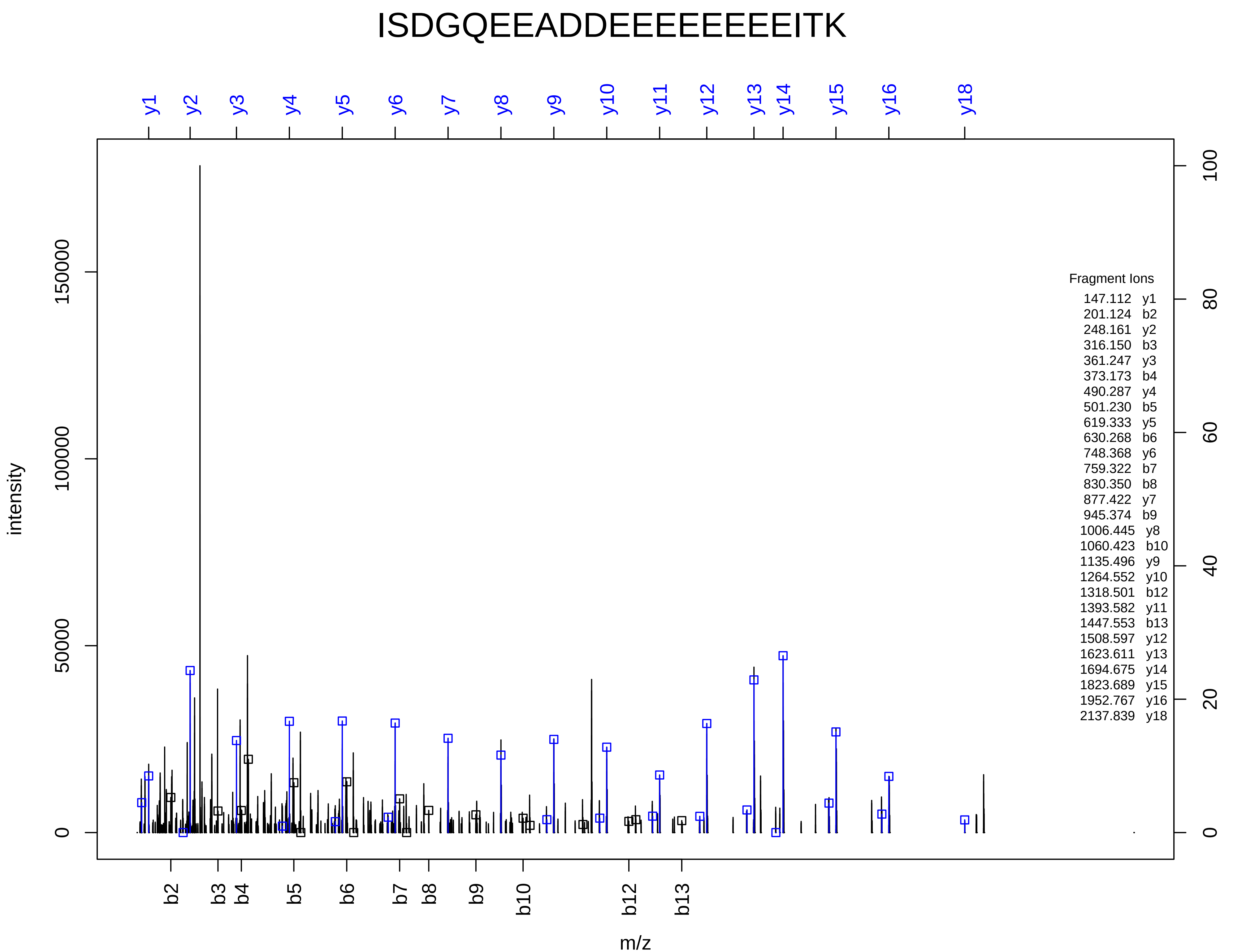

ISDGQEEADDEEEEEEEEEITK
y1 y2 y3 y4 y5 y6 y7 y8 y9 y10 y11 y12 y13 y14 y15 y16 y18
b2 b3 b4 b5 b6 b7 b8 b9 b10 b12 b13
intensity
0
50000
100000
150000
0
20
40
60
80
100
m/z
Fragment Ions
147.112 y1
201.124 b2
248.161 y2
316.150 b3
361.247 y3
373.173 b4
490.287 y4
501.230 b5
619.333 y5
630.268 b6
748.368 y6
759.322 b7
830.350 b8
877.422 y7
945.374 b9
1006.445 y8
1060.423 b10
1135.496 y9
1264.552 y10
1318.501 b12
1393.582 y11
1447.553 b13
1508.597 y12
1623.611 y13
1694.675 y14
1823.689 y15
1952.767 y16
2137.839 y18
ISDGQEEADDEEEEEEEEEITK / File: christine_10pools_02.raw; SpectrumID: ; scans: 9053

DAVSPFSFSLLK

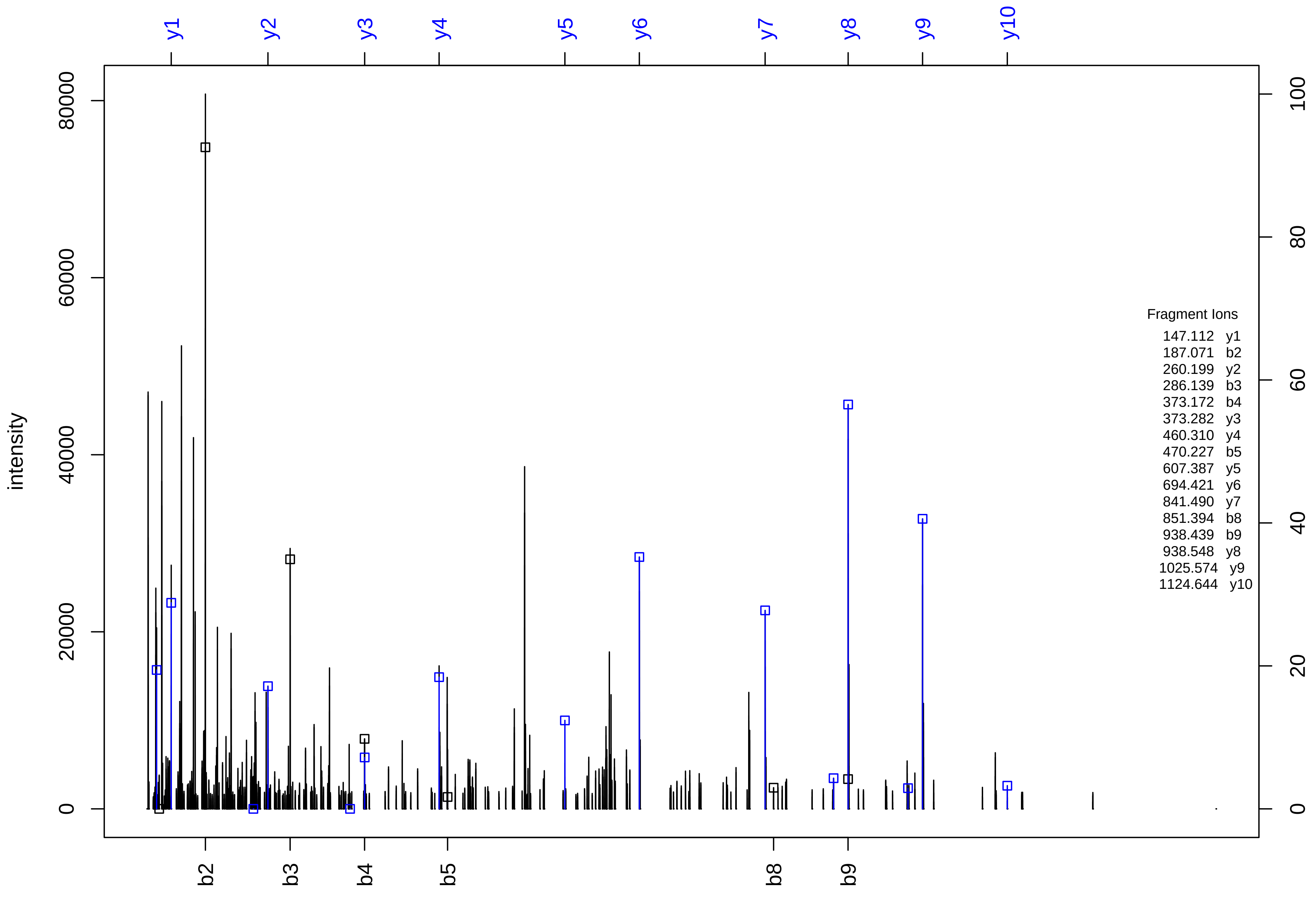

DAVSPFSFSLLK / File: christine_10pools_02.raw; SpectrumID: ; scans: 32750

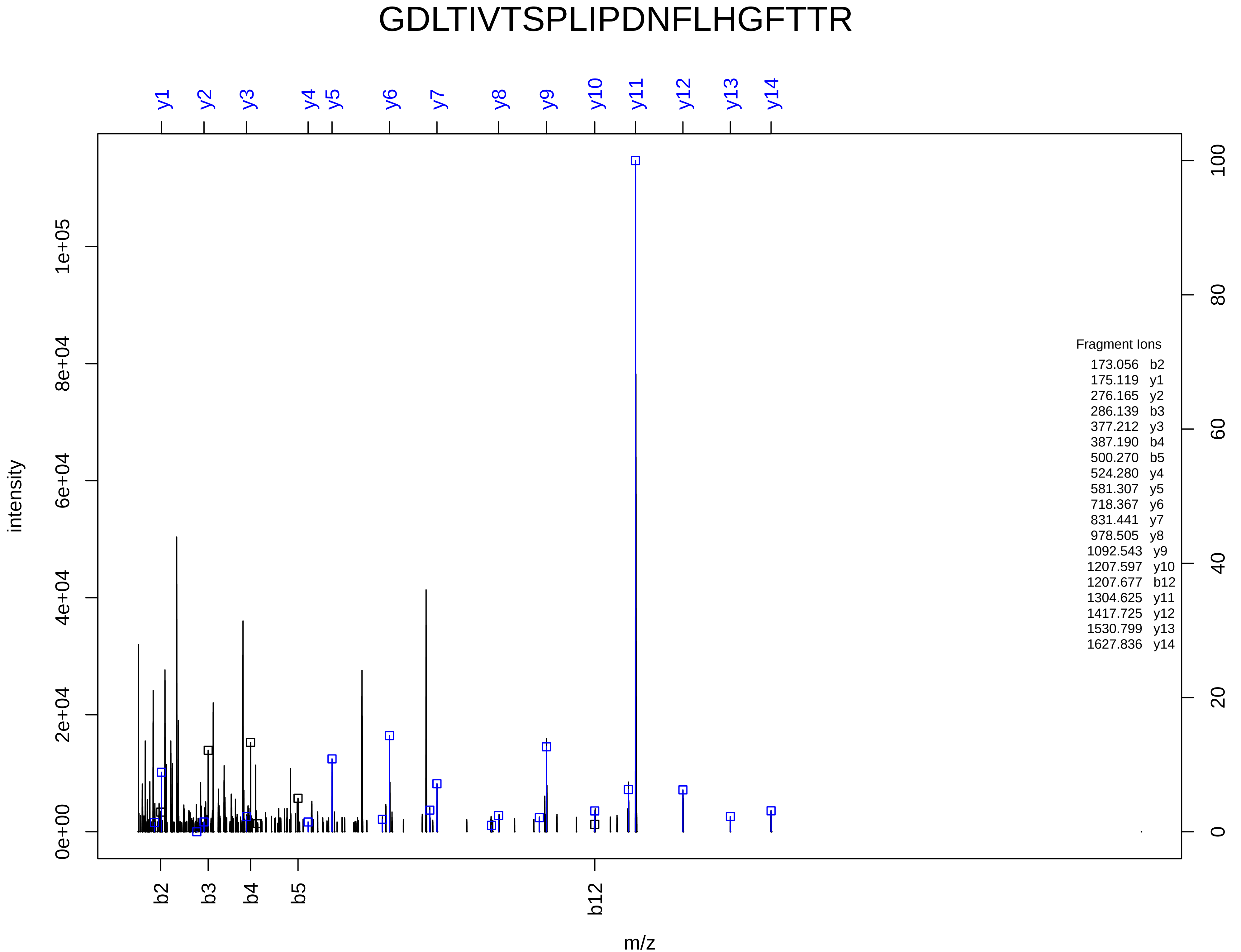

GDLTIVTSPLIPDNFLHGFTTR
y1
y2
y3
y4
y5
y6
y7
y8
y9
y10
y11
y12
y13
y14
intensity
0e+00
2e+04
4e+04
6e+04
8e+04
1e+05
0
20
40
60
80
100
Fragment Ions
173.056 b2
175.119 y1
276.165 y2
286.139 b3
377.212 y3
387.190 b4
500.270 b5
524.280 y4
581.307 y5
718.367 y6
831.441 y7
978.505 y8
1092.543 y9
1207.597 y10
1207.677 b12
1304.625 y11
1417.725 y12
1530.799 y13
1627.836 y14
b2
b3
b4
b5
b12
m/z
GDLTIVTSPLIPDNFLHGFTTR / File: christine_10pools_02.raw; SpectrumID: ; scans: 36651

DSPPQEVEAVSWASGPGSSENLK

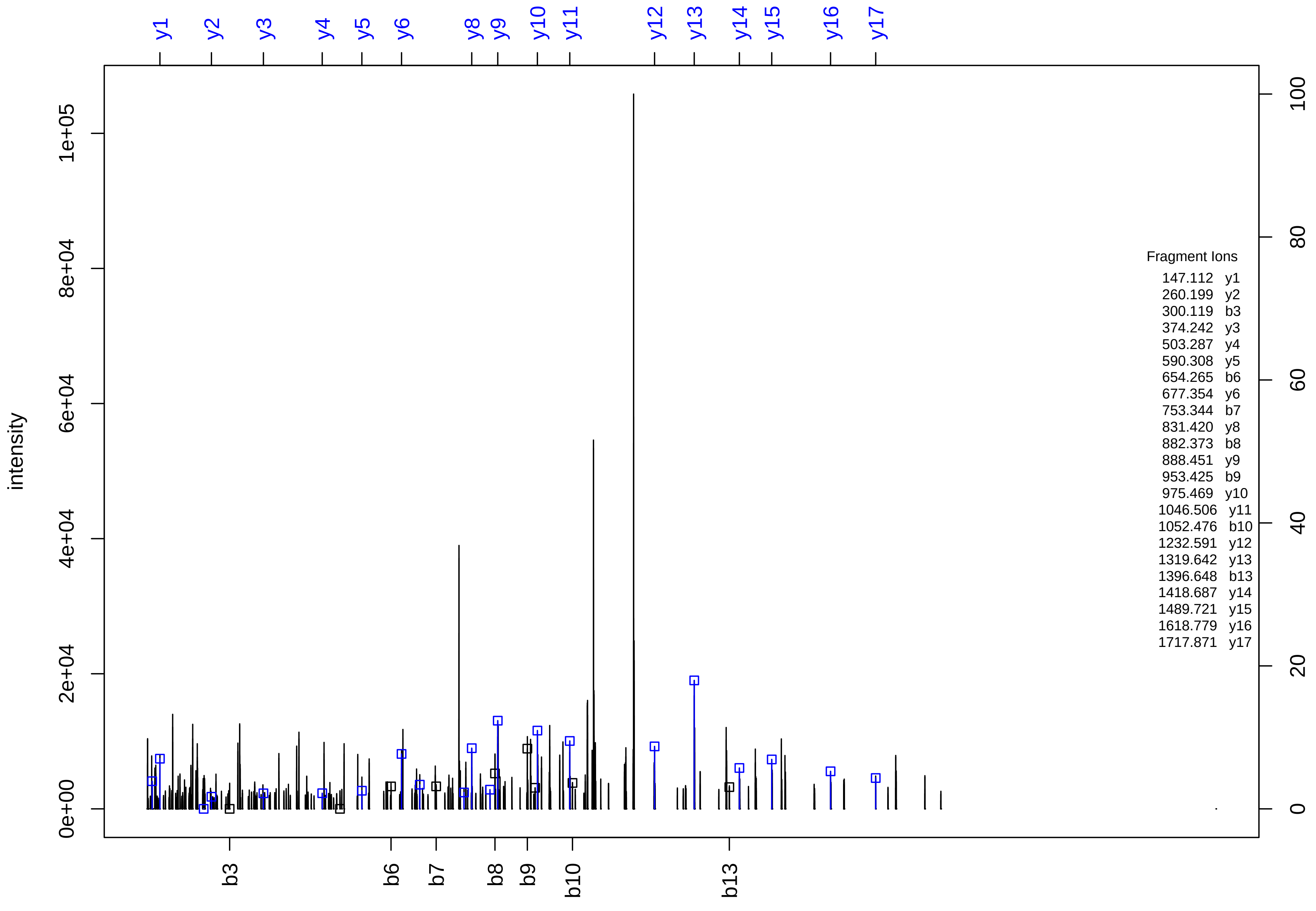

DSPPQEVEAVSWASGPGSSENLK / File: christine_10pools_02.raw; SpectrumID: ; scans: 18962

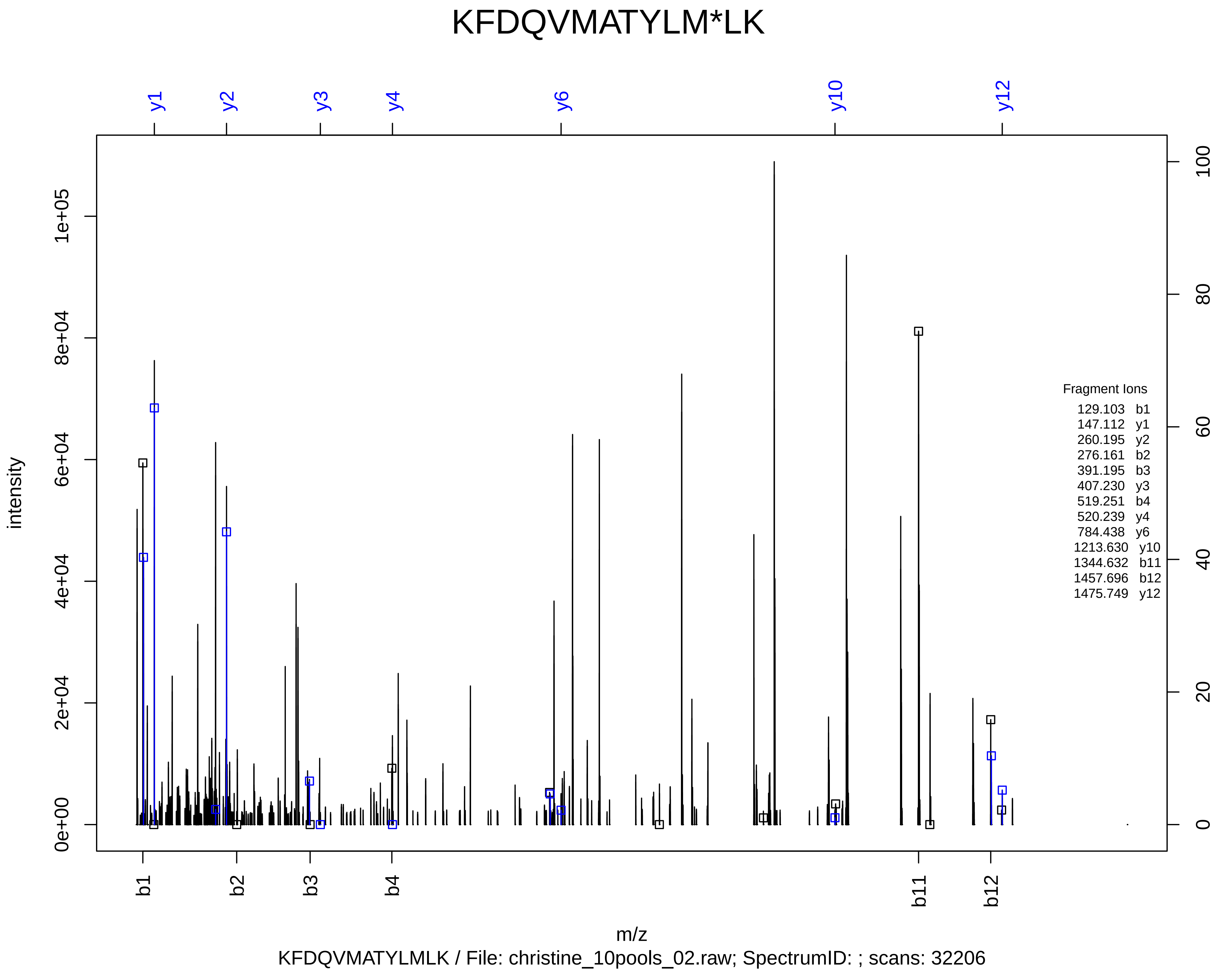

KFDQVMATYLM*LK
y1
y2
y3
y4
y6
y10
y12
b1
b2
b3
b4
b11
b12
intensity
m/z
Fragment Ions
129.103 b1
147.112 y1
260.195 y2
276.161 b2
391.195 b3
407.230 y3
519.251 b4
520.239 y4
784.438 y6
1213.630 y10
1344.632 b11
1457.696 b12
1475.749 y12
KFDQVMATYLMLK / File: christine_10pools_02.raw; SpectrumID: ; scans: 32206

FIADGGPEVETIALQNNR

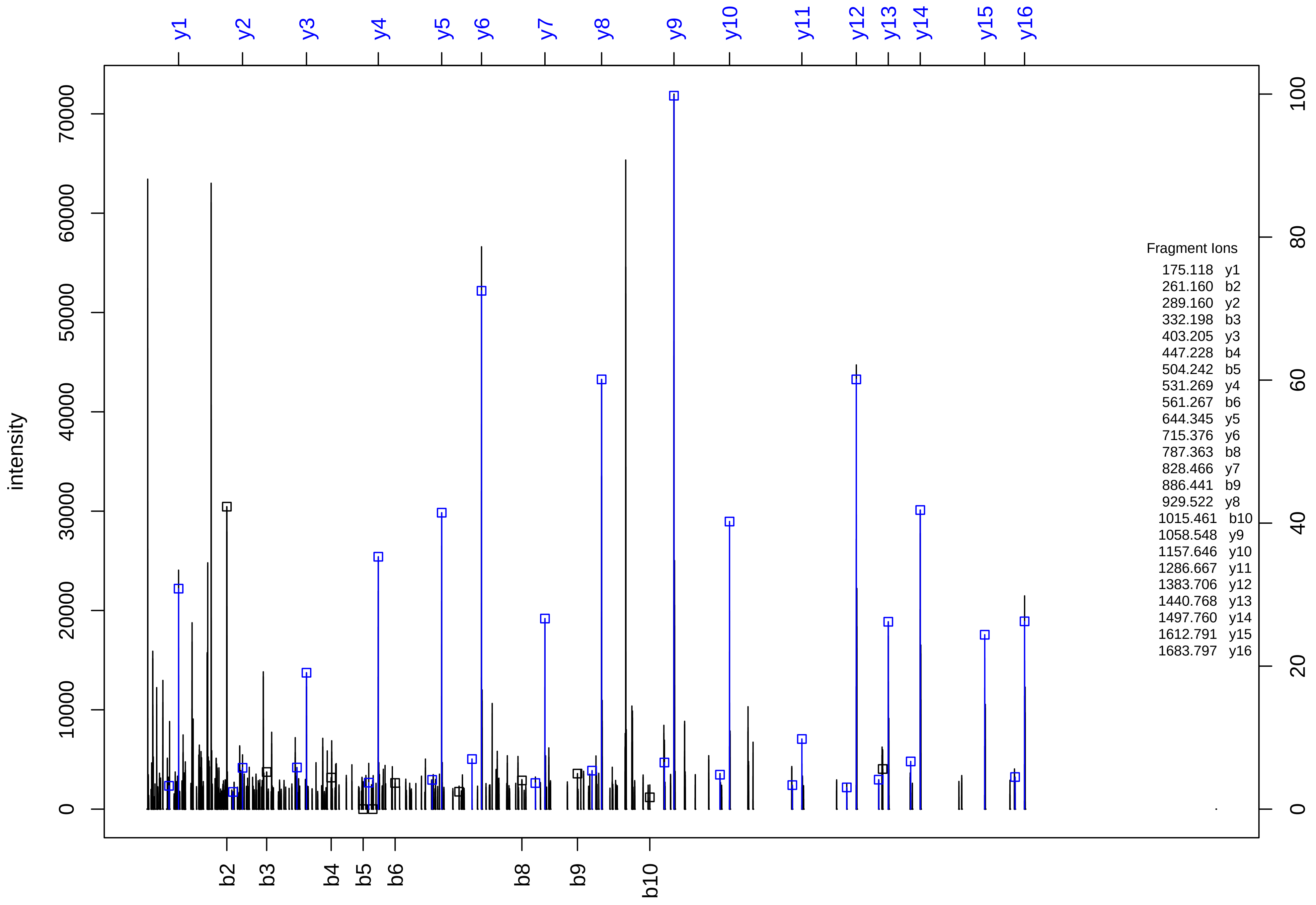

FIADGGPEVETIALQNNR / File: christine_10pools_02.raw; SpectrumID: ; scans: 19046

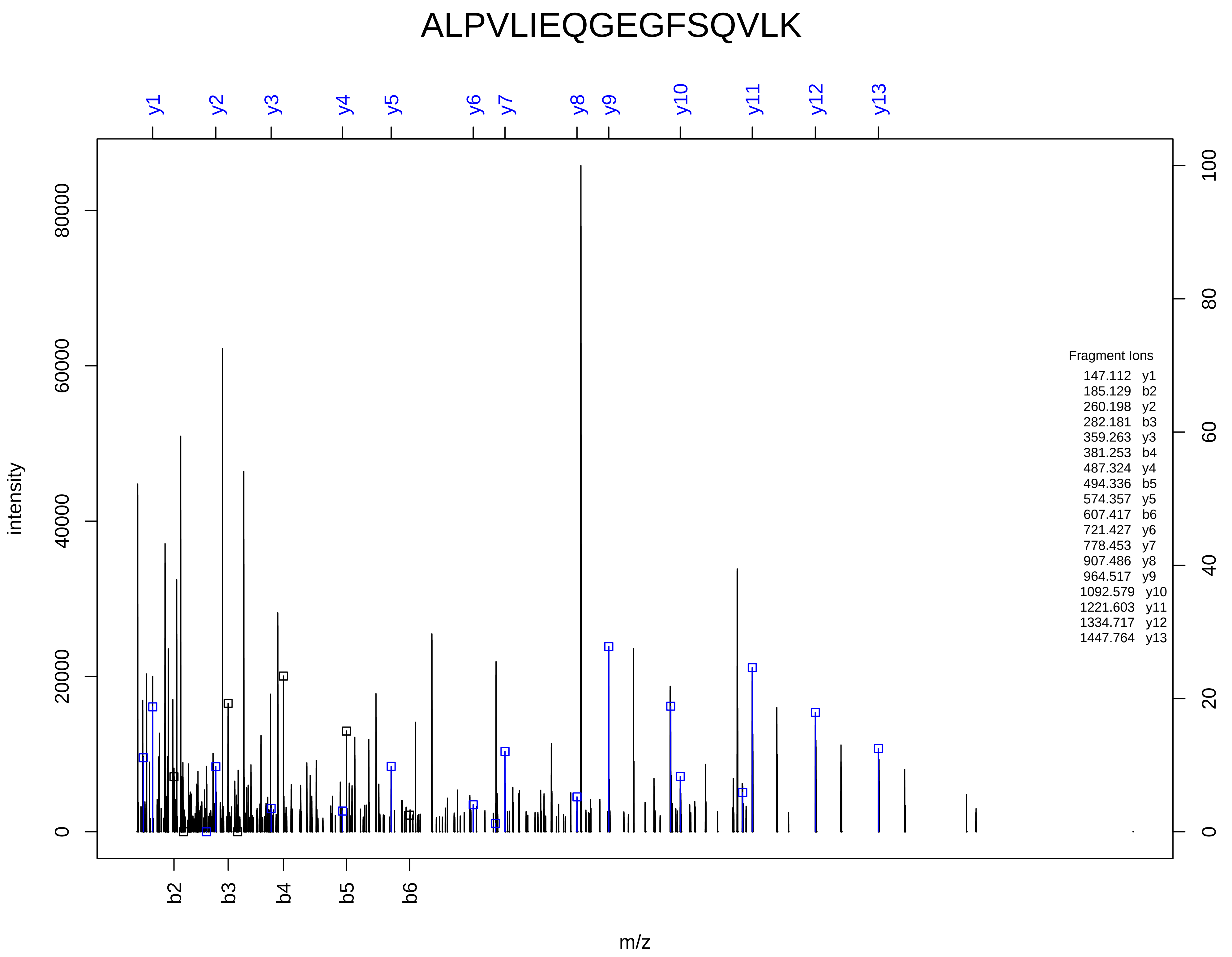
ALPVLIEQGEGFSQVLK
y1 y2 y3 y4 y5 y6 y7 y8 y9 y10 y11 y12 y13
b2 b3 b4 b5 b6
intensity
m/z
Fragment Ions
147.112 y1
185.129 b2
260.198 y2
282.181 b3
359.263 y3
381.253 b4
487.324 y4
494.336 b5
574.357 y5
607.417 b6
721.427 y6
778.453 y7
907.486 y8
964.517 y9
1092.579 y10
1221.603 y11
1334.717 y12
1447.764 y13
ALPVLIEQGEGFSQVLK / File: christine_10pools_02.raw; SpectrumID: ; scans: 29934

SALDEIMELEEEK

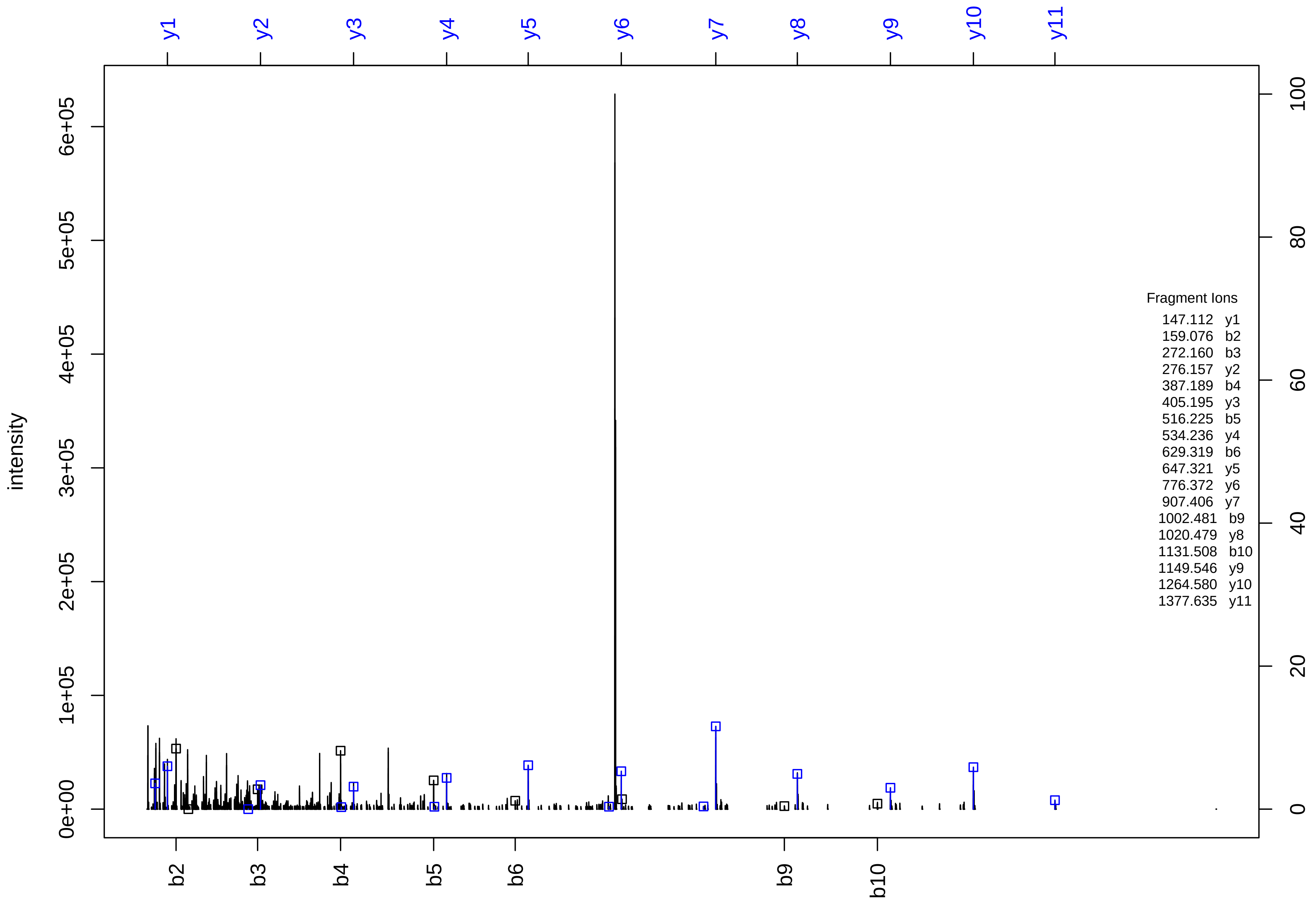

SALDEIMELEEEK / File: christine_10pools_02.raw; SpectrumID: ; scans: 28402

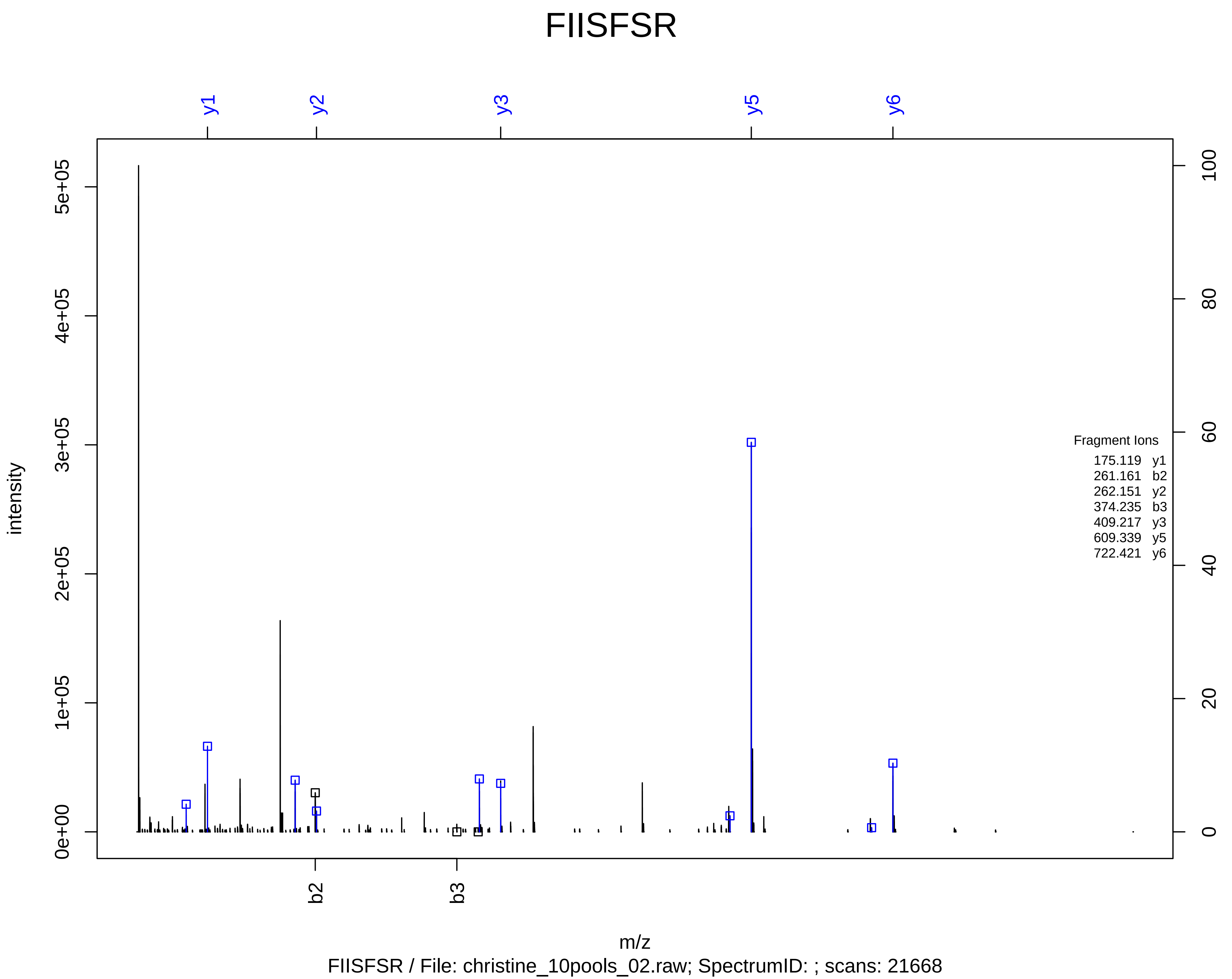

FIISFSR
y1
y2
y3
y5
y6
intensity
0e+00
1e+05
2e+05
3e+05
4e+05
5e+05
0
20
40
60
80
100
Fragment Ions
175.119 y1
261.161 b2
262.151 y2
374.235 b3
409.217 y3
609.339 y5
722.421 y6
b2
b3
m/z
FIISFSR / File: christine_10pools_02.raw; SpectrumID: ; scans: 21668

LAVLVPFR

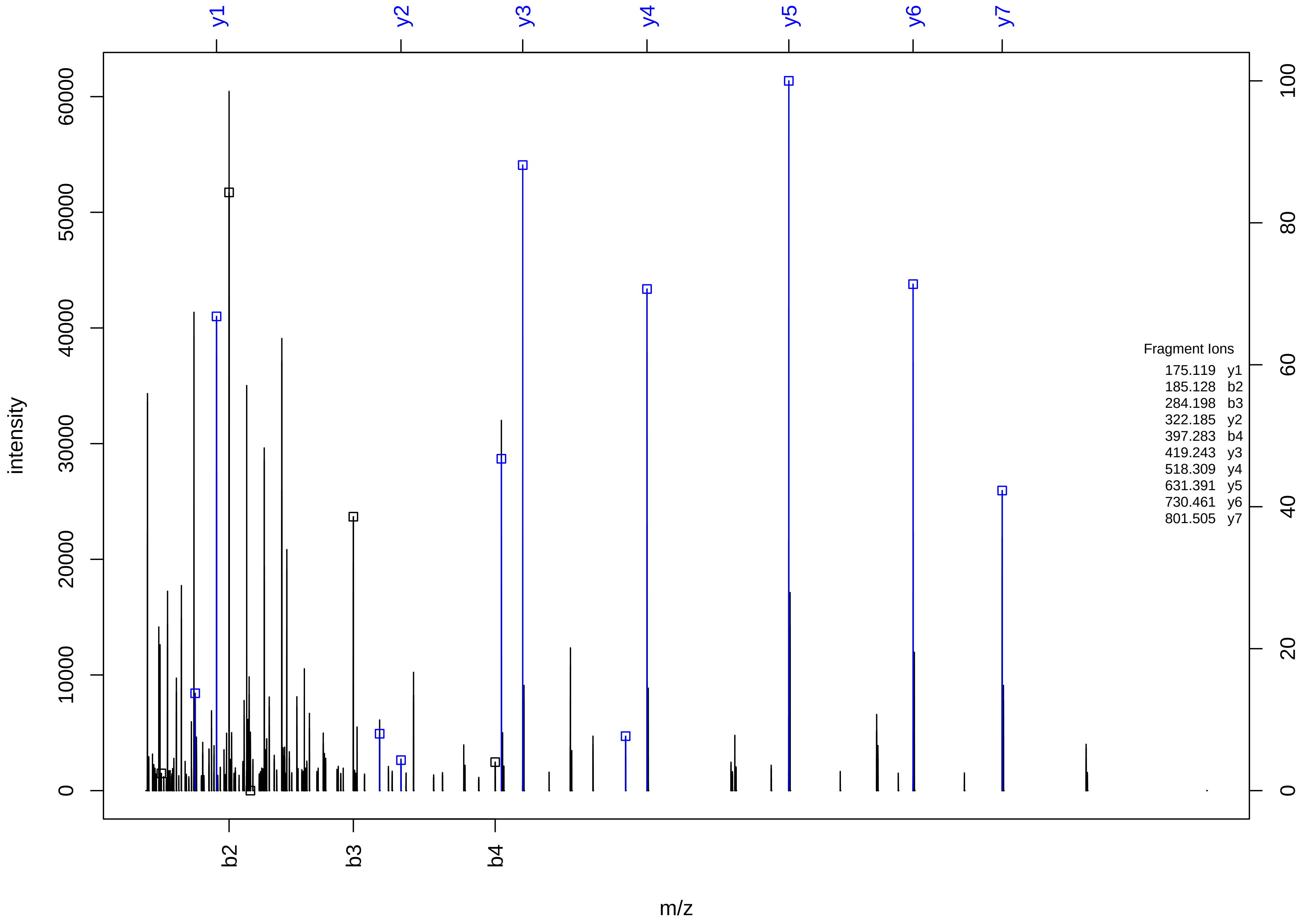

LAVLVPFR / File: christine_10pools_02.raw; SpectrumID: ; scans: 22424

SQNTPDSSASNLGFR

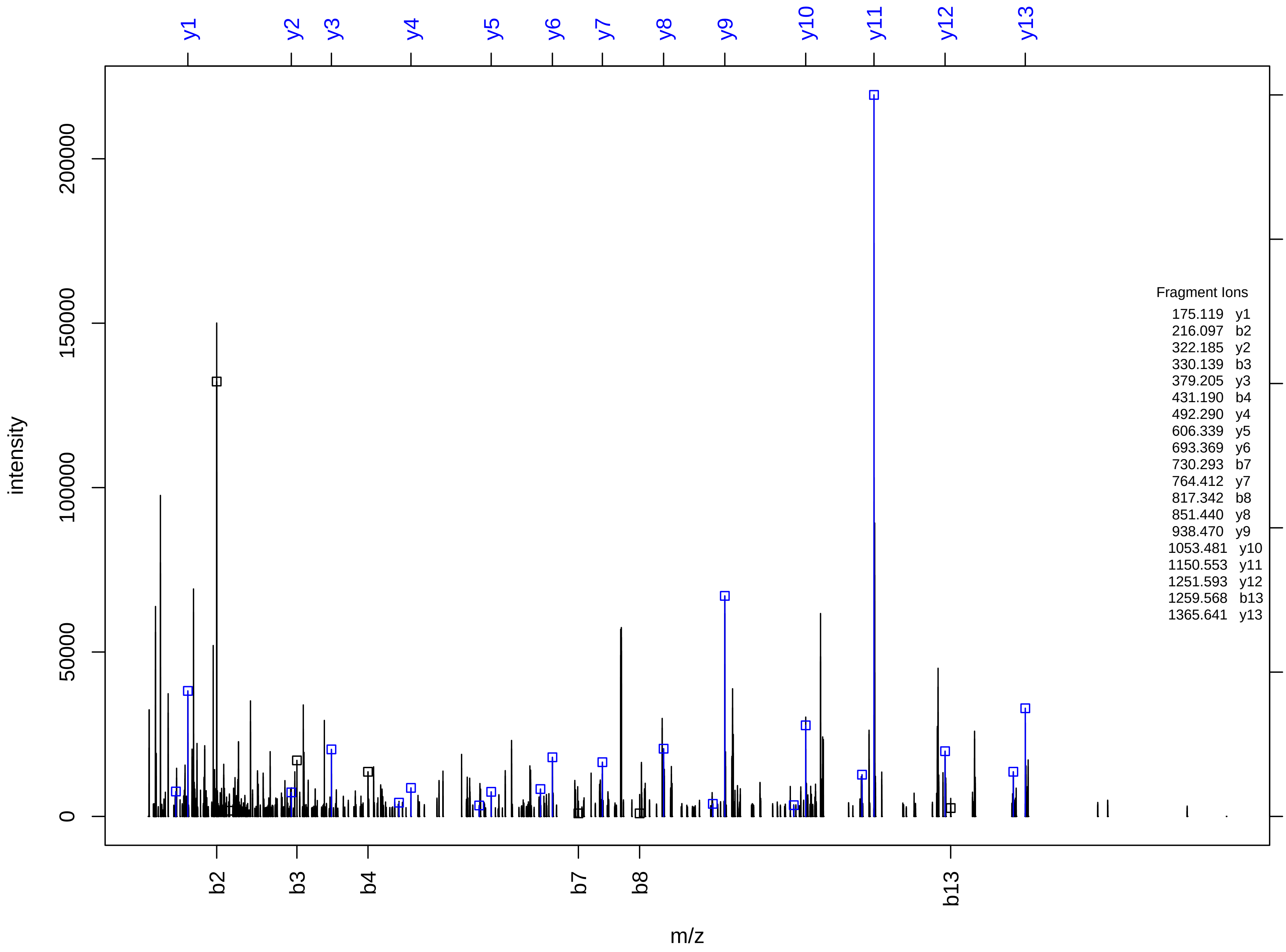

SQNTPDSSASNLGFR / File: christine_10pools_02.raw; SpectrumID: ; scans: 5325

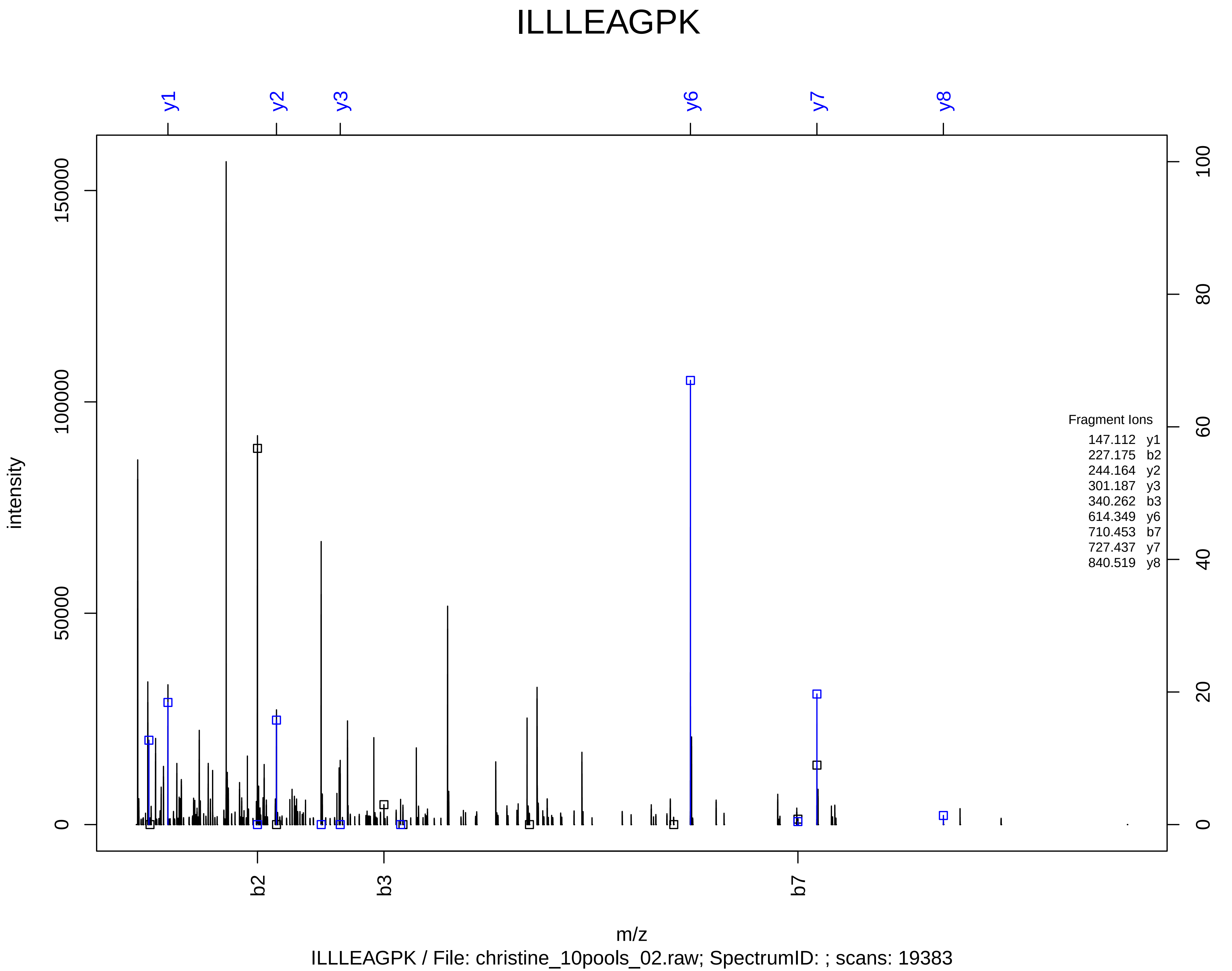
ILLLEAGPK
y1
y2
y3
y6
y7
y8
intensity
0
50000
100000
150000
0
20
40
60
80
100
Fragment Ions
147.112 y1
227.175 b2
244.164 y2
301.187 y3
340.262 b3
614.349 y6
710.453 b7
727.437 y7
840.519 y8
b2
b3
b7
m/z
ILLLEAGPK / File: christine_10pools_02.raw; SpectrumID: ; scans: 19383

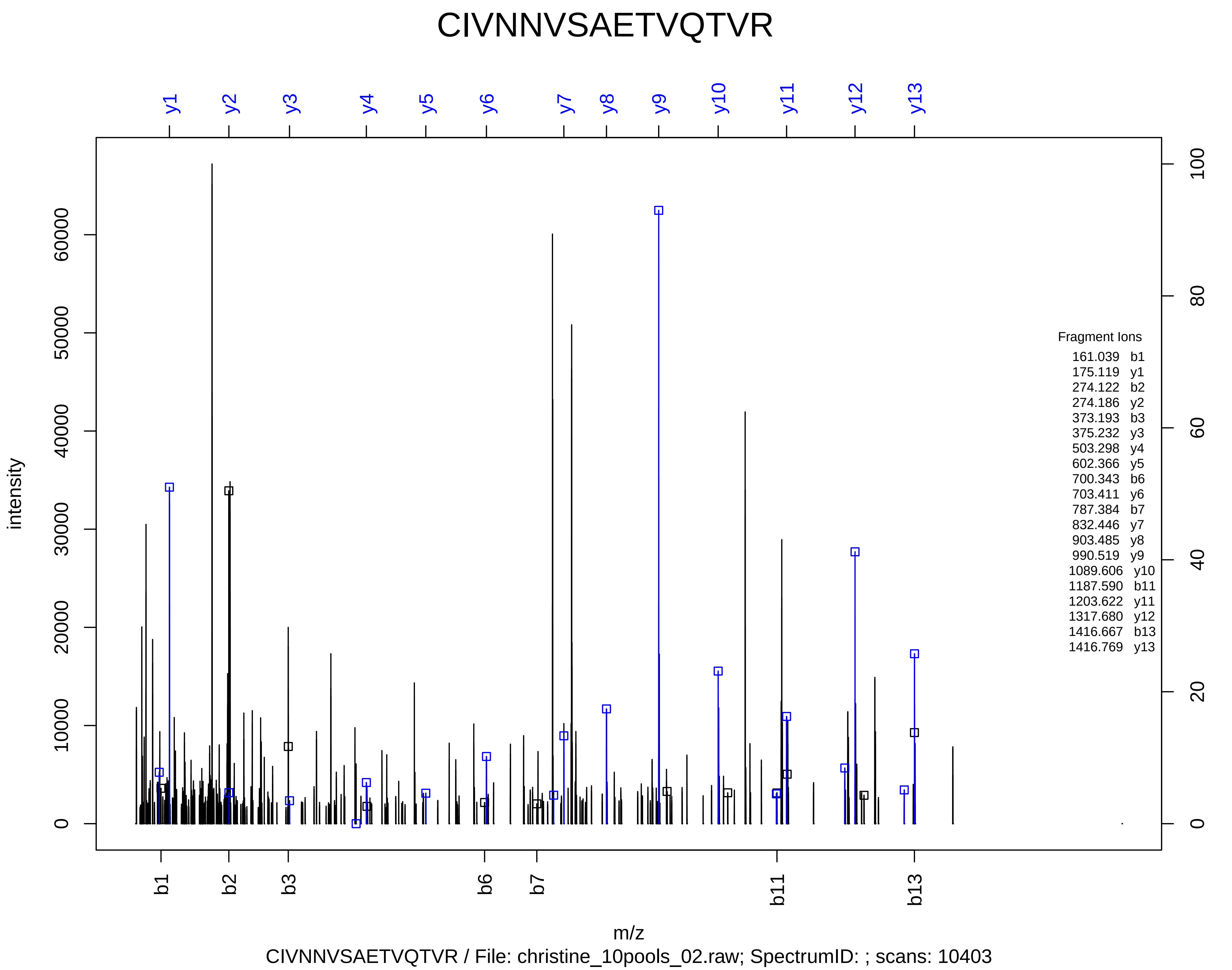

CIVNNVSAETVQTVR
y1 y2 y3 y4 y5 y6 y7 y8 y9 y10 y11 y12 y13
b1 b2 b3 b6 b7 b11 b13
intensity
m/z
Fragment Ions
161.039 b1
175.119 y1
274.122 b2
274.186 y2
373.193 b3
375.232 y3
503.298 y4
602.366 y5
700.343 b6
703.411 y6
787.384 b7
832.446 y7
903.485 y8
990.519 y9
1089.606 y10
1187.590 b11
1203.622 y11
1317.680 y12
1416.667 b13
1416.769 y13
CIVNNVSAETVQTVR / File: christine_10pools_02.raw; SpectrumID: ; scans: 10403

AAVQQAPDESQMAQTPDGTQLPSGHPSPATSQGSGSK

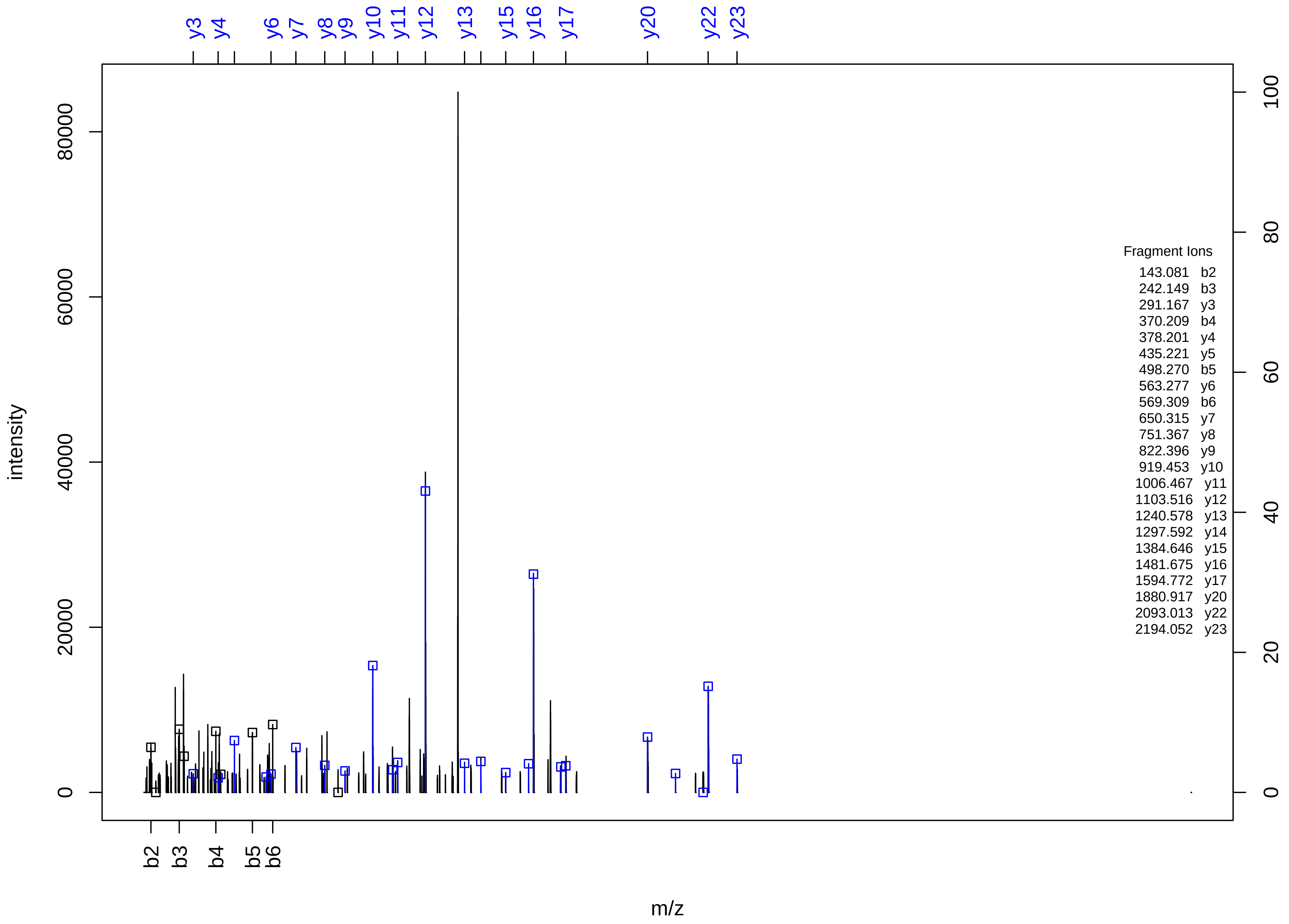

AAVQQAPDESQMAQTPDGTQLPSGHPSPATSQGSGSK / File: christine_10pools_02.raw; SpectrumID: ; scans: 6348

(Ac)ASPAPEEHATQGCPATEEQPPRPGVPGEEAGPEGAGPQVEEAAGR

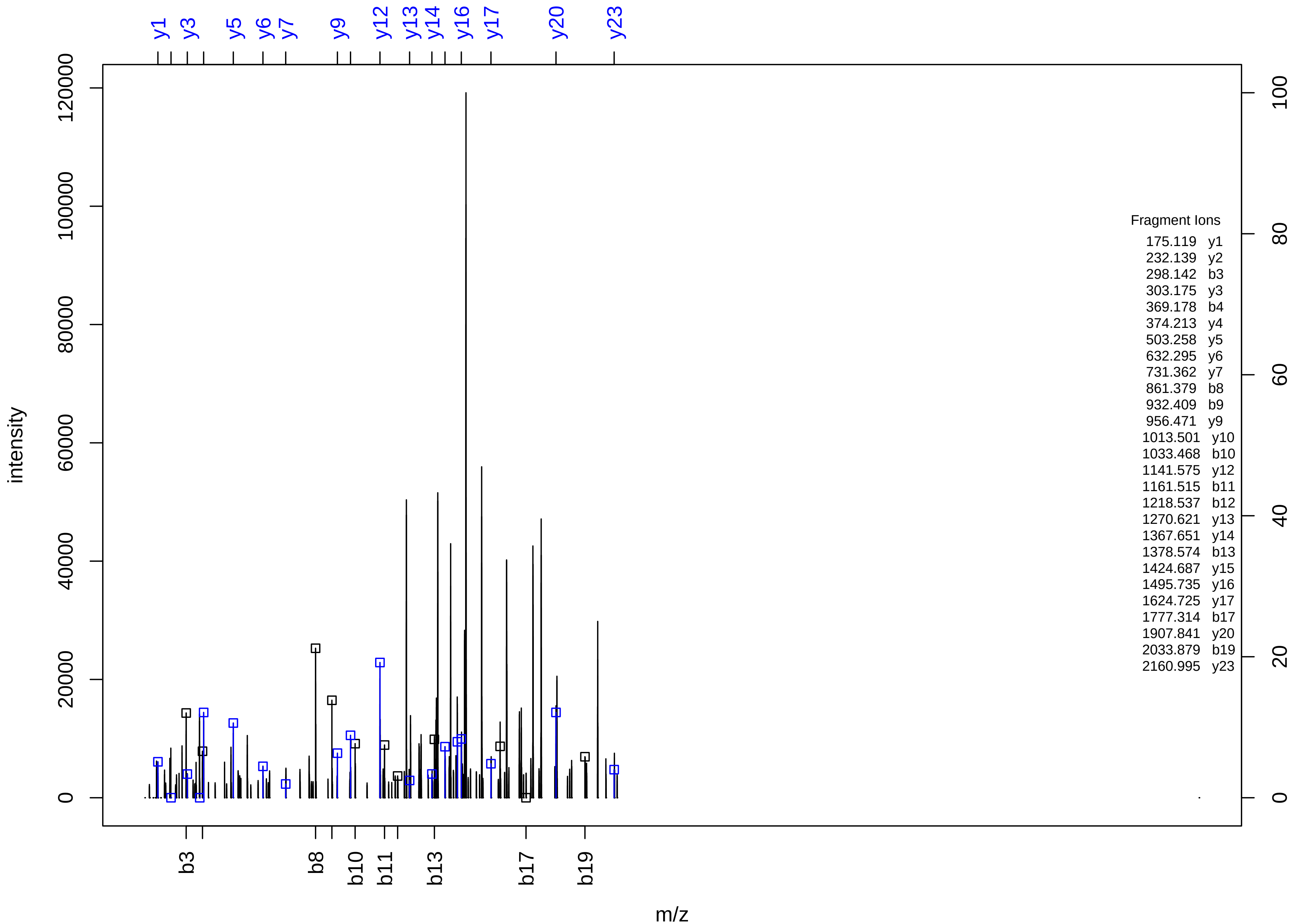

ASPAPEEHATQGCPATEEQPPRPGVPGEEAGPEGAGPQVEEAAGR / File: christine_10pools_02.raw; SpectrumID: ; scans: 10592

ALADILSESLHSLATSLPR

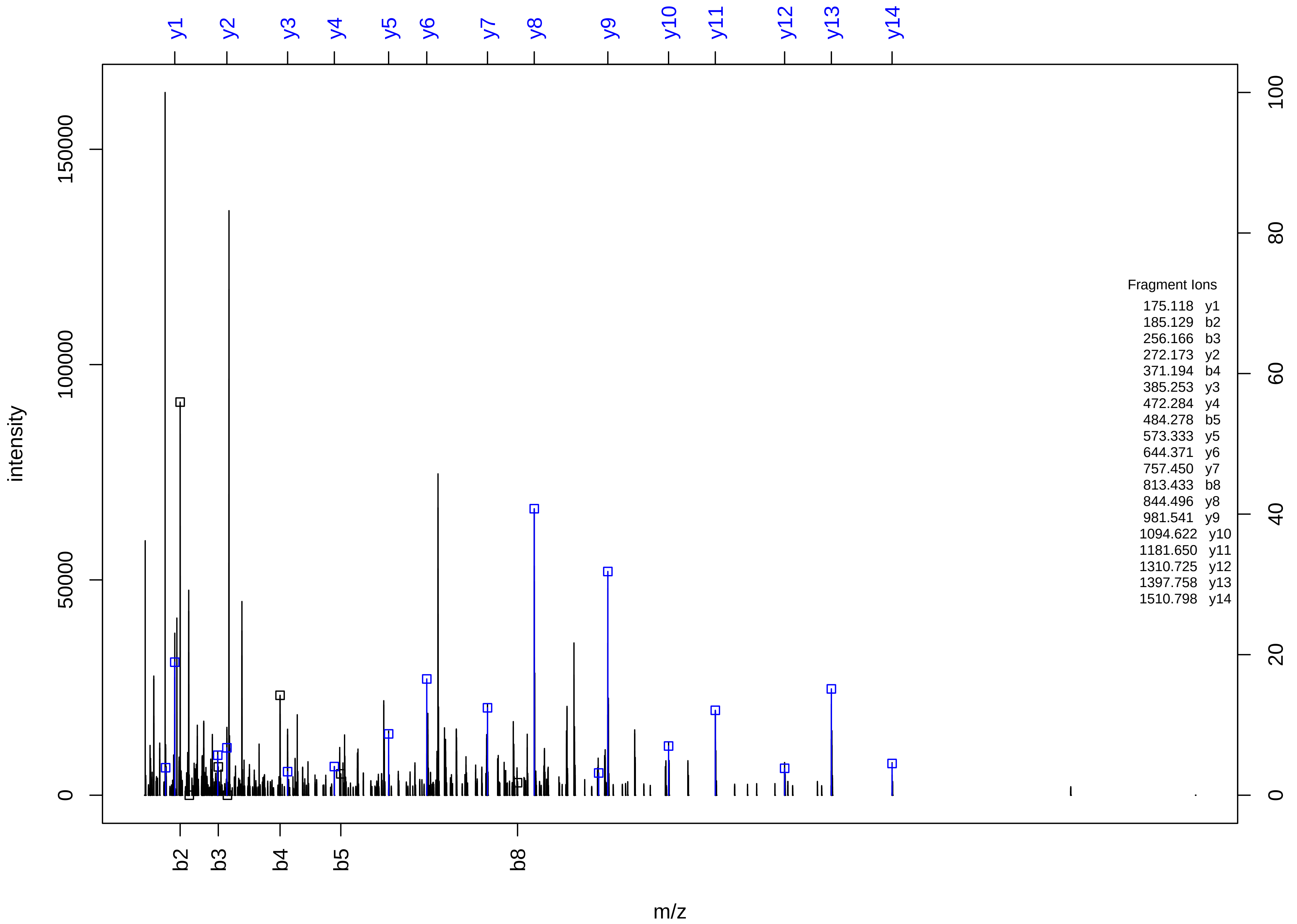

ALADILSESLHSLATSLPR / File: christine_10pools_02.raw; SpectrumID: ; scans: 41380

FLEPYIYPVVSR

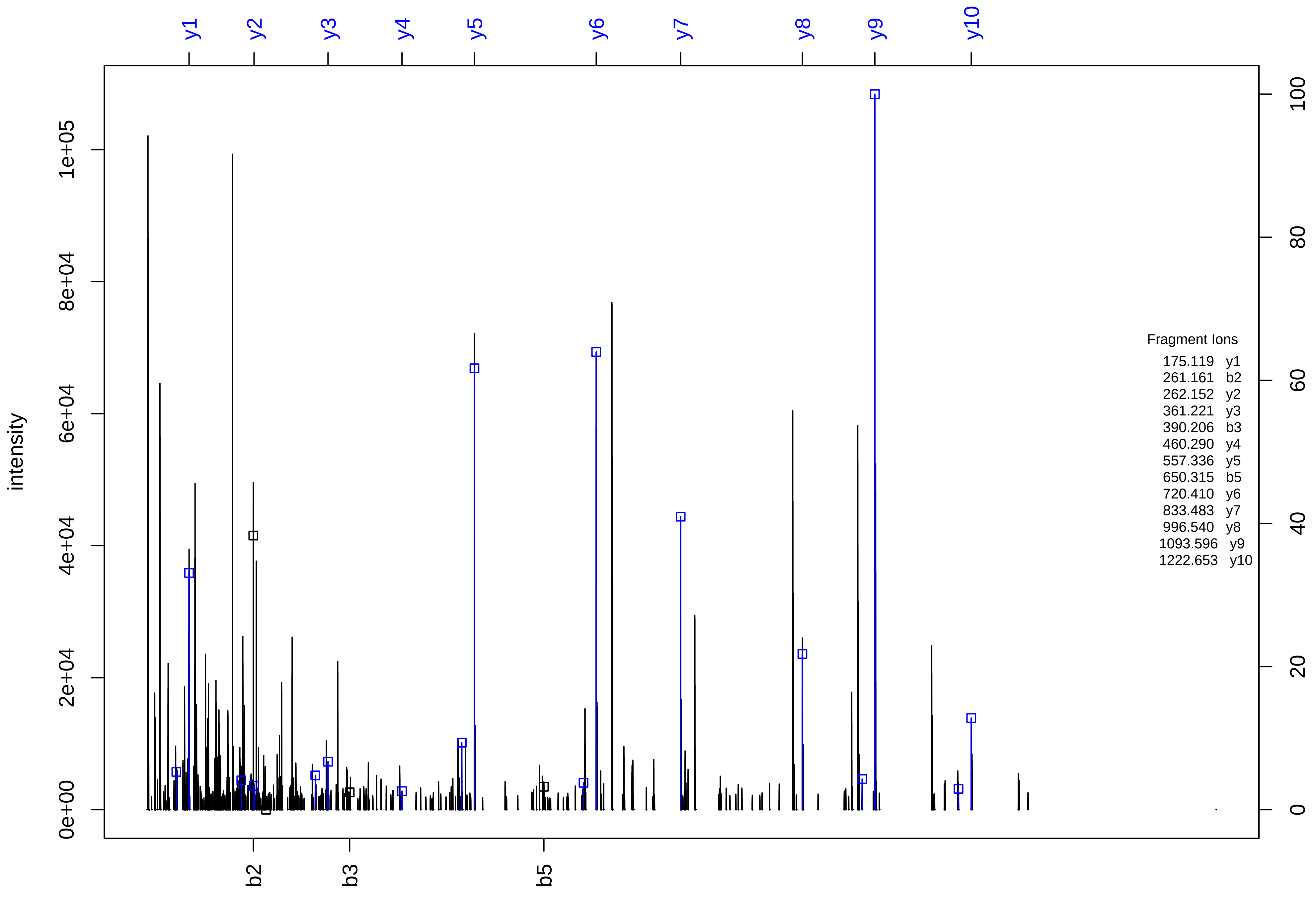

FLEPYIYPVVSR / File: christine_10pools_02.raw; SpectrumID: ; scans: 25781

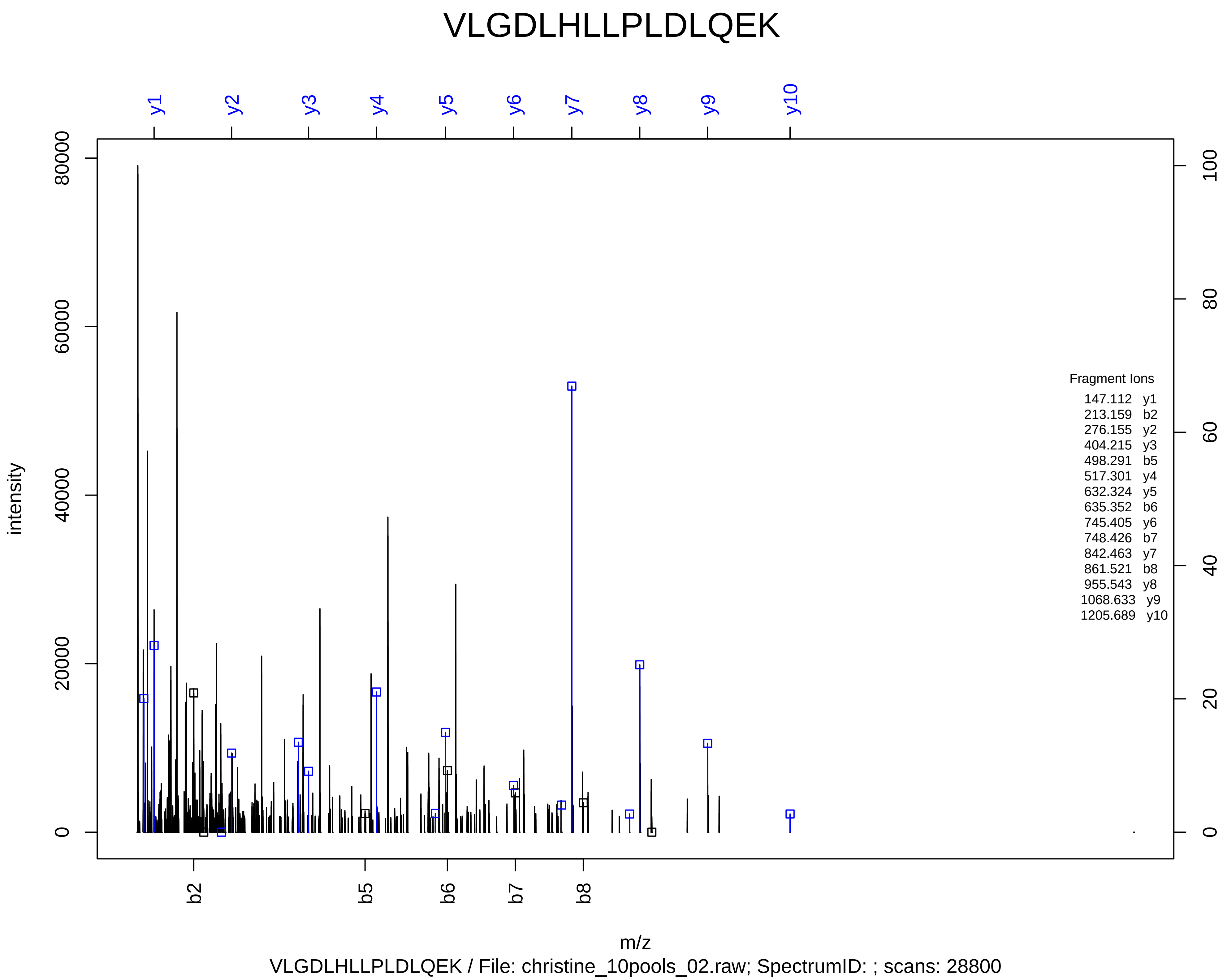
VLGDLHLLPLDLQEK
y1
y2
y3
y4
y5
y6
y7
y8
y9
y10
intensity
0
20000
40000
60000
80000
0
20
40
60
80
100
b2
b5
b6
b7
b8
m/z
Fragment Ions
147.112 y1
213.159 b2
276.155 y2
404.215 y3
498.291 b5
517.301 y4
632.324 y5
635.352 b6
745.405 y6
748.426 b7
842.463 y7
861.521 b8
955.543 y8
1068.633 y9
1205.689 y10
VLGDLHLLPLDLQEK / File: christine_10pools_02.raw; SpectrumID: ; scans: 28800

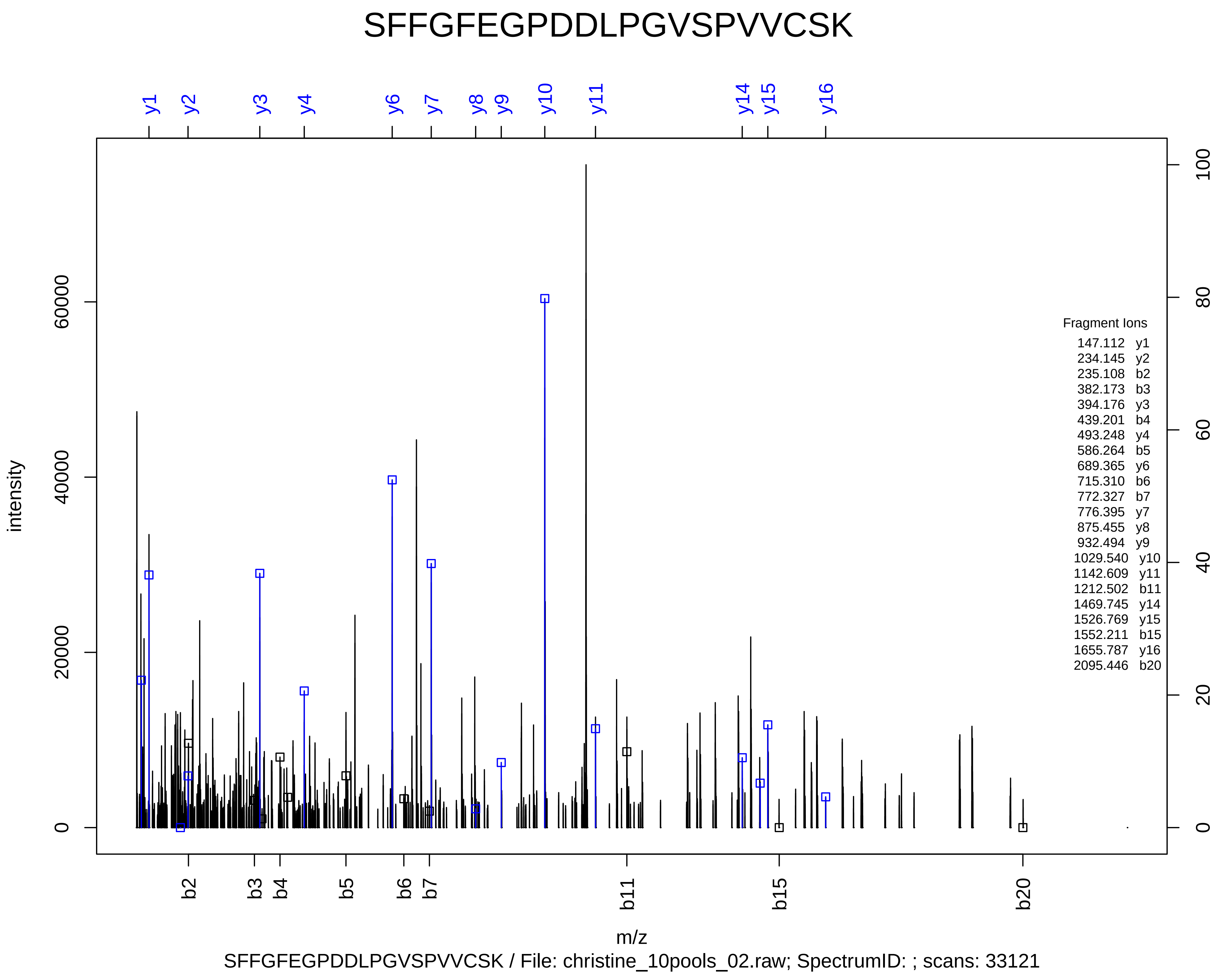
SFFGFEGPDDLPGVSPVVCSK
y1
y2
y3
y4
y6
y7
y8
y9
y10
y11
y14
y15
y16
intensity
0
20000
40000
60000
0
20
40
60
80
100
Fragment Ions
147.112 y1
234.145 y2
235.108 b2
382.173 b3
394.176 y3
439.201 b4
493.248 y4
586.264 b5
689.365 y6
715.310 b6
772.327 b7
776.395 y7
875.455 y8
932.494 y9
1029.540 y10
1142.609 y11
1212.502 b11
1469.745 y14
1526.769 y15
1552.211 b15
1655.787 y16
2095.446 b20
b2
b3
b4
b5
b6
b7
b11
b15
b20
m/z
SFFGFEGPDDLPGVSPVVCSK / File: christine_10pools_02.raw; SpectrumID: ; scans: 33121

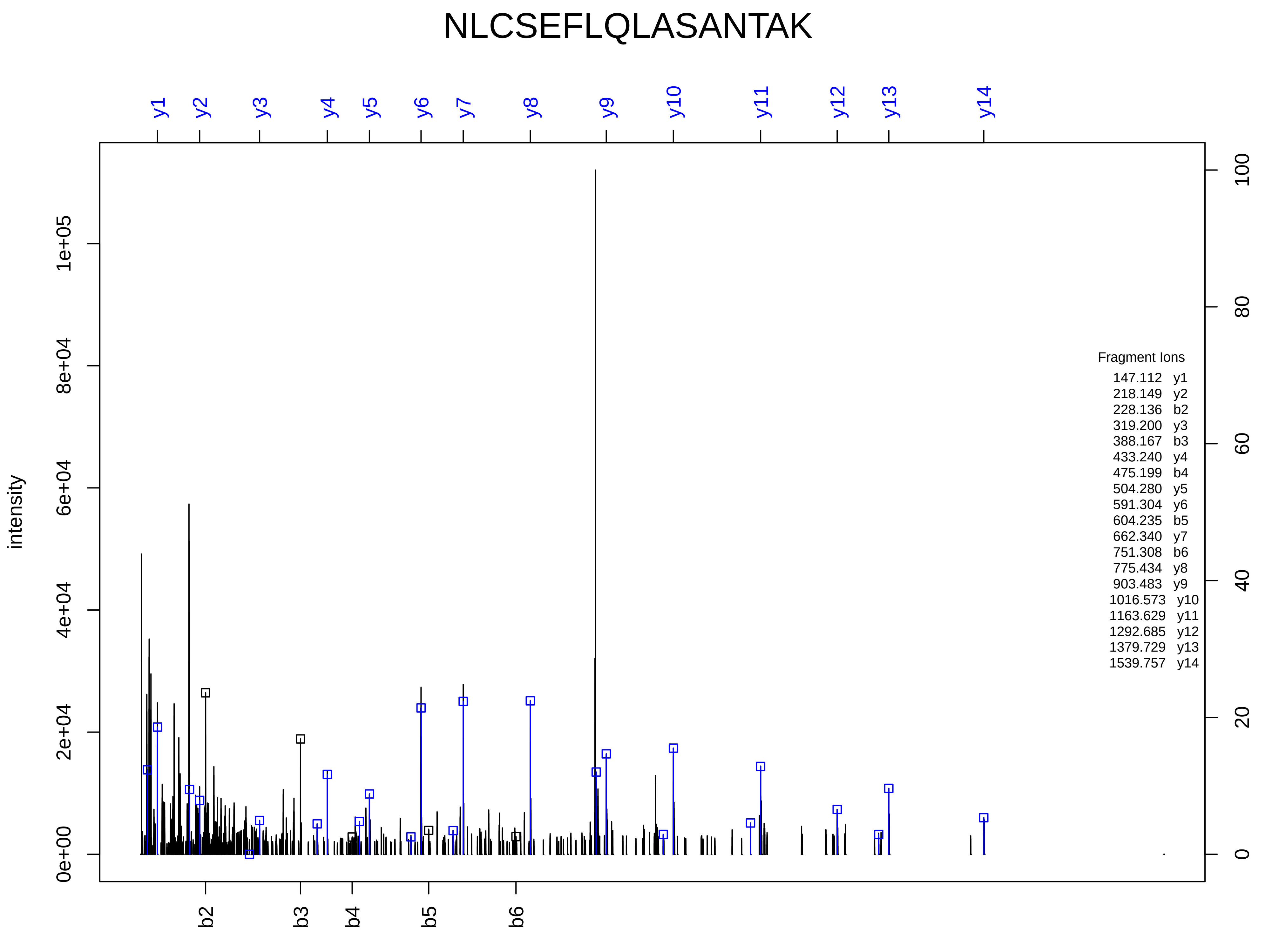

NLCSEFLQLASANTAK / File: christine_10pools_02.raw; SpectrumID: ; scans: 27948

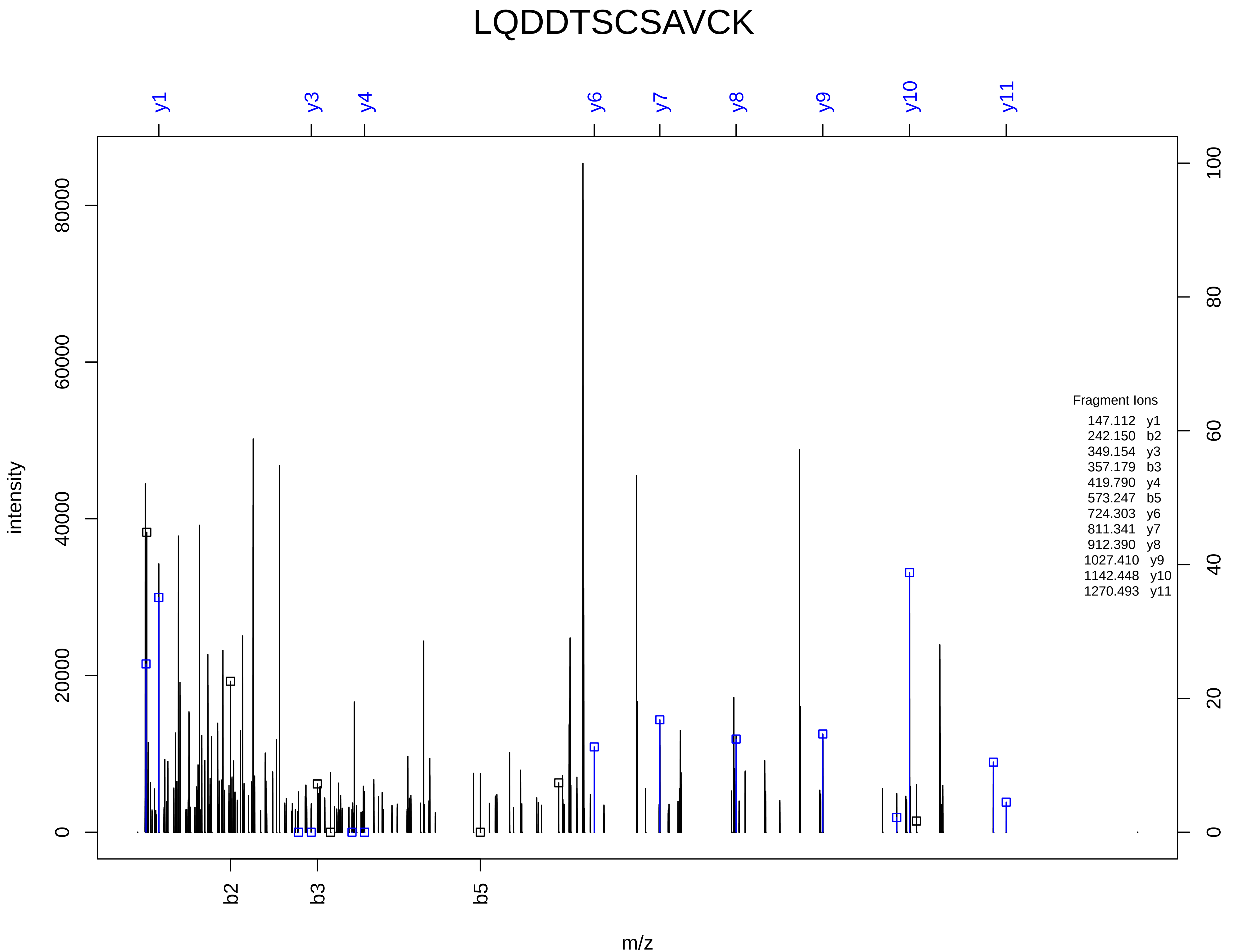

LQDDTSCSAVCK / File: christine_10pools_02.raw; SpectrumID: ; scans: 1335

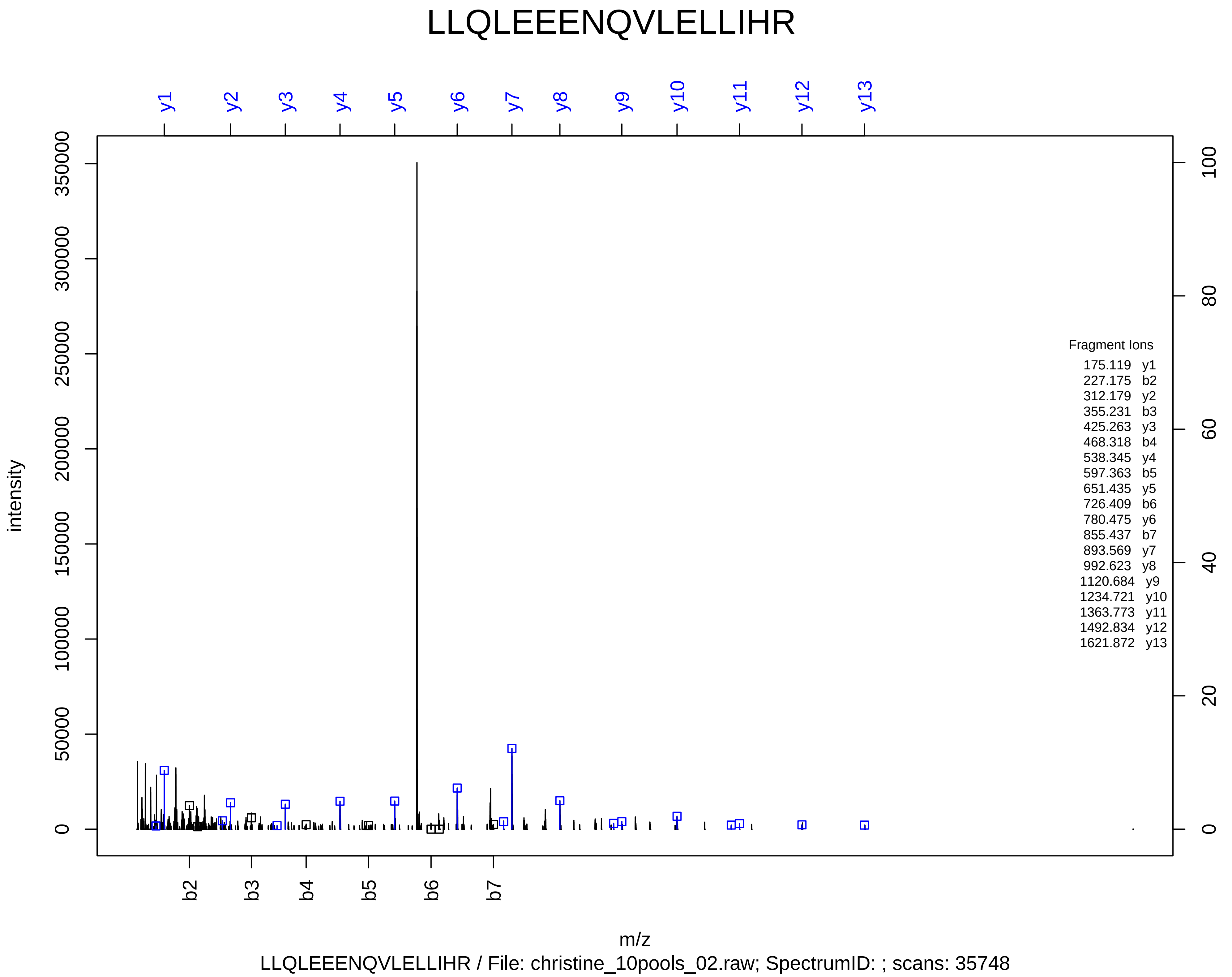

LLQLEEEENQVLELLIHR
y1
y2
y3
y4
y5
y6
y7
y8
y9
y10
y11
y12
y13
intensity
0
50000
100000
150000
200000
250000
300000
350000
0
20
40
60
80
100
Fragment Ions
175.119 y1
227.175 b2
312.179 y2
355.231 b3
425.263 y3
468.318 b4
538.345 y4
597.363 b5
651.435 y5
726.409 b6
780.475 y6
855.437 b7
893.569 y7
992.623 y8
1120.684 y9
1234.721 y10
1363.773 y11
1492.834 y12
1621.872 y13
b2
b3
b4
b5
b6
b7
m/z
LLQLEEEENQVLELLIHR / File: christine_10pools_02.raw; SpectrumID: ; scans: 35748

FVSEAWQQVER

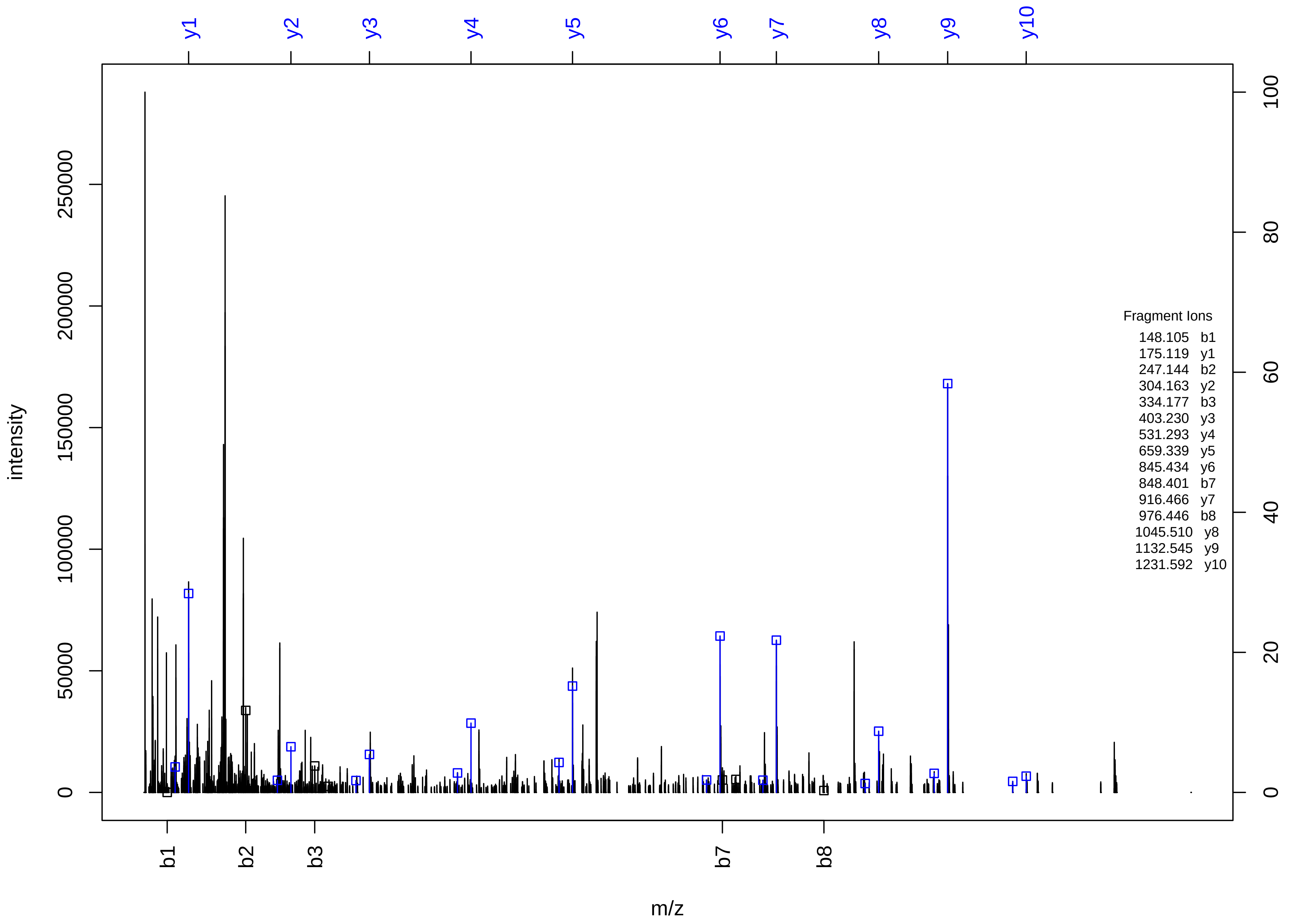

FVSEAWQQVER / File: christine_10pools_02.raw; SpectrumID: ; scans: 11599

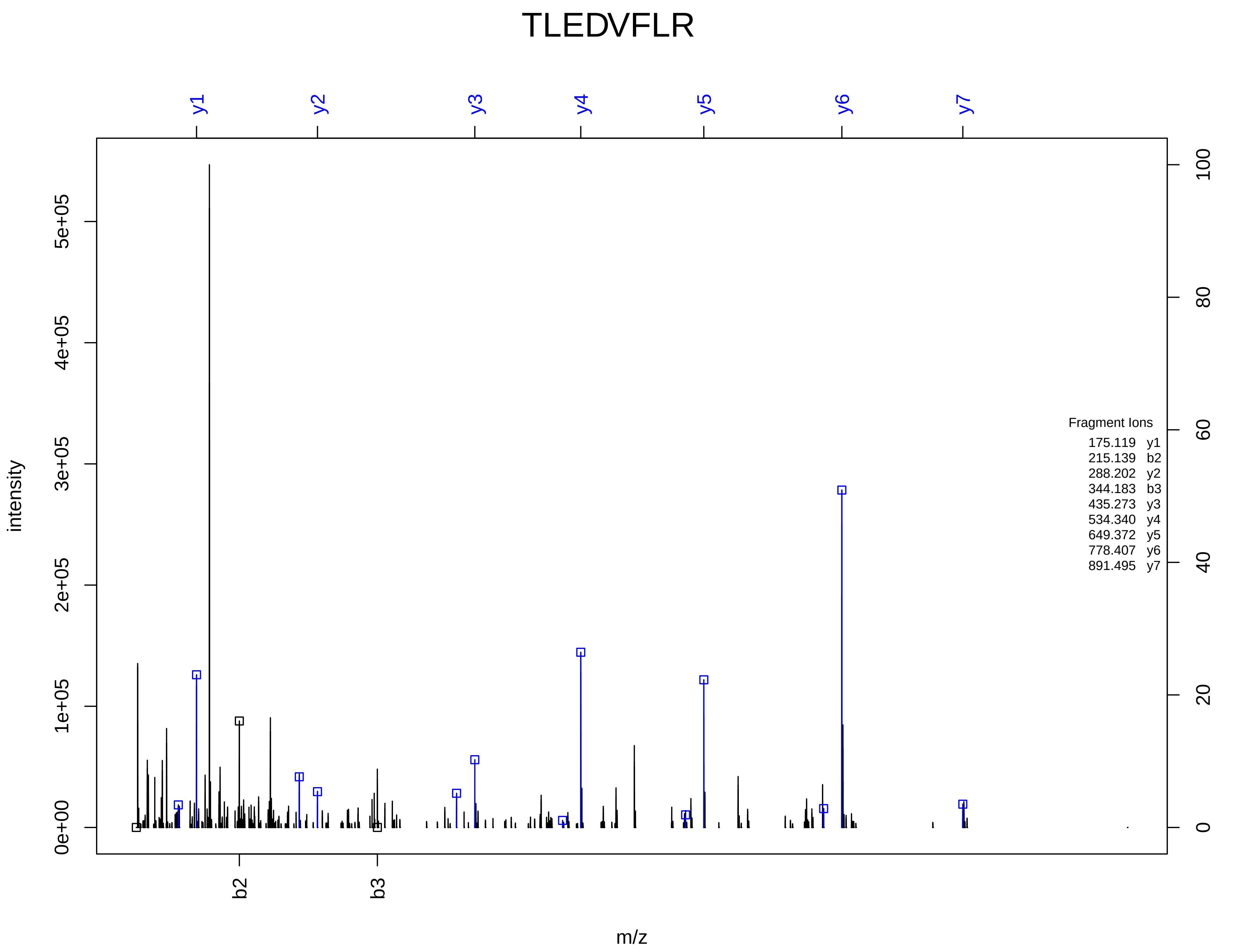

TLEDVFLR
y1
y2
y3
y4
y5
y6
y7
intensity
0e+00
1e+05
2e+05
3e+05
4e+05
5e+05
0
20
40
60
80
100
b2
b3
Fragment Ions
175.119 y1
215.139 b2
288.202 y2
344.183 b3
435.273 y3
534.340 y4
649.372 y5
778.407 y6
891.495 y7
m/z
TLEDVFLR / File: christine_10pools_02.raw; SpectrumID: ; scans: 20129

NIADLMTQAGVEELDPENK

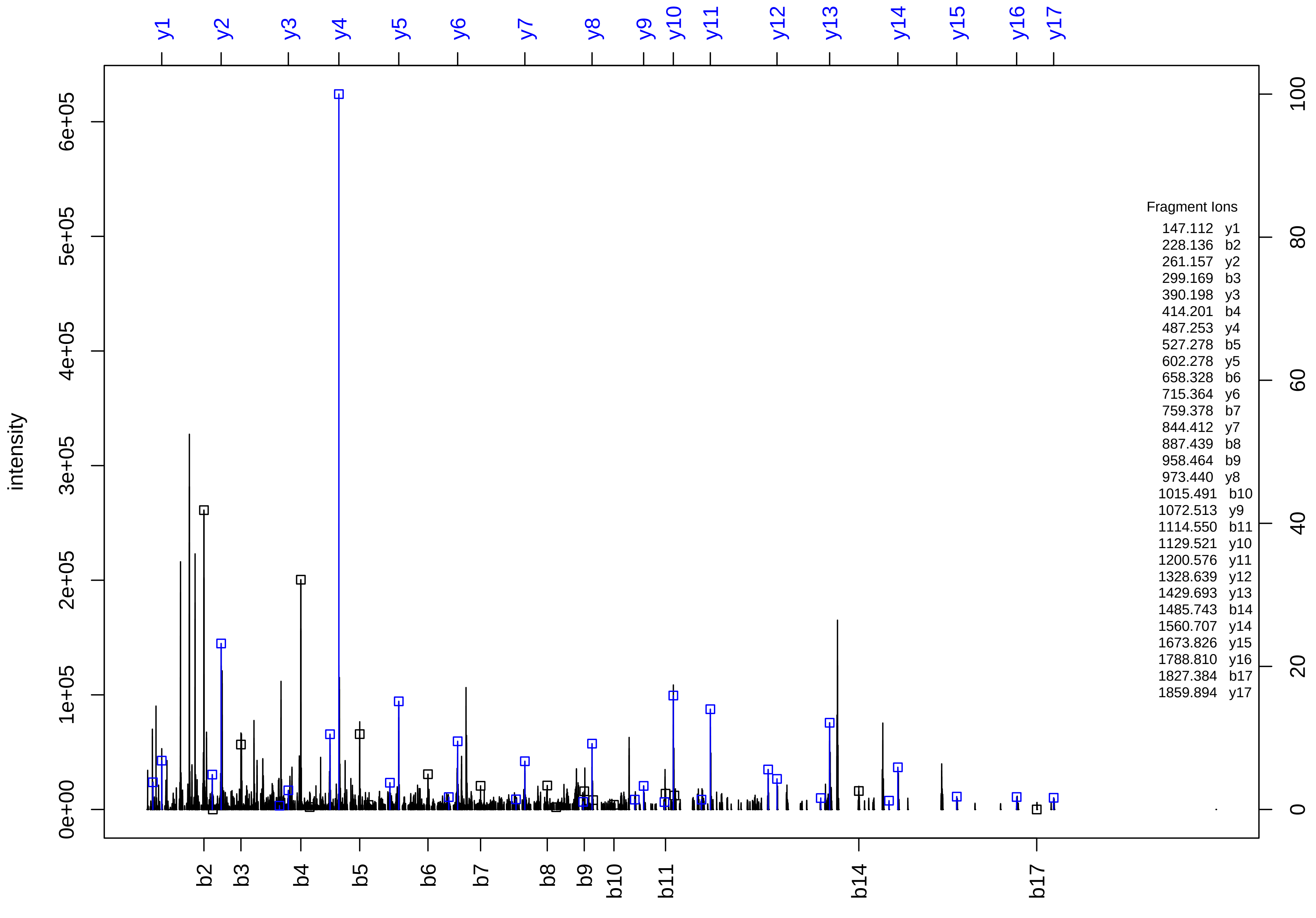

NIADLMTQAGVEELDPENK / File: christine_10pools_02.raw; SpectrumID: ; scans: 28794

VSGGSPLVAPCPSPR

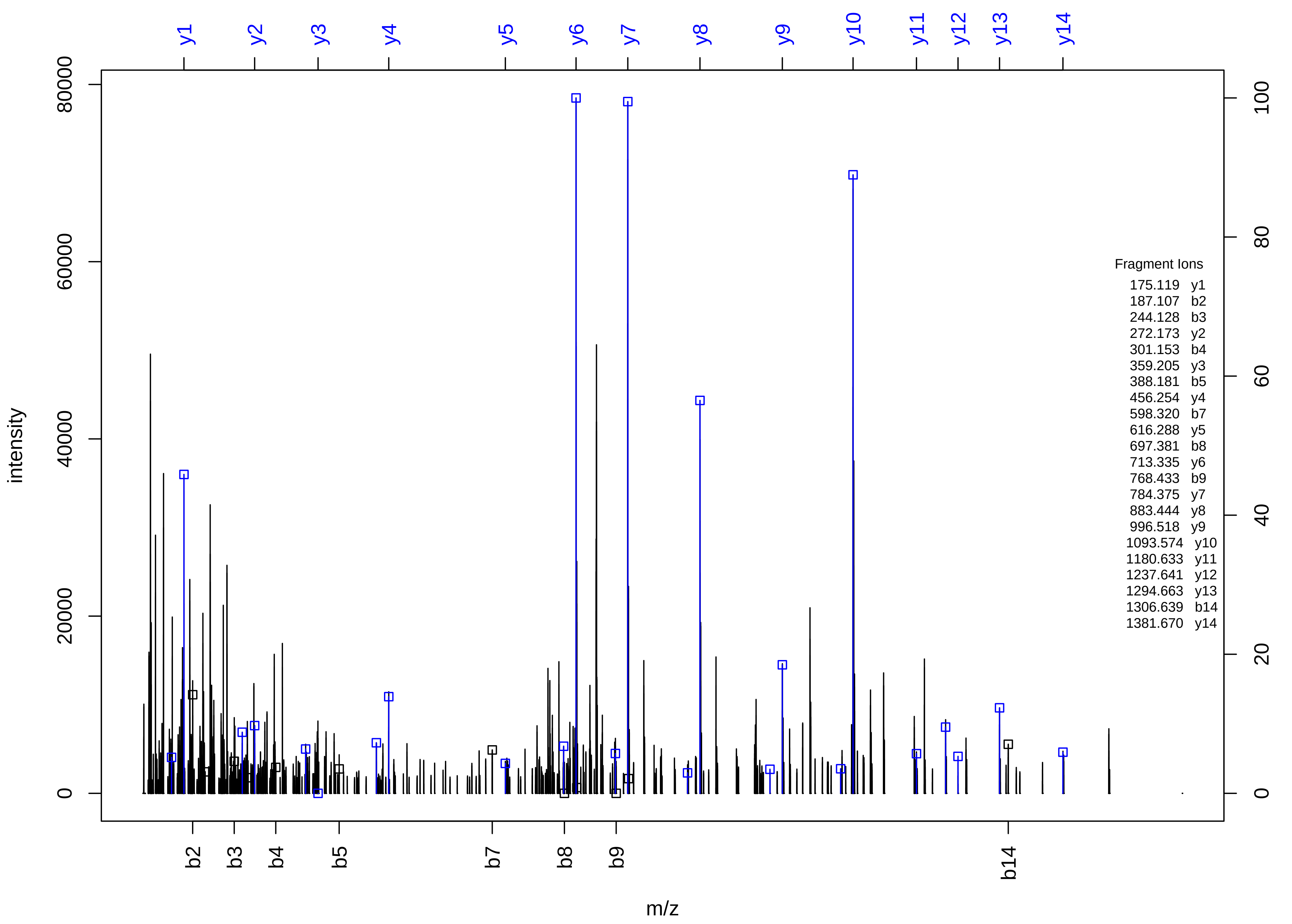

VSGGSPLVAPCPSPR / File: christine_10pools_02.raw; SpectrumID: ; scans: 6113

(Ac)ASYFDEHDCEPLNPER

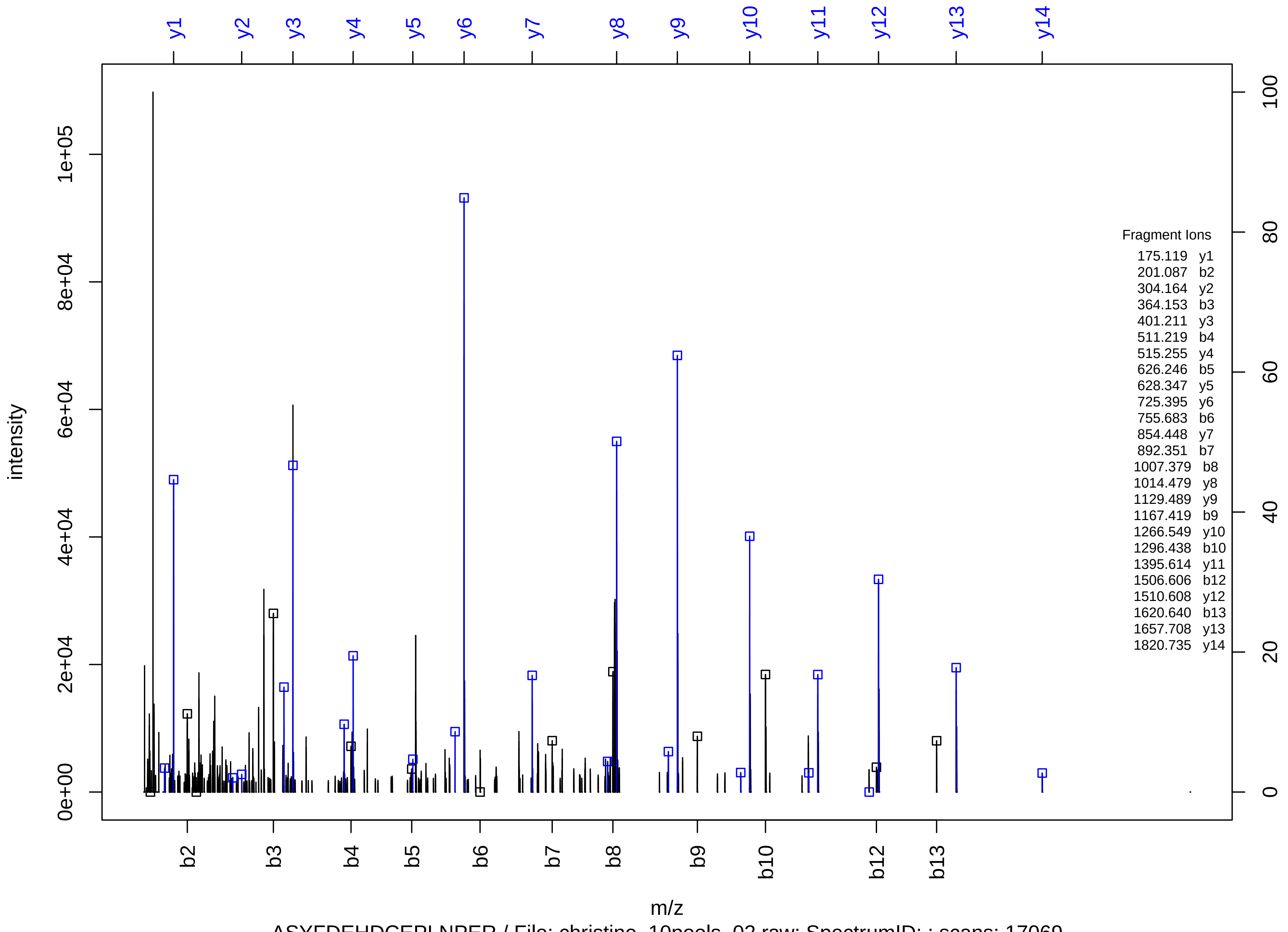

ASYFDEHDCEPLNPER / File: christine_10pools_02.raw; SpectrumID: ; scans: 17069

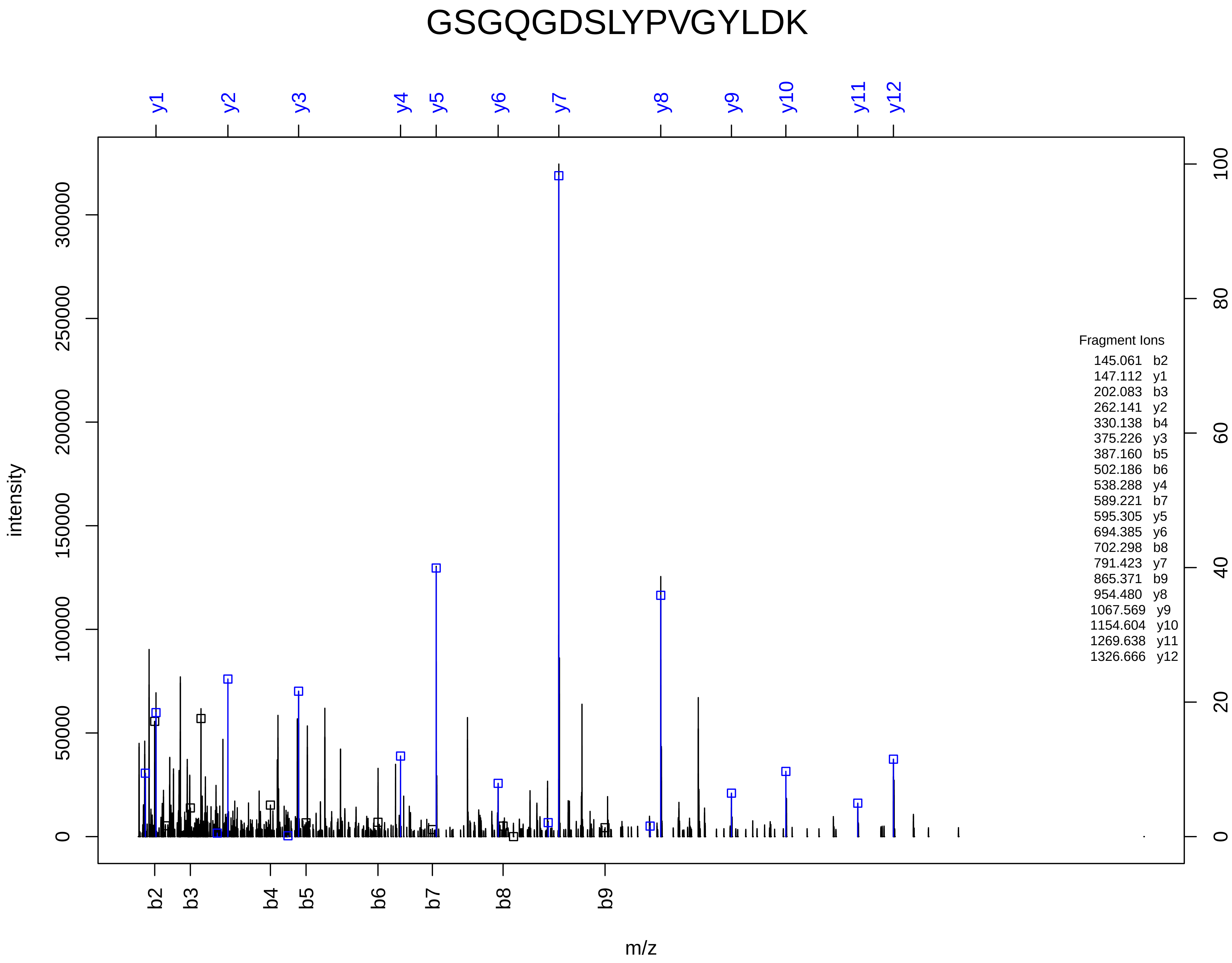
GSGQGDSLYPVGYLDK
y1 y2 y3 y4 y5 y6 y7 y8 y9 y10 y11 y12
b2 b3 b4 b5 b6 b7 b8 b9
intensity
0
50000
100000
150000
200000
250000
300000
0
20
40
60
80
100
m/z
Fragment Ions
145.061 b2
147.112 y1
202.083 b3
262.141 y2
330.138 b4
375.226 y3
387.160 b5
502.186 b6
538.288 y4
589.221 b7
595.305 y5
694.385 y6
702.298 b8
791.423 y7
865.371 b9
954.480 y8
1067.569 y9
1154.604 y10
1269.638 y11
1326.666 y12
GSGQGDSLYPVGYLDK / File: christine_10pools_02.raw; SpectrumID: ; scans: 17204

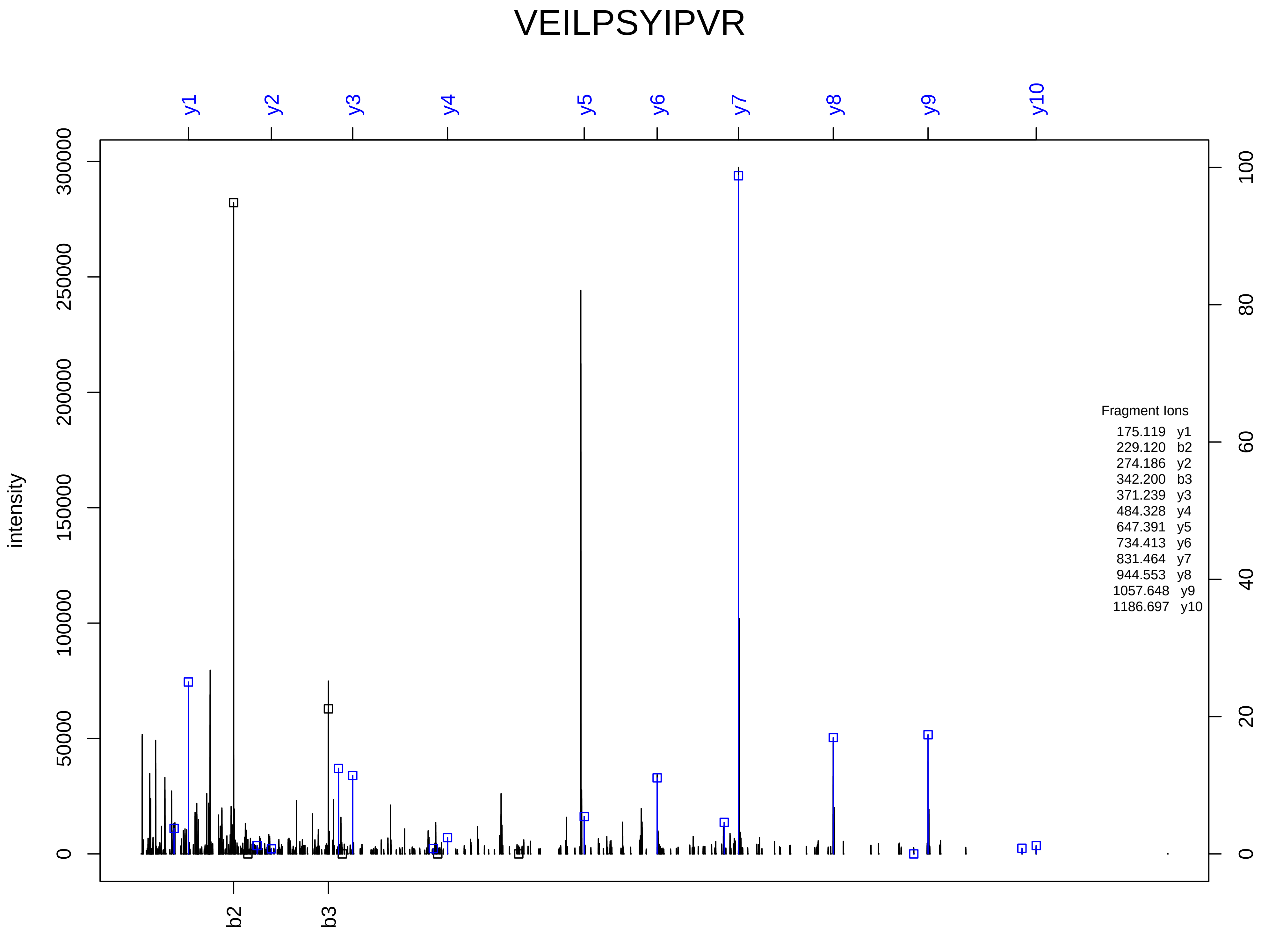

VEILPSYIPVR / File: christine_10pools_02.raw; SpectrumID: ; scans: 23558

ESPVVPLAESELLAEILEEARK

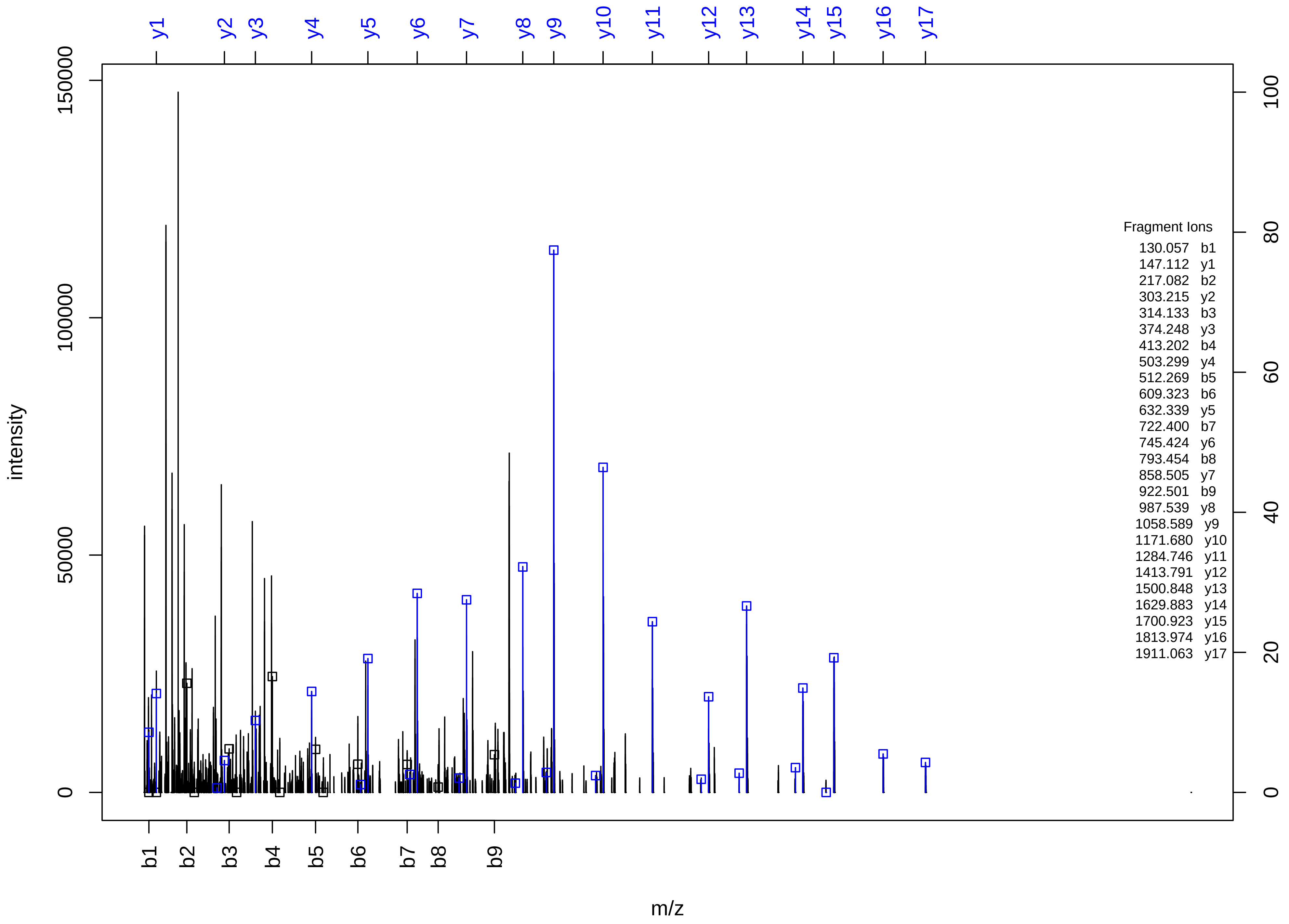

ESPVVPLAESELLAEILEEARK / File: christine_10pools_02.raw; SpectrumID: ; scans: 40784

AVEPSEVQHVTAASGPASAAAEPPSTGK

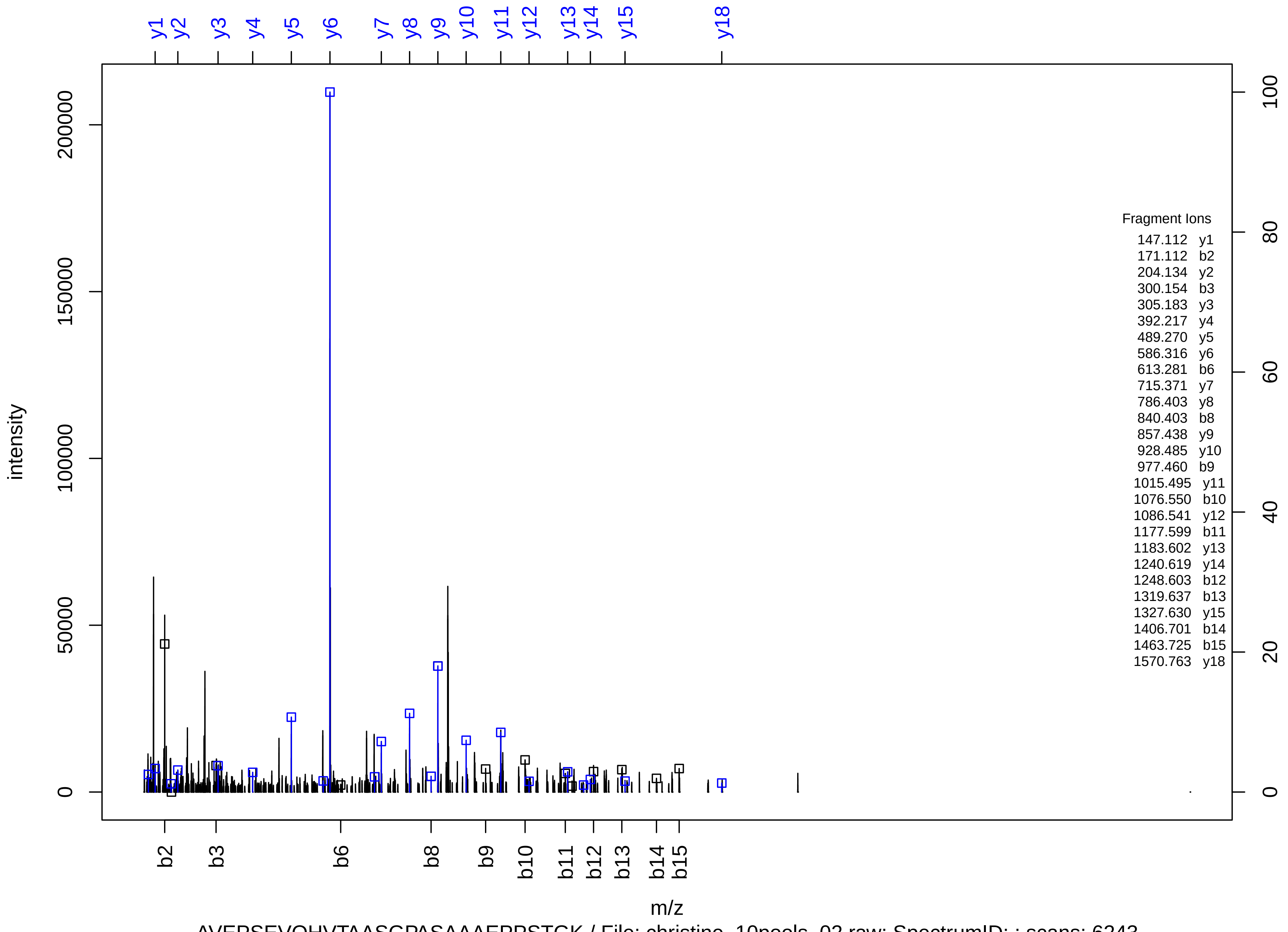

AVEPSEVQHVTAASGPASAAAEPPSTGK / File: christine_10pools_02.raw; SpectrumID: ; scans: 6243

AVEAVLTGLVEAAPRPEVLSR

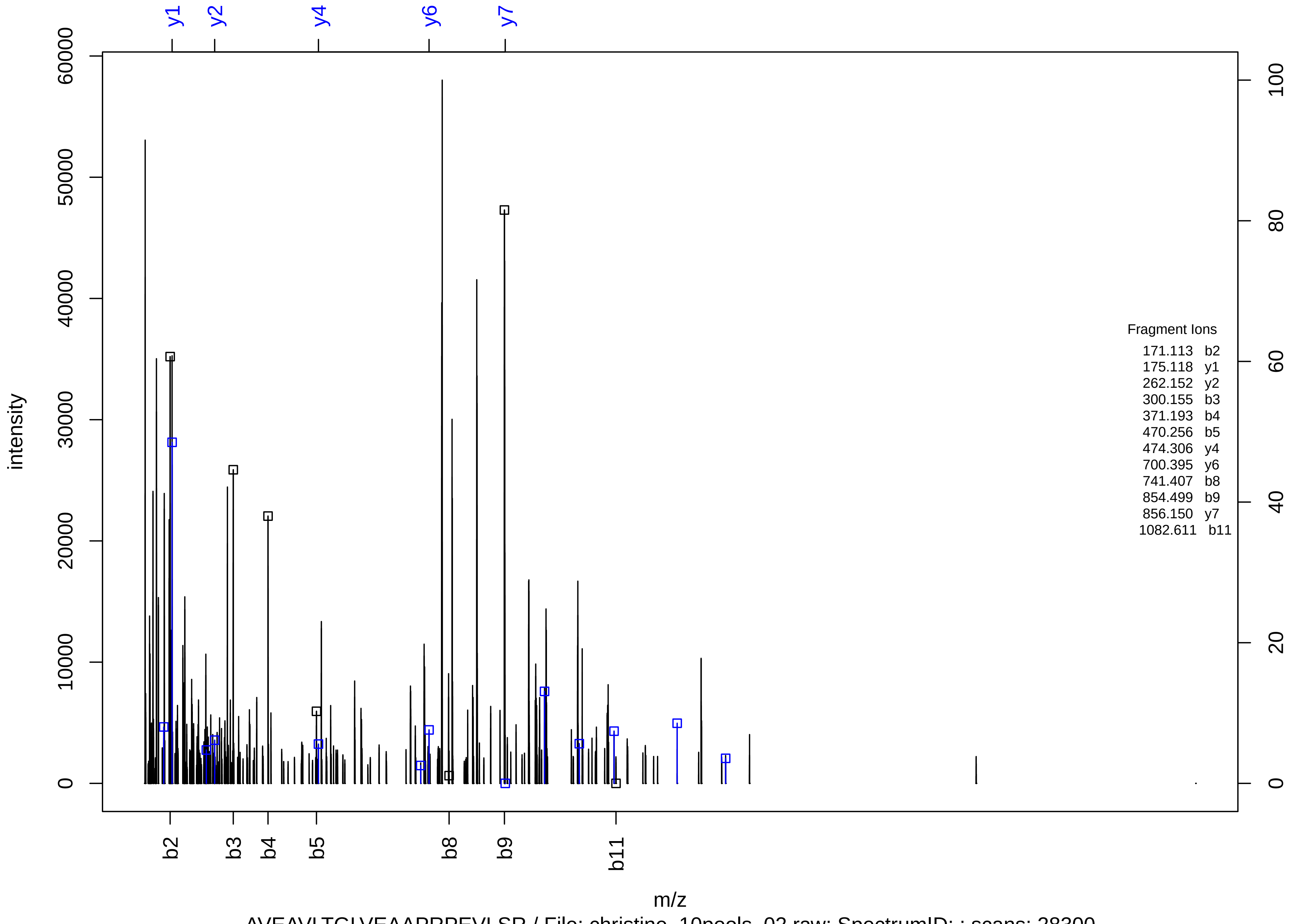

AVEAVLTGLVEAAPRPEVLSR / File: christine_10pools_02.raw; SpectrumID: ; scans: 28300

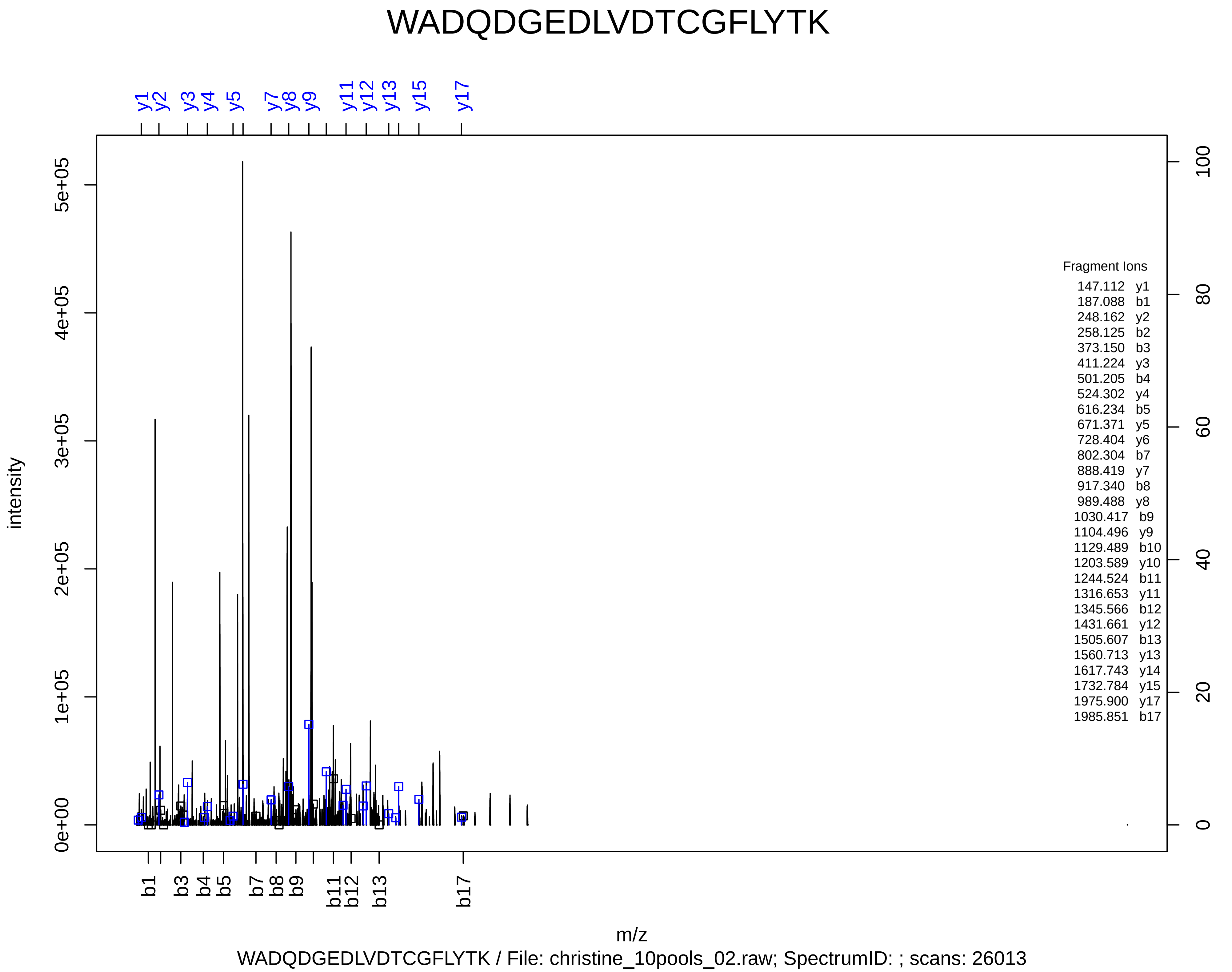

WADQDGEDLVDTCGFLYTK
y1 y2 y3 y4 y5 y7 y8 y9 y11 y12 y13 y15 y17
b1 b3 b4 b5 b7 b8 b9 b11 b12 b13 b17
intensity
m/z
0e+00
1e+05
2e+05
3e+05
4e+05
5e+05
0
20
40
60
80
100
Fragment Ions
147.112 y1
187.088 b1
248.162 y2
258.125 b2
373.150 b3
411.224 y3
501.205 b4
524.302 y4
616.234 b5
671.371 y5
728.404 y6
802.304 b7
888.419 y7
917.340 b8
989.488 y8
1030.417 b9
1104.496 y9
1129.489 b10
1203.589 y10
1244.524 b11
1316.653 y11
1345.566 b12
1431.661 y12
1505.607 b13
1560.713 y13
1617.743 y14
1732.784 y15
1975.900 y17
1985.851 b17
WADQDGEDLVDTCGFLYTK / File: christine_10pools_02.raw; SpectrumID: ; scans: 26013

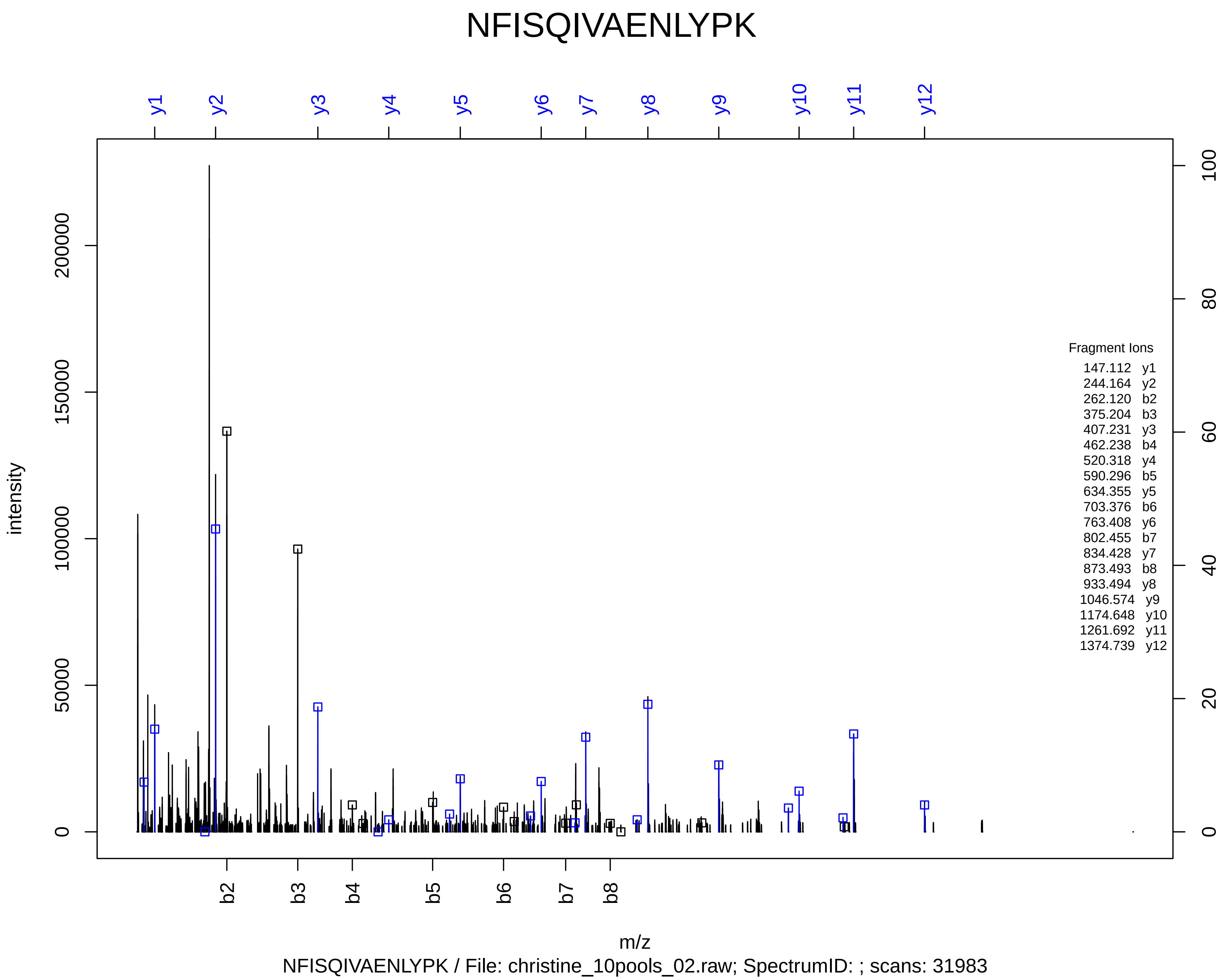
NFISQIVAENLYPK
y1
y2
y3
y4
y5
y6
y7
y8
y9
y10
y11
y12
intensity
0
50000
100000
150000
200000
0
20
40
60
80
100
b2
b3
b4
b5
b6
b7
b8
m/z
Fragment Ions
147.112 y1
244.164 y2
262.120 b2
375.204 b3
407.231 y3
462.238 b4
520.318 y4
590.296 b5
634.355 y5
703.376 b6
763.408 y6
802.455 b7
834.428 y7
873.493 b8
933.494 y8
1046.574 y9
1174.648 y10
1261.692 y11
1374.739 y12
NFISQIVAENLYPK / File: christine_10pools_02.raw; SpectrumID: ; scans: 31983

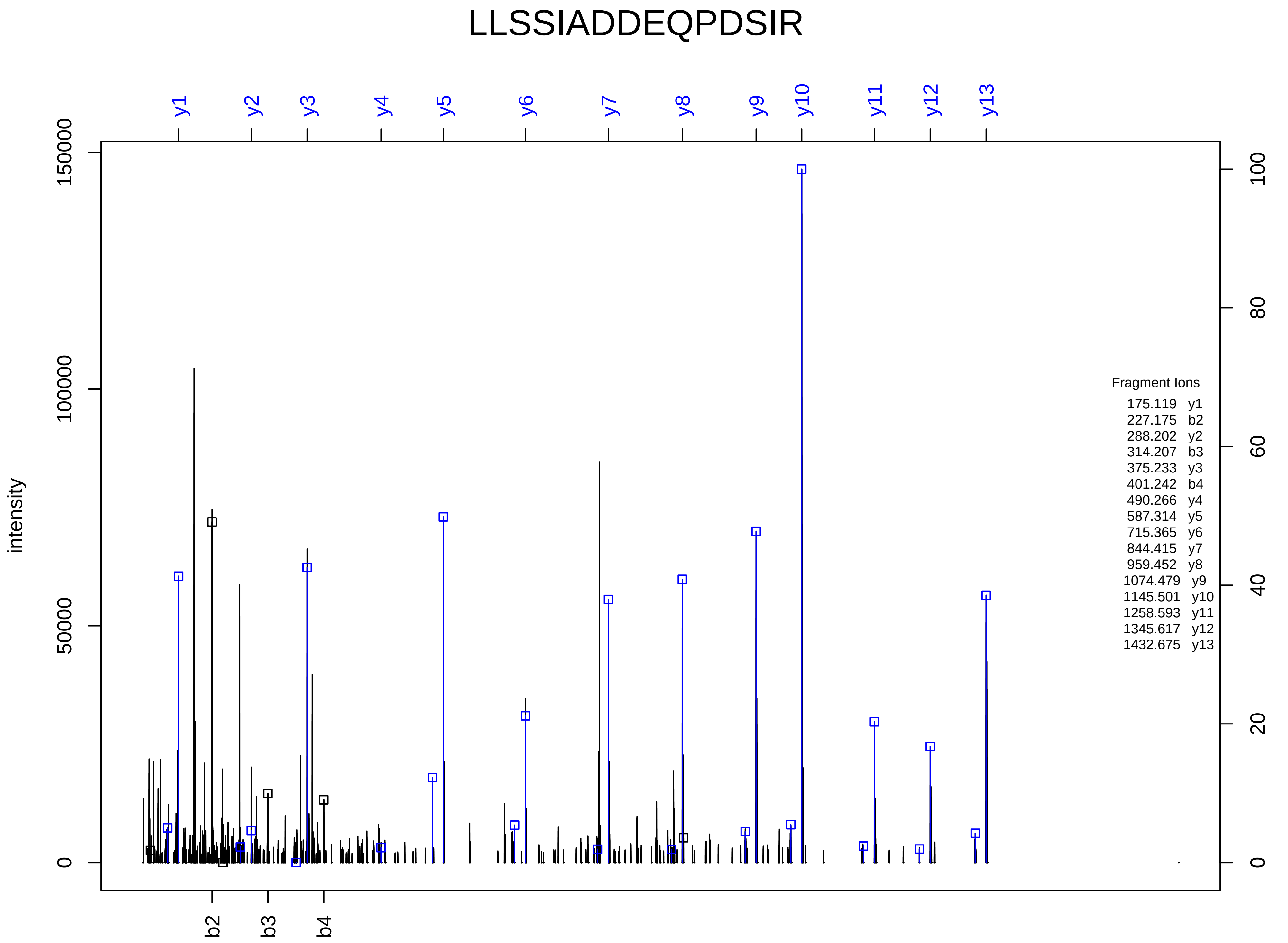

LLSSIADDEQPDSIR / File: christine_10pools_02.raw; SpectrumID: ; scans: 12775

ILADLNQQAPAPAPAER

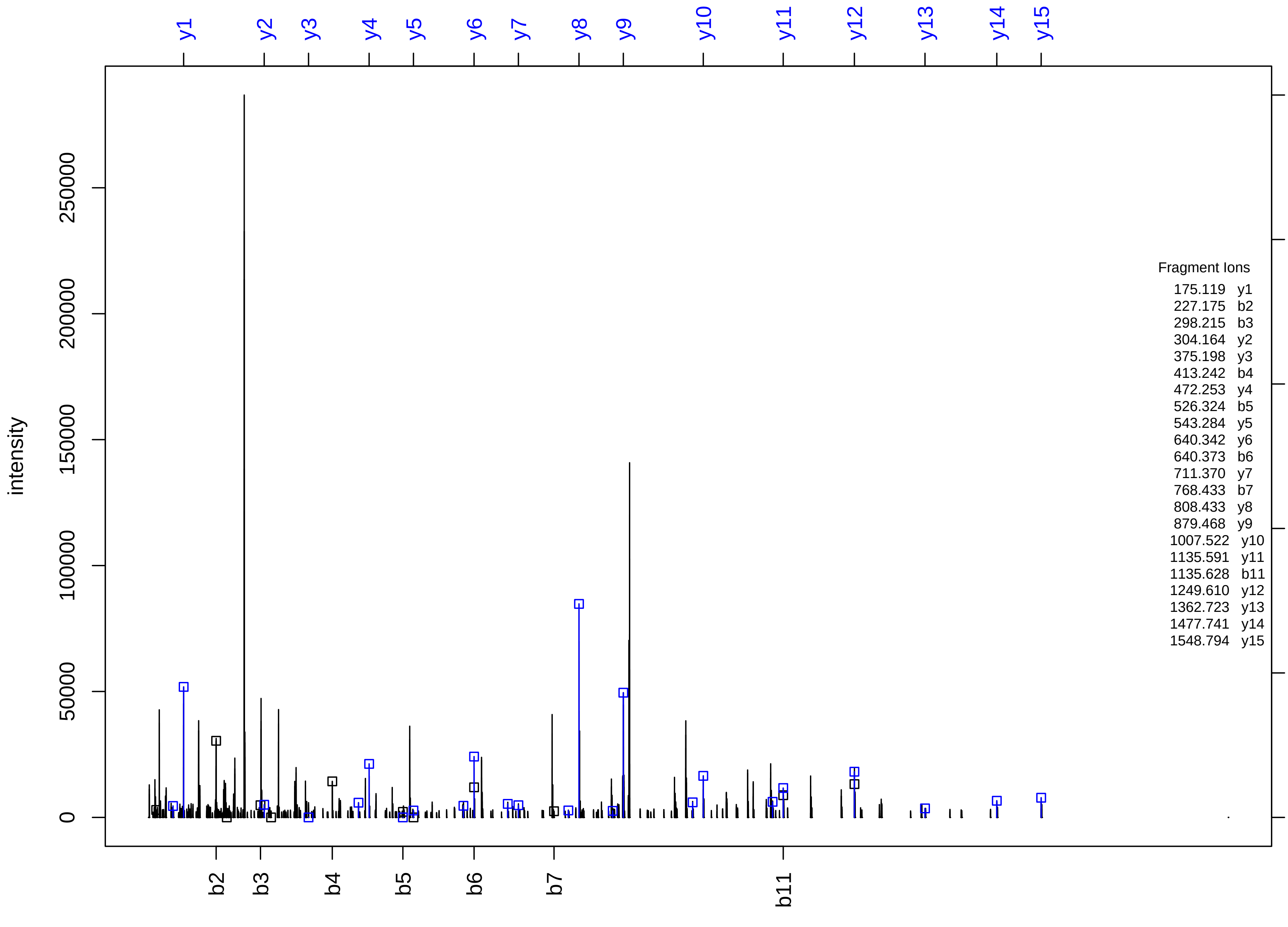

ILADLNQQAPAPAPAER / File: christine_10pools_02.raw; SpectrumID: ; scans: 10324

GTTVSAPMVPSSASQGACPK

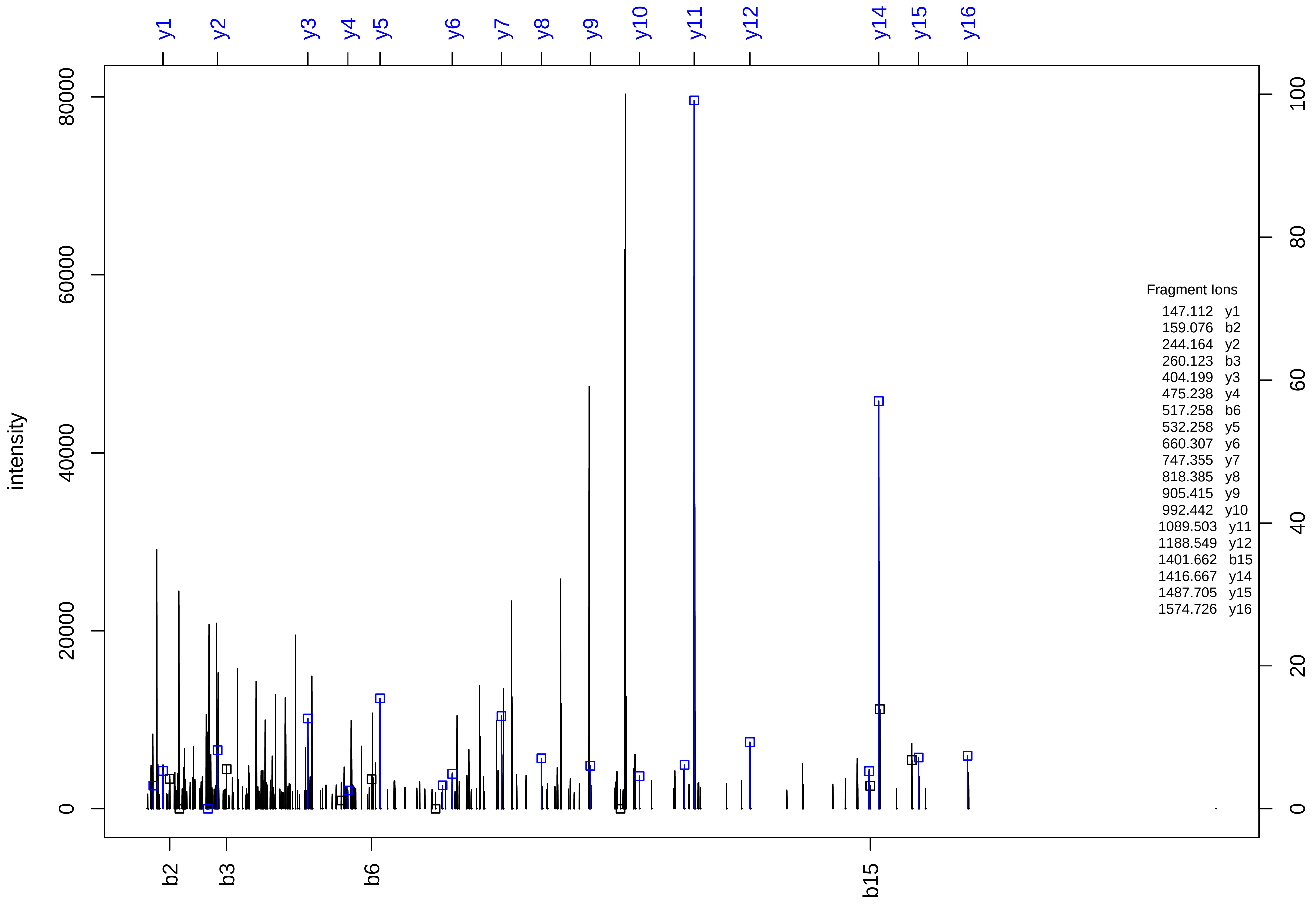

GTTVSAPMVPSSASQGACPK / File: christine_10pools_02.raw; SpectrumID: ; scans: 6681

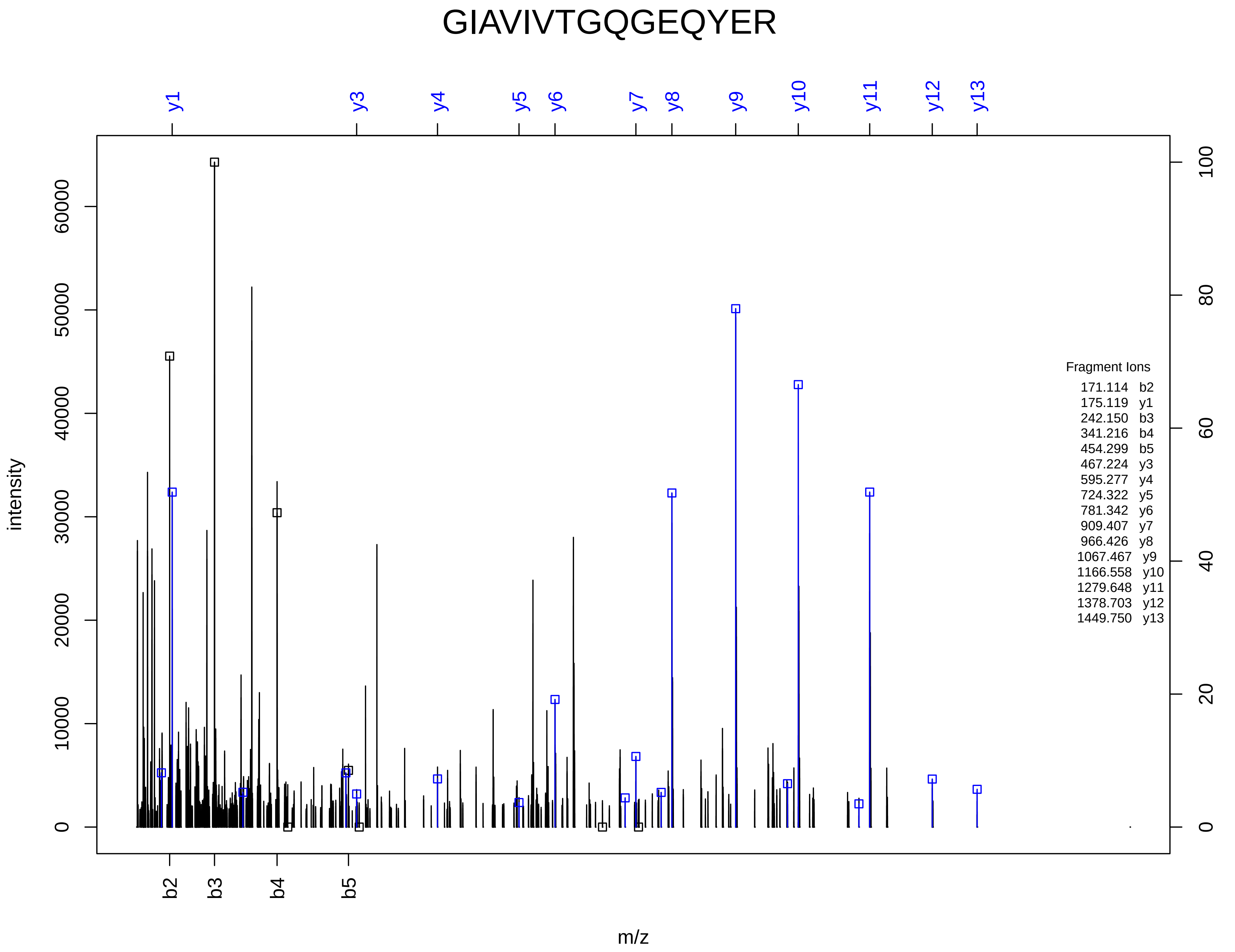

GIAVIVTGQGEQYER / File: christine_10pools_02.raw; SpectrumID: ; scans: 12277

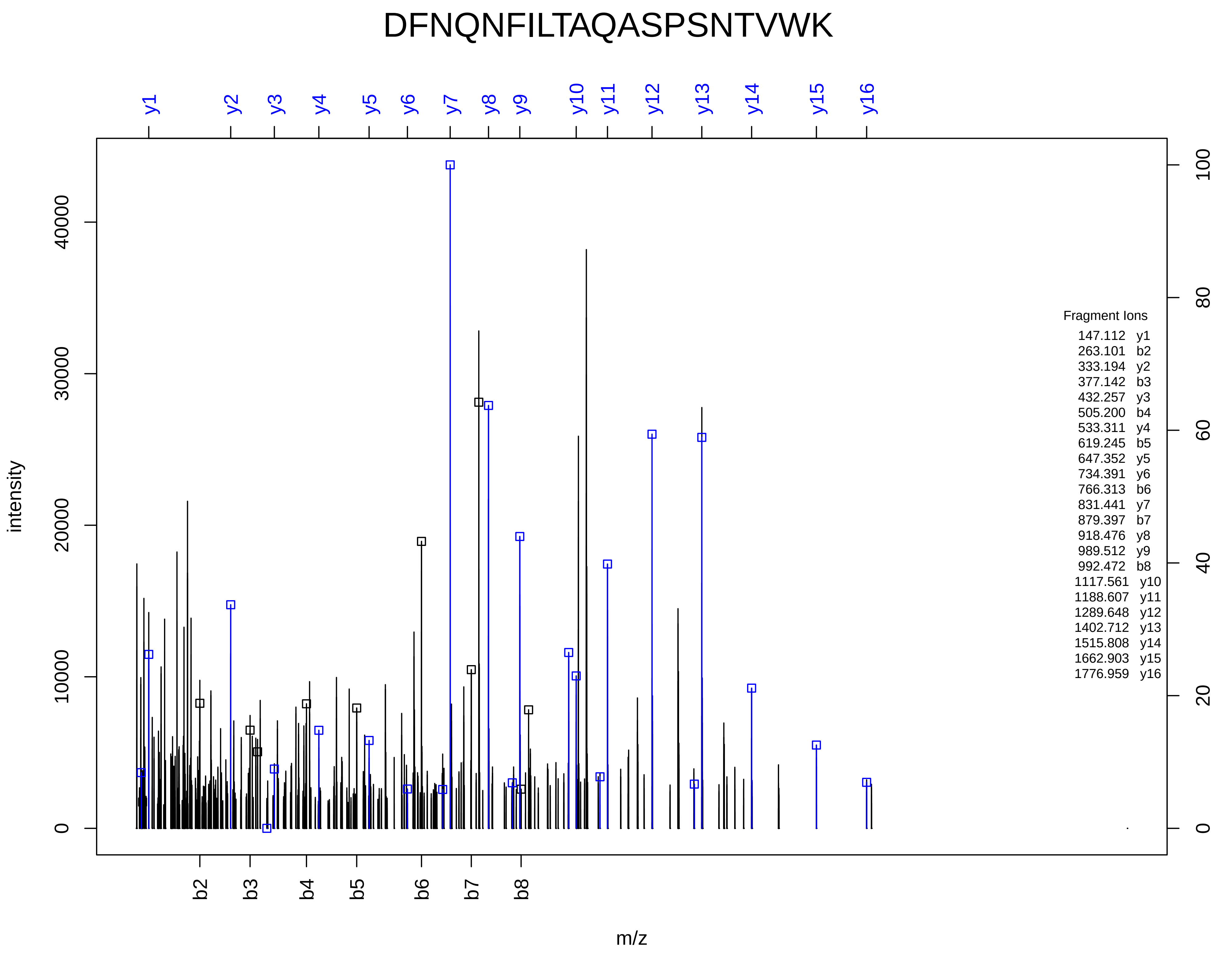

DFNQNFILTAQASPSNTVWK / File: christine_10pools_02.raw; SpectrumID: ; scans: 28276

IFDQLLLDR

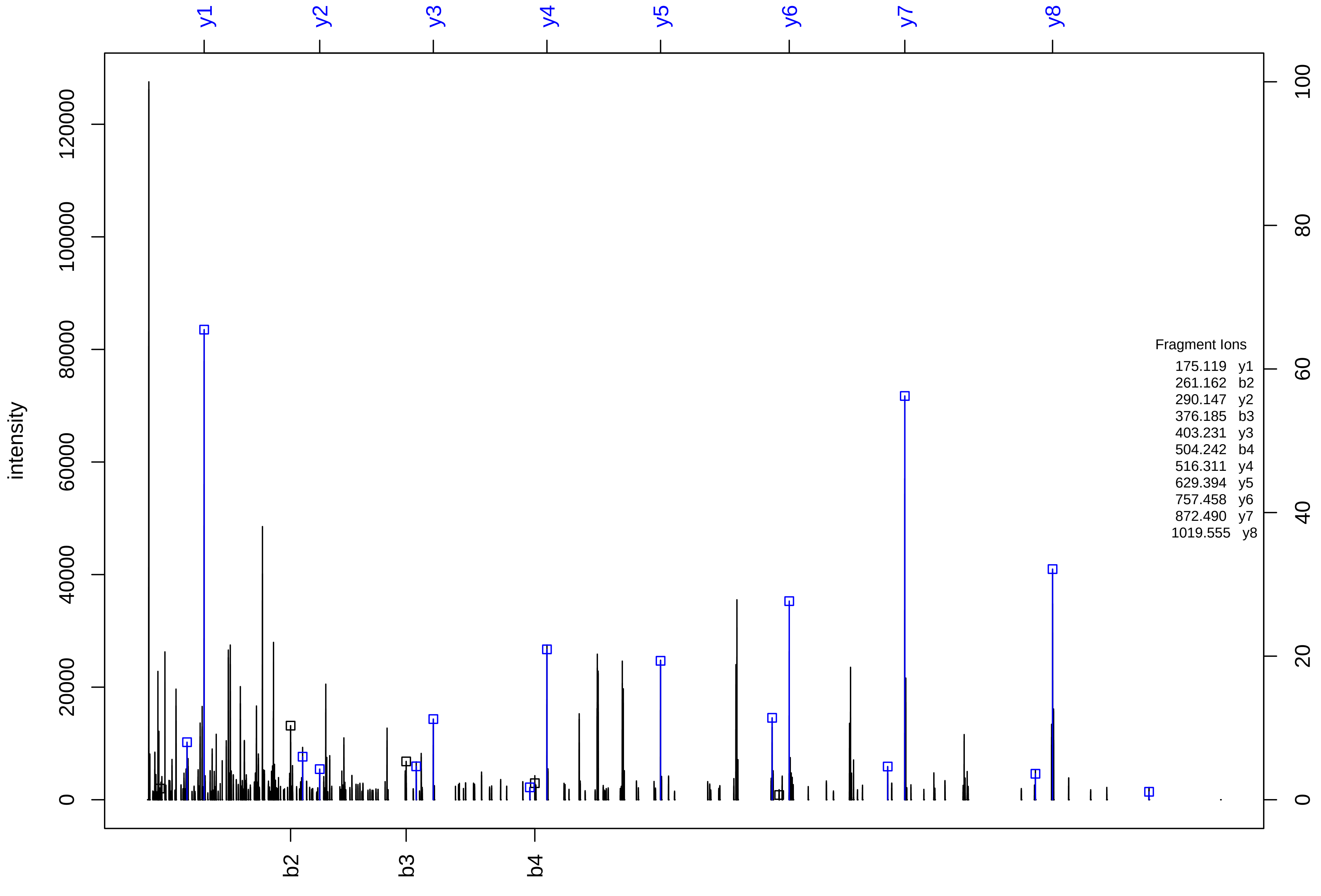

IFDQLLLDR / File: christine_10pools_02.raw; SpectrumID: ; scans: 24356

SALEQLLGAVGSLK

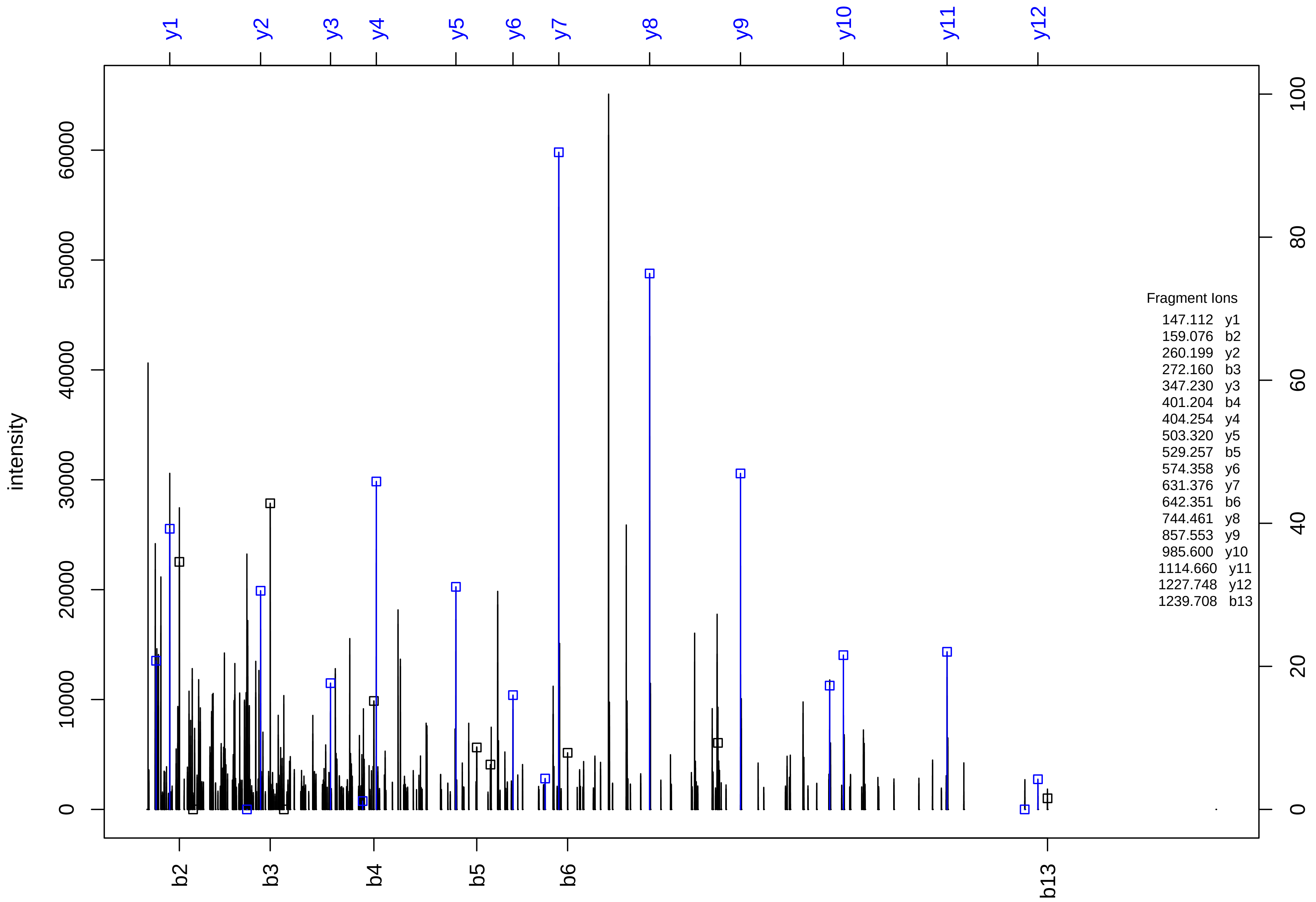

SALEQLLGAVGSLK / File: christine_10pools_02.raw; SpectrumID: ; scans: 36228

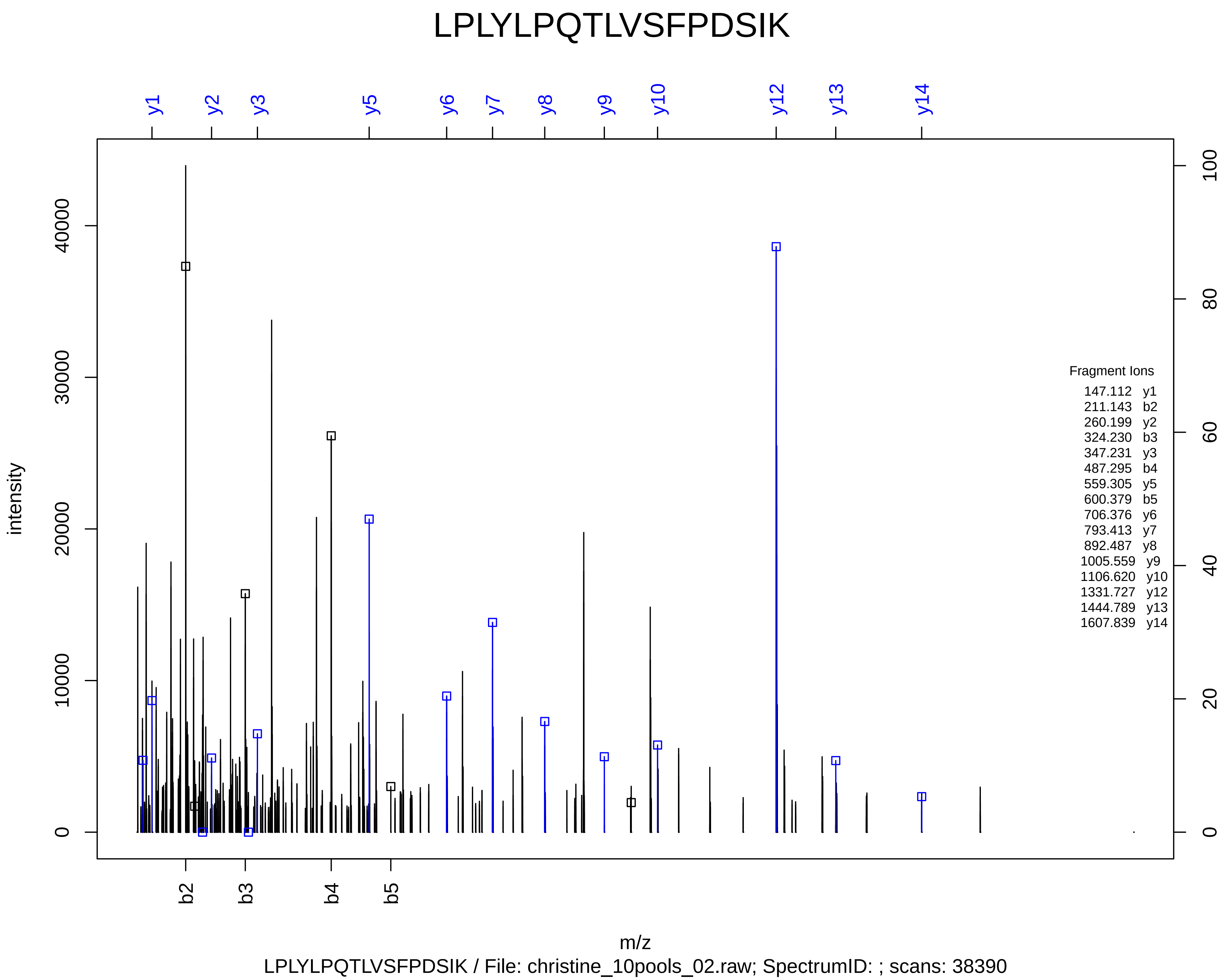

LPLYLPQTLVSFPDSIK
y1
y2
y3
y5
y6
y7
y8
y9
y10
y12
y13
y14
intensity
0
10000
20000
30000
40000
0
20
40
60
80
100
b2
b3
b4
b5
m/z
Fragment Ions
147.112 y1
211.143 b2
260.199 y2
324.230 b3
347.231 y3
487.295 b4
559.305 y5
600.379 b5
706.376 y6
793.413 y7
892.487 y8
1005.559 y9
1106.620 y10
1331.727 y12
1444.789 y13
1607.839 y14
LPLYLPQTLVSFPDSIK / File: christine_10pools_02.raw; SpectrumID: ; scans: 38390

TENSSFSSATLPRPGDPGAPPLPPDLR

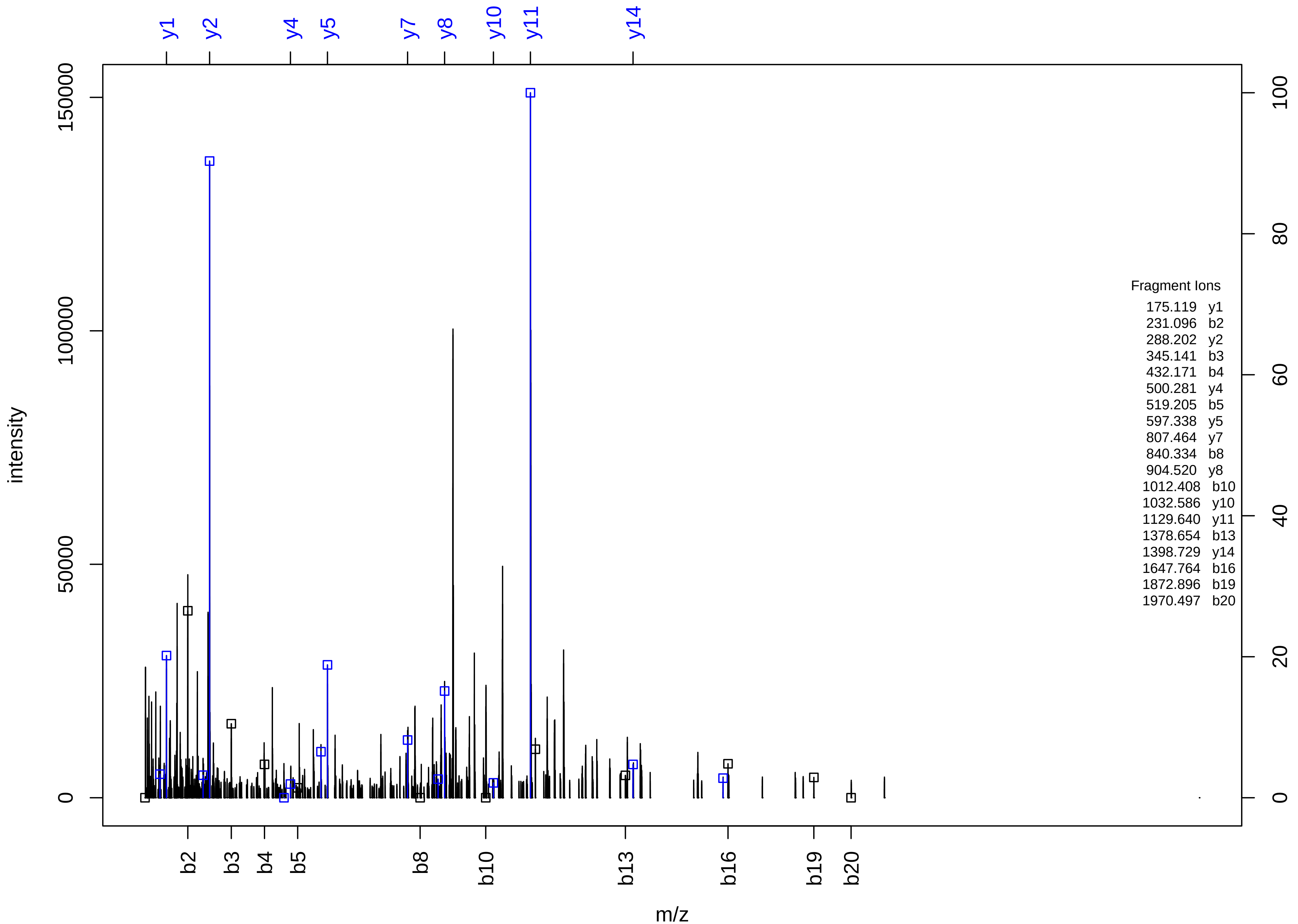

TENSSFSSATLPRPGDPGAPPLPPDLR / File: christine_10pools_02.raw; SpectrumID: ; scans: 20497

(Ac)M*SAPLLQRYPVEK

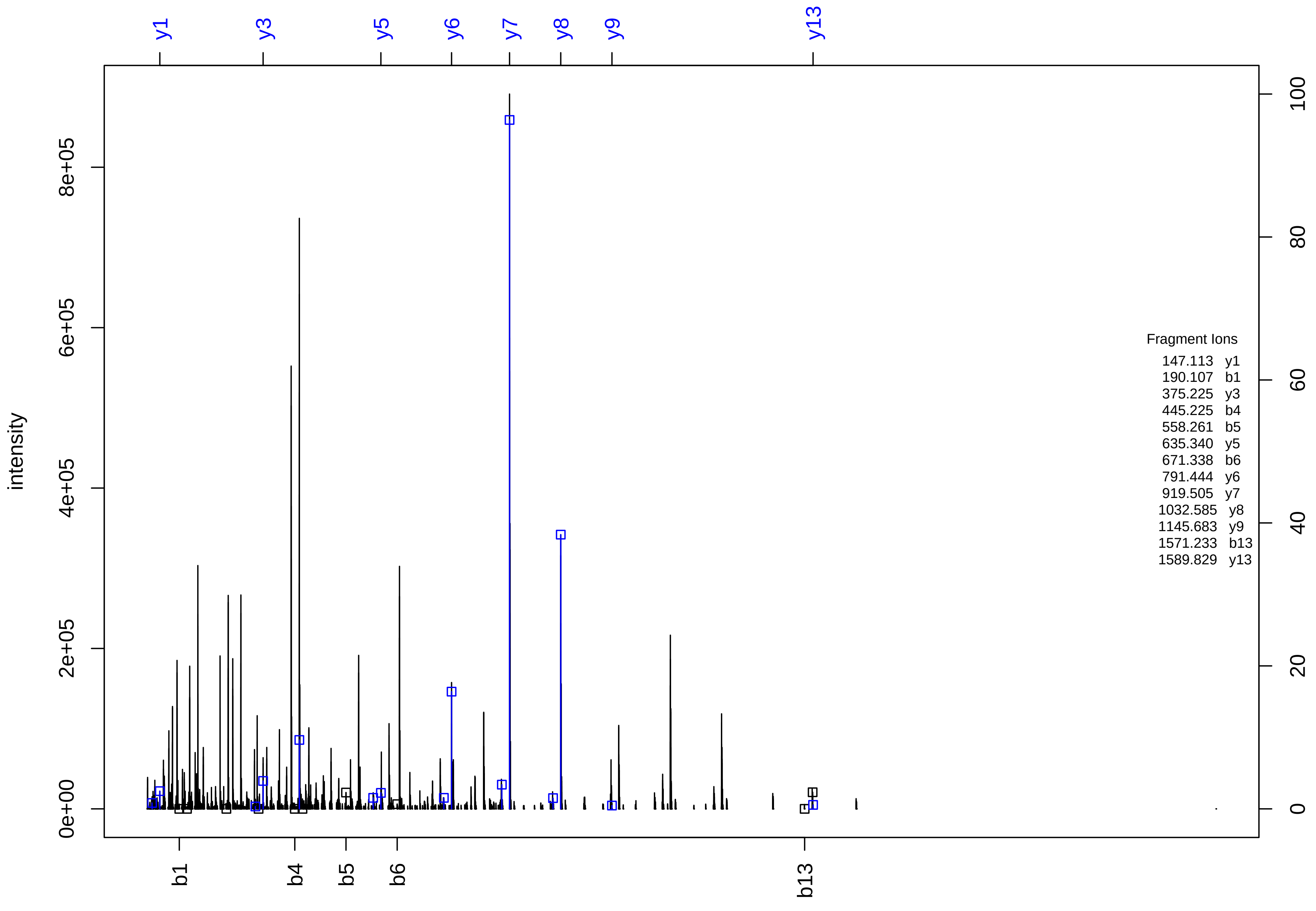

MSAPLLQRYPVEK / File: christine_10pools_02.raw; SpectrumID: ; scans: 42103

NPLRYPVLM*SRR

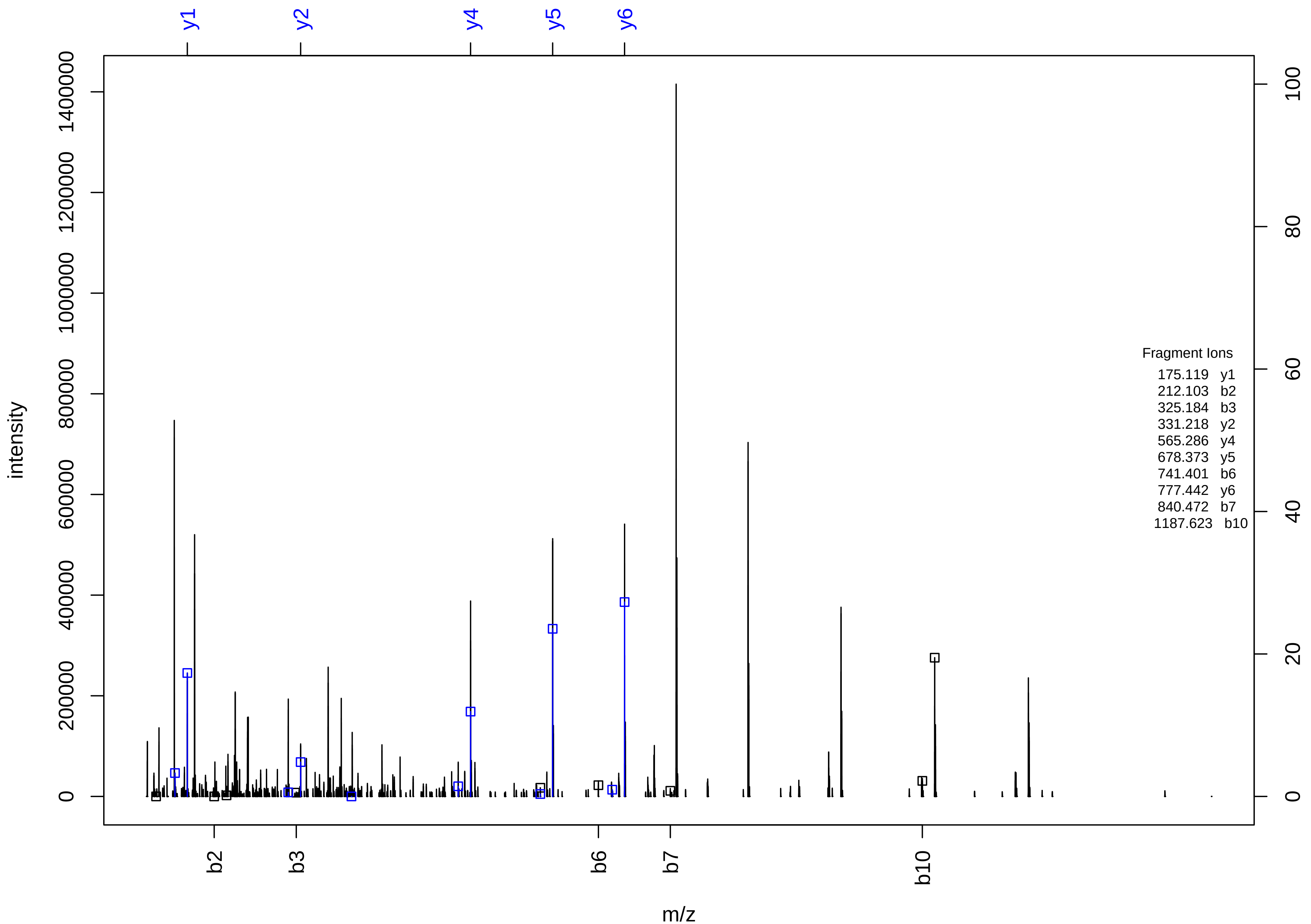

NPLRYPVLMSRR / File: christine_10pools_02.raw; SpectrumID: ; scans: 20628

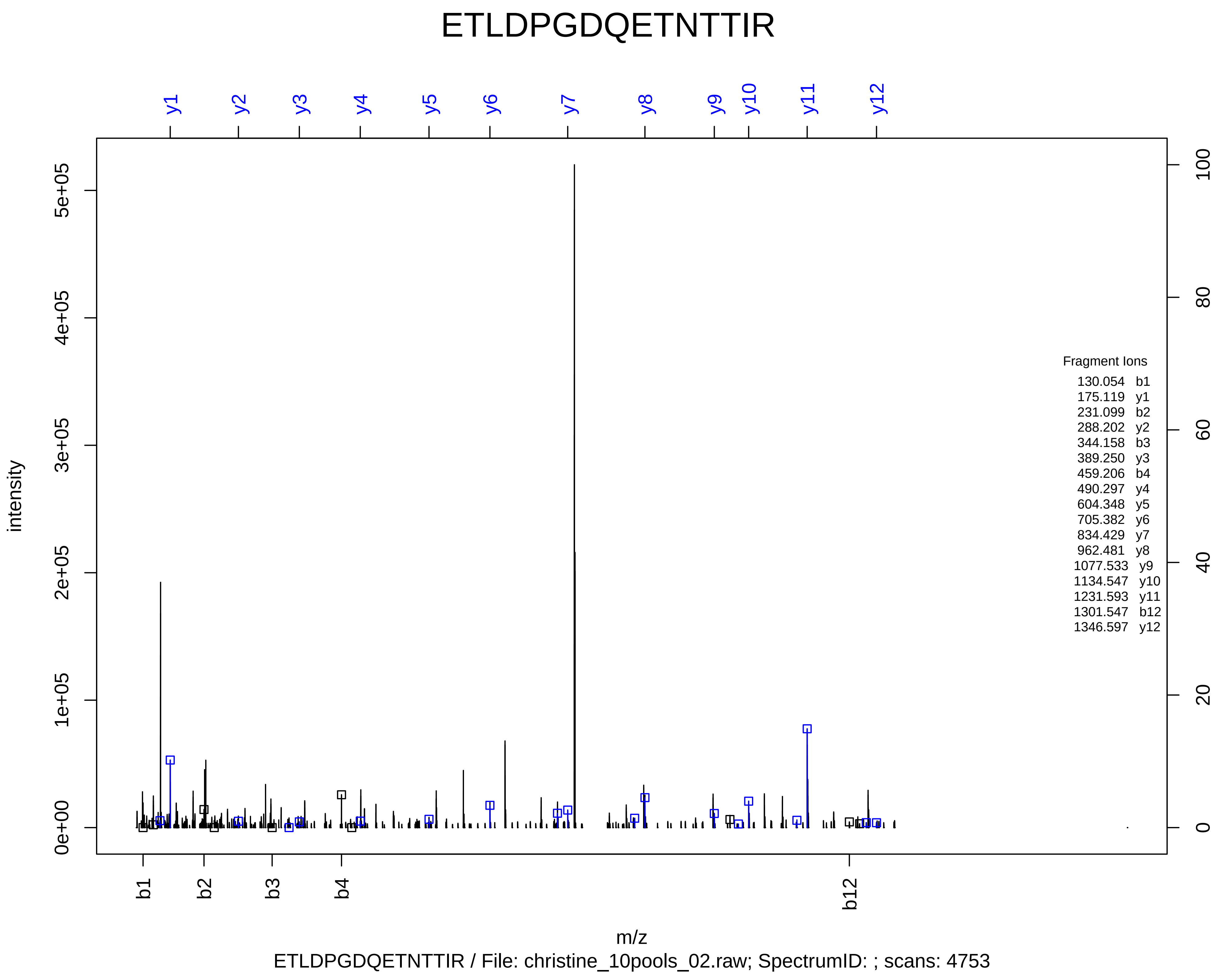

ETLDPGDQETNTTIR
y1
y2
y3
y4
y5
y6
y7
y8
y9
y10
y11
y12
intensity
0e+00
1e+05
2e+05
3e+05
4e+05
5e+05
0
20
40
60
80
100
Fragment Ions
130.054 b1
175.119 y1
231.099 b2
288.202 y2
344.158 b3
389.250 y3
459.206 b4
490.297 y4
604.348 y5
705.382 y6
834.429 y7
962.481 y8
1077.533 y9
1134.547 y10
1231.593 y11
1301.547 b12
1346.597 y12
b1
b2
b3
b4
b12
m/z
ETLDPGDQETNTTIR / File: christine_10pools_02.raw; SpectrumID: ; scans: 4753

VPGVTDCVAEVQK

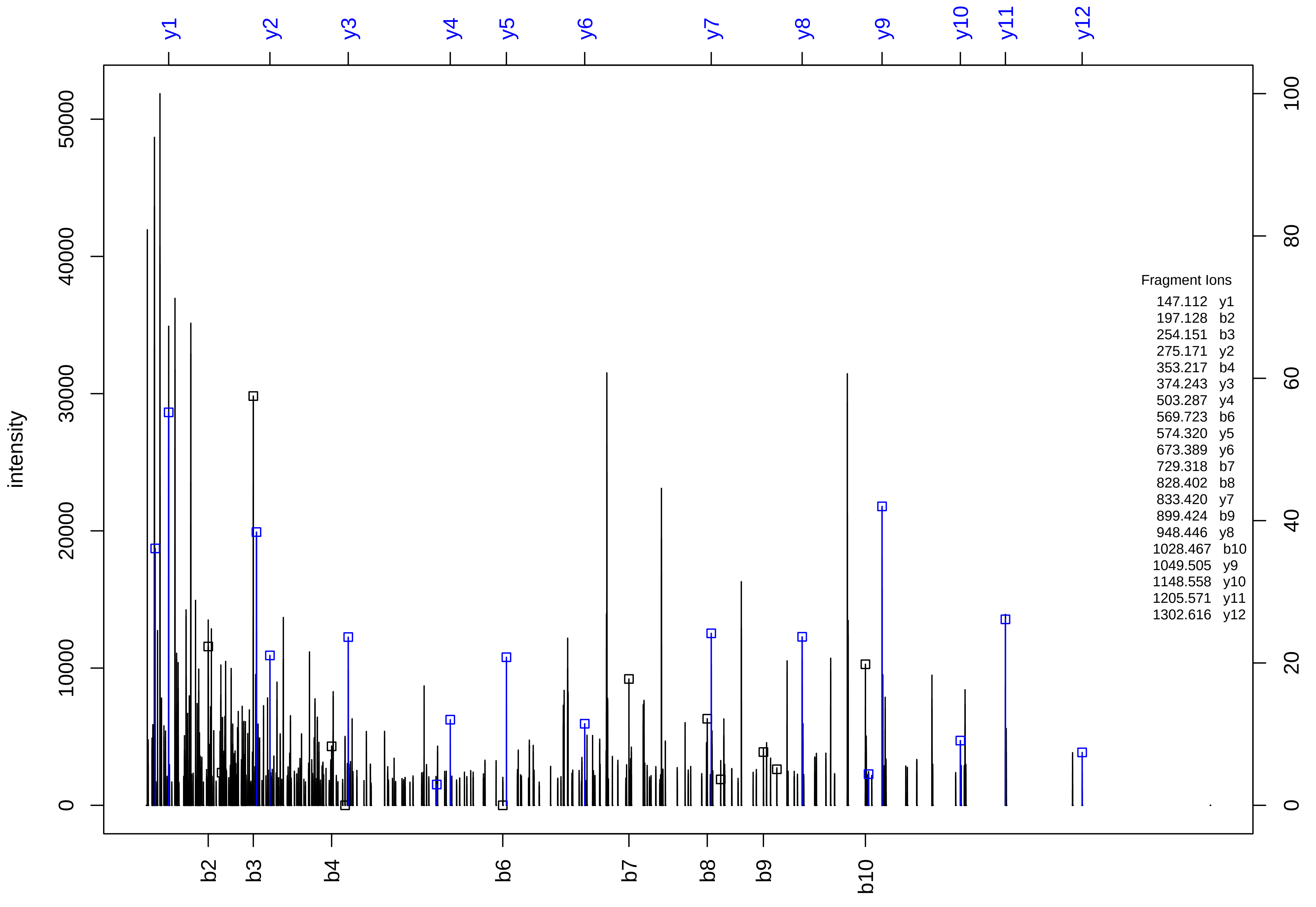

VPGVTDCVAEVQK / File: christine_10pools_02.raw; SpectrumID: ; scans: 6462

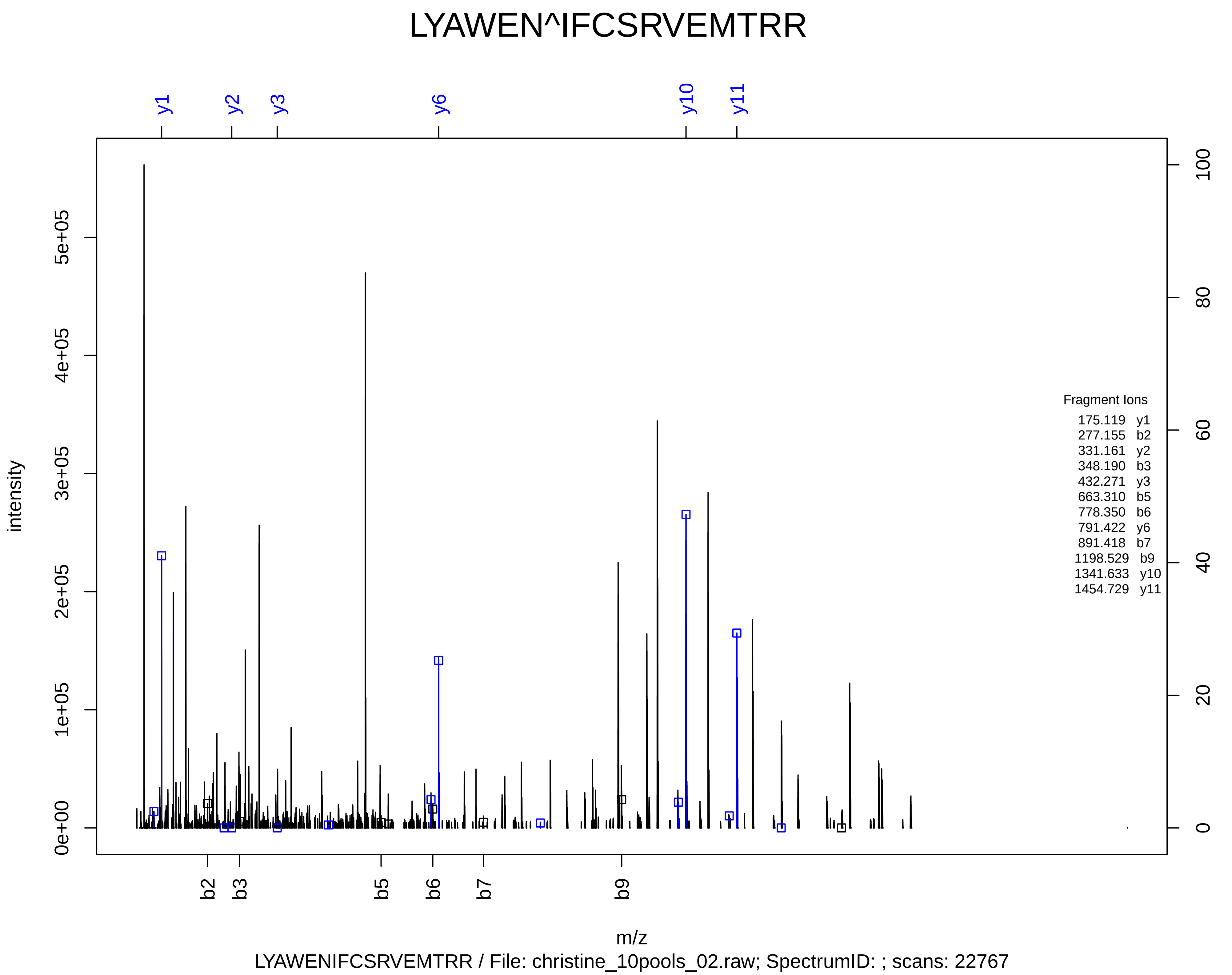

LYAWEN^IFCSRVEMTRR
y1
y2
y3
y6
y10
y11
5e+05
4e+05
3e+05
2e+05
1e+05
0e+00
intensity
100
80
60
40
20
0
Fragment Ions
175.119 y1
277.155 b2
331.161 y2
348.190 b3
432.271 y3
663.310 b5
778.350 b6
791.422 y6
891.418 b7
1198.529 b9
1341.633 y10
1454.729 y11
b2
b3
b5
b6
b7
b9
m/z
LYAWENIFCSRVEMTRR / File: christine_10pools_02.raw; SpectrumID: ; scans: 22767

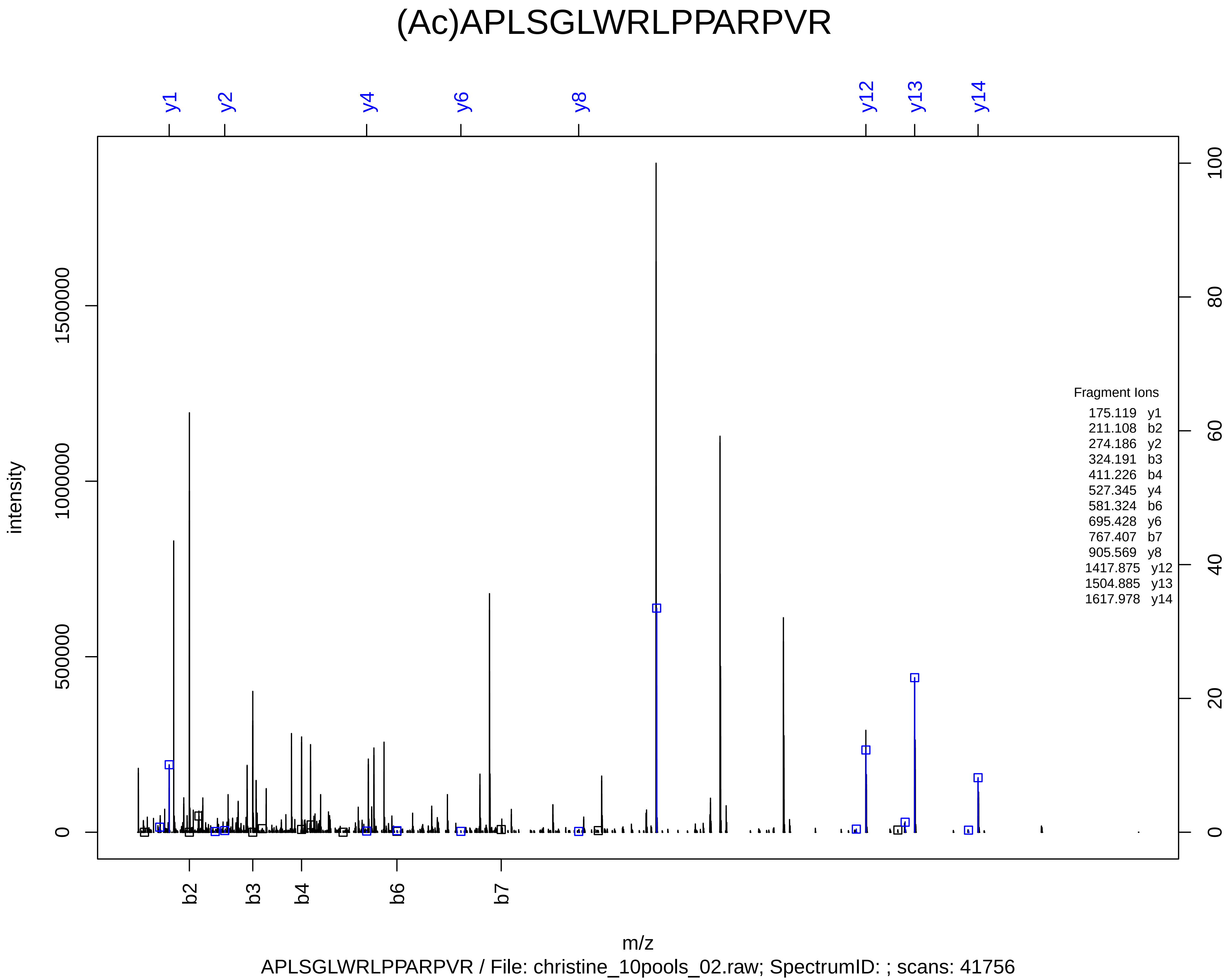
(Ac)APLSGLWRLPPARPVR
y1
y2
y4
y6
y8
y12
y13
y14
intensity
1500000
1000000
500000
0
100
80
60
40
20
0
Fragment Ions
175.119 y1
211.108 b2
274.186 y2
324.191 b3
411.226 b4
527.345 y4
581.324 b6
695.428 y6
767.407 b7
905.569 y8
1417.875 y12
1504.885 y13
1617.978 y14
b2
b3
b4
b6
b7
m/z
APLSGLWRLPPARPVR / File: christine_10pools_02.raw; SpectrumID: ; scans: 41756

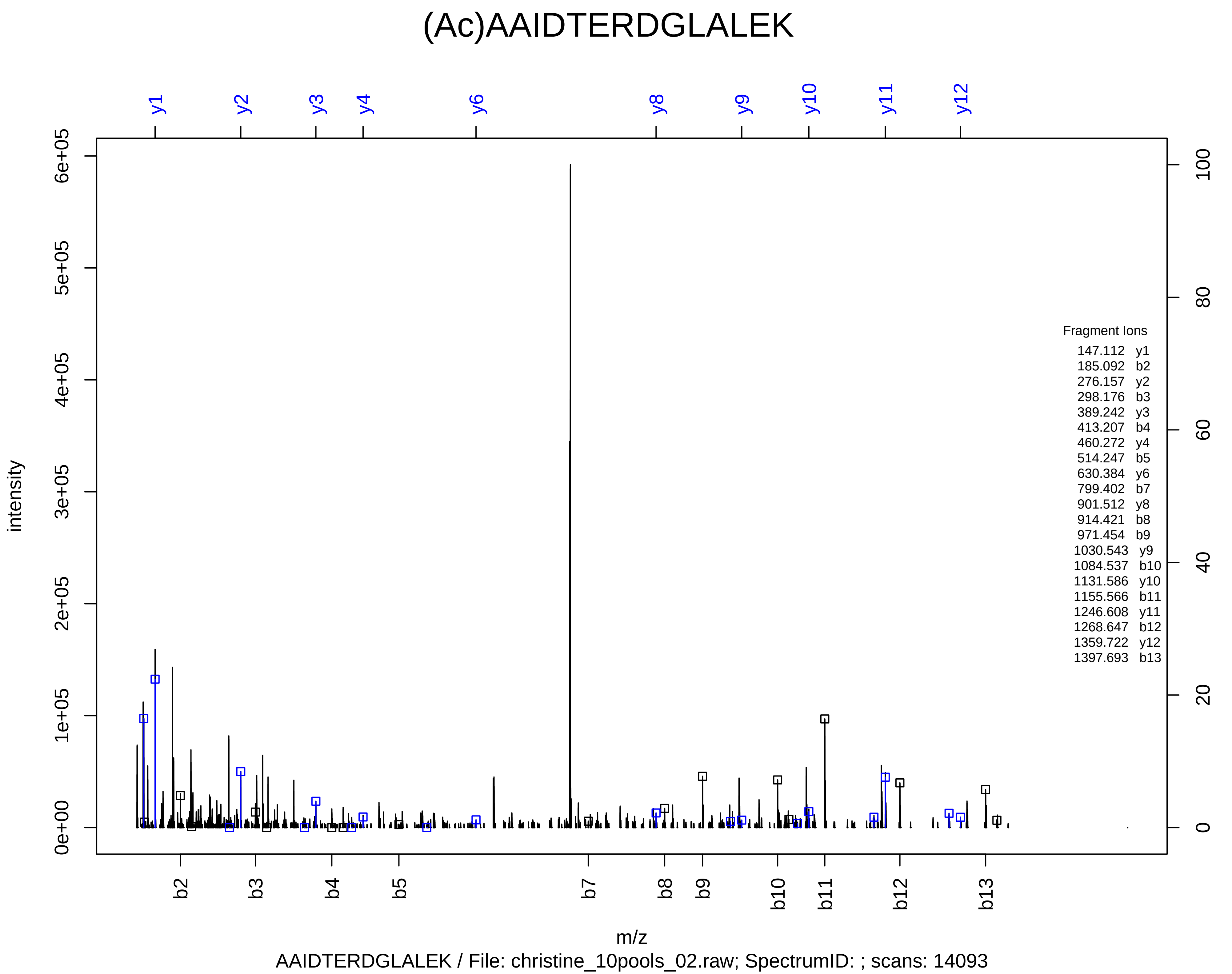

(Ac)AAIDTERDGLALEK
y1
y2
y3
y4
y6
y8
y9
y10
y11
y12
intensity
0e+00
1e+05
2e+05
3e+05
4e+05
5e+05
6e+05
0
20
40
60
80
100
Fragment Ions
147.112 y1
185.092 b2
276.157 y2
298.176 b3
389.242 y3
413.207 b4
460.272 y4
514.247 b5
630.384 y6
799.402 b7
901.512 y8
914.421 b8
971.454 b9
1030.543 y9
1084.537 b10
1131.586 y10
1155.566 b11
1246.608 y11
1268.647 b12
1359.722 y12
1397.693 b13
b2
b3
b4
b5
b7
b8
b9
b10
b11
b12
b13
m/z
AAIDTERDGLALEK / File: christine_10pools_02.raw; SpectrumID: ; scans: 14093

LQGGLSQLSFPLSVEPALGPYK

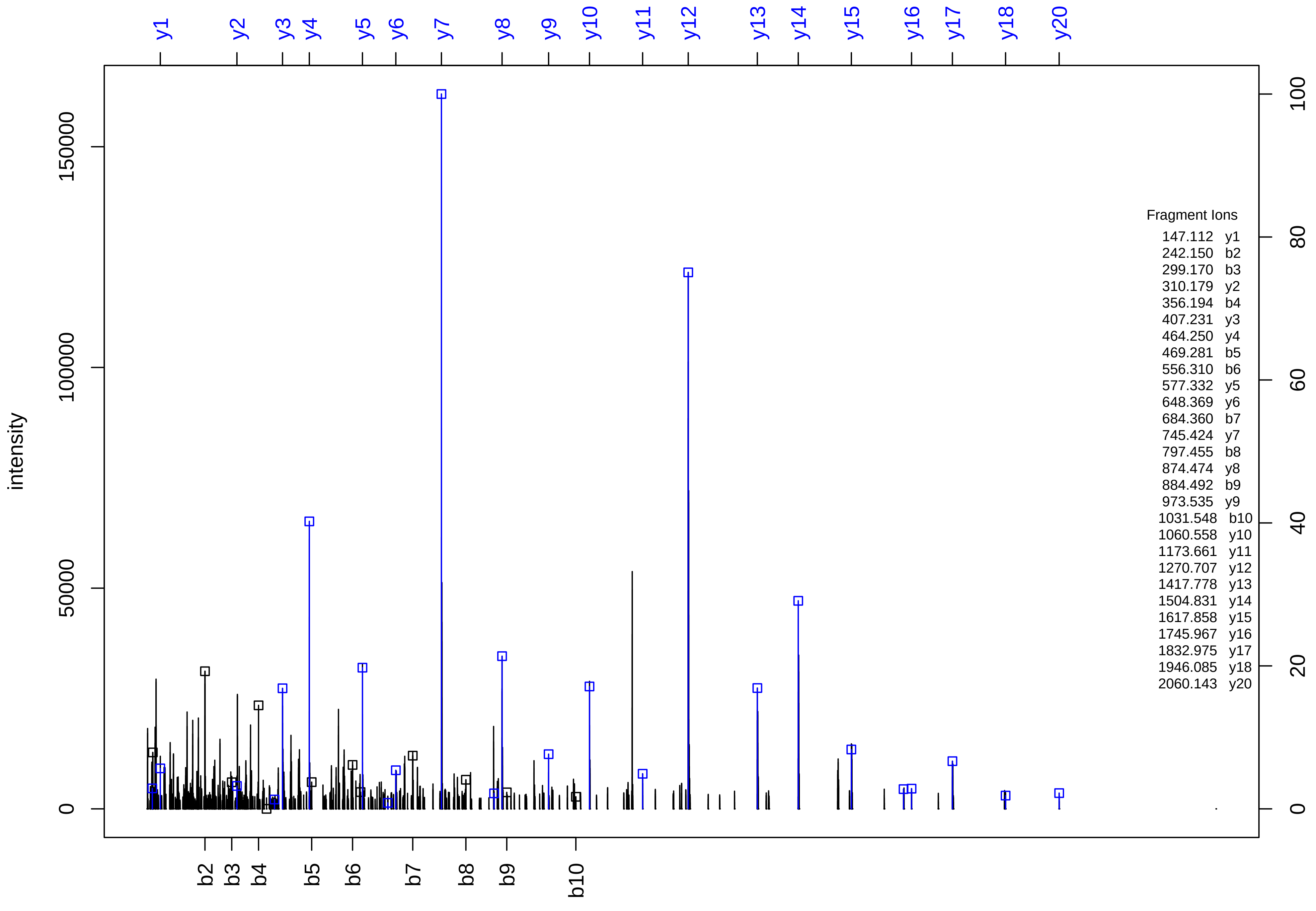

LQGGLSQLSFPLSVEPALGPYK / File: christine_10pools_03.raw; SpectrumID: ; scans: 34572

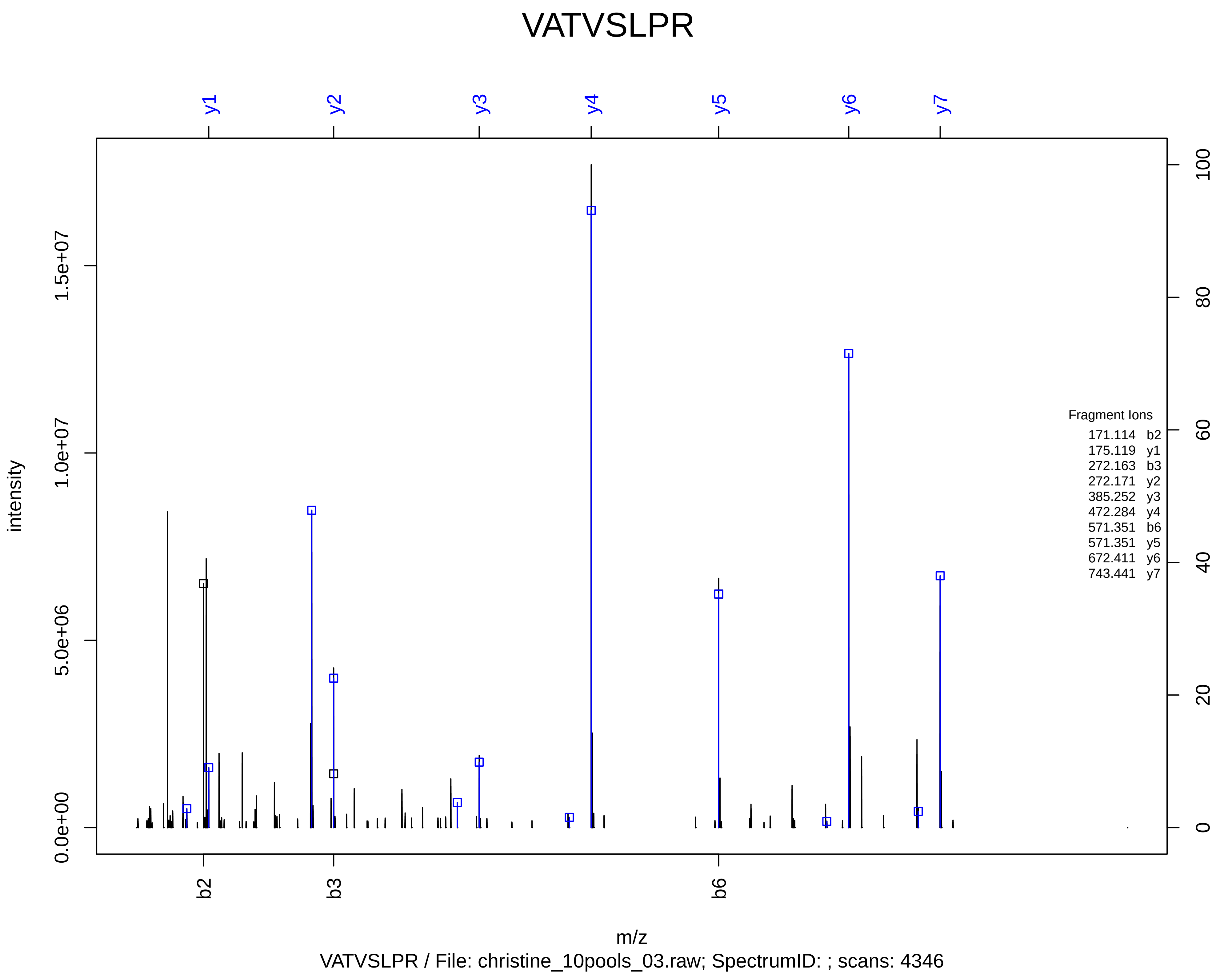

VATVSLPR
y1
y2
y3
y4
y5
y6
y7
intensity
0.0e+00
5.0e+06
1.0e+07
1.5e+07
0
20
40
60
80
100
Fragment Ions
171.114 b2
175.119 y1
272.163 b3
272.171 y2
385.252 y3
472.284 y4
571.351 b6
571.351 y5
672.411 y6
743.441 y7
b2
b3
b6
m/z
VATVSLPR / File: christine_10pools_03.raw; SpectrumID: ; scans: 4346

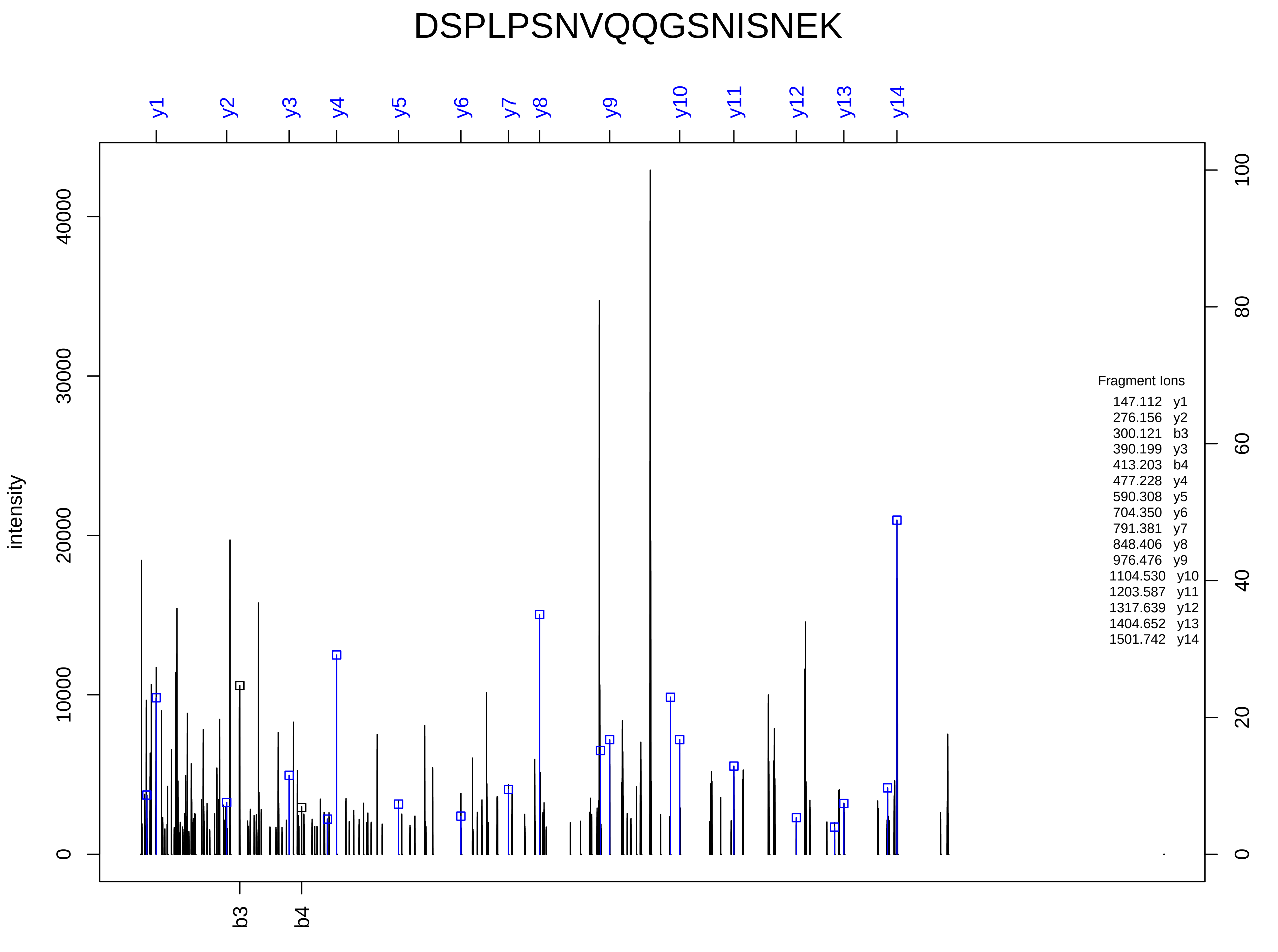

DSPLPSNVQQGSNISNEK / File: christine_10pools_03.raw; SpectrumID: ; scans: 6192

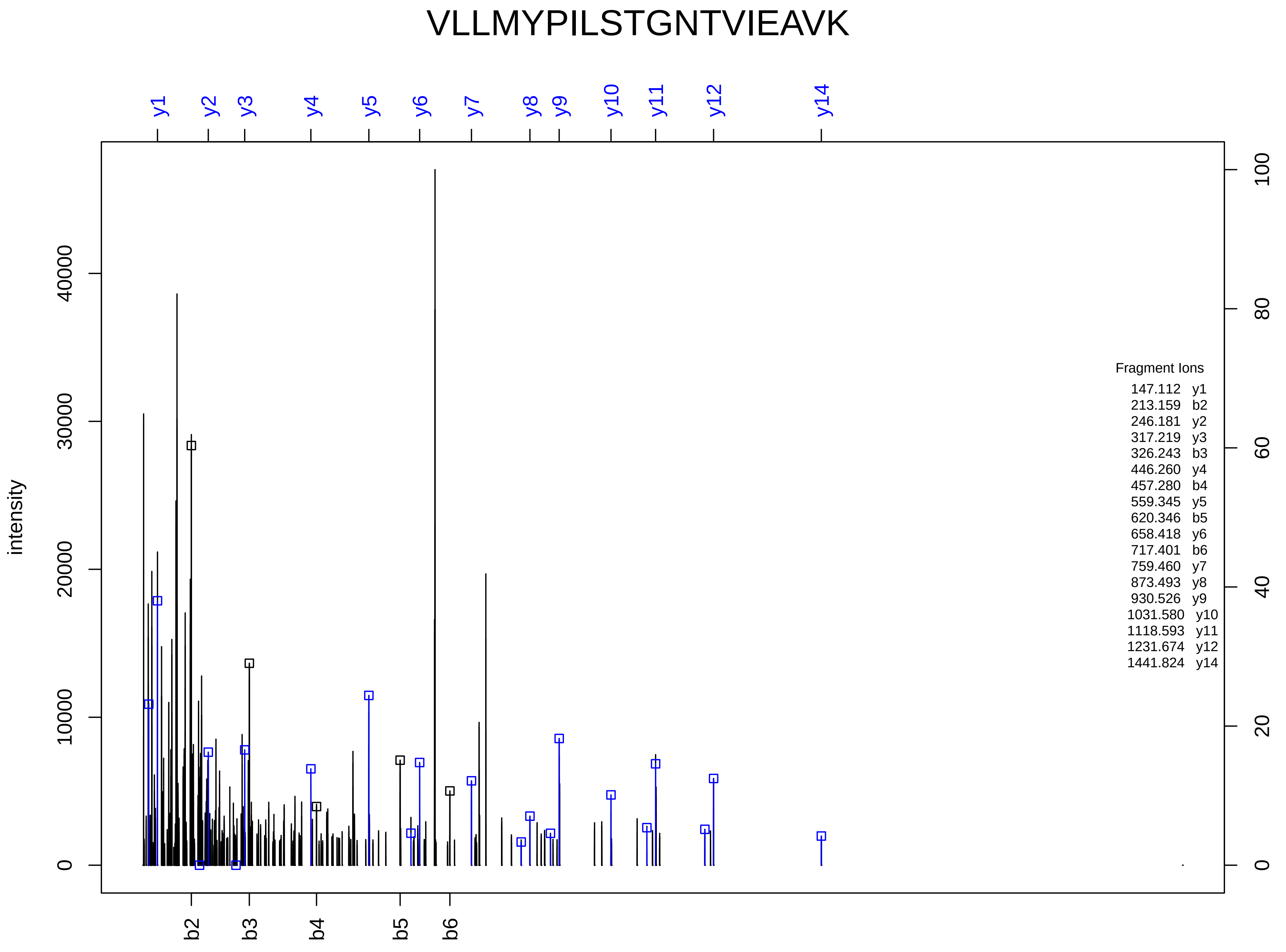

VLLMYPILSTGNTVIEAVK / File: christine_10pools_03.raw; SpectrumID: ; scans: 38491

SEVFYPGETVVHGPGEATALEWGPNTWMVEYGR

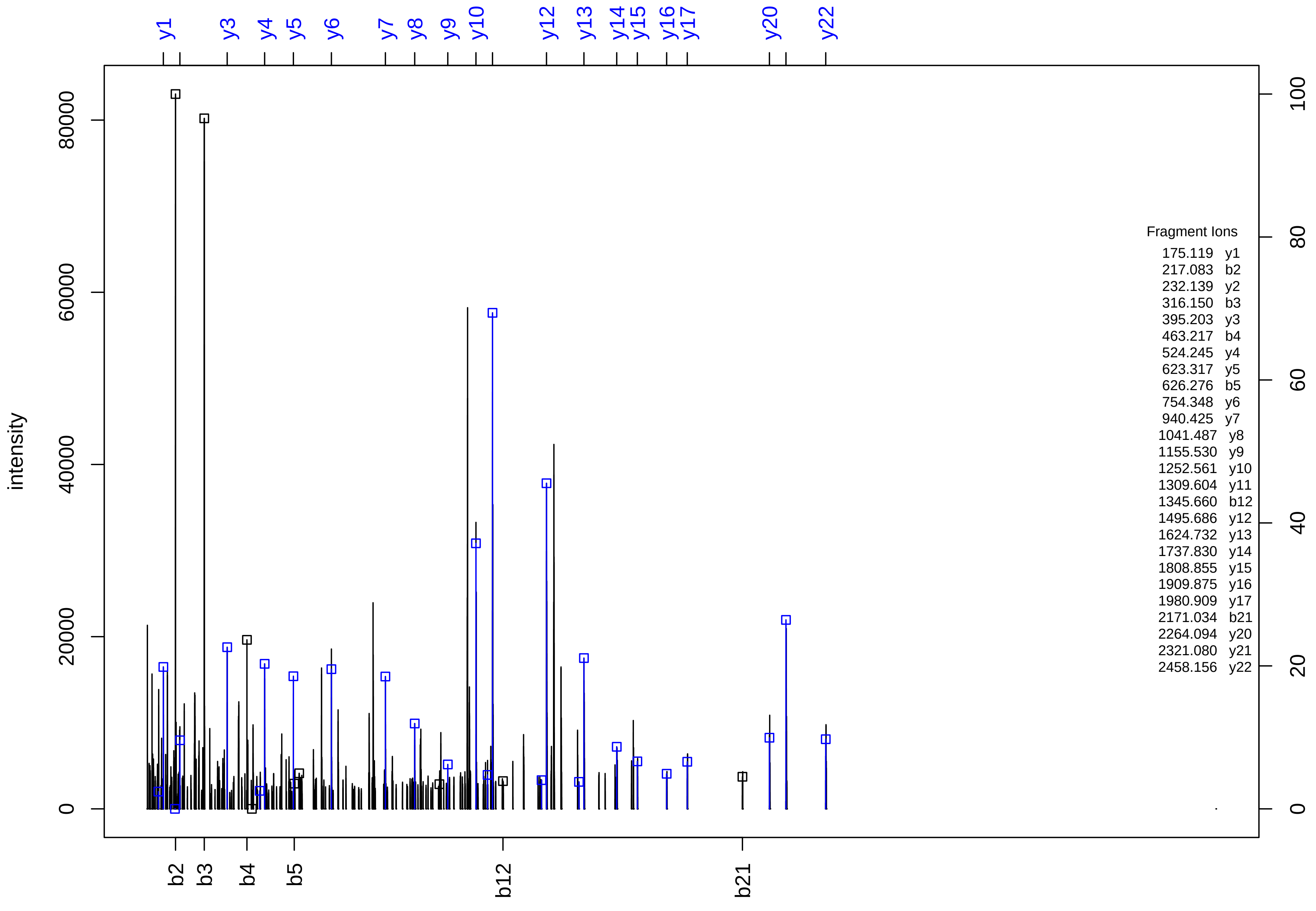

SEVFYPGETVVHGPGEATALEWGPNTWMVEYGR / File: christine_10pools_03.raw; SpectrumID: ; scans: 31951

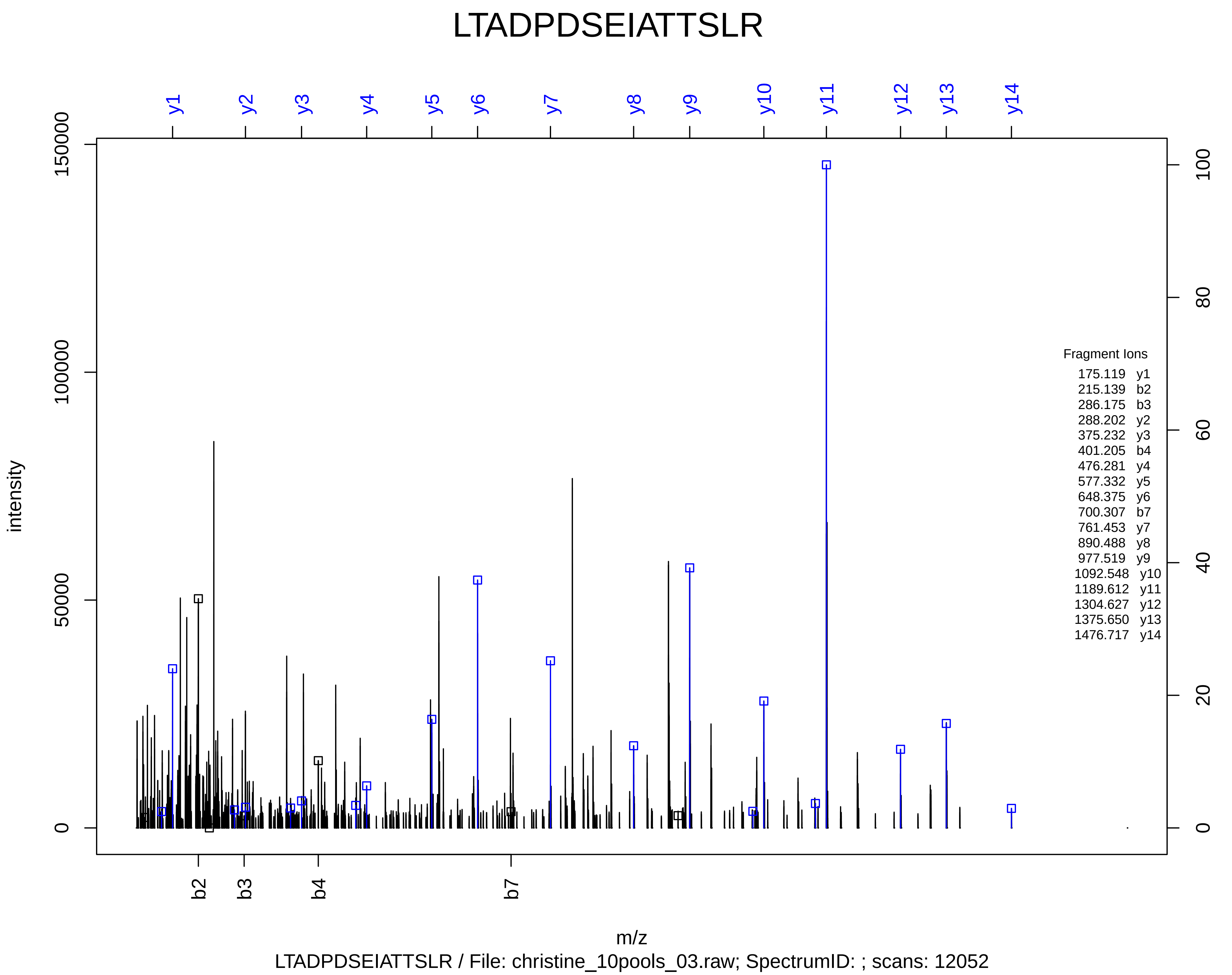
LTADPDSEIATTSLR
y1 y2 y3 y4 y5 y6 y7 y8 y9 y10 y11 y12 y13 y14
b2 b3 b4 b7
intensity
m/z
0
50000
100000
150000
0
20
40
60
80
100
Fragment Ions
175.119 y1
215.139 b2
286.175 b3
288.202 y2
375.232 y3
401.205 b4
476.281 y4
577.332 y5
648.375 y6
700.307 b7
761.453 y7
890.488 y8
977.519 y9
1092.548 y10
1189.612 y11
1304.627 y12
1375.650 y13
1476.717 y14
LTADPDSEIATTSLR / File: christine_10pools_03.raw; SpectrumID: ; scans: 12052

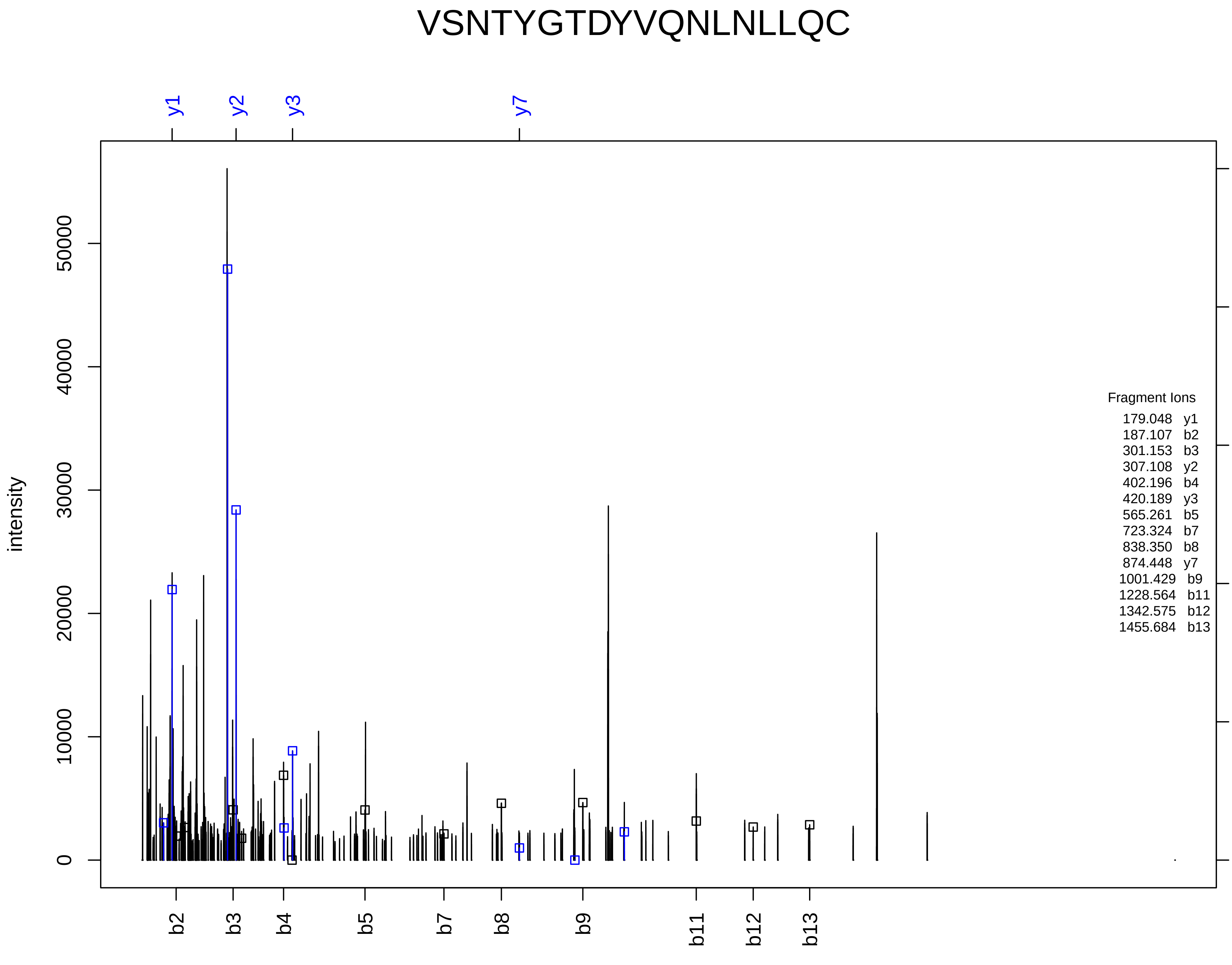
VSNTYGTDYVQNLNLLQC
y1
y2
y3
y7
intensity
0
10000
20000
30000
40000
50000
0
20
40
60
80
100
b2
b3
b4
b5
b7
b8
b9
b11
b12
b13
m/z
Fragment Ions
179.048 y1
187.107 b2
301.153 b3
307.108 y2
402.196 b4
420.189 y3
565.261 b5
723.324 b7
838.350 b8
874.448 y7
1001.429 b9
1228.564 b11
1342.575 b12
1455.684 b13
VSNTYGTDYVQNLNLLQC / File: christine_10pools_03.raw; SpectrumID: ; scans: 29131

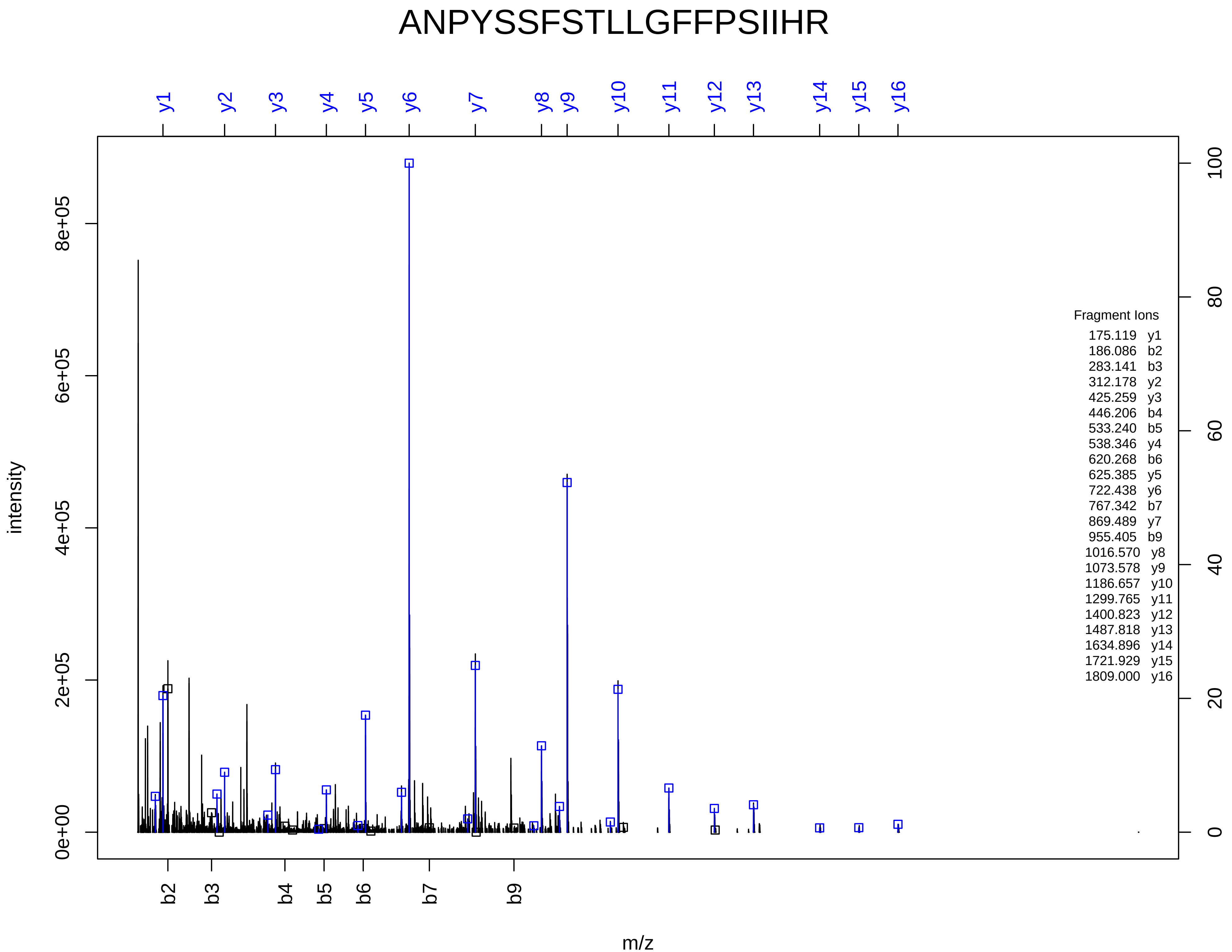

ANPYSSFSTLLGFFPSIIHR / File: christine_10pools_03.raw; SpectrumID: ; scans: 41290

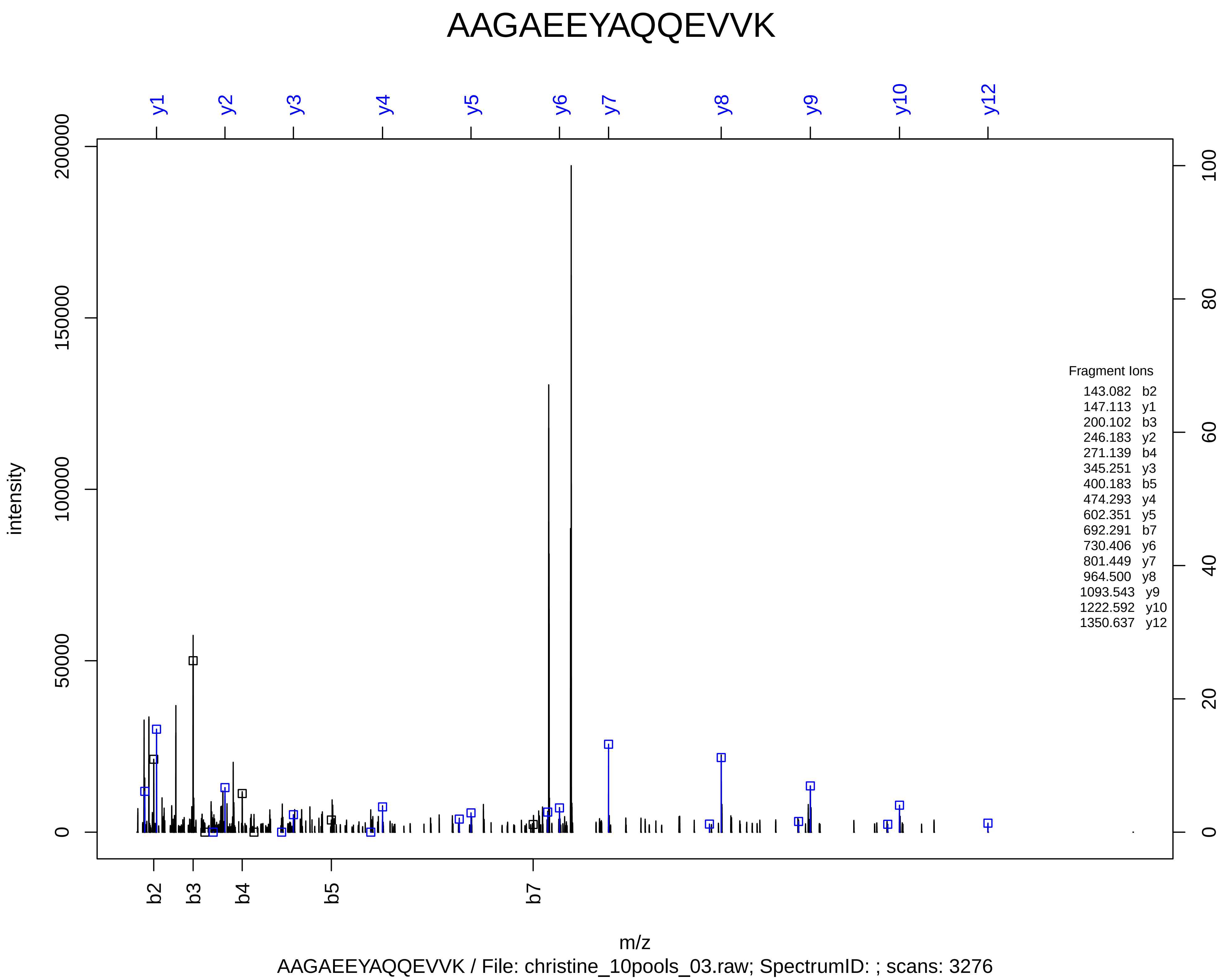
AAGAEEYAQQEVVK
y1
y2
y3
y4
y5
y6
y7
y8
y9
y10
y12
intensity
0
50000
100000
150000
200000
0
20
40
60
80
100
Fragment Ions
143.082 b2
147.113 y1
200.102 b3
246.183 y2
271.139 b4
345.251 y3
400.183 b5
474.293 y4
602.351 y5
692.291 b7
730.406 y6
801.449 y7
964.500 y8
1093.543 y9
1222.592 y10
1350.637 y12
b2
b3
b4
b5
b7
m/z
AAGAEEYAQQEVVK / File: christine_10pools_03.raw; SpectrumID: ; scans: 3276

TFTFDTVFGPESK

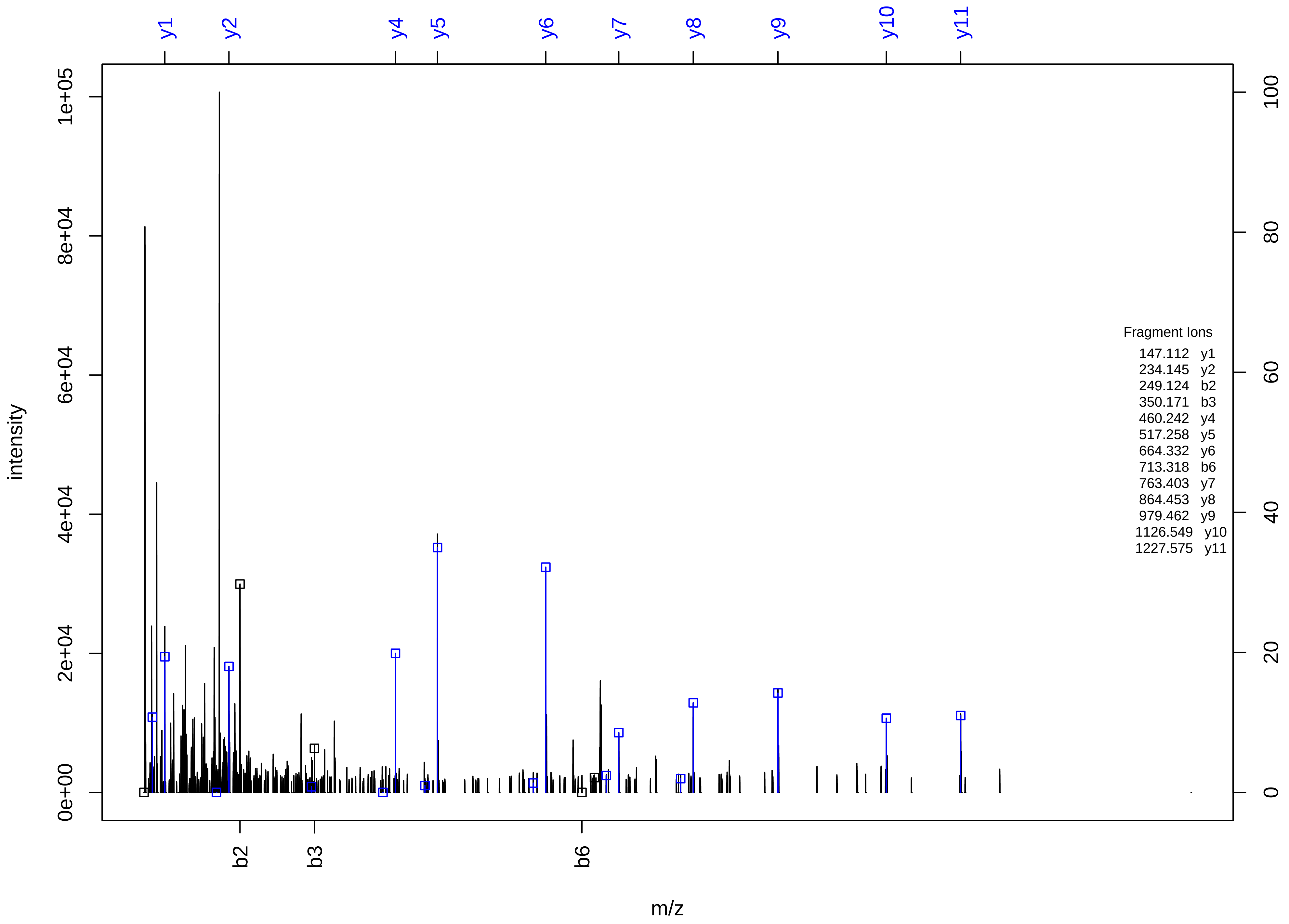

TFTFDTVFGPESK / File: christine_10pools_03.raw; SpectrumID: ; scans: 24961

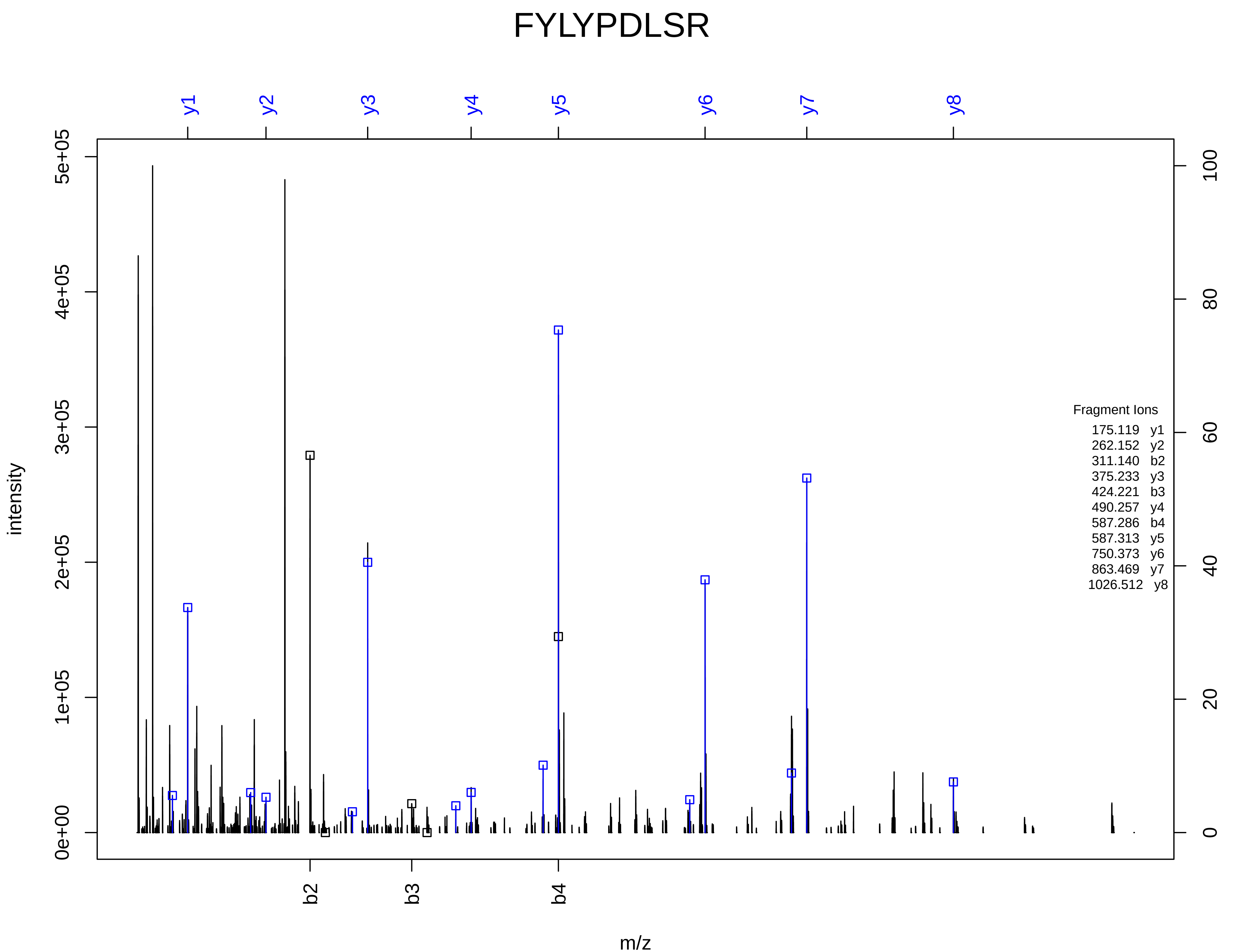
FYLYPDLSR
y1
y2
y3
y4
y5
y6
y7
y8
5e+05
4e+05
3e+05
2e+05
1e+05
0e+00
intensity
100
80
60
40
20
0
Fragment Ions
175.119 y1
262.152 y2
311.140 b2
375.233 y3
424.221 b3
490.257 y4
587.286 b4
587.313 y5
750.373 y6
863.469 y7
1026.512 y8
b2
b3
b4
m/z
FYLYPDLSR / File: christine_10pools_03.raw; SpectrumID: ; scans: 19899

LGALTNYLQPR

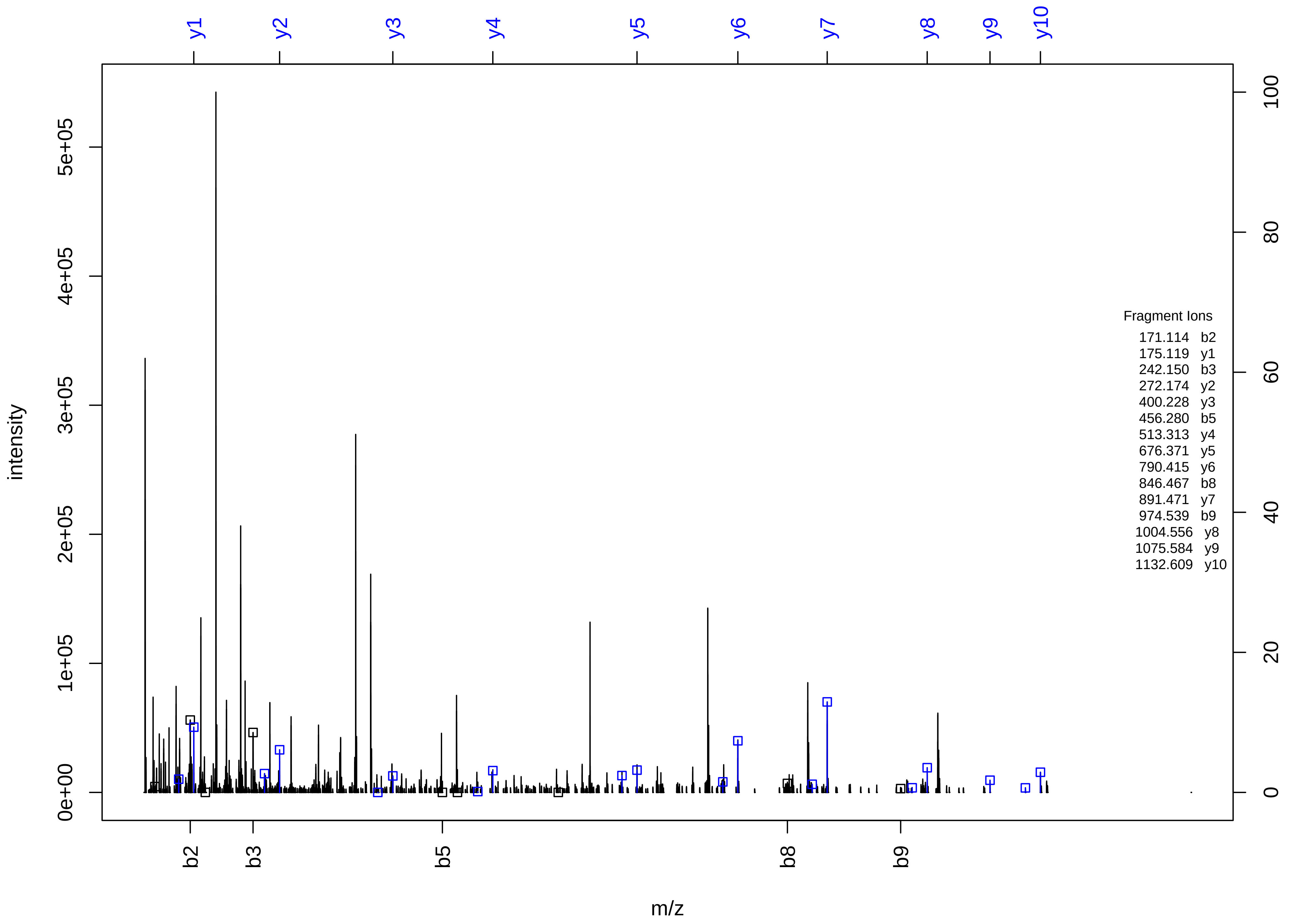

LGALTNYLQPR / File: christine_10pools_03.raw; SpectrumID: ; scans: 16624

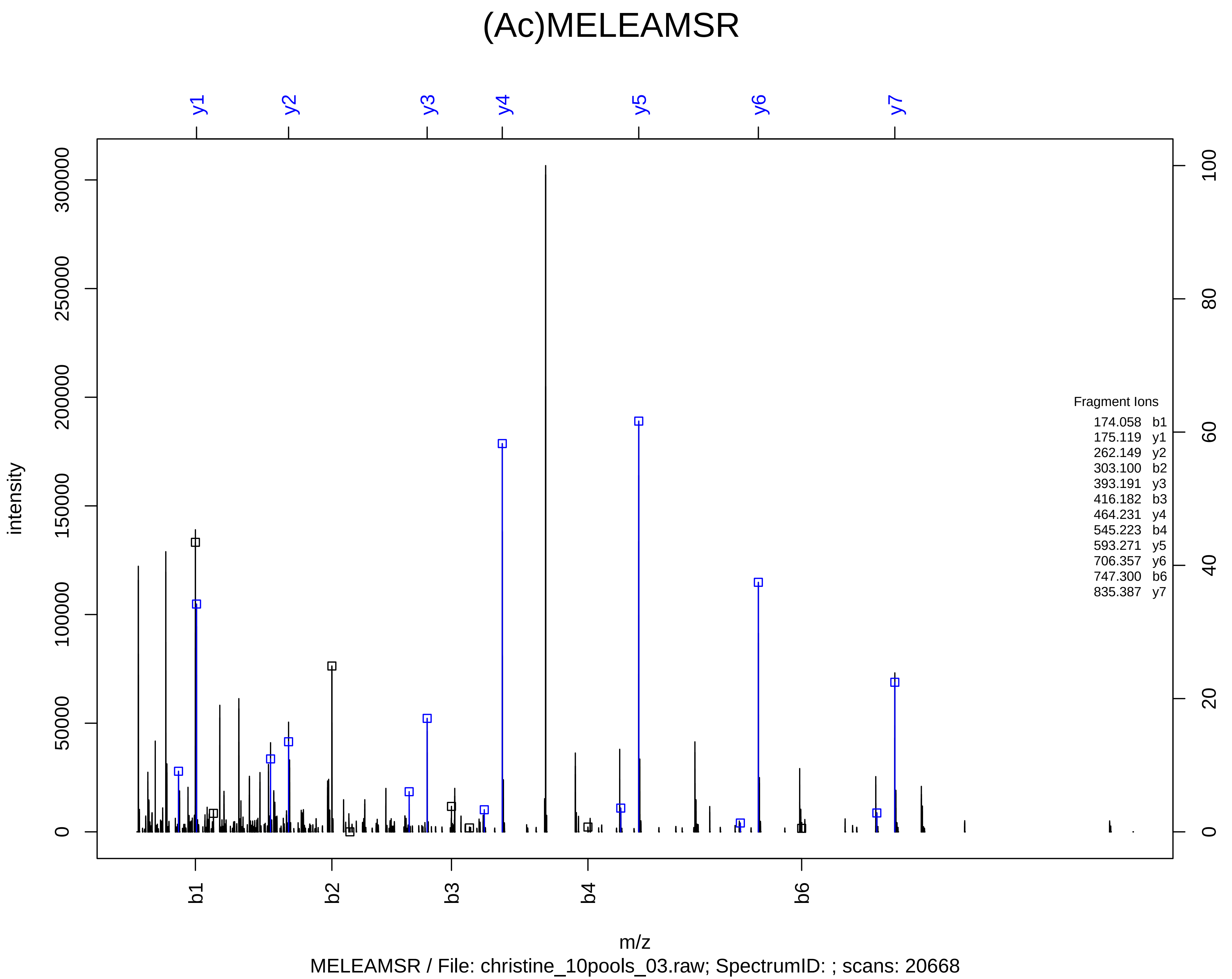

(Ac)MELEAMSR
y1
y2
y3
y4
y5
y6
y7
intensity
0
50000
100000
150000
200000
250000
300000
0
20
40
60
80
100
Fragment Ions
174.058 b1
175.119 y1
262.149 y2
303.100 b2
393.191 y3
416.182 b3
464.231 y4
545.223 b4
593.271 y5
706.357 y6
747.300 b6
835.387 y7
b1
b2
b3
b4
b6
m/z
MELEAMSR / File: christine_10pools_03.raw; SpectrumID: ; scans: 20668

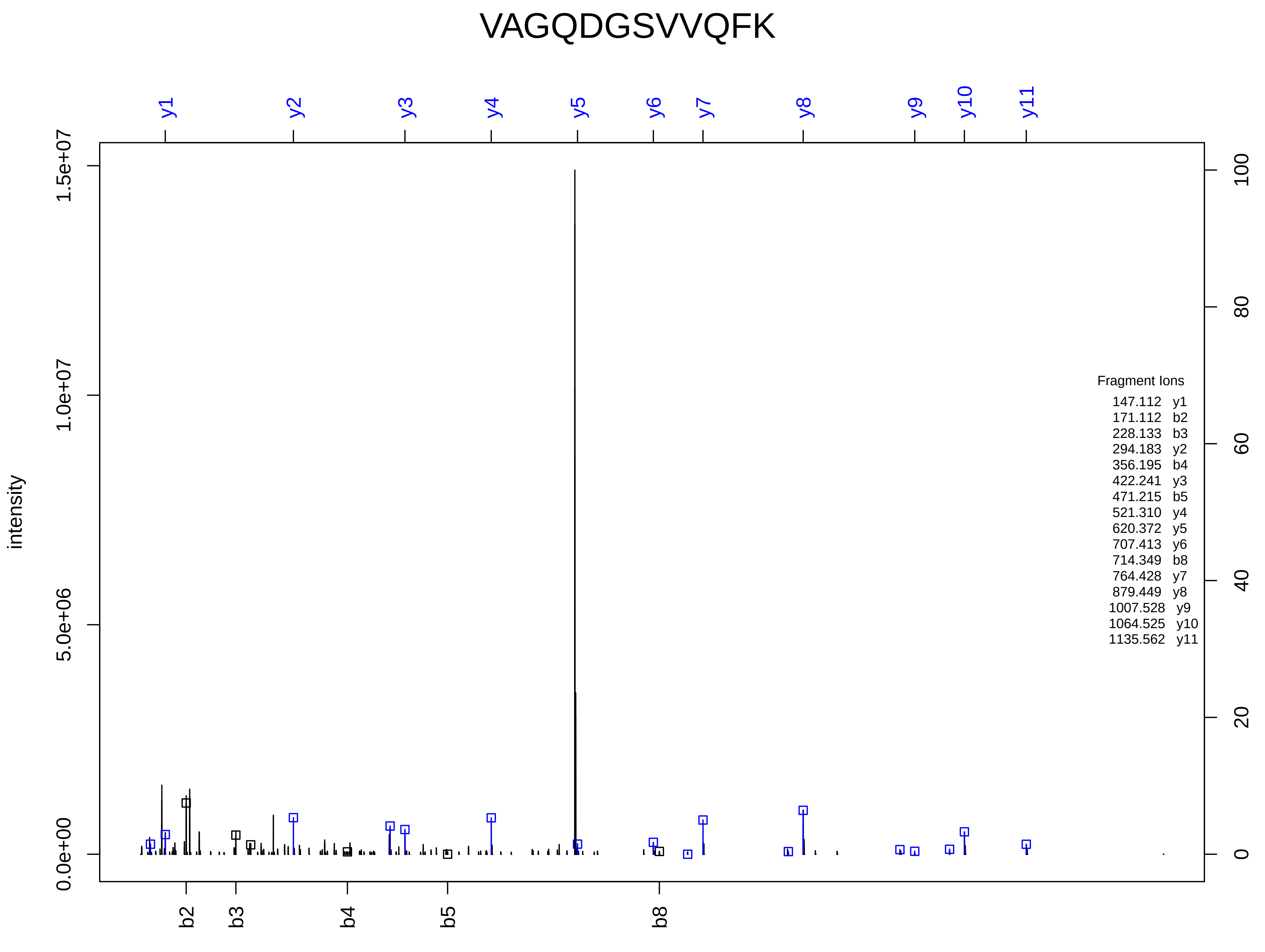

VAGQDGSVVQFK / File: christine_10pools_03.raw; SpectrumID: ; scans: 4912

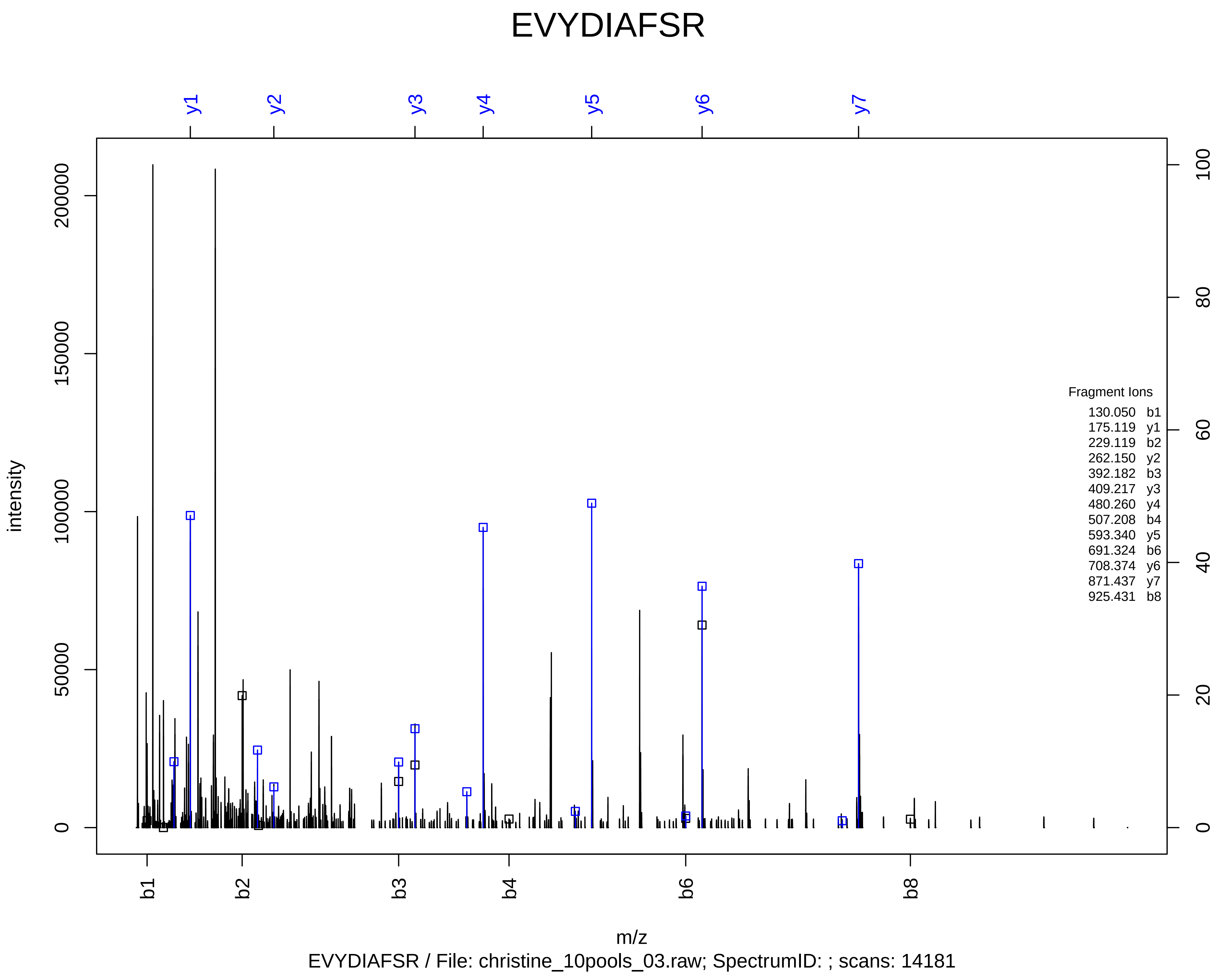

EVYDIAFSR
y1
y2
y3
y4
y5
y6
y7
intensity
0
50000
100000
150000
200000
0
20
40
60
80
100
b1
b2
b3
b4
b6
b8
m/z
Fragment Ions
130.050 b1
175.119 y1
229.119 b2
262.150 y2
392.182 b3
409.217 y3
480.260 y4
507.208 b4
593.340 y5
691.324 b6
708.374 y6
871.437 y7
925.431 b8
EVYDIAFSR / File: christine_10pools_03.raw; SpectrumID: ; scans: 14181

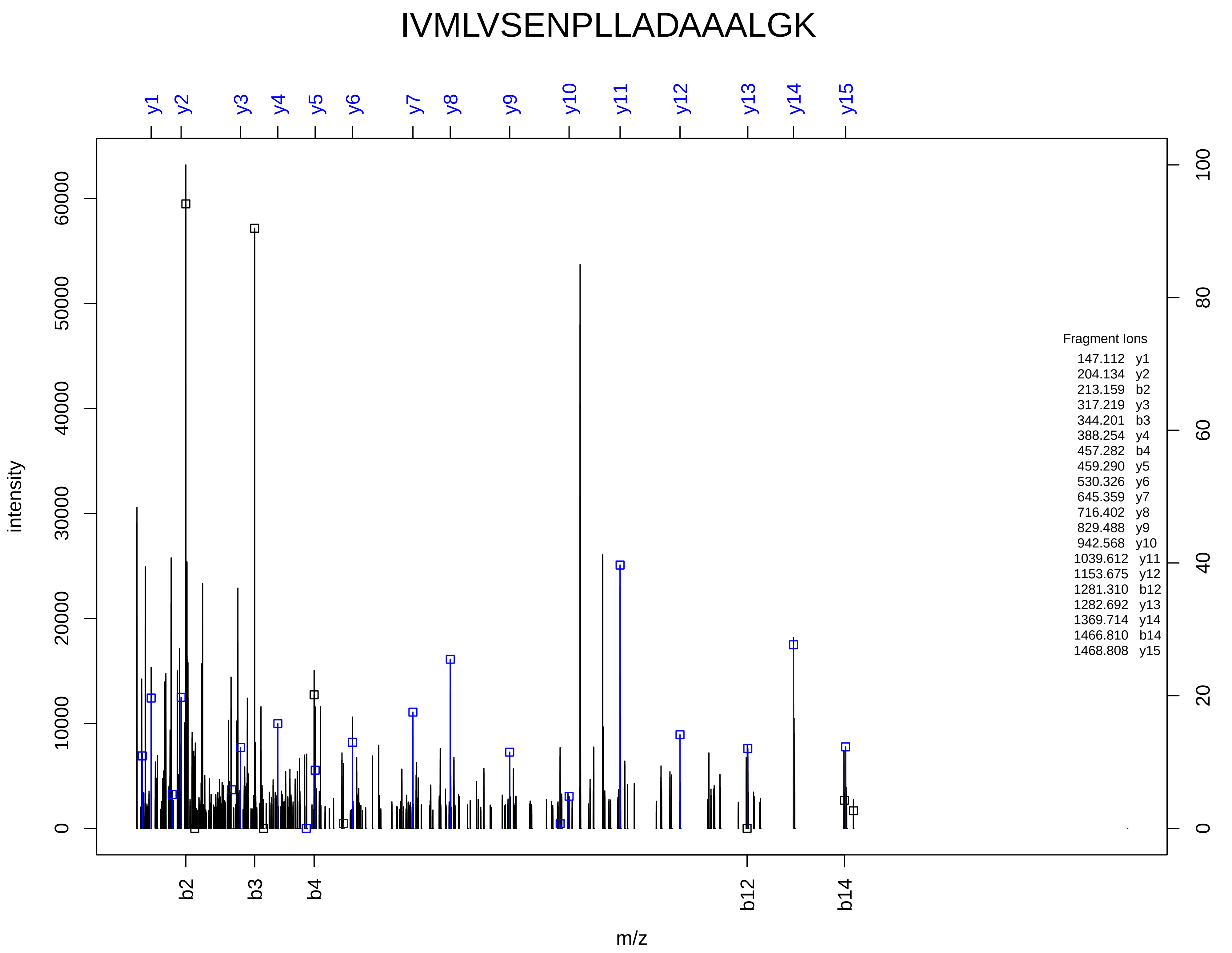
IVMLVSENPLLADAAALGK
y1 y2 y3 y4 y5 y6 y7 y8 y9 y10 y11 y12 y13 y14 y15
intensity
0
10000
20000
30000
40000
50000
60000
0 20 40 60 80 100
b2 b3 b4 b12 b14
m/z
Fragment Ions
147.112 y1
204.134 y2
213.159 b2
317.219 y3
344.201 b3
388.254 y4
457.282 b4
459.290 y5
530.326 y6
645.359 y7
716.402 y8
829.488 y9
942.568 y10
1039.612 y11
1153.675 y12
1281.310 b12
1282.692 y13
1369.714 y14
1466.810 b14
1468.808 y15
IVMLVSENPLLADAAALGK / File: christine_10pools_03.raw; SpectrumID: ; scans: 35586

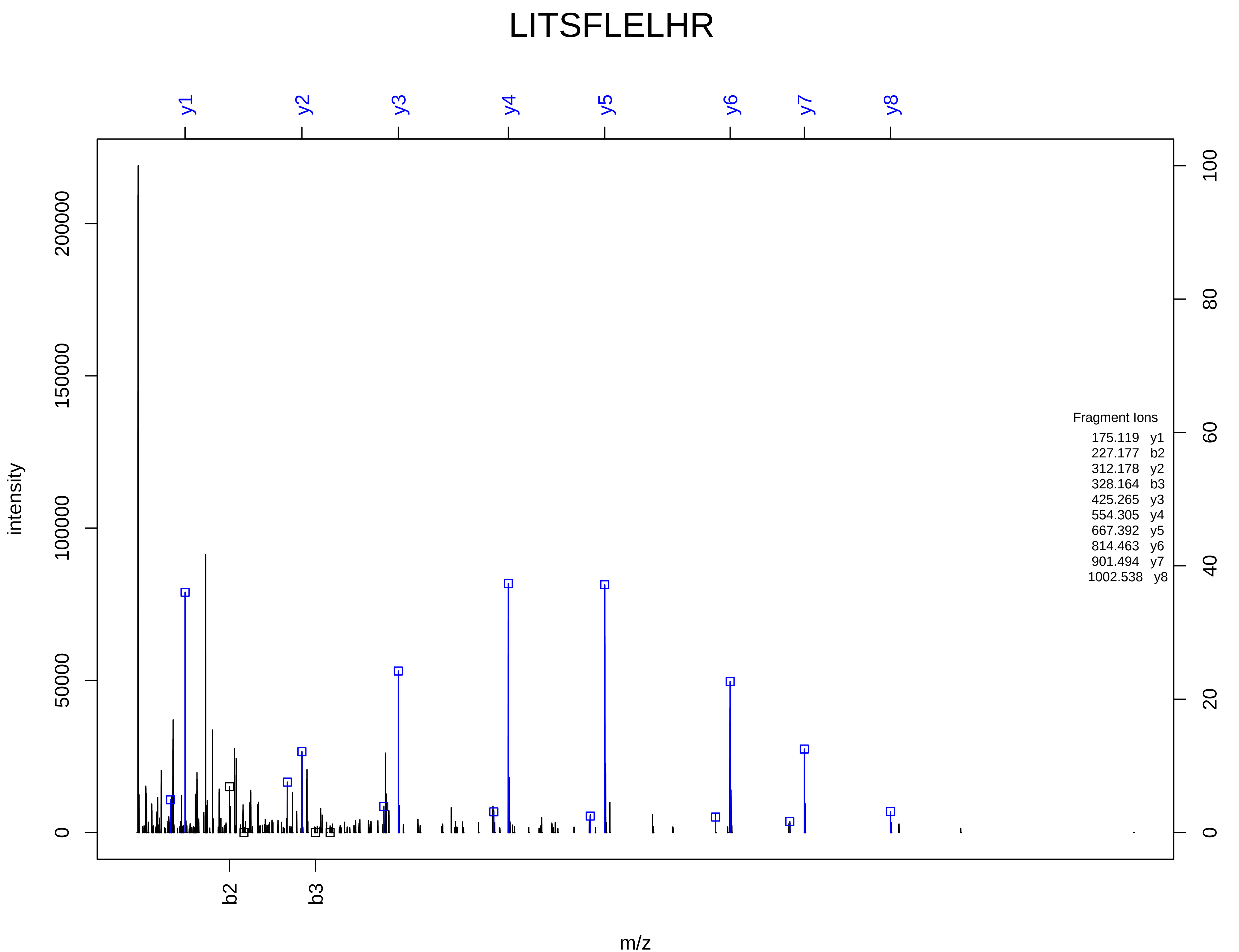
LITSFLELHR
y1
y2
y3
y4
y5
y6
y7
y8
intensity
0
50000
100000
150000
200000
0
20
40
60
80
100
Fragment Ions
175.119 y1
227.177 b2
312.178 y2
328.164 b3
425.265 y3
554.305 y4
667.392 y5
814.463 y6
901.494 y7
1002.538 y8
b2
b3
m/z
LITSFLELHR / File: christine_10pools_03.raw; SpectrumID: ; scans: 19035

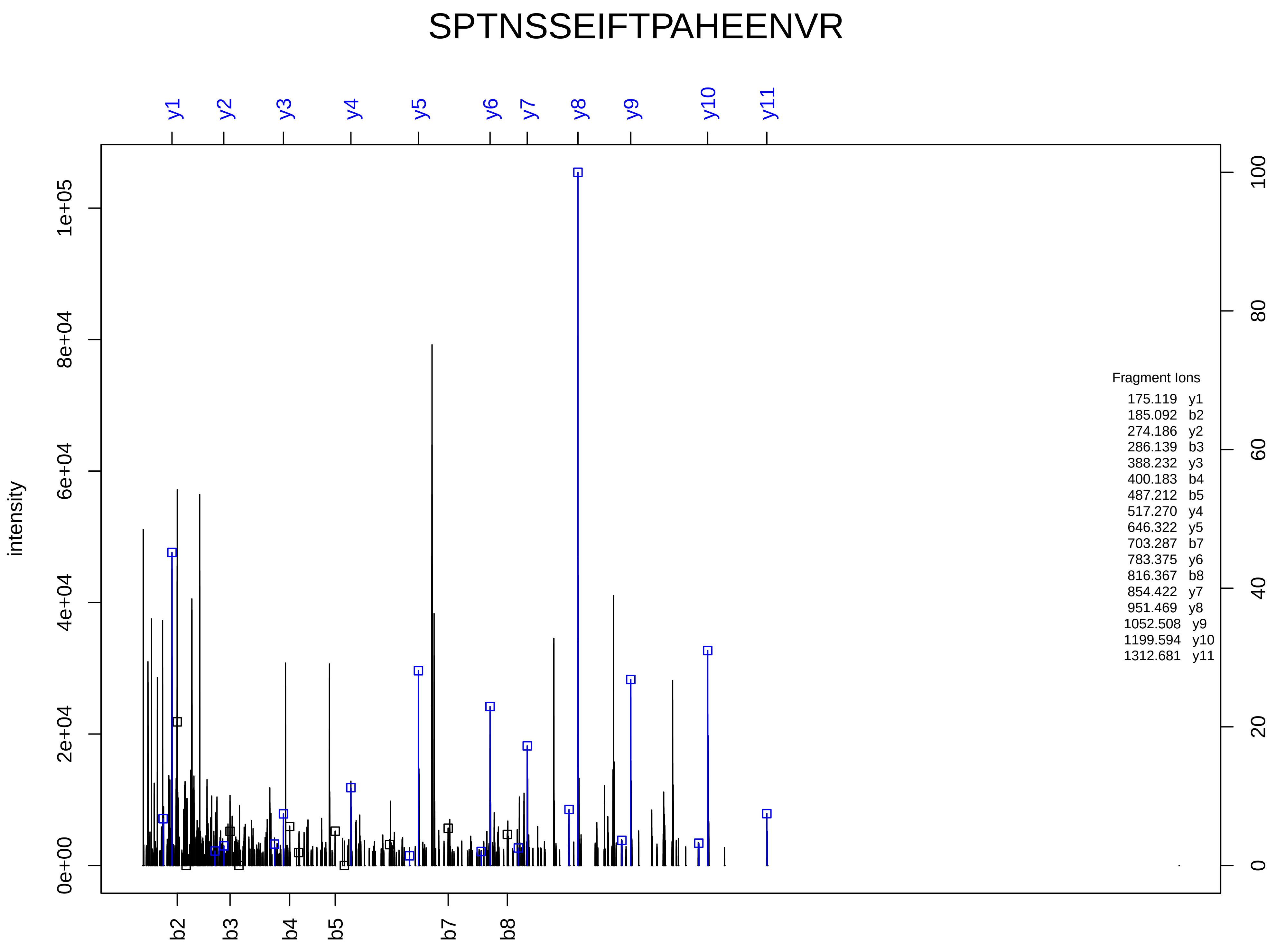

SPTNSSEIFTPAHEENVR / File: christine_10pools_03.raw; SpectrumID: ; scans: 4603

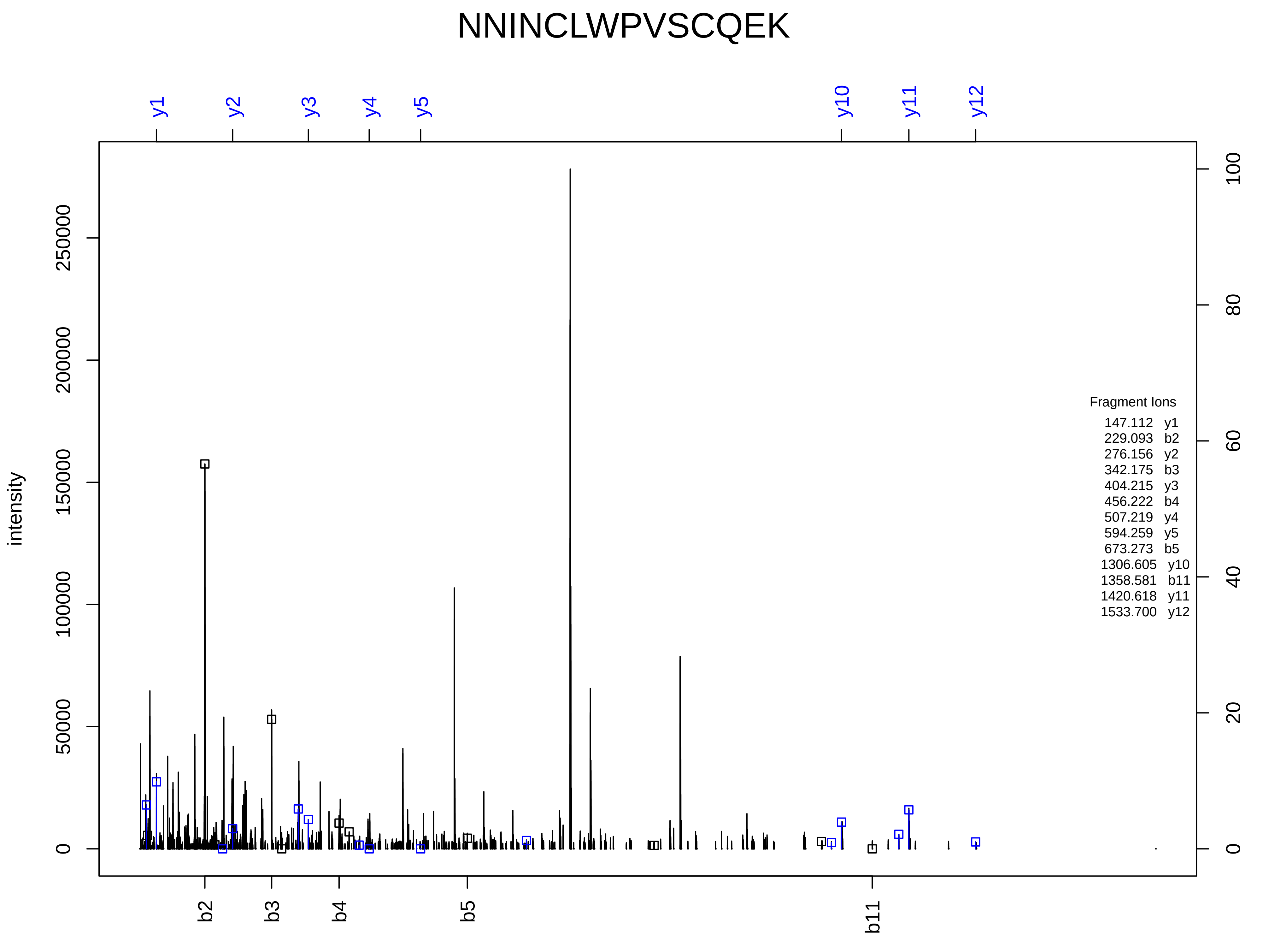

NNINCLWPVSCQEK / File: christine_10pools_03.raw; SpectrumID: ; scans: 16687

FLAQLSEFATSNQISLGPLK

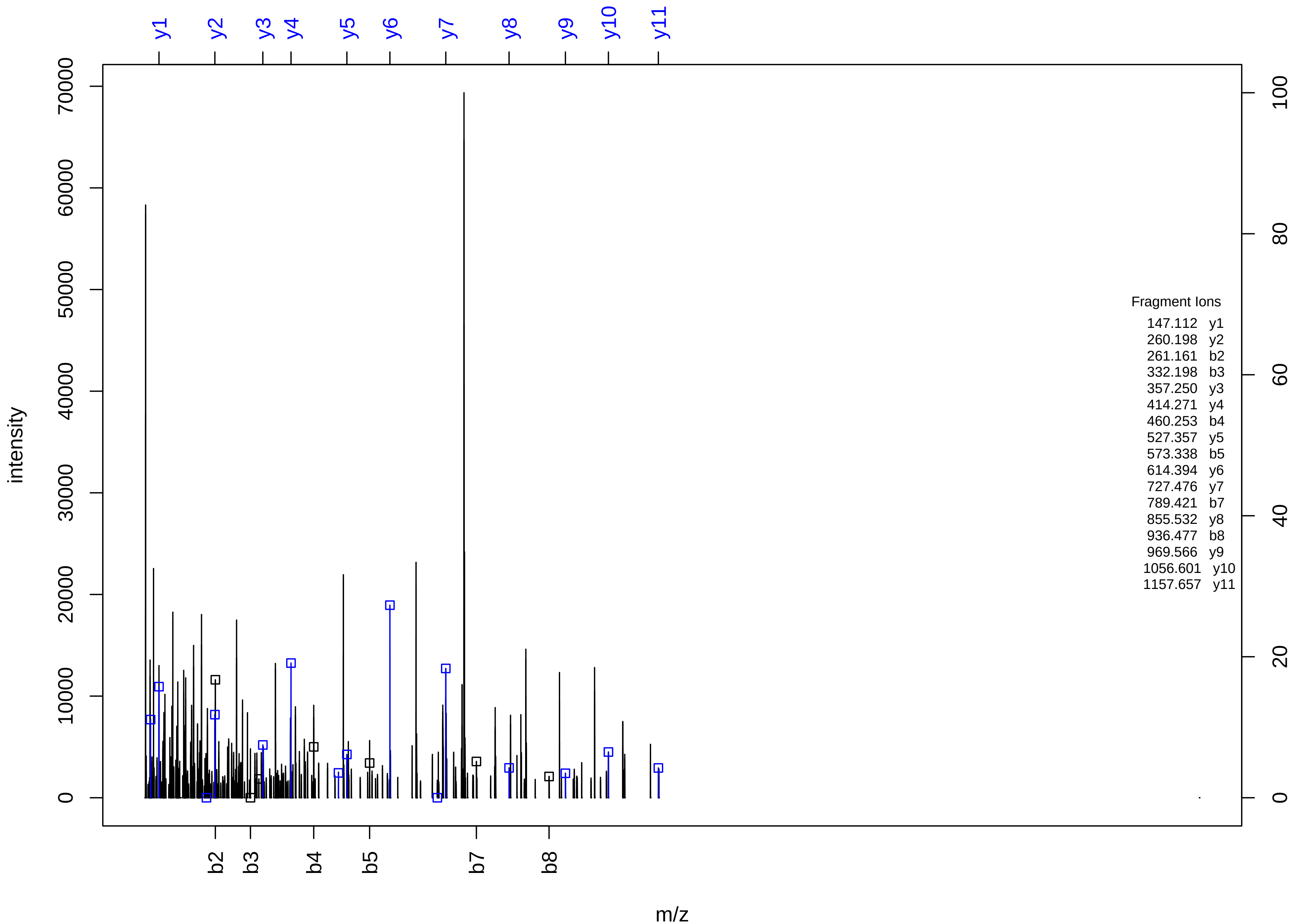

FLAQLSEFATSNQISLGPLK / File: christine_10pools_03.raw; SpectrumID: ; scans: 33565

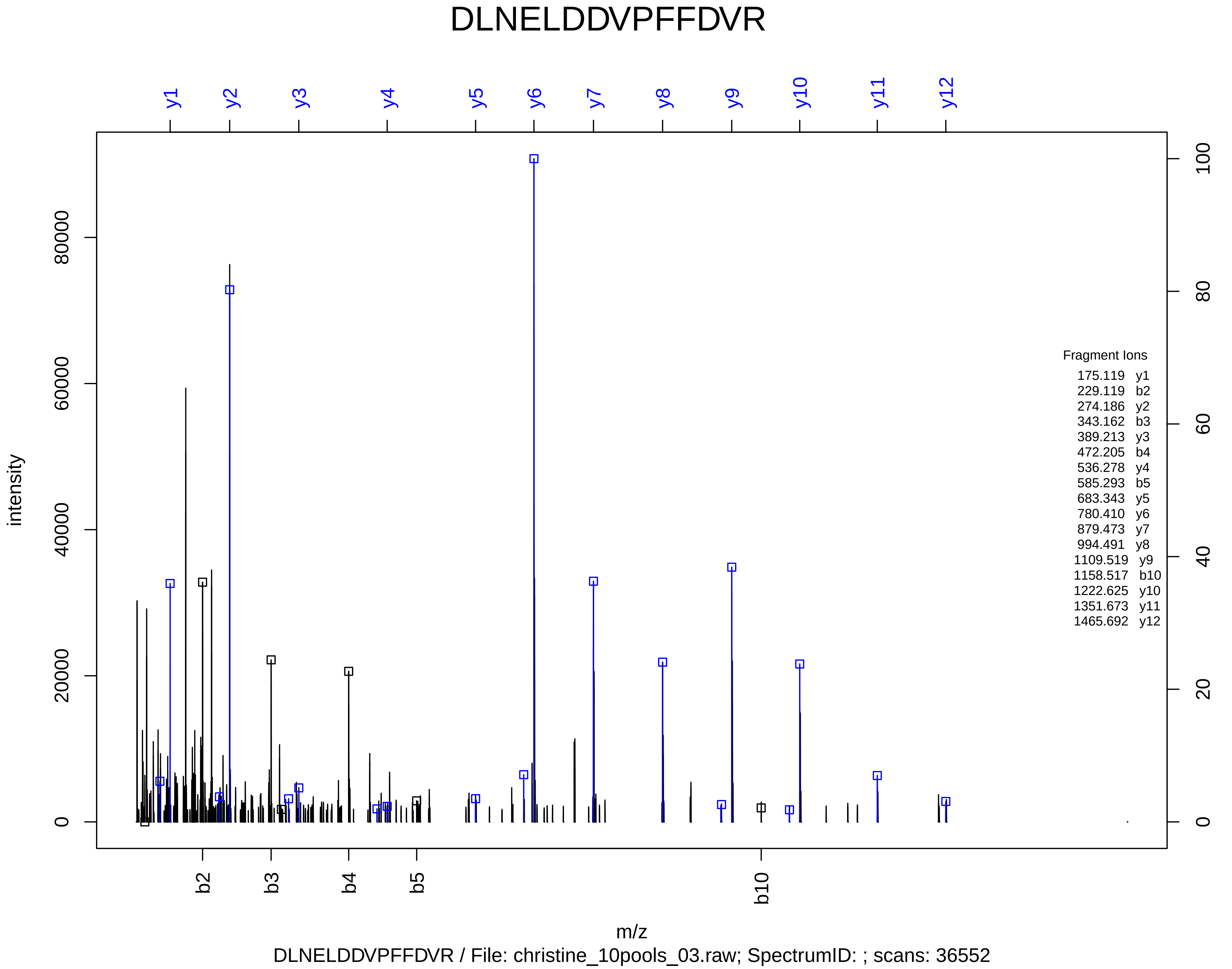

DLNELDDVPFFDVR
y1
y2
y3
y4
y5
y6
y7
y8
y9
y10
y11
y12
intensity
0
20000
40000
60000
80000
0
20
40
60
80
100
b2
b3
b4
b5
b10
Fragment Ions
175.119 y1
229.119 b2
274.186 y2
343.162 b3
389.213 y3
472.205 b4
536.278 y4
585.293 b5
683.343 y5
780.410 y6
879.473 y7
994.491 y8
1109.519 y9
1158.517 b10
1222.625 y10
1351.673 y11
1465.692 y12
m/z
DLNELDDVPFFDVR / File: christine_10pools_03.raw; SpectrumID: ; scans: 36552

ILVISADSGMIEPVVNAVSIHQVK

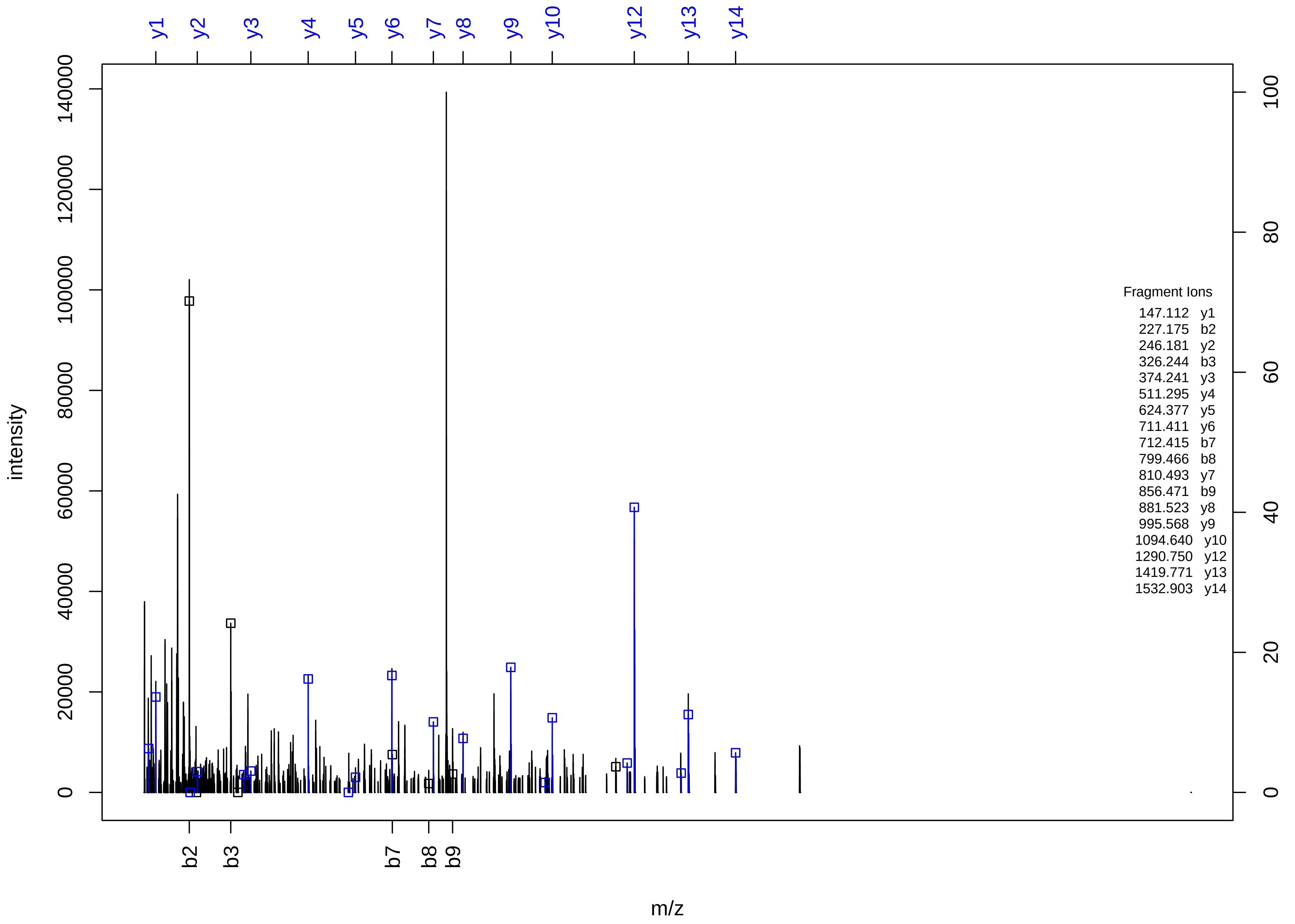

ILVISADSGMIEPVVNAVSIHQVK / File: christine_10pools_03.raw; SpectrumID: ; scans: 29786

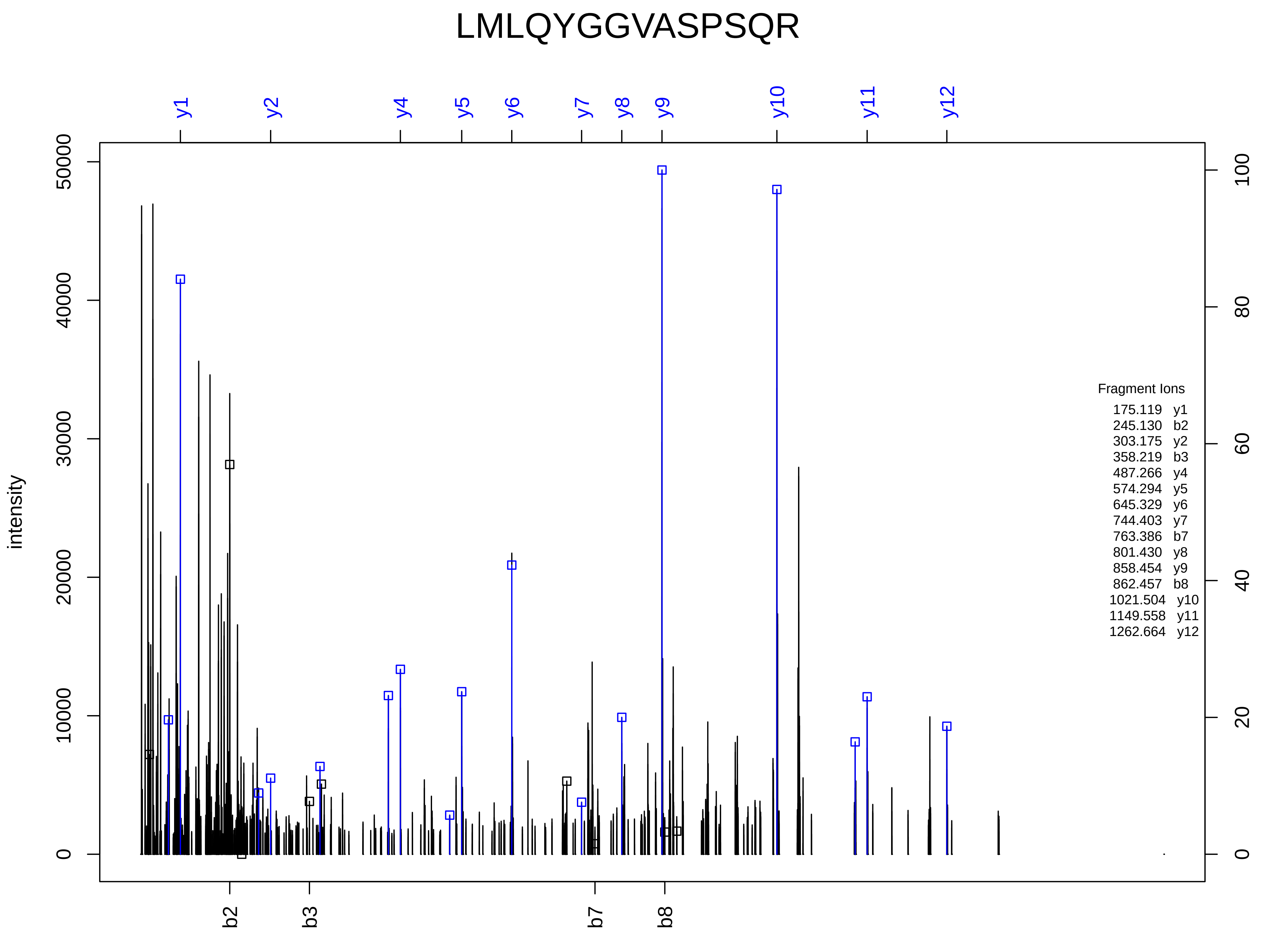

LMLQYGGVASPSQR
y1
y2
y4
y5
y6
y7
y8
y9
y10
y11
y12
intensity
0
10000
20000
30000
40000
50000
0
20
40
60
80
100
b2
b3
b7
b8
Fragment Ions
175.119 y1
245.130 b2
303.175 y2
358.219 b3
487.266 y4
574.294 y5
645.329 y6
744.403 y7
763.386 b7
801.430 y8
858.454 y9
862.457 b8
1021.504 y10
1149.558 y11
1262.664 y12
m/z
LMLQYGGVASPSQR / File: christine_10pools_03.raw; SpectrumID: ; scans: 11880

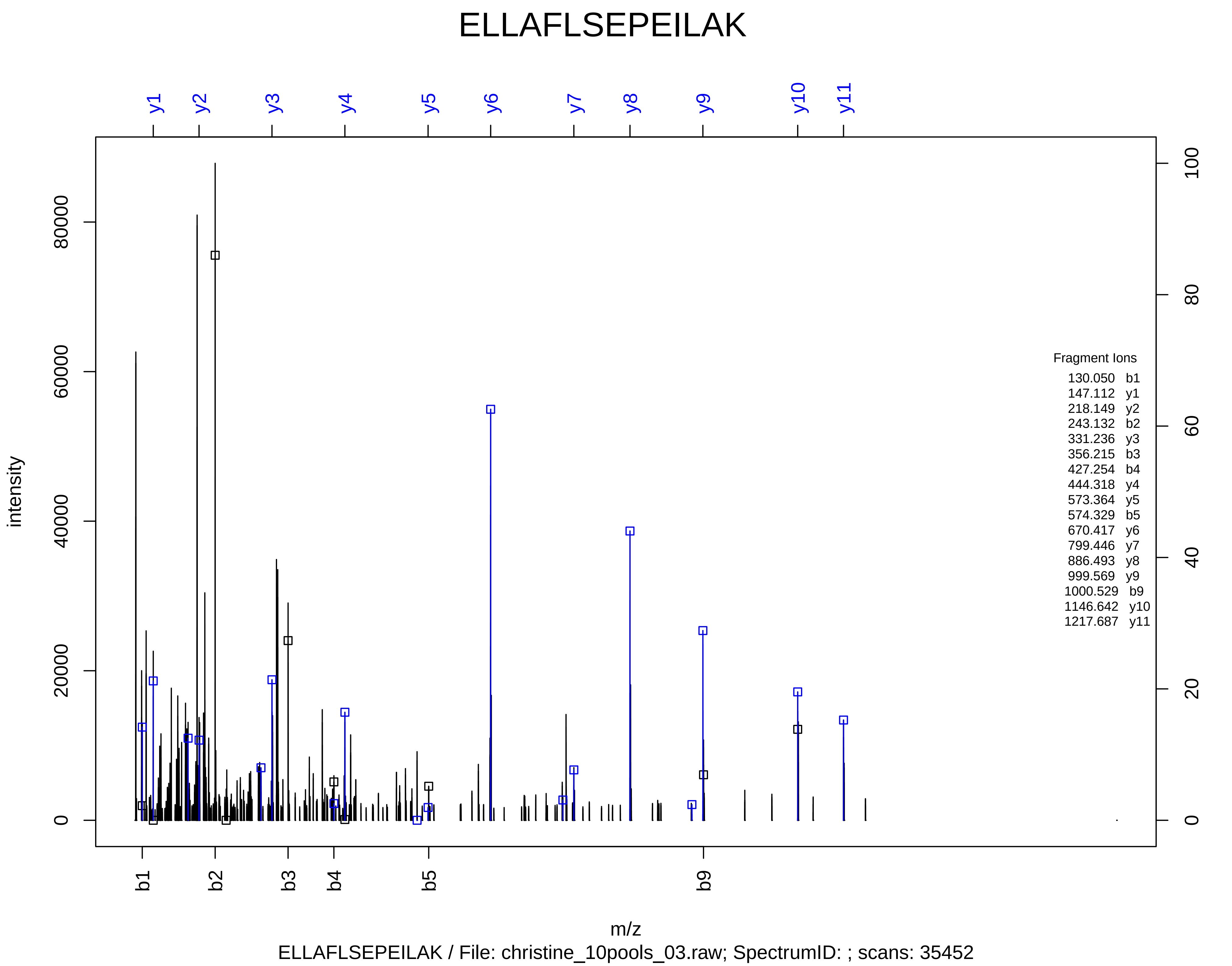
ELLAFLSEPEILAK
y1 y2 y3 y4 y5 y6 y7 y8 y9 y10 y11
b1 b2 b3 b4 b5 b9
intensity
m/z
Fragment Ions
130.050 b1
147.112 y1
218.149 y2
243.132 b2
331.236 y3
356.215 b3
427.254 b4
444.318 y4
573.364 y5
574.329 b5
670.417 y6
799.446 y7
886.493 y8
999.569 y9
1000.529 b9
1146.642 y10
1217.687 y11
ELLAFLSEPEILAK / File: christine_10pools_03.raw; SpectrumID: ; scans: 35452

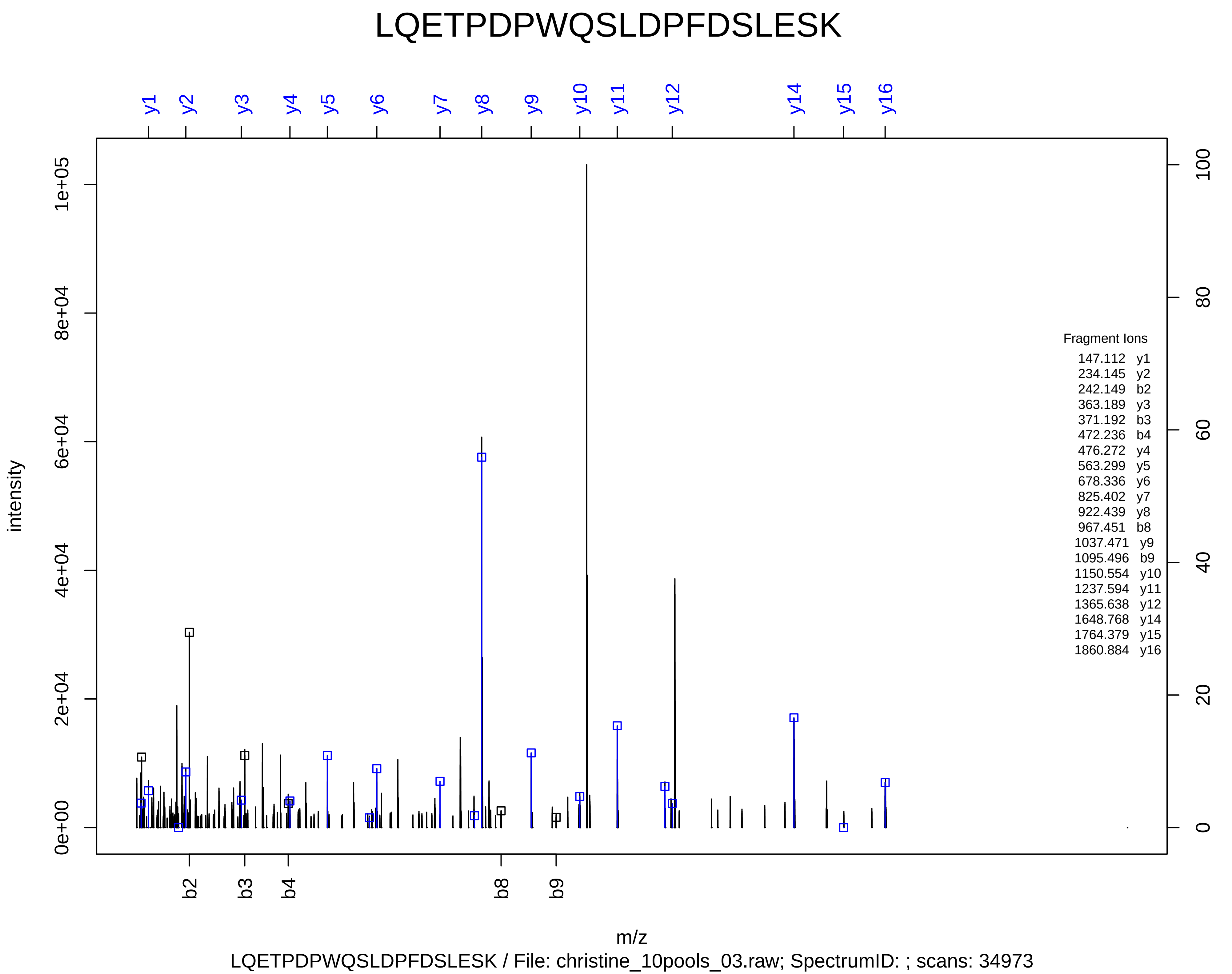
LQETPDPWQSLDPFDSLESK
y1 y2 y3 y4 y5 y6 y7 y8 y9 y10 y11 y12 y14 y15 y16
intensity
0e+00 2e+04 4e+04 6e+04 8e+04 1e+05
0 20 40 60 80 100
b2 b3 b4 b8 b9
m/z
Fragment Ions
147.112 y1
234.145 y2
242.149 b2
363.189 y3
371.192 b3
472.236 b4
476.272 y4
563.299 y5
678.336 y6
825.402 y7
922.439 y8
967.451 b8
1037.471 y9
1095.496 b9
1150.554 y10
1237.594 y11
1365.638 y12
1648.768 y14
1764.379 y15
1860.884 y16
LQETPDPWQSLDPFDSLESK / File: christine_10pools_03.raw; SpectrumID: ; scans: 34973

LDPSQPAVGNLTPVVLGPEELWPAR

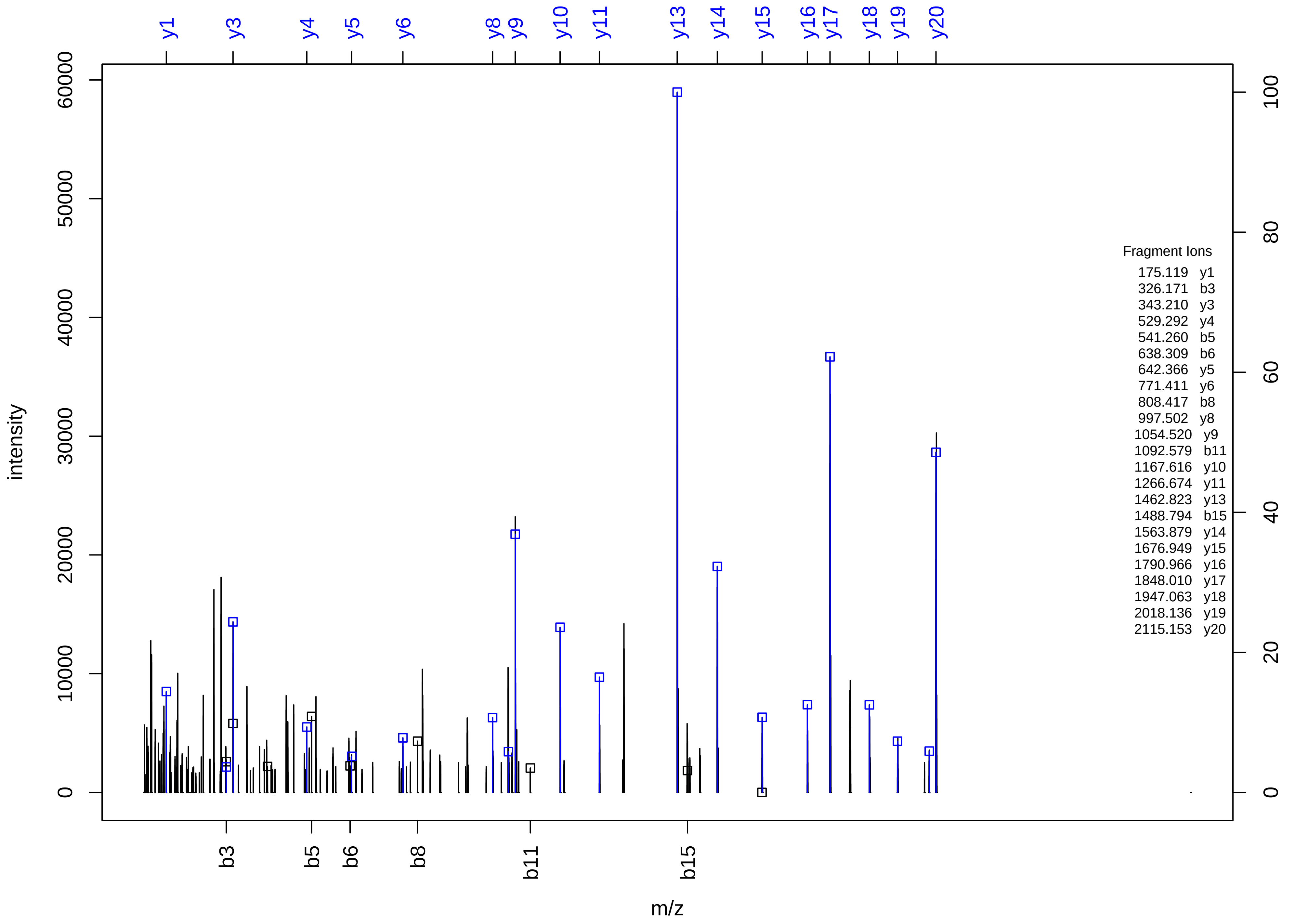

LDPSQPAVGNLTPVVLGPEELWPAR / File: christine_10pools_03.raw; SpectrumID: ; scans: 36802

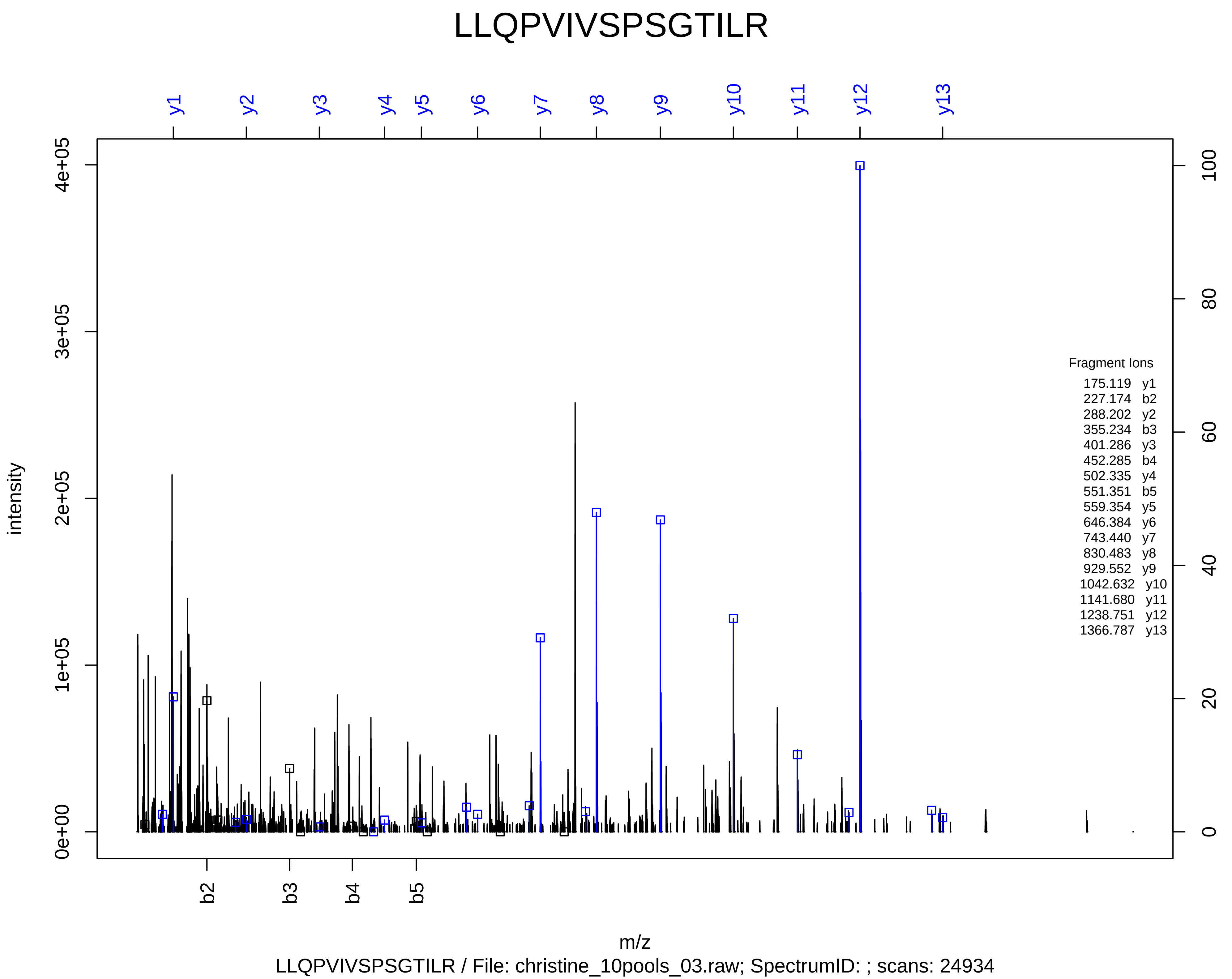

LLQPVIVSPSGTILR
y1
y2
y3
y4
y5
y6
y7
y8
y9
y10
y11
y12
y13
b2
b3
b4
b5
intensity
0e+00
1e+05
2e+05
3e+05
4e+05
0
20
40
60
80
100
Fragment Ions
175.119 y1
227.174 b2
288.202 y2
355.234 b3
401.286 y3
452.285 b4
502.335 y4
551.351 b5
559.354 y5
646.384 y6
743.440 y7
830.483 y8
929.552 y9
1042.632 y10
1141.680 y11
1238.751 y12
1366.787 y13
m/z
LLQPVIVSPSGTILR / File: christine_10pools_03.raw; SpectrumID: ; scans: 24934

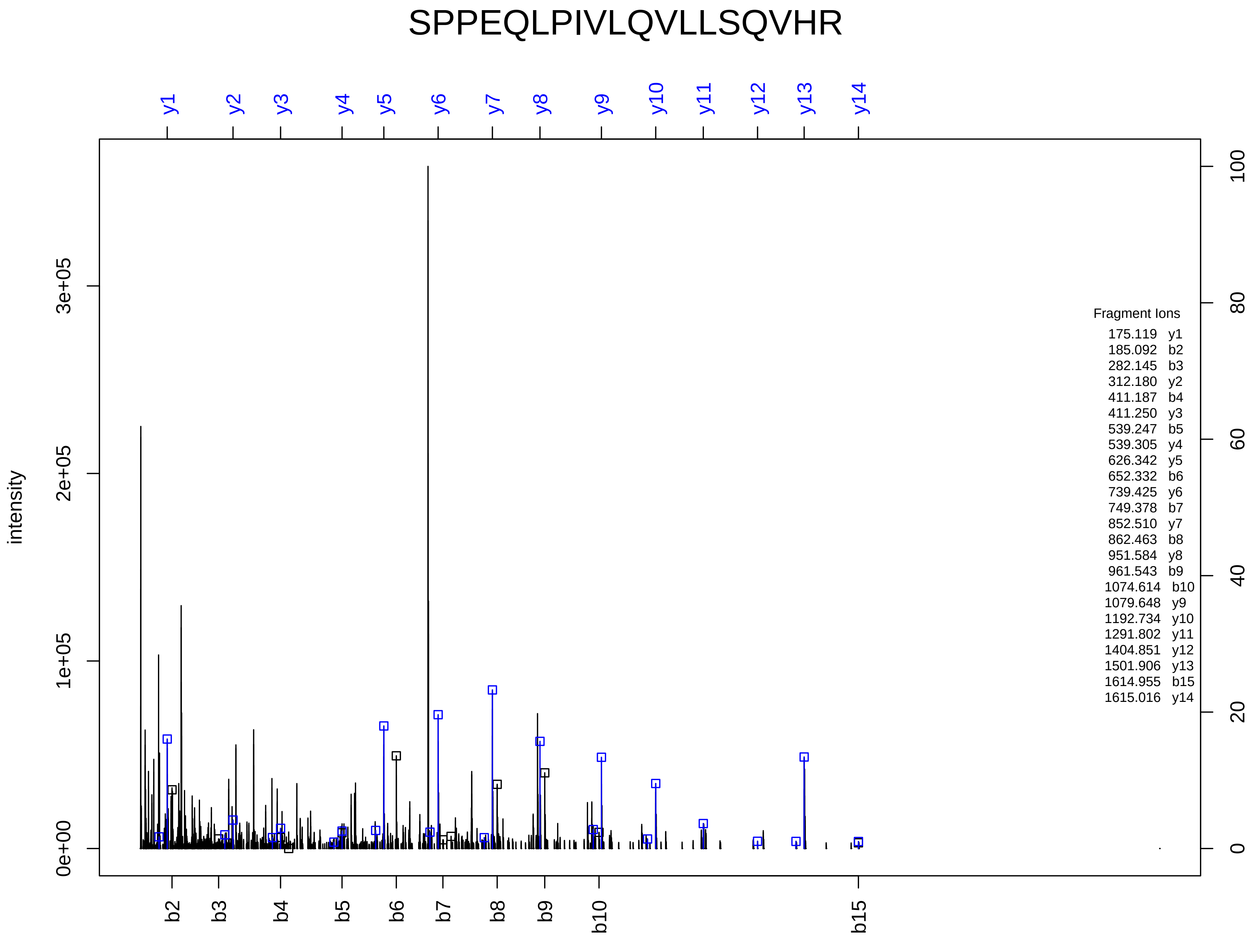

SPPEQLPIVLQVLLSQVHR / File: christine_10pools_03.raw; SpectrumID: ; scans: 41207

NVQLLSQFISPFTGCIYGR

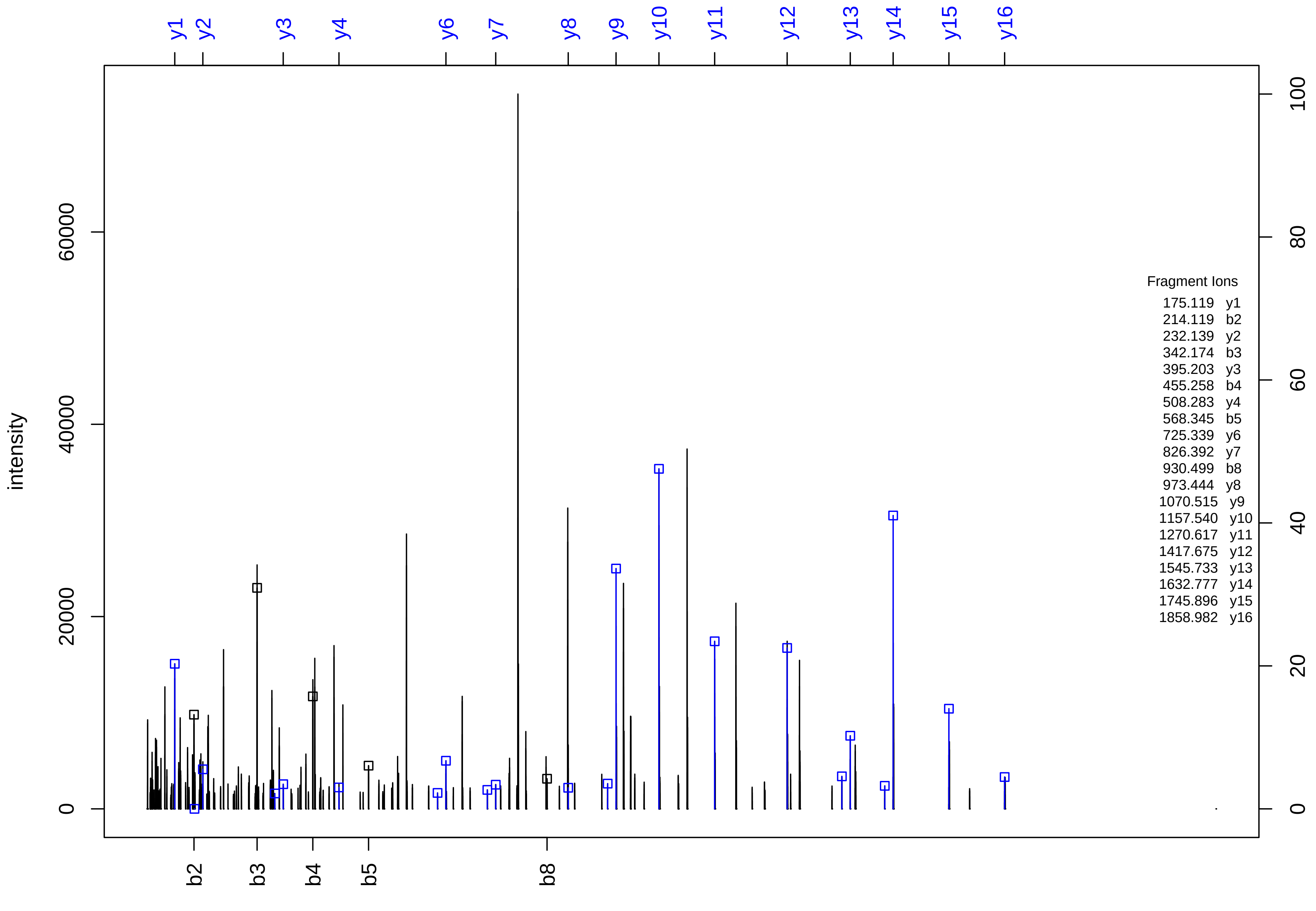

NVQLLSQFISPFTGCIYGR / File: christine_10pools_03.raw; SpectrumID: ; scans: 39459

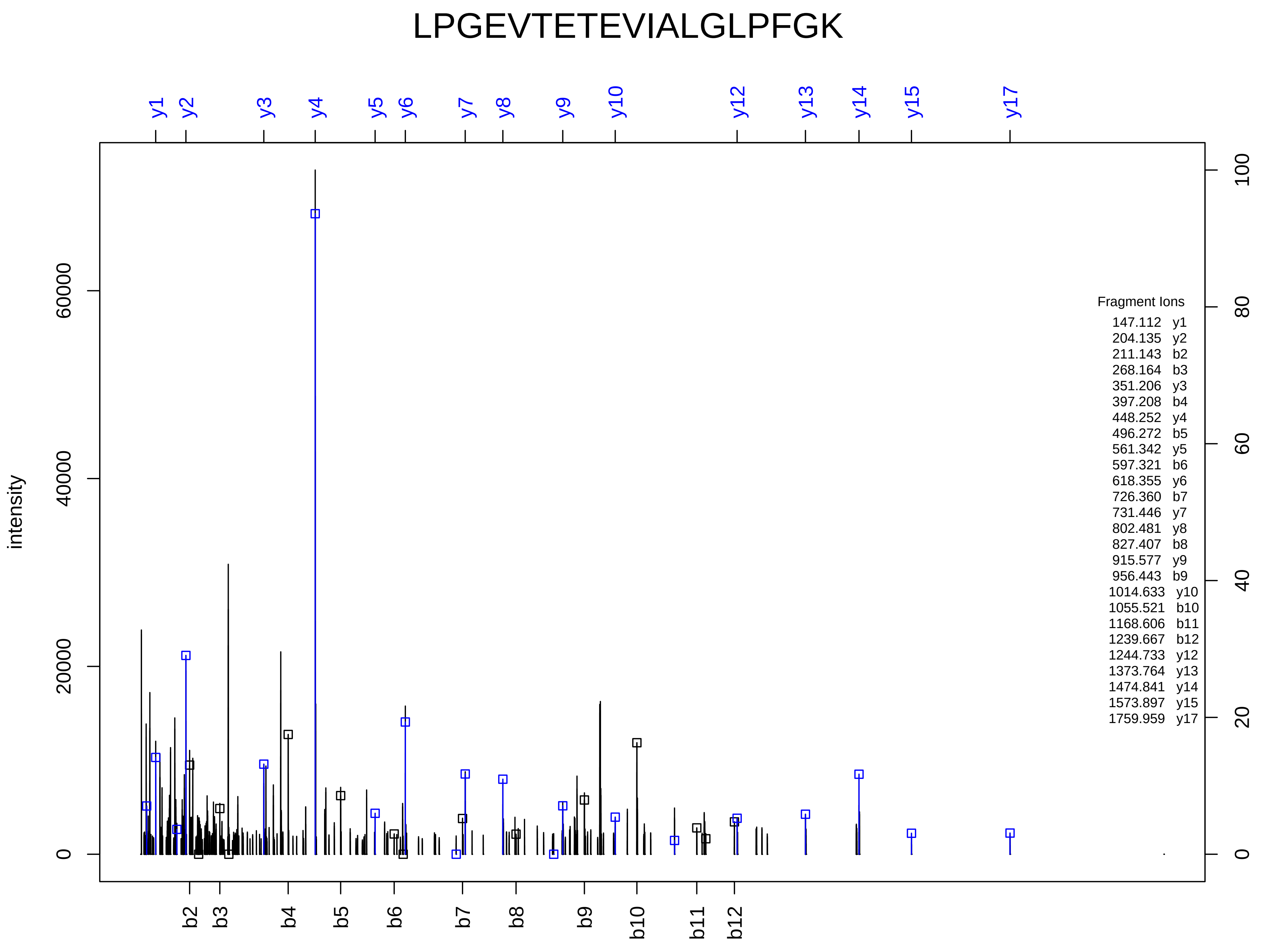

LPGEVTETEVIALGLPFGK / File: christine_10pools_03.raw; SpectrumID: ; scans: 35511

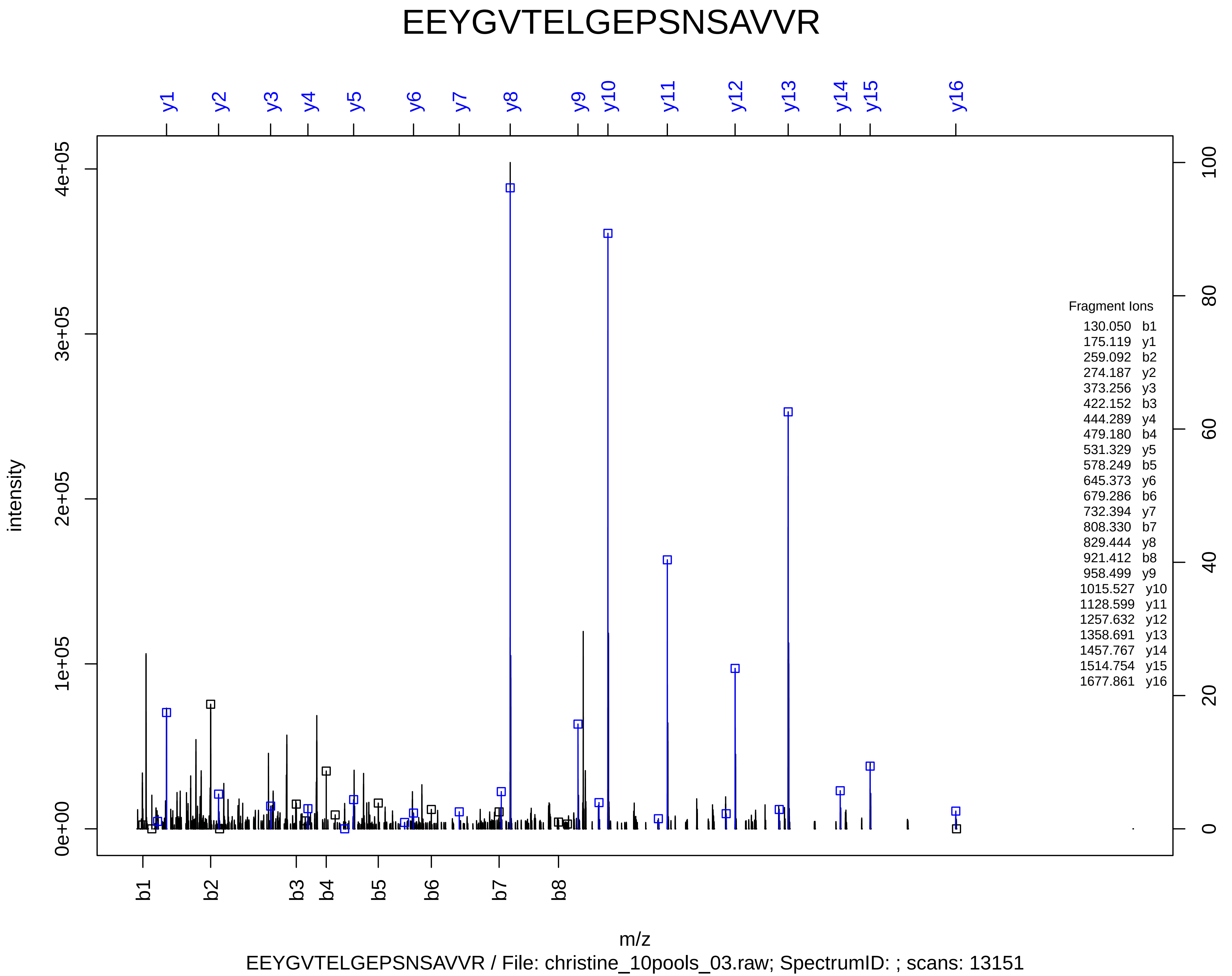
EEYGVTELGEPSNSAVVR
y1 y2 y3 y4 y5 y6 y7 y8 y9 y10 y11 y12 y13 y14 y15 y16
b1 b2 b3 b4 b5 b6 b7 b8
intensity
m/z
Fragment Ions
130.050 b1
175.119 y1
259.092 b2
274.187 y2
373.256 y3
422.152 b3
444.289 y4
479.180 b4
531.329 y5
578.249 b5
645.373 y6
679.286 b6
732.394 y7
808.330 b7
829.444 y8
921.412 b8
958.499 y9
1015.527 y10
1128.599 y11
1257.632 y12
1358.691 y13
1457.767 y14
1514.754 y15
1677.861 y16
EEYGVTELGEPSNSAVVR / File: christine_10pools_03.raw; SpectrumID: ; scans: 13151

DNVGEEVDAEQLIQEACR

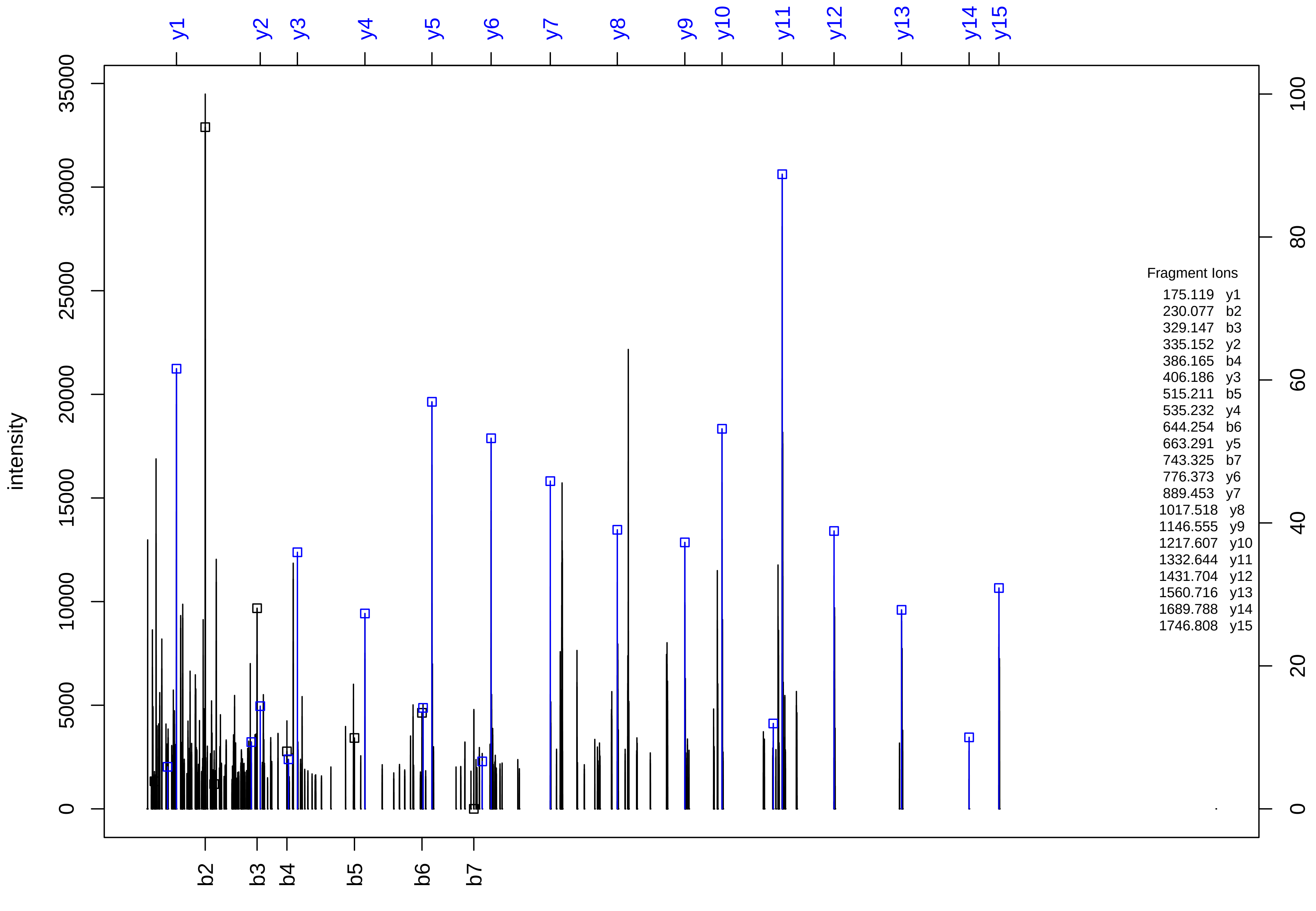

DNVGEEVDAEQLIQEACR / File: christine_10pools_03.raw; SpectrumID: ; scans: 23237

NQVAVVTGGGTGIGK

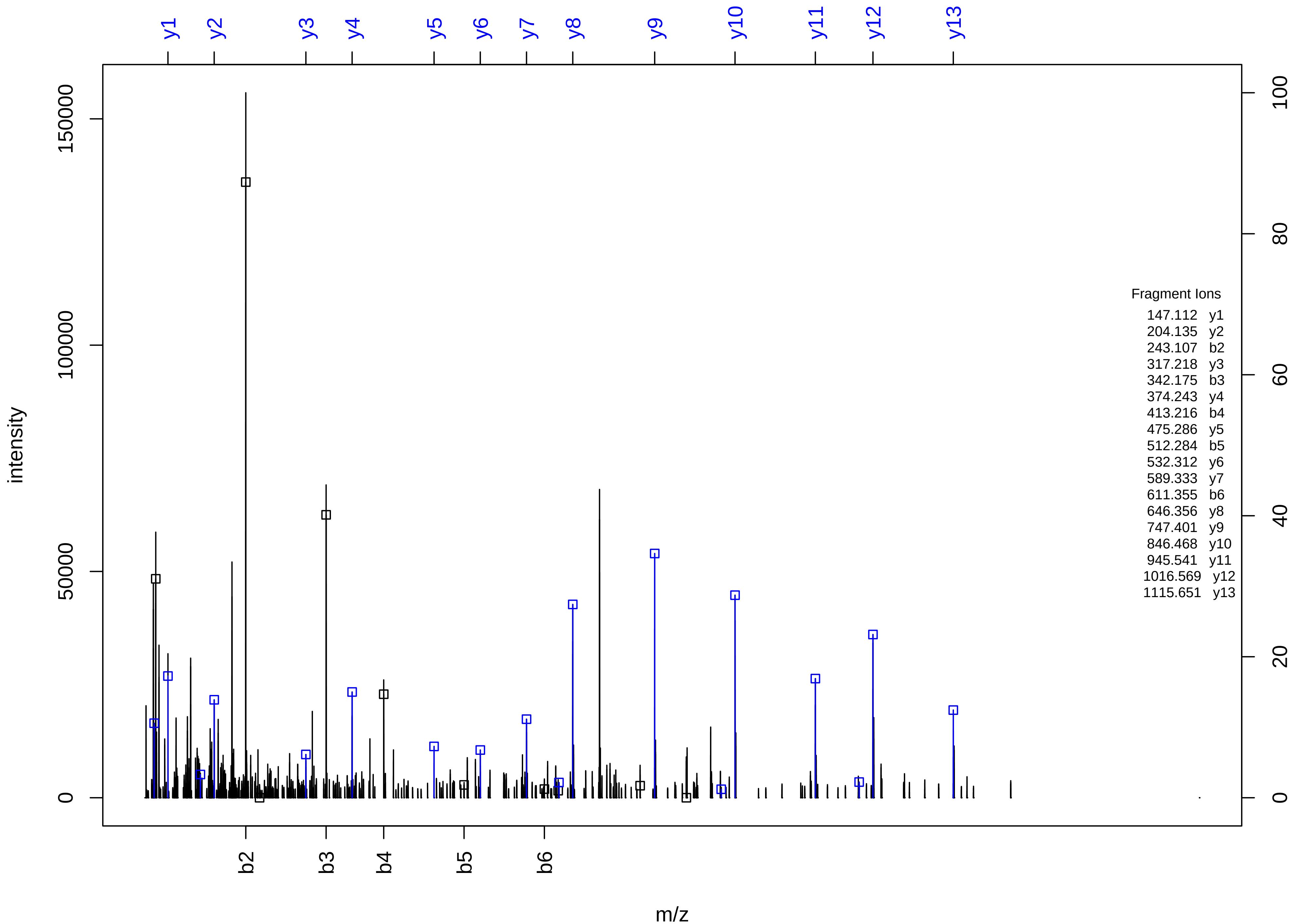

NQVAVVTGGGTGIGK / File: christine_10pools_03.raw; SpectrumID: ; scans: 4142

FVEQLGSYDPLPNSHGEK

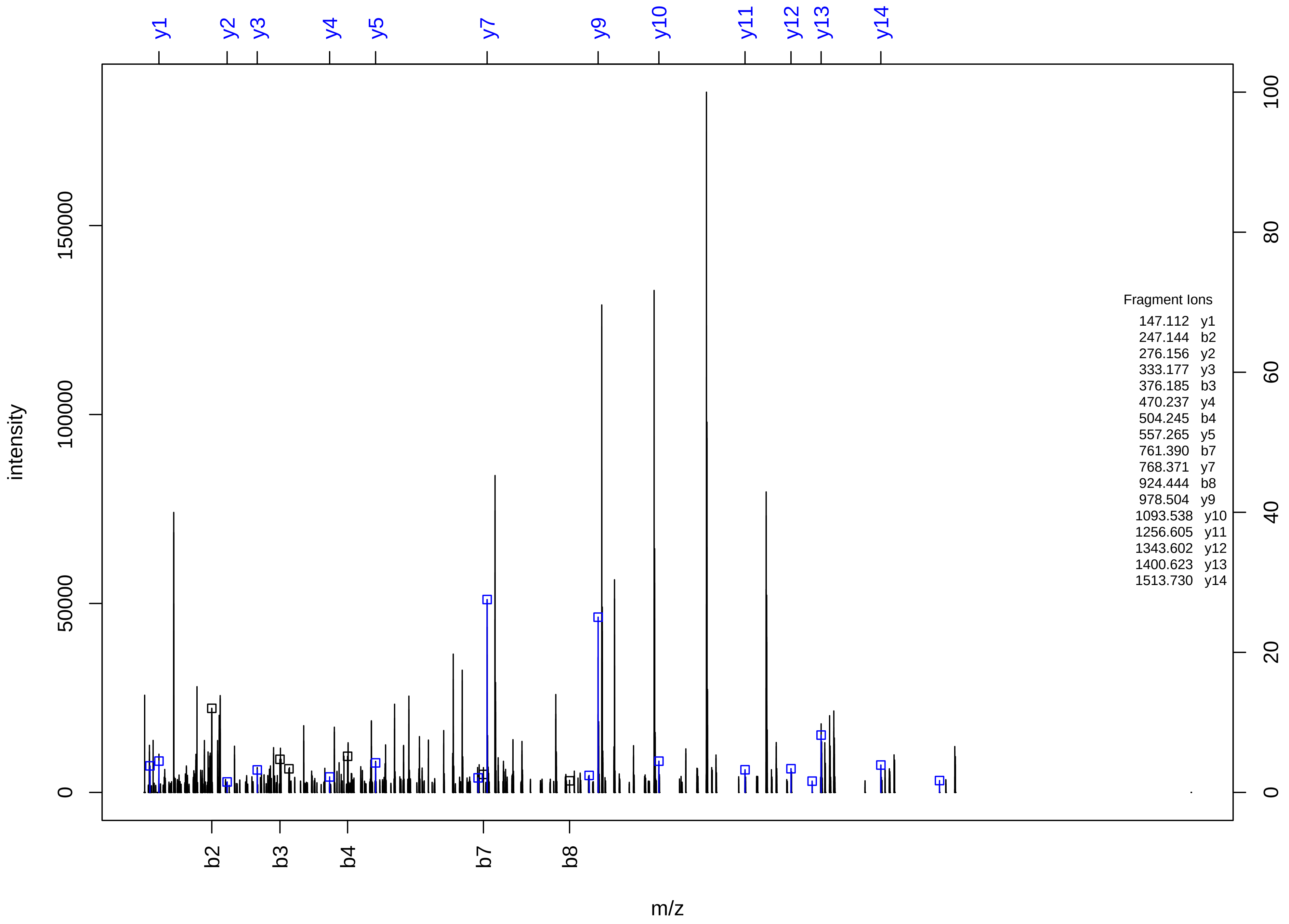

FVEQLGSYDPLPNSHGEK / File: christine_10pools_03.raw; SpectrumID: ; scans: 13742

VGDTLDLITGENK

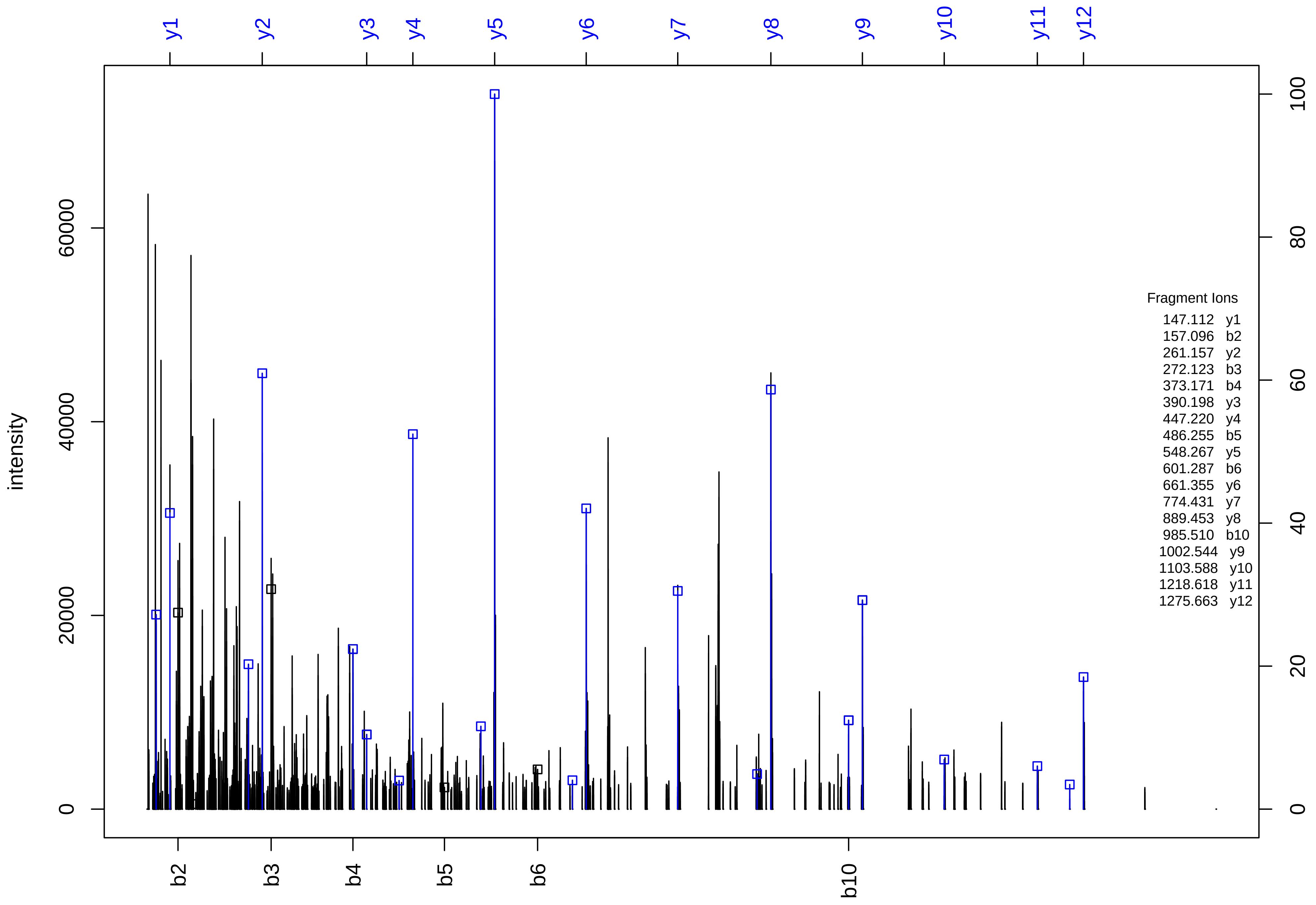

VGDTLDLITGENK / File: christine_10pools_03.raw; SpectrumID: ; scans: 16932

NIGWGTDQGIGGFGEEPGIK

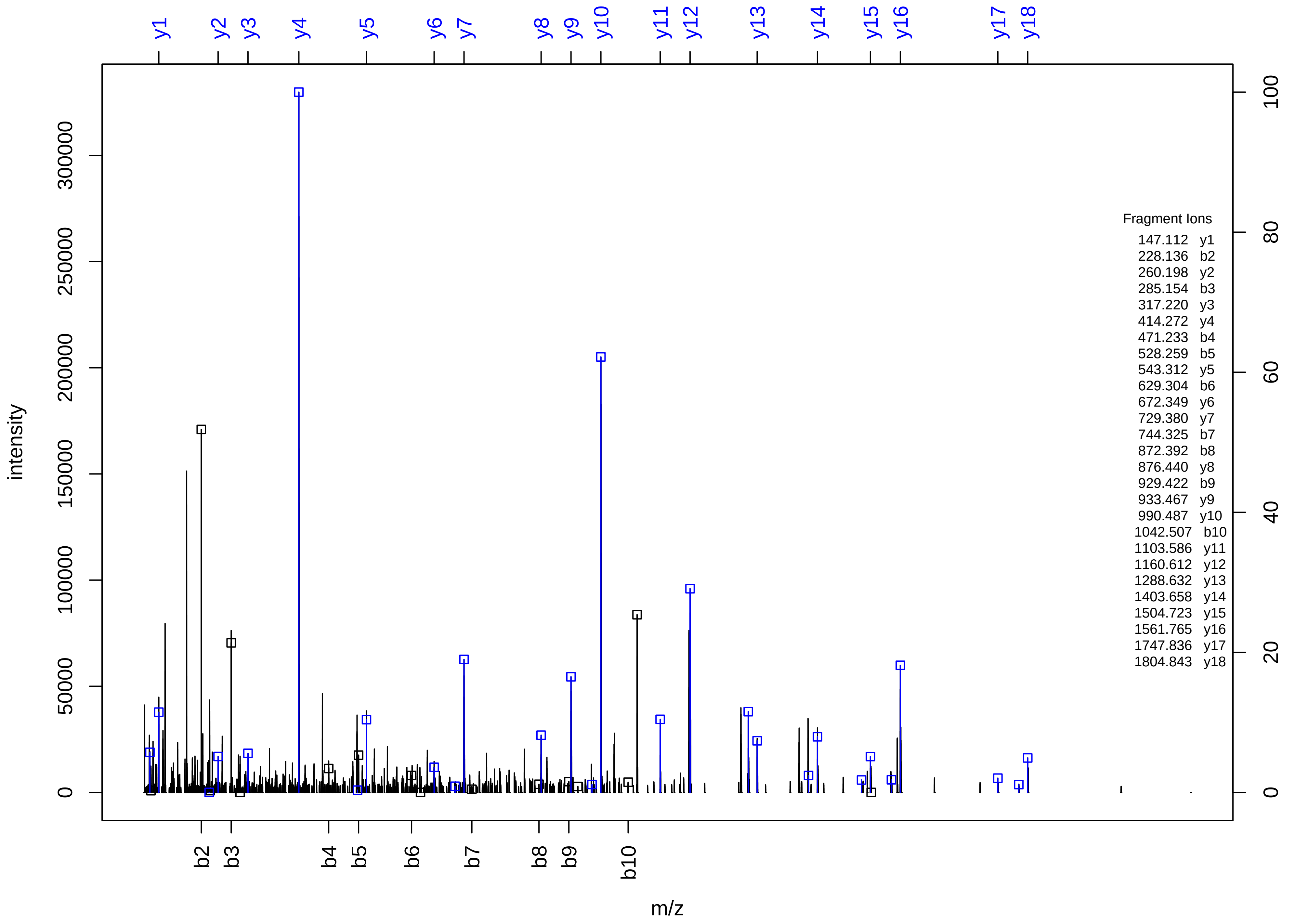

NIGWGTDQGIGGFGEEPGIK / File: christine_10pools_03.raw; SpectrumID: ; scans: 22985

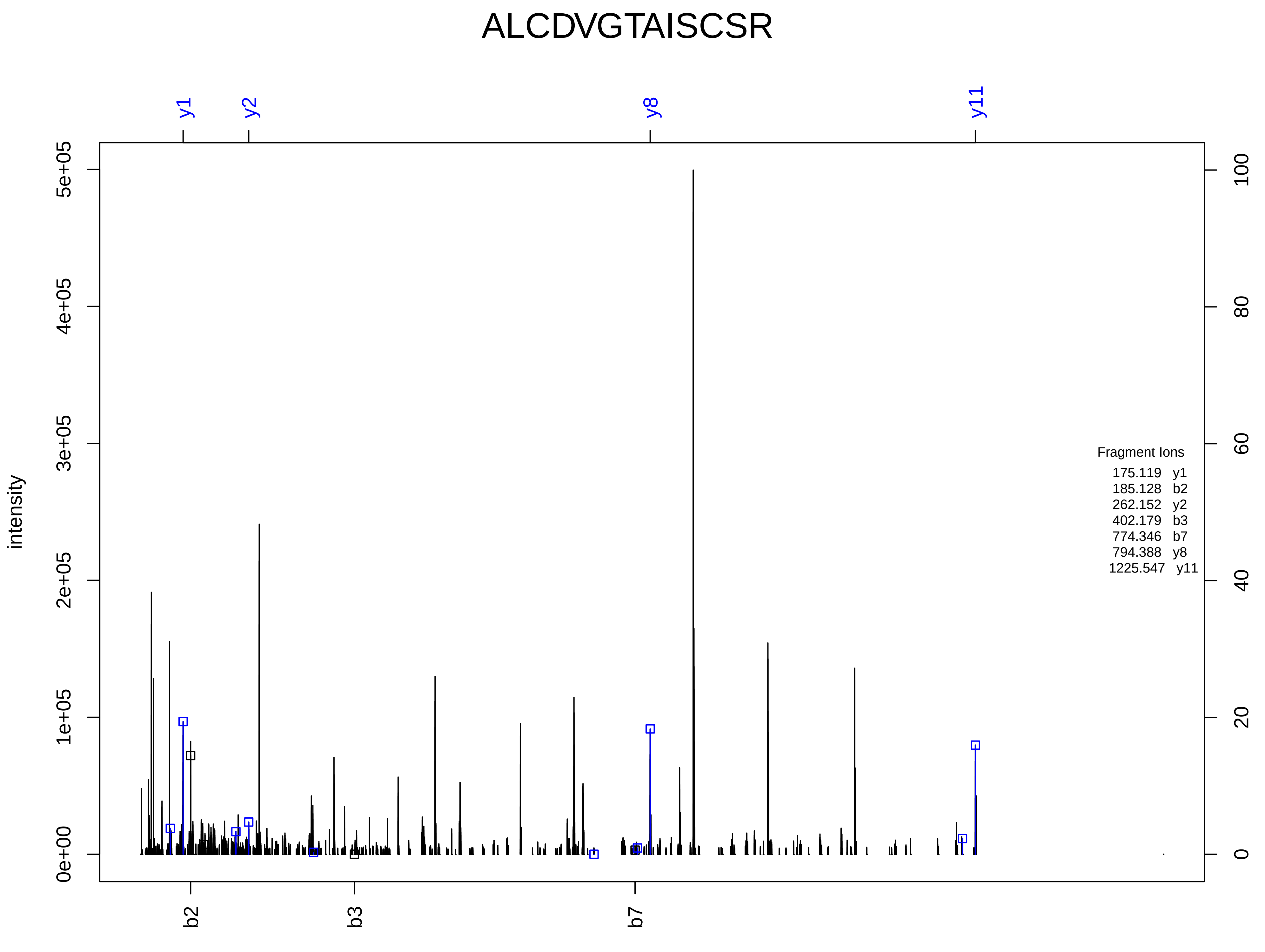

ALCDVGTAISCSR / File: christine_10pools_03.raw; SpectrumID: ; scans: 7681

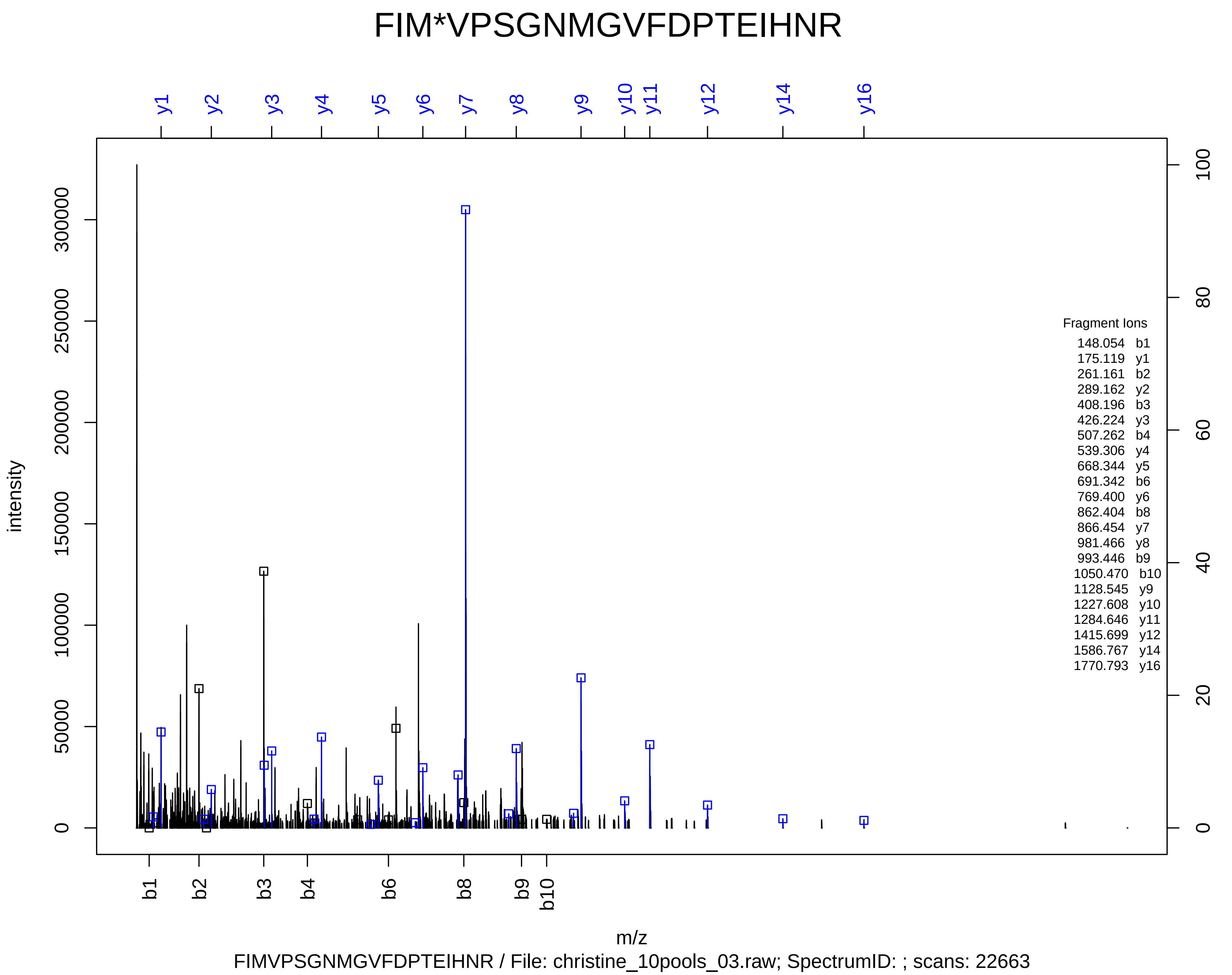
FIM*VPSGNMGVFDPTEIHNR
y1
y2
y3
y4
y5
y6
y7
y8
y9
y10
y11
y12
y14
y16
intensity
0
50000
100000
150000
200000
250000
300000
0
20
40
60
80
100
Fragment Ions
148.054 b1
175.119 y1
261.161 b2
289.162 y2
408.196 b3
426.224 y3
507.262 b4
539.306 y4
668.344 y5
691.342 b6
769.400 y6
862.404 b8
866.454 y7
981.466 y8
993.446 b9
1050.470 b10
1128.545 y9
1227.608 y10
1284.646 y11
1415.699 y12
1586.767 y14
1770.793 y16
b1
b2
b3
b4
b6
b8
b9
b10
m/z
FIMVPSGNMGVFDPTEIHNR / File: christine_10pools_03.raw; SpectrumID: ; scans: 22663

EQLLYLYAR

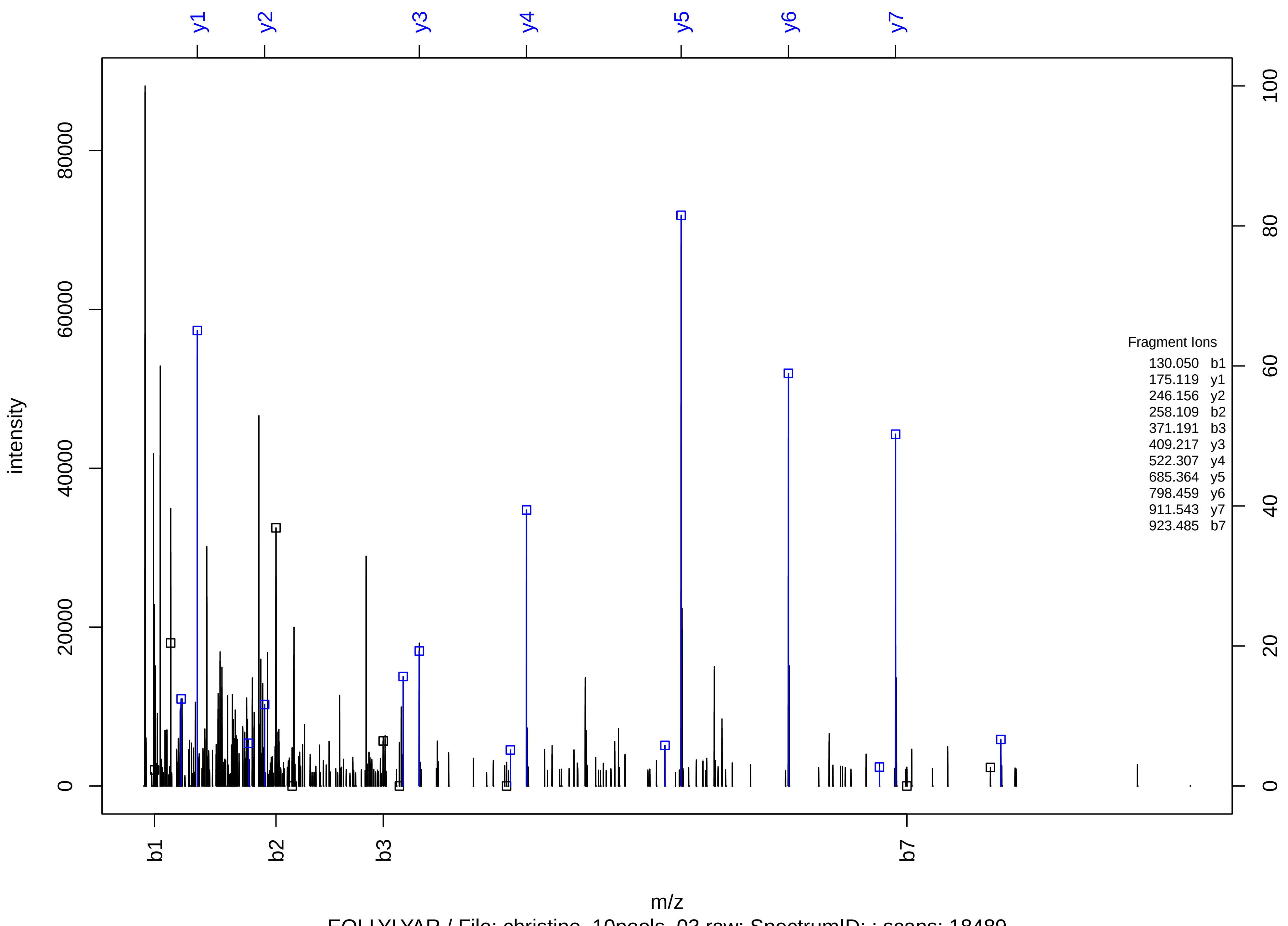

EQLLYLYAR / File: christine_10pools_03.raw; SpectrumID: ; scans: 18489

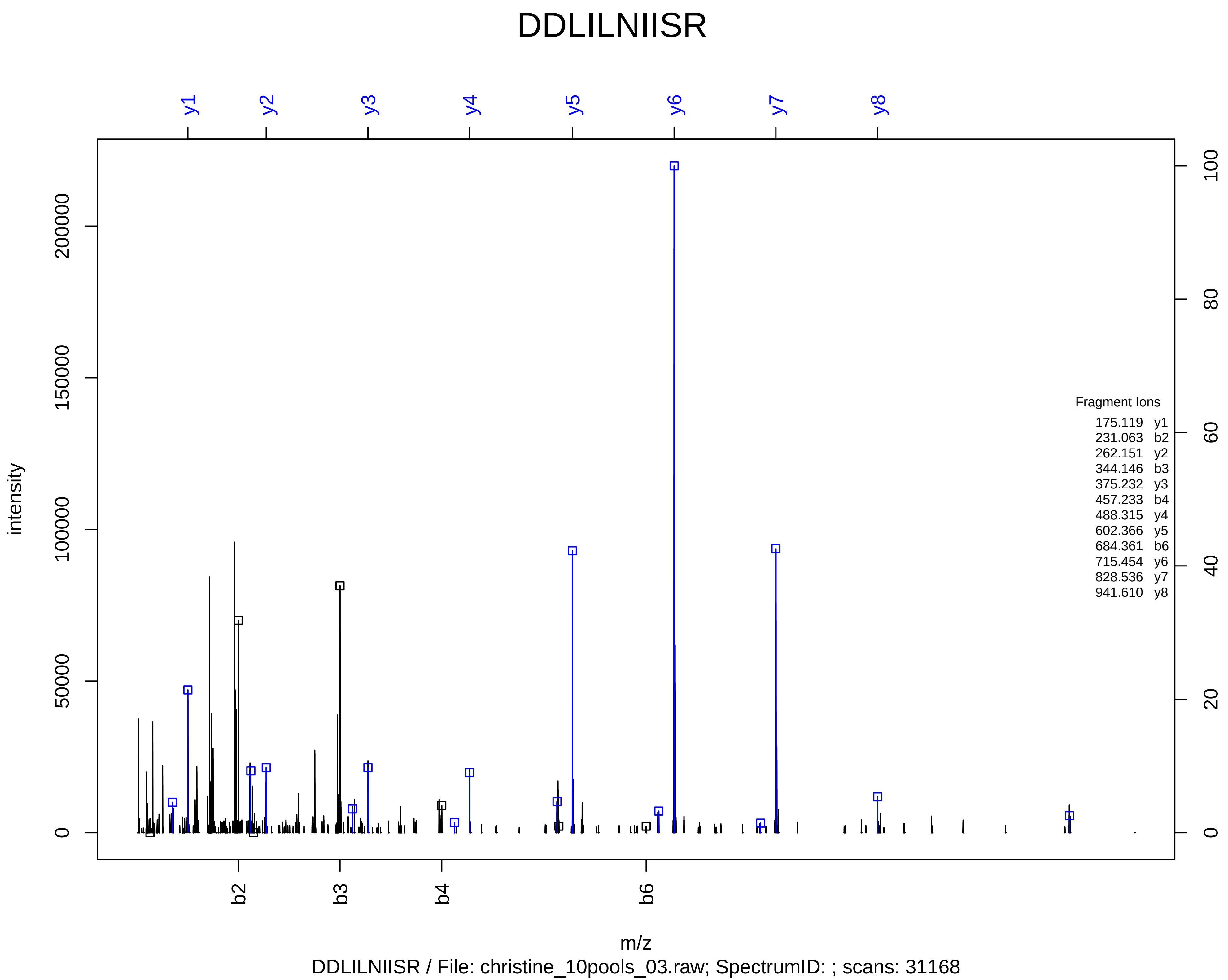

DDLILNIISR
y1
y2
y3
y4
y5
y6
y7
y8
intensity
0
50000
100000
150000
200000
0
20
40
60
80
100
b2
b3
b4
b6
m/z
Fragment Ions
175.119 y1
231.063 b2
262.151 y2
344.146 b3
375.232 y3
457.233 b4
488.315 y4
602.366 y5
684.361 b6
715.454 y6
828.536 y7
941.610 y8
DDLILNIISR / File: christine_10pools_03.raw; SpectrumID: ; scans: 31168

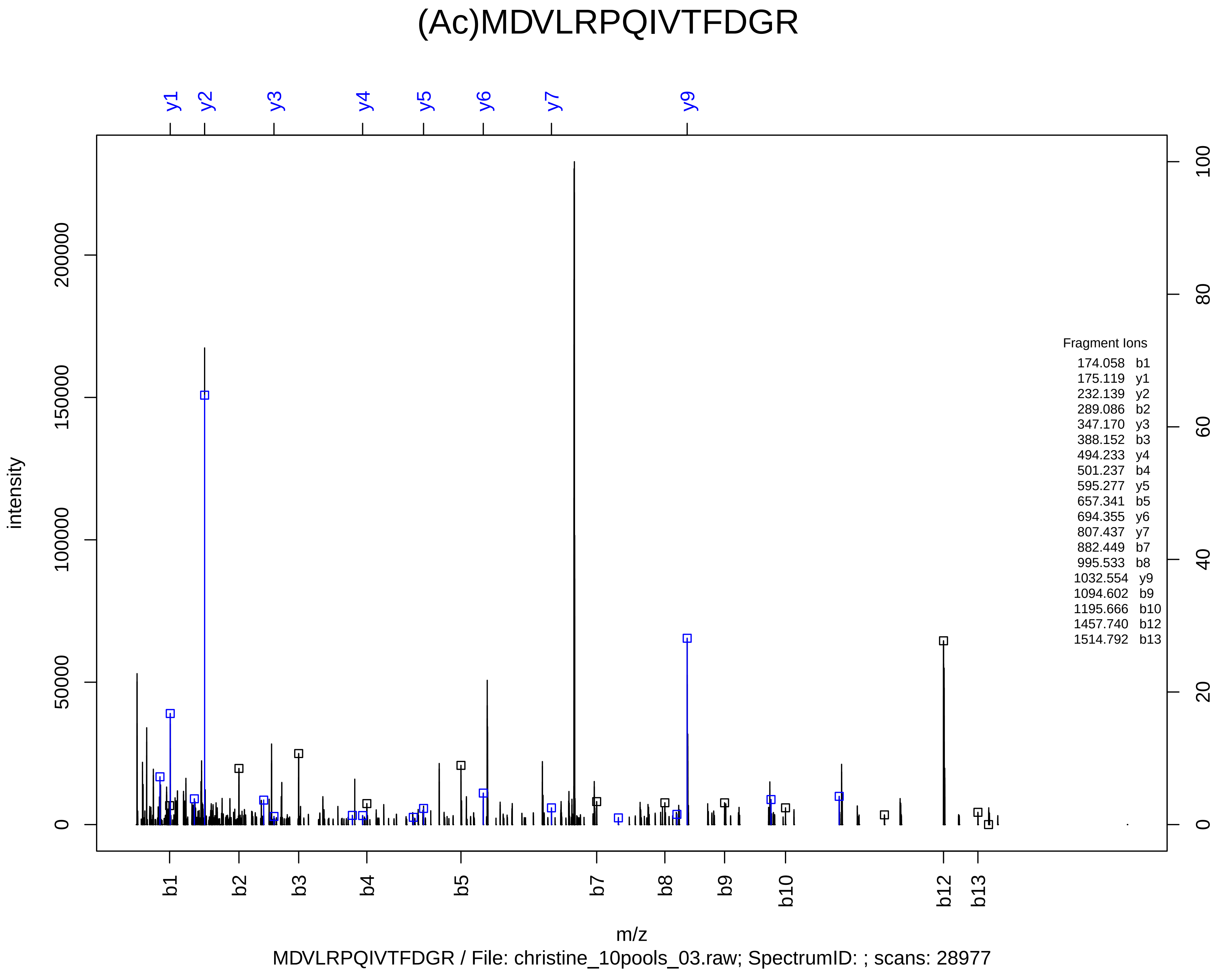

(Ac)MDVLRPQIVTFDGR
y1
y2
y3
y4
y5
y6
y7
y9
intensity
0
50000
100000
150000
200000
0
20
40
60
80
100
Fragment Ions
174.058 b1
175.119 y1
232.139 y2
289.086 b2
347.170 y3
388.152 b3
494.233 y4
501.237 b4
595.277 y5
657.341 b5
694.355 y6
807.437 y7
882.449 b7
995.533 b8
1032.554 y9
1094.602 b9
1195.666 b10
1457.740 b12
1514.792 b13
b1
b2
b3
b4
b5
b7
b8
b9
b10
b12
b13
m/z
MDVLRPQIVTFDGR / File: christine_10pools_03.raw; SpectrumID: ; scans: 28977

SDPLLMGIPTSENPFK

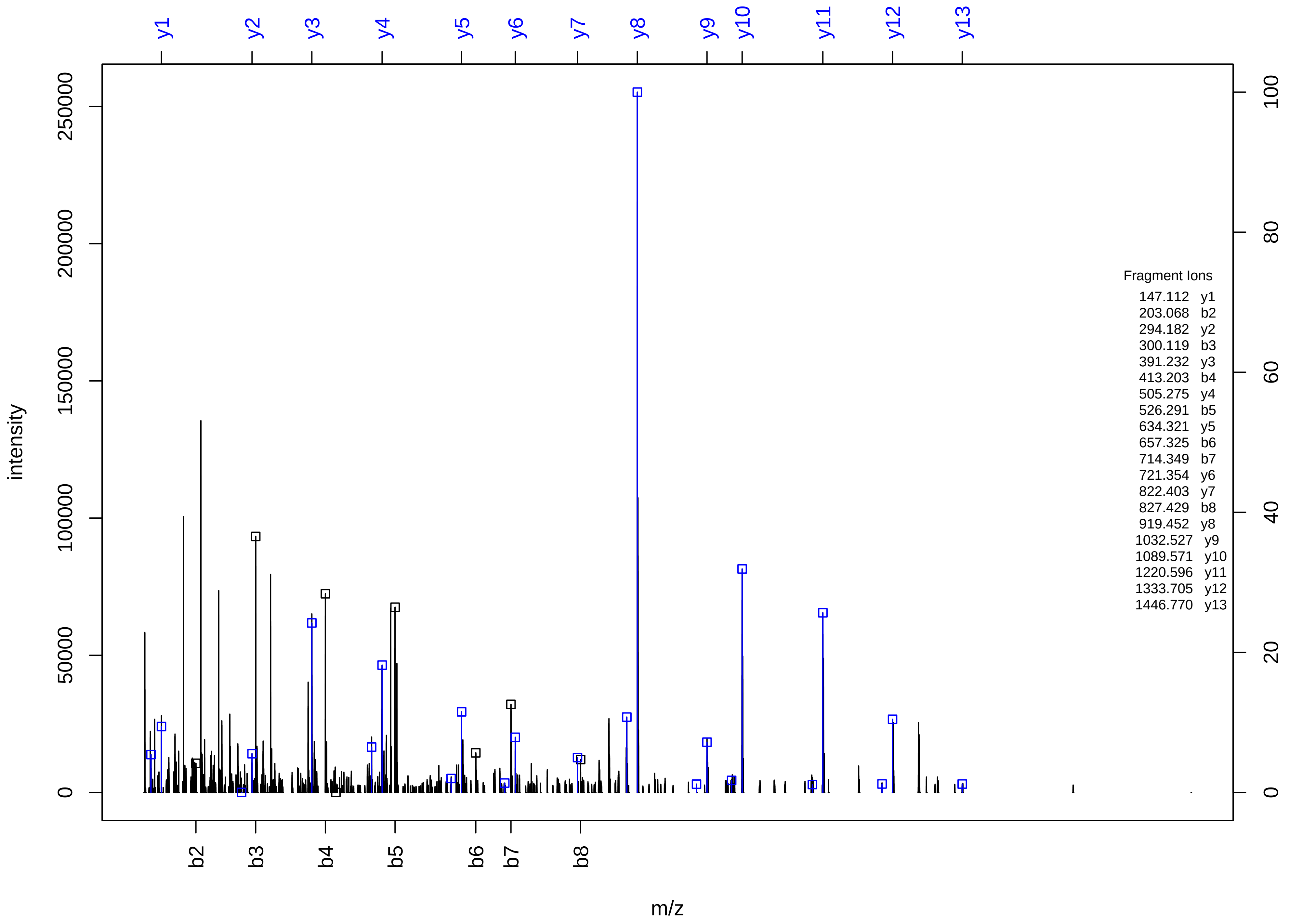

SDPLLMGIPTSENPFK / File: christine_10pools_03.raw; SpectrumID: ; scans: 29106

(Ac)M*FEEPEWVEAAPAIVGLGAATAQVRPATAPPVK

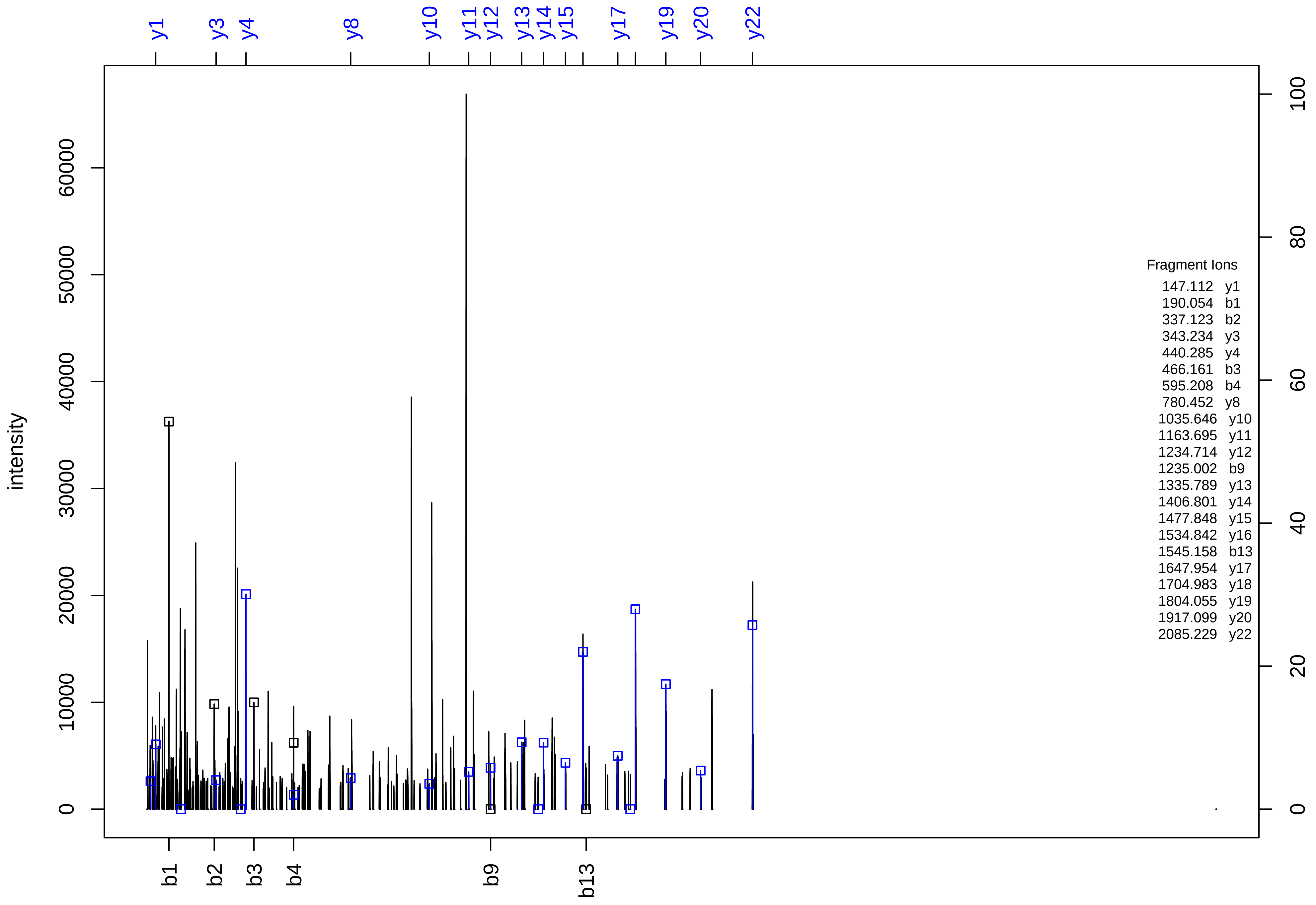

MFEEPEWVEAAPAIVGLGAATAQVRPATAPPVK / File: christine_10pools_03.raw; SpectrumID: ; scans: 39502

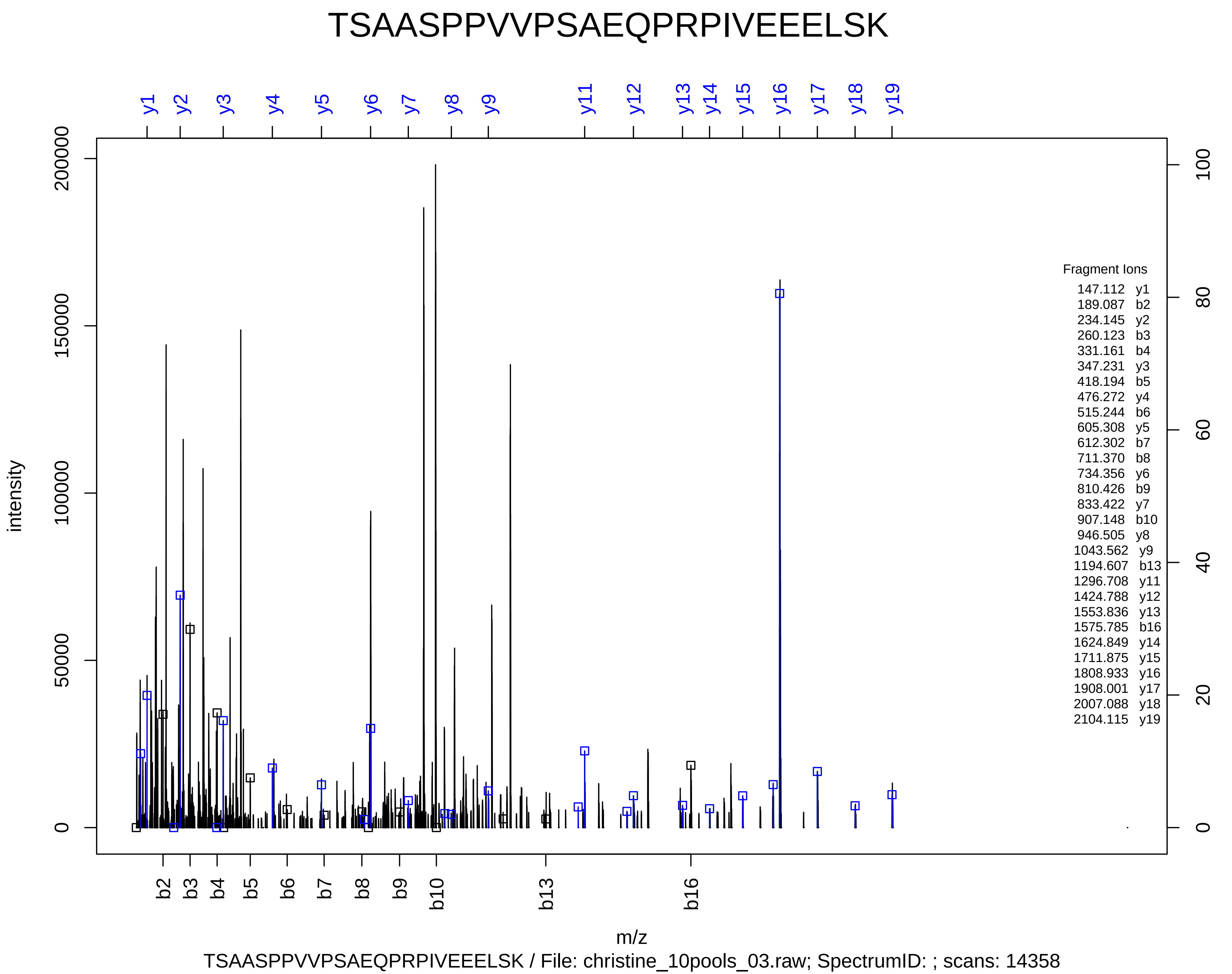
TSAASPPVVPSAEQPRPIVEEELSK
y1 y2 y3 y4 y5 y6 y7 y8 y9 y11 y12 y13 y14 y15 y16 y17 y18 y19
intensity
0
50000
100000
150000
200000
0
20
40
60
80
100
b2 b3 b4 b5 b6 b7 b8 b9 b10 b13 b16
m/z
Fragment Ions
147.112 y1
189.087 b2
234.145 y2
260.123 b3
331.161 b4
347.231 y3
418.194 b5
476.272 y4
515.244 b6
605.308 y5
612.302 b7
711.370 b8
734.356 y6
810.426 b9
833.422 y7
907.148 b10
946.505 y8
1043.562 y9
1194.607 b13
1296.708 y11
1424.788 y12
1553.836 y13
1575.785 b16
1624.849 y14
1711.875 y15
1808.933 y16
1908.001 y17
2007.088 y18
2104.115 y19
TSAASPPVVPSAEQPRPIVEEELSK / File: christine_10pools_03.raw; SpectrumID: ; scans: 14358

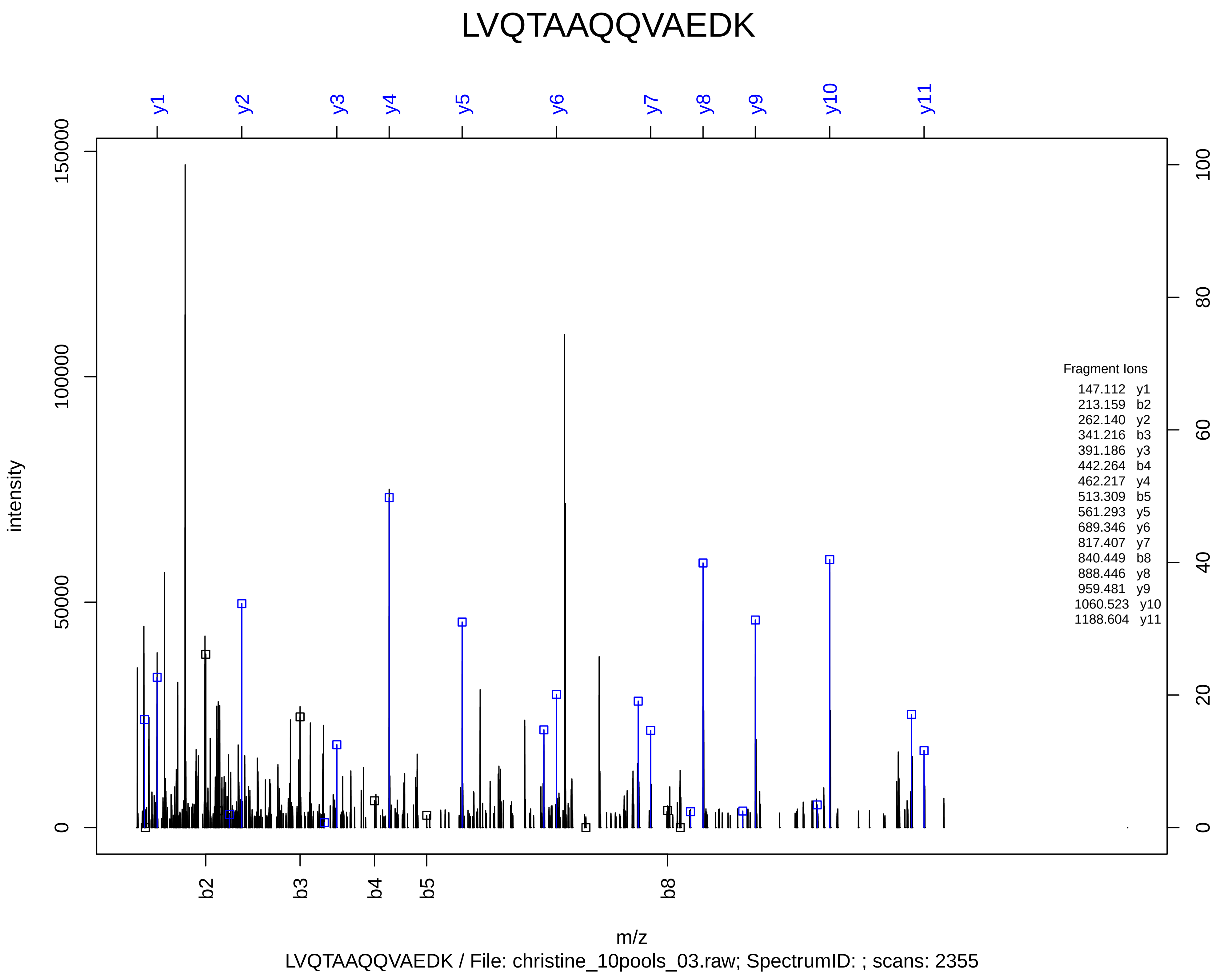

LVQTAAQQVAEDK
y1
y2
y3
y4
y5
y6
y7
y8
y9
y10
y11
intensity
150000
100000
50000
0
100
80
60
40
20
0
b2
b3
b4
b5
b8
m/z
Fragment Ions
147.112 y1
213.159 b2
262.140 y2
341.216 b3
391.186 y3
442.264 b4
462.217 y4
513.309 b5
561.293 y5
689.346 y6
817.407 y7
840.449 b8
888.446 y8
959.481 y9
1060.523 y10
1188.604 y11
LVQTAAQQVAEDK / File: christine_10pools_03.raw; SpectrumID: ; scans: 2355

EEM*LAIM*K

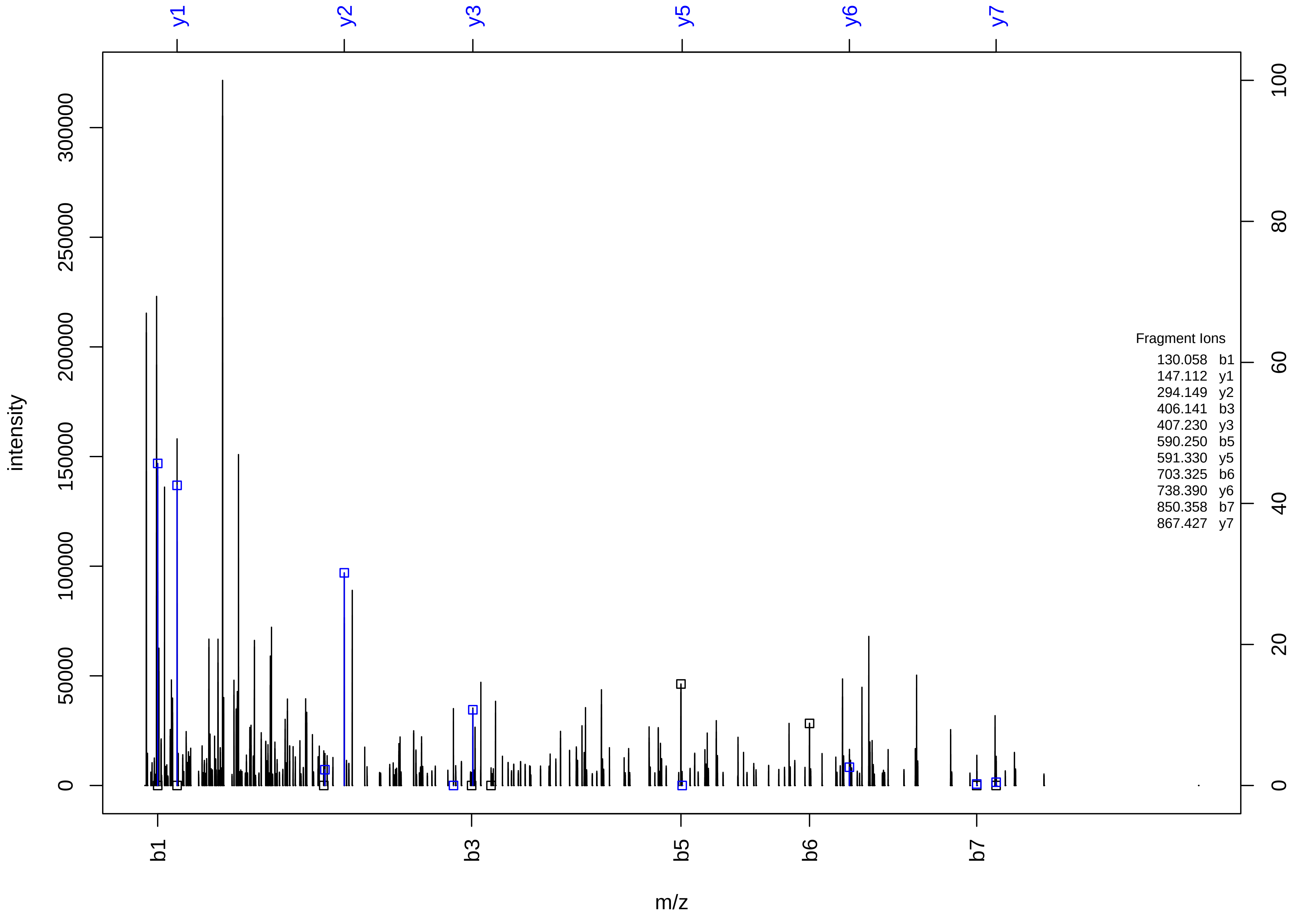

EEMLAIMK / File: christine_10pools_03.raw; SpectrumID: ; scans: 15463

VLTLAALLLNR

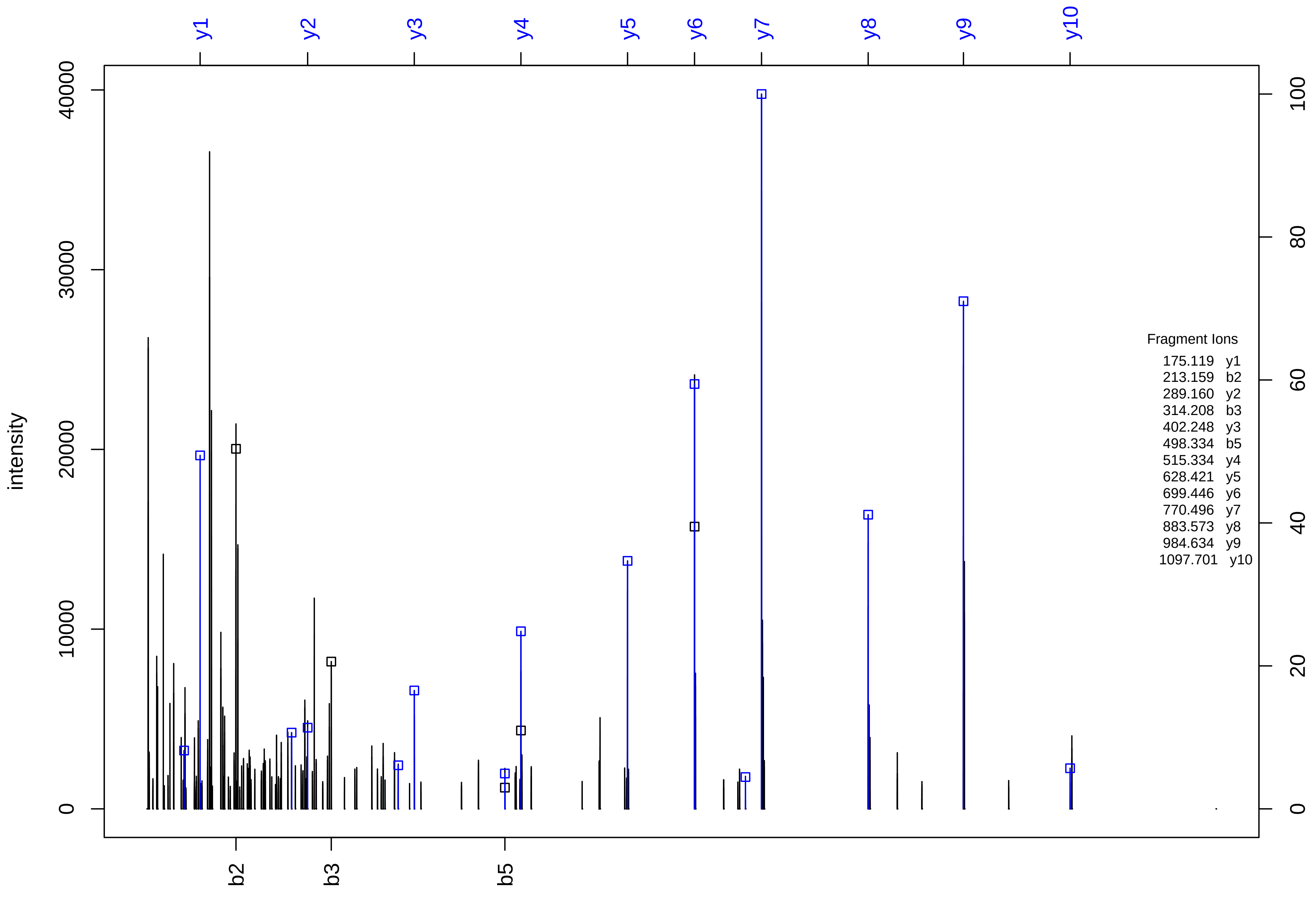

VLTLAALLLNR / File: christine_10pools_03.raw; SpectrumID: ; scans: 35114

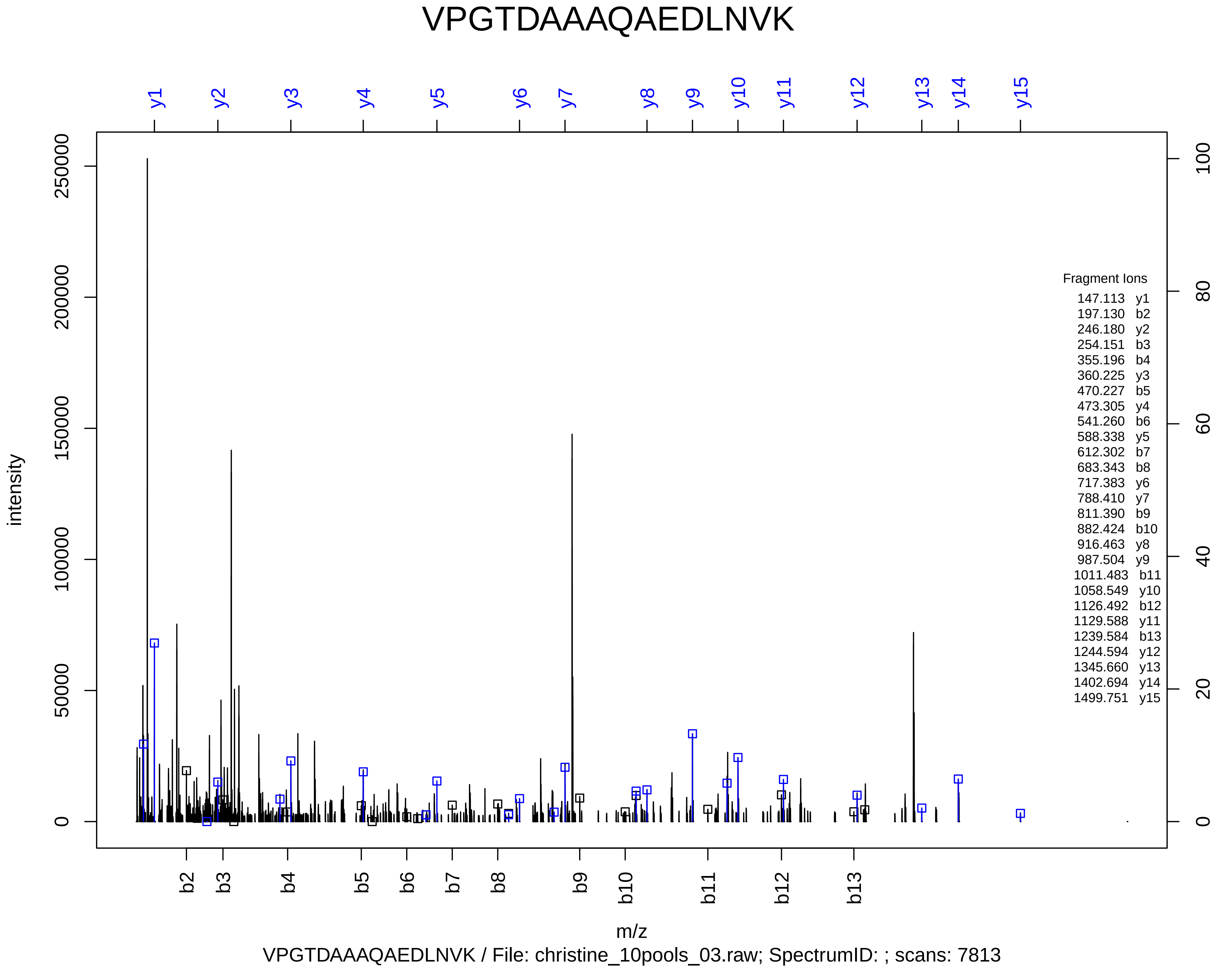
VPGTDAAAQAEDLNVK
y1 y2 y3 y4 y5 y6 y7 y8 y9 y10 y11 y12 y13 y14 y15
b2 b3 b4 b5 b6 b7 b8 b9 b10 b11 b12 b13
intensity
0
50000
100000
150000
200000
250000
0
20
40
60
80
100
m/z
Fragment Ions
147.113 y1
197.130 b2
246.180 y2
254.151 b3
355.196 b4
360.225 y3
470.227 b5
473.305 y4
541.260 b6
588.338 y5
612.302 b7
683.343 b8
717.383 y6
788.410 y7
811.390 b9
882.424 b10
916.463 y8
987.504 y9
1011.483 b11
1058.549 y10
1126.492 b12
1129.588 y11
1239.584 b13
1244.594 y12
1345.660 y13
1402.694 y14
1499.751 y15
VPGTDAAAQAEDLNVK / File: christine_10pools_03.raw; SpectrumID: ; scans: 7813

YAVSSHIPPEN^IR

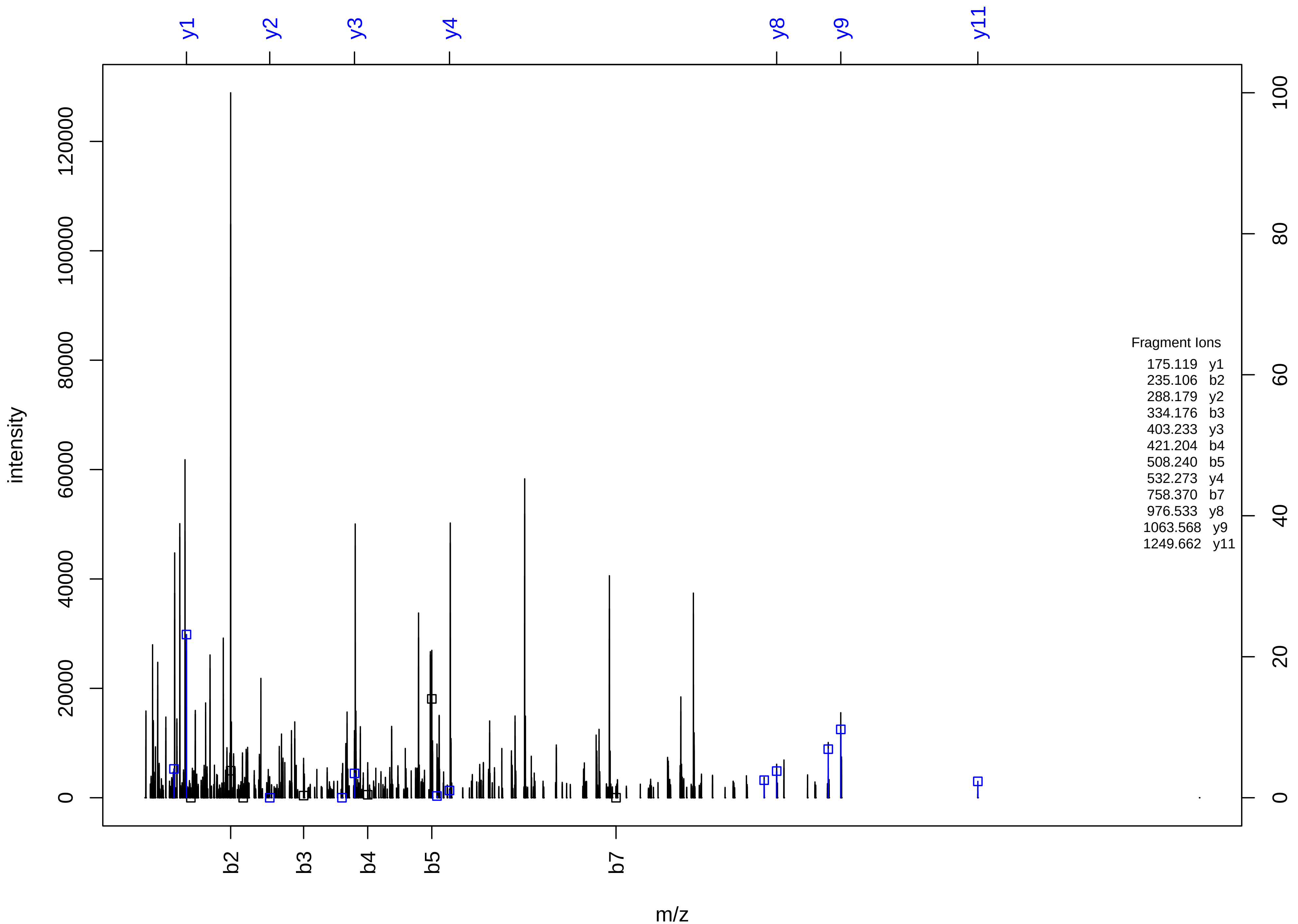

YAVSSHIPPENIR / File: christine_10pools_03.raw; SpectrumID: ; scans: 864

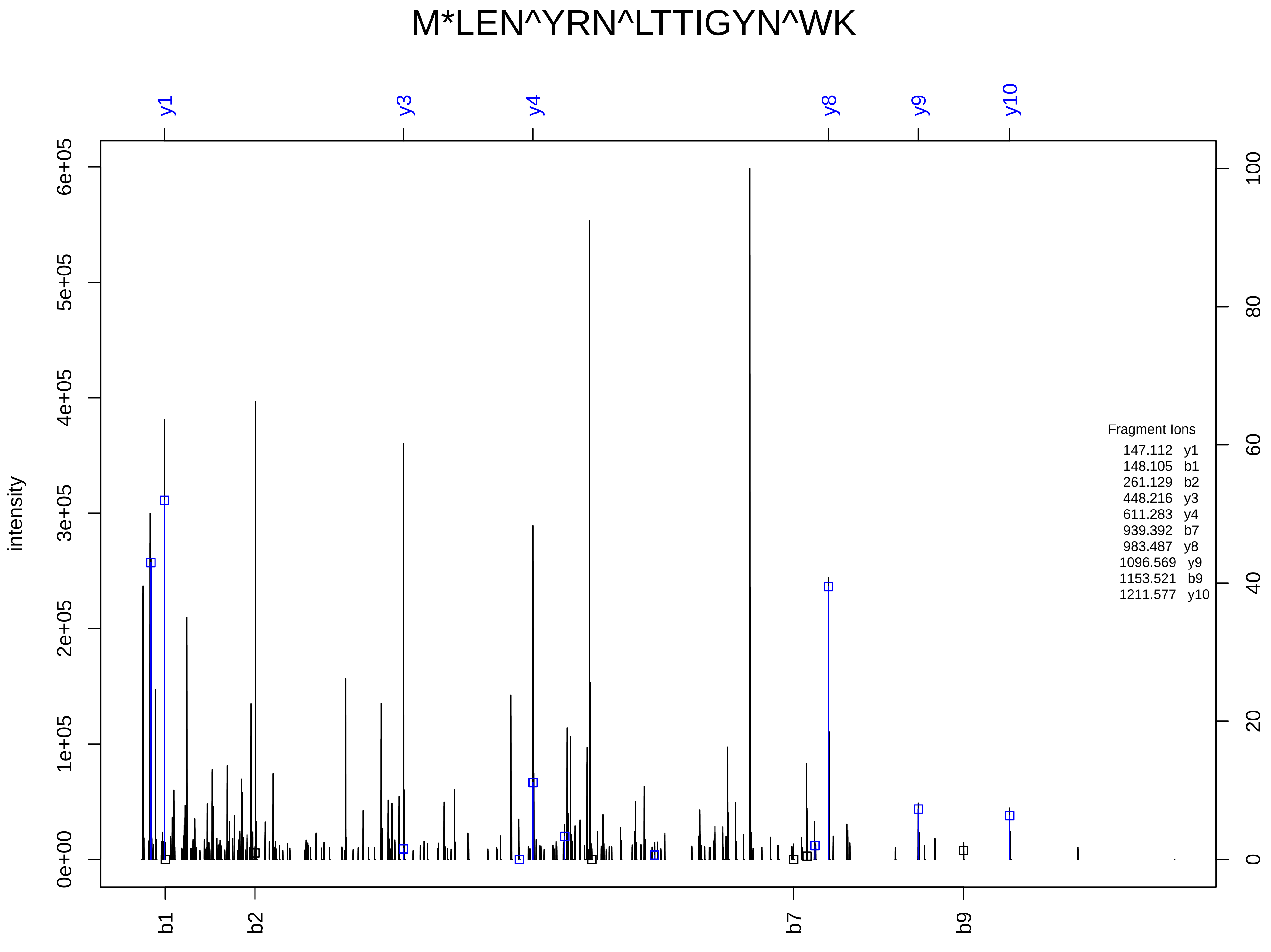

MLENYRNLTTIGYNWK / File: christine_10pools_03.raw; SpectrumID: ; scans: 12623

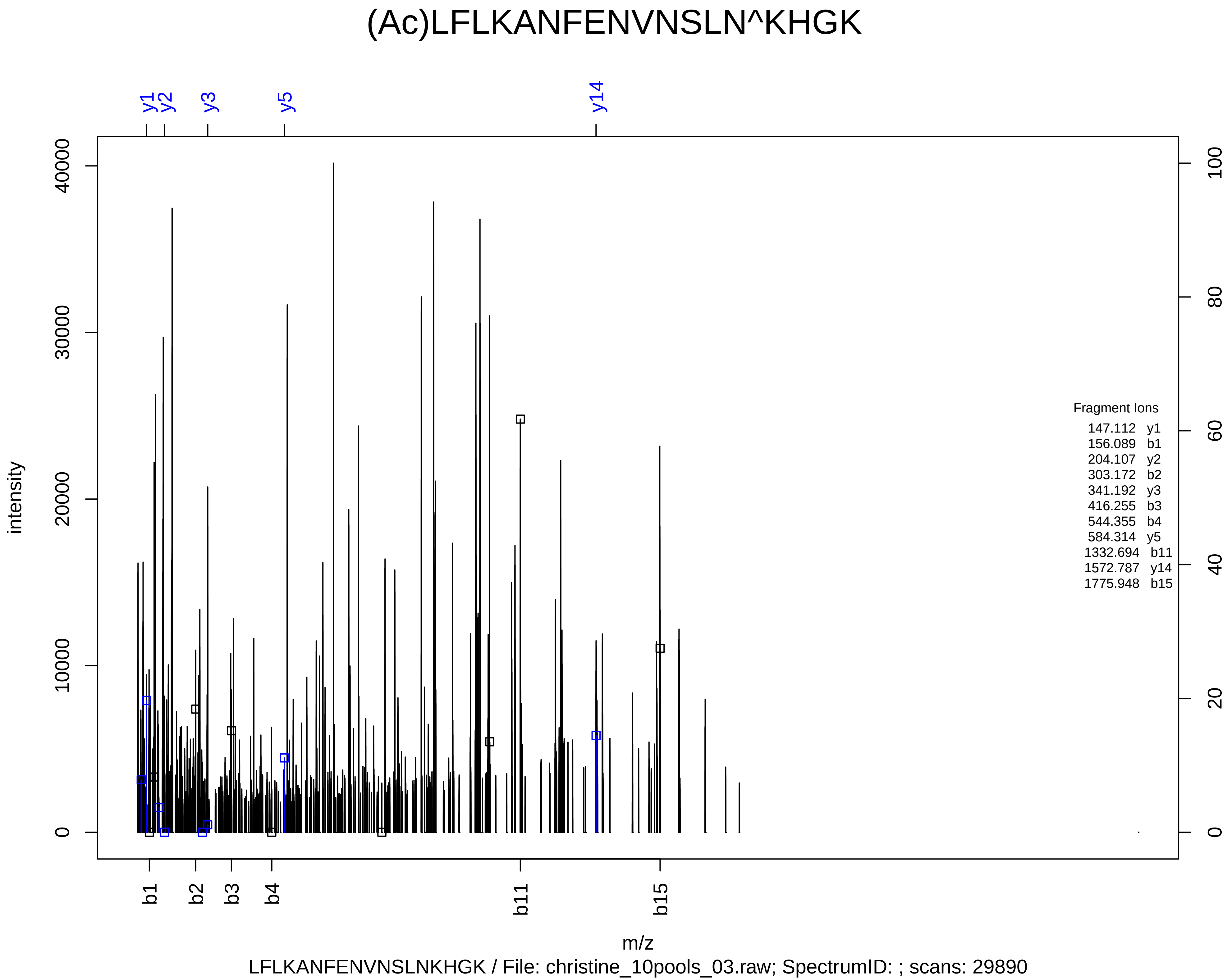

(Ac)LFLKANFENVNSLN^KHGK
y1
y2
y3
y5
y14
intensity
0
10000
20000
30000
40000
0
20
40
60
80
100
b1
b2
b3
b4
b11
b15
m/z
Fragment Ions
147.112 y1
156.089 b1
204.107 y2
303.172 b2
341.192 y3
416.255 b3
544.355 b4
584.314 y5
1332.694 b11
1572.787 y14
1775.948 b15
LFLKANFENVNSLNKHGK / File: christine_10pools_03.raw; SpectrumID: ; scans: 29890

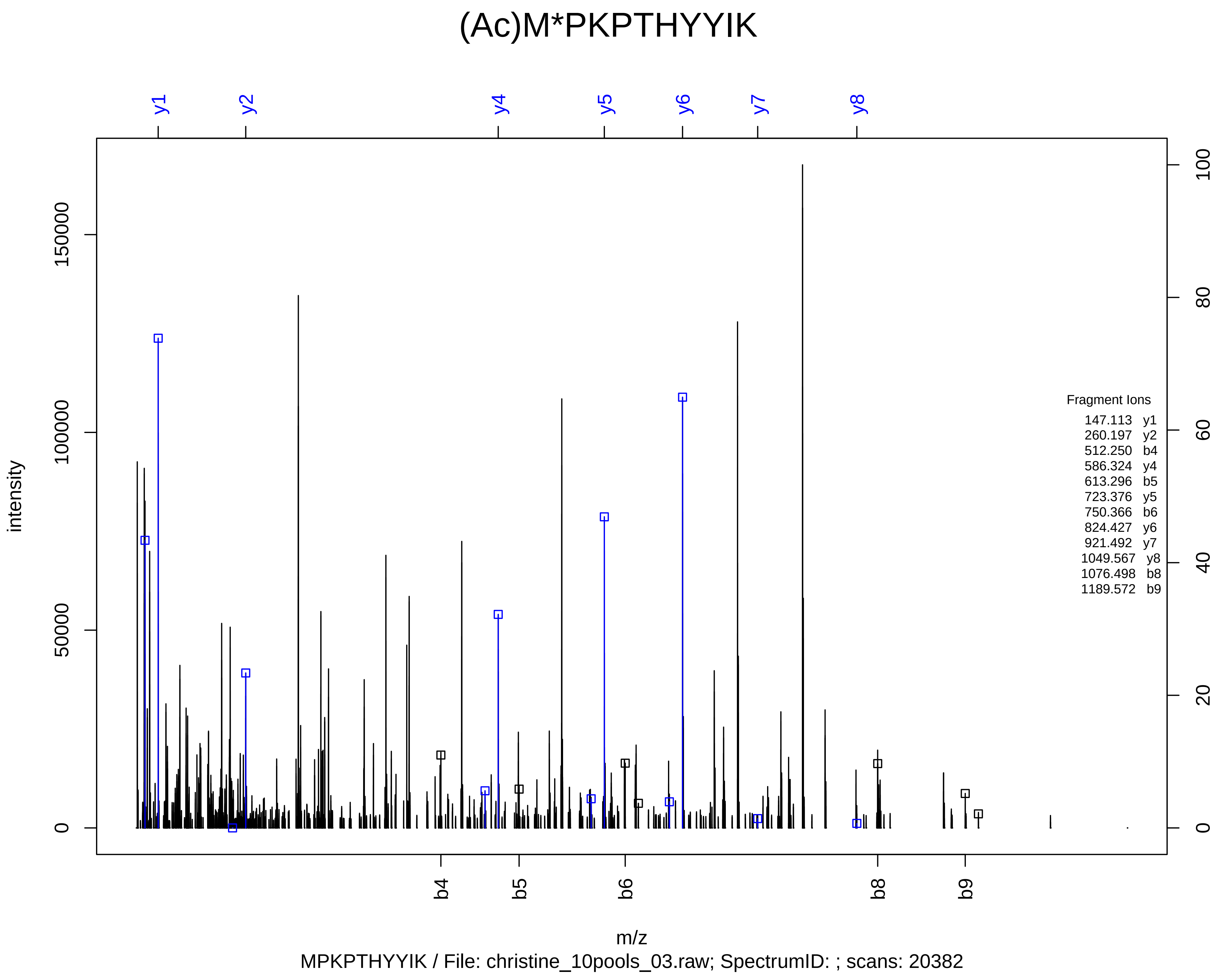

(Ac)M*PKPTHYYIK
y1
y2
y4
y5
y6
y7
y8
intensity
0
50000
100000
150000
0
20
40
60
80
100
Fragment Ions
147.113 y1
260.197 y2
512.250 b4
586.324 y4
613.296 b5
723.376 y5
750.366 b6
824.427 y6
921.492 y7
1049.567 y8
1076.498 b8
1189.572 b9
b4
b5
b6
b8
b9
m/z
MPKPTHYYIK / File: christine_10pools_03.raw; SpectrumID: ; scans: 20382

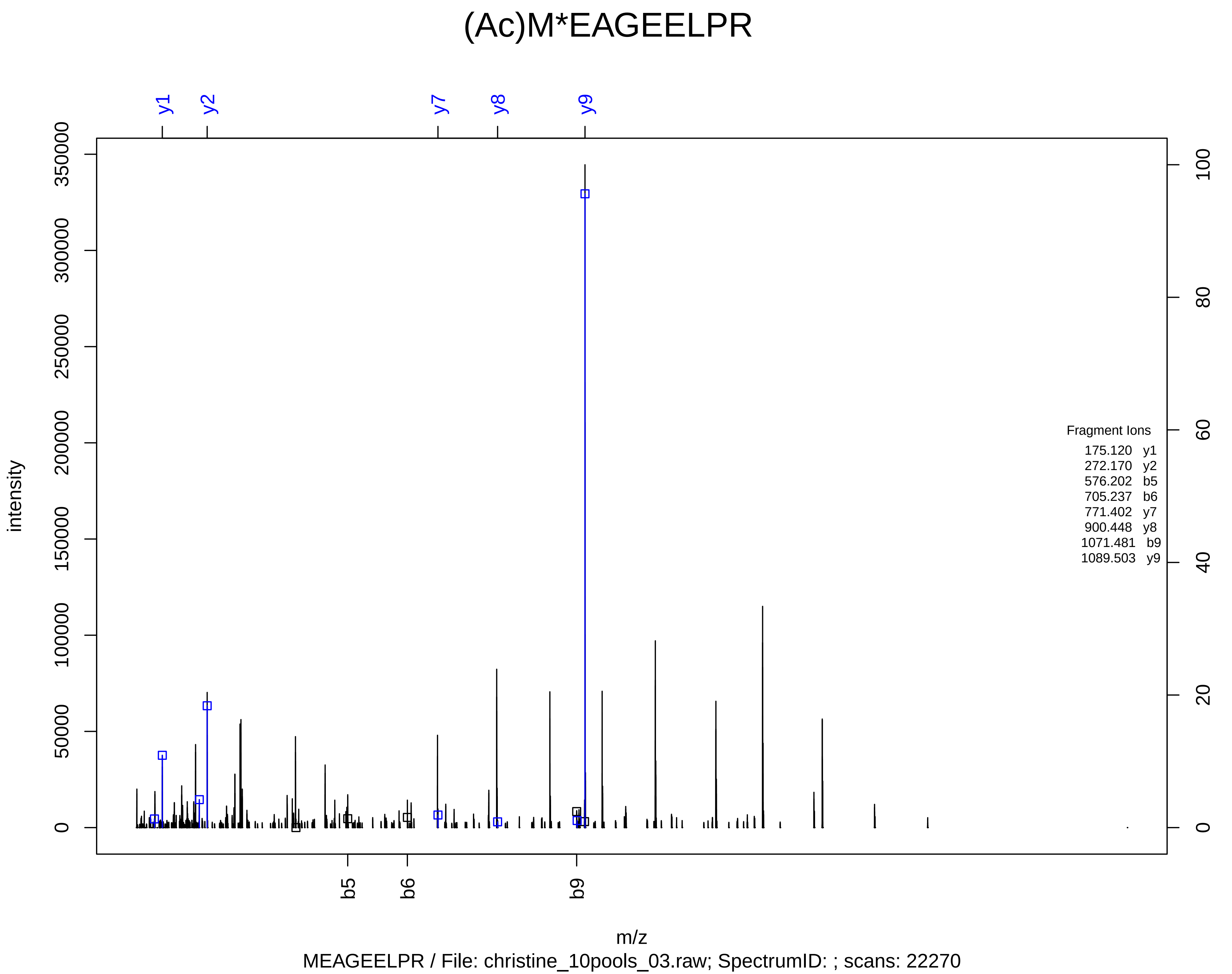

(Ac)M*EAGEELPR
y1
y2
y7
y8
y9
intensity
0
50000
100000
150000
200000
250000
300000
350000
0
20
40
60
80
100
Fragment Ions
175.120 y1
272.170 y2
576.202 b5
705.237 b6
771.402 y7
900.448 y8
1071.481 b9
1089.503 y9
b5
b6
b9
m/z
MEAGEELPR / File: christine_10pools_03.raw; SpectrumID: ; scans: 22270

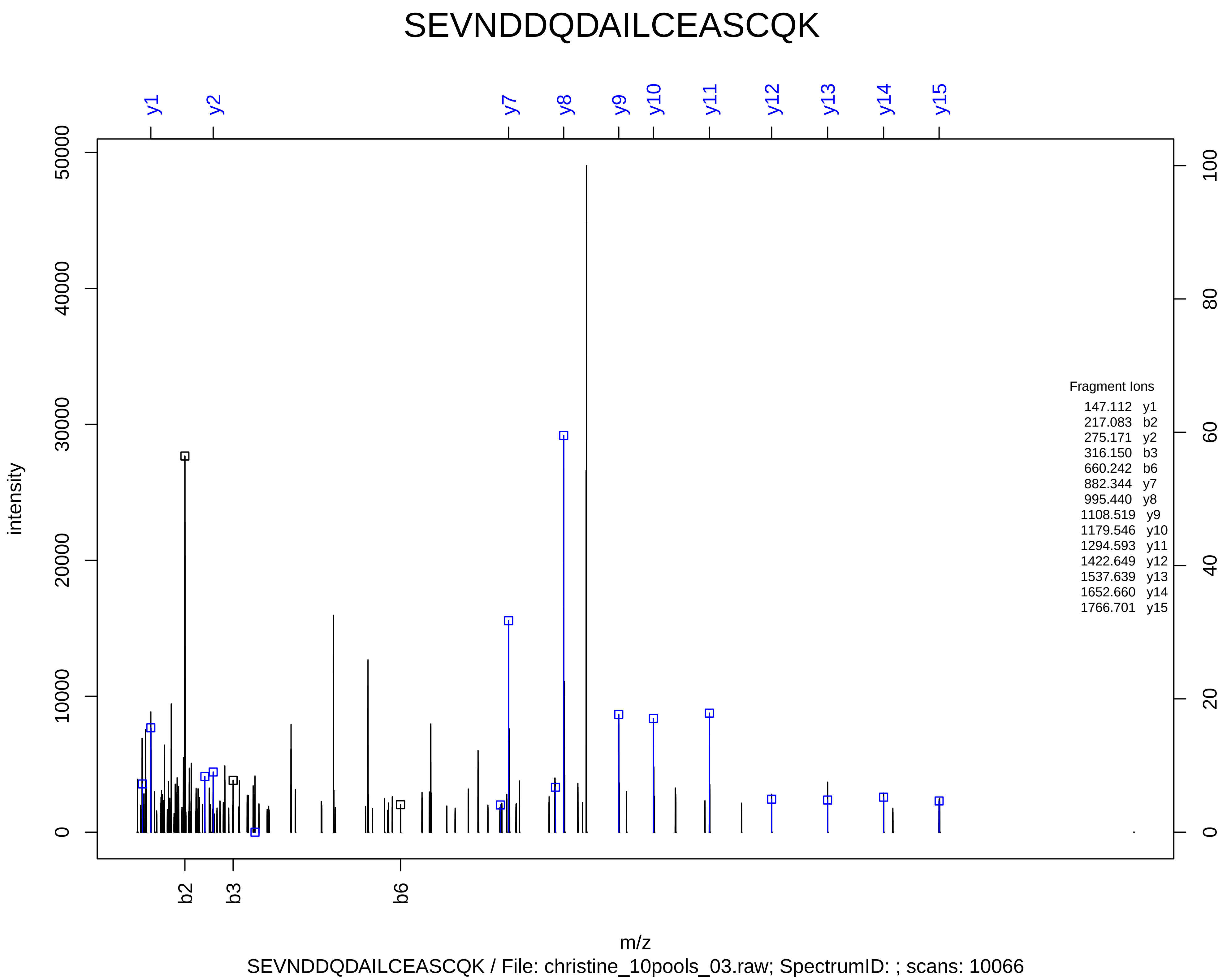
SEVNDDQDAILCEASCQK
y1
y2
y7
y8
y9
y10
y11
y12
y13
y14
y15
intensity
50000
40000
30000
20000
10000
0
100
80
60
40
20
0
Fragment Ions
147.112 y1
217.083 b2
275.171 y2
316.150 b3
660.242 b6
882.344 y7
995.440 y8
1108.519 y9
1179.546 y10
1294.593 y11
1422.649 y12
1537.639 y13
1652.660 y14
1766.701 y15
b2
b3
b6
m/z
SEVNDDQDAILCEASCQK / File: christine_10pools_03.raw; SpectrumID: ; scans: 10066

FPGIPFPLDAAVECHR

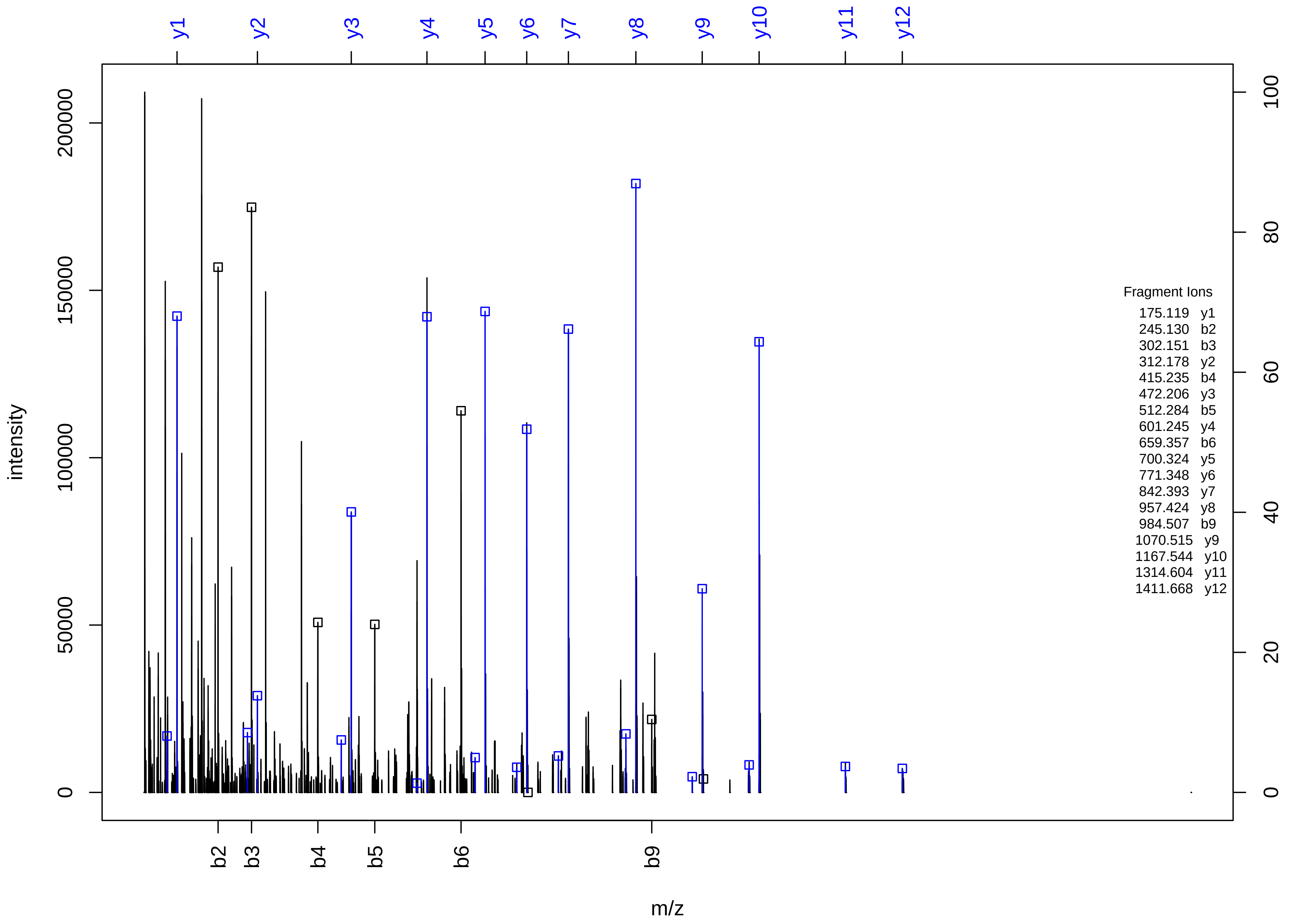

FPGIPFPLDAAVECHR / File: christine_10pools_04.raw; SpectrumID: ; scans: 28399

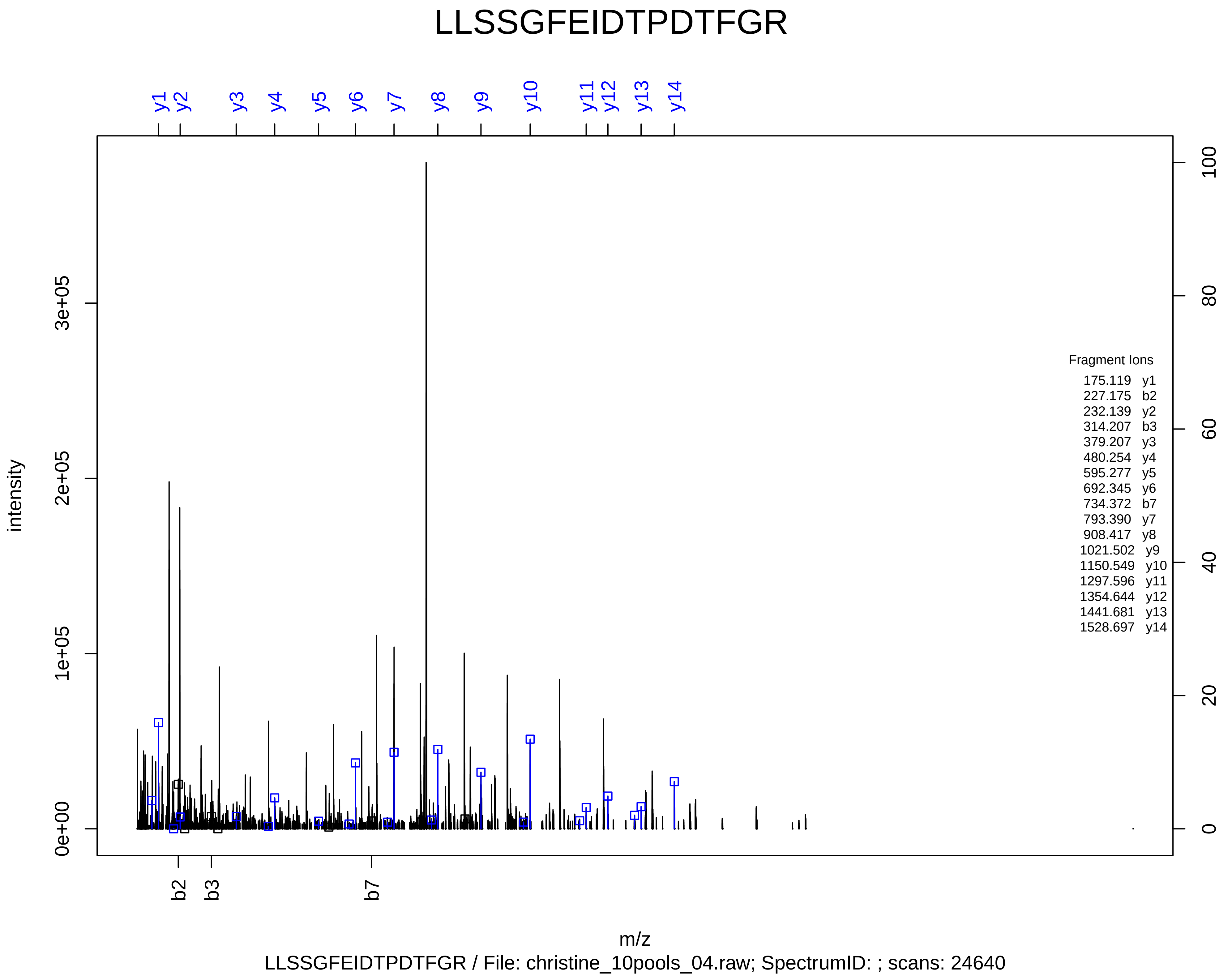

LLSSGFEIDTPDTFGR
y1 y2 y3 y4 y5 y6 y7 y8 y9 y10 y11 y12 y13 y14
intensity
0e+00
1e+05
2e+05
3e+05
0
20
40
60
80
100
b2 b3 b7
m/z
Fragment Ions
175.119 y1
227.175 b2
232.139 y2
314.207 b3
379.207 y3
480.254 y4
595.277 y5
692.345 y6
734.372 b7
793.390 y7
908.417 y8
1021.502 y9
1150.549 y10
1297.596 y11
1354.644 y12
1441.681 y13
1528.697 y14
LLSSGFEIDTPDTFGR / File: christine_10pools_04.raw; SpectrumID: ; scans: 24640

TIADSLINNLLR

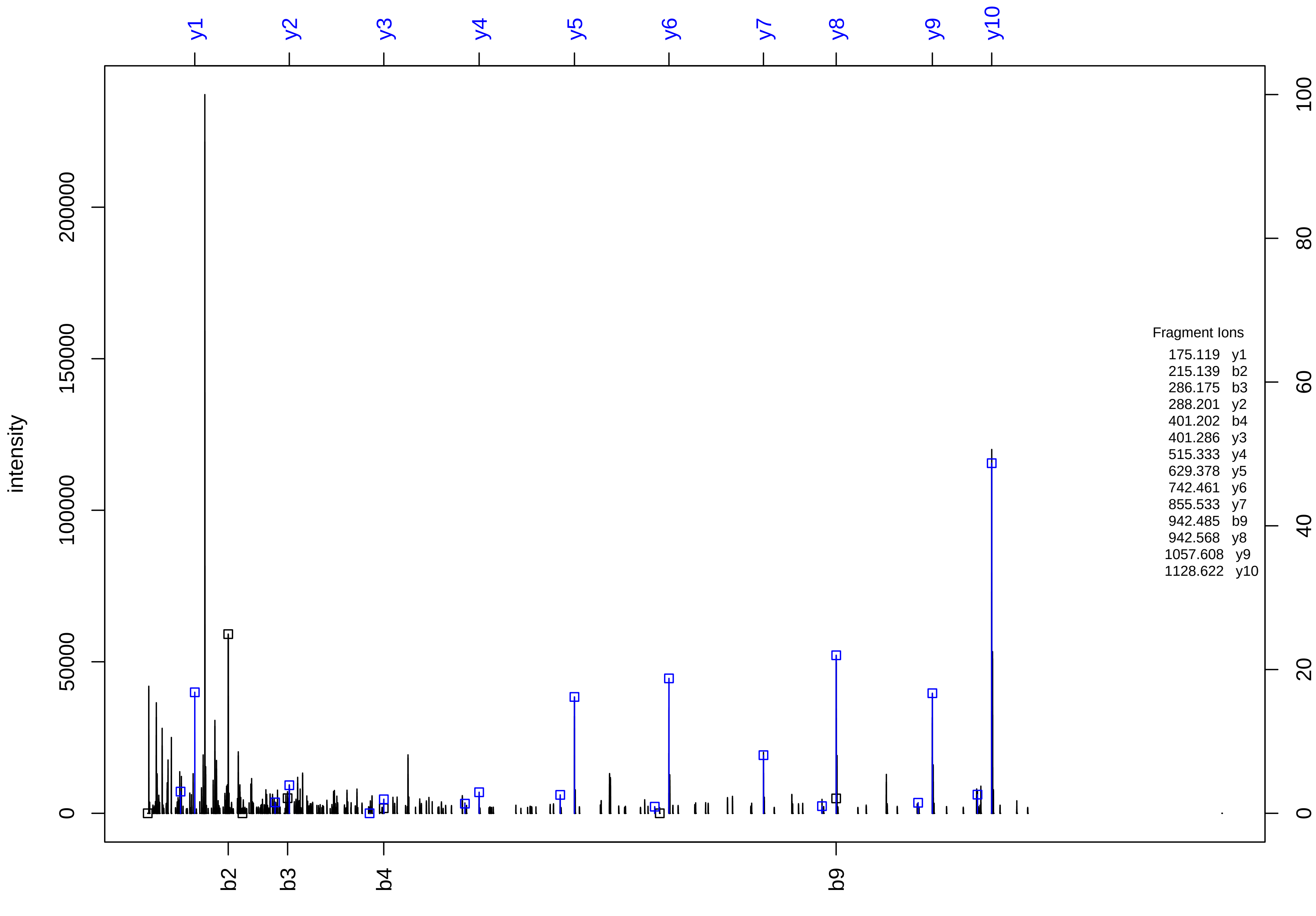

TIADSLINNLLR / File: christine_10pools_04.raw; SpectrumID: ; scans: 33966

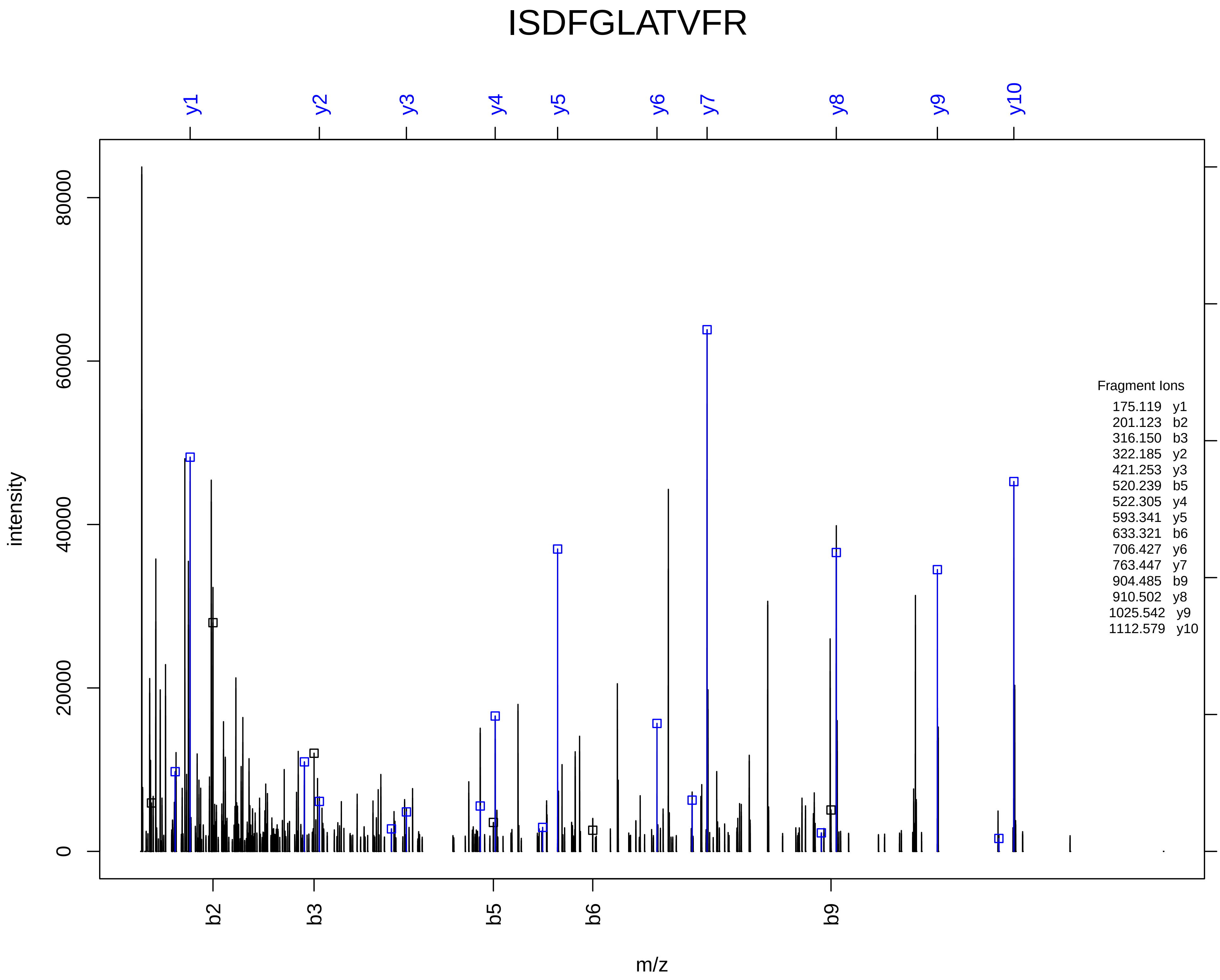

ISDFGLATVFR / File: christine_10pools_04.raw; SpectrumID: ; scans: 26998

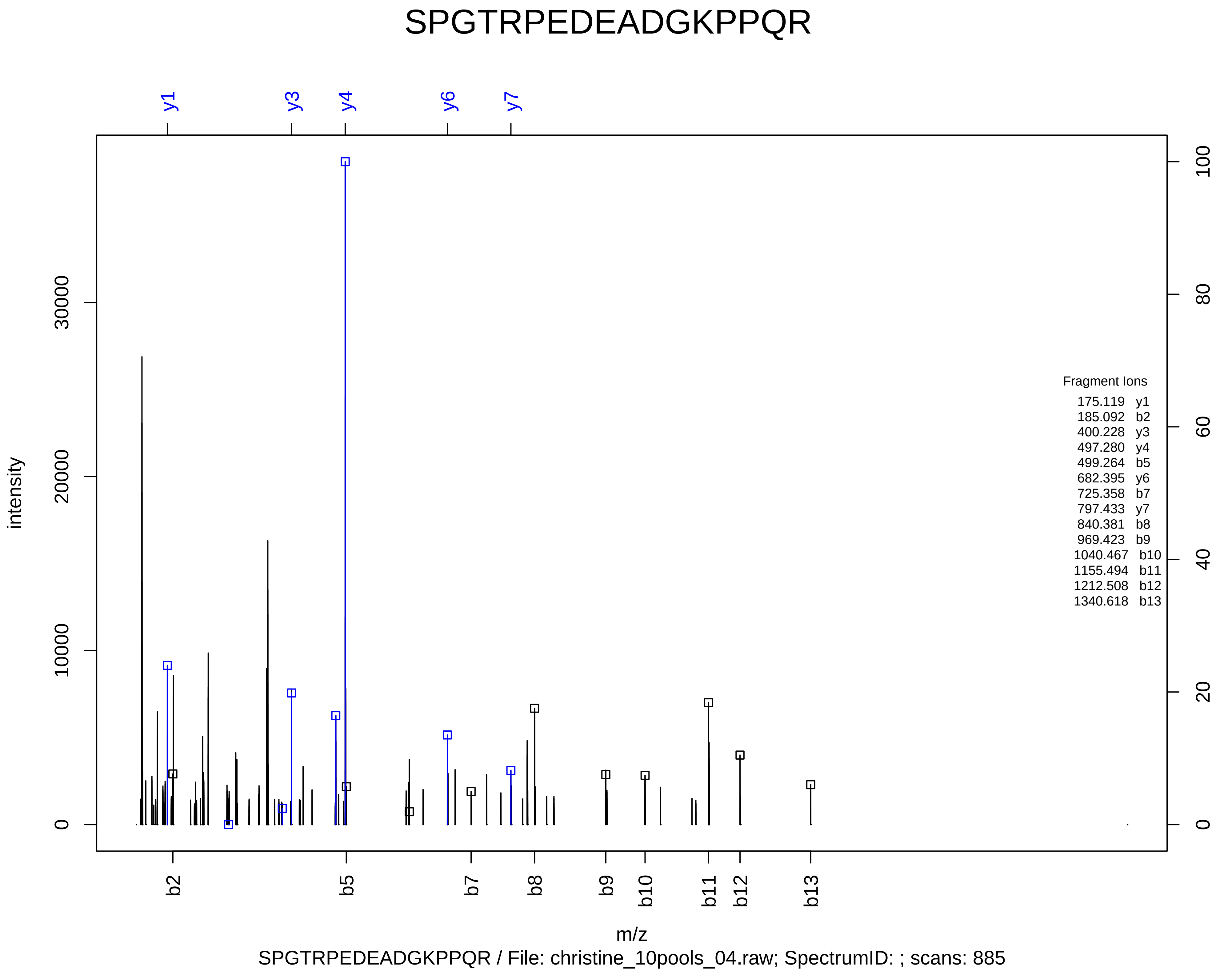
SPGTRPEDEADGKPPQR
y1
y3
y4
y6
y7
intensity
0
10000
20000
30000
0
20
40
60
80
100
Fragment Ions
175.119 y1
185.092 b2
400.228 y3
497.280 y4
499.264 b5
682.395 y6
725.358 b7
797.433 y7
840.381 b8
969.423 b9
1040.467 b10
1155.494 b11
1212.508 b12
1340.618 b13
b2
b5
b7
b8
b9
b10
b11
b12
b13
m/z
SPGTRPEDEADGKPPQR / File: christine_10pools_04.raw; SpectrumID: ; scans: 885

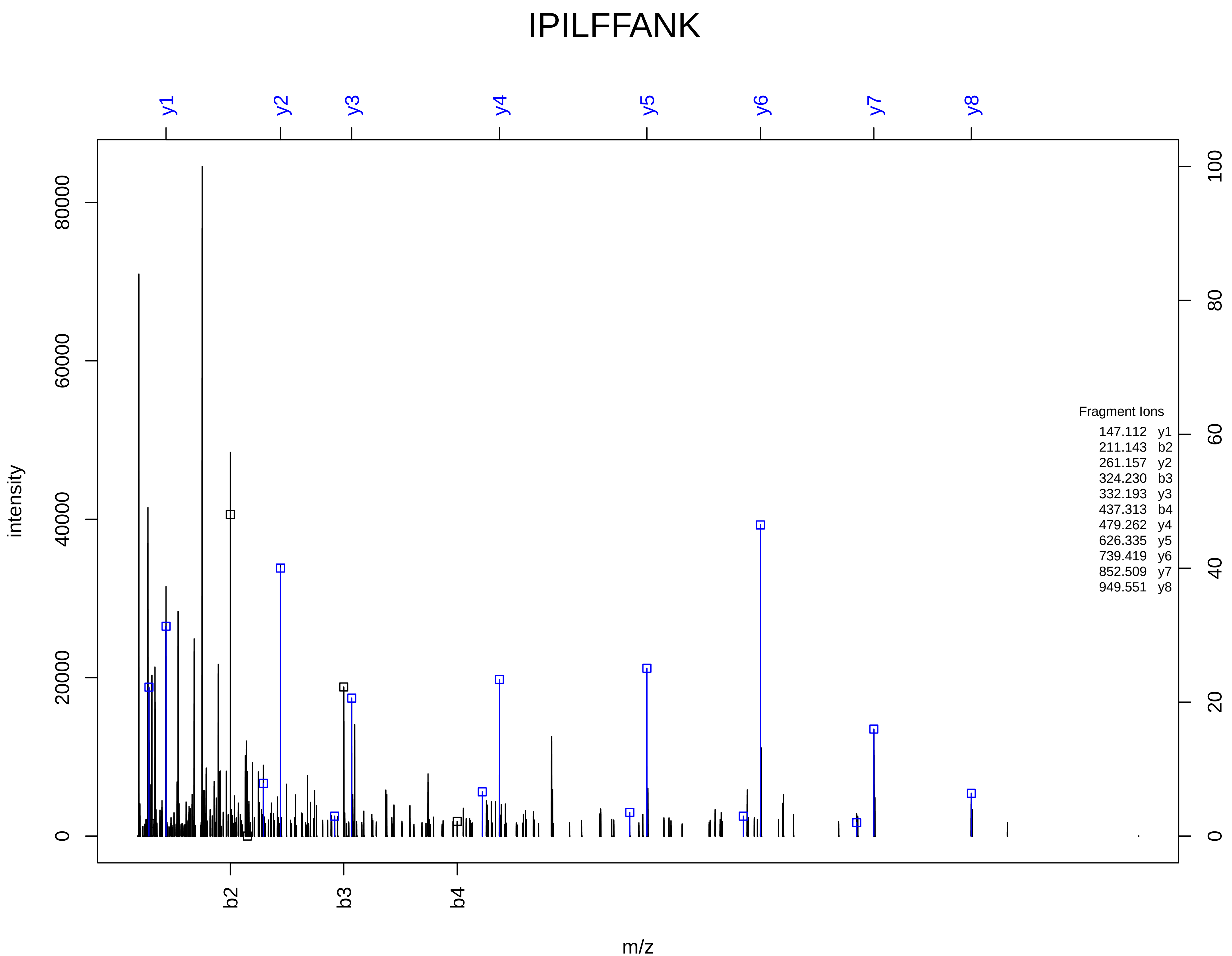
IPILFFANK
y1
y2
y3
y4
y5
y6
y7
y8
b2
b3
b4
intensity
m/z
Fragment Ions
147.112 y1
211.143 b2
261.157 y2
324.230 b3
332.193 y3
437.313 b4
479.262 y4
626.335 y5
739.419 y6
852.509 y7
949.551 y8
IPILFFANK / File: christine_10pools_04.raw; SpectrumID: ; scans: 27816

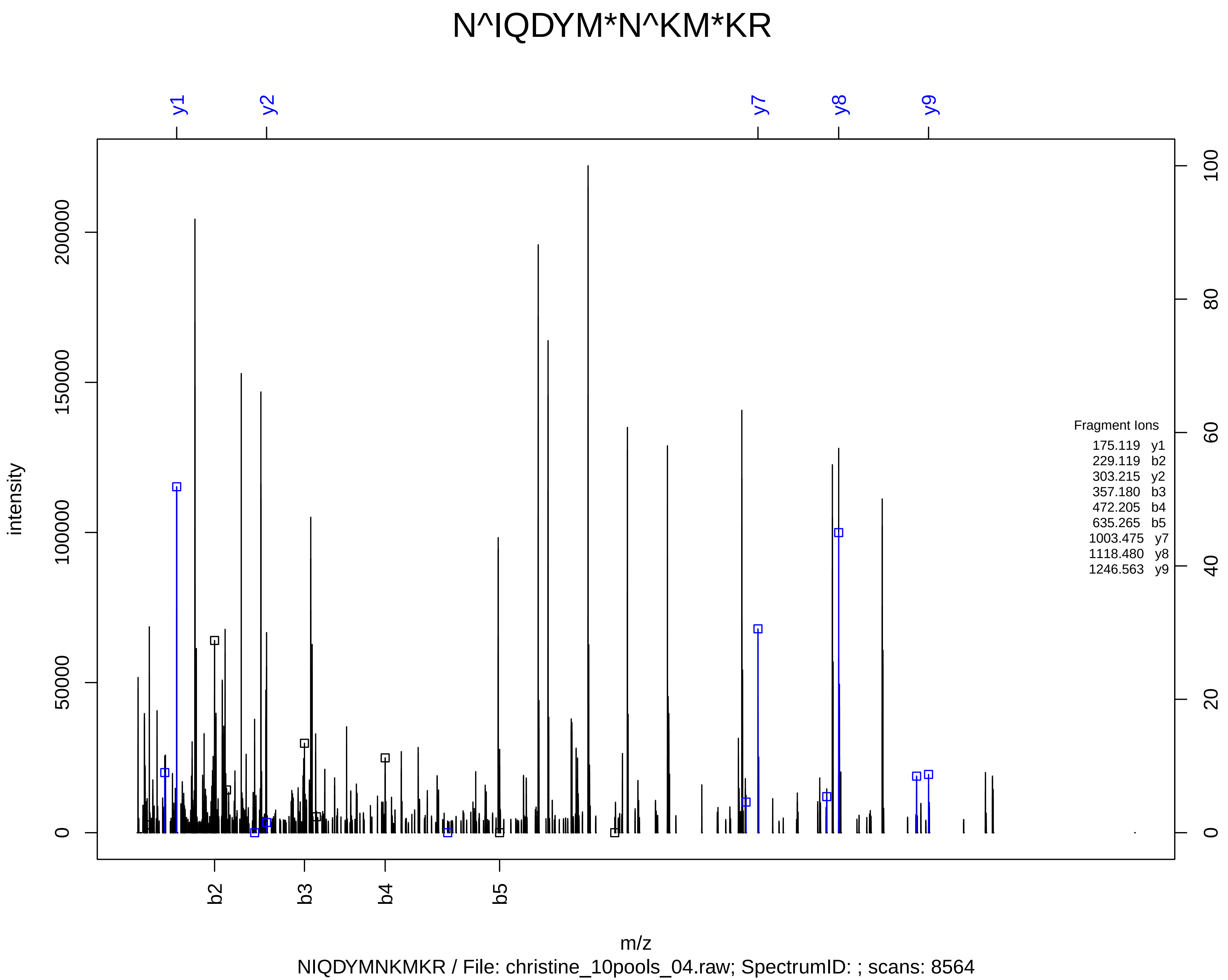

N^IQDYM*N^KM*KR
y1
y2
y7
y8
y9
intensity
0
50000
100000
150000
200000
0
20
40
60
80
100
b2
b3
b4
b5
Fragment Ions
175.119 y1
229.119 b2
303.215 y2
357.180 b3
472.205 b4
635.265 b5
1003.475 y7
1118.480 y8
1246.563 y9
m/z
NIQDYMNKMKR / File: christine_10pools_04.raw; SpectrumID: ; scans: 8564

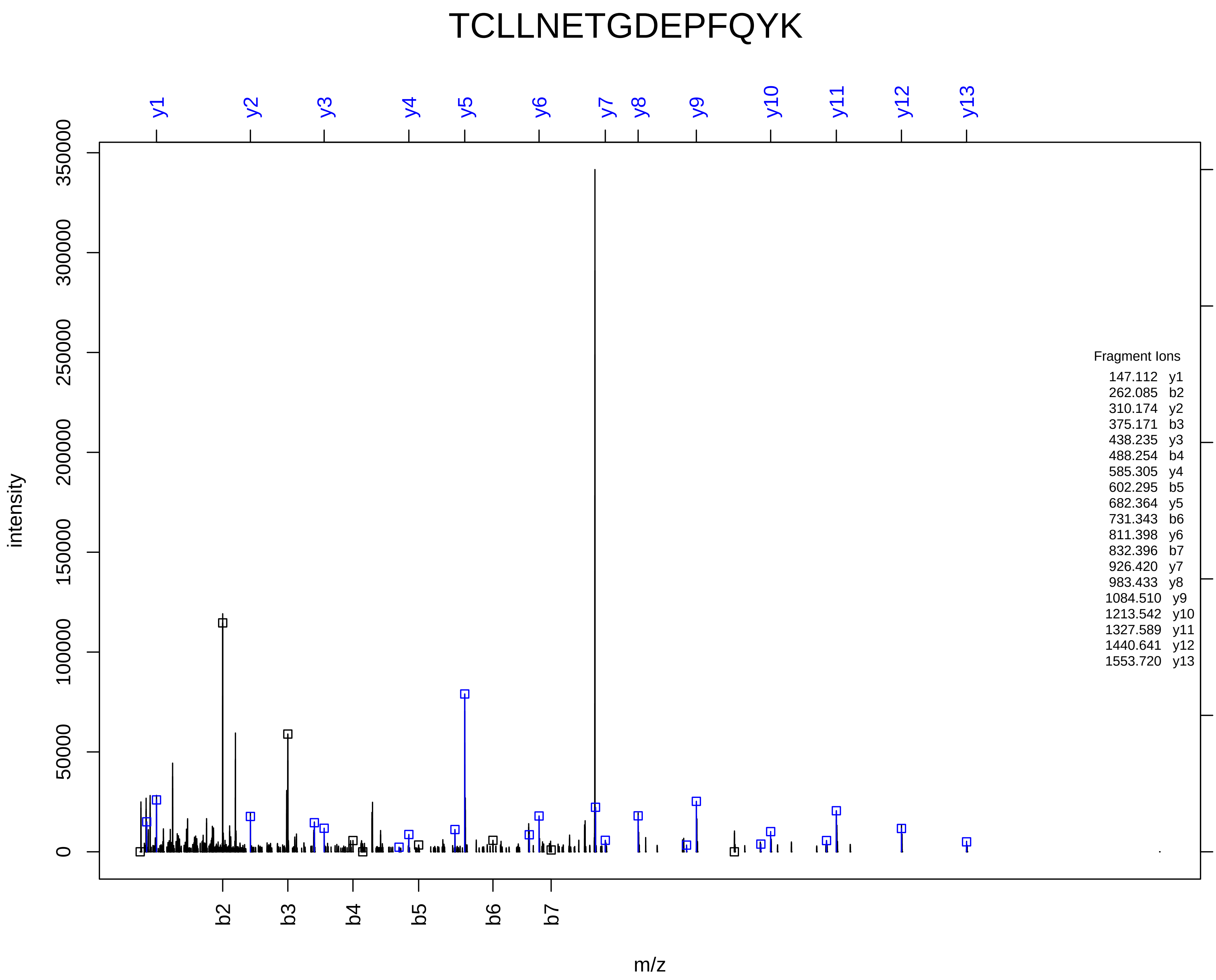

TCLLNETGDEPFQYK
y1 y2 y3 y4 y5 y6 y7 y8 y9 y10 y11 y12 y13
b2 b3 b4 b5 b6 b7
intensity
0
50000
100000
150000
200000
250000
300000
350000
0
20
40
60
80
100
m/z
Fragment Ions
147.112 y1
262.085 b2
310.174 y2
375.171 b3
438.235 y3
488.254 b4
585.305 y4
602.295 b5
682.364 y5
731.343 b6
811.398 y6
832.396 b7
926.420 y7
983.433 y8
1084.510 y9
1213.542 y10
1327.589 y11
1440.641 y12
1553.720 y13
TCLLNETGDEPFQYK / File: christine_10pools_04.raw; SpectrumID: ; scans: 17004

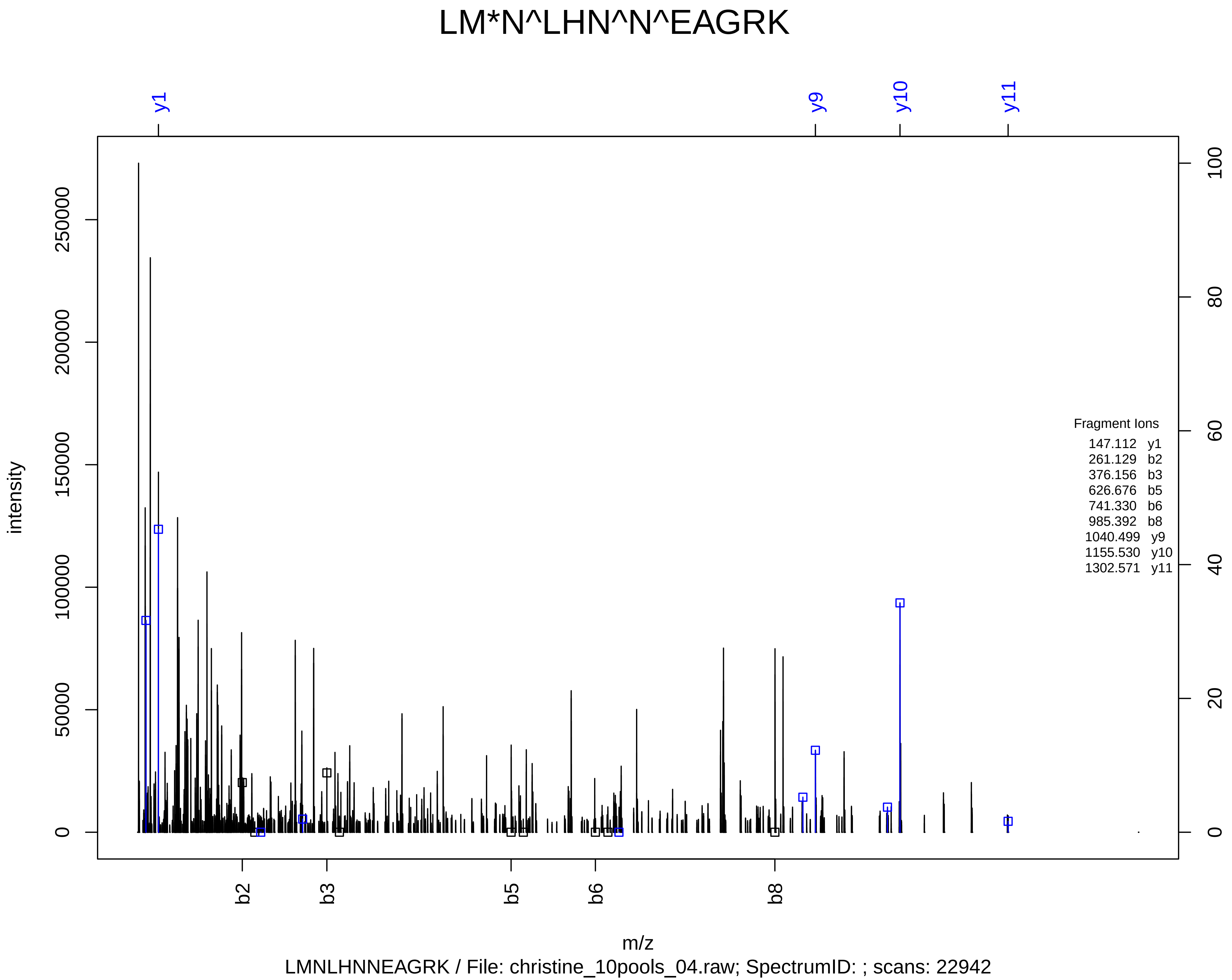

LM*N^LHN^N^EAGRK
y1
y9
y10
y11
intensity
0
50000
100000
150000
200000
250000
0
20
40
60
80
100
b2
b3
b5
b6
b8
m/z
Fragment Ions
147.112 y1
261.129 b2
376.156 b3
626.676 b5
741.330 b6
985.392 b8
1040.499 y9
1155.530 y10
1302.571 y11
LMNLHNNEAGRK / File: christine_10pools_04.raw; SpectrumID: ; scans: 22942

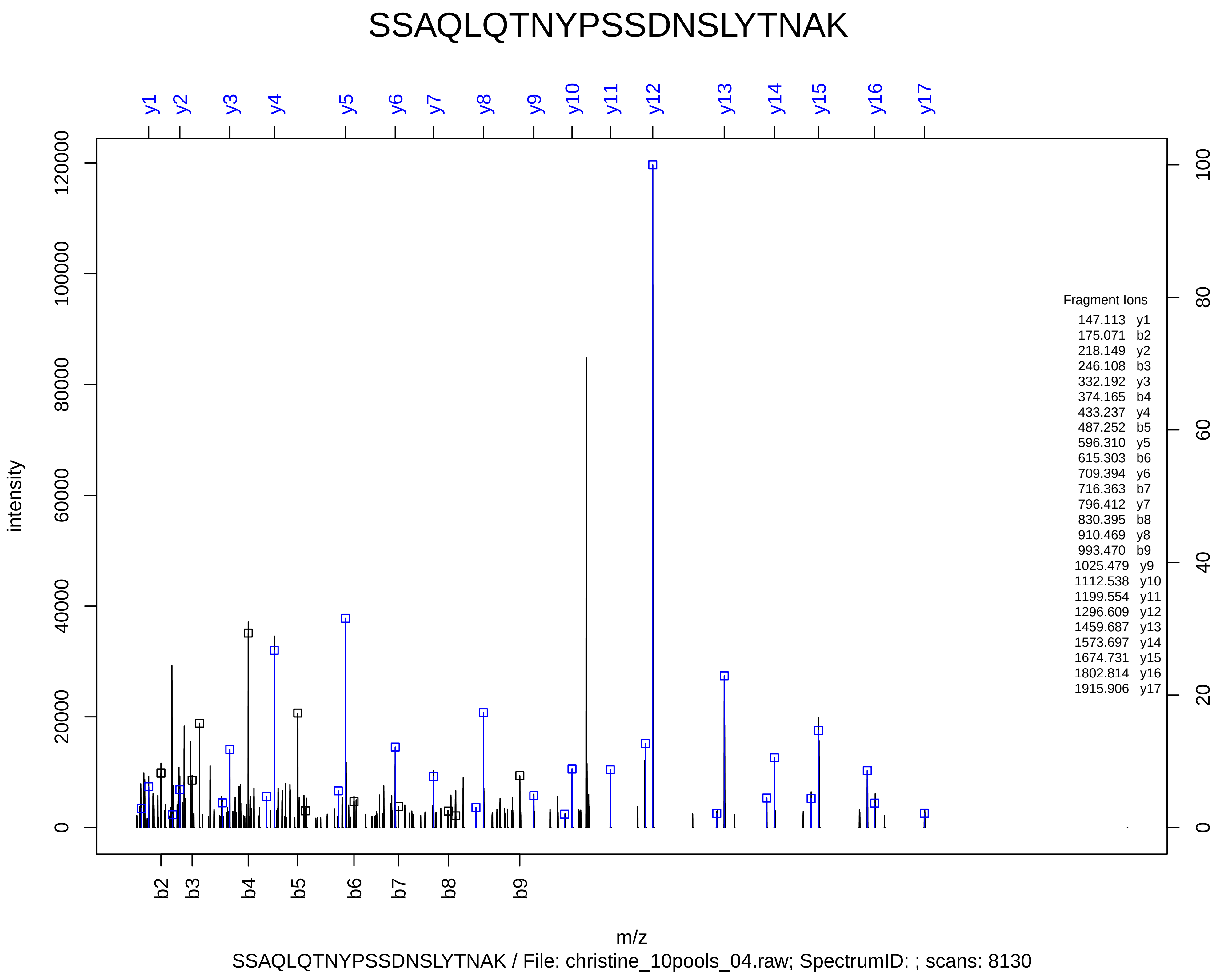
SSAQLQTNYPSSDNSLYTNAK
y1 y2 y3 y4 y5 y6 y7 y8 y9 y10 y11 y12 y13 y14 y15 y16 y17
b2 b3 b4 b5 b6 b7 b8 b9
intensity
0 20000 40000 60000 80000 100000 120000
0 20 40 60 80 100
m/z
Fragment Ions
147.113 y1
175.071 b2
218.149 y2
246.108 b3
332.192 y3
374.165 b4
433.237 y4
487.252 b5
596.310 y5
615.303 b6
709.394 y6
716.363 b7
796.412 y7
830.395 b8
910.469 y8
993.470 b9
1025.479 y9
1112.538 y10
1199.554 y11
1296.609 y12
1459.687 y13
1573.697 y14
1674.731 y15
1802.814 y16
1915.906 y17
SSAQLQTNYPSSDNSLYTNAK / File: christine_10pools_04.raw; SpectrumID: ; scans: 8130

AQASAPAQAPK

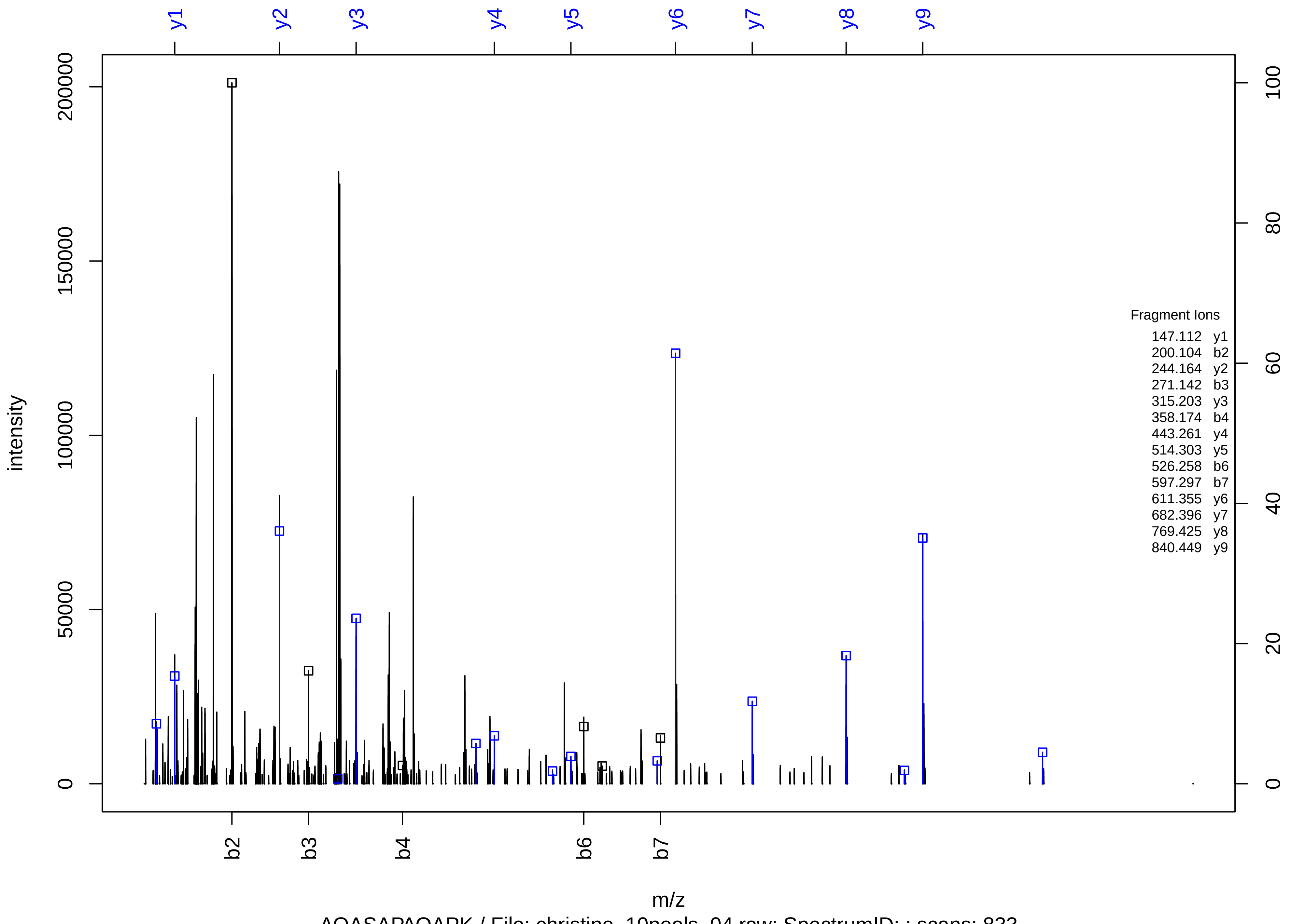

AQASAPAQAPK / File: christine_10pools_04.raw; SpectrumID: ; scans: 833

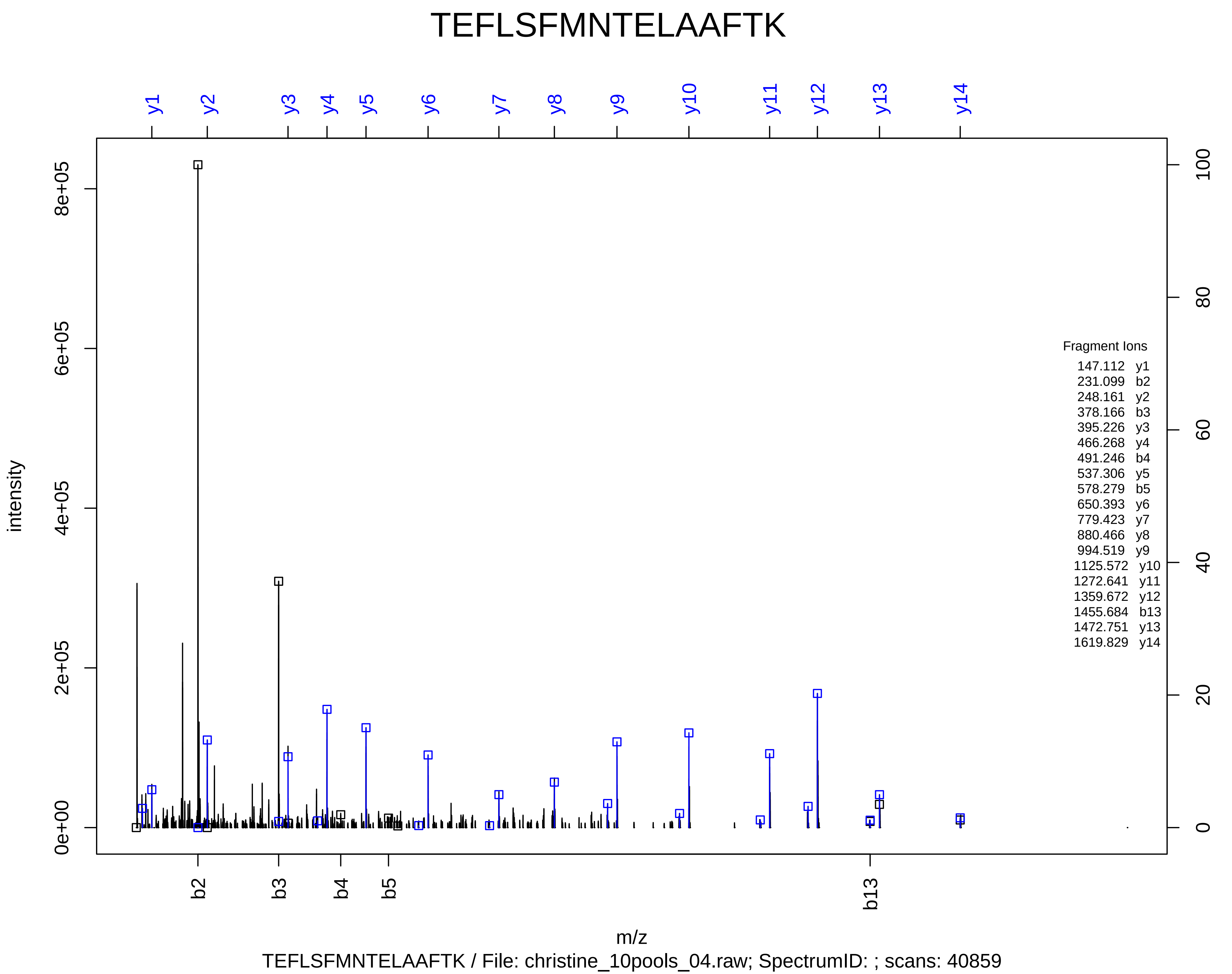

TEFLSFMNTELAAFTK
y1
y2
y3
y4
y5
y6
y7
y8
y9
y10
y11
y12
y13
y14
intensity
0e+00
2e+05
4e+05
6e+05
8e+05
0
20
40
60
80
100
b2
b3
b4
b5
b13
Fragment Ions
147.112 y1
231.099 b2
248.161 y2
378.166 b3
395.226 y3
466.268 y4
491.246 b4
537.306 y5
578.279 b5
650.393 y6
779.423 y7
880.466 y8
994.519 y9
1125.572 y10
1272.641 y11
1359.672 y12
1455.684 b13
1472.751 y13
1619.829 y14
m/z
TEFLSFMNTELAAFTK / File: christine_10pools_04.raw; SpectrumID: ; scans: 40859

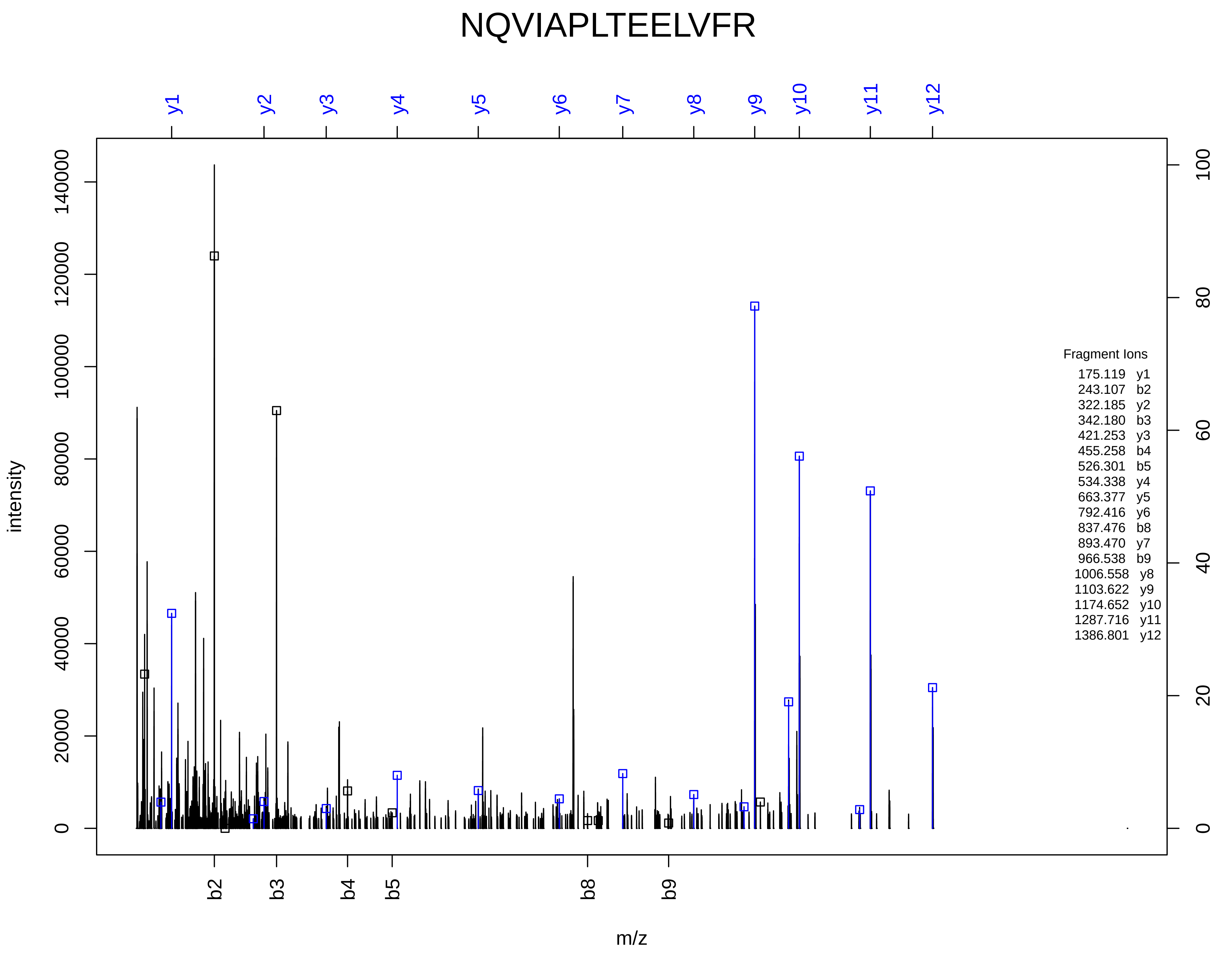

NQVIAPLTEELVFR
y1
y2
y3
y4
y5
y6
y7
y8
y9
y10
y11
y12
intensity
0
20000
40000
60000
80000
100000
120000
140000
0
20
40
60
80
100
Fragment Ions
175.119 y1
243.107 b2
322.185 y2
342.180 b3
421.253 y3
455.258 b4
526.301 b5
534.338 y4
663.377 y5
792.416 y6
837.476 b8
893.470 y7
966.538 b9
1006.558 y8
1103.622 y9
1174.652 y10
1287.716 y11
1386.801 y12
b2
b3
b4
b5
b8
b9
m/z
NQVIAPLTEELVFR / File: christine_10pools_04.raw; SpectrumID: ; scans: 28985

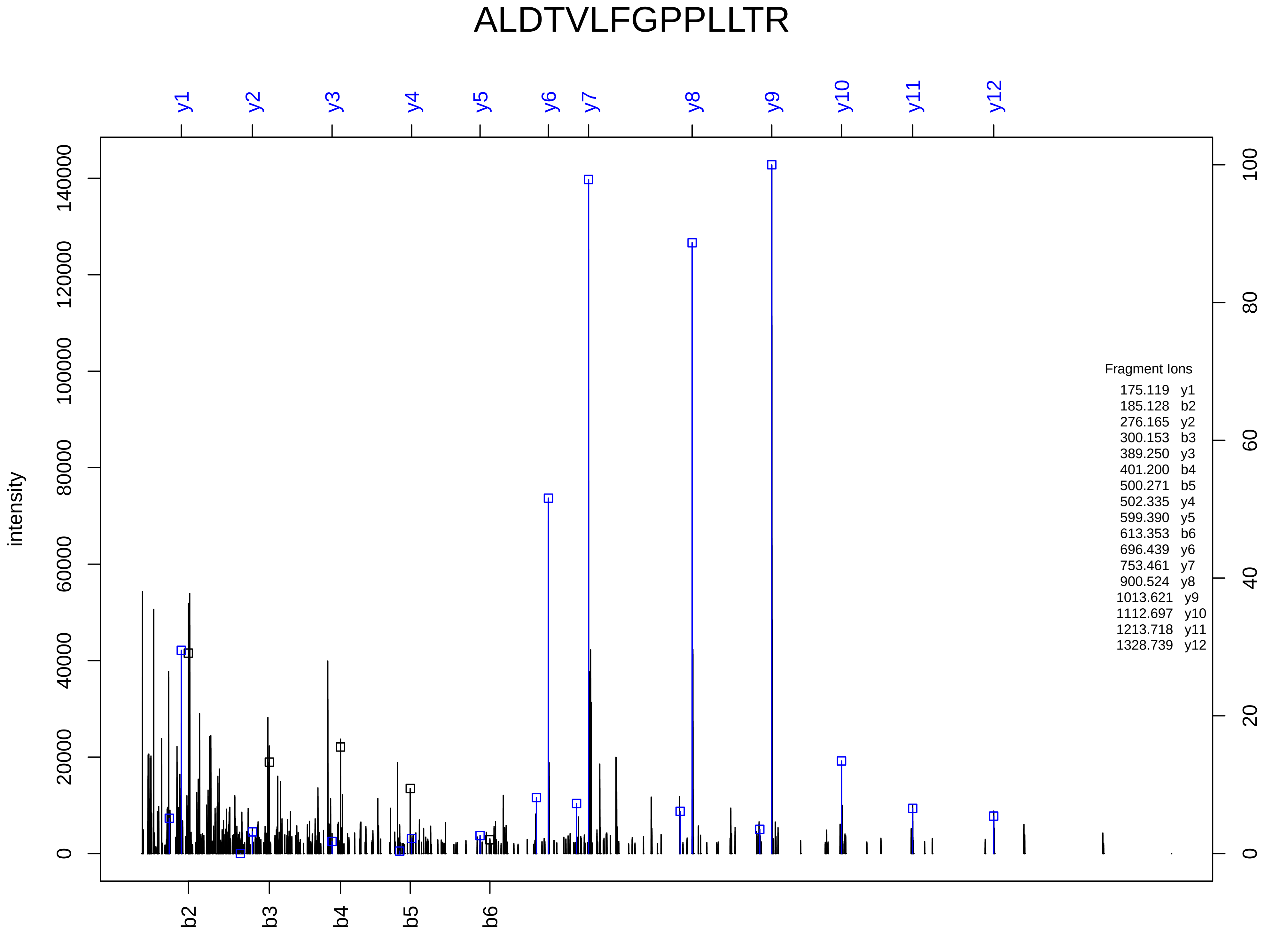

ALDTVLFGPPLLTR / File: christine_10pools_04.raw; SpectrumID: ; scans: 33696

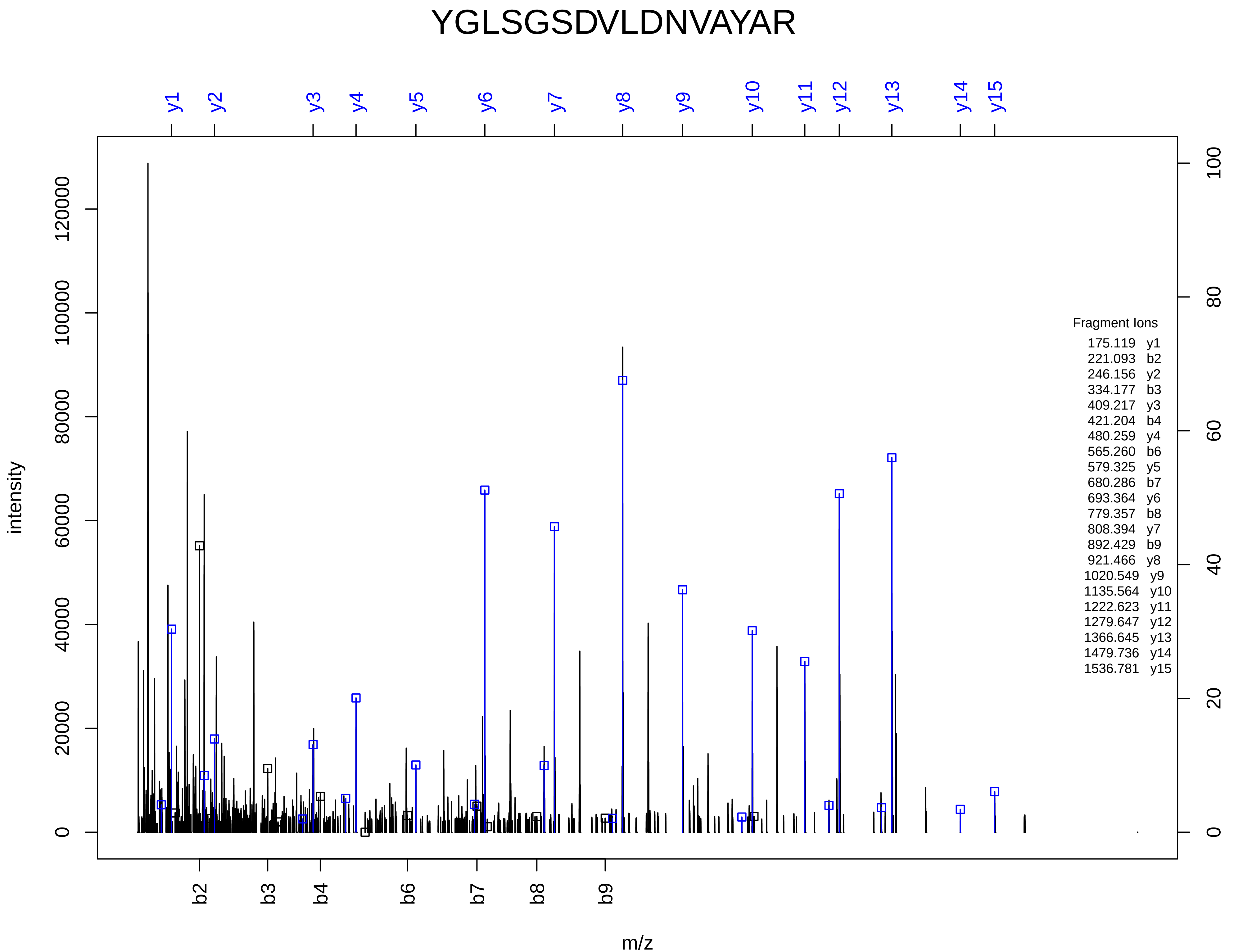

YGLSGSDVLDNVAYAR / File: christine_10pools_04.raw; SpectrumID: ; scans: 20630

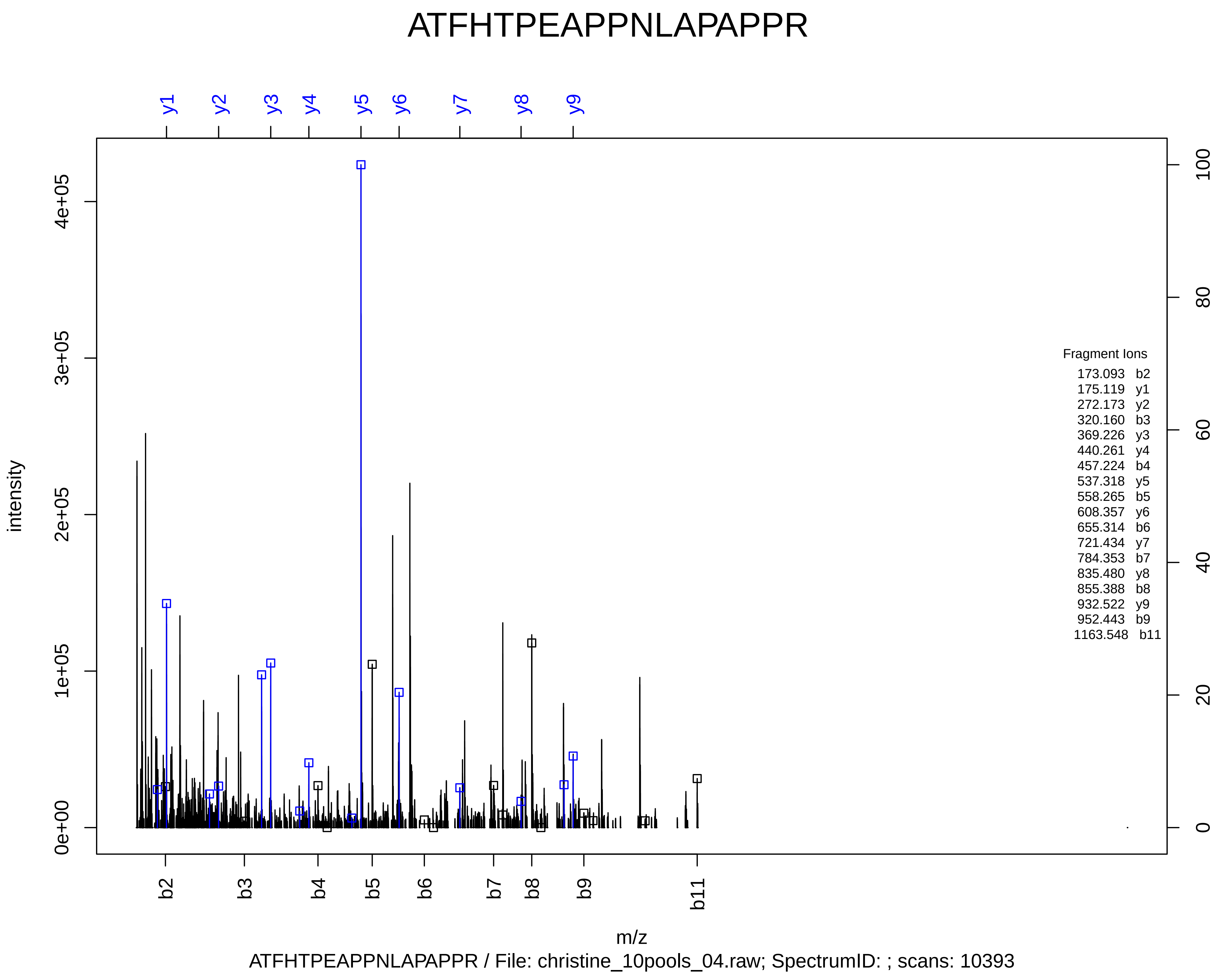
ATFHTPEAPPNLAPAPPR
y1 y2 y3 y4 y5 y6 y7 y8 y9
intensity
0e+00 1e+05 2e+05 3e+05 4e+05
0 20 40 60 80 100
Fragment Ions
173.093 b2
175.119 y1
272.173 y2
320.160 b3
369.226 y3
440.261 y4
457.224 b4
537.318 y5
558.265 b5
608.357 y6
655.314 b6
721.434 y7
784.353 b7
835.480 y8
855.388 b8
932.522 y9
952.443 b9
1163.548 b11
b2 b3 b4 b5 b6 b7 b8 b9 b11
m/z
ATFHTPEAPPNLAPAPPR / File: christine_10pools_04.raw; SpectrumID: ; scans: 10393

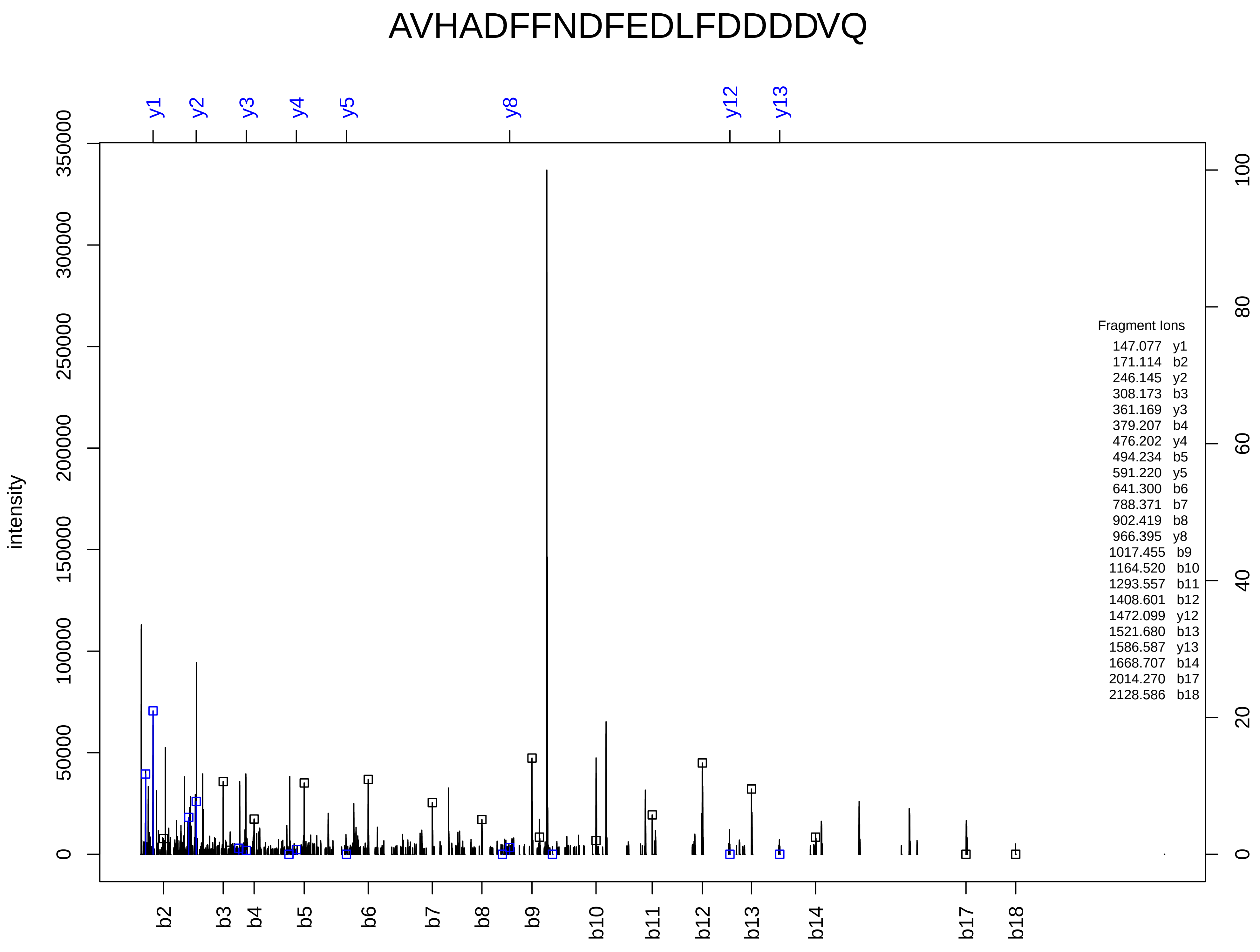

AVHADFFNDFEDLFDDDDVQ / File: christine_10pools_04.raw; SpectrumID: ; scans: 41531

DSASEDLALVEPGSDSLPSPGASHLFPTYLAR

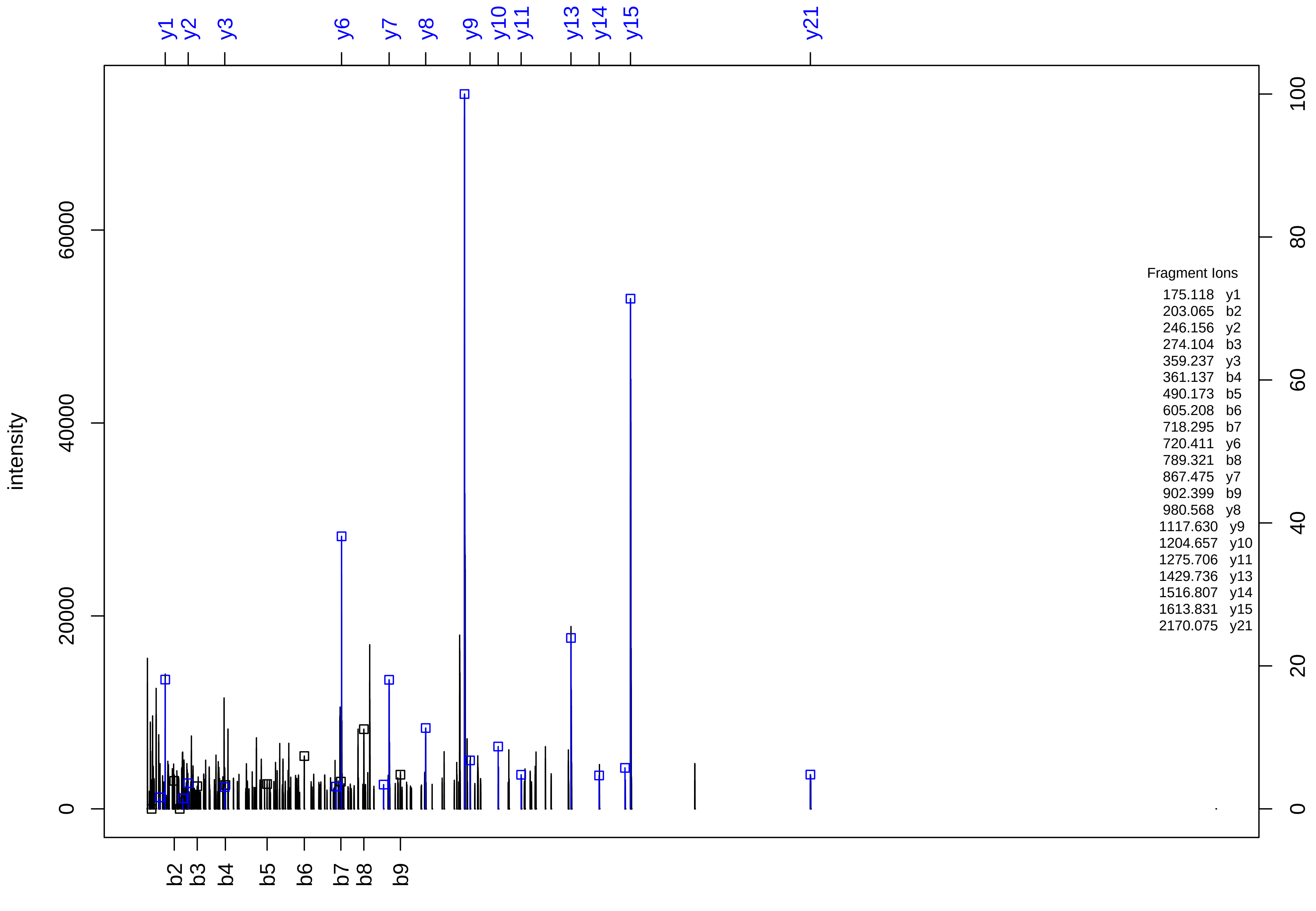

DSASEDLALVEPGSDSLPSPGASHLFPTYLAR / File: christine_10pools_04.raw; SpectrumID: ; scans: 31454

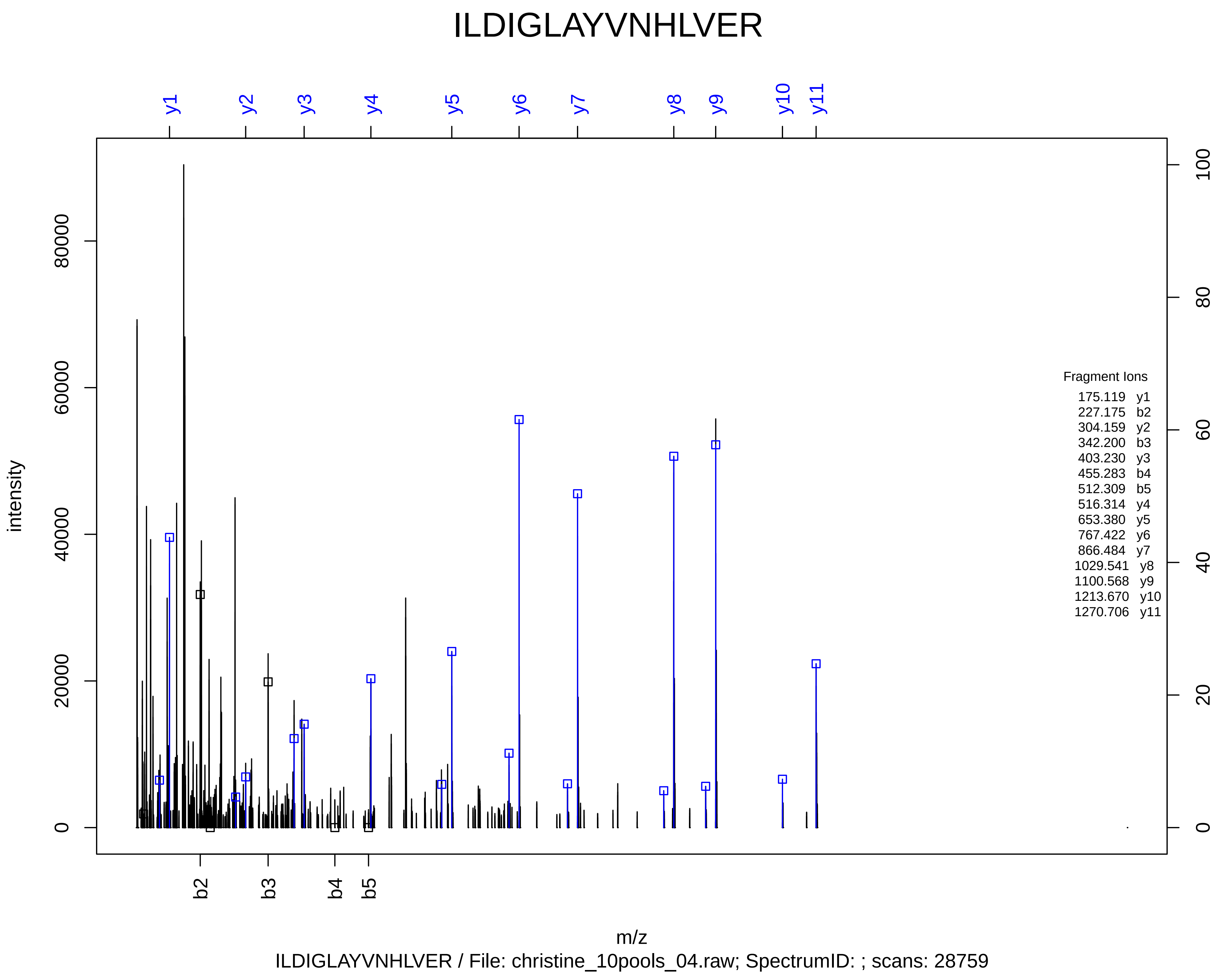
ILDIGLAYVNHLVER
y1
y2
y3
y4
y5
y6
y7
y8
y9
y10
y11
b2
b3
b4
b5
intensity
m/z
Fragment Ions
175.119 y1
227.175 b2
304.159 y2
342.200 b3
403.230 y3
455.283 b4
512.309 b5
516.314 y4
653.380 y5
767.422 y6
866.484 y7
1029.541 y8
1100.568 y9
1213.670 y10
1270.706 y11
ILDIGLAYVNHLVER / File: christine_10pools_04.raw; SpectrumID: ; scans: 28759

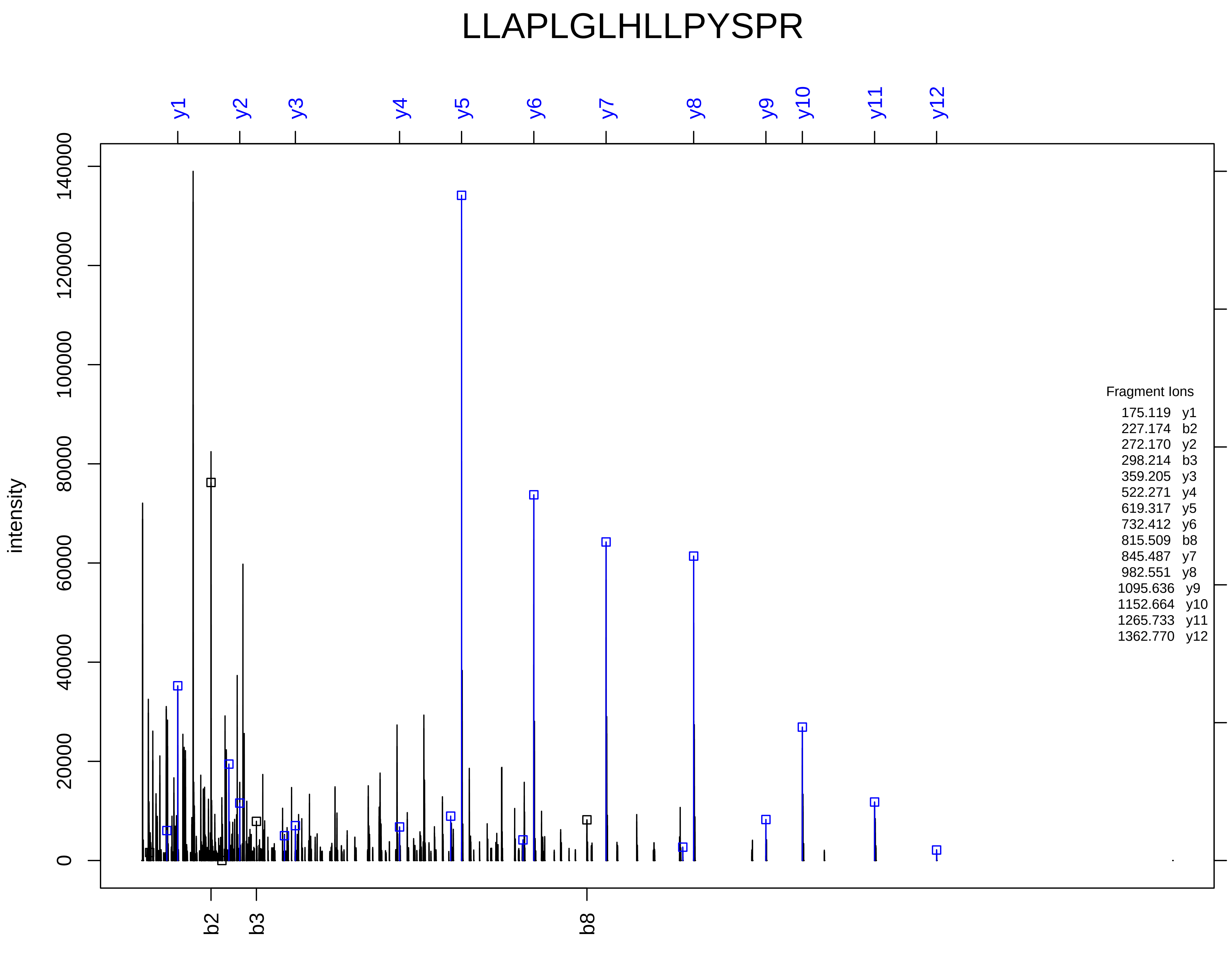

LLAPLGLHLLPYSPR / File: christine_10pools_04.raw; SpectrumID: ; scans: 29285

GLGPSLTEDQPGPYLAPGLLGSNIHQQGR

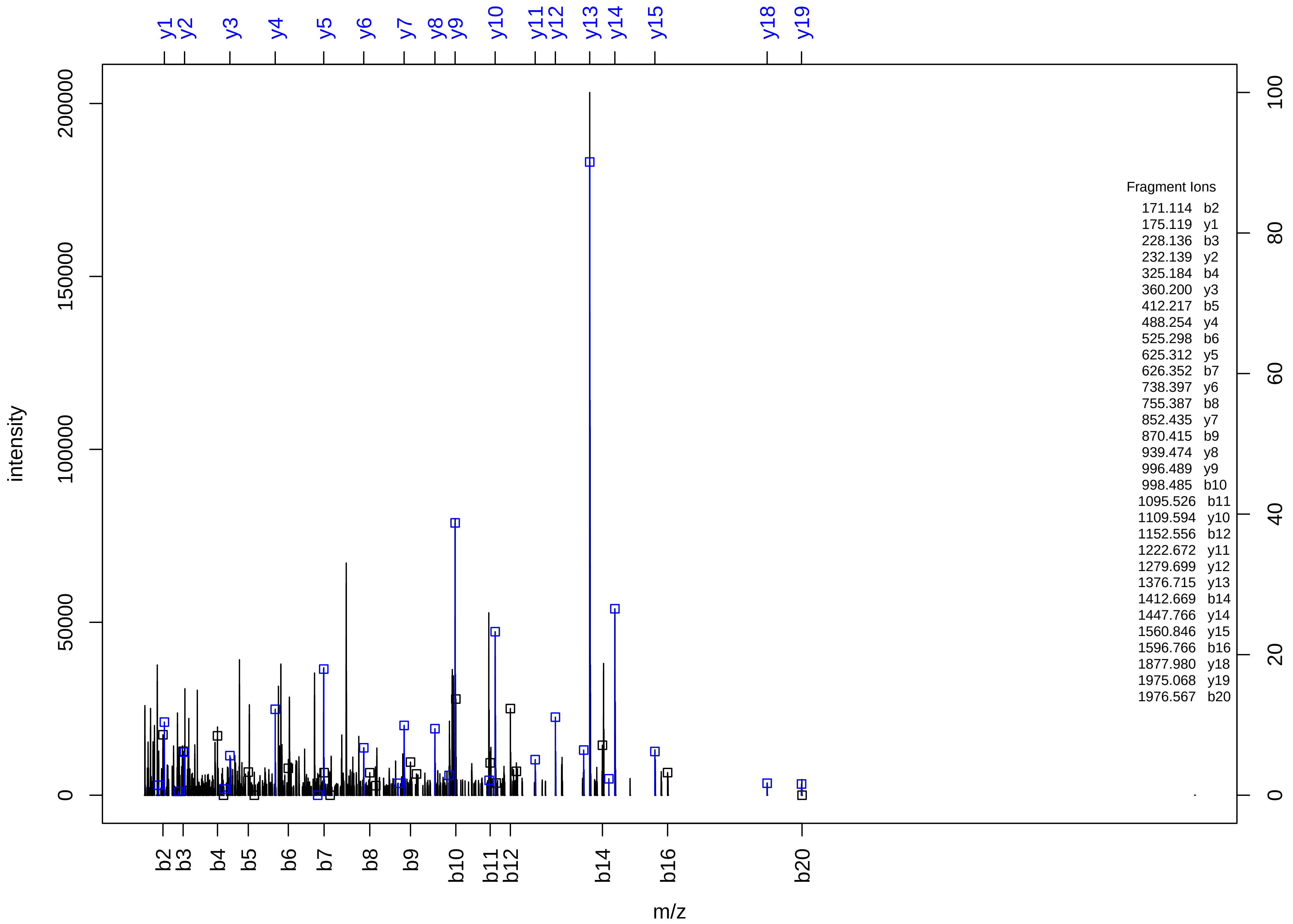

GLGPSLTEDQPGPYLAPGLLGSNIHQQGR / File: christine_10pools_04.raw; SpectrumID: ; scans: 24192

LVLSYVLEGLK

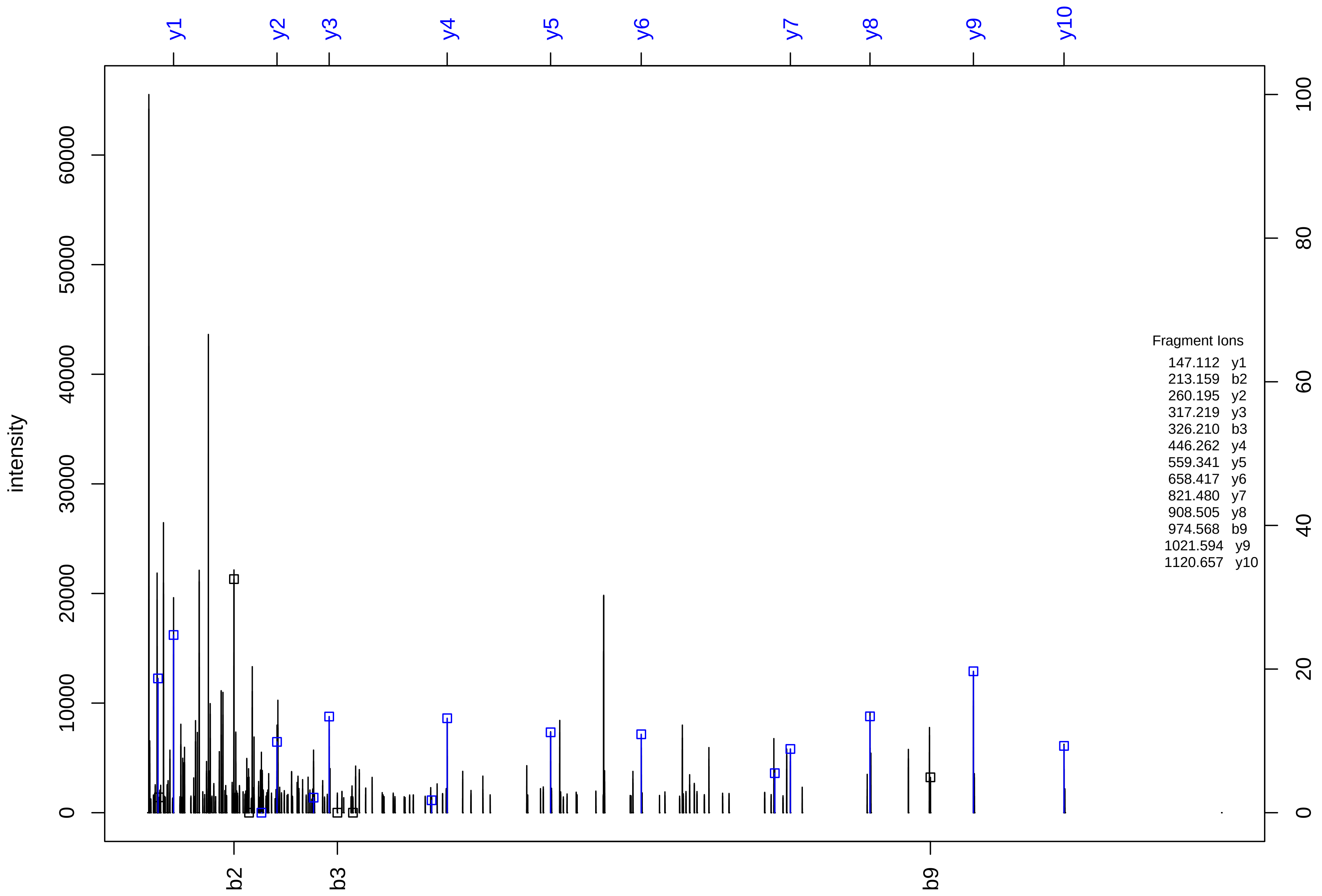

LVLSYVLEGLK / File: christine_10pools_04.raw; SpectrumID: ; scans: 31195

IADFGFSNLFTPGQLLK

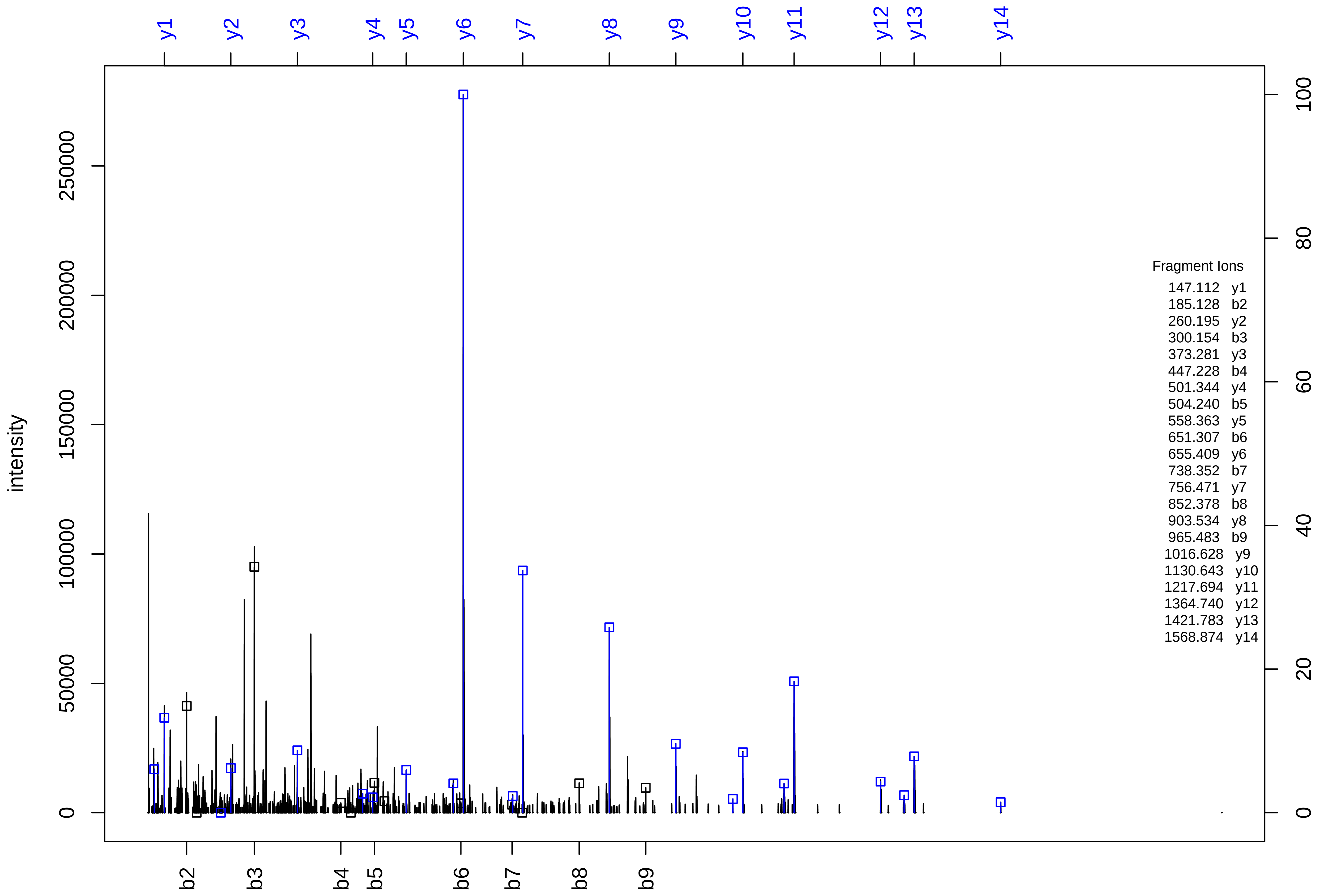

IADFGFSNLFTPGQLLK / File: christine_10pools_04.raw; SpectrumID: ; scans: 40109

LLDPSQIPR

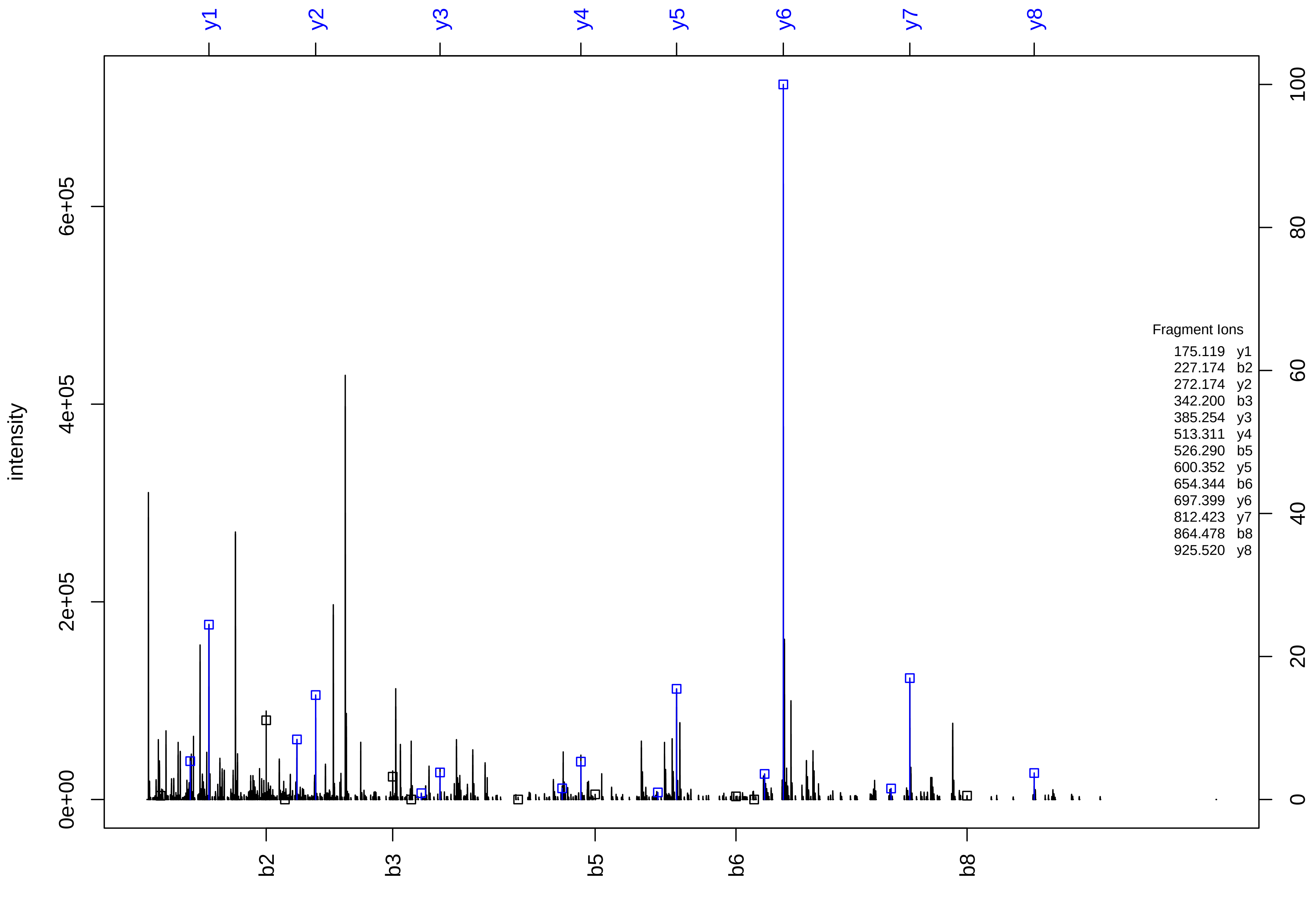

LLDPSQIPR / File: christine_10pools_04.raw; SpectrumID: ; scans: 11493

GLGTPFLVPVPARPPLTR

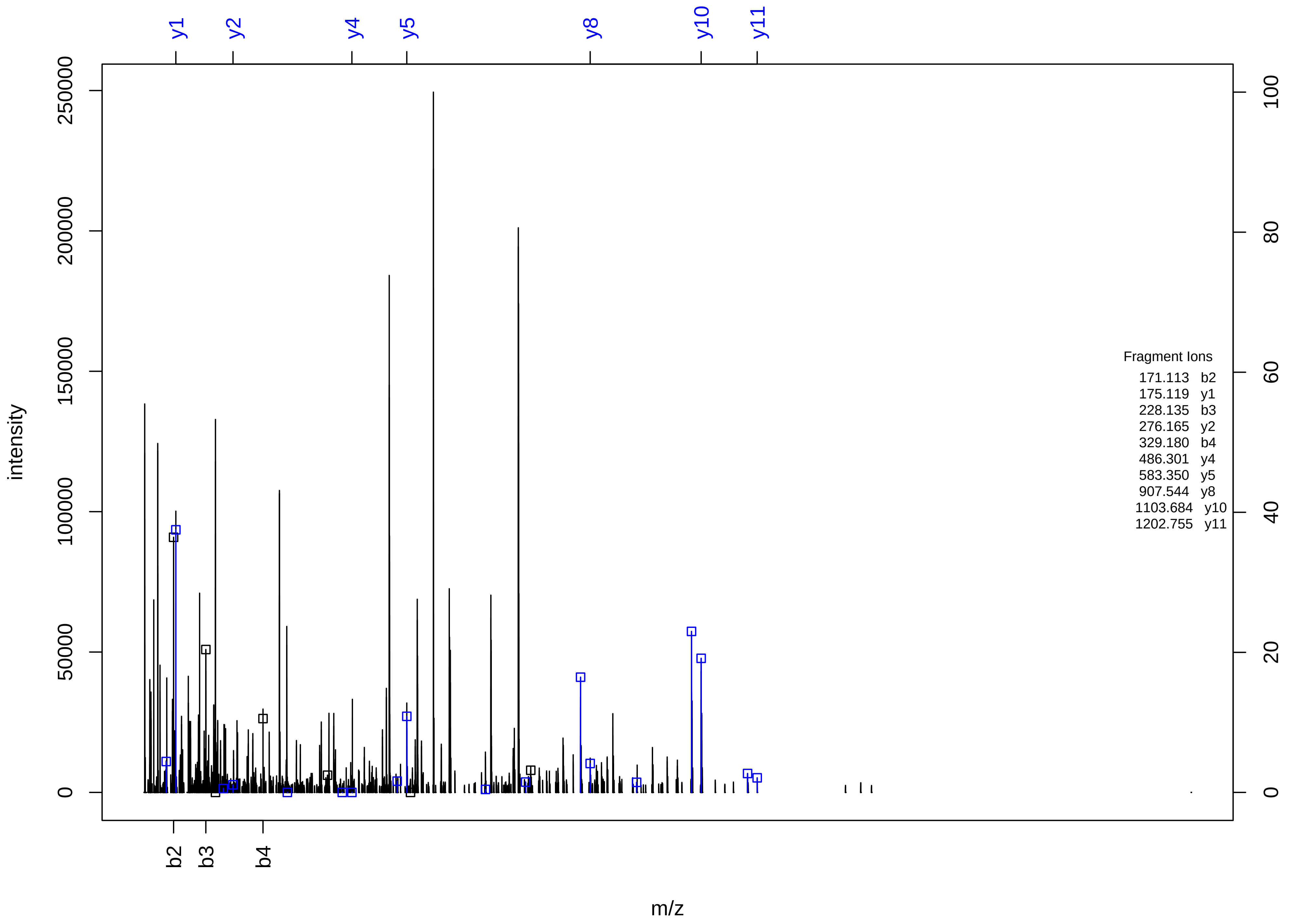

GLGTPFLVPVPARPPLTR / File: christine_10pools_04.raw; SpectrumID: ; scans: 25336

EEPVLPSQVEVVEYSTLALPQEELHYSSVAFNSQR

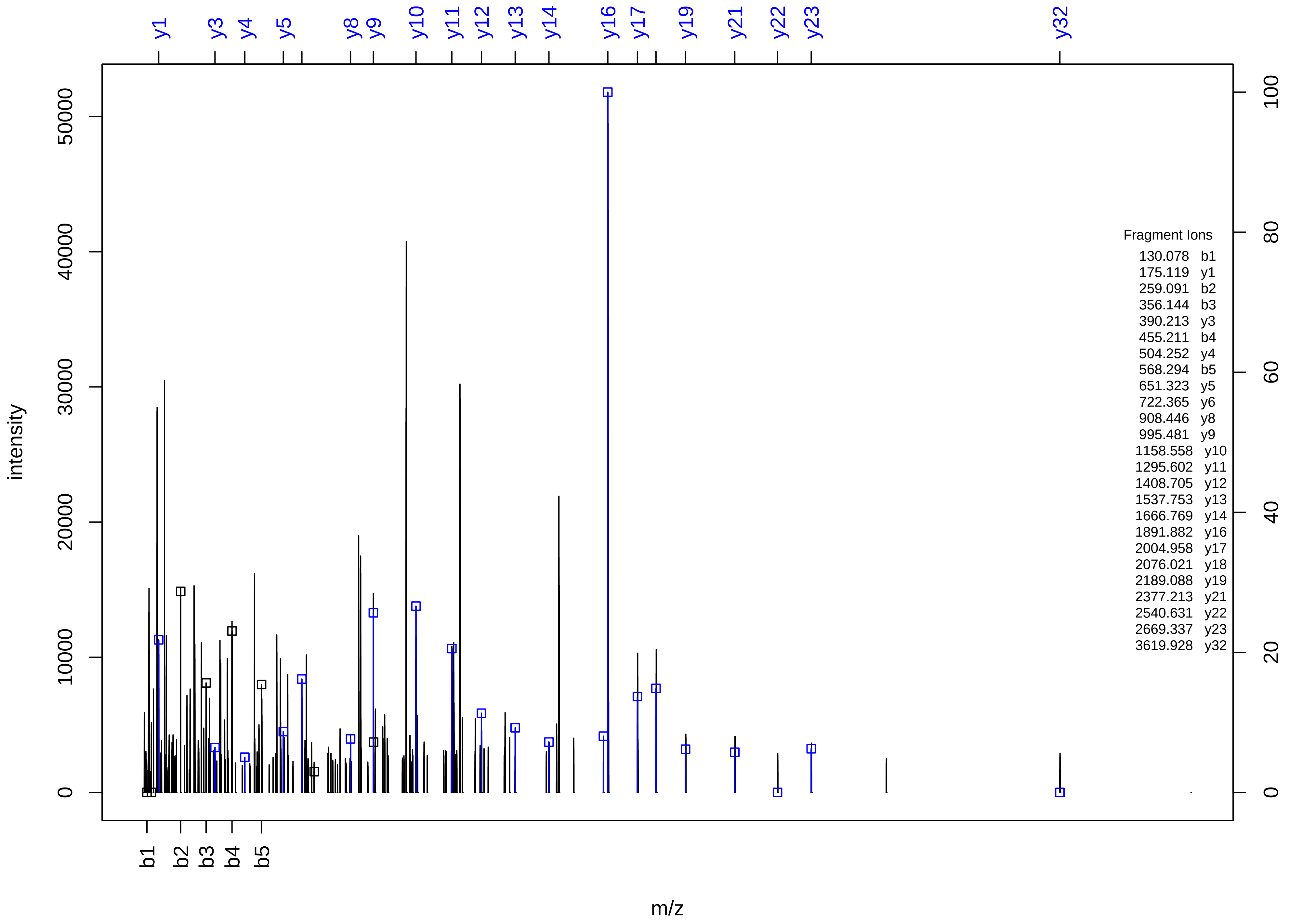

EEPVLPSQVEVVEYSTLALPQEELHYSSVAFNSQR / File: christine_10pools_04.raw; SpectrumID: ; scans: 38274

FDLIWNR

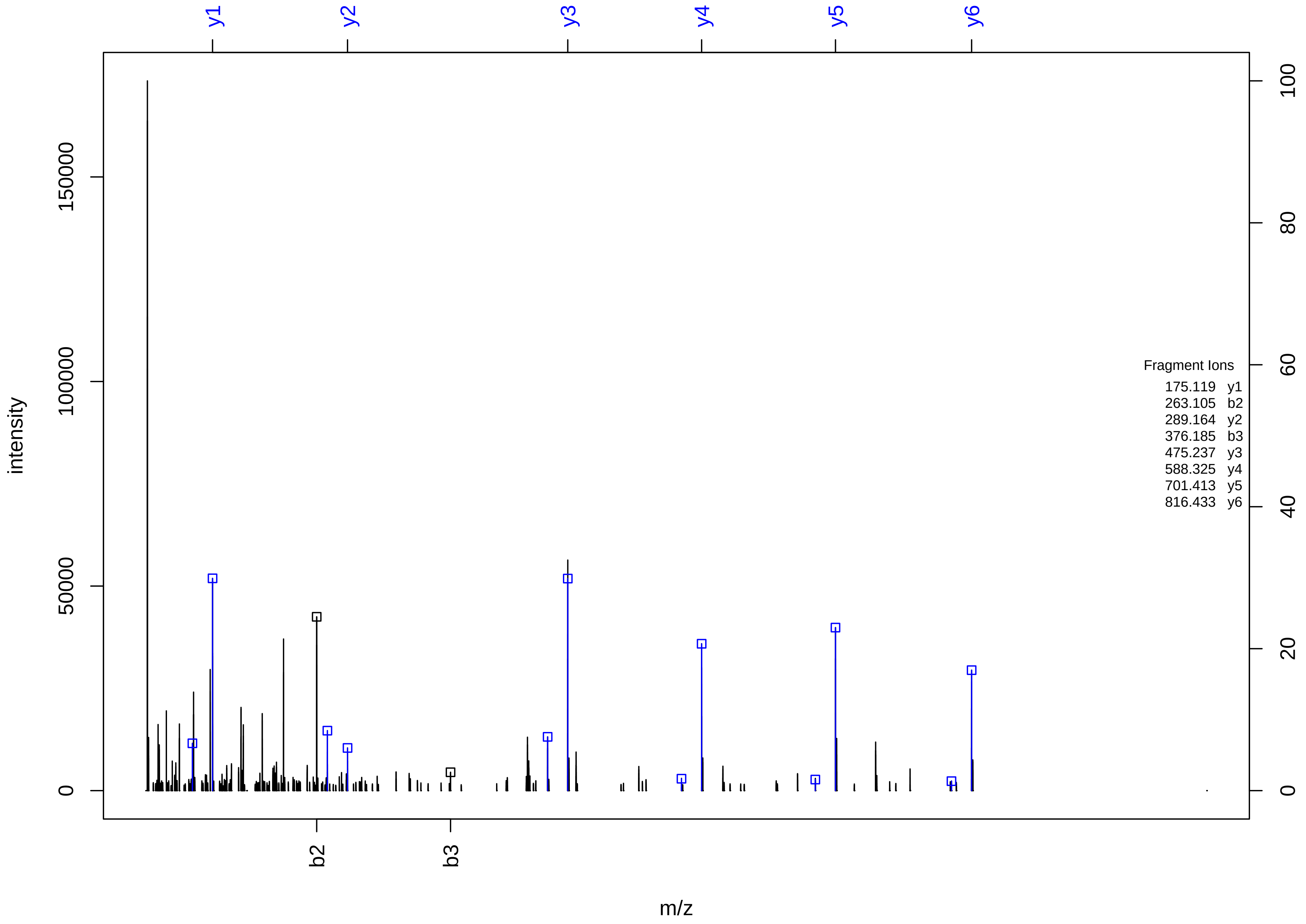

FDLIWNR / File: christine_10pools_04.raw; SpectrumID: ; scans: 21630

DVVDLLR

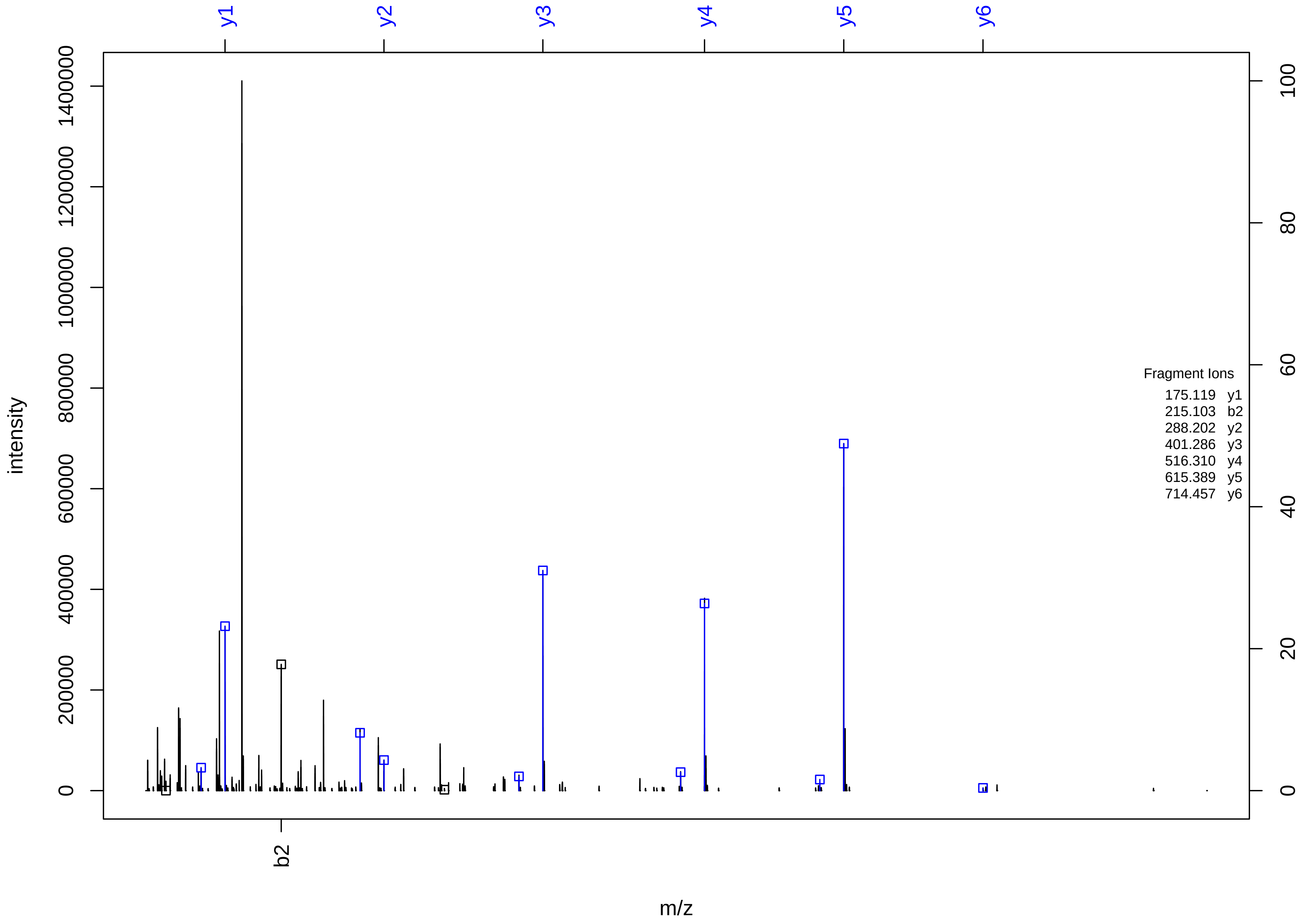

DVVDLLR / File: christine_10pools_04.raw; SpectrumID: ; scans: 16677

DDSNSTQEQAAAQGTTK

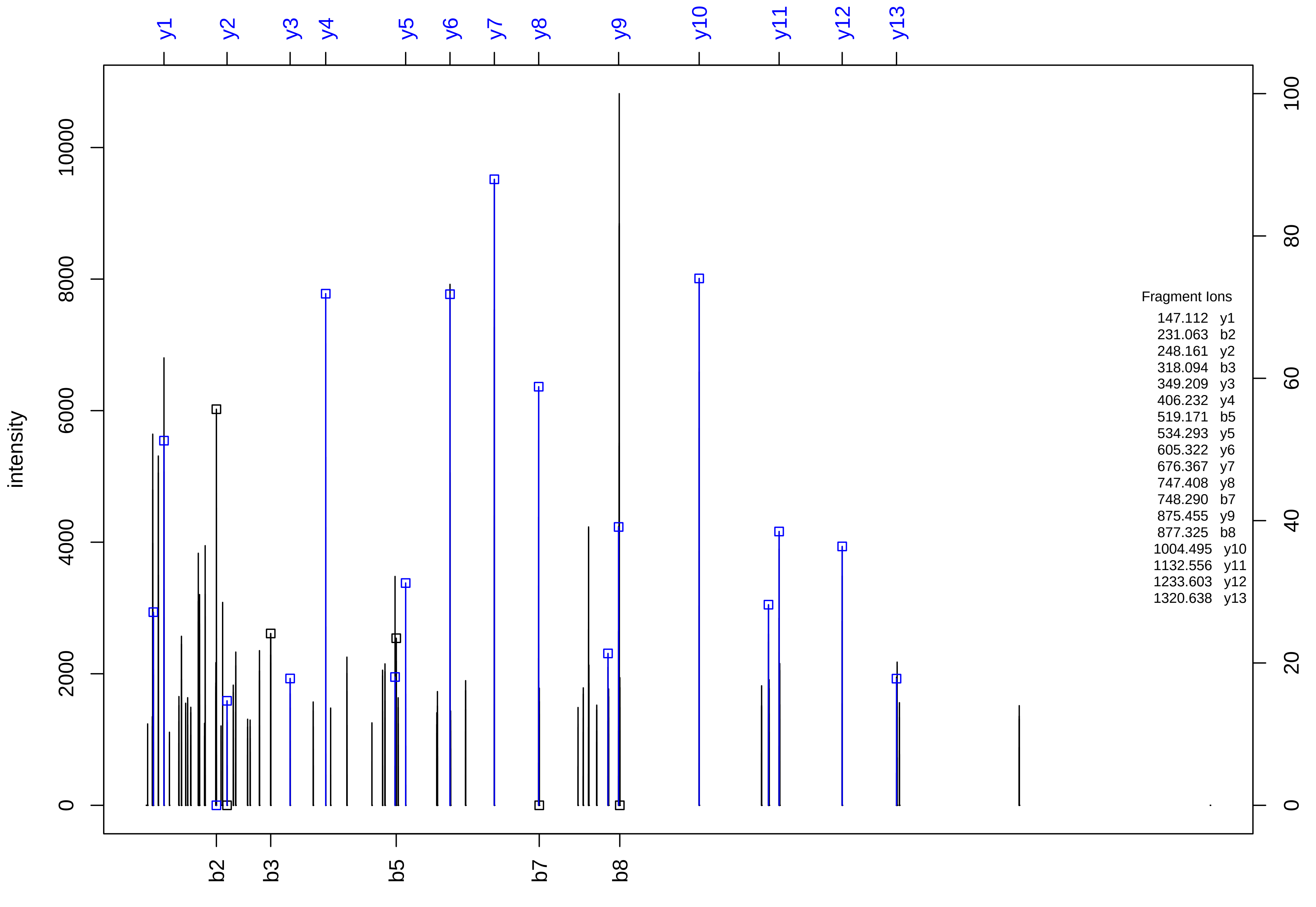

DDSNSTQEQAAAQGTTK / File: christine_10pools_04.raw; SpectrumID: ; scans: 1098

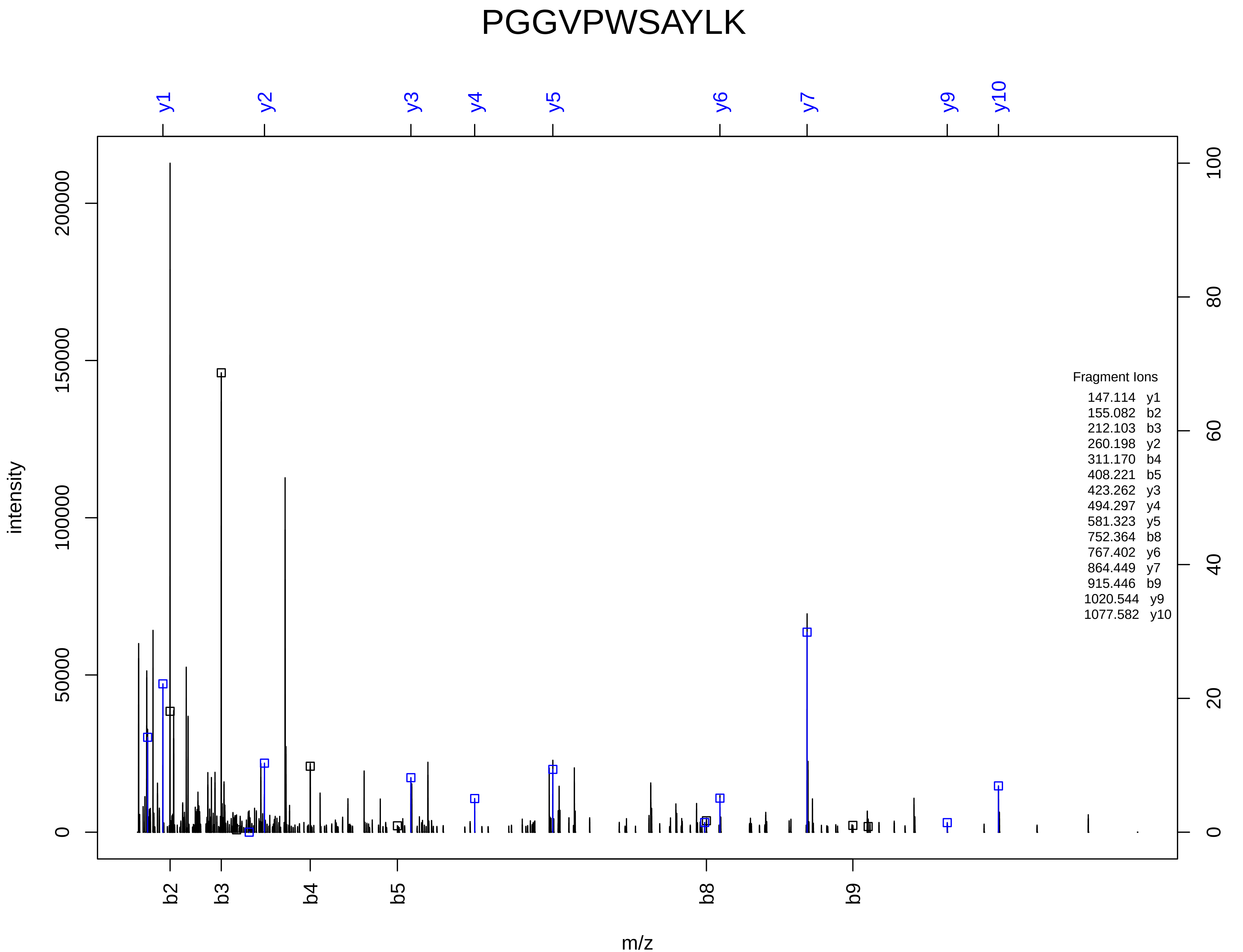

PGGVPWSAYLK / File: christine_10pools_04.raw; SpectrumID: ; scans: 20336

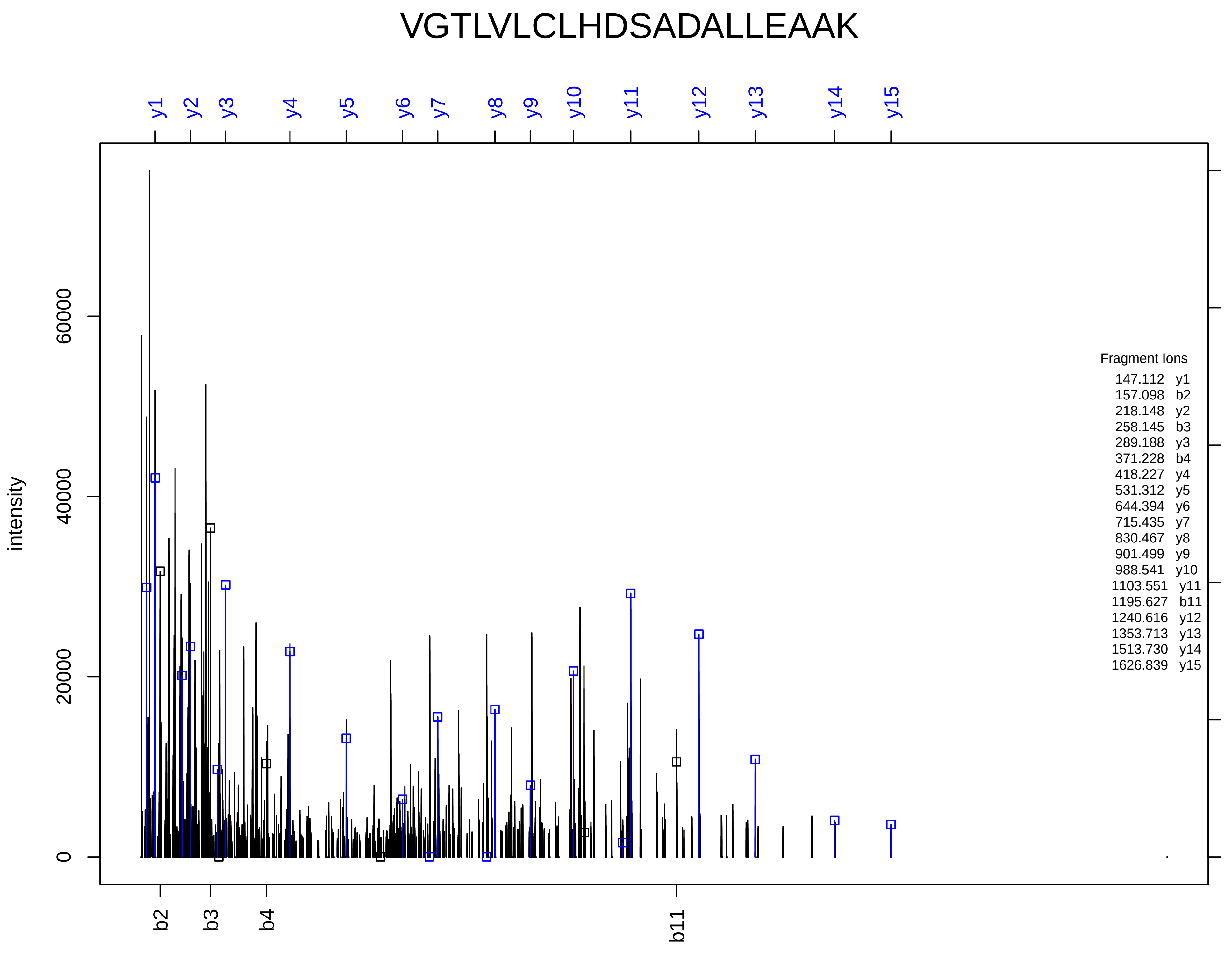

VGTLVLCLHDSADALLEAAK / File: christine_10pools_04.raw; SpectrumID: ; scans: 29958

LLVN^IIR

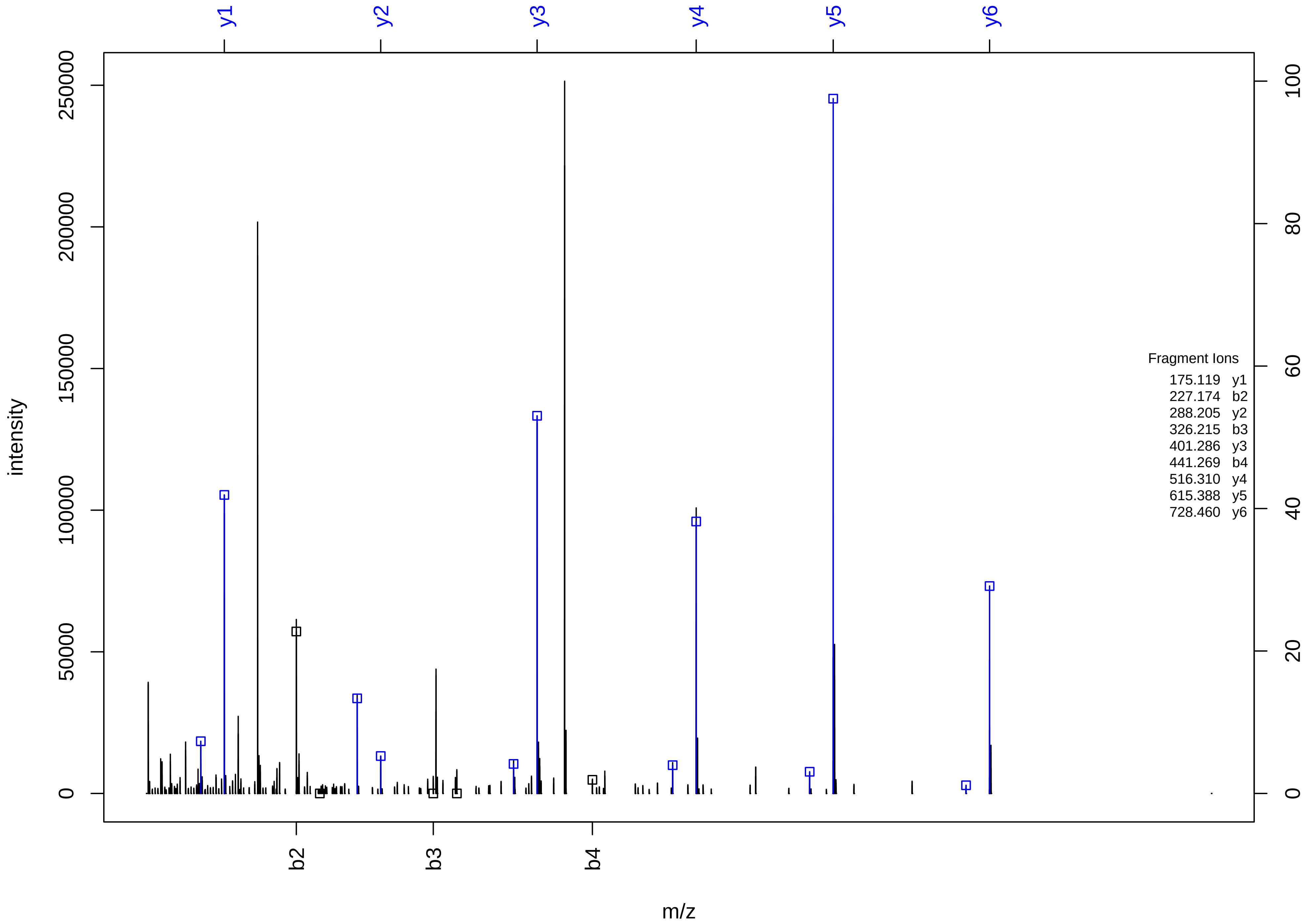

LLVNIIR / File: christine_10pools_04.raw; SpectrumID: ; scans: 22285

(Ac)SDILPLDVIGR

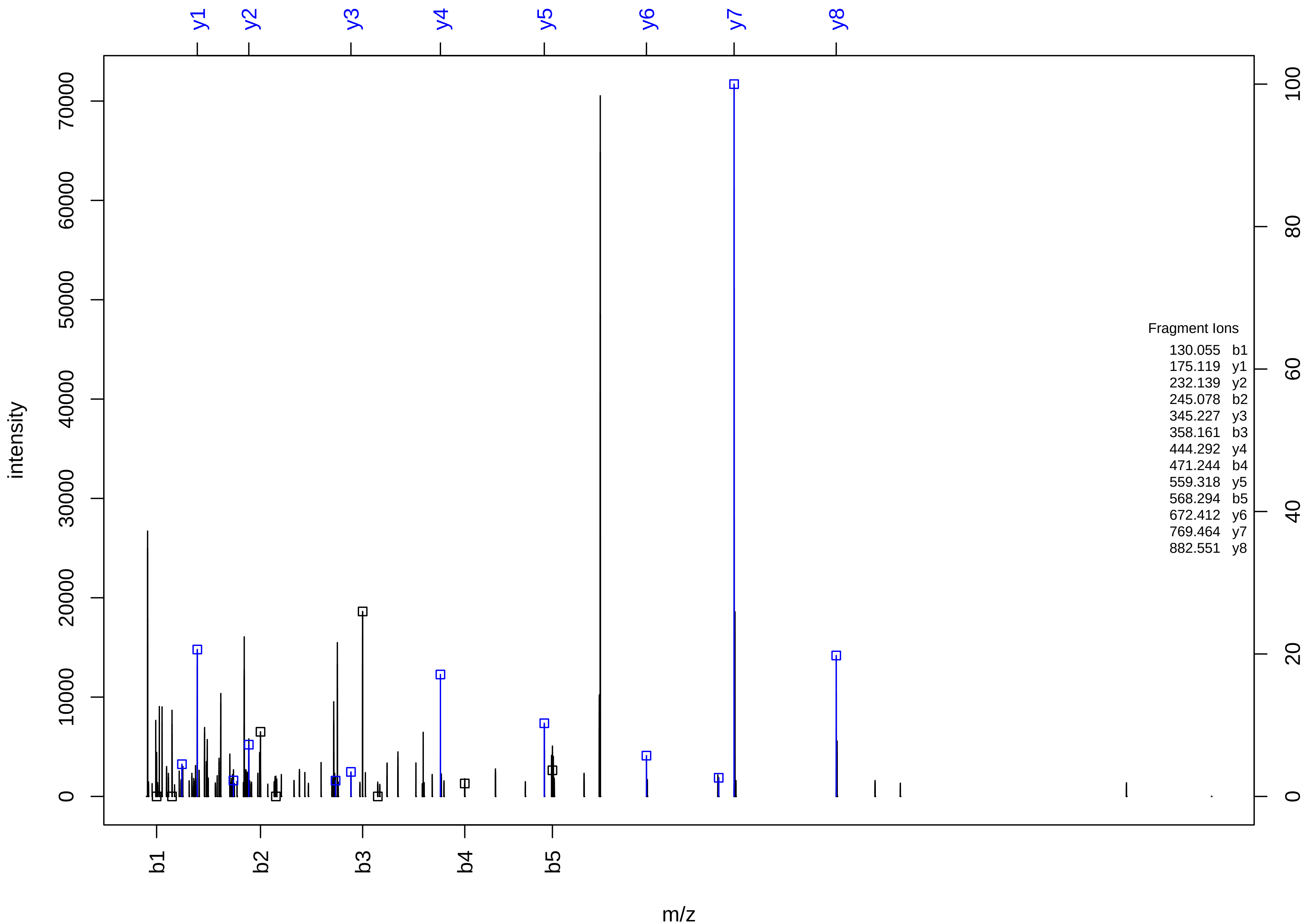

SDILPLDVIGR / File: christine_10pools_04.raw; SpectrumID: ; scans: 38912

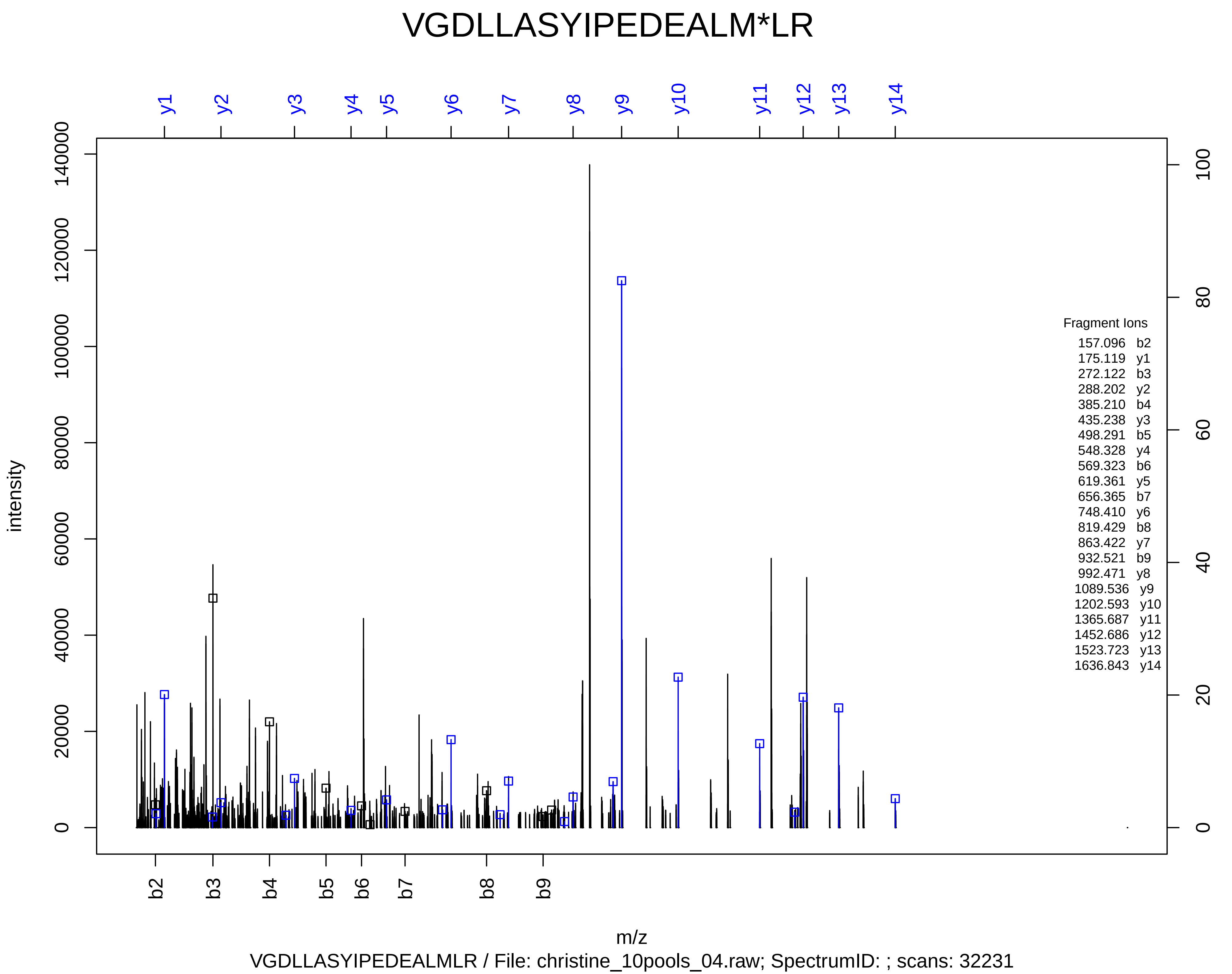

VGDLLASYIPEDEALM*LR
intensity
m/z
y1 y2 y3 y4 y5 y6 y7 y8 y9 y10 y11 y12 y13 y14
b2 b3 b4 b5 b6 b7 b8 b9
Fragment Ions
157.096 b2
175.119 y1
272.122 b3
288.202 y2
385.210 b4
435.238 y3
498.291 b5
548.328 y4
569.323 b6
619.361 y5
656.365 b7
748.410 y6
819.429 b8
863.422 y7
932.521 b9
992.471 y8
1089.536 y9
1202.593 y10
1365.687 y11
1452.686 y12
1523.723 y13
1636.843 y14
VGDLLASYIPEDEALMLR / File: christine_10pools_04.raw; SpectrumID: ; scans: 32231

LEGAEIEAFLR

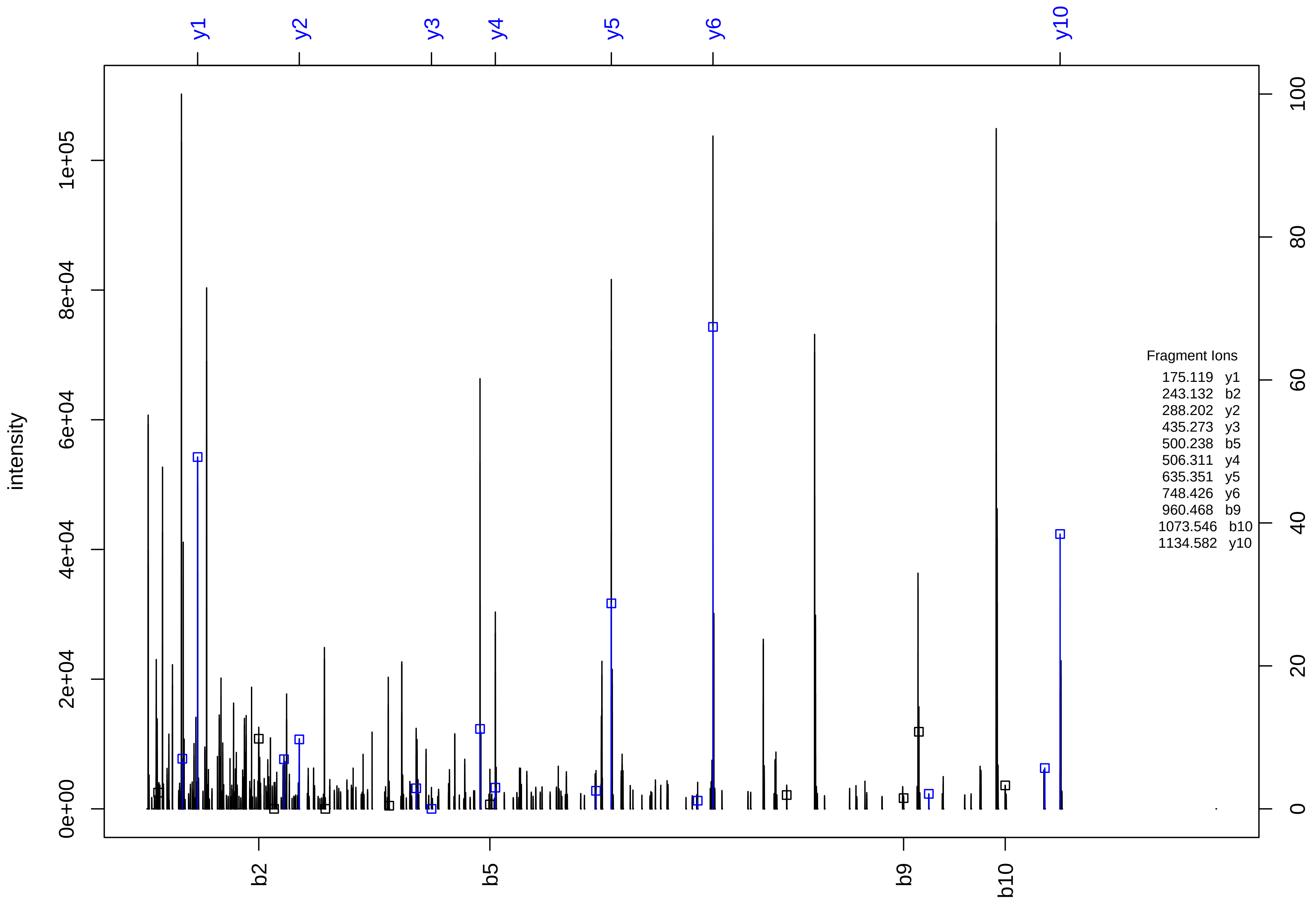

LEGAEIEAFLR / File: christine_10pools_04.raw; SpectrumID: ; scans: 28968

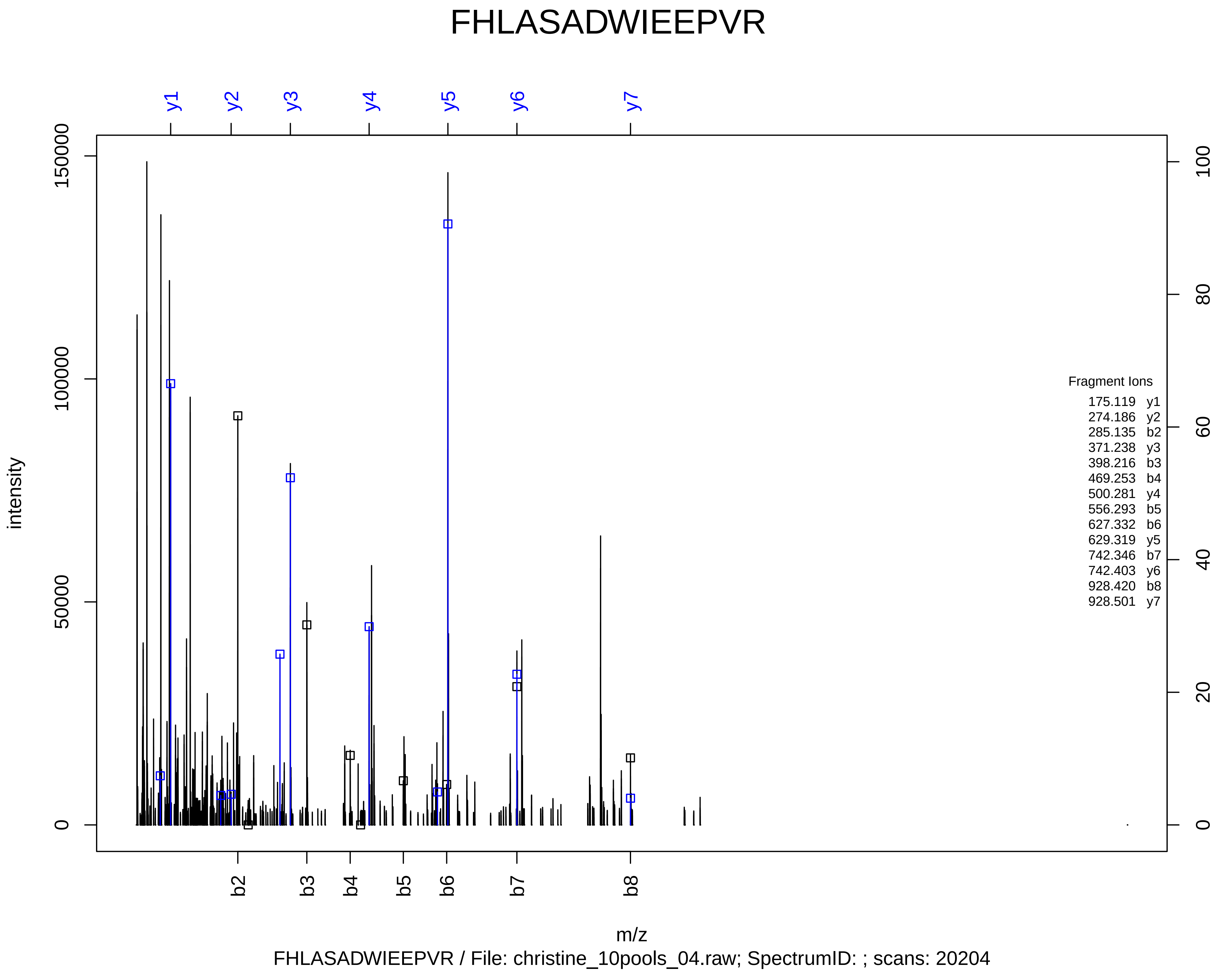

FHLASADWIEEPVR
y1
y2
y3
y4
y5
y6
y7
intensity
150000
100000
50000
0
100
80
60
40
20
0
Fragment Ions
175.119 y1
274.186 y2
285.135 b2
371.238 y3
398.216 b3
469.253 b4
500.281 y4
556.293 b5
627.332 b6
629.319 y5
742.346 b7
742.403 y6
928.420 b8
928.501 y7
b2
b3
b4
b5
b6
b7
b8
m/z
FHLASADWIEEPVR / File: christine_10pools_04.raw; SpectrumID: ; scans: 20204

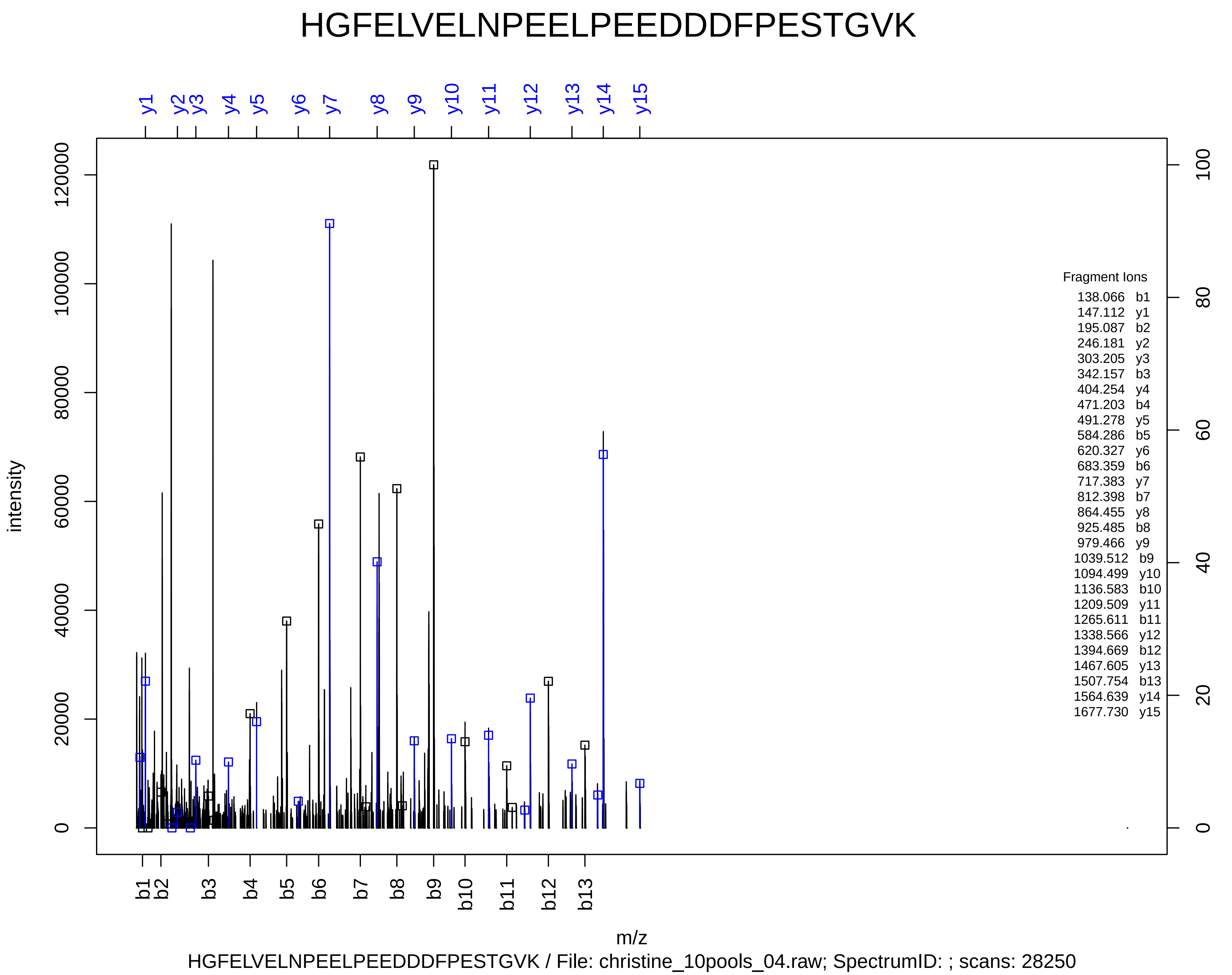

HGFELVELNPEELPEEDDDFPESTGVK
y1 y2 y3 y4 y5 y6 y7 y8 y9 y10 y11 y12 y13 y14 y15
intensity
0 20000 40000 60000 80000 100000 120000
0 20 40 60 80 100
Fragment Ions
138.066 b1
147.112 y1
195.087 b2
246.181 y2
303.205 y3
342.157 b3
404.254 y4
471.203 b4
491.278 y5
584.286 b5
620.327 y6
683.359 b6
717.383 y7
812.398 b7
864.455 y8
925.485 b8
979.466 y9
1039.512 b9
1094.499 y10
1136.583 b10
1209.509 y11
1265.611 b11
1338.566 y12
1394.669 b12
1467.605 y13
1507.754 b13
1564.639 y14
1677.730 y15
b1 b2 b3 b4 b5 b6 b7 b8 b9 b10 b11 b12 b13
m/z
HGFELVELNPEELPEEDDDFPESTGVK / File: christine_10pools_04.raw; SpectrumID: ; scans: 28250

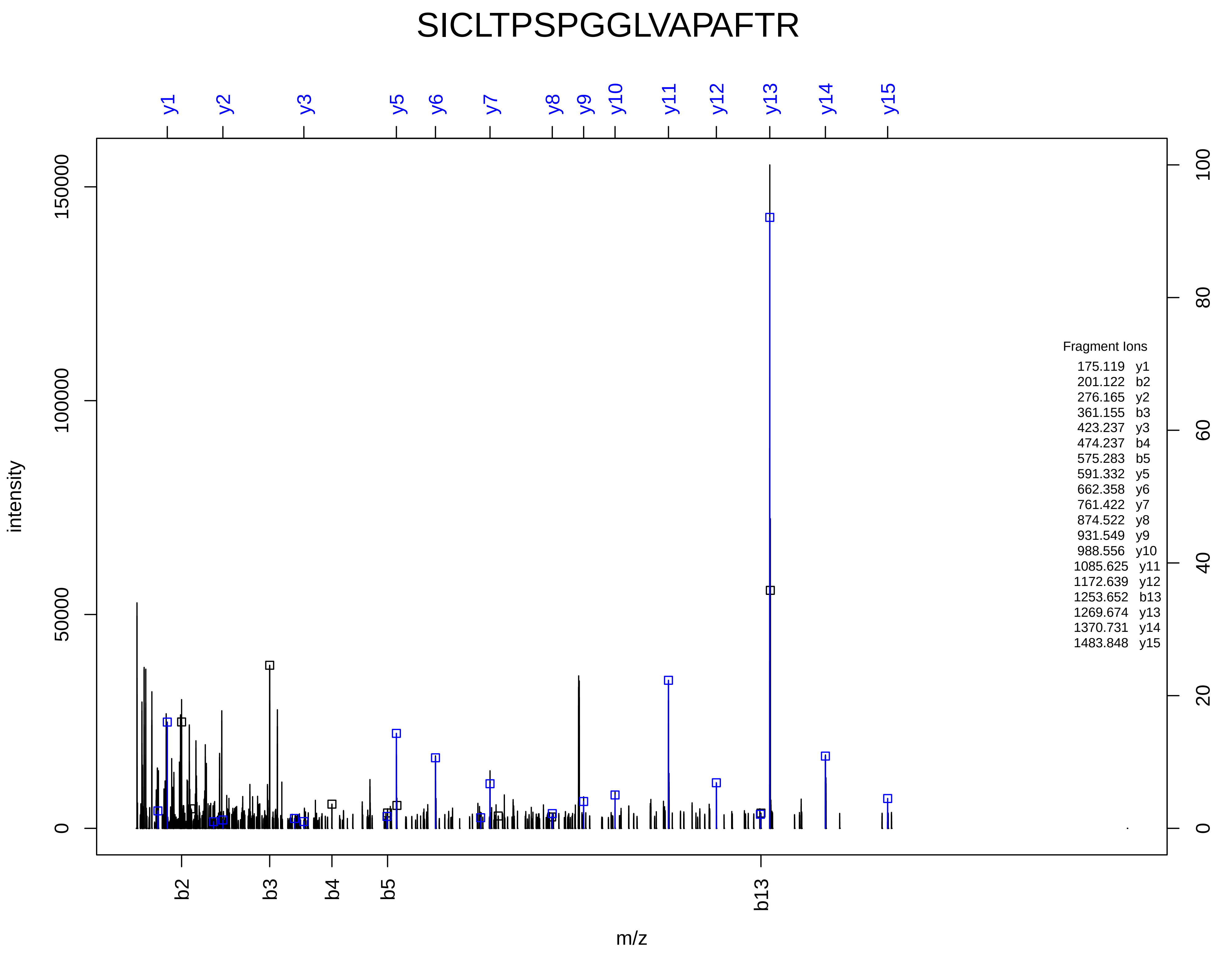

SICLTPSPGGLVAPAFTR / File: christine_10pools_04.raw; SpectrumID: ; scans: 26097

SVISVIHLITAAR

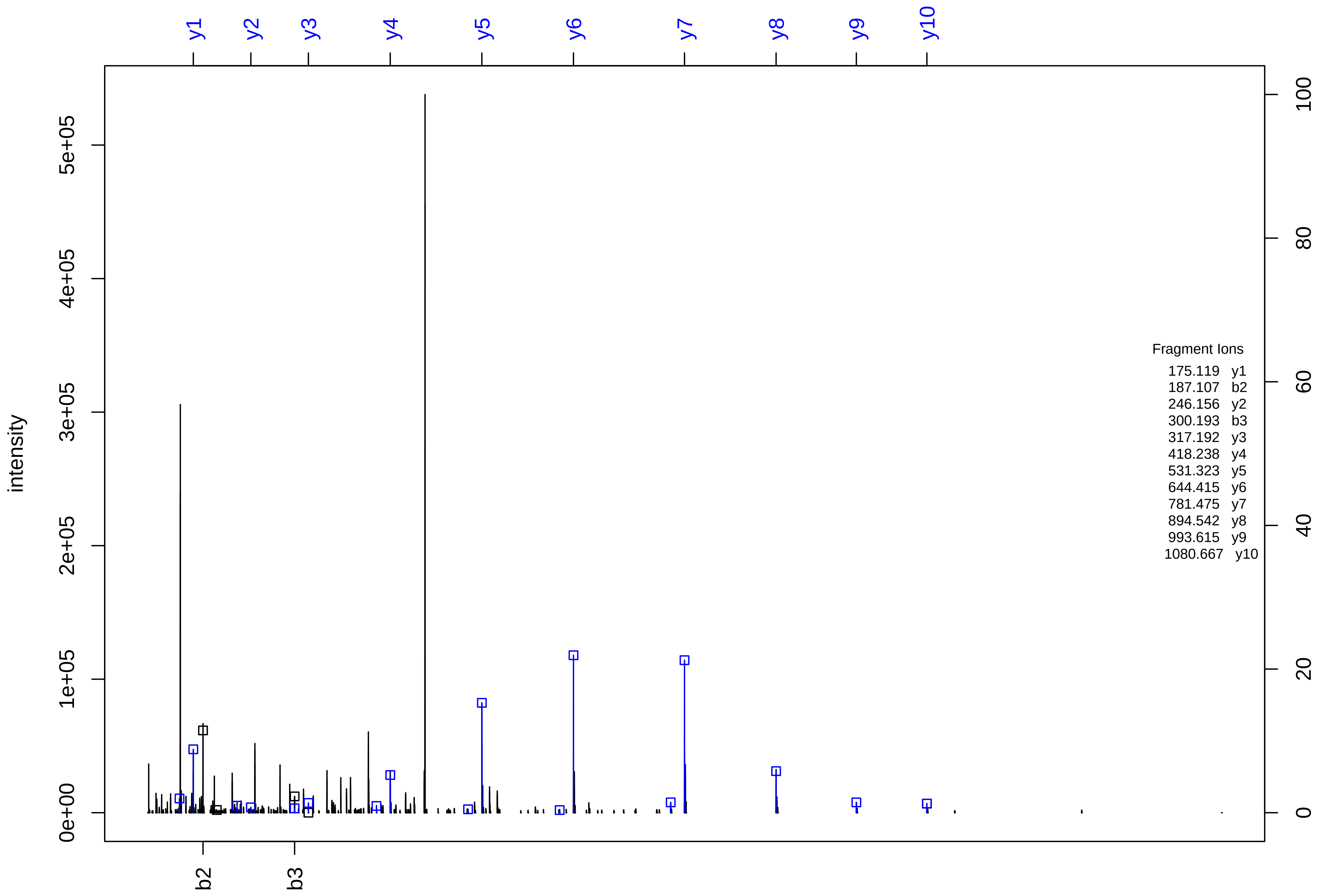

SVISVIHLITAAR / File: christine_10pools_04.raw; SpectrumID: ; scans: 26303

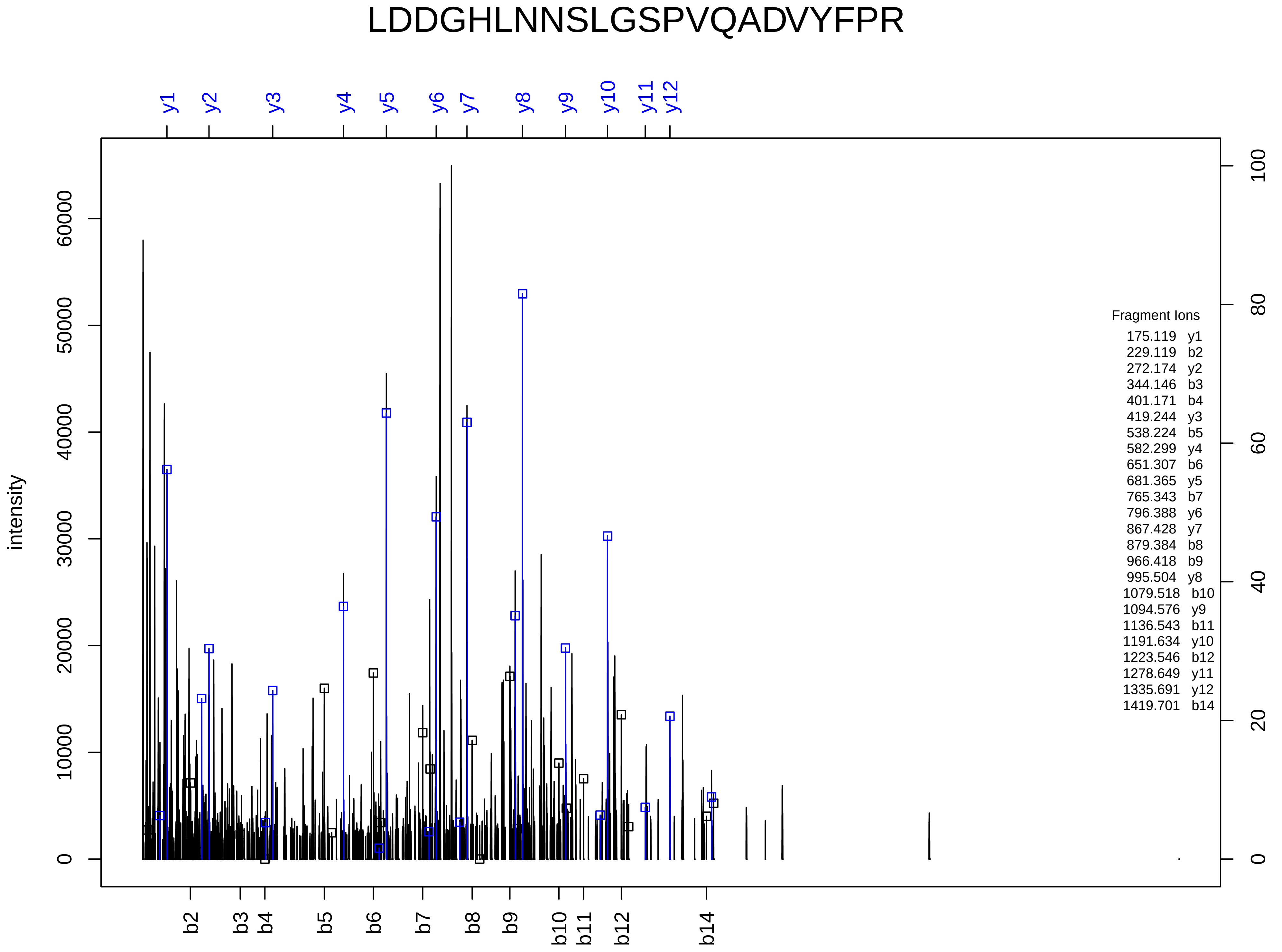

LDDGHLNNSLGSPVQADVYFPR / File: christine_10pools_04.raw; SpectrumID: ; scans: 20538

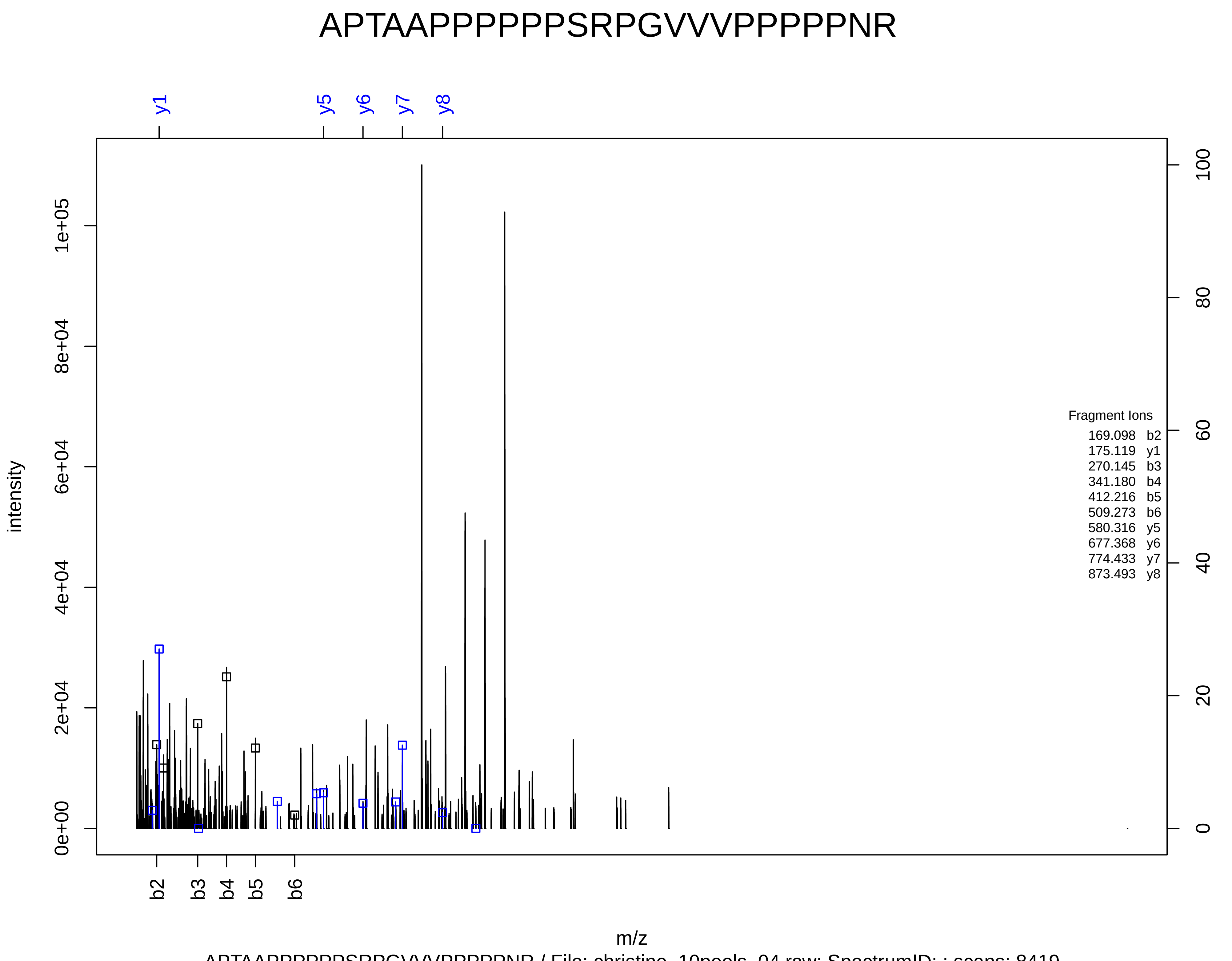
APTAAPPPPPPSRPGVVVPPPPPNR
y1
y5
y6
y7
y8
intensity
0e+00
2e+04
4e+04
6e+04
8e+04
1e+05
0
20
40
60
80
100
b2
b3
b4
b5
b6
m/z
Fragment Ions
169.098 b2
175.119 y1
270.145 b3
341.180 b4
412.216 b5
509.273 b6
580.316 y5
677.368 y6
774.433 y7
873.493 y8
APTAAPPPPPPSRPGVVVPPPPPNR / File: christine_10pools_04.raw; SpectrumID: ; scans: 8419

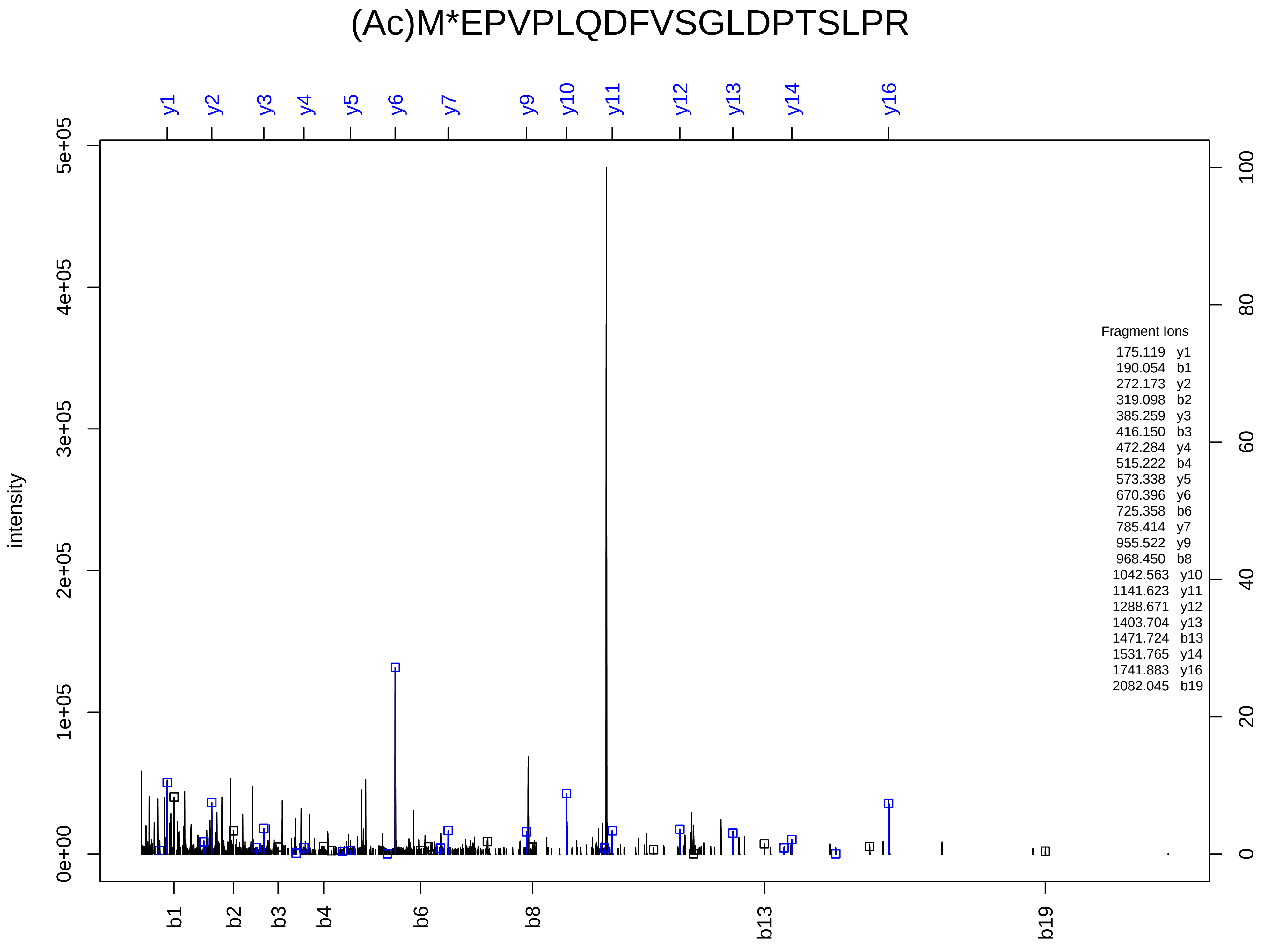

MEPVPLQDFVSGLDPTSLPR / File: christine_10pools_04.raw; SpectrumID: ; scans: 41694

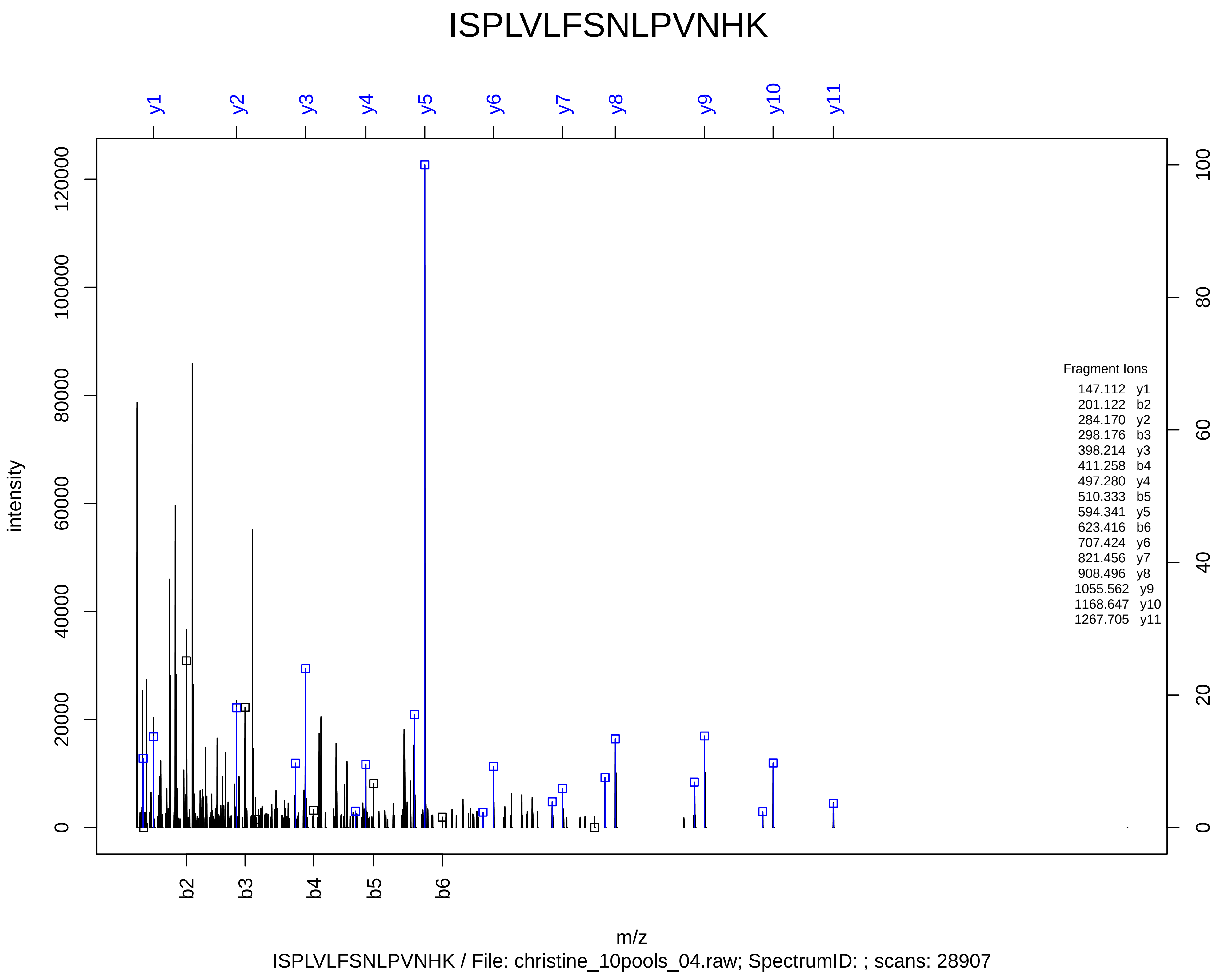

ISPLVLFSNLPVNHK
y1
y2
y3
y4
y5
y6
y7
y8
y9
y10
y11
intensity
0
20000
40000
60000
80000
100000
120000
0
20
40
60
80
100
b2
b3
b4
b5
b6
Fragment Ions
147.112 y1
201.122 b2
284.170 y2
298.176 b3
398.214 y3
411.258 b4
497.280 y4
510.333 b5
594.341 y5
623.416 b6
707.424 y6
821.456 y7
908.496 y8
1055.562 y9
1168.647 y10
1267.705 y11
m/z
ISPLVLFSNLPVNHK / File: christine_10pools_04.raw; SpectrumID: ; scans: 28907

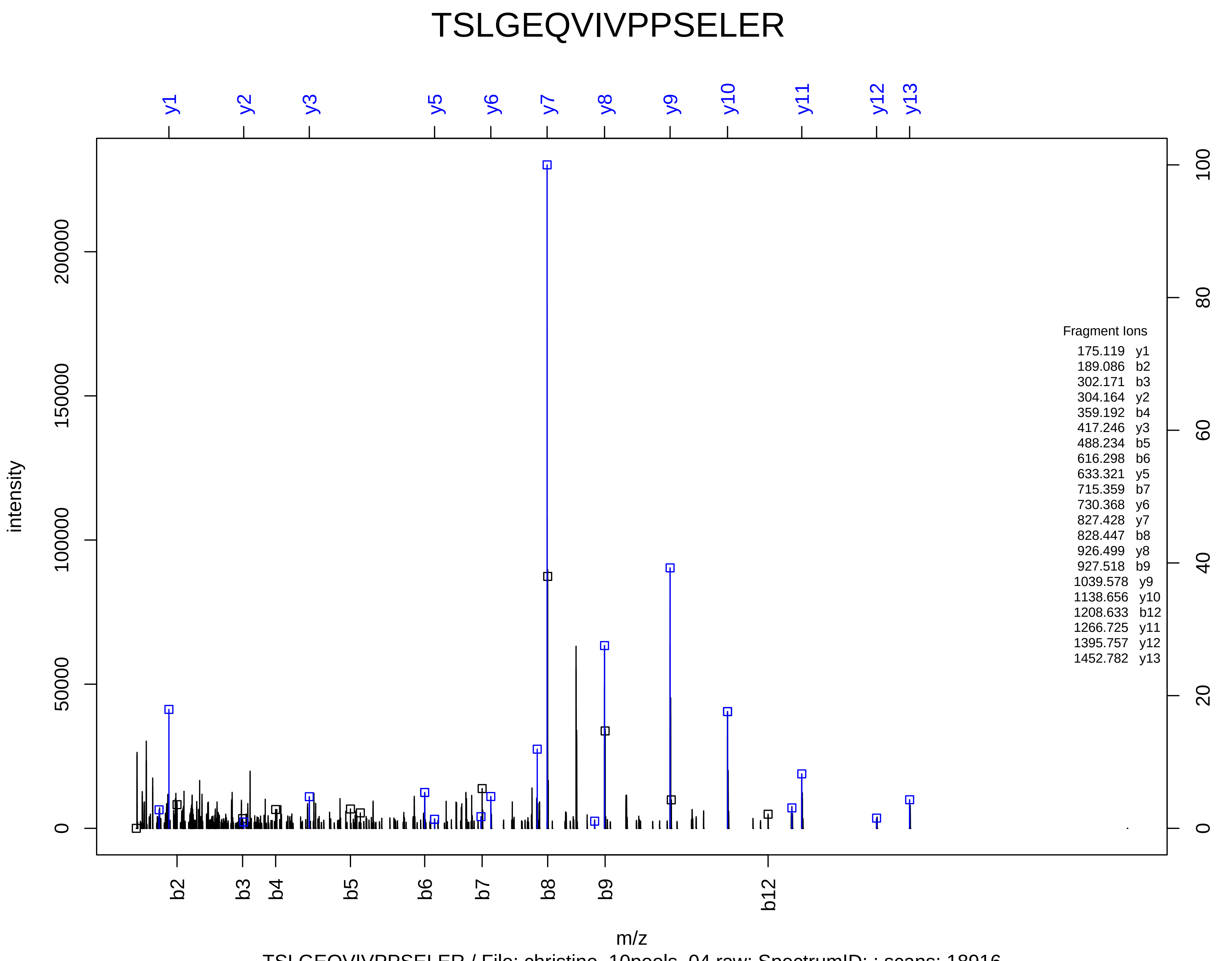
TSLGEQVIVPPSELER
y1
y2
y3
y5
y6
y7
y8
y9
y10
y11
y12
y13
intensity
0
50000
100000
150000
200000
0
20
40
60
80
100
b2
b3
b4
b5
b6
b7
b8
b9
b12
m/z
Fragment Ions
175.119 y1
189.086 b2
302.171 b3
304.164 y2
359.192 b4
417.246 y3
488.234 b5
616.298 b6
633.321 y5
715.359 b7
730.368 y6
827.428 y7
828.447 b8
926.499 y8
927.518 b9
1039.578 y9
1138.656 y10
1208.633 b12
1266.725 y11
1395.757 y12
1452.782 y13
TSLGEQVIVPPSELER / File: christine_10pools_04.raw; SpectrumID: ; scans: 18916

FSVLVPLLAR

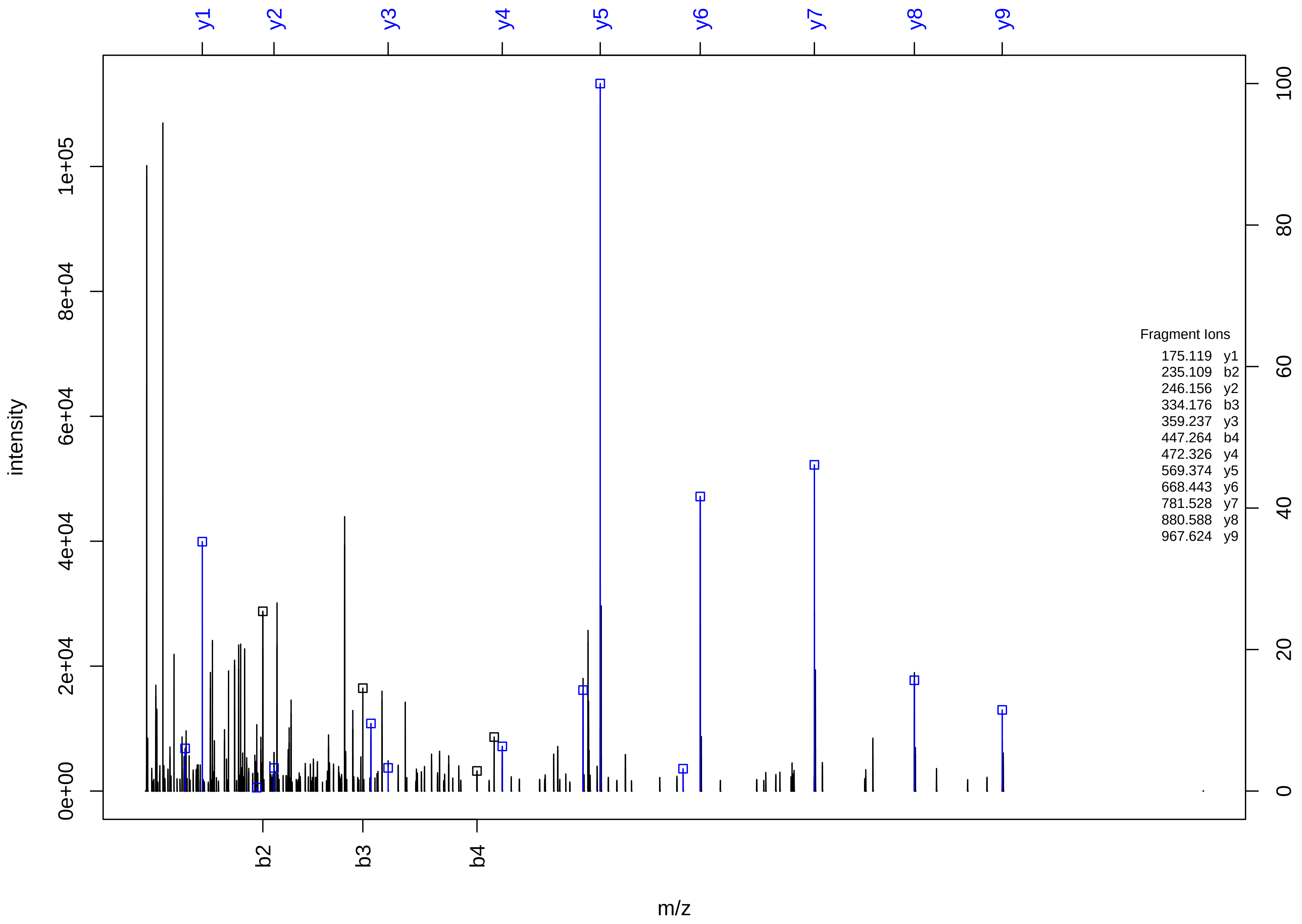

FSVLVPLLAR / File: christine_10pools_04.raw; SpectrumID: ; scans: 31380

LGCQIVLTPELEGVEFALPK

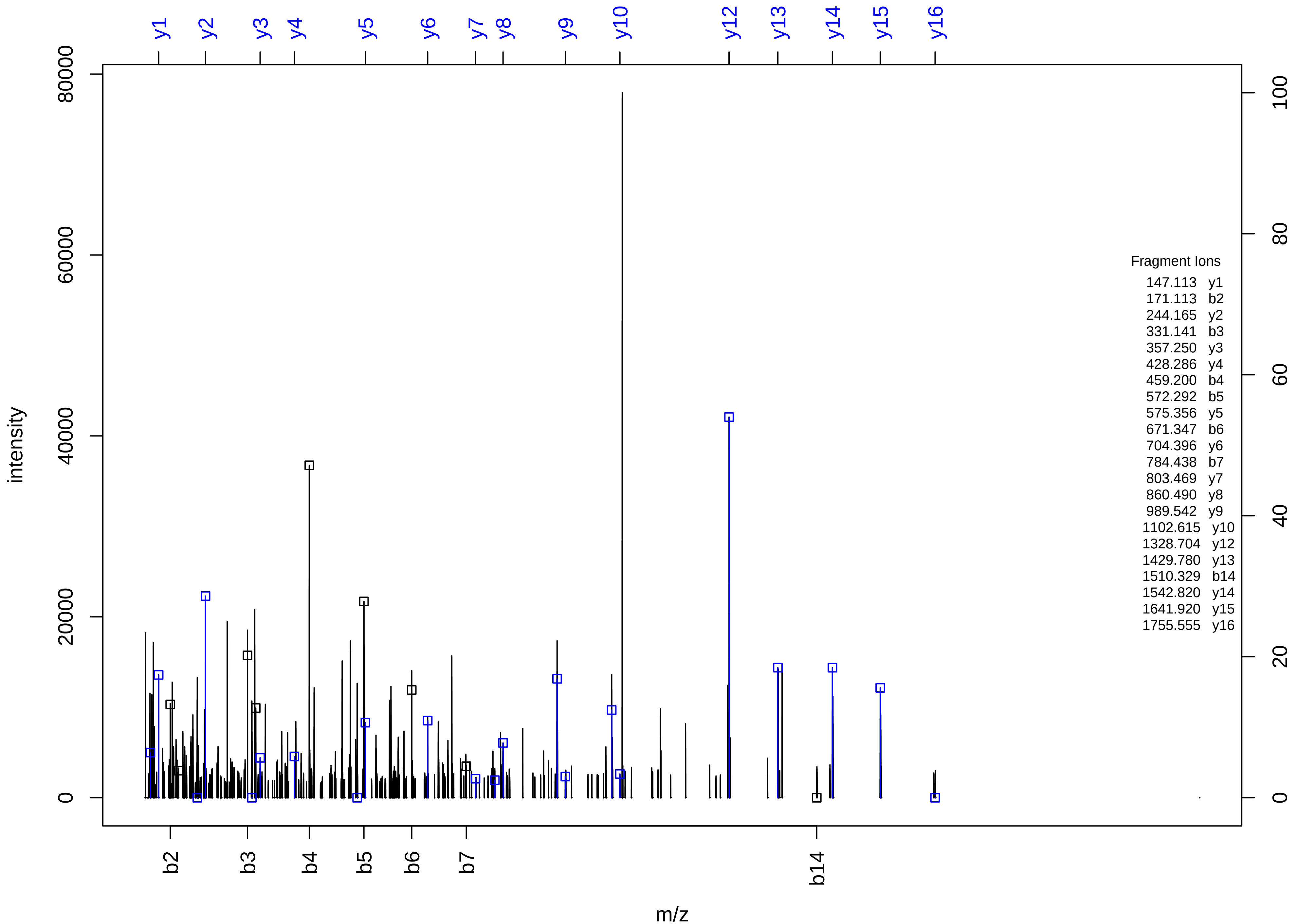

LGCQIVLTPELEGVEFALPK / File: christine_10pools_04.raw; SpectrumID: ; scans: 36217

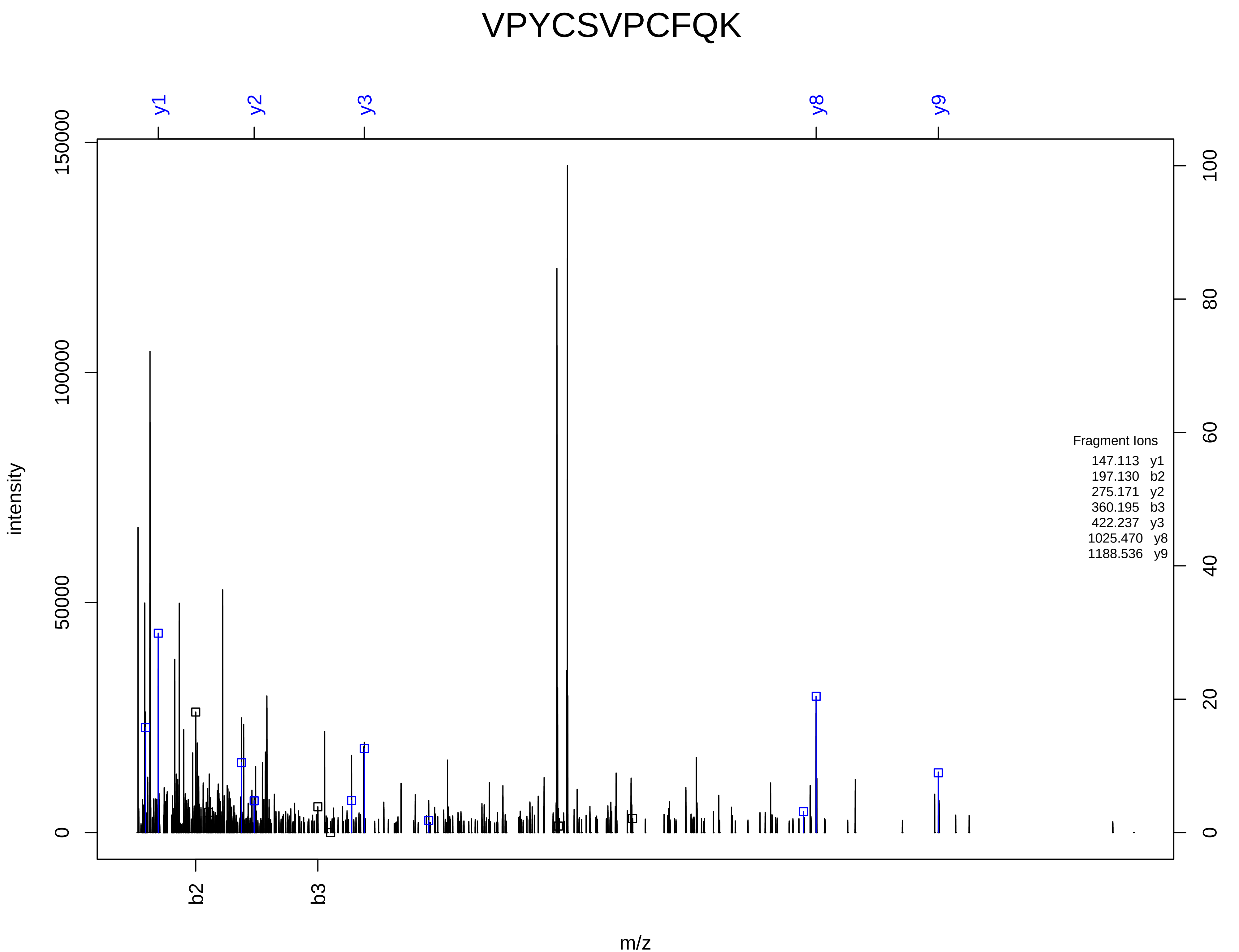
VPYCSVPCFQK
y1
y2
y3
y8
y9
intensity
150000
100000
50000
0
100
80
60
40
20
0
Fragment Ions
147.113 y1
197.130 b2
275.171 y2
360.195 b3
422.237 y3
1025.470 y8
1188.536 y9
b2
b3
m/z
VPYCSVPCFQK / File: christine_10pools_04.raw; SpectrumID: ; scans: 9428

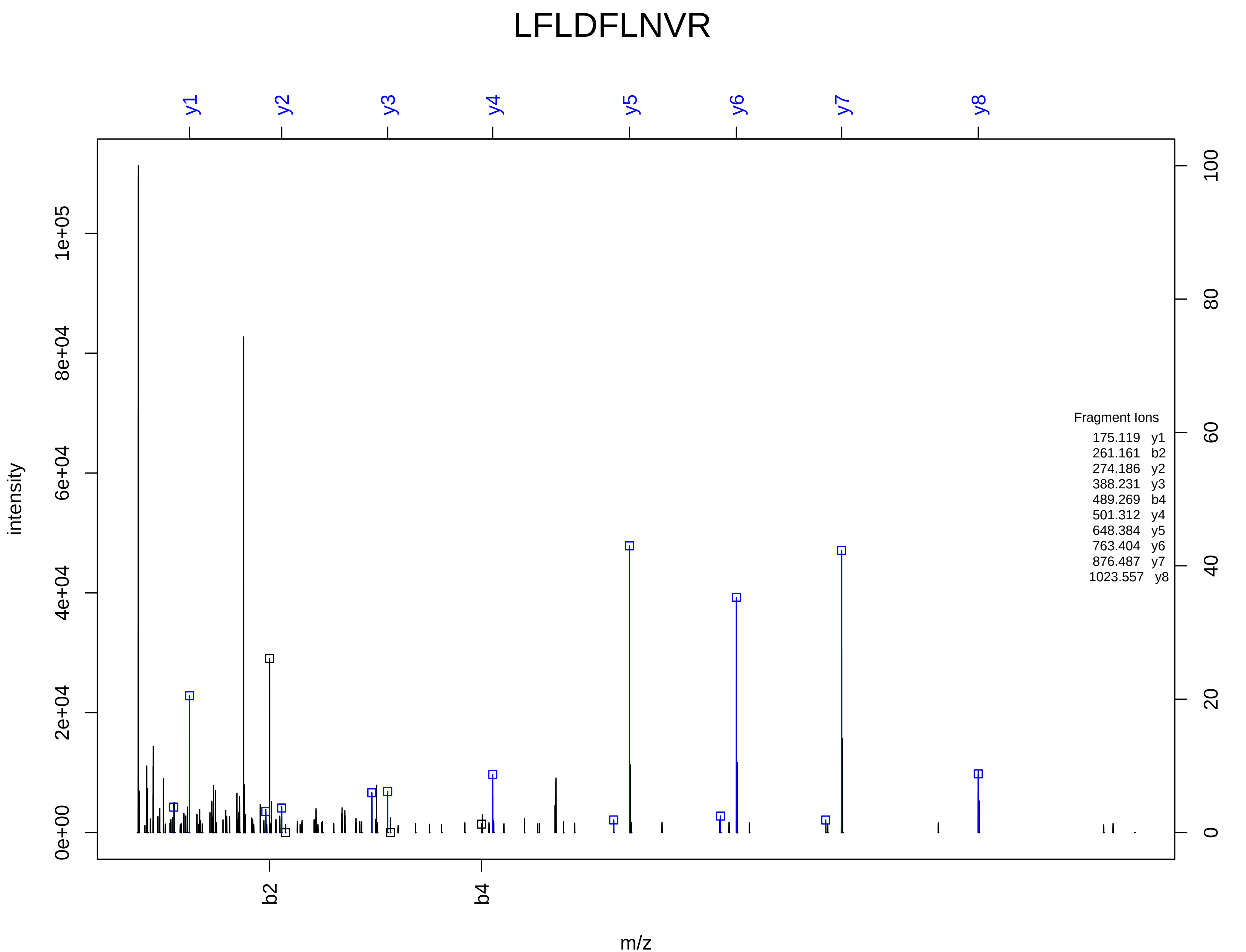

LFLDFLNVR
y1
y2
y3
y4
y5
y6
y7
y8
intensity
0e+00
2e+04
4e+04
6e+04
8e+04
1e+05
0
20
40
60
80
100
Fragment Ions
175.119 y1
261.161 b2
274.186 y2
388.231 y3
489.269 b4
501.312 y4
648.384 y5
763.404 y6
876.487 y7
1023.557 y8
b2
b4
m/z
LFLDFLNVR / File: christine_10pools_04.raw; SpectrumID: ; scans: 35049

FNIFYPDLIR

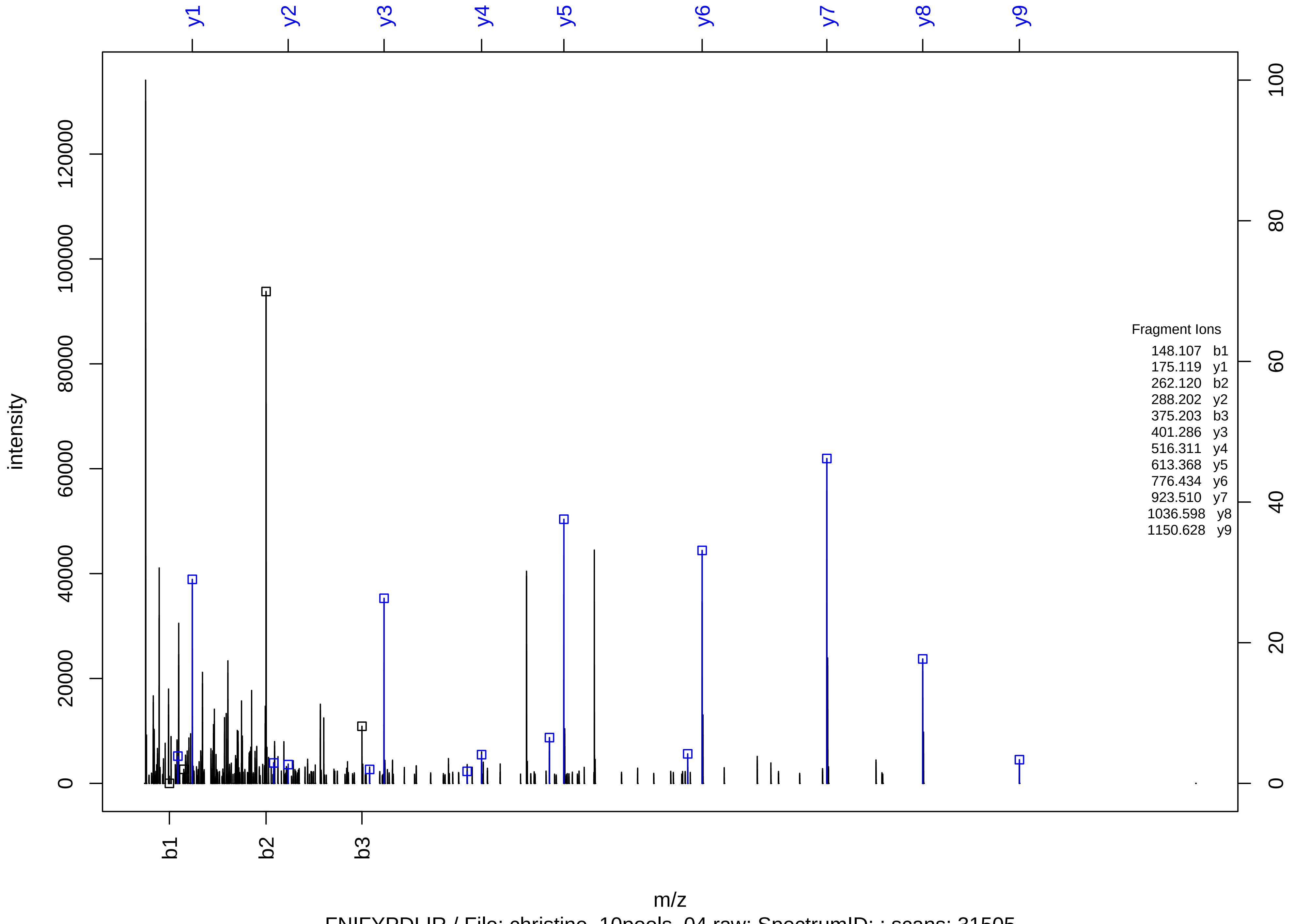

FNIFYPDLIR / File: christine_10pools_04.raw; SpectrumID: ; scans: 31505

VLIPVFALGR

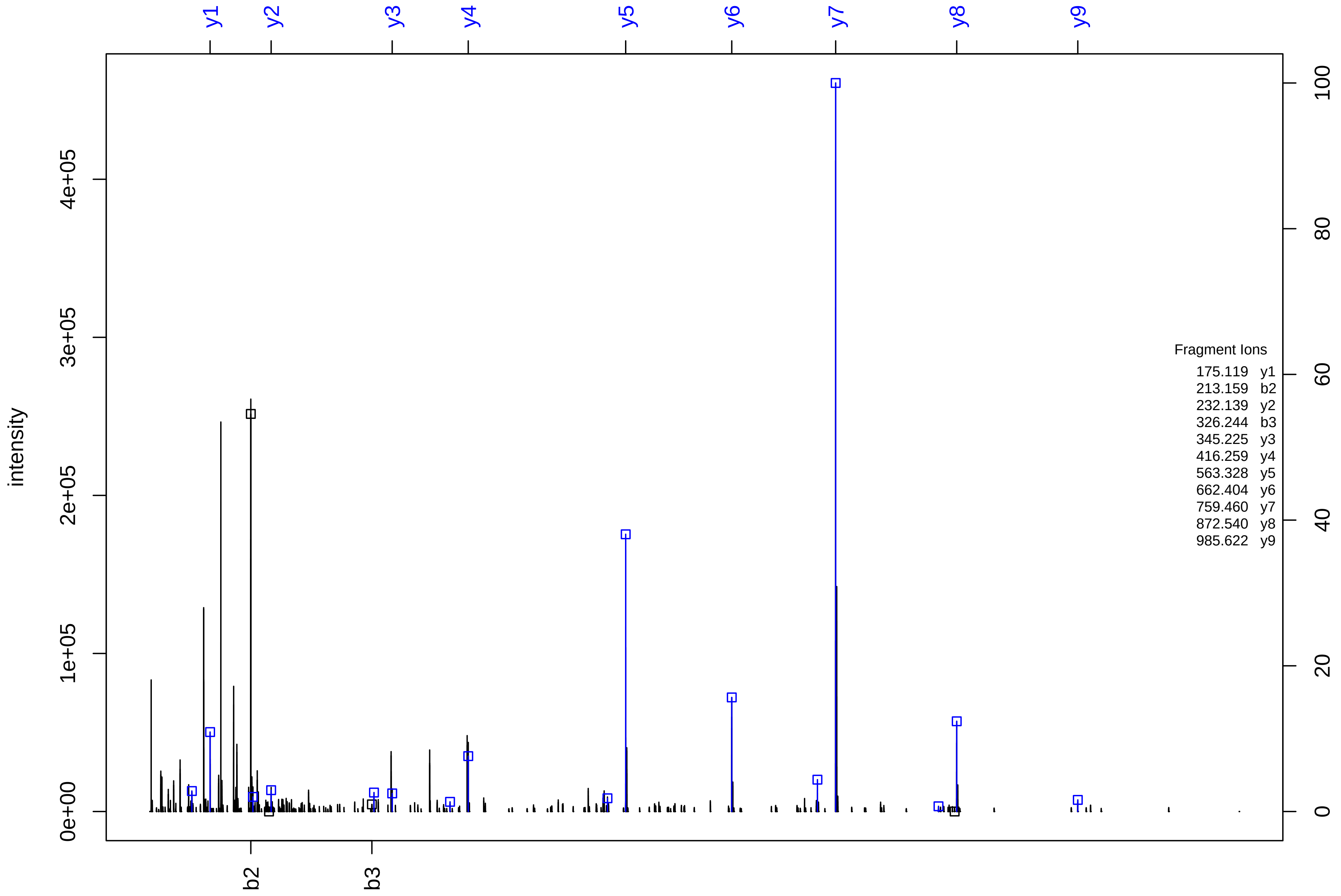

VLIPVFALGR / File: christine_10pools_04.raw; SpectrumID: ; scans: 30484

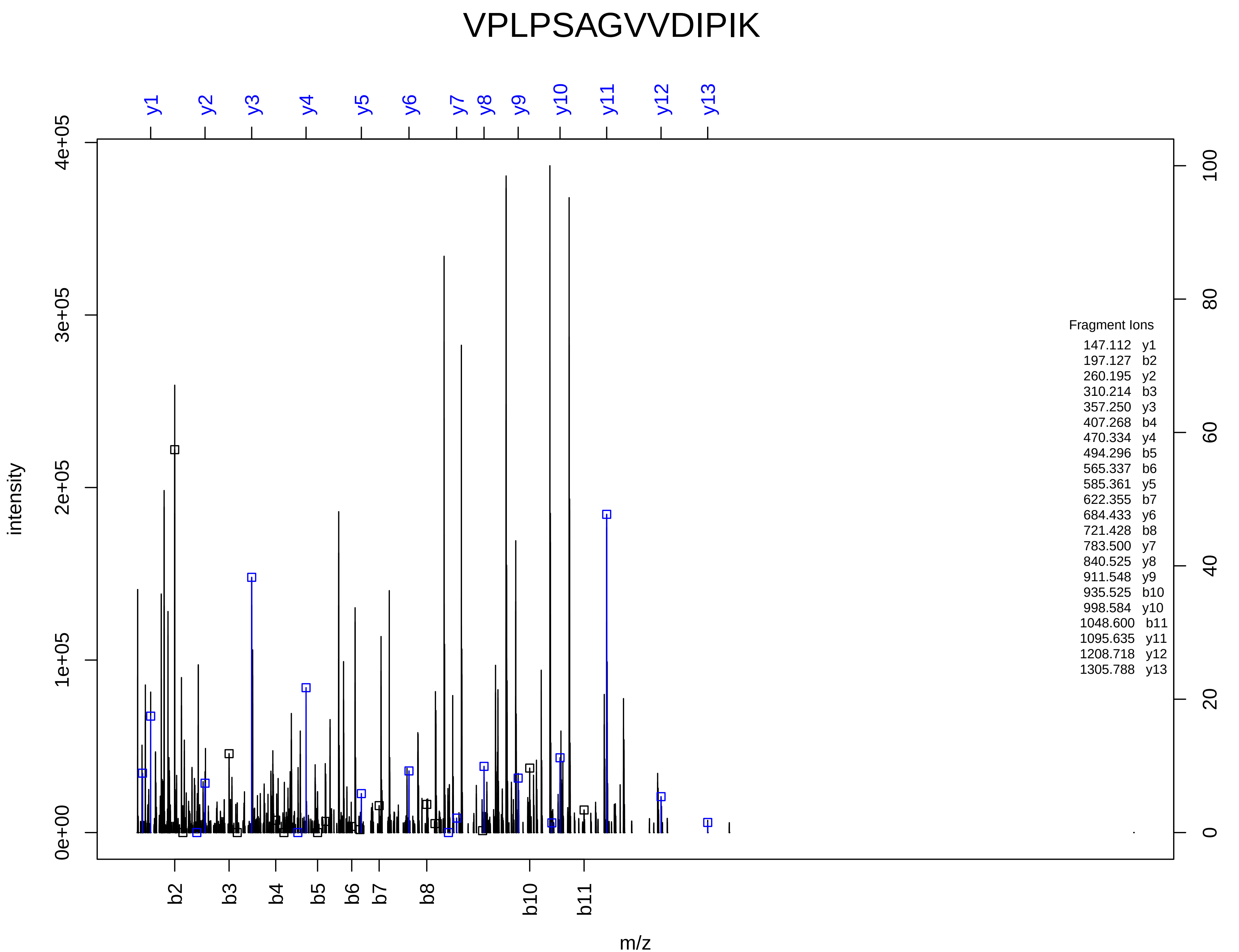
VPLPSAGVVDIPIK
y1 y2 y3 y4 y5 y6 y7 y8 y9 y10 y11 y12 y13
b2 b3 b4 b5 b6 b7 b8 b10 b11
intensity
0e+00 1e+05 2e+05 3e+05 4e+05
0 20 40 60 80 100
m/z
Fragment Ions
147.112 y1
197.127 b2
260.195 y2
310.214 b3
357.250 y3
407.268 b4
470.334 y4
494.296 b5
565.337 b6
585.361 y5
622.355 b7
684.433 y6
721.428 b8
783.500 y7
840.525 y8
911.548 y9
935.525 b10
998.584 y10
1048.600 b11
1095.635 y11
1208.718 y12
1305.788 y13
VPLPSAGVVDIPIK / File: christine_10pools_04.raw; SpectrumID: ; scans: 24448

NVLITDFFGSVR

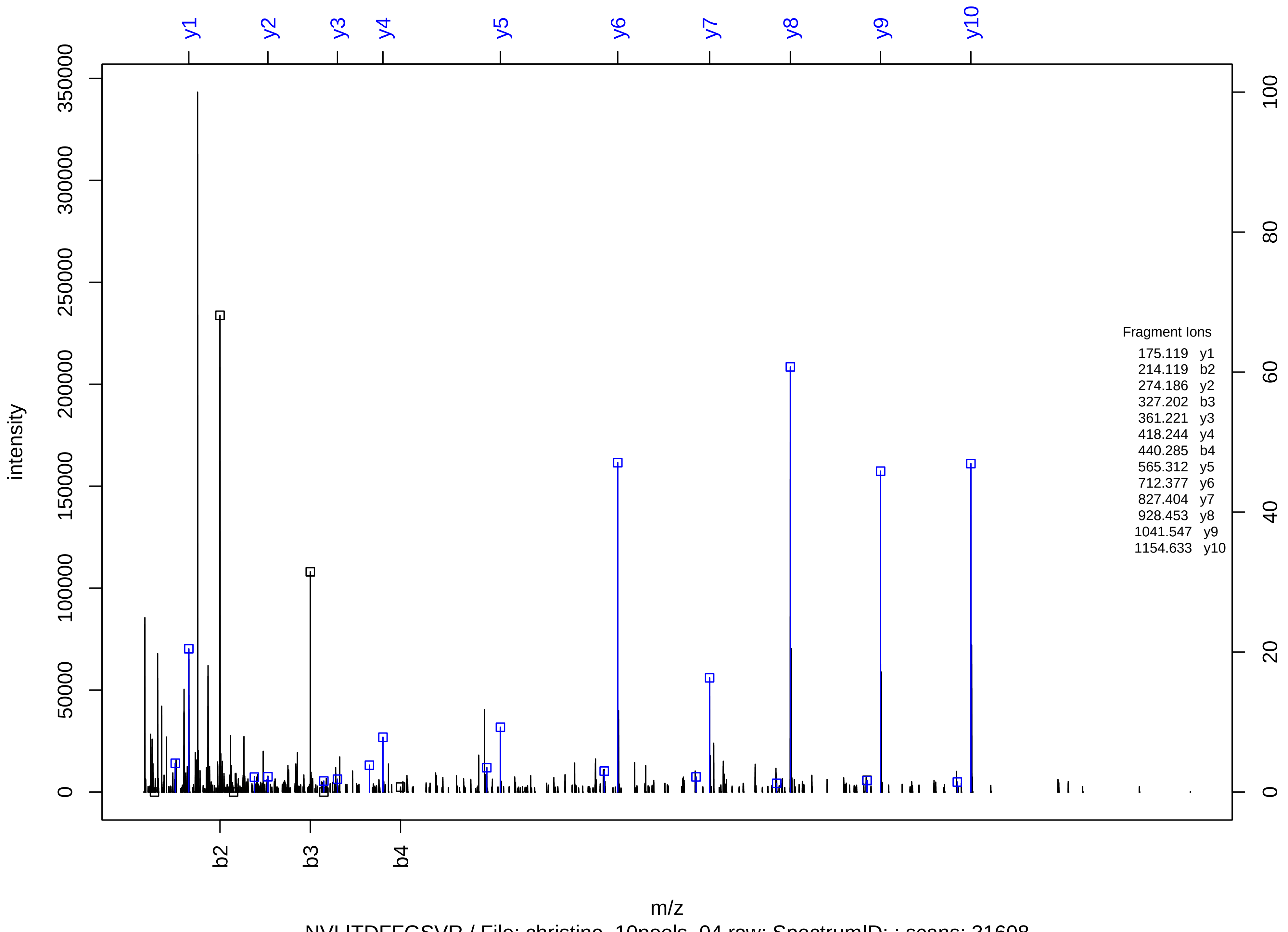

NVLITDFFGSVR / File: christine_10pools_04.raw; SpectrumID: ; scans: 31608

FAQDVSQDDLIR

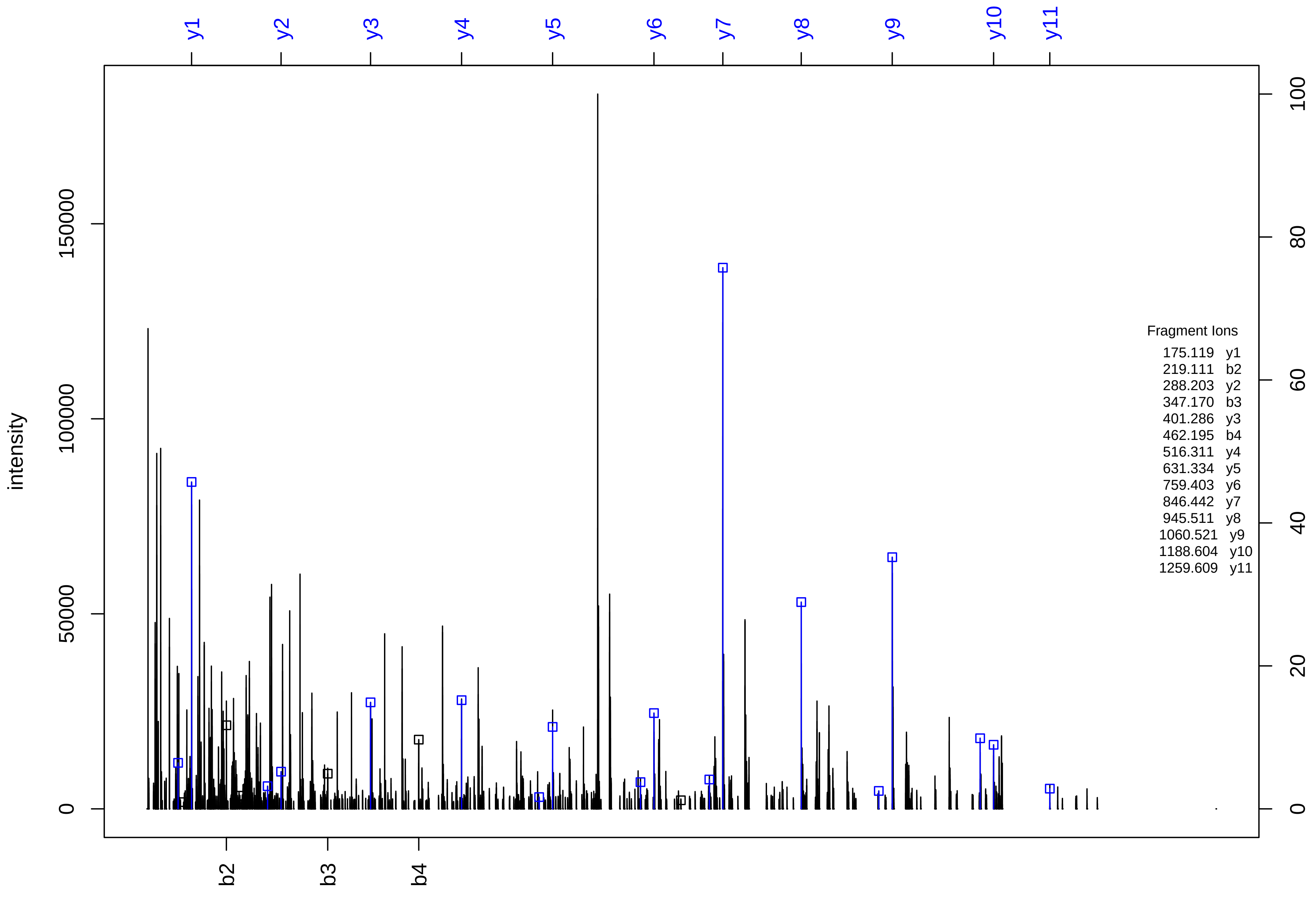

FAQDVSQDDLIR / File: christine_10pools_04.raw; SpectrumID: ; scans: 11204

SLIATAQTQSQQQTEGVK

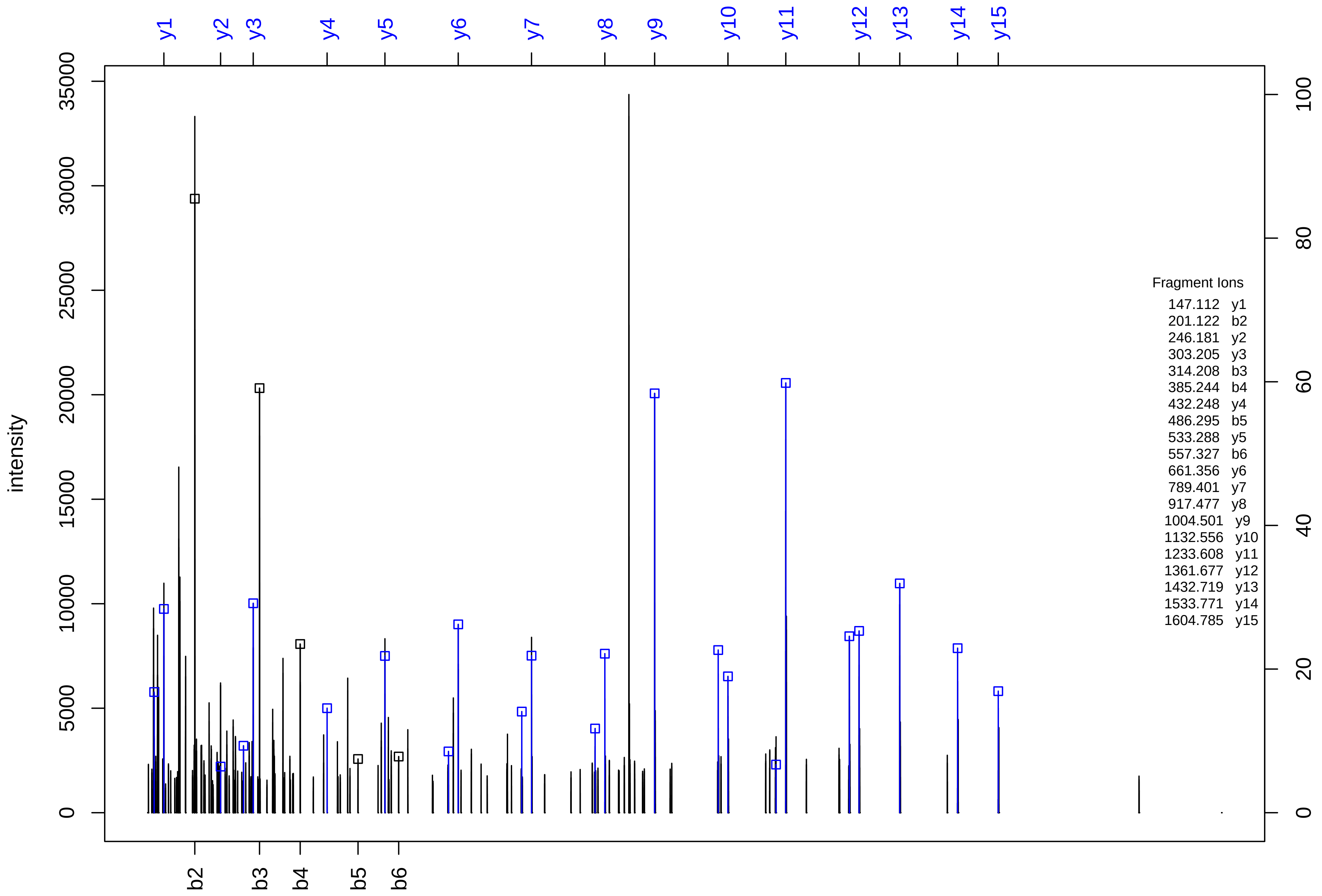

SLIATAQTQSQQQTEGVK / File: christine_10pools_04.raw; SpectrumID: ; scans: 5033

LQQGLAANPSGYGPLTELPDWSFADGRPAPPM*K

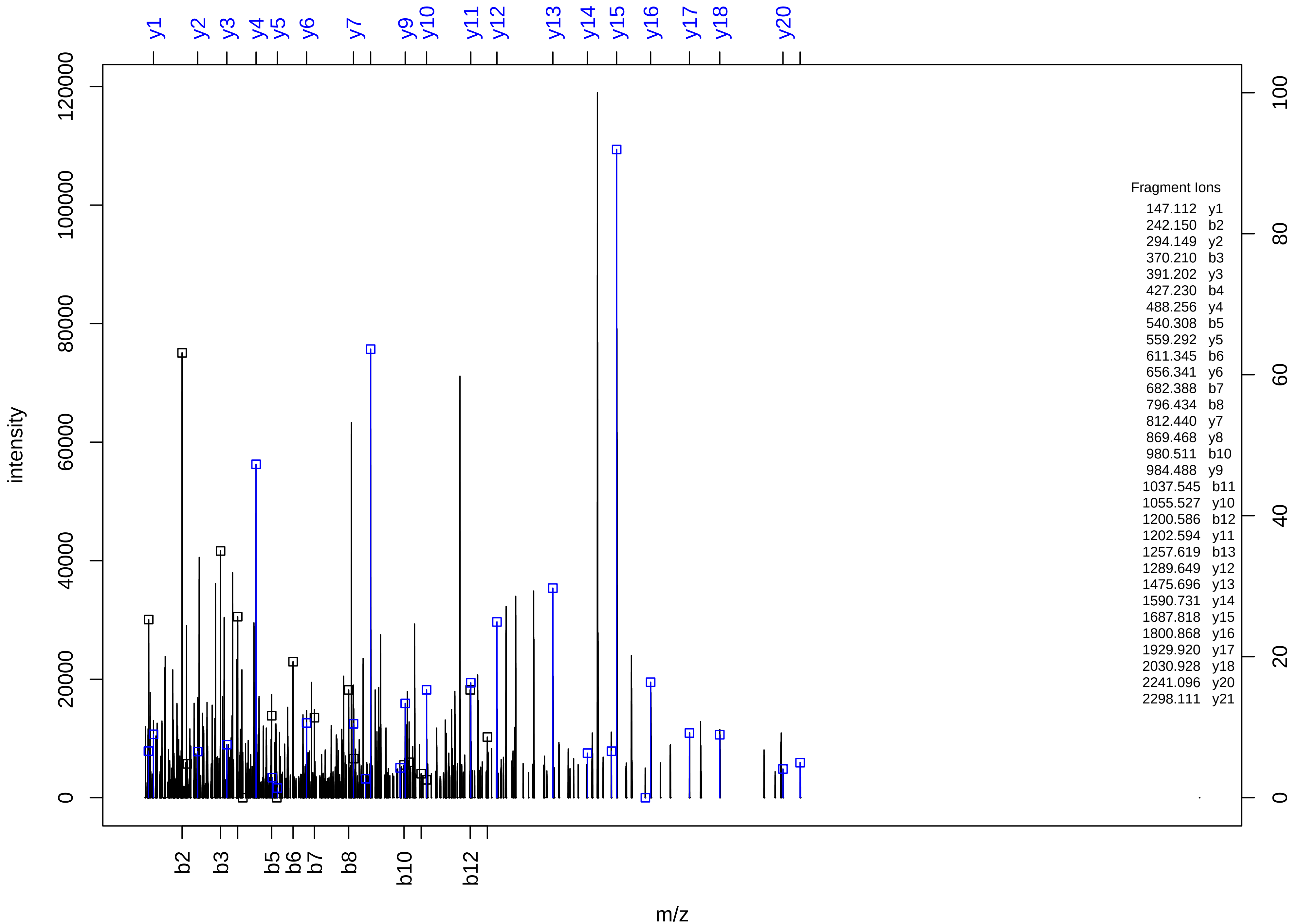

LQQGLAANPSGYGPLTELPDWSFADGRPAPPMK / File: christine_10pools_04.raw; SpectrumID: ; scans: 28177

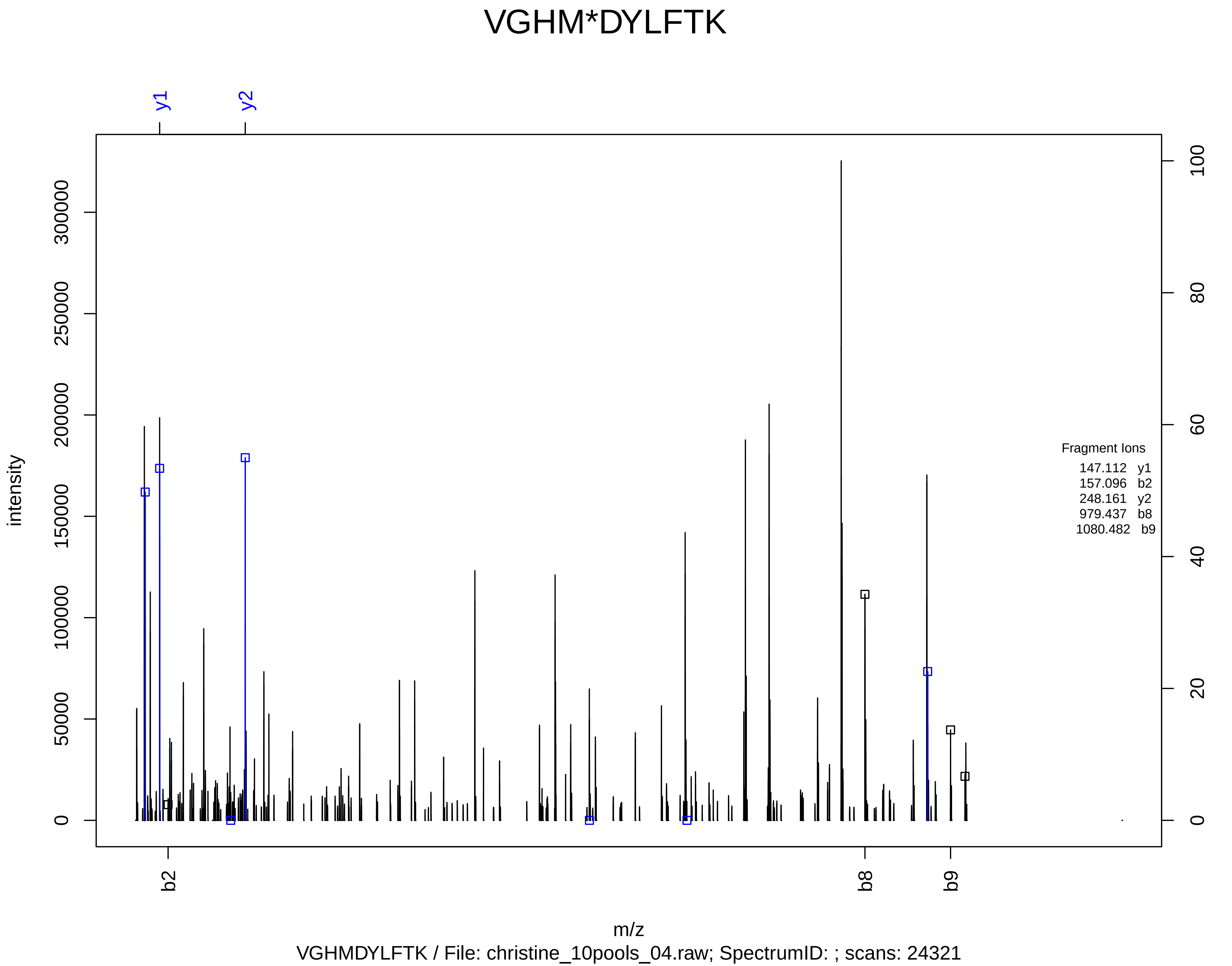

VGHM*DYLFTK
y1
y2
intensity
0
50000
100000
150000
200000
250000
300000
0
20
40
60
80
100
Fragment Ions
147.112 y1
157.096 b2
248.161 y2
979.437 b8
1080.482 b9
b2
b8
b9
m/z
VGHMDYLFTK / File: christine_10pools_04.raw; SpectrumID: ; scans: 24321

LTYTPIFNEYISLVEK

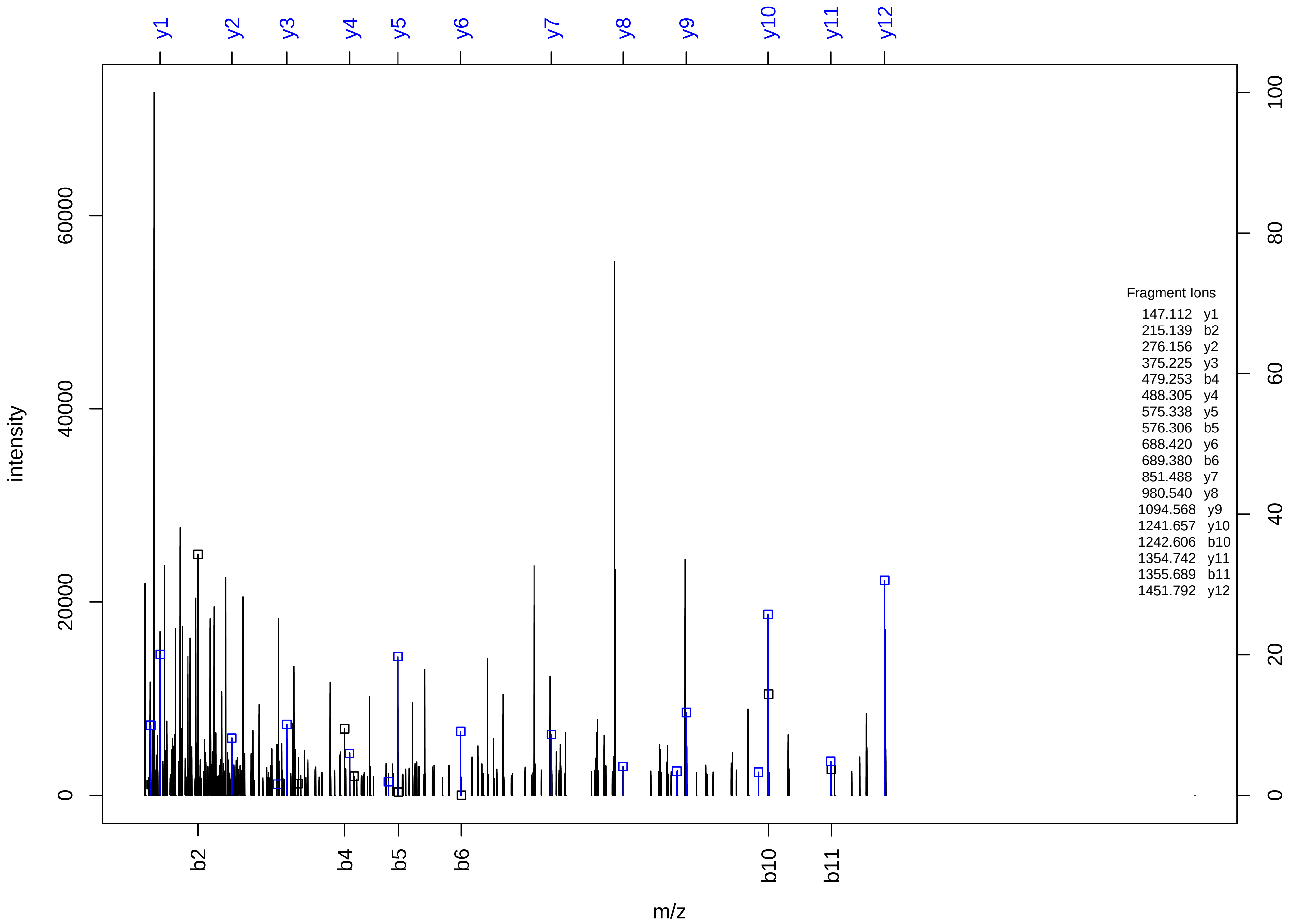

LTYTPIFNEYISLVEK / File: christine_10pools_04.raw; SpectrumID: ; scans: 37094

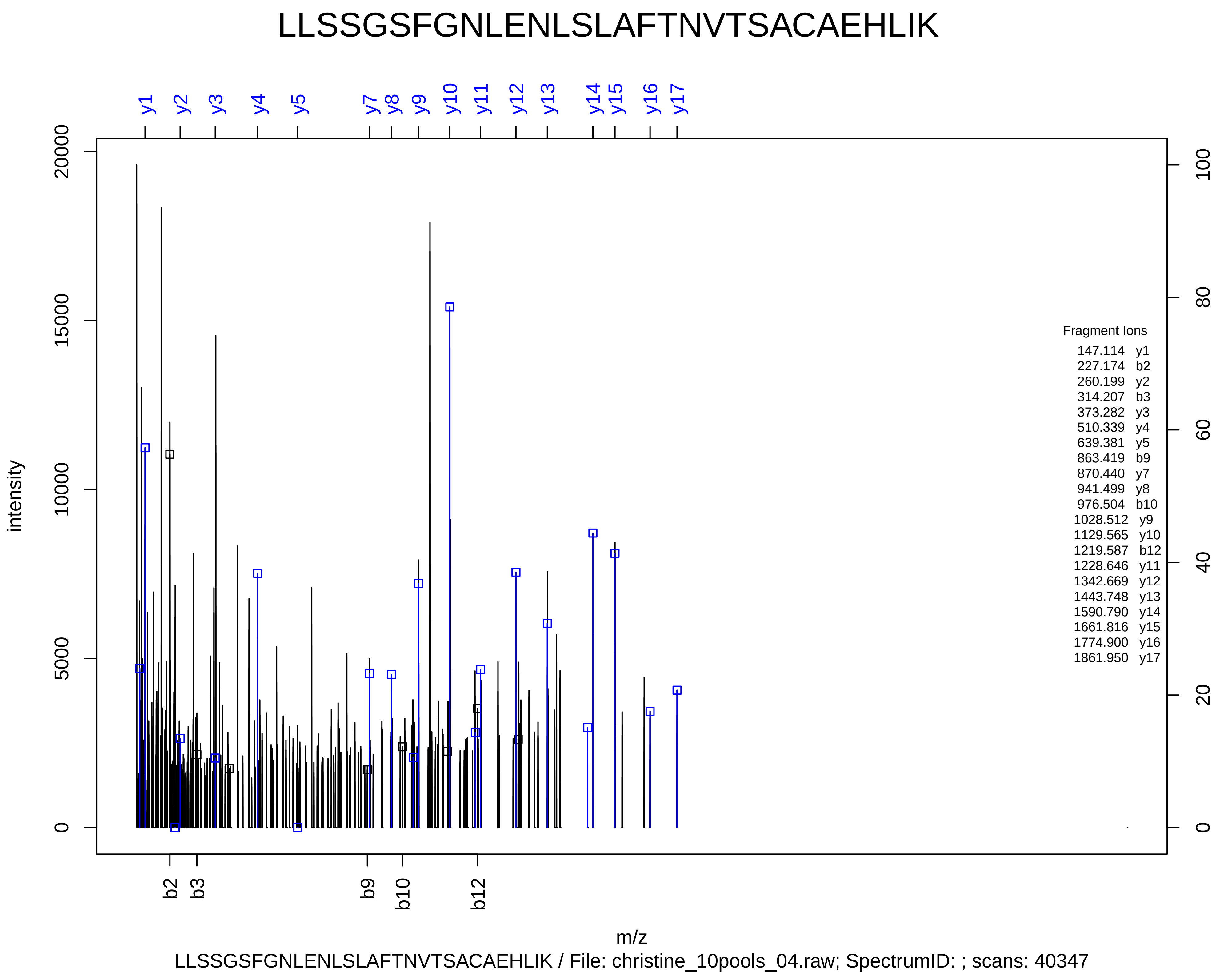

LLSSGSFGNLENLSLAFTNVTSACAEHLIK
y1 y2 y3 y4 y5 y7 y8 y9 y10 y11 y12 y13 y14 y15 y16 y17
intensity
0
5000
10000
15000
20000
0
20
40
60
80
100
Fragment Ions
147.114 y1
227.174 b2
260.199 y2
314.207 b3
373.282 y3
510.339 y4
639.381 y5
863.419 b9
870.440 y7
941.499 y8
976.504 b10
1028.512 y9
1129.565 y10
1219.587 b12
1228.646 y11
1342.669 y12
1443.748 y13
1590.790 y14
1661.816 y15
1774.900 y16
1861.950 y17
b2 b3 b9 b10 b12
m/z
LLSSGSFGNLENLSLAFTNVTSACAEHLIK / File: christine_10pools_04.raw; SpectrumID: ; scans: 40347

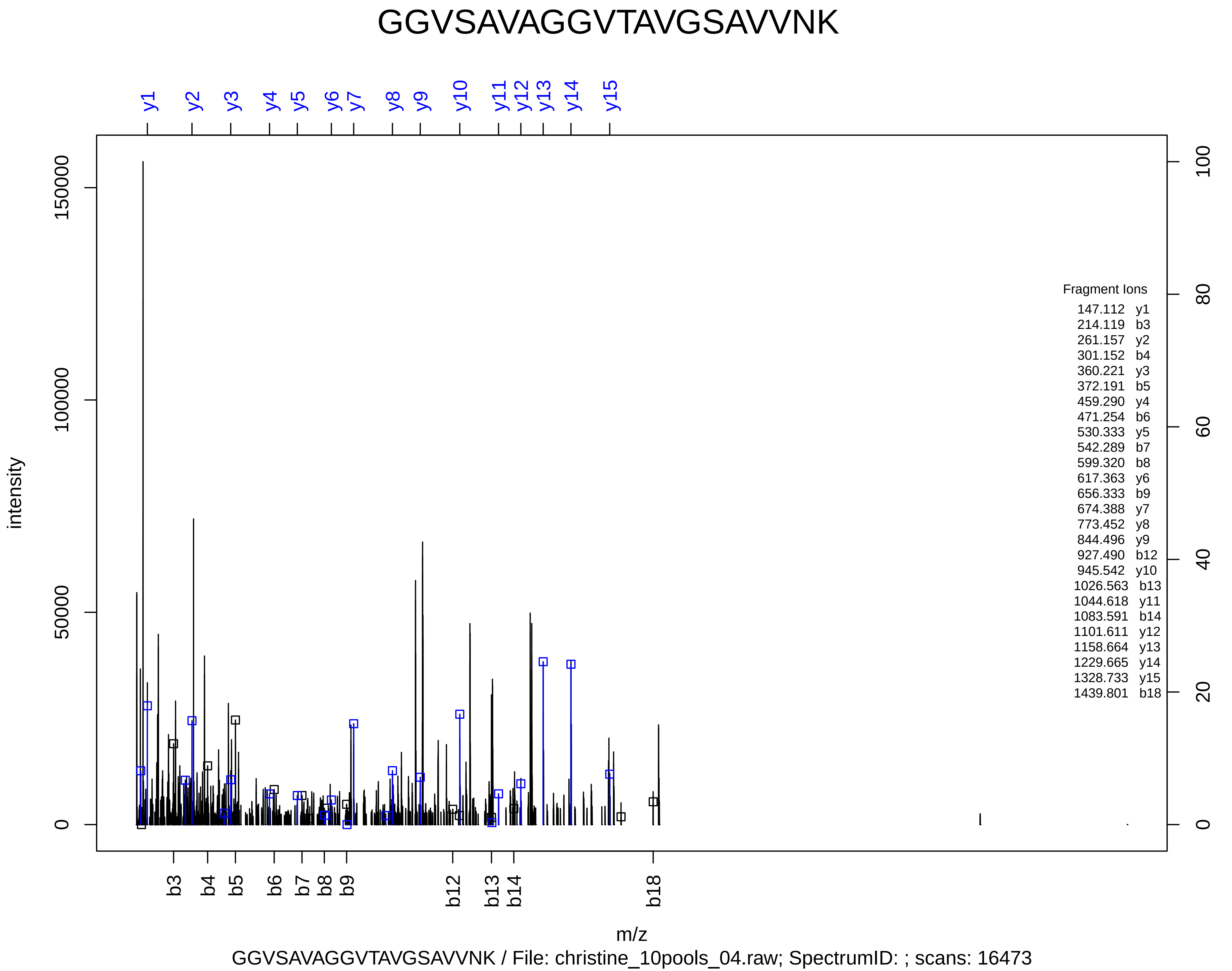
GGVSAVAGGVTAVGSAVVNK
y1
y2
y3
y4
y5
y6
y7
y8
y9
y10
y11
y12
y13
y14
y15
intensity
150000
100000
50000
0
100
80
60
40
20
0
Fragment Ions
147.112 y1
214.119 b3
261.157 y2
301.152 b4
360.221 y3
372.191 b5
459.290 y4
471.254 b6
530.333 y5
542.289 b7
599.320 b8
617.363 y6
656.333 b9
674.388 y7
773.452 y8
844.496 y9
927.490 b12
945.542 y10
1026.563 b13
1044.618 y11
1083.591 b14
1101.611 y12
1158.664 y13
1229.665 y14
1328.733 y15
1439.801 b18
b3
b4
b5
b6
b7
b8
b9
b12
b13
b14
b18
m/z
GGVSAVAGGVTAVGSAVVNK / File: christine_10pools_04.raw; SpectrumID: ; scans: 16473

SVFDIPIFTEEFLNHSK

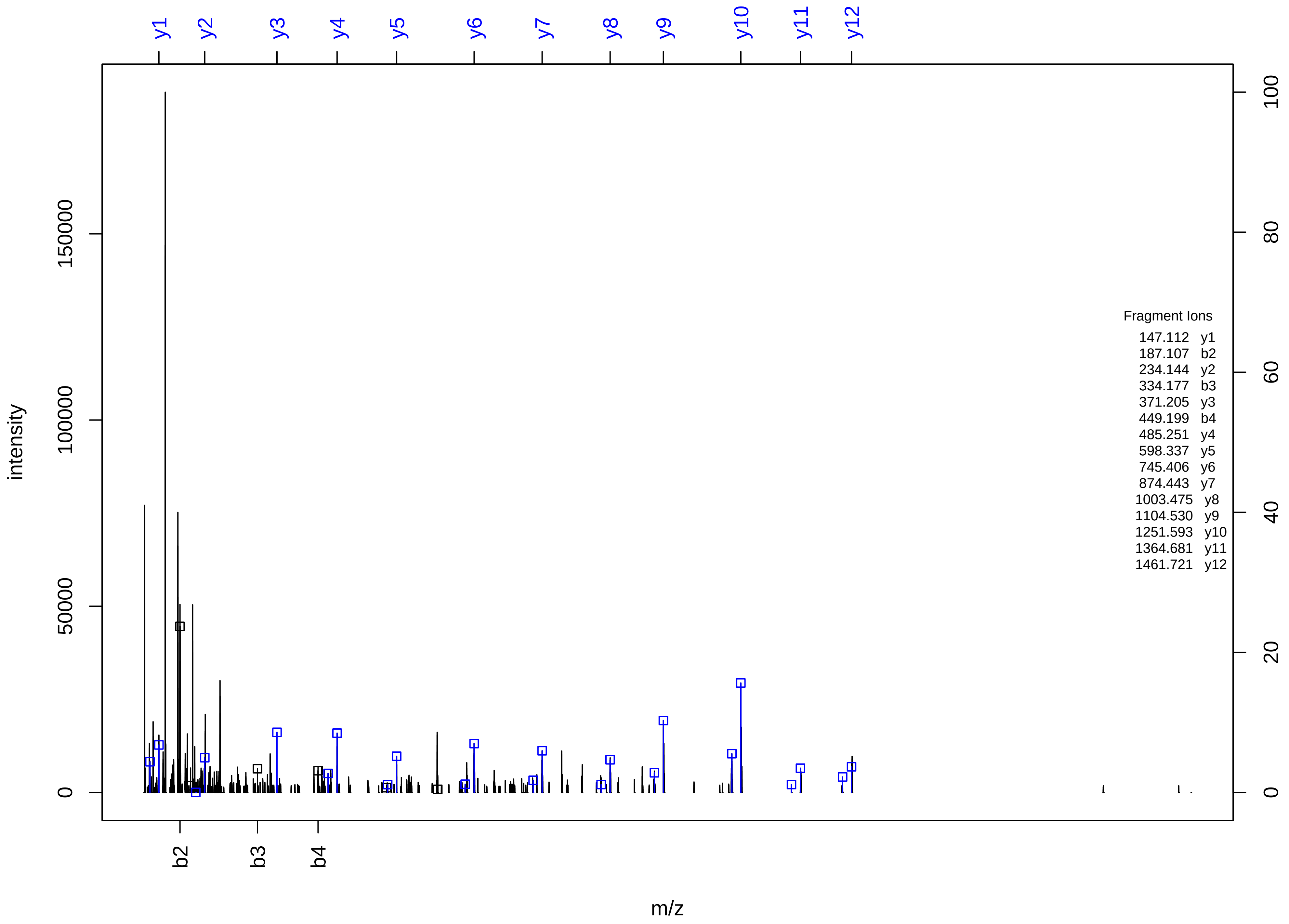

SVFDIPIFTEEFLNHSK / File: christine_10pools_04.raw; SpectrumID: ; scans: 38098

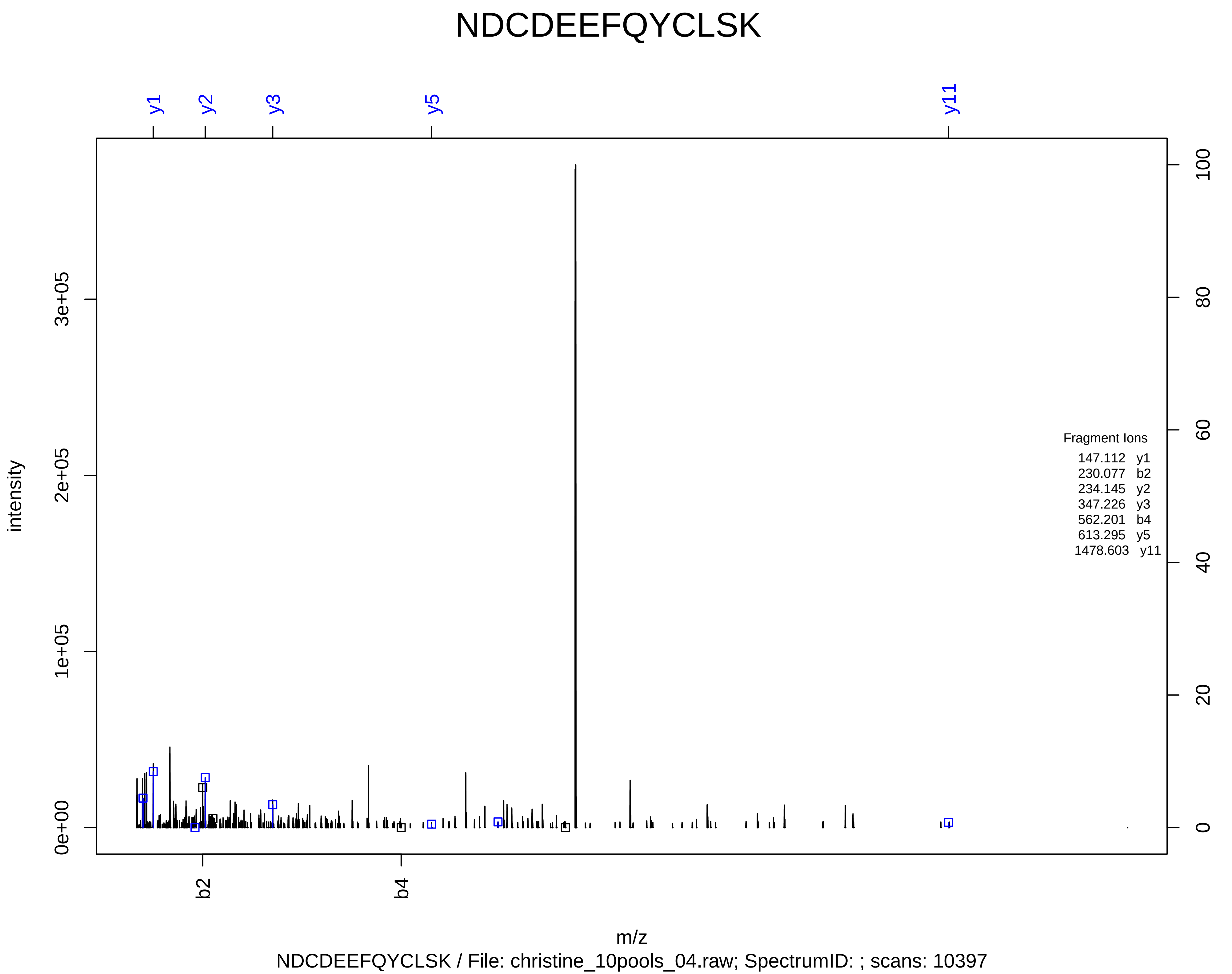

NDCDEEFQYCLSK
y1
y2
y3
y5
y11
intensity
3e+05
2e+05
1e+05
0e+00
100
80
60
40
20
0
Fragment Ions
147.112 y1
230.077 b2
234.145 y2
347.226 y3
562.201 b4
613.295 y5
1478.603 y11
b2
b4
m/z
NDCDEEFQYCLSK / File: christine_10pools_04.raw; SpectrumID: ; scans: 10397

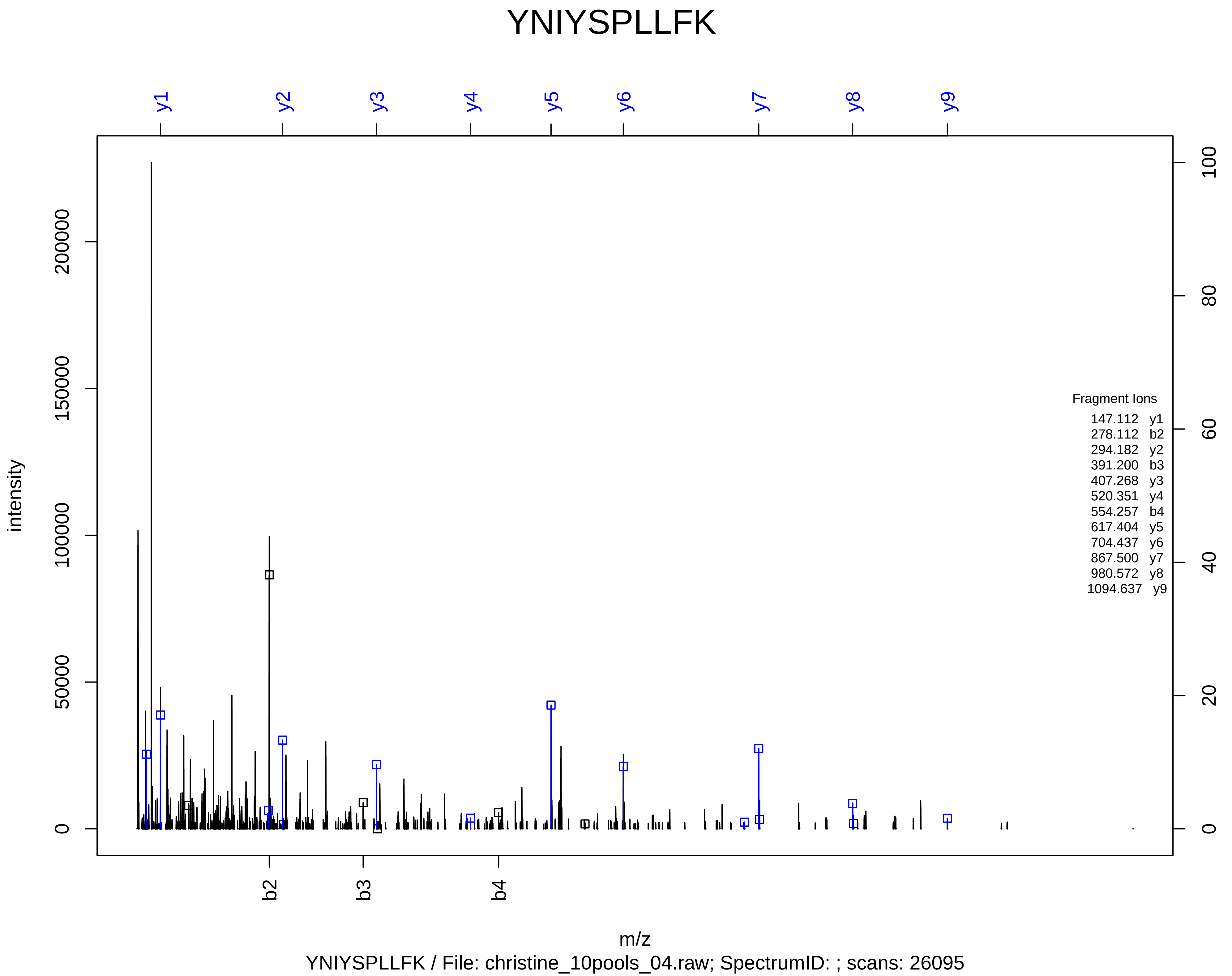
YNIYSPLLFK
y1
y2
y3
y4
y5
y6
y7
y8
y9
b2
b3
b4
intensity
m/z
0
50000
100000
150000
200000
0
20
40
60
80
100
Fragment Ions
147.112 y1
278.112 b2
294.182 y2
391.200 b3
407.268 y3
520.351 y4
554.257 b4
617.404 y5
704.437 y6
867.500 y7
980.572 y8
1094.637 y9
YNIYSPLLFK / File: christine_10pools_04.raw; SpectrumID: ; scans: 26095

NSIAM*QEQMLACK

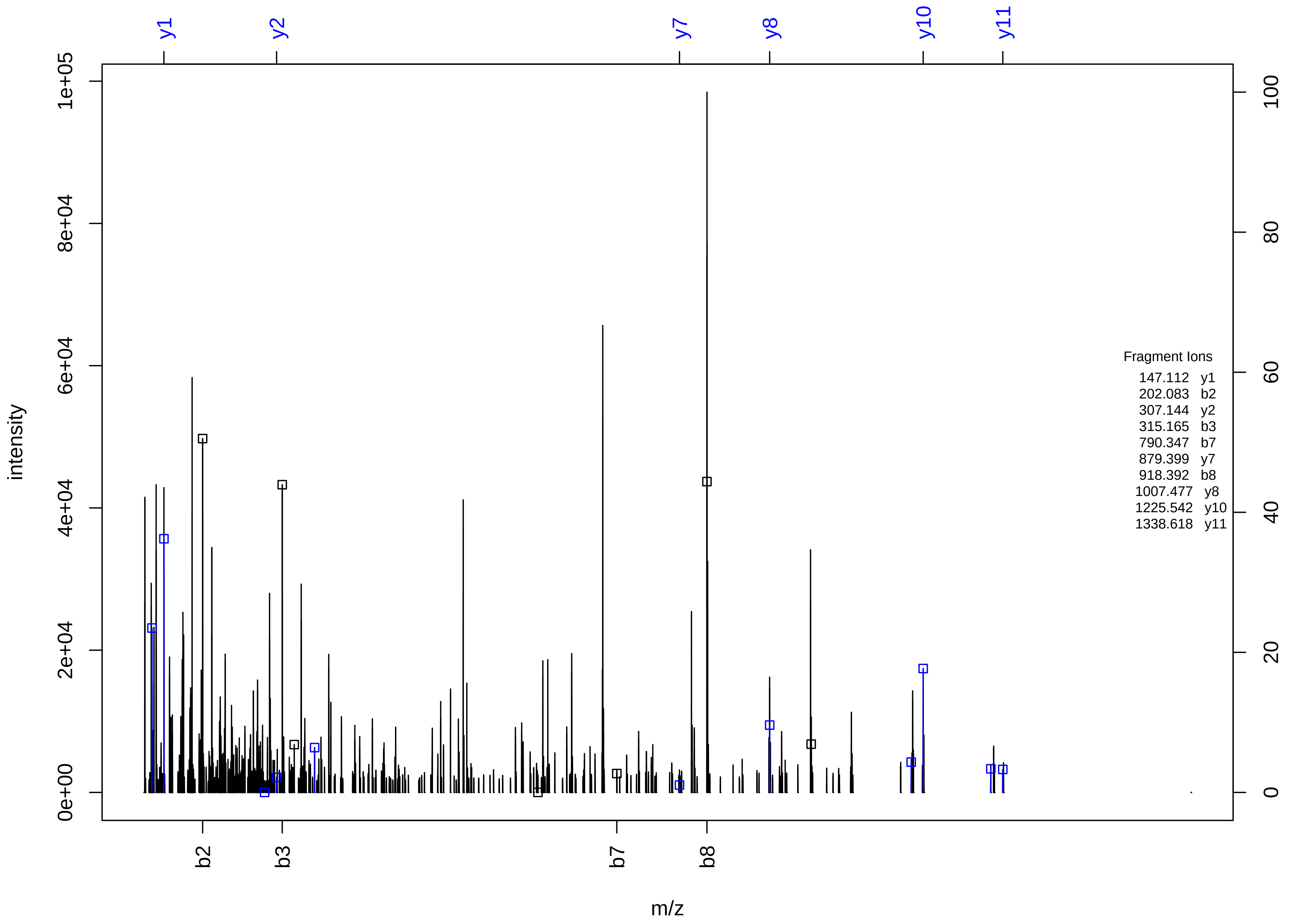

NSIAMQEQMLACK / File: christine_10pools_04.raw; SpectrumID: ; scans: 13406

ILM*DSPEDADIFHSEEIK

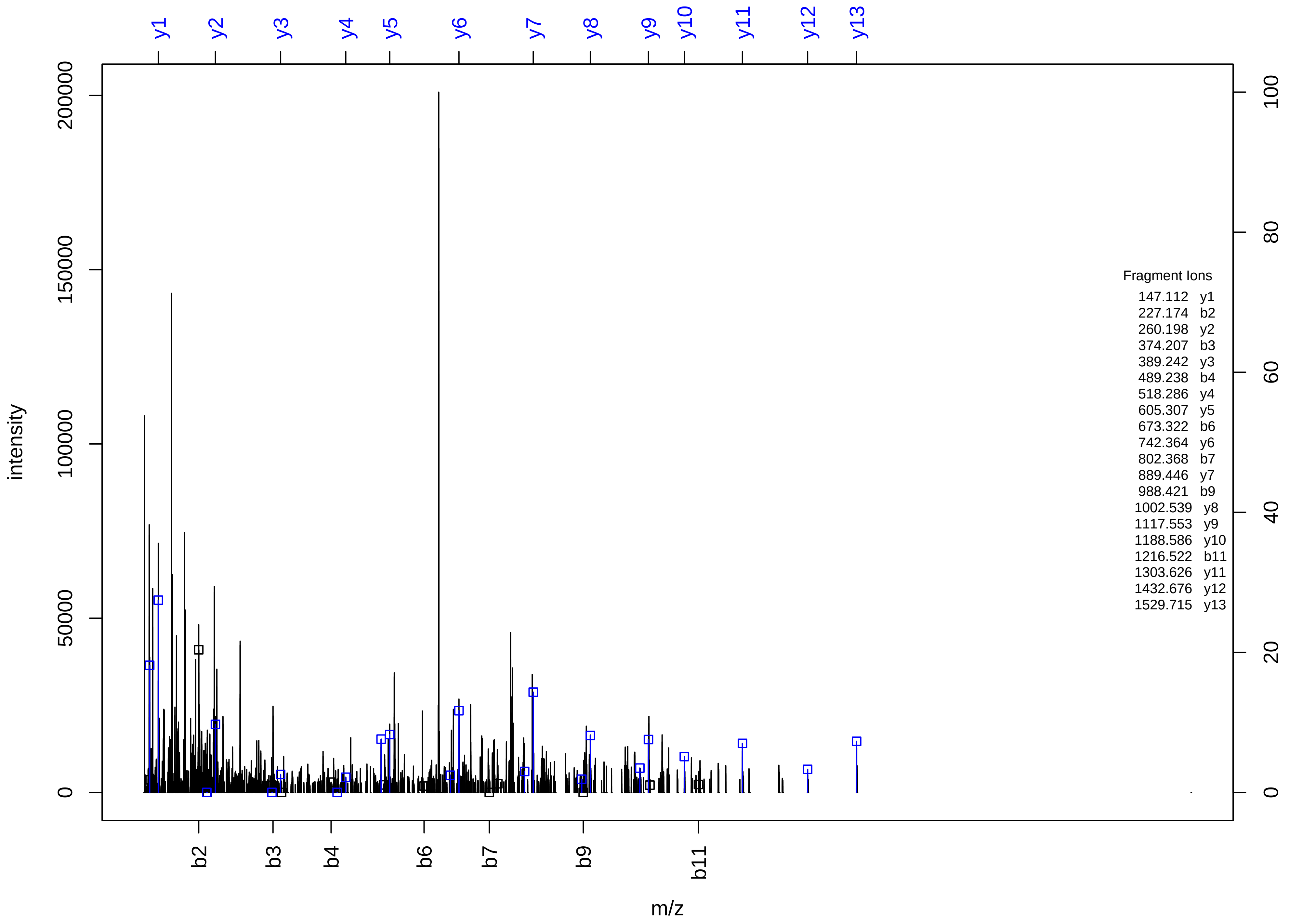

ILMDSPEDADIFHSEEIK / File: christine_10pools_04.raw; SpectrumID: ; scans: 16522

SSLLTELSDSLTK

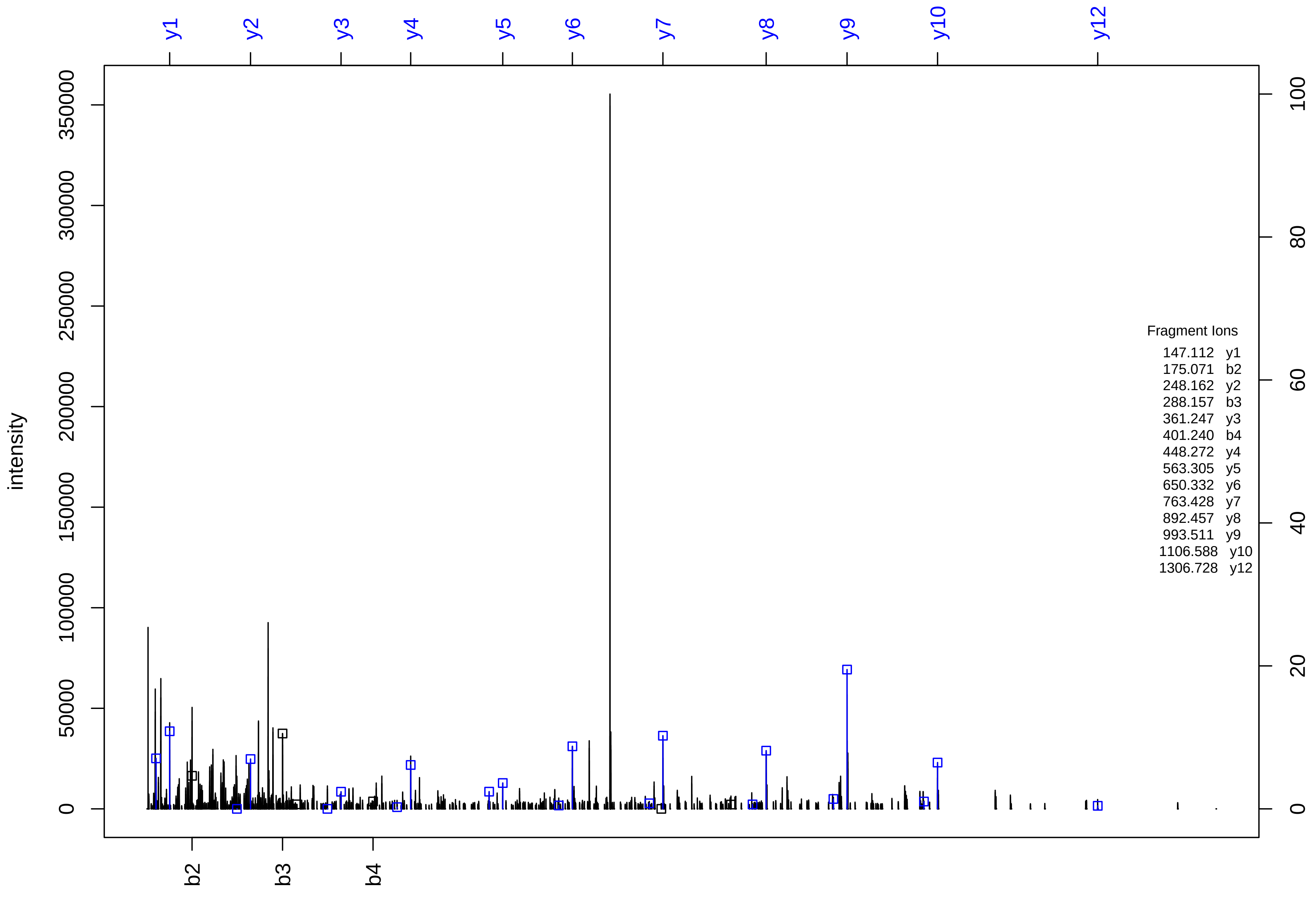

SSLLTELSDSLTK / File: christine_10pools_04.raw; SpectrumID: ; scans: 28743

IVSSNQELIYEGR

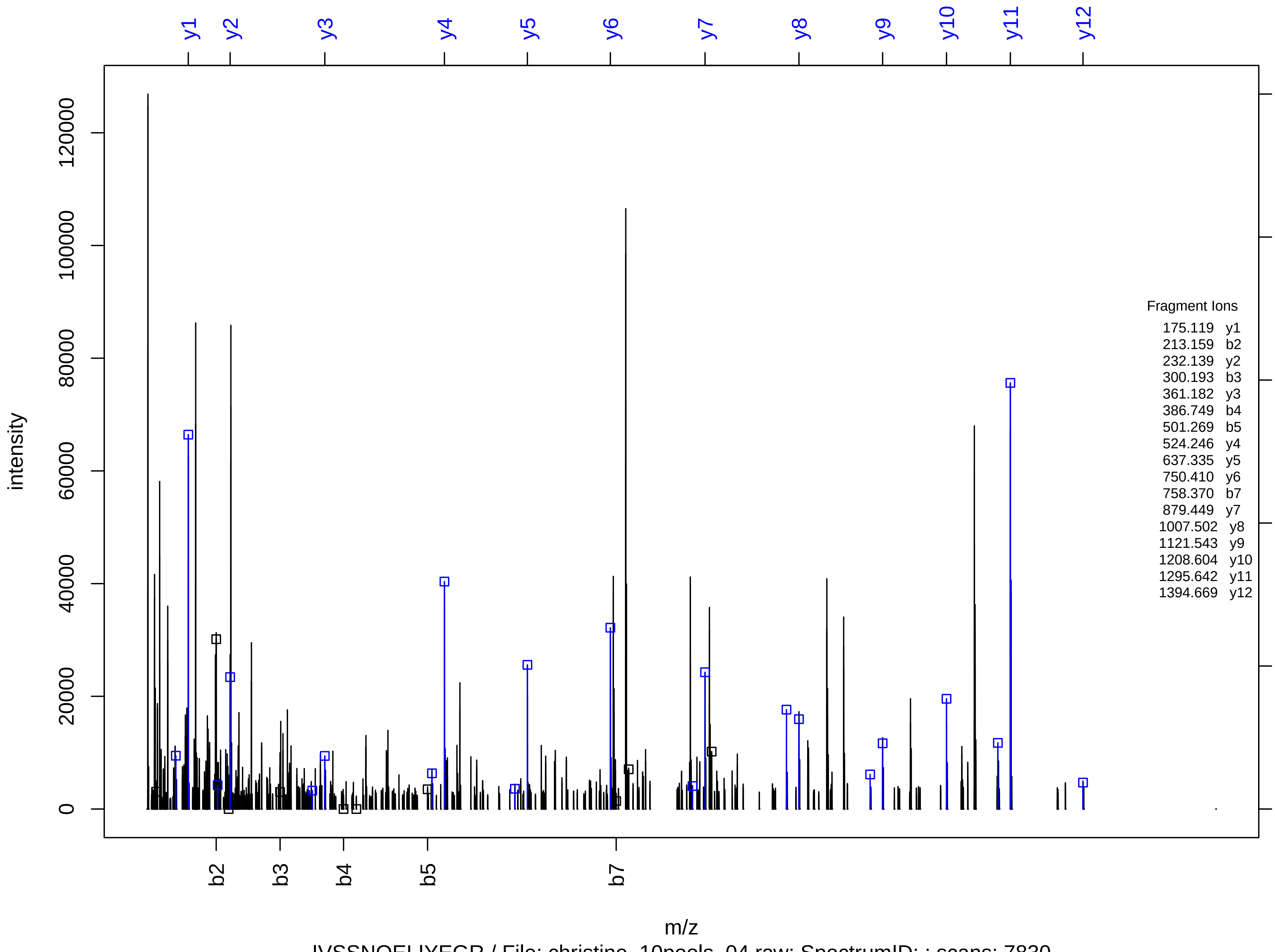

IVSSNQELIYEGR / File: christine_10pools_04.raw; SpectrumID: ; scans: 7830

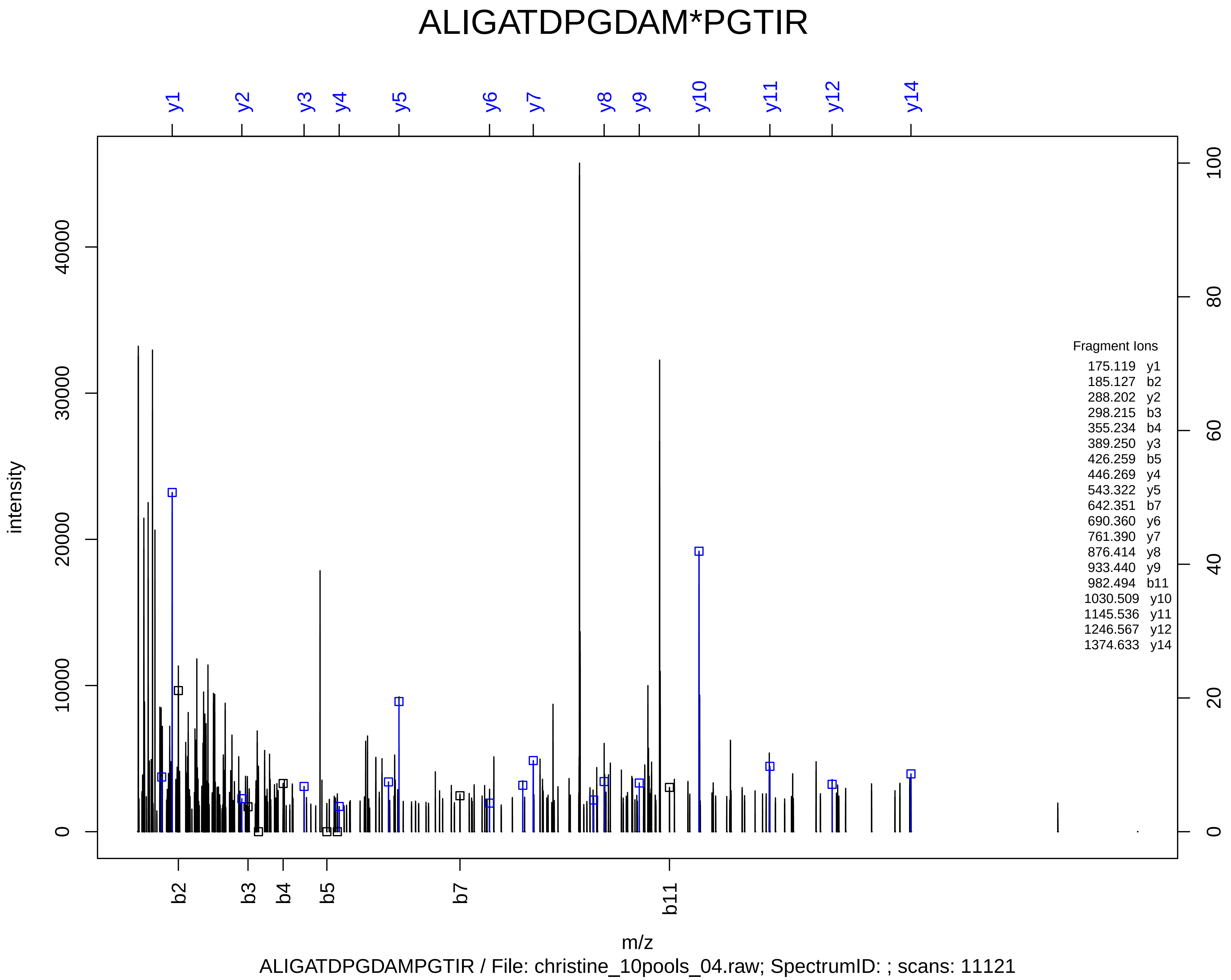

ALIGATDPGDAM*PGTIR
y1
y2
y3
y4
y5
y6
y7
y8
y9
y10
y11
y12
y14
intensity
0
10000
20000
30000
40000
0
20
40
60
80
100
b2
b3
b4
b5
b7
b11
m/z
Fragment Ions
175.119 y1
185.127 b2
288.202 y2
298.215 b3
355.234 b4
389.250 y3
426.259 b5
446.269 y4
543.322 y5
642.351 b7
690.360 y6
761.390 y7
876.414 y8
933.440 y9
982.494 b11
1030.509 y10
1145.536 y11
1246.567 y12
1374.633 y14
ALIGATDPGDAMPGTIR / File: christine_10pools_04.raw; SpectrumID: ; scans: 11121

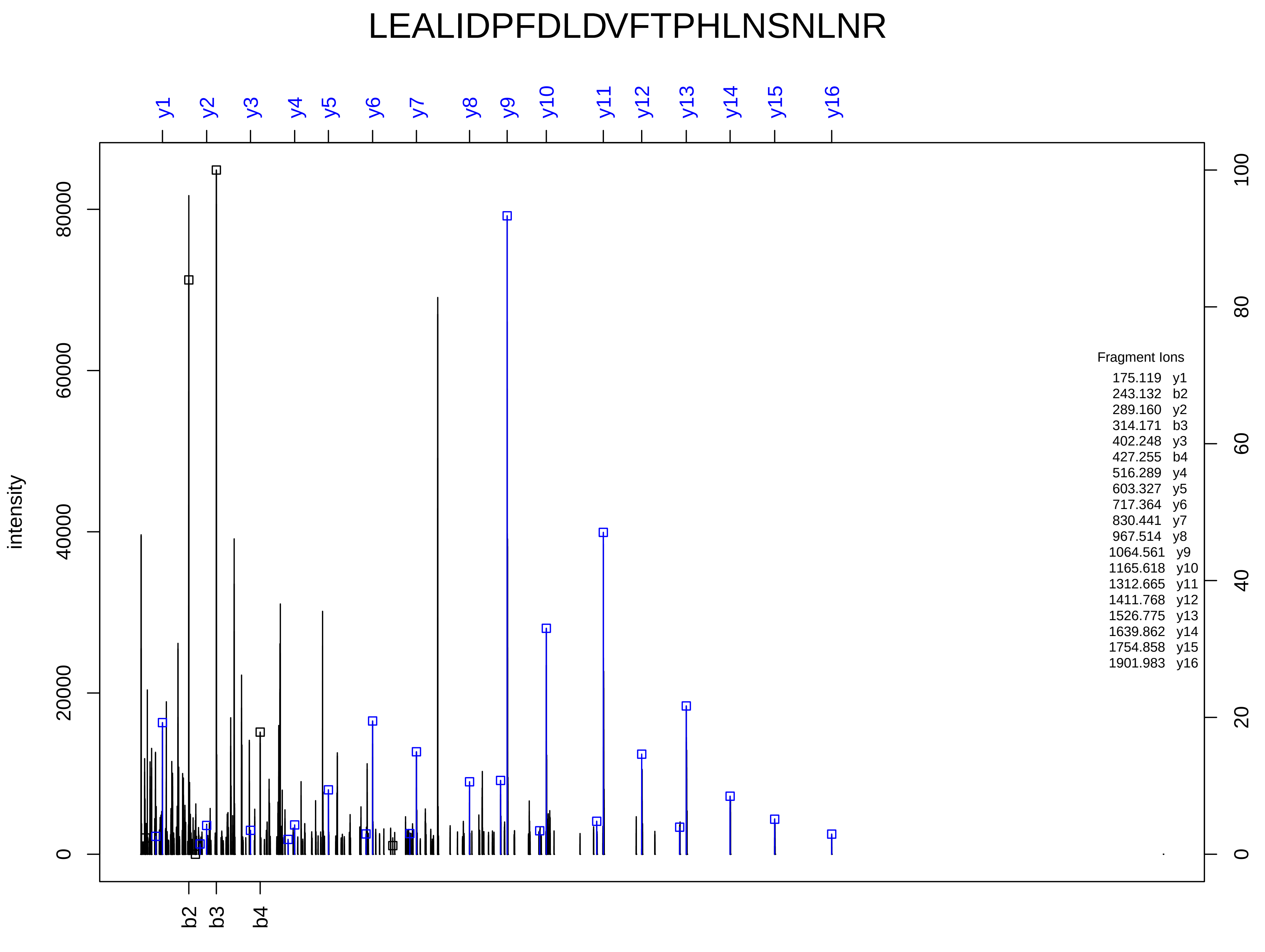

LEALIDPFDLDVFTPHLNSNLNR / File: christine_10pools_04.raw; SpectrumID: ; scans: 40641

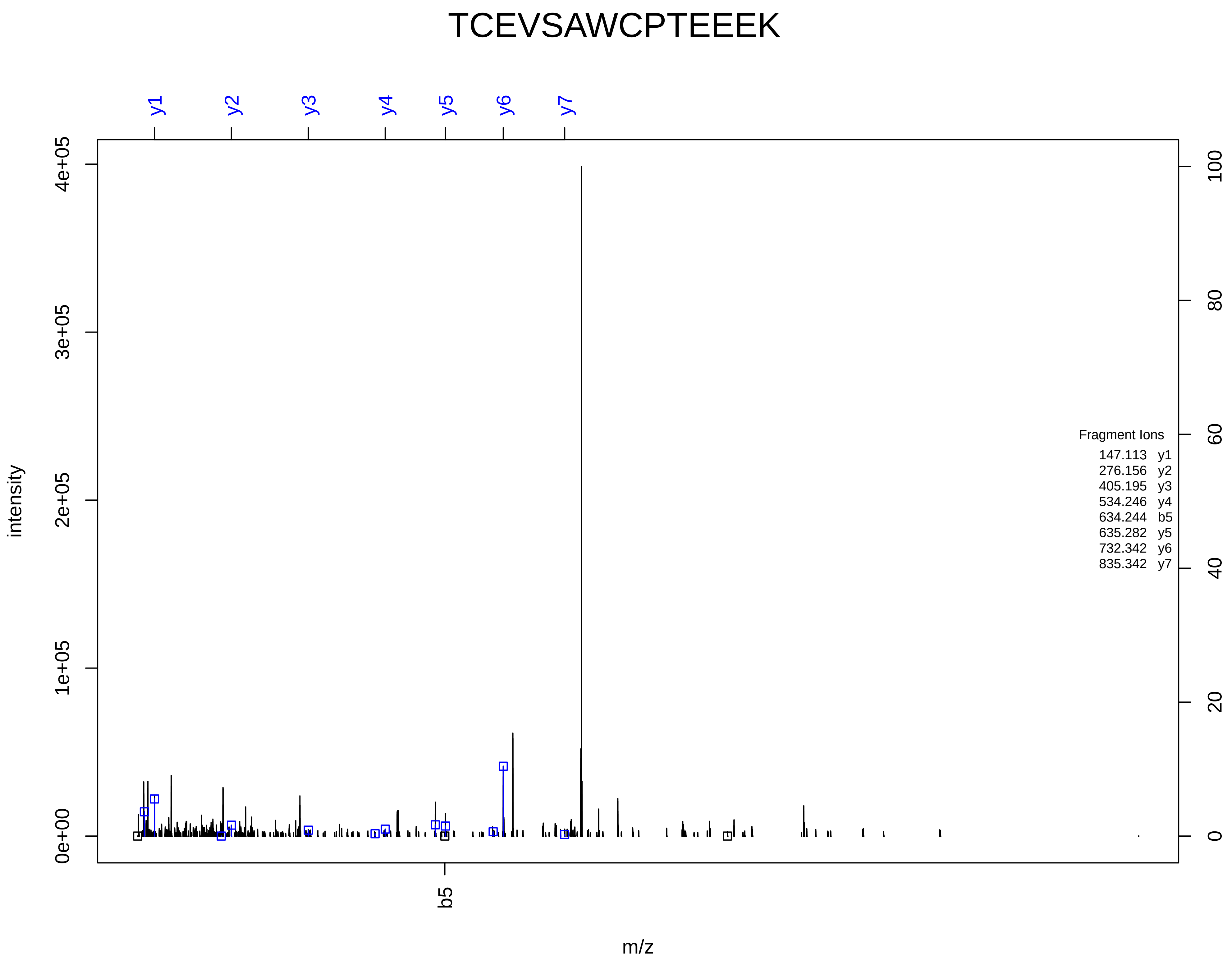
TCEVSAWCPTEEEK
y1
y2
y3
y4
y5
y6
y7
intensity
0e+00
1e+05
2e+05
3e+05
4e+05
0
20
40
60
80
100
Fragment Ions
147.113 y1
276.156 y2
405.195 y3
534.246 y4
634.244 b5
635.282 y5
732.342 y6
835.342 y7
b5
m/z
TCEVSAWCPTEEEK / File: christine_10pools_04.raw; SpectrumID: ; scans: 7532

M*VLPVM*AYGLILN^SM*LWR

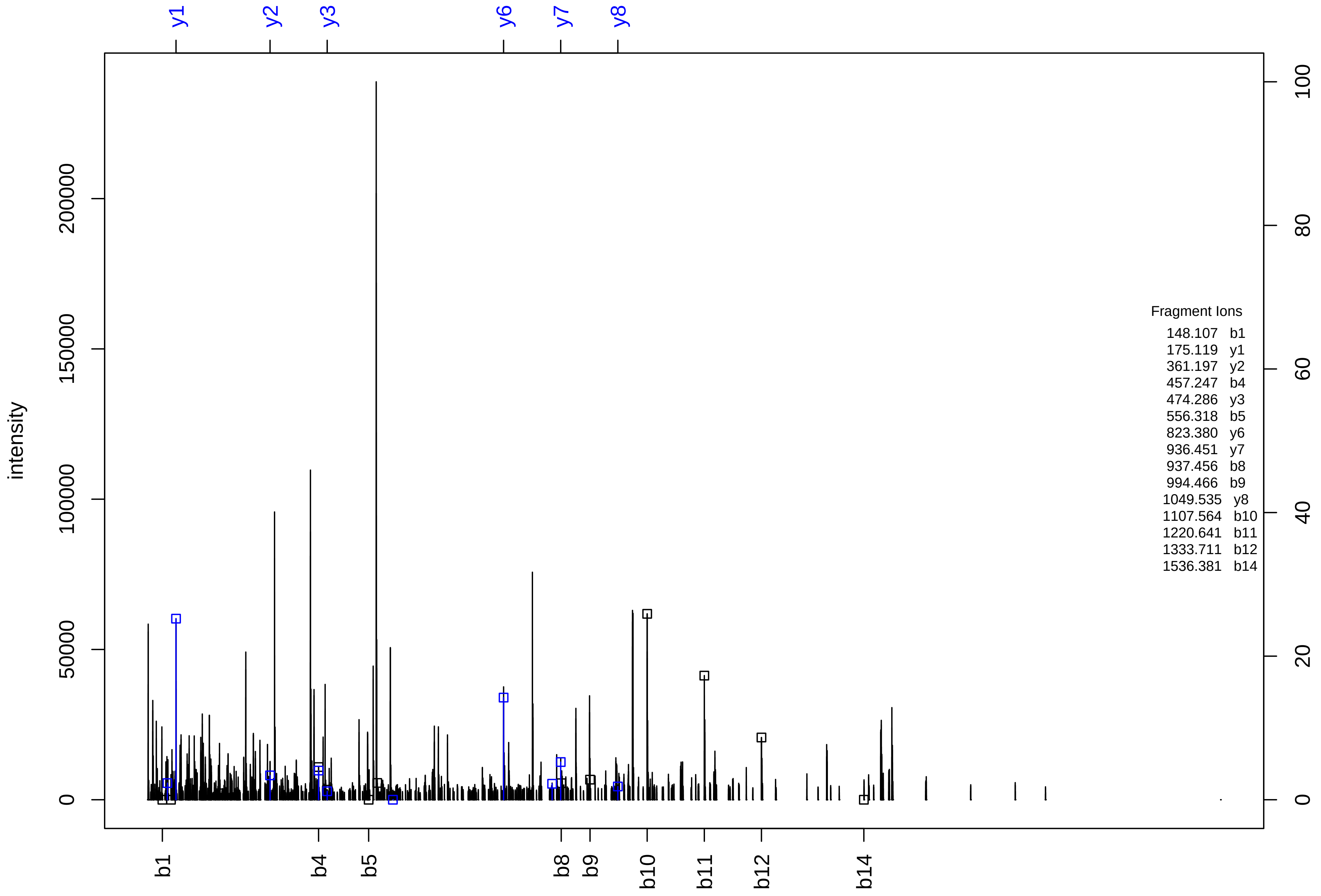

MVLPVMAYGLILNSMLWR / File: christine_10pools_04.raw; SpectrumID: ; scans: 41343

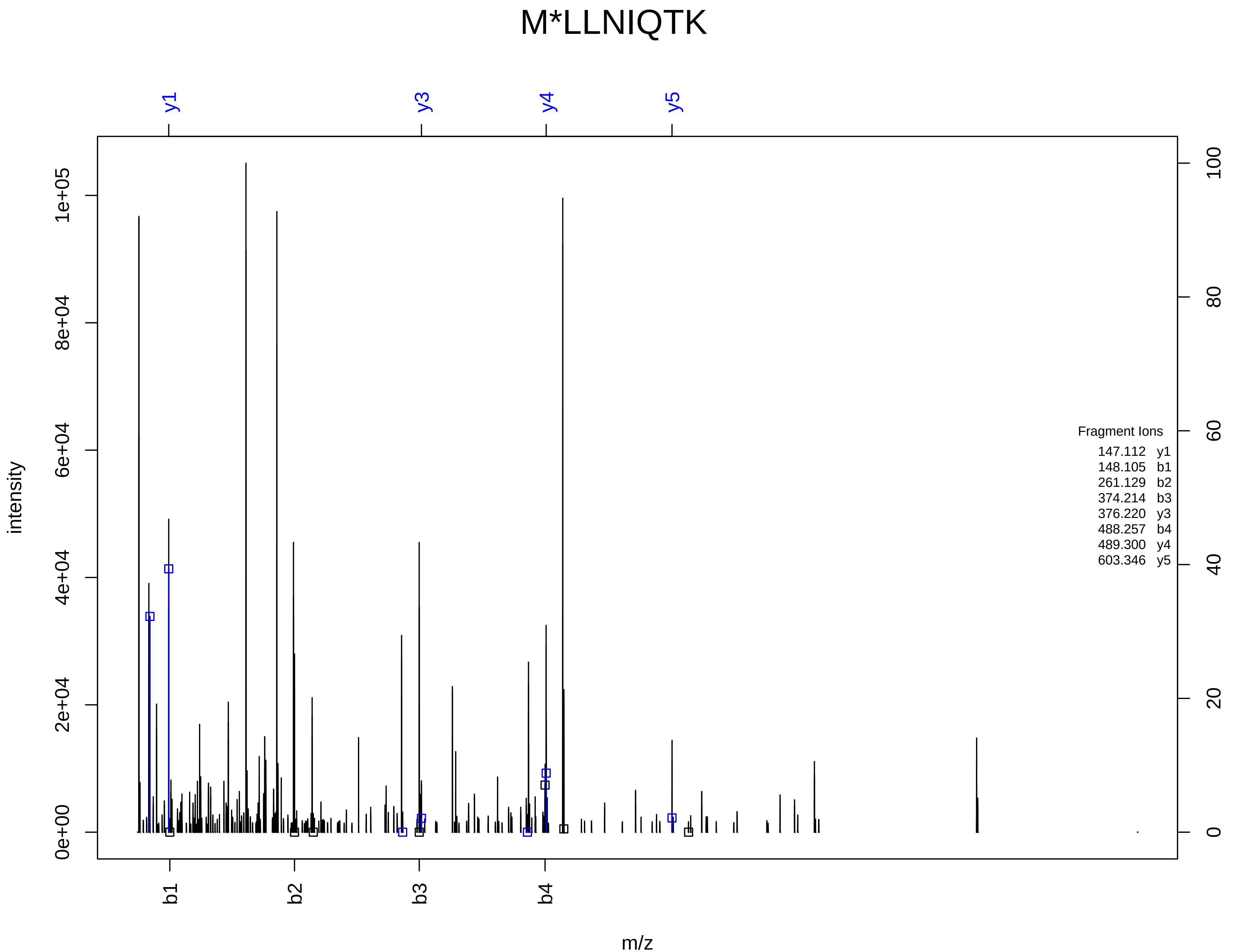
M*LLNIQTK
y1
y3
y4
y5
intensity
0e+00
2e+04
4e+04
6e+04
8e+04
1e+05
0
20
40
60
80
100
Fragment Ions
147.112 y1
148.105 b1
261.129 b2
374.214 b3
376.220 y3
488.257 b4
489.300 y4
603.346 y5
b1
b2
b3
b4
m/z
MLLNIQTK / File: christine_10pools_04.raw; SpectrumID: ; scans: 25683

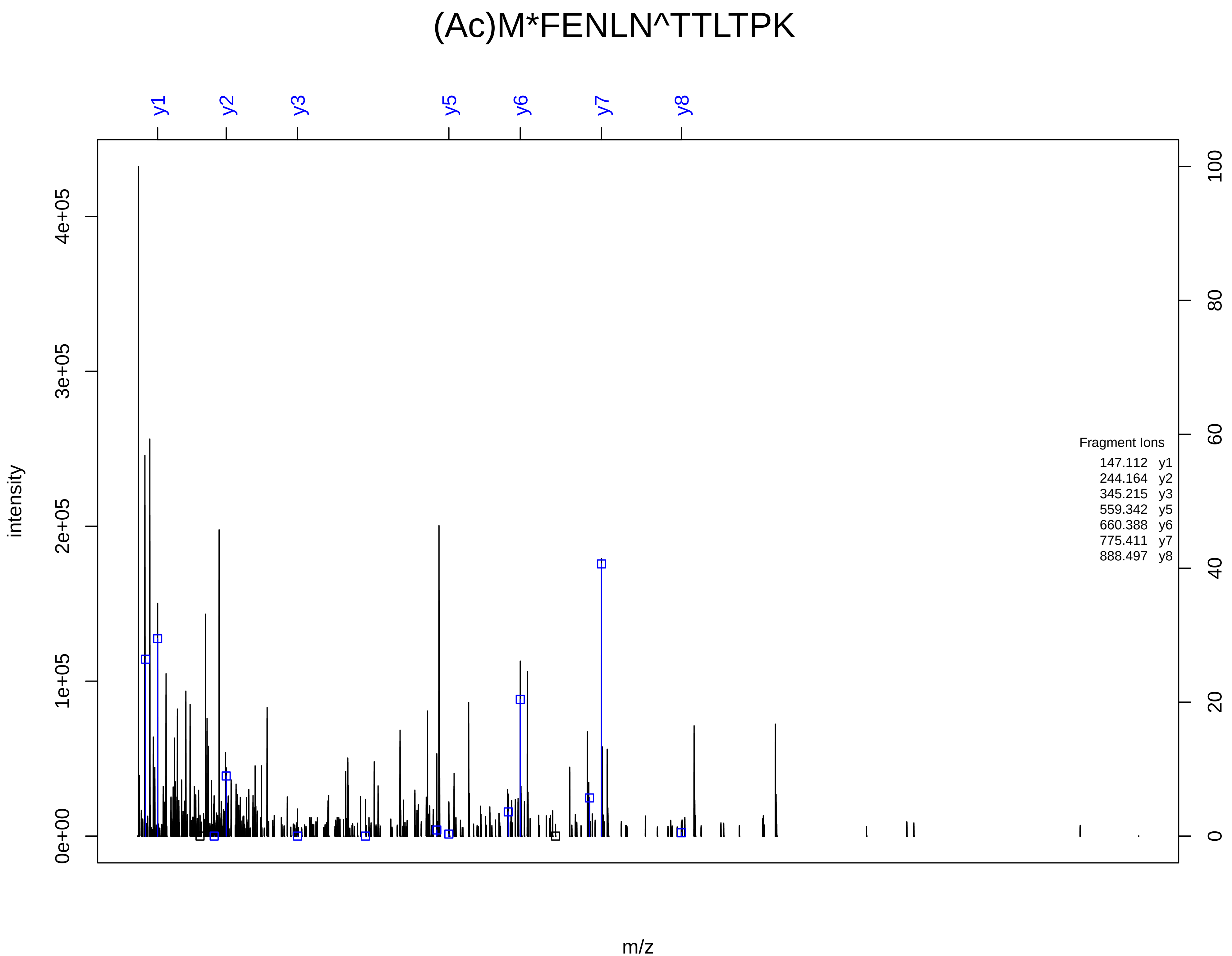

(Ac)M*FENLN^TTLTPK
y1
y2
y3
y5
y6
y7
y8
intensity
0e+00
1e+05
2e+05
3e+05
4e+05
0
20
40
60
80
100
Fragment Ions
147.112 y1
244.164 y2
345.215 y3
559.342 y5
660.388 y6
775.411 y7
888.497 y8
m/z
MFENLNTTLTPK / File: christine_10pools_04.raw; SpectrumID: ; scans: 4453

PLIPILR

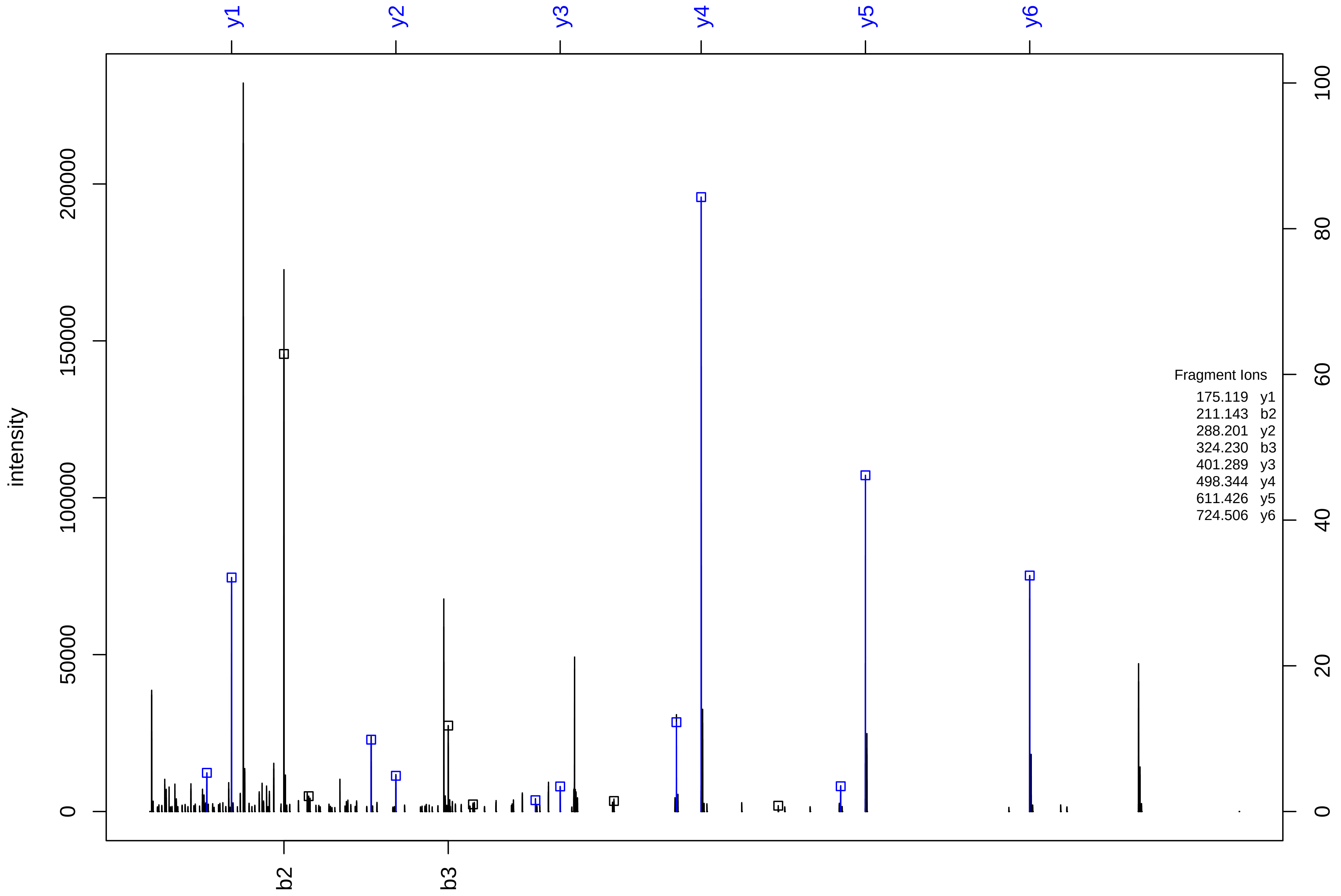

m/z

PLIPILR / File: christine_10pools_04.raw; SpectrumID: ; scans: 22319

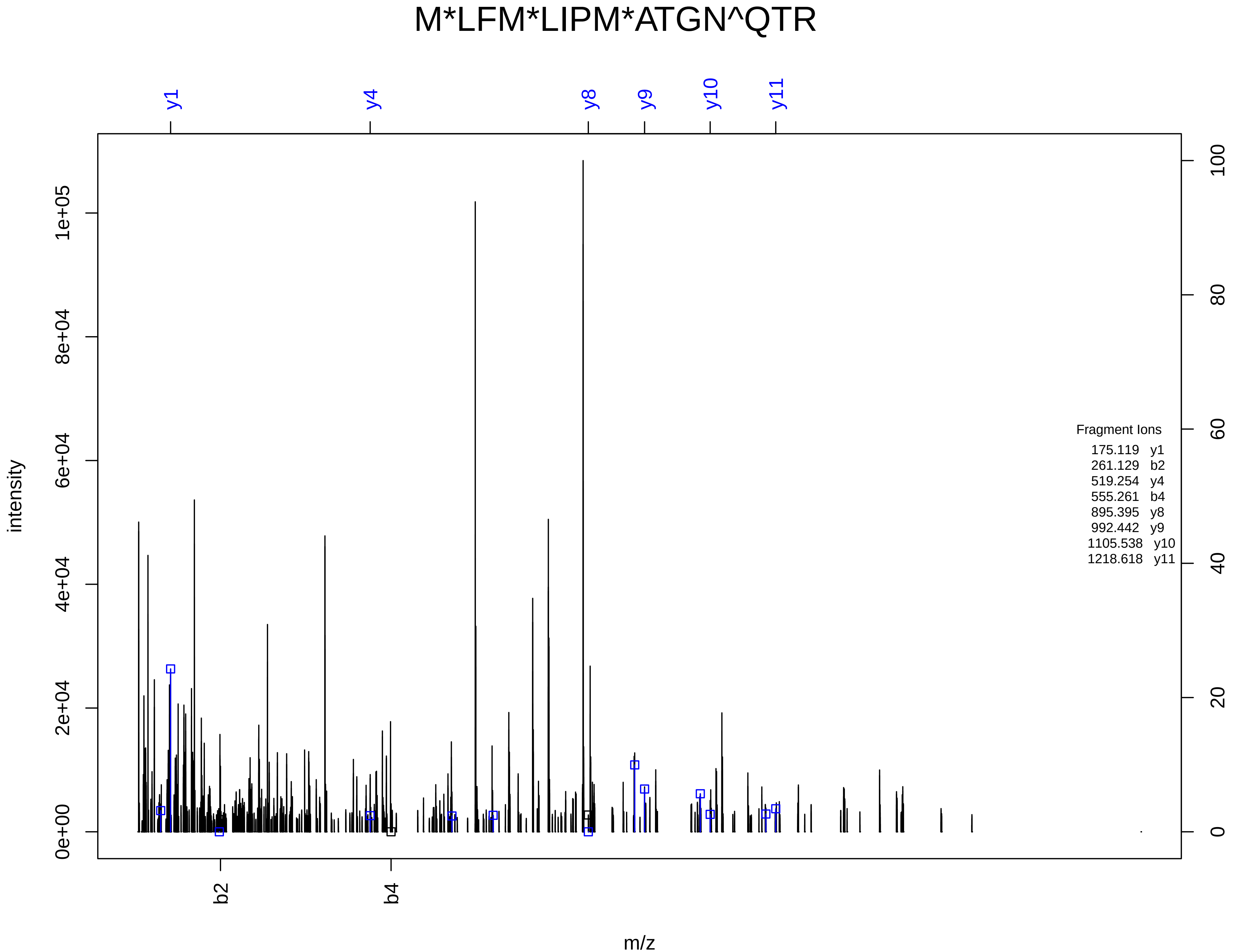

MLFMLIPMATGNQTR / File: christine_10pools_04.raw; SpectrumID: ; scans: 33138
